# Supplementary material for: Global burden of 369 diseases and injuries in 204 countries and territories, 1990–2019: a systematic analysis for the Global Burden of Disease Study 2019
Source: Lancet. 2020 Oct 17;396(10258):1204–22. doi: 10.1016/S0140-6736(20)30925-9 (PMC7567026; doi:10.1016/S0140-6736(20)30925-9)
Supplement: Supplementary appendix 2 [file mmc2c.pdf]

THE LANCET

Supplementary appendix 2c (Part 3 of 5)

This appendix formed part of the original submission and has been peer reviewed. We post it as supplied by the authors.

Supplement to: GBD 2019 Diseases and Injuries Collaborators. Global burden of 369 diseases and injuries in 204 countries and territories, 1990–2019: a systematic analysis for the Global Burden of Disease Study 2019. *Lancet* 2020; **396:** 1204–22.

Appendix 2C: supplementary results appendix to “Global burden of 369 diseases and injuries in 204 countries and territories, 1990–2019: a systematic analysis for the Global Burden of Disease Study 2019”

This supplement provides additional figures and more detailed results for “Global burden of 369 diseases and injuries in 204 countries and territories, 1990–2019: a systematic analysis for the Global Burden of Disease Study 2019."

List of supplementary results appendix figures and tables:

Appendix 2A

Figure S1: Leading GBD Level 3 causes of age-standardised YLDs rates by location for a) females and b) males, 2019. 1

Figure S2: Leading GBD Level 3 causes of age-standardised prevalence rates by location for a) females and b) males, 2019. 3

Figure S3: Trends of age-standardised YLD rates per 100,000 for the top eight Level 3 causes of non-fatal burden in 2019 by SDI quintile for both sexes, from 1990 to 2019. 5

Figure S4: Death counts due to fatal discontinuities by type, 1980–2019. 6

Figure S5: Leading level 3 causes of all-age DALY counts by location for females in 1990 (A) males in 1990 (B) females in 2019 (C) males in 2019 (D) 10

Figure S6: Leading 20 GBD cause hierarchy Level 3 causes of YLLs by SDI grouping and sex for 1990, 2010, and 2019, with percentage change in number of YLLs, and all-age and age-standardised rates. 14

Figure S7: Global distribution of total YLDs by 23 GBD age groups for Level 2 causes of non-fatal burden in 1990 and 2019 for a) females and b) males. 24

Figure S8: Global leading 30 level 3 causes of DALYs for 1990, 2010, and 2019, with percentage change in number of DALYs, and age-standardised rates, by sex and SDI quintile 26

Table S1: All-age DALYs counts and age standardised DALY rates per 100,000 individuals for all causes and level 1 cause groups by location for 1990, 2010, 2019 , both sexes combined. 36

Appendix 2B

Table S2a: Global DALYS by cause and age group for males with 95% uncertainty intervals, by location for 2019. 4734

Appendix 2C

Table S2b: Global DALYS by cause and age group for females with 95% uncertainty intervals, by location for 2019. 6708

Appendix 2D

Table S2c: Global DALYS by cause and age group for both sexes combined with 95% uncertainty intervals, by location for 2019. 8680

Appendix 2E

Table S3: Global deaths and YLL numbers, age-standardised rates per 100,000, and percentage change between 2010 and 2019 for males for all GBD causes and Levels 1 through 4 of the cause hierarchy 10630

Table S4: Global deaths by cause and age group for females (1990, 2010, 2019) 10635

Table S5: Global deaths by cause and age group for males (1990, 2010, 2019) 10642

Table S6: Global deaths by cause and age group for both sexes combined (1990, 2010, 2019) 10648

Table S7: Global YLLs by cause and age group for females. 10654

Table S8: Global YLLs by cause and age group for males. 10656

Table S9: Global YLLs by cause and age group for both sexes combined 10658

Table S10: Deaths due to fatal discontinuities by country, all ages, for both sexes combined 10660

Table S11: Diarrhoeal diseases and lower respiratory infection aetiologies deaths, mortality rates, and annual percentage change from 1990 to 2019. 10676

Table S12: Deaths by location, cause, and age group for males. 10677

Table S13: Deaths by location, cause, and age group for females. 13007

Table S14: Deaths by location, cause, and age group for both sexes combined. 15354

Table S15: Distribution of percentage change in age-standardised deaths for Level 1 causes by location from 1990-2010 and 2010-2019, both sexes. 17543

Table S16: Global deaths and YLL numbers, age-standardised rates per 100,000, and percentage change between 2010 and 2019 for females for all GBD causes and Levels 1 through 4 of the cause hierarchy. 17561

Table S17: Global prevalence, incidence and YLDs for 2019, percentage change of YLDs counts, and percentage change of YLD rates between 2010 and 2019 by cause and by age 17566

Table S18: Prevalence, incidence, and YLDs for 2019, percentage change of YLD counts, and percentage change of age-standardised YLD rates between 2010 and 2019 by cause and GBD location. 17707

SR Table 2b: Global DALYS by cause and age group for females with 95% uncertainty intervals, 2019

Cause	Early Neonatal	Late Neonatal	Post Neonatal	1 to 4	5 to 9	10 to 14	15 to 19	20 to 24	25 to 29	30 to 34	35 to 39	40 to 44	45 to 49	50 to 54	55 to 59	60 to 64	65 to 69	70 to 74	75 to 79	80 to 84	85 to 89	90 to 94	95 plus	Total
All causes	72,400,000 (62.300.000 to 84.400.000)	20,200,000 (17.400.000 to 23.700.000)	58,600,000 (49.500.000 to 70.100.000)	63,400,000 (55.400.000 to 73.500.000)	27,500,000 (23.200.000 to 32.500.000)	27,800,000 (22.600.000 to 34.000.000)	37,200,000 (30.600.000 to 45.100.000)	44,200,000 (36.800.000 to 52.800.000)	50,100,000 (41.700.000 to 60.600.000)	55,400,000 (46.000.000 to 66.900.000)	57,300,000 (48.000.000 to 68.400.000)	59,900,000 (50.600.000 to 70.900.000)	64,200,000 (54.600.000 to 74.900.000)	72,000,000 (62.400.000 to 83.300.000)	73,400,000 (64.100.000 to 84.000.000)	77,100,000 (68.300.000 to 86.800.000)	78,600,000 (70.300.000 to 87.700.000)	71,900,000 (65.200.000 to 79.200.000)	61,900,000 (56.700.000 to 67.600.000)	55,300,000 (51.100.000 to 59.600.000)	35,600,000 (33.100.000 to 38.200.000)	17,900,000 (16.800.000 to 19.000.000)	6,500,000 (6.180.000 to 6.830.000)	1.190.000.000
Communicable, maternal, neonatal, and nutritional diseases	65,000,000 (55.900.000 to 75.800.000)	17,100,000 (14.700.000 to 20.300.000)	45,300,000 (37.700.000 to 55.200.000)	46,800,000 (40.500.000 to 54.600.000)	13,900,000 (11.800.000 to 16.300.000)	9,360,000 (7.920.000 to 11.100.000)	10,200,000 (8.750.000 to 11.800.000)	11,200,000 (9.930.000 to 12.800.000)	11,900,000 (10.600.000 to 13.300.000)	12,300,000 (11.000.000 to 13.800.000)	11,800,000 (10.600.000 to 13.300.000)	10,300,000 (9.140.000 to 11.600.000)	8,450,000 (7.450.000 to 9.610.000)	7,670,000 (6.660.000 to 8.850.000)	6,590,000 (5.710.000 to 7.660.000)	6,230,000 (5.340.000 to 7.380.000)	5,870,000 (4.980.000 to 7.100.000)	5,500,000 (4.590.000 to 6.850.000)	4,570,000 (3.750.000 to 5.770.000)	3,990,000 (3.240.000 to 5.190.000)	2,430,000 (1.980.000 to 3.000.000)	1,240,000 (986.000 to 1.490.000)	500,000 (378.000 to 580.000)	318.000.000
HIV/AIDS and sexually transmitted infections	2,020,000 (702.000 to 4.170.000)	491,000 (168.000 to 1.020.000)	1,370,000 (906.000 to 2.060.000)	1,300,000 (1.170.000 to 1.460.000)	530,000 (490.000 to 574.000)	587,000 (552.000 to 622.000)	1,170,000 (1.060.000 to 1.300.000)	1,770,000 (1.630.000 to 1.940.000)	2,870,000 (2.620.000 to 3.140.000)	3,720,000 (3.470.000 to 4.020.000)	3,710,000 (3.480.000 to 3.980.000)	3,120,000 (2.950.000 to 3.310.000)	2,350,000 (2.210.000 to 2.500.000)	1,560,000 (1.480.000 to 1.650.000)	902,000 (846.000 to 964.000)	492,000 (457.000 to 533.000)	261,000 (239.000 to 288.000)	118,000 (105.000 to 133.000)	55,100 (48.600 to 64.100)	16,600 (13.500 to 21.400)	7,920 (6.420 to 10.200)	2,950 (2.340 to 3.800)	862 (693 to 1.080)	28.400.000
HIV/AIDS	75·7 (52·3 to 104)	273 (190 to 376)	701,000 (617.000 to 809.000)	1,270,000 (1.150.000 to 1.410.000)	513,000 (475.000 to 552.000)	578,000 (544.000 to 613.000)	1,130,000 (1.030.000 to 1.260.000)	1,700,000 (1.570.000 to 1.850.000)	2,740,000 (2.510.000 to 2.990.000)	3,580,000 (3.350.000 to 3.840.000)	3,580,000 (3.370.000 to 3.800.000)	2,990,000 (2.840.000 to 3.140.000)	2,260,000 (2.140.000 to 2.380.000)	1,500,000 (1.430.000 to 1.570.000)	851,000 (806.000 to 898.000)	461,000 (433.000 to 490.000)	235,000 (219.000 to 253.000)	101,000 (92.200 to 110.000)	43,500 (39.600 to 47.400)	8,990 (7.900 to 10.400)	3,730 (3.220 to 4.380)	1,110 (940 to 1.330)	273 (229 to 329)	24.200.000
HIV/AIDS - Drug-susceptible Tuberculosis	2·8 (1·7 to 4·2)	27·3 (17·7 to 37·7)	176,000 (129.000 to 224.000)	337,000 (252.000 to 413.000)	137,000 (106.000 to 166.000)	156,000 (120.000 to 190.000)	281,000 (203.000 to 360.000)	411,000 (297.000 to 517.000)	630,000 (456.000 to 802.000)	811,000 (586.000 to 1.030.000)	808,000 (589.000 to 1.030.000)	685,000 (500.000 to 872.000)	507,000 (370.000 to 653.000)	341,000 (249.000 to 438.000)	192,000 (141.000 to 247.000)	102,000 (73.900 to 131.000)	49,700 (36.600 to 63.600)	22,700 (17.000 to 28.400)	9,950 (7.420 to 12.500)	1,830 (1.400 to 2.270)	706 (536 to 881)	198 (151 to 245)	43·9 (34·2 to 54·1)	5.660.000
HIV/AIDS - Multidrug-resistant Tuberculosis wi	0·1 (0·1 to 0·2)	1·3 (0·7 to 2·1)	21,300 (9.140 to 39.500)	39,200 (15.700 to 75.400)	14,800 (6.390 to 27.500)	15,500 (7.000 to 29.100)	29,600 (12.800 to 56.800)	41,500 (18.100 to 78.800)	59,200 (26.400 to 114.000)	73,600 (33.300 to 138.000)	72,400 (32.900 to 135.000)	61,100 (28.200 to 113.000)	45,000 (20.700 to 83.400)	30,500 (13.900 to 56.400)	17,100 (7.730 to 31.700)	9,000 (4.080 to 16.700)	4,350 (1.990 to 8.080)	1,910 (878 to 3.550)	826 (379 to 1.520)	150 (72 to 274)	55·3 (26·7 to 101)	14·8 (7·3 to 26·8)	3·1 (1·6 to 5·5)	537.000
HIV/AIDS - Extensively drug-resistant Tuberc	0 (0 to 0)	0 (0 to 0)	574 (256 to 1.060)	703 (288 to 1.360)	314 (142 to 601)	312 (139 to 590)	623 (266 to 1.160)	1,010 (442 to 1.860)	1,880 (875 to 3.500)	2,800 (1.360 to 5.100)	2,750 (1.340 to 5.010)	2,190 (1.060 to 3.960)	1,530 (729 to 2.790)	965 (454 to 1.780)	546 (259 to 1.000)	281 (133 to 532)	142 (64·2 to 281)	47·5 (20·7 to 94·8)	19·1 (8·4 to 38)	4·6 (1·9 to 9·6)	1·8 (0·8 to 3·8)	0·5 (0·2 to 1)	0·1 (0·1 to 0·3)	16.700
HIV/AIDS resulting in other diseases	72·7 (50·6 to 100)	244 (166 to 341)	503,000 (430.000 to 610.000)	892,000 (773.000 to 1.060.000)	361,000 (322.000 to 405.000)	406,000 (362.000 to 453.000)	823,000 (712.000 to 963.000)	1,240,000 (1.080.000 to 1.430.000)	2,050,000 (1.790.000 to 2.340.000)	2,700,000 (2.380.000 to 3.020.000)	2,690,000 (2.400.000 to 3.010.000)	2,240,000 (2.000.000 to 2.500.000)	1,710,000 (1.520.000 to 1.880.000)	1,130,000 (1.010.000 to 1.240.000)	642,000 (573.000 to 711.000)	350,000 (312.000 to 389.000)	181,000 (161.000 to 203.000)	76,200 (66.900 to 86.500)	32,700 (28.600 to 37.100)	7,010 (5.960 to 8.230)	2,960 (2.480 to 3.540)	901 (736 to 1.100)	225 (185 to 275)	18.000.000
Sexually transmitted infections excluding HIV	2,020,000 (702.000 to 4.170.000)	491,000 (167.000 to 1.020.000)	666,000 (221.000 to 1.410.000)	35,600 (11.900 to 77.200)	17,000 (5.340 to 37.400)	8,380 (6.320 to 11.100)	33,600 (23.400 to 51.300)	75,800 (48.500 to 126.000)	121,000 (75.000 to 201.000)	140,000 (85.600 to 237.000)	135,000 (80.100 to 234.000)	128,000 (75.700 to 231.000)	92,700 (52.300 to 164.000)	63,700 (39.200 to 112.000)	50,800 (32.500 to 84.700)	31,500 (19.000 to 56.500)	25,800 (15.800 to 43.900)	16,900 (10.600 to 28.800)	11,600 (7.780 to 18.900)	7,570 (5.100 to 12.000)	4,190 (2.960 to 6.300)	1,840 (1.310 to 2.620)	589 (434 to 800)	4.180.000
Syphilis	2,020,000 (702.000 to 4.170.000)	491,000 (167.000 to 1.020.000)	666,000 (221.000 to 1.410.000)	35,600 (11.900 to 77.200)	17,000 (5.340 to 37.400)	3,090 (2.350 to 3.850)	3,580 (2.760 to 4.680)	2,730 (1.940 to 4.130)	5,960 (4.470 to 7.760)	6,230 (4.740 to 7.860)	3,390 (2.600 to 4.380)	1,570 (1.190 to 2.190)	2,020 (1.640 to 2.510)	2,380 (1.770 to 3.040)	1,110 (842 to 1.400)	1,040 (816 to 1.270)	1,160 (914 to 1.420)	1,040 (831 to 1.250)	708 (573 to 849)	751 (588 to 895)	441 (323 to 536)	272 (192 to 350)	87·1 (60·2 to 112)	3.260.000
Chlamydial infection	0 (0 to 0)	0 (0 to 0)	0 (0 to 0)	0 (0 to 0)	0 (0 to 0)	953 (709 to 1.290)	3,780 (2.640 to 5.420)	10,500 (7.760 to 14.400)	14,100 (9.540 to 20.700)	14,400 (9.090 to 22.900)	16,100 (10.300 to 25.500)	16,100 (10.300 to 26.400)	9,290 (5.750 to 15.500)	4,310 (3.290 to 5.650)	3,890 (2.950 to 4.970)	1,860 (1.400 to 2.300)	1,120 (872 to 1.390)	1,010 (808 to 1.190)	835 (695 to 969)	741 (585 to 865)	426 (326 to 500)	205 (152 to 247)	81·2 (58·6 to 97·3)	99.800
Gonococcal infection	0 (0 to 0)	0 (0 to 0)	0 (0 to 0)	0 (0 to 0)	0 (0 to 0)	964 (759 to 1.180)	5,050 (4.080 to 6.230)	9,550 (7.560 to 11.500)	13,400 (10.400 to 16.400)	13,400 (10.500 to 16.400)	11,300 (8.950 to 14.000)	13,100 (10.300 to 16.400)	8,980 (7.260 to 10.900)	9,000 (6.620 to 11.400)	9,050 (6.460 to 11.900)	4,940 (3.770 to 6.070)	6,100 (4.760 to 7.540)	3,470 (2.790 to 4.100)	3,010 (2.510 to 3.490)	1,770 (1.420 to 2.060)	1,120 (865 to 1.320)	542 (404 to 652)	217 (157 to 255)	115.000
Trichomoniasis	0 (0 to 0)	0 (0 to 0)	0 (0 to 0)	0 (0 to 0)	0 (0 to 0)	515 (154 to 1.290)	6,760 (2.170 to 15.700)	24,900 (8.880 to 58.100)	40,800 (13.800 to 94.300)	43,400 (14.200 to 102.000)	37,600 (12.300 to 86.900)	32,900 (11.200 to 77.400)	28,300 (9.000 to 64.600)	21,500 (6.710 to 50.500)	14,600 (4.780 to 34.500)	9,620 (3.310 to 22.500)	6,910 (2.450 to 16.100)	4,290 (1.610 to 9.760)	2,500 (949 to 5.530)	1,390 (541 to 3.060)	580 (223 to 1.310)	168 (63·4 to 377)	26·7 (8·7 to 64·8)	277.000
Genital herpes	0 (0 to 0)	0 (0 to 0)	0 (0 to 0)	0 (0 to 0)	0 (0 to 0)	1,180 (383 to 2.770)	4,830 (1.610 to 11.400)	9,480 (3.190 to 23.400)	14,400 (4.870 to 34.900)	18,100 (5.980 to 44.500)	18,300 (5.900 to 44.900)	17,400 (5.540 to 43.500)	16,700 (5.290 to 42.300)	15,300 (4.740 to 38.100)	12,700 (3.890 to 31.500)	10,200 (3.070 to 25.000)	7,880 (2.380 to 19.300)	5,340 (1.620 to 13.100)	3,480 (1.050 to 8.510)	2,240 (676 to 5.570)	1,140 (340 to 2.840)	449 (135 to 1.090)	128 (38·9 to 311)	159.000
Other sexually transmitted infections	0 (0 to 0)	0 (0 to 0)	0 (0 to 0)	0 (0 to 0)	0 (0 to 0)	1,680 (1.270 to 2.230)	9,590 (7.420 to 12.500)	18,600 (13.700 to 24.900)	32,400 (21.700 to 48.000)	44,800 (30.300 to 67.300)	47,800 (30.400 to 72.300)	[illegible]	27,500 (16.500 to 44.700)	11,300 (8.220 to 15.500)	9,420 (7.280 to 11.900)	3,850 (2.810 to 4.830)	2,620 (2.030 to 3.250)	1,740 (1.390 to 2.060)	1,080 (891 to 1.260)	671 (529 to 784)	482 (359 to 572)	199 (146 to 243)	49·4 (35·4 to 60·4)	261.000
Respiratory infections and tuberculosis	4,590,000 (3.660.000 to 5.600.000)	3,450,000 (2.840.000 to 4.140.000)	14,800,000 (12.100.000 to 18.300.000)	8,980,000 (7.110.000 to 11.100.000)	2,080,000 (1.700.000 to 2.510.000)	1,310,000 (1.070.000 to 1.590.000)	1,500,000 (1.260.000 to 1.810.000)	1,920,000 (1.630.000 to 2.260.000)	1,990,000 (1.710.000 to 2.330.000)	2,010,000 (1.730.000 to 2.350.000)	2,000,000 (1.730.000 to 2.310.000)	1,950,000 (1.700.000 to 2.250.000)	1,870,000 (1.630.000 to 2.130.000)	2,290,000 (1.980.000 to 2.650.000)	2,250,000 (1.970.000 to 2.580.000)	2,380,000 (2.110.000 to 2.690.000)	2,410,000 (2.130.000 to 2.690.000)	2,440,000 (2.170.000 to 2.730.000)	2,180,000 (1.930.000 to 2.410.000)	2,030,000 (1.750.000 to 2.260.000)	1,380,000 (1.120.000 to 1.540.000)	796,000 (607.000 to 894.000)	354,000 (261.000 to 403.000)	67.000.000
Tuberculosis	0·6 (0·3 to 1)	20·7 (9·8 to 36·8)	1,130,000 (859.000 to 1.470.000)	1,150,000 (907.000 to 1.460.000)	486,000 (384.000 to 606.000)	384,000 (310.000 to 469.000)	705,000 (596.000 to 848.000)	1,130,000 (941.000 to 1.330.000)	1,190,000 (1.010.000 to 1.400.000)	1,240,000 (1.050.000 to 1.450.000)	1,210,000 (1.030.000 to 1.420.000)	1,150,000 (976.000 to 1.360.000)	1,060,000 (913.000 to 1.250.000)	1,290,000 (1.080.000 to 1.600.000)	1,170,000 (990.000 to 1.430.000)	1,040,000 (897.000 to 1.260.000)	885,000 (769.000 to 1.080.000)	799,000 (695.000 to 987.000)	586,000 (503.000 to 723.000)	409,000 (344.000 to 512.000)	166,000 (136.000 to 208.000)	49,000 (38.600 to 62.600)	13,900 (10.600 to 18.200)	17.200.000
Latent tuberculosis infection	0 (0 to 0)	0 (0 to 0)	0 (0 to 0)	0 (0 to 0)	0 (0 to 0)	0 (0 to 0)	0 (0 to 0)	0 (0 to 0)	0 (0 to 0)	0 (0 to 0)	0 (0 to 0)	0 (0 to 0)	0 (0 to 0)	0 (0 to 0)	0 (0 to 0)	0 (0 to 0)	0 (0 to 0)	0 (0 to 0)	0 (0 to 0)	0 (0 to 0)	0 (0 to 0)	0 (0 to 0)	0 (0 to 0)	0
[illegible]	[illegible]	[illegible]	[illegible]	[illegible]	[illegible]	[illegible]	[illegible]	[illegible]	[illegible]	[illegible]	[illegible]	[illegible]	[illegible]	[illegible]	[illegible]	[illegible]	[illegible]	[illegible]	[illegible]	[illegible]	[illegible]	[illegible]	[illegible]	[illegible]

Anorexia nervosa	0 (0 to 0)	0 (0 to 0)	0 (0 to 0)	0 (0 to 0)	5,150 (1,580 to 11,200)
Bulimia nervosa	0 (0 to 0)	0 (0 to 0)	0 (0 to 0)	0 (0 to 0)	617 (189 to 1,410)
Autism spectrum disorders	432 (279 to 633)	1,260 (831 to 1,860)	20,200 (13,100 to 29,700)	86,400 (56,200 to 127,000)	103,000 (67,000 to 152,000)
Attention-deficit/hyperactivity disorder	0 (0 to 0)	0 (0 to 0)	0 (0 to 0)	4,280 (2,240 to 7,620)	44,300 (23,600 to 78,600)
Conduct disorder	0 (0 to 0)	0 (0 to 0)	0 (0 to 0)	0 (0 to 0)	266,000 (136,000 to 485,000)
Idiopathic developmental intellectual disability	698 (310 to 1,230)	2,150 (953 to 3,780)	39,500 (19,000 to 67,600)	201,000 (107,000 to 328,000)	267,000 (147,000 to 432,000)
Other mental disorders	0 (0 to 0)	0 (0 to 0)	0 (0 to 0)	0 (0 to 0)	0 (0 to 0)
Substance use disorders	4 (2·5 to 6·5)	12·3 (7·5 to 19·7)	209 (126 to 331)	1,070 (652 to 1,670)	1,360 (835 to 2,080)
Alcohol use disorders	4 (2·5 to 6·5)	12·3 (7·5 to 19·7)	209 (126 to 331)	1,070 (652 to 1,670)	1,360 (835 to 2,080)
Drug use disorders	0 (0 to 0)	0 (0 to 0)	0 (0 to 0)	0 (0 to 0)	0 (0 to 0)
Opioid use disorders	0 (0 to 0)	0 (0 to 0)	0 (0 to 0)	0 (0 to 0)	0 (0 to 0)
Cocaine use disorders	0 (0 to 0)	0 (0 to 0)	0 (0 to 0)	0 (0 to 0)	0 (0 to 0)
Amphetamine use disorders	0 (0 to 0)	0 (0 to 0)	0 (0 to 0)	0 (0 to 0)	0 (0 to 0)
Cannabis use disorders	0 (0 to 0)	0 (0 to 0)	0 (0 to 0)	0 (0 to 0)	0 (0 to 0)
Other drug use disorders	0 (0 to 0)	0 (0 to 0)	0 (0 to 0)	0 (0 to 0)	0 (0 to 0)
Diabetes and kidney diseases	753 (539 to 958)	2,140 (1,530 to 2,850)	206,000 (167,000 to 254,000)	323,000 (258,000 to 391,000)	274,000 (224,000 to 339,000)
Diabetes mellitus	751 (538 to 955)	2,110 (1,500 to 2,830)	48,200 (34,100 to 64,300)	51,000 (37,300 to 68,100)	69,600 (52,200 to 89,000)
Diabetes mellitus type 1	751 (538 to 955)	2,110 (1,500 to 2,830)	48,200 (34,100 to 64,300)	51,000 (37,300 to 68,100)	69,600 (52,200 to 89,000)
Diabetes mellitus type 2	0 (0 to 0)	0 (0 to 0)	0 (0 to 0)	0 (0 to 0)	0 (0 to 0)
Chronic kidney disease	1·5 (0·7 to 2·9)	31·3 (17·5 to 52·1)	154,000 (124,000 to 190,000)	264,000 (211,000 to 322,000)	196,000 (154,000 to 253,000)
Chronic kidney disease due to diabetes mellit	0 (0 to 0)	0 (0 to 0)	1,030 (490 to 2,030)	3,270 (2,000 to 5,150)	4,640 (2,720 to 7,520)
Chronic kidney disease due to diabetes mellit	0 (0 to 0)	0 (0 to 0)	0 (0 to 0)	0 (0 to 0)	0 (0 to 0)
Chronic kidney disease due to hypertension	0 (0 to 0)	0 (0 to 0)	0 (0 to 0)	0 (0 to 0)	0 (0 to 0)
Chronic kidney disease due to glomerulonephr	0·4 (0·2 to 0·9)	8 (4·1 to 14·7)	46,300 (26,100 to 74,200)	88,500 (53,100 to 136,000)	63,700 (39,700 to 92,600)
Chronic kidney disease due to other and unsp	1·1 (0·5 to 2·1)	23·3 [illegible]	107,000 [illegible]	172,000 [illegible]	128,000 [illegible]
Acute glomerulonephritis	0 (0 to 0)	[illegible]	[illegible]	[illegible]	[illegible]
Skin and subcutaneous diseases	89·6 (53·4 to 149)	[illegible]	[illegible]	[illegible]	[illegible]
Dermatitis	0 (0 to 0)	[illegible]	[illegible]	[illegible]	[illegible]
Atopic dermatitis	0 (0 to 0)	[illegible]	[illegible]	[illegible]	[illegible]
Contact dermatitis	0 (0 to 0)	[illegible]	[illegible]	[illegible]	[illegible]
Seborrhoeic dermatitis	0 (0 to 0)	[illegible]	[illegible]	[illegible]	[illegible]
Psoriasis	0 (0 to 0)	[illegible]	[illegible]	[illegible]	[illegible]
Bacterial skin diseases	13·5 (6·2 to 26·2)	[illegible]	[illegible]	[illegible]	[illegible]
Cellulitis	1·9 (1·2 to 2·9)	[illegible]	[illegible]	[illegible]	[illegible]
Pyoderma	11·6 (4·7 to 24·1)	[illegible]	[illegible]	[illegible]	[illegible]
Scabies	25·5 (13·1 to 44·4)	[illegible]	[illegible]	[illegible]	[illegible]
Fungal skin diseases	9·1 (3·4 to 20)	[illegible]	[illegible]	[illegible]	[illegible]
Viral skin diseases	0 (0 to 0)	[illegible]	[illegible]	[illegible]	[illegible]
Acne vulgaris	0 (0 to 0)	[illegible]	[illegible]	[illegible]	[illegible]
Alopecia areata	0 (0 to 0)	[illegible]	[illegible]	[illegible]	[illegible]
Pruritus	0·6 (0·2 to 1·1)	[illegible]	[illegible]	[illegible]	[illegible]
Urticaria	35 (20·7 to 55·2)	[illegible]	[illegible]	[illegible]	[illegible]
Decubitus ulcer	0 (0 to 0)	[illegible]	[illegible]	[illegible]	[illegible]
Other skin and subcutaneous diseases	6 (2·8 to 11·1)	[illegible]	[illegible]	[illegible]	[illegible]
Sense organ diseases	418 (255 to 642)	[illegible]	[illegible]	[illegible]	[illegible]
Blindness and vision loss	204 (109 to 342)	[illegible]	[illegible]	[illegible]	[illegible]
Glaucoma	0 (0 to 0)	[illegible]	[illegible]	[illegible]	[illegible]
Cataract	0 (0 to 0)	[illegible]	[illegible]	[illegible]	[illegible]
Age-related macular degeneration	0 (0 to 0)	[illegible]	[illegible]	[illegible]	[illegible]
Refraction disorders	11·2 (5·1 to 22·6)	[illegible]	[illegible]	[illegible]	[illegible]
Near vision loss	0 (0 to 0)	[illegible]	[illegible]	[illegible]	[illegible]
Other vision loss	193 (105 to 321)	[illegible]	[illegible]	[illegible]	[illegible]
Age-related and other hearing loss	104 (42·8 to 214)	[illegible]	[illegible]	[illegible]	[illegible]
Other sense organ diseases	110 (51·7 to 201)	[illegible]	[illegible]	[illegible]	[illegible]
Musculoskeletal disorders	0 (0 to 0)	[illegible]	[illegible]	[illegible]	[illegible]
Rheumatoid arthritis	0 (0 to 0)	[illegible]	[illegible]	[illegible]	[illegible]
Osteoarthritis	0 (0 to 0)	[illegible]	[illegible]	[illegible]	[illegible]
Osteoarthritis hip	0 (0 to 0)	[illegible]	[illegible]	[illegible]	[illegible]
Osteoarthritis knee	0 (0 to 0)	[illegible]	[illegible]	[illegible]	[illegible]
Osteoarthritis hand	0 (0 to 0)	[illegible]	[illegible]	[illegible]	[illegible]
Osteoarthritis other	0 (0 to 0)	[illegible]	[illegible]	[illegible]	[illegible]
Low back pain	0 (0 to 0)	[illegible]	[illegible]	[illegible]	[illegible]
Neck pain	0 (0 to 0)	[illegible]	[illegible]	[illegible]	[illegible]
Gout	0 (0 to 0)	[illegible]	[illegible]	[illegible]	[illegible]
Other musculoskeletal disorders	0 (0 to 0)	[illegible]	[illegible]	[illegible]	[illegible]
Other non-communicable diseases	6,560,000 (5,450,000 to 7,990,000)	[illegible]	[illegible]	[illegible]	[illegible]
Congenital birth defects	6,480,000 (5,380,000 to 7,920,000)	[illegible]	[illegible]	[illegible]	[illegible]
Neural tube defects	1,440,000 (995,000 to 2,020,000)	[illegible]	[illegible]	[illegible]	[illegible]
Congenital heart anomalies	1,770,000 (1,340,000 to 2,280,000)	[illegible]	[illegible]	[illegible]	[illegible]
Orofacial clefts	50,900 (27,500 to 110,000)	[illegible]	[illegible]	[illegible]	[illegible]
Down syndrome	130,000 (88,900 to 207,000)	[illegible]	[illegible]	[illegible]	[illegible]
Turner syndrome	9 (3·4 to 17·4)	[illegible]	[illegible]	[illegible]	[illegible]
Klinefelter syndrome	0 (0 to 0)	[illegible]	[illegible]	[illegible]	[illegible]
Other chromosomal abnormalities	277,000 (177,000 to 489,000)	[illegible]	[illegible]	[illegible]	[illegible]
Congenital musculoskeletal and limb anomalie	172,000 (107,000 to 257,000)	[illegible]	[illegible]	[illegible]	[illegible]
Urogenital congenital anomalies	177,000 (101,000 to 271,000)	[illegible]	[illegible]	[illegible]	[illegible]
Digestive congenital anomalies	672,000 (418,000 to 1,040,000)	[illegible]	[illegible]	[illegible]	[illegible]
Other congenital birth defects	1,790,000 (1,230,000 to 2,720,000)	[illegible]	[illegible]	[illegible]	[illegible]
Urinary diseases and male infertility	14,200 (9,900 to 18,700)	[illegible]	[illegible]	[illegible]	[illegible]
Urolithiasis	0 (0 to 0)	[illegible]	[illegible]	[illegible]	[illegible]
Benign prostatic hyperplasia	0 (0 to 0)	[illegible]	[illegible]	[illegible]	[illegible]
Male infertility	0 (0 to 0)	[illegible]	[illegible]	[illegible]	[illegible]
Other urinary diseases	9,410 (6,040 to 12,900)	[illegible]	[illegible]	[illegible]	[illegible]
Gynecological diseases	0 (0 to 0)	[illegible]	[illegible]	[illegible]	[illegible]
Uterine fibroids	0 (0 to 0)	[illegible]	[illegible]	[illegible]	[illegible]
Polycystic ovarian syndrome	0 (0 to 0)	[illegible]	[illegible]	[illegible]	[illegible]
Female infertility	0 (0 to 0)	[illegible]	[illegible]	[illegible]	[illegible]
Endometriosis	0 (0 to 0)	[illegible]	[illegible]	[illegible]	[illegible]
Genital prolapse	0 (0 to 0)	[illegible]	[illegible]	[illegible]	[illegible]
Premenstrual syndrome	0 (0 to 0)	[illegible]	[illegible]	[illegible]	[illegible]
Other gynecological diseases	0 (0 to 0)	[illegible]	[illegible]	[illegible]	[illegible]
Hemoglobinopathies and hemolytic anemias	22,800 (18,800 to 27,800)	[illegible]	[illegible]	[illegible]	[illegible]
Thalassemias	9,970 (6,810 to 14,300)	[illegible]	[illegible]	[illegible]	[illegible]
Thalassemias trait	337 (201 to 556)	[illegible]	[illegible]	[illegible]	[illegible]
Sickle cell disorders	10,800 (6,970 to 15,000)	[illegible]	[illegible]	[illegible]	[illegible]
Sickle cell trait	757 (458 to 1,190)	[illegible]	[illegible]	[illegible]	[illegible]
G6PD deficiency	208 (123 to 307)	[illegible]	[illegible]	[illegible]	[illegible]
G6PD trait	0·3 (0·1 to 0·9)	[illegible]	[illegible]	[illegible]	[illegible]
Other hemoglobinopathies and hemolytic anem	649 (472 to 878)	[illegible]	[illegible]	[illegible]	[illegible]
Endocrine, metabolic, blood, and immune disorders	46,900 (33,200 to 58,900)	[illegible]	[illegible]	[illegible]	[illegible]
Oral disorders	0 (0 to 0)	[illegible]	[illegible]	[illegible]	[illegible]
Caries of deciduous teeth	0 (0 to 0)	[illegible]	[illegible]	[illegible]	[illegible]
Caries of permanent teeth	0 (0 to 0)	[illegible]	[illegible]	[illegible]	[illegible]
Periodontal diseases	0 (0 to 0)	[illegible]	[illegible]	[illegible]	[illegible]
Other oral disorders	0 (0 to 0)	[illegible]	[illegible]	[illegible]	[illegible]
Sudden infant death syndrome	--	[illegible]	[illegible]	[illegible]	[illegible]
Injuries	506,000 (402,000 to 616,000)	[illegible]	[illegible]	[illegible]	[illegible]
Transport injuries	22,100 (17,900 to 28,500)	[illegible]	[illegible]	[illegible]	[illegible]
Road injuries	20,600 (16,700 to 26,800)	[illegible]	[illegible]	[illegible]	[illegible]
Pedestrian road injuries	8,720 (6,810 to 11,800)	[illegible]	[illegible]	[illegible]	[illegible]
Cyclist road injuries	0 (0 to 0)	[illegible]	[illegible]	[illegible]	[illegible]
Motorcyclist road injuries	1,840 (1,460 to 2,530)	[illegible]	[illegible]	[illegible]	[illegible]
Motor vehicle road injuries	9,780 (7,470 to 12,400)	[illegible]	[illegible]	[illegible]	[illegible]
Other road injuries	304 (230 to 406)	[illegible]	[illegible]	[illegible]	[illegible]
Other transport injuries	1,440 (1,130 to 1,870)	[illegible]	[illegible]	[illegible]	[illegible]
Unintentional injuries	309,000 (219,000 to 404,000)	[illegible]	[illegible]	[illegible]	[illegible]
Falls	60,800 (25,900 to 89,500)	[illegible]	[illegible]	[illegible]	[illegible]
Drowning	14,000 (11,700 to 17,300)	[illegible]	[illegible]	[illegible]	[illegible]
Fire, heat, and hot substances	9,590 (6,070 to 13,800)	[illegible]	[illegible]	[illegible]	[illegible]
Poisonings	9,140 (7,110 to 12,000)	[illegible]	[illegible]	[illegible]	[illegible]
Poisoning by carbon monoxide	2,460 (1,590 to 3,710)	[illegible]	[illegible]	[illegible]	[illegible]
Poisoning by other means	6,690 (4,990 to 8,670)	[illegible]	[illegible]	[illegible]	[illegible]
Exposure to mechanical forces	45,000 (11,700 to 91,300)	[illegible]	[illegible]	[illegible]	[illegible]
Unintentional firearm injuries	6,930 (1,160 to 18,500)	[illegible]	[illegible]	[illegible]	[illegible]
Other exposure to mechanical forces	38,000 (10,300 to 80,100)	[illegible]	[illegible]	[illegible]	[illegible]
Adverse effects of medical treatment	35,000 (23,400 to 46,400)	[illegible]	[illegible]	[illegible]	[illegible]
Animal contact	10,500 (5,560 to 15,600)	[illegible]	[illegible]	[illegible]	[illegible]
Venomous animal contact	8,620 (4,110 to 13,000)	[illegible]	[illegible]	[illegible]	[illegible]
Non-venomous animal contact	1,910 (1,330 to 2,770)	[illegible]	[illegible]	[illegible]	[illegible]
Foreign body	108,000 (89,600 to 133,000)	[illegible]	[illegible]	[illegible]	[illegible]
Pulmonary aspiration and foreign body in airw	107,000 (88,600 to 132,000)	[illegible]	[illegible]	[illegible]	[illegible]
Foreign body in eyes	14·3 (3·8 to 32·8)	[illegible]	[illegible]	[illegible]	[illegible]
Foreign body in other body part	1,140 (770 to 1,550)	[illegible]	[illegible]	[illegible]	[illegible]
Environmental heat and cold exposure	4,940 (1,230 to 9,420)	[illegible]	[illegible]	[illegible]	[illegible]
Exposure to forces of nature	638 (579 to 702)	[illegible]	[illegible]	[illegible]	[illegible]
Other unintentional injuries	11,300 (9,140 to 14,000)	[illegible]	[illegible]	[illegible]	[illegible]
Self-harm and interpersonal violence	175,000 (159,000 to 193,000)	[illegible]	[illegible]	[illegible]	[illegible]
Self-harm	0 (0 to 0)	[illegible]	[illegible]	[illegible]	[illegible]
Self-harm by firearm	0 (0 to 0)	[illegible]	[illegible]	[illegible]	[illegible]
Self-harm by other specified means	0 (0 to 0)	[illegible]	[illegible]	[illegible]	[illegible]
Interpersonal violence	55,900 (43,200 to 69,100)	[illegible]	[illegible]	[illegible]	[illegible]
Physical violence by firearm	6,230 (4,720 to 7,970)	[illegible]	[illegible]	[illegible]	[illegible]
Physical violence by sharp object	3,780 (2,970 to 4,870)	[illegible]	[illegible]	[illegible]	[illegible]
Sexual violence	0 (0 to 0)	[illegible]	[illegible]	[illegible]	[illegible]
Physical violence by other means	45,900 (35,600 to 57,100)	[illegible]	[illegible]	[illegible]	[illegible]
Conflict and terrorism	119,000 (108,000 to 130,000)	[illegible]	[illegible]	[illegible]	[illegible]

SR Table 2b: Lao People's Democratic Republic DALYS by cause and age group for females with 95% uncertainty intervals, 2019

Cause	Early Neonatal	Late Neonatal	Post Neonatal	1 to 4	5 to 9	10 to 14	15 to 19	20 to 24	25 to 29	30 to 34	35 to 39	40 to 44	45 to 49	50 to 54	55 to 59	60 to 64	65 to 69	70 to 74	75 to 79	80 to 84	85 to 89	90 to 94	95 plus	Total
All causes	110,000 (90,400 to 131,000)	23,400 (16,700 to 31,100)	111,000 (78,500 to 147,000)	45,000 (28,600 to 66,800)	28,800 (22,800 to 36,000)	[illegible]	[illegible]	[illegible]	[illegible]	[illegible]	[illegible]	[illegible]	[illegible]	[illegible]	[illegible]	[illegible]	[illegible]	[illegible]	[illegible]	[illegible]	[illegible]	[illegible]	[illegible]	[illegible]
Communicable, maternal, neonatal, and nutritional diseases	95,400 (76,900 to 116,000)	19,200 (13,400 to 25,600)	85,100 (57,300 to 117,000)	29,700 (17,400 to 46,400)	11,900 (8,760 to 15,900)	[illegible]	[illegible]	[illegible]	[illegible]	[illegible]	[illegible]	[illegible]	[illegible]	[illegible]	[illegible]	[illegible]	[illegible]	[illegible]	[illegible]	[illegible]	[illegible]	[illegible]	[illegible]	[illegible]
HIV/AIDS and sexually transmitted infections	793 (226 to 1,900)	208 (57 to 504)	577 (89·1 to 2,590)	1,230 (13·6 to 9,700)	181 (14·1 to 981)	[illegible]	[illegible]	[illegible]	[illegible]	[illegible]	[illegible]	[illegible]	[illegible]	[illegible]	[illegible]	[illegible]	[illegible]	[illegible]	[illegible]	[illegible]	[illegible]	[illegible]	[illegible]	[illegible]
HIV/AIDS	0 (0 to 0·1)	0·1 (0 to 0·5)	316 (1·9 to 2,380)	1,220 (7·9 to 9,690)	174 (9·7 to 977)	[illegible]	[illegible]	[illegible]	[illegible]	[illegible]	[illegible]	[illegible]	[illegible]	[illegible]	[illegible]	[illegible]	[illegible]	[illegible]	[illegible]	[illegible]	[illegible]	[illegible]	[illegible]	[illegible]
HIV/AIDS - Drug-susceptible Tuberculosis	0 (0 to 0)	0 (0 to 0·1)	97·7 (1·7 to 630)	391 (7 to 2,470)	59·5 (8·4 to 248)	[illegible]	[illegible]	[illegible]	[illegible]	[illegible]	[illegible]	[illegible]	[illegible]	[illegible]	[illegible]	[illegible]	[illegible]	[illegible]	[illegible]	[illegible]	[illegible]	[illegible]	[illegible]	[illegible]
HIV/AIDS - Multidrug-resistant Tuberculosis without exten	0 (0 to 0)	0 (0 to 0)	1·4 (0 to 12·5)	5·6 (0 to 49·2)	0·7 (0 to 4·8)	[illegible]	[illegible]	[illegible]	[illegible]	[illegible]	[illegible]	[illegible]	[illegible]	[illegible]	[illegible]	[illegible]	[illegible]	[illegible]	[illegible]	[illegible]	[illegible]	[illegible]	[illegible]	[illegible]
HIV/AIDS - Extensively drug-resistant Tuberculosis	0 (0 to 0)	0 (0 to 0)	0·3 (0 to 2·5)	1·1 (0 to 9·8)	0·1 (0 to 1)	[illegible]	[illegible]	[illegible]	[illegible]	[illegible]	[illegible]	[illegible]	[illegible]	[illegible]	[illegible]	[illegible]	[illegible]	[illegible]	[illegible]	[illegible]	[illegible]	[illegible]	[illegible]	[illegible]
HIV/AIDS resulting in other diseases	0 (0 to 0·1)	0·1 (0 to 0·5)	216 (0·1 to 1,800)	823 (0·2 to 7,430)	114 (0 to 783)	[illegible]	[illegible]	[illegible]	[illegible]	[illegible]	[illegible]	[illegible]	[illegible]	[illegible]	[illegible]	[illegible]	[illegible]	[illegible]	[illegible]	[illegible]	[illegible]	[illegible]	[illegible]	[illegible]
Sexually transmitted infections excluding HIV	793 (226 to 1,900)	208 (56·9 to 504)	261 (67·6 to 635)	8·8 (1·8 to 22·5)	6·5 (1·7 to 15·4)	[illegible]	[illegible]	[illegible]	[illegible]	[illegible]	[illegible]	[illegible]	[illegible]	[illegible]	[illegible]	[illegible]	[illegible]	[illegible]	[illegible]	[illegible]	[illegible]	[illegible]	[illegible]	[illegible]
Syphilis	793 (226 to 1,900)	208 (56·9 to 504)	261 (67·6 to 635)	8·8 (1·8 to 22·5)	6·5 (1·7 to 15·4)	[illegible]	[illegible]	[illegible]	[illegible]	[illegible]	[illegible]	[illegible]	[illegible]	[illegible]	[illegible]	[illegible]	[illegible]	[illegible]	[illegible]	[illegible]	[illegible]	[illegible]	[illegible]	[illegible]
Chlamydial infection	0 (0 to 0)	0 (0 to 0)	0 (0 to 0)	0 (0 to 0)	0 (0 to 0)	[illegible]	[illegible]	[illegible]	[illegible]	[illegible]	[illegible]	[illegible]	[illegible]	[illegible]	[illegible]	[illegible]	[illegible]	[illegible]	[illegible]	[illegible]	[illegible]	[illegible]	[illegible]	[illegible]
Gonococcal infection	0 (0 to 0)	0 (0 to 0)	0 (0 to 0)	0 (0 to 0)	0 (0 to 0)	[illegible]	[illegible]	[illegible]	[illegible]	[illegible]	[illegible]	[illegible]	[illegible]	[illegible]	[illegible]	[illegible]	[illegible]	[illegible]	[illegible]	[illegible]	[illegible]	[illegible]	[illegible]	[illegible]
Trichomoniasis	0 (0 to 0)	0 (0 to 0)	0 (0 to 0)	0 (0 to 0)	0 (0 to 0)	[illegible]	[illegible]	[illegible]	[illegible]	[illegible]	[illegible]	[illegible]	[illegible]	[illegible]	[illegible]	[illegible]	[illegible]	[illegible]	[illegible]	[illegible]	[illegible]	[illegible]	[illegible]	[illegible]
Genital herpes	0 (0 to 0)	0 (0 to 0)	0 (0 to 0)	0 (0 to 0)	0 (0 to 0)	[illegible]	[illegible]	[illegible]	[illegible]	[illegible]	[illegible]	[illegible]	[illegible]	[illegible]	[illegible]	[illegible]	[illegible]	[illegible]	[illegible]	[illegible]	[illegible]	[illegible]	[illegible]	[illegible]
Other sexually transmitted infections	0 (0 to 0)	0 (0 to 0)	0 (0 to 0)	0 (0 to 0)	0 (0 to 0)	[illegible]	[illegible]	[illegible]	[illegible]	[illegible]	[illegible]	[illegible]	[illegible]	[illegible]	[illegible]	[illegible]	[illegible]	[illegible]	[illegible]	[illegible]	[illegible]	[illegible]	[illegible]	[illegible]
Respiratory infections and tuberculosis	5,490 (2,750 to 10,200)	3,540 (1,790 to 5,930)	43,500 (25,300 to 66,400)	7,640 (3,730 to 13,200)	2,890 (2,010 to 4,000)	[illegible]	[illegible]	[illegible]	[illegible]	[illegible]	[illegible]	[illegible]	[illegible]	[illegible]	[illegible]	[illegible]	[illegible]	[illegible]	[illegible]	[illegible]	[illegible]	[illegible]	[illegible]	[illegible]
[illegible]	[illegible]	[illegible]	[illegible]	[illegible]	[illegible]	[illegible]	[illegible]	[illegible]	[illegible]	[illegible]	[illegible]	[illegible]	[illegible]	[illegible]	[illegible]	[illegible]	[illegible]	[illegible]	[illegible]	[illegible]	[illegible]	[illegible]	[illegible]	[illegible]

Non-rheumatic degenerative mitral valve disease	0 (0 to 0)	0 (0 to 0)	0 (0 to 0)	0 (0 to 0)	0 (0 to 0)	0 (0 to 0)	[illegible]	0·4 (0·2 to 0·6)	103
Other non-rheumatic valve diseases	0 (0 to 0)	0 (0 to 0)	0 (0 to 0)	0 (0 to 0)	0 (0 to 0)	0 (0 to 0)	[illegible]	0·1 (0 to 0·3)	26·6
Cardiomyopathy and myocarditis	9·7 (1·9 to 29·3)	22·1 (6·2 to 53·4)	309 (63·6 to 880)	59·5 (15·2 to 149)	42·4 (14 to 104)	52·3 (18·3 to 116)	[illegible]	11·1 (4·3 to 23·7)	1,980
Myocarditis	3 (0·5 to 9·9)	5·5 (1·1 to 16·6)	135 (24·4 to 437)	27·1 (6·1 to 73·7)	21·8 (6·8 to 56·3)	13·9 (4·2 to 34)	[illegible]	1·4 (0·2 to 4·3)	365
Alcoholic cardiomyopathy	0 (0 to 0)	0 (0 to 0)	0 (0 to 0)	0 (0 to 0)	0 (0 to 0)	0 (0 to 0)	[illegible]	0·3 (0·1 to 0·8)	54·8
Other cardiomyopathy	6·7 (1·4 to 20·7)	16·6 (4·4 to 43·4)	174 (29·8 to 514)	32·4 (7·6 to 92·9)	20·6 (5·5 to 51·3)	38·4 (12·4 to 88·7)	[illegible]	9·4 (3·8 to 19·5)	1,560
Atrial fibrillation and flutter	0 (0 to 0)	0 (0 to 0)	0 (0 to 0)	0 (0 to 0)	0 (0 to 0)	0 (0 to 0)	[illegible]	20·4 (13·8 to 28·4)	1,840
Aortic aneurysm	--	--	--	--	--	--	[illegible]	1·1 (0·6 to 1·9)	NA
Endocarditis	0·5 (0·1 to 1·2)	4 (1·1 to 10·1)	69·5 (15·5 to 185)	11 (3·2 to 27·5)	12·2 (4·1 to 26·5)	9·3 (3·1 to 20·5)	[illegible]	0·6 (0·3 to 1·1)	869
Other cardiovascular and circulatory diseases	6·7 (1·8 to 19·1)	11·5 (4 to 26·4)	195 (54·4 to 449)	59·2 (22 to 126)	67·1 (31·5 to 116)	50·7 (24·5 to 92·3)	[illegible]	4 (2·6 to 5·9)	2,050
Chronic respiratory diseases	0 (0 to 0)	0 (0 to 0)	0·8 (0·6 to 1)	1,690 (815 to 3,610)	946 (644 to 1,370)	826 (588 to 1,130)	[illegible]	105 (72 to 164)	34,000
Chronic obstructive pulmonary disease	0 (0 to 0)	0 (0 to 0)	0·8 (0·6 to 1)	119 (44·6 to 292)	82·9 (45·8 to 134)	129 (77·4 to 191)	[illegible]	85·8 (58·8 to 135)	18,700
Pneumoconiosis	0 (0 to 0)	0 (0 to 0)	0 (0 to 0)	0 (0 to 0)	0 (0 to 0)	0 (0 to 0)	[illegible]	0 (0 to 0·1)	9·2
Silicosis	0 (0 to 0)	0 (0 to 0)	0 (0 to 0)	0 (0 to 0)	0 (0 to 0)	0 (0 to 0)	[illegible]	0 (0 to 0)	1·3
Asbestosis	0 (0 to 0)	0 (0 to 0)	0 (0 to 0)	0 (0 to 0)	0 (0 to 0)	0 (0 to 0)	[illegible]	0 (0 to 0)	3
Coal workers pneumoconiosis	0 (0 to 0)	0 (0 to 0)	0 (0 to 0)	0 (0 to 0)	0 (0 to 0)	0 (0 to 0)	[illegible]	0 (0 to 0)	0·8
Other pneumoconiosis	0 (0 to 0)	0 (0 to 0)	0 (0 to 0)	0 (0 to 0)	0 (0 to 0)	0 (0 to 0)	[illegible]	0 (0 to 0)	4
Asthma	0 (0 to 0)	0 (0 to 0)	0 (0 to 0)	1,460 (692 to 3,130)	739 (476 to 1,120)	554 (365 to 810)	[illegible]	18 (7·9 to 41·2)	13,600
Interstitial lung disease and pulmonary sarcoidosis	0 (0 to 0)	0 (0 to 0)	0 (0 to 0)	5·6 (1·8 to 14·7)	3·2 (1·4 to 6·9)	2·8 (1·4 to 5·2)	[illegible]	0·8 (0·2 to 2·1)	415
Other chronic respiratory diseases	0 (0 to 0)	0 (0 to 0)	0 (0 to 0)	104 (36·7 to 254)	120 (60·1 to 198)	140 (78·7 to 211)	[illegible]	0·7 (0·3 to 1·4)	1,340
Digestive diseases	23·8 (11·9 to 44·1)	44·6 (20·5 to 90·2)	467 (130 to 1,110)	680 (287 to 1,460)	572 (356 to 908)	534 (365 to 753)	[illegible]	60 (35·1 to 112)	29,500
Cirrhosis and other chronic liver diseases	0 (0 to 0)	[illegible]	[illegible]	[illegible]	[illegible]	[illegible]	[illegible]	12 (5·9 to 23·2)	12,600
Cirrhosis and other chronic liver diseases due to hepatitis	0 (0 to 0)	[illegible]	[illegible]	[illegible]	[illegible]	[illegible]	[illegible]	2·7 (0·9 to 6·6)	3,060
Cirrhosis and other chronic liver diseases due to hepatitis	0 (0 to 0)	[illegible]	[illegible]	[illegible]	[illegible]	[illegible]	[illegible]	2·8 (0·9 to 6·6)	3,180
Cirrhosis and other chronic liver diseases due to alcohol u	0 (0 to 0)	0 (0 to 0)	[illegible]	[illegible]	[illegible]	[illegible]	[illegible]	1·3 (0·4 to 3·3)	2,030
Cirrhosis and other chronic liver diseases due to NAFLD	0 (0 to 0)	0 (0 to 0)	[illegible]	[illegible]	[illegible]	[illegible]	[illegible]	2·2 (0·7 to 4·8)	1,270
Cirrhosis and other chronic liver diseases due to other ca	0 (0 to 0)	0 (0 to 0)	[illegible]	[illegible]	[illegible]	[illegible]	[illegible]	3·1 (1 to 6·7)	3,020
Upper digestive system diseases	0 (0 to 0)	0 (0 to 0)	[illegible]	[illegible]	[illegible]	[illegible]	[illegible]	29·6 (15·8 to 57)	9,180
Peptic ulcer disease	0 (0 to 0)	0 (0 to 0)	[illegible]	[illegible]	[illegible]	[illegible]	[illegible]	26·7 (14·3 to 52·5)	6,470
Gastritis and duodenitis	0 (0 to 0)	0 (0 to 0)	[illegible]	[illegible]	[illegible]	[illegible]	[illegible]	2·4 (0·7 to 6·2)	1,340
Gastroesophageal reflux disease	0 (0 to 0)	0 (0 to 0)	[illegible]	[illegible]	[illegible]	[illegible]	[illegible]	0·5 (0·2 to 1)	1,360
Appendicitis	0 (0 to 0)	0 (0 to 0)	[illegible]	[illegible]	[illegible]	[illegible]	[illegible]	0·3 (0·1 to 0·7)	655
Paralytic ileus and intestinal obstruction	17·9 (9·4 to 33·8)	40·9 (19·1 to 83·1)	[illegible]	[illegible]	[illegible]	[illegible]	[illegible]	6·9 (2·7 to 15·8)	2,200
Inguinal, femoral, and abdominal hernia	5·9 (1·5 to 14·2)	3·7 (0·8 to 10·1)	[illegible]	[illegible]	[illegible]	[illegible]	[illegible]	0·5 (0·2 to 1)	344
Inflammatory bowel disease	0 (0 to 0)	0 (0 to 0)	[illegible]	[illegible]	[illegible]	[illegible]	[illegible]	0·3 (0·1 to 0·8)	178
Vascular intestinal disorders	0 (0 to 0)	0 (0 to 0)	[illegible]	[illegible]	[illegible]	[illegible]	[illegible]	1·1 (0·5 to 2·1)	234
Gallbladder and biliary diseases	0 (0 to 0)	0 (0 to 0)	[illegible]	[illegible]	[illegible]	[illegible]	[illegible]	5·6 (2·6 to 10·9)	2,940
Pancreatitis	0 (0 to 0)	0 (0 to 0)	[illegible]	[illegible]	[illegible]	[illegible]	[illegible]	0·7 (0·3 to 1·4)	521
Other digestive diseases	0 (0 to 0)	0 (0 to 0)	[illegible]	[illegible]	[illegible]	[illegible]	[illegible]	3·1 (1·2 to 7·2)	721
Neurological disorders	1 (0·2 to 2·3)	2·9 (0·6 to 6·4)	[illegible]	[illegible]	[illegible]	[illegible]	[illegible]	158 (73·6 to 354)	40,500
Alzheimer's disease and other dementias	0 (0 to 0)	0 (0 to 0)	[illegible]	[illegible]	[illegible]	[illegible]	[illegible]	150 (64·1 to 348)	5,840
Parkinson's disease	0 (0 to 0)	0 (0 to 0)	[illegible]	[illegible]	[illegible]	[illegible]	[illegible]	5·3 (3·9 to 7)	1,340
Idiopathic epilepsy	1 (0·2 to 2·2)	2·8 (0·5 to 6·3)	[illegible]	[illegible]	[illegible]	[illegible]	[illegible]	1·6 (0·5 to 3·2)	4,320
Multiple sclerosis	0 (0 to 0)	0 (0 to 0)	[illegible]	[illegible]	[illegible]	[illegible]	[illegible]	0 (0 to 0)	183
Motor neuron disease	0 (0 to 0·1)	0·1 (0 to 0·1)	[illegible]	[illegible]	[illegible]	[illegible]	[illegible]	0 (0 to 0)	77·2
Headache disorders	0 (0 to 0)	0 (0 to 0)	[illegible]	[illegible]	[illegible]	[illegible]	[illegible]	1·3 (0·2 to 3·3)	27,400
Migraine	0 (0 to 0)	0 (0 to 0)	[illegible]	[illegible]	[illegible]	[illegible]	[illegible]	1·1 (0·1 to 2·8)	25,600
Tension-type headache	0 (0 to 0)	0 (0 to 0)	[illegible]	[illegible]	[illegible]	[illegible]	[illegible]	0·2 (0 to 1·4)	1,850
Other neurological disorders	0 (0 to 0)	0 (0 to 0)	[illegible]	[illegible]	[illegible]	[illegible]	[illegible]	0·4 (0·2 to 0·6)	1,290
Mental disorders	0·8 (0·4 to 1·4)	2·5 (1·2 to 4·4)	[illegible]	[illegible]	[illegible]	[illegible]	[illegible]	8 (5·4 to 11·2)	49,800
Schizophrenia	0 (0 to 0)	0 (0 to 0)	[illegible]	[illegible]	[illegible]	[illegible]	[illegible]	0·2 (0·1 to 0·3)	5,820
Depressive disorders	0 (0 to 0)	0 (0 to 0)	[illegible]	[illegible]	[illegible]	[illegible]	[illegible]	3·4 (1·9 to 5·2)	13,900
Major depressive disorder	0 (0 to 0)	0 (0 to 0)	[illegible]	[illegible]	[illegible]	[illegible]	[illegible]	2·5 (1·2 to 4·2)	8,740
Dysthymia	0 (0 to 0)	0 (0 to 0)	[illegible]	[illegible]	[illegible]	[illegible]	[illegible]	0·8 (0·4 to 1·5)	5,150
Bipolar disorder	0 (0 to 0)	0 (0 to 0)	[illegible]	[illegible]	[illegible]	[illegible]	[illegible]	0·2 (0·1 to 0·5)	2,450
Anxiety disorders	0 (0 to 0)	0 (0 to 0)	[illegible]	[illegible]	[illegible]	[illegible]	[illegible]	2·8 (1·6 to 4·5)	18,900
Eating disorders	0 (0 to 0)	0 (0 to 0)	[illegible]	[illegible]	[illegible]	[illegible]	[illegible]	0 (0 to 0)	1,040
Anorexia nervosa	0 (0 to 0)	0 (0 to 0)	[illegible]	[illegible]	[illegible]	[illegible]	[illegible]	0 (0 to 0)	433
Bulimia nervosa	0 (0 to 0)	0 (0 to 0)	[illegible]	[illegible]	[illegible]	[illegible]	[illegible]	0 (0 to 0)	610
Autism spectrum disorders	0·5 (0·3 to 0·7)	1·4 (0·8 to 2·2)	[illegible]	[illegible]	[illegible]	[illegible]	[illegible]	0·1 (0 to 0·1)	824
Attention-deficit/hyperactivity disorder	0 (0 to 0)	0 (0 to 0)	[illegible]	[illegible]	[illegible]	[illegible]	[illegible]	0 (0 to 0)	249
Conduct disorder	0 (0 to 0)	0 (0 to 0)	[illegible]	[illegible]	[illegible]	[illegible]	[illegible]	0 (0 to 0)	1,880
Idiopathic developmental intellectual disability	0·3 (0·1 to 0·9)	1·2 (0·2 to 2·7)	[illegible]	[illegible]	[illegible]	[illegible]	[illegible]	0·1 (0 to 0·1)	1,880
Other mental disorders	0 (0 to 0)	0 (0 to 0)	[illegible]	[illegible]	[illegible]	[illegible]	[illegible]	1·3 (0·8 to 1·8)	2,870
Substance use disorders	0 (0 to 0)	0 (0 to 0)	[illegible]	[illegible]	[illegible]	[illegible]	[illegible]	0·5 (0·3 to 0·8)	3,960
Alcohol use disorders	0 (0 to 0)	0 (0 to 0)	[illegible]	[illegible]	[illegible]	[illegible]	[illegible]	0·3 (0·1 to 0·4)	1,180
Drug use disorders	0 (0 to 0)	0 (0 to 0)	[illegible]	[illegible]	[illegible]	[illegible]	[illegible]	0·3 (0·2 to 0·5)	2,780
Opioid use disorders	0 (0 to 0)	0 (0 to 0)	[illegible]	[illegible]	[illegible]	[illegible]	[illegible]	0·2 (0·1 to 0·4)	1,290
Cocaine use disorders	0 (0 to 0)	0 (0 to 0)	[illegible]	[illegible]	[illegible]	[illegible]	[illegible]	0 (0 to 0)	32·8
Amphetamine use disorders	0 (0 to 0)	0 (0 to 0)	[illegible]	[illegible]	[illegible]	[illegible]	[illegible]	0 (0 to 0)	670
Cannabis use disorders	0 (0 to 0)	0 (0 to 0)	[illegible]	[illegible]	[illegible]	[illegible]	[illegible]	0 (0 to 0)	250
Other drug use disorders	0 (0 to 0)	0 (0 to 0)	[illegible]	[illegible]	[illegible]	[illegible]	[illegible]	0 (0 to 0·1)	535
Diabetes and kidney diseases	1 (0·5 to 1·8)	3·3 (1·4 to 6·7)	[illegible]	[illegible]	[illegible]	[illegible]	[illegible]	100 (74·8 to 132)	68,000
Diabetes mellitus	1 (0·5 to 1·7)	3·2 (1·4 to 6·7)	[illegible]	[illegible]	[illegible]	[illegible]	[illegible]	57·6 (38·5 to 82·5)	37,400
Diabetes mellitus type 1	1 (0·5 to 1·7)	3·2 (1·4 to 6·7)	[illegible]	[illegible]	[illegible]	[illegible]	[illegible]	2 (0·9 to 4)	3,040
Diabetes mellitus type 2	0 (0 to 0)	0 (0 to 0)	[illegible]	[illegible]	[illegible]	[illegible]	[illegible]	55·6 (37·3 to 79·9)	34,400
Chronic kidney disease	0 (0 to 0)	0·1 (0 to 0·1)	[illegible]	[illegible]	[illegible]	[illegible]	[illegible]	42·1 (29·5 to 61·8)	30,000
Chronic kidney disease due to diabetes mellitus type 1	0 (0 to 0)	0 (0 to 0)	[illegible]	[illegible]	[illegible]	[illegible]	[illegible]	0 (0 to 0)	5,090
Chronic kidney disease due to diabetes mellitus type 2	0 (0 to 0)	0 (0 to 0)	[illegible]	[illegible]	[illegible]	[illegible]	[illegible]	13 (7·1 to 20·7)	8,740
Chronic kidney disease due to hypertension	0 (0 to 0)	0 (0 to 0)	[illegible]	[illegible]	[illegible]	[illegible]	[illegible]	18·6 (11·7 to 28·9)	8,060
Chronic kidney disease due to glomerulonephritis	0 (0 to 0)	0 (0 to 0)	[illegible]	[illegible]	[illegible]	[illegible]	[illegible]	1·1 (0·4 to 2·5)	2,580
Chronic kidney disease due to other and unspecified caus	0 (0 to 0)	0·1 (0 to 0·1)	[illegible]	[illegible]	[illegible]	[illegible]	[illegible]	9·3 (5·9 to 13·9)	5,540
Acute glomerulonephritis	0 (0 to 0)	0 (0 to 0)	[illegible]	[illegible]	[illegible]	[illegible]	[illegible]	0·4 (0 to 1)	549
Skin and subcutaneous diseases	0·1 (0·1 to 0·2)	1·7 (0·9 to 2·9)	[illegible]	[illegible]	[illegible]	[illegible]	[illegible]	15·4 (9 to 27·5)	24,100
Dermatitis	0 (0 to 0)	0 (0 to 0)	[illegible]	[illegible]	[illegible]	[illegible]	[illegible]	1·4 (0·8 to 2·3)	7,810
Atopic dermatitis	0 (0 to 0)	0 (0 to 0)	[illegible]	[illegible]	[illegible]	[illegible]	[illegible]	1·1 (0·6 to 1·8)	6,610
Contact dermatitis	0 (0 to 0)	0 (0 to 0)	[illegible]	[illegible]	[illegible]	[illegible]	[illegible]	0·3 (0·1 to 0·6)	1,080
Seborrhoeic dermatitis	0 (0 to 0)	0 (0 to 0)	[illegible]	[illegible]	[illegible]	[illegible]	[illegible]	0 (0 to 0·1)	111
Psoriasis	0 (0 to 0)	0 (0 to 0)	[illegible]	[illegible]	[illegible]	[illegible]	[illegible]	0·1 (0·1 to 0·2)	536
Bacterial skin diseases	0 (0 to 0)	0·2 (0·1 to 0·3)	[illegible]	[illegible]	[illegible]	[illegible]	[illegible]	4·5 (1·1 to 11·2)	787
Cellulitis	0 (0 to 0)	0 (0 to 0·1)	[illegible]	[illegible]	[illegible]	[illegible]	[illegible]	0·7 (0·1 to 2·1)	197
Pyoderma	0 (0 to 0)	0·1 (0·1 to 0·3)	[illegible]	[illegible]	[illegible]	[illegible]	[illegible]	3·8 (0·9 to 9·2)	590
Scabies	0·1 (0 to 0·2)	1·1 (0·5 to 1·9)	[illegible]	[illegible]	[illegible]	[illegible]	[illegible]	0·9 (0·5 to 1·6)	4,330
Fungal skin diseases	0 (0 to 0)	0·1 (0 to 0·2)	[illegible]	[illegible]	[illegible]	[illegible]	[illegible]	1·8 (0·7 to 3·7)	1,610
Viral skin diseases	0 (0 to 0)	0 (0 to 0)	[illegible]	[illegible]	[illegible]	[illegible]	[illegible]	0·1 (0 to 0·1)	1,990
Acne vulgaris	0 (0 to 0)	0 (0 to 0)	[illegible]	[illegible]	[illegible]	[illegible]	[illegible]	0 (0 to 0)	2,980
Alopecia areata	0 (0 to 0)	0 (0 to 0)	[illegible]	[illegible]	[illegible]	[illegible]	[illegible]	0 (0 to 0)	381
Pruritus	0 (0 to 0)	0 (0 to 0)	[illegible]	[illegible]	[illegible]	[illegible]	[illegible]	0·2 (0·1 to 0·4)	420
Urticaria	0 (0 to 0)	0·2 (0·1 to 0·4)	[illegible]	[illegible]	[illegible]	[illegible]	[illegible]	0·2 (0·1 to 0·3)	1,530
Decubitus ulcer	0 (0 to 0)	0 (0 to 0)	[illegible]	[illegible]	[illegible]	[illegible]	[illegible]	5·7 (1·4 to 13·2)	372
Other skin and subcutaneous diseases	0 (0 to 0)	0·1 (0·1 to 0·2)	[illegible]	[illegible]	[illegible]	[illegible]	[illegible]	0·5 (0·2 to 0·8)	1,360
Sense organ diseases	0·5 (0·3 to 0·9)	1·8 (1 to 3)	[illegible]	[illegible]	[illegible]	[illegible]	[illegible]	64·2 (46·5 to 84·4)	22,900
Blindness and vision loss	0·1 (0 to 0·2)	0·3 (0·1 to 0·7)	[illegible]	[illegible]	[illegible]	[illegible]	[illegible]	34·8 (24 to 47·7)	6,420
Glaucoma	0 (0 to 0)	0 (0 to 0)	[illegible]	[illegible]	[illegible]	[illegible]	[illegible]	1·3 (0·8 to 2·1)	131
Cataract	0 (0 to 0)	0 (0 to 0)	[illegible]	[illegible]	[illegible]	[illegible]	[illegible]	9·5 (6 to 14·1)	1,090
Age-related macular degeneration	0 (0 to 0)	0 (0 to 0)	[illegible]	[illegible]	[illegible]	[illegible]	[illegible]	0·8 (0·5 to 1·2)	200
Refraction disorders	0 (0 to 0)	0·1 (0 to 0·2)	[illegible]	[illegible]	[illegible]	[illegible]	[illegible]	1·4 (0·8 to 2·2)	1,970
Near vision loss	0 (0 to 0)	0 (0 to 0)	[illegible]	[illegible]	[illegible]	[illegible]	[illegible]	3·6 (1·4 to 7·3)	1,360
Other vision loss	0·1 (0 to 0·2)	0·3 (0·1 to 0·6)	[illegible]	[illegible]	[illegible]	[illegible]	[illegible]	18·2 (12·1 to 25·9)	1,670
Age-related and other hearing loss	0·3 (0·1 to 0·6)	0·9 (0·3 to 1·9)	[illegible]	[illegible]	[illegible]	[illegible]	[illegible]	28·1 (20·3 to 37·4)	15,400
Other sense organ diseases	0·1 (0 to 0·2)	0·5 (0·2 to 1)	[illegible]	[illegible]	[illegible]	[illegible]	[illegible]	1·3 (0·6 to 2·3)	1,090
Musculoskeletal disorders	0 (0 to 0)	0 (0 to 0)	[illegible]	[illegible]	[illegible]	[illegible]	[illegible]	30 (20·7 to 42·1)	63,200
Rheumatoid arthritis	0 (0 to 0)	0 (0 to 0)	[illegible]	[illegible]	[illegible]	[illegible]	[illegible]	0·2 (0·1 to 0·2)	640
Osteoarthritis	0 (0 to 0)	0 (0 to 0)	[illegible]	[illegible]	[illegible]	[illegible]	[illegible]	5·6 (2·8 to 11)	3,730
Osteoarthritis hip	0 (0 to 0)	0 (0 to 0)	[illegible]	[illegible]	[illegible]	[illegible]	[illegible]	0·3 (0·1 to 0·6)	160
Osteoarthritis knee	0 (0 to 0)	0 (0 to 0)	[illegible]	[illegible]	[illegible]	[illegible]	[illegible]	3·4 (1·7 to 6·9)	3,000
Osteoarthritis hand	0 (0 to 0)	0 (0 to 0)	[illegible]	[illegible]	[illegible]	[illegible]	[illegible]	0·5 (0·2 to 1)	121
Osteoarthritis other	0 (0 to 0)	0 (0 to 0)	[illegible]	[illegible]	[illegible]	[illegible]	[illegible]	1·4 (0·7 to 2·8)	454
Low back pain	0 (0 to 0)	0 (0 to 0)	[illegible]	[illegible]	[illegible]	[illegible]	[illegible]	19·3 (12·6 to 28·5)	30,200
Neck pain	0 (0 to 0)	0 (0 to 0)	[illegible]	[illegible]	[illegible]	[illegible]	[illegible]	2·2 (1·2 to 3·9)	12,300
Gout	0 (0 to 0)	0 (0 to 0)	[illegible]	[illegible]	[illegible]	[illegible]	[illegible]	0·4 (0·2 to 0·7)	218
Other musculoskeletal disorders	0 (0 to 0)	0 (0 to 0)	[illegible]	[illegible]	[illegible]	[illegible]	[illegible]	2·3 (0·2 to 5·2)	16,200
Other non-communicable diseases	14,000 (5,970 to 24,000)	3,880 (2,090 to 6,270)	[illegible]	[illegible]	[illegible]	[illegible]	[illegible]	19·8 (14·1 to 27·5)	88,800
Congenital birth defects	13,900 (5,930 to 23,900)	3,490 (1,800 to 5,880)	[illegible]	[illegible]	[illegible]	[illegible]	[illegible]	0·3 (0·2 to 0·5)	42,800
Neural tube defects	2,760 (738 to 6,800)	275 (54·9 to 803)	[illegible]	[illegible]	[illegible]	[illegible]	[illegible]	0 (0 to 0)	4,710
Congenital heart anomalies	4,730 (1,640 to 9,210)	1,990 (999 to 3,500)	[illegible]	[illegible]	[illegible]	[illegible]	[illegible]	0 (0 to 0)	19,400
Orofacial clefts	78·3 (19·9 to 228)	28·4 (7·5 to 81·6)	[illegible]	[illegible]	[illegible]	[illegible]	[illegible]	0 (0 to 0)	238
Down syndrome	93·2 (26 to 252)	33 (10·3 to 76·2)	[illegible]	[illegible]	[illegible]	[illegible]	[illegible]	0 (0 to 0)	582
Turner syndrome	0 (0 to 0)	0 (0 to 0·1)	[illegible]	[illegible]	[illegible]	[illegible]	[illegible]	0 (0 to 0)	9·2
Klinefelter syndrome	0 (0 to 0)	0 (0 to 0)	[illegible]	[illegible]	[illegible]	[illegible]	[illegible]	0 (0 to 0)	0
Other chromosomal abnormalities	200 (58·1 to 492)	86·6 (30·7 to 191)	[illegible]	[illegible]	[illegible]	[illegible]	[illegible]	0 (0 to 0)	638
Congenital musculoskeletal and limb anomalies	183 (57·1 to 423)	47·5 (26·8 to 83·8)	[illegible]	[illegible]	[illegible]	[illegible]	[illegible]	0 (0 to 0)	1,940
Urogenital congenital anomalies	260 (66·2 to 690)	29·6 (9·7 to 70)	[illegible]	[illegible]	[illegible]	[illegible]	[illegible]	0 (0 to 0)	472
Digestive congenital anomalies	1,550 (404 to 3,810)	340 (115 to 710)	[illegible]	[illegible]	[illegible]	[illegible]	[illegible]	0 (0 to 0)	4,640
Other congenital birth defects	4,070 (1,080 to 9,790)	667 (225 to 1,410)	[illegible]	[illegible]	[illegible]	[illegible]	[illegible]	0·3 (0·1 to 0·5)	10,100
Urinary diseases and male infertility	0·6 (0·2 to 1·3)	2·1 (0·9 to 4·6)	[illegible]	[illegible]	[illegible]	[illegible]	[illegible]	5·3 (2·5 to 10·3)	2,300
Urolithiasis	0 (0 to 0)	0 (0 to 0)	[illegible]	[illegible]	[illegible]	[illegible]	[illegible]	0·2 (0 to 0·5)	214
Benign prostatic hyperplasia	0 (0 to 0)	0 (0 to 0)	[illegible]	[illegible]	[illegible]	[illegible]	[illegible]	0 (0 to 0)	0
Male infertility	--	--	[illegible]	[illegible]	[illegible]	[illegible]	[illegible]	--	NA
Other urinary diseases	0·2 (0 to 0·4)	1·3 (0·4 to 3·2)	[illegible]	[illegible]	[illegible]	[illegible]	[illegible]	0·9 (0·2 to 2)	290
Gynecological diseases	0 (0 to 0)	0 (0 to 0)	[illegible]	[illegible]	[illegible]	[illegible]	[illegible]	1·8 (1 to 2·8)	19,600
Uterine fibroids	0 (0 to 0)	0 (0 to 0)	[illegible]	[illegible]	[illegible]	[illegible]	[illegible]	0·2 (0 to 0·5)	697
Polycystic ovarian syndrome	0 (0 to 0)	0 (0 to 0)	[illegible]	[illegible]	[illegible]	[illegible]	[illegible]	0 (0 to 0)	771
Female infertility	0 (0 to 0)	0 (0 to 0)	[illegible]	[illegible]	[illegible]	[illegible]	[illegible]	0 (0 to 0)	412
Endometriosis	0 (0 to 0)	0 (0 to 0)	[illegible]	[illegible]	[illegible]	[illegible]	[illegible]	0 (0 to 0)	2,520
Genital prolapse	0 (0 to 0)	0 (0 to 0)	[illegible]	[illegible]	[illegible]	[illegible]	[illegible]	0·2 (0·1 to 0·4)	179
Premenstrual syndrome	0 (0 to 0)	0 (0 to 0)	[illegible]	[illegible]	[illegible]	[illegible]	[illegible]	0 (0 to 0)	7,990
Other gynecological diseases	0 (0 to 0)	0 (0 to 0)	[illegible]	[illegible]	[illegible]	[illegible]	[illegible]	1·4 (0·7 to 2·3)	7,020
Hemoglobinopathies and hemolytic anemias	12·6 (6·6 to 21·2)	31 (20·2 to 45·7)	[illegible]	[illegible]	[illegible]	[illegible]	[illegible]	2·2 (1·4 to 3·1)	7,500
Thalassemias	11 (5·3 to 19·4)	11 (5·7 to 19·7)	[illegible]	[illegible]	[illegible]	[illegible]	[illegible]	0 (0 to 0)	931
Thalassemias trait	1·4 (0·6 to 2·8)	16·3 (9·8 to 25·4)	[illegible]	[illegible]	[illegible]	[illegible]	[illegible]	0·7 (0·4 to 1)	4,690
Sickle cell disorders	0 (0 to 0)	0 (0 to 0)	[illegible]	[illegible]	[illegible]	[illegible]	[illegible]	0 (0 to 0)	0·6
Sickle cell trait	0·1 (0 to 0·1)	0·6 (0·3 to 1·1)	[illegible]	[illegible]	[illegible]	[illegible]	[illegible]	0 (0 to 0)	243

G6PD deficiency	0 (0 to 0)	0·1 (0 to 0·2)	0·2 (0·1 to 0·3)	0·4 (0·2 to 0·7)	1·1 (0·5 to 2)	3·1 (1·2 to 6·4)	15·5 (7·1 to 27·1)	21·8 (8·9 to 40·8)	34·7 (15·1 to 63·7)	39·2 (17·7 to 68·3)	31·6 (15·1 to 53·8)	56·8 (35·5 to 84·2)
G6PD trait	0 (0 to 0)	0 (0 to 0)	0 (0 to 0)	0 (0 to 0)	0 (0 to 0)	0 (0 to 0)	0·3 (0·2 to 0·5)	0·1 (0·1 to 0·2)	0·1 (0·1 to 0·2)	0·1 (0·1 to 0·2)	0·2 (0·1 to 0·3)	6·8 (3·1 to 12·4)
Other hemoglobinopathies and hemolytic anemias	0·1 (0·1 to 0·3)	3·1 (1·7 to 4·8)	24·3 (12·4 to 41·8)	51·6 (20·1 to 102)	73·6 (24·2 to 153)	57·6 (19·8 to 117)	70·7 (37·4 to 117)	56 (29·9 to 95)	63·2 (35·5 to 102)	67·8 (39·9 to 108)	70·2 (41·1 to 107)	105 (66·1 to 155)
Endocrine, metabolic, blood, and immune disorders	32·3 (12·6 to 66·8)	29·4 (14·9 to 55·1)	476 (156 to 1.180)	194 (102 to 320)	197 (110 to 314)	189 (115 to 288)	283 (184 to 405)	331 (209 to 488)	492 (308 to 747)	698 (429 to 1.120)	832 (496 to 1.290)	911 (554 to 1.400)
Oral disorders	0 (0 to 0)	0 (0 to 0)	0 (0 to 0)	198 (112 to 329)	225 (121 to 385)	249 (132 to 424)	298 (164 to 492)	365 (213 to 597)	404 (227 to 647)	404 (230 to 639)	374 (213 to 596)	354 (202 to 588)
Caries of deciduous teeth	0 (0 to 0)	0 (0 to 0)	0 (0 to 0)	61·2 (22·4 to 128)	56·6 (22·1 to 118)	3 (0·4 to 7·8)	0 (0 to 0)	0 (0 to 0)	0 (0 to 0)	0 (0 to 0)	0 (0 to 0)	0 (0 to 0)
Caries of permanent teeth	0 (0 to 0)	0 (0 to 0)	0 (0 to 0)	0 (0 to 0)	53·1 (19·2 to 114)	126 (52·5 to 260)	118 (47·5 to 241)	118 (50·1 to 242)	107 (42·5 to 220)	88·5 (36·3 to 185)	71·9 (28·9 to 153)	54·6 (22 to 112)
Periodontal diseases	0 (0 to 0)	0 (0 to 0)	0 (0 to 0)	0 (0 to 0)	0 (0 to 0)	0 (0 to 0)	4·3 (1 to 11·7)	11·9 (3·3 to 31·3)	25·2 (7 to 61·1)	40·3 (11·9 to 107)	53·3 (16·8 to 137)	67·2 (20·5 to 174)
Other oral disorders	0 (0 to 0)	0 (0 to 0)	0 (0 to 0)	137 (81 to 216)	115 (65·1 to 179)	120 (68·6 to 188)	175 (104 to 270)	210 (128 to 319)	222 (133 to 337)	209 (123 to 312)	175 (106 to 268)	147 (90·3 to 225)
Sudden infant death syndrome	--	319 (124 to 709)	2,620 (525 to 7.080)	--	--	--	--	--	--	--	--	--
Injuries	249 (152 to 397)	142 (76·4 to 260)	3,110 (1.800 to 5.020)	3,760 (1.780 to 6.610)	4,060 (2.830 to 5.530)	3,540 (2.760 to 4.420)	5,900 (4.360 to 7.370)	6,170 (4.330 to 7.830)	5,250 (3.800 to 6.810)	4,700 (3.450 to 6.070)	3,810 (2.900 to 5.010)	3,400 (2.620 to 4.390)
Transport injuries	27·9 (9·8 to 62·1)	28·6 (11 to 58·6)	640 (238 to 1,270)	723 (285 to 1,500)	1,340 (742 to 2,190)	1,040 (641 to 1,510)	2,130 (1,260 to 3,250)	2,220 (1,380 to 3,250)	1,800 (1,160 to 2,680)	1,580 (1,000 to 2,330)	1,290 (858 to 1,850)	1,260 (855 to 1,790)
Road injuries	24·9 (8·7 to 56·9)	27·2 (10·5 to 56·3)	588 (218 to 1.190)	666 (260 to 1.390)	1,260 (683 to 2.060)	979 (600 to 1.430)	2,020 (1.180 to 3.070)	2,110 (1.300 to 3.110)	1,710 (1.100 to 2.560)	1,500 (955 to 2.200)	1,230 (817 to 1.770)	1,190 (815 to 1.710)
Pedestrian road injuries	12 (3·4 to 28·2)	10·4 (3·4 to 23·7)	317 (91·1 to 794)	330 (120 to 695)	667 (308 to 1.210)	486 (271 to 776)	649 (302 to 1.210)	729 (357 to 1.330)	564 (270 to 1.040)	530 (277 to 910)	445 (239 to 775)	378 (204 to 628)
Cyclist road injuries	0 (0 to 0)	0 (0 to 0)	0 (0 to 0)	42·1 (12·4 to 107)	73·3 (28·9 to 147)	63·8 (34 to 119)	96·2 (52·9 to 171)	105 (62·1 to 174)	98·8 (61 to 158)	117 (70·6 to 181)	98·9 (63·3 to 155)	99·3 (63·7 to 151)
Motorcyclist road injuries	5·4 (1·2 to 15)	2·3 (0·6 to 6·1)	102 (18·1 to 279)	94·7 (25·8 to 235)	178 (61·9 to 396)	175 (81·8 to 328)	544 (252 to 1.010)	561 (297 to 1.000)	447 (253 to 764)	367 (218 to 563)	308 (198 to 465)	307 (206 to 463)
Motor vehicle road injuries	7 (1·7 to 18·8)	14·4 (4·7 to 34·2)	156 (31·8 to 464)	189 (55·9 to 461)	326 (118 to 726)	244 (120 to 453)	699 (335 to 1.270)	681 (319 to 1.250)	574 (300 to 1.010)	463 (242 to 836)	356 (192 to 619)	390 (219 to 672)
Other road injuries	0·5 (0·1 to 1·6)	0·2 (0 to 0·6)	12·6 (2·5 to 36·8)	9·6 (2·6 to 24)	15·6 (4·5 to 37·8)	10·1 (4·3 to 21·2)	29·4 (12·3 to 65·4)	32·1 (13·5 to 64·8)	21·8 (10·3 to 42·9)	23·6 (11·7 to 46·4)	17·9 (9·2 to 34·8)	17·5 (9·6 to 31·8)
Other transport injuries	2·9 (0·7 to 8·2)	1·4 (0·4 to 3·4)	52·6 (14·8 to 136)	56·6 (22·1 to 124)	82·4 (38·8 to 167)	61·5 (34·5 to 110)	115 (53·5 to 230)	115 (59·3 to 215)	93·8 (47·6 to 175)	83·7 (42·7 to 156)	63 (33·7 to 114)	63·9 (33·9 to 116)
Unintentional injuries	150 (94·6 to 236)	100 (50·6 to 207)	2,290 (1.230 to 3.880)	2,830 (1.320 to 5.270)	2,460 (1.650 to 3.510)	1,960 (1.420 to 2.660)	1,660 (1.230 to 2.140)	1,710 (1.230 to 2.190)	1,570 (1.170 to 2.060)	1,490 (1.100 to 1.930)	1,300 (996 to 1.660)	1,170 (899 to 1.480)
Falls	27·1 (7 to 68·3)	9 (3·1 to 20·8)	316 (77·9 to 823)	382 (137 to 843)	359 (202 to 574)	307 (201 to 435)	385 (266 to 526)	380 (270 to 519)	427 (302 to 583)	407 (292 to 546)	408 (301 to 539)	384 (283 to 508)
Drowning	22·4 (11 to 41·2)	9·5 (4·1 to 20·1)	469 (164 to 1,030)	1,680 (612 to 3,740)	1,400 (704 to 2,410)	1,030 (565 to 1,670)	511 (276 to 861)	441 (223 to 727)	294 (153 to 498)	262 (142 to 434)	203 (104 to 351)	187 (104 to 310)
Fire, heat, and hot substances	5·6 (1·9 to 10·9)	7·3 (2·8 to 13·9)	143 (32 to 354)	208 (70·1 to 447)	185 (92·4 to 312)	174 (106 to 268)	289 (161 to 465)	345 (179 to 584)	332 (175 to 587)	303 (166 to 503)	233 (143 to 362)	191 (123 to 285)
Poisonings	4·4 (1·3 to 10·5)	1·8 (0·4 to 4·7)	44·5 (9·2 to 128)	96·4 (27·4 to 225)	57·5 (24·6 to 114)	58·7 (26·4 to 119)	62·2 (27·8 to 127)	63 (30·3 to 115)	54·3 (25 to 102)	40 (19·5 to 74·4)	37·1 (17·1 to 70·2)	37·4 (18 to 70·1)
Poisoning by carbon monoxide	2·4 (0·5 to 6·6)	0·2 (0 to 0·9)	4·7 (0·6 to 19·4)	11·2 (1·7 to 37·3)	12·1 (3·2 to 30·9)	9 (2·9 to 22·6)	19·5 (6·1 to 49·7)	18·3 (6·2 to 42·1)	15·2 (5·2 to 34·7)	9·9 (3·7 to 22·4)	8·3 (2·9 to 21·6)	11·7 (4 to 27·3)
Poisoning by other means	2 (0·3 to 5·9)	1·6 (0·4 to 4·2)	39·9 (7·5 to 116)	85·2 (23·1 to 202)	45·4 (17·5 to 91·9)	49·7 (21·3 to 101)	42·6 (15·8 to 89·7)	44·7 (18·9 to 88·4)	39·1 (15·8 to 78·5)	30·2 (13·6 to 59·2)	28·8 (12·8 to 56)	25·7 (11·2 to 51·7)
Exposure to mechanical forces	5·3 (0·2 to 29·6)	18·3 (0·3 to 119)	88·6 (6·2 to 475)	74 (31·3 to 171)	92·1 (56·6 to 145)	83 (55·5 to 121)	108 (70·5 to 163)	134 (90·6 to 202)	140 (93·3 to 202)	149 (102 to 212)	132 (92·5 to 186)	120 (85·2 to 165)
Unintentional firearm injuries	1·8 (0 to 13)	4·2 (0·1 to 37)	25 (1·1 to 187)	14·9 (6 to 39·9)	16·3 (8·6 to 33·4)	18·2 (10·9 to 32·3)	24·4 (14·2 to 45·8)	28 (16·3 to 53·9)	31·3 (18·7 to 56·9)	28·9 (18 to 50·3)	25·7 (16·4 to 40·5)	23·1 (15·4 to 34·8)
Other exposure to mechanical forces	3·5 (0·1 to 21·2)	14·1 (0·2 to 90·2)	63·6 (4·2 to 362)	59·1 (24·6 to 139)	75·8 (45 to 122)	64·8 (41·9 to 95·8)	83·3 (53·4 to 129)	106 (69·1 to 164)	109 (70·9 to 158)	120 (80·4 to 175)	107 (72·1 to 156)	96·9 (67·5 to 138)
Adverse effects of medical treatment	32·1 (14·5 to 59·3)	18·3 (7·8 to 35·6)	204 (61·4 to 442)	87 (36·2 to 178)	64·1 (30·4 to 108)	58·5 (28·9 to 97·4)	59·8 (29·7 to 105)	81·1 (40 to 142)	64·5 (28·3 to 126)	78·6 (31·7 to 160)	58·6 (26 to 119)	58·8 (26·9 to 122)
Animal contact	2·8 (1·1 to 8)	2·3 (0·7 to 6·9)	54·5 (18·2 to 138)	69·9 (22·4 to 227)	78·2 (32·2 to 209)	52·9 (24·4 to 136)	48·1 (22·2 to 112)	48·1 (22·8 to 120)	46·8 (23·7 to 113)	44·7 (21·3 to 114)	44·4 (21·2 to 106)	35·4 (18·6 to 76·7)
Venomous animal contact	0·6 (0·1 to 2·4)	0·2 (0·1 to 0·8)	16·7 (3·2 to 56·3)	31·5 (8·5 to 106)	33·5 (10·9 to 98·4)	25 (9·7 to 70)	19·7 (8 to 50·1)	19·1 (7·8 to 52·6)	17·3 (7·6 to 44·2)	16·1 (7 to 42·7)	20·8 (8·5 to 55·7)	12 (5·5 to 27·9)
Non-venomous animal contact	2·1 (0·8 to 6·1)	2 (0·6 to 6·3)	37·8 (12 to 97·6)	38·3 (10·3 to 130)	44·6 (16·1 to 126)	27·9 (11·1 to 77)	28·4 (12·2 to 72·7)	29 (12·9 to 77·5)	29·6 (12·7 to 75·9)	28·6 (12·6 to 71·4)	23·6 (11 to 54·3)	23·4 (11·3 to 53)
Foreign body	46·9 (20·4 to 102)	31 (14·5 to 58·9)	911 (360 to 1,960)	111 (48·9 to 225)	73·6 (43·1 to 116)	51·4 (33 to 76·8)	41·3 (27·4 to 58·6)	45·6 (30·1 to 65·2)	42·7 (30 to 60·4)	39·4 (26·9 to 54·4)	34·5 (24·2 to 45·7)	36·3 (25·5 to 51·3)
Pulmonary aspiration and foreign body in airway	46·4 (20·1 to 101)	30·8 (14·3 to 58·8)	882 (348 to 1.920)	101 (40·4 to 211)	61·9 (32·8 to 103)	38·5 (22·7 to 62·7)	26·4 (15·4 to 42·7)	28·9 (16·6 to 47·7)	25·1 (15·1 to 39·8)	21·9 (12·4 to 35·9)	18 (10·9 to 27·5)	21·1 (12·5 to 34)
Foreign body in eyes	0 (0 to 0)	0 (0 to 0·1)	0·5 (0·1 to 1)	2·2 (0·6 to 5·1)	2·9 (0·8 to 6·5)	3·2 (1 to 7·6)	3·9 (1·2 to 9·1)	4·7 (1·5 to 11·1)	5·1 (1·8 to 10·7)	4·9 (1·9 to 10·2)	4·4 (1·8 to 9·5)	4·1 (1·7 to 8·4)
Foreign body in other body part	0·5 (0·1 to 2·1)	0·2 (0·1 to 0·5)	28·1 (3·3 to 118)	7·7 (4·2 to 13·7)	8·8 (5·7 to 13)	9·7 (6·3 to 14·4)	11 (7·7 to 15·6)	12 (8·3 to 17·1)	12·6 (8·8 to 17·6)	12·6 (8·9 to 17·5)	12·1 (8·5 to 16·1)	11·1 (7·9 to 14·9)
Environmental heat and cold exposure	0·7 (0·2 to 1·9)	0·4 (0·1 to 1·2)	19 (4·6 to 58·4)	15·7 (7·3 to 35·3)	17·7 (10·8 to 29·8)	18·9 (12·3 to 29·1)	23·2 (15·6 to 36·5)	28·5 (19·2 to 44·2)	25·7 (18 to 36·7)	24·7 (16·9 to 35)	21·5 (15·3 to 30)	18·9 (13·5 to 26·7)
Exposure to forces of nature	0·3 (0·3 to 0·4)	1·3 (1·2 to 1·4)	10·6 (9·6 to 11·7)	54·2 (49·1 to 60·1)	48·4 (43·5 to 53·8)	52 (46·1 to 58·9)	41·2 (36·4 to 47·1)	36·4 (32 to 41·7)	34·2 (30 to 39·2)	29·7 (25·8 to 34·6)	24·2 (20·6 to 28·8)	18·9 (16·4 to 22)
Other unintentional injuries	2·8 (1·2 to 6·3)	1 (0·4 to 2·1)	30·3 (14·5 to 58·7)	51·8 (29·5 to 86·9)	81·9 (51·6 to 128)	78·9 (52·3 to 114)	91·2 (61·7 to 134)	102 (68·2 to 148)	108 (72·8 to 156)	111 (75·4 to 161)	99·3 (66 to 143)	84·5 (57·3 to 119)
Self-harm and interpersonal violence	71 (23·8 to 169)	13·1 (5 to 26·8)	178 (52·6 to 459)	214 (75 to 493)	257 (145 to 424)	540 (369 to 778)	2,110 (1.370 to 3.030)	2,240 (1.470 to 3.260)	1,880 (1.200 to 2.700)	1,630 (1.070 to 2.330)	1,230 (845 to 1.810)	975 (681 to 1.430)
Self-harm	0 (0 to 0)	0 (0 to 0)	0 (0 to 0)	0 (0 to 0)	0 (0 to 0)	201 (107 to 350)	1,240 (666 to 2.050)	1,260 (699 to 2.010)	975 (543 to 1.580)	791 (455 to 1.210)	551 (341 to 883)	424 (264 to 643)
Self-harm by firearm	0 (0 to 0)	0 (0 to 0)	0 (0 to 0)	0 (0 to 0)	0 (0 to 0)	2·6 (1 to 5·4)	4 (1·3 to 9·8)	8·9 (2·6 to 24·9)	4·9 (1·5 to 12·6)	6·1 (2·3 to 13·8)	3 (1·1 to 7·4)	3·9 (1·4 to 9·4)
Self-harm by other specified means	0 (0 to 0)	0 (0 to 0)	0 (0 to 0)	0 (0 to 0)	0 (0 to 0)	199 (105 to 346)	1,240 (664 to 2,040)	1,250 (694 to 2,000)	970 (542 to 1,570)	785 (452 to 1,200)	548 (340 to 881)	420 (261 to 638)
Interpersonal violence	71 (23·8 to 169)	13·1 (5 to 26·8)	178 (52 to 458)	210 (72·5 to 487)	252 (141 to 419)	333 (207 to 520)	854 (495 to 1.370)	966 (543 to 1.560)	889 (501 to 1.450)	797 (465 to 1.320)	655 (385 to 1.110)	533 (332 to 832)
Physical violence by firearm	3·8 (0·9 to 10·5)	1 (0·2 to 2·7)	17·6 (3·6 to 54·2)	25·7 (5·6 to 72)	25·4 (8·8 to 55·9)	28·5 (9·6 to 70·4)	181 (71·1 to 369)	177 (64·4 to 374)	187 (71·4 to 382)	185 (75·8 to 362)	146 (53·6 to 318)	127 (50·2 to 265)
Physical violence by sharp object	8·9 (1·9 to 24·8)	0·4 (0·1 to 1·2)	19 (3·5 to 60)	44·9 (12·6 to 116)	60·7 (24·3 to 118)	104 (40·3 to 217)	198 (82·7 to 407)	398 (171 to 754)	370 (157 to 693)	302 (135 to 558)	242 (117 to 472)	188 (88 to 348)
Sexual violence	0 (0 to 0)	0 (0 to 0)	0 (0 to 0)	3·9 (2·3 to 6·4)	31·5 (18·8 to 50·4)	78·3 (44·9 to 123)	160 (90·3 to 253)	157 (90·6 to 240)	118 (68·3 to 190)	98·9 (54·7 to 158)	79·1 (43·5 to 127)	64·1 (36·8 to 106)
Physical violence by other means	58·3 (19 to 137)	11·7 (4·5 to 24·1)	141 (42·3 to 368)	136 (45·7 to 332)	135 (65·2 to 251)	122 (60·8 to 223)	315 (149 to 574)	233 (115 to 440)	214 (111 to 391)	212 (119 to 354)	188 (106 to 317)	154 (96·9 to 251)
Conflict and terrorism	0 (0 to 0)	0 (0 to 0)	0 (0 to 0)	0·2 (0·1 to 0·3)	0·9 (0·5 to 1·6)	2·6 (1·3 to 5·1)	8·2 (4·3 to 15·1)	6·9 (3·6 to 12·5)	9·9 (5·3 to 18·2)	34·8 (15·2 to 70·5)	19·1 (8·7 to 38·6)	17·1 (7·6 to 35·3)

G6PD deficiency	37·7 (22·1 to 60·7)	25·1 (11·6 to 46)	19·6 (8·9 to 34·4)	13·3 (6 to 23·9)	6·7 (2·9 to 12·5)	3·4 (1·5 to 6)	1·5 (0·6 to 2·8)	0·6 (0·3 to 1·1)	0·1 (0·1 to 0·2)	0 (0 to 0)	0 (0 to 0)	312
G6PD trait	6·7 (3·3 to 11·4)	0·1 (0·1 to 0·2)	0 (0 to 0)	0 (0 to 0)	0 (0 to 0)	0 (0 to 0)	0 (0 to 0)	0 (0 to 0)	0 (0 to 0)	0 (0 to 0)	0 (0 to 0)	14·6
Other hemoglobinopathies and hemolytic anemias	109 (71·3 to 162)	93·1 (53·1 to 147)	86·9 (52·9 to 136)	109 (65·2 to 167)	87·8 (52·4 to 134)	72·1 (46·2 to 105)	51·2 (32·8 to 74·7)	36·3 (23·7 to 52·7)	16·4 (11·1 to 22·9)	5·3 (3·6 to 7·4)	1·5 (1 to 2·1)	1,310
Endocrine, metabolic, blood, and immune disorders	877 (519 to 1.320)	818 (494 to 1.270)	691 (420 to 1.090)	458 (283 to 712)	305 (201 to 463)	173 (117 to 252)	120 (79·6 to 171)	65·7 (42 to 96·9)	32·4 (19·9 to 49·8)	11·9 (6·6 to 19·7)	4 (2·1 to 6·9)	8.220
Oral disorders	367 (216 to 596)	399 (231 to 648)	417 (248 to 666)	393 (232 to 630)	353 (211 to 572)	258 (157 to 406)	211 (130 to 321)	132 (83·1 to 199)	61·3 (37 to 93·3)	21·9 (13·3 to 32·7)	6·2 (3·8 to 9·4)	5,490
Caries of deciduous teeth	0 (0 to 0)	0 (0 to 0)	0 (0 to 0)	0 (0 to 0)	0 (0 to 0)	0 (0 to 0)	0 (0 to 0)	0 (0 to 0)	0 (0 to 0)	0 (0 to 0)	0 (0 to 0)	121
Caries of permanent teeth	43 (17·6 to 90·6)	36·1 (14·4 to 75·6)	29·4 (11·4 to 66·6)	22·3 (7·8 to 49)	16·4 (6·3 to 34·2)	10·1 (4·1 to 20·4)	6·8 (2·8 to 13·6)	3·6 (1·4 to 7·4)	1·4 (0·5 to 2·9)	0·4 (0·1 to 0·9)	0·1 (0 to 0·2)	906
Periodontal diseases	82·4 (25·8 to 202)	95 (31·4 to 221)	97 (32·4 to 228)	83·9 (29·3 to 199)	66·1 (22·7 to 144)	42·3 (15·5 to 94·3)	31·3 (12·1 to 71·4)	18·3 (6·9 to 41·1)	8·2 (2·9 to 18·3)	2·8 (1 to 6·1)	0·8 (0·2 to 1·6)	730
Other oral disorders	130 (79·6 to 199)	112 (67·2 to 169)	89·5 (54·6 to 135)	65·2 (39·4 to 97·1)	46·3 (27·5 to 69·1)	25·4 (15·3 to 38·3)	14·7 (8·9 to 22·3)	6·7 (4·1 to 10·1)	2·3 (1·4 to 3·5)	0·7 (0·4 to 1·1)	0·2 (0·1 to 0·3)	2,000
Sudden infant death syndrome	--	--	--	--	--	--	--	--	--	--	--	NA
Injuries	3,000 (2.320 to 3.840)	2,690 (2.080 to 3.400)	2,310 (1.820 to 2.920)	1,870 (1.490 to 2.320)	1,480 (1.200 to 1.780)	1,070 (868 to 1.310)	906 (731 to 1.100)	613 (487 to 755)	331 (262 to 417)	127 (99·3 to 162)	37·5 (27·9 to 51)	58.500
Transport injuries	1,170 (821 to 1,650)	1,080 (744 to 1,490)	936 (672 to 1,290)	722 (532 to 995)	542 (395 to 724)	377 (274 to 514)	266 (196 to 363)	141 (105 to 180)	58·4 (44·1 to 75·1)	17·8 (13·3 to 22·6)	5 (3·7 to 6·5)	19,400
Road injuries	1,110 (785 to 1.570)	1,020 (714 to 1.420)	894 (644 to 1.220)	692 (510 to 952)	519 (381 to 699)	363 (263 to 492)	255 (188 to 345)	134 (99·5 to 172)	55·4 (42 to 71·3)	16·5 (12·4 to 21·1)	4·5 (3·4 to 5·9)	18.400
Pedestrian road injuries	391 (217 to 647)	410 (236 to 667)	360 (221 to 568)	346 (212 to 546)	252 (162 to 372)	189 (127 to 281)	144 (96·2 to 214)	72·7 (50·6 to 98·9)	28·2 (20·4 to 37·7)	7·4 (5·4 to 9·8)	2·1 (1·5 to 2·8)	7.320
Cyclist road injuries	94 (62·3 to 139)	89·5 (60·9 to 131)	80·1 (53·9 to 120)	62·5 (42·8 to 91·4)	51·3 (34·2 to 75·8)	31·9 (22·3 to 46·5)	16·8 (12 to 22·5)	10·3 (7·4 to 13·7)	4·9 (3·4 to 6·6)	1·7 (1·2 to 2·4)	0·3 (0·2 to 0·5)	1,240
Motorcyclist road injuries	294 (193 to 443)	253 (175 to 363)	223 (156 to 313)	133 (96·6 to 177)	100 (70·8 to 133)	60·9 (43·9 to 81·2)	40·8 (29·3 to 53·5)	23·4 (16·5 to 31·3)	10·7 (7·6 to 14·3)	3·6 (2·6 to 4·9)	1·1 (0·8 to 1·4)	4.230
Motor vehicle road injuries	317 (186 to 508)	258 (155 to 419)	219 (141 to 348)	147 (91·2 to 229)	112 (72·5 to 170)	79·5 (49·8 to 118)	49·7 (32·3 to 75·8)	25·2 (17·1 to 36·5)	10·8 (7·3 to 15·6)	3·6 (2·5 to 5·2)	1 (0·6 to 1·4)	5.320
Other road injuries	16·4 (8·8 to 31·2)	14·8 (8·1 to 27·4)	12·2 (6·6 to 23·7)	3·8 (2·6 to 5·3)	3·1 (2·2 to 4·3)	1·8 (1·3 to 2·5)	3·4 (1·9 to 6·3)	2 (1·2 to 3·4)	0·8 (0·5 to 1·3)	0·3 (0·2 to 0·4)	0·1 (0 to 0·1)	250
Other transport injuries	53·4 (29·1 to 87·4)	50·7 (27·4 to 88·5)	41·7 (24 to 73)	30 (16·7 to 51·4)	23·4 (13·1 to 41)	14·2 (8 to 24·4)	11·6 (6·5 to 20·7)	7 (4 to 11·8)	3·1 (2 to 4·9)	1·3 (0·8 to 2·1)	0·5 (0·3 to 0·7)	1,030
Unintentional injuries	1,080 (831 to 1.380)	1,020 (794 to 1.290)	935 (743 to 1.170)	855 (669 to 1.080)	742 (585 to 927)	581 (458 to 733)	570 (440 to 725)	440 (337 to 570)	258 (198 to 340)	106 (79 to 139)	31·5 (22·5 to 43·7)	25.300
Falls	361 (272 to 476)	405 (301 to 536)	396 (296 to 520)	439 (318 to 588)	416 (313 to 555)	368 (265 to 494)	392 (279 to 537)	333 (240 to 454)	205 (150 to 282)	87·9 (63·6 to 120)	26·2 (18·1 to 37·9)	7,220
Drowning	184 (101 to 317)	144 (82·4 to 239)	127 (72 to 205)	87·2 (48 to 153)	79·3 (47·9 to 128)	48·7 (28·4 to 80·1)	43·4 (26·7 to 69·6)	22·5 (13·4 to 36·5)	9·5 (5·7 to 15·3)	2·3 (1·3 to 3·9)	0·5 (0·3 to 0·8)	7,260
Fire, heat, and hot substances	165 (109 to 240)	136 (91·2 to 186)	107 (76 to 146)	80·2 (55·6 to 110)	59·2 (40·4 to 82·7)	36·5 (24·2 to 52·8)	32·4 (20·5 to 49·2)	21·8 (13·9 to 33·7)	12 (7·5 to 18·8)	4·1 (2·4 to 6·6)	1·1 (0·6 to 2)	3.070
Poisonings	23·6 (11·6 to 43·5)	14·6 (7·7 to 26·4)	11·7 (6 to 20·6)	9·2 (4·9 to 16·1)	7·3 (3·9 to 12·3)	4·4 (2·4 to 7·5)	2·8 (1·6 to 5)	2·6 (1·3 to 5)	1·3 (0·7 to 2·4)	0·5 (0·3 to 0·9)	0·2 (0·1 to 0·4)	636
Poisoning by carbon monoxide	6·7 (2·4 to 16·1)	3·6 (1·5 to 7·9)	3 (1·2 to 6·8)	1·9 (0·8 to 4·3)	2·1 (0·8 to 4·7)	1·4 (0·6 to 3·2)	1 (0·4 to 2·1)	0·6 (0·2 to 1·5)	0·3 (0·1 to 0·8)	0·1 (0 to 0·3)	0·1 (0 to 0·2)	143
Poisoning by other means	17 (7·9 to 31·6)	11 (5·7 to 20·6)	8·8 (4·3 to 16·4)	7·3 (3·8 to 13·2)	5·2 (2·7 to 9·4)	3 (1·5 to 5·2)	1·8 (0·9 to 3·5)	2 (0·9 to 3·9)	1 (0·5 to 2)	0·4 (0·2 to 0·7)	0·1 (0 to 0·3)	493
Exposure to mechanical forces	115 (82·4 to 159)	110 (78·2 to 150)	101 (73·3 to 137)	81·3 (57·7 to 111)	60·4 (43·5 to 81·6)	38 (27·3 to 52·4)	28·7 (20·7 to 38·6)	17·2 (12·1 to 23·4)	7·7 (5·5 to 10·7)	2·8 (2 to 3·8)	0·9 (0·6 to 1·3)	1,710
Unintentional firearm injuries	19·9 (14 to 27·7)	17·9 (12·8 to 24·5)	15·4 (10·9 to 20·6)	11·2 (7·9 to 15·2)	7·8 (5·5 to 10·6)	4·4 (3·1 to 6)	3·1 (2·2 to 4·4)	1·9 (1·3 to 2·9)	0·9 (0·6 to 1·4)	0·4 (0·2 to 0·5)	0·1 (0·1 to 0·2)	325
Other exposure to mechanical forces	95·1 (66 to 134)	91·8 (65 to 127)	85·5 (61·4 to 118)	70·2 (49·3 to 97)	52·6 (36·8 to 71·9)	33·6 (23·6 to 47·1)	25·6 (18·3 to 35·1)	15·3 (10·6 to 21·1)	6·8 (4·8 to 9·6)	2·5 (1·7 to 3·4)	0·8 (0·5 to 1·1)	1.380
Adverse effects of medical treatment	61·3 (25·4 to 136)	50·4 (20·4 to 112)	52·6 (21·5 to 119)	50·3 (21·7 to 109)	40·6 (19·7 to 87·8)	28·2 (14·4 to 58·5)	24·7 (12·6 to 49·1)	16·2 (8·8 to 32·4)	8·1 (4·7 to 15·5)	2·9 (1·7 to 5·4)	0·9 (0·5 to 1·5)	1.200
Animal contact	32·4 (17·3 to 70)	38·7 (18·8 to 94·2)	33·3 (16·9 to 70·7)	24·6 (12·1 to 56·1)	15·8 (8·1 to 35·5)	13·4 (6·7 to 32·1)	10 (4·9 to 22·3)	4·7 (2·4 to 10·2)	2·6 (1·3 to 6·5)	0·9 (0·5 to 1·9)	0·3 (0·2 to 0·7)	705
Venomous animal contact	8·8 (4·2 to 20)	9·4 (4·2 to 22·3)	9·8 (4·1 to 24·1)	8·7 (3·5 to 21·9)	5·1 (2·1 to 12·4)	3·9 (1·6 to 9·6)	3·8 (1·5 to 9·4)	1·6 (0·6 to 4·2)	0·6 (0·2 to 1·7)	0·2 (0·1 to 0·5)	0·1 (0 to 0·2)	265
Non-venomous animal contact	23·6 (11·9 to 52·1)	29·3 (13·5 to 74·1)	23·5 (11·1 to 51·4)	16 (7·5 to 36·4)	10·7 (5·4 to 25·4)	9·5 (4·3 to 24·3)	6·1 (2·8 to 15)	3·1 (1·5 to 7·5)	2 (1 to 5·1)	0·7 (0·3 to 1·5)	0·2 (0·1 to 0·6)	440
Foreign body	30·5 (22·4 to 40·9)	27·5 (20·5 to 37·4)	26·6 (19·7 to 35·5)	22 (16·6 to 28·5)	17·7 (13·5 to 22·9)	13·8 (10·2 to 18·6)	13·2 (9·4 to 18·4)	8·6 (6·2 to 11·9)	5·2 (3·4 to 7·6)	2·2 (1·5 to 3·1)	0·8 (0·5 to 1·1)	1,630
Pulmonary aspiration and foreign body in airway	16 (10 to 25·2)	13·7 (8·6 to 21)	13·9 (8·7 to 21·5)	12·2 (8·2 to 17·6)	10 (6·8 to 14)	8·7 (5·7 to 13·1)	8·9 (5·4 to 13·8)	6 (3·8 to 9·2)	3·7 (2·2 to 6)	1·6 (1 to 2·5)	0·6 (0·3 to 0·9)	1.400
Foreign body in eyes	3·7 (1·7 to 7·3)	3·2 (1·6 to 5·9)	2·6 (1·4 to 4·4)	2 (1·1 to 3·3)	1·6 (0·9 to 2·4)	1 (0·6 to 1·5)	0·8 (0·5 to 1·1)	0·5 (0·3 to 0·7)	0·2 (0·1 to 0·3)	0·1 (0 to 0·1)	0 (0 to 0)	51·4
Foreign body in other body part	10·7 (8 to 14·3)	10·6 (7·8 to 14·3)	10·1 (7·4 to 13·5)	7·8 (5·7 to 10·5)	6·2 (4·4 to 8·3)	4·2 (3 to 5·5)	3·5 (2·6 to 4·7)	2·2 (1·6 to 3)	1·2 (0·9 to 1·6)	0·5 (0·4 to 0·7)	0·2 (0·1 to 0·3)	184
Environmental heat and cold exposure	17·8 (12·5 to 25·5)	17·1 (11·2 to 26·9)	12·9 (9·2 to 19·2)	10·8 (7·3 to 17·9)	8·5 (5·4 to 14·1)	5·7 (3·7 to 9·5)	4·2 (2·7 to 7·2)	2·8 (1·7 to 4·8)	1·4 (0·8 to 2·3)	0·5 (0·3 to 1)	0·2 (0·1 to 0·3)	297
Exposure to forces of nature	15·7 (13·6 to 18·4)	13·8 (11·9 to 16·2)	11·7 (10·1 to 13·8)	9 (7·7 to 10·6)	6·3 (5·3 to 7·4)	4·6 (4 to 5·4)	3·1 (2·6 to 3·7)	1·8 (1·6 to 2·1)	0·8 (0·6 to 0·9)	0·2 (0·2 to 0·3)	0 (0 to 0·1)	419
Other unintentional injuries	73·1 (49·5 to 102)	64·3 (43·6 to 91)	54·3 (36·9 to 76·7)	41 (27·7 to 57·9)	30·8 (21·1 to 43·4)	19·8 (13·8 to 28)	14·7 (10·2 to 20·6)	8·9 (6·2 to 12·4)	4·2 (3 to 5·8)	1·5 (1·1 to 2·1)	0·4 (0·3 to 0·6)	1,160
Self-harm and interpersonal violence	751 (531 to 1.050)	592 (428 to 804)	438 (317 to 625)	293 (217 to 399)	194 (149 to 256)	115 (84·7 to 149)	69·5 (53·3 to 90·3)	33 (25·6 to 43·6)	14·5 (10·9 to 18·7)	3·8 (2·8 to 4·8)	1 (0·8 to 1·3)	13.800
Self-harm	321 (198 to 524)	249 (161 to 371)	196 (125 to 292)	124 (79·4 to 182)	86·3 (55·5 to 129)	51·5 (33·8 to 74·6)	27·1 (18 to 40)	15·6 (10·1 to 24·2)	7·8 (4·9 to 11·6)	1·8 (1·2 to 2·6)	0·5 (0·3 to 0·7)	6.530
Self-harm by firearm	2·3 (0·9 to 5·7)	2·2 (0·8 to 5·2)	1·4 (0·6 to 3·2)	1·2 (0·5 to 3)	0·9 (0·4 to 1·9)	0·6 (0·3 to 1·2)	0·5 (0·3 to 1·1)	0·4 (0·2 to 0·8)	0·2 (0·1 to 0·5)	0·1 (0 to 0·2)	0 (0 to 0·1)	43·2
Self-harm by other specified means	319 (197 to 520)	247 (159 to 369)	195 (124 to 290)	122 (78·8 to 180)	85·5 (55 to 128)	50·9 (33·3 to 73·9)	26·5 (17·6 to 39·2)	15·2 (9·8 to 23·6)	7·6 (4·8 to 11·3)	1·7 (1·1 to 2·4)	0·5 (0·3 to 0·7)	6,490
Interpersonal violence	418 (277 to 639)	335 (225 to 491)	237 (160 to 365)	167 (112 to 247)	106 (75·2 to 146)	62·4 (44·5 to 86·2)	41·8 (30·7 to 58·5)	17·1 (13 to 23·1)	6·5 (4·9 to 8·6)	2 (1·5 to 2·6)	0·5 (0·4 to 0·7)	7.140
Physical violence by firearm	91·6 (40 to 176)	68·7 (30·9 to 132)	42·5 (18 to 84·8)	20·1 (8·4 to 39·6)	13·9 (6·4 to 25·6)	4·6 (2·1 to 9·3)	2·5 (1·1 to 4·8)	1·1 (0·5 to 2·3)	0·4 (0·2 to 0·7)	0·1 (0 to 0·1)	0 (0 to 0·1)	1.350
Physical violence by sharp object	131 (68 to 231)	114 (63·1 to 196)	80·5 (44·4 to 141)	67·8 (37·8 to 116)	28·3 (17 to 45·6)	22·8 (13·3 to 36·9)	12·6 (7·8 to 20·5)	4·6 (2·9 to 7·2)	2·2 (1·5 to 3·2)	0·7 (0·5 to 1·1)	0·2 (0·1 to 0·2)	2,400
Sexual violence	53·4 (31 to 89·5)	36·2 (19·9 to 60·1)	18·9 (10·8 to 31·2)	11·4 (6·8 to 17·3)	7·9 (4·9 to 12·2)	4·7 (2·9 to 7)	3·2 (2·1 to 4·8)	1·8 (1·2 to 2·7)	0·7 (0·4 to 1)	0·1 (0·1 to 0·2)	0 (0 to 0)	929
Physical violence by other means	141 (92 to 221)	116 (79·2 to 162)	95·3 (66·6 to 139)	67·3 (47·5 to 94·5)	55·5 (39·2 to 78)	30·3 (21·4 to 41·6)	23·5 (16·7 to 32·7)	9·6 (6·9 to 12·7)	3·3 (2·4 to 4·5)	1·1 (0·8 to 1·5)	0·3 (0·2 to 0·4)	2.460
Conflict and terrorism	10·4 (4·8 to 19·8)	6·6 (3·1 to 12·9)	4·1 (2 to 8)	2·7 (1·3 to 5·1)	1·5 (0·8 to 2·9)	0·7 (0·4 to 1·4)	0·5 (0·3 to 0·9)	0·3 (0·2 to 0·5)	0·1 (0·1 to 0·2)	0 (0 to 0·1)	0 (0 to 0)	127

SR Table 2b: United States of America DALYS by cause and age group for females with 95% uncertainty intervals, 2019

Cause	Early Neonatal	Late Neonatal	Post Neonatal	1 to 4	5 to 9	10 to 14	15 to 19	20 to 24	25 to 29	30 to 34	35 to 39	40 to 44	45 to 49	50 to 54	55 to 59	60 to 64	65 to 69	70 to 74	75 to 79	80 to 84	85 to 89	90 to 94	95 plus	Total
All causes	440,000 (412 000 to 471 000)	113,000 (106 000 to 121 000)	295,000 (279 000 to 311 000)	328,000 (270 000 to 397 000)	392,000 (302 000 to 504 000)	725,000 (545 000 to 945 000)	1,370,000 (1 060 000 to 1 750 000)	1,820,000 (1 410 000 to 2 270 000)	2,200,000 (1 730 000 to 2 730 000)	2,290,000 (1 820 000 to 2 810 000)	2,460,000 (1 980 000 to 2 990 000)	2,580,000 (2 110 000 to 3 100 000)	3,020,000 (2 510 000 to 3 580 000)	3,560,000 (3 040 000 to 4 170 000)	4,460,000 (3 840 000 to 5 140 000)	4,970,000 (4 340 000 to 5 690 000)	4,990,000 (4 400 000 to 5 630 000)	4,900,000 (4 380 000 to 5 460 000)	4,120,000 (3 710 000 to 4 560 000)	3,450,000 (3 150 000 to 3 770 000)	2,730,000 (2 510 000 to 2 960 000)	1,910,000 (1 780 000 to 2 040 000)	1,030,000 (970 000 to 1 090 000)	54 200 000
Communicable, maternal, neonatal, and nutritional diseases	333,000 (304 000 to 362 000)	64,700 (58 000 to 70 200)	67,200 (60 100 to 74 600)	70,000 (55 600 to 88 000)	69,800 (50 600 to 98 500)	63,400 (47 900 to 84 500)	77,400 (58 400 to 106 000)	80,800 (64 000 to 103 000)	98,500 (79 800 to 122 000)	106,000 (85 900 to 131 000)	117,000 (93 100 to 150 000)	118,000 (93 800 to 155 000)	115,000 (92 500 to 148 000)	114,000 (94 900 to 142 000)	125,000 (106 000 to 149 000)	128,000 (112 000 to 149 000)	123,000 (110 000 to 140 000)	121,000 (109 000 to 135 000)	112,000 (102 000 to 124 000)	104,000 (90 500 to 114 000)	93,900 (76 900 to 105 000)	74,800 (58 600 to 84 000)	47,700 (34 300 to 55 200)	2 430 000
HIV/AIDS and sexually transmitted infections	37·5 (32·7 to 43)	23·3 (19·3 to 28·3)	611 (590 to 633)	493 (474 to 513)	286 (274 to 298)	494 (447 to 563)	1,540 (1 200 to 2 060)	5,420 (4 210 to 7 340)	11,400 (8 660 to 15 400)	17,300 (13 600 to 23 000)	21,800 (17 300 to 28 300)	21,400 (17 300 to 27 200)	20,600 (16 700 to 25 700)	17,500 (14 200 to 22 000)	14,300 (11 300 to 18 400)	9,910 (7 540 to 13 200)	6,810 (5 190 to 8 980)	2,870 (1 920 to 4 240)	1,590 (1 080 to 2 360)	752 (521 to 1 160)	476 (332 to 725)	254 (181 to 372)	109 (81·2 to 153)	156 000
HIV/AIDS	0 (0 to 0·1)	0·1 (0·1 to 0·2)	599 (577 to 622)	492 (473 to 512)	284 (272 to 296)	325 (311 to 339)	907 (781 to 1 080)	3,490 (3 020 to 4 120)	7,430 (6 160 to 9 280)	12,300 (10 200 to 14 900)	16,500 (14 000 to 20 100)	16,500 (14 100 to 20 000)	17,100 (14 500 to 20 900)	14,900 (12 400 to 18 100)	12,000 (9 640 to 15 100)	8,100 (6 270 to 10 500)	5,250 (4 090 to 6 820)	1,610 (1 040 to 2 420)	687 (415 to 1 050)	148 (97·9 to 216)	80·2 (46·1 to 127)	41·7 (23·1 to 67·4)	21·8 (13·8 to 33·3)	119 000
HIV/AIDS - Drug-susceptible Tuberculosis	0 (0 to 0)	0 (0 to 0)	11·8 (7·4 to 18·1)	9·4 (5·9 to 14·5)	5·2 (3·3 to 8)	6 (3·8 to 9·2)	13 (8·5 to 19·6)	50·1 (32·5 to 75·1)	106 (69·4 to 158)	179 (117 to 268)	244 (158 to 367)	246 (160 to 370)	250 (162 to 375)	206 (134 to 308)	153 (100 to 228)	97·5 (64·8 to 144)	64·9 (43 to 96·1)	13·2 (9·2 to 19)	5·2 (3·7 to 7·4)	1·4 (0·9 to 2)	0·6 (0·4 to 0·8)	0·3 (0·2 to 0·4)	0·2 (0·1 to 0·3)	1 660
HIV/AIDS - Multidrug-resistant Tuberculosis without	0 (0 to 0)	0 (0 to 0)	0·4 (0·1 to 1·2)	0·3 (0·1 to 1)	0·2 (0 to 0·5)	0·2 (0·1 to 0·6)	0·5 (0·1 to 1·3)	1·8 (0·4 to 4·8)	3·7 (0·9 to 9·9)	6·2 (1·5 to 16·8)	8·3 (2 to 22·8)	8·3 (2·1 to 22·6)	8·4 (2·1 to 22·8)	6·9 (1·7 to 18·8)	5·1 (1·3 to 13·8)	3·2 (0·8 to 8·7)	2·2 (0·6 to 5·9)	0·4 (0·1 to 1·1)	0·2 (0 to 0·4)	0 (0 to 0·1)	0 (0 to 0)	0 (0 to 0)	0 (0 to 0)	56·3
HIV/AIDS - Extensively drug-resistant Tuberculosis	0 (0 to 0)	0 (0 to 0)	0·1 (0 to 0·3)	0·1 (0 to 0·2)	0·1 (0 to 0·1)	0·1 (0 to 0·2)	0·1 (0 to 0·3)	0·5 (0·1 to 1·2)	1 (0·2 to 2·6)	1·6 (0·4 to 4·3)	2·2 (0·5 to 5·8)	2·2 (0·5 to 5·8)	2·2 (0·5 to 5·9)	1·8 (0·4 to 4·8)	1·3 (0·3 to 3·5)	0·8 (0·2 to 2·2)	0·6 (0·1 to 1·5)	0·1 (0 to 0·3)	0 (0 to 0·1)	0 (0 to 0)	0 (0 to 0)	0 (0 to 0)	0 (0 to 0)	14·6
HIV/AIDS resulting in other diseases	0 (0 to 0·1)	0·1 (0·1 to 0·2)	587 (563 to 609)	482 (462 to 503)	278 (265 to 291)	319 (304 to 333)	894 (767 to 1 060)	3,440 (2 960 to 4 080)	7,320 (6 050 to 9 170)	12,100 (10 000 to 14 700)	16,200 (13 700 to 19 900)	16,200 (13 800 to 19 600)	16,800 (14 200 to 20 600)	14,700 (12 200 to 17 900)	11,800 (9 460 to 14 900)	7,990 (6 160 to 10 400)	5,180 (4 040 to 6 760)	1,600 (1 020 to 2 410)	682 (409 to 1 050)	146 (96·2 to 215)	79·6 (45·6 to 127)	41·4 (22·9 to 67·1)	21·6 (13·6 to 33·1)	117 000
Sexually transmitted infections excluding HIV	37·4 (32·6 to 43)	23·2 (19·1 to 28·1)	12·4 (10·2 to 14·9)	1·3 (1·1 to 1·5)	2·1 (1·8 to 2·5)	169 (127 to 232)	629 (368 to 1 120)	1,930 (1 030 to 3 520)	3,960 (2 020 to 7 290)	5,030 (2 660 to 9 350)	5,380 (2 800 to 10 100)	4,910 (2 480 to 9 500)	3,530 (1 720 to 7 060)	2,570 (1 160 to 5 310)	2,300 (1 090 to 4 550)	1,820 (887 to 3 600)	1,560 (829 to 2 950)	1,260 (718 to 2 270)	898 (561 to 1 550)	604 (395 to 994)	395 (271 to 633)	212 (148 to 322)	87·4 (63·2 to 127)	37 300
Syphilis	37·4 (32·6 to 43)	23·2 (19·1 to 28·1)	12·4 (10·2 to 14·9)	1·3 (1·1 to 1·5)	2·1 (1·8 to 2·5)	43·2 (40·9 to 45·2)	4·4 (1·5 to 10·6)	56·4 (48·9 to 69·5)	24·6 (14·7 to 42·9)	35·6 (23·4 to 54·8)	45·5 (30·5 to 65·7)	48 (32·7 to 67·6)	56·5 (38·1 to 79·2)	64·6 (43·8 to 92·5)	88·8 (58·8 to 125)	118 (76·2 to 173)	144 (92 to 211)	147 (94·8 to 208)	120 (76 to 171)	86·3 (55·4 to 122)	61·5 (39·7 to 86)	37·2 (24·1 to 52·2)	16·8 (10·7 to 23·9)	1,270
Chlamydial infection	0 (0 to 0)	0 (0 to 0)	0 (0 to 0)	0 (0 to 0)	0 (0 to 0)	22·4 (17·6 to 29·6)	60·9 (38·4 to 93·6)	146 (90·6 to 227)	263 (171 to 396)	268 (155 to 453)	329 (197 to 548)	304 (168 to 545)	138 (87·2 to 229)	61 (47·1 to 81·7)	55·1 (46·9 to 65·3)	42·1 (38·7 to 45·8)	27·3 (24·8 to 30)	42·2 (38·3 to 46·7)	41·5 (37·2 to 45·9)	42·3 (35·8 to 47·2)	31·4 (25·4 to 35·6)	18·1 (13·7 to 21)	8·4 (5·9 to 9·9)	1,900
Gonococcal infection	0 (0 to 0)	0 (0 to 0)	0 (0 to 0)	0 (0 to 0)	0 (0 to 0)	19·1 (17·4 to 21·5)	48·2 (39·8 to 61·1)	81 (59 to 114)	152 (104 to 224)	266 (202 to 377)	259 (190 to 375)	227 (157 to 344)	152 (127 to 200)	104 (95·2 to 114)	111 (103 to 120)	138 (127 to 150)	166 (151 to 182)	152 (138 to 169)	156 (139 to 172)	115 (97·9 to 129)	85 (68·8 to 96·5)	49·4 (37·4 to 57·3)	27 (19·1 to 32)	2,310
Trichomoniasis	0 (0 to 0)	0 (0 to 0)	0 (0 to 0)	0 (0 to 0)	0 (0 to 0)	9·1 (2·4 to 23·7)	165 (49·6 to 388)	835 (281 to 1 970)	1,850 (623 to 4 330)	2,150 (706 to 5 130)	2,070 (658 to 4 810)	1,830 (598 to 4 350)	1,660 (516 to 3 860)	1,270 (392 to 2 990)	996 (326 to 2 370)	699 (245 to 1 640)	538 (198 to 1 240)	383 (142 to 866)	236 (92·1 to 529)	134 (50·7 to 298)	72 (28·7 to 166)	29·7 (11·2 to 67·2)	6·9 (2·2 to 17)	14 900

Gastroesophageal reflux disease	0 (0 to 0)	0 (0 to 0)	0 (0 to 0)	0 (0 to 0)	15·8 (6 to 34·3)	356	2,180	5,900	10,100	12,300	13,000	12,700	13,900	14,800	16,600	16,600	14,700	11,600	7,530	4,540	2,720	1,460	612	162,000
Appendicitis	0 (0 to 0)	0 (0 to 0)	0 (0 to 0)	143 (117 to 171)	292 (237 to 359)	[illegible]	[illegible]	[illegible]	[illegible]	[illegible]	[illegible]	[illegible]	[illegible]	[illegible]	[illegible]	[illegible]	[illegible]	[illegible]	[illegible]	[illegible]	[illegible]	[illegible]	[illegible]	8,610
Paralytic ileus and intestinal obstruction	295 (242 to 378)	587 (489 to 693)	9,070 (7 990 to 10 200)	920 (752 to 1 200)	539 (445 to 661)	[illegible]	[illegible]	[illegible]	[illegible]	[illegible]	[illegible]	[illegible]	[illegible]	[illegible]	[illegible]	[illegible]	[illegible]	[illegible]	[illegible]	[illegible]	[illegible]	[illegible]	[illegible]	106,000
Inguinal, femoral, and abdominal hernia	419 (324 to 481)	334 (259 to 404)	647 (402 to 838)	767 (441 to 1 260)	296 (160 to 502)	[illegible]	[illegible]	[illegible]	[illegible]	[illegible]	[illegible]	[illegible]	[illegible]	[illegible]	[illegible]	[illegible]	[illegible]	[illegible]	[illegible]	[illegible]	[illegible]	[illegible]	[illegible]	27,100
Inflammatory bowel disease	0 (0 to 0)	0 (0 to 0)	0 (0 to 0)	845 (677 to 967)	321 (267 to 363)	[illegible]	[illegible]	[illegible]	[illegible]	[illegible]	[illegible]	[illegible]	[illegible]	[illegible]	[illegible]	[illegible]	[illegible]	[illegible]	[illegible]	[illegible]	[illegible]	[illegible]	[illegible]	123,000
Vascular intestinal disorders	0 (0 to 0)	0 (0 to 0)	0 (0 to 0)	218 (167 to 262)	66·5 (54·5 to 82·7)	[illegible]	[illegible]	[illegible]	[illegible]	[illegible]	[illegible]	[illegible]	[illegible]	[illegible]	[illegible]	[illegible]	[illegible]	[illegible]	[illegible]	[illegible]	[illegible]	[illegible]	[illegible]	112,000
Gallbladder and biliary diseases	0 (0 to 0)	0 (0 to 0)	0 (0 to 0)	229 (154 to 339)	424 (242 to 683)	[illegible]	[illegible]	[illegible]	[illegible]	[illegible]	[illegible]	[illegible]	[illegible]	[illegible]	[illegible]	[illegible]	[illegible]	[illegible]	[illegible]	[illegible]	[illegible]	[illegible]	[illegible]	179,000
Pancreatitis	0 (0 to 0)	0 (0 to 0)	0 (0 to 0)	90·1 (70·1 to 119)	170 (132 to 227)	[illegible]	[illegible]	[illegible]	[illegible]	[illegible]	[illegible]	[illegible]	[illegible]	[illegible]	[illegible]	[illegible]	[illegible]	[illegible]	[illegible]	[illegible]	[illegible]	[illegible]	[illegible]	54,100
Other digestive diseases	0 (0 to 0)	0 (0 to 0)	0 (0 to 0)	690 (506 to 935)	302 (234 to 409)	[illegible]	[illegible]	[illegible]	[illegible]	[illegible]	[illegible]	[illegible]	[illegible]	[illegible]	[illegible]	[illegible]	[illegible]	[illegible]	[illegible]	[illegible]	[illegible]	[illegible]	[illegible]	117,000
Neurological disorders	58·7 (49·3 to 72·3)	201 (168 to 245)	6,130 (5 210 to 7 230)	10,200 (7 050 to 14 600)	22,700 (10 100 to 40 700)	[illegible]	[illegible]	[illegible]	[illegible]	[illegible]	[illegible]	[illegible]	[illegible]	[illegible]	[illegible]	[illegible]	[illegible]	[illegible]	[illegible]	[illegible]	[illegible]	[illegible]	[illegible]	3,590,000
Alzheimer's disease and other dementias	0 (0 to 0)	0 (0 to 0)	0 (0 to 0)	0 (0 to 0)	0 (0 to 0)	[illegible]	[illegible]	[illegible]	[illegible]	[illegible]	[illegible]	[illegible]	[illegible]	[illegible]	[illegible]	[illegible]	[illegible]	[illegible]	[illegible]	[illegible]	[illegible]	[illegible]	[illegible]	[illegible]
Parkinson's disease	0 (0 to 0)	0 (0 to 0)	0 (0 to 0)	0 (0 to 0)	0 (0 to 0)	[illegible]	[illegible]	[illegible]	[illegible]	[illegible]	[illegible]	[illegible]	[illegible]	[illegible]	[illegible]	[illegible]	[illegible]	[illegible]	[illegible]	[illegible]	[illegible]	[illegible]	[illegible]	[illegible]
Idiopathic epilepsy	11·8 (4·5 to 24·5)	35·8 (14·1 to 74·5)	1,300 (905 to 1 940)	5,910 (3 580 to 9 180)	8,170 (4 330 to 13 800)	[illegible]	[illegible]	[illegible]	[illegible]	[illegible]	[illegible]	[illegible]	[illegible]	[illegible]	[illegible]	[illegible]	[illegible]	[illegible]	[illegible]	[illegible]	[illegible]	[illegible]	[illegible]	[illegible]
Multiple sclerosis	0 (0 to 0)	0 (0 to 0)	0 (0 to 0)	0 (0 to 0)	19·4 (12·3 to 28)	[illegible]	[illegible]	[illegible]	[illegible]	[illegible]	[illegible]	[illegible]	[illegible]	[illegible]	[illegible]	[illegible]	[illegible]	[illegible]	[illegible]	[illegible]	[illegible]	[illegible]	[illegible]	[illegible]
Motor neuron disease	47 (41·5 to 52·4)	166 (143 to 188)	2,790 (2 410 to 3 160)	1,220 (1 090 to 1 350)	366 (327 to 405)	[illegible]	[illegible]	[illegible]	[illegible]	[illegible]	[illegible]	[illegible]	[illegible]	[illegible]	[illegible]	[illegible]	[illegible]	[illegible]	[illegible]	[illegible]	[illegible]	[illegible]	[illegible]	[illegible]
Headache disorders	0 (0 to 0)	0 (0 to 0)	0 (0 to 0)	0 (0 to 0)	10,800 (208 to 28 900)	[illegible]	[illegible]	[illegible]	[illegible]	[illegible]	[illegible]	[illegible]	[illegible]	[illegible]	[illegible]	[illegible]	[illegible]	[illegible]	[illegible]	[illegible]	[illegible]	[illegible]	[illegible]	[illegible]
Migraine	0 (0 to 0)	0 (0 to 0)	0 (0 to 0)	0 (0 to 0)	9,750 (141 to 25 900)	[illegible]	[illegible]	[illegible]	[illegible]	[illegible]	[illegible]	[illegible]	[illegible]	[illegible]	[illegible]	[illegible]	[illegible]	[illegible]	[illegible]	[illegible]	[illegible]	[illegible]	[illegible]	[illegible]
Tension-type headache	0 (0 to 0)	0 (0 to 0)	0 (0 to 0)	0 (0 to 0)	1,080 (26 to 6 930)	[illegible]	[illegible]	[illegible]	[illegible]	[illegible]	[illegible]	[illegible]	[illegible]	[illegible]	[illegible]	[illegible]	[illegible]	[illegible]	[illegible]	[illegible]	[illegible]	[illegible]	[illegible]	[illegible]
Other neurological disorders	0 (0 to 0)	0 (0 to 0)	2,040 (1 720 to 2 440)	3,070 (2 230 to 4 230)	3,350 (2 110 to 5 210)	[illegible]	[illegible]	[illegible]	[illegible]	[illegible]	[illegible]	[illegible]	[illegible]	[illegible]	[illegible]	[illegible]	[illegible]	[illegible]	[illegible]	[illegible]	[illegible]	[illegible]	[illegible]	[illegible]
Mental disorders	20·1 (12·8 to 29·7)	61 (38·4 to 90·2)	1,010 (637 to 1 510)	5,990 (3 930 to 8 550)	33,000 (22 200 to 47 200)	[illegible]	[illegible]	[illegible]	[illegible]	[illegible]	[illegible]	[illegible]	[illegible]	[illegible]	[illegible]	[illegible]	[illegible]	[illegible]	[illegible]	[illegible]	[illegible]	[illegible]	[illegible]	[illegible]
Schizophrenia	0 (0 to 0)	[illegible]	[illegible]	[illegible]	[illegible]	[illegible]	[illegible]	[illegible]	[illegible]	[illegible]	[illegible]	[illegible]	[illegible]	[illegible]	[illegible]	[illegible]	[illegible]	[illegible]	[illegible]	[illegible]	[illegible]	[illegible]	[illegible]	[illegible]
Depressive disorders	0 (0 to 0)	[illegible]	[illegible]	[illegible]	[illegible]	[illegible]	[illegible]	[illegible]	[illegible]	[illegible]	[illegible]	[illegible]	[illegible]	[illegible]	[illegible]	[illegible]	[illegible]	[illegible]	[illegible]	[illegible]	[illegible]	[illegible]	[illegible]	[illegible]
Major depressive disorder	0 (0 to 0)	[illegible]	[illegible]	[illegible]	[illegible]	[illegible]	[illegible]	[illegible]	[illegible]	[illegible]	[illegible]	[illegible]	[illegible]	[illegible]	[illegible]	[illegible]	[illegible]	[illegible]	[illegible]	[illegible]	[illegible]	[illegible]	[illegible]	[illegible]
Dysthymia	0 (0 to 0)	[illegible]	[illegible]	[illegible]	[illegible]	[illegible]	[illegible]	[illegible]	[illegible]	[illegible]	[illegible]	[illegible]	[illegible]	[illegible]	[illegible]	[illegible]	[illegible]	[illegible]	[illegible]	[illegible]	[illegible]	[illegible]	[illegible]	[illegible]
Bipolar disorder	0 (0 to 0)	[illegible]	[illegible]	[illegible]	[illegible]	[illegible]	[illegible]	[illegible]	[illegible]	[illegible]	[illegible]	[illegible]	[illegible]	[illegible]	[illegible]	[illegible]	[illegible]	[illegible]	[illegible]	[illegible]	[illegible]	[illegible]	[illegible]	[illegible]
Anxiety disorders	0 (0 to 0)	[illegible]	[illegible]	[illegible]	[illegible]	[illegible]	[illegible]	[illegible]	[illegible]	[illegible]	[illegible]	[illegible]	[illegible]	[illegible]	[illegible]	[illegible]	[illegible]	[illegible]	[illegible]	[illegible]	[illegible]	[illegible]	[illegible]	[illegible]
Eating disorders	0 (0 to 0)	[illegible]	[illegible]	[illegible]	[illegible]	[illegible]	[illegible]	[illegible]	[illegible]	[illegible]	[illegible]	[illegible]	[illegible]	[illegible]	[illegible]	[illegible]	[illegible]	[illegible]	[illegible]	[illegible]	[illegible]	[illegible]	[illegible]	[illegible]
Anorexia nervosa	0 (0 to 0)	[illegible]	[illegible]	[illegible]	[illegible]	[illegible]	[illegible]	[illegible]	[illegible]	[illegible]	[illegible]	[illegible]	[illegible]	[illegible]	[illegible]	[illegible]	[illegible]	[illegible]	[illegible]	[illegible]	[illegible]	[illegible]	[illegible]	[illegible]
Bulimia nervosa	0 (0 to 0)	[illegible]	[illegible]	[illegible]	[illegible]	[illegible]	[illegible]	[illegible]	[illegible]	[illegible]	[illegible]	[illegible]	[illegible]	[illegible]	[illegible]	[illegible]	[illegible]	[illegible]	[illegible]	[illegible]	[illegible]	[illegible]	[illegible]	[illegible]
Autism spectrum disorders	16·8 [illegible]	[illegible]	[illegible]	[illegible]	[illegible]	[illegible]	[illegible]	[illegible]	[illegible]	[illegible]	[illegible]	[illegible]	[illegible]	[illegible]	[illegible]	[illegible]	[illegible]	[illegible]	[illegible]	[illegible]	[illegible]	[illegible]	[illegible]	[illegible]
Attention-deficit/hyperactivity disorder	0 (0 to 0)	[illegible]	[illegible]	[illegible]	[illegible]	[illegible]	[illegible]	[illegible]	[illegible]	[illegible]	[illegible]	[illegible]	[illegible]	[illegible]	[illegible]	[illegible]	[illegible]	[illegible]	[illegible]	[illegible]	[illegible]	[illegible]	[illegible]	[illegible]
Conduct disorder	0 (0 to 0)	[illegible]	[illegible]	[illegible]	[illegible]	[illegible]	[illegible]	[illegible]	[illegible]	[illegible]	[illegible]	[illegible]	[illegible]	[illegible]	[illegible]	[illegible]	[illegible]	[illegible]	[illegible]	[illegible]	[illegible]	[illegible]	[illegible]	[illegible]
Idiopathic developmental intellectual disability	3·3 (0·2 to 8·7)	[illegible]	[illegible]	[illegible]	[illegible]	[illegible]	[illegible]	[illegible]	[illegible]	[illegible]	[illegible]	[illegible]	[illegible]	[illegible]	[illegible]	[illegible]	[illegible]	[illegible]	[illegible]	[illegible]	[illegible]	[illegible]	[illegible]	[illegible]
Other mental disorders	0 (0 to 0)	[illegible]	[illegible]	[illegible]	[illegible]	[illegible]	[illegible]	[illegible]	[illegible]	[illegible]	[illegible]	[illegible]	[illegible]	[illegible]	[illegible]	[illegible]	[illegible]	[illegible]	[illegible]	[illegible]	[illegible]	[illegible]	[illegible]	[illegible]
Substance use disorders	0·2 (0·1 to 0·4)	[illegible]	[illegible]	[illegible]	[illegible]	[illegible]	[illegible]	[illegible]	[illegible]	[illegible]	[illegible]	[illegible]	[illegible]	[illegible]	[illegible]	[illegible]	[illegible]	[illegible]	[illegible]	[illegible]	[illegible]	[illegible]	[illegible]	[illegible]
Alcohol use disorders	0·2 (0·1 to 0·4)	[illegible]	[illegible]	[illegible]	[illegible]	[illegible]	[illegible]	[illegible]	[illegible]	[illegible]	[illegible]	[illegible]	[illegible]	[illegible]	[illegible]	[illegible]	[illegible]	[illegible]	[illegible]	[illegible]	[illegible]	[illegible]	[illegible]	[illegible]
Drug use disorders	0 (0 to 0)	[illegible]	[illegible]	[illegible]	[illegible]	[illegible]	[illegible]	[illegible]	[illegible]	[illegible]	[illegible]	[illegible]	[illegible]	[illegible]	[illegible]	[illegible]	[illegible]	[illegible]	[illegible]	[illegible]	[illegible]	[illegible]	[illegible]	[illegible]
Opioid use disorders	0 (0 to 0)	[illegible]	[illegible]	[illegible]	[illegible]	[illegible]	[illegible]	[illegible]	[illegible]	[illegible]	[illegible]	[illegible]	[illegible]	[illegible]	[illegible]	[illegible]	[illegible]	[illegible]	[illegible]	[illegible]	[illegible]	[illegible]	[illegible]	[illegible]
Cocaine use disorders	0 (0 to 0)	[illegible]	[illegible]	[illegible]	[illegible]	[illegible]	[illegible]	[illegible]	[illegible]	[illegible]	[illegible]	[illegible]	[illegible]	[illegible]	[illegible]	[illegible]	[illegible]	[illegible]	[illegible]	[illegible]	[illegible]	[illegible]	[illegible]	[illegible]
Amphetamine use disorders	0 (0 to 0)	[illegible]	[illegible]	[illegible]	[illegible]	[illegible]	[illegible]	[illegible]	[illegible]	[illegible]	[illegible]	[illegible]	[illegible]	[illegible]	[illegible]	[illegible]	[illegible]	[illegible]	[illegible]	[illegible]	[illegible]	[illegible]	[illegible]	[illegible]
Cannabis use disorders	0 (0 to 0)	[illegible]	[illegible]	[illegible]	[illegible]	[illegible]	[illegible]	[illegible]	[illegible]	[illegible]	[illegible]	[illegible]	[illegible]	[illegible]	[illegible]	[illegible]	[illegible]	[illegible]	[illegible]	[illegible]	[illegible]	[illegible]	[illegible]	[illegible]
Other drug use disorders	0 (0 to 0)	[illegible]	[illegible]	[illegible]	[illegible]	[illegible]	[illegible]	[illegible]	[illegible]	[illegible]	[illegible]	[illegible]	[illegible]	[illegible]	[illegible]	[illegible]	[illegible]	[illegible]	[illegible]	[illegible]	[illegible]	[illegible]	[illegible]	[illegible]
Diabetes and kidney diseases	7·9 [illegible]	[illegible]	[illegible]	[illegible]	[illegible]	[illegible]	[illegible]	[illegible]	[illegible]	[illegible]	[illegible]	[illegible]	[illegible]	[illegible]	[illegible]	[illegible]	[illegible]	[illegible]	[illegible]	[illegible]	[illegible]	[illegible]	[illegible]	[illegible]
Diabetes mellitus	7·9 (6·4 to 13·9)	[illegible]	[illegible]	[illegible]	[illegible]	[illegible]	[illegible]	[illegible]	[illegible]	[illegible]	[illegible]	[illegible]	[illegible]	[illegible]	[illegible]	[illegible]	[illegible]	[illegible]	[illegible]	[illegible]	[illegible]	[illegible]	[illegible]	[illegible]
Diabetes mellitus type 1	7·9 [illegible]	[illegible]	[illegible]	[illegible]	[illegible]	[illegible]	[illegible]	[illegible]	[illegible]	[illegible]	[illegible]	[illegible]	[illegible]	[illegible]	[illegible]	[illegible]	[illegible]	[illegible]	[illegible]	[illegible]	[illegible]	[illegible]	[illegible]	[illegible]
Diabetes mellitus type 2	0 (0 to 0)	[illegible]	[illegible]	[illegible]	[illegible]	[illegible]	[illegible]	[illegible]	[illegible]	[illegible]	[illegible]	[illegible]	[illegible]	[illegible]	[illegible]	[illegible]	[illegible]	[illegible]	[illegible]	[illegible]	[illegible]	[illegible]	[illegible]	[illegible]
Chronic kidney disease	0 (0 to 0)	[illegible]	[illegible]	[illegible]	[illegible]	[illegible]	[illegible]	[illegible]	[illegible]	[illegible]	[illegible]	[illegible]	[illegible]	[illegible]	[illegible]	[illegible]	[illegible]	[illegible]	[illegible]	[illegible]	[illegible]	[illegible]	[illegible]	[illegible]
Chronic kidney disease due to diabetes mellitus type [illegible]	0 (0 to 0)	[illegible]	[illegible]	[illegible]	[illegible]	[illegible]	[illegible]	[illegible]	[illegible]	[illegible]	[illegible]	[illegible]	[illegible]	[illegible]	[illegible]	[illegible]	[illegible]	[illegible]	[illegible]	[illegible]	[illegible]	[illegible]	[illegible]	[illegible]
Chronic kidney disease due to diabetes mellitus type [illegible]	0 (0 to 0)	[illegible]	[illegible]	[illegible]	[illegible]	[illegible]	[illegible]	[illegible]	[illegible]	[illegible]	[illegible]	[illegible]	[illegible]	[illegible]	[illegible]	[illegible]	[illegible]	[illegible]	[illegible]	[illegible]	[illegible]	[illegible]	[illegible]	[illegible]
Chronic kidney disease due to hypertension	0 (0 to 0)	[illegible]	[illegible]	[illegible]	[illegible]	[illegible]	[illegible]	[illegible]	[illegible]	[illegible]	[illegible]	[illegible]	[illegible]	[illegible]	[illegible]	[illegible]	[illegible]	[illegible]	[illegible]	[illegible]	[illegible]	[illegible]	[illegible]	[illegible]
Chronic kidney disease due to glomerulonephritis	0 (0 to 0)	[illegible]	[illegible]	[illegible]	[illegible]	[illegible]	[illegible]	[illegible]	[illegible]	[illegible]	[illegible]	[illegible]	[illegible]	[illegible]	[illegible]	[illegible]	[illegible]	[illegible]	[illegible]	[illegible]	[illegible]	[illegible]	[illegible]	[illegible]
Chronic kidney disease due to other and unspecifie[illegible]	0 (0 to 0)	[illegible]	[illegible]	[illegible]	[illegible]	[illegible]	[illegible]	[illegible]	[illegible]	[illegible]	[illegible]	[illegible]	[illegible]	[illegible]	[illegible]	[illegible]	[illegible]	[illegible]	[illegible]	[illegible]	[illegible]	[illegible]	[illegible]	[illegible]
Acute glomerulonephritis	0 (0 to 0)	[illegible]	[illegible]	[illegible]	[illegible]	[illegible]	[illegible]	[illegible]	[illegible]	[illegible]	[illegible]	[illegible]	[illegible]	[illegible]	[illegible]	[illegible]	[illegible]	[illegible]	[illegible]	[illegible]	[illegible]	[illegible]	[illegible]	[illegible]
Skin and subcutaneous diseases	1·5 (0·9 to 2·2)	[illegible]	[illegible]	[illegible]	[illegible]	[illegible]	[illegible]	[illegible]	[illegible]	[illegible]	[illegible]	[illegible]	[illegible]	[illegible]	[illegible]	[illegible]	[illegible]	[illegible]	[illegible]	[illegible]	[illegible]	[illegible]	[illegible]	[illegible]
Dermatitis	0 (0 to 0)	[illegible]	[illegible]	[illegible]	[illegible]	[illegible]	[illegible]	[illegible]	[illegible]	[illegible]	[illegible]	[illegible]	[illegible]	[illegible]	[illegible]	[illegible]	[illegible]	[illegible]	[illegible]	[illegible]	[illegible]	[illegible]	[illegible]	[illegible]
Atopic dermatitis	0 (0 to 0)	[illegible]	[illegible]	[illegible]	[illegible]	[illegible]	[illegible]	[illegible]	[illegible]	[illegible]	[illegible]	[illegible]	[illegible]	[illegible]	[illegible]	[illegible]	[illegible]	[illegible]	[illegible]	[illegible]	[illegible]	[illegible]	[illegible]	[illegible]
Contact dermatitis	0 (0 to 0)	[illegible]	[illegible]	[illegible]	[illegible]	[illegible]	[illegible]	[illegible]	[illegible]	[illegible]	[illegible]	[illegible]	[illegible]	[illegible]	[illegible]	[illegible]	[illegible]	[illegible]	[illegible]	[illegible]	[illegible]	[illegible]	[illegible]	[illegible]
Seborrhoeic dermatitis	0 (0 to 0)	[illegible]	[illegible]	[illegible]	[illegible]	[illegible]	[illegible]	[illegible]	[illegible]	[illegible]	[illegible]	[illegible]	[illegible]	[illegible]	[illegible]	[illegible]	[illegible]	[illegible]	[illegible]	[illegible]	[illegible]	[illegible]	[illegible]	[illegible]
Psoriasis	0 (0 to 0)	[illegible]	[illegible]	[illegible]	[illegible]	[illegible]	[illegible]	[illegible]	[illegible]	[illegible]	[illegible]	[illegible]	[illegible]	[illegible]	[illegible]	[illegible]	[illegible]	[illegible]	[illegible]	[illegible]	[illegible]	[illegible]	[illegible]	[illegible]
Bacterial skin diseases	0·2 (0·1 to 0·3)	[illegible]	[illegible]	[illegible]	[illegible]	[illegible]	[illegible]	[illegible]	[illegible]	[illegible]	[illegible]	[illegible]	[illegible]	[illegible]	[illegible]	[illegible]	[illegible]	[illegible]	[illegible]	[illegible]	[illegible]	[illegible]	[illegible]	[illegible]
Cellulitis	0·1 (0·1 to 0·2)	[illegible]	[illegible]	[illegible]	[illegible]	[illegible]	[illegible]	[illegible]	[illegible]	[illegible]	[illegible]	[illegible]	[illegible]	[illegible]	[illegible]	[illegible]	[illegible]	[illegible]	[illegible]	[illegible]	[illegible]	[illegible]	[illegible]	[illegible]
Pyoderma	0 (0 to 0·1)	[illegible]	[illegible]	[illegible]	[illegible]	[illegible]	[illegible]	[illegible]	[illegible]	[illegible]	[illegible]	[illegible]	[illegible]	[illegible]	[illegible]	[illegible]	[illegible]	[illegible]	[illegible]	[illegible]	[illegible]	[illegible]	[illegible]	[illegible]
Scabies	0 (0 to 0·1)	[illegible]	[illegible]	[illegible]	[illegible]	[illegible]	[illegible]	[illegible]	[illegible]	[illegible]	[illegible]	[illegible]	[illegible]	[illegible]	[illegible]	[illegible]	[illegible]	[illegible]	[illegible]	[illegible]	[illegible]	[illegible]	[illegible]	[illegible]
Fungal skin diseases	0 (0 to 0·1)	[illegible]	[illegible]	[illegible]	[illegible]	[illegible]	[illegible]	[illegible]	[illegible]	[illegible]	[illegible]	[illegible]	[illegible]	[illegible]	[illegible]	[illegible]	[illegible]	[illegible]	[illegible]	[illegible]	[illegible]	[illegible]	[illegible]	[illegible]
Viral skin diseases	0 (0 to 0)	[illegible]	[illegible]	[illegible]	[illegible]	[illegible]	[illegible]	[illegible]	[illegible]	[illegible]	[illegible]	[illegible]	[illegible]	[illegible]	[illegible]	[illegible]	[illegible]	[illegible]	[illegible]	[illegible]	[illegible]	[illegible]	[illegible]	[illegible]
Acne vulgaris	0 (0 to 0)	[illegible]	[illegible]	[illegible]	[illegible]	[illegible]	[illegible]	[illegible]	[illegible]	[illegible]	[illegible]	[illegible]	[illegible]	[illegible]	[illegible]	[illegible]	[illegible]	[illegible]	[illegible]	[illegible]	[illegible]	[illegible]	[illegible]	[illegible]
Alopecia areata	0 (0 to 0)	[illegible]	[illegible]	[illegible]	[illegible]	[illegible]	[illegible]	[illegible]	[illegible]	[illegible]	[illegible]	[illegible]	[illegible]	[illegible]	[illegible]	[illegible]	[illegible]	[illegible]	[illegible]	[illegible]	[illegible]	[illegible]	[illegible]	[illegible]
Pruritus	0 (0 to 0)	[illegible]	[illegible]	[illegible]	[illegible]	[illegible]	[illegible]	[illegible]	[illegible]	[illegible]	[illegible]	[illegible]	[illegible]	[illegible]	[illegible]	[illegible]	[illegible]	[illegible]	[illegible]	[illegible]	[illegible]	[illegible]	[illegible]	[illegible]
Urticaria	0·9 (0·6 to 1·3)	[illegible]	[illegible]	[illegible]	[illegible]	[illegible]	[illegible]	[illegible]	[illegible]	[illegible]	[illegible]	[illegible]	[illegible]	[illegible]	[illegible]	[illegible]	[illegible]	[illegible]	[illegible]	[illegible]	[illegible]	[illegible]	[illegible]	[illegible]
Decubitus ulcer	0 (0 to 0)	[illegible]	[illegible]	[illegible]	[illegible]	[illegible]	[illegible]	[illegible]	[illegible]	[illegible]	[illegible]	[illegible]	[illegible]	[illegible]	[illegible]	[illegible]	[illegible]	[illegible]	[illegible]	[illegible]	[illegible]	[illegible]	[illegible]	[illegible]
Other skin and subcutaneous diseases	0·3 (0·1 to 0·5)	[illegible]	[illegible]	[illegible]	[illegible]	[illegible]	[illegible]	[illegible]	[illegible]	[illegible]	[illegible]	[illegible]	[illegible]	[illegible]	[illegible]	[illegible]	[illegible]	[illegible]	[illegible]	[illegible]	[illegible]	[illegible]	[illegible]	[illegible]
Sense organ diseases	5·2 (2·7 to 8·9)	[illegible]	[illegible]	[illegible]	[illegible]	[illegible]	[illegible]	[illegible]	[illegible]	[illegible]	[illegible]	[illegible]	[illegible]	[illegible]	[illegible]	[illegible]	[illegible]	[illegible]	[illegible]	[illegible]	[illegible]	[illegible]	[illegible]	[illegible]
Blindness and vision loss	0·9 (0·2 to 2·3)	[illegible]	[illegible]	[illegible]	[illegible]	[illegible]	[illegible]	[illegible]	[illegible]	[illegible]	[illegible]	[illegible]	[illegible]	[illegible]	[illegible]	[illegible]	[illegible]	[illegible]	[illegible]	[illegible]	[illegible]	[illegible]	[illegible]	[illegible]
Glaucoma	0 (0 to 0)	[illegible]	[illegible]	[illegible]	[illegible]	[illegible]	[illegible]	[illegible]	[illegible]	[illegible]	[illegible]	[illegible]	[illegible]	[illegible]	[illegible]	[illegible]	[illegible]	[illegible]	[illegible]	[illegible]	[illegible]	[illegible]	[illegible]	[illegible]
Cataract	0 (0 to 0)	[illegible]	[illegible]	[illegible]	[illegible]	[illegible]	[illegible]	[illegible]	[illegible]	[illegible]	[illegible]	[illegible]	[illegible]	[illegible]	[illegible]	[illegible]	[illegible]	[illegible]	[illegible]	[illegible]	[illegible]	[illegible]	[illegible]	[illegible]
Age-related macular degeneration	0 (0 to 0)	[illegible]	[illegible]	[illegible]	[illegible]	[illegible]	[illegible]	[illegible]	[illegible]	[illegible]	[illegible]	[illegible]	[illegible]	[illegible]	[illegible]	[illegible]	[illegible]	[illegible]	[illegible]	[illegible]	[illegible]	[illegible]	[illegible]	[illegible]
Refraction disorders	0 (0 to 0·1)	[illegible]	[illegible]	[illegible]	[illegible]	[illegible]	[illegible]	[illegible]	[illegible]	[illegible]	[illegible]	[illegible]	[illegible]	[illegible]	[illegible]	[illegible]	[illegible]	[illegible]	[illegible]	[illegible]	[illegible]	[illegible]	[illegible]	[illegible]
Near vision loss	0 (0 to 0)	[illegible]	[illegible]	[illegible]	[illegible]	[illegible]	[illegible]	[illegible]	[illegible]	[illegible]	[illegible]	[illegible]	[illegible]	[illegible]	[illegible]	[illegible]	[illegible]	[illegible]	[illegible]	[illegible]	[illegible]	[illegible]	[illegible]	[illegible]
Other vision loss	0·8 (0·2 to 2·2)	[illegible]	[illegible]	[illegible]	[illegible]	[illegible]	[illegible]	[illegible]	[illegible]	[illegible]	[illegible]	[illegible]	[illegible]	[illegible]	[illegible]	[illegible]	[illegible]	[illegible]	[illegible]	[illegible]	[illegible]	[illegible]	[illegible]	[illegible]
Age-related and other hearing loss	0·4 (0·1 to 1·2)	[illegible]	[illegible]	[illegible]	[illegible]	[illegible]	[illegible]	[illegible]	[illegible]	[illegible]	[illegible]	[illegible]	[illegible]	[illegible]	[illegible]	[illegible]	[illegible]	[illegible]	[illegible]	[illegible]	[illegible]	[illegible]	[illegible]	[illegible]
Other sense organ diseases	4 (1·9 to 7·2)	[illegible]	[illegible]	[illegible]	[illegible]	[illegible]	[illegible]	[illegible]	[illegible]	[illegible]	[illegible]	[illegible]	[illegible]	[illegible]	[illegible]	[illegible]	[illegible]	[illegible]	[illegible]	[illegible]	[illegible]	[illegible]	[illegible]	[illegible]
Musculoskeletal disorders	0 (0 to 0)	[illegible]	[illegible]	[illegible]	[illegible]	[illegible]	[illegible]	[illegible]	[illegible]	[illegible]	[illegible]	[illegible]	[illegible]	[illegible]	[illegible]	[illegible]	[illegible]	[illegible]	[illegible]	[illegible]	[illegible]	[illegible]	[illegible]	[illegible]
Rheumatoid arthritis	0 (0 to 0)	[illegible]	[illegible]	[illegible]	[illegible]	[illegible]	[illegible]	[illegible]	[illegible]	[illegible]	[illegible]	[illegible]	[illegible]	[illegible]	[illegible]	[illegible]	[illegible]	[illegible]	[illegible]	[illegible]	[illegible]	[illegible]	[illegible]	[illegible]
Osteoarthritis	0 (0 to 0)	[illegible]	[illegible]	[illegible]	[illegible]	[illegible]	[illegible]	[illegible]	[illegible]	[illegible]	[illegible]	[illegible]	[illegible]	[illegible]	[illegible]	[illegible]	[illegible]	[illegible]	[illegible]	[illegible]	[illegible]	[illegible]	[illegible]	[illegible]
Osteoarthritis hip	0 (0 to 0)	[illegible]	[illegible]	[illegible]	[illegible]	[illegible]	[illegible]	[illegible]	[illegible]	[illegible]	[illegible]	[illegible]	[illegible]	[illegible]	[illegible]	[illegible]	[illegible]	[illegible]	[illegible]	[illegible]	[illegible]	[illegible]	[illegible]	[illegible]
Osteoarthritis knee	0 (0 to 0)	[illegible]	[illegible]	[illegible]	[illegible]	[illegible]	[illegible]	[illegible]	[illegible]	[illegible]	[illegible]	[illegible]	[illegible]	[illegible]	[illegible]	[illegible]	[illegible]	[illegible]	[illegible]	[illegible]	[illegible]	[illegible]	[illegible]	[illegible]
Osteoarthritis hand	0 (0 to 0)	[illegible]	[illegible]	[illegible]	[illegible]	[illegible]	[illegible]	[illegible]	[illegible]	[illegible]	[illegible]	[illegible]	[illegible]	[illegible]	[illegible]	[illegible]	[illegible]	[illegible]	[illegible]	[illegible]	[illegible]	[illegible]	[illegible]	[illegible]
Osteoarthritis other	0 (0 to 0)	[illegible]	[illegible]	[illegible]	[illegible]	[illegible]	[illegible]	[illegible]	[illegible]	[illegible]	[illegible]	[illegible]	[illegible]	[illegible]	[illegible]	[illegible]	[illegible]	[illegible]	[illegible]	[illegible]	[illegible]	[illegible]	[illegible]	[illegible]
Low back pain	0 (0 to 0)	[illegible]	[illegible]	[illegible]	[illegible]	[illegible]	[illegible]	[illegible]	[illegible]	[illegible]	[illegible]	[illegible]	[illegible]	[illegible]	[illegible]	[illegible]	[illegible]	[illegible]	[illegible]	[illegible]	[illegible]	[illegible]	[illegible]	[illegible]
Neck pain	0 (0 to 0)	[illegible]	[illegible]	[illegible]	[illegible]	[illegible]	[illegible]	[illegible]	[illegible]	[illegible]	[illegible]	[illegible]	[illegible]	[illegible]	[illegible]	[illegible]	[illegible]	[illegible]	[illegible]	[illegible]	[illegible]	[illegible]	[illegible]	[illegible]
Gout	0 (0 to 0)	[illegible]	[illegible]	[illegible]	[illegible]	[illegible]	[illegible]	[illegible]	[illegible]	[illegible]	[illegible]	[illegible]	[illegible]	[illegible]	[illegible]	[illegible]	[illegible]	[illegible]	[illegible]	[illegible]	[illegible]	[illegible]	[illegible]	[illegible]
Other musculoskeletal disorders	0 (0 to 0)	[illegible]	[illegible]	[illegible]	[illegible]	[illegible]	[illegible]	[illegible]	[illegible]	[illegible]	[illegible]	[illegible]	[illegible]	[illegible]	[illegible]	[illegible]	[illegible]	[illegible]	[illegible]	[illegible]	[illegible]	[illegible]	[illegible]	[illegible]
Other non-communicable diseases	100,000 [illegible]	[illegible]	[illegible]	[illegible]	[illegible]	[illegible]	[illegible]	[illegible]	[illegible]	[illegible]	[illegible]	[illegible]	[illegible]	[illegible]	[illegible]	[illegible]	[illegible]	[illegible]	[illegible]	[illegible]	[illegible]	[illegible]	[illegible]	[illegible]
Congenital birth defects	97,900 [illegible]	[illegible]	[illegible]	[illegible]	[illegible]	[illegible]	[illegible]	[illegible]	[illegible]	[illegible]	[illegible]	[illegible]	[illegible]	[illegible]	[illegible]	[illegible]	[illegible]	[illegible]	[illegible]	[illegible]	[illegible]	[illegible]	[illegible]	[illegible]
Neural tube defects	14,700 [illegible]	[illegible]	[illegible]	[illegible]	[illegible]	[illegible]	[illegible]	[illegible]	[illegible]	[illegible]	[illegible]	[illegible]	[illegible]	[illegible]	[illegible]	[illegible]	[illegible]	[illegible]	[illegible]	[illegible]	[illegible]	[illegible]	[illegible]	[illegible]
Congenital heart anomalies	13,300 [illegible]	[illegible]	[illegible]	[illegible]	[illegible]	[illegible]	[illegible]	[illegible]	[illegible]	[illegible]	[illegible]	[illegible]	[illegible]	[illegible]	[illegible]	[illegible]	[illegible]	[illegible]	[illegible]	[illegible]	[illegible]	[illegible]	[illegible]	[illegible]
Orofacial clefts	35·8 [illegible]	[illegible]	[illegible]	[illegible]	[illegible]	[illegible]	[illegible]	[illegible]	[illegible]	[illegible]	[illegible]	[illegible]	[illegible]	[illegible]	[illegible]	[illegible]	[illegible]	[illegible]	[illegible]	[illegible]	[illegible]	[illegible]	[illegible]	[illegible]
Down syndrome	1,190 [illegible]	[illegible]	[illegible]	[illegible]	[illegible]	[illegible]	[illegible]	[illegible]	[illegible]	[illegible]	[illegible]	[illegible]	[illegible]	[illegible]	[illegible]	[illegible]	[illegible]	[illegible]	[illegible]	[illegible]	[illegible]	[illegible]	[illegible]	[illegible]
Turner syndrome	0·5 (0·2 to 1)	[illegible]	[illegible]	[illegible]	[illegible]	[illegible]	[illegible]	[illegible]	[illegible]	[illegible]	[illegible]	[illegible]	[illegible]	[illegible]	[illegible]	[illegible]	[illegible]	[illegible]	[illegible]	[illegible]	[illegible]	[illegible]	[illegible]	[illegible]
Klinefelter syndrome	0 (0 to 0)	[illegible]	[illegible]	[illegible]	[illegible]	[illegible]	[illegible]	[illegible]	[illegible]	[illegible]	[illegible]	[illegible]	[illegible]	[illegible]	[illegible]	[illegible]	[illegible]	[illegible]	[illegible]	[illegible]	[illegible]	[illegible]	[illegible]	[illegible]
Other chromosomal abnormalities	23,000 [illegible]	[illegible]	[illegible]	[illegible]	[illegible]	[illegible]	[illegible]	[illegible]	[illegible]	[illegible]	[illegible]	[illegible]	[illegible]	[illegible]	[illegible]	[illegible]	[illegible]	[illegible]	[illegible]	[illegible]	[illegible]	[illegible]	[illegible]	[illegible]
Congenital musculoskeletal and limb anomalies	5,380 [illegible]	[illegible]	[illegible]	[illegible]	[illegible]	[illegible]	[illegible]	[illegible]	[illegible]	[illegible]	[illegible]	[illegible]	[illegible]	[illegible]	[illegible]	[illegible]	[illegible]	[illegible]	[illegible]	[illegible]	[illegible]	[illegible]	[illegible]	[illegible]
Urogenital congenital anomalies	6,040 [illegible]	[illegible]	[illegible]	[illegible]	[illegible]	[illegible]	[illegible]	[illegible]	[illegible]	[illegible]	[illegible]	[illegible]	[illegible]	[illegible]	[illegible]	[illegible]	[illegible]	[illegible]	[illegible]	[illegible]	[illegible]	[illegible]	[illegible]	[illegible]
Digestive congenital anomalies	7,080 [illegible]	[illegible]	[illegible]	[illegible]	[illegible]	[illegible]	[illegible]	[illegible]	[illegible]	[illegible]	[illegible]	[illegible]	[illegible]	[illegible]	[illegible]	[illegible]	[illegible]	[illegible]	[illegible]	[illegible]	[illegible]	[illegible]	[illegible]	[illegible]
Other congenital birth defects	27,200 [illegible]	[illegible]	[illegible]	[illegible]	[illegible]	[illegible]	[illegible]	[illegible]	[illegible]	[illegible]	[illegible]	[illegible]	[illegible]	[illegible]	[illegible]	[illegible]	[illegible]	[illegible]	[illegible]	[illegible]	[illegible]	[illegible]	[illegible]	[illegible]
Urinary diseases and male infertility	234 [illegible]	[illegible]	[illegible]	[illegible]	[illegible]	[illegible]	[illegible]	[illegible]	[illegible]	[illegible]	[illegible]	[illegible]	[illegible]	[illegible]	[illegible]	[illegible]	[illegible]	[illegible]	[illegible]	[illegible]	[illegible]	[illegible]	[illegible]	[illegible]
Urolithiasis	0 (0 to 0)	[illegible]	[illegible]	[illegible]	[illegible]	[illegible]	[illegible]	[illegible]	[illegible]	[illegible]	[illegible]	[illegible]	[illegible]	[illegible]	[illegible]	[illegible]	[illegible]	[illegible]	[illegible]	[illegible]	[illegible]	[illegible]	[illegible]	[illegible]
Benign prostatic hyperplasia	0 (0 to 0)	[illegible]	[illegible]	[illegible]	[illegible]	[illegible]	[illegible]	[illegible]	[illegible]	[illegible]	[illegible]	[illegible]	[illegible]	[illegible]	[illegible]	[illegible]	[illegible]	[illegible]	[illegible]	[illegible]	[illegible]	[illegible]	[illegible]	[illegible]
Male infertility	0 (0 to 0)	[illegible]	[illegible]	[illegible]	[illegible]	[illegible]	[illegible]	[illegible]	[illegible]	[illegible]	[illegible]	[illegible]	[illegible]	[illegible]	[illegible]	[illegible]	[illegible]	[illegible]	[illegible]	[illegible]	[illegible]	[illegible]	[illegible]	[illegible]
Other urinary diseases	149 [illegible]	[illegible]	[illegible]	[illegible]	[illegible]	[illegible]	[illegible]	[illegible]	[illegible]	[illegible]	[illegible]	[illegible]	[illegible]	[illegible]	[illegible]	[illegible]	[illegible]	[illegible]	[illegible]	[illegible]	[illegible]	[illegible]	[illegible]	[illegible]
Gynecological diseases	0 (0 to 0)	[illegible]	[illegible]	[illegible]	[illegible]	[illegible]	[illegible]	[illegible]	[illegible]	[illegible]	[illegible]	[illegible]	[illegible]	[illegible]	[illegible]	[illegible]	[illegible]	[illegible]	[illegible]	[illegible]	[illegible]	[illegible]	[illegible]	[illegible]
Uterine fibroids	0 (0 to 0)	[illegible]	[illegible]	[illegible]	[illegible]	[illegible]	[illegible]	[illegible]	[illegible]	[illegible]	[illegible]	[illegible]	[illegible]	[illegible]	[illegible]	[illegible]	[illegible]	[illegible]	[illegible]	[illegible]	[illegible]	[illegible]	[illegible]	[illegible]
Polycystic ovarian syndrome	0 (0 to 0)	[illegible]	[illegible]	[illegible]	[illegible]	[illegible]	[illegible]	[illegible]	[illegible]	[illegible]	[illegible]	[illegible]	[illegible]	[illegible]	[illegible]	[illegible]	[illegible]	[illegible]	[illegible]	[illegible]	[illegible]	[illegible]	[illegible]	[illegible]
Female infertility	0 (0 to 0)	[illegible]	[illegible]	[illegible]	[illegible]	[illegible]	[illegible]	[illegible]	[illegible]	[illegible]	[illegible]	[illegible]	[illegible]	[illegible]	[illegible]	[illegible]	[illegible]	[illegible]	[illegible]	[illegible]	[illegible]	[illegible]	[illegible]	[illegible]
Endometriosis	0 (0 to 0)	[illegible]	[illegible]	[illegible]	[illegible]	[illegible]	[illegible]	[illegible]	[illegible]	[illegible]	[illegible]	[illegible]	[illegible]	[illegible]	[illegible]	[illegible]	[illegible]	[illegible]	[illegible]	[illegible]	[illegible]	[illegible]	[illegible]	[illegible]
Genital prolapse	0 (0 to 0)	[illegible]	[illegible]	[illegible]	[illegible]	[illegible]	[illegible]	[illegible]	[illegible]	[illegible]	[illegible]	[illegible]	[illegible]	[illegible]	[illegible]	[illegible]	[illegible]	[illegible]	[illegible]	[illegible]	[illegible]	[illegible]	[illegible]	[illegible]
Premenstrual syndrome	0 (0 to 0)	[illegible]	[illegible]	[illegible]	[illegible]	[illegible]	[illegible]	[illegible]	[illegible]	[illegible]	[illegible]	[illegible]	[illegible]	[illegible]	[illegible]	[illegible]	[illegible]	[illegible]	[illegible]	[illegible]	[illegible]	[illegible]	[illegible]	[illegible]
Other gynecological diseases	0 (0 to 0)	[illegible]	[illegible]	[illegible]	[illegible]	[illegible]	[illegible]	[illegible]	[illegible]	[illegible]	[illegible]	[illegible]	[illegible]	[illegible]	[illegible]	[illegible]	[illegible]	[illegible]	[illegible]	[illegible]	[illegible]	[illegible]	[illegible]	[illegible]
Hemoglobinopathies and hemolytic anemias	173 [illegible]	[illegible]	[illegible]	[illegible]	[illegible]	[illegible]	[illegible]	[illegible]	[illegible]	[illegible]	[illegible]	[illegible]	[illegible]	[illegible]	[illegible]	[illegible]	[illegible]	[illegible]	[illegible]	[illegible]	[illegible]	[illegible]	[illegible]	[illegible]
Thalassemias	51·6 (35 to 73·4)	[illegible]	[illegible]	[illegible]	[illegible]	[illegible]	[illegible]	[illegible]	[illegible]	[illegible]	[illegible]	[illegible]	[illegible]	[illegible]	[illegible]	[illegible]	[illegible]	[illegible]	[illegible]	[illegible]	[illegible]	[illegible]	[illegible]	[illegible]
Thalassemias trait	0 (0 to 0)	[illegible]	[illegible]	[illegible]	[illegible]	[illegible]	[illegible]	[illegible]	[illegible]	[illegible]	[illegible]	[illegible]	[illegible]	[illegible]	[illegible]	[illegible]	[illegible]	[illegible]	[illegible]	[illegible]	[illegible]	[illegible]	[illegible]	[illegible]
Sickle cell disorders	113 [illegible]	[illegible]	[illegible]	[illegible]	[illegible]	[illegible]	[illegible]	[illegible]	[illegible]	[illegible]	[illegible]	[illegible]	[illegible]	[illegible]	[illegible]	[illegible]	[illegible]	[illegible]	[illegible]	[illegible]	[illegible]	[illegible]	[illegible]	[illegible]
Sickle cell trait	0 (0 to 0)	[illegible]	[illegible]	[illegible]	[illegible]	[illegible]	[illegible]	[illegible]	[illegible]	[illegible]	[illegible]	[illegible]	[illegible]	[illegible]	[illegible]	[illegible]	[illegible]	[illegible]	[illegible]	[illegible]	[illegible]	[illegible]	[illegible]	[illegible]
G6PD deficiency	1·6 (0·9 to 2·5)	[illegible]	[illegible]	[illegible]	[illegible]	[illegible]	[illegible]	[illegible]	[illegible]	[illegible]	[illegible]	[illegible]	[illegible]	[illegible]	[illegible]	[illegible]	[illegible]	[illegible]	[illegible]	[illegible]	[illegible]	[illegible]	[illegible]	[illegible]
G6PD trait	0 (0 to 0)	[illegible]	[illegible]	[illegible]	[illegible]	[illegible]	[illegible]	[illegible]	[illegible]	[illegible]	[illegible]	[illegible]	[illegible]	[illegible]	[illegible]	[illegible]	[illegible]	[illegible]	[illegible]	[illegible]	[illegible]	[illegible]	[illegible]	[illegible]
Other hemoglobinopathies and hemolytic anemias	7·4 [illegible]	[illegible]	[illegible]	[illegible]	[illegible]	[illegible]	[illegible]	[illegible]	[illegible]	[illegible]	[illegible]	[illegible]	[illegible]	[illegible]	[illegible]	[illegible]	[illegible]	[illegible]	[illegible]	[illegible]	[illegible]	[illegible]	[illegible]	[illegible]
Endocrine, metabolic, blood, and immune disorders	2,090 [illegible]	[illegible]	[illegible]	[illegible]	[illegible]	[illegible]	[illegible]	[illegible]	[illegible]	[illegible]	[illegible]	[illegible]	[illegible]	[illegible]	[illegible]	[illegible]	[illegible]	[illegible]	[illegible]	[illegible]	[illegible]	[illegible]	[illegible]	[illegible]
Oral disorders	0 (0 to 0)	[illegible]	[illegible]	[illegible]	[illegible]	[illegible]	[illegible]	[illegible]	[illegible]	[illegible]	[illegible]	[illegible]	[illegible]	[illegible]	[illegible]	[illegible]	[illegible]	[illegible]	[illegible]	[illegible]	[illegible]	[illegible]	[illegible]	[illegible]
Caries of deciduous teeth	0 (0 to 0)	[illegible]	[illegible]	[illegible]	[illegible]	[illegible]	[illegible]	[illegible]	[illegible]	[illegible]	[illegible]	[illegible]	[illegible]	[illegible]	[illegible]	[illegible]	[illegible]	[illegible]	[illegible]	[illegible]	[illegible]	[illegible]	[illegible]	[illegible]
Caries of permanent teeth	0 (0 to 0)	[illegible]	[illegible]	[illegible]	[illegible]	[illegible]	[illegible]	[illegible]	[illegible]	[illegible]	[illegible]	[illegible]	[illegible]	[illegible]	[illegible]	[illegible]	[illegible]	[illegible]	[illegible]	[illegible]	[illegible]	[illegible]	[illegible]	[illegible]
Periodontal diseases	0 (0 to 0)	[illegible]	[illegible]	[illegible]	[illegible]	[illegible]	[illegible]	[illegible]	[illegible]	[illegible]	[illegible]	[illegible]	[illegible]	[illegible]	[illegible]	[illegible]	[illegible]	[illegible]	[illegible]	[illegible]	[illegible]	[illegible]	[illegible]	[illegible]
Other oral disorders	0 (0 to 0)	[illegible]	[illegible]	[illegible]	[illegible]	[illegible]	[illegible]	[illegible]	[illegible]	[illegible]	[illegible]	[illegible]	[illegible]	[illegible]	[illegible]	[illegible]	[illegible]	[illegible]	[illegible]	[illegible]	[illegible]	[illegible]	[illegible]	[illegible]
Sudden infant death syndrome	..	[illegible]	[illegible]	[illegible]	[illegible]	[illegible]	[illegible]	[illegible]	[illegible]	[illegible]	[illegible]	[illegible]	[illegible]	[illegible]	[illegible]	[illegible]	[illegible]	[illegible]	[illegible]	[illegible]	[illegible]	[illegible]	[illegible]	[illegible]
Injuries	2,580 [illegible]	[illegible]	[illegible]	[illegible]	[illegible]	[illegible]	[illegible]	[illegible]	[illegible]	[illegible]	[illegible]	[illegible]	[illegible]	[illegible]	[illegible]	[illegible]	[illegible]	[illegible]	[illegible]	[illegible]	[illegible]	[illegible]	[illegible]	[illegible]
Transport injuries	271 [illegible]	[illegible]	[illegible]	[illegible]	[illegible]	[illegible]	[illegible]	[illegible]	[illegible]	[illegible]	[illegible]	[illegible]	[illegible]	[illegible]	[illegible]	[illegible]	[illegible]	[illegible]	[illegible]	[illegible]	[illegible]	[illegible]	[illegible]	[illegible]
Road injuries	219 [illegible]	[illegible]	[illegible]	[illegible]	[illegible]	[illegible]	[illegible]	[illegible]	[illegible]	[illegible]	[illegible]	[illegible]	[illegible]	[illegible]	[illegible]	[illegible]	[illegible]	[illegible]	[illegible]	[illegible]	[illegible]	[illegible]	[illegible]	[illegible]
Pedestrian road injuries	29·9 [illegible]	[illegible]	[illegible]	[illegible]	[illegible]	[illegible]	[illegible]	[illegible]	[illegible]	[illegible]	[illegible]	[illegible]	[illegible]	[illegible]	[illegible]	[illegible]	[illegible]	[illegible]	[illegible]	[illegible]	[illegible]	[illegible]	[illegible]	[illegible]
Cyclist road injuries	0 (0 to 0)	[illegible]	[illegible]	[illegible]	[illegible]	[illegible]	[illegible]	[illegible]	[illegible]	[illegible]	[illegible]	[illegible]	[illegible]	[illegible]	[illegible]	[illegible]	[illegible]	[illegible]	[illegible]	[illegible]	[illegible]	[illegible]	[illegible]	[illegible]
Motorcyclist road injuries	16·6 (15 to 18·7)	[illegible]	[illegible]	[illegible]	[illegible]	[illegible]	[illegible]	[illegible]	[illegible]	[illegible]	[illegible]	[illegible]	[illegible]	[illegible]	[illegible]	[illegible]	[illegible]	[illegible]	[illegible]	[illegible]	[illegible]	[illegible]	[illegible]	[illegible]
Motor vehicle road injuries	157 [illegible]	[illegible]	[illegible]	[illegible]	[illegible]	[illegible]	[illegible]	[illegible]	[illegible]	[illegible]	[illegible]	[illegible]	[illegible]	[illegible]	[illegible]	[illegible]	[illegible]	[illegible]	[illegible]	[illegible]	[illegible]	[illegible]	[illegible]	[illegible]
Other road injuries	15·3 [illegible]	[illegible]	[illegible]	[illegible]	[illegible]	[illegible]	[illegible]	[illegible]	[illegible]	[illegible]	[illegible]	[illegible]	[illegible]	[illegible]	[illegible]	[illegible]	[illegible]	[illegible]	[illegible]	[illegible]	[illegible]	[illegible]	[illegible]	[illegible]
Other transport injuries	52·4 [illegible]	[illegible]	[illegible]	[illegible]	[illegible]	[illegible]	[illegible]	[illegible]	[illegible]	[illegible]	[illegible]	[illegible]	[illegible]	[illegible]	[illegible]	[illegible]	[illegible]	[illegible]	[illegible]	[illegible]	[illegible]	[illegible]	[illegible]	[illegible]
Unintentional injuries	1,400 [illegible]	[illegible]	[illegible]	[illegible]	[illegible]	[illegible]	[illegible]	[illegible]	[illegible]	[illegible]	[illegible]	[illegible]	[illegible]	[illegible]	[illegible]	[illegible]	[illegible]	[illegible]	[illegible]	[illegible]	[illegible]	[illegible]	[illegible]	[illegible]
Falls	60·2 [illegible]	[illegible]	[illegible]	[illegible]	[illegible]	[illegible]	[illegible]	[illegible]	[illegible]	[illegible]	[illegible]	[illegible]	[illegible]	[illegible]	[illegible]	[illegible]	[illegible]	[illegible]	[illegible]	[illegible]	[illegible]	[illegible]	[illegible]	[illegible]
Drowning	80·5 [illegible]	[illegible]	[illegible]	[illegible]	[illegible]	[illegible]	[illegible]	[illegible]	[illegible]	[illegible]	[illegible]	[illegible]	[illegible]	[illegible]	[illegible]	[illegible]	[illegible]	[illegible]	[illegible]	[illegible]	[illegible]	[illegible]	[illegible]	[illegible]
Fire, heat, and hot substances	28·4 [illegible]	[illegible]	[illegible]	[illegible]	[illegible]	[illegible]	[illegible]	[illegible]	[illegible]	[illegible]	[illegible]	[illegible]	[illegible]	[illegible]	[illegible]	[illegible]	[illegible]	[illegible]	[illegible]	[illegible]	[illegible]	[illegible]	[illegible]	[illegible]
Poisonings	74·9 (67·8 to 82)	[illegible]	[illegible]	[illegible]	[illegible]	[illegible]	[illegible]	[illegible]	[illegible]	[illegible]	[illegible]	[illegible]	[illegible]	[illegible]	[illegible]	[illegible]	[illegible]	[illegible]	[illegible]	[illegible]	[illegible]	[illegible]	[illegible]	[illegible]
Poisoning by carbon monoxide	4·7 (4·2 to 5·3)	[illegible]	[illegible]	[illegible]	[illegible]	[illegible]	[illegible]	[illegible]	[illegible]	[illegible]	[illegible]	[illegible]	[illegible]	[illegible]	[illegible]	[illegible]	[illegible]	[illegible]	[illegible]	[illegible]	[illegible]	[illegible]	[illegible]	[illegible]
Poisoning by other means	70·2 [illegible]	[illegible]	[illegible]	[illegible]	[illegible]	[illegible]	[illegible]	[illegible]	[illegible]	[illegible]	[illegible]	[illegible]	[illegible]	[illegible]	[illegible]	[illegible]	[illegible]	[illegible]	[illegible]	[illegible]	[illegible]	[illegible]	[illegible]	[illegible]
Exposure to mechanical forces	56·6 [illegible]	[illegible]	[illegible]	[illegible]	[illegible]	[illegible]	[illegible]	[illegible]	[illegible]	[illegible]	[illegible]	[illegible]	[illegible]	[illegible]	[illegible]	[illegible]	[illegible]	[illegible]	[illegible]	[illegible]	[illegible]	[illegible]	[illegible]	[illegible]
Unintentional firearm injuries	9·7 [illegible]	[illegible]	[illegible]	[illegible]	[illegible]	[illegible]	[illegible]	[illegible]	[illegible]	[illegible]	[illegible]	[illegible]	[illegible]	[illegible]	[illegible]	[illegible]	[illegible]	[illegible]	[illegible]	[illegible]	[illegible]	[illegible]	[illegible]	[illegible]
Other exposure to mechanical forces	46·9 [illegible]	[illegible]	[illegible]	[illegible]	[illegible]	[illegible]	[illegible]	[illegible]	[illegible]	[illegible]	[illegible]	[illegible]	[illegible]	[illegible]	[illegible]	[illegible]	[illegible]	[illegible]	[illegible]	[illegible]	[illegible]	[illegible]	[illegible]	[illegible]
Adverse effects of medical treatment	258 [illegible]	[illegible]	[illegible]	[illegible]	[illegible]	[illegible]	[illegible]	[illegible]	[illegible]	[illegible]	[illegible]	[illegible]	[illegible]	[illegible]	[illegible]	[illegible]	[illegible]	[illegible]	[illegible]	[illegible]	[illegible]	[illegible]	[illegible]	[illegible]
Animal contact	26·9 [illegible]	[illegible]	[illegible]	[illegible]	[illegible]	[illegible]	[illegible]	[illegible]	[illegible]	[illegible]	[illegible]	[illegible]	[illegible]	[illegible]	[illegible]	[illegible]	[illegible]	[illegible]	[illegible]	[illegible]	[illegible]	[illegible]	[illegible]	[illegible]
Venomous animal contact	3·5 (2·9 to 4·2)	[illegible]	[illegible]	[illegible]	[illegible]	[illegible]	[illegible]	[illegible]	[illegible]	[illegible]	[illegible]	[illegible]	[illegible]	[illegible]	[illegible]	[illegible]	[illegible]	[illegible]	[illegible]	[illegible]	[illegible]	[illegible]	[illegible]	[illegible]
Non-venomous animal contact	23·3 [illegible]	[illegible]	[illegible]	[illegible]	[illegible]	[illegible]	[illegible]	[illegible]	[illegible]	[illegible]	[illegible]	[illegible]	[illegible]	[illegible]	[illegible]	[illegible]	[illegible]	[illegible]	[illegible]	[illegible]	[illegible]	[illegible]	[illegible]	[illegible]
Foreign body	761 [illegible]	[illegible]	[illegible]	[illegible]	[illegible]	[illegible]	[illegible]	[illegible]	[illegible]	[illegible]	[illegible]	[illegible]	[illegible]	[illegible]	[illegible]	[illegible]	[illegible]	[illegible]	[illegible]	[illegible]	[illegible]	[illegible]	[illegible]	[illegible]
Pulmonary aspiration and foreign body in airway	758 [illegible]	[illegible]	[illegible]	[illegible]	[illegible]	[illegible]	[illegible]	[illegible]	[illegible]	[illegible]	[illegible]	[illegible]	[illegible]	[illegible]	[illegible]	[illegible]	[illegible]	[illegible]	[illegible]	[illegible]	[illegible]	[illegible]	[illegible]	[illegible]
Foreign body in eyes	0·7 (0·2 to 1·7)	[illegible]	[illegible]	[illegible]	[illegible]	[illegible]	[illegible]	[illegible]	[illegible]	[illegible]	[illegible]	[illegible]	[illegible]	[illegible]	[illegible]	[illegible]	[illegible]	[illegible]	[illegible]	[illegible]	[illegible]	[illegible]	[illegible]	[illegible]
Foreign body in other body part	2·9 (2 to 4·5)	[illegible]	[illegible]	[illegible]	[illegible]	[illegible]	[illegible]	[illegible]	[illegible]	[illegible]	[illegible]	[illegible]	[illegible]	[illegible]	[illegible]	[illegible]	[illegible]	[illegible]	[illegible]	[illegible]	[illegible]	[illegible]	[illegible]	[illegible]
Environmental heat and cold exposure	17·9 [illegible]	[illegible]	[illegible]	[illegible]	[illegible]	[illegible]	[illegible]	[illegible]	[illegible]	[illegible]	[illegible]	[illegible]	[illegible]	[illegible]	[illegible]	[illegible]	[illegible]	[illegible]	[illegible]	[illegible]	[illegible]	[illegible]	[illegible]	[illegible]
Exposure to forces of nature	0·9 (0·8 to 1)	[illegible]	[illegible]	[illegible]	[illegible]	[illegible]	[illegible]	[illegible]	[illegible]	[illegible]	[illegible]	[illegible]	[illegible]	[illegible]	[illegible]	[illegible]	[illegible]	[illegible]	[illegible]	[illegible]	[illegible]	[illegible]	[illegible]	[illegible]
Other unintentional injuries	29·8 [illegible]	[illegible]	[illegible]	[illegible]	[illegible]	[illegible]	[illegible]	[illegible]	[illegible]	[illegible]	[illegible]	[illegible]	[illegible]	[illegible]	[illegible]	[illegible]	[illegible]	[illegible]	[illegible]	[illegible]	[illegible]	[illegible]	[illegible]	[illegible]
Self-harm and interpersonal violence	909 [illegible]	[illegible]	[illegible]	[illegible]	[illegible]	[illegible]	[illegible]	[illegible]	[illegible]	[illegible]	[illegible]	[illegible]	[illegible]	[illegible]	[illegible]	[illegible]	[illegible]	[illegible]	[illegible]	[illegible]	[illegible]	[illegible]	[illegible]	[illegible]
Self-harm	0 (0 to 0)	[illegible]	[illegible]	[illegible]	[illegible]	[illegible]	[illegible]	[illegible]	[illegible]	[illegible]	[illegible]	[illegible]	[illegible]	[illegible]	[illegible]	[illegible]	[illegible]	[illegible]	[illegible]	[illegible]	[illegible]	[illegible]	[illegible]	[illegible]
Self-harm by firearm	0 (0 to 0)	[illegible]	[illegible]	[illegible]	[illegible]	[illegible]	[illegible]	[illegible]	[illegible]	[illegible]	[illegible]	[illegible]	[illegible]	[illegible]	[illegible]	[illegible]	[illegible]	[illegible]	[illegible]	[illegible]	[illegible]	[illegible]	[illegible]	[illegible]
Self-harm by other specified means	0 (0 to 0)	[illegible]	[illegible]	[illegible]	[illegible]	[illegible]	[illegible]	[illegible]	[illegible]	[illegible]	[illegible]	[illegible]	[illegible]	[illegible]	[illegible]	[illegible]	[illegible]	[illegible]	[illegible]	[illegible]	[illegible]	[illegible]	[illegible]	[illegible]
Interpersonal violence	903 [illegible]	[illegible]	[illegible]	[illegible]	[illegible]	[illegible]	[illegible]	[illegible]	[illegible]	[illegible]	[illegible]	[illegible]	[illegible]	[illegible]	[illegible]	[illegible]	[illegible]	[illegible]	[illegible]	[illegible]	[illegible]	[illegible]	[illegible]	[illegible]
Physical violence by firearm	48·1 [illegible]	[illegible]	[illegible]	[illegible]	[illegible]	[illegible]	[illegible]	[illegible]	[illegible]	[illegible]	[illegible]	[illegible]	[illegible]	[illegible]	[illegible]	[illegible]	[illegible]	[illegible]	[illegible]	[illegible]	[illegible]	[illegible]	[illegible]	[illegible]
Physical violence by sharp object	46·9 (41 to 54·8)	[illegible]	[illegible]	[illegible]	[illegible]	[illegible]	[illegible]	[illegible]	[illegible]	[illegible]	[illegible]	[illegible]	[illegible]	[illegible]	[illegible]	[illegible]	[illegible]	[illegible]	[illegible]	[illegible]	[illegible]	[illegible]	[illegible]	[illegible]
Sexual violence	0 (0 to 0)	[illegible]	[illegible]	[illegible]	[illegible]	[illegible]	[illegible]	[illegible]	[illegible]	[illegible]	[illegible]	[illegible]	[illegible]	[illegible]	[illegible]	[illegible]	[illegible]	[illegible]	[illegible]	[illegible]	[illegible]	[illegible]	[illegible]	[illegible]
Physical violence by other means	808 [illegible]	[illegible]	[illegible]	[illegible]	[illegible]	[illegible]	[illegible]	[illegible]	[illegible]	[illegible]	[illegible]	[illegible]	[illegible]	[illegible]	[illegible]	[illegible]	[illegible]	[illegible]	[illegible]	[illegible]	[illegible]	[illegible]	[illegible]	[illegible]
Conflict and terrorism	5·7 (5·2 to 6·3)	[illegible]	[illegible]	[illegible]	[illegible]	[illegible]	[illegible]	[illegible]	[illegible]	[illegible]	[illegible]	[illegible]	[illegible]	[illegible]	[illegible]	[illegible]	[illegible]	[illegible]	[illegible]	[illegible]	[illegible]	[illegible]	[illegible]	[illegible]

SR Table 2b: Latin America and Caribbean DALYS by cause and age group for females with 95% uncertainty intervals, 2019

Cause	Early Neonatal	Late Neonatal	Post Neonatal	1 to 4	5 to 9	10 to 14	15 to 19	20 to 24	25 to 29	30 to 34	35 to 39	40 to 44	45 to 49	50 to 54	55 to 59	60 to 64	65 to 69	70 to 74	75 to 79	80 to 84	85 to 89	90 to 94	95 plus	Total
All causes	2,700,000 (2,190,000 to 3,300,000)	826,000 (678,000 to 1,000,000)	2,460,000 (1,970,000 to 3,050,000)	1,900,000 (1,600,000 to 2,230,000)	1,450,000 (1,180,000 to 1,750,000)	1,910,000 (1,490,000 to 2,420,000)	2,770,000 (2,210,000 to 3,470,000)	3,360,000 (2,730,000 to 4,100,000)	3,730,000 (3,040,000 to 4,550,000)	4,070,000 (3,320,000 to 4,940,000)	4,490,000 (3,720,000 to 5,460,000)	4,710,000 (3,940,000 to 5,640,000)	4,910,000 (4,160,000 to 5,750,000)	5,290,000 (4,550,000 to 6,140,000)	5,540,000 (4,830,000 to 6,390,000)	5,520,000 (4,890,000 to 6,330,000)	5,130,000 (4,580,000 to 5,800,000)	4,640,000 (4,190,000 to 5,160,000)	3,950,000 (3,600,000 to 4,370,000)	3,240,000 (2,970,000 to 3,530,000)	2,220,000 (2,050,000 to 2,410,000)	1,150,000 (1,070,000 to 1,230,000)	488,000 (459,000 to 519,000)	76,500,000
Communicable, maternal, neonatal, and nutritional diseases	2,120,000 (1,690,000 to 2,630,000)	604,000 (488,000 to 740,000)	1,350,000 (1,070,000 to 1,680,000)	829,000 (701,000 to 969,000)	415,000 (334,000 to 516,000)	315,000 (253,000 to 392,000)	399,000 (329,000 to 487,000)	468,000 (397,000 to 556,000)	513,000 (436,000 to 602,000)	534,000 (460,000 to 627,000)	553,000 (474,000 to 654,000)	487,000 (417,000 to 579,000)	414,000 (351,000 to 489,000)	363,000 (309,000 to 431,000)	340,000 (294,000 to 397,000)	310,000 (274,000 to 355,000)	282,000 (252,000 to 317,000)	269,000 (240,000 to 299,000)	257,000 (230,000 to 282,000)	236,000 (204,000 to 261,000)	193,000 (160,000 to 216,000)	113,000 (89,800 to 128,000)	53,400 (40,200 to 61,200)	11,400,000
HIV/AIDS and sexually transmitted infections	46,900 (19,900 to 92,200)	13,200 (5,200 to 26,600)	34,700 (23,900 to 52,300)	23,900 (16,600 to 61,300)	11,700 (9,680 to 14,500)	11,800 (10,200 to 13,700)	24,800 (20,300 to 30,800)	50,900 (43,900 to 60,700)	92,600 (79,000 to 112,000)	122,000 (105,000 to 145,000)	135,000 (118,000 to 157,000)	115,000 (102,000 to 134,000)	91,200 (80,900 to 105,000)	58,000 (51,700 to 66,300)	38,900 (34,300 to 45,000)	25,800 (22,700 to 30,300)	15,600 (13,600 to 18,800)	5,640 (4,530 to 7,500)	2,790 (2,180 to 3,790)	1,230 (921 to 1,800)	669 (508 to 950)	239 (176 to 347)	83·1 (63·6 to 117)	922,000
HIV/AIDS	1·9 (1·2 to 3)	6·3 (4·2 to 10·1)	18,200 (16,100 to 26,900)	23,100 (15,900 to 59,400)	11,400 (9,370 to 14,000)	11,100 (9,570 to 13,000)	21,800 (17,700 to 27,400)	43,700 (38,300 to 51,400)	80,800 (70,100 to 97,700)	107,000 (93,800 to 127,000)	120,000 (106,000 to 137,000)	101,000 (90,900 to 113,000)	79,900 (72,300 to 89,000)	50,900 (46,500 to 56,100)	33,800 (30,800 to 37,200)	22,300 (20,100 to 25,200)	13,000 (11,600 to 14,800)	3,750 (3,140 to 4,670)	1,540 (1,300 to 1,840)	433 (376 to 493)	197 (168 to 228)	69·6 (57·4 to 82·5)	30·4 (25·6 to 35·8)	744,000
HIV/AIDS - Drug-susceptible Tuberculosis	0 (0 to 0·1)	0·3 (0·2 to 0·4)	1,940 (1,210 to 2,950)	2,160 (1,340 to 3,300)	1,140 (730 to 1,720)	1,070 (687 to 1,600)	2,040 (1,340 to 3,020)	4,380 (2,930 to 6,440)	7,860 (5,200 to 11,500)	10,300 (6,770 to 15,300)	11,600 (7,630 to 17,400)	9,910 (6,460 to 14,900)	7,680 (5,020 to 11,400)	4,960 (3,270 to 7,390)	3,290 (2,180 to 4,840)	2,120 (1,420 to 3,090)	1,210 (815 to 1,760)	337 (239 to 470)	138 (98·3 to 193)	39·7 (28 to 56·5)	18·1 (13 to 25·4)	6·2 (4·5 to 8·5)	2·6 (1·9 to 3·7)	72,200
HIV/AIDS - Multidrug-resistant Tuberculosis wit[illegible]	0 (0 to 0)	0 (0 to 0)	155 (48·8 to 383)	141 (46·7 to 328)	71·1 (24·3 to 171)	63·7 (20·8 to 158)	124 (42 to 299)	303 (107 to 714)	551 (195 to 1,300)	689 (238 to 1,680)	751 (239 to 1,840)	618 (195 to 1,520)	472 (149 to 1,180)	289 (87·3 to 711)	202 (62·6 to 496)	135 (43·6 to 331)	78 (25·2 to 193)	17·9 (5·8 to 43·5)	7·4 (2·5 to 17·6)	2·6 (0·9 to 6)	1·4 (0·6 to 2·9)	0·5 (0·2 to 1·1)	0·2 (0·1 to 0·5)	4,670
HIV/AIDS - Extensively drug-resistant Tubercul[illegible]	0 (0 to 0)	0 (0 to 0)	25·6 (7·9 to 62·3)	23·3 (7·2 to 53·4)	11·6 (3·9 to 26·8)	10·4 (3·2 to 25·2)	20·1 (6·5 to 48·8)	49·1 (16·5 to 113)	89·3 (29·6 to 205)	112 (36·7 to 262)	122 (36·7 to 298)	101 (29·9 to 245)	76·8 (23·3 to 187)	46·8 (13·6 to 116)	32·6 (9·6 to 80·5)	21·6 (6·6 to 52·4)	12·4 (3·8 to 30·1)	2·7 (0·8 to 6·8)	1·1 (0·4 to 2·8)	0·4 (0·1 to 1)	0·2 (0·1 to 0·5)	0·1 (0 to 0·2)	0 (0 to 0·1)	759
HIV/AIDS resulting in other diseases	1·8 (1·2 to 3)	6 (3·9 to 9·7)	16,000 (13,600 to 25,000)	20,800 (13,600 to 57,100)	10,100 (8,140 to 12,900)	10,000 (8,450 to 11,800)	19,700 (15,400 to 25,300)	39,000 (33,400 to 47,100)	72,300 (61,100 to 90,100)	96,300 (82,300 to 116,000)	107,000 (92,600 to 125,000)	90,000 (79,100 to 104,000)	71,600 (63,300 to 81,900)	45,600 (40,700 to 51,100)	30,300 (26,800 to 34,000)	20,000 (17,700 to 22,900)	11,700 (10,300 to 13,700)	3,400 (2,790 to 4,370)	1,390 (1,150 to 1,690)	390 (333 to 454)	177 (148 to 209)	62·8 (51 to 75·6)	27·5 (22·5 to 33·1)	666,000
Sexually transmitted infections excluding HIV	46,900 (19,900 to 92,200)	13,200 (5,190 to 26,600)	16,500 (6,920 to 32,700)	765 (302 to 1,590)	382 (140 to 775)	667 (517 to 903)	2,930 (2,050 to 4,570)	7,210 (4,270 to 12,600)	11,900 (6,420 to 21,000)	14,500 (7,870 to 26,600)	15,000 (8,120 to 27,200)	14,300 (7,820 to 26,600)	11,400 (6,210 to 20,900)	7,070 (3,560 to 14,100)	5,060 (2,540 to 9,750)	3,540 (1,880 to 6,730)	2,670 (1,500 to 4,870)	1,890 (1,110 to 3,410)	1,250 (758 to 2,220)	799 (506 to 1,350)	472 (318 to 734)	169 (112 to 275)	52·7 (34·6 to 85·1)	178,000
Syphilis	46,900 (19,900 to 92,200)	13,200 (5,190 to 26,600)	16,500 (6,920 to 32,700)	765 (302 to 1,590)	382 (140 to 775)	226 (187 to 280)	295 (245 to 368)	197 (136 to 320)	418 (333 to 557)	539 (451 to 673)	388 (328 to 489)	194 (156 to 263)	304 (262 to 362)	203 (172 to 251)	102 (78·5 to 135)	116 (92·2 to 146)	117 (96·2 to 142)	115 (97 to 139)	75·6 (62·8 to 91·1)	87·3 (74·3 to 100)	58·1 (47·6 to 67·7)	30·8 (24·4 to 36·4)	11 (8·3 to 13·5)	81,200
Chlamydial infection	0 (0 to 0)	0 (0 to 0)	0 (0 to 0)	0 (0 to 0)	0 (0 to 0)	74·6 (55·3 to 102)	324 (229 to 463)	856 (638 to 1,160)	1,030 (687 to 1,550)	1,130 (703 to 1,810)	1,370 (867 to 2,190)	1,410 (898 to 2,260)	955 (639 to 1,590)	293 (233 to 381)	198 (167 to 239)	142 (126 to 163)	84 (74·2 to 93·9)	88 (77·4 to 100)	68·3 (59 to 77·6)	61·8 (51·3 to 70·6)	39·2 (31·9 to 45·7)	12 (9·1 to 14·3)	4·9 (3·6 to 6·2)	8,140
Gonococcal infection	0 (0 to 0)	0 (0 to 0)	0 (0 to 0)	0 (0 to 0)	0 (0 to 0)	70·2 (58·4 to 87)	402 (342 to 481)	719 (609 to 846)	876 (729 to 1,060)	1,000 (835 to 1,240)	1,040 (881 to 1,270)	1,240 (1,060 to 1,500)	1,120 (967 to 1,320)	548 (481 to 639)	395 (351 to 455)	364 (322 to 417)	450 (397 to 502)	300 (264 to 343)	244 (211 to 277)	138 (115 to 158)	102 (82·6 to 119)	30·5 (23·1 to 36·3)	8·7 (6·4 to 11)	9,050
Trichomoniasis	0 (0 to 0)	0 (0 to 0)	0 (0 to 0)	0 (0 to 0)	0 (0 to 0)	25·9 (7·4 to 66·9)	501 (156 to 1,210)	2,620 (922 to 6,150)	5,210 (1,740 to 12,100)	5,960 (1,970 to 13,900)	5,630 (1,850 to 12,900)	5,020 (1,720 to 11,800)	4,200 (1,350 to 9,570)	3,160 (997 to 7,400)	2,070 (675 to 4,790)	1,210 (407 to 2,870)	798 (281 to 1,850)	515 (192 to 1,170)	303 (117 to 680)	164 (62·9 to 367)	69·6 (27·3 to 155)	20·5 (7·6 to 45·4)	3·6 (1·2 to 8·5)	37,500
Genital herpes	0 (0 to 0)	0 (0 to 0)	0 (0 to 0)	0 (0 to 0)	0 (0 to 0)	133 (42·7 to 324)	594 (198 to 1,480)	1,310 (438 to 3,170)	2,070 (728 to 5,010)	2,620 (893 to 6,360)	2,820 (929 to 6,920)	2,660 (854 to 6,620)	2,380 (754 to 5,980)	2,120 (660 to 5,180)	1,790 (544 to 4,290)	1,390 (428 to 3,410)	1,020 (312 to 2,500)	716 (217 to 1,760)	467 (143 to 1,140)	291 (89·3 to 711)	152 (46·5 to 369)	62 (18·8 to 151)	20·7 (6·3 to 50·5)	22,600
Other sexually transmitted infections	0 (0 to 0)	0 (0 to 0)	0 (0 to 0)	0 (0 to 0)	0 (0 to 0)	137 (104 to 184)	814 (639 to 1,050)	1,510 (1,110 to 2,010)	2,250 (1,500 to 3,360)	3,210 (2,150 to 4,730)	3,730 (2,400 to 5,500)	3,760 (2,390 to 5,790)	2,420 (1,580 to 4,030)	745 (556 to 1,000)	503 (403 to 636)	314 (278 to 360)	199 (176 to 222)	154 (136 to 176)	94·7 (81·8 to 108)	57 (47·3 to 65·1)	51·1 (41·5 to 59·6)	13·3 (10·1 to 15·8)	3·8 (2·8 to 4·8)	20,000
Respiratory infections and tuberculosis	66,900 (49,300 to 87,600)	69,500 (52,200 to 90,800)	450,000 (343,000 to 571,000)	247,000 (199,000 to 301,000)	92,700 (73,500 to 120,000)	70,400 (54,600 to 92,800)	71,000 (56,500 to 91,800)	86,600 (70,700 to 107,000)	89,100 (73,400 to 109,000)	87,600 (73,500 to 107,000)	92,500 (77,800 to 112,000)	95,800 (83,100 to 112,000)	98,100 (85,500 to 115,000)	110,000 (97,900 to 124,000)	121,000 (108,000 to 136,000)	132,000 (119,000 to 146,000)	140,000 (126,000 to 154,000)	153,000 (137,000 to 168,000)	162,000 (144,000 to 178,000)	161,000 (137,000 to 180,000)	138,000 (111,000 to 155,000)	82,800 (63,600 to 94,900)	40,000 (29,300 to 46,200)	2,860,000
Tuberculosis	0 (0 to 0)	0·2 (0·1 to 0·4)	9,010 (6,670 to 12,000)	11,300 (8,360 to 15,700)	5,540 (4,350 to 7,080)	5,650 (4,590 to 7,020)	10,200 (8,400 to 12,600)	21,500 (17,100 to 26,900)	24,100 (19,000 to 30,300)	21,900 (17,600 to 28,100)	20,500 (16,400 to 26,100)	20,100 (16,000 to 25,400)	17,700 (14,600 to 22,200)	18,100 (14,900 to 22,400)	16,300 (13,500 to 20,000)	14,600 (12,200 to 17,700)	13,100 (10,900 to 15,800)	11,400 (9,600 to 13,700)	8,570 (7,250 to 10,200)	5,370 (4,420 to 6,300)	2,910 (2,330 to 3,450)	993 (755 to 1,220)	331 (239 to 407)	259,000
Latent tuberculosis infection	0 (0 to 0)	0 (0 to 0)	0 (0 to 0)	0 (0 to 0)	0 (0 to 0)	0 (0 to 0)	0 (0 to 0)	0 (0 to 0)	0 (0 to 0)	0 (0 to 0)	0 (0 to 0)	0 (0 to 0)	0 (0 to 0)	0 (0 to 0)	0 (0 to 0)	0 (0 to 0)	0 (0 to 0)	0 (0 to 0)	0 (0 to 0)	0 (0 to 0)	0 (0 to 0)	0 (0 to 0)	0 (0 to 0)	0
Drug-susceptible tuberculosis	0 (0 to 0)	0·2 (0·1 to 0·4)	8,400 (6,140 to 11,400)	10,700 (7,840 to 15,100)	5,220 (4,050 to 6,730)	5,280 (4,210 to 6,620)	9,460 (7,650 to 11,800)	20,200 (16,000 to 25,300)	22,700 (17,800 to 28,900)	20,600 (16,400 to 26,200)	19,300 (15,000 to 24,600)	18,700 (15,000 to 23,700)	16,400 (13,500 to 20,600)	16,800 (13,700 to 20,700)	15,100 (12,400 to 18,400)	13,500 (11,100 to 16,400)	12,100 (9,990 to 14,700)	10,500 (8,740 to 12,500)	7,880 (6,510 to 9,390)	4,940 (4,010 to 5,850)	2,670 (2,120 to 3,180)	911 (685 to 1,120)	303 (214 to 375)	242,000
Multidrug-resistant tuberculosis without extensiv[illegible]	0 (0 to 0)	0 (0 to 0)	521 (197 to 1,110)	507 (201 to 1,080)	284 (129 to 532)	323 (142 to 633)	645 (269 to 1,250)	1,170 (519 to 2,310)	1,250 (547 to 2,480)	1,130 (481 to 2,250)	1,100 (456 to 2,220)	1,160 (479 to 2,430)	1,090 (445 to 2,200)	1,120 (439 to 2,350)	1,030 (415 to 2,030)	925 (391 to 1,880)	878 (367 to 1,800)	764 (327 to 1,520)	592 (255 to 1,190)	376 (160 to 778)	208 (86 to 423)	70·9 (30·5 to 145)	24 (10 to 52·1)	15,200
Extensively drug-resistant tuberculosis	0 (0 to 0)	0 (0 to 0)	86 (31·6 to 185)	80·9 (30·9 to 170)	42·7 (17·8 to 83·5)	50 (20·4 to 99·6)	103 (41·2 to 206)	178 (73·8 to 352)	191 (77·6 to 395)	174 (71·6 to 363)	173 (67·2 to 360)	185 (71·7 to 384)	175 (68·9 to 360)	179 (67·7 to 381)	166 (65·1 to 337)	150 (59·9 to 305)	143 (55·5 to 286)	123 (50·6 to 253)	95·2 (38·9 to 199)	60·4 (24·6 to 124)	33·5 (13·3 to 68·9)	11·3 (4·5 to 23·1)	3·8 (1·5 to 8·1)	2,410
Lower respiratory infections	66,600 (49,200 to 87,300)	68,800 (51,500 to 90,300)	431,000 (326,000 to 548,000)	198,000 (153,000 to 247,000)	45,400 (38,700 to 52,800)	31,200 (27,200 to 36,100)	30,100 (26,300 to 34,400)	36,000 (31,000 to 41,800)	38,400 (32,800 to 44,800)	41,100 (35,800 to 47,200)	48,200 (42,300 to 55,000)	54,900 (48,300 to 62,100)	62,700 (55,800 to 70,500)	76,300 (67,900 to 85,000)	92,000 (82,700 to 102,000)	107,000 (97,000 to 119,000)	119,000 (108,000 to 132,000)	136,000 (122,000 to 151,000)	150,000 (133,000 to 165,000)	154,000 (130,000 to 172,000)	134,000 (107,000 to 150,000)	81,200 (62,400 to 93,100)	39,400 (28,900 to 45,700)	2,240,000
Upper respiratory infections	154 (114 to 199)	569 (404 to 801)	8,730 (5,960 to 12,600)	27,700 (16,500 to 44,200)	29,600 (16,500 to 50,000)	25,100 (13,400 to 42,900)	23,400 (12,500 to 41,100)	23,000 (12,500 to 39,100)	21,500 (12,100 to 35,600)	20,400 (11,100 to 34,200)	19,500 (10,200 to 32,000)	16,700 (9,150 to 29,200)	13,900 (7,480 to 24,000)	12,200 (6,730 to 20,800)	10,100 (5,590 to 16,700)	7,900 (4,320 to 12,900)	5,990 (3,240 to 10,000)	4,310 (2,410 to 7,100)	2,830 (1,560 to 4,610)	1,770 (978 to 2,920)	949 (575 to 1,510)	442 (272 to 691)	173 (107 to 273)	277,000
Otitis media	98·2 (10·6 to 531)	47·5 (29 to 75·2)	1,400 (792 to 2,390)	9,950 (5,340 to 17,400)	12,200 (6,920 to 20,300)	8,400 (4,660 to 14,400)	7,290 (3,970 to 12,500)	6,060 (2,730 to 11,600)	5,040 (2,430 to 9,350)	4,280 (1,920 to 8,190)	4,220 (2,140 to 7,800)	4,130 (1,990 to 7,370)	3,880 (1,990 to 6,780)	3,600 (1,760 to 6,330)	2,920 (1,520 to 5,130)	2,130 (1,050 to 4,030)	1,320 (683 to 2,460)	817 (382 to 1,570)	567 (289 to 1,030)	404 (191 to 739)	245 (123 to 427)	115 (56·1 to 204)	45·1 (22·4 to 79·8)	79,100
Enteric infections	7,160 (3,410 to 13,200)	23,300 (10,600 to 45,800)	237,000 (166,000 to 337,000)	181,000 (129,000 to 249,000)	62,100 (44,400 to 87,600)	40,100 (27,600 to 57,900)	25,500 (18,600 to 34,200)	22,000 (16,300 to 29,300)	23,200 (17,000 to 30,700)	24,700 (18,400 to 32,600)	26,900 (20,200 to 34,900)	27,400 (20,800 to 35,100)	28,100 (21,600 to 36,000)	29,500 (23,000 to 37,100)	30,700 (24,400 to 38,100)	32,400 (26,200 to 40,100)	33,600 (27,300 to 41,700)	33,900 (27,700 to 42,000)	31,400 (25,800 to 38,300)	27,300 (22,800 to 33,100)	19,200 (15,900 to 23,300)	10,100 (8,140 to 12,000)	4,610 (3,620 to 5,620)	982,000
Diarrheal diseases	7,160 (3,410 to 13,200)	23,300 (10,600 to 45,800)	236,000 (164,000 to 336,000)	180,000 (128,000 to 247,000)	61,000 (43,100 to 86,700)	39,300 (26,900 to 57,100)	25,100 (18,200 to 33,700)	21,600 (15,900 to 28,900)	22,900 (16,800 to 30,400)	24,500 (18,200 to 32,100)	26,600 (19,900 to 34,600)	27,100 (20,600 to 34,900)	27,900 (21,400 to 35,800)	29,300 (22,800 to 36,900)	30,400 (24,100 to 37,900)	32,200 (26,000 to 39,900)	33,400 (27,000 to 41,600)	33,700 (27,600 to 41,800)	31,300 (25,700 to 38,200)	27,200 (22,700 to 32,900)	19,100 (15,900 to 23,200)	10,000 (8,120 to 12,000)	4,600 (3,610 to 5,590)	974,000
Typhoid and paratyphoid	0 (0 to 0)	0 (0 to 0)	153 (57·9 to 546)	334 (167 to 680)	185 (94·6 to 349)	218 (106 to 562)	179 (79·5 to 415)	144 (68·7 to 350)	130 (60 to 341)	113 (50·2 to 319)	128 (57·2 to 434)	146 (58·5 to 632)	117 (54·4 to 443)	145 (64·5 to 658)	132 (63·4 to 599)	111 (54 to 424)	98·2 (47·6 to 409)	86·5 (40·7 to 370)	75·5 (39·7 to 294)	71·2 (35·7 to 277)	52·7 (22·6 to 272)	32 (11·9 to 182)	8·5 (3·7 to 37·2)	2,660
Typhoid fever	0 (0 to 0)	0 (0 to 0)	149 (53·2 to 540)	316 (158 to 657)	173 (88 to 329)	208 (99·9 to 541)	172 (74·4 to 406)	139 (64·6 to 344)	126 (58·5 to 338)	110 (47·2 to 316)	126 (54·9 to 433)	143 (56·4 to 629)	115 (53 to 441)	143 (62·3 to 656)	128 (60·4 to 597)	108 (51·8 to 422)	96·1 (45·7 to 407)	84·7 (39 to 368)	73·7 (38·2 to 290)	69·7 (34·4 to 276)	51·5 (21·6 to 271)	31·4 (11·6 to 181)	8·2 (3·4 to 36·9)	2,570
Paratyphoid fever	0 (0 to 0)	0 (0 to 0)	3·8 (1·6 to 10·7)	18·2 (6·5 to 42)	12·6 (4·5 to 29·4)	10·3 (3·9 to 23·2)	7·2 (2·8 to 15·4)	5 (1·9 to 10·7)	3·7 (1·5 to 8)	3·4 (1·5 to 7)	2·7 (1·1 to 5·7)	2·8 (1·5 to 5·4)	2 (0·9 to 4·4)	2·6 (1·4 to 4·9)	3·6 (1·9 to 7·1)	3·2 (1·8 to 6)	2·1 (1·2 to 3·6)	1·9 (1·1 to 3·3)	1·9 (1·2 to 3·1)	1·5 (0·9 to 2·5)	1·2 (0·8 to 2·1)	0·6 (0·3 to 1·1)	0·4 (0·2 to 0·7)	90·6
Invasive Non-typhoidal Salmonella (iNTS)	0 (0 to 0)	18·1 (9 to 40·9)	367 (191 to 833)	700 (311 to 1,290)	643 (272 to 1,450)	440 (168 to 1,140)	214 (81·4 to 474)	162 (57 to 391)	121 (43·7 to 279)	97·1 (35·9 to 214)	90·4 (36·6 to 196)	78·9 (31·6 to 173)	77·1 (32·1 to 153)	80·8 (33·4 to 157)	96·8 (41·5 to 199)	83·2 (31 to 173)	78·1 (32·3 to 154)	65·2 (29·1 to 126)	51·7 (23·6 to 113)	33·9 (16·5 to 72·7)	15·9 (8 to 30·7)	8·9 (4·5 to 18·3)	3·3 (1·5 to 8·4)	3,530
Other intestinal infectious diseases	0 (0 to 0)	0 (0 to 0)	431 (15 to 1,480)	533 (81·6 to 1,470)	200 (42·4 to 586)	144 (27·4 to 436)	71·8 (12·1 to 221)	62·5 (10·7 to 191)	30·4 (6·7 to 87·9)	75·1 (19·5 to 196)	41·4 (10·8 to 119)	44·9 (11·9 to 126)	27·4 (6·8 to 72·9)	26·4 (5·7 to 76·7)	20·1 (3·9 to 56·9)	16·5 (2·6 to 54·6)	24·1 (3·8 to 77·7)	15·9 (2·2 to 49·1)	10·5 (1·3 to 34·8)	10·9 (1·2 to 34·3)	14 (1·6 to 48·6)	6·5 (0·8 to 21·4)	4·1 (0·5 to 12·9)	1,810
Neglected tropical diseases and malaria	1,490 (846 to 2,520)	1,070 (666 to 1,730)	14,000 (9,230 to 20,800)	31,900 (20,700 to 50,400)	32,800 (22,400 to 51,200)	24,400 (16,500 to 35,400)	37,900 (26,000 to 54,100)	41,400 (28,600 to 57,900)	42,600 (29,700 to 58,400)	41,700 (28,800 to 57,000)	39,900 (28,900 to 54,300)	35,300 (25,800 to 48,000)	33,800 (24,900 to 45,200)	35,100 (25,300 to 49,400)	37,700 (27,400 to 51,300)	36,100 (25,800 to 51,700)	33,600 (23,600 to 47,100)	29,200 (19,900 to 40,300)	23,100 (15,700 to 31,800)	15,800 (10,400 to 21,800)	9,910 (6,570 to 14,400)	4,250 (2,800 to 6,300)	1,530 (959 to 2,270)	605,000
Malaria	0·5 (0·2 to 1)	375 (95·4 to 979)	3,530 (1,120 to 8,130)	4,660 (1,740 to 10,500)	2,730 (1,210 to 5,630)	2,670 (1,070 to 5,710)	7,320 (2,910 to 15,200)	6,060 (2,400 to 13,200)	4,650 (1,900 to 10,100)	3,520 (1,500 to 7,270)	3,150 (1,400 to 6,540)	1,830 (816 to 3,820)	1,500 (640 to 3,220)	1,400 (578 to 3,020)	1,420 (556 to 3,150)	1,110 (438 to 2,520)	739 (293 to 1,630)	491 (200 to 1,070)	144 (70·2 to 277)	66·3 (34·6 to 124)	22 (12·1 to 35·4)	9 (4·9 to 15·1)	2·7 (1·2 to 5·2)	47,400
Chagas disease	0 (0 to 0)	0 (0 to 0)	37 (15·8 to 110)	21·6 (10·8 to 68·9)	75 (46·8 to 152)	282 (185 to 449)	647 (426 to 1,000)	1,100 (741 to 1,770)	1,570 (1,030 to 2,690)	2,680 (1,780 to 5,070)	4,160 (2,700 to 7,870)	5,310 (3,160 to 10,400)	6,900 (4,020 to 13,300)	9,100 (4,580 to 19,800)	11,700 (5,360 to 21,500)	12,200 (5,540 to 24,900)	12,300 (5,250 to 22,300)	11,100 (4,300 to 19,500)	9,060 (3,680 to 14,700)	5,740 (2,230 to 8,980)	4,100 (1,930 to 7,780)	1,680 (787 to 3,460)	572 (268 to 1,180)	100,000
Leishmaniasis	0 (0 to 0)	0 (0 to 0)	1,350 (20·2 to 4,460)	5,220 (177 to 21,100)	4,010 (214 to 20,500)	1,960 (176 to 9,330)	1,460 (162 to 5,960)	1,340 (181 to 4,920)	1,590 (202 to 5,230)	1,710 (226 to 5,300)	1,620 (242 to 4,600)	1,300 (240 to 3,320)	997 (238 to 2,170)	841 (230 to 1,740)	732 (219 to 1,450)	628 (213 to 1,170)	515 (204 to 898)	380 (164 to 626)	251 (116 to 405)	153 (76·7 to 241)	78·3 (41·8 to 122)	32 (17 to 50·6)	12·1 (6·3 to 18·9)	26,200
Visceral leishmaniasis	0 (0 to 0)	0 (0 to 0)	1,330 (1·1 to 4,450)	5,000 (7·5 to 21,000)	3,740 (6·4 to 20,200)	1,720 (2·6 to 9,110)	1,240 (1·8 to 5,730)	1,100 (1·5 to 4,670)	1,320 (1·8 to 4,960)	1,420 (2 to 5,010)	1,310 (1·9 to 4,280)	975 (1·4 to 3,000)	684 (1·1 to 1,860)	533 (1 to 1,410)	435 (0·9 to 1,120)	341 (0·8 to 832)	242 (0·6 to 577)	155 (0·5 to 362)	86·4 (0·3 to 208)	46·2 (0·2 to 111)	20·3 (0·1 to 51·2)	8·2 (0·1 to 20·9)	2·8 (0 to 7·3)	21,700
Cutaneous and mucocutaneous leishmaniasis	0 (0 to 0)	0 (0 to 0)	23·2 (11·4 to 44·3)	219 (122 to 377)	276 (163 to 443)	237 (144 to 388)	225 (135 to 361)	242 (149 to 380)	269 (168 to 419)	294 (178 to 454)	316 (197 to 488)	322 (197 to 497)	313 (192 to 481)	309 (189 to 477)	297 (180 to 459)	287 (178 to 446)	273 (166 to 425)	225 (140 to 352)	165 (102 to 256)	107 (66·5 to 165)	58·1 (36 to 90·8)	23·8 (14·5 to 36·9)	9·3 (5·5 to 15)	4,490

Multiple sclerosis	0 (0 to 0)	[illegible]
Motor neuron disease	33 (25·8 to 42·4)	[illegible]
Headache disorders	0 (0 to 0)	[illegible]
Migraine	0 (0 to 0)	[illegible]
Tension-type headache	0 (0 to 0)	[illegible]
Other neurological disorders	0 (0 to 0)	[illegible]
Mental disorders	38·9 (24·6 to 58·3)	[illegible]
Schizophrenia	0 (0 to 0)	[illegible]
Depressive disorders	0 (0 to 0)	[illegible]
Major depressive disorder	0 (0 to 0)	[illegible]
Dysthymia	0 (0 to 0)	[illegible]
Bipolar disorder	0 (0 to 0)	[illegible]
Anxiety disorders	0 (0 to 0)	[illegible]
Eating disorders	0 (0 to 0)	[illegible]
Anorexia nervosa	0 (0 to 0)	[illegible]
Bulimia nervosa	0 (0 to 0)	[illegible]
Autism spectrum disorders	31·7 (20·5 to 45·9)	[illegible]
Attention-deficit/hyperactivity disorder	0 (0 to 0)	[illegible]
Conduct disorder	0 (0 to 0)	[illegible]
Idiopathic developmental intellectual disability	7·2 (0·7 to 18·3)	[illegible]
Other mental disorders	0 (0 to 0)	[illegible]
Substance use disorders	0·1 (0 to 0·1)	[illegible]
Alcohol use disorders	0·1 (0 to 0·1)	[illegible]
Drug use disorders	0 (0 to 0)	[illegible]
Opioid use disorders	0 (0 to 0)	[illegible]
Cocaine use disorders	0 (0 to 0)	[illegible]
Amphetamine use disorders	0 (0 to 0)	[illegible]
Cannabis use disorders	0 (0 to 0)	[illegible]
Other drug use disorders	0 (0 to 0)	[illegible]
Diabetes and kidney diseases	24·9 [illegible]	[illegible]
Diabetes mellitus	24·8 [illegible]	[illegible]
Diabetes mellitus type 1	24·8 [illegible]	[illegible]
Diabetes mellitus type 2	0 (0 to 0)	[illegible]
Chronic kidney disease	0·1 [illegible]	[illegible]
Chronic kidney disease due to diabetes mellitu[illegible]	0 (0 to 0)	[illegible]
Chronic kidney disease due to diabetes mellitu[illegible]	0 (0 to 0)	[illegible]
Chronic kidney disease due to hypertension	0 (0 to 0)	[illegible]
Chronic kidney disease due to glomerulonephri[illegible]	0 [illegible]	[illegible]
Chronic kidney disease due to other and unspe[illegible]	0·1 [illegible]	[illegible]
Acute glomerulonephritis	0 (0 to 0)	[illegible]
Skin and subcutaneous diseases	7·3 [illegible]	[illegible]
Dermatitis	0 (0 to 0)	[illegible]
Atopic dermatitis	0 (0 to 0)	[illegible]
Contact dermatitis	0 (0 to 0)	[illegible]
Seborrhoeic dermatitis	0 (0 to 0)	[illegible]
Psoriasis	0 (0 to 0)	[illegible]
Bacterial skin diseases	0·9 [illegible]	[illegible]
Cellulitis	0·2 [illegible]	[illegible]
Pyoderma	0·7 [illegible]	[illegible]
Scabies	3·1 [illegible]	[illegible]
Fungal skin diseases	0·4 [illegible]	[illegible]
Viral skin diseases	0 (0 to 0)	[illegible]
Acne vulgaris	0 (0 to 0)	[illegible]
Alopecia areata	0 (0 to 0)	[illegible]
Pruritus	0 [illegible]	[illegible]
Urticaria	2·5 [illegible]	[illegible]
Decubitus ulcer	0 (0 to 0)	[illegible]
Other skin and subcutaneous diseases	0·4 [illegible]	[illegible]
Sense organ diseases	20·6 [illegible]	[illegible]
Blindness and vision loss	10·2 [illegible]	[illegible]
Glaucoma	0 (0 to 0)	[illegible]
Cataract	0 (0 to 0)	[illegible]
Age-related macular degeneration	0 (0 to 0)	[illegible]
Refraction disorders	0·6 [illegible]	[illegible]
Near vision loss	0 (0 to 0)	[illegible]
Other vision loss	9·6 [illegible]	[illegible]
Age-related and other hearing loss	2·5 [illegible]	[illegible]
Other sense organ diseases	8 [illegible]	[illegible]
Musculoskeletal disorders	0 (0 to 0)	[illegible]
Rheumatoid arthritis	0 (0 to 0)	[illegible]
Osteoarthritis	0 (0 to 0)	[illegible]
Osteoarthritis hip	0 (0 to 0)	[illegible]
Osteoarthritis knee	0 (0 to 0)	[illegible]
Osteoarthritis hand	0 (0 to 0)	[illegible]
Osteoarthritis other	0 (0 to 0)	[illegible]
Low back pain	0 (0 to 0)	[illegible]
Neck pain	0 (0 to 0)	[illegible]
Gout	0 (0 to 0)	[illegible]
Other musculoskeletal disorders	0 (0 to 0)	[illegible]
Other non-communicable diseases	545,000 [illegible]	[illegible]
Congenital birth defects	541,000 [illegible]	[illegible]
Neural tube defects	82,600 [illegible]	[illegible]
Congenital heart anomalies	171,000 [illegible]	[illegible]
Orofacial clefts	1,280 [illegible]	[illegible]
Down syndrome	9,030 [illegible]	[illegible]
Turner syndrome	0·8 [illegible]	[illegible]
Klinefelter syndrome	0 (0 to 0)	[illegible]
Other chromosomal abnormalities	23,900 [illegible]	[illegible]
Congenital musculoskeletal and limb anomalie[illegible]	11,000 [illegible]	[illegible]
Urogenital congenital anomalies	22,300 [illegible]	[illegible]
Digestive congenital anomalies	72,600 [illegible]	[illegible]
Other congenital birth defects	147,000 [illegible]	[illegible]
Urinary diseases and male infertility	313 [illegible]	[illegible]
Urolithiasis	0 (0 to 0)	[illegible]
Benign prostatic hyperplasia	0 (0 to 0)	[illegible]
Male infertility	0 (0 to 0)	[illegible]
Other urinary diseases	110 [illegible]	[illegible]
Gynecological diseases	0 (0 to 0)	[illegible]
Uterine fibroids	0 (0 to 0)	[illegible]
Polycystic ovarian syndrome	0 (0 to 0)	[illegible]
Female infertility	0 (0 to 0)	[illegible]
Endometriosis	0 (0 to 0)	[illegible]
Genital prolapse	0 (0 to 0)	[illegible]
Premenstrual syndrome	0 (0 to 0)	[illegible]
Other gynecological diseases	0 (0 to 0)	[illegible]
Hemoglobinopathies and hemolytic anemias	500 [illegible]	[illegible]
Thalassemias	30 [illegible]	[illegible]
Thalassemias trait	3·4 [illegible]	[illegible]
Sickle cell disorders	372 [illegible]	[illegible]
Sickle cell trait	9·5 [illegible]	[illegible]
G6PD deficiency	13·5 [illegible]	[illegible]
G6PD trait	0 (0 to 0)	[illegible]
Other hemoglobinopathies and hemolytic anem[illegible]	72·5 [illegible]	[illegible]
Endocrine, metabolic, blood, and immune disorders	3,390 [illegible]	[illegible]
Oral disorders	0 (0 to 0)	[illegible]
Caries of deciduous teeth	0 (0 to 0)	[illegible]
Caries of permanent teeth	0 (0 to 0)	[illegible]
Periodontal diseases	0 (0 to 0)	[illegible]
Other oral disorders	0 (0 to 0)	[illegible]
Sudden infant death syndrome	–	[illegible]
Injuries	22,000 [illegible]	[illegible]
Transport injuries	1,610 [illegible]	[illegible]
Road injuries	1,430 [illegible]	[illegible]
Pedestrian road injuries	654 [illegible]	[illegible]
Cyclist road injuries	0 (0 to 0)	[illegible]
Motorcyclist road injuries	144 [illegible]	[illegible]
Motor vehicle road injuries	572 [illegible]	[illegible]
Other road injuries	56·8 [illegible]	[illegible]
Other transport injuries	188 [illegible]	[illegible]
Unintentional injuries	12,700 [illegible]	[illegible]
Falls	660 [illegible]	[illegible]
Drowning	629 [illegible]	[illegible]
Fire, heat, and hot substances	334 [illegible]	[illegible]
Poisonings	389 [illegible]	[illegible]
Poisoning by carbon monoxide	50·6 [illegible]	[illegible]
Poisoning by other means	339 [illegible]	[illegible]
Exposure to mechanical forces	1,300 [illegible]	[illegible]
Unintentional firearm injuries	587 [illegible]	[illegible]
Other exposure to mechanical forces	716 [illegible]	[illegible]
Adverse effects of medical treatment	1,990 [illegible]	[illegible]
Animal contact	108 [illegible]	[illegible]
Venomous animal contact	73 [illegible]	[illegible]
Non-venomous animal contact	34·6 [illegible]	[illegible]
Foreign body	6,340 [illegible]	[illegible]
Pulmonary aspiration and foreign body in airw[illegible]	6,290 [illegible]	[illegible]
Foreign body in eyes	1·2 [illegible]	[illegible]
Foreign body in other body part	49·3 [illegible]	[illegible]
Environmental heat and cold exposure	126 [illegible]	[illegible]
Exposure to forces of nature	4·2 [illegible]	[illegible]
Other unintentional injuries	854 [illegible]	[illegible]
Self-harm and interpersonal violence	7,680 [illegible]	[illegible]
Self-harm	0 (0 to 0)	[illegible]
Self-harm by firearm	0 (0 to 0)	[illegible]
Self-harm by other specified means	0 (0 to 0)	[illegible]
Interpersonal violence	7,680 [illegible]	[illegible]
Physical violence by firearm	835 [illegible]	[illegible]
Physical violence by sharp object	788 [illegible]	[illegible]
Sexual violence	0 (0 to 0)	[illegible]
Physical violence by other means	6,060 [illegible]	[illegible]
Conflict and terrorism	0 (0 to 0)	[illegible]

SR Table 2b: Caribbean DALYS by cause and age group for females with 95% uncertainty intervals, 2019

Cause	Early Neonatal	Late Neonatal	Post Neonatal	1 to 4	5 to 9	10 to 14	15 to 19	20 to 24	25 to 29	30 to 34	35 to 39
All causes	441,000 (346,000 to 553,000)	182,000 (144,000 to 226,000)	319,000 (243,000 to 413,000)	349,000 (297,000 to 405,000)	176,000 (145,000 to 208,000)	182,000 (147,000 to 223,000)	252,000 (206,000 to 307,000)	313,000 (261,000 to 373,000)	361,000 (300,000 to 429,000)	400,000 (335,000 to 471,000)	413,000 (349,000 to 485,000)
Communicable, maternal, neonatal, and nutritional diseases	367,000 (277,000 to 464,000)	138,000 (104,000 to 176,000)	223,000 (165,000 to 301,000)	190,000 (149,000 to 238,000)	59,300 (47,200 to 73,300)	41,400 (33,100 to 51,300)	53,400 (42,900 to 65,400)	70,100 (58,500 to 83,000)	86,100 (70,300 to 105,000)	95,300 (77,900 to 116,000)	91,300 (74,300 to 111,000)
HIV/AIDS and sexually transmitted infections	22,900 (7,820 to 48,200)	6,540 (2,200 to 13,800)	10,500 (5,830 to 18,800)	9,300 (6,720 to 13,000)	4,880 (3,220 to 6,930)	4,690 (3,280 to 6,310)	9,310 (5,450 to 14,800)	13,600 (9,430 to 20,100)	25,300 (16,600 to 38,300)	35,200 (24,900 to 50,400)	38,000 (27,400 to 53,000)
HIV/AIDS	0·4 (0·2 to 0·6)	1·3 (0·8 to 2·1)	3,760 (2,900 to 4,950)	8,980 (6,420 to 12,500)	4,700 (3,080 to 6,740)	4,610 (3,190 to 6,220)	8,980 (5,120 to 14,400)	12,900 (8,690 to 19,600)	24,300 (15,900 to 37,300)	34,000 (23,500 to 49,000)	36,800 (26,200 to 51,900)
HIV/AIDS - Drug-susceptible Tuberculosis	0 (0 to 0)	0·1 (0·1 to 0·1)	230 (140 to 361)	831 (502 to 1,320)	402 (248 to 631)	377 (233 to 591)	639 (399 to 992)	963 (628 to 1,460)	1,680 (1,080 to 2,550)	2,380 (1,540 to 3,630)	2,740 (1,760 to 4,230)
HIV/AIDS - Multidrug-resistant Tuberculosis without exte	0 (0 to 0)	0 (0 to 0)	3 (0·6 to 9·7)	9·8 (1·2 to 37·3)	4·7 (0·7 to 17·5)	4·4 (0·6 to 16·1)	7·6 (1·2 to 26·5)	12 (2·5 to 40·7)	20·7 (4·4 to 65·2)	28·8 (6 to 91·3)	32·3 (6·2 to 108)
HIV/AIDS - Extensively drug-resistant Tuberculosis	0 (0 to 0)	0 (0 to 0)	0·5 (0·1 to 1·7)	1·6 (0·2 to 6·1)	0·8 (0·1 to 2·9)	0·7 (0·1 to 2·7)	1·2 (0·2 to 4·4)	2 (0·4 to 6·5)	3·4 (0·6 to 11)	4·7 (0·9 to 15·4)	5·3 (0·9 to 17·7)
HIV/AIDS resulting in other diseases	0·4 (0·2 to 0·6)	1·3 (0·8 to 2)	3,520 (2,650 to 4,740)	8,130 (5,550 to 11,700)	4,300 (2,690 to 6,270)	4,230 (2,850 to 5,850)	8,340 (4,450 to 13,700)	11,900 (7,670 to 18,500)	22,600 (14,200 to 35,800)	31,600 (21,100 to 47,000)	34,000 (23,500 to 49,000)
Sexually transmitted infections excluding HIV	22,900 (7,820 to 48,200)	6,540 (2,200 to 13,800)	6,730 (2,300 to 14,600)	326 (112 to 718)	179 (61·2 to 362)	82·7 (44·5 to 150)	327 (199 to 520)	701 (422 to 1,130)	989 (584 to 1,650)	1,260 (756 to 2,060)	1,240 (721 to 2,060)
Syphilis	22,900 (7,820 to 48,200)	6,540 (2,200 to 13,800)	6,730 (2,300 to 14,600)	326 (112 to 718)	179 (61·2 to 362)	35·3 (16 to 74·1)	48 (26·6 to 81·1)	33·9 (19·2 to 57)	62·2 (36·6 to 105)	92·4 (55·3 to 163)	57 (34·4 to 96·9)
Chlamydial infection	0 (0 to 0)	0 (0 to 0)	0 (0 to 0)	0 (0 to 0)	0 (0 to 0)	8·4 (4·7 to 15·3)	34·6 (21·9 to 53·2)	104 (68·2 to 163)	103 (63·8 to 155)	110 (66·7 to 173)	137 (88·4 to 210)
Gonococcal infection	0 (0 to 0)	0 (0 to 0)	0 (0 to 0)	0 (0 to 0)	0 (0 to 0)	10·4 (5 to 21·1)	60·9 (34·3 to 106)	121 (71·6 to 209)	127 (78·5 to 212)	168 (105 to 285)	159 (100 to 264)
Trichomoniasis	0 (0 to 0)	0 (0 to 0)	0 (0 to 0)	0 (0 to 0)	0 (0 to 0)	2·2 (0·5 to 5·5)	36·3 (11·4 to 88·7)	166 (59 to 395)	317 (108 to 723)	359 (117 to 856)	316 (104 to 738)
Genital herpes	0 (0 to 0)	0 (0 to 0)	0 (0 to 0)	0 (0 to 0)	0 (0 to 0)	10·6 (3·5 to 24·9)	47·9 (14·7 to 112)	101 (33·1 to 244)	158 (55·1 to 383)	194 (65·7 to 471)	191 (61·4 to 474)
Other sexually transmitted infections	0 (0 to 0)	0 (0 to 0)	0 (0 to 0)	0 (0 to 0)	0 (0 to 0)	15·8 (8·8 to 29·6)	99·5 (61·9 to 162)	175 (114 to 272)	222 (144 to 332)	341 (223 to 496)	376 (245 to 563)
Respiratory infections and tuberculosis	22,400 (11,800 to 37,500)	23,700 (12,900 to 38,800)	59,400 (33,500 to 95,100)	48,100 (29,400 to 71,500)	12,800 (9,190 to 17,200)	7,810 (5,870 to 10,200)	7,320 (5,520 to 9,530)	11,100 (8,560 to 14,300)	13,000 (9,790 to 17,100)	12,900 (9,680 to 17,100)	11,400 (8,360 to 15,900)
Tuberculosis	0 (0 to 0)	0·1 (0 to 0·1)	2,590 (1,340 to 4,950)	5,070 (2,910 to 8,640)	1,710 (1,110 to 2,650)	1,520 (1,020 to 2,370)	2,070 (1,310 to 3,230)	4,950 (3,360 to 7,370)	6,320 (4,280 to 9,410)	5,940 (4,090 to 8,920)	4,740 (3,040 to 7,950)
Latent tuberculosis infection	0 (0 to 0)	0 (0 to 0)	0 (0 to 0)	0 (0 to 0)	0 (0 to 0)	0 (0 to 0)	0 (0 to 0)	0 (0 to 0)	0 (0 to 0)	0 (0 to 0)	0 (0 to 0)
Drug-susceptible tuberculosis	0 (0 to 0)	0·1 (0 to 0·1)	2,560 (1,320 to 4,930)	5,020 (2,880 to 8,620)	1,700 (1,110 to 2,640)	1,500 (1,010 to 2,350)	2,040 (1,290 to 3,210)	4,900 (3,340 to 7,300)	6,250 (4,210 to 9,260)	5,880 (4,060 to 8,870)	4,690 (2,970 to 7,870)
Multidrug-resistant tuberculosis without extensive drug r	0 (0 to 0)	0 (0 to 0)	24·7 (3·1 to 96·6)	46·5 (5·5 to 193)	14·6 (2·4 to 54·1)	13·9 (2·3 to 54·3)	20·1 (3·4 to 68·2)	45·2 (8·7 to 148)	57·1 (11·3 to 202)	53·3 (10·5 to 175)	42·5 (7·9 to 145)
Extensively drug-resistant tuberculosis	0 (0 to 0)	0 (0 to 0)	4·1 (0·5 to 15·6)	7·7 (0·8 to 31·2)	2·2 (0·3 to 9)	2·2 (0·3 to 8·4)	3·3 (0·5 to 12)	7·1 (1·2 to 24·4)	8·9 (1·5 to 32·9)	8·4 (1·4 to 29·9)	6·8 (1·1 to 24·4)
Lower respiratory infections	22,300 (11,700 to 37,400)	23,700 (12,900 to 38,800)	56,100 (30,800 to 91,200)	40,000 (22,200 to 62,400)	7,980 (5,010 to 11,700)	3,890 (2,640 to 5,700)	2,980 (2,070 to 4,260)	4,030 (2,720 to 5,670)	4,730 (3,190 to 6,670)	5,140 (3,430 to 7,410)	5,030 (3,400 to 7,330)
Upper respiratory infections	6·6 (3·2 to 15·2)	27·3 (15 to 44·4)	607 (320 to 1,130)	2,180 (1,210 to 3,520)	2,090 (1,180 to 3,490)	1,740 (930 to 2,970)	1,670 (889 to 2,960)	1,560 (849 to 2,650)	1,510 (838 to 2,530)	1,400 (752 to 2,390)	1,250 (655 to 2,100)
Otitis media	85·4 (0·6 to 515)	2·6 (1·1 to 5·6)	95·5 (49·3 to 174)	800 (433 to 1,410)	991 (555 to 1,610)	662 (363 to 1,150)	606 (331 to 1,030)	564 (258 to 1,090)	484 (235 to 916)	398 (181 to 736)	358 (175 to 650)
Enteric infections	4,430 (1,180 to 10,100)	16,300 (4,090 to 39,000)	78,000 (27,100 to 152,000)	55,400 (19,500 to 111,000)	6,440 (3,810 to 10,400)	3,890 (2,490 to 5,690)	2,310 (1,530 to 3,360)	2,160 (1,380 to 3,370)	2,510 (1,620 to 3,770)	2,760 (1,840 to 4,030)	3,030 (1,960 to 4,500)
Diarrheal diseases	4,430 (1,180 to 10,100)	16,300 (4,090 to 39,000)	77,900 (26,900 to 151,000)	55,100 (19,200 to 111,000)	6,270 (3,680 to 10,200)	3,770 (2,400 to 5,600)	2,250 (1,490 to 3,290)	2,120 (1,330 to 3,320)	2,480 (1,600 to 3,740)	2,740 (1,810 to 4,000)	3,010 (1,940 to 4,480)
Typhoid and paratyphoid	0 (0 to 0)	0 (0 to 0)	4·3 (1·4 to 10·3)	93·6 (35·8 to 193)	68·2 (25 to 145)	51·9 (20·3 to 102)	30·2 (12·9 to 62·2)	19·1 (8·2 to 37·6)	12·3 (4·9 to 24·9)	8·4 (3·5 to 17·6)	6·4 (2·7 to 13·3)
Typhoid fever	0 (0 to 0)	0 (0 to 0)	3·8 (1·3 to 9·3)	82·4 (31·8 to 169)	58·9 (21·4 to 124)	44 (17·1 to 86·9)	25·2 (10·3 to 53)	16·1 (6·5 to 32·5)	10·6 (4·2 to 21·7)	7·3 (3 to 15·7)	5·6 (2·4 to 11·8)
Paratyphoid fever	0 (0 to 0)	0 (0 to 0)	0·4 (0·1 to 1·1)	11·1 (3·4 to 26·5)	9·3 (3 to 23)	7·9 (2·7 to 18·8)	5·1 (1·8 to 11·1)	3 (1 to 6·8)	1·7 (0·6 to 3·8)	1·1 (0·4 to 2·5)	0·8 (0·3 to 1·8)
Invasive Non-typhoidal Salmonella (iNTS)	0 (0 to 0)	1·1 (0·1 to 3)	10 (1·2 to 27·8)	53·2 (16·3 to 116)	51·7 (15·4 to 109)	37·2 (11·1 to 86·7)	20·7 (3·6 to 53·7)	13·9 (1·9 to 42·4)	9·7 (2·3 to 25·9)	6·9 (1·4 to 19·8)	5·7 (0·8 to 16·2)
Other intestinal infectious diseases	0 (0 to 0)	0 (0 to 0)	78·3 (2·1 to 250)	142 (9·4 to 393)	42·1 (3 to 141)	22·8 (2·1 to 75·8)	10 (1 to 32·9)	9·1 (0·9 to 29·6)	4·5 (0·6 to 14·5)	10·8 (1·8 to 31)	5·4 (1 to 17·3)
Neglected tropical diseases and malaria	16 (7·3 to 35·6)	316 (89·9 to 874)	1,420 (687 to 3,140)	3,500 (1,880 to 6,640)	3,970 (2,450 to 6,360)	3,380 (1,980 to 5,880)	7,120 (3,690 to 13,900)	5,430 (3,120 to 9,350)	4,880 (2,950 to 8,340)	4,240 (2,610 to 7,080)	3,620 (2,210 to 6,220)
Malaria	0·1 (0 to 0·2)	249 (32 to 823)	617 (87·1 to 2,180)	1,380 (219 to 4,360)	931 (164 to 3,010)	954 (169 to 3,080)	3,170 (516 to 9,390)	1,700 (299 to 5,190)	1,360 (234 to 4,220)	1,120 (190 to 3,510)	1,030 (173 to 3,240)
Chagas disease	0 (0 to 0)	0 (0 to 0)	0 (0 to 0)	0 (0 to 0)	0 (0 to 0)	0 (0 to 0)	0·1 (0 to 0·1)	0·1 (0·1 to 0·2)	0·2 (0·1 to 0·3)	0·4 (0·2 to 0·6)	0·5 (0·3 to 0·7)
Leishmaniasis	0 (0 to 0)	0 (0 to 0)	0·4 (0·1 to 0·8)	2·5 (1 to 5·7)	2 (0·8 to 5·1)	0·9 (0·3 to 2·2)	0·7 (0·2 to 1·5)	0·6 (0·1 to 1·2)	0·6 (0·1 to 1·3)	0·5 (0·1 to 1·1)	0·5 (0·1 to 1·1)
Visceral leishmaniasis	0 (0 to 0)	0 (0 to 0)	0·1 (0 to 0·5)	0·7 (0 to 3·8)	0·5 (0 to 3·5)	0·2 (0 to 1·4)	0·1 (0 to 0·8)	0·1 (0 to 0·6)	0·2 (0 to 0·7)	0·1 (0 to 0·6)	0·1 (0 to 0·5)
Cutaneous and mucocutaneous leishmaniasis	0 (0 to 0)	0 (0 to 0)	0·2 (0·1 to 0·5)	1·8 (0·8 to 3·3)	1·6 (0·7 to 2·7)	0·7 (0·2 to 1·5)	0·5 (0·2 to 1·1)	0·4 (0·1 to 0·9)	0·4 (0·1 to 0·9)	0·4 (0·1 to 0·9)	0·4 (0·1 to 0·8)
African trypanosomiasis	0 (0 to 0)	0 (0 to 0)	0 (0 to 0)	0 (0 to 0)	0 (0 to 0)	0 (0 to 0)	0 (0 to 0)	0 (0 to 0)	0 (0 to 0)	0 (0 to 0)	0 (0 to 0)
Schistosomiasis	0 (0 to 0)	0 (0 to 0)	0 (0 to 0)	31·4 (7·6 to 77·5)	258 (74·8 to 672)	503 (146 to 1,230)	1,010 (399 to 2,080)	1,160 (471 to 2,310)	1,180 (489 to 2,300)	922 (388 to 1,730)	440 (170 to 971)
Cysticercosis	0 (0 to 0)	0 (0 to 0)	0 (0 to 0)	10·8 (0 to 39·7)	42·3 (0·2 to 132)	53·4 (5·4 to 132)	155 (36·3 to 339)	427 (167 to 870)	710 (314 to 1,320)	789 (350 to 1,450)	768 (347 to 1,390)
Cystic echinococcosis	0 (0 to 0)	0 (0 to 0)	0 (0 to 0)	0·8 (0 to 3)	0·8 (0 to 3·2)	0·6 (0·1 to 1·7)	0·5 (0·2 to 1·3)	0·6 (0·2 to 1·3)	0·7 (0·2 to 2)	0·8 (0·1 to 2·1)	0·7 (0·1 to 2·3)
Lymphatic filariasis	0 (0 to 0)	0 (0 to 0)	0 (0 to 0)	0 (0 to 0)	251 (157 to 379)	244 (152 to 365)	243 (154 to 372)	247 (159 to 362)	243 (152 to 362)	227 (144 to 331)	200 (126 to 298)
Onchocerciasis	0 (0 to 0)	0 (0 to 0)	0 (0 to 0)	0 (0 to 0)	0 (0 to 0)	0 (0 to 0)	0 (0 to 0)	0 (0 to 0)	0 (0 to 0)	0 (0 to 0)	0 (0 to 0)
Trachoma	0 (0 to 0)	0 (0 to 0)	0 (0 to 0)	0 (0 to 0)	0 (0 to 0)	0 (0 to 0)	0 (0 to 0)	0 (0 to 0)	0 (0 to 0)	0 (0 to 0)	0 (0 to 0)
Dengue	0 (0 to 0)	18·9 (3·3 to 71·4)	330 (65·3 to 941)	405 (92·5 to 1,210)	476 (135 to 1,000)	264 (94 to 488)	240 (101 to 436)	215 (95 to 376)	227 (101 to 417)	181 (78·7 to 323)	167 (78·4 to 283)
Yellow fever	0 (0 to 0)	0 (0 to 0)	0 (0 to 0·1)	0·2 (0 to 0·7)	0·4 (0·1 to 1·5)	0·4 (0 to 1·3)	0·3 (0 to 1·2)	0·3 (0 to 1·1)	0·3 (0 to 0·9)	0·2 (0 to 0·7)	0·2 (0 to 0·6)

Other cardiovascular and circulatory diseases	691 (282 to 1,600)	483 (190 to 1,090)	1,560 (503 to 3,850)	1,780 (619 to 3,980)	[illegible]	[illegible]	[illegible]	[illegible]	[illegible]	[illegible]	[illegible]	[illegible]
Chronic respiratory diseases	0 (0 to 0)	0 (0 to 0)	2·1 (1·4 to 2·9)	17,900 (10,300 to 27,800)	[illegible]	[illegible]	[illegible]	[illegible]	[illegible]	[illegible]	[illegible]	[illegible]
Chronic obstructive pulmonary disease	0 (0 to 0)	0 (0 to 0)	2·1 (1·4 to 2·9)	709 (196 to 1,940)	327 (190 to 554)	[illegible] (243 to 532)	[illegible] (522 to 1,060)	[illegible] (781 to 1,480)	[illegible] (985 to 1,900)	[illegible] (1,240 to 2,210)	[illegible] (1,520 to 2,680)	[illegible] (1,950 to 3,510)
Pneumoconiosis	0 (0 to 0)	0 (0 to 0)	0 (0 to 0)	0 (0 to 0)	0 (0 to 0)	0 (0 to 0)	7·5 (3·2 to 15·3)	3·5 (1·7 to 7·3)	3·2 (1·5 to 6·8)	3 (1·6 to 6·6)	8·3 (4·3 to 15·1)	8·7 (3·9 to 20·6)
Silicosis	0 (0 to 0)	0 (0 to 0)	0 (0 to 0)	0 (0 to 0)	0 (0 to 0)	0 (0 to 0)	0·6 (0·2 to 2)	0·5 (0·2 to 1·5)	0·4 (0·1 to 1·3)	0·4 (0·1 to 1·3)	1·3 (0·4 to 3·5)	1·1 (0·3 to 4)
Asbestosis	0 (0 to 0)	0 (0 to 0)	0 (0 to 0)	0 (0 to 0)	0 (0 to 0)	0 (0 to 0)	0·7 (0·3 to 1·8)	0·7 (0·3 to 1·4)	0·8 (0·3 to 1·5)	0·8 (0·3 to 1·6)	1·4 (0·6 to 3)	1·1 (0·4 to 2·8)
Coal workers pneumoconiosis	0 (0 to 0)	0 (0 to 0)	0 (0 to 0)	0 (0 to 0)	0 (0 to 0)	0 (0 to 0)	0·5 (0·1 to 2·1)	0·4 (0·1 to 2)	0·3 (0·1 to 1·5)	0·4 (0·1 to 1·6)	1·1 (0·2 to 3·9)	0·7 (0·1 to 2·6)
Other pneumoconiosis	0 (0 to 0)	0 (0 to 0)	0 (0 to 0)	0 (0 to 0)	0 (0 to 0)	0 (0 to 0)	5·7 (2·2 to 12·2)	1·8 (0·7 to 4·8)	1·7 (0·6 to 4·1)	1·4 (0·6 to 3·5)	4·5 (2 to 9·6)	5·9 (2·5 to 14·1)
Asthma	0 (0 to 0)	0 (0 to 0)	0 (0 to 0)	15,000 (8,470 to 24,100)	9,710 (6,230 to 14,700)	7,630 (4,910 to 11,500)	7,000 (4,560 to 10,700)	6,660 (4,410 to 9,410)	6,630 (4,450 to 9,330)	6,730 (4,750 to 9,070)	6,140 (4,450 to 8,250)	5,440 (3,920 to 7,250)
Interstitial lung disease and pulmonary sarcoidosis	0 (0 to 0)	0 (0 to 0)	0 (0 to 0)	245 (135 to 464)	99·9 (60·3 to 179)	67·1 (42·1 to 109)	69 (44·2 to 119)	106 (62·7 to 159)	182 (114 to 282)	283 (168 to 446)	342 (212 to 516)	322 (203 to 467)
Other chronic respiratory diseases	0 (0 to 0)	0 (0 to 0)	0 (0 to 0)	1,950 (569 to 5,720)	728 (389 to 1,340)	675 (443 to 1,030)	244 (167 to 349)	425 (300 to 624)	692 (458 to 1,110)	478 (333 to 728)	459 (320 to 701)	494 (332 to 742)
Digestive diseases	982 (467 to 1,880)	1,060 (416 to 2,310)	5,650 (2,140 to 12,400)	10,300 (3,940 to 20,600)	4,790 (3,020 to 6,950)	4,220 (3,020 to 5,630)	4,940 (3,650 to 6,390)	7,850 (5,550 to 10,500)	9,860 (6,910 to 13,400)	12,100 (8,550 to 16,300)	13,300 (9,560 to 17,800)	14,800 (11,200 to 19,400)
Cirrhosis and other chronic liver diseases	0 (0 to 0)	0 (0 to 0)	2·4 (1·2 to 4)	2,320 (560 to 5,510)	1,120 (529 to 2,010)	1,160 (646 to 1,820)	1,340 (780 to 2,120)	2,030 (1,110 to 3,110)	2,370 (1,240 to 3,730)	3,370 (1,930 to 5,080)	4,250 (2,530 to 6,370)	5,340 (3,520 to 7,690)
Cirrhosis and other chronic liver diseases due to hepati[illegible]	0 (0 to 0)	0 (0 to 0)	0 (0 to 0)	2·8 (0·4 to 8·9)	3·4 (0·7 to 8·7)	6·2 (1·5 to 14·4)	10·8 (2·7 to 25)	56·8 (18·2 to 134)	130 (35·4 to 317)	273 (107 to 546)	435 (167 to 910)	568 (243 to 1,050)
Cirrhosis and other chronic liver diseases due to hepati[illegible]	0 (0 to 0)	0 (0 to 0)	0 (0 to 0)	8·2 (1·5 to 23·8)	10·7 (3·5 to 23·8)	20·4 (7·4 to 46·1)	36·4 (12·8 to 82·5)	187 (69·8 to 382)	414 (147 to 871)	742 (358 to 1,310)	1,000 (460 to 1,880)	1,250 (654 to 2,180)
Cirrhosis and other chronic liver diseases due to alcoh[illegible]	0 (0 to 0)	0 (0 to 0)	0 (0 to 0)	0 (0 to 0)	0 (0 to 0)	0 (0 to 0)	3·5 (0·8 to 10·7)	91·9 (30·1 to 227)	244 (85·8 to 562)	641 (295 to 1,170)	1,150 (508 to 2,060)	1,670 (889 to 2,690)
Cirrhosis and other chronic liver diseases due to NAFL[illegible]	0 (0 to 0)	0 (0 to 0)	0 (0 to 0)	0 (0 to 0)	0 (0 to 0)	0 (0 to 0)	46·9 (17·8 to 104)	181 (68·3 to 368)	366 (128 to 753)	608 (250 to 1,180)	767 (321 to 1,540)	1,000 (466 to 1,810)
Cirrhosis and other chronic liver diseases due to other [illegible]	0 (0 to 0)	0 (0 to 0)	2·4 (1·2 to 4)	2,310 (558 to 5,490)	1,110 (524 to 1,990)	1,130 (629 to 1,780)	1,250 (719 to 1,950)	1,520 (804 to 2,370)	1,220 (559 to 2,070)	1,110 (523 to 2,030)	898 (391 to 1,690)	856 (372 to 1,590)
Upper digestive system diseases	0 (0 to 0)	0 (0 to 0)	0 (0 to 0)	1,130 (381 to 2,420)	522 (298 to 849)	648 (417 to 967)	1,360 (821 to 2,190)	2,940 (1,700 to 5,060)	4,050 (2,330 to 6,840)	4,740 (2,760 to 8,010)	4,610 (2,670 to 7,510)	4,550 (2,740 to 7,310)
Peptic ulcer disease	0 (0 to 0)	0 (0 to 0)	0 (0 to 0)	820 (191 to 1,930)	226 (90·8 to 440)	266 (135 to 470)	199 (115 to 336)	389 (215 to 629)	421 (220 to 690)	647 (357 to 1,030)	759 (438 to 1,270)	814 (475 to 1,340)
Gastritis and duodenitis	0 (0 to 0)	0 (0 to 0)	0 (0 to 0)	311 (137 to 615)	291 (138 to 495)	249 (119 to 466)	306 (145 to 568)	383 (199 to 664)	521 (278 to 831)	639 (347 to 1,070)	667 (366 to 1,080)	707 (374 to 1,230)
Gastroesophageal reflux disease	0 (0 to 0)	0 (0 to 0)	0 (0 to 0)	0 (0 to 0)	4·5 (1·6 to 9·8)	133 (58·5 to 253)	855 (414 to 1,570)	2,170 (1,070 to 4,170)	3,110 (1,520 to 5,770)	3,450 (1,620 to 6,640)	3,180 (1,490 to 5,870)	3,020 (1,440 to 5,460)
Appendicitis	0 (0 to 0)	0 (0 to 0)	0 (0 to 0)	1,380 (377 to 3,380)	929 (433 to 1,760)	785 (472 to 1,230)	566 (345 to 887)	544 (300 to 910)	428 (234 to 712)	397 (220 to 677)	565 (312 to 939)	343 (198 to 561)
Paralytic ileus and intestinal obstruction	533 (257 to 1,040)	836 (343 to 1,800)	4,640 (1,910 to 9,820)	1,380 (554 to 3,020)	472 (261 to 792)	382 (225 to 611)	215 (137 to 333)	191 (125 to 281)	251 (162 to 390)	269 (179 to 415)	465 (309 to 694)	522 (362 to 771)
Inguinal, femoral, and abdominal hernia	449 (159 to 1,030)	226 (52·5 to 713)	1,000 (182 to 3,120)	1,020 (470 to 2,110)	884 (489 to 1,430)	593 (322 to 954)	475 (280 to 752)	425 (251 to 662)	446 (256 to 718)	521 (314 to 824)	534 (313 to 824)	592 (374 to 888)
Inflammatory bowel disease	0 (0 to 0)	0 (0 to 0)	0 (0 to 0)	585 (84 to 1,730)	111 (34·2 to 250)	30·3 (14 to 57·4)	67·1 (37·9 to 115)	137 (78·4 to 231)	261 (146 to 442)	281 (175 to 448)	335 (217 to 522)	465 (306 to 719)
Vascular intestinal disorders	0 (0 to 0)	0 (0 to 0)	0 (0 to 0)	35·4 (10·8 to 91·8)	40 (17 to 90·1)	3·6 (1·7 to 7·2)	8·3 (4·6 to 15)	36·1 (18·9 to 67·9)	60 (33·5 to 106)	53·1 (31·1 to 90·6)	92·1 (58·1 to 151)	155 (105 to 232)
Gallbladder and biliary diseases	0 (0 to 0)	0 (0 to 0)	0 (0 to 0)	240 (89·4 to 546)	220 (120 to 353)	296 (163 to 488)	538 (331 to 822)	905 (560 to 1,350)	1,250 (788 to 1,830)	1,680 (1,100 to 2,450)	1,840 (1,220 to 2,640)	2,000 (1,340 to 2,840)
Pancreatitis	0 (0 to 0)	0 (0 to 0)	0 (0 to 0)	129 (35·1 to 311)	32·5 (16·5 to 61·4)	55·2 (33·2 to 85·4)	135 (88·2 to 204)	402 (254 to 609)	382 (242 to 610)	411 (271 to 639)	314 (214 to 469)	444 (307 to 624)
Other digestive diseases	0 (0 to 0)	0 (0 to 0)	0 (0 to 0)	2,030 (672 to 5,570)	456 (232 to 943)	268 (159 to 466)	228 (130 to 411)	244 (136 to 430)	348 (185 to 633)	363 (203 to 633)	342 (201 to 572)	417 (247 to 704)
Neurological disorders	6 (2·5 to 11·4)	45·3 (23·7 to 82·7)	1,720 (931 to 2,800)	5,680 (3,100 to 8,630)	7,540 (4,150 to 12,200)	17,600 (5,750 to 36,800)	22,500 (7,420 to 48,200)	23,500 (8,140 to 48,300)	24,000 (8,070 to 48,300)	23,600 (8,150 to 47,200)	21,600 (7,730 to 42,100)	20,500 (7,730 to 40,700)
Alzheimer's disease and other dementias	0 (0 to 0)	0 (0 to 0)	0 (0 to 0)	0 (0 to 0)	0 (0 to 0)	0 (0 to 0)	0 (0 to 0)	0 (0 to 0)	0 (0 to 0)	0 (0 to 0)	0 (0 to 0)	105 (23·5 to 335)
Parkinson's disease	0 (0 to 0)	0 (0 to 0)	0 (0 to 0)	0 (0 to 0)	0 (0 to 0)	0 (0 to 0)	0 (0 to 0)	7·6 (5 to 10·5)	12·3 (7·8 to 19)	28·5 (16·9 to 45·4)	46·4 (31 to 68·8)	109 (81·1 to 147)
Idiopathic epilepsy	5·4 (2 to 10·8)	15·8 (5·9 to 31·9)	839 (424 to 1,420)	3,770 (1,920 to 6,190)	3,740 (2,230 to 5,800)	3,990 (2,390 to 6,220)	4,410 (2,730 to 6,700)	4,630 (2,930 to 6,910)	4,390 (2,710 to 6,580)	4,100 (2,690 to 6,050)	3,420 (2,250 to 4,870)	2,840 (1,940 to 4,070)
Multiple sclerosis	0 (0 to 0)	0 (0 to 0)	0 (0 to 0)	0 (0 to 0)	0·7 (0·3 to 1·6)	9·4 (3·8 to 28·5)	38·9 (23·7 to 75·7)	113 (76·6 to 196)	193 (134 to 302)	264 (193 to 382)	416 (310 to 592)	436 (325 to 620)
Motor neuron disease	0·6 (0·3 to 1·2)	29·5 (12·8 to 66·9)	202 (78·2 to 472)	224 (90·2 to 520)	32·3 (24·6 to 51)	39 (30·4 to 51·3)	23·7 (19·1 to 29·4)	29·1 (23·3 to 36·7)	32·5 (25·1 to 42·6)	43·5 (32·6 to 58·3)	72·9 (54 to 96·7)	143 (109 to 187)
Headache disorders	0 (0 to 0)	0 (0 to 0)	0 (0 to 0)	0 (0 to 0)	2,540 (39·5 to 6,800)	12,000 (548 to 30,200)	16,800 (1,800 to 41,800)	17,700 (2,340 to 41,800)	18,500 (2,700 to 41,900)	18,300 (3,180 to 41,100)	16,800 (3,050 to 37,700)	16,000 (3,290 to 35,800)
Migraine	0 (0 to 0)	0 (0 to 0)	0 (0 to 0)	0 (0 to 0)	2,360 (27·6 to 6,300)	11,300 (353 to 30,000)	15,700 (1,220 to 41,100)	16,500 (1,590 to 40,100)	17,100 (1,880 to 40,700)	16,900 (2,180 to 39,700)	15,400 (2,230 to 35,900)	14,700 (2,410 to 34,200)
Tension-type headache	0 (0 to 0)	0 (0 to 0)	0 (0 to 0)	0 (0 to 0)	182 (5 to 1,180)	642 (56·8 to 4,220)	1,040 (217 to 4,520)	1,230 (289 to 4,970)	1,360 (323 to 5,080)	1,400 (366 to 4,700)	1,380 (391 to 4,340)	1,350 (412 to 3,850)
Other neurological disorders	0 (0 to 0)	0 (0 to 0)	683 (364 to 1,320)	1,680 (878 to 3,020)	1,230 (725 to 1,910)	1,570 (961 to 2,430)	1,280 (780 to 2,030)	942 (598 to 1,430)	865 (589 to 1,230)	851 (612 to 1,150)	824 (608 to 1,110)	856 (657 to 1,120)
Mental disorders	3·9 (2·2 to 6·1)	11·7 (6·6 to 18·1)	204 (121 to 316)	1,300 (799 to 1,910)	7,650 (5,060 to 11,300)	24,700 (16,400 to 35,500)	41,200 (28,300 to 57,100)	47,600 (32,800 to 65,900)	49,300 (33,600 to 68,100)	48,100 (33,800 to 65,700)	44,200 (31,700 to 59,200)	41,100 (29,500 to 56,200)
Schizophrenia	0 (0 to 0)	0 (0 to 0)	0 (0 to 0)	0 (0 to 0)	0 (0 to 0)	100 (42·6 to 195)	764 (392 to 1,310)	2,640 (1,500 to 4,300)	4,640 (2,760 to 6,770)	5,650 (3,550 to 8,160)	5,470 (3,490 to 7,850)	5,030 (3,390 to 6,840)
Depressive disorders	0 (0 to 0)	0 (0 to 0)	0 (0 to 0)	4·7 (1·9 to 10·2)	558 (231 to 1,060)	6,080 (3,200 to 10,800)	15,800 (9,220 to 25,200)	19,800 (11,600 to 30,700)	18,900 (11,200 to 28,700)	18,100 (10,900 to 28,100)	17,400 (11,100 to 25,600)	17,000 (10,800 to 25,500)
Major depressive disorder	0 (0 to 0)	0 (0 to 0)	0 (0 to 0)	3·9 (1·4 to 9·6)	504 (188 to 1,010)	5,690 (2,870 to 10,300)	14,700 (8,410 to 23,600)	17,800 (9,820 to 28,500)	16,400 (9,490 to 25,700)	15,400 (8,840 to 25,200)	14,800 (9,080 to 22,400)	14,500 (8,790 to 22,500)
Dysthymia	0 (0 to 0)	0 (0 to 0)	0 (0 to 0)	0·7 (0·2 to 1·9)	54·3 (14·7 to 122)	388 (167 to 722)	1,130 (570 to 1,980)	1,950 (1,020 to 3,480)	2,480 (1,320 to 4,230)	2,690 (1,520 to 4,380)	2,570 (1,440 to 4,260)	2,450 (1,310 to 4,190)
Bipolar disorder	0 (0 to 0)	0 (0 to 0)	0 (0 to 0)	0 (0 to 0)	0 (0 to 0)	1,450 (635 to 2,540)	5,270 (2,580 to 9,250)	6,370 (3,290 to 10,600)	6,300 (3,290 to 10,600)	5,690 (3,100 to 9,280)	4,910 (2,750 to 8,000)	4,490 (2,570 to 7,190)
Anxiety disorders	0 (0 to 0)	0 (0 to 0)	233 (104 to 426)	3,460 (1,820 to 5,930)	8,730 (4,910 to 14,400)	11,800 (7,200 to 18,000)	12,700 (7,310 to 20,500)	13,100 (7,300 to 21,500)	12,800 (7,560 to 20,200)	11,500 (6,880 to 17,600)	10,600 (6,190 to 16,600)	[illegible]
Eating disorders	0 (0 to 0)	0 (0 to 0)	0 (0 to 0)	0 (0 to 0)	35·9 (12·1 to 72·4)	529 (283 to 859)	2,000 (1,050 to 3,490)	3,200 (1,720 to 5,320)	2,970 (1,660 to 4,820)	2,240 (1,260 to 3,530)	1,480 (814 to 2,360)	864 (493 to 1,440)
Anorexia nervosa	0 (0 to 0)	0 (0 to 0)	0 (0 to 0)	0 (0 to 0)	32·7 (9·9 to 70·1)	285 (151 to 471)	795 (402 to 1,400)	807 (428 to 1,420)	621 (344 to 1,030)	387 (203 to 655)	254 (133 to 432)	184 (94·1 to 305)
Bulimia nervosa	0 (0 to 0)	0 (0 to 0)	0 (0 to 0)	0 (0 to 0)	3·2 (1 to 7·4)	243 (91 to 481)	1,210 (543 to 2,420)	2,390 (1,120 to 4,270)	2,350 (1,180 to 4,070)	1,850 (977 to 3,070)	1,220 (633 to 2,070)	681 (364 to 1,170)
Autism spectrum disorders	2·5 (1·6 to 3·7)	7·4 (4·7 to 11·2)	119 (75 to 177)	505 (324 to 756)	605 (387 to 910)	565 (368 to 846)	550 (352 to 823)	521 (331 to 764)	501 (319 to 741)	461 (296 to 690)	393 (255 to 585)	344 (221 to 505)
Attention-deficit/hyperactivity disorder	0 (0 to 0)	0 (0 to 0)	0 (0 to 0)	67·7 (34·1 to 124)	715 (381 to 1,300)	939 (511 to 1,670)	769 (420 to 1,380)	553 (302 to 994)	415 (221 to 742)	314 (161 to 550)	226 (115 to 389)	168 (86·4 to 292)
Conduct disorder	0 (0 to 0)	0 (0 to 0)	0 (0 to 0)	0 (0 to 0)	1,610 (787 to 2,910)	5,600 (2,870 to 9,640)	3,170 (1,580 to 5,600)	0 (0 to 0)	0 (0 to 0)	0 (0 to 0)	0 (0 to 0)	0 (0 to 0)
Idiopathic developmental intellectual disability	1·4 (0·2 to 3)	4·3 (0·6 to 9)	84·6 (18 to 170)	492 (197 to 885)	666 (307 to 1,170)	593 (250 to 1,060)	550 (234 to 981)	506 (203 to 903)	475 (197 to 846)	429 (174 to 768)	352 (148 to 629)	289 (118 to 514)
Other mental disorders	0 (0 to 0)	0 (0 to 0)	0 (0 to 0)	0 (0 to 0)	0 (0 to 0)	77·7 (38·1 to 133)	515 (248 to 886)	1,270 (662 to 2,170)	1,960 (1,110 to 3,140)	2,400 (1,440 to 3,710)	2,420 (1,470 to 3,770)	2,320 (1,440 to 3,540)
Substance use disorders	0 (0 to 0)	0 (0 to 0)	0·3 (0·2 to 0·5)	1·9 (1·1 to 3)	2·4 (1·4 to 3·8)	182 (97·8 to 308)	3,270 (2,130 to 4,630)	6,740 (4,410 to 9,520)	7,170 (4,900 to 9,850)	6,420 (4,360 to 8,970)	5,410 (3,860 to 7,380)	4,360 (3,180 to 5,850)

Other transport injuries	96·3 (26·1 to 249)	111 (35·7 to 261)	149 (62·5 to 346)	448 (208 to 990)	412 (243 to 800)	420 (282 to 779)	520 (369 to 851)	572 (418 to 926)
Unintentional injuries	3,860 (2,030 to 9,110)	4,130 (1,670 to 19,800)	14,300 (7,890 to 23,400)	37,000 (21,600 to 55,600)	19,800 (14,600 to 25,500)	17,900 (14,000 to 22,600)	15,300 (12,000 to 19,200)	17,700 (13,700 to 22,800)
Falls	52·9 (23·2 to 118)	35·8 (14·8 to 84)	378 (160 to 948)	1,180 (529 to 2,920)	911 (617 to 1,360)	982 (718 to 1,310)	1,130 (810 to 1,530)	1,360 (963 to 1,880)
Drowning	171 (97·5 to 289)	154 (70·2 to 291)	1,570 (591 to 3,540)	14,900 (5,740 to 26,800)	7,220 (3,790 to 11,900)	4,860 (2,920 to 7,250)	2,350 (1,430 to 3,560)	1,380 (919 to 2,080)
Fire, heat, and hot substances	98·3 (40·3 to 179)	163 (55·2 to 341)	1,410 (462 to 3,030)	6,450 (1,790 to 13,400)	3,220 (1,680 to 5,300)	2,300 (1,550 to 3,130)	2,550 (1,780 to 3,450)	2,860 (2,000 to 3,840)
Poisonings	67 (28·1 to 136)	141 (51·6 to 345)	408 (138 to 1,030)	2,040 (645 to 4,320)	544 (294 to 919)	286 (190 to 417)	329 (220 to 477)	380 (263 to 531)
Poisoning by carbon monoxide	29·8 (8·1 to 82·2)	24·9 (4·4 to 82·4)	72 (14·1 to 254)	643 (165 to 1,770)	185 (73 to 420)	97·9 (53·4 to 186)	142 (79·1 to 248)	178 (105 to 288)
Poisoning by other means	37·3 (12·9 to 84·3)	116 (40·3 to 280)	336 (100 to 858)	1,390 (311 to 3,290)	360 (157 to 658)	188 (117 to 287)	187 (123 to 280)	202 (132 to 293)
Exposure to mechanical forces	1,010 (37·8 to 6,020)	1,880 (21·7 to 18,000)	862 (126 to 4,200)	2,600 (998 to 5,370)	2,510 (1,240 to 4,600)	2,080 (1,140 to 3,810)	1,890 (1,160 to 2,950)	2,140 (1,420 to 3,260)
Unintentional firearm injuries	492 (11·7 to 2,890)	533 (2 to 4,450)	302 (20·1 to 1,790)	653 (121 to 2,120)	667 (197 to 1,770)	795 (305 to 2,020)	814 (409 to 1,750)	947 (531 to 1,870)
Other exposure to mechanical forces	516 (12·7 to 3,560)	1,350 (13·3 to 13,900)	561 (89 to 2,840)	1,950 (666 to 4,140)	1,840 (800 to 3,470)	1,290 (699 to 2,310)	1,080 (688 to 1,680)	1,190 (781 to 1,810)
Adverse effects of medical treatment	581 (336 to 971)	532 (273 to 929)	1,750 (727 to 3,310)	1,780 (748 to 3,330)	827 (458 to 1,340)	506 (342 to 739)	545 (375 to 768)	1,010 (653 to 1,460)
Animal contact	5·6 (2·4 to 10)	5·7 (1·8 to 11·9)	70·3 (25·9 to 150)	224 (94·3 to 441)	217 (124 to 348)	181 (121 to 255)	187 (127 to 257)	203 (138 to 275)
Venomous animal contact	3·3 (1·4 to 6·1)	1·5 (0·6 to 3·3)	38·4 (15·8 to 82)	107 (52 to 212)	117 (71·3 to 187)	131 (86·1 to 189)	130 (85·8 to 182)	149 (101 to 204)
Non-venomous animal contact	2·3 (0·8 to 4·7)	4·2 (1·1 to 9·1)	31·9 (8·2 to 81·4)	116 (33·9 to 270)	100 (44·1 to 187)	50·2 (31 to 77·6)	57·8 (36·6 to 85·5)	54·4 (34·8 to 81·4)
Foreign body	1,310 (700 to 2,360)	890 (490 to 1,530)	7,220 (3,610 to 12,900)	5,600 (3,310 to 9,390)	1,830 (1,270 to 2,560)	1,100 (805 to 1,420)	539 (415 to 685)	695 (523 to 902)
Pulmonary aspiration and foreign body in airway	1,300 (697 to 2,350)	885 (488 to 1,520)	7,170 (3,580 to 12,800)	5,460 (3,150 to 9,250)	1,670 (1,120 to 2,390)	932 (668 to 1,230)	362 (272 to 479)	494 (366 to 661)
Foreign body in eyes	0·1 (0 to 0·2)	0·3 (0·1 to 0·6)	4·5 (1·2 to 10)	23·8 (7·1 to 54)	37·3 (10·6 to 85·3)	45·5 (14·2 to 103)	60·2 (19·8 to 136)	76·1 (25·2 to 167)
Foreign body in other body part	5·9 (2 to 16·4)	3·9 (1·6 to 10·2)	48·1 (17·7 to 131)	125 (70·8 to 234)	124 (85·8 to 176)	120 (87·1 to 162)	117 (85·3 to 156)	125 (92·1 to 165)
Environmental heat and cold exposure	85·1 (16 to 250)	120 (38·8 to 305)	188 (65·2 to 460)	719 (328 to 1,530)	761 (410 to 1,580)	599 (378 to 966)	578 (401 to 813)	658 (459 to 914)
Exposure to forces of nature	0·6 (0·5 to 0·6)	2·2 (2 to 2·4)	18·1 (16·5 to 20)	103 (91·5 to 123)	497 (334 to 728)	3,840 (2,360 to 6,370)	4,090 (2,440 to 6,630)	5,730 (3,390 to 9,570)
Other unintentional injuries	482 (212 to 1,000)	205 (78·2 to 445)	447 (249 to 811)	1,400 (779 to 2,650)	1,310 (870 to 2,050)	1,160 (806 to 1,690)	1,070 (748 to 1,500)	1,240 (874 to 1,730)
Self-harm and interpersonal violence	1,620 (792 to 3,150)	409 (194 to 798)	1,300 (709 to 2,330)	3,130 (1,610 to 5,960)	2,650 (1,560 to 4,360)	4,830 (3,320 to 6,760)	15,300 (11,400 to 20,200)	20,000 (15,300 to 25,900)
Self-harm	0 (0 to 0)	0 (0 to 0)	0 (0 to 0)	0 (0 to 0)	0 (0 to 0)	1,510 (1,020 to 2,160)	7,000 (4,770 to 10,100)	7,880 (5,310 to 11,400)
Self-harm by firearm	0 (0 to 0)	0 (0 to 0)	0 (0 to 0)	0 (0 to 0)	0 (0 to 0)	26·2 (16·6 to 42·4)	237 (104 to 505)	323 (159 to 696)
Self-harm by other specified means	0 (0 to 0)	0 (0 to 0)	0 (0 to 0)	0 (0 to 0)	0 (0 to 0)	1,480 (1,000 to 2,130)	6,760 (4,650 to 9,820)	7,560 (5,120 to 10,900)
Interpersonal violence	1,620 (792 to 3,150)	409 (194 to 798)	1,290 (705 to 2,330)	3,100 (1,580 to 5,930)	2,630 (1,540 to 4,330)	3,290 (1,990 to 5,110)	8,220 (5,420 to 12,300)	12,100 (8,380 to 17,400)
Physical violence by firearm	56·8 (24·1 to 121)	15·3 (5·2 to 39·2)	53·7 (25·6 to 116)	394 (168 to 933)	420 (205 to 884)	758 (365 to 1,410)	3,040 (1,750 to 5,060)	4,450 (2,760 to 7,060)
Physical violence by sharp object	89·6 (32·9 to 213)	5·7 (2·3 to 14·4)	53·5 (26 to 105)	199 (104 to 428)	259 (150 to 471)	513 (296 to 904)	2,290 (1,450 to 3,770)	3,810 (2,590 to 5,680)
Sexual violence	0 (0 to 0)	0 (0 to 0)	0 (0 to 0)	19·9 (11·7 to 31·9)	162 (98·4 to 254)	357 (208 to 562)	835 (477 to 1,320)	1,110 (645 to 1,680)
Physical violence by other means	1,480 (717 to 2,830)	388 (183 to 765)	1,190 (644 to 2,140)	2,490 (1,210 to 4,820)	1,790 (966 to 3,120)	1,670 (896 to 2,890)	2,050 (1,180 to 3,350)	2,730 (1,650 to 4,340)
Conflict and terrorism	0 (0 to 0)	0 (0 to 0)	0 (0 to 0)	1·8 (0·9 to 3·2)	2 (1 to 3·9)	7·5 (4·2 to 13)	39·5 (19 to 74·7)	28 (14·3 to 51·7)

Other transport injuries	557 (401 to 869)	469 (339 to 719)	393 (289 to 627)	423 (320 to 657)	325 (252 to 447)	321 (252 to 420)	288 (225 to 382)	208 (165 to 275)
Unintentional injuries	15,500 (11,900 to 19,400)	15,300 (12,200 to 19,100)	14,600 (11,500 to 18,400)	13,300 (10,500 to 16,900)	12,900 (10,200 to 16,400)	12,800 (10,100 to 16,100)	12,200 (9,680 to 15,300)	11,200 (9,000 to 13,900)
Falls	1,600 (1,130 to 2,190)	1,740 (1,240 to 2,380)	1,800 (1,310 to 2,430)	1,900 (1,380 to 2,580)	2,350 (1,730 to 3,140)	2,900 (2,190 to 3,820)	3,240 (2,470 to 4,190)	3,680 (2,880 to 4,570)
Drowning	1,270 (788 to 1,850)	1,050 (708 to 1,530)	886 (582 to 1,300)	636 (434 to 885)	505 (367 to 700)	364 (279 to 487)	287 (219 to 373)	221 (173 to 284)
Fire, heat, and hot substances	3,250 (2,200 to 4,440)	3,270 (2,250 to 4,350)	3,070 (2,150 to 4,120)	2,650 (1,850 to 3,560)	2,470 (1,760 to 3,340)	2,360 (1,690 to 3,290)	2,120 (1,500 to 2,920)	1,650 (1,170 to 2,290)
Poisonings	342 (232 to 492)	311 (217 to 438)	288 (195 to 415)	252 (175 to 345)	209 (149 to 285)	181 (132 to 239)	157 (114 to 207)	118 (87 to 157)
Poisoning by carbon monoxide	144 (83·8 to 250)	135 (81 to 230)	107 (61·6 to 189)	104 (60·6 to 176)	72·9 (45·3 to 118)	57 (37 to 85·1)	59 (39·3 to 85·2)	42·7 (28·5 to 63·3)
Poisoning by other means	198 (132 to 288)	176 (118 to 250)	181 (121 to 264)	148 (101 to 206)	136 (94·4 to 191)	124 (86 to 169)	98·4 (69 to 136)	75·7 (52·8 to 105)
Exposure to mechanical forces	2,120 (1,470 to 3,100)	2,110 (1,480 to 3,000)	2,020 (1,370 to 2,940)	1,890 (1,300 to 2,710)	1,840 (1,260 to 2,600)	1,820 (1,290 to 2,610)	1,610 (1,130 to 2,290)	1,290 (910 to 1,840)
Unintentional firearm injuries	777 (452 to 1,450)	761 (449 to 1,360)	657 (391 to 1,240)	541 (329 to 974)	415 (274 to 668)	331 (232 to 507)	274 (195 to 400)	202 (146 to 284)
Other exposure to mechanical forces	1,340 (902 to 1,980)	1,350 (908 to 1,990)	1,360 (914 to 2,000)	1,350 (917 to 1,960)	1,430 (961 to 2,090)	1,490 (1,020 to 2,170)	1,330 (923 to 1,970)	1,090 (750 to 1,610)
Adverse effects of medical treatment	1,010 (644 to 1,440)	1,210 (759 to 1,830)	1,000 (676 to 1,420)	1,060 (740 to 1,520)	1,190 (834 to 1,690)	1,110 (799 to 1,590)	1,150 (822 to 1,620)	1,140 (822 to 1,560)
Animal contact	222 (154 to 302)	208 (144 to 287)	199 (139 to 274)	190 (136 to 255)	207 (148 to 278)	194 (137 to 265)	201 (146 to 268)	164 (122 to 218)
Venomous animal contact	170 (116 to 236)	155 (105 to 218)	139 (94·5 to 191)	134 (94·8 to 182)	133 (93·2 to 181)	121 (82·8 to 167)	120 (84·5 to 164)	104 (76·4 to 138)
Non-venomous animal contact	52·3 (34·2 to 77·8)	52·7 (34·1 to 79·7)	59·9 (39·5 to 88·5)	55·7 (36·7 to 84·2)	73·8 (51·1 to 104)	72·9 (49·8 to 104)	80 (56·9 to 112)	60 (41·6 to 86·3)
Foreign body	709 (550 to 901)	652 (506 to 859)	628 (482 to 824)	596 (465 to 762)	589 (460 to 737)	637 (505 to 797)	616 (501 to 744)	593 (488 to 722)
Pulmonary aspiration and foreign body in airway	500 (373 to 658)	442 (328 to 597)	431 (318 to 580)	405 (304 to 537)	386 (297 to 494)	443 (340 to 573)	450 (358 to 564)	449 (357 to 564)
Foreign body in eyes	86·2 (31·5 to 181)	89·3 (33·1 to 181)	85·1 (34·5 to 173)	83·4 (37 to 165)	83·8 (42·2 to 153)	75·5 (40·6 to 131)	63·2 (37·1 to 101)	50·9 (31·2 to 77·4)
Foreign body in other body part	123 (90·4 to 161)	121 (89·9 to 163)	112 (82 to 147)	108 (80·9 to 141)	119 (88·7 to 156)	118 (88·4 to 154)	103 (76·5 to 137)	92·8 (69·3 to 121)
Environmental heat and cold exposure	702 (493 to 985)	752 (525 to 1,050)	679 (482 to 956)	646 (457 to 908)	676 (470 to 940)	678 (473 to 946)	657 (451 to 944)	571 (389 to 818)
Exposure to forces of nature	2,900 (1,740 to 4,740)	2,710 (1,630 to 4,180)	2,800 (1,670 to 4,350)	2,340 (1,420 to 3,770)	1,710 (1,040 to 2,720)	1,370 (833 to 2,200)	1,100 (693 to 1,740)	932 (588 to 1,510)
Other unintentional injuries	1,320 (929 to 1,830)	1,270 (895 to 1,760)	1,180 (848 to 1,660)	1,130 (797 to 1,560)	1,190 (838 to 1,660)	1,180 (814 to 1,650)	1,070 (738 to 1,500)	851 (599 to 1,190)
Self-harm and interpersonal violence	17,800 (13,400 to 23,700)	15,500 (11,800 to 20,400)	11,900 (9,040 to 16,100)	9,060 (6,830 to 12,100)	7,480 (5,890 to 9,440)	5,740 (4,660 to 7,140)	4,470 (3,660 to 5,450)	3,160 (2,640 to 3,830)
Self-harm	6,060 (4,300 to 8,370)	5,370 (3,970 to 7,030)	4,240 (3,150 to 5,650)	3,390 (2,660 to 4,390)	3,310 (2,660 to 4,200)	2,990 (2,370 to 3,730)	2,610 (2,110 to 3,240)	1,890 (1,550 to 2,340)
Self-harm by firearm	190 (102 to 337)	247 (152 to 404)	146 (89 to 260)	79·4 (53·4 to 122)	67·5 (47·6 to 96·7)	59·5 (44·1 to 80·5)	45·3 (34·8 to 61·6)	19·8 (15·1 to 26·8)
Self-harm by other specified means	5,870 (4,170 to 8,150)	5,120 (3,790 to 6,680)	4,090 (3,040 to 5,420)	3,310 (2,590 to 4,270)	3,240 (2,610 to 4,100)	2,930 (2,320 to 3,660)	2,560 (2,080 to 3,190)	1,870 (1,530 to 2,310)
Interpersonal violence	11,700 (8,160 to 16,600)	10,000 (6,910 to 14,300)	7,630 (5,070 to 11,200)	5,610 (3,820 to 8,260)	4,130 (2,920 to 5,730)	2,720 (2,000 to 3,700)	1,840 (1,350 to 2,440)	1,250 (958 to 1,650)
Physical violence by firearm	4,110 (2,530 to 6,430)	3,350 (2,060 to 5,320)	2,490 (1,490 to 3,970)	1,540 (949 to 2,420)	1,150 (749 to 1,730)	621 (397 to 949)	387 (259 to 571)	228 (161 to 324)
Physical violence by sharp object	3,840 (2,610 to 5,600)	3,180 (2,140 to 4,830)	2,590 (1,680 to 3,970)	1,990 (1,300 to 3,120)	1,270 (868 to 1,810)	758 (556 to 1,050)	491 (361 to 673)	281 (208 to 373)
Sexual violence	1,030 (583 to 1,660)	919 (516 to 1,500)	733 (406 to 1,170)	538 (312 to 872)	420 (246 to 693)	289 (166 to 470)	186 (109 to 298)	131 (82 to 196)
Physical violence by other means	2,680 (1,670 to 4,510)	2,560 (1,580 to 4,070)	1,810 (1,070 to 3,030)	1,540 (927 to 2,470)	1,290 (834 to 2,010)	1,050 (712 to 1,550)	773 (544 to 1,090)	611 (438 to 858)
Conflict and terrorism	68·5 (36·6 to 119)	93 (46·4 to 167)	70·3 (37·4 to 125)	51 (28 to 88·1)	32·1 (18·1 to 55·8)	25·3 (14·8 to 42·4)	18·2 (10·9 to 29·8)	11·9 (7·2 to 19·4)

Other transport injuries	154 (120 to 198)	127 (99·6 to 161)	111 (87·7 to 142)	63·9 (49·1 to 81·3)	39 (30·1 to 48·8)	21·8 (17·3 to 27·2)	12·4 (9·6 to 15·4)	6,240
Unintentional injuries	11,100 (9,000 to 13,600)	12,300 (10,200 to 14,800)	13,100 (10,900 to 15,700)	14,300 (12,000 to 17,100)	12,800 (10,600 to 15,100)	8,540 (6,950 to 10,200)	4,690 (3,790 to 5,540)	315,000
Falls	4,750 (3,770 to 5,820)	6,850 (5,650 to 8,240)	8,780 (7,230 to 10,700)	11,100 (9,080 to 13,400)	10,700 (8,710 to 12,700)	7,220 (5,810 to 8,660)	3,940 (3,170 to 4,690)	78,500
Drowning	176 (141 to 217)	124 (102 to 152)	103 (84·3 to 123)	63·2 (51·8 to 75·4)	52·5 (42·3 to 63·1)	40·2 (31·8 to 48·5)	22·9 (17·7 to 27·7)	38,500
Fire, heat, and hot substances	1,310 (932 to 1,820)	1,070 (767 to 1,490)	788 (565 to 1,080)	562 (414 to 759)	386 (292 to 518)	205 (158 to 270)	105 (82·4 to 133)	44,300
Poisonings	110 (83·8 to 142)	88·8 (66·7 to 116)	66·9 (50·2 to 85·4)	49·1 (37·8 to 62·6)	27·4 (21·2 to 35)	11·2 (8·5 to 14·1)	4·9 (3·8 to 6·2)	6,410
Poisoning by carbon monoxide	45·9 (32·6 to 64)	32 (22·2 to 45·7)	23·2 (16·3 to 31·9)	14·8 (10·3 to 20·9)	7·9 (5·4 to 10·9)	3·1 (2·2 to 4·2)	1·1 (0·7 to 1·4)	2,220
Poisoning by other means	63·8 (45·6 to 86·9)	56·8 (41·1 to 77·5)	43·7 (31·5 to 57·2)	34·4 (25·4 to 45·3)	19·6 (14·5 to 25·7)	8·1 (6 to 10·4)	3·8 (2·9 to 4·9)	4,190
Exposure to mechanical forces	1,100 (773 to 1,560)	940 (659 to 1,330)	734 (523 to 1,030)	537 (382 to 740)	355 (260 to 480)	226 (171 to 293)	131 (99·3 to 169)	33,700
Unintentional firearm injuries	156 (110 to 217)	147 (106 to 204)	116 (83·3 to 160)	103 (75·9 to 140)	80·6 (60·2 to 104)	67·5 (51 to 85·3)	44·9 (34 to 58·6)	9,880
Other exposure to mechanical forces	946 (656 to 1,360)	793 (546 to 1,150)	618 (437 to 882)	433 (302 to 615)	275 (194 to 386)	158 (114 to 214)	85·9 (62·8 to 115)	23,800
Adverse effects of medical treatment	913 (676 to 1,210)	890 (658 to 1,140)	765 (566 to 982)	561 (415 to 706)	404 (296 to 507)	183 (133 to 230)	87·2 (60·1 to 110)	20,200
Animal contact	143 (104 to 190)	150 (114 to 197)	153 (118 to 194)	193 (143 to 258)	179 (131 to 245)	192 (139 to 260)	126 (86·7 to 180)	3,810
Venomous animal contact	89·9 (65·8 to 118)	98·3 (75·1 to 126)	109 (83·2 to 138)	144 (102 to 197)	141 (101 to 199)	161 (115 to 223)	106 (71 to 151)	2,600
Non-venomous animal contact	52·8 (35·8 to 76·6)	51·4 (36·2 to 74·1)	44·2 (31·3 to 62·1)	49·3 (34·5 to 68·5)	37·8 (26·2 to 53·7)	30·5 (20·4 to 43·5)	20·1 (13·2 to 29·7)	1,210
Foreign body	591 (496 to 712)	622 (521 to 736)	567 (470 to 681)	522 (429 to 621)	328 (267 to 389)	234 (183 to 282)	152 (116 to 181)	27,200
Pulmonary aspiration and foreign body in airway	449 (363 to 556)	493 (404 to 595)	444 (358 to 547)	409 (326 to 497)	249 (193 to 305)	171 (127 to 212)	114 (83·3 to 141)	24,100
Foreign body in eyes	44·2 (27·4 to 66·3)	37·1 (23·7 to 53·7)	28·1 (18·2 to 41·1)	19·3 (12·7 to 27·9)	11·3 (7·6 to 16·2)	5·5 (3·7 to 7·8)	2·5 (1·7 to 3·5)	1,010
Foreign body in other body part	98·4 (74·1 to 128)	91·3 (70·2 to 118)	94·7 (69·7 to 130)	93·5 (65·8 to 121)	67·9 (48·7 to 93·2)	57·2 (40·4 to 76·5)	35·5 (24·7 to 48·5)	2,110
Environmental heat and cold exposure	542 (363 to 788)	472 (316 to 696)	389 (267 to 593)	306 (213 to 451)	200 (144 to 276)	130 (96·4 to 178)	75·6 (55·6 to 101)	11,200
Exposure to forces of nature	747 (466 to 1,150)	491 (294 to 758)	295 (181 to 455)	153 (96·2 to 243)	52 (32·9 to 83)	15·9 (9·7 to 28·3)	4·5 (2·4 to 10)	31,900
Other unintentional injuries	726 (508 to 1,020)	607 (424 to 851)	478 (338 to 659)	318 (221 to 442)	188 (132 to 262)	90·1 (63·3 to 127)	42·1 (29·9 to 57·7)	19,000
Self-harm and interpersonal violence	2,480 (2,080 to 2,970)	1,780 (1,510 to 2,110)	1,160 (985 to 1,350)	615 (518 to 726)	325 (270 to 383)	94·7 (78·1 to 112)	37·9 (30·5 to 45·7)	131,000
Self-harm	1,510 (1,230 to 1,830)	1,100 (891 to 1,350)	692 (559 to 859)	381 (309 to 475)	194 (152 to 240)	45·1 (35·8 to 54·6)	16·4 (13·1 to 19·6)	50,200
Self-harm by firearm	21·9 (16·7 to 29·6)	21·4 (16·7 to 26·9)	19·5 (15·3 to 24·8)	23·3 (17·8 to 30·2)	18·4 (13·4 to 24·7)	1·6 (1·2 to 2)	0·8 (0·6 to 1)	1,550
Self-harm by other specified means	1,490 (1,210 to 1,810)	1,070 (875 to 1,320)	673 (544 to 834)	357 (288 to 446)	176 (138 to 217)	43·5 (34·4 to 52·8)	15·6 (12·4 to 18·7)	48,600
Interpersonal violence	965 (756 to 1,230)	681 (544 to 837)	460 (376 to 563)	232 (190 to 279)	129 (106 to 158)	49·1 (38·6 to 60·8)	21·3 (16·2 to 27·4)	80,100
Physical violence by firearm	139 (100 to 194)	78·5 (58 to 104)	42·3 (33·1 to 55·8)	15·4 (12·1 to 19·7)	13·3 (9·9 to 17·8)	3·7 (2·7 to 4·7)	2·5 (1·8 to 3·2)	23,400
Physical violence by sharp object	208 (159 to 269)	149 (118 to 188)	79·6 (64·4 to 98·9)	46·9 (36·5 to 59·4)	24·4 (18·4 to 31·9)	12·3 (9 to 16·5)	7·1 (5·2 to 9·5)	22,100
Sexual violence	103 (63·6 to 153)	76·9 (48·4 to 114)	54 (34·8 to 78·8)	36·1 (23·4 to 52·5)	17·6 (10·5 to 27·4)	3·3 (2 to 5·1)	0·3 (0·1 to 0·4)	7,020
Physical violence by other means	515 (380 to 703)	377 (290 to 486)	284 (224 to 356)	133 (105 to 167)	74 (58·7 to 92·3)	29·9 (22·8 to 37·5)	11·5 (8·4 to 15·2)	27,500
Conflict and terrorism	8·2 (4·9 to 13·3)	4·8 (2·9 to 8)	3·7 (2·3 to 6·1)	2 (1·2 to 3·2)	1·2 (0·8 to 1·9)	0·4 (0·3 to 0·7)	0·2 (0·1 to 0·3)	470

SR Table 2b: Antigua and Barbuda DALYS by cause and age group for females with 95% uncertainty intervals, 2019

Cause	Early Neonatal	Late Neonatal	Post Neonatal	1 to 4	5 to 9	10 to 14	15 to 19	20 to 24	25 to 29	30 to 34	35 to 39	40 to 44	45 to 49	50 to 54	55 to 59	60 to 64	65 to 69	70 to 74	75 to 79	80 to 84	85 to 89	90 to 94	95 plus	Total
All causes	138 (104 to 179)	59.2 (44.9 to 75.9)	116 (91.1 to 145)	184 (129 to 271)	141 (108 to 180)	206 (156 to 272)	336 (265 to 433)	403 (316 to 511)	460 (358 to 579)	532 (418 to 664)	612 (496 to 756)	731 (602 to 890)	879 (730 to 1,050)	1,030 (867 to 1,210)	1,110 (935 to 1,290)	1,080 (920 to 1,230)	992 (876 to 1,120)	899 (798 to 1,000)	750 (675 to 825)	588 (541 to 637)	409 (384 to 435)	198 (189 to 208)	72.3 (69.4 to 75.4)	11,900
Communicable, maternal, neonatal, and nutritional diseases	110 (81.5 to 142)	41.5 (30.7 to 54.2)	54.9 (41.5 to 69.9)	61.3 (42.5 to 88.1)	38.6 (25.6 to 59.7)	32.6 (21.4 to 48.7)	43 (29.7 to 61.6)	48.6 (36.6 to 66.5)	55.7 (42.8 to 75)	59.6 (46.2 to 80.4)	63.7 (48.5 to 85.4)	66.3 (50.5 to 90.8)	61 (47 to 81.9)	58.6 (44.3 to 78.7)	53.4 (40.7 to 69.3)	45.6 (35.5 to 59.4)	40.7 (32.6 to 50.9)	41.3 (32.3 to 52.1)	41.8 (31.9 to 53.9)	38.9 (30.8 to 49.4)	32.5 (24.1 to 40.8)	17.7 (13.3 to 22.8)	7.6 (5.3 to 10)	1,110
HIV/AIDS and sexually transmitted infections	[illegible]	[illegible]	[illegible]	[illegible]	[illegible]	[illegible]	[illegible]	[illegible]	[illegible]	[illegible]	[illegible]	[illegible]	[illegible]	[illegible]	[illegible]	[illegible]	[illegible]	[illegible]	[illegible]	[illegible]	[illegible]	[illegible]	[illegible]	125
HIV/AIDS	[illegible]	[illegible]	[illegible]	[illegible]	[illegible]	[illegible]	[illegible]	[illegible]	[illegible]	[illegible]	[illegible]	[illegible]	[illegible]	[illegible]	[illegible]	[illegible]	[illegible]	[illegible]	[illegible]	[illegible]	[illegible]	[illegible]	[illegible]	112
HIV/AIDS - Drug-susceptible Tuberculosis	[illegible]	[illegible]	[illegible]	[illegible]	[illegible]	[illegible]	[illegible]	[illegible]	[illegible]	[illegible]	[illegible]	[illegible]	[illegible]	[illegible]	[illegible]	[illegible]	[illegible]	[illegible]	[illegible]	[illegible]	[illegible]	[illegible]	[illegible]	1.9
HIV/AIDS - Multidrug-resistant Tuberculosis without extensive drug resistance	[illegible]	[illegible]	[illegible]	[illegible]	[illegible]	[illegible]	[illegible]	[illegible]	[illegible]	[illegible]	[illegible]	[illegible]	[illegible]	[illegible]	[illegible]	[illegible]	[illegible]	[illegible]	[illegible]	[illegible]	[illegible]	[illegible]	[illegible]	0
HIV/AIDS - Extensively drug-resistant Tuberculosis	[illegible]	[illegible]	[illegible]	[illegible]	[illegible]	[illegible]	[illegible]	[illegible]	[illegible]	[illegible]	[illegible]	[illegible]	[illegible]	[illegible]	[illegible]	[illegible]	[illegible]	[illegible]	[illegible]	[illegible]	[illegible]	[illegible]	[illegible]	0
HIV/AIDS resulting in other diseases	[illegible]	[illegible]	[illegible]	[illegible]	[illegible]	[illegible]	[illegible]	[illegible]	[illegible]	[illegible]	[illegible]	[illegible]	[illegible]	[illegible]	[illegible]	[illegible]	[illegible]	[illegible]	[illegible]	[illegible]	[illegible]	[illegible]	[illegible]	110
Sexually transmitted infections excluding HIV	[illegible]	[illegible]	[illegible]	[illegible]	[illegible]	[illegible]	[illegible]	[illegible]	[illegible]	[illegible]	[illegible]	[illegible]	[illegible]	[illegible]	[illegible]	[illegible]	[illegible]	[illegible]	[illegible]	[illegible]	[illegible]	[illegible]	[illegible]	13.5
Syphilis	[illegible]	[illegible]	[illegible]	[illegible]	[illegible]	[illegible]	[illegible]	[illegible]	[illegible]	[illegible]	[illegible]	[illegible]	[illegible]	[illegible]	[illegible]	[illegible]	[illegible]	[illegible]	[illegible]	[illegible]	[illegible]	[illegible]	[illegible]	0.3
Chlamydial infection	[illegible]	[illegible]	[illegible]	[illegible]	[illegible]	[illegible]	[illegible]	[illegible]	[illegible]	[illegible]	[illegible]	[illegible]	[illegible]	[illegible]	[illegible]	[illegible]	[illegible]	[illegible]	[illegible]	[illegible]	[illegible]	[illegible]	[illegible]	1.1
Gonococcal infection	[illegible]	[illegible]	[illegible]	[illegible]	[illegible]	[illegible]	[illegible]	[illegible]	[illegible]	[illegible]	[illegible]	[illegible]	[illegible]	[illegible]	[illegible]	[illegible]	[illegible]	[illegible]	[illegible]	[illegible]	[illegible]	[illegible]	[illegible]	0.9
Trichomoniasis	[illegible]	[illegible]	[illegible]	[illegible]	[illegible]	[illegible]	[illegible]	[illegible]	[illegible]	[illegible]	[illegible]	[illegible]	[illegible]	[illegible]	[illegible]	[illegible]	[illegible]	[illegible]	[illegible]	[illegible]	[illegible]	[illegible]	[illegible]	4.8
Genital herpes	[illegible]	[illegible]	[illegible]	[illegible]	[illegible]	[illegible]	[illegible]	[illegible]	[illegible]	[illegible]	[illegible]	[illegible]	[illegible]	[illegible]	[illegible]	[illegible]	[illegible]	[illegible]	[illegible]	[illegible]	[illegible]	[illegible]	[illegible]	3.7
Other sexually transmitted infections	[illegible]	[illegible]	[illegible]	[illegible]	[illegible]	[illegible]	[illegible]	[illegible]	[illegible]	[illegible]	[illegible]	[illegible]	[illegible]	[illegible]	[illegible]	[illegible]	[illegible]	[illegible]	[illegible]	[illegible]	[illegible]	[illegible]	[illegible]	2.7
Respiratory infections and tuberculosis	[illegible]	[illegible]	[illegible]	[illegible]	[illegible]	[illegible]	[illegible]	[illegible]	[illegible]	[illegible]	[illegible]	[illegible]	[illegible]	[illegible]	[illegible]	[illegible]	[illegible]	[illegible]	[illegible]	[illegible]	[illegible]	[illegible]	[illegible]	331
Tuberculosis	[illegible]	[illegible]	[illegible]	[illegible]	[illegible]	[illegible]	[illegible]	[illegible]	[illegible]	[illegible]	[illegible]	[illegible]	[illegible]	[illegible]	[illegible]	[illegible]	[illegible]	[illegible]	[illegible]	[illegible]	[illegible]	[illegible]	[illegible]	10.8
Latent tuberculosis infection	[illegible]	[illegible]	[illegible]	[illegible]	[illegible]	[illegible]	[illegible]	[illegible]	[illegible]	[illegible]	[illegible]	[illegible]	[illegible]	[illegible]	[illegible]	[illegible]	[illegible]	[illegible]	[illegible]	[illegible]	[illegible]	[illegible]	[illegible]	0
Drug-susceptible tuberculosis	[illegible]	[illegible]	[illegible]	[illegible]	[illegible]	[illegible]	[illegible]	[illegible]	[illegible]	[illegible]	[illegible]	[illegible]	[illegible]	[illegible]	[illegible]	[illegible]	[illegible]	[illegible]	[illegible]	[illegible]	[illegible]	[illegible]	[illegible]	10.7
Multidrug-resistant tuberculosis without extensive drug resistance	[illegible]	[illegible]	[illegible]	[illegible]	[illegible]	[illegible]	[illegible]	[illegible]	[illegible]	[illegible]	[illegible]	[illegible]	[illegible]	[illegible]	[illegible]	[illegible]	[illegible]	[illegible]	[illegible]	[illegible]	[illegible]	[illegible]	[illegible]	0.1
Extensively drug-resistant tuberculosis	[illegible]	[illegible]	[illegible]	[illegible]	[illegible]	[illegible]	[illegible]	[illegible]	[illegible]	[illegible]	[illegible]	[illegible]	[illegible]	[illegible]	[illegible]	[illegible]	[illegible]	[illegible]	[illegible]	[illegible]	[illegible]	[illegible]	[illegible]	0
Lower respiratory infections	[illegible]	[illegible]	[illegible]	[illegible]	[illegible]	[illegible]	[illegible]	[illegible]	[illegible]	[illegible]	[illegible]	[illegible]	[illegible]	[illegible]	[illegible]	[illegible]	[illegible]	[illegible]	[illegible]	[illegible]	[illegible]	[illegible]	[illegible]	276
Upper respiratory infections	[illegible]	[illegible]	[illegible]	[illegible]	[illegible]	[illegible]	[illegible]	[illegible]	[illegible]	[illegible]	[illegible]	[illegible]	[illegible]	[illegible]	[illegible]	[illegible]	[illegible]	[illegible]	[illegible]	[illegible]	[illegible]	[illegible]	[illegible]	34.5
Otitis media	[illegible]	[illegible]	[illegible]	[illegible]	[illegible]	[illegible]	[illegible]	[illegible]	[illegible]	[illegible]	[illegible]	[illegible]	[illegible]	[illegible]	[illegible]	[illegible]	[illegible]	[illegible]	[illegible]	[illegible]	[illegible]	[illegible]	[illegible]	9.4
Enteric infections	[illegible]	[illegible]	[illegible]	[illegible]	[illegible]	[illegible]	[illegible]	[illegible]	[illegible]	[illegible]	[illegible]	[illegible]	[illegible]	[illegible]	[illegible]	[illegible]	[illegible]	[illegible]	[illegible]	[illegible]	[illegible]	[illegible]	[illegible]	96.6
Diarrheal diseases	[illegible]	[illegible]	[illegible]	[illegible]	[illegible]	[illegible]	[illegible]	[illegible]	[illegible]	[illegible]	[illegible]	[illegible]	[illegible]	[illegible]	[illegible]	[illegible]	[illegible]	[illegible]	[illegible]	[illegible]	[illegible]	[illegible]	[illegible]	96.4
Typhoid and paratyphoid	[illegible]	[illegible]	[illegible]	[illegible]	[illegible]	[illegible]	[illegible]	[illegible]	[illegible]	[illegible]	[illegible]	[illegible]	[illegible]	[illegible]	[illegible]	[illegible]	[illegible]	[illegible]	[illegible]	[illegible]	[illegible]	[illegible]	[illegible]	0
Typhoid fever	[illegible]	[illegible]	[illegible]	[illegible]	[illegible]	[illegible]	[illegible]	[illegible]	[illegible]	[illegible]	[illegible]	[illegible]	[illegible]	[illegible]	[illegible]	[illegible]	[illegible]	[illegible]	[illegible]	[illegible]	[illegible]	[illegible]	[illegible]	0
Paratyphoid fever	[illegible]	[illegible]	[illegible]	[illegible]	[illegible]	[illegible]	[illegible]	[illegible]	[illegible]	[illegible]	[illegible]	[illegible]	[illegible]	[illegible]	[illegible]	[illegible]	[illegible]	[illegible]	[illegible]	[illegible]	[illegible]	[illegible]	[illegible]	0
Invasive Non-typhoidal Salmonella (iNTS)	[illegible]	[illegible]	[illegible]	[illegible]	[illegible]	[illegible]	[illegible]	[illegible]	[illegible]	[illegible]	[illegible]	[illegible]	[illegible]	[illegible]	[illegible]	[illegible]	[illegible]	[illegible]	[illegible]	[illegible]	[illegible]	[illegible]	[illegible]	0.1
Other intestinal infectious diseases	[illegible]	[illegible]	[illegible]	[illegible]	[illegible]	[illegible]	[illegible]	[illegible]	[illegible]	[illegible]	[illegible]	[illegible]	[illegible]	[illegible]	[illegible]	[illegible]	[illegible]	[illegible]	[illegible]	[illegible]	[illegible]	[illegible]	[illegible]	0.2
Neglected tropical diseases and malaria	[illegible]	[illegible]	[illegible]	[illegible]	[illegible]	[illegible]	[illegible]	[illegible]	[illegible]	[illegible]	[illegible]	[illegible]	[illegible]	[illegible]	[illegible]	[illegible]	[illegible]	[illegible]	[illegible]	[illegible]	[illegible]	[illegible]	[illegible]	48.2
Malaria	[illegible]	[illegible]	[illegible]	[illegible]	[illegible]	[illegible]	[illegible]	[illegible]	[illegible]	[illegible]	[illegible]	[illegible]	[illegible]	[illegible]	[illegible]	[illegible]	[illegible]	[illegible]	[illegible]	[illegible]	[illegible]	[illegible]	[illegible]	0
Chagas disease	[illegible]	[illegible]	[illegible]	[illegible]	[illegible]	[illegible]	[illegible]	[illegible]	[illegible]	[illegible]	[illegible]	[illegible]	[illegible]	[illegible]	[illegible]	[illegible]	[illegible]	[illegible]	[illegible]	[illegible]	[illegible]	[illegible]	[illegible]	0
Leishmaniasis	[illegible]	[illegible]	[illegible]	[illegible]	[illegible]	[illegible]	[illegible]	[illegible]	[illegible]	[illegible]	[illegible]	[illegible]	[illegible]	[illegible]	[illegible]	[illegible]	[illegible]	[illegible]	[illegible]	[illegible]	[illegible]	[illegible]	[illegible]	0
Visceral leishmaniasis	[illegible]	[illegible]	[illegible]	[illegible]	[illegible]	[illegible]	[illegible]	[illegible]	[illegible]	[illegible]	[illegible]	[illegible]	[illegible]	[illegible]	[illegible]	[illegible]	[illegible]	[illegible]	[illegible]	[illegible]	[illegible]	[illegible]	[illegible]	0
Cutaneous and mucocutaneous leishmaniasis	[illegible]	[illegible]	[illegible]	[illegible]	[illegible]	[illegible]	[illegible]	[illegible]	[illegible]	[illegible]	[illegible]	[illegible]	[illegible]	[illegible]	[illegible]	[illegible]	[illegible]	[illegible]	[illegible]	[illegible]	[illegible]	[illegible]	[illegible]	0
African trypanosomiasis	[illegible]	[illegible]	[illegible]	[illegible]	[illegible]	[illegible]	[illegible]	[illegible]	[illegible]	[illegible]	[illegible]	[illegible]	[illegible]	[illegible]	[illegible]	[illegible]	[illegible]	[illegible]	[illegible]	[illegible]	[illegible]	[illegible]	[illegible]	0
Schistosomiasis	[illegible]	[illegible]	[illegible]	[illegible]	[illegible]	[illegible]	[illegible]	[illegible]	[illegible]	[illegible]	[illegible]	[illegible]	[illegible]	[illegible]	[illegible]	[illegible]	[illegible]	[illegible]	[illegible]	[illegible]	[illegible]	[illegible]	[illegible]	31.4
Cysticercosis	[illegible]	[illegible]	[illegible]	[illegible]	[illegible]	[illegible]	[illegible]	[illegible]	[illegible]	[illegible]	[illegible]	[illegible]	[illegible]	[illegible]	[illegible]	[illegible]	[illegible]	[illegible]	[illegible]	[illegible]	[illegible]	[illegible]	[illegible]	0.5
Cystic echinococcosis	[illegible]	[illegible]	[illegible]	[illegible]	[illegible]	[illegible]	[illegible]	[illegible]	[illegible]	[illegible]	[illegible]	[illegible]	[illegible]	[illegible]	[illegible]	[illegible]	[illegible]	[illegible]	[illegible]	[illegible]	[illegible]	[illegible]	[illegible]	0
Lymphatic filariasis	[illegible]	[illegible]	[illegible]	[illegible]	[illegible]	[illegible]	[illegible]	[illegible]	[illegible]	[illegible]	[illegible]	[illegible]	[illegible]	[illegible]	[illegible]	[illegible]	[illegible]	[illegible]	[illegible]	[illegible]	[illegible]	[illegible]	[illegible]	0
Onchocerciasis	[illegible]	[illegible]	[illegible]	[illegible]	[illegible]	[illegible]	[illegible]	[illegible]	[illegible]	[illegible]	[illegible]	[illegible]	[illegible]	[illegible]	[illegible]	[illegible]	[illegible]	[illegible]	[illegible]	[illegible]	[illegible]	[illegible]	[illegible]	0
Trachoma	[illegible]	[illegible]	[illegible]	[illegible]	[illegible]	[illegible]	[illegible]	[illegible]	[illegible]	[illegible]	[illegible]	[illegible]	[illegible]	[illegible]	[illegible]	[illegible]	[illegible]	[illegible]	[illegible]	[illegible]	[illegible]	[illegible]	[illegible]	0
Dengue	[illegible]	[illegible]	[illegible]	[illegible]	[illegible]	[illegible]	[illegible]	[illegible]	[illegible]	[illegible]	[illegible]	[illegible]	[illegible]	[illegible]	[illegible]	[illegible]	[illegible]	[illegible]	[illegible]	[illegible]	[illegible]	[illegible]	[illegible]	3.2
Yellow fever	[illegible]	[illegible]	[illegible]	[illegible]	[illegible]	[illegible]	[illegible]	[illegible]	[illegible]	[illegible]	[illegible]	[illegible]	[illegible]	[illegible]	[illegible]	[illegible]	[illegible]	[illegible]	[illegible]	[illegible]	[illegible]	[illegible]	[illegible]	0
Rabies	[illegible]	[illegible]	[illegible]	[illegible]	[illegible]	[illegible]	[illegible]	[illegible]	[illegible]	[illegible]	[illegible]	[illegible]	[illegible]	[illegible]	[illegible]	[illegible]	[illegible]	[illegible]	[illegible]	[illegible]	[illegible]	[illegible]	[illegible]	[illegible]
Intestinal nematode infections	[illegible]	[illegible]	[illegible]	[illegible]	[illegible]	[illegible]	[illegible]	[illegible]	[illegible]	[illegible]	[illegible]	[illegible]	[illegible]	[illegible]	[illegible]	[illegible]	[illegible]	[illegible]	[illegible]	[illegible]	[illegible]	[illegible]	[illegible]	2.4
Ascariasis	[illegible]	[illegible]	[illegible]	[illegible]	[illegible]	[illegible]	[illegible]	[illegible]	[illegible]	[illegible]	[illegible]	[illegible]	[illegible]	[illegible]	[illegible]	[illegible]	[illegible]	[illegible]	[illegible]	[illegible]	[illegible]	[illegible]	[illegible]	[illegible]
Trichuriasis	[illegible]	[illegible]	[illegible]	[illegible]	[illegible]	[illegible]	[illegible]	[illegible]	[illegible]	[illegible]	[illegible]	[illegible]	[illegible]	[illegible]	[illegible]	[illegible]	[illegible]	[illegible]	[illegible]	[illegible]	[illegible]	[illegible]	[illegible]	[illegible]
Hookworm disease	[illegible]	[illegible]	[illegible]	[illegible]	[illegible]	[illegible]	[illegible]	[illegible]	[illegible]	[illegible]	[illegible]	[illegible]	[illegible]	[illegible]	[illegible]	[illegible]	[illegible]	[illegible]	[illegible]	[illegible]	[illegible]	[illegible]	[illegible]	2.3
Food-borne trematodiases	[illegible]	[illegible]	[illegible]	[illegible]	[illegible]	[illegible]	[illegible]	[illegible]	[illegible]	[illegible]	[illegible]	[illegible]	[illegible]	[illegible]	[illegible]	[illegible]	[illegible]	[illegible]	[illegible]	[illegible]	[illegible]	[illegible]	[illegible]	0
Leprosy	[illegible]	[illegible]	[illegible]	[illegible]	[illegible]	[illegible]	[illegible]	[illegible]	[illegible]	[illegible]	[illegible]	[illegible]	[illegible]	[illegible]	[illegible]	[illegible]	[illegible]	[illegible]	[illegible]	[illegible]	[illegible]	[illegible]	[illegible]	0
Ebola	[illegible]	[illegible]	[illegible]	[illegible]	[illegible]	[illegible]	[illegible]	[illegible]	[illegible]	[illegible]	[illegible]	[illegible]	[illegible]	[illegible]	[illegible]	[illegible]	[illegible]	[illegible]	[illegible]	[illegible]	[illegible]	[illegible]	[illegible]	0
Zika virus	[illegible]	[illegible]	[illegible]	[illegible]	[illegible]	[illegible]	[illegible]	[illegible]	[illegible]	[illegible]	[illegible]	[illegible]	[illegible]	[illegible]	[illegible]	[illegible]	[illegible]	[illegible]	[illegible]	[illegible]	[illegible]	[illegible]	[illegible]	[illegible]
Guinea worm disease	[illegible]	[illegible]	[illegible]	[illegible]	[illegible]	[illegible]	[illegible]	[illegible]	[illegible]	[illegible]	[illegible]	[illegible]	[illegible]	[illegible]	[illegible]	[illegible]	[illegible]	[illegible]	[illegible]	[illegible]	[illegible]	[illegible]	[illegible]	[illegible]
Other neglected tropical diseases	[illegible]	[illegible]	[illegible]	[illegible]	[illegible]	[illegible]	[illegible]	[illegible]	[illegible]	[illegible]	[illegible]	[illegible]	[illegible]	[illegible]	[illegible]	[illegible]	[illegible]	[illegible]	[illegible]	[illegible]	[illegible]	[illegible]	[illegible]	9.8
Other infectious diseases	[illegible]	[illegible]	[illegible]	[illegible]	[illegible]	[illegible]	[illegible]	[illegible]	[illegible]	[illegible]	[illegible]	[illegible]	[illegible]	[illegible]	[illegible]	[illegible]	[illegible]	[illegible]	[illegible]	[illegible]	[illegible]	[illegible]	[illegible]	44.5
Meningitis	[illegible]	[illegible]	[illegible]	[illegible]	[illegible]	[illegible]	[illegible]	[illegible]	[illegible]	[illegible]	[illegible]	[illegible]	[illegible]	[illegible]	[illegible]	[illegible]	[illegible]	[illegible]	[illegible]	[illegible]	[illegible]	[illegible]	[illegible]	13.9
Encephalitis	[illegible]	[illegible]	[illegible]	[illegible]	[illegible]	[illegible]	[illegible]	[illegible]	[illegible]	[illegible]	[illegible]	[illegible]	[illegible]	[illegible]	[illegible]	[illegible]	[illegible]	[illegible]	[illegible]	[illegible]	[illegible]	[illegible]	[illegible]	10
Diphtheria	[illegible]	[illegible]	[illegible]	[illegible]	[illegible]	[illegible]	[illegible]	[illegible]	[illegible]	[illegible]	[illegible]	[illegible]	[illegible]	[illegible]	[illegible]	[illegible]	[illegible]	[illegible]	[illegible]	[illegible]	[illegible]	[illegible]	[illegible]	0
Tetanus	[illegible]	[illegible]	[illegible]	[illegible]	[illegible]	[illegible]	[illegible]	[illegible]	[illegible]	[illegible]	[illegible]	[illegible]	[illegible]	[illegible]	[illegible]	[illegible]	[illegible]	[illegible]	[illegible]	[illegible]	[illegible]	[illegible]	[illegible]	0
Measles	[illegible]	[illegible]	[illegible]	[illegible]	[illegible]	[illegible]	[illegible]	[illegible]	[illegible]	[illegible]	[illegible]	[illegible]	[illegible]	[illegible]	[illegible]	[illegible]	[illegible]	[illegible]	[illegible]	[illegible]	[illegible]	[illegible]	[illegible]	0.3
Varicella and herpes zoster	[illegible]	[illegible]	[illegible]	[illegible]	[illegible]	[illegible]	[illegible]	[illegible]	[illegible]	[illegible]	[illegible]	[illegible]	[illegible]	[illegible]	[illegible]	[illegible]	[illegible]	[illegible]	[illegible]	[illegible]	[illegible]	[illegible]	[illegible]	3.5
Acute hepatitis	[illegible]	[illegible]	[illegible]	[illegible]	[illegible]	[illegible]	[illegible]	[illegible]	[illegible]	[illegible]	[illegible]	[illegible]	[illegible]	[illegible]	[illegible]	[illegible]	[illegible]	[illegible]	[illegible]	[illegible]	[illegible]	[illegible]	[illegible]	2.8
Acute hepatitis A	[illegible]	[illegible]	[illegible]	[illegible]	[illegible]	[illegible]	[illegible]	[illegible]	[illegible]	[illegible]	[illegible]	[illegible]	[illegible]	[illegible]	[illegible]	[illegible]	[illegible]	[illegible]	[illegible]	[illegible]	[illegible]	[illegible]	[illegible]	1.6
Acute hepatitis B	[illegible]	[illegible]	[illegible]	[illegible]	[illegible]	[illegible]	[illegible]	[illegible]	[illegible]	[illegible]	[illegible]	[illegible]	[illegible]	[illegible]	[illegible]	[illegible]	[illegible]	[illegible]	[illegible]	[illegible]	[illegible]	[illegible]	[illegible]	0.8
Acute hepatitis C	[illegible]	[illegible]	[illegible]	[illegible]	[illegible]	[illegible]	[illegible]	[illegible]	[illegible]	[illegible]	[illegible]	[illegible]	[illegible]	[illegible]	[illegible]	[illegible]	[illegible]	[illegible]	[illegible]	[illegible]	[illegible]	[illegible]	[illegible]	0.1
Acute hepatitis E	[illegible]	[illegible]	[illegible]	[illegible]	[illegible]	[illegible]	[illegible]	[illegible]	[illegible]	[illegible]	[illegible]	[illegible]	[illegible]	[illegible]	[illegible]	[illegible]	[illegible]	[illegible]	[illegible]	[illegible]	[illegible]	[illegible]	[illegible]	0.3
Other unspecified infectious diseases	[illegible]	[illegible]	[illegible]	[illegible]	[illegible]	[illegible]	[illegible]	[illegible]	[illegible]	[illegible]	[illegible]	[illegible]	[illegible]	[illegible]	[illegible]	[illegible]	[illegible]	[illegible]	[illegible]	[illegible]	[illegible]	[illegible]	[illegible]	13.7
Maternal and neonatal disorders	[illegible]	[illegible]	[illegible]	[illegible]	[illegible]	[illegible]	[illegible]	[illegible]	[illegible]	[illegible]	[illegible]	[illegible]	[illegible]	[illegible]	[illegible]	[illegible]	[illegible]	[illegible]	[illegible]	[illegible]	[illegible]	[illegible]	[illegible]	259
Maternal disorders	[illegible]	[illegible]	[illegible]	[illegible]	[illegible]	[illegible]	[illegible]	[illegible]	[illegible]	[illegible]	[illegible]	[illegible]	[illegible]	[illegible]	[illegible]	[illegible]	[illegible]	[illegible]	[illegible]	[illegible]	[illegible]	[illegible]	[illegible]	30
Maternal hemorrhage	[illegible]	[illegible]	[illegible]	[illegible]	[illegible]	[illegible]	[illegible]	[illegible]	[illegible]	[illegible]	[illegible]	[illegible]	[illegible]	[illegible]	[illegible]	[illegible]	[illegible]	[illegible]	[illegible]	[illegible]	[illegible]	[illegible]	[illegible]	4.5
Maternal sepsis and other maternal infections	[illegible]	[illegible]	[illegible]	[illegible]	[illegible]	[illegible]	[illegible]	[illegible]	[illegible]	[illegible]	[illegible]	[illegible]	[illegible]	[illegible]	[illegible]	[illegible]	[illegible]	[illegible]	[illegible]	[illegible]	[illegible]	[illegible]	[illegible]	3.9
Maternal hypertensive disorders	[illegible]	[illegible]	[illegible]	[illegible]	[illegible]	[illegible]	[illegible]	[illegible]	[illegible]	[illegible]	[illegible]	[illegible]	[illegible]	[illegible]	[illegible]	[illegible]	[illegible]	[illegible]	[illegible]	[illegible]	[illegible]	[illegible]	[illegible]	5.2
Maternal obstructed labor and uterine rupture	[illegible]	[illegible]	[illegible]	[illegible]	[illegible]	[illegible]	[illegible]	[illegible]	[illegible]	[illegible]	[illegible]	[illegible]	[illegible]	[illegible]	[illegible]	[illegible]	[illegible]	[illegible]	[illegible]	[illegible]	[illegible]	[illegible]	[illegible]	1
Maternal abortion and miscarriage	[illegible]	[illegible]	[illegible]	[illegible]	[illegible]	[illegible]	[illegible]	[illegible]	[illegible]	[illegible]	[illegible]	[illegible]	[illegible]	[illegible]	[illegible]	[illegible]	[illegible]	[illegible]	[illegible]	[illegible]	[illegible]	[illegible]	[illegible]	2.2
Ectopic pregnancy	[illegible]	[illegible]	[illegible]	[illegible]	[illegible]	[illegible]	[illegible]	[illegible]	[illegible]	[illegible]	[illegible]	[illegible]	[illegible]	[illegible]	[illegible]	[illegible]	[illegible]	[illegible]	[illegible]	[illegible]	[illegible]	[illegible]	[illegible]	3.5
Indirect maternal deaths	[illegible]	[illegible]	[illegible]	[illegible]	[illegible]	[illegible]	[illegible]	[illegible]	[illegible]	[illegible]	[illegible]	[illegible]	[illegible]	[illegible]	[illegible]	[illegible]	[illegible]	[illegible]	[illegible]	[illegible]	[illegible]	[illegible]	[illegible]	NA
Late maternal deaths	[illegible]	[illegible]	[illegible]	[illegible]	[illegible]	[illegible]	[illegible]	[illegible]	[illegible]	[illegible]	[illegible]	[illegible]	[illegible]	[illegible]	[illegible]	[illegible]	[illegible]	[illegible]	[illegible]	[illegible]	[illegible]	[illegible]	[illegible]	NA
Maternal deaths aggravated by HIV/AIDS	[illegible]	[illegible]	[illegible]	[illegible]	[illegible]	[illegible]	[illegible]	[illegible]	[illegible]	[illegible]	[illegible]	[illegible]	[illegible]	[illegible]	[illegible]	[illegible]	[illegible]	[illegible]	[illegible]	[illegible]	[illegible]	[illegible]	[illegible]	NA
Neonatal disorders	[illegible]	[illegible]	[illegible]	[illegible]	[illegible]	[illegible]	[illegible]	[illegible]	[illegible]	[illegible]	[illegible]	[illegible]	[illegible]	[illegible]	[illegible]	[illegible]	[illegible]	[illegible]	[illegible]	[illegible]	[illegible]	[illegible]	[illegible]	229
Neonatal preterm birth	[illegible]	[illegible]	[illegible]	[illegible]	[illegible]	[illegible]	[illegible]	[illegible]	[illegible]	[illegible]	[illegible]	[illegible]	[illegible]	[illegible]	[illegible]	[illegible]	[illegible]	[illegible]	[illegible]	[illegible]	[illegible]	[illegible]	[illegible]	153
Neonatal encephalopathy due to birth asphyxia and trauma	[illegible]	[illegible]	[illegible]	[illegible]	[illegible]	[illegible]	[illegible]	[illegible]	[illegible]	[illegible]	[illegible]	[illegible]	[illegible]	[illegible]	[illegible]	[illegible]	[illegible]	[illegible]	[illegible]	[illegible]	[illegible]	[illegible]	[illegible]	30.8
Neonatal sepsis and other neonatal infections	[illegible]	[illegible]	[illegible]	[illegible]	[illegible]	[illegible]	[illegible]	[illegible]	[illegible]	[illegible]	[illegible]	[illegible]	[illegible]	[illegible]	[illegible]	[illegible]	[illegible]	[illegible]	[illegible]	[illegible]	[illegible]	[illegible]	[illegible]	12
Hemolytic disease and other neonatal jaundice	[illegible]	[illegible]	[illegible]	[illegible]	[illegible]	[illegible]	[illegible]	[illegible]	[illegible]	[illegible]	[illegible]	[illegible]	[illegible]	[illegible]	[illegible]	[illegible]	[illegible]	[illegible]	[illegible]	[illegible]	[illegible]	[illegible]	[illegible]	3.5
Other neonatal disorders	[illegible]	[illegible]	[illegible]	[illegible]	[illegible]	[illegible]	[illegible]	[illegible]	[illegible]	[illegible]	[illegible]	[illegible]	[illegible]	[illegible]	[illegible]	[illegible]	[illegible]	[illegible]	[illegible]	[illegible]	[illegible]	[illegible]	[illegible]	30.2
Nutritional deficiencies	[illegible]	[illegible]	[illegible]	[illegible]	[illegible]	[illegible]	[illegible]	[illegible]	[illegible]	[illegible]	[illegible]	[illegible]	[illegible]	[illegible]	[illegible]	[illegible]	[illegible]	[illegible]	[illegible]	[illegible]	[illegible]	[illegible]	[illegible]	210
Protein-energy malnutrition	[illegible]	[illegible]	[illegible]	[illegible]	[illegible]	[illegible]	[illegible]	[illegible]	[illegible]	[illegible]	[illegible]	[illegible]	[illegible]	[illegible]	[illegible]	[illegible]	[illegible]	[illegible]	[illegible]	[illegible]	[illegible]	[illegible]	[illegible]	39.5
Iodine deficiency	[illegible]	[illegible]	[illegible]	[illegible]	[illegible]	[illegible]	[illegible]	[illegible]	[illegible]	[illegible]	[illegible]	[illegible]	[illegible]	[illegible]	[illegible]	[illegible]	[illegible]	[illegible]	[illegible]	[illegible]	[illegible]	[illegible]	[illegible]	2
Vitamin A deficiency	[illegible]	[illegible]	[illegible]	[illegible]	[illegible]	[illegible]	[illegible]	[illegible]	[illegible]	[illegible]	[illegible]	[illegible]	[illegible]	[illegible]	[illegible]	[illegible]	[illegible]	[illegible]	[illegible]	[illegible]	[illegible]	[illegible]	[illegible]	[illegible]
Dietary iron deficiency	[illegible]	[illegible]	[illegible]	[illegible]	[illegible]	[illegible]	[illegible]	[illegible]	[illegible]	[illegible]	[illegible]	[illegible]	[illegible]	[illegible]	[illegible]	[illegible]	[illegible]	[illegible]	[illegible]	[illegible]	[illegible]	[illegible]	[illegible]	162
Other nutritional deficiencies	[illegible]	[illegible]	[illegible]	[illegible]	[illegible]	[illegible]	[illegible]	[illegible]	[illegible]	[illegible]	[illegible]	[illegible]	[illegible]	[illegible]	[illegible]	[illegible]	[illegible]	[illegible]	[illegible]	[illegible]	[illegible]	[illegible]	[illegible]	4.7
Non-communicable diseases	[illegible]	[illegible]	[illegible]	[illegible]	[illegible]	[illegible]	[illegible]	[illegible]	[illegible]	[illegible]	[illegible]	[illegible]	[illegible]	[illegible]	[illegible]	[illegible]	[illegible]	[illegible]	[illegible]	[illegible]	[illegible]	[illegible]	[illegible]	10,300
Neoplasms	[illegible]	[illegible]	[illegible]	[illegible]	[illegible]	[illegible]	[illegible]	[illegible]	[illegible]	[illegible]	[illegible]	[illegible]	[illegible]	[illegible]	[illegible]	[illegible]	[illegible]	[illegible]	[illegible]	[illegible]	[illegible]	[illegible]	[illegible]	1,630
Lip and oral cavity cancer	[illegible]	[illegible]	[illegible]	[illegible]	[illegible]	[illegible]	[illegible]	[illegible]	[illegible]	[illegible]	[illegible]	[illegible]	[illegible]	[illegible]	[illegible]	[illegible]	[illegible]	[illegible]	[illegible]	[illegible]	[illegible]	[illegible]	[illegible]	7.5
Nasopharynx cancer	[illegible]	[illegible]	[illegible]	[illegible]	[illegible]	[illegible]	[illegible]	[illegible]	[illegible]	[illegible]	[illegible]	[illegible]	[illegible]	[illegible]	[illegible]	[illegible]	[illegible]	[illegible]	[illegible]	[illegible]	[illegible]	[illegible]	[illegible]	2.8
Other pharynx cancer	[illegible]	[illegible]	[illegible]	[illegible]	[illegible]	[illegible]	[illegible]	[illegible]	[illegible]	[illegible]	[illegible]	[illegible]	[illegible]	[illegible]	[illegible]	[illegible]	[illegible]	[illegible]	[illegible]	[illegible]	[illegible]	[illegible]	[illegible]	3.1
Esophageal cancer	[illegible]	[illegible]	[illegible]	[illegible]	[illegible]	[illegible]	[illegible]	[illegible]	[illegible]	[illegible]	[illegible]	[illegible]	[illegible]	[illegible]	[illegible]	[illegible]	[illegible]	[illegible]	[illegible]	[illegible]	[illegible]	[illegible]	[illegible]	22.2
Stomach cancer	[illegible]	[illegible]	[illegible]	[illegible]	[illegible]	[illegible]	[illegible]	[illegible]	[illegible]	[illegible]	[illegible]	[illegible]	[illegible]	[illegible]	[illegible]	[illegible]	[illegible]	[illegible]	[illegible]	[illegible]	[illegible]	[illegible]	[illegible]	84.7
Colon and rectum cancer	[illegible]	[illegible]	[illegible]	[illegible]	[illegible]	[illegible]	[illegible]	[illegible]	[illegible]	[illegible]	[illegible]	[illegible]	[illegible]	[illegible]	[illegible]	[illegible]	[illegible]	[illegible]	[illegible]	[illegible]	[illegible]	[illegible]	[illegible]	193
Liver cancer	[illegible]	[illegible]	[illegible]	[illegible]	[illegible]	[illegible]	[illegible]	[illegible]	[illegible]	[illegible]	[illegible]	[illegible]	[illegible]	[illegible]	[illegible]	[illegible]	[illegible]	[illegible]	[illegible]	[illegible]	[illegible]	[illegible]	[illegible]	24.2
Liver cancer due to hepatitis B	[illegible]	[illegible]	[illegible]	[illegible]	[illegible]	[illegible]	[illegible]	[illegible]	[illegible]	[illegible]	[illegible]	[illegible]	[illegible]	[illegible]	[illegible]	[illegible]	[illegible]	[illegible]	[illegible]	[illegible]	[illegible]	[illegible]	[illegible]	5.3
Liver cancer due to hepatitis C	[illegible]	[illegible]	[illegible]	[illegible]	[illegible]	[illegible]	[illegible]	[illegible]	[illegible]	[illegible]	[illegible]	[illegible]	[illegible]	[illegible]	[illegible]	[illegible]	[illegible]	[illegible]	[illegible]	[illegible]	[illegible]	[illegible]	[illegible]	7.3
Liver cancer due to alcohol use	[illegible]	[illegible]	[illegible]	[illegible]	[illegible]	[illegible]	[illegible]	[illegible]	[illegible]	[illegible]	[illegible]	[illegible]	[illegible]	[illegible]	[illegible]	[illegible]	[illegible]	[illegible]	[illegible]	[illegible]	[illegible]	[illegible]	[illegible]	5.9
Liver cancer due to NASH	[illegible]	[illegible]	[illegible]	[illegible]	[illegible]	[illegible]	[illegible]	[illegible]	[illegible]	[illegible]	[illegible]	[illegible]	[illegible]	[illegible]	[illegible]	[illegible]	[illegible]	[illegible]	[illegible]	[illegible]	[illegible]	[illegible]	[illegible]	3.1
Liver cancer due to other causes	[illegible]	[illegible]	[illegible]	[illegible]	[illegible]	[illegible]	[illegible]	[illegible]	[illegible]	[illegible]	[illegible]	[illegible]	[illegible]	[illegible]	[illegible]	[illegible]	[illegible]	[illegible]	[illegible]	[illegible]	[illegible]	[illegible]	[illegible]	2.6
Gallbladder and biliary tract cancer	[illegible]	[illegible]	[illegible]	[illegible]	[illegible]	[illegible]	[illegible]	[illegible]	[illegible]	[illegible]	[illegible]	[illegible]	[illegible]	[illegible]	[illegible]	[illegible]	[illegible]	[illegible]	[illegible]	[illegible]	[illegible]	[illegible]	[illegible]	18.1
Pancreatic cancer	[illegible]	[illegible]	[illegible]	[illegible]	[illegible]	[illegible]	[illegible]	[illegible]	[illegible]	[illegible]	[illegible]	[illegible]	[illegible]	[illegible]	[illegible]	[illegible]	[illegible]	[illegible]	[illegible]	[illegible]	[illegible]	[illegible]	[illegible]	60.1
Larynx cancer	[illegible]	[illegible]	[illegible]	[illegible]	[illegible]	[illegible]	[illegible]	[illegible]	[illegible]	[illegible]	[illegible]	[illegible]	[illegible]	[illegible]	[illegible]	[illegible]	[illegible]	[illegible]	[illegible]	[illegible]	[illegible]	[illegible]	[illegible]	1.1
Tracheal, bronchus, and lung cancer	[illegible]	[illegible]	[illegible]	[illegible]	[illegible]	[illegible]	[illegible]	[illegible]	[illegible]	[illegible]	[illegible]	[illegible]	[illegible]	[illegible]	[illegible]	[illegible]	[illegible]	[illegible]	[illegible]	[illegible]	[illegible]	[illegible]	[illegible]	81.7
Malignant skin melanoma	[illegible]	[illegible]	[illegible]	[illegible]	[illegible]	[illegible]	[illegible]	[illegible]	[illegible]	[illegible]	[illegible]	[illegible]	[illegible]	[illegible]	[illegible]	[illegible]	[illegible]	[illegible]	[illegible]	[illegible]	[illegible]	[illegible]	[illegible]	11.3
Non-melanoma skin cancer	[illegible]	[illegible]	[illegible]	[illegible]	[illegible]	[illegible]	[illegible]	[illegible]	[illegible]	[illegible]	[illegible]	[illegible]	[illegible]	[illegible]	[illegible]	[illegible]	[illegible]	[illegible]	[illegible]	[illegible]	[illegible]	[illegible]	[illegible]	4.6
Non-melanoma skin cancer (squamous-cell carcinoma)	[illegible]	[illegible]	[illegible]	[illegible]	[illegible]	[illegible]	[illegible]	[illegible]	[illegible]	[illegible]	[illegible]	[illegible]	[illegible]	[illegible]	[illegible]	[illegible]	[illegible]	[illegible]	[illegible]	[illegible]	[illegible]	[illegible]	[illegible]	4.6
Non-melanoma skin cancer (basal-cell carcinoma)	[illegible]	[illegible]	[illegible]	[illegible]	[illegible]	[illegible]	[illegible]	[illegible]	[illegible]	[illegible]	[illegible]	[illegible]	[illegible]	[illegible]	[illegible]	[illegible]	[illegible]	[illegible]	[illegible]	[illegible]	[illegible]	[illegible]	[illegible]	0
Breast cancer	[illegible]	[illegible]	[illegible]	[illegible]	[illegible]	[illegible]	[illegible]	[illegible]	[illegible]	[illegible]	[illegible]	[illegible]	[illegible]	[illegible]	[illegible]	[illegible]	[illegible]	[illegible]	[illegible]	[illegible]	[illegible]	[illegible]	[illegible]	457
Cervical cancer	[illegible]	[illegible]	[illegible]	[illegible]	[illegible]	[illegible]	[illegible]	[illegible]	[illegible]	[illegible]	[illegible]	[illegible]	[illegible]	[illegible]	[illegible]	[illegible]	[illegible]	[illegible]	[illegible]	[illegible]	[illegible]	[illegible]	[illegible]	174
Uterine cancer	[illegible]	[illegible]	[illegible]	[illegible]	[illegible]	[illegible]	[illegible]	[illegible]	[illegible]	[illegible]	[illegible]	[illegible]	[illegible]	[illegible]	[illegible]	[illegible]	[illegible]	[illegible]	[illegible]	[illegible]	[illegible]	[illegible]	[illegible]	72
Ovarian cancer	[illegible]	[illegible]	[illegible]	[illegible]	[illegible]	[illegible]	[illegible]	[illegible]	[illegible]	[illegible]	[illegible]	[illegible]	[illegible]	[illegible]	[illegible]	[illegible]	[illegible]	[illegible]	[illegible]	[illegible]	[illegible]	[illegible]	[illegible]	98.3
Prostate cancer	[illegible]	[illegible]	[illegible]	[illegible]	[illegible]	[illegible]	[illegible]	[illegible]	[illegible]	[illegible]	[illegible]	[illegible]	[illegible]	[illegible]	[illegible]	[illegible]	[illegible]	[illegible]	[illegible]	[illegible]	[illegible]	[illegible]	[illegible]	[illegible]
Testicular cancer	[illegible]	[illegible]	[illegible]	[illegible]	[illegible]	[illegible]	[illegible]	[illegible]	[illegible]	[illegible]	[illegible]	[illegible]	[illegible]	[illegible]	[illegible]	[illegible]	[illegible]	[illegible]	[illegible]	[illegible]	[illegible]	[illegible]	[illegible]	NA
Kidney cancer	[illegible]	[illegible]	[illegible]	[illegible]	[illegible]	[illegible]	[illegible]	[illegible]	[illegible]	[illegible]	[illegible]	[illegible]	[illegible]	[illegible]	[illegible]	[illegible]	[illegible]	[illegible]	[illegible]	[illegible]	[illegible]	[illegible]	[illegible]	21.4
Bladder cancer	[illegible]	[illegible]	[illegible]	[illegible]	[illegible]	[illegible]	[illegible]	[illegible]	[illegible]	[illegible]	[illegible]	[illegible]	[illegible]	[illegible]	[illegible]	[illegible]	[illegible]	[illegible]	[illegible]	[illegible]	[illegible]	[illegible]	[illegible]	23.5
Brain and central nervous system cancer	[illegible]	[illegible]	[illegible]	[illegible]	[illegible]	[illegible]	[illegible]	[illegible]	[illegible]	[illegible]	[illegible]	[illegible]	[illegible]	[illegible]	[illegible]	[illegible]	[illegible]	[illegible]	[illegible]	[illegible]	[illegible]	[illegible]	[illegible]	42.4
Thyroid cancer	[illegible]	[illegible]	[illegible]	[illegible]	[illegible]	[illegible]	[illegible]	[illegible]	[illegible]	[illegible]	[illegible]	[illegible]	[illegible]	[illegible]	[illegible]	[illegible]	[illegible]	[illegible]	[illegible]	[illegible]	[illegible]	[illegible]	[illegible]	15.1
Mesothelioma	[illegible]	[illegible]	[illegible]	[illegible]	[illegible]	[illegible]	[illegible]	[illegible]	[illegible]	[illegible]	[illegible]	[illegible]	[illegible]	[illegible]	[illegible]	[illegible]	[illegible]	[illegible]	[illegible]	[illegible]	[illegible]	[illegible]	[illegible]	1
Hodgkin lymphoma	[illegible]	[illegible]	[illegible]	[illegible]	[illegible]	[illegible]	[illegible]	[illegible]	[illegible]	[illegible]	[illegible]	[illegible]	[illegible]	[illegible]	[illegible]	[illegible]	[illegible]	[illegible]	[illegible]	[illegible]	[illegible]	[illegible]	[illegible]	[illegible]
Non-Hodgkin lymphoma	[illegible]	[illegible]	[illegible]	[illegible]	[illegible]	[illegible]	[illegible]	[illegible]	[illegible]	[illegible]	[illegible]	[illegible]	[illegible]	[illegible]	[illegible]	[illegible]	[illegible]	[illegible]	[illegible]	[illegible]	[illegible]	[illegible]	[illegible]	44.2
Multiple myeloma	[illegible]	[illegible]	[illegible]	[illegible]	[illegible]	[illegible]	[illegible]	[illegible]	[illegible]	[illegible]	[illegible]	[illegible]	[illegible]	[illegible]	[illegible]	[illegible]	[illegible]	[illegible]	[illegible]	[illegible]	[illegible]	[illegible]	[illegible]	31.9
Leukemia	[illegible]	[illegible]	[illegible]	[illegible]	[illegible]	[illegible]	[illegible]	[illegible]	[illegible]	[illegible]	[illegible]	[illegible]	[illegible]	[illegible]	[illegible]	[illegible]	[illegible]	[illegible]	[illegible]	[illegible]	[illegible]	[illegible]	[illegible]	61.7
Acute lymphoid leukemia	[illegible]	[illegible]	[illegible]	[illegible]	[illegible]	[illegible]	[illegible]	[illegible]	[illegible]	[illegible]	[illegible]	[illegible]	[illegible]	[illegible]	[illegible]	[illegible]	[illegible]	[illegible]	[illegible]	[illegible]	[illegible]	[illegible]	[illegible]	9.5
Chronic lymphoid leukemia	[illegible]	[illegible]	[illegible]	[illegible]	[illegible]	[illegible]	[illegible]	[illegible]	[illegible]	[illegible]	[illegible]	[illegible]	[illegible]	[illegible]	[illegible]	[illegible]	[illegible]	[illegible]	[illegible]	[illegible]	[illegible]	[illegible]	[illegible]	[illegible]
Acute myeloid leukemia	[illegible]	[illegible]	[illegible]	[illegible]	[illegible]	[illegible]	[illegible]	[illegible]	[illegible]	[illegible]	[illegible]	[illegible]	[illegible]	[illegible]	[illegible]	[illegible]	[illegible]	[illegible]	[illegible]	[illegible]	[illegible]	[illegible]	[illegible]	16.7

Chronic myeloid leukemia	0 (0 to 0)	[illegible]	0 (0 to 0)	5·5
Other leukemia	0 (0 to 0·1)	[illegible]	0·1 (0 to 0·1)	29·9
Other malignant neoplasms	0·1 (0 to 0·2)	[illegible]	0·2 (0·1 to 0·3)	66·2
Other neoplasms	0 (0 to 0)	[illegible]	0 (0 to 0)	4·5
Myelodysplastic, myeloproliferative, and other hematopoietic neo	0 (0 to 0)	[illegible]	0 (0 to 0)	4·5
Benign and in situ intestinal neoplasms	0 (0 to 0)	[illegible]	0 (0 to 0)	0
Benign and in situ cervical and uterine neoplasms	0 (0 to 0)	[illegible]	0 (0 to 0)	0
Other benign and in situ neoplasms	0 (0 to 0)	[illegible]	0 (0 to 0)	0
Cardiovascular diseases	0·6 (0·3 to 1)	[illegible]	28·7 (22·5 to 32·9)	1,970
Rheumatic heart disease	0 (0 to 0)	[illegible]	0·1 (0 to 0·1)	44·9
Ischemic heart disease	0 (0 to 0)	[illegible]	11·6 (8·8 to 14·1)	653
Stroke	0·3 (0·1 to 0·5)	[illegible]	7·5 (5·3 to 9·8)	682
Ischemic stroke	0·1 (0 to 0·1)	[illegible]	6·1 (4·3 to 7·9)	316
Intracerebral hemorrhage	0·1 (0 to 0·2)	[illegible]	1·2 (0·8 to 1·6)	290
Subarachnoid hemorrhage	0·1 (0·1 to 0·2)	[illegible]	0·2 (0·1 to 0·2)	75·9
Hypertensive heart disease	0 (0 to 0)	[illegible]	4·6 (2·9 to 6·3)	284
Non-rheumatic valvular heart disease	0 (0 to 0)	[illegible]	0·1 (0·1 to 0·1)	13·7
Non-rheumatic calcific aortic valve disease	0 (0 to 0)	[illegible]	0·1 (0 to 0·1)	7
Non-rheumatic degenerative mitral valve disease	0 (0 to 0)	[illegible]	0 (0 to 0)	5·3
Other non-rheumatic valve diseases	0 (0 to 0)	[illegible]	0 (0 to 0)	1·4
Cardiomyopathy and myocarditis	0·1 (0·1 to 0·2)	[illegible]	0·4 (0·2 to 0·6)	50·9
Myocarditis	0 (0 to 0·1)	[illegible]	0·2 (0·1 to 0·3)	12·9
Alcoholic cardiomyopathy	0 (0 to 0)	[illegible]	0 (0 to 0)	1
Other cardiomyopathy	0·1 (0 to 0·2)	[illegible]	0·2 (0·1 to 0·4)	37·1
Atrial fibrillation and flutter	0 (0 to 0)	[illegible]	2·2 (1·6 to 2·9)	55·5
Aortic aneurysm	--	[illegible]	0·2 (0·1 to 0·2)	NA
Endocarditis	0 (0 to 0)	[illegible]	0·1 (0 to 0·1)	9·6
Other cardiovascular and circulatory diseases	0·2 (0·1 to 0·3)	[illegible]	1·5 (1·1 to 2)	142
Chronic respiratory diseases	0 (0 to 0)	[illegible]	1·2 (0·9 to 1·6)	213
Chronic obstructive pulmonary disease	0 (0 to 0)	[illegible]	1 (0·7 to 1·3)	72·8
Pneumoconiosis	0 (0 to 0)	[illegible]	0 (0 to 0)	0·2
Silicosis	0 (0 to 0)	[illegible]	0 (0 to 0)	0
Asbestosis	0 (0 to 0)	[illegible]	0 (0 to 0)	0·1
Coal workers pneumoconiosis	0 (0 to 0)	[illegible]	0 (0 to 0)	0
Other pneumoconiosis	0 (0 to 0)	[illegible]	0 (0 to 0)	0
Asthma	0 (0 to 0)	[illegible]	0·2 (0·1 to 0·2)	116
Interstitial lung disease and pulmonary sarcoidosis	0 (0 to 0)	[illegible]	0·1 (0 to 0·1)	11·1
Other chronic respiratory diseases	0 (0 to 0)	[illegible]	0 (0 to 0)	12·5
Digestive diseases	0·2 (0·2 to 0·4)	[illegible]	1·4 (1·1 to 1·7)	349
Cirrhosis and other chronic liver diseases	0 (0 to 0)	[illegible]	0·3 (0·2 to 0·5)	119
Cirrhosis and other chronic liver diseases due to hepatitis B	0 (0 to 0)	[illegible]	0 (0 to 0)	6
Cirrhosis and other chronic liver diseases due to hepatitis C	0 (0 to 0)	[illegible]	0·1 (0 to 0·1)	20·6
Cirrhosis and other chronic liver diseases due to alcohol use	0 (0 to 0)	[illegible]	0·1 (0 to 0·1)	38·6
Cirrhosis and other chronic liver diseases due to NAFLD	0 (0 to 0)	[illegible]	0·1 (0 to 0·2)	26·4
Cirrhosis and other chronic liver diseases due to other causes	0 (0 to 0)	[illegible]	0·1 (0 to 0·2)	26·8
Upper digestive system diseases	0 (0 to 0)	[illegible]	0·2 (0·2 to 0·3)	99·3
Peptic ulcer disease	0 (0 to 0)	[illegible]	0·1 (0·1 to 0·2)	11·5
Gastritis and duodenitis	0 (0 to 0)	[illegible]	0·1 (0 to 0·1)	20·3
Gastroesophageal reflux disease	0 (0 to 0)	[illegible]	0·1 (0 to 0·1)	67·5
Appendicitis	0 (0 to 0)	[illegible]	0 (0 to 0)	5·1
Paralytic ileus and intestinal obstruction	0·2 (0·1 to 0·3)	[illegible]	0·3 (0·2 to 0·5)	30·3
Inguinal, femoral, and abdominal hernia	0 (0 to 0·1)	[illegible]	0 (0 to 0)	14·1
Inflammatory bowel disease	0 (0 to 0)	[illegible]	0 (0 to 0)	8·5
Vascular intestinal disorders	0 (0 to 0)	[illegible]	0 (0 to 0·1)	4·3
Gallbladder and biliary diseases	0 (0 to 0)	[illegible]	0·1 (0·1 to 0·2)	42·4
Pancreatitis	0 (0 to 0)	[illegible]	0 (0 to 0)	5·3
Other digestive diseases	0 (0 to 0)	[illegible]	0·3 (0·2 to 0·4)	21·5
Neurological disorders	0 (0 to 0)	[illegible]	9·6 (4·3 to 21·4)	711
Alzheimer's disease and other dementias	0 (0 to 0)	[illegible]	8·9 (3·6 to 20·7)	157
Parkinson's disease	0 (0 to 0)	[illegible]	0·4 (0·4 to 0·5)	36
Idiopathic epilepsy	0 (0 to 0)	[illegible]	0·1 (0·1 to 0·3)	111
Multiple sclerosis	0 (0 to 0)	[illegible]	0 (0 to 0)	16·1
Motor neuron disease	0 (0 to 0)	[illegible]	0 (0 to 0)	3·5
Headache disorders	0 (0 to 0)	[illegible]	0·1 (0 to 0·1)	356
Migraine	0 (0 to 0)	[illegible]	0 (0 to 0·1)	325
Tension-type headache	0 (0 to 0)	[illegible]	0 (0 to 0·1)	30·8
Other neurological disorders	0 (0 to 0)	[illegible]	0 (0 to 0)	31·2
Mental disorders	0 (0 to 0)	[illegible]	0·6 (0·4 to 0·9)	952
Schizophrenia	0 (0 to 0)	[illegible]	0 (0 to 0)	95·9
Depressive disorders	0 (0 to 0)	[illegible]	0·4 (0·2 to 0·7)	360
Major depressive disorder	0 (0 to 0)	[illegible]	0·4 (0·2 to 0·6)	308
Dysthymia	0 (0 to 0)	[illegible]	0 (0 to 0·1)	52
Bipolar disorder	0 (0 to 0)	[illegible]	0 (0 to 0)	112
Anxiety disorders	0 (0 to 0)	[illegible]	0·1 (0·1 to 0·2)	256
Eating disorders	0 (0 to 0)	[illegible]	0 (0 to 0)	37·9
Anorexia nervosa	0 (0 to 0)	[illegible]	0 (0 to 0)	9·1
Bulimia nervosa	0 (0 to 0)	[illegible]	0 (0 to 0)	28·8
Autism spectrum disorders	0 (0 to 0)	[illegible]	0 (0 to 0)	11·4
Attention-deficit/hyperactivity disorder	0 (0 to 0)	[illegible]	0 (0 to 0)	8·1
Conduct disorder	0 (0 to 0)	[illegible]	0 (0 to 0)	17
Idiopathic developmental intellectual disability	0 (0 to 0)	[illegible]	0 (0 to 0)	5
Other mental disorders	0 (0 to 0)	[illegible]	0·1 (0 to 0·1)	47·9
Substance use disorders	0 (0 to 0)	[illegible]	0·2 (0·1 to 0·3)	145
Alcohol use disorders	0 (0 to 0)	[illegible]	0·2 (0·1 to 0·3)	99·2
Drug use disorders	0 (0 to 0)	[illegible]	0 (0 to 0)	45·7
Opioid use disorders	0 (0 to 0)	[illegible]	0 (0 to 0)	27
Cocaine use disorders	0 (0 to 0)	[illegible]	0 (0 to 0)	6·6
Amphetamine use disorders	0 (0 to 0)	[illegible]	0 (0 to 0)	2·3
Cannabis use disorders	0 (0 to 0)	[illegible]	0 (0 to 0)	4·4
Other drug use disorders	0 (0 to 0)	[illegible]	0 (0 to 0)	5·5
Diabetes and kidney diseases	0 (0 to 0)	[illegible]	7·6 (5·9 to 9·3)	1,580
Diabetes mellitus	0 (0 to 0)	[illegible]	4·8 (3·5 to 6·2)	1,090
Diabetes mellitus type 1	0 (0 to 0)	[illegible]	0·1 (0·1 to 0·2)	46·6
Diabetes mellitus type 2	0 (0 to 0)	[illegible]	4·7 (3·5 to 6)	1,040
Chronic kidney disease	0 (0 to 0)	[illegible]	2·8 (2 to 3·7)	498
Chronic kidney disease due to diabetes mellitus type 1	0 (0 to 0)	[illegible]	0 (0 to 0)	34·1
Chronic kidney disease due to diabetes mellitus type 2	0 (0 to 0)	[illegible]	0·8 (0·4 to 1·2)	124
Chronic kidney disease due to hypertension	0 (0 to 0)	[illegible]	1·1 (0·7 to 1·7)	102
Chronic kidney disease due to glomerulonephritis	0 (0 to 0)	[illegible]	0·2 (0·1 to 0·4)	95·2
Chronic kidney disease due to other and unspecified causes	0 (0 to 0)	[illegible]	0·7 (0·4 to 1·1)	143
Acute glomerulonephritis	0 (0 to 0)	[illegible]	0 (0 to 0)	0·1
Skin and subcutaneous diseases	0 (0 to 0)	[illegible]	1·8 (1 to 2·5)	287
Dermatitis	0 (0 to 0)	[illegible]	0 (0 to 0·1)	51·8
Atopic dermatitis	0 (0 to 0)	[illegible]	0 (0 to 0)	35·3
Contact dermatitis	0 (0 to 0)	[illegible]	0 (0 to 0)	14·9
Seborrhoeic dermatitis	0 (0 to 0)	[illegible]	0 (0 to 0)	1·6
Psoriasis	0 (0 to 0)	[illegible]	0 (0 to 0)	15·6
Bacterial skin diseases	0 (0 to 0)	[illegible]	0·1 (0 to 0·2)	13·7
Cellulitis	0 (0 to 0)	[illegible]	0 (0 to 0·1)	4·8
Pyoderma	0 (0 to 0)	[illegible]	0·1 (0 to 0·2)	9
Scabies	0 (0 to 0)	[illegible]	0 (0 to 0)	35·2
Fungal skin diseases	0 (0 to 0)	[illegible]	0·1 (0 to 0·2)	20·4
Viral skin diseases	0 (0 to 0)	[illegible]	0 (0 to 0)	17·2
Acne vulgaris	0 (0 to 0)	[illegible]	0 (0 to 0)	23·5
Alopecia areata	0 (0 to 0)	[illegible]	0 (0 to 0)	4·6
Pruritus	0 (0 to 0)	[illegible]	0 (0 to 0)	4·8
Urticaria	0 (0 to 0)	[illegible]	0 (0 to 0)	22·6
Decubitus ulcer	0 (0 to 0)	[illegible]	1·4 (0·7 to 2·1)	49·4
Other skin and subcutaneous diseases	0 (0 to 0)	[illegible]	0 (0 to 0·1)	27·9
Sense organ diseases	0 (0 to 0)	[illegible]	2·7 (1·9 to 3·6)	374
Blindness and vision loss	0 (0 to 0)	[illegible]	1 (0·7 to 1·4)	98·5
Glaucoma	0 (0 to 0)	[illegible]	0·1 (0·1 to 0·2)	4·7
Cataract	0 (0 to 0)	[illegible]	0·4 (0·2 to 0·5)	17·4
Age-related macular degeneration	0 (0 to 0)	[illegible]	0 (0 to 0)	0·7
Refraction disorders	0 (0 to 0)	[illegible]	0 (0 to 0·1)	28·6
Near vision loss	0 (0 to 0)	[illegible]	0·1 (0 to 0·3)	23
Other vision loss	0 (0 to 0)	[illegible]	0·4 (0·2 to 0·6)	24·1
Age-related and other hearing loss	0 (0 to 0)	[illegible]	1·6 (1·1 to 2·1)	255
Other sense organ diseases	0 (0 to 0)	[illegible]	0·1 (0 to 0·1)	20·3
Musculoskeletal disorders	0 (0 to 0)	[illegible]	1·5 (1 to 2·1)	970
Rheumatoid arthritis	0 (0 to 0)	[illegible]	0·1 (0·1 to 0·2)	28·6
Osteoarthritis	0 (0 to 0)	[illegible]	0·4 (0·2 to 0·8)	126
Osteoarthritis hip	0 (0 to 0)	[illegible]	0 (0 to 0)	5·3
Osteoarthritis knee	0 (0 to 0)	[illegible]	0·2 (0·1 to 0·4)	91·2
Osteoarthritis hand	0 (0 to 0)	[illegible]	0·1 (0 to 0·2)	17·6

Osteoarthritis other	0 (0 to 0)	0 (0 to 0)	0 (0 to 0)	0 (0 to 0)	0 (0 to 0)	0 (0 to 0)	0 (0 to 0)	0 (0 to 0)	0 (0 to 0)	0 (0 to 0)	0·1 (0 to 0·3)	0·4 (0·1 to 0·9)	0·8 (0·3 to 1·8)	1·3 (0·6 to 2·8)	1·7 (0·8 to 3·6)	1·8 (0·8 to 3·7)	1·6 (0·8 to 3·4)	1·4 (0·7 to 2·9)	1·1 (0·5 to 2·2)	0·8 (0·4 to 1·6)	0·5 (0·2 to 1)	0·2 (0·1 to 0·4)	0·1 (0 to 0·1)	11·8
Low back pain	0 (0 to 0)	0 (0 to 0)	0 (0 to 0)	0 (0 to 0)	1·6 (0·7 to 3·1)	8·6 (5 to 14)	16·7 (9·7 to 26·9)	21·4 (12·9 to 34·2)	26 (15·1 to 39·6)	31·7 (17·8 to 49·4)	37 (22·2 to 56·5)	41·4 (24·5 to 63·8)	45·1 (27·5 to 70·6)	47 (28·4 to 72·9)	43·3 (27·4 to 64·4)	35·4 (22·3 to 52·8)	27·4 (17·1 to 42·2)	21 (12·9 to 31·7)	14·9 (9·3 to 22·4)	9·9 (6·1 to 14·8)	5·2 (3·2 to 7·7)	1·8 (1·1 to 2·7)	0·5 (0·3 to 0·8)	436
Neck pain	0 (0 to 0)	0 (0 to 0)	0 (0 to 0)	0 (0 to 0)	0·1 (0 to 0·2)	0·5 (0·2 to 1·1)	1·3 (0·5 to 2·4)	2·1 (0·9 to 4·2)	3·4 (1·5 to 6·7)	5·6 (2·6 to 10·7)	7·6 (3·8 to 13·6)	9·7 (4·8 to 17)	10·9 (5·4 to 19·4)	9·8 (4·9 to 18·4)	8·4 (4 to 15·4)	6·5 (3·2 to 11·1)	4·7 (2·4 to 8)	3·4 (1·8 to 6)	2·2 (1·1 to 4·1)	1·2 (0·6 to 2·2)	0·6 (0·3 to 1·1)	0·2 (0·1 to 0·4)	0·1 (0 to 0·1)	78·4
Gout	0 (0 to 0)	0 (0 to 0)	0 (0 to 0)	0 (0 to 0)	0 (0 to 0)	0 (0 to 0)	0 (0 to 0)	0 (0 to 0·1)	0·1 (0 to 0·1)	0·1 (0 to 0·3)	0·2 (0 to 0·4)	0·3 (0·1 to 0·6)	0·3 (0·1 to 0·6)	0·4 (0·1 to 0·7)	0·4 (0·1 to 0·7)	0·3 (0·1 to 0·6)	0·3 (0·1 to 0·5)	0·2 (0·1 to 0·4)	0·2 (0·1 to 0·3)	0·1 (0 to 0·2)	0·1 (0 to 0·1)	0 (0 to 0·1)	0 (0 to 0)	2·9
Other musculoskeletal disorders	0 (0 to 0)	0 (0 to 0)	0 (0 to 0)	0 (0 to 0)	0·1 (0·1 to 0·2)	0·7 (0·3 to 1·2)	5·1 (3·2 to 7·6)	11·2 (7·5 to 15·8)	19·9 (12·8 to 29·5)	27 (17·6 to 40·4)	29·6 (19·4 to 41·9)	32·7 (21 to 47·4)	35·1 (23·6 to 51·3)	35·2 (22·8 to 51·7)	31·6 (20·3 to 45·9)	24·4 (15 to 36·7)	17·9 (11·3 to 26·5)	12·4 (7·2 to 19·1)	7 (4·2 to 10·6)	3·7 (1·9 to 6)	3·1 (1·8 to 4·3)	1·2 (0·6 to 1·7)	0·3 (0·2 to 0·6)	298
Other non-communicable diseases	25·9 (16·9 to 39·8)	15·9 (10·6 to 22·5)	44·1 (32·6 to 59·3)	37 (22·3 to 60·1)	15·9 (12·3 to 20·5)	19·9 (15·2 to 26·1)	34·9 (25·8 to 47·9)	52·5 (36·9 to 75·1)	69·8 (48·3 to 98·1)	82·6 (57·4 to 117)	92 (65·1 to 127)	99 (69·6 to 138)	97·2 (68·9 to 131)	89·2 (64·4 to 120)	77·7 (58·1 to 103)	63·2 (46·8 to 83·9)	51·4 (38·3 to 68)	41·4 (31·4 to 54)	30·7 (24 to 38·8)	20·8 (16·6 to 26·2)	14·2 (11·3 to 17·7)	6·4 (5·1 to 7·9)	2·2 (1·7 to 2·8)	1,080
Congenital birth defects	25·2 (16·3 to 38·8)	14·6 (9·5 to 20·8)	31·6 (21·8 to 44·7)	21·2 (11·4 to 36·5)	6·4 (4·8 to 8·5)	6·1 (4·7 to 8)	6·2 (4·7 to 8·2)	4·8 (3·6 to 6·2)	4·2 (3·2 to 5·5)	3·8 (2·8 to 4·9)	3·8 (2·9 to 5)	3·8 (2·9 to 4·9)	3·8 (2·9 to 4·9)	3·7 (2·8 to 4·8)	3·1 (2·3 to 4)	1·8 (1·4 to 2·4)	1·2 (0·9 to 1·6)	0·4 (0·2 to 0·6)	0·2 (0·1 to 0·3)	0·1 (0·1 to 0·2)	0·1 (0 to 0·1)	0 (0 to 0)	0 (0 to 0)	146
Neural tube defects	1·9 (1 to 3·3)	0·7 (0·3 to 1·2)	2 (0·9 to 4·2)	1·2 (0·5 to 2·7)	0·2 (0·1 to 0·2)	0·3 (0·2 to 0·4)	0·3 (0·2 to 0·4)	0·1 (0·1 to 0·2)	0·1 (0·1 to 0·2)	0·1 (0·1 to 0·2)	0·1 (0·1 to 0·2)	0·1 (0·1 to 0·2)	0·1 (0·1 to 0·1)	0·1 (0 to 0·1)	0·1 (0 to 0·1)	0 (0 to 0)	0 (0 to 0)	0 (0 to 0)	0 (0 to 0)	0 (0 to 0)	0 (0 to 0)	0 (0 to 0)	0 (0 to 0)	7·4
Congenital heart anomalies	10·3 (5·8 to 17·2)	7·5 (4·6 to 11·4)	16·4 (10·7 to 24·2)	10·9 (4·7 to 19·9)	2·1 (1·4 to 3·1)	1·8 (1·2 to 2·5)	2 (1·3 to 2·8)	1·3 (0·9 to 1·9)	1·3 (0·9 to 1·8)	0·9 (0·6 to 1·2)	1·1 (0·7 to 1·6)	1·1 (0·8 to 1·5)	1·1 (0·7 to 1·5)	1 (0·7 to 1·5)	0·8 (0·5 to 1·2)	0·4 (0·3 to 0·6)	0·3 (0·2 to 0·4)	0 (0 to 0·1)	0 (0 to 0)	0 (0 to 0)	0 (0 to 0)	0 (0 to 0)	0 (0 to 0)	60·2
Orofacial clefts	0 (0 to 0)	0 (0 to 0)	0 (0 to 0·1)	0·1 (0 to 0·1)	0·1 (0 to 0·1)	0·1 (0 to 0·2)	0·1 (0 to 0·2)	0·1 (0 to 0·2)	0·1 (0 to 0·2)	0·1 (0 to 0·2)	0·1 (0 to 0·2)	0·1 (0 to 0·2)	0·1 (0 to 0·2)	0·1 (0 to 0·2)	0·1 (0 to 0·1)	0 (0 to 0·1)	0 (0 to 0·1)	0 (0 to 0·1)	0 (0 to 0)	0 (0 to 0)	0 (0 to 0)	0 (0 to 0)	0 (0 to 0)	1·1
Down syndrome	0·2 (0·1 to 0·4)	0·1 (0·1 to 0·2)	0·7 (0·3 to 1·2)	0·8 (0·4 to 1·6)	0·2 (0·1 to 0·3)	0·2 (0·1 to 0·3)	0·2 (0·2 to 0·3)	0·2 (0·2 to 0·4)	0·2 (0·1 to 0·3)	0·2 (0·1 to 0·3)	0·3 (0·2 to 0·4)	0·3 (0·2 to 0·4)	0·4 (0·3 to 0·6)	0·5 (0·3 to 0·8)	0·5 (0·3 to 0·8)	0·2 (0·1 to 0·3)	0·1 (0 to 0·1)	0 (0 to 0)	0 (0 to 0)	0 (0 to 0)	0 (0 to 0)	0 (0 to 0)	0 (0 to 0)	5·4
Turner syndrome	0 (0 to 0)	0 (0 to 0)	0 (0 to 0)	0 (0 to 0)	0 (0 to 0)	0 (0 to 0)	0 (0 to 0)	0 (0 to 0)	0 (0 to 0)	0 (0 to 0)	0 (0 to 0)	0 (0 to 0)	0 (0 to 0)	0 (0 to 0)	0 (0 to 0)	0 (0 to 0)	0 (0 to 0)	0 (0 to 0)	0 (0 to 0)	0 (0 to 0)	0 (0 to 0)	0 (0 to 0)	0 (0 to 0)	0·1
Klinefelter syndrome	0 (0 to 0)	0 (0 to 0)	0 (0 to 0)	0 (0 to 0)	0 (0 to 0)	0 (0 to 0)	0 (0 to 0)	0 (0 to 0)	0 (0 to 0)	0 (0 to 0)	0 (0 to 0)	0 (0 to 0)	0 (0 to 0)	0 (0 to 0)	0 (0 to 0)	0 (0 to 0)	0 (0 to 0)	0 (0 to 0)	0 (0 to 0)	0 (0 to 0)	0 (0 to 0)	0 (0 to 0)	0 (0 to 0)	0
Other chromosomal abnormalities	2·8 (1·4 to 5·2)	2 (1 to 3·5)	4·5 (2·4 to 7·5)	1·3 (0·6 to 2·5)	0·4 (0·3 to 0·7)	0·4 (0·3 to 0·6)	0·5 (0·3 to 0·8)	0·4 (0·3 to 0·6)	0·3 (0·2 to 0·5)	0·2 (0·2 to 0·4)	0·1 (0·1 to 0·2)	0·1 (0·1 to 0·2)	0·1 (0·1 to 0·1)	0·1 (0·1 to 0·2)	0 (0 to 0·1)	0 (0 to 0)	0 (0 to 0)	0 (0 to 0)	0 (0 to 0)	0 (0 to 0)	0 (0 to 0)	0 (0 to 0)	0 (0 to 0)	13·4
Congenital musculoskeletal and limb anomalies	0·3 (0·1 to 0·5)	0·1 (0·1 to 0·2)	0·8 (0·5 to 1·2)	1·5 (0·8 to 2·4)	1·7 (1 to 2·7)	1·9 (1·1 to 2·9)	1·9 (1·1 to 2·9)	1·5 (0·9 to 2·4)	1·4 (0·8 to 2·2)	1·4 (0·8 to 2·2)	1·4 (0·8 to 2·1)	1·4 (0·8 to 2·1)	1·3 (0·8 to 2·2)	1·2 (0·7 to 1·9)	1 (0·6 to 1·7)	0·8 (0·4 to 1·2)	0·5 (0·3 to 0·8)	0·3 (0·2 to 0·5)	0·2 (0·1 to 0·3)	0·1 (0 to 0·1)	0 (0 to 0·1)	0 (0 to 0)	0 (0 to 0)	20·7
Urogenital congenital anomalies	0·3 (0·1 to 0·6)	0·1 (0·1 to 0·3)	0·1 (0·1 to 0·2)	0·2 (0·1 to 0·3)	0·1 (0·1 to 0·2)	0·1 (0·1 to 0·2)	0·1 (0·1 to 0·1)	0·1 (0 to 0·1)	0·1 (0 to 0·1)	0·1 (0 to 0·1)	0 (0 to 0·1)	0 (0 to 0·1)	0 (0 to 0·1)	0 (0 to 0)	0 (0 to 0)	0 (0 to 0)	0 (0 to 0)	0 (0 to 0)	0 (0 to 0)	0 (0 to 0)	0 (0 to 0)	0 (0 to 0)	0 (0 to 0)	1·4
Digestive congenital anomalies	2·4 (1·2 to 4·9)	1·6 (0·8 to 2·7)	2 (1·1 to 3·5)	0·9 (0·4 to 1·7)	0·1 (0·1 to 0·2)	0·1 (0·1 to 0·1)	0 (0 to 0·1)	0 (0 to 0·1)	0 (0 to 0)	0 (0 to 0)	0 (0 to 0)	0 (0 to 0)	0 (0 to 0)	0 (0 to 0)	0 (0 to 0)	0 (0 to 0)	0 (0 to 0)	0 (0 to 0)	0 (0 to 0)	0 (0 to 0)	0 (0 to 0)	0 (0 to 0)	0 (0 to 0)	7·5
Other congenital birth defects	7·1 (4 to 11·9)	2·5 (1·3 to 4·2)	5·1 (2·9 to 8·2)	4·3 (2 to 8·3)	1·4 (0·9 to 2·1)	1·3 (0·8 to 1·8)	1·2 (0·8 to 1·8)	0·9 (0·6 to 1·4)	0·7 (0·5 to 1·1)	0·7 (0·5 to 1·1)	0·7 (0·4 to 1)	0·6 (0·4 to 1)	0·6 (0·4 to 1)	0·6 (0·4 to 0·9)	0·5 (0·3 to 0·8)	0·3 (0·2 to 0·5)	0·3 (0·2 to 0·4)	0 (0 to 0·1)	0 (0 to 0·1)	0 (0 to 0·1)	0 (0 to 0)	0 (0 to 0)	0 (0 to 0)	29
Urinary diseases and male infertility	0 (0 to 0)	0·1 (0 to 0·1)	0·4 (0·2 to 0·6)	0·6 (0·3 to 1)	0·3 (0·2 to 0·5)	0·5 (0·3 to 0·7)	0·9 (0·6 to 1·2)	1 (0·7 to 1·4)	1·3 (0·9 to 1·7)	1·7 (1·2 to 2·3)	2·1 (1·5 to 2·9)	2·7 (1·9 to 3·6)	3·2 (2·2 to 4·5)	4·2 (2·8 to 5·7)	4·9 (3·1 to 7·1)	4·6 (3 to 6·6)	4·6 (3 to 6·7)	4·6 (3 to 6·7)	4·4 (2·9 to 6·2)	4·1 (2·6 to 5·9)	3·1 (2 to 4·5)	1·6 (1 to 2·2)	0·6 (0·4 to 0·9)	51·3
Urolithiasis	0 (0 to 0)	0 (0 to 0)	0 (0 to 0)	0 (0 to 0)	0 (0 to 0)	0 (0 to 0)	0 (0 to 0)	0·1 (0 to 0·1)	0·1 (0 to 0·1)	0·2 (0·1 to 0·3)	0·3 (0·2 to 0·4)	0·3 (0·2 to 0·4)	0·5 (0·2 to 0·8)	0·4 (0·2 to 0·8)	0·6 (0·3 to 0·9)	0·4 (0·2 to 0·7)	0·4 (0·2 to 0·6)	0·2 (0·1 to 0·3)	0·1 (0·1 to 0·2)	0·1 (0 to 0·1)	0 (0 to 0·1)	0 (0 to 0)	0 (0 to 0)	3·6
Benign prostatic hyperplasia	0 (0 to 0)	0 (0 to 0)	0 (0 to 0)	0 (0 to 0)	0 (0 to 0)	0 (0 to 0)	0 (0 to 0)	0 (0 to 0)	0 (0 to 0)	0 (0 to 0)	0 (0 to 0)	0 (0 to 0)	0 (0 to 0)	0 (0 to 0)	0 (0 to 0)	0 (0 to 0)	0 (0 to 0)	0 (0 to 0)	0 (0 to 0)	0 (0 to 0)	0 (0 to 0)	0 (0 to 0)	0 (0 to 0)	0
Male infertility	--	--	--	--	--	--	--	--	--	--	--	--	--	--	--	--	--	--	--	--	--	--	--	NA
Other urinary diseases	0 (0 to 0)	0 (0 to 0)	0 (0 to 0)	0·1 (0·1 to 0·2)	0·1 (0 to 0·1)	0·1 (0·1 to 0·2)	0·1 (0·1 to 0·2)	0·1 (0·1 to 0·1)	0·1 (0·1 to 0·2)	0·2 (0·1 to 0·3)	0·2 (0·1 to 0·3)	0·2 (0·1 to 0·2)	0·2 (0·1 to 0·3)	0·1 (0·1 to 0·2)	0·1 (0·1 to 0·2)	0·1 (0 to 0·1)	0·1 (0 to 0·1)	0 (0 to 0·1)	0 (0 to 0)	0 (0 to 0)	0 (0 to 0)	0 (0 to 0)	0 (0 to 0)	1·8
Gynecological diseases	0 (0 to 0)	0 (0 to 0)	0 (0 to 0)	0 (0 to 0)	0 (0 to 0)	4·4 (2·1 to 7·5)	12·4 (7 to 21·2)	28·2 (17·8 to 44·3)	41·9 (27·2 to 61·5)	51·6 (33·5 to 74·6)	55·4 (36·9 to 80·2)	56·3 (36·9 to 83)	47·2 (30·7 to 67·4)	31·7 (19·4 to 48·2)	17·6 (10·6 to 25·5)	10·3 (6 to 15·6)	7 (4·2 to 10·2)	4·6 (2·9 to 6·6)	2·8 (1·8 to 4·1)	1·6 (1 to 2·4)	0·9 (0·6 to 1·4)	0·4 (0·2 to 0·6)	0·1 (0·1 to 0·2)	374
Uterine fibroids	0 (0 to 0)	0 (0 to 0)	0 (0 to 0)	0 (0 to 0)	0 (0 to 0)	0·1 (0 to 0·2)	0·2 (0·1 to 0·4)	0·5 (0·2 to 1·1)	1·3 (0·5 to 2·6)	2·8 (1·3 to 5·6)	4·7 (2·3 to 9·2)	5·5 (2·8 to 10·1)	5·2 (2·7 to 9·3)	3·6 (1·8 to 6·5)	2·3 (1·2 to 4·1)	1·2 (0·6 to 2·2)	0·7 (0·3 to 1·3)	0·4 (0·2 to 0·7)	0·2 (0·1 to 0·3)	0·1 (0·1 to 0·2)	0 (0 to 0·1)	0 (0 to 0)	0 (0 to 0)	28·7
Polycystic ovarian syndrome	0 (0 to 0)	0 (0 to 0)	0 (0 to 0)	0 (0 to 0)	0 (0 to 0)	0·2 (0·1 to 0·4)	0·8 (0·3 to 1·6)	1 (0·4 to 2)	0·9 (0·4 to 2)	0·9 (0·4 to 1·9)	0·9 (0·4 to 1·8)	0·8 (0·4 to 1·7)	0·7 (0·3 to 1·4)	0·2 (0·1 to 0·4)	0 (0 to 0)	0 (0 to 0)	0 (0 to 0)	0 (0 to 0)	0 (0 to 0)	0 (0 to 0)	0 (0 to 0)	0 (0 to 0)	0 (0 to 0)	6·4
Female infertility	0 (0 to 0)	0 (0 to 0)	0 (0 to 0)	0 (0 to 0)	0 (0 to 0)	0 (0 to 0)	0 (0 to 0)	1 (0·2 to 2·7)	1·5 (0·4 to 3·9)	1·8 (0·4 to 5·5)	1·9 (0·3 to 5·6)	1·8 (0·2 to 5)	0 (0 to 0·2)	0 (0 to 0)	0 (0 to 0)	0 (0 to 0)	0 (0 to 0)	0 (0 to 0)	0 (0 to 0)	0 (0 to 0)	0 (0 to 0)	0 (0 to 0)	0 (0 to 0)	8
Endometriosis	0 (0 to 0)	0 (0 to 0)	0 (0 to 0)	0 (0 to 0)	0 (0 to 0)	0 (0 to 0)	1 (0·4 to 2)	4·2 (1·9 to 8·1)	4·9 (2·4 to 8·9)	4 (1·9 to 7·4)	3·5 (1·8 to 6·3)	3·4 (1·7 to 6)	3 (1·6 to 4·9)	1·4 (0·7 to 2·5)	0 (0 to 0)	0 (0 to 0)	0 (0 to 0)	0 (0 to 0)	0 (0 to 0)	0 (0 to 0)	0 (0 to 0)	0 (0 to 0)	0 (0 to 0)	25·4
Genital prolapse	0 (0 to 0)	0 (0 to 0)	0 (0 to 0)	0 (0 to 0)	0 (0 to 0)	0 (0 to 0)	0 (0 to 0)	0 (0 to 0·1)	0·1 (0 to 0·2)	0·2 (0·1 to 0·4)	0·4 (0·1 to 0·8)	0·6 (0·2 to 1·2)	0·8 (0·3 to 1·6)	0·9 (0·4 to 1·8)	0·8 (0·4 to 1·6)	0·6 (0·3 to 1·3)	0·5 (0·2 to 1)	0·4 (0·2 to 0·7)	0·2 (0·1 to 0·5)	0·2 (0·1 to 0·3)	0·1 (0 to 0·2)	0 (0 to 0·1)	0 (0 to 0)	5·8
Premenstrual syndrome	0 (0 to 0)	0 (0 to 0)	0 (0 to 0)	0 (0 to 0)	0 (0 to 0)	4 (1·9 to 7·1)	9·9 (4·9 to 17·8)	13·4 (6·5 to 23·2)	13·5 (7·8 to 21·7)	14 (7·1 to 22·9)	15·3 (8·6 to 24·4)	15·2 (8·5 to 24·7)	10·6 (5·8 to 17·9)	3·5 (1·7 to 6·5)	0 (0 to 0)	0 (0 to 0)	0 (0 to 0)	0 (0 to 0)	0 (0 to 0)	0 (0 to 0)	0 (0 to 0)	0 (0 to 0)	0 (0 to 0)	99·3
Other gynecological diseases	0 (0 to 0)	0 (0 to 0)	0 (0 to 0)	0 (0 to 0)	0 (0 to 0)	0·2 (0 to 0·4)	0·5 (0·2 to 1·1)	8·1 (4·5 to 13·3)	19·7 (11 to 31·6)	27·9 (16·5 to 43·5)	28·8 (16·6 to 46·1)	29 (16·2 to 46·1)	26·9 (14·9 to 42·1)	22·1 (10·4 to 35·7)	14·5 (8·5 to 21·8)	8·5 (4·5 to 13·6)	5·8 (3·2 to 9)	3·8 (2·4 to 5·8)	2·3 (1·4 to 3·6)	1·4 (0·8 to 2·1)	0·8 (0·5 to 1·2)	0·3 (0·2 to 0·5)	0·1 (0 to 0·2)	201
Hemoglobinopathies and hemolytic anemias	0·1 (0·1 to 0·2)	0·1 (0·1 to 0·2)	1 (0·7 to 1·5)	4·3 (2·4 to 7·3)	3·8 (2·5 to 5·4)	3·6 (2·2 to 5·1)	6·5 (4·4 to 9·1)	7·5 (4 to 12·7)	7·3 (3·8 to 12·7)	5·3 (2·9 to 10·1)	5·2 (3·2 to 7·6)	5·1 (3·3 to 7·4)	5·1 (3·3 to 7·3)	5·4 (3·5 to 7·8)	4·6 (3 to 6·4)	3·7 (2·5 to 5·5)	2·4 (1·6 to 3·6)	1·9 (1·3 to 2·7)	1·4 (1 to 2·1)	0·9 (0·7 to 1·4)	1 (0·7 to 1·4)	0·5 (0·3 to 0·6)	0·1 (0·1 to 0·2)	76·9
Thalassemias	0 (0 to 0)	0 (0 to 0)	0 (0 to 0)	0 (0 to 0·1)	0 (0 to 0)	0 (0 to 0)	0 (0 to 0)	0 (0 to 0)	0 (0 to 0)	0 (0 to 0)	0 (0 to 0)	0 (0 to 0)	0 (0 to 0)	0 (0 to 0)	0 (0 to 0)	0 (0 to 0)	0 (0 to 0)	0 (0 to 0)	0 (0 to 0)	0 (0 to 0)	0 (0 to 0)	0 (0 to 0)	0 (0 to 0)	0·1
Thalassemias trait	0 (0 to 0)	0 (0 to 0)	0 (0 to 0)	0·1 (0 to 0·2)	0·1 (0 to 0·2)	0·1 (0 to 0·2)	0·3 (0·1 to 0·5)	0·3 (0·2 to 0·6)	0·3 (0·2 to 0·6)	0·4 (0·2 to 0·6)	0·3 (0·1 to 0·6)	0·2 (0·1 to 0·4)	0·1 (0 to 0·2)	0·1 (0 to 0·2)	0·1 (0 to 0·2)	0 (0 to 0·1)	0 (0 to 0·1)	0 (0 to 0·1)	0 (0 to 0)	0 (0 to 0)	0 (0 to 0)	0 (0 to 0)	0 (0 to 0)	2·5
Sickle cell disorders	0·1 (0·1 to 0·2)	0·1 (0 to 0·1)	0·7 (0·5 to 1·2)	3 (1·3 to 5·8)	2·2 (1·5 to 3·2)	2·5 (1·6 to 3·7)	4·5 (3 to 6·5)	3·4 (2·2 to 5)	2·7 (1·8 to 4·1)	2·1 (1·3 to 3·2)	2·5 (1·4 to 4)	2·4 (1·5 to 3·6)	2·7 (1·7 to 4·2)	2·6 (1·6 to 4·1)	1·5 (0·9 to 2·5)	0·5 (0·2 to 0·9)	0·1 (0 to 0·2)	0 (0 to 0)	0 (0 to 0)	0 (0 to 0)	0 (0 to 0)	0 (0 to 0)	0 (0 to 0)	33·6
Sickle cell trait	0 (0 to 0)	0 (0 to 0·1)	0·2 (0·1 to 0·3)	0·7 (0·3 to 1·3)	0·9 (0·4 to 1·8)	0·6 (0·2 to 1·2)	0·2 (0 to 0·5)	3·1 (0·4 to 7·3)	3·5 (0·6 to 7·8)	2 (0·3 to 5·9)	0·4 (0·1 to 0·8)	0·8 (0·3 to 1·4)	0·8 (0·3 to 1·5)	0·8 (0·4 to 1·5)	0·7 (0·3 to 1·2)	0·4 (0·2 to 0·8)	0·2 (0·1 to 0·5)	0·2 (0·1 to 0·4)	0·1 (0 to 0·3)	0·1 (0 to 0·2)	0·1 (0 to 0·1)	0 (0 to 0)	0 (0 to 0)	15·9
G6PD deficiency	0 (0 to 0)	0 (0 to 0)	0 (0 to 0)	0 (0 to 0·1)	0 (0 to 0·1)	0 (0 to 0·1)	0·7 (0·3 to 1·3)	0·1 (0 to 0·2)	0·2 (0·1 to 0·4)	0·2 (0·1 to 0·4)	0·7 (0·3 to 1·3)	0·3 (0·2 to 0·6)	0·2 (0·1 to 0·5)	0·3 (0·1 to 0·6)	0·3 (0·1 to 0·5)	0·3 (0·1 to 0·5)	0·2 (0·1 to 0·3)	0·1 (0 to 0·1)	0 (0 to 0·1)	0 (0 to 0)	0 (0 to 0)	0 (0 to 0)	0 (0 to 0)	3·7
G6PD trait	0 (0 to 0)	0 (0 to 0)	0 (0 to 0)	0 (0 to 0)	0 (0 to 0)	0 (0 to 0)	0·1 (0 to 0·1)	0 (0 to 0)	0 (0 to 0)	0 (0 to 0)	0·1 (0 to 0·1)	0·1 (0 to 0·2)	0 (0 to 0)	0 (0 to 0)	0 (0 to 0)	0 (0 to 0)	0 (0 to 0)	0 (0 to 0)	0 (0 to 0)	0 (0 to 0)	0 (0 to 0)	0 (0 to 0)	0 (0 to 0)	0·2
Other hemoglobinopathies and hemolytic anemias	0 (0 to 0)	0 (0 to 0)	0·1 (0 to 0·2)	0·5 (0·2 to 0·9)	0·5 (0·2 to 1·1)	0·3 (0·1 to 0·8)	0·8 (0·3 to 1·5)	0·5 (0·2 to 1·1)	0·6 (0·3 to 1·2)	0·7 (0·3 to 1·3)	1·2 (0·5 to 2·2)	1·3 (0·6 to 2·4)	1·2 (0·6 to 2)	1·6 (0·9 to 2·6)	2 (1·2 to 2·9)	2·5 (1·6 to 3·8)	1·9 (1·3 to 2·8)	1·6 (1·1 to 2·3)	1·2 (0·8 to 1·8)	0·8 (0·6 to 1·2)	0·9 (0·6 to 1·3)	0·4 (0·3 to 0·6)	0·1 (0·1 to 0·2)	20·8
Endocrine, metabolic, blood, and immune disorders	0·6 (0·3 to 1)	1 (0·5 to 1·6)	10·2 (6·1 to 15·9)	9·6 (5·2 to 16·9)	3·7 (2·5 to 5·3)	3·4 (2·3 to 4·9)	5·8 (4 to 8·2)	5·6 (4 to 8)	7·2 (5·2 to 10·6)	9·9 (6·9 to 14·5)	13·5 (9·6 to 18·9)	17·4 (11·7 to 25)	21·8 (14·6 to 30·9)	25·5 (17·3 to 36·1)	27·6 (19·2 to 39·2)	23·7 (16 to 33·6)	19·4 (13·5 to 28·6)	16·1 (11·5 to 22·2)	12·1 (8·5 to 16·9)	7·9 (5·7 to 11)	5·7 (4 to 7·7)	2·6 (1·9 to 3·7)	1 (0·6 to 1·4)	251
Oral disorders	0 (0 to 0)	0 (0 to 0)	0 (0 to 0)	1·3 (0·7 to 2·2)	1·7 (0·9 to 2·9)	2 (1 to 3·4)	3·2 (1·7 to 5·5)	5·4 (2·8 to 10)	7·9 (4 to 15)	10·3 (5 to 19·4)	12 (5·7 to 22·4)	13·9 (6·7 to 25·2)	16·2 (8·5 to 28·3)	18·7 (10·5 to 32·1)	19·9 (11·3 to 32·5)	19 (11·6 to 29·9)	16·8 (10·2 to 25·9)	13·8 (8·5 to 20·9)	9·7 (6·1 to 14·5)	6·2 (3·9 to 9·1)	3·5 (2·1 to 5·1)	1·4 (0·9 to 2)	0·4 (0·3 to 0·6)	183
Caries of deciduous teeth	0 (0 to 0)	0 (0 to 0)	0 (0 to 0)	0·4 (0·2 to 0·8)	0·4 (0·2 to 0·9)	0 (0 to 0·1)	0 (0 to 0)	0 (0 to 0)	0 (0 to 0)	0 (0 to 0)	0 (0 to 0)	0 (0 to 0)	0 (0 to 0)	0 (0 to 0)	0 (0 to 0)	0 (0 to 0)	0 (0 to 0)	0 (0 to 0)	0 (0 to 0)	0 (0 to 0)	0 (0 to 0)	0 (0 to 0)	0 (0 to 0)	0·8
Caries of permanent teeth	0 (0 to 0)	0 (0 to 0)	0 (0 to 0)	0 (0 to 0)	0·4 (0·1 to 0·9)	0·9 (0·4 to 1·9)	1 (0·4 to 2·1)	1·3 (0·5 to 2·6)	1·2 (0·5 to 2·5)	1·2 (0·5 to 2·5)	1·2 (0·5 to 2·3)	1·1 (0·4 to 2·2)	1 (0·4 to 2·2)	0·9 (0·4 to 2)	0·7 (0·3 to 1·6)	0·5 (0·2 to 1·1)	0·3 (0·1 to 0·7)	0·2 (0·1 to 0·5)	0·1 (0·1 to 0·3)	0·1 (0 to 0·2)	0 (0 to 0·1)	0 (0 to 0)	0 (0 to 0)	12·3
Periodontal diseases	0 (0 to 0)	0 (0 to 0)	0 (0 to 0)	0 (0 to 0)	0 (0 to 0)	0 (0 to 0)	0·4 (0·1 to 1·1)	1·7 (0·5 to 4·4)	3·8 (1·2 to 9·1)	5·8 (2 to 13·4)	7·2 (2·6 to 15·9)	8·3 (3 to 17·7)	8·8 (3·4 to 17·9)	8·2 (3·2 to 16·7)	6·5 (2·6 to 13·4)	4·4 (1·8 to 9)	2·9 (1·1 to 6)	1·9 (0·7 to 3·9)	1·2 (0·5 to 2·4)	0·7 (0·3 to 1·4)	0·4 (0·1 to 0·8)	0·2 (0·1 to 0·3)	0 (0 to 0·1)	62·5
Other oral disorders	0 (0 to 0)	0 (0 to 0)	0 (0 to 0)	0·9 (0·5 to 1·4)	0·9 (0·5 to 1·4)	1·1 (0·6 to 1·6)	1·7 (1 to 2·6)	2·2 (1·3 to 3·4)	2·4 (1·4 to 3·5)	2·5 (1·5 to 3·9)	2·5 (1·5 to 3·8)	2·5 (1·5 to 3·9)	2·6 (1·6 to 3·9)	2·4 (1·5 to 3·6)	2 (1·2 to 3·1)	1·5 (0·9 to 2·3)	1·1 (0·7 to 1·7)	0·7 (0·4 to 1·1)	0·4 (0·2 to 0·6)	0·2 (0·1 to 0·3)	0·1 (0·1 to 0·1)	0 (0 to 0)	0 (0 to 0)	27·7
Sudden infant death syndrome	--	0·1 (0·1 to 0·2)	0·9 (0·5 to 1·5)	--	--	--	--	--	--	--	--	--	--	--	--	--	--	--	--	--	--	--	--	NA
Injuries	1·1 (0·8 to 1·6)	0·9 (0·6 to 1·3)	7·2 (5 to 10·7)	29·5 (16·9 to 49·3)	16·5 (13·8 to 19·6)	20·8 (17·7 to 24·4)	37·3 (32·1 to 43·7)	37·5 (31·8 to 44·9)	35·3 (29·9 to 42·3)	35·7 (29·5 to 43·3)	37·1 (30·6 to 45·3)	38·4 (31·4 to 46·4)	39·4 (32·2 to 48·5)	38·4 (30·9 to 47)	35·2 (28·4 to 43·3)	29·4 (23·8 to 36·1)	24·5 (20·2 to 29·8)	21·5 (17·7 to 25·7)	18 (14·9 to 21·3)	15 (12·5 to 17·7)	11·5 (9·6 to 13·4)	6·6 (5·5 to 7·6)	2·8 (2·3 to 3·2)	539
Transport injuries	0·1 (0 to 0·1)	0 (0 to 0·1)	0·5 (0·3 to 0·7)	3·9 (1·9 to 7·3)	3·2 (2·1 to 4·5)	4·4 (3·1 to 5·8)	10·8 (7·4 to 14·6)	9·1 (6·6 to 11·9)	7·7 (5·7 to 10·2)	6·9 (5·2 to 9)	7·6 (5·7 to 9·8)	8 (6·2 to 10·1)	8·6 (6·5 to 10·9)	8·2 (6·3 to 10·3)	7·6 (5·8 to 9·8)	6·1 (4·8 to 7·8)	5·1 (3·9 to 6·3)	3·7 (2·9 to 4·7)	2·6 (2·1 to 3·2)	1·6 (1·2 to 2)	0·9 (0·7 to 1·1)	0·4 (0·3 to 0·5)	0·1 (0·1 to 0·2)	107
Road injuries	0 (0 to 0·1)	0 (0 to 0)	0·4 (0·2 to 0·6)	3·2 (1·5 to 6·2)	2·8 (1·8 to 3·9)	3·8 (2·7 to 5·1)	9·8 (6·6 to 13·2)	8·1 (5·8 to 10·7)	6·8 (5 to 9)	6·1 (4·6 to 8)	6·8 (5·1 to 8·9)	7 (5·3 to 8·8)	7·7 (5·8 to 9·8)	7·3 (5·5 to 9·2)	6·7 (5 to 8·6)	5·5 (4·3 to 7·1)	4·7 (3·6 to 5·8)	3·4 (2·7 to 4·3)	2·3 (1·8 to 2·8)	1·4 (1·1 to 1·8)	0·8 (0·6 to 1)	0·3 (0·3 to 0·4)	0·1 (0·1 to 0·1)	94·9
Pedestrian road injuries	0 (0 to 0)	0 (0 to 0)	0·1 (0 to 0·1)	1 (0·5 to 1·9)	0·7 (0·4 to 1)	0·8 (0·5 to 1·1)	1 (0·7 to 1·3)	0·8 (0·6 to 1·1)	0·8 (0·6 to 1)	1 (0·7 to 1·2)	1·1 (0·8 to 1·5)	1·2 (0·9 to 1·6)	1·5 (1·1 to 1·9)	1·6 (1·2 to 2·1)	1·6 (1·2 to 2)	1·5 (1·1 to 1·9)	1·4 (1·1 to 1·8)	1·1 (0·9 to 1·4)	0·8 (0·6 to 1)	0·5 (0·4 to 0·7)	0·3 (0·2 to 0·4)	0·1 (0·1 to 0·1)	0 (0 to 0·1)	19·1
Cyclist road injuries	0 (0 to 0)	0 (0 to 0)	0 (0 to 0)	0·3 (0·1 to 0·5)	0·3 (0·2 to 0·4)	0·6 (0·3 to 0·9)	0·9 (0·6 to 1·5)	0·8 (0·6 to 1·1)	0·8 (0·6 to 1·1)	0·9 (0·6 to 1·2)	1 (0·7 to 1·3)	1·1 (0·8 to 1·4)	1·2 (0·8 to 1·5)	1·2 (0·9 to 1·6)	1 (0·7 to 1·3)	0·8 (0·6 to 1)	0·6 (0·4 to 0·8)	0·4 (0·3 to 0·6)	0·3 (0·2 to 0·4)	0·2 (0·1 to 0·2)	0·1 (0·1 to 0·2)	0 (0 to 0·1)	0 (0 to 0)	12·4
Motorcyclist road injuries	0 (0 to 0)	0 (0 to 0)	0·1 (0 to 0·1)	0·3 (0·1 to 0·6)	0·3 (0·1 to 0·5)	0·4 (0·2 to 0·7)	2·3 (1·2 to 3·8)	2 (1·1 to 3·1)	1·5 (0·9 to 2·4)	1·1 (0·7 to 1·7)	1·2 (0·8 to 1·8)	1 (0·6 to 1·5)	1·1 (0·8 to 1·6)	0·8 (0·5 to 1·1)	0·7 (0·5 to 1·1)	0·5 (0·4 to 0·7)	0·5 (0·3 to 0·6)	0·3 (0·2 to 0·4)	0·2 (0·1 to 0·3)	0·2 (0·1 to 0·2)	0·1 (0·1 to 0·1)	0 (0 to 0·1)	0 (0 to 0)	14·5
Motor vehicle road injuries	0 (0 to 0)	0 (0 to 0)	0·2 (0·2 to 0·4)	1·5 (0·7 to 2·9)	1·4 (0·9 to 2)	1·8 (1·2 to 2·5)	5·3 (3·4 to 7·6)	4·3 (2·9 to 5·9)	3·4 (2·3 to 4·7)	2·9 (2·1 to 4)	3·1 (2·2 to 4·3)	3·4 (2·4 to 4·4)	3·6 (2·6 to 4·8)	3·3 (2·5 to 4·4)	3·1 (2·2 to 4·1)	2·5 (1·9 to 3·2)	2 (1·5 to 2·6)	1·4 (1·1 to 1·8)	0·9 (0·7 to 1·1)	0·5 (0·4 to 0·6)	0·2 (0·2 to 0·3)	0·1 (0·1 to 0·1)	0 (0 to 0)	45
Other road injuries	0 (0 to 0)	0 (0 to 0)	0 (0 to 0)	0·2 (0·1 to 0·4)	0·1 (0·1 to 0·3)	0·1 (0·1 to 0·2)	0·2 (0·1 to 0·4)	0·2 (0·1 to 0·3)	0·3 (0·2 to 0·5)	0·3 (0·2 to 0·4)	0·3 (0·2 to 0·4)	0·3 (0·2 to 0·4)	0·3 (0·2 to 0·5)	0·3 (0·2 to 0·5)	0·3 (0·2 to 0·5)	0·3 (0·2 to 0·4)	0·2 (0·1 to 0·3)	0·1 (0·1 to 0·1)	0·1 (0·1 to 0·1)	0·1 (0 to 0·1)	0 (0 to 0)	0 (0 to 0)	0 (0 to 0)	3·9
Other transport injuries	0 (0 to 0)	0 (0 to 0)	0·1 (0·1 to 0·2)	0·7 (0·3 to 1·3)	0·4 (0·3 to 0·6)	0·6 (0·4 to 0·9)	1·1 (0·6 to 1·8)	1 (0·6 to 1·5)	0·9 (0·5 to 1·3)	0·8 (0·5 to 1·2)	0·8 (0·5 to 1·2)	1·1 (0·8 to 1·6)	0·8 (0·6 to 1·2)	1 (0·7 to 1·4)	1 (0·6 to 1·4)	0·6 (0·4 to 0·9)	0·4 (0·3 to 0·5)	0·3 (0·2 to 0·4)	0·3 (0·2 to 0·4)	0·1 (0·1 to 0·2)	0·1 (0·1 to 0·1)	0 (0 to 0·1)	0 (0 to 0)	12·2
Unintentional injuries	0·8 (0·5 to 1·2)	0·8 (0·5 to 1·1)	6·2 (4·1 to 9·4)	23·8 (13·6 to 39·8)	12·2 (10 to 14·9)	14·4 (11·8 to 17·5)	17·8 (14·5 to 21·6)	17·7 (14 to 22·6)	18 (14 to 23·3)	19·6 (15·1 to 25·5)	21·3 (16·6 to 27·7)	22·9 (17·7 to 29·9)	24·4 (18·8 to 31·3)	25·2 (19·6 to 32·3)	23·9 (18·8 to 30·6)	20·7 (16·2 to 26·2)	17·5 (14·1 to 21·7)	16·4 (13·3 to 19·9)	14·6 (12·1 to 17·5)	13 (10·8 to 15·4)	10·4 (8·7 to 12·1)	6·1 (5·1 to 7·1)	2·6 (2·1 to 3)	350
Falls	0 (0 to 0)	0 (0 to 0)	0·1 (0·1 to 0·1)	0·5 (0·3 to 0·8)	0·7 (0·5 to 1)	1·1 (0·7 to 1·6)	1·6 (1·1 to 2·3)	2 (1·3 to 2·9)	2·3 (1·5 to 3·4)	2·7 (1·9 to 3·9)	3·1 (2·2 to 4·5)	3·6 (2·5 to 5)	4·2 (3 to 5·8)	4·8 (3·4 to 6·5)	4·9 (3·5 to 6·6)	4·8 (3·6 to 6·3)	4·6 (3·5 to 5·9)	5 (3·9 to 6·4)	5·2 (4 to 6·4)	5·7 (4·5 to 7·1)	4·9 (3·9 to 6·1)	2·7 (2·1 to 3·3)	1·1 (0·8 to 1·3)	65·4
Drowning	0·1 (0 to 0·1)	0·1 (0 to 0·1)	0·7 (0·4 to 1)	7·5 (3·6 to 13·6)	3·2 (2·4 to 4·4)	3·8 (2·9 to 5)	3·3 (2·3 to 4·7)	1·5 (1·1 to 2·1)	1·3 (0·9 to 1·7)	1·2 (0·9 to 1·6)	1·2 (0·9 to 1·6)	1·1 (0·8 to 1·5)	1·2 (0·9 to 1·6)	1 (0·8 to 1·4)	0·9 (0·7 to 1·3)	0·7 (0·5 to 1)	0·6 (0·4 to 0·7)	0·4 (0·3 to 0·4)	0·3 (0·2 to 0·4)	0·2 (0·1 to 0·2)	0·1 (0·1 to 0·2)	0·1 (0·1 to 0·1)	0 (0 to 0·1)	30·6
Fire, heat, and hot substances	0 (0 to 0·1)	0·1 (0 to 0·1)	0·8 (0·5 to 1·2)	4·5 (2·3 to 7·8)	2 (1·5 to 2·6)	2·2 (1·6 to 2·9)	3·5 (2·6 to 4·7)	3·8 (2·7 to 5·1)	4·2 (3·1 to 5·6)	4·8 (3·4 to 6·5)	5·2 (3·9 to 7·2)	5·3 (3·9 to 7·3)	5·3 (3·9 to 7·3)	5·5 (4 to 7·4)	5 (3·6 to 6·6)	4 (3 to 5·3)	3 (2·3 to 4)	2·5 (2 to 3·2)	1·9 (1·4 to 2·4)	1·3 (1·1 to 1·7)	1 (0·8 to 1·2)	0·5 (0·4 to 0·6)	0·2 (0·2 to 0·3)	66·8
Poisonings	0 (0 to 0)	0·1 (0 to 0·1)	0·3 (0·2 to 0·4)	1·7 (0·9 to 3·1)	0·4 (0·3 to 0·6)	0·3 (0·2 to 0·5)	0·6 (0·4 to 0·8)	0·5 (0·4 to 0·8)	0·5 (0·3 to 0·7)	0·5 (0·4 to 0·7)	0·5 (0·4 to 0·7)	0·5 (0·4 to 0·7)	0·5 (0·4 to 0·7)	0·5 (0·4 to 0·7)	0·5 (0·4 to 0·6)	0·3 (0·3 to 0·4)	0·3 (0·3 to 0·4)	0·3 (0·2 to 0·4)	0·2 (0·1 to 0·3)	0·2 (0·1 to 0·2)	0·1 (0·1 to 0·1)	0 (0 to 0)	0 (0 to 0)	8·9
Poisoning by carbon monoxide	0 (0 to 0)	0 (0 to 0)	0 (0 to 0·1)	0·5 (0·2 to 1)	0·1 (0·1 to 0·2)	0·1 (0·1 to 0·2)	0·2 (0·2 to 0·4)	0·2 (0·2 to 0·3)	0·2 (0·1 to 0·3)	0·2 (0·1 to 0·3)	0·2 (0·1 to 0·3)	0·2 (0·2 to 0·3)	0·2 (0·1 to 0·3)	0·2 (0·1 to 0·3)	0·2 (0·2 to 0·3)	0·2 (0·1 to 0·2)	0·2 (0·1 to 0·3)	0·1 (0·1 to 0·2)	0·1 (0·1 to 0·2)	0·1 (0 to 0·1)	0 (0 to 0)	0 (0 to 0)	0 (0 to 0)	3·4
Poisoning by other means	0 (0 to 0)	0·1 (0 to 0·1)	0·2 (0·1 to 0·4)	1·2 (0·6 to 2·3)	0·3 (0·2 to 0·4)	0·2 (0·1 to 0·3)	0·3 (0·2 to 0·5)	0·3 (0·2 to 0·4)	0·3 (0·2 to 0·4)	0·3 (0·2 to 0·4)	0·3 (0·2 to 0·5)	0·3 (0·2 to 0·4)	0·3 (0·2 to 0·4)	0·3 (0·2 to 0·4)	0·2 (0·2 to 0·3)	0·2 (0·1 to 0·2)	0·1 (0·1 to 0·2)	0·1 (0·1 to 0·2)	0·1 (0·1 to 0·2)	0·1 (0·1 to 0·1)	0·1 (0 to 0·1)	0 (0 to 0)	0 (0 to 0)	5·5
Exposure to mechanical forces	0·1 (0 to 0·1)	0·1 (0·1 to 0·1)	0·5 (0·4 to 0·8)	4·1 (2·2 to 7·2)	2·9 (2·2 to 3·8)	3·4 (2·6 to 4·6)	4·7 (3·4 to 6·5)	5 (3·6 to 6·8)	4·6 (3·4 to 6·2)	4·8 (3·6 to 6·5)	5·3 (3·9 to 7·2)	5·7 (4·2 to 7·7)	5·7 (4·2 to 7·7)	5·8 (4·3 to 7·8)	5 (3·6 to 6·7)	4 (2·9 to 5·4)	3·1 (2·3 to 4·2)	2·7 (2 to 3·6)	2·2 (1·6 to 2·9)	1·7 (1·3 to 2·2)	1·3 (0·9 to 1·6)	0·8 (0·7 to 1·1)	0·4 (0·3 to 0·5)	74
Unintentional firearm injuries	0 (0 to 0)	0 (0 to 0)	0·1 (0·1 to 0·2)	0·7 (0·3 to 1·4)	0·6 (0·4 to 1)	1·3 (0·8 to 1·9)	2·4 (1·5 to 3·7)	2·5 (1·7 to 3·6)	1·8 (1·2 to 2·5)	1·9 (1·3 to 2·6)	2 (1·4 to 2·8)	2 (1·4 to 2·8)	1·7 (1·2 to 2·3)	1·4 (1 to 1·8)	1·2 (0·9 to 1·6)	0·8 (0·6 to 1)	0·6 (0·4 to 0·7)	0·6 (0·5 to 0·9)	0·5 (0·4 to 0·7)	0·6 (0·4 to 0·7)	0·5 (0·4 to 0·7)	0·4 (0·3 to 0·5)	0·2 (0·2 to 0·3)	23·8
Other exposure to mechanical forces	0 (0 to 0·1)	0·1 (0 to 0·1)	0·4 (0·3 to 0·6)	3·4 (1·8 to 6)	2·3 (1·7 to 3·1)	2·1 (1·5 to 3)	2·2 (1·5 to 3·3)	2·5 (1·7 to 3·6)	2·8 (2 to 4·1)	3 (2 to 4·3)	3·4 (2·2 to 4·9)	3·8 (2·6 to 5·4)	4 (2·8 to 5·7)	4·4 (3·2 to 6·2)	3·8 (2·7 to 5·3)	3·2 (2·2 to 4·4)	2·6 (1·9 to 3·6)	2·1 (1·4 to 2·8)	1·6 (1·2 to 2·2)	1·1 (0·8 to 1·5)	0·8 (0·6 to 1)	0·4 (0·3 to 0·6)	0·2 (0·1 to 0·3)	50·2
Adverse effects of medical treatment	0·2 (0·1 to 0·3)	0·1 (0·1 to 0·2)	0·7 (0·3 to 1·2)	0·7 (0·3 to 1·4)	0·3 (0·2 to 0·4)	0·3 (0·2 to 0·4)	0·4 (0·3 to 0·6)	0·7 (0·5 to 1·1)	0·7 (0·4 to 1·1)	0·9 (0·6 to 1·3)	0·9 (0·6 to 1·2)	1·2 (0·8 to 1·6)	1·6 (1·1 to 2·3)	1·7 (1·1 to 2·5)	2 (1·3 to 2·9)	2 (1·2 to 3)	1·7 (1·1 to 2·5)	1·8 (1·1 to 2·6)	1·6 (0·9 to 2·3)	1·1 (0·7 to 1·6)	0·9 (0·5 to 1·3)	0·4 (0·2 to 0·6)	0·2 (0·1 to 0·2)	21·9
Animal contact	0 (0 to 0)	0 (0 to 0)	0·1 (0·1 to 0·2)	0·3 (0·2 to 0·6)	0·2 (0·1 to 0·3)	0·3 (0·2 to 0·4)	0·3 (0·2 to 0·4)	0·3 (0·2 to 0·4)	0·3 (0·2 to 0·4)	0·3 (0·2 to 0·5)	0·4 (0·3 to 0·5)	0·4 (0·3 to 0·5)	0·4 (0·3 to 0·6)	0·4 (0·3 to 0·6)	0·5 (0·4 to 0·8)	0·4 (0·3 to 0·6)	0·3 (0·2 to 0·5)	0·4 (0·3 to 0·6)	0·5 (0·3 to 0·6)	0·7 (0·4 to 1)	0·7 (0·4 to 1)	0·6 (0·4 to 0·9)	0·3 (0·2 to 0·4)	8·2
Venomous animal contact	0 (0 to 0)	0 (0 to 0)	0 (0 to 0·1)	0·1 (0·1 to 0·3)	0·1 (0·1 to 0·2)	0·2 (0·1 to 0·3)	0·2 (0·1 to 0·2)	0·2 (0·1 to 0·2)	0·2 (0·2 to 0·3)	0·2 (0·2 to 0·3)	0·2 (0·2 to 0·3)	0·3 (0·2 to 0·4)	0·2 (0·2 to 0·3)	0·2 (0·1 to 0·3)	0·3 (0·2 to 0·4)	0·2 (0·2 to 0·3)	0·2 (0·1 to 0·3)	0·2 (0·1 to 0·4)	0·3 (0·2 to 0·4)	0·4 (0·2 to 0·6)	0·4 (0·3 to 0·7)	0·4 (0·3 to 0·6)	0·2 (0·1 to 0·3)	4·9
Non-venomous animal contact	0 (0 to 0)	0 (0 to 0)	0·1 (0 to 0·1)	0·2 (0·1 to 0·4)	0·1 (0·1 to 0·2)	0·1 (0 to 0·1)	0·1 (0·1 to 0·2)	0·1 (0·1 to 0·2)	0·1 (0·1 to 0·1)	0·1 (0·1 to 0·1)	0·1 (0·1 to 0·2)	0·1 (0·1 to 0·2)	0·2 (0·1 to 0·3)	0·2 (0·1 to 0·3)	0·3 (0·2 to 0·4)	0·2 (0·1 to 0·3)	0·1 (0·1 to 0·2)	0·2 (0·1 to 0·3)	0·2 (0·1 to 0·3)	0·3 (0·1 to 0·5)	0·2 (0·1 to 0·4)	0·2 (0·1 to 0·3)	0·1 (0·1 to 0·2)	3·3
Foreign body	0·4 (0·2 to 0·7)	0·3 (0·2 to 0·5)	2·7 (1·4 to 5·1)	2·7 (1·4 to 4·8)	0·7 (0·6 to 0·9)	0·7 (0·5 to 0·9)	0·5 (0·4 to 0·7)	0·6 (0·5 to 0·9)	0·6 (0·5 to 0·9)	0·6 (0·5 to 0·9)	0·7 (0·5 to 1)	0·8 (0·6 to 1·1)	0·9 (0·7 to 1·2)	1 (0·8 to 1·4)	1 (0·8 to 1·3)	1 (0·8 to 1·3)	1 (0·8 to 1·3)	1·1 (0·8 to 1·4)	1 (0·7 to 1·4)	0·9 (0·7 to 1·1)	0·5 (0·4 to 0·6)	0·3 (0·2 to 0·4)	0·1 (0·1 to 0·2)	20·3
Pulmonary aspiration and foreign body in airway	0·4 (0·2 to 0·7)	0·3 (0·2 to 0·5)	2·7 (1·3 to 5·1)	2·5 (1·3 to 4·7)	0·6 (0·4 to 0·8)	0·5 (0·4 to 0·7)	0·3 (0·2 to 0·4)	0·3 (0·3 to 0·4)	0·3 (0·2 to 0·4)	0·3 (0·2 to 0·4)	0·4 (0·3 to 0·5)	0·4 (0·3 to 0·6)	0·5 (0·4 to 0·7)	0·7 (0·5 to 0·9)	0·7 (0·5 to 1)	0·8 (0·5 to 1)	0·8 (0·6 to 1)	0·9 (0·6 to 1·2)	0·9 (0·6 to 1·2)	0·7 (0·6 to 1)	0·4 (0·3 to 0·5)	0·2 (0·2 to 0·3)	0·1 (0·1 to 0·2)	15·8
Foreign body in eyes	0 (0 to 0)	0 (0 to 0)	0 (0 to 0)	0 (0 to 0·1)	0·1 (0 to 0·1)	0·1 (0 to 0·2)	0·1 (0 to 0·2)	0·1 (0 to 0·3)	0·2 (0·1 to 0·3)	0·2 (0·1 to 0·4)	0·2 (0·1 to 0·4)	0·2 (0·1 to 0·4)	0·2 (0·1 to 0·4)	0·2 (0·1 to 0·3)	0·1 (0·1 to 0·2)	0·1 (0·1 to 0·2)	0·1 (0 to 0·1)	0·1 (0 to 0·1)	0 (0 to 0·1)	0 (0 to 0)	0 (0 to 0)	0 (0 to 0)	0 (0 to 0)	1·9
Foreign body in other body part	0 (0 to 0)	0 (0 to 0)	0 (0 to 0)	0·1 (0·1 to 0·1)	0·1 (0·1 to 0·2)	0·1 (0·1 to 0·2)	0·1 (0·1 to 0·2)	0·2 (0·1 to 0·2)	0·2 (0·1 to 0·2)	0·2 (0·1 to 0·2)	0·2 (0·1 to 0·2)	0·2 (0·1 to 0·2)	0·2 (0·1 to 0·3)	0·2 (0·1 to 0·3)	0·2 (0·1 to 0·2)	0·1 (0·1 to 0·2)	0·1 (0·1 to 0·2)	0·1 (0·1 to 0·2)	0·1 (0·1 to 0·2)	0·1 (0·1 to 0·2)	0·1 (0 to 0·1)	0 (0 to 0·1)	0 (0 to 0)	2·6
Environmental heat and cold exposure	0 (0 to 0)	0 (0 to 0)	0·1 (0 to 0·1)	0·3 (0·2 to 0·5)	0·5 (0·3 to 0·8)	0·7 (0·4 to 1)	0·9 (0·6 to 1·3)	1·1 (0·7 to 1·5)	1·2 (0·8 to 1·7)	1·4 (0·9 to 2)	1·4 (1 to 2·1)	1·5 (1 to 2·2)	1·6 (1·1 to 2·3)	1·7 (1·2 to 2·3)	1·6 (1·1 to 2·1)	1·3 (0·9 to 1·8)	1·1 (0·8 to 1·5)	1 (0·7 to 1·3)	0·8 (0·6 to 1·1)	0·7 (0·5 to 0·9)	0·5 (0·4 to 0·7)	0·4 (0·3 to 0·6)	0·2 (0·1 to 0·3)	19·9
Exposure to forces of nature	0 (0 to 0)	0 (0 to 0)	0 (0 to 0)	0 (0 to 0)	0 (0 to 0)	0 (0 to 0)	0 (0 to 0)	0·1 (0·1 to 0·2)	0·1 (0·1 to 0·1)	0·1 (0·1 to 0·2)	0·1 (0·1 to 0·2)	0·1 (0·1 to 0·2)	0·1 (0·1 to 0·2)	0·2 (0·1 to 0·2)	0·2 (0·1 to 0·2)	0·1 (0·1 to 0·2)	0·1 (0·1 to 0·2)	0·1 (0·1 to 0·2)	0·1 (0·1 to 0·2)	0·1 (0·1 to 0·1)	0·1 (0 to 0·1)	0 (0 to 0)	0 (0 to 0)	1·8
Other unintentional injuries	0·1 (0 to 0·1)	0 (0 to 0)	0·3 (0·2 to 0·4)	1·5 (0·9 to 2·3)	1·3 (0·9 to 1·8)	1·6 (1·1 to 2·1)	1·9 (1·4 to 2·6)	2 (1·4 to 2·9)	2·1 (1·5 to 2·9)	2·2 (1·6 to 3·2)	2·4 (1·7 to 3·3)	2·5 (1·8 to 3·5)	2·7 (1·9 to 3·7)	2·6 (1·9 to 3·7)	2·3 (1·6 to 3·2)	1·9 (1·4 to 2·6)	1·5 (1·1 to 2)	1·2 (0·8 to 1·6)	0·9 (0·7 to 1·2)	0·6 (0·4 to 0·8)	0·3 (0·2 to 0·4)	0·1 (0·1 to 0·2)	0 (0 to 0·1)	32·2
Self-harm and interpersonal violence	0·3 (0·2 to 0·4)	0·1 (0 to 0·1)	0·5 (0·3 to 0·8)	1·8 (0·9 to 3·2)	1·1 (0·8 to 1·4)	2 (1·6 to 2·6)	8·7 (6·7 to 11)	10·7 (8·2 to 13·7)	9·7 (7·6 to 12·5)	9·2 (7·2 to 11·7)	8·2 (6·5 to 10·4)	7·4 (5·8 to 9·4)	6·4 (5·1 to 8·1)	4·9 (3·8 to 6·2)	3·7 (3 to 4·6)	2·6 (2·1 to 3·2)	1·9 (1·6 to 2·3)	1·3 (1·1 to 1·6)	0·8 (0·7 to 1)	0·4 (0·3 to 0·5)	0·2 (0·2 to 0·3)	0·1 (0·1 to 0·1)	0 (0 to 0)	82·1
Self-harm	0 (0 to 0)	0 (0 to 0)	0 (0 to 0)	0 (0 to 0)	0 (0 to 0)	0·2 (0·1 to 0·3)	2 (1·2 to 2·9)	2·4 (1·6 to 3·6)	2·4 (1·5 to 3·4)	2·5 (1·6 to 3·7)	2·4 (1·6 to 3·5)	2·2 (1·5 to 3·2)	2·1 (1·4 to 2·9)	1·7 (1·2 to 2·5)	1·4 (1 to 1·9)	1 (0·7 to 1·3)	0·7 (0·5 to 0·9)	0·4 (0·4 to 0·6)	0·2 (0·2 to 0·3)	0·1 (0·1 to 0·2)	0·1 (0·1 to 0·1)	0 (0 to 0)	0 (0 to 0)	21·8
Self-harm by firearm	0 (0 to 0)	0 (0 to 0)	0 (0 to 0)	0 (0 to 0)	0 (0 to 0)	0 (0 to 0)	0·1 (0 to 0·1)	0·1 (0 to 0·1)	0 (0 to 0·1)	0·1 (0 to 0·1)	0·1 (0 to 0·1)	0 (0 to 0·1)	0 (0 to 0·1)	0·1 (0 to 0·1)	0 (0 to 0·1)	0 (0 to 0)	0 (0 to 0)	0 (0 to 0)	0 (0 to 0)	0 (0 to 0)	0 (0 to 0)	0 (0 to 0)	0 (0 to 0)	0·6
Self-harm by other specified means	0 (0 to 0)	0 (0 to 0)	0 (0 to 0)	0 (0 to 0)	0 (0 to 0)	0·2 (0·1 to 0·3)	1·9 (1·2 to 2·8)	2·4 (1·5 to 3·5)	2·3 (1·5 to 3·3)	2·5 (1·6 to 3·6)	2·4 (1·6 to 3·4)	2·2 (1·5 to 3·1)	2 (1·4 to 2·8)	1·7 (1·2 to 2·4)	1·3 (0·9 to 1·8)	1 (0·7 to 1·3)	0·7 (0·5 to 0·9)	0·4 (0·3 to 0·5)	0·2 (0·2 to 0·3)	0·1 (0·1 to 0·1)	0·1 (0·1 to 0·1)	0 (0 to 0)	0 (0 to 0)	21·2
Interpersonal violence	0·3 (0·2 to 0·4)	0·1 (0 to 0·1)	0·5 (0·3 to 0·8)	1·8 (0·8 to 3·1)	1 (0·8 to 1·4)	1·8 (1·4 to 2·4)	6·8 (4·9 to 8·8)	8·3 (6·1 to 10·7)	7·3 (5·5 to 9·7)	6·7 (5 to 8·7)	5·8 (4·4 to 7·5)	5·2 (3·8 to 6·9)	4·3 (3·3 to 5·6)	3·2 (2·4 to 4·2)	2·3 (1·8 to 3)	1·6 (1·3 to 2)	1·2 (1 to 1·6)	0·9 (0·7 to 1·1)	0·6 (0·5 to 0·7)	0·3 (0·2 to 0·4)	0·2 (0·1 to 0·2)	0·1 (0 to 0·1)	0 (0 to 0)	60·1
Physical violence by firearm	0 (0 to 0)	0 (0 to 0)	0 (0 to 0)	0·1 (0·1 to 0·3)	0·1 (0·1 to 0·2)	0·3 (0·2 to 0·4)	2·4 (1·5 to 3·4)	2·9 (1·9 to 4·2)	2·3 (1·6 to 3·4)	1·9 (1·2 to 2·7)	1·6 (1·1 to 2·3)	1·1 (0·8 to 1·6)	1 (0·7 to 1·5)	0·6 (0·4 to 0·9)	0·4 (0·3 to 0·6)	0·2 (0·1 to 0·3)	0·1 (0·1 to 0·1)	0·1 (0 to 0·1)	0 (0 to 0)	0 (0 to 0)	0 (0 to 0)	0 (0 to 0)	0 (0 to 0)	15·2
Physical violence by sharp object	0 (0 to 0)	0 (0 to 0)	0 (0 to 0)	0 (0 to 0·1)	0·1 (0 to 0·1)	0·1 (0·1 to 0·1)	0·4 (0·3 to 0·5)	0·6 (0·4 to 0·8)	0·5 (0·4 to 0·7)	0·5 (0·4 to 0·6)	0·5 (0·4 to 0·6)	0·4 (0·3 to 0·6)	0·3 (0·2 to 0·4)	0·3 (0·2 to 0·3)	0·2 (0·2 to 0·3)	0·1 (0·1 to 0·2)	0·1 (0·1 to 0·1)	0·1 (0·1 to 0·1)	0·1 (0 to 0·1)	0 (0 to 0)	0 (0 to 0)	0 (0 to 0)	0 (0 to 0)	4·3
Sexual violence	0 (0 to 0)	0 (0 to 0)	0 (0 to 0)	0 (0 to 0)	0·2 (0·1 to 0·3)	0·4 (0·2 to 0·7)	1·1 (0·6 to 1·8)	1·6 (0·9 to 2·5)	1·5 (0·9 to 2·4)	1·4 (0·8 to 2·4)	1·3 (0·7 to 2)	1·1 (0·6 to 1·7)	0·8 (0·5 to 1·4)	0·6 (0·3 to 1)	0·4 (0·2 to 0·6)	0·3 (0·2 to 0·4)	0·2 (0·1 to 0·3)	0·1 (0·1 to 0·2)	0·1 (0·1 to 0·1)	0·1 (0 to 0·1)	0 (0 to 0)	0 (0 to 0)	0 (0 to 0)	11·2
Physical violence by other means	0·3 (0·2 to 0·4)	0·1 (0 to 0·1)	0·5 (0·3 to 0·8)	1·6 (0·7 to 2·8)	0·7 (0·5 to 1)	1 (0·7 to 1·4)	2·8 (1·9 to 3·9)	3·2 (2·2 to 4·5)	2·9 (2·1 to 4·2)	2·8 (2 to 3·9)	2·4 (1·7 to 3·3)	2·5 (1·8 to 3·5)	2·2 (1·5 to 2·9)	1·8 (1·3 to 2·4)	1·4 (1 to 1·8)	1 (0·8 to 1·3)	0·8 (0·6 to 1·1)	0·6 (0·5 to 0·8)	0·4 (0·3 to 0·5)	0·2 (0·1 to 0·2)	0·1 (0·1 to 0·1)	0 (0 to 0)	0 (0 to 0)	29·3
Conflict and terrorism	0 (0 to 0)	0 (0 to 0)	0 (0 to 0)	0 (0 to 0)	0 (0 to 0)	0 (0 to 0)	0 (0 to 0)	0 (0 to 0)	0 (0 to 0)	0 (0 to 0)	0 (0 to 0)	0 (0 to 0)	0 (0 to 0)	0 (0 to 0)	0 (0 to 0)	0 (0 to 0)	0 (0 to 0)	0 (0 to 0)	0 (0 to 0)	0 (0 to 0)	0 (0 to 0)	0 (0 to 0)	0 (0 to 0)	0

SR Table 2b: Bahamas DALYS by cause and age group for females with 95% uncertainty intervals, 2019

Cause	Early Neonatal	Late Neonatal	Post Neonatal	1 to 4	5 to 9	10 to 14	15 to 19	20 to 24	25 to 29	30 to 34	35 to 39	40 to 44	45 to 49	50 to 54	55 to 59	60 to 64	65 to 69	70 to 74	75 to 79	80 to 84	85 to 89	90 to 94	95 plus	Total
All causes	678 (544 to 837)	353 (217 to 567)	569 (462 to 697)	689 (566 to 845)	874 (708 to 1,080)	1,310 (1,050 to 1,660)	1,940 (1,570 to 2,390)	2,420 (1,980 to 2,930)	2,970 (2,450 to 3,590)	3,380 (2,810 to 4,030)	3,860 (3,210 to 4,590)	4,160 (3,480 to 5,000)	4,620 (3,840 to 5,540)	4,890 (4,100 to 5,890)	4,790 (4,020 to 5,690)	4,470 (3,810 to 5,270)	3,680 (3,160 to 4,280)	2,890 (2,490 to 3,340)	2,600 (2,270 to 3,000)	2,050 (1,820 to 2,330)	1,160 (1,020 to 1,310)	587 (527 to 656)	311 (287 to 338)	55,300
Communicable, maternal, neonatal, and nutritional diseases	571 (452 to 709)	264 (162 to 430)	253 (196 to 316)	210 (161 to 280)	223 (154 to 324)	212 (155 to 292)	272 (205 to 371)	370 (303 to 461)	595 (521 to 686)	693 (616 to 789)	711 (626 to 816)	654 (571 to 769)	545 (471 to 645)	413 (342 to 502)	315 (256 to 395)	228 (184 to 280)	165 (133 to 203)	119 (93·8 to 150)	119 (92·4 to 153)	108 (84·9 to 135)	71·8 (56·5 to 89·5)	42·1 (31·5 to 52·6)	22·8 (15·6 to 29·7)	7,180
HIV/AIDS and sexually transmitted infections	0·1 (0·1 to 0·2)	0·1 (0 to 0·1)	18·8 (17·4 to 20·1)	15·2 (14·2 to 16·4)	17·1 (15·9 to 18·3)	17·7 (16·4 to 19·1)	39·4 (35·7 to 44·4)	130 (119 to 145)	339 (313 to 371)	439 (406 to 480)	447 (414 to 481)	401 (372 to 437)	313 (289 to 338)	185 (171 to 199)	108 (99·4 to 118)	54·6 (49·9 to 59·4)	33·2 (30·5 to 36·1)	8·2 (7·2 to 9·5)	4·8 (4·1 to 5·5)	2·6 (2·3 to 2·9)	1·2 (1·1 to 1·4)	0·3 (0·3 to 0·4)	0·2 (0·1 to 0·2)	2,570
HIV/AIDS	0 (0 to 0)	0 (0 to 0)	18·7 (17·4 to 20·1)	15·2 (14·2 to 16·4)	17·1 (15·9 to 18·3)	17·4 (16 to 18·6)	37·6 (34·2 to 42·3)	126 (115 to 139)	332 (308 to 362)	430 (398 to 470)	437 (406 to 469)	391 (364 to 426)	305 (282 to 329)	180 (166 to 193)	105 (96·6 to 113)	52·5 (48·3 to 56·9)	31·8 (29·3 to 34·3)	7·3 (6·5 to 8·2)	4·2 (3·7 to 4·6)	2·2 (2 to 2·5)	1·1 (1 to 1·2)	0·3 (0·2 to 0·3)	0·1 (0·1 to 0·2)	2,510
HIV/AIDS - Drug-susceptible Tuberculosis	0 (0 to 0)	0 (0 to 0)	0·6 (0·3 to 0·8)	0·5 (0·3 to 0·7)	0·5 (0·3 to 0·8)	0·6 (0·4 to 0·9)	1·2 (0·8 to 1·7)	3·9 (2·5 to 5·7)	10·1 (6·6 to 15)	12·9 (8·2 to 19·1)	13·4 (8·5 to 19·9)	11·9 (7·5 to 17·9)	9·3 (5·8 to 13·7)	5·5 (3·6 to 8·2)	3·2 (2·1 to 4·7)	1·6 (1·1 to 2·4)	1 (0·6 to 1·4)	0·2 (0·2 to 0·3)	0·1 (0·1 to 0·2)	0·1 (0 to 0·1)	0 (0 to 0)	0 (0 to 0)	0 (0 to 0)	76·6
HIV/AIDS - Multidrug-resistant Tuberculosis without extensive dru	0 (0 to 0)	0 (0 to 0)	0 (0 to 0·1)	0 (0 to 0·1)	0 (0 to 0·1)	0 (0 to 0·1)	0 (0 to 0·2)	0·2 (0 to 0·6)	0·4 (0 to 1·6)	0·5 (0·1 to 2)	0·5 (0·1 to 2·1)	0·5 (0·1 to 1·8)	0·4 (0 to 1·4)	0·2 (0 to 0·9)	0·1 (0 to 0·5)	0·1 (0 to 0·3)	0 (0 to 0·2)	0 (0 to 0)	0 (0 to 0)	0 (0 to 0)	0 (0 to 0)	0 (0 to 0)	0 (0 to 0)	2·9
HIV/AIDS - Extensively drug-resistant Tuberculosis	0 (0 to 0)	0 (0 to 0)	0 (0 to 0)	0 (0 to 0)	0 (0 to 0)	0 (0 to 0)	0 (0 to 0)	0 (0 to 0·1)	0·1 (0 to 0·3)	0·1 (0 to 0·3)	0·1 (0 to 0·3)	0·1 (0 to 0·3)	0·1 (0 to 0·2)	0 (0 to 0·1)	0 (0 to 0·1)	0 (0 to 0)	0 (0 to 0)	0 (0 to 0)	0 (0 to 0)	0 (0 to 0)	0 (0 to 0)	0 (0 to 0)	0 (0 to 0)	0·5
HIV/AIDS resulting in other diseases	0 (0 to 0)	0 (0 to 0)	18·1 (16·7 to 19·5)	14·7 (13·6 to 15·9)	16·5 (15·3 to 17·8)	16·8 (15·5 to 18·1)	36·4 (33 to 41·1)	122 (111 to 135)	321 (297 to 351)	416 (384 to 455)	423 (392 to 456)	379 (350 to 415)	295 (271 to 319)	174 (160 to 187)	101 (93·2 to 110)	50·7 (46·5 to 55·1)	30·8 (28·3 to 33·4)	7·1 (6·3 to 7·9)	4·1 (3·6 to 4·5)	2·2 (1·9 to 2·4)	1·1 (0·9 to 1·2)	0·3 (0·2 to 0·3)	0·1 (0·1 to 0·1)	2,430
Sexually transmitted infections excluding HIV	0·1 (0·1 to 0·2)	0·1 (0 to 0·1)	0 (0 to 0·1)	0 (0 to 0)	0 (0 to 0)	0·4 (0·2 to 0·6)	1·8 (1·1 to 2·9)	4·4 (2·5 to 7·7)	7 (3·9 to 12·2)	8·8 (4·9 to 15·3)	9·7 (5·7 to 16·3)	9·9 (5·6 to 17·7)	8·2 (4·5 to 14·8)	5 (2·6 to 9·4)	3·4 (1·7 to 6·6)	2·1 (1 to 4·3)	1·4 (0·6 to 2·8)	0·9 (0·4 to 1·7)	0·6 (0·3 to 1·1)	0·3 (0·2 to 0·6)	0·2 (0·1 to 0·3)	0·1 (0 to 0·1)	0 (0 to 0)	64·2
Syphilis	0·1 (0·1 to 0·2)	0·1 (0 to 0·1)	0 (0 to 0·1)	0 (0 to 0)	0 (0 to 0)	0·1 (0·1 to 0·2)	0·2 (0·1 to 0·3)	0·2 (0·1 to 0·3)	0·3 (0·2 to 0·5)	0·5 (0·3 to 0·7)	0·4 (0·2 to 0·5)	0·2 (0·1 to 0·2)	0·2 (0·1 to 0·3)	0·1 (0·1 to 0·2)	0 (0 to 0·1)	0 (0 to 0·1)	0 (0 to 0·1)	0 (0 to 0)	0 (0 to 0)	0 (0 to 0)	0 (0 to 0)	0 (0 to 0)	0 (0 to 0)	2·6
Chlamydial infection	0 (0 to 0)	0 (0 to 0)	0 (0 to 0)	0 (0 to 0)	0 (0 to 0)	0 (0 to 0·1)	0·2 (0·1 to 0·3)	0·5 (0·4 to 0·8)	0·6 (0·3 to 1·1)	0·7 (0·4 to 1·2)	1 (0·6 to 1·5)	1·1 (0·7 to 1·8)	0·8 (0·5 to 1·3)	0·2 (0·1 to 0·3)	0·1 (0·1 to 0·2)	0·1 (0 to 0·1)	0 (0 to 0)	0·1 (0 to 0·1)	0·1 (0 to 0·1)	0 (0 to 0)	0 (0 to 0)	0 (0 to 0)	0 (0 to 0)	5·5
Gonococcal infection	0 (0 to 0)	0 (0 to 0)	0 (0 to 0)	0 (0 to 0)	0 (0 to 0)	0 (0 to 0)	0·3 (0·2 to 0·4)	0·5 (0·3 to 0·8)	0·6 (0·4 to 0·9)	0·9 (0·6 to 1·3)	1 (0·7 to 1·5)	1·2 (0·8 to 1·7)	1 (0·6 to 1·4)	0·5 (0·3 to 0·8)	0·2 (0·2 to 0·4)	0·2 (0·1 to 0·3)	0·2 (0·1 to 0·3)	0·1 (0·1 to 0·1)	0·1 (0 to 0·1)	0 (0 to 0)	0 (0 to 0)	0 (0 to 0)	0 (0 to 0)	6·7
Trichomoniasis	0 (0 to 0)	0 (0 to 0)	0 (0 to 0)	0 (0 to 0)	0 (0 to 0)	0 (0 to 0·1)	0·3 (0·1 to 0·7)	1·4 (0·5 to 3·2)	2·6 (0·9 to 5·8)	2·9 (0·9 to 6·9)	2·8 (0·9 to 6·5)	2·7 (0·9 to 6·7)	2·5 (0·8 to 5·7)	1·9 (0·6 to 4·5)	1·3 (0·4 to 3·1)	0·8 (0·3 to 1·8)	0·4 (0·1 to 0·9)	0·3 (0·1 to 0·6)	0·2 (0·1 to 0·3)	0·1 (0 to 0·2)	0 (0 to 0·1)	0 (0 to 0)	0 (0 to 0)	20·1
Genital herpes	0 (0 to 0)	0 (0 to 0)	0 (0 to 0)	0 (0 to 0)	0 (0 to 0)	0·1 (0 to 0·2)	0·4 (0·1 to 1·1)	0·9 (0·3 to 2·1)	1·4 (0·5 to 3·4)	1·7 (0·5 to 4·2)	1·8 (0·6 to 4·5)	1·8 (0·6 to 4·6)	1·8 (0·6 to 4·4)	1·6 (0·5 to 4)	1·3 (0·4 to 3·2)	1 (0·3 to 2·4)	0·6 (0·2 to 1·6)	0·4 (0·1 to 1)	0·3 (0·1 to 0·7)	0·2 (0 to 0·4)	0·1 (0 to 0·2)	0 (0 to 0·1)	0 (0 to 0)	15·4
Other sexually transmitted infections	0 (0 to 0)	0 (0 to 0)	0 (0 to 0)	0 (0 to 0)	0 (0 to 0)	0·1 (0 to 0·1)	0·5 (0·3 to 0·7)	0·9 (0·5 to 1·4)	1·4 (0·8 to 2·3)	2·2 (1·4 to 3·4)	2·7 (1·7 to 4·1)	2·9 (1·8 to 4·6)	2 (1·1 to 3·4)	0·6 (0·3 to 1)	0·4 (0·2 to 0·5)	0·1 (0·1 to 0·2)	0·1 (0 to 0·1)	0 (0 to 0·1)	0 (0 to 0·1)	0 (0 to 0)	0 (0 to 0)	0 (0 to 0)	0 (0 to 0)	13·9
Respiratory infections and tuberculosis	16·1 (11·1 to 22·9)	24·8 (13·8 to 40·2)	85·4 (57·4 to 120)	48·1 (34·4 to 66·4)	34·9 (25 to 48·9)	31·8 (21·9 to 44·6)	35·8 (25·5 to 50·4)	45·6 (33·4 to 62·3)	52·2 (37·6 to 69·4)	56·1 (40·5 to 76)	59·9 (43 to 82·4)	56·6 (39·9 to 78·4)	61·9 (45 to 85·5)	67·8 (48·7 to 92·4)	69·5 (48·1 to 95·7)	76·5 (53·9 to 104)	70·6 (48·8 to 99·4)	65·5 (47·1 to 88·4)	76·7 (54·5 to 105)	79 (57·5 to 105)	55·4 (41·7 to 71·9)	34 (24·1 to 43·9)	18·7 (12·2 to 25)	1,220
Tuberculosis	0 (0 to 0)	0 (0 to 0)	0·7 (0·4 to 1)	1·2 (0·7 to 2)	1·7 (0·7 to 3)	1·5 (0·8 to 2·6)	3 (2·1 to 4·2)	7·8 (5 to 11·4)	10·7 (6·8 to 15·8)	10·8 (6·6 to 15·9)	9·7 (6 to 14·7)	8 (5 to 12)	6·6 (4·1 to 9·8)	6·5 (4 to 9·9)	5·4 (3·4 to 8·2)	4·8 (2·9 to 7·1)	3·1 (2 to 4·7)	2·5 (1·6 to 3·6)	2·1 (1·4 to 3·1)	1·5 (1 to 2·2)	0·8 (0·5 to 1·2)	0·3 (0·2 to 0·4)	0·1 (0·1 to 0·2)	88·8
Latent tuberculosis infection	0 (0 to 0)	0 (0 to 0)	0 (0 to 0)	0 (0 to 0)	0 (0 to 0)	0 (0 to 0)	0 (0 to 0)	0 (0 to 0)	0 (0 to 0)	0 (0 to 0)	0 (0 to 0)	0 (0 to 0)	0 (0 to 0)	0 (0 to 0)	0 (0 to 0)	0 (0 to 0)	0 (0 to 0)	0 (0 to 0)	0 (0 to 0)	0 (0 to 0)	0 (0 to 0)	0 (0 to 0)	0 (0 to 0)	0
Drug-susceptible tuberculosis	0 (0 to 0)	0 (0 to 0)	0·6 (0·4 to 1)	1·2 (0·7 to 2)	1·6 (0·7 to 2·9)	1·5 (0·8 to 2·5)	2·9 (2 to 4·1)	7·5 (4·9 to 11)	10·4 (6·5 to 15·4)	10·4 (6·4 to 15·6)	9·4 (5·8 to 14·2)	7·8 (4·8 to 11·6)	6·4 (4 to 9·7)	6·3 (3·9 to 9·7)	5·2 (3·3 to 7·9)	4·6 (2·7 to 6·9)	3 (1·8 to 4·5)	2·4 (1·5 to 3·5)	2·1 (1·3 to 3)	1·5 (0·9 to 2·1)	0·8 (0·5 to 1·2)	0·3 (0·2 to 0·4)	0·1 (0·1 to 0·2)	85·9
Multidrug-resistant tuberculosis without extensive drug resistanc	0 (0 to 0)	0 (0 to 0)	0 (0 to 0·1)	0 (0 to 0·1)	0 (0 to 0·1)	0 (0 to 0·2)	0·1 (0 to 0·4)	0·2 (0 to 0·8)	0·3 (0 to 1·1)	0·3 (0 to 1·1)	0·3 (0 to 1)	0·2 (0 to 0·8)	0·2 (0 to 0·8)	0·2 (0 to 0·7)	0·2 (0 to 0·7)	0·1 (0 to 0·6)	0·1 (0 to 0·4)	0·1 (0 to 0·3)	0·1 (0 to 0·3)	0 (0 to 0·2)	0 (0 to 0·1)	0 (0 to 0)	0 (0 to 0)	2·5
Extensively drug-resistant tuberculosis	0 (0 to 0)	0 (0 to 0)	0 (0 to 0)	0 (0 to 0)	0 (0 to 0)	0 (0 to 0)	0 (0 to 0·1)	0 (0 to 0·1)	0 (0 to 0·2)	0 (0 to 0·2)	0 (0 to 0·2)	0 (0 to 0·1)	0 (0 to 0·1)	0 (0 to 0·1)	0 (0 to 0·1)	0 (0 to 0·1)	0 (0 to 0·1)	0 (0 to 0·1)	0 (0 to 0)	0 (0 to 0)	0 (0 to 0)	0 (0 to 0)	0 (0 to 0)	0·4
Lower respiratory infections	16·1 (11·1 to 22·8)	24·6 (13·7 to 40·1)	80·8 (52·7 to 116)	31 (19·5 to 45·8)	13·1 (9·1 to 18·2)	11·1 (7·5 to 15·9)	15·4 (10·2 to 23·2)	22 (14 to 32·6)	26·1 (16·1 to 39·5)	31·5 (19·9 to 47)	35·1 (22·4 to 55·4)	35·5 (21·7 to 53·2)	42·8 (27·4 to 63·3)	50·5 (33 to 73·4)	55·1 (34·9 to 80·5)	65·1 (43·8 to 92)	63·2 (41·6 to 90·9)	60·2 (42·1 to 83)	72·7 (50·3 to 101)	76·4 (55 to 102)	54·1 (40·4 to 70·8)	33·5 (23·6 to 43·2)	18·5 (12 to 24·6)	935
Upper respiratory infections	0 (0 to 0)	0·1 (0·1 to 0·2)	3·5 (2·2 to 5·5)	11·7 (6·9 to 19·2)	14·4 (8 to 23·8)	14·8 (7·8 to 25·1)	13·8 (7·3 to 24·3)	12·9 (6·7 to 21·9)	12·9 (7·2 to 21·2)	11·8 (6·3 to 19·7)	12 (6·3 to 20)	10·8 (5·9 to 19·4)	10·1 (5·5 to 17·2)	8·4 (4·6 to 14·6)	7·1 (3·8 to 11·6)	5·3 (2·8 to 9·1)	3·7 (2 to 6·1)	2·4 (1·4 to 4·1)	1·6 (0·9 to 2·6)	1 (0·5 to 1·6)	0·4 (0·2 to 0·7)	0·2 (0·1 to 0·3)	0·1 (0·1 to 0·2)	159
Otitis media	0 (0 to 0)	0 (0 to 0)	0·5 (0·2 to 0·9)	4·2 (2·2 to 7·4)	5·8 (3·1 to 9·7)	4·4 (2·2 to 7·8)	3·6 (1·8 to 6·3)	2·9 (1·3 to 5·6)	2·5 (1·1 to 4·8)	2·1 (0·9 to 4·3)	2·1 (1 to 4·1)	2·3 (1 to 4·3)	2·4 (1·1 to 4·4)	2·2 (1 to 4·2)	1·8 (0·9 to 3·4)	1·2 (0·6 to 2·5)	0·7 (0·3 to 1·3)	0·4 (0·1 to 0·8)	0·2 (0·1 to 0·5)	0·1 (0 to 0·3)	0·1 (0 to 0·2)	0 (0 to 0·1)	0 (0 to 0)	39·8
Enteric infections	0·7 (0·5 to 1·1)	2·9 (1·6 to 5·1)	23·9 (15·1 to 37·1)	24·1 (15·9 to 35·9)	22·2 (13·3 to 35·4)	21·8 (12·4 to 35·2)	13·8 (8·6 to 20·8)	11·2 (6·8 to 17·2)	13·2 (8·6 to 19·4)	14·2 (9·6 to 20·1)	16 (10·9 to 22·3)	17·2 (11·7 to 23·8)	18·7 (13 to 25·9)	18·8 (12·8 to 26·8)	18·1 (12·5 to 25·7)	18 (12·8 to 24·8)	17·2 (12·3 to 23·2)	15·5 (11·1 to 20·9)	14·4 (10·4 to 19·5)	10·7 (7·7 to 14·5)	5·7 (4·2 to 7·5)	2·8 (2 to 3·8)	1·4 (1 to 1·8)	323
Diarrheal diseases	0·7 (0·5 to 1·1)	2·9 (1·6 to 5)	23·8 (15 to 37)	23·8 (15·6 to 35·7)	21·9 (13 to 35·1)	21·3 (12 to 34·8)	13·6 (8·4 to 20·6)	11 (6·7 to 17)	13 (8·4 to 19·2)	14·1 (9·5 to 20)	15·9 (10·9 to 22·3)	17 (11·5 to 23·6)	18·6 (12·9 to 25·8)	18·8 (12·7 to 26·7)	17·9 (12·3 to 25·6)	17·9 (12·8 to 24·7)	17·1 (12·1 to 23)	15·5 (11 to 20·9)	14·3 (10·3 to 19·4)	10·7 (7·6 to 14·4)	5·7 (4·2 to 7·5)	2·8 (2 to 3·8)	1·4 (1 to 1·8)	320
Typhoid and paratyphoid	0 (0 to 0)	0 (0 to 0)	0 (0 to 0)	0 (0 to 0)	0 (0 to 0)	0 (0 to 0)	0 (0 to 0)	0 (0 to 0)	0 (0 to 0)	0 (0 to 0)	0 (0 to 0)	0 (0 to 0)	0 (0 to 0)	0 (0 to 0)	0 (0 to 0)	0 (0 to 0)	0 (0 to 0)	0 (0 to 0)	0 (0 to 0)	0 (0 to 0)	0 (0 to 0)	0 (0 to 0)	0 (0 to 0)	0
Typhoid fever	0 (0 to 0)	0 (0 to 0)	0 (0 to 0)	0 (0 to 0)	0 (0 to 0)	0 (0 to 0)	0 (0 to 0)	0 (0 to 0)	0 (0 to 0)	0 (0 to 0)	0 (0 to 0)	0 (0 to 0)	0 (0 to 0)	0 (0 to 0)	0 (0 to 0)	0 (0 to 0)	0 (0 to 0)	0 (0 to 0)	0 (0 to 0)	0 (0 to 0)	0 (0 to 0)	0 (0 to 0)	0 (0 to 0)	0
Paratyphoid fever	0 (0 to 0)	0 (0 to 0)	0 (0 to 0)	0 (0 to 0)	0 (0 to 0)	0 (0 to 0)	0 (0 to 0)	0 (0 to 0)	0 (0 to 0)	0 (0 to 0)	0 (0 to 0)	0 (0 to 0)	0 (0 to 0)	0 (0 to 0)	0 (0 to 0)	0 (0 to 0)	0 (0 to 0)	0 (0 to 0)	0 (0 to 0)	0 (0 to 0)	0 (0 to 0)	0 (0 to 0)	0 (0 to 0)	0
Invasive Non-typhoidal Salmonella (iNTS)	0 (0 to 0)	0 (0 to 0)	0 (0 to 0·1)	0·3 (0·1 to 0·7)	0·3 (0·1 to 0·6)	0·4 (0·1 to 0·9)	0 (0 to 0·2)	0·1 (0 to 0·2)	0·2 (0·1 to 0·6)	0·1 (0 to 0·2)	0 (0 to 0)	0·2 (0·1 to 0·5)	0 (0 to 0)	0·1 (0 to 0·2)	0·1 (0 to 0·3)	0·1 (0 to 0·1)	0·1 (0 to 0·3)	0 (0 to 0·1)	0 (0 to 0·1)	0 (0 to 0·1)	0 (0 to 0)	0 (0 to 0·1)	0 (0 to 0)	2·3
Other intestinal infectious diseases	0 (0 to 0)	0 (0 to 0)	0·1 (0 to 0·3)	0·1 (0 to 0·3)	0·1 (0 to 0·2)	0 (0 to 0·2)	0 (0 to 0·1)	0 (0 to 0·1)	0 (0 to 0·1)	0·1 (0 to 0·2)	0 (0 to 0·1)	0 (0 to 0·1)	0 (0 to 0)	0 (0 to 0)	0 (0 to 0)	0 (0 to 0)	0 (0 to 0·1)	0 (0 to 0)	0 (0 to 0)	0 (0 to 0)	0 (0 to 0)	0 (0 to 0)	0 (0 to 0)	0·7
Neglected tropical diseases and malaria	0 (0 to 0)	0·2 (0·1 to 0·3)	2·1 (1 to 3·8)	5·5 (2·5 to 10·4)	8·6 (3·5 to 16·4)	6·9 (3 to 13·3)	7·3 (3·5 to 12·8)	8·3 (4 to 14·5)	9·5 (4·9 to 16·1)	9·5 (4·4 to 16·5)	11·4 (6·1 to 19·1)	12·3 (6·7 to 21·1)	11·7 (5·8 to 21)	12·8 (6·2 to 22·4)	13·1 (6·5 to 23·7)	11·5 (5·2 to 21·7)	9·3 (4 to 17·2)	7·5 (3·2 to 13·8)	6·1 (2·5 to 11·4)	4 (1·7 to 7·4)	2 (0·9 to 3·8)	0·9 (0·4 to 1·8)	0·5 (0·2 to 0·9)	161
Malaria	0 (0 to 0)	0 (0 to 0)	0 (0 to 0)	0 (0 to 0)	0 (0 to 0)	0 (0 to 0)	0 (0 to 0)	0 (0 to 0)	0 (0 to 0)	0 (0 to 0)	0 (0 to 0)	0 (0 to 0)	0 (0 to 0)	0 (0 to 0)	0 (0 to 0)	0 (0 to 0)	0 (0 to 0)	0 (0 to 0)	0 (0 to 0)	0 (0 to 0)	0 (0 to 0)	0 (0 to 0)	0 (0 to 0)	0
Chagas disease	0 (0 to 0)	0 (0 to 0)	0 (0 to 0)	0 (0 to 0)	0 (0 to 0)	0 (0 to 0)	0 (0 to 0)	0 (0 to 0)	0 (0 to 0)	0 (0 to 0)	0 (0 to 0)	0 (0 to 0)	0 (0 to 0)	0 (0 to 0)	0 (0 to 0)	0 (0 to 0)	0 (0 to 0)	0 (0 to 0)	0 (0 to 0)	0 (0 to 0)	0 (0 to 0)	0 (0 to 0)	0 (0 to 0)	0
Leishmaniasis	0 (0 to 0)	0 (0 to 0)	0 (0 to 0)	0 (0 to 0)	0 (0 to 0)	0 (0 to 0)	0 (0 to 0)	0 (0 to 0)	0 (0 to 0)	0 (0 to 0)	0 (0 to 0)	0 (0 to 0)	0 (0 to 0)	0 (0 to 0)	0 (0 to 0)	0 (0 to 0)	0 (0 to 0)	0 (0 to 0)	0 (0 to 0)	0 (0 to 0)	0 (0 to 0)	0 (0 to 0)	0 (0 to 0)	0
Visceral leishmaniasis	0 (0 to 0)	0 (0 to 0)	0 (0 to 0)	0 (0 to 0)	0 (0 to 0)	0 (0 to 0)	0 (0 to 0)	0 (0 to 0)	0 (0 to 0)	0 (0 to 0)	0 (0 to 0)	0 (0 to 0)	0 (0 to 0)	0 (0 to 0)	0 (0 to 0)	0 (0 to 0)	0 (0 to 0)	0 (0 to 0)	0 (0 to 0)	0 (0 to 0)	0 (0 to 0)	0 (0 to 0)	0 (0 to 0)	0
Cutaneous and mucocutaneous leishmaniasis	0 (0 to 0)	0 (0 to 0)	0 (0 to 0)	0 (0 to 0)	0 (0 to 0)	0 (0 to 0)	0 (0 to 0)	0 (0 to 0)	0 (0 to 0)	0 (0 to 0)	0 (0 to 0)	0 (0 to 0)	0 (0 to 0)	0 (0 to 0)	0 (0 to 0)	0 (0 to 0)	0 (0 to 0)	0 (0 to 0)	0 (0 to 0)	0 (0 to 0)	0 (0 to 0)	0 (0 to 0)	0 (0 to 0)	0
African trypanosomiasis	0 (0 to 0)	0 (0 to 0)	0 (0 to 0)	0 (0 to 0)	0 (0 to 0)	0 (0 to 0)	0 (0 to 0)	0 (0 to 0)	0 (0 to 0)	0 (0 to 0)	0 (0 to 0)	0 (0 to 0)	0 (0 to 0)	0 (0 to 0)	0 (0 to 0)	0 (0 to 0)	0 (0 to 0)	0 (0 to 0)	0 (0 to 0)	0 (0 to 0)	0 (0 to 0)	0 (0 to 0)	0 (0 to 0)	0
Schistosomiasis	0 (0 to 0)	0 (0 to 0)	0 (0 to 0)	0 (0 to 0)	0 (0 to 0)	0 (0 to 0)	0 (0 to 0)	0 (0 to 0)	0 (0 to 0)	0 (0 to 0)	0 (0 to 0)	0 (0 to 0)	0 (0 to 0)	0 (0 to 0)	0 (0 to 0)	0 (0 to 0)	0 (0 to 0)	0 (0 to 0)	0 (0 to 0)	0 (0 to 0)	0 (0 to 0)	0 (0 to 0)	0 (0 to 0)	0
Cysticercosis	0 (0 to 0)	0 (0 to 0)	0 (0 to 0)	0 (0 to 0)	0 (0 to 0)	0·1 (0 to 0·3)	0·6 (0 to 2)	2·1 (0·4 to 5·5)	3·8 (1·1 to 8·5)	4·5 (1·3 to 10·2)	5·2 (1·5 to 12·1)	6·2 (1·9 to 14·1)	7·3 (2·5 to 16·3)	8·2 (2·8 to 17·2)	9·4 (3·6 to 19)	9·7 (3·5 to 19·8)	8·4 (3·1 to 16·2)	6·9 (2·7 to 13·3)	5·6 (2·1 to 10·9)	3·7 (1·4 to 7)	1·8 (0·7 to 3·5)	0·8 (0·3 to 1·7)	0·4 (0·1 to 0·8)	84·8
Cystic echinococcosis	0 (0 to 0)	0 (0 to 0)	0 (0 to 0)	0 (0 to 0)	0 (0 to 0)	0 (0 to 0)	0 (0 to 0)	0 (0 to 0)	0 (0 to 0)	0 (0 to 0)	0 (0 to 0)	0 (0 to 0)	0 (0 to 0)	0 (0 to 0)	0 (0 to 0)	0 (0 to 0)	0 (0 to 0)	0 (0 to 0)	0 (0 to 0)	0 (0 to 0)	0 (0 to 0)	0 (0 to 0)	0 (0 to 0)	0
Lymphatic filariasis	0 (0 to 0)	0 (0 to 0)	0 (0 to 0)	0 (0 to 0)	0 (0 to 0)	0 (0 to 0)	0 (0 to 0)	0 (0 to 0)	0 (0 to 0)	0 (0 to 0)	0 (0 to 0)	0 (0 to 0)	0 (0 to 0)	0 (0 to 0)	0 (0 to 0)	0 (0 to 0)	0 (0 to 0)	0 (0 to 0)	0 (0 to 0)	0 (0 to 0)	0 (0 to 0)	0 (0 to 0)	0 (0 to 0)	0
Onchocerciasis	0 (0 to 0)	0 (0 to 0)	0 (0 to 0)	0 (0 to 0)	0 (0 to 0)	0 (0 to 0)	0 (0 to 0)	0 (0 to 0)	0 (0 to 0)	0 (0 to 0)	0 (0 to 0)	0 (0 to 0)	0 (0 to 0)	0 (0 to 0)	0 (0 to 0)	0 (0 to 0)	0 (0 to 0)	0 (0 to 0)	0 (0 to 0)	0 (0 to 0)	0 (0 to 0)	0 (0 to 0)	0 (0 to 0)	0
Trachoma	0 (0 to 0)	0 (0 to 0)	0 (0 to 0)	0 (0 to 0)	0 (0 to 0)	0 (0 to 0)	0 (0 to 0)	0 (0 to 0)	0 (0 to 0)	0 (0 to 0)	0 (0 to 0)	0 (0 to 0)	0 (0 to 0)	0 (0 to 0)	0 (0 to 0)	0 (0 to 0)	0 (0 to 0)	0 (0 to 0)	0 (0 to 0)	0 (0 to 0)	0 (0 to 0)	0 (0 to 0)	0 (0 to 0)	0
Dengue	0 (0 to 0)	0 (0 to 0·1)	0·8 (0·2 to 2·1)	0·9 (0·3 to 2·1)	1·9 (0·5 to 3·9)	1·6 (0·4 to 3·4)	1·6 (0·5 to 3·4)	1·4 (0·4 to 3·1)	1·6 (0·5 to 3·4)	1·3 (0·4 to 3)	1·4 (0·4 to 3)	1·4 (0·4 to 2·8)	1·1 (0·3 to 2·4)	1·2 (0·3 to 2·8)	0·9 (0·2 to 2)	0·8 (0·2 to 1·8)	0·5 (0·1 to 1·1)	0·3 (0·1 to 0·8)	0·3 (0·1 to 0·6)	0·2 (0 to 0·4)	0·1 (0 to 0·2)	0·1 (0 to 0·1)	0·1 (0 to 0·1)	19·7
Yellow fever	0 (0 to 0)	0 (0 to 0)	0 (0 to 0)	0 (0 to 0)	0 (0 to 0)	0 (0 to 0)	0 (0 to 0)	0 (0 to 0)	0 (0 to 0)	0 (0 to 0)	0 (0 to 0)	0 (0 to 0)	0 (0 to 0)	0 (0 to 0)	0 (0 to 0)	0 (0 to 0)	0 (0 to 0)	0 (0 to 0)	0 (0 to 0)	0 (0 to 0)	0 (0 to 0)	0 (0 to 0)	0 (0 to 0)	0
Rabies	0 (0 to 0)	0 (0 to 0)	0 (0 to 0)	0 (0 to 0)	0 (0 to 0)	0 (0 to 0)	0 (0 to 0)	0 (0 to 0)	0 (0 to 0)	0 (0 to 0)	0 (0 to 0)	0 (0 to 0)	0 (0 to 0)	0 (0 to 0)	0 (0 to 0)	0 (0 to 0)	0 (0 to 0)	0 (0 to 0)	0 (0 to 0)	0 (0 to 0)	0 (0 to 0)	0 (0 to 0)	0 (0 to 0)	0·1
Intestinal nematode infections	0 (0 to 0)	0 (0 to 0)	0 (0 to 0)	0·1 (0 to 0·3)	0·3 (0·1 to 0·6)	0·2 (0 to 0·5)	1·9 (0·7 to 3·9)	2·4 (0·7 to 4·8)	1·7 (0·6 to 3·4)	1·3 (0·4 to 2·8)	1·6 (0·7 to 3)	1 (0·4 to 2)	0·1 (0 to 0·1)	0·1 (0 to 0·3)	0·1 (0 to 0·2)	0·1 (0 to 0·2)	0 (0 to 0·1)	0 (0 to 0·1)	0 (0 to 0·1)	0 (0 to 0)	0 (0 to 0)	0 (0 to 0)	0 (0 to 0)	11·1
Ascariasis	0 (0 to 0)	0 (0 to 0)	0 (0 to 0)	0 (0 to 0·2)	0 (0 to 0·2)	0 (0 to 0·1)	0 (0 to 0)	0 (0 to 0)	0 (0 to 0)	0 (0 to 0)	0 (0 to 0)	0 (0 to 0)	0 (0 to 0)	0 (0 to 0)	0 (0 to 0)	0 (0 to 0)	0 (0 to 0)	0 (0 to 0)	0 (0 to 0)	0 (0 to 0)	0 (0 to 0)	0 (0 to 0)	0 (0 to 0)	0·2
Trichuriasis	0 (0 to 0)	0 (0 to 0)	0 (0 to 0)	0 (0 to 0)	0 (0 to 0)	0 (0 to 0)	0 (0 to 0)	0 (0 to 0)	0 (0 to 0)	0 (0 to 0)	0 (0 to 0)	0 (0 to 0)	0 (0 to 0)	0 (0 to 0)	0 (0 to 0)	0 (0 to 0)	0 (0 to 0)	0 (0 to 0)	0 (0 to 0)	0 (0 to 0)	0 (0 to 0)	0 (0 to 0)	0 (0 to 0)	0
Hookworm disease	0 (0 to 0)	0 (0 to 0)	0 (0 to 0)	0 (0 to 0·2)	0·2 (0 to 0·6)	0·2 (0 to 0·5)	1·9 (0·7 to 3·8)	2·4 (0·7 to 4·8)	1·7 (0·6 to 3·4)	1·3 (0·4 to 2·8)	1·6 (0·7 to 3)	1 (0·4 to 2)	0 (0 to 0·1)	0·1 (0 to 0·3)	0·1 (0 to 0·2)	0 (0 to 0·1)	0 (0 to 0·1)	0 (0 to 0·1)	0 (0 to 0)	0 (0 to 0)	0 (0 to 0)	0 (0 to 0)	0 (0 to 0)	10·9
Food-borne trematodiases	0 (0 to 0)	0 (0 to 0)	0 (0 to 0)	0 (0 to 0)	0 (0 to 0)	0 (0 to 0)	0 (0 to 0)	0 (0 to 0)	0 (0 to 0)	0 (0 to 0)	0 (0 to 0)	0 (0 to 0)	0 (0 to 0)	0 (0 to 0)	0 (0 to 0)	0 (0 to 0)	0 (0 to 0)	0 (0 to 0)	0 (0 to 0)	0 (0 to 0)	0 (0 to 0)	0 (0 to 0)	0 (0 to 0)	0
Leprosy	0 (0 to 0)	0 (0 to 0)	0 (0 to 0)	0 (0 to 0)	0 (0 to 0)	0 (0 to 0)	0 (0 to 0)	0 (0 to 0)	0 (0 to 0)	0 (0 to 0)	0 (0 to 0)	0 (0 to 0)	0 (0 to 0)	0 (0 to 0)	0 (0 to 0)	0 (0 to 0)	0 (0 to 0)	0 (0 to 0)	0 (0 to 0)	0 (0 to 0)	0 (0 to 0)	0 (0 to 0)	0 (0 to 0)	0·2
Ebola	0 (0 to 0)	0 (0 to 0)	0 (0 to 0)	0 (0 to 0)	0 (0 to 0)	0 (0 to 0)	0 (0 to 0)	0 (0 to 0)	0 (0 to 0)	0 (0 to 0)	0 (0 to 0)	0 (0 to 0)	0 (0 to 0)	0 (0 to 0)	0 (0 to 0)	0 (0 to 0)	0 (0 to 0)	0 (0 to 0)	0 (0 to 0)	0 (0 to 0)	0 (0 to 0)	0 (0 to 0)	0 (0 to 0)	0
Zika virus	0 (0 to 0)	0 (0 to 0)	0 (0 to 0·1)	0 (0 to 0·2)	0·1 (0 to 0·3)	0·1 (0 to 0·3)	0·1 (0 to 0·4)	0·1 (0 to 0·4)	0·1 (0 to 0·4)	0·1 (0 to 0·4)	0·1 (0 to 0·3)	0 (0 to 0·3)	0 (0 to 0·2)	0 (0 to 0·2)	0 (0 to 0·1)	0 (0 to 0·1)	0 (0 to 0)	0 (0 to 0)	0 (0 to 0)	0 (0 to 0)	0 (0 to 0)	0 (0 to 0)	0 (0 to 0)	0·7
Guinea worm disease	0 (0 to 0)	0 (0 to 0)	0 (0 to 0)	0 (0 to 0)	0 (0 to 0)	0 (0 to 0)	0 (0 to 0)	0 (0 to 0)	0 (0 to 0)	0 (0 to 0)	0 (0 to 0)	0 (0 to 0)	0 (0 to 0)	0 (0 to 0)	0 (0 to 0)	0 (0 to 0)	0 (0 to 0)	0 (0 to 0)	0 (0 to 0)	0 (0 to 0)	0 (0 to 0)	0 (0 to 0)	0 (0 to 0)	0
Other neglected tropical diseases	0 (0 to 0)	0·1 (0 to 0·3)	1·3 (0·5 to 2·4)	4·4 (1·7 to 9)	6·4 (1·9 to 13·7)	5 (1·4 to 11·1)	3 (1·1 to 6·4)	2·3 (0·6 to 5·2)	2·3 (0·7 to 4·7)	2·3 (0·6 to 5·1)	3 (1·1 to 6·1)	3·7 (1·4 to 7·2)	3·2 (1·2 to 6·1)	3·1 (1·1 to 6·1)	2·6 (0·9 to 5·2)	0·8 (0·3 to 1·8)	0·3 (0·1 to 0·7)	0·2 (0·1 to 0·5)	0·2 (0 to 0·4)	0·1 (0 to 0·3)	0·1 (0 to 0·2)	0 (0 to 0·1)	0 (0 to 0)	44·5
Other infectious diseases	1·3 (0·9 to 1·8)	3·4 (1·8 to 5·9)	16·3 (11·4 to 23·1)	15·2 (9·9 to 22·5)	13·6 (9·5 to 19)	13·2 (9·3 to 18·3)	14 (10·2 to 18·4)	12·9 (9·3 to 17·8)	12·1 (8·8 to 16·3)	12·5 (8·8 to 16·9)	13·8 (9·8 to 18·5)	14·2 (10·2 to 19·4)	13·9 (10·2 to 18·7)	13·6 (9·9 to 18·5)	12·4 (8·8 to 16·5)	9·1 (6·9 to 11·7)	6·5 (4·9 to 8·4)	4·5 (3·3 to 5·7)	3·3 (2·4 to 4·1)	2·4 (1·7 to 3·2)	1·5 (1·1 to 2·1)	0·6 (0·4 to 0·8)	0·3 (0·2 to 0·4)	210
Meningitis	0·7 (0·4 to 1·1)	2·3 (1·1 to 4·2)	6·1 (3·7 to 9·6)	3·4 (2·1 to 5·1)	2·6 (1·9 to 3·5)	2·3 (1·7 to 3·1)	2·6 (1·8 to 3·7)	2·5 (1·7 to 3·8)	2·2 (1·5 to 3·3)	2·7 (1·9 to 4)	2·7 (1·8 to 3·9)	2·3 (1·6 to 3·3)	2·6 (1·8 to 3·7)	2·7 (1·8 to 3·8)	2·3 (1·5 to 3·3)	1·9 (1·3 to 2·6)	1·5 (1 to 2·2)	0·9 (0·6 to 1·2)	0·8 (0·5 to 1·2)	0·6 (0·4 to 0·8)	0·3 (0·2 to 0·4)	0·1 (0·1 to 0·1)	0 (0 to 0)	46·1
Encephalitis	0·4 (0·2 to 0·6)	0·5 (0·3 to 1)	6·8 (3·8 to 11·7)	6·5 (3·4 to 12·3)	4·6 (2·8 to 7·3)	4·8 (2·9 to 7·6)	4·9 (3 to 7·5)	4·4 (2·8 to 6·8)	3·6 (2·2 to 5·9)	3·3 (2 to 5·2)	3·1 (2 to 4·6)	2·7 (1·9 to 4·5)	2·9 (1·9 to 4·5)	2·1 (1·4 to 3·2)	2 (1·3 to 3)	1·7 (1·1 to 2·6)	1·1 (0·7 to 1·6)	0·7 (0·4 to 1·1)	0·4 (0·3 to 0·6)	0·3 (0·2 to 0·4)	0·1 (0·1 to 0·2)	0 (0 to 0)	0 (0 to 0)	57·4
Diphtheria	0 (0 to 0)	0 (0 to 0)	0 (0 to 0)	0 (0 to 0)	0 (0 to 0)	0 (0 to 0)	0 (0 to 0)	0 (0 to 0)	0 (0 to 0)	0 (0 to 0)	0 (0 to 0)	0 (0 to 0)	0 (0 to 0)	0 (0 to 0)	0 (0 to 0)	0 (0 to 0)	0 (0 to 0)	0 (0 to 0)	0 (0 to 0)	0 (0 to 0)	0 (0 to 0)	0 (0 to 0)	0 (0 to 0)	0
Tetanus	0 (0 to 0)	0 (0 to 0)	0 (0 to 0·1)	0 (0 to 0)	0 (0 to 0·1)	0 (0 to 0)	0 (0 to 0)	0 (0 to 0)	0 (0 to 0·1)	0 (0 to 0·1)	0 (0 to 0·3)	0·1 (0 to 0·2)	0·1 (0 to 0·2)	0·2 (0·1 to 0·4)	0·1 (0 to 0·3)	0·1 (0 to 0·3)	0·1 (0 to 0·2)	0 (0 to 0·2)	0·1 (0 to 0·2)	0 (0 to 0·1)	0 (0 to 0·1)	0 (0 to 0)	0 (0 to 0)	1·2
Measles	0 (0 to 0)	0 (0 to 0)	0 (0 to 0)	0 (0 to 0)	0 (0 to 0)	0 (0 to 0)	0 (0 to 0)	0 (0 to 0)	0 (0 to 0)	0 (0 to 0)	0 (0 to 0)	0 (0 to 0)	0 (0 to 0)	0 (0 to 0)	0 (0 to 0)	0 (0 to 0)	0 (0 to 0)	0 (0 to 0)	0 (0 to 0)	0 (0 to 0)	0 (0 to 0)	0 (0 to 0)	0 (0 to 0)	0
Varicella and herpes zoster	0 (0 to 0)	0 (0 to 0)	0·1 (0 to 0·2)	0·1 (0·1 to 0·2)	0·1 (0·1 to 0·2)	0·3 (0 to 0·4)	0·2 (0·1 to 0·5)	0·3 (0·1 to 0·6)	0·3 (0·1 to 0·7)	0·3 (0·1 to 0·7)	0·3 (0·1 to 0·7)	0·4 (0·2 to 0·8)	0·4 (0·2 to 0·9)	0·5 (0·2 to 0·9)	0·5 (0·2 to 1)	0·4 (0·2 to 1)	0·4 (0·2 to 0·7)	0·2 (0·1 to 0·5)	0·2 (0·1 to 0·4)	0·1 (0·1 to 0·3)	0·1 (0 to 0·1)	0 (0 to 0·1)	0 (0 to 0)	5·7
Acute hepatitis	0 (0 to 0)	0 (0 to 0)	0·1 (0·1 to 0·3)	0·3 (0·1 to 0·5)	1·1 (0·6 to 1·7)	1·5 (0·9 to 2·3)	1·6 (1 to 2·4)	1·6 (1·1 to 2·3)	1·6 (1·1 to 2·4)	1·3 (0·9 to 1·9)	1·2 (0·8 to 1·6)	1·2 (0·8 to 1·7)	0·9 (0·7 to 1·3)	0·8 (0·5 to 1·1)	0·6 (0·4 to 0·9)	0·4 (0·3 to 0·6)	0·3 (0·2 to 0·3)	0·2 (0·1 to 0·3)	0·1 (0·1 to 0·2)	0·1 (0 to 0·1)	0 (0 to 0·1)	0 (0 to 0)	0 (0 to 0)	14·9
Acute hepatitis A	0 (0 to 0)	0 (0 to 0)	0 (0 to 0·1)	0·2 (0·1 to 0·4)	1 (0·4 to 1·5)	1·3 (0·7 to 2)	1·3 (0·8 to 2)	1·2 (0·6 to 1·7)	0·9 (0·5 to 1·5)	0·8 (0·5 to 1·4)	0·6 (0·3 to 0·9)	0·6 (0·3 to 1)	0·4 (0·2 to 0·7)	0·3 (0·1 to 0·6)	0·2 (0·1 to 0·5)	0·1 (0·1 to 0·3)	0·1 (0 to 0·1)	0·1 (0 to 0·1)	0 (0 to 0)	0 (0 to 0)	0 (0 to 0)	0 (0 to 0)	0 (0 to 0)	9
Acute hepatitis B	0 (0 to 0)	0 (0 to 0)	0·1 (0 to 0·2)	0 (0 to 0·1)	0·1 (0 to 0·2)	0·1 (0 to 0·2)	0·1 (0 to 0·4)	0·4 (0·2 to 0·8)	0·6 (0·3 to 1)	0·4 (0·2 to 0·7)	0·5 (0·3 to 0·8)	0·6 (0·3 to 0·9)	0·5 (0·3 to 0·7)	0·4 (0·2 to 0·6)	0·3 (0·1 to 0·5)	0·3 (0·1 to 0·4)	0·1 (0·1 to 0·2)	0·1 (0·1 to 0·2)	0·1 (0 to 0·1)	0 (0 to 0·1)	0 (0 to 0)	0 (0 to 0)	0 (0 to 0)	4·7
Acute hepatitis C	0 (0 to 0)	0 (0 to 0)	0 (0 to 0)	0 (0 to 0)	0 (0 to 0)	0 (0 to 0)	0 (0 to 0)	0 (0 to 0)	0 (0 to 0)	0 (0 to 0)	0 (0 to 0)	0 (0 to 0·1)	0 (0 to 0)	0 (0 to 0·1)	0 (0 to 0·1)	0 (0 to 0)	0 (0 to 0)	0 (0 to 0)	0 (0 to 0)	0 (0 to 0)	0 (0 to 0)	0 (0 to 0)	0 (0 to 0)	0·3
Acute hepatitis E	0 (0 to 0)	0 (0 to 0)	0 (0 to 0)	0 (0 to 0)	0·1 (0 to 0·1)	0·1 (0 to 0·3)	0·1 (0 to 0·3)	0·1 (0 to 0·3)	0·1 (0 to 0·2)	0·1 (0 to 0·2)	0·1 (0 to 0·1)	0·1 (0 to 0·1)	0·1 (0 to 0·1)	0 (0 to 0·1)	0 (0 to 0·1)	0 (0 to 0)	0 (0 to 0)	0 (0 to 0)	0 (0 to 0)	0 (0 to 0)	0 (0 to 0)	0 (0 to 0)	0 (0 to 0)	0·9
Other unspecified infectious diseases	0·3 (0·2 to 0·5)	0·5 (0·3 to 0·9)	2·5 (1·6 to 3·8)	4·1 (2·1 to 7·1)	5 (2 to 8)	4·3 (1·9 to 8·3)	4·7 (2·5 to 8·2)	4·1 (2 to 7·2)	4·2 (2·3 to 6·9)	4·7 (2·5 to 7·9)	6·4 (3·5 to 10·1)	7·1 (4·1 to 11·7)	7 (4·1 to 11)	7·3 (4·5 to 11·3)	6·7 (4 to 10·3)	4·4 (2·6 to 6·5)	3·1 (1·9 to 4·7)	2·3 (1·3 to 3·3)	1·6 (0·9 to 2·3)	1·3 (0·7 to 2)	1 (0·5 to 1·5)	0·4 (0·2 to 0·6)	0·2 (0·1 to 0·3)	83·2
Maternal and neonatal disorders	553 (437 to 684)	231 (142 to 379)	79·6 (53·9 to 112)	48·8 (36·3 to 63·3)	48·4 (34·8 to 64)	58·1 (42·6 to 76·5)	80·7 (63·9 to 98)	97·2 (78·2 to 119)	103 (82·4 to 128)	97·9 (78 to 124)	82·5 (66·3 to 103)	56·8 (44·2 to 70·6)	41·5 (31·1 to 53·8)	31·6 (22·8 to 41·6)	23·8 (17 to 31·2)	16 (11·5 to 21·4)	8·5 (6·1 to 11·3)	4 (2·7 to 5·5)	1·7 (1·2 to 2·5)	0·5 (0·3 to 0·7)	0·1 (0·1 to 0·1)	0 (0 to 0)	0 (0 to 0)	1,660
Maternal disorders	0 (0 to 0)	0 (0 to 0)	0 (0 to 0)	0 (0 to 0)	0 (0 to 0)	0·8 (0·6 to 1·1)	27·1 (20·4 to 34·8)	45·3 (32·8 to 60·1)	51·4 (36·2 to 69·7)	50·7 (36·7 to 70·3)	38·2 (27·7 to 50·8)	15·5 (11·5 to 20·7)	3·8 (2·8 to 5·2)	0·5 (0·4 to 0·6)	0 (0 to 0)	0 (0 to 0)	0 (0 to 0)	0 (0 to 0)	0 (0 to 0)	0 (0 to 0)	0 (0 to 0)	0 (0 to 0)	0 (0 to 0)	233
Maternal hemorrhage	0 (0 to 0)	0 (0 to 0)	0 (0 to 0)	0 (0 to 0)	0 (0 to 0)	0 (0 to 0)	1·8 (1·1 to 2·9)	3·4 (1·9 to 5·7)	4·7 (2·8 to 7·3)	4·2 (2·3 to 6·5)	3·3 (2 to 5·2)	1·3 (0·7 to 2)	0·1 (0 to 0·2)	0 (0 to 0)	0 (0 to 0)	0 (0 to 0)	0 (0 to 0)	0 (0 to 0)	0 (0 to 0)	0 (0 to 0)	0 (0 to 0)	0 (0 to 0)	0 (0 to 0)	18·8
Maternal sepsis and other maternal infections	0 (0 to 0)	0 (0 to 0)	0 (0 to 0)	0 (0 to 0)	0 (0 to 0)	0 (0 to 0)	1 (0·6 to 1·8)	2·1 (1·2 to 3·2)	2·3 (1·3 to 3·8)	2 (1·1 to 3·3)	1·4 (0·8 to 2·4)	0·5 (0·3 to 0·9)	0·1 (0 to 0·2)	0 (0 to 0)	0 (0 to 0)	0 (0 to 0)	0 (0 to 0)	0 (0 to 0)	0 (0 to 0)	0 (0 to 0)	0 (0 to 0)	0 (0 to 0)	0 (0 to 0)	9·7
Maternal hypertensive disorders	0 (0 to 0)	0 (0 to 0)	0 (0 to 0)	0 (0 to 0)	0 (0 to 0)	0·2 (0·1 to 0·2)	4·8 (3·3 to 6·8)	6 (4 to 8·5)	6·8 (4·5 to 9·9)	6 (4 to 8·7)	4·2 (2·8 to 6·1)	1·6 (1 to 2·3)	0·3 (0·2 to 0·5)	0 (0 to 0)	0 (0 to 0)	0 (0 to 0)	0 (0 to 0)	0 (0 to 0)	0 (0 to 0)	0 (0 to 0)	0 (0 to 0)	0 (0 to 0)	0 (0 to 0)	29·8
Maternal obstructed labor and uterine rupture	0 (0 to 0)	0 (0 to 0)	0 (0 to 0)	0 (0 to 0)	0 (0 to 0)	0 (0 to 0)	0·6 (0·3 to 1)	1 (0·5 to 1·7)	1·2 (0·6 to 2·2)	1·2 (0·7 to 2·2)	1·1 (0·6 to 2)	0·4 (0·2 to 0·8)	0·1 (0 to 0·1)	0 (0 to 0)	0 (0 to 0)	0 (0 to 0)	0 (0 to 0)	0 (0 to 0)	0 (0 to 0)	0 (0 to 0)	0 (0 to 0)	0 (0 to 0)	0 (0 to 0)	5·7
Maternal abortion and miscarriage	0 (0 to 0)	0 (0 to 0)	0 (0 to 0)	0 (0 to 0)	0 (0 to 0)	0 (0 to 0·1)	1·9 (1 to 3·4)	3·1 (1·7 to 5·7)	3·3 (1·7 to 5·8)	3·1 (1·6 to 5·5)	2·4 (1·2 to 4·5)	1·1 (0·6 to 2·1)	0·4 (0·2 to 0·8)	0 (0 to 0)	0 (0 to 0)	0 (0 to 0)	0 (0 to 0)	0 (0 to 0)	0 (0 to 0)	0 (0 to 0)	0 (0 to 0)	0 (0 to 0)	0 (0 to 0)	15·7
Ectopic pregnancy	0 (0 to 0)	0 (0 to 0)	0 (0 to 0)	0 (0 to 0)	0 (0 to 0)	0 (0 to 0)	1·8 (1 to 2·9)	4·6 (2·6 to 7·2)	5·6 (3·2 to 9·1)	5·9 (3·5 to 9·3)	4 (2·4 to 6·4)	1·3 (0·8 to 2·1)	0·4 (0·2 to 0·7)	0 (0 to 0)	0 (0 to 0)	0 (0 to 0)	0 (0 to 0)	0 (0 to 0)	0 (0 to 0)	0 (0 to 0)	0 (0 to 0)	0 (0 to 0)	0 (0 to 0)	23·7
Indirect maternal deaths	--	--	--	--	--	0·2 (0·1 to 0·2)	3·7 (2·4 to 6·3)	6·6 (3·8 to 10·6)	6·6 (3·8 to 10·9)	5·9 (3·4 to 9·7)	4 (2·1 to 6·1)	1·2 (0·7 to 1·9)	0·2 (0·1 to 0·3)	0 (0 to 0)	--	--	--	--	--	--	--	--	--	NA
Late maternal deaths	--	--	--	--	--	0·1 (0 to 0·1)	2·2 (1·3 to 3·3)	3·5 (2 to 5·4)	3·6 (2 to 5·7)	3·5 (2 to 5·6)	2·6 (1·5 to 4·3)	1 (0·6 to 1·7)	0·3 (0·2 to 0·4)	0 (0 to 0)	--	--	--	--	--	--	--	--	--	NA
Maternal deaths aggravated by HIV/AIDS	--	--	--	--	--	0 (0 to 0)	0·1 (0 to 0·1)	0·3 (0·1 to 0·5)	0·4 (0·2 to 0·8)	0·5 (0·3 to 1)	0·4 (0·2 to 0·6)	0·2 (0·1 to 0·2)	0 (0 to 0·1)	0 (0 to 0)	--	--	--	--	--	--	--	--	--	NA
Neonatal disorders	553 (437 to 684)	231 (142 to 379)	79·6 (53·9 to 112)	48·8 (36·3 to 63·3)	48·4 (34·8 to 64)	57·3 (41·7 to 75·8)	53·5 (38·5 to 70·1)	51·9 (37·2 to 68·1)	51·3 (37·7 to 67·4)	47·2 (34·3 to 62·4)	44·4 (31·3 to 58·5)	41·3 (29·8 to 54·3)	37·6 (27·1 to 49·5)	31·1 (22·3 to 41·2)	23·8 (17 to 31·2)	16 (11·5 to 21·4)	8·5 (6·1 to 11·3)	4 (2·7 to 5·5)	1·7 (1·2 to 2·5)	0·5 (0·3 to 0·7)	0·1 (0·1 to 0·1)	0 (0 to 0)	0 (0 to 0)	1,430
Neonatal preterm birth	189 (136 to 254)	60·6 (33 to 107)	24 (15 to 36·6)	15·5 (10·7 to 21·1)	22·2 (15·4 to 30·1)	26 (18·1 to 35·2)	24·2 (16·8 to 33·3)	22·9 (15·6 to 31·2)	22·8 (16 to 31·4)	20·5 (13·9 to 27·9)	19·1 (12·8 to 25·9)	17·3 (11·9 to 23·8)	15·3 (10·5 to 21·1)	12·4 (8·6 to 17)	8·9 (6·1 to 12·3)	5·6 (3·9 to 7·5)	2·8 (2·1 to 3·5)	1·2 (0·9 to 1·5)	0·5 (0·3 to 0·7)	0·1 (0·1 to 0·2)	0 (0 to 0)	0 (0 to 0)	0 (0 to 0)	511
Neonatal encephalopathy due to birth asphyxia and trauma	141 (91 to 211)	27·1 (12·6 to 50·2)	11·2 (6 to 18·7)	5 (3·2 to 7·5)	4·8 (2·3 to 8)	5·7 (2·8 to 9·3)	5·4 (2·7 to 9·1)	5·3 (2·7 to 8·6)	5·1 (2·6 to 8·5)	4·9 (2·4 to 8·2)	4·6 (2·2 to 7·5)	4·4 (2·1 to 7·4)	3·6 (1·8 to 6·8)	3·3 (1·5 to 5·5)	2·6 (1·2 to 4·7)	1·9 (1·1 to 3·1)	0·9 (0·6 to 1·4)	0·3 (0·2 to 0·5)	0·1 (0 to 0·1)	0 (0 to 0)	0 (0 to 0)	0 (0 to 0)	0 (0 to 0)	238
Neonatal sepsis and other neonatal infections	99·6 (65·5 to 141)	87·8 (51 to 144)	26·5 (16·1 to 40·8)	12·7 (7·8 to 19)	19 (11·1 to 28·8)	22·7 (13·7 to 35)	21·2 (12·4 to 32·2)	21 (12·1 to 31·9)	20·7 (12·1 to 31·6)	19·2 (11·4 to 29·4)	18·2 (10·6 to 27·5)	17·3 (10·2 to 26)	16·1 (9·7 to 24·7)	13·6 (7·8 to 20·7)	10·7 (6·3 to 16·2)	7·4 (4·5 to 11·5)	4·2 (2·2 to 6·3)	2·2 (1·1 to 3·5)	1 (0·5 to 1·8)	0·3 (0·2 to 0·5)	0·1 (0 to 0·1)	0 (0 to 0)	0 (0 to 0)	441
Hemolytic disease and other neonatal jaundice	0·4 (0·2 to 0·5)	0·3 (0·2 to 0·5)	0·4 (0·2 to 0·5)	1·6 (1·1 to 2·4)	2·4 (1·8 to 3·1)	2·9 (2·1 to 3·7)	2·7 (2 to 3·6)	2·7 (2 to 3·5)	2·7 (2 to 3·5)	2·6 (1·9 to 3·3)	2·5 (1·8 to 3·2)	2·4 (1·7 to 3·1)	2·2 (1·6 to 2·9)	1·9 (1·4 to 2·5)	1·5 (1 to 2)	1·1 (0·8 to 1·4)	0·6 (0·4 to 0·8)	0·3 (0·2 to 0·4)	0·1 (0·1 to 0·2)	0 (0 to 0·1)	0 (0 to 0)	0 (0 to 0)	0 (0 to 0)	31·3
Other neonatal disorders	123 (79 to 176)	54·9 (31·8 to 92·2)	17·5 (10·4 to 25·9)	14 (10·6 to 18)	0 (0 to 0)	0 (0 to 0)	0 (0 to 0)	0 (0 to 0)	0 (0 to 0)	0 (0 to 0)	0 (0 to 0)	0 (0 to 0)	0 (0 to 0)	0 (0 to 0)	0 (0 to 0)	0 (0 to 0)	0 (0 to 0)	0 (0 to 0)	0 (0 to 0)	0 (0 to 0)	0 (0 to 0)	0 (0 to 0)	0 (0 to 0)	209
Nutritional deficiencies	0·1 (0 to 0·1)	1·5 (0·8 to 2·7)	26·7 (17·2 to 40·6)	52·9 (23·7 to 102)	78·2 (32 to 158)	62·5 (25·3 to 130)	81·1 (34·2 to 157)	64·7 (24·1 to 129)	66·5 (28·8 to 117)	63·7 (23·7 to 128)	81 (36·5 to 149)	96 (43·9 to 175)	84·1 (37·8 to 153)	83·9 (37·9 to 148)	69·7 (31·8 to 126)	42·3 (19·6 to 78·1)	19·4 (10·4 to 33·8)	14·1 (8 to 26·1)	11·9 (7·2 to 19·3)	8·9 (5·5 to 14·3)	5·9 (3·6 to 9·5)	3·4 (2·2 to 5·1)	1·7 (1·2 to 2·5)	1,020
Protein-energy malnutrition	0·1 (0 to 0·1)	0·2 (0·1 to 0·3)	13·7 (9 to 19·7)	6·9 (4·1 to 13·2)	8·8 (5·3 to 13·8)	8 (4·7 to 13)	8·1 (4·8 to 12·7)	8·8 (5·5 to 13·4)	9 (5·5 to 13·6)	8·5 (5·3 to 13)	9·3 (5·9 to 13·9)	9·1 (5·8 to 13·5)	9·1 (5·9 to 13·6)	8·8 (5·7 to 12·7)	8·4 (5·7 to 12·2)	7·5 (5·3 to 10·4)	5·7 (3·9 to 7·7)	5 (3·5 to 6·7)	4·8 (3·5 to 6·5)	4 (2·8 to 5·4)	2·8 (1·9 to 3·9)	1·8 (1·2 to 2·5)	1·2 (0·8 to 1·8)	150
Iodine deficiency	0 (0 to 0)	0 (0 to 0)	0 (0 to 0)	0 (0 to 0)	0·2 (0·1 to 0·3)	0·4 (0·2 to 0·8)	0·6 (0·3 to 1·1)	0·7 (0·3 to 1·2)	0·7 (0·3 to 1·3)	0·7 (0·3 to 1·3)	0·7 (0·4 to 1·3)	0·7 (0·4 to 1·2)	0·7 (0·4 to 1·1)	0·6 (0·3 to 1)	0·5 (0·3 to 0·8)	0·4 (0·2 to 0·6)	0·2 (0·1 to 0·4)	0·1 (0·1 to 0·2)	0·1 (0·1 to 0·2)	0·1 (0 to 0·1)	0 (0 to 0)	0 (0 to 0)	0 (0 to 0)	7·5
Vitamin A deficiency	0 (0 to 0)	0 (0 to 0·1)	0·1 (0 to 0·4)	1·3 (0·6 to 2·2)	1·8 (0·8 to 3·3)	1·5 (0·6 to 2·8)	0·7 (0·2 to 1·5)	0·6 (0·1 to 1·2)	0·5 (0·2 to 0·9)	0·4 (0·2 to 0·7)	0·3 (0·2 to 0·6)	0·3 (0·1 to 0·5)	0·2 (0·1 to 0·5)	0·2 (0·1 to 0·4)	0·1 (0·1 to 0·3)	0·1 (0 to 0·2)	0·1 (0 to 0·1)	0·1 (0 to 0·1)	0 (0 to 0·1)	0 (0 to 0)	0 (0 to 0)	0 (0 to 0)	0 (0 to 0)	8·6
Dietary iron deficiency	0 (0 to 0)	1·3 (0·6 to 2·5)	12 (4·7 to 23·4)	44·2 (16·1 to 93·1)	65·7 (20·3 to 142)	50·6 (14·8 to 116)	67·9 (21·9 to 144)	50·7 (12 to 116)	51·3 (15 to 101)	50·6 (11·2 to 114)	67·1 (24·6 to 136)	82·4 (30·8 to 157)	70·5 (25·3 to 136)	70·4 (25·9 to 134)	57·3 (20·4 to 112)	31·8 (10·1 to 67·6)	11·2 (3 to 25·1)	7·4 (1·9 to 19·3)	5·6 (1·5 to 13·1)	3·9 (1 to 9)	2·4 (0·6 to 5·8)	1·2 (0·4 to 2·7)	0·3 (0·1 to 0·8)	806
Other nutritional deficiencies	0 (0 to 0)	0 (0 to 0)	0·8 (0·4 to 1·4)	0·6 (0·2 to 1·2)	1·8 (0·9 to 3)	2 (1·1 to 3·2)	3·8 (2·2 to 6)	3·9 (2·2 to 6·2)	5 (2·8 to 8·1)	3·5 (2 to 5·3)	3·6 (2·1 to 5·4)	3·4 (2 to 5·3)	3·5 (2·3 to 5·2)	3·9 (2·5 to 5·8)	3·2 (2 to 4·6)	2·5 (1·6 to 3·7)	2·2 (1·4 to 3·1)	1·5 (0·9 to 2·2)	1·2 (0·7 to 1·9)	0·8 (0·5 to 1·5)	0·6 (0·3 to 1·1)	0·3 (0·2 to 0·6)	0·1 (0 to 0·2)	48·5
Non-communicable diseases	97·2 (64·7 to 152)	80·8 (46·3 to 135)	254 (190 to 326)	302 (226 to 391)	437 (334 to 564)	823 (610 to 1,100)	1,260 (963 to 1,640)	1,600 (1,240 to 2,030)	1,950 (1,520 to 2,460)	2,300 (1,830 to 2,830)	2,780 (2,240 to 3,390)	3,190 (2,580 to 3,910)	3,780 (3,090 to 4,600)	4,220 (3,480 to 5,100)	4,260 (3,560 to 5,090)	4,070 (3,450 to 4,810)	3,390 (2,890 to 3,960)	2,670 (2,300 to 3,100)	2,400 (2,080 to 2,770)	1,870 (1,650 to 2,130)	1,040 (913 to 1,180)	520 (466 to 583)	276 (253 to 301)	43,600
Neoplasms	0·9 (0·6 to 1·2)	1·3 (0·7 to 2·2)	6·4 (4·4 to 8·9)	26·6 (18 to 38·6)	37·5 (29·3 to 47·8)	46·1 (36·3 to 57·9)	72·2 (57·7 to 89·2)	102 (77·7 to 130)	161 (115 to 224)	306 (222 to 416)	504 (373 to 666)	640 (481 to 845)	869 (670 to 1,120)	1,000 (788 to 1,270)	1,000 (791 to 1,270)	926 (755 to 1,130)	707 (586 to 863)	505 (418 to 613)	381 (314 to 456)	245 (203 to 289)	107 (85·7 to 129)	41·9 (32·1 to 49·8)	15·3 (10·4 to 19)	7,700
Lip and oral cavity cancer	0 (0 to 0)	0 (0 to 0)	0 (0 to 0)	0 (0 to 0)	0·1 (0 to 0·2)	0·2 (0·1 to 0·4)	0·6 (0·4 to 0·7)	0·7 (0·4 to 0·9)	1·3 (0·8 to 2)	1·8 (1·1 to 2·6)	2·5 (1·4 to 3·8)	3·5 (2·4 to 5·2)	5·2 (3·6 to 7·5)	7·6 (5·2 to 10·7)	8·2 (5·8 to 11·5)	8·7 (6·2 to 12·1)	7·6 (5·6 to 10·5)	6·1 (4·5 to 8·2)	5·3 (3·8 to 7·2)	3·6 (2·7 to 4·9)	1·6 (1·1 to 2·2)	0·7 (0·5 to 0·9)	0·3 (0·2 to 0·4)	66
Nasopharynx cancer	0 (0 to 0)	0 (0 to 0)	0 (0 to 0)	0 (0 to 0)	0·2 (0·1 to 0·2)	0·1 (0·1 to 0·2)	0·5 (0·4 to 0·7)	0·7 (0·5 to 0·9)	0·8 (0·5 to 1·2)	0·9 (0·6 to 1·3)	1 (0·6 to 1·4)	1·4 (0·9 to 1·9)	2 (1·4 to 3)	2·2 (1·5 to 3·1)	2·1 (1·5 to 2·9)	1·9 (1·3 to 2·7)	1·4 (0·9 to 1·9)	1 (0·7 to 1·5)	0·6 (0·4 to 0·8)	0·4 (0·3 to 0·6)	0·3 (0·2 to 0·5)	0·1 (0·1 to 0·2)	0 (0 to 0·1)	17·7
Other pharynx cancer	0 (0 to 0)	0 (0 to 0)	0 (0 to 0)	0 (0 to 0)	0 (0 to 0)	0 (0 to 0)	0 (0 to 0)	0·3 (0·2 to 0·5)	0·4 (0·3 to 0·6)	0·6 (0·4 to 0·8)	0·8 (0·5 to 1·2)	1·7 (1·1 to 2·6)	2·8 (1·9 to 4)	3·4 (2·4 to 4·9)	3·9 (2·7 to 5·5)	4·1 (3 to 5·5)	2·7 (1·8 to 3·9)	2·2 (1·5 to 3·2)	1·6 (1 to 2·3)	1·1 (0·7 to 1·5)	0·5 (0·4 to 0·7)	0·3 (0·2 to 0·3)	0·1 (0·1 to 0·1)	26·4
Esophageal cancer	0 (0 to 0)	0 (0 to 0)	0 (0 to 0)	0 (0 to 0)	0 (0 to 0)	0 (0 to 0)	0 (0 to 0)	0·3 (0·2 to 0·4)	0·4 (0·3 to 0·6)	0·8 (0·5 to 1·2)	1·6 (1 to 2·5)	3·2 (2·1 to 4·8)	5·8 (3·8 to 8·4)	9·3 (6·7 to 12·7)	9·8 (6·7 to 13·8)	11·2 (8 to 15·2)	8·7 (6·3 to 11·9)	6·7 (4·6 to 9·2)	5·7 (3·9 to 8·1)	3·8 (2·7 to 5·3)	1·7 (1·2 to 2·2)	0·7 (0·5 to 0·8)	0·2 (0·1 to 0·3)	69·8
Stomach cancer	0 (0 to 0)	0 (0 to 0)	0 (0 to 0)	0 (0 to 0)	0 (0 to 0)	0 (0 to 0)	1 (0·8 to 1·2)	2·5 (1·8 to 3·3)	5 (3·6 to 6·7)	10·8 (7·3 to 15·4)	16·5 (10·6 to 23·9)	21·3 (15·1 to 29·2)	30 (20·3 to 43·3)	38 (28 to 51·2)	39·9 (29·9 to 53·1)	44·7 (33·2 to 58·7)	40·1 (29·1 to 53·6)	30·8 (21·2 to 41)	28·6 (21 to 38)	19·7 (14·6 to 26·1)	9·2 (6·9 to 12)	3·7 (2·8 to 4·7)	1·3 (0·9 to 1·7)	344
Colon and rectum cancer	0 (0 to 0)	0 (0 to 0)	0 (0 to 0)	0 (0 to 0)	0·3 (0·2 to 0·5)	0·5 (0·3 to 0·7)	2·5 (1·8 to 3·3)	4·3 (2·9 to 6·1)	8·2 (5 to 13·4)	17·2 (10·9 to 26)	27·8 (18·2 to 40·1)	37·4 (25·6 to 52·2)	57·5 (39·4 to 79·1)	84·3 (60·7 to 113)	94 (68·3 to 129)	96 (68 to 132)	84·1 (60·7 to 115)	71·9 (52·3 to 96·3)	62·9 (46·4 to 84·2)	44·2 (33·7 to 56·3)	20 (14·5 to 26·5)	8·5 (6·2 to 11)	3·1 (2 to 4)	725
Liver cancer	0 (0 to 0)	0 (0 to 0·1)	0·1 (0·1 to 0·2)	0·4 (0·3 to 0·6)	0·5 (0·4 to 0·7)	0·8 (0·6 to 1·1)	0·5 (0·4 to 0·8)	1·2 (0·7 to 1·8)	1·5 (0·9 to 2·4)	2·3 (1·4 to 3·7)	4·1 (2·5 to 6·2)	6·5 (4·1 to 9·7)	9·5 (6·4 to 13·5)	13·8 (9·7 to 19·4)	16·4 (11·3 to 23·4)	15·9 (10·7 to 22·2)	13·7 (9·8 to 18·8)	10·8 (7·4 to 15·4)	8·7 (6 to 12·5)	5·8 (4·1 to 8·3)	1·7 (1·2 to 2·5)	0·6 (0·4 to 0·8)	0·1 (0·1 to 0·1)	115
Liver cancer due to hepatitis B	0 (0 to 0)	0 (0 to 0)	0 (0 to 0)	0 (0 to 0)	0 (0 to 0)	0·1 (0 to 0·1)	0·1 (0 to 0·2)	0·5 (0·2 to 0·7)	0·7 (0·3 to 1·2)	1·1 (0·6 to 1·9)	1·8 (1 to 3·2)	2·7 (1·4 to 4·4)	3·4 (1·8 to 5·7)	4·2 (2·3 to 7·3)	4·5 (2·3 to 7·6)	3·7 (1·8 to 6·6)	2·5 (1·2 to 4·4)	1·6 (0·7 to 3·2)	1·1 (0·6 to 2·1)	0·6 (0·3 to 1·2)	0·2 (0·1 to 0·3)	0·1 (0 to 0·1)	0 (0 to 0)	29
Liver cancer due to hepatitis C	0 (0 to 0)	0 (0 to 0)	0 (0 to 0)	0 (0 to 0)	0 (0 to 0)	0 (0 to 0)	0 (0 to 0)	0 (0 to 0·1)	0·1 (0 to 0·2)	0·2 (0·1 to 0·3)	0·4 (0·2 to 0·9)	0·9 (0·4 to 1·7)	1·8 (0·8 to 3·1)	3·2 (1·7 to 5·6)	4·5 (2·2 to 7·9)	4·8 (2·3 to 8·3)	4·4 (2·4 to 7·1)	3·7 (2·1 to 6)	3·4 (1·8 to 5·5)	2·6 (1·5 to 4·1)	0·9 (0·5 to 1·3)	0·3 (0·2 to 0·5)	0·1 (0 to 0·1)	31·2
Liver cancer due to alcohol use	0 (0 to 0)	0 (0 to 0)	0 (0 to 0)	0 (0 to 0)	0 (0 to 0)	0 (0 to 0)	0 (0 to 0)	0·1 (0 to 0·1)	0·1 (0 to 0·3)	0·3 (0·1 to 0·5)	0·7 (0·3 to 1·3)	1·2 (0·6 to 2·4)	1·7 (0·9 to 3·7)	3·1 (1·5 to 5·6)	3·8 (1·8 to 6·9)	4 (2 to 7)	3·8 (2·1 to 6·1)	3 (1·6 to 5)	2·1 (1·1 to 3·5)	1·1 (0·6 to 1·8)	0·3 (0·2 to 0·5)	0·1 (0·1 to 0·2)	0 (0 to 0)	25·7
Liver cancer due to NASH	0 (0 to 0)	0 (0 to 0)	0 (0 to 0)	0 (0 to 0)	0 (0 to 0)	0 (0 to 0)	0·2 (0·1 to 0·3)	0·3 (0·1 to 0·5)	0·2 (0·1 to 0·4)	0·3 (0·1 to 0·6)	0·5 (0·2 to 0·9)	0·7 (0·3 to 1·3)	1·1 (0·5 to 2)	1·7 (0·9 to 2·9)	2 (1 to 3·6)	2 (1·1 to 3·6)	1·9 (0·9 to 3·4)	1·6 (0·9 to 2·8)	1·5 (0·8 to 2·6)	1·1 (0·6 to 1·8)	0·3 (0·2 to 0·5)	0·1 (0·1 to 0·2)	0 (0 to 0)	15·5
Liver cancer due to other causes	0 (0 to 0)	0 (0 to 0·1)	0·1 (0·1 to 0·2)	0·4 (0·3 to 0·6)	0·5 (0·4 to 0·7)	0·7 (0·5 to 0·9)	0·2 (0·1 to 0·4)	0·4 (0·2 to 0·6)	0·4 (0·2 to 0·7)	0·5 (0·2 to 0·8)	0·7 (0·3 to 1·3)	0·9 (0·4 to 1·6)	1·2 (0·6 to 2·2)	1·6 (0·8 to 2·9)	1·6 (0·8 to 2·9)	1·4 (0·7 to 2·5)	1·1 (0·5 to 1·9)	0·8 (0·4 to 1·4)	0·6 (0·3 to 1·1)	0·4 (0·2 to 0·8)	0·1 (0·1 to 0·2)	0 (0 to 0·1)	0 (0 to 0)	13·7
Gallbladder and biliary tract cancer	0 (0 to 0)	0 (0 to 0)	0 (0 to 0)	0 (0 to 0)	0 (0 to 0)	0 (0 to 0)	0 (0 to 0)	0·2 (0·1 to 0·3)	0·4 (0·2 to 0·6)	0·7 (0·5 to 1·3)	2·1 (1·3 to 3·5)	3·9 (2·4 to 6·6)	6·8 (4·4 to 11·4)	10·7 (6·7 to 17·1)	12·9 (8·3 to 21·4)	13·3 (9·2 to 22·5)	11 (7·7 to 18·1)	8·3 (5·8 to 14·4)	6·5 (4·6 to 10·8)	4 (2·8 to 6·5)	1·4 (1 to 2·4)	0·5 (0·4 to 0·9)	0·2 (0·1 to 0·3)	82·9
Pancreatic cancer	0 (0 to 0)	0 (0 to 0)	0 (0 to 0)	0 (0 to 0)	0 (0 to 0)	0 (0 to 0)	0·2 (0·2 to 0·4)	0·4 (0·3 to 0·6)	1·2 (0·8 to 1·9)	2·3 (1·3 to 3·1)	4·3 (2·7 to 6·8)	8 (5·5 to 11·4)	13·7 (9·6 to 18·8)	20·3 (14·5 to 27·6)	25·3 (18·1 to 33·5)	29·5 (20·9 to 38·8)	25·3 (18·2 to 34·3)	19·6 (14·2 to 27·4)	15·3 (10·7 to 21·1)	10·2 (7·2 to 13·8)	4·4 (3·3 to 5·8)	1·6 (1·2 to 2)	0·5 (0·3 to 0·7)	182
Larynx cancer	0 (0 to 0)	0 (0 to 0)	0 (0 to 0)	0 (0 to 0)	0 (0 to 0)	0 (0 to 0)	0 (0 to 0)	0·1 (0 to 0·1)	0·2 (0·1 to 0·3)	0·3 (0·2 to 0·4)	0·5 (0·3 to 0·8)	1 (0·5 to 1·2)	1·6 (1·1 to 2·3)	2·5 (1·7 to 3·5)	3 (2 to 4·1)	3·5 (2·5 to 4·7)	2·6 (1·7 to 3·8)	1·7 (1·2 to 2·4)	1·2 (0·9 to 1·7)	0·7 (0·5 to 1)	0·3 (0·2 to 0·5)	0·1 (0 to 0·1)	0 (0 to 0)	19·2
Tracheal, bronchus, and lung cancer	0 (0 to 0)	0 (0 to 0)	0 (0 to 0)	0 (0 to 0)	0 (0 to 0)	0·2 (0·1 to 0·4)	1·4 (1 to 1·9)	1·5 (1 to 2·2)	2·5 (1·4 to 3·8)	6·3 (3·8 to 9·4)	13·5 (8·6 to 20)	23·4 (16·1 to 32·8)	39·2 (27·1 to 53·9)	55·9 (39·7 to 77·5)	67 (46·2 to 95·3)	73·7 (51 to 103)	58·3 (41·1 to 83·8)	39·5 (27·2 to 54·1)	27·6 (19·8 to 38)	15·9 (11·1 to 22)	6·6 (4·6 to 9·1)	2·3 (1·6 to 2·9)	0·8 (0·5 to 1·1)	436
Malignant skin melanoma	0 (0 to 0)	0 (0 to 0)	0·1 (0 to 0·3)	0·4 (0·1 to 1·1)	0·1 (0·1 to 0·2)	0·2 (0·1 to 0·3)	0·5 (0·3 to 0·7)	1·3 (0·7 to 2)	1·5 (0·8 to 2·4)	2 (1·2 to 3·2)	4·6 (2·6 to 7·4)	3·4 (2 to 5·2)	4·3 (2·6 to 6·3)	4·5 (2·9 to 6·5)	4 (2·5 to 5·8)	3·9 (2·5 to 5·7)	3·1 (2·1 to 4·6)	2·3 (1·5 to 3·5)	1·5 (1 to 2·2)	1·2 (0·8 to 1·8)	0·7 (0·5 to 1·1)	0·3 (0·2 to 0·5)	0·2 (0·1 to 0·2)	40·1
Non-melanoma skin cancer	0 (0 to 0)	0 (0 to 0)	0 (0 to 0)	0 (0 to 0)	0 (0 to 0)	0 (0 to 0)	0 (0 to 0)	0·2 (0·2 to 0·4)	0·4 (0·2 to 0·6)	0·4 (0·3 to 0·7)	0·6 (0·3 to 0·9)	1 (0·6 to 1·6)	0·8 (0·5 to 1·3)	1·4 (0·9 to 2)	1·2 (0·8 to 1·9)	1·1 (0·7 to 1·6)	1 (0·7 to 1·5)	0·9 (0·6 to 1·2)	1·1 (0·8 to 1·5)	1 (0·7 to 1·3)	0·7 (0·5 to 0·9)	0·5 (0·3 to 0·7)	0·3 (0·2 to 0·4)	12·6
Non-melanoma skin cancer (squamous-cell carcinoma)	0 (0 to 0)	0 (0 to 0)	0 (0 to 0)	0 (0 to 0)	0 (0 to 0)	0 (0 to 0)	0 (0 to 0)	0·2 (0·2 to 0·4)	0·4 (0·2 to 0·6)	0·4 (0·3 to 0·7)	0·6 (0·3 to 0·9)	1 (0·6 to 1·6)	0·8 (0·5 to 1·3)	1·4 (0·9 to 2)	1·2 (0·8 to 1·9)	1·1 (0·7 to 1·6)	1 (0·7 to 1·5)	0·9 (0·6 to 1·2)	1·1 (0·8 to 1·5)	1 (0·7 to 1·3)	0·7 (0·5 to 0·9)	0·5 (0·3 to 0·7)	0·3 (0·2 to 0·4)	12·6
Non-melanoma skin cancer (basal-cell carcinoma)	0 (0 to 0)	0 (0 to 0)	0 (0 to 0)	0 (0 to 0)	0 (0 to 0)	0 (0 to 0)	0 (0 to 0)	0 (0 to 0)	0 (0 to 0)	0 (0 to 0)	0 (0 to 0)	0 (0 to 0)	0 (0 to 0)	0 (0 to 0)	0 (0 to 0)	0 (0 to 0)	0 (0 to 0)	0 (0 to 0)	0 (0 to 0)	0 (0 to 0)	0 (0 to 0)	0 (0 to 0)	0 (0 to 0)	0
Breast cancer	0 (0 to 0)	0 (0 to 0)	0 (0 to 0)	0 (0 to 0)	0 (0 to 0)	0 (0 to 0)	3·2 (2·5 to 4·2)	7·2 (4·9 to 10·1)	31·1 (20·6 to 46·8)	112 (75·5 to 159)	202 (142 to 273)	270 (188 to 374)	362 (257 to 486)	374 (268 to 505)	346 (244 to 478)	283 (214 to 374)	189 (140 to 248)	126 (93·1 to 168)	90·2 (66 to 119)	58 (42·9 to 75·2)	27·4 (20·6 to 35·4)	10·6 (7·9 to 13·4)	4·4 (2·9 to 5·8)	2,500
Cervical cancer	0 (0 to 0)	0 (0 to 0)	0 (0 to 0)	0 (0 to 0)	0 (0 to 0)	0 (0 to 0)	2 (1·4 to 2·9)	8·2 (5·2 to 12·7)	24·6 (15 to 38·7)	52·2 (33·1 to 79·4)	89·7 (60·2 to 129)	92·4 (62·8 to 135)	116 (80·7 to 168)	119 (82 to 167)	103 (73·8 to 141)	75·3 (54·5 to 102)	53·2 (37·6 to 72·5)	33·6 (24·8 to 45)	22·4 (17 to 29)	13·6 (9·6 to 18·7)	5 (3·5 to 6·6)	1·9 (1·3 to 2·7)	0·7 (0·4 to 1·1)	813
Uterine cancer	0 (0 to 0)	0 (0 to 0)	0 (0 to 0)	0 (0 to 0)	0 (0 to 0)	0 (0 to 0)	0 (0 to 0)	1·8 (1·3 to 2·4)	4·4 (2·8 to 6·5)	9·2 (6 to 13·3)	15·7 (9·9 to 23·4)	20·4 (13·4 to 30·2)	31·2 (20·8 to 44·8)	38·1 (25·9 to 54·2)	42·2 (30·3 to 58·6)	50 (35·9 to 67·9)	40·4 (29·4 to 54)	26·9 (19·9 to 35·7)	16·8 (12·2 to 22·3)	10 (7·1 to 13·5)	3·6 (2·6 to 4·7)	1·4 (1 to 1·8)	0·5 (0·3 to 0·7)	313
Ovarian cancer	0 (0 to 0)	0 (0 to 0)	0 (0 to 0)	0 (0 to 0)	1·4 (0·6 to 2·4)	2·5 (1·3 to 4·2)	7·1 (3·8 to 10·7)	10·3 (5·8 to 16·2)	10·7 (6·1 to 16·4)	17·5 (9·7 to 27·4)	29 (16·9 to 43·4)	44·1 (27·5 to 62·2)	60·7 (38·4 to 86)	69·6 (46·3 to 96·4)	71·4 (47·9 to 101)	61·2 (39·7 to 85·5)	45·5 (30·1 to 63·2)	28·2 (19·1 to 38·7)	18·4 (12·3 to 25·2)	10·8 (7·2 to 14·6)	4·4 (3 to 5·7)	1·6 (1·1 to 2·1)	0·4 (0·2 to 0·5)	495
Prostate cancer	0 (0 to 0)	0 (0 to 0)	0 (0 to 0)	0 (0 to 0)	0 (0 to 0)	0 (0 to 0)	0 (0 to 0)	0 (0 to 0)	0 (0 to 0)	0 (0 to 0)	0 (0 to 0)	0 (0 to 0)	0 (0 to 0)	0 (0 to 0)	0 (0 to 0)	0 (0 to 0)	0 (0 to 0)	0 (0 to 0)	0 (0 to 0)	0 (0 to 0)	0 (0 to 0)	0 (0 to 0)	0 (0 to 0)	0
Testicular cancer	--	--	--	--	--	--	--	--	--	--	--	--	--	--	--	--	--	--	--	--	--	--	--	NA
Kidney cancer	0 (0 to 0)	0 (0 to 0·1)	0·3 (0·2 to 0·5)	1·4 (0·8 to 2·4)	1·7 (1·1 to 2·5)	1·2 (0·8 to 1·8)	1·6 (1·1 to 2·3)	2·4 (1·6 to 3·5)	2·6 (1·6 to 3·8)	3·4 (2·1 to 5·1)	5 (3 to 7·3)	7·4 (4·8 to 10·6)	10·6 (7·3 to 15·1)	12·2 (8·3 to 16·9)	13·3 (9·2 to 19·1)	12·4 (8·7 to 16·5)	9·1 (6·7 to 12)	6·1 (4·5 to 8)	4 (2·9 to 5·2)	2·5 (1·8 to 3·2)	0·8 (0·6 to 1·1)	0·3 (0·2 to 0·4)	0·1 (0·1 to 0·2)	98·4
Bladder cancer	0 (0 to 0)	0 (0 to 0)	0 (0 to 0)	0 (0 to 0)	0 (0 to 0)	0 (0 to 0)	0·1 (0·1 to 0·2)	0·1 (0·1 to 0·2)	0·2 (0·1 to 0·3)	0·7 (0·5 to 1·1)	1 (0·6 to 1·4)	1·6 (1 to 2·4)	2·4 (1·6 to 3·6)	4·8 (3·4 to 6·8)	5·8 (4·2 to 7·9)	6 (4·5 to 7·9)	6·2 (4·7 to 8·1)	5·2 (3·8 to 6·9)	5·2 (3·8 to 6·9)	4 (2·9 to 5·4)	1·9 (1·4 to 2·6)	0·9 (0·6 to 1·2)	0·3 (0·2 to 0·4)	46·6
Brain and central nervous system cancer	0·2 (0·1 to 0·4)	0·3 (0·1 to 0·7)	1·4 (0·5 to 2·5)	5 (1·7 to 9)	9·1 (3·9 to 14·8)	8·8 (3·7 to 14·4)	6·9 (2·8 to 11·9)	7·9 (3 to 13·9)	9·1 (3·1 to 16·8)	9·6 (3·3 to 16·8)	12·9 (4·5 to 22·1)	13·2 (4·9 to 21·6)	14·2 (5·9 to 22·1)	16·2 (6·7 to 25·1)	15·9 (6·9 to 25)	12·9 (5·4 to 20·1)	9·6 (4·1 to 14·7)	5·6 (2·2 to 8·4)	3·9 (1·6 to 6·1)	1·8 (0·7 to 2·9)	0·8 (0·3 to 1·2)	0·2 (0·1 to 0·4)	0·1 (0 to 0·1)	166
Thyroid cancer	0 (0 to 0)	0 (0 to 0)	0 (0 to 0)	0 (0 to 0)	0·1 (0·1 to 0·2)	0·1 (0·1 to 0·2)	0·4 (0·3 to 0·6)	0·6 (0·4 to 0·8)	0·9 (0·6 to 1·3)	1·2 (0·8 to 1·8)	2·2 (1·4 to 3·3)	2·8 (1·8 to 4·1)	3·6 (2·4 to 5·2)	5·4 (3·4 to 8)	5·6 (3·9 to 8·2)	5·1 (3·8 to 6·7)	4·5 (3·3 to 6·5)	3·5 (2·6 to 4·6)	2·5 (1·8 to 3·2)	1·6 (1·1 to 2·2)	0·6 (0·4 to 0·8)	0·2 (0·1 to 0·3)	0·1 (0 to 0·1)	41
Mesothelioma	0 (0 to 0)	0 (0 to 0)	0 (0 to 0)	0 (0 to 0)	0 (0 to 0)	0 (0 to 0)	0 (0 to 0)	0·5 (0·3 to 0·8)	0·3 (0·2 to 0·5)	0·4 (0·2 to 0·6)	0·9 (0·5 to 1·5)	1 (0·6 to 1·5)	1·1 (0·7 to 1·6)	1·4 (0·9 to 2·1)	1·4 (0·9 to 2)	0·5 (0·3 to 0·7)	0·3 (0·2 to 0·5)	0·4 (0·2 to 0·6)	0·2 (0·1 to 0·2)	0·1 (0·1 to 0·2)	0 (0 to 0·1)	0 (0 to 0)	0 (0 to 0)	8·4
Hodgkin lymphoma	0 (0 to 0)	0 (0 to 0)	0 (0 to 0)	0·1 (0·1 to 0·1)	0·1 (0·1 to 0·2)	0·4 (0·2 to 0·5)	2·7 (1·6 to 4·2)	4·5 (2·7 to 7·1)	3·7 (2 to 6·1)	3·1 (1·6 to 5·2)	2·6 (1·5 to 4·2)	2·5 (1·5 to 3·8)	2 (1·2 to 3)	1·8 (1·1 to 2·6)	1·8 (1·1 to 2·8)	1·5 (1 to 2·4)	1·3 (0·9 to 2)	0·8 (0·6 to 1·3)	0·8 (0·5 to 1·2)	0·5 (0·3 to 0·7)	0·2 (0·1 to 0·3)	0 (0 to 0·1)	0 (0 to 0)	30·3
Non-Hodgkin lymphoma	0 (0 to 0)	0 (0 to 0)	0 (0 to 0)	2·3 (1·4 to 3·5)	3·3 (2·2 to 4·7)	4·6 (3·2 to 6·3)	8·2 (5·7 to 11·3)	12·7 (8·6 to 18·6)	14·5 (9·2 to 21·6)	16·1 (10·6 to 23·9)	17·9 (11·8 to 25·7)	15·6 (10·5 to 22·4)	20·2 (14·6 to 27·8)	22·7 (16·2 to 30·7)	22·9 (16·5 to 30·4)	22·7 (16·2 to 30·2)	18·5 (13·5 to 24·9)	13 (9·4 to 17·7)	9·6 (7 to 13)	5·4 (4 to 7·4)	1·8 (1·4 to 2·5)	0·6 (0·5 to 0·8)	0·2 (0·1 to 0·3)	233
Multiple myeloma	0 (0 to 0)	0 (0 to 0)	0 (0 to 0)	0 (0 to 0)	0 (0 to 0)	0 (0 to 0)	0 (0 to 0)	0·4 (0·3 to 0·6)	0·8 (0·5 to 1·2)	1·9 (1·2 to 2·7)	5 (2·9 to 8)	10 (6·3 to 14·5)	17·1 (10·8 to 26·2)	28·1 (19·3 to 39·6)	37·8 (26·4 to 52·1)	40·5 (29·1 to 57·3)	31·9 (23·6 to 42·7)	25·4 (18·4 to 35)	18·5 (13·5 to 24·8)	10 (7 to 13·9)	3·7 (2·6 to 5·2)	1·1 (0·8 to 1·6)	0·3 (0·2 to 0·4)	233
Leukemia	0·2 (0·1 to 0·3)	0·3 (0·2 to 0·5)	1·9 (1·1 to 2·8)	7·1 (4·4 to 11·3)	11·7 (7·9 to 16·3)	13·1 (8·6 to 19)	16·1 (11·4 to 22·1)	16·1 (11·1 to 22·9)	17·2 (11·1 to 25·6)	15·6 (10·3 to 22)	18·7 (12·1 to 27·4)	17·4 (11·9 to 25·1)	18·9 (13·4 to 26·3)	18·4 (13·5 to 24·2)	18·3 (13 to 24·8)	16·8 (12·8 to 21·8)	12·6 (9·7 to 16·3)	9·5 (7·1 to 12·7)	7·2 (5·4 to 9·3)	4·8 (3·5 to 6·5)	2 (1·5 to 2·6)	0·8 (0·6 to 1)	0·3 (0·2 to 0·4)	245
Acute lymphoid leukemia	0·1 (0 to 0·1)	0 (0 to 0·1)	0·5 (0·3 to 0·9)	2·9 (1·5 to 5)	5·1 (2·9 to 8·1)	4·3 (2·4 to 7)	4·5 (2·5 to 7·1)	2·3 (1·2 to 3·6)	1·5 (0·8 to 2·6)	1·2 (0·7 to 2)	0·9 (0·5 to 1·6)	1 (0·6 to 1·6)	0·9 (0·6 to 1·5)	1 (0·6 to 1·6)	0·8 (0·5 to 1·2)	0·6 (0·3 to 0·9)	0·4 (0·2 to 0·6)	0·2 (0·1 to 0·4)	0·2 (0·1 to 0·4)	0·1 (0·1 to 0·2)	0 (0 to 0)	0 (0 to 0)	0 (0 to 0)	28·6
Chronic lymphoid leukemia	0 (0 to 0)	0 (0 to 0)	0 (0 to 0)	0 (0 to 0)	0 (0 to 0)	0 (0 to 0)	0 (0 to 0)	0·6 (0·4 to 1)	0·4 (0·2 to 0·6)	0·4 (0·2 to 0·7)	1·2 (0·7 to 1·8)	1·4 (0·8 to 2·1)	0·8 (0·5 to 1·4)	3 (1·9 to 4·4)	4 (2·5 to 5·9)	4·9 (3·4 to 7·1)	4·6 (3·2 to 6·5)	3·3 (2·3 to 4·7)	2·8 (1·9 to 3·9)	1·8 (1·2 to 2·6)	0·8 (0·5 to 1·1)	0·3 (0·2 to 0·4)	0·1 (0·1 to 0·1)	30·5
Acute myeloid leukemia	0·1 (0 to 0·1)	0·2 (0·1 to 0·4)	0·7 (0·4 to 1·2)	3·4 (1·8 to 5·7)	5·2 (3·1 to 8·3)	7·3 (4·5 to 11·6)	9·4 (6·1 to 13·6)	8·7 (5·6 to 13)	10·1 (6·2 to 15·4)	8 (4·9 to 11·9)	10·2 (6·1 to 15·4)	8·4 (5·4 to 12·5)	10·6 (7·1 to 15)	8·5 (5·9 to 11·6)	7·7 (5·1 to 11)	6·3 (4·4 to 9)	3·7 (2·5 to 5·2)	2·7 (1·8 to 3·9)	1·6 (1 to 2·3)	1 (0·6 to 1·5)	0·3 (0·2 to 0·5)	0·1 (0·1 to 0·1)	0 (0 to 0·1)	114

Chronic myeloid leukemia	0 (0 to 0)	0 (0 to 0)	0 (0 to 0·1)	0·2 (0·1 to 0·3)	0·2 (0·1 to 0·4)	0·2 (0·1 to 0·4)	0·4 (0·2 to 0·8)	1·5 (0·8 to 2·7)	1·8 (1 to 3·1)	2·3 (1·3 to 3·9)	2·3 (1·2 to 3·9)	2·2 (1·1 to 3·7)	2·2 (1·2 to 3·5)	2·6 (1·6 to 4)	2·5 (1·5 to 3·7)	2·1 (1·3 to 3·2)	1·4 (0·9 to 2·2)	1·2 (0·8 to 1·8)	0·9 (0·6 to 1·3)	0·6 (0·4 to 1)	0·2 (0·1 to 0·4)	0·1 (0·1 to 0·1)	0 (0 to 0·1)	25·1
Other leukemia	0·1 (0·1 to 0·1)	0 (0 to 0·1)	0·6 (0·3 to 1·1)	0·7 (0·3 to 1·2)	1·2 (0·6 to 2)	1·2 (0·6 to 2·2)	1·8 (1·1 to 3)	2·9 (1·7 to 4·8)	3·4 (1·9 to 5·6)	3·6 (2·1 to 5·7)	4·1 (2·5 to 6·5)	4·3 (2·7 to 6·7)	4·4 (2·8 to 6·5)	3·3 (2·2 to 4·6)	3·4 (2·2 to 5)	2·8 (1·9 to 4)	2·5 (1·7 to 3·5)	2·1 (1·4 to 3·1)	1·7 (1·1 to 2·5)	1·2 (0·8 to 1·8)	0·6 (0·4 to 0·9)	0·2 (0·2 to 0·3)	0·1 (0·1 to 0·1)	46·4
Other malignant neoplasms	0·4 (0·2 to 0·7)	0·5 (0·3 to 0·8)	2·1 (1·5 to 3·1)	8·7 (5·7 to 12·8)	8·6 (6·8 to 11·1)	12·6 (9·5 to 16·8)	15·5 (11·9 to 19·8)	14·3 (10·7 to 18·9)	14·7 (10·3 to 20·9)	16·4 (11·5 to 23·2)	19·9 (14 to 27·5)	23·2 (16·7 to 31·4)	27 (20 to 35·6)	31·7 (24 to 42·1)	28·9 (22 to 37·7)	26·8 (20·5 to 34·4)	20·7 (15·9 to 26·1)	14·9 (11·3 to 18·9)	11·8 (9·2 to 15)	8·2 (6·3 to 10·4)	4·3 (3·1 to 5·7)	1·8 (1·3 to 2·3)	0·6 (0·4 to 0·9)	314
Other neoplasms	0 (0 to 0·1)	0·1 (0 to 0·3)	0·5 (0·2 to 0·9)	1·1 (0·6 to 1·9)	0·3 (0·2 to 0·5)	0·7 (0·4 to 1·1)	1 (0·5 to 1·6)	0·9 (0·6 to 1·4)	1·5 (0·9 to 2·4)	1·6 (0·9 to 2·4)	1·1 (0·7 to 1·6)	2·7 (1·7 to 4·1)	2·4 (1·6 to 3·6)	3·2 (2 to 4·7)	3·2 (2 to 4·7)	3·8 (2·6 to 5·4)	3·6 (2·6 to 4·8)	3·4 (2·4 to 4·9)	3·2 (2·3 to 4·6)	2·1 (1·5 to 3)	0·9 (0·6 to 1·3)	0·4 (0·2 to 0·5)	0·1 (0·1 to 0·2)	37·6
Myelodysplastic, myeloproliferative, and other hematopoietic neo	0 (0 to 0·1)	0·1 (0 to 0·3)	0·5 (0·2 to 0·9)	1·1 (0·6 to 1·9)	0·3 (0·2 to 0·5)	0·7 (0·4 to 1·1)	1 (0·5 to 1·6)	0·9 (0·6 to 1·4)	1·5 (0·9 to 2·4)	1·6 (0·9 to 2·4)	1·1 (0·7 to 1·6)	2·7 (1·7 to 4·1)	2·4 (1·6 to 3·6)	3·2 (2 to 4·7)	3·2 (2 to 4·7)	3·8 (2·6 to 5·4)	3·6 (2·6 to 4·8)	3·4 (2·4 to 4·9)	3·2 (2·3 to 4·6)	2·1 (1·5 to 3)	0·9 (0·6 to 1·3)	0·4 (0·2 to 0·5)	0·1 (0·1 to 0·2)	37·6
Benign and in situ intestinal neoplasms	0 (0 to 0)	0 (0 to 0)	0 (0 to 0)	0 (0 to 0)	0 (0 to 0)	0 (0 to 0)	0 (0 to 0)	0 (0 to 0)	0 (0 to 0)	0 (0 to 0)	0 (0 to 0)	0 (0 to 0)	0 (0 to 0)	0 (0 to 0)	0 (0 to 0)	0 (0 to 0)	0 (0 to 0)	0 (0 to 0)	0 (0 to 0)	0 (0 to 0)	0 (0 to 0)	0 (0 to 0)	0 (0 to 0)	0
Benign and in situ cervical and uterine neoplasms	0 (0 to 0)	0 (0 to 0)	0 (0 to 0)	0 (0 to 0)	0 (0 to 0)	0 (0 to 0)	0 (0 to 0)	0 (0 to 0)	0 (0 to 0)	0 (0 to 0)	0 (0 to 0)	0 (0 to 0)	0 (0 to 0)	0 (0 to 0)	0 (0 to 0)	0 (0 to 0)	0 (0 to 0)	0 (0 to 0)	0 (0 to 0)	0 (0 to 0)	0 (0 to 0)	0 (0 to 0)	0 (0 to 0)	0
Other benign and in situ neoplasms	0 (0 to 0)	0 (0 to 0)	0 (0 to 0)	0 (0 to 0)	0 (0 to 0)	0 (0 to 0)	0 (0 to 0)	0 (0 to 0)	0 (0 to 0)	0 (0 to 0)	0 (0 to 0)	0 (0 to 0)	0 (0 to 0)	0 (0 to 0)	0 (0 to 0)	0 (0 to 0)	0 (0 to 0)	0 (0 to 0)	0 (0 to 0)	0 (0 to 0)	0 (0 to 0)	0 (0 to 0)	0 (0 to 0)	0
Cardiovascular diseases	2·7 (1·9 to 3·9)	1·5 (0·9 to 2·6)	7·8 (5·3 to 11·1)	5·5 (4 to 7·5)	11·4 (9·1 to 14·3)	23·1 (17·4 to 29·8)	57·2 (45·4 to 70·3)	86 (66·1 to 113)	112 (82·7 to 151)	173 (126 to 232)	270 (202 to 360)	395 (299 to 516)	575 (440 to 748)	789 (625 to 999)	873 (704 to 1,090)	950 (769 to 1,170)	944 (787 to 1,120)	839 (711 to 984)	854 (727 to 1,010)	765 (650 to 889)	446 (362 to 524)	225 (178 to 263)	114 (80·1 to 138)	8,520
Rheumatic heart disease	0 (0 to 0)	0 (0 to 0)	0 (0 to 0)	0·5 (0·2 to 0·9)	3 (1·7 to 5·1)	7·6 (4·3 to 12·6)	12·3 (7·9 to 19)	14·5 (9·5 to 21·4)	17·2 (11·5 to 24·8)	18·8 (13 to 26·5)	20·5 (14·3 to 28·7)	20·5 (14·3 to 28·9)	18·4 (12·8 to 26·2)	15·5 (10·9 to 21·7)	11·5 (8·2 to 16)	7·2 (5·2 to 9·8)	4·8 (3·5 to 6·4)	2·9 (2·1 to 3·9)	2·2 (1·6 to 2·9)	1·4 (1 to 1·9)	0·7 (0·5 to 0·9)	0·3 (0·2 to 0·4)	0·2 (0·1 to 0·2)	180
Ischemic heart disease	0 (0 to 0)	0 (0 to 0)	0 (0 to 0)	0 (0 to 0)	0 (0 to 0)	0 (0 to 0)	3·8 (2·7 to 5·1)	7·8 (5·1 to 11·5)	11·8 (7·6 to 18·1)	27 (17·6 to 40·3)	50 (35·1 to 71·4)	83·3 (59·3 to 114)	134 (92·2 to 187)	213 (161 to 283)	269 (204 to 351)	332 (256 to 427)	334 (271 to 411)	292 (235 to 357)	275 (218 to 342)	250 (199 to 301)	155 (123 to 189)	81 (61·5 to 99·5)	41·5 (28·3 to 52·2)	2,560
Stroke	0·8 (0·4 to 1·4)	0·2 (0·1 to 0·3)	0·7 (0·4 to 1·4)	0·9 (0·6 to 1·1)	2·6 (1·9 to 3·3)	5 (3·6 to 6·9)	13·5 (9·8 to 18·1)	21·6 (14·9 to 31·9)	29·6 (20·5 to 42·5)	50·1 (33·8 to 71·6)	80·1 (55·5 to 113)	123 (85·5 to 168)	179 (133 to 240)	249 (180 to 342)	249 (183 to 335)	240 (187 to 308)	262 (197 to 336)	242 (188 to 304)	269 (212 to 332)	225 (175 to 279)	121 (94·9 to 150)	53·2 (39·5 to 67·4)	24·3 (15·9 to 32·3)	2,440
Ischemic stroke	0·2 (0·1 to 0·4)	0 (0 to 0)	0 (0 to 0·1)	0·4 (0·3 to 0·6)	1·3 (0·9 to 1·8)	2·2 (1·2 to 3·5)	3·8 (2·5 to 5·5)	5·8 (4 to 8·2)	7 (4·8 to 10·1)	10·8 (7·3 to 15)	15·1 (10·6 to 20·3)	23·6 (17 to 30·8)	33·9 (25·6 to 43·8)	49·6 (37·5 to 65·1)	59·5 (45·1 to 76·8)	83·2 (66·1 to 105)	92·3 (71·4 to 117)	135 (107 to 169)	161 (129 to 199)	170 (133 to 210)	95·6 (74·5 to 117)	42·1 (31·1 to 53·2)	19·7 (12·9 to 26·1)	1,010
Intracerebral hemorrhage	0·3 (0·1 to 0·5)	0·1 (0 to 0·1)	0·4 (0·2 to 0·8)	0·2 (0·1 to 0·3)	0·7 (0·5 to 0·9)	1·5 (1 to 2)	5·7 (3·9 to 7·9)	9·9 (6·3 to 15)	13·9 (8·8 to 20·6)	24·9 (15·5 to 37·9)	42·1 (27·7 to 62·4)	66·5 (44·3 to 94·8)	108 (77·2 to 148)	155 (109 to 218)	150 (107 to 208)	133 (101 to 174)	148 (109 to 194)	94·8 (72 to 121)	97 (75·8 to 120)	49·5 (38 to 62)	23·1 (17·9 to 28·9)	10·1 (7·4 to 12·7)	4·1 (2·6 to 5·5)	1,140
Subarachnoid hemorrhage	0·3 (0·2 to 0·6)	0·1 (0 to 0·1)	0·3 (0·1 to 0·6)	0·3 (0·2 to 0·4)	0·6 (0·4 to 0·9)	1·4 (0·9 to 2·1)	4 (2·4 to 6·1)	6 (3·5 to 9·5)	8·7 (5·1 to 13·8)	14·5 (8·8 to 22·5)	23 (14·2 to 35·6)	32·5 (20·9 to 49·4)	37·4 (24·7 to 53·9)	44·2 (28·6 to 65·2)	39 (25 to 56·7)	24·5 (16·2 to 35·1)	21·3 (14·1 to 31)	11·6 (7·8 to 16·4)	10·2 (7 to 14·5)	5·8 (3·9 to 8·3)	2·5 (1·7 to 3·4)	1·1 (0·7 to 1·5)	0·5 (0·3 to 0·8)	290
Hypertensive heart disease	0 (0 to 0)	0 (0 to 0)	0 (0 to 0)	0 (0 to 0)	0 (0 to 0)	0 (0 to 0)	3·2 (1·8 to 4·9)	7·5 (4·9 to 11·3)	10·2 (6·3 to 15·9)	24·4 (14·9 to 36·4)	48·7 (30·6 to 70·9)	74·8 (49·9 to 106)	132 (89 to 190)	191 (132 to 265)	211 (146 to 287)	230 (165 to 317)	214 (156 to 296)	194 (142 to 254)	198 (145 to 264)	187 (137 to 243)	102 (73·3 to 139)	53·6 (37·9 to 71·4)	28·6 (17·8 to 38·1)	1,910
Non-rheumatic valvular heart disease	0 (0 to 0)	0 (0 to 0)	0 (0 to 0)	0 (0 to 0)	0 (0 to 0)	0 (0 to 0)	2·4 (1·6 to 3·5)	2·1 (1·4 to 3·2)	2·3 (1·4 to 3·6)	3·3 (2 to 5)	4·3 (2·8 to 6·6)	5·2 (3·2 to 8)	7·3 (4·5 to 10·8)	7 (4·5 to 10·5)	7·1 (4·6 to 10·2)	7·5 (4·9 to 10·8)	6 (4·2 to 8·3)	4·4 (3 to 6)	4·1 (3 to 5·6)	3·2 (2·3 to 4·4)	1·8 (1·2 to 2·4)	0·8 (0·6 to 1·1)	0·3 (0·2 to 0·4)	69·1
Non-rheumatic calcific aortic valve disease	0 (0 to 0)	0 (0 to 0)	0 (0 to 0)	0 (0 to 0)	0 (0 to 0)	0 (0 to 0)	0·9 (0·5 to 1·4)	1·1 (0·7 to 1·8)	0·9 (0·5 to 1·6)	1·1 (0·5 to 2·1)	1·7 (0·9 to 2·9)	2·1 (1·1 to 3·6)	3·2 (1·8 to 5·3)	3·4 (1·9 to 5·5)	3·7 (2·2 to 5·7)	4·7 (2·9 to 7·1)	4 (2·7 to 5·8)	3·1 (2 to 4·4)	3·1 (2·1 to 4·3)	2·5 (1·7 to 3·5)	1·5 (1 to 2)	0·6 (0·4 to 0·9)	0·2 (0·1 to 0·3)	37·8
Non-rheumatic degenerative mitral valve disease	0 (0 to 0)	0 (0 to 0)	0 (0 to 0)	0 (0 to 0)	0 (0 to 0)	0 (0 to 0)	1·2 (0·7 to 2)	0·8 (0·4 to 1·4)	1·1 (0·6 to 1·8)	1·6 (0·9 to 2·6)	2·2 (1·1 to 3·5)	2·8 (1·5 to 4·5)	3·4 (1·8 to 5·4)	3·1 (1·7 to 5·2)	2·9 (1·6 to 4·7)	2·6 (1·4 to 4·2)	1·7 (1 to 2·8)	1 (0·6 to 1·7)	0·8 (0·5 to 1·4)	0·6 (0·3 to 0·9)	0·3 (0·1 to 0·4)	0·2 (0·1 to 0·3)	0·1 (0 to 0·1)	26·3
Other non-rheumatic valve diseases	0 (0 to 0)	0 (0 to 0)	0 (0 to 0)	0 (0 to 0)	0 (0 to 0)	0 (0 to 0)	0·3 (0·1 to 0·7)	0·1 (0·1 to 0·3)	0·3 (0·1 to 0·6)	0·6 (0·2 to 1·1)	0·5 (0·2 to 1)	0·3 (0·1 to 0·8)	0·7 (0·3 to 1·6)	0·4 (0·2 to 0·9)	0·5 (0·2 to 1)	0·2 (0·1 to 0·5)	0·2 (0·1 to 0·5)	0·3 (0·1 to 0·5)	0·2 (0·1 to 0·4)	0·2 (0·1 to 0·3)	0·1 (0 to 0·1)	0 (0 to 0·1)	0 (0 to 0)	4·9
Cardiomyopathy and myocarditis	0·5 (0·3 to 0·9)	0·5 (0·3 to 1)	3·2 (2 to 4·8)	2·2 (1·4 to 3·2)	2 (1·4 to 2·8)	3·9 (2·7 to 5·4)	5·8 (3·7 to 8·2)	9 (5·6 to 13·2)	9·9 (5·9 to 15·8)	14·2 (8·3 to 21·8)	17·7 (10·5 to 27·5)	21·4 (13·2 to 32·2)	27·3 (16·9 to 41·7)	32·4 (20·1 to 48·2)	32·5 (20·9 to 47·5)	32 (21·7 to 46·5)	26·7 (18 to 38·7)	18·6 (12·9 to 25·9)	17·5 (11·7 to 25·3)	13·6 (9 to 19·3)	9·2 (6·1 to 13·3)	4·2 (2·6 to 6·2)	2·1 (1·1 to 3·5)	306
Myocarditis	0 (0 to 0·1)	0 (0 to 0·1)	0·3 (0·2 to 0·6)	0·3 (0·2 to 0·6)	0·5 (0·3 to 0·7)	0·5 (0·3 to 0·7)	0·5 (0·3 to 0·8)	0·6 (0·3 to 1)	0·6 (0·3 to 1·1)	1·2 (0·6 to 2·1)	0·9 (0·5 to 1·5)	0·5 (0·3 to 0·9)	1·2 (0·7 to 2·1)	1 (0·5 to 1·8)	0·6 (0·4 to 1)	0·6 (0·3 to 0·9)	0·3 (0·2 to 0·4)	0·4 (0·2 to 0·7)	0·4 (0·2 to 0·7)	0·5 (0·2 to 0·9)	0·4 (0·2 to 0·8)	0·2 (0·1 to 0·4)	0·2 (0·1 to 0·3)	11·8
Alcoholic cardiomyopathy	0 (0 to 0)	0 (0 to 0)	0 (0 to 0)	0 (0 to 0)	0 (0 to 0)	0 (0 to 0)	0·2 (0·1 to 0·5)	0·6 (0·2 to 1)	0·8 (0·3 to 1·6)	0·6 (0·3 to 1·2)	0·8 (0·3 to 1·4)	0·8 (0·3 to 1·4)	3·4 (1·4 to 5·9)	3·7 (1·4 to 7·2)	2·4 (1·1 to 4·4)	2·1 (0·9 to 3·7)	1·8 (0·8 to 3·2)	0·2 (0·1 to 0·4)	0·3 (0·1 to 0·7)	0·3 (0·1 to 0·7)	0·1 (0 to 0·1)	0 (0 to 0·1)	0 (0 to 0)	18
Other cardiomyopathy	0·5 (0·3 to 0·8)	0·5 (0·2 to 0·9)	2·9 (1·7 to 4·3)	1·9 (1·1 to 2·9)	1·5 (0·9 to 2·3)	3·4 (2·2 to 5)	5 (3 to 7·5)	7·9 (4·7 to 11·9)	8·5 (4·8 to 13·9)	12·4 (6·8 to 19·4)	16·1 (9·2 to 25·5)	20·1 (12 to 30·7)	22·7 (13·3 to 35·8)	27·7 (17 to 41)	29·4 (18·5 to 43·7)	29·4 (19·4 to 43·6)	24·7 (16·2 to 36·6)	17·9 (12·2 to 25·2)	16·8 (11·2 to 24·3)	12·9 (8·6 to 18·2)	8·7 (5·7 to 12·5)	3·9 (2·4 to 5·9)	1·9 (1 to 3·3)	277
Atrial fibrillation and flutter	0 (0 to 0)	0 (0 to 0)	0 (0 to 0)	0 (0 to 0)	0 (0 to 0)	0 (0 to 0)	0 (0 to 0)	0 (0 to 0)	0 (0 to 0)	0·4 (0·2 to 0·6)	0·7 (0·4 to 1)	3·1 (2·1 to 4·4)	4·9 (3·3 to 7·2)	7·1 (5 to 10)	11·3 (8·3 to 15·6)	15·3 (11·5 to 20·5)	18·2 (14·2 to 23·7)	21·5 (16·9 to 27·4)	26·2 (20·3 to 34·1)	30·6 (24 to 38·7)	22·7 (16·9 to 30·9)	15·1 (11 to 19·9)	7·9 (5·4 to 11·3)	185
Aortic aneurysm	--	--	--	--	--	--	0·3 (0·2 to 0·4)	0·6 (0·4 to 1)	1·4 (0·9 to 2·3)	1·6 (0·9 to 2·5)	3·3 (2·1 to 5)	4·8 (3·3 to 7)	6·9 (4·4 to 10·3)	9·6 (6·4 to 13·7)	12·8 (8·5 to 18·2)	15·5 (10·8 to 21·5)	17·5 (12·5 to 24·3)	14·6 (10 to 20·2)	14·2 (9·9 to 19·9)	11·2 (7·7 to 15·7)	5·4 (3·8 to 7·4)	2·2 (1·5 to 2·9)	1 (0·6 to 1·4)	NA
Endocarditis	0·1 (0 to 0·1)	0·1 (0 to 0·2)	0·6 (0·3 to 1·1)	0·4 (0·2 to 0·7)	0·6 (0·3 to 1)	0·9 (0·5 to 1·4)	3 (1·7 to 4·5)	5·9 (3·2 to 9·4)	6 (3·2 to 10·2)	5·6 (3 to 9·3)	9·1 (5 to 14·6)	8·6 (5 to 13·5)	9·4 (5·1 to 15·8)	8·1 (4·8 to 12·5)	8·1 (4·8 to 12·3)	7·9 (4·7 to 12·2)	5·1 (3·2 to 7·8)	4 (2·6 to 6·4)	3·3 (2·1 to 5·2)	3·5 (2·2 to 5·4)	1·8 (1·1 to 3)	0·9 (0·5 to 1·4)	0·5 (0·3 to 1·1)	93·3
Other cardiovascular and circulatory diseases	1·3 (0·8 to 2)	0·7 (0·4 to 1·3)	3·2 (1·7 to 5·2)	1·7 (1 to 2·6)	3·2 (2·1 to 4·6)	5·6 (3·5 to 8·3)	13 (8·8 to 19)	16·9 (11·6 to 24)	23·3 (14·7 to 34·7)	27·3 (17·8 to 39·8)	35·8 (23·5 to 51·7)	49·7 (34·3 to 69·1)	53·8 (39·2 to 73·5)	52 (36·9 to 71·2)	54·4 (39·8 to 72)	52·2 (36·9 to 71·2)	44·7 (33·5 to 57·6)	36·7 (27·6 to 47·7)	34·4 (25·3 to 46·2)	28·3 (20·7 to 37·1)	18·6 (12·4 to 25·3)	9·1 (6·1 to 11·8)	4·6 (2·9 to 6·3)	570
Chronic respiratory diseases	0 (0 to 0)	0 (0 to 0)	0 (0 to 0)	53·1 (27·8 to 95·7)	60·4 (35 to 101)	60·2 (36·6 to 99)	52 (32·8 to 80·9)	49·4 (33·1 to 71·1)	50·7 (34·3 to 72·8)	51·3 (36·3 to 69)	54·5 (39·8 to 71·7)	53·9 (40·6 to 69·9)	60·1 (45·6 to 77·4)	68·2 (53·1 to 87·8)	70·3 (55·1 to 91)	70·9 (54·7 to 92·8)	60·7 (46·6 to 80·2)	48·9 (37·6 to 64)	43·2 (32·9 to 56·6)	32·8 (24·8 to 42·7)	18·6 (14·2 to 23·9)	9·8 (7·4 to 12·5)	5·3 (3·7 to 7·3)	974
Chronic obstructive pulmonary disease	0 (0 to 0)	0 (0 to 0)	0 (0 to 0)	0·4 (0·3 to 0·6)	1·1 (0·3 to 2·3)	2·1 (0·9 to 3·9)	3·6 (1·9 to 5·8)	5 (2·9 to 7·7)	6·3 (3·8 to 9·5)	7·9 (5 to 11·5)	10·1 (6·7 to 14·3)	12·7 (8·6 to 17·3)	17·7 (12·7 to 23·4)	25·5 (19 to 33·5)	28·7 (21·5 to 37·6)	38·9 (29·1 to 50·8)	35·5 (26·4 to 48)	29 (21·7 to 38)	27·4 (20·3 to 36·6)	22·2 (16·5 to 29·9)	13·5 (10·3 to 17·6)	7·4 (5·6 to 9·4)	4 (2·8 to 5·5)	299
Pneumoconiosis	0 (0 to 0)	0 (0 to 0)	0 (0 to 0)	0 (0 to 0)	0 (0 to 0)	0 (0 to 0)	0 (0 to 0·1)	0 (0 to 0)	0 (0 to 0)	0 (0 to 0)	0 (0 to 0·1)	0 (0 to 0·1)	0·1 (0 to 0·1)	0·1 (0·1 to 0·2)	0 (0 to 0·1)	0 (0 to 0·1)	0 (0 to 0·1)	0 (0 to 0·1)	0·1 (0 to 0·1)	0 (0 to 0·1)	0 (0 to 0·1)	0 (0 to 0)	0 (0 to 0)	0·7
Silicosis	0 (0 to 0)	0 (0 to 0)	0 (0 to 0)	0 (0 to 0)	0 (0 to 0)	0 (0 to 0)	0 (0 to 0)	0 (0 to 0)	0 (0 to 0)	0 (0 to 0)	0 (0 to 0)	0 (0 to 0)	0 (0 to 0)	0 (0 to 0)	0 (0 to 0)	0 (0 to 0)	0 (0 to 0)	0 (0 to 0)	0 (0 to 0)	0 (0 to 0)	0 (0 to 0)	0 (0 to 0)	0 (0 to 0)	0·1
Asbestosis	0 (0 to 0)	0 (0 to 0)	0 (0 to 0)	0 (0 to 0)	0 (0 to 0)	0 (0 to 0)	0 (0 to 0)	0 (0 to 0)	0 (0 to 0)	0 (0 to 0)	0 (0 to 0)	0 (0 to 0)	0 (0 to 0)	0 (0 to 0·1)	0 (0 to 0·1)	0 (0 to 0)	0 (0 to 0)	0 (0 to 0)	0 (0 to 0·1)	0 (0 to 0)	0 (0 to 0)	0 (0 to 0)	0 (0 to 0)	0·3
Coal workers pneumoconiosis	0 (0 to 0)	0 (0 to 0)	0 (0 to 0)	0 (0 to 0)	0 (0 to 0)	0 (0 to 0)	0 (0 to 0)	0 (0 to 0)	0 (0 to 0)	0 (0 to 0)	0 (0 to 0)	0 (0 to 0)	0 (0 to 0)	0 (0 to 0)	0 (0 to 0)	0 (0 to 0)	0 (0 to 0)	0 (0 to 0)	0 (0 to 0)	0 (0 to 0)	0 (0 to 0)	0 (0 to 0)	0 (0 to 0)	0
Other pneumoconiosis	0 (0 to 0)	0 (0 to 0)	0 (0 to 0)	0 (0 to 0)	0 (0 to 0)	0 (0 to 0)	0 (0 to 0)	0 (0 to 0)	0 (0 to 0)	0 (0 to 0)	0 (0 to 0)	0 (0 to 0)	0 (0 to 0)	0 (0 to 0·1)	0 (0 to 0)	0 (0 to 0)	0 (0 to 0)	0 (0 to 0)	0 (0 to 0)	0 (0 to 0)	0 (0 to 0)	0 (0 to 0)	0 (0 to 0)	0·3
Asthma	0 (0 to 0)	0 (0 to 0)	0 (0 to 0)	47·5 (22·8 to 89)	55·5 (30·7 to 95·7)	52·8 (29·6 to 91)	44·4 (26 to 73·1)	38 (23·1 to 58·5)	34·5 (20·8 to 54·3)	33·1 (21 to 49·2)	31·7 (21 to 46·8)	28·2 (19·1 to 40·3)	27·5 (19 to 38·1)	26 (18·3 to 36·1)	20 (14 to 27·6)	16·8 (12 to 23)	12·8 (9·1 to 17·5)	8·3 (5·8 to 11·5)	6·2 (4·3 to 8·5)	3·8 (2·6 to 5·3)	1·7 (1·1 to 2·5)	0·8 (0·5 to 1·1)	0·4 (0·3 to 0·7)	490
Interstitial lung disease and pulmonary sarcoidosis	0 (0 to 0)	0 (0 to 0)	0 (0 to 0)	1·1 (0·6 to 2·1)	0·6 (0·3 to 1)	0·6 (0·3 to 1·1)	1·4 (0·7 to 2·4)	1·8 (0·9 to 2·9)	2·4 (1·1 to 4·1)	4·6 (2·1 to 7·8)	5·9 (2·8 to 10)	5·5 (3 to 8·7)	8·3 (4·9 to 12·7)	9·7 (5·7 to 14·8)	10·4 (6·1 to 15·6)	13 (7·9 to 19·3)	11·3 (7 to 16·9)	10·4 (6·4 to 15·5)	8·7 (5·3 to 13·2)	6 (3·6 to 9·1)	3 (1·7 to 4·8)	1·5 (0·9 to 2·3)	0·8 (0·4 to 1·4)	107
Other chronic respiratory diseases	0 (0 to 0)	0 (0 to 0)	0 (0 to 0)	4·1 (2·3 to 6·7)	3·1 (1·7 to 4·9)	4·7 (2·8 to 7·2)	2·7 (1·6 to 4·3)	4·6 (3 to 6·7)	7·4 (4·6 to 11·1)	5·6 (3·3 to 8·8)	6·7 (3·9 to 10·3)	7·5 (4·5 to 11·8)	6·5 (3·9 to 10·2)	6·9 (3·9 to 11·4)	11·2 (6·4 to 18·3)	2·3 (1·1 to 4·1)	1·1 (0·5 to 1·9)	1·2 (0·5 to 2·1)	0·9 (0·4 to 1·5)	0·8 (0·4 to 1·5)	0·4 (0·2 to 0·7)	0·2 (0·1 to 0·3)	0·1 (0 to 0·3)	77·9
Digestive diseases	1·3 (0·9 to 1·8)	1·8 (1 to 3·2)	10·5 (6·1 to 17·3)	15·8 (11 to 22·3)	16·1 (12 to 21·9)	20·3 (15·4 to 26·1)	33·9 (25·8 to 44·1)	56·3 (41·7 to 75·6)	73·9 (52·7 to 102)	93·9 (67·8 to 126)	115 (86·5 to 152)	127 (97 to 164)	154 (116 to 199)	183 (141 to 233)	181 (143 to 226)	171 (135 to 212)	134 (107 to 166)	98·2 (78·5 to 120)	85·8 (65·7 to 110)	61·6 (46·7 to 78·2)	29·6 (23·1 to 36·4)	14·4 (10·7 to 17·9)	6 (4·2 to 7·7)	1,680
Cirrhosis and other chronic liver diseases	0 (0 to 0)	0 (0 to 0)	0 (0 to 0)	1·6 (0·9 to 2·7)	2·1 (1·4 to 3·1)	3·7 (2·4 to 5·3)	7·5 (4·8 to 10·8)	11·3 (7·7 to 16·2)	14·1 (8·6 to 21·4)	22·8 (14·3 to 33·6)	33·7 (22·9 to 46·4)	38·7 (26·7 to 53·3)	53·8 (36·7 to 77·5)	73·5 (52·8 to 101)	76·4 (56·6 to 102)	71·1 (52 to 94·8)	55·4 (41·6 to 71)	36·9 (27·7 to 47·6)	28·9 (20·5 to 39·7)	18·7 (13·3 to 25·3)	7·3 (5·3 to 9·5)	3·1 (2·1 to 4·2)	1·2 (0·7 to 1·7)	562
Cirrhosis and other chronic liver diseases due to hepatitis B	0 (0 to 0)	0 (0 to 0)	0 (0 to 0)	0 (0 to 0)	0 (0 to 0)	0 (0 to 0)	0 (0 to 0·1)	0·2 (0·1 to 0·5)	0·5 (0·2 to 1·2)	1·3 (0·6 to 2·5)	2·4 (1 to 4·8)	2·9 (1·4 to 5·4)	4 (1·9 to 7·8)	5 (2·3 to 9·9)	4·4 (2 to 7·9)	3·5 (1·6 to 6·9)	2·4 (1·1 to 4·4)	1·5 (0·7 to 2·8)	1·2 (0·6 to 2·2)	0·8 (0·4 to 1·4)	0·3 (0·2 to 0·5)	0·1 (0·1 to 0·2)	0 (0 to 0·1)	30·9
Cirrhosis and other chronic liver diseases due to hepatitis C	0 (0 to 0)	0 (0 to 0)	0 (0 to 0)	0 (0 to 0)	0 (0 to 0)	0·1 (0 to 0·1)	0·2 (0·1 to 0·4)	1 (0·4 to 2·1)	2·5 (1·1 to 4·8)	5·1 (2·6 to 8·6)	8·2 (4·2 to 14·4)	9·3 (4·8 to 15·7)	11·9 (6·2 to 20·6)	15 (8 to 25·8)	14·3 (7·3 to 25·6)	12·3 (5·8 to 22·6)	9 (5 to 15·2)	5·7 (3·3 to 9·2)	4·2 (2·1 to 7·4)	2·7 (1·4 to 4·6)	1·1 (0·6 to 1·8)	0·5 (0·3 to 0·8)	0·2 (0·1 to 0·4)	103
Cirrhosis and other chronic liver diseases due to alcohol use	0 (0 to 0)	0 (0 to 0)	0 (0 to 0)	0 (0 to 0)	0 (0 to 0)	0 (0 to 0)	0 (0 to 0·1)	0·5 (0·2 to 1·1)	1·5 (0·6 to 3)	4·5 (2·3 to 7·9)	9·4 (4·5 to 16·4)	12·4 (6·8 to 20·5)	18·6 (9·8 to 31·2)	26·8 (14·9 to 42)	29·2 (16·6 to 43·7)	26·9 (15·6 to 40·3)	19·4 (11·1 to 28·9)	11·5 (6·4 to 17·7)	7·7 (4·3 to 12·4)	4·5 (2·4 to 7·7)	1·7 (1 to 2·7)	0·7 (0·4 to 1·2)	0·2 (0·1 to 0·5)	176
Cirrhosis and other chronic liver diseases due to NAFLD	0 (0 to 0)	0 (0 to 0)	0 (0 to 0)	0 (0 to 0)	0 (0 to 0)	0 (0 to 0)	0·3 (0·1 to 0·6)	1·1 (0·5 to 2·1)	2·3 (1 to 4·4)	4·4 (2 to 7·9)	6·5 (2·9 to 12·4)	7·8 (3·8 to 13·5)	11·6 (5·4 to 20·8)	16 (7·9 to 27·6)	16·1 (8 to 28·1)	15·3 (7·4 to 27·3)	12·9 (7·5 to 20·5)	9·2 (5·4 to 14)	7·7 (4·1 to 12·4)	5·2 (2·6 to 8·4)	2 (1·2 to 3·1)	0·9 (0·5 to 1·4)	0·3 (0·1 to 0·6)	120
Cirrhosis and other chronic liver diseases due to other causes	0 (0 to 0)	0 (0 to 0)	0 (0 to 0)	1·6 (0·9 to 2·7)	2·1 (1·4 to 3)	3·6 (2·4 to 5·2)	7 (4·4 to 10)	8·4 (5·5 to 12·4)	7·2 (3·9 to 12·2)	7·5 (3·8 to 12·6)	7·2 (3·4 to 12·2)	6·3 (3 to 11·1)	7·8 (3·8 to 14)	10·7 (5·1 to 19·3)	12·4 (5·8 to 21·5)	13·2 (6·7 to 22·3)	11·6 (5·7 to 20)	8·9 (4·8 to 14·3)	8·1 (4·4 to 12·9)	5·6 (3 to 9·2)	2·2 (1·3 to 3·3)	1 (0·5 to 1·5)	0·4 (0·1 to 0·6)	133
Upper digestive system diseases	0 (0 to 0)	0 (0 to 0)	0 (0 to 0)	1·1 (0·6 to 2)	1·8 (0·8 to 3·2)	3·2 (1·8 to 5·2)	9·5 (5·2 to 15·8)	21·4 (11·8 to 38·1)	30·8 (16·6 to 53·6)	35·2 (19·2 to 61·7)	37·9 (21·1 to 64·1)	38·3 (21·6 to 63)	42·5 (24·9 to 68·1)	43·2 (26·4 to 69·4)	41 (25·8 to 64)	35·5 (23·2 to 54·8)	26·2 (17·9 to 38·7)	19·4 (13·6 to 27·9)	15·9 (11·1 to 22·2)	11 (7·7 to 15·8)	5·4 (3·9 to 7·5)	2·7 (1·9 to 3·6)	1·1 (0·7 to 1·5)	423
Peptic ulcer disease	0 (0 to 0)	0 (0 to 0)	0 (0 to 0)	0·2 (0·1 to 0·3)	0·2 (0·1 to 0·2)	0·4 (0·3 to 0·6)	0·6 (0·4 to 0·9)	1 (0·7 to 1·4)	1·3 (0·8 to 2)	2·4 (1·6 to 3·5)	3·3 (2·3 to 4·5)	3·6 (2·5 to 4·9)	4·8 (3·2 to 7·2)	6·2 (4·4 to 8·5)	6·8 (4·9 to 9·2)	7·6 (5 to 10·8)	6·6 (4·9 to 8·6)	5·6 (4 to 7·3)	5·7 (4 to 8)	4·9 (3·1 to 7·3)	2·4 (1·6 to 3·2)	1·3 (0·8 to 1·8)	0·5 (0·3 to 0·8)	65·4
Gastritis and duodenitis	0 (0 to 0)	0 (0 to 0)	0 (0 to 0)	0·9 (0·4 to 1·8)	1·6 (0·7 to 3)	1·6 (0·7 to 3·2)	2 (0·8 to 3·9)	2·6 (1·3 to 4·7)	3·5 (1·8 to 6·1)	4·4 (2·2 to 7·8)	5·6 (3 to 9·5)	5·1 (2·6 to 9·3)	7·7 (4 to 13·9)	9 (5·1 to 15)	9·4 (5·4 to 15·5)	7·9 (4·5 to 12·8)	5·8 (3·5 to 9)	4·3 (2·7 to 6·5)	3·3 (2 to 4·8)	1·9 (1·2 to 2·9)	1 (0·7 to 1·5)	0·5 (0·3 to 0·8)	0·2 (0·1 to 0·3)	78·4
Gastroesophageal reflux disease	0 (0 to 0)	0 (0 to 0)	0 (0 to 0)	0 (0 to 0)	0 (0 to 0·1)	1·1 (0·5 to 2·2)	6·9 (3·3 to 12·7)	17·8 (8·8 to 34·1)	26 (12·5 to 48·2)	28·4 (13·4 to 53·6)	29 (13·5 to 53·4)	29·7 (14·2 to 54)	29·9 (14·7 to 52·1)	28·1 (13·6 to 50)	24·8 (11·7 to 45·6)	20 (9·9 to 36·4)	13·8 (6·9 to 24·8)	9·5 (4·7 to 16·9)	6·8 (3·4 to 12·4)	4·2 (2 to 7·5)	2 (1 to 3·6)	0·9 (0·4 to 1·6)	0·4 (0·2 to 0·7)	279
Appendicitis	0 (0 to 0)	0 (0 to 0)	0 (0 to 0)	0·6 (0·3 to 1·1)	1·3 (0·9 to 2)	2·3 (1·2 to 3·8)	2·8 (1·5 to 4·5)	2·6 (1·4 to 4·4)	2 (1 to 3·5)	2·1 (1·1 to 3·3)	3 (1·7 to 4·7)	2·1 (1·2 to 3·4)	2·3 (1·3 to 3·8)	2·1 (1·2 to 3·4)	2·1 (1·2 to 3·6)	1·5 (0·9 to 2·3)	1·4 (0·9 to 2·2)	1 (0·6 to 1·5)	0·8 (0·4 to 1·2)	0·5 (0·3 to 0·8)	0·3 (0·2 to 0·5)	0·2 (0·1 to 0·3)	0·1 (0 to 0·1)	31·1
Paralytic ileus and intestinal obstruction	0·9 (0·6 to 1·2)	1·6 (0·9 to 2·8)	9·3 (5·3 to 15·4)	3·4 (1·8 to 5·7)	1·9 (1·2 to 2·8)	2·4 (1·5 to 3·6)	2·2 (1·2 to 3·7)	1·9 (1·1 to 3)	2·4 (1·3 to 4·1)	2·6 (1·4 to 4·7)	5 (2·7 to 8·4)	5·2 (3 to 8·4)	6·9 (3·9 to 11·1)	9·5 (5·5 to 15·2)	8·7 (5·3 to 13·2)	10·6 (6·8 to 15·3)	9·6 (6·2 to 14)	8·9 (5·7 to 13)	9·9 (6·4 to 14·7)	8·6 (5·6 to 12·6)	4·6 (3·1 to 6·5)	2·5 (1·6 to 3·8)	1·3 (0·7 to 2·3)	120
Inguinal, femoral, and abdominal hernia	0·4 (0·2 to 0·7)	0·2 (0·1 to 0·5)	1·1 (0·6 to 2·1)	3·7 (1·9 to 6·3)	5·1 (2·4 to 9)	3·9 (1·8 to 7)	2·9 (1·3 to 5·1)	2·8 (1·2 to 5)	3·2 (1·5 to 5·7)	4 (2·1 to 6·8)	4·7 (2·4 to 7·9)	5·5 (3·1 to 8·9)	6·1 (3·5 to 10)	6·5 (4 to 9·8)	5·7 (3·6 to 8·8)	4·9 (3·1 to 7·2)	3·1 (2·1 to 4·4)	2·2 (1·5 to 3·2)	2 (1·3 to 3)	1·5 (1 to 2·3)	0·8 (0·5 to 1·2)	0·4 (0·3 to 0·6)	0·2 (0·1 to 0·3)	71·1
Inflammatory bowel disease	0 (0 to 0)	0 (0 to 0)	0 (0 to 0)	0·9 (0·4 to 1·5)	0·4 (0·2 to 0·6)	0·3 (0·2 to 0·4)	0·9 (0·6 to 1·4)	1·7 (1·1 to 2·4)	2·8 (1·7 to 4·3)	3·4 (2·1 to 5·2)	4·1 (2·6 to 6·2)	5·4 (3·4 to 8·1)	4·4 (2·9 to 6·6)	4·3 (2·9 to 6·3)	4·2 (2·8 to 6·2)	4 (2·8 to 5·7)	2·4 (1·7 to 3·4)	2·2 (1·5 to 3)	1·9 (1·3 to 2·9)	1·1 (0·7 to 1·6)	0·6 (0·4 to 0·8)	0·4 (0·2 to 0·6)	0·1 (0·1 to 0·1)	45·4
Vascular intestinal disorders	0 (0 to 0)	0 (0 to 0)	0 (0 to 0)	0·1 (0 to 0·1)	0·2 (0·1 to 0·4)	0 (0 to 0·1)	0·1 (0·1 to 0·2)	0·6 (0·4 to 0·9)	0·8 (0·5 to 1·3)	0·8 (0·4 to 1·2)	1·6 (0·9 to 2·5)	2·4 (1·4 to 3·7)	3·9 (2·3 to 6·5)	4·8 (2·9 to 7·4)	5·2 (3·4 to 7·6)	7 (4·5 to 10·2)	6·5 (4·4 to 9·1)	5·5 (3·7 to 7·7)	5·7 (3·7 to 8·3)	4·4 (2·8 to 6·3)	2·3 (1·5 to 3·2)	0·9 (0·6 to 1·3)	0·3 (0·2 to 0·5)	53·1
Gallbladder and biliary diseases	0 (0 to 0)	0 (0 to 0)	0 (0 to 0)	0·5 (0·3 to 0·8)	1·2 (0·4 to 2·4)	2·2 (0·8 to 4·2)	3·9 (2 to 6·8)	6·8 (3·9 to 10·6)	9·6 (5·7 to 14·8)	13·1 (8·2 to 19·7)	16 (10·3 to 23·9)	18·6 (12·1 to 27·2)	21·3 (13·9 to 31·2)	23·1 (15·8 to 33·2)	21·9 (15·2 to 31)	19·5 (13·7 to 27)	14·5 (10·4 to 19·9)	10·1 (7·1 to 14)	8·8 (6·1 to 12·4)	6·1 (4·2 to 8·5)	2·7 (1·9 to 3·9)	1·4 (1 to 2)	0·6 (0·4 to 0·9)	202
Pancreatitis	0 (0 to 0)	0 (0 to 0)	0 (0 to 0)	0·2 (0·1 to 0·4)	0·2 (0·1 to 0·3)	0·4 (0·3 to 0·7)	1·6 (1·1 to 2·5)	4·8 (3·1 to 7·5)	4·6 (2·7 to 7·5)	5·7 (3·3 to 9·3)	4·8 (2·9 to 7·6)	6·2 (3·7 to 9·7)	7 (4·2 to 11·2)	7·6 (4·7 to 11·7)	7·5 (4·8 to 11·2)	7·1 (4·6 to 10·5)	6·1 (4·1 to 8·6)	4·6 (3·2 to 6·7)	4·1 (2·7 to 6·2)	2·7 (1·7 to 4)	1·3 (0·8 to 1·8)	0·5 (0·3 to 0·7)	0·2 (0·1 to 0·3)	77·4
Other digestive diseases	0 (0 to 0)	0 (0 to 0)	0 (0 to 0)	3·6 (2 to 6)	1·9 (1·2 to 2·7)	1·7 (1 to 2·5)	2·4 (1·2 to 3·7)	2·4 (1·2 to 3·9)	3·5 (1·8 to 6·1)	4·2 (2 to 7·1)	4·2 (2 to 6·9)	4·7 (2·5 to 7·8)	5·8 (3 to 9·4)	8·4 (4·5 to 13·5)	8·5 (4·8 to 13·6)	9·6 (5·5 to 14·4)	8·9 (5·2 to 13·4)	7·3 (4·2 to 10·8)	7·8 (4·5 to 11·8)	7 (3·9 to 10·9)	4·3 (2·5 to 6·5)	2·2 (1·2 to 3·3)	1 (0·5 to 1·6)	99·4
Neurological disorders	0 (0 to 0·1)	0·2 (0·1 to 0·4)	7·5 (4·4 to 11·2)	20·3 (9·7 to 35·6)	46·3 (18·8 to 83·3)	147 (44·2 to 314)	187 (58·7 to 393)	198 (66·6 to 406)	204 (68·5 to 416)	199 (69·6 to 398)	203 (77·2 to 394)	208 (81·6 to 402)	204 (88·8 to 387)	180 (83·6 to 326)	155 (79·5 to 265)	135 (78·4 to 218)	113 (71·1 to 183)	111 (70·6 to 193)	128 (79·1 to 236)	155 (85·9 to 312)	117 (58·3 to 240)	75·8 (36 to 157)	61·3 (26·6 to 125)	2,850
Alzheimer's disease and other dementias	0 (0 to 0)	0 (0 to 0)	0 (0 to 0)	0 (0 to 0)	0 (0 to 0)	0 (0 to 0)	0 (0 to 0)	0 (0 to 0)	0 (0 to 0)	0 (0 to 0)	0 (0 to 0)	0·9 (0·2 to 2·9)	5·2 (1·6 to 14·2)	10·3 (3·6 to 25·4)	17·8 (7 to 41·8)	29·2 (11·8 to 68·9)	39·8 (15·6 to 97·6)	56·2 (24·8 to 133)	77·5 (33·9 to 182)	116 (48·5 to 274)	97·8 (39·7 to 220)	68·2 (28·1 to 151)	58·5 (23·6 to 122)	577
Parkinson's disease	0 (0 to 0)	0 (0 to 0)	0 (0 to 0)	0 (0 to 0)	0 (0 to 0)	0 (0 to 0)	0 (0 to 0)	0·1 (0 to 0·1)	0·1 (0·1 to 0·2)	0·3 (0·2 to 0·4)	0·5 (0·3 to 0·7)	1·1 (0·8 to 1·6)	2·4 (1·7 to 3·4)	4·2 (3·1 to 5·6)	6·2 (4·5 to 8·2)	9·8 (7·7 to 12·3)	12·9 (10·3 to 15·9)	18·3 (15 to 22)	26·4 (21·7 to 31·6)	25·4 (20·6 to 30·7)	12·8 (10·2 to 15·6)	5·1 (3·9 to 6·2)	1·8 (1·2 to 2·2)	127
Idiopathic epilepsy	0 (0 to 0·1)	0·1 (0 to 0·2)	2·6 (1·2 to 4·7)	11·7 (4 to 22·6)	20·1 (6·7 to 40·5)	29·1 (10·2 to 57·9)	35·3 (15·6 to 65·7)	38·2 (17·2 to 68·4)	36·8 (16·1 to 66·7)	34·6 (15·7 to 59·5)	32·8 (15·2 to 57)	29·2 (14·1 to 49·7)	28 (13·3 to 48·7)	23·6 (10·4 to 44)	19·9 (7·7 to 37·1)	17·1 (6·6 to 32·1)	12·9 (4·5 to 24)	9·8 (3·5 to 18·4)	7·7 (2·7 to 14·2)	5 (1·7 to 9·2)	2·5 (0·9 to 4·7)	1·2 (0·4 to 2·3)	0·5 (0·2 to 1·1)	399
Multiple sclerosis	0 (0 to 0)	0 (0 to 0)	0 (0 to 0)	0 (0 to 0)	0 (0 to 0)	0·1 (0 to 0·2)	0·5 (0·2 to 1·1)	1·4 (0·8 to 2·1)	2·2 (1·3 to 3·4)	2·9 (1·8 to 4·1)	5·3 (3·1 to 7·6)	5·4 (3·2 to 7·7)	5·8 (3·6 to 8·6)	5 (3·1 to 7)	4·4 (2·8 to 6·1)	3·2 (2 to 4·5)	1·4 (1 to 2)	1 (0·7 to 1·4)	0·5 (0·3 to 0·7)	0·2 (0·2 to 0·3)	0·1 (0·1 to 0·2)	0 (0 to 0·1)	0 (0 to 0)	39·4
Motor neuron disease	0 (0 to 0)	0·1 (0·1 to 0·3)	0·8 (0·4 to 1·6)	0·8 (0·5 to 1·3)	0·2 (0·1 to 0·3)	0·4 (0·3 to 0·6)	0·2 (0·2 to 0·3)	0·4 (0·3 to 0·5)	0·4 (0·3 to 0·6)	0·6 (0·4 to 0·8)	1 (0·6 to 1·6)	1·6 (1 to 2·5)	2·8 (1·8 to 4)	3·8 (2·4 to 5·6)	3·8 (2·5 to 5·7)	3·6 (2·4 to 5·1)	2·8 (1·8 to 4·2)	1·8 (1·2 to 2·5)	1·1 (0·7 to 1·6)	0·4 (0·3 to 0·7)	0·1 (0·1 to 0·2)	0 (0 to 0·1)	0 (0 to 0)	27
Headache disorders	0 (0 to 0)	0 (0 to 0)	0 (0 to 0)	0 (0 to 0)	17·4 (0·3 to 46·2)	102 (4 to 259)	136 (14·5 to 339)	147 (19·7 to 352)	154 (22·8 to 352)	151 (25·2 to 347)	153 (28·2 to 342)	158 (32·8 to 354)	145 (34·1 to 334)	120 (30 to 260)	91·5 (24·7 to 194)	63·6 (19·2 to 136)	37·3 (11·8 to 76·7)	20·8 (6·9 to 43·6)	12·1 (3·8 to 25·1)	6·3 (2 to 13·1)	2·7 (0·9 to 5·7)	1 (0·3 to 2·1)	0·3 (0·1 to 0·7)	1,520
Migraine	0 (0 to 0)	0 (0 to 0)	0 (0 to 0)	0 (0 to 0)	16·1 (0·2 to 43·6)	96·8 (2·9 to 256)	128 (9·9 to 335)	136 (13·5 to 343)	143 (15·6 to 339)	139 (18·1 to 331)	140 (19·6 to 327)	144 (25·3 to 333)	132 (23·6 to 306)	108 (21·1 to 240)	81·8 (17 to 180)	55·8 (13·3 to 128)	31·9 (8·5 to 69·5)	17·4 (4·8 to 39)	10·1 (2·7 to 22·2)	5·2 (1·5 to 11·7)	2·3 (0·6 to 4·9)	0·8 (0·2 to 1·8)	0·3 (0·1 to 0·6)	1,390
Tension-type headache	0 (0 to 0)	0 (0 to 0)	0 (0 to 0)	0 (0 to 0)	1·3 (0 to 7·9)	5·5 (0·3 to 35)	8·4 (1·6 to 36)	10·2 (2·1 to 39·3)	11·4 (2·5 to 42·8)	11·5 (2·6 to 39·4)	12·5 (3·3 to 38·8)	13·2 (4 to 38)	13·3 (4·1 to 34·5)	12·3 (3·7 to 33)	9·7 (2·9 to 24·6)	7·8 (2·4 to 21·9)	5·4 (1·6 to 16·6)	3·4 (0·8 to 11·6)	2 (0·4 to 7·2)	1·1 (0·2 to 3·8)	0·5 (0·1 to 1·7)	0·2 (0 to 0·7)	0·1 (0 to 0·4)	130
Other neurological disorders	0 (0 to 0)	0 (0 to 0)	4 (2·4 to 6·2)	7·7 (4·2 to 12·3)	8·6 (3·8 to 15·6)	15·2 (6·8 to 27·7)	14·5 (7·5 to 24·6)	11 (5·7 to 17·6)	10·2 (5·9 to 15·6)	10 (6 to 15)	10·9 (6·8 to 16)	11·7 (7·8 to 16·9)	14·2 (9·3 to 20)	12·5 (8·8 to 17·4)	11·1 (7·7 to 15·3)	8·5 (6·2 to 11·4)	5·5 (4 to 7·4)	3·3 (2·5 to 4·4)	2·6 (2 to 3·5)	1·6 (1·2 to 2·1)	0·6 (0·4 to 0·9)	0·2 (0·2 to 0·3)	0·1 (0·1 to 0·2)	164
Mental disorders	0 (0 to 0)	0 (0 to 0·1)	0·8 (0·4 to 1·2)	5·5 (3·4 to 8·4)	49·5 (31·4 to 74·7)	203 (135 to 294)	319 (217 to 449)	373 (251 to 524)	399 (270 to 556)	385 (270 to 530)	386 (278 to 521)	383 (271 to 529)	369 (266 to 503)	326 (233 to 449)	275 (196 to 374)	207 (143 to 282)	131 (90·7 to 173)	84 (59 to 112)	55·7 (38·4 to 75·2)	32·1 (21·7 to 44·2)	15·6 (10·8 to 20·9)	7·1 (4·8 to 9·8)	3·2 (2·1 to 4·7)	4,010
Schizophrenia	0 (0 to 0)	0 (0 to 0)	0 (0 to 0)	0 (0 to 0)	0 (0 to 0)	0·9 (0·4 to 1·7)	6·4 (2·1 to 12·7)	22·9 (11·5 to 39·2)	41 (23·4 to 64·3)	49·3 (29·6 to 72·5)	52·8 (32·5 to 76·7)	52 (33·2 to 72·8)	48·2 (31·7 to 68·2)	40·9 (26·9 to 56·3)	31·1 (19·8 to 43·1)	20·9 (13·6 to 30·2)	11·8 (7·4 to 17·2)	6·7 (4·1 to 9·5)	3·8 (2·3 to 5·8)	1·8 (1 to 2·8)	0·7 (0·3 to 1·1)	0·2 (0·1 to 0·4)	0·1 (0 to 0·1)	392
Depressive disorders	0 (0 to 0)	0 (0 to 0)	0 (0 to 0)	0 (0 to 0·1)	3·8 (1·3 to 7·6)	47·6 (24 to 86·5)	111 (62·6 to 179)	134 (76·1 to 216)	134 (78·1 to 207)	128 (74·9 to 209)	136 (82·8 to 206)	143 (85·2 to 221)	146 (92·8 to 216)	138 (89·8 to 205)	124 (76·1 to 187)	98·2 (57·4 to 149)	63·7 (39·7 to 93·7)	41·9 (26·8 to 60)	28·4 (16·9 to 42·9)	17·2 (9·9 to 26·8)	9 (5·7 to 13·2)	4·4 (2·6 to 6·5)	2·1 (1·2 to 3·4)	1,510
Major depressive disorder	0 (0 to 0)	0 (0 to 0)	0 (0 to 0)	0 (0 to 0·1)	3·4 (1 to 7·2)	44·3 (20·6 to 82·4)	101 (56·4 to 169)	118 (62·1 to 194)	113 (62·7 to 181)	106 (57·4 to 176)	112 (65·1 to 178)	118 (67·9 to 191)	122 (74·6 to 186)	117 (73·6 to 176)	106 (61·2 to 168)	84·6 (46·9 to 130)	55·1 (33·1 to 82·8)	36·3 (22·6 to 53·8)	24·9 (14·3 to 39·3)	15·2 (8·4 to 24·2)	8·1 (4·9 to 12)	4 (2·3 to 6)	1·9 (1 to 3·2)	1,290
Dysthymia	0 (0 to 0)	0 (0 to 0)	0 (0 to 0)	0 (0 to 0)	0·4 (0 to 1)	3·3 (1·3 to 6·6)	9·2 (4·4 to 16)	16 (7·9 to 28·6)	20·8 (11 to 35·4)	22·2 (12·3 to 36·8)	23·4 (12·7 to 39)	24 (12·8 to 42)	23·6 (12·8 to 40·1)	21·1 (12·2 to 35·3)	17·9 (10·5 to 29)	13·6 (7·3 to 23·3)	8·7 (4·6 to 14·5)	5·5 (3·2 to 8·7)	3·6 (2·1 to 5·9)	2 (1·1 to 3·4)	0·9 (0·5 to 1·6)	0·4 (0·2 to 0·7)	0·2 (0·1 to 0·4)	217
Bipolar disorder	0 (0 to 0)	0 (0 to 0)	0 (0 to 0)	0 (0 to 0)	0 (0 to 0)	12·5 (4·8 to 23·7)	43 (20 to 76·3)	53 (26·8 to 92)	53·3 (27·5 to 91·5)	47·1 (25·3 to 78·1)	44·5 (23·3 to 71·9)	43·8 (23·4 to 69·6)	41·6 (23 to 67·2)	36·2 (19·5 to 60·6)	28·6 (15·7 to 47·3)	20 (11·2 to 32·6)	11·6 (6·4 to 18·8)	6·6 (3·5 to 11)	3·7 (2 to 6·1)	1·7 (0·8 to 2·8)	0·6 (0·3 to 1·1)	0·2 (0·1 to 0·4)	0·1 (0 to 0·2)	448
Anxiety disorders	0 (0 to 0)	0 (0 to 0)	0 (0 to 0)	1·3 (0·4 to 2·8)	23·4 (11·7 to 40·7)	72·4 (39·4 to 123)	93·3 (55·2 to 149)	102 (57·3 to 170)	107 (56·4 to 177)	104 (60·6 to 165)	103 (60·4 to 157)	103 (55·4 to 163)	97·8 (55·4 to 158)	85·4 (49 to 133)	69·4 (43·1 to 105)	51·2 (30·9 to 79·9)	32·7 (18·9 to 51·4)	21·3 (12·8 to 34·8)	14·3 (8·8 to 22·8)	8 (4·8 to 12·5)	3·6 (2·1 to 5·5)	1·4 (0·8 to 2·3)	0·5 (0·3 to 0·9)	1,100
Eating disorders	0 (0 to 0)	0 (0 to 0)	0 (0 to 0)	0 (0 to 0)	0·3 (0·1 to 0·7)	6·3 (2·9 to 10·8)	23·4 (11·9 to 41·4)	39·9 (21·3 to 66·4)	38·5 (20·9 to 63·9)	28·8 (15·7 to 45·9)	20·6 (10·9 to 33·1)	13 (6·7 to 21·2)	7·3 (3·8 to 12·2)	0 (0 to 0)	0 (0 to 0)	0 (0 to 0)	0 (0 to 0)	0 (0 to 0)	0 (0 to 0)	0 (0 to 0)	0 (0 to 0)	0 (0 to 0)	0 (0 to 0)	178
Anorexia nervosa	0 (0 to 0)	0 (0 to 0)	0 (0 to 0)	0 (0 to 0)	0·3 (0·1 to 0·6)	3·3 (1·4 to 5·9)	8·9 (4·2 to 16·1)	9·5 (4·6 to 17·7)	7·7 (3·6 to 13·5)	5 (2·3 to 9)	3·5 (1·6 to 6·7)	2·6 (1·1 to 4·6)	1·9 (0·8 to 3·5)	0 (0 to 0)	0 (0 to 0)	0 (0 to 0)	0 (0 to 0)	0 (0 to 0)	0 (0 to 0)	0 (0 to 0)	0 (0 to 0)	0 (0 to 0)	0 (0 to 0)	42·7
Bulimia nervosa	0 (0 to 0)	0 (0 to 0)	0 (0 to 0)	0 (0 to 0)	0 (0 to 0·1)	3 (0·9 to 6·4)	14·4 (6·1 to 28·7)	30·4 (14 to 54·8)	30·8 (15 to 54·6)	23·8 (12 to 40·2)	17·1 (8·5 to 29·1)	10·4 (5 to 18·2)	5·4 (2·5 to 9·7)	0 (0 to 0)	0 (0 to 0)	0 (0 to 0)	0 (0 to 0)	0 (0 to 0)	0 (0 to 0)	0 (0 to 0)	0 (0 to 0)	0 (0 to 0)	0 (0 to 0)	135
Autism spectrum disorders	0 (0 to 0)	0 (0 to 0·1)	0·6 (0·3 to 1)	2·8 (1·6 to 4·5)	4·2 (2·4 to 6·8)	4·9 (2·8 to 7·8)	4·5 (2·5 to 6·9)	4·3 (2·4 to 7)	4·2 (2·4 to 6·5)	3·8 (2·1 to 6)	3·6 (2 to 5·8)	3·4 (2 to 5·3)	3·1 (1·7 to 5·1)	2·7 (1·5 to 4·5)	2·2 (1·2 to 3·7)	1·7 (1 to 2·7)	1·1 (0·6 to 1·8)	0·7 (0·4 to 1·2)	0·5 (0·3 to 0·8)	0·3 (0·2 to 0·5)	0·1 (0·1 to 0·2)	0·1 (0 to 0·1)	0 (0 to 0)	49·1
Attention-deficit/hyperactivity disorder	0 (0 to 0)	0 (0 to 0)	0 (0 to 0)	0·4 (0·2 to 0·8)	4·9 (2·5 to 9)	8 (4·1 to 14·4)	6·2 (3·4 to 11·1)	4·6 (2·4 to 8·3)	3·5 (1·8 to 6·1)	2·6 (1·3 to 4·5)	2 (1 to 3·6)	1·7 (0·8 to 3)	1·3 (0·6 to 2·4)	0·9 (0·4 to 1·8)	0·6 (0·2 to 1·1)	0·3 (0·1 to 0·6)	0·1 (0 to 0·3)	0 (0 to 0·1)	0 (0 to 0)	0 (0 to 0)	0 (0 to 0)	0 (0 to 0)	0 (0 to 0)	37·2
Conduct disorder	0 (0 to 0)	0 (0 to 0)	0 (0 to 0)	0 (0 to 0)	11 (5·1 to 20·9)	47·8 (24·1 to 80·9)	25·7 (12·5 to 45·3)	0 (0 to 0)	0 (0 to 0)	0 (0 to 0)	0 (0 to 0)	0 (0 to 0)	0 (0 to 0)	0 (0 to 0)	0 (0 to 0)	0 (0 to 0)	0 (0 to 0)	0 (0 to 0)	0 (0 to 0)	0 (0 to 0)	0 (0 to 0)	0 (0 to 0)	0 (0 to 0)	84·5
Idiopathic developmental intellectual disability	0 (0 to 0)	0 (0 to 0)	0·1 (0 to 0·3)	1 (0·2 to 2)	1·8 (0·6 to 3·4)	2·1 (0·6 to 4)	1·9 (0·6 to 3·6)	1·9 (0·6 to 3·6)	1·8 (0·5 to 3·4)	1·6 (0·5 to 3·2)	1·5 (0·4 to 2·9)	1·4 (0·4 to 2·7)	1·3 (0·4 to 2·4)	1·1 (0·3 to 2·1)	0·9 (0·3 to 1·6)	0·6 (0·2 to 1·2)	0·4 (0·1 to 0·7)	0·2 (0·1 to 0·4)	0·1 (0 to 0·3)	0·1 (0 to 0·2)	0 (0 to 0·1)	0 (0 to 0)	0 (0 to 0)	19·7
Other mental disorders	0 (0 to 0)	0 (0 to 0)	0 (0 to 0)	0 (0 to 0)	0 (0 to 0)	0·7 (0·3 to 1·1)	4·2 (1·6 to 7·9)	10·4 (5 to 18·1)	16·5 (8·6 to 26·5)	19·8 (11·4 to 31·7)	22·1 (13·1 to 35·1)	22·7 (13 to 35·4)	22·6 (13·3 to 35·5)	20·6 (12·1 to 32·4)	17·8 (10·7 to 27·1)	13·9 (8·3 to 21·1)	9·4 (5·9 to 14·6)	6·6 (4 to 9·8)	4·8 (3 to 7)	3 (1·9 to 4·4)	1·6 (1 to 2·3)	0·7 (0·5 to 1·1)	0·4 (0·2 to 0·5)	198
Substance use disorders	0 (0 to 0)	0 (0 to 0)	0 (0 to 0)	0 (0 to 0)	0 (0 to 0)	1·8 (0·9 to 3·1)	31·5 (20·4 to 45·2)	70·1 (46·8 to 99·5)	82·5 (55·6 to 115)	79·2 (54·6 to 113)	77·2 (55 to 106)	67·1 (48·1 to 90·3)	60·2 (44·1 to 79·4)	45 (32·7 to 59·7)	34·1 (25·2 to 45·2)	23·8 (17·3 to 32)	13·3 (9·6 to 17·6)	8 (5·8 to 10·8)	5·5 (4 to 7·5)	3·7 (2·6 to 5·1)	2·2 (1·5 to 3)	1·4 (0·9 to 2)	0·9 (0·5 to 1·4)	607
Alcohol use disorders	0 (0 to 0)	0 (0 to 0)	0 (0 to 0)	0 (0 to 0)	0 (0 to 0)	0·9 (0·3 to 1·9)	12·5 (7 to 19·7)	31·7 (18 to 52·7)	42·8 (25·6 to 65·4)	48·8 (30·9 to 73·9)	52·7 (35·8 to 74·9)	48·4 (33·1 to 67·5)	44·5 (30·8 to 60·1)	32·6 (23 to 45)	24·4 (17·4 to 33·8)	17·4 (12·5 to 23·7)	9·6 (6·9 to 12·9)	5·8 (4·1 to 8)	4·1 (2·9 to 5·8)	2·9 (2 to 4·2)	1·8 (1·2 to 2·6)	1·2 (0·8 to 1·8)	0·8 (0·5 to 1·3)	383
Drug use disorders	0 (0 to 0)	0 (0 to 0)	0 (0 to 0)	0 (0 to 0)	0 (0 to 0)	0·9 (0·4 to 1·6)	19 (11·6 to 29·5)	38·4 (24·2 to 57·3)	39·7 (24·4 to 59·6)	30·4 (19·2 to 47·1)	24·5 (15·5 to 37·4)	18·7 (11·4 to 28·3)	15·7 (9·6 to 23·2)	12·4 (7·1 to 18·5)	9·6 (5·6 to 14·6)	6·4 (3·5 to 10·1)	3·7 (2·1 to 5·8)	2·2 (1·1 to 3·6)	1·4 (0·7 to 2·2)	0·8 (0·4 to 1·2)	0·3 (0·2 to 0·6)	0·2 (0·1 to 0·2)	0·1 (0 to 0·1)	224
Opioid use disorders	0 (0 to 0)	0 (0 to 0)	0 (0 to 0)	0 (0 to 0)	0 (0 to 0)	0 (0 to 0)	4·8 (1·7 to 9·3)	20·7 (10·3 to 37·1)	25·8 (14·2 to 43)	21·1 (11·6 to 35·5)	16·7 (8·6 to 28·2)	12·9 (6·5 to 20·9)	10·8 (5·3 to 17·8)	9·5 (4·6 to 15·6)	7·6 (3·8 to 12·5)	5·2 (2·4 to 8·8)	3 (1·4 to 4·9)	1·7 (0·7 to 3·1)	1 (0·4 to 1·8)	0·6 (0·2 to 1)	0·2 (0·1 to 0·5)	0·1 (0 to 0·2)	0·1 (0 to 0·1)	142
Cocaine use disorders	0 (0 to 0)	0 (0 to 0)	0 (0 to 0)	0 (0 to 0)	0 (0 to 0)	0·1 (0 to 0·2)	3·5 (1·2 to 7·3)	4 (1·7 to 7·3)	3·1 (1·4 to 5·5)	2·3 (0·9 to 4·2)	2·1 (1 to 3·6)	1·5 (0·6 to 3)	1·2 (0·8 to 1·9)	0·8 (0·5 to 1·2)	0·7 (0·4 to 1)	0·4 (0·3 to 0·7)	0·3 (0·2 to 0·4)	0·2 (0·1 to 0·3)	0·1 (0·1 to 0·2)	0·1 (0 to 0·1)	0 (0 to 0)	0 (0 to 0)	0 (0 to 0)	20·4
Amphetamine use disorders	0 (0 to 0)	0 (0 to 0)	0 (0 to 0)	0 (0 to 0)	0 (0 to 0)	0 (0 to 0)	0·6 (0·3 to 1)	2·6 (0·7 to 5·5)	2·5 (0·7 to 5·3)	1·2 (0·2 to 3)	0·7 (0·4 to 1·2)	0·4 (0·2 to 0·7)	0·3 (0·1 to 0·4)	0·2 (0·1 to 0·3)	0·1 (0·1 to 0·2)	0·1 (0 to 0·1)	0 (0 to 0·1)	0 (0 to 0)	0 (0 to 0)	0 (0 to 0)	0 (0 to 0)	0 (0 to 0)	0 (0 to 0)	8·8
Cannabis use disorders	0 (0 to 0)	0 (0 to 0)	0 (0 to 0)	0 (0 to 0)	0 (0 to 0)	0·7 (0·3 to 1·4)	6·8 (3·1 to 13)	5·5 (2·4 to 10·3)	3·1 (1·2 to 6)	1·8 (0·7 to 3·5)	1·3 (0·4 to 2·8)	1·1 (0·3 to 2·2)	0·9 (0·3 to 1·8)	0·6 (0·3 to 1·1)	0·3 (0·2 to 0·6)	0·2 (0·1 to 0·3)	0·1 (0 to 0·1)	0 (0 to 0·1)	0 (0 to 0)	0 (0 to 0)	0 (0 to 0)	0 (0 to 0)	0 (0 to 0)	22·5
Other drug use disorders	0 (0 to 0)	0 (0 to 0)	0 (0 to 0)	0 (0 to 0)	0 (0 to 0)	0·1 (0 to 0·1)	3·3 (1·6 to 5·9)	5·6 (3·2 to 8·6)	5·2 (3·1 to 8·1)	3·8 (2·3 to 6·2)	3·7 (2·4 to 5·3)	2·9 (1·8 to 4·4)	2·5 (1·6 to 3·5)	1·3 (0·8 to 2)	0·9 (0·5 to 1·3)	0·5 (0·3 to 0·7)	0·3 (0·2 to 0·5)	0·2 (0·2 to 0·4)	0·2 (0·2 to 0·3)	0·1 (0·1 to 0·2)	0·1 (0 to 0·1)	0 (0 to 0)	0 (0 to 0)	30·8
Diabetes and kidney diseases	0 (0 to 0)	0 (0 to 0·1)	4·3 (2·8 to 6·4)	6·2 (4·3 to 8·7)	11·6 (8·3 to 15·9)	18·8 (13·5 to 26)	58·1 (44·4 to 73·3)	94·3 (73·2 to 121)	133 (98·1 to 172)	174 (132 to 222)	233 (181 to 299)	299 (230 to 380)	419 (323 to 530)	559 (439 to 718)	637 (502 to 799)	680 (543 to 842)	594 (480 to 722)	439 (352 to 533)	398 (325 to 478)	264 (212 to 323)	122 (98·9 to 148)	56·5 (44·9 to 67·8)	23·8 (18 to 29·1)	5,220
Diabetes mellitus	0 (0 to 0)	0 (0 to 0·1)	1·2 (0·6 to 2·3)	0·9 (0·5 to 1·5)	1·7 (1·1 to 2·5)	3·5 (2·3 to 4·9)	21·6 (16 to 27·7)	39·2 (29·2 to 52·4)	57·9 (41·1 to 77·6)	81·5 (59·3 to 108)	123 (89·9 to 165)	177 (130 to 235)	274 (202 to 362)	386 (291 to 506)	445 (338 to 577)	472 (358 to 600)	422 (324 to 535)	316 (244 to 398)	292 (229 to 369)	188 (146 to 240)	82·9 (63·9 to 104)	37·9 (29·4 to 46·9)	15·1 (11·3 to 19·5)	3,440
Diabetes mellitus type 1	0 (0 to 0)	0 (0 to 0·1)	1·2 (0·6 to 2·3)	0·9 (0·5 to 1·5)	1·7 (1·1 to 2·5)	3·5 (2·3 to 4·9)	7·2 (4·8 to 10·4)	12 (7·8 to 18·2)	14·8 (9 to 23·2)	14·7 (8·9 to 23·8)	22·2 (14·1 to 33·2)	27·8 (17·3 to 42·4)	11·3 (7·2 to 17·4)	14·8 (8·9 to 23)	16·7 (10 to 26·3)	14·3 (8·5 to 22·4)	12·7 (7·5 to 20·8)	8 (4·7 to 12·8)	7·2 (4·3 to 12·4)	3·2 (2 to 5·3)	1·4 (0·9 to 2·3)	0·7 (0·4 to 1·2)	0·3 (0·2 to 0·6)	196
Diabetes mellitus type 2	0 (0 to 0)	0 (0 to 0)	0 (0 to 0)	0 (0 to 0)	0 (0 to 0)	0 (0 to 0)	14·4 (9·7 to 19·5)	27·2 (18·5 to 37·6)	43·1 (29·4 to 59·3)	66·8 (47·3 to 90·9)	101 (71 to 139)	149 (107 to 205)	263 (193 to 349)	371 (280 to 489)	428 (325 to 555)	458 (345 to 583)	409 (313 to 519)	308 (237 to 388)	285 (224 to 360)	185 (143 to 236)	81·5 (62·7 to 102)	37·1 (28·9 to 46·1)	14·8 (10·9 to 19·1)	3,240
Chronic kidney disease	0 (0 to 0)	0 (0 to 0)	3·1 (2 to 4·7)	5·3 (3·7 to 7·6)	9·6 (6·6 to 13·7)	15·1 (10·2 to 22·2)	36·2 (25·9 to 49·8)	54·7 (39·7 to 74·8)	74·5 (51·7 to 104)	91·9 (64·5 to 127)	110 (77·2 to 152)	122 (86·2 to 165)	145 (103 to 198)	173 (126 to 237)	191 (141 to 258)	208 (156 to 271)	172 (130 to 226)	123 (94·3 to 158)	106 (79·5 to 133)	75·8 (58·3 to 98·6)	39·3 (30·2 to 49·8)	18·6 (14·2 to 23·3)	8·7 (6 to 11·4)	1,780
Chronic kidney disease due to diabetes mellitus type 1	0 (0 to 0)	0 (0 to 0)	0 (0 to 0·1)	0·1 (0 to 0·2)	0·2 (0·1 to 0·6)	0·4 (0·2 to 1)	1·4 (0·6 to 2·8)	3 (1·3 to 6·1)	7·6 (3·2 to 14·6)	14 (6·6 to 25·4)	19·5 (9·6 to 33·1)	20·5 (10 to 35·7)	20·3 (9·2 to 36)	18·3 (7 to 34)	14 (4·7 to 29·7)	11·6 (4 to 25)	7·6 (2·8 to 17·1)	4 (1·5 to 8·3)	2·2 (0·8 to 5)	0·8 (0·3 to 1·8)	0·2 (0 to 0·4)	0 (0 to 0)	0 (0 to 0)	146
Chronic kidney disease due to diabetes mellitus type 2	0 (0 to 0)	0 (0 to 0)	0 (0 to 0)	0 (0 to 0)	0 (0 to 0)	0 (0 to 0)	0·6 (0·2 to 1·4)	1·4 (0·5 to 3)	3·1 (1·2 to 6·3)	5·7 (2·5 to 11·1)	9·4 (4·3 to 17·3)	15 (7·3 to 25·6)	25·9 (14·3 to 42·5)	41·5 (23·7 to 64·7)	55·2 (31·3 to 84·7)	64·8 (39·2 to 95·7)	55·2 (34·5 to 79·2)	39·9 (25·4 to 56·2)	33·5 (21·9 to 47)	23·3 (14·8 to 33)	11·3 (7·3 to 16·3)	5 (3·1 to 7·2)	2·2 (1·1 to 3·4)	393
Chronic kidney disease due to hypertension	0 (0 to 0)	0 (0 to 0)	0 (0 to 0)	0 (0 to 0)	0 (0 to 0)	0 (0 to 0)	2·4 (1 to 5·1)	4 (1·6 to 7·6)	6·2 (2·9 to 10·8)	8·5 (4·5 to 14·1)	11 (5·9 to 18·5)	14·2 (8·6 to 22·3)	19·2 (12 to 30)	26·4 (15·7 to 40·8)	34·4 (22 to 52)	44·1 (27·5 to 67)	41·6 (27·3 to 61·6)	32·5 (21·5 to 46·9)	30·7 (20·8 to 43·1)	24·2 (16·9 to 33·8)	13·8 (9·8 to 18·8)	7·1 (4·9 to 9·6)	3·4 (2·2 to 4·9)	324
Chronic kidney disease due to glomerulonephritis	0 (0 to 0)	0 (0 to 0)	0·9 (0·4 to 1·6)	1·6 (0·9 to 2·6)	3·2 (1·9 to 5)	5·8 (3·4 to 9)	14·3 (8·5 to 21·7)	20·7 (12·2 to 31·9)	26·5 (15·7 to 40·3)	29·8 (17·4 to 45)	34 (20 to 51·7)	34·8 (19·6 to 53·3)	36·1 (21·6 to 55·7)	38·3 (21·9 to 61·7)	38·2 (19·7 to 64·5)	36·5 (18·7 to 60·1)	25·8 (12·5 to 47·9)	15·4 (8·3 to 27·3)	11·5 (5·6 to 20·1)	7 (3·1 to 13)	2·9 (1·3 to 5·1)	1·1 (0·5 to 2·1)	0·5 (0·2 to 1)	385
Chronic kidney disease due to other and unspecified causes	0 (0 to 0)	0 (0 to 0)	2·2 (1·4 to 3·5)	3·6 (2·4 to 5·2)	6·2 (3·9 to 9·4)	8·9 (5·3 to 14·3)	17·5 (11·1 to 26)	25·6 (16·3 to 37·7)	31·2 (20 to 45·2)	34 (20·7 to 51·6)	35·9 (21·1 to 55·6)	37·3 (21·7 to 57·1)	43·3 (25·9 to 64·6)	48 (29·4 to 73·9)	49·5 (30·3 to 77·8)	50·6 (30·4 to 80·5)	42 (25·2 to 64·7)	31·7 (20 to 47·8)	27·6 (16·9 to 40·8)	20·4 (12·8 to 30·7)	11·1 (7·4 to 16·2)	5·4 (3·8 to 7·6)	2·6 (1·6 to 3·8)	534
Acute glomerulonephritis	0 (0 to 0)	0 (0 to 0)	0 (0 to 0)	0 (0 to 0)	0·3 (0·1 to 0·5)	0·2 (0·1 to 0·5)	0·3 (0·1 to 0·6)	0·3 (0·1 to 0·8)	0·3 (0·1 to 0·8)	0·4 (0·2 to 1)	0·3 (0·1 to 0·7)	0·2 (0·1 to 0·6)	0·2 (0·1 to 0·4)	0·1 (0 to 0·1)	0·3 (0·1 to 0·6)	0·1 (0·1 to 0·3)	0·1 (0 to 0·1)	0·1 (0 to 0·3)	0 (0 to 0·1)	0·1 (0 to 0·2)	0 (0 to 0·1)	0 (0 to 0·1)	0 (0 to 0)	3·5
Skin and subcutaneous diseases	0 (0 to 0)	0 (0 to 0·1)	9·6 (5·9 to 14·4)	62·8 (40 to 92·5)	93·4 (58·3 to 138)	112 (71·7 to 166)	116 (75·2 to 174)	77·2 (50·3 to 114)	68·1 (44·1 to 98·8)	65·3 (43·4 to 93·3)	66·3 (44 to 93·4)	72·1 (49·8 to 105)	71·9 (49·1 to 105)	81 (55·6 to 116)	82·3 (57·1 to 119)	76·6 (53 to 111)	61 (43·1 to 88·6)	53·7 (37·3 to 82·9)	57·5 (40·3 to 91·3)	48·4 (31·7 to 80·4)	31·4 (21·3 to 56·6)	16·7 (11 to 30)	10·5 (6·1 to 24·3)	1,330
Dermatitis	0 (0 to 0)	0 (0 to 0)	0 (0 to 0)	22·6 (11·8 to 39·8)	32·2 (17·4 to 56·4)	26·6 (14·5 to 44·9)	18·8 (10·6 to 31·1)	15·5 (8·8 to 25·5)	14 (7·9 to 22·3)	12·7 (7 to 20·5)	12 (6·8 to 19·7)	11·7 (6·7 to 19·5)	11·6 (6·3 to 19·2)	11·4 (6·2 to 18·7)	10·8 (6 to 17·8)	9 (4·9 to 14·8)	6·7 (3·7 to 10·7)	4·9 (2·7 to 8)	3·5 (2 to 5·6)	2·1 (1·2 to 3·3)	1 (0·6 to 1·6)	0·4 (0·2 to 0·7)	0·2 (0·1 to 0·3)	228
Atopic dermatitis	0 (0 to 0)	0 (0 to 0)	0 (0 to 0)	22·5 (11·7 to 39·5)	31·6 (17 to 55·5)	24·1 (12·8 to 41·6)	14·8 (7·8 to 26·2)	10·6 (5·4 to 18·3)	8·3 (4·1 to 14·9)	6·8 (3·4 to 12·2)	6·2 (3 to 10·9)	5·9 (3 to 10·5)	5·7 (2·8 to 10·3)	5·2 (2·6 to 9·4)	4·9 (2·4 to 9)	4 (2 to 7·1)	2·8 (1·4 to 4·7)	2 (1 to 3·4)	1·5 (0·8 to 2·5)	0·9 (0·5 to 1·6)	0·5 (0·2 to 0·8)	0·2 (0·1 to 0·4)	0·1 (0 to 0·2)	159
Contact dermatitis	0 (0 to 0)	0 (0 to 0)	0 (0 to 0)	0 (0 to 0)	0·4 (0·1 to 0·8)	2·1 (0·8 to 4·2)	3·5 (1·4 to 7)	4·4 (1·8 to 8·7)	5·1 (2·3 to 9·7)	5·3 (2·2 to 10·2)	5·3 (2·2 to 10·3)	5·2 (2·4 to 10·7)	5·4 (2·4 to 10·6)	5·7 (2·5 to 10·4)	5·5 (2·4 to 11·1)	4·7 (2 to 8·7)	3·6 (1·6 to 7)	2·6 (1·2 to 5·1)	1·8 (0·8 to 3·5)	1 (0·5 to 1·9)	0·4 (0·2 to 0·9)	0·2 (0·1 to 0·3)	0·1 (0 to 0·2)	62·4
Seborrhoeic dermatitis	0 (0 to 0)	0 (0 to 0)	0 (0 to 0)	0·2 (0·1 to 0·3)	0·2 (0·1 to 0·4)	0·4 (0·2 to 0·7)	0·4 (0·2 to 0·8)	0·5 (0·2 to 0·9)	0·6 (0·3 to 1·1)	0·6 (0·3 to 1·1)	0·6 (0·2 to 1)	0·5 (0·2 to 1)	0·5 (0·2 to 0·9)	0·4 (0·2 to 0·8)	0·4 (0·2 to 0·7)	0·4 (0·2 to 0·6)	0·3 (0·2 to 0·6)	0·3 (0·1 to 0·5)	0·2 (0·1 to 0·4)	0·1 (0·1 to 0·2)	0·1 (0 to 0·1)	0 (0 to 0·1)	0 (0 to 0)	6·7
Psoriasis	0 (0 to 0)	0 (0 to 0)	0 (0 to 0)	0·8 (0·4 to 1·1)	2·3 (1 to 4·2)	3·4 (1·5 to 6)	3·6 (1·6 to 6·1)	3·8 (1·8 to 6·4)	4·4 (2·2 to 7)	4·7 (2·5 to 7·9)	5·2 (2·9 to 8·5)	5·7 (3·1 to 8·8)	6·2 (3·5 to 9·9)	6·1 (3·5 to 9·5)	5·8 (3·4 to 8·8)	4·8 (3 to 7·2)	3·3 (2 to 4·9)	2·1 (1·3 to 3·2)	1·3 (0·8 to 2)	0·6 (0·3 to 1)	0·2 (0·1 to 0·4)	0·1 (0 to 0·1)	0 (0 to 0·1)	64·8
Bacterial skin diseases	0 (0 to 0)	0 (0 to 0)	4·9 (2·3 to 8·9)	2·3 (1·1 to 4)	2·1 (1·1 to 3·5)	1·7 (0·9 to 2·9)	1·3 (0·7 to 2·2)	2·5 (1·2 to 4·6)	2·1 (1 to 4·1)	4·2 (1·9 to 8·1)	3·1 (1·3 to 5·9)	5·7 (2·5 to 10·8)	4·4 (2 to 8·5)	5·8 (2·4 to 10·8)	5·6 (2·3 to 9·8)	6·2 (2·5 to 11·2)	4·3 (1·9 to 8·9)	2·7 (1·1 to 5·7)	2·8 (1·2 to 5·6)	2·4 (0·9 to 4·9)	1·4 (0·5 to 2·9)	0·7 (0·3 to 1·5)	0·5 (0·2 to 1·2)	66·6
Cellulitis	0 (0 to 0)	0 (0 to 0)	1·4 (0·4 to 3·2)	0·7 (0·3 to 1·4)	0·9 (0·4 to 1·7)	0·5 (0·2 to 0·9)	0·2 (0·1 to 0·5)	0·6 (0·2 to 1·1)	0·7 (0·3 to 1·7)	0·9 (0·3 to 1·9)	0·8 (0·3 to 1·8)	1·2 (0·5 to 2·6)	0·9 (0·4 to 2)	1·5 (0·6 to 3·3)	1·5 (0·6 to 3·3)	1·5 (0·6 to 3·4)	1·2 (0·4 to 2·8)	0·6 (0·2 to 1·4)	0·6 (0·2 to 1·5)	0·4 (0·2 to 1·2)	0·2 (0·1 to 0·6)	0·1 (0 to 0·3)	0·1 (0 to 0·4)	16·7
Pyoderma	0 (0 to 0)	0 (0 to 0)	3·5 (1·4 to 6·5)	1·6 (0·7 to 2·9)	1·1 (0·6 to 2·1)	1·2 (0·6 to 2·1)	1·1 (0·5 to 1·9)	2 (0·9 to 3·6)	1·3 (0·6 to 2·6)	3·4 (1·5 to 6·7)	2·3 (1 to 4·6)	4·5 (2 to 8·3)	3·5 (1·5 to 7)	4·2 (1·7 to 7·9)	4 (1·6 to 7·1)	4·7 (1·9 to 8·7)	3·1 (1·2 to 6)	2·1 (0·8 to 4·3)	2·2 (0·9 to 4·2)	2 (0·7 to 3·8)	1·2 (0·4 to 2·5)	0·6 (0·2 to 1·2)	0·4 (0·1 to 0·9)	49·9
Scabies	0 (0 to 0)	0 (0 to 0)	1·3 (0·7 to 2·3)	9·6 (4·8 to 17·1)	15 (7·2 to 26·7)	17·8 (8·7 to 30·8)	17·8 (8·9 to 30·5)	16 (7·8 to 28·4)	12 (5·9 to 21)	8·7 (4 to 15·6)	8·3 (4·1 to 14·2)	9·1 (4·7 to 15·5)	9·1 (4·6 to 15·4)	8 (4 to 13·7)	6·5 (3·3 to 11·3)	4·7 (2·5 to 8·2)	3·2 (1·6 to 5·7)	2·3 (1·1 to 4)	1·9 (0·9 to 3·2)	1·3 (0·7 to 2·3)	0·7 (0·4 to 1·3)	0·4 (0·2 to 0·6)	0·2 (0·1 to 0·3)	154
Fungal skin diseases	0 (0 to 0)	0 (0 to 0)	0·2 (0·1 to 0·5)	1·8 (0·7 to 4)	3·5 (1·3 to 7·4)	4·6 (1·7 to 10·3)	4·5 (1·6 to 10)	4·7 (1·8 to 10·4)	5·1 (1·9 to 11·2)	5 (1·9 to 11·5)	5·1 (2 to 11·1)	5·2 (2 to 10·9)	5·3 (2 to 11·6)	5·5 (2·1 to 11·9)	5·9 (2·4 to 12·8)	5·5 (2·1 to 12·1)	5·2 (2·1 to 11·1)	5·2 (2 to 11)	4·8 (1·8 to 10·1)	3·8 (1·5 to 8·2)	2·4 (1 to 5·1)	1·3 (0·5 to 2·7)	0·7 (0·3 to 1·4)	85·4
Viral skin diseases	0 (0 to 0)	0 (0 to 0)	0 (0 to 0)	10·5 (6·6 to 16·3)	19·3 (11·9 to 29·3)	11·5 (7·2 to 17·8)	6·6 (4·1 to 10·1)	5·3 (3·2 to 8·2)	4·1 (2·4 to 6·3)	3·2 (1·8 to 5)	2·9 (1·7 to 4·6)	2·8 (1·6 to 4·5)	2·6 (1·6 to 4·2)	2·4 (1·3 to 3·8)	2 (1·2 to 3·2)	1·6 (1 to 2·6)	1·1 (0·7 to 1·7)	0·7 (0·4 to 1·1)	0·4 (0·3 to 0·7)	0·2 (0·1 to 0·4)	0·1 (0·1 to 0·2)	0 (0 to 0·1)	0 (0 to 0)	77·6
Acne vulgaris	0 (0 to 0)	0 (0 to 0)	0 (0 to 0)	0 (0 to 0)	1·2 (0·6 to 1·9)	29·4 (16·9 to 49·4)	46·8 (26·6 to 76·4)	12·2 (6·7 to 20·3)	6 (3 to 9·8)	4 (2·1 to 6·9)	3 (1·5 to 5·2)	2·1 (1 to 3·8)	1·6 (0·8 to 2·5)	1 (0·5 to 1·6)	0·6 (0·3 to 1·1)	0·4 (0·2 to 0·7)	0·2 (0·1 to 0·4)	0·2 (0·1 to 0·3)	0·1 (0 to 0·2)	0 (0 to 0·1)	0 (0 to 0)	0 (0 to 0)	0 (0 to 0)	109
Alopecia areata	0 (0 to 0)	0 (0 to 0)	0 (0 to 0)	0·3 (0·1 to 0·5)	0·4 (0·2 to 0·8)	0·7 (0·3 to 1·2)	1·1 (0·6 to 1·9)	1·8 (1 to 2·9)	2·5 (1·4 to 3·9)	2·5 (1·4 to 4)	2·3 (1·3 to 3·6)	1·9 (1·1 to 3·1)	1·6 (0·9 to 2·5)	1·3 (0·7 to 2·2)	1·1 (0·6 to 1·7)	0·9 (0·5 to 1·4)	0·5 (0·3 to 0·9)	0·4 (0·2 to 0·6)	0·2 (0·1 to 0·3)	0·1 (0·1 to 0·2)	0 (0 to 0·1)	0 (0 to 0)	0 (0 to 0)	19·5
Pruritus	0 (0 to 0)	0 (0 to 0)	0 (0 to 0·1)	0·5 (0·2 to 0·9)	0·8 (0·3 to 1·6)	0·9 (0·4 to 1·8)	1 (0·4 to 2)	1·2 (0·5 to 2·4)	1·4 (0·6 to 2·8)	1·5 (0·6 to 3)	1·6 (0·7 to 3)	1·6 (0·7 to 3)	1·6 (0·7 to 3·1)	1·6 (0·7 to 3·2)	1·6 (0·7 to 3·2)	1·5 (0·7 to 3·1)	1·3 (0·6 to 2·7)	1 (0·4 to 2)	0·7 (0·3 to 1·5)	0·5 (0·2 to 0·9)	0·2 (0·1 to 0·4)	0·1 (0 to 0·2)	0 (0 to 0·1)	20·5
Urticaria	0 (0 to 0)	0 (0 to 0)	1·6 (0·9 to 2·5)	11·2 (6·5 to 17·6)	11·5 (6·4 to 19·5)	9·3 (4·9 to 15·5)	7·6 (4 to 12·7)	7·1 (3·7 to 12)	6·7 (3·5 to 11·4)	6·6 (3·5 to 11)	6·6 (3·8 to 11·1)	6·2 (3·4 to 9·9)	5·5 (2·9 to 9·3)	5 (2·8 to 8·2)	4·3 (2·2 to 7)	3·4 (1·7 to 5·7)	2·3 (1·3 to 3·9)	1·6 (0·9 to 2·7)	1·1 (0·6 to 1·8)	0·6 (0·3 to 1)	0·3 (0·1 to 0·4)	0·1 (0·1 to 0·2)	0 (0 to 0·1)	98·5
Decubitus ulcer	0 (0 to 0)	0 (0 to 0)	0 (0 to 0)	0·9 (0·2 to 2)	2·2 (1 to 3·7)	1·9 (0·7 to 3·5)	2·5 (0·9 to 4·2)	0·7 (0·3 to 1·4)	4·1 (1·7 to 7·5)	2·4 (0·9 to 5)	7·6 (3·3 to 14·1)	8·4 (3·7 to 14·9)	11·3 (5·5 to 19·2)	20 (10·4 to 35·2)	24·9 (13·2 to 43·4)	26 (14·8 to 44·9)	23·6 (13·9 to 42·1)	25·6 (15·4 to 49·8)	34·8 (21·2 to 64·1)	33·1 (19·6 to 61·7)	23·4 (14·5 to 45·3)	12·9 (8 to 25·4)	8·6 (4·6 to 21·6)	275
Other skin and subcutaneous diseases	0 (0 to 0)	0 (0 to 0)	1·5 (0·6 to 3·1)	2·4 (1·1 to 4·6)	3 (1·5 to 5·3)	3·9 (1·8 to 7·1)	4·8 (2·5 to 8·6)	6·2 (3·5 to 10·3)	5·7 (2·8 to 10·1)	9·7 (5·3 to 16·1)	8·6 (4·6 to 14·6)	11·7 (6·4 to 19·7)	11·1 (5·9 to 19·5)	13·1 (7·3 to 22·5)	13·4 (7·5 to 23·4)	12·4 (6·5 to 21·7)	9·1 (4·7 to 16·5)	6·9 (3·6 to 12·3)	5·7 (3·1 to 10)	3·6 (1·9 to 6·3)	1·6 (0·8 to 2·8)	0·7 (0·4 to 1·3)	0·3 (0·1 to 0·5)	135
Sense organ diseases	0 (0 to 0)	0 (0 to 0)	0·5 (0·3 to 0·8)	6 (3·6 to 8·9)	15·5 (9·7 to 23)	23·4 (15 to 33·9)	27·1 (17·3 to 39·2)	33·7 (21·3 to 50·4)	42·9 (27·4 to 63·8)	51 (32·4 to 77·1)	59·9 (36·7 to 91)	74·6 (47 to 116)	103 (64·4 to 163)	132 (84·1 to 208)	157 (100 to 243)	163 (103 to 246)	146 (95·9 to 219)	131 (87·2 to 189)	124 (84·3 to 175)	95·7 (66·6 to 133)	57·5 (40·4 to 77·5)	30·4 (21·5 to 40·7)	15·6 (11·1 to 20·5)	1,490
Blindness and vision loss	0 (0 to 0)	0 (0 to 0)	0·1 (0 to 0·2)	1·7 (0·9 to 2·9)	4·7 (2·7 to 7·5)	7·1 (4·2 to 11·5)	8·2 (4·9 to 12·9)	9·5 (5·5 to 15)	10·4 (6·5 to 15·5)	11·1 (6·8 to 16·9)	13·4 (8·4 to 20·2)	17·7 (11 to 27)	23·4 (14·6 to 36·8)	29·4 (18·5 to 47·2)	36·1 (23·4 to 56·8)	40·1 (26·1 to 61)	39·3 (25·7 to 57·8)	38·3 (25·7 to 55·8)	37·5 (25·7 to 53·5)	29·8 (20·2 to 41)	18·5 (12·8 to 25·2)	10·4 (7·2 to 14·2)	6 (4·1 to 8·3)	393
Glaucoma	0 (0 to 0)	0 (0 to 0)	0 (0 to 0)	0 (0 to 0)	0 (0 to 0)	0 (0 to 0)	0 (0 to 0)	0 (0 to 0)	0 (0 to 0)	0 (0 to 0)	0 (0 to 0)	0 (0 to 0)	0 (0 to 0·1)	0·3 (0·1 to 0·8)	1 (0·4 to 1·9)	1·7 (0·8 to 3)	2·2 (1·2 to 3·6)	2·8 (1·6 to 4·3)	3·4 (2 to 5·5)	3·1 (1·9 to 4·8)	2·2 (1·3 to 3·4)	1·2 (0·7 to 1·8)	0·7 (0·4 to 1·1)	18·7
Cataract	0 (0 to 0)	0 (0 to 0)	0 (0 to 0)	0 (0 to 0)	0 (0 to 0)	0 (0 to 0)	0 (0 to 0)	0 (0 to 0·1)	0·2 (0·1 to 0·4)	0·5 (0·2 to 0·9)	1 (0·4 to 1·8)	1·7 (0·8 to 2·8)	2·6 (1·3 to 4·4)	3·4 (1·7 to 5·6)	4·3 (2·4 to 6·9)	5·5 (3·2 to 8·5)	6·7 (4·1 to 10·2)	8·6 (5·6 to 12·3)	10·9 (7 to 15·9)	9·9 (6·7 to 14)	6·7 (4·5 to 9·4)	3·6 (2·4 to 5·1)	2 (1·2 to 3)	67·5
Age-related macular degeneration	0 (0 to 0)	0 (0 to 0)	0 (0 to 0)	0 (0 to 0)	0 (0 to 0)	0 (0 to 0)	0 (0 to 0)	0 (0 to 0)	0 (0 to 0)	0 (0 to 0)	0 (0 to 0)	0 (0 to 0)	0 (0 to 0·1)	0·1 (0·1 to 0·3)	0·3 (0·2 to 0·5)	0·4 (0·1 to 0·8)	0·4 (0·2 to 0·8)	0·5 (0·2 to 0·8)	0·5 (0·2 to 0·8)	0·4 (0·2 to 0·6)	0·2 (0·1 to 0·4)	0·1 (0·1 to 0·2)	0·1 (0 to 0·1)	3·1
Refraction disorders	0 (0 to 0)	0 (0 to 0)	0·1 (0 to 0·1)	1·4 (0·8 to 2·4)	4·1 (2·3 to 6·7)	6·2 (3·6 to 10·2)	7·1 (4·2 to 11·6)	8·1 (4·6 to 13·1)	8·4 (5·2 to 13·2)	8 (4·7 to 12·3)	8·4 (5 to 13)	9 (5·4 to 14·1)	9·6 (5·8 to 14·9)	9·4 (5·6 to 14·8)	9·3 (5·7 to 14·5)	8·2 (4·9 to 12·7)	6·5 (3·9 to 9·6)	5·5 (3·3 to 8·3)	4·9 (3·1 to 7·4)	3·1 (1·9 to 4·8)	1·5 (0·9 to 2·2)	0·6 (0·3 to 0·8)	0·2 (0·1 to 0·4)	119
Near vision loss	0 (0 to 0)	0 (0 to 0)	0 (0 to 0)	0 (0 to 0)	0 (0 to 0·1)	0 (0 to 0·1)	0·1 (0 to 0·2)	0·2 (0·1 to 0·4)	0·4 (0·1 to 0·8)	0·9 (0·4 to 2·1)	1·8 (0·6 to 4·1)	3·7 (1·4 to 7·8)	6·4 (2·5 to 13·8)	9·4 (3·8 to 20·1)	12 (4·9 to 25·6)	12·9 (5·2 to 26·5)	11·4 (4·7 to 22·4)	9·8 (4 to 19·2)	8·4 (3·5 to 17·2)	5·8 (2·5 to 11·8)	3·1 (1·4 to 6·3)	1·5 (0·6 to 3)	0·7 (0·3 to 1·6)	88·6
Other vision loss	0 (0 to 0)	0 (0 to 0)	0 (0 to 0·1)	0·3 (0·1 to 0·5)	0·6 (0·2 to 1·2)	0·9 (0·3 to 1·8)	1 (0·3 to 2·1)	1·2 (0·4 to 2·4)	1·4 (0·5 to 2·6)	1·6 (0·7 to 2·9)	2·3 (1·1 to 3·9)	3·3 (1·7 to 5·7)	4·7 (2·4 to 7·5)	6·8 (4 to 10·6)	9·2 (5·5 to 13·9)	11·4 (7·3 to 17·1)	12·1 (7·7 to 17·6)	11·3 (7·2 to 16·3)	9·5 (5·9 to 13·8)	7·4 (4·8 to 10·8)	4·8 (3 to 7·2)	3·4 (2·3 to 4·8)	2·3 (1·4 to 3·4)	95·4
Age-related and other hearing loss	0 (0 to 0)	0 (0 to 0)	0·2 (0·1 to 0·3)	3 (1·5 to 5)	9·1 (5·1 to 14·3)	14·2 (8·2 to 21·5)	16·7 (10·2 to 25)	21·8 (13 to 33·7)	29·4 (18·1 to 45·4)	35·9 (22·1 to 55·4)	41·7 (24·5 to 65·2)	51·8 (30·2 to 83·5)	74·1 (44·2 to 121)	95·4 (57·2 to 154)	113 (68·6 to 177)	114 (69 to 178)	98·4 (61·3 to 152)	86·4 (55·1 to 128)	80·3 (53·2 to 118)	62·3 (41·8 to 88·3)	37·5 (25·4 to 50·9)	19·4 (13·4 to 26·3)	9·1 (6·4 to 12·2)	1,010
Other sense organ diseases	0 (0 to 0)	0 (0 to 0)	0·3 (0·1 to 0·4)	1·4 (0·6 to 2·4)	1·8 (0·8 to 3·3)	2 (0·9 to 3·6)	2·2 (1·1 to 4·2)	2·5 (1 to 4·8)	3·1 (1·3 to 5·9)	4 (1·8 to 7·9)	4·8 (2·1 to 9)	5·2 (2·2 to 10)	5·8 (2·6 to 11·3)	7·6 (3·5 to 14·7)	7·9 (3·6 to 15·5)	8·6 (4·1 to 16·2)	7·9 (3·6 to 14·7)	6·6 (3 to 12·3)	5·8 (2·6 to 10·6)	3·6 (1·8 to 6·7)	1·6 (0·7 to 2·9)	0·6 (0·3 to 1·1)	0·4 (0·2 to 0·7)	83·7
Musculoskeletal disorders	0 (0 to 0)	0 (0 to 0)	0 (0 to 0)	0 (0 to 0)	9·7 (5·1 to 16·7)	53·9 (33·9 to 82·7)	119 (79·8 to 167)	179 (124 to 248)	253 (175 to 347)	308 (213 to 410)	357 (249 to 480)	407 (287 to 549)	456 (321 to 629)	470 (330 to 659)	453 (311 to 618)	384 (265 to 541)	275 (192 to 389)	195 (135 to 273)	141 (95·7 to 203)	85·4 (58·5 to 120)	45·2 (32 to 61·4)	18·7 (13·3 to 26·2)	8·3 (5·8 to 11·8)	4,220
Rheumatoid arthritis	0 (0 to 0)	0 (0 to 0)	0 (0 to 0)	0 (0 to 0)	0·2 (0·1 to 0·4)	0·6 (0·3 to 1·1)	1·9 (1·1 to 3)	3·6 (1·6 to 6·3)	5·3 (2·7 to 9)	7·3 (3·9 to 11·5)	9·6 (5·7 to 14·6)	11·6 (7·2 to 17·7)	13·4 (8·6 to 19·9)	13·8 (9·2 to 20·2)	13·8 (9·1 to 19·5)	11·4 (7·9 to 16)	8·5 (5·9 to 11·9)	5·7 (3·9 to 8·4)	4·6 (3·2 to 6·7)	3 (2·1 to 4·3)	2·6 (1·6 to 4·1)	1·1 (0·7 to 1·9)	0·5 (0·3 to 0·9)	119
Osteoarthritis	0 (0 to 0)	0 (0 to 0)	0 (0 to 0)	0 (0 to 0)	0 (0 to 0)	0 (0 to 0)	0 (0 to 0)	0 (0 to 0)	0 (0 to 0)	1·2 (0·5 to 2·8)	8·3 (4 to 17·3)	23·5 (11·4 to 48·2)	46·5 (22·6 to 91·6)	69·2 (33·6 to 139)	84·4 (41·2 to 171)	84·5 (42 to 168)	67 (33·8 to 135)	51·1 (25·6 to 105)	38·8 (19·2 to 77)	24·1 (12·1 to 48·2)	11·8 (6 to 23·5)	5·2 (2·6 to 10·4)	2·3 (1·2 to 4·6)	518
Osteoarthritis hip	0 (0 to 0)	0 (0 to 0)	0 (0 to 0)	0 (0 to 0)	0 (0 to 0)	0 (0 to 0)	0 (0 to 0)	0 (0 to 0)	0 (0 to 0)	0·4 (0·1 to 0·9)	0·6 (0·1 to 1·4)	1 (0·3 to 2·3)	1·7 (0·7 to 3·9)	2·5 (1·1 to 5·9)	3·3 (1·4 to 7·2)	3·6 (1·6 to 7·9)	3·1 (1·4 to 6·5)	2·5 (1·1 to 5·2)	1·9 (0·9 to 4·1)	1·2 (0·6 to 2·5)	0·6 (0·3 to 1·2)	0·3 (0·1 to 0·6)	0·1 (0·1 to 0·3)	22·8
Osteoarthritis knee	0 (0 to 0)	0 (0 to 0)	0 (0 to 0)	0 (0 to 0)	0 (0 to 0)	0 (0 to 0)	0 (0 to 0)	0 (0 to 0)	0 (0 to 0)	0·7 (0·2 to 1·7)	6·2 (2·8 to 13·2)	18 (8·6 to 37·8)	35·3 (16·8 to 71·9)	52 (24·3 to 105)	62·5 (29·5 to 129)	61·8 (29·4 to 124)	48·5 (23·8 to 97·9)	36·3 (17·7 to 73·6)	26·9 (13·1 to 54·9)	16·2 (8 to 33·1)	7·5 (3·7 to 15·2)	3·1 (1·5 to 6·3)	1·3 (0·6 to 2·6)	376
Osteoarthritis hand	0 (0 to 0)	0 (0 to 0)	0 (0 to 0)	0 (0 to 0)	0 (0 to 0)	0 (0 to 0)	0 (0 to 0)	0 (0 to 0)	0 (0 to 0)	0·1 (0 to 0·4)	0·9 (0·2 to 2·5)	2·8 (1 to 6·2)	6 (2·4 to 12·7)	9·1 (4·1 to 19·9)	11·3 (5·4 to 24·7)	11·4 (5·4 to 23·8)	9·1 (4·2 to 19·2)	7 (3·4 to 14·2)	5·5 (2·7 to 11·2)	3·7 (1·8 to 7·7)	2 (1 to 4·1)	1 (0·5 to 2·1)	0·5 (0·2 to 1)	70·4

Osteoarthritis other	0 (0 to 0)	0 (0 to 0)	0 (0 to 0)	0 (0 to 0)	0 (0 to 0)	0 (0 to 0)	0 (0 to 0)	0 (0 to 0)	0 (0 to 0)	0·1 (0 to 0·2)	0·5 (0·1 to 1·3)	1·7 (0·6 to 3·8)	3·5 (1·5 to 8·1)	5·5 (2·4 to 11·8)	7·2 (3·2 to 16·1)	7·7 (3·6 to 16·5)	6·5 (3·1 to 13·6)	5·3 (2·6 to 11·1)	4·4 (2·2 to 9·1)	3·1 (1·5 to 6·3)	1·7 (0·8 to 3·4)	0·9 (0·4 to 1·7)	0·4 (0·2 to 0·9)	48·4
Low back pain	0 (0 to 0)	0 (0 to 0)	0 (0 to 0)	0 (0 to 0)	7·9 (3·5 to 14·7)	45 (26·1 to 72·9)	76·9 (44·4 to 123)	94·6 (55·7 to 146)	116 (68·3 to 183)	131 (74·1 to 200)	153 (90·5 to 239)	171 (103 to 260)	185 (112 to 295)	188 (115 to 293)	177 (111 to 267)	149 (94·9 to 227)	106 (67·4 to 166)	77·4 (48 to 114)	59·3 (36·8 to 88·9)	38·2 (24 to 56)	17·7 (10·9 to 25·9)	7 (4·4 to 10·4)	3 (1·8 to 4·5)	1,800
Neck pain	0 (0 to 0)	0 (0 to 0)	0 (0 to 0)	0 (0 to 0)	0·4 (0·1 to 1)	2·7 (1·1 to 5·6)	5·9 (2·4 to 11·4)	9·3 (4 to 18·4)	15·2 (6·9 to 29·7)	23·1 (10·6 to 43·6)	31·8 (16 to 56·9)	39·5 (19·9 to 69·8)	44·5 (21·8 to 79·1)	39·2 (20·1 to 73·5)	34·5 (16·2 to 60·6)	27·3 (13·5 to 46·9)	18·3 (9·6 to 31·3)	12·6 (6·5 to 21·9)	8·8 (4·2 to 16·3)	4·8 (2·3 to 8·9)	2·2 (1·2 to 3·9)	1 (0·5 to 1·6)	0·5 (0·3 to 0·9)	321
Gout	0 (0 to 0)	0 (0 to 0)	0 (0 to 0)	0 (0 to 0)	0 (0 to 0)	0 (0 to 0)	0 (0 to 0)	0·1 (0·1 to 0·3)	0·4 (0·2 to 0·6)	0·6 (0·1 to 1·4)	0·8 (0·2 to 1·8)	1·1 (0·4 to 2·4)	1·3 (0·5 to 2·6)	1·5 (0·5 to 3)	1·5 (0·6 to 2·9)	1·4 (0·5 to 2·5)	1·1 (0·4 to 1·9)	0·8 (0·3 to 1·4)	0·7 (0·3 to 1·2)	0·4 (0·2 to 0·9)	0·3 (0·1 to 0·5)	0·1 (0·1 to 0·2)	0·1 (0 to 0·1)	12·2
Other musculoskeletal disorders	0 (0 to 0)	0 (0 to 0)	0 (0 to 0)	0 (0 to 0)	1·2 (0·7 to 1·9)	5·7 (3·2 to 9·4)	33·9 (21·4 to 49·7)	71·9 (49·7 to 102)	116 (78·7 to 163)	145 (98 to 204)	153 (105 to 214)	161 (107 to 229)	165 (113 to 230)	158 (107 to 226)	142 (93·8 to 203)	111 (70·3 to 167)	73·8 (46·7 to 107)	47·6 (28·7 to 74·1)	28·9 (16·9 to 44·1)	14·8 (7·8 to 24·4)	10·5 (6·4 to 15·3)	4·2 (2·1 to 6·6)	1·9 (0·9 to 3·6)	1,450
Other non-communicable diseases	92·2 (60·3 to 148)	75·8 (42·9 to 127)	206 (148 to 270)	100 (72·2 to 136)	85·9 (66·4 to 110)	113 (84·3 to 147)	187 (141 to 252)	281 (205 to 390)	369 (270 to 506)	412 (296 to 556)	455 (335 to 605)	461 (335 to 625)	442 (321 to 586)	390 (288 to 516)	335 (244 to 445)	279 (208 to 377)	208 (156 to 274)	152 (115 to 199)	122 (93 to 159)	84·6 (64·8 to 109)	48·1 (37·3 to 62·2)	22·8 (17·6 to 29·2)	10·9 (7·7 to 14·8)	4,930
Congenital birth defects	88·2 (56·7 to 144)	63·4 (35·1 to 110)	117 (77·7 to 166)	48·4 (32·6 to 74·7)	32 (23·6 to 41·9)	32·5 (23·8 to 42·9)	31·7 (23·2 to 41·1)	25·9 (19·6 to 34·5)	24·4 (17·6 to 33·5)	19·7 (14·3 to 26·8)	19·7 (14·3 to 27)	16·7 (12·3 to 22·1)	15·5 (11·5 to 20·8)	14 (10·4 to 18·3)	10·8 (8 to 14·4)	6·5 (4·6 to 8·6)	3·9 (2·9 to 5·3)	1·4 (0·8 to 2)	0·8 (0·5 to 1·2)	0·4 (0·2 to 0·6)	0·2 (0·1 to 0·3)	0·1 (0 to 0·1)	0 (0 to 0)	573
Neural tube defects	1·8 (0·8 to 3·3)	1·1 (0·4 to 2·2)	3·1 (1·2 to 6·4)	2·1 (1 to 4·2)	0·5 (0·4 to 0·7)	0·7 (0·5 to 1)	0·6 (0·4 to 0·8)	0·5 (0·3 to 0·7)	0·5 (0·3 to 0·6)	0·4 (0·3 to 0·5)	0·3 (0·2 to 0·4)	0·3 (0·2 to 0·4)	0·2 (0·2 to 0·3)	0·2 (0·1 to 0·2)	0·1 (0·1 to 0·2)	0·1 (0·1 to 0·1)	0·1 (0 to 0·1)	0 (0 to 0)	0 (0 to 0)	0 (0 to 0)	0 (0 to 0)	0 (0 to 0)	0 (0 to 0)	12·5
Congenital heart anomalies	31·4 (16 to 62·3)	28·7 (14·7 to 49·5)	51·7 (30·3 to 82·5)	18·1 (10 to 33·8)	8·4 (5·4 to 13)	7·6 (4·8 to 12)	8·1 (5 to 12·3)	6·4 (4·1 to 9·5)	7 (4·1 to 11·2)	4·5 (2·5 to 7)	5·3 (3 to 8·3)	4 (2·5 to 6)	3·2 (2 to 5)	2·6 (1·6 to 3·8)	1·6 (1 to 2·6)	0·9 (0·6 to 1·4)	0·6 (0·4 to 0·9)	0·1 (0 to 0·2)	0·1 (0 to 0·1)	0 (0 to 0·1)	0 (0 to 0)	0 (0 to 0)	0 (0 to 0)	190
Orofacial clefts	0 (0 to 0)	0 (0 to 0)	0·1 (0 to 0·3)	0·3 (0·1 to 0·7)	0·3 (0·1 to 0·7)	0·4 (0·1 to 0·8)	0·4 (0·1 to 0·8)	0·4 (0·1 to 0·8)	0·4 (0·1 to 0·8)	0·3 (0·1 to 0·7)	0·3 (0·1 to 0·7)	0·3 (0·1 to 0·7)	0·3 (0·1 to 0·7)	0·3 (0·1 to 0·6)	0·2 (0·1 to 0·5)	0·2 (0 to 0·4)	0·1 (0 to 0·3)	0·1 (0 to 0·2)	0·1 (0 to 0·1)	0 (0 to 0·1)	0 (0 to 0)	0 (0 to 0)	0 (0 to 0)	4·7
Down syndrome	1·5 (0·8 to 2·8)	1·4 (0·6 to 2·6)	5·7 (2·8 to 9·7)	4·1 (2 to 7·2)	1·8 (1·1 to 2·8)	1·9 (1·1 to 3)	2·2 (1·4 to 3·5)	2·5 (1·5 to 3·8)	2·2 (1·3 to 3·6)	2·2 (1·3 to 3·7)	3 (1·7 to 4·9)	2·6 (1·6 to 4)	3·2 (2 to 5)	3·6 (2·1 to 5·4)	2·8 (1·6 to 4·4)	1·1 (0·7 to 1·8)	0·4 (0·2 to 0·6)	0 (0 to 0)	0 (0 to 0)	0 (0 to 0)	0 (0 to 0)	0 (0 to 0)	0 (0 to 0)	42·1
Turner syndrome	0 (0 to 0)	0 (0 to 0)	0 (0 to 0)	0 (0 to 0·1)	0 (0 to 0·1)	0 (0 to 0·1)	0·1 (0 to 0·1)	0·1 (0 to 0·1)	0·1 (0 to 0·1)	0 (0 to 0·1)	0 (0 to 0·1)	0 (0 to 0)	0 (0 to 0)	0 (0 to 0)	0 (0 to 0)	0 (0 to 0)	0 (0 to 0)	0 (0 to 0)	0 (0 to 0)	0 (0 to 0)	0 (0 to 0)	0 (0 to 0)	0 (0 to 0)	0·5
Klinefelter syndrome	0 (0 to 0)	0 (0 to 0)	0 (0 to 0)	0 (0 to 0)	0 (0 to 0)	0 (0 to 0)	0 (0 to 0)	0 (0 to 0)	0 (0 to 0)	0 (0 to 0)	0 (0 to 0)	0 (0 to 0)	0 (0 to 0)	0 (0 to 0)	0 (0 to 0)	0 (0 to 0)	0 (0 to 0)	0 (0 to 0)	0 (0 to 0)	0 (0 to 0)	0 (0 to 0)	0 (0 to 0)	0 (0 to 0)	0
Other chromosomal abnormalities	15·9 (7·9 to 31·9)	13·2 (6·5 to 25)	24·2 (13·6 to 40·3)	4·5 (2·5 to 8·1)	2·8 (1·8 to 4·5)	3 (1·7 to 4·8)	3·5 (1·9 to 5·7)	3 (1·7 to 4·8)	2·4 (1·2 to 4)	1·8 (0·9 to 3·1)	0·8 (0·5 to 1·2)	0·7 (0·4 to 1·1)	0·3 (0·2 to 0·4)	0·4 (0·2 to 0·7)	0·1 (0·1 to 0·2)	0 (0 to 0·1)	0·1 (0 to 0·1)	0 (0 to 0)	0 (0 to 0)	0 (0 to 0)	0 (0 to 0)	0 (0 to 0)	0 (0 to 0)	76·5
Congenital musculoskeletal and limb anomalies	5·8 (2·7 to 10·2)	3 (1·4 to 5·7)	7·7 (4·3 to 12·7)	8·2 (4·9 to 13)	10·5 (6·2 to 15·8)	11·9 (7 to 18·4)	10·5 (6·5 to 15·6)	7·6 (4·9 to 11·5)	7 (4·3 to 10·7)	6 (3·5 to 9·4)	6 (3·5 to 9·1)	6 (3·6 to 8·8)	5·7 (3·4 to 8·4)	5 (3 to 7·9)	4·3 (2·6 to 6·8)	3·2 (1·9 to 4·8)	2 (1·2 to 3)	1 (0·5 to 1·6)	0·5 (0·3 to 0·9)	0·2 (0·1 to 0·4)	0·1 (0·1 to 0·2)	0 (0 to 0·1)	0 (0 to 0)	112
Urogenital congenital anomalies	1 (0·4 to 2·2)	0·6 (0·3 to 1·2)	0·5 (0·3 to 1)	0·7 (0·4 to 1·1)	0·6 (0·4 to 1)	0·6 (0·3 to 0·9)	0·4 (0·3 to 0·6)	0·3 (0·2 to 0·5)	0·3 (0·2 to 0·4)	0·2 (0·1 to 0·3)	0·2 (0·1 to 0·3)	0·1 (0·1 to 0·2)	0·1 (0·1 to 0·2)	0·1 (0·1 to 0·1)	0·1 (0 to 0·1)	0 (0 to 0)	0 (0 to 0)	0 (0 to 0)	0 (0 to 0)	0 (0 to 0)	0 (0 to 0)	0 (0 to 0)	0 (0 to 0)	5·9
Digestive congenital anomalies	5·2 (2·3 to 13·2)	4 (1·8 to 8·3)	4·3 (2·2 to 8·9)	1·3 (0·7 to 2·6)	0·5 (0·2 to 0·8)	0·3 (0·2 to 0·5)	0·2 (0·2 to 0·3)	0·2 (0·1 to 0·2)	0·1 (0·1 to 0·2)	0·1 (0·1 to 0·2)	0·1 (0·1 to 0·2)	0·1 (0·1 to 0·1)	0·1 (0·1 to 0·1)	0·1 (0 to 0·1)	0·1 (0 to 0·1)	0 (0 to 0·1)	0 (0 to 0)	0 (0 to 0)	0 (0 to 0)	0 (0 to 0)	0 (0 to 0)	0 (0 to 0)	0 (0 to 0)	16·8
Other congenital birth defects	25·6 (13·8 to 43·7)	11·4 (5·4 to 21·3)	19·5 (11·1 to 32·9)	9 (5·1 to 15·8)	6·4 (4·1 to 10)	6·2 (3·9 to 9·6)	5·8 (3·7 to 8·7)	5 (3·1 to 8)	4·4 (2·7 to 7)	4·1 (2·4 to 6·4)	3·6 (2·1 to 5·6)	2·6 (1·6 to 4)	2·3 (1·4 to 3·5)	1·8 (1·1 to 2·9)	1·5 (0·9 to 2·4)	0·8 (0·5 to 1·5)	0·7 (0·4 to 1·2)	0·2 (0 to 0·5)	0·1 (0 to 0·4)	0·1 (0 to 0·2)	0 (0 to 0·1)	0 (0 to 0·1)	0 (0 to 0)	111
Urinary diseases and male infertility	0·2 (0·1 to 0·3)	0·6 (0·3 to 1·1)	2·8 (1·7 to 4·3)	2·2 (1·4 to 3·2)	2·1 (1·5 to 3·1)	3·6 (2·6 to 5)	6·4 (4·6 to 8·7)	9·1 (6·4 to 12·7)	12·2 (8·3 to 17·3)	16·7 (11 to 25)	20·7 (13·6 to 29·8)	22·3 (15 to 31·3)	23·3 (15·7 to 32·3)	26·9 (18·3 to 37·8)	27·3 (17·6 to 39·3)	25·2 (17·8 to 34·9)	22·5 (15·4 to 31·9)	19·6 (13·5 to 27·1)	20·6 (13·8 to 29·5)	19·2 (12·6 to 27·4)	11·8 (7·6 to 16·8)	6 (3·8 to 8·7)	2·9 (1·7 to 4·3)	304
Urolithiasis	0 (0 to 0)	0 (0 to 0)	0 (0 to 0)	0 (0 to 0)	0 (0 to 0)	0·1 (0 to 0·1)	0·1 (0 to 0·2)	0·2 (0·1 to 0·4)	0·4 (0·2 to 0·6)	1 (0·5 to 1·6)	1·4 (0·8 to 2·4)	1·1 (0·6 to 1·8)	1·7 (0·9 to 2·8)	1·4 (0·7 to 2·3)	1·5 (0·7 to 2·5)	1·1 (0·6 to 1·7)	0·8 (0·4 to 1·2)	0·4 (0·2 to 0·6)	0·2 (0·1 to 0·4)	0·1 (0·1 to 0·2)	0·1 (0 to 0·1)	0 (0 to 0)	0 (0 to 0)	11·5
Benign prostatic hyperplasia	0 (0 to 0)	0 (0 to 0)	0 (0 to 0)	0 (0 to 0)	0 (0 to 0)	0 (0 to 0)	0 (0 to 0)	0 (0 to 0)	0 (0 to 0)	0 (0 to 0)	0 (0 to 0)	0 (0 to 0)	0 (0 to 0)	0 (0 to 0)	0 (0 to 0)	0 (0 to 0)	0 (0 to 0)	0 (0 to 0)	0 (0 to 0)	0 (0 to 0)	0 (0 to 0)	0 (0 to 0)	0 (0 to 0)	0
Male infertility	--	--	--	--	--	--	--	--	--	--	--	--	--	--	--	--	--	--	--	--	--	--	--	NA
Other urinary diseases	0·1 (0 to 0·1)	0·4 (0·2 to 0·7)	0·3 (0·1 to 0·7)	0·5 (0·3 to 0·8)	0·4 (0·3 to 0·7)	1·1 (0·6 to 1·6)	0·9 (0·5 to 1·4)	1·5 (0·8 to 2·5)	0·9 (0·5 to 1·4)	1·3 (0·8 to 2·3)	1·1 (0·7 to 1·8)	1·5 (0·9 to 2·7)	1·4 (0·9 to 2·1)	1·7 (1 to 2·8)	1·4 (0·8 to 2·3)	1·3 (0·8 to 2·3)	0·7 (0·4 to 1·2)	0·4 (0·2 to 0·7)	0·4 (0·2 to 0·7)	0·3 (0·1 to 0·6)	0·1 (0·1 to 0·3)	0·1 (0 to 0·1)	0 (0 to 0)	17·9
Gynecological diseases	0 (0 to 0)	0 (0 to 0)	0 (0 to 0)	0 (0 to 0)	0 (0 to 0)	22·5 (11·3 to 39·6)	57·1 (32·2 to 96·3)	126 (78·5 to 192)	190 (125 to 279)	218 (144 to 313)	235 (158 to 336)	235 (155 to 344)	195 (128 to 277)	128 (77·3 to 190)	72·7 (44·7 to 105)	43·9 (25·9 to 67·1)	27 (16·4 to 39·9)	16·9 (10·9 to 24·7)	11 (6·9 to 16·5)	6·3 (3·8 to 9·6)	3·1 (1·9 to 4·6)	1·4 (0·9 to 2·2)	0·7 (0·4 to 1·1)	1,590
Uterine fibroids	0 (0 to 0)	0 (0 to 0)	0 (0 to 0)	0 (0 to 0)	0 (0 to 0)	0·4 (0·1 to 0·8)	0·9 (0·4 to 1·9)	2·2 (0·9 to 4·6)	5·7 (2·4 to 12·2)	11·7 (5·3 to 24·2)	20·4 (10 to 38·7)	23·1 (11·6 to 43·8)	21·2 (10·8 to 38·6)	14·1 (6·8 to 26·2)	8·9 (4·3 to 16·9)	4·8 (2·2 to 9·3)	2·4 (1·1 to 4·5)	1·2 (0·5 to 2·3)	0·6 (0·3 to 1·2)	0·3 (0·1 to 0·5)	0·1 (0 to 0·2)	0 (0 to 0·1)	0 (0 to 0)	118
Polycystic ovarian syndrome	0 (0 to 0)	0 (0 to 0)	0 (0 to 0)	0 (0 to 0)	0 (0 to 0)	1 (0·4 to 2·1)	4·2 (1·7 to 8·6)	5 (2 to 10·3)	4·8 (2 to 10·1)	4·4 (1·8 to 9·1)	4·2 (1·7 to 8·5)	4 (1·6 to 8·2)	3·3 (1·4 to 6·7)	0·8 (0·3 to 1·7)	0 (0 to 0)	0 (0 to 0)	0 (0 to 0)	0 (0 to 0)	0 (0 to 0)	0 (0 to 0)	0 (0 to 0)	0 (0 to 0)	0 (0 to 0)	31·6
Female infertility	0 (0 to 0)	0 (0 to 0)	0 (0 to 0)	0 (0 to 0)	0 (0 to 0)	0 (0 to 0)	0 (0 to 0)	4·2 (0·8 to 11·6)	6·4 (1·5 to 17·3)	7·7 (1·6 to 22·8)	8·2 (1·1 to 25)	7·3 (0·9 to 20·8)	0·1 (0 to 1·1)	0 (0 to 0)	0 (0 to 0)	0 (0 to 0)	0 (0 to 0)	0 (0 to 0)	0 (0 to 0)	0 (0 to 0)	0 (0 to 0)	0 (0 to 0)	0 (0 to 0)	33·9
Endometriosis	0 (0 to 0)	0 (0 to 0)	0 (0 to 0)	0 (0 to 0)	0 (0 to 0)	0 (0 to 0)	4·3 (1·7 to 9·3)	17·3 (8 to 31·6)	20·7 (10·2 to 37·4)	16 (8 to 28·5)	13·9 (7 to 24·9)	13·4 (6·9 to 22·9)	11·8 (6·3 to 19·6)	5·4 (2·7 to 9·1)	0 (0 to 0)	0 (0 to 0)	0 (0 to 0)	0 (0 to 0)	0 (0 to 0)	0 (0 to 0)	0 (0 to 0)	0 (0 to 0)	0 (0 to 0)	103
Genital prolapse	0 (0 to 0)	0 (0 to 0)	0 (0 to 0)	0 (0 to 0)	0 (0 to 0)	0 (0 to 0)	0 (0 to 0)	0·1 (0 to 0·3)	0·4 (0·1 to 0·9)	0·8 (0·3 to 1·7)	1·5 (0·6 to 3·1)	2·3 (0·9 to 5)	3·1 (1·4 to 6·4)	3·4 (1·5 to 6·8)	3·3 (1·4 to 6·5)	2·7 (1·2 to 5·5)	1·9 (0·9 to 3·8)	1·3 (0·6 to 2·6)	1 (0·4 to 1·9)	0·6 (0·3 to 1·1)	0·3 (0·1 to 0·6)	0·2 (0·1 to 0·3)	0·1 (0 to 0·2)	23
Premenstrual syndrome	0 (0 to 0)	0 (0 to 0)	0 (0 to 0)	0 (0 to 0)	0 (0 to 0)	20·2 (9·5 to 37·3)	45·3 (23 to 81·4)	58·7 (28·7 to 104)	59·8 (34·8 to 97·6)	57·7 (30·2 to 93·4)	62·1 (34·4 to 98·3)	61·5 (33·9 to 99)	42·8 (24·4 to 72·8)	14 (7·1 to 25·4)	0 (0 to 0)	0 (0 to 0)	0 (0 to 0)	0 (0 to 0)	0 (0 to 0)	0 (0 to 0)	0 (0 to 0)	0 (0 to 0)	0 (0 to 0)	422
Other gynecological diseases	0 (0 to 0)	0 (0 to 0)	0 (0 to 0)	0 (0 to 0)	0 (0 to 0)	0·9 (0·3 to 2·2)	2·5 (1·2 to 4·2)	38·4 (21·5 to 62)	92·1 (51·6 to 144)	119 (71·2 to 185)	125 (74·4 to 194)	124 (70·5 to 195)	113 (64·5 to 175)	89·8 (44·6 to 144)	60·5 (36·2 to 92)	36·4 (19·6 to 57·8)	22·8 (13·1 to 35·3)	14·4 (8·9 to 21·8)	9·5 (5·8 to 14·5)	5·4 (3·1 to 8·4)	2·7 (1·6 to 4·1)	1·2 (0·7 to 2)	0·6 (0·3 to 1)	857
Hemoglobinopathies and hemolytic anemias	0·3 (0·2 to 0·5)	0·6 (0·4 to 1)	4 (2·7 to 5·7)	11·6 (7·1 to 17·3)	19·7 (12·6 to 29·2)	19·5 (12·5 to 27·7)	34·3 (23 to 48·2)	45·4 (27·3 to 69·4)	39·3 (21·9 to 66·1)	35 (20·4 to 56·1)	30·4 (19·4 to 44·1)	25·8 (16·9 to 36·8)	23 (15 to 33·5)	21·9 (14·4 to 31·9)	16·9 (11·2 to 24·1)	13·8 (8·6 to 20·5)	7·6 (4·7 to 11·1)	5·2 (3·3 to 7·9)	4·2 (2·7 to 6)	2·8 (1·9 to 4·1)	2·2 (1·4 to 3·2)	1·1 (0·7 to 1·5)	0·4 (0·2 to 0·5)	365
Thalassemias	0 (0 to 0)	0 (0 to 0)	0 (0 to 0)	0 (0 to 0·1)	0 (0 to 0·1)	0 (0 to 0)	0 (0 to 0)	0 (0 to 0)	0 (0 to 0)	0 (0 to 0)	0 (0 to 0)	0 (0 to 0)	0 (0 to 0)	0 (0 to 0)	0 (0 to 0)	0 (0 to 0)	0 (0 to 0)	0 (0 to 0)	0 (0 to 0)	0 (0 to 0)	0 (0 to 0)	0 (0 to 0)	0 (0 to 0)	0·1
Thalassemias trait	0 (0 to 0)	0 (0 to 0)	0·1 (0 to 0·1)	0·3 (0·1 to 0·6)	0·5 (0·1 to 1·2)	0·4 (0·1 to 0·9)	1·4 (0·7 to 2·5)	1·6 (0·6 to 3)	1·8 (0·8 to 3·2)	1·6 (0·6 to 2·9)	1·5 (0·7 to 2·5)	1·4 (0·6 to 2·5)	0·3 (0·1 to 0·6)	0·4 (0·1 to 0·9)	0·4 (0·1 to 0·7)	0·2 (0·1 to 0·5)	0·1 (0 to 0·3)	0·1 (0 to 0·2)	0·1 (0 to 0·2)	0·1 (0 to 0·1)	0 (0 to 0·1)	0 (0 to 0)	0 (0 to 0)	12·2
Sickle cell disorders	0·3 (0·2 to 0·5)	0·3 (0·2 to 0·7)	2·6 (1·7 to 4·1)	6 (3·3 to 9·9)	10·8 (7·4 to 15·5)	12·7 (8·2 to 18·9)	23 (15·1 to 34·5)	21·9 (13·9 to 34·6)	21·2 (12·1 to 33·9)	15·8 (9·2 to 25·8)	17·1 (9·8 to 28·5)	12·1 (7·3 to 18·9)	11·3 (6·7 to 17·8)	9 (5·4 to 14·7)	4·4 (2·5 to 7·2)	1·4 (0·7 to 2·4)	0·3 (0·1 to 0·5)	0 (0 to 0·1)	0 (0 to 0)	0 (0 to 0)	0 (0 to 0)	0 (0 to 0)	0 (0 to 0)	170
Sickle cell trait	0 (0 to 0)	0·2 (0·1 to 0·4)	0·8 (0·4 to 1·4)	3·6 (1·6 to 6·7)	5·8 (2 to 11·3)	4·4 (1·5 to 8·8)	3·3 (1·5 to 6·2)	18·3 (4 to 38·6)	12·1 (1·8 to 36·4)	13·4 (2·9 to 30·3)	1·8 (0·7 to 3·5)	4·3 (2 to 7·2)	4·8 (2·1 to 8·6)	4·8 (2·2 to 8·4)	4·1 (1·8 to 7)	2·6 (1·1 to 4·7)	1·3 (0·4 to 2·6)	1 (0·4 to 1·9)	0·8 (0·3 to 1·5)	0·6 (0·3 to 1·1)	0·4 (0·2 to 0·7)	0·2 (0·1 to 0·3)	0 (0 to 0·1)	88·7
G6PD deficiency	0 (0 to 0)	0 (0 to 0)	0 (0 to 0)	0 (0 to 0·1)	0·1 (0 to 0·1)	0·1 (0 to 0·1)	2·2 (0·9 to 4·3)	0·5 (0·2 to 1)	0·8 (0·3 to 1·6)	0·8 (0·3 to 1·5)	4·3 (2·1 to 7·1)	1·8 (0·9 to 3·1)	1 (0·4 to 1·9)	1·1 (0·4 to 2·1)	0·9 (0·4 to 1·8)	0·7 (0·3 to 1·2)	0·4 (0·2 to 0·7)	0·1 (0·1 to 0·2)	0·1 (0 to 0·1)	0 (0 to 0)	0 (0 to 0)	0 (0 to 0)	0 (0 to 0)	14·7
G6PD trait	0 (0 to 0)	0 (0 to 0)	0 (0 to 0)	0 (0 to 0)	0 (0 to 0)	0 (0 to 0)	0·3 (0·1 to 0·7)	0 (0 to 0)	0 (0 to 0)	0 (0 to 0)	0·3 (0·1 to 0·7)	0·4 (0·1 to 0·7)	0·1 (0 to 0·1)	0 (0 to 0)	0 (0 to 0)	0 (0 to 0)	0 (0 to 0)	0 (0 to 0)	0 (0 to 0)	0 (0 to 0)	0 (0 to 0)	0 (0 to 0)	0 (0 to 0)	1·1
Other hemoglobinopathies and hemolytic anemias	0 (0 to 0)	0 (0 to 0·1)	0·4 (0·2 to 0·8)	1·6 (0·6 to 3·3)	2·4 (0·7 to 5·1)	1·9 (0·5 to 4·2)	4 (1·3 to 8·4)	3·1 (0·9 to 6·8)	3·5 (1·4 to 6·5)	3·4 (1·2 to 6·8)	5·3 (2·4 to 9·4)	5·9 (2·9 to 10·3)	5·5 (2·7 to 9·5)	6·7 (3·6 to 10·8)	7·2 (4·5 to 10·9)	8·8 (5·4 to 13·8)	5·5 (3·5 to 8·3)	4 (2·6 to 5·8)	3·2 (2·1 to 4·7)	2·2 (1·5 to 3·1)	1·8 (1·2 to 2·6)	0·9 (0·6 to 1·3)	0·3 (0·2 to 0·4)	77·8
Endocrine, metabolic, blood, and immune disorders	3·5 (2·1 to 6·3)	6·8 (3·3 to 12·8)	59·4 (36·5 to 93·6)	32·5 (20·8 to 53·2)	24·1 (17 to 33·9)	25 (17·4 to 37·2)	43 (30·1 to 60·7)	50·5 (34·4 to 72·2)	67·7 (45·1 to 101)	79·9 (53·7 to 118)	99·1 (68·1 to 136)	104 (73·1 to 148)	119 (83·7 to 166)	125 (88·3 to 172)	127 (85·3 to 182)	111 (74·5 to 163)	82·7 (57·4 to 122)	59·6 (41·7 to 85·3)	48 (33·2 to 68·7)	32·3 (22·5 to 46·1)	19·3 (13 to 28·3)	9·1 (6·1 to 13·8)	4·6 (2·6 to 7·9)	1,330
Oral disorders	0 (0 to 0)	0 (0 to 0)	0 (0 to 0)	5·6 (3·2 to 9·1)	8 (4·3 to 13·6)	10·2 (5·4 to 17·6)	14·6 (7·8 to 25·5)	23·8 (12·5 to 43·6)	35·4 (17·5 to 66·3)	43·1 (20·8 to 80·9)	50·1 (23·4 to 94·9)	57·2 (27·7 to 103)	65·8 (35 to 117)	74 (41·1 to 129)	80·5 (47 to 130)	78·8 (47·8 to 124)	63·8 (38·7 to 98·6)	49·6 (30·7 to 76·3)	37·9 (23·5 to 56·7)	23·6 (14·9 to 34·9)	11·6 (7·2 to 17·1)	5·2 (3·2 to 7·7)	2·4 (1·5 to 3·6)	741
Caries of deciduous teeth	0 (0 to 0)	0 (0 to 0)	0 (0 to 0)	1·7 (0·7 to 3·6)	2 (0·8 to 4·1)	0·1 (0 to 0·3)	0 (0 to 0)	0 (0 to 0)	0 (0 to 0)	0 (0 to 0)	0 (0 to 0)	0 (0 to 0)	0 (0 to 0)	0 (0 to 0)	0 (0 to 0)	0 (0 to 0)	0 (0 to 0)	0 (0 to 0)	0 (0 to 0)	0 (0 to 0)	0 (0 to 0)	0 (0 to 0)	0 (0 to 0)	3·8
Caries of permanent teeth	0 (0 to 0)	0 (0 to 0)	0 (0 to 0)	0 (0 to 0)	1·8 (0·6 to 4·1)	4·7 (1·9 to 10·4)	4·8 (1·8 to 10·8)	5·6 (2·3 to 11·7)	5·6 (2·2 to 11·7)	5 (2 to 10·7)	4·8 (1·9 to 9·7)	4·5 (1·8 to 9·3)	4·2 (1·7 to 9·2)	3·7 (1·4 to 7·8)	3 (1·1 to 6·5)	2·1 (0·7 to 4·7)	1·2 (0·5 to 2·6)	0·8 (0·3 to 1·6)	0·5 (0·2 to 1·1)	0·3 (0·1 to 0·7)	0·1 (0·1 to 0·3)	0·1 (0 to 0·1)	0 (0 to 0·1)	52·9
Periodontal diseases	0 (0 to 0)	0 (0 to 0)	0 (0 to 0)	0 (0 to 0)	0 (0 to 0)	0 (0 to 0)	2 (0·6 to 5·4)	7·7 (2·3 to 19·8)	17·1 (5·4 to 39·8)	24·6 (8·4 to 56·1)	30·4 (11 to 69·5)	34·5 (12·7 to 74·5)	35·9 (14 to 74·1)	32·7 (12·8 to 67·9)	26·7 (10·7 to 56·3)	18·9 (7·5 to 37·9)	11·4 (4·5 to 22·7)	7·2 (2·8 to 14·5)	4·8 (1·9 to 9·6)	2·9 (1·1 to 5·7)	1·4 (0·5 to 2·8)	0·6 (0·2 to 1·3)	0·3 (0·1 to 0·6)	259
Other oral disorders	0 (0 to 0)	0 (0 to 0)	0 (0 to 0)	3·9 (2·3 to 6·1)	4·2 (2·4 to 6·6)	5·5 (3·1 to 8·6)	7·8 (4·6 to 12·3)	9·6 (5·7 to 14·8)	10·6 (6·4 to 15·8)	10·5 (6·3 to 15·9)	10·4 (6·2 to 16)	10·6 (6·3 to 16)	10·6 (6·5 to 16·2)	9·7 (5·9 to 14·7)	8·3 (5 to 12·4)	6·4 (3·9 to 9·6)	4·3 (2·6 to 6·5)	2·7 (1·6 to 4·1)	1·6 (1 to 2·3)	0·8 (0·5 to 1·2)	0·3 (0·2 to 0·4)	0·1 (0·1 to 0·2)	0·1 (0 to 0·1)	118
Sudden infant death syndrome	--	4·5 (2·2 to 8·3)	23·3 (12·1 to 41)	--	--	--	--	--	--	--	--	--	--	--	--	--	--	--	--	--	--	--	--	NA
Injuries	9·1 (6·7 to 12·2)	8·3 (5·5 to 12·4)	62·9 (48 to 81·4)	177 (153 to 207)	214 (195 to 235)	272 (246 to 303)	404 (357 to 458)	454 (382 to 545)	428 (352 to 534)	395 (320 to 478)	364 (302 to 437)	320 (270 to 378)	293 (250 to 344)	258 (221 to 301)	216 (184 to 255)	178 (151 to 208)	132 (112 to 154)	103 (88·1 to 119)	90·4 (77·4 to 105)	72·9 (62·1 to 85)	44·4 (37·4 to 52·6)	24·4 (20 to 28·8)	12·6 (9·9 to 15)	4,530
Transport injuries	0·5 (0·4 to 0·7)	0·4 (0·3 to 0·7)	4·8 (3·5 to 6·5)	24·5 (19·4 to 32·1)	41·2 (32·7 to 51·3)	58 (47·2 to 71·5)	136 (106 to 171)	137 (105 to 183)	121 (91·3 to 163)	102 (78 to 132)	99 (76·6 to 127)	87·7 (69·6 to 110)	83 (67·5 to 102)	71·3 (58·9 to 85·9)	57·4 (46·9 to 69·6)	44·5 (37 to 53·5)	32·2 (26·5 to 38·5)	21·4 (17·4 to 25·8)	16 (13·2 to 19)	9·3 (7·6 to 11·3)	4·2 (3·4 to 5·1)	1·9 (1·5 to 2·3)	0·9 (0·7 to 1·1)	1,150
Road injuries	0·2 (0·2 to 0·4)	0·2 (0·1 to 0·4)	2·6 (1·6 to 4)	10·5 (6·6 to 16·6)	23·9 (16·3 to 33)	32·9 (23·7 to 45·3)	94·2 (67 to 128)	95·2 (66·2 to 135)	83·9 (58·1 to 120)	68·2 (48 to 94·3)	68·4 (48·4 to 92·4)	56·1 (41·6 to 74·6)	56·7 (43·4 to 72·8)	47 (35·8 to 59)	39·7 (30·6 to 50·3)	32·6 (25·8 to 40·3)	25 (20 to 31·2)	16·1 (12·6 to 20)	11·5 (9·1 to 14·1)	7·2 (5·7 to 9)	3·3 (2·6 to 4·1)	1·5 (1·2 to 1·9)	0·7 (0·6 to 0·9)	778
Pedestrian road injuries	0·1 (0 to 0·1)	0·1 (0 to 0·1)	0·5 (0·3 to 0·7)	3·7 (2·2 to 6·1)	7·6 (5 to 10·8)	8·4 (5·7 to 12·3)	10·9 (7·8 to 15·1)	11·2 (7·8 to 16·1)	10·7 (7·5 to 15·6)	11·6 (8·1 to 16·2)	12·4 (8·7 to 17·1)	10·2 (7·3 to 13·6)	11·2 (8·5 to 14·7)	10·7 (8 to 13·8)	9·2 (6·9 to 12)	8·6 (6·7 to 11·1)	7·7 (6 to 9·7)	5·2 (4 to 6·7)	4·1 (3·2 to 5·2)	2·8 (2·1 to 3·6)	1·2 (1 to 1·6)	0·5 (0·4 to 0·7)	0·3 (0·2 to 0·3)	149
Cyclist road injuries	0 (0 to 0)	0 (0 to 0)	0 (0 to 0)	0·3 (0·2 to 0·5)	1 (0·6 to 1·5)	2·5 (1·6 to 3·8)	4·2 (2·5 to 6·4)	4·3 (2·9 to 6·1)	4·7 (3·2 to 6·4)	4·5 (3·1 to 6·1)	5·1 (3·6 to 6·8)	5 (3·6 to 6·8)	5·3 (3·7 to 7·1)	5 (3·6 to 6·8)	4·3 (3·1 to 5·9)	3·4 (2·5 to 4·7)	2·4 (1·7 to 3·2)	1·7 (1·2 to 2·2)	1·2 (0·8 to 1·6)	0·7 (0·5 to 1)	0·4 (0·3 to 0·5)	0·2 (0·1 to 0·2)	0·1 (0·1 to 0·1)	56·3
Motorcyclist road injuries	0 (0 to 0)	0 (0 to 0·1)	0·1 (0·1 to 0·2)	0·3 (0·2 to 0·5)	1 (0·7 to 1·3)	1·7 (1·2 to 2·3)	8·3 (5·9 to 11·4)	9·8 (7·1 to 13·8)	9 (6·5 to 12·5)	6·6 (4·8 to 8·8)	6·9 (5·1 to 9·2)	5·2 (3·8 to 6·9)	5·3 (4·1 to 6·9)	4·2 (3·2 to 5·5)	3·9 (2·8 to 5·2)	3 (2·2 to 4)	2·3 (1·7 to 3·1)	1·6 (1·1 to 2·2)	1·3 (0·9 to 1·7)	0·9 (0·6 to 1·2)	0·5 (0·3 to 0·6)	0·2 (0·2 to 0·3)	0·1 (0·1 to 0·2)	72·3
Motor vehicle road injuries	0·1 (0·1 to 0·2)	0·1 (0 to 0·2)	1·9 (1·2 to 3)	5·9 (3·6 to 9·6)	13·8 (9·3 to 19·3)	19·7 (13·7 to 27·7)	69·8 (49 to 95·8)	68·5 (46·8 to 98·8)	57·5 (38·1 to 84·6)	43·7 (29·9 to 62·3)	42·2 (28·6 to 59)	34·2 (24·1 to 47)	33·5 (24·8 to 44·1)	25·7 (19·2 to 33·6)	21·1 (15·8 to 27·4)	16·5 (12·9 to 21)	11·8 (9·2 to 14·9)	7·2 (5·6 to 9·1)	4·6 (3·6 to 5·8)	2·6 (2 to 3·3)	1·1 (0·9 to 1·4)	0·5 (0·4 to 0·6)	0·2 (0·2 to 0·3)	482
Other road injuries	0 (0 to 0)	0 (0 to 0)	0·1 (0 to 0·1)	0·2 (0·1 to 0·5)	0·6 (0·3 to 1·1)	0·5 (0·3 to 0·9)	1·1 (0·6 to 1·8)	1·4 (0·8 to 2·2)	2 (1·1 to 3·3)	1·9 (1·1 to 3·2)	1·7 (1·1 to 2·6)	1·5 (1 to 2·1)	1·4 (1 to 2)	1·4 (1 to 1·9)	1·2 (0·9 to 1·6)	1 (0·7 to 1·4)	0·7 (0·5 to 1)	0·4 (0·3 to 0·5)	0·3 (0·2 to 0·4)	0·2 (0·1 to 0·2)	0·1 (0·1 to 0·1)	0 (0 to 0·1)	0 (0 to 0)	17·7
Other transport injuries	0·3 (0·2 to 0·3)	0·2 (0·1 to 0·4)	2·2 (1·8 to 2·9)	14 (12·1 to 16·3)	17·3 (15·2 to 20)	25·1 (21·8 to 29·2)	41·7 (35·8 to 49·6)	42 (35 to 52·9)	37·5 (31·1 to 46·7)	33·3 (27·3 to 41·6)	30·7 (25·6 to 38)	31·7 (26·3 to 38·8)	26·3 (22·6 to 31·4)	24·3 (21 to 28·9)	17·8 (15·2 to 21·4)	11·9 (10·4 to 14·1)	7·2 (6·3 to 8·4)	5·2 (4·6 to 6·1)	4·5 (3·8 to 5·3)	2·1 (1·8 to 2·4)	0·9 (0·8 to 1·1)	0·4 (0·3 to 0·5)	0·2 (0·1 to 0·2)	377
Unintentional injuries	5·8 (4·2 to 7·7)	7·1 (4·8 to 10·4)	52·9 (40·1 to 69·3)	144 (124 to 169)	162 (147 to 180)	193 (174 to 216)	172 (151 to 195)	170 (146 to 197)	171 (145 to 204)	168 (144 to 199)	167 (142 to 199)	161 (138 to 189)	157 (132 to 187)	150 (127 to 177)	136 (114 to 162)	117 (99 to 139)	88·5 (75 to 105)	75 (64·2 to 87)	69·6 (59·3 to 81·5)	61·4 (51·9 to 71·5)	39·1 (32·9 to 46·7)	22·1 (18·1 to 26·1)	11·5 (9·1 to 13·8)	2,500
Falls	0·1 (0 to 0·1)	0·1 (0 to 0·1)	0·8 (0·6 to 1·2)	2·1 (1·4 to 3)	3·9 (2·7 to 5·4)	6·2 (4·2 to 8·8)	7·5 (5·2 to 10·7)	9·2 (6·4 to 13)	11 (7·6 to 15·6)	12·1 (8·4 to 17)	13·8 (9·7 to 19·1)	15·3 (10·8 to 21·3)	17·8 (13 to 24·2)	20·3 (15 to 27·2)	21·4 (16 to 28·2)	22·9 (17·4 to 29)	21·4 (16·7 to 26·9)	23·5 (18·6 to 29·2)	27·8 (22·3 to 34·4)	31·7 (25·4 to 39·4)	23·1 (18·3 to 28·5)	13·5 (10·7 to 16·5)	6·8 (5·2 to 8·4)	312
Drowning	0·4 (0·3 to 0·6)	0·4 (0·2 to 0·7)	4·3 (2·6 to 6·4)	22·2 (14·1 to 32)	24·9 (17·7 to 33·2)	30·5 (21·5 to 41·2)	28·7 (20·8 to 38·7)	17·4 (12·1 to 25·4)	16·3 (10·7 to 24·2)	14·7 (9·8 to 21)	13·8 (9·4 to 19·7)	9·8 (6·8 to 13·8)	8·9 (5·9 to 12·9)	6·5 (4·7 to 8·9)	4·9 (3·6 to 6·5)	3·5 (2·7 to 4·6)	2·6 (1·9 to 3·4)	1·4 (1·1 to 1·8)	1·3 (0·9 to 1·8)	0·7 (0·5 to 0·9)	0·5 (0·4 to 0·6)	0·4 (0·3 to 0·5)	0·2 (0·2 to 0·3)	214
Fire, heat, and hot substances	0·2 (0·2 to 0·3)	0·4 (0·2 to 0·7)	3·9 (2·5 to 5·7)	10·7 (6·9 to 15·7)	12·1 (9·6 to 15)	14 (10·5 to 18)	21·2 (16·3 to 27·4)	25·8 (19·2 to 34·5)	30·3 (22·2 to 40·2)	31·2 (23 to 42·1)	33·1 (25 to 44·1)	29·3 (21·7 to 38·8)	27 (19·7 to 36·7)	25·8 (18·8 to 34·1)	22·4 (16·6 to 29·8)	17·9 (13·2 to 23·9)	12·1 (9·1 to 16·4)	9 (6·8 to 11·9)	6·9 (5·2 to 9·1)	4·9 (3·8 to 6·3)	3 (2·3 to 3·8)	1·6 (1·3 to 2)	0·9 (0·6 to 1·1)	344
Poisonings	0 (0 to 0)	0·1 (0·1 to 0·2)	0·3 (0·2 to 0·5)	1·1 (0·7 to 1·7)	1 (0·7 to 1·5)	1·2 (0·7 to 1·9)	1·6 (1·1 to 2·3)	1·8 (1·2 to 2·6)	1·7 (1·1 to 2·5)	1·7 (1·1 to 2·5)	1·7 (1·2 to 2·4)	1·5 (1·1 to 2·2)	1·4 (0·9 to 2)	1·2 (0·8 to 1·8)	1 (0·7 to 1·5)	0·8 (0·5 to 1·1)	0·6 (0·4 to 0·9)	0·4 (0·3 to 0·6)	0·3 (0·2 to 0·5)	0·2 (0·2 to 0·3)	0·1 (0·1 to 0·2)	0 (0 to 0·1)	0 (0 to 0)	20·1
Poisoning by carbon monoxide	0 (0 to 0)	0 (0 to 0)	0 (0 to 0)	0·1 (0·1 to 0·2)	0·3 (0·2 to 0·5)	0·3 (0·2 to 0·6)	0·4 (0·2 to 0·6)	0·5 (0·3 to 0·7)	0·4 (0·3 to 0·7)	0·4 (0·3 to 0·7)	0·4 (0·2 to 0·6)	0·4 (0·2 to 0·6)	0·3 (0·2 to 0·5)	0·3 (0·2 to 0·5)	0·3 (0·2 to 0·4)	0·2 (0·1 to 0·3)	0·2 (0·1 to 0·3)	0·1 (0·1 to 0·2)	0·1 (0 to 0·1)	0 (0 to 0·1)	0 (0 to 0)	0 (0 to 0)	0 (0 to 0)	4·9
Poisoning by other means	0 (0 to 0)	0·1 (0·1 to 0·2)	0·3 (0·2 to 0·5)	1 (0·6 to 1·5)	0·7 (0·5 to 1·1)	0·9 (0·5 to 1·4)	1·2 (0·8 to 1·8)	1·3 (0·9 to 1·9)	1·3 (0·8 to 1·9)	1·3 (0·8 to 1·9)	1·3 (0·9 to 1·9)	1·1 (0·8 to 1·6)	1·1 (0·7 to 1·5)	0·9 (0·6 to 1·3)	0·8 (0·5 to 1·1)	0·6 (0·4 to 0·9)	0·4 (0·3 to 0·6)	0·3 (0·2 to 0·4)	0·3 (0·2 to 0·4)	0·2 (0·1 to 0·2)	0·1 (0·1 to 0·1)	0 (0 to 0·1)	0 (0 to 0)	15·2
Exposure to mechanical forces	0·1 (0 to 0·1)	0·1 (0·1 to 0·2)	0·7 (0·5 to 1)	3·6 (2·4 to 5·1)	6·4 (4·7 to 8·7)	8·8 (6·3 to 12·1)	10·8 (8·1 to 14·4)	12·9 (9·5 to 17·6)	13·2 (9·5 to 17·7)	13·2 (9·6 to 17·9)	14 (10·2 to 19·2)	14 (10·1 to 19)	13·7 (9·7 to 18·9)	13·1 (9·5 to 18·2)	11·5 (8·1 to 16·2)	9·5 (6·5 to 13·3)	6·8 (4·7 to 9·5)	5 (3·6 to 7·1)	4 (2·8 to 5·5)	2·8 (2 to 3·8)	1·7 (1·2 to 2·2)	1 (0·8 to 1·3)	0·6 (0·5 to 0·8)	168
Unintentional firearm injuries	0 (0 to 0)	0 (0 to 0)	0·1 (0 to 0·1)	0·3 (0·2 to 0·5)	0·5 (0·3 to 0·8)	1 (0·7 to 1·4)	1·8 (1·2 to 2·6)	2·4 (1·7 to 3·4)	2 (1·4 to 2·6)	2·2 (1·6 to 3)	2·1 (1·6 to 2·9)	2 (1·4 to 2·6)	1·9 (1·4 to 2·5)	1·7 (1·2 to 2·2)	1·5 (1·1 to 2)	1·2 (0·9 to 1·6)	0·8 (0·6 to 1·1)	0·6 (0·5 to 0·8)	0·5 (0·3 to 0·7)	0·4 (0·3 to 0·5)	0·2 (0·2 to 0·3)	0·1 (0·1 to 0·2)	0·1 (0·1 to 0·1)	23·3
Other exposure to mechanical forces	0·1 (0 to 0·1)	0·1 (0·1 to 0·2)	0·7 (0·4 to 0·9)	3·3 (2·2 to 4·7)	5·9 (4·3 to 8·1)	7·8 (5·5 to 11)	9 (6·5 to 12·2)	10·6 (7·7 to 14·7)	11·2 (7·9 to 15·5)	11 (7·8 to 15·2)	11·9 (8·5 to 16·4)	12 (8·6 to 16·7)	11·8 (8·3 to 16·5)	11·5 (8·1 to 16·1)	10 (7 to 14·3)	8·3 (5·7 to 11·7)	5·9 (4·1 to 8·4)	4·4 (3·1 to 6·2)	3·5 (2·5 to 4·9)	2·5 (1·8 to 3·4)	1·4 (1 to 2)	0·9 (0·6 to 1·2)	0·5 (0·4 to 0·7)	144
Adverse effects of medical treatment	0·9 (0·6 to 1·4)	0·9 (0·5 to 1·6)	3·8 (2·2 to 6·4)	2·7 (1·6 to 4·3)	2 (1·3 to 3)	2·2 (1·4 to 3·3)	3·8 (2·1 to 6)	7·4 (4·2 to 11·4)	7·2 (4 to 12·1)	8·8 (4·7 to 13·6)	8·3 (4·6 to 12·8)	9·4 (5·6 to 14·8)	11·4 (6·9 to 17·2)	10·8 (6·8 to 16·5)	10·7 (6·7 to 16·3)	10·4 (6·9 to 15·3)	7·7 (5·1 to 10·6)	6·6 (4·4 to 9·2)	6·3 (4·3 to 8·9)	4·7 (3·2 to 6·4)	2·8 (1·9 to 4·1)	1·3 (0·9 to 2)	0·7 (0·4 to 1·4)	131
Animal contact	0 (0 to 0)	0 (0 to 0)	0·2 (0·1 to 0·3)	0·5 (0·3 to 0·8)	0·7 (0·5 to 1·1)	1 (0·6 to 1·4)	1·1 (0·7 to 1·5)	1·2 (0·8 to 1·6)	1·4 (0·9 to 1·9)	1·3 (0·9 to 1·8)	1·4 (0·9 to 1·9)	1·3 (0·9 to 1·8)	1·4 (0·9 to 1·9)	1·2 (0·8 to 1·7)	1·3 (0·9 to 1·8)	1 (0·7 to 1·4)	0·8 (0·5 to 1)	0·7 (0·5 to 1)	0·7 (0·5 to 1)	0·9 (0·6 to 1·3)	0·7 (0·4 to 1)	0·6 (0·4 to 0·9)	0·4 (0·2 to 0·6)	19·6
Venomous animal contact	0 (0 to 0)	0 (0 to 0)	0 (0 to 0·1)	0·2 (0·1 to 0·3)	0·3 (0·2 to 0·6)	0·6 (0·4 to 1)	0·6 (0·4 to 1)	0·7 (0·4 to 1·1)	0·8 (0·5 to 1·2)	0·8 (0·5 to 1·2)	0·8 (0·5 to 1·1)	0·8 (0·5 to 1·1)	0·7 (0·4 to 1·1)	0·7 (0·4 to 1)	0·6 (0·4 to 0·9)	0·5 (0·4 to 0·8)	0·4 (0·3 to 0·6)	0·3 (0·2 to 0·5)	0·3 (0·2 to 0·5)	0·3 (0·2 to 0·5)	0·3 (0·2 to 0·4)	0·3 (0·2 to 0·4)	0·2 (0·1 to 0·3)	10·3
Non-venomous animal contact	0 (0 to 0)	0 (0 to 0)	0·1 (0·1 to 0·2)	0·3 (0·2 to 0·6)	0·4 (0·2 to 0·6)	0·4 (0·2 to 0·5)	0·4 (0·3 to 0·6)	0·4 (0·3 to 0·7)	0·5 (0·3 to 0·8)	0·5 (0·3 to 0·7)	0·6 (0·4 to 0·9)	0·5 (0·3 to 0·8)	0·6 (0·4 to 0·9)	0·6 (0·4 to 0·8)	0·7 (0·4 to 0·9)	0·5 (0·4 to 0·7)	0·4 (0·3 to 0·5)	0·4 (0·3 to 0·6)	0·4 (0·3 to 0·6)	0·6 (0·4 to 0·9)	0·4 (0·2 to 0·6)	0·4 (0·2 to 0·6)	0·2 (0·1 to 0·4)	9·3
Foreign body	3·4 (2·3 to 4·9)	3 (1·5 to 5·1)	21·7 (13·2 to 33·3)	10·6 (7 to 15·3)	6·8 (5·5 to 8·5)	6·3 (4·7 to 8·2)	4·5 (3·5 to 5·8)	6·1 (4·5 to 8·2)	6·5 (4·8 to 8·8)	6·3 (4·6 to 8·6)	6·9 (5·2 to 9·4)	6·5 (4·9 to 8·6)	6·8 (5·2 to 9)	7·2 (5·3 to 9·4)	6·7 (5·1 to 8·8)	6·5 (4·8 to 8·6)	6·1 (4·7 to 7·8)	5·5 (4·3 to 7)	5·7 (4·4 to 7·5)	5·1 (3·9 to 6·7)	2·2 (1·7 to 2·8)	1·4 (1 to 1·8)	0·8 (0·6 to 1·1)	143
Pulmonary aspiration and foreign body in airway	3·4 (2·3 to 4·9)	3 (1·5 to 5·1)	21·6 (13·1 to 33·1)	10·1 (6·5 to 14·8)	6 (4·7 to 7·6)	5·3 (3·8 to 7)	3·4 (2·6 to 4·3)	4·7 (3·5 to 6·6)	5 (3·5 to 7)	4·8 (3·3 to 7)	5·4 (3·9 to 7·6)	5 (3·5 to 6·9)	5·3 (3·8 to 7·1)	5·7 (4·2 to 7·9)	5·5 (4 to 7·5)	5·5 (3·9 to 7·6)	5·3 (4 to 6·9)	5 (3·7 to 6·4)	5·2 (3·9 to 6·9)	4·7 (3·5 to 6·2)	2 (1·5 to 2·6)	1·3 (0·9 to 1·7)	0·8 (0·5 to 1)	124
Foreign body in eyes	0 (0 to 0)	0 (0 to 0)	0 (0 to 0·1)	0·1 (0 to 0·3)	0·3 (0·1 to 0·6)	0·4 (0·1 to 0·9)	0·5 (0·2 to 1·1)	0·6 (0·2 to 1·4)	0·7 (0·3 to 1·5)	0·7 (0·3 to 1·5)	0·7 (0·3 to 1·5)	0·8 (0·3 to 1·6)	0·8 (0·4 to 1·4)	0·7 (0·4 to 1·1)	0·5 (0·3 to 0·9)	0·4 (0·3 to 0·6)	0·3 (0·2 to 0·5)	0·2 (0·1 to 0·3)	0·2 (0·1 to 0·2)	0·1 (0·1 to 0·2)	0·1 (0 to 0·1)	0 (0 to 0)	0 (0 to 0)	8·1
Foreign body in other body part	0 (0 to 0)	0 (0 to 0)	0·1 (0 to 0·1)	0·4 (0·3 to 0·6)	0·6 (0·4 to 0·8)	0·7 (0·5 to 1)	0·7 (0·5 to 1)	0·8 (0·5 to 1·1)	0·8 (0·5 to 1·1)	0·8 (0·5 to 1·1)	0·8 (0·5 to 1)	0·8 (0·5 to 1·1)	0·8 (0·6 to 1·1)	0·8 (0·6 to 1)	0·7 (0·5 to 0·9)	0·6 (0·4 to 0·8)	0·5 (0·4 to 0·6)	0·4 (0·3 to 0·5)	0·4 (0·2 to 0·5)	0·3 (0·2 to 0·4)	0·2 (0·1 to 0·2)	0·1 (0·1 to 0·2)	0·1 (0 to 0·1)	11
Environmental heat and cold exposure	0 (0 to 0)	0 (0 to 0·1)	0·2 (0·1 to 0·2)	0·9 (0·6 to 1·3)	1·9 (1·3 to 2·9)	2·9 (1·8 to 4·2)	3·4 (2·3 to 4·9)	3·9 (2·6 to 5·7)	4·3 (2·9 to 6·3)	4·5 (3·1 to 6·5)	4·7 (3·2 to 6·7)	4·9 (3·4 to 6·9)	5·1 (3·5 to 7·2)	5 (3·5 to 7)	4·7 (3·3 to 6·6)	4·1 (2·8 to 5·7)	3·1 (2·2 to 4·3)	2·4 (1·7 to 3·3)	2 (1·4 to 2·7)	1·5 (1 to 2)	0·9 (0·6 to 1·2)	0·5 (0·4 to 0·7)	0·3 (0·2 to 0·4)	61·5
Exposure to forces of nature	0·5 (0·5 to 0·6)	2 (1·8 to 2·2)	16·2 (14·7 to 17·8)	86·5 (78·6 to 95·2)	97 (88·2 to 107)	114 (103 to 125)	82·1 (74·6 to 90·5)	75·6 (68·7 to 83·3)	70·1 (63·7 to 77·2)	65·2 (59·2 to 71·9)	59·3 (53·9 to 65·3)	59·8 (54·4 to 65·9)	53·8 (48·8 to 59·2)	50·1 (45·5 to 55·2)	43·1 (39·2 to 47·5)	34 (30·9 to 37·6)	22·7 (20·6 to 25·1)	17 (15·5 to 18·8)	11·8 (10·7 to 13)	7·1 (6·5 to 7·9)	3·3 (3 to 3·7)	1·2 (1·1 to 1·4)	0·4 (0·3 to 0·4)	972
Other unintentional injuries	0·1 (0·1 to 0·2)	0·1 (0·1 to 0·2)	0·8 (0·6 to 1·2)	2·9 (2 to 4·2)	5·1 (3·7 to 7·1)	6·7 (4·7 to 9·4)	7·6 (5·4 to 10·6)	8·8 (6·2 to 12·2)	9·5 (6·7 to 13)	9·3 (6·5 to 12·9)	9·7 (6·8 to 13·3)	9·5 (6·6 to 13·2)	9·5 (6·5 to 13·2)	8·9 (6·1 to 12·4)	7·8 (5·4 to 11·2)	6·5 (4·4 to 9·1)	4·6 (3·2 to 6·4)	3·4 (2·4 to 4·7)	2·7 (1·9 to 3·7)	1·7 (1·2 to 2·3)	0·9 (0·6 to 1·2)	0·4 (0·3 to 0·6)	0·2 (0·1 to 0·3)	117
Self-harm and interpersonal violence	2·8 (1·6 to 4·5)	0·7 (0·4 to 1·4)	5·1 (3·1 to 8·3)	8·8 (5·5 to 14·7)	11 (8·3 to 14·6)	21·1 (15·6 to 28·5)	95·2 (71·3 to 125)	147 (106 to 200)	136 (94·8 to 190)	125 (85·8 to 173)	98·4 (71 to 138)	70·9 (50·9 to 96·7)	53·6 (38·7 to 71·6)	36 (26·8 to 48·5)	23·3 (17·9 to 30·3)	16·1 (12·6 to 20·3)	10·9 (8·7 to 13·9)	6·6 (5·2 to 8·2)	4·8 (3·9 to 5·9)	2·3 (1·8 to 2·8)	1·1 (0·9 to 1·3)	0·4 (0·3 to 0·5)	0·2 (0·1 to 0·2)	877
Self-harm	0 (0 to 0)	0 (0 to 0)	0 (0 to 0)	0 (0 to 0)	0 (0 to 0)	1·2 (0·8 to 1·9)	10·3 (6·9 to 14·5)	14·9 (10·2 to 22·1)	15·2 (9·7 to 22·8)	15·5 (10·1 to 22·7)	13·1 (8·7 to 18·6)	9·4 (6·5 to 13)	7·8 (5·5 to 10·9)	5·9 (4·3 to 8·1)	4·2 (3 to 5·8)	3·1 (2·3 to 4·2)	2 (1·6 to 2·7)	1·2 (0·9 to 1·5)	0·7 (0·6 to 0·9)	0·4 (0·3 to 0·5)	0·2 (0·1 to 0·2)	0·1 (0·1 to 0·1)	0 (0 to 0)	105
Self-harm by firearm	0 (0 to 0)	0 (0 to 0)	0 (0 to 0)	0 (0 to 0)	0 (0 to 0)	0·1 (0 to 0·1)	0·2 (0·1 to 0·2)	0·2 (0·2 to 0·4)	0·2 (0·1 to 0·3)	0·3 (0·2 to 0·4)	0·2 (0·2 to 0·3)	0·2 (0·1 to 0·2)	0·2 (0·1 to 0·2)	0·2 (0·1 to 0·2)	0·1 (0·1 to 0·2)	0·1 (0·1 to 0·1)	0·1 (0·1 to 0·1)	0·1 (0 to 0·1)	0 (0 to 0)	0 (0 to 0)	0 (0 to 0)	0 (0 to 0)	0 (0 to 0)	2·1
Self-harm by other specified means	0 (0 to 0)	0 (0 to 0)	0 (0 to 0)	0 (0 to 0)	0 (0 to 0)	1·1 (0·7 to 1·8)	10·1 (6·8 to 14·3)	14·6 (10 to 21·7)	15 (9·6 to 22·5)	15·2 (9·9 to 22·3)	12·9 (8·5 to 18·3)	9·2 (6·3 to 12·7)	7·7 (5·4 to 10·7)	5·8 (4·2 to 7·9)	4·1 (2·9 to 5·6)	3 (2·2 to 4)	2 (1·5 to 2·6)	1·1 (0·8 to 1·5)	0·7 (0·5 to 0·9)	0·4 (0·3 to 0·5)	0·2 (0·1 to 0·2)	0·1 (0 to 0·1)	0 (0 to 0)	103
Interpersonal violence	2·8 (1·6 to 4·5)	0·7 (0·4 to 1·4)	5·1 (3·1 to 8·2)	8·7 (5·3 to 14·5)	10·9 (8·2 to 14·5)	19·8 (14·5 to 26·9)	84·8 (61·7 to 114)	132 (92·6 to 181)	120 (81·6 to 172)	110 (72·7 to 154)	85·2 (59·9 to 122)	61·5 (42·7 to 86)	45·7 (32·5 to 62·6)	30·1 (21·7 to 41·5)	19·1 (13·8 to 25·3)	12·9 (9·9 to 16·8)	8·9 (6·8 to 11·6)	5·4 (4·2 to 6·9)	3·9 (3 to 4·8)	1·9 (1·5 to 2·3)	0·9 (0·7 to 1·1)	0·3 (0·3 to 0·4)	0·2 (0·1 to 0·2)	770
Physical violence by firearm	0·1 (0·1 to 0·3)	0 (0 to 0·1)	0·4 (0·2 to 0·7)	1·7 (0·9 to 3·2)	2·7 (1·7 to 4·1)	6·5 (4·2 to 9·7)	42·2 (28 to 60·5)	62·9 (40·4 to 91·8)	53·7 (33·9 to 80·9)	47·8 (30 to 68·7)	35·1 (22·7 to 52·9)	21·1 (13·5 to 31·7)	17·6 (11·4 to 26·1)	9·9 (6·4 to 15)	6 (3·9 to 8·6)	3·2 (2·1 to 4·6)	1·6 (1·1 to 2·3)	0·8 (0·6 to 1·2)	0·4 (0·3 to 0·6)	0·1 (0·1 to 0·2)	0·1 (0·1 to 0·1)	0 (0 to 0)	0 (0 to 0)	314
Physical violence by sharp object	0·2 (0·1 to 0·3)	0 (0 to 0)	0·3 (0·2 to 0·4)	0·7 (0·4 to 1)	1·1 (0·8 to 1·5)	2·8 (2 to 4)	20·7 (14·2 to 29)	38·5 (25·8 to 54·7)	36·9 (24·1 to 55)	32·9 (20·6 to 48)	27·3 (17·9 to 39·9)	20 (13 to 28·9)	11·4 (7·4 to 16·2)	6·8 (4·7 to 9·4)	4 (2·9 to 5·6)	2·4 (1·8 to 3·1)	1·6 (1·2 to 2·1)	1 (0·8 to 1·2)	0·6 (0·5 to 0·7)	0·3 (0·3 to 0·4)	0·2 (0·1 to 0·2)	0·1 (0·1 to 0·1)	0 (0 to 0·1)	210
Sexual violence	0 (0 to 0)	0 (0 to 0)	0 (0 to 0)	0·1 (0 to 0·1)	0·8 (0·5 to 1·3)	2·3 (1·3 to 3·6)	5·2 (2·8 to 8·4)	7·2 (4·2 to 11)	6·7 (3·8 to 10·9)	6 (3·3 to 10)	5·3 (3 to 8·5)	4·4 (2·5 to 7·2)	3·5 (2·1 to 5·8)	2·4 (1·3 to 4)	1·5 (0·9 to 2·4)	1·1 (0·7 to 1·6)	0·7 (0·4 to 1·1)	0·5 (0·3 to 0·7)	0·3 (0·2 to 0·5)	0·2 (0·1 to 0·3)	0·1 (0·1 to 0·2)	0 (0 to 0)	0 (0 to 0)	48·3
Physical violence by other means	2·5 (1·4 to 4·1)	0·7 (0·3 to 1·3)	4·4 (2·6 to 7·2)	6·2 (3·6 to 10·5)	6·2 (4·3 to 8·5)	8·2 (5·7 to 11·5)	16·8 (11·8 to 23·2)	23·3 (16·2 to 32·9)	22·9 (15·5 to 33·8)	23 (14·9 to 32·9)	17·4 (12 to 24·9)	16 (10·9 to 22·6)	13·2 (9 to 18·1)	11 (7·8 to 15·2)	7·6 (5·5 to 10·3)	6·3 (4·8 to 8·4)	4·9 (3·7 to 6·5)	3·1 (2·4 to 4·1)	2·5 (1·9 to 3·2)	1·1 (0·9 to 1·5)	0·5 (0·4 to 0·7)	0·2 (0·2 to 0·3)	0·1 (0·1 to 0·1)	198
Conflict and terrorism	0 (0 to 0)	0 (0 to 0)	0 (0 to 0)	0 (0 to 0)	0 (0 to 0)	0 (0 to 0)	0 (0 to 0)	0 (0 to 0)	0 (0 to 0)	0 (0 to 0·1)	0 (0 to 0·1)	0 (0 to 0·1)	0 (0 to 0)	0 (0 to 0)	0 (0 to 0)	0 (0 to 0)	0 (0 to 0)	0 (0 to 0)	0 (0 to 0)	0·2 (0·1 to 0·3)	0 (0 to 0)	0 (0 to 0)	0 (0 to 0)	0·4

SR Table 2b: Barbados DALYS by cause and age group for females with 95% uncertainty intervals, 2019

Cause	Early Neonatal	Late Neonatal	Post Neonatal	1 to 4	5 to 9	10 to 14	15 to 19	20 to 24	25 to 29	30 to 34	35 to 39
All causes	730 (511 to 1,020)	292 (205 to 408)	328 (242 to 440)	323 (253 to 407)	532 (407 to 694)	670 (520 to 855)	1,060 (846 to 1,320)	1,240 (1,000 to 1,520)	1,440 (1,150 to 1,790)	1,610 (1,300 to 1,970)	2,080 (1,690 to 2,530)
Communicable, maternal, neonatal, and nutritional diseases	592 (409 to 829)	217 (151 to 304)	150 (107 to 206)	116 (88·9 to 153)	203 (138 to 292)	138 (99·5 to 190)	140 (110 to 181)	175 (145 to 215)	193 (158 to 238)	200 (167 to 245)	226 (187 to 280)
HIV/AIDS and sexually transmitted infections	0·8 (0·5 to 1·2)	0·3 (0·1 to 0·6)	3·6 (3·3 to 3·9)	2·2 (2·1 to 2·4)	2·6 (2·4 to 2·8)	5·5 (5·1 to 5·9)	8·3 (7·4 to 9·2)	44·3 (40·4 to 49)	58·1 (52·4 to 64·8)	68·7 (62·6 to 76·3)	73·7 (67·2 to 81·5)
HIV/AIDS	0 (0 to 0)	0 (0 to 0)	3·4 (3·2 to 3·7)	2·2 (2·1 to 2·4)	2·6 (2·4 to 2·8)	5·3 (4·9 to 5·7)	7 (6·4 to 7·7)	41·6 (38·2 to 45·7)	53·7 (49·3 to 58·7)	63·1 (58·1 to 68·7)	66·8 (61·6 to 72)
HIV/AIDS - Drug-susceptible Tuberculosis	0 (0 to 0)	0 (0 to 0)	0·1 (0 to 0·1)	0 (0 to 0·1)	0·1 (0 to 0·1)	0·1 (0 to 0·1)	0·1 (0·1 to 0·2)	0·7 (0·5 to 1)	0·9 (0·6 to 1·3)	1·1 (0·7 to 1·6)	1·2 (0·8 to 1·8)
HIV/AIDS - Multidrug-resistant Tuberculosis without extensive dru	0 (0 to 0)	0 (0 to 0)	0 (0 to 0)	0 (0 to 0)	0 (0 to 0)	0 (0 to 0)	0 (0 to 0)	0 (0 to 0)	0 (0 to 0)	0 (0 to 0)	0 (0 to 0)
HIV/AIDS - Extensively drug-resistant Tuberculosis	0 (0 to 0)	0 (0 to 0)	0 (0 to 0)	0 (0 to 0)	0 (0 to 0)	0 (0 to 0)	0 (0 to 0)	0 (0 to 0)	0 (0 to 0)	0 (0 to 0)	0 (0 to 0)
HIV/AIDS resulting in other diseases	0 (0 to 0)	0 (0 to 0)	3·4 (3·1 to 3·6)	2·2 (2 to 2·4)	2·6 (2·4 to 2·8)	5·2 (4·8 to 5·7)	6·9 (6·3 to 7·5)	40·9 (37·4 to 44·9)	52·8 (48·4 to 57·9)	62 (57 to 67·7)	65·6 (60·5 to 70·9)
Sexually transmitted infections excluding HIV	0·8 (0·5 to 1·2)	0·3 (0·1 to 0·6)	0·2 (0·1 to 0·3)	0 (0 to 0)	0 (0 to 0)	0·2 (0·1 to 0·4)	1·2 (0·8 to 1·9)	2·7 (1·6 to 4·6)	4·3 (2·3 to 8)	5·6 (3·1 to 9·8)	6·8 (3·9 to 12·3)
Syphilis	0·8 (0·5 to 1·2)	0·3 (0·1 to 0·6)	0·2 (0·1 to 0·3)	0 (0 to 0)	0 (0 to 0)	0·1 (0 to 0·1)	0·1 (0·1 to 0·2)	0·1 (0 to 0·1)	0·1 (0·1 to 0·2)	0·2 (0·1 to 0·3)	0·2 (0·1 to 0·3)
Chlamydial infection	0 (0 to 0)	0 (0 to 0)	0 (0 to 0)	0 (0 to 0)	0 (0 to 0)	0 (0 to 0)	0·1 (0·1 to 0·2)	0·3 (0·2 to 0·5)	0·4 (0·2 to 0·7)	0·5 (0·2 to 0·8)	0·7 (0·4 to 1·2)
Gonococcal infection	0 (0 to 0)	0 (0 to 0)	0 (0 to 0)	0 (0 to 0)	0 (0 to 0)	0 (0 to 0)	0·2 (0·1 to 0·3)	0·3 (0·2 to 0·5)	0·3 (0·2 to 0·5)	0·5 (0·3 to 0·7)	0·7 (0·4 to 0·9)
Trichomoniasis	0 (0 to 0)	0 (0 to 0)	0 (0 to 0)	0 (0 to 0)	0 (0 to 0)	0 (0 to 0)	0·2 (0·1 to 0·5)	0·8 (0·3 to 2)	1·7 (0·6 to 4)	1·9 (0·6 to 4·6)	2 (0·7 to 5)
Genital herpes	0 (0 to 0)	0 (0 to 0)	0 (0 to 0)	0 (0 to 0)	0 (0 to 0)	0·1 (0 to 0·1)	0·3 (0·1 to 0·7)	0·5 (0·2 to 1·3)	0·9 (0·3 to 2·2)	1·1 (0·4 to 2·7)	1·3 (0·4 to 3·2)
Other sexually transmitted infections	0 (0 to 0)	0 (0 to 0)	0 (0 to 0)	0 (0 to 0)	0 (0 to 0)	0 (0 to 0·1)	0·3 (0·2 to 0·5)	0·6 (0·4 to 0·9)	0·9 (0·5 to 1·5)	1·4 (0·8 to 2·1)	1·9 (1·2 to 2·9)
Respiratory infections and tuberculosis	16·4 (9·9 to 25·6)	18 (10·8 to 28·3)	48·3 (30·1 to 72·6)	24 (16·9 to 32·3)	20·9 (14·9 to 29·4)	17·5 (12·3 to 24·8)	19·9 (13·9 to 27·7)	21·6 (15·5 to 29·4)	22·4 (16·2 to 30·3)	23 (16·9 to 31·1)	28·7 (20·8 to 38·5)
Tuberculosis	0 (0 to 0)	0 (0 to 0)	0·1 (0·1 to 0·2)	0·3 (0·1 to 0·8)	0·6 (0·2 to 1·3)	0·4 (0·3 to 0·6)	0·6 (0·4 to 0·8)	1·9 (1 to 3·2)	2·8 (1·5 to 4·8)	2·5 (1·3 to 4·2)	2·4 (1·3 to 4)
Latent tuberculosis infection	0 (0 to 0)	0 (0 to 0)	0 (0 to 0)	0 (0 to 0)	0 (0 to 0)	0 (0 to 0)	0 (0 to 0)	0 (0 to 0)	0 (0 to 0)	0 (0 to 0)	0 (0 to 0)
Drug-susceptible tuberculosis	0 (0 to 0)	0 (0 to 0)	0·1 (0·1 to 0·2)	0·3 (0·1 to 0·8)	0·6 (0·1 to 1·3)	0·4 (0·3 to 0·5)	0·6 (0·4 to 0·8)	1·8 (1 to 3·2)	2·8 (1·5 to 4·7)	2·5 (1·3 to 4·2)	2·4 (1·2 to 4)
Multidrug-resistant tuberculosis without extensive drug resistanc	0 (0 to 0)	0 (0 to 0)	0 (0 to 0)	0 (0 to 0)	0 (0 to 0)	0 (0 to 0)	0 (0 to 0)	0 (0 to 0)	0 (0 to 0·1)	0 (0 to 0·1)	0 (0 to 0·1)
Extensively drug-resistant tuberculosis	0 (0 to 0)	0 (0 to 0)	0 (0 to 0)	0 (0 to 0)	0 (0 to 0)	0 (0 to 0)	0 (0 to 0)	0 (0 to 0)	0 (0 to 0)	0 (0 to 0)	0 (0 to 0)
Lower respiratory infections	16·4 (9·9 to 25·5)	17·9 (10·7 to 28·2)	46 (27·6 to 70·2)	13·6 (8·8 to 20)	8·3 (5·7 to 11·4)	6·3 (4·2 to 8·8)	8·3 (5·5 to 11·9)	9·9 (6·5 to 14·8)	9·6 (6·4 to 14·4)	11·2 (7·3 to 16·5)	16·3 (10·5 to 24·1)
Upper respiratory infections	0 (0 to 0)	0·1 (0·1 to 0·2)	1·9 (1·2 to 3)	7·2 (4 to 12·1)	8·3 (4·6 to 14·1)	8·2 (4·3 to 14·1)	8·4 (4·4 to 14·9)	7·8 (4·2 to 13·3)	8·1 (4·4 to 13·1)	7·6 (4·1 to 12·7)	8·2 (4·2 to 13·7)
Otitis media	0 (0 to 0)	0 (0 to 0)	0·3 (0·2 to 0·6)	2·8 (1·5 to 5)	3·7 (2 to 6·4)	2·7 (1·4 to 4·7)	2·6 (1·3 to 4·5)	2·1 (0·9 to 4)	1·9 (0·9 to 3·6)	1·6 (0·7 to 3·2)	1·8 (0·8 to 3·4)
Enteric infections	0·9 (0·5 to 1·4)	2·6 (1·5 to 4·2)	17·5 (11·2 to 26·7)	17·7 (11·2 to 26·9)	16·2 (9·7 to 26·4)	14·7 (8·6 to 24)	10·6 (6·5 to 16·1)	8·1 (4·8 to 12·7)	10 (6·3 to 15)	11·1 (7·2 to 16·4)	13·8 (9 to 19·8)
Diarrheal diseases	0·9 (0·5 to 1·4)	2·6 (1·5 to 4·2)	17·4 (11·2 to 26·7)	17·6 (11·1 to 26·8)	16·1 (9·6 to 26·3)	14·6 (8·6 to 24)	10·5 (6·4 to 16)	8·1 (4·8 to 12·6)	10 (6·3 to 14·9)	11·1 (7·2 to 16·3)	13·8 (9 to 19·8)
Typhoid and paratyphoid	0 (0 to 0)	0 (0 to 0)	0 (0 to 0)	0 (0 to 0)	0 (0 to 0)	0 (0 to 0)	0 (0 to 0)	0 (0 to 0)	0 (0 to 0)	0 (0 to 0)	0 (0 to 0)
Typhoid fever	0 (0 to 0)	0 (0 to 0)	0 (0 to 0)	0 (0 to 0)	0 (0 to 0)	0 (0 to 0)	0 (0 to 0)	0 (0 to 0)	0 (0 to 0)	0 (0 to 0)	0 (0 to 0)
Paratyphoid fever	0 (0 to 0)	0 (0 to 0)	0 (0 to 0)	0 (0 to 0)	0 (0 to 0)	0 (0 to 0)	0 (0 to 0)	0 (0 to 0)	0 (0 to 0)	0 (0 to 0)	0 (0 to 0)
Invasive Non-typhoidal Salmonella (iNTS)	0 (0 to 0)	0 (0 to 0)	0 (0 to 0)	0 (0 to 0)	0 (0 to 0)	0 (0 to 0)	0 (0 to 0)	0 (0 to 0)	0 (0 to 0)	0 (0 to 0)	0 (0 to 0)
Other intestinal infectious diseases	0 (0 to 0)	0 (0 to 0)	0·1 (0 to 0·2)	0·1 (0 to 0·2)	0 (0 to 0·1)	0 (0 to 0·1)	0 (0 to 0·1)	0 (0 to 0·1)	0 (0 to 0)	0 (0 to 0·1)	0 (0 to 0·1)
Neglected tropical diseases and malaria	0 (0 to 0·1)	0·2 (0·1 to 0·5)	3·4 (1·1 to 7·8)	3·6 (1·8 to 6·7)	13·9 (7·3 to 23·6)	6·6 (3·3 to 11·5)	5·4 (2·8 to 9·6)	5·7 (3 to 9·7)	6·5 (3·6 to 10·8)	6·2 (3·2 to 10·8)	7·9 (4·3 to 13)
Malaria	0 (0 to 0)	0 (0 to 0)	0 (0 to 0)	0 (0 to 0)	0 (0 to 0)	0 (0 to 0)	0 (0 to 0)	0 (0 to 0)	0 (0 to 0)	0 (0 to 0)	0 (0 to 0)
Chagas disease	0 (0 to 0)	0 (0 to 0)	0 (0 to 0)	0 (0 to 0)	0 (0 to 0)	0 (0 to 0)	0 (0 to 0)	0 (0 to 0)	0 (0 to 0)	0 (0 to 0)	0 (0 to 0)
Leishmaniasis	0 (0 to 0)	0 (0 to 0)	0 (0 to 0)	0 (0 to 0)	0 (0 to 0)	0 (0 to 0)	0 (0 to 0)	0 (0 to 0)	0 (0 to 0)	0 (0 to 0)	0 (0 to 0)
Visceral leishmaniasis	0 (0 to 0)	0 (0 to 0)	0 (0 to 0)	0 (0 to 0)	0 (0 to 0)	0 (0 to 0)	0 (0 to 0)	0 (0 to 0)	0 (0 to 0)	0 (0 to 0)	0 (0 to 0)
Cutaneous and mucocutaneous leishmaniasis	0 (0 to 0)	0 (0 to 0)	0 (0 to 0)	0 (0 to 0)	0 (0 to 0)	0 (0 to 0)	0 (0 to 0)	0 (0 to 0)	0 (0 to 0)	0 (0 to 0)	0 (0 to 0)
African trypanosomiasis	0 (0 to 0)	0 (0 to 0)	0 (0 to 0)	0 (0 to 0)	0 (0 to 0)	0 (0 to 0)	0 (0 to 0)	0 (0 to 0)	0 (0 to 0)	0 (0 to 0)	0 (0 to 0)
Schistosomiasis	0 (0 to 0)	0 (0 to 0)	0 (0 to 0)	0 (0 to 0)	0 (0 to 0)	0 (0 to 0)	0 (0 to 0)	0 (0 to 0)	0 (0 to 0)	0 (0 to 0)	0 (0 to 0)
Cysticercosis	0 (0 to 0)	0 (0 to 0)	0 (0 to 0)	0 (0 to 0)	0 (0 to 0)	0 (0 to 0·1)	0·4 (0 to 1·3)	1·3 (0·2 to 3·6)	2·3 (0·6 to 5·3)	2·5 (0·6 to 5·9)	2·9 (0·8 to 6·9)
Cystic echinococcosis	0 (0 to 0)	0 (0 to 0)	0 (0 to 0)	0 (0 to 0)	0 (0 to 0)	0 (0 to 0)	0 (0 to 0)	0 (0 to 0)	0 (0 to 0)	0 (0 to 0)	0 (0 to 0)
Lymphatic filariasis	0 (0 to 0)	0 (0 to 0)	0 (0 to 0)	0 (0 to 0)	0 (0 to 0)	0 (0 to 0)	0 (0 to 0)	0 (0 to 0)	0 (0 to 0)	0 (0 to 0)	0 (0 to 0)
Onchocerciasis	0 (0 to 0)	0 (0 to 0)	0 (0 to 0)	0 (0 to 0)	0 (0 to 0)	0 (0 to 0)	0 (0 to 0)	0 (0 to 0)	0 (0 to 0)	0 (0 to 0)	0 (0 to 0)
Trachoma	0 (0 to 0)	0 (0 to 0)	0 (0 to 0)	0 (0 to 0)	0 (0 to 0)	0 (0 to 0)	0 (0 to 0)	0 (0 to 0)	0 (0 to 0)	0 (0 to 0)	0 (0 to 0)
Dengue	0 (0 to 0)	0·1 (0 to 0·4)	2·8 (0·6 to 7·2)	2·1 (0·7 to 4·7)	4·6 (1·3 to 11·3)	2·9 (1 to 6·2)	3·2 (1·2 to 6·8)	2·5 (0·9 to 5·1)	2·5 (1 to 4·9)	2·1 (0·8 to 4·5)	2·7 (1 to 5·6)
Yellow fever	0 (0 to 0)	0 (0 to 0)	0 (0 to 0)	0 (0 to 0)	0 (0 to 0)	0 (0 to 0)	0 (0 to 0)	0 (0 to 0)	0 (0 to 0)	0 (0 to 0)	0 (0 to 0)
Rabies	0 (0 to 0)	0 (0 to 0)	0 (0 to 0)	0 (0 to 0)	0 (0 to 0·1)	0 (0 to 0)	0 (0 to 0)	0 (0 to 0)	0 (0 to 0)	0 (0 to 0)	0 (0 to 0)
Intestinal nematode infections	0 (0 to 0)	0 (0 to 0)	0 (0 to 0)	0·1 (0 to 0·2)	0·3 (0·1 to 0·5)	0·1 (0 to 0·3)	0·8 (0·2 to 1·9)	1·2 (0·3 to 2·6)	0·9 (0·2 to 2·1)	0·7 (0·1 to 1·8)	1 (0·3 to 2)
Ascariasis	0 (0 to 0)	0 (0 to 0)	0 (0 to 0)	0 (0 to 0·1)	0 (0 to 0·1)	0 (0 to 0·1)	0 (0 to 0)	0 (0 to 0)	0 (0 to 0)	0 (0 to 0)	0 (0 to 0)
Trichuriasis	0 (0 to 0)	0 (0 to 0)	0 (0 to 0)	0 (0 to 0)	0 (0 to 0)	0 (0 to 0)	0 (0 to 0)	0 (0 to 0)	0 (0 to 0)	0 (0 to 0)	0 (0 to 0)
Hookworm disease	0 (0 to 0)	0 (0 to 0)	0 (0 to 0)	0 (0 to 0·1)	0·2 (0·1 to 0·5)	0·1 (0 to 0·3)	0·8 (0·2 to 1·9)	1·2 (0·3 to 2·6)	0·9 (0·2 to 2·1)	0·7 (0·1 to 1·8)	1 (0·3 to 2)
Food-borne trematodiases	0 (0 to 0)	0 (0 to 0)	0 (0 to 0)	0 (0 to 0)	0 (0 to 0)	0 (0 to 0)	0 (0 to 0)	0 (0 to 0)	0 (0 to 0)	0 (0 to 0)	0 (0 to 0)
Leprosy	0 (0 to 0)	0 (0 to 0)	0 (0 to 0)	0 (0 to 0)	0 (0 to 0)	0 (0 to 0)	0 (0 to 0)	0 (0 to 0)	0 (0 to 0)	0 (0 to 0)	0 (0 to 0)
Ebola	0 (0 to 0)	0 (0 to 0)	0 (0 to 0)	0 (0 to 0)	0 (0 to 0)	0 (0 to 0)	0 (0 to 0)	0 (0 to 0)	0 (0 to 0)	0 (0 to 0)	0 (0 to 0)
Zika virus	0 (0 to 0)	0 (0 to 0)	0·1 (0 to 0·4)	0 (0 to 0·2)	0·1 (0 to 0·3)	0·1 (0 to 0·3)	0·1 (0 to 0·4)	0·1 (0 to 0·4)	0·1 (0 to 0·4)	0·1 (0 to 0·4)	0·1 (0 to 0·4)
Guinea worm disease	0 (0 to 0)	0 (0 to 0)	0 (0 to 0)	0 (0 to 0)	0 (0 to 0)	0 (0 to 0)	0 (0 to 0)	0 (0 to 0)	0 (0 to 0)	0 (0 to 0)	0 (0 to 0)
Other neglected tropical diseases	0 (0 to 0·1)	0·1 (0 to 0·1)	0·4 (0·1 to 0·8)	1·4 (0·4 to 3·5)	8·9 (4·6 to 15·5)	3·4 (1·1 to 7·2)	0·9 (0·2 to 2)	0·7 (0·1 to 1·7)	0·8 (0·1 to 2)	0·8 (0·2 to 2·1)	1·2 (0·3 to 2·7)
Other infectious diseases	1·9 (1·1 to 2·8)	3·6 (2·3 to 5·4)	14·3 (8·5 to 23·1)	10·7 (6·7 to 18·2)	14·4 (10·2 to 20·1)	9·4 (6·9 to 12·7)	9·6 (7 to 12·6)	8·3 (6·2 to 11·2)	7·5 (5·5 to 10·1)	7·5 (5·6 to 10)	9·4 (7 to 12·1)
Meningitis	1 (0·6 to 1·5)	2·4 (1·4 to 3·8)	4·9 (2·7 to 8)	2·1 (1·4 to 3·1)	2·2 (1·6 to 3)	1·7 (1·3 to 2·4)	1·9 (1·4 to 2·6)	1·6 (1·1 to 2·4)	1·3 (0·9 to 1·8)	1·5 (1·1 to 2)	1·8 (1·3 to 2·4)
Encephalitis	0·6 (0·3 to 1)	0·7 (0·3 to 1·1)	7 (3·3 to 14·3)	6·1 (2·9 to 13·2)	4·7 (2·7 to 8·9)	3·8 (2·4 to 5·8)	4·6 (2·8 to 7)	3·6 (2·3 to 5·7)	3·1 (1·8 to 4·9)	2·7 (1·8 to 4·3)	3 (1·9 to 4·8)
Diphtheria	0 (0 to 0)	0 (0 to 0)	0 (0 to 0)	0 (0 to 0)	0 (0 to 0)	0 (0 to 0)	0 (0 to 0)	0 (0 to 0)	0 (0 to 0)	0 (0 to 0)	0 (0 to 0)
Tetanus	0·1 (0 to 0·2)	0·1 (0 to 0·2)	0·1 (0 to 0·4)	0 (0 to 0·1)	0·1 (0 to 0·3)	0 (0 to 0·1)	0·1 (0 to 0·2)	0·1 (0 to 0·3)	0·1 (0 to 0·2)	0·1 (0 to 0·3)	0·3 (0·1 to 0·8)
Measles	0 (0 to 0)	0 (0 to 0)	0 (0 to 0)	0 (0 to 0)	0 (0 to 0)	0 (0 to 0)	0 (0 to 0)	0 (0 to 0)	0 (0 to 0)	0 (0 to 0)	0 (0 to 0)
Varicella and herpes zoster	0 (0 to 0)	0·1 (0 to 0·2)	0·5 (0·2 to 1)	0·2 (0·1 to 0·5)	0·4 (0·2 to 0·8)	0·4 (0·2 to 0·6)	0·3 (0·2 to 0·6)	0·5 (0·3 to 0·8)	0·5 (0·3 to 0·9)	0·5 (0·3 to 0·9)	0·5 (0·3 to 0·9)
Acute hepatitis	0 (0 to 0)	0 (0 to 0)	0·1 (0 to 0·2)	0·2 (0·1 to 0·3)	0·6 (0·3 to 1)	0·8 (0·5 to 1·2)	0·9 (0·6 to 1·4)	0·9 (0·6 to 1·3)	0·8 (0·5 to 1·2)	0·7 (0·4 to 1)	0·6 (0·4 to 0·9)
Acute hepatitis A	0 (0 to 0)	0 (0 to 0)	0 (0 to 0·1)	0·1 (0·1 to 0·3)	0·5 (0·3 to 0·9)	0·7 (0·4 to 1·1)	0·8 (0·5 to 1·2)	0·6 (0·4 to 1)	0·6 (0·3 to 0·9)	0·5 (0·3 to 0·8)	0·4 (0·2 to 0·7)
Acute hepatitis B	0 (0 to 0)	0 (0 to 0)	0 (0 to 0·1)	0 (0 to 0)	0 (0 to 0·1)	0 (0 to 0·1)	0 (0 to 0·1)	0·1 (0·1 to 0·3)	0·2 (0·1 to 0·3)	0·1 (0 to 0·2)	0·2 (0·1 to 0·3)
Acute hepatitis C	0 (0 to 0)	0 (0 to 0)	0 (0 to 0)	0 (0 to 0)	0 (0 to 0)	0 (0 to 0)	0 (0 to 0)	0 (0 to 0)	0 (0 to 0)	0 (0 to 0)	0 (0 to 0)
Acute hepatitis E	0 (0 to 0)	0 (0 to 0)	0 (0 to 0)	0 (0 to 0)	0 (0 to 0·1)	0·1 (0 to 0·2)	0·1 (0 to 0·2)	0·1 (0 to 0·2)	0·1 (0 to 0·1)	0 (0 to 0·1)	0 (0 to 0·1)
Other unspecified infectious diseases	0·2 (0·1 to 0·4)	0·3 (0·2 to 0·5)	1·2 (0·6 to 2·1)	1·5 (0·7 to 2·9)	6·2 (3·3 to 10·9)	2·7 (1·1 to 5)	1·8 (1 to 3)	1·6 (0·8 to 2·6)	1·8 (1 to 3·2)	2 (1·1 to 3·4)	3·1 (1·9 to 4·9)
Maternal and neonatal disorders	572 (394 to 800)	192 (134 to 267)	51·4 (34·3 to 73·8)	40·2 (29·4 to 52·2)	39·7 (28·7 to 53·4)	46·3 (33·6 to 61·7)	64·5 (50·4 to 81·6)	68·7 (54 to 85·3)	67·1 (52·1 to 84·7)	62·8 (49 to 79·8)	62 (48·9 to 77·9)
Maternal disorders	0 (0 to 0)	0 (0 to 0)	0 (0 to 0)	0 (0 to 0)	0 (0 to 0)	0·5 (0·4 to 0·6)	16·1 (12·4 to 20·6)	23·2 (17·4 to 30·7)	19·8 (14·8 to 26·3)	19·1 (14·6 to 25)	17·3 (13·1 to 21·8)
Maternal hemorrhage	0 (0 to 0)	0 (0 to 0)	0 (0 to 0)	0 (0 to 0)	0 (0 to 0)	0 (0 to 0·1)	1·9 (1·2 to 2·9)	3·3 (1·9 to 5)	3·3 (2 to 5)	3·2 (2 to 4·8)	2·9 (1·8 to 4·4)
Maternal sepsis and other maternal infections	0 (0 to 0)	0 (0 to 0)	0 (0 to 0)	0 (0 to 0)	0 (0 to 0)	0 (0 to 0·1)	1·8 (1·1 to 2·7)	2·7 (1·6 to 4)	2·4 (1·3 to 3·6)	2·1 (1·2 to 3·3)	1·8 (1·1 to 2·8)
Maternal hypertensive disorders	0 (0 to 0)	0 (0 to 0)	0 (0 to 0)	0 (0 to 0)	0 (0 to 0)	0·1 (0·1 to 0·1)	2·8 (2 to 3·8)	3 (2 to 4·1)	2·7 (1·8 to 3·8)	2·3 (1·6 to 3·3)	1·9 (1·3 to 2·6)
Maternal obstructed labor and uterine rupture	0 (0 to 0)	0 (0 to 0)	0 (0 to 0)	0 (0 to 0)	0 (0 to 0)	0 (0 to 0)	0·4 (0·2 to 0·7)	0·6 (0·3 to 1)	0·5 (0·3 to 0·9)	0·6 (0·3 to 1)	0·6 (0·3 to 1)
Maternal abortion and miscarriage	0 (0 to 0)	0 (0 to 0)	0 (0 to 0)	0 (0 to 0)	0 (0 to 0)	0 (0 to 0·1)	1·1 (0·6 to 1·8)	1·6 (0·9 to 2·6)	1·2 (0·6 to 2)	1 (0·5 to 1·7)	0·9 (0·5 to 1·7)
Ectopic pregnancy	0 (0 to 0)	0 (0 to 0)	0 (0 to 0)	0 (0 to 0)	0 (0 to 0)	0 (0 to 0)	1·3 (0·8 to 2)	2·6 (1·6 to 4·2)	2·3 (1·4 to 3·6)	2·4 (1·4 to 3·6)	2 (1·2 to 3)
Indirect maternal deaths	--	--	--	--	--	0 (0 to 0·1)	1·3 (0·8 to 1·9)	1·8 (1 to 2·8)	1·3 (0·7 to 2)	1·1 (0·6 to 1·8)	0·9 (0·5 to 1·4)
Late maternal deaths	--	--	--	--	--	0·1 (0 to 0·1)	1·6 (1 to 2·5)	2·2 (1·3 to 3·4)	1·7 (1 to 2·8)	1·6 (0·9 to 2·7)	1·5 (0·9 to 2·5)
Maternal deaths aggravated by HIV/AIDS	--	--	--	--	--	0 (0 to 0)	0 (0 to 0)	0·1 (0 to 0·1)	0·1 (0 to 0·1)	0·1 (0 to 0·1)	0·1 (0 to 0·1)
Neonatal disorders	572 (394 to 800)	192 (134 to 267)	51·4 (34·3 to 73·8)	40·2 (29·4 to 52·2)	39·7 (28·7 to 53·4)	45·8 (33·1 to 61·2)	48·4 (34·8 to 64·7)	45·5 (32·9 to 61·1)	47·2 (33·8 to 62·8)	43·7 (30·9 to 59·1)	44·7 (32·2 to 60·1)
Neonatal preterm birth	241 (164 to 355)	64·2 (40·7 to 95·6)	19·6 (12·4 to 29·7)	9·2 (6·3 to 12·8)	12·4 (8·2 to 17·2)	14·3 (9·6 to 19·8)	14·7 (10·1 to 20·5)	13·7 (9·2 to 19·1)	14·2 (9·7 to 19·7)	13 (8·7 to 18·1)	13·2 (9 to 18·7)
Neonatal encephalopathy due to birth asphyxia and trauma	172 (105 to 255)	29·1 (15·8 to 48)	8·8 (5·2 to 14)	3·4 (2·2 to 5)	3·2 (1·6 to 5·5)	3·7 (1·9 to 6·2)	4 (2 to 6·7)	3·8 (2 to 6·3)	4 (2 to 6·5)	3·6 (1·7 to 6)	3·7 (1·8 to 6)
Neonatal sepsis and other neonatal infections	92·4 (55·4 to 143)	72·2 (45·1 to 109)	15·6 (10·3 to 22·5)	15·3 (9·8 to 21·8)	22 (14·3 to 31·9)	25·4 (16·1 to 36·7)	27·1 (16·7 to 40·1)	25·6 (16·4 to 37·3)	26·6 (17·4 to 39·5)	24·8 (16·1 to 35·9)	25·4 (16·5 to 36·8)
Hemolytic disease and other neonatal jaundice	0·4 (0·3 to 0·7)	0·4 (0·3 to 0·6)	0·3 (0·2 to 0·5)	1·4 (0·7 to 2·3)	2 (0·9 to 3·4)	2·3 (1·1 to 3·9)	2·5 (1·2 to 4·4)	2·4 (1·1 to 4·3)	2·4 (1·1 to 4·1)	2·3 (1 to 3·9)	2·4 (1·2 to 3·9)
Other neonatal disorders	66·6 (37·6 to 107)	25·7 (15·9 to 38·1)	7·2 (5·1 to 10)	10·8 (7·9 to 14·1)	0 (0 to 0)	0 (0 to 0)	0 (0 to 0)	0 (0 to 0)	0 (0 to 0)	0 (0 to 0)	0 (0 to 0)
Nutritional deficiencies	0 (0 to 0·1)	0·6 (0·3 to 1·2)	11·1 (7 to 16·7)	17·6 (7·2 to 39·3)	95·4 (49·7 to 163)	38·1 (14·2 to 75·8)	22·1 (7·8 to 47)	18·3 (6·5 to 41·4)	21·1 (6·8 to 47·8)	20·7 (5·8 to 47·4)	30·9 (11·4 to 62·2)
Protein-energy malnutrition	0 (0 to 0·1)	0·1 (0·1 to 0·2)	7·3 (4·7 to 11·1)	3·4 (1·9 to 5·3)	2·2 (1·4 to 3·2)	1·6 (1 to 2·5)	1·6 (0·8 to 2·7)	1·8 (1 to 2·8)	1·8 (1·1 to 2·9)	1·8 (1·1 to 2·8)	2·4 (1·5 to 3·5)
Iodine deficiency	0 (0 to 0)	0 (0 to 0)	0 (0 to 0)	0 (0 to 0·1)	0·3 (0·1 to 0·6)	0·7 (0·2 to 1·6)	1 (0·3 to 2)	1·1 (0·4 to 2·1)	1·3 (0·5 to 2·4)	1·2 (0·5 to 2·4)	1·4 (0·5 to 2·6)
Vitamin A deficiency	0 (0 to 0)	0 (0 to 0)	0·1 (0 to 0·2)	0·6 (0·3 to 1·2)	1·1 (0·5 to 2·1)	0·7 (0·3 to 1·5)	0·3 (0·1 to 0·7)	0·2 (0·1 to 0·4)	0·2 (0·1 to 0·4)	0·2 (0·1 to 0·3)	0·1 (0·1 to 0·2)
Dietary iron deficiency	0 (0 to 0)	0·5 (0·2 to 1·1)	2·8 (0·6 to 6·7)	12·9 (3 to 34·2)	91·2 (45·4 to 158)	34·5 (11·2 to 70·8)	17·9 (4·4 to 42·7)	14·2 (2·6 to 36·5)	16·6 (3 to 43·1)	16·6 (2·1 to 43·2)	25·9 (6·4 to 56·7)
Other nutritional deficiencies	0 (0 to 0)	0 (0 to 0)	1 (0·4 to 1·9)	0·6 (0·3 to 1·3)	0·6 (0·3 to 1)	0·5 (0·2 to 0·8)	1·2 (0·7 to 2)	1 (0·6 to 1·6)	1·2 (0·7 to 1·9)	0·9 (0·5 to 1·4)	1·2 (0·7 to 1·7)
Non-communicable diseases	131 (83·1 to 200)	72 (47·4 to 107)	163 (115 to 227)	180 (137 to 232)	281 (218 to 356)	470 (352 to 618)	781 (602 to 1,010)	919 (714 to 1,170)	1,120 (866 to 1,430)	1,290 (1,010 to 1,610)	1,720 (1,390 to 2,120)
Neoplasms	1·3 (0·8 to 2)	1·4 (0·9 to 2·1)	6·1 (3·9 to 8·8)	20·4 (13·9 to 28·6)	36·3 (28·9 to 45)	36·3 (29·3 to 44·3)	56·4 (44·9 to 70·7)	65·2 (51·3 to 84·6)	83·7 (64·5 to 113)	144 (111 to 188)	291 (229 to 369)
Lip and oral cavity cancer	0 (0 to 0)	0 (0 to 0)	0 (0 to 0)	0 (0 to 0)	0·1 (0·1 to 0·1)	0·1 (0 to 0·2)	0·2 (0·2 to 0·3)	0·2 (0·2 to 0·3)	0·4 (0·2 to 0·6)	0·5 (0·3 to 0·7)	0·8 (0·5 to 1·2)
Nasopharynx cancer	0 (0 to 0)	0 (0 to 0)	0 (0 to 0)	0 (0 to 0)	0·1 (0·1 to 0·2)	0·1 (0 to 0·1)	0·3 (0·2 to 0·5)	0·3 (0·2 to 0·5)	0·3 (0·2 to 0·5)	0·4 (0·3 to 0·5)	0·5 (0·3 to 0·7)
Other pharynx cancer	0 (0 to 0)	0 (0 to 0)	0 (0 to 0)	0 (0 to 0)	0 (0 to 0)	0 (0 to 0)	0 (0 to 0)	0·1 (0·1 to 0·2)	0·1 (0·1 to 0·2)	0·2 (0·1 to 0·2)	0·3 (0·2 to 0·4)
Esophageal cancer	0 (0 to 0)	0 (0 to 0)	0 (0 to 0)	0 (0 to 0)	0 (0 to 0)	0 (0 to 0)	0 (0 to 0)	0·2 (0·1 to 0·3)	0·3 (0·2 to 0·4)	0·5 (0·3 to 0·7)	1·2 (0·8 to 1·8)
Stomach cancer	0 (0 to 0)	0 (0 to 0)	0 (0 to 0)	0 (0 to 0)	0 (0 to 0)	0 (0 to 0)	0·7 (0·5 to 0·8)	1·4 (1 to 1·9)	2·5 (1·7 to 3·8)	4·5 (3·1 to 6·4)	8·8 (6·1 to 12·4)
Colon and rectum cancer	0 (0 to 0)	0 (0 to 0)	0 (0 to 0)	0 (0 to 0)	0·3 (0·2 to 0·4)	0·4 (0·3 to 0·6)	2 (1·5 to 2·6)	2·8 (1·9 to 4)	4·1 (2·3 to 6·3)	8·5 (5·5 to 12·2)	17·3 (11·9 to 24·3)
Liver cancer	0·1 (0 to 0·1)	0·1 (0 to 0·1)	0·5 (0·3 to 0·7)	1·5 (0·8 to 2·7)	1·5 (0·6 to 3·1)	1·2 (0·6 to 2·3)	0·3 (0·2 to 0·4)	0·6 (0·4 to 0·9)	0·6 (0·4 to 1)	0·9 (0·6 to 1·4)	2 (1·3 to 3)
Liver cancer due to hepatitis B	0 (0 to 0)	0 (0 to 0)	0 (0 to 0)	0 (0 to 0)	0 (0 to 0)	0·1 (0 to 0·1)	0·1 (0 to 0·1)	0·2 (0·1 to 0·4)	0·3 (0·1 to 0·5)	0·4 (0·2 to 0·7)	0·9 (0·4 to 1·4)
Liver cancer due to hepatitis C	0 (0 to 0)	0 (0 to 0)	0 (0 to 0)	0 (0 to 0)	0 (0 to 0)	0 (0 to 0)	0 (0 to 0)	0 (0 to 0)	0 (0 to 0·1)	0·1 (0 to 0·1)	0·2 (0·1 to 0·4)
Liver cancer due to alcohol use	0 (0 to 0)	0 (0 to 0)	0 (0 to 0)	0 (0 to 0)	0 (0 to 0)	0 (0 to 0)	0 (0 to 0)	0 (0 to 0·1)	0·1 (0 to 0·1)	0·1 (0·1 to 0·3)	0·4 (0·2 to 0·8)
Liver cancer due to NASH	0 (0 to 0)	0 (0 to 0)	0 (0 to 0)	0 (0 to 0)	0 (0 to 0)	0 (0 to 0)	0·1 (0 to 0·1)	0·1 (0·1 to 0·2)	0·1 (0 to 0·2)	0·1 (0·1 to 0·2)	0·2 (0·1 to 0·4)
Liver cancer due to other causes	0·1 (0 to 0·1)	0·1 (0 to 0·1)	0·5 (0·3 to 0·7)	1·5 (0·8 to 2·7)	1·5 (0·6 to 3·1)	1·1 (0·6 to 2·2)	0·1 (0·1 to 0·2)	0·2 (0·1 to 0·3)	0·1 (0·1 to 0·3)	0·2 (0·1 to 0·3)	0·3 (0·2 to 0·6)
Gallbladder and biliary tract cancer	0 (0 to 0)	0 (0 to 0)	0 (0 to 0)	0 (0 to 0)	0 (0 to 0)	0 (0 to 0)	0 (0 to 0)	0·1 (0·1 to 0·1)	0·2 (0·1 to 0·3)	0·3 (0·2 to 0·6)	1·1 (0·7 to 2)
Pancreatic cancer	0 (0 to 0)	0 (0 to 0)	0 (0 to 0)	0 (0 to 0)	0 (0 to 0)	0 (0 to 0)	0·3 (0·2 to 0·4)	0·4 (0·3 to 0·6)	0·9 (0·6 to 1·3)	1·4 (0·9 to 2·1)	3·8 (2·5 to 5·8)
Larynx cancer	0 (0 to 0)	0 (0 to 0)	0 (0 to 0)	0 (0 to 0)	0 (0 to 0)	0 (0 to 0)	0 (0 to 0)	0·1 (0·1 to 0·1)	0·1 (0·1 to 0·1)	0·1 (0·1 to 0·1)	0·2 (0·1 to 0·3)
Tracheal, bronchus, and lung cancer	0 (0 to 0)	0 (0 to 0)	0 (0 to 0)	0 (0 to 0)	0 (0 to 0)	0·1 (0 to 0·1)	0·5 (0·4 to 0·7)	0·5 (0·4 to 0·8)	0·8 (0·5 to 1·2)	1·9 (1·2 to 2·8)	5·1 (3·5 to 7·3)
Malignant skin melanoma	0 (0 to 0)	0 (0 to 0)	0 (0 to 0·1)	0·1 (0 to 0·3)	0·1 (0·1 to 0·1)	0 (0 to 0·1)	0·2 (0·1 to 0·2)	0·4 (0·2 to 0·6)	0·4 (0·2 to 0·6)	0·5 (0·3 to 0·8)	1·4 (0·9 to 2·2)
Non-melanoma skin cancer	0 (0 to 0)	0 (0 to 0)	0 (0 to 0)	0 (0 to 0)	0 (0 to 0)	0 (0 to 0)	0 (0 to 0)	0·2 (0·1 to 0·2)	0·2 (0·2 to 0·4)	0·3 (0·2 to 0·4)	0·4 (0·3 to 0·6)
Non-melanoma skin cancer (squamous-cell carcinoma)	0 (0 to 0)	0 (0 to 0)	0 (0 to 0)	0 (0 to 0)	0 (0 to 0)	0 (0 to 0)	0 (0 to 0)	0·2 (0·1 to 0·2)	0·2 (0·2 to 0·4)	0·3 (0·2 to 0·4)	0·4 (0·3 to 0·6)
Non-melanoma skin cancer (basal-cell carcinoma)	0 (0 to 0)	0 (0 to 0)	0 (0 to 0)	0 (0 to 0)	0 (0 to 0)	0 (0 to 0)	0 (0 to 0)	0 (0 to 0)	0 (0 to 0)	0 (0 to 0)	0 (0 to 0)
Breast cancer	0 (0 to 0)	0 (0 to 0)	0 (0 to 0)	0 (0 to 0)	0 (0 to 0)	0 (0 to 0)	2 (1·5 to 2·5)	3·7 (2·6 to 5·1)	12·6 (8·6 to 18·7)	42·8 (30·5 to 60)	104 (76·6 to 135)
Cervical cancer	0 (0 to 0)	0 (0 to 0)	0 (0 to 0)	0 (0 to 0)	0 (0 to 0)	0 (0 to 0)	1·6 (1·1 to 2·3)	5·6 (3·6 to 8·5)	13·9 (8·8 to 21·4)	28·6 (19·3 to 40·5)	56·4 (39·7 to 78·1)
Uterine cancer	0 (0 to 0)	0 (0 to 0)	0 (0 to 0)	0 (0 to 0)	0 (0 to 0)	0 (0 to 0)	0 (0 to 0)	1·3 (0·9 to 1·7)	2·4 (1·7 to 3·5)	4·9 (3·4 to 7)	10·5 (6·9 to 15·8)
Ovarian cancer	0 (0 to 0)	0 (0 to 0)	0 (0 to 0)	0 (0 to 0)	0·7 (0·3 to 1·1)	0·7 (0·3 to 1·3)	3·3 (1·4 to 4·9)	4·5 (1·9 to 6·9)	4·4 (2·3 to 6·8)	7 (3·5 to 10·7)	13·8 (6·9 to 19·9)
Prostate cancer	0 (0 to 0)	0 (0 to 0)	0 (0 to 0)	0 (0 to 0)	0 (0 to 0)	0 (0 to 0)	0 (0 to 0)	0 (0 to 0)	0 (0 to 0)	0 (0 to 0)	0 (0 to 0)
Testicular cancer	--	--	--	--	--	--	--	--	--	--	--
Kidney cancer	0 (0 to 0·1)	0 (0 to 0·1)	0·3 (0·1 to 0·5)	1 (0·6 to 1·6)	1·5 (1 to 2·2)	1 (0·6 to 1·4)	1·3 (0·9 to 1·9)	1·7 (1·1 to 2·4)	1·6 (1 to 2·4)	2 (1·3 to 2·9)	3·5 (2·2 to 5·1)
Bladder cancer	0 (0 to 0)	0 (0 to 0)	0 (0 to 0)	0 (0 to 0)	0 (0 to 0)	0 (0 to 0)	0·1 (0·1 to 0·1)	0·1 (0·1 to 0·1)	0·1 (0·1 to 0·2)	0·4 (0·3 to 0·6)	0·7 (0·5 to 1)
Brain and central nervous system cancer	0·3 (0·1 to 0·5)	0·3 (0·1 to 0·6)	0·9 (0·3 to 1·8)	2·6 (0·7 to 4·9)	6·3 (2·1 to 10·5)	5·2 (1·6 to 8·7)	4·3 (1·4 to 7·8)	4·2 (1·2 to 8·1)	4 (1·1 to 7·3)	4·2 (1·2 to 7·4)	6·9 (2·1 to 12·1)
Thyroid cancer	0 (0 to 0)	0 (0 to 0)	0 (0 to 0)	0 (0 to 0)	0·1 (0·1 to 0·1)	0·1 (0·1 to 0·1)	0·3 (0·2 to 0·4)	0·3 (0·2 to 0·4)	0·4 (0·3 to 0·6)	0·5 (0·4 to 0·8)	1·3 (0·8 to 1·8)
Mesothelioma	0 (0 to 0)	0 (0 to 0)	0 (0 to 0)	0 (0 to 0)	0 (0 to 0)	0 (0 to 0)	0 (0 to 0)	0·3 (0·2 to 0·5)	0·2 (0·1 to 0·3)	0·2 (0·1 to 0·3)	0·6 (0·4 to 1)
Hodgkin lymphoma	0 (0 to 0)	0 (0 to 0)	0 (0 to 0)	0·1 (0 to 0·1)	0·1 (0·1 to 0·1)	0·1 (0·1 to 0·2)	1·2 (0·8 to 1·9)	1·8 (1·1 to 2·9)	1·5 (0·9 to 2·5)	1·2 (0·7 to 1·9)	1·3 (0·8 to 2)
Non-Hodgkin lymphoma	0 (0 to 0)	0 (0 to 0)	0 (0 to 0)	2 (1·3 to 2·9)	4·1 (2·7 to 5·8)	5·3 (3·8 to 7·3)	8·8 (6·2 to 12·3)	11·1 (7·4 to 16·3)	10 (6·8 to 14·7)	10·7 (7·2 to 15·3)	15 (10·5 to 21·3)
Multiple myeloma	0 (0 to 0)	0 (0 to 0)	0 (0 to 0)	0 (0 to 0)	0 (0 to 0)	0 (0 to 0)	0 (0 to 0)	0·3 (0·2 to 0·4)	0·5 (0·4 to 0·8)	1·2 (0·8 to 1·7)	3·7 (2·3 to 5·6)
Leukemia	0·4 (0·2 to 0·5)	0·4 (0·2 to 0·5)	1·8 (1 to 2·9)	5·3 (3·2 to 8·1)	12 (8·3 to 16·5)	12 (8·2 to 16·8)	14·4 (10·3 to 19·9)	12 (8·3 to 16·9)	10·3 (6·7 to 14·9)	9·2 (6·4 to 12·6)	13·8 (9·5 to 19·2)
Acute lymphoid leukemia	0·1 (0 to 0·2)	0·1 (0 to 0·2)	0·4 (0·2 to 0·7)	1·8 (0·9 to 3)	4·3 (2·2 to 7·2)	2·9 (1·4 to 5·2)	3·1 (1·6 to 5·4)	1·4 (0·7 to 2·6)	0·8 (0·3 to 1·4)	0·6 (0·3 to 1·1)	0·6 (0·3 to 1)
Chronic lymphoid leukemia	0 (0 to 0)	0 (0 to 0)	0 (0 to 0)	0 (0 to 0)	0 (0 to 0)	0 (0 to 0)	0 (0 to 0)	0·1 (0·1 to 0·2)	0·1 (0 to 0·1)	0·1 (0 to 0·1)	0·2 (0·1 to 0·3)
Acute myeloid leukemia	0·1 (0 to 0·2)	0·3 (0·2 to 0·4)	0·7 (0·4 to 1·2)	2·8 (1·6 to 5)	5·9 (3·5 to 8·9)	7 (4·4 to 10·6)	8·9 (5·9 to 12·7)	7 (4·6 to 10·4)	6·8 (4·4 to 9·9)	5·5 (3·8 to 7·9)	8·6 (5·6 to 12·6)

Cause	40 to 44	45 to 49	50 to 54	55 to 59	60 to 64	65 to 69	70 to 74	75 to 79	80 to 84	85 to 89	90 to 94	95 plus	Total
All causes	2,320 (1,920 to 2,810)	2,910 (2,410 to 3,480)	3,600 (3,000 to 4,270)	4,310 (3,570 to 5,060)	5,180 (4,390 to 6,020)	4,800 (4,120 to 5,520)	4,900 (4,260 to 5,580)	4,220 (3,700 to 4,760)	3,500 (3,120 to 3,880)	2,710 (2,450 to 2,960)	1,360 (1,260 to 1,470)	404 (381 to 426)	50,500
Communicable, maternal, neonatal, and nutritional diseases	203 (167 to 251)	198 (162 to 245)	190 (152 to 237)	193 (157 to 245)	213 (168 to 264)	199 (158 to 249)	220 (172 to 284)	235 (180 to 298)	223 (175 to 279)	221 (172 to 276)	129 (98·1 to 161)	42·1 (31·6 to 53·3)	4,620
HIV/AIDS and sexually transmitted infections	63·5 (57·8 to 70·9)	56·1 (50·5 to 63·4)	36·2 (32·1 to 41·7)	26·6 (23·2 to 31)	18·2 (15·9 to 21·3)	11·2 (9·6 to 13·5)	5 (4 to 6·6)	3 (2·4 to 3·9)	1·9 (1·5 to 2·5)	1·3 (1·1 to 1·6)	0·4 (0·3 to 0·6)	0·1 (0·1 to 0·1)	492
HIV/AIDS	56·4 (52·1 to 60·8)	49·5 (45·7 to 53·6)	31·7 (29 to 34·9)	23 (20·9 to 25·3)	15·5 (14 to 17·1)	9·2 (8·3 to 10·4)	3·5 (3 to 4·2)	2 (1·8 to 2·4)	1·3 (1·1 to 1·6)	0·9 (0·8 to 1·1)	0·2 (0·2 to 0·3)	0·1 (0·1 to 0·1)	439
HIV/AIDS - Drug-susceptible Tuberculosis	1 (0·7 to 1·5)	0·9 (0·6 to 1·3)	0·6 (0·4 to 0·9)	0·5 (0·3 to 0·7)	0·3 (0·2 to 0·5)	0·2 (0·1 to 0·3)	0·1 (0·1 to 0·1)	0 (0 to 0·1)	0 (0 to 0)	0 (0 to 0)	0 (0 to 0)	0 (0 to 0)	7·9
HIV/AIDS - Multidrug-resistant Tuberculosis without extensive dru	0 (0 to 0)	0 (0 to 0)	0 (0 to 0)	0 (0 to 0)	0 (0 to 0)	0 (0 to 0)	0 (0 to 0)	0 (0 to 0)	0 (0 to 0)	0 (0 to 0)	0 (0 to 0)	0 (0 to 0)	0·1
HIV/AIDS - Extensively drug-resistant Tuberculosis	0 (0 to 0)	0 (0 to 0)	0 (0 to 0)	0 (0 to 0)	0 (0 to 0)	0 (0 to 0)	0 (0 to 0)	0 (0 to 0)	0 (0 to 0)	0 (0 to 0)	0 (0 to 0)	0 (0 to 0)	0
HIV/AIDS resulting in other diseases	55·4 (51·1 to 59·8)	48·6 (44·7 to 52·7)	31·1 (28·4 to 34·3)	22·5 (20·4 to 24·8)	15·1 (13·7 to 16·8)	9 (8·1 to 10·2)	3·4 (2·9 to 4·2)	2 (1·7 to 2·4)	1·3 (1·1 to 1·5)	0·9 (0·8 to 1·1)	0·2 (0·2 to 0·3)	0·1 (0·1 to 0·1)	431
Sexually transmitted infections excluding HIV	7·1 (4·2 to 12·4)	6·6 (3·7 to 11·6)	4·5 (2·4 to 8·5)	3·5 (1·8 to 6·9)	2·7 (1·4 to 5·3)	2 (1 to 3·9)	1·5 (0·8 to 2·8)	1 (0·5 to 1·8)	0·6 (0·4 to 1·1)	0·4 (0·2 to 0·7)	0·2 (0·1 to 0·3)	0 (0 to 0·1)	52·4
Syphilis	0·1 (0·1 to 0·1)	0·2 (0·1 to 0·3)	0·1 (0·1 to 0·2)	0 (0 to 0·1)	0·1 (0 to 0·1)	0·1 (0 to 0·1)	0·1 (0 to 0·1)	0 (0 to 0·1)	0·1 (0 to 0·1)	0 (0 to 0·1)	0 (0 to 0·1)	0 (0 to 0)	2·9
Chlamydial infection	0·8 (0·5 to 1·3)	0·6 (0·4 to 1·1)	0·2 (0·2 to 0·3)	0·2 (0·1 to 0·2)	0·1 (0·1 to 0·2)	0·1 (0 to 0·1)	0·1 (0 to 0·1)	0·1 (0 to 0·1)	0 (0 to 0·1)	0 (0 to 0·1)	0 (0 to 0)	0 (0 to 0)	4·2
Gonococcal infection	0·8 (0·6 to 1·2)	0·8 (0·5 to 1·2)	0·5 (0·3 to 0·7)	0·3 (0·2 to 0·5)	0·3 (0·2 to 0·4)	0·3 (0·2 to 0·5)	0·2 (0·1 to 0·3)	0·2 (0·1 to 0·3)	0·1 (0·1 to 0·2)	0·1 (0·1 to 0·1)	0 (0 to 0·1)	0 (0 to 0)	5·6
Trichomoniasis	1·9 (0·6 to 4·6)	2 (0·6 to 4·6)	1·7 (0·6 to 4)	1·3 (0·4 to 3·2)	0·9 (0·3 to 2·1)	0·6 (0·2 to 1·4)	0·4 (0·1 to 0·9)	0·2 (0·1 to 0·5)	0·1 (0 to 0·3)	0·1 (0 to 0·1)	0 (0 to 0)	0 (0 to 0)	16
Genital herpes	1·3 (0·4 to 3·2)	1·4 (0·5 to 3·6)	1·4 (0·4 to 3·5)	1·3 (0·4 to 3·3)	1·2 (0·4 to 2·8)	0·8 (0·2 to 2·1)	0·6 (0·2 to 1·6)	0·4 (0·1 to 1·1)	0·3 (0·1 to 0·6)	0·1 (0 to 0·3)	0·1 (0 to 0·1)	0 (0 to 0)	13·2
Other sexually transmitted infections	2·1 (1·3 to 3·4)	1·6 (0·9 to 2·7)	0·6 (0·3 to 0·9)	0·4 (0·3 to 0·6)	0·2 (0·1 to 0·4)	0·1 (0·1 to 0·2)	0·1 (0·1 to 0·2)	0·1 (0 to 0·1)	0 (0 to 0·1)	0 (0 to 0·1)	0 (0 to 0)	0 (0 to 0)	10·5
Respiratory infections and tuberculosis	29 (21·3 to 39·5)	36·4 (26·6 to 49·1)	45·7 (31·6 to 63·5)	56·9 (40·6 to 80·7)	90·8 (61 to 126)	97·6 (65·5 to 136)	131 (89·4 to 185)	167 (116 to 226)	171 (126 to 224)	184 (138 to 236)	112 (83·2 to 142)	36·8 (26·8 to 47·4)	1,420
Tuberculosis	2 (1·1 to 3·2)	1·8 (1 to 3)	2 (1·1 to 3·1)	1·8 (1·1 to 2·9)	1·9 (1·1 to 2·9)	1·3 (0·8 to 2)	1·4 (0·9 to 2·1)	1·2 (0·8 to 1·7)	0·9 (0·6 to 1·3)	0·6 (0·4 to 0·9)	0·2 (0·2 to 0·3)	0·1 (0 to 0·1)	26·9
Latent tuberculosis infection	0 (0 to 0)	0 (0 to 0)	0 (0 to 0)	0 (0 to 0)	0 (0 to 0)	0 (0 to 0)	0 (0 to 0)	0 (0 to 0)	0 (0 to 0)	0 (0 to 0)	0 (0 to 0)	0 (0 to 0)	0
Drug-susceptible tuberculosis	2 (1·1 to 3·2)	1·8 (0·9 to 3)	2 (1 to 3·1)	1·8 (1·1 to 2·9)	1·9 (1·1 to 2·9)	1·3 (0·8 to 1·9)	1·4 (0·9 to 2·1)	1·2 (0·8 to 1·7)	0·9 (0·6 to 1·3)	0·6 (0·4 to 0·9)	0·2 (0·1 to 0·3)	0·1 (0 to 0·1)	26·7
Multidrug-resistant tuberculosis without extensive drug resistanc	0 (0 to 0·1)	0 (0 to 0)	0 (0 to 0·1)	0 (0 to 0·1)	0 (0 to 0·1)	0 (0 to 0)	0 (0 to 0)	0 (0 to 0)	0 (0 to 0)	0 (0 to 0)	0 (0 to 0)	0 (0 to 0)	0·2
Extensively drug-resistant tuberculosis	0 (0 to 0)	0 (0 to 0)	0 (0 to 0)	0 (0 to 0)	0 (0 to 0)	0 (0 to 0)	0 (0 to 0)	0 (0 to 0)	0 (0 to 0)	0 (0 to 0)	0 (0 to 0)	0 (0 to 0)	0
Lower respiratory infections	17·7 (11·2 to 26·1)	24·7 (16·1 to 36·2)	34·2 (21·8 to 50·9)	45·9 (29·8 to 68·3)	81 (52·1 to 116)	90·5 (58·6 to 129)	125 (84·2 to 179)	163 (113 to 222)	168 (123 to 221)	182 (137 to 235)	112 (82·7 to 141)	36·6 (26·6 to 47·2)	1,240
Upper respiratory infections	7·5 (3·9 to 13)	7·7 (4·2 to 13·3)	7·2 (4 to 12·3)	7 (3·8 to 11·9)	6·2 (3·3 to 10·3)	4·8 (2·5 to 8)	3·6 (1·9 to 6·2)	2·3 (1·2 to 3·8)	1·4 (0·8 to 2·5)	0·8 (0·4 to 1·3)	0·3 (0·2 to 0·6)	0·1 (0·1 to 0·2)	115
Otitis media	1·9 (0·8 to 3·6)	2·3 (1·1 to 4·1)	2·4 (1·1 to 4·4)	2·2 (1·1 to 4)	1·7 (0·8 to 3·4)	1 (0·5 to 1·9)	0·7 (0·3 to 1·4)	0·5 (0·2 to 0·8)	0·3 (0·1 to 0·7)	0·2 (0·1 to 0·4)	0·1 (0 to 0·2)	0 (0 to 0·1)	32·8
Enteric infections	14·7 (10 to 21)	18 (12·2 to 25·7)	20 (13·5 to 28·6)	22·3 (15·3 to 31·2)	26·9 (19·2 to 37)	30 (21·3 to 41)	33 (23·6 to 44)	29·2 (21·3 to 39)	23·7 (17 to 31·5)	15·2 (11 to 19·6)	7 (5·2 to 9·1)	1·9 (1·4 to 2·5)	365
Diarrheal diseases	14·7 (10 to 21)	18 (12·1 to 25·7)	20 (13·5 to 28·6)	22·3 (15·3 to 31·2)	26·8 (19·2 to 37)	30 (21·3 to 41)	33 (23·5 to 44)	29·2 (21·3 to 38·9)	23·7 (17 to 31·5)	15·2 (11 to 19·5)	7 (5·2 to 9·1)	1·9 (1·4 to 2·4)	364
Typhoid and paratyphoid	0 (0 to 0)	0 (0 to 0)	0 (0 to 0)	0 (0 to 0)	0 (0 to 0)	0 (0 to 0·1)	0 (0 to 0)	0 (0 to 0)	0 (0 to 0)	0 (0 to 0)	0 (0 to 0)	0 (0 to 0)	0·2
Typhoid fever	0 (0 to 0)	0 (0 to 0)	0 (0 to 0)	0 (0 to 0)	0 (0 to 0)	0 (0 to 0·1)	0 (0 to 0)	0 (0 to 0)	0 (0 to 0)	0 (0 to 0)	0 (0 to 0)	0 (0 to 0)	0·2
Paratyphoid fever	0 (0 to 0)	0 (0 to 0)	0 (0 to 0)	0 (0 to 0)	0 (0 to 0)	0 (0 to 0)	0 (0 to 0)	0 (0 to 0)	0 (0 to 0)	0 (0 to 0)	0 (0 to 0)	0 (0 to 0)	0
Invasive Non-typhoidal Salmonella (iNTS)	0 (0 to 0)	0 (0 to 0)	0 (0 to 0)	0 (0 to 0)	0 (0 to 0)	0 (0 to 0)	0 (0 to 0)	0 (0 to 0)	0 (0 to 0)	0 (0 to 0)	0 (0 to 0)	0 (0 to 0)	0·1
Other intestinal infectious diseases	0 (0 to 0·1)	0 (0 to 0)	0 (0 to 0)	0 (0 to 0)	0 (0 to 0·1)	0 (0 to 0·1)	0 (0 to 0·1)	0 (0 to 0)	0 (0 to 0)	0 (0 to 0·1)	0 (0 to 0)	0 (0 to 0)	0·5
Neglected tropical diseases and malaria	7·7 (4·2 to 13·3)	7·7 (3·8 to 13·5)	9·9 (4·6 to 18·4)	11·4 (5·7 to 20·3)	14 (7 to 24·3)	13·4 (6·7 to 23·6)	13·2 (6·5 to 22·8)	10·3 (4·9 to 18·3)	7·7 (3·8 to 13·7)	4·8 (2·4 to 8·5)	2·1 (1 to 3·8)	0·6 (0·3 to 1·1)	162
Malaria	0 (0 to 0)	0 (0 to 0)	0 (0 to 0)	0 (0 to 0)	0 (0 to 0)	0 (0 to 0)	0 (0 to 0)	0 (0 to 0)	0 (0 to 0)	0 (0 to 0)	0 (0 to 0)	0 (0 to 0)	0
Chagas disease	0 (0 to 0)	0 (0 to 0)	0 (0 to 0)	0 (0 to 0)	0 (0 to 0)	0 (0 to 0)	0 (0 to 0)	0 (0 to 0)	0 (0 to 0)	0 (0 to 0)	0 (0 to 0)	0 (0 to 0)	0
Leishmaniasis	0 (0 to 0)	0 (0 to 0)	0 (0 to 0)	0 (0 to 0)	0 (0 to 0)	0 (0 to 0)	0 (0 to 0)	0 (0 to 0)	0 (0 to 0)	0 (0 to 0)	0 (0 to 0)	0 (0 to 0)	0
Visceral leishmaniasis	0 (0 to 0)	0 (0 to 0)	0 (0 to 0)	0 (0 to 0)	0 (0 to 0)	0 (0 to 0)	0 (0 to 0)	0 (0 to 0)	0 (0 to 0)	0 (0 to 0)	0 (0 to 0)	0 (0 to 0)	0
Cutaneous and mucocutaneous leishmaniasis	0 (0 to 0)	0 (0 to 0)	0 (0 to 0)	0 (0 to 0)	0 (0 to 0)	0 (0 to 0)	0 (0 to 0)	0 (0 to 0)	0 (0 to 0)	0 (0 to 0)	0 (0 to 0)	0 (0 to 0)	0
African trypanosomiasis	0 (0 to 0)	0 (0 to 0)	0 (0 to 0)	0 (0 to 0)	0 (0 to 0)	0 (0 to 0)	0 (0 to 0)	0 (0 to 0)	0 (0 to 0)	0 (0 to 0)	0 (0 to 0)	0 (0 to 0)	0
Schistosomiasis	0 (0 to 0)	0 (0 to 0)	0 (0 to 0)	0 (0 to 0)	0 (0 to 0)	0 (0 to 0)	0 (0 to 0)	0 (0 to 0)	0 (0 to 0)	0 (0 to 0)	0 (0 to 0)	0 (0 to 0)	0
Cysticercosis	3·3 (0·9 to 7·7)	4·2 (1·1 to 9·2)	5·2 (1·6 to 11·5)	7·3 (2·7 to 14·8)	9·5 (3·7 to 19·2)	10·3 (4·4 to 20·3)	10·6 (4·3 to 20·1)	8·4 (3·3 to 16·4)	6·2 (2·5 to 12·2)	3·7 (1·4 to 7·4)	1·6 (0·6 to 3·2)	0·4 (0·1 to 0·8)	80·1
Cystic echinococcosis	0 (0 to 0)	0 (0 to 0)	0 (0 to 0)	0 (0 to 0)	0 (0 to 0)	0 (0 to 0)	0 (0 to 0)	0 (0 to 0)	0 (0 to 0)	0 (0 to 0)	0 (0 to 0)	0 (0 to 0)	0
Lymphatic filariasis	0 (0 to 0)	0 (0 to 0)	0 (0 to 0)	0 (0 to 0)	0 (0 to 0)	0 (0 to 0)	0 (0 to 0)	0 (0 to 0)	0 (0 to 0)	0 (0 to 0)	0 (0 to 0)	0 (0 to 0)	0
Onchocerciasis	0 (0 to 0)	0 (0 to 0)	0 (0 to 0)	0 (0 to 0)	0 (0 to 0)	0 (0 to 0)	0 (0 to 0)	0 (0 to 0)	0 (0 to 0)	0 (0 to 0)	0 (0 to 0)	0 (0 to 0)	0
Trachoma	0 (0 to 0)	0 (0 to 0)	0 (0 to 0)	0 (0 to 0)	0 (0 to 0)	0 (0 to 0)	0 (0 to 0)	0 (0 to 0)	0 (0 to 0)	0 (0 to 0)	0 (0 to 0)	0 (0 to 0)	0
Dengue	2·9 (1·1 to 6·2)	2·4 (0·9 to 5·1)	3·6 (1·1 to 9·9)	3·1 (1 to 7·4)	3·9 (1·4 to 8·4)	2·6 (1 to 5·7)	2·2 (0·9 to 4·1)	1·6 (0·6 to 3)	1·2 (0·5 to 2·3)	0·9 (0·4 to 1·7)	0·4 (0·1 to 0·7)	0·1 (0 to 0·3)	50·5
Yellow fever	0 (0 to 0)	0 (0 to 0)	0 (0 to 0)	0 (0 to 0)	0 (0 to 0)	0 (0 to 0)	0 (0 to 0)	0 (0 to 0)	0 (0 to 0)	0 (0 to 0)	0 (0 to 0)	0 (0 to 0)	0
Rabies	0 (0 to 0)	0 (0 to 0)	0 (0 to 0)	0 (0 to 0)	0 (0 to 0·1)	0 (0 to 0·1)	0 (0 to 0)	0 (0 to 0)	0 (0 to 0)	0 (0 to 0)	0 (0 to 0)	0 (0 to 0)	0·1
Intestinal nematode infections	0·3 (0·1 to 0·7)	0·1 (0 to 0·2)	0·1 (0 to 0·2)	0·1 (0 to 0·2)	0·1 (0 to 0·2)	0·1 (0 to 0·1)	0·1 (0 to 0·1)	0 (0 to 0·1)	0 (0 to 0·1)	0 (0 to 0)	0 (0 to 0)	0 (0 to 0)	6
Ascariasis	0 (0 to 0)	0 (0 to 0)	0 (0 to 0)	0 (0 to 0)	0 (0 to 0·1)	0 (0 to 0)	0 (0 to 0)	0 (0 to 0)	0 (0 to 0)	0 (0 to 0)	0 (0 to 0)	0 (0 to 0)	0·1
Trichuriasis	0 (0 to 0)	0 (0 to 0)	0 (0 to 0)	0 (0 to 0)	0 (0 to 0)	0 (0 to 0)	0 (0 to 0)	0 (0 to 0)	0 (0 to 0)	0 (0 to 0)	0 (0 to 0)	0 (0 to 0)	0
Hookworm disease	0·3 (0·1 to 0·7)	0·1 (0 to 0·2)	0·1 (0 to 0·2)	0·1 (0 to 0·2)	0·1 (0 to 0·1)	0·1 (0 to 0·1)	0·1 (0 to 0·1)	0 (0 to 0·1)	0 (0 to 0·1)	0 (0 to 0)	0 (0 to 0)	0 (0 to 0)	5·8
Food-borne trematodiases	0 (0 to 0)	0 (0 to 0)	0 (0 to 0)	0 (0 to 0)	0 (0 to 0)	0 (0 to 0)	0 (0 to 0)	0 (0 to 0)	0 (0 to 0)	0 (0 to 0)	0 (0 to 0)	0 (0 to 0)	0
Leprosy	0 (0 to 0)	0 (0 to 0)	0 (0 to 0)	0 (0 to 0)	0 (0 to 0)	0 (0 to 0)	0 (0 to 0)	0 (0 to 0)	0 (0 to 0)	0 (0 to 0)	0 (0 to 0)	0 (0 to 0)	0·2
Ebola	0 (0 to 0)	0 (0 to 0)	0 (0 to 0)	0 (0 to 0)	0 (0 to 0)	0 (0 to 0)	0 (0 to 0)	0 (0 to 0)	0 (0 to 0)	0 (0 to 0)	0 (0 to 0)	0 (0 to 0)	0
Zika virus	0·1 (0 to 0·3)	0 (0 to 0·2)	0 (0 to 0·2)	0 (0 to 0·1)	0 (0 to 0·1)	0 (0 to 0·1)	0 (0 to 0)	0 (0 to 0)	0 (0 to 0)	0 (0 to 0)	0 (0 to 0)	0 (0 to 0)	0·9
Guinea worm disease	0 (0 to 0)	0 (0 to 0)	0 (0 to 0)	0 (0 to 0)	0 (0 to 0)	0 (0 to 0)	0 (0 to 0)	0 (0 to 0)	0 (0 to 0)	0 (0 to 0)	0 (0 to 0)	0 (0 to 0)	0
Other neglected tropical diseases	1·1 (0·3 to 2·7)	0·9 (0·3 to 2·4)	1 (0·3 to 2·5)	1 (0·3 to 2·3)	0·4 (0·1 to 0·9)	0·3 (0·1 to 0·8)	0·3 (0·1 to 0·7)	0·3 (0·1 to 0·6)	0·2 (0·1 to 0·4)	0·2 (0·1 to 0·3)	0·1 (0 to 0·2)	0 (0 to 0·1)	24·5
Other infectious diseases	9·3 (7 to 12·2)	10·1 (7·7 to 13·1)	11 (8·4 to 14·5)	12·5 (9·3 to 16·4)	13 (9·8 to 16·7)	10·9 (8·3 to 13·8)	9·7 (7·3 to 12·5)	7 (5·2 to 8·9)	5·2 (3·7 to 6·9)	4·4 (2·9 to 6·1)	1·6 (1·1 to 2·2)	0·5 (0·3 to 0·7)	192
Meningitis	1·7 (1·2 to 2·3)	2·1 (1·6 to 2·9)	2·5 (1·8 to 3·5)	2·7 (1·9 to 3·8)	3 (2·1 to 4·2)	2·6 (1·8 to 3·6)	2·1 (1·4 to 2·9)	1·8 (1·2 to 2·5)	1·3 (0·8 to 1·8)	0·9 (0·6 to 1·3)	0·3 (0·2 to 0·3)	0·1 (0 to 0·1)	43·3
Encephalitis	2·9 (1·9 to 4·7)	3·2 (2 to 5)	2·7 (1·7 to 4·2)	3·2 (2 to 5·4)	3·5 (2·2 to 5·7)	2·5 (1·6 to 4·2)	2·1 (1·3 to 3·5)	1·1 (0·7 to 1·8)	0·8 (0·4 to 1·2)	0·4 (0·3 to 0·7)	0·2 (0·1 to 0·2)	0 (0 to 0·1)	62·5
Diphtheria	0 (0 to 0)	0 (0 to 0)	0 (0 to 0)	0 (0 to 0)	0 (0 to 0)	0 (0 to 0)	0 (0 to 0)	0 (0 to 0)	0 (0 to 0)	0 (0 to 0)	0 (0 to 0)	0 (0 to 0)	0
Tetanus	0·2 (0·1 to 0·6)	0·3 (0·1 to 0·6)	0·7 (0·2 to 1·6)	0·7 (0·2 to 1·7)	0·9 (0·3 to 2·1)	0·8 (0·2 to 2)	0·8 (0·3 to 1·9)	0·8 (0·3 to 2)	0·4 (0·1 to 1·1)	0·4 (0·1 to 0·9)	0·1 (0 to 0·3)	0 (0 to 0)	7·3
Measles	0 (0 to 0)	0 (0 to 0)	0 (0 to 0)	0 (0 to 0)	0 (0 to 0)	0 (0 to 0)	0 (0 to 0)	0 (0 to 0)	0 (0 to 0)	0 (0 to 0)	0 (0 to 0)	0 (0 to 0)	0
Varicella and herpes zoster	0·7 (0·4 to 1·2)	0·6 (0·3 to 1)	0·6 (0·3 to 1·1)	0·6 (0·3 to 1·1)	0·8 (0·4 to 1·4)	0·7 (0·4 to 1·2)	0·7 (0·4 to 1·1)	0·6 (0·3 to 0·9)	0·5 (0·3 to 0·8)	0·4 (0·2 to 0·8)	0·2 (0·1 to 0·4)	0·1 (0·1 to 0·3)	10·7
Acute hepatitis	0·7 (0·5 to 1)	0·6 (0·4 to 0·8)	0·5 (0·3 to 0·7)	0·5 (0·4 to 0·8)	0·5 (0·3 to 0·6)	0·3 (0·2 to 0·4)	0·3 (0·2 to 0·4)	0·2 (0·1 to 0·3)	0·1 (0·1 to 0·1)	0·1 (0·1 to 0·1)	0 (0 to 0)	0 (0 to 0)	9·3
Acute hepatitis A	0·4 (0·2 to 0·6)	0·3 (0·1 to 0·5)	0·3 (0·1 to 0·5)	0·3 (0·1 to 0·5)	0·2 (0·1 to 0·4)	0·1 (0 to 0·2)	0·1 (0 to 0·2)	0·1 (0 to 0·2)	0 (0 to 0·1)	0 (0 to 0·1)	0 (0 to 0)	0 (0 to 0)	6·1
Acute hepatitis B	0·2 (0·1 to 0·3)	0·2 (0·1 to 0·3)	0·2 (0·1 to 0·3)	0·2 (0·1 to 0·3)	0·2 (0·1 to 0·4)	0·1 (0·1 to 0·2)	0·1 (0 to 0·2)	0·1 (0 to 0·1)	0 (0 to 0·1)	0 (0 to 0·1)	0 (0 to 0)	0 (0 to 0)	2·1
Acute hepatitis C	0 (0 to 0·1)	0 (0 to 0)	0 (0 to 0·1)	0 (0 to 0·1)	0 (0 to 0·1)	0 (0 to 0·1)	0 (0 to 0·1)	0 (0 to 0)	0 (0 to 0)	0 (0 to 0)	0 (0 to 0)	0 (0 to 0)	0·4
Acute hepatitis E	0 (0 to 0·1)	0·1 (0 to 0·1)	0 (0 to 0·1)	0 (0 to 0·1)	0 (0 to 0·1)	0 (0 to 0)	0 (0 to 0·1)	0 (0 to 0)	0 (0 to 0)	0 (0 to 0·1)	0 (0 to 0)	0 (0 to 0)	0·8
Other unspecified infectious diseases	3·1 (1·9 to 4·9)	3·3 (2 to 5)	3·9 (2·5 to 6)	4·7 (2·8 to 7)	4·4 (2·5 to 6·5)	4 (2·3 to 5·6)	3·8 (2·1 to 5·8)	2·5 (1·3 to 3·6)	2·1 (1 to 3·5)	2·3 (1 to 3·6)	0·9 (0·4 to 1·4)	0·3 (0·1 to 0·4)	57·7
Maternal and neonatal disorders	48·8 (37·5 to 62·8)	44·9 (32·8 to 59·3)	40·9 (29·9 to 55·2)	37·7 (27·4 to 50·4)	31·2 (22·2 to 42·4)	20·2 (14·2 to 27·7)	12·2 (8·3 to 17·2)	5 (3·3 to 7·2)	1·4 (0·8 to 2·2)	0·3 (0·2 to 0·5)	0 (0 to 0·1)	0 (0 to 0)	1,510
Maternal disorders	7·5 (5·8 to 9·7)	2·1 (1·4 to 3)	0·3 (0·2 to 0·4)	0 (0 to 0)	0 (0 to 0)	0 (0 to 0)	0 (0 to 0)	0 (0 to 0)	0 (0 to 0)	0 (0 to 0)	0 (0 to 0)	0 (0 to 0)	106
Maternal hemorrhage	1·1 (0·7 to 1·7)	0·2 (0·1 to 0·3)	0 (0 to 0)	0 (0 to 0)	0 (0 to 0)	0 (0 to 0)	0 (0 to 0)	0 (0 to 0)	0 (0 to 0)	0 (0 to 0)	0 (0 to 0)	0 (0 to 0)	15·9
Maternal sepsis and other maternal infections	0·7 (0·4 to 1·1)	0·2 (0·1 to 0·3)	0 (0 to 0)	0 (0 to 0)	0 (0 to 0)	0 (0 to 0)	0 (0 to 0)	0 (0 to 0)	0 (0 to 0)	0 (0 to 0)	0 (0 to 0)	0 (0 to 0)	11·7
Maternal hypertensive disorders	0·8 (0·5 to 1·2)	0·2 (0·1 to 0·2)	0 (0 to 0)	0 (0 to 0)	0 (0 to 0)	0 (0 to 0)	0 (0 to 0)	0 (0 to 0)	0 (0 to 0)	0 (0 to 0)	0 (0 to 0)	0 (0 to 0)	13·8
Maternal obstructed labor and uterine rupture	0·2 (0·1 to 0·4)	0·1 (0 to 0·1)	0 (0 to 0)	0 (0 to 0)	0 (0 to 0)	0 (0 to 0)	0 (0 to 0)	0 (0 to 0)	0 (0 to 0)	0 (0 to 0)	0 (0 to 0)	0 (0 to 0)	3
Maternal abortion and miscarriage	0·5 (0·3 to 0·8)	0·2 (0·1 to 0·4)	0 (0 to 0)	0 (0 to 0)	0 (0 to 0)	0 (0 to 0)	0 (0 to 0)	0 (0 to 0)	0 (0 to 0)	0 (0 to 0)	0 (0 to 0)	0 (0 to 0)	6·5
Ectopic pregnancy	0·7 (0·4 to 1·2)	0·3 (0·2 to 0·5)	0 (0 to 0)	0 (0 to 0)	0 (0 to 0)	0 (0 to 0)	0 (0 to 0)	0 (0 to 0)	0 (0 to 0)	0 (0 to 0)	0 (0 to 0)	0 (0 to 0)	11·6
Indirect maternal deaths	0·3 (0·2 to 0·5)	0·1 (0 to 0·1)	0 (0 to 0)	--	--	--	--	--	--	--	--	--	NA
Late maternal deaths	0·7 (0·4 to 1·1)	0·2 (0·1 to 0·3)	0 (0 to 0)	--	--	--	--	--	--	--	--	--	NA
Maternal deaths aggravated by HIV/AIDS	0 (0 to 0)	0 (0 to 0)	0 (0 to 0)	--	--	--	--	--	--	--	--	--	NA
Neonatal disorders	41·3 (30·1 to 55)	42·8 (30·7 to 57·1)	40·6 (29·6 to 55)	37·7 (27·4 to 50·4)	31·2 (22·2 to 42·4)	20·2 (14·2 to 27·7)	12·2 (8·3 to 17·2)	5 (3·3 to 7·2)	1·4 (0·8 to 2·2)	0·3 (0·2 to 0·5)	0 (0 to 0·1)	0 (0 to 0)	1,400
Neonatal preterm birth	12 (8·2 to 16·6)	12·1 (8 to 16·9)	11·1 (7·5 to 15·6)	9·8 (6·5 to 13·7)	7·4 (4·9 to 10·3)	4·5 (3·2 to 6)	2·4 (1·8 to 3)	0·8 (0·6 to 1)	0·1 (0·1 to 0·2)	0 (0 to 0·1)	0 (0 to 0)	0 (0 to 0)	490
Neonatal encephalopathy due to birth asphyxia and trauma	3·4 (1·8 to 5·7)	3·6 (1·7 to 5·8)	3·4 (1·6 to 5·5)	3·3 (1·6 to 5·5)	2·8 (1·4 to 5)	1·6 (0·8 to 2·8)	0·7 (0·4 to 1·1)	0·2 (0·1 to 0·3)	0 (0 to 0·1)	0 (0 to 0)	0 (0 to 0)	0 (0 to 0)	258
Neonatal sepsis and other neonatal infections	23·6 (14·9 to 33·9)	24·8 (15·4 to 36·3)	23·7 (15·6 to 35)	22·4 (14·4 to 32·7)	19 (11·9 to 28·4)	12·8 (8 to 19·4)	8·3 (4·9 to 12·8)	3·6 (2 to 5·7)	1·1 (0·6 to 2)	0·2 (0·1 to 0·4)	0 (0 to 0)	0 (0 to 0)	512
Hemolytic disease and other neonatal jaundice	2·3 (1·1 to 3·9)	2·4 (1·2 to 4)	2·4 (1·5 to 3·5)	2·2 (1·4 to 3·3)	1·9 (1·2 to 2·8)	1·4 (1 to 1·8)	0·9 (0·6 to 1·1)	0·4 (0·3 to 0·5)	0·1 (0·1 to 0·2)	0 (0 to 0)	0 (0 to 0)	0 (0 to 0)	33
Other neonatal disorders	0 (0 to 0)	0 (0 to 0)	0 (0 to 0)	0 (0 to 0)	0 (0 to 0)	0 (0 to 0)	0 (0 to 0)	0 (0 to 0)	0 (0 to 0)	0 (0 to 0)	0 (0 to 0)	0 (0 to 0)	110
Nutritional deficiencies	29·4 (10·9 to 63·5)	24·5 (9·8 to 54·8)	26·4 (10·8 to 60·2)	25·9 (10·9 to 56·8)	18·9 (9·4 to 38·6)	16·1 (8·2 to 32·8)	16·2 (9·2 to 30·1)	14 (8·2 to 24)	11·6 (7 to 19·2)	10·4 (6·7 to 16·3)	5·9 (3·8 to 8·6)	2·2 (1·4 to 3·1)	478
Protein-energy malnutrition	2·3 (1·4 to 3·5)	2·6 (1·5 to 3·9)	3 (1·9 to 4·4)	3·7 (2·4 to 5·2)	4·3 (2·9 to 6)	3·9 (2·5 to 5·6)	4·6 (3·1 to 6·6)	5 (3·1 to 7·2)	4·6 (3 to 6·8)	4·7 (2·8 to 7)	3·2 (2 to 4·8)	1·5 (0·9 to 2·2)	67·3
Iodine deficiency	1·3 (0·5 to 2·4)	1·3 (0·6 to 2·6)	1·3 (0·5 to 2·4)	1·2 (0·5 to 2·4)	1 (0·4 to 1·9)	0·7 (0·3 to 1·4)	0·5 (0·2 to 1)	0·3 (0·2 to 0·5)	0·2 (0·1 to 0·3)	0·1 (0·1 to 0·2)	0 (0 to 0·1)	0 (0 to 0)	15·1
Vitamin A deficiency	0·1 (0 to 0·2)	0·1 (0 to 0·2)	0·1 (0 to 0·2)	0·1 (0 to 0·2)	0·1 (0 to 0·1)	0·1 (0 to 0·1)	0 (0 to 0·1)	0 (0 to 0·1)	0 (0 to 0)	0 (0 to 0)	0 (0 to 0)	0 (0 to 0)	4·3
Dietary iron deficiency	24·6 (6·3 to 58)	18·8 (4·5 to 48·2)	20 (4·6 to 53·3)	18·9 (4·7 to 49·9)	11 (2·3 to 30)	8·8 (1·9 to 25·3)	8·2 (2 to 21·6)	6 (1·3 to 15·7)	4·5 (0·8 to 11·5)	3·3 (0·7 to 8·5)	1·1 (0·2 to 3)	0·3 (0·1 to 0·8)	358
Other nutritional deficiencies	1·2 (0·7 to 1·7)	1·7 (1 to 2·5)	2 (1·2 to 3)	2 (1·2 to 3)	2·6 (1·5 to 3·8)	2·6 (1·5 to 4)	2·9 (1·6 to 4·3)	2·7 (1·3 to 4·2)	2·3 (1·1 to 3·8)	2·4 (1·1 to 4·1)	1·5 (0·6 to 2·6)	0·4 (0·2 to 0·7)	32·4
Non-communicable diseases	2,000 (1,640 to 2,430)	2,590 (2,120 to 3,100)	3,280 (2,720 to 3,900)	3,980 (3,300 to 4,680)	4,830 (4,080 to 5,620)	4,480 (3,850 to 5,170)	4,570 (3,950 to 5,190)	3,880 (3,410 to 4,400)	3,190 (2,840 to 3,530)	2,410 (2,180 to 2,640)	1,190 (1,090 to 1,290)	349 (327 to 372)	43,900
Neoplasms	399 (313 to 510)	586 (454 to 744)	855 (674 to 1,060)	1,070 (832 to 1,320)	1,340 (1,080 to 1,600)	1,140 (939 to 1,360)	1,060 (881 to 1,240)	801 (660 to 958)	528 (437 to 609)	326 (267 to 380)	128 (102 to 148)	29·1 (22·7 to 33·3)	9,010
Lip and oral cavity cancer	1·3 (0·9 to 1·9)	2·3 (1·6 to 3·2)	3·8 (2·6 to 5·3)	5·2 (3·7 to 7·3)	7·6 (5·4 to 10·4)	7·4 (5·2 to 10)	7·9 (5·7 to 10·6)	6·6 (4·7 to 9·2)	4·6 (3·3 to 6·2)	2·9 (2·2 to 3·8)	1·3 (1 to 1·6)	0·3 (0·2 to 0·5)	53·7
Nasopharynx cancer	0·8 (0·5 to 1)	1·3 (0·9 to 1·9)	1·6 (1·1 to 2·3)	2·1 (1·5 to 2·8)	2·6 (1·8 to 3·5)	2·1 (1·4 to 2·9)	2 (1·3 to 2·9)	1·2 (0·8 to 1·6)	0·8 (0·6 to 1·2)	0·9 (0·7 to 1·3)	0·4 (0·3 to 0·5)	0·1 (0 to 0·1)	17·9
Other pharynx cancer	0·6 (0·4 to 0·9)	1·2 (0·8 to 1·7)	1·7 (1·2 to 2·4)	2·5 (1·7 to 3·5)	3·5 (2·5 to 4·7)	2·6 (1·7 to 3·6)	2·8 (1·9 to 4)	2 (1·3 to 2·9)	1·3 (0·9 to 1·8)	0·9 (0·7 to 1·2)	0·5 (0·3 to 0·6)	0·1 (0·1 to 0·1)	20·3
Esophageal cancer	2·6 (1·7 to 3·7)	5·3 (3·4 to 7·8)	9·9 (6·7 to 13·7)	13 (9·1 to 17·9)	20·1 (13·7 to 27·1)	17·7 (12·3 to 23·9)	18·1 (12·5 to 24·8)	15·2 (10·1 to 21·2)	10·2 (7·1 to 13·9)	6·4 (4·5 to 8·4)	2·6 (1·9 to 3·3)	0·6 (0·4 to 0·8)	124
Stomach cancer	12·8 (9·3 to 17·3)	20·1 (13·8 to 27·9)	30·2 (21·9 to 41·1)	38·4 (27·9 to 49·9)	60·6 (45·4 to 78·2)	56·7 (41·9 to 73·8)	58·3 (43·1 to 76·7)	53 (38·6 to 69·4)	39·1 (29·4 to 51·4)	25·8 (19·1 to 32·6)	10·6 (8·1 to 12·9)	2·3 (1·8 to 2·8)	426
Colon and rectum cancer	26·8 (18·9 to 36·5)	45·9 (32·6 to 63·6)	80·9 (60·1 to 109)	121 (85·4 to 162)	177 (125 to 238)	166 (121 to 217)	169 (123 to 229)	159 (115 to 214)	114 (82·1 to 148)	74·7 (55·5 to 97·5)	29·6 (22·1 to 37·2)	6·3 (4·7 to 7·7)	1,210
Liver cancer	3·4 (2·1 to 5·3)	5·7 (3·8 to 8·2)	9·5 (6·5 to 13·3)	14·2 (9·6 to 19·9)	18·3 (12·6 to 25·8)	18·2 (12·9 to 24·8)	18·7 (13 to 26·5)	14·9 (10·3 to 21·1)	10 (7 to 13·7)	4·4 (3 to 6·2)	1·6 (1·2 to 2·2)	0·3 (0·2 to 0·4)	129
Liver cancer due to hepatitis B	1·4 (0·7 to 2·4)	1·9 (1 to 3·2)	2·8 (1·5 to 4·9)	3·7 (1·9 to 6·4)	4 (2 to 7·1)	3·1 (1·5 to 5·5)	2·6 (1·3 to 4·9)	1·8 (0·9 to 3·2)	1 (0·4 to 2)	0·4 (0·2 to 0·8)	0·1 (0·1 to 0·3)	0 (0 to 0·1)	24·9
Liver cancer due to hepatitis C	0·5 (0·2 to 0·9)	1 (0·5 to 1·8)	2·1 (1·1 to 3·5)	3·7 (1·7 to 6·6)	5·2 (2·5 to 8·9)	5·5 (3 to 8·8)	6·1 (3·4 to 10·2)	5·4 (2·9 to 8·9)	4·2 (2·5 to 6·5)	2·1 (1·3 to 3·2)	0·9 (0·5 to 1·3)	0·2 (0·1 to 0·3)	37·1
Liver cancer due to alcohol use	0·8 (0·4 to 1·5)	1·4 (0·6 to 2·5)	2·5 (1·2 to 4·4)	3·8 (1·8 to 6·6)	5·3 (2·7 to 8·9)	5·8 (3·3 to 9·4)	5·9 (3·1 to 10)	4 (2·2 to 6·9)	2·2 (1·2 to 3·6)	0·8 (0·5 to 1·4)	0·3 (0·1 to 0·5)	0 (0 to 0·1)	33·5
Liver cancer due to NASH	0·4 (0·2 to 0·7)	0·7 (0·3 to 1·2)	1·2 (0·6 to 2)	1·8 (0·9 to 3·3)	2·3 (1·2 to 4)	2·5 (1·3 to 4·4)	2·8 (1·5 to 5)	2·6 (1·4 to 4·6)	1·8 (1 to 3·1)	0·8 (0·4 to 1·3)	0·3 (0·1 to 0·5)	0·1 (0 to 0·1)	17·8
Liver cancer due to other causes	0·5 (0·2 to 0·8)	0·7 (0·3 to 1·2)	1 (0·5 to 1·8)	1·3 (0·6 to 2·4)	1·5 (0·7 to 2·6)	1·3 (0·6 to 2·3)	1·3 (0·6 to 2·3)	1 (0·5 to 1·8)	0·7 (0·3 to 1·2)	0·3 (0·1 to 0·5)	0·1 (0 to 0·2)	0 (0 to 0)	15·2
Gallbladder and biliary tract cancer	2·2 (1·4 to 4·1)	4·5 (2·8 to 8)	8·3 (5·1 to 14·1)	12·4 (7·9 to 22·2)	17·6 (11·8 to 31·6)	16·1 (11·2 to 29·1)	16·5 (11 to 29·7)	12·4 (8·6 to 22·1)	7·7 (5·4 to 13·6)	4 (2·8 to 7·2)	1·5 (1 to 2·7)	0·3 (0·2 to 0·6)	105
Pancreatic cancer	7·6 (5·4 to 10·6)	14·7 (10·3 to 19·9)	25·9 (18·8 to 35·5)	39·7 (28·5 to 53·1)	65·4 (47 to 87·5)	64·6 (46·8 to 86·9)	66·7 (48·3 to 88·6)	49·4 (34·2 to 67·7)	30·8 (22·3 to 41·4)	19 (13·8 to 24·6)	7·2 (5·4 to 8·8)	1·5 (1·1 to 1·8)	399
Larynx cancer	0·3 (0·2 to 0·5)	0·8 (0·6 to 1·1)	1·4 (1 to 2·1)	2·2 (1·5 to 3·1)	3·5 (2·5 to 4·7)	2·9 (2 to 4·2)	2·5 (1·8 to 3·5)	1·8 (1·3 to 2·4)	1·1 (0·8 to 1·4)	0·7 (0·5 to 1)	0·3 (0·2 to 0·4)	0·1 (0 to 0·1)	18·1
Tracheal, bronchus, and lung cancer	9·5 (6·7 to 12·9)	18·5 (13·1 to 25·2)	31·7 (22·3 to 44·5)	47 (32·5 to 65·6)	66·2 (44·4 to 92·5)	58·3 (41 to 80·9)	53·3 (37·9 to 74·6)	36·9 (26·4 to 51·3)	21·5 (15·1 to 29·4)	12·7 (9·1 to 17·1)	4·4 (3·2 to 5·5)	1 (0·7 to 1·3)	370
Malignant skin melanoma	1·1 (0·7 to 1·7)	1·6 (1 to 2·4)	2 (1·2 to 2·8)	2·1 (1·3 to 3·2)	2·8 (1·8 to 4·2)	2·6 (1·7 to 3·7)	2·5 (1·6 to 3·7)	1·6 (1 to 2·2)	1·3 (0·9 to 1·8)	1·1 (0·7 to 1·5)	0·5 (0·3 to 0·6)	0·2 (0·1 to 0·2)	22·4
Non-melanoma skin cancer	0·7 (0·5 to 1)	0·7 (0·4 to 1)	1·3 (0·8 to 1·9)	1·4 (0·9 to 2·1)	1·7 (1·1 to 2·4)	1·8 (1·1 to 2·6)	2 (1·4 to 2·8)	2·3 (1·6 to 3·2)	2·2 (1·6 to 3·1)	2·2 (1·6 to 2·9)	1·7 (1·1 to 2·2)	0·6 (0·4 to 0·9)	19·6
Non-melanoma skin cancer (squamous-cell carcinoma)	0·7 (0·5 to 1)	0·7 (0·4 to 1)	1·3 (0·8 to 1·9)	1·4 (0·9 to 2·1)	1·7 (1·1 to 2·4)	1·8 (1·1 to 2·6)	2 (1·4 to 2·8)	2·3 (1·6 to 3·2)	2·2 (1·6 to 3·1)	2·2 (1·6 to 2·9)	1·7 (1·1 to 2·2)	0·6 (0·4 to 0·9)	19·6
Non-melanoma skin cancer (basal-cell carcinoma)	0 (0 to 0)	0 (0 to 0)	0 (0 to 0)	0 (0 to 0)	0 (0 to 0)	0 (0 to 0)	0 (0 to 0)	0 (0 to 0)	0 (0 to 0)	0 (0 to 0)	0 (0 to 0)	0 (0 to 0)	0
Breast cancer	145 (105 to 194)	205 (146 to 275)	304 (212 to 406)	343 (243 to 463)	362 (262 to 470)	277 (208 to 354)	232 (173 to 296)	161 (118 to 214)	106 (78·8 to 139)	70 (52·6 to 89·5)	27·9 (21·5 to 34·5)	7·5 (5·6 to 9·3)	2,410
Cervical cancer	71·2 (51·3 to 98·2)	90·3 (63·1 to 128)	105 (73·7 to 141)	112 (79·7 to 151)	120 (87·4 to 163)	90·2 (66·2 to 125)	77·1 (56·7 to 102)	51·4 (37·7 to 67·9)	33·1 (23·9 to 44·1)	17·5 (12·9 to 23·2)	6·9 (4·9 to 9·4)	1·6 (1·1 to 2·3)	882
Uterine cancer	15·5 (10·3 to 22·5)	27·1 (18·5 to 37·9)	40 (27·1 to 57·8)	54·3 (37·5 to 74·3)	86·7 (62·2 to 116)	76·5 (55·9 to 103)	70·1 (52·1 to 93·4)	42·8 (31·5 to 57·9)	25·2 (18 to 33·7)	13·2 (9·8 to 16·8)	5 (3·7 to 6·5)	1·1 (0·8 to 1·5)	477
Ovarian cancer	22·2 (11·7 to 31·4)	36·4 (17·5 to 52·4)	48·1 (24·3 to 67·3)	59·4 (30·6 to 82·3)	66·3 (33·8 to 93·1)	56·5 (28·5 to 78·1)	45·4 (22·9 to 62·9)	28·5 (13·4 to 39·6)	17·3 (8·1 to 23·3)	10 (4·4 to 13·5)	3·9 (1·6 to 5)	0·6 (0·2 to 0·8)	429
Prostate cancer	0 (0 to 0)	0 (0 to 0)	0 (0 to 0)	0 (0 to 0)	0 (0 to 0)	0 (0 to 0)	0 (0 to 0)	0 (0 to 0)	0 (0 to 0)	0 (0 to 0)	0 (0 to 0)	0 (0 to 0)	0
Testicular cancer	--	--	--	--	--	--	--	--	--	--	--	--	NA
Kidney cancer	5·6 (3·8 to 7·8)	9 (6 to 12·9)	12·1 (8·5 to 17·1)	16·7 (11·4 to 23·1)	20·6 (14·4 to 28·4)	17 (12·2 to 22·5)	15 (10·8 to 20·4)	9·6 (6·8 to 13·3)	6·2 (4·5 to 8·3)	2·9 (2·1 to 4·1)	1·2 (0·8 to 1·6)	0·3 (0·2 to 0·4)	130
Bladder cancer	1·2 (0·8 to 1·8)	2·1 (1·4 to 3)	4·9 (3·4 to 6·8)	7·5 (5·3 to 9·9)	10·5 (7·9 to 13·6)	12 (9 to 15·5)	13·5 (10 to 17·5)	13·1 (9·6 to 17)	10·2 (7·6 to 13·4)	7·2 (5·2 to 9·4)	3·3 (2·4 to 4·1)	0·8 (0·5 to 1)	87·7
Brain and central nervous system cancer	7·7 (2·3 to 13·2)	9·4 (2·9 to 15·9)	12·5 (4 to 20·5)	15·4 (4·7 to 25·1)	16·8 (5·2 to 27·4)	14 (4·6 to 22)	10·8 (3·2 to 17·8)	7·2 (2·2 to 12·3)	3·5 (1 to 6)	2·1 (0·6 to 3·6)	0·7 (0·2 to 1·1)	0·1 (0 to 0·2)	139
Thyroid cancer	1·7 (1·1 to 2·5)	2·5 (1·6 to 3·7)	4·5 (2·8 to 6·8)	5·8 (3·8 to 8·3)	7·3 (5·3 to 9·5)	6·9 (4·7 to 9·7)	7·1 (5·2 to 9·3)	4·9 (3·6 to 6·5)	3·2 (2·4 to 4·2)	1·7 (1·2 to 2·2)	0·6 (0·4 to 0·8)	0·1 (0·1 to 0·2)	49·3
Mesothelioma	0·7 (0·4 to 1·1)	0·8 (0·5 to 1·3)	1·3 (0·9 to 2)	1·6 (1 to 2·4)	0·7 (0·4 to 1·1)	0·5 (0·4 to 0·8)	0·8 (0·5 to 1·2)	0·3 (0·2 to 0·5)	0·3 (0·2 to 0·5)	0·1 (0·1 to 0·2)	0·1 (0 to 0·1)	0 (0 to 0)	8·6
Hodgkin lymphoma	1·2 (0·7 to 1·8)	1·1 (0·7 to 1·6)	1·1 (0·7 to 1·7)	1·4 (0·9 to 2·1)	1·5 (0·9 to 2·3)	1·5 (1 to 2·3)	1·2 (0·8 to 1·8)	1 (0·7 to 1·5)	0·7 (0·4 to 1)	0·3 (0·2 to 0·5)	0·1 (0·1 to 0·1)	0 (0 to 0)	18·4
Non-Hodgkin lymphoma	14·9 (10·3 to 21·5)	21·8 (15·5 to 29·9)	29·1 (20·8 to 39·5)	37·7 (27 to 51·3)	51·7 (37·4 to 70·1)	44·8 (33·5 to 58·6)	41·1 (30·6 to 54·9)	30·3 (22·2 to 41)	17·3 (12·6 to 23)	8·8 (6·5 to 11·5)	3·1 (2·4 to 3·9)	0·6 (0·5 to 0·8)	368
Multiple myeloma	7·7 (5·1 to 10·9)	14·7 (9·3 to 20·9)	28·6 (20·1 to 39·4)	46·9 (32·7 to 63·3)	66·7 (46·4 to 93·4)	61·6 (44·3 to 83·3)	61·6 (45·3 to 83·7)	42·9 (31·4 to 57·5)	24 (17·3 to 33)	12·2 (8·7 to 16·7)	3·9 (2·8 to 5)	0·7 (0·5 to 1)	377
Leukemia	14·4 (10·1 to 19·7)	17·5 (12·4 to 24·6)	20·6 (15 to 28·1)	25·3 (18·5 to 34·1)	31·6 (23·5 to 41·5)	26·3 (19·8 to 33·8)	26·7 (19·6 to 35·6)	19·6 (14·5 to 25·6)	13·5 (9·8 to 17·8)	8·2 (6 to 10·7)	3·2 (2·4 to 4)	0·7 (0·5 to 0·9)	299
Acute lymphoid leukemia	0·8 (0·4 to 1·3)	0·7 (0·4 to 1·2)	1 (0·5 to 1·7)	1 (0·5 to 1·8)	1·1 (0·5 to 2)	0·8 (0·4 to 1·5)	0·7 (0·4 to 1·2)	0·7 (0·4 to 1·4)	0·4 (0·2 to 0·8)	0·1 (0·1 to 0·2)	0·1 (0 to 0·1)	0 (0 to 0)	23·5
Chronic lymphoid leukemia	0·2 (0·1 to 0·4)	0·2 (0·1 to 0·3)	0·8 (0·5 to 1·4)	1·4 (0·7 to 2·5)	2·6 (1·4 to 4·5)	2·9 (1·5 to 5)	2·8 (1·5 to 4·9)	2·4 (1·4 to 3·9)	1·5 (0·8 to 2·5)	1 (0·5 to 1·7)	0·4 (0·2 to 0·7)	0·1 (0 to 0·1)	16·9
Acute myeloid leukemia	7·9 (5·2 to 11·3)	10·6 (7·2 to 15)	11·7 (8·2 to 16·4)	13·7 (9·3 to 19)	16·1 (11·5 to 21·8)	11·3 (7·7 to 15·5)	10·6 (7·1 to 15·1)	6·5 (4·3 to 9·4)	4·2 (2·7 to 6)	1·8 (1·1 to 2·7)	0·6 (0·4 to 0·9)	0·1 (0·1 to 0·2)	149

Chronic myeloid leukemia	0 (0 to 0)	0 (0 to 0)	0 (0 to 0)	0·1 (0 to 0·1)	0·1 (0·1 to 0·2)	0·1 (0·1 to 0·2)	0·2 (0·1 to 0·5)	0·6 (0·3 to 1·2)	0·5 (0·2 to 1·1)	0·6 (0·3 to 1·2)	0·8 (0·4 to 1·6)	1 (0·5 to 1·8)	1·1 (0·5 to 1·9)	1·8 (1 to 3·1)	2·2 (1·1 to 3·8)	3 (1·5 to 5)	2·2 (1·2 to 3·6)	2·5 (1·3 to 4·1)	1·9 (1 to 3·2)	1·4 (0·7 to 2·3)	0·8 (0·4 to 1·3)	0·3 (0·2 to 0·6)	0·1 (0 to 0·1)	21·4
Other leukemia	0·2 (0·1 to 0·3)	0 (0 to 0·1)	0·7 (0·4 to 1·2)	0·6 (0·3 to 1)	1·7 (1 to 2·9)	1·9 (0·9 to 3·2)	2·2 (1·3 to 3·6)	2·8 (1·6 to 4·4)	2·1 (1·2 to 3·4)	2·3 (1·4 to 3·6)	3·5 (2 to 5·5)	4·5 (2·8 to 6·8)	5 (3·1 to 7·5)	5·2 (3·4 to 7·6)	7 (4·7 to 10·2)	8·9 (5·9 to 13)	9 (6·2 to 12·6)	10·1 (7 to 14·2)	8 (5·4 to 11·4)	5·9 (3·8 to 8·2)	4·4 (3 to 6·2)	1·8 (1·3 to 2·3)	0·4 (0·3 to 0·5)	88·2
Other malignant neoplasms	0·5 (0·3 to 0·8)	0·6 (0·4 to 0·9)	2·4 (1·5 to 3·5)	7·5 (4·7 to 11·2)	9·4 (7·1 to 12·3)	9·8 (7·6 to 12·6)	14·1 (10·7 to 18·9)	10·8 (8 to 14·3)	10·2 (7·5 to 14·1)	11 (8 to 14·9)	15·9 (11·8 to 21·3)	19 (13·8 to 25·9)	24·6 (18·4 to 33)	33·8 (24·9 to 45·4)	37·4 (27·9 to 49·4)	44·8 (33·4 to 58·7)	38·6 (29·2 to 51)	36·4 (28·1 to 46·7)	27·8 (21·3 to 36)	19·6 (15·2 to 24·5)	14 (10·8 to 17·6)	5·7 (4·3 to 7·1)	1·2 (0·9 to 1·6)	395
Other neoplasms	0 (0 to 0·1)	0·1 (0 to 0·2)	0·2 (0·1 to 0·5)	0·5 (0·2 to 0·8)	0·1 (0·1 to 0·2)	0·3 (0·2 to 0·5)	0·5 (0·3 to 0·7)	0·4 (0·2 to 0·5)	0·6 (0·3 to 0·9)	0·6 (0·4 to 0·9)	0·5 (0·3 to 0·7)	1·3 (0·8 to 1·9)	1·3 (0·9 to 1·9)	2 (1·2 to 2·9)	2·5 (1·7 to 3·8)	3·8 (2·7 to 5·3)	4·1 (2·9 to 5·6)	5·1 (3·6 to 7·2)	4·5 (3·3 to 6·4)	3·2 (2·2 to 4·5)	1·9 (1·3 to 2·6)	0·8 (0·5 to 1·1)	0·1 (0·1 to 0·2)	34·3
Myelodysplastic, myeloproliferative, and other hematopoietic neo	0 (0 to 0·1)	0·1 (0 to 0·2)	0·2 (0·1 to 0·5)	0·5 (0·2 to 0·8)	0·1 (0·1 to 0·2)	0·3 (0·2 to 0·5)	0·5 (0·3 to 0·7)	0·4 (0·2 to 0·5)	0·6 (0·3 to 0·9)	0·6 (0·4 to 0·9)	0·5 (0·3 to 0·7)	1·3 (0·8 to 1·9)	1·3 (0·9 to 1·9)	2 (1·2 to 2·9)	2·5 (1·7 to 3·8)	3·8 (2·7 to 5·3)	4·1 (2·9 to 5·6)	5·1 (3·6 to 7·2)	4·5 (3·3 to 6·4)	3·2 (2·2 to 4·5)	1·9 (1·3 to 2·6)	0·8 (0·5 to 1·1)	0·1 (0·1 to 0·2)	34·3
Benign and in situ intestinal neoplasms	0 (0 to 0)	0 (0 to 0)	0 (0 to 0)	0 (0 to 0)	0 (0 to 0)	0 (0 to 0)	0 (0 to 0)	0 (0 to 0)	0 (0 to 0)	0 (0 to 0)	0 (0 to 0)	0 (0 to 0)	0 (0 to 0)	0 (0 to 0)	0 (0 to 0)	0 (0 to 0)	0 (0 to 0)	0 (0 to 0)	0 (0 to 0)	0 (0 to 0)	0 (0 to 0)	0 (0 to 0)	0 (0 to 0)	0
Benign and in situ cervical and uterine neoplasms	0 (0 to 0)	0 (0 to 0)	0 (0 to 0)	0 (0 to 0)	0 (0 to 0)	0 (0 to 0)	0 (0 to 0)	0 (0 to 0)	0 (0 to 0)	0 (0 to 0)	0 (0 to 0)	0 (0 to 0)	0 (0 to 0)	0 (0 to 0)	0 (0 to 0)	0 (0 to 0)	0 (0 to 0)	0 (0 to 0)	0 (0 to 0)	0 (0 to 0)	0 (0 to 0)	0 (0 to 0)	0 (0 to 0)	0
Other benign and in situ neoplasms	0 (0 to 0)	0 (0 to 0)	0 (0 to 0)	0 (0 to 0)	0 (0 to 0)	0 (0 to 0)	0 (0 to 0)	0 (0 to 0)	0 (0 to 0)	0 (0 to 0)	0 (0 to 0)	0 (0 to 0)	0 (0 to 0)	0 (0 to 0)	0 (0 to 0)	0 (0 to 0)	0 (0 to 0)	0 (0 to 0)	0 (0 to 0)	0 (0 to 0)	0 (0 to 0)	0 (0 to 0)	0 (0 to 0)	0
Cardiovascular diseases	2·7 (1·6 to 4·1)	1·1 (0·7 to 1·6)	4·2 (2·6 to 6·4)	2·5 (1·8 to 3·3)	6·8 (5·2 to 8·7)	12·7 (9·6 to 16·3)	30·9 (24·5 to 38·5)	38·3 (29·5 to 48·9)	42·9 (33·2 to 55·2)	59·7 (46·5 to 75·6)	104 (79·3 to 133)	166 (131 to 209)	254 (198 to 324)	369 (290 to 462)	539 (419 to 683)	832 (670 to 1,020)	935 (775 to 1,120)	1,140 (960 to 1,350)	1,110 (942 to 1,300)	1,040 (875 to 1,210)	887 (737 to 1,020)	469 (383 to 531)	148 (116 to 168)	8,200
Rheumatic heart disease	0 (0 to 0)	0 (0 to 0)	0 (0 to 0)	0·3 (0·1 to 0·6)	2 (1·2 to 3·3)	4·8 (3 to 7·5)	8·5 (5·8 to 12·6)	9·1 (5·9 to 13·2)	10·4 (7 to 14·8)	11·2 (7·8 to 15·5)	14·1 (10·1 to 19·1)	14·7 (10·3 to 19·8)	15·1 (10·5 to 21·2)	15 (10·6 to 20·3)	14·2 (10·1 to 19·3)	11·7 (8·4 to 15·7)	8·8 (6·6 to 11·6)	7·1 (5·1 to 9·3)	5·3 (3·9 to 7·1)	3·7 (2·7 to 4·9)	2·5 (1·9 to 3·4)	1·3 (0·9 to 1·6)	0·4 (0·3 to 0·6)	160
Ischemic heart disease	0 (0 to 0)	0 (0 to 0)	0 (0 to 0)	0 (0 to 0)	0 (0 to 0)	0 (0 to 0)	1·8 (1·2 to 2·3)	3 (2 to 4·4)	3·4 (2·2 to 5)	7·6 (5·3 to 10·8)	17·4 (12·1 to 23·6)	33·1 (24·3 to 44·1)	61·5 (43·6 to 85·2)	96·1 (71·3 to 126)	160 (121 to 206)	314 (238 to 408)	350 (280 to 424)	402 (329 to 482)	382 (315 to 456)	352 (288 to 421)	317 (259 to 382)	172 (136 to 206)	53·3 (41·1 to 62·7)	2,730
Stroke	1 (0·4 to 1·8)	0·1 (0·1 to 0·3)	0·5 (0·2 to 1)	0·5 (0·4 to 0·7)	1·7 (1·2 to 2·5)	3·2 (2·2 to 4·4)	8·6 (6·1 to 11·7)	11·5 (7·8 to 16·5)	13·1 (9·2 to 18·4)	21 (14·7 to 28·2)	39·4 (27·7 to 53·8)	61·6 (43·8 to 84·8)	100 (74·1 to 131)	150 (108 to 205)	205 (144 to 282)	273 (208 to 353)	327 (242 to 435)	449 (342 to 574)	448 (348 to 573)	420 (323 to 521)	336 (267 to 403)	163 (124 to 198)	50·8 (37·9 to 62·9)	3,080
Ischemic stroke	0·3 (0·1 to 0·6)	0 (0 to 0)	0 (0 to 0·1)	0·3 (0·2 to 0·4)	0·9 (0·5 to 1·4)	1·4 (0·8 to 2·2)	2·7 (1·8 to 3·8)	3·7 (2·5 to 5·2)	4·2 (2·6 to 6·2)	6·2 (4·2 to 8·7)	10 (7·1 to 13·7)	15·8 (11·2 to 21·2)	25·5 (19·1 to 33·1)	41·3 (30·5 to 55·3)	62·7 (46·8 to 85·3)	116 (88·3 to 149)	141 (105 to 184)	285 (216 to 365)	304 (236 to 391)	340 (262 to 421)	280 (223 to 335)	139 (106 to 169)	44·6 (33 to 55·2)	1,830
Intracerebral hemorrhage	0·2 (0·1 to 0·4)	0 (0 to 0·1)	0·2 (0·1 to 0·5)	0·1 (0·1 to 0·1)	0·4 (0·3 to 0·6)	0·9 (0·6 to 1·2)	3 (2·1 to 4·2)	4·2 (2·7 to 6·3)	4·7 (3·1 to 6·8)	7·7 (5·1 to 10·9)	15·7 (10·3 to 22·8)	26 (17·2 to 36·4)	48·4 (34·1 to 66·5)	74·9 (52·2 to 106)	102 (69·4 to 146)	124 (90·3 to 165)	152 (108 to 206)	139 (103 to 180)	125 (94·5 to 162)	68·5 (51·1 to 87·8)	48·5 (38 to 59·1)	20·6 (15·4 to 25·9)	5·4 (4 to 6·7)	971
Subarachnoid hemorrhage	0·4 (0·2 to 0·8)	0·1 (0 to 0·1)	0·3 (0·1 to 0·5)	0·1 (0·1 to 0·3)	0·5 (0·3 to 0·8)	1 (0·6 to 1·5)	2·9 (1·7 to 4·5)	3·5 (2 to 5·9)	4·3 (2·6 to 6·8)	7 (4·5 to 10·8)	13·6 (8·4 to 20·3)	19·9 (12·5 to 30)	26·2 (17·7 to 38·1)	34·2 (21·9 to 50·4)	40·1 (25·9 to 60·2)	33 (21·3 to 49)	33·6 (22 to 49·5)	24·9 (16·7 to 35·9)	19·4 (12·8 to 28·3)	11·4 (7·7 to 16·3)	7·2 (5 to 10·1)	3·2 (2·2 to 4·6)	0·9 (0·6 to 1·2)	288
Hypertensive heart disease	0 (0 to 0)	0 (0 to 0)	0 (0 to 0)	0 (0 to 0)	0 (0 to 0)	0 (0 to 0)	0·4 (0·2 to 0·7)	0·9 (0·5 to 1·4)	1 (0·6 to 1·6)	2·4 (1·5 to 3·7)	5·9 (3·7 to 8·8)	9·7 (6·4 to 14)	21·1 (13 to 31·6)	34·7 (23·2 to 49·9)	51·3 (33·5 to 73·4)	76 (49·9 to 108)	83·5 (58·6 to 116)	95·9 (67·9 to 132)	89·5 (62·2 to 124)	86·6 (61·4 to 116)	65·2 (46·5 to 89·2)	38·2 (25·8 to 50·8)	12·7 (8·5 to 17·1)	675
Non-rheumatic valvular heart disease	0 (0 to 0)	0 (0 to 0)	0 (0 to 0)	0 (0 to 0)	0 (0 to 0)	0 (0 to 0)	1 (0·6 to 1·4)	0·7 (0·5 to 1·1)	0·6 (0·4 to 1)	0·9 (0·6 to 1·3)	1·4 (0·9 to 2·1)	1·8 (1·2 to 2·8)	3 (1·9 to 4·5)	3·6 (2·2 to 5·3)	4·8 (3·2 to 7·1)	7·1 (4·8 to 10·2)	6·4 (4·5 to 8·9)	6·4 (4·7 to 8·8)	5·8 (4·2 to 8)	4·7 (3·3 to 6·3)	3·8 (2·6 to 5·2)	1·8 (1·3 to 2·4)	0·4 (0·3 to 0·5)	54·4
Non-rheumatic calcific aortic valve disease	0 (0 to 0)	0 (0 to 0)	0 (0 to 0)	0 (0 to 0)	0 (0 to 0)	0 (0 to 0)	0·5 (0·3 to 0·8)	0·6 (0·3 to 0·8)	0·4 (0·2 to 0·6)	0·4 (0·2 to 0·7)	0·8 (0·5 to 1·3)	1·2 (0·7 to 1·8)	1·9 (1·1 to 3)	2·5 (1·5 to 3·8)	3·5 (2·2 to 5·3)	5·8 (3·9 to 8·5)	5·4 (3·7 to 7·6)	5·4 (3·8 to 7·6)	5·1 (3·6 to 6·9)	4·1 (2·9 to 5·6)	3·5 (2·4 to 4·8)	1·6 (1·1 to 2·1)	0·3 (0·2 to 0·5)	42·9
Non-rheumatic degenerative mitral valve disease	0 (0 to 0)	0 (0 to 0)	0 (0 to 0)	0 (0 to 0)	0 (0 to 0)	0 (0 to 0)	0·2 (0·1 to 0·4)	0·1 (0·1 to 0·2)	0·1 (0·1 to 0·2)	0·2 (0·1 to 0·3)	0·3 (0·2 to 0·5)	0·4 (0·2 to 0·8)	0·6 (0·3 to 1·1)	0·7 (0·3 to 1·2)	0·8 (0·4 to 1·4)	0·9 (0·5 to 1·6)	0·7 (0·4 to 1·1)	0·5 (0·3 to 0·9)	0·4 (0·2 to 0·7)	0·3 (0·2 to 0·5)	0·2 (0·1 to 0·3)	0·1 (0·1 to 0·2)	0 (0 to 0)	6·6
Other non-rheumatic valve diseases	0 (0 to 0)	0 (0 to 0)	0 (0 to 0)	0 (0 to 0)	0 (0 to 0)	0 (0 to 0)	0·2 (0·1 to 0·4)	0·1 (0 to 0·2)	0·1 (0·1 to 0·3)	0·3 (0·1 to 0·5)	0·3 (0·1 to 0·6)	0·2 (0·1 to 0·5)	0·5 (0·2 to 1·1)	0·4 (0·2 to 0·8)	0·5 (0·2 to 1)	0·4 (0·1 to 0·7)	0·4 (0·2 to 0·8)	0·5 (0·2 to 1)	0·3 (0·1 to 0·7)	0·3 (0·1 to 0·6)	0·2 (0·1 to 0·3)	0·1 (0 to 0·2)	0 (0 to 0)	4·9
Cardiomyopathy and myocarditis	0·4 (0·2 to 0·7)	0·3 (0·2 to 0·5)	1·5 (0·8 to 2·4)	0·8 (0·5 to 1·1)	0·9 (0·6 to 1·4)	1·6 (1 to 2·4)	2·4 (1·6 to 3·5)	3·1 (2 to 4·9)	2·9 (1·8 to 4·4)	4 (2·6 to 6)	6·2 (4 to 9·3)	7·5 (5·1 to 11·3)	11·6 (7·6 to 17·4)	16·7 (10·8 to 25)	22·3 (14·8 to 32·2)	29·7 (19·5 to 43·5)	28·1 (19 to 39·4)	27·3 (18·6 to 38·8)	24 (16·1 to 34·4)	18·8 (12·9 to 27·4)	17·5 (11·1 to 25·7)	8·5 (5·4 to 12·3)	2·5 (1·6 to 3·7)	239
Myocarditis	0 (0 to 0·1)	0 (0 to 0·1)	0·3 (0·1 to 0·5)	0·2 (0·1 to 0·4)	0·4 (0·2 to 0·5)	0·3 (0·2 to 0·5)	0·4 (0·2 to 0·6)	0·4 (0·2 to 0·6)	0·3 (0·2 to 0·5)	0·6 (0·3 to 0·9)	0·5 (0·3 to 0·8)	0·4 (0·2 to 0·6)	0·9 (0·5 to 1·4)	0·8 (0·5 to 1·3)	0·8 (0·5 to 1·3)	0·9 (0·5 to 1·5)	0·5 (0·3 to 0·8)	1·1 (0·7 to 1·8)	1·1 (0·6 to 2)	1·3 (0·6 to 2·6)	1·5 (0·7 to 3)	0·9 (0·5 to 1·4)	0·3 (0·2 to 0·5)	14
Alcoholic cardiomyopathy	0 (0 to 0)	0 (0 to 0)	0 (0 to 0)	0 (0 to 0)	0 (0 to 0)	0 (0 to 0)	0·2 (0·1 to 0·3)	0·4 (0·2 to 0·7)	0·5 (0·2 to 0·9)	0·4 (0·2 to 0·7)	0·6 (0·3 to 1)	0·6 (0·3 to 1)	2·7 (1·3 to 4·6)	3·5 (1·7 to 5·7)	3·4 (1·7 to 5·8)	4 (1·9 to 7)	3·8 (1·8 to 6·7)	0·7 (0·3 to 1·3)	0·9 (0·3 to 2)	0·7 (0·3 to 1·6)	0·2 (0·1 to 0·5)	0·1 (0·1 to 0·2)	0 (0 to 0·1)	22·7
Other cardiomyopathy	0·3 (0·2 to 0·6)	0·3 (0·1 to 0·4)	1·2 (0·6 to 2)	0·6 (0·3 to 0·9)	0·6 (0·3 to 0·9)	1·3 (0·7 to 2)	1·9 (1·1 to 2·9)	2·4 (1·4 to 3·9)	2·1 (1·2 to 3·4)	3·1 (1·8 to 4·8)	5·1 (3·1 to 7·9)	6·6 (4·2 to 10·2)	8 (4·7 to 12·7)	12·3 (7·2 to 19·5)	18·1 (11·1 to 27·5)	24·8 (15·6 to 37·9)	23·8 (15·3 to 34·9)	25·4 (17·2 to 36·9)	21·9 (14·6 to 31·5)	16·8 (11·4 to 24·8)	15·8 (10 to 23·2)	7·5 (4·5 to 11·3)	2·2 (1·3 to 3·3)	202
Atrial fibrillation and flutter	0 (0 to 0)	0 (0 to 0)	0 (0 to 0)	0 (0 to 0)	0 (0 to 0)	0 (0 to 0)	0 (0 to 0)	0 (0 to 0)	0 (0 to 0)	0·1 (0·1 to 0·2)	0·3 (0·2 to 0·4)	1·2 (0·7 to 1·8)	2·3 (1·5 to 3·5)	4·2 (2·9 to 6)	8·5 (6 to 11·9)	15 (10·8 to 20·1)	20·3 (15·5 to 26·4)	30·8 (23·9 to 40·9)	35·6 (27·6 to 45·2)	44·2 (35·4 to 55·3)	44·8 (33·9 to 57·2)	29·8 (23 to 38·2)	9·3 (6·9 to 11·9)	246
Aortic aneurysm	--	--	--	--	--	--	0·1 (0 to 0·1)	0·1 (0·1 to 0·2)	0·3 (0·2 to 0·5)	0·3 (0·2 to 0·5)	0·8 (0·5 to 1·2)	1·1 (0·7 to 1·6)	1·8 (1·2 to 2·7)	3 (2·1 to 4·2)	5·4 (3·7 to 7·6)	8·7 (5·9 to 12·6)	11·7 (8·2 to 16·5)	13·6 (9·5 to 18·7)	12·4 (8·6 to 17·5)	10·3 (7·2 to 14·3)	7·2 (5·1 to 9·9)	3·2 (2·3 to 4·3)	0·8 (0·6 to 1·1)	NA
Endocarditis	0 (0 to 0·1)	0·1 (0 to 0·1)	0·3 (0·2 to 0·7)	0·2 (0·1 to 0·3)	0·4 (0·2 to 0·6)	0·4 (0·2 to 0·7)	1·5 (0·9 to 2·3)	2·5 (1·5 to 3·9)	2·1 (1·2 to 3·5)	1·9 (1·1 to 3·2)	3·9 (2·3 to 6·5)	3·8 (2·3 to 6·2)	4·9 (2·6 to 8·2)	5 (3·1 to 7·9)	6·7 (4 to 10·6)	8·8 (5·3 to 14·4)	6·7 (4·3 to 10·5)	7·1 (4·4 to 11·2)	5·6 (3·7 to 8·6)	6 (3·8 to 9·5)	4·3 (2·7 to 6·8)	2·2 (1·4 to 3·4)	0·7 (0·5 to 1·1)	75·3
Other cardiovascular and circulatory diseases	1·3 (0·6 to 2·1)	0·5 (0·3 to 0·9)	1·9 (0·9 to 3·1)	0·7 (0·4 to 1·1)	1·7 (1·2 to 2·4)	2·6 (1·8 to 3·8)	6·6 (4·5 to 9·2)	7·3 (5·1 to 10)	9·1 (6·1 to 12·7)	10·1 (7 to 13·9)	14·7 (10·5 to 20·1)	29·6 (20·6 to 42·2)	28·9 (20·6 to 38·3)	31·9 (24·1 to 41·7)	44·3 (33·3 to 56·5)	57 (42·3 to 74)	54·5 (41·9 to 68·6)	60·1 (45·9 to 78·9)	53·2 (40·1 to 69·2)	46·3 (34·7 to 60·5)	43·8 (31·2 to 58)	21·2 (15·3 to 27·6)	6·2 (4·4 to 8)	534
Chronic respiratory diseases	0 (0 to 0)	0 (0 to 0)	0 (0 to 0)	33·6 (17·7 to 60·9)	36·7 (21·8 to 62·2)	35·4 (21·2 to 56·6)	35·8 (21·8 to 57·1)	33·6 (20·9 to 50·3)	35·2 (22·1 to 53·8)	34·9 (23 to 50·8)	39·7 (27·7 to 55·8)	39·3 (28·9 to 52·5)	47 (35·8 to 61)	57·4 (44·3 to 73·2)	68·5 (52·7 to 86·7)	84·2 (65·5 to 105)	80·7 (62·8 to 100)	83·2 (64·5 to 104)	68 (51·6 to 89·1)	55·1 (42·1 to 70·1)	46·8 (36 to 58·8)	24 (18·6 to 29·8)	7·3 (5·6 to 9)	946
Chronic obstructive pulmonary disease	0 (0 to 0)	0 (0 to 0)	0 (0 to 0)	0·3 (0·2 to 0·4)	0·7 (0·2 to 1·4)	1·2 (0·4 to 2·2)	2·1 (1·1 to 3·2)	2·8 (1·6 to 4·4)	3·7 (2·1 to 5·8)	4·4 (2·5 to 6·7)	6·1 (3·8 to 8·8)	7·6 (5·1 to 10·3)	11·1 (7·9 to 15·1)	17·6 (13·1 to 23·5)	24·7 (18·4 to 31·6)	39·9 (29·6 to 52·6)	40·6 (30·4 to 52·2)	44·7 (33·6 to 58·5)	40·9 (29·8 to 55·5)	37·4 (27 to 48·9)	34·8 (25·9 to 44·4)	18·2 (13·8 to 22·8)	5·6 (4·2 to 7)	344
Pneumoconiosis	0 (0 to 0)	0 (0 to 0)	0 (0 to 0)	0 (0 to 0)	0 (0 to 0)	0 (0 to 0)	0 (0 to 0)	0 (0 to 0)	0 (0 to 0)	0 (0 to 0)	0 (0 to 0)	0 (0 to 0)	0 (0 to 0)	0 (0 to 0·1)	0 (0 to 0)	0 (0 to 0)	0 (0 to 0·1)	0 (0 to 0·1)	0·1 (0 to 0·1)	0 (0 to 0·1)	0 (0 to 0·1)	0 (0 to 0)	0 (0 to 0)	0·4
Silicosis	0 (0 to 0)	0 (0 to 0)	0 (0 to 0)	0 (0 to 0)	0 (0 to 0)	0 (0 to 0)	0 (0 to 0)	0 (0 to 0)	0 (0 to 0)	0 (0 to 0)	0 (0 to 0)	0 (0 to 0)	0 (0 to 0)	0 (0 to 0)	0 (0 to 0)	0 (0 to 0)	0 (0 to 0)	0 (0 to 0)	0 (0 to 0)	0 (0 to 0)	0 (0 to 0)	0 (0 to 0)	0 (0 to 0)	0
Asbestosis	0 (0 to 0)	0 (0 to 0)	0 (0 to 0)	0 (0 to 0)	0 (0 to 0)	0 (0 to 0)	0 (0 to 0)	0 (0 to 0)	0 (0 to 0)	0 (0 to 0)	0 (0 to 0)	0 (0 to 0)	0 (0 to 0)	0 (0 to 0)	0 (0 to 0)	0 (0 to 0)	0 (0 to 0)	0 (0 to 0)	0 (0 to 0)	0 (0 to 0)	0 (0 to 0)	0 (0 to 0)	0 (0 to 0)	0·1
Coal workers pneumoconiosis	0 (0 to 0)	0 (0 to 0)	0 (0 to 0)	0 (0 to 0)	0 (0 to 0)	0 (0 to 0)	0 (0 to 0)	0 (0 to 0)	0 (0 to 0)	0 (0 to 0)	0 (0 to 0)	0 (0 to 0)	0 (0 to 0)	0 (0 to 0)	0 (0 to 0)	0 (0 to 0)	0 (0 to 0)	0 (0 to 0)	0 (0 to 0)	0 (0 to 0)	0 (0 to 0)	0 (0 to 0)	0 (0 to 0)	0
Other pneumoconiosis	0 (0 to 0)	0 (0 to 0)	0 (0 to 0)	0 (0 to 0)	0 (0 to 0)	0 (0 to 0)	0 (0 to 0)	0 (0 to 0)	0 (0 to 0)	0 (0 to 0)	0 (0 to 0)	0 (0 to 0)	0 (0 to 0)	0 (0 to 0)	0 (0 to 0)	0 (0 to 0)	0 (0 to 0)	0 (0 to 0)	0 (0 to 0·1)	0 (0 to 0)	0 (0 to 0)	0 (0 to 0)	0 (0 to 0)	0·2
Asthma	0 (0 to 0)	0 (0 to 0)	0 (0 to 0)	31·8 (16·1 to 59)	34·4 (19·8 to 58·9)	32·1 (18·2 to 53)	32·3 (18·3 to 52·9)	28·8 (16·3 to 45)	28·8 (16·4 to 47·5)	27·5 (16·3 to 42·6)	29·6 (19·1 to 44)	27·6 (18·2 to 39·3)	30·4 (20·8 to 42·5)	32·9 (22·9 to 45·7)	32·9 (22·7 to 45·7)	33·2 (23·4 to 44·7)	29·2 (20·7 to 38·7)	25·4 (17·9 to 33·7)	17·3 (12·2 to 23·4)	10·8 (7·7 to 14·4)	7·1 (5 to 9·7)	3·4 (2·5 to 4·6)	1·1 (0·8 to 1·5)	496
Interstitial lung disease and pulmonary sarcoidosis	0 (0 to 0)	0 (0 to 0)	0 (0 to 0)	0·6 (0·3 to 1·1)	0·3 (0·2 to 0·5)	0·2 (0·1 to 0·5)	0·5 (0·2 to 0·9)	0·6 (0·3 to 1·1)	1 (0·6 to 1·6)	1·4 (0·8 to 2·3)	2·3 (1·4 to 3·7)	2·3 (1·3 to 3·5)	3·8 (2·3 to 5·6)	5·1 (3·2 to 7·6)	7·3 (4·5 to 11)	10·3 (6 to 15·7)	10·4 (6·4 to 15·7)	12·4 (7·5 to 19)	9·2 (5·6 to 14)	6·4 (3·7 to 9·9)	4·6 (2·4 to 7·5)	2·2 (1·2 to 3·6)	0·5 (0·3 to 0·9)	81·4
Other chronic respiratory diseases	0 (0 to 0)	0 (0 to 0)	0 (0 to 0)	1 (0·5 to 1·6)	1·3 (0·6 to 2·4)	1·9 (0·9 to 3·2)	0·9 (0·5 to 1·4)	1·4 (0·9 to 2·2)	1·8 (1·1 to 2·7)	1·4 (0·9 to 2·1)	1·7 (1·1 to 2·5)	1·8 (1·2 to 2·6)	1·7 (1·1 to 2·5)	1·8 (1·1 to 2·7)	3·5 (1·9 to 5·9)	0·9 (0·4 to 1·5)	0·5 (0·2 to 0·8)	0·6 (0·3 to 1·1)	0·4 (0·2 to 0·8)	0·4 (0·2 to 0·8)	0·3 (0·1 to 0·5)	0·1 (0·1 to 0·2)	0·1 (0 to 0·1)	23·5
Digestive diseases	1·1 (0·7 to 1·7)	1·1 (0·7 to 1·8)	5·2 (2·9 to 9)	7·1 (5 to 9·9)	8·8 (6·3 to 11·8)	10·2 (7·5 to 13·4)	17·7 (13·2 to 23·4)	27·3 (19·6 to 38·4)	35·7 (24·4 to 51·8)	44·1 (31 to 61·7)	60 (43·8 to 81·7)	68·6 (51·6 to 90·2)	89·4 (68 to 116)	112 (84 to 145)	134 (104 to 171)	162 (127 to 202)	143 (114 to 179)	142 (112 to 176)	109 (87 to 136)	93·7 (72·1 to 119)	66·9 (53·4 to 80·6)	30·9 (23·9 to 37·1)	7·6 (5·8 to 8·9)	1,380
Cirrhosis and other chronic liver diseases	0 (0 to 0)	0 (0 to 0)	0 (0 to 0)	0·4 (0·2 to 0·5)	0·7 (0·4 to 1·1)	1·3 (0·7 to 2)	2·3 (1·4 to 3·4)	3·1 (2 to 4·4)	2·8 (1·8 to 4·3)	4·6 (3·1 to 6·6)	8·8 (5·9 to 12·5)	11·8 (8·1 to 16·2)	18·6 (12·6 to 26)	27·8 (19·1 to 38·3)	37·6 (26·2 to 51·6)	48·1 (33·8 to 65·5)	42 (30·4 to 55·4)	36·9 (26·7 to 48·8)	24·7 (18 to 32·8)	17·8 (12·2 to 24·9)	10·5 (7·5 to 14·2)	4 (3 to 5)	0·9 (0·6 to 1·2)	305
Cirrhosis and other chronic liver diseases due to hepatitis B	0 (0 to 0)	0 (0 to 0)	0 (0 to 0)	0 (0 to 0)	0 (0 to 0)	0 (0 to 0)	0 (0 to 0)	0·1 (0 to 0·1)	0·1 (0 to 0·3)	0·3 (0·1 to 0·5)	0·6 (0·3 to 1·2)	0·9 (0·4 to 1·7)	1·4 (0·7 to 2·6)	1·9 (0·9 to 3·7)	2·2 (1 to 4·2)	2·4 (1·1 to 4·5)	1·9 (0·9 to 3·5)	1·6 (0·7 to 2·9)	1·1 (0·5 to 1·9)	0·8 (0·3 to 1·4)	0·4 (0·2 to 0·8)	0·2 (0·1 to 0·3)	0 (0 to 0·1)	15·9
Cirrhosis and other chronic liver diseases due to hepatitis C	0 (0 to 0)	0 (0 to 0)	0 (0 to 0)	0 (0 to 0)	0 (0 to 0)	0 (0 to 0)	0·1 (0 to 0·1)	0·3 (0·1 to 0·5)	0·5 (0·2 to 0·9)	1 (0·5 to 1·7)	2 (1 to 3·6)	2·7 (1·4 to 4·5)	3·9 (1·9 to 6·8)	5·4 (2·7 to 9·6)	6·7 (3·3 to 11·8)	7·9 (3·5 to 15)	6·5 (3·4 to 11·5)	5·4 (3 to 9·3)	3·4 (1·8 to 5·8)	2·5 (1·2 to 4·3)	1·5 (0·8 to 2·5)	0·6 (0·3 to 1)	0·1 (0·1 to 0·3)	50·5
Cirrhosis and other chronic liver diseases due to alcohol use	0 (0 to 0)	0 (0 to 0)	0 (0 to 0)	0 (0 to 0)	0 (0 to 0)	0 (0 to 0)	0 (0 to 0)	0·2 (0·1 to 0·3)	0·3 (0·1 to 0·7)	1 (0·5 to 1·7)	2·6 (1·3 to 4·5)	4 (2·3 to 6·2)	6·7 (3·6 to 10·7)	10·6 (5·7 to 16·4)	15 (8·2 to 23·2)	19 (10·4 to 28·7)	15·3 (8·8 to 23·3)	12·1 (6·5 to 18·6)	7 (3·9 to 11)	4·5 (2·5 to 7·8)	2·5 (1·4 to 4)	0·9 (0·5 to 1·6)	0·2 (0·1 to 0·4)	102
Cirrhosis and other chronic liver diseases due to NAFLD	0 (0 to 0)	0 (0 to 0)	0 (0 to 0)	0 (0 to 0)	0 (0 to 0)	0 (0 to 0)	0·1 (0 to 0·2)	0·3 (0·1 to 0·6)	0·5 (0·2 to 1)	0·9 (0·5 to 1·6)	1·7 (0·8 to 3·2)	2·4 (1·2 to 4·1)	4 (1·9 to 7·5)	6·1 (2·8 to 11·3)	8 (3·9 to 14)	10·4 (5 to 18·3)	9·9 (5·7 to 15·3)	9·3 (5·4 to 14·5)	6·7 (3·4 to 10·8)	5 (2·6 to 8·1)	3 (1·7 to 4·5)	1·2 (0·6 to 1·8)	0·3 (0·1 to 0·4)	69·6
Cirrhosis and other chronic liver diseases due to other causes	0 (0 to 0)	0 (0 to 0)	0 (0 to 0)	0·3 (0·2 to 0·5)	0·7 (0·4 to 1·1)	1·2 (0·7 to 1·9)	2·1 (1·3 to 3·2)	2·3 (1·5 to 3·3)	1·4 (0·8 to 2·3)	1·5 (0·8 to 2·6)	1·8 (0·9 to 3·2)	1·8 (0·8 to 3·4)	2·6 (1·2 to 4·8)	3·8 (1·8 to 6·8)	5·7 (2·9 to 10·4)	8·4 (4·2 to 14·9)	8·3 (4·2 to 14·8)	8·5 (4·5 to 14·3)	6·6 (3·5 to 10·9)	5·1 (2·7 to 8·7)	3 (1·8 to 4·7)	1·2 (0·7 to 1·8)	0·3 (0·1 to 0·4)	66·8
Upper digestive system diseases	0 (0 to 0)	0 (0 to 0)	0 (0 to 0)	0·6 (0·3 to 1)	1 (0·5 to 1·8)	1·8 (1·1 to 2·9)	5·9 (3·3 to 10)	12·9 (7 to 23·4)	19·3 (10·3 to 34·1)	22·4 (11·7 to 40·2)	26·2 (14·3 to 44·9)	27·1 (15·5 to 45·5)	33 (19·5 to 52·9)	36·9 (22·8 to 58·6)	41·4 (26·4 to 65·3)	44·2 (28·9 to 67·2)	37·5 (26 to 55·6)	35·5 (25·2 to 49·6)	26·6 (19·4 to 36·7)	21·5 (15·2 to 30)	15 (11·2 to 19·7)	6·9 (5·2 to 8·9)	1·7 (1·2 to 2·1)	418
Peptic ulcer disease	0 (0 to 0)	0 (0 to 0)	0 (0 to 0)	0·1 (0 to 0·1)	0·1 (0·1 to 0·2)	0·3 (0·2 to 0·5)	0·5 (0·3 to 0·7)	0·8 (0·5 to 1·1)	0·7 (0·5 to 1·1)	1·3 (0·9 to 1·9)	2·2 (1·5 to 3·1)	2·7 (1·9 to 3·7)	4·3 (2·9 to 6·2)	6·1 (4·4 to 8·3)	8·8 (6·2 to 11·8)	13·1 (8·6 to 18·5)	12·4 (8·9 to 16·6)	14·9 (10·6 to 20)	12·9 (9·2 to 17·4)	12·3 (7·8 to 18·5)	9·3 (6·6 to 12·7)	4·5 (3·3 to 6)	1·1 (0·8 to 1·5)	109
Gastritis and duodenitis	0 (0 to 0)	0 (0 to 0)	0 (0 to 0)	0·5 (0·2 to 0·9)	0·9 (0·3 to 1·7)	0·9 (0·3 to 1·8)	1·1 (0·4 to 2·2)	1·3 (0·6 to 2·3)	1·7 (0·8 to 3·1)	2·2 (1·1 to 4·1)	3 (1·4 to 5)	3·2 (1·6 to 6·3)	4·8 (2·4 to 8·7)	6 (3·2 to 10·8)	7·4 (4 to 13·1)	7·4 (4·2 to 12·3)	6·4 (3·7 to 10·4)	5·6 (3·5 to 8·8)	3·6 (2·3 to 5·4)	2·4 (1·4 to 3·6)	1·8 (1·1 to 2·6)	0·8 (0·5 to 1·2)	0·2 (0·1 to 0·3)	61
Gastroesophageal reflux disease	0 (0 to 0)	0 (0 to 0)	0 (0 to 0)	0 (0 to 0)	0 (0 to 0)	0·6 (0·3 to 1·2)	4·4 (2·1 to 8·2)	10·9 (5·5 to 21·1)	16·9 (8·1 to 31·1)	18·9 (8·8 to 36·1)	21 (10 to 38·7)	21·2 (10·1 to 38·5)	23·8 (11·6 to 41·8)	24·7 (11·9 to 45)	25·2 (12 to 47·5)	23·7 (11·5 to 42·9)	18·7 (9·4 to 33·8)	15 (7·4 to 26·8)	10·1 (5·1 to 18·4)	6·8 (3·4 to 12·2)	3·9 (2 to 6·9)	1·6 (0·8 to 2·8)	0·4 (0·2 to 0·7)	248
Appendicitis	0 (0 to 0)	0 (0 to 0)	0 (0 to 0)	0·2 (0·1 to 0·3)	0·6 (0·4 to 1)	1·2 (0·6 to 2)	1·5 (0·7 to 2·5)	1·3 (0·7 to 2·2)	0·9 (0·3 to 1·6)	0·9 (0·4 to 1·6)	1·3 (0·7 to 2·1)	1·1 (0·7 to 1·6)	1·3 (0·8 to 2)	1·3 (0·8 to 2)	1·6 (0·9 to 2·7)	1·5 (0·9 to 2·4)	1·6 (1 to 2·4)	1·5 (1 to 2·4)	1 (0·6 to 1·6)	0·8 (0·5 to 1·2)	0·7 (0·4 to 1·2)	0·3 (0·2 to 0·6)	0·1 (0 to 0·1)	20·6
Paralytic ileus and intestinal obstruction	0·8 (0·5 to 1·2)	1 (0·6 to 1·6)	4·7 (2·6 to 8·3)	1·8 (1 to 3)	1·5 (0·9 to 2·1)	1·5 (0·9 to 2·2)	1·7 (1·1 to 2·6)	1·4 (0·9 to 2·3)	1·7 (1 to 2·8)	1·9 (1·1 to 3)	4·4 (2·6 to 6·6)	4·9 (3·1 to 7·3)	7·3 (4·4 to 11·3)	10·6 (6·5 to 16)	12·3 (7·8 to 18·9)	18·8 (11·6 to 27·5)	19·2 (12·4 to 28·1)	23·8 (15·5 to 34)	21·3 (14·1 to 30·4)	21·9 (14·9 to 32·6)	16·3 (11·4 to 22·4)	8·3 (5·7 to 11·3)	2·2 (1·5 to 2·9)	189
Inguinal, femoral, and abdominal hernia	0·3 (0·2 to 0·5)	0·1 (0 to 0·2)	0·4 (0·2 to 0·8)	2·2 (1·1 to 3·8)	2·8 (1·3 to 4·8)	2 (0·9 to 3·5)	1·6 (0·7 to 2·8)	1·4 (0·6 to 2·7)	1·8 (0·8 to 3·3)	2·3 (1·1 to 4·2)	3 (1·5 to 5·3)	3·6 (2 to 5·8)	4·5 (2·5 to 7·3)	5·2 (3·1 to 7·9)	5·5 (3·4 to 8·8)	5·9 (3·8 to 8·6)	4·3 (2·8 to 6·2)	4 (2·7 to 5·7)	3·1 (2 to 4·5)	2·8 (1·8 to 4·2)	2·1 (1·3 to 3·2)	1 (0·6 to 1·5)	0·2 (0·2 to 0·3)	60·1
Inflammatory bowel disease	0 (0 to 0)	0 (0 to 0)	0 (0 to 0)	0·2 (0·1 to 0·4)	0·2 (0·1 to 0·3)	0·2 (0·1 to 0·2)	0·6 (0·4 to 0·8)	1·1 (0·6 to 1·6)	1·7 (1 to 2·6)	2·3 (1·4 to 3·6)	3·5 (2·2 to 5·2)	4·5 (3 to 6·4)	4·3 (2·8 to 6·3)	4·7 (3 to 6·8)	5·5 (3·8 to 7·7)	6·3 (4·3 to 8·9)	4·2 (2·8 to 5·7)	4·6 (3·2 to 6·4)	3·4 (2·3 to 4·7)	2·1 (1·4 to 3)	1·6 (1 to 2·3)	0·9 (0·6 to 1·3)	0·1 (0·1 to 0·2)	51·9
Vascular intestinal disorders	0 (0 to 0)	0 (0 to 0)	0 (0 to 0)	0 (0 to 0)	0·1 (0 to 0·1)	0 (0 to 0)	0 (0 to 0·1)	0·1 (0·1 to 0·2)	0·2 (0·1 to 0·3)	0·2 (0·1 to 0·3)	0·4 (0·2 to 0·7)	0·7 (0·4 to 1)	1·3 (0·7 to 2·1)	1·6 (1 to 2·5)	2·3 (1·5 to 3·4)	4 (2·5 to 5·8)	4·2 (2·7 to 6·1)	4·9 (3·2 to 7·2)	4·2 (2·8 to 6·2)	3·8 (2·5 to 5·7)	2·9 (1·9 to 4·3)	1·1 (0·7 to 1·6)	0·2 (0·2 to 0·4)	32·3
Gallbladder and biliary diseases	0 (0 to 0)	0 (0 to 0)	0 (0 to 0)	0·2 (0·1 to 0·4)	0·7 (0·2 to 1·4)	1·2 (0·4 to 2·3)	2·1 (1 to 4)	3·3 (1·8 to 5·4)	4·7 (2·6 to 8)	6·4 (3·7 to 10·3)	8·7 (5·1 to 13·4)	10·1 (6·1 to 15·3)	12·6 (8·1 to 18·9)	14·3 (9·2 to 21·3)	15·6 (10·3 to 22·4)	16·4 (11 to 23·1)	13·3 (9·1 to 18·8)	11·7 (8·3 to 16·3)	8·8 (6 to 12·2)	6·8 (4·5 to 9·7)	4·2 (2·9 to 6)	2 (1·4 to 2·9)	0·5 (0·3 to 0·7)	144
Pancreatitis	0 (0 to 0)	0 (0 to 0)	0 (0 to 0)	0·1 (0 to 0·1)	0·1 (0 to 0·1)	0·1 (0·1 to 0·2)	0·5 (0·3 to 0·8)	1·3 (0·8 to 2)	1 (0·6 to 1·6)	1·2 (0·8 to 2)	1·3 (0·8 to 2)	2 (1·2 to 3)	2·5 (1·6 to 4)	3 (1·8 to 4·5)	3·8 (2·5 to 5·6)	4·8 (3 to 7·3)	4·5 (2·9 to 6·3)	4·7 (3·1 to 6·9)	3·5 (2·3 to 5)	2·6 (1·6 to 3·9)	1·8 (1·2 to 2·6)	0·7 (0·4 to 1)	0·2 (0·1 to 0·2)	39·7
Other digestive diseases	0 (0 to 0)	0 (0 to 0)	0 (0 to 0)	1·4 (0·7 to 2·3)	1·1 (0·6 to 1·6)	1 (0·5 to 1·5)	1·4 (0·7 to 2·2)	1·3 (0·6 to 2·2)	1·5 (0·7 to 2·5)	1·8 (0·9 to 3)	2·3 (1·1 to 4)	3 (1·4 to 4·9)	4·1 (2·1 to 6·8)	6·4 (3 to 10·4)	8·4 (4 to 13·7)	12·5 (5·7 to 19·4)	12·7 (5·9 to 19·7)	14·3 (6·6 to 21·8)	12·8 (6 to 20·2)	13·6 (6·2 to 21·2)	11·8 (5·8 to 17·5)	5·7 (2·7 to 8·3)	1·5 (0·7 to 2·1)	119
Neurological disorders	0 (0 to 0·1)	0·4 (0·2 to 0·6)	5·8 (3·5 to 8·8)	12·9 (6·3 to 24·4)	28·2 (12·1 to 50·6)	82·9 (23·8 to 174)	118 (35·9 to 252)	119 (37·1 to 246)	129 (40·3 to 265)	129 (43·2 to 255)	146 (51·7 to 281)	148 (56·4 to 284)	163 (72·6 to 304)	162 (77·2 to 291)	165 (89·1 to 279)	174 (106 to 272)	166 (105 to 268)	196 (123 to 337)	209 (131 to 378)	260 (146 to 524)	240 (125 to 495)	148 (69·6 to 311)	53·1 (23·9 to 113)	2,860
Alzheimer's disease and other dementias	0 (0 to 0)	0 (0 to 0)	0 (0 to 0)	0 (0 to 0)	0 (0 to 0)	0 (0 to 0)	0 (0 to 0)	0 (0 to 0)	0 (0 to 0)	0 (0 to 0)	0 (0 to 0)	0·6 (0·2 to 2)	4·2 (1·3 to 11·2)	9·4 (3·3 to 23·8)	19·2 (7·5 to 46·4)	37·6 (14·7 to 89·1)	59·2 (23·3 to 141)	98 (41 to 230)	124 (52·8 to 291)	193 (81·7 to 461)	195 (80·1 to 452)	131 (52·9 to 294)	49·5 (20·3 to 110)	921
Parkinson's disease	0 (0 to 0)	0 (0 to 0)	0 (0 to 0)	0 (0 to 0)	0 (0 to 0)	0 (0 to 0)	0 (0 to 0)	0 (0 to 0)	0·1 (0 to 0·1)	0·1 (0·1 to 0·2)	0·3 (0·2 to 0·4)	0·6 (0·4 to 0·9)	1·5 (1 to 2·2)	3 (2·2 to 4·2)	5·3 (3·8 to 7·3)	11·4 (8·7 to 14·6)	16·8 (13·1 to 20·7)	31·1 (25·5 to 37·3)	43·6 (35·4 to 52·7)	42·7 (34·5 to 51·3)	31 (25·2 to 37·2)	11·9 (9·6 to 14·3)	2·4 (1·9 to 2·9)	202
Idiopathic epilepsy	0 (0 to 0·1)	0·1 (0 to 0·1)	1·8 (0·8 to 3·4)	7·4 (2·6 to 15·5)	12·3 (4·4 to 24·5)	16·6 (6·3 to 31·9)	22·4 (10·2 to 40·6)	22·1 (10·2 to 38·6)	20·8 (9·2 to 36·8)	19·5 (9·1 to 34·7)	20·7 (9·9 to 36·2)	18·8 (8·7 to 32·9)	20·2 (9·5 to 35·1)	19·4 (9·3 to 34·2)	19·3 (8·2 to 36·3)	20·1 (8·4 to 37·5)	17·3 (7·1 to 32·5)	16 (6·3 to 29·3)	12·1 (4·9 to 22·3)	8·7 (3·5 to 16·2)	5·5 (2·3 to 10)	2·4 (1 to 4·4)	0·6 (0·2 to 1·1)	304
Multiple sclerosis	0 (0 to 0)	0 (0 to 0)	0 (0 to 0)	0 (0 to 0)	0 (0 to 0)	0·1 (0 to 0·2)	0·5 (0·2 to 1·1)	1·3 (0·7 to 2·1)	2 (1·2 to 3·1)	2·6 (1·7 to 3·7)	5·7 (3·5 to 8·2)	6·1 (3·8 to 8·6)	7·4 (4·6 to 10·7)	7·3 (4·6 to 10·3)	7·8 (4·8 to 10·8)	7·3 (4·2 to 10·4)	3·6 (2·3 to 5·2)	3 (1·9 to 4·3)	1·4 (0·9 to 2)	0·7 (0·4 to 1)	0·4 (0·3 to 0·5)	0·1 (0·1 to 0·2)	0 (0 to 0·1)	57·2
Motor neuron disease	0 (0 to 0)	0·3 (0·2 to 0·5)	1·4 (0·7 to 2·8)	1 (0·5 to 1·6)	0·3 (0·2 to 0·5)	0·5 (0·3 to 0·9)	0·2 (0·2 to 0·3)	0·4 (0·3 to 0·6)	0·4 (0·3 to 0·5)	0·5 (0·3 to 0·7)	1·2 (0·8 to 1·8)	2·3 (1·4 to 3·3)	4·4 (2·8 to 6·5)	7·2 (4·6 to 10·4)	9·2 (5·9 to 13·2)	12·1 (8·1 to 16·5)	10·2 (6·7 to 14·8)	8·4 (5·5 to 12·1)	5·2 (3·3 to 7·7)	2·1 (1·3 to 3)	0·8 (0·5 to 1·1)	0·2 (0·2 to 0·3)	0·1 (0 to 0·1)	68·6
Headache disorders	0 (0 to 0)	0 (0 to 0)	0 (0 to 0)	0 (0 to 0)	10·4 (0·2 to 27·9)	57·5 (2·6 to 145)	86·5 (9·5 to 218)	89·7 (12·2 to 209)	100 (14·5 to 232)	101 (17·3 to 228)	111 (20·4 to 247)	112 (23·6 to 250)	116 (27·6 to 258)	106 (27·3 to 229)	93·3 (25 to 202)	75·3 (22·5 to 159)	50·6 (15·6 to 104)	32·8 (10·6 to 68·5)	17·9 (5·6 to 37·3)	10·3 (3·4 to 21·7)	5·4 (1·7 to 11)	1·8 (0·6 to 3·8)	0·3 (0·1 to 0·7)	1,180
Migraine	0 (0 to 0)	0 (0 to 0)	0 (0 to 0)	0 (0 to 0)	9·7 (0·1 to 26)	54·4 (1·7 to 143)	81·2 (6·3 to 214)	83·5 (8 to 206)	93 (10·4 to 224)	92·9 (11·9 to 216)	102 (14·8 to 236)	103 (16·6 to 237)	105 (19·1 to 240)	95·1 (18·4 to 213)	83·4 (17·5 to 189)	66·1 (16 to 146)	43·3 (10·9 to 94·1)	27·5 (7·5 to 61·4)	14·9 (3·9 to 32·6)	8·5 (2·4 to 18·9)	4·4 (1·2 to 9·7)	1·5 (0·4 to 3·2)	0·2 (0·1 to 0·6)	1,070
Tension-type headache	0 (0 to 0)	0 (0 to 0)	0 (0 to 0)	0 (0 to 0)	0·8 (0 to 4·6)	3·1 (0·2 to 19·5)	5·4 (0·9 to 23·1)	6·2 (1·3 to 24)	7·4 (1·6 to 27·6)	7·7 (1·8 to 25·8)	9·1 (2·4 to 27·6)	9·4 (2·8 to 26·2)	10·6 (3·5 to 27·5)	10·9 (3·2 to 30·2)	9·9 (3·1 to 25·9)	9·2 (2·8 to 25·7)	7·3 (2·1 to 22·6)	5·3 (1·3 to 18·6)	3 (0·7 to 10·7)	1·7 (0·4 to 6·2)	0·9 (0·2 to 3·4)	0·3 (0·1 to 1·2)	0·1 (0 to 0·4)	108
Other neurological disorders	0 (0 to 0)	0 (0 to 0)	2·5 (1·4 to 4·1)	4·5 (2·4 to 7·7)	5·1 (2·5 to 9·3)	8·2 (3·7 to 14·9)	8·5 (4·2 to 14·8)	5·9 (3·2 to 9·5)	5·5 (3·2 to 8·3)	5·4 (3·2 to 8·1)	6·6 (4·1 to 9·9)	7·2 (4·6 to 10·7)	10 (6·7 to 14)	10 (7·1 to 13·7)	11 (7·6 to 15)	10·6 (7·6 to 14·5)	8·1 (5·9 to 10·9)	6·3 (4·7 to 8·3)	4·8 (3·4 to 6·5)	3 (2·1 to 4·1)	1·6 (1 to 2·3)	0·6 (0·5 to 0·9)	0·2 (0·1 to 0·3)	126
Mental disorders	0 (0 to 0)	0 (0 to 0·1)	0·6 (0·3 to 0·9)	3·9 (2·4 to 6)	29·6 (19·3 to 44·1)	114 (76·3 to 164)	203 (138 to 288)	228 (155 to 315)	257 (176 to 355)	253 (178 to 346)	279 (201 to 378)	272 (193 to 379)	295 (212 to 405)	288 (206 to 391)	281 (199 to 386)	246 (173 to 336)	179 (125 to 242)	134 (93·7 to 178)	83·6 (57·4 to 113)	53·3 (36·2 to 72·2)	30·9 (21·6 to 41·8)	12·9 (8·7 to 18)	3·2 (2 to 4·6)	3,250
Schizophrenia	0 (0 to 0)	0 (0 to 0)	0 (0 to 0)	0 (0 to 0)	0 (0 to 0)	0·5 (0·2 to 0·9)	4·1 (1·4 to 8)	14·1 (7·2 to 24·1)	26·8 (15·4 to 41·5)	33·1 (19·7 to 48·6)	38·8 (23·3 to 56·9)	37 (23·6 to 52)	38·5 (24·3 to 54)	35·5 (23·1 to 50·2)	31·4 (20·6 to 44·7)	24·5 (15·7 to 35·4)	15·9 (10·1 to 22·4)	10·2 (6·3 to 14·9)	5·4 (3·1 to 8·1)	2·7 (1·5 to 4·1)	1·2 (0·6 to 1·9)	0·4 (0·2 to 0·6)	0·1 (0 to 0·1)	320
Depressive disorders	0 (0 to 0)	0 (0 to 0)	0 (0 to 0)	0 (0 to 0)	2·2 (0·8 to 4·7)	27·3 (13·3 to 49·1)	72·6 (42 to 118)	84·8 (46·9 to 132)	87·5 (51·5 to 135)	84·1 (50·3 to 131)	97·9 (59·8 to 146)	101 (61·6 to 157)	116 (76·2 to 177)	122 (80 to 183)	127 (78 to 192)	118 (72·3 to 181)	87·7 (55·1 to 126)	67·7 (43·5 to 96·3)	43·5 (26 to 65·5)	29·1 (17·1 to 43·6)	18·1 (11·4 to 26·4)	8·1 (4·9 to 12·3)	2·1 (1·1 to 3·4)	1,300
Major depressive disorder	0 (0 to 0)	0 (0 to 0)	0 (0 to 0)	0 (0 to 0)	2 (0·6 to 4·5)	25·4 (11·7 to 46·8)	66·7 (37·7 to 110)	74·9 (38·4 to 120)	74 (40·9 to 117)	69·3 (38 to 113)	81 (47·4 to 125)	84·3 (47·8 to 134)	97·5 (59·9 to 151)	103 (64·8 to 156)	109 (63·1 to 168)	101 (59·6 to 158)	75·9 (47·1 to 110)	59 (37·3 to 86·2)	38·2 (22·1 to 59·6)	25·8 (14·7 to 40)	16·3 (10 to 24·3)	7·3 (4·3 to 11·2)	1·9 (1 to 3·2)	1,110
Dysthymia	0 (0 to 0)	0 (0 to 0)	0 (0 to 0)	0 (0 to 0)	0·2 (0 to 0·6)	1·8 (0·7 to 3·7)	5·8 (2·8 to 10·5)	9·8 (5 to 17·3)	13·5 (7 to 23·2)	14·7 (8·1 to 24·3)	16·9 (9·2 to 28)	17·1 (9·2 to 30)	18·8 (10·3 to 32·9)	18·7 (10·7 to 31·1)	18·2 (10·6 to 29·3)	16·1 (8·6 to 27)	11·8 (6·4 to 19·8)	8·7 (4·9 to 14·3)	5·3 (3·1 to 8·8)	3·2 (1·8 to 5·4)	1·8 (1 to 3·1)	0·8 (0·4 to 1·3)	0·2 (0·1 to 0·3)	184
Bipolar disorder	0 (0 to 0)	0 (0 to 0)	0 (0 to 0)	0 (0 to 0)	0 (0 to 0)	7·1 (2·7 to 13·2)	27·3 (12·8 to 47·7)	33 (16·8 to 55·5)	35·6 (19·3 to 59)	32·7 (18 to 54·3)	34·2 (18·6 to 55·6)	32·7 (17·7 to 53·2)	34·9 (19·7 to 56·2)	33·2 (18·3 to 54·5)	30·5 (16·9 to 50·3)	24·7 (13·2 to 39·6)	16·5 (8·8 to 27·3)	10·8 (5·8 to 17·5)	5·6 (3 to 9·2)	2·8 (1·4 to 4·8)	1·2 (0·6 to 2·1)	0·4 (0·2 to 0·8)	0·1 (0 to 0·2)	363
Anxiety disorders	0 (0 to 0)	0 (0 to 0)	0 (0 to 0)	0·8 (0·2 to 1·8)	13·8 (7 to 25·1)	40·7 (22·1 to 67·1)	59·3 (36 to 90·6)	62·6 (35 to 102)	69·9 (38·9 to 116)	68·9 (40·2 to 107)	74·8 (44·5 to 115)	72·7 (41·8 to 116)	77·4 (43·8 to 124)	74·3 (44·2 to 114)	70·2 (43·8 to 107)	59·9 (36·5 to 92·6)	43·8 (26·2 to 68·8)	33·2 (19·8 to 51·5)	21 (12·8 to 33·2)	13·1 (7·8 to 20·7)	7 (4·1 to 11)	2·5 (1·4 to 4·1)	0·5 (0·3 to 0·9)	867
Eating disorders	0 (0 to 0)	0 (0 to 0)	0 (0 to 0)	0 (0 to 0)	0·2 (0·1 to 0·4)	3 (1·4 to 5·3)	12·6 (6·6 to 21·9)	20·1 (10·7 to 33·6)	20·4 (10·8 to 34·3)	15·5 (8·5 to 24·8)	12·2 (6·8 to 19·3)	7·4 (3·7 to 12·6)	4·7 (2·4 to 8)	0 (0 to 0)	0 (0 to 0)	0 (0 to 0)	0 (0 to 0)	0 (0 to 0)	0 (0 to 0)	0 (0 to 0)	0 (0 to 0)	0 (0 to 0)	0 (0 to 0)	96·1
Anorexia nervosa	0 (0 to 0)	0 (0 to 0)	0 (0 to 0)	0 (0 to 0)	0·2 (0 to 0·3)	1·6 (0·7 to 3·1)	4·9 (2·3 to 8·8)	4·9 (2·1 to 8·9)	4·1 (2·1 to 7·3)	2·6 (1·2 to 4·8)	2·1 (0·9 to 3·9)	1·5 (0·6 to 2·8)	1·2 (0·4 to 2·4)	0 (0 to 0)	0 (0 to 0)	0 (0 to 0)	0 (0 to 0)	0 (0 to 0)	0 (0 to 0)	0 (0 to 0)	0 (0 to 0)	0 (0 to 0)	0 (0 to 0)	23·1
Bulimia nervosa	0 (0 to 0)	0 (0 to 0)	0 (0 to 0)	0 (0 to 0)	0 (0 to 0)	1·4 (0·4 to 3·1)	7·7 (3·3 to 15·6)	15·2 (6·8 to 27·7)	16·3 (8 to 28·8)	12·9 (6·6 to 21·3)	10·1 (5·3 to 17·3)	5·9 (2·7 to 10·7)	3·4 (1·5 to 6·2)	0 (0 to 0)	0 (0 to 0)	0 (0 to 0)	0 (0 to 0)	0 (0 to 0)	0 (0 to 0)	0 (0 to 0)	0 (0 to 0)	0 (0 to 0)	0 (0 to 0)	73
Autism spectrum disorders	0 (0 to 0)	0 (0 to 0)	0·4 (0·2 to 0·7)	1·9 (1·1 to 3)	2·5 (1·4 to 4)	2·7 (1·5 to 4·2)	2·8 (1·6 to 4·5)	2·6 (1·5 to 4·2)	2·8 (1·6 to 4·4)	2·6 (1·4 to 4·1)	2·6 (1·5 to 4·3)	2·4 (1·4 to 3·8)	2·5 (1·4 to 4)	2·5 (1·3 to 3·9)	2·3 (1·3 to 3·7)	2 (1·1 to 3·2)	1·5 (0·8 to 2·5)	1·2 (0·6 to 1·9)	0·8 (0·4 to 1·2)	0·5 (0·3 to 0·8)	0·3 (0·2 to 0·4)	0·1 (0·1 to 0·2)	0 (0 to 0)	37·2
Attention-deficit/hyperactivity disorder	0 (0 to 0)	0 (0 to 0)	0 (0 to 0)	0·2 (0·1 to 0·5)	3 (1·6 to 5·4)	4·5 (2·4 to 8)	4 (2·1 to 7)	2·8 (1·4 to 5)	2·2 (1·2 to 4·1)	1·7 (0·9 to 3·1)	1·5 (0·7 to 2·6)	1·2 (0·5 to 2·1)	1 (0·5 to 1·9)	0·8 (0·3 to 1·5)	0·6 (0·3 to 1·1)	0·3 (0·1 to 0·7)	0·1 (0 to 0·3)	0·1 (0 to 0·2)	0 (0 to 0)	0 (0 to 0)	0 (0 to 0)	0 (0 to 0)	0 (0 to 0)	24·1
Conduct disorder	0 (0 to 0)	0 (0 to 0)	0 (0 to 0)	0 (0 to 0)	6·6 (3·1 to 12)	26·9 (13·6 to 47)	16·4 (7·8 to 28·5)	0 (0 to 0)	0 (0 to 0)	0 (0 to 0)	0 (0 to 0)	0 (0 to 0)	0 (0 to 0)	0 (0 to 0)	0 (0 to 0)	0 (0 to 0)	0 (0 to 0)	0 (0 to 0)	0 (0 to 0)	0 (0 to 0)	0 (0 to 0)	0 (0 to 0)	0 (0 to 0)	49·9
Idiopathic developmental intellectual disability	0 (0 to 0)	0 (0 to 0)	0·2 (0 to 0·3)	0·9 (0·3 to 1·8)	1·4 (0·4 to 2·6)	1·4 (0·5 to 2·7)	1·4 (0·4 to 2·8)	1·3 (0·4 to 2·5)	1·4 (0·4 to 2·6)	1·3 (0·4 to 2·5)	1·3 (0·4 to 2·5)	1·2 (0·3 to 2·4)	1·2 (0·4 to 2·3)	1·1 (0·3 to 2·2)	1 (0·3 to 2·1)	0·9 (0·3 to 1·7)	0·6 (0·2 to 1·2)	0·4 (0·1 to 0·8)	0·2 (0·1 to 0·5)	0·2 (0 to 0·3)	0·1 (0 to 0·2)	0 (0 to 0·1)	0 (0 to 0)	17·5
Other mental disorders	0 (0 to 0)	0 (0 to 0)	0 (0 to 0)	0 (0 to 0)	0 (0 to 0)	0·4 (0·2 to 0·6)	2·7 (1·1 to 5)	6·4 (3 to 11·5)	10·7 (6 to 17·6)	13·2 (7·7 to 21)	16 (9·4 to 25·4)	16·2 (9·6 to 25·7)	17·9 (10·5 to 27·9)	18·2 (11·3 to 28·3)	18·1 (10·9 to 27·5)	16·5 (10 to 24·6)	12·8 (7·8 to 19·1)	10·3 (6·4 to 15·5)	7·1 (4·5 to 10·7)	4·9 (3·1 to 7·3)	3 (2 to 4·5)	1·4 (0·8 to 2)	0·4 (0·2 to 0·5)	176
Substance use disorders	0 (0 to 0)	0 (0 to 0)	0 (0 to 0)	0 (0 to 0)	0 (0 to 0)	1·1 (0·5 to 1·9)	18·3 (11·6 to 26·4)	39·7 (25·2 to 58·4)	45·8 (29·9 to 65·6)	38·7 (24·1 to 57·1)	35·3 (23·6 to 50·1)	28·1 (18·7 to 40)	26·8 (18·3 to 38·5)	22·9 (15·2 to 33·5)	20·2 (13·7 to 28·6)	16·3 (10·7 to 23)	10·6 (7 to 15·2)	7·5 (4·9 to 10·9)	4·8 (3·2 to 6·7)	3·5 (2·3 to 5)	2·6 (1·8 to 3·8)	1·6 (1 to 2·3)	0·6 (0·3 to 0·9)	324
Alcohol use disorders	0 (0 to 0)	0 (0 to 0)	0 (0 to 0)	0 (0 to 0)	0 (0 to 0)	0·5 (0·2 to 1·1)	6 (2·9 to 10·3)	14·7 (7·1 to 25·5)	18 (9·5 to 28·9)	17·5 (9·2 to 29·6)	17·4 (10·5 to 27)	14·6 (8·5 to 22·6)	14 (8·8 to 20·9)	11·5 (7 to 18·7)	10 (6·2 to 15·1)	8·2 (5·4 to 12·7)	5·3 (3·3 to 8·1)	3·9 (2·4 to 6·2)	2·7 (1·6 to 4)	2·2 (1·3 to 3·4)	1·9 (1·2 to 2·9)	1·2 (0·8 to 1·9)	0·5 (0·3 to 0·8)	150
Drug use disorders	0 (0 to 0)	0 (0 to 0)	0 (0 to 0)	0 (0 to 0)	0 (0 to 0)	0·6 (0·2 to 1·2)	12·3 (7·5 to 18·4)	25·1 (15·5 to 37·9)	27·8 (17·1 to 41·9)	21·2 (12·9 to 33·1)	17·9 (10·7 to 26·8)	13·5 (8·3 to 20·7)	12·8 (7·3 to 20)	11·4 (6·5 to 18·3)	10·3 (5·7 to 16·7)	8·1 (4·1 to 13·1)	5·3 (2·8 to 8·4)	3·5 (1·8 to 6)	2·1 (1·1 to 3·5)	1·3 (0·7 to 2·1)	0·7 (0·4 to 1·2)	0·3 (0·2 to 0·5)	0·1 (0 to 0·1)	174
Opioid use disorders	0 (0 to 0)	0 (0 to 0)	0 (0 to 0)	0 (0 to 0)	0 (0 to 0)	0 (0 to 0)	3·6 (1·3 to 6·7)	15 (7·3 to 26·4)	19·8 (10·6 to 33)	16·2 (8·7 to 27·3)	13·6 (7·2 to 22)	10·2 (5·2 to 17)	9·9 (4·9 to 16·3)	9·4 (4·6 to 16)	8·7 (4·2 to 15)	7 (3·1 to 11·9)	4·5 (2·1 to 7·7)	3 (1·3 to 5·5)	1·7 (0·7 to 3)	1·1 (0·4 to 1·8)	0·6 (0·3 to 1·1)	0·2 (0·1 to 0·4)	0·1 (0 to 0·1)	125
Cocaine use disorders	0 (0 to 0)	0 (0 to 0)	0 (0 to 0)	0 (0 to 0)	0 (0 to 0)	0 (0 to 0)	1·2 (0·4 to 2·4)	1·6 (0·7 to 2·8)	1·2 (0·6 to 2·3)	1 (0·7 to 1·4)	1·2 (0·8 to 1·8)	1 (0·6 to 1·4)	0·8 (0·5 to 1·2)	0·5 (0·3 to 0·7)	0·5 (0·4 to 0·8)	0·4 (0·2 to 0·5)	0·3 (0·2 to 0·4)	0·1 (0·1 to 0·2)	0·1 (0·1 to 0·1)	0 (0 to 0·1)	0 (0 to 0)	0 (0 to 0)	0 (0 to 0)	10
Amphetamine use disorders	0 (0 to 0)	0 (0 to 0)	0 (0 to 0)	0 (0 to 0)	0 (0 to 0)	0 (0 to 0)	0·4 (0·2 to 0·7)	1·8 (0·6 to 3·8)	1·9 (0·6 to 3·9)	0·9 (0·2 to 2·1)	0·6 (0·3 to 1)	0·3 (0·2 to 0·5)	0·2 (0·1 to 0·4)	0·2 (0·1 to 0·3)	0·1 (0·1 to 0·3)	0·1 (0·1 to 0·2)	0·1 (0 to 0·1)	0 (0 to 0·1)	0 (0 to 0)	0 (0 to 0)	0 (0 to 0)	0 (0 to 0)	0 (0 to 0)	6·8
Cannabis use disorders	0 (0 to 0)	0 (0 to 0)	0 (0 to 0)	0 (0 to 0)	0 (0 to 0)	0·6 (0·2 to 1·1)	6 (3·1 to 9·9)	4·5 (2·3 to 8)	2·8 (1·2 to 5·2)	1·6 (0·7 to 3)	1·2 (0·5 to 2·3)	0·9 (0·3 to 1·8)	0·9 (0·3 to 1·7)	0·6 (0·2 to 1·3)	0·4 (0·2 to 0·7)	0·2 (0·1 to 0·5)	0·1 (0·1 to 0·2)	0·1 (0 to 0·1)	0 (0 to 0·1)	0 (0 to 0)	0 (0 to 0)	0 (0 to 0)	0 (0 to 0)	20
Other drug use disorders	0 (0 to 0)	0 (0 to 0)	0 (0 to 0)	0 (0 to 0)	0 (0 to 0)	0 (0 to 0)	1·1 (0·5 to 2)	2·2 (1·1 to 3·9)	2·1 (1 to 3·8)	1·4 (0·8 to 2·4)	1·3 (0·8 to 2)	1·1 (0·7 to 1·7)	1 (0·6 to 1·6)	0·7 (0·3 to 1·3)	0·5 (0·3 to 0·8)	0·3 (0·2 to 0·5)	0·3 (0·2 to 0·4)	0·3 (0·2 to 0·4)	0·3 (0·2 to 0·4)	0·2 (0·1 to 0·3)	0·1 (0·1 to 0·2)	0 (0 to 0·1)	0 (0 to 0)	13
Diabetes and kidney diseases	0 (0 to 0)	0 (0 to 0·1)	2·7 (1·6 to 4·2)	3 (1·9 to 4·3)	7 (5·1 to 9·3)	10·7 (7·7 to 15)	36·4 (29 to 45·3)	52·8 (40·7 to 67·3)	67·8 (51·2 to 87·9)	88·9 (69·7 to 113)	138 (110 to 170)	189 (144 to 237)	313 (242 to 392)	502 (392 to 622)	679 (533 to 832)	902 (732 to 1,100)	888 (712 to 1,080)	924 (745 to 1,100)	792 (634 to 952)	600 (487 to 723)	390 (320 to 466)	179 (147 to 214)	42 (32·4 to 50·8)	6,810
Diabetes mellitus	0 (0 to 0)	0 (0 to 0·1)	1 (0·4 to 2)	0·6 (0·3 to 1)	1·5 (0·9 to 2·1)	2·8 (1·6 to 4·1)	17·6 (13·2 to 22·7)	27·3 (21 to 35·3)	36 (25·9 to 48·1)	49·5 (37·1 to 66)	83·6 (63·6 to 108)	124 (93·6 to 161)	222 (165 to 284)	378 (288 to 484)	517 (391 to 645)	675 (526 to 848)	687 (538 to 860)	735 (570 to 900)	632 (492 to 783)	487 (388 to 601)	308 (245 to 378)	140 (109 to 174)	31 (23·4 to 38·3)	5,160
Diabetes mellitus type 1	0 (0 to 0)	0 (0 to 0·1)	1 (0·4 to 2)	0·6 (0·3 to 1)	1·5 (0·9 to 2·1)	2·8 (1·6 to 4·1)	5·3 (3·5 to 8)	7·7 (4·9 to 11·7)	7·6 (4·7 to 11·5)	7 (4·5 to 10·8)	12·7 (7·9 to 19·2)	17·3 (11 to 26·8)	7·5 (4·7 to 11·6)	11·9 (7·2 to 19·1)	16·5 (9·2 to 25·5)	18·8 (10·3 to 29·6)	18·8 (10·3 to 29·1)	15·7 (9·2 to 24·9)	13·5 (7·4 to 23·2)	6·2 (3·5 to 11·9)	4 (2·1 to 7·3)	2 (1·1 to 3·6)	0·6 (0·3 to 1)	179
Diabetes mellitus type 2	0 (0 to 0)	0 (0 to 0)	0 (0 to 0)	0 (0 to 0)	0 (0 to 0)	0 (0 to 0)	12·2 (8·6 to 16·3)	19·6 (14 to 26·9)	28·3 (19·8 to 39)	42·4 (30·7 to 58)	70·9 (51·7 to 93·9)	107 (78·7 to 143)	214 (159 to 275)	366 (278 to 470)	500 (379 to 623)	657 (508 to 828)	668 (521 to 836)	719 (558 to 882)	618 (480 to 764)	481 (383 to 594)	304 (242 to 374)	138 (108 to 171)	30·4 (23 to 37·5)	4,980
Chronic kidney disease	0 (0 to 0)	0 (0 to 0)	1·6 (1 to 2·6)	2·4 (1·5 to 3·6)	5·5 (3·7 to 7·7)	7·9 (5·2 to 11·9)	18·8 (13·4 to 25·8)	25·5 (17·8 to 35·2)	31·8 (22·2 to 45)	39·4 (27·3 to 54·1)	54·2 (39·4 to 74·5)	65·1 (45·8 to 90·2)	91·5 (67·6 to 125)	124 (90·5 to 166)	162 (121 to 214)	227 (170 to 295)	201 (148 to 268)	189 (146 to 243)	160 (124 to 203)	112 (85·8 to 141)	82·1 (63·7 to 102)	39·2 (29·2 to 48·8)	11 (8·3 to 14)	1,650
Chronic kidney disease due to diabetes mellitus type 1	0 (0 to 0)	0 (0 to 0)	0 (0 to 0)	0 (0 to 0·1)	0·1 (0·1 to 0·3)	0·2 (0·1 to 0·5)	0·7 (0·3 to 1·5)	1·3 (0·6 to 2·7)	2·8 (1·2 to 5·3)	5·2 (2·5 to 9·3)	8·8 (4·5 to 14·7)	10·2 (5·3 to 17·3)	12·3 (5·5 to 21·8)	13 (5·2 to 25·3)	12 (4 to 27·2)	13 (4·5 to 29·4)	8·8 (3·2 to 20·1)	6·2 (2·3 to 12·7)	3·4 (1·3 to 7·3)	1·2 (0·4 to 2·7)	0·3 (0·1 to 0·8)	0 (0 to 0·1)	0 (0 to 0)	99·8
Chronic kidney disease due to diabetes mellitus type 2	0 (0 to 0)	0 (0 to 0)	0 (0 to 0)	0 (0 to 0)	0 (0 to 0)	0 (0 to 0)	0·3 (0·1 to 0·8)	0·6 (0·2 to 1·4)	1·3 (0·6 to 2·5)	2·4 (1·1 to 4·4)	4·7 (2·2 to 8·6)	8·2 (3·9 to 14·1)	16·6 (9·3 to 26·7)	30·4 (18·2 to 46·1)	47·8 (28·1 to 69·9)	72·4 (44·1 to 105)	65·6 (42·1 to 97·9)	62 (41·4 to 86)	51·7 (33·3 to 72·4)	34·8 (23·3 to 48·9)	24·2 (15·7 to 33·9)	10·9 (6·8 to 16)	2·9 (1·6 to 4·3)	437
Chronic kidney disease due to hypertension	0 (0 to 0)	0 (0 to 0)	0 (0 to 0)	0 (0 to 0)	0 (0 to 0)	0 (0 to 0)	1·4 (0·4 to 2·9)	2·1 (0·8 to 4·2)	3·2 (1·3 to 6·1)	4·2 (2 to 7·2)	6 (3·3 to 9·9)	8 (4·4 to 12·5)	12·6 (7·4 to 19·7)	19·2 (11·7 to 29·9)	29·2 (18·5 to 44)	48·3 (31 to 71·3)	48 (30·9 to 71)	49·5 (33·5 to 70·9)	46·4 (31·1 to 67)	35·3 (24·5 to 48·8)	28·8 (20·3 to 39·7)	14·9 (10·5 to 20)	4·3 (3 to 5·9)	361
Chronic kidney disease due to glomerulonephritis	0 (0 to 0)	0 (0 to 0)	0·5 (0·2 to 0·8)	0·7 (0·4 to 1·2)	1·8 (1·1 to 3)	3 (1·8 to 4·7)	7·2 (4·4 to 10·8)	9·2 (5·6 to 14)	10·7 (6·4 to 16·5)	12·3 (7·4 to 18·2)	16·5 (10 to 24·5)	18·3 (10·2 to 28·1)	22·6 (12·9 to 35·7)	27·4 (16 to 44·6)	32·3 (16·6 to 55·3)	40·1 (20 to 68)	29·6 (14 to 55)	23·4 (11·9 to 40·3)	17·3 (8·3 to 31·8)	10·1 (4·8 to 19·1)	6 (2·8 to 10·7)	2·4 (1·1 to 4·3)	0·6 (0·2 to 1·3)	292
Chronic kidney disease due to other and unspecified causes	0 (0 to 0)	0 (0 to 0)	1·2 (0·7 to 1·9)	1·6 (0·9 to 2·6)	3·5 (2·2 to 5·3)	4·7 (2·6 to 7·6)	9·1 (5·4 to 13·4)	12·1 (7·5 to 18)	13·9 (8·6 to 21·1)	15·4 (9·4 to 23·2)	18·1 (10·8 to 27·7)	20·5 (12·1 to 30·8)	27·3 (16·5 to 42·1)	34 (20·4 to 52·4)	40·9 (24·7 to 64·1)	53·1 (30·7 to 86·5)	48·7 (29·2 to 76·5)	48 (30·1 to 72·1)	41·2 (25·5 to 61·3)	30·9 (19·6 to 45)	22·9 (15·2 to 32·9)	10·8 (7·1 to 15·3)	3·2 (2 to 4·6)	461
Acute glomerulonephritis	0 (0 to 0)	0 (0 to 0)	0 (0 to 0)	0 (0 to 0)	0 (0 to 0)	0 (0 to 0)	0 (0 to 0)	0 (0 to 0)	0 (0 to 0)	0 (0 to 0)	0 (0 to 0)	0 (0 to 0)	0 (0 to 0)	0 (0 to 0)	0 (0 to 0)	0 (0 to 0)	0 (0 to 0)	0 (0 to 0)	0 (0 to 0)	0 (0 to 0)	0 (0 to 0)	0 (0 to 0)	0 (0 to 0)	0·2
Skin and subcutaneous diseases	0 (0 to 0)	0 (0 to 0)	8·5 (5 to 13·4)	41·9 (26·7 to 61·8)	57·7 (37·2 to 84·6)	62·3 (40·2 to 93·2)	71·9 (46·7 to 107)	47·2 (31·7 to 68·6)	44·8 (30 to 64·8)	44·2 (29·4 to 64·1)	50·5 (34·7 to 72·9)	55·8 (38·3 to 80·9)	62·6 (42·9 to 89·4)	82·1 (56·9 to 117)	103 (70·3 to 148)	119 (83·2 to 187)	111 (77·3 to 183)	127 (86 to 220)	140 (93·6 to 239)	126 (81·8 to 226)	118 (73·3 to 201)	64·9 (38·6 to 108)	22·5 (12·9 to 38)	1,560
Dermatitis	0 (0 to 0)	0 (0 to 0)	0 (0 to 0)	14·9 (7·9 to 26·1)	19·2 (10·2 to 32·8)	15 (8·2 to 25·5)	11·9 (6·7 to 19·3)	9·4 (5·4 to 15)	9·1 (5·1 to 15)	8·4 (4·7 to 13·8)	8·7 (4·8 to 14·1)	8·3 (4·6 to 13·6)	9·2 (5·1 to 15)	10 (5·2 to 16·5)	11 (6·1 to 18·4)	10·8 (6 to 17·2)	9·1 (5·1 to 14·6)	7·7 (4·4 to 12·6)	5·2 (3 to 8·4)	3·4 (2 to 5·5)	1·9 (1·1 to 3·1)	0·7 (0·4 to 1·2)	0·2 (0·1 to 0·3)	174
Atopic dermatitis	0 (0 to 0)	0 (0 to 0)	0 (0 to 0)	14·8 (7·9 to 26)	18·9 (9·9 to 32·6)	13·6 (7·1 to 23·9)	9·4 (5 to 16·5)	6·5 (3·3 to 11·7)	5·5 (2·7 to 10)	4·5 (2·2 to 8·3)	4·5 (2·2 to 7·9)	4·2 (2·1 to 7·4)	4·5 (2·4 to 7·9)	4·6 (2·3 to 8·3)	5 (2·5 to 8·9)	4·7 (2·4 to 8·4)	3·7 (1·8 to 6·7)	3·1 (1·6 to 5·4)	2·2 (1·2 to 3·8)	1·5 (0·8 to 2·7)	0·9 (0·5 to 1·6)	0·4 (0·2 to 0·6)	0·1 (0 to 0·2)	113
Contact dermatitis	0 (0 to 0)	0 (0 to 0)	0 (0 to 0)	0 (0 to 0)	0·2 (0·1 to 0·5)	1·2 (0·5 to 2·4)	2·2 (1 to 4·4)	2·7 (1·1 to 5·2)	3·3 (1·4 to 6·4)	3·5 (1·5 to 6·6)	3·8 (1·6 to 7·4)	3·7 (1·7 to 7·4)	4·3 (1·8 to 8·4)	5 (2 to 9·1)	5·6 (2·3 to 11·3)	5·7 (2·4 to 10·5)	4·9 (2·2 to 9·7)	4·2 (1·9 to 8)	2·7 (1·3 to 5·3)	1·7 (0·8 to 3·1)	0·9 (0·4 to 1·6)	0·3 (0·1 to 0·6)	0·1 (0 to 0·2)	55·9
Seborrhoeic dermatitis	0 (0 to 0)	0 (0 to 0)	0 (0 to 0)	0·1 (0 to 0·2)	0·1 (0·1 to 0·3)	0·2 (0·1 to 0·4)	0·3 (0·1 to 0·5)	0·3 (0·1 to 0·5)	0·4 (0·2 to 0·7)	0·4 (0·2 to 0·7)	0·4 (0·2 to 0·7)	0·4 (0·2 to 0·7)	0·4 (0·2 to 0·7)	0·4 (0·2 to 0·7)	0·4 (0·2 to 0·7)	0·4 (0·2 to 0·8)	0·5 (0·2 to 0·8)	0·4 (0·2 to 0·8)	0·3 (0·2 to 0·6)	0·2 (0·1 to 0·4)	0·1 (0·1 to 0·2)	0·1 (0 to 0·1)	0 (0 to 0)	5·9
Psoriasis	0 (0 to 0)	0 (0 to 0)	0 (0 to 0)	0·5 (0·3 to 0·7)	1·4 (0·5 to 2·5)	1·9 (0·9 to 3·3)	2·3 (1·1 to 3·8)	2·3 (1·1 to 3·9)	2·8 (1·4 to 4·7)	3·1 (1·7 to 5)	3·7 (1·9 to 6)	4 (2·2 to 6·3)	4·8 (2·7 to 7·6)	5·3 (3 to 8·3)	5·8 (3·5 to 8·7)	5·5 (3·3 to 8·3)	4·3 (2·6 to 6·4)	3·3 (1·9 to 4·9)	1·9 (1·1 to 2·8)	1 (0·6 to 1·6)	0·4 (0·2 to 0·7)	0·1 (0·1 to 0·3)	0 (0 to 0)	54·6
Bacterial skin diseases	0 (0 to 0)	0 (0 to 0)	6 (2·8 to 10·7)	2·4 (1·3 to 4·1)	3·1 (1·6 to 5·2)	1·7 (0·8 to 2·9)	2·1 (0·9 to 3·7)	3·5 (1·6 to 5·8)	3·2 (1·5 to 5·8)	6·4 (3·1 to 11·5)	6 (2·6 to 11·4)	10·7 (5 to 19·1)	9·3 (4·1 to 17·3)	15 (6·3 to 28·6)	18·4 (7·3 to 34·8)	25·6 (11·6 to 48·5)	21·7 (9·4 to 46·2)	18·8 (7·6 to 38·8)	20·2 (9·1 to 42·1)	19·5 (7·8 to 40·9)	15·6 (5·8 to 31·5)	8·6 (3 to 15·7)	2·9 (1 to 5·5)	221
Cellulitis	0 (0 to 0)	0 (0 to 0)	1·3 (0·3 to 3·2)	0·6 (0·2 to 1·1)	1·3 (0·5 to 2·3)	0·4 (0·1 to 0·8)	0·3 (0·1 to 0·6)	0·6 (0·2 to 1·2)	1 (0·4 to 2·1)	0·9 (0·4 to 2)	1·1 (0·4 to 2·5)	1·6 (0·6 to 3·8)	1·4 (0·6 to 3·1)	3 (1·1 to 7·4)	3·8 (1·4 to 9·8)	4·6 (1·7 to 12·4)	4·6 (1·7 to 12)	2·9 (1 to 8·1)	3 (1·1 to 7·4)	2·3 (0·8 to 6)	1·6 (0·6 to 4·2)	0·8 (0·3 to 2)	0·3 (0·1 to 0·8)	37·2
Pyoderma	0 (0 to 0)	0 (0 to 0)	4·7 (2 to 8·7)	1·8 (0·9 to 3·2)	1·8 (0·9 to 3·3)	1·3 (0·5 to 2·3)	1·8 (0·8 to 3·2)	2·9 (1·4 to 4·8)	2·3 (1 to 4·1)	5·5 (2·6 to 9·8)	4·9 (2·1 to 9·6)	9·2 (4·2 to 16·5)	8 (3·4 to 14·5)	12 (4·7 to 22)	14·6 (5·5 to 27·9)	21 (9·3 to 38·8)	17·1 (7·3 to 33·9)	15·8 (6·4 to 32·1)	17·2 (7·5 to 33·6)	17·1 (6·5 to 34·3)	14 (5·1 to 28·2)	7·8 (2·8 to 14·1)	2·5 (0·8 to 4·8)	183
Scabies	0 (0 to 0)	0 (0 to 0)	0·9 (0·5 to 1·6)	6·3 (3·2 to 11·3)	8·9 (4·3 to 15·9)	9·9 (4·8 to 17·7)	11·2 (5·8 to 19·5)	9·7 (4·9 to 17·1)	7·7 (3·9 to 13·7)	5·7 (2·8 to 10·4)	6 (2·9 to 10·4)	6·5 (3·3 to 11·2)	7·2 (3·7 to 12·5)	7 (3·6 to 12)	6·5 (3·3 to 11·4)	5·6 (2·9 to 10)	4·4 (2·2 to 7·8)	3·6 (1·8 to 6·4)	2·8 (1·4 to 4·8)	2·1 (1·1 to 3·7)	1·4 (0·7 to 2·4)	0·7 (0·3 to 1·1)	0·2 (0·1 to 0·3)	114
Fungal skin diseases	0 (0 to 0)	0 (0 to 0)	0·2 (0·1 to 0·3)	1·2 (0·4 to 2·5)	2 (0·7 to 4·3)	2·5 (0·9 to 5·5)	2·8 (1 to 6·5)	2·9 (1·1 to 6·1)	3·3 (1·2 to 7·2)	3·3 (1·3 to 7·5)	3·7 (1·4 to 8)	3·7 (1·4 to 7·9)	4·2 (1·6 to 9·2)	4·8 (1·9 to 10·5)	6 (2·4 to 13·2)	6·5 (2·5 to 14·5)	7 (2·8 to 14·9)	8·2 (3·2 to 17·6)	7 (2·6 to 15)	6·2 (2·4 to 13·3)	4·8 (1·9 to 10·1)	2·3 (1 to 4·9)	0·6 (0·3 to 1·3)	83·3
Viral skin diseases	0 (0 to 0)	0 (0 to 0)	0 (0 to 0)	6·9 (4·3 to 10·6)	11·5 (7·2 to 17·6)	6·5 (4 to 9·8)	4·2 (2·5 to 6·4)	3·3 (2 to 5·1)	2·6 (1·6 to 4·2)	2·1 (1·2 to 3·3)	2·1 (1·2 to 3·4)	2 (1·2 to 3·2)	2·1 (1·2 to 3·3)	2·1 (1·2 to 3·3)	2 (1·1 to 3·3)	2 (1·1 to 3·1)	1·6 (0·9 to 2·4)	1·2 (0·7 to 1·8)	0·7 (0·4 to 1)	0·4 (0·2 to 0·6)	0·2 (0·1 to 0·3)	0·1 (0 to 0·1)	0 (0 to 0)	53·5
Acne vulgaris	0 (0 to 0)	0 (0 to 0)	0 (0 to 0)	0 (0 to 0)	0·6 (0·3 to 1·1)	15·3 (8·4 to 25·4)	26·5 (15 to 42·6)	6·5 (3·6 to 10·7)	3·4 (1·7 to 5·6)	2·4 (1·2 to 4·1)	2 (0·9 to 3·4)	1·4 (0·6 to 2·4)	1·1 (0·6 to 1·8)	0·8 (0·4 to 1·3)	0·6 (0·3 to 1)	0·4 (0·2 to 0·7)	0·3 (0·1 to 0·5)	0·2 (0·1 to 0·4)	0·1 (0·1 to 0·2)	0·1 (0 to 0·2)	0 (0 to 0·1)	0 (0 to 0)	0 (0 to 0)	61·6
Alopecia areata	0 (0 to 0)	0 (0 to 0)	0 (0 to 0)	0·2 (0·1 to 0·3)	0·3 (0·1 to 0·5)	0·4 (0·2 to 0·7)	0·7 (0·4 to 1·2)	1·1 (0·6 to 1·8)	1·6 (1 to 2·6)	1·7 (1 to 2·6)	1·7 (0·9 to 2·7)	1·4 (0·8 to 2·2)	1·3 (0·7 to 2)	1·1 (0·6 to 1·8)	1·1 (0·6 to 1·7)	1 (0·6 to 1·6)	0·7 (0·4 to 1·2)	0·6 (0·3 to 0·9)	0·3 (0·2 to 0·5)	0·2 (0·1 to 0·3)	0·1 (0 to 0·1)	0 (0 to 0)	0 (0 to 0)	15·3
Pruritus	0 (0 to 0)	0 (0 to 0)	0 (0 to 0)	0·3 (0·1 to 0·6)	0·4 (0·2 to 0·9)	0·5 (0·2 to 1)	0·6 (0·2 to 1·2)	0·7 (0·3 to 1·4)	0·9 (0·4 to 1·8)	1 (0·4 to 1·9)	1·1 (0·5 to 2·1)	1·1 (0·5 to 2)	1·2 (0·5 to 2·4)	1·4 (0·6 to 2·8)	1·6 (0·6 to 3·2)	1·8 (0·8 to 3·7)	1·7 (0·7 to 3·4)	1·5 (0·7 to 3)	1·1 (0·5 to 2·1)	0·7 (0·3 to 1·4)	0·4 (0·2 to 0·9)	0·2 (0·1 to 0·3)	0 (0 to 0·1)	18·3
Urticaria	0 (0 to 0)	0 (0 to 0)	1·1 (0·6 to 1·7)	7·3 (4·4 to 11·5)	6·9 (3·9 to 11·4)	5·2 (2·8 to 8·7)	4·8 (2·5 to 8·2)	4·4 (2·4 to 7·3)	4·4 (2·3 to 7·3)	4·4 (2·3 to 7·3)	4·8 (2·7 to 7·9)	4·4 (2·5 to 7·1)	4·4 (2·4 to 7·5)	4·3 (2·4 to 7·3)	4·4 (2·3 to 7·1)	4 (2·1 to 6·6)	3·1 (1·7 to 5·2)	2·5 (1·4 to 4·1)	1·6 (0·8 to 2·7)	1 (0·6 to 1·6)	0·5 (0·3 to 0·8)	0·2 (0·1 to 0·3)	0 (0 to 0·1)	73·9
Decubitus ulcer	0 (0 to 0)	0 (0 to 0)	0 (0 to 0)	0·4 (0·1 to 1·1)	1·7 (0·6 to 3·1)	1·4 (0·4 to 2·5)	2 (0·6 to 3·4)	0·4 (0·1 to 0·8)	2·3 (0·9 to 4·5)	1·2 (0·4 to 2·6)	5·6 (2·4 to 10·8)	6·3 (2·8 to 12)	10·3 (4·7 to 19·3)	21·1 (10·8 to 37·4)	34 (17·9 to 68)	43·9 (24·7 to 90·4)	46 (26·4 to 91·8)	69·8 (40·9 to 142)	91·7 (54·4 to 174)	86·5 (50·2 to 166)	90·1 (53·2 to 162)	50·9 (29·3 to 91·6)	18·3 (10·3 to 31·2)	584
Other skin and subcutaneous diseases	0 (0 to 0)	0 (0 to 0)	0·4 (0·2 to 0·7)	1·5 (0·7 to 2·9)	1·6 (0·8 to 3)	2·1 (1 to 3·9)	2·8 (1·4 to 5·2)	3 (1·5 to 5·4)	3·4 (1·6 to 6·2)	4·4 (2·4 to 8·1)	5·1 (2·5 to 9)	6·1 (3·2 to 10·8)	7·3 (3·8 to 13·2)	9·2 (4·8 to 16·2)	11·3 (5·8 to 19·9)	12·3 (6·3 to 21·9)	10·8 (5·5 to 19·8)	9·9 (5 to 17·9)	7·5 (3·8 to 12·9)	5 (2·7 to 8·8)	2·7 (1·4 to 4·5)	1·1 (0·6 to 2)	0·2 (0·1 to 0·4)	108
Sense organ diseases	0 (0 to 0)	0 (0 to 0)	0·3 (0·2 to 0·5)	3·6 (2·1 to 5·2)	8 (4·9 to 11·8)	11·3 (7 to 16·6)	14·8 (9·4 to 21·8)	17·7 (10·9 to 26)	24·6 (15·4 to 37·3)	30·5 (18·9 to 46·8)	39·3 (23·5 to 60·6)	48 (29·5 to 74·8)	75·5 (45·6 to 120)	108 (67·1 to 170)	148 (91 to 233)	178 (111 to 278)	179 (114 to 273)	185 (120 to 271)	160 (108 to 231)	136 (92·4 to 187)	96·3 (66·5 to 131)	46·4 (33 to 62·4)	12·8 (9·2 to 17·3)	1,520
Blindness and vision loss	0 (0 to 0)	0 (0 to 0)	0 (0 to 0·1)	0·7 (0·4 to 1·1)	1·6 (0·8 to 2·6)	2·2 (1·2 to 3·6)	2·8 (1·6 to 4·3)	3 (1·8 to 4·8)	3·6 (2·1 to 5·6)	4 (2·3 to 6·4)	5·5 (3·3 to 8·9)	7·5 (4·3 to 12·3)	11·7 (6·7 to 20)	16·9 (9·7 to 29·3)	24·4 (14 to 41·4)	31·2 (18·4 to 52·1)	33·9 (20·7 to 55)	37 (23 to 57·6)	32·8 (21 to 50·8)	28·2 (18·6 to 42·1)	20·5 (13·8 to 29·4)	10·7 (7·2 to 15·1)	3·6 (2·4 to 5·1)	282
Glaucoma	0 (0 to 0)	0 (0 to 0)	0 (0 to 0)	0 (0 to 0)	0 (0 to 0)	0 (0 to 0)	0 (0 to 0)	0 (0 to 0)	0 (0 to 0)	0 (0 to 0)	0 (0 to 0)	0 (0 to 0)	0 (0 to 0)	0·1 (0·1 to 0·2)	0·4 (0·1 to 0·9)	0·9 (0·4 to 1·6)	1·3 (0·6 to 2·3)	1·9 (1·1 to 3·1)	2·3 (1·4 to 3·7)	2·4 (1·4 to 3·7)	2 (1·2 to 3·1)	1·1 (0·6 to 1·6)	0·4 (0·2 to 0·6)	12·8
Cataract	0 (0 to 0)	0 (0 to 0)	0 (0 to 0)	0 (0 to 0)	0 (0 to 0)	0 (0 to 0)	0 (0 to 0)	0 (0 to 0)	0·1 (0 to 0·1)	0·1 (0·1 to 0·2)	0·3 (0·1 to 0·5)	0·5 (0·2 to 0·9)	0·9 (0·4 to 1·6)	1·3 (0·6 to 2·2)	2 (1 to 3·3)	2·9 (1·7 to 4·7)	4·1 (2·5 to 6·3)	6·2 (4 to 9·4)	7·6 (5 to 11·3)	7·8 (5 to 11·1)	6·3 (4 to 8·8)	3·3 (2·2 to 4·6)	1·1 (0·7 to 1·6)	44·4
Age-related macular degeneration	0 (0 to 0)	0 (0 to 0)	0 (0 to 0)	0 (0 to 0)	0 (0 to 0)	0 (0 to 0)	0 (0 to 0)	0 (0 to 0)	0 (0 to 0)	0 (0 to 0)	0 (0 to 0)	0 (0 to 0)	0 (0 to 0)	0 (0 to 0·1)	0·1 (0 to 0·2)	0·2 (0·1 to 0·3)	0·2 (0·1 to 0·3)	0·3 (0·1 to 0·4)	0·2 (0·1 to 0·4)	0·2 (0·1 to 0·4)	0·2 (0·1 to 0·3)	0·1 (0 to 0·1)	0 (0 to 0)	1·4
Refraction disorders	0 (0 to 0)	0 (0 to 0)	0 (0 to 0)	0·6 (0·3 to 1)	1·4 (0·7 to 2·2)	1·9 (1 to 3·2)	2·4 (1·4 to 3·9)	2·6 (1·4 to 4·2)	2·9 (1·7 to 4·6)	2·9 (1·6 to 4·6)	3·3 (1·9 to 5·1)	3·4 (2 to 5·3)	4·1 (2·4 to 6·2)	4·4 (2·6 to 6·7)	4·9 (2·9 to 7·8)	4·9 (2·9 to 7·7)	4·4 (2·5 to 6·8)	4·3 (2·5 to 6·8)	3·6 (2·2 to 5·5)	2·6 (1·6 to 4)	1·5 (0·9 to 2·3)	0·5 (0·3 to 0·9)	0·1 (0·1 to 0·2)	56·7
Near vision loss	0 (0 to 0)	0 (0 to 0)	0 (0 to 0)	0 (0 to 0)	0 (0 to 0)	0 (0 to 0·1)	0 (0 to 0·1)	0·1 (0 to 0·3)	0·3 (0·1 to 0·6)	0·6 (0·2 to 1·4)	1·4 (0·5 to 3·1)	2·7 (1·1 to 5·9)	5·3 (2 to 11·5)	8·6 (3·3 to 18·4)	12·8 (4·9 to 26·8)	15·9 (6·7 to 33)	16 (6·7 to 31·4)	15·8 (6·6 to 32·3)	12·7 (5·4 to 26·3)	9·8 (4·1 to 20)	6·4 (2·7 to 12·6)	2·9 (1·2 to 5·9)	0·7 (0·3 to 1·6)	112
Other vision loss	0 (0 to 0)	0 (0 to 0)	0 (0 to 0)	0·1 (0·1 to 0·2)	0·2 (0 to 0·5)	0·3 (0·1 to 0·6)	0·3 (0·1 to 0·7)	0·3 (0·1 to 0·7)	0·4 (0·1 to 0·8)	0·4 (0·2 to 0·8)	0·6 (0·2 to 1·2)	0·9 (0·4 to 1·6)	1·4 (0·7 to 2·5)	2·5 (1·3 to 4)	4·2 (2·3 to 6·6)	6·3 (3·8 to 9·5)	7·8 (5 to 11·6)	8·4 (5·3 to 12·4)	6·3 (3·9 to 9·6)	5·4 (3·5 to 7·9)	4·3 (2·7 to 6·4)	2·9 (1·9 to 4·2)	1·2 (0·8 to 1·8)	54·4
Age-related and other hearing loss	0 (0 to 0)	0 (0 to 0)	0·1 (0·1 to 0·2)	2 (1 to 3·2)	5·4 (2·9 to 8·6)	7·9 (4·5 to 12)	10·6 (6·3 to 15·9)	13·2 (7·9 to 20·1)	18·9 (11·6 to 28·7)	23·8 (14·6 to 36·6)	30·2 (17·7 to 47·7)	36·8 (21·8 to 58)	59·1 (34·5 to 95·9)	84·1 (51·3 to 134)	115 (70·2 to 183)	136 (83·3 to 218)	134 (83·9 to 210)	137 (85·5 to 205)	119 (78 to 173)	102 (67·8 to 144)	72·7 (49·1 to 100)	34·6 (24·1 to 47·2)	8·8 (6·2 to 12·1)	1,150
Other sense organ diseases	0 (0 to 0)	0 (0 to 0)	0·2 (0·1 to 0·3)	0·9 (0·4 to 1·6)	1·1 (0·5 to 1·9)	1·1 (0·5 to 2·1)	1·4 (0·6 to 2·7)	1·5 (0·6 to 2·8)	2 (0·9 to 3·9)	2·7 (1·1 to 5·5)	3·5 (1·6 to 6·4)	3·7 (1·6 to 7·1)	4·7 (2·1 to 8·9)	6·7 (3 to 12·9)	8 (3·6 to 15·7)	10·2 (4·9 to 18·8)	10·7 (5 to 19·9)	10·4 (4·8 to 18·9)	8·5 (4 to 15·3)	5·9 (2·9 to 11)	3 (1·4 to 5·4)	1·1 (0·5 to 2)	0·4 (0·2 to 0·7)	87·5
Musculoskeletal disorders	0 (0 to 0)	0 (0 to 0)	0 (0 to 0)	0 (0 to 0)	6·2 (3·4 to 10·3)	31·2 (20·1 to 48)	76·9 (51·7 to 108)	110 (75 to 153)	160 (109 to 219)	197 (134 to 267)	255 (181 to 348)	291 (207 to 397)	364 (257 to 505)	417 (294 to 575)	466 (326 to 636)	465 (322 to 647)	379 (265 to 534)	316 (219 to 440)	215 (144 to 305)	146 (102 to 203)	96·3 (69·8 to 130)	38·3 (28·2 to 53·4)	9·6 (6·9 to 13·2)	4,040
Rheumatoid arthritis	0 (0 to 0)	0 (0 to 0)	0 (0 to 0)	0 (0 to 0)	0·2 (0·1 to 0·4)	0·4 (0·2 to 0·7)	1·4 (0·8 to 2·1)	2·4 (1·2 to 4·3)	3·9 (2 to 6·3)	5·5 (2·9 to 8·7)	8·1 (4·8 to 12·3)	10·3 (6·1 to 15·1)	13·5 (8·7 to 19·4)	16·1 (10·8 to 22·6)	19·2 (13·3 to 27·1)	19·5 (13·4 to 26·8)	16·8 (11·6 to 23·2)	14·1 (9·9 to 20·1)	11·3 (7·9 to 15·8)	8·2 (5·6 to 11·7)	8·8 (5·5 to 14·8)	4 (2·4 to 6·8)	1·1 (0·6 to 1·8)	165
Osteoarthritis	0 (0 to 0)	0 (0 to 0)	0 (0 to 0)	0 (0 to 0)	0 (0 to 0)	0 (0 to 0)	0 (0 to 0)	0 (0 to 0)	0 (0 to 0)	0·8 (0·3 to 1·8)	6·1 (2·9 to 12·8)	16·7 (8·1 to 34·3)	36·9 (18·2 to 75·2)	61 (29·2 to 123)	86·3 (41·7 to 173)	101 (50·6 to 199)	91·4 (45·8 to 179)	81 (40·2 to 161)	57·5 (28·8 to 116)	39·6 (20 to 79·9)	23·1 (11·8 to 46)	9·4 (4·8 to 18·9)	2·3 (1·2 to 4·5)	613
Osteoarthritis hip	0 (0 to 0)	0 (0 to 0)	0 (0 to 0)	0 (0 to 0)	0 (0 to 0)	0 (0 to 0)	0 (0 to 0)	0 (0 to 0)	0 (0 to 0)	0·3 (0·1 to 0·6)	0·4 (0·1 to 1·1)	0·7 (0·2 to 1·7)	1·4 (0·5 to 3)	2·2 (0·9 to 4·9)	3·4 (1·4 to 7·6)	4·3 (1·9 to 9·4)	4·2 (1·8 to 8·8)	3·9 (1·8 to 8)	2·9 (1·3 to 5·9)	2 (0·9 to 4·2)	1·2 (0·5 to 2·4)	0·5 (0·2 to 1·1)	0·1 (0·1 to 0·3)	27·5
Osteoarthritis knee	0 (0 to 0)	0 (0 to 0)	0 (0 to 0)	0 (0 to 0)	0 (0 to 0)	0 (0 to 0)	0 (0 to 0)	0 (0 to 0)	0 (0 to 0)	0·4 (0·1 to 1)	4·6 (2·1 to 9·7)	12·8 (6 to 27·3)	28 (13·3 to 58·8)	45·9 (21·5 to 95·1)	64 (30·6 to 131)	73·8 (35·8 to 148)	66·1 (32·5 to 134)	57·7 (28·3 to 116)	39·9 (19·9 to 80·4)	26·5 (13·1 to 54·7)	14·7 (7·3 to 29·9)	5·6 (2·8 to 11·4)	1·3 (0·6 to 2·5)	441
Osteoarthritis hand	0 (0 to 0)	0 (0 to 0)	0 (0 to 0)	0 (0 to 0)	0 (0 to 0)	0 (0 to 0)	0 (0 to 0)	0 (0 to 0)	0 (0 to 0)	0·1 (0 to 0·2)	0·7 (0·1 to 1·8)	2 (0·7 to 4·5)	4·7 (1·9 to 10)	8 (3·6 to 17·1)	11·5 (5·5 to 26)	13·4 (6·4 to 28·9)	12·3 (5·8 to 25·8)	11 (5·2 to 23)	8·2 (4 to 16·8)	6 (2·9 to 12·5)	3·9 (1·9 to 8)	1·8 (0·9 to 3·6)	0·5 (0·2 to 1)	84·1

Osteoarthritis other	0 (0 to 0)	0 (0 to 0)	0 (0 to 0)	0 (0 to 0)	0 (0 to 0)	0 (0 to 0)	0 (0 to 0)	0 (0 to 0)	0 (0 to 0)	0 (0 to 0·1)	0·4 (0·1 to 0·9)	1·2 (0·4 to 2·8)	2·8 (1·1 to 6·5)	4·9 (2·2 to 10·4)	7·4 (3·4 to 16·1)	9·1 (4·3 to 18·9)	8·8 (4·1 to 18·3)	8·3 (4·1 to 17·1)	6·5 (3·2 to 13·4)	5 (2·5 to 10·2)	3·3 (1·7 to 6·7)	1·5 (0·8 to 3·1)	0·4 (0·2 to 0·8)	59·8
Low back pain	0 (0 to 0)	0 (0 to 0)	0 (0 to 0)	0 (0 to 0)	4·8 (2·1 to 8·8)	25·5 (15 to 41·6)	49·2 (28·1 to 78·1)	58·8 (33·1 to 93·4)	77·1 (44·9 to 120)	88·3 (49·1 to 136)	113 (67·3 to 173)	124 (73·3 to 192)	149 (88·8 to 232)	167 (103 to 260)	181 (111 to 270)	178 (110 to 265)	145 (90·6 to 218)	122 (77·7 to 182)	88·1 (53·9 to 131)	63·9 (39·8 to 95·1)	34·9 (21·4 to 51)	12·7 (7·9 to 18·7)	2·9 (1·8 to 4·4)	1,690
Neck pain	0 (0 to 0)	0 (0 to 0)	0 (0 to 0)	0 (0 to 0)	0·2 (0·1 to 0·6)	1·5 (0·6 to 3·1)	3·7 (1·5 to 6·9)	5·7 (2·4 to 11·2)	9·9 (4·3 to 19·8)	15·4 (7·3 to 29·2)	23·1 (11·9 to 40·2)	28·4 (14·4 to 50·2)	35·5 (17·9 to 64·4)	34·5 (17·7 to 64·9)	35·1 (16·8 to 62·9)	32·3 (16·6 to 55·9)	24·8 (12·8 to 42·2)	19·9 (10·3 to 34)	12·9 (6·3 to 23·9)	7·8 (3·9 to 14·3)	4·3 (2·2 to 7·4)	1·7 (0·9 to 3)	0·5 (0·2 to 0·8)	297
Gout	0 (0 to 0)	0 (0 to 0)	0 (0 to 0)	0 (0 to 0)	0 (0 to 0)	0 (0 to 0)	0 (0 to 0)	0·1 (0 to 0·2)	0·2 (0·1 to 0·4)	0·4 (0·1 to 1)	0·6 (0·1 to 1·3)	0·8 (0·2 to 1·6)	1·1 (0·4 to 2·1)	1·3 (0·5 to 2·7)	1·6 (0·6 to 3)	1·6 (0·7 to 3·1)	1·4 (0·6 to 2·6)	1·3 (0·6 to 2·4)	1 (0·4 to 1·8)	0·7 (0·3 to 1·3)	0·5 (0·2 to 0·9)	0·2 (0·1 to 0·4)	0·1 (0 to 0·1)	13
Other musculoskeletal disorders	0 (0 to 0)	0 (0 to 0)	0 (0 to 0)	0 (0 to 0)	1 (0·5 to 1·5)	3·8 (2 to 6·2)	22·6 (15·2 to 33·4)	43·2 (30 to 60·4)	68·4 (46·4 to 97·8)	86·9 (58·4 to 123)	105 (71·7 to 148)	111 (74·5 to 156)	128 (86·3 to 182)	137 (91·1 to 196)	143 (94·4 to 203)	133 (85 to 196)	100 (65·9 to 147)	76·8 (47·6 to 121)	44·4 (27·9 to 67·2)	25·8 (14·4 to 41·5)	24·7 (16 to 35·4)	10·2 (6·2 to 15·5)	2·8 (1·5 to 4·5)	1,270
Other non-communicable diseases	126 (79·2 to 194)	67·9 (44·5 to 102)	129 (87·7 to 185)	51·2 (37·4 to 68·9)	55·8 (43·7 to 70·7)	60·9 (45·1 to 79·9)	101 (75·3 to 134)	140 (101 to 197)	198 (140 to 281)	229 (160 to 319)	284 (204 to 390)	291 (205 to 401)	310 (221 to 417)	305 (220 to 410)	308 (225 to 410)	313 (233 to 416)	268 (200 to 349)	245 (187 to 317)	193 (148 to 243)	146 (113 to 184)	109 (85·1 to 136)	49·5 (38·5 to 61·8)	13·7 (10·3 to 17·5)	4,000
Congenital birth defects	123 (77 to 190)	62 (39·4 to 95·1)	94·3 (60·9 to 139)	30·7 (20·4 to 45·1)	22·7 (17·2 to 29·5)	19·8 (13·8 to 26·4)	21·3 (15·8 to 27·7)	16 (11·7 to 21·7)	14·1 (10·6 to 18·8)	11·6 (8·6 to 15·4)	13·5 (9·9 to 18)	11·9 (9 to 15·7)	12·4 (9·1 to 15·9)	12·7 (9·5 to 16·8)	11·9 (8·8 to 15·9)	8·7 (6·3 to 11·5)	6·1 (4·5 to 8·1)	2·3 (1·4 to 3·5)	1·3 (0·8 to 2)	0·7 (0·4 to 1·2)	0·4 (0·2 to 0·6)	0·1 (0·1 to 0·2)	0 (0 to 0·1)	498
Neural tube defects	5·4 (2·5 to 10·5)	1·7 (0·8 to 3·3)	3·1 (1·2 to 6·8)	1·4 (0·6 to 2·9)	0·5 (0·3 to 0·7)	0·7 (0·4 to 1)	0·6 (0·4 to 0·9)	0·3 (0·2 to 0·4)	0·4 (0·2 to 0·5)	0·3 (0·2 to 0·5)	0·3 (0·2 to 0·4)	0·3 (0·2 to 0·4)	0·2 (0·1 to 0·3)	0·2 (0·1 to 0·2)	0·2 (0·1 to 0·2)	0·1 (0·1 to 0·2)	0·1 (0·1 to 0·2)	0 (0 to 0·1)	0 (0 to 0)	0 (0 to 0)	0 (0 to 0)	0 (0 to 0)	0 (0 to 0)	16·1
Congenital heart anomalies	50·6 (28·5 to 80·6)	30·8 (18·2 to 48·9)	50·3 (30 to 78·4)	13·4 (7·5 to 24·6)	8·8 (5·8 to 12·7)	6·3 (3·1 to 9·6)	7·5 (4·7 to 10·9)	5·2 (3·2 to 7·9)	4·6 (3·1 to 6·9)	3 (1·8 to 4·5)	4·1 (2·6 to 6·2)	3·4 (2·2 to 5·1)	2·9 (1·8 to 4·2)	2·8 (1·8 to 4·2)	2·3 (1·4 to 3·5)	1·6 (1 to 2·3)	1·2 (0·8 to 1·8)	0·2 (0 to 0·3)	0·1 (0 to 0·2)	0·1 (0 to 0·1)	0 (0 to 0·1)	0 (0 to 0)	0 (0 to 0)	199
Orofacial clefts	0·1 (0 to 1)	0 (0 to 0·2)	0·1 (0·1 to 0·4)	0·2 (0·1 to 0·4)	0·2 (0·1 to 0·4)	0·2 (0 to 0·5)	0·2 (0·1 to 0·5)	0·2 (0 to 0·5)	0·2 (0·1 to 0·5)	0·2 (0 to 0·5)	0·2 (0 to 0·5)	0·2 (0 to 0·5)	0·2 (0·1 to 0·5)	0·2 (0·1 to 0·5)	0·2 (0·1 to 0·5)	0·2 (0 to 0·5)	0·2 (0 to 0·4)	0·1 (0 to 0·3)	0·1 (0 to 0·2)	0·1 (0 to 0·1)	0 (0 to 0·1)	0 (0 to 0)	0 (0 to 0)	3·7
Down syndrome	2·4 (1·2 to 4·1)	1·3 (0·7 to 2·4)	4·9 (2·3 to 8·8)	2·8 (1·4 to 4·9)	1·4 (0·8 to 2·5)	1·3 (0·7 to 2·2)	1·6 (1 to 2·7)	1·8 (1·1 to 2·9)	1·4 (0·8 to 2·3)	1·5 (0·9 to 2·3)	2·2 (1·3 to 3·5)	2·1 (1·3 to 3·2)	2·7 (1·7 to 4·1)	3·5 (2·1 to 5·2)	3·5 (2·1 to 5·5)	1·8 (1·1 to 2·9)	0·7 (0·4 to 1·1)	0 (0 to 0)	0 (0 to 0)	0 (0 to 0)	0 (0 to 0)	0 (0 to 0)	0 (0 to 0)	36·9
Turner syndrome	0 (0 to 0)	0 (0 to 0)	0 (0 to 0)	0 (0 to 0)	0 (0 to 0)	0 (0 to 0)	0 (0 to 0·1)	0 (0 to 0·1)	0 (0 to 0·1)	0 (0 to 0)	0 (0 to 0)	0 (0 to 0)	0 (0 to 0)	0 (0 to 0)	0 (0 to 0)	0 (0 to 0)	0 (0 to 0)	0 (0 to 0)	0 (0 to 0)	0 (0 to 0)	0 (0 to 0)	0 (0 to 0)	0 (0 to 0)	0·3
Klinefelter syndrome	0 (0 to 0)	0 (0 to 0)	0 (0 to 0)	0 (0 to 0)	0 (0 to 0)	0 (0 to 0)	0 (0 to 0)	0 (0 to 0)	0 (0 to 0)	0 (0 to 0)	0 (0 to 0)	0 (0 to 0)	0 (0 to 0)	0 (0 to 0)	0 (0 to 0)	0 (0 to 0)	0 (0 to 0)	0 (0 to 0)	0 (0 to 0)	0 (0 to 0)	0 (0 to 0)	0 (0 to 0)	0 (0 to 0)	0
Other chromosomal abnormalities	13 (6·5 to 23·2)	7·2 (3·7 to 12·2)	12·3 (6·5 to 21·5)	2·1 (1·2 to 3·5)	1·5 (1 to 2·4)	1·3 (0·8 to 2·2)	1·7 (0·9 to 2·7)	1·4 (0·7 to 2·2)	1 (0·6 to 1·6)	0·8 (0·5 to 1·2)	0·4 (0·3 to 0·6)	0·4 (0·3 to 0·6)	0·2 (0·2 to 0·3)	0·3 (0·2 to 0·5)	0·1 (0·1 to 0·2)	0·1 (0 to 0·1)	0·1 (0 to 0·1)	0 (0 to 0)	0 (0 to 0)	0 (0 to 0)	0 (0 to 0)	0 (0 to 0)	0 (0 to 0)	44
Congenital musculoskeletal and limb anomalies	3·9 (1·8 to 7·2)	1·3 (0·6 to 2·3)	3·4 (2 to 5·3)	4·4 (2·6 to 7)	5·4 (3·3 to 8·1)	5·7 (3·4 to 8·7)	5·7 (3·3 to 8·6)	4·2 (2·5 to 6·5)	4·1 (2·5 to 6·3)	3·8 (2·1 to 5·9)	4·1 (2·4 to 6·4)	4 (2·3 to 6·1)	4·3 (2·5 to 6·7)	4·2 (2·5 to 6·5)	4·2 (2·4 to 6·5)	3·7 (2·2 to 5·7)	2·7 (1·6 to 4·1)	1·7 (0·9 to 2·7)	0·9 (0·5 to 1·4)	0·5 (0·2 to 0·8)	0·2 (0·1 to 0·4)	0·1 (0 to 0·1)	0 (0 to 0)	72·2
Urogenital congenital anomalies	11·7 (5·4 to 22)	5·1 (2·4 to 9·4)	3·2 (1·5 to 5·9)	1·1 (0·7 to 1·8)	0·9 (0·6 to 1·5)	1·3 (0·7 to 2·2)	1·1 (0·5 to 2)	0·6 (0·3 to 1·1)	0·7 (0·3 to 1·2)	0·3 (0·2 to 0·6)	0·4 (0·2 to 0·7)	0·1 (0·1 to 0·2)	0·5 (0·3 to 0·9)	0·2 (0·1 to 0·3)	0·1 (0 to 0·2)	0·2 (0·1 to 0·3)	0·3 (0·2 to 0·5)	0 (0 to 0)	0 (0 to 0)	0 (0 to 0)	0 (0 to 0)	0 (0 to 0)	0 (0 to 0)	27·8
Digestive congenital anomalies	18·1 (8·9 to 30·9)	9·3 (4·9 to 16·4)	8·7 (4·5 to 15·1)	2 (1·1 to 3·7)	0·8 (0·4 to 1·2)	0·4 (0·2 to 0·6)	0·2 (0·1 to 0·3)	0·2 (0·1 to 0·3)	0·1 (0·1 to 0·2)	0·1 (0·1 to 0·1)	0·1 (0·1 to 0·1)	0·1 (0·1 to 0·1)	0·1 (0·1 to 0·2)	0·1 (0·1 to 0·1)	0·1 (0·1 to 0·2)	0·2 (0·1 to 0·3)	0·1 (0 to 0·1)	0 (0 to 0)	0 (0 to 0)	0 (0 to 0)	0 (0 to 0)	0 (0 to 0)	0 (0 to 0)	40·6
Other congenital birth defects	18·2 (9·8 to 30·6)	5·2 (2·9 to 9)	8·2 (4·4 to 13·8)	3·3 (1·8 to 5·9)	3·3 (2 to 4·9)	2·6 (1·6 to 4)	2·6 (1·6 to 4)	2·1 (1·2 to 3·4)	1·6 (1 to 2·7)	1·5 (0·9 to 2·5)	1·6 (0·9 to 2·6)	1·3 (0·8 to 2·2)	1·3 (0·7 to 2·3)	1·2 (0·7 to 2·1)	1·2 (0·7 to 2·1)	0·9 (0·5 to 1·8)	0·8 (0·5 to 1·5)	0·3 (0·1 to 0·8)	0·2 (0 to 0·6)	0·1 (0 to 0·4)	0·1 (0 to 0·2)	0 (0 to 0·1)	0 (0 to 0)	57·5
Urinary diseases and male infertility	0·3 (0·1 to 0·4)	0·6 (0·3 to 1)	2·3 (1·3 to 3·7)	1·4 (0·9 to 2·1)	1·6 (1·2 to 2·2)	2·5 (1·8 to 3·4)	4·6 (3·5 to 6·2)	5·8 (4 to 8)	6·8 (4·8 to 9·4)	8·9 (6 to 12·9)	13·4 (9·1 to 18·6)	15·7 (11 to 21·8)	18·7 (13 to 26·1)	26·2 (18·2 to 37·6)	32·3 (21·1 to 46·2)	41·3 (28·4 to 57·5)	39·7 (27·1 to 54·9)	47·6 (31·4 to 66·8)	50 (32 to 69·9)	49·9 (31·7 to 71·8)	43·2 (26·4 to 62·7)	20·5 (12·7 to 29·3)	6 (3·7 to 8·7)	439
Urolithiasis	0 (0 to 0)	0 (0 to 0)	0 (0 to 0)	0 (0 to 0)	0 (0 to 0)	0·1 (0 to 0·1)	0·1 (0 to 0·1)	0·2 (0·1 to 0·2)	0·2 (0·1 to 0·4)	0·5 (0·3 to 0·8)	0·9 (0·5 to 1·4)	0·8 (0·4 to 1·3)	1·3 (0·7 to 2·1)	1·3 (0·6 to 2·2)	1·6 (0·8 to 2·8)	1·6 (0·9 to 2·6)	1·3 (0·7 to 2·2)	0·8 (0·4 to 1·3)	0·5 (0·3 to 0·9)	0·3 (0·2 to 0·5)	0·2 (0·1 to 0·5)	0·1 (0 to 0·1)	0 (0 to 0)	11·7
Benign prostatic hyperplasia	0 (0 to 0)	0 (0 to 0)	0 (0 to 0)	0 (0 to 0)	0 (0 to 0)	0 (0 to 0)	0 (0 to 0)	0 (0 to 0)	0 (0 to 0)	0 (0 to 0)	0 (0 to 0)	0 (0 to 0)	0 (0 to 0)	0 (0 to 0)	0 (0 to 0)	0 (0 to 0)	0 (0 to 0)	0 (0 to 0)	0 (0 to 0)	0 (0 to 0)	0 (0 to 0)	0 (0 to 0)	0 (0 to 0)	0
Male infertility	--	--	--	--	--	--	--	--	--	--	--	--	--	--	--	--	--	--	--	--	--	--	--	NA
Other urinary diseases	0 (0 to 0·1)	0·2 (0·1 to 0·3)	0·1 (0 to 0·1)	0·3 (0·2 to 0·5)	0·2 (0·1 to 0·4)	0·4 (0·2 to 0·6)	0·3 (0·2 to 0·5)	0·3 (0·2 to 0·5)	0·3 (0·2 to 0·5)	0·5 (0·3 to 0·8)	0·5 (0·3 to 0·8)	0·5 (0·3 to 0·8)	0·7 (0·4 to 1·1)	0·6 (0·4 to 0·9)	0·6 (0·4 to 1)	0·6 (0·4 to 1)	0·4 (0·2 to 0·5)	0·3 (0·2 to 0·4)	0·2 (0·1 to 0·4)	0·2 (0·1 to 0·3)	0·1 (0 to 0·2)	0 (0 to 0·1)	0 (0 to 0)	7·4
Gynecological diseases	0 (0 to 0)	0 (0 to 0)	0 (0 to 0)	0 (0 to 0)	0 (0 to 0)	12·6 (6 to 22·2)	35·6 (19·8 to 59·8)	73·3 (45·6 to 114)	120 (76·7 to 178)	141 (90·9 to 203)	167 (111 to 242)	166 (108 to 241)	153 (99·2 to 219)	111 (65·4 to 167)	73·1 (45 to 106)	51·6 (29·6 to 79·5)	36·3 (21·9 to 53·8)	26·6 (16·6 to 38·5)	16·3 (10·3 to 24·2)	10·2 (6·1 to 15·4)	6 (3·8 to 8·9)	2·6 (1·6 to 4)	0·7 (0·4 to 1·1)	1,200
Uterine fibroids	0 (0 to 0)	0 (0 to 0)	0 (0 to 0)	0 (0 to 0)	0 (0 to 0)	0·2 (0·1 to 0·5)	0·6 (0·3 to 1·2)	1·4 (0·6 to 2·8)	3·7 (1·5 to 7·6)	7·7 (3·5 to 16·1)	14·5 (7 to 28)	16·5 (8·2 to 29·9)	16·8 (8·8 to 30·4)	12·5 (6·2 to 23·7)	9·2 (4·5 to 17·1)	5·8 (2·6 to 10·9)	3·3 (1·5 to 6·3)	1·9 (0·9 to 3·7)	1 (0·5 to 1·9)	0·5 (0·3 to 0·9)	0·2 (0·1 to 0·4)	0·1 (0·1 to 0·2)	0 (0 to 0)	95·9
Polycystic ovarian syndrome	0 (0 to 0)	0 (0 to 0)	0 (0 to 0)	0 (0 to 0)	0 (0 to 0)	0·5 (0·2 to 1·1)	2·6 (1·1 to 5·3)	3 (1·2 to 6·3)	3 (1·3 to 6·3)	2·8 (1·2 to 5·9)	2·9 (1·2 to 6)	2·8 (1·1 to 5·5)	2·6 (1·1 to 5·2)	0·7 (0·3 to 1·5)	0 (0 to 0)	0 (0 to 0)	0 (0 to 0)	0 (0 to 0)	0 (0 to 0)	0 (0 to 0)	0 (0 to 0)	0 (0 to 0)	0 (0 to 0)	21
Female infertility	0 (0 to 0)	0 (0 to 0)	0 (0 to 0)	0 (0 to 0)	0 (0 to 0)	0 (0 to 0)	0 (0 to 0)	2·5 (0·5 to 7)	4·2 (1 to 11·2)	5 (1·1 to 14·7)	5·8 (0·8 to 17·3)	5·2 (0·6 to 14·6)	0·1 (0 to 0·8)	0 (0 to 0)	0 (0 to 0)	0 (0 to 0)	0 (0 to 0)	0 (0 to 0)	0 (0 to 0)	0 (0 to 0)	0 (0 to 0)	0 (0 to 0)	0 (0 to 0)	22·8
Endometriosis	0 (0 to 0)	0 (0 to 0)	0 (0 to 0)	0 (0 to 0)	0 (0 to 0)	0 (0 to 0)	2·7 (1·1 to 5·8)	10·9 (5·3 to 19·8)	13·7 (6·6 to 25·2)	10·9 (5·3 to 19·8)	10·3 (5·5 to 18·2)	9·8 (5 to 17)	9·5 (5·1 to 15·9)	4·9 (2·4 to 8·2)	0 (0 to 0)	0 (0 to 0)	0 (0 to 0)	0 (0 to 0)	0 (0 to 0)	0 (0 to 0)	0 (0 to 0)	0 (0 to 0)	0 (0 to 0)	72·6
Genital prolapse	0 (0 to 0)	0 (0 to 0)	0 (0 to 0)	0 (0 to 0)	0 (0 to 0)	0 (0 to 0)	0 (0 to 0)	0·1 (0 to 0·2)	0·3 (0·1 to 0·5)	0·5 (0·2 to 1·1)	1·1 (0·4 to 2·2)	1·6 (0·6 to 3·6)	2·5 (1·1 to 5)	3·1 (1·3 to 6)	3·5 (1·6 to 7)	3·4 (1·6 to 6·5)	2·9 (1·5 to 5·3)	2·3 (1·2 to 4·3)	1·6 (0·8 to 2·9)	1·1 (0·5 to 2)	0·7 (0·3 to 1·2)	0·3 (0·2 to 0·6)	0·1 (0 to 0·2)	25
Premenstrual syndrome	0 (0 to 0)	0 (0 to 0)	0 (0 to 0)	0 (0 to 0)	0 (0 to 0)	11·4 (5·1 to 20·6)	28·6 (14·1 to 51·7)	35·6 (17·6 to 63·6)	39·2 (22·1 to 64·7)	38·3 (19·7 to 63·3)	45·8 (26·2 to 72·5)	44·4 (25 to 70·2)	34·2 (18·7 to 56·7)	12·3 (6 to 22·3)	0 (0 to 0)	0 (0 to 0)	0 (0 to 0)	0 (0 to 0)	0 (0 to 0)	0 (0 to 0)	0 (0 to 0)	0 (0 to 0)	0 (0 to 0)	290
Other gynecological diseases	0 (0 to 0)	0 (0 to 0)	0 (0 to 0)	0 (0 to 0)	0 (0 to 0)	0·5 (0·1 to 1)	1·1 (0·5 to 1·9)	19·8 (11·4 to 32·2)	56·1 (31·1 to 90·9)	76·1 (44·4 to 118)	87·1 (51·6 to 137)	85·8 (48·6 to 137)	87·8 (50·1 to 137)	77·7 (37·1 to 124)	60·4 (35·4 to 91·2)	42·5 (22·7 to 68·1)	30·2 (17·6 to 46·5)	22·4 (13·5 to 33·5)	13·7 (8·2 to 21·2)	8·6 (4·8 to 13·6)	5·1 (3·1 to 7·8)	2·2 (1·2 to 3·5)	0·6 (0·3 to 1)	678
Hemoglobinopathies and hemolytic anemias	0·3 (0·2 to 0·5)	0·3 (0·2 to 0·5)	1·7 (1·1 to 2·7)	4·2 (2·2 to 7·3)	13 (8·9 to 18·4)	9·5 (6·1 to 14·2)	14·2 (9·5 to 20·1)	13·9 (8·2 to 23·4)	12·7 (7 to 23·8)	11·5 (6·4 to 19·8)	12·3 (7·6 to 18·5)	10 (6 to 15·3)	9·7 (6 to 14·7)	10·6 (6·7 to 16·1)	9·9 (6·2 to 15)	9·9 (6·2 to 15·6)	7·6 (4·7 to 11·6)	7 (4·4 to 10·4)	5·5 (3·6 to 8·1)	4 (2·5 to 5·8)	4·3 (2·9 to 6·1)	2 (1·3 to 2·8)	0·5 (0·3 to 0·6)	174
Thalassemias	0 (0 to 0)	0 (0 to 0)	0 (0 to 0)	0 (0 to 0·1)	0 (0 to 0·1)	0 (0 to 0)	0 (0 to 0)	0 (0 to 0)	0 (0 to 0)	0 (0 to 0)	0 (0 to 0)	0 (0 to 0)	0 (0 to 0)	0 (0 to 0)	0 (0 to 0)	0 (0 to 0)	0 (0 to 0)	0 (0 to 0)	0 (0 to 0)	0 (0 to 0)	0 (0 to 0)	0 (0 to 0)	0 (0 to 0)	0·1
Thalassemias trait	0 (0 to 0)	0 (0 to 0)	0 (0 to 0·1)	0·1 (0 to 0·3)	0·4 (0·2 to 0·7)	0·2 (0·1 to 0·4)	0·7 (0·3 to 1·2)	0·7 (0·3 to 1·3)	0·8 (0·3 to 1·4)	0·7 (0·3 to 1·3)	0·7 (0·3 to 1·4)	0·4 (0·1 to 0·9)	0·2 (0 to 0·4)	0·2 (0 to 0·5)	0·2 (0 to 0·5)	0·1 (0 to 0·3)	0·1 (0 to 0·3)	0·1 (0 to 0·3)	0·1 (0 to 0·2)	0·1 (0 to 0·2)	0·1 (0 to 0·1)	0 (0 to 0·1)	0 (0 to 0)	5·9
Sickle cell disorders	0·3 (0·2 to 0·5)	0·2 (0·1 to 0·4)	1·3 (0·8 to 2·2)	2·3 (1·3 to 3·9)	5·6 (3·8 to 8·2)	6 (3·7 to 9·7)	10·6 (6·9 to 15·8)	8·6 (5·2 to 13·3)	6·6 (4·1 to 10·2)	4·8 (2·9 to 7·7)	6·3 (3·6 to 10·4)	5 (3 to 7·8)	5·4 (3·3 to 8·6)	5·1 (2·9 to 8·2)	3 (1·7 to 5)	1·3 (0·7 to 2·5)	0·3 (0·1 to 0·6)	0 (0 to 0·1)	0 (0 to 0)	0 (0 to 0)	0 (0 to 0)	0 (0 to 0)	0 (0 to 0)	72·9
Sickle cell trait	0 (0 to 0)	0·1 (0 to 0·1)	0·3 (0·1 to 0·6)	1·2 (0·3 to 2·8)	3·5 (2 to 5·9)	1·9 (0·7 to 3·6)	0·7 (0·2 to 1·6)	3·4 (0·4 to 10·3)	3·7 (0·4 to 12·7)	4·3 (0·9 to 11·4)	1·1 (0·3 to 2·2)	1·6 (0·5 to 3·5)	1·6 (0·4 to 3·6)	1·7 (0·4 to 3·9)	1·8 (0·5 to 4)	1·2 (0·2 to 2·9)	1 (0·2 to 2·4)	1·1 (0·3 to 2·2)	0·9 (0·2 to 1·8)	0·7 (0·2 to 1·4)	0·5 (0·2 to 1)	0·2 (0·1 to 0·4)	0·1 (0 to 0·1)	32·4
G6PD deficiency	0 (0 to 0)	0 (0 to 0)	0 (0 to 0)	0 (0 to 0·1)	0·1 (0 to 0·1)	0 (0 to 0·1)	1 (0·3 to 2·1)	0·3 (0·1 to 0·5)	0·4 (0·2 to 0·8)	0·4 (0·2 to 0·8)	1·7 (0·7 to 3·2)	0·7 (0·3 to 1·4)	0·5 (0·2 to 1·1)	0·7 (0·3 to 1·4)	0·7 (0·3 to 1·4)	0·7 (0·3 to 1·3)	0·4 (0·2 to 0·8)	0·2 (0·1 to 0·4)	0·1 (0·1 to 0·2)	0 (0 to 0·1)	0 (0 to 0)	0 (0 to 0)	0 (0 to 0)	8·2
G6PD trait	0 (0 to 0)	0 (0 to 0)	0 (0 to 0)	0 (0 to 0)	0 (0 to 0)	0 (0 to 0)	0·2 (0 to 0·3)	0 (0 to 0)	0 (0 to 0)	0 (0 to 0)	0·2 (0·1 to 0·4)	0·2 (0 to 0·4)	0 (0 to 0)	0 (0 to 0)	0 (0 to 0)	0 (0 to 0)	0 (0 to 0)	0 (0 to 0)	0 (0 to 0)	0 (0 to 0)	0 (0 to 0)	0 (0 to 0)	0 (0 to 0)	0·5
Other hemoglobinopathies and hemolytic anemias	0 (0 to 0)	0 (0 to 0)	0·1 (0 to 0·3)	0·5 (0·1 to 1·2)	3·4 (1·7 to 6)	1·3 (0·4 to 2·7)	1·1 (0·3 to 2·6)	1 (0·3 to 2·3)	1·3 (0·5 to 2·8)	1·3 (0·4 to 2·7)	2·2 (1 to 4·3)	2·1 (0·9 to 4·1)	2 (1 to 3·8)	3 (1·6 to 5)	4·2 (2·5 to 6·7)	6·5 (4·1 to 10·3)	5·8 (3·7 to 8·7)	5·6 (3·6 to 7·8)	4·5 (3 to 6·5)	3·1 (2·1 to 4·4)	3·7 (2·5 to 5·4)	1·7 (1·1 to 2·5)	0·4 (0·3 to 0·6)	54·5
Endocrine, metabolic, blood, and immune disorders	2·2 (1·3 to 3·9)	3·3 (1·9 to 5·7)	24·2 (14·1 to 42·8)	11·2 (7 to 19·1)	13·6 (9·5 to 19·6)	10·6 (7·3 to 15·3)	16 (10·9 to 22)	16·8 (11·5 to 24·3)	21·9 (14·8 to 31·6)	27·8 (18·5 to 40·4)	41·4 (29·4 to 58·4)	47·3 (31·9 to 69·2)	63·3 (42·7 to 92·5)	77·8 (50·7 to 113)	95·9 (63·4 to 143)	104 (68·8 to 154)	88·2 (60·4 to 131)	80 (53·7 to 115)	61·6 (42 to 88·3)	41·5 (28·4 to 59·8)	32·1 (22·1 to 47)	14·5 (9·7 to 21·6)	4·2 (2·8 to 6·5)	900
Oral disorders	0 (0 to 0)	0 (0 to 0)	0 (0 to 0)	3·7 (2 to 6)	4·9 (2·6 to 8·4)	5·8 (3·1 to 10·1)	9·2 (5 to 15·7)	14·4 (7·5 to 25·6)	22·7 (11·3 to 43·3)	28·3 (13·7 to 52·5)	36·1 (17·2 to 67·4)	40·6 (19·9 to 74·2)	52·9 (28 to 93·8)	66·3 (36·7 to 113)	84·5 (48·6 to 133)	97 (59·3 to 153)	90 (54·7 to 138)	81·4 (50·9 to 124)	58·2 (36·6 to 85·9)	40 (25·4 to 58·8)	23·4 (14·4 to 34·4)	9·7 (6 to 14·3)	2·4 (1·5 to 3·6)	771
Caries of deciduous teeth	0 (0 to 0)	0 (0 to 0)	0 (0 to 0)	1·2 (0·4 to 2·4)	1·2 (0·5 to 2·6)	0·1 (0 to 0·2)	0 (0 to 0)	0 (0 to 0)	0 (0 to 0)	0 (0 to 0)	0 (0 to 0)	0 (0 to 0)	0 (0 to 0)	0 (0 to 0)	0 (0 to 0)	0 (0 to 0)	0 (0 to 0)	0 (0 to 0)	0 (0 to 0)	0 (0 to 0)	0 (0 to 0)	0 (0 to 0)	0 (0 to 0)	2·4
Caries of permanent teeth	0 (0 to 0)	0 (0 to 0)	0 (0 to 0)	0 (0 to 0)	1·1 (0·4 to 2·6)	2·7 (1·1 to 5·8)	3·1 (1·2 to 6·6)	3·4 (1·4 to 7·2)	3·7 (1·4 to 7·4)	3·3 (1·3 to 6·8)	3·6 (1·4 to 7·4)	3·2 (1·3 to 6·6)	3·4 (1·4 to 7)	3·4 (1·3 to 7)	3·1 (1·2 to 7·2)	2·5 (0·9 to 5·6)	1·7 (0·7 to 3·6)	1·3 (0·5 to 2·8)	0·8 (0·3 to 1·6)	0·6 (0·2 to 1·1)	0·3 (0·1 to 0·6)	0·1 (0 to 0·3)	0 (0 to 0·1)	41·4
Periodontal diseases	0 (0 to 0)	0 (0 to 0)	0 (0 to 0)	0 (0 to 0)	0 (0 to 0)	0 (0 to 0)	1·2 (0·4 to 3·1)	4·5 (1·4 to 11·5)	10·7 (3·4 to 25·5)	15·8 (5·4 to 34)	21·5 (7·7 to 48·1)	24 (8·9 to 51·5)	28·1 (10·8 to 58·2)	28·3 (11·2 to 60·2)	26·5 (10·6 to 55·1)	21·7 (8·7 to 44·4)	15 (5·7 to 30·7)	10·9 (4·3 to 21·8)	6·9 (2·8 to 13·6)	4·5 (1·7 to 9·1)	2·6 (1 to 5·5)	1·1 (0·4 to 2·3)	0·3 (0·1 to 0·6)	223
Other oral disorders	0 (0 to 0)	0 (0 to 0)	0 (0 to 0)	2·5 (1·5 to 4)	2·5 (1·5 to 4·1)	3·1 (1·8 to 4·8)	4·9 (3 to 7·7)	5·8 (3·5 to 8·9)	6·9 (4·1 to 10·4)	7 (4·2 to 10·7)	7·6 (4·7 to 11·6)	7·5 (4·3 to 11·5)	8·4 (5·1 to 12·8)	8·6 (5·2 to 13·1)	8·5 (5 to 12·6)	7·6 (4·6 to 11·4)	5·9 (3·5 to 8·9)	4·3 (2·6 to 6·6)	2·3 (1·4 to 3·5)	1·3 (0·8 to 1·9)	0·6 (0·4 to 0·9)	0·2 (0·1 to 0·3)	0·1 (0 to 0·1)	95·5
Sudden infant death syndrome	--	1·6 (0·9 to 2·8)	7 (3·5 to 12·7)	--	--	--	--	--	--	--	--	--	--	--	--	--	--	--	--	--	--	--	--	NA
Injuries	5·9 (3·8 to 8·8)	2·9 (1·9 to 4·2)	15·8 (10·4 to 23·2)	26·7 (19·5 to 35·5)	47·9 (40 to 57·2)	62·9 (51·9 to 74·8)	136 (113 to 165)	147 (119 to 181)	125 (99·9 to 156)	117 (95·4 to 143)	130 (105 to 160)	121 (98·4 to 148)	128 (103 to 158)	129 (104 to 161)	133 (106 to 164)	140 (112 to 173)	120 (96 to 146)	117 (95·6 to 141)	101 (83·2 to 120)	88·9 (73·6 to 107)	75·1 (61·9 to 89·2)	40·5 (33·2 to 47·7)	13·1 (10·8 to 15·4)	2,020
Transport injuries	0·3 (0·2 to 0·4)	0·2 (0·1 to 0·3)	1·4 (0·8 to 2·4)	4·2 (2·6 to 6·5)	12·3 (8·1 to 17·6)	16·1 (11·2 to 21·7)	40·5 (28·1 to 57·4)	37·4 (25·2 to 51·9)	28·5 (20·1 to 40·1)	24·3 (17·6 to 32·8)	29·8 (21·4 to 40·6)	28·2 (21·2 to 36·5)	30·4 (22·6 to 39·3)	30·6 (23·1 to 39·2)	31·2 (23·9 to 39·7)	31·7 (24·2 to 40)	26·5 (20·6 to 33·2)	21·6 (16·6 to 26·8)	15·4 (12 to 19·2)	10 (7·8 to 12·3)	5·9 (4·6 to 7·3)	2·6 (2 to 3·2)	0·7 (0·6 to 0·9)	430
Road injuries	0·2 (0·1 to 0·4)	0·2 (0·1 to 0·3)	1·3 (0·7 to 2·1)	3·8 (2·3 to 6)	11·7 (7·5 to 16·7)	15·1 (10·3 to 20·4)	38·9 (27 to 55·6)	35·9 (24·2 to 49·8)	27·2 (19 to 39)	23 (16·6 to 31)	28·4 (20·2 to 38·7)	26·5 (19·9 to 34·2)	28·9 (21·5 to 37·7)	28·9 (21·6 to 37·4)	29·4 (22·5 to 37·7)	30·1 (23 to 38)	25·5 (19·7 to 32·1)	20·6 (15·9 to 25·8)	14·6 (11·3 to 18·1)	9·5 (7·4 to 11·7)	5·6 (4·3 to 6·9)	2·4 (1·9 to 3)	0·7 (0·5 to 0·8)	408
Pedestrian road injuries	0·1 (0 to 0·1)	0·1 (0 to 0·1)	0·2 (0·1 to 0·4)	1·1 (0·7 to 1·8)	3·4 (2·2 to 5)	3·7 (2·5 to 5·1)	4·5 (3·1 to 6·3)	4·1 (2·8 to 5·7)	3·3 (2·3 to 4·5)	3·6 (2·6 to 4·8)	4·8 (3·5 to 6·7)	4·7 (3·5 to 6·1)	5·6 (4·1 to 7·2)	6·4 (4·7 to 8·4)	6·7 (5 to 8·7)	8 (5·9 to 10·4)	7·5 (5·7 to 9·8)	6·5 (4·8 to 8·4)	5·2 (3·9 to 6·6)	3·5 (2·7 to 4·5)	2·1 (1·6 to 2·6)	0·8 (0·6 to 1·1)	0·2 (0·2 to 0·3)	86·2
Cyclist road injuries	0 (0 to 0)	0 (0 to 0)	0 (0 to 0)	0·2 (0·1 to 0·3)	0·6 (0·4 to 1·1)	1·5 (0·9 to 2·3)	2·5 (1·6 to 3·9)	2·3 (1·6 to 3·3)	2·5 (1·8 to 3·5)	2·5 (1·8 to 3·3)	3·1 (2·2 to 4·2)	3·2 (2·3 to 4·3)	3·7 (2·7 to 5)	4 (2·8 to 5·3)	4 (2·8 to 5·5)	3·8 (2·6 to 5·1)	3·1 (2·2 to 4·1)	2·5 (1·8 to 3·3)	1·7 (1·2 to 2·3)	1·2 (0·8 to 1·6)	0·7 (0·5 to 1)	0·3 (0·2 to 0·4)	0·1 (0·1 to 0·1)	43·5
Motorcyclist road injuries	0 (0 to 0)	0 (0 to 0)	0·1 (0 to 0·1)	0·1 (0·1 to 0·2)	0·4 (0·2 to 0·6)	0·6 (0·4 to 0·9)	2·5 (1·4 to 4·4)	2·6 (1·4 to 4·4)	2 (1·3 to 3·2)	1·7 (1·1 to 2·4)	2·1 (1·4 to 3)	1·8 (1·3 to 2·5)	2·1 (1·5 to 2·8)	2 (1·4 to 2·7)	2·2 (1·6 to 2·9)	2 (1·5 to 2·8)	1·8 (1·3 to 2·4)	1·5 (1·1 to 2·1)	1·1 (0·8 to 1·6)	0·9 (0·6 to 1·2)	0·5 (0·4 to 0·8)	0·3 (0·2 to 0·4)	0·1 (0·1 to 0·1)	28·5
Motor vehicle road injuries	0·2 (0·1 to 0·2)	0·1 (0 to 0·1)	1 (0·6 to 1·7)	2·2 (1·3 to 3·6)	7·1 (4·4 to 10·4)	9·1 (5·9 to 12·6)	29 (19·3 to 42·1)	26·3 (17·2 to 37·2)	18·8 (12·7 to 27·8)	14·7 (10 to 20·8)	17·6 (12 to 25·2)	16·1 (11·6 to 21·5)	16·7 (12 to 23)	15·6 (11·3 to 21·2)	15·6 (11·6 to 20·6)	15·4 (11·3 to 20)	12·3 (9·2 to 15·9)	9·5 (7·1 to 12·1)	6·2 (4·6 to 7·8)	3·6 (2·8 to 4·6)	2·1 (1·6 to 2·6)	0·9 (0·7 to 1·1)	0·3 (0·2 to 0·3)	240
Other road injuries	0 (0 to 0)	0 (0 to 0)	0 (0 to 0)	0·1 (0·1 to 0·2)	0·2 (0·1 to 0·3)	0·2 (0·1 to 0·3)	0·4 (0·2 to 0·6)	0·4 (0·3 to 0·7)	0·6 (0·4 to 0·8)	0·6 (0·4 to 0·8)	0·7 (0·5 to 0·9)	0·7 (0·5 to 1)	0·8 (0·6 to 1·1)	0·9 (0·6 to 1·2)	1 (0·7 to 1·3)	1 (0·7 to 1·3)	0·8 (0·6 to 1·1)	0·6 (0·4 to 0·8)	0·4 (0·3 to 0·6)	0·3 (0·2 to 0·4)	0·2 (0·1 to 0·2)	0·1 (0 to 0·1)	0 (0 to 0)	9·8
Other transport injuries	0 (0 to 0·1)	0 (0 to 0·1)	0·1 (0·1 to 0·2)	0·4 (0·2 to 0·6)	0·7 (0·4 to 1)	1 (0·6 to 1·6)	1·6 (0·9 to 2·6)	1·6 (1 to 2·5)	1·4 (0·9 to 2·1)	1·3 (0·9 to 1·8)	1·4 (1 to 2·1)	1·7 (1·1 to 2·6)	1·4 (1 to 2·1)	1·6 (1·1 to 2·3)	1·8 (1·2 to 2·6)	1·6 (1·1 to 2·2)	1·1 (0·8 to 1·4)	0·9 (0·7 to 1·3)	0·9 (0·6 to 1·2)	0·5 (0·4 to 0·6)	0·3 (0·2 to 0·4)	0·2 (0·1 to 0·2)	0·1 (0 to 0·1)	21·4
Unintentional injuries	2·8 (1·8 to 4·1)	2·1 (1·4 to 3·1)	11·3 (7·4 to 17·2)	18·4 (13·2 to 24·9)	29·2 (23·4 to 36·1)	35·1 (27·7 to 44)	41·7 (32·8 to 53·2)	41·3 (32 to 52·5)	43·6 (33·1 to 56·7)	46·3 (34·8 to 61·7)	55·2 (41·8 to 72·4)	56·4 (42·3 to 73·6)	65·9 (49·4 to 87·2)	72·5 (55·3 to 95·1)	80·2 (61·2 to 104)	87·7 (68·1 to 112)	77·8 (60·8 to 99)	83·1 (67·1 to 102)	77·3 (63·2 to 93·2)	74·9 (61·5 to 90·6)	66·8 (54·9 to 79·8)	37·1 (30·4 to 43·8)	12·2 (10 to 14·4)	1,120
Falls	0·1 (0 to 0·1)	0 (0 to 0·1)	0·4 (0·3 to 0·7)	1·1 (0·8 to 1·6)	2·4 (1·6 to 3·4)	3·6 (2·4 to 5·2)	4·9 (3·4 to 7)	5·6 (3·8 to 8)	6·9 (4·7 to 10)	7·8 (5·3 to 10·9)	9·8 (6·8 to 14)	10·9 (7·6 to 15·2)	14 (9·9 to 19·5)	17·5 (12·8 to 24)	21·4 (15·7 to 28·9)	26·7 (20 to 34·8)	27·5 (21 to 35)	35·9 (28·7 to 44·5)	39 (31·5 to 48·2)	45·5 (36·3 to 56·6)	44·3 (35·1 to 54·4)	24·9 (19·9 to 30·2)	8·2 (6·6 to 10)	358
Drowning	0·2 (0·2 to 0·4)	0·2 (0·1 to 0·3)	1·5 (0·9 to 2·5)	5·8 (3·6 to 8·6)	9·4 (6·7 to 12·7)	11·3 (7·9 to 15·2)	9·6 (6·8 to 12·9)	4·8 (3·2 to 6·9)	3·6 (2·5 to 5·3)	3·2 (2·3 to 4·4)	3·8 (2·6 to 5·3)	3·1 (2·2 to 4·2)	3·2 (2·3 to 4·5)	2·8 (2·1 to 3·8)	2·7 (2 to 3·6)	2·7 (2 to 3·5)	2·1 (1·6 to 2·8)	1·5 (1·2 to 2)	1·4 (1 to 1·8)	0·8 (0·6 to 1)	0·7 (0·6 to 0·9)	0·6 (0·4 to 0·7)	0·2 (0·2 to 0·2)	75·3
Fire, heat, and hot substances	0·1 (0·1 to 0·2)	0·2 (0·1 to 0·3)	1·3 (0·8 to 2)	3 (2 to 4·5)	4·9 (3·8 to 6·5)	5·7 (4 to 7·9)	8·6 (6·2 to 11·6)	9·3 (6·6 to 12·9)	10·4 (7·4 to 14·5)	11·4 (7·9 to 16)	13·7 (9·7 to 19·2)	13·1 (9·1 to 18)	14·1 (9·9 to 20·2)	15 (10·6 to 21·2)	15·5 (10·9 to 22·2)	15 (10·5 to 20·8)	11·4 (8·1 to 15·9)	10·1 (7·3 to 13·9)	7·3 (5·4 to 9·8)	5·4 (4 to 7·2)	4·3 (3·3 to 5·4)	2·2 (1·8 to 2·7)	0·7 (0·6 to 0·9)	183
Poisonings	0 (0 to 0·1)	0·1 (0·1 to 0·1)	0·2 (0·1 to 0·4)	0·6 (0·4 to 0·9)	0·7 (0·4 to 1)	0·7 (0·4 to 1·1)	1 (0·7 to 1·5)	1 (0·7 to 1·5)	1 (0·6 to 1·4)	1 (0·6 to 1·4)	1·1 (0·7 to 1·6)	1 (0·7 to 1·5)	1·1 (0·7 to 1·5)	1 (0·7 to 1·5)	1·1 (0·7 to 1·5)	1 (0·6 to 1·4)	0·9 (0·6 to 1·2)	0·7 (0·5 to 1)	0·5 (0·4 to 0·7)	0·4 (0·3 to 0·5)	0·3 (0·2 to 0·3)	0·1 (0·1 to 0·1)	0 (0 to 0)	15·6
Poisoning by carbon monoxide	0 (0 to 0)	0 (0 to 0)	0 (0 to 0)	0·1 (0 to 0·1)	0·2 (0·1 to 0·3)	0·2 (0·1 to 0·3)	0·2 (0·1 to 0·4)	0·2 (0·1 to 0·4)	0·2 (0·1 to 0·4)	0·2 (0·1 to 0·4)	0·2 (0·1 to 0·4)	0·2 (0·1 to 0·4)	0·2 (0·1 to 0·4)	0·2 (0·1 to 0·4)	0·3 (0·2 to 0·4)	0·2 (0·1 to 0·4)	0·2 (0·1 to 0·3)	0·2 (0·1 to 0·3)	0·1 (0·1 to 0·2)	0·1 (0 to 0·1)	0 (0 to 0·1)	0 (0 to 0)	0 (0 to 0)	3·5
Poisoning by other means	0 (0 to 0·1)	0·1 (0·1 to 0·1)	0·2 (0·1 to 0·3)	0·5 (0·3 to 0·8)	0·5 (0·3 to 0·8)	0·5 (0·3 to 0·9)	0·8 (0·5 to 1·2)	0·8 (0·5 to 1·1)	0·7 (0·5 to 1·1)	0·7 (0·5 to 1·1)	0·8 (0·6 to 1·2)	0·8 (0·5 to 1·2)	0·8 (0·5 to 1·2)	0·8 (0·5 to 1·2)	0·8 (0·5 to 1·2)	0·7 (0·5 to 1·1)	0·6 (0·4 to 0·9)	0·6 (0·4 to 0·8)	0·4 (0·3 to 0·6)	0·3 (0·2 to 0·5)	0·2 (0·2 to 0·3)	0·1 (0·1 to 0·1)	0 (0 to 0)	12·1
Exposure to mechanical forces	0·1 (0·1 to 0·1)	0·1 (0·1 to 0·2)	0·5 (0·4 to 0·7)	2·2 (1·5 to 3·2)	4·4 (3·1 to 6·1)	5·5 (3·9 to 7·7)	7 (5·1 to 9·6)	7·7 (5·7 to 10·4)	7·8 (5·5 to 10·9)	8 (5·7 to 11·4)	9·6 (6·9 to 13·5)	9·8 (7 to 13·7)	11 (7·7 to 15·6)	11·7 (8·3 to 16·9)	12·1 (8·5 to 17·3)	11·8 (8·3 to 16·6)	9·6 (6·7 to 13·5)	8·5 (5·9 to 11·9)	6·4 (4·6 to 8·8)	4·9 (3·6 to 6·7)	3·5 (2·6 to 4·7)	2·2 (1·6 to 2·8)	0·8 (0·6 to 1)	145
Unintentional firearm injuries	0 (0 to 0)	0 (0 to 0)	0·1 (0 to 0·1)	0·2 (0·1 to 0·4)	0·5 (0·3 to 0·8)	1·1 (0·7 to 1·6)	2 (1·3 to 2·8)	2·3 (1·6 to 3·2)	1·6 (1·2 to 2·2)	1·8 (1·3 to 2·3)	2 (1·5 to 2·6)	1·9 (1·4 to 2·4)	1·8 (1·4 to 2·5)	1·7 (1·2 to 2·3)	1·8 (1·3 to 2·4)	1·6 (1·2 to 2·1)	1·3 (0·9 to 1·7)	1·2 (0·9 to 1·6)	0·9 (0·7 to 1·2)	0·8 (0·6 to 1·1)	0·7 (0·5 to 1)	0·6 (0·4 to 0·7)	0·2 (0·2 to 0·3)	26·1
Other exposure to mechanical forces	0·1 (0 to 0·1)	0·1 (0·1 to 0·2)	0·5 (0·3 to 0·7)	2 (1·3 to 2·9)	4 (2·8 to 5·5)	4·4 (3·1 to 6·4)	5 (3·4 to 7·4)	5·4 (3·7 to 7·9)	6·1 (4·2 to 9)	6·3 (4·2 to 9·3)	7·6 (5·2 to 11·2)	8 (5·4 to 11·4)	9·1 (6·2 to 13·2)	10 (7 to 14·6)	10·3 (7·1 to 15)	10·2 (7 to 14·6)	8·4 (5·7 to 11·9)	7·3 (5 to 10·4)	5·5 (3·8 to 7·6)	4 (2·9 to 5·6)	2·8 (2 to 3·8)	1·6 (1·2 to 2·1)	0·6 (0·4 to 0·7)	119
Adverse effects of medical treatment	1 (0·6 to 1·6)	0·7 (0·4 to 1·1)	2·6 (1·4 to 4·6)	1·7 (1 to 2·9)	1·4 (0·9 to 2)	1·1 (0·7 to 1·7)	2·3 (1·4 to 3·6)	3·9 (2·4 to 6·2)	3·8 (2·3 to 6)	4·4 (2·8 to 6·5)	4·9 (3·1 to 7·3)	5·8 (3·6 to 8·8)	7·9 (5·1 to 11·9)	8·7 (5·7 to 12·9)	10·6 (6·8 to 16·1)	13·3 (8·7 to 19·5)	11·4 (7·8 to 16·1)	12·7 (8·5 to 18)	11·7 (8·1 to 16·4)	9·2 (6·3 to 12·4)	7·8 (5·1 to 11·4)	3·8 (2·5 to 5·3)	1·1 (0·7 to 1·6)	132
Animal contact	0 (0 to 0)	0 (0 to 0)	0 (0 to 0·1)	0·2 (0·2 to 0·3)	0·4 (0·2 to 0·6)	0·5 (0·3 to 0·7)	0·6 (0·4 to 0·9)	0·6 (0·4 to 0·9)	0·7 (0·4 to 1)	0·7 (0·4 to 1)	0·8 (0·5 to 1·2)	0·8 (0·5 to 1·2)	0·9 (0·6 to 1·4)	1 (0·7 to 1·4)	1·1 (0·7 to 1·6)	1·1 (0·7 to 1·6)	0·9 (0·6 to 1·3)	0·8 (0·6 to 1·2)	0·7 (0·5 to 0·9)	0·7 (0·5 to 1)	0·7 (0·5 to 0·9)	0·5 (0·4 to 0·7)	0·2 (0·1 to 0·3)	14·1
Venomous animal contact	0 (0 to 0)	0 (0 to 0)	0 (0 to 0)	0·1 (0·1 to 0·2)	0·2 (0·1 to 0·4)	0·4 (0·2 to 0·6)	0·5 (0·3 to 0·7)	0·5 (0·3 to 0·7)	0·5 (0·3 to 0·8)	0·5 (0·3 to 0·8)	0·6 (0·4 to 0·9)	0·6 (0·4 to 0·9)	0·7 (0·4 to 1)	0·7 (0·4 to 1)	0·7 (0·5 to 1·1)	0·7 (0·5 to 1)	0·6 (0·4 to 0·9)	0·5 (0·3 to 0·8)	0·4 (0·3 to 0·6)	0·4 (0·3 to 0·6)	0·4 (0·3 to 0·6)	0·4 (0·2 to 0·5)	0·1 (0·1 to 0·2)	9·6
Non-venomous animal contact	0 (0 to 0)	0 (0 to 0)	0 (0 to 0)	0·1 (0·1 to 0·1)	0·1 (0·1 to 0·2)	0·1 (0·1 to 0·2)	0·2 (0·1 to 0·2)	0·2 (0·1 to 0·2)	0·2 (0·1 to 0·3)	0·2 (0·1 to 0·3)	0·2 (0·1 to 0·3)	0·2 (0·1 to 0·3)	0·3 (0·2 to 0·4)	0·3 (0·2 to 0·5)	0·4 (0·3 to 0·6)	0·4 (0·2 to 0·6)	0·3 (0·2 to 0·5)	0·3 (0·2 to 0·5)	0·3 (0·2 to 0·4)	0·3 (0·2 to 0·4)	0·2 (0·2 to 0·4)	0·2 (0·1 to 0·3)	0·1 (0 to 0·1)	4·5
Foreign body	1·1 (0·7 to 1·8)	0·7 (0·4 to 1·1)	4·1 (2·4 to 6·8)	1·7 (1·1 to 2·4)	1·7 (1·3 to 2·1)	1·6 (1·2 to 2)	1·4 (1 to 1·9)	1·6 (1·1 to 2·2)	1·6 (1·1 to 2·2)	1·6 (1·1 to 2·2)	1·9 (1·4 to 2·7)	2 (1·4 to 2·6)	2·3 (1·7 to 3)	2·6 (1·9 to 3·4)	2·8 (2·1 to 3·5)	3·3 (2·5 to 4·2)	3·2 (2·5 to 4)	3·5 (2·8 to 4·4)	3·4 (2·5 to 4·5)	3 (2·3 to 3·9)	1·9 (1·5 to 2·3)	1·1 (0·9 to 1·4)	0·4 (0·3 to 0·5)	48·4
Pulmonary aspiration and foreign body in airway	1·1 (0·7 to 1·8)	0·7 (0·4 to 1·1)	4 (2·4 to 6·7)	1·4 (0·9 to 2)	1·2 (0·9 to 1·6)	1 (0·8 to 1·3)	0·6 (0·5 to 0·8)	0·8 (0·6 to 1)	0·7 (0·5 to 0·9)	0·7 (0·5 to 0·9)	0·9 (0·7 to 1·2)	0·9 (0·7 to 1·2)	1·1 (0·8 to 1·4)	1·4 (1 to 1·8)	1·6 (1·2 to 2·2)	2·2 (1·6 to 3)	2·2 (1·7 to 3)	2·7 (2 to 3·5)	2·8 (1·9 to 3·8)	2·5 (1·8 to 3·3)	1·6 (1·2 to 2)	1 (0·7 to 1·2)	0·4 (0·3 to 0·4)	33·4
Foreign body in eyes	0 (0 to 0)	0 (0 to 0)	0 (0 to 0)	0·1 (0 to 0·2)	0·2 (0 to 0·4)	0·2 (0·1 to 0·5)	0·3 (0·1 to 0·7)	0·4 (0·1 to 0·8)	0·5 (0·2 to 1)	0·5 (0·2 to 1)	0·5 (0·2 to 1·1)	0·6 (0·2 to 1·1)	0·6 (0·3 to 1·1)	0·6 (0·3 to 1)	0·5 (0·3 to 0·9)	0·5 (0·3 to 0·8)	0·4 (0·2 to 0·6)	0·3 (0·2 to 0·5)	0·2 (0·2 to 0·4)	0·2 (0·1 to 0·2)	0·1 (0·1 to 0·2)	0 (0 to 0·1)	0 (0 to 0)	6·7
Foreign body in other body part	0 (0 to 0)	0 (0 to 0)	0 (0 to 0·1)	0·2 (0·1 to 0·3)	0·3 (0·2 to 0·5)	0·4 (0·2 to 0·5)	0·4 (0·3 to 0·6)	0·4 (0·3 to 0·6)	0·5 (0·3 to 0·6)	0·5 (0·3 to 0·6)	0·5 (0·4 to 0·7)	0·5 (0·4 to 0·7)	0·6 (0·4 to 0·8)	0·6 (0·4 to 0·8)	0·6 (0·5 to 0·9)	0·6 (0·5 to 0·9)	0·5 (0·4 to 0·7)	0·5 (0·3 to 0·6)	0·4 (0·3 to 0·5)	0·3 (0·2 to 0·4)	0·2 (0·2 to 0·3)	0·1 (0·1 to 0·2)	0 (0 to 0·1)	8·3
Environmental heat and cold exposure	0 (0 to 0)	0 (0 to 0)	0·1 (0·1 to 0·1)	0·6 (0·4 to 0·9)	1·1 (0·7 to 1·7)	1·6 (1 to 2·4)	2·1 (1·4 to 3·1)	2·4 (1·6 to 3·4)	2·8 (1·9 to 4·2)	3 (2·1 to 4·4)	3·5 (2·4 to 5)	3·6 (2·4 to 5·2)	4·2 (2·9 to 6)	4·6 (3·2 to 6·5)	5 (3·5 to 6·9)	5·1 (3·5 to 7·1)	4·4 (3 to 6)	4 (2·7 to 5·4)	3 (2·1 to 4·1)	2·3 (1·6 to 3·1)	1·6 (1·1 to 2·1)	0·8 (0·6 to 1·1)	0·3 (0·2 to 0·4)	56·1
Exposure to forces of nature	0 (0 to 0)	0 (0 to 0)	0 (0 to 0)	0 (0 to 0)	0 (0 to 0)	0 (0 to 0)	0 (0 to 0)	0 (0 to 0)	0 (0 to 0)	0 (0 to 0)	0 (0 to 0)	0 (0 to 0)	0 (0 to 0)	0 (0 to 0)	0 (0 to 0)	0 (0 to 0)	0 (0 to 0)	0 (0 to 0)	0 (0 to 0)	0 (0 to 0)	0 (0 to 0)	0 (0 to 0)	0 (0 to 0)	0·2
Other unintentional injuries	0·1 (0·1 to 0·2)	0·1 (0 to 0·1)	0·4 (0·3 to 0·6)	1·6 (1 to 2·3)	2·8 (2 to 4·1)	3·5 (2·4 to 5)	4·2 (2·8 to 6·1)	4·5 (3 to 6·6)	4·9 (3·3 to 7·3)	5·2 (3·4 to 7·8)	6 (4 to 8·9)	6·2 (4·1 to 9)	7·1 (4·7 to 10·4)	7·5 (5 to 11)	7·9 (5·3 to 11·4)	7·7 (5·2 to 11·2)	6·3 (4·3 to 9·1)	5·4 (3·7 to 7·7)	4 (2·7 to 5·6)	2·7 (1·9 to 3·9)	1·7 (1·2 to 2·4)	0·7 (0·5 to 1)	0·2 (0·1 to 0·3)	90·5
Self-harm and interpersonal violence	2·8 (1·6 to 4·8)	0·6 (0·3 to 0·9)	3 (1·7 to 5)	4·1 (2·4 to 6·5)	6·4 (4·8 to 8·6)	11·8 (8·9 to 15·4)	54·1 (41·6 to 70·5)	68·2 (49·8 to 90·5)	52·6 (38·6 to 71·4)	46·2 (34·6 to 62·5)	45·1 (33·7 to 59·7)	36·3 (27·3 to 48·8)	32·1 (24·2 to 42·3)	26·2 (19·8 to 33·5)	21·8 (16·8 to 27·8)	20·3 (16·1 to 25·3)	15·7 (12·6 to 19·1)	11·9 (9·4 to 14·4)	7·9 (6·5 to 9·8)	4 (3·2 to 4·9)	2·4 (2 to 2·9)	0·8 (0·6 to 1)	0·2 (0·2 to 0·3)	475
Self-harm	0 (0 to 0)	0 (0 to 0)	0 (0 to 0)	0 (0 to 0)	0 (0 to 0)	1·5 (1 to 2·4)	10·2 (6·9 to 14·8)	10 (6·8 to 14·4)	7·8 (5·2 to 11·4)	7·5 (5·1 to 10·7)	8·8 (6·4 to 12)	7·2 (5 to 10)	7 (5 to 9·9)	7·1 (5 to 9·6)	6·9 (4·9 to 9·7)	7·1 (5·1 to 9·4)	5·4 (3·9 to 7·2)	3·9 (2·9 to 5·2)	2·4 (1·7 to 3·3)	1·3 (1 to 1·6)	0·7 (0·6 to 1)	0·2 (0·2 to 0·2)	0·1 (0 to 0·1)	95·1
Self-harm by firearm	0 (0 to 0)	0 (0 to 0)	0 (0 to 0)	0 (0 to 0)	0 (0 to 0)	0·1 (0 to 0·1)	0·8 (0·5 to 1·2)	0·8 (0·5 to 1·1)	0·4 (0·3 to 0·6)	0·6 (0·4 to 0·8)	0·5 (0·4 to 0·7)	0·3 (0·2 to 0·5)	0·3 (0·2 to 0·5)	0·4 (0·3 to 0·5)	0·4 (0·3 to 0·5)	0·2 (0·1 to 0·2)	0·2 (0·2 to 0·3)	0·2 (0·2 to 0·3)	0·2 (0·1 to 0·2)	0·2 (0·1 to 0·3)	0·2 (0·1 to 0·2)	0 (0 to 0)	0 (0 to 0)	5·7
Self-harm by other specified means	0 (0 to 0)	0 (0 to 0)	0 (0 to 0)	0 (0 to 0)	0 (0 to 0)	1·5 (0·9 to 2·3)	9·4 (6·3 to 13·6)	9·2 (6·3 to 13·3)	7·3 (4·9 to 10·8)	7 (4·7 to 9·9)	8·3 (6 to 11·3)	6·8 (4·7 to 9·6)	6·7 (4·7 to 9·4)	6·7 (4·7 to 9·1)	6·5 (4·6 to 9·2)	6·9 (4·9 to 9·2)	5·2 (3·7 to 6·9)	3·7 (2·7 to 5)	2·2 (1·6 to 3)	1·1 (0·8 to 1·4)	0·6 (0·4 to 0·7)	0·2 (0·1 to 0·2)	0 (0 to 0·1)	89·3
Interpersonal violence	2·8 (1·6 to 4·8)	0·6 (0·3 to 0·9)	3 (1·7 to 5)	4 (2·4 to 6·3)	6·3 (4·7 to 8·5)	10·2 (7·5 to 13·7)	43·8 (31·9 to 59·1)	58·1 (41·6 to 78·5)	44·7 (32·1 to 61·7)	38·5 (28 to 53·2)	36·2 (25·9 to 48·8)	29·1 (21 to 40·4)	25 (18·2 to 34·1)	19 (14 to 25·6)	14·9 (11·3 to 19·8)	13·2 (9·9 to 16·9)	10·3 (8 to 13·2)	7·9 (6·2 to 9·9)	5·5 (4·3 to 6·8)	2·7 (2·1 to 3·5)	1·7 (1·3 to 2·1)	0·6 (0·5 to 0·8)	0·2 (0·1 to 0·2)	378
Physical violence by firearm	0 (0 to 0·1)	0 (0 to 0)	0·1 (0 to 0·1)	0·2 (0·1 to 0·3)	0·4 (0·3 to 0·7)	1 (0·6 to 1·4)	7·7 (4·7 to 11·8)	9·3 (5·7 to 13·9)	6·2 (3·8 to 9·7)	5 (3·1 to 7·8)	4·6 (3 to 7)	2·8 (1·8 to 4·3)	2·9 (1·8 to 4·3)	1·8 (1·1 to 2·7)	1·4 (0·9 to 2)	0·9 (0·6 to 1·3)	0·5 (0·4 to 0·7)	0·3 (0·3 to 0·5)	0·2 (0·1 to 0·2)	0·1 (0·1 to 0·1)	0·1 (0 to 0·1)	0 (0 to 0)	0 (0 to 0)	45·4
Physical violence by sharp object	0·1 (0·1 to 0·2)	0 (0 to 0)	0·1 (0·1 to 0·2)	0·3 (0·2 to 0·5)	0·7 (0·5 to 1)	1·8 (1·3 to 2·4)	16·6 (11·9 to 23·1)	25·7 (17·7 to 36·1)	19·7 (13·4 to 27·7)	16 (11 to 23)	15·7 (10·6 to 22·3)	11·8 (8 to 17·1)	8·1 (5·5 to 11·5)	5·2 (3·7 to 7·1)	3·8 (2·7 to 5·1)	2·6 (2 to 3·4)	1·9 (1·5 to 2·4)	1·4 (1·1 to 1·8)	0·8 (0·6 to 1)	0·5 (0·4 to 0·6)	0·3 (0·2 to 0·4)	0·1 (0·1 to 0·2)	0 (0 to 0·1)	133
Sexual violence	0 (0 to 0)	0 (0 to 0)	0 (0 to 0)	0·1 (0 to 0·1)	0·5 (0·3 to 0·8)	1·3 (0·7 to 2)	3·3 (1·8 to 5·3)	4·4 (2·5 to 6·7)	4·4 (2·5 to 7·1)	4 (2·2 to 6·6)	3·9 (2·1 to 6·2)	3·1 (1·8 to 5·1)	2·8 (1·6 to 4·6)	2·1 (1·2 to 3·5)	1·5 (0·9 to 2·5)	1·3 (0·8 to 1·9)	1 (0·6 to 1·4)	0·8 (0·5 to 1·1)	0·5 (0·3 to 0·7)	0·4 (0·2 to 0·5)	0·2 (0·1 to 0·3)	0 (0 to 0·1)	0 (0 to 0)	35·3
Physical violence by other means	2·7 (1·5 to 4·5)	0·6 (0·3 to 0·9)	2·8 (1·5 to 4·7)	3·4 (1·9 to 5·5)	4·7 (3·3 to 6·6)	6·2 (4·4 to 8·6)	16·2 (11·8 to 22·4)	18·8 (13·1 to 26·1)	14·5 (10 to 20·3)	13·6 (9·5 to 19·4)	12·1 (8·3 to 16·8)	11·3 (8 to 16)	11·2 (7·8 to 15·7)	9·9 (7·2 to 13·7)	8·2 (6 to 11·3)	8·3 (6 to 11)	6·9 (5·1 to 9·2)	5·4 (4·1 to 7)	4·1 (3·1 to 5·2)	1·8 (1·4 to 2·4)	1·1 (0·9 to 1·4)	0·4 (0·3 to 0·6)	0·1 (0·1 to 0·1)	164
Conflict and terrorism	0 (0 to 0)	0 (0 to 0)	0 (0 to 0)	0 (0 to 0)	0 (0 to 0)	0 (0 to 0)	0 (0 to 0)	0 (0 to 0)	0·1 (0 to 0·1)	0·1 (0 to 0·2)	0·1 (0 to 0·1)	0·1 (0 to 0·1)	0·1 (0 to 0·1)	0 (0 to 0·1)	0 (0 to 0·1)	0 (0 to 0)	0 (0 to 0)	0 (0 to 0)	0 (0 to 0)	0 (0 to 0)	0 (0 to 0)	0 (0 to 0)	0 (0 to 0)	0·5

SR Table 2b: Belize DALYS by cause and age group for females with 95% uncertainty intervals, 2019

Cause	Early Neonatal	Late Neonatal	Post Neonatal	1 to 4	5 to 9	10 to 14	15 to 19	20 to 24	25 to 29	30 to 34	35 to 39
All causes	2,150 (1,760 to 2,630)	745 (548 to 976)	1,070 (736 to 1,560)	1,200 (990 to 1,450)	1,230 (963 to 1,540)	1,620 (1,240 to 2,080)	2,670 (2,150 to 3,320)	2,870 (2,350 to 3,470)	3,200 (2,660 to 3,850)	3,310 (2,770 to 3,940)	3,060 (2,560 to 3,660)
Communicable, maternal, neonatal, and nutritional diseases	1,840 (1,500 to 2,230)	578 (423 to 762)	581 (404 to 845)	485 (381 to 619)	388 (270 to 555)	326 (231 to 456)	424 (331 to 565)	514 (429 to 636)	635 (552 to 743)	631 (552 to 735)	565 (486 to 674)
HIV/AIDS and sexually transmitted infections	0·6 (0·4 to 0·9)	0·2 (0·1 to 0·4)	24·9 (23·1 to 26·6)	26·3 (24·4 to 28·4)	21·1 (18·9 to 24)	14·3 (12·9 to 15·9)	47·7 (43·9 to 52·5)	148 (134 to 165)	270 (245 to 299)	286 (262 to 315)	287 (266 to 312)
HIV/AIDS	0 (0 to 0)	0 (0 to 0)	24·8 (23 to 26·5)	26·3 (24·3 to 28·4)	21·1 (18·9 to 24)	13·9 (12·6 to 15·5)	45·5 (41·9 to 49·9)	143 (130 to 159)	262 (238 to 291)	277 (254 to 304)	279 (258 to 302)
HIV/AIDS - Drug-susceptible Tuberculosis	0 (0 to 0)	0 (0 to 0)	1·8 (1·1 to 2·7)	1·9 (1·2 to 2·8)	1·5 (1 to 2·3)	1·1 (0·7 to 1·6)	3·4 (2·2 to 5)	10·7 (7·1 to 15·7)	19·2 (12·4 to 28·6)	20·3 (13 to 30·6)	20·3 (13·1 to 30·7)
HIV/AIDS - Multidrug-resistant Tuberculosis without extensive dru	0 (0 to 0)	0 (0 to 0)	0 (0 to 0·1)	0 (0 to 0·1)	0 (0 to 0·1)	0 (0 to 0·1)	0 (0 to 0·2)	0·1 (0 to 0·5)	0·2 (0 to 1)	0·2 (0 to 1)	0·2 (0 to 1)
HIV/AIDS - Extensively drug-resistant Tuberculosis	0 (0 to 0)	0 (0 to 0)	0 (0 to 0)	0 (0 to 0)	0 (0 to 0)	0 (0 to 0)	0 (0 to 0)	0 (0 to 0·1)	0 (0 to 0·2)	0 (0 to 0·2)	0 (0 to 0·2)
HIV/AIDS resulting in other diseases	0 (0 to 0)	0 (0 to 0)	23 (21·1 to 25)	24·4 (22·3 to 26·6)	19·5 (17·2 to 22·5)	12·8 (11·4 to 14·5)	42·1 (38·1 to 46·5)	132 (118 to 148)	243 (219 to 271)	256 (232 to 284)	258 (235 to 283)
Sexually transmitted infections excluding HIV	0·6 (0·4 to 0·9)	0·2 (0·1 to 0·4)	0·1 (0 to 0·2)	0 (0 to 0)	0 (0 to 0)	0·4 (0·3 to 0·6)	2·2 (1·3 to 3·8)	5 (2·8 to 8·9)	7·6 (3·9 to 13·7)	9 (4·8 to 16·2)	8·6 (4·5 to 16)
Syphilis	0·6 (0·4 to 0·9)	0·2 (0·1 to 0·4)	0·1 (0 to 0·2)	0 (0 to 0)	0 (0 to 0)	0·1 (0·1 to 0·1)	0·2 (0·1 to 0·2)	0·1 (0·1 to 0·2)	0·2 (0·1 to 0·3)	0·3 (0·2 to 0·5)	0·2 (0·1 to 0·2)
Chlamydial infection	0 (0 to 0)	0 (0 to 0)	0 (0 to 0)	0 (0 to 0)	0 (0 to 0)	0 (0 to 0·1)	0·2 (0·1 to 0·4)	0·6 (0·4 to 0·8)	0·7 (0·4 to 1·3)	0·8 (0·4 to 1·4)	0·9 (0·5 to 1·5)
Gonococcal infection	0 (0 to 0)	0 (0 to 0)	0 (0 to 0)	0 (0 to 0)	0 (0 to 0)	0 (0 to 0)	0·2 (0·2 to 0·3)	0·4 (0·3 to 0·6)	0·5 (0·3 to 0·8)	0·7 (0·5 to 1)	0·5 (0·4 to 0·7)
Trichomoniasis	0 (0 to 0)	0 (0 to 0)	0 (0 to 0)	0 (0 to 0)	0 (0 to 0)	0 (0 to 0·1)	0·4 (0·1 to 1·1)	1·8 (0·6 to 4·2)	3 (1 to 6·9)	3·1 (1 to 7·3)	2·8 (0·9 to 6·6)
Genital herpes	0 (0 to 0)	0 (0 to 0)	0 (0 to 0)	0 (0 to 0)	0 (0 to 0)	0·1 (0 to 0·3)	0·6 (0·2 to 1·5)	1·1 (0·4 to 2·7)	1·6 (0·5 to 3·9)	1·8 (0·6 to 4·4)	1·8 (0·6 to 4·4)
Other sexually transmitted infections	0 (0 to 0)	0 (0 to 0)	0 (0 to 0)	0 (0 to 0)	0 (0 to 0)	0·1 (0·1 to 0·1)	0·5 (0·4 to 0·8)	1 (0·6 to 1·7)	1·6 (0·9 to 2·7)	2·3 (1·4 to 3·5)	2·5 (1·4 to 3·9)
Respiratory infections and tuberculosis	72·7 (49·3 to 102)	76·3 (50·1 to 110)	206 (127 to 326)	130 (95·4 to 165)	68·5 (51·3 to 90·5)	52·9 (38·6 to 73·2)	69·5 (51·7 to 91)	78·1 (60·6 to 100)	90·7 (70·9 to 116)	91·1 (70·5 to 116)	67·3 (51·1 to 87·5)
Tuberculosis	0 (0 to 0)	0 (0 to 0)	2·9 (1·7 to 4·8)	5·3 (3·7 to 7·5)	4·1 (2·3 to 6·4)	4 (2·6 to 5·8)	10·6 (7·6 to 14·6)	19·9 (13·7 to 27)	26·8 (18·8 to 37·3)	26 (18·2 to 36·6)	15·9 (10·9 to 22·5)
Latent tuberculosis infection	0 (0 to 0)	0 (0 to 0)	0 (0 to 0)	0 (0 to 0)	0 (0 to 0)	0 (0 to 0)	0 (0 to 0)	0 (0 to 0)	0 (0 to 0)	0 (0 to 0)	0 (0 to 0)
Drug-susceptible tuberculosis	0 (0 to 0)	0 (0 to 0)	2·8 (1·7 to 4·7)	5·2 (3·6 to 7·4)	4·1 (2·3 to 6·4)	3·9 (2·5 to 5·8)	10·5 (7·5 to 14·4)	19·7 (13·6 to 26·9)	26·5 (18·6 to 36·5)	25·8 (18 to 36·2)	15·7 (10·8 to 22·4)
Multidrug-resistant tuberculosis without extensive drug resistanc	0 (0 to 0)	0 (0 to 0)	0 (0 to 0·1)	0·1 (0 to 0·2)	0 (0 to 0·1)	0 (0 to 0·1)	0·1 (0 to 0·5)	0·2 (0 to 0·7)	0·2 (0 to 0·9)	0·2 (0 to 1)	0·1 (0 to 0·5)
Extensively drug-resistant tuberculosis	0 (0 to 0)	0 (0 to 0)	0 (0 to 0)	0 (0 to 0)	0 (0 to 0)	0 (0 to 0)	0 (0 to 0·1)	0 (0 to 0·1)	0 (0 to 0·1)	0 (0 to 0·2)	0 (0 to 0·1)
Lower respiratory infections	72·7 (49·3 to 102)	76 (49·9 to 109)	198 (119 to 317)	98·8 (69·8 to 133)	33·8 (23·9 to 46·4)	21·6 (15 to 30·7)	32·7 (22·4 to 46)	36·3 (24·8 to 51·3)	45·7 (31·4 to 63·3)	49·8 (34 to 69·7)	37·8 (25·7 to 53·1)
Upper respiratory infections	0 (0 to 0·1)	0·2 (0·1 to 0·4)	4·4 (2·5 to 7·3)	18·2 (10·2 to 29·9)	20·6 (11·4 to 34·4)	19·9 (10·4 to 34·9)	19·4 (10 to 34·4)	16·5 (8·8 to 28·7)	14·1 (7·7 to 23·6)	12·1 (6·4 to 20·3)	10·8 (5·3 to 18·4)
Otitis media	0 (0 to 0)	0 (0 to 0)	0·9 (0·4 to 1·6)	7·5 (3·9 to 13·4)	10 (5·4 to 16·8)	7·4 (3·9 to 12·9)	6·8 (3·5 to 11·5)	5·4 (2·3 to 10·8)	4·1 (1·9 to 7·6)	3·1 (1·3 to 6)	2·9 (1·3 to 5·4)
Enteric infections	6·9 (4·5 to 10·2)	17·3 (9·9 to 27·8)	104 (58·1 to 179)	61·3 (42·6 to 85·8)	35·8 (23·4 to 53·1)	35·7 (21·5 to 56·7)	24·5 (16·1 to 35·3)	17·9 (11·7 to 26·3)	19·1 (13·3 to 26·9)	19 (13·3 to 26·5)	18·3 (13·1 to 25)
Diarrheal diseases	6·9 (4·5 to 10·2)	17·3 (9·9 to 27·8)	104 (58·1 to 178)	60·7 (42 to 85·2)	35·5 (23·2 to 52·6)	35·3 (21·1 to 56·1)	24·2 (15·9 to 35·1)	17·7 (11·5 to 26·1)	18·9 (13·2 to 26·7)	18·9 (13·2 to 26·3)	18·2 (13 to 24·9)
Typhoid and paratyphoid	0 (0 to 0)	0 (0 to 0)	0 (0 to 0·1)	0 (0 to 0·1)	0 (0 to 0)	0 (0 to 0·1)	0 (0 to 0·1)	0 (0 to 0·1)	0 (0 to 0·1)	0 (0 to 0·1)	0 (0 to 0·1)
Typhoid fever	0 (0 to 0)	0 (0 to 0)	0 (0 to 0·1)	0 (0 to 0·1)	0 (0 to 0)	0 (0 to 0·1)	0 (0 to 0·1)	0 (0 to 0·1)	0 (0 to 0·1)	0 (0 to 0·1)	0 (0 to 0·1)
Paratyphoid fever	0 (0 to 0)	0 (0 to 0)	0 (0 to 0)	0 (0 to 0)	0 (0 to 0)	0 (0 to 0)	0 (0 to 0)	0 (0 to 0)	0 (0 to 0)	0 (0 to 0)	0 (0 to 0)
Invasive Non-typhoidal Salmonella (iNTS)	0 (0 to 0)	0 (0 to 0)	0 (0 to 0·1)	0·3 (0·1 to 0·7)	0·2 (0·1 to 0·5)	0·3 (0·1 to 0·6)	0·1 (0 to 0·2)	0·1 (0 to 0·2)	0·1 (0 to 0·3)	0 (0 to 0·1)	0 (0 to 0)
Other intestinal infectious diseases	0 (0 to 0)	0 (0 to 0)	0·2 (0 to 0·8)	0·3 (0 to 0·8)	0·1 (0 to 0·4)	0·1 (0 to 0·3)	0·1 (0 to 0·3)	0·1 (0 to 0·2)	0 (0 to 0·1)	0·1 (0 to 0·2)	0 (0 to 0·1)
Neglected tropical diseases and malaria	0·1 (0 to 0·1)	0·4 (0·2 to 0·6)	4·2 (2·4 to 6·7)	12·9 (6·8 to 22·2)	18·7 (9·1 to 32·5)	16·1 (7·6 to 31·1)	18·9 (9·1 to 35·1)	18·3 (9 to 34·5)	19 (9·4 to 34·9)	18·4 (8·9 to 32·6)	17·3 (8·6 to 30·6)
Malaria	0 (0 to 0)	0 (0 to 0)	0 (0 to 0)	0·1 (0 to 0·4)	0·2 (0 to 0·5)	0·1 (0 to 0·2)	0·2 (0 to 0·5)	0·1 (0 to 0·4)	0·1 (0 to 0·2)	0·1 (0 to 0·2)	0·1 (0 to 0·2)
Chagas disease	0 (0 to 0)	0 (0 to 0)	0 (0 to 0)	0 (0 to 0)	0 (0 to 0)	0 (0 to 0)	0 (0 to 0)	0 (0 to 0)	0 (0 to 0)	0 (0 to 0)	0 (0 to 0)
Leishmaniasis	0 (0 to 0)	0 (0 to 0)	0 (0 to 0)	0 (0 to 0)	0 (0 to 0)	0 (0 to 0)	0 (0 to 0)	0 (0 to 0)	0 (0 to 0)	0 (0 to 0)	0 (0 to 0)
Visceral leishmaniasis	0 (0 to 0)	0 (0 to 0)	0 (0 to 0)	0 (0 to 0)	0 (0 to 0)	0 (0 to 0)	0 (0 to 0)	0 (0 to 0)	0 (0 to 0)	0 (0 to 0)	0 (0 to 0)
Cutaneous and mucocutaneous leishmaniasis	0 (0 to 0)	0 (0 to 0)	0 (0 to 0)	0 (0 to 0)	0 (0 to 0)	0 (0 to 0)	0 (0 to 0)	0 (0 to 0)	0 (0 to 0)	0 (0 to 0)	0 (0 to 0)
African trypanosomiasis	0 (0 to 0)	0 (0 to 0)	0 (0 to 0)	0 (0 to 0)	0 (0 to 0)	0 (0 to 0)	0 (0 to 0)	0 (0 to 0)	0 (0 to 0)	0 (0 to 0)	0 (0 to 0)
Schistosomiasis	0 (0 to 0)	0 (0 to 0)	0 (0 to 0)	0 (0 to 0)	0 (0 to 0)	0 (0 to 0)	0 (0 to 0)	0 (0 to 0)	0 (0 to 0)	0 (0 to 0)	0 (0 to 0)
Cysticercosis	0 (0 to 0)	0 (0 to 0)	0 (0 to 0)	0 (0 to 0·1)	0·2 (0 to 0·8)	0·5 (0·1 to 1·2)	2·1 (0·2 to 5·2)	5·1 (1·3 to 11·8)	8 (2·5 to 16·8)	8·6 (2·7 to 17·4)	8·3 (2·9 to 16·9)
Cystic echinococcosis	0 (0 to 0)	0 (0 to 0)	0 (0 to 0)	0 (0 to 0)	0 (0 to 0)	0 (0 to 0)	0 (0 to 0)	0 (0 to 0)	0 (0 to 0)	0 (0 to 0)	0 (0 to 0)
Lymphatic filariasis	0 (0 to 0)	0 (0 to 0)	0 (0 to 0)	0 (0 to 0)	0 (0 to 0)	0 (0 to 0)	0 (0 to 0)	0 (0 to 0)	0 (0 to 0)	0 (0 to 0)	0 (0 to 0)
Onchocerciasis	0 (0 to 0)	0 (0 to 0)	0 (0 to 0)	0 (0 to 0)	0 (0 to 0)	0 (0 to 0)	0 (0 to 0)	0 (0 to 0)	0 (0 to 0)	0 (0 to 0)	0 (0 to 0)
Trachoma	0 (0 to 0)	0 (0 to 0)	0 (0 to 0)	0 (0 to 0)	0 (0 to 0)	0 (0 to 0)	0 (0 to 0)	0 (0 to 0)	0 (0 to 0)	0 (0 to 0)	0 (0 to 0)
Dengue	0 (0 to 0)	0 (0 to 0·1)	1·2 (0·5 to 2·5)	2·3 (0·9 to 4·8)	4·6 (1·7 to 10·6)	5·6 (1·7 to 16·9)	6·2 (1·6 to 19·6)	5·8 (1·1 to 19·7)	5·4 (1·1 to 18·1)	4·4 (0·8 to 14·8)	3·7 (0·8 to 12·5)
Yellow fever	0 (0 to 0)	0 (0 to 0)	0 (0 to 0)	0 (0 to 0)	0 (0 to 0)	0 (0 to 0)	0 (0 to 0)	0 (0 to 0)	0 (0 to 0)	0 (0 to 0)	0 (0 to 0)
Rabies	0 (0 to 0)	0 (0 to 0)	0·1 (0 to 0·2)	0·1 (0 to 0·2)	0·3 (0·1 to 0·8)	0·2 (0·1 to 0·4)	0 (0 to 0·1)	0 (0 to 0)	0 (0 to 0)	0 (0 to 0)	0 (0 to 0)
Intestinal nematode infections	0 (0 to 0)	0 (0 to 0)	0·1 (0 to 0·4)	0·9 (0·3 to 2·1)	1 (0·4 to 2)	0·7 (0·2 to 1·4)	6·5 (2·6 to 12·3)	4 (1·7 to 7·6)	2·6 (1·1 to 4·8)	2·1 (1 to 3·9)	2 (0·8 to 4)
Ascariasis	0 (0 to 0)	0 (0 to 0)	0·1 (0 to 0·4)	0·5 (0·1 to 1·7)	0·2 (0 to 1·2)	0·1 (0 to 0·3)	0·4 (0·1 to 0·8)	0·3 (0·1 to 0·6)	0·2 (0·1 to 0·5)	0·2 (0·1 to 0·4)	0·2 (0·1 to 0·4)
Trichuriasis	0 (0 to 0)	0 (0 to 0)	0 (0 to 0)	0 (0 to 0)	0 (0 to 0)	0 (0 to 0)	0·1 (0 to 0·3)	0·1 (0 to 0·3)	0·1 (0 to 0·2)	0·1 (0 to 0·1)	0 (0 to 0·1)
Hookworm disease	0 (0 to 0)	0 (0 to 0)	0 (0 to 0)	0·4 (0·1 to 0·8)	0·7 (0·3 to 1·4)	0·6 (0·2 to 1·3)	6 (2·2 to 11·6)	3·6 (1·4 to 7·1)	2·3 (0·9 to 4·5)	1·9 (0·8 to 3·5)	1·8 (0·6 to 3·7)
Food-borne trematodiases	0 (0 to 0)	0 (0 to 0)	0 (0 to 0)	0 (0 to 0)	0 (0 to 0)	0 (0 to 0)	0 (0 to 0)	0 (0 to 0)	0 (0 to 0)	0 (0 to 0)	0 (0 to 0)
Leprosy	0 (0 to 0)	0 (0 to 0)	0 (0 to 0)	0 (0 to 0)	0 (0 to 0)	0 (0 to 0)	0 (0 to 0)	0 (0 to 0)	0 (0 to 0)	0 (0 to 0)	0 (0 to 0·1)
Ebola	0 (0 to 0)	0 (0 to 0)	0 (0 to 0)	0 (0 to 0)	0 (0 to 0)	0 (0 to 0)	0 (0 to 0)	0 (0 to 0)	0 (0 to 0)	0 (0 to 0)	0 (0 to 0)
Zika virus	0 (0 to 0)	0 (0 to 0)	0 (0 to 0·1)	0·1 (0 to 0·3)	0·1 (0 to 0·3)	0·1 (0 to 0·3)	0·1 (0 to 0·4)	0·1 (0 to 0·4)	0·1 (0 to 0·4)	0·1 (0 to 0·5)	0·1 (0 to 0·4)
Guinea worm disease	0 (0 to 0)	0 (0 to 0)	0 (0 to 0)	0 (0 to 0)	0 (0 to 0)	0 (0 to 0)	0 (0 to 0)	0 (0 to 0)	0 (0 to 0)	0 (0 to 0)	0 (0 to 0)
Other neglected tropical diseases	0·1 (0 to 0·1)	0·3 (0·2 to 0·5)	2·8 (1·3 to 5)	9·4 (4 to 18)	12·3 (4·4 to 24·3)	8·9 (2·9 to 18·7)	3·8 (1·1 to 8·5)	3 (0·9 to 6·9)	2·8 (0·9 to 6·5)	3 (1·1 to 5·7)	3 (0·9 to 6·9)
Other infectious diseases	4 (2·8 to 5·3)	7·6 (5 to 11·4)	34·6 (21·7 to 54·4)	33·5 (23·6 to 46·3)	24 (16·7 to 33·6)	19·2 (13·4 to 27)	19·2 (14·5 to 25·6)	16·1 (12·4 to 21·4)	14·8 (11·3 to 19·3)	14 (10·6 to 18)	11·9 (8·6 to 16·3)
Meningitis	2·5 (1·7 to 3·6)	5·7 (3·4 to 8·8)	12·8 (7 to 21·8)	8·8 (5·9 to 13)	4·9 (3·7 to 6·4)	3·5 (2·7 to 4·4)	4·3 (3·2 to 5·7)	3·3 (2·4 to 4·5)	3 (2·2 to 3·9)	3·3 (2·5 to 4·4)	2·3 (1·7 to 3)
Encephalitis	0·9 (0·5 to 1·4)	1 (0·6 to 1·7)	13·2 (6·3 to 24)	13·1 (7·1 to 22)	7·2 (4·4 to 11·3)	6·1 (3·8 to 9·8)	6·3 (4·2 to 9·1)	5·2 (3·4 to 7·9)	4·3 (2·8 to 6·1)	3·5 (2·4 to 5)	2·7 (1·9 to 4)
Diphtheria	0 (0 to 0)	0 (0 to 0)	0 (0 to 0)	0 (0 to 0)	0 (0 to 0)	0 (0 to 0)	0 (0 to 0)	0 (0 to 0)	0 (0 to 0)	0 (0 to 0)	0 (0 to 0)
Tetanus	0 (0 to 0)	0 (0 to 0·1)	0 (0 to 0·1)	0 (0 to 0)	0 (0 to 0·1)	0 (0 to 0)	0 (0 to 0)	0 (0 to 0·1)	0 (0 to 0)	0 (0 to 0·1)	0 (0 to 0·1)
Measles	0 (0 to 0)	0 (0 to 0)	0 (0 to 0)	0 (0 to 0)	0 (0 to 0)	0 (0 to 0)	0 (0 to 0)	0 (0 to 0)	0 (0 to 0)	0 (0 to 0)	0 (0 to 0)
Varicella and herpes zoster	0 (0 to 0)	0·1 (0·1 to 0·2)	0·7 (0·3 to 1·4)	0·4 (0·2 to 0·7)	0·5 (0·3 to 0·9)	0·3 (0·1 to 0·7)	0·5 (0·2 to 0·9)	0·6 (0·3 to 1)	0·6 (0·3 to 1)	0·6 (0·3 to 1)	0·5 (0·2 to 0·8)
Acute hepatitis	0 (0 to 0)	0 (0 to 0)	0·4 (0·2 to 0·8)	0·7 (0·4 to 1·1)	1·9 (1·1 to 3)	2·2 (1·3 to 3·3)	2·6 (1·6 to 3·9)	2·3 (1·5 to 3·2)	2·2 (1·6 to 3·1)	1·7 (1·2 to 2·4)	1·2 (0·8 to 1·6)
Acute hepatitis A	0 (0 to 0)	0 (0 to 0)	0·1 (0 to 0·3)	0·6 (0·3 to 1)	1·5 (0·8 to 2·5)	1·9 (1·1 to 2·9)	2 (1·2 to 3·2)	1·4 (0·8 to 2·2)	1·1 (0·6 to 1·7)	1 (0·6 to 1·6)	0·5 (0·2 to 0·8)
Acute hepatitis B	0 (0 to 0)	0 (0 to 0)	0·2 (0·1 to 0·5)	0·1 (0 to 0·2)	0·2 (0·1 to 0·4)	0·1 (0 to 0·2)	0·3 (0·1 to 0·6)	0·6 (0·3 to 1)	1 (0·5 to 1·6)	0·5 (0·2 to 0·9)	0·5 (0·3 to 0·8)
Acute hepatitis C	0 (0 to 0)	0 (0 to 0)	0 (0 to 0·1)	0·1 (0 to 0·1)	0·1 (0 to 0·1)	0 (0 to 0·1)	0·1 (0 to 0·3)	0·1 (0 to 0·2)	0·1 (0 to 0·2)	0·1 (0 to 0·1)	0·1 (0 to 0·1)
Acute hepatitis E	0 (0 to 0)	0 (0 to 0)	0 (0 to 0·1)	0 (0 to 0)	0·1 (0·1 to 0·2)	0·2 (0 to 0·4)	0·2 (0·1 to 0·5)	0·2 (0 to 0·4)	0·1 (0 to 0·3)	0·1 (0 to 0·2)	0·1 (0 to 0·1)
Other unspecified infectious diseases	0·5 (0·3 to 0·8)	0·8 (0·5 to 1·2)	4 (2·4 to 6·2)	7·7 (4 to 13·3)	9 (3·7 to 17·6)	6·9 (2·9 to 13·2)	5·6 (2·7 to 10·4)	4·8 (2·4 to 8·7)	4·6 (2·5 to 8·2)	4·8 (2·8 to 7·9)	5·2 (2·7 to 9·3)
Maternal and neonatal disorders	1,760 (1,420 to 2,130)	473 (341 to 625)	130 (81·2 to 202)	88·4 (66·6 to 114)	78·5 (57·3 to 105)	87·2 (63·9 to 114)	148 (121 to 181)	159 (128 to 190)	149 (123 to 180)	131 (109 to 156)	87·9 (71 to 106)
Maternal disorders	0 (0 to 0)	0 (0 to 0)	0 (0 to 0)	0 (0 to 0)	0 (0 to 0)	1·6 (1·3 to 2)	63·2 (50·7 to 78·9)	84·9 (66 to 105)	85·1 (67·3 to 106)	75·8 (60·6 to 93·8)	40·7 (32·2 to 50·1)
Maternal hemorrhage	0 (0 to 0)	0 (0 to 0)	0 (0 to 0)	0 (0 to 0)	0 (0 to 0)	0·1 (0·1 to 0·2)	10 (6·4 to 14·8)	16·1 (10·1 to 23·9)	17·2 (11·2 to 26·1)	15·9 (10·4 to 22·9)	9·2 (5·9 to 13·6)
Maternal sepsis and other maternal infections	0 (0 to 0)	0 (0 to 0)	0 (0 to 0)	0 (0 to 0)	0 (0 to 0)	0·1 (0·1 to 0·2)	5·5 (3·4 to 8·2)	8·5 (5·2 to 13)	8·3 (5·2 to 13·1)	7·1 (4·2 to 11·1)	3·6 (2·1 to 5·7)
Maternal hypertensive disorders	0 (0 to 0)	0 (0 to 0)	0 (0 to 0)	0 (0 to 0)	0 (0 to 0)	0·5 (0·3 to 0·6)	16·5 (11·9 to 22·1)	16·7 (11·9 to 22·6)	16·3 (11·5 to 21·8)	13·2 (9·3 to 17·7)	6·3 (4·5 to 8·5)
Maternal obstructed labor and uterine rupture	0 (0 to 0)	0 (0 to 0)	0 (0 to 0)	0 (0 to 0)	0 (0 to 0)	0·1 (0 to 0·1)	2 (1·1 to 3·4)	2·8 (1·5 to 4·8)	3 (1·6 to 5)	2·9 (1·6 to 5·2)	1·8 (0·9 to 3)
Maternal abortion and miscarriage	0 (0 to 0)	0 (0 to 0)	0 (0 to 0)	0 (0 to 0)	0 (0 to 0)	0·1 (0·1 to 0·2)	4·4 (2·5 to 7·1)	6·1 (3·5 to 10)	5·2 (2·9 to 8·6)	4·3 (2·2 to 7·6)	2·4 (1·3 to 4)
Ectopic pregnancy	0 (0 to 0)	0 (0 to 0)	0 (0 to 0)	0 (0 to 0)	0 (0 to 0)	0 (0 to 0)	1 (0·6 to 1·5)	2 (1·2 to 3·2)	2·3 (1·4 to 3·5)	2·2 (1·3 to 3·2)	1 (0·6 to 1·6)
Indirect maternal deaths	--	--	--	--	--	0·1 (0·1 to 0·2)	3·6 (2·2 to 5·5)	4·7 (2·9 to 7·2)	4·3 (2·6 to 6·8)	3·5 (2·1 to 5·6)	1·5 (0·9 to 2·4)
Late maternal deaths	--	--	--	--	--	0·1 (0·1 to 0·2)	3·9 (2·4 to 5·7)	4·9 (3 to 7·4)	4·9 (2·9 to 7·7)	4·3 (2·5 to 6·7)	2·2 (1·3 to 3·5)
Maternal deaths aggravated by HIV/AIDS	--	--	--	--	--	0 (0 to 0)	0·1 (0 to 0·2)	0·3 (0·1 to 0·5)	0·6 (0·2 to 1·1)	0·4 (0·2 to 0·7)	0·2 (0·1 to 0·3)
Neonatal disorders	1,760 (1,420 to 2,130)	473 (341 to 625)	130 (81·2 to 202)	88·4 (66·6 to 114)	78·5 (57·3 to 105)	85·6 (62·2 to 112)	85 (61·4 to 114)	73·8 (51·7 to 97·3)	64 (47·1 to 84·8)	55 (40·5 to 73·2)	47·2 (34·6 to 62·4)
Neonatal preterm birth	872 (657 to 1,110)	166 (112 to 236)	49·1 (29 to 78)	29 (20·6 to 39·8)	36·5 (25 to 49·8)	39·5 (27·7 to 54·5)	39·1 (27·8 to 53·5)	33·5 (22·7 to 45·7)	28·9 (20·2 to 40)	24·5 (17 to 33·4)	20·6 (14·3 to 28·4)
Neonatal encephalopathy due to birth asphyxia and trauma	497 (350 to 658)	72·7 (46·6 to 109)	24·3 (13·4 to 39·7)	11·3 (7·5 to 16·2)	8·6 (4·2 to 14·7)	9·3 (4·8 to 15·8)	9·3 (4·7 to 16)	8·1 (4 to 13·9)	7·1 (3·3 to 12·2)	6·3 (3·1 to 10·6)	5·4 (2·6 to 9·1)
Neonatal sepsis and other neonatal infections	191 (132 to 269)	154 (96·5 to 221)	35·2 (19·5 to 60·1)	21·3 (12·9 to 31·6)	29·6 (17·5 to 46·1)	32·6 (19·8 to 49·4)	32·4 (19·9 to 49·2)	28·4 (16 to 43·5)	24·8 (14·8 to 37·7)	21·3 (13·2 to 32·6)	18·7 (11·3 to 28·2)
Hemolytic disease and other neonatal jaundice	20·2 (11·9 to 31·3)	6·9 (3·3 to 13·2)	2·2 (1·1 to 4·6)	3·1 (1·9 to 4·8)	3·8 (2·4 to 5·5)	4·2 (2·3 to 7·1)	4·1 (2·3 to 6·7)	3·8 (2·8 to 4·9)	3·3 (2·4 to 4·3)	2·9 (2·1 to 3·8)	2·5 (1·9 to 3·4)
Other neonatal disorders	176 (115 to 260)	72·6 (47·7 to 102)	19·2 (11·6 to 32·3)	23·7 (17·7 to 30·8)	0 (0 to 0)	0 (0 to 0)	0 (0 to 0)	0 (0 to 0)	0 (0 to 0)	0 (0 to 0)	0 (0 to 0)
Nutritional deficiencies	0·1 (0·1 to 0·2)	3·3 (1·7 to 5·6)	76·9 (49·6 to 116)	133 (71·8 to 219)	141 (55·9 to 271)	101 (36·8 to 199)	96 (33·6 to 205)	77·1 (29·9 to 163)	72 (30·5 to 144)	71·9 (31·4 to 135)	75·2 (26·6 to 166)
Protein-energy malnutrition	0·1 (0·1 to 0·2)	0·3 (0·2 to 0·5)	48·1 (27·8 to 81·5)	34·7 (22·4 to 53·4)	10·3 (6·7 to 15·1)	5·5 (3·7 to 7·8)	7 (4·1 to 10·5)	6·7 (4·6 to 9·2)	6·8 (4·7 to 9·3)	5·9 (4·2 to 7·8)	5·5 (3·9 to 7·5)
Iodine deficiency	0 (0 to 0)	0 (0 to 0)	0 (0 to 0)	0 (0 to 0)	0·2 (0·1 to 0·3)	0·4 (0·2 to 0·8)	0·6 (0·3 to 1·1)	0·6 (0·3 to 1·1)	0·6 (0·3 to 1)	0·5 (0·3 to 0·9)	0·5 (0·3 to 0·8)
Vitamin A deficiency	0 (0 to 0)	0·2 (0·1 to 0·4)	0·8 (0·4 to 1·6)	4·2 (2 to 7·8)	4·8 (2 to 9·2)	3·6 (1·6 to 6·6)	1 (0·3 to 2·1)	0·7 (0·3 to 1·3)	0·5 (0·2 to 1)	0·4 (0·2 to 0·7)	0·3 (0·1 to 0·5)
Dietary iron deficiency	0 (0 to 0)	2·8 (1·3 to 5)	26·1 (11·6 to 48·4)	92·2 (36·1 to 178)	125 (44·1 to 254)	90·6 (28·2 to 187)	84·7 (23·3 to 192)	67·7 (21 to 151)	62·4 (21·4 to 134)	63·4 (23·5 to 127)	67·6 (19·9 to 159)
Other nutritional deficiencies	0 (0 to 0)	0 (0 to 0)	1·9 (0·8 to 3·8)	1·6 (0·6 to 3·2)	0·8 (0·4 to 1·7)	0·6 (0·3 to 1·1)	2·7 (1·2 to 4·8)	1·5 (0·7 to 2·9)	1·7 (0·9 to 2·9)	1·6 (0·9 to 2·6)	1·3 (0·7 to 2)
Non-communicable diseases	279 (195 to 390)	153 (105 to 218)	408 (258 to 607)	540 (432 to 668)	660 (503 to 842)	1,100 (802 to 1,460)	1,810 (1,390 to 2,380)	1,940 (1,500 to 2,470)	2,160 (1,700 to 2,680)	2,330 (1,870 to 2,860)	2,250 (1,820 to 2,750)
Neoplasms	2·3 (1·6 to 3·2)	2·3 (1·5 to 3·4)	10·2 (5·9 to 16·5)	48·9 (35·4 to 65·1)	53·9 (41·1 to 68·1)	46·4 (36·9 to 56·3)	79·4 (65·7 to 93·8)	90·9 (74·6 to 111)	152 (121 to 190)	256 (206 to 309)	276 (222 to 332)
Lip and oral cavity cancer	0 (0 to 0)	0 (0 to 0)	0 (0 to 0)	0 (0 to 0)	0·2 (0·1 to 0·2)	0·2 (0·1 to 0·4)	0·6 (0·4 to 0·7)	0·5 (0·4 to 0·6)	1·1 (0·8 to 1·6)	1·4 (0·9 to 1·8)	1·2 (0·8 to 1·7)
Nasopharynx cancer	0 (0 to 0)	0 (0 to 0)	0 (0 to 0)	0 (0 to 0)	0·2 (0·1 to 0·3)	0·2 (0·1 to 0·2)	0·5 (0·4 to 0·7)	0·5 (0·3 to 0·6)	0·6 (0·4 to 0·8)	0·6 (0·4 to 0·9)	0·4 (0·3 to 0·6)
Other pharynx cancer	0 (0 to 0)	0 (0 to 0)	0 (0 to 0)	0 (0 to 0)	0 (0 to 0)	0 (0 to 0)	0 (0 to 0)	0·2 (0·2 to 0·3)	0·3 (0·2 to 0·4)	0·4 (0·3 to 0·5)	0·3 (0·2 to 0·5)
Esophageal cancer	0 (0 to 0)	0 (0 to 0)	0 (0 to 0)	0 (0 to 0)	0 (0 to 0)	0 (0 to 0)	0 (0 to 0)	0·2 (0·2 to 0·3)	0·4 (0·2 to 0·5)	0·6 (0·4 to 0·8)	0·9 (0·6 to 1·2)
Stomach cancer	0 (0 to 0)	0 (0 to 0)	0 (0 to 0)	0 (0 to 0)	0 (0 to 0)	0 (0 to 0)	1·6 (1·4 to 2)	2·9 (2·2 to 3·8)	7·5 (5 to 10·8)	13·3 (9·5 to 18·1)	12·6 (8·8 to 17·1)
Colon and rectum cancer	0 (0 to 0)	0 (0 to 0)	0 (0 to 0)	0 (0 to 0)	0·4 (0·3 to 0·5)	0·4 (0·3 to 0·6)	2·3 (1·7 to 3)	2·9 (2 to 3·9)	5·8 (3·6 to 8·5)	11·8 (8·2 to 16·6)	11·6 (8·3 to 15·8)
Liver cancer	0·1 (0·1 to 0·1)	0·1 (0·1 to 0·2)	0·3 (0·2 to 0·5)	0·7 (0·5 to 1)	0·8 (0·6 to 1)	1·1 (0·8 to 1·4)	0·8 (0·5 to 1·1)	1·5 (1 to 2·3)	2 (1·3 to 2·8)	2·8 (1·8 to 4)	3·5 (2·3 to 5)
Liver cancer due to hepatitis B	0 (0 to 0)	0 (0 to 0)	0 (0 to 0)	0 (0 to 0)	0 (0 to 0)	0·2 (0·1 to 0·3)	0·2 (0·1 to 0·4)	0·6 (0·3 to 0·9)	0·9 (0·5 to 1·5)	1·3 (0·7 to 2·1)	1·6 (0·9 to 2·6)
Liver cancer due to hepatitis C	0 (0 to 0)	0 (0 to 0)	0 (0 to 0)	0 (0 to 0)	0 (0 to 0)	0 (0 to 0)	0 (0 to 0)	0 (0 to 0·1)	0·1 (0 to 0·2)	0·2 (0·1 to 0·4)	0·4 (0·1 to 0·7)
Liver cancer due to alcohol use	0 (0 to 0)	0 (0 to 0)	0 (0 to 0)	0 (0 to 0)	0 (0 to 0)	0 (0 to 0)	0 (0 to 0)	0·1 (0 to 0·1)	0·2 (0·1 to 0·4)	0·3 (0·2 to 0·6)	0·6 (0·2 to 1·1)
Liver cancer due to NASH	0 (0 to 0)	0 (0 to 0)	0 (0 to 0)	0 (0 to 0)	0 (0 to 0)	0 (0 to 0)	0·2 (0·1 to 0·4)	0·3 (0·2 to 0·6)	0·3 (0·2 to 0·6)	0·4 (0·2 to 0·6)	0·4 (0·2 to 0·7)
Liver cancer due to other causes	0·1 (0·1 to 0·1)	0·1 (0·1 to 0·2)	0·3 (0·2 to 0·5)	0·7 (0·5 to 1)	0·8 (0·6 to 1)	0·9 (0·7 to 1·2)	0·3 (0·2 to 0·5)	0·5 (0·3 to 0·8)	0·5 (0·2 to 0·9)	0·5 (0·3 to 1)	0·6 (0·3 to 1)
Gallbladder and biliary tract cancer	0 (0 to 0)	0 (0 to 0)	0 (0 to 0)	0 (0 to 0)	0 (0 to 0)	0 (0 to 0)	0 (0 to 0)	0·2 (0·1 to 0·4)	0·5 (0·3 to 0·8)	0·9 (0·6 to 1·6)	1·7 (1·1 to 3)
Pancreatic cancer	0 (0 to 0)	0 (0 to 0)	0 (0 to 0)	0 (0 to 0)	0 (0 to 0)	0 (0 to 0)	0·9 (0·7 to 1·1)	1 (0·7 to 1·3)	2·8 (1·9 to 3·9)	4·3 (2·8 to 6·4)	6 (3·9 to 8·7)
Larynx cancer	0 (0 to 0)	0 (0 to 0)	0 (0 to 0)	0 (0 to 0)	0 (0 to 0)	0 (0 to 0)	0 (0 to 0)	0·2 (0·1 to 0·2)	0·2 (0·2 to 0·3)	0·3 (0·2 to 0·4)	0·3 (0·2 to 0·4)
Tracheal, bronchus, and lung cancer	0 (0 to 0)	0 (0 to 0)	0 (0 to 0)	0 (0 to 0)	0 (0 to 0)	0·3 (0·1 to 0·5)	1·7 (1·3 to 2·1)	1·5 (1·1 to 2·1)	2·6 (1·7 to 3·8)	6 (3·9 to 8·8)	9·1 (6·6 to 12·6)
Malignant skin melanoma	0 (0 to 0)	0 (0 to 0)	0·1 (0 to 0·2)	0·3 (0·1 to 0·7)	0·2 (0·1 to 0·2)	0·2 (0·1 to 0·2)	0·3 (0·2 to 0·5)	0·7 (0·4 to 1)	0·8 (0·5 to 1·2)	1 (0·6 to 1·5)	1·6 (1 to 2·5)
Non-melanoma skin cancer	0 (0 to 0)	0 (0 to 0)	0 (0 to 0)	0 (0 to 0)	0 (0 to 0)	0 (0 to 0)	0 (0 to 0)	0·6 (0·4 to 0·9)	1·1 (0·7 to 1·6)	1·1 (0·7 to 1·5)	1·1 (0·7 to 1·6)
Non-melanoma skin cancer (squamous-cell carcinoma)	0 (0 to 0)	0 (0 to 0)	0 (0 to 0)	0 (0 to 0)	0 (0 to 0)	0 (0 to 0)	0 (0 to 0)	0·6 (0·4 to 0·9)	1·1 (0·7 to 1·6)	1·1 (0·7 to 1·5)	1·1 (0·7 to 1·6)
Non-melanoma skin cancer (basal-cell carcinoma)	0 (0 to 0)	0 (0 to 0)	0 (0 to 0)	0 (0 to 0)	0 (0 to 0)	0 (0 to 0)	0 (0 to 0)	0 (0 to 0)	0 (0 to 0)	0 (0 to 0)	0 (0 to 0)
Breast cancer	0 (0 to 0)	0 (0 to 0)	0 (0 to 0)	0 (0 to 0)	0 (0 to 0)	0 (0 to 0)	1·6 (1·3 to 1·9)	2·6 (1·8 to 3·6)	12·1 (8·3 to 16·9)	41 (29·9 to 56·9)	48 (34·5 to 63·4)
Cervical cancer	0 (0 to 0)	0 (0 to 0)	0 (0 to 0)	0 (0 to 0)	0 (0 to 0)	0 (0 to 0)	4·8 (3·4 to 6·3)	15·1 (10·2 to 21·6)	46·9 (30·8 to 68·7)	98·6 (68·3 to 134)	112 (79·8 to 149)
Uterine cancer	0 (0 to 0)	0 (0 to 0)	0 (0 to 0)	0 (0 to 0)	0 (0 to 0)	0 (0 to 0)	0 (0 to 0)	1·8 (1·4 to 2·3)	4·5 (3·3 to 6·1)	9·1 (6·5 to 12·5)	9·6 (6·5 to 13·5)
Ovarian cancer	0 (0 to 0)	0 (0 to 0)	0 (0 to 0)	0 (0 to 0)	0·7 (0·3 to 1·2)	1·1 (0·6 to 1·9)	3·3 (1·8 to 4·9)	4·3 (2·5 to 6·4)	4·6 (2·8 to 6·8)	6·9 (4 to 9·9)	8·5 (5·6 to 12·2)
Prostate cancer	0 (0 to 0)	0 (0 to 0)	0 (0 to 0)	0 (0 to 0)	0 (0 to 0)	0 (0 to 0)	0 (0 to 0)	0 (0 to 0)	0 (0 to 0)	0 (0 to 0)	0 (0 to 0)
Testicular cancer	--	--	--	--	--	--	--	--	--	--	--
Kidney cancer	0·1 (0·1 to 0·1)	0·1 (0 to 0·1)	0·6 (0·3 to 1·1)	3·4 (2·1 to 5·2)	3·2 (2·1 to 4·5)	1·7 (1·1 to 2·4)	2·3 (1·5 to 3·1)	2·8 (2 to 4)	3·1 (2·2 to 4·3)	3·7 (2·4 to 5·3)	3·8 (2·6 to 5·4)
Bladder cancer	0 (0 to 0)	0 (0 to 0)	0 (0 to 0)	0 (0 to 0)	0 (0 to 0)	0 (0 to 0)	0·2 (0·2 to 0·3)	0·2 (0·2 to 0·3)	0·3 (0·2 to 0·4)	1·2 (0·8 to 1·8)	1·1 (0·7 to 1·5)
Brain and central nervous system cancer	0·7 (0·2 to 1·1)	0·6 (0·2 to 1·2)	2·3 (0·7 to 4·5)	9·7 (3·4 to 16·8)	14·1 (5·6 to 23·4)	10 (3·9 to 16·6)	8·5 (3·3 to 13·9)	7·8 (3 to 13·6)	9·2 (3·3 to 16·2)	9 (3·2 to 14·9)	8·4 (3 to 13·8)
Thyroid cancer	0 (0 to 0)	0 (0 to 0)	0 (0 to 0)	0 (0 to 0)	0·2 (0·1 to 0·2)	0·2 (0·1 to 0·2)	0·4 (0·3 to 0·5)	0·4 (0·3 to 0·5)	0·6 (0·5 to 0·9)	0·8 (0·6 to 1·1)	1 (0·7 to 1·4)
Mesothelioma	0 (0 to 0)	0 (0 to 0)	0 (0 to 0)	0 (0 to 0)	0 (0 to 0)	0 (0 to 0)	0 (0 to 0)	0·9 (0·6 to 1·5)	0·6 (0·4 to 1)	0·7 (0·4 to 1·1)	1·1 (0·7 to 1·8)
Hodgkin lymphoma	0 (0 to 0)	0 (0 to 0)	0 (0 to 0)	0·2 (0·1 to 0·3)	0·2 (0·1 to 0·3)	0·5 (0·3 to 0·8)	4 (2·5 to 6·1)	6·1 (3·9 to 9·3)	5 (3 to 7·7)	3·8 (2·2 to 5·9)	2·6 (1·6 to 4·2)
Non-Hodgkin lymphoma	0 (0 to 0)	0 (0 to 0)	0 (0 to 0)	2·4 (1·6 to 3·4)	2·6 (1·7 to 3·8)	2·7 (1·9 to 3·6)	5·5 (3·8 to 7·5)	6·1 (4·2 to 8·3)	7·5 (5·1 to 10·3)	7·8 (5·4 to 10·7)	5·3 (3·8 to 7·1)
Multiple myeloma	0 (0 to 0)	0 (0 to 0)	0 (0 to 0)	0 (0 to 0)	0 (0 to 0)	0 (0 to 0)	0 (0 to 0)	0·1 (0·1 to 0·1)	0·2 (0·1 to 0·3)	0·4 (0·3 to 0·5)	0·8 (0·5 to 1·1)
Leukemia	0·6 (0·5 to 0·9)	0·6 (0·4 to 0·9)	3·2 (1·8 to 5·7)	15·5 (10·3 to 22·4)	19·7 (13·4 to 27·5)	15·6 (11 to 21·7)	21·9 (15·8 to 28·9)	16·2 (11·9 to 21·6)	18·6 (13·3 to 25·9)	15·8 (11·5 to 20·8)	12·1 (8·5 to 16·7)
Acute lymphoid leukemia	0·1 (0 to 0·1)	0·2 (0·1 to 0·4)	0·9 (0·4 to 1·8)	9·2 (5·8 to 13·6)	12·1 (7·7 to 17·5)	8·5 (5·5 to 12·7)	9·8 (6·4 to 14·1)	4 (2·4 to 6·3)	2·9 (1·7 to 4·6)	1·9 (1·1 to 3)	1·2 (0·7 to 1·9)
Chronic lymphoid leukemia	0 (0 to 0)	0 (0 to 0)	0 (0 to 0)	0 (0 to 0)	0 (0 to 0)	0 (0 to 0)	0 (0 to 0)	0·2 (0·1 to 0·2)	0·1 (0·1 to 0·2)	0·1 (0·1 to 0·2)	0·2 (0·1 to 0·3)
Acute myeloid leukemia	0·1 (0 to 0·3)	0·2 (0 to 0·4)	0·2 (0·1 to 0·4)	1·4 (0·7 to 2·6)	1·4 (0·7 to 2·7)	1·6 (0·8 to 3·1)	2·3 (1·1 to 4·1)	1·8 (1 to 3)	2·2 (1·2 to 3·8)	1·5 (0·8 to 2·4)	1·5 (0·8 to 2·6)

Cause	40 to 44	45 to 49	50 to 54	55 to 59	60 to 64	65 to 69	70 to 74	75 to 79	80 to 84	85 to 89	90 to 94	95 plus	Total
All causes	2,970 (2,490 to 3,550)	3,240 (2,750 to 3,770)	3,240 (2,790 to 3,740)	3,040 (2,630 to 3,500)	2,640 (2,310 to 3,010)	2,080 (1,840 to 2,350)	1,670 (1,490 to 1,870)	1,340 (1,200 to 1,480)	1,050 (951 to 1,160)	747 (675 to 820)	419 (384 to 453)	186 (174 to 198)	45,800
Communicable, maternal, neonatal, and nutritional diseases	455 (378 to 547)	369 (308 to 452)	272 (219 to 341)	218 (174 to 273)	162 (130 to 201)	119 (94·7 to 148)	107 (85·4 to 136)	95·7 (75·3 to 120)	90·7 (71·8 to 111)	76·6 (60 to 93·2)	50·2 (38 to 61·6)	25·1 (17·9 to 31·7)	9,010
HIV/AIDS and sexually transmitted infections	196 (181 to 213)	142 (131 to 154)	68·3 (62·8 to 74)	41 (37·4 to 45)	21·3 (19·1 to 23·9)	11·3 (10·2 to 12·8)	3·5 (3 to 4·2)	1·6 (1·4 to 2)	1 (0·8 to 1·2)	0·6 (0·5 to 0·7)	0·2 (0·1 to 0·2)	0·1 (0·1 to 0·1)	1,610
HIV/AIDS	188 (173 to 204)	136 (127 to 147)	65·1 (60·3 to 70·2)	38·9 (35·7 to 42·4)	20·1 (18·3 to 22·2)	10·6 (9·6 to 11·7)	3 (2·6 to 3·5)	1·4 (1·2 to 1·6)	0·8 (0·7 to 0·9)	0·5 (0·4 to 0·5)	0·1 (0·1 to 0·2)	0 (0 to 0·1)	1,560
HIV/AIDS - Drug-susceptible Tuberculosis	13·9 (8·7 to 21)	10·1 (6·5 to 15)	4·8 (3 to 7·2)	2·9 (1·8 to 4·3)	1·5 (1 to 2·2)	0·8 (0·6 to 1·2)	0·2 (0·2 to 0·3)	0·1 (0·1 to 0·1)	0·1 (0 to 0·1)	0 (0 to 0·1)	0 (0 to 0)	0 (0 to 0)	115
HIV/AIDS - Multidrug-resistant Tuberculosis without extensive dru	0·2 (0 to 0·7)	0·1 (0 to 0·5)	0·1 (0 to 0·3)	0 (0 to 0·2)	0 (0 to 0·1)	0 (0 to 0)	0 (0 to 0)	0 (0 to 0)	0 (0 to 0)	0 (0 to 0)	0 (0 to 0)	0 (0 to 0)	1·4
HIV/AIDS - Extensively drug-resistant Tuberculosis	0 (0 to 0·1)	0 (0 to 0·1)	0 (0 to 0)	0 (0 to 0)	0 (0 to 0)	0 (0 to 0)	0 (0 to 0)	0 (0 to 0)	0 (0 to 0)	0 (0 to 0)	0 (0 to 0)	0 (0 to 0)	0·2
HIV/AIDS resulting in other diseases	174 (158 to 190)	126 (115 to 137)	60·3 (54·8 to 65·6)	36 (32·6 to 39·5)	18·5 (16·6 to 20·7)	9·7 (8·7 to 10·9)	2·8 (2·4 to 3·2)	1·3 (1·1 to 1·5)	0·7 (0·6 to 0·8)	0·4 (0·4 to 0·5)	0·1 (0·1 to 0·1)	0 (0 to 0·1)	1,440
Sexually transmitted infections excluding HIV	7·9 (4·2 to 14·6)	5·5 (3 to 10)	3·2 (1·6 to 6·3)	2·1 (1 to 4·2)	1·2 (0·5 to 2·5)	0·8 (0·3 to 1·6)	0·5 (0·2 to 1)	0·3 (0·1 to 0·6)	0·2 (0·1 to 0·3)	0·1 (0·1 to 0·2)	0 (0 to 0·1)	0 (0 to 0)	55·4
Syphilis	0·1 (0 to 0·1)	0·1 (0·1 to 0·2)	0·1 (0 to 0·1)	0 (0 to 0)	0 (0 to 0)	0 (0 to 0)	0 (0 to 0)	0 (0 to 0)	0 (0 to 0)	0 (0 to 0)	0 (0 to 0)	0 (0 to 0)	2·3
Chlamydial infection	0·9 (0·5 to 1·5)	0·4 (0·3 to 0·8)	0·1 (0·1 to 0·2)	0·1 (0·1 to 0·1)	0 (0 to 0)	0 (0 to 0)	0 (0 to 0)	0 (0 to 0)	0 (0 to 0)	0 (0 to 0)	0 (0 to 0)	0 (0 to 0)	4·8
Gonococcal infection	0·6 (0·4 to 0·8)	0·5 (0·3 to 0·7)	0·2 (0·1 to 0·3)	0·1 (0·1 to 0·2)	0·1 (0 to 0·1)	0·1 (0 to 0·1)	0 (0 to 0·1)	0 (0 to 0)	0 (0 to 0)	0 (0 to 0)	0 (0 to 0)	0 (0 to 0)	4
Trichomoniasis	2·4 (0·8 to 5·7)	1·9 (0·6 to 4·5)	1·3 (0·4 to 3·1)	0·8 (0·3 to 2)	0·5 (0·2 to 1·1)	0·3 (0·1 to 0·6)	0·2 (0·1 to 0·3)	0·1 (0 to 0·2)	0 (0 to 0·1)	0 (0 to 0)	0 (0 to 0)	0 (0 to 0)	18·6
Genital herpes	1·6 (0·5 to 4)	1·4 (0·4 to 3·3)	1·1 (0·3 to 2·6)	0·8 (0·3 to 2)	0·6 (0·2 to 1·5)	0·4 (0·1 to 1)	0·2 (0·1 to 0·6)	0·1 (0 to 0·3)	0·1 (0 to 0·2)	0·1 (0 to 0·1)	0 (0 to 0·1)	0 (0 to 0)	13·5
Other sexually transmitted infections	2·4 (1·4 to 4)	1·2 (0·7 to 1·9)	0·4 (0·2 to 0·6)	0·2 (0·1 to 0·3)	0·1 (0 to 0·1)	0 (0 to 0)	0 (0 to 0)	0 (0 to 0)	0 (0 to 0)	0 (0 to 0)	0 (0 to 0)	0 (0 to 0)	12·2
Respiratory infections and tuberculosis	61·7 (46·6 to 80·5)	71·9 (55·1 to 92·5)	72·9 (54·3 to 96·9)	73 (54·3 to 96·5)	71·4 (52 to 94·5)	62·9 (45·4 to 82)	69·2 (51·6 to 92·1)	67·5 (49·9 to 87·9)	68·8 (51·6 to 88·4)	60·4 (45·9 to 74·7)	41·2 (29·9 to 51·6)	21·1 (14·4 to 27·4)	1,750
Tuberculosis	13·1 (9 to 18·5)	12·3 (8·4 to 17)	11·7 (8 to 16·9)	9·3 (6·3 to 13·1)	6·9 (4·7 to 9·8)	4·2 (2·9 to 5·7)	3·5 (2·5 to 4·7)	2·7 (1·9 to 3·8)	1·8 (1·3 to 2·6)	1·3 (0·8 to 1·8)	0·5 (0·3 to 0·7)	0·2 (0·1 to 0·2)	183
Latent tuberculosis infection	0 (0 to 0)	0 (0 to 0)	0 (0 to 0)	0 (0 to 0)	0 (0 to 0)	0 (0 to 0)	0 (0 to 0)	0 (0 to 0)	0 (0 to 0)	0 (0 to 0)	0 (0 to 0)	0 (0 to 0)	0
Drug-susceptible tuberculosis	13 (8·9 to 18·4)	12·2 (8·3 to 16·9)	11·5 (7·9 to 16·7)	9·2 (6·2 to 13)	6·8 (4·7 to 9·7)	4·1 (2·8 to 5·6)	3·5 (2·5 to 4·7)	2·6 (1·8 to 3·7)	1·8 (1·3 to 2·5)	1·2 (0·8 to 1·8)	0·5 (0·3 to 0·7)	0·2 (0·1 to 0·2)	181
Multidrug-resistant tuberculosis without extensive drug resistanc	0·1 (0 to 0·5)	0·1 (0 to 0·5)	0·1 (0 to 0·5)	0·1 (0 to 0·4)	0·1 (0 to 0·3)	0 (0 to 0·2)	0 (0 to 0·2)	0 (0 to 0·1)	0 (0 to 0·1)	0 (0 to 0·1)	0 (0 to 0)	0 (0 to 0)	1·7
Extensively drug-resistant tuberculosis	0 (0 to 0·1)	0 (0 to 0·1)	0 (0 to 0·1)	0 (0 to 0·1)	0 (0 to 0)	0 (0 to 0)	0 (0 to 0)	0 (0 to 0)	0 (0 to 0)	0 (0 to 0)	0 (0 to 0)	0 (0 to 0)	0·3
Lower respiratory infections	36·8 (24·6 to 53·4)	49·7 (34·8 to 69)	53·5 (35·9 to 74·7)	57·8 (39·9 to 81·2)	60·3 (41·4 to 82·7)	56·1 (38·2 to 76)	64·1 (46·7 to 86·4)	63·9 (46·3 to 84·6)	66·3 (49·4 to 85·8)	58·8 (44·3 to 73)	40·5 (29·3 to 50·8)	20·8 (14·2 to 27·1)	1,330
Upper respiratory infections	9 (4·7 to 15·9)	7·3 (3·9 to 12·8)	5·6 (2·9 to 9·5)	4·3 (2·3 to 7·1)	3·1 (1·6 to 5·1)	2·1 (1·1 to 3·6)	1·3 (0·7 to 2·2)	0·8 (0·4 to 1·3)	0·5 (0·3 to 0·8)	0·3 (0·1 to 0·5)	0·1 (0·1 to 0·2)	0·1 (0 to 0·1)	171
Otitis media	2·8 (1·2 to 5·2)	2·6 (1·2 to 4·7)	2·1 (1 to 3·9)	1·6 (0·8 to 2·9)	1·1 (0·5 to 2)	0·6 (0·3 to 1·1)	0·3 (0·1 to 0·6)	0·2 (0·1 to 0·4)	0·1 (0·1 to 0·3)	0·1 (0 to 0·2)	0 (0 to 0·1)	0 (0 to 0)	59·6
Enteric infections	17·4 (12·3 to 23·7)	18·3 (13·1 to 24·6)	17 (12·5 to 22·6)	15·8 (11·3 to 21)	14·9 (10·8 to 19·8)	14·4 (10·4 to 18·9)	13·3 (9·9 to 17·7)	11·4 (8·5 to 15)	9·5 (6·8 to 12·8)	6·4 (4·7 to 8·3)	3·4 (2·5 to 4·5)	1·5 (1·1 to 1·9)	503
Diarrheal diseases	17·3 (12·2 to 23·6)	18·3 (13·1 to 24·6)	17 (12·4 to 22·5)	15·7 (11·2 to 20·9)	14·9 (10·7 to 19·7)	14·3 (10·4 to 18·8)	13·3 (9·9 to 17·6)	11·4 (8·5 to 15)	9·5 (6·8 to 12·7)	6·3 (4·7 to 8·3)	3·4 (2·5 to 4·5)	1·4 (1·1 to 1·9)	501
Typhoid and paratyphoid	0 (0 to 0·1)	0 (0 to 0)	0 (0 to 0·1)	0 (0 to 0)	0 (0 to 0)	0 (0 to 0)	0 (0 to 0)	0 (0 to 0)	0 (0 to 0)	0 (0 to 0)	0 (0 to 0)	0 (0 to 0)	0·3
Typhoid fever	0 (0 to 0·1)	0 (0 to 0)	0 (0 to 0·1)	0 (0 to 0)	0 (0 to 0)	0 (0 to 0)	0 (0 to 0)	0 (0 to 0)	0 (0 to 0)	0 (0 to 0)	0 (0 to 0)	0 (0 to 0)	0·3
Paratyphoid fever	0 (0 to 0)	0 (0 to 0)	0 (0 to 0)	0 (0 to 0)	0 (0 to 0)	0 (0 to 0)	0 (0 to 0)	0 (0 to 0)	0 (0 to 0)	0 (0 to 0)	0 (0 to 0)	0 (0 to 0)	0
Invasive Non-typhoidal Salmonella (iNTS)	0·1 (0 to 0·2)	0 (0 to 0)	0 (0 to 0·1)	0·1 (0 to 0·1)	0 (0 to 0)	0 (0 to 0·1)	0 (0 to 0)	0 (0 to 0)	0 (0 to 0)	0 (0 to 0)	0 (0 to 0)	0 (0 to 0)	1·4
Other intestinal infectious diseases	0 (0 to 0·1)	0 (0 to 0·1)	0 (0 to 0·1)	0 (0 to 0)	0 (0 to 0)	0 (0 to 0)	0 (0 to 0)	0 (0 to 0)	0 (0 to 0)	0 (0 to 0)	0 (0 to 0)	0 (0 to 0)	1·1
Neglected tropical diseases and malaria	16·5 (8·9 to 28·4)	14·1 (7·2 to 23·3)	13·1 (6·8 to 21·4)	11·6 (6·3 to 18·9)	9·1 (4·7 to 15·1)	7·1 (3·7 to 11·9)	5·4 (2·9 to 9·1)	3·9 (2 to 6·7)	2·9 (1·4 to 5)	2·1 (1·1 to 3·6)	1 (0·5 to 1·6)	0·4 (0·2 to 0·6)	232
Malaria	0·1 (0 to 0·2)	0·1 (0 to 0·1)	0 (0 to 0)	0 (0 to 0)	0 (0 to 0)	0 (0 to 0)	0 (0 to 0)	0 (0 to 0)	0 (0 to 0)	0 (0 to 0)	0 (0 to 0)	0 (0 to 0)	1·2
Chagas disease	0 (0 to 0·1)	0 (0 to 0·1)	0 (0 to 0·1)	0 (0 to 0·1)	0 (0 to 0·1)	0 (0 to 0·1)	0 (0 to 0·1)	0 (0 to 0·1)	0 (0 to 0·1)	0·4 (0·2 to 1·3)	0·1 (0·1 to 0·4)	0 (0 to 0·1)	1
Leishmaniasis	0 (0 to 0)	0 (0 to 0)	0 (0 to 0)	0 (0 to 0)	0 (0 to 0)	0 (0 to 0)	0 (0 to 0)	0 (0 to 0)	0 (0 to 0)	0 (0 to 0)	0 (0 to 0)	0 (0 to 0)	0
Visceral leishmaniasis	0 (0 to 0)	0 (0 to 0)	0 (0 to 0)	0 (0 to 0)	0 (0 to 0)	0 (0 to 0)	0 (0 to 0)	0 (0 to 0)	0 (0 to 0)	0 (0 to 0)	0 (0 to 0)	0 (0 to 0)	0
Cutaneous and mucocutaneous leishmaniasis	0 (0 to 0)	0 (0 to 0)	0 (0 to 0)	0 (0 to 0)	0 (0 to 0)	0 (0 to 0)	0 (0 to 0)	0 (0 to 0)	0 (0 to 0)	0 (0 to 0)	0 (0 to 0)	0 (0 to 0)	0
African trypanosomiasis	0 (0 to 0)	0 (0 to 0)	0 (0 to 0)	0 (0 to 0)	0 (0 to 0)	0 (0 to 0)	0 (0 to 0)	0 (0 to 0)	0 (0 to 0)	0 (0 to 0)	0 (0 to 0)	0 (0 to 0)	0
Schistosomiasis	0 (0 to 0)	0 (0 to 0)	0 (0 to 0)	0 (0 to 0)	0 (0 to 0)	0 (0 to 0)	0 (0 to 0)	0 (0 to 0)	0 (0 to 0)	0 (0 to 0)	0 (0 to 0)	0 (0 to 0)	0
Cysticercosis	7·9 (2·6 to 16·7)	8 (3 to 16·3)	7·7 (2·8 to 14·6)	7·6 (3·2 to 14·1)	7 (3·1 to 12·5)	6 (2·7 to 10·4)	4·6 (2·2 to 8)	3·3 (1·5 to 6·1)	2·5 (1·1 to 4·5)	1·5 (0·6 to 2·8)	0·7 (0·3 to 1·3)	0·3 (0·1 to 0·5)	89·8
Cystic echinococcosis	0 (0 to 0)	0 (0 to 0)	0 (0 to 0)	0 (0 to 0)	0 (0 to 0)	0 (0 to 0)	0 (0 to 0)	0 (0 to 0)	0 (0 to 0)	0 (0 to 0)	0 (0 to 0)	0 (0 to 0)	0
Lymphatic filariasis	0 (0 to 0)	0 (0 to 0)	0 (0 to 0)	0 (0 to 0)	0 (0 to 0)	0 (0 to 0)	0 (0 to 0)	0 (0 to 0)	0 (0 to 0)	0 (0 to 0)	0 (0 to 0)	0 (0 to 0)	0
Onchocerciasis	0 (0 to 0)	0 (0 to 0)	0 (0 to 0)	0 (0 to 0)	0 (0 to 0)	0 (0 to 0)	0 (0 to 0)	0 (0 to 0)	0 (0 to 0)	0 (0 to 0)	0 (0 to 0)	0 (0 to 0)	0
Trachoma	0 (0 to 0)	0 (0 to 0)	0 (0 to 0)	0 (0 to 0)	0 (0 to 0)	0 (0 to 0)	0 (0 to 0)	0 (0 to 0)	0 (0 to 0)	0 (0 to 0)	0 (0 to 0)	0 (0 to 0)	0
Dengue	3·2 (0·6 to 10·1)	2·5 (0·5 to 8·6)	2·2 (0·4 to 6·9)	1·6 (0·4 to 4·7)	1·1 (0·3 to 3·4)	0·7 (0·2 to 1·8)	0·5 (0·1 to 1·4)	0·4 (0·1 to 1·1)	0·2 (0·1 to 0·7)	0·2 (0·1 to 0·4)	0·1 (0 to 0·2)	0·1 (0 to 0·1)	51·8
Yellow fever	0 (0 to 0)	0 (0 to 0)	0 (0 to 0)	0 (0 to 0)	0 (0 to 0)	0 (0 to 0)	0 (0 to 0)	0 (0 to 0)	0 (0 to 0)	0 (0 to 0)	0 (0 to 0)	0 (0 to 0)	0
Rabies	0 (0 to 0)	0 (0 to 0)	0·1 (0 to 0·2)	0 (0 to 0)	0·1 (0 to 0·1)	0 (0 to 0·1)	0 (0 to 0)	0 (0 to 0)	0 (0 to 0)	0 (0 to 0)	0 (0 to 0)	0 (0 to 0)	0·9
Intestinal nematode infections	1·3 (0·6 to 2·2)	0·3 (0·2 to 0·6)	0·3 (0·1 to 0·6)	0·3 (0·1 to 0·5)	0·2 (0·1 to 0·4)	0·1 (0·1 to 0·2)	0·1 (0 to 0·1)	0·1 (0 to 0·1)	0 (0 to 0·1)	0 (0 to 0)	0 (0 to 0)	0 (0 to 0)	22·6
Ascariasis	0·2 (0·1 to 0·3)	0·1 (0 to 0·3)	0·1 (0 to 0·2)	0·1 (0 to 0·2)	0·1 (0 to 0·2)	0 (0 to 0·1)	0 (0 to 0·1)	0 (0 to 0)	0 (0 to 0)	0 (0 to 0)	0 (0 to 0)	0 (0 to 0)	2·8
Trichuriasis	0 (0 to 0)	0 (0 to 0)	0 (0 to 0)	0 (0 to 0)	0 (0 to 0)	0 (0 to 0)	0 (0 to 0)	0 (0 to 0)	0 (0 to 0)	0 (0 to 0)	0 (0 to 0)	0 (0 to 0)	0·5
Hookworm disease	1·1 (0·5 to 1·9)	0·2 (0·1 to 0·4)	0·2 (0·1 to 0·4)	0·2 (0·1 to 0·3)	0·1 (0 to 0·3)	0·1 (0 to 0·2)	0·1 (0 to 0·1)	0 (0 to 0·1)	0 (0 to 0)	0 (0 to 0)	0 (0 to 0)	0 (0 to 0)	19·2
Food-borne trematodiases	0 (0 to 0)	0 (0 to 0)	0 (0 to 0)	0 (0 to 0)	0 (0 to 0)	0 (0 to 0)	0 (0 to 0)	0 (0 to 0)	0 (0 to 0)	0 (0 to 0)	0 (0 to 0)	0 (0 to 0)	0
Leprosy	0 (0 to 0·1)	0 (0 to 0·1)	0 (0 to 0·1)	0 (0 to 0·1)	0 (0 to 0·1)	0 (0 to 0·1)	0 (0 to 0)	0 (0 to 0)	0 (0 to 0)	0 (0 to 0)	0 (0 to 0)	0 (0 to 0)	0·4
Ebola	0 (0 to 0)	0 (0 to 0)	0 (0 to 0)	0 (0 to 0)	0 (0 to 0)	0 (0 to 0)	0 (0 to 0)	0 (0 to 0)	0 (0 to 0)	0 (0 to 0)	0 (0 to 0)	0 (0 to 0)	0
Zika virus	0·1 (0 to 0·3)	0·1 (0 to 0·2)	0·1 (0 to 0·2)	0 (0 to 0·1)	0 (0 to 0·1)	0 (0 to 0)	0 (0 to 0)	0 (0 to 0)	0 (0 to 0)	0 (0 to 0)	0 (0 to 0)	0 (0 to 0)	1·1
Guinea worm disease	0 (0 to 0)	0 (0 to 0)	0 (0 to 0)	0 (0 to 0)	0 (0 to 0)	0 (0 to 0)	0 (0 to 0)	0 (0 to 0)	0 (0 to 0)	0 (0 to 0)	0 (0 to 0)	0 (0 to 0)	0
Other neglected tropical diseases	4 (1·7 to 7·3)	3 (1·2 to 5·9)	2·7 (1·1 to 4·9)	2 (0·8 to 3·9)	0·6 (0·2 to 1·2)	0·3 (0·1 to 0·6)	0·2 (0·1 to 0·4)	0·1 (0 to 0·3)	0·1 (0 to 0·2)	0·1 (0 to 0·1)	0 (0 to 0·1)	0 (0 to 0)	62·7
Other infectious diseases	12·2 (9 to 16·2)	11·1 (8·5 to 14·5)	9·7 (7·2 to 12·8)	8·2 (6·3 to 10·6)	5·2 (4·1 to 6·3)	3·5 (2·8 to 4·3)	2·4 (1·9 to 3)	1·6 (1·2 to 1·9)	1·1 (0·9 to 1·4)	0·9 (0·7 to 1·2)	0·4 (0·3 to 0·5)	0·2 (0·1 to 0·2)	255
Meningitis	2 (1·5 to 2·6)	2·3 (1·7 to 2·9)	2·1 (1·6 to 2·9)	1·7 (1·3 to 2·3)	1·2 (0·9 to 1·6)	0·9 (0·7 to 1·2)	0·6 (0·4 to 0·8)	0·4 (0·3 to 0·6)	0·3 (0·2 to 0·4)	0·2 (0·1 to 0·3)	0·1 (0 to 0·1)	0 (0 to 0)	66·1
Encephalitis	2·4 (1·7 to 3·6)	2·2 (1·5 to 3·3)	1·6 (1·1 to 2·2)	1·4 (0·9 to 2·1)	1 (0·7 to 1·5)	0·6 (0·4 to 1)	0·4 (0·3 to 0·7)	0·2 (0·1 to 0·3)	0·1 (0·1 to 0·2)	0·1 (0 to 0·1)	0 (0 to 0·1)	0 (0 to 0)	73·6
Diphtheria	0 (0 to 0)	0 (0 to 0)	0 (0 to 0)	0 (0 to 0)	0 (0 to 0)	0 (0 to 0)	0 (0 to 0)	0 (0 to 0)	0 (0 to 0)	0 (0 to 0)	0 (0 to 0)	0 (0 to 0)	0
Tetanus	0 (0 to 0·1)	0 (0 to 0·1)	0·1 (0 to 0·2)	0·1 (0 to 0·1)	0·1 (0 to 0·1)	0 (0 to 0·1)	0 (0 to 0·1)	0 (0 to 0·1)	0 (0 to 0)	0 (0 to 0)	0 (0 to 0)	0 (0 to 0)	0·7
Measles	0 (0 to 0)	0 (0 to 0)	0 (0 to 0)	0 (0 to 0)	0 (0 to 0)	0 (0 to 0)	0 (0 to 0)	0 (0 to 0)	0 (0 to 0)	0 (0 to 0)	0 (0 to 0)	0 (0 to 0)	0
Varicella and herpes zoster	0·5 (0·3 to 0·9)	0·4 (0·2 to 0·8)	0·4 (0·2 to 0·7)	0·3 (0·1 to 0·6)	0·3 (0·2 to 0·6)	0·3 (0·1 to 0·5)	0·2 (0·1 to 0·3)	0·1 (0·1 to 0·2)	0·1 (0·1 to 0·2)	0·1 (0 to 0·1)	0 (0 to 0·1)	0 (0 to 0)	7·7
Acute hepatitis	1·2 (0·9 to 1·6)	0·9 (0·7 to 1·2)	0·7 (0·5 to 1)	0·6 (0·4 to 0·8)	0·4 (0·3 to 0·5)	0·2 (0·2 to 0·3)	0·1 (0·1 to 0·2)	0·1 (0·1 to 0·1)	0 (0 to 0·1)	0 (0 to 0·1)	0 (0 to 0)	0 (0 to 0)	19·5
Acute hepatitis A	0·5 (0·2 to 0·8)	0·3 (0·2 to 0·5)	0·2 (0·1 to 0·4)	0·2 (0·1 to 0·4)	0·1 (0 to 0·2)	0 (0 to 0·1)	0 (0 to 0·1)	0 (0 to 0·1)	0 (0 to 0)	0 (0 to 0)	0 (0 to 0)	0 (0 to 0)	11·6
Acute hepatitis B	0·5 (0·3 to 0·8)	0·4 (0·2 to 0·6)	0·3 (0·2 to 0·5)	0·2 (0·1 to 0·4)	0·2 (0·1 to 0·3)	0·1 (0·1 to 0·1)	0·1 (0 to 0·1)	0 (0 to 0·1)	0 (0 to 0)	0 (0 to 0)	0 (0 to 0)	0 (0 to 0)	5·4
Acute hepatitis C	0·1 (0 to 0·2)	0·1 (0 to 0·1)	0·1 (0 to 0·2)	0·1 (0 to 0·2)	0·1 (0 to 0·1)	0 (0 to 0·1)	0 (0 to 0·1)	0 (0 to 0)	0 (0 to 0)	0 (0 to 0)	0 (0 to 0)	0 (0 to 0)	1·1
Acute hepatitis E	0·1 (0 to 0·1)	0·1 (0·1 to 0·2)	0 (0 to 0·1)	0 (0 to 0·1)	0 (0 to 0)	0 (0 to 0)	0 (0 to 0)	0 (0 to 0)	0 (0 to 0)	0 (0 to 0)	0 (0 to 0)	0 (0 to 0)	1·5
Other unspecified infectious diseases	6 (3·5 to 9·5)	5·2 (3·1 to 8)	4·8 (2·9 to 7·5)	4 (2·5 to 6·1)	2·1 (1·4 to 3)	1·4 (0·9 to 2)	1·1 (0·7 to 1·6)	0·6 (0·4 to 0·9)	0·5 (0·3 to 0·8)	0·5 (0·3 to 0·8)	0·2 (0·1 to 0·3)	0·1 (0·1 to 0·1)	80·6
Maternal and neonatal disorders	55·4 (43·6 to 69·4)	37 (28·3 to 47·7)	24·1 (17·6 to 32)	16·7 (12·3 to 22·4)	10·6 (7·6 to 14·2)	5·9 (4·2 to 7·9)	2·7 (1·9 to 3·7)	1 (0·7 to 1·5)	0·3 (0·2 to 0·5)	0·1 (0·1 to 0·1)	0 (0 to 0)	0 (0 to 0)	3,440
Maternal disorders	16·3 (12·9 to 20·4)	4·9 (3·6 to 6·5)	0·6 (0·5 to 0·7)	0 (0 to 0)	0 (0 to 0)	0 (0 to 0)	0 (0 to 0)	0 (0 to 0)	0 (0 to 0)	0 (0 to 0)	0 (0 to 0)	0 (0 to 0)	373
Maternal hemorrhage	3·2 (2·1 to 4·7)	0·6 (0·3 to 0·9)	0 (0 to 0)	0 (0 to 0)	0 (0 to 0)	0 (0 to 0)	0 (0 to 0)	0 (0 to 0)	0 (0 to 0)	0 (0 to 0)	0 (0 to 0)	0 (0 to 0)	72·4
Maternal sepsis and other maternal infections	1·3 (0·7 to 2)	0·4 (0·2 to 0·6)	0 (0 to 0)	0 (0 to 0)	0 (0 to 0)	0 (0 to 0)	0 (0 to 0)	0 (0 to 0)	0 (0 to 0)	0 (0 to 0)	0 (0 to 0)	0 (0 to 0)	34·8
Maternal hypertensive disorders	2·3 (1·6 to 3·1)	0·6 (0·4 to 0·9)	0·1 (0 to 0·1)	0 (0 to 0)	0 (0 to 0)	0 (0 to 0)	0 (0 to 0)	0 (0 to 0)	0 (0 to 0)	0 (0 to 0)	0 (0 to 0)	0 (0 to 0)	72·4
Maternal obstructed labor and uterine rupture	0·7 (0·3 to 1·2)	0·2 (0·1 to 0·4)	0 (0 to 0)	0 (0 to 0)	0 (0 to 0)	0 (0 to 0)	0 (0 to 0)	0 (0 to 0)	0 (0 to 0)	0 (0 to 0)	0 (0 to 0)	0 (0 to 0)	13·4
Maternal abortion and miscarriage	1·2 (0·6 to 2·1)	0·6 (0·3 to 1)	0 (0 to 0·1)	0 (0 to 0)	0 (0 to 0)	0 (0 to 0)	0 (0 to 0)	0 (0 to 0)	0 (0 to 0)	0 (0 to 0)	0 (0 to 0)	0 (0 to 0)	24·4
Ectopic pregnancy	0·3 (0·2 to 0·5)	0·1 (0·1 to 0·2)	0 (0 to 0)	0 (0 to 0)	0 (0 to 0)	0 (0 to 0)	0 (0 to 0)	0 (0 to 0)	0 (0 to 0)	0 (0 to 0)	0 (0 to 0)	0 (0 to 0)	8·9
Indirect maternal deaths	0·5 (0·3 to 0·8)	0·1 (0·1 to 0·2)	0 (0 to 0)	--	--	--	--	--	--	--	--	--	NA
Late maternal deaths	0·9 (0·5 to 1·4)	0·3 (0·2 to 0·4)	0 (0 to 0)	--	--	--	--	--	--	--	--	--	NA
Maternal deaths aggravated by HIV/AIDS	0·1 (0 to 0·1)	0 (0 to 0)	0 (0 to 0)	--	--	--	--	--	--	--	--	--	NA
Neonatal disorders	39 (28 to 53·2)	32·1 (23·1 to 42·6)	23·5 (17·1 to 31·5)	16·7 (12·3 to 22·4)	10·6 (7·6 to 14·2)	5·9 (4·2 to 7·9)	2·7 (1·9 to 3·7)	1 (0·7 to 1·5)	0·3 (0·2 to 0·5)	0·1 (0·1 to 0·1)	0 (0 to 0)	0 (0 to 0)	3,070
Neonatal preterm birth	16·9 (11·5 to 24)	13·6 (9·4 to 18·5)	9·7 (6·9 to 13·7)	6·5 (4·5 to 9)	3·8 (2·6 to 5·3)	2 (1·6 to 2·7)	0·9 (0·7 to 1·1)	0·3 (0·2 to 0·4)	0·1 (0 to 0·1)	0 (0 to 0·1)	0 (0 to 0)	0 (0 to 0)	1,390
Neonatal encephalopathy due to birth asphyxia and trauma	4·5 (2·2 to 7·7)	3·7 (1·8 to 6·3)	2·7 (1·3 to 4·8)	2·1 (1 to 3·6)	1·4 (0·6 to 2·4)	0·7 (0·5 to 1·1)	0·2 (0·1 to 0·4)	0·1 (0 to 0·1)	0 (0 to 0)	0 (0 to 0)	0 (0 to 0)	0 (0 to 0)	675
Neonatal sepsis and other neonatal infections	15·5 (9·6 to 23·5)	13 (7·8 to 19·5)	9·7 (5·7 to 15)	7·2 (4·3 to 11·1)	4·7 (2·8 to 7·2)	2·8 (1·6 to 4·4)	1·4 (0·8 to 2·3)	0·6 (0·3 to 1)	0·2 (0·1 to 0·4)	0·1 (0 to 0·1)	0 (0 to 0)	0 (0 to 0)	645
Hemolytic disease and other neonatal jaundice	2·1 (1·6 to 2·8)	1·8 (1·3 to 2·4)	1·3 (1 to 1·8)	1 (0·7 to 1·3)	0·7 (0·5 to 0·9)	0·4 (0·3 to 0·5)	0·2 (0·1 to 0·3)	0·1 (0·1 to 0·1)	0 (0 to 0)	0 (0 to 0)	0 (0 to 0)	0 (0 to 0)	64·6
Other neonatal disorders	0 (0 to 0)	0 (0 to 0)	0 (0 to 0)	0 (0 to 0)	0 (0 to 0)	0 (0 to 0)	0 (0 to 0)	0 (0 to 0)	0 (0 to 0)	0 (0 to 0)	0 (0 to 0)	0 (0 to 0)	292
Nutritional deficiencies	95·5 (42·9 to 167)	74·7 (34·5 to 131)	67·4 (31·5 to 118)	51·9 (23·6 to 91)	29·9 (14·9 to 52·4)	14·1 (7 to 25·7)	10·8 (6·2 to 18·9)	8·6 (5·1 to 13·8)	7·1 (4·5 to 11·3)	6·1 (3·9 to 8·9)	4 (2·7 to 5·8)	2 (1·4 to 2·8)	1,220
Protein-energy malnutrition	4·8 (3·4 to 6·5)	5·4 (3·8 to 7·5)	5·2 (3·6 to 7·3)	5·3 (3·6 to 7·1)	4·8 (3·4 to 6·4)	3·8 (2·6 to 5·1)	4 (2·8 to 5·4)	4 (2·8 to 5·7)	3·6 (2·5 to 5)	3·4 (2·2 to 4·8)	2·6 (1·6 to 3·6)	1·6 (1·1 to 2·3)	179
Iodine deficiency	0·4 (0·2 to 0·7)	0·4 (0·2 to 0·6)	0·3 (0·1 to 0·5)	0·2 (0·1 to 0·4)	0·2 (0·1 to 0·2)	0·1 (0·1 to 0·2)	0·1 (0 to 0·1)	0 (0 to 0·1)	0 (0 to 0)	0 (0 to 0)	0 (0 to 0)	0 (0 to 0)	5·1
Vitamin A deficiency	0·2 (0·1 to 0·4)	0·2 (0·1 to 0·3)	0·1 (0·1 to 0·2)	0·1 (0 to 0·2)	0·1 (0 to 0·1)	0 (0 to 0·1)	0 (0 to 0·1)	0 (0 to 0)	0 (0 to 0)	0 (0 to 0)	0 (0 to 0)	0 (0 to 0)	17·3
Dietary iron deficiency	89 (37·4 to 159)	66·8 (27·5 to 122)	60·1 (24·3 to 110)	45·1 (17·2 to 85·4)	23·9 (9 to 46·5)	9·2 (2·5 to 20·6)	6 (1·5 to 13·8)	3·9 (1 to 8·8)	3 (0·9 to 7)	2·2 (0·7 to 4·7)	1·1 (0·4 to 2·4)	0·2 (0·1 to 0·6)	993
Other nutritional deficiencies	1·1 (0·6 to 1·8)	1·9 (1 to 3·2)	1·7 (1 to 2·8)	1·2 (0·6 to 2)	1·1 (0·6 to 1·7)	1 (0·5 to 1·5)	0·8 (0·4 to 1·2)	0·6 (0·3 to 1·1)	0·5 (0·2 to 0·9)	0·5 (0·2 to 0·9)	0·3 (0·1 to 0·6)	0·1 (0 to 0·2)	24·5
Non-communicable diseases	2,320 (1,910 to 2,800)	2,680 (2,260 to 3,140)	2,820 (2,410 to 3,250)	2,710 (2,330 to 3,100)	2,390 (2,100 to 2,730)	1,890 (1,670 to 2,140)	1,510 (1,350 to 1,700)	1,200 (1,080 to 1,340)	927 (836 to 1,020)	643 (581 to 710)	352 (320 to 385)	153 (141 to 166)	33,200
Neoplasms	345 (287 to 418)	489 (408 to 578)	532 (451 to 627)	493 (418 to 574)	409 (349 to 473)	291 (254 to 334)	220 (189 to 254)	153 (130 to 175)	95·3 (78·6 to 111)	53·5 (42·8 to 62·9)	23·5 (17·7 to 27·8)	8 (5·7 to 9·4)	4,130
Lip and oral cavity cancer	1·8 (1·2 to 2·5)	2·9 (2·2 to 3·9)	4 (2·9 to 5·3)	4·1 (3 to 5·4)	3·8 (2·7 to 5)	3 (2·2 to 4)	2·5 (1·9 to 3·3)	2 (1·4 to 2·6)	1·3 (1 to 1·7)	0·7 (0·5 to 1)	0·4 (0·3 to 0·5)	0·1 (0·1 to 0·2)	31·6
Nasopharynx cancer	0·6 (0·4 to 0·8)	1 (0·7 to 1·4)	1·1 (0·7 to 1·4)	1 (0·7 to 1·3)	0·8 (0·6 to 1)	0·5 (0·3 to 0·7)	0·4 (0·3 to 0·6)	0·2 (0·2 to 0·3)	0·1 (0·1 to 0·2)	0·1 (0·1 to 0·2)	0·1 (0 to 0·1)	0 (0 to 0)	8·8
Other pharynx cancer	0·7 (0·5 to 1)	1·3 (0·9 to 1·8)	1·5 (1·1 to 2)	1·6 (1·2 to 2·2)	1·5 (1·1 to 2)	0·9 (0·6 to 1·3)	0·8 (0·5 to 1·1)	0·5 (0·3 to 0·7)	0·3 (0·2 to 0·4)	0·2 (0·1 to 0·3)	0·1 (0·1 to 0·1)	0 (0 to 0)	10·7
Esophageal cancer	1·6 (1·2 to 2·3)	3·2 (2·2 to 4·5)	4·9 (3·7 to 6·4)	4·9 (3·7 to 6·3)	4·9 (3·8 to 6·3)	3·6 (2·7 to 4·6)	2·9 (2·1 to 3·8)	2·2 (1·6 to 2·9)	1·4 (1 to 1·9)	0·8 (0·6 to 1·1)	0·3 (0·2 to 0·4)	0·1 (0·1 to 0·2)	32·9
Stomach cancer	16·4 (12·5 to 20·9)	26·8 (19·2 to 35·7)	33·4 (26 to 41·7)	32·4 (25·9 to 40·4)	30·5 (23·7 to 38·1)	24·5 (18·9 to 31·3)	19·8 (15·3 to 25·2)	16·4 (12·6 to 20·9)	10·8 (8·2 to 13·8)	6·8 (5·1 to 8·8)	3 (2·2 to 3·8)	1 (0·7 to 1·2)	260
Colon and rectum cancer	16·5 (12 to 21·9)	27·7 (20·6 to 36·6)	39·4 (29·9 to 49·3)	41·5 (31·6 to 53·7)	37·2 (27·6 to 48·8)	30·1 (22 to 39·2)	24·9 (18·5 to 32·6)	20·9 (15·5 to 27·5)	14·6 (10·7 to 18·9)	9·1 (6·5 to 11·5)	4·1 (3·1 to 5·2)	1·3 (0·9 to 1·6)	302
Liver cancer	5·3 (3·4 to 7·7)	8·2 (5·7 to 11)	11·1 (8 to 14·9)	12·6 (8·6 to 18·1)	10·7 (7·5 to 14·7)	8·8 (6·4 to 11·5)	7·2 (5 to 9·9)	5·1 (3·5 to 7)	3·4 (2·4 to 4·5)	1·3 (0·9 to 1·8)	0·5 (0·4 to 0·7)	0·1 (0·1 to 0·2)	87·9
Liver cancer due to hepatitis B	2·2 (1·1 to 3·5)	2·9 (1·6 to 4·6)	3·4 (1·9 to 5·6)	3·4 (1·9 to 6)	2·5 (1·2 to 4·4)	1·6 (0·8 to 2·9)	1·1 (0·5 to 2)	0·7 (0·3 to 1·1)	0·4 (0·2 to 0·7)	0·1 (0·1 to 0·2)	0 (0 to 0·1)	0 (0 to 0)	23
Liver cancer due to hepatitis C	0·7 (0·3 to 1·3)	1·5 (0·7 to 2·7)	2·6 (1·3 to 4·2)	3·5 (1·6 to 6·2)	3·2 (1·6 to 5·5)	2·8 (1·7 to 4·3)	2·5 (1·4 to 3·9)	2 (1·1 to 3·1)	1·5 (0·9 to 2·3)	0·7 (0·4 to 1)	0·3 (0·2 to 0·4)	0·1 (0 to 0·1)	21·9
Liver cancer due to alcohol use	1 (0·5 to 1·9)	1·8 (0·8 to 3·2)	2·6 (1·3 to 4·5)	3 (1·5 to 5·1)	2·7 (1·4 to 4·5)	2·5 (1·4 to 4)	2 (1·1 to 3·5)	1·2 (0·7 to 2)	0·7 (0·4 to 1·1)	0·2 (0·1 to 0·4)	0·1 (0 to 0·1)	0 (0 to 0)	18·9
Liver cancer due to NASH	0·6 (0·3 to 1·1)	1 (0·5 to 1·7)	1·3 (0·7 to 2·4)	1·5 (0·8 to 2·7)	1·4 (0·7 to 2·3)	1·2 (0·6 to 2)	1·1 (0·6 to 1·9)	0·9 (0·5 to 1·5)	0·6 (0·3 to 1)	0·2 (0·1 to 0·4)	0·1 (0 to 0·2)	0 (0 to 0)	11·4
Liver cancer due to other causes	0·8 (0·4 to 1·3)	1·1 (0·5 to 1·8)	1·3 (0·7 to 2·3)	1·3 (0·6 to 2·4)	1 (0·5 to 1·7)	0·7 (0·4 to 1·2)	0·5 (0·3 to 1)	0·4 (0·2 to 0·7)	0·2 (0·1 to 0·4)	0·1 (0 to 0·2)	0 (0 to 0·1)	0 (0 to 0)	12·7
Gallbladder and biliary tract cancer	3·1 (2 to 5·5)	6 (3·9 to 10·1)	9 (6 to 15·2)	10·2 (6·9 to 17·9)	9·2 (6·5 to 16·3)	7 (5 to 12·2)	5·6 (3·8 to 9·7)	4 (2·7 to 6·8)	2·3 (1·6 to 4·2)	1·1 (0·7 to 1·9)	0·4 (0·3 to 0·8)	0·1 (0·1 to 0·3)	61·1
Pancreatic cancer	10·6 (7·6 to 14·2)	20·5 (15·6 to 26·6)	29 (22 to 37)	33·4 (25·8 to 42·7)	33·1 (24·9 to 44·2)	26·5 (19·6 to 34·7)	22·2 (16·2 to 29·6)	15·5 (11·2 to 20·4)	9·7 (7·1 to 12·9)	5·5 (4 to 7·1)	2·2 (1·6 to 2·9)	0·7 (0·5 to 0·9)	224
Larynx cancer	0·5 (0·3 to 0·7)	1·1 (0·8 to 1·5)	1·6 (1·2 to 2·2)	1·9 (1·3 to 2·6)	1·9 (1·4 to 2·5)	1·3 (0·9 to 1·8)	0·9 (0·7 to 1·2)	0·6 (0·4 to 0·8)	0·3 (0·2 to 0·5)	0·2 (0·1 to 0·3)	0·1 (0·1 to 0·1)	0 (0 to 0)	11·5
Tracheal, bronchus, and lung cancer	15·4 (11·3 to 20·8)	28·2 (21 to 36·6)	39·7 (28·9 to 53·1)	43·1 (31·2 to 58·2)	41·1 (29·8 to 55·2)	28·7 (20·3 to 38·9)	20·8 (14·9 to 27·9)	13 (9·6 to 17·7)	7·4 (5·2 to 10·3)	3·9 (2·8 to 5·2)	1·5 (1·1 to 1·9)	0·5 (0·3 to 0·7)	264
Malignant skin melanoma	1·2 (0·7 to 1·7)	1·6 (1 to 2·3)	1·6 (0·9 to 2·2)	1·3 (0·8 to 1·9)	1·1 (0·7 to 1·6)	0·8 (0·5 to 1·2)	0·7 (0·4 to 0·9)	0·4 (0·2 to 0·6)	0·3 (0·2 to 0·4)	0·2 (0·1 to 0·3)	0·1 (0·1 to 0·2)	0·1 (0 to 0·1)	14·4
Non-melanoma skin cancer	1·7 (1·1 to 2·5)	1·4 (0·9 to 2)	2·1 (1·5 to 3)	1·9 (1·3 to 2·7)	1·5 (1 to 2·1)	1·3 (0·9 to 1·8)	1·2 (0·8 to 1·6)	1·2 (0·8 to 1·6)	1·1 (0·8 to 1·5)	1 (0·7 to 1·3)	0·8 (0·6 to 1·1)	0·4 (0·3 to 0·6)	19·4
Non-melanoma skin cancer (squamous-cell carcinoma)	1·7 (1·1 to 2·5)	1·4 (0·9 to 2)	2·1 (1·5 to 3)	1·9 (1·3 to 2·7)	1·5 (1 to 2·1)	1·3 (0·9 to 1·8)	1·2 (0·8 to 1·6)	1·2 (0·8 to 1·6)	1·1 (0·8 to 1·5)	1 (0·7 to 1·3)	0·8 (0·6 to 1·1)	0·4 (0·3 to 0·6)	19·4
Non-melanoma skin cancer (basal-cell carcinoma)	0 (0 to 0)	0 (0 to 0)	0 (0 to 0)	0 (0 to 0)	0 (0 to 0)	0 (0 to 0)	0 (0 to 0)	0 (0 to 0)	0 (0 to 0)	0 (0 to 0)	0 (0 to 0)	0 (0 to 0)	0
Breast cancer	65·9 (47·1 to 88·4)	93·4 (68 to 125)	96 (69·9 to 131)	80·7 (58·6 to 108)	58·3 (43·6 to 77·5)	34·9 (26 to 46·1)	24·2 (17·9 to 31·8)	15·7 (11·8 to 20·8)	9·6 (7·1 to 12·8)	6 (4·3 to 7·8)	2·6 (1·9 to 3·4)	1 (0·7 to 1·3)	594
Cervical cancer	127 (98·9 to 164)	161 (121 to 207)	139 (104 to 176)	107 (80·7 to 138)	74·9 (55·7 to 99·7)	46·7 (35·7 to 59·5)	32·9 (25·1 to 42·6)	20·4 (15·9 to 26·1)	11·7 (8·3 to 15·6)	5·5 (4 to 7·3)	2·4 (1·6 to 3·2)	0·8 (0·6 to 1·2)	1,010
Uterine cancer	12·9 (9 to 18·2)	22·3 (15·7 to 29·6)	26·4 (19·1 to 35·9)	26·7 (19·9 to 35·8)	26·6 (19·8 to 34·9)	19·2 (14·7 to 24·6)	13·7 (10·2 to 17·6)	8·3 (6·1 to 10·9)	4·5 (3·3 to 6)	2·1 (1·6 to 2·7)	0·9 (0·7 to 1·2)	0·3 (0·2 to 0·4)	189
Ovarian cancer	12·8 (8·4 to 17·7)	18·3 (11·9 to 24·8)	20·5 (13·3 to 27·2)	19·1 (12·3 to 25·9)	14·9 (9·5 to 20·5)	10·7 (6·9 to 14·6)	6·7 (4·6 to 9·1)	3·6 (2·5 to 4·9)	2·1 (1·4 to 2·8)	1 (0·7 to 1·4)	0·4 (0·3 to 0·6)	0·1 (0·1 to 0·1)	140
Prostate cancer	0 (0 to 0)	0 (0 to 0)	0 (0 to 0)	0 (0 to 0)	0 (0 to 0)	0 (0 to 0)	0 (0 to 0)	0 (0 to 0)	0 (0 to 0)	0 (0 to 0)	0 (0 to 0)	0 (0 to 0)	0
Testicular cancer	--	--	--	--	--	--	--	--	--	--	--	--	NA
Kidney cancer	5·5 (3·8 to 7·5)	8·3 (5·8 to 11·5)	9·2 (6·6 to 12·8)	9·5 (6·6 to 12·9)	7·7 (5·6 to 10·2)	5·2 (4 to 6·7)	3·7 (2·8 to 4·8)	2·1 (1·5 to 2·8)	1·3 (0·9 to 1·7)	0·5 (0·4 to 0·7)	0·2 (0·2 to 0·3)	0·1 (0·1 to 0·1)	78
Bladder cancer	1·7 (1·1 to 2·4)	2·8 (1·9 to 3·8)	5·2 (3·8 to 6·8)	6 (4·7 to 7·8)	5·4 (4·2 to 6·9)	5·1 (4 to 6·6)	4·6 (3·5 to 6·1)	4·1 (3·2 to 5·3)	3 (2·2 to 3·9)	1·9 (1·3 to 2·5)	1 (0·7 to 1·2)	0·3 (0·2 to 0·5)	44·1
Brain and central nervous system cancer	8·5 (3 to 13·6)	9·8 (3·8 to 15)	10·6 (4·2 to 16·2)	9·8 (3·7 to 14·9)	6·9 (2·5 to 10·6)	4·8 (1·8 to 7·3)	3 (1 to 4·6)	1·8 (0·7 to 2·9)	0·8 (0·3 to 1·4)	0·4 (0·2 to 0·7)	0·2 (0·1 to 0·2)	0 (0 to 0·1)	137
Thyroid cancer	1·2 (0·8 to 1·7)	1·8 (1·2 to 2·5)	2·5 (1·7 to 3·7)	2·5 (1·7 to 3·4)	2 (1·5 to 2·5)	1·6 (1·1 to 2·2)	1·3 (1 to 1·7)	0·8 (0·6 to 1·1)	0·5 (0·4 to 0·7)	0·2 (0·2 to 0·3)	0·1 (0·1 to 0·1)	0 (0 to 0)	18·1
Mesothelioma	1·2 (0·7 to 1·9)	1·4 (0·9 to 2·1)	1·8 (1·2 to 2·5)	1·6 (1·1 to 2·4)	0·5 (0·3 to 0·7)	0·3 (0·2 to 0·5)	0·4 (0·2 to 0·6)	0·1 (0·1 to 0·2)	0·1 (0·1 to 0·2)	0 (0 to 0·1)	0 (0 to 0)	0 (0 to 0)	10·8
Hodgkin lymphoma	2·3 (1·4 to 3·5)	1·8 (1·1 to 2·6)	1·4 (1 to 2·1)	1·4 (0·9 to 2·1)	1·1 (0·7 to 1·6)	0·9 (0·6 to 1·4)	0·6 (0·4 to 0·9)	0·4 (0·3 to 0·7)	0·3 (0·2 to 0·5)	0·1 (0·1 to 0·2)	0 (0 to 0·1)	0 (0 to 0)	32·8
Non-Hodgkin lymphoma	4·8 (3·4 to 6·6)	7 (5·2 to 9·1)	7·6 (5·8 to 10)	7·2 (5·5 to 9·4)	6·2 (4·7 to 8·2)	4·6 (3·5 to 5·9)	3·4 (2·5 to 4·4)	2·3 (1·7 to 3)	1·2 (0·9 to 1·6)	0·5 (0·4 to 0·7)	0·2 (0·1 to 0·3)	0·1 (0 to 0·1)	84·8
Multiple myeloma	1·5 (1 to 2·1)	2·6 (1·7 to 3·8)	4·1 (3 to 5·3)	5·2 (3·8 to 6·9)	4·9 (3·5 to 6·7)	3·7 (2·8 to 4·9)	3 (2·2 to 4·1)	1·9 (1·4 to 2·5)	1 (0·7 to 1·5)	0·5 (0·3 to 0·7)	0·2 (0·1 to 0·2)	0 (0 to 0·1)	30·1
Leukemia	11·3 (8·3 to 15·1)	13·8 (10·3 to 18·3)	12·9 (10·1 to 16·7)	11·9 (8·9 to 15·2)	9·5 (7·6 to 12·1)	6·5 (5·2 to 8·3)	5·2 (4 to 6·7)	3·6 (2·7 to 4·6)	2·3 (1·7 to 3)	1·2 (0·9 to 1·6)	0·5 (0·4 to 0·7)	0·2 (0·1 to 0·2)	219
Acute lymphoid leukemia	1·1 (0·6 to 1·8)	1·1 (0·7 to 1·6)	1·3 (0·8 to 2)	0·9 (0·6 to 1·4)	0·7 (0·4 to 1)	0·4 (0·2 to 0·6)	0·2 (0·1 to 0·4)	0·2 (0·1 to 0·3)	0·1 (0·1 to 0·2)	0 (0 to 0)	0 (0 to 0)	0 (0 to 0)	56·8
Chronic lymphoid leukemia	0·2 (0·1 to 0·3)	0·1 (0·1 to 0·2)	0·5 (0·3 to 0·8)	0·6 (0·3 to 1)	0·7 (0·4 to 1·1)	0·6 (0·3 to 0·9)	0·4 (0·2 to 0·7)	0·3 (0·2 to 0·5)	0·2 (0·1 to 0·3)	0·1 (0 to 0·1)	0 (0 to 0·1)	0 (0 to 0)	4·2
Acute myeloid leukemia	1·4 (0·7 to 2·3)	1·6 (0·9 to 2·7)	1·3 (0·7 to 2)	1·1 (0·6 to 1·8)	0·8 (0·5 to 1·3)	0·4 (0·2 to 0·7)	0·3 (0·1 to 0·5)	0·1 (0·1 to 0·3)	0·1 (0 to 0·2)	0 (0 to 0·1)	0 (0 to 0)	0 (0 to 0)	21·5

Chronic myeloid leukemia	0 (0 to 0)	0 (0 to 0)	0 (0 to 0·1)	0·2 (0·1 to 0·3)	0·2 (0·1 to 0·3)	0·2 (0·1 to 0·3)	0·2 (0·2 to 0·4)	0·4 (0·2 to 0·8)	0·6 (0·3 to 1·1)	0·6 (0·3 to 1·1)	0·4 (0·2 to 0·8)	0·3 (0·2 to 0·6)	0·4 (0·2 to 0·8)	0·6 (0·3 to 1)	0·5 (0·3 to 0·9)	0·4 (0·2 to 0·8)	0·2 (0·1 to 0·4)	0·2 (0·1 to 0·3)	0·1 (0·1 to 0·2)	0·1 (0 to 0·1)	0 (0 to 0·1)	0 (0 to 0)	0 (0 to 0)	6
Other leukemia	0·4 (0·2 to 0·6)	0·2 (0·1 to 0·4)	2 (1·1 to 3·6)	4·7 (2·6 to 7·8)	5·9 (3·5 to 9·3)	5·3 (3·2 to 8·2)	9·5 (6·2 to 13·5)	9·7 (6·8 to 13·3)	12·7 (8·8 to 17·5)	11·7 (8·3 to 16)	8·9 (6·2 to 12·4)	8·3 (5·9 to 11·3)	10·6 (7·8 to 14)	9·2 (7·1 to 12·2)	8·7 (6·5 to 11·4)	6·9 (5·2 to 8·9)	4·9 (3·8 to 6·3)	4·1 (3·1 to 5·4)	2·8 (2·1 to 3·7)	1·8 (1·3 to 2·5)	1·1 (0·8 to 1·4)	0·5 (0·3 to 0·6)	0·1 (0·1 to 0·2)	130
Other malignant neoplasms	0·8 (0·5 to 1·1)	0·8 (0·5 to 1·1)	3·2 (1·9 to 5·3)	15·6 (11 to 22·6)	11·3 (8·9 to 14·4)	11·9 (9·3 to 15·2)	17·6 (13·8 to 22·4)	13 (9·9 to 16·4)	12·6 (9·9 to 16·1)	12·3 (9·7 to 15·5)	10·6 (8·2 to 13·6)	12 (9 to 16)	14·4 (11·3 to 18·5)	15·6 (12·2 to 19·8)	13·9 (10·9 to 17·6)	11·5 (8·8 to 14·6)	8·8 (6·9 to 10·9)	6·7 (5·3 to 8·4)	4·8 (3·8 to 5·9)	3·3 (2·5 to 4·2)	2 (1·4 to 2·8)	0·9 (0·6 to 1·2)	0·3 (0·2 to 0·4)	204
Other neoplasms	0 (0 to 0·1)	0·1 (0 to 0·3)	0·4 (0·2 to 0·9)	1·1 (0·7 to 1·8)	0·3 (0·2 to 0·5)	0·5 (0·2 to 0·8)	0·7 (0·4 to 1)	0·5 (0·3 to 0·8)	0·8 (0·5 to 1·3)	0·8 (0·5 to 1·2)	0·4 (0·3 to 0·7)	1 (0·7 to 1·5)	1 (0·7 to 1·4)	1·2 (0·8 to 1·7)	1·2 (0·8 to 1·6)	1·2 (0·9 to 1·7)	1·1 (0·8 to 1·5)	1·1 (0·8 to 1·5)	0·8 (0·6 to 1·1)	0·6 (0·4 to 0·8)	0·3 (0·2 to 0·4)	0·1 (0·1 to 0·2)	0 (0 to 0·1)	15·4
Myelodysplastic, myeloproliferative, and other hematopoietic neo	0 (0 to 0·1)	0·1 (0 to 0·3)	0·4 (0·2 to 0·9)	1·1 (0·7 to 1·8)	0·3 (0·2 to 0·5)	0·5 (0·2 to 0·8)	0·7 (0·4 to 1)	0·5 (0·3 to 0·8)	0·8 (0·5 to 1·3)	0·8 (0·5 to 1·2)	0·4 (0·3 to 0·7)	1 (0·7 to 1·5)	1 (0·7 to 1·4)	1·2 (0·8 to 1·7)	1·2 (0·8 to 1·6)	1·2 (0·9 to 1·7)	1·1 (0·8 to 1·5)	1·1 (0·8 to 1·5)	0·8 (0·6 to 1·1)	0·6 (0·4 to 0·8)	0·3 (0·2 to 0·4)	0·1 (0·1 to 0·2)	0 (0 to 0·1)	15·4
Benign and in situ intestinal neoplasms	0 (0 to 0)	0 (0 to 0)	0 (0 to 0)	0 (0 to 0)	0 (0 to 0)	0 (0 to 0)	0 (0 to 0)	0 (0 to 0)	0 (0 to 0)	0 (0 to 0)	0 (0 to 0)	0 (0 to 0)	0 (0 to 0)	0 (0 to 0)	0 (0 to 0)	0 (0 to 0)	0 (0 to 0)	0 (0 to 0)	0 (0 to 0)	0 (0 to 0)	0 (0 to 0)	0 (0 to 0)	0 (0 to 0)	0
Benign and in situ cervical and uterine neoplasms	0 (0 to 0)	0 (0 to 0)	0 (0 to 0)	0 (0 to 0)	0 (0 to 0)	0 (0 to 0)	0 (0 to 0)	0 (0 to 0)	0 (0 to 0)	0 (0 to 0)	0 (0 to 0)	0 (0 to 0)	0 (0 to 0)	0 (0 to 0)	0 (0 to 0)	0 (0 to 0)	0 (0 to 0)	0 (0 to 0)	0 (0 to 0)	0 (0 to 0)	0 (0 to 0)	0 (0 to 0)	0 (0 to 0)	0
Other benign and in situ neoplasms	0 (0 to 0)	0 (0 to 0)	0 (0 to 0)	0 (0 to 0)	0 (0 to 0)	0 (0 to 0)	0 (0 to 0)	0 (0 to 0)	0 (0 to 0)	0 (0 to 0)	0 (0 to 0)	0 (0 to 0)	0 (0 to 0)	0 (0 to 0)	0 (0 to 0)	0 (0 to 0)	0 (0 to 0)	0 (0 to 0)	0 (0 to 0)	0 (0 to 0)	0 (0 to 0)	0 (0 to 0)	0 (0 to 0)	0
Cardiovascular diseases	8·2 (5·5 to 11·2)	3 (1·9 to 4·3)	12·4 (7·3 to 20·3)	11·1 (8·5 to 14·4)	17·5 (13·6 to 22·3)	29·2 (22 to 38·2)	76·9 (62·1 to 93·4)	84·3 (67·2 to 105)	117 (92 to 145)	154 (122 to 188)	139 (111 to 172)	201 (164 to 241)	339 (274 to 414)	412 (340 to 492)	462 (386 to 550)	483 (418 to 560)	427 (365 to 497)	380 (333 to 434)	370 (325 to 417)	316 (269 to 358)	237 (190 to 272)	138 (106 to 158)	62·3 (46·2 to 72·9)	4,480
Rheumatic heart disease	0 (0 to 0)	0 (0 to 0)	0 (0 to 0)	0·9 (0·5 to 1·6)	4·8 (2·9 to 7·8)	10·7 (6·2 to 17·6)	19·9 (13·2 to 29·5)	19·1 (12·8 to 27·5)	22·1 (15·4 to 30·6)	22·4 (16·1 to 30)	17·5 (12·3 to 23·5)	16·5 (11·7 to 22·1)	16·1 (11·5 to 22·1)	12·8 (9·2 to 17·3)	9·3 (6·7 to 12·7)	5·2 (3·9 to 6·9)	3·3 (2·5 to 4·2)	2 (1·6 to 2·6)	1·4 (1·1 to 1·8)	0·9 (0·7 to 1·2)	0·6 (0·4 to 0·8)	0·3 (0·2 to 0·4)	0·2 (0·1 to 0·2)	186
Ischemic heart disease	0 (0 to 0)	0 (0 to 0)	0 (0 to 0)	0 (0 to 0)	0 (0 to 0)	0 (0 to 0)	6·2 (4·8 to 7·8)	8·7 (6 to 11·9)	14·7 (10·5 to 19·9)	29·6 (21 to 40·2)	29·4 (21·9 to 38·6)	51·6 (39·5 to 64·8)	108 (79·9 to 142)	150 (120 to 185)	178 (139 to 229)	209 (170 to 253)	184 (156 to 218)	160 (135 to 185)	159 (135 to 186)	131 (106 to 156)	98·8 (78·2 to 118)	59·7 (44·7 to 70·7)	29 (21·4 to 35·4)	1,610
Stroke	2·9 (1·5 to 4·7)	0·3 (0·2 to 0·6)	1·3 (0·6 to 2·5)	1·7 (1·3 to 2·3)	4 (2·7 to 5·5)	6·6 (4·7 to 9)	19·3 (14·2 to 25·4)	22 (15·9 to 29·9)	32·5 (23·6 to 44)	47·4 (34·6 to 63·7)	46 (33·6 to 60·7)	66·3 (49 to 86·7)	111 (86·1 to 142)	133 (97·1 to 177)	148 (117 to 181)	147 (119 to 182)	132 (99·7 to 173)	122 (96 to 152)	118 (96·7 to 141)	103 (83·6 to 121)	73·2 (57 to 87·6)	39·4 (29·8 to 47·9)	15·9 (11·3 to 19·6)	1,390
Ischemic stroke	0·7 (0·4 to 1·2)	0 (0 to 0·1)	0·1 (0 to 0·1)	0·7 (0·5 to 1)	2 (1·1 to 3·3)	3 (1·7 to 5·1)	5·3 (3·6 to 7·4)	6·3 (4·4 to 9)	7·3 (4·7 to 10·6)	10·2 (6·9 to 14·3)	10·6 (7·5 to 14·9)	14·4 (10·3 to 19·1)	20·9 (16 to 26·1)	26·9 (20·7 to 34·5)	32·7 (26·5 to 39·4)	45·4 (36·6 to 55·8)	44·4 (34·7 to 55·9)	63·2 (50·6 to 78·1)	65·6 (53·8 to 78·7)	72·6 (59·1 to 85·4)	54·6 (42·6 to 65·6)	30·2 (23 to 36·8)	12·5 (8·9 to 15·5)	530
Intracerebral hemorrhage	0·9 (0·5 to 1·6)	0·2 (0·1 to 0·3)	0·6 (0·2 to 1·3)	0·4 (0·3 to 0·6)	1·1 (0·7 to 1·5)	1·9 (1·3 to 2·6)	8·5 (6 to 11·6)	9·6 (6·5 to 13·8)	15·2 (10·3 to 21·4)	23·4 (16 to 33)	21·7 (14·9 to 30·4)	33 (23·3 to 45·3)	65·8 (48·8 to 85·2)	80·2 (56 to 110)	90·4 (69·7 to 113)	86·5 (68·6 to 109)	76·1 (56·3 to 101)	52·1 (39·7 to 65·9)	47·6 (37·8 to 58·9)	27·5 (21·8 to 32·8)	16·7 (12·8 to 20·3)	8·2 (6·2 to 10·1)	3 (2·1 to 3·8)	671
Subarachnoid hemorrhage	1·2 (0·7 to 2)	0·2 (0·1 to 0·3)	0·6 (0·2 to 1·2)	0·6 (0·4 to 0·9)	1 (0·6 to 1·4)	1·6 (1·1 to 2·3)	5·5 (3·5 to 8·1)	6·1 (3·7 to 9·2)	9·9 (6·2 to 14·9)	13·8 (9 to 20·1)	13·7 (8·9 to 20·2)	19 (12·5 to 27·2)	24·5 (16·7 to 34·7)	25·9 (17·4 to 37·5)	24·7 (16·9 to 34·6)	15·4 (10·8 to 21·8)	11·9 (7·8 to 17·4)	7 (4·8 to 9·9)	5·2 (3·6 to 7·3)	3·2 (2·3 to 4·6)	1·8 (1·2 to 2·6)	0·9 (0·6 to 1·3)	0·3 (0·2 to 0·5)	194
Hypertensive heart disease	0 (0 to 0)	0 (0 to 0)	0 (0 to 0)	0 (0 to 0)	0 (0 to 0)	0 (0 to 0)	1·8 (0·9 to 2·6)	3 (1·8 to 4·4)	4·7 (3 to 6·8)	9·5 (6 to 13·4)	11·6 (7·5 to 16·6)	17·9 (12·3 to 25·6)	38·2 (24·3 to 54)	49·4 (33·4 to 68·7)	55·3 (36·5 to 78·8)	53·9 (37·1 to 74·2)	50·4 (36·3 to 66·8)	45·7 (32·7 to 60·4)	44·7 (31 to 59·3)	39·2 (27·8 to 53·3)	28·7 (19·4 to 38·7)	16·6 (11 to 22·2)	8 (5·1 to 11)	479
Non-rheumatic valvular heart disease	0 (0 to 0)	0 (0 to 0)	0 (0 to 0)	0 (0 to 0)	0 (0 to 0)	0 (0 to 0)	2·2 (1·5 to 3)	1·4 (0·9 to 1·9)	1·7 (1·1 to 2·4)	2 (1·4 to 2·9)	1·5 (1 to 2·1)	1·8 (1·2 to 2·6)	3 (2 to 4·5)	2·8 (1·9 to 4·1)	2·8 (1·8 to 4·1)	2·7 (1·9 to 3·8)	2 (1·5 to 2·7)	1·5 (1·1 to 2·1)	1·3 (1 to 1·8)	1 (0·7 to 1·4)	0·7 (0·5 to 1)	0·4 (0·3 to 0·5)	0·1 (0·1 to 0·2)	29·1
Non-rheumatic calcific aortic valve disease	0 (0 to 0)	0 (0 to 0)	0 (0 to 0)	0 (0 to 0)	0 (0 to 0)	0 (0 to 0)	0·3 (0·1 to 0·6)	0·3 (0·2 to 0·6)	0·2 (0·1 to 0·5)	0·2 (0·1 to 0·5)	0·2 (0·1 to 0·4)	0·3 (0·1 to 0·5)	0·5 (0·2 to 1)	0·6 (0·3 to 1·1)	0·6 (0·3 to 1·2)	0·9 (0·5 to 1·4)	0·7 (0·4 to 1·1)	0·6 (0·4 to 0·9)	0·6 (0·4 to 0·9)	0·5 (0·3 to 0·7)	0·4 (0·3 to 0·6)	0·2 (0·1 to 0·3)	0·1 (0 to 0·1)	7·2
Non-rheumatic degenerative mitral valve disease	0 (0 to 0)	0 (0 to 0)	0 (0 to 0)	0 (0 to 0)	0 (0 to 0)	0 (0 to 0)	1·4 (0·8 to 2)	0·8 (0·5 to 1·2)	1 (0·6 to 1·6)	1·2 (0·7 to 1·9)	0·9 (0·6 to 1·4)	1·3 (0·8 to 1·9)	1·9 (1·1 to 3)	1·8 (1·1 to 2·7)	1·8 (1·1 to 2·7)	1·6 (1 to 2·3)	1·1 (0·7 to 1·5)	0·7 (0·4 to 1)	0·5 (0·3 to 0·8)	0·4 (0·2 to 0·6)	0·2 (0·1 to 0·4)	0·1 (0·1 to 0·2)	0 (0 to 0·1)	16·7
Other non-rheumatic valve diseases	0 (0 to 0)	0 (0 to 0)	0 (0 to 0)	0 (0 to 0)	0 (0 to 0)	0 (0 to 0)	0·6 (0·3 to 1)	0·2 (0·1 to 0·5)	0·4 (0·2 to 0·8)	0·6 (0·3 to 1·2)	0·4 (0·1 to 0·7)	0·3 (0·1 to 0·5)	0·6 (0·3 to 1·2)	0·4 (0·2 to 0·8)	0·4 (0·2 to 0·9)	0·2 (0·1 to 0·5)	0·2 (0·1 to 0·5)	0·3 (0·1 to 0·5)	0·2 (0·1 to 0·4)	0·2 (0·1 to 0·3)	0·1 (0 to 0·2)	0·1 (0 to 0·1)	0 (0 to 0)	5·2
Cardiomyopathy and myocarditis	1·7 (1·1 to 2·5)	1·1 (0·7 to 1·7)	6·1 (3·4 to 10·2)	4·9 (3·3 to 6·8)	3·4 (2·5 to 4·4)	4·6 (3·4 to 6·2)	8 (5·5 to 11·2)	9·2 (6·4 to 13·3)	11·1 (7·5 to 15·8)	13·7 (8·8 to 19·7)	10·2 (6·8 to 14·5)	11·9 (8·1 to 16·8)	18·3 (12·1 to 26·1)	20·3 (13·6 to 29)	20·1 (13·5 to 28·9)	18·1 (12·7 to 24·7)	13·8 (9·8 to 19·1)	10·4 (7·4 to 14·2)	9·1 (6·3 to 12·7)	7·1 (4·6 to 10·1)	6·2 (3·9 to 9·1)	3·2 (2 to 4·7)	1·3 (0·8 to 1·9)	214
Myocarditis	0·2 (0·1 to 0·3)	0·2 (0·1 to 0·3)	1·1 (0·5 to 2·2)	1·2 (0·6 to 1·9)	1·3 (0·7 to 1·8)	1 (0·5 to 1·5)	1·3 (0·7 to 2·1)	1·2 (0·7 to 1·8)	1·4 (0·7 to 2·3)	2·1 (1·2 to 3·3)	1 (0·5 to 1·6)	0·6 (0·4 to 1)	1·6 (0·9 to 2·6)	1·2 (0·6 to 2)	0·8 (0·5 to 1·2)	0·6 (0·3 to 1·1)	0·3 (0·2 to 0·4)	0·4 (0·3 to 0·7)	0·4 (0·2 to 0·7)	0·5 (0·2 to 0·9)	0·5 (0·2 to 1)	0·3 (0·2 to 0·5)	0·2 (0·1 to 0·3)	19·2
Alcoholic cardiomyopathy	0 (0 to 0)	0 (0 to 0)	0 (0 to 0)	0 (0 to 0)	0 (0 to 0)	0 (0 to 0)	0·2 (0·1 to 0·4)	0·4 (0·2 to 0·7)	0·6 (0·2 to 1·2)	0·4 (0·2 to 0·7)	0·3 (0·1 to 0·6)	0·3 (0·1 to 0·5)	1·5 (0·6 to 2·5)	1·7 (0·6 to 3·1)	1·1 (0·4 to 1·9)	0·8 (0·3 to 1·6)	0·7 (0·3 to 1·2)	0·1 (0 to 0·2)	0·1 (0 to 0·2)	0·1 (0 to 0·2)	0 (0 to 0·1)	0 (0 to 0)	0 (0 to 0)	8·3
Other cardiomyopathy	1·5 (0·9 to 2·3)	1 (0·6 to 1·5)	5 (2·7 to 8·4)	3·7 (2·4 to 5·5)	2·1 (1·3 to 3·1)	3·7 (2·4 to 5·3)	6·5 (4·2 to 9·6)	7·7 (5 to 11·7)	9·2 (5·8 to 13·3)	11·2 (6·8 to 16·9)	8·9 (5·8 to 12·9)	11 (7·2 to 15·6)	15·1 (9·6 to 22·6)	17·5 (11·6 to 25)	18·2 (11·8 to 26·7)	16·6 (11·6 to 22·7)	12·8 (8·9 to 18)	9·9 (6·9 to 13·6)	8·6 (5·9 to 12·1)	6·6 (4·3 to 9·3)	5·6 (3·5 to 8·4)	2·8 (1·7 to 4·2)	1·1 (0·6 to 1·6)	186
Atrial fibrillation and flutter	0 (0 to 0)	0 (0 to 0)	0 (0 to 0)	0 (0 to 0)	0 (0 to 0)	0 (0 to 0)	0 (0 to 0)	0 (0 to 0)	0 (0 to 0)	0·3 (0·1 to 0·4)	0·4 (0·2 to 0·5)	1·4 (0·9 to 2·2)	2·7 (1·8 to 4·1)	3·8 (2·7 to 5·5)	6 (4·3 to 8·2)	7·5 (5·5 to 9·8)	8·5 (6·5 to 11·3)	10·1 (7·8 to 13·2)	11·1 (8·7 to 14)	13·1 (10·2 to 16·6)	12·1 (9·4 to 15·9)	9 (6·6 to 12)	3·9 (2·7 to 5·2)	89·8
Aortic aneurysm	--	--	--	--	--	--	0·2 (0·1 to 0·3)	0·3 (0·2 to 0·5)	0·9 (0·6 to 1·3)	0·8 (0·5 to 1·2)	1 (0·7 to 1·6)	1·5 (1 to 2)	2·3 (1·6 to 3·3)	3 (2·1 to 4)	4 (2·8 to 5·7)	4·5 (3·2 to 6)	4·8 (3·5 to 6·5)	4·3 (3·1 to 5·9)	3·8 (2·6 to 5·2)	3 (2 to 4·2)	1·8 (1·3 to 2·5)	0·9 (0·6 to 1·2)	0·3 (0·2 to 0·4)	NA
Endocarditis	0·1 (0 to 0·1)	0·1 (0 to 0·1)	0·3 (0·1 to 0·7)	0·2 (0·1 to 0·4)	0·3 (0·2 to 0·5)	0·3 (0·2 to 0·5)	1·2 (0·8 to 1·8)	1·8 (1·1 to 2·8)	2 (1·1 to 3·2)	1·6 (0·9 to 2·5)	1·6 (0·9 to 2·6)	1·5 (0·9 to 2·3)	1·9 (1·1 to 3·1)	1·5 (0·9 to 2·3)	1·5 (0·9 to 2·3)	1·3 (0·8 to 2·1)	0·8 (0·5 to 1·3)	0·7 (0·4 to 1·1)	0·5 (0·3 to 0·8)	0·5 (0·3 to 0·9)	0·3 (0·2 to 0·6)	0·2 (0·1 to 0·3)	0·1 (0·1 to 0·2)	20·1
Other cardiovascular and circulatory diseases	3·5 (2·1 to 5·3)	1·4 (0·8 to 2·2)	4·7 (2·2 to 8·4)	3·4 (2·2 to 4·9)	5 (3·1 to 7·2)	7 (4·5 to 10·3)	18·1 (13 to 24·5)	18·8 (13·1 to 25·6)	27·1 (19 to 38)	26·4 (18·1 to 35·9)	20·4 (13·6 to 28·5)	30·5 (20·6 to 43)	37·1 (28·3 to 48)	34·3 (26·4 to 43·7)	35·7 (27·9 to 43·7)	31·7 (23·6 to 42)	24·9 (18·7 to 32·7)	20·8 (15·7 to 26·5)	18·3 (13·6 to 23·7)	15·2 (11·7 to 18·8)	13·2 (9·3 to 17·4)	7·5 (5 to 10·1)	3·2 (2·2 to 4·2)	408
Chronic respiratory diseases	0 (0 to 0)	0 (0 to 0)	0 (0 to 0)	94·5 (56·7 to 150)	90 (54·6 to 144)	85·6 (53 to 134)	85 (54·8 to 126)	73·9 (51·4 to 103)	73 (52·9 to 98·6)	72·1 (53 to 93·5)	62·2 (47·8 to 78·7)	58·3 (46 to 72·4)	68·8 (54·2 to 85·3)	75·1 (59·4 to 92·7)	73·5 (58·3 to 90·4)	69 (54 to 87·7)	56·7 (44·5 to 71·5)	49·6 (38·3 to 63·9)	40·5 (30·8 to 51·9)	28·9 (22·3 to 37·1)	20·5 (15·6 to 26·1)	11·5 (8·6 to 14·6)	5·1 (3·7 to 6·6)	1,190
Chronic obstructive pulmonary disease	0 (0 to 0)	0 (0 to 0)	0 (0 to 0)	2·4 (1·5 to 3·6)	2·2 (0·9 to 4·1)	3·5 (1·7 to 6)	8·4 (5·4 to 12·2)	10·4 (7·3 to 14·1)	12·8 (8·9 to 17·3)	15·1 (10·4 to 20·7)	16·1 (11·6 to 21·2)	19·9 (14·9 to 25·5)	30 (22·6 to 39·1)	41 (31·8 to 51)	44·6 (34 to 57)	49·3 (37·6 to 63·1)	41·7 (32·3 to 53·7)	37·3 (28·1 to 48·6)	31·7 (23·7 to 41)	23·1 (17·8 to 29·8)	16·8 (12·6 to 21·8)	9·5 (7 to 12·2)	4·3 (3 to 5·6)	420
Pneumoconiosis	0 (0 to 0)	0 (0 to 0)	0 (0 to 0)	0 (0 to 0)	0 (0 to 0)	0 (0 to 0)	0·1 (0 to 0·2)	0 (0 to 0)	0 (0 to 0·1)	0 (0 to 0)	0·1 (0 to 0·1)	0·1 (0 to 0·1)	0·1 (0 to 0·1)	0·1 (0·1 to 0·2)	0 (0 to 0·1)	0 (0 to 0)	0 (0 to 0·1)	0 (0 to 0)	0 (0 to 0·1)	0 (0 to 0)	0 (0 to 0)	0 (0 to 0)	0 (0 to 0)	0·7
Silicosis	0 (0 to 0)	0 (0 to 0)	0 (0 to 0)	0 (0 to 0)	0 (0 to 0)	0 (0 to 0)	0 (0 to 0)	0 (0 to 0)	0 (0 to 0)	0 (0 to 0)	0 (0 to 0)	0 (0 to 0)	0 (0 to 0)	0 (0 to 0)	0 (0 to 0)	0 (0 to 0)	0 (0 to 0)	0 (0 to 0)	0 (0 to 0)	0 (0 to 0)	0 (0 to 0)	0 (0 to 0)	0 (0 to 0)	0·1
Asbestosis	0 (0 to 0)	0 (0 to 0)	0 (0 to 0)	0 (0 to 0)	0 (0 to 0)	0 (0 to 0)	0 (0 to 0)	0 (0 to 0)	0 (0 to 0)	0 (0 to 0)	0 (0 to 0)	0 (0 to 0)	0 (0 to 0)	0 (0 to 0)	0 (0 to 0)	0 (0 to 0)	0 (0 to 0)	0 (0 to 0)	0 (0 to 0)	0 (0 to 0)	0 (0 to 0)	0 (0 to 0)	0 (0 to 0)	0·2
Coal workers pneumoconiosis	0 (0 to 0)	0 (0 to 0)	0 (0 to 0)	0 (0 to 0)	0 (0 to 0)	0 (0 to 0)	0 (0 to 0)	0 (0 to 0)	0 (0 to 0)	0 (0 to 0)	0 (0 to 0)	0 (0 to 0)	0 (0 to 0)	0 (0 to 0)	0 (0 to 0)	0 (0 to 0)	0 (0 to 0)	0 (0 to 0)	0 (0 to 0)	0 (0 to 0)	0 (0 to 0)	0 (0 to 0)	0 (0 to 0)	0·1
Other pneumoconiosis	0 (0 to 0)	0 (0 to 0)	0 (0 to 0)	0 (0 to 0)	0 (0 to 0)	0 (0 to 0)	0·1 (0 to 0·1)	0 (0 to 0)	0 (0 to 0)	0 (0 to 0)	0 (0 to 0)	0 (0 to 0·1)	0 (0 to 0·1)	0·1 (0 to 0·1)	0 (0 to 0)	0 (0 to 0)	0 (0 to 0)	0 (0 to 0)	0 (0 to 0)	0 (0 to 0)	0 (0 to 0)	0 (0 to 0)	0 (0 to 0)	0·3
Asthma	0 (0 to 0)	0 (0 to 0)	0 (0 to 0)	78·9 (43·1 to 135)	80·8 (46·5 to 135)	74·1 (42·8 to 121)	70·7 (42·9 to 111)	55·4 (34·6 to 83·8)	47·6 (30·4 to 70·4)	44·4 (30·5 to 62·9)	35 (24 to 49·5)	28·4 (19·9 to 39·4)	27·3 (20 to 36·1)	23·7 (17·4 to 31·5)	17·2 (12·5 to 23·8)	11·8 (8·3 to 16·2)	8·6 (6·2 to 11·5)	6 (4·2 to 8·1)	4·1 (2·9 to 5·7)	2·5 (1·7 to 3·5)	1·5 (1 to 2)	0·8 (0·5 to 1·1)	0·4 (0·2 to 0·5)	619
Interstitial lung disease and pulmonary sarcoidosis	0 (0 to 0)	0 (0 to 0)	0 (0 to 0)	5·2 (2·6 to 8·6)	2·1 (1·1 to 3·3)	1·7 (0·9 to 2·7)	2·7 (1·3 to 4·6)	3·2 (1·7 to 5·3)	5·4 (2·8 to 8·7)	7·6 (3·9 to 12·3)	7·1 (4·2 to 10·8)	6·1 (3·6 to 9·2)	8·1 (4·7 to 12·1)	7·7 (4·5 to 11·9)	7·8 (4·7 to 11·7)	7·4 (4·4 to 11·3)	6·1 (3·8 to 9·4)	5·9 (3·5 to 9·2)	4·5 (2·7 to 6·9)	3·1 (1·8 to 4·8)	2 (1·2 to 3·3)	1·1 (0·6 to 1·8)	0·4 (0·2 to 0·6)	95·1
Other chronic respiratory diseases	0 (0 to 0)	0 (0 to 0)	0 (0 to 0)	8 (4·5 to 13·1)	4·9 (2·5 to 7·8)	6·2 (3·5 to 9·8)	3·1 (1·9 to 4·8)	4·7 (3·1 to 6·9)	7·1 (4·7 to 10·5)	4·9 (3·2 to 7·2)	4 (2·7 to 5·6)	3·8 (2·6 to 5·7)	3·4 (2·2 to 4·9)	2·7 (1·8 to 4·1)	3·8 (2·2 to 6·2)	0·6 (0·3 to 1)	0·3 (0·2 to 0·4)	0·3 (0·1 to 0·5)	0·2 (0·1 to 0·4)	0·2 (0·1 to 0·3)	0·1 (0·1 to 0·2)	0 (0 to 0·1)	0 (0 to 0·1)	58·3
Digestive diseases	4·9 (3·4 to 6·6)	4·5 (2·6 to 7·2)	22 (11·3 to 39·1)	38·2 (27·2 to 51·9)	29·1 (21·8 to 37·9)	30·4 (23·4 to 39·7)	55·3 (43·2 to 70·9)	73·5 (55·7 to 99·4)	93·3 (70·2 to 125)	114 (85·1 to 149)	105 (80·3 to 138)	110 (86·5 to 139)	142 (112 to 178)	158 (123 to 198)	151 (119 to 186)	127 (106 to 154)	93·7 (75·1 to 115)	69·7 (57·4 to 83·8)	52·1 (40·9 to 65)	36·4 (28·5 to 44·8)	23·5 (17·5 to 28·9)	12·1 (9·2 to 14·7)	4·6 (3·3 to 5·8)	1,550
Cirrhosis and other chronic liver diseases	0 (0 to 0)	0 (0 to 0)	0 (0 to 0)	5·5 (3·2 to 8·7)	5 (3·3 to 6·9)	6·3 (4·4 to 8·6)	15·4 (10·7 to 20·8)	17·9 (12·9 to 24·3)	24·2 (16·4 to 33·9)	36·6 (25·7 to 49·3)	31·8 (22·8 to 42·5)	38·5 (28·7 to 49·9)	62·8 (45·9 to 82·7)	79·4 (58·2 to 105)	81 (59·9 to 104)	67·1 (52·9 to 84·5)	48·4 (36·7 to 60·5)	33·4 (26·3 to 41·5)	22·5 (16·9 to 29)	13·5 (9·7 to 17·9)	7·5 (5·2 to 9·8)	3·3 (2·4 to 4·3)	1·2 (0·8 to 1·6)	601
Cirrhosis and other chronic liver diseases due to hepatitis B	0 (0 to 0)	0 (0 to 0)	0 (0 to 0)	0 (0 to 0)	0 (0 to 0)	0 (0 to 0·1)	0·1 (0 to 0·2)	0·4 (0·1 to 0·7)	1 (0·3 to 2)	2·2 (1·1 to 3·8)	2·4 (1·1 to 4·5)	3·1 (1·6 to 5·6)	4·9 (2·4 to 9·4)	5·7 (2·7 to 10·6)	4·9 (2·2 to 9)	3·5 (1·6 to 6·7)	2·2 (1·1 to 3·9)	1·4 (0·7 to 2·6)	1 (0·5 to 1·7)	0·6 (0·3 to 1)	0·3 (0·2 to 0·5)	0·1 (0·1 to 0·3)	0·1 (0 to 0·1)	33·7
Cirrhosis and other chronic liver diseases due to hepatitis C	0 (0 to 0)	0 (0 to 0)	0 (0 to 0)	0 (0 to 0)	0 (0 to 0·1)	0·1 (0·1 to 0·2)	0·4 (0·2 to 0·9)	1·6 (0·8 to 3·2)	4·3 (1·9 to 7·9)	8·2 (4·4 to 13·2)	7·7 (4·1 to 13·2)	9·3 (5·2 to 15)	14 (7·7 to 23·1)	16·4 (8·5 to 27·6)	15·3 (8·2 to 27·5)	11·7 (5·8 to 20·9)	8 (4·5 to 13)	5·2 (3 to 8·3)	3·3 (1·8 to 5·6)	2 (1 to 3·4)	1·1 (0·6 to 1·9)	0·5 (0·3 to 0·9)	0·2 (0·1 to 0·4)	110
Cirrhosis and other chronic liver diseases due to alcohol use	0 (0 to 0)	0 (0 to 0)	0 (0 to 0)	0 (0 to 0)	0 (0 to 0)	0 (0 to 0)	0 (0 to 0·1)	0·8 (0·3 to 1·7)	2·5 (1 to 4·9)	7 (3·6 to 11·7)	8·6 (4·2 to 14·2)	12·1 (7·1 to 18·5)	21·1 (11·6 to 33·7)	28·3 (16 to 42·4)	30·5 (17·4 to 45)	24·9 (14·6 to 37·6)	16·5 (9·4 to 24·6)	10·2 (5·8 to 16)	5·9 (3·4 to 9·2)	3·1 (1·7 to 5·3)	1·7 (1 to 2·6)	0·7 (0·4 to 1·2)	0·2 (0·1 to 0·5)	174
Cirrhosis and other chronic liver diseases due to NAFLD	0 (0 to 0)	0 (0 to 0)	0 (0 to 0)	0 (0 to 0)	0 (0 to 0)	0 (0 to 0)	0·6 (0·2 to 1·1)	1·7 (0·8 to 3·2)	3·9 (1·8 to 7·4)	7 (3·6 to 11·7)	6·1 (2·9 to 10·9)	7·8 (3·8 to 13·4)	13·5 (6·7 to 23·9)	17·3 (8·1 to 30·7)	17·1 (8·3 to 30)	14·5 (7·1 to 25·4)	11·3 (6·3 to 17·9)	8·4 (5·1 to 12·9)	6 (3·2 to 9·7)	3·7 (2 to 5·9)	2·1 (1·2 to 3·1)	0·9 (0·5 to 1·4)	0·3 (0·2 to 0·6)	122
Cirrhosis and other chronic liver diseases due to other causes	0 (0 to 0)	0 (0 to 0)	0 (0 to 0)	5·5 (3·2 to 8·7)	4·9 (3·3 to 6·8)	6·2 (4·3 to 8·4)	14·3 (9·9 to 19·5)	13·4 (9·3 to 18·6)	12·6 (7·6 to 19·4)	12·3 (6·7 to 19·8)	6·9 (3·4 to 12)	6·4 (3 to 11·2)	9·3 (4·6 to 16·3)	11·7 (5·5 to 20·6)	13·2 (6·4 to 23·4)	12·5 (6·7 to 20·2)	10·3 (5·2 to 17·7)	8·2 (4·5 to 13·2)	6·4 (3·4 to 10·1)	4·1 (2·3 to 6·6)	2·2 (1·3 to 3·3)	1 (0·6 to 1·5)	0·4 (0·2 to 0·6)	162
Upper digestive system diseases	0 (0 to 0)	0 (0 to 0)	0 (0 to 0)	2·6 (1·4 to 4·2)	3·4 (1·6 to 6·2)	5·2 (2·9 to 9)	15 (8·1 to 25·1)	28·8 (16 to 51·3)	36·4 (19·9 to 62·5)	39·5 (22 to 67·7)	38 (20·9 to 65·2)	33·9 (19 to 56)	34·7 (20·9 to 56·2)	32 (20·1 to 50·2)	28·5 (18·1 to 43·6)	23 (15 to 34)	16·6 (11·3 to 24·9)	12·3 (8·6 to 17·3)	9 (6·5 to 12·2)	6·5 (4·6 to 9·1)	4·1 (3 to 5·6)	2·1 (1·6 to 2·8)	0·8 (0·6 to 1·1)	373
Peptic ulcer disease	0 (0 to 0)	0 (0 to 0)	0 (0 to 0)	0·4 (0·2 to 0·7)	0·3 (0·2 to 0·4)	0·6 (0·4 to 0·8)	1 (0·7 to 1·4)	1·2 (0·8 to 1·7)	1·6 (1 to 2·4)	2·8 (1·9 to 3·9)	2·3 (1·6 to 3·2)	2·6 (1·9 to 3·5)	3·9 (2·7 to 5·3)	4·7 (3·3 to 6·2)	4·9 (3·5 to 6·4)	4·8 (3·3 to 6·6)	3·8 (2·7 to 4·9)	3·4 (2·5 to 4·3)	3·1 (2·2 to 4·2)	2·6 (1·7 to 3·9)	1·7 (1·1 to 2·3)	1 (0·6 to 1·3)	0·4 (0·3 to 0·6)	47
Gastritis and duodenitis	0 (0 to 0)	0 (0 to 0)	0 (0 to 0)	2·1 (1·1 to 3·8)	3·1 (1·3 to 5·8)	3 (1·2 to 6·2)	3·9 (1·6 to 7·8)	4·4 (2·4 to 7·8)	5·2 (2·7 to 8·8)	6·3 (3·4 to 10·9)	7·3 (3·5 to 13·3)	5·7 (2·8 to 11·1)	7·8 (4·2 to 14·2)	8·2 (4·7 to 13·4)	8 (4·8 to 12·6)	6·2 (3·7 to 9·8)	4·5 (2·9 to 6·6)	3·4 (2·2 to 4·9)	2·4 (1·6 to 3·4)	1·5 (0·9 to 2·2)	1 (0·7 to 1·4)	0·5 (0·4 to 0·8)	0·2 (0·1 to 0·3)	84·8
Gastroesophageal reflux disease	0 (0 to 0)	0 (0 to 0)	0 (0 to 0)	0 (0 to 0)	0 (0 to 0·1)	1·6 (0·7 to 3·1)	10·1 (4·8 to 19)	23·1 (11·5 to 44·4)	29·6 (14·4 to 55)	30·4 (14 to 57·5)	28·4 (13·3 to 52·2)	25·6 (12·3 to 46)	23 (11·2 to 40·2)	19·1 (9·3 to 34·2)	15·7 (7·5 to 29·5)	12 (5·8 to 21·5)	8·4 (4·2 to 15·3)	5·5 (2·7 to 9·7)	3·5 (1·7 to 6·4)	2·4 (1·2 to 4·4)	1·4 (0·7 to 2·6)	0·6 (0·3 to 1·1)	0·2 (0·1 to 0·4)	241
Appendicitis	0 (0 to 0)	0 (0 to 0)	0 (0 to 0)	2·3 (1·2 to 3·9)	3 (2 to 4·5)	3·8 (2·1 to 5·8)	5·1 (3 to 7·8)	4·1 (2·3 to 6·3)	3·2 (1·8 to 5·2)	3·1 (1·8 to 4·9)	2·9 (1·8 to 4·5)	2·1 (1·3 to 3·2)	2·6 (1·6 to 4·1)	2·2 (1·2 to 3·6)	2·2 (1·2 to 3·7)	1·3 (0·8 to 2·1)	1·2 (0·7 to 1·9)	0·9 (0·6 to 1·4)	0·6 (0·3 to 1)	0·4 (0·2 to 0·6)	0·4 (0·2 to 0·6)	0·2 (0·1 to 0·3)	0·1 (0 to 0·1)	41·6
Paralytic ileus and intestinal obstruction	3·1 (2·1 to 4·3)	3·9 (2·3 to 6·5)	19·7 (9·9 to 35·6)	9·1 (5·5 to 14·4)	3·8 (2·3 to 5·8)	3·6 (2·3 to 5·1)	3·5 (2 to 5·5)	2·9 (1·8 to 4·5)	3·8 (2·2 to 5·8)	3·7 (2·1 to 6)	5 (3 to 7·7)	5·2 (3·2 to 8·1)	6·8 (4·2 to 10·4)	8·3 (5 to 12·8)	7·1 (4·5 to 10·9)	7·8 (5·1 to 11·5)	6·8 (4·3 to 10)	6·7 (4·4 to 9·7)	6·3 (4·1 to 9·4)	5·7 (3·6 to 8·7)	4·2 (2·7 to 6·4)	2·4 (1·6 to 3·6)	1 (0·6 to 1·6)	130
Inguinal, femoral, and abdominal hernia	1·8 (1·1 to 2·8)	0·6 (0·2 to 1·1)	2·3 (1·1 to 4·6)	6·6 (3·7 to 10·6)	7·8 (4 to 13·3)	5·4 (2·5 to 10)	4·5 (2·2 to 7·8)	3·6 (1·8 to 6·5)	3·7 (1·7 to 6·6)	4·5 (2·4 to 7·5)	4·4 (2·3 to 7·2)	4·7 (2·7 to 7·5)	4·9 (2·9 to 7·7)	4·8 (3 to 7·1)	4 (2·6 to 5·9)	3·3 (2·2 to 4·8)	2·1 (1·4 to 2·9)	1·6 (1·1 to 2·2)	1·2 (0·8 to 1·7)	0·9 (0·6 to 1·3)	0·7 (0·4 to 1)	0·4 (0·2 to 0·5)	0·1 (0·1 to 0·2)	73·8
Inflammatory bowel disease	0 (0 to 0)	0 (0 to 0)	0 (0 to 0)	1·8 (1 to 3)	0·5 (0·3 to 0·9)	0·3 (0·2 to 0·4)	1·1 (0·7 to 1·7)	1·6 (1·1 to 2·4)	2·8 (1·8 to 4·2)	3·2 (2·1 to 4·7)	2·5 (1·7 to 3·7)	3·3 (2·2 to 4·9)	3 (2·1 to 4·3)	2·6 (1·7 to 3·7)	2·4 (1·6 to 3·4)	2 (1·4 to 2·8)	1·1 (0·8 to 1·6)	1·1 (0·7 to 1·5)	0·8 (0·6 to 1·2)	0·5 (0·3 to 0·7)	0·3 (0·2 to 0·5)	0·2 (0·2 to 0·3)	0·1 (0 to 0·1)	31·4
Vascular intestinal disorders	0 (0 to 0)	0 (0 to 0)	0 (0 to 0)	0·1 (0·1 to 0·2)	0·2 (0·1 to 0·3)	0 (0 to 0·1)	0·1 (0·1 to 0·2)	0·3 (0·2 to 0·5)	0·4 (0·3 to 0·7)	0·4 (0·2 to 0·6)	0·5 (0·3 to 0·8)	0·7 (0·5 to 1·1)	1·3 (0·8 to 2·1)	1·5 (0·9 to 2·2)	1·5 (1 to 2·2)	1·8 (1·3 to 2·6)	1·6 (1·1 to 2·3)	1·5 (1 to 2·1)	1·3 (0·8 to 1·9)	1 (0·6 to 1·4)	0·7 (0·5 to 1)	0·3 (0·2 to 0·4)	0·1 (0·1 to 0·2)	15·4
Gallbladder and biliary diseases	0 (0 to 0)	0 (0 to 0)	0 (0 to 0)	1 (0·6 to 1·6)	1·9 (0·6 to 3·7)	3·2 (1·3 to 6·1)	6·1 (3·3 to 10·4)	9·1 (5·5 to 13·8)	12 (7·3 to 17·9)	15·4 (9·8 to 22·5)	15·4 (9·6 to 22·8)	16·5 (10·9 to 24)	18·3 (12 to 25·7)	18 (12·1 to 25·3)	16 (11·3 to 21·9)	13·2 (9·4 to 17·5)	9·5 (6·8 to 13)	6·7 (4·8 to 9·2)	5·2 (3·6 to 7)	3·6 (2·5 to 5)	2·2 (1·5 to 3)	1·2 (0·8 to 1·7)	0·5 (0·3 to 0·6)	175
Pancreatitis	0 (0 to 0)	0 (0 to 0)	0 (0 to 0)	0·3 (0·2 to 0·4)	0·2 (0·1 to 0·3)	0·3 (0·2 to 0·4)	1 (0·7 to 1·5)	2·3 (1·4 to 3·4)	2·4 (1·5 to 3·6)	2·7 (1·6 to 4·1)	1·4 (0·9 to 2·1)	1·8 (1·2 to 2·7)	2·3 (1·5 to 3·5)	2·3 (1·5 to 3·5)	2·2 (1·4 to 3·2)	1·8 (1·2 to 2·6)	1·4 (1 to 2)	1·1 (0·8 to 1·6)	0·9 (0·6 to 1·3)	0·6 (0·4 to 0·8)	0·4 (0·2 to 0·5)	0·2 (0·1 to 0·2)	0·1 (0 to 0·1)	25·6
Other digestive diseases	0 (0 to 0)	0 (0 to 0)	0 (0 to 0)	8·9 (4·9 to 14·5)	3·3 (2 to 4·9)	2·3 (1·4 to 3·4)	3·6 (1·8 to 5·5)	2·9 (1·6 to 4·5)	4·4 (2·2 to 7·3)	4·9 (2·3 to 7·8)	3·1 (1·7 to 4·8)	3·6 (2 to 5·5)	4·8 (2·6 to 7·7)	6·4 (3·2 to 10·3)	6 (3·1 to 9·7)	6 (3·3 to 9·1)	5·2 (2·9 to 7·9)	4·5 (2·5 to 6·8)	4·2 (2·2 to 6·5)	3·8 (2 to 5·8)	3·1 (1·8 to 4·6)	1·7 (1 to 2·6)	0·7 (0·4 to 1·1)	83·5
Neurological disorders	0·1 (0 to 0·1)	0·3 (0·1 to 0·5)	11·6 (6·6 to 18·9)	35·1 (17·5 to 59·3)	70·7 (29·3 to 126)	202 (57·2 to 423)	274 (90·2 to 568)	257 (87·3 to 514)	236 (82·5 to 466)	216 (80·7 to 421)	193 (71·2 to 384)	174 (64·7 to 340)	153 (66·8 to 290)	120 (58 to 218)	96 (49·8 to 162)	78 (45·2 to 126)	65·6 (40·4 to 107)	65·1 (39·9 to 115)	67·2 (41·1 to 123)	88·3 (48·4 to 176)	83·3 (41·1 to 165)	56·8 (26·7 to 119)	30·9 (13·6 to 64·7)	2,570
Alzheimer's disease and other dementias	0 (0 to 0)	0 (0 to 0)	0 (0 to 0)	0 (0 to 0)	0 (0 to 0)	0 (0 to 0)	0 (0 to 0)	0 (0 to 0)	0 (0 to 0)	0 (0 to 0)	0 (0 to 0)	0·9 (0·2 to 2·7)	4·5 (1·3 to 12·3)	7·6 (2·6 to 19·3)	12·1 (4·7 to 29·2)	18 (7·2 to 41·4)	24·6 (10 to 59·8)	34·4 (14·8 to 80·1)	41·9 (17·8 to 97·9)	68·2 (28·6 to 156)	70·7 (29·4 to 153)	51·4 (21·3 to 113)	29·2 (12 to 63·4)	364
Parkinson's disease	0 (0 to 0)	0 (0 to 0)	0 (0 to 0)	0 (0 to 0)	0 (0 to 0)	0 (0 to 0)	0 (0 to 0)	0·1 (0 to 0·1)	0·1 (0·1 to 0·2)	0·3 (0·2 to 0·4)	0·4 (0·2 to 0·6)	0·8 (0·6 to 1·1)	1·8 (1·3 to 2·4)	3·1 (2·4 to 3·9)	4·1 (3·2 to 5·4)	5·9 (4·7 to 7·3)	7·2 (5·9 to 8·8)	10·7 (8·9 to 12·6)	13·9 (11·5 to 16·3)	12·8 (10·3 to 15·3)	8·4 (6·6 to 10·1)	3·6 (2·8 to 4·4)	1·1 (0·8 to 1·4)	74·2
Idiopathic epilepsy	0·1 (0 to 0·1)	0·2 (0 to 0·4)	5·5 (2·8 to 9·7)	22·7 (10·1 to 41·5)	32·8 (12·5 to 62·6)	42·3 (15·4 to 83)	58 (28·7 to 104)	54·1 (27 to 98·3)	49·4 (26·1 to 84·4)	44·7 (24 to 71·4)	33·1 (16·8 to 55·2)	26·5 (13·8 to 45·5)	24·8 (13·3 to 40·9)	18·7 (10·1 to 32)	14 (7·2 to 24·8)	10·7 (4·9 to 19·2)	7·8 (3·3 to 14·4)	5·8 (2·5 to 10·8)	4 (1·6 to 7·2)	2·8 (1·2 to 5·2)	1·8 (0·7 to 3·3)	0·9 (0·3 to 1·6)	0·3 (0·1 to 0·6)	461
Multiple sclerosis	0 (0 to 0)	0 (0 to 0)	0 (0 to 0)	0 (0 to 0)	0 (0 to 0)	0·1 (0 to 0·1)	0·3 (0·2 to 0·6)	0·8 (0·5 to 1·2)	1·2 (0·8 to 1·8)	1·5 (1·1 to 2·2)	2·1 (1·5 to 3·3)	2·1 (1·5 to 3·2)	2·2 (1·6 to 3·2)	1·8 (1·3 to 2·5)	1·4 (1 to 2)	1 (0·7 to 1·4)	0·5 (0·3 to 0·7)	0·3 (0·2 to 0·4)	0·1 (0·1 to 0·2)	0·1 (0·1 to 0·1)	0·1 (0 to 0·1)	0 (0 to 0)	0 (0 to 0)	15·6
Motor neuron disease	0 (0 to 0)	0·1 (0·1 to 0·2)	0·5 (0·2 to 0·9)	0·6 (0·4 to 1)	0·2 (0·1 to 0·2)	0·2 (0·2 to 0·3)	0·2 (0·1 to 0·2)	0·2 (0·2 to 0·3)	0·2 (0·2 to 0·3)	0·3 (0·2 to 0·3)	0·3 (0·2 to 0·4)	0·4 (0·3 to 0·6)	0·7 (0·5 to 1)	0·9 (0·7 to 1·3)	0·9 (0·6 to 1·2)	0·7 (0·5 to 0·9)	0·5 (0·3 to 0·7)	0·3 (0·2 to 0·4)	0·2 (0·1 to 0·3)	0·1 (0 to 0·1)	0 (0 to 0)	0 (0 to 0)	0 (0 to 0)	7·5
Headache disorders	0 (0 to 0)	0 (0 to 0)	0 (0 to 0)	0 (0 to 0)	26·3 (0·4 to 68·5)	142 (6·5 to 363)	199 (20·5 to 493)	191 (25·8 to 453)	175 (25·1 to 399)	161 (27 to 366)	150 (27 to 340)	136 (29·4 to 304)	111 (26 to 254)	81·8 (21 to 177)	58 (15·7 to 125)	38·2 (11·6 to 81·1)	22·7 (7·3 to 46·7)	12·1 (4 to 25)	6·2 (2 to 12·8)	3·6 (1·2 to 7·7)	2 (0·7 to 4)	0·7 (0·2 to 1·5)	0·2 (0 to 0·4)	1,520
Migraine	0 (0 to 0)	0 (0 to 0)	0 (0 to 0)	0 (0 to 0)	24·4 (0·3 to 65)	134 (3·7 to 358)	186 (14·5 to 489)	177 (17·8 to 443)	162 (17·2 to 389)	149 (18·2 to 351)	138 (20·1 to 322)	125 (20·1 to 293)	101 (18·6 to 232)	73·4 (14·4 to 164)	51·9 (11 to 116)	33·5 (8·2 to 75·4)	19·4 (5·2 to 43·1)	10·1 (2·8 to 22)	5·2 (1·4 to 11·4)	3 (0·9 to 6·7)	1·6 (0·5 to 3·6)	0·6 (0·2 to 1·3)	0·1 (0 to 0·3)	1,400
Tension-type headache	0 (0 to 0)	0 (0 to 0)	0 (0 to 0)	0 (0 to 0)	1·9 (0·1 to 11·8)	7·6 (0·4 to 49)	12·3 (2·1 to 55·8)	13·2 (2·7 to 53·6)	13 (3 to 50·7)	12·3 (2·9 to 41·6)	12·3 (3·1 to 38·7)	11·4 (3·5 to 32·3)	10·2 (3·4 to 26·3)	8·4 (2·4 to 22·8)	6·2 (1·9 to 15·9)	4·7 (1·4 to 12·8)	3·3 (0·9 to 9·8)	2 (0·5 to 6·7)	1·1 (0·3 to 3·7)	0·6 (0·1 to 2·2)	0·3 (0·1 to 1·2)	0·1 (0 to 0·5)	0 (0 to 0·2)	121
Other neurological disorders	0 (0 to 0)	0 (0 to 0)	5·5 (3 to 9)	11·8 (6·5 to 19)	11·4 (4·8 to 21·6)	17·8 (6·7 to 34·8)	16·9 (7·3 to 31·8)	11·1 (5·1 to 20·1)	9·2 (5 to 15·2)	8 (4·7 to 12·2)	7·2 (4·2 to 11·3)	6·9 (4·2 to 10·5)	7·8 (5·3 to 11·6)	6·3 (4·3 to 8·7)	5·4 (3·9 to 7·5)	3·7 (2·7 to 4·9)	2·3 (1·8 to 3)	1·5 (1·1 to 1·9)	1 (0·7 to 1·3)	0·6 (0·5 to 0·8)	0·3 (0·2 to 0·4)	0·1 (0·1 to 0·2)	0·1 (0 to 0·1)	135
Mental disorders	0 (0 to 0·1)	0·1 (0·1 to 0·2)	1·7 (0·9 to 2·6)	10·8 (6·5 to 16·4)	75·6 (49·4 to 113)	280 (185 to 405)	464 (316 to 649)	477 (327 to 672)	444 (301 to 606)	401 (286 to 552)	371 (263 to 502)	325 (232 to 448)	280 (200 to 379)	220 (159 to 296)	174 (123 to 235)	124 (86·6 to 169)	79·3 (56·6 to 106)	49 (34·4 to 65·5)	28·7 (19·9 to 39·2)	18·8 (12·6 to 25·9)	11·2 (7·7 to 15·3)	5·2 (3·5 to 7·3)	1·8 (1·2 to 2·7)	3,840
Schizophrenia	0 (0 to 0)	0 (0 to 0)	0 (0 to 0)	0 (0 to 0)	0 (0 to 0)	1·2 (0·5 to 2·3)	9·3 (3·2 to 18·7)	28·9 (15·1 to 46·5)	45·4 (25·8 to 69·5)	50·7 (30·8 to 74·6)	49·9 (30·2 to 72·6)	42·9 (27·8 to 60·5)	35·7 (23·2 to 50)	26·4 (17·3 to 36·7)	18·8 (12·4 to 25·8)	11·9 (7·6 to 16·5)	6·7 (4·2 to 9·6)	3·6 (2·1 to 5·3)	1·8 (1 to 2·6)	0·9 (0·5 to 1·4)	0·4 (0·2 to 0·6)	0·1 (0 to 0·2)	0 (0 to 0·1)	335
Depressive disorders	0 (0 to 0)	0 (0 to 0)	0 (0 to 0)	0 (0 to 0·1)	5·6 (1·8 to 12·2)	67·6 (33·6 to 122)	170 (98·1 to 276)	186 (109 to 291)	159 (94·7 to 246)	141 (83·3 to 221)	136 (84·7 to 203)	126 (77·3 to 196)	114 (73·4 to 169)	95·6 (61·7 to 139)	79·8 (49·5 to 121)	59·7 (35·8 to 90·4)	39·4 (25·1 to 57·5)	25·1 (15·9 to 35·8)	15·1 (9·3 to 22·9)	10·4 (6·2 to 15·8)	6·7 (4·2 to 9·7)	3·3 (2 to 4·9)	1·2 (0·7 to 2)	1,440
Major depressive disorder	0 (0 to 0)	0 (0 to 0)	0 (0 to 0)	0 (0 to 0·1)	5 (1·4 to 11·3)	63 (30·3 to 117)	157 (88·1 to 262)	165 (89·6 to 269)	135 (77·2 to 214)	117 (66·3 to 193)	114 (66·7 to 173)	105 (60·2 to 166)	95·7 (59·8 to 145)	81·2 (51 to 121)	68·5 (40·2 to 105)	51·5 (29·1 to 78·7)	34·2 (21·2 to 50·3)	21·9 (13·6 to 31·9)	13·3 (7·8 to 20·9)	9·3 (5·4 to 14·3)	6 (3·7 to 9)	3 (1·8 to 4·6)	1·1 (0·6 to 1·8)	1,250
Dysthymia	0 (0 to 0)	0 (0 to 0)	0 (0 to 0)	0 (0 to 0)	0·6 (0 to 1·5)	4·6 (1·9 to 9)	13·4 (6·5 to 23·8)	20·9 (10·6 to 36·2)	23·7 (12·3 to 40·2)	23·6 (12·9 to 39·1)	22·7 (12·9 to 37·1)	20·7 (11·3 to 36)	18 (9·9 to 31·2)	14·4 (8·4 to 24·4)	11·4 (6·4 to 18·4)	8·2 (4·3 to 13·8)	5·3 (2·8 to 8·9)	3·2 (1·8 to 5·3)	1·8 (1·1 to 3)	1·1 (0·6 to 1·9)	0·7 (0·4 to 1·1)	0·3 (0·2 to 0·5)	0·1 (0·1 to 0·2)	195
Bipolar disorder	0 (0 to 0)	0 (0 to 0)	0 (0 to 0)	0 (0 to 0)	0 (0 to 0)	17·2 (7·2 to 31·5)	62·8 (28·5 to 109)	69 (35·8 to 117)	60·7 (31·5 to 101)	51·3 (27·8 to 81·8)	44·8 (24·1 to 72)	38·8 (21·7 to 63)	32·9 (18·3 to 54·5)	25 (13·4 to 41·5)	18·6 (10·2 to 30·9)	12·3 (7 to 20·4)	7·1 (3·8 to 11·6)	3·9 (2·1 to 6·5)	1·9 (1 to 3·2)	1 (0·5 to 1·7)	0·4 (0·2 to 0·8)	0·2 (0·1 to 0·3)	0 (0 to 0·1)	448
Anxiety disorders	0 (0 to 0)	0 (0 to 0)	0 (0 to 0)	2·1 (0·6 to 4·4)	34·1 (17 to 57·5)	97·5 (52·6 to 159)	133 (79·6 to 207)	129 (74 to 216)	119 (66·3 to 196)	107 (63·5 to 171)	97·2 (57·3 to 152)	84·3 (48·2 to 135)	71·6 (39·9 to 115)	55·2 (32·7 to 84·6)	42·2 (26·1 to 64·2)	29·5 (17·7 to 45·4)	19·2 (11·3 to 30·5)	12 (7·2 to 19·1)	7·1 (4·3 to 11·2)	4·5 (2·7 to 7)	2·5 (1·4 to 3·9)	1 (0·6 to 1·6)	0·3 (0·2 to 0·5)	1,050
Eating disorders	0 (0 to 0)	0 (0 to 0)	0 (0 to 0)	0 (0 to 0)	0·4 (0·1 to 0·8)	6·4 (3 to 11·3)	24·2 (12·4 to 42·3)	34·6 (17·9 to 59·2)	28·6 (15·5 to 47·4)	20 (11 to 32·6)	13·3 (7·1 to 22·2)	7·2 (3·5 to 12·2)	3·5 (1·8 to 6·1)	0 (0 to 0)	0 (0 to 0)	0 (0 to 0)	0 (0 to 0)	0 (0 to 0)	0 (0 to 0)	0 (0 to 0)	0 (0 to 0)	0 (0 to 0)	0 (0 to 0)	138
Anorexia nervosa	0 (0 to 0)	0 (0 to 0)	0 (0 to 0)	0 (0 to 0)	0·4 (0·1 to 0·8)	3·5 (1·4 to 6·5)	9·7 (4·3 to 17·4)	8·8 (4·1 to 15·8)	6 (2·9 to 11·2)	3·4 (1·4 to 6·3)	2·3 (0·9 to 4·4)	1·5 (0·5 to 2·9)	1 (0·3 to 1·9)	0 (0 to 0)	0 (0 to 0)	0 (0 to 0)	0 (0 to 0)	0 (0 to 0)	0 (0 to 0)	0 (0 to 0)	0 (0 to 0)	0 (0 to 0)	0 (0 to 0)	36·5
Bulimia nervosa	0 (0 to 0)	0 (0 to 0)	0 (0 to 0)	0 (0 to 0)	0 (0 to 0·1)	3 (0·7 to 6·3)	14·5 (6·3 to 30)	25·8 (11·3 to 48·2)	22·6 (11 to 39·5)	16·6 (8·4 to 28)	11 (5·4 to 19·1)	5·6 (2·5 to 10·3)	2·5 (1 to 4·7)	0 (0 to 0)	0 (0 to 0)	0 (0 to 0)	0 (0 to 0)	0 (0 to 0)	0 (0 to 0)	0 (0 to 0)	0 (0 to 0)	0 (0 to 0)	0 (0 to 0)	102
Autism spectrum disorders	0 (0 to 0)	0·1 (0 to 0·1)	1·1 (0·6 to 1·8)	4·8 (2·8 to 7·7)	6·3 (3·6 to 9·9)	6·7 (3·7 to 10·6)	6·5 (3·4 to 10·4)	5·6 (3·1 to 9·1)	4·8 (2·8 to 7·5)	4 (2·3 to 6·3)	3·5 (1·9 to 5·5)	2·9 (1·6 to 4·5)	2·4 (1·4 to 3·8)	1·9 (1 to 3)	1·4 (0·8 to 2·2)	1 (0·5 to 1·7)	0·7 (0·4 to 1·1)	0·4 (0·2 to 0·7)	0·3 (0·1 to 0·4)	0·2 (0·1 to 0·3)	0·1 (0·1 to 0·2)	0 (0 to 0·1)	0 (0 to 0)	54·7
Attention-deficit/hyperactivity disorder	0 (0 to 0)	0 (0 to 0)	0 (0 to 0)	0·6 (0·3 to 1·2)	7·4 (3·9 to 13·9)	11·1 (5·8 to 19·7)	9·1 (4·7 to 16·6)	5·9 (3·1 to 10·6)	3·9 (2 to 6·9)	2·8 (1·4 to 5)	2 (1 to 3·6)	1·4 (0·7 to 2·6)	1 (0·4 to 1·8)	0·6 (0·3 to 1·2)	0·4 (0·2 to 0·7)	0·2 (0·1 to 0·4)	0·1 (0 to 0·1)	0 (0 to 0·1)	0 (0 to 0·1)	0 (0 to 0)	0 (0 to 0)	0 (0 to 0)	0 (0 to 0)	46·6
Conduct disorder	0 (0 to 0)	0 (0 to 0)	0 (0 to 0)	0 (0 to 0)	16·8 (7·8 to 31·3)	66·1 (32·9 to 115)	37·6 (18·1 to 65·2)	0 (0 to 0)	0 (0 to 0)	0 (0 to 0)	0 (0 to 0)	0 (0 to 0)	0 (0 to 0)	0 (0 to 0)	0 (0 to 0)	0 (0 to 0)	0 (0 to 0)	0 (0 to 0)	0 (0 to 0)	0 (0 to 0)	0 (0 to 0)	0 (0 to 0)	0 (0 to 0)	120
Idiopathic developmental intellectual disability	0 (0 to 0)	0 (0 to 0·1)	0·6 (0·1 to 1·2)	3·2 (1 to 6)	5·1 (2 to 9·2)	5·4 (2 to 9·7)	5·3 (2 to 9·8)	4·6 (1·7 to 8·5)	3·8 (1·4 to 7·2)	3·2 (1·3 to 6)	2·8 (1 to 5·1)	2·2 (0·9 to 4)	1·8 (0·7 to 3·3)	1·4 (0·6 to 2·6)	1 (0·4 to 1·9)	0·7 (0·2 to 1·3)	0·4 (0·2 to 0·8)	0·2 (0·1 to 0·4)	0·1 (0·1 to 0·2)	0·1 (0 to 0·2)	0·1 (0 to 0·1)	0 (0 to 0)	0 (0 to 0)	42·2
Other mental disorders	0 (0 to 0)	0 (0 to 0)	0 (0 to 0)	0 (0 to 0)	0 (0 to 0)	0·9 (0·4 to 1·6)	6·2 (2·4 to 11·5)	13·6 (6·4 to 23·6)	18·9 (10 to 30·7)	21·1 (12·2 to 33·6)	21·7 (12·5 to 33·4)	19·6 (11·3 to 30·6)	17·2 (10·4 to 27·2)	14 (8·3 to 21·5)	11·3 (6·9 to 17·5)	8·3 (5·2 to 12·7)	5·7 (3·6 to 8·6)	3·8 (2·4 to 5·7)	2·5 (1·6 to 3·6)	1·8 (1·1 to 2·6)	1·1 (0·7 to 1·6)	0·5 (0·4 to 0·8)	0·2 (0·1 to 0·3)	168
Substance use disorders	0 (0 to 0)	0 (0 to 0)	0 (0 to 0)	0 (0 to 0)	0 (0 to 0)	2·4 (1·3 to 4)	37·8 (23·6 to 55·5)	69·7 (43·9 to 100)	66·1 (43·8 to 93·5)	53·6 (34 to 79·1)	41·9 (28·1 to 60·2)	31·3 (21 to 44·8)	24·9 (17·5 to 33·7)	17 (11·7 to 23·7)	12 (8·5 to 16·5)	7·6 (5·2 to 10·9)	4·4 (2·9 to 6·1)	2·6 (1·8 to 3·7)	1·6 (1·1 to 2·3)	1·2 (0·8 to 1·7)	0·9 (0·6 to 1·3)	0·5 (0·3 to 0·8)	0·3 (0·2 to 0·5)	376
Alcohol use disorders	0 (0 to 0)	0 (0 to 0)	0 (0 to 0)	0 (0 to 0)	0 (0 to 0)	1·3 (0·5 to 2·6)	14·2 (7·2 to 24·7)	32 (16·2 to 55)	32·7 (18·4 to 51·5)	29·9 (16·4 to 47·9)	25·5 (15·7 to 38·9)	20·1 (12·3 to 30·9)	16·3 (10·8 to 22·9)	10·9 (7·2 to 16·2)	7·6 (5 to 11)	4·9 (3·1 to 7·1)	2·7 (1·7 to 4·1)	1·6 (1·1 to 2·5)	1·1 (0·7 to 1·6)	0·8 (0·5 to 1·3)	0·7 (0·4 to 1)	0·5 (0·3 to 0·7)	0·2 (0·1 to 0·4)	203
Drug use disorders	0 (0 to 0)	0 (0 to 0)	0 (0 to 0)	0 (0 to 0)	0 (0 to 0)	1·1 (0·6 to 1·9)	23·6 (14 to 36·4)	37·7 (23·2 to 56·5)	33·4 (20·9 to 49·6)	23·7 (14·2 to 36·7)	16·4 (10 to 25·6)	11·2 (6·6 to 17·1)	8·6 (5·2 to 12·8)	6·1 (3·5 to 9·3)	4·4 (2·5 to 6·9)	2·7 (1·5 to 4·3)	1·7 (0·9 to 2·8)	1 (0·5 to 1·6)	0·6 (0·3 to 0·9)	0·4 (0·2 to 0·6)	0·2 (0·1 to 0·3)	0·1 (0·1 to 0·2)	0 (0 to 0·1)	173
Opioid use disorders	0 (0 to 0)	0 (0 to 0)	0 (0 to 0)	0 (0 to 0)	0 (0 to 0)	0 (0 to 0)	4·4 (1·1 to 9·3)	16·6 (7·6 to 30·8)	18·2 (9·1 to 30·9)	14·3 (6·4 to 24·4)	10·3 (4·9 to 18·5)	7 (3·3 to 12·4)	5·4 (2·4 to 9·3)	4·2 (1·8 to 7·3)	3·1 (1·4 to 5·5)	2 (0·9 to 3·5)	1·2 (0·5 to 2·3)	0·7 (0·3 to 1·3)	0·4 (0·1 to 0·7)	0·2 (0·1 to 0·4)	0·1 (0 to 0·3)	0·1 (0 to 0·1)	0 (0 to 0·1)	88·3
Cocaine use disorders	0 (0 to 0)	0 (0 to 0)	0 (0 to 0)	0 (0 to 0)	0 (0 to 0)	0·1 (0·1 to 0·2)	5·3 (2 to 10·4)	5·4 (2·3 to 9·6)	4 (2 to 6·9)	3 (1·6 to 5·2)	2 (1 to 3·7)	1·4 (0·7 to 2·6)	1·1 (0·8 to 1·6)	0·6 (0·4 to 0·9)	0·5 (0·3 to 0·7)	0·3 (0·2 to 0·4)	0·2 (0·1 to 0·3)	0·1 (0·1 to 0·1)	0·1 (0 to 0·1)	0 (0 to 0·1)	0 (0 to 0)	0 (0 to 0)	0 (0 to 0)	24·1
Amphetamine use disorders	0 (0 to 0)	0 (0 to 0)	0 (0 to 0)	0 (0 to 0)	0 (0 to 0)	0 (0 to 0)	0·9 (0·5 to 1·6)	3·4 (1 to 7)	3 (1 to 6·3)	1·4 (0·2 to 3·3)	0·7 (0·4 to 1·2)	0·4 (0·2 to 0·6)	0·2 (0·1 to 0·4)	0·1 (0·1 to 0·2)	0·1 (0 to 0·1)	0 (0 to 0·1)	0 (0 to 0·1)	0 (0 to 0)	0 (0 to 0)	0 (0 to 0)	0 (0 to 0)	0 (0 to 0)	0 (0 to 0)	10·3
Cannabis use disorders	0 (0 to 0)	0 (0 to 0)	0 (0 to 0)	0 (0 to 0)	0 (0 to 0)	0·9 (0·4 to 1·6)	8·9 (4·5 to 16·2)	6·6 (3 to 12·2)	3·5 (1·4 to 6·9)	2 (0·7 to 3·9)	1·3 (0·5 to 2·5)	0·9 (0·3 to 1·8)	0·7 (0·2 to 1·4)	0·4 (0·2 to 0·7)	0·2 (0·1 to 0·4)	0·1 (0 to 0·2)	0 (0 to 0·1)	0 (0 to 0)	0 (0 to 0)	0 (0 to 0)	0 (0 to 0)	0 (0 to 0)	0 (0 to 0)	25·6
Other drug use disorders	0 (0 to 0)	0 (0 to 0)	0 (0 to 0)	0 (0 to 0)	0 (0 to 0)	0·1 (0 to 0·2)	4·2 (1·8 to 7·7)	5·8 (2·9 to 9·7)	4·7 (2·5 to 8·1)	3·1 (1·6 to 5·1)	2 (1·1 to 3·4)	1·5 (0·8 to 2·5)	1·2 (0·8 to 1·8)	0·7 (0·4 to 1·2)	0·4 (0·3 to 0·7)	0·3 (0·2 to 0·4)	0·2 (0·1 to 0·3)	0·2 (0·1 to 0·2)	0·1 (0·1 to 0·2)	0·1 (0·1 to 0·1)	0 (0 to 0·1)	0 (0 to 0)	0 (0 to 0)	24·6
Diabetes and kidney diseases	0 (0 to 0)	0·1 (0 to 0·1)	10·9 (6·1 to 18·3)	17·5 (12·5 to 23·3)	26·5 (19·6 to 35·2)	32·4 (24·2 to 42·5)	110 (87·5 to 135)	135 (108 to 169)	189 (150 to 234)	230 (183 to 282)	227 (183 to 279)	272 (218 to 330)	412 (336 to 500)	588 (488 to 703)	623 (514 to 739)	576 (484 to 675)	483 (408 to 567)	381 (326 to 443)	280 (236 to 326)	181 (151 to 213)	102 (82·9 to 121)	48·4 (38·3 to 58·2)	18·4 (14·1 to 22·5)	4,940
Diabetes mellitus	0 (0 to 0)	0·1 (0 to 0·1)	2·8 (1·2 to 5·8)	2·6 (1·5 to 4·1)	3·6 (2·3 to 5·4)	5·7 (3·7 to 8·1)	37·8 (28·9 to 48·2)	52·6 (39·7 to 67·4)	73·5 (55 to 96·7)	95·9 (72·9 to 126)	110 (82·9 to 143)	149 (114 to 193)	256 (203 to 321)	388 (309 to 479)	420 (333 to 519)	390 (312 to 474)	348 (285 to 422)	279 (229 to 334)	204 (167 to 249)	128 (104 to 156)	69·2 (54·7 to 86·3)	30·9 (23·6 to 38·6)	11·2 (8·3 to 13·9)	3,060
Diabetes mellitus type 1	0 (0 to 0)	0·1 (0 to 0·1)	2·8 (1·2 to 5·8)	2·6 (1·5 to 4·1)	3·6 (2·3 to 5·4)	5·7 (3·7 to 8·1)	11 (7 to 16·4)	14·1 (9·1 to 21·6)	18 (11·5 to 27·3)	16·2 (10·2 to 26·2)	16·3 (10·2 to 26)	19·9 (12·8 to 33)	8·2 (5·3 to 13·8)	10·5 (6·8 to 18·5)	11·1 (6·8 to 19)	8·2 (5·1 to 14·6)	7 (4·5 to 12·7)	4·5 (2·8 to 8·6)	3·5 (2·1 to 6·9)	1·5 (0·9 to 2·8)	0·9 (0·5 to 1·6)	0·5 (0·3 to 0·7)	0·2 (0·1 to 0·3)	166
Diabetes mellitus type 2	0 (0 to 0)	0 (0 to 0)	0 (0 to 0)	0 (0 to 0)	0 (0 to 0)	0 (0 to 0)	26·8 (19·2 to 35)	38·5 (27·2 to 51)	55·5 (39·7 to 75·3)	79·8 (58·5 to 106)	93·5 (68·2 to 123)	129 (97·4 to 170)	248 (195 to 313)	378 (300 to 468)	408 (322 to 503)	381 (304 to 463)	341 (279 to 413)	275 (224 to 330)	201 (163 to 244)	127 (103 to 154)	68·3 (54·1 to 84·9)	30·5 (23·2 to 38·1)	11 (8·2 to 13·7)	2,890
Chronic kidney disease	0 (0 to 0)	0 (0 to 0)	8 (4·5 to 13·5)	14·9 (10·3 to 20·3)	21 (14·7 to 29·8)	25·7 (17·7 to 35·5)	70·8 (52·3 to 92·2)	81·3 (59·7 to 107)	114 (84 to 148)	132 (95 to 175)	116 (87·2 to 156)	123 (92·5 to 158)	156 (119 to 200)	200 (154 to 255)	203 (154 to 259)	186 (144 to 233)	135 (103 to 169)	102 (80·5 to 127)	76 (58·3 to 96·6)	52·3 (41·4 to 65·6)	32·3 (25·1 to 39·9)	17·3 (12·7 to 21·4)	7·2 (5·2 to 9·3)	1,870
Chronic kidney disease due to diabetes mellitus type 1	0 (0 to 0)	0 (0 to 0)	0·1 (0 to 0·2)	0·2 (0·1 to 0·5)	0·5 (0·2 to 1·1)	0·8 (0·3 to 1·6)	3 (1·2 to 5·8)	4·7 (1·9 to 9·6)	12·9 (5·7 to 25)	21·9 (10·6 to 37·4)	19·9 (10·5 to 33)	21·2 (10·9 to 35·3)	23·3 (10·9 to 40·4)	23 (9·3 to 42·7)	16 (5 to 33·6)	11 (3·8 to 24·9)	6·1 (2·2 to 13·6)	3·5 (1·4 to 7·1)	1·7 (0·6 to 3·7)	0·6 (0·2 to 1·3)	0·1 (0 to 0·3)	0 (0 to 0)	0 (0 to 0)	170
Chronic kidney disease due to diabetes mellitus type 2	0 (0 to 0)	0 (0 to 0)	0 (0 to 0)	0 (0 to 0)	0 (0 to 0)	0 (0 to 0)	1·2 (0·4 to 2·9)	2·2 (0·9 to 4·5)	5 (2 to 10·6)	8·6 (3·7 to 16·7)	10 (4·8 to 18·3)	15·4 (7·7 to 26·3)	28·1 (15·3 to 44·5)	48·6 (28·8 to 72·7)	59·1 (35·4 to 86·4)	58·8 (35·1 to 83·8)	43·8 (28·6 to 62)	33·2 (22·5 to 46·1)	24·5 (15·7 to 33·9)	16·3 (11 to 23·1)	9·5 (6·3 to 13·2)	4·9 (2·9 to 7)	2 (1·1 to 2·9)	371
Chronic kidney disease due to hypertension	0 (0 to 0)	0 (0 to 0)	0 (0 to 0)	0 (0 to 0)	0 (0 to 0)	0 (0 to 0)	4·2 (1·7 to 8)	6 (2·6 to 11·4)	9 (4·4 to 15·7)	11·5 (6 to 18·9)	11·8 (6·6 to 18·8)	14·1 (8·7 to 21·6)	20·1 (12·8 to 30·8)	29·9 (18·4 to 45·9)	36·3 (23 to 54·6)	39·5 (25 to 57·9)	32·4 (20·9 to 46·3)	26·8 (18·2 to 37·8)	22·2 (14·9 to 31·4)	16·6 (11·9 to 22·9)	11·2 (7·9 to 14·8)	6·6 (4·4 to 8·9)	2·9 (1·9 to 4·1)	301
Chronic kidney disease due to glomerulonephritis	0 (0 to 0)	0 (0 to 0)	2·2 (1 to 4·6)	4·5 (2·6 to 7·4)	7·2 (4·2 to 11·2)	9·8 (5·9 to 14·6)	27·5 (16·9 to 40·7)	30·3 (18 to 45·1)	40·5 (26·1 to 60·9)	42·5 (24·9 to 66·6)	34·4 (22·1 to 50·5)	34·7 (20·2 to 52·7)	39·2 (23 to 59·9)	45·3 (24·2 to 73·5)	41·5 (21·8 to 73)	33·2 (16·7 to 55·1)	20·1 (9·8 to 36·2)	12·9 (6·5 to 21·8)	8·4 (4 to 14·7)	4·9 (2·2 to 9·2)	2·4 (1 to 4·2)	1·1 (0·5 to 2)	0·4 (0·2 to 0·9)	443
Chronic kidney disease due to other and unspecified causes	0 (0 to 0)	0 (0 to 0)	5·8 (3·1 to 9·7)	10·1 (6·5 to 14·4)	13·3 (8·7 to 19·9)	15·1 (9·1 to 23·5)	34·8 (22·3 to 48·8)	38·2 (24·5 to 54)	46·3 (30·1 to 66)	47·4 (30·6 to 70·1)	40·1 (25·1 to 59·1)	37·4 (23·2 to 55·5)	45·1 (28·4 to 66·3)	52·8 (32·2 to 81·4)	50 (31 to 79·3)	43·9 (26·8 to 68·4)	32·5 (19·3 to 48·9)	25·5 (15·8 to 37·1)	19·2 (11·8 to 28·7)	13·9 (8·7 to 21)	9·1 (6 to 12·6)	4·8 (3·2 to 6·7)	1·9 (1·2 to 2·8)	587
Acute glomerulonephritis	0 (0 to 0)	0 (0 to 0)	0 (0 to 0·1)	0 (0 to 0·1)	1·8 (0·8 to 3·6)	0·9 (0·4 to 2)	1·5 (0·6 to 3)	1·4 (0·5 to 3·1)	1·3 (0·5 to 3·1)	1·7 (0·7 to 3·5)	0·8 (0·4 to 1·7)	0·6 (0·2 to 1·2)	0·5 (0·2 to 1·1)	0·2 (0·1 to 0·4)	0·6 (0·2 to 1·4)	0·3 (0·1 to 0·7)	0·1 (0 to 0·2)	0·3 (0·1 to 0·6)	0·1 (0 to 0·2)	0·1 (0·1 to 0·3)	0·1 (0 to 0·2)	0·1 (0 to 0·2)	0 (0 to 0)	12·6
Skin and subcutaneous diseases	0 (0 to 0)	0·1 (0 to 0·1)	10·2 (6·4 to 15·2)	104 (65 to 155)	138 (84·9 to 206)	148 (94·4 to 221)	157 (99·2 to 236)	94·2 (58·9 to 140)	72·3 (46·2 to 107)	62·3 (39·3 to 91·5)	56·4 (36·4 to 82·6)	51·5 (34·1 to 74·8)	46·3 (30·7 to 68·1)	42·6 (29 to 63)	38·7 (26·5 to 55·3)	32·2 (22·3 to 46·7)	24·9 (17 to 37·6)	20·2 (13·3 to 30·1)	16·2 (11·4 to 23·9)	13·2 (9 to 20·2)	9·9 (6·8 to 15·8)	5·5 (3·8 to 8·9)	2·4 (1·6 to 4·3)	1,150
Dermatitis	0 (0 to 0)	0 (0 to 0)	0 (0 to 0)	38·2 (21·2 to 65)	48·5 (25·8 to 84·3)	36·7 (19·8 to 61·6)	27·5 (15·1 to 46)	20·1 (11·2 to 31·9)	16 (9·1 to 26·5)	13·6 (7·5 to 22·3)	11·8 (6·5 to 19·2)	10 (5·7 to 16·4)	8·8 (4·9 to 14·2)	7·7 (4·2 to 12·5)	6·8 (3·7 to 11·2)	5·4 (3 to 8·7)	4·1 (2·2 to 6·5)	2·8 (1·6 to 4·5)	1·8 (1 to 2·9)	1·2 (0·7 to 2)	0·7 (0·4 to 1·1)	0·3 (0·2 to 0·5)	0·1 (0·1 to 0·2)	262
Atopic dermatitis	0 (0 to 0)	0 (0 to 0)	0 (0 to 0)	37·9 (20·9 to 64·6)	47·6 (25·1 to 83·2)	33·3 (17·3 to 56·9)	21·6 (11·4 to 38·1)	13·7 (7·1 to 23·3)	9·6 (4·7 to 17·1)	7·3 (3·6 to 13·6)	6·1 (3·1 to 11·1)	5 (2·5 to 8·8)	4·3 (2·2 to 7·6)	3·6 (1·8 to 6·1)	3·1 (1·5 to 5·4)	2·4 (1·2 to 4·1)	1·7 (0·9 to 2·9)	1·1 (0·6 to 2)	0·8 (0·4 to 1·3)	0·5 (0·3 to 0·9)	0·3 (0·2 to 0·6)	0·2 (0·1 to 0·3)	0·1 (0 to 0·1)	200
Contact dermatitis	0 (0 to 0)	0 (0 to 0)	0 (0 to 0)	0 (0 to 0)	0·6 (0·2 to 1·2)	2·9 (1·1 to 5·8)	5·2 (2·1 to 10·2)	5·7 (2·5 to 10·7)	5·8 (2·6 to 11)	5·7 (2·3 to 11·1)	5·2 (2·2 to 10·2)	4·5 (2 to 9·3)	4·1 (1·7 to 8·1)	3·8 (1·7 to 7·2)	3·5 (1·5 to 6·8)	2·8 (1·2 to 5·3)	2·2 (1 to 4·3)	1·5 (0·7 to 2·9)	0·9 (0·4 to 1·8)	0·6 (0·2 to 1·1)	0·3 (0·1 to 0·6)	0·1 (0·1 to 0·2)	0 (0 to 0·1)	55·5
Seborrhoeic dermatitis	0 (0 to 0)	0 (0 to 0)	0 (0 to 0)	0·3 (0·1 to 0·6)	0·4 (0·1 to 0·7)	0·5 (0·2 to 1)	0·6 (0·3 to 1·2)	0·6 (0·3 to 1·1)	0·6 (0·3 to 1·2)	0·6 (0·3 to 1·1)	0·6 (0·3 to 1)	0·5 (0·2 to 0·8)	0·4 (0·2 to 0·7)	0·3 (0·1 to 0·6)	0·3 (0·1 to 0·5)	0·2 (0·1 to 0·4)	0·2 (0·1 to 0·4)	0·2 (0·1 to 0·3)	0·1 (0·1 to 0·2)	0·1 (0 to 0·1)	0·1 (0 to 0·1)	0 (0 to 0)	0 (0 to 0)	6·5
Psoriasis	0 (0 to 0)	0 (0 to 0)	0 (0 to 0)	1·5 (0·9 to 2·2)	4 (1·6 to 7·1)	5·7 (2·5 to 9·8)	6·3 (3 to 10·7)	6 (2·9 to 9·9)	6 (3·1 to 9·7)	6 (3·3 to 9·3)	6·1 (3·3 to 9·5)	5·8 (3·4 to 8·9)	5·5 (3·2 to 8·3)	5 (2·9 to 7·5)	4·3 (2·6 to 6·4)	3·5 (2·1 to 5·2)	2·4 (1·5 to 3·5)	1·5 (0·9 to 2·2)	0·8 (0·5 to 1·2)	0·4 (0·3 to 0·7)	0·2 (0·1 to 0·3)	0·1 (0 to 0·1)	0 (0 to 0)	71·1
Bacterial skin diseases	0 (0 to 0)	0 (0 to 0)	3·6 (1·5 to 6·6)	2·8 (1·6 to 4·2)	2·8 (1·6 to 4·3)	1·9 (1·1 to 3)	1·6 (0·9 to 2·5)	1·7 (1 to 2·6)	2 (1·2 to 3·3)	2·6 (1·4 to 4·6)	2·3 (1·3 to 4)	3·2 (1·7 to 5·6)	3 (1·7 to 5·1)	3·7 (1·9 to 6·2)	3·7 (1·8 to 6·2)	3·7 (2 to 6·3)	2·7 (1·4 to 4·9)	2·2 (1 to 4·1)	2 (0·9 to 3·9)	1·8 (0·8 to 3·5)	1·4 (0·6 to 3·1)	0·8 (0·3 to 1·7)	0·4 (0·1 to 0·9)	49·7
Cellulitis	0 (0 to 0)	0 (0 to 0)	0·7 (0·2 to 1·9)	0·8 (0·3 to 1·4)	1·1 (0·5 to 1·8)	0·5 (0·3 to 0·7)	0·2 (0·1 to 0·4)	0·3 (0·1 to 0·5)	0·6 (0·3 to 1·1)	0·4 (0·2 to 0·8)	0·4 (0·2 to 0·9)	0·5 (0·2 to 1·2)	0·5 (0·2 to 1)	0·7 (0·3 to 1·4)	0·8 (0·3 to 1·8)	0·7 (0·3 to 1·4)	0·6 (0·2 to 1·3)	0·4 (0·1 to 0·9)	0·3 (0·1 to 0·8)	0·2 (0·1 to 0·6)	0·2 (0·1 to 0·4)	0·1 (0 to 0·2)	0 (0 to 0·1)	9·8
Pyoderma	0 (0 to 0)	0 (0 to 0)	2·8 (1·2 to 5·5)	2 (1·1 to 3·2)	1·7 (0·9 to 2·9)	1·4 (0·8 to 2·4)	1·3 (0·7 to 2·2)	1·4 (0·8 to 2·3)	1·5 (0·8 to 2·4)	2·2 (1·1 to 3·9)	1·9 (1 to 3·2)	2·6 (1·4 to 4·5)	2·5 (1·3 to 4·3)	3 (1·5 to 4·9)	2·9 (1·3 to 4·8)	3·1 (1·5 to 4·9)	2·1 (1 to 3·8)	1·8 (0·8 to 3·4)	1·7 (0·7 to 3·2)	1·6 (0·7 to 3)	1·2 (0·5 to 2·7)	0·7 (0·3 to 1·6)	0·3 (0·1 to 0·7)	39·9
Scabies	0 (0 to 0)	0 (0 to 0)	2·4 (1·2 to 4·3)	16·6 (8·4 to 29·1)	23·1 (11·1 to 40·9)	25·1 (12·4 to 44)	26·4 (13·7 to 45·5)	21·2 (10·6 to 37·4)	13·9 (7 to 24·3)	9·4 (4·7 to 17·3)	8·2 (4·1 to 14·7)	8 (4·2 to 14)	7·1 (3·7 to 12·2)	5·5 (2·8 to 9·8)	4·2 (2·1 to 7·5)	2·9 (1·5 to 5·2)	2 (1 to 3·5)	1·4 (0·7 to 2·4)	1 (0·5 to 1·7)	0·8 (0·4 to 1·4)	0·5 (0·3 to 0·9)	0·3 (0·1 to 0·5)	0·1 (0 to 0·2)	180
Fungal skin diseases	0 (0 to 0)	0 (0 to 0)	0·4 (0·1 to 0·9)	3 (1·1 to 6·6)	5·1 (1·9 to 11)	6·3 (2·3 to 13·9)	6·6 (2·2 to 15)	6·2 (2·3 to 13·3)	5·8 (2·2 to 12·9)	5·4 (2·1 to 12)	5 (1·9 to 11·1)	4·5 (1·8 to 9·6)	4 (1·6 to 8·9)	3·7 (1·5 to 8)	3·7 (1·5 to 8·2)	3·3 (1·2 to 7·3)	3·1 (1·2 to 6·8)	3 (1·2 to 6·4)	2·5 (0·9 to 5·2)	2·2 (0·8 to 4·7)	1·7 (0·7 to 3·7)	0·9 (0·4 to 2)	0·4 (0·2 to 0·8)	76·9
Viral skin diseases	0 (0 to 0)	0 (0 to 0)	0 (0 to 0)	17·7 (11·3 to 27·4)	29·1 (17·6 to 44·4)	16 (10 to 24)	9·7 (5·9 to 15·1)	7 (4·1 to 10·9)	4·7 (2·7 to 7·4)	3·4 (2 to 5·3)	2·9 (1·7 to 4·6)	2·4 (1·4 to 3·9)	2 (1·1 to 3·3)	1·6 (0·9 to 2·5)	1·3 (0·7 to 2·1)	1 (0·6 to 1·6)	0·7 (0·4 to 1·1)	0·4 (0·2 to 0·7)	0·2 (0·1 to 0·4)	0·1 (0·1 to 0·2)	0·1 (0 to 0·1)	0 (0 to 0)	0 (0 to 0)	100
Acne vulgaris	0 (0 to 0)	0 (0 to 0)	0 (0 to 0)	0 (0 to 0)	1·6 (0·8 to 2·6)	36·4 (20·5 to 60·3)	58 (32·2 to 95·9)	13·1 (6·9 to 21·6)	5·7 (2·9 to 9·8)	3·6 (1·9 to 6·5)	2·5 (1·3 to 4·3)	1·6 (0·9 to 2·5)	1 (0·5 to 1·7)	0·6 (0·3 to 0·9)	0·3 (0·2 to 0·6)	0·2 (0·1 to 0·3)	0·1 (0 to 0·2)	0·1 (0 to 0·1)	0 (0 to 0·1)	0 (0 to 0)	0 (0 to 0)	0 (0 to 0)	0 (0 to 0)	125
Alopecia areata	0 (0 to 0)	0 (0 to 0)	0 (0 to 0)	0·5 (0·2 to 0·9)	0·7 (0·3 to 1·2)	0·9 (0·4 to 1·6)	1·6 (0·9 to 2·8)	2·3 (1·3 to 3·7)	2·8 (1·6 to 4·6)	2·7 (1·6 to 4·2)	2·2 (1·3 to 3·7)	1·6 (0·9 to 2·7)	1·2 (0·7 to 1·9)	0·9 (0·5 to 1·4)	0·7 (0·4 to 1·1)	0·5 (0·3 to 0·8)	0·3 (0·2 to 0·5)	0·2 (0·1 to 0·3)	0·1 (0·1 to 0·2)	0·1 (0 to 0·1)	0 (0 to 0)	0 (0 to 0)	0 (0 to 0)	19·4
Pruritus	0 (0 to 0)	0 (0 to 0)	0·1 (0 to 0·1)	0·7 (0·3 to 1·4)	1·1 (0·5 to 2·1)	1·2 (0·5 to 2·4)	1·4 (0·6 to 2·8)	1·5 (0·6 to 2·9)	1·5 (0·6 to 3)	1·5 (0·6 to 3·1)	1·4 (0·6 to 2·9)	1·3 (0·5 to 2·5)	1·1 (0·5 to 2·3)	1 (0·4 to 2·1)	1 (0·4 to 1·9)	0·9 (0·4 to 1·7)	0·7 (0·3 to 1·4)	0·6 (0·2 to 1·1)	0·4 (0·2 to 0·7)	0·2 (0·1 to 0·5)	0·1 (0·1 to 0·3)	0·1 (0 to 0·1)	0 (0 to 0)	17·7
Urticaria	0 (0 to 0)	0 (0 to 0)	2·9 (1·7 to 4·5)	18·8 (10·9 to 29·6)	17·4 (9·7 to 29·1)	12·7 (6·8 to 21·7)	11·1 (5·7 to 19·1)	9·3 (5·1 to 15·5)	7·7 (3·9 to 12·9)	7 (3·8 to 11·5)	6·5 (3·6 to 10·5)	5·4 (3 to 8·6)	4·3 (2·4 to 7)	3·4 (1·8 to 5·4)	2·7 (1·5 to 4·4)	2 (1 to 3·4)	1·4 (0·8 to 2·3)	0·9 (0·5 to 1·5)	0·6 (0·3 to 1)	0·4 (0·2 to 0·6)	0·2 (0·1 to 0·3)	0·1 (0 to 0·1)	0 (0 to 0)	115
Decubitus ulcer	0 (0 to 0)	0 (0 to 0)	0 (0 to 0)	0·2 (0 to 0·4)	0·4 (0·2 to 0·8)	0·3 (0·1 to 0·6)	0·4 (0·1 to 0·8)	0·1 (0 to 0·1)	0·6 (0·2 to 1·1)	0·2 (0·1 to 0·5)	0·7 (0·3 to 1·7)	0·7 (0·3 to 1·4)	1·2 (0·6 to 2·2)	2·2 (1·1 to 4)	2·8 (1·5 to 5·1)	2·5 (1·5 to 4·8)	2·4 (1·3 to 4·7)	3·3 (2 to 6·1)	4·2 (2·5 to 7·5)	4·1 (2·5 to 7·7)	3·9 (2·5 to 7·7)	2·5 (1·6 to 4·6)	1·2 (0·7 to 2·6)	34
Other skin and subcutaneous diseases	0 (0 to 0)	0 (0 to 0)	0·9 (0·5 to 1·6)	3·7 (1·8 to 6·9)	4 (2 to 7·5)	5 (2·3 to 9·6)	6·2 (3·1 to 11·7)	5·9 (3 to 10·8)	5·7 (2·8 to 10·5)	6·8 (3·6 to 12·3)	6·7 (3·4 to 12·1)	7·2 (3·9 to 12·8)	7 (3·6 to 12·7)	7·3 (4 to 12·7)	7·2 (3·7 to 12·9)	6·3 (3·3 to 11·4)	4·9 (2·5 to 8·7)	3·7 (1·9 to 6·7)	2·7 (1·4 to 4·7)	1·8 (1 to 3·3)	1 (0·5 to 1·8)	0·5 (0·3 to 0·8)	0·1 (0·1 to 0·3)	94·7
Sense organ diseases	0 (0 to 0)	0 (0 to 0·1)	1 (0·6 to 1·7)	11·2 (7 to 16·3)	25·5 (15·7 to 37·5)	35·2 (21·6 to 51·7)	42·6 (26·9 to 61·8)	46·4 (28·6 to 67·9)	51 (33 to 75·8)	56·8 (36·1 to 85·9)	60·9 (38·1 to 93·2)	66·8 (42·2 to 102)	83·3 (51·8 to 131)	95·5 (59·7 to 148)	106 (67 to 165)	104 (67·2 to 159)	94·3 (61·8 to 140)	81·7 (54·5 to 119)	67·6 (46·5 to 96·2)	59·3 (41·3 to 81·4)	43·6 (30·7 to 58·3)	23·4 (16·6 to 31·1)	9·4 (6·6 to 12·4)	1,170
Blindness and vision loss	0 (0 to 0)	0 (0 to 0)	0·2 (0·1 to 0·3)	3 (1·7 to 5·2)	7·5 (4·3 to 11·9)	10·5 (6 to 16·9)	12·7 (7·5 to 19·8)	13·1 (7·9 to 20·1)	12·5 (7·5 to 18·9)	12·6 (7·6 to 18·9)	14·1 (9·1 to 21)	16·4 (10·1 to 24·9)	19·4 (12 to 30·6)	21·8 (13·5 to 34·9)	25 (16·1 to 41·3)	26·3 (16·7 to 40·7)	26·2 (16·9 to 38·6)	24·7 (16·6 to 35·5)	21·4 (14·7 to 30·3)	19·4 (13·3 to 26·9)	14·8 (10·2 to 20)	8·5 (5·9 to 11·7)	3·8 (2·6 to 5·4)	314
Glaucoma	0 (0 to 0)	0 (0 to 0)	0 (0 to 0)	0 (0 to 0)	0 (0 to 0)	0 (0 to 0)	0 (0 to 0)	0 (0 to 0)	0 (0 to 0)	0 (0 to 0)	0 (0 to 0)	0 (0 to 0)	0 (0 to 0·1)	0·3 (0 to 0·6)	0·7 (0·3 to 1·3)	1·2 (0·6 to 2)	1·6 (0·8 to 2·5)	1·9 (1·1 to 3)	2·1 (1·2 to 3·3)	2·1 (1·3 to 3·3)	1·8 (1·1 to 2·8)	1 (0·6 to 1·6)	0·4 (0·2 to 0·7)	13
Cataract	0 (0 to 0)	0 (0 to 0)	0 (0 to 0)	0 (0 to 0)	0 (0 to 0)	0 (0 to 0)	0 (0 to 0)	0·1 (0 to 0·1)	0·3 (0·1 to 0·6)	0·6 (0·2 to 1·4)	1·1 (0·5 to 2·1)	1·7 (0·7 to 3)	2·3 (1·1 to 4)	2·6 (1·4 to 4·3)	3·1 (1·7 to 4·9)	3·7 (2·3 to 5·7)	4·6 (2·9 to 7)	5·8 (3·7 to 8·5)	6·4 (4·2 to 9·4)	6·6 (4·4 to 9·5)	5·5 (3·7 to 7·8)	3·1 (2 to 4·3)	1·3 (0·8 to 2)	48·8
Age-related macular degeneration	0 (0 to 0)	0 (0 to 0)	0 (0 to 0)	0 (0 to 0)	0 (0 to 0)	0 (0 to 0)	0 (0 to 0)	0 (0 to 0)	0 (0 to 0)	0 (0 to 0)	0 (0 to 0)	0 (0 to 0)	0 (0 to 0)	0·1 (0·1 to 0·2)	0·2 (0·1 to 0·3)	0·3 (0·1 to 0·5)	0·3 (0·1 to 0·5)	0·3 (0·1 to 0·5)	0·3 (0·1 to 0·4)	0·2 (0·1 to 0·4)	0·2 (0·1 to 0·3)	0·1 (0 to 0·1)	0 (0 to 0·1)	1·9
Refraction disorders	0 (0 to 0)	0 (0 to 0)	0·1 (0 to 0·2)	2·5 (1·3 to 4·3)	6·4 (3·5 to 10·4)	8·9 (5·1 to 14·9)	10·8 (6·3 to 17·1)	11 (6·4 to 17·3)	10·5 (5·9 to 15)	8·8 (5·2 to 13·6)	8·5 (5 to 12·8)	8 (4·6 to 12·5)	7·7 (4·6 to 11·5)	6·6 (4 to 10·3)	6 (3·7 to 9·5)	5·1 (3·1 to 7·8)	4 (2·4 to 6·1)	3·3 (2 to 5·1)	2·6 (1·6 to 4)	1·9 (1·2 to 2·9)	1·1 (0·7 to 1·7)	0·4 (0·3 to 0·6)	0·1 (0·1 to 0·2)	114
Near vision loss	0 (0 to 0)	0 (0 to 0)	0 (0 to 0)	0 (0 to 0)	0 (0 to 0·1)	0·1 (0 to 0·2)	0·1 (0 to 0·3)	0·2 (0·1 to 0·6)	0·5 (0·1 to 1)	1·1 (0·4 to 2·4)	2 (0·7 to 4·4)	3·5 (1·4 to 7·6)	5·4 (2·1 to 12·1)	7·1 (2·9 to 16)	8·6 (3·4 to 19)	8·7 (3·6 to 18·3)	7·7 (3·2 to 15·7)	6·3 (2·7 to 12·5)	4·7 (2 to 9·9)	3·7 (1·6 to 7·5)	2·5 (1·1 to 5)	1·3 (0·5 to 2·6)	0·5 (0·2 to 1)	64·1
Other vision loss	0 (0 to 0)	0 (0 to 0)	0·1 (0 to 0·1)	0·5 (0·3 to 0·9)	1·1 (0·4 to 2·3)	1·5 (0·5 to 3)	1·8 (0·7 to 3·4)	1·8 (0·7 to 3·4)	1·8 (0·8 to 3·4)	2 (0·9 to 3·7)	2·5 (1·2 to 4·3)	3·2 (1·8 to 5·2)	4 (2·1 to 6·4)	5·1 (3·1 to 7·8)	6·3 (3·8 to 9·6)	7·4 (4·8 to 10·7)	8 (5·1 to 11·5)	7·2 (4·6 to 10·5)	5·3 (3·4 to 7·9)	4·7 (3 to 6·9)	3·7 (2·4 to 5·7)	2·7 (1·8 to 3·9)	1·4 (0·9 to 2·1)	72·3
Age-related and other hearing loss	0 (0 to 0)	0 (0 to 0)	0·4 (0·2 to 0·7)	5·9 (3 to 9·6)	15·3 (7·9 to 24·6)	21·9 (12·2 to 33·6)	26·8 (16 to 40·7)	30·2 (17·9 to 46·8)	35·2 (22·1 to 53·9)	40 (24·2 to 61·5)	42·3 (25·7 to 66·5)	46·1 (26·6 to 72·9)	59·5 (35 to 96·5)	68·7 (41·2 to 110)	75·9 (47·1 to 120)	72·8 (44·5 to 114)	63·4 (40·1 to 97·4)	53·3 (34·5 to 79·3)	43·3 (28·7 to 62·5)	37·8 (25·6 to 52·8)	27·7 (18·8 to 37·7)	14·5 (10 to 19·6)	5·3 (3·8 to 7·1)	786
Other sense organ diseases	0 (0 to 0)	0 (0 to 0)	0·5 (0·2 to 0·8)	2·2 (1·1 to 3·9)	2·6 (1·1 to 4·7)	2·7 (1·2 to 5·1)	3·1 (1·4 to 6·2)	3·1 (1·3 to 5·7)	3·4 (1·4 to 6·3)	4·2 (1·8 to 8·1)	4·5 (2 to 8·8)	4·3 (1·9 to 8·3)	4·3 (1·9 to 8·2)	5·1 (2·3 to 10)	4·8 (2·2 to 9·2)	5 (2·4 to 9·3)	4·7 (2·1 to 8·6)	3·7 (1·8 to 7)	2·8 (1·3 to 5·1)	2 (1 to 3·8)	1·1 (0·5 to 2)	0·4 (0·2 to 0·8)	0·2 (0·1 to 0·4)	64·9
Musculoskeletal disorders	0 (0 to 0)	0 (0 to 0)	0 (0 to 0)	0 (0 to 0)	14·7 (7·8 to 24·9)	74·1 (45·8 to 115)	164 (108 to 240)	214 (143 to 304)	270 (181 to 376)	308 (205 to 421)	322 (225 to 438)	331 (231 to 454)	336 (236 to 469)	310 (216 to 439)	280 (194 to 385)	225 (154 to 319)	162 (112 to 228)	111 (76·2 to 155)	70·8 (48 to 99·6)	48·7 (33·8 to 68·5)	30·2 (21·4 to 42)	12·8 (9·1 to 17·9)	4·4 (3·1 to 6·2)	3,290
Rheumatoid arthritis	0 (0 to 0)	0 (0 to 0)	0 (0 to 0)	0 (0 to 0)	0·5 (0·2 to 0·8)	0·9 (0·4 to 1·5)	3 (1·7 to 4·7)	5 (2·2 to 8·6)	6·4 (3·1 to 10·8)	8·3 (4·8 to 13)	9·9 (6·1 to 15)	11·1 (7·1 to 16·4)	11·7 (7·8 to 16·7)	11 (7·6 to 15·8)	10·5 (7·3 to 14·4)	7·9 (5·5 to 10·9)	5·7 (4 to 7·7)	3·7 (2·7 to 5)	2·6 (1·8 to 3·5)	1·8 (1·3 to 2·5)	2·1 (1·3 to 3·1)	1 (0·6 to 1·6)	0·4 (0·2 to 0·6)	104
Osteoarthritis	0 (0 to 0)	0 (0 to 0)	0 (0 to 0)	0 (0 to 0)	0 (0 to 0)	0 (0 to 0)	0 (0 to 0)	0 (0 to 0)	0 (0 to 0)	1·4 (0·6 to 2·9)	8·3 (3·8 to 17·4)	20·6 (10 to 42·9)	36·2 (17·7 to 73·5)	47·9 (22·8 to 95·7)	54·4 (26·2 to 109)	51·6 (25·4 to 101)	41·3 (20·9 to 82·4)	30·3 (15·2 to 60·9)	20·1 (10·1 to 40·8)	14·2 (7·1 to 28·2)	8·5 (4·4 to 16·9)	3·9 (2 to 7·6)	1·4 (0·7 to 2·7)	340
Osteoarthritis hip	0 (0 to 0)	0 (0 to 0)	0 (0 to 0)	0 (0 to 0)	0 (0 to 0)	0 (0 to 0)	0 (0 to 0)	0 (0 to 0)	0 (0 to 0)	0·4 (0·1 to 1)	0·6 (0·2 to 1·5)	0·9 (0·3 to 2)	1·4 (0·5 to 3·1)	1·8 (0·7 to 4·3)	2·2 (0·9 to 4·9)	2·3 (1 to 5)	2 (0·9 to 4·3)	1·5 (0·7 to 3·2)	1 (0·5 to 2·1)	0·7 (0·3 to 1·5)	0·4 (0·2 to 1)	0·2 (0·1 to 0·4)	0·1 (0 to 0·2)	15·5
Osteoarthritis knee	0 (0 to 0)	0 (0 to 0)	0 (0 to 0)	0 (0 to 0)	0 (0 to 0)	0 (0 to 0)	0 (0 to 0)	0 (0 to 0)	0 (0 to 0)	0·7 (0·2 to 1·7)	6·3 (2·8 to 13·6)	15·8 (7·2 to 32·7)	27·5 (13 to 57·3)	36 (16·8 to 74)	40·4 (18·8 to 82·3)	37·8 (18·2 to 76·4)	29·9 (14·5 to 60)	21·6 (10·6 to 43·7)	14 (6·8 to 28·1)	9·5 (4·7 to 19·3)	5·4 (2·7 to 11)	2·3 (1·1 to 4·6)	0·7 (0·4 to 1·5)	248
Osteoarthritis hand	0 (0 to 0)	0 (0 to 0)	0 (0 to 0)	0 (0 to 0)	0 (0 to 0)	0 (0 to 0)	0 (0 to 0)	0 (0 to 0)	0 (0 to 0)	0·1 (0 to 0·4)	0·9 (0·2 to 2·4)	2·4 (0·9 to 5·8)	4·5 (1·9 to 9·9)	6·2 (2·7 to 13·6)	7·2 (3·3 to 15·4)	6·8 (3·2 to 14·3)	5·5 (2·6 to 11·7)	4·1 (2 to 8·3)	2·8 (1·4 to 5·7)	2·1 (1·1 to 4·4)	1·4 (0·7 to 2·9)	0·7 (0·3 to 1·5)	0·3 (0·1 to 0·6)	45·1

Osteoarthritis other	0 (0 to 0)	0 (0 to 0)	0 (0 to 0)	0 (0 to 0)	0 (0 to 0)	0 (0 to 0)	0 (0 to 0)	0 (0 to 0)	0 (0 to 0)	0·1 (0 to 0·2)	0·5 (0·1 to 1·3)	1·5 (0·5 to 3·5)	2·8 (1·1 to 6·2)	3·9 (1·6 to 8·2)	4·7 (2·1 to 10)	4·7 (2·2 to 10·2)	4 (1·9 to 8·4)	3·2 (1·5 to 6·4)	2·3 (1·2 to 4·8)	1·8 (0·9 to 3·7)	1·2 (0·6 to 2·4)	0·6 (0·3 to 1·3)	0·2 (0·1 to 0·5)	31·6
Low back pain	0 (0 to 0)	0 (0 to 0)	0 (0 to 0)	0 (0 to 0)	12·4 (5·8 to 22·5)	64·8 (38·3 to 104)	116 (67·6 to 185)	128 (74·6 to 198)	138 (81 to 212)	145 (83·2 to 222)	156 (92·4 to 236)	153 (94·4 to 235)	147 (89·4 to 230)	133 (81·6 to 206)	116 (71·7 to 173)	92·2 (56·6 to 136)	66·2 (42 to 101)	46·4 (28·9 to 69·2)	31·2 (19·2 to 46·9)	23·1 (14·8 to 34·5)	13·1 (8 to 18·9)	5·3 (3·3 to 7·9)	1·7 (1·1 to 2·6)	1,590
Neck pain	0 (0 to 0)	0 (0 to 0)	0 (0 to 0)	0 (0 to 0)	0·6 (0·1 to 1·4)	3·7 (1·4 to 7·8)	8·6 (3·6 to 16·4)	12 (5·2 to 23·6)	17·3 (8·1 to 33·1)	24·7 (11·5 to 46·9)	31·2 (15·8 to 54·1)	34·2 (17·5 to 60·5)	34 (16·8 to 60·5)	26·5 (13·4 to 49·4)	21·9 (10·5 to 39·2)	16·4 (8·2 to 28·6)	11·1 (5·7 to 18·8)	7·3 (3·8 to 12·8)	4·5 (2·2 to 8·3)	2·8 (1·4 to 4·9)	1·6 (0·8 to 2·7)	0·7 (0·4 to 1·2)	0·3 (0·2 to 0·5)	259
Gout	0 (0 to 0)	0 (0 to 0)	0 (0 to 0)	0 (0 to 0)	0 (0 to 0)	0 (0 to 0)	0 (0 to 0·1)	0·2 (0·1 to 0·3)	0·4 (0·2 to 0·7)	0·6 (0·1 to 1·6)	0·9 (0·2 to 1·9)	1 (0·3 to 2)	1·1 (0·3 to 2·1)	1·1 (0·4 to 2)	1 (0·4 to 1·9)	0·9 (0·4 to 1·6)	0·7 (0·3 to 1·2)	0·5 (0·2 to 0·9)	0·4 (0·2 to 0·6)	0·3 (0·1 to 0·5)	0·2 (0·1 to 0·3)	0·1 (0 to 0·2)	0 (0 to 0·1)	9·2
Other musculoskeletal disorders	0 (0 to 0)	0 (0 to 0)	0 (0 to 0)	0 (0 to 0)	1·2 (0·6 to 1·9)	4·7 (2·5 to 8·5)	36·7 (23·5 to 56·3)	68·9 (46·6 to 97)	108 (73 to 156)	128 (85·8 to 182)	116 (75·3 to 164)	111 (71·1 to 166)	106 (69·4 to 151)	90·9 (59·4 to 136)	76·3 (48·3 to 112)	56·2 (34·2 to 86·2)	37·2 (23 to 56·5)	23 (13·1 to 36·6)	12 (6·9 to 19·2)	6·5 (3 to 11·1)	4·7 (2·7 to 7·2)	1·9 (0·9 to 3·1)	0·7 (0·3 to 1·3)	990
Other non-communicable diseases	264 (181 to 370)	142 (96 to 202)	328 (203 to 509)	169 (130 to 218)	119 (89·8 to 155)	135 (98·7 to 176)	267 (191 to 378)	328 (228 to 466)	396 (286 to 551)	407 (289 to 553)	393 (279 to 544)	354 (247 to 487)	311 (226 to 416)	247 (178 to 333)	197 (145 to 262)	153 (113 to 205)	111 (82·3 to 149)	81 (59·4 to 108)	56·4 (43 to 73·1)	40·6 (31·1 to 53·2)	27·6 (21·4 to 35·5)	13·8 (10·5 to 17·4)	5·5 (4 to 7·2)	4,550
Congenital birth defects	257 (174 to 362)	120 (77·7 to 175)	205 (120 to 335)	92·8 (67·5 to 126)	48·3 (36·2 to 63)	40·4 (29·2 to 52·8)	41·4 (31·3 to 53)	28·4 (21 to 37·4)	25·4 (19·5 to 33·3)	19·4 (14·8 to 25·5)	15·2 (11·5 to 19·9)	12·3 (9·1 to 16·2)	11 (8·3 to 14·3)	9·1 (6·9 to 11·7)	6·7 (5 to 8·7)	3·7 (2·7 to 5)	2·2 (1·6 to 3)	0·8 (0·5 to 1·2)	0·4 (0·2 to 0·6)	0·2 (0·1 to 0·4)	0·1 (0·1 to 0·2)	0·1 (0 to 0·1)	0 (0 to 0)	940
Neural tube defects	20·3 (10·3 to 36·4)	6·6 (3·1 to 12)	16·8 (6·8 to 36·3)	10·6 (5·1 to 19·9)	1·4 (1 to 2·1)	1·8 (1·2 to 2·7)	1·8 (1·2 to 2·6)	0·8 (0·5 to 1)	0·9 (0·6 to 1·3)	0·8 (0·5 to 1·1)	0·4 (0·3 to 0·6)	0·3 (0·2 to 0·4)	0·2 (0·1 to 0·3)	0·1 (0·1 to 0·2)	0·1 (0·1 to 0·2)	0·1 (0 to 0·1)	0 (0 to 0·1)	0 (0 to 0)	0 (0 to 0)	0 (0 to 0)	0 (0 to 0)	0 (0 to 0)	0 (0 to 0)	63·1
Congenital heart anomalies	82 (49·3 to 124)	50·5 (30·6 to 76·7)	88·9 (46·8 to 161)	30·5 (18·9 to 47·5)	12·4 (8·1 to 19)	8·2 (5·2 to 13·2)	10 (6·6 to 14·2)	6·4 (4·2 to 9·4)	6·9 (4·6 to 9·9)	4 (2·5 to 6·1)	3·1 (2 to 4·7)	2·4 (1·4 to 3·7)	2 (1·2 to 2·8)	1·5 (0·9 to 2·2)	0·9 (0·5 to 1·3)	0·4 (0·3 to 0·7)	0·3 (0·2 to 0·4)	0 (0 to 0·1)	0 (0 to 0·1)	0 (0 to 0)	0 (0 to 0)	0 (0 to 0)	0 (0 to 0)	311
Orofacial clefts	0·6 (0·2 to 1·3)	0·2 (0·1 to 0·4)	1 (0·3 to 2·5)	0·7 (0·2 to 1·3)	0·5 (0·1 to 1·1)	0·5 (0·1 to 1·1)	0·5 (0·1 to 1·1)	0·5 (0·1 to 1)	0·4 (0·1 to 0·9)	0·4 (0·1 to 0·8)	0·3 (0·1 to 0·7)	0·3 (0·1 to 0·6)	0·2 (0·1 to 0·5)	0·2 (0 to 0·4)	0·2 (0 to 0·3)	0·1 (0 to 0·2)	0·1 (0 to 0·2)	0·1 (0 to 0·1)	0 (0 to 0·1)	0 (0 to 0·1)	0 (0 to 0)	0 (0 to 0)	0 (0 to 0)	6·7
Down syndrome	5·2 (2·6 to 8·7)	3·2 (1·6 to 5·7)	12·9 (5·7 to 24·7)	8·8 (4·7 to 15·1)	3·1 (1·9 to 5)	2·5 (1·6 to 3·9)	3·1 (1·9 to 4·6)	3·2 (2 to 5)	2·8 (1·7 to 4·5)	2·5 (1·6 to 3·9)	2·4 (1·5 to 3·6)	2 (1·2 to 2·9)	2·4 (1·6 to 3·5)	2·5 (1·6 to 3·5)	1·8 (1·1 to 2·7)	0·6 (0·4 to 0·9)	0·2 (0·1 to 0·3)	0 (0 to 0)	0 (0 to 0)	0 (0 to 0)	0 (0 to 0)	0 (0 to 0)	0 (0 to 0)	59·1
Turner syndrome	0 (0 to 0)	0 (0 to 0)	0 (0 to 0)	0·1 (0 to 0·1)	0·1 (0 to 0·1)	0·1 (0 to 0·1)	0·1 (0 to 0·2)	0·1 (0 to 0·2)	0·1 (0 to 0·1)	0 (0 to 0·1)	0 (0 to 0·1)	0 (0 to 0)	0 (0 to 0)	0 (0 to 0)	0 (0 to 0)	0 (0 to 0)	0 (0 to 0)	0 (0 to 0)	0 (0 to 0)	0 (0 to 0)	0 (0 to 0)	0 (0 to 0)	0 (0 to 0)	0·6
Klinefelter syndrome	0 (0 to 0)	0 (0 to 0)	0 (0 to 0)	0 (0 to 0)	0 (0 to 0)	0 (0 to 0)	0 (0 to 0)	0 (0 to 0)	0 (0 to 0)	0 (0 to 0)	0 (0 to 0)	0 (0 to 0)	0 (0 to 0)	0 (0 to 0)	0 (0 to 0)	0 (0 to 0)	0 (0 to 0)	0 (0 to 0)	0 (0 to 0)	0 (0 to 0)	0 (0 to 0)	0 (0 to 0)	0 (0 to 0)	0
Other chromosomal abnormalities	13 (6·7 to 21·9)	7·8 (4 to 13·8)	14·8 (7 to 27·2)	3·6 (2·3 to 5·4)	2·1 (1·4 to 3·1)	1·8 (1·2 to 2·6)	2·1 (1·4 to 2·9)	1·6 (1·1 to 2·4)	1·2 (0·8 to 1·8)	0·8 (0·5 to 1·2)	0·4 (0·3 to 0·6)	0·3 (0·2 to 0·4)	0·2 (0·1 to 0·2)	0·1 (0·1 to 0·2)	0·1 (0 to 0·1)	0 (0 to 0)	0 (0 to 0)	0 (0 to 0)	0 (0 to 0)	0 (0 to 0)	0 (0 to 0)	0 (0 to 0)	0 (0 to 0)	50
Congenital musculoskeletal and limb anomalies	8·3 (4 to 13·7)	3·2 (1·8 to 5·5)	10·7 (6·4 to 16·8)	14·4 (8·5 to 22·2)	15·3 (8·4 to 23·6)	15·9 (8·7 to 25·4)	14·4 (8·1 to 22)	9 (5·5 to 13·8)	7·3 (4·4 to 11)	6·1 (3·4 to 9·6)	5·5 (3·2 to 8·6)	4·8 (2·8 to 7·3)	4·1 (2·4 to 6·3)	3·3 (1·9 to 5)	2·6 (1·5 to 4)	1·8 (1 to 2·8)	1·1 (0·6 to 1·7)	0·5 (0·3 to 0·9)	0·3 (0·1 to 0·4)	0·1 (0·1 to 0·2)	0·1 (0 to 0·1)	0 (0 to 0)	0 (0 to 0)	129
Urogenital congenital anomalies	5·5 (2·6 to 10·1)	2·5 (1·2 to 4·4)	1·9 (1 to 3·7)	1·4 (0·9 to 2·3)	1 (0·6 to 1·6)	1 (0·6 to 1·6)	0·8 (0·5 to 1·2)	0·5 (0·3 to 0·8)	0·4 (0·3 to 0·7)	0·2 (0·2 to 0·4)	0·2 (0·1 to 0·3)	0·1 (0·1 to 0·2)	0·1 (0·1 to 0·2)	0·1 (0 to 0·1)	0 (0 to 0)	0 (0 to 0)	0 (0 to 0)	0 (0 to 0)	0 (0 to 0)	0 (0 to 0)	0 (0 to 0)	0 (0 to 0)	0 (0 to 0)	15·9
Digestive congenital anomalies	47·6 (26·2 to 75·2)	24·4 (13·2 to 41)	23·4 (11·2 to 51·4)	6·1 (3·4 to 10·9)	1·6 (0·9 to 2·6)	0·7 (0·4 to 1·2)	0·4 (0·3 to 0·7)	0·3 (0·2 to 0·5)	0·2 (0·1 to 0·2)	0·1 (0·1 to 0·2)	0·1 (0·1 to 0·1)	0·1 (0·1 to 0·2)	0·1 (0·1 to 0·2)	0·1 (0 to 0·1)	0·1 (0 to 0·1)	0·1 (0 to 0·1)	0 (0 to 0)	0 (0 to 0)	0 (0 to 0)	0 (0 to 0)	0 (0 to 0)	0 (0 to 0)	0 (0 to 0)	105
Other congenital birth defects	74 (43·1 to 116)	22·1 (11·9 to 35·1)	34·6 (17·6 to 65·6)	16·7 (10 to 25·9)	10·7 (6·9 to 15·8)	7·8 (5 to 11·8)	8·2 (5·5 to 12·3)	6·1 (3·8 to 9·2)	5·2 (3·2 to 8)	4·3 (2·8 to 6·5)	2·8 (1·7 to 4·4)	2 (1·2 to 3·4)	1·8 (1 to 3)	1·3 (0·8 to 2·2)	1 (0·5 to 1·7)	0·5 (0·3 to 1·1)	0·4 (0·2 to 0·8)	0·1 (0 to 0·4)	0·1 (0 to 0·2)	0·1 (0 to 0·2)	0 (0 to 0·1)	0 (0 to 0·1)	0 (0 to 0)	200
Urinary diseases and male infertility	0·6 (0·3 to 0·8)	1·1 (0·6 to 1·8)	4·7 (2·5 to 7·9)	4·1 (2·8 to 5·8)	3·2 (2·2 to 4·4)	4·4 (3·1 to 6·2)	8·3 (6·2 to 11·1)	9·4 (6·9 to 12·6)	13·1 (9·4 to 18)	16·4 (11·3 to 22·6)	14 (10·2 to 19·1)	14·7 (10·6 to 20)	16·6 (12 to 22·3)	18·4 (13·3 to 24·9)	17·5 (12·1 to 24)	14·2 (10·1 to 19·2)	11·5 (8·1 to 15·4)	10·2 (7·1 to 13·9)	9·3 (6·2 to 13)	8·4 (5·5 to 12·4)	6·7 (4·4 to 9·8)	3·8 (2·4 to 5·4)	1·7 (1 to 2·5)	212
Urolithiasis	0 (0 to 0)	0 (0 to 0)	0 (0 to 0)	0 (0 to 0)	0 (0 to 0·1)	0·1 (0·1 to 0·2)	0·2 (0·1 to 0·3)	0·4 (0·2 to 0·6)	0·6 (0·4 to 1)	1·6 (0·9 to 2·6)	1·6 (0·9 to 2·5)	1·2 (0·7 to 1·9)	2 (1·1 to 3·4)	1·6 (0·9 to 2·6)	1·7 (0·8 to 2·7)	1·1 (0·6 to 1·7)	0·7 (0·4 to 1·2)	0·4 (0·2 to 0·6)	0·2 (0·1 to 0·4)	0·1 (0·1 to 0·2)	0·1 (0 to 0·2)	0 (0 to 0)	0 (0 to 0)	13·6
Benign prostatic hyperplasia	0 (0 to 0)	0 (0 to 0)	0 (0 to 0)	0 (0 to 0)	0 (0 to 0)	0 (0 to 0)	0 (0 to 0)	0 (0 to 0)	0 (0 to 0)	0 (0 to 0)	0 (0 to 0)	0 (0 to 0)	0 (0 to 0)	0 (0 to 0)	0 (0 to 0)	0 (0 to 0)	0 (0 to 0)	0 (0 to 0)	0 (0 to 0)	0 (0 to 0)	0 (0 to 0)	0 (0 to 0)	0 (0 to 0)	0
Male infertility	--	--	--	--	--	--	--	--	--	--	--	--	--	--	--	--	--	--	--	--	--	--	--	NA
Other urinary diseases	0·3 (0·2 to 0·5)	0·8 (0·4 to 1·4)	0·7 (0·3 to 1·6)	1·1 (0·6 to 1·6)	0·7 (0·4 to 1)	1·4 (0·9 to 2·2)	1·4 (0·8 to 2·2)	1·8 (1 to 2·9)	1·1 (0·6 to 1·6)	1·5 (0·9 to 2·5)	0·9 (0·6 to 1·4)	1·2 (0·7 to 2)	1·1 (0·7 to 1·7)	1·4 (0·8 to 2·2)	1·1 (0·6 to 1·7)	0·9 (0·5 to 1·5)	0·4 (0·2 to 0·7)	0·3 (0·1 to 0·5)	0·2 (0·1 to 0·4)	0·2 (0·1 to 0·3)	0·1 (0 to 0·2)	0 (0 to 0·1)	0 (0 to 0)	18·6
Gynecological diseases	0 (0 to 0)	0 (0 to 0)	0 (0 to 0)	0 (0 to 0)	0 (0 to 0)	31·4 (16·2 to 53·9)	102 (58·3 to 168)	160 (103 to 244)	213 (139 to 311)	230 (151 to 333)	229 (154 to 331)	199 (129 to 287)	150 (96·8 to 212)	87·5 (53 to 131)	46·1 (28·3 to 67·1)	26·4 (15·5 to 41)	16·4 (10·1 to 24·1)	9·9 (6·2 to 14·6)	5·7 (3·6 to 8·3)	3·6 (2·2 to 5·5)	2·2 (1·4 to 3·3)	1·1 (0·6 to 1·6)	0·4 (0·2 to 0·6)	1,510
Uterine fibroids	0 (0 to 0)	0 (0 to 0)	0 (0 to 0)	0 (0 to 0)	0 (0 to 0)	0·5 (0·2 to 1·1)	1·1 (0·4 to 2·5)	2·6 (1 to 5·7)	6·1 (2·4 to 12·9)	12·1 (5·2 to 25·3)	18·4 (8·1 to 37·2)	18·3 (8·4 to 34·9)	14·4 (6·6 to 27·5)	9·1 (4·2 to 17·2)	5·3 (2·4 to 10·2)	2·8 (1·2 to 5·6)	1·4 (0·6 to 2·6)	0·6 (0·3 to 1·3)	0·3 (0·1 to 0·6)	0·1 (0·1 to 0·3)	0·1 (0 to 0·1)	0 (0 to 0)	0 (0 to 0)	93·2
Polycystic ovarian syndrome	0 (0 to 0)	0 (0 to 0)	0 (0 to 0)	0 (0 to 0)	0 (0 to 0)	1·3 (0·5 to 2·9)	5·5 (2·3 to 11·4)	6·3 (2·6 to 12·9)	5·3 (2·2 to 10·9)	4·5 (1·9 to 9·3)	3·9 (1·6 to 7·8)	3·3 (1·4 to 6·7)	2·3 (0·9 to 4·7)	0·5 (0·2 to 1·1)	0 (0 to 0)	0 (0 to 0)	0 (0 to 0)	0 (0 to 0)	0 (0 to 0)	0 (0 to 0)	0 (0 to 0)	0 (0 to 0)	0 (0 to 0)	33
Female infertility	0 (0 to 0)	0 (0 to 0)	0 (0 to 0)	0 (0 to 0)	0 (0 to 0)	0 (0 to 0)	0 (0 to 0)	3·1 (0·4 to 9·4)	4 (0·7 to 11·4)	4·1 (0·5 to 13·1)	3·6 (0·1 to 12·1)	2·8 (0·1 to 9·8)	0 (0 to 0·1)	0 (0 to 0)	0 (0 to 0)	0 (0 to 0)	0 (0 to 0)	0 (0 to 0)	0 (0 to 0)	0 (0 to 0)	0 (0 to 0)	0 (0 to 0)	0 (0 to 0)	17·8
Endometriosis	0 (0 to 0)	0 (0 to 0)	0 (0 to 0)	0 (0 to 0)	0 (0 to 0)	0 (0 to 0)	7 (2·8 to 14·9)	25·8 (12·5 to 47)	27 (13·3 to 47·7)	19·8 (9·6 to 35·3)	15·7 (8 to 28)	13·4 (7 to 23·1)	10·4 (5·6 to 17)	4·3 (2·1 to 7·3)	0 (0 to 0)	0 (0 to 0)	0 (0 to 0)	0 (0 to 0)	0 (0 to 0)	0 (0 to 0)	0 (0 to 0)	0 (0 to 0)	0 (0 to 0)	124
Genital prolapse	0 (0 to 0)	0 (0 to 0)	0 (0 to 0)	0 (0 to 0)	0 (0 to 0)	0 (0 to 0)	0 (0 to 0·1)	0·2 (0·1 to 0·4)	0·5 (0·2 to 1·1)	1 (0·4 to 2)	1·8 (0·8 to 3·6)	2·4 (1·1 to 5·2)	3·1 (1·5 to 6·2)	2·9 (1·4 to 5·6)	2·7 (1·3 to 5·1)	2 (1 to 3·9)	1·4 (0·7 to 2·8)	1 (0·5 to 1·8)	0·6 (0·3 to 1·1)	0·4 (0·2 to 0·8)	0·3 (0·1 to 0·5)	0·1 (0·1 to 0·3)	0·1 (0 to 0·1)	20·5
Premenstrual syndrome	0 (0 to 0)	0 (0 to 0)	0 (0 to 0)	0 (0 to 0)	0 (0 to 0)	28·1 (13·2 to 50·2)	64·7 (32·3 to 116)	71·4 (35·7 to 126)	66 (37·7 to 108)	60·5 (30·7 to 99·3)	60·8 (33·8 to 97·5)	53·4 (30·2 to 85·1)	32·9 (18·2 to 55·2)	9·6 (5 to 17·7)	0 (0 to 0)	0 (0 to 0)	0 (0 to 0)	0 (0 to 0)	0 (0 to 0)	0 (0 to 0)	0 (0 to 0)	0 (0 to 0)	0 (0 to 0)	447
Other gynecological diseases	0 (0 to 0)	0 (0 to 0)	0 (0 to 0)	0 (0 to 0)	0 (0 to 0)	1·6 (0·5 to 3·3)	23·8 (8·6 to 47·4)	50·4 (28·4 to 81·8)	104 (58·4 to 165)	128 (76·4 to 198)	125 (74·3 to 192)	105 (60·1 to 166)	86·4 (49·7 to 133)	61·1 (30·5 to 97·9)	38 (22·3 to 58·1)	21·6 (11·6 to 35)	13·6 (7·9 to 21·2)	8·3 (5 to 12·6)	4·8 (2·9 to 7·2)	3·1 (1·8 to 4·8)	1·9 (1·1 to 2·9)	0·9 (0·5 to 1·4)	0·3 (0·2 to 0·6)	778
Hemoglobinopathies and hemolytic anemias	0·6 (0·4 to 0·9)	1 (0·6 to 1·5)	5·6 (3·6 to 8·3)	18·7 (11·7 to 28·8)	26·5 (16 to 40·2)	20·4 (12 to 32)	54 (29·8 to 93·3)	62·2 (32·4 to 103)	54·7 (27·9 to 89·3)	36·6 (15·4 to 64·4)	29·4 (15·4 to 52·1)	17·3 (10·6 to 25·7)	14 (8·8 to 20·1)	12·5 (8·2 to 18·2)	9·1 (5·9 to 13·6)	7·2 (4·4 to 11·3)	3·6 (2 to 5·9)	2·5 (1·4 to 4·1)	1·8 (1 to 2·7)	1·3 (0·7 to 2)	1·1 (0·7 to 1·7)	0·6 (0·4 to 0·8)	0·2 (0·1 to 0·2)	381
Thalassemias	0 (0 to 0)	0 (0 to 0)	0 (0 to 0)	0 (0 to 0·1)	0 (0 to 0·1)	0 (0 to 0)	0 (0 to 0)	0 (0 to 0)	0 (0 to 0)	0 (0 to 0)	0 (0 to 0)	0 (0 to 0)	0 (0 to 0)	0 (0 to 0)	0 (0 to 0)	0 (0 to 0)	0 (0 to 0)	0 (0 to 0)	0 (0 to 0)	0 (0 to 0)	0 (0 to 0)	0 (0 to 0)	0 (0 to 0)	0·1
Thalassemias trait	0 (0 to 0)	0 (0 to 0)	0·2 (0·1 to 0·3)	0·7 (0·2 to 1·3)	1 (0·4 to 1·8)	0·7 (0·2 to 1·5)	2·5 (1 to 4·6)	2·4 (1·2 to 4·2)	2·1 (1·1 to 3·6)	1·9 (0·9 to 3·2)	1·6 (0·8 to 2·8)	1·2 (0·5 to 2·3)	0·2 (0·1 to 0·4)	0·3 (0·1 to 0·6)	0·3 (0·1 to 0·5)	0·2 (0·1 to 0·4)	0·1 (0 to 0·2)	0·1 (0 to 0·1)	0 (0 to 0·1)	0 (0 to 0·1)	0 (0 to 0·1)	0 (0 to 0)	0 (0 to 0)	15·5
Sickle cell disorders	0·6 (0·4 to 0·9)	0·4 (0·2 to 0·7)	2·9 (1·6 to 4·7)	7·7 (4·6 to 12·5)	10·9 (7·4 to 15·7)	9·1 (5·9 to 13·6)	17·8 (11·6 to 26·4)	13·1 (8·8 to 19·5)	13·2 (8·3 to 20·1)	9·1 (5·5 to 14·3)	6·4 (3·9 to 10·3)	4·5 (2·8 to 6·7)	4·7 (3·1 to 7·1)	3·5 (2·2 to 5·5)	1·6 (0·9 to 2·5)	0·4 (0·2 to 0·7)	0·1 (0 to 0·1)	0 (0 to 0)	0 (0 to 0)	0 (0 to 0)	0 (0 to 0)	0 (0 to 0)	0 (0 to 0)	106
Sickle cell trait	0 (0 to 0)	0·5 (0·2 to 0·8)	1·6 (0·8 to 2·7)	6·8 (3·2 to 11·8)	9·9 (4·4 to 18·1)	7·2 (2·8 to 13·6)	28·4 (5·8 to 65·2)	42·5 (16 to 75·4)	34·8 (10·9 to 64·9)	20·9 (3·4 to 45·1)	14·5 (0·4 to 39·3)	4·3 (2·2 to 7·5)	4 (2 to 7)	3·6 (1·8 to 6·1)	2·9 (1·4 to 4·9)	1·8 (0·9 to 3)	1 (0·3 to 1·8)	0·7 (0·3 to 1·3)	0·5 (0·2 to 0·9)	0·4 (0·2 to 0·7)	0·3 (0·1 to 0·5)	0·1 (0·1 to 0·2)	0 (0 to 0·1)	187
G6PD deficiency	0 (0 to 0)	0 (0 to 0)	0 (0 to 0)	0 (0 to 0·1)	0·1 (0 to 0·1)	0·1 (0 to 0·1)	0·4 (0·1 to 0·8)	0·4 (0·2 to 0·7)	0·6 (0·3 to 1·2)	0·6 (0·2 to 1·2)	2·2 (0·4 to 7·4)	1·3 (0·7 to 2·2)	0·5 (0·2 to 1)	0·5 (0·2 to 0·9)	0·4 (0·2 to 0·6)	0·2 (0·1 to 0·4)	0·1 (0·1 to 0·2)	0 (0 to 0·1)	0 (0 to 0)	0 (0 to 0)	0 (0 to 0)	0 (0 to 0)	0 (0 to 0)	7·5
G6PD trait	0 (0 to 0)	0 (0 to 0)	0 (0 to 0)	0 (0 to 0)	0 (0 to 0)	0 (0 to 0)	0 (0 to 0)	0 (0 to 0)	0 (0 to 0)	0 (0 to 0)	0·3 (0 to 0·6)	0·3 (0·1 to 0·6)	0·1 (0 to 0·2)	0 (0 to 0)	0 (0 to 0)	0 (0 to 0)	0 (0 to 0)	0 (0 to 0)	0 (0 to 0)	0 (0 to 0)	0 (0 to 0)	0 (0 to 0)	0 (0 to 0)	0·8
Other hemoglobinopathies and hemolytic anemias	0 (0 to 0)	0·1 (0 to 0·2)	1 (0·4 to 1·8)	3·4 (1·3 to 6·8)	4·6 (1·6 to 9·5)	3·4 (1 to 7)	4·9 (1·3 to 10·8)	4 (1·3 to 8·6)	4 (1·6 to 8·3)	4 (1·8 to 7·5)	4·5 (1·6 to 10)	5·6 (2·6 to 9·7)	4·4 (2·1 to 7·9)	4·5 (2·5 to 7·4)	4 (2·4 to 6·4)	4·6 (2·7 to 7·5)	2·4 (1·4 to 3·9)	1·7 (1 to 2·7)	1·2 (0·7 to 1·8)	0·8 (0·5 to 1·3)	0·8 (0·5 to 1·2)	0·4 (0·3 to 0·6)	0·1 (0·1 to 0·2)	64·2
Endocrine, metabolic, blood, and immune disorders	6·1 (3·8 to 9·8)	8·3 (4·9 to 14·1)	65·8 (34·4 to 117)	44·1 (30·2 to 67·9)	28·6 (19·3 to 42·3)	23·6 (16·1 to 35·1)	40·1 (27·5 to 58·5)	38·4 (26·5 to 55·7)	51·4 (35·4 to 72·9)	60·1 (42·2 to 83·1)	57·5 (39·6 to 81·4)	62 (41·4 to 89·7)	68·7 (47·9 to 94·8)	67·5 (47·2 to 97·7)	63·6 (44·1 to 91·1)	50·2 (33·8 to 73·7)	35·9 (24·5 to 54·5)	26·3 (17·4 to 39)	18·3 (12·7 to 26·3)	12·2 (8·4 to 17·7)	8·6 (5·8 to 13)	4·3 (2·9 to 6·5)	1·8 (1·1 to 2·8)	843
Oral disorders	0 (0 to 0)	0 (0 to 0)	0 (0 to 0)	9·4 (5·2 to 15·6)	12·4 (6·7 to 21·1)	14·5 (7·7 to 24·6)	21·2 (11·4 to 36·4)	30 (16 to 54·7)	38·6 (19·4 to 73·4)	44·3 (21·1 to 80·9)	47·7 (22·7 to 89·1)	48·5 (24·2 to 88·7)	50·8 (27·1 to 89·1)	51·9 (29 to 87·8)	54 (31·6 to 86·5)	50·8 (30·9 to 79)	41·8 (25·6 to 65)	31·3 (19·8 to 47·6)	21 (13 to 31·3)	14·8 (9·3 to 21·6)	8·8 (5·5 to 13)	4·1 (2·5 to 5·9)	1·5 (0·9 to 2·2)	597
Caries of deciduous teeth	0 (0 to 0)	0 (0 to 0)	0 (0 to 0)	3 (1·1 to 6)	3 (1·2 to 6·6)	0·2 (0 to 0·5)	0 (0 to 0)	0 (0 to 0)	0 (0 to 0)	0 (0 to 0)	0 (0 to 0)	0 (0 to 0)	0 (0 to 0)	0 (0 to 0)	0 (0 to 0)	0 (0 to 0)	0 (0 to 0)	0 (0 to 0)	0 (0 to 0)	0 (0 to 0)	0 (0 to 0)	0 (0 to 0)	0 (0 to 0)	6·2
Caries of permanent teeth	0 (0 to 0)	0 (0 to 0)	0 (0 to 0)	0 (0 to 0)	3 (1·1 to 6·7)	6·8 (2·8 to 14·7)	7·3 (2·8 to 15·7)	7·4 (3·1 to 15·9)	6·6 (2·6 to 13·6)	5·5 (2·2 to 11·8)	5 (2 to 9·8)	4·1 (1·6 to 8·4)	3·4 (1·4 to 7·3)	2·7 (1·1 to 5·8)	2 (0·8 to 4·5)	1·4 (0·5 to 3)	0·8 (0·3 to 1·7)	0·5 (0·2 to 1·1)	0·3 (0·1 to 0·6)	0·2 (0·1 to 0·4)	0·1 (0 to 0·2)	0 (0 to 0·1)	0 (0 to 0)	57·1
Periodontal diseases	0 (0 to 0)	0 (0 to 0)	0 (0 to 0)	0 (0 to 0)	0 (0 to 0)	0 (0 to 0)	2·6 (0·8 to 6·8)	8·7 (2·6 to 21·5)	17·3 (5·4 to 40·9)	23·7 (8·2 to 53·2)	27·5 (9·8 to 61·3)	27·8 (10·2 to 60·5)	26 (10 to 55·9)	21·1 (8·2 to 44·1)	15·9 (6·3 to 33·6)	10·5 (4·2 to 21·5)	6·3 (2·4 to 12·8)	3·8 (1·4 to 7·7)	2·2 (0·9 to 4·5)	1·5 (0·6 to 3)	0·9 (0·3 to 1·9)	0·4 (0·1 to 0·9)	0·1 (0·1 to 0·3)	196
Other oral disorders	0 (0 to 0)	0 (0 to 0)	0 (0 to 0)	6·5 (3·7 to 10)	6·3 (3·5 to 9·8)	7·5 (4·3 to 12)	11·3 (6·7 to 17·5)	12·4 (7·4 to 19·2)	12 (7·3 to 18·4)	11·2 (6·8 to 17)	10·2 (6 to 15·8)	9·1 (5·4 to 13·6)	8·1 (4·9 to 12·5)	6·6 (4 to 9·9)	5·3 (3·1 to 7·8)	3·9 (2·4 to 5·9)	2·6 (1·5 to 4)	1·6 (1 to 2·4)	0·8 (0·5 to 1·2)	0·5 (0·3 to 0·7)	0·2 (0·1 to 0·3)	0·1 (0·1 to 0·1)	0 (0 to 0)	116
Sudden infant death syndrome	--	11·7 (6·8 to 18·6)	46·8 (22 to 87·5)	--	--	--	--	--	--	--	--	--	--	--	--	--	--	--	--	--	--	--	--	NA
Injuries	27·2 (20·3 to 36·4)	14·2 (9·6 to 19·7)	81·4 (50·6 to 133)	177 (138 to 224)	185 (158 to 215)	194 (169 to 219)	431 (384 to 483)	414 (354 to 476)	410 (343 to 491)	352 (294 to 418)	242 (203 to 288)	200 (166 to 238)	189 (157 to 223)	151 (128 to 181)	120 (99·3 to 142)	90·9 (75·4 to 109)	66 (55 to 78·5)	50·8 (42·7 to 59·8)	40·4 (34·1 to 47·3)	34·6 (29 to 40·6)	27·4 (22·6 to 32·7)	16·9 (13·9 to 20)	7·9 (6·4 to 9·4)	3,520
Transport injuries	1·3 (1 to 1·8)	1 (0·6 to 1·4)	7 (4 to 11·7)	32·2 (22 to 46·9)	51·3 (37·1 to 68·9)	50·9 (37·6 to 65·1)	144 (107 to 186)	124 (90·9 to 160)	117 (88·3 to 153)	95·3 (72·6 to 123)	65·2 (50·3 to 83)	54·5 (43·1 to 69)	52·9 (41·5 to 67·3)	42·3 (33·8 to 51·7)	32·9 (26·1 to 40·2)	23·4 (18·9 to 28·1)	16·6 (13·4 to 20)	10·8 (8·7 to 13·4)	7·2 (5·8 to 8·6)	4·5 (3·6 to 5·5)	2·6 (2·1 to 3·3)	1·3 (1 to 1·6)	0·5 (0·4 to 0·7)	938
Road injuries	1 (0·7 to 1·4)	0·6 (0·4 to 0·8)	5·5 (3·1 to 9·5)	27·3 (18·4 to 40)	45·6 (31·9 to 62·4)	44·1 (32·5 to 57·7)	131 (96·5 to 170)	112 (81·7 to 147)	105 (78·8 to 139)	84·7 (63·8 to 111)	59 (45·1 to 75·3)	47·4 (37·1 to 60·3)	48 (37·4 to 61·1)	37·5 (30·1 to 46·5)	29 (22·9 to 35·8)	21·2 (17 to 25·8)	15·4 (12·5 to 18·7)	9·9 (8 to 12·4)	6·4 (5·1 to 7·7)	4·1 (3·3 to 5)	2·4 (1·9 to 2·9)	1·1 (0·9 to 1·4)	0·5 (0·4 to 0·6)	838
Pedestrian road injuries	0·3 (0·2 to 0·5)	0·2 (0·1 to 0·3)	1·1 (0·6 to 1·8)	10·3 (6·7 to 15·3)	15·6 (9·2 to 24·1)	12·1 (7·2 to 18·4)	18·8 (11·2 to 29·3)	14·2 (9·7 to 21·5)	14·3 (9 to 21·5)	15·6 (11·6 to 20·5)	10·8 (8·1 to 14·4)	9 (6·8 to 11·8)	10·4 (7·8 to 14·1)	9·7 (7 to 12·9)	7·6 (5·9 to 9·6)	6·2 (5 to 7·8)	5·1 (4·1 to 6·2)	3·5 (2·8 to 4·4)	2·5 (2 to 3·2)	1·7 (1·3 to 2·1)	1 (0·7 to 1·2)	0·4 (0·3 to 0·5)	0·2 (0·1 to 0·2)	171
Cyclist road injuries	0 (0 to 0)	0 (0 to 0)	0 (0 to 0)	1·3 (0·7 to 2·2)	2·5 (1·6 to 3·8)	4·9 (3·1 to 7·7)	8·8 (5·5 to 13·3)	7·6 (5·2 to 11)	7·9 (5·6 to 11·3)	6·8 (4·9 to 9·2)	6·4 (4·7 to 8·5)	5·5 (4 to 7·3)	5·3 (3·8 to 7)	4·5 (3·3 to 5·9)	3·5 (2·5 to 4·6)	2·5 (1·8 to 3·4)	1·8 (1·3 to 2·4)	1·2 (0·8 to 1·6)	0·7 (0·5 to 1)	0·5 (0·4 to 0·7)	0·3 (0·2 to 0·4)	0·2 (0·1 to 0·2)	0·1 (0 to 0·1)	72·2
Motorcyclist road injuries	0·1 (0 to 0·1)	0·1 (0 to 0·1)	0·3 (0·2 to 0·5)	0·7 (0·5 to 1)	1·6 (1 to 2·5)	1·9 (1·4 to 2·5)	13 (9·1 to 17·4)	11·3 (6·9 to 16·8)	10·4 (7·7 to 14·1)	7·3 (4·8 to 11·1)	5·1 (3·5 to 7·3)	3·5 (2·6 to 4·5)	3·5 (2·7 to 4·6)	2·3 (1·7 to 3)	1·9 (1·4 to 2·5)	1·3 (1 to 1·7)	1 (0·7 to 1·3)	0·6 (0·5 to 0·8)	0·4 (0·3 to 0·6)	0·3 (0·2 to 0·5)	0·2 (0·2 to 0·3)	0·1 (0·1 to 0·2)	0 (0 to 0·1)	67
Motor vehicle road injuries	0·6 (0·4 to 0·8)	0·2 (0·2 to 0·4)	4·1 (2·3 to 7·3)	14·3 (9·5 to 21)	24·7 (16 to 36)	24·5 (16·9 to 34·4)	88 (62·8 to 117)	77 (54·8 to 104)	69·4 (50·6 to 93·5)	52·5 (38 to 70·3)	35 (25·9 to 46·4)	28·1 (21·2 to 37·3)	27·5 (20·8 to 35·9)	19·9 (15·3 to 26·1)	15·1 (11·7 to 19·1)	10·4 (8·4 to 12·8)	7 (5·7 to 8·6)	4·4 (3·4 to 5·5)	2·5 (2 to 3)	1·4 (1·1 to 1·8)	0·8 (0·6 to 1)	0·4 (0·3 to 0·5)	0·2 (0·1 to 0·2)	508
Other road injuries	0 (0 to 0·1)	0 (0 to 0·1)	0·1 (0 to 0·3)	0·7 (0·3 to 1·5)	1·2 (0·6 to 2·1)	0·8 (0·5 to 1·2)	1·9 (1·1 to 3)	1·8 (1·1 to 2·9)	2·8 (1·7 to 4·7)	2·6 (1·6 to 4·2)	1·7 (1·1 to 2·4)	1·4 (1 to 1·9)	1·3 (0·9 to 1·8)	1·2 (0·8 to 1·6)	1 (0·7 to 1·3)	0·7 (0·5 to 1)	0·5 (0·4 to 0·7)	0·3 (0·2 to 0·4)	0·2 (0·2 to 0·3)	0·1 (0·1 to 0·2)	0·1 (0·1 to 0·1)	0 (0 to 0)	0 (0 to 0)	20·5
Other transport injuries	0·4 (0·2 to 0·5)	0·4 (0·2 to 0·6)	1·5 (0·8 to 2·5)	4·9 (2·7 to 8·1)	5·7 (3·5 to 8·5)	6·8 (4·4 to 10)	13·3 (8·2 to 21·6)	11·8 (7 to 18·5)	12 (7·3 to 19·1)	10·5 (6·8 to 15·7)	6·3 (3·9 to 9·8)	7·1 (4·6 to 10·4)	4·9 (3·3 to 7·2)	4·7 (3·1 to 6·8)	3·8 (2·6 to 5·7)	2·2 (1·6 to 3)	1·2 (0·8 to 1·6)	0·8 (0·6 to 1·1)	0·8 (0·5 to 1·1)	0·4 (0·3 to 0·5)	0·3 (0·2 to 0·4)	0·2 (0·1 to 0·2)	0·1 (0·1 to 0·1)	99·9
Unintentional injuries	17·3 (12·6 to 23·3)	11·6 (7·8 to 16·4)	65 (38·8 to 106)	125 (95·2 to 165)	115 (92·4 to 139)	114 (93·4 to 137)	142 (114 to 171)	125 (101 to 152)	128 (103 to 157)	122 (97·8 to 154)	100 (79·4 to 126)	91·5 (72·1 to 114)	89·9 (72 to 111)	78·7 (62·8 to 99)	67·6 (54·1 to 83·3)	55·1 (44·4 to 68)	41·5 (33·3 to 50·4)	35·3 (29·2 to 42·6)	30·4 (25·4 to 36·4)	28·6 (23·9 to 33·9)	23·9 (19·7 to 28·7)	15·3 (12·6 to 18·2)	7·2 (5·8 to 8·6)	1,630
Falls	0·2 (0·1 to 0·3)	0·1 (0·1 to 0·2)	1·3 (0·8 to 2·2)	4·1 (3 to 5·7)	6·4 (4·5 to 9·1)	9·2 (6·2 to 13)	11·9 (8·3 to 17·1)	12·7 (8·6 to 18·4)	13·5 (9·5 to 19)	13·9 (9·9 to 19·5)	14·1 (10 to 19·9)	14·1 (10·2 to 19·6)	14·8 (10·8 to 20·1)	14·9 (11 to 19·9)	14·5 (10·7 to 19)	13·8 (10·5 to 18)	12·1 (9·4 to 15·2)	12·5 (9·9 to 15·4)	12·7 (10·2 to 15·8)	14·5 (11·7 to 17·8)	13·3 (10·7 to 16·6)	8·4 (6·7 to 10·4)	3·8 (3 to 4·6)	237
Drowning	1·3 (0·9 to 1·9)	0·9 (0·6 to 1·4)	7·8 (4·3 to 13·6)	49·8 (34·6 to 69·9)	43·1 (30·2 to 57·3)	39·1 (29 to 49·9)	41·4 (30·8 to 54·6)	18·3 (12·7 to 25·9)	18·8 (13·1 to 26·4)	16 (11·7 to 21·2)	9·2 (6·5 to 12·5)	6·8 (5 to 9)	6·8 (4·9 to 9·3)	4·9 (3·5 to 6·6)	3·4 (2·5 to 4·5)	2·1 (1·7 to 2·7)	1·4 (1·1 to 1·9)	0·8 (0·6 to 1)	0·7 (0·5 to 0·9)	0·4 (0·3 to 0·5)	0·3 (0·2 to 0·4)	0·3 (0·2 to 0·4)	0·1 (0·1 to 0·2)	274
Fire, heat, and hot substances	0·3 (0·2 to 0·4)	0·3 (0·2 to 0·5)	2·8 (1·6 to 4·5)	10·5 (7·3 to 14·7)	12·8 (9·6 to 16·9)	15·9 (11 to 22)	23·2 (16·7 to 30·8)	23·1 (16·9 to 30·8)	24·7 (18·4 to 32)	24·2 (17·5 to 32·1)	21·6 (15·6 to 29·2)	18·8 (13·5 to 25·4)	16·8 (12·2 to 22·6)	14·1 (10·3 to 19·2)	11·3 (8·3 to 15·3)	8·1 (5·9 to 11·1)	5·2 (3·8 to 7·2)	3·5 (2·6 to 4·8)	2·2 (1·6 to 3)	1·5 (1·1 to 2·1)	1·1 (0·8 to 1·4)	0·6 (0·4 to 0·8)	0·3 (0·2 to 0·3)	243
Poisonings	0·3 (0·2 to 0·4)	0·6 (0·4 to 0·9)	1·6 (0·9 to 2·8)	6·1 (4 to 9)	3 (2·1 to 4·1)	2·3 (1·5 to 3·4)	4 (2·8 to 5·4)	3·6 (2·6 to 4·8)	3·4 (2·5 to 4·5)	3·2 (2·3 to 4·2)	2·4 (1·7 to 3·2)	2 (1·4 to 2·6)	1·7 (1·2 to 2·3)	1·4 (1 to 1·8)	1·1 (0·8 to 1·5)	0·7 (0·5 to 1)	0·6 (0·4 to 0·8)	0·4 (0·3 to 0·5)	0·3 (0·2 to 0·4)	0·2 (0·2 to 0·3)	0·1 (0·1 to 0·2)	0·1 (0 to 0·1)	0 (0 to 0)	39·1
Poisoning by carbon monoxide	0·3 (0·2 to 0·4)	0·3 (0·2 to 0·5)	0·8 (0·4 to 1·4)	4·2 (2·6 to 6·3)	1·9 (1·3 to 2·6)	1·2 (0·8 to 1·8)	2·6 (1·9 to 3·5)	2·4 (1·8 to 3·3)	2·2 (1·7 to 3)	2·2 (1·6 to 2·9)	1·5 (1·1 to 1·9)	1·3 (0·9 to 1·7)	1·1 (0·8 to 1·5)	0·8 (0·6 to 1·1)	0·7 (0·5 to 1)	0·5 (0·3 to 0·6)	0·4 (0·3 to 0·6)	0·3 (0·2 to 0·4)	0·2 (0·1 to 0·3)	0·2 (0·1 to 0·2)	0·1 (0·1 to 0·1)	0 (0 to 0)	0 (0 to 0)	25·2
Poisoning by other means	0 (0 to 0·1)	0·3 (0·1 to 0·4)	0·8 (0·4 to 1·5)	1·9 (1 to 3·5)	1·2 (0·7 to 1·8)	1·1 (0·6 to 1·8)	1·4 (0·8 to 2·1)	1·1 (0·7 to 1·8)	1·1 (0·7 to 1·7)	1 (0·6 to 1·5)	0·9 (0·6 to 1·4)	0·7 (0·4 to 1·1)	0·6 (0·4 to 0·9)	0·5 (0·3 to 0·8)	0·4 (0·2 to 0·6)	0·3 (0·2 to 0·4)	0·2 (0·1 to 0·3)	0·1 (0·1 to 0·2)	0·1 (0·1 to 0·1)	0·1 (0 to 0·1)	0·1 (0 to 0·1)	0 (0 to 0)	0 (0 to 0)	13·9
Exposure to mechanical forces	0·7 (0·4 to 1)	0·8 (0·5 to 1·1)	3·2 (2 to 5·2)	16·5 (11·3 to 24)	21·1 (15·1 to 28)	20·7 (15·6 to 26·9)	28·7 (21·3 to 36·5)	28·4 (21·7 to 36·5)	27·2 (20·3 to 35·2)	25 (19·3 to 32)	20·7 (15·8 to 27·3)	18·5 (14·2 to 24·6)	16·6 (12·5 to 22)	14·4 (10·9 to 18·9)	11·2 (8·2 to 15)	8·4 (6·1 to 11·3)	5·9 (4·3 to 8)	4·4 (3·2 to 5·9)	3·2 (2·4 to 4·2)	2·6 (1·9 to 3·4)	2 (1·5 to 2·5)	1·4 (1·1 to 1·7)	0·7 (0·6 to 0·9)	282
Unintentional firearm injuries	0·4 (0·3 to 0·6)	0·2 (0·1 to 0·4)	1·1 (0·6 to 1·9)	4·2 (2·2 to 6·9)	6·4 (3·7 to 10·1)	9 (6 to 12·8)	16·5 (11·7 to 21·7)	16·1 (11·5 to 21·5)	14 (9·9 to 19·3)	12·7 (9·3 to 16·7)	8·7 (6·4 to 11·5)	7 (5·3 to 9·1)	5·7 (4·3 to 7·4)	4·1 (3·1 to 5·2)	3 (2·3 to 3·8)	1·8 (1·4 to 2·4)	1·2 (0·9 to 1·5)	1·1 (0·9 to 1·4)	0·9 (0·7 to 1·1)	0·8 (0·7 to 1·1)	0·8 (0·6 to 1)	0·7 (0·5 to 0·9)	0·4 (0·3 to 0·5)	117
Other exposure to mechanical forces	0·3 (0·2 to 0·4)	0·5 (0·3 to 0·8)	2·1 (1·3 to 3·4)	12·3 (8 to 18)	14·7 (10·1 to 20·5)	11·7 (8 to 16·8)	12·2 (8·1 to 17·8)	12·3 (8·4 to 17·9)	13·2 (9·2 to 18·8)	12·3 (8·5 to 17·6)	12 (8·3 to 17·7)	11·5 (8·1 to 16·4)	10·9 (7·7 to 15·4)	10·3 (7·4 to 14·4)	8·2 (5·8 to 11·6)	6·5 (4·6 to 9·2)	4·8 (3·4 to 6·6)	3·3 (2·3 to 4·6)	2·4 (1·7 to 3·2)	1·7 (1·2 to 2·4)	1·2 (0·8 to 1·6)	0·7 (0·5 to 0·9)	0·3 (0·2 to 0·4)	165
Adverse effects of medical treatment	4·3 (2·7 to 6·1)	3·1 (1·8 to 4·8)	12 (6 to 22·2)	8·5 (5 to 13·5)	5·3 (3·4 to 7·8)	4·5 (2·9 to 6·5)	8 (4·7 to 12)	13·7 (8·3 to 20·7)	13·8 (7·9 to 21·7)	14·9 (8·8 to 22·9)	11 (6·8 to 15·9)	11·8 (7·7 to 17·1)	14·2 (9·1 to 20·2)	12·3 (7·9 to 18)	11·5 (7·3 to 16·8)	10·1 (6·8 to 14·4)	7·2 (5 to 10·1)	6·4 (4·4 to 8·7)	5·2 (3·7 to 7·3)	4 (2·9 to 5·5)	3·1 (2·1 to 4·4)	1·6 (1·1 to 2·4)	0·7 (0·4 to 1)	187
Animal contact	0·1 (0·1 to 0·2)	0·1 (0·1 to 0·2)	1·1 (0·6 to 1·9)	2·7 (1·6 to 4·5)	3 (1·9 to 4·3)	2·7 (1·9 to 3·8)	3·1 (2·2 to 4·3)	2·9 (2·1 to 4·1)	3·5 (2·4 to 5)	2·9 (2·1 to 3·9)	2·4 (1·8 to 3·4)	2·1 (1·6 to 2·8)	2·2 (1·6 to 2·9)	1·8 (1·3 to 2·3)	1·9 (1·3 to 2·6)	1·4 (1 to 1·8)	1 (0·7 to 1·3)	1 (0·7 to 1·4)	1 (0·7 to 1·4)	1·4 (0·9 to 2)	1·4 (0·9 to 2·1)	1·5 (1 to 2·2)	0·9 (0·5 to 1·3)	42·1
Venomous animal contact	0·1 (0 to 0·1)	0 (0 to 0·1)	0·6 (0·2 to 1·1)	1·4 (0·7 to 2·6)	1·4 (0·8 to 2·3)	2 (1·3 to 2·8)	1·8 (1·1 to 2·7)	2 (1·3 to 2·8)	2·6 (1·8 to 3·8)	2·2 (1·6 to 3)	1·6 (1·1 to 2·3)	1·5 (1·1 to 2)	1·2 (0·8 to 1·7)	0·9 (0·6 to 1·3)	1·1 (0·7 to 1·5)	0·8 (0·6 to 1·2)	0·6 (0·4 to 0·8)	0·6 (0·4 to 0·9)	0·7 (0·5 to 1)	0·9 (0·5 to 1·4)	1 (0·6 to 1·5)	1·1 (0·7 to 1·7)	0·7 (0·4 to 1)	26·7
Non-venomous animal contact	0 (0 to 0·1)	0·1 (0 to 0·1)	0·5 (0·2 to 1)	1·3 (0·6 to 2·5)	1·5 (0·8 to 2·6)	0·8 (0·4 to 1·2)	1·3 (0·8 to 1·9)	0·9 (0·6 to 1·5)	0·9 (0·5 to 1·4)	0·7 (0·5 to 1·1)	0·8 (0·5 to 1·3)	0·7 (0·4 to 1)	1 (0·7 to 1·5)	0·9 (0·6 to 1·2)	0·9 (0·5 to 1·3)	0·5 (0·3 to 0·8)	0·4 (0·2 to 0·6)	0·4 (0·2 to 0·6)	0·3 (0·2 to 0·5)	0·5 (0·3 to 0·8)	0·4 (0·2 to 0·7)	0·4 (0·2 to 0·6)	0·2 (0·1 to 0·4)	15·4
Foreign body	9·9 (6·9 to 13·9)	5·4 (3·4 to 8·3)	33·9 (18·6 to 58·9)	21 (14·8 to 29·2)	10·3 (8·1 to 12·8)	7·4 (5·8 to 9·2)	6·3 (4·9 to 8)	6·3 (4·9 to 8·1)	6·8 (5·4 to 8·6)	6·2 (4·7 to 7·9)	4·8 (3·6 to 6)	4·4 (3·4 to 5·5)	4·8 (3·8 to 6)	4·7 (3·5 to 6)	4 (3 to 5·1)	3·4 (2·7 to 4·3)	2·9 (2·3 to 3·6)	2·7 (2·1 to 3·3)	2·5 (2 to 3·1)	2·1 (1·6 to 2·8)	1·2 (0·9 to 1·6)	0·9 (0·6 to 1·1)	0·5 (0·3 to 0·6)	152
Pulmonary aspiration and foreign body in airway	9·9 (6·9 to 13·9)	5·4 (3·3 to 8·3)	33·8 (18·5 to 58·7)	20·1 (14 to 28·4)	9·1 (7 to 11·4)	5·9 (4·5 to 7·4)	4·5 (3·5 to 5·6)	4·5 (3·4 to 5·9)	5·1 (3·9 to 6·6)	4·6 (3·4 to 6)	3·2 (2·4 to 4·2)	3 (2·3 to 3·8)	3·5 (2·7 to 4·6)	3·7 (2·6 to 5)	3·2 (2·3 to 4·3)	2·8 (2·1 to 3·6)	2·4 (1·9 to 3·2)	2·4 (1·8 to 3)	2·3 (1·8 to 2·9)	2 (1·4 to 2·6)	1·1 (0·8 to 1·4)	0·8 (0·6 to 1)	0·4 (0·3 to 0·6)	134
Foreign body in eyes	0 (0 to 0)	0 (0 to 0)	0 (0 to 0·1)	0·2 (0·1 to 0·5)	0·4 (0·1 to 0·9)	0·5 (0·2 to 1·2)	0·7 (0·2 to 1·6)	0·8 (0·3 to 1·8)	0·8 (0·3 to 1·7)	0·8 (0·3 to 1·6)	0·7 (0·3 to 1·5)	0·7 (0·3 to 1·4)	0·6 (0·3 to 1·1)	0·4 (0·2 to 0·8)	0·3 (0·2 to 0·6)	0·3 (0·2 to 0·4)	0·2 (0·1 to 0·3)	0·1 (0·1 to 0·2)	0·1 (0·1 to 0·1)	0·1 (0 to 0·1)	0 (0 to 0·1)	0 (0 to 0)	0 (0 to 0)	7·8
Foreign body in other body part	0 (0 to 0)	0 (0 to 0)	0·1 (0·1 to 0·2)	0·6 (0·4 to 0·9)	0·8 (0·5 to 1·2)	1 (0·7 to 1·4)	1·1 (0·7 to 1·5)	1 (0·7 to 1·4)	0·9 (0·7 to 1·3)	0·9 (0·6 to 1·2)	0·8 (0·6 to 1·1)	0·7 (0·5 to 1)	0·6 (0·5 to 0·9)	0·5 (0·4 to 0·7)	0·4 (0·3 to 0·6)	0·4 (0·3 to 0·5)	0·3 (0·2 to 0·4)	0·2 (0·1 to 0·3)	0·1 (0·1 to 0·2)	0·1 (0·1 to 0·2)	0·1 (0·1 to 0·1)	0 (0 to 0·1)	0 (0 to 0)	10·7
Environmental heat and cold exposure	0 (0 to 0)	0·1 (0 to 0·1)	0·3 (0·2 to 0·4)	1·5 (1 to 2·3)	3·1 (2 to 4·6)	4·2 (2·7 to 6·2)	5·2 (3·5 to 7·5)	5·4 (3·6 to 7·7)	5·3 (3·6 to 7·5)	5·2 (3·6 to 7·3)	4·9 (3·4 to 7)	4·6 (3·2 to 6·5)	4·3 (3 to 5·9)	3·7 (2·6 to 5·1)	3·2 (2·3 to 4·5)	2·6 (1·8 to 3·6)	2 (1·4 to 2·8)	1·5 (1·1 to 2)	1·1 (0·8 to 1·4)	0·9 (0·6 to 1·1)	0·6 (0·4 to 0·8)	0·4 (0·3 to 0·5)	0·2 (0·1 to 0·2)	60·2
Exposure to forces of nature	0 (0 to 0)	0 (0 to 0)	0 (0 to 0)	0 (0 to 0)	0 (0 to 0)	0·1 (0 to 0·1)	0·3 (0·2 to 0·5)	1·1 (0·7 to 1·8)	1·1 (0·7 to 1·8)	1·3 (0·8 to 2·2)	0·6 (0·3 to 0·9)	0·5 (0·3 to 0·8)	0·6 (0·3 to 0·9)	0·5 (0·3 to 0·8)	0·3 (0·2 to 0·6)	0·3 (0·2 to 0·4)	0·2 (0·1 to 0·3)	0·1 (0·1 to 0·2)	0·1 (0·1 to 0·1)	0·1 (0 to 0·1)	0 (0 to 0)	0 (0 to 0)	0 (0 to 0)	7·2
Other unintentional injuries	0·3 (0·2 to 0·4)	0·1 (0·1 to 0·2)	1 (0·7 to 1·5)	4·5 (3·1 to 6·5)	7·1 (4·9 to 10·3)	8·3 (5·7 to 12·1)	10 (6·9 to 14·7)	9·8 (6·5 to 14·2)	9·9 (6·8 to 14)	9·4 (6·4 to 13·3)	8·5 (5·8 to 12·3)	7·8 (5·3 to 11·2)	7·3 (5 to 10·4)	6·2 (4·2 to 9·1)	5·2 (3·5 to 7·5)	4·1 (2·7 to 5·8)	2·9 (2 to 4·2)	2 (1·4 to 2·9)	1·4 (1 to 2)	1 (0·7 to 1·4)	0·6 (0·4 to 0·9)	0·3 (0·2 to 0·4)	0·1 (0·1 to 0·2)	108
Self-harm and interpersonal violence	8·6 (5·1 to 13·5)	1·6 (0·9 to 2·6)	9·4 (5·1 to 16·7)	19·2 (12·4 to 29·8)	18·4 (13·7 to 24·5)	28·8 (22·3 to 36·6)	145 (115 to 180)	165 (129 to 209)	165 (126 to 215)	134 (101 to 172)	77 (59·2 to 98·5)	53·8 (42 to 70·1)	45·9 (36·3 to 57·9)	30·5 (24·4 to 38)	19·3 (15·5 to 24)	12·4 (10·2 to 15·2)	8 (6·7 to 9·7)	4·7 (3·8 to 5·7)	2·8 (2·3 to 3·4)	1·5 (1·2 to 1·8)	0·9 (0·7 to 1)	0·3 (0·2 to 0·4)	0·1 (0·1 to 0·2)	953
Self-harm	0 (0 to 0)	0 (0 to 0)	0 (0 to 0)	0 (0 to 0)	0 (0 to 0)	4·5 (2·9 to 6·7)	36 (25·3 to 50·3)	29·7 (21 to 41·4)	26·5 (17·9 to 38·8)	24·3 (16·9 to 33·1)	14·7 (10·8 to 19·3)	11·6 (8·6 to 15·7)	12·2 (8·9 to 16)	10·2 (7·6 to 13·6)	7·4 (5·5 to 9·9)	4·9 (3·6 to 6·4)	3 (2·3 to 4)	1·8 (1·3 to 2·4)	1 (0·7 to 1·3)	0·5 (0·4 to 0·6)	0·3 (0·2 to 0·3)	0·1 (0·1 to 0·1)	0 (0 to 0)	189
Self-harm by firearm	0 (0 to 0)	0 (0 to 0)	0 (0 to 0)	0 (0 to 0)	0 (0 to 0)	0·2 (0·1 to 0·4)	2·5 (1·7 to 3·5)	2·5 (1·8 to 3·5)	1·9 (1 to 3·1)	2·4 (1·7 to 3·3)	1·1 (0·6 to 1·7)	0·6 (0·4 to 0·8)	0·6 (0·4 to 0·8)	0·5 (0·4 to 0·7)	0·4 (0·3 to 0·5)	0·1 (0·1 to 0·2)	0·1 (0·1 to 0·1)	0·1 (0·1 to 0·1)	0·1 (0·1 to 0·1)	0·1 (0·1 to 0·1)	0·1 (0 to 0·1)	0 (0 to 0)	0 (0 to 0)	13·2
Self-harm by other specified means	0 (0 to 0)	0 (0 to 0)	0 (0 to 0)	0 (0 to 0)	0 (0 to 0)	4·2 (2·8 to 6·4)	33·5 (23·6 to 46·8)	27·2 (19·4 to 37·9)	24·6 (16·5 to 36)	21·9 (15·3 to 29·8)	13·6 (10 to 18)	11 (8·2 to 14·9)	11·6 (8·5 to 15·3)	9·7 (7·2 to 12·9)	7 (5·2 to 9·4)	4·8 (3·5 to 6·3)	2·9 (2·2 to 3·8)	1·7 (1·3 to 2·3)	0·9 (0·7 to 1·2)	0·4 (0·3 to 0·5)	0·2 (0·2 to 0·3)	0·1 (0·1 to 0·1)	0 (0 to 0)	175
Interpersonal violence	8·6 (5·1 to 13·5)	1·6 (0·9 to 2·6)	9·3 (5·1 to 16·6)	18·9 (12·2 to 29·5)	18·3 (13·5 to 24·3)	24·2 (17·9 to 32·1)	109 (82·1 to 142)	135 (101 to 180)	137 (99·9 to 182)	110 (79·5 to 145)	60·3 (44·8 to 80·5)	41·9 (31·1 to 57·4)	32·8 (24·7 to 43·3)	19·7 (14·6 to 25·9)	11·7 (9 to 15·4)	7·1 (5·5 to 9·1)	4·6 (3·6 to 5·8)	2·8 (2·2 to 3·5)	1·8 (1·4 to 2·2)	0·9 (0·7 to 1·1)	0·5 (0·4 to 0·7)	0·2 (0·2 to 0·3)	0·1 (0·1 to 0·1)	756
Physical violence by firearm	0·3 (0·2 to 0·6)	0·1 (0 to 0·1)	0·6 (0·3 to 1·1)	2·9 (1·6 to 5)	3·6 (2·1 to 5·6)	6·2 (4 to 9·4)	43·9 (30·4 to 61·5)	50·5 (34 to 70·1)	48·7 (32 to 69·9)	37·5 (24·2 to 52·9)	19·2 (12·6 to 27·8)	10·8 (7·2 to 16)	9·9 (6·7 to 14·6)	5·1 (3·4 to 7·5)	2·9 (2 to 4·2)	1·3 (0·9 to 1·8)	0·6 (0·4 to 0·9)	0·3 (0·2 to 0·4)	0·1 (0·1 to 0·2)	0 (0 to 0·1)	0 (0 to 0·1)	0 (0 to 0)	0 (0 to 0)	245
Physical violence by sharp object	0·6 (0·3 to 0·9)	0 (0 to 0)	0·6 (0·3 to 1)	1·6 (1·1 to 2·5)	2·2 (1·6 to 3)	4·1 (3 to 5·7)	33·6 (23·6 to 45·7)	49·1 (35·2 to 68)	52 (36·2 to 73·4)	40·2 (27·5 to 54·3)	22·7 (15·9 to 31·3)	15·9 (11·4 to 22·9)	10 (7 to 13·6)	5·3 (3·8 to 7)	3 (2·2 to 4)	1·5 (1·2 to 2)	1 (0·7 to 1·2)	0·6 (0·4 to 0·7)	0·3 (0·2 to 0·4)	0·2 (0·1 to 0·2)	0·1 (0·1 to 0·1)	0·1 (0 to 0·1)	0 (0 to 0)	244
Sexual violence	0 (0 to 0)	0 (0 to 0)	0 (0 to 0)	0·1 (0·1 to 0·2)	1·3 (0·8 to 2)	3·1 (1·8 to 5)	7·6 (4·2 to 12·2)	9·3 (5·4 to 14·3)	7·7 (4·3 to 12·4)	6·4 (3·5 to 10·7)	5·2 (2·9 to 8·3)	3·8 (2·2 to 6·2)	2·7 (1·6 to 4·5)	1·6 (0·9 to 2·7)	1 (0·6 to 1·6)	0·6 (0·4 to 1)	0·4 (0·3 to 0·6)	0·3 (0·2 to 0·4)	0·2 (0·1 to 0·3)	0·1 (0·1 to 0·2)	0·1 (0 to 0·1)	0 (0 to 0)	0 (0 to 0)	51·6
Physical violence by other means	7·7 (4·6 to 12·2)	1·5 (0·8 to 2·5)	8·2 (4·4 to 14·8)	14·2 (9 to 22·4)	11·2 (7·8 to 15·4)	10·6 (7·7 to 14·7)	24 (17·2 to 32·3)	26·2 (19 to 35·7)	28·1 (20 to 39·3)	25·6 (17·9 to 34·5)	13·3 (9·8 to 17·8)	11·5 (8·5 to 15·9)	10·2 (7·5 to 13·6)	7·6 (5·6 to 10·2)	5 (3·7 to 6·6)	3·6 (2·7 to 4·7)	2·6 (2 to 3·3)	1·6 (1·3 to 2·1)	1·2 (0·9 to 1·5)	0·5 (0·4 to 0·7)	0·3 (0·2 to 0·4)	0·1 (0·1 to 0·2)	0 (0 to 0·1)	215
Conflict and terrorism	0 (0 to 0)	0 (0 to 0)	0 (0 to 0)	0 (0 to 0)	0 (0 to 0)	0 (0 to 0)	0 (0 to 0)	0 (0 to 0)	2·3 (1·2 to 4·3)	0·2 (0·1 to 0·4)	1·9 (1 to 3·4)	0·3 (0·1 to 0·5)	0·8 (0·5 to 1·5)	0·6 (0·3 to 1·1)	0·1 (0·1 to 0·2)	0·5 (0·3 to 0·8)	0·4 (0·2 to 0·6)	0·1 (0 to 0·1)	0 (0 to 0)	0·1 (0 to 0·1)	0·1 (0 to 0·1)	0 (0 to 0)	0 (0 to 0)	7·3

SR Table 2b: Cuba DALYS by cause and age group for females with 95% uncertainty intervals, 2019

Cause	Early Neonatal	Late Neonatal	Post Neonatal	1 to 4	5 to 9	10 to 14	15 to 19	20 to 24	25 to 29	30 to 34	35 to 39	40 to 44	45 to 49	50 to 54	55 to 59	60 to 64	65 to 69	70 to 74	75 to 79	80 to 84	85 to 89	90 to 94	95 plus	Total
All causes	5,790 (4,650 to 7,020)	3,060 (2,500 to 3,680)	7,470 (6,340 to 8,760)	11,100 (8,830 to 14,100)	14,100 (10,600 to 18,700)	18,700 (14,200 to 24,200)	31,000 (24,100 to 39,800)	38,000 (29,400 to 47,900)	47,500 (36,700 to 59,500)	56,700 (44,400 to 70,300)	55,000 (43,900 to 68,000)	69,200 (56,000 to 85,300)	112,000 (91,800 to 137,000)	144,000 (118,000 to 173,000)	160,000 (134,000 to 190,000)	140,000 (118,000 to 164,000)	150,000 (127,000 to 174,000)	151,000 (129,000 to 175,000)	145,000 (125,000 to 166,000)	119,000 (104,000 to 135,000)	82,700 (72,100 to 93,900)	49,400 (43,700 to 55,200)	25,900 (23,800 to 28,200)	1,640,000
Communicable, maternal, neonatal, and nutritional diseases	4,390 (2,660 to 5,490)	1,980 (1,330 to 2,480)	3,180 (2,390 to 3,900)	3,470 (2,570 to 4,860)	3,490 (2,260 to 5,420)	2,520 (1,790 to 3,620)	3,560 (2,510 to 5,190)	3,800 (2,740 to 5,360)	4,500 (3,390 to 6,300)	5,070 (3,830 to 6,950)	4,570 (3,460 to 6,370)	4,930 (3,530 to 7,140)	6,660 (4,850 to 9,210)	7,470 (5,450 to 10,100)	7,430 (5,550 to 10,000)	6,020 (4,660 to 7,640)	6,540 (5,100 to 8,230)	7,680 (5,950 to 9,740)	8,820 (6,940 to 11,400)	8,120 (6,550 to 10,000)	6,460 (5,050 to 7,930)	4,380 (3,390 to 5,340)	2,740 (2,020 to 3,450)	118,000
HIV/AIDS and sexually transmitted infections	0·2 (0·1 to 0·4)	0·1 (0 to 0·2)	27·7 (25·7 to 30·1)	20·8 (17·8 to 27·5)	27·4 (23·7 to 33·5)	27·9 (24·3 to 35·1)	85·4 (66·9 to 112)	227 (178 to 303)	559 (457 to 713)	751 (607 to 955)	679 (544 to 865)	648 (507 to 859)	771 (601 to 1,010)	437 (321 to 618)	351 (255 to 498)	202 (138 to 294)	161 (110 to 237)	71·3 (41·3 to 121)	41·2 (24·3 to 73·5)	21·1 (11·5 to 40·1)	10·7 (5·9 to 20)	4·5 (2·4 to 8·3)	1·6 (0·9 to 3·2)	5,130
HIV/AIDS	0 (0 to 0)	0 (0 to 0·1)	27·7 (25·6 to 30·1)	20·8 (17·8 to 27·5)	27·4 (23·7 to 33·5)	22·2 (19·4 to 28·8)	34·3 (31·2 to 38·4)	109 (98·4 to 124)	363 (322 to 429)	483 (423 to 581)	413 (360 to 511)	336 (293 to 411)	404 (354 to 489)	218 (185 to 271)	195 (158 to 269)	118 (87·7 to 180)	95·4 (69·8 to 151)	20·2 (9·9 to 42·1)	6·4 (3·6 to 10·8)	1·7 (1·4 to 2·1)	0·8 (0·6 to 1)	0·2 (0·1 to 0·3)	0·1 (0 to 0·1)	2,900
HIV/AIDS - Drug-susceptible Tuberculosis	0 (0 to 0)	0 (0 to 0)	0·4 (0·2 to 0·6)	0·3 (0·2 to 0·4)	0·4 (0·2 to 0·5)	0·3 (0·2 to 0·4)	0·5 (0·3 to 0·7)	1·6 (1·1 to 2·3)	5·2 (3·5 to 7·6)	7 (4·8 to 10·1)	6·1 (4·2 to 8·7)	5 (3·5 to 7·2)	6 (4·1 to 8·5)	3·3 (2·4 to 4·7)	3·1 (2·2 to 4·2)	2 (1·5 to 2·6)	1·6 (1·2 to 2·2)	0·4 (0·3 to 0·5)	0·1 (0·1 to 0·2)	0 (0 to 0)	0 (0 to 0)	0 (0 to 0)	0 (0 to 0)	43·4
HIV/AIDS - Multidrug-resistant Tuberculosis without exten	0 (0 to 0)	0 (0 to 0)	0 (0 to 0·1)	0 (0 to 0·1)	0 (0 to 0·1)	0 (0 to 0·1)	0 (0 to 0·1)	0·1 (0 to 0·3)	0·3 (0 to 0·9)	0·4 (0·1 to 1·2)	0·3 (0·1 to 1)	0·3 (0 to 0·8)	0·3 (0·1 to 1)	0·2 (0 to 0·6)	0·1 (0 to 0·5)	0·1 (0 to 0·3)	0·1 (0 to 0·2)	0 (0 to 0)	0 (0 to 0)	0 (0 to 0)	0 (0 to 0)	0 (0 to 0)	0 (0 to 0)	2·3
HIV/AIDS - Extensively drug-resistant Tuberculosis	0 (0 to 0)	0 (0 to 0)	0 (0 to 0)	0 (0 to 0)	0 (0 to 0)	0 (0 to 0)	0 (0 to 0)	0 (0 to 0)	0 (0 to 0·2)	0·1 (0 to 0·2)	0 (0 to 0·2)	0 (0 to 0·1)	0 (0 to 0·2)	0 (0 to 0·1)	0 (0 to 0·1)	0 (0 to 0)	0 (0 to 0)	0 (0 to 0)	0 (0 to 0)	0 (0 to 0)	0 (0 to 0)	0 (0 to 0)	0 (0 to 0)	0·3
HIV/AIDS resulting in other diseases	0 (0 to 0)	0 (0 to 0·1)	27·3 (25·2 to 29·7)	20·5 (17·5 to 27·3)	27 (23·3 to 33·1)	21·9 (19·1 to 28·5)	33·8 (30·7 to 37·9)	107 (96·6 to 122)	357 (316 to 424)	476 (416 to 576)	407 (352 to 504)	330 (287 to 406)	398 (348 to 483)	215 (182 to 267)	192 (155 to 266)	116 (85·8 to 177)	93·7 (68 to 149)	19·8 (9·5 to 41·7)	6·3 (3·5 to 10·6)	1·6 (1·4 to 2·1)	0·8 (0·6 to 1)	0·2 (0·1 to 0·3)	0·1 (0 to 0·1)	2,850
Sexually transmitted infections excluding HIV	0·2 (0·1 to 0·3)	0·1 (0 to 0·1)	0 (0 to 0)	0 (0 to 0)	0 (0 to 0)	5·6 (3·8 to 8·7)	51·1 (33·2 to 77·1)	118 (73·6 to 189)	197 (113 to 328)	268 (156 to 436)	265 (164 to 434)	312 (197 to 515)	367 (227 to 605)	219 (117 to 402)	156 (83·6 to 285)	83·6 (42·7 to 160)	65·1 (32·5 to 130)	51·1 (25·9 to 99·9)	34·8 (18·8 to 67·1)	19·5 (9·9 to 38·3)	10 (5·2 to 19·3)	4·3 (2·2 to 8·1)	1·6 (0·8 to 3·1)	2,230
Syphilis	0·2 (0·1 to 0·3)	[illegible]	[illegible]	[illegible]	[illegible]	[illegible]	[illegible]	[illegible]	[illegible]	[illegible]	[illegible]	[illegible]	[illegible]	[illegible]	[illegible]	[illegible]	[illegible]	[illegible]	[illegible]	[illegible]	[illegible]	[illegible]	0·2 (0·1 to 0·3)	90·5
Chlamydial infection	0 (0 to 0)	[illegible]	[illegible]	[illegible]	[illegible]	[illegible]	[illegible]	[illegible]	[illegible]	[illegible]	[illegible]	[illegible]	[illegible]	[illegible]	[illegible]	[illegible]	[illegible]	[illegible]	[illegible]	[illegible]	[illegible]	[illegible]	0·1 (0·1 to 0·1)	210
Gonococcal infection	0 (0 to 0)	[illegible]	[illegible]	[illegible]	[illegible]	[illegible]	[illegible]	[illegible]	[illegible]	[illegible]	[illegible]	[illegible]	[illegible]	[illegible]	[illegible]	[illegible]	[illegible]	[illegible]	[illegible]	[illegible]	[illegible]	[illegible]	0·2 (0·1 to 0·2)	317
Trichomoniasis	0 (0 to 0)	[illegible]	[illegible]	[illegible]	[illegible]	[illegible]	[illegible]	[illegible]	[illegible]	[illegible]	[illegible]	[illegible]	[illegible]	[illegible]	[illegible]	[illegible]	[illegible]	[illegible]	[illegible]	[illegible]	[illegible]	[illegible]	0·1 (0 to 0·3)	593
Genital herpes	0 (0 to 0)	[illegible]	[illegible]	[illegible]	[illegible]	[illegible]	[illegible]	[illegible]	[illegible]	[illegible]	[illegible]	[illegible]	[illegible]	[illegible]	[illegible]	[illegible]	[illegible]	[illegible]	[illegible]	[illegible]	[illegible]	[illegible]	0·9 (0·3 to 2·3)	493
Other sexually transmitted infections	0 (0 to 0)	[illegible]	[illegible]	[illegible]	[illegible]	[illegible]	[illegible]	[illegible]	[illegible]	[illegible]	[illegible]	[illegible]	[illegible]	[illegible]	[illegible]	[illegible]	[illegible]	[illegible]	[illegible]	[illegible]	[illegible]	[illegible]	0·1 (0 to 0·1)	525
Respiratory infections and tuberculosis	229 (135 to 324)	[illegible]	[illegible]	[illegible]	[illegible]	[illegible]	[illegible]	[illegible]	[illegible]	[illegible]	[illegible]	[illegible]	[illegible]	[illegible]	[illegible]	[illegible]	[illegible]	[illegible]	[illegible]	[illegible]	[illegible]	[illegible]	2,550 (1,850 to 3,220)	52,700
Tuberculosis	0 (0 to 0)	[illegible]	[illegible]	[illegible]	[illegible]	[illegible]	[illegible]	[illegible]	[illegible]	[illegible]	[illegible]	[illegible]	[illegible]	[illegible]	[illegible]	[illegible]	[illegible]	[illegible]	[illegible]	[illegible]	[illegible]	[illegible]	3 (2 to 4·1)	518
Latent tuberculosis infection	0 (0 to 0)	[illegible]	[illegible]	[illegible]	[illegible]	[illegible]	[illegible]	[illegible]	[illegible]	[illegible]	[illegible]	[illegible]	[illegible]	[illegible]	[illegible]	[illegible]	[illegible]	[illegible]	[illegible]	[illegible]	[illegible]	[illegible]	0 (0 to 0)	0
Drug-susceptible tuberculosis	0 (0 to 0)	[illegible]	[illegible]	[illegible]	[illegible]	[illegible]	[illegible]	[illegible]	[illegible]	[illegible]	[illegible]	[illegible]	[illegible]	[illegible]	[illegible]	[illegible]	[illegible]	[illegible]	[illegible]	[illegible]	[illegible]	[illegible]	2·8 (1·9 to 3·9)	499
Multidrug-resistant tuberculosis without extensive drug res	0 (0 to 0)	[illegible]	[illegible]	[illegible]	[illegible]	[illegible]	[illegible]	[illegible]	[illegible]	[illegible]	[illegible]	[illegible]	[illegible]	[illegible]	[illegible]	[illegible]	[illegible]	[illegible]	[illegible]	[illegible]	[illegible]	[illegible]	0·1 (0 to 0·4)	17
Extensively drug-resistant tuberculosis	0 (0 to 0)	[illegible]	[illegible]	[illegible]	[illegible]	[illegible]	[illegible]	[illegible]	[illegible]	[illegible]	[illegible]	[illegible]	[illegible]	[illegible]	[illegible]	[illegible]	[illegible]	[illegible]	[illegible]	[illegible]	[illegible]	[illegible]	0 (0 to 0·1)	2·2
Lower respiratory infections	229 (134 to 324)	[illegible]	[illegible]	[illegible]	[illegible]	[illegible]	[illegible]	[illegible]	[illegible]	[illegible]	[illegible]	[illegible]	[illegible]	[illegible]	[illegible]	[illegible]	[illegible]	[illegible]	[illegible]	[illegible]	[illegible]	[illegible]	2,540 (1,840 to 3,210)	46,500
Upper respiratory infections	0·5 (0·3 to 0·9)	[illegible]	[illegible]	[illegible]	[illegible]	[illegible]	[illegible]	[illegible]	[illegible]	[illegible]	[illegible]	[illegible]	[illegible]	[illegible]	[illegible]	[illegible]	[illegible]	[illegible]	[illegible]	[illegible]	[illegible]	[illegible]	6·2 (3·3 to 10·8)	4,240
Otitis media	0 (0 to 0·1)	[illegible]	[illegible]	[illegible]	[illegible]	[illegible]	[illegible]	[illegible]	[illegible]	[illegible]	[illegible]	[illegible]	[illegible]	[illegible]	[illegible]	[illegible]	[illegible]	[illegible]	[illegible]	[illegible]	[illegible]	[illegible]	2·3 (1·1 to 4·3)	1,410
Enteric infections	5·5 (3·6 to 8)	[illegible]	[illegible]	[illegible]	[illegible]	[illegible]	[illegible]	[illegible]	[illegible]	[illegible]	[illegible]	[illegible]	[illegible]	[illegible]	[illegible]	[illegible]	[illegible]	[illegible]	[illegible]	[illegible]	[illegible]	[illegible]	138 (106 to 174)	13,300
Diarrheal diseases	5·5 (3·6 to 8)	[illegible]	[illegible]	[illegible]	[illegible]	[illegible]	[illegible]	[illegible]	[illegible]	[illegible]	[illegible]	[illegible]	[illegible]	[illegible]	[illegible]	[illegible]	[illegible]	[illegible]	[illegible]	[illegible]	[illegible]	[illegible]	138 (105 to 174)	13,300
Typhoid and paratyphoid	0 (0 to 0)	[illegible]	[illegible]	[illegible]	[illegible]	[illegible]	[illegible]	[illegible]	[illegible]	[illegible]	[illegible]	[illegible]	[illegible]	[illegible]	[illegible]	[illegible]	[illegible]	[illegible]	[illegible]	[illegible]	[illegible]	[illegible]	0 (0 to 0)	5·8
Typhoid fever	0 (0 to 0)	[illegible]	[illegible]	[illegible]	[illegible]	[illegible]	[illegible]	[illegible]	[illegible]	[illegible]	[illegible]	[illegible]	[illegible]	[illegible]	[illegible]	[illegible]	[illegible]	[illegible]	[illegible]	[illegible]	[illegible]	[illegible]	0 (0 to 0)	5·6
Paratyphoid fever	0 (0 to 0)	[illegible]	[illegible]	[illegible]	[illegible]	[illegible]	[illegible]	[illegible]	[illegible]	[illegible]	[illegible]	[illegible]	[illegible]	[illegible]	[illegible]	[illegible]	[illegible]	[illegible]	[illegible]	[illegible]	[illegible]	[illegible]	0 (0 to 0)	0·3
Invasive Non-typhoidal Salmonella (iNTS)	0 (0 to 0)	[illegible]	[illegible]	[illegible]	[illegible]	[illegible]	[illegible]	[illegible]	[illegible]	[illegible]	[illegible]	[illegible]	[illegible]	[illegible]	[illegible]	[illegible]	[illegible]	[illegible]	[illegible]	[illegible]	[illegible]	[illegible]	0 (0 to 0)	5·7
Other intestinal infectious diseases	0 (0 to 0)	[illegible]	[illegible]	[illegible]	[illegible]	[illegible]	[illegible]	[illegible]	[illegible]	[illegible]	[illegible]	[illegible]	[illegible]	[illegible]	[illegible]	[illegible]	[illegible]	[illegible]	[illegible]	[illegible]	[illegible]	[illegible]	0·2 (0 to 0·7)	15·6
Neglected tropical diseases and malaria	0·2 (0·1 to 0·4)	[illegible]	[illegible]	[illegible]	[illegible]	[illegible]	[illegible]	[illegible]	[illegible]	[illegible]	[illegible]	[illegible]	[illegible]	[illegible]	[illegible]	[illegible]	[illegible]	[illegible]	[illegible]	[illegible]	[illegible]	[illegible]	5·2 (0·7 to 13·3)	1,740
Malaria	0 (0 to 0)	[illegible]	[illegible]	[illegible]	[illegible]	[illegible]	[illegible]	[illegible]	[illegible]	[illegible]	[illegible]	[illegible]	[illegible]	[illegible]	[illegible]	[illegible]	[illegible]	[illegible]	[illegible]	[illegible]	[illegible]	[illegible]	0 (0 to 0)	0
Chagas disease	0 (0 to 0)	[illegible]	[illegible]	[illegible]	[illegible]	[illegible]	[illegible]	[illegible]	[illegible]	[illegible]	[illegible]	[illegible]	[illegible]	[illegible]	[illegible]	[illegible]	[illegible]	[illegible]	[illegible]	[illegible]	[illegible]	[illegible]	0 (0 to 0)	0·1
Leishmaniasis	0 (0 to 0)	[illegible]	[illegible]	[illegible]	[illegible]	[illegible]	[illegible]	[illegible]	[illegible]	[illegible]	[illegible]	[illegible]	[illegible]	[illegible]	[illegible]	[illegible]	[illegible]	[illegible]	[illegible]	[illegible]	[illegible]	[illegible]	0 (0 to 0)	0
Visceral leishmaniasis	0 (0 to 0)	[illegible]	[illegible]	[illegible]	[illegible]	[illegible]	[illegible]	[illegible]	[illegible]	[illegible]	[illegible]	[illegible]	[illegible]	[illegible]	[illegible]	[illegible]	[illegible]	[illegible]	[illegible]	[illegible]	[illegible]	[illegible]	0 (0 to 0)	0
Cutaneous and mucocutaneous leishmaniasis	0 (0 to 0)	[illegible]	[illegible]	[illegible]	[illegible]	[illegible]	[illegible]	[illegible]	[illegible]	[illegible]	[illegible]	[illegible]	[illegible]	[illegible]	[illegible]	[illegible]	[illegible]	[illegible]	[illegible]	[illegible]	[illegible]	[illegible]	0 (0 to 0)	0
African trypanosomiasis	0 (0 to 0)	[illegible]	[illegible]	[illegible]	[illegible]	[illegible]	[illegible]	[illegible]	[illegible]	[illegible]	[illegible]	[illegible]	[illegible]	[illegible]	[illegible]	[illegible]	[illegible]	[illegible]	[illegible]	[illegible]	[illegible]	[illegible]	0 (0 to 0)	0
Schistosomiasis	0 (0 to 0)	[illegible]	[illegible]	[illegible]	[illegible]	[illegible]	[illegible]	[illegible]	[illegible]	[illegible]	[illegible]	[illegible]	[illegible]	[illegible]	[illegible]	[illegible]	[illegible]	[illegible]	[illegible]	[illegible]	[illegible]	[illegible]	0 (0 to 0)	0
Cysticercosis	0 (0 to 0)	[illegible]	[illegible]	[illegible]	[illegible]	[illegible]	[illegible]	[illegible]	[illegible]	[illegible]	[illegible]	[illegible]	[illegible]	[illegible]	[illegible]	[illegible]	[illegible]	[illegible]	[illegible]	[illegible]	[illegible]	[illegible]	0 (0 to 0)	0
Cystic echinococcosis	0 (0 to 0)	[illegible]	[illegible]	[illegible]	[illegible]	[illegible]	[illegible]	[illegible]	[illegible]	[illegible]	[illegible]	[illegible]	[illegible]	[illegible]	[illegible]	[illegible]	[illegible]	[illegible]	[illegible]	[illegible]	[illegible]	[illegible]	0 (0 to 0)	0·8
Lymphatic filariasis	0 (0 to 0)	[illegible]	[illegible]	[illegible]	[illegible]	[illegible]	[illegible]	[illegible]	[illegible]	[illegible]	[illegible]	[illegible]	[illegible]	[illegible]	[illegible]	[illegible]	[illegible]	[illegible]	[illegible]	[illegible]	[illegible]	[illegible]	0 (0 to 0)	0
Onchocerciasis	0 (0 to 0)	[illegible]	[illegible]	[illegible]	[illegible]	[illegible]	[illegible]	[illegible]	[illegible]	[illegible]	[illegible]	[illegible]	[illegible]	[illegible]	[illegible]	[illegible]	[illegible]	[illegible]	[illegible]	[illegible]	[illegible]	[illegible]	0 (0 to 0)	0
Trachoma	0 (0 to 0)	[illegible]	[illegible]	[illegible]	[illegible]	[illegible]	[illegible]	[illegible]	[illegible]	[illegible]	[illegible]	[illegible]	[illegible]	[illegible]	[illegible]	[illegible]	[illegible]	[illegible]	[illegible]	[illegible]	[illegible]	[illegible]	0 (0 to 0)	0
Dengue	0 (0 to 0)	[illegible]	[illegible]	[illegible]	[illegible]	[illegible]	[illegible]	[illegible]	[illegible]	[illegible]	[illegible]	[illegible]	[illegible]	[illegible]	[illegible]	[illegible]	[illegible]	[illegible]	[illegible]	[illegible]	[illegible]	[illegible]	4·2 (0 to 12·3)	373
Yellow fever	0 (0 to 0)	[illegible]	[illegible]	[illegible]	[illegible]	[illegible]	[illegible]	[illegible]	[illegible]	[illegible]	[illegible]	[illegible]	[illegible]	[illegible]	[illegible]	[illegible]	[illegible]	[illegible]	[illegible]	[illegible]	[illegible]	[illegible]	0 (0 to 0)	0
Rabies	0 (0 to 0)	[illegible]	[illegible]	[illegible]	[illegible]	[illegible]	[illegible]	[illegible]	[illegible]	[illegible]	[illegible]	[illegible]	[illegible]	[illegible]	[illegible]	[illegible]	[illegible]	[illegible]	[illegible]	[illegible]	[illegible]	[illegible]	0 (0 to 0)	0·2
Intestinal nematode infections	0 (0 to 0)	[illegible]	[illegible]	[illegible]	[illegible]	[illegible]	[illegible]	[illegible]	[illegible]	[illegible]	[illegible]	[illegible]	[illegible]	[illegible]	[illegible]	[illegible]	[illegible]	[illegible]	[illegible]	[illegible]	[illegible]	[illegible]	0·3 (0·1 to 0·6)	577
Ascariasis	0 (0 to 0)	[illegible]	[illegible]	[illegible]	[illegible]	[illegible]	[illegible]	[illegible]	[illegible]	[illegible]	[illegible]	[illegible]	[illegible]	[illegible]	[illegible]	[illegible]	[illegible]	[illegible]	[illegible]	[illegible]	[illegible]	[illegible]	0·2 (0 to 0·4)	177
Trichuriasis	0 (0 to 0)	[illegible]	[illegible]	[illegible]	[illegible]	[illegible]	[illegible]	[illegible]	[illegible]	[illegible]	[illegible]	[illegible]	[illegible]	[illegible]	[illegible]	[illegible]	[illegible]	[illegible]	[illegible]	[illegible]	[illegible]	[illegible]	0 (0 to 0)	21·3
Hookworm disease	0 (0 to 0)	[illegible]	[illegible]	[illegible]	[illegible]	[illegible]	[illegible]	[illegible]	[illegible]	[illegible]	[illegible]	[illegible]	[illegible]	[illegible]	[illegible]	[illegible]	[illegible]	[illegible]	[illegible]	[illegible]	[illegible]	[illegible]	0·2 (0·1 to 0·3)	379
Food-borne trematodiases	0 (0 to 0)	[illegible]	[illegible]	[illegible]	[illegible]	[illegible]	[illegible]	[illegible]	[illegible]	[illegible]	[illegible]	[illegible]	[illegible]	[illegible]	[illegible]	[illegible]	[illegible]	[illegible]	[illegible]	[illegible]	[illegible]	[illegible]	0 (0 to 0)	0
Leprosy	0 (0 to 0)	[illegible]	[illegible]	[illegible]	[illegible]	[illegible]	[illegible]	[illegible]	[illegible]	[illegible]	[illegible]	[illegible]	[illegible]	[illegible]	[illegible]	[illegible]	[illegible]	[illegible]	[illegible]	[illegible]	[illegible]	[illegible]	0·1 (0 to 0·1)	6·3
Ebola	0 (0 to 0)	[illegible]	[illegible]	[illegible]	[illegible]	[illegible]	[illegible]	[illegible]	[illegible]	[illegible]	[illegible]	[illegible]	[illegible]	[illegible]	[illegible]	[illegible]	[illegible]	[illegible]	[illegible]	[illegible]	[illegible]	[illegible]	0 (0 to 0)	0
Zika virus	0 (0 to 0)	[illegible]	[illegible]	[illegible]	[illegible]	[illegible]	[illegible]	[illegible]	[illegible]	[illegible]	[illegible]	[illegible]	[illegible]	[illegible]	[illegible]	[illegible]	[illegible]	[illegible]	[illegible]	[illegible]	[illegible]	[illegible]	0 (0 to 0)	1·1
Guinea worm disease	0 (0 to 0)	[illegible]	[illegible]	[illegible]	[illegible]	[illegible]	[illegible]	[illegible]	[illegible]	[illegible]	[illegible]	[illegible]	[illegible]	[illegible]	[illegible]	[illegible]	[illegible]	[illegible]	[illegible]	[illegible]	[illegible]	[illegible]	0 (0 to 0)	0
Other neglected tropical diseases	0·2 (0·1 to 0·4)	[illegible]	[illegible]	[illegible]	[illegible]	[illegible]	[illegible]	[illegible]	[illegible]	[illegible]	[illegible]	[illegible]	[illegible]	[illegible]	[illegible]	[illegible]	[illegible]	[illegible]	[illegible]	[illegible]	[illegible]	[illegible]	0·6 (0·2 to 1·4)	783
Other infectious diseases	21·2 (12·7 to 29·8)	[illegible]	[illegible]	[illegible]	[illegible]	[illegible]	[illegible]	[illegible]	[illegible]	[illegible]	[illegible]	[illegible]	[illegible]	[illegible]	[illegible]	[illegible]	[illegible]	[illegible]	[illegible]	[illegible]	[illegible]	[illegible]	11·8 (7·8 to 20·4)	5,040
Meningitis	9·6 (5·5 to 14·2)	[illegible]	[illegible]	[illegible]	[illegible]	[illegible]	[illegible]	[illegible]	[illegible]	[illegible]	[illegible]	[illegible]	[illegible]	[illegible]	[illegible]	[illegible]	[illegible]	[illegible]	[illegible]	[illegible]	[illegible]	[illegible]	3·1 (2·3 to 4)	1,730
Encephalitis	3·5 (1·4 to 10·4)	[illegible]	[illegible]	[illegible]	[illegible]	[illegible]	[illegible]	[illegible]	[illegible]	[illegible]	[illegible]	[illegible]	[illegible]	[illegible]	[illegible]	[illegible]	[illegible]	[illegible]	[illegible]	[illegible]	[illegible]	[illegible]	1 (0·6 to 1·8)	1,190
Diphtheria	0 (0 to 0)	[illegible]	[illegible]	[illegible]	[illegible]	[illegible]	[illegible]	[illegible]	[illegible]	[illegible]	[illegible]	[illegible]	[illegible]	[illegible]	[illegible]	[illegible]	[illegible]	[illegible]	[illegible]	[illegible]	[illegible]	[illegible]	0 (0 to 0)	0·3
Tetanus	0 (0 to 0)	[illegible]	[illegible]	[illegible]	[illegible]	[illegible]	[illegible]	[illegible]	[illegible]	[illegible]	[illegible]	[illegible]	[illegible]	[illegible]	[illegible]	[illegible]	[illegible]	[illegible]	[illegible]	[illegible]	[illegible]	[illegible]	0 (0 to 0)	0·6
Measles	0 (0 to 0)	[illegible]	[illegible]	[illegible]	[illegible]	[illegible]	[illegible]	[illegible]	[illegible]	[illegible]	[illegible]	[illegible]	[illegible]	[illegible]	[illegible]	[illegible]	[illegible]	[illegible]	[illegible]	[illegible]	[illegible]	[illegible]	0 (0 to 0)	0·7
Varicella and herpes zoster	0·1 (0 to 0·3)	[illegible]	[illegible]	[illegible]	[illegible]	[illegible]	[illegible]	[illegible]	[illegible]	[illegible]	[illegible]	[illegible]	[illegible]	[illegible]	[illegible]	[illegible]	[illegible]	[illegible]	[illegible]	[illegible]	[illegible]	[illegible]	2·8 (1·5 to 7·5)	312
Acute hepatitis	0 (0 to 0)	[illegible]	[illegible]	[illegible]	[illegible]	[illegible]	[illegible]	[illegible]	[illegible]	[illegible]	[illegible]	[illegible]	[illegible]	[illegible]	[illegible]	[illegible]	[illegible]	[illegible]	[illegible]	[illegible]	[illegible]	[illegible]	0·2 (0·1 to 0·4)	260
Acute hepatitis A	0 (0 to 0)	[illegible]	[illegible]	[illegible]	[illegible]	[illegible]	[illegible]	[illegible]	[illegible]	[illegible]	[illegible]	[illegible]	[illegible]	[illegible]	[illegible]	[illegible]	[illegible]	[illegible]	[illegible]	[illegible]	[illegible]	[illegible]	0 (0 to 0)	180
Acute hepatitis B	0 (0 to 0)	[illegible]	[illegible]	[illegible]	[illegible]	[illegible]	[illegible]	[illegible]	[illegible]	[illegible]	[illegible]	[illegible]	[illegible]	[illegible]	[illegible]	[illegible]	[illegible]	[illegible]	[illegible]	[illegible]	[illegible]	[illegible]	0 (0 to 0·1)	45·2
Acute hepatitis C	0 (0 to 0)	[illegible]	[illegible]	[illegible]	[illegible]	[illegible]	[illegible]	[illegible]	[illegible]	[illegible]	[illegible]	[illegible]	[illegible]	[illegible]	[illegible]	[illegible]	[illegible]	[illegible]	[illegible]	[illegible]	[illegible]	[illegible]	0 (0 to 0·1)	13·3
Acute hepatitis E	0 (0 to 0)	[illegible]	[illegible]	[illegible]	[illegible]	[illegible]	[illegible]	[illegible]	[illegible]	[illegible]	[illegible]	[illegible]	[illegible]	[illegible]	[illegible]	[illegible]	[illegible]	[illegible]	[illegible]	[illegible]	[illegible]	[illegible]	0·1 (0 to 0·3)	21·1
Other unspecified infectious diseases	8 (2·5 to 14·4)	[illegible]	[illegible]	[illegible]	[illegible]	[illegible]	[illegible]	[illegible]	[illegible]	[illegible]	[illegible]	[illegible]	[illegible]	[illegible]	[illegible]	[illegible]	[illegible]	[illegible]	[illegible]	[illegible]	[illegible]	[illegible]	4·6 (2·1 to 9·9)	1,530
Maternal and neonatal disorders	4,130 (2,500 to 5,200)	[illegible]	[illegible]	[illegible]	[illegible]	[illegible]	[illegible]	[illegible]	[illegible]	[illegible]	[illegible]	[illegible]	[illegible]	[illegible]	[illegible]	[illegible]	[illegible]	[illegible]	[illegible]	[illegible]	[illegible]	[illegible]	0·7 (0·2 to 1·4)	21,400
Maternal disorders	0 (0 to 0)	[illegible]	[illegible]	[illegible]	[illegible]	[illegible]	[illegible]	[illegible]	[illegible]	[illegible]	[illegible]	[illegible]	[illegible]	[illegible]	[illegible]	[illegible]	[illegible]	[illegible]	[illegible]	[illegible]	[illegible]	[illegible]	0 (0 to 0)	3,760
Maternal hemorrhage	0 (0 to 0)	[illegible]	[illegible]	[illegible]	[illegible]	[illegible]	[illegible]	[illegible]	[illegible]	[illegible]	[illegible]	[illegible]	[illegible]	[illegible]	[illegible]	[illegible]	[illegible]	[illegible]	[illegible]	[illegible]	[illegible]	[illegible]	0 (0 to 0)	528
Maternal sepsis and other maternal infections	0 (0 to 0)	[illegible]	[illegible]	[illegible]	[illegible]	[illegible]	[illegible]	[illegible]	[illegible]	[illegible]	[illegible]	[illegible]	[illegible]	[illegible]	[illegible]	[illegible]	[illegible]	[illegible]	[illegible]	[illegible]	[illegible]	[illegible]	0 (0 to 0)	221
Maternal hypertensive disorders	0 (0 to 0)	[illegible]	[illegible]	[illegible]	[illegible]	[illegible]	[illegible]	[illegible]	[illegible]	[illegible]	[illegible]	[illegible]	[illegible]	[illegible]	[illegible]	[illegible]	[illegible]	[illegible]	[illegible]	[illegible]	[illegible]	[illegible]	0 (0 to 0)	377
Maternal obstructed labor and uterine rupture	0 (0 to 0)	[illegible]	[illegible]	[illegible]	[illegible]	[illegible]	[illegible]	[illegible]	[illegible]	[illegible]	[illegible]	[illegible]	[illegible]	[illegible]	[illegible]	[illegible]	[illegible]	[illegible]	[illegible]	[illegible]	[illegible]	[illegible]	0 (0 to 0)	52·5
Maternal abortion and miscarriage	0 (0 to 0)	[illegible]	[illegible]	[illegible]	[illegible]	[illegible]	[illegible]	[illegible]	[illegible]	[illegible]	[illegible]	[illegible]	[illegible]	[illegible]	[illegible]	[illegible]	[illegible]	[illegible]	[illegible]	[illegible]	[illegible]	[illegible]	0 (0 to 0)	283
Ectopic pregnancy	0 (0 to 0)	[illegible]	[illegible]	[illegible]	[illegible]	[illegible]	[illegible]	[illegible]	[illegible]	[illegible]	[illegible]	[illegible]	[illegible]	[illegible]	[illegible]	[illegible]	[illegible]	[illegible]	[illegible]	[illegible]	[illegible]	[illegible]	0 (0 to 0)	183
Indirect maternal deaths	--	[illegible]	[illegible]	[illegible]	[illegible]	[illegible]	[illegible]	[illegible]	[illegible]	[illegible]	[illegible]	[illegible]	[illegible]	[illegible]	[illegible]	[illegible]	[illegible]	[illegible]	[illegible]	[illegible]	[illegible]	[illegible]	--	NA
Late maternal deaths	--	[illegible]	[illegible]	[illegible]	[illegible]	[illegible]	[illegible]	[illegible]	[illegible]	[illegible]	[illegible]	[illegible]	[illegible]	[illegible]	[illegible]	[illegible]	[illegible]	[illegible]	[illegible]	[illegible]	[illegible]	[illegible]	--	NA
Maternal deaths aggravated by HIV/AIDS	--	[illegible]	[illegible]	[illegible]	[illegible]	[illegible]	[illegible]	[illegible]	[illegible]	[illegible]	[illegible]	[illegible]	[illegible]	[illegible]	[illegible]	[illegible]	[illegible]	[illegible]	[illegible]	[illegible]	[illegible]	[illegible]	--	NA
Neonatal disorders	4,130 (2,500 to 5,200)	[illegible]	[illegible]	[illegible]	[illegible]	[illegible]	[illegible]	[illegible]	[illegible]	[illegible]	[illegible]	[illegible]	[illegible]	[illegible]	[illegible]	[illegible]	[illegible]	[illegible]	[illegible]	[illegible]	[illegible]	[illegible]	0·7 (0·2 to 1·4)	17,600
Neonatal preterm birth	1,170 (686 to 1,580)	[illegible]	[illegible]	[illegible]	[illegible]	[illegible]	[illegible]	[illegible]	[illegible]	[illegible]	[illegible]	[illegible]	[illegible]	[illegible]	[illegible]	[illegible]	[illegible]	[illegible]	[illegible]	[illegible]	[illegible]	[illegible]	0·7 (0·2 to 1·4)	6,890
Neonatal encephalopathy due to birth asphyxia and traum	1,000 (585 to 1,400)	[illegible]	[illegible]	[illegible]	[illegible]	[illegible]	[illegible]	[illegible]	[illegible]	[illegible]	[illegible]	[illegible]	[illegible]	[illegible]	[illegible]	[illegible]	[illegible]	[illegible]	[illegible]	[illegible]	[illegible]	[illegible]	0 (0 to 0)	2,690
Neonatal sepsis and other neonatal infections	659 (389 to 917)	[illegible]	[illegible]	[illegible]	[illegible]	[illegible]	[illegible]	[illegible]	[illegible]	[illegible]	[illegible]	[illegible]	[illegible]	[illegible]	[illegible]	[illegible]	[illegible]	[illegible]	[illegible]	[illegible]	[illegible]	[illegible]	0 (0 to 0)	5,440
Hemolytic disease and other neonatal jaundice	75·7 (37·9 to 123)	[illegible]	[illegible]	[illegible]	[illegible]	[illegible]	[illegible]	[illegible]	[illegible]	[illegible]	[illegible]	[illegible]	[illegible]	[illegible]	[illegible]	[illegible]	[illegible]	[illegible]	[illegible]	[illegible]	[illegible]	[illegible]	0 (0 to 0)	271
Other neonatal disorders	1,220 (680 to 1,730)	[illegible]	[illegible]	[illegible]	[illegible]	[illegible]	[illegible]	[illegible]	[illegible]	[illegible]	[illegible]	[illegible]	[illegible]	[illegible]	[illegible]	[illegible]	[illegible]	[illegible]	[illegible]	[illegible]	[illegible]	[illegible]	0 (0 to 0)	2,360
Nutritional deficiencies	0·6 (0·3 to 1·1)	[illegible]	[illegible]	[illegible]	[illegible]	[illegible]	[illegible]	[illegible]	[illegible]	[illegible]	[illegible]	[illegible]	[illegible]	[illegible]	[illegible]	[illegible]	[illegible]	[illegible]	[illegible]	[illegible]	[illegible]	[illegible]	33·2 (20·6 to 56·4)	18,500
Protein-energy malnutrition	0·6 (0·3 to 1)	[illegible]	[illegible]	[illegible]	[illegible]	[illegible]	[illegible]	[illegible]	[illegible]	[illegible]	[illegible]	[illegible]	[illegible]	[illegible]	[illegible]	[illegible]	[illegible]	[illegible]	[illegible]	[illegible]	[illegible]	[illegible]	16·6 (11·5 to 22·4)	2,110
Iodine deficiency	0 (0 to 0)	[illegible]	[illegible]	[illegible]	[illegible]	[illegible]	[illegible]	[illegible]	[illegible]	[illegible]	[illegible]	[illegible]	[illegible]	[illegible]	[illegible]	[illegible]	[illegible]	[illegible]	[illegible]	[illegible]	[illegible]	[illegible]	0·8 (0·4 to 1·3)	479
Vitamin A deficiency	0 (0 to 0·1)	[illegible]	[illegible]	[illegible]	[illegible]	[illegible]	[illegible]	[illegible]	[illegible]	[illegible]	[illegible]	[illegible]	[illegible]	[illegible]	[illegible]	[illegible]	[illegible]	[illegible]	[illegible]	[illegible]	[illegible]	[illegible]	0 (0 to 0)	39·8
Dietary iron deficiency	0 (0 to 0·1)	[illegible]	[illegible]	[illegible]	[illegible]	[illegible]	[illegible]	[illegible]	[illegible]	[illegible]	[illegible]	[illegible]	[illegible]	[illegible]	[illegible]	[illegible]	[illegible]	[illegible]	[illegible]	[illegible]	[illegible]	[illegible]	13·1 (3 to 34)	15,100
Other nutritional deficiencies	0 (0 to 0)	[illegible]	[illegible]	[illegible]	[illegible]	[illegible]	[illegible]	[illegible]	[illegible]	[illegible]	[illegible]	[illegible]	[illegible]	[illegible]	[illegible]	[illegible]	[illegible]	[illegible]	[illegible]	[illegible]	[illegible]	[illegible]	2·6 (1·4 to 4·2)	769
Non-communicable diseases	1,320 (856 to 3,080)	[illegible]	[illegible]	[illegible]	[illegible]	[illegible]	[illegible]	[illegible]	[illegible]	[illegible]	[illegible]	[illegible]	[illegible]	[illegible]	[illegible]	[illegible]	[illegible]	[illegible]	[illegible]	[illegible]	[illegible]	[illegible]	19,600 (17,800 to 21,800)	1,410,000
Neoplasms	14·2 (6·7 to 20·6)	[illegible]	[illegible]	[illegible]	[illegible]	[illegible]	[illegible]	[illegible]	[illegible]	[illegible]	[illegible]	[illegible]	[illegible]	[illegible]	[illegible]	[illegible]	[illegible]	[illegible]	[illegible]	[illegible]	[illegible]	[illegible]	1,540 (1,150 to 1,840)	267,000
Lip and oral cavity cancer	0 (0 to 0)	[illegible]	[illegible]	[illegible]	[illegible]	[illegible]	[illegible]	[illegible]	[illegible]	[illegible]	[illegible]	[illegible]	[illegible]	[illegible]	[illegible]	[illegible]	[illegible]	[illegible]	[illegible]	[illegible]	[illegible]	[illegible]	30 (19·8 to 40·8)	2,780
Nasopharynx cancer	0 (0 to 0)	[illegible]	[illegible]	[illegible]	[illegible]	[illegible]	[illegible]	[illegible]	[illegible]	[illegible]	[illegible]	[illegible]	[illegible]	[illegible]	[illegible]	[illegible]	[illegible]	[illegible]	[illegible]	[illegible]	[illegible]	[illegible]	3·3 (1·9 to 5·4)	473
Other pharynx cancer	0 (0 to 0)	[illegible]	[illegible]	[illegible]	[illegible]	[illegible]	[illegible]	[illegible]	[illegible]	[illegible]	[illegible]	[illegible]	[illegible]	[illegible]	[illegible]	[illegible]	[illegible]	[illegible]	[illegible]	[illegible]	[illegible]	[illegible]	4·4 (2·6 to 6·9)	691
Esophageal cancer	0 (0 to 0)	[illegible]	[illegible]	[illegible]	[illegible]	[illegible]	[illegible]	[illegible]	[illegible]	[illegible]	[illegible]	[illegible]	[illegible]	[illegible]	[illegible]	[illegible]	[illegible]	[illegible]	[illegible]	[illegible]	[illegible]	[illegible]	23·8 (15·1 to 35·3)	3,440
Stomach cancer	0 (0 to 0)	[illegible]	[illegible]	[illegible]	[illegible]	[illegible]	[illegible]	[illegible]	[illegible]	[illegible]	[illegible]	[illegible]	[illegible]	[illegible]	[illegible]	[illegible]	[illegible]	[illegible]	[illegible]	[illegible]	[illegible]	[illegible]	68·1 (46·6 to 92·1)	8,540
Colon and rectum cancer	0 (0 to 0)	[illegible]	[illegible]	[illegible]	[illegible]	[illegible]	[illegible]	[illegible]	[illegible]	[illegible]	[illegible]	[illegible]	[illegible]	[illegible]	[illegible]	[illegible]	[illegible]	[illegible]	[illegible]	[illegible]	[illegible]	[illegible]	370 (260 to 490)	37,000
Liver cancer	0·2 (0·1 to 0·3)	[illegible]	[illegible]	[illegible]	[illegible]	[illegible]	[illegible]	[illegible]	[illegible]	[illegible]	[illegible]	[illegible]	[illegible]	[illegible]	[illegible]	[illegible]	[illegible]	[illegible]	[illegible]	[illegible]	[illegible]	[illegible]	11·8 (7·7 to 17·4)	3,020
Liver cancer due to hepatitis B	0 (0 to 0)	[illegible]	[illegible]	[illegible]	[illegible]	[illegible]	[illegible]	[illegible]	[illegible]	[illegible]	[illegible]	[illegible]	[illegible]	[illegible]	[illegible]	[illegible]	[illegible]	[illegible]	[illegible]	[illegible]	[illegible]	[illegible]	1 (0·3 to 2·2)	614
Liver cancer due to hepatitis C	0 (0 to 0)	[illegible]	[illegible]	[illegible]	[illegible]	[illegible]	[illegible]	[illegible]	[illegible]	[illegible]	[illegible]	[illegible]	[illegible]	[illegible]	[illegible]	[illegible]	[illegible]	[illegible]	[illegible]	[illegible]	[illegible]	[illegible]	6·8 (3·6 to 10·8)	950
Liver cancer due to alcohol use	0 (0 to 0)	[illegible]	[illegible]	[illegible]	[illegible]	[illegible]	[illegible]	[illegible]	[illegible]	[illegible]	[illegible]	[illegible]	[illegible]	[illegible]	[illegible]	[illegible]	[illegible]	[illegible]	[illegible]	[illegible]	[illegible]	[illegible]	1·4 (0·6 to 2·6)	723
Liver cancer due to NASH	0 (0 to 0)	[illegible]	[illegible]	[illegible]	[illegible]	[illegible]	[illegible]	[illegible]	[illegible]	[illegible]	[illegible]	[illegible]	[illegible]	[illegible]	[illegible]	[illegible]	[illegible]	[illegible]	[illegible]	[illegible]	[illegible]	[illegible]	2 (0·8 to 4)	440
Liver cancer due to other causes	0·2 (0·1 to 0·3)	[illegible]	[illegible]	[illegible]	[illegible]	[illegible]	[illegible]	[illegible]	[illegible]	[illegible]	[illegible]	[illegible]	[illegible]	[illegible]	[illegible]	[illegible]	[illegible]	[illegible]	[illegible]	[illegible]	[illegible]	[illegible]	0·7 (0·3 to 1·4)	295
Gallbladder and biliary tract cancer	0 (0 to 0)	[illegible]	[illegible]	[illegible]	[illegible]	[illegible]	[illegible]	[illegible]	[illegible]	[illegible]	[illegible]	[illegible]	[illegible]	[illegible]	[illegible]	[illegible]	[illegible]	[illegible]	[illegible]	[illegible]	[illegible]	[illegible]	15·1 (9·1 to 28·1)	2,750
Pancreatic cancer	0 (0 to 0)	[illegible]	[illegible]	[illegible]	[illegible]	[illegible]	[illegible]	[illegible]	[illegible]	[illegible]	[illegible]	[illegible]	[illegible]	[illegible]	[illegible]	[illegible]	[illegible]	[illegible]	[illegible]	[illegible]	[illegible]	[illegible]	74·7 (51·4 to 100)	11,700
Larynx cancer	0 (0 to 0)	[illegible]	[illegible]	[illegible]	[illegible]	[illegible]	[illegible]	[illegible]	[illegible]	[illegible]	[illegible]	[illegible]	[illegible]	[illegible]	[illegible]	[illegible]	[illegible]	[illegible]	[illegible]	[illegible]	[illegible]	[illegible]	11·8 (6·7 to 20·1)	2,620
Tracheal, bronchus, and lung cancer	0 (0 to 0)	[illegible]	[illegible]	[illegible]	[illegible]	[illegible]	[illegible]	[illegible]	[illegible]	[illegible]	[illegible]	[illegible]	[illegible]	[illegible]	[illegible]	[illegible]	[illegible]	[illegible]	[illegible]	[illegible]	[illegible]	[illegible]	249 (167 to 348)	52,400
Malignant skin melanoma	0 (0 to 0·1)	[illegible]	[illegible]	[illegible]	[illegible]	[illegible]	[illegible]	[illegible]	[illegible]	[illegible]	[illegible]	[illegible]	[illegible]	[illegible]	[illegible]	[illegible]	[illegible]	[illegible]	[illegible]	[illegible]	[illegible]	[illegible]	10·3 (5·9 to 16·5)	975
Non-melanoma skin cancer	0 (0 to 0)	[illegible]	[illegible]	[illegible]	[illegible]	[illegible]	[illegible]	[illegible]	[illegible]	[illegible]	[illegible]	[illegible]	[illegible]	[illegible]	[illegible]	[illegible]	[illegible]	[illegible]	[illegible]	[illegible]	[illegible]	[illegible]	120 (75·2 to 163)	2,100
Non-melanoma skin cancer (squamous-cell carcinoma)	0 (0 to 0)	[illegible]	[illegible]	[illegible]	[illegible]	[illegible]	[illegible]	[illegible]	[illegible]	[illegible]	[illegible]	[illegible]	[illegible]	[illegible]	[illegible]	[illegible]	[illegible]	[illegible]	[illegible]	[illegible]	[illegible]	[illegible]	120 (75·2 to 163)	2,100
Non-melanoma skin cancer (basal-cell carcinoma)	0 (0 to 0)	[illegible]	[illegible]	[illegible]	[illegible]	[illegible]	[illegible]	[illegible]	[illegible]	[illegible]	[illegible]	[illegible]	[illegible]	[illegible]	[illegible]	[illegible]	[illegible]	[illegible]	[illegible]	[illegible]	[illegible]	[illegible]	0 (0 to 0)	0·4
Breast cancer	0 (0 to 0)	[illegible]	[illegible]	[illegible]	[illegible]	[illegible]	[illegible]	[illegible]	[illegible]	[illegible]	[illegible]	[illegible]	[illegible]	[illegible]	[illegible]	[illegible]	[illegible]	[illegible]	[illegible]	[illegible]	[illegible]	[illegible]	245 (159 to 345)	44,300
Cervical cancer	0 (0 to 0)	[illegible]	[illegible]	[illegible]	[illegible]	[illegible]	[illegible]	[illegible]	[illegible]	[illegible]	[illegible]	[illegible]	[illegible]	[illegible]	[illegible]	[illegible]	[illegible]	[illegible]	[illegible]	[illegible]	[illegible]	[illegible]	40·8 (26·1 to 59·9)	20,300
Uterine cancer	0 (0 to 0)	[illegible]	[illegible]	[illegible]	[illegible]	[illegible]	[illegible]	[illegible]	[illegible]	[illegible]	[illegible]	[illegible]	[illegible]	[illegible]	[illegible]	[illegible]	[illegible]	[illegible]	[illegible]	[illegible]	[illegible]	[illegible]	61·2 (41·6 to 83·2)	15,600
Ovarian cancer	0 (0 to 0)	[illegible]	[illegible]	[illegible]	[illegible]	[illegible]	[illegible]	[illegible]	[illegible]	[illegible]	[illegible]	[illegible]	[illegible]	[illegible]	[illegible]	[illegible]	[illegible]	[illegible]	[illegible]	[illegible]	[illegible]	[illegible]	14·6 (7·5 to 22·3)	9,700
Prostate cancer	0 (0 to 0)	[illegible]	[illegible]	[illegible]	[illegible]	[illegible]	[illegible]	[illegible]	[illegible]	[illegible]	[illegible]	[illegible]	[illegible]	[illegible]	[illegible]	[illegible]	[illegible]	[illegible]	[illegible]	[illegible]	[illegible]	[illegible]	0 (0 to 0)	0
Testicular cancer	--	[illegible]	[illegible]	[illegible]	[illegible]	[illegible]	[illegible]	[illegible]	[illegible]	[illegible]	[illegible]	[illegible]	[illegible]	[illegible]	[illegible]	[illegible]	[illegible]	[illegible]	[illegible]	[illegible]	[illegible]	[illegible]	--	NA
Kidney cancer	0·7 (0·3 to 0·9)	[illegible]	[illegible]	[illegible]	[illegible]	[illegible]	[illegible]	[illegible]	[illegible]	[illegible]	[illegible]	[illegible]	[illegible]	[illegible]	[illegible]	[illegible]	[illegible]	[illegible]	[illegible]	[illegible]	[illegible]	[illegible]	10·5 (6·4 to 15·3)	3,080
Bladder cancer	0 (0 to 0)	[illegible]	[illegible]	[illegible]	[illegible]	[illegible]	[illegible]	[illegible]	[illegible]	[illegible]	[illegible]	[illegible]	[illegible]	[illegible]	[illegible]	[illegible]	[illegible]	[illegible]	[illegible]	[illegible]	[illegible]	[illegible]	55·4 (37·7 to 75·3)	3,960
Brain and central nervous system cancer	4·8 (0·9 to 8·3)	[illegible]	[illegible]	[illegible]	[illegible]	[illegible]	[illegible]	[illegible]	[illegible]	[illegible]	[illegible]	[illegible]	[illegible]	[illegible]	[illegible]	[illegible]	[illegible]	[illegible]	[illegible]	[illegible]	[illegible]	[illegible]	8·6 (2·8 to 16·3)	7,210
Thyroid cancer	0 (0 to 0)	[illegible]	[illegible]	[illegible]	[illegible]	[illegible]	[illegible]	[illegible]	[illegible]	[illegible]	[illegible]	[illegible]	[illegible]	[illegible]	[illegible]	[illegible]	[illegible]	[illegible]	[illegible]	[illegible]	[illegible]	[illegible]	6·1 (3·8 to 9·2)	1,440
Mesothelioma	0 (0 to 0)	[illegible]	[illegible]	[illegible]	[illegible]	[illegible]	[illegible]	[illegible]	[illegible]	[illegible]	[illegible]	[illegible]	[illegible]	[illegible]	[illegible]	[illegible]	[illegible]	[illegible]	[illegible]	[illegible]	[illegible]	[illegible]	0·7 (0·5 to 1·1)	246
Hodgkin lymphoma	0 (0 to 0)	[illegible]	[illegible]	[illegible]	[illegible]	[illegible]	[illegible]	[illegible]	[illegible]	[illegible]	[illegible]	[illegible]	[illegible]	[illegible]	[illegible]	[illegible]	[illegible]	[illegible]	[illegible]	[illegible]	[illegible]	[illegible]	2·2 (0·4 to 5·5)	1,160
Non-Hodgkin lymphoma	0 (0 to 0)	[illegible]	[illegible]	[illegible]	[illegible]	[illegible]	[illegible]	[illegible]	[illegible]	[illegible]	[illegible]	[illegible]	[illegible]	[illegible]	[illegible]	[illegible]	[illegible]	[illegible]	[illegible]	[illegible]	[illegible]	[illegible]	13·7 (10·1 to 17·1)	7,070
Multiple myeloma	0 (0 to 0)	[illegible]	[illegible]	[illegible]	[illegible]	[illegible]	[illegible]	[illegible]	[illegible]	[illegible]	[illegible]	[illegible]	[illegible]	[illegible]	[illegible]	[illegible]	[illegible]	[illegible]	[illegible]	[illegible]	[illegible]	[illegible]	8 (4·6 to 14·1)	4,320
Leukemia	2·2 (1·2 to 3·2)	[illegible]	[illegible]	[illegible]	[illegible]	[illegible]	[illegible]	[illegible]	[illegible]	[illegible]	[illegible]	[illegible]	[illegible]	[illegible]	[illegible]	[illegible]	[illegible]	[illegible]	[illegible]	[illegible]	[illegible]	[illegible]	24·6 (15·6 to 36·2)	8,150
Acute lymphoid leukemia	0·6 (0·3 to 1)	[illegible]	[illegible]	[illegible]	[illegible]	[illegible]	[illegible]	[illegible]	[illegible]	[illegible]	[illegible]	[illegible]	[illegible]	[illegible]	[illegible]	[illegible]	[illegible]	[illegible]	[illegible]	[illegible]	[illegible]	[illegible]	1 (0·5 to 1·6)	1,540
Chronic lymphoid leukemia	0 (0 to 0)	[illegible]	[illegible]	[illegible]	[illegible]	[illegible]	[illegible]	[illegible]	[illegible]	[illegible]	[illegible]	[illegible]	[illegible]	[illegible]	[illegible]	[illegible]	[illegible]	[illegible]	[illegible]	[illegible]	[illegible]	[illegible]	4·9 (2·8 to 7·8)	970
Acute myeloid leukemia	0·6 (0·3 to 1)	[illegible]	[illegible]	[illegible]	[illegible]	[illegible]	[illegible]	[illegible]	[illegible]	[illegible]	[illegible]	[illegible]	[illegible]	[illegible]	[illegible]	[illegible]	[illegible]	[illegible]	[illegible]	[illegible]	[illegible]	[illegible]	2·4 (1·4 to 4·2)	3,120
Chronic myeloid leukemia	0·2 (0 to 0·4)	[illegible]	[illegible]	[illegible]	[illegible]	[illegible]	[illegible]	[illegible]	[illegible]	[illegible]	[illegible]	[illegible]	[illegible]	[illegible]	[illegible]	[illegible]	[illegible]	[illegible]	[illegible]	[illegible]	[illegible]	[illegible]	7·8 (4·7 to 11·8)	1,200
Other leukemia	0·8 (0·4 to 1·2)	[illegible]	[illegible]	[illegible]	[illegible]	[illegible]	[illegible]	[illegible]	[illegible]	[illegible]	[illegible]	[illegible]	[illegible]	[illegible]	[illegible]	[illegible]	[illegible]	[illegible]	[illegible]	[illegible]	[illegible]	[illegible]	8·5 (5·1 to 14·2)	1,310
Other malignant neoplasms	6·4 (2·9 to 14·8)	[illegible]	[illegible]	[illegible]	[illegible]	[illegible]	[illegible]	[illegible]	[illegible]	[illegible]	[illegible]	[illegible]	[illegible]	[illegible]	[illegible]	[illegible]	[illegible]	[illegible]	[illegible]	[illegible]	[illegible]	[illegible]	53·9 (35·5 to 75·9)	11,100
Other neoplasms	0 (0 to 0·1)	[illegible]	[illegible]	[illegible]	[illegible]	[illegible]	[illegible]	[illegible]	[illegible]	[illegible]	[illegible]	[illegible]	[illegible]	[illegible]	[illegible]	[illegible]	[illegible]	[illegible]	[illegible]	[illegible]	[illegible]	[illegible]	0·9 (0·5 to 1·7)	596
Myelodysplastic, myeloproliferative, and other hematopoie	0 (0 to 0·1)	[illegible]	[illegible]	[illegible]	[illegible]	[illegible]	[illegible]	[illegible]	[illegible]	[illegible]	[illegible]	[illegible]	[illegible]	[illegible]	[illegible]	[illegible]	[illegible]	[illegible]	[illegible]	[illegible]	[illegible]	[illegible]	0·9 (0·5 to 1·7)	596
Benign and in situ intestinal neoplasms	0 (0 to 0)	[illegible]	[illegible]	[illegible]	[illegible]	[illegible]	[illegible]	[illegible]	[illegible]	[illegible]	[illegible]	[illegible]	[illegible]	[illegible]	[illegible]	[illegible]	[illegible]	[illegible]	[illegible]	[illegible]	[illegible]	[illegible]	0 (0 to 0)	0
Benign and in situ cervical and uterine neoplasms	0 (0 to 0)	[illegible]	[illegible]	[illegible]	[illegible]	[illegible]	[illegible]	[illegible]	[illegible]	[illegible]	[illegible]	[illegible]	[illegible]	[illegible]	[illegible]	[illegible]	[illegible]	[illegible]	[illegible]	[illegible]	[illegible]	[illegible]	0 (0 to 0)	0
Other benign and in situ neoplasms	0 (0 to 0)	[illegible]	[illegible]	[illegible]	[illegible]	[illegible]	[illegible]	[illegible]	[illegible]	[illegible]	[illegible]	[illegible]	[illegible]	[illegible]	[illegible]	[illegible]	[illegible]	[illegible]	[illegible]	[illegible]	[illegible]	[illegible]	0 (0 to 0)	0
Cardiovascular diseases	8·1 (4·7 to 11·4)	[illegible]	[illegible]	[illegible]	[illegible]	[illegible]	[illegible]	[illegible]	[illegible]	[illegible]	[illegible]	[illegible]	[illegible]	[illegible]	[illegible]	[illegible]	[illegible]	[illegible]	[illegible]	[illegible]	[illegible]	[illegible]	9,880 (7,720 to 11,400)	304,000
Rheumatic heart disease	0 (0 to 0)	[illegible]	[illegible]	[illegible]	[illegible]	[illegible]	[illegible]	[illegible]	[illegible]	[illegible]	[illegible]	[illegible]	[illegible]	[illegible]	[illegible]	[illegible]	[illegible]	[illegible]	[illegible]	[illegible]	[illegible]	[illegible]	15·7 (11·4 to 20·3)	6,070
Ischemic heart disease	0 (0 to 0)	[illegible]	[illegible]	[illegible]	[illegible]	[illegible]	[illegible]	[illegible]	[illegible]	[illegible]	[illegible]	[illegible]	[illegible]	[illegible]	[illegible]	[illegible]	[illegible]	[illegible]	[illegible]	[illegible]	[illegible]	[illegible]	5,920 (4,640 to 6,870)	153,000
Stroke	0·3 (0·1 to 0·7)	[illegible]	[illegible]	[illegible]	[illegible]	[illegible]	[illegible]	[illegible]	[illegible]	[illegible]	[illegible]	[illegible]	[illegible]	[illegible]	[illegible]	[illegible]	[illegible]	[illegible]	[illegible]	[illegible]	[illegible]	[illegible]	2,310 (1,700 to 2,920)	92,200
Ischemic stroke	0·1 (0 to 0·2)	[illegible]	[illegible]	[illegible]	[illegible]	[illegible]	[illegible]	[illegible]	[illegible]	[illegible]	[illegible]	[illegible]	[illegible]	[illegible]	[illegible]	[illegible]	[illegible]	[illegible]	[illegible]	[illegible]	[illegible]	[illegible]	2,000 (1,460 to 2,530)	55,300
Intracerebral hemorrhage	0 (0 to 0·1)	[illegible]	[illegible]	[illegible]	[illegible]	[illegible]	[illegible]	[illegible]	[illegible]	[illegible]	[illegible]	[illegible]	[illegible]	[illegible]	[illegible]	[illegible]	[illegible]	[illegible]	[illegible]	[illegible]	[illegible]	[illegible]	275 (203 to 350)	28,700
Subarachnoid hemorrhage	0·2 (0·1 to 0·5)	[illegible]	[illegible]	[illegible]	[illegible]	[illegible]	[illegible]	[illegible]	[illegible]	[illegible]	[illegible]	[illegible]	[illegible]	[illegible]	[illegible]	[illegible]	[illegible]	[illegible]	[illegible]	[illegible]	[illegible]	[illegible]	35·4 (23·6 to 49·1)	8,300
Hypertensive heart disease	0 (0 to 0)	[illegible]	[illegible]	[illegible]	[illegible]	[illegible]	[illegible]	[illegible]	[illegible]	[illegible]	[illegible]	[illegible]	[illegible]	[illegible]	[illegible]	[illegible]	[illegible]	[illegible]	[illegible]	[illegible]	[illegible]	[illegible]	583 (251 to 783)	18,900
Non-rheumatic valvular heart disease	0 (0 to 0)	[illegible]	[illegible]	[illegible]	[illegible]	[illegible]	[illegible]	[illegible]	[illegible]	[illegible]	[illegible]	[illegible]	[illegible]	[illegible]	[illegible]	[illegible]	[illegible]	[illegible]	[illegible]	[illegible]	[illegible]	[illegible]	11 (7·4 to 15·2)	2,650
Non-rheumatic calcific aortic valve disease	0 (0 to 0)	[illegible]	[illegible]	[illegible]	[illegible]	[illegible]	[illegible]	[illegible]	[illegible]	[illegible]	[illegible]	[illegible]	[illegible]	[illegible]	[illegible]	[illegible]	[illegible]	[illegible]	[illegible]	[illegible]	[illegible]	[illegible]	5·8 (3·5 to 9·5)	1,500

Non-rheumatic degenerative mitral valve disease	0 (0 to 0)	0 (0 to 0)	0 (0 to 0)	0 (0 to 0)	0 (0 to 0)	0 (0 to 0)	[illegible]
Other non-rheumatic valve diseases	0 (0 to 0)	0 (0 to 0)	0 (0 to 0)	0 (0 to 0)	0 (0 to 0)	0 (0 to 0)	[illegible]
Cardiomyopathy and myocarditis	5 (1·6 to 7·3)	7·3 (4·2 to 10·6)	109 (71·6 to 155)	63·3 (43·3 to 83·6)	34·4 (24·7 to 44·6)	53·3 (37·3 to 74·4)	[illegible]
Myocarditis	0·8 (0·1 to 1·6)	1·6 (0·3 to 3·3)	22·5 (8 to 42·6)	16·9 (9·1 to 26·7)	14·8 (9·8 to 20·3)	11·8 (7 to 19·2)	[illegible]
Alcoholic cardiomyopathy	0 (0 to 0)	0 (0 to 0)	0 (0 to 0)	0 (0 to 0)	0 (0 to 0)	0 (0 to 0)	[illegible]
Other cardiomyopathy	4·2 (1·5 to 6·5)	5·8 (3·4 to 8·7)	87 (56·3 to 126)	46·4 (29 to 64·8)	19·7 (11·8 to 30·7)	41·5 (25·5 to 58·8)	[illegible]
Atrial fibrillation and flutter	0 (0 to 0)	0 (0 to 0)	0 (0 to 0)	0 (0 to 0)	0 (0 to 0)	0 (0 to 0)	[illegible]
Aortic aneurysm	--	--	--	--	--	--	[illegible]
Endocarditis	0·9 (0·1 to 1·9)	0·9 (0·3 to 1·9)	5·1 (2·4 to 10·7)	3·8 (2·2 to 6·1)	3·4 (1·9 to 6·1)	4·6 (2·6 to 8·6)	[illegible]
Other cardiovascular and circulatory diseases	1·9 (0·8 to 5·4)	2 (0·9 to 4·7)	17·3 (7·5 to 44·5)	14·5 (7·7 to 25·3)	19·6 (11·9 to 30·3)	34·8 (20·2 to 53·8)	[illegible]
Chronic respiratory diseases	0 (0 to 0)	0 (0 to 0)	0·3 (0·2 to 0·4)	1,410 (700 to 2,570)	1,510 (874 to 2,530)	1,220 (715 to 1,880)	[illegible]
Chronic obstructive pulmonary disease	0 (0 to 0)	0 (0 to 0)	0·3 (0·2 to 0·4)	21 (12 to 48·2)	35·3 (14·4 to 66·3)	48·1 (22 to 79·2)	[illegible]
Pneumoconiosis	0 (0 to 0)	0 (0 to 0)	0 (0 to 0)	0 (0 to 0)	0 (0 to 0)	0 (0 to 0)	[illegible]
Silicosis	0 (0 to 0)	0 (0 to 0)	0 (0 to 0)	0 (0 to 0)	0 (0 to 0)	0 (0 to 0)	[illegible]
Asbestosis	0 (0 to 0)	0 (0 to 0)	0 (0 to 0)	0 (0 to 0)	0 (0 to 0)	0 (0 to 0)	[illegible]
Coal workers pneumoconiosis	0 (0 to 0)	0 (0 to 0)	0 (0 to 0)	0 (0 to 0)	0 (0 to 0)	0 (0 to 0)	[illegible]
Other pneumoconiosis	0 (0 to 0)	0 (0 to 0)	0 (0 to 0)	0 (0 to 0)	0 (0 to 0)	0 (0 to 0)	[illegible]
Asthma	0 (0 to 0)	[illegible]	[illegible]	[illegible]	[illegible]	[illegible]	[illegible]
Interstitial lung disease and pulmonary sarcoidosis	0 (0 to 0)	[illegible]	[illegible]	[illegible]	[illegible]	[illegible]	[illegible]
Other chronic respiratory diseases	0 (0 to 0)	[illegible]	[illegible]	[illegible]	[illegible]	[illegible]	[illegible]
Digestive diseases	15·9 (11 to 21·3)	[illegible]	[illegible]	[illegible]	[illegible]	[illegible]	[illegible]
Cirrhosis and other chronic liver diseases	0 (0 to 0)	[illegible]	[illegible]	[illegible]	[illegible]	[illegible]	[illegible]
Cirrhosis and other chronic liver diseases due to hepatitis [illegible]	0 (0 to 0)	[illegible]	[illegible]	[illegible]	[illegible]	[illegible]	[illegible]
Cirrhosis and other chronic liver diseases due to hepatitis [illegible]	0 (0 to 0)	[illegible]	[illegible]	[illegible]	[illegible]	[illegible]	[illegible]
Cirrhosis and other chronic liver diseases due to alcohol u[illegible]	0 (0 to 0)	[illegible]	[illegible]	[illegible]	[illegible]	[illegible]	[illegible]
Cirrhosis and other chronic liver diseases due to NAFLD	0 (0 to 0)	[illegible]	[illegible]	[illegible]	[illegible]	[illegible]	[illegible]
Cirrhosis and other chronic liver diseases due to other ca[illegible]	0 (0 to 0)	[illegible]	[illegible]	[illegible]	[illegible]	[illegible]	[illegible]
Upper digestive system diseases	0 (0 to 0)	[illegible]	[illegible]	[illegible]	[illegible]	[illegible]	[illegible]
Peptic ulcer disease	0 (0 to 0)	[illegible]	[illegible]	[illegible]	[illegible]	[illegible]	[illegible]
Gastritis and duodenitis	0 (0 to 0)	[illegible]	[illegible]	[illegible]	[illegible]	[illegible]	[illegible]
Gastroesophageal reflux disease	0 (0 to 0)	[illegible]	[illegible]	[illegible]	[illegible]	[illegible]	[illegible]
Appendicitis	0 (0 to 0)	[illegible]	[illegible]	[illegible]	[illegible]	[illegible]	[illegible]
Paralytic ileus and intestinal obstruction	10·9 (7·6 to 16·2)	[illegible]	[illegible]	[illegible]	[illegible]	[illegible]	[illegible]
Inguinal, femoral, and abdominal hernia	5 (1·5 to 9)	[illegible]	[illegible]	[illegible]	[illegible]	[illegible]	[illegible]
Inflammatory bowel disease	0 (0 to 0)	[illegible]	[illegible]	[illegible]	[illegible]	[illegible]	[illegible]
Vascular intestinal disorders	0 (0 to 0)	[illegible]	[illegible]	[illegible]	[illegible]	[illegible]	[illegible]
Gallbladder and biliary diseases	0 (0 to 0)	[illegible]	[illegible]	[illegible]	[illegible]	[illegible]	[illegible]
Pancreatitis	0 (0 to 0)	[illegible]	[illegible]	[illegible]	[illegible]	[illegible]	[illegible]
Other digestive diseases	0 (0 to 0)	[illegible]	[illegible]	[illegible]	[illegible]	[illegible]	[illegible]
Neurological disorders	0·5 (0·1 to 1·1)	[illegible]	[illegible]	[illegible]	[illegible]	[illegible]	[illegible]
Alzheimer's disease and other dementias	0 (0 to 0)	[illegible]	[illegible]	[illegible]	[illegible]	[illegible]	[illegible]
Parkinson's disease	0 (0 to 0)	[illegible]	[illegible]	[illegible]	[illegible]	[illegible]	[illegible]
Idiopathic epilepsy	0·5 (0·1 to 1·1)	[illegible]	[illegible]	[illegible]	[illegible]	[illegible]	[illegible]
Multiple sclerosis	0 (0 to 0)	[illegible]	[illegible]	[illegible]	[illegible]	[illegible]	[illegible]
Motor neuron disease	0 (0 to 0·1)	[illegible]	[illegible]	[illegible]	[illegible]	[illegible]	[illegible]
Headache disorders	0 (0 to 0)	[illegible]	[illegible]	[illegible]	[illegible]	[illegible]	[illegible]
Migraine	0 (0 to 0)	[illegible]	[illegible]	[illegible]	[illegible]	[illegible]	[illegible]
Tension-type headache	0 (0 to 0)	[illegible]	[illegible]	[illegible]	[illegible]	[illegible]	[illegible]
Other neurological disorders	0 (0 to 0)	[illegible]	[illegible]	[illegible]	[illegible]	[illegible]	[illegible]
Mental disorders	0·5 (0·3 to 0·8)	[illegible]	[illegible]	[illegible]	[illegible]	[illegible]	[illegible]
Schizophrenia	0 (0 to 0)	[illegible]	[illegible]	[illegible]	[illegible]	[illegible]	[illegible]
Depressive disorders	0 (0 to 0)	[illegible]	[illegible]	[illegible]	[illegible]	[illegible]	[illegible]
Major depressive disorder	0 (0 to 0)	[illegible]	[illegible]	[illegible]	[illegible]	[illegible]	[illegible]
Dysthymia	0 (0 to 0)	[illegible]	[illegible]	[illegible]	[illegible]	[illegible]	[illegible]
Bipolar disorder	0 (0 to 0)	[illegible]	[illegible]	[illegible]	[illegible]	[illegible]	[illegible]
Anxiety disorders	0 (0 to 0)	[illegible]	[illegible]	[illegible]	[illegible]	[illegible]	[illegible]
Eating disorders	0 (0 to 0)	[illegible]	[illegible]	[illegible]	[illegible]	[illegible]	[illegible]
Anorexia nervosa	0 (0 to 0)	[illegible]	[illegible]	[illegible]	[illegible]	[illegible]	[illegible]
Bulimia nervosa	0 (0 to 0)	[illegible]	[illegible]	[illegible]	[illegible]	[illegible]	[illegible]
Autism spectrum disorders	0·3 (0·2 to 0·5)	[illegible]	[illegible]	[illegible]	[illegible]	[illegible]	[illegible]
Attention-deficit/hyperactivity disorder	0 (0 to 0)	[illegible]	[illegible]	[illegible]	[illegible]	[illegible]	[illegible]
Conduct disorder	0 (0 to 0)	[illegible]	[illegible]	[illegible]	[illegible]	[illegible]	[illegible]
Idiopathic developmental intellectual disability	0·2 (0 to 0·3)	[illegible]	[illegible]	[illegible]	[illegible]	[illegible]	[illegible]
Other mental disorders	0 (0 to 0)	[illegible]	[illegible]	[illegible]	[illegible]	[illegible]	[illegible]
Substance use disorders	0 (0 to 0)	[illegible]	[illegible]	[illegible]	[illegible]	[illegible]	[illegible]
Alcohol use disorders	0 (0 to 0)	[illegible]	[illegible]	[illegible]	[illegible]	[illegible]	[illegible]
Drug use disorders	0 (0 to 0)	[illegible]	[illegible]	[illegible]	[illegible]	[illegible]	[illegible]
Opioid use disorders	0 (0 to 0)	[illegible]	[illegible]	[illegible]	[illegible]	[illegible]	[illegible]
Cocaine use disorders	0 (0 to 0)	[illegible]	[illegible]	[illegible]	[illegible]	[illegible]	[illegible]
Amphetamine use disorders	0 (0 to 0)	[illegible]	[illegible]	[illegible]	[illegible]	[illegible]	[illegible]
Cannabis use disorders	0 (0 to 0)	[illegible]	[illegible]	[illegible]	[illegible]	[illegible]	[illegible]
Other drug use disorders	0 (0 to 0)	[illegible]	[illegible]	[illegible]	[illegible]	[illegible]	[illegible]
Diabetes and kidney diseases	0·1 (0 to 0·2)	[illegible]	[illegible]	[illegible]	[illegible]	[illegible]	[illegible]
Diabetes mellitus	0·1 (0 to 0·2)	[illegible]	[illegible]	[illegible]	[illegible]	[illegible]	[illegible]
Diabetes mellitus type 1	0·1 (0 to 0·2)	[illegible]	[illegible]	[illegible]	[illegible]	[illegible]	[illegible]
Diabetes mellitus type 2	0 (0 to 0)	[illegible]	[illegible]	[illegible]	[illegible]	[illegible]	[illegible]
Chronic kidney disease	0 (0 to 0)	[illegible]	[illegible]	[illegible]	[illegible]	[illegible]	[illegible]
Chronic kidney disease due to diabetes mellitus type 1	0 (0 to 0)	[illegible]	[illegible]	[illegible]	[illegible]	[illegible]	[illegible]
Chronic kidney disease due to diabetes mellitus type 2	0 (0 to 0)	[illegible]	[illegible]	[illegible]	[illegible]	[illegible]	[illegible]
Chronic kidney disease due to hypertension	0 (0 to 0)	[illegible]	[illegible]	[illegible]	[illegible]	[illegible]	[illegible]
Chronic kidney disease due to glomerulonephritis	0 (0 to 0)	[illegible]	[illegible]	[illegible]	[illegible]	[illegible]	[illegible]
Chronic kidney disease due to other and unspecified caus[illegible]	0 (0 to 0)	[illegible]	[illegible]	[illegible]	[illegible]	[illegible]	[illegible]
Acute glomerulonephritis	0 (0 to 0)	[illegible]	[illegible]	[illegible]	[illegible]	[illegible]	[illegible]
Skin and subcutaneous diseases	0·1 (0 to 0·1)	[illegible]	[illegible]	[illegible]	[illegible]	[illegible]	[illegible]
Dermatitis	0 (0 to 0)	[illegible]	[illegible]	[illegible]	[illegible]	[illegible]	[illegible]
Atopic dermatitis	0 (0 to 0)	[illegible]	[illegible]	[illegible]	[illegible]	[illegible]	[illegible]
Contact dermatitis	0 (0 to 0)	[illegible]	[illegible]	[illegible]	[illegible]	[illegible]	[illegible]
Seborrhoeic dermatitis	0 (0 to 0)	[illegible]	[illegible]	[illegible]	[illegible]	[illegible]	[illegible]
Psoriasis	0 (0 to 0)	[illegible]	[illegible]	[illegible]	[illegible]	[illegible]	[illegible]
Bacterial skin diseases	0 (0 to 0)	[illegible]	[illegible]	[illegible]	[illegible]	[illegible]	[illegible]
Cellulitis	0 (0 to 0)	[illegible]	[illegible]	[illegible]	[illegible]	[illegible]	[illegible]
Pyoderma	0 (0 to 0)	[illegible]	[illegible]	[illegible]	[illegible]	[illegible]	[illegible]
Scabies	0 (0 to 0·1)	[illegible]	[illegible]	[illegible]	[illegible]	[illegible]	[illegible]
Fungal skin diseases	0 (0 to 0)	[illegible]	[illegible]	[illegible]	[illegible]	[illegible]	[illegible]
Viral skin diseases	0 (0 to 0)	[illegible]	[illegible]	[illegible]	[illegible]	[illegible]	[illegible]
Acne vulgaris	0 (0 to 0)	[illegible]	[illegible]	[illegible]	[illegible]	[illegible]	[illegible]
Alopecia areata	0 (0 to 0)	[illegible]	[illegible]	[illegible]	[illegible]	[illegible]	[illegible]
Pruritus	0 (0 to 0)	[illegible]	[illegible]	[illegible]	[illegible]	[illegible]	[illegible]
Urticaria	0 (0 to 0)	[illegible]	[illegible]	[illegible]	[illegible]	[illegible]	[illegible]
Decubitus ulcer	0 (0 to 0)	[illegible]	[illegible]	[illegible]	[illegible]	[illegible]	[illegible]
Other skin and subcutaneous diseases	0 (0 to 0)	[illegible]	[illegible]	[illegible]	[illegible]	[illegible]	[illegible]
Sense organ diseases	0·2 (0·1 to 0·3)	[illegible]	[illegible]	[illegible]	[illegible]	[illegible]	[illegible]
Blindness and vision loss	0·1 (0 to 0·2)	[illegible]	[illegible]	[illegible]	[illegible]	[illegible]	[illegible]
Glaucoma	0 (0 to 0)	[illegible]	[illegible]	[illegible]	[illegible]	[illegible]	[illegible]
Cataract	0 (0 to 0)	[illegible]	[illegible]	[illegible]	[illegible]	[illegible]	[illegible]
Age-related macular degeneration	0 (0 to 0)	[illegible]	[illegible]	[illegible]	[illegible]	[illegible]	[illegible]
Refraction disorders	0 (0 to 0)	[illegible]	[illegible]	[illegible]	[illegible]	[illegible]	[illegible]
Near vision loss	0 (0 to 0)	[illegible]	[illegible]	[illegible]	[illegible]	[illegible]	[illegible]
Other vision loss	0·1 (0 to 0·2)	[illegible]	[illegible]	[illegible]	[illegible]	[illegible]	[illegible]
Age-related and other hearing loss	0 (0 to 0)	[illegible]	[illegible]	[illegible]	[illegible]	[illegible]	[illegible]
Other sense organ diseases	0·1 (0 to 0·2)	[illegible]	[illegible]	[illegible]	[illegible]	[illegible]	[illegible]
Musculoskeletal disorders	0 (0 to 0)	[illegible]	[illegible]	[illegible]	[illegible]	[illegible]	[illegible]
Rheumatoid arthritis	0 (0 to 0)	[illegible]	[illegible]	[illegible]	[illegible]	[illegible]	[illegible]
Osteoarthritis	0 (0 to 0)	[illegible]	[illegible]	[illegible]	[illegible]	[illegible]	[illegible]
Osteoarthritis hip	0 (0 to 0)	[illegible]	[illegible]	[illegible]	[illegible]	[illegible]	[illegible]
Osteoarthritis knee	0 (0 to 0)	[illegible]	[illegible]	[illegible]	[illegible]	[illegible]	[illegible]
Osteoarthritis hand	0 (0 to 0)	[illegible]	[illegible]	[illegible]	[illegible]	[illegible]	[illegible]
Osteoarthritis other	0 (0 to 0)	[illegible]	[illegible]	[illegible]	[illegible]	[illegible]	[illegible]
Low back pain	0 (0 to 0)	[illegible]	[illegible]	[illegible]	[illegible]	[illegible]	[illegible]
Neck pain	0 (0 to 0)	[illegible]	[illegible]	[illegible]	[illegible]	[illegible]	[illegible]
Gout	0 (0 to 0)	[illegible]	[illegible]	[illegible]	[illegible]	[illegible]	[illegible]
Other musculoskeletal disorders	0 (0 to 0)	[illegible]	[illegible]	[illegible]	[illegible]	[illegible]	[illegible]
Other non-communicable diseases	1,280 (819 to 3,050)	[illegible]	[illegible]	[illegible]	[illegible]	[illegible]	[illegible]
Congenital birth defects	1,270 (803 to 3,030)	[illegible]	[illegible]	[illegible]	[illegible]	[illegible]	[illegible]
Neural tube defects	38·6 (15·4 to 73)	[illegible]	[illegible]	[illegible]	[illegible]	[illegible]	[illegible]
Congenital heart anomalies	455 (165 to 1,440)	[illegible]	[illegible]	[illegible]	[illegible]	[illegible]	[illegible]
Orofacial clefts	0·2 (0·1 to 0·3)	[illegible]	[illegible]	[illegible]	[illegible]	[illegible]	[illegible]
Down syndrome	15·5 (6·3 to 30·8)	[illegible]	[illegible]	[illegible]	[illegible]	[illegible]	[illegible]
Turner syndrome	0 (0 to 0)	[illegible]	[illegible]	[illegible]	[illegible]	[illegible]	[illegible]
Klinefelter syndrome	0 (0 to 0)	[illegible]	[illegible]	[illegible]	[illegible]	[illegible]	[illegible]
Other chromosomal abnormalities	78·1 (21·5 to 188)	[illegible]	[illegible]	[illegible]	[illegible]	[illegible]	[illegible]
Congenital musculoskeletal and limb anomalies	30·4 (12·8 to 55·8)	[illegible]	[illegible]	[illegible]	[illegible]	[illegible]	[illegible]
Urogenital congenital anomalies	26·1 (8·4 to 82·2)	[illegible]	[illegible]	[illegible]	[illegible]	[illegible]	[illegible]
Digestive congenital anomalies	288 (94·1 to 1,020)	[illegible]	[illegible]	[illegible]	[illegible]	[illegible]	[illegible]
Other congenital birth defects	337 (167 to 756)	[illegible]	[illegible]	[illegible]	[illegible]	[illegible]	[illegible]
Urinary diseases and male infertility	0·1 (0·1 to 0·3)	[illegible]	[illegible]	[illegible]	[illegible]	[illegible]	[illegible]
Urolithiasis	0 (0 to 0)	[illegible]	[illegible]	[illegible]	[illegible]	[illegible]	[illegible]
Benign prostatic hyperplasia	0 (0 to 0)	[illegible]	[illegible]	[illegible]	[illegible]	[illegible]	[illegible]
Male infertility	--	[illegible]	[illegible]	[illegible]	[illegible]	[illegible]	[illegible]
Other urinary diseases	0·1 (0 to 0·2)	[illegible]	[illegible]	[illegible]	[illegible]	[illegible]	[illegible]
Gynecological diseases	0 (0 to 0)	[illegible]	[illegible]	[illegible]	[illegible]	[illegible]	[illegible]
Uterine fibroids	0 (0 to 0)	[illegible]	[illegible]	[illegible]	[illegible]	[illegible]	[illegible]
Polycystic ovarian syndrome	0 (0 to 0)	[illegible]	[illegible]	[illegible]	[illegible]	[illegible]	[illegible]
Female infertility	0 (0 to 0)	[illegible]	[illegible]	[illegible]	[illegible]	[illegible]	[illegible]
Endometriosis	0 (0 to 0)	[illegible]	[illegible]	[illegible]	[illegible]	[illegible]	[illegible]
Genital prolapse	0 (0 to 0)	[illegible]	[illegible]	[illegible]	[illegible]	[illegible]	[illegible]
Premenstrual syndrome	0 (0 to 0)	[illegible]	[illegible]	[illegible]	[illegible]	[illegible]	[illegible]
Other gynecological diseases	0 (0 to 0)	[illegible]	[illegible]	[illegible]	[illegible]	[illegible]	[illegible]
Hemoglobinopathies and hemolytic anemias	0·3 (0·1 to 0·5)	[illegible]	[illegible]	[illegible]	[illegible]	[illegible]	[illegible]
Thalassemias	0 (0 to 0)	[illegible]	[illegible]	[illegible]	[illegible]	[illegible]	[illegible]
Thalassemias trait	0 (0 to 0)	[illegible]	[illegible]	[illegible]	[illegible]	[illegible]	[illegible]
Sickle cell disorders	0·2 (0·1 to 0·4)	[illegible]	[illegible]	[illegible]	[illegible]	[illegible]	[illegible]
Sickle cell trait	0 (0 to 0·2)	[illegible]	[illegible]	[illegible]	[illegible]	[illegible]	[illegible]

G6PD deficiency	0 (0 to 0)	0·1 (0 to 0·2)	0 (0 to 0·1)	0·9 (0·4 to 1·8)	1·3 (0·5 to 2·4)	1·1 (0·5 to 2·4)	52·2 (16·3 to 110)	7·9 (3·4 to 14·8)	17·1 (7·3 to 32·5)	20·2 (8·4 to 37·9)	49·2 (22·6 to 88·7)	23·6 (11·3 to 40·4)	22 (9·5 to 40·6)	24·6 (10·8 to 45·4)	20·4 (9·1 to 36·4)	11·4 (5·1 to 20·5)	6·4 (2·9 to 11·5)	2·8 (1·4 to 4·8)	1·6 (0·7 to 2·8)	0·4 (0·2 to 0·7)	0·2 (0·1 to 0·3)	0·1 (0 to 0·1)	0 (0 to 0)	263
G6PD trait	0 (0 to 0)	0 (0 to 0·1)	0 (0 to 0)	0 (0 to 0)	0 (0 to 0)	0 (0 to 0)	6·3 (1·6 to 15·1)	0·1 (0 to 0·3)	0·1 (0 to 0·3)	0·3 (0·1 to 0·4)	6·3 (1·9 to 12·6)	6·6 (1·4 to 14·1)	0·3 (0·1 to 0·6)	0 (0 to 0)	0 (0 to 0)	0 (0 to 0)	0 (0 to 0)	0 (0 to 0)	0 (0 to 0)	0 (0 to 0)	0 (0 to 0)	0 (0 to 0)	0 (0 to 0)	20·1
Other hemoglobinopathies and hemolytic anemias	0 (0 to 0)	0·9 (0·3 to 1·9)	7 (2·2 to 14·8)	29·7 (8·9 to 64·5)	39·1 (8·7 to 90·4)	18·7 (4 to 46·7)	53·7 (16·9 to 124)	41·4 (11·1 to 109)	54·4 (19·9 to 124)	65·9 (24·6 to 142)	86·4 (36·5 to 170)	102 (46·4 to 199)	127 (59·7 to 238)	173 (88·6 to 298)	172 (96·1 to 287)	162 (88 to 283)	104 (59·5 to 171)	84·2 (52·2 to 140)	74·6 (46·8 to 119)	42·2 (24·8 to 73·8)	34·5 (19·4 to 59·3)	19·3 (11·6 to 31·4)	7 (4·5 to 10·9)	1,500
Endocrine, metabolic, blood, and immune disorders	14·9 (7·2 to 27·6)	36·1 (17·4 to 57·3)	358 (212 to 541)	282 (186 to 409)	247 (157 to 372)	197 (130 to 293)	285 (172 to 456)	293 (174 to 477)	424 (253 to 689)	622 (367 to 983)	777 (480 to 1,170)	1,160 (693 to 1,760)	2,090 (1,270 to 3,180)	2,690 (1,650 to 4,200)	2,850 (1,780 to 4,400)	2,130 (1,300 to 3,420)	1,940 (1,160 to 3,180)	1,700 (1,040 to 2,750)	1,320 (805 to 2,060)	790 (479 to 1,250)	415 (261 to 714)	190 (119 to 314)	79·2 (45·5 to 147)	20,900
Oral disorders	0 (0 to 0)	0 (0 to 0)	0 (0 to 0)	142 (80·6 to 233)	197 (105 to 347)	197 (103 to 344)	319 (174 to 559)	520 (277 to 943)	815 (418 to 1,550)	1,100 (545 to 2,050)	1,110 (535 to 2,080)	1,410 (698 to 2,550)	2,380 (1,270 to 4,140)	3,210 (1,790 to 5,350)	3,670 (2,190 to 5,790)	3,180 (2,030 to 4,720)	3,330 (2,150 to 4,870)	3,140 (2,050 to 4,530)	2,490 (1,640 to 3,520)	1,610 (1,080 to 2,250)	908 (595 to 1,260)	434 (288 to 608)	181 (118 to 253)	30,400
Caries of deciduous teeth	0 (0 to 0)	0 (0 to 0)	0 (0 to 0)	44·6 (16·8 to 89)	46·8 (19·3 to 107)	2·3 (0·3 to 6·5)	0 (0 to 0)	0 (0 to 0)	0 (0 to 0)	0 (0 to 0)	0 (0 to 0)	0 (0 to 0)	0 (0 to 0)	0 (0 to 0)	0 (0 to 0)	0 (0 to 0)	0 (0 to 0)	0 (0 to 0)	0 (0 to 0)	0 (0 to 0)	0 (0 to 0)	0 (0 to 0)	0 (0 to 0)	93·8
Caries of permanent teeth	0 (0 to 0)	0 (0 to 0)	0 (0 to 0)	0 (0 to 0)	51·4 (18·3 to 113)	96·7 (38·6 to 208)	115 (46·1 to 244)	134 (55·1 to 278)	146 (58·6 to 294)	146 (56·8 to 304)	124 (48·7 to 256)	127 (52·8 to 261)	172 (69·5 to 364)	183 (73·6 to 383)	161 (64 to 348)	106 (40·4 to 221)	79·7 (32·4 to 156)	60·4 (25·5 to 124)	42·9 (18·5 to 86·2)	26 (10·8 to 53·1)	14 (5·4 to 27·9)	6·3 (2·3 to 13·8)	2·3 (0·8 to 5·2)	1,790
Periodontal diseases	0 (0 to 0)	0 (0 to 0)	0 (0 to 0)	0 (0 to 0)	0 (0 to 0)	0 (0 to 0)	37·6 (11 to 94·6)	146 (43·6 to 366)	354 (111 to 858)	577 (199 to 1,310)	624 (222 to 1,470)	791 (285 to 1,700)	1,180 (449 to 2,480)	1,240 (489 to 2,630)	1,020 (401 to 2,100)	612 (247 to 1,280)	464 (181 to 965)	350 (139 to 684)	252 (101 to 499)	159 (62·2 to 320)	91·4 (35 to 182)	44·2 (16·5 to 89·5)	18 (6·8 to 36·6)	7,960
Other oral disorders	0 (0 to 0)	0 (0 to 0)	0 (0 to 0)	97·7 (57·5 to 152)	98·3 (56·8 to 157)	98·3 (55·8 to 153)	166 (99 to 258)	211 (126 to 330)	249 (149 to 378)	275 (168 to 425)	232 (138 to 350)	257 (155 to 386)	369 (226 to 559)	392 (240 to 589)	339 (203 to 511)	228 (134 to 339)	197 (120 to 295)	151 (90·8 to 229)	93·6 (56·4 to 143)	48·3 (29·1 to 73·8)	21·9 (13·5 to 32·7)	9·3 (5·7 to 13·9)	4 (2·4 to 6·1)	3,540
Sudden infant death syndrome	–	15·7 (8·8 to 24·6)	117 (60 to 201)	–	–	–	–	–	–	–	–	–	–	–	–	–	–	–	–	–	–	–	–	NA
Injuries	78·5 (45·4 to 111)	52·7 (35·2 to 72·7)	503 (359 to 673)	1,080 (868 to 1,310)	1,430 (1,170 to 1,730)	2,000 (1,660 to 2,390)	4,020 (3,380 to 4,800)	4,570 (3,770 to 5,550)	4,910 (3,870 to 6,090)	5,080 (4,040 to 6,350)	4,400 (3,530 to 5,470)	4,700 (3,760 to 5,890)	6,640 (5,230 to 8,280)	7,230 (5,740 to 9,130)	7,060 (5,600 to 8,850)	5,680 (4,540 to 7,050)	6,400 (5,240 to 7,870)	7,660 (6,370 to 9,280)	9,030 (7,390 to 10,900)	10,200 (8,350 to 12,400)	9,390 (7,580 to 11,300)	6,540 (5,300 to 7,910)	3,620 (2,910 to 4,300)	112,000
Transport injuries	1·8 (1·1 to 2·4)	1·5 (1 to 2)	33·5 (21·7 to 45·9)	200 (141 to 274)	351 (261 to 453)	510 (378 to 663)	1,250 (922 to 1,610)	1,220 (909 to 1,590)	1,180 (860 to 1,580)	1,040 (775 to 1,330)	926 (699 to 1,180)	999 (777 to 1,290)	1,280 (1,010 to 1,620)	1,370 (1,070 to 1,680)	1,270 (1,000 to 1,580)	884 (691 to 1,110)	832 (657 to 1,030)	706 (558 to 878)	568 (453 to 700)	364 (290 to 451)	200 (155 to 248)	102 (79·4 to 129)	48·3 (37·3 to 60·4)	15,300
Road injuries	1·6 (1 to 2·3)	1·3 (0·8 to 1·8)	30·4 (19·1 to 43)	181 (125 to 252)	321 (237 to 419)	469 (341 to 607)	1,200 (873 to 1,550)	1,160 (857 to 1,530)	1,110 (806 to 1,510)	974 (724 to 1,250)	871 (659 to 1,120)	921 (712 to 1,190)	1,190 (938 to 1,500)	1,260 (983 to 1,560)	1,170 (929 to 1,460)	820 (640 to 1,020)	777 (615 to 960)	657 (521 to 813)	525 (417 to 646)	339 (269 to 421)	185 (143 to 230)	94·7 (73·6 to 118)	44·5 (34·3 to 56)	14,300
Pedestrian road injuries	0·3 (0·2 to 0·4)	0·5 (0·3 to 0·7)	4·5 (2·3 to 7·5)	72·2 (47·3 to 106)	123 (86·7 to 174)	180 (125 to 242)	212 (132 to 314)	172 (109 to 249)	172 (118 to 243)	185 (135 to 245)	171 (127 to 226)	205 (156 to 272)	268 (191 to 357)	333 (255 to 422)	339 (265 to 431)	285 (218 to 363)	307 (236 to 384)	290 (225 to 369)	243 (189 to 307)	165 (125 to 212)	87·7 (66·9 to 114)	45·3 (34·2 to 57·4)	22·7 (17·4 to 29)	3,880
Cyclist road injuries	0 (0 to 0)	0 (0 to 0)	0 (0 to 0)	14·5 (8·7 to 22·3)	32·8 (21·5 to 49·6)	74·8 (47·1 to 115)	128 (81·3 to 195)	121 (82·8 to 169)	136 (95 to 194)	136 (97·4 to 186)	132 (95·1 to 178)	143 (104 to 191)	208 (148 to 275)	238 (170 to 314)	206 (145 to 278)	141 (97·8 to 189)	126 (87·9 to 170)	104 (72·6 to 142)	80·3 (56·2 to 109)	52·6 (36·6 to 70·6)	30·8 (21·5 to 41·3)	15·2 (10·7 to 20·6)	6·7 (4·7 to 9·2)	2,130
Motorcyclist road injuries	0 (0 to 0)	0·1 (0·1 to 0·1)	2·8 (1·7 to 4·1)	9·3 (5·7 to 14·2)	23·9 (15 to 36·4)	32·9 (24·8 to 41·9)	236 (165 to 317)	261 (186 to 354)	190 (123 to 283)	149 (111 to 195)	145 (104 to 194)	132 (100 to 174)	177 (137 to 228)	163 (121 to 213)	161 (119 to 212)	106 (76·3 to 142)	105 (78·3 to 138)	87·3 (62·4 to 118)	71·8 (50·5 to 97·2)	49·8 (34·9 to 69·1)	29·8 (20·7 to 41·8)	15·8 (11 to 22·1)	7·6 (5·2 to 10·6)	2,160
Motor vehicle road injuries	1·3 (0·8 to 1·8)	0·6 (0·4 to 0·9)	22·2 (13·7 to 32·2)	70·3 (44·7 to 102)	123 (87 to 164)	163 (111 to 219)	594 (421 to 793)	573 (398 to 783)	554 (378 to 780)	442 (314 to 599)	377 (270 to 515)	393 (287 to 533)	480 (364 to 628)	455 (347 to 582)	399 (304 to 509)	235 (180 to 296)	192 (147 to 248)	148 (112 to 191)	103 (79 to 130)	58·5 (44·1 to 75)	29·7 (22·1 to 38·5)	14·7 (11 to 19)	6·2 (4·6 to 8·1)	5,430
Other road injuries	0·1 (0 to 0·1)	0·1 (0 to 0·1)	0·9 (0·5 to 1·4)	15·1 (6·7 to 30·3)	18 (9·8 to 31·2)	18·1 (10·1 to 31·8)	28·4 (16·3 to 46·2)	35·2 (21·1 to 56)	62·2 (36·2 to 101)	60·9 (37·2 to 99·2)	46·3 (29·7 to 68·8)	48·2 (32·7 to 67·8)	60·6 (42·8 to 83·5)	72·3 (49·8 to 96·9)	68·8 (47·9 to 96·9)	52·2 (35·4 to 73·5)	46·7 (32·5 to 66·4)	28·2 (20·1 to 38·4)	26·4 (18·9 to 35·1)	13·1 (9·4 to 17·7)	7·3 (5·1 to 9·9)	3·7 (2·6 to 5·1)	1·3 (0·9 to 1·8)	714
Other transport injuries	0·1 (0·1 to 0·2)	0·2 (0·1 to 0·4)	3 (1·9 to 4·5)	18·8 (12·4 to 26·6)	29·8 (19·9 to 42·2)	41 (27·7 to 56·8)	53·6 (36·8 to 76·4)	57·9 (40·4 to 79)	62·2 (44·6 to 85·1)	65·5 (46·8 to 87·9)	54·9 (38·6 to 74·6)	77·6 (55·1 to 106)	90·6 (64·9 to 120)	105 (76·9 to 138)	100 (74·9 to 131)	64 (47·3 to 84·5)	55 (40·8 to 73·4)	48·5 (35·4 to 64·5)	43·7 (32·2 to 57·2)	25 (18·3 to 32·8)	14·7 (10·8 to 19·5)	7·7 (5·6 to 10·3)	3·7 (2·7 to 5)	1,020
Unintentional injuries	30·4 (18·5 to 42)	44·1 (29·4 to 62·5)	418 (293 to 571)	786 (608 to 975)	971 (759 to 1,230)	1,150 (891 to 1,470)	1,320 (983 to 1,780)	1,440 (1,050 to 1,980)	1,740 (1,260 to 2,420)	2,040 (1,490 to 2,810)	1,870 (1,350 to 2,620)	2,150 (1,550 to 2,970)	3,290 (2,360 to 4,520)	3,960 (2,910 to 5,370)	4,070 (3,040 to 5,440)	3,600 (2,750 to 4,780)	4,490 (3,530 to 5,640)	6,090 (4,940 to 7,460)	7,850 (6,390 to 9,560)	9,490 (7,760 to 11,600)	9,030 (7,260 to 10,900)	6,400 (5,170 to 7,740)	3,550 (2,850 to 4,240)	75,800
Falls	1·1 (0·7 to 1·6)	0·9 (0·6 to 1·3)	28·1 (18·8 to 39·5)	95 (68·7 to 128)	139 (99·6 to 191)	178 (131 to 243)	230 (163 to 327)	273 (187 to 396)	351 (241 to 505)	424 (298 to 599)	422 (303 to 593)	522 (371 to 722)	895 (640 to 1,220)	1,270 (958 to 1,700)	1,500 (1,140 to 1,940)	1,710 (1,330 to 2,160)	2,730 (2,150 to 3,400)	4,430 (3,570 to 5,420)	6,370 (5,130 to 7,880)	8,350 (6,760 to 10,300)	8,210 (6,590 to 9,930)	5,780 (4,630 to 7,050)	3,170 (2,510 to 3,800)	47,100
Drowning	0·9 (0·5 to 1·2)	0·4 (0·2 to 0·5)	12·7 (8·2 to 18·4)	163 (109 to 220)	171 (120 to 234)	230 (166 to 308)	147 (106 to 196)	69·3 (48·6 to 92·5)	74·2 (52·7 to 101)	69 (52 to 89·9)	56·6 (42·4 to 75·4)	54·6 (41·6 to 70·5)	77·3 (57·9 to 100)	75·7 (58 to 96·8)	63·2 (49·3 to 79·8)	44·5 (34·8 to 55·9)	43·4 (33·7 to 54·3)	36 (28 to 45)	38·3 (30·2 to 47·8)	27 (20·8 to 33·9)	23·4 (18·3 to 29·7)	23·9 (18·7 to 29·5)	16·5 (12·2 to 20·6)	1,520
Fire, heat, and hot substances	0·3 (0·2 to 0·4)	0·7 (0·5 to 0·9)	11·1 (7·8 to 14·8)	74·6 (53·3 to 100)	139 (93·5 to 207)	177 (117 to 268)	277 (184 to 408)	321 (206 to 475)	403 (266 to 592)	505 (330 to 762)	455 (295 to 686)	482 (305 to 738)	685 (433 to 1,060)	764 (487 to 1,180)	711 (461 to 1,090)	498 (319 to 764)	437 (288 to 657)	374 (247 to 556)	297 (199 to 440)	204 (138 to 291)	133 (96·1 to 187)	80·6 (60·2 to 108)	44·3 (33·6 to 56·7)	7,070
Poisonings	0·2 (0·1 to 0·3)	1·1 (0·7 to 1·6)	6·1 (3·8 to 9)	25·9 (16·7 to 38·2)	24·2 (13·9 to 39·8)	28 (15·8 to 43·8)	38·8 (24 to 58)	41 (26·1 to 61·3)	42·4 (26·1 to 64·7)	43·3 (27 to 65·1)	37·2 (22·8 to 57·1)	40·3 (26·1 to 59·8)	55·2 (34·8 to 83·6)	58·5 (36·8 to 87·3)	54·8 (35·4 to 82·6)	35·7 (22·9 to 53·2)	35 (23·3 to 50·6)	30·8 (21·5 to 44·1)	24 (16·3 to 33·4)	16·9 (11·9 to 23·1)	9·8 (6·9 to 13·4)	4·4 (3·1 to 6·1)	1·7 (1·2 to 2·4)	655
Poisoning by carbon monoxide	0 (0 to 0)	0 (0 to 0)	0·4 (0·2 to 0·6)	4·2 (2·1 to 7·2)	8·1 (4·5 to 13·5)	8·7 (5 to 14·7)	11·7 (7·2 to 18)	13·5 (8·4 to 20·2)	13·5 (8·2 to 20·5)	13·5 (8·3 to 20·7)	11·1 (6·6 to 17·1)	13·2 (8·5 to 19·7)	16·8 (10·6 to 25·7)	16·8 (10·3 to 25·8)	18·6 (11·9 to 27·7)	11·4 (7·1 to 17·4)	13·4 (9·3 to 18·9)	9·8 (6·8 to 14)	8·6 (5·6 to 12·4)	6·1 (4·1 to 8·3)	3·4 (2·3 to 4·8)	1·5 (1 to 2)	0·5 (0·3 to 0·7)	205
Poisoning by other means	0·2 (0·1 to 0·2)	1·1 (0·7 to 1·6)	5·8 (3·5 to 8·5)	21·7 (13·8 to 32·9)	16·1 (8·5 to 27·8)	19·3 (10·5 to 32)	27·1 (16·3 to 41·8)	27·5 (16 to 42·1)	28·9 (16·5 to 46·8)	29·8 (17·1 to 47·2)	26·1 (15·3 to 42·2)	27 (16·1 to 43·3)	38·4 (22·7 to 60·4)	41·6 (25·3 to 65·4)	36·2 (22·1 to 57·1)	24·3 (14·7 to 38·1)	21·5 (12·9 to 33·4)	20·9 (14 to 30·6)	15·4 (9·8 to 22·6)	10·9 (7·2 to 16·1)	6·4 (4·3 to 9·2)	3 (2 to 4·3)	1·2 (0·8 to 1·7)	450
Exposure to mechanical forces	0·7 (0·5 to 1)	1·2 (0·8 to 1·6)	17·7 (12·1 to 24·8)	121 (85·3 to 171)	187 (132 to 261)	206 (147 to 290)	244 (166 to 351)	278 (190 to 405)	317 (220 to 461)	367 (250 to 533)	336 (227 to 493)	396 (266 to 580)	581 (390 to 854)	670 (460 to 975)	627 (432 to 921)	452 (307 to 660)	420 (282 to 604)	384 (265 to 550)	321 (227 to 454)	239 (170 to 329)	168 (123 to 226)	123 (91·9 to 158)	77·4 (59·6 to 99·3)	6,530
Unintentional firearm injuries	0·1 (0·1 to 0·2)	0·1 (0·1 to 0·2)	2·2 (1·4 to 3·4)	11·9 (7·1 to 18·8)	18·3 (11·5 to 28·4)	28 (18·5 to 41·1)	38·1 (26·6 to 52·9)	42·2 (29·7 to 58·2)	43·1 (30·2 to 58·8)	52·3 (37·4 to 70·4)	44·8 (31·9 to 60·4)	52·3 (37·2 to 70·6)	74·7 (52 to 102)	84·6 (60·2 to 116)	79·4 (55·7 to 108)	56·2 (39·4 to 75·8)	51·4 (36·3 to 69·7)	51·2 (36·8 to 68·3)	46·9 (34·5 to 61·1)	46·3 (34·9 to 59·8)	41 (30·9 to 52·7)	42·5 (31·8 to 55)	31·7 (23·8 to 41·2)	939
Other exposure to mechanical forces	0·6 (0·4 to 0·9)	1·1 (0·7 to 1·5)	15·5 (10·5 to 21·8)	110 (76·3 to 154)	169 (119 to 236)	178 (124 to 262)	206 (136 to 309)	236 (156 to 355)	274 (184 to 411)	315 (209 to 469)	291 (196 to 435)	344 (227 to 515)	506 (338 to 759)	585 (395 to 861)	548 (371 to 823)	396 (264 to 588)	369 (246 to 538)	333 (226 to 483)	274 (189 to 393)	192 (132 to 274)	127 (89·3 to 177)	80·1 (57·1 to 108)	45·7 (32·9 to 60·7)	5,600
Adverse effects of medical treatment	9·2 (5·1 to 15)	8·6 (5·6 to 12·7)	36·4 (20·6 to 60·9)	33·6 (20·8 to 53·2)	15·1 (8·3 to 30·8)	11 (6 to 22·9)	18·1 (10·7 to 35)	39·1 (23·3 to 69·8)	51·9 (32·1 to 83·5)	73·2 (46·5 to 112)	55·6 (35·9 to 83·8)	73·7 (48·3 to 112)	131 (88·2 to 188)	147 (98·4 to 212)	163 (107 to 246)	140 (94·4 to 206)	127 (86·3 to 188)	149 (102 to 209)	138 (96·7 to 198)	77·8 (50·9 to 138)	56·8 (36·5 to 104)	26·6 (16·6 to 54·1)	14·1 (8·3 to 28·8)	1,600
Animal contact	0·1 (0 to 0·1)	0·1 (0·1 to 0·2)	1·8 (1·1 to 2·5)	13·4 (8·9 to 19·2)	24·7 (15·9 to 37·5)	25·4 (15·5 to 38·3)	32·5 (20·7 to 47·1)	36 (22·9 to 51·8)	40·8 (26 to 59·5)	43 (28·1 to 64·3)	40·6 (26·5 to 59·5)	46·8 (31·4 to 66·7)	73·2 (49·4 to 104)	75·2 (50·3 to 107)	79·2 (54·9 to 108)	63 (45·7 to 86·9)	59·9 (42·4 to 82·1)	71·8 (52·3 to 96·9)	86·5 (62·1 to 116)	130 (87·3 to 185)	133 (89 to 192)	154 (106 to 220)	107 (69·7 to 158)	1,340
Venomous animal contact	0 (0 to 0·1)	0·1 (0 to 0·1)	0·9 (0·6 to 1·4)	7·3 (4·3 to 11·6)	14·9 (8·8 to 24·1)	18·4 (10·5 to 28·3)	24 (14·8 to 36·1)	26·9 (16·3 to 40)	30·3 (18·4 to 45)	31·7 (20·1 to 47·8)	28·3 (17·1 to 42·1)	32·9 (21·6 to 48)	49·3 (32·6 to 71)	48·9 (31·1 to 71·2)	46·5 (30·1 to 67·7)	40·5 (28·2 to 56·8)	39·1 (27·3 to 53·3)	52·2 (36·9 to 71·6)	68·7 (48·9 to 94·3)	103 (66·9 to 153)	110 (71·3 to 164)	133 (89·6 to 192)	92·5 (59 to 135)	1,000
Non-venomous animal contact	0 (0 to 0·1)	0·1 (0 to 0·1)	0·8 (0·5 to 1·3)	6·1 (4 to 9·3)	9·8 (5·9 to 15·2)	7 (4 to 12)	8·5 (5·2 to 14)	9·2 (5·5 to 14·7)	10·6 (6·4 to 16·8)	11·3 (6·8 to 18·6)	12·3 (7·9 to 19·8)	13·9 (8·6 to 22)	23·8 (15·2 to 36)	26·3 (16·6 to 39·5)	32·7 (22·1 to 47·1)	22·5 (14·8 to 33·8)	20·8 (13·4 to 31)	19·6 (12·5 to 30·1)	17·8 (11·4 to 26·7)	26·5 (16·1 to 41·4)	23·2 (13·6 to 38·2)	20·7 (11·9 to 33·7)	14·2 (8 to 23·2)	338
Foreign body	14·9 (8·6 to 21·3)	28 (17 to 42·6)	272 (177 to 397)	126 (88 to 169)	64·2 (49·3 to 81·7)	62·6 (48·1 to 81·4)	53·3 (39·4 to 72·2)	64·3 (46·9 to 88·8)	77·1 (56·9 to 106)	81·9 (61 to 109)	77·1 (56·6 to 103)	90·7 (67·9 to 119)	137 (106 to 178)	167 (129 to 212)	184 (144 to 233)	156 (119 to 200)	177 (139 to 218)	199 (155 to 248)	219 (165 to 286)	195 (151 to 246)	138 (107 to 174)	109 (84·4 to 134)	68 (50·6 to 82·7)	2,760
Pulmonary aspiration and foreign body in airway	14·9 (8·5 to 21·2)	27·9 (16·9 to 42·4)	268 (173 to 392)	111 (74·7 to 151)	42·4 (32 to 54·5)	39·3 (29·3 to 51·4)	24 (18·6 to 30·2)	29·4 (21·9 to 39·3)	38·3 (28·1 to 50·5)	39·3 (29·3 to 51·8)	37·8 (28·1 to 50·3)	44·5 (33·6 to 58·4)	67·2 (50·3 to 89·9)	92·2 (66·5 to 123)	119 (86·8 to 159)	106 (78·3 to 143)	119 (88·7 to 157)	147 (109 to 190)	159 (111 to 215)	135 (97·9 to 178)	91·1 (65·4 to 120)	64·2 (43·6 to 86·1)	38·9 (26·2 to 52·3)	1,850
Foreign body in eyes	0 (0 to 0)	0 (0 to 0·1)	0·6 (0·2 to 1·3)	3·4 (1 to 7·7)	5·9 (1·7 to 13·6)	6·9 (2·1 to 15·5)	10·3 (3·3 to 23·4)	13·5 (4·3 to 29·8)	16·6 (5·9 to 35·1)	18·6 (6·5 to 38·3)	16·6 (6·5 to 34)	19·1 (8·2 to 38·9)	26·5 (12·7 to 49·8)	26·2 (13·7 to 46·3)	21·5 (12·4 to 34·8)	14·9 (9 to 22·9)	13·5 (8·4 to 20·3)	11·9 (7·8 to 17·6)	9·6 (6·3 to 14·2)	6·5 (4·2 to 9·6)	3·8 (2·6 to 5·5)	2 (1·3 to 2·8)	0·9 (0·6 to 1·2)	249
Foreign body in other body part	0·1 (0 to 0·1)	0·1 (0·1 to 0·2)	3·3 (2 to 5·2)	11·9 (7·9 to 17·9)	15·9 (10·5 to 23)	16·3 (11·1 to 23·2)	19 (13·2 to 26·8)	21·4 (14·7 to 29·7)	22·2 (15·5 to 30·3)	24 (16·7 to 34)	22·7 (16 to 31·2)	27·1 (19 to 37·1)	43·7 (31·3 to 60·4)	48·8 (35·3 to 65·7)	43·7 (30·6 to 59·6)	34·5 (24·7 to 46·5)	44·3 (30·5 to 61·5)	40·5 (28·7 to 54·6)	51·1 (33·6 to 79·5)	53·9 (35·2 to 78·8)	43·2 (28·8 to 63·9)	42·4 (27·6 to 59·3)	28·2 (18·1 to 39·9)	659
Environmental heat and cold exposure	0·1 (0·1 to 0·1)	1·1 (0·7 to 1·7)	3·1 (2 to 4·5)	19·7 (12·6 to 29·5)	37·4 (22·9 to 57·8)	44·1 (28·5 to 65·1)	61·8 (40·8 to 90·3)	73·2 (48·3 to 106)	88·1 (57·9 to 129)	102 (68·7 to 152)	91·6 (61·7 to 133)	107 (72·9 to 153)	159 (108 to 231)	182 (124 to 261)	175 (119 to 247)	134 (91·6 to 189)	131 (90·1 to 183)	124 (87·1 to 172)	110 (77·5 to 151)	87·8 (62·7 to 116)	65·5 (48·6 to 86·1)	54·6 (40·4 to 70·9)	39·9 (28·2 to 57·4)	1,890
Exposure to forces of nature	0 (0 to 0)	0·2 (0·1 to 0·2)	1·3 (1·2 to 1·5)	7·6 (6·9 to 8·3)	7·3 (6·6 to 8·1)	7·5 (6·7 to 8·2)	7 (6·3 to 7·7)	6·3 (5·7 to 7)	7·4 (6·6 to 8·3)	6·7 (6 to 7·6)	5·6 (4·9 to 6·4)	7·3 (6·5 to 8·2)	8·7 (7·7 to 10)	9·2 (8·1 to 10·4)	7·1 (6·3 to 8·1)	5·7 (5 to 6·4)	4·9 (4·3 to 5·6)	4·2 (3·8 to 4·8)	3·1 (2·8 to 3·5)	2 (1·8 to 2·3)	1·1 (1 to 1·2)	0·5 (0·4 to 0·6)	0·2 (0·2 to 0·2)	111
Other unintentional injuries	2·9 (1·7 to 4·4)	1·8 (1·2 to 2·6)	28·4 (20·5 to 38·4)	106 (72·2 to 151)	162 (106 to 240)	176 (121 to 257)	210 (142 to 311)	239 (157 to 359)	287 (193 to 420)	326 (218 to 486)	293 (195 to 429)	332 (219 to 489)	484 (321 to 706)	540 (355 to 785)	501 (330 to 737)	358 (238 to 524)	332 (227 to 486)	296 (201 to 424)	246 (170 to 345)	159 (110 to 226)	92·5 (62·9 to 132)	45·3 (30·6 to 65·1)	20 (13·7 to 28·4)	5,240
Self-harm and interpersonal violence	46·3 (24·5 to 77·1)	7·1 (4·1 to 11·7)	51·2 (31·1 to 76·9)	93·9 (64·9 to 145)	106 (83·3 to 136)	346 (273 to 428)	1,450 (1,140 to 1,790)	1,900 (1,470 to 2,400)	1,990 (1,500 to 2,580)	2,000 (1,490 to 2,580)	1,610 (1,230 to 2,090)	1,550 (1,200 to 2,010)	2,070 (1,580 to 2,670)	1,900 (1,440 to 2,470)	1,720 (1,300 to 2,270)	1,190 (903 to 1,560)	1,080 (839 to 1,360)	862 (674 to 1,090)	606 (478 to 767)	328 (260 to 416)	162 (125 to 204)	40·9 (32·4 to 50)	14·8 (11·8 to 18·3)	21,100
Self-harm	0 (0 to 0)	0 (0 to 0)	0 (0 to 0)	0 (0 to 0)	0 (0 to 0)	151 (102 to 217)	700 (492 to 944)	757 (542 to 1,040)	735 (507 to 1,070)	793 (549 to 1,120)	715 (504 to 997)	804 (578 to 1,090)	1,300 (932 to 1,750)	1,370 (984 to 1,890)	1,350 (981 to 1,850)	967 (703 to 1,310)	858 (641 to 1,110)	687 (513 to 887)	466 (351 to 621)	259 (199 to 342)	126 (91·6 to 167)	26·3 (20·1 to 33·4)	8·7 (6·7 to 11)	12,100
Self-harm by firearm	0 (0 to 0)	0 (0 to 0)	0 (0 to 0)	0 (0 to 0)	0 (0 to 0)	2·8 (1·6 to 4·8)	9·5 (6·5 to 13·2)	7·4 (4·2 to 11·9)	4·9 (3 to 7·8)	14·4 (8·7 to 23)	12 (7 to 20·1)	6·7 (4·6 to 9·3)	10 (7·7 to 12·7)	12·8 (8·7 to 18·2)	11·4 (7·9 to 16·3)	5·1 (3·8 to 6·7)	6·6 (4·8 to 8·8)	8·6 (6·2 to 12·2)	10·7 (7·4 to 14·9)	14·8 (10·4 to 21·1)	11·3 (7·6 to 16·2)	0·7 (0·5 to 0·8)	0·3 (0·2 to 0·3)	150
Self-harm by other specified means	[illegible]	[illegible]	[illegible]	[illegible]	[illegible]	[illegible]	[illegible]	[illegible]	[illegible]	[illegible]	[illegible]	[illegible]	[illegible]	[illegible]	[illegible]	[illegible]	[illegible]	[illegible]	[illegible]	[illegible]	[illegible]	[illegible]	[illegible]	[illegible]
Interpersonal violence	[illegible]	[illegible]	[illegible]	[illegible]	[illegible]	[illegible]	[illegible]	[illegible]	[illegible]	[illegible]	[illegible]	[illegible]	[illegible]	[illegible]	[illegible]	[illegible]	[illegible]	[illegible]	[illegible]	[illegible]	[illegible]	[illegible]	[illegible]	[illegible]
Physical violence by firearm	[illegible]	[illegible]	[illegible]	[illegible]	[illegible]	[illegible]	[illegible]	[illegible]	[illegible]	[illegible]	[illegible]	[illegible]	[illegible]	[illegible]	[illegible]	[illegible]	[illegible]	[illegible]	[illegible]	[illegible]	[illegible]	[illegible]	[illegible]	[illegible]
Physical violence by sharp object	[illegible]	[illegible]	[illegible]	[illegible]	[illegible]	[illegible]	[illegible]	[illegible]	[illegible]	[illegible]	[illegible]	[illegible]	[illegible]	[illegible]	[illegible]	[illegible]	[illegible]	[illegible]	[illegible]	[illegible]	[illegible]	[illegible]	[illegible]	[illegible]
Sexual violence	[illegible]	[illegible]	[illegible]	[illegible]	[illegible]	[illegible]	[illegible]	[illegible]	[illegible]	[illegible]	[illegible]	[illegible]	[illegible]	[illegible]	[illegible]	[illegible]	[illegible]	[illegible]	[illegible]	[illegible]	[illegible]	[illegible]	[illegible]	[illegible]
Physical violence by other means	[illegible]	[illegible]	[illegible]	[illegible]	[illegible]	[illegible]	[illegible]	[illegible]	[illegible]	[illegible]	[illegible]	[illegible]	[illegible]	[illegible]	[illegible]	[illegible]	[illegible]	[illegible]	[illegible]	[illegible]	[illegible]	[illegible]	[illegible]	[illegible]
Conflict and terrorism	[illegible]	[illegible]	[illegible]	[illegible]	[illegible]	[illegible]	[illegible]	[illegible]	[illegible]	[illegible]	[illegible]	[illegible]	[illegible]	[illegible]	[illegible]	[illegible]	[illegible]	[illegible]	[illegible]	[illegible]	[illegible]	[illegible]	[illegible]	[illegible]

SR Table 2b: Dominica DALYS by cause and age group for females with 95% uncertainty intervals, 2019

Cause	Early Neonatal	Late Neonatal	Post Neonatal	90 to 94	95 plus	Total
All causes	384 (274 to 528)	129 (92·4 to 177)	225 (167 to 304)	270 (244 to 298)	100 (89·6 to 110)	11,200
Communicable, maternal, neonatal, and nutritional diseases	330 (234 to 453)	99·6 (70·7 to 141)	118 (81·5 to 162)	18·9 (13·4 to 24·7)	8 (5·7 to 10·7)	1,410
HIV/AIDS and sexually transmitted infections	14·5 (4 to 32·9)	4·2 (1·2 to 10)	8·7 (4 to 17·5)	0 (0 to 0·1)	0 (0 to 0)	107
HIV/AIDS	0 (0 to 0)	0 (0 to 0)	2·1 (2 to 2·3)	0 (0 to 0)	0 (0 to 0)	71·8
HIV/AIDS - Drug-susceptible Tuberculosis	0 (0 to 0)	0 (0 to 0)	0·3 (0·2 to 0·4)	0 (0 to 0)	0 (0 to 0)	9·2
HIV/AIDS - Multidrug-resistant Tuberculosis without extensive dru	0 (0 to 0)	0 (0 to 0)	0 (0 to 0)	0 (0 to 0)	0 (0 to 0)	0·1
HIV/AIDS - Extensively drug-resistant Tuberculosis	0 (0 to 0)	0 (0 to 0)	0 (0 to 0)	0 (0 to 0)	0 (0 to 0)	0
HIV/AIDS resulting in other diseases	0 (0 to 0)	0 (0 to 0)	1·8 (1·6 to 2)	0 (0 to 0)	0 (0 to 0)	62·4
Sexually transmitted infections excluding HIV	14·5 (4 to 32·9)	4·2 (1·2 to 10)	6·5 (1·9 to 15·5)	0 (0 to 0)	0 (0 to 0)	34·7
Syphilis	14·5 (4 to 32·9)	4·2 (1·2 to 10)	6·5 (1·9 to 15·5)	0 (0 to 0)	0 (0 to 0)	25·9
Chlamydial infection	0 (0 to 0)	0 (0 to 0)	0 (0 to 0)	0 (0 to 0)	0 (0 to 0)	0·7
Gonococcal infection	0 (0 to 0)	0 (0 to 0)	0 (0 to 0)	0 (0 to 0)	0 (0 to 0)	0·5
Trichomoniasis	0 (0 to 0)	0 (0 to 0)	0 (0 to 0)	0 (0 to 0)	0 (0 to 0)	3·3
Genital herpes	0 (0 to 0)	0 (0 to 0)	0 (0 to 0)	0 (0 to 0)	0 (0 to 0)	2·6
Other sexually transmitted infections	0 (0 to 0)	0 (0 to 0)	0 (0 to 0)	0 (0 to 0)	0 (0 to 0)	1·7
Respiratory infections and tuberculosis	7·7 (3·8 to 14·2)	7·5 (3·6 to 13·7)	35·1 (19·5 to 57·7)	14·7 (9·5 to 20·3)	6·4 (4·2 to 9·1)	317
Tuberculosis	0 (0 to 0)	0 (0 to 0)	0·5 (0·3 to 0·9)	0·3 (0·2 to 0·4)	0·1 (0 to 0·1)	27·7
Latent tuberculosis infection	0 (0 to 0)	0 (0 to 0)	0 (0 to 0)	0 (0 to 0)	0 (0 to 0)	0
Drug-susceptible tuberculosis	0 (0 to 0)	0 (0 to 0)	0·5 (0·3 to 0·9)	0·2 (0·2 to 0·4)	0·1 (0 to 0·1)	27·3
Multidrug-resistant tuberculosis without extensive drug resistanc	0 (0 to 0)	0 (0 to 0)	0 (0 to 0)	0 (0 to 0)	0 (0 to 0)	0·3
Extensively drug-resistant tuberculosis	0 (0 to 0)	0 (0 to 0)	0 (0 to 0)	0 (0 to 0)	0 (0 to 0)	0·1
Lower respiratory infections	7·7 (3·8 to 14·2)	7·4 (3·6 to 13·7)	33·5 (18 to 56)	14·4 (9·2 to 20)	6·3 (4·1 to 9)	253
Upper respiratory infections	0 (0 to 0)	0 (0 to 0·1)	1 (0·5 to 2)	0·1 (0 to 0·1)	0 (0 to 0)	28·4
Otitis media	0 (0 to 0)	0 (0 to 0)	0·1 (0 to 0·2)	0 (0 to 0)	0 (0 to 0)	8·4
Enteric infections	0·5 (0·2 to 0·9)	1·2 (0·5 to 2·8)	11·3 (5·1 to 23·5)	1·5 (0·9 to 2·3)	0·5 (0·3 to 0·9)	81·5
Diarrheal diseases	0·5 (0·2 to 0·9)	1·2 (0·5 to 2·8)	11·2 (5 to 23·5)	1·5 (0·9 to 2·3)	0·5 (0·3 to 0·9)	80·7
Typhoid and paratyphoid	0 (0 to 0)	0 (0 to 0)	0 (0 to 0)	0 (0 to 0)	0 (0 to 0)	0·3
Typhoid fever	0 (0 to 0)	0 (0 to 0)	0 (0 to 0)	0 (0 to 0)	0 (0 to 0)	0·2
Paratyphoid fever	0 (0 to 0)	0 (0 to 0)	0 (0 to 0)	0 (0 to 0)	0 (0 to 0)	0
Invasive Non-typhoidal Salmonella (iNTS)	0 (0 to 0)	0 (0 to 0)	0 (0 to 0)	0 (0 to 0)	0 (0 to 0)	0·4
Other intestinal infectious diseases	0 (0 to 0)	0 (0 to 0)	0 (0 to 0·2)	0 (0 to 0)	0 (0 to 0)	0·2
Neglected tropical diseases and malaria	0 (0 to 0·1)	0·1 (0 to 0·2)	1·3 (0·5 to 2·9)	0·5 (0·2 to 0·8)	0·2 (0·1 to 0·3)	42·5
Malaria	0 (0 to 0)	0 (0 to 0)	0 (0 to 0)	0 (0 to 0)	0 (0 to 0)	0
Chagas disease	0 (0 to 0)	0 (0 to 0)	0 (0 to 0)	0 (0 to 0)	0 (0 to 0)	0
Leishmaniasis	0 (0 to 0)	0 (0 to 0)	0 (0 to 0)	0 (0 to 0)	0 (0 to 0)	0
Visceral leishmaniasis	0 (0 to 0)	0 (0 to 0)	0 (0 to 0)	0 (0 to 0)	0 (0 to 0)	0
Cutaneous and mucocutaneous leishmaniasis	0 (0 to 0)	0 (0 to 0)	0 (0 to 0)	0 (0 to 0)	0 (0 to 0)	0
African trypanosomiasis	0 (0 to 0)	0 (0 to 0)	0 (0 to 0)	0 (0 to 0)	0 (0 to 0)	0
Schistosomiasis	0 (0 to 0)	0 (0 to 0)	0 (0 to 0)	0 (0 to 0)	0 (0 to 0)	0
Cysticercosis	0 (0 to 0)	0 (0 to 0)	0 (0 to 0)	0·4 (0·1 to 0·7)	0·1 (0 to 0·2)	22·3
Cystic echinococcosis	0 (0 to 0)	0 (0 to 0)	0 (0 to 0)	0 (0 to 0)	0 (0 to 0)	0
Lymphatic filariasis	0 (0 to 0)	0 (0 to 0)	0 (0 to 0)	0 (0 to 0)	0 (0 to 0)	0
Onchocerciasis	0 (0 to 0)	0 (0 to 0)	0 (0 to 0)	0 (0 to 0)	0 (0 to 0)	0
Trachoma	0 (0 to 0)	0 (0 to 0)	0 (0 to 0)	0 (0 to 0)	0 (0 to 0)	0
Dengue	0 (0 to 0)	0 (0 to 0·1)	0·7 (0·1 to 2·2)	0 (0 to 0·1)	0 (0 to 0)	6·8
Yellow fever	0 (0 to 0)	0 (0 to 0)	0 (0 to 0)	0 (0 to 0)	0 (0 to 0)	0
Rabies	0 (0 to 0)	0 (0 to 0)	0 (0 to 0·1)	0 (0 to 0)	0 (0 to 0)	0·1
Intestinal nematode infections	0 (0 to 0)	0 (0 to 0)	0 (0 to 0)	0 (0 to 0)	0 (0 to 0)	2·5
Ascariasis	0 (0 to 0)	0 (0 to 0)	0 (0 to 0)	0 (0 to 0)	0 (0 to 0)	0·1
Trichuriasis	0 (0 to 0)	0 (0 to 0)	0 (0 to 0)	0 (0 to 0)	0 (0 to 0)	0
Hookworm disease	0 (0 to 0)	0 (0 to 0)	0 (0 to 0)	0 (0 to 0)	0 (0 to 0)	2·4
Food-borne trematodiases	0 (0 to 0)	0 (0 to 0)	0 (0 to 0)	0 (0 to 0)	0 (0 to 0)	0
Leprosy	0 (0 to 0)	0 (0 to 0)	0 (0 to 0)	0 (0 to 0)	0 (0 to 0)	0·2
Ebola	0 (0 to 0)	0 (0 to 0)	0 (0 to 0)	0 (0 to 0)	0 (0 to 0)	0
Zika virus	0 (0 to 0)	0 (0 to 0)	0 (0 to 0·1)	0 (0 to 0)	0 (0 to 0)	1·2
Guinea worm disease	0 (0 to 0)	0 (0 to 0)	0 (0 to 0)	0 (0 to 0)	0 (0 to 0)	0
Other neglected tropical diseases	0 (0 to 0·1)	0·1 (0 to 0·1)	0·5 (0·2 to 1)	0·1 (0 to 0·1)	0 (0 to 0·1)	9·4
Other infectious diseases	1 (0·5 to 1·8)	2 (1 to 4·1)	16·1 (7·1 to 31·7)	0·5 (0·3 to 0·9)	0·2 (0·1 to 0·4)	76·3
Meningitis	0·8 (0·3 to 1·5)	1·7 (0·7 to 3·6)	5·8 (2·4 to 10·4)	0·1 (0 to 0·1)	0 (0 to 0)	22·5
Encephalitis	0·1 (0 to 0·3)	0·1 (0 to 0·3)	1·9 (0·8 to 4·3)	0 (0 to 0)	0 (0 to 0)	12·2
Diphtheria	0 (0 to 0)	0 (0 to 0)	0 (0 to 0)	0 (0 to 0)	0 (0 to 0)	0
Tetanus	0 (0 to 0·1)	0·1 (0 to 0·2)	0·1 (0 to 0·2)	0 (0 to 0·1)	0 (0 to 0)	2·1
Measles	0 (0 to 0)	0 (0 to 0)	0 (0 to 0)	0 (0 to 0)	0 (0 to 0)	0
Varicella and herpes zoster	0 (0 to 0)	0 (0 to 0·1)	0·3 (0 to 1)	0·1 (0 to 0·4)	0·1 (0 to 0·2)	3
Acute hepatitis	0 (0 to 0)	0 (0 to 0)	0·1 (0 to 0·1)	0 (0 to 0)	0 (0 to 0)	2·9
Acute hepatitis A	0 (0 to 0)	0 (0 to 0)	0 (0 to 0)	0 (0 to 0)	0 (0 to 0)	1·5
Acute hepatitis B	0 (0 to 0)	0 (0 to 0)	0 (0 to 0·1)	0 (0 to 0)	0 (0 to 0)	1·1
Acute hepatitis C	0 (0 to 0)	0 (0 to 0)	0 (0 to 0)	0 (0 to 0)	0 (0 to 0)	0
Acute hepatitis E	0 (0 to 0)	0 (0 to 0)	0 (0 to 0)	0 (0 to 0)	0 (0 to 0)	0·3
Other unspecified infectious diseases	0·1 (0 to 0·3)	0·1 (0·1 to 0·3)	0·8 (0·3 to 1·7)	0·2 (0·1 to 0·5)	0·1 (0 to 0·2)	17·1
Maternal and neonatal disorders	306 (219 to 422)	84·3 (57·8 to 120)	36·2 (19·7 to 59)	0 (0 to 0)	0 (0 to 0)	614
Maternal disorders	0 (0 to 0)	0 (0 to 0)	0 (0 to 0)	0 (0 to 0)	0 (0 to 0)	57·4
Maternal hemorrhage	0 (0 to 0)	0 (0 to 0)	0 (0 to 0)	0 (0 to 0)	0 (0 to 0)	16·7
Maternal sepsis and other maternal infections	0 (0 to 0)	0 (0 to 0)	0 (0 to 0)	0 (0 to 0)	0 (0 to 0)	10·3
Maternal hypertensive disorders	0 (0 to 0)	0 (0 to 0)	0 (0 to 0)	0 (0 to 0)	0 (0 to 0)	1·7
Maternal obstructed labor and uterine rupture	0 (0 to 0)	0 (0 to 0)	0 (0 to 0)	0 (0 to 0)	0 (0 to 0)	0·9
Maternal abortion and miscarriage	0 (0 to 0)	0 (0 to 0)	0 (0 to 0)	0 (0 to 0)	0 (0 to 0)	8·3
Ectopic pregnancy	0 (0 to 0)	0 (0 to 0)	0 (0 to 0)	0 (0 to 0)	0 (0 to 0)	8·5
Indirect maternal deaths	--	--	--	--	--	NA
Late maternal deaths	--	--	--	--	--	NA
Maternal deaths aggravated by HIV/AIDS	--	--	--	--	--	NA
Neonatal disorders	306 (219 to 422)	84·3 (57·8 to 120)	36·2 (19·7 to 59)	0 (0 to 0)	0 (0 to 0)	556
Neonatal preterm birth	104 (61·3 to 159)	22 (11·3 to 35·6)	12·3 (5·8 to 22·7)	0 (0 to 0)	0 (0 to 0)	180
Neonatal encephalopathy due to birth asphyxia and trauma	131 (73·8 to 198)	17·3 (8·5 to 29·6)	9·3 (3·4 to 18·9)	0 (0 to 0)	0 (0 to 0)	172
Neonatal sepsis and other neonatal infections	46·8 (25 to 82)	36·9 (22·1 to 56·6)	10·9 (5·2 to 19·9)	0 (0 to 0)	0 (0 to 0)	160
Hemolytic disease and other neonatal jaundice	4·9 (2·4 to 9·1)	1·4 (0·7 to 2·6)	0·7 (0·2 to 2)	0 (0 to 0)	0 (0 to 0)	11·6
Other neonatal disorders	20·1 (9·8 to 36·7)	6·7 (3·3 to 11·8)	3 (1·6 to 5·4)	0 (0 to 0)	0 (0 to 0)	33·1
Nutritional deficiencies	0 (0 to 0)	0·3 (0·1 to 0·5)	9·4 (4·9 to 15·4)	1·7 (1·1 to 2·6)	0·7 (0·4 to 1·1)	176
Protein-energy malnutrition	0 (0 to 0)	0 (0 to 0·1)	6·3 (2·6 to 12)	1 (0·6 to 1·7)	0·5 (0·3 to 0·9)	29·3
Iodine deficiency	0 (0 to 0)	0 (0 to 0)	0 (0 to 0)	0 (0 to 0)	0 (0 to 0)	2·3
Vitamin A deficiency	0 (0 to 0)	0 (0 to 0)	0·1 (0 to 0·1)	0 (0 to 0)	0 (0 to 0)	1·5
Dietary iron deficiency	0 (0 to 0)	0·2 (0·1 to 0·5)	2·6 (1·2 to 4·7)	0·5 (0·2 to 1)	0·1 (0 to 0·2)	137
Other nutritional deficiencies	0 (0 to 0)	0 (0 to 0)	0·4 (0·1 to 1·1)	0·2 (0·1 to 0·4)	0·1 (0 to 0·1)	6·7
Non-communicable diseases	51·9 (31·1 to 83)	27·9 (17·2 to 42·7)	96·3 (64·3 to 138)	245 (220 to 272)	90 (79·8 to 99·3)	9,260
Neoplasms	0·5 (0·2 to 1)	0·5 (0·2 to 1·1)	3·1 (1·8 to 5)	23·4 (18 to 28·8)	6·8 (5·1 to 8·5)	1,590
Lip and oral cavity cancer	0 (0 to 0)	0 (0 to 0)	0 (0 to 0)	0·4 (0·3 to 0·6)	0·2 (0·1 to 0·2)	17·1
Nasopharynx cancer	0 (0 to 0)	0 (0 to 0)	0 (0 to 0)	0·1 (0 to 0·1)	0 (0 to 0)	3·4
Other pharynx cancer	0 (0 to 0)	0 (0 to 0)	0 (0 to 0)	0·1 (0·1 to 0·2)	0 (0 to 0·1)	4·7
Esophageal cancer	0 (0 to 0)	0 (0 to 0)	0 (0 to 0)	0·5 (0·3 to 0·8)	0·2 (0·1 to 0·2)	23·6
Stomach cancer	0 (0 to 0)	0 (0 to 0)	0 (0 to 0)	3·8 (2·5 to 5·4)	1·1 (0·7 to 1·6)	152
Colon and rectum cancer	0 (0 to 0)	0 (0 to 0)	0 (0 to 0)	3·5 (2·3 to 5·3)	0·9 (0·6 to 1·5)	125
Liver cancer	0 (0 to 0)	0 (0 to 0·1)	0 (0 to 0·1)	0·3 (0·2 to 0·5)	0·1 (0·1 to 0·2)	23·8
Liver cancer due to hepatitis B	0 (0 to 0)	0 (0 to 0)	0 (0 to 0)	0 (0 to 0·1)	0 (0 to 0)	5
Liver cancer due to hepatitis C	0 (0 to 0)	0 (0 to 0)	0 (0 to 0)	0·2 (0·1 to 0·3)	0·1 (0 to 0·1)	7·2
Liver cancer due to alcohol use	0 (0 to 0)	0 (0 to 0)	0 (0 to 0)	0 (0 to 0·1)	0 (0 to 0)	5·6
Liver cancer due to NASH	0 (0 to 0)	0 (0 to 0)	0 (0 to 0)	0·1 (0 to 0·1)	0 (0 to 0)	3·3
Liver cancer due to other causes	0 (0 to 0)	0 (0 to 0·1)	0 (0 to 0·1)	0 (0 to 0)	0 (0 to 0)	2·7
Gallbladder and biliary tract cancer	0 (0 to 0)	0 (0 to 0)	0 (0 to 0)	0·3 (0·2 to 0·6)	0·1 (0 to 0·2)	19·6
Pancreatic cancer	0 (0 to 0)	0 (0 to 0)	0 (0 to 0)	1·3 (0·8 to 1·9)	0·3 (0·2 to 0·5)	64·1
Larynx cancer	0 (0 to 0)	0 (0 to 0)	0 (0 to 0)	0·1 (0·1 to 0·2)	0 (0 to 0·1)	6
Tracheal, bronchus, and lung cancer	0 (0 to 0)	0 (0 to 0)	0 (0 to 0)	1·3 (0·8 to 2·1)	0·4 (0·2 to 0·6)	103
Malignant skin melanoma	0 (0 to 0)	0 (0 to 0)	0 (0 to 0)	0·1 (0 to 0·1)	0 (0 to 0·1)	3·8
Non-melanoma skin cancer	0 (0 to 0)	0 (0 to 0)	0 (0 to 0)	0·2 (0·1 to 0·4)	0·1 (0·1 to 0·2)	3
Non-melanoma skin cancer (squamous-cell carcinoma)	0 (0 to 0)	0 (0 to 0)	0 (0 to 0)	0·2 (0·1 to 0·4)	0·1 (0·1 to 0·2)	3
Non-melanoma skin cancer (basal-cell carcinoma)	0 (0 to 0)	0 (0 to 0)	0 (0 to 0)	0 (0 to 0)	0 (0 to 0)	0
Breast cancer	0 (0 to 0)	0 (0 to 0)	0 (0 to 0)	4·5 (3 to 6·2)	1·5 (0·9 to 2·2)	357
Cervical cancer	0 (0 to 0)	0 (0 to 0)	0 (0 to 0)	1·5 (0·9 to 2·3)	0·4 (0·3 to 0·7)	213
Uterine cancer	0 (0 to 0)	0 (0 to 0)	0 (0 to 0)	0·6 (0·4 to 0·8)	0·2 (0·1 to 0·2)	48·8
Ovarian cancer	0 (0 to 0)	0 (0 to 0)	0 (0 to 0)	0·3 (0·2 to 0·5)	0·1 (0 to 0·1)	38·9
Prostate cancer	0 (0 to 0)	0 (0 to 0)	0 (0 to 0)	0 (0 to 0)	0 (0 to 0)	0
Testicular cancer	--	--	--	--	--	NA
Kidney cancer	0 (0 to 0)	0 (0 to 0)	0·1 (0 to 0·2)	0·2 (0·1 to 0·3)	0 (0 to 0·1)	18·8
Bladder cancer	0 (0 to 0)	0 (0 to 0)	0 (0 to 0)	1·1 (0·7 to 1·6)	0·3 (0·2 to 0·5)	26·9
Brain and central nervous system cancer	0·1 (0 to 0·2)	0·1 (0 to 0·2)	0·3 (0·1 to 0·6)	0·1 (0 to 0·1)	0 (0 to 0)	18
Thyroid cancer	0 (0 to 0)	0 (0 to 0)	0 (0 to 0)	0·1 (0·1 to 0·2)	0 (0 to 0)	7·1
Mesothelioma	0 (0 to 0)	0 (0 to 0)	0 (0 to 0)	0 (0 to 0)	0 (0 to 0)	0·4
Hodgkin lymphoma	0 (0 to 0)	0 (0 to 0)	0 (0 to 0)	0 (0 to 0)	0 (0 to 0)	3·8
Non-Hodgkin lymphoma	0 (0 to 0)	0 (0 to 0)	0 (0 to 0)	0·5 (0·3 to 0·8)	0·1 (0·1 to 0·2)	73·4
Multiple myeloma	0 (0 to 0)	0 (0 to 0)	0 (0 to 0)	0·6 (0·3 to 0·9)	0·1 (0·1 to 0·2)	53
Leukemia	0·2 (0 to 0·6)	0·2 (0 to 0·6)	1·3 (0·6 to 2·4)	0·7 (0·4 to 1·1)	0·2 (0·1 to 0·3)	84·2
Acute lymphoid leukemia	0 (0 to 0·1)	0 (0 to 0·2)	0·4 (0·1 to 0·8)	0 (0 to 0·1)	0 (0 to 0)	17·8
Chronic lymphoid leukemia	0 (0 to 0)	0 (0 to 0)	0 (0 to 0)	0 (0 to 0·1)	0 (0 to 0)	2
Acute myeloid leukemia	0 (0 to 0·1)	0·1 (0 to 0·3)	0·3 (0·1 to 0·6)	0·1 (0 to 0·2)	0 (0 to 0·1)	28·9

Cause	1 to 4	5 to 9	10 to 14	15 to 19	20 to 24	25 to 29	30 to 34	35 to 39	40 to 44	45 to 49	50 to 54	55 to 59	60 to 64	65 to 69	70 to 74	75 to 79	80 to 84	85 to 89
All causes	183 (145 to 226)	138 (112 to 171)	262 (213 to 324)	359 (292 to 438)	407 (331 to 497)	431 (346 to 530)	499 (404 to 609)	477 (390 to 579)	489 (400 to 591)	661 (543 to 790)	692 (572 to 826)	728 (604 to 862)	847 (715 to 1,000)	854 (732 to 994)	791 (692 to 909)	878 (779 to 992)	845 (774 to 937)	560 (516 to 616)
Communicable, maternal, neonatal, and nutritional diseases	65·9 (49·6 to 86·7)	36·7 (27·3 to 51·1)	43·3 (32·5 to 58·7)	53·3 (41 to 69)	61·5 (49·4 to 77·2)	63·7 (51·8 to 77·1)	64·2 (52·2 to 78·9)	52·9 (42 to 67·1)	46·2 (36·4 to 60·5)	45·5 (35·9 to 58·3)	42·1 (30·9 to 56·6)	38 (28·4 to 51·3)	37·1 (29·1 to 48·1)	33·9 (26·1 to 42·8)	33·2 (25·7 to 43·2)	40·2 (30·5 to 52·9)	45·9 (34·2 to 61)	36·4 (26·4 to 48·9)
HIV/AIDS and sexually transmitted infections	1·3 (1·1 to 1·7)	0·8 (0·7 to 1)	1 (0·9 to 1·1)	2·1 (1·9 to 2·4)	9·4 (8·3 to 10·6)	14·5 (12·9 to 16·8)	14·3 (12·6 to 16·6)	9·3 (8·2 to 10·8)	8·5 (7·7 to 9·8)	6·7 (5·9 to 7·8)	3·9 (3·4 to 4·7)	2·5 (2·1 to 3·1)	2 (1·7 to 2·5)	1·4 (1·1 to 1·7)	0·6 (0·4 to 0·8)	0·4 (0·3 to 0·6)	0·3 (0·2 to 0·4)	0·2 (0·1 to 0·2)
HIV/AIDS	1 (1 to 1·1)	0·7 (0·6 to 0·7)	0·9 (0·9 to 1)	1·9 (1·7 to 2·1)	8·7 (7·9 to 9·8)	13·5 (12·1 to 15·7)	12·9 (11·7 to 14·9)	8·1 (7·3 to 9)	7·4 (6·8 to 8)	5·6 (5·2 to 6·1)	3·2 (2·9 to 3·6)	2 (1·8 to 2·1)	1·6 (1·4 to 1·9)	1·1 (0·9 to 1·3)	0·4 (0·3 to 0·5)	0·3 (0·2 to 0·3)	0·2 (0·2 to 0·2)	0·1 (0·1 to 0·1)
HIV/AIDS - Drug-susceptible Tuberculosis	0·1 (0·1 to 0·2)	0·1 (0 to 0·1)	0·1 (0·1 to 0·2)	0·3 (0·2 to 0·4)	1·2 (0·8 to 1·7)	1·6 (1·1 to 2·4)	1·7 (1·1 to 2·5)	0·9 (0·6 to 1·3)	1 (0·7 to 1·6)	0·8 (0·5 to 1·2)	0·4 (0·3 to 0·6)	0·2 (0·2 to 0·3)	0·2 (0·1 to 0·3)	0·1 (0·1 to 0·2)	0 (0 to 0·1)	0 (0 to 0)	0 (0 to 0)	0 (0 to 0)
HIV/AIDS - Multidrug-resistant Tuberculosis without extensive dru	0 (0 to 0)	0 (0 to 0)	0 (0 to 0)	0 (0 to 0)	0 (0 to 0·1)	0 (0 to 0·1)	0 (0 to 0·1)	0 (0 to 0·1)	0 (0 to 0·1)	0 (0 to 0·1)	0 (0 to 0)	0 (0 to 0)	0 (0 to 0)	0 (0 to 0)	0 (0 to 0)	0 (0 to 0)	0 (0 to 0)	0 (0 to 0)
HIV/AIDS - Extensively drug-resistant Tuberculosis	0 (0 to 0)	0 (0 to 0)	0 (0 to 0)	0 (0 to 0)	0 (0 to 0)	0 (0 to 0)	0 (0 to 0)	0 (0 to 0)	0 (0 to 0)	0 (0 to 0)	0 (0 to 0)	0 (0 to 0)	0 (0 to 0)	0 (0 to 0)	0 (0 to 0)	0 (0 to 0)	0 (0 to 0)	0 (0 to 0)
HIV/AIDS resulting in other diseases	0·9 (0·8 to 1)	0·6 (0·5 to 0·7)	0·8 (0·7 to 0·9)	1·6 (1·4 to 1·8)	7·5 (6·6 to 8·7)	11·9 (10·3 to 14)	11·2 (9·7 to 13·2)	7·2 (6·3 to 8·2)	6·3 (5·5 to 7·1)	4·8 (4·2 to 5·3)	2·8 (2·5 to 3·2)	1·7 (1·5 to 1·9)	1·4 (1·2 to 1·7)	0·9 (0·8 to 1·1)	0·3 (0·3 to 0·4)	0·2 (0·2 to 0·3)	0·2 (0·1 to 0·2)	0·1 (0·1 to 0·1)
Sexually transmitted infections excluding HIV	0·3 (0·1 to 0·7)	0·1 (0 to 0·3)	0·1 (0 to 0·1)	0·3 (0·1 to 0·5)	0·6 (0·3 to 1·1)	1 (0·5 to 1·8)	1·3 (0·7 to 2·5)	1·2 (0·6 to 2·3)	1·1 (0·6 to 2·1)	1 (0·5 to 2)	0·7 (0·3 to 1·5)	0·5 (0·2 to 1·1)	0·3 (0·1 to 0·7)	0·3 (0·1 to 0·5)	0·2 (0·1 to 0·4)	0·1 (0·1 to 0·3)	0·1 (0 to 0·2)	0·1 (0 to 0·1)
Syphilis	0·3 (0·1 to 0·7)	0·1 (0 to 0·3)	0 (0 to 0)	0 (0 to 0)	0 (0 to 0·1)	0 (0 to 0·1)	0 (0 to 0·1)	0 (0 to 0)	0 (0 to 0)	0 (0 to 0)	0 (0 to 0)	0 (0 to 0)	0 (0 to 0)	0 (0 to 0)	0 (0 to 0)	0 (0 to 0)	0 (0 to 0)	0 (0 to 0)
Chlamydial infection	0 (0 to 0)	0 (0 to 0)	0 (0 to 0)	0 (0 to 0)	0·1 (0 to 0·1)	0·1 (0 to 0·2)	0·1 (0·1 to 0·2)	0·1 (0·1 to 0·2)	0·1 (0·1 to 0·2)	0·1 (0 to 0·2)	0 (0 to 0)	0 (0 to 0)	0 (0 to 0)	0 (0 to 0)	0 (0 to 0)	0 (0 to 0)	0 (0 to 0)	0 (0 to 0)
Gonococcal infection	0 (0 to 0)	0 (0 to 0)	0 (0 to 0)	0 (0 to 0·1)	0·1 (0 to 0·1)	0·1 (0 to 0·1)	0·1 (0 to 0·1)	0·1 (0 to 0·1)	0·1 (0 to 0·1)	0·1 (0 to 0·1)	0 (0 to 0·1)	0 (0 to 0)	0 (0 to 0)	0 (0 to 0)	0 (0 to 0)	0 (0 to 0)	0 (0 to 0)	0 (0 to 0)
Trichomoniasis	0 (0 to 0)	0 (0 to 0)	0 (0 to 0)	0·1 (0 to 0·1)	0·2 (0·1 to 0·5)	0·4 (0·1 to 0·9)	0·5 (0·2 to 1·2)	0·4 (0·1 to 1)	0·4 (0·1 to 0·9)	0·4 (0·1 to 0·9)	0·3 (0·1 to 0·8)	0·2 (0·1 to 0·5)	0·1 (0 to 0·3)	0·1 (0 to 0·2)	0·1 (0 to 0·1)	0 (0 to 0·1)	0 (0 to 0·1)	0 (0 to 0)
Genital herpes	[illegible]	[illegible]	[illegible]	[illegible]	[illegible]	[illegible]	[illegible]	[illegible]	[illegible]	[illegible]	[illegible]	[illegible]	[illegible]	[illegible]	[illegible]	[illegible]	[illegible]	[illegible]
Other sexually transmitted infections	[illegible]	[illegible]	[illegible]	[illegible]	[illegible]	[illegible]	[illegible]	[illegible]	[illegible]	[illegible]	[illegible]	[illegible]	[illegible]	[illegible]	[illegible]	[illegible]	[illegible]	[illegible]
Respiratory infections and tuberculosis	[illegible]	[illegible]	[illegible]	[illegible]	[illegible]	[illegible]	[illegible]	[illegible]	[illegible]	[illegible]	[illegible]	[illegible]	[illegible]	[illegible]	[illegible]	[illegible]	[illegible]	[illegible]
Tuberculosis	[illegible]	[illegible]	[illegible]	[illegible]	[illegible]	[illegible]	[illegible]	[illegible]	[illegible]	[illegible]	[illegible]	[illegible]	[illegible]	[illegible]	[illegible]	[illegible]	[illegible]	[illegible]
Latent tuberculosis infection	[illegible]	[illegible]	[illegible]	[illegible]	[illegible]	[illegible]	[illegible]	[illegible]	[illegible]	[illegible]	[illegible]	[illegible]	[illegible]	[illegible]	[illegible]	[illegible]	[illegible]	[illegible]
Drug-susceptible tuberculosis	[illegible]	[illegible]	[illegible]	[illegible]	[illegible]	[illegible]	[illegible]	[illegible]	[illegible]	[illegible]	[illegible]	[illegible]	[illegible]	[illegible]	[illegible]	[illegible]	[illegible]	[illegible]
Multidrug-resistant tuberculosis without extensive drug resistanc	[illegible]	[illegible]	[illegible]	[illegible]	[illegible]	[illegible]	[illegible]	[illegible]	[illegible]	[illegible]	[illegible]	[illegible]	[illegible]	[illegible]	[illegible]	[illegible]	[illegible]	[illegible]
Extensively drug-resistant tuberculosis	[illegible]	[illegible]	[illegible]	[illegible]	[illegible]	[illegible]	[illegible]	[illegible]	[illegible]	[illegible]	[illegible]	[illegible]	[illegible]	[illegible]	[illegible]	[illegible]	[illegible]	[illegible]
Lower respiratory infections	[illegible]	[illegible]	[illegible]	[illegible]	[illegible]	[illegible]	[illegible]	[illegible]	[illegible]	[illegible]	[illegible]	[illegible]	[illegible]	[illegible]	[illegible]	[illegible]	[illegible]	[illegible]
Upper respiratory infections	[illegible]	[illegible]	[illegible]	[illegible]	[illegible]	[illegible]	[illegible]	[illegible]	[illegible]	[illegible]	[illegible]	[illegible]	[illegible]	[illegible]	[illegible]	[illegible]	[illegible]	[illegible]
Otitis media	[illegible]	[illegible]	[illegible]	[illegible]	[illegible]	[illegible]	[illegible]	[illegible]	[illegible]	[illegible]	[illegible]	[illegible]	[illegible]	[illegible]	[illegible]	[illegible]	[illegible]	[illegible]
Enteric infections	[illegible]	[illegible]	[illegible]	[illegible]	[illegible]	[illegible]	[illegible]	[illegible]	[illegible]	[illegible]	[illegible]	[illegible]	[illegible]	[illegible]	[illegible]	[illegible]	[illegible]	[illegible]
Diarrheal diseases	[illegible]	[illegible]	[illegible]	[illegible]	[illegible]	[illegible]	[illegible]	[illegible]	[illegible]	[illegible]	[illegible]	[illegible]	[illegible]	[illegible]	[illegible]	[illegible]	[illegible]	[illegible]
Typhoid and paratyphoid	[illegible]	[illegible]	[illegible]	[illegible]	[illegible]	[illegible]	[illegible]	[illegible]	[illegible]	[illegible]	[illegible]	[illegible]	[illegible]	[illegible]	[illegible]	[illegible]	[illegible]	[illegible]
Typhoid fever	[illegible]	[illegible]	[illegible]	[illegible]	[illegible]	[illegible]	[illegible]	[illegible]	[illegible]	[illegible]	[illegible]	[illegible]	[illegible]	[illegible]	[illegible]	[illegible]	[illegible]	[illegible]
Paratyphoid fever	[illegible]	[illegible]	[illegible]	[illegible]	[illegible]	[illegible]	[illegible]	[illegible]	[illegible]	[illegible]	[illegible]	[illegible]	[illegible]	[illegible]	[illegible]	[illegible]	[illegible]	[illegible]
Invasive Non-typhoidal Salmonella (iNTS)	[illegible]	[illegible]	[illegible]	[illegible]	[illegible]	[illegible]	[illegible]	[illegible]	[illegible]	[illegible]	[illegible]	[illegible]	[illegible]	[illegible]	[illegible]	[illegible]	[illegible]	[illegible]
Other intestinal infectious diseases	[illegible]	[illegible]	[illegible]	[illegible]	[illegible]	[illegible]	[illegible]	[illegible]	[illegible]	[illegible]	[illegible]	[illegible]	[illegible]	[illegible]	[illegible]	[illegible]	[illegible]	[illegible]
Neglected tropical diseases and malaria	[illegible]	[illegible]	[illegible]	[illegible]	[illegible]	[illegible]	[illegible]	[illegible]	[illegible]	[illegible]	[illegible]	[illegible]	[illegible]	[illegible]	[illegible]	[illegible]	[illegible]	[illegible]
Malaria	[illegible]	[illegible]	[illegible]	[illegible]	[illegible]	[illegible]	[illegible]	[illegible]	[illegible]	[illegible]	[illegible]	[illegible]	[illegible]	[illegible]	[illegible]	[illegible]	[illegible]	[illegible]
Chagas disease	[illegible]	[illegible]	[illegible]	[illegible]	[illegible]	[illegible]	[illegible]	[illegible]	[illegible]	[illegible]	[illegible]	[illegible]	[illegible]	[illegible]	[illegible]	[illegible]	[illegible]	[illegible]
Leishmaniasis	[illegible]	[illegible]	[illegible]	[illegible]	[illegible]	[illegible]	[illegible]	[illegible]	[illegible]	[illegible]	[illegible]	[illegible]	[illegible]	[illegible]	[illegible]	[illegible]	[illegible]	[illegible]
Visceral leishmaniasis	[illegible]	[illegible]	[illegible]	[illegible]	[illegible]	[illegible]	[illegible]	[illegible]	[illegible]	[illegible]	[illegible]	[illegible]	[illegible]	[illegible]	[illegible]	[illegible]	[illegible]	[illegible]
Cutaneous and mucocutaneous leishmaniasis	[illegible]	[illegible]	[illegible]	[illegible]	[illegible]	[illegible]	[illegible]	[illegible]	[illegible]	[illegible]	[illegible]	[illegible]	[illegible]	[illegible]	[illegible]	[illegible]	[illegible]	[illegible]
African trypanosomiasis	[illegible]	[illegible]	[illegible]	[illegible]	[illegible]	[illegible]	[illegible]	[illegible]	[illegible]	[illegible]	[illegible]	[illegible]	[illegible]	[illegible]	[illegible]	[illegible]	[illegible]	[illegible]
Schistosomiasis	[illegible]	[illegible]	[illegible]	[illegible]	[illegible]	[illegible]	[illegible]	[illegible]	[illegible]	[illegible]	[illegible]	[illegible]	[illegible]	[illegible]	[illegible]	[illegible]	[illegible]	[illegible]
Cysticercosis	[illegible]	[illegible]	[illegible]	[illegible]	[illegible]	[illegible]	[illegible]	[illegible]	[illegible]	[illegible]	[illegible]	[illegible]	[illegible]	[illegible]	[illegible]	[illegible]	[illegible]	[illegible]
Cystic echinococcosis	[illegible]	[illegible]	[illegible]	[illegible]	[illegible]	[illegible]	[illegible]	[illegible]	[illegible]	[illegible]	[illegible]	[illegible]	[illegible]	[illegible]	[illegible]	[illegible]	[illegible]	[illegible]
Lymphatic filariasis	[illegible]	[illegible]	[illegible]	[illegible]	[illegible]	[illegible]	[illegible]	[illegible]	[illegible]	[illegible]	[illegible]	[illegible]	[illegible]	[illegible]	[illegible]	[illegible]	[illegible]	[illegible]
Onchocerciasis	[illegible]	[illegible]	[illegible]	[illegible]	[illegible]	[illegible]	[illegible]	[illegible]	[illegible]	[illegible]	[illegible]	[illegible]	[illegible]	[illegible]	[illegible]	[illegible]	[illegible]	[illegible]
Trachoma	[illegible]	[illegible]	[illegible]	[illegible]	[illegible]	[illegible]	[illegible]	[illegible]	[illegible]	[illegible]	[illegible]	[illegible]	[illegible]	[illegible]	[illegible]	[illegible]	[illegible]	[illegible]
Dengue	[illegible]	[illegible]	[illegible]	[illegible]	[illegible]	[illegible]	[illegible]	[illegible]	[illegible]	[illegible]	[illegible]	[illegible]	[illegible]	[illegible]	[illegible]	[illegible]	[illegible]	[illegible]
Yellow fever	[illegible]	[illegible]	[illegible]	[illegible]	[illegible]	[illegible]	[illegible]	[illegible]	[illegible]	[illegible]	[illegible]	[illegible]	[illegible]	[illegible]	[illegible]	[illegible]	[illegible]	[illegible]
Rabies	[illegible]	[illegible]	[illegible]	[illegible]	[illegible]	[illegible]	[illegible]	[illegible]	[illegible]	[illegible]	[illegible]	[illegible]	[illegible]	[illegible]	[illegible]	[illegible]	[illegible]	[illegible]
Intestinal nematode infections	[illegible]	[illegible]	[illegible]	[illegible]	[illegible]	[illegible]	[illegible]	[illegible]	[illegible]	[illegible]	[illegible]	[illegible]	[illegible]	[illegible]	[illegible]	[illegible]	[illegible]	[illegible]
Ascariasis	[illegible]	[illegible]	[illegible]	[illegible]	[illegible]	[illegible]	[illegible]	[illegible]	[illegible]	[illegible]	[illegible]	[illegible]	[illegible]	[illegible]	[illegible]	[illegible]	[illegible]	[illegible]
Trichuriasis	[illegible]	[illegible]	[illegible]	[illegible]	[illegible]	[illegible]	[illegible]	[illegible]	[illegible]	[illegible]	[illegible]	[illegible]	[illegible]	[illegible]	[illegible]	[illegible]	[illegible]	[illegible]
Hookworm disease	[illegible]	[illegible]	[illegible]	[illegible]	[illegible]	[illegible]	[illegible]	[illegible]	[illegible]	[illegible]	[illegible]	[illegible]	[illegible]	[illegible]	[illegible]	[illegible]	[illegible]	[illegible]
Food-borne trematodiases	[illegible]	[illegible]	[illegible]	[illegible]	[illegible]	[illegible]	[illegible]	[illegible]	[illegible]	[illegible]	[illegible]	[illegible]	[illegible]	[illegible]	[illegible]	[illegible]	[illegible]	[illegible]
Leprosy	[illegible]	[illegible]	[illegible]	[illegible]	[illegible]	[illegible]	[illegible]	[illegible]	[illegible]	[illegible]	[illegible]	[illegible]	[illegible]	[illegible]	[illegible]	[illegible]	[illegible]	[illegible]
Ebola	[illegible]	[illegible]	[illegible]	[illegible]	[illegible]	[illegible]	[illegible]	[illegible]	[illegible]	[illegible]	[illegible]	[illegible]	[illegible]	[illegible]	[illegible]	[illegible]	[illegible]	[illegible]
Zika virus	[illegible]	[illegible]	[illegible]	[illegible]	[illegible]	[illegible]	[illegible]	[illegible]	[illegible]	[illegible]	[illegible]	[illegible]	[illegible]	[illegible]	[illegible]	[illegible]	[illegible]	[illegible]
Guinea worm disease	[illegible]	[illegible]	[illegible]	[illegible]	[illegible]	[illegible]	[illegible]	[illegible]	[illegible]	[illegible]	[illegible]	[illegible]	[illegible]	[illegible]	[illegible]	[illegible]	[illegible]	[illegible]
Other neglected tropical diseases	[illegible]	[illegible]	[illegible]	[illegible]	[illegible]	[illegible]	[illegible]	[illegible]	[illegible]	[illegible]	[illegible]	[illegible]	[illegible]	[illegible]	[illegible]	[illegible]	[illegible]	[illegible]
Other infectious diseases	[illegible]	[illegible]	[illegible]	[illegible]	[illegible]	[illegible]	[illegible]	[illegible]	[illegible]	[illegible]	[illegible]	[illegible]	[illegible]	[illegible]	[illegible]	[illegible]	[illegible]	[illegible]
Meningitis	[illegible]	[illegible]	[illegible]	[illegible]	[illegible]	[illegible]	[illegible]	[illegible]	[illegible]	[illegible]	[illegible]	[illegible]	[illegible]	[illegible]	[illegible]	[illegible]	[illegible]	[illegible]
Encephalitis	[illegible]	[illegible]	[illegible]	[illegible]	[illegible]	[illegible]	[illegible]	[illegible]	[illegible]	[illegible]	[illegible]	[illegible]	[illegible]	[illegible]	[illegible]	[illegible]	[illegible]	[illegible]
Diphtheria	[illegible]	[illegible]	[illegible]	[illegible]	[illegible]	[illegible]	[illegible]	[illegible]	[illegible]	[illegible]	[illegible]	[illegible]	[illegible]	[illegible]	[illegible]	[illegible]	[illegible]	[illegible]
Tetanus	[illegible]	[illegible]	[illegible]	[illegible]	[illegible]	[illegible]	[illegible]	[illegible]	[illegible]	[illegible]	[illegible]	[illegible]	[illegible]	[illegible]	[illegible]	[illegible]	[illegible]	[illegible]
Measles	[illegible]	[illegible]	[illegible]	[illegible]	[illegible]	[illegible]	[illegible]	[illegible]	[illegible]	[illegible]	[illegible]	[illegible]	[illegible]	[illegible]	[illegible]	[illegible]	[illegible]	[illegible]
Varicella and herpes zoster	[illegible]	[illegible]	[illegible]	[illegible]	[illegible]	[illegible]	[illegible]	[illegible]	[illegible]	[illegible]	[illegible]	[illegible]	[illegible]	[illegible]	[illegible]	[illegible]	[illegible]	[illegible]
Acute hepatitis	[illegible]	[illegible]	[illegible]	[illegible]	[illegible]	[illegible]	[illegible]	[illegible]	[illegible]	[illegible]	[illegible]	[illegible]	[illegible]	[illegible]	[illegible]	[illegible]	[illegible]	[illegible]
Acute hepatitis A	[illegible]	[illegible]	[illegible]	[illegible]	[illegible]	[illegible]	[illegible]	[illegible]	[illegible]	[illegible]	[illegible]	[illegible]	[illegible]	[illegible]	[illegible]	[illegible]	[illegible]	[illegible]
Acute hepatitis B	[illegible]	[illegible]	[illegible]	[illegible]	[illegible]	[illegible]	[illegible]	[illegible]	[illegible]	[illegible]	[illegible]	[illegible]	[illegible]	[illegible]	[illegible]	[illegible]	[illegible]	[illegible]
Acute hepatitis C	[illegible]	[illegible]	[illegible]	[illegible]	[illegible]	[illegible]	[illegible]	[illegible]	[illegible]	[illegible]	[illegible]	[illegible]	[illegible]	[illegible]	[illegible]	[illegible]	[illegible]	[illegible]
Acute hepatitis E	[illegible]	[illegible]	[illegible]	[illegible]	[illegible]	[illegible]	[illegible]	[illegible]	[illegible]	[illegible]	[illegible]	[illegible]	[illegible]	[illegible]	[illegible]	[illegible]	[illegible]	[illegible]
Other unspecified infectious diseases	[illegible]	[illegible]	[illegible]	[illegible]	[illegible]	[illegible]	[illegible]	[illegible]	[illegible]	[illegible]	[illegible]	[illegible]	[illegible]	[illegible]	[illegible]	[illegible]	[illegible]	[illegible]
Maternal and neonatal disorders	[illegible]	[illegible]	[illegible]	[illegible]	[illegible]	[illegible]	[illegible]	[illegible]	[illegible]	[illegible]	[illegible]	[illegible]	[illegible]	[illegible]	[illegible]	[illegible]	[illegible]	[illegible]
Maternal disorders	[illegible]	[illegible]	[illegible]	[illegible]	[illegible]	[illegible]	[illegible]	[illegible]	[illegible]	[illegible]	[illegible]	[illegible]	[illegible]	[illegible]	[illegible]	[illegible]	[illegible]	[illegible]
Maternal hemorrhage	[illegible]	[illegible]	[illegible]	[illegible]	[illegible]	[illegible]	[illegible]	[illegible]	[illegible]	[illegible]	[illegible]	[illegible]	[illegible]	[illegible]	[illegible]	[illegible]	[illegible]	[illegible]
Maternal sepsis and other maternal infections	[illegible]	[illegible]	[illegible]	[illegible]	[illegible]	[illegible]	[illegible]	[illegible]	[illegible]	[illegible]	[illegible]	[illegible]	[illegible]	[illegible]	[illegible]	[illegible]	[illegible]	[illegible]
Maternal hypertensive disorders	[illegible]	[illegible]	[illegible]	[illegible]	[illegible]	[illegible]	[illegible]	[illegible]	[illegible]	[illegible]	[illegible]	[illegible]	[illegible]	[illegible]	[illegible]	[illegible]	[illegible]	[illegible]
Maternal obstructed labor and uterine rupture	[illegible]	[illegible]	[illegible]	[illegible]	[illegible]	[illegible]	[illegible]	[illegible]	[illegible]	[illegible]	[illegible]	[illegible]	[illegible]	[illegible]	[illegible]	[illegible]	[illegible]	[illegible]
Maternal abortion and miscarriage	[illegible]	[illegible]	[illegible]	[illegible]	[illegible]	[illegible]	[illegible]	[illegible]	[illegible]	[illegible]	[illegible]	[illegible]	[illegible]	[illegible]	[illegible]	[illegible]	[illegible]	[illegible]
Ectopic pregnancy	[illegible]	[illegible]	[illegible]	[illegible]	[illegible]	[illegible]	[illegible]	[illegible]	[illegible]	[illegible]	[illegible]	[illegible]	[illegible]	[illegible]	[illegible]	[illegible]	[illegible]	[illegible]
Indirect maternal deaths	[illegible]	[illegible]	[illegible]	[illegible]	[illegible]	[illegible]	[illegible]	[illegible]	[illegible]	[illegible]	[illegible]	[illegible]	[illegible]	[illegible]	[illegible]	[illegible]	[illegible]	[illegible]
Late maternal deaths	[illegible]	[illegible]	[illegible]	[illegible]	[illegible]	[illegible]	[illegible]	[illegible]	[illegible]	[illegible]	[illegible]	[illegible]	[illegible]	[illegible]	[illegible]	[illegible]	[illegible]	[illegible]
Maternal deaths aggravated by HIV/AIDS	[illegible]	[illegible]	[illegible]	[illegible]	[illegible]	[illegible]	[illegible]	[illegible]	[illegible]	[illegible]	[illegible]	[illegible]	[illegible]	[illegible]	[illegible]	[illegible]	[illegible]	[illegible]
Neonatal disorders	[illegible]	[illegible]	[illegible]	[illegible]	[illegible]	[illegible]	[illegible]	[illegible]	[illegible]	[illegible]	[illegible]	[illegible]	[illegible]	[illegible]	[illegible]	[illegible]	[illegible]	[illegible]
Neonatal preterm birth	[illegible]	[illegible]	[illegible]	[illegible]	[illegible]	[illegible]	[illegible]	[illegible]	[illegible]	[illegible]	[illegible]	[illegible]	[illegible]	[illegible]	[illegible]	[illegible]	[illegible]	[illegible]
Neonatal encephalopathy due to birth asphyxia and trauma	[illegible]	[illegible]	[illegible]	[illegible]	[illegible]	[illegible]	[illegible]	[illegible]	[illegible]	[illegible]	[illegible]	[illegible]	[illegible]	[illegible]	[illegible]	[illegible]	[illegible]	[illegible]
Neonatal sepsis and other neonatal infections	[illegible]	[illegible]	[illegible]	[illegible]	[illegible]	[illegible]	[illegible]	[illegible]	[illegible]	[illegible]	[illegible]	[illegible]	[illegible]	[illegible]	[illegible]	[illegible]	[illegible]	[illegible]
Hemolytic disease and other neonatal jaundice	[illegible]	[illegible]	[illegible]	[illegible]	[illegible]	[illegible]	[illegible]	[illegible]	[illegible]	[illegible]	[illegible]	[illegible]	[illegible]	[illegible]	[illegible]	[illegible]	[illegible]	[illegible]
Other neonatal disorders	[illegible]	[illegible]	[illegible]	[illegible]	[illegible]	[illegible]	[illegible]	[illegible]	[illegible]	[illegible]	[illegible]	[illegible]	[illegible]	[illegible]	[illegible]	[illegible]	[illegible]	[illegible]
Nutritional deficiencies	[illegible]	[illegible]	[illegible]	[illegible]	[illegible]	[illegible]	[illegible]	[illegible]	[illegible]	[illegible]	[illegible]	[illegible]	[illegible]	[illegible]	[illegible]	[illegible]	[illegible]	[illegible]
Protein-energy malnutrition	[illegible]	[illegible]	[illegible]	[illegible]	[illegible]	[illegible]	[illegible]	[illegible]	[illegible]	[illegible]	[illegible]	[illegible]	[illegible]	[illegible]	[illegible]	[illegible]	[illegible]	[illegible]
Iodine deficiency	[illegible]	[illegible]	[illegible]	[illegible]	[illegible]	[illegible]	[illegible]	[illegible]	[illegible]	[illegible]	[illegible]	[illegible]	[illegible]	[illegible]	[illegible]	[illegible]	[illegible]	[illegible]
Vitamin A deficiency	[illegible]	[illegible]	[illegible]	[illegible]	[illegible]	[illegible]	[illegible]	[illegible]	[illegible]	[illegible]	[illegible]	[illegible]	[illegible]	[illegible]	[illegible]	[illegible]	[illegible]	[illegible]
Dietary iron deficiency	[illegible]	[illegible]	[illegible]	[illegible]	[illegible]	[illegible]	[illegible]	[illegible]	[illegible]	[illegible]	[illegible]	[illegible]	[illegible]	[illegible]	[illegible]	[illegible]	[illegible]	[illegible]
Other nutritional deficiencies	[illegible]	[illegible]	[illegible]	[illegible]	[illegible]	[illegible]	[illegible]	[illegible]	[illegible]	[illegible]	[illegible]	[illegible]	[illegible]	[illegible]	[illegible]	[illegible]	[illegible]	[illegible]
Non-communicable diseases	[illegible]	[illegible]	[illegible]	[illegible]	[illegible]	[illegible]	[illegible]	[illegible]	[illegible]	[illegible]	[illegible]	[illegible]	[illegible]	[illegible]	[illegible]	[illegible]	[illegible]	[illegible]
Neoplasms	[illegible]	[illegible]	[illegible]	[illegible]	[illegible]	[illegible]	[illegible]	[illegible]	[illegible]	[illegible]	[illegible]	[illegible]	[illegible]	[illegible]	[illegible]	[illegible]	[illegible]	[illegible]
Lip and oral cavity cancer	[illegible]	[illegible]	[illegible]	[illegible]	[illegible]	[illegible]	[illegible]	[illegible]	[illegible]	[illegible]	[illegible]	[illegible]	[illegible]	[illegible]	[illegible]	[illegible]	[illegible]	[illegible]
Nasopharynx cancer	[illegible]	[illegible]	[illegible]	[illegible]	[illegible]	[illegible]	[illegible]	[illegible]	[illegible]	[illegible]	[illegible]	[illegible]	[illegible]	[illegible]	[illegible]	[illegible]	[illegible]	[illegible]
Other pharynx cancer	[illegible]	[illegible]	[illegible]	[illegible]	[illegible]	[illegible]	[illegible]	[illegible]	[illegible]	[illegible]	[illegible]	[illegible]	[illegible]	[illegible]	[illegible]	[illegible]	[illegible]	[illegible]
Esophageal cancer	[illegible]	[illegible]	[illegible]	[illegible]	[illegible]	[illegible]	[illegible]	[illegible]	[illegible]	[illegible]	[illegible]	[illegible]	[illegible]	[illegible]	[illegible]	[illegible]	[illegible]	[illegible]
Stomach cancer	[illegible]	[illegible]	[illegible]	[illegible]	[illegible]	[illegible]	[illegible]	[illegible]	[illegible]	[illegible]	[illegible]	[illegible]	[illegible]	[illegible]	[illegible]	[illegible]	[illegible]	[illegible]
Colon and rectum cancer	[illegible]	[illegible]	[illegible]	[illegible]	[illegible]	[illegible]	[illegible]	[illegible]	[illegible]	[illegible]	[illegible]	[illegible]	[illegible]	[illegible]	[illegible]	[illegible]	[illegible]	[illegible]
Liver cancer	[illegible]	[illegible]	[illegible]	[illegible]	[illegible]	[illegible]	[illegible]	[illegible]	[illegible]	[illegible]	[illegible]	[illegible]	[illegible]	[illegible]	[illegible]	[illegible]	[illegible]	[illegible]
Liver cancer due to hepatitis B	[illegible]	[illegible]	[illegible]	[illegible]	[illegible]	[illegible]	[illegible]	[illegible]	[illegible]	[illegible]	[illegible]	[illegible]	[illegible]	[illegible]	[illegible]	[illegible]	[illegible]	[illegible]
Liver cancer due to hepatitis C	[illegible]	[illegible]	[illegible]	[illegible]	[illegible]	[illegible]	[illegible]	[illegible]	[illegible]	[illegible]	[illegible]	[illegible]	[illegible]	[illegible]	[illegible]	[illegible]	[illegible]	[illegible]
Liver cancer due to alcohol use	[illegible]	[illegible]	[illegible]	[illegible]	[illegible]	[illegible]	[illegible]	[illegible]	[illegible]	[illegible]	[illegible]	[illegible]	[illegible]	[illegible]	[illegible]	[illegible]	[illegible]	[illegible]
Liver cancer due to NASH	[illegible]	[illegible]	[illegible]	[illegible]	[illegible]	[illegible]	[illegible]	[illegible]	[illegible]	[illegible]	[illegible]	[illegible]	[illegible]	[illegible]	[illegible]	[illegible]	[illegible]	[illegible]
Liver cancer due to other causes	[illegible]	[illegible]	[illegible]	[illegible]	[illegible]	[illegible]	[illegible]	[illegible]	[illegible]	[illegible]	[illegible]	[illegible]	[illegible]	[illegible]	[illegible]	[illegible]	[illegible]	[illegible]
Gallbladder and biliary tract cancer	[illegible]	[illegible]	[illegible]	[illegible]	[illegible]	[illegible]	[illegible]	[illegible]	[illegible]	[illegible]	[illegible]	[illegible]	[illegible]	[illegible]	[illegible]	[illegible]	[illegible]	[illegible]
Pancreatic cancer	[illegible]	[illegible]	[illegible]	[illegible]	[illegible]	[illegible]	[illegible]	[illegible]	[illegible]	[illegible]	[illegible]	[illegible]	[illegible]	[illegible]	[illegible]	[illegible]	[illegible]	[illegible]
Larynx cancer	[illegible]	[illegible]	[illegible]	[illegible]	[illegible]	[illegible]	[illegible]	[illegible]	[illegible]	[illegible]	[illegible]	[illegible]	[illegible]	[illegible]	[illegible]	[illegible]	[illegible]	[illegible]
Tracheal, bronchus, and lung cancer	[illegible]	[illegible]	[illegible]	[illegible]	[illegible]	[illegible]	[illegible]	[illegible]	[illegible]	[illegible]	[illegible]	[illegible]	[illegible]	[illegible]	[illegible]	[illegible]	[illegible]	[illegible]
Malignant skin melanoma	[illegible]	[illegible]	[illegible]	[illegible]	[illegible]	[illegible]	[illegible]	[illegible]	[illegible]	[illegible]	[illegible]	[illegible]	[illegible]	[illegible]	[illegible]	[illegible]	[illegible]	[illegible]
Non-melanoma skin cancer	[illegible]	[illegible]	[illegible]	[illegible]	[illegible]	[illegible]	[illegible]	[illegible]	[illegible]	[illegible]	[illegible]	[illegible]	[illegible]	[illegible]	[illegible]	[illegible]	[illegible]	[illegible]
Non-melanoma skin cancer (squamous-cell carcinoma)	[illegible]	[illegible]	[illegible]	[illegible]	[illegible]	[illegible]	[illegible]	[illegible]	[illegible]	[illegible]	[illegible]	[illegible]	[illegible]	[illegible]	[illegible]	[illegible]	[illegible]	[illegible]
Non-melanoma skin cancer (basal-cell carcinoma)	[illegible]	[illegible]	[illegible]	[illegible]	[illegible]	[illegible]	[illegible]	[illegible]	[illegible]	[illegible]	[illegible]	[illegible]	[illegible]	[illegible]	[illegible]	[illegible]	[illegible]	[illegible]
Breast cancer	[illegible]	[illegible]	[illegible]	[illegible]	[illegible]	[illegible]	[illegible]	[illegible]	[illegible]	[illegible]	[illegible]	[illegible]	[illegible]	[illegible]	[illegible]	[illegible]	[illegible]	[illegible]
Cervical cancer	[illegible]	[illegible]	[illegible]	[illegible]	[illegible]	[illegible]	[illegible]	[illegible]	[illegible]	[illegible]	[illegible]	[illegible]	[illegible]	[illegible]	[illegible]	[illegible]	[illegible]	[illegible]
Uterine cancer	[illegible]	[illegible]	[illegible]	[illegible]	[illegible]	[illegible]	[illegible]	[illegible]	[illegible]	[illegible]	[illegible]	[illegible]	[illegible]	[illegible]	[illegible]	[illegible]	[illegible]	[illegible]
Ovarian cancer	[illegible]	[illegible]	[illegible]	[illegible]	[illegible]	[illegible]	[illegible]	[illegible]	[illegible]	[illegible]	[illegible]	[illegible]	[illegible]	[illegible]	[illegible]	[illegible]	[illegible]	[illegible]
Prostate cancer	[illegible]	[illegible]	[illegible]	[illegible]	[illegible]	[illegible]	[illegible]	[illegible]	[illegible]	[illegible]	[illegible]	[illegible]	[illegible]	[illegible]	[illegible]	[illegible]	[illegible]	[illegible]
Testicular cancer	[illegible]	[illegible]	[illegible]	[illegible]	[illegible]	[illegible]	[illegible]	[illegible]	[illegible]	[illegible]	[illegible]	[illegible]	[illegible]	[illegible]	[illegible]	[illegible]	[illegible]	[illegible]
Kidney cancer	[illegible]	[illegible]	[illegible]	[illegible]	[illegible]	[illegible]	[illegible]	[illegible]	[illegible]	[illegible]	[illegible]	[illegible]	[illegible]	[illegible]	[illegible]	[illegible]	[illegible]	[illegible]
Bladder cancer	[illegible]	[illegible]	[illegible]	[illegible]	[illegible]	[illegible]	[illegible]	[illegible]	[illegible]	[illegible]	[illegible]	[illegible]	[illegible]	[illegible]	[illegible]	[illegible]	[illegible]	[illegible]
Brain and central nervous system cancer	[illegible]	[illegible]	[illegible]	[illegible]	[illegible]	[illegible]	[illegible]	[illegible]	[illegible]	[illegible]	[illegible]	[illegible]	[illegible]	[illegible]	[illegible]	[illegible]	[illegible]	[illegible]
Thyroid cancer	[illegible]	[illegible]	[illegible]	[illegible]	[illegible]	[illegible]	[illegible]	[illegible]	[illegible]	[illegible]	[illegible]	[illegible]	[illegible]	[illegible]	[illegible]	[illegible]	[illegible]	[illegible]
Mesothelioma	[illegible]	[illegible]	[illegible]	[illegible]	[illegible]	[illegible]	[illegible]	[illegible]	[illegible]	[illegible]	[illegible]	[illegible]	[illegible]	[illegible]	[illegible]	[illegible]	[illegible]	[illegible]
Hodgkin lymphoma	[illegible]	[illegible]	[illegible]	[illegible]	[illegible]	[illegible]	[illegible]	[illegible]	[illegible]	[illegible]	[illegible]	[illegible]	[illegible]	[illegible]	[illegible]	[illegible]	[illegible]	[illegible]
Non-Hodgkin lymphoma	[illegible]	[illegible]	[illegible]	[illegible]	[illegible]	[illegible]	[illegible]	[illegible]	[illegible]	[illegible]	[illegible]	[illegible]	[illegible]	[illegible]	[illegible]	[illegible]	[illegible]	[illegible]
Multiple myeloma	[illegible]	[illegible]	[illegible]	[illegible]	[illegible]	[illegible]	[illegible]	[illegible]	[illegible]	[illegible]	[illegible]	[illegible]	[illegible]	[illegible]	[illegible]	[illegible]	[illegible]	[illegible]
Leukemia	[illegible]	[illegible]	[illegible]	[illegible]	[illegible]	[illegible]	[illegible]	[illegible]	[illegible]	[illegible]	[illegible]	[illegible]	[illegible]	[illegible]	[illegible]	[illegible]	[illegible]	[illegible]
Acute lymphoid leukemia	[illegible]	[illegible]	[illegible]	[illegible]	[illegible]	[illegible]	[illegible]	[illegible]	[illegible]	[illegible]	[illegible]	[illegible]	[illegible]	[illegible]	[illegible]	[illegible]	[illegible]	[illegible]
Chronic lymphoid leukemia	[illegible]	[illegible]	[illegible]	[illegible]	[illegible]	[illegible]	[illegible]	[illegible]	[illegible]	[illegible]	[illegible]	[illegible]	[illegible]	[illegible]	[illegible]	[illegible]	[illegible]	[illegible]
Acute myeloid leukemia	[illegible]	[illegible]	[illegible]	[illegible]	[illegible]	[illegible]	[illegible]	[illegible]	[illegible]	[illegible]	[illegible]	[illegible]	[illegible]	[illegible]	[illegible]	[illegible]	[illegible]	[illegible]

Chronic myeloid leukemia	0 (0 to 0)	0 (0 to 0)	0 (0 to 0)	0 (0 to 0·1)	0 (0 to 0)	0·1 (0 to 0·1)	0 (0 to 0·1)	0 (0 to 0·1)	0 (0 to 0·1)	0 (0 to 0·1)	0 (0 to 0·1)	0 (0 to 0·1)	0 (0 to 0·1)	0 (0 to 0·1)	0 (0 to 0·1)	0·1 (0 to 0·1)	0 (0 to 0·1)	0 (0 to 0·1)	0 (0 to 0·1)	0 (0 to 0·1)	0 (0 to 0)	0 (0 to 0)	0 (0 to 0)	0·6
Other leukemia	0·1 (0 to 0·5)	0 (0 to 0·1)	0·7 (0·3 to 1·4)	1·1 (0·4 to 2·1)	0·7 (0·3 to 1·4)	1·8 (0·7 to 3·5)	1·4 (0·6 to 2·7)	2 (0·8 to 3·5)	1·7 (0·8 to 3)	1·8 (0·9 to 3)	1·6 (0·8 to 2·8)	1·8 (1 to 2·9)	2·3 (1·3 to 4)	1·6 (0·8 to 2·8)	1·9 (1 to 3·2)	2·5 (1·4 to 4·2)	2·6 (1·4 to 4·2)	2·5 (1·4 to 4)	2·6 (1·6 to 4·1)	2·2 (1·3 to 3·6)	1·3 (0·8 to 2·1)	0·5 (0·3 to 0·8)	0·1 (0·1 to 0·2)	35
Other malignant neoplasms	0·2 (0·1 to 0·4)	0·2 (0·1 to 0·6)	1·2 (0·6 to 2·1)	5 (2·9 to 8·3)	2·7 (1·9 to 3·7)	6 (3·9 to 8·9)	5·5 (3·5 to 8·8)	4·7 (3 to 7)	3·8 (2·4 to 5·6)	3·9 (2·7 to 5·8)	3·5 (2·4 to 5)	3·9 (2·5 to 5·8)	5·4 (3·6 to 7·8)	5·5 (3·6 to 7·9)	5·6 (3·7 to 8·3)	6·7 (4·5 to 9·9)	6·4 (4·3 to 9·3)	5·5 (3·6 to 7·8)	5·2 (3·6 to 7·4)	4·4 (2·9 to 6·2)	2·8 (1·8 to 4)	1·1 (0·7 to 1·7)	0·3 (0·2 to 0·5)	89·6
Other neoplasms	0 (0 to 0)	0 (0 to 0·1)	0·2 (0·1 to 0·4)	0·4 (0·2 to 0·7)	0·1 (0 to 0·1)	0·2 (0·1 to 0·5)	0·2 (0·1 to 0·4)	0·2 (0·1 to 0·3)	0·3 (0·1 to 0·4)	0·3 (0·2 to 0·5)	0·2 (0·1 to 0·2)	0·4 (0·2 to 0·6)	0·4 (0·3 to 0·6)	0·4 (0·3 to 0·7)	0·5 (0·3 to 0·7)	0·8 (0·5 to 1·1)	0·9 (0·6 to 1·3)	1 (0·7 to 1·4)	1·2 (0·8 to 1·6)	1 (0·6 to 1·4)	0·5 (0·3 to 0·7)	0·2 (0·1 to 0·3)	0 (0 to 0·1)	9·3
Myelodysplastic, myeloproliferative, and other hematopoietic neo	0 (0 to 0)	0 (0 to 0·1)	0·2 (0·1 to 0·4)	0·4 (0·2 to 0·7)	0·1 (0 to 0·1)	0·2 (0·1 to 0·5)	0·2 (0·1 to 0·4)	0·2 (0·1 to 0·3)	0·3 (0·1 to 0·4)	0·3 (0·2 to 0·5)	0·2 (0·1 to 0·2)	0·4 (0·2 to 0·6)	0·4 (0·3 to 0·6)	0·4 (0·3 to 0·7)	0·5 (0·3 to 0·7)	0·8 (0·5 to 1·1)	0·9 (0·6 to 1·3)	1 (0·7 to 1·4)	1·2 (0·8 to 1·6)	1 (0·6 to 1·4)	0·5 (0·3 to 0·7)	0·2 (0·1 to 0·3)	0 (0 to 0·1)	9·3
Benign and in situ intestinal neoplasms	0 (0 to 0)	0 (0 to 0)	0 (0 to 0)	0 (0 to 0)	0 (0 to 0)	0 (0 to 0)	0 (0 to 0)	0 (0 to 0)	0 (0 to 0)	0 (0 to 0)	0 (0 to 0)	0 (0 to 0)	0 (0 to 0)	0 (0 to 0)	0 (0 to 0)	0 (0 to 0)	0 (0 to 0)	0 (0 to 0)	0 (0 to 0)	0 (0 to 0)	0 (0 to 0)	0 (0 to 0)	0 (0 to 0)	0
Benign and in situ cervical and uterine neoplasms	0 (0 to 0)	0 (0 to 0)	0 (0 to 0)	0 (0 to 0)	0 (0 to 0)	0 (0 to 0)	0 (0 to 0)	0 (0 to 0)	0 (0 to 0)	0 (0 to 0)	0 (0 to 0)	0 (0 to 0)	0 (0 to 0)	0 (0 to 0)	0 (0 to 0)	0 (0 to 0)	0 (0 to 0)	0 (0 to 0)	0 (0 to 0)	0 (0 to 0)	0 (0 to 0)	0 (0 to 0)	0 (0 to 0)	0
Other benign and in situ neoplasms	0 (0 to 0)	0 (0 to 0)	0 (0 to 0)	0 (0 to 0)	0 (0 to 0)	0 (0 to 0)	0 (0 to 0)	0 (0 to 0)	0 (0 to 0)	0 (0 to 0)	0 (0 to 0)	0 (0 to 0)	0 (0 to 0)	0 (0 to 0)	0 (0 to 0)	0 (0 to 0)	0 (0 to 0)	0 (0 to 0)	0 (0 to 0)	0 (0 to 0)	0 (0 to 0)	0 (0 to 0)	0 (0 to 0)	0
Cardiovascular diseases	1·5 (0·7 to 3·1)	0·5 (0·3 to 1·1)	3·6 (1·6 to 6·6)	2·4 (1·4 to 4)	2·3 (1·7 to 3)	7·5 (5 to 11)	12·9 (8·9 to 17·8)	16·3 (11·1 to 23)	17·7 (12 to 25·2)	26·6 (17·9 to 37·2)	30·9 (22 to 42·5)	44 (30·4 to 59·2)	81·6 (57·4 to 111)	88 (64·8 to 116)	108 (82 to 140)	168 (125 to 215)	207 (161 to 260)	227 (185 to 277)	296 (245 to 355)	317 (263 to 374)	219 (180 to 260)	112 (87·2 to 134)	44·4 (34·6 to 53·2)	2,030
Rheumatic heart disease	0 (0 to 0)	0 (0 to 0)	0 (0 to 0)	0·2 (0·1 to 0·3)	0·6 (0·4 to 1)	2·1 (1·3 to 3·4)	3·4 (2·2 to 5)	3·4 (2·2 to 4·9)	3·7 (2·4 to 5·4)	4·3 (2·9 to 6·2)	4 (2·6 to 5·6)	3·9 (2·5 to 5·9)	4·5 (2·9 to 6·5)	3·5 (2·4 to 5)	2·8 (1·9 to 4·2)	2·4 (1·5 to 3·5)	2·1 (1·4 to 3)	1·6 (1·1 to 2·4)	1·6 (1·1 to 2·3)	1·3 (0·9 to 1·9)	0·8 (0·5 to 1·1)	0·4 (0·2 to 0·6)	0·2 (0·1 to 0·3)	46·8
Ischemic heart disease	0 (0 to 0)	0 (0 to 0)	0 (0 to 0)	0 (0 to 0)	0 (0 to 0)	0 (0 to 0)	0·7 (0·4 to 1·2)	1·2 (0·6 to 2·1)	1·5 (0·8 to 2·4)	3·4 (1·9 to 5·5)	4·6 (2·8 to 7·1)	8·3 (5 to 12·5)	18·5 (11·7 to 28)	22 (14·4 to 31·4)	29·9 (20·9 to 41·9)	52·6 (35·6 to 73·9)	68·7 (49·6 to 92·8)	77·5 (55·4 to 105)	99·9 (73 to 130)	103 (76·2 to 134)	75·9 (56·8 to 98·3)	39·9 (28·5 to 52·5)	16·4 (11·7 to 21·4)	624
Stroke	0·5 (0·2 to 1)	0 (0 to 0·1)	0·3 (0·1 to 0·7)	0·3 (0·2 to 0·4)	0·4 (0·3 to 0·6)	1·2 (0·8 to 1·8)	2·6 (1·7 to 3·8)	3·4 (2·2 to 5·1)	3·9 (2·5 to 5·6)	6·5 (4·2 to 9·4)	8·3 (5·6 to 11·9)	12·4 (8·1 to 17·7)	23·6 (16·1 to 33·8)	26·7 (19·1 to 37·1)	31·2 (22 to 43·5)	47·3 (31·8 to 66·8)	59·1 (41·7 to 83)	68·3 (48·8 to 91·2)	95·3 (69 to 124)	102 (77·4 to 132)	63·8 (48·2 to 83·1)	29·8 (20·9 to 39)	10·9 (7·7 to 14·6)	598
Ischemic stroke	0·2 (0·1 to 0·3)	0 (0 to 0)	0 (0 to 0·1)	0·1 (0·1 to 0·1)	0·2 (0·1 to 0·4)	0·4 (0·3 to 0·7)	0·8 (0·5 to 1·2)	1·1 (0·7 to 1·5)	1·1 (0·7 to 1·7)	1·8 (1·1 to 2·6)	2·1 (1·4 to 3)	3·1 (2·1 to 4·4)	5·6 (3·8 to 8·1)	7·4 (5 to 10·5)	9·8 (6·8 to 13·6)	19·4 (12·9 to 28)	26·3 (17·8 to 37·3)	43·2 (30·7 to 59·7)	63·9 (45·9 to 84·2)	81·6 (62·1 to 106)	52·8 (39·3 to 69·4)	25 (17·5 to 33)	9·3 (6·5 to 12·7)	355
Intracerebral hemorrhage	0·1 (0 to 0·3)	0 (0 to 0)	0·2 (0 to 0·4)	0·1 (0 to 0·1)	0·1 (0·1 to 0·2)	0·4 (0·2 to 0·7)	1·1 (0·6 to 1·6)	1·4 (0·8 to 2·3)	1·6 (1 to 2·5)	2·8 (1·7 to 4·5)	3·8 (2·3 to 5·8)	5·9 (3·5 to 8·9)	13 (8·2 to 19·2)	14·5 (9·5 to 21·1)	16·6 (11·1 to 24·4)	23·6 (14·5 to 34·8)	28·2 (18 to 41·7)	22·1 (13·6 to 32·6)	28·1 (17·9 to 41·2)	18·3 (11·3 to 27·2)	9·8 (6·2 to 14·5)	4·3 (2·7 to 6·1)	1·4 (0·8 to 2·1)	198
Subarachnoid hemorrhage	0·2 (0·1 to 0·4)	0 (0 to 0·1)	0·1 (0 to 0·3)	0·1 (0 to 0·2)	0·1 (0 to 0·2)	0·4 (0·2 to 0·7)	0·8 (0·4 to 1·3)	0·9 (0·5 to 1·6)	1·1 (0·6 to 1·9)	1·9 (1·1 to 3·2)	2·4 (1·4 to 3·9)	3·4 (1·9 to 5·5)	5 (2·9 to 8·2)	4·8 (3 to 7·5)	4·8 (2·9 to 7·7)	4·3 (2·5 to 7)	4·6 (2·8 to 7·6)	3 (1·8 to 4·8)	3·3 (1·9 to 5·4)	2·3 (1·3 to 3·7)	1·2 (0·7 to 1·9)	0·5 (0·3 to 0·8)	0·2 (0·1 to 0·3)	45·2
Hypertensive heart disease	0 (0 to 0)	0 (0 to 0)	0 (0 to 0)	0 (0 to 0)	0 (0 to 0)	0 (0 to 0)	0·5 (0·2 to 0·8)	0·9 (0·5 to 1·6)	1·1 (0·6 to 1·9)	2·6 (1·4 to 4·4)	3·9 (2·2 to 6·6)	6·1 (3·3 to 9·7)	14·5 (8·3 to 22·5)	16·6 (10·4 to 25·3)	20·6 (12·4 to 32·4)	32·2 (19·9 to 47·7)	38·2 (23·9 to 56·9)	39·3 (24·8 to 57·5)	49·8 (31·4 to 72·5)	56 (36·6 to 82·7)	35·9 (23·8 to 50·7)	19·3 (12 to 28·2)	8·6 (5·4 to 12·7)	346
Non-rheumatic valvular heart disease	0 (0 to 0)	0 (0 to 0)	0 (0 to 0)	0 (0 to 0)	0 (0 to 0)	0 (0 to 0)	0·1 (0·1 to 0·2)	0·1 (0 to 0·2)	0·1 (0 to 0·1)	0·1 (0·1 to 0·2)	0·1 (0·1 to 0·2)	0·1 (0·1 to 0·2)	0·2 (0·1 to 0·4)	0·2 (0·1 to 0·3)	0·2 (0·2 to 0·4)	0·4 (0·2 to 0·6)	0·4 (0·2 to 0·6)	0·4 (0·2 to 0·5)	0·4 (0·3 to 0·6)	0·4 (0·2 to 0·6)	0·3 (0·1 to 0·4)	0·1 (0·1 to 0·2)	0 (0 to 0·1)	3·7
Non-rheumatic calcific aortic valve disease	0 (0 to 0)	0 (0 to 0)	0 (0 to 0)	0 (0 to 0)	0 (0 to 0)	0 (0 to 0)	0·1 (0 to 0·1)	0·1 (0 to 0·1)	0 (0 to 0·1)	0 (0 to 0·1)	0·1 (0 to 0·1)	0·1 (0 to 0·1)	0·1 (0·1 to 0·3)	0·1 (0·1 to 0·2)	0·2 (0·1 to 0·3)	0·3 (0·2 to 0·4)	0·3 (0·2 to 0·5)	0·3 (0·2 to 0·4)	0·3 (0·2 to 0·5)	0·3 (0·2 to 0·5)	0·2 (0·1 to 0·4)	0·1 (0·1 to 0·2)	0 (0 to 0)	2·6
Non-rheumatic degenerative mitral valve disease	0 (0 to 0)	0 (0 to 0)	0 (0 to 0)	0 (0 to 0)	0 (0 to 0)	0 (0 to 0)	0 (0 to 0·1)	0 (0 to 0)	0 (0 to 0)	0 (0 to 0)	0 (0 to 0)	0 (0 to 0·1)	0·1 (0 to 0·1)	0 (0 to 0·1)	0·1 (0 to 0·1)	0·1 (0 to 0·1)	0·1 (0 to 0·1)	0·1 (0 to 0·1)	0·1 (0 to 0·1)	0·1 (0 to 0·1)	0 (0 to 0·1)	0 (0 to 0)	0 (0 to 0)	0·7
Other non-rheumatic valve diseases	0 (0 to 0)	0 (0 to 0)	0 (0 to 0)	0 (0 to 0)	0 (0 to 0)	0 (0 to 0)	0 (0 to 0·1)	0 (0 to 0)	0 (0 to 0)	0 (0 to 0·1)	0 (0 to 0·1)	0 (0 to 0·1)	0·1 (0 to 0·1)	0 (0 to 0·1)	0 (0 to 0·1)	0 (0 to 0·1)	0 (0 to 0·1)	0 (0 to 0·1)	0 (0 to 0·1)	0 (0 to 0·1)	0 (0 to 0)	0 (0 to 0)	0 (0 to 0)	0·4
Cardiomyopathy and myocarditis	0·5 (0·2 to 1·1)	0·3 (0·1 to 0·6)	2·1 (0·9 to 4·2)	1·3 (0·7 to 2·4)	0·6 (0·3 to 0·9)	2·1 (1 to 3·8)	2·2 (1·1 to 3·9)	2·8 (1·4 to 5·1)	2·8 (1·4 to 4·9)	4 (2·2 to 6·9)	3·6 (2 to 6·2)	4·5 (2·4 to 7·5)	7·5 (4·1 to 12·4)	7·7 (4·3 to 13)	8·6 (4·9 to 14·4)	11·8 (6·7 to 19·8)	13 (7·7 to 20·7)	11·9 (6·8 to 19·4)	14 (8·4 to 21·7)	13·3 (7·8 to 20·7)	10·7 (6·2 to 17·5)	4·9 (2·9 to 7·9)	1·7 (0·9 to 2·9)	132
Myocarditis	0 (0 to 0·1)	0 (0 to 0·1)	0·3 (0·1 to 0·8)	0·3 (0·1 to 0·6)	0·2 (0·1 to 0·4)	0·3 (0·1 to 0·7)	0·3 (0·1 to 0·6)	0·3 (0·1 to 0·7)	0·3 (0·1 to 0·6)	0·5 (0·2 to 1)	0·3 (0·1 to 0·5)	0·2 (0·1 to 0·4)	0·6 (0·2 to 1·2)	0·4 (0·1 to 0·8)	0·3 (0·1 to 0·6)	0·3 (0·1 to 0·7)	0·2 (0·1 to 0·4)	0·4 (0·2 to 0·9)	0·5 (0·2 to 1·1)	0·7 (0·3 to 1·4)	0·7 (0·3 to 1·5)	0·4 (0·2 to 0·9)	0·2 (0·1 to 0·4)	7·8
Alcoholic cardiomyopathy	0 (0 to 0)	0 (0 to 0)	0 (0 to 0)	0 (0 to 0)	0 (0 to 0)	0 (0 to 0)	0 (0 to 0·1)	0·1 (0 to 0·2)	0·1 (0 to 0·2)	0·1 (0 to 0·2)	0·1 (0 to 0·2)	0·1 (0 to 0·1)	0·4 (0·1 to 0·8)	0·4 (0·1 to 0·8)	0·3 (0·1 to 0·6)	0·3 (0·1 to 0·7)	0·4 (0·1 to 0·8)	0·1 (0 to 0·1)	0·1 (0 to 0·2)	0·1 (0 to 0·2)	0 (0 to 0·1)	0 (0 to 0)	0 (0 to 0)	2·4
Other cardiomyopathy	0·5 (0·2 to 1)	0·2 (0·1 to 0·5)	1·8 (0·8 to 3·7)	1·1 (0·5 to 2)	0·4 (0·2 to 0·7)	1·8 (0·8 to 3·3)	1·9 (0·9 to 3·3)	2·5 (1·3 to 4·4)	2·4 (1·2 to 4·2)	3·5 (1·9 to 5·9)	3·3 (1·8 to 5·7)	4·2 (2·2 to 7·2)	6·5 (3·6 to 10·8)	7 (3·8 to 11·7)	8·1 (4·5 to 13·4)	11·2 (6·3 to 18·7)	12·4 (7·4 to 19·8)	11·4 (6·5 to 18·8)	13·4 (8 to 20·5)	12·5 (7·4 to 19·6)	10 (5·8 to 16·6)	4·5 (2·6 to 7·2)	1·5 (0·8 to 2·6)	122
Atrial fibrillation and flutter	0 (0 to 0)	0 (0 to 0)	0 (0 to 0)	0 (0 to 0)	0 (0 to 0)	0 (0 to 0)	0 (0 to 0)	0 (0 to 0)	0 (0 to 0)	0 (0 to 0·1)	0·1 (0 to 0·1)	0·3 (0·1 to 0·4)	0·5 (0·3 to 0·8)	0·8 (0·5 to 1·1)	1·3 (0·9 to 1·8)	2·3 (1·6 to 3·1)	3·4 (2·5 to 4·5)	4·9 (3·7 to 6·3)	7·3 (5·6 to 9·2)	11 (8·5 to 13·7)	9·8 (7·5 to 12·5)	6·7 (4·8 to 8·9)	2·5 (1·6 to 3·7)	50·8
Aortic aneurysm	--	--	--	--	--	--	0·1 (0 to 0·1)	0·1 (0·1 to 0·2)	0·2 (0·1 to 0·4)	0·3 (0·1 to 0·5)	0·4 (0·2 to 0·7)	0·6 (0·3 to 0·9)	1 (0·6 to 1·5)	1·1 (0·7 to 1·8)	1·6 (1 to 2·5)	2·8 (1·7 to 4·4)	4·5 (2·9 to 6·7)	4·8 (3·1 to 7·1)	5·9 (4 to 8·6)	5·7 (3·8 to 8)	3·4 (2·2 to 4·9)	1·5 (0·9 to 2·1)	0·5 (0·2 to 0·8)	NA
Endocarditis	0 (0 to 0·1)	0 (0 to 0·1)	0·3 (0·1 to 0·7)	0·1 (0·1 to 0·3)	0·1 (0 to 0·2)	0·3 (0·1 to 0·7)	0·7 (0·3 to 1·3)	1·2 (0·5 to 2·2)	1 (0·5 to 1·9)	1 (0·5 to 1·8)	1·1 (0·6 to 2·1)	1·1 (0·6 to 2)	1·6 (0·8 to 2·7)	1·1 (0·6 to 1·9)	1·2 (0·7 to 2·2)	1·7 (0·9 to 2·8)	1·5 (0·9 to 2·4)	1·4 (0·9 to 2·3)	1·5 (0·9 to 2·5)	2 (1·1 to 3·1)	1·2 (0·7 to 1·9)	0·6 (0·3 to 1)	0·2 (0·1 to 0·4)	21
Other cardiovascular and circulatory diseases	0·5 (0·2 to 1·1)	0·2 (0·1 to 0·4)	0·8 (0·3 to 1·7)	0·6 (0·2 to 1·1)	0·5 (0·3 to 0·8)	1·7 (0·8 to 2·8)	2·5 (1·6 to 3·8)	3·2 (1·9 to 5)	3·5 (2·2 to 5·5)	4·3 (2·7 to 6·6)	4·7 (3 to 7)	6·7 (4·3 to 9·4)	9·6 (6·2 to 14·1)	8 (5·5 to 11·7)	9·6 (6·9 to 13)	13·2 (9·2 to 18·9)	15 (10·2 to 21·1)	15·2 (10·7 to 21·1)	18·3 (12·7 to 25·5)	18·8 (13·4 to 25·9)	15·5 (11·1 to 21)	7·8 (5·3 to 10·9)	2·9 (1·9 to 4·1)	163
Chronic respiratory diseases	0 (0 to 0)	0 (0 to 0)	0 (0 to 0)	11·2 (6·3 to 18·1)	10·4 (6·2 to 16·7)	11·6 (7·5 to 17·5)	11·1 (7·1 to 16·6)	9·8 (6·7 to 13·8)	9·3 (6·4 to 13·1)	9·8 (6·9 to 13·1)	8·6 (6·1 to 11·4)	7·8 (5·8 to 10)	10·3 (7·7 to 13·4)	11·1 (8·3 to 14·5)	12·2 (8·8 to 16·3)	15·7 (10·7 to 21·8)	17 (11·6 to 23·5)	16·2 (11·2 to 22·8)	18·1 (11·9 to 25·4)	17·3 (10·9 to 24)	11·5 (7·7 to 16·1)	5·5 (3·7 to 7·4)	2 (1·3 to 2·8)	226
Chronic obstructive pulmonary disease	0 (0 to 0)	0 (0 to 0)	0 (0 to 0)	0·2 (0·1 to 0·3)	0·2 (0·1 to 0·4)	0·4 (0·2 to 0·7)	0·7 (0·4 to 1·1)	0·9 (0·5 to 1·4)	1·1 (0·7 to 1·7)	1·4 (0·9 to 2)	1·6 (1 to 2·2)	1·8 (1·2 to 2·5)	3 (2 to 4·3)	4·2 (2·8 to 5·7)	5·4 (3·6 to 7·6)	9·1 (5·6 to 13·3)	10·4 (6·7 to 15·1)	10·4 (6·7 to 15)	12·4 (7·9 to 17·9)	12·7 (7·9 to 18·2)	8·7 (5·8 to 12·2)	4·2 (2·8 to 5·7)	1·6 (1 to 2·2)	90·4
Pneumoconiosis	0 (0 to 0)	0 (0 to 0)	0 (0 to 0)	0 (0 to 0)	0 (0 to 0)	0 (0 to 0)	0 (0 to 0)	0 (0 to 0)	0 (0 to 0)	0 (0 to 0)	0 (0 to 0)	0 (0 to 0)	0 (0 to 0)	0 (0 to 0)	0 (0 to 0)	0 (0 to 0)	0 (0 to 0)	0 (0 to 0)	0 (0 to 0)	0 (0 to 0)	0 (0 to 0)	0 (0 to 0)	0 (0 to 0)	0·1
Silicosis	0 (0 to 0)	0 (0 to 0)	0 (0 to 0)	0 (0 to 0)	0 (0 to 0)	0 (0 to 0)	0 (0 to 0)	0 (0 to 0)	0 (0 to 0)	0 (0 to 0)	0 (0 to 0)	0 (0 to 0)	0 (0 to 0)	0 (0 to 0)	0 (0 to 0)	0 (0 to 0)	0 (0 to 0)	0 (0 to 0)	0 (0 to 0)	0 (0 to 0)	0 (0 to 0)	0 (0 to 0)	0 (0 to 0)	0
Asbestosis	0 (0 to 0)	0 (0 to 0)	0 (0 to 0)	0 (0 to 0)	0 (0 to 0)	0 (0 to 0)	0 (0 to 0)	0 (0 to 0)	0 (0 to 0)	0 (0 to 0)	0 (0 to 0)	0 (0 to 0)	0 (0 to 0)	0 (0 to 0)	0 (0 to 0)	0 (0 to 0)	0 (0 to 0)	0 (0 to 0)	0 (0 to 0)	0 (0 to 0)	0 (0 to 0)	0 (0 to 0)	0 (0 to 0)	0·1
Coal workers pneumoconiosis	0 (0 to 0)	0 (0 to 0)	0 (0 to 0)	0 (0 to 0)	0 (0 to 0)	0 (0 to 0)	0 (0 to 0)	0 (0 to 0)	0 (0 to 0)	0 (0 to 0)	0 (0 to 0)	0 (0 to 0)	0 (0 to 0)	0 (0 to 0)	0 (0 to 0)	0 (0 to 0)	0 (0 to 0)	0 (0 to 0)	0 (0 to 0)	0 (0 to 0)	0 (0 to 0)	0 (0 to 0)	0 (0 to 0)	0
Other pneumoconiosis	0 (0 to 0)	0 (0 to 0)	0 (0 to 0)	0 (0 to 0)	0 (0 to 0)	0 (0 to 0)	0 (0 to 0)	0 (0 to 0)	0 (0 to 0)	0 (0 to 0)	0 (0 to 0)	0 (0 to 0)	0 (0 to 0)	0 (0 to 0)	0 (0 to 0)	0 (0 to 0)	0 (0 to 0)	0 (0 to 0)	0 (0 to 0)	0 (0 to 0)	0 (0 to 0)	0 (0 to 0)	0 (0 to 0)	0
Asthma	0 (0 to 0)	0 (0 to 0)	0 (0 to 0)	9·7 (5·3 to 16·2)	9·6 (5·6 to 15·9)	10·2 (6·1 to 16·2)	9·9 (6 to 15·3)	8·1 (5·1 to 11·9)	7·2 (4·6 to 10·5)	7·3 (4·8 to 10·5)	6·1 (4 to 8·5)	5·1 (3·5 to 7)	6·1 (4·3 to 8·4)	5·8 (4 to 7·9)	5·1 (3·5 to 7·2)	5·4 (3·6 to 7·9)	5·3 (3·5 to 7·8)	4·4 (2·8 to 6·7)	4·2 (2·5 to 6·6)	3·3 (2 to 5·2)	1·9 (1·1 to 3)	0·9 (0·5 to 1·3)	0·3 (0·2 to 0·6)	116
Interstitial lung disease and pulmonary sarcoidosis	0 (0 to 0)	0 (0 to 0)	0 (0 to 0)	0·2 (0·1 to 0·4)	0·1 (0 to 0·1)	0·1 (0 to 0·2)	0·1 (0 to 0·3)	0·1 (0·1 to 0·3)	0·2 (0·1 to 0·4)	0·4 (0·2 to 0·7)	0·3 (0·2 to 0·6)	0·3 (0·2 to 0·5)	0·6 (0·3 to 0·9)	0·6 (0·4 to 1·1)	0·7 (0·4 to 1·2)	1 (0·5 to 1·8)	1·1 (0·6 to 1·8)	1·2 (0·6 to 2·1)	1·2 (0·6 to 2·2)	1·1 (0·5 to 1·9)	0·7 (0·3 to 1·2)	0·3 (0·2 to 0·6)	0·1 (0 to 0·2)	10·5
Other chronic respiratory diseases	0 (0 to 0)	0 (0 to 0)	0 (0 to 0)	1·2 (0·4 to 2·5)	0·5 (0·2 to 0·8)	0·9 (0·5 to 1·5)	0·4 (0·2 to 0·6)	0·6 (0·3 to 1)	0·9 (0·5 to 1·5)	0·6 (0·4 to 1)	0·6 (0·3 to 1)	0·6 (0·3 to 1·1)	0·6 (0·3 to 1·1)	0·6 (0·3 to 1)	1 (0·5 to 1·8)	0·2 (0·1 to 0·5)	0·1 (0·1 to 0·3)	0·2 (0·1 to 0·4)	0·2 (0·1 to 0·4)	0·2 (0·1 to 0·4)	0·1 (0 to 0·3)	0 (0 to 0·1)	0 (0 to 0)	9·6
Digestive diseases	0·5 (0·2 to 1·1)	0·5 (0·2 to 0·9)	3·7 (1·5 to 7·8)	5·1 (2·7 to 9·3)	2·9 (2 to 4)	5·3 (3·8 to 7·1)	6·5 (4·8 to 8·9)	9·7 (6·8 to 13·8)	11·1 (7·8 to 15·5)	14·6 (10·4 to 19·4)	14·4 (10·2 to 19·6)	15·4 (11·3 to 20·9)	22·2 (16 to 29·6)	23·5 (17·3 to 31·1)	24·8 (18·6 to 32·7)	29·3 (21·7 to 39)	27·9 (20·4 to 37·1)	24·5 (18 to 32·9)	25·7 (18·9 to 34·3)	22·9 (15·8 to 31·4)	13·9 (9·6 to 19·1)	6·3 (4·3 to 8·7)	2 (1·3 to 3·1)	313
Cirrhosis and other chronic liver diseases	0 (0 to 0)	0 (0 to 0)	0 (0 to 0)	0·6 (0·2 to 1·4)	0·3 (0·2 to 0·6)	1·1 (0·5 to 2)	1·5 (0·9 to 2·4)	2 (1·1 to 3·2)	1·9 (1·1 to 3)	3·1 (1·8 to 4·6)	3·4 (2 to 5·1)	4·3 (2·6 to 6·8)	7·3 (4·6 to 11·2)	7·8 (4·8 to 11·6)	9·3 (6 to 13·5)	11·8 (7·6 to 17)	11·2 (7·2 to 16·2)	8·9 (5·5 to 13·4)	8·3 (5·1 to 12·5)	6·4 (3·8 to 9·5)	3·1 (1·8 to 4·8)	1·2 (0·7 to 1·9)	0·4 (0·2 to 0·6)	94
Cirrhosis and other chronic liver diseases due to hepatitis B	0 (0 to 0)	0 (0 to 0)	0 (0 to 0)	0 (0 to 0)	0 (0 to 0)	0 (0 to 0)	0 (0 to 0)	0 (0 to 0·1)	0·1 (0 to 0·2)	0·2 (0·1 to 0·3)	0·2 (0·1 to 0·5)	0·3 (0·1 to 0·6)	0·5 (0·2 to 1·1)	0·5 (0·2 to 1·1)	0·5 (0·2 to 1)	0·6 (0·3 to 1·1)	0·5 (0·2 to 0·9)	0·4 (0·2 to 0·7)	0·3 (0·1 to 0·7)	0·3 (0·1 to 0·5)	0·1 (0·1 to 0·2)	0 (0 to 0·1)	0 (0 to 0)	4·7
Cirrhosis and other chronic liver diseases due to hepatitis C	0 (0 to 0)	0 (0 to 0)	0 (0 to 0)	0 (0 to 0)	0 (0 to 0)	0 (0 to 0)	0 (0 to 0·1)	0·2 (0·1 to 0·4)	0·3 (0·1 to 0·7)	0·7 (0·3 to 1·2)	0·8 (0·3 to 1·5)	1 (0·5 to 1·8)	1·6 (0·8 to 2·9)	1·6 (0·7 to 2·9)	1·7 (0·8 to 3·1)	2 (0·9 to 3·9)	1·8 (0·9 to 3·2)	1·4 (0·7 to 2·5)	1·2 (0·6 to 2·2)	0·9 (0·4 to 1·7)	0·5 (0·2 to 0·8)	0·2 (0·1 to 0·4)	0·1 (0 to 0·1)	15·8
Cirrhosis and other chronic liver diseases due to alcohol use	0 (0 to 0)	0 (0 to 0)	0 (0 to 0)	0 (0 to 0)	0 (0 to 0)	0 (0 to 0)	0 (0 to 0)	0·1 (0 to 0·2)	0·2 (0·1 to 0·4)	0·6 (0·3 to 1·1)	1 (0·4 to 1·7)	1·4 (0·7 to 2·4)	2·6 (1·3 to 4·3)	2·9 (1·6 to 4·8)	3·6 (1·9 to 5·9)	4·6 (2·4 to 7·4)	4 (2·1 to 6·8)	2·9 (1·4 to 4·9)	2·3 (1·2 to 3·9)	1·6 (0·8 to 2·8)	0·7 (0·4 to 1·2)	0·3 (0·1 to 0·5)	0·1 (0 to 0·2)	28·7
Cirrhosis and other chronic liver diseases due to NAFLD	0 (0 to 0)	0 (0 to 0)	0 (0 to 0)	0 (0 to 0)	0 (0 to 0)	0 (0 to 0)	0·1 (0 to 0·1)	0·2 (0·1 to 0·4)	0·3 (0·1 to 0·7)	0·6 (0·3 to 1·1)	0·7 (0·3 to 1·3)	0·9 (0·4 to 1·7)	1·6 (0·6 to 3·1)	1·7 (0·7 to 3·2)	2 (0·9 to 3·8)	2·5 (1·1 to 4·8)	2·6 (1·4 to 4·5)	2·2 (1·1 to 3·7)	2·2 (1·1 to 3·8)	1·8 (0·9 to 3·1)	0·9 (0·4 to 1·5)	0·3 (0·2 to 0·6)	0·1 (0 to 0·2)	20·6
Cirrhosis and other chronic liver diseases due to other causes	0 (0 to 0)	0 (0 to 0)	0 (0 to 0)	0·6 (0·2 to 1·4)	0·3 (0·2 to 0·6)	1·1 (0·5 to 1·9)	1·4 (0·8 to 2·2)	1·4 (0·8 to 2·4)	1 (0·5 to 1·6)	1 (0·5 to 1·9)	0·7 (0·3 to 1·4)	0·7 (0·3 to 1·4)	1·1 (0·5 to 2)	1·1 (0·5 to 2·1)	1·5 (0·7 to 2·7)	2·1 (1·1 to 3·8)	2·3 (1·1 to 4·1)	2·1 (1 to 3·8)	2·3 (1·1 to 4·1)	1·9 (0·9 to 3·3)	0·9 (0·5 to 1·6)	0·4 (0·2 to 0·6)	0·1 (0 to 0·2)	24·2
Upper digestive system diseases	0 (0 to 0)	0 (0 to 0)	0 (0 to 0)	0·3 (0·1 to 0·5)	0·3 (0·1 to 0·6)	0·7 (0·4 to 1·1)	1·8 (1 to 2·9)	3·6 (2 to 6·4)	4·9 (2·7 to 8·7)	6 (3·2 to 10·6)	5·6 (3 to 9·5)	5 (2·9 to 8·4)	6·3 (3·8 to 10·2)	7 (4·1 to 11·4)	6·7 (4 to 10·6)	6·3 (4 to 9·8)	5·4 (3·5 to 8·4)	4·6 (3 to 6·8)	4·3 (2·9 to 6·4)	3·8 (2·5 to 5·7)	2·2 (1·5 to 3·3)	1 (0·6 to 1·6)	0·3 (0·2 to 0·5)	76·2
Peptic ulcer disease	0 (0 to 0)	0 (0 to 0)	0 (0 to 0)	0·1 (0 to 0·2)	0 (0 to 0·1)	0·2 (0·1 to 0·3)	0·1 (0·1 to 0·2)	0·2 (0·1 to 0·4)	0·2 (0·1 to 0·4)	0·4 (0·2 to 0·7)	0·4 (0·2 to 0·7)	0·5 (0·3 to 0·8)	0·8 (0·4 to 1·5)	0·8 (0·4 to 1·4)	1 (0·6 to 1·7)	1·5 (0·8 to 2·4)	1·5 (0·8 to 2·5)	1·6 (0·9 to 2·8)	2 (1·1 to 3·3)	2 (1·1 to 3·4)	1·2 (0·7 to 2)	0·6 (0·3 to 1)	0·2 (0·1 to 0·4)	15·6
Gastritis and duodenitis	0 (0 to 0)	0 (0 to 0)	0 (0 to 0)	0·2 (0·1 to 0·3)	0·3 (0·1 to 0·5)	0·3 (0·1 to 0·6)	0·4 (0·2 to 0·8)	0·4 (0·2 to 0·8)	0·5 (0·3 to 1)	0·7 (0·3 to 1·4)	0·8 (0·4 to 1·4)	0·7 (0·3 to 1·3)	1·1 (0·5 to 2)	1·4 (0·7 to 2·5)	1·4 (0·7 to 2·5)	1·2 (0·7 to 2·1)	1 (0·6 to 1·7)	0·8 (0·4 to 1·2)	0·6 (0·3 to 0·9)	0·4 (0·2 to 0·6)	0·2 (0·1 to 0·4)	0·1 (0·1 to 0·2)	0 (0 to 0·1)	12·6
Gastroesophageal reflux disease	0 (0 to 0)	0 (0 to 0)	0 (0 to 0)	0 (0 to 0)	0 (0 to 0)	0·2 (0·1 to 0·4)	1·2 (0·6 to 2·3)	2·9 (1·4 to 5·6)	4·1 (2 to 7·6)	4·8 (2·3 to 9·4)	4·4 (2·1 to 8·1)	3·9 (1·8 to 7)	4·4 (2·2 to 7·7)	4·8 (2·4 to 8·7)	4·2 (2 to 8)	3·5 (1·7 to 6·4)	2·9 (1·4 to 5·3)	2·2 (1·1 to 3·9)	1·8 (0·9 to 3·3)	1·4 (0·7 to 2·4)	0·8 (0·4 to 1·4)	0·3 (0·1 to 0·5)	0·1 (0 to 0·2)	48
Appendicitis	0 (0 to 0)	0 (0 to 0)	0 (0 to 0)	0·2 (0·1 to 0·6)	0·2 (0·1 to 0·4)	0·7 (0·3 to 1·2)	0·5 (0·3 to 0·9)	0·4 (0·2 to 0·8)	0·3 (0·1 to 0·5)	0·3 (0·1 to 0·5)	0·3 (0·1 to 0·6)	0·2 (0·1 to 0·4)	0·3 (0·2 to 0·6)	0·2 (0·1 to 0·4)	0·3 (0·1 to 0·5)	0·2 (0·1 to 0·4)	0·3 (0·1 to 0·5)	0·2 (0·1 to 0·4)	0·2 (0·1 to 0·4)	0·2 (0·1 to 0·3)	0·1 (0·1 to 0·3)	0·1 (0 to 0·1)	0 (0 to 0)	5·4
Paralytic ileus and intestinal obstruction	0·3 (0·1 to 0·7)	0·4 (0·2 to 0·8)	3·3 (1·3 to 7)	1·3 (0·5 to 3)	0·4 (0·2 to 0·7)	0·9 (0·5 to 1·6)	0·5 (0·3 to 1)	0·5 (0·2 to 0·9)	0·5 (0·3 to 0·9)	0·6 (0·3 to 1·1)	0·8 (0·4 to 1·4)	0·9 (0·5 to 1·5)	1·4 (0·8 to 2·3)	1·5 (0·8 to 2·6)	1·5 (0·8 to 2·4)	2·2 (1·3 to 3·8)	2·5 (1·4 to 4)	2·8 (1·7 to 4·8)	3·7 (2 to 5·8)	3·9 (2·2 to 6·2)	2·6 (1·5 to 4·1)	1·3 (0·8 to 2·1)	0·4 (0·2 to 0·8)	34·2
Inguinal, femoral, and abdominal hernia	0·2 (0·1 to 0·5)	0·1 (0 to 0·2)	0·4 (0·1 to 1·1)	0·8 (0·4 to 1·3)	0·9 (0·5 to 1·6)	0·7 (0·3 to 1·2)	0·6 (0·3 to 1)	0·5 (0·2 to 0·9)	0·5 (0·2 to 0·9)	0·7 (0·3 to 1·2)	0·7 (0·4 to 1·1)	0·7 (0·4 to 1·2)	0·9 (0·5 to 1·5)	1 (0·6 to 1·6)	0·9 (0·6 to 1·4)	0·9 (0·6 to 1·5)	0·7 (0·4 to 1·1)	0·6 (0·4 to 1)	0·7 (0·4 to 1·2)	0·7 (0·3 to 1·1)	0·4 (0·2 to 0·8)	0·2 (0·1 to 0·4)	0·1 (0 to 0·1)	14
Inflammatory bowel disease	0 (0 to 0)	0 (0 to 0)	0 (0 to 0)	0·2 (0·1 to 0·4)	0 (0 to 0·1)	0 (0 to 0·1)	0·1 (0 to 0·2)	0·2 (0·1 to 0·3)	0·2 (0·1 to 0·4)	0·3 (0·2 to 0·5)	0·3 (0·2 to 0·5)	0·4 (0·2 to 0·6)	0·4 (0·2 to 0·7)	0·4 (0·2 to 0·6)	0·4 (0·2 to 0·6)	0·4 (0·2 to 0·7)	0·3 (0·2 to 0·5)	0·3 (0·2 to 0·5)	0·3 (0·2 to 0·5)	0·2 (0·1 to 0·3)	0·1 (0·1 to 0·2)	0·1 (0 to 0·1)	0 (0 to 0)	4·8
Vascular intestinal disorders	0 (0 to 0)	0 (0 to 0)	0 (0 to 0)	0 (0 to 0·1)	0·1 (0 to 0·1)	0 (0 to 0)	0 (0 to 0·1)	0·2 (0·1 to 0·3)	0·2 (0·1 to 0·4)	0·2 (0·1 to 0·3)	0·3 (0·1 to 0·5)	0·4 (0·3 to 0·7)	0·9 (0·5 to 1·5)	0·8 (0·5 to 1·3)	1 (0·6 to 1·6)	1·7 (1·1 to 2·7)	2 (1·3 to 3·1)	2·2 (1·3 to 3·3)	2·6 (1·7 to 4)	2·5 (1·6 to 3·7)	1·7 (1 to 2·6)	0·7 (0·4 to 1)	0·2 (0·1 to 0·4)	17·7
Gallbladder and biliary diseases	0 (0 to 0)	0 (0 to 0)	0 (0 to 0)	0·1 (0·1 to 0·2)	0·2 (0·1 to 0·4)	0·4 (0·2 to 0·7)	0·7 (0·3 to 1·2)	1 (0·6 to 1·6)	1·3 (0·8 to 2·1)	1·9 (1·1 to 2·8)	2 (1·2 to 3)	2 (1·3 to 3·1)	2·6 (1·7 to 3·9)	2·9 (1·9 to 4·3)	2·7 (1·7 to 3·9)	2·6 (1·7 to 3·7)	2·2 (1·5 to 3·2)	1·8 (1·2 to 2·5)	1·8 (1·1 to 2·7)	1·5 (1 to 2·2)	0·8 (0·5 to 1·2)	0·4 (0·2 to 0·6)	0·1 (0·1 to 0·2)	28·9
Pancreatitis	0 (0 to 0)	0 (0 to 0)	0 (0 to 0)	0·1 (0 to 0·2)	0 (0 to 0·1)	0·1 (0·1 to 0·3)	0·3 (0·2 to 0·5)	0·8 (0·4 to 1·4)	0·6 (0·3 to 1·1)	0·8 (0·4 to 1·3)	0·5 (0·3 to 0·8)	0·7 (0·4 to 1·2)	1 (0·5 to 1·7)	0·8 (0·5 to 1·4)	0·9 (0·5 to 1·5)	1·1 (0·6 to 2)	1·1 (0·6 to 1·9)	1·1 (0·6 to 1·7)	1·1 (0·6 to 2)	0·9 (0·5 to 1·6)	0·5 (0·3 to 0·9)	0·2 (0·1 to 0·4)	0·1 (0 to 0·1)	12·9
Other digestive diseases	0 (0 to 0)	0 (0 to 0)	0 (0 to 0)	1·4 (0·5 to 2·9)	0·4 (0·2 to 0·7)	0·6 (0·3 to 1·2)	0·6 (0·2 to 1)	0·5 (0·2 to 1)	0·6 (0·3 to 1·1)	0·7 (0·4 to 1·4)	0·6 (0·2 to 1)	0·7 (0·3 to 1·2)	1 (0·5 to 1·9)	1·1 (0·6 to 1·9)	1·2 (0·6 to 2·2)	1·9 (1 to 3·2)	2 (1 to 3·6)	2 (1 to 3·6)	2·6 (1·3 to 4·5)	2·9 (1·4 to 5·1)	2·3 (1·1 to 3·9)	1·1 (0·5 to 1·9)	0·4 (0·2 to 0·7)	24·7
Neurological disorders	0 (0 to 0)	0·1 (0 to 0·3)	3·6 (2 to 5·8)	7·6 (4·7 to 11·5)	10 (4·7 to 17·4)	31·4 (13·2 to 57·8)	40 (16·6 to 78·7)	38·5 (16·4 to 72·2)	37 (15·3 to 69)	38·2 (16 to 70·6)	33 (13·3 to 61·4)	29·4 (12·5 to 54)	33·2 (16·5 to 59·3)	31·5 (15 to 55·7)	27·2 (14·5 to 45·8)	25·9 (15·5 to 40)	26·2 (17·1 to 42)	28·3 (18·1 to 49)	38·5 (23·7 to 69·6)	56·1 (31 to 112)	49·3 (24·7 to 101)	29·1 (13·6 to 61·1)	11·5 (5·3 to 25)	626
Alzheimer's disease and other dementias	0 (0 to 0)	0 (0 to 0)	0 (0 to 0)	0 (0 to 0)	0 (0 to 0)	0 (0 to 0)	0 (0 to 0)	0 (0 to 0)	0 (0 to 0)	0 (0 to 0)	0 (0 to 0)	0·1 (0 to 0·4)	0·9 (0·3 to 2·6)	1·7 (0·6 to 4·3)	3 (1·1 to 7·1)	5·3 (2·1 to 12·8)	9·1 (3·6 to 22·3)	13·8 (5·9 to 32·7)	22·4 (9·1 to 53·4)	40·8 (16·6 to 97·9)	40·4 (15·9 to 91·9)	25·7 (10 to 58·5)	10·6 (4·3 to 24·2)	174
Parkinson's disease	0 (0 to 0)	0 (0 to 0)	0 (0 to 0)	0 (0 to 0)	0 (0 to 0)	0 (0 to 0)	0 (0 to 0)	0 (0 to 0)	0 (0 to 0)	0 (0 to 0·1)	0·1 (0 to 0·1)	0·1 (0·1 to 0·2)	0·3 (0·2 to 0·5)	0·5 (0·4 to 0·8)	0·8 (0·6 to 1·1)	1·6 (1·2 to 2·1)	2·6 (2 to 3·4)	4·4 (3·4 to 5·5)	8·1 (6·1 to 10·2)	9·5 (7·1 to 12)	5·9 (4·3 to 7·5)	2·2 (1·6 to 2·8)	0·6 (0·4 to 0·7)	36·8
Idiopathic epilepsy	0 (0 to 0)	0 (0 to 0)	1·3 (0·7 to 2·1)	4·2 (2·2 to 6·7)	5 (2·1 to 9·4)	9·7 (5·2 to 16·4)	11·7 (6 to 19·4)	11·8 (6·3 to 18·9)	10 (5·1 to 16·6)	10 (5·5 to 15·7)	7·4 (4·2 to 11·7)	6 (3·3 to 9·7)	6·7 (3·8 to 10·4)	5·3 (2·6 to 9·1)	4·5 (2 to 8)	4·3 (2 to 7·3)	3·8 (1·8 to 6·5)	3·3 (1·5 to 5·7)	3·1 (1·4 to 5·3)	2·5 (1·1 to 4·5)	1·5 (0·7 to 2·7)	0·6 (0·3 to 1·1)	0·2 (0·1 to 0·3)	113
Multiple sclerosis	0 (0 to 0)	0 (0 to 0)	0 (0 to 0)	0 (0 to 0)	0 (0 to 0)	0 (0 to 0)	0 (0 to 0·1)	0·1 (0·1 to 0·2)	0·2 (0·1 to 0·3)	0·2 (0·2 to 0·3)	0·3 (0·2 to 0·4)	0·3 (0·2 to 0·5)	0·4 (0·3 to 0·6)	0·3 (0·3 to 0·5)	0·3 (0·2 to 0·4)	0·3 (0·2 to 0·4)	0·2 (0·1 to 0·2)	0·1 (0·1 to 0·2)	0·1 (0·1 to 0·1)	0·1 (0 to 0·1)	0 (0 to 0)	0 (0 to 0)	0 (0 to 0)	3
Motor neuron disease	0 (0 to 0)	0·1 (0 to 0·3)	0·8 (0·2 to 2)	0·7 (0·2 to 1·7)	0·1 (0 to 0·1)	0·3 (0·1 to 0·6)	0·1 (0 to 0·2)	0·1 (0·1 to 0·3)	0·1 (0·1 to 0·2)	0·2 (0·1 to 0·3)	0·2 (0·1 to 0·4)	0·4 (0·2 to 0·6)	0·8 (0·4 to 1·4)	0·9 (0·5 to 1·3)	1 (0·6 to 1·6)	1·3 (0·7 to 2·2)	1·2 (0·6 to 2·1)	0·9 (0·4 to 1·8)	0·7 (0·3 to 1·2)	0·3 (0·2 to 0·7)	0·1 (0 to 0·2)	0 (0 to 0·1)	0 (0 to 0)	10·2
Headache disorders	0 (0 to 0)	0 (0 to 0)	0 (0 to 0)	0 (0 to 0)	3·1 (0 to 7·7)	17·1 (0·8 to 43)	24·6 (2·5 to 61·6)	24 (3·2 to 56·3)	24·5 (3·6 to 55·3)	25·5 (4·3 to 57·7)	23·1 (4·3 to 51·8)	20·5 (4·3 to 45·6)	21·5 (5·2 to 47·2)	20·7 (5·4 to 44)	15·7 (4·2 to 34·2)	11·2 (3·3 to 23·7)	7·8 (2·5 to 16·3)	4·8 (1·5 to 10)	3·1 (1 to 6·6)	2·1 (0·7 to 4·4)	1·1 (0·4 to 2·1)	0·3 (0·1 to 0·7)	0·1 (0 to 0·2)	251
Migraine	0 (0 to 0)	0 (0 to 0)	0 (0 to 0)	0 (0 to 0)	2·9 (0 to 7·6)	16·1 (0·5 to 42·6)	23 (1·7 to 60·5)	22·3 (2·3 to 54·5)	22·7 (2·5 to 53·7)	23·6 (2·9 to 55·6)	21·2 (2·9 to 48·9)	18·8 (3·1 to 42·9)	19·5 (3·7 to 44·3)	18·5 (3·8 to 42·1)	14 (2·8 to 31·5)	9·8 (2·3 to 21·9)	6·7 (1·7 to 14·6)	4 (1·1 to 8·7)	2·6 (0·7 to 5·8)	1·7 (0·5 to 3·9)	0·9 (0·3 to 1·9)	0·3 (0·1 to 0·6)	0·1 (0 to 0·1)	229
Tension-type headache	0 (0 to 0)	0 (0 to 0)	0 (0 to 0)	0 (0 to 0)	0·2 (0 to 1·4)	0·9 (0·1 to 5·8)	1·5 (0·3 to 6·5)	1·7 (0·3 to 6·5)	1·8 (0·4 to 6·6)	2 (0·5 to 6·6)	1·9 (0·5 to 5·8)	1·7 (0·5 to 4·8)	2 (0·7 to 5)	2·1 (0·6 to 5·7)	1·7 (0·5 to 4·3)	1·4 (0·4 to 3·8)	1·1 (0·3 to 3·5)	0·8 (0·2 to 2·7)	0·5 (0·1 to 1·9)	0·3 (0·1 to 1·3)	0·2 (0 to 0·7)	0·1 (0 to 0·2)	0 (0 to 0·1)	21·9
Other neurological disorders	0 (0 to 0)	0 (0 to 0)	1·6 (0·8 to 2·9)	2·7 (1·5 to 4·4)	1·9 (0·9 to 3·3)	4·3 (2·3 to 7·1)	3·6 (1·9 to 6·1)	2·6 (1·4 to 4·1)	2·2 (1·2 to 3·4)	2·2 (1·3 to 3·5)	1·8 (1·1 to 2·8)	1·8 (1·1 to 2·7)	2·5 (1·6 to 3·6)	2 (1·3 to 2·9)	1·9 (1·3 to 2·7)	1·8 (1·2 to 2·6)	1·5 (1 to 2·2)	1·1 (0·7 to 1·5)	1 (0·7 to 1·6)	0·8 (0·5 to 1·2)	0·3 (0·2 to 0·5)	0·1 (0·1 to 0·2)	0·1 (0 to 0·1)	38
Mental disorders	0 (0 to 0)	0 (0 to 0)	0·2 (0·1 to 0·3)	1·2 (0·7 to 1·8)	8·9 (5·8 to 13·4)	33·7 (22·2 to 48·7)	56·4 (38·4 to 79)	58·8 (40·1 to 82)	61·3 (41·5 to 84·4)	63·5 (43·1 to 88·2)	57·2 (41 to 78·4)	49·2 (34·8 to 68)	54 (39·2 to 73·6)	55·7 (39·7 to 75·8)	46·9 (33·1 to 63·6)	36·5 (25·6 to 49·6)	27·4 (19·3 to 36·7)	19·3 (13·5 to 25·8)	14·4 (10 to 19·6)	10·5 (7·2 to 14·3)	6 (4·1 to 8·2)	2·4 (1·6 to 3·3)	0·7 (0·5 to 1·1)	664
Schizophrenia	0 (0 to 0)	0 (0 to 0)	0 (0 to 0)	0 (0 to 0)	0 (0 to 0)	0·1 (0·1 to 0·3)	1·1 (0·4 to 2·2)	3·6 (1·9 to 6·1)	6·3 (3·5 to 9·7)	8 (4·7 to 11·7)	7·7 (4·7 to 11·3)	6·6 (4·2 to 9·3)	6·9 (4·5 to 9·5)	6·7 (4·4 to 9·3)	5·1 (3·3 to 7·2)	3·5 (2·3 to 5)	2·4 (1·5 to 3·4)	1·4 (0·8 to 2·1)	0·9 (0·5 to 1·3)	0·5 (0·3 to 0·8)	0·2 (0·1 to 0·4)	0·1 (0 to 0·1)	0 (0 to 0)	61·2
Depressive disorders	0 (0 to 0)	0 (0 to 0)	0 (0 to 0)	0 (0 to 0)	0·7 (0·2 to 1·4)	7·8 (3·8 to 14·2)	19·7 (11·1 to 31·8)	21·7 (12·3 to 34·8)	21·2 (12·5 to 33·1)	21·6 (12·7 to 33·8)	20·4 (12·5 to 31)	18·5 (11·1 to 29·3)	21·5 (14 to 31·9)	23·7 (15·4 to 35·4)	21·2 (12·9 to 32)	17·4 (10·1 to 26·1)	13·3 (8·3 to 19·2)	9·6 (6·2 to 13·8)	7·4 (4·4 to 11·3)	5·6 (3·2 to 8·6)	3·5 (2·2 to 5·2)	1·5 (0·9 to 2·2)	0·5 (0·3 to 0·8)	257
Major depressive disorder	0 (0 to 0)	0 (0 to 0)	0 (0 to 0)	0 (0 to 0)	0·6 (0·2 to 1·3)	7·3 (3·5 to 13·3)	18 (10 to 29·8)	19·1 (10·1 to 31·8)	17·9 (9·9 to 28·7)	17·9 (9·9 to 29·7)	16·9 (9·8 to 26·6)	15·4 (8·8 to 25·1)	18 (11·3 to 27·4)	20·1 (12·6 to 30·2)	18·2 (10·6 to 27·8)	15 (8·3 to 22·7)	11·5 (7 to 16·9)	8·3 (5·2 to 12·4)	6·5 (3·7 to 10·3)	5 (2·7 to 7·9)	3·1 (1·9 to 4·7)	1·3 (0·8 to 2)	0·4 (0·2 to 0·7)	220
Dysthymia	0 (0 to 0)	0 (0 to 0)	0 (0 to 0)	0 (0 to 0)	0·1 (0 to 0·2)	0·6 (0·2 to 1·1)	1·7 (0·8 to 3)	2·6 (1·3 to 4·6)	3·3 (1·7 to 5·6)	3·8 (2·1 to 6·1)	3·5 (2 to 5·9)	3·1 (1·7 to 5·5)	3·5 (1·9 to 5·9)	3·6 (2·1 to 5·9)	3·1 (1·8 to 5·1)	2·4 (1·3 to 4·1)	1·8 (1 to 3·1)	1·3 (0·7 to 2·1)	0·9 (0·5 to 1·5)	0·7 (0·4 to 1·1)	0·4 (0·2 to 0·6)	0·1 (0·1 to 0·2)	0 (0 to 0·1)	36·4
Bipolar disorder	0 (0 to 0)	0 (0 to 0)	0 (0 to 0)	0 (0 to 0)	0 (0 to 0)	2 (0·9 to 3·8)	7·6 (3·5 to 13·4)	8·5 (4·5 to 14·6)	8·4 (4·5 to 14·4)	8 (4·3 to 13·2)	6·8 (3·7 to 11)	5·8 (3·2 to 9·2)	6·2 (3·5 to 10·1)	6·3 (3·3 to 10·3)	5 (2·8 to 8·2)	3·6 (2 to 5·8)	2·5 (1·4 to 4)	1·6 (0·8 to 2·6)	1 (0·5 to 1·6)	0·6 (0·3 to 0·9)	0·2 (0·1 to 0·4)	0·1 (0 to 0·1)	0 (0 to 0)	74·1
Anxiety disorders	0 (0 to 0)	0 (0 to 0)	0 (0 to 0)	0·2 (0·1 to 0·5)	4·2 (2 to 7·2)	12·1 (6·7 to 20·4)	16·9 (10 to 26·3)	16·7 (9·5 to 26·8)	17 (9·5 to 27·8)	17·6 (10·2 to 28·1)	15·6 (9·2 to 23·7)	13·3 (7·5 to 21·5)	14·4 (8·1 to 23·1)	14·6 (8·6 to 22·9)	11·8 (7·4 to 17·9)	9·1 (5·5 to 14)	6·9 (4 to 11)	4·9 (2·9 to 7·9)	3·7 (2·3 to 5·9)	2·6 (1·6 to 4·1)	1·4 (0·8 to 2·2)	0·5 (0·3 to 0·7)	0·1 (0·1 to 0·2)	184
Eating disorders	0 (0 to 0)	0 (0 to 0)	0 (0 to 0)	0 (0 to 0)	0 (0 to 0·1)	0·8 (0·4 to 1·4)	3·2 (1·6 to 5·7)	4·7 (2·4 to 7·9)	4·3 (2·3 to 7·2)	3·4 (1·8 to 5·5)	2·2 (1·2 to 3·7)	1·1 (0·6 to 2)	0·7 (0·4 to 1·2)	0 (0 to 0)	0 (0 to 0)	0 (0 to 0)	0 (0 to 0)	0 (0 to 0)	0 (0 to 0)	0 (0 to 0)	0 (0 to 0)	0 (0 to 0)	0 (0 to 0)	20·5
Anorexia nervosa	0 (0 to 0)	0 (0 to 0)	0 (0 to 0)	0 (0 to 0)	0 (0 to 0·1)	0·4 (0·2 to 0·8)	1·3 (0·6 to 2·3)	1·2 (0·6 to 2·1)	0·9 (0·4 to 1·6)	0·6 (0·3 to 1·1)	0·4 (0·1 to 0·7)	0·2 (0·1 to 0·5)	0·2 (0·1 to 0·4)	0 (0 to 0)	0 (0 to 0)	0 (0 to 0)	0 (0 to 0)	0 (0 to 0)	0 (0 to 0)	0 (0 to 0)	0 (0 to 0)	0 (0 to 0)	0 (0 to 0)	5·2
Bulimia nervosa	0 (0 to 0)	0 (0 to 0)	0 (0 to 0)	0 (0 to 0)	0 (0 to 0)	0·4 (0·1 to 0·9)	1·9 (0·8 to 4)	3·5 (1·6 to 6·4)	3·4 (1·7 to 6)	2·8 (1·4 to 4·9)	1·8 (0·9 to 3·2)	0·9 (0·4 to 1·6)	0·5 (0·2 to 1)	0 (0 to 0)	0 (0 to 0)	0 (0 to 0)	0 (0 to 0)	0 (0 to 0)	0 (0 to 0)	0 (0 to 0)	0 (0 to 0)	0 (0 to 0)	0 (0 to 0)	15·3
Autism spectrum disorders	0 (0 to 0)	0 (0 to 0)	0·1 (0·1 to 0·2)	0·5 (0·3 to 0·9)	0·7 (0·4 to 1·2)	0·8 (0·5 to 1·3)	0·8 (0·5 to 1·3)	0·7 (0·4 to 1·1)	0·7 (0·4 to 1)	0·6 (0·4 to 1·1)	0·5 (0·3 to 0·8)	0·4 (0·2 to 0·7)	0·5 (0·3 to 0·7)	0·5 (0·3 to 0·7)	0·4 (0·2 to 0·6)	0·3 (0·2 to 0·5)	0·2 (0·1 to 0·4)	0·2 (0·1 to 0·3)	0·1 (0·1 to 0·2)	0·1 (0·1 to 0·2)	0·1 (0 to 0·1)	0 (0 to 0)	0 (0 to 0)	8·4
Attention-deficit/hyperactivity disorder	0 (0 to 0)	0 (0 to 0)	0 (0 to 0)	0·1 (0 to 0·1)	0·9 (0·5 to 1·6)	1·3 (0·7 to 2·4)	1·1 (0·6 to 2)	0·7 (0·4 to 1·3)	0·6 (0·3 to 1)	0·4 (0·2 to 0·8)	0·3 (0·1 to 0·5)	0·2 (0·1 to 0·4)	0·2 (0·1 to 0·3)	0·2 (0·1 to 0·3)	0·1 (0 to 0·2)	0·1 (0 to 0·1)	0 (0 to 0)	0 (0 to 0)	0 (0 to 0)	0 (0 to 0)	0 (0 to 0)	0 (0 to 0)	0 (0 to 0)	6·2
Conduct disorder	0 (0 to 0)	0 (0 to 0)	0 (0 to 0)	0 (0 to 0)	2 (0·9 to 3·7)	8 (4 to 13·9)	4·6 (2·2 to 8·1)	0 (0 to 0)	0 (0 to 0)	0 (0 to 0)	0 (0 to 0)	0 (0 to 0)	0 (0 to 0)	0 (0 to 0)	0 (0 to 0)	0 (0 to 0)	0 (0 to 0)	0 (0 to 0)	0 (0 to 0)	0 (0 to 0)	0 (0 to 0)	0 (0 to 0)	0 (0 to 0)	14·6
Idiopathic developmental intellectual disability	0 (0 to 0)	0 (0 to 0)	0·1 (0 to 0·1)	0·3 (0·1 to 0·6)	0·5 (0·2 to 0·9)	0·5 (0·2 to 1)	0·5 (0·2 to 1)	0·5 (0·2 to 0·9)	0·4 (0·1 to 0·8)	0·4 (0·1 to 0·8)	0·3 (0·1 to 0·7)	0·3 (0·1 to 0·5)	0·3 (0·1 to 0·5)	0·3 (0·1 to 0·5)	0·2 (0·1 to 0·4)	0·2 (0·1 to 0·3)	0·1 (0 to 0·2)	0·1 (0 to 0·2)	0·1 (0 to 0·1)	0 (0 to 0·1)	0 (0 to 0)	0 (0 to 0)	0 (0 to 0)	5·2
Other mental disorders	0 (0 to 0)	0 (0 to 0)	0 (0 to 0)	0 (0 to 0)	0 (0 to 0)	0·1 (0·1 to 0·2)	0·8 (0·3 to 1·4)	1·7 (0·8 to 3·1)	2·6 (1·5 to 4·3)	3·4 (1·9 to 5·3)	3·3 (1·9 to 5·2)	3 (1·8 to 4·7)	3·3 (2 to 5·2)	3·5 (2·1 to 5·4)	3·1 (1·9 to 4·7)	2·5 (1·5 to 3·8)	2 (1·2 to 2·9)	1·5 (1 to 2·2)	1·3 (0·8 to 1·9)	1 (0·6 to 1·5)	0·6 (0·4 to 0·9)	0·3 (0·2 to 0·4)	0·1 (0·1 to 0·1)	33·9
Substance use disorders	0 (0 to 0)	0 (0 to 0)	0 (0 to 0)	0 (0 to 0)	0 (0 to 0)	0·3 (0·2 to 0·6)	7 (4·8 to 10)	13·6 (9·3 to 19·1)	13·5 (9·3 to 18·6)	13·2 (9·2 to 17·9)	11·5 (8·4 to 15·6)	9·4 (6·7 to 12·7)	9·9 (6·9 to 13·4)	6·7 (4·7 to 9·2)	5·5 (4 to 7·4)	4·7 (3·4 to 6·5)	3·1 (2·2 to 4·2)	2·1 (1·5 to 2·8)	1·8 (1·3 to 2·4)	1·6 (1·2 to 2·2)	1 (0·7 to 1·4)	0·5 (0·4 to 0·8)	0·2 (0·1 to 0·3)	106
Alcohol use disorders	0 (0 to 0)	0 (0 to 0)	0 (0 to 0)	0 (0 to 0)	0 (0 to 0)	0·2 (0·1 to 0·4)	3·1 (1·8 to 4·8)	6·9 (4·1 to 11)	7·4 (4·5 to 11·4)	8·2 (5·3 to 12·3)	8 (5·5 to 11·1)	6·9 (4·7 to 9·9)	7·5 (4·9 to 10·6)	5 (3·3 to 7·3)	4·1 (2·8 to 5·8)	3·7 (2·6 to 5·3)	2·3 (1·5 to 3·4)	1·6 (1·1 to 2·2)	1·4 (1 to 2)	1·3 (0·9 to 1·8)	0·9 (0·6 to 1·2)	0·5 (0·3 to 0·7)	0·2 (0·1 to 0·3)	69·1
Drug use disorders	0 (0 to 0)	0 (0 to 0)	0 (0 to 0)	0 (0 to 0)	0 (0 to 0)	0·2 (0·1 to 0·3)	3·9 (2·5 to 6·1)	6·6 (4·4 to 9·5)	6·1 (4 to 8·5)	5 (3·2 to 7·2)	3·6 (2·3 to 5·2)	2·5 (1·5 to 3·6)	2·4 (1·5 to 3·5)	1·7 (1·1 to 2·5)	1·4 (0·9 to 2·1)	1 (0·6 to 1·5)	0·7 (0·4 to 1·1)	0·5 (0·3 to 0·7)	0·4 (0·3 to 0·6)	0·3 (0·2 to 0·5)	0·2 (0·1 to 0·2)	0·1 (0 to 0·1)	0 (0 to 0)	36·6
Opioid use disorders	0 (0 to 0)	0 (0 to 0)	0 (0 to 0)	0 (0 to 0)	0 (0 to 0)	0 (0 to 0)	0·8 (0·3 to 1·4)	2·7 (1·4 to 4·5)	3 (1·7 to 4·8)	2·7 (1·4 to 4·2)	1·8 (1 to 3)	1·3 (0·6 to 2)	1·2 (0·6 to 2)	1·1 (0·5 to 1·9)	0·9 (0·4 to 1·6)	0·7 (0·3 to 1·2)	0·5 (0·2 to 0·8)	0·3 (0·1 to 0·6)	0·3 (0·1 to 0·4)	0·2 (0·1 to 0·3)	0·1 (0 to 0·2)	0 (0 to 0·1)	0 (0 to 0)	17·6
Cocaine use disorders	0 (0 to 0)	0 (0 to 0)	0 (0 to 0)	0 (0 to 0)	0 (0 to 0)	0 (0 to 0)	1 (0·5 to 1·8)	1·5 (0·8 to 2·5)	1·2 (0·6 to 2·1)	1·1 (0·5 to 1·8)	0·9 (0·5 to 1·7)	0·6 (0·3 to 1·2)	0·6 (0·3 to 1·2)	0·3 (0·2 to 0·4)	0·2 (0·1 to 0·4)	0·2 (0·1 to 0·3)	0·1 (0·1 to 0·2)	0·1 (0 to 0·1)	0·1 (0 to 0·1)	0 (0 to 0)	0 (0 to 0)	0 (0 to 0)	0 (0 to 0)	8
Amphetamine use disorders	0 (0 to 0)	0 (0 to 0)	0 (0 to 0)	0 (0 to 0)	0 (0 to 0)	0 (0 to 0)	0·2 (0·1 to 0·2)	0·5 (0·2 to 1)	0·5 (0·2 to 1)	0·3 (0·1 to 0·6)	0·2 (0·1 to 0·3)	0·1 (0·1 to 0·2)	0·1 (0·1 to 0·2)	0 (0 to 0·1)	0 (0 to 0·1)	0 (0 to 0)	0 (0 to 0)	0 (0 to 0)	0 (0 to 0)	0 (0 to 0)	0 (0 to 0)	0 (0 to 0)	0 (0 to 0)	2·1
Cannabis use disorders	0 (0 to 0)	0 (0 to 0)	0 (0 to 0)	0 (0 to 0)	0 (0 to 0)	0·1 (0·1 to 0·3)	1·3 (0·6 to 2·5)	1 (0·4 to 1·8)	0·5 (0·2 to 1·1)	0·3 (0·1 to 0·7)	0·2 (0·1 to 0·4)	0·2 (0 to 0·3)	0·1 (0 to 0·3)	0·1 (0 to 0·2)	0·1 (0 to 0·1)	0 (0 to 0·1)	0 (0 to 0)	0 (0 to 0)	0 (0 to 0)	0 (0 to 0)	0 (0 to 0)	0 (0 to 0)	0 (0 to 0)	4·1
Other drug use disorders	0 (0 to 0)	0 (0 to 0)	0 (0 to 0)	0 (0 to 0)	0 (0 to 0)	0 (0 to 0)	0·6 (0·3 to 1·1)	0·9 (0·5 to 1·5)	0·8 (0·4 to 1·3)	0·6 (0·3 to 0·9)	0·4 (0·2 to 0·7)	0·3 (0·2 to 0·5)	0·3 (0·2 to 0·5)	0·2 (0·1 to 0·3)	0·1 (0·1 to 0·2)	0·1 (0·1 to 0·1)	0·1 (0 to 0·1)	0·1 (0 to 0·1)	0·1 (0 to 0·1)	0·1 (0·1 to 0·1)	0 (0 to 0·1)	0 (0 to 0)	0 (0 to 0)	4·8
Diabetes and kidney diseases	0 (0 to 0)	0 (0 to 0)	2·6 (1·4 to 4·4)	3·2 (2·1 to 4·8)	3·1 (2·2 to 4·4)	8·1 (5·8 to 10·7)	16·9 (12·6 to 21·9)	24·1 (17·6 to 32·4)	28·3 (21·2 to 37·2)	38·4 (29·1 to 49·8)	40·6 (31·1 to 51·2)	49·4 (37·7 to 62·2)	84·8 (66·5 to 107)	106 (82·5 to 134)	133 (105 to 165)	171 (136 to 215)	178 (142 to 224)	158 (127 to 192)	168 (137 to 204)	142 (112 to 173)	79·2 (62·5 to 99)	34·5 (26·6 to 44)	11·2 (8·2 to 14·8)	1,480
Diabetes mellitus	0 (0 to 0)	0 (0 to 0)	0·5 (0·2 to 1·2)	0·4 (0·2 to 0·8)	0·4 (0·2 to 0·6)	1·4 (0·8 to 2·5)	5·7 (3·8 to 8·1)	8·9 (6·2 to 12·7)	11 (7·6 to 15·2)	15·8 (11·4 to 21·2)	19·6 (14 to 25·8)	26·8 (19·5 to 35·7)	50·5 (37·3 to 67·2)	71·5 (52·9 to 94·2)	92·6 (70·2 to 120)	118 (89·4 to 152)	127 (96·3 to 164)	115 (87·7 to 147)	120 (93·1 to 151)	99·7 (74·3 to 127)	54 (40·9 to 69·8)	22·5 (15·8 to 30·8)	6·8 (4·6 to 9·8)	968
Diabetes mellitus type 1	0 (0 to 0)	0 (0 to 0)	0·5 (0·2 to 1·2)	0·4 (0·2 to 0·8)	0·4 (0·2 to 0·6)	1·4 (0·8 to 2·5)	1·8 (1 to 2·9)	2·6 (1·6 to 4·2)	2·5 (1·5 to 4·2)	2·5 (1·4 to 3·9)	2·8 (1·7 to 4·3)	3·6 (2·2 to 5·6)	1·7 (1·1 to 2·6)	2 (1·3 to 3·1)	2·4 (1·5 to 3·7)	2·7 (1·6 to 4·3)	2·9 (1·7 to 4·7)	2·1 (1·3 to 3·3)	2·3 (1·4 to 3·7)	1·2 (0·8 to 2)	0·7 (0·4 to 1·1)	0·3 (0·2 to 0·5)	0·1 (0·1 to 0·2)	37
Diabetes mellitus type 2	0 (0 to 0)	0 (0 to 0)	0 (0 to 0)	0 (0 to 0)	0 (0 to 0)	0 (0 to 0)	3·9 (2·6 to 5·6)	6·4 (4·4 to 9)	8·5 (5·8 to 11·8)	13·4 (9·6 to 18)	16·8 (11·7 to 22·5)	23·2 (16·8 to 31·1)	48·8 (36·1 to 65·2)	69·5 (51·3 to 92·1)	90·1 (68·1 to 117)	116 (87·4 to 149)	124 (94 to 161)	112 (86·2 to 144)	117 (91·8 to 148)	98·5 (73·3 to 125)	53·3 (40·4 to 68·9)	22·2 (15·6 to 30·4)	6·7 (4·6 to 9·7)	931
Chronic kidney disease	0 (0 to 0)	0 (0 to 0)	2 (1·1 to 3·5)	2·8 (1·8 to 4·2)	2·7 (1·8 to 3·9)	6·6 (4·5 to 8·9)	11·2 (7·7 to 15·4)	15·2 (10·2 to 21·3)	17·3 (12 to 23·5)	22·5 (15·8 to 31·7)	20·9 (14·9 to 28·8)	22·5 (16 to 30·5)	34·3 (24·4 to 46·3)	34·6 (25·1 to 47)	40·1 (29·5 to 54·1)	53·3 (37·4 to 74·8)	50·9 (36 to 69·4)	43·5 (30·9 to 58)	48·4 (34·9 to 65·4)	41·8 (30·3 to 56·4)	25·3 (18·5 to 34·8)	12 (8·6 to 16·2)	4·3 (3 to 6·3)	512
Chronic kidney disease due to diabetes mellitus type 1	0 (0 to 0)	0 (0 to 0)	0 (0 to 0)	0 (0 to 0·1)	0·1 (0 to 0·1)	0·2 (0·1 to 0·4)	0·5 (0·2 to 1)	1 (0·3 to 2)	1·9 (0·8 to 3·6)	3·6 (1·7 to 6·4)	3·8 (1·9 to 6·9)	4 (2 to 6·9)	5·3 (2·3 to 9·6)	3·8 (1·6 to 7·4)	3·1 (0·9 to 6·8)	3·1 (1 to 6·9)	2·3 (0·8 to 5·3)	1·5 (0·6 to 3·1)	1·1 (0·4 to 2·5)	0·5 (0·2 to 1·1)	0·1 (0 to 0·3)	0 (0 to 0)	0 (0 to 0)	35·9
Chronic kidney disease due to diabetes mellitus type 2	0 (0 to 0)	0 (0 to 0)	0 (0 to 0)	0 (0 to 0)	0 (0 to 0)	0 (0 to 0)	0·2 (0·1 to 0·5)	0·4 (0·1 to 0·9)	0·8 (0·3 to 1·6)	1·4 (0·6 to 2·9)	1·8 (0·8 to 3·5)	2·8 (1·3 to 4·9)	6·2 (3·4 to 10·2)	8·5 (4·9 to 12·9)	11·7 (6·7 to 17·7)	16·8 (9·8 to 26)	16·5 (10·3 to 24·6)	14·2 (8·9 to 21)	15·7 (9·6 to 23·6)	13·3 (8·4 to 19·9)	7·7 (4·7 to 11·4)	3·5 (2 to 5·3)	1·2 (0·7 to 1·9)	123
Chronic kidney disease due to hypertension	0 (0 to 0)	0 (0 to 0)	0 (0 to 0)	0 (0 to 0)	0 (0 to 0)	0 (0 to 0)	0·7 (0·3 to 1·2)	1 (0·5 to 1·8)	1·4 (0·7 to 2·4)	2 (1·1 to 3·3)	2·1 (1·2 to 3·3)	2·5 (1·5 to 4)	4·4 (2·6 to 7)	5·2 (3·2 to 8·2)	7·2 (4·4 to 11)	11·3 (6·5 to 17·5)	12·3 (7·5 to 18·9)	11·5 (7·2 to 16·9)	14·3 (9·1 to 21·3)	13·6 (8·8 to 19·6)	9·1 (6·1 to 13·2)	4·7 (3 to 7)	1·8 (1·1 to 2·8)	105
Chronic kidney disease due to glomerulonephritis	0 (0 to 0)	0 (0 to 0)	0·6 (0·2 to 1·1)	0·9 (0·5 to 1·4)	0·9 (0·5 to 1·4)	2·7 (1·5 to 4·2)	4·5 (2·6 to 6·8)	5·8 (3·3 to 9·2)	6·1 (3·7 to 9·4)	7·2 (4·2 to 11·4)	6·5 (3·8 to 9·9)	6·4 (3·5 to 10·2)	8·7 (4·8 to 13·8)	7·7 (4·2 to 12·9)	8·1 (4·1 to 14·1)	9·5 (4·7 to 17·7)	7·7 (3·5 to 14·2)	5·5 (2·8 to 9·8)	5·5 (2·4 to 10·1)	4 (1·8 to 7·7)	1·9 (0·9 to 3·6)	0·8 (0·3 to 1·5)	0·3 (0·1 to 0·6)	101
Chronic kidney disease due to other and unspecified causes	0 (0 to 0)	0 (0 to 0)	1·5 (0·8 to 2·6)	1·9 (1·1 to 3)	1·7 (1 to 2·6)	3·7 (2·2 to 5·6)	5·4 (3·2 to 8·2)	6·9 (4·2 to 10·5)	7·1 (4·4 to 10·2)	8·2 (5 to 12·2)	6·8 (4·1 to 10·6)	6·7 (3·9 to 10·8)	9·7 (5·7 to 15·2)	9·5 (5·8 to 14·7)	10·1 (6·1 to 16·3)	12·5 (7·2 to 20·8)	12·1 (7·2 to 19·2)	10·7 (6·5 to 16·6)	11·8 (7·1 to 18·3)	10·4 (6·1 to 15·8)	6·5 (4 to 9·7)	3 (1·9 to 4·4)	1·1 (0·6 to 1·7)	147
Acute glomerulonephritis	0 (0 to 0)	0 (0 to 0)	0 (0 to 0)	0 (0 to 0)	0 (0 to 0·1)	0 (0 to 0·1)	0 (0 to 0·1)	0 (0 to 0·1)	0 (0 to 0)	0 (0 to 0·1)	0 (0 to 0)	0 (0 to 0)	0 (0 to 0)	0 (0 to 0)	0 (0 to 0)	0 (0 to 0)	0 (0 to 0)	0 (0 to 0)	0 (0 to 0)	0 (0 to 0)	0 (0 to 0)	0 (0 to 0)	0 (0 to 0)	0·3
Skin and subcutaneous diseases	0 (0 to 0)	0 (0 to 0)	3 (1·6 to 5·2)	12·6 (8·2 to 18·6)	16·7 (10·4 to 24·5)	19 (12·5 to 27·7)	20·4 (13·7 to 30·4)	12·7 (8·2 to 18·6)	10·9 (7·3 to 15·8)	11·3 (7·6 to 16·3)	10 (6·9 to 14·3)	9·7 (6·6 to 13·8)	11·1 (7·6 to 15·9)	12·9 (9·1 to 18·6)	13·4 (9·4 to 19·1)	14·4 (10 to 20·7)	14·1 (9·8 to 20·2)	14·4 (9·6 to 20·7)	19·1 (12·6 to 29·3)	20·2 (12·7 to 31·5)	16·2 (10·1 to 25·6)	8·6 (5·2 to 13·1)	3·5 (2 to 6)	274
Dermatitis	0 (0 to 0)	0 (0 to 0)	0 (0 to 0)	4·3 (2·3 to 7·5)	5·7 (3·1 to 10·1)	4·4 (2·4 to 7·6)	3·4 (1·8 to 5·5)	2·5 (1·4 to 4·1)	2·2 (1·3 to 3·6)	2·2 (1·2 to 3·5)	1·8 (1 to 2·9)	1·5 (0·9 to 2·5)	1·7 (0·9 to 2·8)	1·9 (1 to 3·2)	1·8 (1 to 3)	1·6 (0·9 to 2·6)	1·4 (0·8 to 2·3)	1·1 (0·6 to 1·8)	0·9 (0·5 to 1·5)	0·7 (0·4 to 1·1)	0·4 (0·2 to 0·6)	0·1 (0·1 to 0·2)	0 (0 to 0·1)	39·9
Atopic dermatitis	0 (0 to 0)	0 (0 to 0)	0 (0 to 0)	4·3 (2·3 to 7·5)	5·6 (3 to 10)	4 (2·1 to 7)	2·7 (1·4 to 4·5)	1·7 (0·9 to 3)	1·3 (0·6 to 2·3)	1·1 (0·6 to 2·1)	0·9 (0·5 to 1·7)	0·8 (0·4 to 1·3)	0·8 (0·4 to 1·5)	0·9 (0·5 to 1·6)	0·8 (0·4 to 1·5)	0·7 (0·4 to 1·3)	0·6 (0·3 to 1)	0·5 (0·2 to 0·8)	0·4 (0·2 to 0·7)	0·3 (0·2 to 0·5)	0·2 (0·1 to 0·3)	0·1 (0 to 0·1)	0 (0 to 0)	27·8
Contact dermatitis	0 (0 to 0)	0 (0 to 0)	0 (0 to 0)	0 (0 to 0)	0·1 (0 to 0·1)	0·3 (0·1 to 0·7)	0·6 (0·2 to 1·2)	0·7 (0·3 to 1·4)	0·8 (0·4 to 1·6)	0·9 (0·4 to 1·8)	0·8 (0·3 to 1·5)	0·7 (0·3 to 1·3)	0·8 (0·3 to 1·5)	1 (0·4 to 1·8)	0·9 (0·4 to 1·8)	0·8 (0·4 to 1·5)	0·8 (0·3 to 1·5)	0·6 (0·3 to 1·2)	0·5 (0·2 to 0·9)	0·3 (0·2 to 0·6)	0·2 (0·1 to 0·3)	0·1 (0 to 0·1)	0 (0 to 0)	10·9
Seborrhoeic dermatitis	0 (0 to 0)	0 (0 to 0)	0 (0 to 0)	0 (0 to 0·1)	0 (0 to 0·1)	0·1 (0 to 0·1)	0·1 (0 to 0·1)	0·1 (0 to 0·1)	0·1 (0 to 0·2)	0·1 (0 to 0·2)	0·1 (0 to 0·2)	0·1 (0 to 0·1)	0·1 (0 to 0·1)	0·1 (0 to 0·1)	0·1 (0 to 0·1)	0·1 (0 to 0·1)	0·1 (0 to 0·1)	0·1 (0 to 0·1)	0·1 (0 to 0·1)	0 (0 to 0·1)	0 (0 to 0)	0 (0 to 0)	0 (0 to 0)	1·2
Psoriasis	0 (0 to 0)	0 (0 to 0)	0 (0 to 0)	0·1 (0·1 to 0·2)	0·4 (0·2 to 0·8)	0·6 (0·3 to 1)	0·7 (0·3 to 1·1)	0·6 (0·3 to 1·1)	0·7 (0·4 to 1·2)	0·8 (0·4 to 1·3)	0·8 (0·4 to 1·3)	0·8 (0·4 to 1·2)	0·9 (0·5 to 1·4)	1·1 (0·6 to 1·6)	1 (0·6 to 1·5)	0·9 (0·5 to 1·2)	0·7 (0·4 to 1·1)	0·5 (0·3 to 0·8)	0·3 (0·2 to 0·5)	0·2 (0·1 to 0·3)	0·1 (0 to 0·2)	0 (0 to 0)	0 (0 to 0)	11·3
Bacterial skin diseases	0 (0 to 0)	0 (0 to 0)	2·3 (1 to 4·6)	1 (0·4 to 2)	0·6 (0·3 to 1·2)	0·8 (0·3 to 1·7)	0·6 (0·2 to 1·3)	1 (0·5 to 2)	0·8 (0·3 to 1·7)	1·7 (0·7 to 3·3)	1 (0·3 to 2·1)	1·6 (0·7 to 3·2)	1·6 (0·6 to 3)	1·6 (0·5 to 3·4)	1·7 (0·6 to 3·4)	2·7 (0·9 to 5·8)	2·3 (0·7 to 5·1)	1·9 (0·6 to 4·3)	2·6 (0·7 to 6)	2·7 (0·7 to 6·2)	2·1 (0·5 to 5·1)	1·1 (0·3 to 2·6)	0·4 (0·1 to 1·2)	32
Cellulitis	0 (0 to 0)	0 (0 to 0)	0·6 (0·1 to 1·6)	0·3 (0·1 to 0·6)	0·3 (0·1 to 0·6)	0·2 (0·1 to 0·5)	0·1 (0 to 0·3)	0·2 (0·1 to 0·5)	0·3 (0·1 to 0·7)	0·3 (0·1 to 0·7)	0·2 (0·1 to 0·5)	0·3 (0·1 to 0·8)	0·3 (0·1 to 0·7)	0·4 (0·1 to 1·1)	0·5 (0·1 to 1·1)	0·6 (0·2 to 1·6)	0·7 (0·2 to 1·6)	0·4 (0·1 to 1)	0·5 (0·1 to 1·4)	0·4 (0·1 to 1·2)	0·3 (0·1 to 0·9)	0·1 (0 to 0·4)	0·1 (0 to 0·2)	7·2
Pyoderma	0 (0 to 0)	0 (0 to 0)	1·7 (0·7 to 3·6)	0·7 (0·3 to 1·6)	0·3 (0·1 to 0·7)	0·6 (0·2 to 1·3)	0·5 (0·2 to 1·1)	0·8 (0·4 to 1·6)	0·6 (0·2 to 1·2)	1·4 (0·6 to 2·7)	0·8 (0·3 to 1·6)	1·3 (0·6 to 2·6)	1·2 (0·5 to 2·5)	1·1 (0·4 to 2·5)	1·2 (0·4 to 2·6)	2 (0·6 to 4·5)	1·6 (0·5 to 3·8)	1·5 (0·4 to 3·4)	2·1 (0·5 to 4·8)	2·2 (0·6 to 5·3)	1·8 (0·4 to 4·4)	1 (0·2 to 2·3)	0·4 (0·1 to 1)	24·8
Scabies	0 (0 to 0)	0 (0 to 0)	0·3 (0·1 to 0·5)	1·8 (0·9 to 3·3)	2·7 (1·3 to 4·8)	3 (1·5 to 5·3)	3·2 (1·7 to 5·6)	2·6 (1·3 to 4·6)	1·9 (1 to 3·4)	1·5 (0·7 to 2·7)	1·3 (0·6 to 2·2)	1·2 (0·6 to 2·1)	1·4 (0·7 to 2·4)	1·4 (0·7 to 2·4)	1·1 (0·6 to 2)	0·8 (0·4 to 1·5)	0·7 (0·3 to 1·2)	0·5 (0·3 to 0·9)	0·5 (0·2 to 0·9)	0·4 (0·2 to 0·7)	0·3 (0·1 to 0·5)	0·1 (0·1 to 0·2)	0 (0 to 0·1)	26·8
Fungal skin diseases	0 (0 to 0)	0 (0 to 0)	0 (0 to 0·1)	0·4 (0·1 to 0·8)	0·6 (0·2 to 1·4)	0·8 (0·3 to 1·7)	0·8 (0·3 to 1·9)	0·8 (0·3 to 1·7)	0·8 (0·3 to 1·8)	0·9 (0·3 to 1·9)	0·8 (0·3 to 1·7)	0·7 (0·3 to 1·4)	0·8 (0·3 to 1·7)	0·9 (0·4 to 2·1)	1 (0·4 to 2·2)	1 (0·4 to 2·2)	1·1 (0·4 to 2·3)	1·2 (0·5 to 2·6)	1·2 (0·5 to 2·7)	1·3 (0·5 to 2·7)	0·9 (0·4 to 2)	0·4 (0·2 to 0·9)	0·2 (0·1 to 0·3)	16·6
Viral skin diseases	0 (0 to 0)	0 (0 to 0)	0 (0 to 0)	2 (1·3 to 3·1)	3·4 (2·1 to 5·3)	1·9 (1·2 to 3)	1·2 (0·8 to 1·9)	0·9 (0·5 to 1·3)	0·6 (0·4 to 1)	0·5 (0·3 to 0·8)	0·4 (0·2 to 0·7)	0·4 (0·2 to 0·7)	0·4 (0·2 to 0·6)	0·4 (0·2 to 0·7)	0·3 (0·2 to 0·5)	0·3 (0·2 to 0·5)	0·2 (0·1 to 0·4)	0·2 (0·1 to 0·3)	0·1 (0·1 to 0·2)	0·1 (0 to 0·1)	0 (0 to 0·1)	0 (0 to 0)	0 (0 to 0)	13·5
Acne vulgaris	0 (0 to 0)	0 (0 to 0)	0 (0 to 0)	0 (0 to 0)	0·2 (0·1 to 0·3)	4·5 (2·6 to 7·5)	7·5 (4·2 to 12·3)	1·7 (0·9 to 2·8)	0·8 (0·4 to 1·4)	0·6 (0·3 to 1·1)	0·4 (0·2 to 0·7)	0·4 (0·2 to 0·7)	0·2 (0·1 to 0·4)	0·2 (0·1 to 0·3)	0·2 (0·1 to 0·2)	0·1 (0·1 to 0·2)	0·1 (0 to 0·1)	0 (0 to 0·1)	0 (0 to 0·1)	0 (0 to 0)	0 (0 to 0)	0 (0 to 0)	0 (0 to 0)	16·7
Alopecia areata	0 (0 to 0)	0 (0 to 0)	0 (0 to 0)	0·1 (0 to 0·1)	0·1 (0 to 0·1)	0·1 (0 to 0·2)	0·2 (0·1 to 0·4)	0·3 (0·2 to 0·5)	0·4 (0·2 to 0·6)	0·4 (0·2 to 0·7)	0·3 (0·2 to 0·6)	0·3 (0·2 to 0·6)	0·2 (0·1 to 0·4)	0·2 (0·1 to 0·4)	0·2 (0·1 to 0·4)	0·2 (0·1 to 0·3)	0·2 (0·1 to 0·2)	0·1 (0·1 to 0·2)	0·1 (0 to 0·1)	0·1 (0 to 0·1)	0 (0 to 0·1)	0 (0 to 0)	0 (0 to 0)	3·2
Pruritus	0 (0 to 0)	0 (0 to 0)	0 (0 to 0)	0·1 (0 to 0·2)	0·1 (0·1 to 0·3)	0·1 (0·1 to 0·3)	0·2 (0·1 to 0·3)	0·2 (0·1 to 0·4)	0·2 (0·1 to 0·4)	0·2 (0·1 to 0·5)	0·2 (0·1 to 0·4)	0·2 (0·1 to 0·4)	0·2 (0·1 to 0·4)	0·2 (0·1 to 0·5)	0·3 (0·1 to 0·5)	0·3 (0·1 to 0·5)	0·3 (0·1 to 0·5)	0·3 (0·1 to 0·5)	0·2 (0·1 to 0·4)	0·2 (0·1 to 0·4)	0·1 (0·1 to 0·3)	0·1 (0 to 0·2)	0 (0 to 0·1)	0 (0 to 0)	3·5
Urticaria	0 (0 to 0)	0 (0 to 0)	0·3 (0·2 to 0·5)	2·1 (1·3 to 3·4)	2 (1·2 to 3·4)	1·6 (0·8 to 2·6)	1·4 (0·7 to 2·4)	1·2 (0·6 to 1·9)	1·1 (0·6 to 1·7)	1·1 (0·6 to 1·8)	1 (0·6 to 1·7)	0·8 (0·4 to 1·3)	0·8 (0·4 to 1·4)	0·8 (0·5 to 1·4)	0·7 (0·4 to 1·2)	0·6 (0·3 to 1)	0·5 (0·3 to 0·8)	0·4 (0·2 to 0·6)	0·3 (0·2 to 0·5)	0·2 (0·1 to 0·3)	0·1 (0·1 to 0·2)	0 (0 to 0·1)	0 (0 to 0)	17·1
Decubitus ulcer	0 (0 to 0)	0 (0 to 0)	0 (0 to 0)	0·2 (0 to 0·7)	0·3 (0·1 to 0·7)	0·5 (0·1 to 1·3)	0·5 (0·2 to 1·1)	0·1 (0 to 0·3)	0·5 (0·1 to 1·2)	0·3 (0·1 to 0·9)	0·9 (0·3 to 1·8)	0·8 (0·2 to 2)	1·5 (0·5 to 3·1)	2·3 (0·9 to 4·8)	3·1 (1·5 to 5·7)	4·2 (1·8 to 8·2)	4·9 (2·3 to 8·9)	6·7 (3·4 to 11·9)	11·4 (6·5 to 19·3)	13·4 (7·3 to 23·2)	11·7 (6·3 to 20·1)	6·4 (3·5 to 10·6)	2·7 (1·4 to 4·9)	72·5
Other skin and subcutaneous diseases	0 (0 to 0)	0 (0 to 0)	0·1 (0 to 0·2)	0·5 (0·2 to 0·9)	0·5 (0·2 to 0·8)	0·6 (0·3 to 1·2)	0·8 (0·4 to 1·4)	0·8 (0·4 to 1·4)	0·8 (0·4 to 1·6)	1·1 (0·5 to 2)	1·1 (0·5 to 1·9)	1·2 (0·6 to 2·2)	1·4 (0·7 to 2·6)	1·8 (0·9 to 3·2)	2 (1 to 3·5)	1·9 (0·9 to 3·4)	1·8 (0·9 to 3·3)	1·5 (0·8 to 2·7)	1·4 (0·7 to 2·4)	1·1 (0·6 to 1·8)	0·5 (0·3 to 0·9)	0·2 (0·1 to 0·4)	0·1 (0 to 0·1)	21
Sense organ diseases	0 (0 to 0)	0 (0 to 0)	0·1 (0·1 to 0·2)	1·2 (0·7 to 1·7)	2·8 (1·7 to 4·1)	3·9 (2·5 to 5·8)	5 (3·1 to 7·3)	5·5 (3·4 to 8·1)	6·8 (4·3 to 10·2)	8·7 (5·4 to 13·3)	9·1 (5·7 to 13·8)	9·8 (6·3 to 15·2)	15·5 (9·8 to 24·7)	23·1 (14·7 to 36·2)	27·4 (17·2 to 42·4)	29·6 (18·9 to 44·9)	31·4 (20·4 to 46·8)	31·3 (21 to 45·5)	33·3 (22·8 to 47·6)	32·6 (22·6 to 44·9)	22·9 (16·1 to 31)	10·5 (7·5 to 13·9)	3·7 (2·6 to 4·9)	314
Blindness and vision loss	0 (0 to 0)	0 (0 to 0)	0 (0 to 0)	0·3 (0·2 to 0·6)	0·9 (0·5 to 1·4)	1·2 (0·7 to 2)	1·5 (0·9 to 2·4)	1·6 (0·9 to 2·4)	1·7 (1 to 2·5)	1·9 (1·1 to 2·9)	2·1 (1·3 to 3·1)	2·4 (1·5 to 3·6)	3·5 (2·2 to 5·6)	5·2 (3·2 to 8·3)	6·4 (4 to 10)	7·3 (4·8 to 11·3)	8·6 (5·6 to 12·6)	9·3 (6·2 to 13·4)	10·4 (7·1 to 14·8)	10·5 (7·2 to 14·4)	7·6 (5·3 to 10·3)	3·7 (2·6 to 5)	1·5 (1 to 2)	87·5
Glaucoma	0 (0 to 0)	0 (0 to 0)	0 (0 to 0)	0 (0 to 0)	0 (0 to 0)	0 (0 to 0)	0 (0 to 0)	0 (0 to 0)	0 (0 to 0)	0 (0 to 0)	0 (0 to 0)	0 (0 to 0)	0 (0 to 0)	0·1 (0 to 0·1)	0·2 (0·1 to 0·3)	0·3 (0·2 to 0·5)	0·5 (0·3 to 0·8)	0·7 (0·4 to 1·1)	1 (0·6 to 1·6)	1·1 (0·7 to 1·8)	0·9 (0·6 to 1·4)	0·4 (0·3 to 0·7)	0·2 (0·1 to 0·3)	5·4
Cataract	0 (0 to 0)	0 (0 to 0)	0 (0 to 0)	0 (0 to 0)	0 (0 to 0)	0 (0 to 0)	0 (0 to 0)	0 (0 to 0)	0 (0 to 0·1)	0·1 (0 to 0·2)	0·2 (0·1 to 0·3)	0·2 (0·1 to 0·4)	0·4 (0·2 to 0·7)	0·6 (0·3 to 1)	0·8 (0·4 to 1·2)	1 (0·6 to 1·6)	1·5 (0·9 to 2·2)	2·1 (1·4 to 3·1)	3·1 (2 to 4·4)	3·5 (2·3 to 5)	2·8 (1·8 to 3·9)	1·3 (0·9 to 1·8)	0·5 (0·3 to 0·8)	18
Age-related macular degeneration	0 (0 to 0)	0 (0 to 0)	0 (0 to 0)	0 (0 to 0)	0 (0 to 0)	0 (0 to 0)	0 (0 to 0)	0 (0 to 0)	0 (0 to 0)	0 (0 to 0)	0 (0 to 0)	0 (0 to 0)	0 (0 to 0)	0 (0 to 0)	0·1 (0 to 0·1)	0·1 (0 to 0·1)	0·1 (0 to 0·2)	0·1 (0 to 0·2)	0·1 (0·1 to 0·2)	0·1 (0·1 to 0·2)	0·1 (0 to 0·1)	0 (0 to 0·1)	0 (0 to 0)	0·8
Refraction disorders	0 (0 to 0)	0 (0 to 0)	0 (0 to 0)	0·3 (0·2 to 0·5)	0·7 (0·4 to 1·2)	1·1 (0·6 to 1·8)	1·3 (0·8 to 2·1)	1·3 (0·8 to 2·1)	1·4 (0·8 to 2·1)	1·4 (0·8 to 2·1)	1·3 (0·8 to 2)	1·2 (0·7 to 1·8)	1·4 (0·9 to 2·2)	1·6 (1 to 2·5)	1·6 (1 to 2·5)	1·5 (0·9 to 2·2)	1·4 (0·8 to 2·1)	1·3 (0·8 to 2)	1·3 (0·8 to 2)	1·1 (0·6 to 1·6)	0·6 (0·4 to 0·9)	0·2 (0·1 to 0·3)	0·1 (0 to 0·1)	22
Near vision loss	0 (0 to 0)	0 (0 to 0)	0 (0 to 0)	0 (0 to 0)	0 (0 to 0)	0 (0 to 0)	0 (0 to 0)	0 (0 to 0·1)	0·1 (0 to 0·1)	0·2 (0·1 to 0·4)	0·3 (0·1 to 0·6)	0·5 (0·2 to 1·1)	1 (0·4 to 2·1)	1·7 (0·7 to 3·7)	2·2 (0·9 to 4·7)	2·4 (1 to 5)	2·5 (1 to 5)	2·4 (1 to 4·6)	2·3 (1 to 4·6)	2 (0·8 to 4)	1·3 (0·5 to 2·5)	0·5 (0·2 to 1·1)	0·2 (0·1 to 0·4)	19·5
Other vision loss	0 (0 to 0)	0 (0 to 0)	0 (0 to 0)	0·1 (0 to 0·1)	0·1 (0 to 0·2)	0·2 (0·1 to 0·3)	0·2 (0·1 to 0·4)	0·2 (0·1 to 0·4)	0·2 (0·1 to 0·4)	0·3 (0·1 to 0·5)	0·3 (0·2 to 0·6)	0·4 (0·2 to 0·7)	0·7 (0·4 to 1·1)	1·2 (0·7 to 1·8)	1·6 (1 to 2·5)	2·1 (1·3 to 3)	2·6 (1·7 to 3·8)	2·7 (1·7 to 4)	2·6 (1·7 to 3·9)	2·6 (1·6 to 3·8)	2 (1·2 to 3)	1·2 (0·8 to 1·7)	0·6 (0·3 to 0·8)	21·8
Age-related and other hearing loss	0 (0 to 0)	0 (0 to 0)	0 (0 to 0·1)	0·6 (0·3 to 1)	1·6 (0·9 to 2·6)	2·4 (1·4 to 3·6)	3 (1·8 to 4·6)	3·5 (2·1 to 5·4)	4·7 (2·8 to 7·3)	6·1 (3·7 to 9·5)	6·3 (3·8 to 9·9)	6·8 (4 to 11)	11·1 (6·6 to 18·1)	16·6 (10·1 to 26·6)	19·7 (12·1 to 31)	20·7 (12·5 to 32·9)	21·2 (13·1 to 32·3)	20·5 (13·1 to 30·6)	21·4 (14·2 to 31·3)	21 (14 to 29·6)	14·7 (10 to 20)	6·6 (4·6 to 8·9)	2·1 (1·5 to 2·8)	211
Other sense organ diseases	0 (0 to 0)	0 (0 to 0)	0·1 (0 to 0·1)	0·3 (0·1 to 0·5)	0·3 (0·2 to 0·6)	0·3 (0·1 to 0·6)	0·4 (0·2 to 0·8)	0·4 (0·2 to 0·8)	0·5 (0·2 to 0·9)	0·7 (0·3 to 1·3)	0·7 (0·3 to 1·3)	0·7 (0·3 to 1·3)	0·9 (0·4 to 1·6)	1·3 (0·6 to 2·6)	1·3 (0·6 to 2·6)	1·5 (0·7 to 2·8)	1·6 (0·8 to 3·1)	1·5 (0·7 to 2·8)	1·5 (0·7 to 2·7)	1·2 (0·6 to 2·2)	0·6 (0·3 to 1·1)	0·2 (0·1 to 0·4)	0·1 (0 to 0·2)	16
Musculoskeletal disorders	0 (0 to 0)	0 (0 to 0)	0 (0 to 0)	0 (0 to 0)	1·6 (0·8 to 2·8)	9·2 (5·8 to 13·8)	20 (13·1 to 29·1)	27·1 (18·4 to 37·8)	36·9 (25·1 to 52·7)	47·7 (32·1 to 65·9)	49·4 (34·3 to 67·8)	49·8 (34·9 to 68·1)	64 (44·1 to 88)	76 (53·3 to 106)	73·6 (49·8 to 102)	65·5 (45 to 93)	55·9 (38·4 to 79)	44·2 (30·2 to 62·5)	36·6 (24·9 to 51·3)	28·2 (19·4 to 39·6)	17·1 (12·1 to 23·2)	6·3 (4·4 to 8·6)	1·9 (1·3 to 2·6)	711
Rheumatoid arthritis	0 (0 to 0)	0 (0 to 0)	0 (0 to 0)	0 (0 to 0)	0 (0 to 0·1)	0·1 (0·1 to 0·2)	0·3 (0·2 to 0·5)	0·6 (0·2 to 1)	0·8 (0·4 to 1·3)	1·1 (0·6 to 1·8)	1·2 (0·7 to 1·9)	1·3 (0·8 to 2)	1·8 (1·1 to 2·6)	2 (1·2 to 2·9)	2 (1·3 to 2·8)	1·7 (1·2 to 2·4)	1·5 (1·1 to 2·1)	1·2 (0·8 to 1·7)	1·2 (0·8 to 1·6)	1 (0·7 to 1·4)	1·2 (0·7 to 1·9)	0·5 (0·3 to 0·8)	0·2 (0·1 to 0·3)	19·7
Osteoarthritis	0 (0 to 0)	0 (0 to 0)	0 (0 to 0)	0 (0 to 0)	0 (0 to 0)	0 (0 to 0)	0 (0 to 0)	0 (0 to 0)	0 (0 to 0)	0·2 (0·1 to 0·4)	1·2 (0·6 to 2·6)	3 (1·4 to 6·1)	6·7 (3·3 to 13·4)	11·6 (5·6 to 23·4)	14·1 (7 to 28·1)	14·6 (7·2 to 29·3)	13·8 (7 to 27·3)	11·6 (5·8 to 23·4)	9·9 (5 to 20·1)	7·8 (3·9 to 15·4)	4·5 (2·3 to 8·8)	1·7 (0·9 to 3·4)	0·5 (0·3 to 1)	101
Osteoarthritis hip	0 (0 to 0)	0 (0 to 0)	0 (0 to 0)	0 (0 to 0)	0 (0 to 0)	0 (0 to 0)	0 (0 to 0)	0 (0 to 0)	0 (0 to 0)	0·1 (0 to 0·1)	0·1 (0 to 0·2)	0·1 (0 to 0·3)	0·2 (0·1 to 0·5)	0·4 (0·2 to 0·9)	0·5 (0·2 to 1·2)	0·6 (0·3 to 1·3)	0·6 (0·3 to 1·2)	0·5 (0·2 to 1·1)	0·5 (0·2 to 1)	0·4 (0·2 to 0·8)	0·2 (0·1 to 0·4)	0·1 (0 to 0·2)	0 (0 to 0·1)	4·3
Osteoarthritis knee	0 (0 to 0)	0 (0 to 0)	0 (0 to 0)	0 (0 to 0)	0 (0 to 0)	0 (0 to 0)	0 (0 to 0)	0 (0 to 0)	0 (0 to 0)	0·1 (0 to 0·3)	0·9 (0·4 to 2)	2·3 (1·1 to 4·8)	5·1 (2·4 to 10·3)	8·7 (4·1 to 17·7)	10·4 (4·9 to 21·4)	10·7 (5·2 to 21·7)	9·9 (4·9 to 20·2)	8·2 (4·1 to 16·6)	6·9 (3·3 to 13·7)	5·2 (2·6 to 10·5)	2·8 (1·4 to 5·6)	1 (0·5 to 2)	0·3 (0·1 to 0·6)	72·6
Osteoarthritis hand	0 (0 to 0)	0 (0 to 0)	0 (0 to 0)	0 (0 to 0)	0 (0 to 0)	0 (0 to 0)	0 (0 to 0)	0 (0 to 0)	0 (0 to 0)	0 (0 to 0·1)	0·1 (0 to 0·4)	0·4 (0·1 to 0·9)	0·9 (0·4 to 2)	1·6 (0·7 to 3·3)	1·9 (0·9 to 4·2)	2 (0·9 to 4·3)	1·9 (0·9 to 4)	1·6 (0·8 to 3·4)	1·4 (0·7 to 3)	1·2 (0·6 to 2·5)	0·8 (0·4 to 1·6)	0·3 (0·2 to 0·7)	0·1 (0·1 to 0·2)	14·3

Osteoarthritis other	0 (0 to 0)	0 (0 to 0)	0 (0 to 0)	0 (0 to 0)	0 (0 to 0)	0 (0 to 0)	0 (0 to 0)	0 (0 to 0)	0 (0 to 0)	0 (0 to 0)	0·1 (0 to 0·2)	0·2 (0·1 to 0·5)	0·5 (0·2 to 1·1)	0·9 (0·4 to 2)	1·2 (0·5 to 2·5)	1·3 (0·6 to 2·9)	1·3 (0·6 to 2·8)	1·2 (0·6 to 2·5)	1·1 (0·6 to 2·4)	1 (0·5 to 2)	0·6 (0·3 to 1·3)	0·3 (0·1 to 0·6)	0·1 (0 to 0·2)	9·9
Low back pain	0 (0 to 0)	0 (0 to 0)	0 (0 to 0)	0 (0 to 0)	1·4 (0·6 to 2·5)	7·7 (4·5 to 12·2)	14·2 (8·2 to 22·6)	15·8 (9·1 to 24·8)	18·9 (11·1 to 29·5)	22·6 (12·6 to 34·8)	23·7 (14·2 to 36·4)	22·9 (13·9 to 35·2)	28·1 (16·8 to 45·2)	33 (20·2 to 50·5)	30·9 (18·8 to 46·3)	26·9 (16·9 to 39·9)	22·8 (14·4 to 34·7)	18·3 (11·3 to 27·1)	15·9 (9·9 to 23·5)	13 (8·2 to 19·4)	7 (4·3 to 10·1)	2·4 (1·5 to 3·5)	0·7 (0·4 to 1·1)	326
Neck pain	0 (0 to 0)	0 (0 to 0)	0 (0 to 0)	0 (0 to 0)	0·1 (0 to 0·2)	0·4 (0·2 to 0·9)	1·1 (0·4 to 2)	1·5 (0·6 to 3·1)	2·4 (1 to 4·8)	3·9 (1·9 to 7·5)	4·8 (2·5 to 8·6)	5·2 (2·6 to 9·2)	6·6 (3·2 to 11·8)	6·7 (3·5 to 12·6)	5·9 (2·8 to 10·7)	4·8 (2·4 to 8·4)	3·9 (2 to 6·5)	2·9 (1·6 to 5·1)	2·3 (1·1 to 4·3)	1·6 (0·8 to 2·9)	0·9 (0·4 to 1·5)	0·3 (0·2 to 0·5)	0·1 (0·1 to 0·2)	55·3
Gout	0 (0 to 0)	0 (0 to 0)	0 (0 to 0)	0 (0 to 0)	0 (0 to 0)	0 (0 to 0)	0 (0 to 0)	0 (0 to 0)	0·1 (0 to 0·1)	0·1 (0 to 0·2)	0·1 (0 to 0·3)	0·1 (0 to 0·3)	0·2 (0·1 to 0·4)	0·2 (0·1 to 0·5)	0·2 (0·1 to 0·5)	0·2 (0·1 to 0·5)	0·2 (0·1 to 0·4)	0·2 (0·1 to 0·3)	0·2 (0·1 to 0·3)	0·1 (0·1 to 0·3)	0·1 (0 to 0·2)	0 (0 to 0·1)	0 (0 to 0)	2·2
Other musculoskeletal disorders	0 (0 to 0)	0 (0 to 0)	0 (0 to 0)	0 (0 to 0)	0·1 (0·1 to 0·2)	1 (0·5 to 1·5)	4·4 (2·8 to 6·7)	9·2 (6·3 to 12·9)	14·8 (9·8 to 21·5)	19·8 (13·2 to 28·6)	18·3 (12 to 26·3)	17·2 (11·3 to 25·3)	20·6 (13·7 to 30)	22·5 (14·3 to 34·1)	20·5 (12·5 to 30·2)	17·2 (10·1 to 27)	13·8 (8·6 to 20·9)	10 (5·7 to 16·1)	7·1 (4·2 to 10·6)	4·7 (2·6 to 7·8)	3·5 (2·2 to 5)	1·3 (0·8 to 2)	0·4 (0·2 to 0·6)	206
Other non-communicable diseases	49·3 (29 to 79·5)	26·2 (15·8 to 40·5)	76·4 (47·7 to 111)	34·1 (24 to 47·4)	16·6 (12·8 to 20·8)	31·1 (23·7 to 39·8)	38·3 (28·5 to 50·3)	49·6 (36·6 to 67·4)	59·5 (43·7 to 81·5)	66·4 (48 to 90·6)	61·3 (44·5 to 83·9)	55·9 (40·3 to 75·6)	60·6 (43·6 to 81·9)	56·9 (40·1 to 76·8)	48·5 (34·8 to 66·4)	43·7 (31·9 to 59·7)	38 (27·7 to 51·4)	30·7 (22·6 to 40·9)	27·5 (20·4 to 36·1)	22·2 (16·9 to 28·5)	14·7 (11·4 to 18·9)	6·4 (4·9 to 8·2)	2·1 (1·5 to 2·9)	916
Congenital birth defects	48·2 (28 to 77·9)	24·2 (14·5 to 38·1)	57·1 (33 to 87·2)	21·1 (13·3 to 31·9)	7·4 (5·4 to 9·9)	11·2 (7·5 to 15·6)	8 (5·4 to 12·2)	6 (4 to 8·7)	4·8 (3·2 to 7)	3·9 (2·7 to 5·7)	3·1 (2·2 to 4·2)	2·5 (1·8 to 3·5)	2·7 (1·9 to 3·7)	2·3 (1·6 to 3·2)	1·9 (1·4 to 2·6)	1·3 (0·9 to 1·7)	1 (0·7 to 1·3)	0·3 (0·2 to 0·5)	0·2 (0·1 to 0·3)	0·1 (0·1 to 0·2)	0·1 (0 to 0·1)	0 (0 to 0)	0 (0 to 0)	207
Neural tube defects	4·5 (1·4 to 10·7)	1·5 (0·4 to 4·1)	3 (0·7 to 8·2)	2·2 (0·5 to 6·7)	0·2 (0·1 to 0·4)	0·6 (0·3 to 1·3)	0·4 (0·2 to 0·9)	0·1 (0·1 to 0·2)	0·2 (0·1 to 0·3)	0·2 (0·1 to 0·3)	0·1 (0·1 to 0·2)	0·1 (0 to 0·1)	0 (0 to 0·1)	0 (0 to 0·1)	0 (0 to 0·1)	0 (0 to 0)	0 (0 to 0·1)	0 (0 to 0)	0 (0 to 0)	0 (0 to 0)	0 (0 to 0)	0 (0 to 0)	0 (0 to 0)	13·4
Congenital heart anomalies	16 (7·9 to 29·8)	10·3 (5·5 to 17·9)	25·8 (12·6 to 45·2)	8·4 (4·2 to 15)	2·1 (1·2 to 3·3)	3·4 (1·8 to 5·9)	2·4 (1·2 to 4·2)	1·7 (0·9 to 3)	1·4 (0·8 to 2·5)	0·9 (0·5 to 1·6)	0·8 (0·5 to 1·3)	0·6 (0·4 to 1)	0·6 (0·3 to 1)	0·4 (0·2 to 0·7)	0·3 (0·2 to 0·5)	0·2 (0·1 to 0·3)	0·2 (0·1 to 0·3)	0 (0 to 0)	0 (0 to 0)	0 (0 to 0)	0 (0 to 0)	0 (0 to 0)	0 (0 to 0)	75·8
Orofacial clefts	0 (0 to 0)	0 (0 to 0)	0 (0 to 0·1)	0·1 (0 to 0·1)	0·1 (0 to 0·1)	0·1 (0 to 0·1)	0·1 (0 to 0·1)	0·1 (0 to 0·1)	0·1 (0 to 0·1)	0·1 (0 to 0·1)	0 (0 to 0·1)	0 (0 to 0·1)	0 (0 to 0·1)	0 (0 to 0·1)	0 (0 to 0·1)	0 (0 to 0·1)	0 (0 to 0·1)	0 (0 to 0)	0 (0 to 0)	0 (0 to 0)	0 (0 to 0)	0 (0 to 0)	0 (0 to 0)	0·9
Down syndrome	0·7 (0·3 to 1·6)	0·5 (0·2 to 1)	2·7 (1 to 5·8)	1·7 (0·7 to 3·5)	0·4 (0·2 to 0·8)	0·7 (0·3 to 1·3)	0·5 (0·3 to 0·9)	0·6 (0·3 to 1·1)	0·5 (0·2 to 0·9)	0·5 (0·3 to 0·9)	0·5 (0·2 to 0·8)	0·4 (0·2 to 0·7)	0·6 (0·3 to 1·1)	0·5 (0·3 to 0·9)	0·5 (0·2 to 0·8)	0·3 (0·1 to 0·5)	0·1 (0 to 0·2)	0 (0 to 0)	0 (0 to 0)	0 (0 to 0)	0 (0 to 0)	0 (0 to 0)	0 (0 to 0)	11·6
Turner syndrome	0 (0 to 0)	0 (0 to 0)	0 (0 to 0)	0 (0 to 0)	0 (0 to 0)	0 (0 to 0)	0 (0 to 0)	0 (0 to 0)	0 (0 to 0)	0 (0 to 0)	0 (0 to 0)	0 (0 to 0)	0 (0 to 0)	0 (0 to 0)	0 (0 to 0)	0 (0 to 0)	0 (0 to 0)	0 (0 to 0)	0 (0 to 0)	0 (0 to 0)	0 (0 to 0)	0 (0 to 0)	0 (0 to 0)	0·1
Klinefelter syndrome	0 (0 to 0)	0 (0 to 0)	0 (0 to 0)	0 (0 to 0)	0 (0 to 0)	0 (0 to 0)	0 (0 to 0)	0 (0 to 0)	0 (0 to 0)	0 (0 to 0)	0 (0 to 0)	0 (0 to 0)	0 (0 to 0)	0 (0 to 0)	0 (0 to 0)	0 (0 to 0)	0 (0 to 0)	0 (0 to 0)	0 (0 to 0)	0 (0 to 0)	0 (0 to 0)	0 (0 to 0)	0 (0 to 0)	0
Other chromosomal abnormalities	5·6 (2·2 to 11·3)	3·6 (1·6 to 6·8)	8·3 (3·4 to 16·6)	1·3 (0·6 to 2·5)	0·5 (0·3 to 0·9)	0·9 (0·4 to 1·8)	0·7 (0·3 to 1·6)	0·6 (0·3 to 1·1)	0·4 (0·2 to 0·7)	0·3 (0·2 to 0·6)	0·1 (0·1 to 0·2)	0·1 (0·1 to 0·2)	0 (0 to 0·1)	0·1 (0 to 0·1)	0 (0 to 0)	0 (0 to 0)	0 (0 to 0)	0 (0 to 0)	0 (0 to 0)	0 (0 to 0)	0 (0 to 0)	0 (0 to 0)	0 (0 to 0)	22·5
Congenital musculoskeletal and limb anomalies	2·6 (1·1 to 5·2)	1·1 (0·5 to 2·2)	3 (1·5 to 5·7)	2·1 (1·3 to 3·1)	2·2 (1·3 to 3·4)	2·4 (1·4 to 3·7)	2·1 (1·3 to 3·2)	1·4 (0·9 to 2·1)	1·2 (0·8 to 1·8)	1 (0·6 to 1·6)	0·9 (0·5 to 1·4)	0·8 (0·5 to 1·2)	0·9 (0·5 to 1·2)	0·8 (0·5 to 1·3)	0·7 (0·4 to 1·1)	0·6 (0·3 to 0·9)	0·4 (0·3 to 0·7)	0·2 (0·1 to 0·4)	0·1 (0·1 to 0·2)	0·1 (0 to 0·1)	0 (0 to 0·1)	0 (0 to 0)	0 (0 to 0)	24·6
Urogenital congenital anomalies	0·3 (0·1 to 0·5)	0·1 (0·1 to 0·2)	0·1 (0·1 to 0·3)	0·1 (0·1 to 0·2)	0·1 (0·1 to 0·1)	0·1 (0·1 to 0·2)	0·1 (0 to 0·1)	0·1 (0 to 0·1)	0 (0 to 0·1)	0 (0 to 0·1)	0 (0 to 0)	0 (0 to 0)	0 (0 to 0)	0 (0 to 0)	0 (0 to 0)	0 (0 to 0)	0 (0 to 0)	0 (0 to 0)	0 (0 to 0)	0 (0 to 0)	0 (0 to 0)	0 (0 to 0)	0 (0 to 0)	1·2
Digestive congenital anomalies	4·8 (2·1 to 9·4)	2·6 (1·2 to 4·7)	3·9 (1·6 to 8)	0·9 (0·4 to 1·6)	0·2 (0·1 to 0·3)	0·2 (0·1 to 0·3)	0·1 (0 to 0·1)	0 (0 to 0·1)	0 (0 to 0)	0 (0 to 0)	0 (0 to 0)	0 (0 to 0)	0 (0 to 0)	0 (0 to 0)	0 (0 to 0)	0 (0 to 0)	0 (0 to 0)	0 (0 to 0)	0 (0 to 0)	0 (0 to 0)	0 (0 to 0)	0 (0 to 0)	0 (0 to 0)	12·8
Other congenital birth defects	13·6 (6·7 to 23·7)	4·5 (2·2 to 8)	10·2 (4·3 to 20·6)	4·2 (1·9 to 7·7)	1·6 (0·9 to 2·6)	2·8 (1·6 to 4·7)	1·7 (0·8 to 2·9)	1·4 (0·7 to 2·5)	1 (0·5 to 1·7)	0·9 (0·5 to 1·6)	0·6 (0·3 to 1)	0·4 (0·3 to 0·7)	0·5 (0·2 to 0·8)	0·3 (0·2 to 0·6)	0·3 (0·2 to 0·5)	0·2 (0·1 to 0·3)	0·2 (0·1 to 0·3)	0 (0 to 0·1)	0 (0 to 0·1)	0 (0 to 0·1)	0 (0 to 0)	0 (0 to 0)	0 (0 to 0)	44·5
Urinary diseases and male infertility	0 (0 to 0)	0 (0 to 0·1)	0·3 (0·1 to 0·5)	0·3 (0·2 to 0·4)	0·3 (0·2 to 0·4)	0·5 (0·3 to 0·7)	0·5 (0·3 to 0·8)	0·6 (0·4 to 0·9)	0·7 (0·5 to 1)	0·9 (0·6 to 1·3)	0·9 (0·6 to 1·2)	0·9 (0·6 to 1·3)	1·1 (0·7 to 1·7)	1·1 (0·7 to 1·6)	1·1 (0·7 to 1·6)	1·2 (0·7 to 2)	1·2 (0·7 to 1·9)	1·2 (0·7 to 1·9)	1·5 (0·9 to 2·4)	1·7 (1 to 2·8)	1·2 (0·7 to 2·1)	0·6 (0·4 to 1)	0·2 (0·1 to 0·4)	17·9
Urolithiasis	0 (0 to 0)	0 (0 to 0)	0 (0 to 0)	0 (0 to 0)	0 (0 to 0)	0 (0 to 0)	0 (0 to 0)	0 (0 to 0·1)	0 (0 to 0·1)	0·1 (0·1 to 0·2)	0·1 (0·1 to 0·2)	0·1 (0·1 to 0·2)	0·2 (0·1 to 0·3)	0·1 (0 to 0·3)	0·1 (0·1 to 0·3)	0·1 (0·1 to 0·2)	0·1 (0·1 to 0·2)	0·1 (0 to 0·1)	0 (0 to 0·1)	0 (0 to 0·1)	0 (0 to 0)	0 (0 to 0)	0 (0 to 0)	1·3
Benign prostatic hyperplasia	0 (0 to 0)	0 (0 to 0)	0 (0 to 0)	0 (0 to 0)	0 (0 to 0)	0 (0 to 0)	0 (0 to 0)	0 (0 to 0)	0 (0 to 0)	0 (0 to 0)	0 (0 to 0)	0 (0 to 0)	0 (0 to 0)	0 (0 to 0)	0 (0 to 0)	0 (0 to 0)	0 (0 to 0)	0 (0 to 0)	0 (0 to 0)	0 (0 to 0)	0 (0 to 0)	0 (0 to 0)	0 (0 to 0)	0
Male infertility	--	--	--	--	--	--	--	--	--	--	--	--	--	--	--	--	--	--	--	--	--	--	--	NA
Other urinary diseases	0 (0 to 0)	0 (0 to 0·1)	0 (0 to 0·1)	0·1 (0·1 to 0·2)	0·1 (0 to 0·1)	0·1 (0·1 to 0·2)	0·1 (0·1 to 0·2)	0·1 (0·1 to 0·2)	0·1 (0 to 0·1)	0·1 (0·1 to 0·2)	0·1 (0·1 to 0·2)	0·1 (0·1 to 0·2)	0·1 (0·1 to 0·2)	0·1 (0·1 to 0·2)	0·1 (0·1 to 0·2)	0·1 (0·1 to 0·2)	0·1 (0 to 0·1)	0 (0 to 0·1)	0 (0 to 0·1)	0 (0 to 0·1)	0 (0 to 0)	0 (0 to 0)	0 (0 to 0)	1·7
Gynecological diseases	0 (0 to 0)	0 (0 to 0)	0 (0 to 0)	0 (0 to 0)	0 (0 to 0)	3·8 (1·9 to 6·7)	10·4 (6 to 17·6)	21 (13·3 to 32·5)	30·3 (19·7 to 44·2)	36·9 (24·3 to 53·3)	35·3 (23·7 to 51·3)	30·6 (19·9 to 43·9)	28·8 (18·8 to 41)	21·8 (13 to 32·8)	12·3 (7·5 to 18·1)	7·8 (4·5 to 12)	5·7 (3·5 to 8·5)	4 (2·5 to 5·7)	2·9 (1·9 to 4·3)	2·1 (1·3 to 3·1)	1·2 (0·8 to 1·8)	0·5 (0·3 to 0·7)	0·2 (0·1 to 0·3)	256
Uterine fibroids	0 (0 to 0)	0 (0 to 0)	0 (0 to 0)	0 (0 to 0)	0 (0 to 0)	0·1 (0 to 0·1)	0·2 (0·1 to 0·3)	0·4 (0·1 to 0·8)	0·9 (0·4 to 1·9)	2 (0·9 to 4·1)	2·9 (1·4 to 5·8)	2·9 (1·4 to 5·2)	3·1 (1·5 to 5·5)	2·3 (1·1 to 4·2)	1·5 (0·7 to 2·7)	0·8 (0·4 to 1·6)	0·5 (0·2 to 0·9)	0·3 (0·1 to 0·5)	0·2 (0·1 to 0·3)	0·1 (0 to 0·2)	0 (0 to 0·1)	0 (0 to 0)	0 (0 to 0)	18
Polycystic ovarian syndrome	0 (0 to 0)	0 (0 to 0)	0 (0 to 0)	0 (0 to 0)	0 (0 to 0)	0·1 (0·1 to 0·3)	0·7 (0·3 to 1·4)	0·7 (0·3 to 1·5)	0·7 (0·3 to 1·4)	0·7 (0·3 to 1·4)	0·6 (0·2 to 1·1)	0·5 (0·2 to 0·9)	0·4 (0·2 to 0·9)	0·1 (0 to 0·3)	0 (0 to 0)	0 (0 to 0)	0 (0 to 0)	0 (0 to 0)	0 (0 to 0)	0 (0 to 0)	0 (0 to 0)	0 (0 to 0)	0 (0 to 0)	4·5
Female infertility	0 (0 to 0)	0 (0 to 0)	0 (0 to 0)	0 (0 to 0)	0 (0 to 0)	0 (0 to 0)	0 (0 to 0)	0·7 (0·1 to 1·9)	1 (0·3 to 2·7)	1·3 (0·3 to 3·7)	1·2 (0·2 to 3·6)	1 (0·1 to 2·7)	0 (0 to 0·2)	0 (0 to 0)	0 (0 to 0)	0 (0 to 0)	0 (0 to 0)	0 (0 to 0)	0 (0 to 0)	0 (0 to 0)	0 (0 to 0)	0 (0 to 0)	0 (0 to 0)	5·2
Endometriosis	0 (0 to 0)	0 (0 to 0)	0 (0 to 0)	0 (0 to 0)	0 (0 to 0)	0 (0 to 0)	0·8 (0·3 to 1·6)	3 (1·4 to 5·5)	3·4 (1·7 to 6·1)	2·9 (1·4 to 5·1)	2·2 (1·1 to 4)	1·9 (0·9 to 3·2)	1·8 (1 to 3)	1 (0·5 to 1·8)	0 (0 to 0)	0 (0 to 0)	0 (0 to 0)	0 (0 to 0)	0 (0 to 0)	0 (0 to 0)	0 (0 to 0)	0 (0 to 0)	0 (0 to 0)	17
Genital prolapse	0 (0 to 0)	0 (0 to 0)	0 (0 to 0)	0 (0 to 0)	0 (0 to 0)	0 (0 to 0)	0·1 (0 to 0·2)	0·1 (0 to 0·3)	0·1 (0·1 to 0·3)	0·2 (0·1 to 0·4)	0·3 (0·1 to 0·6)	0·4 (0·2 to 0·8)	0·6 (0·3 to 1·1)	0·7 (0·3 to 1·3)	0·6 (0·3 to 1·2)	0·5 (0·3 to 1)	0·5 (0·2 to 0·9)	0·4 (0·2 to 0·7)	0·3 (0·1 to 0·6)	0·2 (0·1 to 0·4)	0·1 (0·1 to 0·3)	0·1 (0 to 0·1)	0 (0 to 0)	5·3
Premenstrual syndrome	0 (0 to 0)	0 (0 to 0)	0 (0 to 0)	0 (0 to 0)	0 (0 to 0)	3·4 (1·6 to 6·3)	8·2 (4·1 to 14·9)	9·5 (4·8 to 16·7)	9·5 (5·5 to 15·6)	9·8 (5·1 to 15·8)	9·5 (5·3 to 15·1)	8·1 (4·6 to 13·4)	6·4 (3·7 to 10·7)	2·4 (1·2 to 4·4)	0 (0 to 0)	0 (0 to 0)	0 (0 to 0)	0 (0 to 0)	0 (0 to 0)	0 (0 to 0)	0 (0 to 0)	0 (0 to 0)	0 (0 to 0)	66·8
Other gynecological diseases	0 (0 to 0)	0 (0 to 0)	0 (0 to 0)	0 (0 to 0)	0 (0 to 0)	0·2 (0 to 0·4)	0·6 (0·3 to 1)	6·6 (3·9 to 10·3)	14·6 (8·5 to 22·9)	20·2 (12·2 to 31·3)	18·6 (11·1 to 29·5)	15·9 (8·9 to 25·1)	16·5 (9·4 to 25·2)	15·2 (7·6 to 24·3)	10·2 (5·9 to 15·6)	6·4 (3·4 to 10·4)	4·7 (2·7 to 7·3)	3·3 (2 to 4·9)	2·5 (1·5 to 3·8)	1·8 (1 to 2·8)	1 (0·6 to 1·6)	0·4 (0·2 to 0·7)	0·1 (0·1 to 0·2)	139
Hemoglobinopathies and hemolytic anemias	0·3 (0·2 to 0·5)	0·2 (0·1 to 0·4)	2·1 (1 to 3·9)	4·8 (2·6 to 8)	4·6 (2·8 to 6·9)	9·3 (5·6 to 14·3)	11 (7·1 to 16·3)	12·2 (7·4 to 18·1)	11·3 (6·7 to 17·2)	8·5 (4·7 to 13·4)	5·6 (3·7 to 8·1)	4·7 (3 to 6·9)	5·1 (3·3 to 7·5)	4·2 (2·7 to 6)	3·4 (2·3 to 4·9)	3·6 (2·4 to 5·3)	2·6 (1·6 to 4)	2·1 (1·4 to 3)	2·1 (1·3 to 3·1)	1·7 (1·1 to 2·4)	1·7 (1·1 to 2·4)	0·8 (0·5 to 1·1)	0·2 (0·1 to 0·3)	102
Thalassemias	0 (0 to 0)	0 (0 to 0)	0 (0 to 0)	0 (0 to 0·1)	0 (0 to 0)	0 (0 to 0)	0 (0 to 0)	0 (0 to 0)	0 (0 to 0)	0 (0 to 0)	0 (0 to 0)	0 (0 to 0)	0 (0 to 0)	0 (0 to 0)	0 (0 to 0)	0 (0 to 0)	0 (0 to 0)	0 (0 to 0)	0 (0 to 0)	0 (0 to 0)	0 (0 to 0)	0 (0 to 0)	0 (0 to 0)	0·1
Thalassemias trait	0 (0 to 0)	0 (0 to 0)	0 (0 to 0)	0 (0 to 0·1)	0·1 (0 to 0·1)	0·1 (0 to 0·1)	0·3 (0·1 to 0·5)	0·3 (0·2 to 0·5)	0·3 (0·2 to 0·5)	0·3 (0·1 to 0·5)	0·2 (0·1 to 0·4)	0·2 (0·1 to 0·3)	0·1 (0 to 0·1)	0·1 (0 to 0·1)	0·1 (0 to 0·1)	0 (0 to 0·1)	0 (0 to 0·1)	0 (0 to 0·1)	0 (0 to 0)	0 (0 to 0)	0 (0 to 0)	0 (0 to 0)	0 (0 to 0)	2·1
Sickle cell disorders	0·3 (0·1 to 0·5)	0·2 (0·1 to 0·4)	1·8 (0·8 to 3·6)	4 (2 to 7)	3·6 (2·1 to 5·8)	8·2 (4·6 to 13)	8·9 (5·4 to 13·8)	7·7 (4·6 to 11·8)	5·9 (3·1 to 10·1)	4·3 (2·4 to 7·1)	3·2 (1·8 to 4·9)	2·4 (1·3 to 4)	2·8 (1·6 to 4·7)	1·7 (1 to 2·8)	0·9 (0·5 to 1·5)	0·4 (0·2 to 0·7)	0·1 (0 to 0·2)	0 (0 to 0)	0 (0 to 0)	0 (0 to 0)	0 (0 to 0)	0 (0 to 0)	0 (0 to 0)	56·3
Sickle cell trait	0 (0 to 0)	0 (0 to 0·1)	0·2 (0·1 to 0·3)	0·5 (0·2 to 0·8)	0·6 (0·2 to 1·2)	0·6 (0·2 to 1·3)	0·2 (0·1 to 0·5)	3·4 (0·6 to 7)	4·1 (1·1 to 7·6)	2·8 (0·4 to 6·6)	0·2 (0 to 0·4)	0·5 (0·3 to 0·9)	0·6 (0·3 to 1·1)	0·7 (0·3 to 1·2)	0·6 (0·3 to 1·1)	0·5 (0·2 to 0·8)	0·3 (0·1 to 0·6)	0·2 (0·1 to 0·5)	0·2 (0·1 to 0·4)	0·2 (0·1 to 0·4)	0·1 (0·1 to 0·2)	0 (0 to 0·1)	0 (0 to 0)	16·6
G6PD deficiency	0 (0 to 0)	0 (0 to 0)	0 (0 to 0)	0 (0 to 0·1)	0 (0 to 0·1)	0·1 (0 to 0·1)	0·7 (0·3 to 1·2)	0·2 (0·1 to 0·4)	0·3 (0·1 to 0·7)	0·3 (0·1 to 0·7)	0·9 (0·5 to 1·4)	0·4 (0·2 to 0·7)	0·4 (0·1 to 0·7)	0·3 (0·1 to 0·6)	0·2 (0·1 to 0·5)	0·2 (0·1 to 0·5)	0·2 (0·1 to 0·3)	0·1 (0 to 0·1)	0 (0 to 0·1)	0 (0 to 0)	0 (0 to 0)	0 (0 to 0)	0 (0 to 0)	4·3
G6PD trait	0 (0 to 0)	0 (0 to 0)	0 (0 to 0)	0 (0 to 0)	0 (0 to 0)	0 (0 to 0)	0·1 (0 to 0·1)	0 (0 to 0)	0 (0 to 0)	0 (0 to 0)	0·1 (0 to 0·1)	0·1 (0 to 0·1)	0 (0 to 0)	0 (0 to 0)	0 (0 to 0)	0 (0 to 0)	0 (0 to 0)	0 (0 to 0)	0 (0 to 0)	0 (0 to 0)	0 (0 to 0)	0 (0 to 0)	0 (0 to 0)	0·2
Other hemoglobinopathies and hemolytic anemias	0 (0 to 0)	0 (0 to 0)	0·1 (0 to 0·2)	0·2 (0·1 to 0·5)	0·3 (0·1 to 0·6)	0·4 (0·1 to 0·8)	0·7 (0·3 to 1·4)	0·6 (0·3 to 1·1)	0·8 (0·4 to 1·3)	0·8 (0·4 to 1·3)	1·1 (0·6 to 1·7)	1·1 (0·6 to 1·7)	1·2 (0·7 to 1·9)	1·4 (0·8 to 2·2)	1·6 (1 to 2·5)	2·5 (1·6 to 3·7)	2·1 (1·3 to 3·1)	1·7 (1·1 to 2·5)	1·8 (1·1 to 2·7)	1·4 (0·9 to 2·1)	1·5 (1 to 2·2)	0·7 (0·5 to 1)	0·2 (0·1 to 0·3)	22·2
Endocrine, metabolic, blood, and immune disorders	0·8 (0·4 to 1·6)	1·1 (0·5 to 2·1)	12·9 (5·9 to 23·5)	6·8 (3·6 to 11·6)	3 (1·9 to 4·4)	4·5 (3 to 6·7)	5·7 (3·7 to 8·3)	6 (3·9 to 9)	7 (4·5 to 10·7)	9 (5·7 to 13·2)	9·1 (6·1 to 13)	9·9 (6·6 to 14·2)	13·1 (8·4 to 18·9)	14·5 (9·2 to 21·4)	15·4 (10·1 to 22·6)	15 (9·8 to 22·6)	13·3 (8·9 to 19·9)	11 (7·4 to 15·7)	10·3 (7·1 to 14·7)	8·3 (5·8 to 11·4)	5·8 (3·8 to 8·8)	2·6 (1·7 to 4)	0·9 (0·5 to 1·5)	186
Oral disorders	0 (0 to 0)	0 (0 to 0)	0 (0 to 0)	1·1 (0·6 to 1·8)	1·4 (0·8 to 2·5)	1·7 (0·9 to 3)	2·6 (1·4 to 4·5)	3·8 (2 to 7)	5·4 (2·8 to 10·2)	7·1 (3·4 to 13·3)	7·4 (3·6 to 14·1)	7·4 (3·6 to 13·5)	9·8 (5·2 to 17·2)	13 (7·3 to 22·3)	14·4 (8·4 to 23·4)	14·8 (8·9 to 23·4)	14·2 (8·7 to 22)	12·2 (7·7 to 18·8)	10·5 (6·6 to 15·7)	8·3 (5·3 to 12·1)	4·7 (3 to 7)	1·8 (1·1 to 2·7)	0·6 (0·4 to 0·9)	142
Caries of deciduous teeth	0 (0 to 0)	0 (0 to 0)	0 (0 to 0)	0·3 (0·1 to 0·7)	0·4 (0·1 to 0·8)	0 (0 to 0·1)	0 (0 to 0)	0 (0 to 0)	0 (0 to 0)	0 (0 to 0)	0 (0 to 0)	0 (0 to 0)	0 (0 to 0)	0 (0 to 0)	0 (0 to 0)	0 (0 to 0)	0 (0 to 0)	0 (0 to 0)	0 (0 to 0)	0 (0 to 0)	0 (0 to 0)	0 (0 to 0)	0 (0 to 0)	0·7
Caries of permanent teeth	0 (0 to 0)	0 (0 to 0)	0 (0 to 0)	0 (0 to 0)	0·3 (0·1 to 0·8)	0·8 (0·3 to 1·7)	0·9 (0·3 to 1·9)	0·9 (0·4 to 2)	0·9 (0·4 to 1·9)	0·9 (0·4 to 1·8)	0·8 (0·3 to 1·6)	0·6 (0·2 to 1·3)	0·6 (0·3 to 1·4)	0·7 (0·3 to 1·4)	0·5 (0·2 to 1·2)	0·4 (0·1 to 0·9)	0·3 (0·1 to 0·6)	0·2 (0·1 to 0·4)	0·2 (0·1 to 0·3)	0·1 (0 to 0·2)	0·1 (0 to 0·1)	0 (0 to 0)	0 (0 to 0)	9·2
Periodontal diseases	0 (0 to 0)	0 (0 to 0)	0 (0 to 0)	0 (0 to 0)	0 (0 to 0)	0 (0 to 0)	0·3 (0·1 to 0·9)	1·1 (0·3 to 2·8)	2·5 (0·8 to 6)	3·8 (1·3 to 8·8)	4·3 (1·5 to 9·8)	4·3 (1·5 to 9·3)	5·1 (2 to 10·7)	5·4 (2·1 to 11·3)	4·4 (1·7 to 9)	3·2 (1·3 to 6·4)	2·2 (0·9 to 4·5)	1·5 (0·6 to 3)	1·2 (0·5 to 2·4)	0·9 (0·3 to 1·8)	0·5 (0·2 to 1·1)	0·2 (0·1 to 0·4)	0·1 (0 to 0·1)	41
Other oral disorders	0 (0 to 0)	0 (0 to 0)	0 (0 to 0)	0·7 (0·4 to 1·2)	0·7 (0·4 to 1·2)	0·9 (0·5 to 1·4)	1·4 (0·8 to 2·1)	1·6 (0·9 to 2·5)	1·7 (1 to 2·6)	1·8 (1·1 to 2·7)	1·6 (0·9 to 2·5)	1·4 (0·8 to 2·1)	1·6 (0·9 to 2·4)	1·7 (1 to 2·5)	1·4 (0·8 to 2·2)	1·1 (0·7 to 1·7)	0·9 (0·5 to 1·4)	0·6 (0·4 to 1)	0·4 (0·3 to 0·6)	0·3 (0·2 to 0·4)	0·1 (0·1 to 0·2)	0 (0 to 0·1)	0 (0 to 0)	19·9
Sudden infant death syndrome	--	0·6 (0·2 to 1·2)	4·1 (1·3 to 9)	--	--	--	--	--	--	--	--	--	--	--	--	--	--	--	--	--	--	--	--	NA
Injuries	2·2 (1·3 to 3·6)	1·4 (0·8 to 2·5)	11 (6·8 to 17·3)	24 (16·4 to 33·7)	16·4 (13·3 to 20·1)	36·6 (28·8 to 45·8)	50·6 (38·7 to 64·1)	55·5 (42·4 to 72·5)	44·5 (33·5 to 58·6)	42·2 (32·6 to 54·3)	32·3 (25·5 to 40·7)	27·4 (21·8 to 34)	30·5 (24·2 to 37·7)	26·5 (21·3 to 33)	24·2 (19·3 to 29·9)	23·1 (18·8 to 28·4)	20·5 (16·4 to 25)	17·1 (14·1 to 20·6)	17·2 (14·2 to 20·6)	16·3 (13·5 to 19·5)	11·7 (9·5 to 14)	6·1 (4·9 to 7·4)	2·4 (1·9 to 3)	540
Transport injuries	0·2 (0·1 to 0·4)	0·1 (0·1 to 0·3)	1·5 (0·7 to 2·8)	5·4 (2·9 to 9·1)	5·3 (3·5 to 7·8)	14·2 (9·7 to 20)	20·8 (13·2 to 31·7)	20·6 (13·3 to 29·9)	14·5 (9·7 to 21·5)	12·6 (8·6 to 18·3)	9·7 (6·7 to 13·4)	8·4 (6 to 11·5)	9·4 (6·9 to 12·6)	7·4 (5·7 to 9·5)	6·7 (5·1 to 8·6)	6·4 (4·8 to 8·6)	5·7 (4·2 to 7·4)	4·2 (3·1 to 5·4)	3·7 (2·8 to 4·9)	2·8 (2·1 to 3·5)	1·5 (1·2 to 1·9)	0·6 (0·5 to 0·8)	0·2 (0·2 to 0·3)	162
Road injuries	0·2 (0·1 to 0·3)	0·1 (0 to 0·2)	1·2 (0·6 to 2·5)	4·9 (2·6 to 8·2)	4·9 (3·2 to 7·2)	13 (8·8 to 18·4)	19·6 (12·2 to 30·1)	19·2 (12·4 to 28·1)	13·4 (9·1 to 20·1)	11·6 (8 to 17·1)	9·1 (6·3 to 12·5)	7·6 (5·5 to 10·5)	8·8 (6·4 to 11·8)	6·9 (5·3 to 8·8)	6·2 (4·7 to 8)	6 (4·5 to 7·9)	5·4 (4 to 7·1)	3·9 (3 to 5·1)	3·4 (2·5 to 4·5)	2·6 (2 to 3·3)	1·4 (1·1 to 1·8)	0·6 (0·4 to 0·7)	0·2 (0·2 to 0·3)	150
Pedestrian road injuries	0·1 (0 to 0·2)	0 (0 to 0·1)	0·4 (0·2 to 0·9)	2·6 (1·2 to 4·8)	2·3 (1·3 to 3·7)	5·4 (3·2 to 8·4)	3·8 (1·9 to 7)	3·5 (1·9 to 5·8)	2·4 (1·4 to 4·1)	2·7 (1·6 to 4·4)	2·3 (1·4 to 3·7)	1·9 (1·2 to 3)	2·5 (1·6 to 3·7)	2 (1·4 to 2·9)	1·9 (1·3 to 2·7)	2·2 (1·5 to 3·3)	2·2 (1·5 to 3·2)	1·7 (1·2 to 2·4)	1·7 (1·1 to 2·4)	1·3 (0·9 to 1·8)	0·7 (0·5 to 1)	0·3 (0·2 to 0·4)	0·1 (0·1 to 0·1)	44·2
Cyclist road injuries	0 (0 to 0)	0 (0 to 0)	0 (0 to 0)	0·2 (0·1 to 0·4)	0·3 (0·1 to 0·4)	1·1 (0·6 to 1·9)	1·3 (0·8 to 2·2)	1·2 (0·7 to 1·9)	1·1 (0·7 to 1·6)	1·1 (0·8 to 1·4)	1 (0·7 to 1·4)	0·9 (0·6 to 1·2)	1 (0·7 to 1·4)	1 (0·7 to 1·4)	0·9 (0·6 to 1·2)	0·7 (0·5 to 1)	0·6 (0·4 to 0·8)	0·4 (0·3 to 0·6)	0·4 (0·3 to 0·5)	0·3 (0·2 to 0·4)	0·2 (0·1 to 0·2)	0·1 (0 to 0·1)	0 (0 to 0)	13·7
Motorcyclist road injuries	0 (0 to 0)	0 (0 to 0)	0·1 (0 to 0·3)	0·2 (0·1 to 0·4)	0·3 (0·1 to 0·5)	0·8 (0·4 to 1·5)	3 (1·4 to 5·5)	3·2 (1·7 to 5·7)	2·2 (1·2 to 3·7)	1·5 (0·9 to 2·5)	1·2 (0·8 to 1·9)	0·9 (0·6 to 1·3)	1 (0·7 to 1·5)	0·7 (0·5 to 1)	0·6 (0·5 to 0·9)	0·5 (0·4 to 0·7)	0·5 (0·3 to 0·6)	0·3 (0·2 to 0·4)	0·3 (0·2 to 0·4)	0·3 (0·2 to 0·3)	0·1 (0·1 to 0·2)	0·1 (0 to 0·1)	0 (0 to 0)	17·9
Motor vehicle road injuries	0·1 (0 to 0·1)	0 (0 to 0·1)	0·7 (0·3 to 1·4)	1·8 (0·8 to 3·3)	2 (1·1 to 3·3)	5·5 (3·2 to 8·7)	11·2 (6·5 to 17·7)	11 (6·7 to 17·2)	7·5 (4·6 to 11·7)	6 (3·8 to 9·1)	4·3 (2·7 to 6·4)	3·7 (2·5 to 5·5)	4·1 (2·7 to 5·9)	2·9 (2·1 to 3·9)	2·5 (1·8 to 3·5)	2·3 (1·6 to 3·4)	2 (1·3 to 2·7)	1·4 (1 to 1·9)	1 (0·7 to 1·5)	0·7 (0·5 to 0·9)	0·4 (0·3 to 0·5)	0·2 (0·1 to 0·2)	0·1 (0 to 0·1)	71·2
Other road injuries	0 (0 to 0)	0 (0 to 0)	0 (0 to 0·1)	0·1 (0 to 0·2)	0·1 (0 to 0·2)	0·2 (0·1 to 0·3)	0·2 (0·1 to 0·4)	0·3 (0·1 to 0·4)	0·3 (0·2 to 0·5)	0·3 (0·2 to 0·4)	0·2 (0·1 to 0·3)	0·2 (0·1 to 0·3)	0·2 (0·2 to 0·3)	0·2 (0·2 to 0·3)	0·2 (0·1 to 0·3)	0·2 (0·1 to 0·3)	0·2 (0·1 to 0·2)	0·1 (0·1 to 0·1)	0·1 (0·1 to 0·1)	0·1 (0 to 0·1)	0 (0 to 0)	0 (0 to 0)	0 (0 to 0)	3·2
Other transport injuries	0 (0 to 0·1)	0 (0 to 0·1)	0·2 (0·1 to 0·5)	0·6 (0·2 to 1·1)	0·4 (0·2 to 0·8)	1·2 (0·7 to 2·1)	1·2 (0·6 to 2·4)	1·3 (0·7 to 2·3)	1 (0·5 to 1·8)	1 (0·5 to 1·8)	0·7 (0·4 to 1·2)	0·8 (0·4 to 1·3)	0·6 (0·4 to 1)	0·6 (0·4 to 0·9)	0·5 (0·3 to 0·8)	0·4 (0·3 to 0·7)	0·3 (0·2 to 0·4)	0·2 (0·2 to 0·4)	0·3 (0·2 to 0·5)	0·2 (0·1 to 0·3)	0·1 (0·1 to 0·2)	0·1 (0 to 0·1)	0 (0 to 0)	11·8
Unintentional injuries	1·3 (0·8 to 2·2)	1·1 (0·7 to 2·1)	8·4 (5 to 13·7)	16·5 (10·9 to 23·7)	9·7 (7·6 to 12·2)	17·5 (13·5 to 22·6)	15·3 (11·8 to 19·4)	15·4 (11·9 to 19·5)	14·6 (11·2 to 18·7)	15·8 (12·3 to 20·3)	13·8 (10·8 to 17·6)	12·6 (9·8 to 16·1)	15 (11·5 to 19·2)	15·4 (11·7 to 19·9)	14·7 (11·4 to 19·1)	14·3 (11·3 to 18·1)	12·9 (10·1 to 16·3)	11·7 (9·4 to 14·2)	12·4 (10·1 to 14·9)	12·9 (10·5 to 15·7)	9·8 (7·9 to 11·9)	5·3 (4·3 to 6·5)	2·2 (1·7 to 2·7)	269
Falls	0 (0 to 0)	0 (0 to 0)	0·1 (0·1 to 0·2)	0·4 (0·2 to 0·5)	0·6 (0·4 to 0·9)	1·1 (0·7 to 1·5)	1·4 (0·9 to 1·9)	1·5 (1 to 2·2)	1·8 (1·2 to 2·5)	2 (1·4 to 2·9)	2 (1·4 to 2·9)	2 (1·4 to 2·8)	2·6 (1·9 to 3·6)	3·3 (2·4 to 4·6)	3·5 (2·5 to 4·8)	3·5 (2·6 to 4·6)	3·4 (2·6 to 4·5)	3·4 (2·6 to 4·3)	4 (3 to 5·1)	5 (3·8 to 6·7)	4·3 (3·2 to 5·5)	2·3 (1·7 to 3)	0·9 (0·7 to 1·3)	49·3
Drowning	0·1 (0 to 0·2)	0·1 (0 to 0·1)	0·9 (0·4 to 1·8)	4·5 (2·1 to 8·4)	2·4 (1·3 to 4·2)	6 (3·5 to 9·7)	2·9 (1·6 to 4·7)	1·5 (0·9 to 2·5)	1·1 (0·6 to 1·8)	1 (0·6 to 1·6)	0·7 (0·4 to 1·1)	0·5 (0·3 to 0·8)	0·6 (0·4 to 0·9)	0·4 (0·2 to 0·5)	0·3 (0·2 to 0·5)	0·3 (0·2 to 0·5)	0·3 (0·2 to 0·4)	0·2 (0·1 to 0·3)	0·2 (0·1 to 0·3)	0·1 (0·1 to 0·2)	0·1 (0·1 to 0·2)	0·1 (0·1 to 0·1)	0 (0 to 0·1)	24·3
Fire, heat, and hot substances	0·1 (0 to 0·2)	0·1 (0·1 to 0·2)	1·5 (0·6 to 2·7)	4·4 (2·1 to 7·5)	2·5 (1·6 to 3·7)	3·7 (2·6 to 5·3)	4·3 (3 to 6)	4·7 (3·2 to 6·5)	4·7 (3·4 to 6·5)	5·1 (3·6 to 6·9)	4·5 (3·3 to 6)	3·8 (2·9 to 5·2)	4·2 (3·1 to 5·7)	4·2 (3 to 5·8)	3·8 (2·8 to 5·2)	3·3 (2·4 to 4·5)	2·7 (1·9 to 3·7)	2·2 (1·6 to 3·1)	2 (1·4 to 2·8)	1·8 (1·2 to 2·6)	1·4 (0·9 to 2·1)	0·7 (0·5 to 1·1)	0·3 (0·2 to 0·4)	66·2
Poisonings	0·1 (0 to 0·3)	0·2 (0·1 to 0·6)	0·9 (0·3 to 2·3)	2·6 (1·1 to 5·8)	0·7 (0·4 to 1·3)	0·9 (0·4 to 1·6)	1 (0·5 to 1·9)	1·1 (0·6 to 2)	0·8 (0·4 to 1·6)	0·8 (0·5 to 1·4)	0·7 (0·4 to 1·2)	0·6 (0·3 to 1·1)	0·6 (0·3 to 1)	0·4 (0·3 to 0·7)	0·4 (0·2 to 0·7)	0·4 (0·2 to 0·6)	0·4 (0·2 to 0·7)	0·3 (0·2 to 0·6)	0·3 (0·2 to 0·6)	0·3 (0·2 to 0·6)	0·2 (0·1 to 0·3)	0·1 (0 to 0·1)	0 (0 to 0)	14
Poisoning by carbon monoxide	0 (0 to 0·1)	0 (0 to 0·1)	0·1 (0 to 0·2)	0·4 (0·1 to 1·1)	0·1 (0 to 0·2)	0·1 (0·1 to 0·3)	0·3 (0·1 to 0·6)	0·4 (0·2 to 0·7)	0·2 (0·1 to 0·5)	0·3 (0·1 to 0·5)	0·2 (0·1 to 0·4)	0·2 (0·1 to 0·4)	0·2 (0·1 to 0·4)	0·1 (0·1 to 0·2)	0·1 (0·1 to 0·3)	0·1 (0·1 to 0·2)	0·2 (0·1 to 0·3)	0·1 (0·1 to 0·2)	0·1 (0 to 0·2)	0·1 (0 to 0·2)	0 (0 to 0·1)	0 (0 to 0)	0 (0 to 0)	3·4
Poisoning by other means	0·1 (0 to 0·2)	0·2 (0·1 to 0·5)	0·9 (0·3 to 2·2)	2·2 (0·9 to 4·8)	0·6 (0·3 to 1·1)	0·7 (0·3 to 1·4)	0·7 (0·4 to 1·4)	0·7 (0·4 to 1·3)	0·6 (0·3 to 1·1)	0·6 (0·3 to 1)	0·5 (0·3 to 0·9)	0·4 (0·2 to 0·7)	0·4 (0·2 to 0·7)	0·3 (0·2 to 0·6)	0·3 (0·2 to 0·4)	0·2 (0·1 to 0·4)	0·2 (0·1 to 0·4)	0·2 (0·1 to 0·4)	0·2 (0·1 to 0·4)	0·3 (0·1 to 0·5)	0·2 (0·1 to 0·3)	0·1 (0 to 0·1)	0 (0 to 0)	10·6
Exposure to mechanical forces	0·1 (0 to 0·3)	0·1 (0 to 0·7)	0·4 (0·1 to 1·5)	1·1 (0·5 to 2·4)	1·2 (0·8 to 1·8)	2·1 (1·4 to 3·3)	2·1 (1·4 to 3)	2·3 (1·5 to 3·2)	2·1 (1·4 to 3)	2·2 (1·5 to 3·1)	2 (1·4 to 2·8)	1·8 (1·3 to 2·6)	2·1 (1·4 to 2·9)	2·2 (1·6 to 3·1)	2 (1·4 to 2·8)	1·7 (1·2 to 2·4)	1·4 (1 to 2)	1·2 (0·8 to 1·6)	1·1 (0·8 to 1·4)	0·9 (0·7 to 1·3)	0·6 (0·5 to 0·9)	0·4 (0·3 to 0·5)	0·2 (0·1 to 0·2)	31·3
Unintentional firearm injuries	0 (0 to 0·1)	0 (0 to 0·1)	0 (0 to 0·2)	0·1 (0 to 0·2)	0·1 (0·1 to 0·2)	0·3 (0·2 to 0·6)	0·5 (0·2 to 0·9)	0·6 (0·3 to 1)	0·4 (0·2 to 0·7)	0·4 (0·3 to 0·7)	0·4 (0·2 to 0·5)	0·3 (0·2 to 0·4)	0·3 (0·2 to 0·4)	0·3 (0·2 to 0·4)	0·3 (0·2 to 0·4)	0·2 (0·2 to 0·3)	0·2 (0·1 to 0·2)	0·2 (0·1 to 0·2)	0·1 (0·1 to 0·2)	0·1 (0·1 to 0·2)	0·1 (0·1 to 0·1)	0·1 (0 to 0·1)	0 (0 to 0·1)	5
Other exposure to mechanical forces	0·1 (0 to 0·3)	0·1 (0 to 0·7)	0·3 (0·1 to 1·4)	1·1 (0·5 to 2·3)	1·1 (0·7 to 1·6)	1·8 (1·1 to 2·8)	1·6 (1·1 to 2·4)	1·7 (1·1 to 2·5)	1·7 (1·1 to 2·4)	1·8 (1·2 to 2·5)	1·6 (1·1 to 2·4)	1·5 (1·1 to 2·2)	1·7 (1·2 to 2·5)	1·9 (1·3 to 2·7)	1·7 (1·2 to 2·4)	1·5 (1·1 to 2·1)	1·3 (0·9 to 1·7)	1 (0·7 to 1·4)	0·9 (0·6 to 1·3)	0·8 (0·6 to 1·1)	0·5 (0·4 to 0·7)	0·3 (0·2 to 0·4)	0·1 (0·1 to 0·2)	26·3
Adverse effects of medical treatment	0·3 (0·2 to 0·6)	0·3 (0·1 to 0·5)	1·2 (0·6 to 2·3)	0·8 (0·5 to 1·3)	0·4 (0·2 to 0·6)	0·7 (0·4 to 1·2)	0·8 (0·4 to 1·3)	1·4 (0·8 to 2·2)	1·1 (0·7 to 1·8)	1·5 (0·8 to 2·6)	1 (0·6 to 1·7)	1·1 (0·7 to 1·8)	1·7 (1 to 2·8)	1·3 (0·8 to 2·1)	1·4 (0·9 to 2·3)	1·8 (1·1 to 2·8)	1·7 (1·1 to 2·5)	1·7 (1·1 to 2·5)	2 (1·3 to 3·1)	1·9 (1·2 to 2·9)	1·4 (0·9 to 2)	0·7 (0·4 to 0·9)	0·2 (0·1 to 0·3)	26·6
Animal contact	0 (0 to 0)	0 (0 to 0)	0·1 (0 to 0·1)	0·1 (0·1 to 0·2)	0·2 (0·1 to 0·2)	0·2 (0·1 to 0·3)	0·2 (0·1 to 0·3)	0·2 (0·2 to 0·3)	0·2 (0·2 to 0·3)	0·2 (0·2 to 0·4)	0·2 (0·1 to 0·3)	0·2 (0·1 to 0·3)	0·2 (0·2 to 0·3)	0·2 (0·2 to 0·3)	0·2 (0·2 to 0·3)	0·2 (0·2 to 0·3)	0·2 (0·1 to 0·3)	0·2 (0·1 to 0·3)	0·2 (0·1 to 0·3)	0·3 (0·1 to 0·6)	0·3 (0·1 to 0·5)	0·3 (0·1 to 0·5)	0·1 (0 to 0·2)	4·5
Venomous animal contact	0 (0 to 0)	0 (0 to 0)	0 (0 to 0·1)	0·1 (0 to 0·1)	0·1 (0 to 0·1)	0·2 (0·1 to 0·2)	0·1 (0·1 to 0·2)	0·2 (0·1 to 0·2)	0·2 (0·1 to 0·3)	0·2 (0·1 to 0·3)	0·1 (0·1 to 0·2)	0·1 (0·1 to 0·2)	0·1 (0·1 to 0·2)	0·1 (0·1 to 0·2)	0·1 (0·1 to 0·2)	0·1 (0·1 to 0·2)	0·1 (0·1 to 0·2)	0·1 (0·1 to 0·2)	0·1 (0·1 to 0·2)	0·2 (0·1 to 0·3)	0·2 (0·1 to 0·3)	0·2 (0 to 0·3)	0·1 (0 to 0·2)	2·8
Non-venomous animal contact	0 (0 to 0)	0 (0 to 0)	0 (0 to 0·1)	0·1 (0 to 0·2)	0·1 (0 to 0·1)	0·1 (0 to 0·1)	0·1 (0 to 0·1)	0·1 (0 to 0·1)	0·1 (0 to 0·1)	0·1 (0 to 0·1)	0·1 (0 to 0·1)	0·1 (0 to 0·1)	0·1 (0·1 to 0·2)	0·1 (0·1 to 0·1)	0·1 (0·1 to 0·1)	0·1 (0·1 to 0·1)	0·1 (0 to 0·1)	0·1 (0 to 0·1)	0·1 (0 to 0·2)	0·2 (0·1 to 0·3)	0·1 (0 to 0·2)	0·1 (0 to 0·2)	0·1 (0 to 0·1)	1·8
Foreign body	0·6 (0·2 to 1·1)	0·3 (0·1 to 0·5)	3·1 (1·3 to 6·2)	1·6 (0·9 to 2·7)	0·5 (0·4 to 0·8)	0·9 (0·6 to 1·3)	0·5 (0·3 to 0·7)	0·5 (0·4 to 0·7)	0·5 (0·3 to 0·7)	0·5 (0·4 to 0·7)	0·4 (0·3 to 0·6)	0·4 (0·3 to 0·6)	0·5 (0·4 to 0·7)	0·5 (0·4 to 0·6)	0·5 (0·4 to 0·6)	0·5 (0·4 to 0·7)	0·5 (0·4 to 0·7)	0·5 (0·4 to 0·7)	0·6 (0·4 to 0·9)	0·7 (0·5 to 1)	0·4 (0·2 to 0·6)	0·2 (0·1 to 0·3)	0·1 (0·1 to 0·1)	14·8
Pulmonary aspiration and foreign body in airway	0·6 (0·2 to 1·1)	0·3 (0·1 to 0·5)	3 (1·2 to 6·2)	1·4 (0·8 to 2·5)	0·4 (0·3 to 0·6)	0·7 (0·4 to 1·1)	0·2 (0·2 to 0·4)	0·3 (0·2 to 0·4)	0·3 (0·2 to 0·4)	0·2 (0·2 to 0·4)	0·2 (0·2 to 0·3)	0·2 (0·1 to 0·3)	0·3 (0·2 to 0·4)	0·2 (0·2 to 0·3)	0·2 (0·2 to 0·3)	0·3 (0·2 to 0·5)	0·4 (0·2 to 0·5)	0·4 (0·3 to 0·6)	0·5 (0·3 to 0·8)	0·5 (0·3 to 0·8)	0·3 (0·2 to 0·5)	0·2 (0·1 to 0·3)	0·1 (0 to 0·1)	11·3
Foreign body in eyes	0 (0 to 0)	0 (0 to 0)	0 (0 to 0)	0 (0 to 0·1)	0 (0 to 0·1)	0·1 (0 to 0·1)	0·1 (0 to 0·2)	0·1 (0 to 0·2)	0·1 (0 to 0·2)	0·1 (0 to 0·3)	0·1 (0 to 0·2)	0·1 (0 to 0·2)	0·1 (0·1 to 0·2)	0·1 (0·1 to 0·2)	0·1 (0·1 to 0·1)	0·1 (0 to 0·1)	0·1 (0 to 0·1)	0·1 (0 to 0·1)	0 (0 to 0·1)	0 (0 to 0·1)	0 (0 to 0)	0 (0 to 0)	0 (0 to 0)	1·4
Foreign body in other body part	0 (0 to 0)	0 (0 to 0)	0 (0 to 0·1)	0·1 (0·1 to 0·1)	0·1 (0·1 to 0·2)	0·1 (0·1 to 0·2)	0·1 (0·1 to 0·2)	0·1 (0·1 to 0·2)	0·1 (0·1 to 0·2)	0·1 (0·1 to 0·2)	0·1 (0·1 to 0·2)	0·1 (0·1 to 0·1)	0·1 (0·1 to 0·2)	0·1 (0·1 to 0·2)	0·1 (0·1 to 0·2)	0·1 (0·1 to 0·1)	0·1 (0·1 to 0·1)	0·1 (0·1 to 0·1)	0·1 (0·1 to 0·1)	0·1 (0·1 to 0·1)	0·1 (0 to 0·1)	0 (0 to 0·1)	0 (0 to 0)	2·1
Environmental heat and cold exposure	0 (0 to 0)	0 (0 to 0)	0·1 (0 to 0·1)	0·2 (0·1 to 0·3)	0·4 (0·2 to 0·6)	0·5 (0·4 to 0·8)	0·6 (0·4 to 0·9)	0·7 (0·4 to 0·9)	0·7 (0·5 to 1)	0·8 (0·6 to 1·2)	0·7 (0·5 to 1·1)	0·7 (0·5 to 1)	0·8 (0·6 to 1·1)	0·9 (0·6 to 1·2)	0·9 (0·6 to 1·2)	0·8 (0·6 to 1·1)	0·7 (0·5 to 1)	0·6 (0·4 to 0·8)	0·6 (0·4 to 0·8)	0·5 (0·4 to 0·7)	0·4 (0·3 to 0·5)	0·2 (0·1 to 0·4)	0·1 (0·1 to 0·2)	12
Exposure to forces of nature	0 (0 to 0)	0 (0 to 0)	0 (0 to 0)	0·1 (0·1 to 0·2)	0 (0 to 0·1)	0·1 (0·1 to 0·2)	0·2 (0·1 to 0·3)	0·2 (0·1 to 0·3)	0·2 (0·1 to 0·3)	0·2 (0·1 to 0·3)	0·2 (0·1 to 0·3)	0·2 (0·1 to 0·4)	0·3 (0·2 to 0·5)	0·4 (0·3 to 0·7)	0·5 (0·3 to 0·8)	0·5 (0·3 to 0·9)	0·6 (0·4 to 0·9)	0·6 (0·4 to 0·9)	0·6 (0·4 to 1)	0·6 (0·4 to 0·9)	0·5 (0·3 to 0·7)	0·3 (0·2 to 0·4)	0·1 (0·1 to 0·2)	6·6
Other unintentional injuries	0 (0 to 0·1)	0 (0 to 0)	0·2 (0·1 to 0·3)	0·6 (0·4 to 1)	0·8 (0·5 to 1·2)	1·3 (0·8 to 1·9)	1·2 (0·8 to 1·8)	1·3 (0·8 to 1·8)	1·3 (0·9 to 1·9)	1·4 (0·9 to 2)	1·2 (0·8 to 1·8)	1·1 (0·7 to 1·6)	1·3 (0·9 to 1·9)	1·4 (0·9 to 2)	1·3 (0·9 to 1·9)	1·1 (0·8 to 1·6)	1 (0·7 to 1·4)	0·8 (0·5 to 1·1)	0·7 (0·5 to 1)	0·5 (0·4 to 0·8)	0·3 (0·2 to 0·4)	0·1 (0·1 to 0·2)	0 (0 to 0·1)	19·1
Self-harm and interpersonal violence	0·7 (0·3 to 1·6)	0·1 (0 to 0·3)	1·1 (0·5 to 2·3)	2·1 (0·9 to 4·2)	1·4 (0·9 to 2·1)	4·9 (3·2 to 7·5)	14·5 (9·2 to 22)	19·6 (12·3 to 30·4)	15·4 (9·7 to 22·9)	13·9 (8·7 to 21·4)	8·7 (5·8 to 12·8)	6·5 (4·4 to 9·4)	6 (4·2 to 8·4)	3·7 (2·8 to 5)	2·7 (2·1 to 3·6)	2·3 (1·8 to 3)	1·9 (1·5 to 2·6)	1·3 (1 to 1·7)	1·1 (0·8 to 1·4)	0·7 (0·5 to 0·8)	0·4 (0·3 to 0·5)	0·1 (0·1 to 0·2)	0 (0 to 0·1)	109
Self-harm	0 (0 to 0)	0 (0 to 0)	0 (0 to 0)	0 (0 to 0)	0 (0 to 0)	0·8 (0·4 to 1·3)	3·2 (1·8 to 5·5)	3·9 (2·1 to 6·5)	3 (1·8 to 5)	3 (1·8 to 4·6)	2 (1·2 to 3)	1·5 (0·9 to 2·3)	1·6 (1 to 2·4)	1 (0·7 to 1·5)	0·8 (0·6 to 1·2)	0·8 (0·5 to 1·1)	0·7 (0·4 to 1)	0·4 (0·3 to 0·6)	0·3 (0·2 to 0·5)	0·2 (0·1 to 0·3)	0·1 (0·1 to 0·2)	0 (0 to 0·1)	0 (0 to 0)	23·5
Self-harm by firearm	0 (0 to 0)	0 (0 to 0)	0 (0 to 0)	0 (0 to 0)	0 (0 to 0)	0 (0 to 0)	0·1 (0 to 0·2)	0·1 (0 to 0·2)	0·1 (0 to 0·1)	0·1 (0 to 0·2)	0 (0 to 0·1)	0 (0 to 0)	0 (0 to 0·1)	0 (0 to 0)	0 (0 to 0)	0 (0 to 0)	0 (0 to 0)	0 (0 to 0)	0 (0 to 0)	0 (0 to 0)	0 (0 to 0)	0 (0 to 0)	0 (0 to 0)	0·6
Self-harm by other specified means	0 (0 to 0)	0 (0 to 0)	0 (0 to 0)	0 (0 to 0)	0 (0 to 0)	0·7 (0·4 to 1·2)	3·1 (1·8 to 5·4)	3·8 (2·1 to 6·4)	3 (1·7 to 4·9)	2·9 (1·8 to 4·5)	1·9 (1·2 to 3)	1·5 (0·9 to 2·3)	1·6 (1 to 2·3)	1 (0·7 to 1·4)	0·8 (0·5 to 1·2)	0·8 (0·5 to 1·1)	0·6 (0·4 to 1)	0·4 (0·3 to 0·6)	0·3 (0·2 to 0·5)	0·2 (0·1 to 0·3)	0·1 (0·1 to 0·2)	0 (0 to 0)	0 (0 to 0)	22·9
Interpersonal violence	0·7 (0·3 to 1·6)	0·1 (0 to 0·3)	1·1 (0·5 to 2·3)	2·1 (0·9 to 4·2)	1·4 (0·8 to 2·1)	4·2 (2·5 to 6·7)	11·2 (6·3 to 18·4)	15·6 (8·9 to 26·4)	12·3 (7·3 to 19·4)	10·9 (6·3 to 17·9)	6·5 (4 to 10·2)	4·8 (2·9 to 7·5)	4·2 (2·8 to 6·3)	2·5 (1·7 to 3·8)	1·8 (1·3 to 2·6)	1·4 (1·1 to 2)	1·2 (0·9 to 1·8)	0·9 (0·6 to 1·2)	0·7 (0·5 to 1)	0·4 (0·3 to 0·6)	0·3 (0·2 to 0·3)	0·1 (0·1 to 0·1)	0 (0 to 0)	84·5
Physical violence by firearm	0 (0 to 0)	0 (0 to 0)	0 (0 to 0·1)	0·2 (0·1 to 0·5)	0·2 (0·1 to 0·3)	0·8 (0·3 to 1·8)	3·3 (1·4 to 6·5)	4·3 (1·9 to 8·3)	3 (1·3 to 5·8)	2·5 (1·1 to 4·6)	1·4 (0·6 to 2·7)	0·8 (0·4 to 1·6)	0·8 (0·4 to 1·6)	0·4 (0·2 to 0·7)	0·3 (0·1 to 0·5)	0·2 (0·1 to 0·3)	0·1 (0·1 to 0·2)	0·1 (0 to 0·1)	0 (0 to 0·1)	0 (0 to 0)	0 (0 to 0)	0 (0 to 0)	0 (0 to 0)	18·5
Physical violence by sharp object	0 (0 to 0·1)	0 (0 to 0)	0·1 (0 to 0·1)	0·2 (0·1 to 0·4)	0·2 (0·1 to 0·3)	0·8 (0·4 to 1·6)	4 (1·8 to 7·5)	6·4 (3·1 to 12)	5·2 (2·6 to 9·2)	4·4 (2·2 to 8·2)	2·6 (1·3 to 4·6)	1·9 (1 to 3·4)	1·4 (0·7 to 2·3)	0·7 (0·4 to 1·1)	0·5 (0·3 to 0·7)	0·3 (0·2 to 0·5)	0·3 (0·2 to 0·4)	0·2 (0·1 to 0·3)	0·1 (0·1 to 0·2)	0·1 (0·1 to 0·1)	0·1 (0 to 0·1)	0 (0 to 0)	0 (0 to 0)	29·4
Sexual violence	0 (0 to 0)	0 (0 to 0)	0 (0 to 0)	0 (0 to 0)	0·2 (0·1 to 0·2)	0·4 (0·2 to 0·6)	0·9 (0·5 to 1·5)	1·2 (0·7 to 1·8)	1·1 (0·6 to 1·7)	1 (0·6 to 1·7)	0·8 (0·4 to 1·3)	0·6 (0·3 to 0·9)	0·5 (0·3 to 0·9)	0·4 (0·2 to 0·7)	0·3 (0·1 to 0·4)	0·2 (0·1 to 0·3)	0·2 (0·1 to 0·2)	0·1 (0·1 to 0·2)	0·1 (0·1 to 0·1)	0·1 (0 to 0·1)	0 (0 to 0·1)	0 (0 to 0)	0 (0 to 0)	8
Physical violence by other means	0·7 (0·2 to 1·5)	0·1 (0 to 0·3)	1 (0·4 to 2·1)	1·7 (0·7 to 3·4)	0·9 (0·5 to 1·4)	2·2 (1·2 to 3·8)	3 (1·4 to 5·5)	3·7 (1·8 to 7·1)	3 (1·5 to 5·5)	3 (1·5 to 5·3)	1·7 (0·9 to 2·8)	1·5 (0·8 to 2·5)	1·5 (0·9 to 2·4)	1 (0·7 to 1·7)	0·8 (0·5 to 1·2)	0·8 (0·5 to 1·1)	0·7 (0·5 to 1·1)	0·5 (0·3 to 0·7)	0·5 (0·3 to 0·7)	0·3 (0·2 to 0·4)	0·2 (0·1 to 0·2)	0·1 (0 to 0·1)	0 (0 to 0)	28·6
Conflict and terrorism	0 (0 to 0)	0 (0 to 0)	0 (0 to 0)	0 (0 to 0)	0 (0 to 0)	0 (0 to 0)	0 (0 to 0)	0 (0 to 0)	0 (0 to 0)	0 (0 to 0·1)	0·2 (0·1 to 0·4)	0·2 (0·1 to 0·4)	0·2 (0·1 to 0·3)	0·2 (0·1 to 0·3)	0·1 (0·1 to 0·2)	0·1 (0 to 0·1)	0 (0 to 0·1)	0 (0 to 0)	0 (0 to 0)	0 (0 to 0)	0 (0 to 0)	0 (0 to 0)	0 (0 to 0)	1·1

SR Table 2b: Dominican Republic DALYS by cause and age group for females with 95% uncertainty intervals, 2019

Cause	Early Neonatal	Late Neonatal	Post Neonatal	1 to 4	5 to 9	10 to 14	15 to 19	20 to 24	25 to 29	30 to 34	35 to 39	40 to 44	45 to 49	50 to 54	55 to 59	60 to 64	65 to 69	70 to 74	75 to 79	80 to 84	85 to 89	90 to 94	95 plus	Total
All causes	111,000 (80,300 to 150,000)	30,300 (22,000 to 40,900)	52,000 (39,200 to 68,500)	37,500 (30,400 to 45,400)	30,700 (25,000 to 37,800)	37,600 (29,800 to 47,200)	54,000 (42,900 to 67,700)	73,100 (59,100 to 89,000)	79,100 (63,300 to 96,800)	81,500 (66,100 to 99,500)	83,100 (67,900 to 101,000)	84,200 (68,900 to 102,000)	86,300 (70,600 to 104,000)	93,600 (76,800 to 113,000)	86,700 (71,300 to 105,000)	87,400 (72,400 to 105,000)	75,500 (63,600 to 89,700)	75,500 (64,100 to 88,600)	57,200 (50,000 to 66,500)	51,500 (46,100 to 58,300)	40,300 (36,000 to 45,200)	16,600 (14,800 to 18,500)	6,170 (5,580 to 6,680)	1,430,000
Communicable, maternal, neonatal, and nutritional diseases	99,100 (70,700 to 134,000)	24,900 (17,600 to 34,300)	31,800 (21,100 to 44,600)	17,900 (12,900 to 23,700)	9,490 (7,310 to 12,100)	7,980 (6,030 to 10,300)	9,750 (7,540 to 12,500)	14,200 (11,000 to 17,700)	15,300 (11,500 to 20,100)	15,100 (11,500 to 20,100)	13,600 (10,200 to 17,700)	11,600 (8,590 to 15,200)	9,410 (6,880 to 12,200)	8,010 (6,010 to 10,400)	6,190 (4,730 to 8,100)	4,980 (3,890 to 6,360)	3,770 (2,950 to 4,790)	3,630 (2,850 to 4,710)	2,920 (2,250 to 3,730)	2,710 (2,060 to 3,570)	2,650 (1,970 to 3,480)	1,220 (850 to 1,650)	502 (363 to 663)	317,000
HIV/AIDS and sexually transmitted infections	4,160 (1,300 to 9,140)	1,260 (391 to 2,810)	1,860 (709 to 3,910)	404 (170 to 954)	209 (142 to 289)	162 (99·3 to 242)	632 (306 to 1,250)	1,670 (781 to 3,400)	2,970 (1,270 to 6,470)	3,880 (1,810 to 7,890)	4,100 (1,970 to 7,540)	3,880 (1,960 to 6,590)	3,460 (1,620 to 5,960)	2,500 (1,220 to 4,130)	1,490 (731 to 2,500)	784 (383 to 1,360)	415 (211 to 718)	226 (116 to 397)	110 (58·2 to 190)	29·6 (16·4 to 48·3)	21·9 (12·6 to 35·4)	9 (5 to 15·1)	3·4 (1·8 to 5·7)	34,200
HIV/AIDS	0 (0 to 0)	0·1 (0 to 0·1)	280 (151 to 449)	341 (128 to 883)	173 (114 to 246)	134 (77·5 to 206)	553 (229 to 1,180)	1,480 (596 to 3,220)	2,740 (1,070 to 6,260)	3,620 (1,520 to 7,640)	3,860 (1,750 to 7,280)	3,660 (1,760 to 6,370)	3,300 (1,460 to 5,770)	2,390 (1,140 to 4,030)	1,420 (669 to 2,440)	738 (340 to 1,310)	377 (173 to 675)	196 (90·6 to 362)	91 (42·7 to 168)	20·3 (9·1 to 37·6)	10·2 (4·7 to 18·6)	3·9 (1·7 to 7·1)	1·7 (0·7 to 3·3)	25,400
HIV/AIDS - Drug-susceptible Tuberculosis	0 (0 to 0)	0 (0 to 0)	42·2 (26·4 to 66·7)	54·5 (34 to 83·6)	26·7 (17·5 to 40·8)	23 (15·2 to 34·7)	87·5 (57·6 to 133)	251 (162 to 381)	395 (256 to 594)	532 (343 to 808)	609 (387 to 930)	590 (385 to 895)	538 (343 to 815)	418 (270 to 641)	236 (152 to 356)	124 (79·1 to 192)	58·6 (36·9 to 88·9)	31·4 (19·7 to 48)	13·9 (9·5 to 20·3)	3·5 (2·4 to 5·1)	1·9 (1·2 to 2·7)	0·5 (0·4 to 0·8)	0·2 (0·1 to 0·2)	4,040
HIV/AIDS - Multidrug-resistant Tuberculosis without exten	0 (0 to 0)	0 (0 to 0)	0·6 (0 to 3·1)	0·8 (0·1 to 3·8)	0·4 (0 to 1·8)	0·3 (0 to 1·5)	1·2 (0·1 to 6)	3·5 (0·3 to 16·4)	5·3 (0·4 to 25)	7 (0·6 to 33·2)	8 (0·6 to 38·5)	7·8 (0·6 to 37·2)	7·2 (0·6 to 34·5)	5·6 (0·4 to 26·6)	3·2 (0·3 to 15)	1·7 (0·1 to 7·9)	0·8 (0·1 to 3·6)	0·4 (0 to 1·9)	0·2 (0 to 0·8)	0 (0 to 0·2)	0 (0 to 0·1)	0 (0 to 0)	0 (0 to 0)	54
HIV/AIDS - Extensively drug-resistant Tuberculosis	0 (0 to 0)	0 (0 to 0)	0·1 (0 to 0·5)	0·1 (0 to 0·7)	0·1 (0 to 0·3)	0·1 (0 to 0·2)	0·2 (0 to 1)	0·6 (0 to 2·7)	0·9 (0·1 to 4·2)	1·2 (0·1 to 5·7)	1·3 (0·1 to 6·4)	1·3 (0·1 to 6·2)	1·2 (0·1 to 5·8)	0·9 (0·1 to 4·5)	0·5 (0 to 2·5)	0·3 (0 to 1·3)	0·1 (0 to 0·6)	0·1 (0 to 0·3)	0 (0 to 0·1)	0 (0 to 0)	0 (0 to 0)	0 (0 to 0)	0 (0 to 0)	8·9
HIV/AIDS resulting in other diseases	0 (0 to 0)	0·1 (0 to 0·1)	237 (106 to 405)	286 (75·2 to 829)	146 (85·7 to 218)	111 (54·3 to 185)	464 (154 to 1,100)	1,230 (395 to 2,970)	2,340 (696 to 5,840)	3,080 (995 to 7,060)	3,240 (1,050 to 6,660)	3,060 (1,150 to 5,700)	2,750 (962 to 5,190)	1,970 (719 to 3,650)	1,180 (437 to 2,230)	611 (232 to 1,200)	318 (120 to 617)	165 (59·9 to 332)	76·9 (29·3 to 152)	16·7 (6·2 to 34)	8·3 (3·2 to 16·6)	3·3 (1·2 to 6·6)	1·5 (0·5 to 3·1)	21,300
Sexually transmitted infections excluding HIV	4,160 (1,300 to 9,140)	1,260 (391 to 2,810)	1,580 (447 to 3,670)	62·4 (17·6 to 151)	35·8 (11·3 to 77·7)	27·5 (9·8 to 70)	79 (39·9 to 148)	183 (97 to 323)	232 (127 to 399)	262 (143 to 456)	242 (128 to 430)	219 (119 to 408)	159 (82 to 294)	108 (52·9 to 202)	68·2 (31·5 to 136)	45·7 (20·4 to 91·7)	37·5 (18·2 to 71·9)	29·6 (14 to 56·6)	18·8 (9·2 to 35·2)	9·3 (4·4 to 17)	11·6 (5·1 to 23·3)	5·2 (2 to 10·1)	1·7 (0·5 to 3·3)	8,830
Syphilis	4,160 (1,300 to 9,140)	1,260 (391 to 2,810)	1,580 (447 to 3,670)	62·4 (17·6 to 151)	35·8 (11·3 to 77·7)	12·6 (3·2 to 36·8)	11·1 (3·9 to 25·2)	8·7 (3·8 to 18·3)	13·2 (5·3 to 28·7)	15·3 (6·2 to 30·9)	7·7 (3·4 to 15·3)	2·9 (1·5 to 5·5)	4 (1·9 to 8·1)	3·5 (1·5 to 7)	1 (0·5 to 1·8)	1·1 (0·6 to 2·3)	1·4 (0·7 to 3)	1·8 (0·8 to 3·8)	1·1 (0·5 to 2·2)	0·8 (0·4 to 1·6)	1·8 (0·6 to 4·1)	1·4 (0·4 to 2·9)	0·5 (0·1 to 1)	7,180
Chlamydial infection	0 (0 to 0)	0 (0 to 0)	0 (0 to 0)	0 (0 to 0)	0 (0 to 0)	2·7 (1 to 6·8)	8·2 (4·3 to 15·1)	27·1 (13·5 to 53)	23·4 (11·5 to 39·2)	22·4 (11·4 to 39)	24·9 (13·6 to 42·1)	23·9 (12·8 to 41·4)	13·6 (7·5 to 24·5)	5·9 (2·8 to 11·1)	2·9 (1·4 to 6)	1·8 (0·4 to 5·1)	1·3 (0·4 to 3·3)	1·8 (0·6 to 4·1)	1·2 (0·4 to 2·9)	0·5 (0·2 to 1·2)	1·4 (0·4 to 3·4)	0·6 (0·2 to 1·4)	0·3 (0·1 to 0·6)	164

Non-rheumatic degenerative mitral valve disease	0 (0 to 0)	[illegible]	343
Other non-rheumatic valve diseases	0 (0 to 0)	[illegible]	4·2
Cardiomyopathy and myocarditis	40·2 (12·3 to 102)	[illegible]	2,840
Myocarditis	2·9 (0·6 to 12·4)	[illegible]	236
Alcoholic cardiomyopathy	0 (0 to 0)	[illegible]	54·9
Other cardiomyopathy	37·3 (10·9 to 98)	[illegible]	2,550
Atrial fibrillation and flutter	0 (0 to 0)	[illegible]	3,950
Aortic aneurysm	--	[illegible]	NA
Endocarditis	8·2 (2·3 to 20·7)	[illegible]	1,430
Other cardiovascular and circulatory diseases	88·6 (34 to 181)	[illegible]	8,290
Chronic respiratory diseases	0 (0 to 0)	[illegible]	31,500
Chronic obstructive pulmonary disease	0 (0 to 0)	[illegible]	13,200
Pneumoconiosis	0 (0 to 0)	[illegible]	8·4
Silicosis	0 (0 to 0)	[illegible]	0·9
Asbestosis	0 (0 to 0)	[illegible]	1·7
Coal workers pneumoconiosis	0 (0 to 0)	[illegible]	0·7
Other pneumoconiosis	0 (0 to 0)	[illegible]	5·1
Asthma	0 (0 to 0)	[illegible]	16,200
Interstitial lung disease and pulmonary sarcoidosis	0 (0 to 0)	[illegible]	884
Other chronic respiratory diseases	0 (0 to 0)	[illegible]	1,260
Digestive diseases	172 (77·5 to 359)	[illegible]	53,500
Cirrhosis and other chronic liver diseases	0 (0 to 0)	[illegible]	28,000
Cirrhosis and other chronic liver diseases due to hepatitis	0 (0 to 0)	[illegible]	1,720
Cirrhosis and other chronic liver diseases due to hepatitis	0 (0 to 0)	[illegible]	4,870
Cirrhosis and other chronic liver diseases due to alcohol u	0 (0 to 0)	[illegible]	8,140
Cirrhosis and other chronic liver diseases due to NAFLD	0 (0 to 0)	[illegible]	5,620
Cirrhosis and other chronic liver diseases due to other ca	0 (0 to 0)	[illegible]	7,670
Upper digestive system diseases	0 (0 to 0)	[illegible]	11,100
Peptic ulcer disease	0 (0 to 0)	[illegible]	2,440
Gastritis and duodenitis	0 (0 to 0)	[illegible]	1,890
Gastroesophageal reflux disease	0 (0 to 0)	[illegible]	6,780
Appendicitis	0 (0 to 0)	[illegible]	2,050
Paralytic ileus and intestinal obstruction	111 (40·3 to 248)	[illegible]	3,110
Inguinal, femoral, and abdominal hernia	61 (17 to 140)	[illegible]	1,910
Inflammatory bowel disease	0 (0 to 0)	[illegible]	549
Vascular intestinal disorders	0 (0 to 0)	[illegible]	637
Gallbladder and biliary diseases	0 (0 to 0)	[illegible]	3,770
Pancreatitis	0 (0 to 0)	[illegible]	979
Other digestive diseases	0 (0 to 0)	[illegible]	1,360
Neurological disorders	1·8 (0·5 to 4)	[illegible]	72,400
Alzheimer's disease and other dementias	0 (0 to 0)	[illegible]	16,400
Parkinson's disease	0 (0 to 0)	[illegible]	2,740
Idiopathic epilepsy	1·5 (0·3 to 3·6)	[illegible]	10,400
Multiple sclerosis	0 (0 to 0)	[illegible]	441
Motor neuron disease	0·3 (0·1 to 0·8)	[illegible]	456
Headache disorders	0 (0 to 0)	[illegible]	39,200
Migraine	0 (0 to 0)	[illegible]	36,000
Tension-type headache	0 (0 to 0)	[illegible]	3,250
Other neurological disorders	0 (0 to 0)	[illegible]	2,670
Mental disorders	0·9 (0·5 to 1·4)	[illegible]	113,000
Schizophrenia	0 (0 to 0)	[illegible]	9,350
Depressive disorders	0 (0 to 0)	[illegible]	47,900
Major depressive disorder	0 (0 to 0)	[illegible]	42,600
Dysthymia	0 (0 to 0)	[illegible]	5,340
Bipolar disorder	0 (0 to 0)	[illegible]	11,700
Anxiety disorders	0 (0 to 0)	[illegible]	29,600
Eating disorders	0 (0 to 0)	[illegible]	4,030
Anorexia nervosa	0 (0 to 0)	[illegible]	1,020
Bulimia nervosa	0 (0 to 0)	[illegible]	3,010
Autism spectrum disorders	0·7 (0·4 to 1·1)	[illegible]	1,400
Attention-deficit/hyperactivity disorder	0 (0 to 0)	[illegible]	1,110
Conduct disorder	0 (0 to 0)	[illegible]	2,620
Idiopathic developmental intellectual disability	0·2 (0 to 0·4)	[illegible]	727
Other mental disorders	0 (0 to 0)	[illegible]	4,760
Substance use disorders	0 (0 to 0)	[illegible]	8,770
Alcohol use disorders	0 (0 to 0)	[illegible]	4,950
Drug use disorders	0 (0 to 0)	[illegible]	3,820
Opioid use disorders	0 (0 to 0)	[illegible]	1,950
Cocaine use disorders	0 (0 to 0)	[illegible]	458
Amphetamine use disorders	0 (0 to 0)	[illegible]	244
Cannabis use disorders	0 (0 to 0)	[illegible]	415
Other drug use disorders	0 (0 to 0)	[illegible]	747
Diabetes and kidney diseases	0·8 (0·4 to 1·6)	[illegible]	89,300
Diabetes mellitus	0·8 (0·4 to 1·6)	[illegible]	54,400
Diabetes mellitus type 1	0·8 (0·4 to 1·6)	[illegible]	3,960
Diabetes mellitus type 2	0 (0 to 0)	[illegible]	50,500
Chronic kidney disease	0 (0 to 0)	[illegible]	34,900
Chronic kidney disease due to diabetes mellitus type 1	0 (0 to 0)	[illegible]	2,570
Chronic kidney disease due to diabetes mellitus type 2	0 (0 to 0)	[illegible]	6,580
Chronic kidney disease due to hypertension	0 (0 to 0)	[illegible]	6,270
Chronic kidney disease due to glomerulonephritis	0 (0 to 0)	[illegible]	8,170
Chronic kidney disease due to other and unspecified caus	0 (0 to 0)	[illegible]	11,300
Acute glomerulonephritis	0 (0 to 0)	[illegible]	2·4
Skin and subcutaneous diseases	0·2 (0·1 to 0·3)	[illegible]	28,400
Dermatitis	0 (0 to 0)	[illegible]	6,810
Atopic dermatitis	0 (0 to 0)	[illegible]	5,090
Contact dermatitis	0 (0 to 0)	[illegible]	1,540
Seborrhoeic dermatitis	0 (0 to 0)	[illegible]	178
Psoriasis	0 (0 to 0)	[illegible]	1,970
Bacterial skin diseases	0 (0 to 0)	[illegible]	731
Cellulitis	0 (0 to 0)	[illegible]	84·4
Pyoderma	0 (0 to 0)	[illegible]	646
Scabies	0·1 (0 to 0·1)	[illegible]	4,580
Fungal skin diseases	0 (0 to 0)	[illegible]	2,210
Viral skin diseases	0 (0 to 0)	[illegible]	2,520
Acne vulgaris	0 (0 to 0)	[illegible]	2,850
Alopecia areata	0 (0 to 0)	[illegible]	513
Pruritus	0 (0 to 0)	[illegible]	510
Urticaria	0·1 (0 to 0·1)	[illegible]	2,990
Decubitus ulcer	0 (0 to 0)	[illegible]	217
Other skin and subcutaneous diseases	0 (0 to 0)	[illegible]	2,500
Sense organ diseases	0·4 (0·2 to 0·7)	[illegible]	39,500
Blindness and vision loss	0·2 (0·1 to 0·4)	[illegible]	12,200
Glaucoma	0 (0 to 0)	[illegible]	583
Cataract	0 (0 to 0)	[illegible]	1,560
Age-related macular degeneration	0 (0 to 0)	[illegible]	92·6
Refraction disorders	0 (0 to 0)	[illegible]	4,580
Near vision loss	0 (0 to 0)	[illegible]	2,250
Other vision loss	0·2 (0·1 to 0·4)	[illegible]	3,110
Age-related and other hearing loss	0 (0 to 0·1)	[illegible]	25,300
Other sense organ diseases	0·2 (0·1 to 0·4)	[illegible]	1,980
Musculoskeletal disorders	0 (0 to 0)	[illegible]	86,800
Rheumatoid arthritis	0 (0 to 0)	[illegible]	1,730
Osteoarthritis	0 (0 to 0)	[illegible]	11,200
Osteoarthritis hip	0 (0 to 0)	[illegible]	485
Osteoarthritis knee	0 (0 to 0)	[illegible]	8,090
Osteoarthritis hand	0 (0 to 0)	[illegible]	1,550
Osteoarthritis other	0 (0 to 0)	[illegible]	1,060
Low back pain	0 (0 to 0)	[illegible]	40,600
Neck pain	0 (0 to 0)	[illegible]	7,490
Gout	0 (0 to 0)	[illegible]	275
Other musculoskeletal disorders	0 (0 to 0)	[illegible]	25,500
Other non-communicable diseases	10,000 (5,020 to 19,900)	[illegible]	125,000
Congenital birth defects	9,780 (4,890 to 19,500)	[illegible]	31,700
Neural tube defects	662 (194 to 1,750)	[illegible]	1,390
Congenital heart anomalies	4,020 (1,810 to 7,990)	[illegible]	13,200
Orofacial clefts	9·6 (3·5 to 20·8)	[illegible]	167
Down syndrome	299 (93·7 to 754)	[illegible]	2,220
Turner syndrome	0 (0 to 0)	[illegible]	14·9
Klinefelter syndrome	0 (0 to 0)	[illegible]	0
Other chromosomal abnormalities	126 (48·6 to 265)	[illegible]	757
Congenital musculoskeletal and limb anomalies	86·9 (38·4 to 176)	[illegible]	2,950
Urogenital congenital anomalies	143 (21 to 337)	[illegible]	362
Digestive congenital anomalies	2,160 (889 to 4,310)	[illegible]	4,540
Other congenital birth defects	2,280 (726 to 4,770)	[illegible]	6,100
Urinary diseases and male infertility	1·2 (0·5 to 2·7)	[illegible]	1,510
Urolithiasis	0 (0 to 0)	[illegible]	128
Benign prostatic hyperplasia	0 (0 to 0)	[illegible]	0
Male infertility	--	[illegible]	NA
Other urinary diseases	0·5 (0·2 to 1)	[illegible]	218
Gynecological diseases	0 (0 to 0)	[illegible]	39,900
Uterine fibroids	0 (0 to 0)	[illegible]	2,640
Polycystic ovarian syndrome	0 (0 to 0)	[illegible]	766
Female infertility	0 (0 to 0)	[illegible]	732
Endometriosis	0 (0 to 0)	[illegible]	3,440
Genital prolapse	0 (0 to 0)	[illegible]	742
Premenstrual syndrome	0 (0 to 0)	[illegible]	11,100
Other gynecological diseases	0 (0 to 0)	[illegible]	20,400
Hemoglobinopathies and hemolytic anemias	47·9 (24·7 to 90·1)	[illegible]	9,200
Thalassemias	0 (0 to 0·1)	[illegible]	8·4
Thalassemias trait	0 (0 to 0·1)	[illegible]	699
Sickle cell disorders	47·7 (24·3 to 89·1)	[illegible]	3,500
Sickle cell trait	0·1 (0 to 0·4)	[illegible]	3,400

G6PD deficiency	0 (0 to 0)	0·1 (0 to 0·8)	0·1 (0·1 to 0·3)	1·3 (0·5 to 2·8)	2·6 (1 to 5·1)	2·4 (1 to 4·8)	5·6 (2·7 to 9·9)	11·2 (4·5 to 22·9)	16·4 (6 to 34·7)	15·7 (6·3 to 31·1)	79·4 (38 to 134)	22·7 (12·1 to 38·7)	12·9 (5·4 to 26·2)	12·8 (4·6 to 26·9)	9·2 (3·8 to 17·6)	7·4 (3·2 to 14·2)	3·7 (1·5 to 7)	1·6 (0·7 to 2·9)	0·7 (0·3 to 1·3)	0·3 (0·1 to 0·5)	0·3 (0·1 to 0·5)	0·1 (0 to 0·1)	0 (0 to 0)	206
G6PD trait	0 (0 to 0)	0 (0 to 0·3)	0 (0 to 0)	0 (0 to 0)	0 (0 to 0)	0 (0 to 0)	0·1 (0·1 to 0·3)	0·1 (0·1 to 0·2)	0·1 (0·1 to 0·2)	0·2 (0·1 to 0·3)	9 (3·4 to 16·7)	8·2 (3·1 to 15·6)	1·2 (0·5 to 2·1)	0 (0 to 0)	0 (0 to 0)	0 (0 to 0)	0 (0 to 0)	0 (0 to 0)	0 (0 to 0)	0 (0 to 0)	0 (0 to 0)	0 (0 to 0)	0 (0 to 0)	19·1
Other hemoglobinopathies and hemolytic anemias	0·1 (0 to 0·1)	1·9 (0·7 to 3·8)	20·1 (8·2 to 37·8)	43·5 (13 to 92·2)	44·2 (10·8 to 105)	46·6 (13·8 to 105)	49·5 (17·5 to 99)	55·8 (22·2 to 106)	64·7 (30·5 to 116)	77·5 (31·8 to 139)	128 (62 to 223)	124 (59·1 to 215)	93·4 (47 to 165)	102 (51·7 to 177)	97·9 (55·5 to 156)	135 (71·6 to 235)	75·9 (41·6 to 130)	60·4 (33·8 to 102)	41·3 (22·5 to 73·6)	33·3 (18·8 to 54·5)	42·9 (26·6 to 62·4)	19·6 (12·6 to 28·3)	5·8 (3·8 to 8·3)	1,360
Endocrine, metabolic, blood, and immune disorders	202 (54·4 to 459)	254 (83·8 to 563)	2,900 (902 to 6.100)	1,320 (570 to 2.850)	690 (365 to 1.100)	515 (301 to 767)	565 (346 to 855)	738 (433 to 1.160)	883 (525 to 1.410)	1,020 (631 to 1.560)	1,270 (808 to 1.910)	1,410 (875 to 2.080)	1,490 (940 to 2.250)	1,670 (1.050 to 2.520)	1,640 (1.030 to 2.520)	1,420 (893 to 2.210)	1,140 (710 to 1.780)	971 (613 to 1.480)	665 (413 to 997)	494 (310 to 741)	404 (215 to 659)	157 (75·1 to 256)	48·7 (21·3 to 85·9)	21.900
Oral disorders	0 (0 to 0)	0 (0 to 0)	0 (0 to 0)	274 (153 to 460)	300 (159 to 529)	302 (162 to 532)	453 (246 to 775)	749 (394 to 1,370)	1,050 (526 to 1,980)	1,210 (597 to 2,220)	1,290 (631 to 2,420)	1,360 (666 to 2,410)	1,480 (794 to 2,550)	1,690 (958 to 2,840)	1,860 (1,100 to 2,910)	1,890 (1,170 to 2,930)	1,780 (1,100 to 2,670)	1,530 (963 to 2,280)	1,110 (701 to 1,630)	754 (479 to 1,100)	403 (256 to 591)	140 (87·7 to 205)	44·3 (27·9 to 65·1)	19,700
Caries of deciduous teeth	0 (0 to 0)	0 (0 to 0)	0 (0 to 0)	85·7 (32·4 to 178)	73·1 (29·7 to 161)	3·7 (0·5 to 10·2)	0 (0 to 0)	0 (0 to 0)	0 (0 to 0)	0 (0 to 0)	0 (0 to 0)	0 (0 to 0)	0 (0 to 0)	0 (0 to 0)	0 (0 to 0)	0 (0 to 0)	0 (0 to 0)	0 (0 to 0)	0 (0 to 0)	0 (0 to 0)	0 (0 to 0)	0 (0 to 0)	0 (0 to 0)	163
Caries of permanent teeth	0 (0 to 0)	0 (0 to 0)	0 (0 to 0)	0 (0 to 0)	71 (25·4 to 165)	139 (54·9 to 312)	152 (59·7 to 334)	177 (74·4 to 377)	168 (67·2 to 345)	140 (56·2 to 294)	125 (49·5 to 255)	106 (42·9 to 221)	90·5 (37·3 to 191)	78·6 (30·7 to 170)	60·8 (22·6 to 138)	43·3 (15·5 to 99·9)	29·8 (11·4 to 62·5)	21·1 (7·9 to 43·7)	13·8 (5·6 to 27·4)	8·9 (3·4 to 18·7)	4·5 (1·7 to 9·7)	1·4 (0·5 to 3·2)	0·4 (0·1 to 0·8)	1.430
Periodontal diseases	0 (0 to 0)	0 (0 to 0)	0 (0 to 0)	0 (0 to 0)	0 (0 to 0)	0 (0 to 0)	59·1 (17·5 to 149)	225 (68·5 to 564)	477 (150 to 1.120)	652 (226 to 1.490)	737 (268 to 1.630)	763 (278 to 1.640)	724 (281 to 1.530)	629 (248 to 1.340)	480 (192 to 1.020)	330 (129 to 670)	222 (86·8 to 456)	150 (58·5 to 304)	96 (37·4 to 195)	62·8 (24·3 to 129)	33·8 (12·8 to 69·5)	11·8 (4·2 to 25)	3·6 (1·3 to 7·6)	5.660
Other oral disorders	0 (0 to 0)	0 (0 to 0)	0 (0 to 0)	188 (109 to 296)	156 (88·7 to 247)	159 (94·5 to 250)	242 (144 to 375)	300 (182 to 468)	312 (187 to 471)	293 (180 to 447)	265 (161 to 401)	243 (141 to 371)	222 (134 to 335)	196 (120 to 293)	162 (97·4 to 245)	127 (77 to 192)	98·3 (60·1 to 148)	68·4 (40·6 to 104)	38·4 (22·9 to 59·1)	20·5 (12·7 to 31·3)	8·8 (5·4 to 13·2)	2·7 (1·6 to 4)	0·9 (0·5 to 1·4)	3,100
Sudden infant death syndrome	--	162 (77·2 to 319)	901 (314 to 1.960)	--	--	--	--	--	--	--	--	--	--	--	--	--	--	--	--	--	--	--	--	NA
Injuries	824 (467 to 1.420)	374 (222 to 634)	3,070 (1.630 to 5.030)	3,680 (2.260 to 5.630)	4,130 (3.290 to 4.960)	4,530 (3.680 to 5.440)	6,890 (5.470 to 8.560)	10,100 (7.660 to 13.100)	9,200 (6.690 to 12.500)	7,730 (5.830 to 10.200)	6,730 (5.100 to 8.800)	5,560 (4.290 to 7.220)	4,810 (3.790 to 6.070)	4,340 (3.410 to 5.500)	3,520 (2.810 to 4.410)	3,190 (2.560 to 3.990)	2,550 (2.070 to 3.150)	2,320 (1.870 to 2.830)	1,690 (1.390 to 2.070)	1,550 (1.250 to 1.870)	1,290 (1.010 to 1.670)	560 (433 to 719)	199 (155 to 251)	88.800
Transport injuries	50 (16·1 to 113)	23·6 (9·2 to 49·7)	209 (84·5 to 425)	521 (225 to 1,070)	1,130 (697 to 1,720)	1,230 (845 to 1,750)	2,100 (1,360 to 3,080)	3,080 (2,060 to 4,520)	2,670 (1,680 to 4,010)	2,040 (1,370 to 3,020)	1,930 (1,320 to 2,820)	1,610 (1,160 to 2,200)	1,430 (1,040 to 1,900)	1,290 (959 to 1,790)	1,080 (821 to 1,460)	990 (734 to 1,350)	793 (601 to 1,060)	672 (500 to 885)	411 (313 to 543)	277 (215 to 349)	169 (130 to 218)	56·9 (43·8 to 73·5)	18·9 (14·3 to 24·8)	23,800
Road injuries	47·3 (15·5 to 109)	21·3 (8·4 to 45·7)	198 (77·5 to 405)	494 (206 to 1.020)	1,090 (669 to 1.680)	1,180 (812 to 1.700)	2,040 (1.320 to 3.020)	3,010 (2.000 to 4.410)	2,590 (1.630 to 3.900)	1,980 (1.330 to 2.930)	1,870 (1.280 to 2.740)	1,560 (1.120 to 2.130)	1,380 (1.010 to 1.850)	1,260 (926 to 1.750)	1,050 (796 to 1.430)	963 (708 to 1.320)	774 (584 to 1.040)	656 (485 to 867)	399 (304 to 528)	269 (210 to 339)	164 (126 to 213)	55·1 (42·4 to 71·4)	18·2 (13·6 to 23·9)	23.100
Pedestrian road injuries	14·1 (4·1 to 33)	5·8 (1·9 to 13·9)	27·3 (7·7 to 69·2)	99·9 (26·3 to 233)	177 (78 to 333)	155 (83·1 to 262)	135 (75·2 to 242)	220 (123 to 374)	216 (121 to 383)	207 (125 to 340)	212 (128 to 354)	171 (111 to 266)	179 (118 to 270)	204 (128 to 318)	177 (116 to 261)	181 (115 to 277)	163 (108 to 247)	151 (98·2 to 230)	98·5 (65·7 to 144)	67·3 (47·3 to 93·7)	45·9 (32 to 65·4)	14·1 (10 to 19·5)	5·2 (3·6 to 7·3)	2.930
Cyclist road injuries	0 (0 to 0)	0 (0 to 0)	0 (0 to 0)	10·8 (4·5 to 23·2)	23·7 (14·9 to 36·7)	39·7 (26·1 to 56·3)	61·2 (42·2 to 84·2)	91·7 (64·2 to 124)	107 (73·2 to 148)	105 (75·2 to 142)	110 (77·3 to 150)	109 (76·2 to 148)	109 (76·2 to 148)	105 (74·6 to 142)	89·6 (63·7 to 122)	74·6 (53·6 to 101)	60·9 (43·5 to 81·9)	48·4 (34·6 to 63·9)	32·6 (23 to 43·5)	22·4 (15·9 to 29·8)	13·2 (9·4 to 17·6)	5 (3·6 to 6·7)	1·7 (1·2 to 2·3)	1,220
Motorcyclist road injuries	2·9 (0·5 to 7·7)	5·2 (1·2 to 13·3)	10 (2·6 to 26·5)	15 (6·2 to 32·6)	41·5 (22·1 to 76·2)	63·9 (37·4 to 105)	161 (90·5 to 283)	245 (146 to 404)	239 (141 to 401)	186 (118 to 293)	190 (122 to 298)	160 (108 to 234)	148 (103 to 208)	119 (86·8 to 161)	103 (75·8 to 136)	91·6 (67 to 126)	77·9 (56·8 to 105)	59·6 (42·8 to 80·1)	43·8 (30·9 to 58·9)	36·2 (26·3 to 49·1)	20·2 (14·3 to 27·6)	7·9 (5·6 to 11)	2·9 (2 to 4)	2.030
Motor vehicle road injuries	30·1 (9·3 to 74·4)	10·3 (3·2 to 23·8)	160 (63·3 to 311)	364 (148 to 756)	839 (490 to 1.300)	916 (615 to 1.350)	1,670 (1.040 to 2.500)	2,430 (1.500 to 3.680)	2,010 (1.210 to 3.100)	1,460 (924 to 2.210)	1,330 (864 to 2.050)	1,090 (726 to 1.590)	924 (643 to 1.290)	804 (565 to 1.160)	662 (477 to 952)	600 (415 to 861)	459 (337 to 637)	387 (269 to 551)	217 (159 to 299)	138 (102 to 182)	82·1 (59·4 to 115)	27·2 (19·7 to 38)	8·1 (5·5 to 11·3)	16.600
Other road injuries	0·3 (0·1 to 0·8)	0·1 (0 to 0·3)	1·1 (0·3 to 2·8)	3·6 (1·4 to 7·5)	8·7 (3·9 to 19·7)	7·5 (5·1 to 11·2)	12·8 (7·8 to 19·8)	20·4 (13 to 30·3)	24·5 (15·9 to 35·9)	23·6 (16·2 to 32·8)	24·3 (16·8 to 33·7)	24·3 (16·8 to 33·3)	24·4 (16·8 to 33·8)	23·8 (16·5 to 32·7)	20·4 (13·9 to 28·1)	16·4 (11·4 to 22·7)	13·5 (9·4 to 18·5)	10·3 (7·2 to 14)	7·3 (5·2 to 10·1)	5·2 (3·6 to 7)	2·6 (1·8 to 3·6)	0·8 (0·6 to 1·2)	0·3 (0·2 to 0·4)	276
Other transport injuries	2·6 (0·7 to 6·8)	2·2 (0·7 to 5·8)	11·1 (4·7 to 24)	27·6 (14·5 to 52·6)	38·1 (23·4 to 61·8)	42·2 (28·7 to 62·1)	53 (34·8 to 81)	75·7 (49·3 to 118)	76·9 (47 to 128)	66·5 (40·9 to 108)	55·7 (36 to 91)	52·7 (35·1 to 79·4)	42·6 (30 to 60)	39·2 (27·3 to 56·7)	31·4 (23 to 42·2)	26·9 (19·5 to 36·6)	19·2 (13·9 to 25·4)	15·6 (11·3 to 20·7)	12 (8·8 to 15·7)	7·8 (5·7 to 10)	4·7 (3·5 to 6)	1·9 (1·4 to 2·4)	0·7 (0·5 to 1)	706
Unintentional injuries	431 (243 to 760)	289 (167 to 505)	2,410 (1.230 to 4.230)	2,540 (1.530 to 3.970)	2,400 (1.890 to 2.970)	2,290 (1.820 to 2.850)	2,050 (1.590 to 2.610)	2,600 (2.010 to 3.290)	2,600 (1.960 to 3.300)	2,490 (1.920 to 3.190)	2,360 (1.820 to 3.030)	2,210 (1.700 to 2.810)	2,100 (1.630 to 2.690)	2,070 (1.590 to 2.660)	1,810 (1.390 to 2.320)	1,720 (1.360 to 2.190)	1,410 (1.120 to 1.770)	1,400 (1.110 to 1.760)	1,140 (918 to 1.400)	1,180 (942 to 1.470)	1,060 (815 to 1.420)	486 (370 to 641)	175 (133 to 226)	39.200
Falls	12·3 (3·9 to 31·6)	5·3 (2 to 12)	72·8 (29·1 to 159)	142 (69 to 277)	184 (127 to 260)	221 (157 to 309)	269 (180 to 380)	337 (236 to 468)	370 (257 to 511)	372 (264 to 519)	382 (271 to 527)	391 (279 to 532)	413 (302 to 559)	460 (336 to 612)	448 (331 to 592)	474 (359 to 621)	446 (339 to 574)	527 (396 to 694)	498 (382 to 646)	649 (483 to 857)	686 (492 to 989)	333 (239 to 471)	118 (84·5 to 164)	7,810
Drowning	24·7 (11·7 to 47·9)	15·8 (6·5 to 34·8)	143 (52·7 to 333)	439 (177 to 921)	436 (234 to 772)	449 (248 to 747)	227 (127 to 384)	210 (115 to 356)	193 (104 to 342)	155 (86·6 to 261)	131 (73·5 to 228)	91·7 (54·9 to 148)	75·4 (44·2 to 127)	51·5 (32 to 82)	34·1 (21·4 to 54·2)	31·5 (18·9 to 52·5)	24·4 (15·7 to 37·5)	22·2 (14 to 35·3)	16 (10·5 to 24·5)	11·6 (7·4 to 18·1)	13·4 (8·1 to 21·4)	6·6 (3·7 to 11·2)	2·1 (1·2 to 3·5)	2,810
Fire, heat, and hot substances	19 (8 to 52·9)	25·4 (10·4 to 72·8)	261 (88·2 to 711)	592 (200 to 1.590)	469 (281 to 888)	401 (268 to 587)	465 (314 to 650)	569 (385 to 806)	594 (401 to 834)	584 (408 to 807)	556 (383 to 769)	498 (345 to 699)	451 (318 to 627)	420 (299 to 585)	355 (251 to 481)	289 (206 to 401)	214 (151 to 299)	174 (123 to 243)	119 (84·6 to 168)	94·6 (63 to 143)	76·2 (47·2 to 121)	31·6 (19·5 to 50·9)	10·7 (7·1 to 15·2)	7.270
Poisonings	21·6 (6·6 to 53·4)	31 (8·3 to 82·4)	95·1 (24·4 to 256)	174 (62·8 to 408)	101 (54·4 to 176)	73·1 (43·1 to 120)	68·5 (40·4 to 107)	95·3 (56·9 to 146)	72·5 (43·7 to 113)	63·4 (38·8 to 100)	63·8 (37·9 to 107)	59·8 (34·8 to 95·8)	46·8 (28·7 to 76·1)	38·6 (24·6 to 59·2)	27·7 (17·7 to 42)	25·3 (15·8 to 39·7)	21·6 (13·7 to 31·8)	18·6 (11·7 to 28·6)	14·9 (8·9 to 24·1)	11·9 (6·9 to 20·2)	6·6 (3·9 to 10·6)	1·9 (1·2 to 2·9)	0·6 (0·4 to 1)	1,130
Poisoning by carbon monoxide	5·6 (0·8 to 18·6)	2·7 (0·3 to 9·6)	6·1 (0·8 to 21·1)	24·1 (6 to 72·9)	20 (9·9 to 37·6)	16·4 (8·9 to 27·7)	19 (9·9 to 34·2)	31·5 (16·4 to 56·5)	20·8 (11·5 to 36·8)	18 (9·7 to 31)	14·4 (8 to 24·4)	16·2 (8·1 to 29·2)	11·2 (6·2 to 19·3)	8·2 (4·9 to 13·1)	7·2 (4·1 to 12·3)	6·7 (3·6 to 11·7)	7 (3·8 to 11·6)	5·4 (2·9 to 9·3)	3·6 (1·7 to 6·8)	2·2 (1·1 to 4·4)	1·5 (0·7 to 3)	0·5 (0·2 to 1·1)	0·1 (0·1 to 0·2)	248
Poisoning by other means	16 (4·5 to 40·6)	28·3 (7·3 to 76·4)	89 (22·6 to 242)	150 (48·8 to 334)	80·7 (41·3 to 143)	56·7 (32·3 to 94·5)	49·5 (29·3 to 80)	63·8 (36·1 to 103)	51·7 (30·5 to 83·2)	45·3 (27·3 to 73·4)	49·4 (28·7 to 83·8)	43·6 (24·9 to 72·6)	35·6 (21·1 to 59·5)	30·4 (18·4 to 48·1)	20·5 (12·6 to 31·9)	18·6 (11·5 to 29·9)	14·5 (8·8 to 22·4)	13·3 (8·2 to 21·3)	11·3 (6·6 to 18·6)	9·7 (5·5 to 17·2)	5·1 (2·8 to 8·5)	1·4 (0·8 to 2·3)	0·5 (0·3 to 0·8)	885
Exposure to mechanical forces	14·1 (0·7 to 83)	22·2 (0·7 to 150)	64·6 (12·7 to 253)	184 (94 to 365)	249 (166 to 361)	269 (182 to 385)	306 (218 to 432)	385 (270 to 535)	388 (274 to 538)	369 (256 to 504)	353 (249 to 502)	333 (233 to 461)	305 (216 to 433)	290 (201 to 402)	246 (171 to 340)	209 (144 to 293)	169 (116 to 235)	138 (95·6 to 192)	100 (69·9 to 138)	74·7 (53·8 to 102)	47·2 (33·8 to 64·4)	21·7 (15·9 to 29·3)	8·8 (6·3 to 11·7)	4,550
Unintentional firearm injuries	8·6 (0·2 to 60·8)	7·8 (0·1 to 62)	24·5 (2 to 125)	47·5 (15·8 to 122)	65·2 (30·8 to 131)	82 (45·7 to 136)	97·6 (60·7 to 158)	143 (85·6 to 240)	126 (74·8 to 209)	114 (69·1 to 184)	95·2 (60·1 to 154)	83·1 (55·5 to 124)	66·9 (47·4 to 93·1)	59 (41·9 to 84·3)	44·8 (32·9 to 58·8)	35 (25 to 46·6)	25·9 (18·5 to 34·1)	22·8 (16·6 to 30·1)	16·6 (12 to 21·9)	14·3 (10·2 to 19)	10·9 (7·8 to 15)	6·2 (4·3 to 8·8)	2·7 (1·8 to 3·8)	1,200
Other exposure to mechanical forces	5·5 (0·2 to 32·3)	14·4 (0·5 to 93·8)	40·1 (9 to 166)	137 (67·9 to 274)	184 (116 to 275)	187 (121 to 286)	209 (136 to 318)	242 (158 to 365)	262 (172 to 389)	256 (167 to 381)	258 (173 to 383)	250 (170 to 368)	238 (161 to 349)	231 (155 to 329)	201 (136 to 286)	174 (117 to 250)	143 (96·3 to 202)	115 (78·7 to 163)	83·6 (57·2 to 118)	60·5 (42·2 to 85·6)	36·4 (25·4 to 51)	15·4 (10·7 to 21·1)	6·2 (4·2 to 8·5)	3.350
Adverse effects of medical treatment	78·7 (38·8 to 145)	54·6 (26·2 to 101)	224 (91·1 to 447)	139 (65·3 to 259)	104 (58·9 to 167)	83·5 (47·3 to 139)	101 (58·7 to 169)	208 (106 to 355)	191 (90·1 to 317)	228 (104 to 406)	186 (97·2 to 317)	194 (109 to 321)	225 (117 to 423)	216 (116 to 375)	198 (107 to 355)	218 (121 to 391)	154 (83·7 to 254)	167 (94·2 to 268)	134 (74·4 to 220)	121 (73·7 to 192)	105 (64·3 to 160)	37·9 (24·1 to 56·9)	12·4 (7·8 to 18·2)	3.380
Animal contact	0·1 (0·1 to 0·5)	0·2 (0·1 to 0·4)	3·4 (1·9 to 7·4)	16·9 (10·6 to 24·8)	23·9 (14·5 to 37·6)	30·4 (19·1 to 45·1)	33·8 (21·3 to 48·9)	37·4 (24·4 to 55)	36·1 (23·8 to 52·6)	33·6 (21·5 to 49·4)	31·7 (20·6 to 46)	29·6 (19·4 to 42·4)	28 (18·2 to 40·7)	26·2 (17·1 to 37·4)	23·5 (15·8 to 34·3)	19·7 (13·2 to 28·2)	16·2 (10·9 to 23)	13·8 (9·3 to 19·7)	10·3 (7·1 to 14·9)	8·5 (5·7 to 12·7)	5·9 (4 to 9·3)	3·1 (2 to 5·6)	1·1 (0·7 to 2·3)	433
Venomous animal contact	0·1 (0 to 0·3)	0·1 (0·1 to 0·2)	2·5 (1·4 to 5·6)	10·6 (6 to 16·7)	17 (9·7 to 28·4)	22 (12·7 to 34·7)	25 (14·6 to 38·3)	28·5 (17·5 to 43·7)	27·9 (17·6 to 42·4)	25·5 (15·8 to 39·3)	23·5 (14·5 to 35)	21·6 (13·5 to 31·3)	20 (12·5 to 29·9)	18·3 (11·7 to 27·3)	16·1 (10·4 to 24)	13·4 (8·7 to 19·5)	10·8 (7 to 15·8)	9·2 (6 to 13·2)	6·7 (4·5 to 9·7)	5·5 (3·6 to 8·3)	4 (2·6 to 6·9)	2·2 (1·4 to 4·5)	0·8 (0·5 to 1·8)	311
Non-venomous animal contact	0·1 (0 to 0·2)	0·1 (0 to 0·2)	0·9 (0·4 to 1·9)	6·3 (3·7 to 9·6)	6·9 (4·1 to 11·6)	8·4 (5·3 to 13·3)	8·8 (5·7 to 13·8)	8·9 (5·6 to 13·7)	8·2 (4·9 to 13·2)	8·1 (5 to 12·8)	8·2 (5·2 to 13·1)	8 (5 to 12·6)	8 (4·9 to 12·3)	7·9 (4·9 to 12·1)	7·4 (4·5 to 11·2)	6·3 (4 to 9·5)	5·4 (3·4 to 8·3)	4·6 (2·9 to 7)	3·6 (2·3 to 5·5)	3 (2 to 4·5)	1·9 (1·3 to 2·9)	0·9 (0·6 to 1·4)	0·3 (0·2 to 0·5)	122
Foreign body	240 (107 to 473)	124 (58·8 to 236)	1,470 (566 to 3,060)	624 (273 to 1,190)	458 (270 to 693)	370 (237 to 542)	165 (108 to 240)	246 (154 to 369)	237 (153 to 364)	210 (137 to 317)	210 (136 to 318)	198 (126 to 304)	180 (119 to 267)	204 (128 to 316)	170 (111 to 254)	185 (126 to 275)	155 (106 to 225)	166 (112 to 246)	114 (71·6 to 183)	116 (73·3 to 173)	61·4 (37·8 to 96)	28·2 (16·6 to 43·7)	12·3 (7·8 to 18·7)	5,940
Pulmonary aspiration and foreign body in airway	240 (106 to 472)	124 (58·5 to 236)	1,460 (563 to 3,060)	594 (253 to 1.150)	422 (243 to 649)	330 (202 to 492)	121 (73·6 to 190)	194 (113 to 302)	185 (109 to 305)	161 (94·6 to 263)	165 (97·8 to 268)	155 (91·3 to 253)	142 (84·1 to 224)	170 (99·1 to 276)	143 (87·7 to 225)	159 (100 to 247)	132 (85·3 to 197)	142 (89·9 to 214)	92·9 (54 to 154)	95·5 (57·5 to 148)	48·8 (27·7 to 76·4)	22·2 (11·9 to 36·5)	9·8 (5·9 to 15·5)	5.310
Foreign body in eyes	0 (0 to 0·1)	0·1 (0 to 0·2)	1·3 (0·3 to 2·8)	6·5 (1·9 to 14·9)	9·4 (2·7 to 21·8)	11·3 (3·4 to 25·6)	15·1 (4·9 to 34·3)	19·4 (6·3 to 42·4)	20·9 (7·7 to 43·9)	20·1 (7·2 to 41·3)	19 (7·5 to 39·6)	18·1 (7·7 to 37)	16·2 (7·8 to 30·3)	13·3 (7 to 23·4)	10·4 (6 to 17)	8·4 (5 to 13·3)	6·9 (4·1 to 10·4)	5·5 (3·5 to 8·1)	4 (2·6 to 5·9)	2·8 (1·8 to 4·2)	1·6 (1·1 to 2·3)	0·6 (0·4 to 0·8)	0·2 (0·1 to 0·3)	211
Foreign body in other body part	0·3 (0·1 to 0·9)	0·3 (0·1 to 0·5)	4·2 (2·1 to 7·6)	22·9 (12·1 to 42·6)	27·4 (16·5 to 44·7)	29·1 (18·9 to 43·7)	28·7 (19·2 to 39·9)	32·9 (21·9 to 46·4)	30·7 (21·5 to 43)	28·4 (20·2 to 39)	26·1 (18·3 to 36·1)	24·6 (17·7 to 33·6)	21·9 (15·9 to 28·9)	20·6 (14·7 to 27·5)	16·4 (12·1 to 21·3)	17·8 (11·9 to 27·2)	16·7 (10·9 to 25·7)	19 (11·4 to 33·3)	16·8 (9·4 to 28·7)	17·7 (8·6 to 33·8)	11 (5·4 to 21·1)	5·5 (2·7 to 10·3)	2·2 (1·1 to 4·4)	421
Environmental heat and cold exposure	0·4 (0·2 to 0·9)	0·9 (0·4 to 2)	8·5 (5·1 to 13·9)	47·2 (31·1 to 71·4)	80·4 (51·5 to 117)	96·8 (64 to 140)	120 (79·7 to 171)	139 (92·6 to 197)	147 (99·8 to 210)	144 (99·5 to 204)	136 (95·3 to 193)	130 (89·8 to 182)	124 (87·2 to 173)	118 (81·6 to 161)	106 (74·9 to 145)	93·2 (64·9 to 128)	80·9 (55·7 to 110)	69·2 (48·3 to 93·9)	53·2 (37·4 to 71·5)	42·2 (30·5 to 56·1)	26·3 (18·6 to 35·4)	11·1 (8 to 15)	4·4 (3 to 5·9)	1.780
Exposure to forces of nature	0 (0 to 0)	0 (0 to 0)	0 (0 to 0)	0·1 (0 to 0·1)	0·3 (0·1 to 0·4)	1·5 (0·9 to 2·4)	9·8 (5·8 to 16·2)	14·3 (8·5 to 22·8)	21·8 (13·1 to 36)	15·1 (9·3 to 24·1)	11·3 (6·9 to 17·7)	10·4 (6·5 to 16·2)	10·4 (6·3 to 16·6)	8·9 (5·5 to 13·9)	7·1 (4·4 to 11·2)	5·7 (3·6 to 9·1)	4·7 (3 to 7·4)	3·7 (2·4 to 5·7)	2·7 (1·7 to 4)	1·8 (1·1 to 2·6)	0·9 (0·6 to 1·4)	0·3 (0·2 to 0·5)	0·1 (0·1 to 0·1)	131
Other unintentional injuries	19·8 (7·9 to 43·1)	9·6 (3·6 to 21·9)	71·7 (35·8 to 138)	184 (98·9 to 325)	289 (169 to 484)	296 (176 to 487)	281 (179 to 438)	357 (219 to 560)	351 (222 to 538)	311 (201 to 466)	295 (192 to 457)	271 (180 to 397)	245 (167 to 346)	234 (156 to 334)	193 (129 to 269)	166 (114 to 233)	129 (89·7 to 180)	101 (69·4 to 141)	73·5 (50·9 to 102)	51·7 (36·2 to 71·3)	29·6 (20·8 to 40·3)	10·7 (7·6 to 14·7)	3·9 (2·8 to 5·3)	3,970
Self-harm and interpersonal violence	343 (127 to 800)	61·3 (23·4 to 131)	449 (168 to 1.030)	614 (227 to 1.380)	607 (345 to 1.010)	1,010 (645 to 1.510)	2,740 (1.790 to 3.930)	4,390 (2.770 to 6.560)	3,930 (2.450 to 6.090)	3,200 (2.030 to 4.780)	2,450 (1.560 to 3.940)	1,740 (1.130 to 2.660)	1,280 (856 to 1.970)	981 (641 to 1.460)	630 (429 to 912)	481 (340 to 674)	338 (248 to 477)	244 (184 to 325)	147 (110 to 196)	87·5 (66·3 to 114)	61·2 (45·1 to 80·9)	16·7 (12·3 to 21·6)	5·1 (3·9 to 6·3)	25.800
Self-harm	0 (0 to 0)	0 (0 to 0)	0 (0 to 0)	0 (0 to 0)	0 (0 to 0)	196 (101 to 335)	794 (448 to 1.300)	1,050 (592 to 1.730)	855 (466 to 1.440)	726 (429 to 1.190)	523 (293 to 823)	411 (250 to 651)	351 (214 to 544)	311 (187 to 492)	221 (139 to 343)	192 (121 to 301)	138 (86·7 to 217)	99·1 (66·6 to 148)	59 (38·6 to 87·6)	38·7 (25·3 to 59·8)	30 (19·1 to 45·8)	7·1 (4·7 to 10·5)	1·9 (1·3 to 2·7)	6.010
Self-harm by firearm	0 (0 to 0)	0 (0 to 0)	0 (0 to 0)	0 (0 to 0)	0 (0 to 0)	11·2 (4·7 to 20·8)	60·3 (18·1 to 144)	85·9 (30·1 to 175)	61·7 (22·3 to 133)	65·1 (27·3 to 130)	32·5 (13·1 to 69·3)	18·4 (8·9 to 37·5)	15·8 (7·2 to 31·6)	11 (5·2 to 22·1)	6·8 (3·6 to 12·3)	5·1 (2·7 to 10·6)	4·4 (2·2 to 8·3)	3·7 (1·8 to 6·7)	2·2 (1·3 to 3·9)	2·6 (1·4 to 4·7)	3·3 (1·5 to 6·2)	0·3 (0·2 to 0·5)	0·1 (0 to 0·1)	390
Self-harm by other specified means	0 (0 to 0)	0 (0 to 0)	0 (0 to 0)	0 (0 to 0)	0 (0 to 0)	185 (94·8 to 317)	733 (416 to 1,200)	966 (535 to 1,610)	793 (427 to 1,340)	661 (390 to 1,080)	491 (276 to 780)	392 (237 to 623)	335 (204 to 523)	300 (181 to 478)	214 (135 to 331)	187 (117 to 291)	134 (83·2 to 210)	95·4 (63·7 to 142)	56·8 (37·1 to 84·7)	36·1 (23·3 to 55·3)	26·7 (16·7 to 40·8)	6·8 (4·5 to 9·9)	1·8 (1·3 to 2·6)	5,620
Interpersonal violence	343 (127 to 800)	61·3 (23·4 to 131)	448 (167 to 1.030)	610 (225 to 1.370)	603 (341 to 1.010)	808 (475 to 1.280)	1,940 (1.110 to 3.040)	3,340 (1.920 to 5.450)	3,070 (1.730 to 5.130)	2,470 (1.380 to 3.980)	1,920 (1.080 to 3.280)	1,330 (774 to 2.140)	929 (572 to 1.550)	667 (409 to 1.090)	406 (257 to 624)	288 (188 to 435)	199 (138 to 290)	144 (99·6 to 209)	87·2 (62·8 to 121)	48·6 (36·4 to 65·6)	30·9 (22·1 to 42·6)	9·5 (7 to 12·8)	3·1 (2·3 to 4·1)	19.800
Physical violence by firearm	24·1 (6·3 to 64·1)	3 (0·8 to 8·5)	18·6 (5·7 to 48)	82·8 (22·9 to 220)	98·5 (41·5 to 200)	214 (94·4 to 406)	879 (421 to 1.550)	1,500 (715 to 2.700)	1,390 (671 to 2.510)	1,090 (514 to 1.950)	795 (365 to 1.530)	489 (226 to 919)	323 (152 to 641)	188 (85·2 to 360)	98·1 (45·5 to 180)	60·2 (29·3 to 110)	40·7 (20·1 to 73)	22·3 (11·4 to 40·8)	9 (4·6 to 16·9)	2·7 (1·7 to 4·5)	4·7 (2·4 to 8·3)	0·7 (0·4 to 1·3)	0·5 (0·3 to 0·9)	7.330
Physical violence by sharp object	20·4 (4·9 to 58·5)	1·2 (0·4 to 3)	15·9 (5 to 38·8)	31·9 (13·2 to 70·4)	49 (24·6 to 92·5)	111 (46·4 to 233)	426 (183 to 841)	880 (393 to 1,670)	840 (366 to 1,660)	640 (287 to 1,200)	569 (251 to 1,150)	430 (184 to 819)	252 (129 to 484)	172 (91 to 306)	98·8 (54·8 to 173)	70·4 (40·5 to 124)	47·7 (28·6 to 75·5)	33·4 (21·4 to 53·3)	13·7 (9·8 to 19·2)	7·7 (5·5 to 10·4)	4·6 (3·3 to 6·2)	1·9 (1·3 to 2·6)	0·8 (0·6 to 1·1)	4,720
Sexual violence	0 (0 to 0)	0 (0 to 0)	0 (0 to 0)	4·3 (2·5 to 6·9)	32·1 (19·4 to 50·2)	68·5 (38·7 to 110)	167 (95·9 to 264)	232 (136 to 353)	206 (117 to 337)	173 (95·4 to 277)	139 (79·4 to 220)	103 (59·6 to 164)	75·2 (42·6 to 123)	49·7 (27·7 to 82·9)	30·3 (17·5 to 48)	21·7 (13 to 33·5)	16·6 (10·1 to 25·4)	12·5 (7·6 to 18·9)	8·6 (5·4 to 12·7)	6 (3·8 to 8·9)	2·9 (1·7 to 4·7)	0·4 (0·2 to 0·7)	0 (0 to 0)	1.350
Physical violence by other means	298 (113 to 684)	57·1 (21·7 to 124)	414 (153 to 937)	491 (178 to 1.120)	423 (212 to 722)	414 (205 to 719)	472 (226 to 861)	729 (351 to 1.340)	637 (303 to 1.210)	567 (277 to 1.020)	417 (208 to 810)	306 (161 to 532)	278 (154 to 493)	258 (145 to 451)	179 (107 to 290)	135 (84 to 212)	94·3 (61·1 to 142)	75·9 (49·7 to 114)	55·9 (37 to 80·8)	32·1 (22 to 46·5)	18·8 (12·8 to 26·8)	6·5 (4·5 to 9·2)	1·8 (1·2 to 2·4)	6.360
Conflict and terrorism	0 (0 to 0)	0 (0 to 0)	0 (0 to 0)	0 (0 to 0)	0 (0 to 0·1)	0 (0 to 0·1)	0·1 (0 to 0·2)	0·2 (0·1 to 0·4)	0·6 (0·3 to 1·1)	2·4 (1·4 to 4·2)	4 (2·2 to 7·2)	2·4 (1·3 to 4·3)	0·8 (0·4 to 1·3)	1·9 (1·1 to 3·3)	2·8 (1·6 to 4·7)	0·5 (0·3 to 0·8)	0·6 (0·3 to 0·9)	0·8 (0·5 to 1·2)	0·7 (0·4 to 1·1)	0·2 (0·1 to 0·4)	0·3 (0·2 to 0·5)	0 (0 to 0·1)	0 (0 to 0)	18·2

SR Table 2b: Malaysia DALYS by cause and age group for females with 95% uncertainty intervals, 2019

Cause	Early Neonatal	Late Neonatal	Post Neonatal	1 to 4	5 to 9	10 to 14	15 to 19	20 to 24	25 to 29	30 to 34	35 to 39	40 to 44	45 to 49	50 to 54	55 to 59	60 to 64	65 to 69	70 to 74	75 to 79	80 to 84	85 to 89	90 to 94	95 plus	Total
All causes	53,900 (44,100 to 64,600)	19,400 (16,000 to 23,100)	40,300 (33,800 to 47,300)	42,700 (30,900 to 58,000)	58,200 (45,600 to 72,900)	83,600 (64,400 to 106,000)	128,000 (99,500 to 165,000)	153,000 (119,000 to 192,000)	174,000 (136,000 to 220,000)	190,000 (149,000 to 236,000)	191,000 (152,000 to 238,000)	192,000 (155,000 to 237,000)	205,000 (167,000 to 249,000)	233,000 (191,000 to 278,000)	266,000 (220,000 to 316,000)	288,000 (242,000 to 340,000)	300,000 (256,000 to 349,000)	270,000 (231,000 to 311,000)	197,000 (171,000 to 227,000)	150,000 (131,000 to 170,000)	72,900 (63,900 to 82,500)	33,000 (29,700 to 36,600)	4,170 (3,840 to 4,530)	3,350,000
Communicable, maternal, neonatal, and nutritional diseases	36,300 (27,700 to 45,300)	12,400 (9,560 to 15,300)	20,100 (15,400 to 25,500)	22,700 (15,900 to 31,700)	20,100 (14,800 to 27,300)	16,300 (11,900 to 21,700)	21,300 (15,100 to 29,400)	22,000 (16,700 to 29,100)	24,500 (18,900 to 32,200)	25,000 (19,700 to 31,900)	23,900 (18,500 to 31,400)	21,900 (16,900 to 28,500)	20,400 (15,600 to 26,400)	21,000 (15,500 to 27,100)	24,000 (16,400 to 31,800)	27,900 (17,900 to 37,000)	32,600 (19,900 to 43,700)	34,000 (20,900 to 45,700)	28,000 (17,700 to 37,800)	22,500 (14,400 to 29,700)	12,000 (8,020 to 15,700)	5,890 (3,970 to 7,680)	752 (500 to 1,010)	495,000
HIV/AIDS and sexually transmitted infections	417 (134 to 941)	119 (34·7 to 266)	415 (298 to 592)	346 (275 to 432)	334 (266 to 415)	395 (284 to 542)	466 (370 to 578)	1,020 (810 to 1,290)	1,910 (1,520 to 2,370)	2,400 (1,940 to 2,980)	2,250 (1,830 to 2,800)	2,400 (1,930 to 3,010)	2,000 (1,610 to 2,480)	1,730 (1,390 to 2,120)	1,330 (1,070 to 1,650)	1,220 (960 to 1,520)	541 (433 to 669)	94·3 (65·2 to 140)	46·6 (26·6 to 81·3)	13·4 (6·5 to 25·3)	4·3 (2 to 9·1)	1·4 (0·6 to 2·9)	0·1 (0·1 to 0·3)	19,500
HIV/AIDS	0·2 (0·1 to 0·3)	0·7 (0·4 to 1·1)	290 (229 to 363)	342 (271 to 430)	332 (264 to 414)	372 (265 to 516)	395 (313 to 497)	824 (649 to 1,020)	1,600 (1,270 to 2,010)	2,060 (1,650 to 2,540)	1,910 (1,530 to 2,380)	2,090 (1,650 to 2,640)	1,750 (1,400 to 2,170)	1,570 (1,250 to 1,950)	1,200 (965 to 1,500)	1,130 (885 to 1,400)	467 (370 to 579)	45·5 (30 to 65·6)	9·9 (5·8 to 15·2)	0·5 (0·3 to 0·8)	0·2 (0·1 to 0·3)	0·1 (0·1 to 0·1)	0 (0 to 0)	16,400
HIV/AIDS - Drug-susceptible Tuberculosis	0 (0 to 0)	0 (0 to 0·1)	51·2 (33·1 to 76·2)	60·7 (40·8 to 88·7)	53·3 (36·5 to 77·4)	56 (38·8 to 80·5)	69·1 (46·5 to 101)	149 (102 to 217)	290 (201 to 416)	369 (250 to 536)	337 (227 to 492)	365 (245 to 540)	305 (205 to 452)	272 (181 to 404)	208 (140 to 308)	196 (132 to 291)	82·8 (56·9 to 120)	8·6 (6·2 to 11·3)	2 (1·4 to 2·7)	0·1 (0·1 to 0·1)	0 (0 to 0·1)	0 (0 to 0)	0 (0 to 0)	2,880
HIV/AIDS - Multidrug-resistant Tuberculosis without exte	0 (0 to 0)	0 (0 to 0)	1·1 (0·2 to 3·6)	1·3 (0·2 to 4)	1·1 (0·2 to 3·4)	1·1 (0·2 to 3·5)	1·4 (0·3 to 4·6)	3 (0·6 to 9·6)	5·8 (1·1 to 18·2)	7·5 (1·4 to 23·5)	6·9 (1·3 to 21·9)	7·7 (1·4 to 24·5)	6·5 (1·2 to 20·6)	5·8 (1·1 to 18·6)	4·4 (0·8 to 14)	4·2 (0·8 to 13·2)	1·7 (0·3 to 5·3)	0·1 (0 to 0·3)	0 (0 to 0·1)	0 (0 to 0)	0 (0 to 0)	0 (0 to 0)	0 (0 to 0)	59·8
HIV/AIDS - Extensively drug-resistant Tuberculosis	0 (0 to 0)	0 (0 to 0)	0·2 (0 to 0·7)	0·2 (0 to 0·8)	0·2 (0 to 0·6)	0·2 (0 to 0·7)	0·3 (0 to 0·9)	0·6 (0·1 to 1·8)	1·1 (0·2 to 3·4)	1·4 (0·2 to 4·4)	1·3 (0·2 to 4·1)	1·4 (0·2 to 4·7)	1·2 (0·2 to 3·9)	1·1 (0·2 to 3·6)	0·8 (0·1 to 2·7)	0·8 (0·1 to 2·5)	0·3 (0·1 to 1)	0 (0 to 0)	0 (0 to 0)	0 (0 to 0)	0 (0 to 0)	0 (0 to 0)	0 (0 to 0)	11·1
HIV/AIDS resulting in other diseases	0·2 (0·1 to 0·3)	0·7 (0·3 to 1·1)	237 (174 to 312)	280 (201 to 367)	277 (205 to 356)	315 (205 to 462)	324 (234 to 432)	671 (491 to 876)	1,310 (948 to 1,710)	1,680 (1,270 to 2,200)	1,560 (1,160 to 2,060)	1,720 (1,250 to 2,280)	1,440 (1,060 to 1,870)	1,290 (949 to 1,690)	988 (735 to 1,290)	927 (677 to 1,210)	382 (279 to 494)	36·8 (22 to 55·5)	7·9 (4·1 to 13)	0·4 (0·2 to 0·7)	0·2 (0·1 to 0·3)	0·1 (0 to 0·1)	0 (0 to 0)	13,400
Sexually transmitted infections excluding HIV	417 (133 to 940)	118 (34·5 to 265)	125 (34·7 to 292)	3·9 (0·9 to 11)	2·8 (0·8 to 6·3)	22·6 (12·7 to 44)	70·8 (35·9 to 135)	196 (95·5 to 381)	305 (148 to 594)	346 (160 to 662)	340 (170 to 633)	306 (153 to 609)	255 (123 to 480)	165 (74·9 to 339)	129 (55·7 to 260)	93·2 (39·9 to 191)	73·6 (35·1 to 141)	48·8 (24·6 to 89·1)	36·7 (17·1 to 71·8)	12·9 (6 to 24·6)	4·1 (1·8 to 8·8)	1·3 (0·5 to 2·8)	0·1 (0·1 to 0·3)	3,070
Syphilis	417 (133 to 940)	118 (34·5 to 265)	125 (34·7 to 292)	3·9 (0·9 to 11)	2·8 (0·8 to 6·3)	8 (4 to 19·3)	3 (1·9 to 6·2)	1·9 (0·9 to 4)	5·2 (2·1 to 11·4)	6 (2·4 to 13·7)	6·4 (2·5 to 13·6)	2·2 (0·9 to 4·7)	4·6 (1·8 to 9·8)	2·3 (0·9 to 4·7)	0·8 (0·3 to 1·7)	1 (0·4 to 2·5)	1·6 (0·6 to 3·8)	2·1 (0·8 to 4·4)	2 (0·7 to 4·8)	0·8 (0·3 to 1·7)	0·2 (0·1 to 0·5)	0·1 (0 to 0·3)	0 (0 to 0)	715
Chlamydial infection	0 (0 to 0)	0 (0 to 0)	0 (0 to 0)	0 (0 to 0)	0 (0 to 0)	1·9 (1·1 to 3·8)	4·5 (2·7 to 7·3)	14·1 (8·5 to 22·2)	19 (11·2 to 30·8)	20·2 (11·5 to 34·1)	24·3 (13·8 to 39·7)	23·3 (12·9 to 39·5)	18·2 (9·3 to 32·4)	5 (2·6 to 8·7)	4 (1·9 to 8)	2·5 (0·8 to 6·5)	2 (0·7 to 5)	2·4 (0·9 to 5·3)	3·1 (1·1 to 7·3)	0·7 (0·2 to 1·5)	0·1 (0 to 0·4)	0 (0 to 0·1)	0 (0 to 0)	145
Gonococcal infection	0 (0 to 0)	0 (0 to 0)	0 (0 to 0)	0 (0 to 0)	0 (0 to 0)	2·4 (1·3 to 5·6)	4·7 (3 to 8·8)	9·6 (4·8 to 20·2)	13 (6·4 to 25·3)	13·2 (6·7 to 26)	19·6 (9·2 to 38·8)	21·6 (9·4 to 45·7)	22·1 (9·3 to 46·2)	9·4 (3·5 to 19·8)	8·6 (3 to 20·1)	6·4 (1·9 to 16·5)	10·8 (3·6 to 26·9)	8·3 (3·1 to 18·1)	11 (3·9 to 26·2)	1·6 (0·6 to 3·5)	0·3 (0·1 to 1·1)	0·1 (0 to 0·4)	0 (0 to 0)	163
Trichomoniasis	0 (0 to 0)	0 (0 to 0)	0 (0 to 0)	0 (0 to 0)	0 (0 to 0)	1·8 (0 to 5·7)	25 (7·2 to 61·3)	95 (31·5 to 231)	148 (47·7 to 335)	153 (49·5 to 358)	131 (42·7 to 319)	114 (37·7 to 286)	95·8 (30·7 to 224)	77·1 (25·2 to 180)	56·3 (18 to 139)	38 (12·9 to 87·7)	25·5 (8·7 to 60·7)	14·2 (5·1 to 32)	6·9 (2·5 to 15·6)	3·5 (1·2 to 7·5)	1·1 (0·4 to 2·6)	0·3 (0·1 to 0·6)	0 (0 to 0)	987
Genital herpes	0 (0 to 0)	0 (0 to 0)	0 (0 to 0)	0 (0 to 0)	0 (0 to 0)	4·7 (1·3 to 11·6)	22·6 (5·5 to 55·1)	49·5 (15·6 to 126)	73·5 (21·1 to 174)	88·9 (28 to 218)	83·7 (25 to 205)	73·6 (24 to 185)	64·2 (20·1 to 156)	57·7 (17·5 to 143)	49·9 (15 to 123)	39·6 (11·7 to 101)	29 (8·8 to 71·4)	17·6 (5·3 to 44·1)	9·5 (2·9 to 23·9)	5·6 (1·7 to 13·7)	2·2 (0·7 to 5·8)	0·8 (0·2 to 1·9)	0·1 (0 to 0·2)	673
Other sexually transmitted infections	0 (0 to 0)	0 (0 to 0)	0 (0 to 0)	0 (0 to 0)	0 (0 to 0)	3·8 (2·2 to 7·5)	11·1 (7 to 18·4)	26 (16·2 to 39·5)	46·3 (20·9 to 87·9)	64·7 (31·9 to 116)	75·1 (39·4 to 126)	71·1 (37·5 to 120)	50·3 (22·7 to 89·8)	13·2 (7·2 to 21·8)	9·9 (5·2 to 18·6)	5·5 (1·6 to 14·2)	4·8 (1·6 to 11·9)	4·3 (1·6 to 9·3)	4·2 (1·5 to 10·2)	0·7 (0·2 to 1·4)	0·2 (0 to 0·5)	0 (0 to 0·2)	0 (0 to 0)	391
Respiratory infections and tuberculosis	1,040 (562 to 1,750)	899 (484 to 1,500)	4,970 (2,940 to 7,540)	2,690 (1,700 to 4,000)	3,700 (2,700 to 5,040)	3,200 (2,360 to 4,290)	4,100 (3,070 to 5,410)	5,780 (4,290 to 7,540)	6,100 (4,570 to 8,030)	6,460 (4,610 to 8,700)	6,710 (4,910 to 9,280)	6,650 (4,600 to 9,200)	8,290 (5,460 to 11,700)	10,800 (6,380 to 15,300)	15,100 (7,950 to 21,800)	20,100 (10,500 to 29,000)	25,800 (13,400 to 36,200)	28,600 (15,200 to 40,300)	24,100 (13,600 to 33,600)	19,500 (11,500 to 26,700)	10,400 (6,350 to 14,100)	5,160 (3,250 to 6,980)	646 (412 to 901)	221,000
Tuberculosis	0 (0 to 0)	0 (0 to 0)	185 (103 to 295)	115 (44·1 to 216)	308 (156 to 503)	312 (170 to 501)	575 (342 to 894)	1,680 (1,110 to 2,460)	1,880 (1,210 to 2,760)	1,490 (961 to 2,170)	1,520 (996 to 2,220)	1,260 (805 to 1,810)	1,420 (927 to 2,080)	1,270 (802 to 1,970)	1,290 (806 to 2,000)	1,670 (1,110 to 2,480)	2,270 (1,510 to 3,230)	2,150 (1,470 to 3,090)	1,370 (916 to 2,010)	952 (616 to 1,410)	403 (260 to 599)	147 (97·6 to 220)	17·4 (11·2 to 25·9)	22,300
Latent tuberculosis infection	0 (0 to 0)	0 (0 to 0)	0 (0 to 0)	0 (0 to 0)	0 (0 to 0)	0 (0 to 0)	0 (0 to 0)	0 (0 to 0)	0 (0 to 0)	0 (0 to 0)	0 (0 to 0)	0 (0 to 0)	0 (0 to 0)	0 (0 to 0)	0 (0 to 0)	0 (0 to 0)	0 (0 to 0)	0 (0 to 0)	0 (0 to 0)	0 (0 to 0)	0 (0 to 0)	0 (0 to 0)	0 (0 to 0)	0
Drug-susceptible tuberculosis	0 (0 to 0)	0 (0 to 0)	181 (101 to 289)	113 (42·9 to 213)	304 (150 to 499)	307 (168 to 495)	564 (337 to 882)	1,650 (1,080 to 2,420)	1,850 (1,190 to 2,720)	1,460 (947 to 2,130)	1,490 (981 to 2,170)	1,240 (792 to 1,770)	1,390 (905 to 2,050)	1,240 (785 to 1,910)	1,260 (785 to 1,950)	1,640 (1,090 to 2,450)	2,220 (1,480 to 3,150)	2,110 (1,420 to 3,040)	1,340 (885 to 1,980)	933 (606 to 1,380)	395 (256 to 592)	145 (94·5 to 217)	17 (11·1 to 25·2)	21,900
Multidrug-resistant tuberculosis without extensive drug r	0 (0 to 0)	0 (0 to 0)	3·3 (0·6 to 11·3)	1·4 (0·3 to 4·7)	3·8 (0·8 to 11·7)	4·3 (0·9 to 13·6)	9·4 (1·8 to 30·7)	23·7 (4·9 to 69·1)	26·2 (5·4 to 78·3)	22·2 (4·3 to 64·4)	23·7 (4·5 to 71·2)	19·8 (3·6 to 65·3)	22·7 (4·4 to 71·9)	21·4 (4 to 69·9)	22·7 (4 to 70·6)	27·6 (5·5 to 86·8)	38·4 (7·2 to 117)	35·8 (7·6 to 111)	22·8 (4·5 to 70·2)	16 (3 to 52)	6·8 (1·3 to 20·7)	2·5 (0·5 to 7·1)	0·3 (0·1 to 1)	355
Extensively drug-resistant tuberculosis	0 (0 to 0)	0 (0 to 0)	0·6 (0·1 to 2·1)	0·2 (0 to 0·8)	0·6 (0·1 to 1·9)	0·7 (0·1 to 2·4)	1·7 (0·3 to 5·8)	3·9 (0·7 to 13)	4·4 (0·9 to 13·8)	3·8 (0·7 to 12·1)	4·2 (0·7 to 13·1)	3·5 (0·6 to 12)	4·1 (0·8 to 13·6)	4 (0·7 to 12·6)	4·3 (0·7 to 14·8)	5 (0·9 to 17·4)	7·1 (1·2 to 22·9)	6·5 (1·2 to 21·4)	4·1 (0·7 to 13·7)	2·9 (0·5 to 9·2)	1·2 (0·2 to 4)	0·4 (0·1 to 1·4)	0·1 (0 to 0·2)	63·5
Lower respiratory infections	1,040 (559 to 1,740)	883 (466 to 1,490)	4,450 (2,460 to 7,010)	903 (392 to 1,790)	1,540 (1,060 to 2,200)	1,510 (1,010 to 2,160)	2,130 (1,420 to 3,020)	2,710 (1,730 to 3,970)	2,920 (1,840 to 4,380)	3,800 (2,280 to 5,720)	4,220 (2,600 to 6,320)	4,600 (2,750 to 6,800)	6,220 (3,580 to 9,230)	9,000 (4,680 to 13,000)	13,300 (6,210 to 19,800)	18,000 (8,470 to 26,900)	23,200 (10,700 to 33,000)	26,200 (13,100 to 37,600)	22,600 (12,100 to 31,900)	18,500 (10,500 to 25,500)	10,000 (5,950 to 13,600)	5,010 (3,100 to 6,810)	628 (393 to 880)	183,000
Upper respiratory infections	2·4 (1·3 to 4)	15·1 (8·3 to 25·3)	289 (163 to 477)	1,220 (669 to 2,060)	1,300 (687 to 2,220)	1,080 (561 to 1,830)	1,120 (575 to 2,010)	1,150 (598 to 2,030)	1,090 (591 to 1,830)	992 (522 to 1,700)	822 (416 to 1,400)	656 (337 to 1,170)	531 (269 to 930)	455 (244 to 799)	395 (213 to 664)	321 (171 to 549)	239 (122 to 417)	151 (82·8 to 250)	81·5 (43·5 to 136)	48·1 (25·5 to 81·4)	18·8 (10 to 31·6)	7·2 (4 to 12·1)	0·8 (0·4 to 1·4)	12,000
Otitis media	0·1 (0 to 0·2)	0·9 (0·4 to 1·6)	43·1 (22·7 to 75·5)	451 (248 to 778)	553 (293 to 946)	286 (136 to 536)	267 (130 to 481)	244 (97·8 to 484)	213 (95·3 to 404)	179 (73·5 to 365)	149 (68·6 to 288)	135 (55·7 to 264)	125 (59·8 to 224)	121 (53·3 to 221)	108 (53·6 to 195)	75·6 (33·6 to 147)	52·9 (25 to 100)	30·2 (13·7 to 57·7)	14·6 (6·7 to 27·2)	9·7 (3·9 to 18·7)	4·3 (1·8 to 7·9)	1·7 (0·7 to 3·3)	0·2 (0·1 to 0·4)	3,070
Enteric infections	51·4 (27·3 to 94·7)	192 (99·6 to 367)	2,340 (1,360 to 3,970)	4,560 (2,870 to 6,930)	3,630 (2,150 to 5,690)	2,990 (1,620 to 5,000)	2,550 (1,290 to 4,490)	1,960 (1,060 to 3,470)	1,650 (1,000 to 2,650)	1,450 (925 to 2,280)	1,190 (790 to 1,800)	1,030 (691 to 1,520)	1,060 (689 to 1,640)	1,210 (736 to 2,010)	1,400 (801 to 2,540)	1,970 (1,130 to 3,480)	2,560 (1,510 to 4,610)	2,570 (1,450 to 4,950)	2,200 (1,190 to 4,140)	1,850 (996 to 3,650)	979 (494 to 1,970)	470 (221 to 1,030)	69 (30·3 to 145)	39,900
Diarrheal diseases	51·4 (27·3 to 94·7)	189 (97·1 to 363)	2,280 (1,290 to 3,920)	3,390 (2,060 to 5,250)	1,510 (907 to 2,500)	855 (498 to 1,360)	705 (429 to 1,070)	795 (463 to 1,290)	885 (542 to 1,390)	983 (637 to 1,540)	944 (607 to 1,460)	887 (566 to 1,340)	948 (591 to 1,510)	1,110 (637 to 1,940)	1,310 (720 to 2,460)	1,890 (1,050 to 3,400)	2,500 (1,460 to 4,530)	2,540 (1,410 to 4,930)	2,190 (1,180 to 4,130)	1,840 (988 to 3,630)	975 (492 to 1,970)	469 (220 to 1,030)	68·8 (30·2 to 145)	29,300
Typhoid and paratyphoid	0 (0 to 0)	0 (0 to 0)	16·6 (5·1 to 41·2)	1,080 (273 to 2,750)	2,020 (708 to 4,140)	2,070 (787 to 4,070)	1,800 (577 to 3,830)	1,130 (377 to 2,570)	737 (266 to 1,670)	447 (155 to 1,030)	231 (84·2 to 492)	134 (51 to 280)	102 (37 to 222)	86·2 (32·2 to 185)	76·3 (25·9 to 177)	59·5 (20·8 to 137)	41·4 (15·6 to 90)	22·1 (8·2 to 48·9)	10·5 (3·7 to 20·8)	5·4 (1·8 to 11·5)	2 (0·8 to 3·8)	0·6 (0·3 to 1·4)	0·1 (0 to 0·2)	10,100
Typhoid fever	0 (0 to 0)	0 (0 to 0)	15·9 (4·9 to 39·5)	1,040 (263 to 2,670)	1,930 (686 to 3,940)	1,990 (753 to 3,950)	1,730 (561 to 3,720)	1,090 (363 to 2,490)	711 (258 to 1,630)	432 (150 to 1,000)	224 (81·5 to 480)	130 (49·4 to 271)	99·1 (36 to 218)	83·8 (31·2 to 179)	74·2 (25 to 172)	57·9 (20·2 to 134)	40·3 (15·2 to 87·6)	21·5 (7·9 to 47·4)	10·3 (3·6 to 20·6)	5·3 (1·7 to 11·3)	1·9 (0·8 to 3·7)	0·6 (0·2 to 1·3)	0·1 (0 to 0·2)	9,680
Paratyphoid fever	0 (0 to 0)	0 (0 to 0)	0·7 (0·2 to 2)	47·6 (8·4 to 139)	87·7 (22·1 to 237)	86·5 (22·3 to 216)	71·2 (16·9 to 185)	42·4 (9·9 to 118)	26·2 (6·8 to 70·9)	15·2 (3·5 to 41·6)	7·5 (2 to 20·4)	4·2 (1·1 to 10·9)	3 (0·7 to 8·1)	2·5 (0·6 to 6·7)	2·1 (0·5 to 5·9)	1·6 (0·4 to 4·5)	1·1 (0·3 to 3·1)	0·6 (0·1 to 1·7)	0·3 (0·1 to 0·8)	0·1 (0 to 0·4)	0 (0 to 0·1)	0 (0 to 0)	0 (0 to 0)	401
Invasive Non-typhoidal Salmonella (iNTS)	0 (0 to 0)	2·6 (1·4 to 4·4)	36·9 (18·8 to 64·1)	79·7 (24·8 to 178)	97·4 (40·5 to 198)	61·8 (22·7 to 132)	47·1 (15 to 110)	36·3 (9·7 to 87·5)	25·7 (6·9 to 61·9)	18·6 (4·5 to 43·9)	13·2 (3·4 to 29·7)	10·4 (2·8 to 24)	9·4 (2·6 to 21·2)	10·4 (2·9 to 22·5)	13 (3·5 to 29·5)	15·3 (4·5 to 33·4)	15·3 (4·3 to 32·8)	11·6 (3·6 to 24·3)	7·1 (2·3 to 15)	4·2 (1·4 to 9·5)	1·5 (0·5 to 3·3)	0·5 (0·1 to 1·3)	0·1 (0 to 0·2)	518

Other cardiovascular and circulatory diseases	8·8 (2·4 to 35·6)	17·5 (7·7 to 33·6)	129 (48·9 to 272)	[illegible]
Chronic respiratory diseases	0 (0 to 0)	0 (0 to 0)	2 (0·9 to 3)	[illegible]
Chronic obstructive pulmonary disease	0 (0 to 0)	0 (0 to 0)	2 (0·9 to 3)	[illegible]
Pneumoconiosis	0 (0 to 0)	0 (0 to 0)	0 (0 to 0)	[illegible]
Silicosis	0 (0 to 0)	0 (0 to 0)	0 (0 to 0)	[illegible]
Asbestosis	0 (0 to 0)	0 (0 to 0)	0 (0 to 0)	[illegible]
Coal workers pneumoconiosis	0 (0 to 0)	0 (0 to 0)	0 (0 to 0)	[illegible]
Other pneumoconiosis	0 (0 to 0)	0 (0 to 0)	0 (0 to 0)	[illegible]
Asthma	0 (0 to 0)	0 (0 to 0)	0 (0 to 0)	[illegible]
Interstitial lung disease and pulmonary sarcoidosis	0 (0 to 0)	0 (0 to 0)	0 (0 to 0)	[illegible]
Other chronic respiratory diseases	0 (0 to 0)	0 (0 to 0)	0 (0 to 0)	[illegible]
Digestive diseases	69·6 (46·4 to 106)	125 (79·5 to 200)	697 (352 to 1,290)	[illegible]
Cirrhosis and other chronic liver diseases	0 (0 to 0)	0 (0 to 0)	1·4 (0·7 to 2·4)	[illegible]
Cirrhosis and other chronic liver diseases due to hepati	0 (0 to 0)	0 (0 to 0)	0 (0 to 0)	[illegible]
Cirrhosis and other chronic liver diseases due to hepati	0 (0 to 0)	0 (0 to 0)	0 (0 to 0)	[illegible]
Cirrhosis and other chronic liver diseases due to alcoho	0 (0 to 0)	0 (0 to 0)	0 (0 to 0)	[illegible]
Cirrhosis and other chronic liver diseases due to NAFL[	0 (0 to 0)	0 (0 to 0)	0 (0 to 0)	[illegible]
Cirrhosis and other chronic liver diseases due to other c	0 (0 to 0)	0 (0 to 0)	1·4 (0·7 to 2·4)	[illegible]
Upper digestive system diseases	0 (0 to 0)	0 (0 to 0)	0 (0 to 0)	[illegible]
Peptic ulcer disease	0 (0 to 0)	0 (0 to 0)	0 (0 to 0)	[illegible]
Gastritis and duodenitis	0 (0 to 0)	0 (0 to 0)	0 (0 to 0)	[illegible]
Gastroesophageal reflux disease	0 (0 to 0)	0 (0 to 0)	0 (0 to 0)	[illegible]
Appendicitis	0 (0 to 0)	0 (0 to 0)	0 (0 to 0)	[illegible]
Paralytic ileus and intestinal obstruction	61·5 (40·9 to 90·7)	120 (76·3 to 192)	667 (341 to 1,230)	[illegible]
Inguinal, femoral, and abdominal hernia	8·2 (2 to 21·6)	4·6 (1·2 to 13·1)	28·6 (8·8 to 78·6)	[illegible]
Inflammatory bowel disease	0 (0 to 0)	0 (0 to 0)	0 (0 to 0)	[illegible]
Vascular intestinal disorders	0 (0 to 0)	0 (0 to 0)	0 (0 to 0)	[illegible]
Gallbladder and biliary diseases	0 (0 to 0)	0 (0 to 0)	0 (0 to 0)	[illegible]
Pancreatitis	0 (0 to 0)	0 (0 to 0)	0 (0 to 0)	[illegible]
Other digestive diseases	0 (0 to 0)	0 (0 to 0)	0 (0 to 0)	[illegible]
Neurological disorders	3·3 (0·7 to 7·8)	9·9 (2·3 to 23)	826 (472 to 1,320)	[illegible]
Alzheimer's disease and other dementias	0 (0 to 0)	0 (0 to 0)	0 (0 to 0)	[illegible]
Parkinson's disease	0 (0 to 0)	0 (0 to 0)	0 (0 to 0)	[illegible]
Idiopathic epilepsy	3·2 (0·7 to 7·7)	9·8 (2·1 to 22·9)	284 (127 to 518)	[illegible]
Multiple sclerosis	0 (0 to 0)	0 (0 to 0)	0 (0 to 0)	[illegible]
Motor neuron disease	0·1 (0 to 0·1)	0·2 (0·1 to 0·2)	2·6 (1·8 to 3·4)	[illegible]
Headache disorders	0 (0 to 0)	0 (0 to 0)	0 (0 to 0)	[illegible]
Migraine	0 (0 to 0)	0 (0 to 0)	0 (0 to 0)	[illegible]
Tension-type headache	0 (0 to 0)	0 (0 to 0)	0 (0 to 0)	[illegible]
Other neurological disorders	0 (0 to 0)	0 (0 to 0)	539 (307 to 881)	[illegible]
Mental disorders	2 (1·1 to 3·4)	6·3 (3·2 to 10·3)	111 (58·9 to 170)	[illegible]
Schizophrenia	0 (0 to 0)	0 (0 to 0)	0 (0 to 0)	[illegible]
Depressive disorders	0 (0 to 0)	0 (0 to 0)	0 (0 to 0)	[illegible]
Major depressive disorder	0 (0 to 0)	0 (0 to 0)	0 (0 to 0)	[illegible]
Dysthymia	0 (0 to 0)	0 (0 to 0)	0 (0 to 0)	[illegible]
Bipolar disorder	0 (0 to 0)	0 (0 to 0)	0 (0 to 0)	[illegible]
Anxiety disorders	0 (0 to 0)	0 (0 to 0)	0 (0 to 0)	[illegible]
Eating disorders	0 (0 to 0)	0 (0 to 0)	0 (0 to 0)	[illegible]
Anorexia nervosa	0 (0 to 0)	0 (0 to 0)	0 (0 to 0)	[illegible]
Bulimia nervosa	0 (0 to 0)	0 (0 to 0)	0 (0 to 0)	[illegible]
Autism spectrum disorders	1·4 (0·8 to 2·3)	4·2 (2·4 to 6·8)	67·6 (38·8 to 105)	[illegible]
Attention-deficit/hyperactivity disorder	0 (0 to 0)	0 (0 to 0)	0 (0 to 0)	[illegible]
Conduct disorder	0 (0 to 0)	0 (0 to 0)	0 (0 to 0)	[illegible]
Idiopathic developmental intellectual disability	0·6 (0·1 to 1·6)	2·1 (0·2 to 4·8)	43 (7·5 to 87·6)	[illegible]
Other mental disorders	0 (0 to 0)	0 (0 to 0)	0 (0 to 0)	[illegible]
Substance use disorders	0 (0 to 0)	0 (0 to 0·1)	0·6 (0·3 to 0·9)	[illegible]
Alcohol use disorders	0 (0 to 0)	0 (0 to 0·1)	0·6 (0·3 to 0·9)	[illegible]
Drug use disorders	0 (0 to 0)	0 (0 to 0)	0 (0 to 0)	[illegible]
Opioid use disorders	0 (0 to 0)	0 (0 to 0)	0 (0 to 0)	[illegible]
Cocaine use disorders	0 (0 to 0)	0 (0 to 0)	0 (0 to 0)	[illegible]
Amphetamine use disorders	0 (0 to 0)	0 (0 to 0)	0 (0 to 0)	[illegible]
Cannabis use disorders	0 (0 to 0)	0 (0 to 0)	0 (0 to 0)	[illegible]
Other drug use disorders	0 (0 to 0)	0 (0 to 0)	0 (0 to 0)	[illegible]
Diabetes and kidney diseases	1 (0·6 to 1·6)	2·9 (1·5 to 5·1)	171 (102 to 269)	[illegible]
Diabetes mellitus	0·9 (0·6 to 1·6)	2·8 (1·4 to 5)	13·2 (4·6 to 27·5)	[illegible]
Diabetes mellitus type 1	0·9 (0·6 to 1·6)	2·8 (1·4 to 5)	13·2 (4·6 to 27·5)	[illegible]
Diabetes mellitus type 2	0 (0 to 0)	0 (0 to 0)	0 (0 to 0)	[illegible]
Chronic kidney disease	0 (0 to 0)	0·1 (0·1 to 0·2)	158 (91 to 252)	[illegible]
Chronic kidney disease due to diabetes mellitus type 1	0 (0 to 0)	0 (0 to 0)	3·2 (1·2 to 7·2)	[illegible]
Chronic kidney disease due to diabetes mellitus type 2	0 (0 to 0)	0 (0 to 0)	0 (0 to 0)	[illegible]
Chronic kidney disease due to hypertension	0 (0 to 0)	0 (0 to 0)	0 (0 to 0)	[illegible]
Chronic kidney disease due to glomerulonephritis	0 (0 to 0)	0 (0 to 0·1)	28·3 (13·2 to 57·9)	[illegible]
Chronic kidney disease due to other and unspecified ca	0 (0 to 0)	0·1 (0 to 0·2)	126 (71 to 203)	[illegible]
Acute glomerulonephritis	0 (0 to 0)	0 (0 to 0)	0·2 (0·1 to 0·3)	[illegible]
Skin and subcutaneous diseases	0·4 (0·2 to 0·7)	5·4 (2·9 to 9·2)	507 (298 to 818)	[illegible]
Dermatitis	0 (0 to 0)	0 (0 to 0)	0 (0 to 0)	[illegible]
Atopic dermatitis	0 (0 to 0)	0 (0 to 0)	0 (0 to 0)	[illegible]
Contact dermatitis	0 (0 to 0)	0 (0 to 0)	0 (0 to 0)	[illegible]
Seborrhoeic dermatitis	0 (0 to 0)	0 (0 to 0)	0 (0 to 0)	[illegible]
Psoriasis	0 (0 to 0)	0 (0 to 0)	0 (0 to 0)	[illegible]
Bacterial skin diseases	0·1 (0 to 0·1)	0·5 (0·3 to 1)	55·8 (29·3 to 104)	[illegible]
Cellulitis	0 (0 to 0)	0·1 (0·1 to 0·2)	6·5 (3 to 17)	[illegible]
Pyoderma	0 (0 to 0·1)	0·4 (0·2 to 0·9)	49·3 (24·2 to 94·4)	[illegible]
Scabies	0·3 (0·1 to 0·5)	3·5 (1·8 to 6)	298 (158 to 506)	[illegible]
Fungal skin diseases	0 (0 to 0·1)	0·3 (0·1 to 0·7)	28·6 (10·7 to 63)	[illegible]
Viral skin diseases	0 (0 to 0)	0 (0 to 0)	0 (0 to 0)	[illegible]
Acne vulgaris	0 (0 to 0)	0 (0 to 0)	0 (0 to 0)	[illegible]
Alopecia areata	0 (0 to 0)	0 (0 to 0)	0 (0 to 0)	[illegible]
Pruritus	0 (0 to 0)	0 (0 to 0·1)	5·2 (2 to 10·9)	[illegible]
Urticaria	0 (0 to 0·1)	0·6 (0·2 to 1·3)	71·2 (38 to 119)	[illegible]
Decubitus ulcer	0 (0 to 0)	0 (0 to 0)	0 (0 to 0)	[illegible]
Other skin and subcutaneous diseases	0 (0 to 0·1)	0·4 (0·2 to 0·8)	48·6 (22·4 to 90·5)	[illegible]
Sense organ diseases	1·5 (0·9 to 2·6)	5·2 (3·1 to 8·4)	127 (79 to 193)	[illegible]
Blindness and vision loss	0·6 (0·2 to 1·1)	1·7 (0·8 to 3·4)	39·4 (20·7 to 68·7)	[illegible]
Glaucoma	0 (0 to 0)	0 (0 to 0)	0 (0 to 0)	[illegible]
Cataract	0 (0 to 0)	0 (0 to 0)	0 (0 to 0)	[illegible]
Age-related macular degeneration	0 (0 to 0)	0 (0 to 0)	0 (0 to 0)	[illegible]
Refraction disorders	0 (0 to 0·1)	0·4 (0·1 to 0·9)	28·7 (14·3 to 51·3)	[illegible]
Near vision loss	0 (0 to 0)	0 (0 to 0)	0 (0 to 0·1)	[illegible]
Other vision loss	0·5 (0·2 to 1·1)	1·3 (0·6 to 2·6)	10·7 (4·5 to 20·4)	[illegible]
Age-related and other hearing loss	0·5 (0·2 to 1·1)	1·7 (0·8 to 3·2)	52·9 (27·4 to 89·3)	[illegible]
Other sense organ diseases	0·4 (0·2 to 0·9)	1·8 (0·8 to 3·4)	34·3 (16·5 to 61·2)	[illegible]
Musculoskeletal disorders	0 (0 to 0)	0 (0 to 0)	0 (0 to 0)	[illegible]
Rheumatoid arthritis	0 (0 to 0)	0 (0 to 0)	0 (0 to 0)	[illegible]
Osteoarthritis	0 (0 to 0)	0 (0 to 0)	0 (0 to 0)	[illegible]
Osteoarthritis hip	0 (0 to 0)	0 (0 to 0)	0 (0 to 0)	[illegible]
Osteoarthritis knee	0 (0 to 0)	0 (0 to 0)	0 (0 to 0)	[illegible]
Osteoarthritis hand	0 (0 to 0)	0 (0 to 0)	0 (0 to 0)	[illegible]
Osteoarthritis other	0 (0 to 0)	0 (0 to 0)	0 (0 to 0)	[illegible]
Low back pain	0 (0 to 0)	0 (0 to 0)	0 (0 to 0)	[illegible]
Neck pain	0 (0 to 0)	0 (0 to 0)	0 (0 to 0)	[illegible]
Gout	0 (0 to 0)	0 (0 to 0)	0 (0 to 0)	[illegible]
Other musculoskeletal disorders	0 (0 to 0)	0 (0 to 0)	0 (0 to 0)	[illegible]
Other non-communicable diseases	17,100 (11,300 to 24,500)	6,440 (4,590 to 8,880)	13,700 (9,380 to 18,900)	[illegible]
Congenital birth defects	17,100 (11,200 to 24,500)	5,840 (4,020 to 8,210)	11,400 (7,280 to 16,500)	[illegible]
Neural tube defects	2,070 (695 to 4,240)	242 (69·1 to 604)	650 (175 to 1,550)	[illegible]
Congenital heart anomalies	4,130 (2,180 to 7,630)	2,410 (1,380 to 3,950)	5,160 (2,820 to 8,700)	[illegible]
Orofacial clefts	136 (24·9 to 387)	58·8 (10·5 to 183)	114 (24·4 to 328)	[illegible]
Down syndrome	117 (44·9 to 252)	45·2 (18·1 to 97·2)	208 (81 to 426)	[illegible]
Turner syndrome	0 (0 to 0)	0 (0 to 0·1)	0·6 (0·2 to 1·2)	[illegible]
Klinefelter syndrome	0 (0 to 0)	0 (0 to 0)	0 (0 to 0)	[illegible]
Other chromosomal abnormalities	4,300 (1,670 to 8,030)	1,800 (854 to 3,050)	2,080 (915 to 3,970)	[illegible]
Congenital musculoskeletal and limb anomalies	327 (141 to 639)	98·2 (51 to 176)	526 (288 to 885)	[illegible]
Urogenital congenital anomalies	368 (85·7 to 801)	30·2 (11·9 to 57)	53 (29·2 to 99·1)	[illegible]
Digestive congenital anomalies	405 (172 to 1,130)	187 (90·1 to 377)	700 (288 to 1,480)	[illegible]
Other congenital birth defects	5,200 (2,440 to 8,740)	972 (504 to 1,670)	1,890 (920 to 3,430)	[illegible]
Urinary diseases and male infertility	0·2 (0·1 to 0·9)	1·8 (0·6 to 8·4)	23·6 (9·5 to 58·4)	[illegible]
Urolithiasis	0 (0 to 0)	0 (0 to 0)	0 (0 to 0)	[illegible]
Benign prostatic hyperplasia	0 (0 to 0)	0 (0 to 0)	0 (0 to 0)	[illegible]
Male infertility	--	--	--	[illegible]
Other urinary diseases	0·1 (0 to 0·5)	1·3 (0·2 to 8)	16·9 (5·2 to 50·1)	[illegible]
Gynecological diseases	0 (0 to 0)	0 (0 to 0)	0 (0 to 0)	[illegible]
Uterine fibroids	0 (0 to 0)	0 (0 to 0)	0 (0 to 0)	[illegible]
Polycystic ovarian syndrome	0 (0 to 0)	0 (0 to 0)	0 (0 to 0)	[illegible]
Female infertility	0 (0 to 0)	0 (0 to 0)	0 (0 to 0)	[illegible]
Endometriosis	0 (0 to 0)	0 (0 to 0)	0 (0 to 0)	[illegible]
Genital prolapse	0 (0 to 0)	0 (0 to 0)	0 (0 to 0)	[illegible]
Premenstrual syndrome	0 (0 to 0)	0 (0 to 0)	0 (0 to 0)	[illegible]
Other gynecological diseases	0 (0 to 0)	0 (0 to 0)	0 (0 to 0)	[illegible]
Hemoglobinopathies and hemolytic anemias	6·9 (3·4 to 13·4)	21 (10·7 to 34·5)	164 (86·4 to 268)	[illegible]
Thalassemias	5·9 (2·8 to 11·7)	7·3 (3·2 to 14·9)	57·2 (23·9 to 123)	[illegible]
Thalassemias trait	0·3 (0 to 1)	6·9 (2·3 to 12·7)	57·1 (26·2 to 104)	[illegible]
Sickle cell disorders	0 (0 to 0·1)	0 (0 to 0·1)	0·3 (0·1 to 0·8)	[illegible]
Sickle cell trait	0 (0 to 0·1)	0·8 (0·2 to 1·5)	2·8 (0·4 to 6·4)	[illegible]
G6PD deficiency	0·1 (0 to 0·2)	0·4 (0·1 to 1)	0·9 (0·3 to 2)	[illegible]
G6PD trait	0 (0 to 0)	0 (0 to 0·6)	0 (0 to 0)	[illegible]
Other hemoglobinopathies and hemolytic anemias	0·5 (0·2 to 1·1)	5·5 (2·1 to 10·4)	45·5 (16·3 to 95)	[illegible]
Endocrine, metabolic, blood, and immune disorders	46 (19·3 to 100)	60·5 (33·1 to 110)	642 (333 to 1,100)	[illegible]
Oral disorders	0 (0 to 0)	0 (0 to 0)	0 (0 to 0)	[illegible]
Caries of deciduous teeth	0 (0 to 0)	0 (0 to 0)	0 (0 to 0)	[illegible]
Caries of permanent teeth	0 (0 to 0)	0 (0 to 0)	0 (0 to 0)	[illegible]
Periodontal diseases	0 (0 to 0)	0 (0 to 0)	0 (0 to 0)	[illegible]
Other oral disorders	0 (0 to 0)	0 (0 to 0)	0 (0 to 0)	[illegible]
Sudden infant death syndrome	--	511 (215 to 1,210)	1,540 (645 to 3,300)	[illegible]
Injuries	259 (147 to 442)	247 (147 to 386)	2,500 (1,540 to 3,820)	[illegible]
Transport injuries	23·4 (8·5 to 49·1)	32·7 (13·9 to 64·3)	424 (224 to 730)	[illegible]
Road injuries	21·3 (7·5 to 44·6)	31 (12·9 to 61·5)	386 (200 to 674)	[illegible]
Pedestrian road injuries	8·7 (2·9 to 19·3)	8·3 (2·6 to 18)	129 (51·1 to 267)	[illegible]
Cyclist road injuries	0 (0 to 0)	0 (0 to 0)	0 (0 to 0)	[illegible]
Motorcyclist road injuries	4·4 (1·3 to 11·9)	2 (0·6 to 5)	70·3 (23·1 to 161)	[illegible]
Motor vehicle road injuries	7·7 (2·2 to 17·9)	20·5 (7·8 to 42·2)	179 (77·8 to 345)	[illegible]
Other road injuries	0·4 (0·1 to 1)	0·3 (0·1 to 0·7)	8·4 (2·3 to 21)	[illegible]

Other transport injuries	2·1 (0·7 to 4·9)	1·7 (0·8 to 3·5)	37·7 (18·9 to 72·5)	67·4 (39·8 to 110)	160 (93·8 to 261)	182 (117 to 274)	430 (237 to 750)	500 (279 to 829)
Unintentional injuries	209 (107 to 382)	208 (116 to 339)	2,010 (1,160 to 3,270)	1,450 (848 to 2,470)	3,200 (2,440 to 4,090)	3,680 (2,840 to 4,650)	3,780 (2,980 to 4,720)	4,290 (3,310 to 5,470)
Falls	11·3 (2·9 to 38·6)	4·8 (1·8 to 11·5)	113 (47·9 to 248)	219 (115 to 463)	509 (355 to 719)	671 (478 to 935)	947 (680 to 1,310)	1,110 (797 to 1,520)
Drowning	12·2 (6·5 to 21·6)	7·4 (3·6 to 14·1)	133 (60 to 275)	422 (158 to 932)	1,180 (662 to 1,880)	1,370 (843 to 2,120)	650 (381 to 1,050)	501 (299 to 813)
Fire, heat, and hot substances	4·2 (1·7 to 10·8)	6·9 (2·8 to 17·2)	66·8 (29·4 to 153)	145 (54·8 to 423)	276 (175 to 431)	324 (210 to 464)	563 (336 to 880)	704 (385 to 1,170)
Poisonings	1·4 (0·4 to 3·8)	0·9 (0·2 to 2·8)	11·1 (3·5 to 29·2)	23·2 (10 to 58·2)	46·6 (23·5 to 92·3)	95·8 (49·9 to 173)	247 (127 to 434)	315 (174 to 552)
Poisoning by carbon monoxide	0·5 (0·1 to 2·5)	0·3 (0 to 0·7)	2·5 (0·5 to 7·8)	8·9 (2·3 to 23·8)	19·8 (6·5 to 37·7)	23·4 (9·4 to 49·1)	55·3 (21·7 to 117)	62·5 (27·5 to 130)
Poisoning by other means	0·9 (0·1 to 2·9)	0·7 (0·2 to 2·3)	8·6 (2·5 to 23·1)	14·3 (5·3 to 41·6)	26·8 (11·3 to 61·6)	72·5 (35·4 to 142)	192 (94·9 to 348)	253 (137 to 447)
Exposure to mechanical forces	5·5 (0·6 to 28·9)	14·1 (1·1 to 82·8)	93·8 (21·5 to 366)	139 (79 to 242)	248 (159 to 366)	277 (187 to 394)	376 (254 to 535)	475 (326 to 672)
Unintentional firearm injuries	0·6 (0·1 to 3·9)	1·8 (0·1 to 12·2)	8·7 (2·6 to 35)	24 (12·9 to 43·6)	32·7 (20·6 to 50·2)	42·7 (27·5 to 63·7)	56·3 (37·1 to 86·6)	64·6 (43·6 to 91·7)
Other exposure to mechanical forces	4·9 (0·5 to 25·8)	12·3 (0·9 to 74·3)	85·1 (17·8 to 335)	115 (64·6 to 207)	215 (135 to 317)	234 (156 to 343)	320 (215 to 466)	411 (278 to 594)
Adverse effects of medical treatment	8·9 (2·4 to 31·6)	10 (4·2 to 26·3)	89·7 (47·9 to 158)	65·6 (34·8 to 130)	71·4 (44·8 to 113)	77·4 (44·1 to 122)	94·8 (57·6 to 152)	125 (68·1 to 198)
Animal contact	1·5 (0·8 to 2·9)	1·6 (0·8 to 3·4)	22·5 (10·6 to 47·3)	44·6 (22·9 to 90·2)	126 (70·3 to 217)	109 (64·4 to 172)	107 (71·3 to 163)	127 (81·2 to 189)
Venomous animal contact	1·3 (0·7 to 2·5)	1 (0·4 to 2)	16·1 (7·2 to 35·8)	26·1 (12·6 to 57·5)	98·1 (50 to 178)	80·1 (44·4 to 133)	73·1 (43·7 to 114)	89·2 (52·2 to 143)
Non-venomous animal contact	0·3 (0·1 to 0·5)	0·6 (0·3 to 1·4)	6·3 (2·8 to 13·9)	18·4 (9·2 to 36·5)	28·3 (15·7 to 48·7)	29·1 (16·6 to 46·9)	34·1 (20·6 to 53·1)	37·9 (23·5 to 57·3)
Foreign body	159 (69·9 to 322)	157 (80·4 to 266)	1,410 (664 to 2,580)	180 (83·8 to 368)	325 (210 to 477)	318 (209 to 486)	247 (165 to 361)	288 (196 to 423)
Pulmonary aspiration and foreign body in airway	159 (69·7 to 321)	157 (80·1 to 266)	1,400 (657 to 2,570)	151 (61 to 324)	284 (177 to 434)	271 (165 to 428)	192 (119 to 298)	223 (137 to 345)
Foreign body in eyes	0 (0 to 0·1)	0·1 (0 to 0·2)	1·4 (0·4 to 3·2)	6·9 (1·9 to 16)	9·9 (2·7 to 22·2)	11·3 (3·4 to 27·1)	14·7 (4·5 to 34·8)	18·9 (6·1 to 45)
Foreign body in other body part	0·2 (0·1 to 0·6)	0·4 (0·2 to 0·7)	8·7 (3·9 to 20·6)	22·5 (12·7 to 36·8)	31·1 (20·6 to 44·6)	35·2 (23·8 to 51)	40·3 (27·2 to 55·7)	46·2 (32 to 64·2)
Environmental heat and cold exposure	0·2 (0·1 to 0·5)	0·3 (0·2 to 0·5)	8·4 (4 to 18·5)	27·7 (16·4 to 44·9)	55 (34·1 to 89·6)	62·2 (40·6 to 93·7)	77·6 (53·2 to 112)	89·2 (61·6 to 126)
Exposure to forces of nature	0 (0 to 0)	0·1 (0 to 0·1)	0·4 (0·4 to 0·5)	2·3 (2·1 to 2·6)	2·9 (2·5 to 3·4)	3·9 (3·1 to 5)	6·5 (4·7 to 9·3)	9·1 (6·4 to 13·1)
Other unintentional injuries	4·2 (2·2 to 7·7)	4·8 (2·7 to 8·2)	69·9 (43·2 to 108)	181 (107 to 285)	355 (225 to 540)	367 (234 to 547)	465 (298 to 696)	545 (353 to 822)
Self-harm and interpersonal violence	27·2 (10·3 to 59·4)	6·7 (3 to 13·6)	66 (31 to 132)	108 (54·8 to 229)	264 (188 to 373)	591 (436 to 785)	2,370 (1,690 to 3,270)	2,980 (2,110 to 4,060)
Self-harm	0 (0 to 0)	0 (0 to 0)	0 (0 to 0)	0 (0 to 0)	0 (0 to 0)	83 (49·3 to 142)	1,150 (652 to 1,850)	1,530 (900 to 2,390)
Self-harm by firearm	0 (0 to 0)	0 (0 to 0)	0 (0 to 0)	0 (0 to 0)	0 (0 to 0)	7·4 (1·2 to 15·2)	8·8 (2·5 to 15·8)	10·5 (4·3 to 20)
Self-harm by other specified means	0 (0 to 0)	0 (0 to 0)	0 (0 to 0)	0 (0 to 0)	0 (0 to 0)	75·6 (45·1 to 128)	1,140 (645 to 1,840)	1,520 (895 to 2,370)
Interpersonal violence	27·1 (10·2 to 59·3)	6·7 (3 to 13·5)	64 (29·3 to 130)	102 (50·4 to 221)	252 (177 to 361)	496 (355 to 675)	1,200 (809 to 1,700)	1,440 (987 to 1,990)
Physical violence by firearm	0·4 (0·1 to 1·1)	0·3 (0·1 to 0·8)	1·3 (0·6 to 2·6)	4·4 (1·9 to 9·9)	6 (3·8 to 9·1)	8·2 (5·2 to 13)	34·3 (14·5 to 68·9)	49·6 (24·1 to 97·8)
Physical violence by sharp object	2·2 (0·6 to 5·6)	0·2 (0·1 to 0·4)	2·3 (1·2 to 4·2)	8·2 (4·3 to 15·8)	13·7 (8·4 to 21·4)	28·9 (16·4 to 49·1)	64·3 (36·2 to 121)	172 (88·2 to 302)
Sexual violence	0 (0 to 0)	0 (0 to 0)	0 (0 to 0)	12·3 (7·2 to 20·1)	108 (64·5 to 173)	278 (160 to 438)	606 (342 to 958)	635 (367 to 969)
Physical violence by other means	24·5 (9·3 to 53·3)	6·2 (2·7 to 12·8)	60·5 (26·9 to 125)	77·4 (30·8 to 183)	125 (75·1 to 201)	181 (116 to 277)	497 (296 to 828)	582 (342 to 964)
Conflict and terrorism	0·1 (0·1 to 0·1)	0 (0 to 0)	0·1 (0·1 to 0·2)	0·9 (0·8 to 1·1)	3 (1·8 to 5·5)	2·8 (1·6 to 5·4)	3·7 (2·1 to 7)	3·6 (2·2 to 6·6)

Other transport injuries	414 (236 to 661)	358 (205 to 602)	288 (169 to 479)	301 (184 to 489)	291 (169 to 472)	268 (164 to 434)	249 (146 to 419)	217 (133 to 351)
Unintentional injuries	4,320 (3,320 to 5,550)	4,450 (3,400 to 5,770)	4,100 (3,130 to 5,240)	3,760 (2,940 to 4,850)	3,680 (2,850 to 4,650)	3,950 (3,050 to 5,020)	4,280 (3,360 to 5,430)	4,790 (3,660 to 6,020)
Falls	1,280 (916 to 1,760)	1,340 (944 to 1,840)	1,330 (951 to 1,790)	1,290 (923 to 1,740)	1,280 (931 to 1,710)	1,610 (1,190 to 2,120)	1,810 (1,340 to 2,370)	2,440 (1,760 to 3,190)
Drowning	360 (205 to 579)	341 (208 to 537)	281 (169 to 448)	249 (156 to 386)	261 (162 to 392)	233 (145 to 358)	224 (141 to 339)	198 (124 to 323)
Fire, heat, and hot substances	706 (398 to 1,160)	713 (430 to 1,090)	592 (391 to 838)	507 (353 to 711)	486 (343 to 678)	454 (329 to 626)	440 (314 to 637)	416 (289 to 629)
Poisonings	281 (147 to 519)	231 (130 to 390)	216 (112 to 377)	186 (93·5 to 334)	144 (75·3 to 260)	103 (56·9 to 176)	103 (57·6 to 171)	99·8 (54·5 to 172)
Poisoning by carbon monoxide	54·7 (23·8 to 119)	40·2 (17·7 to 80·5)	38 (14·9 to 86·9)	44·6 (17·3 to 97·2)	32·4 (13·3 to 65·5)	17·8 (8·9 to 34·3)	13·2 (6 to 25·9)	11·4 (5·4 to 21·9)
Poisoning by other means	227 (117 to 421)	191 (103 to 318)	178 (87·1 to 312)	141 (68·4 to 261)	112 (56·1 to 204)	84·8 (46·2 to 146)	90·2 (48·9 to 157)	88·4 (46·9 to 157)
Exposure to mechanical forces	515 (357 to 734)	572 (396 to 812)	539 (373 to 779)	517 (355 to 744)	515 (363 to 727)	549 (384 to 775)	582 (407 to 811)	556 (390 to 780)
Unintentional firearm injuries	81·9 (55·3 to 120)	80·4 (56 to 110)	78·6 (54·2 to 108)	72·3 (49·4 to 99)	68·8 (49·2 to 93·3)	69 (49·6 to 94·5)	67·5 (48·8 to 92·1)	57·3 (41 to 76·9)
Other exposure to mechanical forces	433 (294 to 631)	491 (333 to 711)	461 (313 to 674)	445 (302 to 653)	447 (311 to 633)	480 (330 to 687)	514 (357 to 729)	498 (347 to 709)
Adverse effects of medical treatment	112 (66·3 to 173)	137 (69·2 to 236)	118 (68·7 to 182)	127 (75·5 to 199)	172 (97 to 270)	156 (86·7 to 251)	217 (117 to 344)	259 (140 to 418)
Animal contact	115 (74·1 to 170)	102 (65 to 156)	106 (70·1 to 161)	89·3 (57 to 135)	94·8 (61·8 to 142)	133 (81·7 to 219)	144 (86·2 to 225)	124 (71·6 to 204)
Venomous animal contact	75·6 (44·1 to 118)	62·3 (35·1 to 104)	71·2 (41·6 to 124)	56·5 (31·2 to 92·1)	62·5 (35·1 to 106)	90·6 (49·3 to 161)	105 (55·2 to 180)	96·3 (49·7 to 169)
Non-venomous animal contact	39·2 (24·7 to 59)	40·1 (24·7 to 62·8)	34·7 (21·7 to 55·9)	32·8 (20·7 to 52)	32·3 (20·1 to 48·6)	42·5 (25·2 to 70·8)	39 (23·8 to 63·6)	27·5 (16·9 to 42·1)
Foreign body	280 (192 to 400)	281 (187 to 416)	242 (170 to 345)	222 (157 to 318)	199 (141 to 269)	207 (148 to 290)	263 (185 to 376)	264 (188 to 366)
Pulmonary aspiration and foreign body in airway	211 (135 to 322)	210 (124 to 337)	174 (111 to 270)	160 (101 to 251)	138 (90·6 to 204)	142 (89·1 to 216)	199 (124 to 308)	210 (139 to 313)
Foreign body in eyes	21 (7·3 to 44·4)	21·4 (7·9 to 45·8)	20·1 (7·6 to 42·8)	18·9 (8 to 38·5)	17·4 (8 to 34·4)	15·9 (7·8 to 29·3)	13·9 (7·7 to 23·8)	12·5 (7·1 to 20·6)
Foreign body in other body part	48 (33·5 to 66·6)	50 (35·1 to 68·9)	48·1 (34 to 65·2)	43·8 (30·9 to 61·4)	43·7 (31·1 to 60·1)	49·3 (35·6 to 67·1)	50·3 (35·5 to 69)	40·6 (28·7 to 55·2)
Environmental heat and cold exposure	94·5 (65·6 to 133)	96·3 (67·8 to 138)	86·5 (59·1 to 121)	75·2 (51·1 to 106)	68·8 (48·4 to 97·2)	68·5 (48·2 to 95·9)	64·5 (45·6 to 90·3)	60·5 (42·6 to 85·1)
Exposure to forces of nature	17·3 (11·7 to 25·7)	17·2 (11·7 to 25·9)	17·3 (11·7 to 26·3)	10·1 (6·8 to 14·5)	8·5 (5·8 to 12·1)	7·9 (5·4 to 11·5)	7·1 (4·8 to 10·4)	6 (4·2 to 8·5)
Other unintentional injuries	565 (382 to 823)	618 (415 to 890)	574 (380 to 826)	493 (339 to 706)	448 (307 to 632)	431 (290 to 609)	425 (281 to 618)	367 (251 to 512)
Self-harm and interpersonal violence	2,710 (1,930 to 3,700)	2,560 (1,850 to 3,480)	2,190 (1,640 to 2,940)	1,820 (1,370 to 2,430)	1,680 (1,240 to 2,240)	1,520 (1,130 to 2,020)	1,440 (1,060 to 1,940)	1,230 (931 to 1,650)
Self-harm	1,410 (825 to 2,220)	1,380 (831 to 2,110)	1,180 (753 to 1,770)	978 (632 to 1,460)	956 (611 to 1,430)	910 (595 to 1,350)	948 (603 to 1,420)	827 (561 to 1,220)
Self-harm by firearm	9·1 (4·4 to 15·7)	8·1 (3·8 to 14·7)	6·3 (3·2 to 12·4)	5·5 (2·8 to 10·3)	4 (2·4 to 6·7)	3·5 (2·1 to 6)	3 (1·8 to 4·9)	2·9 (1·9 to 4·8)
Self-harm by other specified means	1,400 (820 to 2,200)	1,370 (827 to 2,100)	1,170 (750 to 1,760)	973 (629 to 1,460)	952 (608 to 1,420)	906 (592 to 1,340)	945 (601 to 1,410)	824 (559 to 1,220)
Interpersonal violence	1,290 (895 to 1,850)	1,180 (851 to 1,620)	1,000 (722 to 1,380)	826 (612 to 1,120)	712 (535 to 961)	603 (459 to 801)	483 (360 to 622)	403 (311 to 520)
Physical violence by firearm	47·3 (21·5 to 93·2)	42·9 (22·9 to 78·5)	40·1 (19·2 to 77·6)	31·4 (16·6 to 57·8)	23·7 (13·7 to 40·9)	21·1 (12·5 to 34·7)	15·8 (10·2 to 25)	10·9 (7·5 to 16·4)
Physical violence by sharp object	167 (93·2 to 306)	140 (84 to 234)	130 (79·6 to 214)	113 (69·7 to 185)	88 (57·1 to 133)	84·1 (56 to 122)	70·2 (47·6 to 99·9)	72·4 (48 to 108)
Sexual violence	497 (287 to 798)	442 (244 to 708)	362 (199 to 583)	301 (172 to 495)	252 (147 to 423)	183 (101 to 304)	105 (59·7 to 173)	71·6 (43·1 to 109)
Physical violence by other means	582 (362 to 935)	551 (360 to 847)	469 (305 to 710)	381 (266 to 551)	348 (245 to 500)	315 (220 to 433)	292 (208 to 393)	248 (181 to 329)
Conflict and terrorism	2·8 (1·7 to 5)	3·1 (2 to 5·2)	5·4 (2·4 to 13·7)	8·3 (2·1 to 31·7)	5·2 (1·6 to 16·9)	5·1 (1·6 to 16·8)	3·4 (1·1 to 10·7)	2·1 (0·7 to 6·6)

Other transport injuries	233 (130 to 388)	147 (83·8 to 252)	97·2 (58 to 163)	53 (33·2 to 82·9)	18 (11·9 to 26·9)	9·3 (5·8 to 14·5)	1·3 (0·8 to 2)	4,320
Unintentional injuries	4,820 (3,720 to 5,960)	4,380 (3,380 to 5,470)	3,490 (2,630 to 4,450)	2,910 (2,200 to 3,730)	1,630 (1,240 to 2,060)	800 (592 to 1,020)	103 (76 to 137)	70,300
Falls	2,610 (1,850 to 3,410)	2,580 (1,760 to 3,510)	2,250 (1,540 to 3,030)	2,060 (1,430 to 2,770)	1,190 (846 to 1,580)	596 (410 to 805)	75·7 (52·4 to 105)	27,300
Drowning	208 (132 to 316)	150 (96 to 237)	120 (76·7 to 179)	69·3 (43·7 to 107)	28·8 (18·4 to 42·9)	10·1 (6·3 to 15·3)	1 (0·7 to 1·5)	7,010
Fire, heat, and hot substances	373 (248 to 561)	279 (167 to 456)	202 (113 to 341)	141 (82·8 to 228)	66·8 (41·9 to 107)	25·8 (15·5 to 38·6)	3 (1·8 to 5·2)	7,490
Poisonings	91·5 (51·5 to 150)	76·8 (42·3 to 127)	42·2 (22·2 to 75·6)	31·6 (16·2 to 56·7)	15·7 (8·2 to 27·9)	6·6 (3·6 to 11·7)	0·9 (0·4 to 1·8)	2,370
Poisoning by carbon monoxide	16·5 (7·2 to 32·2)	15·3 (6 to 30·7)	10·2 (3·4 to 23)	3·3 (1·3 to 7·5)	1·8 (0·7 to 4)	0·9 (0·4 to 2·1)	0·2 (0·1 to 0·4)	474
Poisoning by other means	75 (41·2 to 127)	61·5 (33 to 105)	32 (16 to 58·5)	28·2 (14·4 to 51·8)	13·9 (7·3 to 25·2)	5·7 (3·1 to 10)	0·8 (0·3 to 1·5)	1,900
Exposure to mechanical forces	474 (329 to 656)	336 (233 to 458)	211 (148 to 295)	134 (95·7 to 182)	58·5 (42 to 79·4)	24·4 (18 to 32·8)	3·2 (2·3 to 4·5)	7,210
Unintentional firearm injuries	45·5 (32·1 to 60·7)	29·1 (21 to 39·2)	16·6 (11·7 to 22·2)	10·7 (7·6 to 14·7)	4·9 (3·5 to 6·7)	2·1 (1·5 to 2·9)	0·3 (0·2 to 0·4)	916
Other exposure to mechanical forces	429 (293 to 600)	307 (211 to 420)	194 (136 to 273)	123 (87·3 to 169)	53·6 (38·4 to 73·3)	22·2 (16·3 to 29·8)	2·9 (2·1 to 4)	6,300
Adverse effects of medical treatment	251 (132 to 389)	190 (98·2 to 297)	128 (64·6 to 197)	87·4 (44·7 to 135)	38·5 (21·7 to 57·9)	17·7 (9·6 to 26·7)	2 (1·1 to 3·2)	2,560
Animal contact	98 (57·5 to 163)	112 (58·6 to 191)	69·8 (35·2 to 115)	32·3 (17·5 to 54·8)	16·2 (9 to 27·4)	5·7 (3·4 to 8·7)	0·8 (0·4 to 1·3)	1,780
Venomous animal contact	77·8 (39·6 to 144)	94·7 (44 to 169)	60·5 (26·9 to 103)	26·1 (12 to 48)	10·8 (5·3 to 20·4)	3·5 (1·9 to 5·9)	0·5 (0·2 to 0·9)	1,280
Non-venomous animal contact	20·2 (13·1 to 31·1)	17·5 (11·2 to 27·1)	9·2 (5·9 to 14·1)	6·2 (4 to 9·2)	5·3 (3·1 to 8·9)	2·2 (1·3 to 3·3)	0·3 (0·2 to 0·5)	504
Foreign body	323 (234 to 434)	357 (249 to 517)	285 (180 to 439)	236 (155 to 343)	152 (95·4 to 234)	87·6 (52·6 to 135)	13·2 (7·6 to 20·2)	6,490
Pulmonary aspiration and foreign body in airway	275 (186 to 387)	324 (215 to 481)	260 (157 to 409)	220 (139 to 327)	140 (86·4 to 218)	81·4 (47·4 to 129)	12·3 (6·9 to 19·1)	5,590
Foreign body in eyes	10·4 (6·1 to 16)	7 (4·4 to 10·8)	4·4 (2·8 to 6·5)	2·8 (1·9 to 4·2)	1·2 (0·9 to 1·8)	0·5 (0·3 to 0·7)	0·1 (0 to 0·1)	231
Foreign body in other body part	37·3 (26·9 to 50·1)	26·2 (18·7 to 34·5)	21·4 (14·8 to 29·3)	13·1 (9·4 to 17·5)	10·4 (6·4 to 17·4)	5·7 (3·5 to 10·5)	0·9 (0·5 to 1·6)	673
Environmental heat and cold exposure	56·3 (38·3 to 79·7)	38·2 (26·4 to 53·3)	22·2 (15·5 to 31·2)	14·4 (9·6 to 21·4)	6·6 (4·5 to 10·3)	2·8 (1·9 to 4·7)	0·3 (0·2 to 0·6)	1,080
Exposure to forces of nature	5 (3·5 to 7)	3·6 (2·5 to 5)	2·2 (1·6 to 3·1)	1·4 (1 to 2)	0·6 (0·4 to 0·9)	0·2 (0·2 to 0·3)	0 (0 to 0)	130
Other unintentional injuries	335 (222 to 473)	256 (176 to 362)	154 (105 to 218)	111 (75·3 to 156)	53·3 (37·5 to 75·1)	23·7 (16·2 to 34·1)	3 (2·1 to 4·3)	6,850
Self-harm and interpersonal violence	1,090 (798 to 1,490)	785 (584 to 1,040)	414 (296 to 568)	263 (185 to 366)	115 (78·5 to 164)	40 (28·3 to 54·7)	4·7 (3·2 to 6·6)	24,300
Self-harm	778 (512 to 1,170)	580 (392 to 813)	296 (190 to 443)	202 (128 to 298)	91·9 (57·3 to 141)	32·2 (21·1 to 46·3)	3·9 (2·4 to 5·7)	13,300
Self-harm by firearm	3 (1·8 to 5·5)	2·5 (1·4 to 4·8)	1·6 (0·9 to 2·8)	1·5 (0·9 to 2·6)	0·8 (0·4 to 1·4)	0·5 (0·2 to 1)	0·1 (0 to 0·2)	79
Self-harm by other specified means	775 (510 to 1,160)	578 (390 to 810)	295 (189 to 441)	200 (127 to 297)	91·1 (56·7 to 141)	31·7 (20·9 to 45·8)	3·8 (2·4 to 5·6)	13,300
Interpersonal violence	306 (233 to 387)	203 (156 to 260)	117 (89·5 to 153)	60·4 (47·2 to 76·9)	23·3 (17·9 to 30·1)	7·8 (5·8 to 10·2)	0·8 (0·6 to 1·1)	10,800
Physical violence by firearm	8 (5·5 to 11·3)	3·8 (2·8 to 5)	1·9 (1·4 to 2·5)	1·1 (0·8 to 1·5)	0·5 (0·4 to 0·7)	0·2 (0·1 to 0·3)	0 (0 to 0)	353
Physical violence by sharp object	41·7 (29 to 57·3)	32·7 (22·8 to 46·8)	16·9 (11·7 to 25·1)	8·1 (5·7 to 11·2)	3·8 (2·7 to 5·2)	1·4 (1 to 1·9)	0·1 (0·1 to 0·2)	1,260
Sexual violence	54·1 (33·5 to 82·9)	34·2 (21 to 51·1)	19·3 (12·3 to 28·3)	11·9 (7·6 to 17·7)	4·2 (2·4 to 6·6)	0·7 (0·4 to 1·1)	0 (0 to 0)	3,980
Physical violence by other means	202 (148 to 266)	132 (97·4 to 174)	79·2 (57·9 to 108)	39·3 (28·8 to 53·1)	14·8 (10·6 to 20·1)	5·4 (3·9 to 7·3)	0·6 (0·4 to 0·8)	5,210
Conflict and terrorism	1·6 (0·7 to 4·6)	0·9 (0·4 to 2·6)	0·4 (0·2 to 1·1)	0·2 (0·1 to 0·6)	0·1 (0 to 0·3)	0 (0 to 0·1)	0 (0 to 0)	53

SR Table 2b: Grenada DALYS by cause and age group for females with 95% uncertainty intervals, 2019

Cause	Early Neonatal	Late Neonatal	Post Neonatal	1 to 4	5 to 9	10 to 14	15 to 19	20 to 24	25 to 29	30 to 34	35 to 39	40 to 44	45 to 49	50 to 54	55 to 59	60 to 64	65 to 69	70 to 74	75 to 79	80 to 84	85 to 89	90 to 94	95 plus	Total
All causes	368 (263 to 503)	108 (64·7 to 166)	208 (155 to 279)	235 (181 to 297)	221 (174 to 282)	280 (217 to 359)	460 (369 to 564)	598 (481 to 731)	656 (525 to 803)	662 (547 to 794)	667 (557 to 788)	749 (626 to 888)	966 (816 to 1,130)	1,240 (1,070 to 1,430)	1,280 (1,120 to 1,460)	1,220 (1,090 to 1,370)	1,170 (1,060 to 1,300)	1,200 (1,100 to 1,310)	1,090 (1,000 to 1,190)	694 (643 to 744)	418 (391 to 448)	245 (231 to 260)	104 (98·6 to 109)	14,900
Communicable, maternal, neonatal, and nutritional diseases	322 (231 to 443)	84·1 (50·2 to 131)	111 (79·3 to 150)	91·4 (67·4 to 119)	68·9 (47·4 to 101)	49·3 (36·2 to 68·9)	63 (47·2 to 84·4)	78·6 (62·7 to 100)	83·2 (66·3 to 107)	77·1 (63·2 to 94·9)	67·8 (54·5 to 85·3)	65·2 (50·3 to 85·4)	64·7 (50·7 to 85)	69·8 (53·4 to 91)	65·7 (50·5 to 85·2)	59·3 (47·1 to 73·1)	56·8 (44·9 to 72·2)	65·6 (50·1 to 84·5)	75·1 (57·7 to 95·6)	56·9 (44·5 to 71·2)	42·2 (33·3 to 51·3)	26·4 (19·8 to 32·4)	14 (10 to 17·5)	1,760
HIV/AIDS and sexually transmitted infections	0 (0 to 0)	0 (0 to 0)	0·7 (0·6 to 0·7)	0·8 (0·8 to 0·9)	0·8 (0·7 to 0·8)	0·7 (0·6 to 0·7)	2 (1·8 to 2·3)	8·3 (7·3 to 9·7)	14·6 (12·9 to 17·2)	14·8 (13·1 to 17·1)	13·6 (12·1 to 15·4)	12·2 (10·9 to 14·4)	9·5 (8·3 to 11·3)	7 (6·1 to 8·3)	4·1 (3·6 to 5)	2·3 (2 to 2·9)	1·6 (1·4 to 2)	0·8 (0·6 to 1·1)	0·5 (0·4 to 0·7)	0·2 (0·2 to 0·3)	0·1 (0·1 to 0·1)	0 (0 to 0·1)	0 (0 to 0)	94·8
HIV/AIDS	0 (0 to 0)	0 (0 to 0)	0·7 (0·6 to 0·7)	0·8 (0·8 to 0·9)	0·8 (0·7 to 0·8)	0·6 (0·6 to 0·7)	1·6 (1·4 to 1·7)	7·2 (6·5 to 8·2)	12·9 (11·7 to 14·7)	13 (11·7 to 14·5)	11·8 (10·8 to 12·9)	10·4 (9·6 to 11·4)	7·8 (7·2 to 8·5)	5·8 (5·3 to 6·2)	3·3 (3 to 3·6)	1·9 (1·7 to 2·1)	1·3 (1·1 to 1·5)	0·5 (0·4 to 0·6)	0·3 (0·2 to 0·4)	0·1 (0·1 to 0·1)	0·1 (0·1 to 0·1)	0 (0 to 0)	0 (0 to 0)	80·9
HIV/AIDS - Drug-susceptible Tuberculosis	0 (0 to 0)	0 (0 to 0)	0 (0 to 0)	0 (0 to 0)	0 (0 to 0)	0 (0 to 0)	0 (0 to 0·1)	0·2 (0·1 to 0·2)	0·3 (0·2 to 0·4)	0·3 (0·2 to 0·4)	0·3 (0·2 to 0·4)	0·3 (0·2 to 0·4)	0·2 (0·1 to 0·3)	0·1 (0·1 to 0·2)	0·1 (0·1 to 0·1)	0·1 (0 to 0·1)	0 (0 to 0·1)	0 (0 to 0)	0 (0 to 0)	0 (0 to 0)	0 (0 to 0)	0 (0 to 0)	0 (0 to 0)	2
HIV/AIDS - Multidrug-resistant Tuberculosis without extensive dru	0 (0 to 0)	0 (0 to 0)	0 (0 to 0)	0 (0 to 0)	0 (0 to 0)	0 (0 to 0)	0 (0 to 0)	0 (0 to 0)	0 (0 to 0)	0 (0 to 0)	0 (0 to 0)	0 (0 to 0)	0 (0 to 0)	0 (0 to 0)	0 (0 to 0)	0 (0 to 0)	0 (0 to 0)	0 (0 to 0)	0 (0 to 0)	0 (0 to 0)	0 (0 to 0)	0 (0 to 0)	0 (0 to 0)	0
HIV/AIDS - Extensively drug-resistant Tuberculosis	0 (0 to 0)	0 (0 to 0)	0 (0 to 0)	0 (0 to 0)	0 (0 to 0)	0 (0 to 0)	0 (0 to 0)	0 (0 to 0)	0 (0 to 0)	0 (0 to 0)	0 (0 to 0)	0 (0 to 0)	0 (0 to 0)	0 (0 to 0)	0 (0 to 0)	0 (0 to 0)	0 (0 to 0)	0 (0 to 0)	0 (0 to 0)	0 (0 to 0)	0 (0 to 0)	0 (0 to 0)	0 (0 to 0)	0
HIV/AIDS resulting in other diseases	0 (0 to 0)	0 (0 to 0)	0·7 (0·6 to 0·7)	0·8 (0·8 to 0·9)	0·8 (0·7 to 0·8)	0·6 (0·5 to 0·6)	1·5 (1·4 to 1·7)	7·1 (6·3 to 8)	12·6 (11·4 to 14·4)	12·7 (11·4 to 14·2)	11·5 (10·5 to 12·6)	10·1 (9·3 to 11·2)	7·6 (7 to 8·3)	5·6 (5·2 to 6·1)	3·2 (3 to 3·5)	1·8 (1·6 to 2)	1·2 (1·1 to 1·4)	0·5 (0·4 to 0·6)	0·3 (0·2 to 0·3)	0·1 (0·1 to 0·1)	0·1 (0·1 to 0·1)	0 (0 to 0)	0 (0 to 0)	78·9
Sexually transmitted infections excluding HIV	0 (0 to 0)	0 (0 to 0)	0 (0 to 0)	0 (0 to 0)	0 (0 to 0)	0·1 (0·1 to 0·1)	0·4 (0·3 to 0·7)	1·1 (0·6 to 2)	1·8 (0·9 to 3·2)	1·8 (0·9 to 3·4)	1·7 (0·9 to 3·2)	1·8 (0·9 to 3·6)	1·7 (0·8 to 3·3)	1·2 (0·5 to 2·4)	0·8 (0·4 to 1·6)	0·5 (0·2 to 1)	0·3 (0·2 to 0·7)	0·3 (0·1 to 0·5)	0·2 (0·1 to 0·4)	0·1 (0 to 0·2)	0 (0 to 0·1)	0 (0 to 0)	0 (0 to 0)	13·9
Syphilis	0 (0 to 0)	0 (0 to 0)	0 (0 to 0)	0 (0 to 0)	0 (0 to 0)	0 (0 to 0)	0 (0 to 0·1)	0 (0 to 0·1)	0·1 (0 to 0·1)	0·1 (0 to 0·1)	0 (0 to 0·1)	0 (0 to 0)	0 (0 to 0·1)	0 (0 to 0)	0 (0 to 0)	0 (0 to 0)	0 (0 to 0)	0 (0 to 0)	0 (0 to 0)	0 (0 to 0)	0 (0 to 0)	0 (0 to 0)	0 (0 to 0)	0·4
Chlamydial infection	0 (0 to 0)	0 (0 to 0)	0 (0 to 0)	0 (0 to 0)	0 (0 to 0)	0 (0 to 0)	0 (0 to 0·1)	0·1 (0·1 to 0·2)	0·2 (0·1 to 0·3)	0·2 (0·1 to 0·3)	0·2 (0·1 to 0·3)	0·2 (0·1 to 0·3)	0·1 (0·1 to 0·3)	0 (0 to 0·1)	0 (0 to 0)	0 (0 to 0)	0 (0 to 0)	0 (0 to 0)	0 (0 to 0)	0 (0 to 0)	0 (0 to 0)	0 (0 to 0)	0 (0 to 0)	1·1
Gonococcal infection	0 (0 to 0)	0 (0 to 0)	0 (0 to 0)	0 (0 to 0)	0 (0 to 0)	0 (0 to 0)	0·1 (0 to 0·1)	0·1 (0·1 to 0·1)	0·1 (0·1 to 0·1)	0·1 (0·1 to 0·1)	0·1 (0·1 to 0·1)	0·1 (0·1 to 0·2)	0·1 (0·1 to 0·1)	0·1 (0 to 0·1)	0 (0 to 0)	0 (0 to 0)	0 (0 to 0)	0 (0 to 0)	0 (0 to 0)	0 (0 to 0)	0 (0 to 0)	0 (0 to 0)	0 (0 to 0)	0·8
Trichomoniasis	0 (0 to 0)	0 (0 to 0)	0 (0 to 0)	0 (0 to 0)	0 (0 to 0)	0 (0 to 0)	0·1 (0 to 0·2)	0·4 (0·1 to 1)	0·7 (0·2 to 1·6)	0·7 (0·2 to 1·6)	0·6 (0·2 to 1·4)	0·6 (0·2 to 1·5)	0·6 (0·2 to 1·5)	0·5 (0·2 to 1·2)	0·3 (0·1 to 0·8)	0·2 (0·1 to 0·4)	0·1 (0 to 0·3)	0·1 (0 to 0·2)	0·1 (0 to 0·1)	0 (0 to 0)	0 (0 to 0)	0 (0 to 0)	0 (0 to 0)	5
Genital herpes	0 (0 to 0)	0 (0 to 0)	0 (0 to 0)	0 (0 to 0)	0 (0 to 0)	0 (0 to 0·1)	0·1 (0 to 0·3)	0·3 (0·1 to 0·6)	0·4 (0·1 to 0·9)	0·4 (0·1 to 1)	0·4 (0·1 to 0·9)	0·4 (0·1 to 1)	0·4 (0·1 to 1·1)	0·4 (0·1 to 1·1)	0·3 (0·1 to 0·8)	0·2 (0·1 to 0·6)	0·2 (0 to 0·4)	0·1 (0 to 0·3)	0·1 (0 to 0·2)	0 (0 to 0·1)	0 (0 to 0·1)	0 (0 to 0)	0 (0 to 0)	3·8
Other sexually transmitted infections	0 (0 to 0)	0 (0 to 0)	0 (0 to 0)	0 (0 to 0)	0 (0 to 0)	0 (0 to 0)	0·1 (0·1 to 0·2)	0·2 (0·1 to 0·4)	0·3 (0·2 to 0·6)	0·4 (0·2 to 0·7)	0·5 (0·3 to 0·8)	0·5 (0·3 to 0·9)	0·4 (0·2 to 0·8)	0·1 (0·1 to 0·2)	0·1 (0 to 0·1)	0 (0 to 0)	0 (0 to 0)	0 (0 to 0)	0 (0 to 0)	0 (0 to 0)	0 (0 to 0)	0 (0 to 0)	0 (0 to 0)	2·8
Respiratory infections and tuberculosis	12·9 (8·3 to 19·4)	11·2 (6 to 18·3)	49·7 (31·6 to 72·6)	29·3 (19·9 to 41·1)	12·4 (9·2 to 16·5)	9·6 (7 to 13·1)	11·7 (8·4 to 15·9)	15·3 (11·2 to 20·6)	15·4 (11·6 to 20·3)	15·6 (11·5 to 20·6)	14·6 (10·7 to 19·5)	14·4 (10·4 to 19·4)	18·4 (13·4 to 24·6)	24·7 (17·8 to 33·5)	28·1 (20·5 to 38·7)	32·1 (24·2 to 40·9)	36·9 (26·7 to 50·6)	47·7 (33·2 to 65·2)	59·7 (43·7 to 78·1)	47·8 (36·1 to 61·6)	36·7 (28·5 to 45·5)	23·2 (16·7 to 28·8)	12·6 (8·7 to 16)	580
Tuberculosis	0 (0 to 0)	0 (0 to 0)	0·1 (0 to 0·1)	0·3 (0·1 to 0·6)	0·5 (0·1 to 0·9)	0·2 (0·1 to 0·5)	0·3 (0·2 to 0·4)	1·2 (0·7 to 2·1)	1·8 (1 to 3·1)	1·4 (0·8 to 2·3)	0·9 (0·5 to 1·5)	0·8 (0·4 to 1·4)	0·8 (0·4 to 1·3)	0·8 (0·4 to 1·3)	0·6 (0·4 to 1)	0·4 (0·3 to 0·6)	0·3 (0·2 to 0·4)	0·3 (0·2 to 0·5)	0·3 (0·2 to 0·4)	0·2 (0·1 to 0·2)	0·1 (0·1 to 0·1)	0 (0 to 0·1)	0 (0 to 0)	11·5
Latent tuberculosis infection	0 (0 to 0)	0 (0 to 0)	0 (0 to 0)	0 (0 to 0)	0 (0 to 0)	0 (0 to 0)	0 (0 to 0)	0 (0 to 0)	0 (0 to 0)	0 (0 to 0)	0 (0 to 0)	0 (0 to 0)	0 (0 to 0)	0 (0 to 0)	0 (0 to 0)	0 (0 to 0)	0 (0 to 0)	0 (0 to 0)	0 (0 to 0)	0 (0 to 0)	0 (0 to 0)	0 (0 to 0)	0 (0 to 0)	0
Drug-susceptible tuberculosis	0 (0 to 0)	0 (0 to 0)	0·1 (0 to 0·1)	0·3 (0·1 to 0·6)	0·5 (0·1 to 0·9)	0·2 (0·1 to 0·5)	0·3 (0·2 to 0·4)	1·2 (0·7 to 2·1)	1·8 (1 to 3·1)	1·4 (0·8 to 2·3)	0·9 (0·5 to 1·5)	0·8 (0·4 to 1·4)	0·7 (0·4 to 1·2)	0·8 (0·4 to 1·3)	0·6 (0·4 to 1)	0·4 (0·3 to 0·6)	0·3 (0·2 to 0·4)	0·3 (0·2 to 0·4)	0·3 (0·2 to 0·4)	0·2 (0·1 to 0·2)	0·1 (0·1 to 0·1)	0 (0 to 0·1)	0 (0 to 0)	11·3
Multidrug-resistant tuberculosis without extensive drug resistanc	0 (0 to 0)	0 (0 to 0)	0 (0 to 0)	0 (0 to 0)	0 (0 to 0)	0 (0 to 0)	0 (0 to 0)	0 (0 to 0·1)	0 (0 to 0·1)	0 (0 to 0·1)	0 (0 to 0·1)	0 (0 to 0)	0 (0 to 0)	0 (0 to 0)	0 (0 to 0)	0 (0 to 0)	0 (0 to 0)	0 (0 to 0)	0 (0 to 0)	0 (0 to 0)	0 (0 to 0)	0 (0 to 0)	0 (0 to 0)	0·1
Extensively drug-resistant tuberculosis	0 (0 to 0)	0 (0 to 0)	0 (0 to 0)	0 (0 to 0)	0 (0 to 0)	0 (0 to 0)	0 (0 to 0)	0 (0 to 0)	0 (0 to 0)	0 (0 to 0)	0 (0 to 0)	0 (0 to 0)	0 (0 to 0)	0 (0 to 0)	0 (0 to 0)	0 (0 to 0)	0 (0 to 0)	0 (0 to 0)	0 (0 to 0)	0 (0 to 0)	0 (0 to 0)	0 (0 to 0)	0 (0 to 0)	0
Lower respiratory infections	12·9 (8·2 to 19·4)	11·1 (6 to 18·3)	48·7 (30·5 to 71·3)	24·3 (15·4 to 36·3)	6·6 (4·6 to 9·1)	4·7 (3·3 to 6·6)	6·8 (4·5 to 9·4)	9·2 (6·2 to 13·2)	9·3 (6·4 to 13)	10·8 (7·5 to 15·2)	10·9 (7·4 to 15·2)	10·7 (7·2 to 15·3)	14·6 (9·9 to 20·4)	20·9 (14·3 to 29·8)	25·2 (17·9 to 35·8)	30·1 (22·3 to 38·9)	35·4 (25·3 to 49·1)	46·5 (32·2 to 64·3)	58·8 (42·9 to 77·3)	47·3 (35·6 to 61·1)	36·5 (28·2 to 45·3)	23·1 (16·6 to 28·7)	12·5 (8·7 to 16)	517
Upper respiratory infections	0 (0 to 0)	0 (0 to 0·1)	0·8 (0·5 to 1·3)	3·4 (1·9 to 5·5)	3·7 (2 to 6·2)	3·4 (1·8 to 6)	3·5 (1·8 to 6·2)	3·7 (2 to 6·5)	3·4 (1·9 to 5·6)	2·7 (1·4 to 4·5)	2·2 (1·1 to 3·9)	2·2 (1·2 to 4)	2·3 (1·2 to 4)	2·2 (1·2 to 3·7)	1·7 (0·9 to 2·9)	1·2 (0·6 to 2)	0·9 (0·5 to 1·5)	0·7 (0·4 to 1·2)	0·5 (0·3 to 0·8)	0·2 (0·1 to 0·4)	0·1 (0·1 to 0·2)	0·1 (0 to 0·1)	0 (0 to 0)	39·2
Otitis media	0 (0 to 0)	0 (0 to 0)	0·2 (0·1 to 0·3)	1·4 (0·7 to 2·4)	1·7 (0·9 to 2·9)	1·2 (0·6 to 2·1)	1·1 (0·6 to 2)	1·1 (0·4 to 2)	0·9 (0·4 to 1·7)	0·6 (0·3 to 1·2)	0·5 (0·2 to 1)	0·6 (0·3 to 1·1)	0·7 (0·3 to 1·3)	0·8 (0·3 to 1·4)	0·6 (0·3 to 1)	0·4 (0·2 to 0·7)	0·2 (0·1 to 0·4)	0·1 (0·1 to 0·3)	0·1 (0 to 0·2)	0·1 (0 to 0·1)	0 (0 to 0·1)	0 (0 to 0)	0 (0 to 0)	12·2
Enteric infections	0·4 (0·2 to 0·7)	0·9 (0·5 to 1·5)	10 (6·2 to 15·6)	9·9 (6·8 to 14·5)	7·9 (4·7 to 12·7)	7·1 (4·2 to 11·4)	5·1 (3·1 to 7·6)	4·6 (2·8 to 6·9)	4·9 (3·2 to 7)	4·6 (3 to 6·4)	4·3 (2·9 to 6)	4·8 (3·3 to 6·6)	5·8 (3·9 to 8·1)	6·4 (4·4 to 9)	5·9 (4·1 to 8·1)	5·4 (3·9 to 7·3)	5·8 (4·2 to 7·8)	6·4 (4·6 to 8·3)	6·2 (4·5 to 8·1)	3·8 (2·8 to 5·1)	2·2 (1·6 to 2·8)	1·2 (0·9 to 1·5)	0·5 (0·4 to 0·6)	114
Diarrheal diseases	0·4 (0·2 to 0·7)	0·9 (0·5 to 1·5)	9·9 (6·2 to 15·5)	9·9 (6·7 to 14·4)	7·9 (4·7 to 12·7)	7·1 (4·2 to 11·4)	5·1 (3·1 to 7·6)	4·5 (2·7 to 6·8)	4·9 (3·2 to 7)	4·5 (3 to 6·4)	4·3 (2·9 to 5·9)	4·7 (3·2 to 6·6)	5·7 (3·9 to 8·1)	6·4 (4·4 to 9)	5·8 (4·1 to 8·1)	5·4 (3·9 to 7·3)	5·8 (4·1 to 7·8)	6·4 (4·6 to 8·3)	6·2 (4·5 to 8·1)	3·8 (2·8 to 5·1)	2·2 (1·6 to 2·8)	1·2 (0·9 to 1·5)	0·5 (0·4 to 0·6)	113
Typhoid and paratyphoid	0 (0 to 0)	0 (0 to 0)	0 (0 to 0)	0 (0 to 0)	0 (0 to 0)	0 (0 to 0)	0 (0 to 0)	0 (0 to 0)	0 (0 to 0)	0 (0 to 0)	0 (0 to 0)	0 (0 to 0)	0 (0 to 0)	0 (0 to 0)	0 (0 to 0)	0 (0 to 0)	0 (0 to 0)	0 (0 to 0)	0 (0 to 0)	0 (0 to 0)	0 (0 to 0)	0 (0 to 0)	0 (0 to 0)	0
Typhoid fever	0 (0 to 0)	0 (0 to 0)	0 (0 to 0)	0 (0 to 0)	0 (0 to 0)	0 (0 to 0)	0 (0 to 0)	0 (0 to 0)	0 (0 to 0)	0 (0 to 0)	0 (0 to 0)	0 (0 to 0)	0 (0 to 0)	0 (0 to 0)	0 (0 to 0)	0 (0 to 0)	0 (0 to 0)	0 (0 to 0)	0 (0 to 0)	0 (0 to 0)	0 (0 to 0)	0 (0 to 0)	0 (0 to 0)	0
Paratyphoid fever	0 (0 to 0)	0 (0 to 0)	0 (0 to 0)	0 (0 to 0)	0 (0 to 0)	0 (0 to 0)	0 (0 to 0)	0 (0 to 0)	0 (0 to 0)	0 (0 to 0)	0 (0 to 0)	0 (0 to 0)	0 (0 to 0)	0 (0 to 0)	0 (0 to 0)	0 (0 to 0)	0 (0 to 0)	0 (0 to 0)	0 (0 to 0)	0 (0 to 0)	0 (0 to 0)	0 (0 to 0)	0 (0 to 0)	0
Invasive Non-typhoidal Salmonella (iNTS)	0 (0 to 0)	0 (0 to 0)	0 (0 to 0)	0 (0 to 0·1)	0 (0 to 0)	0 (0 to 0)	0 (0 to 0)	0 (0 to 0)	0 (0 to 0)	0 (0 to 0)	0 (0 to 0)	0 (0 to 0)	0 (0 to 0)	0 (0 to 0)	0 (0 to 0)	0 (0 to 0)	0 (0 to 0)	0 (0 to 0)	0 (0 to 0)	0 (0 to 0)	0 (0 to 0)	0 (0 to 0)	0 (0 to 0)	0·1
Other intestinal infectious diseases	0 (0 to 0)	0 (0 to 0)	0 (0 to 0·1)	0·1 (0 to 0·2)	0 (0 to 0·1)	0 (0 to 0·1)	0 (0 to 0)	0 (0 to 0)	0 (0 to 0)	0 (0 to 0)	0 (0 to 0)	0 (0 to 0)	0 (0 to 0)	0 (0 to 0)	0 (0 to 0)	0 (0 to 0)	0 (0 to 0)	0 (0 to 0)	0 (0 to 0)	0 (0 to 0)	0 (0 to 0)	0 (0 to 0)	0 (0 to 0)	0·3
Neglected tropical diseases and malaria	0·1 (0 to 0·2)	0·2 (0·1 to 0·3)	1·3 (0·7 to 2·4)	3·5 (1·9 to 6·3)	3·8 (2 to 6·3)	2·2 (1·2 to 3·9)	2·5 (1·4 to 4·4)	3·6 (1·9 to 5·9)	3·7 (2 to 6·4)	3·5 (1·9 to 5·7)	3 (1·6 to 5)	3·2 (1·8 to 5·5)	3·4 (1·8 to 5·9)	3·9 (2 to 6·8)	4 (2·1 to 6·5)	3·4 (1·8 to 5·8)	3·1 (1·6 to 5·4)	3·1 (1·6 to 5·4)	2·7 (1·3 to 4·7)	1·5 (0·8 to 2·6)	0·8 (0·4 to 1·4)	0·4 (0·2 to 0·8)	0·2 (0·1 to 0·4)	57·1
Malaria	0 (0 to 0)	0 (0 to 0)	0 (0 to 0)	0 (0 to 0)	0 (0 to 0)	0 (0 to 0)	0 (0 to 0)	0 (0 to 0)	0 (0 to 0)	0 (0 to 0)	0 (0 to 0)	0 (0 to 0)	0 (0 to 0)	0 (0 to 0)	0 (0 to 0)	0 (0 to 0)	0 (0 to 0)	0 (0 to 0)	0 (0 to 0)	0 (0 to 0)	0 (0 to 0)	0 (0 to 0)	0 (0 to 0)	0
Chagas disease	0 (0 to 0)	0 (0 to 0)	0 (0 to 0)	0 (0 to 0)	0 (0 to 0)	0 (0 to 0)	0 (0 to 0)	0 (0 to 0)	0 (0 to 0)	0 (0 to 0)	0 (0 to 0)	0 (0 to 0)	0 (0 to 0)	0 (0 to 0)	0 (0 to 0)	0 (0 to 0)	0 (0 to 0)	0 (0 to 0)	0 (0 to 0)	0 (0 to 0)	0 (0 to 0)	0 (0 to 0)	0 (0 to 0)	0·1
Leishmaniasis	0 (0 to 0)	0 (0 to 0)	0 (0 to 0)	0 (0 to 0)	0 (0 to 0)	0 (0 to 0)	0 (0 to 0)	0 (0 to 0)	0 (0 to 0)	0 (0 to 0)	0 (0 to 0)	0 (0 to 0)	0 (0 to 0)	0 (0 to 0)	0 (0 to 0)	0 (0 to 0)	0 (0 to 0)	0 (0 to 0)	0 (0 to 0)	0 (0 to 0)	0 (0 to 0)	0 (0 to 0)	0 (0 to 0)	0
Visceral leishmaniasis	0 (0 to 0)	0 (0 to 0)	0 (0 to 0)	0 (0 to 0)	0 (0 to 0)	0 (0 to 0)	0 (0 to 0)	0 (0 to 0)	0 (0 to 0)	0 (0 to 0)	0 (0 to 0)	0 (0 to 0)	0 (0 to 0)	0 (0 to 0)	0 (0 to 0)	0 (0 to 0)	0 (0 to 0)	0 (0 to 0)	0 (0 to 0)	0 (0 to 0)	0 (0 to 0)	0 (0 to 0)	0 (0 to 0)	0
Cutaneous and mucocutaneous leishmaniasis	0 (0 to 0)	0 (0 to 0)	0 (0 to 0)	0 (0 to 0)	0 (0 to 0)	0 (0 to 0)	0 (0 to 0)	0 (0 to 0)	0 (0 to 0)	0 (0 to 0)	0 (0 to 0)	0 (0 to 0)	0 (0 to 0)	0 (0 to 0)	0 (0 to 0)	0 (0 to 0)	0 (0 to 0)	0 (0 to 0)	0 (0 to 0)	0 (0 to 0)	0 (0 to 0)	0 (0 to 0)	0 (0 to 0)	0
African trypanosomiasis	0 (0 to 0)	0 (0 to 0)	0 (0 to 0)	0 (0 to 0)	0 (0 to 0)	0 (0 to 0)	0 (0 to 0)	0 (0 to 0)	0 (0 to 0)	0 (0 to 0)	0 (0 to 0)	0 (0 to 0)	0 (0 to 0)	0 (0 to 0)	0 (0 to 0)	0 (0 to 0)	0 (0 to 0)	0 (0 to 0)	0 (0 to 0)	0 (0 to 0)	0 (0 to 0)	0 (0 to 0)	0 (0 to 0)	0
Schistosomiasis	0 (0 to 0)	0 (0 to 0)	0 (0 to 0)	0 (0 to 0)	0 (0 to 0)	0 (0 to 0)	0 (0 to 0)	0 (0 to 0)	0 (0 to 0)	0 (0 to 0)	0 (0 to 0)	0 (0 to 0)	0 (0 to 0)	0 (0 to 0)	0 (0 to 0)	0 (0 to 0)	0 (0 to 0)	0 (0 to 0)	0 (0 to 0)	0 (0 to 0)	0 (0 to 0)	0 (0 to 0)	0 (0 to 0)	0
Cysticercosis	0 (0 to 0)	0 (0 to 0)	0 (0 to 0)	0 (0 to 0)	0 (0 to 0)	0 (0 to 0·1)	0·2 (0 to 0·7)	0·9 (0·2 to 2·3)	1·5 (0·4 to 3·4)	1·5 (0·5 to 3·3)	1·4 (0·5 to 3·2)	1·6 (0·5 to 3·6)	2·1 (0·8 to 4·4)	2·6 (0·9 to 5·1)	2·8 (1·1 to 5·3)	2·7 (1·1 to 5·1)	2·6 (1·1 to 4·9)	2·6 (1·2 to 4·9)	2·3 (0·9 to 4·2)	1·3 (0·5 to 2·4)	0·7 (0·3 to 1·2)	0·3 (0·1 to 0·6)	0·1 (0 to 0·2)	27·5
Cystic echinococcosis	0 (0 to 0)	0 (0 to 0)	0 (0 to 0)	0 (0 to 0)	0 (0 to 0)	0 (0 to 0)	0 (0 to 0)	0 (0 to 0)	0 (0 to 0)	0 (0 to 0)	0 (0 to 0)	0 (0 to 0)	0 (0 to 0)	0 (0 to 0)	0 (0 to 0)	0 (0 to 0)	0 (0 to 0)	0 (0 to 0)	0 (0 to 0)	0 (0 to 0)	0 (0 to 0)	0 (0 to 0)	0 (0 to 0)	0
Lymphatic filariasis	0 (0 to 0)	0 (0 to 0)	0 (0 to 0)	0 (0 to 0)	0 (0 to 0)	0 (0 to 0)	0 (0 to 0)	0 (0 to 0)	0 (0 to 0)	0 (0 to 0)	0 (0 to 0)	0 (0 to 0)	0 (0 to 0)	0 (0 to 0)	0 (0 to 0)	0 (0 to 0)	0 (0 to 0)	0 (0 to 0)	0 (0 to 0)	0 (0 to 0)	0 (0 to 0)	0 (0 to 0)	0 (0 to 0)	0
Onchocerciasis	0 (0 to 0)	0 (0 to 0)	0 (0 to 0)	0 (0 to 0)	0 (0 to 0)	0 (0 to 0)	0 (0 to 0)	0 (0 to 0)	0 (0 to 0)	0 (0 to 0)	0 (0 to 0)	0 (0 to 0)	0 (0 to 0)	0 (0 to 0)	0 (0 to 0)	0 (0 to 0)	0 (0 to 0)	0 (0 to 0)	0 (0 to 0)	0 (0 to 0)	0 (0 to 0)	0 (0 to 0)	0 (0 to 0)	0
Trachoma	0 (0 to 0)	0 (0 to 0)	0 (0 to 0)	0 (0 to 0)	0 (0 to 0)	0 (0 to 0)	0 (0 to 0)	0 (0 to 0)	0 (0 to 0)	0 (0 to 0)	0 (0 to 0)	0 (0 to 0)	0 (0 to 0)	0 (0 to 0)	0 (0 to 0)	0 (0 to 0)	0 (0 to 0)	0 (0 to 0)	0 (0 to 0)	0 (0 to 0)	0 (0 to 0)	0 (0 to 0)	0 (0 to 0)	0
Dengue	0 (0 to 0)	0 (0 to 0)	0·1 (0 to 0·2)	0·3 (0·1 to 0·7)	0·6 (0·2 to 1·6)	0·6 (0·1 to 1·2)	0·6 (0·2 to 1·5)	0·8 (0·2 to 2·1)	0·8 (0·2 to 2·3)	0·6 (0·2 to 1·5)	0·4 (0·1 to 0·8)	0·3 (0·1 to 0·8)	0·3 (0·1 to 0·8)	0·4 (0·1 to 0·8)	0·3 (0·1 to 0·6)	0·2 (0 to 0·4)	0·2 (0 to 0·3)	0·1 (0 to 0·3)	0·1 (0 to 0·2)	0·1 (0 to 0·1)	0 (0 to 0·1)	0 (0 to 0·1)	0 (0 to 0·1)	6·8
Yellow fever	0 (0 to 0)	0 (0 to 0)	0 (0 to 0)	0 (0 to 0)	0 (0 to 0)	0 (0 to 0)	0 (0 to 0)	0 (0 to 0)	0 (0 to 0)	0 (0 to 0)	0 (0 to 0)	0 (0 to 0)	0 (0 to 0)	0 (0 to 0)	0 (0 to 0)	0 (0 to 0)	0 (0 to 0)	0 (0 to 0)	0 (0 to 0)	0 (0 to 0)	0 (0 to 0)	0 (0 to 0)	0 (0 to 0)	0
Rabies	0 (0 to 0)	0 (0 to 0)	0 (0 to 0)	0 (0 to 0)	0 (0 to 0)	0 (0 to 0)	0 (0 to 0)	0 (0 to 0)	0 (0 to 0)	0 (0 to 0)	0 (0 to 0)	0 (0 to 0)	0 (0 to 0)	0 (0 to 0)	0 (0 to 0)	0 (0 to 0)	0 (0 to 0)	0 (0 to 0)	0 (0 to 0)	0 (0 to 0)	0 (0 to 0)	0 (0 to 0)	0 (0 to 0)	0·1
Intestinal nematode infections	0 (0 to 0)	0 (0 to 0)	0 (0 to 0)	0·1 (0 to 0·3)	0·3 (0·1 to 0·5)	0·2 (0·1 to 0·4)	0·8 (0·3 to 1·5)	1 (0·4 to 1·8)	0·6 (0·3 to 1·2)	0·4 (0·2 to 0·8)	0·4 (0·2 to 0·8)	0·4 (0·2 to 0·7)	0·1 (0 to 0·2)	0·1 (0 to 0·2)	0·1 (0 to 0·1)	0·1 (0 to 0·1)	0 (0 to 0·1)	0 (0 to 0·1)	0 (0 to 0·1)	0 (0 to 0)	0 (0 to 0)	0 (0 to 0)	0 (0 to 0)	4·6
Ascariasis	0 (0 to 0)	0 (0 to 0)	0 (0 to 0)	0·1 (0 to 0·2)	0·1 (0 to 0·2)	0 (0 to 0·1)	0 (0 to 0·1)	0 (0 to 0·1)	0 (0 to 0·1)	0 (0 to 0·1)	0 (0 to 0)	0 (0 to 0)	0 (0 to 0)	0 (0 to 0)	0 (0 to 0)	0 (0 to 0)	0 (0 to 0)	0 (0 to 0)	0 (0 to 0)	0 (0 to 0)	0 (0 to 0)	0 (0 to 0)	0 (0 to 0)	0·4
Trichuriasis	0 (0 to 0)	0 (0 to 0)	0 (0 to 0)	0 (0 to 0)	0 (0 to 0)	0 (0 to 0)	0 (0 to 0)	0 (0 to 0)	0 (0 to 0)	0 (0 to 0)	0 (0 to 0)	0 (0 to 0)	0 (0 to 0)	0 (0 to 0)	0 (0 to 0)	0 (0 to 0)	0 (0 to 0)	0 (0 to 0)	0 (0 to 0)	0 (0 to 0)	0 (0 to 0)	0 (0 to 0)	0 (0 to 0)	0
Hookworm disease	0 (0 to 0)	0 (0 to 0)	0 (0 to 0)	0·1 (0 to 0·2)	0·2 (0·1 to 0·3)	0·2 (0·1 to 0·3)	0·7 (0·3 to 1·4)	0·9 (0·4 to 1·8)	0·6 (0·2 to 1·2)	0·4 (0·2 to 0·8)	0·4 (0·1 to 0·8)	0·4 (0·1 to 0·7)	0·1 (0 to 0·1)	0·1 (0 to 0·1)	0·1 (0 to 0·1)	0 (0 to 0·1)	0 (0 to 0·1)	0 (0 to 0)	0 (0 to 0)	0 (0 to 0)	0 (0 to 0)	0 (0 to 0)	0 (0 to 0)	4·2
Food-borne trematodiases	0 (0 to 0)	0 (0 to 0)	0 (0 to 0)	0 (0 to 0)	0 (0 to 0)	0 (0 to 0)	0 (0 to 0)	0 (0 to 0)	0 (0 to 0)	0 (0 to 0)	0 (0 to 0)	0 (0 to 0)	0 (0 to 0)	0 (0 to 0)	0 (0 to 0)	0 (0 to 0)	0 (0 to 0)	0 (0 to 0)	0 (0 to 0)	0 (0 to 0)	0 (0 to 0)	0 (0 to 0)	0 (0 to 0)	0
Leprosy	0 (0 to 0)	0 (0 to 0)	0 (0 to 0)	0 (0 to 0)	0 (0 to 0)	0 (0 to 0)	0 (0 to 0)	0 (0 to 0)	0 (0 to 0)	0 (0 to 0)	0 (0 to 0)	0 (0 to 0)	0 (0 to 0)	0 (0 to 0·1)	0 (0 to 0·1)	0 (0 to 0·1)	0 (0 to 0)	0 (0 to 0)	0 (0 to 0)	0 (0 to 0)	0 (0 to 0)	0 (0 to 0)	0 (0 to 0)	0·2
Ebola	0 (0 to 0)	0 (0 to 0)	0 (0 to 0)	0 (0 to 0)	0 (0 to 0)	0 (0 to 0)	0 (0 to 0)	0 (0 to 0)	0 (0 to 0)	0 (0 to 0)	0 (0 to 0)	0 (0 to 0)	0 (0 to 0)	0 (0 to 0)	0 (0 to 0)	0 (0 to 0)	0 (0 to 0)	0 (0 to 0)	0 (0 to 0)	0 (0 to 0)	0 (0 to 0)	0 (0 to 0)	0 (0 to 0)	0
Zika virus	0 (0 to 0)	0 (0 to 0)	0·2 (0 to 0·5)	0·1 (0 to 0·3)	0·1 (0 to 0·4)	0·1 (0 to 0·3)	0·1 (0 to 0·5)	0·1 (0 to 0·5)	0·1 (0 to 0·4)	0·1 (0 to 0·4)	0·1 (0 to 0·4)	0·1 (0 to 0·3)	0 (0 to 0·2)	0 (0 to 0·2)	0 (0 to 0·1)	0 (0 to 0·1)	0 (0 to 0·1)	0 (0 to 0)	0 (0 to 0)	0 (0 to 0)	0 (0 to 0)	0 (0 to 0)	0 (0 to 0)	1
Guinea worm disease	0 (0 to 0)	0 (0 to 0)	0 (0 to 0)	0 (0 to 0)	0 (0 to 0)	0 (0 to 0)	0 (0 to 0)	0 (0 to 0)	0 (0 to 0)	0 (0 to 0)	0 (0 to 0)	0 (0 to 0)	0 (0 to 0)	0 (0 to 0)	0 (0 to 0)	0 (0 to 0)	0 (0 to 0)	0 (0 to 0)	0 (0 to 0)	0 (0 to 0)	0 (0 to 0)	0 (0 to 0)	0 (0 to 0)	0
Other neglected tropical diseases	0·1 (0 to 0·2)	0·2 (0·1 to 0·3)	1 (0·5 to 2·1)	3 (1·4 to 5·5)	2·8 (1·2 to 5·2)	1·3 (0·5 to 2·8)	0·8 (0·4 to 1·6)	0·8 (0·3 to 1·6)	0·7 (0·3 to 1·4)	0·9 (0·4 to 1·6)	0·7 (0·2 to 1·3)	0·8 (0·3 to 1·4)	0·8 (0·4 to 1·5)	0·8 (0·3 to 1·5)	0·8 (0·4 to 1·4)	0·4 (0·2 to 0·7)	0·3 (0·1 to 0·6)	0·3 (0·1 to 0·6)	0·3 (0·1 to 0·7)	0·1 (0·1 to 0·3)	0·1 (0 to 0·2)	0·1 (0 to 0·2)	0·1 (0 to 0·2)	17
Other infectious diseases	0·4 (0·3 to 0·7)	1 (0·6 to 1·7)	5·9 (3·8 to 9·5)	5·8 (3·6 to 9·2)	3·9 (2·6 to 5·7)	2·9 (2 to 4·1)	3·5 (2·6 to 4·5)	3·1 (2·3 to 4·4)	2·7 (2 to 3·7)	2·6 (2 to 3·5)	2·4 (1·7 to 3·2)	2·4 (1·8 to 3·4)	2·5 (1·9 to 3·5)	3 (2·2 to 3·9)	2·8 (2·1 to 3·6)	2·1 (1·7 to 2·6)	1·7 (1·4 to 2·1)	1·6 (1·2 to 1·9)	1·2 (0·9 to 1·5)	0·7 (0·5 to 0·9)	0·5 (0·4 to 0·6)	0·2 (0·2 to 0·3)	0·1 (0·1 to 0·2)	53
Meningitis	0·3 (0·2 to 0·4)	0·7 (0·3 to 1·2)	2 (1·2 to 3·3)	1·4 (0·8 to 2·1)	0·7 (0·5 to 0·9)	0·5 (0·4 to 0·7)	0·8 (0·6 to 1)	0·6 (0·4 to 0·9)	0·5 (0·4 to 0·6)	0·6 (0·4 to 0·7)	0·4 (0·3 to 0·6)	0·4 (0·3 to 0·5)	0·5 (0·4 to 0·6)	0·6 (0·4 to 0·8)	0·5 (0·4 to 0·7)	0·5 (0·4 to 0·6)	0·4 (0·3 to 0·6)	0·3 (0·2 to 0·5)	0·3 (0·2 to 0·4)	0·2 (0·1 to 0·2)	0·1 (0·1 to 0·1)	0 (0 to 0)	0 (0 to 0)	12·1
Encephalitis	0·1 (0 to 0·2)	0·1 (0·1 to 0·3)	2 (0·8 to 5·1)	2 (0·9 to 4·9)	1 (0·5 to 2)	0·9 (0·5 to 1·7)	1·1 (0·7 to 1·8)	1 (0·6 to 1·7)	0·7 (0·5 to 1·2)	0·6 (0·4 to 1)	0·5 (0·3 to 0·9)	0·5 (0·3 to 0·9)	0·5 (0·3 to 0·9)	0·5 (0·3 to 0·8)	0·5 (0·3 to 0·8)	0·4 (0·2 to 0·6)	0·3 (0·2 to 0·5)	0·2 (0·1 to 0·4)	0·1 (0·1 to 0·2)	0·1 (0 to 0·1)	0 (0 to 0·1)	0 (0 to 0)	0 (0 to 0)	13·4
Diphtheria	0 (0 to 0)	0 (0 to 0)	0 (0 to 0)	0 (0 to 0)	0 (0 to 0)	0 (0 to 0)	0 (0 to 0)	0 (0 to 0)	0 (0 to 0)	0 (0 to 0)	0 (0 to 0)	0 (0 to 0)	0 (0 to 0)	0 (0 to 0)	0 (0 to 0)	0 (0 to 0)	0 (0 to 0)	0 (0 to 0)	0 (0 to 0)	0 (0 to 0)	0 (0 to 0)	0 (0 to 0)	0 (0 to 0)	0
Tetanus	0 (0 to 0)	0 (0 to 0)	0 (0 to 0·1)	0 (0 to 0)	0 (0 to 0)	0 (0 to 0)	0 (0 to 0)	0 (0 to 0)	0 (0 to 0)	0 (0 to 0·1)	0·1 (0 to 0·2)	0 (0 to 0·1)	0·1 (0 to 0·1)	0·1 (0·1 to 0·3)	0·1 (0·1 to 0·3)	0·1 (0·1 to 0·3)	0·1 (0 to 0·3)	0·1 (0 to 0·3)	0·1 (0 to 0·3)	0·1 (0 to 0·1)	0 (0 to 0)	0 (0 to 0)	0 (0 to 0)	1·2
Measles	0 (0 to 0)	0 (0 to 0)	0 (0 to 0)	0 (0 to 0)	0 (0 to 0)	0 (0 to 0)	0 (0 to 0)	0 (0 to 0)	0 (0 to 0)	0 (0 to 0)	0 (0 to 0)	0 (0 to 0)	0 (0 to 0)	0 (0 to 0)	0 (0 to 0)	0 (0 to 0)	0 (0 to 0)	0 (0 to 0)	0 (0 to 0)	0 (0 to 0)	0 (0 to 0)	0 (0 to 0)	0 (0 to 0)	0
Varicella and herpes zoster	0 (0 to 0)	0·1 (0 to 0·1)	0·5 (0·2 to 0·9)	0·2 (0·1 to 0·5)	0·2 (0·1 to 0·6)	0·2 (0·1 to 0·3)	0·2 (0·1 to 0·3)	0·2 (0·1 to 0·4)	0·2 (0·1 to 0·5)	0·1 (0·1 to 0·4)	0·1 (0·1 to 0·3)	0·1 (0·1 to 0·4)	0·1 (0·1 to 0·3)	0·3 (0·1 to 0·4)	0·2 (0·1 to 0·3)	0·2 (0·1 to 0·3)	0·1 (0·1 to 0·2)	0·1 (0·1 to 0·3)	0·1 (0·1 to 0·3)	0·1 (0 to 0·2)	0·1 (0 to 0·2)	0 (0 to 0·1)	0 (0 to 0·1)	3·4
Acute hepatitis	0 (0 to 0)	0 (0 to 0)	0·1 (0 to 0·1)	0·1 (0·1 to 0·2)	0·3 (0·1 to 0·5)	0·3 (0·2 to 0·5)	0·4 (0·2 to 0·6)	0·4 (0·2 to 0·6)	0·3 (0·2 to 0·5)	0·2 (0·2 to 0·4)	0·2 (0·1 to 0·3)	0·2 (0·1 to 0·3)	0·2 (0·1 to 0·2)	0·1 (0·1 to 0·2)	0·1 (0·1 to 0·2)	0·1 (0·1 to 0·1)	0·1 (0 to 0·1)	0 (0 to 0·1)	0 (0 to 0)	0 (0 to 0)	0 (0 to 0)	0 (0 to 0)	0 (0 to 0)	3·1
Acute hepatitis A	0 (0 to 0)	0 (0 to 0)	0 (0 to 0)	0·1 (0 to 0·1)	0·2 (0·1 to 0·4)	0·3 (0·1 to 0·5)	0·3 (0·1 to 0·4)	0·2 (0·1 to 0·4)	0·2 (0·1 to 0·3)	0·1 (0·1 to 0·2)	0·1 (0 to 0·2)	0·1 (0 to 0·1)	0·1 (0 to 0·1)	0·1 (0 to 0·1)	0 (0 to 0·1)	0 (0 to 0)	0 (0 to 0)	0 (0 to 0)	0 (0 to 0)	0 (0 to 0)	0 (0 to 0)	0 (0 to 0)	0 (0 to 0)	1·8
Acute hepatitis B	0 (0 to 0)	0 (0 to 0)	0 (0 to 0·1)	0 (0 to 0·1)	0 (0 to 0)	0 (0 to 0)	0·1 (0 to 0·1)	0·1 (0·1 to 0·1)	0·1 (0·1 to 0·1)	0·1 (0 to 0·1)	0·1 (0·1 to 0·1)	0·1 (0·1 to 0·1)	0·1 (0 to 0·1)	0·1 (0 to 0·1)	0·1 (0 to 0·1)	0·1 (0 to 0·1)	0 (0 to 0)	0 (0 to 0)	0 (0 to 0)	0 (0 to 0)	0 (0 to 0)	0 (0 to 0)	0 (0 to 0)	1
Acute hepatitis C	0 (0 to 0)	0 (0 to 0)	0 (0 to 0)	0 (0 to 0)	0 (0 to 0)	0 (0 to 0)	0 (0 to 0)	0 (0 to 0)	0 (0 to 0)	0 (0 to 0)	0 (0 to 0)	0 (0 to 0)	0 (0 to 0)	0 (0 to 0)	0 (0 to 0)	0 (0 to 0)	0 (0 to 0)	0 (0 to 0)	0 (0 to 0)	0 (0 to 0)	0 (0 to 0)	0 (0 to 0)	0 (0 to 0)	0·1
Acute hepatitis E	0 (0 to 0)	0 (0 to 0)	0 (0 to 0)	0 (0 to 0)	0 (0 to 0)	0 (0 to 0·1)	0 (0 to 0·1)	0 (0 to 0·1)	0 (0 to 0·1)	0 (0 to 0)	0 (0 to 0)	0 (0 to 0)	0 (0 to 0)	0 (0 to 0)	0 (0 to 0)	0 (0 to 0)	0 (0 to 0)	0 (0 to 0)	0 (0 to 0)	0 (0 to 0)	0 (0 to 0)	0 (0 to 0)	0 (0 to 0)	0·2
Other unspecified infectious diseases	0·1 (0 to 0·1)	0·2 (0·1 to 0·3)	0·9 (0·5 to 1·5)	1·7 (0·9 to 2·8)	1·7 (0·7 to 3·3)	0·9 (0·4 to 1·8)	1 (0·5 to 1·7)	1 (0·5 to 1·7)	1 (0·6 to 1·7)	1 (0·6 to 1·5)	1 (0·6 to 1·6)	1·2 (0·7 to 1·9)	1·2 (0·7 to 1·9)	1·4 (0·8 to 2·1)	1·4 (0·9 to 2)	0·9 (0·6 to 1·2)	0·7 (0·5 to 1)	0·7 (0·4 to 1)	0·5 (0·3 to 0·6)	0·3 (0·2 to 0·4)	0·3 (0·2 to 0·4)	0·1 (0·1 to 0·2)	0 (0 to 0·1)	19
Maternal and neonatal disorders	308 (221 to 425)	70·2 (41·7 to 109)	32·4 (21 to 48)	17·1 (12·9 to 22)	13·8 (10 to 18·1)	14·7 (11 to 19·5)	23·8 (19·2 to 29)	28·4 (22·8 to 34·3)	26·9 (21·7 to 32·7)	23·4 (19·4 to 28·2)	17·1 (14 to 20·8)	12·7 (10 to 15·7)	10·8 (8 to 13·9)	9·4 (6·8 to 12·3)	6·8 (4·8 to 9·1)	4·1 (2·9 to 5·4)	2·4 (1·7 to 3·2)	1·3 (0·9 to 1·7)	0·5 (0·3 to 0·8)	0·1 (0·1 to 0·2)	0 (0 to 0)	0 (0 to 0)	0 (0 to 0)	624
Maternal disorders	0 (0 to 0)	0 (0 to 0)	0 (0 to 0)	0 (0 to 0)	0 (0 to 0)	0·2 (0·2 to 0·3)	8·9 (7 to 11·1)	12·4 (9·7 to 15·7)	11·8 (9 to 15·2)	11·3 (8·8 to 13·9)	7·3 (5·9 to 9·1)	3 (2·4 to 3·8)	0·7 (0·6 to 1)	0·1 (0·1 to 0·1)	0 (0 to 0)	0 (0 to 0)	0 (0 to 0)	0 (0 to 0)	0 (0 to 0)	0 (0 to 0)	0 (0 to 0)	0 (0 to 0)	0 (0 to 0)	55·8
Maternal hemorrhage	0 (0 to 0)	0 (0 to 0)	0 (0 to 0)	0 (0 to 0)	0 (0 to 0)	0 (0 to 0)	0·9 (0·6 to 1·4)	1·6 (1 to 2·5)	1·8 (1·1 to 2·8)	1·8 (1·2 to 2·6)	1·2 (0·8 to 1·8)	0·5 (0·3 to 0·8)	0·1 (0 to 0·1)	0 (0 to 0)	0 (0 to 0)	0 (0 to 0)	0 (0 to 0)	0 (0 to 0)	0 (0 to 0)	0 (0 to 0)	0 (0 to 0)	0 (0 to 0)	0 (0 to 0)	8
Maternal sepsis and other maternal infections	0 (0 to 0)	0 (0 to 0)	0 (0 to 0)	0 (0 to 0)	0 (0 to 0)	0 (0 to 0)	0·2 (0·1 to 0·4)	0·4 (0·2 to 0·7)	0·3 (0·2 to 0·6)	0·3 (0·1 to 0·4)	0·1 (0·1 to 0·2)	0·1 (0 to 0·1)	0 (0 to 0)	0 (0 to 0)	0 (0 to 0)	0 (0 to 0)	0 (0 to 0)	0 (0 to 0)	0 (0 to 0)	0 (0 to 0)	0 (0 to 0)	0 (0 to 0)	0 (0 to 0)	1·4
Maternal hypertensive disorders	0 (0 to 0)	0 (0 to 0)	0 (0 to 0)	0 (0 to 0)	0 (0 to 0)	0·1 (0·1 to 0·1)	4·1 (3·1 to 5·3)	4·7 (3·4 to 6·3)	4·3 (3·1 to 6)	3·7 (2·7 to 5)	2·2 (1·6 to 3)	0·8 (0·6 to 1·1)	0·2 (0·1 to 0·3)	0 (0 to 0)	0 (0 to 0)	0 (0 to 0)	0 (0 to 0)	0 (0 to 0)	0 (0 to 0)	0 (0 to 0)	0 (0 to 0)	0 (0 to 0)	0 (0 to 0)	20·2
Maternal obstructed labor and uterine rupture	0 (0 to 0)	0 (0 to 0)	0 (0 to 0)	0 (0 to 0)	0 (0 to 0)	0 (0 to 0)	0·2 (0·1 to 0·3)	0·3 (0·2 to 0·4)	0·3 (0·2 to 0·4)	0·3 (0·2 to 0·5)	0·2 (0·1 to 0·4)	0·1 (0·1 to 0·2)	0 (0 to 0)	0 (0 to 0)	0 (0 to 0)	0 (0 to 0)	0 (0 to 0)	0 (0 to 0)	0 (0 to 0)	0 (0 to 0)	0 (0 to 0)	0 (0 to 0)	0 (0 to 0)	1·3
Maternal abortion and miscarriage	0 (0 to 0)	0 (0 to 0)	0 (0 to 0)	0 (0 to 0)	0 (0 to 0)	0 (0 to 0)	0·4 (0·2 to 0·7)	0·7 (0·3 to 1·1)	0·5 (0·3 to 0·9)	0·5 (0·3 to 0·9)	0·4 (0·2 to 0·6)	0·2 (0·1 to 0·3)	0·1 (0 to 0·2)	0 (0 to 0)	0 (0 to 0)	0 (0 to 0)	0 (0 to 0)	0 (0 to 0)	0 (0 to 0)	0 (0 to 0)	0 (0 to 0)	0 (0 to 0)	0 (0 to 0)	2·8
Ectopic pregnancy	0 (0 to 0)	0 (0 to 0)	0 (0 to 0)	0 (0 to 0)	0 (0 to 0)	0 (0 to 0)	0·3 (0·2 to 0·4)	0·5 (0·3 to 0·8)	0·5 (0·3 to 0·8)	0·5 (0·3 to 0·7)	0·3 (0·2 to 0·4)	0·1 (0·1 to 0·1)	0 (0 to 0·1)	0 (0 to 0)	0 (0 to 0)	0 (0 to 0)	0 (0 to 0)	0 (0 to 0)	0 (0 to 0)	0 (0 to 0)	0 (0 to 0)	0 (0 to 0)	0 (0 to 0)	2·2
Indirect maternal deaths	--	--	--	--	--	0 (0 to 0)	0·4 (0·2 to 0·6)	0·6 (0·3 to 0·9)	0·5 (0·3 to 0·8)	0·5 (0·3 to 0·8)	0·3 (0·2 to 0·4)	0·1 (0·1 to 0·1)	0 (0 to 0)	0 (0 to 0)	--	--	--	--	--	--	--	--	--	NA
Late maternal deaths	--	--	--	--	--	0 (0 to 0)	0·6 (0·4 to 1)	0·8 (0·5 to 1·3)	0·7 (0·4 to 1·2)	0·7 (0·4 to 1·1)	0·4 (0·3 to 0·7)	0·2 (0·1 to 0·3)	0 (0 to 0·1)	0 (0 to 0)	--	--	--	--	--	--	--	--	--	NA
Maternal deaths aggravated by HIV/AIDS	--	--	--	--	--	0 (0 to 0)	0 (0 to 0)	0 (0 to 0)	0 (0 to 0)	0 (0 to 0)	0 (0 to 0)	0 (0 to 0)	0 (0 to 0)	0 (0 to 0)	--	--	--	--	--	--	--	--	--	NA
Neonatal disorders	308 (221 to 425)	70·2 (41·7 to 109)	32·4 (21 to 48)	17·1 (12·9 to 22)	13·8 (10 to 18·1)	14·4 (10·8 to 19·3)	14·9 (10·8 to 19·5)	16 (11·8 to 20·8)	15·1 (11·2 to 19·9)	12·2 (8·7 to 15·9)	9·8 (7·1 to 12·9)	9·6 (7 to 12·5)	10 (7·3 to 13·1)	9·3 (6·7 to 12·2)	6·8 (4·8 to 9·1)	4·1 (2·9 to 5·4)	2·4 (1·7 to 3·2)	1·3 (0·9 to 1·7)	0·5 (0·3 to 0·8)	0·1 (0·1 to 0·2)	0 (0 to 0)	0 (0 to 0)	0 (0 to 0)	568
Neonatal preterm birth	150 (106 to 207)	23·5 (12·8 to 38·8)	13·5 (8·5 to 19·7)	5·2 (3·7 to 6·8)	6 (4·1 to 8·1)	6·2 (4·3 to 8·7)	6·3 (4·3 to 8·7)	6·7 (4·7 to 9·2)	6·3 (4·4 to 8·6)	5 (3·4 to 6·9)	4 (2·8 to 5·6)	3·9 (2·7 to 5·3)	4 (2·8 to 5·5)	3·6 (2·5 to 5)	2·4 (1·7 to 3·4)	1·4 (0·9 to 1·9)	0·7 (0·6 to 0·9)	0·4 (0·3 to 0·5)	0·1 (0·1 to 0·2)	0 (0 to 0)	0 (0 to 0)	0 (0 to 0)	0 (0 to 0)	249
Neonatal encephalopathy due to birth asphyxia and trauma	87·6 (60·3 to 125)	12·7 (6·2 to 21·8)	6·2 (3·3 to 10·8)	2·5 (1·5 to 3·6)	1·6 (0·8 to 2·8)	1·7 (0·9 to 2·8)	1·8 (0·9 to 3)	2 (0·9 to 3·2)	1·8 (0·9 to 3)	1·5 (0·7 to 2·5)	1·2 (0·5 to 2)	1·2 (0·6 to 2)	1·2 (0·6 to 2)	1·2 (0·6 to 1·9)	0·9 (0·4 to 1·5)	0·6 (0·3 to 0·9)	0·3 (0·2 to 0·5)	0·1 (0·1 to 0·2)	0 (0 to 0)	0 (0 to 0)	0 (0 to 0)	0 (0 to 0)	0 (0 to 0)	126
Neonatal sepsis and other neonatal infections	34·2 (21·6 to 52·7)	23·2 (12·8 to 39·2)	7·5 (4·8 to 11·5)	4·2 (2·6 to 6·3)	5·5 (3·4 to 8·3)	5·8 (3·6 to 8·9)	6 (3·7 to 9)	6·5 (3·9 to 9·6)	6·2 (3·7 to 9·2)	5 (2·9 to 7·5)	4 (2·5 to 6·1)	4 (2·5 to 5·9)	4·3 (2·6 to 6·3)	4 (2·5 to 6·2)	3 (1·9 to 4·5)	1·9 (1·1 to 2·9)	1·1 (0·7 to 1·8)	0·7 (0·4 to 1·1)	0·3 (0·1 to 0·5)	0·1 (0 to 0·1)	0 (0 to 0)	0 (0 to 0)	0 (0 to 0)	128
Hemolytic disease and other neonatal jaundice	2·3 (1·3 to 3·8)	0·6 (0·3 to 1)	0·4 (0·2 to 0·8)	0·6 (0·4 to 0·9)	0·7 (0·3 to 1·1)	0·7 (0·4 to 1·1)	0·8 (0·6 to 1)	0·8 (0·6 to 1·1)	0·8 (0·6 to 1)	0·6 (0·5 to 0·8)	0·5 (0·4 to 0·7)	0·5 (0·4 to 0·7)	0·6 (0·4 to 0·7)	0·5 (0·4 to 0·7)	0·4 (0·3 to 0·5)	0·3 (0·2 to 0·3)	0·2 (0·1 to 0·2)	0·1 (0·1 to 0·1)	0 (0 to 0·1)	0 (0 to 0)	0 (0 to 0)	0 (0 to 0)	0 (0 to 0)	11·4
Other neonatal disorders	34·7 (20·9 to 55·5)	10·2 (5·2 to 17·3)	4·8 (3·2 to 7)	4·6 (3·4 to 6)	0 (0 to 0)	0 (0 to 0)	0 (0 to 0)	0 (0 to 0)	0 (0 to 0)	0 (0 to 0)	0 (0 to 0)	0 (0 to 0)	0 (0 to 0)	0 (0 to 0)	0 (0 to 0)	0 (0 to 0)	0 (0 to 0)	0 (0 to 0)	0 (0 to 0)	0 (0 to 0)	0 (0 to 0)	0 (0 to 0)	0 (0 to 0)	54·4
Nutritional deficiencies	0 (0 to 0)	0·6 (0·3 to 1·1)	10·8 (6·9 to 15·9)	24·9 (12·4 to 42·6)	26·3 (11 to 50·1)	12·2 (4·2 to 26)	14·3 (5·6 to 29·6)	15·3 (6·1 to 30·5)	14·9 (6·1 to 30·3)	12·7 (5 to 24·2)	12·9 (4·5 to 25·1)	15·5 (6·3 to 30)	14·3 (6 to 28·8)	15·5 (6·5 to 29·4)	14·1 (6·2 to 26·6)	9·9 (4·3 to 18·1)	5·3 (2·6 to 10·2)	4·9 (2·6 to 9·1)	4·4 (2·5 to 7·7)	2·8 (1·7 to 4·5)	2 (1·2 to 3·1)	1·3 (0·9 to 1·9)	0·6 (0·4 to 0·8)	235
Protein-energy malnutrition	0 (0 to 0)	0·1 (0 to 0·1)	5·4 (3·4 to 8·1)	3·5 (2·1 to 5·5)	1·2 (0·8 to 1·8)	0·8 (0·5 to 1·2)	1 (0·6 to 1·5)	1·2 (0·8 to 1·7)	1·1 (0·7 to 1·7)	1 (0·7 to 1·4)	1 (0·7 to 1·4)	1 (0·7 to 1·4)	1·2 (0·8 to 1·7)	1·4 (1 to 2)	1·4 (1 to 2)	1·4 (1 to 1·8)	1·3 (0·9 to 1·8)	1·6 (1·1 to 2·1)	1·7 (1·2 to 2·3)	1·2 (0·9 to 1·7)	1 (0·7 to 1·4)	0·8 (0·5 to 1·1)	0·5 (0·3 to 0·6)	30·8
Iodine deficiency	0 (0 to 0)	0 (0 to 0)	0 (0 to 0)	0 (0 to 0)	0·1 (0 to 0·1)	0·2 (0·1 to 0·3)	0·3 (0·1 to 0·4)	0·3 (0·2 to 0·6)	0·3 (0·1 to 0·7)	0·3 (0·1 to 0·6)	0·2 (0·1 to 0·4)	0·2 (0·1 to 0·5)	0·2 (0·1 to 0·5)	0·2 (0·1 to 0·5)	0·2 (0·1 to 0·4)	0·1 (0·1 to 0·2)	0·1 (0 to 0·1)	0·1 (0 to 0·1)	0 (0 to 0·1)	0 (0 to 0)	0 (0 to 0)	0 (0 to 0)	0 (0 to 0)	2·9
Vitamin A deficiency	0 (0 to 0)	0 (0 to 0·1)	0·1 (0·1 to 0·3)	0·7 (0·3 to 1·2)	0·8 (0·3 to 1·5)	0·5 (0·2 to 0·9)	0·2 (0·1 to 0·4)	0·2 (0·1 to 0·3)	0·1 (0·1 to 0·2)	0·1 (0 to 0·2)	0·1 (0 to 0·1)	0·1 (0 to 0·1)	0·1 (0 to 0·1)	0·1 (0 to 0·1)	0 (0 to 0·1)	0 (0 to 0·1)	0 (0 to 0)	0 (0 to 0)	0 (0 to 0)	0 (0 to 0)	0 (0 to 0)	0 (0 to 0)	0 (0 to 0)	3
Dietary iron deficiency	0 (0 to 0)	0·6 (0·2 to 1)	5 (2 to 9·1)	20·5 (8·3 to 37·8)	24·1 (8·9 to 47·5)	10·6 (2·9 to 24·4)	12·4 (3·6 to 27·8)	13·2 (4·2 to 28·3)	12·8 (4·1 to 28·4)	11 (3·4 to 22·5)	11·3 (2·9 to 23·2)	13·9 (4·8 to 28)	12·3 (4·1 to 26·5)	13·2 (4·2 to 26·9)	12 (4·3 to 24·2)	8 (2·5 to 16)	3·5 (0·9 to 8·4)	2·8 (0·6 to 6·8)	2·2 (0·5 to 5·4)	1·3 (0·3 to 2·9)	0·8 (0·2 to 1·7)	0·4 (0·1 to 0·9)	0·1 (0 to 0·2)	192
Other nutritional deficiencies	0 (0 to 0)	0 (0 to 0)	0·3 (0·1 to 0·5)	0·2 (0·1 to 0·5)	0·2 (0·1 to 0·3)	0·2 (0·1 to 0·3)	0·5 (0·2 to 0·8)	0·4 (0·2 to 0·7)	0·5 (0·3 to 0·8)	0·3 (0·2 to 0·5)	0·3 (0·2 to 0·5)	0·3 (0·2 to 0·5)	0·5 (0·3 to 0·7)	0·6 (0·4 to 0·9)	0·5 (0·3 to 0·7)	0·4 (0·3 to 0·6)	0·4 (0·3 to 0·7)	0·4 (0·2 to 0·7)	0·4 (0·2 to 0·6)	0·2 (0·1 to 0·4)	0·2 (0·1 to 0·4)	0·1 (0·1 to 0·2)	0 (0 to 0·1)	6·9
Non-communicable diseases	42·7 (28·5 to 63)	22·3 (13 to 35·4)	84 (59·6 to 118)	114 (86·3 to 148)	126 (97·7 to 161)	201 (150 to 265)	342 (262 to 437)	455 (353 to 572)	516 (400 to 644)	535 (433 to 649)	555 (459 to 665)	645 (537 to 765)	860 (724 to 1,010)	1,130 (974 to 1,300)	1,180 (1,040 to 1,340)	1,130 (1,010 to 1,270)	1,090 (986 to 1,200)	1,110 (1,010 to 1,210)	993 (911 to 1,080)	620 (574 to 668)	365 (339 to 392)	211 (197 to 226)	86·3 (80·6 to 92·9)	12,400
Neoplasms	0·4 (0·3 to 0·6)	0·4 (0·2 to 0·7)	2·6 (1·7 to 3·9)	13·6 (9·1 to 20·1)	12·8 (10·1 to 15·7)	13·1 (10·9 to 15·6)	22·9 (19 to 26·9)	29·4 (23·4 to 35·7)	39·9 (31·4 to 51·1)	69·5 (56·8 to 85·2)	99·8 (83 to 119)	126 (103 to 153)	181 (152 to 215)	265 (222 to 311)	285 (242 to 330)	264 (226 to 300)	234 (205 to 264)	218 (193 to 244)	163 (145 to 184)	83·8 (72·3 to 94·6)	41·7 (34·8 to 47·7)	18·2 (14·7 to 21·2)	5·7 (4·2 to 6·8)	2,190
Lip and oral cavity cancer	0 (0 to 0)	0 (0 to 0)	0 (0 to 0)	0 (0 to 0)	0 (0 to 0)	0 (0 to 0)	0·1 (0·1 to 0·1)	0·1 (0·1 to 0·1)	0·2 (0·1 to 0·3)	0·2 (0·1 to 0·4)	0·3 (0·2 to 0·4)	0·4 (0·3 to 0·6)	0·7 (0·5 to 1)	1·2 (0·8 to 1·6)	1·4 (1 to 2)	1·5 (1·1 to 2·1)	1·5 (1·1 to 2·1)	1·6 (1·1 to 2·2)	1·3 (0·9 to 1·8)	0·7 (0·5 to 1)	0·4 (0·3 to 0·5)	0·2 (0·1 to 0·3)	0·1 (0 to 0·1)	11·9
Nasopharynx cancer	0 (0 to 0)	0 (0 to 0)	0 (0 to 0)	0 (0 to 0)	0 (0 to 0·1)	0 (0 to 0)	0·2 (0·1 to 0·3)	0·2 (0·1 to 0·3)	0·2 (0·1 to 0·3)	0·2 (0·1 to 0·3)	0·2 (0·1 to 0·3)	0·3 (0·2 to 0·4)	0·4 (0·3 to 0·6)	0·6 (0·4 to 0·9)	0·7 (0·5 to 1)	0·6 (0·4 to 0·9)	0·5 (0·3 to 0·7)	0·4 (0·3 to 0·6)	0·2 (0·2 to 0·4)	0·1 (0·1 to 0·2)	0·1 (0·1 to 0·2)	0 (0 to 0·1)	0 (0 to 0)	5·1
Other pharynx cancer	0 (0 to 0)	0 (0 to 0)	0 (0 to 0)	0 (0 to 0)	0 (0 to 0)	0 (0 to 0)	0 (0 to 0)	0·1 (0·1 to 0·2)	0·1 (0·1 to 0·2)	0·1 (0·1 to 0·2)	0·2 (0·1 to 0·3)	0·3 (0·2 to 0·5)	0·6 (0·4 to 0·9)	0·9 (0·6 to 1·4)	1·1 (0·8 to 1·6)	1·2 (0·8 to 1·7)	0·9 (0·6 to 1·3)	1 (0·7 to 1·4)	0·7 (0·5 to 1)	0·4 (0·2 to 0·5)	0·2 (0·1 to 0·3)	0·1 (0·1 to 0·2)	0 (0 to 0·1)	8
Esophageal cancer	0 (0 to 0)	0 (0 to 0)	0 (0 to 0)	0 (0 to 0)	0 (0 to 0)	0 (0 to 0)	0 (0 to 0)	0·1 (0 to 0·1)	0·1 (0·1 to 0·1)	0·2 (0·1 to 0·3)	0·3 (0·2 to 0·5)	0·6 (0·4 to 0·9)	1·2 (0·8 to 1·7)	2·5 (1·7 to 3·5)	2·8 (2 to 3·9)	3·3 (2·3 to 4·4)	3 (2·1 to 4·1)	3 (2·1 to 4·2)	2·5 (1·7 to 3·5)	1·3 (0·9 to 1·9)	0·7 (0·5 to 0·9)	0·3 (0·2 to 0·4)	0·1 (0·1 to 0·1)	22
Stomach cancer	0 (0 to 0)	0 (0 to 0)	0 (0 to 0)	0 (0 to 0)	0 (0 to 0)	0 (0 to 0)	0·3 (0·2 to 0·4)	0·6 (0·4 to 0·9)	1·3 (0·9 to 1·9)	2·4 (1·6 to 3·4)	3·2 (2·2 to 4·6)	4·2 (3 to 5·8)	6·7 (4·7 to 9·2)	10·6 (7·6 to 14·2)	12·1 (8·8 to 15·8)	13·7 (10·3 to 17·7)	14·1 (10·7 to 18·1)	14 (10·7 to 18·1)	12·8 (10·2 to 16·2)	7·2 (5·4 to 9·5)	3·8 (2·8 to 4·9)	1·7 (1·2 to 2·4)	0·5 (0·3 to 0·8)	109
Colon and rectum cancer	0 (0 to 0)	0 (0 to 0)	0 (0 to 0)	0 (0 to 0)	0·1 (0·1 to 0·1)	0·1 (0·1 to 0·2)	0·6 (0·4 to 0·9)	1 (0·6 to 1·5)	1·6 (1·1 to 2·3)	3·4 (2·2 to 4·9)	4·8 (3·3 to 6·6)	6·7 (4·6 to 9·2)	11·2 (7·8 to 15·3)	20 (14 to 27·8)	24·5 (17·5 to 33·5)	27 (19·8 to 36·5)	27·5 (20·3 to 36·7)	27·5 (20·7 to 35·1)	24·7 (18·8 to 31·5)	14·4 (10·7 to 19)	8·2 (5·8 to 11)	3·6 (2·6 to 4·9)	1·1 (0·7 to 1·5)	208
Liver cancer	0 (0 to 0)	0 (0 to 0)	0·1 (0 to 0·1)	0·2 (0·1 to 0·3)	0·1 (0·1 to 0·2)	0·2 (0·2 to 0·3)	0·1 (0·1 to 0·2)	0·3 (0·2 to 0·4)	0·3 (0·2 to 0·5)	0·5 (0·3 to 0·8)	0·7 (0·4 to 1·2)	1·1 (0·7 to 1·7)	1·8 (1·1 to 2·7)	3·2 (2 to 4·8)	4·2 (2·7 to 6·3)	4 (2·7 to 5·9)	4·1 (2·7 to 6)	4·2 (2·8 to 5·9)	3·3 (2·3 to 4·8)	1·7 (1·2 to 2·4)	0·6 (0·4 to 0·8)	0·2 (0·2 to 0·3)	0·1 (0 to 0·1)	31·2
Liver cancer due to hepatitis B	0 (0 to 0)	0 (0 to 0)	0 (0 to 0)	0 (0 to 0)	0 (0 to 0)	0 (0 to 0·1)	0 (0 to 0·1)	0·1 (0·1 to 0·2)	0·2 (0·1 to 0·3)	0·2 (0·1 to 0·4)	0·3 (0·2 to 0·6)	0·4 (0·2 to 0·8)	0·6 (0·3 to 1)	0·9 (0·4 to 1·6)	1·1 (0·6 to 2)	0·9 (0·4 to 1·7)	0·7 (0·3 to 1·4)	0·6 (0·3 to 1·1)	0·4 (0·2 to 0·7)	0·2 (0·1 to 0·3)	0·1 (0 to 0·1)	0 (0 to 0)	0 (0 to 0)	6·9
Liver cancer due to hepatitis C	0 (0 to 0)	0 (0 to 0)	0 (0 to 0)	0 (0 to 0)	0 (0 to 0)	0 (0 to 0)	0 (0 to 0)	0 (0 to 0)	0 (0 to 0)	0 (0 to 0·1)	0·1 (0 to 0·2)	0·2 (0·1 to 0·3)	0·3 (0·1 to 0·6)	0·7 (0·3 to 1·3)	1·1 (0·5 to 2·1)	1·2 (0·6 to 2·1)	1·3 (0·7 to 2·1)	1·4 (0·8 to 2·3)	1·2 (0·7 to 2)	0·8 (0·4 to 1·1)	0·3 (0·2 to 0·4)	0·1 (0·1 to 0·2)	0 (0 to 0·1)	8·8
Liver cancer due to alcohol use	0 (0 to 0)	0 (0 to 0)	0 (0 to 0)	0 (0 to 0)	0 (0 to 0)	0 (0 to 0)	0 (0 to 0)	0 (0 to 0)	0 (0 to 0·1)	0·1 (0 to 0·1)	0·1 (0·1 to 0·3)	0·3 (0·1 to 0·5)	0·4 (0·2 to 0·8)	0·8 (0·4 to 1·5)	1·1 (0·6 to 2)	1·2 (0·6 to 1·9)	1·3 (0·7 to 2·2)	1·3 (0·7 to 2·1)	0·9 (0·5 to 1·5)	0·4 (0·2 to 0·7)	0·1 (0·1 to 0·2)	0 (0 to 0·1)	0 (0 to 0)	8·2
Liver cancer due to NASH	0 (0 to 0)	0 (0 to 0)	0 (0 to 0)	0 (0 to 0)	0 (0 to 0)	0 (0 to 0)	0 (0 to 0·1)	0·1 (0 to 0·1)	0 (0 to 0·1)	0·1 (0 to 0·1)	0·1 (0 to 0·1)	0·1 (0·1 to 0·2)	0·2 (0·1 to 0·3)	0·3 (0·2 to 0·6)	0·4 (0·2 to 0·8)	0·5 (0·2 to 0·8)	0·5 (0·2 to 0·9)	0·5 (0·3 to 0·9)	0·5 (0·3 to 0·9)	0·3 (0·1 to 0·5)	0·1 (0·1 to 0·2)	0 (0 to 0·1)	0 (0 to 0)	3·8
Liver cancer due to other causes	0 (0 to 0)	0 (0 to 0)	0·1 (0 to 0·1)	0·2 (0·1 to 0·3)	0·1 (0·1 to 0·2)	0·2 (0·1 to 0·2)	0·1 (0 to 0·1)	0·1 (0 to 0·2)	0·1 (0 to 0·1)	0·1 (0 to 0·2)	0·1 (0·1 to 0·2)	0·2 (0·1 to 0·3)	0·2 (0·1 to 0·4)	0·3 (0·2 to 0·6)	0·4 (0·2 to 0·7)	0·3 (0·2 to 0·6)	0·3 (0·2 to 0·6)	0·3 (0·1 to 0·6)	0·2 (0·1 to 0·5)	0·1 (0·1 to 0·2)	0 (0 to 0·1)	0 (0 to 0)	0 (0 to 0)	3·6
Gallbladder and biliary tract cancer	0 (0 to 0)	0 (0 to 0)	0 (0 to 0)	0 (0 to 0)	0 (0 to 0)	0 (0 to 0)	0 (0 to 0)	0 (0 to 0·1)	0·1 (0 to 0·1)	0·1 (0·1 to 0·2)	0·4 (0·2 to 0·6)	0·7 (0·4 to 1·1)	1·3 (0·8 to 2·1)	2·5 (1·7 to 4)	3·3 (2·2 to 5·2)	3·5 (2·4 to 5·6)	3·4 (2·3 to 5·2)	3·3 (2·2 to 5·3)	2·6 (1·8 to 4·1)	1·3 (0·8 to 2)	0·5 (0·3 to 0·8)	0·2 (0·1 to 0·3)	0·1 (0 to 0·1)	23·2
Pancreatic cancer	0 (0 to 0)	0 (0 to 0)	0 (0 to 0)	0 (0 to 0)	0 (0 to 0)	0 (0 to 0)	0·2 (0·1 to 0·2)	0·2 (0·1 to 0·3)	0·5 (0·3 to 0·7)	0·7 (0·5 to 1·1)	1·4 (1 to 1·9)	2·6 (1·9 to 3·5)	5 (3·4 to 6·9)	9·1 (6·3 to 12·7)	12·1 (8·5 to 16·1)	14·4 (10·4 to 19·3)	14·6 (10·6 to 19·5)	15·1 (10·8 to 20·2)	11·6 (8·5 to 15·3)	6·1 (4·2 to 8·5)	2·9 (2·1 to 4)	1·2 (0·8 to 1·7)	0·3 (0·2 to 0·5)	97·9
Larynx cancer	0 (0 to 0)	0 (0 to 0)	0 (0 to 0)	0 (0 to 0)	0 (0 to 0)	0 (0 to 0)	0 (0 to 0)	0 (0 to 0)	0 (0 to 0)	0 (0 to 0·1)	0·1 (0 to 0·1)	0·1 (0·1 to 0·2)	0·3 (0·2 to 0·4)	0·5 (0·3 to 0·7)	0·6 (0·4 to 0·9)	0·7 (0·5 to 1·1)	0·6 (0·4 to 0·9)	0·5 (0·4 to 0·8)	0·4 (0·3 to 0·6)	0·2 (0·1 to 0·3)	0·1 (0·1 to 0·1)	0 (0 to 0·1)	0 (0 to 0)	4·2
Tracheal, bronchus, and lung cancer	0 (0 to 0)	0 (0 to 0)	0 (0 to 0)	0 (0 to 0)	0 (0 to 0)	0 (0 to 0·1)	0·3 (0·2 to 0·6)	0·4 (0·2 to 0·6)	0·5 (0·3 to 0·8)	1·3 (0·8 to 1·9)	2·5 (1·7 to 3·5)	4·2 (2·8 to 5·9)	7·9 (5·5 to 11)	14·4 (10·1 to 19·2)	18·1 (12·7 to 24·8)	19·7 (14·4 to 26)	17·7 (13·1 to 23·8)	15·7 (11·7 to 20·4)	10·8 (7·9 to 14·4)	5 (3·6 to 6·8)	2·3 (1·6 to 3·2)	0·9 (0·6 to 1·3)	0·3 (0·2 to 0·4)	122
Malignant skin melanoma	0 (0 to 0)	0 (0 to 0)	0 (0 to 0·1)	0·1 (0 to 0·3)	0 (0 to 0)	0 (0 to 0)	0·1 (0·1 to 0·2)	0·2 (0·1 to 0·4)	0·2 (0·1 to 0·4)	0·3 (0·2 to 0·5)	0·6 (0·4 to 1)	0·4 (0·2 to 0·8)	0·6 (0·4 to 1·1)	0·7 (0·5 to 1·3)	0·7 (0·4 to 1·3)	0·7 (0·4 to 1·2)	0·7 (0·4 to 1·1)	0·6 (0·4 to 1·1)	0·4 (0·3 to 0·8)	0·3 (0·2 to 0·5)	0·2 (0·1 to 0·3)	0·1 (0·1 to 0·2)	0 (0 to 0·1)	7·1
Non-melanoma skin cancer	0 (0 to 0)	0 (0 to 0)	0 (0 to 0)	0 (0 to 0)	0 (0 to 0)	0 (0 to 0)	0 (0 to 0)	0 (0 to 0)	0 (0 to 0·1)	0 (0 to 0·1)	0 (0 to 0·1)	0·1 (0 to 0·1)	0·1 (0 to 0·1)	0·1 (0·1 to 0·2)	0·1 (0·1 to 0·2)	0·1 (0·1 to 0·2)	0·1 (0·1 to 0·2)	0·2 (0·1 to 0·2)	0·2 (0·1 to 0·2)	0·1 (0·1 to 0·2)	0·1 (0·1 to 0·1)	0·1 (0·1 to 0·1)	0 (0 to 0·1)	1·5
Non-melanoma skin cancer (squamous-cell carcinoma)	0 (0 to 0)	0 (0 to 0)	0 (0 to 0)	0 (0 to 0)	0 (0 to 0)	0 (0 to 0)	0 (0 to 0)	0 (0 to 0)	0 (0 to 0·1)	0 (0 to 0·1)	0 (0 to 0·1)	0·1 (0 to 0·1)	0·1 (0 to 0·1)	0·1 (0·1 to 0·2)	0·1 (0·1 to 0·2)	0·1 (0·1 to 0·2)	0·1 (0·1 to 0·2)	0·2 (0·1 to 0·2)	0·2 (0·1 to 0·2)	0·1 (0·1 to 0·2)	0·1 (0·1 to 0·1)	0·1 (0·1 to 0·1)	0 (0 to 0·1)	1·5
Non-melanoma skin cancer (basal-cell carcinoma)	0 (0 to 0)	0 (0 to 0)	0 (0 to 0)	0 (0 to 0)	0 (0 to 0)	0 (0 to 0)	0 (0 to 0)	0 (0 to 0)	0 (0 to 0)	0 (0 to 0)	0 (0 to 0)	0 (0 to 0)	0 (0 to 0)	0 (0 to 0)	0 (0 to 0)	0 (0 to 0)	0 (0 to 0)	0 (0 to 0)	0 (0 to 0)	0 (0 to 0)	0 (0 to 0)	0 (0 to 0)	0 (0 to 0)	0
Breast cancer	0 (0 to 0)	0 (0 to 0)	0 (0 to 0)	0 (0 to 0)	0 (0 to 0)	0 (0 to 0)	0·6 (0·4 to 1)	1·3 (0·9 to 1·9)	5 (3·3 to 7)	17·3 (12·1 to 23·9)	29·4 (21·5 to 39·5)	39·7 (28·4 to 53)	55·7 (42·1 to 72·2)	79·1 (59·9 to 102)	76·7 (57·9 to 98·9)	57·8 (43·6 to 73·6)	44 (33·5 to 57·6)	39·4 (30·1 to 50·7)	27·9 (21 to 35·6)	14·3 (10·5 to 18·9)	7·8 (5·8 to 10)	3·4 (2·5 to 4·5)	1·2 (0·8 to 1·8)	501
Cervical cancer	0 (0 to 0)	0 (0 to 0)	0 (0 to 0)	0 (0 to 0)	0 (0 to 0)	0 (0 to 0)	0·8 (0·4 to 1·3)	3 (1·8 to 4·9)	8 (4·9 to 12·8)	17 (10·8 to 25·1)	25·6 (16·8 to 36·3)	28·7 (19·5 to 39·6)	35·4 (25 to 48·6)	43·1 (30·1 to 58·6)	40·4 (29·3 to 53·5)	31·1 (22·1 to 41·9)	24·3 (17·3 to 32·1)	20·2 (14·5 to 26·9)	13·4 (9·6 to 17·7)	6·3 (4·4 to 8·7)	2·7 (1·9 to 3·6)	1·2 (0·8 to 1·6)	0·4 (0·3 to 0·5)	302
Uterine cancer	0 (0 to 0)	0 (0 to 0)	0 (0 to 0)	0 (0 to 0)	0 (0 to 0)	0 (0 to 0)	0 (0 to 0)	0·9 (0·5 to 1·3)	1·8 (1 to 3)	3·7 (2·3 to 5·9)	5·4 (3·4 to 7·9)	7·2 (4·7 to 10·3)	12·4 (8·4 to 17·2)	18·1 (12·7 to 25·7)	22 (15·2 to 31·4)	25·3 (17·5 to 35·9)	23 (16·1 to 32·9)	21·3 (15 to 29·5)	13·6 (9·3 to 18·8)	6·5 (4·4 to 9)	2·6 (1·8 to 3·6)	1·1 (0·8 to 1·6)	0·3 (0·2 to 0·5)	165
Ovarian cancer	0 (0 to 0)	0 (0 to 0)	0 (0 to 0)	0 (0 to 0)	0·3 (0·1 to 0·5)	0·5 (0·3 to 0·7)	1·6 (0·9 to 2·6)	2·5 (1·4 to 3·9)	2·4 (1·4 to 3·6)	3·8 (2·1 to 5·5)	5·3 (3·1 to 7·5)	8·1 (5·1 to 11·5)	12·2 (7·8 to 16·6)	18·1 (11·2 to 25·3)	19 (11·6 to 27·3)	15·7 (9·3 to 21·3)	13·5 (7·9 to 18·7)	11·1 (6·6 to 15)	6·8 (4·3 to 9·4)	3·2 (2 to 4·6)	1·5 (0·9 to 2·1)	0·6 (0·4 to 0·9)	0·1 (0·1 to 0·2)	126
Prostate cancer	0 (0 to 0)	0 (0 to 0)	0 (0 to 0)	0 (0 to 0)	0 (0 to 0)	0 (0 to 0)	0 (0 to 0)	0 (0 to 0)	0 (0 to 0)	0 (0 to 0)	0 (0 to 0)	0 (0 to 0)	0 (0 to 0)	0 (0 to 0)	0 (0 to 0)	0 (0 to 0)	0 (0 to 0)	0 (0 to 0)	0 (0 to 0)	0 (0 to 0)	0 (0 to 0)	0 (0 to 0)	0 (0 to 0)	0
Testicular cancer	--	--	--	--	--	--	--	--	--	--	--	--	--	--	--	--	--	--	--	--	--	--	--	NA
Kidney cancer	0 (0 to 0)	0 (0 to 0)	0·1 (0 to 0·1)	0·5 (0·3 to 0·8)	0·4 (0·3 to 0·6)	0·2 (0·2 to 0·3)	0·3 (0·2 to 0·4)	0·5 (0·3 to 0·6)	0·5 (0·3 to 0·6)	0·6 (0·4 to 0·8)	0·7 (0·5 to 1)	1·1 (0·7 to 1·5)	1·6 (1·1 to 2·2)	2·3 (1·7 to 3·2)	2·8 (2 to 3·9)	2·6 (1·9 to 3·5)	2·2 (1·7 to 2·9)	1·9 (1·4 to 2·5)	1·2 (0·9 to 1·6)	0·6 (0·5 to 0·8)	0·2 (0·2 to 0·3)	0·1 (0·1 to 0·1)	0 (0 to 0)	20·5
Bladder cancer	0 (0 to 0)	0 (0 to 0)	0 (0 to 0)	0 (0 to 0)	0 (0 to 0)	0 (0 to 0)	0 (0 to 0·1)	0·1 (0 to 0·1)	0·1 (0 to 0·1)	0·2 (0·2 to 0·4)	0·3 (0·2 to 0·5)	0·5 (0·3 to 0·7)	0·8 (0·5 to 1·1)	1·8 (1·3 to 2·5)	2·5 (1·7 to 3·5)	2·6 (1·8 to 3·5)	3·1 (2·2 to 4·2)	3·4 (2·5 to 4·6)	3·4 (2·4 to 4·6)	2 (1·4 to 2·8)	1·1 (0·8 to 1·6)	0·6 (0·4 to 0·8)	0·2 (0·1 to 0·3)	22·6
Brain and central nervous system cancer	0·1 (0 to 0·1)	0·1 (0 to 0·2)	0·4 (0·1 to 0·8)	1·9 (0·7 to 3·5)	2·4 (1 to 3·9)	1·9 (0·8 to 3·1)	1·6 (0·7 to 2·6)	1·7 (0·7 to 3)	1·7 (0·6 to 3·1)	1·8 (0·7 to 3·1)	2·1 (0·8 to 3·5)	2·1 (0·8 to 3·4)	2·5 (1·1 to 4·3)	3·6 (1·5 to 6)	3·8 (1·5 to 6)	3·1 (1·2 to 5·2)	2·7 (1·1 to 4·4)	2·1 (0·8 to 3·5)	1·4 (0·6 to 2·5)	0·5 (0·2 to 1)	0·2 (0·1 to 0·4)	0·1 (0 to 0·1)	0 (0 to 0)	38
Thyroid cancer	0 (0 to 0)	0 (0 to 0)	0 (0 to 0)	0 (0 to 0)	0 (0 to 0·1)	0 (0 to 0)	0·2 (0·1 to 0·3)	0·1 (0·1 to 0·3)	0·2 (0·1 to 0·3)	0·3 (0·1 to 0·5)	0·4 (0·2 to 0·7)	0·6 (0·3 to 0·9)	0·8 (0·5 to 1·2)	1·5 (1 to 2·3)	1·7 (1·2 to 2·5)	1·7 (1·1 to 2·4)	1·7 (1·2 to 2·4)	1·8 (1·2 to 2·4)	1·2 (0·8 to 1·8)	0·6 (0·4 to 1)	0·3 (0·2 to 0·4)	0·1 (0·1 to 0·2)	0 (0 to 0)	13·3
Mesothelioma	0 (0 to 0)	0 (0 to 0)	0 (0 to 0)	0 (0 to 0)	0 (0 to 0)	0 (0 to 0)	0 (0 to 0)	0·2 (0·1 to 0·5)	0·1 (0·1 to 0·3)	0·2 (0·1 to 0·3)	0·4 (0·2 to 0·6)	0·4 (0·3 to 0·7)	0·5 (0·3 to 0·9)	0·7 (0·5 to 1·3)	0·8 (0·5 to 1·3)	0·3 (0·2 to 0·4)	0·2 (0·2 to 0·4)	0·3 (0·2 to 0·6)	0·1 (0·1 to 0·2)	0·1 (0·1 to 0·2)	0 (0 to 0)	0 (0 to 0)	0 (0 to 0)	4·4
Hodgkin lymphoma	0 (0 to 0)	0 (0 to 0)	0 (0 to 0)	0 (0 to 0)	0 (0 to 0)	0 (0 to 0·1)	0·3 (0·2 to 0·5)	0·5 (0·3 to 0·9)	0·4 (0·2 to 0·7)	0·3 (0·2 to 0·5)	0·2 (0·1 to 0·4)	0·2 (0·1 to 0·4)	0·2 (0·1 to 0·3)	0·2 (0·1 to 0·3)	0·2 (0·1 to 0·4)	0·2 (0·1 to 0·3)	0·2 (0·1 to 0·3)	0·2 (0·1 to 0·2)	0·1 (0·1 to 0·2)	0·1 (0 to 0·1)	0 (0 to 0)	0 (0 to 0)	0 (0 to 0)	3·5
Non-Hodgkin lymphoma	0 (0 to 0)	0 (0 to 0)	0 (0 to 0)	2 (1·2 to 3)	1·9 (1·3 to 2·7)	2·3 (1·7 to 3)	4·5 (3·2 to 6·1)	5·9 (4 to 8·1)	5·7 (3·9 to 8)	6 (4·3 to 8·4)	6 (4·5 to 8·1)	5·3 (3·8 to 7·5)	7·7 (5·7 to 10·2)	10·6 (7·9 to 13·9)	11·7 (8·8 to 15·1)	11·7 (8·8 to 15·2)	11·1 (8·6 to 14·2)	10·1 (7·7 to 12·8)	7·4 (5·6 to 9·5)	3·3 (2·5 to 4·3)	1·3 (1 to 1·6)	0·5 (0·4 to 0·6)	0·1 (0·1 to 0·2)	115
Multiple myeloma	0 (0 to 0)	0 (0 to 0)	0 (0 to 0)	0 (0 to 0)	0 (0 to 0)	0 (0 to 0)	0 (0 to 0)	0·1 (0 to 0·1)	0·1 (0·1 to 0·2)	0·2 (0·1 to 0·4)	0·5 (0·3 to 0·8)	1 (0·6 to 1·4)	1·8 (1·2 to 2·6)	3·7 (2·5 to 5·3)	5·3 (3·7 to 7·6)	5·6 (3·8 to 7·8)	5·3 (3·6 to 7·6)	5·3 (3·7 to 7·4)	3·7 (2·7 to 5·1)	1·5 (1 to 2·3)	0·7 (0·4 to 1·1)	0·2 (0·1 to 0·3)	0·1 (0 to 0·1)	35
Leukemia	0·1 (0·1 to 0·2)	0·1 (0·1 to 0·2)	0·9 (0·5 to 1·4)	4·1 (2·5 to 6·5)	4·5 (3·1 to 6·3)	4·2 (3 to 6)	6·1 (4·4 to 8·1)	5 (3·6 to 6·9)	4·5 (3·1 to 6·4)	4 (3 to 5·2)	4·3 (3 to 6)	3·9 (2·9 to 5·3)	4·8 (3·5 to 6·5)	6 (4·6 to 7·7)	6·6 (5 to 8·7)	6·3 (5 to 7·8)	5·5 (4·3 to 6·8)	5·5 (4·2 to 6·9)	4·1 (3·1 to 5·3)	2·1 (1·6 to 2·8)	1 (0·7 to 1·3)	0·4 (0·3 to 0·5)	0·1 (0·1 to 0·2)	84·4
Acute lymphoid leukemia	0 (0 to 0·1)	0 (0 to 0·1)	0·2 (0·1 to 0·3)	1·5 (0·7 to 2·7)	2 (1·1 to 3·2)	1·6 (0·9 to 2·5)	2·1 (1·2 to 3·2)	0·9 (0·5 to 1·5)	0·5 (0·3 to 0·9)	0·4 (0·2 to 0·6)	0·3 (0·1 to 0·5)	0·3 (0·1 to 0·5)	0·3 (0·1 to 0·5)	0·4 (0·2 to 0·6)	0·3 (0·2 to 0·5)	0·3 (0·1 to 0·4)	0·2 (0·1 to 0·3)	0·1 (0·1 to 0·2)	0·1 (0·1 to 0·2)	0·1 (0 to 0·1)	0 (0 to 0)	0 (0 to 0)	0 (0 to 0)	11·5
Chronic lymphoid leukemia	0 (0 to 0)	0 (0 to 0)	0 (0 to 0)	0 (0 to 0)	0 (0 to 0)	0 (0 to 0)	0 (0 to 0)	0·2 (0·1 to 0·3)	0·1 (0·1 to 0·2)	0·1 (0·1 to 0·2)	0·2 (0·1 to 0·4)	0·3 (0·1 to 0·5)	0·2 (0·1 to 0·3)	0·8 (0·5 to 1·3)	1·1 (0·6 to 1·8)	1·3 (0·8 to 2)	1·2 (0·7 to 1·9)	1 (0·6 to 1·6)	0·8 (0·5 to 1·3)	0·3 (0·2 to 0·6)	0·1 (0·1 to 0·2)	0·1 (0 to 0·1)	0 (0 to 0)	8
Acute myeloid leukemia	0 (0 to 0·1)	0·1 (0 to 0·1)	0·1 (0·1 to 0·3)	1·2 (0·6 to 2·2)	1 (0·5 to 1·9)	1·2 (0·7 to 2)	1·8 (1 to 3·1)	1·5 (0·9 to 2·4)	1·5 (0·9 to 2·4)	1·2 (0·7 to 1·8)	1·3 (0·7 to 2)	1·1 (0·6 to 1·6)	1·5 (0·9 to 2·2)	1·5 (0·9 to 2·3)	1·4 (0·8 to 2·2)	1·2 (0·7 to 1·9)	0·8 (0·4 to 1·2)	0·6 (0·3 to 1·1)	0·4 (0·2 to 0·7)	0·2 (0·1 to 0·3)	0·1 (0 to 0·1)	0 (0 to 0)	0 (0 to 0)	19·6

Chronic myeloid leukemia	0 (0 to 0)	0 (0 to 0)	0 (0 to 0)	0·2 (0·1 to 0·3)	0·1 (0·1 to 0·2)	0·1 (0·1 to 0·2)	0·2 (0·1 to 0·3)	0·3 (0·2 to 0·6)	0·3 (0·2 to 0·6)	0·4 (0·2 to 0·6)	0·4 (0·2 to 0·7)
Other leukemia	0·1 (0 to 0·1)	0 (0 to 0·1)	0·5 (0·3 to 0·9)	1·3 (0·6 to 2·2)	1·4 (0·7 to 2·3)	1·3 (0·8 to 2)	2 (1·2 to 3·1)	2·1 (1·4 to 3·1)	2 (1·3 to 3)	2 (1·3 to 2·8)	2·1 (1·4 to 3·2)
Other malignant neoplasms	0·2 (0·1 to 0·3)	0·2 (0·1 to 0·3)	0·9 (0·6 to 1·3)	4 (2·6 to 6·1)	2·8 (2·1 to 3·6)	3·2 (2·5 to 4·2)	4·6 (3·5 to 5·9)	4 (3·1 to 5·1)	3·6 (2·6 to 4·9)	4 (3 to 5·3)	4·3 (3·1 to 5·7)
Other neoplasms	0 (0 to 0)	0 (0 to 0·1)	0·2 (0·1 to 0·5)	0·7 (0·4 to 1·2)	0·1 (0·1 to 0·2)	0·2 (0·1 to 0·4)	0·3 (0·2 to 0·5)	0·3 (0·2 to 0·5)	0·5 (0·3 to 0·8)	0·5 (0·3 to 0·8)	0·3 (0·2 to 0·5)
Myelodysplastic, myeloproliferative, and other hematopoietic neo	0 (0 to 0)	0 (0 to 0·1)	0·2 (0·1 to 0·5)	0·7 (0·4 to 1·2)	0·1 (0·1 to 0·2)	0·2 (0·1 to 0·4)	0·3 (0·2 to 0·5)	0·3 (0·2 to 0·5)	0·5 (0·3 to 0·8)	0·5 (0·3 to 0·8)	0·3 (0·2 to 0·5)
Benign and in situ intestinal neoplasms	0 (0 to 0)	0 (0 to 0)	0 (0 to 0)	0 (0 to 0)	0 (0 to 0)	0 (0 to 0)	0 (0 to 0)	0 (0 to 0)	0 (0 to 0)	0 (0 to 0)	0 (0 to 0)
Benign and in situ cervical and uterine neoplasms	0 (0 to 0)	0 (0 to 0)	0 (0 to 0)	0 (0 to 0)	0 (0 to 0)	0 (0 to 0)	0 (0 to 0)	0 (0 to 0)	0 (0 to 0)	0 (0 to 0)	0 (0 to 0)
Other benign and in situ neoplasms	0 (0 to 0)	0 (0 to 0)	0 (0 to 0)	0 (0 to 0)	0 (0 to 0)	0 (0 to 0)	0 (0 to 0)	0 (0 to 0)	0 (0 to 0)	0 (0 to 0)	0 (0 to 0)
Cardiovascular diseases	1·4 (0·9 to 2·1)	0·4 (0·2 to 0·7)	3 (1·9 to 4·6)	2·6 (1·8 to 3·7)	3·5 (2·8 to 4·3)	5·9 (4·7 to 7·5)	16·2 (12·9 to 19·6)	22·3 (17·6 to 27·5)	25·6 (20·1 to 32·1)	35·8 (28·8 to 43·7)	48 (38·9 to 59·6)
Rheumatic heart disease	0 (0 to 0)	0 (0 to 0)	0 (0 to 0)	0·2 (0·1 to 0·3)	1 (0·6 to 1·6)	2·2 (1·5 to 3·5)	4·8 (3·3 to 6·7)	5·3 (3·7 to 7·3)	5·9 (4·3 to 8·1)	6·2 (4·6 to 8·2)	6·2 (4·6 to 8·1)
Ischemic heart disease	0 (0 to 0)	0 (0 to 0)	0 (0 to 0)	0 (0 to 0)	0 (0 to 0)	0 (0 to 0)	1 (0·7 to 1·3)	1·6 (1·1 to 2·2)	2·3 (1·6 to 3·1)	5 (3·6 to 6·7)	8·2 (6·1 to 10·9)
Stroke	0·7 (0·4 to 1·1)	0·1 (0 to 0·1)	0·4 (0·2 to 0·7)	0·4 (0·3 to 0·6)	0·9 (0·6 to 1·2)	1·4 (1 to 1·8)	4·5 (3·3 to 5·6)	6·4 (4·6 to 8·6)	8 (5·7 to 10·7)	12·5 (9·1 to 16·6)	17·9 (13·4 to 23·9)
Ischemic stroke	0·2 (0·1 to 0·4)	0 (0 to 0)	0 (0 to 0)	0·2 (0·1 to 0·2)	0·4 (0·2 to 0·7)	0·6 (0·4 to 0·9)	1·3 (0·9 to 1·8)	2 (1·4 to 2·8)	2·2 (1·5 to 3·2)	3·2 (2·2 to 4·4)	4 (2·9 to 5·3)
Intracerebral hemorrhage	0·2 (0·1 to 0·3)	0 (0 to 0)	0·2 (0·1 to 0·4)	0·1 (0·1 to 0·2)	0·2 (0·1 to 0·3)	0·4 (0·3 to 0·5)	1·7 (1·2 to 2·3)	2·5 (1·7 to 3·6)	3·2 (2·1 to 4·5)	5·3 (3·7 to 7·3)	8·3 (5·8 to 11·3)
Subarachnoid hemorrhage	0·3 (0·2 to 0·5)	0 (0 to 0·1)	0·2 (0·1 to 0·4)	0·2 (0·1 to 0·3)	0·2 (0·1 to 0·4)	0·4 (0·3 to 0·6)	1·4 (1 to 2)	1·9 (1·1 to 2·9)	2·5 (1·6 to 3·8)	4 (2·6 to 5·8)	5·7 (3·7 to 8·2)
Hypertensive heart disease	0 (0 to 0)	0 (0 to 0)	0 (0 to 0)	0 (0 to 0)	0 (0 to 0)	0 (0 to 0)	0·3 (0·2 to 0·5)	0·8 (0·4 to 1·1)	0·9 (0·6 to 1·4)	2·2 (1·3 to 3·1)	3·8 (2·3 to 5·4)
Non-rheumatic valvular heart disease	0 (0 to 0)	0 (0 to 0)	0 (0 to 0)	0 (0 to 0)	0 (0 to 0)	0 (0 to 0)	0·3 (0·2 to 0·4)	0·2 (0·1 to 0·3)	0·2 (0·1 to 0·3)	0·3 (0·2 to 0·4)	0·3 (0·2 to 0·5)
Non-rheumatic calcific aortic valve disease	0 (0 to 0)	0 (0 to 0)	0 (0 to 0)	0 (0 to 0)	0 (0 to 0)	0 (0 to 0)	0 (0 to 0·1)	0·1 (0 to 0·1)	0 (0 to 0·1)	0 (0 to 0·1)	0·1 (0 to 0·1)
Non-rheumatic degenerative mitral valve disease	0 (0 to 0)	0 (0 to 0)	0 (0 to 0)	0 (0 to 0)	0 (0 to 0)	0 (0 to 0)	0·2 (0·1 to 0·3)	0·1 (0·1 to 0·2)	0·1 (0·1 to 0·2)	0·2 (0·1 to 0·3)	0·2 (0·2 to 0·3)
Other non-rheumatic valve diseases	0 (0 to 0)	0 (0 to 0)	0 (0 to 0)	0 (0 to 0)	0 (0 to 0)	0 (0 to 0)	0 (0 to 0·1)	0 (0 to 0)	0 (0 to 0·1)	0·1 (0 to 0·1)	0 (0 to 0·1)
Cardiomyopathy and myocarditis	0·4 (0·3 to 0·6)	0·2 (0·1 to 0·3)	1·8 (1·1 to 2·7)	1·3 (0·8 to 1·9)	0·7 (0·5 to 1)	1·1 (0·8 to 1·5)	1·9 (1·2 to 2·7)	2·8 (1·8 to 3·9)	2·7 (1·8 to 3·9)	3·8 (2·4 to 5·3)	4·1 (2·7 to 5·7)
Myocarditis	0·1 (0 to 0·1)	0 (0 to 0·1)	0·4 (0·2 to 0·7)	0·4 (0·2 to 0·7)	0·3 (0·2 to 0·5)	0·3 (0·1 to 0·5)	0·4 (0·2 to 0·7)	0·4 (0·2 to 0·8)	0·4 (0·2 to 0·7)	0·7 (0·4 to 1·3)	0·5 (0·2 to 0·8)
Alcoholic cardiomyopathy	0 (0 to 0)	0 (0 to 0)	0 (0 to 0)	0 (0 to 0)	0 (0 to 0)	0 (0 to 0)	0·1 (0 to 0·1)	0·2 (0·1 to 0·3)	0·2 (0·1 to 0·4)	0·2 (0 to 0·3)	0·2 (0·1 to 0·3)
Other cardiomyopathy	0·3 (0·2 to 0·5)	0·2 (0·1 to 0·3)	1·4 (0·8 to 2·1)	0·9 (0·5 to 1·4)	0·4 (0·3 to 0·6)	0·8 (0·6 to 1·2)	1·4 (0·9 to 2·2)	2·2 (1·4 to 3·1)	2·1 (1·3 to 3·1)	2·9 (1·8 to 4·2)	3·4 (2·1 to 4·8)
Atrial fibrillation and flutter	0 (0 to 0)	0 (0 to 0)	0 (0 to 0)	0 (0 to 0)	0 (0 to 0)	0 (0 to 0)	0 (0 to 0)	0 (0 to 0)	0 (0 to 0)	0·1 (0 to 0·1)	0·1 (0·1 to 0·1)
Aortic aneurysm	--	--	--	--	--	--	0 (0 to 0·1)	0·1 (0·1 to 0·2)	0·2 (0·1 to 0·3)	0·2 (0·1 to 0·3)	0·4 (0·2 to 0·5)
Endocarditis	0 (0 to 0·1)	0·1 (0 to 0·1)	0·4 (0·2 to 0·7)	0·2 (0·1 to 0·4)	0·2 (0·2 to 0·4)	0·3 (0·2 to 0·4)	1·2 (0·8 to 1·9)	2 (1·2 to 3·2)	1·8 (1·1 to 3·2)	1·6 (0·9 to 2·7)	2·3 (1·3 to 3·9)
Other cardiovascular and circulatory diseases	0·3 (0·2 to 0·4)	0·1 (0·1 to 0·2)	0·5 (0·2 to 0·9)	0·5 (0·3 to 0·8)	0·6 (0·4 to 0·9)	0·9 (0·6 to 1·2)	2·1 (1·4 to 3·1)	3 (2·1 to 4·2)	3·4 (2·3 to 4·8)	3·9 (2·7 to 5·3)	4·8 (3·4 to 6·6)
Chronic respiratory diseases	0 (0 to 0)	0 (0 to 0)	0 (0 to 0)	19·5 (11·1 to 33·5)	18 (10·6 to 29·7)	16·3 (9·7 to 25·5)	16·9 (10·5 to 26·7)	19 (12 to 29·1)	18·7 (12·2 to 28·4)	16·6 (11·7 to 23·8)	14·9 (10·6 to 20·3)
Chronic obstructive pulmonary disease	0 (0 to 0)	0 (0 to 0)	0 (0 to 0)	0·3 (0·2 to 0·5)	0·4 (0·1 to 0·7)	0·6 (0·3 to 1)	1·2 (0·7 to 1·8)	2 (1·2 to 2·9)	2·4 (1·6 to 3·3)	2·6 (1·8 to 3·6)	3 (2·1 to 4)
Pneumoconiosis	0 (0 to 0)	0 (0 to 0)	0 (0 to 0)	0 (0 to 0)	0 (0 to 0)	0 (0 to 0)	0 (0 to 0)	0 (0 to 0)	0 (0 to 0)	0 (0 to 0)	0 (0 to 0)
Silicosis	0 (0 to 0)	0 (0 to 0)	0 (0 to 0)	0 (0 to 0)	0 (0 to 0)	0 (0 to 0)	0 (0 to 0)	0 (0 to 0)	0 (0 to 0)	0 (0 to 0)	0 (0 to 0)
Asbestosis	0 (0 to 0)	0 (0 to 0)	0 (0 to 0)	0 (0 to 0)	0 (0 to 0)	0 (0 to 0)	0 (0 to 0)	0 (0 to 0)	0 (0 to 0)	0 (0 to 0)	0 (0 to 0)
Coal workers pneumoconiosis	0 (0 to 0)	0 (0 to 0)	0 (0 to 0)	0 (0 to 0)	0 (0 to 0)	0 (0 to 0)	0 (0 to 0)	0 (0 to 0)	0 (0 to 0)	0 (0 to 0)	0 (0 to 0)
Other pneumoconiosis	0 (0 to 0)	0 (0 to 0)	0 (0 to 0)	0 (0 to 0)	0 (0 to 0)	0 (0 to 0)	0 (0 to 0)	0 (0 to 0)	0 (0 to 0)	0 (0 to 0)	0 (0 to 0)
Asthma	0 (0 to 0)	0 (0 to 0)	0 (0 to 0)	17·3 (8·9 to 31·3)	16·7 (9·3 to 28·3)	14·5 (7·9 to 23·8)	14·8 (8·5 to 24·1)	15·7 (8·8 to 25·2)	14·5 (8·4 to 23·9)	12·2 (7·6 to 18·9)	10·1 (6·5 to 15)
Interstitial lung disease and pulmonary sarcoidosis	0 (0 to 0)	0 (0 to 0)	0 (0 to 0)	0·5 (0·2 to 1)	0·2 (0·1 to 0·3)	0·1 (0·1 to 0·3)	0·3 (0·1 to 0·4)	0·4 (0·2 to 0·6)	0·6 (0·3 to 0·9)	0·8 (0·4 to 1·4)	0·9 (0·5 to 1·5)
Other chronic respiratory diseases	0 (0 to 0)	0 (0 to 0)	0 (0 to 0)	1·4 (0·8 to 2·4)	0·8 (0·4 to 1·2)	1·1 (0·6 to 1·6)	0·6 (0·3 to 0·9)	1 (0·7 to 1·5)	1·3 (0·9 to 2)	0·9 (0·6 to 1·5)	0·9 (0·6 to 1·4)
Digestive diseases	0·5 (0·3 to 0·8)	0·4 (0·2 to 0·8)	3·4 (1·9 to 5·6)	5·9 (4·2 to 8·4)	4·4 (3·2 to 5·8)	4·7 (3·6 to 6·1)	8·4 (6·5 to 11·1)	14·4 (10·8 to 20)	17·3 (12·4 to 23·9)	19·6 (14·7 to 26·1)	20·7 (15·9 to 27·5)
Cirrhosis and other chronic liver diseases	0 (0 to 0)	0 (0 to 0)	0 (0 to 0)	0·4 (0·2 to 0·7)	0·4 (0·3 to 0·7)	0·6 (0·4 to 0·9)	1·5 (0·9 to 2·1)	2·1 (1·4 to 2·8)	2·1 (1·4 to 3)	3·4 (2·2 to 4·6)	4·5 (3·1 to 6·2)
Cirrhosis and other chronic liver diseases due to hepatitis B	0 (0 to 0)	0 (0 to 0)	0 (0 to 0)	0 (0 to 0)	0 (0 to 0)	0 (0 to 0)	0 (0 to 0)	0 (0 to 0·1)	0·1 (0 to 0·2)	0·2 (0·1 to 0·3)	0·3 (0·1 to 0·6)
Cirrhosis and other chronic liver diseases due to hepatitis C	0 (0 to 0)	0 (0 to 0)	0 (0 to 0)	0 (0 to 0)	0 (0 to 0)	0 (0 to 0)	0 (0 to 0·1)	0·2 (0·1 to 0·4)	0·4 (0·2 to 0·7)	0·7 (0·4 to 1·2)	1·1 (0·5 to 1·9)
Cirrhosis and other chronic liver diseases due to alcohol use	0 (0 to 0)	0 (0 to 0)	0 (0 to 0)	0 (0 to 0)	0 (0 to 0)	0 (0 to 0)	0 (0 to 0)	0·1 (0 to 0·2)	0·2 (0·1 to 0·5)	0·7 (0·4 to 1·1)	1·3 (0·7 to 2·1)
Cirrhosis and other chronic liver diseases due to NAFLD	0 (0 to 0)	0 (0 to 0)	0 (0 to 0)	0 (0 to 0)	0 (0 to 0)	0 (0 to 0)	0·1 (0 to 0·1)	0·2 (0·1 to 0·4)	0·3 (0·1 to 0·7)	0·6 (0·3 to 1·1)	0·8 (0·4 to 1·5)
Cirrhosis and other chronic liver diseases due to other causes	0 (0 to 0)	0 (0 to 0)	0 (0 to 0)	0·4 (0·2 to 0·7)	0·4 (0·3 to 0·7)	0·6 (0·4 to 0·8)	1·4 (0·8 to 2)	1·5 (1 to 2·2)	1·1 (0·6 to 1·6)	1·1 (0·6 to 1·8)	0·9 (0·5 to 1·6)
Upper digestive system diseases	0 (0 to 0)	0 (0 to 0)	0 (0 to 0)	0·4 (0·2 to 0·7)	0·5 (0·2 to 0·9)	0·8 (0·5 to 1·3)	2·5 (1·4 to 4·3)	6·3 (3·5 to 11·3)	8·5 (4·6 to 15)	8·5 (4·6 to 14·9)	7·8 (4·3 to 13·3)
Peptic ulcer disease	0 (0 to 0)	0 (0 to 0)	0 (0 to 0)	0·1 (0 to 0·1)	0·1 (0 to 0·1)	0·1 (0·1 to 0·2)	0·2 (0·1 to 0·3)	0·3 (0·2 to 0·5)	0·4 (0·2 to 0·5)	0·6 (0·4 to 0·8)	0·8 (0·5 to 1)
Gastritis and duodenitis	0 (0 to 0)	0 (0 to 0)	0 (0 to 0)	0·3 (0·1 to 0·6)	0·4 (0·2 to 0·8)	0·4 (0·2 to 0·8)	0·5 (0·2 to 1·1)	0·8 (0·4 to 1·4)	0·9 (0·4 to 1·6)	1 (0·5 to 1·9)	1·2 (0·6 to 2·1)
Gastroesophageal reflux disease	0 (0 to 0)	0 (0 to 0)	0 (0 to 0)	0 (0 to 0)	0 (0 to 0)	0·3 (0·1 to 0·5)	1·8 (0·9 to 3·3)	5·2 (2·5 to 10)	7·2 (3·5 to 13·3)	6·8 (3·2 to 13·1)	5·9 (2·8 to 10·9)
Appendicitis	0 (0 to 0)	0 (0 to 0)	0 (0 to 0)	0·2 (0·1 to 0·4)	0·4 (0·2 to 0·5)	0·6 (0·3 to 0·9)	0·7 (0·4 to 1·1)	0·7 (0·4 to 1·2)	0·5 (0·2 to 0·8)	0·5 (0·3 to 0·7)	0·6 (0·3 to 0·9)
Paralytic ileus and intestinal obstruction	0·4 (0·2 to 0·5)	0·4 (0·2 to 0·7)	3·2 (1·8 to 5·3)	1·4 (0·8 to 2·3)	0·6 (0·4 to 0·8)	0·6 (0·4 to 0·9)	0·7 (0·4 to 1·1)	0·7 (0·4 to 1)	0·7 (0·4 to 1·2)	0·8 (0·5 to 1·2)	1·3 (0·8 to 2)
Inguinal, femoral, and abdominal hernia	0·2 (0·1 to 0·3)	0 (0 to 0·1)	0·2 (0·1 to 0·4)	1·1 (0·6 to 2)	1·4 (0·7 to 2·4)	0·9 (0·4 to 1·6)	0·7 (0·4 to 1·3)	0·8 (0·3 to 1·4)	0·9 (0·4 to 1·6)	0·9 (0·5 to 1·6)	0·9 (0·5 to 1·6)
Inflammatory bowel disease	0 (0 to 0)	0 (0 to 0)	0 (0 to 0)	0·5 (0·3 to 0·9)	0·2 (0·1 to 0·3)	0·1 (0·1 to 0·1)	0·3 (0·2 to 0·5)	0·6 (0·4 to 0·9)	0·9 (0·6 to 1·3)	1 (0·7 to 1·5)	1·1 (0·7 to 1·5)
Vascular intestinal disorders	0 (0 to 0)	0 (0 to 0)	0 (0 to 0)	0 (0 to 0)	0 (0 to 0)	0 (0 to 0)	0 (0 to 0)	0·1 (0 to 0·1)	0·1 (0 to 0·1)	0·1 (0 to 0·1)	0·1 (0·1 to 0·2)
Gallbladder and biliary diseases	0 (0 to 0)	0 (0 to 0)	0 (0 to 0)	0·1 (0·1 to 0·2)	0·3 (0·1 to 0·7)	0·5 (0·2 to 1)	1 (0·5 to 1·7)	1·8 (1 to 2·9)	2·4 (1·3 to 3·8)	2·8 (1·8 to 4·3)	3 (1·9 to 4·5)
Pancreatitis	0 (0 to 0)	0 (0 to 0)	0 (0 to 0)	0 (0 to 0·1)	0 (0 to 0)	0·1 (0 to 0·1)	0·2 (0·1 to 0·3)	0·5 (0·4 to 0·8)	0·4 (0·3 to 0·7)	0·5 (0·3 to 0·8)	0·4 (0·3 to 0·6)
Other digestive diseases	0 (0 to 0)	0 (0 to 0)	0 (0 to 0)	1·6 (0·8 to 2·6)	0·6 (0·4 to 0·8)	0·5 (0·3 to 0·7)	0·7 (0·4 to 1·1)	0·8 (0·4 to 1·2)	0·9 (0·5 to 1·5)	1·1 (0·6 to 1·8)	1 (0·5 to 1·6)
Neurological disorders	0 (0 to 0)	0 (0 to 0·1)	1·9 (1·1 to 3·1)	6·2 (3 to 10·7)	12·5 (5·5 to 23)	35 (10·9 to 76·5)	49·4 (16·2 to 105)	57·4 (20·4 to 117)	55·9 (19·7 to 115)	47·5 (17·1 to 94)	41·1 (15·6 to 80·5)
Alzheimer's disease and other dementias	0 (0 to 0)	0 (0 to 0)	0 (0 to 0)	0 (0 to 0)	0 (0 to 0)	0 (0 to 0)	0 (0 to 0)	0 (0 to 0)	0 (0 to 0)	0 (0 to 0)	0 (0 to 0)
Parkinson's disease	0 (0 to 0)	0 (0 to 0)	0 (0 to 0)	0 (0 to 0)	0 (0 to 0)	0 (0 to 0)	0 (0 to 0)	0 (0 to 0)	0 (0 to 0)	0·1 (0 to 0·1)	0·1 (0·1 to 0·1)
Idiopathic epilepsy	0 (0 to 0)	0 (0 to 0·1)	1 (0·5 to 1·8)	4·3 (1·9 to 7·7)	5·9 (2·1 to 11·6)	7·5 (2·7 to 14·4)	10·4 (4·5 to 18·9)	12 (5·6 to 21·3)	10·8 (4·9 to 19·5)	9·2 (4·5 to 15·8)	7·3 (3·8 to 12·4)
Multiple sclerosis	0 (0 to 0)	0 (0 to 0)	0 (0 to 0)	0 (0 to 0)	0 (0 to 0)	0 (0 to 0)	0·1 (0·1 to 0·3)	0·4 (0·2 to 0·6)	0·5 (0·4 to 0·8)	0·7 (0·5 to 1·1)	1·1 (0·8 to 1·8)
Motor neuron disease	0 (0 to 0)	0 (0 to 0)	0·1 (0 to 0·2)	0·1 (0·1 to 0·2)	0 (0 to 0)	0 (0 to 0·1)	0 (0 to 0)	0·1 (0 to 0·1)	0 (0 to 0·1)	0·1 (0 to 0·1)	0·1 (0·1 to 0·1)
Headache disorders	0 (0 to 0)	0 (0 to 0)	0 (0 to 0)	0 (0 to 0)	4·7 (0·1 to 12·5)	24·4 (1·1 to 61·3)	36 (4 to 92·8)	42·7 (5·6 to 101)	42·9 (6·3 to 98·6)	36·2 (6 to 82·6)	31·2 (5·5 to 69·8)
Migraine	0 (0 to 0)	0 (0 to 0)	0 (0 to 0)	0 (0 to 0)	4·4 (0·1 to 11·8)	23·1 (0·8 to 60·2)	33·7 (2·7 to 90·4)	39·8 (4 to 97·6)	39·7 (4·3 to 94·6)	33·5 (4·1 to 79·4)	28·7 (3·9 to 66·7)
Tension-type headache	0 (0 to 0)	0 (0 to 0)	0 (0 to 0)	0 (0 to 0)	0·3 (0 to 2·2)	1·3 (0·1 to 8·8)	2·2 (0·4 to 9·6)	3 (0·6 to 11·7)	3·2 (0·7 to 11·9)	2·8 (0·6 to 9·7)	2·5 (0·6 to 8·1)
Other neurological disorders	0 (0 to 0)	0 (0 to 0)	0·8 (0·4 to 1·3)	1·7 (0·8 to 3)	1·9 (0·6 to 3·8)	3 (0·9 to 5·8)	2·9 (1 to 5·6)	2·2 (0·9 to 4·2)	1·7 (0·7 to 3)	1·4 (0·7 to 2·4)	1·3 (0·7 to 2·1)
Mental disorders	0 (0 to 0)	0 (0 to 0)	0·3 (0·2 to 0·4)	1·9 (1·1 to 2·8)	13·5 (8·8 to 20·2)	48·5 (32·1 to 69·6)	84·1 (57 to 118)	107 (73·9 to 148)	109 (75·2 to 152)	91·2 (62·7 to 126)	78·2 (55·8 to 105)
Schizophrenia	0 (0 to 0)	0 (0 to 0)	0 (0 to 0)	0 (0 to 0)	0 (0 to 0)	0·2 (0·1 to 0·4)	1·7 (0·7 to 3·1)	6·6 (3·4 to 11·1)	11·2 (6·3 to 17·3)	11·6 (7 to 17)	10·6 (6·6 to 15·3)
Depressive disorders	0 (0 to 0)	0 (0 to 0)	0 (0 to 0)	0 (0 to 0)	1 (0·3 to 2)	11·3 (5·8 to 20·1)	29·7 (17·1 to 47·8)	40 (23 to 62·7)	37·7 (21·7 to 57·8)	31 (17·8 to 47·7)	27·9 (17·2 to 41·5)
Major depressive disorder	0 (0 to 0)	0 (0 to 0)	0 (0 to 0)	0 (0 to 0)	0·9 (0·2 to 1·9)	10·5 (5·2 to 19)	27·3 (15·3 to 45·1)	35·3 (19·1 to 57·2)	31·9 (17·4 to 50·5)	25·7 (14 to 41·6)	23·1 (13·7 to 34·8)
Dysthymia	0 (0 to 0)	0 (0 to 0)	0 (0 to 0)	0 (0 to 0)	0·1 (0 to 0·3)	0·8 (0·3 to 1·6)	2·4 (1·2 to 4·3)	4·7 (2·3 to 8·4)	5·8 (3 to 10)	5·3 (3 to 8·7)	4·8 (2·7 to 7·8)
Bipolar disorder	0 (0 to 0)	0 (0 to 0)	0 (0 to 0)	0 (0 to 0)	0 (0 to 0)	3 (1·2 to 5·7)	11·4 (5·3 to 20·3)	15·6 (7·7 to 26·4)	15 (7·6 to 25·2)	11·6 (5·9 to 19)	9·4 (5·1 to 15·5)
Anxiety disorders	0 (0 to 0)	0 (0 to 0)	0 (0 to 0)	0·4 (0·1 to 0·9)	6·3 (3·1 to 11·1)	17·3 (9·3 to 29·2)	24·7 (15 to 38·4)	29·7 (16·6 to 48)	29·6 (16·2 to 49·2)	24·9 (14·7 to 39·4)	21·1 (12·7 to 32·6)
Eating disorders	0 (0 to 0)	0 (0 to 0)	0 (0 to 0)	0 (0 to 0)	0·1 (0 to 0·2)	1·2 (0·6 to 2·2)	5 (2·6 to 9)	9·1 (4·7 to 14·8)	8·2 (4·6 to 13·3)	5·3 (2·9 to 8·5)	3·2 (1·7 to 5·3)
Anorexia nervosa	0 (0 to 0)	0 (0 to 0)	0 (0 to 0)	0 (0 to 0)	0·1 (0 to 0·2)	0·7 (0·3 to 1·3)	2 (0·9 to 3·6)	2·2 (1·1 to 4·1)	1·7 (0·8 to 3)	0·9 (0·4 to 1·7)	0·6 (0·2 to 1·1)
Bulimia nervosa	0 (0 to 0)	0 (0 to 0)	0 (0 to 0)	0 (0 to 0)	0 (0 to 0)	0·6 (0·2 to 1·2)	3·1 (1·2 to 6·2)	6·8 (3 to 12·1)	6·5 (3·3 to 11·2)	4·4 (2·2 to 7·3)	2·7 (1·3 to 4·5)
Autism spectrum disorders	0 (0 to 0)	0 (0 to 0)	0·2 (0·1 to 0·3)	0·9 (0·5 to 1·4)	1·1 (0·6 to 1·8)	1·2 (0·7 to 1·8)	1·2 (0·7 to 1·9)	1·3 (0·7 to 1·9)	1·2 (0·7 to 1·8)	0·9 (0·5 to 1·4)	0·7 (0·4 to 1·1)
Attention-deficit/hyperactivity disorder	0 (0 to 0)	0 (0 to 0)	0 (0 to 0)	0·1 (0 to 0·2)	1·3 (0·7 to 2·4)	1·9 (1 to 3·3)	1·6 (0·9 to 2·9)	1·3 (0·7 to 2·4)	1 (0·5 to 1·7)	0·6 (0·3 to 1·1)	0·4 (0·2 to 0·7)
Conduct disorder	0 (0 to 0)	0 (0 to 0)	0 (0 to 0)	0 (0 to 0)	3 (1·4 to 5·8)	11·4 (5·8 to 19·6)	6·8 (3·3 to 11·9)	0 (0 to 0)	0 (0 to 0)	0 (0 to 0)	0 (0 to 0)
Idiopathic developmental intellectual disability	0 (0 to 0)	0 (0 to 0)	0·1 (0 to 0·2)	0·5 (0·1 to 0·9)	0·7 (0·3 to 1·3)	0·7 (0·3 to 1·3)	0·7 (0·3 to 1·4)	0·8 (0·3 to 1·4)	0·7 (0·2 to 1·3)	0·5 (0·2 to 1)	0·4 (0·1 to 0·8)
Other mental disorders	0 (0 to 0)	0 (0 to 0)	0 (0 to 0)	0 (0 to 0)	0 (0 to 0)	0·2 (0·1 to 0·3)	1·1 (0·4 to 2·1)	3 (1·4 to 5·5)	4·5 (2·4 to 7·4)	4·8 (2·7 to 7·6)	4·5 (2·6 to 7·1)
Substance use disorders	0 (0 to 0)	0 (0 to 0)	0 (0 to 0)	0 (0 to 0)	0 (0 to 0)	0·4 (0·2 to 0·8)	7·6 (4·9 to 11·2)	17·2 (11·1 to 24·6)	17·4 (11·5 to 24·3)	13·5 (9·1 to 19·2)	10·4 (7·5 to 14·1)
Alcohol use disorders	0 (0 to 0)	0 (0 to 0)	0 (0 to 0)	0 (0 to 0)	0 (0 to 0)	0·2 (0·1 to 0·4)	2·6 (1·3 to 4·3)	7·3 (3·9 to 12·5)	8·1 (4·6 to 12·7)	7·2 (4·2 to 11·4)	6 (4 to 8·8)
Drug use disorders	0 (0 to 0)	0 (0 to 0)	0 (0 to 0)	0 (0 to 0)	0 (0 to 0)	0·2 (0·1 to 0·4)	5 (3·1 to 7·9)	9·9 (6·4 to 14·6)	9·3 (6 to 13·6)	6·3 (4·1 to 9·1)	4·4 (3 to 6·2)
Opioid use disorders	0 (0 to 0)	0 (0 to 0)	0 (0 to 0)	0 (0 to 0)	0 (0 to 0)	0 (0 to 0)	1 (0·3 to 2)	4·5 (2·3 to 8)	5·4 (2·8 to 8·7)	3·9 (2·1 to 6·3)	2·6 (1·4 to 4·3)
Cocaine use disorders	0 (0 to 0)	0 (0 to 0)	0 (0 to 0)	0 (0 to 0)	0 (0 to 0)	0 (0 to 0)	1 (0·4 to 2)	1·2 (0·6 to 2·2)	0·9 (0·4 to 1·6)	0·6 (0·3 to 1·1)	0·5 (0·3 to 0·8)
Amphetamine use disorders	0 (0 to 0)	0 (0 to 0)	0 (0 to 0)	0 (0 to 0)	0 (0 to 0)	0 (0 to 0)	0·2 (0·1 to 0·3)	0·8 (0·2 to 1·6)	0·7 (0·2 to 1·5)	0·3 (0·1 to 0·7)	0·2 (0·1 to 0·3)
Cannabis use disorders	0 (0 to 0)	0 (0 to 0)	0 (0 to 0)	0 (0 to 0)	0 (0 to 0)	0·2 (0·1 to 0·4)	1·8 (0·8 to 3·5)	1·6 (0·7 to 2·8)	0·8 (0·3 to 1·7)	0·4 (0·2 to 0·8)	0·2 (0·1 to 0·5)
Other drug use disorders	0 (0 to 0)	0 (0 to 0)	0 (0 to 0)	0 (0 to 0)	0 (0 to 0)	0 (0 to 0)	1·1 (0·6 to 1·8)	1·8 (1·1 to 2·7)	1·5 (0·9 to 2·3)	1·1 (0·7 to 1·6)	0·9 (0·6 to 1·2)
Diabetes and kidney diseases	0 (0 to 0)	0 (0 to 0)	2·2 (1·4 to 3·2)	3·4 (2·3 to 4·8)	4·3 (3·2 to 5·7)	5·9 (4·3 to 7·9)	20·2 (16·1 to 25)	32 (25·4 to 39·9)	40·8 (31·8 to 51·4)	50 (39·9 to 62·6)	58·6 (46·8 to 70·9)
Diabetes mellitus	0 (0 to 0)	0 (0 to 0)	0·6 (0·3 to 1·2)	0·6 (0·3 to 0·9)	0·7 (0·4 to 1)	1·1 (0·8 to 1·6)	7·7 (6 to 9·8)	13·4 (10·1 to 17·1)	17·7 (13·1 to 23·4)	22·7 (17·1 to 29·8)	29·6 (22·7 to 37·6)
Diabetes mellitus type 1	0 (0 to 0)	0 (0 to 0)	0·6 (0·3 to 1·2)	0·6 (0·3 to 0·9)	0·7 (0·4 to 1)	1·1 (0·8 to 1·6)	1·9 (1·3 to 3)	3 (2 to 4·4)	3·2 (2 to 4·9)	2·9 (1·9 to 4·4)	3·8 (2·4 to 6)
Diabetes mellitus type 2	0 (0 to 0)	0 (0 to 0)	0 (0 to 0)	0 (0 to 0)	0 (0 to 0)	0 (0 to 0)	5·8 (4·3 to 7·5)	10·3 (7·4 to 13·6)	14·5 (10·4 to 19·6)	19·9 (14·4 to 26·7)	25·8 (19·2 to 33·5)
Chronic kidney disease	0 (0 to 0)	0 (0 to 0)	1·6 (1 to 2·4)	2·8 (1·9 to 4)	3·6 (2·6 to 5)	4·7 (3·2 to 6·9)	12·5 (9 to 16·3)	18·7 (13·7 to 24·5)	23·1 (16·8 to 31·2)	27·3 (20·2 to 35·8)	29 (21·1 to 38·1)
Chronic kidney disease due to diabetes mellitus type 1	0 (0 to 0)	0 (0 to 0)	0 (0 to 0)	0 (0 to 0·1)	0·1 (0 to 0·2)	0·1 (0·1 to 0·3)	0·5 (0·2 to 1·1)	1·1 (0·5 to 2·4)	2·5 (1·1 to 4·5)	4·4 (2·3 to 7·4)	5·4 (2·8 to 9)
Chronic kidney disease due to diabetes mellitus type 2	0 (0 to 0)	0 (0 to 0)	0 (0 to 0)	0 (0 to 0)	0 (0 to 0)	0 (0 to 0)	0·2 (0·1 to 0·6)	0·5 (0·2 to 1·2)	1 (0·4 to 2)	1·8 (0·8 to 3·4)	2·6 (1·1 to 4·8)
Chronic kidney disease due to hypertension	0 (0 to 0)	0 (0 to 0)	0 (0 to 0)	0 (0 to 0)	0 (0 to 0)	0 (0 to 0)	0·8 (0·3 to 1·5)	1·4 (0·6 to 2·7)	2 (0·9 to 3·6)	2·5 (1·2 to 4·1)	2·8 (1·6 to 4·5)
Chronic kidney disease due to glomerulonephritis	0 (0 to 0)	0 (0 to 0)	0·4 (0·2 to 0·8)	0·9 (0·5 to 1·5)	1·2 (0·7 to 1·9)	1·8 (1·1 to 2·8)	4·9 (3·1 to 7·3)	6·9 (4·2 to 10·4)	8 (4·8 to 11·6)	8·7 (5·3 to 12·6)	8·7 (5·2 to 13·1)
Chronic kidney disease due to other and unspecified causes	0 (0 to 0)	0 (0 to 0)	1·1 (0·6 to 1·8)	1·9 (1·3 to 2·8)	2·3 (1·5 to 3·3)	2·8 (1·6 to 4·3)	6 (3·6 to 8·6)	8·7 (5·6 to 12·2)	9·7 (6·4 to 13·7)	9·9 (6·4 to 14·6)	9·6 (5·8 to 13·9)
Acute glomerulonephritis	0 (0 to 0)	0 (0 to 0)	0 (0 to 0)	0 (0 to 0)	0 (0 to 0)	0 (0 to 0)	0 (0 to 0)	0 (0 to 0)	0 (0 to 0)	0 (0 to 0)	0 (0 to 0)
Skin and subcutaneous diseases	0 (0 to 0)	0 (0 to 0)	3·7 (2·2 to 5·7)	20 (12·9 to 29·7)	25·4 (16·1 to 37·8)	26·3 (16·9 to 38·8)	29·6 (19·2 to 44·2)	22 (14 to 32·8)	18·7 (12·3 to 27)	15·8 (10·8 to 23·1)	13·7 (9·3 to 19·8)
Dermatitis	0 (0 to 0)	0 (0 to 0)	0 (0 to 0)	7·1 (3·8 to 12·3)	8·7 (4·5 to 15·2)	6·4 (3·5 to 10·5)	5 (2·8 to 8·1)	4·5 (2·5 to 7·3)	3·9 (2·2 to 6·3)	3·1 (1·7 to 5)	2·5 (1·3 to 3·9)
Atopic dermatitis	0 (0 to 0)	0 (0 to 0)	0 (0 to 0)	7 (3·8 to 12·3)	8·5 (4·4 to 15)	5·8 (3·1 to 9·8)	3·9 (2·1 to 6·9)	3·1 (1·6 to 5·3)	2·3 (1·2 to 4)	1·6 (0·8 to 2·9)	1·3 (0·6 to 2·2)
Contact dermatitis	0 (0 to 0)	0 (0 to 0)	0 (0 to 0)	0 (0 to 0)	0·1 (0 to 0·2)	0·5 (0·2 to 1)	0·9 (0·4 to 1·8)	1·3 (0·5 to 2·4)	1·4 (0·6 to 2·7)	1·3 (0·5 to 2·5)	1·1 (0·5 to 2·2)
Seborrhoeic dermatitis	0 (0 to 0)	0 (0 to 0)	0 (0 to 0)	0·1 (0 to 0·1)	0·1 (0 to 0·1)	0·1 (0 to 0·2)	0·1 (0·1 to 0·2)	0·1 (0·1 to 0·3)	0·2 (0·1 to 0·3)	0·1 (0·1 to 0·3)	0·1 (0·1 to 0·2)
Psoriasis	0 (0 to 0)	0 (0 to 0)	0 (0 to 0)	0·3 (0·2 to 0·4)	0·6 (0·3 to 1·1)	0·9 (0·4 to 1·6)	1 (0·5 to 1·7)	1·2 (0·6 to 2)	1·3 (0·7 to 2·2)	1·2 (0·7 to 2)	1·1 (0·6 to 1·8)
Bacterial skin diseases	0 (0 to 0)	0 (0 to 0)	2·5 (1·1 to 4·4)	1·2 (0·6 to 2)	1 (0·5 to 1·6)	0·7 (0·3 to 1)	0·8 (0·4 to 1·2)	1·3 (0·6 to 2)	1·3 (0·7 to 2·2)	2·3 (1·2 to 4)	1·8 (0·9 to 3·2)
Cellulitis	0 (0 to 0)	0 (0 to 0)	0·2 (0·1 to 0·7)	0·2 (0·1 to 0·3)	0·3 (0·1 to 0·5)	0·1 (0·1 to 0·2)	0·1 (0 to 0·2)	0·1 (0 to 0·2)	0·2 (0·1 to 0·5)	0·1 (0·1 to 0·5)	0·2 (0·1 to 0·4)
Pyoderma	0 (0 to 0)	0 (0 to 0)	2·2 (1 to 3·9)	1·1 (0·5 to 1·8)	0·8 (0·4 to 1·2)	0·5 (0·3 to 0·9)	0·7 (0·4 to 1·1)	1·2 (0·6 to 1·8)	1·1 (0·5 to 1·8)	2·2 (1·1 to 3·8)	1·6 (0·8 to 3)
Scabies	0 (0 to 0)	0 (0 to 0)	0·4 (0·2 to 0·7)	3 (1·5 to 5·4)	4·1 (2 to 7·3)	4·3 (2 to 7·6)	4·7 (2·4 to 8·1)	4·7 (2·4 to 8·3)	3·3 (1·7 to 6)	2·1 (1 to 3·8)	1·7 (0·8 to 2·9)
Fungal skin diseases	0 (0 to 0)	0 (0 to 0)	0·1 (0 to 0·2)	0·6 (0·2 to 1·2)	0·9 (0·3 to 2)	1·1 (0·4 to 2·4)	1·2 (0·4 to 2·7)	1·4 (0·5 to 3)	1·4 (0·5 to 3·1)	1·2 (0·5 to 2·7)	1 (0·4 to 2·3)
Viral skin diseases	0 (0 to 0)	0 (0 to 0)	0 (0 to 0)	3·3 (2·1 to 5·1)	5·2 (3·2 to 8)	2·7 (1·7 to 4·2)	1·8 (1·1 to 2·8)	1·6 (0·9 to 2·4)	1·1 (0·7 to 1·8)	0·8 (0·4 to 1·2)	0·6 (0·4 to 0·9)
Acne vulgaris	0 (0 to 0)	0 (0 to 0)	0 (0 to 0)	0 (0 to 0)	0·3 (0·2 to 0·5)	6·5 (3·7 to 10·7)	11·2 (6·3 to 18·9)	3·2 (1·7 to 5·2)	1·5 (0·8 to 2·5)	0·9 (0·4 to 1·5)	0·6 (0·3 to 1)
Alopecia areata	0 (0 to 0)	0 (0 to 0)	0 (0 to 0)	0·1 (0 to 0·2)	0·1 (0 to 0·2)	0·2 (0·1 to 0·3)	0·3 (0·2 to 0·5)	0·5 (0·3 to 0·9)	0·7 (0·4 to 1·1)	0·6 (0·3 to 1)	0·5 (0·3 to 0·7)
Pruritus	0 (0 to 0)	0 (0 to 0)	0 (0 to 0)	0·1 (0·1 to 0·3)	0·2 (0·1 to 0·4)	0·2 (0·1 to 0·4)	0·2 (0·1 to 0·5)	0·3 (0·1 to 0·7)	0·4 (0·2 to 0·8)	0·4 (0·2 to 0·7)	0·3 (0·1 to 0·6)
Urticaria	0 (0 to 0)	0 (0 to 0)	0·5 (0·3 to 0·8)	3·5 (2 to 5·5)	3·1 (1·8 to 5·1)	2·2 (1·2 to 3·6)	2 (1·1 to 3·4)	2·1 (1·1 to 3·6)	1·9 (1 to 3)	1·6 (0·8 to 2·6)	1·4 (0·8 to 2·2)
Decubitus ulcer	0 (0 to 0)	0 (0 to 0)	0 (0 to 0)	0·2 (0 to 0·4)	0·4 (0·1 to 0·7)	0·3 (0·1 to 0·5)	0·3 (0·1 to 0·6)	0·1 (0 to 0·2)	0·5 (0·2 to 1)	0·3 (0·1 to 0·6)	0·9 (0·4 to 1·8)
Other skin and subcutaneous diseases	0 (0 to 0)	0 (0 to 0)	0·1 (0·1 to 0·2)	0·7 (0·3 to 1·3)	0·7 (0·3 to 1·4)	0·9 (0·4 to 1·7)	1·1 (0·6 to 2·1)	1·3 (0·6 to 2·4)	1·4 (0·7 to 2·5)	1·4 (0·7 to 2·6)	1·3 (0·6 to 2·5)
Sense organ diseases	0 (0 to 0)	0 (0 to 0)	0·2 (0·1 to 0·3)	2 (1·2 to 2·8)	4·4 (2·7 to 6·4)	5·8 (3·6 to 8·6)	7·5 (4·6 to 10·8)	10·1 (6·2 to 15·1)	12·2 (7·8 to 18·3)	12·6 (8 to 19)	12·5 (7·9 to 18·9)
Blindness and vision loss	0 (0 to 0)	0 (0 to 0)	0 (0 to 0·1)	0·6 (0·3 to 0·9)	1·3 (0·8 to 2·2)	1·8 (1·1 to 2·8)	2·2 (1·4 to 3·5)	2·9 (1·7 to 4·4)	3 (1·9 to 4·4)	2·8 (1·6 to 4·2)	2·8 (1·8 to 4·3)
Glaucoma	0 (0 to 0)	0 (0 to 0)	0 (0 to 0)	0 (0 to 0)	0 (0 to 0)	0 (0 to 0)	0 (0 to 0)	0 (0 to 0)	0 (0 to 0)	0 (0 to 0)	0 (0 to 0)
Cataract	0 (0 to 0)	0 (0 to 0)	0 (0 to 0)	0 (0 to 0)	0 (0 to 0)	0 (0 to 0)	0 (0 to 0)	0 (0 to 0)	0·1 (0 to 0·1)	0·1 (0 to 0·3)	0·2 (0·1 to 0·4)
Age-related macular degeneration	0 (0 to 0)	0 (0 to 0)	0 (0 to 0)	0 (0 to 0)	0 (0 to 0)	0 (0 to 0)	0 (0 to 0)	0 (0 to 0)	0 (0 to 0)	0 (0 to 0)	0 (0 to 0)
Refraction disorders	0 (0 to 0)	0 (0 to 0)	0 (0 to 0)	0·5 (0·3 to 0·8)	1·1 (0·6 to 1·9)	1·5 (0·9 to 2·5)	1·9 (1·1 to 3·1)	2·4 (1·4 to 3·9)	2·4 (1·4 to 3·7)	2 (1·1 to 3·1)	1·8 (1 to 2·7)
Near vision loss	0 (0 to 0)	0 (0 to 0)	0 (0 to 0)	0 (0 to 0)	0 (0 to 0)	0 (0 to 0)	0 (0 to 0)	0·1 (0 to 0·1)	0·1 (0 to 0·2)	0·2 (0·1 to 0·5)	0·4 (0·1 to 0·9)
Other vision loss	0 (0 to 0)	0 (0 to 0)	0 (0 to 0)	0·1 (0 to 0·2)	0·2 (0·1 to 0·4)	0·2 (0·1 to 0·5)	0·3 (0·1 to 0·6)	0·4 (0·2 to 0·7)	0·4 (0·2 to 0·8)	0·4 (0·2 to 0·8)	0·5 (0·2 to 0·8)
Age-related and other hearing loss	0 (0 to 0)	0 (0 to 0)	0·1 (0 to 0·1)	1 (0·5 to 1·7)	2·6 (1·4 to 4·1)	3·6 (2 to 5·6)	4·7 (2·7 to 7·1)	6·5 (3·9 to 10·1)	8·4 (5 to 13)	8·9 (5·4 to 13·5)	8·7 (5·2 to 13·7)
Other sense organ diseases	0 (0 to 0)	0 (0 to 0)	0·1 (0 to 0·2)	0·4 (0·2 to 0·7)	0·5 (0·2 to 0·9)	0·5 (0·2 to 0·8)	0·6 (0·3 to 1·1)	0·7 (0·3 to 1·4)	0·8 (0·3 to 1·7)	1 (0·4 to 1·9)	1 (0·4 to 1·8)
Musculoskeletal disorders	0 (0 to 0)	0 (0 to 0)	0 (0 to 0)	0 (0 to 0)	2·6 (1·4 to 4·4)	12·7 (7·9 to 19·6)	30·1 (20·2 to 43)	48·3 (32·8 to 69·4)	64·6 (43 to 91·1)	68·5 (45·1 to 92·8)	67·8 (47·1 to 94·4)
Rheumatoid arthritis	0 (0 to 0)	0 (0 to 0)	0 (0 to 0)	0 (0 to 0)	0 (0 to 0)	0·1 (0·1 to 0·2)	0·4 (0·2 to 0·6)	0·8 (0·4 to 1·4)	1·2 (0·6 to 2·1)	1·3 (0·7 to 2·1)	1·4 (0·8 to 2·1)
Osteoarthritis	0 (0 to 0)	0 (0 to 0)	0 (0 to 0)	0 (0 to 0)	0 (0 to 0)	0 (0 to 0)	0 (0 to 0)	0 (0 to 0)	0 (0 to 0)	0·3 (0·1 to 0·6)	1·6 (0·8 to 3·4)
Osteoarthritis hip	0 (0 to 0)	0 (0 to 0)	0 (0 to 0)	0 (0 to 0)	0 (0 to 0)	0 (0 to 0)	0 (0 to 0)	0 (0 to 0)	0 (0 to 0)	0·1 (0 to 0·2)	0·1 (0 to 0·3)
Osteoarthritis knee	0 (0 to 0)	0 (0 to 0)	0 (0 to 0)	0 (0 to 0)	0 (0 to 0)	0 (0 to 0)	0 (0 to 0)	0 (0 to 0)	0 (0 to 0)	0·2 (0 to 0·4)	1·2 (0·5 to 2·6)
Osteoarthritis hand	0 (0 to 0)	0 (0 to 0)	0 (0 to 0)	0 (0 to 0)	0 (0 to 0)	0 (0 to 0)	0 (0 to 0)	0 (0 to 0)	0 (0 to 0)	0 (0 to 0·1)	0·2 (0 to 0·5)

Chronic myeloid leukemia	0·3 (0·1 to 0·5)	0·4 (0·2 to 0·6)	0·6 (0·3 to 1)	0·6 (0·3 to 0·9)	0·5 (0·3 to 0·9)	0·4 (0·2 to 0·7)	0·4 (0·2 to 0·7)	0·2 (0·1 to 0·4)	0·1 (0·1 to 0·2)	0·1 (0 to 0·1)	0 (0 to 0)	0 (0 to 0)	5·6
Other leukemia	2 (1·4 to 2·9)	2·5 (1·7 to 3·6)	2·7 (1·9 to 3·7)	3·3 (2·2 to 4·6)	3 (2·2 to 4)	2·9 (2·1 to 3·8)	3·3 (2·4 to 4·3)	2·5 (1·8 to 3·4)	1·4 (1 to 1·9)	0·7 (0·5 to 1)	0·3 (0·3 to 0·4)	0·1 (0·1 to 0·1)	39·7
Other malignant neoplasms	4·9 (3·4 to 6·7)	6 (4·3 to 8·2)	8·6 (6·3 to 11·5)	8·4 (6 to 11·2)	7·9 (5·6 to 10·3)	7·1 (5·3 to 9·6)	6·5 (4·7 to 8·6)	5 (3·7 to 6·6)	2·7 (2 to 3·7)	1·6 (1·2 to 2·2)	0·7 (0·5 to 1)	0·2 (0·1 to 0·3)	91·4
Other neoplasms	0·8 (0·5 to 1·1)	0·7 (0·5 to 1)	1·2 (0·8 to 1·7)	1·3 (0·9 to 1·8)	1·5 (1·1 to 2)	1·6 (1·2 to 2·1)	2 (1·5 to 2·7)	1·8 (1·4 to 2·5)	1 (0·7 to 1·3)	0·5 (0·3 to 0·7)	0·2 (0·1 to 0·3)	0·1 (0 to 0·1)	16·1
Myelodysplastic, myeloproliferative, and other hematopoietic neo	0·8 (0·5 to 1·1)	0·7 (0·5 to 1)	1·2 (0·8 to 1·7)	1·3 (0·9 to 1·8)	1·5 (1·1 to 2)	1·6 (1·2 to 2·1)	2 (1·5 to 2·7)	1·8 (1·4 to 2·5)	1 (0·7 to 1·3)	0·5 (0·3 to 0·7)	0·2 (0·1 to 0·3)	0·1 (0 to 0·1)	16·1
Benign and in situ intestinal neoplasms	0 (0 to 0)	0 (0 to 0)	0 (0 to 0)	0 (0 to 0)	0 (0 to 0)	0 (0 to 0)	0 (0 to 0)	0 (0 to 0)	0 (0 to 0)	0 (0 to 0)	0 (0 to 0)	0 (0 to 0)	0
Benign and in situ cervical and uterine neoplasms	0 (0 to 0)	0 (0 to 0)	0 (0 to 0)	0 (0 to 0)	0 (0 to 0)	0 (0 to 0)	0 (0 to 0)	0 (0 to 0)	0 (0 to 0)	0 (0 to 0)	0 (0 to 0)	0 (0 to 0)	0
Other benign and in situ neoplasms	0 (0 to 0)	0 (0 to 0)	0 (0 to 0)	0 (0 to 0)	0 (0 to 0)	0 (0 to 0)	0 (0 to 0)	0 (0 to 0)	0 (0 to 0)	0 (0 to 0)	0 (0 to 0)	0 (0 to 0)	0
Cardiovascular diseases	65·5 (52·8 to 79·8)	113 (92·5 to 138)	185 (154 to 218)	231 (195 to 265)	265 (229 to 299)	299 (263 to 338)	344 (302 to 386)	351 (314 to 386)	242 (213 to 268)	148 (126 to 164)	94·1 (78·2 to 106)	38·4 (29·6 to 44)	2,540
Rheumatic heart disease	6·2 (4·4 to 8·2)	6·7 (5 to 8·7)	7·2 (5·1 to 9·9)	5·9 (4·3 to 8·2)	3·7 (2·8 to 4·9)	3 (2·2 to 4)	2·4 (1·8 to 3·2)	1·9 (1·5 to 2·5)	1·1 (0·8 to 1·4)	0·6 (0·4 to 0·7)	0·3 (0·2 to 0·4)	0·2 (0·1 to 0·2)	71·1
Ischemic heart disease	13·2 (10·2 to 16·8)	28·5 (22·6 to 35·5)	53·8 (42·4 to 65·3)	76·3 (62 to 92·4)	96·8 (81·7 to 113)	110 (91 to 133)	128 (111 to 147)	129 (112 to 147)	82·3 (70·1 to 94·2)	52·1 (42·3 to 61·9)	34·8 (27·9 to 41)	14·7 (11·1 to 17·7)	839
Stroke	25·3 (18·5 to 33·2)	45·1 (32·9 to 59·7)	72·6 (55·8 to 92·2)	85 (64·9 to 107)	96·8 (76·1 to 120)	114 (93·8 to 136)	132 (104 to 162)	139 (113 to 164)	101 (83·1 to 120)	57·5 (47·8 to 66·8)	32·8 (26·4 to 38·8)	12 (9 to 14·6)	966
Ischemic stroke	6·1 (4·6 to 8·1)	10·8 (8 to 14·1)	18·9 (14·7 to 23·7)	25·2 (19·3 to 31·4)	39·7 (31·3 to 50)	49·8 (41 to 59·2)	84·9 (67·2 to 105)	95 (76·4 to 113)	81·5 (67 to 97)	47·9 (39·6 to 55·8)	27·8 (22·2 to 33)	10·3 (7·7 to 12·5)	512
Intracerebral hemorrhage	11·8 (8·4 to 16·1)	24·1 (17·2 to 32·5)	39·5 (29·3 to 50·8)	45·6 (33·7 to 58·5)	47·5 (36·8 to 59·4)	55·1 (44·9 to 66·2)	40·8 (31·9 to 50·7)	39 (31·4 to 46·3)	17·1 (13·9 to 20·7)	8·6 (7 to 10·1)	4·5 (3·6 to 5·3)	1·5 (1·1 to 1·9)	357
Subarachnoid hemorrhage	7·4 (4·8 to 10·5)	10·2 (6·6 to 14·9)	14·2 (9·7 to 20·1)	14·2 (9·8 to 19·8)	9·5 (6·3 to 13·5)	9·2 (6·3 to 12·7)	6 (4 to 8·6)	4·9 (3·4 to 6·8)	2·2 (1·5 to 3)	1 (0·7 to 1·4)	0·6 (0·4 to 0·8)	0·2 (0·1 to 0·3)	96·4
Hypertensive heart disease	5·5 (3·6 to 8)	12·1 (7·9 to 16·8)	21·8 (14·9 to 29·7)	27·7 (18·3 to 38)	30·1 (20·1 to 40·1)	32·8 (22·7 to 44·6)	38·1 (27·2 to 50·2)	38·4 (27·2 to 51·3)	26·7 (18·9 to 35·4)	15·7 (10·9 to 20·8)	11·3 (7·4 to 14·7)	5·1 (3·3 to 7)	273
Non-rheumatic valvular heart disease	0·4 (0·2 to 0·5)	0·6 (0·4 to 0·9)	0·8 (0·5 to 1·1)	0·9 (0·6 to 1·2)	1 (0·7 to 1·3)	0·9 (0·7 to 1·2)	0·9 (0·7 to 1·2)	0·8 (0·6 to 1·1)	0·5 (0·4 to 0·7)	0·3 (0·2 to 0·4)	0·2 (0·1 to 0·2)	0·1 (0 to 0·1)	8·6
Non-rheumatic calcific aortic valve disease	0·1 (0 to 0·1)	0·1 (0·1 to 0·2)	0·2 (0·1 to 0·3)	0·2 (0·1 to 0·4)	0·3 (0·2 to 0·5)	0·4 (0·2 to 0·6)	0·4 (0·2 to 0·6)	0·4 (0·3 to 0·6)	0·2 (0·2 to 0·4)	0·2 (0·1 to 0·3)	0·1 (0·1 to 0·1)	0 (0 to 0)	2·8
Non-rheumatic degenerative mitral valve disease	0·3 (0·2 to 0·4)	0·4 (0·3 to 0·6)	0·5 (0·3 to 0·8)	0·6 (0·4 to 0·9)	0·6 (0·4 to 0·9)	0·5 (0·3 to 0·7)	0·4 (0·3 to 0·6)	0·4 (0·2 to 0·6)	0·2 (0·1 to 0·3)	0·1 (0·1 to 0·2)	0·1 (0 to 0·1)	0 (0 to 0)	5·1
Other non-rheumatic valve diseases	0 (0 to 0·1)	0·1 (0 to 0·1)	0·1 (0 to 0·1)	0·1 (0 to 0·1)	0 (0 to 0·1)	0·1 (0 to 0·1)	0·1 (0 to 0·2)	0·1 (0 to 0·1)	0 (0 to 0·1)	0 (0 to 0)	0 (0 to 0)	0 (0 to 0)	0·7
Cardiomyopathy and myocarditis	4·4 (3 to 6·2)	7 (4·6 to 9·8)	10·9 (7·2 to 15·4)	12 (8·1 to 16·5)	12·2 (8·2 to 16·4)	11·5 (7·7 to 16)	11 (7·6 to 15)	10 (6·7 to 13·8)	6·1 (4 to 8·8)	4·5 (2·9 to 6·7)	2·6 (1·6 to 3·8)	1 (0·6 to 1·5)	114
Myocarditis	0·3 (0·1 to 0·5)	0·8 (0·4 to 1·3)	0·8 (0·4 to 1·3)	0·6 (0·3 to 1)	0·5 (0·3 to 0·9)	0·3 (0·1 to 0·5)	0·6 (0·3 to 1·1)	0·6 (0·3 to 1·1)	0·5 (0·2 to 1)	0·5 (0·2 to 0·9)	0·3 (0·1 to 0·6)	0·2 (0·1 to 0·3)	9·7
Alcoholic cardiomyopathy	0·2 (0·1 to 0·3)	0·8 (0·3 to 1·5)	1·2 (0·4 to 2·2)	0·9 (0·3 to 1·6)	0·8 (0·3 to 1·5)	0·8 (0·3 to 1·6)	0·1 (0 to 0·2)	0·2 (0·1 to 0·3)	0·1 (0 to 0·3)	0 (0 to 0·1)	0 (0 to 0)	0 (0 to 0)	5·8
Other cardiomyopathy	4 (2·7 to 5·5)	5·4 (3·6 to 7·6)	8·9 (5·9 to 12·8)	10·6 (7·2 to 14·7)	10·9 (7·3 to 14·8)	10·4 (6·9 to 14·6)	10·3 (7·1 to 14·1)	9·3 (6·2 to 12·8)	5·5 (3·6 to 7·9)	4 (2·5 to 6·1)	2·3 (1·4 to 3·4)	0·9 (0·5 to 1·3)	98·4
Atrial fibrillation and flutter	0·4 (0·2 to 0·6)	0·8 (0·5 to 1·1)	1·4 (1 to 2)	2·4 (1·8 to 3·2)	3·2 (2·5 to 4·2)	4·4 (3·4 to 5·8)	6·7 (5·3 to 8·9)	8·3 (6·6 to 10·6)	8 (6·4 to 9·9)	6·2 (5 to 8·2)	5 (3·9 to 7)	2·1 (1·6 to 3)	49·1
Aortic aneurysm	0·5 (0·3 to 0·6)	0·8 (0·5 to 1)	1·4 (1 to 1·9)	2·1 (1·5 to 2·8)	2·6 (2 to 3·4)	3·5 (2·6 to 4·7)	4 (2·9 to 5·3)	3·7 (2·7 to 4·9)	2·3 (1·6 to 3·1)	1·2 (0·9 to 1·7)	0·6 (0·5 to 0·9)	0·2 (0·2 to 0·3)	NA
Endocarditis	2 (1·2 to 3·6)	2·6 (1·6 to 4·5)	2·9 (1·7 to 5·2)	3·3 (2 to 5·9)	3·2 (2 to 5·7)	2·5 (1·6 to 4·3)	2·6 (1·6 to 4·7)	2·1 (1·3 to 3·7)	1·8 (1·1 to 3·1)	1 (0·6 to 1·8)	0·6 (0·3 to 1·1)	0·3 (0·2 to 0·5)	35·1
Other cardiovascular and circulatory diseases	7·4 (5·2 to 10·5)	8·8 (6·4 to 11·8)	10·8 (8·2 to 13·7)	12·9 (9·8 to 16·2)	12·9 (10·1 to 16)	13 (9·3 to 17·7)	14·1 (11 to 17·6)	13·2 (10 to 16·8)	8·7 (6·5 to 11·4)	6·3 (4·9 to 7·8)	3·9 (3 to 4·9)	1·6 (1·1 to 2·2)	134
Chronic respiratory diseases	15·2 (11·2 to 20·1)	19·1 (14·6 to 24·8)	25·2 (20 to 31·2)	26·6 (21·4 to 33)	26 (20·3 to 32·1)	25·6 (19·9 to 31·8)	26·4 (20·3 to 32·4)	23·6 (18·3 to 29·2)	14 (11 to 17·2)	8·8 (6·8 to 10·8)	5·1 (4 to 6·3)	2·2 (1·7 to 2·8)	358
Chronic obstructive pulmonary disease	4 (2·9 to 5·3)	6·5 (4·8 to 8·3)	11·1 (8·5 to 13·9)	13·8 (10·5 to 17·4)	16·8 (12·7 to 21·2)	17·5 (13·2 to 22·2)	18·7 (14·2 to 23·7)	17·5 (13·2 to 22)	10·9 (8·3 to 13·5)	7·1 (5·4 to 8·8)	4·2 (3·2 to 5·2)	1·9 (1·4 to 2·4)	142
Pneumoconiosis	0 (0 to 0)	0 (0 to 0)	0 (0 to 0)	0 (0 to 0)	0 (0 to 0)	0 (0 to 0)	0 (0 to 0)	0 (0 to 0·1)	0 (0 to 0)	0 (0 to 0)	0 (0 to 0)	0 (0 to 0)	0·2
Silicosis	0 (0 to 0)	0 (0 to 0)	0 (0 to 0)	0 (0 to 0)	0 (0 to 0)	0 (0 to 0)	0 (0 to 0)	0 (0 to 0)	0 (0 to 0)	0 (0 to 0)	0 (0 to 0)	0 (0 to 0)	0
Asbestosis	0 (0 to 0)	0 (0 to 0)	0 (0 to 0)	0 (0 to 0)	0 (0 to 0)	0 (0 to 0)	0 (0 to 0)	0 (0 to 0)	0 (0 to 0)	0 (0 to 0)	0 (0 to 0)	0 (0 to 0)	0
Coal workers pneumoconiosis	0 (0 to 0)	0 (0 to 0)	0 (0 to 0)	0 (0 to 0)	0 (0 to 0)	0 (0 to 0)	0 (0 to 0)	0 (0 to 0)	0 (0 to 0)	0 (0 to 0)	0 (0 to 0)	0 (0 to 0)	0
Other pneumoconiosis	0 (0 to 0)	0 (0 to 0)	0 (0 to 0)	0 (0 to 0)	0 (0 to 0)	0 (0 to 0)	0 (0 to 0)	0 (0 to 0)	0 (0 to 0)	0 (0 to 0)	0 (0 to 0)	0 (0 to 0)	0·1
Asthma	9·5 (6·2 to 14)	10·4 (7 to 15·1)	11·4 (8·1 to 16)	9·3 (6·5 to 13·1)	7 (4·9 to 9·7)	6 (4·2 to 8·3)	5·2 (3·7 to 7·1)	3·9 (2·7 to 5·4)	1·9 (1·3 to 2·7)	1 (0·7 to 1·4)	0·5 (0·4 to 0·7)	0·2 (0·2 to 0·3)	182
Interstitial lung disease and pulmonary sarcoidosis	0·8 (0·5 to 1·3)	1·3 (0·8 to 1·9)	1·6 (0·9 to 2·4)	1·9 (1·1 to 2·9)	1·9 (1 to 3)	1·9 (1·1 to 2·9)	2·3 (1·2 to 3·5)	2 (1·1 to 3·1)	1·1 (0·6 to 1·7)	0·6 (0·3 to 1)	0·3 (0·2 to 0·5)	0·1 (0·1 to 0·2)	19·7
Other chronic respiratory diseases	0·9 (0·6 to 1·5)	0·9 (0·6 to 1·4)	1 (0·6 to 1·6)	1·5 (0·9 to 2·6)	0·3 (0·1 to 0·5)	0·2 (0·1 to 0·3)	0·2 (0·1 to 0·4)	0·2 (0·1 to 0·3)	0·1 (0·1 to 0·2)	0·1 (0 to 0·1)	0 (0 to 0·1)	0 (0 to 0)	13·4
Digestive diseases	23·7 (18·3 to 30·5)	31·5 (24·7 to 40·2)	42·1 (33·5 to 52·2)	42·9 (34·5 to 52·1)	41·6 (34·3 to 49·2)	36·9 (30·5 to 44·4)	37 (29·9 to 45·3)	30·5 (25·3 to 36·2)	18·2 (14·4 to 22·4)	9·6 (7·6 to 12)	5·1 (3·9 to 6·3)	1·8 (1·4 to 2·2)	421
Cirrhosis and other chronic liver diseases	5·3 (3·8 to 7·2)	8·5 (6·2 to 11·3)	13·8 (10 to 18)	15·5 (11·7 to 20·1)	15·6 (11·9 to 19·5)	13·9 (10·7 to 17·8)	12·3 (9·1 to 16·2)	8·7 (6·4 to 11·5)	4·4 (3·2 to 6·1)	1·8 (1·3 to 2·4)	0·8 (0·6 to 1·1)	0·3 (0·2 to 0·4)	116
Cirrhosis and other chronic liver diseases due to hepatitis B	0·4 (0·2 to 0·7)	0·6 (0·3 to 1·1)	0·9 (0·4 to 1·8)	0·9 (0·4 to 1·6)	0·8 (0·3 to 1·4)	0·6 (0·3 to 1·1)	0·5 (0·2 to 0·9)	0·4 (0·2 to 0·6)	0·2 (0·1 to 0·3)	0·1 (0 to 0·1)	0 (0 to 0·1)	0 (0 to 0)	6
Cirrhosis and other chronic liver diseases due to hepatitis C	1·2 (0·7 to 2·1)	1·8 (1 to 3·1)	2·8 (1·5 to 4·7)	2·8 (1·5 to 5)	2·6 (1·3 to 4·9)	2·2 (1·2 to 3·8)	1·9 (1·1 to 3·1)	1·2 (0·7 to 2·2)	0·6 (0·3 to 1·1)	0·3 (0·1 to 0·5)	0·1 (0·1 to 0·2)	0 (0 to 0·1)	20·2
Cirrhosis and other chronic liver diseases due to alcohol use	1·8 (1 to 2·8)	3 (1·7 to 4·7)	5·2 (3 to 7·9)	6·1 (3·6 to 9)	6·1 (3·6 to 9)	5 (2·9 to 7·5)	4 (2·3 to 6·2)	2·4 (1·4 to 3·8)	1·1 (0·6 to 1·9)	0·4 (0·3 to 0·7)	0·2 (0·1 to 0·3)	0·1 (0 to 0·1)	37·7
Cirrhosis and other chronic liver diseases due to NAFLD	1·1 (0·5 to 1·9)	1·8 (0·8 to 3·2)	3 (1·4 to 5·2)	3·2 (1·6 to 5·5)	3·3 (1·6 to 5·8)	3·2 (1·8 to 5)	3 (1·8 to 4·7)	2·3 (1·2 to 3·5)	1·2 (0·7 to 1·9)	0·5 (0·3 to 0·8)	0·2 (0·1 to 0·3)	0·1 (0 to 0·1)	24·8
Cirrhosis and other chronic liver diseases due to other causes	0·8 (0·4 to 1·5)	1·2 (0·6 to 2·1)	2 (1 to 3·3)	2·5 (1·2 to 4·2)	2·8 (1·5 to 4·8)	2·9 (1·4 to 4·8)	2·9 (1·5 to 4·8)	2·4 (1·3 to 4)	1·3 (0·7 to 2·2)	0·6 (0·3 to 0·8)	0·2 (0·1 to 0·4)	0·1 (0 to 0·1)	27·2
Upper digestive system diseases	8·3 (4·8 to 13·7)	10·4 (6·3 to 16·5)	11·8 (7·2 to 18·7)	10·9 (7·1 to 17·1)	9·2 (6·2 to 13·7)	7·8 (5·5 to 11·3)	7·5 (5·4 to 10·4)	6·3 (4·5 to 8·7)	3·7 (2·6 to 5)	1·9 (1·4 to 2·6)	1 (0·8 to 1·4)	0·4 (0·3 to 0·5)	114
Peptic ulcer disease	0·8 (0·6 to 1·1)	1·3 (1 to 1·8)	2 (1·5 to 2·5)	2·3 (1·8 to 2·9)	2·7 (1·9 to 3·7)	2·6 (2 to 3·4)	3·1 (2·3 to 4·1)	3 (2 to 4·1)	2 (1·3 to 2·9)	1·1 (0·7 to 1·5)	0·6 (0·4 to 0·8)	0·2 (0·2 to 0·3)	24·4
Gastritis and duodenitis	1·1 (0·6 to 2)	1·8 (0·9 to 3·2)	2·2 (1·2 to 3·9)	2·2 (1·2 to 3·8)	1·8 (1 to 3)	1·5 (0·9 to 2·3)	1·3 (0·8 to 2)	1 (0·6 to 1·4)	0·5 (0·3 to 0·7)	0·3 (0·2 to 0·4)	0·1 (0·1 to 0·2)	0 (0 to 0·1)	19·4
Gastroesophageal reflux disease	6·4 (3 to 11·5)	7·3 (3·6 to 12·8)	7·6 (3·7 to 13·7)	6·3 (3 to 11·8)	4·7 (2·3 to 8·6)	3·6 (1·8 to 6·7)	3 (1·5 to 5·4)	2·3 (1·1 to 4·2)	1·2 (0·6 to 2·2)	0·6 (0·3 to 1·1)	0·3 (0·1 to 0·5)	0·1 (0 to 0·2)	70·7
Appendicitis	0·4 (0·3 to 0·6)	0·5 (0·3 to 0·8)	0·6 (0·3 to 0·9)	0·6 (0·4 to 1)	0·4 (0·3 to 0·7)	0·5 (0·3 to 0·7)	0·5 (0·3 to 0·8)	0·3 (0·2 to 0·5)	0·2 (0·1 to 0·3)	0·1 (0·1 to 0·2)	0·1 (0 to 0·1)	0 (0 to 0)	8·3
Paralytic ileus and intestinal obstruction	1·4 (0·9 to 2·1)	2 (1·3 to 3)	3·1 (2 to 4·7)	3 (2 to 4·4)	3·7 (2·5 to 5·1)	3·7 (2·4 to 5·3)	4·8 (3·1 to 6·8)	4·7 (3·2 to 6·5)	3·3 (2·3 to 4·7)	1·9 (1·3 to 2·7)	1·1 (0·7 to 1·6)	0·4 (0·3 to 0·6)	44
Inguinal, femoral, and abdominal hernia	1·1 (0·7 to 1·9)	1·4 (0·8 to 2·3)	1·7 (1 to 2·6)	1·5 (1 to 2·3)	1·3 (0·9 to 1·9)	0·9 (0·6 to 1·3)	0·9 (0·6 to 1·3)	0·8 (0·5 to 1·1)	0·5 (0·3 to 0·7)	0·3 (0·2 to 0·4)	0·2 (0·1 to 0·2)	0·1 (0 to 0·1)	18·9
Inflammatory bowel disease	1·4 (1 to 2·1)	1·4 (0·9 to 2)	1·5 (1 to 2·2)	1·6 (1·1 to 2·4)	1·6 (1·1 to 2·3)	1 (0·7 to 1·5)	1·4 (0·9 to 2·1)	1·1 (0·7 to 1·6)	0·5 (0·3 to 0·7)	0·3 (0·2 to 0·4)	0·2 (0·1 to 0·3)	0 (0 to 0·1)	16·8
Vascular intestinal disorders	0·2 (0·1 to 0·3)	0·4 (0·2 to 0·6)	0·5 (0·4 to 0·8)	0·6 (0·4 to 0·9)	0·9 (0·6 to 1·3)	1 (0·7 to 1·4)	1·2 (0·8 to 1·8)	1·1 (0·8 to 1·6)	0·7 (0·5 to 1)	0·4 (0·3 to 0·6)	0·2 (0·1 to 0·2)	0·1 (0 to 0·1)	7·7
Gallbladder and biliary diseases	3·6 (2·3 to 5·4)	4·7 (3 to 6·9)	5·5 (3·8 to 8·1)	5·2 (3·6 to 7·2)	4·5 (3·2 to 6)	3·8 (2·7 to 5)	3·5 (2·5 to 4·6)	2·8 (2 to 3·8)	1·6 (1·1 to 2·2)	0·8 (0·5 to 1·1)	0·4 (0·3 to 0·6)	0·2 (0·1 to 0·2)	48·6
Pancreatitis	0·5 (0·3 to 0·8)	0·7 (0·4 to 1)	0·9 (0·6 to 1·3)	0·9 (0·6 to 1·3)	0·9 (0·6 to 1·4)	0·9 (0·6 to 1·2)	0·9 (0·6 to 1·3)	0·7 (0·5 to 1)	0·4 (0·3 to 0·6)	0·2 (0·1 to 0·3)	0·1 (0·1 to 0·1)	0 (0 to 0)	9·4
Other digestive diseases	1·2 (0·6 to 1·8)	1·6 (0·9 to 2·5)	2·7 (1·5 to 4·2)	2·9 (1·6 to 4·7)	3·4 (1·9 to 5·1)	3·5 (2 to 5·2)	4 (2·2 to 6·1)	3·9 (2·3 to 5·8)	2·9 (1·7 to 4·3)	1·9 (1·1 to 2·8)	1 (0·6 to 1·5)	0·4 (0·2 to 0·6)	36·7
Neurological disorders	43·3 (16·1 to 83·9)	47·4 (20 to 93)	46·5 (21·5 to 86·7)	38·2 (20·2 to 65·8)	31 (18·3 to 50·6)	29·4 (18·3 to 48·4)	36·5 (23 to 64·2)	43·8 (26·9 to 80·6)	44·2 (24·4 to 88·3)	35·7 (17·8 to 74·6)	26 (12 to 56·7)	14·5 (6·2 to 31·9)	744
Alzheimer's disease and other dementias	0·2 (0 to 0·6)	1·3 (0·4 to 3·5)	2·8 (1 to 6·8)	4·7 (1·8 to 10·8)	6·9 (2·8 to 16·5)	10·7 (4·3 to 27)	18·6 (7·8 to 44·5)	26·6 (11·5 to 64)	33·3 (14 to 78·4)	29·8 (12·1 to 69·4)	23·4 (9·2 to 53·8)	13·7 (5·4 to 31·2)	172
Parkinson's disease	0·2 (0·1 to 0·3)	0·4 (0·3 to 0·7)	0·9 (0·7 to 1·2)	1·4 (1 to 1·8)	2·2 (1·8 to 2·7)	3·5 (2·9 to 4·2)	6·5 (5·5 to 7·4)	9·3 (8 to 10·9)	7·2 (6 to 8·4)	4·1 (3·4 to 4·8)	1·8 (1·5 to 2·1)	0·5 (0·4 to 0·6)	38·2
Idiopathic epilepsy	6·5 (3·1 to 11·3)	6·9 (3·2 to 12·1)	6·8 (3·2 to 11·8)	5·5 (2·5 to 9·9)	4·5 (1·9 to 7·8)	3·8 (1·6 to 6·7)	3·5 (1·4 to 6·3)	2·9 (1·2 to 5·2)	1·6 (0·6 to 2·8)	0·8 (0·3 to 1·5)	0·4 (0·2 to 0·8)	0·2 (0·1 to 0·3)	112
Multiple sclerosis	1·1 (0·8 to 2)	1·3 (0·9 to 2·3)	1·4 (1 to 2·2)	1·2 (0·9 to 2)	0·9 (0·6 to 1·4)	0·4 (0·3 to 0·7)	0·4 (0·3 to 0·6)	0·2 (0·1 to 0·3)	0·1 (0·1 to 0·1)	0 (0 to 0·1)	0 (0 to 0)	0 (0 to 0)	10
Motor neuron disease	0·1 (0·1 to 0·2)	0·2 (0·2 to 0·3)	0·4 (0·3 to 0·5)	0·4 (0·3 to 0·5)	0·4 (0·3 to 0·5)	0·3 (0·2 to 0·4)	0·3 (0·2 to 0·4)	0·2 (0·1 to 0·2)	0·1 (0 to 0·1)	0 (0 to 0)	0 (0 to 0)	0 (0 to 0)	2·9
Headache disorders	33·8 (7·3 to 74·6)	35·4 (8·3 to 80·2)	32·5 (8·7 to 70·1)	23·3 (6·2 to 49·6)	14·9 (4·6 to 31·6)	9·8 (3·1 to 20·5)	6·6 (2·1 to 13·9)	4·1 (1·3 to 8·6)	1·8 (0·6 to 3·8)	0·8 (0·3 to 1·7)	0·3 (0·1 to 0·7)	0·1 (0 to 0·2)	381
Migraine	30·9 (5 to 71·8)	32·1 (6 to 74·7)	29·1 (5·8 to 66·9)	20·8 (4·5 to 46·4)	13 (3·1 to 28·9)	8·4 (2·2 to 18·4)	5·5 (1·6 to 12·1)	3·4 (0·9 to 7·5)	1·5 (0·4 to 3·3)	0·7 (0·2 to 1·4)	0·3 (0·1 to 0·6)	0·1 (0 to 0·1)	349
Tension-type headache	2·8 (0·8 to 8)	3·2 (1·1 to 8·6)	3·4 (1 to 9·2)	2·5 (0·8 to 6·4)	1·8 (0·5 to 5·1)	1·4 (0·4 to 4·3)	1·1 (0·3 to 3·7)	0·7 (0·2 to 2·5)	0·3 (0·1 to 1)	0·1 (0 to 0·5)	0·1 (0 to 0·2)	0 (0 to 0·1)	32·8
Other neurological disorders	1·4 (0·7 to 2·3)	1·8 (1 to 2·9)	1·8 (1·1 to 2·7)	1·6 (1·1 to 2·3)	1·2 (0·8 to 1·6)	0·9 (0·6 to 1·1)	0·7 (0·5 to 0·9)	0·5 (0·4 to 0·7)	0·2 (0·2 to 0·3)	0·1 (0·1 to 0·1)	0·1 (0 to 0·1)	0 (0 to 0)	27
Mental disorders	81·7 (56·9 to 113)	89·7 (64·2 to 122)	88·2 (63·6 to 120)	70·1 (50·7 to 94·8)	48·6 (33·9 to 65·2)	34·5 (24·1 to 45·8)	26·7 (19·1 to 35·5)	18·9 (13·1 to 25·5)	9·2 (6·4 to 12·3)	4·6 (3·2 to 6·2)	2·3 (1·5 to 3·2)	0·8 (0·5 to 1·2)	1,010
Schizophrenia	10·9 (6·9 to 15·1)	11·6 (7·6 to 15·8)	10·7 (7·1 to 14·9)	7·7 (5·1 to 11·1)	4·8 (3·1 to 6·7)	3 (1·9 to 4·3)	2 (1·3 to 2·9)	1·2 (0·7 to 1·8)	0·5 (0·3 to 0·7)	0·2 (0·1 to 0·3)	0·1 (0 to 0·1)	0 (0 to 0)	94·5
Depressive disorders	30·7 (18·3 to 48·2)	35·5 (22·6 to 52·8)	37·5 (24 to 55·3)	31·7 (19·8 to 46·9)	23·1 (13·9 to 34·9)	16·8 (10·7 to 24·4)	13·4 (8·5 to 19·3)	9·7 (5·9 to 14·6)	5 (3 to 7·5)	2·7 (1·7 to 3·9)	1·4 (0·9 to 2·1)	0·5 (0·3 to 0·9)	387
Major depressive disorder	25·5 (14·4 to 41·9)	29·8 (17·9 to 45·2)	31·8 (19·5 to 48)	27·2 (16 to 41·2)	19·9 (11·1 to 30·5)	14·6 (8·9 to 21·2)	11·7 (7·2 to 17·3)	8·5 (4·8 to 13·3)	4·4 (2·5 to 6·9)	2·4 (1·5 to 3·6)	1·3 (0·8 to 2)	0·5 (0·3 to 0·8)	332
Dysthymia	5·1 (2·7 to 8·9)	5·7 (3·2 to 9·9)	5·7 (3·3 to 9·6)	4·5 (2·6 to 7·5)	3·2 (1·7 to 5·4)	2·3 (1·2 to 3·8)	1·7 (1 to 2·9)	1·2 (0·7 to 2)	0·6 (0·3 to 1)	0·3 (0·2 to 0·5)	0·1 (0·1 to 0·2)	0 (0 to 0·1)	54·4
Bipolar disorder	9·6 (5·3 to 15·5)	10·4 (5·9 to 17·1)	10 (5·6 to 16·4)	7·4 (4·1 to 12·2)	4·8 (2·7 to 7·8)	3·1 (1·7 to 4·9)	2·1 (1·1 to 3·4)	1·2 (0·7 to 2·1)	0·5 (0·2 to 0·8)	0·2 (0·1 to 0·3)	0·1 (0 to 0·1)	0 (0 to 0)	115
Anxiety disorders	22 (12·9 to 35·3)	23·8 (13·9 to 39·2)	23 (13·8 to 36·1)	17·7 (11 to 27)	12 (7·3 to 18·6)	8·6 (5 to 13·7)	6·8 (4·1 to 10·9)	4·8 (3 to 7·5)	2·3 (1·4 to 3·6)	1 (0·6 to 1·6)	0·5 (0·3 to 0·7)	0·1 (0·1 to 0·2)	277
Eating disorders	2·1 (1·1 to 3·5)	1·4 (0·7 to 2·3)	0 (0 to 0)	0 (0 to 0)	0 (0 to 0)	0 (0 to 0)	0 (0 to 0)	0 (0 to 0)	0 (0 to 0)	0 (0 to 0)	0 (0 to 0)	0 (0 to 0)	35·6
Anorexia nervosa	0·4 (0·2 to 0·9)	0·4 (0·1 to 0·7)	0 (0 to 0)	0 (0 to 0)	0 (0 to 0)	0 (0 to 0)	0 (0 to 0)	0 (0 to 0)	0 (0 to 0)	0 (0 to 0)	0 (0 to 0)	0 (0 to 0)	8·9
Bulimia nervosa	1·7 (0·8 to 2·9)	1 (0·4 to 1·8)	0 (0 to 0)	0 (0 to 0)	0 (0 to 0)	0 (0 to 0)	0 (0 to 0)	0 (0 to 0)	0 (0 to 0)	0 (0 to 0)	0 (0 to 0)	0 (0 to 0)	26·6
Autism spectrum disorders	0·7 (0·4 to 1·1)	0·8 (0·4 to 1·2)	0·7 (0·4 to 1·1)	0·6 (0·3 to 1)	0·4 (0·2 to 0·6)	0·3 (0·2 to 0·5)	0·2 (0·1 to 0·4)	0·2 (0·1 to 0·3)	0·1 (0 to 0·1)	0 (0 to 0·1)	0 (0 to 0)	0 (0 to 0)	12·7
Attention-deficit/hyperactivity disorder	0·4 (0·2 to 0·6)	0·3 (0·2 to 0·6)	0·3 (0·1 to 0·5)	0·2 (0·1 to 0·3)	0·1 (0 to 0·1)	0 (0 to 0·1)	0 (0 to 0)	0 (0 to 0)	0 (0 to 0)	0 (0 to 0)	0 (0 to 0)	0 (0 to 0)	9·6
Conduct disorder	0 (0 to 0)	0 (0 to 0)	0 (0 to 0)	0 (0 to 0)	0 (0 to 0)	0 (0 to 0)	0 (0 to 0)	0 (0 to 0)	0 (0 to 0)	0 (0 to 0)	0 (0 to 0)	0 (0 to 0)	21·3
Idiopathic developmental intellectual disability	0·4 (0·1 to 0·8)	0·4 (0·1 to 0·8)	0·4 (0·1 to 0·8)	0·3 (0·1 to 0·6)	0·2 (0·1 to 0·4)	0·1 (0 to 0·3)	0·1 (0 to 0·2)	0·1 (0 to 0·1)	0 (0 to 0·1)	0 (0 to 0)	0 (0 to 0)	0 (0 to 0)	7·2
Other mental disorders	4·9 (2·8 to 7·7)	5·5 (3·3 to 8·4)	5·6 (3·4 to 8·7)	4·5 (2·8 to 6·9)	3·3 (2 to 5)	2·5 (1·5 to 3·8)	2·1 (1·4 to 3·1)	1·6 (1 to 2·5)	0·9 (0·5 to 1·2)	0·5 (0·3 to 0·7)	0·2 (0·2 to 0·4)	0·1 (0·1 to 0·1)	49·6
Substance use disorders	9 (6·4 to 12·4)	8·8 (6·5 to 11·9)	7·5 (5·2 to 10·5)	5·6 (4·2 to 7·6)	3·6 (2·6 to 4·9)	2·4 (1·7 to 3·2)	1·8 (1·2 to 2·4)	1·3 (0·9 to 1·8)	0·7 (0·5 to 1)	0·4 (0·3 to 0·6)	0·3 (0·2 to 0·4)	0·2 (0·1 to 0·2)	108
Alcohol use disorders	5·5 (3·5 to 8·3)	5·5 (3·7 to 7·8)	4·7 (3·1 to 7)	3·5 (2·5 to 5)	2·4 (1·6 to 3·4)	1·5 (1 to 2·1)	1·1 (0·8 to 1·6)	0·8 (0·5 to 1·1)	0·5 (0·3 to 0·7)	0·3 (0·2 to 0·5)	0·2 (0·2 to 0·4)	0·1 (0·1 to 0·2)	57·5
Drug use disorders	3·5 (2·2 to 5·1)	3·3 (2·1 to 4·8)	2·8 (1·6 to 4·2)	2·1 (1·3 to 3·1)	1·3 (0·7 to 2)	0·9 (0·5 to 1·4)	0·6 (0·4 to 1)	0·5 (0·3 to 0·7)	0·2 (0·1 to 0·3)	0·1 (0·1 to 0·2)	0·1 (0 to 0·1)	0 (0 to 0)	50·6
Opioid use disorders	2·1 (1·1 to 3·5)	2 (1 to 3·3)	1·9 (0·9 to 3·3)	1·5 (0·8 to 2·5)	0·9 (0·4 to 1·6)	0·6 (0·3 to 1·1)	0·4 (0·2 to 0·8)	0·3 (0·1 to 0·5)	0·1 (0·1 to 0·3)	0·1 (0 to 0·1)	0 (0 to 0·1)	0 (0 to 0)	27·6
Cocaine use disorders	0·4 (0·2 to 0·7)	0·3 (0·2 to 0·5)	0·2 (0·2 to 0·4)	0·2 (0·1 to 0·3)	0·1 (0·1 to 0·2)	0·1 (0·1 to 0·1)	0·1 (0 to 0·1)	0 (0 to 0·1)	0 (0 to 0)	0 (0 to 0)	0 (0 to 0)	0 (0 to 0)	5·8
Amphetamine use disorders	0·1 (0·1 to 0·2)	0·1 (0 to 0·1)	0·1 (0 to 0·1)	0 (0 to 0·1)	0 (0 to 0)	0 (0 to 0)	0 (0 to 0)	0 (0 to 0)	0 (0 to 0)	0 (0 to 0)	0 (0 to 0)	0 (0 to 0)	2·4
Cannabis use disorders	0·2 (0·1 to 0·4)	0·2 (0·1 to 0·4)	0·1 (0·1 to 0·3)	0·1 (0 to 0·1)	0 (0 to 0·1)	0 (0 to 0)	0 (0 to 0)	0 (0 to 0)	0 (0 to 0)	0 (0 to 0)	0 (0 to 0)	0 (0 to 0)	5·7
Other drug use disorders	0·7 (0·4 to 1)	0·7 (0·5 to 0·9)	0·4 (0·3 to 0·6)	0·3 (0·2 to 0·4)	0·2 (0·1 to 0·2)	0·1 (0·1 to 0·2)	0·1 (0·1 to 0·2)	0·1 (0·1 to 0·2)	0·1 (0·1 to 0·1)	0 (0 to 0)	0 (0 to 0)	0 (0 to 0)	9
Diabetes and kidney diseases	75·2 (59·8 to 91·6)	124 (99·2 to 153)	193 (155 to 232)	227 (185 to 273)	243 (204 to 286)	247 (206 to 292)	246 (210 to 288)	208 (177 to 243)	118 (98·4 to 138)	61·2 (50·9 to 72·1)	30·2 (24·8 to 35·8)	10·7 (8·3 to 12·8)	2,000
Diabetes mellitus	44 (32·9 to 56·5)	78·6 (59·2 to 103)	131 (99·8 to 163)	157 (121 to 198)	174 (140 to 212)	176 (140 to 219)	184 (151 to 222)	155 (126 to 186)	88·9 (71·7 to 108)	43·9 (35 to 53·7)	21·1 (16·6 to 26)	6·9 (5·2 to 8·5)	1,350
Diabetes mellitus type 1	4·7 (3 to 7·3)	2 (1·3 to 3·1)	3·1 (2 to 4·6)	3·6 (2·3 to 5·5)	3·1 (1·9 to 5·2)	3·2 (2 to 5)	2·6 (1·6 to 4·3)	2·3 (1·4 to 3·9)	0·8 (0·5 to 1·5)	0·4 (0·3 to 0·7)	0·2 (0·1 to 0·4)	0·1 (0·1 to 0·1)	44·1
Diabetes mellitus type 2	39·3 (28·2 to 51·8)	76·5 (57·6 to 100)	128 (97·3 to 160)	153 (118 to 194)	170 (137 to 208)	173 (137 to 215)	181 (149 to 220)	153 (124 to 183)	88·1 (70·8 to 107)	43·5 (34·7 to 53·3)	20·8 (16·5 to 25·7)	6·8 (5·2 to 8·4)	1,310
Chronic kidney disease	31·2 (23·1 to 40·5)	45·2 (33·6 to 58·3)	62 (47·2 to 79·6)	70·2 (53·5 to 90·6)	70 (54·1 to 86·7)	71·1 (55·7 to 90·1)	62·7 (48·6 to 78·1)	53·6 (42 to 68·1)	29·2 (22·8 to 36·2)	17·4 (13·7 to 21·6)	9·1 (7 to 11·5)	3·8 (2·8 to 4·8)	649
Chronic kidney disease due to diabetes mellitus type 1	5·5 (2·9 to 9)	6·8 (3 to 11·6)	7 (2·8 to 13)	5·6 (1·8 to 11·7)	4·2 (1·4 to 9)	3·3 (1·2 to 7·1)	2·2 (0·8 to 4·6)	1·2 (0·5 to 2·7)	0·3 (0·1 to 0·7)	0·1 (0 to 0·2)	0 (0 to 0)	0 (0 to 0)	50·4
Chronic kidney disease due to diabetes mellitus type 2	4 (1·9 to 6·9)	8·3 (4·6 to 13)	15·4 (8·8 to 22·9)	21 (12·5 to 30·9)	22·6 (13·8 to 32·5)	23·5 (15 to 33·7)	20·7 (13·9 to 28·5)	17·5 (11·8 to 24·1)	9·3 (6·2 to 12·9)	5·3 (3·5 to 7·3)	2·7 (1·7 to 3·8)	1·1 (0·6 to 1·6)	157
Chronic kidney disease due to hypertension	3·6 (2·2 to 5·5)	5·9 (3·8 to 9·1)	9·3 (5·9 to 14·4)	12·4 (7·8 to 18·9)	14·6 (9·5 to 21·4)	17 (11·1 to 24·6)	16·5 (11·4 to 23·2)	15·6 (10·6 to 22·1)	9·3 (6·5 to 12·8)	6·2 (4·5 to 8·3)	3·5 (2·5 to 4·7)	1·5 (1 to 2·1)	125
Chronic kidney disease due to glomerulonephritis	8·9 (4·9 to 13·6)	11·3 (6·8 to 17·2)	13·8 (7·8 to 22)	14·1 (7·4 to 23·3)	12·2 (6·4 to 20·6)	10·6 (5·2 to 18·5)	8 (4·1 to 14·1)	5·9 (2·8 to 10·5)	2·7 (1·2 to 5·1)	1·3 (0·6 to 2·3)	0·6 (0·3 to 1·1)	0·2 (0·1 to 0·5)	131
Chronic kidney disease due to other and unspecified causes	9·3 (5·6 to 13·7)	13 (8 to 19·6)	16·4 (10·3 to 25·3)	17·1 (10·2 to 27)	16·3 (9·8 to 26·2)	16·6 (9·8 to 25·5)	15·3 (9·6 to 22·7)	13·3 (8·1 to 20)	7·5 (4·8 to 11·1)	4·5 (2·9 to 6·4)	2·4 (1·6 to 3·4)	1 (0·6 to 1·5)	185
Acute glomerulonephritis	0 (0 to 0)	0 (0 to 0)	0 (0 to 0)	0 (0 to 0)	0 (0 to 0)	0 (0 to 0)	0 (0 to 0)	0 (0 to 0)	0 (0 to 0)	0 (0 to 0)	0 (0 to 0)	0 (0 to 0)	0·1
Skin and subcutaneous diseases	15·4 (10·6 to 22·2)	17·2 (11·7 to 25·2)	21·9 (15·3 to 31·1)	22·2 (15·4 to 31·5)	19·8 (13·7 to 28·5)	17·9 (12·2 to 25·6)	20·5 (13·9 to 29·3)	22·5 (14·9 to 33·2)	15·2 (9·9 to 23·2)	10·7 (6·8 to 17·7)	6·8 (4·4 to 10·8)	3·2 (2·1 to 5·8)	368
Dermatitis	2·5 (1·4 to 4·1)	2·8 (1·6 to 4·6)	3·1 (1·7 to 5)	2·7 (1·6 to 4·4)	2·1 (1·1 to 3·4)	1·8 (1 to 2·9)	1·6 (0·9 to 2·6)	1·2 (0·7 to 1·9)	0·6 (0·4 to 1)	0·3 (0·2 to 0·5)	0·1 (0·1 to 0·2)	0 (0 to 0·1)	59·8
Atopic dermatitis	1·3 (0·6 to 2·2)	1·4 (0·7 to 2·4)	1·4 (0·7 to 2·5)	1·2 (0·6 to 2·1)	0·9 (0·5 to 1·6)	0·7 (0·4 to 1·3)	0·6 (0·3 to 1·1)	0·5 (0·3 to 0·9)	0·3 (0·1 to 0·5)	0·1 (0·1 to 0·2)	0·1 (0 to 0·1)	0 (0 to 0)	42·1
Contact dermatitis	1·1 (0·5 to 2·2)	1·3 (0·6 to 2·5)	1·5 (0·7 to 2·9)	1·4 (0·6 to 2·7)	1·1 (0·5 to 2)	1 (0·4 to 1·8)	0·8 (0·4 to 1·7)	0·6 (0·3 to 1·2)	0·3 (0·1 to 0·5)	0·1 (0·1 to 0·2)	0·1 (0 to 0·1)	0 (0 to 0)	16
Seborrhoeic dermatitis	0·1 (0·1 to 0·2)	0·1 (0·1 to 0·2)	0·1 (0·1 to 0·2)	0·1 (0 to 0·2)	0·1 (0 to 0·2)	0·1 (0 to 0·2)	0·1 (0 to 0·2)	0·1 (0 to 0·1)	0 (0 to 0·1)	0 (0 to 0)	0 (0 to 0)	0 (0 to 0)	1·8
Psoriasis	1·3 (0·7 to 2)	1·6 (0·9 to 2·5)	1·8 (1 to 2·6)	1·6 (0·9 to 2·4)	1·2 (0·7 to 1·8)	0·9 (0·6 to 1·4)	0·7 (0·4 to 1·1)	0·5 (0·3 to 0·7)	0·2 (0·1 to 0·3)	0·1 (0 to 0·1)	0 (0 to 0)	0 (0 to 0)	17·6
Bacterial skin diseases	3 (1·6 to 5·7)	2·5 (1·3 to 5)	4·4 (2·1 to 7·9)	4·7 (2·1 to 8·4)	5·3 (2·4 to 9·4)	4·2 (1·8 to 7·1)	4 (1·6 to 6·9)	4·3 (1·8 to 8·2)	2·8 (1 to 5·3)	2 (0·8 to 4)	1·2 (0·5 to 2·4)	0·6 (0·2 to 1·3)	51·8
Cellulitis	0·2 (0·1 to 0·9)	0·2 (0·1 to 0·7)	0·5 (0·2 to 1·5)	0·6 (0·2 to 1·7)	0·5 (0·2 to 1·4)	0·5 (0·2 to 1·2)	0·4 (0·1 to 0·9)	0·3 (0·1 to 0·9)	0·2 (0·1 to 0·5)	0·1 (0 to 0·3)	0·1 (0 to 0·2)	0 (0 to 0·1)	5·2
Pyoderma	2·7 (1·4 to 4·6)	2·3 (1·1 to 4·4)	3·8 (1·8 to 6·9)	4·1 (1·6 to 7·2)	4·8 (2 to 8·2)	3·7 (1·6 to 6·1)	3·6 (1·4 to 6·1)	4 (1·6 to 7·5)	2·7 (1 to 4·8)	1·9 (0·8 to 3·8)	1·2 (0·5 to 2·2)	0·6 (0·2 to 1·2)	46·7
Scabies	2 (1 to 3·3)	2·2 (1·1 to 3·8)	2·1 (1·1 to 3·7)	1·7 (0·9 to 2·9)	1·1 (0·6 to 2)	0·9 (0·4 to 1·5)	0·7 (0·4 to 1·3)	0·6 (0·3 to 1·1)	0·4 (0·2 to 0·7)	0·2 (0·1 to 0·4)	0·1 (0·1 to 0·2)	0 (0 to 0·1)	40·5
Fungal skin diseases	1·1 (0·4 to 2·4)	1·3 (0·5 to 2·8)	1·5 (0·6 to 3·2)	1·5 (0·6 to 3·3)	1·3 (0·5 to 2·9)	1·4 (0·5 to 2·9)	1·7 (0·6 to 3·6)	1·6 (0·6 to 3·4)	1·1 (0·4 to 2·3)	0·7 (0·3 to 1·5)	0·4 (0·2 to 0·9)	0·2 (0·1 to 0·3)	22·6
Viral skin diseases	0·6 (0·3 to 1)	0·6 (0·4 to 1)	0·6 (0·4 to 1)	0·5 (0·3 to 0·8)	0·4 (0·2 to 0·6)	0·3 (0·2 to 0·5)	0·2 (0·1 to 0·4)	0·2 (0·1 to 0·2)	0·1 (0 to 0·1)	0 (0 to 0)	0 (0 to 0)	0 (0 to 0)	20·6
Acne vulgaris	0·4 (0·2 to 0·7)	0·3 (0·2 to 0·6)	0·2 (0·1 to 0·4)	0·1 (0·1 to 0·2)	0·1 (0 to 0·1)	0·1 (0 to 0·1)	0 (0 to 0·1)	0 (0 to 0)	0 (0 to 0)	0 (0 to 0)	0 (0 to 0)	0 (0 to 0)	25·5
Alopecia areata	0·4 (0·2 to 0·6)	0·4 (0·2 to 0·6)	0·3 (0·2 to 0·6)	0·3 (0·1 to 0·4)	0·2 (0·1 to 0·3)	0·1 (0·1 to 0·2)	0·1 (0·1 to 0·2)	0·1 (0 to 0·1)	0 (0 to 0)	0 (0 to 0)	0 (0 to 0)	0 (0 to 0)	4·9
Pruritus	0·3 (0·1 to 0·6)	0·4 (0·2 to 0·7)	0·4 (0·2 to 0·8)	0·4 (0·2 to 0·8)	0·4 (0·2 to 0·7)	0·3 (0·1 to 0·6)	0·3 (0·1 to 0·6)	0·2 (0·1 to 0·5)	0·1 (0·1 to 0·3)	0·1 (0 to 0·1)	0 (0 to 0·1)	0 (0 to 0)	5·1
Urticaria	1·3 (0·7 to 2·2)	1·4 (0·7 to 2·3)	1·3 (0·7 to 2·2)	1·1 (0·6 to 1·8)	0·8 (0·4 to 1·3)	0·6 (0·3 to 1)	0·5 (0·3 to 0·9)	0·4 (0·2 to 0·6)	0·2 (0·1 to 0·3)	0·1 (0 to 0·1)	0 (0 to 0·1)	0 (0 to 0)	25·9
Decubitus ulcer	0·9 (0·4 to 1·8)	1·6 (0·7 to 3·2)	3·5 (1·8 to 6·4)	5 (2·5 to 8·8)	4·7 (2·4 to 8·4)	5·3 (2·7 to 8·8)	8·7 (4·7 to 13·8)	11·8 (6·4 to 18·8)	8·9 (4·9 to 15·1)	6·8 (4 to 11·6)	4·6 (2·7 to 7·8)	2·2 (1·4 to 4·2)	66·8
Other skin and subcutaneous diseases	1·6 (0·8 to 2·9)	2·1 (1 to 3·9)	2·6 (1·3 to 4·7)	2·6 (1·3 to 4·8)	2·3 (1·2 to 4·3)	2 (1 to 3·7)	1·9 (1 to 3·5)	1·6 (0·8 to 2·8)	0·8 (0·4 to 1·5)	0·4 (0·2 to 0·6)	0·2 (0·1 to 0·3)	0·1 (0 to 0·1)	27·3
Sense organ diseases	16·4 (10·2 to 25)	25·9 (16 to 40·7)	37 (23·6 to 58·7)	41·4 (26·3 to 64·9)	39·7 (25·1 to 59·8)	40 (26 to 59·3)	43·6 (29·2 to 62·9)	43·6 (29·8 to 61·4)	28·6 (19·9 to 39·2)	17·6 (12·3 to 23·7)	10·2 (7·1 to 13·5)	4 (2·9 to 5·4)	415
Blindness and vision loss	3·9 (2·4 to 6)	5·8 (3·6 to 9·2)	8·2 (5·1 to 13·1)	9·5 (6 to 14·9)	9·7 (6·3 to 14·7)	10·8 (7·1 to 15·9)	12·8 (8·5 to 18·3)	13·5 (9·3 to 19·2)	9·2 (6·3 to 12·5)	5·8 (4 to 7·9)	3·6 (2·5 to 4·9)	1·6 (1·1 to 2·2)	112
Glaucoma	0 (0 to 0)	0 (0 to 0)	0·1 (0 to 0·2)	0·2 (0·1 to 0·5)	0·4 (0·2 to 0·7)	0·6 (0·3 to 1)	0·9 (0·5 to 1·5)	1·3 (0·7 to 2)	1 (0·6 to 1·6)	0·7 (0·4 to 1·1)	0·4 (0·2 to 0·6)	0·2 (0·1 to 0·3)	5·8
Cataract	0·4 (0·2 to 0·7)	0·6 (0·3 to 1·1)	0·9 (0·5 to 1·5)	1·1 (0·6 to 1·8)	1·3 (0·8 to 2)	1·9 (1·1 to 2·8)	2·9 (1·9 to 4·3)	4 (2·5 to 5·8)	3·1 (2·1 to 4·4)	2·1 (1·5 to 3)	1·3 (0·8 to 1·8)	0·6 (0·3 to 0·8)	20·6
Age-related macular degeneration	0 (0 to 0)	0 (0 to 0)	0 (0 to 0·1)	0·1 (0 to 0·1)	0·1 (0 to 0·2)	0·1 (0·1 to 0·2)	0·1 (0·1 to 0·2)	0·2 (0·1 to 0·3)	0·1 (0·1 to 0·2)	0·1 (0 to 0·1)	0 (0 to 0·1)	0 (0 to 0)	0·8
Refraction disorders	2 (1·1 to 3·1)	2·4 (1·4 to 3·7)	2·6 (1·5 to 4·1)	2·4 (1·5 to 3·7)	2 (1·2 to 2·9)	1·7 (1·1 to 2·7)	1·8 (1·1 to 2·7)	1·7 (1·1 to 2·6)	0·9 (0·6 to 1·4)	0·4 (0·3 to 0·7)	0·2 (0·1 to 0·3)	0·1 (0 to 0·1)	31·8
Near vision loss	0·8 (0·3 to 1·8)	1·7 (0·7 to 3·6)	2·7 (1·1 to 5·7)	3·3 (1·3 to 7)	3·3 (1·4 to 6·8)	3·2 (1·4 to 6·6)	3·3 (1·4 to 6·6)	3 (1·2 to 6·2)	1·8 (0·8 to 3·7)	1 (0·4 to 2)	0·5 (0·2 to 1·1)	0·2 (0·1 to 0·4)	25·8
Other vision loss	0·7 (0·4 to 1·2)	1·1 (0·6 to 1·8)	1·8 (1 to 2·8)	2·3 (1·3 to 3·6)	2·7 (1·7 to 4)	3·3 (2·1 to 4·8)	3·7 (2·4 to 5·3)	3·3 (2·1 to 5)	2·2 (1·4 to 3·2)	1·5 (0·9 to 2·2)	1·1 (0·8 to 1·6)	0·6 (0·4 to 0·9)	27
Age-related and other hearing loss	11·4 (6·7 to 18·2)	18·6 (11·1 to 30·3)	26·8 (16·3 to 43·1)	29·9 (18·5 to 47·6)	28 (16·9 to 43·6)	27·1 (17 to 41·2)	28·7 (18·5 to 42·7)	28·2 (18·8 to 40·9)	18·5 (12·4 to 25·4)	11·3 (7·7 to 15·5)	6·4 (4·4 to 8·6)	2·3 (1·6 to 3·1)	282
Other sense organ diseases	1·1 (0·5 to 2·1)	1·4 (0·6 to 2·8)	2 (0·9 to 3·9)	2 (0·9 to 3·8)	2 (0·9 to 3·7)	2·1 (0·9 to 3·9)	2·1 (1 to 3·9)	1·9 (0·9 to 3·4)	1 (0·5 to 1·9)	0·4 (0·2 to 0·8)	0·2 (0·1 to 0·4)	0·1 (0 to 0·2)	21·7
Musculoskeletal disorders	82·1 (57·7 to 111)	104 (73 to 145)	120 (83·5 to 168)	110 (76·2 to 153)	86·7 (59·4 to 122)	69·6 (48·8 to 98·5)	60·3 (41·8 to 83·8)	46·5 (31·6 to 65·2)	24 (16·7 to 33·8)	13·1 (9·3 to 17·9)	6 (4·3 to 8·2)	2·1 (1·5 to 2·9)	1,020
Rheumatoid arthritis	1·7 (1 to 2·6)	2·1 (1·3 to 3·2)	2·3 (1·4 to 3·4)	2·2 (1·4 to 3·1)	1·7 (1·1 to 2·4)	1·4 (0·9 to 2)	1·1 (0·8 to 1·6)	1 (0·7 to 1·4)	0·5 (0·3 to 0·7)	0·4 (0·3 to 0·7)	0·2 (0·1 to 0·4)	0·1 (0 to 0·1)	19·9
Osteoarthritis	4·8 (2·3 to 9·7)	10·7 (5·3 to 21·9)	17·7 (8·6 to 36·2)	20·5 (10 to 40·9)	18·9 (9·4 to 37·3)	16·9 (8·6 to 33·7)	15·6 (7·8 to 31·5)	12·6 (6·3 to 25·8)	6·7 (3·4 to 13·4)	3·3 (1·7 to 6·7)	1·6 (0·8 to 3·2)	0·6 (0·3 to 1·1)	132
Osteoarthritis hip	0·2 (0·1 to 0·4)	0·4 (0·1 to 0·8)	0·6 (0·2 to 1·4)	0·7 (0·3 to 1·7)	0·7 (0·3 to 1·6)	0·7 (0·3 to 1·5)	0·7 (0·3 to 1·5)	0·6 (0·3 to 1·2)	0·3 (0·1 to 0·6)	0·2 (0·1 to 0·3)	0·1 (0 to 0·2)	0 (0 to 0·1)	5·4
Osteoarthritis knee	3·7 (1·7 to 7·6)	8·1 (3·9 to 16·4)	13·3 (6·2 to 27·3)	15·1 (7·1 to 31·2)	13·8 (6·7 to 27·7)	12·2 (5·8 to 24·8)	11·1 (5·5 to 22·5)	8·7 (4·3 to 17·5)	4·5 (2·2 to 9·1)	2·1 (1 to 4·3)	1 (0·5 to 1·9)	0·3 (0·2 to 0·6)	95·2
Osteoarthritis hand	0·6 (0·2 to 1·3)	1·4 (0·6 to 3·1)	2·5 (1·1 to 5·5)	2·9 (1·4 to 6·3)	2·7 (1·3 to 5·6)	2·4 (1·2 to 5)	2·2 (1·1 to 4·6)	1·9 (0·9 to 3·8)	1·1 (0·5 to 2·1)	0·6 (0·3 to 1·2)	0·3 (0·2 to 0·6)	0·1 (0·1 to 0·3)	18·8

Osteoarthritis other	0 (0 to 0)	0 (0 to 0)	0 (0 to 0)	0 (0 to 0)	0 (0 to 0)	0 (0 to 0)
Low back pain	0 (0 to 0)	0 (0 to 0)	0 (0 to 0)	0 (0 to 0)	2·2 (1 to 4)	10·9 (6·3 to 17·8)
Neck pain	0 (0 to 0)	0 (0 to 0)	0 (0 to 0)	0 (0 to 0)	0·1 (0 to 0·3)	0·6 (0·2 to 1·4)
Gout	0 (0 to 0)	0 (0 to 0)	0 (0 to 0)	0 (0 to 0)	0 (0 to 0)	0 (0 to 0)
Other musculoskeletal disorders	0 (0 to 0)	0 (0 to 0)	0 (0 to 0)	0 (0 to 0)	0·3 (0·2 to 0·5)	1 (0·6 to 1·8)
Other non-communicable diseases	40·3 (26·5 to 60)	21 (12·1 to 33·6)	66·7 (45·1 to 96·3)	39 (27·9 to 53·5)	24·7 (19·6 to 31)	26·9 (20·4 to 34·3)
Congenital birth defects	39 (25·6 to 58·3)	18·9 (10·6 to 31)	45·3 (29 to 70·1)	20·8 (13·9 to 30·8)	9·5 (7·3 to 12·2)	8·3 (6·3 to 11)
Neural tube defects	3·7 (1·8 to 6·7)	1·4 (0·6 to 2·8)	6·7 (2·8 to 13·2)	3·9 (1·6 to 8·1)	0·3 (0·2 to 0·4)	0·4 (0·3 to 0·6)
Congenital heart anomalies	20 (12·2 to 31·8)	11·8 (6·5 to 20·5)	25·9 (15 to 45)	9·6 (5·3 to 17·4)	3·8 (2·7 to 5·3)	2·8 (1·9 to 4·1)
Orofacial clefts	0 (0 to 0)	0 (0 to 0)	0 (0 to 0·1)	0·1 (0 to 0·2)	0·1 (0 to 0·2)	0·1 (0 to 0·2)
Down syndrome	0·4 (0·2 to 0·8)	0·1 (0 to 0·2)	0·5 (0·3 to 1)	0·4 (0·2 to 0·8)	0·2 (0·1 to 0·3)	0·2 (0·1 to 0·3)
Turner syndrome	0 (0 to 0)	0 (0 to 0)	0 (0 to 0)	0 (0 to 0)	0 (0 to 0)	0 (0 to 0)
Klinefelter syndrome	0 (0 to 0)	0 (0 to 0)	0 (0 to 0)	0 (0 to 0)	0 (0 to 0)	0 (0 to 0)
Other chromosomal abnormalities	4 (2·1 to 7)	2·2 (1 to 4·3)	4·8 (2·4 to 9·7)	1·1 (0·6 to 1·9)	0·6 (0·4 to 0·8)	0·5 (0·3 to 0·8)
Congenital musculoskeletal and limb anomalies	0·7 (0·3 to 1·3)	0·3 (0·1 to 0·4)	1·4 (0·8 to 2·1)	2·5 (1·4 to 3·8)	2·7 (1·6 to 4·2)	2·8 (1·6 to 4·5)
Urogenital congenital anomalies	0·7 (0·3 to 1·3)	0·3 (0·1 to 0·6)	0·3 (0·2 to 0·6)	0·3 (0·2 to 0·4)	0·2 (0·1 to 0·3)	0·2 (0·1 to 0·3)
Digestive congenital anomalies	2·7 (1·4 to 4·8)	1·1 (0·5 to 2·2)	1·7 (0·8 to 3·3)	0·6 (0·3 to 1)	0·2 (0·1 to 0·3)	0·1 (0·1 to 0·2)
Other congenital birth defects	6·9 (3·7 to 11·5)	1·8 (0·8 to 3·4)	4 (2·1 to 7·4)	2·3 (1·2 to 4)	1·4 (0·9 to 2·2)	1·2 (0·7 to 1·8)
Urinary diseases and male infertility	0·1 (0 to 0·1)	0·1 (0·1 to 0·2)	0·7 (0·4 to 1·2)	0·7 (0·5 to 1)	0·5 (0·4 to 0·7)	0·7 (0·5 to 1)
Urolithiasis	0 (0 to 0)	0 (0 to 0)	0 (0 to 0)	0 (0 to 0)	0 (0 to 0)	0 (0 to 0)
Benign prostatic hyperplasia	0 (0 to 0)	0 (0 to 0)	0 (0 to 0)	0 (0 to 0)	0 (0 to 0)	0 (0 to 0)
Male infertility	--	--	--	--	--	--
Other urinary diseases	0 (0 to 0)	0·1 (0 to 0·1)	0·1 (0 to 0·2)	0·2 (0·1 to 0·3)	0·1 (0·1 to 0·2)	0·2 (0·1 to 0·3)
Gynecological diseases	0 (0 to 0)	0 (0 to 0)	0 (0 to 0)	0 (0 to 0)	0 (0 to 0)	5·5 (2·7 to 9·5)
Uterine fibroids	0 (0 to 0)	0 (0 to 0)	0 (0 to 0)	0 (0 to 0)	0 (0 to 0)	0·1 (0 to 0·2)
Polycystic ovarian syndrome	0 (0 to 0)	0 (0 to 0)	0 (0 to 0)	0 (0 to 0)	0 (0 to 0)	0·2 (0·1 to 0·4)
Female infertility	0 (0 to 0)	0 (0 to 0)	0 (0 to 0)	0 (0 to 0)	0 (0 to 0)	0 (0 to 0)
Endometriosis	0 (0 to 0)	0 (0 to 0)	0 (0 to 0)	0 (0 to 0)	0 (0 to 0)	0 (0 to 0)
Genital prolapse	0 (0 to 0)	0 (0 to 0)	0 (0 to 0)	0 (0 to 0)	0 (0 to 0)	0 (0 to 0)
Premenstrual syndrome	0 (0 to 0)	0 (0 to 0)	0 (0 to 0)	0 (0 to 0)	0 (0 to 0)	5 (2·3 to 8·9)
Other gynecological diseases	0 (0 to 0)	0 (0 to 0)	0 (0 to 0)	0 (0 to 0)	0 (0 to 0)	0·2 (0·1 to 0·5)
Hemoglobinopathies and hemolytic anemias	0·2 (0·1 to 0·3)	0·2 (0·1 to 0·4)	1·8 (1·2 to 2·6)	5·6 (3·6 to 8·3)	6·9 (4·7 to 9·6)	5·4 (3·6 to 7·8)
Thalassemias	0 (0 to 0)	0 (0 to 0)	0 (0 to 0)	0 (0 to 0·1)	0 (0 to 0)	0 (0 to 0)
Thalassemias trait	0 (0 to 0)	0 (0 to 0)	0 (0 to 0·1)	0·1 (0 to 0·2)	0·2 (0·1 to 0·3)	0·1 (0 to 0·2)
Sickle cell disorders	0·2 (0·1 to 0·3)	0·1 (0·1 to 0·3)	1·3 (0·7 to 2·1)	3·3 (1·8 to 5·7)	4·2 (2·7 to 6)	4 (2·6 to 6)
Sickle cell trait	0 (0 to 0)	0·1 (0 to 0·1)	0·3 (0·2 to 0·5)	1·3 (0·6 to 2·2)	1·6 (0·7 to 2·9)	0·8 (0·2 to 1·7)
G6PD deficiency	0 (0 to 0)	0 (0 to 0)	0 (0 to 0)	0 (0 to 0·1)	0 (0 to 0·1)	0 (0 to 0·1)
G6PD trait	0 (0 to 0)	0 (0 to 0)	0 (0 to 0)	0 (0 to 0)	0 (0 to 0)	0 (0 to 0)
Other hemoglobinopathies and hemolytic anemias	0 (0 to 0)	0 (0 to 0)	0·2 (0·1 to 0·3)	0·8 (0·3 to 1·4)	0·9 (0·3 to 1·8)	0·4 (0·1 to 0·9)
Endocrine, metabolic, blood, and immune disorders	1 (0·6 to 1·7)	1·2 (0·6 to 2·2)	15·6 (9·1 to 26·4)	10·2 (6·6 to 16·5)	5·6 (3·9 to 8·1)	4·5 (3·3 to 6·5)
Oral disorders	0 (0 to 0)	0 (0 to 0)	0 (0 to 0)	1·8 (1 to 2·9)	2·2 (1·2 to 3·8)	2·5 (1·3 to 4·2)
Caries of deciduous teeth	0 (0 to 0)	0 (0 to 0)	0 (0 to 0)	0·6 (0·2 to 1·1)	0·5 (0·2 to 1·2)	0 (0 to 0·1)
Caries of permanent teeth	0 (0 to 0)	0 (0 to 0)	0 (0 to 0)	0 (0 to 0)	0·5 (0·2 to 1·2)	1·1 (0·5 to 2·5)
Periodontal diseases	0 (0 to 0)	0 (0 to 0)	0 (0 to 0)	0 (0 to 0)	0 (0 to 0)	0 (0 to 0)
Other oral disorders	0 (0 to 0)	0 (0 to 0)	0 (0 to 0)	1·2 (0·7 to 1·9)	1·1 (0·7 to 1·8)	1·3 (0·7 to 2)
Sudden infant death syndrome	--	0·5 (0·2 to 0·8)	3·3 (1·6 to 6·1)	--	--	--
Injuries	2·7 (1·8 to 3·9)	1·4 (0·8 to 2·2)	13·3 (8·6 to 20)	29·3 (20·3 to 42·1)	26·2 (22·1 to 31·1)	29·5 (24·9 to 34·5)
Transport injuries	0·1 (0·1 to 0·1)	0·1 (0 to 0·1)	0·7 (0·4 to 1·1)	3·3 (2 to 5)	4·8 (3·4 to 6·5)	5·8 (4·3 to 7·5)
Road injuries	0·1 (0 to 0·1)	0 (0 to 0·1)	0·5 (0·3 to 0·8)	2·6 (1·5 to 4·1)	4 (2·8 to 5·6)	4·8 (3·5 to 6·5)
Pedestrian road injuries	0 (0 to 0)	0 (0 to 0)	0·1 (0 to 0·1)	0·7 (0·4 to 1·1)	1 (0·6 to 1·4)	0·9 (0·6 to 1·2)
Cyclist road injuries	0 (0 to 0)	0 (0 to 0)	0 (0 to 0)	0·2 (0·1 to 0·4)	0·4 (0·2 to 0·6)	0·8 (0·5 to 1·2)
Motorcyclist road injuries	0 (0 to 0)	0 (0 to 0)	0 (0 to 0·1)	0·1 (0·1 to 0·2)	0·3 (0·2 to 0·4)	0·4 (0·3 to 0·5)
Motor vehicle road injuries	0 (0 to 0·1)	0 (0 to 0)	0·4 (0·2 to 0·6)	1·5 (0·9 to 2·4)	2·3 (1·5 to 3·2)	2·6 (1·9 to 3·7)
Other road injuries	0 (0 to 0)	0 (0 to 0)	0 (0 to 0)	0·1 (0 to 0·2)	0·2 (0·1 to 0·3)	0·1 (0·1 to 0·2)
Other transport injuries	0 (0 to 0·1)	0 (0 to 0·1)	0·2 (0·1 to 0·3)	0·7 (0·4 to 1·1)	0·7 (0·5 to 1·1)	1 (0·6 to 1·4)
Unintentional injuries	2·1 (1·3 to 3)	1·2 (0·7 to 2)	11·7 (7·4 to 17·9)	24 (16·2 to 35·5)	19·7 (15·6 to 23·7)	20·4 (16·4 to 24·9)
Falls	0 (0 to 0)	0 (0 to 0)	0·2 (0·2 to 0·3)	0·7 (0·5 to 1·1)	1·1 (0·7 to 1·5)	1·5 (1·1 to 2·2)
Drowning	0·1 (0·1 to 0·2)	0·1 (0 to 0·1)	1·3 (0·8 to 2)	8·7 (5·5 to 13·3)	6·5 (4·7 to 8·5)	6·5 (4·9 to 8·4)
Fire, heat, and hot substances	0·1 (0 to 0·1)	0·1 (0 to 0·1)	1 (0·6 to 1·5)	3·5 (2·3 to 5·4)	3 (2·4 to 3·8)	3·2 (2·4 to 4·2)
Poisonings	0 (0 to 0·1)	0·1 (0 to 0·1)	0·3 (0·2 to 0·4)	1 (0·6 to 1·5)	0·5 (0·3 to 0·6)	0·4 (0·3 to 0·6)
Poisoning by carbon monoxide	0 (0 to 0)	0 (0 to 0)	0 (0 to 0·1)	0·2 (0·1 to 0·4)	0·1 (0·1 to 0·2)	0·1 (0·1 to 0·2)
Poisoning by other means	0 (0 to 0)	0·1 (0 to 0·1)	0·2 (0·1 to 0·4)	0·8 (0·5 to 1·2)	0·3 (0·2 to 0·5)	0·3 (0·2 to 0·4)
Exposure to mechanical forces	0·1 (0·1 to 0·1)	0·1 (0·1 to 0·2)	0·6 (0·4 to 1)	3·2 (2·2 to 4·8)	3·8 (2·9 to 4·8)	4 (3·1 to 5·2)
Unintentional firearm injuries	0 (0 to 0)	0 (0 to 0)	0 (0 to 0)	0·1 (0·1 to 0·2)	0·2 (0·1 to 0·3)	0·4 (0·3 to 0·7)
Other exposure to mechanical forces	0·1 (0·1 to 0·1)	0·1 (0·1 to 0·2)	0·6 (0·4 to 0·9)	3·1 (2·1 to 4·6)	3·6 (2·7 to 4·5)	3·6 (2·8 to 4·7)
Adverse effects of medical treatment	0·4 (0·2 to 0·6)	0·3 (0·1 to 0·5)	1·4 (0·7 to 2·7)	1·2 (0·7 to 1·8)	0·6 (0·4 to 0·8)	0·5 (0·4 to 0·8)
Animal contact	0 (0 to 0)	0 (0 to 0)	0·1 (0 to 0·1)	0·2 (0·1 to 0·3)	0·2 (0·1 to 0·3)	0·3 (0·2 to 0·4)
Venomous animal contact	0 (0 to 0)	0 (0 to 0)	0 (0 to 0)	0·1 (0 to 0·1)	0·1 (0·1 to 0·2)	0·2 (0·1 to 0·3)
Non-venomous animal contact	0 (0 to 0)	0 (0 to 0)	0 (0 to 0·1)	0·1 (0·1 to 0·2)	0·1 (0·1 to 0·2)	0·1 (0 to 0·1)
Foreign body	1·2 (0·8 to 1·9)	0·6 (0·3 to 1)	6·3 (3·7 to 10·1)	3·9 (2·5 to 6)	1·7 (1·3 to 2·1)	1·3 (1 to 1·6)
Pulmonary aspiration and foreign body in airway	1·2 (0·8 to 1·9)	0·6 (0·3 to 1)	6·3 (3·7 to 10·1)	3·8 (2·4 to 5·8)	1·5 (1·1 to 1·8)	1·1 (0·8 to 1·3)
Foreign body in eyes	0 (0 to 0)	0 (0 to 0)	0 (0 to 0)	0 (0 to 0·1)	0·1 (0 to 0·2)	0·1 (0 to 0·2)
Foreign body in other body part	0 (0 to 0)	0 (0 to 0)	0 (0 to 0)	0·1 (0·1 to 0·2)	0·1 (0·1 to 0·2)	0·2 (0·1 to 0·2)
Environmental heat and cold exposure	0 (0 to 0)	0 (0 to 0)	0·1 (0·1 to 0·1)	0·4 (0·2 to 0·5)	0·7 (0·4 to 1)	0·8 (0·6 to 1·2)
Exposure to forces of nature	0 (0 to 0)	0 (0 to 0)	0 (0 to 0)	0 (0 to 0)	0 (0 to 0)	0 (0 to 0)
Other unintentional injuries	0·1 (0 to 0·1)	0 (0 to 0·1)	0·3 (0·2 to 0·5)	1·2 (0·8 to 1·7)	1·6 (1·2 to 2·3)	1·8 (1·3 to 2·5)
Self-harm and interpersonal violence	0·6 (0·3 to 0·9)	0·1 (0 to 0·2)	0·9 (0·5 to 1·5)	2 (1·2 to 3·4)	1·8 (1·4 to 2·3)	3·3 (2·6 to 4·1)
Self-harm	0 (0 to 0)	0 (0 to 0)	0 (0 to 0)	0 (0 to 0)	0 (0 to 0)	0·7 (0·4 to 1)
Self-harm by firearm	0 (0 to 0)	0 (0 to 0)	0 (0 to 0)	0 (0 to 0)	0 (0 to 0)	0 (0 to 0)
Self-harm by other specified means	0 (0 to 0)	0 (0 to 0)	0 (0 to 0)	0 (0 to 0)	0 (0 to 0)	0·7 (0·4 to 1)
Interpersonal violence	0·6 (0·3 to 0·9)	0·1 (0 to 0·2)	0·9 (0·5 to 1·5)	2 (1·2 to 3·3)	1·8 (1·3 to 2·3)	2·6 (2 to 3·4)
Physical violence by firearm	0 (0 to 0)	0 (0 to 0)	0 (0 to 0)	0·1 (0 to 0·1)	0·1 (0·1 to 0·2)	0·2 (0·1 to 0·3)
Physical violence by sharp object	0 (0 to 0)	0 (0 to 0)	0 (0 to 0)	0·1 (0 to 0·1)	0·1 (0·1 to 0·1)	0·2 (0·1 to 0·2)
Sexual violence	0 (0 to 0)	0 (0 to 0)	0 (0 to 0)	0 (0 to 0)	0·2 (0·1 to 0·4)	0·5 (0·3 to 0·9)
Physical violence by other means	0·5 (0·3 to 0·9)	0·1 (0 to 0·2)	0·9 (0·5 to 1·4)	1·8 (1·1 to 3·1)	1·3 (1 to 1·8)	1·7 (1·2 to 2·2)
Conflict and terrorism	0 (0 to 0)	0 (0 to 0)	0 (0 to 0)	0 (0 to 0)	0 (0 to 0)	0 (0 to 0)

Osteoarthritis other	0 (0 to 0)	0 (0 to 0)	0 (0 to 0)	0 (0 to 0)	0·1 (0 to 0·2)	0·3 (0·1 to 0·8)	0·8 (0·3 to 1·8)
Low back pain	20·7 (12·3 to 32·8)	28·1 (16·5 to 46·4)	33 (19·3 to 52·3)	31·9 (18 to 48·7)	31·8 (19·2 to 48·9)	37·4 (22·6 to 57·4)	46 (27·7 to 71·4)
Neck pain	1·6 (0·6 to 3)	2·7 (1·1 to 5·5)	4·2 (1·8 to 8·3)	5·5 (2·6 to 10·5)	6·5 (3·2 to 11·6)	8·5 (4·2 to 15·2)	10·8 (5·3 to 19·6)
Gout	0 (0 to 0)	0 (0 to 0·1)	0·1 (0 to 0·2)	0·1 (0 to 0·3)	0·2 (0 to 0·4)	0·2 (0·1 to 0·5)	0·3 (0·1 to 0·6)
Other musculoskeletal disorders	7·5 (4·9 to 11·3)	16·6 (11·3 to 23·7)	26·1 (17·2 to 37·3)	29·3 (19·9 to 41)	26·4 (18 to 37·2)	29·4 (19·7 to 42·7)	34·5 (23·5 to 50)
Other non-communicable diseases	48·6 (37·3 to 63·6)	75·9 (55·2 to 106)	95·9 (69·2 to 132)	94·1 (67·2 to 129)	89·1 (65·4 to 121)	91·8 (65·8 to 126)	97·9 (70·1 to 131)
Congenital birth defects	8·8 (6·7 to 11·4)	7·3 (5·6 to 9·5)	6·1 (4·5 to 7·9)	4·6 (3·4 to 6)	4 (3 to 5·1)	3·5 (2·5 to 4·5)	3·5 (2·7 to 4·6)
Neural tube defects	0·4 (0·3 to 0·6)	0·2 (0·1 to 0·3)	0·2 (0·2 to 0·3)	0·2 (0·1 to 0·3)	0·1 (0·1 to 0·2)	0·1 (0·1 to 0·1)	0·1 (0·1 to 0·1)
Congenital heart anomalies	3·6 (2·5 to 4·8)	2·9 (2 to 4)	2·5 (1·7 to 3·6)	1·6 (1·1 to 2·3)	1·7 (1·2 to 2·3)	1·3 (0·9 to 1·9)	1·3 (0·9 to 1·9)
Orofacial clefts	0·1 (0 to 0·2)	0·1 (0 to 0·2)	0·1 (0 to 0·2)	0·1 (0 to 0·2)	0·1 (0 to 0·1)	0·1 (0 to 0·2)	0·1 (0 to 0·2)
Down syndrome	0·2 (0·1 to 0·3)	0·3 (0·2 to 0·4)	0·2 (0·2 to 0·3)	0·2 (0·1 to 0·3)	0·2 (0·1 to 0·3)	0·1 (0·1 to 0·2)	0·2 (0·1 to 0·3)
Turner syndrome	0 (0 to 0)	0 (0 to 0)	0 (0 to 0)	0 (0 to 0)	0 (0 to 0)	0 (0 to 0)	0 (0 to 0)
Klinefelter syndrome	0 (0 to 0)	0 (0 to 0)	0 (0 to 0)	0 (0 to 0)	0 (0 to 0)	0 (0 to 0)	0 (0 to 0)
Other chromosomal abnormalities	0·6 (0·4 to 1)	0·5 (0·3 to 0·8)	0·4 (0·2 to 0·6)	0·3 (0·2 to 0·4)	0·1 (0·1 to 0·2)	0·1 (0·1 to 0·2)	0·1 (0 to 0·1)
Congenital musculoskeletal and limb anomalies	2·6 (1·5 to 4)	2 (1·2 to 3·2)	1·8 (1·1 to 2·7)	1·4 (0·8 to 2·2)	1·2 (0·7 to 1·9)	1·2 (0·7 to 1·9)	1·3 (0·8 to 2·1)
Urogenital congenital anomalies	0·1 (0·1 to 0·2)	0·1 (0·1 to 0·2)	0·1 (0·1 to 0·1)	0·1 (0 to 0·1)	0 (0 to 0·1)	0 (0 to 0·1)	0 (0 to 0·1)
Digestive congenital anomalies	0·1 (0·1 to 0·1)	0·1 (0 to 0·1)	0 (0 to 0·1)	0 (0 to 0·1)	0 (0 to 0)	0 (0 to 0)	0 (0 to 0)
Other congenital birth defects	1·1 (0·7 to 1·7)	1 (0·7 to 1·6)	0·8 (0·5 to 1·3)	0·7 (0·4 to 1·1)	0·5 (0·3 to 0·9)	0·4 (0·2 to 0·8)	0·5 (0·3 to 0·8)
Urinary diseases and male infertility	1·2 (0·9 to 1·6)	1·8 (1·3 to 2·4)	2·1 (1·5 to 2·9)	2·6 (1·8 to 3·6)	2·8 (2·1 to 3·8)	3 (2·2 to 4·1)	3·6 (2·6 to 4·8)
Urolithiasis	0 (0 to 0)	0·1 (0·1 to 0·1)	0·1 (0·1 to 0·2)	0·3 (0·2 to 0·4)	0·3 (0·2 to 0·6)	0·3 (0·2 to 0·4)	0·5 (0·3 to 0·8)
Benign prostatic hyperplasia	0 (0 to 0)	0 (0 to 0)	0 (0 to 0)	0 (0 to 0)	0 (0 to 0)	0 (0 to 0)	0 (0 to 0)
Male infertility	--	--	--	--	--	--	--
Other urinary diseases	0·2 (0·1 to 0·3)	0·2 (0·1 to 0·4)	0·2 (0·1 to 0·2)	0·2 (0·1 to 0·3)	0·2 (0·1 to 0·2)	0·2 (0·1 to 0·3)	0·2 (0·2 to 0·4)
Gynecological diseases	15·5 (8·8 to 25·3)	37·7 (24·1 to 58·3)	53·5 (34·6 to 78·9)	52·9 (35·3 to 75·9)	50 (34 to 71·9)	51·5 (33·9 to 74·9)	48·3 (32·3 to 68·3)
Uterine fibroids	0·2 (0·1 to 0·5)	0·6 (0·3 to 1·3)	1·5 (0·6 to 3·1)	2·7 (1·2 to 5·4)	3·7 (1·6 to 7·5)	4·7 (2·2 to 8·9)	4·5 (2·1 to 8·4)
Polycystic ovarian syndrome	0·9 (0·4 to 1·8)	1·1 (0·5 to 2·4)	1·1 (0·4 to 2·1)	0·8 (0·3 to 1·7)	0·7 (0·3 to 1·4)	0·7 (0·3 to 1·3)	0·6 (0·3 to 1·3)
Female infertility	0 (0 to 0)	1·3 (0·3 to 3·5)	1·8 (0·4 to 4·8)	1·8 (0·4 to 5·3)	1·6 (0·2 to 4·7)	1·6 (0·2 to 4·4)	0 (0 to 0·2)
Endometriosis	1·3 (0·5 to 2·6)	5·7 (2·7 to 10·4)	6·4 (3·1 to 11·4)	4·3 (2·2 to 7·7)	3·2 (1·6 to 5·7)	3·3 (1·7 to 5·9)	3·2 (1·8 to 5·2)
Genital prolapse	0 (0 to 0)	0 (0 to 0·1)	0·1 (0 to 0·3)	0·2 (0·1 to 0·5)	0·4 (0·2 to 0·8)	0·6 (0·3 to 1·3)	1 (0·5 to 1·9)
Premenstrual syndrome	12·1 (6 to 21·9)	17·2 (8·8 to 30·5)	16·7 (9·4 to 27·7)	13·7 (7·2 to 22·4)	12·7 (7·2 to 20·7)	13·3 (7·5 to 21·1)	10·6 (5·9 to 17·6)
Other gynecological diseases	1 (0·6 to 1·7)	11·8 (7·1 to 18·7)	25·9 (14·8 to 40·7)	29·4 (17·8 to 44·1)	27·6 (17·1 to 42·7)	27·4 (15·6 to 43·5)	28·4 (16·4 to 43·4)
Hemoglobinopathies and hemolytic anemias	11·6 (7·9 to 16·1)	12·9 (7·6 to 20·9)	12·9 (7 to 21·1)	10·2 (5·5 to 16·4)	7·8 (4·8 to 11·8)	5·6 (3·6 to 7·9)	5·4 (3·6 to 7·8)
Thalassemias	0 (0 to 0)	0 (0 to 0)	0 (0 to 0)	0 (0 to 0)	0 (0 to 0)	0 (0 to 0)	0 (0 to 0)
Thalassemias trait	0·4 (0·2 to 0·6)	0·5 (0·2 to 0·8)	0·5 (0·2 to 0·8)	0·4 (0·2 to 0·6)	0·3 (0·1 to 0·6)	0·3 (0·1 to 0·5)	0·1 (0 to 0·2)
Sickle cell disorders	9·3 (6 to 13·6)	7·4 (4·9 to 11)	6 (3·9 to 8·9)	4·3 (2·7 to 6·7)	4 (2·4 to 6·3)	2·8 (1·8 to 4·4)	2·9 (1·8 to 4·6)
Sickle cell trait	0·4 (0·1 to 0·7)	3·9 (0·6 to 10·2)	5·2 (0·8 to 11·9)	4·4 (0·7 to 9·8)	2·2 (0·4 to 5·4)	0·6 (0·3 to 1·1)	0·9 (0·3 to 1·7)
G6PD deficiency	0·8 (0·3 to 1·6)	0·2 (0·1 to 0·4)	0·3 (0·1 to 0·6)	0·3 (0·1 to 0·5)	0·3 (0·1 to 0·6)	0·6 (0·3 to 1)	0·3 (0·1 to 0·6)
G6PD trait	0·1 (0 to 0·2)	0 (0 to 0)	0 (0 to 0)	0 (0 to 0)	0 (0 to 0)	0·1 (0 to 0·1)	0 (0 to 0)
Other hemoglobinopathies and hemolytic anemias	0·8 (0·3 to 1·7)	0·8 (0·3 to 1·7)	1 (0·4 to 1·9)	0·8 (0·4 to 1·5)	1 (0·4 to 1·6)	1·2 (0·6 to 2·1)	1·2 (0·7 to 2·1)
Endocrine, metabolic, blood, and immune disorders	7·6 (5·2 to 10·7)	9·4 (6·6 to 13·1)	11·6 (7·8 to 16·3)	13·5 (9·5 to 18·5)	14·4 (10·4 to 19·8)	16·1 (11·1 to 23·4)	20·9 (14·1 to 29·3)
Oral disorders	3·9 (2·1 to 6·6)	6·8 (3·5 to 12·6)	9·6 (4·8 to 18·5)	10·2 (4·9 to 18·8)	10·1 (4·8 to 19·2)	12·2 (5·9 to 22·1)	16·2 (8·5 to 28·9)
Caries of deciduous teeth	0 (0 to 0)	0 (0 to 0)	0 (0 to 0)	0 (0 to 0)	0 (0 to 0)	0 (0 to 0)	0 (0 to 0)
Caries of permanent teeth	1·3 (0·5 to 2·8)	1·6 (0·7 to 3·4)	1·6 (0·6 to 3·3)	1·2 (0·5 to 2·5)	1 (0·4 to 2)	1 (0·4 to 1·9)	1 (0·4 to 2·2)
Periodontal diseases	0·5 (0·1 to 1·3)	2·1 (0·6 to 5·4)	4·5 (1·4 to 10·6)	5·6 (1·9 to 12·7)	6 (2·1 to 13·4)	7·2 (2·7 to 15·4)	8·5 (3·2 to 17·9)
Other oral disorders	2 (1·2 to 3·1)	2·8 (1·6 to 4·3)	2·9 (1·8 to 4·5)	2·5 (1·5 to 3·8)	2·1 (1·3 to 3·3)	2·2 (1·4 to 3·4)	2·6 (1·5 to 3·9)
Sudden infant death syndrome	--	--	--	--	--	--	--
Injuries	55·2 (47·5 to 63·5)	64·1 (53·6 to 75·5)	56·3 (46·3 to 67·6)	49·6 (41·4 to 59)	43·9 (37·2 to 52·4)	38·9 (32·4 to 46·8)	41·4 (33·9 to 50·5)
Transport injuries	16·1 (11·6 to 21·2)	16 (11·6 to 21·7)	12·6 (9·5 to 16·5)	10·2 (7·8 to 13)	9·1 (7 to 11·6)	8·1 (6·4 to 10·3)	8·5 (6·8 to 10·6)
Road injuries	14·2 (10·1 to 18·8)	14 (10 to 19·1)	11 (8·1 to 14·5)	8·8 (6·7 to 11·3)	7·9 (6·1 to 10·2)	6·8 (5·3 to 8·7)	7·5 (6 to 9·4)
Pedestrian road injuries	1·3 (0·9 to 1·6)	1·2 (0·9 to 1·6)	1·1 (0·8 to 1·4)	1·1 (0·8 to 1·5)	1·1 (0·8 to 1·4)	1 (0·8 to 1·3)	1·3 (1 to 1·6)
Cyclist road injuries	1·3 (0·8 to 2·1)	1·4 (0·9 to 2·1)	1·3 (0·9 to 1·9)	1·1 (0·8 to 1·6)	1·1 (0·8 to 1·5)	1 (0·7 to 1·4)	1·2 (0·9 to 1·6)
Motorcyclist road injuries	1·9 (1·4 to 2·6)	2 (1·4 to 2·8)	1·6 (1·1 to 2·1)	1·1 (0·8 to 1·5)	1 (0·8 to 1·4)	0·8 (0·6 to 1)	0·9 (0·7 to 1·2)
Motor vehicle road injuries	9·4 (6·5 to 12·7)	9 (6·2 to 12·5)	6·7 (4·7 to 9·1)	5·1 (3·7 to 6·8)	4·4 (3·3 to 5·8)	3·6 (2·7 to 4·8)	3·8 (2·9 to 4·9)
Other road injuries	0·3 (0·2 to 0·5)	0·3 (0·2 to 0·5)	0·4 (0·2 to 0·6)	0·3 (0·2 to 0·5)	0·3 (0·2 to 0·4)	0·3 (0·2 to 0·4)	0·3 (0·2 to 0·4)
Other transport injuries	1·9 (1·1 to 3·1)	2 (1·2 to 3·2)	1·6 (1 to 2·4)	1·4 (0·9 to 2·1)	1·1 (0·7 to 1·7)	1·3 (0·9 to 1·9)	1 (0·7 to 1·4)
Unintentional injuries	24·6 (20·3 to 29·9)	27·5 (21·7 to 34)	26·6 (21 to 33·9)	24·7 (19·3 to 30·8)	22·1 (17·5 to 28)	22·1 (17·2 to 28·3)	25·1 (19·5 to 32·3)
Falls	2·1 (1·4 to 3)	2·7 (1·9 to 3·8)	3 (2·1 to 4·3)	2·9 (2 to 4·1)	2·8 (2 to 3·9)	3·3 (2·4 to 4·7)	4·3 (3·1 to 5·9)
Drowning	5·8 (4·1 to 7·9)	3·5 (2·4 to 4·7)	2·8 (2 to 3·9)	2·4 (1·8 to 3·2)	2 (1·5 to 2·7)	1·5 (1·1 to 1·9)	1·5 (1·1 to 2)
Fire, heat, and hot substances	4·8 (3·5 to 6·2)	6 (4·5 to 7·8)	6·4 (4·8 to 8·4)	6 (4·5 to 7·9)	5·5 (4·2 to 7·2)	5·2 (3·9 to 7)	5·6 (4·1 to 7·5)
Poisonings	0·7 (0·5 to 0·9)	0·7 (0·5 to 1)	0·6 (0·4 to 0·9)	0·6 (0·4 to 0·8)	0·5 (0·4 to 0·7)	0·5 (0·3 to 0·6)	0·4 (0·3 to 0·6)
Poisoning by carbon monoxide	0·2 (0·2 to 0·3)	0·3 (0·2 to 0·4)	0·2 (0·1 to 0·3)	0·2 (0·1 to 0·3)	0·2 (0·1 to 0·2)	0·2 (0·1 to 0·2)	0·2 (0·1 to 0·2)
Poisoning by other means	0·4 (0·3 to 0·6)	0·4 (0·3 to 0·6)	0·4 (0·3 to 0·6)	0·4 (0·2 to 0·5)	0·3 (0·2 to 0·5)	0·3 (0·2 to 0·4)	0·3 (0·2 to 0·4)
Exposure to mechanical forces	5·5 (4·2 to 7·2)	6·8 (5·1 to 8·6)	6 (4·5 to 7·8)	5·4 (4·1 to 7)	4·9 (3·7 to 6·4)	4·8 (3·7 to 6·4)	5·2 (3·8 to 7)
Unintentional firearm injuries	1·1 (0·7 to 1·8)	1·4 (1 to 2·1)	0·9 (0·6 to 1·3)	1 (0·7 to 1·3)	0·8 (0·6 to 1)	0·6 (0·5 to 0·8)	0·6 (0·5 to 0·8)
Other exposure to mechanical forces	4·4 (3·2 to 5·9)	5·3 (4 to 7)	5·1 (3·8 to 6·7)	4·4 (3·3 to 5·9)	4·1 (3·1 to 5·5)	4·2 (3·1 to 5·6)	4·5 (3·3 to 6·2)
Adverse effects of medical treatment	1·1 (0·6 to 1·6)	2 (1·3 to 2·9)	1·8 (1·1 to 2·7)	2·1 (1·4 to 3·1)	1·7 (1·1 to 2·5)	1·9 (1·2 to 2·7)	2·4 (1·6 to 3·5)
Animal contact	0·3 (0·2 to 0·4)	0·4 (0·2 to 0·5)	0·4 (0·3 to 0·5)	0·3 (0·2 to 0·4)	0·3 (0·2 to 0·4)	0·3 (0·2 to 0·4)	0·4 (0·2 to 0·5)
Venomous animal contact	0·2 (0·1 to 0·3)	0·3 (0·2 to 0·4)	0·3 (0·2 to 0·4)	0·2 (0·1 to 0·3)	0·2 (0·1 to 0·3)	0·2 (0·1 to 0·3)	0·2 (0·1 to 0·3)
Non-venomous animal contact	0·1 (0·1 to 0·1)	0·1 (0·1 to 0·2)	0·1 (0·1 to 0·2)	0·1 (0·1 to 0·1)	0·1 (0·1 to 0·2)	0·1 (0·1 to 0·1)	0·1 (0·1 to 0·2)
Foreign body	1 (0·8 to 1·2)	1·3 (1 to 1·7)	1·2 (1 to 1·6)	1·1 (0·9 to 1·4)	1·1 (0·8 to 1·4)	1 (0·8 to 1·3)	1·2 (1 to 1·5)
Pulmonary aspiration and foreign body in airway	0·7 (0·5 to 0·8)	0·9 (0·7 to 1·2)	0·8 (0·6 to 1·1)	0·8 (0·6 to 1)	0·8 (0·6 to 1)	0·7 (0·5 to 0·9)	0·8 (0·6 to 1·1)
Foreign body in eyes	0·1 (0 to 0·3)	0·2 (0·1 to 0·4)	0·2 (0·1 to 0·4)	0·2 (0·1 to 0·4)	0·2 (0·1 to 0·3)	0·2 (0·1 to 0·3)	0·2 (0·1 to 0·3)
Foreign body in other body part	0·2 (0·1 to 0·3)	0·2 (0·2 to 0·3)	0·2 (0·1 to 0·3)	0·2 (0·1 to 0·2)	0·2 (0·1 to 0·2)	0·2 (0·1 to 0·2)	0·2 (0·1 to 0·3)
Environmental heat and cold exposure	1·1 (0·7 to 1·5)	1·4 (0·9 to 2)	1·5 (1 to 2·2)	1·4 (0·9 to 2)	1·2 (0·8 to 1·7)	1·3 (0·9 to 1·9)	1·6 (1·1 to 2·2)
Exposure to forces of nature	0·1 (0·1 to 0·2)	0·1 (0·1 to 0·2)	0·3 (0·2 to 0·4)	0·1 (0·1 to 0·2)	0·1 (0 to 0·1)	0·1 (0·1 to 0·2)	0·1 (0·1 to 0·2)
Other unintentional injuries	2·3 (1·6 to 3·2)	2·6 (1·9 to 3·7)	2·6 (1·8 to 3·7)	2·3 (1·6 to 3·4)	2·1 (1·5 to 2·9)	2·1 (1·5 to 3)	2·4 (1·7 to 3·4)
Self-harm and interpersonal violence	14·5 (11·6 to 18·1)	20·6 (16·2 to 26·6)	17·1 (12·7 to 22·3)	14·8 (11·5 to 18·7)	12·8 (10·1 to 16·2)	8·8 (7 to 11)	7·8 (6·2 to 9·7)
Self-harm	4·6 (3·2 to 6·4)	5·1 (3·6 to 7·1)	4 (2·7 to 5·7)	3·9 (2·7 to 5·3)	3·1 (2·2 to 4·2)	2·4 (1·8 to 3·3)	2·4 (1·8 to 3·3)
Self-harm by firearm	0 (0 to 0·1)	0·1 (0 to 0·1)	0 (0 to 0·1)	0·1 (0 to 0·1)	0 (0 to 0·1)	0 (0 to 0)	0 (0 to 0)
Self-harm by other specified means	4·5 (3·2 to 6·4)	5 (3·5 to 7)	3·9 (2·6 to 5·6)	3·8 (2·7 to 5·2)	3·1 (2·2 to 4·1)	2·4 (1·7 to 3·2)	2·4 (1·8 to 3·2)
Interpersonal violence	9·9 (7·4 to 13)	15·5 (11·6 to 20·7)	13·1 (9·4 to 17·5)	10·9 (8·2 to 14·3)	7·6 (5·7 to 10)	5·5 (4·2 to 7·2)	4·5 (3·5 to 6)
Physical violence by firearm	2 (1·3 to 3)	3 (1·9 to 4·6)	2·2 (1·3 to 3·3)	1·7 (1·1 to 2·5)	1·1 (0·8 to 1·7)	0·6 (0·4 to 0·9)	0·5 (0·3 to 0·7)
Physical violence by sharp object	1·4 (1 to 2·1)	2·7 (1·9 to 3·7)	2·3 (1·6 to 3·2)	1·8 (1·3 to 2·4)	1·3 (1 to 1·8)	0·8 (0·6 to 1·1)	0·5 (0·4 to 0·7)
Sexual violence	1·4 (0·8 to 2·2)	2·1 (1·2 to 3·2)	1·9 (1·1 to 3)	1·4 (0·8 to 2·4)	1·1 (0·6 to 1·7)	0·9 (0·5 to 1·5)	0·8 (0·5 to 1·4)
Physical violence by other means	5 (3·5 to 6·8)	7·7 (5·4 to 10·8)	6·7 (4·8 to 9·3)	6 (4·3 to 7·9)	4 (2·9 to 5·4)	3·1 (2·3 to 4·2)	2·6 (1·9 to 3·6)
Conflict and terrorism	0 (0 to 0)	0 (0 to 0)	0 (0 to 0)	0 (0 to 0)	2·1 (0·9 to 4·1)	0·9 (0·4 to 1·6)	0·8 (0·3 to 1·4)

Osteoarthritis other	1·4 (0·6 to 3)	1·7 (0·8 to 3·8)	1·7 (0·8 to 3·6)	1·6 (0·8 to 3·3)	1·6 (0·8 to 3·2)	1·4 (0·7 to 3)
Low back pain	51·4 (31·4 to 81)	45·8 (28·6 to 68)	35·5 (22·4 to 52·8)	28·2 (18·3 to 42·5)	25·1 (15·4 to 37·5)	20·4 (12·6 to 30·2)
Neck pain	10·5 (5·2 to 19·7)	8·8 (4·2 to 15·6)	6·4 (3·1 to 11·2)	4·8 (2·5 to 8·4)	4 (2·2 to 6·9)	3 (1·4 to 5·7)
Gout	0·4 (0·1 to 0·7)	0·4 (0·1 to 0·7)	0·3 (0·1 to 0·6)	0·3 (0·1 to 0·5)	0·2 (0·1 to 0·4)	0·2 (0·1 to 0·4)
Other musculoskeletal disorders	37·6 (24·7 to 55·3)	32·8 (21·9 to 47·7)	24 (14·8 to 36·4)	18 (11·7 to 26·1)	14·3 (8·6 to 21·8)	9·4 (5·7 to 14·3)
Other non-communicable diseases	96·3 (69·8 to 128)	80·3 (59·7 to 105)	63·1 (47·4 to 82·9)	53 (40 to 69·4)	48 (36·2 to 63·1)	39·7 (30·2 to 51)
Congenital birth defects	3·7 (2·8 to 4·8)	3 (2·2 to 3·9)	1·7 (1·2 to 2·2)	1·1 (0·9 to 1·5)	0·4 (0·3 to 0·7)	0·3 (0·2 to 0·4)
Neural tube defects	0·1 (0 to 0·1)	0·1 (0 to 0·1)	0 (0 to 0)	0 (0 to 0)	0 (0 to 0)	0 (0 to 0)
Congenital heart anomalies	1·4 (1 to 1·9)	1 (0·7 to 1·4)	0·5 (0·3 to 0·7)	0·4 (0·3 to 0·5)	0 (0 to 0·1)	0 (0 to 0)
Orofacial clefts	0·1 (0 to 0·2)	0·1 (0 to 0·1)	0 (0 to 0·1)	0 (0 to 0·1)	0 (0 to 0·1)	0 (0 to 0)
Down syndrome	0·2 (0·1 to 0·4)	0·2 (0·1 to 0·4)	0·1 (0 to 0·1)	0 (0 to 0)	0 (0 to 0)	0 (0 to 0)
Turner syndrome	0 (0 to 0)	0 (0 to 0)	0 (0 to 0)	0 (0 to 0)	0 (0 to 0)	0 (0 to 0)
Klinefelter syndrome	0 (0 to 0)	0 (0 to 0)	0 (0 to 0)	0 (0 to 0)	0 (0 to 0)	0 (0 to 0)
Other chromosomal abnormalities	0·1 (0 to 0·1)	0 (0 to 0)	0 (0 to 0)	0 (0 to 0)	0 (0 to 0)	0 (0 to 0)
Congenital musculoskeletal and limb anomalies	1·3 (0·8 to 2·1)	1·1 (0·7 to 1·7)	0·8 (0·4 to 1·2)	0·5 (0·3 to 0·8)	0·3 (0·2 to 0·5)	0·2 (0·1 to 0·3)
Urogenital congenital anomalies	0 (0 to 0)	0 (0 to 0)	0 (0 to 0)	0 (0 to 0)	0 (0 to 0)	0 (0 to 0)
Digestive congenital anomalies	0 (0 to 0)	0 (0 to 0)	0 (0 to 0)	0 (0 to 0)	0 (0 to 0)	0 (0 to 0)
Other congenital birth defects	0·5 (0·3 to 0·8)	0·4 (0·2 to 0·7)	0·2 (0·1 to 0·4)	0·2 (0·1 to 0·3)	0·1 (0 to 0·2)	0·1 (0 to 0·2)
Urinary diseases and male infertility	5 (3·6 to 6·7)	5·4 (3·7 to 7·3)	4·9 (3·6 to 6·7)	5 (3·5 to 6·8)	5·6 (4 to 7·6)	5·7 (4 to 8·1)
Urolithiasis	0·5 (0·2 to 0·8)	0·6 (0·3 to 0·9)	0·4 (0·2 to 0·7)	0·4 (0·2 to 0·6)	0·2 (0·1 to 0·4)	0·1 (0·1 to 0·3)
Benign prostatic hyperplasia	0 (0 to 0)	0 (0 to 0)	0 (0 to 0)	0 (0 to 0)	0 (0 to 0)	0 (0 to 0)
Male infertility	--	--	--	--	--	--
Other urinary diseases	0·3 (0·2 to 0·5)	0·3 (0·2 to 0·4)	0·2 (0·1 to 0·4)	0·1 (0·1 to 0·2)	0·1 (0·1 to 0·2)	0·1 (0·1 to 0·2)
Gynecological diseases	35·1 (21·3 to 52·6)	18·8 (11·7 to 27·4)	10·5 (6·1 to 15·9)	7·4 (4·6 to 10·8)	5·6 (3·6 to 8·1)	3·9 (2·5 to 5·8)
Uterine fibroids	3·5 (1·6 to 6·7)	2·1 (1 to 4)	1·1 (0·5 to 2·1)	0·6 (0·2 to 1·1)	0·3 (0·1 to 0·7)	0·2 (0·1 to 0·4)
Polycystic ovarian syndrome	0·2 (0·1 to 0·4)	0 (0 to 0)	0 (0 to 0)	0 (0 to 0)	0 (0 to 0)	0 (0 to 0)
Female infertility	0 (0 to 0)	0 (0 to 0)	0 (0 to 0)	0 (0 to 0)	0 (0 to 0)	0 (0 to 0)
Endometriosis	1·7 (0·9 to 2·9)	0 (0 to 0)	0 (0 to 0)	0 (0 to 0)	0 (0 to 0)	0 (0 to 0)
Genital prolapse	1·2 (0·5 to 2·2)	1·1 (0·6 to 2·1)	0·8 (0·4 to 1·5)	0·7 (0·4 to 1·3)	0·6 (0·3 to 1)	0·5 (0·2 to 0·8)
Premenstrual syndrome	3·9 (2 to 6·9)	0 (0 to 0)	0 (0 to 0)	0 (0 to 0)	0 (0 to 0)	0 (0 to 0)
Other gynecological diseases	24·7 (12·2 to 39·1)	15·6 (9·4 to 23·5)	8·7 (4·7 to 13·6)	6·1 (3·5 to 9·4)	4·7 (2·9 to 6·9)	3·3 (2 to 5)
Hemoglobinopathies and hemolytic anemias	6·1 (4 to 8·6)	4·9 (3·4 to 7)	4·2 (2·8 to 6·2)	2·9 (1·8 to 4·1)	2·5 (1·7 to 3·6)	2 (1·4 to 3)
Thalassemias	0 (0 to 0)	0 (0 to 0)	0 (0 to 0)	0 (0 to 0)	0 (0 to 0)	0 (0 to 0)
Thalassemias trait	0·1 (0 to 0·2)	0·1 (0 to 0·2)	0·1 (0 to 0·1)	0 (0 to 0·1)	0 (0 to 0·1)	0 (0 to 0·1)
Sickle cell disorders	2·8 (1·7 to 4·3)	1·4 (0·7 to 2·2)	0·4 (0·2 to 0·7)	0·1 (0 to 0·1)	0 (0 to 0)	0 (0 to 0)
Sickle cell trait	1 (0·4 to 1·8)	0·9 (0·4 to 1·5)	0·6 (0·3 to 1·1)	0·4 (0·1 to 0·7)	0·3 (0·1 to 0·6)	0·3 (0·1 to 0·5)
G6PD deficiency	0·4 (0·2 to 0·8)	0·4 (0·2 to 0·7)	0·3 (0·1 to 0·5)	0·2 (0·1 to 0·3)	0·1 (0 to 0·1)	0 (0 to 0·1)
G6PD trait	0 (0 to 0)	0 (0 to 0)	0 (0 to 0)	0 (0 to 0)	0 (0 to 0)	0 (0 to 0)
Other hemoglobinopathies and hemolytic anemias	1·8 (1·1 to 2·9)	2·3 (1·4 to 3·3)	2·9 (1·9 to 4·2)	2·2 (1·4 to 3·2)	2·1 (1·4 to 2·9)	1·7 (1·2 to 2·4)
Endocrine, metabolic, blood, and immune disorders	26 (18·3 to 38)	26·9 (18·9 to 39)	22·4 (15·9 to 32·3)	19 (13·1 to 27·7)	17·2 (12·1 to 23·9)	14·3 (9·8 to 19·9)
Oral disorders	20·4 (11·4 to 35·4)	21·3 (12·3 to 34·5)	19·3 (11·6 to 30·6)	17·6 (10·9 to 27·2)	16·6 (10·3 to 25)	13·5 (8·6 to 20·1)
Caries of deciduous teeth	0 (0 to 0)	0 (0 to 0)	0 (0 to 0)	0 (0 to 0)	0 (0 to 0)	0 (0 to 0)
Caries of permanent teeth	1 (0·4 to 2·1)	0·8 (0·3 to 1·8)	0·5 (0·2 to 1·2)	0·3 (0·1 to 0·7)	0·3 (0·1 to 0·6)	0·2 (0·1 to 0·4)
Periodontal diseases	8·6 (3·4 to 18·1)	6·5 (2·6 to 13·4)	4·2 (1·7 to 8·6)	2·8 (1·1 to 5·9)	2·1 (0·8 to 4·3)	1·5 (0·6 to 3·1)
Other oral disorders	2·6 (1·6 to 4)	2·1 (1·3 to 3·2)	1·5 (0·9 to 2·2)	1·1 (0·7 to 1·7)	0·9 (0·5 to 1·3)	0·5 (0·3 to 0·8)
Sudden infant death syndrome	--	--	--	--	--	--
Injuries	43·3 (35·5 to 52·4)	37·7 (31 to 45·3)	30·5 (25 to 36·9)	26·3 (21·9 to 31·6)	26 (22·1 to 31)	24·3 (20·5 to 28·6)
Transport injuries	8·7 (6·8 to 10·7)	7·5 (6 to 9·2)	5·8 (4·7 to 7·1)	4·9 (4 to 6)	3·9 (3·1 to 4·8)	3 (2·5 to 3·6)
Road injuries	7·5 (5·8 to 9·4)	6·4 (5·1 to 7·9)	5·1 (4·1 to 6·3)	4·4 (3·6 to 5·5)	3·5 (2·8 to 4·3)	2·6 (2·1 to 3·1)
Pedestrian road injuries	1·4 (1·1 to 1·8)	1·3 (1 to 1·6)	1·2 (0·9 to 1·5)	1·2 (1 to 1·4)	1 (0·8 to 1·3)	0·8 (0·6 to 1)
Cyclist road injuries	1·3 (0·9 to 1·7)	1 (0·7 to 1·4)	0·7 (0·5 to 1)	0·6 (0·4 to 0·8)	0·5 (0·3 to 0·6)	0·3 (0·2 to 0·5)
Motorcyclist road injuries	0·8 (0·6 to 1·1)	0·7 (0·6 to 1)	0·5 (0·4 to 0·7)	0·5 (0·4 to 0·6)	0·4 (0·3 to 0·5)	0·3 (0·2 to 0·4)
Motor vehicle road injuries	3·6 (2·8 to 4·6)	3·1 (2·4 to 3·9)	2·4 (1·9 to 3·1)	2 (1·6 to 2·5)	1·6 (1·2 to 2)	1 (0·8 to 1·2)
Other road injuries	0·3 (0·2 to 0·4)	0·3 (0·2 to 0·4)	0·2 (0·2 to 0·3)	0·2 (0·1 to 0·3)	0·1 (0·1 to 0·1)	0·1 (0·1 to 0·1)
Other transport injuries	1·1 (0·8 to 1·6)	1·1 (0·7 to 1·5)	0·7 (0·5 to 0·9)	0·4 (0·3 to 0·6)	0·4 (0·3 to 0·5)	0·4 (0·3 to 0·6)
Unintentional injuries	27·8 (22 to 34·8)	25·1 (19·6 to 31·3)	20·9 (16·7 to 26·3)	18·4 (14·8 to 22·6)	19·7 (16·4 to 23·8)	19·6 (16·5 to 23·3)
Falls	5·5 (3·9 to 7·4)	5·4 (4 to 7·2)	5·2 (3·9 to 6·9)	5·3 (4·1 to 6·7)	7·1 (5·7 to 8·7)	8·3 (6·7 to 10·3)
Drowning	1·3 (1 to 1·7)	1·1 (0·8 to 1·4)	0·8 (0·6 to 1)	0·7 (0·5 to 0·9)	0·5 (0·4 to 0·6)	0·4 (0·3 to 0·5)
Fire, heat, and hot substances	6 (4·4 to 8·1)	5·1 (3·8 to 6·8)	3·8 (2·8 to 5·1)	2·8 (2·1 to 3·8)	2·6 (1·9 to 3·3)	2 (1·5 to 2·6)
Poisonings	0·4 (0·3 to 0·6)	0·4 (0·3 to 0·5)	0·3 (0·2 to 0·4)	0·3 (0·2 to 0·3)	0·2 (0·2 to 0·3)	0·2 (0·1 to 0·2)
Poisoning by carbon monoxide	0·1 (0·1 to 0·2)	0·2 (0·1 to 0·2)	0·1 (0·1 to 0·2)	0·1 (0·1 to 0·2)	0·1 (0·1 to 0·1)	0·1 (0 to 0·1)
Poisoning by other means	0·3 (0·2 to 0·4)	0·2 (0·2 to 0·3)	0·2 (0·1 to 0·2)	0·1 (0·1 to 0·2)	0·1 (0·1 to 0·2)	0·1 (0·1 to 0·2)
Exposure to mechanical forces	5·6 (4·2 to 7·5)	4·6 (3·3 to 6·2)	3·4 (2·5 to 4·7)	2·7 (2 to 3·7)	2·6 (1·9 to 3·4)	2·3 (1·7 to 3)
Unintentional firearm injuries	0·6 (0·4 to 0·7)	0·5 (0·4 to 0·6)	0·3 (0·2 to 0·4)	0·3 (0·2 to 0·3)	0·2 (0·2 to 0·3)	0·2 (0·2 to 0·3)
Other exposure to mechanical forces	5 (3·7 to 6·9)	4·1 (3 to 5·6)	3·1 (2·2 to 4·3)	2·5 (1·8 to 3·4)	2·3 (1·8 to 3·1)	2 (1·6 to 2·7)
Adverse effects of medical treatment	2·7 (1·9 to 4)	2·9 (2 to 4·1)	2·8 (1·8 to 3·9)	2·4 (1·7 to 3·4)	2·7 (1·8 to 3·7)	2·5 (1·8 to 3·4)
Animal contact	0·4 (0·3 to 0·5)	0·4 (0·3 to 0·5)	0·3 (0·2 to 0·4)	0·2 (0·2 to 0·3)	0·3 (0·2 to 0·3)	0·3 (0·2 to 0·4)
Venomous animal contact	0·2 (0·1 to 0·3)	0·2 (0·1 to 0·3)	0·2 (0·1 to 0·2)	0·1 (0·1 to 0·2)	0·1 (0·1 to 0·2)	0·1 (0·1 to 0·2)
Non-venomous animal contact	0·1 (0·1 to 0·2)	0·2 (0·1 to 0·2)	0·1 (0·1 to 0·2)	0·1 (0·1 to 0·1)	0·1 (0·1 to 0·2)	0·1 (0·1 to 0·2)
Foreign body	1·5 (1·2 to 1·8)	1·4 (1·1 to 1·8)	1·4 (1·1 to 1·7)	1·4 (1·1 to 1·8)	1·7 (1·3 to 2·1)	1·7 (1·4 to 2·1)
Pulmonary aspiration and foreign body in airway	1·1 (0·8 to 1·4)	1·1 (0·8 to 1·4)	1·1 (0·9 to 1·4)	1·3 (1 to 1·6)	1·5 (1·2 to 1·9)	1·6 (1·2 to 2)
Foreign body in eyes	0·2 (0·1 to 0·3)	0·1 (0·1 to 0·2)	0·1 (0·1 to 0·2)	0·1 (0 to 0·1)	0·1 (0 to 0·1)	0·1 (0 to 0·1)
Foreign body in other body part	0·2 (0·1 to 0·3)	0·2 (0·1 to 0·2)	0·1 (0·1 to 0·2)	0·1 (0·1 to 0·1)	0·1 (0·1 to 0·1)	0·1 (0·1 to 0·1)
Environmental heat and cold exposure	1·7 (1·2 to 2·4)	1·5 (1·1 to 2·1)	1·2 (0·9 to 1·7)	1·1 (0·7 to 1·4)	1 (0·7 to 1·3)	0·9 (0·6 to 1·1)
Exposure to forces of nature	0·1 (0·1 to 0·2)	0·1 (0 to 0·1)	0·1 (0 to 0·1)	0·1 (0 to 0·1)	0 (0 to 0·1)	0 (0 to 0·1)
Other unintentional injuries	2·6 (1·8 to 3·6)	2·2 (1·5 to 3·1)	1·7 (1·2 to 2·4)	1·3 (0·9 to 1·9)	1·2 (0·8 to 1·7)	1 (0·7 to 1·4)
Self-harm and interpersonal violence	6·9 (5·6 to 8·4)	5·2 (4·2 to 6·4)	3·8 (3·1 to 4·7)	3 (2·5 to 3·7)	2·3 (1·9 to 2·9)	1·6 (1·3 to 2)
Self-harm	2·6 (1·9 to 3·4)	2·1 (1·6 to 2·8)	1·6 (1·2 to 2·2)	1·3 (1 to 1·7)	0·9 (0·7 to 1·2)	0·6 (0·4 to 0·7)
Self-harm by firearm	0 (0 to 0·1)	0 (0 to 0)	0 (0 to 0)	0 (0 to 0)	0 (0 to 0)	0 (0 to 0)
Self-harm by other specified means	2·5 (1·9 to 3·3)	2·1 (1·5 to 2·8)	1·6 (1·2 to 2·2)	1·3 (0·9 to 1·6)	0·9 (0·7 to 1·2)	0·6 (0·4 to 0·7)
Interpersonal violence	3·5 (2·8 to 4·5)	2·4 (1·9 to 3)	1·6 (1·3 to 2)	1·3 (1 to 1·6)	1 (0·8 to 1·2)	0·7 (0·6 to 0·9)
Physical violence by firearm	0·3 (0·2 to 0·4)	0·2 (0·1 to 0·3)	0·1 (0·1 to 0·1)	0·1 (0 to 0·1)	0 (0 to 0·1)	0 (0 to 0)
Physical violence by sharp object	0·4 (0·3 to 0·5)	0·3 (0·2 to 0·3)	0·2 (0·1 to 0·2)	0·1 (0·1 to 0·2)	0·1 (0·1 to 0·1)	0·1 (0·1 to 0·1)
Sexual violence	0·6 (0·4 to 1·1)	0·4 (0·2 to 0·6)	0·3 (0·2 to 0·4)	0·2 (0·1 to 0·3)	0·2 (0·1 to 0·2)	0·1 (0·1 to 0·2)
Physical violence by other means	2·2 (1·7 to 2·9)	1·6 (1·2 to 2)	1·1 (0·8 to 1·4)	0·9 (0·7 to 1·2)	0·7 (0·5 to 0·9)	0·5 (0·4 to 0·7)
Conflict and terrorism	0·7 (0·4 to 1·4)	0·6 (0·3 to 1·2)	0·5 (0·2 to 1)	0·5 (0·2 to 0·9)	0·4 (0·2 to 0·8)	0·3 (0·2 to 0·7)

Osteoarthritis other	0·9 (0·4 to 1·7)	0·5 (0·2 to 1)	0·3 (0·1 to 0·5)	0·1 (0·1 to 0·2)	12·4
Low back pain	11·2 (7·1 to 16·8)	5·3 (3·3 to 7·8)	2·3 (1·4 to 3·5)	0·8 (0·5 to 1·2)	468
Neck pain	1·4 (0·7 to 2·4)	0·6 (0·3 to 1·1)	0·3 (0·2 to 0·5)	0·1 (0·1 to 0·2)	80·5
Gout	0·1 (0 to 0·2)	0·1 (0 to 0·1)	0 (0 to 0·1)	0 (0 to 0)	3
Other musculoskeletal disorders	4·1 (2·2 to 6·7)	3·3 (2 to 4·7)	1·5 (0·8 to 2·2)	0·5 (0·3 to 0·9)	317
Other non-communicable diseases	22 (17·2 to 28·1)	13·2 (10·3 to 16·7)	7·1 (5·5 to 8·8)	2·7 (2 to 3·5)	1,240
Congenital birth defects	0·1 (0·1 to 0·2)	0·1 (0 to 0·1)	0 (0 to 0)	0 (0 to 0)	190
Neural tube defects	0 (0 to 0)	0 (0 to 0)	0 (0 to 0)	0 (0 to 0)	17·9
Congenital heart anomalies	0 (0 to 0)	0 (0 to 0)	0 (0 to 0)	0 (0 to 0)	92·2
Orofacial clefts	0 (0 to 0)	0 (0 to 0)	0 (0 to 0)	0 (0 to 0)	1·2
Down syndrome	0 (0 to 0)	0 (0 to 0)	0 (0 to 0)	0 (0 to 0)	3·9
Turner syndrome	0 (0 to 0)	0 (0 to 0)	0 (0 to 0)	0 (0 to 0)	0·1
Klinefelter syndrome	0 (0 to 0)	0 (0 to 0)	0 (0 to 0)	0 (0 to 0)	0
Other chromosomal abnormalities	0 (0 to 0)	0 (0 to 0)	0 (0 to 0)	0 (0 to 0)	15·3
Congenital musculoskeletal and limb anomalies	0·1 (0 to 0·1)	0 (0 to 0)	0 (0 to 0)	0 (0 to 0)	26·1
Urogenital congenital anomalies	0 (0 to 0)	0 (0 to 0)	0 (0 to 0)	0 (0 to 0)	2·5
Digestive congenital anomalies	0 (0 to 0)	0 (0 to 0)	0 (0 to 0)	0 (0 to 0)	6·8
Other congenital birth defects	0 (0 to 0·1)	0 (0 to 0)	0 (0 to 0)	0 (0 to 0)	23·9
Urinary diseases and male infertility	4·3 (2·9 to 6)	2·9 (1·9 to 4·2)	1·7 (1·1 to 2·4)	0·7 (0·4 to 1)	61·1
Urolithiasis	0·1 (0 to 0·1)	0 (0 to 0·1)	0 (0 to 0)	0 (0 to 0)	3·9
Benign prostatic hyperplasia	0 (0 to 0)	0 (0 to 0)	0 (0 to 0)	0 (0 to 0)	0
Male infertility	--	--	--	--	NA
Other urinary diseases	0·1 (0 to 0·1)	0 (0 to 0·1)	0 (0 to 0)	0 (0 to 0)	3·3
Gynecological diseases	1·9 (1·1 to 2·8)	1 (0·6 to 1·4)	0·5 (0·3 to 0·7)	0·2 (0·1 to 0·3)	400
Uterine fibroids	0·1 (0 to 0·1)	0 (0 to 0·1)	0 (0 to 0)	0 (0 to 0)	25·8
Polycystic ovarian syndrome	0 (0 to 0)	0 (0 to 0)	0 (0 to 0)	0 (0 to 0)	6·2
Female infertility	0 (0 to 0)	0 (0 to 0)	0 (0 to 0)	0 (0 to 0)	8·2
Endometriosis	0 (0 to 0)	0 (0 to 0)	0 (0 to 0)	0 (0 to 0)	29
Genital prolapse	0·2 (0·1 to 0·4)	0·1 (0·1 to 0·2)	0·1 (0 to 0·1)	0 (0 to 0)	7·7
Premenstrual syndrome	0 (0 to 0)	0 (0 to 0)	0 (0 to 0)	0 (0 to 0)	105
Other gynecological diseases	1·6 (0·9 to 2·4)	0·8 (0·5 to 1·2)	0·4 (0·2 to 0·7)	0·1 (0·1 to 0·3)	218
Hemoglobinopathies and hemolytic anemias	1·1 (0·7 to 1·6)	1 (0·7 to 1·3)	0·5 (0·4 to 0·7)	0·2 (0·1 to 0·2)	112
Thalassemias	0 (0 to 0)	0 (0 to 0)	0 (0 to 0)	0 (0 to 0)	0·1
Thalassemias trait	0 (0 to 0)	0 (0 to 0)	0 (0 to 0)	0 (0 to 0)	3·1
Sickle cell disorders	0 (0 to 0)	0 (0 to 0)	0 (0 to 0)	0 (0 to 0)	54·5
Sickle cell trait	0·2 (0·1 to 0·3)	0·1 (0 to 0·2)	0 (0 to 0·1)	0 (0 to 0)	25·5
G6PD deficiency	0 (0 to 0)	0 (0 to 0)	0 (0 to 0)	0 (0 to 0)	4·2
G6PD trait	0 (0 to 0)	0 (0 to 0)	0 (0 to 0)	0 (0 to 0)	0·2
Other hemoglobinopathies and hemolytic anemias	0·9 (0·6 to 1·3)	0·8 (0·6 to 1·2)	0·5 (0·3 to 0·6)	0·1 (0·1 to 0·2)	24·3
Endocrine, metabolic, blood, and immune disorders	7·5 (5·3 to 11)	4·8 (3·3 to 6·9)	2·6 (1·8 to 3·7)	1 (0·7 to 1·6)	273
Oral disorders	7·1 (4·5 to 10·4)	3·6 (2·2 to 5·2)	1·8 (1·1 to 2·6)	0·6 (0·4 to 0·9)	197
Caries of deciduous teeth	0 (0 to 0)	0 (0 to 0)	0 (0 to 0)	0 (0 to 0)	1·1
Caries of permanent teeth	0·1 (0 to 0·2)	0 (0 to 0·1)	0 (0 to 0)	0 (0 to 0)	13·7
Periodontal diseases	0·8 (0·3 to 1·6)	0·4 (0·1 to 0·8)	0·2 (0·1 to 0·4)	0·1 (0 to 0·1)	61·8
Other oral disorders	0·2 (0·1 to 0·3)	0·1 (0·1 to 0·1)	0 (0 to 0·1)	0 (0 to 0)	30
Sudden infant death syndrome	--	--	--	--	NA
Injuries	16·7 (14·3 to 19·6)	11·6 (9·7 to 13·6)	7·5 (6·3 to 8·8)	3·4 (2·8 to 3·9)	679
Transport injuries	1·5 (1·2 to 1·8)	0·7 (0·6 to 0·9)	0·4 (0·3 to 0·5)	0·2 (0·1 to 0·2)	132
Road injuries	1·3 (1·1 to 1·6)	0·6 (0·5 to 0·8)	0·3 (0·3 to 0·4)	0·1 (0·1 to 0·2)	114
Pedestrian road injuries	0·4 (0·3 to 0·5)	0·2 (0·2 to 0·3)	0·1 (0·1 to 0·1)	0 (0 to 0)	18·5
Cyclist road injuries	0·2 (0·1 to 0·2)	0·1 (0·1 to 0·1)	0·1 (0 to 0·1)	0 (0 to 0)	14·6
Motorcyclist road injuries	0·2 (0·1 to 0·2)	0·1 (0·1 to 0·1)	0·1 (0 to 0·1)	0 (0 to 0)	13·9
Motor vehicle road injuries	0·5 (0·4 to 0·6)	0·2 (0·2 to 0·3)	0·1 (0·1 to 0·1)	0 (0 to 0·1)	63·5
Other road injuries	0 (0 to 0·1)	0 (0 to 0)	0 (0 to 0)	0 (0 to 0)	3·8
Other transport injuries	0·2 (0·1 to 0·2)	0·1 (0·1 to 0·1)	0·1 (0 to 0·1)	0 (0 to 0)	17·5
Unintentional injuries	14·4 (12·2 to 17)	10·4 (8·7 to 12·3)	6·9 (5·8 to 8·1)	3·1 (2·6 to 3·7)	418
Falls	7·5 (6 to 9·4)	6 (4·8 to 7·3)	3·9 (3·1 to 4·8)	1·7 (1·3 to 2·1)	80·8
Drowning	0·2 (0·1 to 0·2)	0·1 (0·1 to 0·2)	0·1 (0·1 to 0·2)	0·1 (0 to 0·1)	47·9
Fire, heat, and hot substances	1·1 (0·9 to 1·5)	0·8 (0·6 to 1)	0·5 (0·4 to 0·6)	0·2 (0·2 to 0·3)	75·3
Poisonings	0·1 (0·1 to 0·2)	0·1 (0 to 0·1)	0 (0 to 0)	0 (0 to 0)	8·2
Poisoning by carbon monoxide	0 (0 to 0·1)	0 (0 to 0)	0 (0 to 0)	0 (0 to 0)	2·7
Poisoning by other means	0·1 (0·1 to 0·1)	0 (0 to 0·1)	0 (0 to 0)	0 (0 to 0)	5·5
Exposure to mechanical forces	1·4 (1·1 to 1·8)	0·9 (0·7 to 1·2)	0·7 (0·5 to 0·9)	0·4 (0·3 to 0·5)	75
Unintentional firearm injuries	0·2 (0·1 to 0·2)	0·1 (0·1 to 0·1)	0·1 (0·1 to 0·1)	0·1 (0 to 0·1)	9·8
Other exposure to mechanical forces	1·2 (0·9 to 1·6)	0·8 (0·6 to 1)	0·6 (0·5 to 0·7)	0·3 (0·2 to 0·4)	65·2
Adverse effects of medical treatment	1·5 (1·1 to 2)	1 (0·7 to 1·4)	0·6 (0·4 to 0·8)	0·2 (0·2 to 0·4)	36·7
Animal contact	0·3 (0·2 to 0·4)	0·2 (0·1 to 0·3)	0·2 (0·2 to 0·3)	0·1 (0·1 to 0·2)	5·7
Venomous animal contact	0·1 (0·1 to 0·2)	0·1 (0·1 to 0·2)	0·1 (0·1 to 0·2)	0·1 (0 to 0·1)	3·4
Non-venomous animal contact	0·1 (0·1 to 0·2)	0·1 (0·1 to 0·2)	0·1 (0·1 to 0·2)	0·1 (0 to 0·1)	2·3
Foreign body	1·2 (1 to 1·6)	0·6 (0·5 to 0·8)	0·4 (0·3 to 0·6)	0·2 (0·2 to 0·3)	34·7
Pulmonary aspiration and foreign body in airway	1·1 (0·9 to 1·5)	0·6 (0·4 to 0·7)	0·4 (0·3 to 0·5)	0·2 (0·2 to 0·3)	29·9
Foreign body in eyes	0 (0 to 0)	0 (0 to 0)	0 (0 to 0)	0 (0 to 0)	2·1
Foreign body in other body part	0·1 (0 to 0·1)	0 (0 to 0)	0 (0 to 0)	0 (0 to 0)	2·7
Environmental heat and cold exposure	0·5 (0·4 to 0·7)	0·3 (0·3 to 0·4)	0·3 (0·2 to 0·4)	0·1 (0·1 to 0·2)	20
Exposure to forces of nature	0 (0 to 0)	0 (0 to 0)	0 (0 to 0)	0 (0 to 0)	1·3
Other unintentional injuries	0·5 (0·4 to 0·7)	0·3 (0·2 to 0·4)	0·1 (0·1 to 0·2)	0·1 (0 to 0·1)	32·4
Self-harm and interpersonal violence	0·8 (0·6 to 1)	0·4 (0·3 to 0·6)	0·2 (0·1 to 0·3)	0·1 (0·1 to 0·1)	129
Self-harm	0·2 (0·2 to 0·3)	0·1 (0·1 to 0·1)	0 (0 to 0)	0 (0 to 0)	35·7
Self-harm by firearm	0 (0 to 0)	0 (0 to 0)	0 (0 to 0)	0 (0 to 0)	0·5
Self-harm by other specified means	0·2 (0·2 to 0·3)	0·1 (0·1 to 0·1)	0 (0 to 0)	0 (0 to 0)	35·2
Interpersonal violence	0·3 (0·2 to 0·4)	0·2 (0·1 to 0·2)	0·1 (0·1 to 0·1)	0 (0 to 0)	86
Physical violence by firearm	0 (0 to 0)	0 (0 to 0)	0 (0 to 0)	0 (0 to 0)	12·3
Physical violence by sharp object	0 (0 to 0·1)	0 (0 to 0)	0 (0 to 0)	0 (0 to 0)	12·4
Sexual violence	0·1 (0 to 0·1)	0 (0 to 0)	0 (0 to 0)	0 (0 to 0)	12·3
Physical violence by other means	0·2 (0·1 to 0·2)	0·1 (0·1 to 0·1)	0 (0 to 0·1)	0 (0 to 0)	48·9
Conflict and terrorism	0·3 (0·1 to 0·5)	0·2 (0·1 to 0·3)	0·1 (0 to 0·1)	0 (0 to 0·1)	7·2

SR Table 2b: Guyana DALYS by cause and age group for females with 95% uncertainty intervals, 2019

Cause	Early Neonatal	Late Neonatal	Post Neonatal	1 to 4	5 to 9	10 to 14	15 to 19	20 to 24	25 to 29	30 to 34	35 to 39	40 to 44
All causes	7,210 (5,200 to 9,870)	623 (456 to 841)	3,330 (2,490 to 4,470)	2,840 (2,260 to 3,530)	2,680 (2,210 to 3,270)	3,190 (2,560 to 3,970)	5,310 (4,360 to 6,440)	7,990 (6,510 to 9,760)	8,090 (6,520 to 9,880)	7,210 (5,830 to 8,770)	7,540 (6,110 to 9,110)	8,510 (6,960 to 10,200)
Communicable, maternal, neonatal, and nutritional diseases	6,390 (4,600 to 8,650)	474 (308 to 669)	2,040 (1,470 to 2,730)	1,270 (994 to 1,620)	917 (695 to 1,230)	725 (529 to 994)	963 (769 to 1,230)	1,650 (1,360 to 1,990)	1,880 (1,600 to 2,220)	1,740 (1,500 to 2,030)	1,540 (1,330 to 1,790)	1,340 (1,140 to 1,600)
HIV/AIDS and sexually transmitted infections	5·2 (2·1 to 10·3)	0·5 (0·2 to 1)	77 (71·8 to 83·1)	71·7 (66·7 to 77·2)	56·3 (49 to 70·2)	42·3 (37 to 50·9)	97 (88·5 to 106)	442 (404 to 486)	729 (662 to 806)	856 (788 to 938)	707 (650 to 769)	557 (511 to 607)
HIV/AIDS	0 (0 to 0)	0 (0 to 0·1)	75·9 (70·8 to 81·7)	71·6 (66·6 to 77·2)	56·3 (49 to 70·2)	40·9 (35·7 to 49·3)	90 (82·6 to 98·1)	424 (388 to 465)	709 (646 to 778)	835 (766 to 913)	684 (630 to 746)	533 (486 to 577)
HIV/AIDS - Drug-susceptible Tuberculosis	0 (0 to 0)	0 (0 to 0)	9·4 (5·7 to 14·5)	8·8 (5·4 to 13·4)	6·7 (4·2 to 10·1)	4·8 (3 to 7·1)	10·7 (6·7 to 16·1)	51·8 (32·6 to 77·3)	85·2 (53·7 to 127)	101 (63·7 to 151)	82·9 (52·9 to 124)	64 (40·8 to 96·7)
HIV/AIDS - Multidrug-resistant Tuberculosis without extensive	0 (0 to 0)	0 (0 to 0)	0·2 (0 to 1)	0·2 (0 to 0·9)	0·1 (0 to 0·7)	0·1 (0 to 0·5)	0·2 (0 to 1·1)	1 (0·1 to 5·4)	1·5 (0·1 to 8·7)	1·8 (0·1 to 10·2)	1·4 (0·1 to 8·2)	1·1 (0·1 to 6·4)
HIV/AIDS - Extensively drug-resistant Tuberculosis	0 (0 to 0)	0 (0 to 0)	0 (0 to 0·2)	0 (0 to 0·2)	0 (0 to 0·1)	0 (0 to 0·1)	0 (0 to 0·2)	0·2 (0 to 0·9)	0·2 (0 to 1·4)	0·3 (0 to 1·7)	0·2 (0 to 1·4)	0·2 (0 to 1·1)
HIV/AIDS resulting in other diseases	0 (0 to 0)	0 (0 to 0·1)	66·3 (59·3 to 72·9)	62·6 (55·9 to 69·3)	49·4 (41·4 to 63·6)	36·1 (30·5 to 44·6)	79·1 (70·1 to 88·8)	371 (326 to 417)	622 (545 to 700)	732 (650 to 823)	600 (534 to 671)	467 (412 to 518)
Sexually transmitted infections excluding HIV	5·2 (2·1 to 10·3)	0·4 (0·2 to 1)	1·1 (0·4 to 2·6)	0·1 (0 to 0·2)	0 (0 to 0)	1·4 (0·9 to 2·1)	7 (4·2 to 11·1)	17·3 (10·3 to 29·2)	20·7 (12·8 to 34·1)	21·6 (13·3 to 34·6)	22·5 (14·2 to 35·6)	24 (15·5 to 38)
Syphilis	5·2 (2·1 to 10·3)	0·4 (0·2 to 1)	1·1 (0·4 to 2·6)	0·1 (0 to 0·2)	0 (0 to 0)	0·6 (0·3 to 0·9)	1 (0·5 to 1·8)	0·8 (0·4 to 1·3)	1·5 (0·8 to 2·5)	2 (1 to 3·2)	1·3 (0·8 to 2·1)	0·5 (0·3 to 0·9)
Chlamydial infection	0 (0 to 0)	0 (0 to 0)	0 (0 to 0)	0 (0 to 0)	0 (0 to 0)	0·1 (0·1 to 0·2)	0·7 (0·5 to 1·1)	2·8 (1·6 to 4·6)	2·2 (1·2 to 3·4)	1·8 (1 to 2·9)	2·5 (1·6 to 3·9)	3 (2 to 4·6)
Gonococcal infection	0 (0 to 0)	0 (0 to 0)	0 (0 to 0)	0 (0 to 0)	0 (0 to 0)	0·2 (0·1 to 0·3)	1·4 (0·8 to 2·5)	3·5 (1·7 to 6·4)	3·3 (1·9 to 5·5)	3·5 (2 to 5·6)	3·7 (2·2 to 5·9)	4·9 (3·1 to 7·7)
Trichomoniasis	0 (0 to 0)	0 (0 to 0)	0 (0 to 0)	0 (0 to 0)	0 (0 to 0)	0 (0 to 0·1)	0·7 (0·2 to 1·7)	3·5 (1·2 to 7·9)	5·9 (2 to 13·9)	5·3 (1·7 to 12·5)	4·9 (1·6 to 11·6)	4·7 (1·6 to 11·2)
Genital herpes	0 (0 to 0)	0 (0 to 0)	0 (0 to 0)	0 (0 to 0)	0 (0 to 0)	0·2 (0·1 to 0·5)	1 (0·3 to 2·4)	2·2 (0·7 to 5·6)	3·1 (1 to 7·7)	3 (1 to 7·6)	3·1 (1 to 7·7)	3·1 (1 to 7·8)
Other sexually transmitted infections	0 (0 to 0)	0 (0 to 0)	0 (0 to 0)	0 (0 to 0)	0 (0 to 0)	0·3 (0·2 to 0·4)	2·2 (1·3 to 3·6)	4·6 (2·6 to 7·6)	4·7 (3 to 7·2)	5·9 (3·9 to 8·6)	6·9 (4·6 to 10)	7·8 (5·1 to 11·2)
Respiratory infections and tuberculosis	175 (96·1 to 287)	47·2 (25·8 to 80·4)	592 (369 to 904)	279 (189 to 397)	151 (113 to 192)	114 (83·4 to 151)	133 (98·9 to 177)	236 (171 to 321)	247 (177 to 345)	202 (143 to 284)	192 (132 to 273)	192 (133 to 272)
Tuberculosis	0 (0 to 0)	0 (0 to 0)	11·7 (6·7 to 20)	17·2 (10·4 to 27·8)	13·6 (8·5 to 20·5)	15·5 (9·7 to 23·2)	30·5 (19·2 to 47·4)	85·7 (53·1 to 132)	99·4 (60·4 to 161)	75·5 (46·2 to 121)	62·2 (36·4 to 103)	58·3 (33·7 to 97·9)
Latent tuberculosis infection	0 (0 to 0)	0 (0 to 0)	0 (0 to 0)	0 (0 to 0)	0 (0 to 0)	0 (0 to 0)	0 (0 to 0)	0 (0 to 0)	0 (0 to 0)	0 (0 to 0)	0 (0 to 0)	0 (0 to 0)
Drug-susceptible tuberculosis	0 (0 to 0)	0 (0 to 0)	11·5 (6·5 to 19·7)	16·9 (10·3 to 27·3)	13·4 (8·4 to 20·4)	15·3 (9·5 to 22·9)	30 (18·7 to 47·2)	84·4 (51·6 to 132)	97·9 (59·4 to 159)	74·4 (45·8 to 119)	61·3 (35·9 to 101)	57·4 (32·8 to 97·1)
Multidrug-resistant tuberculosis without extensive drug resista	0 (0 to 0)	0 (0 to 0)	0·2 (0 to 0·9)	0·2 (0 to 1·2)	0·2 (0 to 0·9)	0·2 (0 to 1·2)	0·4 (0 to 2·4)	1·1 (0·1 to 5·5)	1·3 (0·1 to 6·8)	0·9 (0·1 to 5·2)	0·8 (0·1 to 3·9)	0·8 (0·1 to 4·3)
Extensively drug-resistant tuberculosis	0 (0 to 0)	0 (0 to 0)	0 (0 to 0·2)	0 (0 to 0·2)	0 (0 to 0·1)	0 (0 to 0·2)	0·1 (0 to 0·4)	0·2 (0 to 0·9)	0·2 (0 to 1·1)	0·1 (0 to 0·8)	0·1 (0 to 0·6)	0·1 (0 to 0·7)
Lower respiratory infections	175 (96·1 to 287)	46·7 (25·2 to 79·8)	568 (347 to 876)	211 (128 to 326)	84·5 (58·9 to 114)	56·3 (36·8 to 81·5)	60·7 (39·9 to 86·2)	106 (62·8 to 167)	111 (64·2 to 176)	100 (60·1 to 152)	105 (62·6 to 165)	110 (68·6 to 165)
Upper respiratory infections	0·1 (0·1 to 0·1)	0·4 (0·2 to 0·7)	10·1 (6·4 to 15·6)	35·9 (20·9 to 57·6)	36 (19·9 to 61·2)	30·8 (16·6 to 52·3)	30·9 (16·1 to 53·7)	32·8 (17·7 to 55·7)	27·9 (15·7 to 45·8)	20·5 (11·1 to 34·3)	19·2 (9·7 to 31·3)	17·9 (9·5 to 31·3)
Otitis media	0 (0 to 0)	0 (0 to 0·1)	1·7 (0·8 to 3·1)	14·3 (7·5 to 25·1)	17·1 (9·4 to 28·9)	11·6 (6 to 20)	11 (5·9 to 18·9)	11 (4·9 to 22·4)	8·2 (3·8 to 15·6)	5·5 (2·2 to 10·6)	5·2 (2·3 to 9·8)	5·6 (2·6 to 10·4)
Enteric infections	36·7 (17·1 to 68·4)	26·5 (11·6 to 54·9)	472 (274 to 737)	196 (124 to 284)	92·5 (66·3 to 127)	67·8 (46·8 to 99)	45·5 (32·3 to 63·5)	55·4 (38 to 76·3)	57·7 (39·9 to 80·2)	48·2 (34·1 to 66·8)	56 (40·6 to 78·4)	60·9 (43·7 to 83·2)
Diarrheal diseases	36·7 (17·1 to 68·4)	26·5 (11·6 to 54·9)	471 (273 to 737)	194 (123 to 282)	91·4 (65·6 to 126)	66·5 (45·5 to 97·4)	44·8 (31·6 to 62·9)	54·1 (36·7 to 74·9)	56·7 (39·2 to 78·6)	47·6 (33·5 to 66)	55·5 (40·2 to 77·2)	60·2 (43·1 to 82·2)
Typhoid and paratyphoid	0 (0 to 0)	0 (0 to 0)	0·4 (0·1 to 2)	0·7 (0·1 to 3)	0·3 (0·1 to 1)	0·5 (0·1 to 2·1)	0·5 (0·1 to 1·8)	0·9 (0·2 to 3·6)	0·6 (0·1 to 2·5)	0·3 (0·1 to 1·5)	0·4 (0·1 to 1·6)	0·4 (0·1 to 1·7)
Typhoid fever	0 (0 to 0)	0 (0 to 0)	0·4 (0·1 to 2)	0·7 (0·1 to 3)	0·3 (0·1 to 1)	0·5 (0·1 to 2·1)	0·5 (0·1 to 1·8)	0·8 (0·1 to 3·6)	0·6 (0·1 to 2·5)	0·3 (0·1 to 1·5)	0·4 (0·1 to 1·6)	0·4 (0·1 to 1·7)
Paratyphoid fever	0 (0 to 0)	0 (0 to 0)	0 (0 to 0)	0 (0 to 0)	0 (0 to 0)	0 (0 to 0)	0 (0 to 0)	0 (0 to 0)	0 (0 to 0)	0 (0 to 0)	0 (0 to 0)	0 (0 to 0)
Invasive Non-typhoidal Salmonella (iNTS)	0 (0 to 0)	0 (0 to 0)	0·1 (0 to 0·2)	0·6 (0·2 to 1·5)	0·4 (0·1 to 1·1)	0·5 (0·2 to 1·2)	0·1 (0 to 0·3)	0·2 (0·1 to 0·4)	0·3 (0·1 to 0·6)	0·1 (0 to 0·2)	0 (0 to 0)	0·2 (0·1 to 0·4)
Other intestinal infectious diseases	0 (0 to 0)	0 (0 to 0)	0·6 (0 to 2·6)	0·7 (0·1 to 2·4)	0·4 (0 to 1·5)	0·3 (0 to 1·1)	0·2 (0 to 0·7)	0·2 (0 to 0·8)	0·1 (0 to 0·3)	0·2 (0 to 0·6)	0·1 (0 to 0·3)	0·1 (0 to 0·4)
Neglected tropical diseases and malaria	1·6 (0·5 to 3·8)	5 (1·5 to 15·9)	56·7 (20·9 to 150)	102 (45·5 to 242)	104 (60 to 179)	88·3 (43·7 to 181)	146 (64·4 to 364)	126 (67·2 to 274)	115 (61·1 to 242)	92·4 (52·8 to 180)	95·9 (55·3 to 182)	82·6 (51·1 to 135)
Malaria	0 (0 to 0)	3·7 (0·5 to 14·7)	33·4 (5 to 122)	59·2 (13·4 to 196)	41·2 (13·8 to 119)	42·1 (10·2 to 139)	96·2 (21·8 to 316)	66·5 (17·3 to 215)	55·2 (15 to 177)	39·2 (10·9 to 126)	38·3 (10·7 to 123)	21·1 (5·7 to 70·9)
Chagas disease	0 (0 to 0)	0 (0 to 0)	0 (0 to 0)	0 (0 to 0)	0 (0 to 0)	0 (0 to 0)	0 (0 to 0)	0 (0 to 0)	0 (0 to 0)	0 (0 to 0·1)	0 (0 to 0·1)	0·1 (0 to 0·1)
Leishmaniasis	0 (0 to 0)	0 (0 to 0)	0·1 (0 to 0·3)	0·3 (0 to 1·5)	0·2 (0 to 0·8)	0·1 (0 to 0·4)	0·1 (0 to 0·3)	0·1 (0 to 0·3)	0·1 (0 to 0·3)	0 (0 to 0·2)	0 (0 to 0·2)	0 (0 to 0·2)
Visceral leishmaniasis	0 (0 to 0)	0 (0 to 0)	0 (0 to 0)	0 (0 to 0)	0 (0 to 0)	0 (0 to 0)	0 (0 to 0)	0 (0 to 0)	0 (0 to 0)	0 (0 to 0)	0 (0 to 0)	0 (0 to 0)
Cutaneous and mucocutaneous leishmaniasis	0 (0 to 0)	0 (0 to 0)	0·1 (0 to 0·3)	0·3 (0 to 1·5)	0·2 (0 to 0·8)	0·1 (0 to 0·4)	0·1 (0 to 0·3)	0·1 (0 to 0·3)	0·1 (0 to 0·3)	0 (0 to 0·2)	0 (0 to 0·2)	0 (0 to 0·2)
African trypanosomiasis	0 (0 to 0)	0 (0 to 0)	0 (0 to 0)	0 (0 to 0)	0 (0 to 0)	0 (0 to 0)	0 (0 to 0)	0 (0 to 0)	0 (0 to 0)	0 (0 to 0)	0 (0 to 0)	0 (0 to 0)
Schistosomiasis	0 (0 to 0)	0 (0 to 0)	0 (0 to 0)	0 (0 to 0)	0 (0 to 0)	0 (0 to 0)	0 (0 to 0)	0 (0 to 0)	0 (0 to 0)	0 (0 to 0)	0 (0 to 0)	0 (0 to 0)
Cysticercosis	0 (0 to 0)	0 (0 to 0)	0 (0 to 0)	0 (0 to 0)	0 (0 to 0)	0·3 (0·1 to 0·7)	2·2 (0 to 6·8)	8·8 (1·8 to 22·6)	13·4 (4·2 to 31)	12·4 (3·7 to 27·4)	13·7 (4·6 to 29·1)	16·1 (5·7 to 33·6)
Cystic echinococcosis	0 (0 to 0)	0 (0 to 0)	0 (0 to 0)	0 (0 to 0·1)	0 (0 to 0·2)	0·1 (0 to 0·1)	0·1 (0 to 0·1)	0·1 (0 to 0·2)	0·1 (0 to 0·2)	0·1 (0 to 0·2)	0·1 (0 to 0·2)	0·1 (0 to 0·2)
Lymphatic filariasis	0 (0 to 0)	0 (0 to 0)	0 (0 to 0)	0 (0 to 0)	10·8 (6·7 to 16·5)	13·4 (8·2 to 20·3)	18·6 (11·6 to 27·3)	26 (16·3 to 38·3)	26·3 (16·6 to 38·8)	22·8 (14·7 to 32·7)	23·2 (14·7 to 33·6)	24·6 (15·9 to 35·6)
Onchocerciasis	0 (0 to 0)	0 (0 to 0)	0 (0 to 0)	0 (0 to 0)	0 (0 to 0)	0 (0 to 0)	0 (0 to 0)	0 (0 to 0)	0 (0 to 0)	0 (0 to 0)	0 (0 to 0)	0 (0 to 0)
Trachoma	0 (0 to 0)	0 (0 to 0)	0 (0 to 0)	0 (0 to 0)	0 (0 to 0)	0 (0 to 0)	0 (0 to 0)	0 (0 to 0)	0 (0 to 0)	0 (0 to 0)	0 (0 to 0)	0 (0 to 0)
Dengue	0 (0 to 0)	0·1 (0 to 0·6)	11 (1·8 to 38)	12·7 (2·5 to 40·5)	17·2 (4·6 to 47·8)	9 (2·9 to 18·3)	9·3 (2·4 to 26)	9·5 (2·6 to 25)	9 (2·5 to 23·2)	5·5 (1·8 to 13·6)	5·7 (1·8 to 12·8)	6·1 (1·9 to 14·2)
Yellow fever	0 (0 to 0)	0 (0 to 0)	0 (0 to 0)	0·1 (0 to 0·3)	0·2 (0 to 0·6)	0·1 (0 to 0·5)	0·1 (0 to 0·5)	0·1 (0 to 0·5)	0·1 (0 to 0·3)	0·1 (0 to 0·2)	0·1 (0 to 0·2)	0 (0 to 0·2)
Rabies	0 (0 to 0)	0 (0 to 0)	0 (0 to 0)	0 (0 to 0)	0 (0 to 0·1)	0 (0 to 0·1)	0 (0 to 0)	0 (0 to 0)	0 (0 to 0)	0 (0 to 0)	0 (0 to 0)	0 (0 to 0)
Intestinal nematode infections	0 (0 to 0)	0 (0 to 0)	0·2 (0 to 0·8)	2 (0·7 to 5·1)	3·5 (1·6 to 6·5)	2·3 (1·1 to 4)	10·5 (4·6 to 19·2)	6·4 (3 to 11·9)	4·3 (2 to 7·8)	3·3 (1·5 to 6·1)	4·7 (2·3 to 8)	2·4 (1·2 to 3·9)
Ascariasis	0 (0 to 0)	0 (0 to 0)	0·2 (0 to 0·8)	1·4 (0·3 to 4·4)	1·8 (0·5 to 4·2)	0·9 (0·3 to 1·8)	0·6 (0·2 to 1·2)	0·5 (0·2 to 1·1)	0·4 (0·1 to 0·9)	0·3 (0·1 to 0·7)	0·3 (0·1 to 0·7)	0·3 (0·1 to 0·7)
Trichuriasis	0 (0 to 0)	0 (0 to 0)	0 (0 to 0)	0·1 (0 to 0·1)	0·2 (0 to 0·5)	0·2 (0 to 0·4)	0·1 (0 to 0·2)	0·1 (0 to 0·3)	0·1 (0 to 0·2)	0 (0 to 0·1)	0 (0 to 0·1)	0 (0 to 0·1)
Hookworm disease	0 (0 to 0)	0 (0 to 0)	0 (0 to 0)	0·5 (0·2 to 1·1)	1·4 (0·6 to 2·6)	1·2 (0·5 to 2·4)	9·8 (4 to 18·3)	5·8 (2·5 to 11)	3·8 (1·6 to 7·3)	2·9 (1·2 to 5·6)	4·3 (2·1 to 7·6)	2 (1 to 3·4)
Food-borne trematodiases	0 (0 to 0)	0 (0 to 0)	0 (0 to 0)	0 (0 to 0)	0 (0 to 0)	0 (0 to 0)	0 (0 to 0)	0 (0 to 0)	0 (0 to 0)	0 (0 to 0)	0 (0 to 0)	0 (0 to 0)
Leprosy	0 (0 to 0)	0 (0 to 0)	0 (0 to 0)	0 (0 to 0)	0 (0 to 0)	0 (0 to 0)	0·1 (0 to 0·1)	0·1 (0·1 to 0·1)	0·1 (0·1 to 0·2)	0·1 (0·1 to 0·2)	0·2 (0·1 to 0·2)	0·2 (0 to 0·5)
Ebola	0 (0 to 0)	0 (0 to 0)	0 (0 to 0)	0 (0 to 0)	0 (0 to 0)	0 (0 to 0)	0 (0 to 0)	0 (0 to 0)	0 (0 to 0)	0 (0 to 0)	0 (0 to 0)	0 (0 to 0)
Zika virus	0 (0 to 0)	0 (0 to 0·1)	0·3 (0·1 to 0·9)	0 (0 to 0·3)	0·1 (0 to 0·4)	0·1 (0 to 0·4)	0·1 (0 to 0·4)	0·1 (0 to 0·4)	0·1 (0 to 0·5)	0·1 (0 to 0·5)	0·1 (0 to 0·4)	0·1 (0 to 0·4)
Guinea worm disease	0 (0 to 0)	0 (0 to 0)	0 (0 to 0)	0 (0 to 0)	0 (0 to 0)	0 (0 to 0)	0 (0 to 0)	0 (0 to 0)	0 (0 to 0)	0 (0 to 0)	0 (0 to 0)	0 (0 to 0)
Other neglected tropical diseases	1·6 (0·5 to 3·7)	1·1 (0·5 to 2·3)	11·7 (5·9 to 22·9)	27·8 (13·3 to 53·6)	31 (14·7 to 53·5)	20·8 (8·9 to 41·2)	9 (4·2 to 15·4)	8 (3·7 to 14·5)	6·6 (3·1 to 11·8)	8·7 (4·4 to 15·2)	9·9 (5·3 to 15·8)	11·9 (6·6 to 19·2)
Other infectious diseases	10·2 (6 to 16·1)	7 (3·9 to 12·7)	96·7 (58·4 to 150)	90·7 (54·4 to 144)	64 (46·9 to 85·5)	48·6 (34 to 67·8)	42·2 (31·1 to 56·5)	46·5 (33·2 to 62·9)	38·9 (26·7 to 52·9)	31·7 (22·4 to 43·4)	32·9 (23·7 to 43·5)	36·1 (26·4 to 47·9)
Meningitis	5·6 (2·8 to 9·9)	3·2 (1·5 to 6·2)	29·3 (16·6 to 47·7)	16·9 (10 to 26·6)	11·5 (7·9 to 16·3)	7·6 (5·3 to 10·8)	7·1 (5 to 9·9)	8·4 (5·6 to 12·9)	6·3 (4·2 to 9·2)	5·7 (3·8 to 8·3)	5·1 (3·5 to 7·4)	5 (3·4 to 7·1)
Encephalitis	3·2 (1·6 to 5·8)	2·2 (0·7 to 5·3)	48·8 (23·2 to 93·8)	53·1 (23·4 to 103)	28·3 (17·6 to 43·7)	21·6 (12·7 to 34·2)	18·9 (11·4 to 29·7)	20·2 (12·4 to 31·9)	15·4 (8·8 to 25·4)	11·2 (6·7 to 17·9)	9·6 (5·7 to 14·9)	9·5 (5·8 to 15·1)
Diphtheria	0 (0 to 0)	0 (0 to 0)	0·1 (0 to 0·3)	0·3 (0 to 1·9)	0·1 (0 to 0·5)	0 (0 to 0·1)	0 (0 to 0)	0 (0 to 0·1)	0 (0 to 0)	0 (0 to 0·1)	0 (0 to 0)	0 (0 to 0)
Tetanus	0·1 (0 to 0·2)	0 (0 to 0·1)	0·2 (0·1 to 0·4)	0 (0 to 0·1)	0·1 (0·1 to 0·3)	0 (0 to 0·1)	0·1 (0 to 0·1)	0·1 (0·1 to 0·2)	0·1 (0 to 0·2)	0·1 (0 to 0·2)	0·2 (0·1 to 0·4)	0·2 (0·1 to 0·3)
Measles	0 (0 to 0)	0 (0 to 0)	0 (0 to 0)	0 (0 to 0)	0 (0 to 0)	0 (0 to 0)	0 (0 to 0)	0 (0 to 0)	0 (0 to 0)	0 (0 to 0)	0 (0 to 0)	0 (0 to 0)
Varicella and herpes zoster	0 (0 to 0)	0·1 (0 to 0·2)	0·9 (0·4 to 2)	0·6 (0·3 to 1·2)	0·8 (0·4 to 1·5)	0·5 (0·2 to 1·1)	0·7 (0·2 to 1·3)	1 (0·4 to 1·8)	1 (0·5 to 1·8)	0·8 (0·4 to 1·5)	0·8 (0·3 to 1·4)	1 (0·5 to 1·7)
Acute hepatitis	0 (0 to 0)	0 (0 to 0)	1·3 (0·6 to 2·5)	1·7 (0·9 to 3)	3·9 (2·4 to 6·2)	4 (2·5 to 5·9)	4·9 (2·9 to 7·6)	6·6 (3·8 to 11·1)	6 (3·6 to 10·1)	3·8 (2·2 to 6·5)	3·1 (1·9 to 5)	3·8 (2·2 to 6·1)
Acute hepatitis A	0 (0 to 0)	0 (0 to 0)	0·1 (0 to 0·3)	1 (0·4 to 1·8)	2·5 (1·2 to 4·1)	2·9 (1·6 to 4·5)	3 (1·8 to 5)	2·6 (1·5 to 4·2)	1·8 (1 to 2·9)	1·5 (0·7 to 2·6)	0·8 (0·4 to 1·5)	0·8 (0·4 to 1·4)
Acute hepatitis B	0 (0 to 0)	0 (0 to 0)	1 (0·4 to 2)	0·4 (0·1 to 1·1)	0·9 (0·4 to 1·9)	0·6 (0·2 to 1·3)	1 (0·3 to 2·3)	3·1 (1·3 to 6·4)	3·6 (1·7 to 7·1)	1·9 (0·8 to 4)	1·9 (1 to 3·4)	2·2 (1·1 to 4·2)
Acute hepatitis C	0 (0 to 0)	0 (0 to 0)	0·1 (0 to 0·3)	0·2 (0·1 to 0·3)	0·2 (0·1 to 0·4)	0·1 (0 to 0·3)	0·3 (0·1 to 0·8)	0·3 (0·1 to 0·9)	0·2 (0·1 to 0·7)	0·2 (0·1 to 0·5)	0·2 (0·1 to 0·6)	0·5 (0·1 to 1·1)
Acute hepatitis E	0 (0 to 0)	0 (0 to 0)	0·1 (0 to 0·2)	0·1 (0 to 0·3)	0·3 (0·2 to 0·6)	0·4 (0·1 to 0·8)	0·5 (0·2 to 1)	0·6 (0·2 to 1·1)	0·3 (0·2 to 0·6)	0·2 (0·1 to 0·4)	0·2 (0·1 to 0·4)	0·3 (0·1 to 0·5)
Other unspecified infectious diseases	1·3 (0·6 to 2·3)	1·5 (0·7 to 2·9)	9·6 (5·7 to 15·2)	14·3 (8·1 to 22·8)	18·6 (8·8 to 32·7)	14·3 (6·1 to 27·2)	10·5 (5·7 to 16·5)	10·2 (6 to 16·5)	10 (5·6 to 15·5)	10·1 (6·1 to 15·1)	14 (8·8 to 21)	16·6 (10·6 to 24·4)
Maternal and neonatal disorders	6,160 (4,420 to 8,380)	380 (240 to 543)	453 (305 to 657)	188 (146 to 241)	141 (102 to 185)	146 (111 to 189)	308 (244 to 379)	579 (434 to 765)	537 (387 to 728)	362 (259 to 500)	239 (178 to 318)	149 (113 to 196)
Maternal disorders	0 (0 to 0)	0 (0 to 0)	0 (0 to 0)	0 (0 to 0)	0 (0 to 0)	8·8 (6·4 to 11·9)	171 (119 to 233)	434 (292 to 612)	416 (275 to 603)	272 (175 to 411)	160 (106 to 232)	78 (49·8 to 118)
Maternal hemorrhage	0 (0 to 0)	0 (0 to 0)	0 (0 to 0)	0 (0 to 0)	0 (0 to 0)	0·8 (0·5 to 1·2)	28·2 (18·1 to 42·6)	77·8 (45·7 to 126)	84·6 (48·7 to 137)	60·1 (34·3 to 95·5)	38·7 (22 to 62·2)	17·4 (9·6 to 30·1)
Maternal sepsis and other maternal infections	0 (0 to 0)	0 (0 to 0)	0 (0 to 0)	0 (0 to 0)	0 (0 to 0)	0·2 (0·2 to 0·4)	6·7 (3·9 to 10·4)	17·6 (10·4 to 28·9)	15·7 (8·6 to 26·5)	9·5 (5·3 to 17·1)	5·2 (2·8 to 9)	2·2 (1·1 to 3·7)
Maternal hypertensive disorders	0 (0 to 0)	0 (0 to 0)	0 (0 to 0)	0 (0 to 0)	0 (0 to 0)	2·2 (1·5 to 3·2)	34·8 (22·1 to 50·6)	65·2 (40·4 to 96·6)	59·9 (37·1 to 93·3)	34·2 (20·1 to 53·2)	17·4 (10·3 to 27·6)	7·9 (4·7 to 12·7)
Maternal obstructed labor and uterine rupture	0 (0 to 0)	0 (0 to 0)	0 (0 to 0)	0 (0 to 0)	0 (0 to 0)	0·5 (0·3 to 1)	10·1 (5·1 to 18)	26·9 (13·5 to 53·2)	27·5 (12·2 to 52·4)	19·8 (8·4 to 38·6)	13·3 (6·2 to 25·5)	6·3 (2·9 to 11·4)
Maternal abortion and miscarriage	0 (0 to 0)	0 (0 to 0)	0 (0 to 0)	0 (0 to 0)	0 (0 to 0)	0·8 (0·4 to 1·5)	14·5 (8·1 to 24·3)	35·7 (18·6 to 61·4)	30·3 (14·5 to 54·3)	18·5 (8·4 to 34·6)	11·4 (5·3 to 21·5)	6·9 (3·1 to 13·2)
Ectopic pregnancy	0 (0 to 0)	0 (0 to 0)	0 (0 to 0)	0 (0 to 0)	0 (0 to 0)	0·2 (0·1 to 0·4)	8·9 (5 to 14·2)	36·1 (19·9 to 59·2)	36·9 (20·6 to 62·5)	25 (12·9 to 44·1)	13 (7·2 to 21·5)	5·4 (2·8 to 9·3)
Indirect maternal deaths	--	--	--	--	--	1·3 (0·8 to 2)	20·5 (11·4 to 32·3)	52·1 (27·8 to 87·9)	43 (23·6 to 71·3)	24·4 (12·2 to 41·7)	11·9 (6·1 to 20·9)	4·7 (2·4 to 8·6)
Late maternal deaths	--	--	--	--	--	0·4 (0·2 to 0·8)	8·3 (4·7 to 12·9)	21·2 (11·5 to 35·2)	19·3 (10·3 to 32·9)	12·2 (6·3 to 20·9)	7·1 (3·8 to 12·1)	3·4 (1·8 to 6·1)
Maternal deaths aggravated by HIV/AIDS	--	--	--	--	--	0 (0 to 0)	0·3 (0·1 to 0·6)	2·8 (1·2 to 5·1)	4·1 (1·8 to 7·5)	3·6 (1·6 to 6·5)	2·2 (1·1 to 3·8)	1 (0·4 to 1·7)
Neonatal disorders	6,160 (4,420 to 8,380)	380 (240 to 543)	453 (305 to 657)	188 (146 to 241)	141 (102 to 185)	137 (103 to 180)	137 (102 to 175)	145 (108 to 190)	121 (91·4 to 157)	89·2 (66·4 to 115)	78·5 (58·4 to 101)	71 (53 to 93)
Neonatal preterm birth	3,140 (2,180 to 4,400)	94·3 (53·7 to 149)	169 (111 to 244)	77·5 (57·2 to 102)	87·9 (62 to 117)	85 (59·9 to 115)	83·8 (60·8 to 111)	86·9 (61·7 to 117)	73·1 (53·1 to 97·1)	53 (38·7 to 70·1)	46·1 (33·4 to 60·4)	40·8 (29·5 to 54·7)
Neonatal encephalopathy due to birth asphyxia and trauma	1,460 (935 to 2,170)	57·2 (27 to 102)	70·6 (33·8 to 128)	26·8 (16·5 to 39·3)	15·4 (7·5 to 26·4)	15·2 (7·3 to 26·2)	15·5 (7·6 to 26·5)	17 (8·3 to 28·7)	14·2 (6·7 to 24·2)	10·7 (5·2 to 18)	9·6 (4·7 to 16·4)	9 (4·4 to 15·6)
Neonatal sepsis and other neonatal infections	750 (447 to 1,140)	173 (102 to 269)	141 (90·3 to 209)	26·1 (14·9 to 40·5)	30·5 (17·5 to 48·6)	29·8 (16·7 to 47·5)	30·1 (16·8 to 47·8)	32·4 (17 to 52·8)	27 (14·3 to 43·5)	20·2 (11·3 to 32·7)	17·7 (9·8 to 28·3)	16·5 (8·6 to 27·1)
Hemolytic disease and other neonatal jaundice	31·3 (16·5 to 54)	2·3 (1·5 to 3·4)	3·8 (2·4 to 5·8)	6·5 (3·9 to 9·7)	7·3 (3·4 to 13·1)	7·3 (3·6 to 12·1)	7·4 (3·5 to 12·3)	8·2 (4·8 to 13·6)	7 (4 to 11·4)	5·3 (3 to 8·6)	5 (3·7 to 6·4)	4·7 (3·5 to 6·1)
Other neonatal disorders	768 (428 to 1,180)	52·5 (29·1 to 85·2)	69·2 (46·7 to 101)	51·5 (39·5 to 65·5)	0 (0 to 0)	0 (0 to 0)	0 (0 to 0)	0 (0 to 0)	0 (0 to 0)	0 (0 to 0)	0 (0 to 0)	0 (0 to 0)
Nutritional deficiencies	0·8 (0·5 to 1·2)	8·4 (4·8 to 13·5)	297 (187 to 472)	339 (222 to 508)	308 (155 to 540)	218 (94·1 to 430)	191 (97·3 to 327)	169 (91·3 to 291)	160 (84·8 to 272)	148 (79·6 to 249)	216 (119 to 339)	265 (152 to 418)
Protein-energy malnutrition	0·8 (0·5 to 1·1)	2·4 (1·5 to 3·5)	233 (133 to 396)	177 (110 to 305)	43·9 (28·4 to 66·4)	22·7 (13·8 to 35)	21·2 (13·1 to 32·4)	31·5 (19 to 49)	27·4 (16 to 45·3)	19 (11·3 to 29·2)	23·3 (13·5 to 37·3)	22 (13·9 to 33·1)
Iodine deficiency	0 (0 to 0)	0 (0 to 0)	0 (0 to 0)	0·2 (0·1 to 0·5)	1·4 (0·6 to 2·7)	3·2 (1 to 6·5)	4·5 (1·7 to 8·6)	5·7 (2·4 to 10·9)	5·1 (2 to 9·3)	4 (1·7 to 7·6)	3·7 (1·6 to 6·8)	3·5 (1·5 to 6·4)
Vitamin A deficiency	0 (0 to 0)	0·4 (0·1 to 1)	1·8 (0·8 to 3·3)	7·5 (3·5 to 13·6)	9·4 (4·2 to 17·4)	6·7 (2·8 to 12·8)	1·6 (0·5 to 3·4)	1·4 (0·6 to 2·6)	1·1 (0·5 to 1·9)	0·7 (0·3 to 1·3)	0·6 (0·3 to 1)	0·5 (0·2 to 0·8)
Dietary iron deficiency	0 (0 to 0·1)	5·6 (2·6 to 10·1)	51·9 (24·7 to 92·4)	146 (67·9 to 274)	250 (96·4 to 477)	183 (62·3 to 395)	156 (63·6 to 289)	124 (49·8 to 243)	121 (50·5 to 235)	120 (53·9 to 219)	183 (91·7 to 308)	235 (124 to 381)
Other nutritional deficiencies	0 (0 to 0)	0 (0 to 0)	9·9 (4·2 to 20·4)	8·4 (3·8 to 17·2)	2·9 (1·1 to 7·8)	1·8 (0·8 to 3·4)	7·6 (3·6 to 13·8)	6·2 (3 to 13·2)	5·4 (2·7 to 10·2)	4·5 (2·2 to 8·3)	4·6 (2·1 to 8·2)	4·6 (2·2 to 8·1)
Non-communicable diseases	749 (479 to 1,140)	137 (73·1 to 282)	1,040 (706 to 1,490)	1,170 (904 to 1,500)	1,330 (1,070 to 1,640)	1,970 (1,500 to 2,560)	3,280 (2,510 to 4,210)	4,800 (3,800 to 6,020)	4,990 (3,910 to 6,250)	4,610 (3,640 to 5,750)	5,260 (4,200 to 6,500)	6,500 (5,220 to 7,900)
Neoplasms	7·1 (2·5 to 19·9)	2·4 (0·7 to 6·6)	21·6 (13·6 to 32·4)	95·1 (64·2 to 139)	122 (96·2 to 156)	115 (85·7 to 153)	155 (118 to 201)	281 (201 to 384)	394 (256 to 570)	535 (349 to 762)	807 (547 to 1,140)	1,090 (778 to 1,480)
Lip and oral cavity cancer	0 (0 to 0)	0 (0 to 0)	0 (0 to 0)	0 (0 to 0)	0·4 (0·3 to 0·5)	0·6 (0·3 to 0·9)	1 (0·6 to 1·6)	1·4 (0·8 to 2·2)	2·6 (1·5 to 4·3)	2·5 (1·4 to 4·2)	3·1 (1·8 to 5·1)	5·1 (3·1 to 7·8)
Nasopharynx cancer	0 (0 to 0)	0 (0 to 0)	0 (0 to 0)	0 (0 to 0)	0·5 (0·3 to 0·9)	0·3 (0·2 to 0·4)	0·9 (0·5 to 1·5)	1·4 (0·7 to 2·4)	1·4 (0·8 to 2·3)	1·1 (0·6 to 1·9)	1·1 (0·6 to 1·9)	1·7 (1 to 2·7)
Other pharynx cancer	0 (0 to 0)	0 (0 to 0)	0 (0 to 0)	0 (0 to 0)	0 (0 to 0)	0 (0 to 0)	0 (0 to 0)	0·6 (0·3 to 1·1)	0·6 (0·3 to 1·1)	0·6 (0·3 to 1·1)	0·8 (0·4 to 1·5)	1·8 (1 to 3)
Esophageal cancer	0 (0 to 0)	0 (0 to 0)	0 (0 to 0)	0 (0 to 0)	0 (0 to 0)	0 (0 to 0)	0 (0 to 0)	0·7 (0·4 to 1·1)	0·9 (0·5 to 1·5)	1·2 (0·6 to 2·1)	2·5 (1·4 to 4)	5·2 (2·9 to 8·4)
Stomach cancer	0 (0 to 0)	0 (0 to 0)	0 (0 to 0)	0 (0 to 0)	0 (0 to 0)	0 (0 to 0)	2·6 (1·6 to 3·8)	7·7 (4·7 to 11·4)	15·8 (9·3 to 25·7)	20·9 (12·2 to 32·3)	28·9 (17·5 to 45)	42·2 (25·6 to 62·8)
Colon and rectum cancer	0 (0 to 0)	0 (0 to 0)	0 (0 to 0)	0 (0 to 0)	1·1 (0·8 to 1·6)	1·3 (0·8 to 2)	5·2 (3·3 to 7·7)	11·2 (6·4 to 18·3)	17·5 (10·3 to 27·2)	27·1 (15·6 to 43·3)	39·5 (23·4 to 62·3)	61·6 (38·9 to 95·5)
Liver cancer	0·2 (0·1 to 0·4)	0·1 (0 to 0·2)	0·7 (0·3 to 1·3)	1·7 (0·9 to 2·8)	1·8 (1·3 to 2·6)	2·3 (1·4 to 3·4)	1·4 (0·6 to 2·6)	3·7 (1·9 to 6·4)	4·2 (2·3 to 7·2)	5 (2·5 to 8·9)	8·3 (3·9 to 14·9)	13·4 (7·5 to 22·5)
Liver cancer due to hepatitis B	0 (0 to 0)	0 (0 to 0)	0 (0 to 0)	0 (0 to 0)	0 (0 to 0)	0·4 (0·1 to 0·7)	0·4 (0·2 to 0·8)	1·4 (0·7 to 2·6)	1·9 (0·9 to 3·8)	2·4 (1·1 to 4·5)	3·7 (1·6 to 7·3)	5·5 (2·6 to 10·1)
Liver cancer due to hepatitis C	0 (0 to 0)	0 (0 to 0)	0 (0 to 0)	0 (0 to 0)	0 (0 to 0)	0 (0 to 0)	0 (0 to 0)	0·1 (0 to 0·2)	0·2 (0·1 to 0·4)	0·4 (0·1 to 0·8)	0·9 (0·3 to 2)	1·8 (0·8 to 3·6)
Liver cancer due to alcohol use	0 (0 to 0)	0 (0 to 0)	0 (0 to 0)	0 (0 to 0)	0 (0 to 0)	0 (0 to 0)	0 (0 to 0·1)	0·2 (0·1 to 0·4)	0·4 (0·1 to 0·8)	0·7 (0·3 to 1·4)	1·4 (0·5 to 3·1)	2·7 (1·1 to 5·5)
Liver cancer due to NASH	0 (0 to 0)	0 (0 to 0)	0 (0 to 0)	0 (0 to 0)	0 (0 to 0)	0 (0 to 0)	0·4 (0·1 to 0·7)	0·7 (0·3 to 1·4)	0·6 (0·2 to 1·3)	0·6 (0·2 to 1·2)	0·9 (0·3 to 1·9)	1·4 (0·6 to 2·7)
Liver cancer due to other causes	0·2 (0·1 to 0·4)	0·1 (0 to 0·2)	0·7 (0·3 to 1·3)	1·7 (0·9 to 2·8)	1·8 (1·3 to 2·6)	1·9 (1·2 to 2·8)	0·6 (0·2 to 1·2)	1·2 (0·6 to 2·3)	1·1 (0·4 to 2·2)	1 (0·4 to 2)	1·4 (0·6 to 2·9)	1·9 (0·8 to 3·9)
Gallbladder and biliary tract cancer	0 (0 to 0)	0 (0 to 0)	0 (0 to 0)	0 (0 to 0)	0 (0 to 0)	0 (0 to 0)	0 (0 to 0)	0·4 (0·3 to 0·7)	0·8 (0·5 to 1·5)	1·2 (0·6 to 2·2)	3·2 (1·7 to 5·6)	6·5 (3·7 to 11)
Pancreatic cancer	0 (0 to 0)	0 (0 to 0)	0 (0 to 0)	0 (0 to 0)	0 (0 to 0)	0 (0 to 0)	1·2 (0·8 to 1·9)	2·3 (1·1 to 3·9)	5·3 (2·8 to 9·2)	6·3 (3·6 to 9·8)	12·3 (7·6 to 18·6)	24·6 (14·9 to 36·9)
Larynx cancer	0 (0 to 0)	0 (0 to 0)	0 (0 to 0)	0 (0 to 0)	0 (0 to 0)	0 (0 to 0)	0 (0 to 0)	0·3 (0·3 to 0·5)	0·4 (0·2 to 0·6)	0·4 (0·2 to 0·7)	0·6 (0·3 to 1·1)	1 (0·5 to 1·7)
Tracheal, bronchus, and lung cancer	0 (0 to 0)	0 (0 to 0)	0 (0 to 0)	0 (0 to 0)	0 (0 to 0)	0·5 (0·3 to 0·9)	2·5 (1·3 to 4·1)	3·3 (1·7 to 5·7)	4·7 (2·6 to 7·7)	9·1 (5·3 to 14·2)	17·9 (10·7 to 28·1)	33·8 (21·6 to 51)
Malignant skin melanoma	0 (0 to 0)	0 (0 to 0)	0·2 (0·1 to 0·4)	0·7 (0·2 to 1·8)	0·3 (0·3 to 0·4)	0·3 (0·2 to 0·4)	0·5 (0·3 to 0·9)	1·7 (0·9 to 2·8)	1·8 (0·9 to 3·1)	1·8 (1 to 3)	3·8 (2 to 6·3)	3·1 (1·6 to 5·1)
Non-melanoma skin cancer	0 (0 to 0)	0 (0 to 0)	0 (0 to 0)	0 (0 to 0)	0 (0 to 0)	0 (0 to 0)	0 (0 to 0)	0·9 (0·5 to 1·3)	1·3 (0·6 to 2·2)	1·2 (0·5 to 1·9)	1·4 (0·7 to 2·2)	2·5 (1·3 to 3·9)
Non-melanoma skin cancer (squamous-cell carcinoma)	0 (0 to 0)	0 (0 to 0)	0 (0 to 0)	0 (0 to 0)	0 (0 to 0)	0 (0 to 0)	0 (0 to 0)	0·9 (0·5 to 1·3)	1·3 (0·6 to 2·2)	1·2 (0·5 to 1·9)	1·4 (0·7 to 2·2)	2·5 (1·3 to 3·9)
Non-melanoma skin cancer (basal-cell carcinoma)	0 (0 to 0)	0 (0 to 0)	0 (0 to 0)	0 (0 to 0)	0 (0 to 0)	0 (0 to 0)	0 (0 to 0)	0 (0 to 0)	0 (0 to 0)	0 (0 to 0)	0 (0 to 0)	0 (0 to 0)
Breast cancer	0 (0 to 0)	0 (0 to 0)	0 (0 to 0)	0 (0 to 0)	0 (0 to 0)	0 (0 to 0)	5·1 (2·9 to 8·3)	13·4 (8·2 to 20·4)	49·4 (29·4 to 76·1)	122 (71·9 to 191)	213 (130 to 323)	305 (195 to 452)
Cervical cancer	0 (0 to 0)	0 (0 to 0)	0 (0 to 0)	0 (0 to 0)	0 (0 to 0)	0 (0 to 0)	8·8 (4·6 to 15·7)	42·6 (22·5 to 72·5)	105 (56·3 to 176)	168 (91·2 to 270)	261 (152 to 416)	306 (186 to 470)
Uterine cancer	0 (0 to 0)	0 (0 to 0)	0 (0 to 0)	0 (0 to 0)	0 (0 to 0)	0 (0 to 0)	0 (0 to 0)	6·8 (3·9 to 11·4)	14·2 (7·6 to 25·5)	21·5 (11·3 to 38·8)	32·4 (18·1 to 51·8)	46·4 (27·9 to 72·9)
Ovarian cancer	0 (0 to 0)	0 (0 to 0)	0 (0 to 0)	0 (0 to 0)	4·7 (2·1 to 7·6)	6·6 (3·4 to 11·1)	17·1 (8·3 to 29)	30·2 (15·3 to 49·6)	29 (14·7 to 48·3)	36·6 (20 to 59·3)	53·5 (30 to 83·1)	86·5 (50·9 to 135)
Prostate cancer	0 (0 to 0)	0 (0 to 0)	0 (0 to 0)	0 (0 to 0)	0 (0 to 0)	0 (0 to 0)	0 (0 to 0)	0 (0 to 0)	0 (0 to 0)	0 (0 to 0)	0 (0 to 0)	0 (0 to 0)
Testicular cancer	--	--	--	--	--	--	--	--	--	--	--	--
Kidney cancer	0·3 (0 to 1·1)	0·1 (0 to 0·4)	1·3 (0·7 to 2·3)	6·8 (3·7 to 12·4)	7·1 (3·9 to 12·3)	3·6 (2 to 6·3)	4·1 (1·8 to 8·1)	7 (3·6 to 12·1)	6·8 (3·8 to 11·6)	6·6 (3·4 to 11)	8·8 (4·7 to 15·3)	14 (7·8 to 22·7)
Bladder cancer	0 (0 to 0)	0 (0 to 0)	0 (0 to 0)	0 (0 to 0)	0 (0 to 0)	0 (0 to 0)	0·3 (0·2 to 0·4)	0·4 (0·2 to 0·6)	0·5 (0·2 to 0·8)	1·4 (0·8 to 2·2)	1·7 (0·9 to 2·9)	3 (1·7 to 4·8)
Brain and central nervous system cancer	2 (0·1 to 13·8)	0·5 (0 to 2·5)	2·8 (1·2 to 5·3)	11 (4·5 to 21·1)	18·2 (8·8 to 31)	12·8 (6·5 to 21·8)	8 (3·8 to 13·5)	11·1 (4·8 to 19·1)	11·1 (4·6 to 19·8)	8·8 (3·6 to 15·6)	11·2 (4·8 to 20·8)	12·3 (5·4 to 21·1)
Thyroid cancer	0 (0 to 0)	0 (0 to 0)	0 (0 to 0)	0 (0 to 0)	0·4 (0·3 to 0·8)	0·4 (0·2 to 0·6)	1 (0·5 to 1·8)	1·8 (0·7 to 4·1)	2·3 (0·9 to 4·8)	2·2 (0·9 to 4·4)	3·6 (1·9 to 6·3)	5 (2·6 to 8·9)
Mesothelioma	0 (0 to 0)	0 (0 to 0)	0 (0 to 0)	0 (0 to 0)	0 (0 to 0)	0 (0 to 0)	0 (0 to 0)	4·5 (2·5 to 7·5)	2·6 (1·3 to 4·6)	2·4 (1·3 to 4)	4·9 (2·8 to 8·2)	5·8 (3·3 to 9·4)
Hodgkin lymphoma	0 (0 to 0)	0 (0 to 0)	0 (0 to 0)	0·3 (0·2 to 0·6)	0·4 (0·3 to 1)	0·9 (0·4 to 1·9)	5·8 (3·1 to 10·2)	11·6 (6·1 to 19·8)	8·8 (4·6 to 15·1)	6 (3·1 to 10·8)	4·8 (2·5 to 8·1)	4·5 (2·6 to 7·5)
Non-Hodgkin lymphoma	0 (0 to 0)	0 (0 to 0)	0 (0 to 0)	6·9 (4 to 10·9)	8·5 (5·6 to 12·3)	9·4 (5·6 to 14·7)	13 (8 to 19·2)	24·8 (14·9 to 38·7)	24·2 (14·5 to 37·7)	19 (11·2 to 30·8)	19·1 (11·6 to 30·7)	19·2 (11·8 to 30·1)
Multiple myeloma	0 (0 to 0)	0 (0 to 0)	0 (0 to 0)	0 (0 to 0)	0 (0 to 0)	0 (0 to 0)	0 (0 to 0)	0·4 (0·2 to 0·7)	0·7 (0·3 to 1·2)	1·2 (0·6 to 2·2)	2·9 (1·5 to 5·2)	6·1 (3·3 to 10)
Leukemia	1·7 (0·3 to 5·5)	0·4 (0·1 to 1·3)	7·5 (4·4 to 11·6)	31·4 (18·4 to 52·3)	45·8 (29 to 70·5)	38·9 (23·8 to 59·6)	36·5 (22·9 to 54·1)	45·4 (27·2 to 71·2)	42·1 (24·3 to 66·3)	27·5 (16·4 to 43·7)	29·9 (18 to 47·2)	31·4 (19·3 to 47·7)
Acute lymphoid leukemia	0·4 (0 to 1·4)	0·2 (0 to 0·7)	3 (1·6 to 5·1)	22·8 (13·4 to 38·8)	34·5 (21·2 to 55·4)	25·9 (14·6 to 41·2)	21·7 (12·2 to 34·4)	14·7 (7·4 to 26·1)	9·5 (4·3 to 17·1)	5·3 (2·4 to 10·5)	4·1 (1·9 to 7·6)	4·6 (2·1 to 8·3)
Chronic lymphoid leukemia	0 (0 to 0)	0 (0 to 0)	0 (0 to 0)	0 (0 to 0)	0 (0 to 0)	0 (0 to 0)	0 (0 to 0)	0·8 (0·4 to 1·3)	0·5 (0·2 to 0·9)	0·3 (0·1 to 0·6)	0·9 (0·4 to 1·7)	1·1 (0·5 to 2)
Acute myeloid leukemia	0·2 (0 to 1·2)	0·1 (0 to 0·5)	0·5 (0·2 to 1)	2·8 (1·2 to 5·7)	2·8 (1·1 to 5·7)	3·6 (1·4 to 7·3)	3·8 (1·6 to 7·6)	4·4 (1·9 to 8·6)	5 (2·2 to 9·5)	2·8 (1·2 to 5·3)	3·6 (1·7 to 6·9)	3·4 (1·6 to 6·1)
Chronic myeloid leukemia	0 (0 to 0·1)	0 (0 to 0)	0·1 (0·1 to 0·2)	0·8 (0·4 to 1·7)	0·9 (0·4 to 1·6)	0·9 (0·4 to 1·9)	1·4 (0·6 to 2·8)	6·4 (2·6 to 12·6)	6·7 (2·9 to 13·9)	5·3 (2·3 to 10·4)	5·4 (2·4 to 10·3)	5·2 (2·4 to 10)
Other leukemia	1·1 (0·2 to 3·8)	0·1 (0 to 0·4)	3·9 (2·1 to 6·5)	4·9 (2·3 to 9·4)	7·6 (3·3 to 14·5)	8·5 (3·8 to 15·5)	9·6 (4·8 to 16·9)	19·2 (9·6 to 32·2)	20·4 (10·4 to 34·3)	13·8 (7·2 to 23·2)	15·9 (8 to 26·5)	17·2 (10·1 to 28)
Other malignant neoplasms	2·8 (0·8 to 7·3)	1·2 (0·1 to 4·6)	8·4 (4·9 to 14)	34·3 (20·5 to 54·2)	32·4 (24·7 to 41·4)	36·5 (25·2 to 51·2)	39·5 (27·6 to 55)	44·5 (30·1 to 63·8)	38·8 (25·8 to 57·4)	31·8 (20 to 46·2)	35·2 (22·5 to 51·3)	44·3 (29 to 64·7)

Cause	45 to 49	50 to 54	55 to 59	60 to 64	65 to 69	70 to 74	75 to 79	80 to 84	85 to 89	90 to 94	95 plus	Total
All causes	9,300 (7,580 to 11,300)	10,400 (8,480 to 12,600)	11,300 (9,240 to 13,600)	11,000 (9,100 to 13,100)	9,540 (8,030 to 11,200)	6,890 (5,840 to 8,030)	5,280 (4,510 to 6,120)	3,700 (3,220 to 4,220)	2,000 (1,740 to 2,270)	1,150 (1,040 to 1,250)	309 (285 to 329)	135,000
Communicable, maternal, neonatal, and nutritional diseases	1,060 (885 to 1,290)	911 (732 to 1,140)	808 (637 to 1,030)	670 (538 to 824)	549 (430 to 693)	401 (312 to 511)	334 (255 to 438)	273 (209 to 352)	179 (134 to 225)	126 (91·7 to 161)	36·8 (26·5 to 48·2)	26,300
HIV/AIDS and sexually transmitted infections	368 (338 to 403)	233 (213 to 254)	137 (121 to 157)	88·8 (74 to 108)	58·8 (47·7 to 74·4)	20 (12·4 to 30)	8·5 (6·1 to 11·8)	3·9 (2·9 to 5·3)	1·8 (1·3 to 2·4)	0·6 (0·4 to 0·8)	0·1 (0·1 to 0·2)	4,560
HIV/AIDS	349 (321 to 381)	222 (203 to 243)	130 (115 to 150)	84·3 (70·2 to 102)	55·6 (44·6 to 71)	18·2 (10·9 to 28·4)	7·4 (5·1 to 10·5)	3·3 (2·4 to 4·5)	1·5 (1·1 to 2·1)	0·4 (0·2 to 0·7)	0·1 (0·1 to 0·2)	4,390
HIV/AIDS - Drug-susceptible Tuberculosis	41·2 (25·7 to 62)	26·1 (16·6 to 39·4)	14·7 (9·6 to 21·4)	9·3 (6 to 13·8)	6 (3·8 to 8·7)	1·7 (1 to 2·5)	0·7 (0·4 to 1·1)	0·4 (0·3 to 0·5)	0·2 (0·1 to 0·2)	0 (0 to 0·1)	0 (0 to 0)	525
HIV/AIDS - Multidrug-resistant Tuberculosis without extensive	0·7 (0 to 4·2)	0·5 (0 to 2·7)	0·3 (0 to 1·5)	0·2 (0 to 0·9)	0·1 (0 to 0·6)	0 (0 to 0·1)	0 (0 to 0·1)	0 (0 to 0)	0 (0 to 0)	0 (0 to 0)	0 (0 to 0)	9·3
HIV/AIDS - Extensively drug-resistant Tuberculosis	0·1 (0 to 0·7)	0·1 (0 to 0·4)	0 (0 to 0·2)	0 (0 to 0·1)	0 (0 to 0·1)	0 (0 to 0)	0 (0 to 0)	0 (0 to 0)	0 (0 to 0)	0 (0 to 0)	0 (0 to 0)	1·5
HIV/AIDS resulting in other diseases	307 (272 to 342)	196 (174 to 218)	115 (97·8 to 135)	74·8 (60·4 to 93·5)	49·5 (38·2 to 65·4)	16·5 (9·3 to 26·5)	6·6 (4·4 to 9·7)	3 (2 to 4·2)	1·3 (0·9 to 1·9)	0·4 (0·2 to 0·6)	0·1 (0·1 to 0·1)	3,860
Sexually transmitted infections excluding HIV	19·2 (11·9 to 30·7)	10·6 (6·2 to 18)	7·1 (4 to 12·4)	4·5 (2·5 to 8·1)	3·2 (1·9 to 5·6)	1·8 (1 to 3)	1·1 (0·6 to 1·8)	0·6 (0·4 to 1·1)	0·3 (0·2 to 0·5)	0·2 (0·1 to 0·2)	0 (0 to 0)	170
Syphilis	0·9 (0·5 to 1·4)	0·5 (0·3 to 0·8)	0·1 (0·1 to 0·2)	0·1 (0·1 to 0·2)	0·1 (0·1 to 0·2)	0·1 (0·1 to 0·2)	0·1 (0 to 0·1)	0·1 (0 to 0·1)	0 (0 to 0·1)	0 (0 to 0·1)	0 (0 to 0)	16·7
Chlamydial infection	2·3 (1·4 to 3·5)	0·8 (0·5 to 1·3)	0·5 (0·3 to 0·8)	0·3 (0·2 to 0·5)	0·1 (0·1 to 0·2)	0·1 (0·1 to 0·2)	0·1 (0 to 0·1)	0·1 (0 to 0·1)	0 (0 to 0)	0 (0 to 0)	0 (0 to 0)	17·6
Gonococcal infection	4 (2·4 to 6·1)	2 (1·1 to 3·4)	1·2 (0·6 to 2·1)	0·7 (0·4 to 1·2)	0·8 (0·4 to 1·2)	0·4 (0·2 to 0·6)	0·3 (0·1 to 0·4)	0·1 (0·1 to 0·2)	0·1 (0 to 0·1)	0 (0 to 0·1)	0 (0 to 0)	30
Trichomoniasis	3·9 (1·3 to 9·2)	3 (0·9 to 7)	2·1 (0·7 to 4·8)	1·2 (0·4 to 2·8)	0·8 (0·3 to 1·7)	0·4 (0·1 to 0·9)	0·2 (0·1 to 0·5)	0·1 (0 to 0·2)	0 (0 to 0·1)	0 (0 to 0)	0 (0 to 0)	36·7
Genital herpes	2·8 (0·9 to 6·8)	2·4 (0·7 to 5·8)	2 (0·6 to 4·9)	1·5 (0·5 to 3·7)	1·1 (0·3 to 2·6)	0·6 (0·2 to 1·5)	0·4 (0·1 to 0·9)	0·2 (0·1 to 0·5)	0·1 (0 to 0·2)	0 (0 to 0·1)	0 (0 to 0)	27
Other sexually transmitted infections	5·5 (3·3 to 8·3)	1·9 (1·1 to 2·9)	1·2 (0·6 to 1·9)	0·6 (0·4 to 1·1)	0·3 (0·2 to 0·5)	0·2 (0·1 to 0·3)	0·1 (0·1 to 0·2)	0 (0 to 0·1)	0 (0 to 0·1)	0 (0 to 0)	0 (0 to 0)	42·2
Respiratory infections and tuberculosis	200 (142 to 280)	210 (141 to 301)	228 (156 to 318)	241 (172 to 326)	217 (156 to 293)	203 (143 to 282)	201 (140 to 284)	183 (131 to 246)	126 (87·6 to 169)	94·9 (64·7 to 126)	27·4 (18·5 to 37·1)	4,490
Tuberculosis	47·8 (28·9 to 76·9)	45·2 (27·7 to 68·7)	41·4 (25 to 66)	36 (21·7 to 58·7)	23·2 (14·5 to 35·8)	16·5 (10·2 to 26·3)	11·6 (7·7 to 17)	7·5 (4·7 to 11·2)	4 (2·5 to 6·3)	1·8 (1·1 to 2·8)	0·4 (0·2 to 0·6)	705
Latent tuberculosis infection	0 (0 to 0)	0 (0 to 0)	0 (0 to 0)	0 (0 to 0)	0 (0 to 0)	0 (0 to 0)	0 (0 to 0)	0 (0 to 0)	0 (0 to 0)	0 (0 to 0)	0 (0 to 0)	0
Drug-susceptible tuberculosis	47 (28·2 to 76·4)	44·5 (27·1 to 67·9)	40·7 (24·8 to 65·8)	35·4 (21·1 to 57·3)	22·8 (13·8 to 35·7)	16·3 (10·1 to 26)	11·4 (7·5 to 16·7)	7·4 (4·6 to 11·1)	3·9 (2·4 to 6·2)	1·7 (1·1 to 2·8)	0·4 (0·2 to 0·6)	694
Multidrug-resistant tuberculosis without extensive drug resista	0·7 (0 to 3·7)	0·6 (0·1 to 3·4)	0·6 (0 to 3·1)	0·5 (0 to 2·6)	0·3 (0 to 1·7)	0·2 (0 to 1·2)	0·2 (0 to 0·8)	0·1 (0 to 0·6)	0·1 (0 to 0·3)	0 (0 to 0·1)	0 (0 to 0)	9·4
Extensively drug-resistant tuberculosis	0·1 (0 to 0·6)	0·1 (0 to 0·6)	0·1 (0 to 0·5)	0·1 (0 to 0·4)	0·1 (0 to 0·3)	0 (0 to 0·2)	0 (0 to 0·1)	0 (0 to 0·1)	0 (0 to 0·1)	0 (0 to 0)	0 (0 to 0)	1·5
Lower respiratory infections	132 (84·7 to 197)	148 (88·8 to 229)	172 (105 to 252)	194 (128 to 276)	186 (127 to 261)	182 (124 to 259)	187 (127 to 268)	174 (122 to 237)	122 (82·9 to 164)	92·8 (62·6 to 124)	26·9 (18·1 to 36·7)	3,350
Upper respiratory infections	15·2 (8 to 25·9)	12·5 (6·9 to 21·5)	10·7 (5·9 to 17·7)	8·3 (4·4 to 14·1)	6·1 (3·3 to 10·1)	3·4 (1·9 to 5·8)	2·1 (1·1 to 3·5)	1·2 (0·6 to 2·1)	0·5 (0·3 to 0·9)	0·2 (0·1 to 0·4)	0·1 (0 to 0·1)	323
Otitis media	5·3 (2·7 to 9·7)	4·9 (2·2 to 8·9)	4 (2 to 7·3)	2·8 (1·2 to 5·6)	1·6 (0·8 to 3·1)	0·8 (0·3 to 1·5)	0·5 (0·2 to 1)	0·4 (0·2 to 0·7)	0·2 (0·1 to 0·3)	0·1 (0 to 0·2)	0 (0 to 0)	112
Enteric infections	66 (46·9 to 89·2)	68·6 (49·3 to 92·3)	74·1 (51·6 to 100)	78·8 (57·1 to 107)	79·2 (58·1 to 108)	63 (46 to 87)	54·6 (40·1 to 73·3)	41 (29·8 to 55)	22·1 (16 to 30)	12·3 (8·6 to 16·7)	3·4 (2·4 to 4·8)	1,780
Diarrheal diseases	65·6 (46·7 to 88·8)	68·1 (48·9 to 92)	73·6 (51 to 99·7)	78·4 (56·9 to 107)	78·7 (57·5 to 107)	62·8 (45·8 to 86·9)	54·4 (40 to 73·1)	40·9 (29·7 to 54·9)	22 (15·9 to 29·8)	12·3 (8·6 to 16·6)	3·4 (2·4 to 4·8)	1,770
Typhoid and paratyphoid	0·3 (0·1 to 1·2)	0·4 (0·1 to 1·7)	0·3 (0·1 to 1·6)	0·3 (0·1 to 1·2)	0·4 (0·1 to 1·7)	0·1 (0 to 0·6)	0·1 (0 to 0·5)	0·1 (0 to 0·4)	0·1 (0 to 0·3)	0 (0 to 0·1)	0 (0 to 0)	7·1
Typhoid fever	0·3 (0·1 to 1·2)	0·4 (0·1 to 1·7)	0·3 (0·1 to 1·6)	0·3 (0·1 to 1·2)	0·4 (0·1 to 1·7)	0·1 (0 to 0·6)	0·1 (0 to 0·5)	0·1 (0 to 0·4)	0·1 (0 to 0·3)	0 (0 to 0·1)	0 (0 to 0)	7·1
Paratyphoid fever	0 (0 to 0)	0 (0 to 0)	0 (0 to 0)	0 (0 to 0)	0 (0 to 0)	0 (0 to 0)	0 (0 to 0)	0 (0 to 0)	0 (0 to 0)	0 (0 to 0)	0 (0 to 0)	0
Invasive Non-typhoidal Salmonella (iNTS)	0 (0 to 0)	0·1 (0 to 0·1)	0·1 (0 to 0·3)	0 (0 to 0·1)	0·1 (0 to 0·2)	0 (0 to 0·1)	0 (0 to 0·1)	0 (0 to 0)	0 (0 to 0)	0 (0 to 0)	0 (0 to 0)	2·8
Other intestinal infectious diseases	0·1 (0 to 0·2)	0·1 (0 to 0·2)	0·1 (0 to 0·2)	0 (0 to 0·2)	0·1 (0 to 0·2)	0 (0 to 0·1)	0 (0 to 0·1)	0 (0 to 0·1)	0 (0 to 0·1)	0 (0 to 0)	0 (0 to 0)	3·3
Neglected tropical diseases and malaria	77·2 (48·1 to 129)	74·1 (45·9 to 116)	79·8 (47·8 to 141)	61·9 (38·3 to 102)	50·3 (31 to 82·1)	30·1 (19·1 to 45·6)	19·3 (12 to 28·1)	11·8 (7·5 to 17·4)	6·2 (4·1 to 9)	2·7 (1·8 to 4)	0·7 (0·5 to 1)	1,430
Malaria	20 (4·8 to 66·7)	16·1 (4·4 to 53)	22·6 (4·8 to 79·3)	14·1 (3·4 to 49·7)	11·7 (2·6 to 40·6)	5·2 (1·2 to 18·2)	1·9 (0·6 to 6·1)	0·8 (0·3 to 2·2)	0·2 (0·1 to 0·4)	0·1 (0 to 0·1)	0 (0 to 0)	589
Chagas disease	0·1 (0 to 0·1)	0·1 (0 to 0·2)	0·1 (0 to 0·2)	0·1 (0·1 to 0·3)	0·1 (0·1 to 0·3)	0·1 (0·1 to 0·3)	0·1 (0·1 to 0·2)	0·1 (0·1 to 0·2)	0·6 (0·3 to 1·8)	0·2 (0·1 to 0·6)	0 (0 to 0·1)	1·9
Leishmaniasis	0·1 (0 to 0·3)	0·1 (0 to 0·3)	0·1 (0 to 0·3)	0·1 (0 to 0·3)	0·1 (0 to 0·4)	0 (0 to 0·2)	0 (0 to 0·2)	0 (0 to 0·1)	0 (0 to 0)	0 (0 to 0)	0 (0 to 0)	1·4
Visceral leishmaniasis	0 (0 to 0)	0 (0 to 0)	0 (0 to 0)	0 (0 to 0)	0 (0 to 0)	0 (0 to 0)	0 (0 to 0)	0 (0 to 0)	0 (0 to 0)	0 (0 to 0)	0 (0 to 0)	0
Cutaneous and mucocutaneous leishmaniasis	0·1 (0 to 0·3)	0·1 (0 to 0·3)	0·1 (0 to 0·3)	0·1 (0 to 0·3)	0·1 (0 to 0·4)	0 (0 to 0·2)	0 (0 to 0·2)	0 (0 to 0·1)	0 (0 to 0)	0 (0 to 0)	0 (0 to 0)	1·4
African trypanosomiasis	0 (0 to 0)	0 (0 to 0)	0 (0 to 0)	0 (0 to 0)	0 (0 to 0)	0 (0 to 0)	0 (0 to 0)	0 (0 to 0)	0 (0 to 0)	0 (0 to 0)	0 (0 to 0)	0
Schistosomiasis	0 (0 to 0)	0 (0 to 0)	0 (0 to 0)	0 (0 to 0)	0 (0 to 0)	0 (0 to 0)	0 (0 to 0)	0 (0 to 0)	0 (0 to 0)	0 (0 to 0)	0 (0 to 0)	0
Cysticercosis	17·2 (6·5 to 35)	18·5 (6·5 to 36·7)	20·6 (8·7 to 39·2)	20·6 (8·8 to 36·5)	18·5 (7·4 to 32·9)	12·7 (5·8 to 22·2)	9·3 (4 to 16·2)	6·1 (2·6 to 10·8)	3 (1·3 to 5·5)	1·3 (0·5 to 2·4)	0·3 (0·1 to 0·5)	195
Cystic echinococcosis	0·1 (0 to 0·2)	0·1 (0 to 0·2)	0·1 (0 to 0·2)	0·1 (0 to 0·2)	0 (0 to 0·1)	0 (0 to 0·1)	0 (0 to 0·1)	0 (0 to 0)	0 (0 to 0)	0 (0 to 0)	0 (0 to 0)	0·9
Lymphatic filariasis	23·2 (15·2 to 33·9)	22·1 (14·4 to 31·4)	20·9 (14 to 29·9)	17·9 (11·6 to 26)	13·7 (8·9 to 19·6)	8·2 (5·4 to 11·9)	5·5 (3·6 to 7·8)	3·4 (2·2 to 4·8)	1·6 (1·1 to 2·2)	0·7 (0·5 to 0·9)	0·1 (0·1 to 0·2)	283
Onchocerciasis	0 (0 to 0)	0 (0 to 0)	0 (0 to 0)	0 (0 to 0)	0 (0 to 0)	0 (0 to 0)	0 (0 to 0)	0 (0 to 0)	0 (0 to 0)	0 (0 to 0)	0 (0 to 0)	0
Trachoma	0 (0 to 0)	0 (0 to 0)	0 (0 to 0)	0 (0 to 0)	0 (0 to 0)	0 (0 to 0)	0 (0 to 0)	0 (0 to 0)	0 (0 to 0)	0 (0 to 0)	0 (0 to 0)	0
Dengue	4·3 (1·6 to 8·9)	5 (1·7 to 11·4)	4 (1·6 to 7·7)	3·8 (1·6 to 8·1)	2·3 (0·8 to 4·6)	1·3 (0·5 to 2·5)	0·8 (0·3 to 1·7)	0·5 (0·2 to 1)	0·3 (0·1 to 0·7)	0·1 (0 to 0·3)	0·1 (0 to 0·1)	118
Yellow fever	0 (0 to 0·1)	0 (0 to 0·1)	0 (0 to 0·1)	0 (0 to 0)	0 (0 to 0)	0 (0 to 0)	0 (0 to 0)	0 (0 to 0)	0 (0 to 0)	0 (0 to 0)	0 (0 to 0)	1·1
Rabies	0 (0 to 0)	0 (0 to 0)	0 (0 to 0)	0 (0 to 0)	0 (0 to 0)	0 (0 to 0)	0 (0 to 0)	0 (0 to 0)	0 (0 to 0)	0 (0 to 0)	0 (0 to 0)	0·1
Intestinal nematode infections	0·4 (0·2 to 0·9)	0·5 (0·2 to 1)	0·5 (0·2 to 0·9)	0·4 (0·2 to 0·8)	0·3 (0·1 to 0·5)	0·2 (0·1 to 0·3)	0·1 (0 to 0·2)	0·1 (0 to 0·1)	0 (0 to 0·1)	0 (0 to 0)	0 (0 to 0)	42·2
Ascariasis	0·2 (0·1 to 0·5)	0·2 (0·1 to 0·5)	0·2 (0 to 0·4)	0·2 (0 to 0·5)	0·1 (0 to 0·2)	0·1 (0 to 0·2)	0·1 (0 to 0·1)	0 (0 to 0·1)	0 (0 to 0)	0 (0 to 0)	0 (0 to 0)	7·9
Trichuriasis	0 (0 to 0)	0 (0 to 0)	0 (0 to 0)	0 (0 to 0)	0 (0 to 0)	0 (0 to 0)	0 (0 to 0)	0 (0 to 0)	0 (0 to 0)	0 (0 to 0)	0 (0 to 0)	1
Hookworm disease	0·2 (0·1 to 0·4)	0·3 (0·1 to 0·7)	0·3 (0·1 to 0·6)	0·2 (0·1 to 0·4)	0·2 (0·1 to 0·3)	0·1 (0 to 0·2)	0·1 (0 to 0·1)	0 (0 to 0·1)	0 (0 to 0)	0 (0 to 0)	0 (0 to 0)	33·3
Food-borne trematodiases	0 (0 to 0)	0 (0 to 0)	0 (0 to 0)	0 (0 to 0)	0 (0 to 0)	0 (0 to 0)	0 (0 to 0)	0 (0 to 0)	0 (0 to 0)	0 (0 to 0)	0 (0 to 0)	0
Leprosy	0·2 (0 to 0·5)	0·2 (0 to 0·5)	0·2 (0 to 0·5)	0·2 (0·1 to 0·5)	0·2 (0·1 to 0·4)	0·1 (0 to 0·3)	0·1 (0 to 0·2)	0·1 (0 to 0·1)	0 (0 to 0·1)	0 (0 to 0)	0 (0 to 0)	2·3
Ebola	0 (0 to 0)	0 (0 to 0)	0 (0 to 0)	0 (0 to 0)	0 (0 to 0)	0 (0 to 0)	0 (0 to 0)	0 (0 to 0)	0 (0 to 0)	0 (0 to 0)	0 (0 to 0)	0
Zika virus	0 (0 to 0·2)	0 (0 to 0·2)	0 (0 to 0·1)	0 (0 to 0·1)	0 (0 to 0·1)	0 (0 to 0)	0 (0 to 0)	0 (0 to 0)	0 (0 to 0)	0 (0 to 0)	0 (0 to 0)	1·1
Guinea worm disease	0 (0 to 0)	0 (0 to 0)	0 (0 to 0)	0 (0 to 0)	0 (0 to 0)	0 (0 to 0)	0 (0 to 0)	0 (0 to 0)	0 (0 to 0)	0 (0 to 0)	0 (0 to 0)	0
Other neglected tropical diseases	11·6 (6·5 to 18·5)	11·2 (6·3 to 18·2)	10·7 (5·8 to 17·7)	4·5 (2·4 to 7·6)	3·4 (1·8 to 5·4)	2·1 (1·1 to 3·8)	1·4 (0·6 to 2·9)	0·7 (0·3 to 1·5)	0·4 (0·2 to 0·8)	0·3 (0·1 to 0·7)	0·2 (0·1 to 0·4)	195
Other infectious diseases	32·3 (23·7 to 43·2)	30·5 (22·2 to 40·9)	29·3 (21·4 to 38·7)	19·8 (14·9 to 25·7)	14·9 (11·2 to 19·4)	9 (6·8 to 11·6)	5·3 (4 to 6·9)	3·3 (2·5 to 4·4)	2 (1·5 to 2·7)	0·8 (0·6 to 1·1)	0·2 (0·1 to 0·3)	693
Meningitis	5·2 (3·5 to 7·6)	5·2 (3·5 to 7·6)	4·9 (3·4 to 6·8)	4 (2·8 to 5·3)	3·2 (2·1 to 4·7)	1·7 (1·2 to 2·5)	1·3 (0·9 to 1·9)	0·8 (0·5 to 1·2)	0·4 (0·3 to 0·5)	0·1 (0·1 to 0·2)	0 (0 to 0)	139
Encephalitis	8·2 (4·8 to 13·3)	5·8 (3·7 to 8·8)	5·8 (3·7 to 9·1)	4·7 (3 to 7·3)	3·2 (2 to 4·9)	1·9 (1·1 to 3·1)	1 (0·6 to 1·6)	0·5 (0·3 to 0·9)	0·2 (0·1 to 0·4)	0·1 (0·1 to 0·2)	0 (0 to 0)	274
Diphtheria	0 (0 to 0)	0 (0 to 0)	0 (0 to 0)	0 (0 to 0)	0 (0 to 0)	0 (0 to 0)	0 (0 to 0)	0 (0 to 0)	0 (0 to 0)	0 (0 to 0)	0 (0 to 0)	0·6
Tetanus	0·2 (0·1 to 0·3)	0·4 (0·2 to 0·8)	0·3 (0·1 to 0·7)	0·3 (0·1 to 0·6)	0·2 (0·1 to 0·5)	0·2 (0·1 to 0·4)	0·1 (0·1 to 0·3)	0·1 (0 to 0·1)	0 (0 to 0·1)	0 (0 to 0·1)	0 (0 to 0)	3·1
Measles	0 (0 to 0)	0 (0 to 0)	0 (0 to 0)	0 (0 to 0)	0 (0 to 0)	0 (0 to 0)	0 (0 to 0)	0 (0 to 0)	0 (0 to 0)	0 (0 to 0)	0 (0 to 0)	0
Varicella and herpes zoster	0·8 (0·3 to 1·5)	0·9 (0·4 to 1·6)	0·9 (0·4 to 1·6)	0·9 (0·4 to 1·6)	0·7 (0·3 to 1·3)	0·5 (0·2 to 0·8)	0·3 (0·2 to 0·6)	0·2 (0·1 to 0·4)	0·1 (0·1 to 0·2)	0·1 (0 to 0·1)	0 (0 to 0)	13·7
Acute hepatitis	2·7 (1·5 to 4·6)	2·2 (1·2 to 3·8)	2·1 (1·1 to 3·6)	1·4 (0·8 to 2·5)	0·9 (0·5 to 1·5)	0·5 (0·3 to 0·9)	0·3 (0·2 to 0·5)	0·2 (0·1 to 0·2)	0·1 (0·1 to 0·2)	0 (0 to 0·1)	0 (0 to 0)	49·7
Acute hepatitis A	0·5 (0·2 to 0·9)	0·4 (0·2 to 0·7)	0·3 (0·1 to 0·7)	0·2 (0·1 to 0·3)	0·1 (0 to 0·2)	0 (0 to 0·1)	0 (0 to 0·1)	0 (0 to 0)	0 (0 to 0)	0 (0 to 0)	0 (0 to 0)	18·7
Acute hepatitis B	1·5 (0·7 to 3)	1·3 (0·6 to 2·4)	1·1 (0·5 to 2·2)	0·9 (0·4 to 1·7)	0·5 (0·2 to 1)	0·3 (0·1 to 0·5)	0·2 (0·1 to 0·3)	0·1 (0 to 0·2)	0 (0 to 0·1)	0 (0 to 0)	0 (0 to 0)	22·7
Acute hepatitis C	0·2 (0·1 to 0·6)	0·4 (0·1 to 1)	0·5 (0·1 to 1·1)	0·3 (0·1 to 0·6)	0·2 (0·1 to 0·5)	0·2 (0·1 to 0·4)	0·1 (0 to 0·2)	0 (0 to 0·1)	0 (0 to 0)	0 (0 to 0)	0 (0 to 0)	4·1
Acute hepatitis E	0·5 (0·2 to 1·1)	0·1 (0·1 to 0·4)	0·2 (0·1 to 0·4)	0·1 (0 to 0·2)	0·1 (0 to 0·1)	0·1 (0 to 0·1)	0 (0 to 0·1)	0 (0 to 0)	0·1 (0 to 0·1)	0 (0 to 0)	0 (0 to 0)	4·2
Other unspecified infectious diseases	15·2 (9·6 to 22·2)	16 (10·3 to 23·5)	15·3 (9·8 to 22·1)	8·5 (5·5 to 12·4)	6·7 (4·3 to 9·5)	4·2 (2·7 to 6·3)	2·2 (1·4 to 3·3)	1·6 (1 to 2·3)	1·1 (0·7 to 1·8)	0·5 (0·3 to 0·8)	0·1 (0·1 to 0·2)	202
Maternal and neonatal disorders	79·3 (63 to 99)	45·6 (34 to 58·9)	30·6 (22·3 to 40·2)	18·2 (13 to 24·1)	9·1 (6·6 to 12·1)	3·1 (2·3 to 4·2)	1·1 (0·8 to 1·6)	0·2 (0·1 to 0·4)	0·1 (0 to 0·1)	0 (0 to 0·1)	0 (0 to 0)	9,830
Maternal disorders	23·1 (14·5 to 33·6)	2·9 (1·9 to 4·1)	0 (0 to 0)	0 (0 to 0)	0 (0 to 0)	0 (0 to 0)	0 (0 to 0)	0 (0 to 0)	0 (0 to 0)	0 (0 to 0)	0 (0 to 0)	1,570
Maternal hemorrhage	3·1 (1·7 to 5·5)	0·2 (0·1 to 0·3)	0 (0 to 0)	0 (0 to 0)	0 (0 to 0)	0 (0 to 0)	0 (0 to 0)	0 (0 to 0)	0 (0 to 0)	0 (0 to 0)	0 (0 to 0)	311
Maternal sepsis and other maternal infections	0·6 (0·3 to 1)	0 (0 to 0·1)	0 (0 to 0)	0 (0 to 0)	0 (0 to 0)	0 (0 to 0)	0 (0 to 0)	0 (0 to 0)	0 (0 to 0)	0 (0 to 0)	0 (0 to 0)	57·7
Maternal hypertensive disorders	2·2 (1·3 to 3·4)	0·2 (0·1 to 0·4)	0 (0 to 0)	0 (0 to 0)	0 (0 to 0)	0 (0 to 0)	0 (0 to 0)	0 (0 to 0)	0 (0 to 0)	0 (0 to 0)	0 (0 to 0)	224
Maternal obstructed labor and uterine rupture	2·1 (0·9 to 4·2)	0·3 (0·1 to 0·6)	0 (0 to 0)	0 (0 to 0)	0 (0 to 0)	0 (0 to 0)	0 (0 to 0)	0 (0 to 0)	0 (0 to 0)	0 (0 to 0)	0 (0 to 0)	107
Maternal abortion and miscarriage	3 (1·4 to 5·8)	0·2 (0·1 to 0·4)	0 (0 to 0)	0 (0 to 0)	0 (0 to 0)	0 (0 to 0)	0 (0 to 0)	0 (0 to 0)	0 (0 to 0)	0 (0 to 0)	0 (0 to 0)	121
Ectopic pregnancy	2·1 (1·2 to 3·5)	0·1 (0 to 0·1)	0 (0 to 0)	0 (0 to 0)	0 (0 to 0)	0 (0 to 0)	0 (0 to 0)	0 (0 to 0)	0 (0 to 0)	0 (0 to 0)	0 (0 to 0)	128
Indirect maternal deaths	1 (0·5 to 1·7)	0·1 (0·1 to 0·2)	--	--	--	--	--	--	--	--	--	NA
Late maternal deaths	1 (0·6 to 1·6)	0·1 (0·1 to 0·2)	--	--	--	--	--	--	--	--	--	NA
Maternal deaths aggravated by HIV/AIDS	0·3 (0·1 to 0·5)	0 (0 to 0·1)	--	--	--	--	--	--	--	--	--	NA
Neonatal disorders	56·3 (42·2 to 73·4)	42·8 (31 to 55·9)	30·6 (22·3 to 40·2)	18·2 (13 to 24·1)	9·1 (6·6 to 12·1)	3·1 (2·3 to 4·2)	1·1 (0·8 to 1·6)	0·2 (0·1 to 0·4)	0·1 (0 to 0·1)	0 (0 to 0·1)	0 (0 to 0)	8,260
Neonatal preterm birth	31·7 (22·7 to 41·8)	23·1 (16·3 to 31·4)	15·5 (10·8 to 20·8)	8·5 (5·9 to 11·6)	4 (3 to 5·2)	1·4 (1 to 1·9)	0·6 (0·4 to 0·9)	0·1 (0·1 to 0·2)	0·1 (0 to 0·1)	0 (0 to 0·1)	0 (0 to 0)	4,130
Neonatal encephalopathy due to birth asphyxia and trauma	7·4 (3·5 to 12·3)	5·9 (2·7 to 10·2)	4·7 (2 to 8·3)	3·3 (1·7 to 5·5)	1·6 (0·9 to 2·5)	0·4 (0·2 to 0·7)	0·1 (0 to 0·1)	0 (0 to 0)	0 (0 to 0)	0 (0 to 0)	0 (0 to 0)	1,750
Neonatal sepsis and other neonatal infections	13·2 (6·6 to 21·7)	10·5 (5·2 to 17·1)	7·7 (3·7 to 12·9)	4·6 (1·9 to 8·6)	2·4 (1 to 4·6)	0·9 (0·4 to 1·6)	0·3 (0·1 to 0·6)	0 (0 to 0·1)	0 (0 to 0)	0 (0 to 0)	0 (0 to 0)	1,330
Hemolytic disease and other neonatal jaundice	4 (3 to 5·2)	3·3 (2·4 to 4·3)	2·6 (2 to 3·4)	1·8 (1·3 to 2·3)	1 (0·8 to 1·3)	0·4 (0·3 to 0·6)	0·2 (0·1 to 0·2)	0 (0 to 0·1)	0 (0 to 0)	0 (0 to 0)	0 (0 to 0)	110
Other neonatal disorders	0 (0 to 0)	0 (0 to 0)	0 (0 to 0)	0 (0 to 0)	0 (0 to 0)	0 (0 to 0)	0 (0 to 0)	0 (0 to 0)	0 (0 to 0)	0 (0 to 0)	0 (0 to 0)	941
Nutritional deficiencies	240 (146 to 368)	249 (144 to 390)	229 (131 to 361)	161 (94·7 to 257)	120 (68·4 to 183)	73·5 (45·6 to 112)	44·1 (27·9 to 64·5)	29·5 (19·1 to 42·3)	20·3 (13·2 to 29·7)	14·4 (9·7 to 20·7)	5 (3·1 to 7·5)	3,510
Protein-energy malnutrition	22·3 (13·5 to 34)	22·5 (14 to 32·9)	26·8 (16·7 to 40·8)	27·2 (17·3 to 41·9)	24·1 (15 to 37·7)	21·6 (13·7 to 32·7)	21·6 (13·3 to 32·6)	17·6 (11·1 to 26·1)	13·4 (8·2 to 20·3)	10·6 (6·8 to 15·7)	4·3 (2·6 to 6·7)	837
Iodine deficiency	3·1 (1·4 to 5·6)	2·6 (1·1 to 4·8)	2·1 (0·9 to 3·9)	1·6 (0·7 to 2·9)	1·1 (0·4 to 1·9)	0·6 (0·2 to 1·1)	0·4 (0·2 to 0·8)	0·3 (0·2 to 0·4)	0·1 (0·1 to 0·2)	0 (0 to 0·1)	0 (0 to 0)	43·3
Vitamin A deficiency	0·4 (0·2 to 0·7)	0·3 (0·2 to 0·6)	0·3 (0·1 to 0·5)	0·2 (0·1 to 0·4)	0·1 (0·1 to 0·3)	0·1 (0 to 0·2)	0·1 (0 to 0·1)	0 (0 to 0·1)	0 (0 to 0)	0 (0 to 0)	0 (0 to 0)	33·2
Dietary iron deficiency	206 (115 to 333)	217 (115 to 358)	194 (97·5 to 321)	126 (61 to 224)	88·4 (41·1 to 149)	46·7 (20·9 to 81·4)	18·5 (6·9 to 36·9)	9 (2·6 to 18·3)	4·9 (1·6 to 10·4)	2·3 (0·9 to 4·7)	0·3 (0·1 to 0·6)	2,490
Other nutritional deficiencies	8·2 (3·7 to 15·2)	7·3 (3·5 to 13·3)	5·8 (2·6 to 10·8)	6·1 (2·9 to 11·7)	6 (2·6 to 11·3)	4·4 (1·8 to 8)	3·5 (1·4 to 6·7)	2·6 (1·1 to 4·8)	1·9 (0·6 to 3·5)	1·3 (0·4 to 2·7)	0·3 (0·1 to 0·6)	103
Non-communicable diseases	7,680 (6,180 to 9,380)	9,040 (7,350 to 11,100)	10,000 (8,210 to 12,200)	9,980 (8,260 to 11,900)	8,710 (7,310 to 10,200)	6,290 (5,310 to 7,360)	4,790 (4,070 to 5,550)	3,300 (2,870 to 3,750)	1,740 (1,510 to 1,990)	971 (874 to 1,060)	254 (234 to 275)	98,600
Neoplasms	1,410 (1,020 to 1,880)	1,600 (1,170 to 2,140)	1,710 (1,290 to 2,270)	1,510 (1,160 to 1,970)	1,160 (911 to 1,460)	727 (578 to 899)	464 (368 to 563)	265 (214 to 323)	115 (90·9 to 141)	52·9 (42 to 63·3)	11·2 (8·8 to 13·8)	12,700
Lip and oral cavity cancer	7·2 (4·5 to 10·8)	10·1 (6·4 to 14·9)	11·9 (7·3 to 18)	12·2 (8 to 18·2)	10·8 (7·1 to 15·8)	7·7 (5·2 to 11·1)	5·5 (3·8 to 8)	3·3 (2·2 to 4·9)	1·4 (1 to 2·1)	0·7 (0·5 to 1)	0·2 (0·1 to 0·3)	87·8
Nasopharynx cancer	2·4 (1·4 to 3·8)	2·6 (1·6 to 4·2)	2·8 (1·7 to 4·2)	2·5 (1·4 to 3·8)	1·8 (1·1 to 2·8)	1·1 (0·7 to 1·7)	0·6 (0·4 to 0·9)	0·3 (0·2 to 0·5)	0·3 (0·2 to 0·4)	0·1 (0·1 to 0·2)	0 (0 to 0)	23
Other pharynx cancer	2·9 (1·6 to 4·5)	3·5 (2 to 5·4)	4·3 (2·6 to 6·7)	4·4 (2·8 to 6·9)	2·9 (1·7 to 4·4)	2·1 (1·3 to 3·1)	1·3 (0·8 to 1·9)	0·7 (0·4 to 1·1)	0·3 (0·2 to 0·5)	0·2 (0·1 to 0·3)	0 (0 to 0·1)	27·1
Esophageal cancer	8·8 (5·4 to 13·1)	13·9 (8·6 to 20·8)	15·9 (10 to 24·2)	17·4 (11·5 to 26)	13·9 (9 to 20·5)	9·4 (6·3 to 13·6)	6·8 (4·4 to 9·8)	3·9 (2·5 to 5·7)	1·7 (1·1 to 2·4)	0·8 (0·5 to 1·1)	0·2 (0·1 to 0·3)	103
Stomach cancer	55·9 (36·8 to 82·9)	69·3 (45·5 to 99·9)	77·2 (53 to 110)	81·2 (56·8 to 113)	66·2 (47·1 to 90·7)	49·5 (36·1 to 66·9)	38·4 (28 to 50·6)	23·3 (16·9 to 31·3)	11·2 (8·1 to 15·1)	5·4 (3·9 to 7·4)	1·1 (0·8 to 1·6)	597
Colon and rectum cancer	85·4 (53·2 to 129)	126 (80·2 to 187)	150 (93·8 to 223)	149 (99·4 to 215)	128 (89·4 to 182)	86·9 (60 to 121)	71·9 (50·3 to 96·5)	48·5 (34·1 to 65·6)	23·2 (15·8 to 32·4)	10·9 (7·7 to 14·8)	2·1 (1·4 to 3)	1,050
Liver cancer	18·5 (10·6 to 29·3)	25·8 (15·1 to 40·2)	32·9 (18·4 to 52)	30·6 (18·6 to 48)	27·5 (17·3 to 41·7)	18·8 (12 to 27·1)	13·1 (8·3 to 19·5)	7·5 (5·1 to 10·8)	2·4 (1·6 to 3·4)	0·9 (0·7 to 1·3)	0·2 (0·1 to 0·3)	221
Liver cancer due to hepatitis B	6·5 (3 to 11·6)	7·8 (3·7 to 13·7)	8·9 (4·1 to 16·5)	7 (3·3 to 13·1)	5 (2·3 to 9·3)	2·8 (1·2 to 5·3)	1·7 (0·8 to 3·1)	0·8 (0·3 to 1·5)	0·2 (0·1 to 0·4)	0·1 (0 to 0·2)	0 (0 to 0)	56·5
Liver cancer due to hepatitis C	3·4 (1·4 to 6·5)	6 (2·6 to 11·1)	9·1 (3·8 to 17)	9·3 (4·3 to 17·4)	8·8 (4·6 to 15·3)	6·5 (3·6 to 11·2)	5·1 (2·6 to 8·5)	3·3 (2 to 5·3)	1·2 (0·7 to 1·9)	0·5 (0·3 to 0·8)	0·1 (0·1 to 0·2)	56·7
Liver cancer due to alcohol use	4·2 (1·7 to 7·8)	6·2 (2·8 to 11·4)	8 (3·4 to 14·7)	8·1 (3·6 to 15·2)	8·1 (4·1 to 14·3)	5·5 (2·8 to 9·3)	3·3 (1·7 to 5·7)	1·5 (0·8 to 2·6)	0·4 (0·2 to 0·7)	0·1 (0·1 to 0·2)	0 (0 to 0·1)	50·9
Liver cancer due to NASH	2 (0·9 to 3·8)	2·9 (1·3 to 5·3)	3·7 (1·6 to 7·1)	3·5 (1·6 to 6·5)	3·4 (1·6 to 6·2)	2·6 (1·3 to 4·5)	2·1 (1 to 3·6)	1·2 (0·6 to 2·3)	0·4 (0·2 to 0·7)	0·1 (0·1 to 0·2)	0 (0 to 0·1)	26·6
Liver cancer due to other causes	2·4 (1·1 to 4·6)	2·9 (1·3 to 5·6)	3·2 (1·4 to 6·3)	2·7 (1·3 to 5·2)	2·2 (1 to 4·1)	1·4 (0·6 to 2·6)	1 (0·4 to 1·8)	0·5 (0·3 to 1)	0·2 (0·1 to 0·3)	0·1 (0 to 0·1)	0 (0 to 0)	30·1
Gallbladder and biliary tract cancer	10·7 (6·4 to 18)	16·8 (10·3 to 26·6)	21·6 (13·2 to 34·4)	21·7 (13·5 to 35·9)	18·1 (11·4 to 28·9)	12·1 (7·7 to 20)	8·1 (5·1 to 13·6)	4·3 (2·7 to 7·2)	1·5 (1 to 2·7)	0·7 (0·4 to 1·1)	0·1 (0·1 to 0·2)	128
Pancreatic cancer	39·7 (24·8 to 59·3)	57·4 (36·4 to 85·4)	73·3 (47·8 to 107)	85·6 (56·7 to 126)	75·4 (50·6 to 106)	54·1 (37·4 to 74·3)	33·2 (22·3 to 45·7)	18·9 (12·5 to 27·1)	8·4 (5·7 to 11·9)	3·6 (2·5 to 5·1)	0·7 (0·5 to 1)	502
Larynx cancer	1·9 (1·1 to 3·1)	2·9 (1·7 to 4·6)	3·8 (2·3 to 5·9)	4·3 (2·7 to 6·5)	3·2 (1·9 to 4·9)	1·9 (1·2 to 2·8)	1·1 (0·7 to 1·7)	0·6 (0·4 to 0·8)	0·3 (0·2 to 0·4)	0·1 (0·1 to 0·2)	0 (0 to 0)	22·6
Tracheal, bronchus, and lung cancer	53·4 (34·6 to 79·9)	79·4 (50·4 to 120)	95 (60·2 to 140)	95·5 (63·4 to 136)	77·3 (53·9 to 106)	44·9 (32·4 to 61)	27·5 (19·2 to 37·6)	14 (9·6 to 19·8)	5·7 (3·9 to 8)	2·3 (1·5 to 3·2)	0·5 (0·3 to 0·7)	567
Malignant skin melanoma	3·6 (2 to 5·8)	3·8 (2·1 to 6·4)	3·5 (2 to 5·7)	3·4 (2 to 5·2)	2·8 (1·5 to 4·4)	1·8 (1·1 to 2·9)	1 (0·6 to 1·6)	0·7 (0·4 to 1·1)	0·4 (0·2 to 0·7)	0·2 (0·1 to 0·3)	0·1 (0 to 0·1)	35·6
Non-melanoma skin cancer	1·8 (0·9 to 3)	3 (1·5 to 5·1)	2·8 (1·6 to 4·5)	2·5 (1·4 to 3·9)	2·3 (1·4 to 3·5)	1·8 (1·1 to 2·6)	1·8 (1·1 to 2·6)	1·4 (0·9 to 2·1)	1 (0·6 to 1·5)	0·8 (0·4 to 1·4)	0·3 (0·1 to 0·5)	26·8
Non-melanoma skin cancer (squamous-cell carcinoma)	1·8 (0·9 to 3)	3 (1·5 to 5·1)	2·8 (1·6 to 4·5)	2·5 (1·4 to 3·9)	2·3 (1·4 to 3·5)	1·8 (1·1 to 2·6)	1·8 (1·1 to 2·6)	1·4 (0·9 to 2·1)	1 (0·6 to 1·5)	0·8 (0·4 to 1·4)	0·3 (0·1 to 0·5)	26·8
Non-melanoma skin cancer (basal-cell carcinoma)	0 (0 to 0)	0 (0 to 0)	0 (0 to 0)	0 (0 to 0)	0 (0 to 0)	0 (0 to 0)	0 (0 to 0)	0 (0 to 0)	0 (0 to 0)	0 (0 to 0)	0 (0 to 0)	0
Breast cancer	420 (274 to 600)	441 (291 to 635)	440 (304 to 622)	334 (232 to 462)	222 (159 to 304)	130 (93·7 to 171)	75 (53·3 to 101)	43·7 (31 to 60·7)	20·9 (15·1 to 28·6)	9·8 (7 to 13·1)	2·4 (1·5 to 3·6)	2,850
Cervical cancer	348 (224 to 506)	347 (230 to 507)	342 (225 to 508)	256 (171 to 379)	178 (119 to 251)	107 (75·9 to 151)	58·9 (41·3 to 81·5)	29·6 (20·6 to 41·7)	10·4 (7·3 to 14·4)	4·7 (3·1 to 6·9)	1·1 (0·7 to 1·5)	2,570
Uterine cancer	66·9 (40·9 to 101)	78·4 (49 to 118)	89·8 (56·5 to 139)	113 (71·7 to 168)	90·2 (56·8 to 132)	51·4 (33·6 to 75·4)	29·3 (19·1 to 42·6)	15·4 (10 to 22·9)	5·5 (3·7 to 7·8)	2·5 (1·7 to 3·5)	0·5 (0·4 to 0·7)	664
Ovarian cancer	118 (69 to 179)	129 (77·1 to 193)	131 (79·5 to 203)	110 (70 to 168)	86 (53·1 to 127)	44·8 (29·9 to 64)	25·5 (16·7 to 36)	12·7 (8·1 to 18·9)	5·3 (3·5 to 7·9)	2·2 (1·4 to 3·3)	0·3 (0·2 to 0·5)	929
Prostate cancer	0 (0 to 0)	0 (0 to 0)	0 (0 to 0)	0 (0 to 0)	0 (0 to 0)	0 (0 to 0)	0 (0 to 0)	0 (0 to 0)	0 (0 to 0)	0 (0 to 0)	0 (0 to 0)	0
Testicular cancer	--	--	--	--	--	--	--	--	--	--	--	NA
Kidney cancer	18·5 (11 to 29·1)	20·9 (12·8 to 32·8)	25 (15 to 40·1)	22·4 (13·6 to 34)	16·8 (10·5 to 26·3)	10·1 (6·4 to 15·1)	5·5 (3·4 to 8·5)	2·9 (1·8 to 4·5)	1 (0·6 to 1·5)	0·4 (0·3 to 0·7)	0·1 (0·1 to 0·1)	190
Bladder cancer	4·3 (2·7 to 6·5)	8·2 (5·3 to 12·3)	10·8 (7 to 16·1)	10·8 (7·4 to 15·8)	11·1 (7·3 to 16·1)	8·5 (5·8 to 12·1)	7·1 (4·9 to 10·1)	4·7 (3·1 to 6·7)	2·2 (1·5 to 3·3)	1·2 (0·8 to 1·7)	0·3 (0·2 to 0·4)	76·5
Brain and central nervous system cancer	12·3 (5·7 to 21·3)	13·9 (6·5 to 23·7)	14·6 (6·5 to 24·7)	11·6 (5·3 to 19·2)	8·9 (4·1 to 14·6)	4·9 (2 to 8·3)	3 (1·2 to 5·4)	1·3 (0·5 to 2·5)	0·5 (0·2 to 0·9)	0·2 (0·1 to 0·3)	0 (0 to 0·1)	181
Thyroid cancer	6·2 (3·6 to 10·1)	9·3 (5·4 to 14·9)	10·3 (6·3 to 15·7)	9·4 (6 to 14·4)	8·2 (5·5 to 12·1)	5·7 (3·7 to 8·3)	3·4 (2·1 to 5·1)	1·9 (1·2 to 2·9)	0·7 (0·4 to 1)	0·3 (0·2 to 0·5)	0·1 (0 to 0·1)	72·3
Mesothelioma	5·8 (3·5 to 9·1)	7·8 (4·5 to 11·8)	7·7 (4·9 to 12·4)	2·6 (1·6 to 4)	1·8 (1·2 to 2·7)	1·8 (1·1 to 2·9)	0·6 (0·4 to 1·1)	0·5 (0·3 to 0·9)	0·1 (0·1 to 0·2)	0·1 (0·1 to 0·1)	0 (0 to 0)	49
Hodgkin lymphoma	3·2 (1·8 to 5·2)	2·8 (1·5 to 4·6)	2·9 (1·7 to 4·6)	2·4 (1·5 to 3·7)	2·1 (1·4 to 3·2)	1·2 (0·8 to 1·9)	1 (0·6 to 1·5)	0·5 (0·3 to 0·9)	0·2 (0·1 to 0·3)	0 (0 to 0·1)	0 (0 to 0)	59·7
Non-Hodgkin lymphoma	23·5 (14·9 to 34·9)	26 (16·2 to 39·1)	28·6 (18 to 43·2)	27·8 (18·1 to 40·8)	22·4 (14·6 to 32·5)	14·1 (9·5 to 20·7)	8·6 (5·7 to 12·3)	4·2 (2·8 to 6·1)	1·4 (0·9 to 2)	0·6 (0·4 to 0·8)	0·1 (0·1 to 0·2)	301
Multiple myeloma	9·3 (5·5 to 14·4)	15·7 (8·8 to 24·6)	22·1 (13·1 to 33·5)	22·2 (14·2 to 32·9)	18·4 (11·5 to 26·9)	12·5 (8·3 to 18·2)	7·6 (5 to 10·9)	3·6 (2·3 to 5·6)	1·3 (0·8 to 2·2)	0·4 (0·3 to 0·7)	0·1 (0 to 0·1)	124
Leukemia	32 (20·2 to 47·7)	30·6 (19·5 to 47·6)	33·1 (21·3 to 50)	29·4 (18·9 to 44·9)	22·2 (13·4 to 33·6)	15·1 (9·7 to 22·3)	9·8 (6·1 to 14·4)	5·5 (3·5 to 8·5)	2·3 (1·4 to 3·6)	1·1 (0·6 to 1·6)	0·2 (0·1 to 0·3)	520
Acute lymphoid leukemia	4 (1·9 to 7·2)	4·2 (2·1 to 7·8)	3·6 (1·7 to 6·4)	2·6 (1·2 to 4·8)	1·7 (0·8 to 3·2)	0·9 (0·5 to 1·7)	0·7 (0·3 to 1·4)	0·4 (0·2 to 0·7)	0·1 (0 to 0·1)	0 (0 to 0·1)	0 (0 to 0)	165
Chronic lymphoid leukemia	0·7 (0·3 to 1·3)	2·5 (1·2 to 4·7)	3·6 (1·7 to 7)	4·5 (2·2 to 8·1)	4·1 (1·9 to 7·1)	2·5 (1·2 to 4·5)	1·7 (0·8 to 3)	0·9 (0·4 to 1·6)	0·4 (0·2 to 0·7)	0·2 (0·1 to 0·3)	0 (0 to 0·1)	24·6
Acute myeloid leukemia	4 (1·9 to 7·4)	3·1 (1·5 to 5·6)	2·9 (1·3 to 5·2)	2·3 (1·1 to 4·6)	1·4 (0·6 to 2·6)	0·8 (0·4 to 1·6)	0·4 (0·2 to 0·8)	0·2 (0·1 to 0·4)	0·1 (0 to 0·1)	0 (0 to 0)	0 (0 to 0)	48·3
Chronic myeloid leukemia	5·4 (2·7 to 9·6)	6·8 (3·4 to 12)	7·3 (3·5 to 12·9)	6·4 (3·2 to 11·8)	4 (1·8 to 7·4)	2·8 (1·4 to 4·9)	1·7 (0·8 to 2·9)	1 (0·5 to 1·9)	0·4 (0·2 to 0·7)	0·2 (0·1 to 0·3)	0 (0 to 0·1)	69·1
Other leukemia	17·9 (10 to 28·2)	14 (8·1 to 22·6)	15·8 (9·1 to 25·3)	13·6 (7·7 to 22·8)	11·1 (6·3 to 18·2)	8·1 (4·9 to 12·8)	5·2 (3 to 8·3)	3 (1·8 to 5)	1·4 (0·8 to 2·4)	0·6 (0·4 to 1)	0·1 (0·1 to 0·2)	213
Other malignant neoplasms	47·5 (31·5 to 69·2)	54·5 (37 to 78·2)	53·6 (35·4 to 79·8)	48·1 (32 to 70)	38·7 (27·6 to 54·4)	24·7 (17 to 33·9)	16·1 (10·7 to 22·1)	9·8 (6·7 to 13·6)	5 (3·2 to 7·3)	2·3 (1·6 to 3·3)	0·5 (0·3 to 0·7)	651

Other neoplasms	0·1 (0 to 0·1)	0·1 (0 to 0·2)	0·8 (0·3 to 1·6)	2 (1·1 to 3·4)	0·6 (0·3 to 0·9)	0·8 (0·4 to 1·4)	1 (0·6 to 1·5)	1·1 (0·7 to 1·6)	1·6 (0·9 to 2·5)	1·3 (0·8 to 2·1)	0·9 (0·5 to 1·3)	2·2 (1·4 to 3·4)	1·9 (1·2 to 2·8)	2·4 (1·5 to 3·5)	2·6 (1·7 to 3·8)	3 (2·1 to 4·3)	2·9 (2 to 4)	2·4 (1·7 to 3·4)	1·9 (1·3 to 2·6)	1·1 (0·8 to 1·6)	0·5 (0·3 to 0·7)	0·2 (0·1 to 0·3)	0 (0 to 0·1)	31·1
Myelodysplastic, myeloproliferative, and other hematopoietic r	0·1 (0 to 0·1)	0·1 (0 to 0·2)	0·8 (0·3 to 1·6)	2 (1·1 to 3·4)	0·6 (0·3 to 0·9)	0·8 (0·4 to 1·4)	1 (0·6 to 1·5)	1·1 (0·7 to 1·6)	1·6 (0·9 to 2·5)	1·3 (0·8 to 2·1)	0·9 (0·5 to 1·3)	2·2 (1·4 to 3·4)	1·9 (1·2 to 2·8)	2·4 (1·5 to 3·5)	2·6 (1·7 to 3·8)	3 (2·1 to 4·3)	2·9 (2 to 4)	2·4 (1·7 to 3·4)	1·9 (1·3 to 2·6)	1·1 (0·8 to 1·6)	0·5 (0·3 to 0·7)	0·2 (0·1 to 0·3)	0 (0 to 0·1)	31·1
Benign and in situ intestinal neoplasms	0 (0 to 0)	0 (0 to 0)	0 (0 to 0)	0 (0 to 0)	0 (0 to 0)	0 (0 to 0)	0 (0 to 0)	0 (0 to 0)	0 (0 to 0)	0 (0 to 0)	0 (0 to 0)	0 (0 to 0)	0 (0 to 0)	0 (0 to 0)	0 (0 to 0)	0 (0 to 0)	0 (0 to 0)	0 (0 to 0)	0 (0 to 0)	0 (0 to 0)	0 (0 to 0)	0 (0 to 0)	0 (0 to 0)	0
Benign and in situ cervical and uterine neoplasms	0 (0 to 0)	0 (0 to 0)	0 (0 to 0)	0 (0 to 0)	0 (0 to 0)	0 (0 to 0)	0 (0 to 0)	0 (0 to 0)	0 (0 to 0)	0 (0 to 0)	0 (0 to 0)	0 (0 to 0)	0 (0 to 0)	0 (0 to 0)	0 (0 to 0)	0 (0 to 0)	0 (0 to 0)	0 (0 to 0)	0 (0 to 0)	0 (0 to 0)	0 (0 to 0)	0 (0 to 0)	0 (0 to 0)	0
Other benign and in situ neoplasms	0 (0 to 0)	0 (0 to 0)	0 (0 to 0)	0 (0 to 0)	0 (0 to 0)	0 (0 to 0)	0 (0 to 0)	0 (0 to 0)	0 (0 to 0)	0 (0 to 0)	0 (0 to 0)	0 (0 to 0)	0 (0 to 0)	0 (0 to 0)	0 (0 to 0)	0 (0 to 0)	0 (0 to 0)	0 (0 to 0)	0 (0 to 0)	0 (0 to 0)	0 (0 to 0)	0 (0 to 0)	0 (0 to 0)	0
Cardiovascular diseases	38·8 (20·2 to 66·1)	3·4 (1·8 to 5·8)	63 (35·6 to 106)	42·5 (28 to 61·1)	62·7 (50·2 to 76·5)	93·7 (71·5 to 118)	211 (153 to 276)	388 (266 to 541)	468 (321 to 684)	504 (340 to 722)	674 (469 to 935)	1,150 (829 to 1,550)	1,740 (1,290 to 2,290)	2,460 (1,820 to 3,190)	2,970 (2,280 to 3,850)	3,430 (2,690 to 4,320)	3,430 (2,770 to 4,210)	2,770 (2,280 to 3,340)	2,310 (1,900 to 2,740)	1,670 (1,390 to 1,970)	898 (738 to 1,060)	538 (441 to 613)	145 (117 to 164)	26,100
Rheumatic heart disease	0 (0 to 0)	0 (0 to 0)	0 (0 to 0)	2·4 (1·4 to 3·7)	11·7 (7·8 to 17·2)	24·5 (15·9 to 36·3)	40·5 (26·5 to 60·1)	55·9 (36·8 to 81·2)	62·2 (41·8 to 88·5)	51·8 (34·4 to 74·4)	52·5 (36·4 to 72)	59·3 (41·6 to 80·6)	49 (35·3 to 65·1)	42·3 (28·9 to 59·6)	34 (24·1 to 47·3)	21·1 (15·3 to 28·4)	14·6 (10·5 to 19·5)	8·2 (6·2 to 10·7)	5·4 (4 to 7·1)	3·1 (2·3 to 4·2)	1·5 (1·1 to 2)	0·9 (0·6 to 1·2)	0·3 (0·2 to 0·4)	541
Ischemic heart disease	0 (0 to 0)	0 (0 to 0)	0 (0 to 0)	0 (0 to 0)	0 (0 to 0)	0 (0 to 0)	21·2 (12·9 to 31·6)	48·5 (28·4 to 77·7)	65·9 (39·7 to 105)	95 (55·5 to 149)	157 (97·6 to 228)	329 (217 to 476)	551 (384 to 749)	875 (623 to 1,180)	1,120 (805 to 1,480)	1,360 (1,020 to 1,740)	1,430 (1,100 to 1,820)	1,100 (870 to 1,370)	910 (727 to 1,130)	663 (527 to 803)	369 (289 to 453)	238 (185 to 288)	66·6 (51·9 to 80·4)	9,400
Stroke	20·4 (9·2 to 38·1)	0·5 (0·2 to 1·1)	10·8 (4·6 to 20·8)	8·2 (4·8 to 12·8)	17·2 (12·3 to 22·9)	27·1 (18·7 to 37·5)	68·8 (48·3 to 92·7)	126 (81·7 to 182)	161 (105 to 238)	179 (116 to 263)	223 (144 to 337)	397 (261 to 579)	655 (441 to 941)	951 (657 to 1,320)	1,150 (817 to 1,590)	1,290 (939 to 1,710)	1,280 (978 to 1,660)	1,110 (843 to 1,430)	929 (701 to 1,160)	648 (498 to 819)	306 (228 to 386)	156 (114 to 198)	38·5 (28·7 to 48·5)	9,760
Ischemic stroke	6·5 (2·8 to 12·6)	0·1 (0 to 0·1)	0·5 (0·3 to 1)	2 (1·3 to 2·8)	4·9 (3 to 7·4)	6·4 (4·1 to 9·6)	15·4 (11 to 21·2)	29·1 (19·6 to 41·4)	30·6 (20·5 to 42·8)	35·9 (24·7 to 50·9)	42·7 (29·2 to 60·5)	75·1 (52·8 to 104)	113 (80·2 to 155)	166 (118 to 229)	227 (168 to 309)	392 (282 to 519)	428 (327 to 545)	639 (483 to 820)	563 (426 to 707)	497 (381 to 627)	239 (177 to 302)	121 (88·2 to 155)	30·3 (22·4 to 38·5)	3,670
Intracerebral hemorrhage	8 (3·1 to 16·3)	0·2 (0·1 to 0·5)	6·8 (2·8 to 13·6)	2·9 (1·3 to 5·2)	6·8 (4·5 to 9·6)	11·3 (7·1 to 16·6)	36 (23·8 to 50·8)	69·1 (43·4 to 102)	90·2 (56·9 to 136)	97·8 (61·1 to 149)	123 (76·3 to 193)	233 (148 to 348)	438 (294 to 633)	668 (453 to 936)	803 (562 to 1,110)	825 (589 to 1,110)	793 (594 to 1,040)	445 (332 to 587)	346 (262 to 440)	141 (105 to 180)	62 (44·6 to 79·6)	32·5 (23·4 to 42·4)	7·6 (5·5 to 9·8)	5,250
Subarachnoid hemorrhage	5·8 (2·3 to 11·8)	0·2 (0·1 to 0·6)	3·4 (1·4 to 7·1)	3·3 (1·5 to 5·9)	5·5 (3·4 to 7·9)	9·4 (5·6 to 14·9)	17·4 (10·4 to 26·2)	28 (15·2 to 43·9)	40·4 (22·5 to 65·3)	45 (25·7 to 74·8)	57·3 (31·8 to 96·5)	88·9 (51·1 to 142)	104 (60·1 to 165)	117 (72·6 to 177)	117 (72·2 to 179)	75·5 (46·4 to 115)	63·2 (41·3 to 92·9)	31·2 (19·5 to 45·7)	20·9 (13·2 to 31·9)	10·3 (6·5 to 15·9)	4·4 (2·8 to 6·7)	2·3 (1·4 to 3·4)	0·5 (0·3 to 0·8)	851
Hypertensive heart disease	0 (0 to 0)	0 (0 to 0)	0 (0 to 0)	0 (0 to 0)	0 (0 to 0)	0 (0 to 0)	9·7 (5·3 to 15·3)	28 (15·6 to 43·8)	35·7 (19·2 to 59·6)	58·5 (33 to 93·5)	98·7 (58·2 to 158)	174 (103 to 276)	269 (169 to 393)	359 (230 to 545)	406 (264 to 599)	473 (309 to 670)	453 (313 to 634)	345 (239 to 507)	294 (207 to 421)	228 (160 to 326)	136 (96·3 to 195)	86·8 (58·9 to 124)	24·2 (16·2 to 34·2)	3,480
Non-rheumatic valvular heart disease	0 (0 to 0)	0 (0 to 0)	0 (0 to 0)	0 (0 to 0)	0 (0 to 0)	0 (0 to 0)	8·7 (5·2 to 13·7)	9·4 (4·9 to 16·2)	9·2 (5·1 to 16·3)	8·9 (4·7 to 15·3)	9·8 (5·8 to 15·8)	13·4 (7·8 to 20·7)	16·8 (10·5 to 25·5)	16·6 (10·3 to 25·2)	17·8 (11·6 to 26·3)	18·9 (12·7 to 27·4)	15·1 (10·1 to 21·5)	10 (6·5 to 14·4)	7·6 (5·2 to 10·4)	5·1 (3·5 to 7·3)	2·9 (1·9 to 4·1)	1·6 (1 to 2·3)	0·3 (0·2 to 0·5)	172
Non-rheumatic calcific aortic valve disease	0 (0 to 0)	0 (0 to 0)	0 (0 to 0)	0 (0 to 0)	0 (0 to 0)	0 (0 to 0)	2·5 (1·1 to 4·9)	4·4 (2·1 to 8·4)	3 (1·3 to 5·7)	2·3 (1 to 4·5)	3 (1·5 to 5·6)	4·6 (2·4 to 8·1)	6·1 (3·3 to 11)	7 (3·6 to 12·4)	8·2 (4·7 to 13·8)	10·8 (6·6 to 16·6)	9·2 (5·7 to 13·7)	6·3 (3·9 to 9·7)	5·3 (3·5 to 7·5)	3·5 (2·3 to 5·3)	2·2 (1·4 to 3·3)	1·1 (0·7 to 1·7)	0·2 (0·1 to 0·4)	79·9
Non-rheumatic degenerative mitral valve disease	0 (0 to 0)	0 (0 to 0)	0 (0 to 0)	0 (0 to 0)	0 (0 to 0)	0 (0 to 0)	3·9 (2 to 6·7)	3·7 (1·6 to 7·2)	4·2 (2 to 7·6)	3·9 (1·8 to 7·3)	4·7 (2·5 to 8·1)	6·9 (3·7 to 11·4)	7·4 (3·9 to 12·3)	7·4 (4 to 12·5)	7·2 (3·8 to 11·8)	6·7 (3·8 to 11)	4·5 (2·6 to 7·7)	2·4 (1·2 to 4)	1·6 (0·9 to 2·7)	1 (0·5 to 1·8)	0·4 (0·2 to 0·8)	0·3 (0·2 to 0·6)	0·1 (0 to 0·1)	66·5
Other non-rheumatic valve diseases	0 (0 to 0)	0 (0 to 0)	0 (0 to 0)	0 (0 to 0)	0 (0 to 0)	0 (0 to 0)	2·3 (1·1 to 4·4)	1·3 (0·5 to 2·9)	2 (0·7 to 4·2)	2·7 (1·1 to 5·4)	2·1 (0·9 to 3·9)	1·8 (0·7 to 3·8)	3·2 (1·3 to 6·7)	2·2 (1 to 4·2)	2·3 (0·9 to 4·9)	1·3 (0·6 to 2·6)	1·3 (0·5 to 2·7)	1·3 (0·6 to 2·5)	0·7 (0·3 to 1·4)	0·6 (0·2 to 1·2)	0·2 (0·1 to 0·5)	0·2 (0·1 to 0·4)	0 (0 to 0·1)	25·6
Cardiomyopathy and myocarditis	6·5 (3·1 to 12·3)	1·3 (0·6 to 2·7)	27·9 (14 to 51·2)	19·2 (11·4 to 28·7)	14·1 (9·4 to 19·7)	20·1 (13·1 to 28·7)	21·1 (12·7 to 32·3)	40·1 (23·2 to 65·9)	40·6 (23·1 to 68·9)	40·2 (23 to 67)	43·9 (26·6 to 67·9)	57·6 (35·5 to 88)	63·1 (39·8 to 98·3)	76·4 (47·5 to 115)	79·3 (49·3 to 118)	79·8 (52·4 to 119)	64 (41·1 to 95·3)	43·5 (27·7 to 64·1)	34·9 (23 to 51·4)	24 (16·1 to 34·3)	16 (10·2 to 23·1)	8·6 (5·7 to 12·1)	2·3 (1·3 to 3·6)	825
Myocarditis	1·6 (0·8 to 2·9)	0·5 (0·2 to 1·1)	10·9 (5·4 to 19·6)	9·1 (5·2 to 14·1)	8·9 (6·2 to 11·8)	8·6 (5·6 to 12·3)	7·3 (4 to 11·6)	11·4 (6·3 to 18·8)	10·7 (5·9 to 18·4)	13·2 (7·3 to 22)	9·6 (5·7 to 15·1)	7·2 (4·3 to 11·1)	12·6 (7·5 to 19·8)	10·8 (5·2 to 20·3)	7·8 (4·4 to 12·9)	7·1 (3·9 to 11·4)	3·3 (1·8 to 5·6)	5·1 (2·6 to 9·5)	4·3 (2·1 to 7·7)	4·2 (2·1 to 7·5)	3·4 (1·6 to 6)	2·2 (1·3 to 3·3)	0·7 (0·4 to 1·1)	160
Alcoholic cardiomyopathy	0 (0 to 0)	0 (0 to 0)	0 (0 to 0)	0 (0 to 0)	0 (0 to 0)	0 (0 to 0)	1·1 (0·5 to 2)	3·3 (1·3 to 6·4)	4·3 (1·4 to 9)	2·3 (0·8 to 4·8)	2·8 (1·1 to 5·7)	3·3 (1·2 to 6·5)	10·2 (4·5 to 18·2)	13 (4·4 to 25·1)	9·4 (4·2 to 16·7)	8·4 (3·1 to 17·8)	8·6 (1·6 to 24·3)	1·1 (0·2 to 3·3)	1·3 (0·2 to 4·3)	0·9 (0·1 to 3·1)	0·2 (0 to 0·6)	0·1 (0 to 0·1)	0 (0 to 0)	70·2
Other cardiomyopathy	4·9 (2 to 9·8)	0·9 (0·3 to 2·1)	17 (6·9 to 31·9)	10·1 (4·6 to 17·4)	5·2 (2·5 to 9·1)	11·5 (6·3 to 18·7)	12·7 (6·7 to 21·1)	25·4 (13·2 to 45·9)	25·5 (12·5 to 46·9)	24·8 (12 to 45·9)	31·5 (17·6 to 52)	47·1 (26·9 to 73·8)	40·2 (21·5 to 69·4)	52·6 (30·6 to 81·6)	62·2 (36·8 to 98·7)	64·3 (38·2 to 99·7)	52·1 (28·3 to 82·7)	37·3 (23·6 to 56)	29·4 (19·1 to 43·8)	18·9 (12·7 to 27·1)	12·4 (7·6 to 18·4)	6·3 (3·7 to 9·7)	1·6 (0·7 to 2·8)	594
Atrial fibrillation and flutter	0 (0 to 0)	0 (0 to 0)	0 (0 to 0)	0 (0 to 0)	0 (0 to 0)	0 (0 to 0)	0 (0 to 0)	0 (0 to 0)	0 (0 to 0)	0·8 (0·4 to 1·3)	1·2 (0·7 to 1·8)	6 (3·8 to 8·8)	8·7 (5·9 to 12·6)	12·5 (8·8 to 17·9)	20·8 (14·8 to 28·8)	28·3 (21·3 to 37·1)	34·2 (25·9 to 45·3)	36·5 (28 to 48·8)	37·9 (29 to 49·5)	39·8 (30·9 to 52·3)	28·9 (21·8 to 39·5)	23·4 (17·5 to 32·2)	6·4 (4·5 to 9·1)	285
Aortic aneurysm	--	--	--	--	--	--	0·8 (0·5 to 1·4)	2·3 (1·2 to 3·8)	4·9 (2·5 to 8·5)	3·7 (2 to 6·1)	6·8 (4·2 to 10·5)	10·5 (6·7 to 15·2)	13·2 (8·6 to 19·3)	18·3 (11·5 to 26·5)	25·7 (17·3 to 37·3)	31 (21·8 to 43·8)	36 (25·5 to 49·3)	28 (20·1 to 37·8)	22 (16·1 to 28·8)	14·8 (10·9 to 19·2)	7·2 (5·1 to 9·6)	3·6 (2·6 to 4·8)	0·9 (0·5 to 1·4)	NA
Endocarditis	0·8 (0·4 to 1·4)	0·3 (0·1 to 0·9)	6 (2·5 to 12)	3·6 (1·7 to 6·9)	5 (2·7 to 8·6)	5·3 (3 to 8·8)	12·6 (6·6 to 22)	30·6 (15·9 to 53·8)	28·9 (15 to 53·4)	18·2 (9 to 31·7)	25·8 (14·1 to 43)	26·8 (14·6 to 44·9)	26·5 (14·6 to 43·8)	22·3 (12·3 to 37)	23·9 (13·5 to 38·2)	22·8 (13·2 to 35·5)	15·3 (9·2 to 24·3)	10·9 (7 to 16·8)	7·7 (4·6 to 12·3)	7 (4·2 to 11·5)	3·5 (2 to 6·2)	2 (1·2 to 3·4)	0·6 (0·3 to 1)	306
Other cardiovascular and circulatory diseases	11·1 (4·6 to 21·9)	1·2 (0·5 to 2·3)	18·3 (7·8 to 35·1)	9 (4·5 to 15·3)	14·7 (9·3 to 20·7)	16·7 (10·9 to 24·2)	27·4 (18·3 to 39·1)	47·3 (30·8 to 68·4)	59·4 (37·3 to 91·3)	47·8 (30 to 72·4)	55·4 (34·6 to 83·5)	79·3 (53·1 to 110)	82·8 (55·4 to 119)	80·7 (54·2 to 111)	92·9 (63·9 to 129)	90·1 (62·8 to 122)	80·4 (53·7 to 114)	62·1 (45·5 to 82·7)	49·7 (35·7 to 64·9)	35 (25·1 to 45·9)	22·9 (16·2 to 29·6)	14 (9·8 to 17·7)	3·7 (2·5 to 5)	1,000
Chronic respiratory diseases	0 (0 to 0)	0 (0 to 0)	0 (0 to 0·1)	169 (102 to 271)	152 (94·9 to 240)	136 (86·4 to 209)	138 (91·1 to 208)	160 (107 to 222)	146 (101 to 203)	119 (87·4 to 160)	120 (89 to 159)	128 (95·6 to 168)	133 (99·9 to 179)	149 (110 to 198)	169 (124 to 226)	168 (122 to 226)	136 (99·8 to 182)	103 (78·5 to 137)	80·8 (57·8 to 109)	54·3 (38·9 to 71·9)	32 (22·8 to 43·5)	17·8 (12·7 to 23·9)	5·1 (3·6 to 6·9)	2,320
Chronic obstructive pulmonary disease	0 (0 to 0)	0 (0 to 0)	0 (0 to 0·1)	3 (1·8 to 5·2)	3·4 (1·2 to 6·1)	5 (2 to 8·4)	10·1 (5·7 to 14·9)	17·1 (10·6 to 24·5)	19·6 (12·9 to 28·4)	19·1 (12·5 to 26·6)	23·7 (16·1 to 32·2)	32·3 (22·6 to 44·4)	42·4 (29·4 to 58·7)	62 (44 to 84·8)	82·9 (58·6 to 116)	102 (71·4 to 143)	84·4 (59·1 to 117)	67·7 (48·8 to 91·1)	55·8 (39·1 to 77·5)	40 (29·1 to 54)	24·8 (17·6 to 33·6)	13·9 (9·9 to 18·4)	4·2 (2·9 to 5·7)	713
Pneumoconiosis	0 (0 to 0)	0 (0 to 0)	0 (0 to 0)	0 (0 to 0)	0 (0 to 0)	0 (0 to 0)	0 (0 to 0·1)	0 (0 to 0)	0 (0 to 0)	0 (0 to 0)	0 (0 to 0·1)	0 (0 to 0·1)	0 (0 to 0·1)	0·1 (0 to 0·1)	0 (0 to 0·1)	0 (0 to 0)	0 (0 to 0·1)	0 (0 to 0·1)	0·1 (0 to 0·1)	0 (0 to 0·1)	0 (0 to 0)	0 (0 to 0)	0 (0 to 0)	0·6
Silicosis	0 (0 to 0)	0 (0 to 0)	0 (0 to 0)	0 (0 to 0)	0 (0 to 0)	0 (0 to 0)	0 (0 to 0)	0 (0 to 0)	0 (0 to 0)	0 (0 to 0)	0 (0 to 0)	0 (0 to 0)	0 (0 to 0)	0 (0 to 0)	0 (0 to 0)	0 (0 to 0)	0 (0 to 0)	0 (0 to 0)	0 (0 to 0)	0 (0 to 0)	0 (0 to 0)	0 (0 to 0)	0 (0 to 0)	0·1
Asbestosis	0 (0 to 0)	0 (0 to 0)	0 (0 to 0)	0 (0 to 0)	0 (0 to 0)	0 (0 to 0)	0 (0 to 0)	0 (0 to 0)	0 (0 to 0)	0 (0 to 0)	0 (0 to 0)	0 (0 to 0)	0 (0 to 0)	0 (0 to 0·1)	0 (0 to 0·1)	0 (0 to 0)	0 (0 to 0)	0 (0 to 0)	0 (0 to 0)	0 (0 to 0)	0 (0 to 0)	0 (0 to 0)	0 (0 to 0)	0·3
Coal workers pneumoconiosis	0 (0 to 0)	0 (0 to 0)	0 (0 to 0)	0 (0 to 0)	0 (0 to 0)	0 (0 to 0)	0 (0 to 0)	0 (0 to 0)	0 (0 to 0)	0 (0 to 0)	0 (0 to 0)	0 (0 to 0)	0 (0 to 0)	0 (0 to 0)	0 (0 to 0)	0 (0 to 0)	0 (0 to 0)	0 (0 to 0)	0 (0 to 0)	0 (0 to 0)	0 (0 to 0)	0 (0 to 0)	0 (0 to 0)	0
Other pneumoconiosis	0 (0 to 0)	0 (0 to 0)	0 (0 to 0)	0 (0 to 0)	0 (0 to 0)	0 (0 to 0)	0 (0 to 0)	0 (0 to 0)	0 (0 to 0)	0 (0 to 0)	0 (0 to 0)	0 (0 to 0)	0 (0 to 0)	0 (0 to 0·1)	0 (0 to 0)	0 (0 to 0)	0 (0 to 0)	0 (0 to 0)	0 (0 to 0)	0 (0 to 0)	0 (0 to 0)	0 (0 to 0)	0 (0 to 0)	0·2
Asthma	0 (0 to 0)	0 (0 to 0)	0 (0 to 0)	150 (87 to 249)	139 (83·5 to 229)	121 (72·6 to 194)	122 (75·4 to 190)	131 (82·9 to 193)	110 (72·1 to 159)	86·5 (59 to 122)	82·5 (57 to 113)	80·8 (56·8 to 110)	75·9 (54·4 to 103)	72 (49·9 to 99·6)	64·5 (44·5 to 89·2)	51·8 (34·4 to 73·3)	40·1 (26·4 to 56·8)	25·3 (17·5 to 35·5)	17 (11·2 to 25)	9·2 (6 to 13·7)	4·5 (2·8 to 6·7)	2·5 (1·5 to 3·8)	0·7 (0·4 to 1·1)	1,390
Interstitial lung disease and pulmonary sarcoidosis	0 (0 to 0)	0 (0 to 0)	0 (0 to 0)	5·3 (2·4 to 10·2)	2·1 (1·1 to 3·6)	1·6 (0·8 to 2·8)	2·2 (0·9 to 4·3)	3·5 (1·6 to 6·7)	5·8 (2·6 to 10·3)	6·8 (3·3 to 12·2)	8 (4 to 13·8)	7·9 (4 to 13·2)	9·6 (5·2 to 15·6)	10·3 (5·5 to 17·7)	12·7 (6·5 to 21·4)	13·2 (7 to 22·1)	11·3 (6·1 to 18·8)	9·8 (5·6 to 15·8)	7·6 (4·1 to 12·9)	4·7 (2·7 to 8·1)	2·5 (1·3 to 4·2)	1·4 (0·8 to 2·4)	0·3 (0·1 to 0·5)	127
Other chronic respiratory diseases	0 (0 to 0)	0 (0 to 0)	0 (0 to 0)	11 (5·2 to 22·6)	7·4 (3·9 to 11·8)	8·9 (4·7 to 13·8)	4 (2·4 to 5·9)	8·3 (5·2 to 12·2)	11·2 (6·7 to 17·6)	6·3 (4 to 9·5)	6·3 (4 to 9·5)	7 (4·2 to 11·3)	5·3 (3·3 to 8·5)	4·6 (2·7 to 7·5)	8·3 (4·4 to 14·2)	1·4 (0·7 to 2·4)	0·6 (0·4 to 1·1)	0·6 (0·3 to 1·1)	0·4 (0·2 to 0·7)	0·3 (0·2 to 0·6)	0·2 (0·1 to 0·3)	0·1 (0 to 0·2)	0 (0 to 0·1)	92·2
Digestive diseases	13·9 (8·4 to 21·1)	3·5 (1·9 to 6·1)	63·6 (40·4 to 96·6)	91·7 (62·7 to 132)	70·9 (56·6 to 89·2)	73·8 (58·1 to 91·2)	109 (85·8 to 139)	209 (153 to 282)	230 (166 to 311)	234 (165 to 318)	281 (207 to 371)	351 (258 to 470)	402 (299 to 525)	474 (360 to 616)	508 (387 to 666)	477 (360 to 604)	337 (261 to 430)	244 (187 to 311)	161 (124 to 205)	109 (81·5 to 144)	53 (39·9 to 66·8)	29·5 (22·6 to 35·6)	7 (5·5 to 8·3)	4,530
Cirrhosis and other chronic liver diseases	0 (0 to 0)	0 (0 to 0)	0 (0 to 0·1)	12·4 (6·8 to 20·8)	12·7 (8·6 to 17·1)	17·7 (11·8 to 24·5)	28·9 (18·7 to 39·8)	56·2 (36 to 83·4)	60·5 (35 to 94·8)	72·1 (43·4 to 110)	104 (65·7 to 155)	146 (95·2 to 209)	183 (126 to 260)	242 (170 to 333)	266 (188 to 359)	240 (169 to 322)	154 (115 to 205)	101 (71·6 to 137)	55·9 (38·7 to 76·8)	34·8 (23·3 to 49·4)	14·1 (9·9 to 19)	7·1 (5·1 to 9)	1·6 (1·2 to 2·1)	1,810
Cirrhosis and other chronic liver diseases due to hepatitis B	0 (0 to 0)	0 (0 to 0)	0 (0 to 0)	0 (0 to 0)	0 (0 to 0)	0·1 (0 to 0·1)	0·2 (0 to 0·4)	1·1 (0·4 to 2·5)	2·3 (0·7 to 5·3)	4·1 (1·7 to 7·8)	7·4 (3·1 to 15·1)	11 (5·1 to 19·9)	13·5 (6·2 to 25·4)	16·4 (7·6 to 31·9)	15·3 (6·7 to 28·4)	11·9 (5·2 to 23·2)	6·7 (3·2 to 12·4)	4·2 (1·8 to 7·8)	2·4 (1·1 to 4·3)	1·5 (0·6 to 2·7)	0·6 (0·3 to 1)	0·3 (0·1 to 0·5)	0·1 (0 to 0·1)	98·8
Cirrhosis and other chronic liver diseases due to hepatitis C	0 (0 to 0)	0 (0 to 0)	0 (0 to 0)	0 (0 to 0·1)	0·1 (0·1 to 0·2)	0·3 (0·1 to 0·6)	0·8 (0·3 to 1·6)	5·1 (2·1 to 10·4)	10·5 (4·3 to 20·7)	16·1 (8 to 28·5)	25·2 (12·3 to 46·4)	35·1 (17·9 to 60·5)	40·7 (21·7 to 69·7)	49·7 (24·8 to 84·3)	49·9 (25·7 to 88·3)	41·7 (19·4 to 76·2)	25·3 (13·9 to 42)	15·8 (8·4 to 26·8)	8·3 (4·3 to 14·8)	5·1 (2·5 to 9·2)	2·2 (1·1 to 3·6)	1·1 (0·6 to 1·9)	0·3 (0·1 to 0·5)	334
Cirrhosis and other chronic liver diseases due to alcohol use	0 (0 to 0)	0 (0 to 0)	0 (0 to 0)	0 (0 to 0)	0 (0 to 0)	0 (0 to 0)	0·1 (0 to 0·2)	2·6 (1 to 5·6)	6·4 (2·4 to 13·3)	14·1 (6·7 to 24·8)	28·9 (13·2 to 50·8)	46·9 (24·5 to 76·8)	63·2 (33·7 to 101)	88·5 (49·3 to 138)	102 (56·4 to 161)	90·9 (53·3 to 144)	53·9 (30·2 to 82·1)	31·6 (17·2 to 49·7)	14·9 (8·4 to 24·7)	8·2 (4·4 to 14·6)	3·2 (1·8 to 5·2)	1·6 (0·8 to 2·6)	0·3 (0·2 to 0·6)	557
Cirrhosis and other chronic liver diseases due to NAFLD	0 (0 to 0)	0 (0 to 0)	0 (0 to 0)	0 (0 to 0)	0 (0 to 0)	0 (0 to 0)	1 (0·4 to 2·2)	5·2 (2·1 to 10·2)	9·7 (3·8 to 19·6)	13·6 (6 to 25·1)	19·7 (9 to 37·1)	29 (13·7 to 51·8)	38·7 (17·8 to 69·6)	52·2 (24·1 to 93·6)	55·4 (26·9 to 95·8)	51·2 (23·6 to 92·6)	35·5 (20·9 to 57·6)	24·9 (13·7 to 40)	14·6 (7·6 to 24·4)	9·4 (4·9 to 15·9)	3·9 (2·3 to 6)	2 (1·1 to 3)	0·4 (0·2 to 0·7)	366
Cirrhosis and other chronic liver diseases due to other causes	0 (0 to 0)	0 (0 to 0)	0 (0 to 0·1)	12·3 (6·8 to 20·7)	12·5 (8·6 to 16·9)	17·3 (11·5 to 24·1)	26·8 (17·4 to 36·9)	42·2 (26·3 to 63·7)	31·6 (15·9 to 52·3)	24·2 (11·9 to 44)	22·5 (9·6 to 42·7)	23·9 (10·8 to 44·3)	26·7 (13·2 to 49·4)	35·4 (16·9 to 63·2)	43·3 (22 to 76·4)	44·8 (22·9 to 75·7)	32·6 (16·4 to 57·2)	24·8 (12·6 to 43·3)	15·8 (8·4 to 25·3)	10·5 (5·6 to 17·3)	4·3 (2·5 to 6·5)	2·1 (1·3 to 3·2)	0·5 (0·2 to 0·8)	454
Upper digestive system diseases	0 (0 to 0)	0 (0 to 0)	0 (0 to 0)	6·8 (4 to 10·3)	7·3 (4·3 to 11·8)	10·8 (7 to 16)	26·3 (16·4 to 42·2)	64·1 (38·6 to 110)	77·5 (44·6 to 127)	74·5 (43·9 to 123)	78·1 (46·3 to 124)	81·8 (52·3 to 125)	89·2 (59·2 to 131)	92·9 (64 to 134)	97·7 (69·1 to 136)	92·4 (64·5 to 130)	71·3 (52·4 to 97·3)	53·2 (39 to 71·9)	38 (26·8 to 52·6)	26·3 (18·3 to 37)	12·9 (9·6 to 16·9)	7·5 (5·5 to 9·6)	1·8 (1·3 to 2·3)	1,010
Peptic ulcer disease	0 (0 to 0)	0 (0 to 0)	0 (0 to 0)	2·6 (1·4 to 4·4)	1·8 (1·3 to 2·5)	3·6 (2·5 to 5·2)	3·8 (2·6 to 5·4)	8·6 (5·2 to 13·4)	9·1 (5·4 to 14·2)	12·3 (7·7 to 18·9)	15·7 (9·1 to 25·5)	19·6 (13·3 to 27·6)	25·7 (17·6 to 37)	30·4 (21 to 41·9)	37·4 (26·2 to 51·8)	42·2 (26·1 to 62·1)	32·6 (23·7 to 44·7)	28·4 (19·5 to 40)	21·8 (13·6 to 32·2)	16·6 (10·5 to 24·6)	8·1 (5·7 to 11)	5·2 (3·6 to 6·9)	1·3 (0·9 to 1·7)	327
Gastritis and duodenitis	0 (0 to 0)	0 (0 to 0)	0 (0 to 0)	4·1 (2·1 to 7)	5·4 (2·5 to 9·9)	4·8 (2·1 to 9)	6·3 (2·8 to 12·3)	9·7 (5·5 to 16)	11·2 (6 to 17·7)	11·2 (6 to 18·5)	13·2 (7·2 to 21·2)	12 (6·4 to 21·9)	17·4 (10·2 to 29·1)	20 (12·4 to 32)	21·9 (13·4 to 35·1)	18·8 (11·9 to 28·3)	15·2 (9·8 to 22·3)	10·6 (6·7 to 15·3)	6·8 (4·3 to 10·1)	3·9 (2·5 to 5·9)	2·1 (1·4 to 3·2)	1·2 (0·7 to 2)	0·3 (0·2 to 0·4)	196
Gastroesophageal reflux disease	0 (0 to 0)	0 (0 to 0)	0 (0 to 0)	0 (0 to 0)	0·1 (0 to 0·2)	2·4 (1 to 4·5)	16·1 (7·8 to 30·1)	45·8 (23·1 to 87·7)	57·2 (27·9 to 104)	50·9 (23·6 to 97·9)	49·2 (23·2 to 90·7)	50·2 (24·1 to 91·2)	46·1 (22·9 to 80·3)	42·5 (20·7 to 75·6)	38·3 (18·1 to 71·3)	31·5 (15·3 to 57)	23·6 (11·9 to 43·1)	14·1 (7·1 to 25)	9·5 (4·8 to 17)	5·7 (2·9 to 10·3)	2·7 (1·4 to 4·8)	1·1 (0·6 to 1·9)	0·2 (0·1 to 0·4)	487
Appendicitis	0 (0 to 0)	0 (0 to 0)	0 (0 to 0)	7·2 (3·4 to 12·3)	10·7 (7 to 15·5)	12·9 (8·2 to 19)	13·1 (7·9 to 20)	15·7 (8·4 to 25)	10·4 (5·6 to 16·9)	8·3 (4·6 to 14)	11·9 (6·6 to 19·3)	9·6 (5·5 to 15·2)	10·1 (5·4 to 16·6)	8·5 (4·7 to 14·4)	9·9 (5·5 to 16·1)	7 (3·9 to 11·4)	6·2 (3·5 to 10·2)	4·5 (2·7 to 7·3)	2·5 (1·5 to 4)	1·6 (1 to 2·5)	1 (0·6 to 1·6)	0·6 (0·4 to 0·9)	0·1 (0·1 to 0·2)	152
Paralytic ileus and intestinal obstruction	7 (4·1 to 10·9)	3·1 (1·7 to 5·2)	54·1 (34·4 to 82·2)	19·2 (10·1 to 31·4)	8·7 (5·5 to 12·6)	7·9 (4·8 to 12·5)	6·1 (3·4 to 10·1)	6·6 (3·7 to 11·5)	8 (4 to 13·6)	7 (3·6 to 12·1)	12·2 (6·5 to 20·3)	13·9 (7·8 to 22·8)	16 (8·8 to 26·3)	19·7 (11·2 to 30·8)	18·9 (11·1 to 29·4)	22·6 (13·8 to 33·7)	19·6 (12·5 to 30·3)	18·7 (11·7 to 28·7)	15·9 (9·4 to 25·5)	13 (7·6 to 21·5)	7·2 (4·2 to 11·6)	4·3 (2·6 to 6·8)	1·1 (0·7 to 1·6)	311
Inguinal, femoral, and abdominal hernia	6·9 (3·9 to 11·4)	0·5 (0·1 to 1·2)	9·5 (5·1 to 16·1)	15 (8·8 to 24·2)	15·5 (8·4 to 26·8)	10·3 (5 to 17·8)	8·8 (4·3 to 14·9)	9·3 (5 to 15·1)	8·7 (4·5 to 14·9)	8·9 (5·1 to 14·4)	9·7 (5·7 to 15·4)	12·5 (7·7 to 18·6)	13·2 (8·3 to 20·1)	14·3 (9·2 to 21)	13·9 (9·3 to 19·6)	13·2 (8·7 to 18·5)	8·5 (5·8 to 12·1)	6·4 (4·2 to 9·1)	4·7 (3·2 to 6·8)	3·3 (2·1 to 4·8)	1·8 (1·1 to 2·6)	1·1 (0·7 to 1·5)	0·2 (0·2 to 0·4)	196
Inflammatory bowel disease	0 (0 to 0)	0 (0 to 0)	0 (0 to 0)	5·6 (3 to 9·5)	2 (1·2 to 3)	1 (0·6 to 1·6)	2·7 (1·7 to 4·4)	6·1 (3·5 to 9·3)	8·7 (4·8 to 14·2)	8 (4·8 to 13)	8·5 (5·1 to 13)	12·9 (7·8 to 19·3)	10 (6·1 to 15·5)	8·5 (5·4 to 12·9)	9·3 (5·7 to 14·1)	8·9 (5·7 to 12·9)	4·8 (3·1 to 7)	4·6 (2·8 to 6·8)	3·2 (2·1 to 4·8)	1·6 (1 to 2·4)	0·9 (0·6 to 1·3)	0·7 (0·4 to 1)	0·1 (0·1 to 0·1)	108
Vascular intestinal disorders	0 (0 to 0)	0 (0 to 0)	0 (0 to 0)	0·3 (0·1 to 0·5)	0·6 (0·3 to 1)	0·1 (0 to 0·1)	0·2 (0·1 to 0·4)	1·1 (0·6 to 2)	1·4 (0·7 to 2·4)	1 (0·5 to 1·8)	1·9 (1·1 to 3·1)	3·1 (1·7 to 5·2)	4·8 (2·7 to 7·5)	5·2 (3·1 to 7·9)	6·1 (3·8 to 8·9)	8·2 (5·2 to 12)	7·1 (4·7 to 10·5)	6·2 (4 to 9)	4·8 (3 to 7)	3·3 (2 to 5·1)	1·8 (1·1 to 2·6)	0·8 (0·6 to 1·2)	0·2 (0·1 to 0·3)	58·2
Gallbladder and biliary diseases	0 (0 to 0)	0 (0 to 0)	0 (0 to 0)	2·1 (1·2 to 3·3)	3·7 (1·5 to 7)	5·3 (2·3 to 9·8)	10·8 (6 to 17·9)	21·7 (13·5 to 33·7)	27·2 (17·1 to 40·6)	29·8 (19·1 to 43·9)	34·4 (22·3 to 50)	42·3 (28·7 to 59·4)	44·9 (30·8 to 62·8)	47·1 (31·7 to 64·3)	47·3 (32·7 to 63·8)	43·5 (29·6 to 59·6)	32 (22·6 to 43·3)	21·7 (15 to 29·8)	15·4 (10·7 to 21·4)	9·9 (6·8 to 14·3)	4·4 (3·1 to 6·2)	2·5 (1·7 to 3·6)	0·6 (0·4 to 0·9)	447
Pancreatitis	0 (0 to 0)	0 (0 to 0)	0 (0 to 0)	1·5 (0·8 to 2·4)	0·9 (0·5 to 1·5)	1·5 (0·8 to 2·5)	4·9 (3·1 to 7·3)	18·6 (10·7 to 29·5)	15·4 (8·3 to 25·9)	13·6 (7·5 to 23·2)	10·8 (6·1 to 17·5)	16 (9·3 to 25·2)	16·8 (9·9 to 26·9)	16·9 (10 to 26·8)	18 (11·2 to 27·4)	17·1 (10·5 to 26·2)	13·1 (8·6 to 19·4)	10·3 (6·3 to 15)	6·8 (4·4 to 10)	4·1 (2·5 to 6·2)	1·9 (1·2 to 2·8)	0·9 (0·6 to 1·3)	0·2 (0·2 to 0·3)	189
Other digestive diseases	0 (0 to 0)	0 (0 to 0)	0 (0 to 0)	21·8 (11·2 to 38·9)	8·8 (4·9 to 13·1)	6·3 (3·3 to 9·9)	7·6 (3·6 to 13·1)	9·5 (4·4 to 16·6)	12 (5·4 to 22·1)	10·5 (4·8 to 18·5)	9·9 (4·8 to 17·5)	12·7 (6·3 to 21·5)	14·2 (7·3 to 24·6)	19 (9·8 to 32·2)	21 (10·8 to 34·2)	23·7 (12·2 to 40·5)	19·9 (10·8 to 33·1)	16·9 (8·8 to 28·2)	13·7 (7·4 to 21·9)	11·2 (5·7 to 18·2)	7 (3·7 to 10·9)	4·1 (2·2 to 6·4)	1·1 (0·6 to 1·7)	251
Neurological disorders	0·1 (0 to 0·2)	0·4 (0·2 to 0·8)	34·2 (21·3 to 52·3)	84·9 (47·3 to 134)	136 (64·6 to 231)	331 (108 to 683)	452 (153 to 943)	540 (196 to 1,070)	479 (181 to 957)	377 (143 to 733)	360 (140 to 690)	366 (145 to 689)	330 (149 to 622)	283 (137 to 495)	249 (133 to 417)	217 (128 to 345)	194 (124 to 313)	169 (109 to 285)	176 (109 to 320)	204 (114 to 414)	153 (76·9 to 315)	104 (47·8 to 224)	32·4 (13·6 to 73)	5,270
Alzheimer's disease and other dementias	0 (0 to 0)	0 (0 to 0)	0 (0 to 0)	0 (0 to 0)	0 (0 to 0)	0 (0 to 0)	0 (0 to 0)	0 (0 to 0)	0 (0 to 0)	0 (0 to 0)	0 (0 to 0)	1·8 (0·4 to 5·5)	8·7 (2·6 to 23·5)	15·6 (5·3 to 40·2)	26·6 (9·9 to 64·1)	42·5 (17·8 to 100)	63·7 (25·6 to 152)	79·6 (33·9 to 183)	103 (44·3 to 239)	152 (64·7 to 362)	128 (51·7 to 292)	92·9 (36·2 to 214)	30·3 (11·5 to 71·2)	745
Parkinson's disease	0 (0 to 0)	0 (0 to 0)	0 (0 to 0)	0 (0 to 0)	0 (0 to 0)	0 (0 to 0)	0 (0 to 0)	0·2 (0·2 to 0·3)	0·3 (0·2 to 0·4)	0·5 (0·3 to 0·8)	0·8 (0·6 to 1·2)	2·2 (1·6 to 3·1)	4·3 (3·1 to 5·9)	7·8 (5·7 to 10·3)	11·8 (8·6 to 15·4)	18·9 (14·3 to 24·3)	25·7 (20·3 to 31·8)	32·9 (26·3 to 40·3)	39·6 (31·9 to 48·1)	33 (26·8 to 39·9)	16·8 (13·4 to 20·6)	7·6 (6·1 to 9)	1·4 (1·2 to 1·8)	204
Idiopathic epilepsy	0·1 (0 to 0·2)	0·3 (0·1 to 0·7)	12·4 (7 to 20·1)	46·5 (22·6 to 81·1)	63·1 (29·4 to 111)	77·2 (34·2 to 141)	101 (50·2 to 176)	131 (71·5 to 214)	111 (60·5 to 183)	82·9 (44·8 to 133)	73·7 (42·5 to 118)	67·4 (36·7 to 109)	59·6 (33·8 to 93·2)	47·6 (25 to 78·7)	39·8 (20·2 to 68·3)	32·7 (15·4 to 56·1)	25·1 (11·6 to 44·1)	16·6 (7·4 to 29·3)	11·7 (5 to 20·4)	7·3 (3·1 to 13·2)	3·6 (1·4 to 6·3)	1·6 (0·7 to 2·9)	0·4 (0·1 to 0·6)	1,010
Multiple sclerosis	0 (0 to 0)	0 (0 to 0)	0 (0 to 0)	0 (0 to 0)	0 (0 to 0)	0·1 (0 to 0·1)	0·4 (0·2 to 0·7)	1·5 (0·9 to 2·3)	2·1 (1·3 to 3·5)	2·2 (1·4 to 3·4)	3·8 (2·4 to 5·9)	4 (2·6 to 6·4)	4 (2·6 to 6·3)	3·4 (2·3 to 5·4)	3 (2 to 4·6)	2·2 (1·5 to 3·4)	1·1 (0·7 to 1·6)	0·6 (0·4 to 1)	0·3 (0·2 to 0·4)	0·2 (0·1 to 0·2)	0·1 (0 to 0·1)	0 (0 to 0)	0 (0 to 0)	29·1
Motor neuron disease	0 (0 to 0)	0·1 (0·1 to 0·2)	3·5 (1·6 to 6·9)	3·6 (1·8 to 6·5)	0·7 (0·4 to 1·1)	1·1 (0·6 to 1·8)	0·4 (0·3 to 0·6)	1·1 (0·7 to 1·5)	0·9 (0·6 to 1·4)	1 (0·6 to 1·5)	1·6 (0·9 to 2·5)	2·9 (1·9 to 4·4)	4·7 (3 to 7·1)	6·2 (4·1 to 8·9)	6·8 (4·6 to 9·6)	6·3 (4 to 9·2)	4·8 (3·4 to 6·9)	2·7 (1·8 to 3·8)	1·4 (0·9 to 2·1)	0·5 (0·3 to 0·7)	0·1 (0·1 to 0·2)	0 (0 to 0·1)	0 (0 to 0)	50·6
Headache disorders	0 (0 to 0)	0 (0 to 0)	0 (0 to 0)	0 (0 to 0)	44·9 (0·7 to 119)	215 (9·2 to 558)	316 (32·5 to 800)	375 (52 to 891)	339 (49·8 to 786)	269 (48·5 to 593)	259 (47·3 to 577)	264 (55·5 to 589)	223 (51·6 to 500)	180 (46·8 to 380)	141 (38·6 to 299)	99·1 (27·7 to 207)	63·5 (20·2 to 132)	30·6 (10 to 63·9)	16·6 (5·4 to 34·3)	8·6 (2·7 to 17·8)	3·6 (1·3 to 7·3)	1·3 (0·4 to 2·6)	0·2 (0 to 0·4)	2,850
Migraine	0 (0 to 0)	0 (0 to 0)	0 (0 to 0)	0 (0 to 0)	41·6 (0·5 to 115)	204 (6·4 to 546)	296 (23·2 to 787)	349 (32·8 to 859)	314 (35·5 to 753)	248 (31·4 to 575)	238 (33·9 to 552)	242 (40·7 to 558)	202 (37·3 to 473)	162 (32·2 to 358)	126 (26·5 to 281)	86·9 (20·8 to 192)	54·2 (14·3 to 120)	25·6 (7·4 to 55·9)	13·8 (3·7 to 30·2)	7·1 (2 to 15·7)	3 (0·8 to 6·5)	1 (0·3 to 2·2)	0·1 (0 to 0·3)	2,610
Tension-type headache	0 (0 to 0)	0 (0 to 0)	0 (0 to 0)	0 (0 to 0)	3·2 (0·1 to 20·9)	11·5 (0·7 to 75·9)	19·5 (3·6 to 85·3)	26·1 (5·4 to 104)	25 (5·6 to 95·7)	20·5 (4·9 to 71·1)	21·2 (5·8 to 67·1)	22·2 (6·6 to 64·5)	20·5 (6·7 to 52·4)	18·6 (5·6 to 51·2)	14·9 (4·5 to 37·9)	12·2 (3·8 to 33·2)	9·2 (2·6 to 28·5)	5 (1·3 to 16·8)	2·8 (0·7 to 10·1)	1·4 (0·3 to 5)	0·6 (0·1 to 2·2)	0·2 (0 to 0·8)	0 (0 to 0·2)	235
Other neurological disorders	0 (0 to 0)	0 (0 to 0)	18·2 (10·4 to 29·3)	34·9 (19·9 to 56·2)	26·9 (13·8 to 43·4)	37·1 (18·4 to 63·3)	34·8 (17·5 to 60·3)	31 (17·1 to 49·6)	26·9 (16·3 to 41·4)	21·4 (13·3 to 32·4)	21·2 (12·6 to 32·1)	23·4 (14·6 to 33·8)	25·7 (16·2 to 37·4)	22·3 (14·8 to 31·3)	20·8 (14·3 to 28·6)	15·4 (11 to 21)	10·7 (7·7 to 14·9)	5·7 (4·2 to 7·6)	3·8 (2·7 to 5·1)	2 (1·5 to 2·8)	0·8 (0·5 to 1·1)	0·3 (0·2 to 0·5)	0·1 (0 to 0·2)	383
Mental disorders	0·1 (0 to 0·1)	0·2 (0·1 to 0·3)	3·2 (1·8 to 5·1)	21·2 (12·6 to 31·3)	132 (85 to 197)	468 (305 to 680)	906 (620 to 1,290)	1,250 (852 to 1,780)	1,090 (738 to 1,490)	830 (568 to 1,150)	788 (554 to 1,080)	777 (542 to 1,060)	680 (480 to 923)	591 (417 to 798)	508 (356 to 698)	389 (270 to 525)	273 (192 to 362)	155 (111 to 207)	98·6 (67·6 to 135)	57·2 (39·4 to 79·1)	26·5 (18·3 to 36·2)	11 (7·3 to 15·3)	2·3 (1·4 to 3·3)	9,060
Schizophrenia	0 (0 to 0)	0 (0 to 0)	0 (0 to 0)	0 (0 to 0)	0 (0 to 0)	1·8 (0·7 to 3·5)	14·6 (4·9 to 27·9)	55·3 (27·8 to 92·7)	83·7 (46·3 to 126)	81·5 (49·8 to 121)	83·1 (50·8 to 122)	81·4 (52·2 to 118)	68·9 (43·8 to 96·5)	56·5 (36·8 to 78·3)	44·3 (28·7 to 61·6)	29·9 (19·2 to 42)	18·1 (11·4 to 25·6)	8·6 (5·1 to 12·5)	4·5 (2·6 to 6·8)	2 (1·1 to 3·2)	0·7 (0·3 to 1·1)	0·2 (0·1 to 0·3)	0 (0 to 0·1)	635
Depressive disorders	0 (0 to 0)	0 (0 to 0)	0 (0 to 0)	0·1 (0 to 0·2)	10·5 (3·7 to 23)	139 (70·5 to 249)	438 (268 to 687)	674 (401 to 1,060)	539 (328 to 821)	396 (232 to 624)	382 (237 to 557)	386 (230 to 585)	346 (222 to 508)	313 (208 to 450)	280 (177 to 412)	222 (142 to 331)	161 (108 to 228)	94·4 (63 to 134)	61·7 (38·8 to 92)	37·1 (23·1 to 56·3)	18 (11·4 to 26·1)	7·7 (4·8 to 11·5)	1·7 (0·9 to 2·6)	4,510
Major depressive disorder	0 (0 to 0)	0 (0 to 0)	0 (0 to 0)	0·1 (0 to 0·2)	9·5 (2·7 to 22·2)	132 (65·4 to 235)	416 (249 to 667)	633 (364 to 1,010)	494 (295 to 773)	356 (200 to 574)	343 (206 to 512)	345 (200 to 534)	310 (195 to 458)	281 (181 to 409)	252 (155 to 372)	201 (124 to 301)	146 (96·7 to 209)	86·3 (57·4 to 125)	56·8 (35·1 to 86·4)	34·4 (20·9 to 53·4)	16·7 (10·4 to 24·7)	7·2 (4·3 to 10·8)	1·5 (0·8 to 2·5)	4,120
Dysthymia	0 (0 to 0)	0 (0 to 0)	0 (0 to 0)	0 (0 to 0)	1 (0 to 2·6)	7 (2·7 to 13·6)	21·4 (10·4 to 38·3)	41·1 (20·7 to 71·8)	45·7 (23·5 to 79·1)	39·7 (21·4 to 63·9)	39·3 (21·9 to 65·7)	40·6 (21·7 to 69·9)	36·2 (19·9 to 61·9)	31·9 (18·2 to 53)	27·6 (16·1 to 45·4)	21·2 (11·8 to 35·4)	14·8 (7·9 to 24·4)	8·1 (4·4 to 13)	4·9 (2·8 to 8·3)	2·7 (1·5 to 4·5)	1·2 (0·7 to 2)	0·5 (0·3 to 0·9)	0·1 (0·1 to 0·2)	385
Bipolar disorder	0 (0 to 0)	0 (0 to 0)	0 (0 to 0)	0 (0 to 0)	0 (0 to 0)	25·7 (10·1 to 46·4)	96·3 (45·1 to 168)	131 (64·6 to 224)	112 (56·6 to 196)	81·2 (42·6 to 131)	73·3 (39·5 to 119)	71·9 (39·2 to 120)	62·6 (33·9 to 105)	52·7 (28·7 to 89·2)	42·4 (23·6 to 69·7)	29·5 (16·1 to 47)	18·7 (10·2 to 30·7)	9·2 (4·8 to 15·3)	4·8 (2·5 to 8)	2·2 (1·1 to 3·6)	0·8 (0·4 to 1·4)	0·3 (0·1 to 0·5)	0 (0 to 0·1)	814
Anxiety disorders	0 (0 to 0)	0 (0 to 0)	0 (0 to 0)	4·2 (1 to 8·7)	60·1 (30·2 to 107)	154 (83·1 to 259)	218 (130 to 334)	265 (152 to 427)	239 (134 to 392)	188 (109 to 297)	177 (107 to 281)	174 (102 to 282)	151 (90 to 244)	129 (77·4 to 201)	108 (65·8 to 162)	80·6 (47·9 to 121)	56·2 (33·4 to 88·2)	31·8 (18·9 to 50·2)	19·9 (11·8 to 31·3)	11·1 (6·7 to 17·7)	4·8 (2·8 to 7·6)	1·8 (1 to 2·8)	0·3 (0·2 to 0·5)	2,070
Eating disorders	0 (0 to 0)	0 (0 to 0)	0 (0 to 0)	0 (0 to 0)	0·6 (0·2 to 1·3)	9·6 (4·3 to 17·3)	37·1 (19·1 to 65·1)	65·9 (33·2 to 109)	53 (29 to 86·9)	32 (17·5 to 51·8)	21·8 (12·3 to 36·3)	13·3 (6·8 to 22·4)	6·6 (3·3 to 11·7)	0 (0 to 0)	0 (0 to 0)	0 (0 to 0)	0 (0 to 0)	0 (0 to 0)	0 (0 to 0)	0 (0 to 0)	0 (0 to 0)	0 (0 to 0)	0 (0 to 0)	240
Anorexia nervosa	0 (0 to 0)	0 (0 to 0)	0 (0 to 0)	0 (0 to 0)	0·6 (0·2 to 1·2)	5·2 (1·9 to 9·6)	14·9 (6·7 to 26·8)	16·8 (7·5 to 29)	11·2 (5·2 to 19·6)	5·6 (2·3 to 10·2)	3·8 (1·5 to 7·5)	2·8 (1·1 to 5·5)	1·9 (0·6 to 3·7)	0 (0 to 0)	0 (0 to 0)	0 (0 to 0)	0 (0 to 0)	0 (0 to 0)	0 (0 to 0)	0 (0 to 0)	0 (0 to 0)	0 (0 to 0)	0 (0 to 0)	62·7
Bulimia nervosa	0 (0 to 0)	0 (0 to 0)	0 (0 to 0)	0 (0 to 0)	0·1 (0 to 0·1)	4·4 (1·1 to 10)	22·2 (9·3 to 44·3)	49·1 (21·5 to 89·2)	41·8 (20·4 to 72·3)	26·4 (13·1 to 44·9)	18·1 (9·2 to 32·4)	10·5 (4·9 to 19·2)	4·7 (1·9 to 9·1)	0 (0 to 0)	0 (0 to 0)	0 (0 to 0)	0 (0 to 0)	0 (0 to 0)	0 (0 to 0)	0 (0 to 0)	0 (0 to 0)	0 (0 to 0)	0 (0 to 0)	177
Autism spectrum disorders	0 (0 to 0·1)	0·1 (0·1 to 0·2)	2·1 (1·2 to 3·4)	9·2 (5·1 to 14·8)	10·8 (6 to 17)	10·2 (5·8 to 16)	10·3 (5·9 to 17·1)	11 (6 to 17·7)	9·2 (5·2 to 14·7)	6·8 (3·9 to 11·2)	6 (3·3 to 9·6)	5·6 (3·3 to 8·8)	4·8 (2·5 to 7·5)	4 (2·3 to 6·5)	3·4 (2 to 5·7)	2·6 (1·4 to 4·2)	1·9 (1 to 3)	1·1 (0·6 to 1·7)	0·7 (0·4 to 1·1)	0·4 (0·2 to 0·6)	0·2 (0·1 to 0·3)	0·1 (0 to 0·1)	0 (0 to 0)	101
Attention-deficit/hyperactivity disorder	0 (0 to 0)	0 (0 to 0)	0 (0 to 0)	1·2 (0·5 to 2·4)	12·7 (6·5 to 22·9)	17 (8·8 to 30·8)	14·5 (7·6 to 26·4)	11·7 (6·2 to 21·5)	7·6 (4 to 13·4)	4·6 (2·2 to 8·3)	3·5 (1·7 to 6·3)	2·8 (1·3 to 5)	2 (0·9 to 3·7)	1·4 (0·6 to 2·7)	0·9 (0·4 to 1·7)	0·5 (0·2 to 0·9)	0·2 (0 to 0·4)	0·1 (0 to 0·1)	0 (0 to 0)	0 (0 to 0)	0 (0 to 0)	0 (0 to 0)	0 (0 to 0)	80·7
Conduct disorder	0 (0 to 0)	0 (0 to 0)	0 (0 to 0)	0 (0 to 0)	28·4 (13·1 to 52·9)	101 (50·6 to 176)	59·8 (29·7 to 105)	0 (0 to 0)	0 (0 to 0)	0 (0 to 0)	0 (0 to 0)	0 (0 to 0)	0 (0 to 0)	0 (0 to 0)	0 (0 to 0)	0 (0 to 0)	0 (0 to 0)	0 (0 to 0)	0 (0 to 0)	0 (0 to 0)	0 (0 to 0)	0 (0 to 0)	0 (0 to 0)	190
Idiopathic developmental intellectual disability	0 (0 to 0)	0·1 (0 to 0·1)	1·1 (0·2 to 2·3)	6·6 (2 to 12·4)	9 (3·5 to 16·6)	8·3 (3 to 15·2)	8·2 (2·9 to 15·6)	8·7 (3 to 16·3)	7 (2·3 to 12·9)	5 (1·8 to 9·3)	4·4 (1·5 to 8·2)	4 (1·3 to 7·3)	3·3 (1·1 to 6·1)	2·7 (0·9 to 5·3)	2·2 (0·7 to 4·2)	1·6 (0·5 to 3·1)	1·1 (0·3 to 2·1)	0·7 (0·2 to 1·2)	0·4 (0·1 to 0·7)	0·2 (0·1 to 0·4)	0·1 (0 to 0·2)	0 (0 to 0·1)	0 (0 to 0)	74·7
Other mental disorders	0 (0 to 0)	0 (0 to 0)	0 (0 to 0)	0 (0 to 0)	0 (0 to 0)	1·4 (0·7 to 2·4)	9·8 (3·7 to 18·6)	26·9 (12·4 to 48)	36·1 (19·5 to 59)	35·5 (20·4 to 56·6)	37·3 (22·3 to 58·2)	38·2 (22·8 to 60·7)	34·6 (20·7 to 53·7)	30·9 (18·6 to 47·2)	27·3 (16·8 to 42·6)	21·7 (13·5 to 32·9)	16·1 (9·7 to 24·5)	9·7 (6 to 14·2)	6·6 (4·2 to 9·7)	4·1 (2·6 to 6)	2 (1·3 to 3)	0·9 (0·6 to 1·4)	0·2 (0·1 to 0·3)	339
Substance use disorders	0 (0 to 0)	0 (0 to 0)	0 (0 to 0)	0 (0 to 0)	0 (0 to 0·1)	3·3 (1·6 to 5·9)	65·5 (41·4 to 94·4)	167 (110 to 246)	162 (109 to 226)	125 (84·8 to 174)	119 (85·5 to 160)	111 (80·3 to 151)	91·2 (66·1 to 124)	65·6 (46·4 to 89·7)	52·1 (37·4 to 70·2)	36·2 (25·8 to 48·8)	21·3 (15·1 to 28·8)	11·1 (7·9 to 15·5)	7·1 (5 to 9·5)	4·7 (3·2 to 6·5)	2·6 (1·8 to 3·8)	1·6 (1·1 to 2·3)	0·5 (0·3 to 0·8)	1,050
Alcohol use disorders	0 (0 to 0)	0 (0 to 0)	0 (0 to 0)	0 (0 to 0)	0 (0 to 0·1)	2·2 (0·8 to 4·4)	32·4 (18·4 to 50·9)	93·8 (54·7 to 158)	97·2 (60·5 to 147)	85 (53·1 to 129)	88·7 (61·7 to 124)	86·8 (60·3 to 121)	72·8 (51 to 103)	51·8 (35·2 to 72·9)	41 (28·2 to 56·5)	28·8 (20 to 40·4)	16·5 (11·3 to 23·2)	8·6 (5·8 to 12·2)	5·5 (3·8 to 7·8)	3·8 (2·5 to 5·5)	2·2 (1·5 to 3·3)	1·4 (0·9 to 2·1)	0·5 (0·3 to 0·7)	719
Drug use disorders	0 (0 to 0)	0 (0 to 0)	0 (0 to 0)	0 (0 to 0)	0 (0 to 0)	1·2 (0·5 to 2·4)	33·1 (18·7 to 54·2)	73·6 (45·7 to 110)	64·4 (39·7 to 95)	39·6 (24·8 to 58·9)	30·8 (19·9 to 44·4)	23·9 (15 to 35·1)	18·4 (11·5 to 27·8)	13·8 (8·2 to 22)	11 (6·4 to 16·8)	7·4 (4·1 to 11·8)	4·8 (2·7 to 7·6)	2·5 (1·3 to 3·9)	1·6 (0·9 to 2·5)	0·9 (0·5 to 1·4)	0·4 (0·2 to 0·6)	0·2 (0·1 to 0·3)	0·1 (0 to 0·1)	328
Opioid use disorders	0 (0 to 0)	0 (0 to 0)	0 (0 to 0)	0 (0 to 0)	0 (0 to 0)	0 (0 to 0)	7 (2 to 14·8)	32·6 (15·6 to 57·1)	35·2 (18·4 to 58·8)	24·1 (12·8 to 39·4)	18·4 (9·6 to 30·4)	14·5 (7·1 to 24·1)	11·2 (5·1 to 20)	9·7 (4·5 to 17·4)	7·9 (3·8 to 13·6)	5·5 (2·5 to 9·8)	3·5 (1·6 to 6·2)	1·8 (0·6 to 3·1)	1·1 (0·4 to 1·9)	0·6 (0·2 to 1·1)	0·3 (0·1 to 0·5)	0·1 (0 to 0·2)	0 (0 to 0·1)	173
Cocaine use disorders	0 (0 to 0)	0 (0 to 0)	0 (0 to 0)	0 (0 to 0)	0 (0 to 0)	0·2 (0·1 to 0·4)	8·4 (3·2 to 15·9)	12·1 (5·9 to 21·2)	8·7 (4·4 to 15·6)	5·3 (2·4 to 9)	4·7 (2·3 to 8·1)	3·5 (1·7 to 6·2)	2·7 (1·7 to 4)	1·5 (0·9 to 2·2)	1·3 (0·8 to 2)	0·8 (0·5 to 1·2)	0·5 (0·3 to 0·8)	0·2 (0·1 to 0·4)	0·2 (0·1 to 0·2)	0·1 (0 to 0·1)	0 (0 to 0·1)	0 (0 to 0)	0 (0 to 0)	50·4
Amphetamine use disorders	0 (0 to 0)	0 (0 to 0)	0 (0 to 0)	0 (0 to 0)	0 (0 to 0)	0 (0 to 0)	1·4 (0·6 to 2·4)	6·6 (1·6 to 13·6)	5·4 (1·5 to 11·5)	2·3 (0·4 to 5·5)	1·4 (0·8 to 2·3)	0·8 (0·5 to 1·3)	0·5 (0·3 to 0·8)	0·3 (0·2 to 0·5)	0·2 (0·1 to 0·4)	0·1 (0·1 to 0·2)	0·1 (0 to 0·1)	0 (0 to 0·1)	0 (0 to 0)	0 (0 to 0)	0 (0 to 0)	0 (0 to 0)	0 (0 to 0)	19·1
Cannabis use disorders	0 (0 to 0)	0 (0 to 0)	0 (0 to 0)	0 (0 to 0)	0 (0 to 0)	0·8 (0·2 to 2)	9·3 (3 to 20·4)	9·5 (3·9 to 18·4)	5·1 (1·8 to 10·3)	2·5 (0·8 to 5)	1·7 (0·5 to 3·6)	1·4 (0·4 to 2·9)	1 (0·2 to 2·3)	0·7 (0·3 to 1·2)	0·4 (0·2 to 0·7)	0·2 (0·1 to 0·4)	0·1 (0 to 0·2)	0 (0 to 0·1)	0 (0 to 0)	0 (0 to 0)	0 (0 to 0)	0 (0 to 0)	0 (0 to 0)	32·9
Other drug use disorders	0 (0 to 0)	0 (0 to 0)	0 (0 to 0)	0 (0 to 0)	0 (0 to 0)	0·1 (0·1 to 0·3)	6·9 (3·2 to 12·6)	12·8 (6·7 to 21·2)	9·9 (5·4 to 16·1)	5·5 (2·9 to 9·2)	4·5 (2·6 to 7)	3·7 (2 to 5·8)	3 (1·9 to 4·4)	1·6 (1 to 2·6)	1·2 (0·7 to 1·8)	0·8 (0·5 to 1·1)	0·6 (0·4 to 0·8)	0·4 (0·3 to 0·6)	0·3 (0·2 to 0·5)	0·2 (0·1 to 0·3)	0·1 (0·1 to 0·1)	0 (0 to 0·1)	0 (0 to 0)	51·7
Diabetes and kidney diseases	0 (0 to 0·1)	0·1 (0 to 0·2)	28·3 (17·9 to 42·4)	36·8 (24·8 to 54·5)	55·1 (41·3 to 72·3)	69·8 (49·8 to 93)	198 (152 to 249)	375 (286 to 485)	436 (319 to 580)	451 (332 to 594)	606 (452 to 803)	879 (673 to 1,120)	1,310 (1,000 to 1,690)	1,870 (1,450 to 2,390)	2,360 (1,810 to 2,980)	2,400 (1,900 to 3,000)	2,040 (1,650 to 2,460)	1,360 (1,110 to 1,670)	937 (766 to 1,150)	562 (450 to 686)	257 (204 to 316)	123 (97·8 to 152)	29·3 (22·4 to 36)	16,400
Diabetes mellitus	0 (0 to 0·1)	0·1 (0 to 0·2)	9·9 (4·9 to 17·6)	8·1 (4·4 to 13·8)	12 (7·3 to 17·9)	18·7 (11·4 to 27·2)	88 (63·3 to 117)	183 (137 to 242)	217 (153 to 301)	234 (167 to 316)	346 (251 to 460)	549 (409 to 715)	908 (670 to 1,200)	1,360 (1,030 to 1,760)	1,740 (1,340 to 2,200)	1,830 (1,420 to 2,350)	1,590 (1,260 to 1,940)	1,070 (849 to 1,330)	724 (579 to 916)	428 (330 to 535)	193 (148 to 243)	89·2 (67·6 to 115)	20·5 (15·2 to 26·2)	11,600
Diabetes mellitus type 1	0 (0 to 0·1)	0·1 (0 to 0·2)	9·9 (4·9 to 17·6)	8·1 (4·4 to 13·8)	12 (7·3 to 17·9)	18·7 (11·4 to 27·2)	26·6 (15·2 to 45·9)	55 (33·2 to 88·9)	56·8 (31·5 to 95·4)	40·4 (22·6 to 70·7)	61·5 (33·2 to 103)	85·3 (49 to 146)	27·5 (16 to 53·6)	38·2 (21·8 to 78·6)	47·2 (27·9 to 85·7)	40·2 (23·1 to 78·5)	35·5 (22·1 to 63·8)	19·9 (12·2 to 35·6)	14·6 (8·6 to 25·8)	5·6 (3·3 to 10·3)	2·6 (1·5 to 4·7)	1·4 (0·7 to 2·4)	0·4 (0·2 to 0·7)	608
Diabetes mellitus type 2	0 (0 to 0)	0 (0 to 0)	0 (0 to 0)	0 (0 to 0)	0 (0 to 0)	0 (0 to 0)	61·4 (41·4 to 85·2)	128 (87·9 to 172)	160 (109 to 227)	193 (135 to 263)	285 (206 to 379)	464 (339 to 606)	880 (651 to 1,160)	1,320 (999 to 1,720)	1,700 (1,300 to 2,150)	1,790 (1,390 to 2,290)	1,550 (1,230 to 1,900)	1,050 (834 to 1,310)	710 (568 to 899)	422 (326 to 528)	191 (146 to 241)	87·8 (66·5 to 113)	20·1 (15 to 25·7)	11,000
Chronic kidney disease	0 (0 to 0)	0 (0 to 0)	18·3 (11·3 to 28·5)	28·6 (18·6 to 45·1)	42·1 (30 to 58·3)	50·6 (34·1 to 72·2)	109 (79·8 to 144)	192 (135 to 270)	218 (145 to 314)	217 (147 to 309)	259 (171 to 370)	329 (225 to 466)	401 (276 to 584)	510 (344 to 714)	615 (423 to 866)	573 (401 to 817)	451 (320 to 615)	291 (210 to 394)	213 (153 to 284)	134 (96·8 to 176)	63·5 (47·2 to 83·5)	34 (25·4 to 43·5)	8·8 (6·2 to 11·6)	4,760
Chronic kidney disease due to diabetes mellitus type 1	0 (0 to 0)	0 (0 to 0)	0·2 (0·1 to 0·3)	0·5 (0·2 to 0·9)	0·9 (0·4 to 1·8)	1·5 (0·6 to 3)	4·8 (2 to 9·8)	13 (4·8 to 27·2)	26·5 (11 to 52·8)	37·4 (17·2 to 66·4)	50·6 (24·3 to 88·9)	61·5 (31·1 to 109)	62·7 (27·1 to 115)	59·8 (22·6 to 116)	49·8 (16·2 to 116)	34·4 (10·7 to 80·8)	20·7 (7·5 to 47)	10 (3·7 to 21·7)	4·8 (1·8 to 10·7)	1·5 (0·6 to 3·5)	0·3 (0·1 to 0·6)	0 (0 to 0·1)	0 (0 to 0)	441
Chronic kidney disease due to diabetes mellitus type 2	0 (0 to 0)	0 (0 to 0)	0 (0 to 0)	0 (0 to 0)	0 (0 to 0)	0 (0 to 0)	2·1 (0·7 to 5·1)	5·7 (2·2 to 12·1)	10·2 (4 to 22·1)	14·6 (6·1 to 29·7)	23·5 (10·2 to 46·3)	42 (19·9 to 76·3)	73 (37·4 to 126)	126 (67·5 to 206)	182 (100 to 282)	184 (99·8 to 279)	147 (90·5 to 220)	94·9 (59·3 to 139)	68·8 (43·5 to 100)	42·3 (26·8 to 62·6)	19·2 (12·2 to 27·6)	9·9 (6·3 to 14·3)	2·5 (1·3 to 3·7)	1,050
Chronic kidney disease due to hypertension	0 (0 to 0)	0 (0 to 0)	0 (0 to 0)	0 (0 to 0)	0 (0 to 0)	0 (0 to 0)	6·7 (3 to 13)	12·7 (5·6 to 25·1)	17 (8·2 to 30·8)	18·6 (10·2 to 30·4)	24·5 (13·7 to 39)	35·4 (21·1 to 53·7)	50·6 (30·8 to 79)	75·7 (43·4 to 120)	109 (64·2 to 171)	120 (71·7 to 184)	107 (65·7 to 161)	75·9 (48·4 to 113)	61·7 (39·5 to 91·4)	42·7 (27·9 to 62·3)	22·1 (15 to 30·7)	13 (8·9 to 18·1)	3·5 (2·3 to 5·2)	795
Chronic kidney disease due to glomerulonephritis	0 (0 to 0)	0 (0 to 0)	5 (2·4 to 9·3)	8·7 (4·5 to 15·4)	14·8 (8·2 to 22·9)	20·2 (11·7 to 31·9)	42·4 (25·5 to 64·4)	74·4 (42·7 to 120)	77·7 (45·2 to 126)	69·3 (38·5 to 107)	79·3 (45·4 to 128)	94·5 (48·6 to 156)	101 (56·1 to 164)	115 (59·8 to 204)	126 (60·4 to 233)	101 (48 to 187)	66·2 (31·8 to 117)	36·4 (17·7 to 63·2)	23·5 (10·5 to 42·4)	12·6 (5·4 to 23·9)	4·6 (2·1 to 8·4)	2·1 (0·9 to 4)	0·5 (0·2 to 1·1)	1,080
Chronic kidney disease due to other and unspecified causes	0 (0 to 0)	0 (0 to 0)	13·2 (7·9 to 20·8)	19·4 (11·9 to 31)	26·4 (17·5 to 38·3)	28·9 (16·8 to 44·5)	53·4 (34·3 to 76·6)	86·2 (53·8 to 128)	86·9 (52·5 to 129)	76·8 (48·2 to 117)	81·3 (46·1 to 129)	95·8 (55·5 to 154)	114 (65·2 to 183)	134 (77·2 to 217)	149 (84·5 to 248)	134 (76·6 to 220)	110 (64·6 to 178)	73·8 (45·9 to 114)	53·7 (33 to 81·5)	34·7 (21·6 to 53)	17·2 (11·2 to 25·4)	8·9 (6 to 12·7)	2·2 (1·3 to 3·4)	1,400
Acute glomerulonephritis	0 (0 to 0)	0 (0 to 0)	0 (0 to 0)	0 (0 to 0)	1 (0·4 to 1·9)	0·5 (0·2 to 1)	0·5 (0·2 to 0·8)	0·7 (0·3 to 1·3)	0·6 (0·3 to 1·2)	0·6 (0·3 to 1·1)	0·4 (0·2 to 0·7)	0·3 (0·2 to 0·6)	0·2 (0·1 to 0·4)	0·1 (0 to 0·2)	0·3 (0·2 to 0·6)	0·2 (0·1 to 0·4)	0·1 (0 to 0·2)	0·2 (0·1 to 0·4)	0·1 (0 to 0·1)	0·1 (0 to 0·1)	0 (0 to 0·1)	0 (0 to 0·1)	0 (0 to 0)	5·9
Skin and subcutaneous diseases	0 (0 to 0)	0·1 (0·1 to 0·2)	19·1 (12·2 to 28)	196 (122 to 290)	235 (144 to 352)	226 (142 to 338)	249 (159 to 373)	185 (117 to 274)	138 (89·4 to 203)	103 (67 to 151)	96·9 (63·3 to 144)	99·6 (66·2 to 146)	91·2 (61·3 to 135)	91·3 (60·8 to 135)	91·1 (62 to 133)	81·3 (55·7 to 116)	68 (46 to 102)	48·6 (32·9 to 72·8)	39·7 (26·7 to 58·8)	28·1 (18·7 to 42·8)	16·1 (10·9 to 26)	8·7 (6 to 14)	2·4 (1·5 to 3·8)	2,110
Dermatitis	0 (0 to 0)	0 (0 to 0)	0 (0 to 0)	72·4 (39·1 to 127)	82·6 (44·3 to 140)	55·9 (30·8 to 94·1)	43·8 (24·2 to 72·3)	39·7 (22·1 to 64·6)	30·9 (17·8 to 49·5)	22·8 (12·5 to 36·8)	20·4 (11·2 to 32·9)	19·7 (11 to 32·1)	17·8 (9·7 to 30·3)	17·1 (9·3 to 27·9)	16·6 (9·4 to 27·6)	14·1 (7·8 to 22·5)	11·4 (6·4 to 18·6)	7·2 (4·1 to 11·4)	4·9 (2·9 to 7·8)	2·9 (1·7 to 4·6)	1·3 (0·8 to 2·1)	0·5 (0·3 to 0·8)	0·1 (0·1 to 0·2)	482
Atopic dermatitis	0 (0 to 0)	0 (0 to 0)	0 (0 to 0)	71·9 (38·8 to 126)	81 (43·1 to 138)	50·7 (26·6 to 88·5)	34·6 (17·8 to 60·3)	27·2 (14·1 to 47·2)	18·4 (9·1 to 32·1)	12·2 (6 to 22·2)	10·5 (5·4 to 18·5)	9·9 (5 to 17·2)	8·8 (4·4 to 16·1)	7·9 (4·1 to 13·4)	7·5 (3·9 to 13·7)	6·2 (3·3 to 10·9)	4·7 (2·4 to 8·2)	2·9 (1·5 to 5·2)	2 (1 to 3·6)	1·3 (0·7 to 2·2)	0·6 (0·3 to 1·1)	0·3 (0·1 to 0·4)	0·1 (0 to 0·1)	359
Contact dermatitis	0 (0 to 0)	0 (0 to 0)	0 (0 to 0)	0 (0 to 0)	1 (0·3 to 2·1)	4·4 (1·6 to 8·4)	8·2 (3·3 to 15·9)	11·2 (4·7 to 22·3)	11·3 (5·2 to 21)	9·5 (4 to 18·4)	8·9 (3·8 to 18)	8·8 (4 to 17·7)	8·2 (3·7 to 16·5)	8·5 (3·6 to 16·6)	8·5 (3·7 to 16·6)	7·4 (3·2 to 13·8)	6·1 (2·7 to 12·1)	3·9 (1·8 to 7·3)	2·5 (1·2 to 4·8)	1·4 (0·6 to 2·6)	0·6 (0·3 to 1·1)	0·2 (0·1 to 0·4)	0 (0 to 0·1)	111
Seborrhoeic dermatitis	0 (0 to 0)	0 (0 to 0)	0 (0 to 0)	0·6 (0·2 to 1·1)	0·6 (0·2 to 1·2)	0·8 (0·4 to 1·5)	1 (0·4 to 1·8)	1·2 (0·6 to 2·2)	1·2 (0·6 to 2·2)	1·1 (0·5 to 1·9)	1 (0·4 to 1·7)	0·9 (0·4 to 1·7)	0·8 (0·3 to 1·3)	0·7 (0·3 to 1·3)	0·6 (0·3 to 1·1)	0·6 (0·3 to 1)	0·6 (0·3 to 1)	0·4 (0·2 to 0·7)	0·3 (0·2 to 0·5)	0·2 (0·1 to 0·3)	0·1 (0 to 0·2)	0 (0 to 0·1)	0 (0 to 0)	12·6
Psoriasis	0 (0 to 0)	0 (0 to 0)	0 (0 to 0)	2·5 (1·6 to 3·9)	6·2 (2·6 to 11)	7·7 (3·5 to 12·9)	9 (4·1 to 15·1)	10·6 (5·2 to 17·9)	10·2 (5 to 16·6)	8·8 (4·7 to 13·6)	9·3 (5·1 to 14·6)	10 (5·5 to 15·8)	10 (6 to 15·8)	9·8 (5·8 to 14·7)	9·5 (5·8 to 14·2)	8 (4·8 to 12·2)	6 (3·5 to 9·1)	3·4 (2 to 5)	1·9 (1·1 to 3)	0·9 (0·5 to 1·5)	0·3 (0·2 to 0·5)	0·1 (0·1 to 0·2)	0 (0 to 0)	124
Bacterial skin diseases	0 (0 to 0)	0 (0 to 0)	6·1 (2·4 to 11·2)	5·2 (2·8 to 8·7)	5 (2·9 to 7·8)	3·2 (1·8 to 5)	2·4 (1·3 to 4)	3 (1·6 to 4·9)	3·8 (2 to 6·2)	3·8 (2 to 6·7)	4·2 (2 to 7·5)	6·1 (3 to 10·2)	5·5 (2·7 to 8·9)	7·7 (3·5 to 12·6)	8·4 (3·8 to 13·4)	9·2 (4·3 to 15·4)	7·7 (3·5 to 12·8)	5·3 (2·2 to 9·6)	5 (2·2 to 9·4)	4 (1·4 to 7·9)	2·5 (1 to 5·5)	1·5 (0·5 to 2·9)	0·5 (0·1 to 1)	100
Cellulitis	0 (0 to 0)	0 (0 to 0)	0·9 (0·2 to 2·2)	1·2 (0·4 to 2·3)	1·7 (0·7 to 3)	0·6 (0·4 to 1)	0·3 (0·2 to 0·5)	0·4 (0·2 to 0·8)	0·7 (0·3 to 1·5)	0·4 (0·2 to 0·9)	0·6 (0·3 to 1·2)	0·7 (0·3 to 1·4)	0·6 (0·4 to 1·1)	1·1 (0·5 to 2·2)	1·2 (0·5 to 2·5)	1·1 (0·5 to 2·3)	1·1 (0·4 to 2·5)	0·6 (0·2 to 1·3)	0·5 (0·2 to 1·3)	0·3 (0·1 to 1)	0·2 (0·1 to 0·6)	0·1 (0 to 0·3)	0 (0 to 0·1)	14·5
Pyoderma	0 (0 to 0)	0 (0 to 0)	5·2 (2·1 to 9·7)	4 (1·9 to 7)	3·3 (1·8 to 5·5)	2·5 (1·2 to 4·1)	2·1 (1 to 3·6)	2·7 (1·4 to 4·4)	3 (1·5 to 5·1)	3·4 (1·7 to 6)	3·6 (1·7 to 6·5)	5·4 (2·5 to 9·3)	4·9 (2·2 to 8·3)	6·6 (2·8 to 10·9)	7·2 (3·1 to 11·8)	8 (3·4 to 13·5)	6·6 (2·6 to 10·8)	4·8 (1·8 to 8·6)	4·5 (1·8 to 8·5)	3·6 (1·2 to 7·2)	2·3 (0·9 to 5)	1·4 (0·5 to 2·7)	0·5 (0·1 to 0·9)	85·5
Scabies	0 (0 to 0)	0·1 (0 to 0·1)	4·5 (2·4 to 7·9)	31·3 (15·4 to 54·4)	39·2 (18·4 to 68·4)	38·5 (18·6 to 67·1)	42 (21·5 to 71·8)	41·6 (20·7 to 72·7)	26·8 (13·4 to 46·9)	15·8 (7·7 to 28·1)	14·3 (7 to 24·6)	15·6 (7·8 to 26·6)	14·2 (7·3 to 24·1)	12·2 (6·3 to 20·8)	10·2 (5·1 to 18·1)	7·6 (4 to 13·4)	5·6 (2·8 to 9·8)	3·5 (1·7 to 6)	2·6 (1·3 to 4·6)	1·8 (0·9 to 3·3)	1 (0·5 to 1·7)	0·5 (0·2 to 0·8)	0·1 (0 to 0·2)	329
Fungal skin diseases	0 (0 to 0)	0 (0 to 0)	0·8 (0·3 to 1·7)	5·9 (2·2 to 13·1)	9·1 (3·4 to 19·7)	9·8 (3·6 to 21·8)	10·5 (3·8 to 24)	12·3 (4·8 to 26·9)	11·2 (4·3 to 24·3)	9·1 (3·5 to 20·4)	8·8 (3·4 to 19·4)	8·8 (3·5 to 18·8)	8·2 (3·3 to 17·9)	8·3 (3·3 to 18)	9·2 (3·6 to 20·1)	8·7 (3·3 to 19·5)	8·9 (3·5 to 18·9)	7·8 (3 to 16·7)	6·6 (2·5 to 13·9)	5·3 (2 to 11·2)	3·2 (1·3 to 6·8)	1·6 (0·7 to 3·3)	0·4 (0·2 to 0·8)	154
Viral skin diseases	0 (0 to 0)	0 (0 to 0)	0 (0 to 0)	33·6 (21·2 to 50·1)	49·5 (30·9 to 75·5)	24·2 (15·2 to 37·3)	15·3 (9·6 to 24)	13·7 (8·3 to 21·4)	8·9 (5·3 to 13·7)	5·7 (3·2 to 8·9)	4·9 (2·9 to 8·1)	4·8 (2·8 to 7·6)	4 (2·4 to 6·4)	3·6 (2·1 to 5·7)	3·1 (1·8 to 5)	2·6 (1·4 to 4·1)	2 (1·1 to 3·1)	1·1 (0·6 to 1·7)	0·6 (0·3 to 1)	0·3 (0·2 to 0·5)	0·1 (0·1 to 0·2)	0 (0 to 0·1)	0 (0 to 0)	178
Acne vulgaris	0 (0 to 0)	0 (0 to 0)	0 (0 to 0)	0 (0 to 0)	2·7 (1·5 to 4·5)	55·9 (31 to 91·5)	93·3 (51·8 to 155)	26·2 (14·2 to 43)	11·2 (5·5 to 18·6)	6·2 (3·1 to 10·6)	4·4 (2·1 to 7·8)	3·1 (1·4 to 5·5)	2 (1·1 to 3·3)	1·3 (0·7 to 2·1)	0·8 (0·4 to 1·4)	0·6 (0·3 to 0·9)	0·4 (0·2 to 0·6)	0·2 (0·1 to 0·3)	0·1 (0·1 to 0·2)	0·1 (0 to 0·1)	0 (0 to 0)	0 (0 to 0)	0 (0 to 0)	208
Alopecia areata	0 (0 to 0)	0 (0 to 0)	0 (0 to 0)	0·9 (0·4 to 1·6)	1·1 (0·5 to 2)	1·4 (0·6 to 2·6)	2·6 (1·4 to 4·3)	4·5 (2·5 to 7·6)	5·5 (3·1 to 8·8)	4·5 (2·6 to 7·2)	3·9 (2·3 to 6·1)	3·2 (1·8 to 5·2)	2·5 (1·3 to 4)	2 (1 to 3·3)	1·6 (0·9 to 2·6)	1·4 (0·7 to 2·2)	0·9 (0·5 to 1·5)	0·5 (0·3 to 0·9)	0·3 (0·1 to 0·5)	0·1 (0·1 to 0·2)	0·1 (0 to 0·1)	0 (0 to 0)	0 (0 to 0)	36·9
Pruritus	0 (0 to 0)	0 (0 to 0)	0·1 (0 to 0·2)	1·4 (0·6 to 2·8)	1·8 (0·8 to 3·7)	1·8 (0·7 to 3·7)	2·1 (0·9 to 4·3)	2·9 (1·2 to 5·7)	2·9 (1·2 to 5·8)	2·5 (1 to 5·1)	2·5 (1·1 to 4·9)	2·5 (1·1 to 4·7)	2·3 (0·9 to 4·5)	2·3 (0·9 to 4·5)	2·3 (0·9 to 4·7)	2·3 (1 to 4·5)	2·1 (0·9 to 4·1)	1·4 (0·6 to 2·7)	1 (0·4 to 1·9)	0·6 (0·2 to 1·2)	0·3 (0·1 to 0·6)	0·1 (0 to 0·2)	0 (0 to 0)	35·1
Urticaria	0 (0 to 0)	0 (0 to 0·1)	5·3 (3·2 to 8·4)	35·5 (20·6 to 56·1)	29·6 (16·7 to 49·4)	19·7 (10·5 to 32·6)	17·5 (9·1 to 30·6)	18·3 (9·6 to 30·1)	14·8 (8 to 24·5)	11·8 (6·2 to 19·6)	11·2 (6·1 to 18·7)	10·4 (5·9 to 16·6)	8·6 (4·5 to 14·6)	7·5 (4·1 to 12·6)	6·7 (3·5 to 11·2)	5·3 (2·7 to 9)	4 (2·1 to 6·7)	2·4 (1·2 to 3·9)	1·5 (0·8 to 2·5)	0·8 (0·5 to 1·3)	0·3 (0·2 to 0·6)	0·1 (0·1 to 0·2)	0 (0 to 0)	211
Decubitus ulcer	0 (0 to 0)	0 (0 to 0)	0 (0 to 0)	0·2 (0·1 to 0·6)	0·7 (0·3 to 1·1)	0·5 (0·2 to 0·9)	0·5 (0·2 to 1)	0·1 (0·1 to 0·2)	0·8 (0·3 to 1·8)	0·2 (0·1 to 0·5)	1 (0·4 to 2·4)	1 (0·4 to 1·9)	1·7 (0·7 to 3·1)	3·2 (1·4 to 5·9)	4·8 (2·2 to 8·5)	4·8 (2·6 to 8·5)	5·3 (2·8 to 9)	6·3 (3·3 to 10·9)	7·8 (4·6 to 13·4)	6·7 (4 to 12·4)	5·1 (3 to 10)	3·4 (2 to 6·2)	1·1 (0·6 to 2)	55·3
Other skin and subcutaneous diseases	0 (0 to 0)	0 (0 to 0)	2·2 (0·9 to 4·1)	7 (3·3 to 13·7)	7·1 (3·6 to 12·9)	7·7 (3·6 to 14·6)	10 (5 to 18·7)	11·9 (6 to 21)	11·1 (5·5 to 20·5)	11·7 (6·3 to 20·7)	12 (6·2 to 21·1)	14·6 (8 to 24·9)	14·4 (7·6 to 25)	16·6 (8·8 to 28)	17·9 (9·7 to 31·1)	16·8 (9·2 to 28·6)	13·8 (7·1 to 24·5)	9·6 (5·1 to 16·8)	7·3 (4 to 12·6)	4·5 (2·5 to 7·9)	1·9 (1 to 3·3)	0·9 (0·5 to 1·5)	0·2 (0·1 to 0·3)	199
Sense organ diseases	0 (0 to 0)	0·1 (0 to 0·1)	1·8 (1·1 to 2·9)	20·5 (12·8 to 30·6)	42·5 (26·6 to 63·2)	52·6 (33·5 to 78·5)	66·9 (42·6 to 97·9)	90·5 (55·9 to 133)	98·2 (63 to 148)	94·8 (59·6 to 142)	106 (65·6 to 159)	131 (83·3 to 196)	167 (105 to 264)	211 (136 to 334)	257 (167 to 397)	272 (175 to 410)	266 (176 to 397)	209 (141 to 303)	184 (126 to 258)	141 (99 to 192)	81·8 (57·5 to 109)	40 (28·6 to 53·3)	9·4 (6·8 to 12·3)	2,540
Blindness and vision loss	0 (0 to 0)	0 (0 to 0)	0·3 (0·1 to 0·6)	5·5 (3·1 to 9)	12·7 (7·3 to 20·5)	15·9 (9·3 to 26)	20·1 (12 to 31·4)	25·9 (15 to 39·4)	24·4 (14·9 to 36·1)	21·4 (13·3 to 32·8)	24·8 (15·7 to 37·3)	32·5 (20·7 to 49·4)	39·2 (23·7 to 62)	48·7 (30·8 to 78·1)	61·6 (38·8 to 97·1)	69·6 (45·3 to 106)	75 (49 to 109)	64·1 (43 to 91·3)	59·2 (40·4 to 83·1)	47·1 (32·3 to 65·2)	28·4 (20 to 38·1)	14·8 (10·4 to 20·1)	3·9 (2·7 to 5·5)	695
Glaucoma	0 (0 to 0)	0 (0 to 0)	0 (0 to 0)	0 (0 to 0)	0 (0 to 0)	0 (0 to 0)	0 (0 to 0)	0 (0 to 0)	0 (0 to 0)	0 (0 to 0)	0 (0 to 0)	0 (0 to 0)	0·1 (0 to 0·2)	0·6 (0·1 to 1·4)	1·8 (0·7 to 3·5)	3·2 (1·6 to 5·4)	4·6 (2·5 to 7·6)	5 (2·9 to 8·1)	5·9 (3·5 to 9·7)	5·4 (3·2 to 8·4)	3·6 (2·2 to 5·7)	1·8 (1 to 2·8)	0·5 (0·2 to 0·8)	32·6
Cataract	0 (0 to 0)	0 (0 to 0)	0 (0 to 0)	0 (0 to 0)	0 (0 to 0)	0 (0 to 0)	0 (0 to 0)	0·1 (0·1 to 0·2)	0·6 (0·3 to 1·2)	1·1 (0·3 to 2·4)	2·1 (0·8 to 3·8)	3·5 (1·6 to 6)	4·8 (2·3 to 8·2)	5·9 (3·2 to 9·6)	7·8 (4·4 to 12·1)	9·9 (6 to 15·3)	13·5 (8·4 to 20·2)	15·1 (9·6 to 22·2)	17·8 (11·5 to 25·6)	16·2 (10·7 to 22·8)	10·6 (7·1 to 14·8)	5·4 (3·5 to 7·6)	1·4 (0·9 to 2)	116
Age-related macular degeneration	0 (0 to 0)	0 (0 to 0)	0 (0 to 0)	0 (0 to 0)	0 (0 to 0)	0 (0 to 0)	0 (0 to 0)	0 (0 to 0)	0 (0 to 0)	0 (0 to 0)	0 (0 to 0)	0 (0 to 0)	0 (0 to 0·1)	0·2 (0·1 to 0·4)	0·5 (0·1 to 1·1)	0·7 (0·3 to 1·4)	0·8 (0·3 to 1·5)	0·7 (0·3 to 1·3)	0·7 (0·4 to 1·2)	0·6 (0·3 to 0·9)	0·3 (0·2 to 0·5)	0·1 (0·1 to 0·2)	0 (0 to 0·1)	4·8
Refraction disorders	0 (0 to 0)	0 (0 to 0)	0·2 (0·1 to 0·4)	4·5 (2·4 to 7·6)	10·7 (5·9 to 17·7)	13·4 (7·6 to 22·3)	16·9 (9·9 to 27·2)	21·4 (12·1 to 33·7)	19·1 (11·1 to 29·4)	14·7 (8·7 to 23·2)	14·7 (8·8 to 22·7)	15·5 (9·2 to 24·7)	15·2 (9·1 to 23·7)	14·6 (9 to 22·3)	14·7 (9 to 22·9)	13·2 (8·1 to 20·5)	11·4 (7 to 17·7)	8·5 (5·2 to 12·8)	7·1 (4·3 to 10·7)	4·6 (2·7 to 6·9)	2·1 (1·3 to 3·1)	0·7 (0·5 to 1·1)	0·1 (0·1 to 0·2)	223
Near vision loss	0 (0 to 0)	0 (0 to 0)	0 (0 to 0)	0 (0 to 0·1)	0·1 (0 to 0·2)	0·1 (0 to 0·3)	0·2 (0 to 0·4)	0·5 (0·1 to 1·2)	0·9 (0·3 to 2·1)	1·8 (0·6 to 4)	3·4 (1·3 to 7·6)	6·9 (2·7 to 14·4)	10·9 (4·2 to 23·6)	15·9 (6·3 to 33·6)	21 (8·2 to 45·2)	22·8 (9·3 to 48·9)	21·8 (8·9 to 44·5)	16 (6·8 to 31·9)	12·7 (5·4 to 25·9)	8·8 (3·8 to 17·7)	4·6 (2 to 8·9)	2·1 (0·9 to 4·1)	0·5 (0·2 to 1)	151
Other vision loss	0 (0 to 0)	0 (0 to 0)	0·1 (0 to 0·2)	1 (0·5 to 1·8)	1·9 (0·7 to 3·9)	2·4 (0·8 to 5)	3 (1·1 to 5·8)	3·9 (1·6 to 7·2)	3·8 (1·6 to 7)	3·7 (1·7 to 6·8)	4·6 (2·3 to 8·1)	6·6 (3·6 to 11·1)	8·2 (4·5 to 13·3)	11·5 (6·8 to 17·6)	15·7 (9·3 to 23·7)	19·8 (12·5 to 29)	22·8 (14·5 to 33·1)	18·7 (11·9 to 27·1)	14·9 (9·4 to 22·3)	11·6 (7·3 to 17·2)	7·3 (4·6 to 10·9)	4·7 (3·1 to 7)	1·5 (0·9 to 2·2)	168
Age-related and other hearing loss	0 (0 to 0)	0 (0 to 0)	0·7 (0·3 to 1·2)	10·7 (5·4 to 18·4)	25·4 (13·9 to 40·5)	32·6 (18·9 to 51·4)	41·7 (24·4 to 63·4)	58·4 (33·1 to 89·4)	67·1 (41·6 to 103)	66·3 (40·4 to 101)	72·7 (43·2 to 115)	89·8 (53·5 to 143)	119 (73·1 to 193)	151 (93·3 to 241)	184 (116 to 287)	189 (116 to 292)	178 (112 to 274)	136 (86·2 to 201)	117 (77 to 170)	89·4 (60·8 to 124)	51·3 (35·1 to 69·4)	24·4 (17 to 33·2)	5·3 (3·8 to 7·1)	1,710
Other sense organ diseases	0 (0 to 0)	0 (0 to 0·1)	0·9 (0·4 to 1·5)	4·2 (2·1 to 7·4)	4·5 (2·1 to 7·7)	4·1 (1·9 to 7·8)	5 (2·1 to 9·5)	6·1 (2·5 to 11·4)	6·6 (2·8 to 12·5)	7·1 (2·9 to 13·8)	8 (3·6 to 14·6)	8·5 (3·9 to 16·3)	8·8 (3·8 to 16·8)	11·3 (5·1 to 21·5)	11·9 (5·7 to 23)	13·2 (6·2 to 24·9)	13·2 (6·2 to 24·6)	9·6 (4·4 to 18·3)	7·7 (3·7 to 14·1)	4·8 (2·5 to 8·8)	2 (1 to 3·6)	0·8 (0·4 to 1·4)	0·2 (0·1 to 0·4)	139
Musculoskeletal disorders	0 (0 to 0)	0 (0 to 0)	0 (0 to 0)	0 (0 to 0)	24 (12·9 to 41·9)	109 (65·9 to 168)	244 (158 to 358)	404 (268 to 577)	494 (332 to 684)	487 (321 to 667)	541 (381 to 753)	626 (436 to 863)	645 (457 to 901)	653 (450 to 921)	645 (443 to 895)	558 (382 to 784)	435 (301 to 612)	267 (183 to 378)	178 (119 to 253)	106 (72·1 to 151)	49·1 (33·8 to 69·6)	19 (13·2 to 27)	4 (2·8 to 5·6)	6,490
Rheumatoid arthritis	0 (0 to 0)	0 (0 to 0)	0 (0 to 0)	0 (0 to 0)	0·5 (0·3 to 0·9)	1·1 (0·6 to 1·8)	3·5 (2 to 5·7)	7·5 (3·8 to 12·4)	9·2 (4·5 to 15·3)	9·6 (5 to 15·9)	11·3 (6·4 to 17·5)	13·3 (7·9 to 19·8)	13·6 (8·6 to 20·1)	13·4 (8·6 to 19·3)	13·6 (8·9 to 19·5)	11·1 (7·2 to 15·7)	8·7 (5·6 to 12·3)	5 (3·3 to 7)	3·5 (2·4 to 4·8)	2·1 (1·4 to 3)	1·8 (1·1 to 2·6)	0·9 (0·5 to 1·3)	0·2 (0·1 to 0·3)	130
Osteoarthritis	0 (0 to 0)	0 (0 to 0)	0 (0 to 0)	0 (0 to 0)	0 (0 to 0)	0 (0 to 0)	0 (0 to 0)	0 (0 to 0)	0 (0 to 0)	2·2 (0·9 to 5)	13·7 (6·5 to 28·2)	38·2 (18·5 to 77·2)	68·3 (34·1 to 140)	99·7 (48·2 to 203)	125 (60·6 to 250)	127 (62·4 to 252)	110 (54·9 to 218)	72·9 (36·6 to 145)	51·6 (25·8 to 102)	32 (16·2 to 64·2)	15·1 (7·8 to 30·2)	6·2 (3·2 to 12·2)	1·3 (0·7 to 2·5)	762
Osteoarthritis hip	0 (0 to 0)	0 (0 to 0)	0 (0 to 0)	0 (0 to 0)	0 (0 to 0)	0 (0 to 0)	0 (0 to 0)	0 (0 to 0)	0 (0 to 0)	0·6 (0·1 to 1·5)	1 (0·3 to 2·1)	1·5 (0·5 to 3·6)	2·4 (0·9 to 5·5)	3·5 (1·3 to 7·7)	4·5 (1·9 to 10·2)	5 (2·3 to 10·6)	4·7 (2·1 to 9·9)	3·3 (1·5 to 6·8)	2·4 (1·1 to 5)	1·5 (0·7 to 3·1)	0·7 (0·3 to 1·5)	0·3 (0·1 to 0·6)	0·1 (0 to 0·1)	31·5
Osteoarthritis knee	0 (0 to 0)	0 (0 to 0)	0 (0 to 0)	0 (0 to 0)	0 (0 to 0)	0 (0 to 0)	0 (0 to 0)	0 (0 to 0)	0 (0 to 0)	1·2 (0·4 to 3)	10·3 (4·7 to 22·3)	29·2 (13·4 to 60·9)	51·6 (24·6 to 107)	74·7 (36·1 to 152)	91·9 (44·5 to 191)	92·4 (44·5 to 184)	79·1 (38·6 to 160)	51·8 (25·4 to 105)	35·7 (17·5 to 71·6)	21·4 (10·5 to 44)	9·6 (4·8 to 19·5)	3·7 (1·8 to 7·3)	0·7 (0·3 to 1·4)	553
Osteoarthritis hand	0 (0 to 0)	0 (0 to 0)	0 (0 to 0)	0 (0 to 0)	0 (0 to 0)	0 (0 to 0)	0 (0 to 0)	0 (0 to 0)	0 (0 to 0)	0·2 (0 to 0·7)	1·6 (0·4 to 4·1)	4·8 (1·8 to 10·8)	9·1 (3·7 to 20·2)	13·7 (6·2 to 29·3)	17·5 (8·3 to 38·2)	17·8 (8·4 to 36·9)	15·5 (7·4 to 32·4)	10·4 (5 to 21·2)	7·6 (3·8 to 15·7)	5 (2·5 to 10·4)	2·6 (1·3 to 5·4)	1·2 (0·6 to 2·5)	0·3 (0·1 to 0·6)	107
Osteoarthritis other	0 (0 to 0)	0 (0 to 0)	0 (0 to 0)	0 (0 to 0)	0 (0 to 0)	0 (0 to 0)	0 (0 to 0)	0 (0 to 0)	0 (0 to 0)	0·1 (0 to 0·3)	0·9 (0·3 to 2)	2·7 (0·9 to 6·2)	5·2 (2·1 to 11·7)	7·8 (3·4 to 17·5)	10·5 (4·6 to 22·3)	11·3 (5·3 to 24·1)	10·5 (5 to 21·7)	7·5 (3·7 to 15·5)	5·9 (2·9 to 11·8)	4·1 (2·1 to 8·2)	2·2 (1·1 to 4·3)	1 (0·5 to 2)	0·2 (0·1 to 0·5)	69·8
Low back pain	0 (0 to 0)	0 (0 to 0)	0 (0 to 0)	0 (0 to 0)	20·7 (9·6 to 38·5)	96·7 (56·1 to 155)	180 (106 to 290)	247 (142 to 389)	259 (150 to 400)	237 (135 to 358)	264 (159 to 401)	294 (178 to 447)	290 (179 to 455)	287 (178 to 443)	275 (173 to 418)	236 (148 to 352)	184 (116 to 280)	116 (71·6 to 175)	82·8 (50·9 to 126)	53·7 (33·2 to 79·8)	23·8 (14·6 to 35·1)	8·7 (5·5 to 13)	1·7 (1·1 to 2·6)	3,160
Neck pain	0 (0 to 0)	0 (0 to 0)	0 (0 to 0)	0 (0 to 0)	1 (0·2 to 2·4)	5·6 (2·2 to 11·3)	13·6 (5·3 to 26)	23·7 (10·1 to 47·9)	33·4 (14·8 to 64)	41·3 (20·1 to 78·3)	53·9 (27 to 92·9)	66·8 (33·4 to 119)	68·3 (34 to 121)	58·7 (30·3 to 108)	53·1 (25·5 to 97·1)	42·6 (21·5 to 74·5)	31·1 (16·4 to 51·4)	18·6 (9·8 to 31·7)	12 (5·9 to 22·6)	6·6 (3·2 to 11·7)	2·9 (1·5 to 5·1)	1·2 (0·6 to 2)	0·3 (0·1 to 0·5)	535
Gout	0 (0 to 0)	0 (0 to 0)	0 (0 to 0)	0 (0 to 0)	0 (0 to 0)	0 (0 to 0)	0 (0 to 0·1)	0·3 (0·1 to 0·6)	0·7 (0·4 to 1·3)	1 (0·5 to 1·8)	1·3 (0·3 to 2·9)	1·8 (0·5 to 3·5)	1·9 (0·7 to 4)	2·1 (0·7 to 4·1)	2·2 (0·8 to 4·1)	2 (0·8 to 3·8)	1·7 (0·8 to 3·1)	1·1 (0·5 to 2)	0·9 (0·4 to 1·5)	0·6 (0·2 to 1·1)	0·3 (0·1 to 0·6)	0·2 (0·1 to 0·3)	0 (0 to 0·1)	18·3
Other musculoskeletal disorders	0 (0 to 0)	0 (0 to 0)	0 (0 to 0)	0 (0 to 0)	1·7 (0·9 to 2·7)	5·7 (3·1 to 10·2)	46·1 (27·3 to 71·5)	126 (83·4 to 177)	192 (126 to 277)	195 (131 to 280)	197 (130 to 279)	211 (134 to 317)	203 (135 to 297)	192 (123 to 283)	177 (113 to 261)	140 (85·4 to 212)	99·5 (60·9 to 151)	53·9 (29 to 89·1)	27·7 (14·5 to 44·7)	10·8 (2·3 to 21·2)	5·1 (2·1 to 9·3)	1·8 (0·8 to 3·5)	0·4 (0·2 to 0·8)	1,890
Other non-communicable diseases	689 (434 to 1,060)	127 (65·1 to 275)	804 (526 to 1,210)	413 (289 to 594)	297 (236 to 370)	293 (224 to 387)	479 (353 to 652)	756 (559 to 1,020)	857 (614 to 1,170)	748 (539 to 1,020)	760 (555 to 1,020)	784 (568 to 1,060)	684 (504 to 905)	594 (436 to 778)	531 (391 to 699)	440 (326 to 576)	353 (264 to 461)	225 (167 to 295)	156 (118 to 201)	96·9 (73·8 to 123)	51·6 (39·7 to 66·1)	25·8 (19·9 to 32·9)	6·4 (4·9 to 8·2)	10,200

Congenital birth defects	669 (414 to 1,030)	115 (56·2 to 261)	584 (356 to 902)	236 (154 to 364)	122 (90 to 159)	94 (70·2 to 125)	82 (60·4 to 109)	77·1 (55·9 to 105)	62·4 (44·3 to 87·9)	38·2 (27·4 to 51·6)	35·6 (25·7 to 47·9)	31·6 (23·1 to 42·1)	26·6 (19·5 to 35·7)	23·6 (17·2 to 31·2)	19·1 (13·8 to 25)	10·7 (7·8 to 14·5)	6·8 (5 to 9·1)	1·8 (1 to 2·9)	1 (0·6 to 1·6)	0·5 (0·3 to 0·8)	0·2 (0·1 to 0·3)	0·1 (0 to 0·1)	0 (0 to 0)	2,240
Neural tube defects	57·7 (28·4 to 104)	5·6 (1·7 to 14)	28·6 (10·9 to 68·1)	14·9 (6·5 to 30·4)	3·6 (2·3 to 5·7)	5 (3·1 to 8·2)	4·1 (2·6 to 6·5)	2·1 (1·4 to 3)	2·5 (1·5 to 4)	1·8 (1 to 2·9)	1·2 (0·7 to 1·9)	1 (0·7 to 1·6)	0·4 (0·3 to 0·6)	0·3 (0·2 to 0·5)	0·4 (0·3 to 0·7)	0·2 (0·1 to 0·3)	0·2 (0·1 to 0·3)	0 (0 to 0)	0 (0 to 0)	0 (0 to 0)	0 (0 to 0)	0 (0 to 0)	0 (0 to 0)	130
Congenital heart anomalies	280 (160 to 456)	61·7 (21·8 to 169)	350 (201 to 559)	111 (61 to 194)	46·5 (29·6 to 69·1)	30·5 (19·6 to 45·4)	28 (18·2 to 41·3)	30 (18·1 to 47·1)	26·6 (15·8 to 41·9)	12·9 (7·6 to 19·8)	13·7 (8·4 to 20·6)	11·9 (7·6 to 18·4)	9·2 (6 to 13·7)	8·4 (5·2 to 12·7)	5·6 (3·5 to 8·3)	2·6 (1·7 to 3·8)	1·6 (1 to 2·4)	0·1 (0 to 0·3)	0·1 (0 to 0·2)	0 (0 to 0·1)	0 (0 to 0)	0 (0 to 0)	0 (0 to 0)	1,030
Orofacial clefts	1·8 (0·6 to 4·4)	0·1 (0·1 to 0·3)	1·6 (0·6 to 3·9)	1·4 (0·5 to 2·5)	0·9 (0·3 to 2)	0·8 (0·2 to 1·9)	0·9 (0·2 to 1·8)	0·9 (0·2 to 2)	0·8 (0·2 to 1·6)	0·6 (0·1 to 1·4)	0·6 (0·1 to 1·3)	0·6 (0·1 to 1·1)	0·5 (0·1 to 1)	0·4 (0·1 to 0·9)	0·4 (0·1 to 0·8)	0·3 (0·1 to 0·6)	0·2 (0 to 0·5)	0·1 (0 to 0·3)	0·1 (0 to 0·2)	0·1 (0 to 0·1)	0 (0 to 0·1)	0 (0 to 0)	0 (0 to 0)	13·2
Down syndrome	2·5 (1·2 to 4·8)	0·7 (0·2 to 1·7)	7·4 (3·4 to 13·9)	6·3 (3·2 to 11·8)	2·8 (1·7 to 4·3)	2·2 (1·5 to 3·3)	2·3 (1·5 to 3·3)	2·9 (1·9 to 4·5)	2·2 (1·3 to 3·5)	1·6 (1 to 2·6)	1·8 (1 to 3)	1·7 (0·9 to 2·8)	1·9 (1·1 to 3·1)	2·3 (1·2 to 4)	2·1 (1 to 3·8)	0·7 (0·4 to 1·3)	0·2 (0·1 to 0·3)	0 (0 to 0)	0 (0 to 0)	0 (0 to 0)	0 (0 to 0)	0 (0 to 0)	0 (0 to 0)	41·6
Turner syndrome	0 (0 to 0)	0 (0 to 0)	0 (0 to 0·1)	0·1 (0·1 to 0·3)	0·1 (0·1 to 0·3)	0·1 (0 to 0·2)	0·2 (0·1 to 0·3)	0·2 (0·1 to 0·3)	0·1 (0·1 to 0·2)	0·1 (0 to 0·2)	0·1 (0 to 0·1)	0 (0 to 0·1)	0 (0 to 0·1)	0 (0 to 0)	0 (0 to 0)	0 (0 to 0)	0 (0 to 0)	0 (0 to 0)	0 (0 to 0)	0 (0 to 0)	0 (0 to 0)	0 (0 to 0)	0 (0 to 0)	1·2
Klinefelter syndrome	0 (0 to 0)	0 (0 to 0)	0 (0 to 0)	0 (0 to 0)	0 (0 to 0)	0 (0 to 0)	0 (0 to 0)	0 (0 to 0)	0 (0 to 0)	0 (0 to 0)	0 (0 to 0)	0 (0 to 0)	0 (0 to 0)	0 (0 to 0)	0 (0 to 0)	0 (0 to 0)	0 (0 to 0)	0 (0 to 0)	0 (0 to 0)	0 (0 to 0)	0 (0 to 0)	0 (0 to 0)	0 (0 to 0)	0
Other chromosomal abnormalities	5·1 (2 to 9·7)	2·9 (0·6 to 6·2)	8·8 (4·7 to 14·8)	4·5 (3 to 6·5)	2·5 (1·6 to 3·8)	1·9 (1·2 to 2·8)	2 (1·3 to 2·8)	1·9 (1·3 to 2·7)	1·3 (0·9 to 1·9)	0·8 (0·5 to 1·1)	0·5 (0·3 to 0·7)	0·4 (0·2 to 0·5)	0·2 (0·1 to 0·3)	0·1 (0·1 to 0·2)	0·1 (0 to 0·1)	0 (0 to 0·1)	0 (0 to 0)	0 (0 to 0)	0 (0 to 0)	0 (0 to 0)	0 (0 to 0)	0 (0 to 0)	0 (0 to 0)	33·1
Congenital musculoskeletal and limb anomalies	13·7 (6·8 to 25)	3 (1·6 to 5·6)	23·4 (14·2 to 35·9)	34·7 (20·6 to 54)	32·3 (19·4 to 50·5)	29·7 (17·3 to 46·2)	26 (15·5 to 39·7)	18·4 (11·2 to 27·7)	14 (8·3 to 22·5)	10·2 (5·9 to 16·5)	9·5 (5·6 to 14·9)	9·3 (5·5 to 14·9)	8·2 (4·8 to 12·9)	7·1 (4·2 to 11·3)	6·2 (3·7 to 9·7)	4·5 (2·6 to 7·2)	2·8 (1·6 to 4·4)	1·1 (0·6 to 1·9)	0·6 (0·3 to 1)	0·3 (0·1 to 0·4)	0·1 (0 to 0·1)	0 (0 to 0)	0 (0 to 0)	255
Urogenital congenital anomalies	23·5 (10·9 to 45·9)	4·9 (1·3 to 16·2)	8·5 (4 to 17·4)	4 (2·4 to 6·3)	2·6 (1·6 to 4·1)	3·1 (1·7 to 5)	2 (1·1 to 3·4)	1·6 (0·9 to 2·8)	1·6 (0·8 to 3·1)	0·6 (0·4 to 1)	0·6 (0·3 to 1)	0·3 (0·2 to 0·4)	0·7 (0·4 to 1·3)	0·2 (0·1 to 0·4)	0·1 (0·1 to 0·2)	0·1 (0·1 to 0·2)	0·2 (0·1 to 0·3)	0 (0 to 0)	0 (0 to 0)	0 (0 to 0)	0 (0 to 0)	0 (0 to 0)	0 (0 to 0)	54·6
Digestive congenital anomalies	109 (56·6 to 188)	18·4 (5·7 to 58·8)	62·4 (29·7 to 127)	15·2 (7·4 to 29·8)	3·6 (2 to 6·3)	1·6 (1 to 2·8)	0·8 (0·5 to 1·4)	0·9 (0·5 to 1·5)	0·4 (0·2 to 0·5)	0·3 (0·2 to 0·5)	0·3 (0·2 to 0·4)	0·3 (0·2 to 0·5)	0·4 (0·2 to 0·6)	0·2 (0·1 to 0·3)	0·4 (0·2 to 0·6)	0·3 (0·2 to 0·5)	0·1 (0·1 to 0·1)	0 (0 to 0)	0 (0 to 0)	0 (0 to 0)	0 (0 to 0)	0 (0 to 0)	0 (0 to 0)	215
Other congenital birth defects	174 (97·1 to 298)	17·7 (6·1 to 44·9)	93·2 (47·7 to 168)	43·2 (23·2 to 76·8)	26·8 (16·5 to 40·6)	19 (11·7 to 29·6)	15·7 (9·9 to 24·6)	18·2 (10·8 to 30·2)	13 (7·5 to 21)	9·3 (5·6 to 14·6)	7·5 (4·5 to 12·1)	6·1 (3·6 to 9·6)	5·1 (3 to 8·1)	4·4 (2·6 to 7·1)	3·8 (2·4 to 6·1)	1·9 (1·1 to 3·3)	1·5 (0·9 to 2·6)	0·4 (0·1 to 1)	0·2 (0·1 to 0·7)	0·2 (0 to 0·4)	0·1 (0 to 0·2)	0 (0 to 0·1)	0 (0 to 0)	462
Urinary diseases and male infertility	1 (0·5 to 1·7)	0·6 (0·3 to 1·1)	8·6 (5 to 13·9)	6·9 (4·3 to 10·9)	5·6 (3·7 to 8·2)	7·5 (5·1 to 10·5)	11·9 (8·1 to 16·7)	20·1 (13·3 to 29)	23·4 (15·4 to 33·3)	23·8 (15·3 to 35·5)	27·1 (17·7 to 40·8)	31·8 (21·5 to 47·2)	31·3 (20·5 to 46·7)	35·7 (22·2 to 55·9)	38·4 (24·7 to 57·8)	33·6 (22·1 to 49·3)	30·7 (19·9 to 45·7)	23·9 (14·9 to 36·3)	19·6 (12·9 to 27·9)	15·9 (10·3 to 23·5)	9·5 (6 to 13·9)	5·9 (3·5 to 8·9)	1·7 (1 to 2·6)	414
Urolithiasis	0 (0 to 0)	0 (0 to 0)	0 (0 to 0)	0·1 (0 to 0·1)	0·1 (0·1 to 0·2)	0·3 (0·2 to 0·5)	0·3 (0·1 to 0·4)	1 (0·6 to 1·6)	1·4 (0·8 to 2·2)	2·8 (1·4 to 4·9)	3·7 (2 to 6·8)	3·1 (1·8 to 5)	4·8 (2·4 to 8·4)	3·8 (2 to 6·5)	4·7 (2·4 to 8·4)	3·3 (1·7 to 6·2)	2·6 (1·4 to 4·6)	1·1 (0·6 to 2)	0·6 (0·3 to 1·1)	0·3 (0·2 to 0·5)	0·2 (0·1 to 0·3)	0·1 (0 to 0·1)	0 (0 to 0)	34·1
Benign prostatic hyperplasia	0 (0 to 0)	0 (0 to 0)	0 (0 to 0)	0 (0 to 0)	0 (0 to 0)	0 (0 to 0)	0 (0 to 0)	0 (0 to 0)	0 (0 to 0)	0 (0 to 0)	0 (0 to 0)	0 (0 to 0)	0 (0 to 0)	0 (0 to 0)	0 (0 to 0)	0 (0 to 0)	0 (0 to 0)	0 (0 to 0)	0 (0 to 0)	0 (0 to 0)	0 (0 to 0)	0 (0 to 0)	0 (0 to 0)	0
Male infertility	--	--	--	--	--	--	--	--	--	--	--	--	--	--	--	--	--	--	--	--	--	--	--	NA
Other urinary diseases	0·5 (0·3 to 1)	0·4 (0·2 to 0·8)	1·4 (0·6 to 2·9)	1·8 (1 to 2·9)	1·2 (0·7 to 1·8)	2·6 (1·6 to 3·9)	2 (1·2 to 3·1)	3·9 (2·1 to 6·9)	1·9 (1·1 to 2·9)	2·2 (1·3 to 3·5)	1·7 (1·1 to 2·6)	2·5 (1·5 to 4)	2·1 (1·2 to 3·3)	2·6 (1·4 to 4·7)	2·3 (1·3 to 4·2)	2·2 (1·2 to 3·9)	1·2 (0·6 to 2·1)	0·7 (0·3 to 1·3)	0·5 (0·2 to 1)	0·3 (0·2 to 0·6)	0·1 (0·1 to 0·3)	0·1 (0 to 0·2)	0 (0 to 0)	34·1
Gynecological diseases	0 (0 to 0)	0 (0 to 0)	0 (0 to 0)	0 (0 to 0)	0 (0 to 0)	49·7 (24·5 to 87·2)	168 (99·8 to 273)	325 (205 to 497)	426 (273 to 629)	402 (263 to 580)	411 (281 to 587)	408 (272 to 591)	307 (203 to 431)	196 (118 to 292)	114 (71·5 to 164)	69·9 (41·7 to 106)	47·2 (29·3 to 69·6)	25·8 (16·4 to 37·3)	15·5 (9·8 to 22·6)	8·7 (5·4 to 13·2)	4·2 (2·6 to 6·1)	1·8 (1·1 to 2·8)	0·4 (0·2 to 0·6)	2,980
Uterine fibroids	0 (0 to 0)	0 (0 to 0)	0 (0 to 0)	0 (0 to 0)	0 (0 to 0)	0·8 (0·3 to 1·8)	2·4 (1·1 to 4·7)	6·2 (2·8 to 12·7)	13·3 (6 to 27)	22·3 (10·7 to 43·7)	37·6 (19·1 to 70·4)	43·4 (23·6 to 77·5)	36·7 (19·9 to 63·1)	23 (11·8 to 40·7)	14·8 (7·6 to 26·2)	7·8 (3·7 to 14·7)	4·2 (2 to 7·9)	1·8 (0·9 to 3·5)	0·9 (0·5 to 1·7)	0·4 (0·2 to 0·8)	0·1 (0·1 to 0·3)	0·1 (0 to 0·1)	0 (0 to 0)	216
Polycystic ovarian syndrome	0 (0 to 0)	0 (0 to 0)	0 (0 to 0)	0 (0 to 0)	0 (0 to 0)	1·6 (0·6 to 3·5)	8 (3·2 to 16·7)	10·4 (4·3 to 21·7)	8·6 (3·4 to 17·5)	6·3 (2·7 to 12·9)	5·7 (2·4 to 11·8)	5·4 (2·3 to 11·1)	4·2 (1·8 to 8·4)	1 (0·4 to 2·1)	0 (0 to 0)	0 (0 to 0)	0 (0 to 0)	0 (0 to 0)	0 (0 to 0)	0 (0 to 0)	0 (0 to 0)	0 (0 to 0)	0 (0 to 0)	51·3
Female infertility	0 (0 to 0)	0 (0 to 0)	0 (0 to 0)	0 (0 to 0)	0 (0 to 0)	0 (0 to 0)	0 (0 to 0)	15 (3·6 to 39·5)	19·8 (5·1 to 54)	19·5 (4·5 to 57·4)	18·8 (3·1 to 55·7)	16·7 (2·8 to 45)	0·3 (0 to 2·6)	0 (0 to 0)	0 (0 to 0)	0 (0 to 0)	0 (0 to 0)	0 (0 to 0)	0 (0 to 0)	0 (0 to 0)	0 (0 to 0)	0 (0 to 0)	0 (0 to 0)	90·1
Endometriosis	0 (0 to 0)	0 (0 to 0)	0 (0 to 0)	0 (0 to 0)	0 (0 to 0)	0 (0 to 0)	11·1 (4·5 to 22)	51·3 (24·3 to 93·5)	52·6 (25 to 95·4)	33·4 (16·2 to 60·1)	27·3 (14·1 to 47·7)	26·7 (13·2 to 46·4)	21 (11 to 34·9)	9·5 (4·7 to 16·1)	0 (0 to 0)	0 (0 to 0)	0 (0 to 0)	0 (0 to 0)	0 (0 to 0)	0 (0 to 0)	0 (0 to 0)	0 (0 to 0)	0 (0 to 0)	233
Genital prolapse	0 (0 to 0)	0 (0 to 0)	0 (0 to 0)	0 (0 to 0)	0 (0 to 0)	0 (0 to 0)	0 (0 to 0·1)	0·4 (0·1 to 0·9)	1 (0·3 to 2·1)	1·6 (0·7 to 3·4)	3 (1·3 to 5·9)	4·5 (1·9 to 9·5)	5·6 (2·6 to 11·7)	6·1 (2·7 to 12·1)	6·4 (3 to 12·2)	5·2 (2·5 to 9·9)	4·2 (2·1 to 7·7)	2·5 (1·3 to 4·6)	1·7 (0·8 to 3)	1 (0·5 to 1·9)	0·5 (0·3 to 1)	0·2 (0·1 to 0·4)	0·1 (0 to 0·1)	44·1
Premenstrual syndrome	0 (0 to 0)	0 (0 to 0)	0 (0 to 0)	0 (0 to 0)	0 (0 to 0)	44·3 (20·3 to 79·9)	104 (53 to 183)	144 (70·3 to 256)	129 (74·8 to 209)	103 (54·7 to 169)	108 (61·4 to 171)	106 (59·6 to 167)	67 (36·8 to 111)	21·7 (10·8 to 38·9)	0 (0 to 0)	0 (0 to 0)	0 (0 to 0)	0 (0 to 0)	0 (0 to 0)	0 (0 to 0)	0 (0 to 0)	0 (0 to 0)	0 (0 to 0)	827
Other gynecological diseases	0 (0 to 0)	0 (0 to 0)	0 (0 to 0)	0 (0 to 0)	0 (0 to 0)	2·9 (1·1 to 6)	42 (19·5 to 71·3)	97·8 (56·9 to 153)	201 (115 to 315)	216 (127 to 327)	211 (127 to 329)	205 (118 to 331)	172 (99·1 to 267)	135 (67 to 219)	93·2 (53·9 to 140)	56·9 (31 to 91)	38·7 (22·7 to 59·8)	21·4 (13 to 32)	12·9 (7·4 to 19·7)	7·3 (4·2 to 11·6)	3·5 (2·1 to 5·4)	1·5 (0·9 to 2·4)	0·3 (0·2 to 0·6)	1,520
Hemoglobinopathies and hemolytic anemias	2·8 (1·7 to 4·6)	1·7 (0·9 to 2·8)	19·4 (12·7 to 28·3)	50·5 (31·1 to 75·9)	79·8 (55·3 to 110)	65·8 (42·2 to 95·2)	107 (66·7 to 166)	171 (109 to 241)	146 (89·9 to 221)	89 (50·5 to 141)	65·5 (43·9 to 94·9)	60·6 (42·1 to 86·9)	52·4 (36·9 to 73·8)	50·2 (34·7 to 71·5)	41·6 (29·3 to 57·7)	38·1 (24·8 to 54·1)	26·6 (17·7 to 37·6)	15·1 (10·1 to 21)	8·4 (5·7 to 12·1)	4·7 (3 to 6·6)	3·4 (2·3 to 4·8)	1·8 (1·2 to 2·5)	0·3 (0·2 to 0·4)	1,100
Thalassemias	0 (0 to 0)	0 (0 to 0)	0 (0 to 0·1)	0·3 (0·1 to 0·6)	0·1 (0·1 to 0·3)	0 (0 to 0·1)	0 (0 to 0·1)	0 (0 to 0·1)	0 (0 to 0)	0 (0 to 0)	0 (0 to 0)	0 (0 to 0)	0 (0 to 0)	0 (0 to 0)	0 (0 to 0)	0 (0 to 0)	0 (0 to 0)	0 (0 to 0)	0 (0 to 0)	0 (0 to 0)	0 (0 to 0)	0 (0 to 0)	0 (0 to 0)	0·6
Thalassemias trait	0 (0 to 0)	0 (0 to 0·1)	0·3 (0·1 to 0·6)	1·1 (0·4 to 2·2)	1·8 (0·8 to 3·3)	1·3 (0·4 to 2·6)	4·5 (2·2 to 7·7)	4·8 (2·5 to 7·8)	4·3 (2·3 to 7·1)	3·6 (1·9 to 5·9)	3·4 (1·8 to 5·5)	2·9 (1·5 to 4·9)	0·6 (0·2 to 1·1)	1 (0·4 to 1·7)	0·9 (0·4 to 1·6)	0·6 (0·3 to 1·1)	0·5 (0·2 to 0·8)	0·3 (0·1 to 0·5)	0·2 (0·1 to 0·3)	0·1 (0 to 0·2)	0·1 (0 to 0·1)	0 (0 to 0)	0 (0 to 0)	32·3
Sickle cell disorders	2·8 (1·6 to 4·6)	0·6 (0·3 to 1·1)	13·6 (8·2 to 22·2)	32·3 (17·3 to 57·1)	50·2 (33·7 to 73)	44·3 (26·3 to 67·4)	65·5 (40·1 to 100)	76·7 (43·6 to 124)	64 (36·6 to 107)	35·4 (19·4 to 58·7)	33·4 (18·3 to 56·2)	26·2 (15·3 to 42·8)	22·9 (13·8 to 37·3)	17 (9·1 to 29·2)	7·8 (4 to 13·3)	2 (0·9 to 3·8)	0·3 (0·1 to 0·7)	0 (0 to 0·1)	0 (0 to 0)	0 (0 to 0)	0 (0 to 0)	0 (0 to 0)	0 (0 to 0)	495
Sickle cell trait	0 (0 to 0·1)	0·8 (0·3 to 1·5)	3·4 (1·8 to 5·6)	11·1 (5·6 to 18·8)	18 (9 to 29·7)	12·9 (5·6 to 24·3)	26·6 (5·6 to 64)	79·4 (37·1 to 131)	65·3 (27·2 to 117)	39 (10·4 to 75·5)	2·5 (0·5 to 5·4)	9 (4·9 to 14·3)	9·6 (5·3 to 14·9)	9·5 (5·5 to 14·6)	8·7 (4·9 to 13·5)	6·2 (3·5 to 10)	4·6 (2·5 to 7·3)	2·6 (1·5 to 4·1)	1·5 (0·7 to 2·5)	0·9 (0·4 to 1·6)	0·5 (0·3 to 0·8)	0·2 (0·1 to 0·3)	0 (0 to 0·1)	312
G6PD deficiency	0 (0 to 0)	0 (0 to 0)	0 (0 to 0)	0·2 (0·1 to 0·5)	0·4 (0·1 to 0·7)	0·3 (0·1 to 0·7)	1 (0·4 to 1·9)	1·9 (0·7 to 3·9)	2·9 (1·1 to 6·2)	2·4 (0·9 to 5·2)	11 (6·6 to 16·9)	4·7 (2·6 to 7·8)	3 (1·2 to 5·8)	3·1 (1·1 to 6·1)	2·6 (1·1 to 5)	1·8 (0·8 to 3·2)	0·9 (0·4 to 1·7)	0·3 (0·1 to 0·5)	0·1 (0·1 to 0·2)	0 (0 to 0·1)	0 (0 to 0)	0 (0 to 0)	0 (0 to 0)	36·7
G6PD trait	0 (0 to 0)	0 (0 to 0)	0 (0 to 0)	0 (0 to 0)	0 (0 to 0)	0 (0 to 0)	0 (0 to 0)	0 (0 to 0)	0 (0 to 0)	0 (0 to 0)	0·8 (0·3 to 1·4)	0·8 (0·4 to 1·4)	0·2 (0·1 to 0·5)	0 (0 to 0)	0 (0 to 0)	0 (0 to 0)	0 (0 to 0)	0 (0 to 0)	0 (0 to 0)	0 (0 to 0)	0 (0 to 0)	0 (0 to 0)	0 (0 to 0)	1·9
Other hemoglobinopathies and hemolytic anemias	0 (0 to 0)	0·2 (0·1 to 0·4)	1·9 (0·9 to 3·4)	5·5 (2·6 to 9·8)	9·3 (3·8 to 17·2)	6·9 (2·5 to 14·6)	9·2 (4·1 to 16·2)	8·1 (3·7 to 15)	9·2 (4·7 to 15·8)	8·6 (4·6 to 14·4)	14·4 (8·6 to 22·2)	17·1 (10 to 26·1)	16·1 (10 to 24·1)	19·6 (12·5 to 29·6)	21·6 (14·5 to 31·7)	27·4 (17·4 to 40)	20·3 (13·1 to 29·5)	11·9 (8 to 16·8)	6·6 (4·5 to 9·6)	3·6 (2·4 to 5·2)	2·8 (1·9 to 4·1)	1·5 (1 to 2·2)	0·3 (0·2 to 0·4)	222
Endocrine, metabolic, blood, and immune disorders	16·1 (8·1 to 28·3)	7·7 (3·2 to 20·9)	167 (94·7 to 295)	102 (63·3 to 175)	68·6 (48·4 to 96·2)	53·8 (38·1 to 76·5)	77·6 (55 to 109)	104 (72·8 to 146)	125 (83 to 181)	121 (80·7 to 174)	137 (94·4 to 193)	157 (107 to 220)	165 (111 to 230)	173 (118 to 249)	186 (131 to 258)	155 (107 to 217)	124 (89·4 to 183)	79·2 (54·3 to 111)	54·9 (38·5 to 75·8)	31·9 (21·9 to 44·2)	17·8 (12·7 to 25·6)	9·3 (6·6 to 13·3)	2·5 (1·6 to 3·9)	2,140
Oral disorders	0 (0 to 0)	0 (0 to 0)	0 (0 to 0)	17·9 (10·3 to 30·1)	21 (11·4 to 35·3)	22·1 (11·5 to 38·6)	33·6 (18·4 to 57·9)	59·3 (31·1 to 110)	74·7 (38·1 to 143)	74·3 (36·4 to 142)	82·8 (40·3 to 154)	94·9 (47 to 175)	101 (54 to 179)	115 (65 to 195)	131 (77·9 to 207)	132 (81·4 to 206)	118 (72·8 to 181)	79·7 (50·1 to 120)	56·7 (36 to 84)	35·1 (22·7 to 51·2)	16·6 (10·5 to 24·2)	7 (4·4 to 10·2)	1·5 (0·9 to 2·2)	1,270
Caries of deciduous teeth	0 (0 to 0)	0 (0 to 0)	0 (0 to 0)	5·6 (2·2 to 11·8)	5·1 (2·1 to 10·7)	0·3 (0 to 0·8)	0 (0 to 0)	0 (0 to 0)	0 (0 to 0)	0 (0 to 0)	0 (0 to 0)	0 (0 to 0)	0 (0 to 0)	0 (0 to 0)	0 (0 to 0)	0 (0 to 0)	0 (0 to 0)	0 (0 to 0)	0 (0 to 0)	0 (0 to 0)	0 (0 to 0)	0 (0 to 0)	0 (0 to 0)	11
Caries of permanent teeth	0 (0 to 0)	0 (0 to 0)	0 (0 to 0)	0 (0 to 0)	5·1 (1·8 to 11·2)	10·3 (4·1 to 22·5)	11·5 (4·3 to 24·6)	14·8 (6·2 to 30·5)	12·8 (5·1 to 24·6)	9·3 (3·7 to 19·5)	8·7 (3·5 to 18·1)	8 (3·3 to 16·9)	6·8 (2·8 to 14·4)	6 (2·4 to 12·6)	4·9 (1·9 to 11)	3·5 (1·3 to 7·9)	2·3 (0·9 to 4·9)	1·3 (0·5 to 2·6)	0·8 (0·3 to 1·6)	0·5 (0·2 to 1)	0·2 (0·1 to 0·5)	0·1 (0 to 0·2)	0 (0 to 0)	107
Periodontal diseases	0 (0 to 0)	0 (0 to 0)	0 (0 to 0)	0 (0 to 0)	0 (0 to 0)	0 (0 to 0)	4·1 (1·1 to 10·8)	17 (5·2 to 42·8)	33·2 (10·2 to 80·2)	39·6 (13·2 to 89·3)	47·4 (16·7 to 107)	54·2 (20 to 117)	51·7 (19·5 to 112)	46·3 (18·2 to 99·1)	38·2 (15·4 to 81·7)	26·9 (10·7 to 56)	17·4 (6·8 to 36·9)	9·4 (3·6 to 19·4)	5·9 (2·3 to 12)	3·5 (1·3 to 7)	1·6 (0·6 to 3·5)	0·7 (0·2 to 1·4)	0·1 (0·1 to 0·3)	397
Other oral disorders	0 (0 to 0)	0 (0 to 0)	0 (0 to 0)	12·3 (7·2 to 19·4)	10·8 (6·3 to 16·9)	11·6 (6·8 to 18·1)	18 (10·8 to 28·1)	24·5 (14·7 to 37·9)	23·3 (13·8 to 35·2)	18·8 (11·5 to 28·5)	17·8 (10·9 to 27·2)	17·8 (10·7 to 27)	16·3 (9·8 to 24·8)	14·7 (8·9 to 22·3)	12·8 (7·7 to 19·5)	10·1 (6·1 to 15·2)	7·4 (4·5 to 11·1)	4 (2·4 to 6·2)	2·2 (1·3 to 3·3)	1·1 (0·6 to 1·6)	0·4 (0·2 to 0·6)	0·1 (0·1 to 0·2)	0 (0 to 0)	224
Sudden infant death syndrome	--	1·6 (0·9 to 2·8)	24·4 (13·1 to 42·6)	--	--	--	--	--	--	--	--	--	--	--	--	--	--	--	--	--	--	--	--	NA
Injuries	74·4 (48·1 to 111)	11·4 (6·9 to 18)	251 (151 to 401)	405 (285 to 557)	437 (366 to 512)	496 (403 to 602)	1,070 (852 to 1,310)	1,530 (1,150 to 2,020)	1,220 (888 to 1,660)	865 (625 to 1,160)	743 (558 to 987)	666 (514 to 857)	561 (441 to 712)	479 (376 to 599)	417 (334 to 517)	343 (277 to 414)	273 (226 to 327)	196 (162 to 236)	161 (134 to 190)	130 (107 to 155)	81·9 (67·2 to 98·2)	53·9 (44·6 to 63)	17·3 (14·3 to 20·3)	10,500
Transport injuries	2·5 (1·5 to 3·9)	0·5 (0·3 to 0·9)	14·2 (8·4 to 22·6)	55 (32·4 to 85·6)	101 (68·3 to 142)	106 (72·1 to 148)	207 (138 to 292)	290 (191 to 422)	223 (140 to 336)	136 (89·1 to 202)	129 (86·9 to 186)	122 (86·6 to 168)	102 (73·5 to 137)	85·9 (63·8 to 115)	74·5 (55·6 to 102)	56·9 (42·9 to 73·4)	43·5 (33·7 to 56·2)	24·2 (19·1 to 31·1)	15·6 (12·3 to 19·8)	8·7 (6·9 to 10·8)	3·9 (3 to 4·8)	1·9 (1·5 to 2·3)	0·5 (0·4 to 0·6)	1,800
Road injuries	1·7 (1 to 2·7)	0·3 (0·1 to 0·6)	10·7 (6·2 to 17·5)	45·3 (26·5 to 72·2)	87·6 (58·8 to 124)	89·7 (58·7 to 128)	184 (121 to 263)	256 (166 to 375)	195 (122 to 296)	118 (76·8 to 175)	114 (75·8 to 167)	103 (73·9 to 144)	90·2 (65·1 to 123)	74·6 (55·3 to 99·9)	64 (47·2 to 88·1)	50·3 (37·9 to 65·2)	39·8 (30·7 to 51·5)	21·9 (17·2 to 28·2)	13·6 (10·6 to 17)	7·8 (6·1 to 9·6)	3·5 (2·7 to 4·3)	1·6 (1·3 to 2)	0·4 (0·3 to 0·5)	1,570
Pedestrian road injuries	0·7 (0·4 to 1·2)	0·1 (0·1 to 0·2)	2·8 (1·6 to 4·6)	19·5 (10·8 to 32·7)	35·1 (22·4 to 50·7)	30·6 (19·1 to 44·6)	31·9 (20·6 to 46·1)	44·2 (28·5 to 66)	33·7 (20·8 to 51·8)	25·5 (15·5 to 38·8)	25·8 (16·3 to 39·1)	24·3 (16·3 to 34·9)	23·5 (16·3 to 33·3)	22·3 (15·5 to 31·2)	20 (14 to 28·5)	17·8 (12·7 to 24·2)	15·9 (11·5 to 21·3)	9·2 (6·8 to 12·7)	6·1 (4·6 to 8·1)	3·6 (2·7 to 4·7)	1·5 (1·2 to 1·9)	0·7 (0·5 to 0·9)	0·2 (0·1 to 0·2)	395
Cyclist road injuries	0 (0 to 0)	0 (0 to 0)	0 (0 to 0)	2·6 (1·1 to 5·2)	5·3 (2·8 to 9·5)	10·1 (5·6 to 18·1)	15·5 (9 to 25·8)	18·2 (11·2 to 29·4)	16·2 (9·8 to 25·7)	11·2 (7·5 to 16·2)	11·7 (7·7 to 17·1)	11·1 (7·6 to 15·7)	10·1 (7·4 to 13·8)	9·3 (6·7 to 12·6)	7·4 (5·4 to 10)	5·5 (4 to 7·4)	4 (2·9 to 5·3)	2·3 (1·6 to 3)	1·5 (1·1 to 2)	0·9 (0·6 to 1·2)	0·5 (0·3 to 0·6)	0·2 (0·1 to 0·3)	0 (0 to 0·1)	144
Motorcyclist road injuries	0·1 (0 to 0·1)	0 (0 to 0·1)	0·5 (0·3 to 0·8)	1 (0·6 to 1·6)	2·8 (1·9 to 3·8)	3·8 (2·7 to 5·2)	16·7 (11 to 23·6)	26·4 (17·6 to 38·6)	19·5 (12·9 to 27·8)	10·2 (7·1 to 14·3)	10·1 (7·3 to 14)	8·2 (6·1 to 10·7)	7·5 (5·7 to 9·8)	5·6 (4·2 to 7·4)	5·2 (3·8 to 6·9)	3·9 (2·9 to 5·2)	3·2 (2·4 to 4·2)	1·8 (1·3 to 2·5)	1·3 (0·9 to 1·8)	0·9 (0·6 to 1·2)	0·4 (0·3 to 0·6)	0·2 (0·1 to 0·3)	0·1 (0 to 0·1)	129
Motor vehicle road injuries	0·8 (0·5 to 1·3)	0·1 (0·1 to 0·3)	7·2 (4 to 12·1)	21·1 (11·8 to 35·7)	42·2 (27·7 to 62·9)	43·7 (28·1 to 63·6)	117 (75·6 to 168)	163 (103 to 245)	121 (73·2 to 187)	68 (41·6 to 103)	63·2 (40·2 to 96·4)	56·7 (38·2 to 82·7)	46·7 (32·7 to 66·5)	35·2 (24·8 to 49·4)	29·4 (20·8 to 41·6)	21·5 (15·8 to 28·5)	15·5 (11·7 to 20·5)	8·1 (6·2 to 10·7)	4·3 (3·3 to 5·6)	2·2 (1·7 to 2·8)	0·9 (0·7 to 1·2)	0·5 (0·4 to 0·6)	0·1 (0·1 to 0·1)	868
Other road injuries	0·1 (0 to 0·1)	0 (0 to 0)	0·2 (0·1 to 0·5)	1·1 (0·4 to 2·5)	2·2 (0·8 to 4·9)	1·4 (0·7 to 2·7)	2·6 (1·4 to 4·4)	4·2 (2·4 to 7·3)	5·3 (2·8 to 9·7)	3·4 (1·9 to 6)	3 (1·8 to 4·8)	2·7 (1·8 to 4·1)	2·3 (1·6 to 3·3)	2·2 (1·4 to 3·2)	2 (1·3 to 3)	1·6 (1 to 2·5)	1·1 (0·8 to 1·6)	0·5 (0·4 to 0·7)	0·4 (0·3 to 0·6)	0·2 (0·2 to 0·3)	0·1 (0·1 to 0·1)	0 (0 to 0·1)	0 (0 to 0)	36·7
Other transport injuries	0·8 (0·4 to 1·2)	0·2 (0·1 to 0·4)	3·5 (1·9 to 6)	9·7 (5·3 to 16·5)	13 (7·7 to 20·5)	16 (9·7 to 25)	22·8 (13 to 37·7)	34 (19·6 to 56·3)	27·6 (15·1 to 46·3)	18·1 (10·5 to 29·5)	15 (8·8 to 24·2)	18·8 (11·6 to 28·1)	11·4 (7·2 to 17·5)	11·3 (7·3 to 16·5)	10·5 (6·8 to 15·8)	6·6 (4·5 to 9·3)	3·6 (2·6 to 5·1)	2·2 (1·6 to 3·2)	2·1 (1·4 to 3)	0·9 (0·7 to 1·2)	0·5 (0·3 to 0·6)	0·3 (0·2 to 0·3)	0·1 (0·1 to 0·1)	229
Unintentional injuries	46·6 (28·4 to 72·8)	9·5 (5·4 to 15·2)	207 (114 to 347)	296 (198 to 422)	283 (227 to 349)	260 (205 to 325)	263 (204 to 335)	347 (264 to 453)	320 (245 to 411)	245 (185 to 318)	236 (183 to 300)	234 (181 to 293)	219 (170 to 274)	207 (164 to 263)	202 (162 to 252)	186 (149 to 229)	160 (130 to 195)	137 (110 to 167)	126 (103 to 152)	112 (91·3 to 136)	74·5 (60·8 to 90)	50·9 (42 to 59·9)	16·6 (13·7 to 19·4)	4,240
Falls	1·1 (0·7 to 1·6)	0·2 (0·1 to 0·3)	8·3 (5·4 to 12·1)	18·1 (12·1 to 25·9)	18·9 (14·6 to 23·7)	20·2 (15·4 to 26·7)	23·3 (17·3 to 31·2)	31·9 (23·3 to 42·8)	32·3 (23·5 to 43·2)	27·5 (20·3 to 36·5)	29·7 (22·2 to 39·4)	32·3 (23·9 to 42·9)	35·6 (27 to 46·2)	41·7 (32 to 53·2)	47·3 (37·1 to 59·3)	57·6 (44·7 to 71·5)	61·5 (49·2 to 75·6)	66·3 (50·8 to 84·3)	69·9 (55·3 to 86·4)	69·8 (55·7 to 86·4)	48·5 (38·7 to 59·9)	30·7 (24·8 to 36·8)	9·8 (8 to 11·5)	783
Drowning	2·2 (1·4 to 3·2)	0·4 (0·2 to 0·6)	14·2 (8·1 to 23·1)	78·3 (46·5 to 120)	75·3 (54·1 to 99·4)	71·8 (51·4 to 97·1)	50·7 (33·3 to 73·5)	39·3 (25·9 to 57·5)	31·8 (19 to 48·3)	20·6 (12·8 to 31·5)	17·4 (11·1 to 26·1)	14·2 (9·1 to 20·7)	12·2 (8·4 to 17)	8·7 (6 to 12·5)	7·1 (5 to 10·2)	5·1 (3·4 to 7·4)	3·6 (2·6 to 5)	1·7 (1·2 to 2·4)	1·4 (1 to 1·8)	0·7 (0·5 to 0·9)	0·4 (0·3 to 0·6)	0·4 (0·3 to 0·5)	0·1 (0·1 to 0·2)	458
Fire, heat, and hot substances	1·5 (1 to 2·1)	0·5 (0·3 to 0·8)	16·6 (10·1 to 25·6)	48·3 (31·9 to 70·4)	46·6 (35·8 to 58·1)	43·1 (32·6 to 55·5)	59·4 (45·4 to 75)	82·7 (61·8 to 108)	82·1 (61·3 to 108)	63·8 (48·4 to 82·4)	62·1 (47·2 to 79·1)	58 (44·5 to 73·7)	48·9 (37·7 to 62·2)	44·5 (34·5 to 58·5)	38·8 (30·1 to 49·9)	29·7 (22·9 to 38·7)	20·3 (15·6 to 26·7)	13·2 (10 to 17)	8·9 (6·9 to 11·5)	5·8 (4·5 to 7·5)	3·5 (2·8 to 4·4)	2·1 (1·7 to 2·5)	0·6 (0·5 to 0·7)	781
Poisonings	0·5 (0·3 to 0·7)	0·2 (0·1 to 0·4)	2·8 (1·6 to 4·5)	8·9 (5·3 to 13·5)	5·1 (3·6 to 6·8)	3·8 (2·6 to 5·4)	5·4 (3·9 to 7·5)	7·2 (5·1 to 9·7)	5·9 (4 to 8·1)	4·5 (3·1 to 6·2)	4·2 (3 to 5·6)	3·9 (2·8 to 5·4)	3·1 (2·2 to 4·2)	2·7 (2 to 3·5)	2·4 (1·8 to 3·2)	1·8 (1·3 to 2·3)	1·5 (1·1 to 2)	1 (0·7 to 1·3)	0·7 (0·5 to 0·9)	0·4 (0·3 to 0·6)	0·2 (0·2 to 0·3)	0·1 (0·1 to 0·1)	0 (0 to 0)	66·1
Poisoning by carbon monoxide	0·3 (0·2 to 0·4)	0 (0 to 0·1)	0·4 (0·2 to 0·9)	2·6 (1·3 to 4·7)	1·4 (0·9 to 2·2)	1·1 (0·7 to 1·8)	2·1 (1·4 to 3·1)	3·2 (2·2 to 4·7)	2·4 (1·5 to 3·6)	1·9 (1·2 to 2·8)	1·5 (1 to 2·1)	1·8 (1·1 to 2·6)	1·3 (0·8 to 1·8)	0·9 (0·7 to 1·3)	1·1 (0·8 to 1·5)	0·8 (0·6 to 1·1)	0·8 (0·6 to 1)	0·4 (0·3 to 0·6)	0·3 (0·2 to 0·4)	0·2 (0·1 to 0·2)	0·1 (0 to 0·1)	0 (0 to 0)	0 (0 to 0)	24·8
Poisoning by other means	0·2 (0·1 to 0·3)	0·2 (0·1 to 0·3)	2·3 (1·3 to 3·8)	6·2 (3·5 to 9·9)	3·6 (2·5 to 5)	2·6 (1·8 to 3·9)	3·3 (2·3 to 4·8)	4 (2·7 to 5·7)	3·5 (2·3 to 5)	2·5 (1·7 to 3·6)	2·7 (1·8 to 3·7)	2·2 (1·4 to 3·1)	1·9 (1·2 to 2·6)	1·7 (1·2 to 2·4)	1·3 (0·9 to 1·9)	1 (0·6 to 1·4)	0·7 (0·5 to 1)	0·5 (0·4 to 0·8)	0·4 (0·2 to 0·5)	0·3 (0·2 to 0·4)	0·1 (0·1 to 0·2)	0·1 (0 to 0·1)	0 (0 to 0)	41·3
Exposure to mechanical forces	1 (0·6 to 1·5)	0·4 (0·2 to 0·7)	5·6 (3·7 to 8·3)	25·6 (17·4 to 37·1)	35·1 (26·8 to 44·5)	34 (25·9 to 44·1)	38·9 (28·3 to 53·7)	55·8 (40·5 to 75·8)	46·6 (33·6 to 63·9)	35 (25·6 to 46·6)	34 (25·3 to 45·2)	33·9 (24·9 to 43·9)	28·9 (21·7 to 38·6)	26·8 (19·9 to 36·2)	23 (17·1 to 30·7)	18·4 (13·3 to 24·9)	13·8 (10 to 18·9)	9·1 (6·6 to 12·3)	6·7 (5 to 8·8)	4·7 (3·5 to 6·2)	2·7 (2 to 3·5)	1·8 (1·4 to 2·3)	0·6 (0·5 to 0·7)	482
Unintentional firearm injuries	0·2 (0·1 to 0·3)	0 (0 to 0)	0·5 (0·3 to 0·8)	1·7 (1 to 2·8)	3·1 (2 to 4·7)	6·3 (3·9 to 9·5)	11·8 (7 to 18·8)	19·6 (11·5 to 31·3)	12·2 (7·2 to 20·4)	9·8 (6·1 to 15·1)	8·4 (5·5 to 12·9)	7·1 (4·8 to 10·3)	5·2 (3·7 to 7·2)	3·8 (2·7 to 5)	3·3 (2·5 to 4·3)	2·3 (1·7 to 3·1)	1·6 (1·2 to 2·1)	1·2 (0·9 to 1·6)	0·9 (0·6 to 1·1)	0·7 (0·6 to 1)	0·5 (0·4 to 0·7)	0·4 (0·3 to 0·6)	0·2 (0·1 to 0·2)	101
Other exposure to mechanical forces	0·8 (0·5 to 1·3)	0·4 (0·2 to 0·6)	5·1 (3·4 to 7·6)	23·9 (16·2 to 34·9)	32 (24·4 to 40·9)	27·7 (20·8 to 36·6)	27·1 (19·6 to 37·9)	36·2 (25·7 to 50·5)	34·3 (24·5 to 47·5)	25·2 (18·3 to 34·9)	25·7 (18·2 to 35·5)	26·8 (19·1 to 36·1)	23·7 (17·4 to 32·1)	23 (16·9 to 31·5)	19·7 (14·5 to 26·7)	16 (11·5 to 22)	12·2 (8·7 to 16·7)	7·9 (5·7 to 10·9)	5·8 (4·3 to 7·8)	3·9 (2·9 to 5·2)	2·2 (1·6 to 2·9)	1·4 (1·1 to 1·8)	0·4 (0·3 to 0·6)	382
Adverse effects of medical treatment	6·5 (3·5 to 10·6)	2·8 (1·4 to 5·8)	21·1 (9·9 to 39)	15·2 (7·5 to 28)	9·8 (6·3 to 14·7)	7·8 (4·9 to 11·1)	11·3 (6·7 to 17·2)	26·5 (14·6 to 44·3)	23·6 (12·8 to 39·9)	23 (12·9 to 36·3)	19·8 (11·6 to 31)	23·3 (14·1 to 34·9)	25·5 (15·8 to 38·9)	23·4 (14 to 37·2)	24·6 (15 to 36·8)	22·8 (14·2 to 36)	18·1 (11·5 to 27·9)	13·6 (9 to 20·2)	10·8 (7·2 to 15·8)	7·3 (4·9 to 10·9)	4·5 (2·8 to 7·2)	2·4 (1·6 to 3·8)	0·7 (0·4 to 1·1)	344
Animal contact	0·7 (0·4 to 1·1)	0·2 (0·1 to 0·3)	7·4 (4 to 12·5)	13·3 (7·5 to 23)	16·3 (10·6 to 24·1)	11·6 (7·4 to 18·3)	11·3 (7·5 to 15·7)	15·4 (9·6 to 24·5)	17·4 (10·5 to 27·5)	11·6 (7·3 to 18·4)	11·5 (7·8 to 17·3)	11 (7·2 to 16·4)	11·2 (7·4 to 17·4)	8·8 (5·8 to 12·8)	12·2 (7·5 to 19·8)	9·3 (6·4 to 13·4)	7 (5 to 9·6)	7·3 (4·7 to 11·5)	7·3 (5·1 to 10·5)	8·9 (5·2 to 14·4)	7·1 (4 to 11·5)	8 (5·4 to 11·3)	3 (2 to 4·4)	218
Venomous animal contact	0·6 (0·3 to 0·9)	0·1 (0·1 to 0·2)	6 (3·2 to 10·2)	9·7 (5·1 to 16·8)	11·4 (7 to 17·8)	10·1 (6·3 to 16)	8·1 (5·3 to 11·6)	12·6 (7·8 to 20·5)	15·6 (9·3 to 25·2)	10·3 (6·3 to 16·5)	9·5 (6·3 to 14·3)	9·4 (6 to 14·2)	7·9 (5·2 to 12·5)	5·7 (3·7 to 8·5)	9·3 (5·5 to 15·4)	7·7 (5·1 to 11·3)	5·7 (3·9 to 8)	6·1 (3·9 to 9·8)	6·3 (4·3 to 9·2)	7·8 (4·3 to 12·9)	6·4 (3·5 to 10·5)	7·3 (4·9 to 10·5)	2·7 (1·8 to 4·1)	176
Non-venomous animal contact	0·1 (0·1 to 0·2)	0·1 (0 to 0·2)	1·4 (0·6 to 3)	3·6 (1·4 to 7·5)	4·9 (2·5 to 8·3)	1·5 (0·8 to 2·7)	3·2 (1·8 to 5·4)	2·8 (1·4 to 5)	1·8 (1 to 3)	1·3 (0·8 to 2·3)	2 (1·1 to 3·5)	1·6 (0·9 to 2·5)	3·3 (1·8 to 5·7)	3·1 (1·9 to 4·9)	2·9 (1·5 to 5·4)	1·7 (1·1 to 2·7)	1·3 (0·8 to 2)	1·1 (0·7 to 1·9)	0·9 (0·6 to 1·5)	1·1 (0·6 to 2·1)	0·7 (0·4 to 1·3)	0·6 (0·3 to 1)	0·2 (0·1 to 0·4)	41·3
Foreign body	29·9 (16·4 to 47·8)	4·1 (2 to 7·4)	119 (55·6 to 225)	56·8 (34·7 to 86·2)	31·1 (23·9 to 39)	22·2 (16·5 to 29·2)	13·5 (9·9 to 17·7)	21·2 (15 to 28·9)	19·2 (13·3 to 26·7)	13·7 (9·5 to 18·9)	13·6 (9·9 to 18·4)	14·1 (9·9 to 19·2)	13·7 (10 to 18·7)	14·1 (10·2 to 19·8)	14·1 (10·6 to 19)	13·6 (10·1 to 17·4)	12·7 (9·4 to 16·9)	10·4 (7·5 to 14)	9·1 (6·5 to 12·8)	7 (5·2 to 9·6)	3·1 (2·2 to 4·2)	2·3 (1·7 to 3)	0·8 (0·5 to 1·1)	460
Pulmonary aspiration and foreign body in airway	29·7 (16·3 to 47·5)	4·1 (1·9 to 7·4)	118 (54·4 to 223)	53·6 (32·2 to 82·7)	27·6 (20·7 to 35·4)	18·7 (13·2 to 25·7)	10 (7 to 14)	16·9 (11·4 to 24·2)	15·3 (9·8 to 22·5)	10·5 (6·7 to 15·8)	10·2 (6·9 to 14·8)	10·5 (6·8 to 15·1)	9·9 (6·8 to 14·3)	10·9 (7·2 to 15·9)	11·5 (8·3 to 16·4)	11·2 (8·1 to 14·7)	10·3 (7·3 to 14·1)	8·8 (6·2 to 12·3)	7·6 (5·3 to 11)	5·7 (4 to 7·9)	2·4 (1·6 to 3·3)	1·8 (1·2 to 2·4)	0·6 (0·4 to 0·8)	406
Foreign body in eyes	0 (0 to 0)	0 (0 to 0)	0·1 (0 to 0·2)	0·4 (0·1 to 1)	0·7 (0·2 to 1·5)	0·8 (0·2 to 1·8)	1·1 (0·4 to 2·6)	1·6 (0·5 to 3·5)	1·6 (0·6 to 3·3)	1·3 (0·5 to 2·7)	1·3 (0·5 to 2·7)	1·4 (0·6 to 2·7)	1·2 (0·6 to 2·2)	1 (0·5 to 1·8)	0·8 (0·5 to 1·4)	0·7 (0·4 to 1·1)	0·5 (0·3 to 0·8)	0·3 (0·2 to 0·5)	0·2 (0·2 to 0·3)	0·1 (0·1 to 0·2)	0·1 (0 to 0·1)	0 (0 to 0)	0 (0 to 0)	15·3
Foreign body in other body part	0·2 (0·1 to 0·4)	0 (0 to 0·1)	1·3 (0·6 to 2·9)	2·8 (1·5 to 4·9)	2·8 (1·9 to 4·2)	2·6 (1·8 to 3·8)	2·3 (1·6 to 3·2)	2·7 (1·9 to 3·8)	2·4 (1·7 to 3·2)	1·9 (1·3 to 2·5)	2·1 (1·5 to 2·9)	2·3 (1·6 to 3)	2·6 (1·8 to 3·6)	2·2 (1·6 to 3·1)	1·8 (1·3 to 2·4)	1·7 (1·3 to 2·4)	1·8 (1·2 to 2·6)	1·2 (0·9 to 1·8)	1·3 (0·8 to 2·1)	1·2 (0·7 to 1·9)	0·6 (0·4 to 1)	0·6 (0·4 to 0·9)	0·2 (0·1 to 0·3)	38·8
Environmental heat and cold exposure	0·3 (0·1 to 0·5)	0·2 (0·1 to 0·4)	2·1 (1·3 to 3·4)	6·4 (4·5 to 9)	10·9 (7·3 to 15·5)	11·4 (8 to 15·8)	13·5 (9·2 to 18·7)	17 (11·5 to 24)	16·2 (11·2 to 22·6)	13·8 (9·7 to 18·9)	13·2 (9·2 to 18·1)	13·7 (9·6 to 18·8)	13·1 (9·4 to 17·8)	12·6 (9·1 to 16·8)	12 (8·7 to 16·2)	10·5 (7·5 to 14·1)	8·9 (6·4 to 11·9)	6·2 (4·5 to 8·3)	5·1 (3·7 to 6·8)	4·1 (2·8 to 5·8)	2·6 (1·8 to 3·8)	2·2 (1·4 to 3·3)	0·8 (0·5 to 1·2)	197
Exposure to forces of nature	0 (0 to 0)	0 (0 to 0)	0 (0 to 0)	0 (0 to 0)	0 (0 to 0)	0 (0 to 0·1)	0·4 (0·2 to 0·6)	0·4 (0·3 to 0·7)	0·6 (0·4 to 1·1)	0·2 (0·1 to 0·3)	0·2 (0·1 to 0·3)	0·2 (0·1 to 0·4)	0·2 (0·1 to 0·4)	0·2 (0·1 to 0·3)	0·2 (0·1 to 0·2)	0·1 (0·1 to 0·2)	0·1 (0·1 to 0·1)	0·1 (0 to 0·1)	0 (0 to 0·1)	0 (0 to 0)	0 (0 to 0)	0 (0 to 0)	0 (0 to 0)	3
Other unintentional injuries	3 (2 to 4·4)	0·4 (0·3 to 0·6)	9·3 (6·5 to 13)	25·4 (18·1 to 34·1)	33·3 (25·4 to 42·7)	34·1 (25·6 to 44·3)	34·8 (26 to 46)	49·7 (35·4 to 69·2)	44·6 (31·3 to 61·5)	31·9 (22·7 to 43·2)	30 (22·3 to 39·9)	29·6 (22·2 to 38·7)	26·7 (19·9 to 34·6)	23·4 (17·4 to 31·6)	20·4 (15·2 to 27·2)	16·6 (12·3 to 22·2)	12·5 (9·3 to 16·8)	7·8 (5·7 to 10·5)	6 (4·5 to 7·8)	3·5 (2·6 to 4·7)	1·7 (1·2 to 2·3)	0·8 (0·6 to 1)	0·2 (0·1 to 0·2)	446
Self-harm and interpersonal violence	25·3 (14·4 to 41·3)	1·4 (0·7 to 2·9)	29·6 (16·5 to 49·4)	53·8 (31·9 to 87·5)	54 (39·6 to 73·8)	130 (94 to 180)	599 (438 to 786)	897 (626 to 1,250)	677 (453 to 981)	483 (313 to 689)	378 (254 to 539)	310 (216 to 435)	240 (167 to 339)	186 (125 to 264)	140 (98·6 to 198)	101 (73·4 to 137)	69·3 (51·9 to 92)	34·9 (27 to 45·5)	19·3 (15·1 to 24·4)	8·7 (7 to 10·7)	3·6 (2·8 to 4·4)	1·1 (0·9 to 1·4)	0·2 (0·2 to 0·3)	4,440
Self-harm	0 (0 to 0)	0 (0 to 0)	0 (0 to 0)	0 (0 to 0)	0 (0 to 0)	58·8 (34·6 to 93·2)	377 (254 to 538)	464 (286 to 720)	309 (188 to 494)	216 (129 to 347)	187 (112 to 290)	165 (102 to 255)	141 (89·2 to 214)	126 (78·5 to 191)	99 (64·6 to 148)	74·3 (51·7 to 107)	50·5 (35·6 to 70·7)	25·2 (18·2 to 34·9)	13·3 (9·6 to 17·4)	6·1 (4·6 to 7·9)	2·4 (1·8 to 3)	0·7 (0·5 to 0·8)	0·1 (0·1 to 0·2)	2,320
Self-harm by firearm	0 (0 to 0)	0 (0 to 0)	0 (0 to 0)	0 (0 to 0)	0 (0 to 0)	0·2 (0·1 to 0·4)	1 (0·6 to 1·4)	1·6 (1 to 2·5)	1 (0·6 to 1·6)	1·1 (0·7 to 1·7)	0·7 (0·5 to 1·1)	0·6 (0·4 to 0·8)	0·5 (0·4 to 0·7)	0·5 (0·4 to 0·7)	0·4 (0·3 to 0·6)	0·3 (0·2 to 0·3)	0·2 (0·2 to 0·3)	0·2 (0·1 to 0·2)	0·1 (0·1 to 0·1)	0·1 (0·1 to 0·1)	0 (0 to 0·1)	0 (0 to 0)	0 (0 to 0)	8·6
Self-harm by other specified means	0 (0 to 0)	0 (0 to 0)	0 (0 to 0)	0 (0 to 0)	0 (0 to 0)	58·6 (34·5 to 92·9)	376 (253 to 536)	462 (285 to 717)	308 (187 to 492)	215 (128 to 346)	186 (111 to 289)	164 (102 to 254)	141 (88·7 to 213)	126 (78·1 to 190)	98·6 (64·3 to 147)	74·1 (51·5 to 107)	50·3 (35·3 to 70·4)	25·1 (18·1 to 34·7)	13·2 (9·5 to 17·3)	6 (4·5 to 7·8)	2·3 (1·8 to 3)	0·6 (0·5 to 0·8)	0·1 (0·1 to 0·2)	2,310
Interpersonal violence	25·3 (14·4 to 41·3)	1·4 (0·7 to 2·9)	29·6 (16·4 to 49·2)	53·3 (31·5 to 86·8)	53·7 (39·3 to 73·5)	68·8 (48·3 to 95·5)	219 (149 to 307)	430 (283 to 602)	366 (232 to 553)	266 (171 to 389)	190 (123 to 284)	144 (97·6 to 209)	97·8 (67 to 140)	59·2 (40·2 to 84·9)	40 (28·6 to 56·3)	25·6 (18·9 to 34·5)	18·4 (13·8 to 24·1)	9·6 (7·3 to 12·4)	5·9 (4·5 to 7·6)	2·5 (1·9 to 3·1)	1·1 (0·9 to 1·4)	0·4 (0·3 to 0·6)	0·1 (0·1 to 0·1)	2,110
Physical violence by firearm	0·4 (0·2 to 0·7)	0 (0 to 0·1)	0·8 (0·4 to 1·3)	4 (2·3 to 6·6)	5·5 (3·9 to 7·6)	10·9 (7·5 to 15·9)	59·5 (39·1 to 84·1)	109 (68·2 to 158)	85·3 (52·6 to 131)	58·5 (36·5 to 87)	41·3 (25·9 to 63·9)	24·5 (15·6 to 36·5)	19·4 (12·4 to 29·4)	9·4 (5·9 to 14·2)	6 (4 to 8·9)	2·8 (1·9 to 3·9)	1·3 (0·9 to 1·8)	0·6 (0·4 to 0·8)	0·2 (0·2 to 0·3)	0·1 (0·1 to 0·1)	0 (0 to 0·1)	0 (0 to 0)	0 (0 to 0)	439
Physical violence by sharp object	1·1 (0·6 to 1·8)	0 (0 to 0)	1·1 (0·7 to 1·8)	3·2 (2 to 5·2)	4·9 (3·6 to 6·6)	10·1 (7 to 14·3)	67·4 (45 to 94·7)	160 (101 to 230)	134 (82·8 to 207)	96·2 (60·5 to 142)	72·5 (45·4 to 110)	52·2 (33·7 to 77·5)	27·4 (18·2 to 40·3)	13·9 (9·2 to 20·3)	8·5 (6·1 to 12·1)	4·3 (3·2 to 5·8)	2·8 (2·1 to 3·7)	1·4 (1·1 to 1·9)	0·7 (0·6 to 0·9)	0·4 (0·3 to 0·5)	0·2 (0·1 to 0·2)	0·1 (0·1 to 0·1)	0 (0 to 0)	663
Sexual violence	0 (0 to 0)	0 (0 to 0)	0 (0 to 0)	0·3 (0·2 to 0·4)	2·2 (1·3 to 3·4)	4·8 (2·8 to 7·6)	12·1 (6·6 to 19·5)	18·5 (10·8 to 28·4)	14·9 (8·4 to 24)	10·8 (5·9 to 18)	9·1 (5·1 to 14·5)	7·5 (4·3 to 12·2)	5·4 (3·2 to 9·1)	3·6 (2 to 6)	2·4 (1·4 to 3·8)	1·7 (1·1 to 2·6)	1·2 (0·8 to 1·8)	0·7 (0·5 to 1·1)	0·5 (0·3 to 0·7)	0·3 (0·2 to 0·4)	0·1 (0·1 to 0·2)	0 (0 to 0)	0 (0 to 0)	96·1
Physical violence by other means	23·8 (13·5 to 39)	1·3 (0·6 to 2·8)	27·7 (15·3 to 46·1)	45·8 (26·5 to 75·4)	41 (29·5 to 56·6)	42·9 (29·1 to 61)	79·6 (53·6 to 113)	142 (91·5 to 205)	132 (81·4 to 201)	100 (62·3 to 148)	66·6 (42·9 to 100)	59·8 (40·3 to 88·6)	45·6 (30·8 to 65·4)	32·3 (21·7 to 46·9)	23·1 (16·2 to 32·9)	16·8 (12·1 to 23·4)	12·9 (9·4 to 17·5)	6·8 (5·1 to 9)	4·5 (3·3 to 5·9)	1·7 (1·3 to 2·2)	0·8 (0·6 to 1)	0·3 (0·2 to 0·4)	0·1 (0 to 0·1)	907
Conflict and terrorism	0 (0 to 0)	0 (0 to 0)	0 (0 to 0)	0 (0 to 0)	0 (0 to 0)	2·3 (1·3 to 4·3)	2·5 (1·4 to 4·6)	2·8 (1·5 to 5·4)	2 (1·1 to 3·6)	1·2 (0·7 to 2·2)	2 (1·2 to 3·5)	1·4 (0·8 to 2·3)	0·6 (0·4 to 1·1)	1·1 (0·7 to 1·8)	0·8 (0·5 to 1·4)	0·8 (0·5 to 1·2)	0·4 (0·3 to 0·6)	0·1 (0·1 to 0·2)	0·2 (0·1 to 0·2)	0·2 (0·1 to 0·2)	0·1 (0·1 to 0·1)	0 (0 to 0)	0 (0 to 0)	18·6

SR Table 2b: Haiti DALYS by cause and age group for females with 95% uncertainty intervals, 2019

Cause	Early Neonatal	Late Neonatal	Post Neonatal	1 to 4	5 to 9	10 to 14	15 to 19	20 to 24	25 to 29	30 to 34	35 to 39	40 to 44	45 to 49	50 to 54	55 to 59	60 to 64	65 to 69	70 to 74	75 to 79	80 to 84	85 to 89	90 to 94	95 plus	Total
All causes	271,000 (215,000 to 334,000)	130,000 (103,000 to 160,000)	232,000 (167,000 to 317,000)	270,000 (231,000 to 314,000)	105,000 (84,300 to 122,000)	91,000 (75,600 to 109,000)	114,000 (95,400 to 135,000)	135,000 (113,000 to 157,000)	155,000 (129,000 to 184,000)	174,000 (144,000 to 207,000)	180,000 (147,000 to 217,000)	170,000 (138,000 to 207,000)	149,000 (118,000 to 186,000)	141,000 (113,000 to 176,000)	137,000 (108,000 to 175,000)	132,000 (108,000 to 164,000)	115,000 (96,700 to 143,000)	87,200 (74,800 to 105,000)	64,300 (55,200 to 77,700)	42,600 (37,000 to 49,100)	18,400 (15,500 to 22,500)	6,090 (5,290 to 7,170)	1,500 (1,360 to 1,630)	2,920,000
Communicable, maternal, neonatal, and nutritional diseases	218,000 (164,000 to 273,000)	96,700 (71,800 to 123,000)	172,000 (117,000 to 241,000)	155,000 (118,000 to 201,000)	38,200 (30,000 to 48,100)	24,300 (18,800 to 31,500)	31,700 (24,100 to 41,200)	41,000 (32,400 to 51,200)	52,400 (39,900 to 68,200)	60,000 (45,800 to 77,200)	58,200 (43,700 to 75,300)	48,900 (37,900 to 62,300)	33,700 (26,000 to 42,200)	22,600 (17,900 to 27,900)	16,500 (12,700 to 20,900)	12,400 (9,270 to 16,000)	9,380 (6,880 to 12,800)	7,010 (4,810 to 9,970)	5,520 (3,670 to 8,060)	3,940 (2,650 to 5,720)	1,970 (1,220 to 2,870)	694 (442 to 1,010)	187 (115 to 277)	1,110,000
HIV/AIDS and sexually transmitted infections	17,800 (6,140 to 37,700)	5,010 (1,630 to 10,700)	7,780 (4,230 to 13,800)	8,170 (5,680 to 11,700)	4,180 (2,600 to 6,160)	4,070 (2,720 to 5,610)	7,690 (3,960 to 12,800)	9,220 (5,350 to 15,400)	16,800 (9,020 to 29,400)	24,200 (14,500 to 38,700)	26,700 (17,000 to 40,500)	23,200 (15,900 to 31,700)	18,200 (12,700 to 24,700)	11,800 (8,380 to 15,500)	6,530 (4,500 to 8,850)	3,270 (2,240 to 4,550)	1,600 (1,050 to 2,270)	672 (431 to 983)	298 (186 to 447)	60·8 (36 to 93·5)	22·8 (13·4 to 34·4)	5·8 (3·4 to 8·7)	1·1 (0·7 to 1·6)	197,000
HIV/AIDS	0·3 (0·2 to 0·5)	1·1 (0·6 to 1·8)	2,930 (2,100 to 4,110)	7,920 (5,420 to 11,300)	4,050 (2,470 to 6,000)	4,030 (2,690 to 5,590)	7,540 (3,840 to 12,700)	8,950 (5,160 to 15,300)	16,500 (8,670 to 29,000)	23,800 (13,900 to 38,200)	26,300 (16,600 to 40,000)	22,800 (15,500 to 31,300)	18,000 (12,400 to 24,400)	11,700 (8,270 to 15,400)	6,450 (4,440 to 8,720)	3,220 (2,200 to 4,470)	1,560 (1,020 to 2,220)	649 (407 to 969)	284 (173 to 435)	54·1 (30·6 to 84·9)	19·8 (10·9 to 30·8)	4·8 (2·8 to 7·6)	0·9 (0·5 to 1·4)	167,000
HIV/AIDS - Drug-susceptible Tuberculosis	0 (0 to 0)	0·1 (0 to 0·1)	159 (95·8 to 253)	729 (441 to 1,160)	347 (214 to 543)	330 (203 to 516)	502 (310 to 782)	572 (359 to 879)	1,020 (645 to 1,580)	1,520 (960 to 2,340)	1,810 (1,130 to 2,810)	1,710 (1,060 to 2,650)	1,360 (837 to 2,110)	911 (557 to 1,420)	518 (322 to 810)	252 (157 to 392)	121 (75 to 187)	50·2 (31·7 to 76·2)	20·4 (12·4 to 31·6)	3·7 (2·5 to 5·5)	1·1 (0·8 to 1·6)	0·3 (0·2 to 0·4)	0·1 (0 to 0·1)	11,900
HIV/AIDS - Multidrug-resistant Tuberculosis without exte	0 (0 to 0)	0 (0 to 0)	1·8 (0·1 to 7·3)	8·3 (0·6 to 33·4)	3·8 (0·3 to 15·5)	3·6 (0·3 to 14·7)	5·5 (0·4 to 22·4)	6·1 (0·5 to 24·8)	10·8 (0·8 to 43·6)	15·9 (1·2 to 64)	18·6 (1·4 to 75)	17·6 (1·4 to 71)	14·1 (1·1 to 56·8)	9·6 (0·7 to 38·7)	5·5 (0·4 to 22·2)	2·7 (0·2 to 10·9)	1·3 (0·1 to 5·2)	0·5 (0 to 2·2)	0·2 (0 to 0·9)	0 (0 to 0·2)	0 (0 to 0)	0 (0 to 0)	0 (0 to 0)	126
HIV/AIDS - Extensively drug-resistant Tuberculosis	0 (0 to 0)	0 (0 to 0)	0·3 (0 to 1·2)	1·4 (0·1 to 5·5)	0·6 (0 to 2·5)	0·6 (0 to 2·4)	0·9 (0·1 to 3·7)	1 (0·1 to 4)	1·8 (0·1 to 7)	2·6 (0·2 to 10·3)	3 (0·2 to 12·2)	2·9 (0·2 to 11·6)	2·3 (0·2 to 9·3)	1·6 (0·1 to 6·3)	0·9 (0·1 to 3·6)	0·4 (0 to 1·8)	0·2 (0 to 0·8)	0·1 (0 to 0·3)	0 (0 to 0·1)	0 (0 to 0)	0 (0 to 0)	0 (0 to 0)	0 (0 to 0)	20·7
HIV/AIDS resulting in other diseases	0·3 (0·2 to 0·5)	1 (0·6 to 1·7)	2,770 (1,950 to 3,950)	7,180 (4,600 to 10,500)	3,690 (2,150 to 5,600)	3,700 (2,360 to 5,240)	7,040 (3,290 to 12,100)	8,370 (4,560 to 14,500)	15,400 (7,710 to 28,100)	22,200 (12,400 to 36,800)	24,400 (14,600 to 38,400)	21,000 (13,700 to 29,700)	16,600 (11,100 to 23,100)	10,700 (7,230 to 14,500)	5,930 (3,850 to 8,220)	2,960 (1,930 to 4,230)	1,430 (901 to 2,090)	598 (352 to 924)	264 (150 to 416)	50·4 (26·9 to 81·3)	18·6 (9·8 to 30)	4·6 (2·5 to 7·4)	0·9 (0·5 to 1·4)	154,000
Sexually transmitted infections excluding HIV	17,800 (6,140 to 37,700)	5,010 (1,630 to 10,700)	4,850 (1,580 to 10,800)	250 (85·3 to 542)	136 (45·4 to 279)	37·9 (14·4 to 91·9)	143 (64·2 to 289)	266 (131 to 499)	351 (188 to 627)	468 (250 to 822)	449 (236 to 831)	404 (215 to 771)	270 (126 to 547)	133 (64·1 to 274)	81·8 (37·8 to 172)	55·7 (22·9 to 128)	41 (18·2 to 87·7)	23·2 (10·7 to 49·8)	13·9 (6·2 to 30·1)	6·8 (3·1 to 14·1)	3·1 (1·3 to 7·2)	0·9 (0·4 to 2·2)	0·1 (0·1 to 0·4)	30,800
Syphilis	17,800 (6,140 to 37,700)	5,010 (1,630 to 10,700)	4,850 (1,580 to 10,800)	250 (85·3 to 542)	136 (45·4 to 279)	17·7 (5·1 to 47·7)	25·4 (9·2 to 56·4)	17·2 (7·1 to 34)	30·8 (12·5 to 69·8)	48·4 (18·5 to 112)	29·4 (10·7 to 65·3)	11·3 (4·8 to 25·1)	14·5 (5·1 to 36·7)	7·9 (3 to 19·2)	2 (0·9 to 4·7)	2·2 (0·8 to 5·7)	2·2 (0·8 to 5·6)	1·8 (0·7 to 4·6)	1 (0·4 to 2·4)	0·9 (0·4 to 2·3)	0·4 (0·2 to 1·2)	0·2 (0·1 to 0·6)	0 (0 to 0·1)	28,300
[illegible]	[illegible]	[illegible]	[illegible]	[illegible]	[illegible]	[illegible]	[illegible]	[illegible]	[illegible]	[illegible]	[illegible]	[illegible]	[illegible]	[illegible]	[illegible]	[illegible]	[illegible]	[illegible]	[illegible]	[illegible]	[illegible]	[illegible]	[illegible]	[illegible]

Other cardiovascular and circulatory diseases	522 (152 to 1,380)	392 (115 to 982)
Chronic respiratory diseases	0 (0 to 0)	0 (0 to 0)
Chronic obstructive pulmonary disease	0 (0 to 0)	0 (0 to 0)
Pneumoconiosis	0 (0 to 0)	0 (0 to 0)
Silicosis	0 (0 to 0)	0 (0 to 0)
Asbestosis	0 (0 to 0)	0 (0 to 0)
Coal workers pneumoconiosis	0 (0 to 0)	0 (0 to 0)
Other pneumoconiosis	0 (0 to 0)	0 (0 to 0)
Asthma	0 (0 to 0)	0 (0 to 0)
Interstitial lung disease and pulmonary sarcoidosis	0 (0 to 0)	0 (0 to 0)
Other chronic respiratory diseases	0 (0 to 0)	0 (0 to 0)
Digestive diseases	662 (203 to 1,500)	741 (161 to 1,930)
Cirrhosis and other chronic liver diseases	0 (0 to 0)	0 (0 to 0)
Cirrhosis and other chronic liver diseases due to hepati	0 (0 to 0)	0 (0 to 0)
Cirrhosis and other chronic liver diseases due to hepati	0 (0 to 0)	0 (0 to 0)
Cirrhosis and other chronic liver diseases due to alcoho	0 (0 to 0)	0 (0 to 0)
Cirrhosis and other chronic liver diseases due to NAFLD	0 (0 to 0)	0 (0 to 0)
Cirrhosis and other chronic liver diseases due to other c	0 (0 to 0)	0 (0 to 0)
Upper digestive system diseases	0 (0 to 0)	0 (0 to 0)
Peptic ulcer disease	0 (0 to 0)	0 (0 to 0)
Gastritis and duodenitis	0 (0 to 0)	0 (0 to 0)
Gastroesophageal reflux disease	0 (0 to 0)	0 (0 to 0)
Appendicitis	0 (0 to 0)	0 (0 to 0)
Paralytic ileus and intestinal obstruction	342 (95·3 to 811)	563 (126 to 1,470)
Inguinal, femoral, and abdominal hernia	320 (67 to 880)	178 (17·1 to 643)
Inflammatory bowel disease	0 (0 to 0)	0 (0 to 0)
Vascular intestinal disorders	0 (0 to 0)	0 (0 to 0)
Gallbladder and biliary diseases	0 (0 to 0)	0 (0 to 0)
Pancreatitis	0 (0 to 0)	0 (0 to 0)
Other digestive diseases	0 (0 to 0)	0 (0 to 0)
Neurological disorders	2·4 (0·5 to 5·7)	22·8 (6·8 to 57·3)
Alzheimer's disease and other dementias	0 (0 to 0)	0 (0 to 0)
Parkinson's disease	0 (0 to 0)	0 (0 to 0)
Idiopathic epilepsy	2·2 (0·4 to 5·4)	6·6 (1·1 to 15·7)
Multiple sclerosis	0 (0 to 0)	0 (0 to 0)
Motor neuron disease	0·2 (0·1 to 0·5)	16·2 (2·8 to 51·6)
Headache disorders	0 (0 to 0)	0 (0 to 0)
Migraine	0 (0 to 0)	0 (0 to 0)
Tension-type headache	0 (0 to 0)	0 (0 to 0)
Other neurological disorders	0 (0 to 0)	0 (0 to 0)
Mental disorders	1·9 (1 to 3·1)	5·8 (2·9 to 9·5)
Schizophrenia	0 (0 to 0)	0 (0 to 0)
Depressive disorders	0 (0 to 0)	0 (0 to 0)
Major depressive disorder	0 (0 to 0)	0 (0 to 0)
Dysthymia	0 (0 to 0)	0 (0 to 0)
Bipolar disorder	0 (0 to 0)	0 (0 to 0)
Anxiety disorders	0 (0 to 0)	0 (0 to 0)
Eating disorders	0 (0 to 0)	0 (0 to 0)
Anorexia nervosa	0 (0 to 0)	0 (0 to 0)
Bulimia nervosa	0 (0 to 0)	0 (0 to 0)
Autism spectrum disorders	1 (0·6 to 1·6)	3 (1·8 to 4·8)
Attention-deficit/hyperactivity disorder	0 (0 to 0)	0 (0 to 0)
Conduct disorder	0 (0 to 0)	0 (0 to 0)
Idiopathic developmental intellectual disability	0·9 (0·1 to 1·9)	2·8 (0·4 to 5·9)
Other mental disorders	0 (0 to 0)	0 (0 to 0)
Substance use disorders	0 (0 to 0)	0 (0 to 0)
Alcohol use disorders	0 (0 to 0)	0 (0 to 0)
Drug use disorders	0 (0 to 0)	0 (0 to 0)
Opioid use disorders	0 (0 to 0)	0 (0 to 0)
Cocaine use disorders	0 (0 to 0)	0 (0 to 0)
Amphetamine use disorders	0 (0 to 0)	0 (0 to 0)
Cannabis use disorders	0 (0 to 0)	0 (0 to 0)
Other drug use disorders	0 (0 to 0)	0 (0 to 0)
Diabetes and kidney diseases	4·1 (2·1 to 7·2)	13·5 (5·7 to 26·3)
Diabetes mellitus	4·1 (2·1 to 7·2)	13·4 (5·6 to 26·2)
Diabetes mellitus type 1	4·1 (2·1 to 7·2)	13·4 (5·6 to 26·2)
Diabetes mellitus type 2	0 (0 to 0)	0 (0 to 0)
Chronic kidney disease	0 (0 to 0)	0·1 (0·1 to 0·2)
Chronic kidney disease due to diabetes mellitus type 1	0 (0 to 0)	0 (0 to 0)
Chronic kidney disease due to diabetes mellitus type 2	0 (0 to 0)	0 (0 to 0)
Chronic kidney disease due to hypertension	0 (0 to 0)	0 (0 to 0)
Chronic kidney disease due to glomerulonephritis	0 (0 to 0)	0 (0 to 0·1)
Chronic kidney disease due to other and unspecified ca	0 (0 to 0)	0·1 (0·1 to 0·2)
Acute glomerulonephritis	0 (0 to 0)	0 (0 to 0)
Skin and subcutaneous diseases	0·2 (0·1 to 0·4)	3 (1·7 to 4·7)
Dermatitis	0 (0 to 0)	0 (0 to 0)
Atopic dermatitis	0 (0 to 0)	0 (0 to 0)
Contact dermatitis	0 (0 to 0)	0 (0 to 0)
Seborrhoeic dermatitis	0 (0 to 0)	0 (0 to 0)
Psoriasis	0 (0 to 0)	0 (0 to 0)
Bacterial skin diseases	0 (0 to 0·1)	0·3 (0·2 to 0·6)
Cellulitis	0 (0 to 0)	0·1 (0·1 to 0·1)
Pyoderma	0 (0 to 0·1)	0·2 (0·1 to 0·5)
Scabies	0·1 (0 to 0·2)	1·2 (0·6 to 2)
Fungal skin diseases	0 (0 to 0)	0·2 (0·1 to 0·4)
Viral skin diseases	0 (0 to 0)	0 (0 to 0)
Acne vulgaris	0 (0 to 0)	0 (0 to 0)
Alopecia areata	0 (0 to 0)	0 (0 to 0)
Pruritus	0 (0 to 0)	0 (0 to 0)
Urticaria	0·1 (0 to 0·1)	1·1 (0·5 to 2)
Decubitus ulcer	0 (0 to 0)	0 (0 to 0)
Other skin and subcutaneous diseases	0 (0 to 0)	0·2 (0·1 to 0·4)
Sense organ diseases	0·5 (0·2 to 0·8)	1·7 (0·9 to 3)
Blindness and vision loss	0·1 (0 to 0·3)	0·4 (0·1 to 0·9)
Glaucoma	0 (0 to 0)	0 (0 to 0)
Cataract	0 (0 to 0)	0 (0 to 0)
Age-related macular degeneration	0 (0 to 0)	0 (0 to 0)
Refraction disorders	0 (0 to 0)	0·1 (0 to 0·2)
Near vision loss	0 (0 to 0)	0 (0 to 0)
Other vision loss	0·1 (0 to 0·3)	0·3 (0·1 to 0·8)
Age-related and other hearing loss	0·1 (0 to 0·2)	0·3 (0·1 to 0·7)
Other sense organ diseases	0·2 (0·1 to 0·5)	1 (0·5 to 1·9)
Musculoskeletal disorders	0 (0 to 0)	0 (0 to 0)
Rheumatoid arthritis	0 (0 to 0)	0 (0 to 0)
Osteoarthritis	0 (0 to 0)	0 (0 to 0)
Osteoarthritis hip	0 (0 to 0)	0 (0 to 0)
Osteoarthritis knee	0 (0 to 0)	0 (0 to 0)
Osteoarthritis hand	0 (0 to 0)	0 (0 to 0)
Osteoarthritis other	0 (0 to 0)	0 (0 to 0)
Low back pain	0 (0 to 0)	0 (0 to 0)
Neck pain	0 (0 to 0)	0 (0 to 0)
Gout	0 (0 to 0)	0 (0 to 0)
Other musculoskeletal disorders	0 (0 to 0)	0 (0 to 0)
Other non-communicable diseases	45,400 (24,100 to 77,400)	26,700 (14,500 to 41,800)
Congenital birth defects	44,900 (23,500 to 76,800)	25,500 (13,700 to 40,300)
Neural tube defects	10,100 (1,930 to 29,000)	4,470 (709 to 13,100)
Congenital heart anomalies	15,000 (6,010 to 30,200)	10,700 (3,880 to 20,200)
Orofacial clefts	70·8 (14·2 to 230)	25·9 (4·6 to 83·3)
Down syndrome	523 (116 to 1,380)	338 (68·5 to 1,070)
Turner syndrome	0 (0 to 0·1)	0·1 (0 to 0·2)
Klinefelter syndrome	0 (0 to 0)	0 (0 to 0)
Other chromosomal abnormalities	1,900 (463 to 5,120)	1,340 (344 to 3,780)
Congenital musculoskeletal and limb anomalies	883 (264 to 2,160)	394 (127 to 854)
Urogenital congenital anomalies	836 (245 to 2,110)	439 (132 to 1,020)
Digestive congenital anomalies	6,850 (1,820 to 16,300)	4,680 (1,150 to 11,200)
Other congenital birth defects	8,750 (2,710 to 18,900)	3,180 (776 to 7,600)
Urinary diseases and male infertility	28·2 (9·8 to 67)	74·5 (28·3 to 159)
Urolithiasis	0 (0 to 0)	0 (0 to 0)
Benign prostatic hyperplasia	0 (0 to 0)	0 (0 to 0)
Male infertility	--	--
Other urinary diseases	13·1 (3·9 to 35·3)	47·4 (14·8 to 107)
Gynecological diseases	0 (0 to 0)	0 (0 to 0)
Uterine fibroids	0 (0 to 0)	0 (0 to 0)
Polycystic ovarian syndrome	0 (0 to 0)	0 (0 to 0)
Female infertility	0 (0 to 0)	0 (0 to 0)
Endometriosis	0 (0 to 0)	0 (0 to 0)
Genital prolapse	0 (0 to 0)	0 (0 to 0)
Premenstrual syndrome	0 (0 to 0)	0 (0 to 0)
Other gynecological diseases	0 (0 to 0)	0 (0 to 0)
Hemoglobinopathies and hemolytic anemias	181 (91·3 to 308)	191 (93·5 to 369)
Thalassemias	0·2 (0·1 to 0·3)	0·1 (0 to 0·4)
Thalassemias trait	0·4 (0·2 to 0·8)	2·8 (1·6 to 4·5)
Sickle cell disorders	175 (85·7 to 301)	140 (46·7 to 322)
Sickle cell trait	5·2 (2·4 to 9)	42·1 (25·2 to 65·1)
G6PD deficiency	0 (0 to 0·1)	0·1 (0 to 0·7)
G6PD trait	0 (0 to 0)	0 (0 to 0·5)
Other hemoglobinopathies and hemolytic anemias	0·4 (0·2 to 0·8)	5·8 (3·4 to 9·1)
Endocrine, metabolic, blood, and immune disorders	262 (105 to 533)	412 (145 to 925)
Oral disorders	0 (0 to 0)	0 (0 to 0)
Caries of deciduous teeth	0 (0 to 0)	0 (0 to 0)
Caries of permanent teeth	0 (0 to 0)	0 (0 to 0)
Periodontal diseases	0 (0 to 0)	0 (0 to 0)
Other oral disorders	0 (0 to 0)	0 (0 to 0)
Sudden infant death syndrome	--	479 (141 to 1,510)
Injuries	4,560 (2,360 to 9,820)	4,170 (1,660 to 19,400)
Transport injuries	434 (136 to 959)	336 (129 to 749)
Road injuries	347 (106 to 782)	236 (86·9 to 526)
Pedestrian road injuries	172 (43·9 to 411)	127 (40·2 to 310)
Cyclist road injuries	0 (0 to 0)	0 (0 to 0)
Motorcyclist road injuries	24·2 (3·9 to 75·8)	39·6 (5·8 to 129)
Motor vehicle road injuries	141 (34·5 to 374)	61·5 (12 to 171)
Other road injuries	9·7 (2 to 32·8)	7·4 (1·1 to 24·1)

Other transport injuries	86·8 (20·3 to 232)	99·9 (27·9 to 246)	115 (31·1 to 297)	337 (109 to 851)	263 (112 to 658)	233 (115 to 567)	242 (110 to 564)	248 (126 to 582)	230 (113 to 526)	189 (99·1 to 397)	155 (78·6 to 379)	153 (79 to 364)
Unintentional injuries	3,040 (1.410 to 8.340)	3,530 (1.250 to 18.800)	10,100 (4.730 to 18.600)	31,000 (15.800 to 48.500)	14,300 (9.470 to 19.700)	12,200 (8.910 to 16.100)	9,580 (7.030 to 12.600)	10,900 (7.810 to 15.100)	8,310 (6.050 to 11.000)	7,940 (5.990 to 10.300)	7,480 (5.630 to 9.750)	6,150 (4.600 to 8.100)
Falls	32·1 (7·8 to 92·6)	23·8 (5·2 to 69·2)	223 (34·9 to 777)	804 (179 to 2,510)	414 (215 to 795)	356 (238 to 526)	345 (240 to 474)	389 (259 to 541)	451 (310 to 630)	486 (344 to 656)	492 (352 to 661)	437 (316 to 583)
Drowning	132 (62 to 244)	127 (45·2 to 256)	1,300 (379 to 3.180)	13,500 (4.460 to 25.000)	6,090 (2.760 to 10.600)	3,700 (1.790 to 5.920)	1,640 (777 to 2.730)	897 (442 to 1.530)	816 (401 to 1.320)	667 (353 to 1.120)	549 (285 to 938)	373 (198 to 612)
Fire, heat, and hot substances	70·4 (17·2 to 146)	125 (19·7 to 295)	1,010 (103 to 2.600)	5,330 (677 to 12.300)	2,250 (797 to 4.340)	1,380 (784 to 2.140)	1,360 (806 to 2.050)	1,440 (853 to 2.170)	1,640 (939 to 2.540)	1,540 (945 to 2.310)	1,410 (852 to 2.090)	1,070 (685 to 1.580)
Poisonings	40·7 (12·5 to 104)	101 (20·8 to 296)	280 (44·9 to 855)	1,720 (349 to 3.970)	367 (135 to 718)	140 (72·8 to 256)	160 (77·8 to 279)	172 (86·3 to 294)	156 (76·6 to 276)	139 (73·6 to 243)	126 (65·5 to 231)	97·8 (47·6 to 169)
Poisoning by carbon monoxide	22·2 (3·5 to 70·7)	20·8 (1·9 to 76·5)	60·6 (7·4 to 234)	580 (124 to 1.660)	139 (36 to 370)	59·1 (21·4 to 145)	88·8 (33·6 to 196)	105 (45·3 to 207)	85·3 (34·7 to 184)	78·4 (32·4 to 167)	61·1 (22·5 to 146)	55·7 (20·5 to 123)
Poisoning by other means	18·4 (1·8 to 58·7)	80 (12·8 to 234)	220 (15·4 to 716)	1,140 (83·3 to 2,990)	227 (40·3 to 499)	80·7 (31·8 to 174)	71·6 (27·3 to 147)	67·4 (25·3 to 137)	70·6 (26·4 to 140)	60·4 (24·4 to 118)	64·7 (25·2 to 131)	42·1 (18·3 to 81·5)
Exposure to mechanical forces	954 (23 to 5.810)	1,790 (5·2 to 17.400)	720 (43·8 to 3.750)	2,070 (584 to 4.720)	1,780 (629 to 3.750)	1,300 (480 to 2.890)	975 (434 to 1.930)	1,000 (473 to 1.990)	926 (470 to 1.780)	882 (460 to 1.580)	830 (427 to 1.520)	687 (360 to 1.250)
Unintentional firearm injuries	465 (5 to 2,770)	506 (0·3 to 4,300)	260 (4·7 to 1,560)	552 (46·4 to 1,970)	529 (83·8 to 1,600)	603 (131 to 1,770)	548 (174 to 1,410)	578 (188 to 1,480)	446 (143 to 1,140)	427 (145 to 1,020)	360 (129 to 926)	274 (97·2 to 698)
Other exposure to mechanical forces	489 (3·9 to 3.440)	1,280 (2·5 to 13.500)	460 (27·2 to 2.570)	1,520 (331 to 3.660)	1,250 (306 to 2.860)	695 (237 to 1.670)	427 (218 to 841)	427 (237 to 863)	480 (268 to 911)	455 (273 to 804)	470 (272 to 849)	413 (235 to 720)
Adverse effects of medical treatment	429 (219 to 807)	421 (186 to 785)	1,280 (355 to 2.810)	1,450 (463 to 2.940)	610 (279 to 1.110)	332 (181 to 550)	327 (188 to 526)	571 (300 to 992)	573 (298 to 935)	685 (324 to 1.240)	558 (302 to 940)	560 (303 to 994)
Animal contact	4·1 (1·1 to 8·4)	4·5 (0·9 to 10·5)	49·1 (7·8 to 126)	156 (37·9 to 367)	123 (49·3 to 240)	82 (47·9 to 125)	74·1 (46·2 to 106)	75·3 (44·7 to 108)	84·3 (49·7 to 126)	79 (49·6 to 116)	74·7 (47·9 to 110)	62·4 (40·2 to 89·4)
Venomous animal contact	2·3 (0·5 to 5)	1·1 (0·2 to 2·7)	24·2 (4·2 to 66·1)	68 (18·8 to 166)	57·3 (25·3 to 110)	58 (32·4 to 89·6)	48·6 (30·2 to 71·3)	53·4 (32·6 to 77)	64·5 (37·7 to 99·7)	58·9 (35·8 to 89·2)	50·7 (31·7 to 75·8)	43·5 (27·7 to 62·1)
Non-venomous animal contact	1·8 (0·3 to 4·2)	3·5 (0·5 to 8·2)	25 (1·9 to 72·8)	87·8 (11·3 to 237)	65·7 (15·4 to 150)	24 (11·8 to 42·2)	25·5 (12·6 to 41·3)	21·8 (11·3 to 36·8)	19·8 (10·7 to 32·5)	20·2 (11·8 to 31·6)	24 (12·7 to 39·2)	18·9 (11·1 to 30·1)
Foreign body	862 (348 to 1.830)	644 (293 to 1.180)	4,750 (1.770 to 10.400)	4,420 (2.330 to 8.020)	1,100 (617 to 1.760)	509 (311 to 789)	218 (148 to 314)	252 (171 to 370)	250 (174 to 351)	224 (153 to 327)	203 (137 to 304)	171 (115 to 252)
Pulmonary aspiration and foreign body in airway	857 (347 to 1.830)	641 (292 to 1.170)	4,720 (1.750 to 10.400)	4,340 (2.260 to 7.930)	1,030 (557 to 1.680)	445 (251 to 709)	157 (96·4 to 241)	188 (116 to 296)	184 (116 to 281)	158 (95·1 to 249)	145 (87·3 to 229)	123 (71·4 to 201)
Foreign body in eyes	0 (0 to 0·1)	0·1 (0 to 0·2)	1·8 (0·5 to 4)	9·2 (2·7 to 20·8)	13·6 (3·8 to 31·1)	16·1 (4·9 to 36·4)	19·4 (6·4 to 44)	22·8 (7·5 to 50·3)	25·7 (9·6 to 53·6)	26·6 (9·8 to 54·6)	24·6 (9·8 to 49·9)	20·7 (9·1 to 41·2)
Foreign body in other body part	4·4 (0·7 to 14·2)	2·9 (0·7 to 9)	34·3 (6·6 to 114)	69·4 (26 to 166)	55·6 (32·3 to 90·6)	48 (32·7 to 65·8)	42·2 (29·9 to 57·5)	41 (29·2 to 55·9)	39·8 (28·7 to 53·8)	38·8 (27·5 to 51·8)	33·8 (24·3 to 45·7)	27·4 (20 to 35·9)
Environmental heat and cold exposure	81·3 (14·7 to 240)	113 (34·9 to 292)	164 (45·4 to 424)	597 (236 to 1,390)	557 (252 to 1,310)	361 (203 to 688)	280 (189 to 404)	310 (207 to 454)	320 (224 to 466)	352 (236 to 526)	298 (207 to 423)	257 (176 to 394)
Exposure to forces of nature	0 (0 to 0)	0 (0 to 0)	0 (0 to 0)	3·2 (1·9 to 5·3)	369 (219 to 586)	3,570 (2.150 to 5.960)	3,840 (2.260 to 6.280)	5,430 (3.190 to 9.120)	2,690 (1.590 to 4.440)	2,520 (1.490 to 3.910)	2,610 (1.530 to 4.060)	2,160 (1.290 to 3.530)
Other unintentional injuries	430 (174 to 933)	182 (60 to 411)	303 (122 to 640)	966 (402 to 2.160)	670 (340 to 1.360)	486 (268 to 906)	360 (218 to 619)	381 (247 to 602)	403 (256 to 659)	366 (246 to 547)	334 (213 to 544)	272 (176 to 415)
Self-harm and interpersonal violence	1,090 (392 to 2,470)	307 (113 to 682)	625 (221 to 1,430)	2,040 (723 to 4,730)	1,550 (569 to 3,210)	2,660 (1,300 to 4,400)	8,020 (4,600 to 12,500)	9,470 (5,560 to 13,900)	7,780 (4,470 to 12,400)	6,700 (3,690 to 11,000)	5,070 (2,600 to 8,490)	3,500 (1,730 to 6,170)
Self-harm	0 (0 to 0)	0 (0 to 0)	0 (0 to 0)	0 (0 to 0)	0 (0 to 0)	901 (469 to 1.560)	4,170 (2.220 to 6.960)	4,620 (2.360 to 7.680)	3,250 (1.900 to 5.130)	2,790 (1.660 to 4.250)	2,090 (1.190 to 3.380)	1,400 (828 to 2.190)
Self-harm by firearm	0 (0 to 0)	0 (0 to 0)	0 (0 to 0)	0 (0 to 0)	0 (0 to 0)	8 (2·9 to 19·6)	138 (33·8 to 387)	183 (48·2 to 518)	88·2 (30·5 to 223)	116 (42 to 248)	66·5 (24·9 to 154)	30·9 (12·8 to 63·5)
Self-harm by other specified means	0 (0 to 0)	0 (0 to 0)	0 (0 to 0)	0 (0 to 0)	0 (0 to 0)	893 (466 to 1.550)	4,030 (2.160 to 6.830)	4,440 (2.280 to 7.380)	3,160 (1.850 to 5.030)	2,670 (1.580 to 4.090)	2,020 (1.130 to 3.240)	1,370 (808 to 2.160)
Interpersonal violence	1,090 (392 to 2.470)	307 (113 to 682)	624 (219 to 1.430)	2,020 (708 to 4.720)	1,530 (555 to 3.190)	1,750 (608 to 3.480)	3,810 (1.360 to 7.500)	4,820 (1.870 to 9.000)	4,460 (1.740 to 8.590)	3,830 (1.390 to 7.610)	2,920 (962 to 5.880)	2,060 (629 to 4.360)
Physical violence by firearm	26·9 (6·5 to 77·6)	10·8 (2 to 33·4)	25·1 (6·2 to 75·2)	245 (57·2 to 788)	232 (50·9 to 680)	388 (67·6 to 984)	1,380 (293 to 3,160)	1,680 (415 to 3,800)	1,430 (341 to 3,370)	1,130 (234 to 2,820)	823 (150 to 1,950)	468 (79·4 to 1,190)
Physical violence by sharp object	57·9 (13·3 to 162)	4 (1 to 12)	24·5 (5·4 to 66·1)	114 (28·3 to 333)	138 (44·4 to 333)	249 (62·5 to 598)	957 (251 to 2.310)	1,360 (407 to 3.090)	1,300 (378 to 2.860)	1,090 (294 to 2.520)	918 (207 to 2.200)	668 (144 to 1.660)
Sexual violence	0 (0 to 0)	0 (0 to 0)	0 (0 to 0)	10·4 (6·1 to 16·6)	83 (50·1 to 130)	184 (107 to 291)	402 (227 to 645)	506 (294 to 773)	464 (255 to 750)	411 (226 to 676)	322 (172 to 519)	210 (120 to 347)
Physical violence by other means	1,000 (350 to 2.260)	293 (108 to 653)	574 (200 to 1.300)	1,650 (572 to 3.780)	1,080 (337 to 2.350)	924 (255 to 2.110)	1,070 (308 to 2.380)	1,280 (412 to 2.790)	1,260 (399 to 2.830)	1,190 (361 to 2.480)	857 (234 to 2.020)	712 (183 to 1.610)
Conflict and terrorism	0 (0 to 0)	0 (0 to 0)	0 (0 to 0)	1·7 (0·9 to 3)	1·8 (0·9 to 3·6)	4·7 (2·7 to 8·2)	35·4 (16·8 to 67·6)	23·4 (11·7 to 43·8)	58·8 (31 to 103)	78·9 (39 to 143)	50·1 (25·5 to 91·6)	34·5 (18·1 to 62)

Other transport injuries	85·9 (48·9 to 189)	72·9 (40·5 to 163)	60·9 (34·2 to 144)	42·6 (25·5 to 90)	26·4 (16·9 to 49·1)	17·1 (10·9 to 35·1)	13·8 (8·6 to 29·4)	6·5 (4·4 to 11·1)	2·8 (2 to 4·3)	1 (0·7 to 1·7)	0·3 (0·2 to 0·6)	2,680
Unintentional injuries	4,680 (3.520 to 6.220)	3,840 (2.890 to 5.060)	3,290 (2.500 to 4.390)	2,870 (2.250 to 3.660)	2,360 (1.830 to 3.020)	1,820 (1.410 to 2.320)	1,390 (1.050 to 1.850)	1,020 (776 to 1.350)	508 (367 to 683)	194 (142 to 272)	54·8 (38·6 to 80·4)	147.000
Falls	395 (283 to 528)	393 (281 to 532)	387 (282 to 517)	432 (309 to 591)	428 (303 to 579)	469 (310 to 660)	451 (299 to 659)	452 (307 to 654)	260 (168 to 378)	100 (66·2 to 151)	29·5 (18·7 to 49·6)	8,250
Drowning	251 (130 to 435)	160 (86·6 to 262)	119 (62·8 to 196)	87·9 (47·6 to 143)	63·9 (36·5 to 102)	32·7 (18 to 54·4)	23 (12·5 to 37·5)	9·8 (5·2 to 16·1)	4·9 (2·6 to 7·9)	2·2 (1·1 to 4)	0·5 (0·3 to 0·9)	30.600
Fire, heat, and hot substances	756 (493 to 1.110)	605 (383 to 863)	494 (317 to 734)	377 (226 to 557)	263 (154 to 401)	180 (97·7 to 286)	118 (63 to 192)	72·9 (37·7 to 117)	36·3 (18·7 to 59·5)	12·7 (6·5 to 20)	3·4 (2 to 5·2)	21.500
Poisonings	60·9 (32·9 to 112)	42·7 (24·2 to 72·4)	36·2 (19·6 to 63·1)	25·7 (14·1 to 44)	23·8 (13·9 to 39·6)	15·5 (8·4 to 28)	10·5 (5·6 to 18·4)	6·6 (3·5 to 11·4)	2·4 (1·3 to 4·2)	0·6 (0·3 to 1)	0·1 (0·1 to 0·2)	3.730
Poisoning by carbon monoxide	30·6 (10·8 to 72·9)	19·4 (7·4 to 44·7)	20·4 (8·4 to 44·9)	14·3 (6 to 29·8)	15·2 (7·1 to 29)	9·1 (3·6 to 20)	5·8 (2 to 13)	3·1 (1 to 7·2)	1 (0·3 to 2·7)	0·2 (0·1 to 0·5)	0 (0 to 0·1)	1.480
Poisoning by other means	30·3 (13 to 62·6)	23·3 (11·2 to 43·9)	15·9 (7·2 to 31·8)	11·4 (5·5 to 20·8)	8·6 (4 to 16·3)	6·4 (2·7 to 12·5)	4·8 (1·8 to 9·5)	3·5 (1·3 to 7·4)	1·4 (0·5 to 2·8)	0·4 (0·2 to 0·6)	0·1 (0 to 0·2)	2,250
Exposure to mechanical forces	489 (266 to 830)	407 (228 to 694)	310 (185 to 507)	244 (151 to 393)	185 (116 to 287)	131 (76·7 to 214)	95·6 (53 to 161)	62·1 (34·2 to 104)	30·9 (17·2 to 53·1)	14·3 (7·1 to 26·9)	5 (2·2 to 10·4)	15.900
Unintentional firearm injuries	163 (65·7 to 410)	103 (44·8 to 253)	80·2 (37·1 to 189)	51 (26 to 107)	33·4 (18·5 to 67·7)	31·5 (14·1 to 74·8)	22·4 (10·3 to 49·7)	18·2 (7·5 to 46·3)	10·2 (4·3 to 24·6)	5·4 (2·1 to 13·3)	1·9 (0·7 to 5)	6,070
Other exposure to mechanical forces	325 (188 to 537)	304 (168 to 527)	230 (137 to 364)	193 (117 to 309)	152 (92·2 to 234)	99·6 (58·1 to 162)	73·2 (40 to 123)	43·9 (25·1 to 72·8)	20·7 (11·8 to 34·8)	8·9 (4·6 to 16·7)	3 (1·3 to 6·8)	9.820
Adverse effects of medical treatment	550 (283 to 1.040)	466 (231 to 873)	473 (236 to 912)	447 (228 to 785)	347 (190 to 590)	271 (152 to 481)	209 (112 to 393)	129 (70·7 to 246)	59·1 (30·4 to 110)	17·9 (9·7 to 34·1)	3·8 (2·1 to 7·1)	10.800
Animal contact	52 (34·6 to 76·7)	42·1 (28·2 to 59·5)	41·9 (26·7 to 62·2)	33·2 (21·9 to 46)	26·1 (17·1 to 37·3)	21·9 (13·2 to 35·1)	17·7 (9·8 to 31)	15·6 (7·4 to 28·5)	8·6 (4 to 17·9)	5·1 (2 to 11·2)	1·7 (0·6 to 4·4)	1,130
Venomous animal contact	31·1 (20·2 to 45·8)	24·4 (15·8 to 34·9)	24·1 (15·5 to 36·1)	20·5 (13·4 to 29·5)	16·1 (10·2 to 23·4)	13·3 (7·8 to 22)	11 (5·9 to 19·5)	9·8 (4·3 to 20·3)	5·9 (2·5 to 13·1)	3·7 (1·3 to 8·5)	1·2 (0·4 to 3·4)	692
Non-venomous animal contact	20·9 (12 to 35)	17·7 (10·3 to 30·3)	17·8 (9·9 to 30·3)	12·6 (7·7 to 19·6)	10 (6·2 to 16)	8·6 (4·7 to 14·7)	6·7 (3·6 to 12·4)	5·8 (2·6 to 12·7)	2·7 (1·2 to 6)	1·4 (0·6 to 3)	0·5 (0·2 to 1·1)	443
Foreign body	132 (88·2 to 200)	114 (74·7 to 173)	101 (66·5 to 150)	92·2 (62·3 to 135)	84 (56·2 to 125)	69·7 (45·8 to 102)	56·8 (34·8 to 87·4)	40·8 (25·7 to 62·5)	14·8 (9 to 23)	6 (3·7 to 9·8)	1·7 (1·1 to 2·6)	14.300
Pulmonary aspiration and foreign body in airway	93·2 (53·2 to 158)	83·3 (47·4 to 139)	77·3 (45 to 125)	71·8 (44·3 to 111)	66·5 (40·8 to 105)	58 (35·1 to 89·3)	47·6 (27 to 77·3)	34·4 (20·3 to 55·4)	12 (6·5 to 19·9)	4·9 (2·7 to 8·4)	1·4 (0·8 to 2·3)	13.500
Foreign body in eyes	15·7 (7·7 to 29·4)	11·8 (6·5 to 20·5)	9 (5·4 to 14·6)	7·3 (4·4 to 11·2)	5·7 (3·6 to 8·8)	3·6 (2·3 to 5·3)	2·5 (1·6 to 3·6)	1·4 (0·9 to 2·1)	0·6 (0·4 to 0·9)	0·2 (0·1 to 0·3)	0 (0 to 0·1)	239
Foreign body in other body part	23·1 (15·8 to 33·4)	18·4 (13·2 to 25·1)	14·5 (10·4 to 19·4)	13·1 (9·2 to 18·4)	11·8 (7·8 to 17·9)	8 (5·3 to 12·7)	6·7 (4·1 to 11·6)	5 (2·8 to 9·6)	2·2 (1·3 to 4·1)	0·9 (0·5 to 2)	0·3 (0·1 to 0·5)	542
Environmental heat and cold exposure	235 (150 to 419)	216 (132 to 403)	209 (122 to 416)	184 (108 to 342)	181 (98·6 to 383)	141 (73·6 to 306)	113 (57 to 262)	84·4 (42·3 to 191)	42·9 (20·9 to 97·1)	22·4 (9·7 to 57)	6·7 (2·9 to 16·4)	5,130
Exposure to forces of nature	1,560 (920 to 2.480)	1,240 (734 to 2.020)	989 (600 to 1.590)	836 (518 to 1.380)	672 (410 to 1.050)	433 (254 to 672)	255 (153 to 394)	129 (77·3 to 206)	39 (24·1 to 60·9)	10 (6·3 to 15·4)	1·9 (1·2 to 3)	29.300
Other unintentional injuries	205 (139 to 321)	161 (108 to 236)	133 (91·4 to 192)	110 (75·7 to 158)	83·8 (57·8 to 120)	53·8 (37·1 to 75·6)	37·5 (26·6 to 53·1)	20·8 (14·7 to 28·3)	8·6 (6·1 to 11·8)	2·6 (1·9 to 3·6)	0·6 (0·5 to 0·9)	5.970
Self-harm and interpersonal violence	2,290 (1,130 to 4,060)	1,480 (778 to 2,480)	1,010 (548 to 1,670)	686 (385 to 1,100)	473 (266 to 779)	252 (147 to 389)	134 (76·9 to 215)	54·9 (35·7 to 82·6)	19·3 (12·9 to 29·1)	4·1 (2·9 to 5·7)	0·8 (0·6 to 1·1)	55,200
Self-harm	980 (557 to 1.660)	707 (414 to 1.160)	515 (314 to 826)	359 (218 to 556)	243 (146 to 392)	124 (78·5 to 187)	59·4 (36·8 to 90·5)	25·1 (15·2 to 37·4)	8·2 (5 to 12·8)	1·5 (1 to 2·2)	0·3 (0·2 to 0·4)	22.200
Self-harm by firearm	21·6 (8·9 to 45·5)	16·5 (7·4 to 32·7)	12 (5·5 to 25·3)	4·5 (2·6 to 8·1)	4·9 (2·5 to 9·7)	3·4 (1·7 to 6·5)	2·1 (1·1 to 3·9)	1·8 (0·9 to 3·4)	0·9 (0·4 to 1·8)	0·1 (0 to 0·1)	0 (0 to 0)	699
Self-harm by other specified means	958 (544 to 1.630)	690 (407 to 1.130)	503 (307 to 810)	354 (214 to 550)	238 (143 to 383)	120 (75·9 to 182)	57·3 (35·6 to 87·2)	23·3 (14 to 35)	7·3 (4·4 to 11·3)	1·5 (1 to 2·1)	0·3 (0·2 to 0·4)	21.500
Interpersonal violence	1,290 (401 to 2.750)	757 (240 to 1.610)	486 (156 to 1.010)	320 (114 to 650)	226 (80·5 to 465)	126 (47·5 to 239)	73 (28·6 to 137)	29·1 (14·3 to 51·4)	10·9 (5·8 to 18·7)	2·5 (1·5 to 3·8)	0·5 (0·3 to 0·7)	32.500
Physical violence by firearm	324 (52·3 to 811)	154 (23·7 to 405)	93·3 (14·9 to 236)	46·9 (8 to 115)	23·7 (4·3 to 64·1)	10·6 (2·1 to 26·5)	4 (0·9 to 10·8)	1·1 (0·3 to 2·6)	0·6 (0·2 to 1·5)	0·1 (0 to 0·2)	0 (0 to 0·1)	8,500
Physical violence by sharp object	338 (71·6 to 827)	176 (39·3 to 429)	105 (25·9 to 260)	56·5 (17·2 to 130)	38 (11·7 to 89·4)	21·1 (6·6 to 47·2)	9·1 (3·7 to 19·1)	4·5 (2 to 9·7)	1·6 (0·9 to 2·8)	0·5 (0·3 to 0·7)	0·1 (0·1 to 0·2)	7,630
Sexual violence	132 (75·3 to 215)	78·8 (44·4 to 129)	49 (28·7 to 79·6)	36·5 (22 to 55·3)	26·8 (16·4 to 40·6)	16·2 (9·9 to 24·3)	10·1 (6·5 to 15·2)	5·8 (3·7 to 8·6)	2·1 (1·2 to 3·3)	0·3 (0·2 to 0·4)	0 (0 to 0)	2,950
Physical violence by other means	497 (133 to 1.190)	348 (93·4 to 782)	239 (65·5 to 552)	180 (50·7 to 410)	137 (38·4 to 307)	77·8 (22·9 to 161)	49·7 (14·3 to 99·7)	17·7 (6·8 to 36·5)	6·5 (2·9 to 12·7)	1·7 (0·9 to 2·8)	0·3 (0·2 to 0·5)	13.500
Conflict and terrorism	18·4 (9·6 to 32)	10·8 (6 to 19)	7·4 (4 to 12·8)	5·2 (2·8 to 9·1)	3·2 (1·8 to 5·5)	1·8 (1 to 3·1)	1·1 (0·6 to 1·9)	0·6 (0·3 to 1)	0·2 (0·1 to 0·4)	0·1 (0 to 0·1)	0 (0 to 0)	338

SR Table 2b: Jamaica DALYS by cause and age group for females with 95% uncertainty intervals, 2019

Cause	Early Neonatal	Late Neonatal	Post Neonatal	1 to 4	5 to 9	10 to 14	15 to 19	20 to 24
All causes	13,700 (9,890 to 18,500)	4,350 (3,160 to 5,860)	3,110 (2,080 to 4,590)	5,000 (3,940 to 6,230)	5,520 (4,260 to 7,040)	7,750 (6,000 to 9,990)	12,000 (9,290 to 15,400)	16,800 (13,400 to 20,900)
Communicable, maternal, neonatal, and nutritional diseases	12,100 (8,700 to 16,300)	3,510 (2,540 to 4,740)	1,650 (1,100 to 2,470)	2,120 (1,590 to 2,760)	1,670 (1,170 to 2,410)	1,440 (1,080 to 1,920)	1,920 (1,400 to 2,620)	2,650 (2,100 to 3,380)
HIV/AIDS and sexually transmitted infections	0·4 (0·2 to 0·6)	0·3 (0·1 to 0·5)	125 (116 to 135)	143 (132 to 154)	97·5 (90·7 to 105)	112 (104 to 120)	194 (175 to 216)	558 (505 to 628)
HIV/AIDS	0 (0 to 0)	0 (0 to 0)	125 (116 to 135)	143 (132 to 154)	97·5 (90·7 to 105)	110 (102 to 117)	181 (164 to 200)	526 (479 to 586)
HIV/AIDS - Drug-susceptible Tuberculosis	0 (0 to 0)	0 (0 to 0)	1·6 (1 to 2·5)	1·9 (1·2 to 2·9)	1·3 (0·8 to 2)	1·4 (0·9 to 2·2)	2·6 (1·8 to 3·7)	7·8 (5·4 to 11·3)
HIV/AIDS - Multidrug-resistant Tuberculosis without extensive	0 (0 to 0)	0 (0 to 0)	0·1 (0 to 0·3)	0·1 (0 to 0·3)	0·1 (0 to 0·2)	0·1 (0 to 0·2)	0·1 (0 to 0·4)	0·3 (0 to 1·1)
HIV/AIDS - Extensively drug-resistant Tuberculosis	0 (0 to 0)	0 (0 to 0)	0 (0 to 0)	0 (0 to 0)	0 (0 to 0)	0 (0 to 0)	0 (0 to 0·1)	0 (0 to 0·2)
HIV/AIDS resulting in other diseases	0 (0 to 0)	0 (0 to 0)	123 (114 to 133)	141 (131 to 152)	96·1 (89·3 to 104)	108 (101 to 116)	178 (161 to 197)	517 (472 to 578)
Sexually transmitted infections excluding HIV	0·4 (0·2 to 0·6)	0·2 (0·1 to 0·5)	0·1 (0 to 0·2)	0 (0 to 0)	0 (0 to 0)	2·5 (1·6 to 4)	13·1 (7·6 to 23·2)	32·3 (16·6 to 59·5)
Syphilis	0·4 (0·2 to 0·6)	0·2 (0·1 to 0·5)	0·1 (0 to 0·2)	0 (0 to 0)	0 (0 to 0)	0·6 (0·4 to 1)	1·1 (0·6 to 1·7)	0·9 (0·4 to 1·9)
Chlamydial infection	0 (0 to 0)	0 (0 to 0)	0 (0 to 0)	0 (0 to 0)	0 (0 to 0)	0·2 (0·2 to 0·4)	1·3 (0·8 to 2)	3·1 (2 to 4·6)
Gonococcal infection	0 (0 to 0)	0 (0 to 0)	0 (0 to 0)	0 (0 to 0)	0 (0 to 0)	0·2 (0·1 to 0·3)	1·4 (0·9 to 2)	1·8 (1·2 to 2·7)
Trichomoniasis	0 (0 to 0)	0 (0 to 0)	0 (0 to 0)	0 (0 to 0)	0 (0 to 0)	0·1 (0 to 0·4)	2·3 (0·7 to 5·5)	11·6 (3·9 to 27·8)
Genital herpes	0 (0 to 0)	0 (0 to 0)	0 (0 to 0)	0 (0 to 0)	0 (0 to 0)	0·9 (0·3 to 2·2)	4·1 (1·2 to 10)	9·5 (3·1 to 23·3)
Other sexually transmitted infections	0 (0 to 0)	0 (0 to 0)	0 (0 to 0)	0 (0 to 0)	0 (0 to 0)	0·4 (0·3 to 0·6)	3 (2 to 4·3)	5·5 (2·8 to 9·6)
Respiratory infections and tuberculosis	204 (127 to 311)	195 (123 to 297)	261 (140 to 446)	289 (199 to 392)	254 (178 to 358)	207 (139 to 304)	209 (142 to 310)	276 (188 to 381)
Tuberculosis	0 (0 to 0)	0 (0 to 0)	0·4 (0·3 to 0·7)	2·3 (1·5 to 3·5)	4 (0·1 to 10·3)	1·9 (1·1 to 3·1)	3·5 (2·4 to 4·9)	20·5 (10·3 to 34·4)
Latent tuberculosis infection	0 (0 to 0)	0 (0 to 0)	0 (0 to 0)	0 (0 to 0)	0 (0 to 0)	0 (0 to 0)	0 (0 to 0)	0 (0 to 0)
Drug-susceptible tuberculosis	0 (0 to 0)	0 (0 to 0)	0·4 (0·2 to 0·7)	2·3 (1·5 to 3·4)	4 (0·1 to 10·2)	1·9 (1 to 3·1)	3·4 (2·3 to 4·8)	20 (9·6 to 34·2)
Multidrug-resistant tuberculosis without extensive drug resistar	0 (0 to 0)	0 (0 to 0)	0 (0 to 0)	0 (0 to 0·2)	0·1 (0 to 0·2)	0 (0 to 0·1)	0·1 (0 to 0·4)	0·5 (0·1 to 1·8)
Extensively drug-resistant tuberculosis	0 (0 to 0)	0 (0 to 0)	0 (0 to 0)	0 (0 to 0)	0 (0 to 0)	0 (0 to 0)	0 (0 to 0·1)	0·1 (0 to 0·2)
Lower respiratory infections	204 (127 to 311)	194 (121 to 296)	236 (113 to 423)	160 (96·5 to 243)	93·7 (65·4 to 132)	67·1 (44·7 to 95·6)	66·2 (45 to 94·9)	112 (66·5 to 164)
Upper respiratory infections	0·2 (0·1 to 0·3)	1·1 (0·6 to 1·8)	20·4 (11·3 to 34·1)	89·9 (50·5 to 149)	105 (56·3 to 179)	101 (52·1 to 179)	103 (53·6 to 183)	109 (57 to 184)
Otitis media	0 (0 to 0·1)	0·1 (0 to 0·2)	4·1 (2 to 7·7)	36·9 (19·8 to 65·1)	50·6 (27·6 to 85·9)	37·1 (19·9 to 66·2)	36 (18·9 to 61·5)	35·5 (15 to 70·5)
Enteric infections	28·9 (16·2 to 47·6)	40·4 (22·1 to 67·3)	193 (118 to 302)	209 (136 to 301)	154 (92·6 to 248)	140 (82·7 to 225)	94·5 (58·8 to 145)	87·1 (56·1 to 128)
Diarrheal diseases	28·9 (16·2 to 47·6)	40·4 (22·1 to 67·3)	193 (118 to 300)	208 (135 to 300)	153 (91·8 to 246)	139 (82 to 225)	94·2 (58·7 to 144)	86·8 (55·7 to 128)
Typhoid and paratyphoid	0 (0 to 0)	0 (0 to 0)	0 (0 to 0)	0 (0 to 0)	0 (0 to 0)	0 (0 to 0)	0 (0 to 0)	0 (0 to 0)
Typhoid fever	0 (0 to 0)	0 (0 to 0)	0 (0 to 0)	0 (0 to 0)	0 (0 to 0)	0 (0 to 0)	0 (0 to 0)	0 (0 to 0)
Paratyphoid fever	0 (0 to 0)	0 (0 to 0)	0 (0 to 0)	0 (0 to 0)	0 (0 to 0)	0 (0 to 0)	0 (0 to 0)	0 (0 to 0)
Invasive Non-typhoidal Salmonella (iNTS)	0 (0 to 0)	0 (0 to 0)	0 (0 to 0)	0·2 (0 to 0·6)	0·2 (0 to 0·4)	0·2 (0 to 0·4)	0 (0 to 0·1)	0 (0 to 0·1)
Other intestinal infectious diseases	0 (0 to 0)	0 (0 to 0)	0·5 (0·1 to 1·9)	0·9 (0·2 to 2·7)	0·5 (0·1 to 1·6)	0·5 (0·1 to 1·4)	0·2 (0 to 0·7)	0·3 (0 to 0·9)
Neglected tropical diseases and malaria	0·4 (0·1 to 0·8)	1·7 (0·8 to 3)	15·5 (8·1 to 26·1)	74·3 (37·9 to 127)	105 (52·1 to 187)	78 (39·2 to 135)	91·4 (47·4 to 156)	141 (73·2 to 234)
Malaria	0 (0 to 0)	0 (0 to 0)	0 (0 to 0)	0 (0 to 0)	0 (0 to 0)	0 (0 to 0)	0 (0 to 0)	0 (0 to 0)
Chagas disease	0 (0 to 0)	0 (0 to 0)	0 (0 to 0)	0 (0 to 0)	0 (0 to 0)	0 (0 to 0)	0 (0 to 0)	0 (0 to 0)
Leishmaniasis	0 (0 to 0)	0 (0 to 0)	0 (0 to 0)	0 (0 to 0)	0 (0 to 0)	0 (0 to 0)	0 (0 to 0)	0 (0 to 0)
Visceral leishmaniasis	0 (0 to 0)	0 (0 to 0)	0 (0 to 0)	0 (0 to 0)	0 (0 to 0)	0 (0 to 0)	0 (0 to 0)	0 (0 to 0)
Cutaneous and mucocutaneous leishmaniasis	0 (0 to 0)	0 (0 to 0)	0 (0 to 0)	0 (0 to 0)	0 (0 to 0)	0 (0 to 0)	0 (0 to 0)	0 (0 to 0)
African trypanosomiasis	0 (0 to 0)	0 (0 to 0)	0 (0 to 0)	0 (0 to 0)	0 (0 to 0)	0 (0 to 0)	0 (0 to 0)	0 (0 to 0)
Schistosomiasis	0 (0 to 0)	0 (0 to 0)	0 (0 to 0)	0 (0 to 0)	0 (0 to 0)	0 (0 to 0)	0 (0 to 0)	0 (0 to 0)
Cysticercosis	0 (0 to 0)	0 (0 to 0)	0 (0 to 0)	0 (0 to 0)	0 (0 to 0·1)	0·8 (0·2 to 2·3)	6·2 (0 to 17·6)	22·4 (4·5 to 60·1)
Cystic echinococcosis	0 (0 to 0)	0 (0 to 0)	0 (0 to 0)	0 (0 to 0)	0 (0 to 0)	0 (0 to 0)	0 (0 to 0)	0 (0 to 0)
Lymphatic filariasis	0 (0 to 0)	0 (0 to 0)	0 (0 to 0)	0 (0 to 0)	0 (0 to 0)	0 (0 to 0)	0 (0 to 0)	0 (0 to 0)
Onchocerciasis	0 (0 to 0)	0 (0 to 0)	0 (0 to 0)	0 (0 to 0)	0 (0 to 0)	0 (0 to 0)	0 (0 to 0)	0 (0 to 0)
Trachoma	0 (0 to 0)	0 (0 to 0)	0 (0 to 0)	0 (0 to 0)	0 (0 to 0)	0 (0 to 0)	0 (0 to 0)	0 (0 to 0)
Dengue	0 (0 to 0)	0·2 (0 to 1·1)	2·8 (0·6 to 6·8)	8·6 (2 to 18·7)	11·4 (1·6 to 29·5)	10·3 (0·4 to 29·1)	11·3 (1·2 to 30·4)	14·5 (3 to 32·5)
Yellow fever	0 (0 to 0)	0 (0 to 0)	0 (0 to 0)	0 (0 to 0)	0 (0 to 0)	0 (0 to 0)	0 (0 to 0)	0 (0 to 0)
Rabies	0 (0 to 0)	0 (0 to 0)	0 (0 to 0)	0 (0 to 0)	0 (0 to 0·1)	0 (0 to 0·1)	0 (0 to 0)	0 (0 to 0)
Intestinal nematode infections	0 (0 to 0)	0 (0 to 0)	0·5 (0·2 to 1)	14·2 (7·1 to 25)	51·7 (25·6 to 92·4)	44·5 (21·7 to 79·8)	53·5 (27·1 to 93·9)	84·5 (41·1 to 153)
Ascariasis	0 (0 to 0)	0 (0 to 0)	0·1 (0 to 0·4)	2 (0·7 to 4·2)	4 (1·5 to 7·8)	2·3 (0·8 to 4·7)	1·6 (0·5 to 3·3)	1·5 (0·4 to 3·2)
Trichuriasis	0 (0 to 0)	0 (0 to 0)	0·3 (0·1 to 0·6)	9·2 (4·1 to 17·6)	40·2 (17·9 to 76·2)	35·7 (16·1 to 64·6)	31·4 (14·1 to 58·5)	33·3 (15 to 61·9)
Hookworm disease	0 (0 to 0)	0 (0 to 0)	0·1 (0·1 to 0·2)	3 (1·2 to 5·9)	7·5 (3·2 to 14·6)	6·5 (2·8 to 13·2)	20·5 (7·7 to 41·4)	49·7 (16·5 to 99·7)
Food-borne trematodiases	0 (0 to 0)	0 (0 to 0)	0 (0 to 0)	0 (0 to 0)	0 (0 to 0)	0 (0 to 0)	0 (0 to 0)	0 (0 to 0)
Leprosy	0 (0 to 0)	0 (0 to 0)	0 (0 to 0)	0 (0 to 0)	0 (0 to 0)	0 (0 to 0)	0 (0 to 0)	0 (0 to 0·1)
Ebola	0 (0 to 0)	0 (0 to 0)	0 (0 to 0)	0 (0 to 0)	0 (0 to 0)	0 (0 to 0)	0 (0 to 0)	0 (0 to 0)
Zika virus	0 (0 to 0)	0 (0 to 0)	0 (0 to 0·1)	0·1 (0 to 0·3)	0·1 (0 to 0·4)	0·1 (0 to 0·4)	0·1 (0 to 0·4)	0·1 (0 to 0·5)
Guinea worm disease	0 (0 to 0)	0 (0 to 0)	0 (0 to 0)	0 (0 to 0)	0 (0 to 0)	0 (0 to 0)	0 (0 to 0)	0 (0 to 0)
Other neglected tropical diseases	0·4 (0·1 to 0·8)	1·4 (0·7 to 2·5)	12·1 (5·3 to 21·4)	51·4 (21·6 to 94·8)	41·5 (12 to 94·7)	22·2 (5·8 to 54·5)	20·2 (6·5 to 46)	19 (5·4 to 42·3)
Other infectious diseases	43·2 (27·1 to 65·5)	47·7 (30·4 to 72·9)	111 (57·2 to 222)	168 (105 to 292)	109 (73·5 to 170)	96·7 (67 to 139)	96·7 (68·6 to 135)	123 (89·4 to 168)
Meningitis	29·1 (18·2 to 45·2)	34·6 (20·7 to 55·6)	35·5 (17·4 to 62·2)	29·2 (19 to 42·6)	23 (17·1 to 30·1)	21·4 (14 to 31)	21 (13·6 to 31·1)	30·3 (18·4 to 44·9)
Encephalitis	8·8 (3·8 to 15·9)	6·1 (3·4 to 10·8)	49·3 (17·3 to 137)	81 (33·6 to 200)	43·2 (22·9 to 91·1)	37·6 (20·2 to 74·6)	30 (17·5 to 56·1)	37·6 (22·7 to 64·4)
Diphtheria	0 (0 to 0)	0 (0 to 0)	0 (0 to 0)	0 (0 to 0)	0·1 (0 to 0·2)	0 (0 to 0·1)	0 (0 to 0)	0 (0 to 0)
Tetanus	0 (0 to 0)	0 (0 to 0·1)	0 (0 to 0)	0 (0 to 0)	0·2 (0·1 to 0·4)	0·1 (0 to 0·3)	0 (0 to 0·1)	0·2 (0·1 to 0·4)
Measles	0 (0 to 0)	0 (0 to 0)	0 (0 to 0)	0 (0 to 0)	0 (0 to 0)	0 (0 to 0)	0 (0 to 0)	0 (0 to 0)
Varicella and herpes zoster	0 (0 to 0)	0 (0 to 0·1)	0·3 (0·1 to 0·7)	0·6 (0·3 to 1·2)	0·8 (0·4 to 1·5)	1·2 (0·2 to 2·7)	1·5 (0·3 to 3·7)	2·1 (0·5 to 4·9)
Acute hepatitis	0 (0 to 0)	0 (0 to 0)	0·2 (0·1 to 0·3)	1·8 (0·7 to 3·4)	8 (4·5 to 12·9)	10·5 (6·1 to 16·4)	10·3 (6·1 to 16·4)	12·2 (7·8 to 18·1)
Acute hepatitis A	0 (0 to 0)	0 (0 to 0)	0 (0 to 0)	1·4 (0·4 to 2·9)	6·4 (3·2 to 11)	8·4 (4·7 to 14)	8·1 (4·4 to 13·2)	7·1 (3·5 to 11·9)
Acute hepatitis B	0 (0 to 0)	0 (0 to 0)	0 (0 to 0·1)	0·1 (0·1 to 0·3)	0·8 (0·4 to 1·5)	1·1 (0·4 to 2·2)	1 (0·4 to 2)	4 (2·3 to 6·6)
Acute hepatitis C	0 (0 to 0)	0 (0 to 0)	0·1 (0 to 0·2)	0·2 (0·1 to 0·4)	0·2 (0·1 to 0·4)	0·2 (0·1 to 0·4)	0·4 (0·1 to 1)	0·2 (0·1 to 0·7)
Acute hepatitis E	0 (0 to 0)	0 (0 to 0)	0 (0 to 0)	0·1 (0 to 0·1)	0·5 (0·2 to 0·9)	0·8 (0·1 to 2)	0·8 (0·1 to 2·1)	0·8 (0·1 to 2·2)
Other unspecified infectious diseases	5·3 (1·4 to 10·6)	7 (2·6 to 13·7)	22·1 (12 to 36·8)	51 (26·1 to 80·5)	33·3 (13·6 to 68)	25·7 (10·9 to 48·2)	33·7 (15·9 to 58·9)	40·8 (18·2 to 70·9)
Maternal and neonatal disorders	11,800 (8,500 to 16,000)	3,210 (2,320 to 4,350)	708 (410 to 1,150)	642 (478 to 842)	485 (355 to 644)	535 (385 to 717)	720 (551 to 924)	980 (766 to 1,230)
Maternal disorders	0 (0 to 0)	0 (0 to 0)	0 (0 to 0)	0 (0 to 0)	0 (0 to 0)	4·8 (3·7 to 6·1)	164 (118 to 216)	384 (270 to 521)
Maternal hemorrhage	0 (0 to 0)	0 (0 to 0)	0 (0 to 0)	0 (0 to 0)	0 (0 to 0)	0·2 (0·1 to 0·3)	17·9 (10·3 to 29·3)	42·7 (24·4 to 67·5)
Maternal sepsis and other maternal infections	0 (0 to 0)	0 (0 to 0)	0 (0 to 0)	0 (0 to 0)	0 (0 to 0)	0·2 (0·1 to 0·3)	6·1 (2·8 to 10·8)	13 (6·6 to 22·5)
Maternal hypertensive disorders	0 (0 to 0)	0 (0 to 0)	0 (0 to 0)	0 (0 to 0)	0 (0 to 0)	1·8 (1·2 to 2·5)	52·7 (34·1 to 76·1)	101 (65·4 to 148)
Maternal obstructed labor and uterine rupture	0 (0 to 0)	0 (0 to 0)	0 (0 to 0)	0 (0 to 0)	0 (0 to 0)	0·1 (0·1 to 0·2)	2·3 (1·3 to 3·5)	3·7 (2·2 to 6)
Maternal abortion and miscarriage	0 (0 to 0)	0 (0 to 0)	0 (0 to 0)	0 (0 to 0)	0 (0 to 0)	0·2 (0·1 to 0·3)	10·7 (5·5 to 18)	29·9 (14·3 to 52·5)
Ectopic pregnancy	0 (0 to 0)	0 (0 to 0)	0 (0 to 0)	0 (0 to 0)	0 (0 to 0)	0·1 (0·1 to 0·2)	5·2 (2·9 to 8·2)	14·2 (8 to 23·7)
Indirect maternal deaths	–	–	–	–	–	0 (0 to 0·1)	1·2 (0·6 to 2)	2 (1·1 to 3·5)
Late maternal deaths	–	–	–	–	–	0·4 (0·2 to 0·7)	15 (8·6 to 24·1)	36·2 (19·7 to 58·5)
Maternal deaths aggravated by HIV/AIDS	–	–	–	–	–	0 (0 to 0)	0·1 (0·1 to 0·3)	0·8 (0·3 to 1·6)
Neonatal disorders	11,800 (8,500 to 16,000)	3,210 (2,320 to 4,350)	708 (410 to 1,150)	642 (478 to 842)	485 (355 to 644)	531 (379 to 712)	557 (407 to 759)	596 (426 to 794)
Neonatal preterm birth	7,520 (5,380 to 10,500)	1,650 (1,120 to 2,320)	323 (171 to 544)	165 (117 to 220)	176 (122 to 243)	192 (130 to 263)	199 (137 to 276)	211 (145 to 289)
Neonatal encephalopathy due to birth asphyxia and trauma	2,130 (1,330 to 3,200)	262 (145 to 420)	79·2 (38·7 to 145)	88·3 (52 to 136)	42·1 (20·8 to 70·7)	45·3 (22·5 to 77·2)	48·2 (24·5 to 81·7)	51·5 (24·7 to 87·1)
Neonatal sepsis and other neonatal infections	1,320 (820 to 2,020)	1,010 (651 to 1,490)	223 (116 to 396)	192 (125 to 276)	250 (159 to 367)	274 (173 to 415)	289 (182 to 435)	311 (192 to 469)
Hemolytic disease and other neonatal jaundice	34·6 (18·6 to 58·5)	15·5 (8·5 to 25·6)	4·2 (2·2 to 7·1)	13·4 (8·4 to 20·2)	17·2 (10·2 to 25·8)	19·1 (13·9 to 24·8)	20·4 (15·2 to 26·4)	22·2 (16·1 to 28·9)
Other neonatal disorders	812 (481 to 1,260)	274 (179 to 392)	78·4 (51 to 118)	183 (135 to 241)	0 (0 to 0)	0 (0 to 0)	0 (0 to 0)	0 (0 to 0)
Nutritional deficiencies	0·6 (0·3 to 0·9)	15·4 (7·7 to 26·8)	239 (144 to 371)	598 (286 to 1,040)	470 (158 to 1,040)	268 (92·9 to 614)	510 (189 to 1,070)	487 (184 to 999)
Protein-energy malnutrition	0·5 (0·3 to 0·9)	1·6 (0·9 to 2·5)	118 (66·6 to 192)	79·8 (48·3 to 121)	31·8 (20·8 to 46·4)	25·1 (15·4 to 37·3)	33·3 (21·5 to 48·7)	32·4 (19·9 to 49·1)
Iodine deficiency	0 (0 to 0)	0 (0 to 0)	0 (0 to 0)	0·4 (0·1 to 0·8)	2·9 (1·2 to 5·5)	7·4 (3·2 to 13·7)	10·5 (3·6 to 21·1)	13·5 (4·5 to 27)
Vitamin A deficiency	0 (0 to 0·1)	0·6 (0·2 to 1·3)	2·5 (1·2 to 5·1)	14·8 (7·3 to 26·7)	16·6 (7·1 to 30·2)	11·3 (4·2 to 22·9)	5·3 (1·4 to 11·5)	4·5 (2 to 8·5)
Dietary iron deficiency	0 (0 to 0·2)	13·2 (6·1 to 23·9)	117 (49·7 to 218)	500 (191 to 942)	414 (108 to 978)	220 (52·5 to 565)	453 (140 to 995)	424 (123 to 942)
Other nutritional deficiencies	0 (0 to 0)	0 (0 to 0)	1·3 (0·8 to 2)	3·9 (1·7 to 7·4)	4·9 (2·5 to 8)	4 (1·8 to 6·9)	7·3 (3·4 to 12·8)	12·2 (6·1 to 19·9)
Non-communicable diseases	1,480 (848 to 2,210)	801 (463 to 1,200)	1,360 (785 to 2,210)	2,570 (1,950 to 3,320)	3,350 (2,560 to 4,290)	5,690 (4,220 to 7,560)	8,940 (6,740 to 11,800)	12,500 (9,600 to 15,900)
Neoplasms	14·4 (9·7 to 20·1)	12·8 (8·8 to 18·3)	34 (17·9 to 56·8)	217 (147 to 305)	316 (247 to 397)	345 (269 to 441)	434 (333 to 552)	777 (578 to 1,020)
Lip and oral cavity cancer	0 (0 to 0)	0 (0 to 0)	0 (0 to 0)	0 (0 to 0)	1·6 (0·8 to 2·7)	2·5 (1·3 to 4·2)	4·5 (2·9 to 6·7)	7·9 (4·5 to 12·6)
Nasopharynx cancer	0 (0 to 0)	0 (0 to 0)	0 (0 to 0)	0 (0 to 0)	3·5 (1·5 to 7·3)	2·2 (1·1 to 3·8)	11·6 (6·9 to 18·5)	19·4 (10 to 33·8)
Other pharynx cancer	0 (0 to 0)	0 (0 to 0)	0 (0 to 0)	0 (0 to 0)	0 (0 to 0)	0 (0 to 0)	0 (0 to 0)	5·2 (2·4 to 9·7)
Esophageal cancer	0 (0 to 0)	0 (0 to 0)	0 (0 to 0)	0 (0 to 0)	0 (0 to 0)	0 (0 to 0)	0 (0 to 0)	1·2 (0·8 to 1·9)
Stomach cancer	0 (0 to 0)	0 (0 to 0)	0 (0 to 0)	0 (0 to 0)	0 (0 to 0)	0 (0 to 0)	7·7 (4·9 to 11·4)	16·3 (10·8 to 24·2)
Colon and rectum cancer	0 (0 to 0)	0 (0 to 0)	0 (0 to 0)	0 (0 to 0)	3·8 (2·6 to 5·4)	5·7 (3·6 to 8·5)	19·3 (12·3 to 28·8)	37·6 (22·7 to 59·1)
Liver cancer	0·1 (0·1 to 0·3)	0·2 (0·1 to 0·4)	0·7 (0·3 to 1·3)	5·9 (2·9 to 10)	4·2 (3 to 5·7)	6·7 (4·4 to 10·5)	2·5 (1·2 to 4·4)	12 (6·6 to 19·6)
Liver cancer due to hepatitis B	0 (0 to 0)	0 (0 to 0)	0 (0 to 0)	0 (0 to 0)	0 (0 to 0)	1·2 (0·5 to 2·5)	0·7 (0·3 to 1·4)	4·5 (2·2 to 8·2)
Liver cancer due to hepatitis C	0 (0 to 0)	0 (0 to 0)	0 (0 to 0)	0 (0 to 0)	0 (0 to 0)	0·1 (0 to 0·1)	0 (0 to 0·1)	0·3 (0·1 to 0·8)
Liver cancer due to alcohol use	0 (0 to 0)	0 (0 to 0)	0 (0 to 0)	0 (0 to 0)	0 (0 to 0)	0 (0 to 0)	0 (0 to 0·1)	0·6 (0·2 to 1·2)
Liver cancer due to NASH	0 (0 to 0)	0 (0 to 0)	0 (0 to 0)	0 (0 to 0)	0 (0 to 0)	0 (0 to 0)	0·7 (0·3 to 1·3)	2·5 (1·2 to 4·7)
Liver cancer due to other causes	0·1 (0·1 to 0·3)	0·2 (0·1 to 0·4)	0·7 (0·3 to 1·3)	5·9 (2·9 to 10)	4·2 (3 to 5·7)	5·5 (3·7 to 8·4)	1 (0·5 to 2·1)	4 (1·9 to 7·2)
Gallbladder and biliary tract cancer	0 (0 to 0)	0 (0 to 0)	0 (0 to 0)	0 (0 to 0)	0 (0 to 0)	0 (0 to 0)	0 (0 to 0)	1·5 (0·9 to 2·4)
Pancreatic cancer	0 (0 to 0)	0 (0 to 0)	0 (0 to 0)	0 (0 to 0)	0 (0 to 0)	0 (0 to 0)	1 (0·7 to 1·5)	2·2 (1·2 to 3·8)
Larynx cancer	0 (0 to 0)	0 (0 to 0)	0 (0 to 0)	0 (0 to 0)	0 (0 to 0)	0 (0 to 0)	0 (0 to 0)	1 (0·8 to 1·3)
Tracheal, bronchus, and lung cancer	0 (0 to 0)	0 (0 to 0)	0 (0 to 0)	0 (0 to 0)	0 (0 to 0)	4·2 (2·1 to 7·3)	19·7 (11·3 to 32·5)	26·4 (13·2 to 46·3)
Malignant skin melanoma	0 (0 to 0·1)	0 (0 to 0·1)	0·3 (0·1 to 0·9)	1·6 (0·6 to 4·1)	0·9 (0·7 to 1·1)	0·9 (0·7 to 1·1)	1·3 (0·8 to 2·2)	5·7 (2·5 to 9·7)
Non-melanoma skin cancer	0 (0 to 0)	0 (0 to 0)	0 (0 to 0)	0 (0 to 0)	0 (0 to 0)	0 (0 to 0)	0 (0 to 0)	0·9 (0·7 to 1·2)
Non-melanoma skin cancer (squamous-cell carcinoma)	0 (0 to 0)	0 (0 to 0)	0 (0 to 0)	0 (0 to 0)	0 (0 to 0)	0 (0 to 0)	0 (0 to 0)	0·9 (0·7 to 1·2)
Non-melanoma skin cancer (basal-cell carcinoma)	0 (0 to 0)	0 (0 to 0)	0 (0 to 0)	0 (0 to 0)	0 (0 to 0)	0 (0 to 0)	0 (0 to 0)	0 (0 to 0)
Breast cancer	0 (0 to 0)	0 (0 to 0)	0 (0 to 0)	0 (0 to 0)	0 (0 to 0)	0 (0 to 0)	13·9 (8·2 to 22·6)	49 (30·8 to 71·7)
Cervical cancer	0 (0 to 0)	0 (0 to 0)	0 (0 to 0)	0 (0 to 0)	0 (0 to 0)	0 (0 to 0)	14·4 (7·9 to 23·8)	72·6 (38 to 121)
Uterine cancer	0 (0 to 0)	0 (0 to 0)	0 (0 to 0)	0 (0 to 0)	0 (0 to 0)	0 (0 to 0)	0 (0 to 0)	11·5 (6·8 to 17·9)
Ovarian cancer	0 (0 to 0)	0 (0 to 0)	0 (0 to 0)	0 (0 to 0)	6·5 (3·5 to 10·6)	11·9 (6 to 19·3)	31·2 (14·6 to 51·4)	51·9 (27·5 to 86·6)
Prostate cancer	0 (0 to 0)	0 (0 to 0)	0 (0 to 0)	0 (0 to 0)	0 (0 to 0)	0 (0 to 0)	0 (0 to 0)	0 (0 to 0)
Testicular cancer	–	–	–	–	–	–	–	–
Kidney cancer	0·2 (0·2 to 0·3)	0·1 (0·1 to 0·2)	0·6 (0·3 to 1·1)	10·2 (6·1 to 16·2)	11·7 (6·2 to 19·9)	7·5 (3·9 to 14)	6·7 (2·9 to 12·6)	14·4 (7 to 25·9)
Bladder cancer	0 (0 to 0)	0 (0 to 0)	0 (0 to 0)	0 (0 to 0)	0 (0 to 0)	0 (0 to 0)	0·9 (0·7 to 1·1)	1 (0·7 to 1·2)
Brain and central nervous system cancer	2·2 (0·9 to 3·7)	2·5 (0·9 to 4·9)	9 (1·9 to 19·8)	40·5 (11·5 to 73·4)	67·3 (20·9 to 116)	45·2 (17·6 to 75·4)	29·8 (8·7 to 53·1)	34 (12·5 to 60·6)
Thyroid cancer	0 (0 to 0)	0 (0 to 0)	0 (0 to 0)	0 (0 to 0)	2·3 (1·1 to 4·3)	1 (0·8 to 1·5)	5·9 (2·8 to 10·7)	11·3 (4·2 to 25·1)
Mesothelioma	0 (0 to 0)	0 (0 to 0)	0 (0 to 0)	0 (0 to 0)	0 (0 to 0)	0 (0 to 0)	0 (0 to 0)	1·3 (0·6 to 4·1)
Hodgkin lymphoma	0 (0 to 0)	0 (0 to 0)	0 (0 to 0)	0·8 (0·5 to 1·1)	0·9 (0·7 to 1·1)	0·9 (0·7 to 1·4)	7·2 (3·7 to 12·9)	19·4 (7·7 to 37·9)
Non-Hodgkin lymphoma	0 (0 to 0)	0 (0 to 0)	0 (0 to 0)	16 (9·6 to 24·9)	29·2 (19·3 to 43)	49·5 (30·6 to 77·6)	50·6 (31·7 to 78·6)	119 (72·6 to 180)
Multiple myeloma	0 (0 to 0)	0 (0 to 0)	0 (0 to 0)	0 (0 to 0)	0 (0 to 0)	0 (0 to 0)	0 (0 to 0)	1·2 (0·7 to 2)
Leukemia	5·3 (3·5 to 7·8)	7·1 (4·4 to 10·4)	15·1 (7·3 to 28·1)	93·6 (56·1 to 142)	122 (79·2 to 180)	114 (72·9 to 170)	110 (69 to 162)	143 (85·2 to 217)
Acute lymphoid leukemia	2·4 (1 to 3·7)	6·1 (1·4 to 9·6)	13·1 (5·1 to 24·2)	61·5 (34·7 to 98·7)	81·1 (48·6 to 130)	42·9 (19·7 to 77·7)	56·2 (29·2 to 92·5)	17·8 (7·7 to 36·1)
Chronic lymphoid leukemia	0 (0 to 0)	0 (0 to 0)	0 (0 to 0)	0 (0 to 0)	0 (0 to 0)	0 (0 to 0)	0 (0 to 0)	1·2 (0·7 to 2)
Acute myeloid leukemia	0·7 (0·4 to 1·3)	0·6 (0·1 to 4·2)	0·9 (0·2 to 5·8)	22·4 (11·1 to 38·9)	25·2 (11·3 to 46·3)	50 (25 to 85·2)	37 (17·2 to 64·1)	39·7 (19·2 to 72·9)
Chronic myeloid leukemia	0·1 (0 to 0·4)	0 (0 to 0·1)	0·1 (0 to 0·5)	2·2 (1 to 4·2)	1·1 (0·5 to 2·1)	5 (2·2 to 9·5)	7·2 (3 to 13·8)	28·9 (12·6 to 51·7)
Other leukemia	2 (1·2 to 3·1)	0·4 (0·1 to 1·6)	1 (0·2 to 4·2)	7·6 (3·6 to 14)	14·2 (6·3 to 27)	16·1 (7·4 to 28·6)	9·7 (4·4 to 18·5)	55·2 (27·4 to 95·1)
Other malignant neoplasms	6·5 (4 to 9·6)	2·8 (1·7 to 5)	6·4 (2·7 to 19·3)	47·4 (27·5 to 103)	61 (46·7 to 81·8)	91·2 (65·6 to 123)	94·2 (65·9 to 131)	109 (76·8 to 153)
Other neoplasms	0 (0 to 0·1)	0 (0 to 0·1)	1·8 (0·6 to 4·5)	1·2 (0·7 to 3·5)	1·4 (0·8 to 2·4)	1·4 (0·8 to 2·6)	1·4 (1 to 1·9)	1·5 (1·1 to 2·2)
Myelodysplastic, myeloproliferative, and other hematopoietic n	0 (0 to 0·1)	0 (0 to 0·1)	1·8 (0·6 to 4·5)	1·2 (0·7 to 3·5)	1·4 (0·8 to 2·4)	1·4 (0·8 to 2·6)	1·4 (1 to 1·9)	1·5 (1·1 to 2·2)
Benign and in situ intestinal neoplasms	0 (0 to 0)	0 (0 to 0)	0 (0 to 0)	0 (0 to 0)	0 (0 to 0)	0 (0 to 0)	0 (0 to 0)	0 (0 to 0)
Benign and in situ cervical and uterine neoplasms	0 (0 to 0)	0 (0 to 0)	0 (0 to 0)	0 (0 to 0)	0 (0 to 0)	0 (0 to 0)	0 (0 to 0)	0 (0 to 0)
Other benign and in situ neoplasms	0 (0 to 0)	0 (0 to 0)	0 (0 to 0)	0 (0 to 0)	0 (0 to 0)	0 (0 to 0)	0 (0 to 0)	0 (0 to 0)
Cardiovascular diseases	75·5 (49·5 to 108)	17 (11 to 25·3)	29·8 (15·5 to 52·3)	49·2 (33·2 to 68·8)	114 (88·5 to 146)	213 (166 to 268)	405 (320 to 498)	624 (475 to 808)
Rheumatic heart disease	0 (0 to 0)	0 (0 to 0)	0 (0 to 0)	7·4 (4·5 to 11·3)	42·8 (30·6 to 59·8)	94 (66·1 to 131)	146 (103 to 197)	174 (121 to 240)

Cause	25 to 29	30 to 34	35 to 39	40 to 44	45 to 49	50 to 54	55 to 59	60 to 64	65 to 69	70 to 74	75 to 79	80 to 84	85 to 89	90 to 94	95 plus	Total
All causes	20,500 (16,600 to 25,300)	21,900 (17,800 to 27,000)	22,200 (18,100 to 27,200)	22,800 (18,700 to 27,300)	25,700 (21,100 to 30,800)	26,400 (21,800 to 31,600)	27,300 (22,600 to 32,500)	28,300 (23,600 to 33,400)	25,200 (21,500 to 29,400)	23,200 (19,800 to 26,900)	19,900 (17,100 to 22,900)	17,400 (15,100 to 19,800)	13,900 (12,100 to 15,700)	8,760 (7,770 to 9,780)	6,140 (5,640 to 6,650)	377,000
Communicable, maternal, neonatal, and nutritional diseases	3,360 (2,770 to 4,100)	3,570 (3,010 to 4,310)	3,640 (3,100 to 4,330)	2,600 (2,130 to 3,220)	2,170 (1,740 to 2,720)	1,780 (1,390 to 2,290)	1,540 (1,200 to 1,960)	1,180 (936 to 1,500)	946 (787 to 1,140)	737 (578 to 924)	669 (522 to 842)	631 (494 to 784)	584 (456 to 740)	424 (311 to 531)	321 (221 to 411)	51,200
HIV/AIDS and sexually transmitted infections	1,180 (1,080 to 1,310)	1,560 (1,440 to 1,710)	1,910 (1,760 to 2,070)	1,100 (1,020 to 1,190)	862 (792 to 945)	579 (533 to 628)	430 (391 to 472)	302 (274 to 335)	264 (240 to 291)	53·6 (44·4 to 66·3)	22·6 (18·4 to 28·5)	10 (7·8 to 13·4)	4·6 (3·3 to 6·8)	2·2 (1·5 to 3·3)	1·4 (1·1 to 2)	9,510
HIV/AIDS	1,130 (1,030 to 1,240)	1,500 (1,380 to 1,620)	1,840 (1,710 to 1,990)	1,030 (962 to 1,110)	804 (744 to 869)	546 (507 to 586)	409 (375 to 442)	284 (260 to 311)	252 (230 to 276)	44·8 (37·7 to 53·9)	17·1 (14·4 to 20·2)	5·2 (4·5 to 6)	1·9 (1·4 to 2·4)	0·4 (0·2 to 0·7)	0·9 (0·7 to 1·1)	9,040
HIV/AIDS - Drug-susceptible Tuberculosis	16·9 (11·4 to 24·6)	21·8 (14·6 to 32)	25·8 (17·1 to 38·2)	15·4 (10·3 to 22·3)	12·1 (8·2 to 17·6)	8·5 (5·8 to 12·3)	6·5 (4·5 to 9·3)	5 (3·6 to 7·1)	4·2 (2·8 to 6)	1·1 (0·8 to 1·5)	0·4 (0·3 to 0·5)	0·1 (0·1 to 0·1)	0 (0 to 0·1)	0 (0 to 0)	0 (0 to 0)	135
HIV/AIDS - Multidrug-resistant Tuberculosis without extensive	0·6 (0·1 to 2·2)	0·8 (0·1 to 2·9)	0·9 (0·1 to 3·4)	0·5 (0·1 to 2)	0·4 (0·1 to 1·6)	0·3 (0 to 1·1)	0·2 (0 to 0·8)	0·2 (0 to 0·6)	0·1 (0 to 0·5)	0 (0 to 0·1)	0 (0 to 0)	0 (0 to 0)	0 (0 to 0)	0 (0 to 0)	0 (0 to 0)	4·7
HIV/AIDS - Extensively drug-resistant Tuberculosis	0·1 (0 to 0·4)	0·1 (0 to 0·5)	0·1 (0 to 0·5)	0·1 (0 to 0·3)	0·1 (0 to 0·2)	0 (0 to 0·2)	0 (0 to 0·1)	0 (0 to 0·1)	0 (0 to 0·1)	0 (0 to 0)	0 (0 to 0)	0 (0 to 0)	0 (0 to 0)	0 (0 to 0)	0 (0 to 0)	0·7
HIV/AIDS resulting in other diseases	1,110 (1,010 to 1,220)	1,470 (1,360 to 1,600)	1,810 (1,680 to 1,970)	1,020 (947 to 1,090)	792 (732 to 857)	537 (498 to 577)	402 (367 to 435)	278 (255 to 305)	248 (226 to 271)	43·7 (36·6 to 52·4)	16·7 (14 to 19·8)	5·1 (4·3 to 5·9)	1·8 (1·4 to 2·4)	0·4 (0·2 to 0·7)	0·9 (0·7 to 1)	8,900
Sexually transmitted infections excluding HIV	53·4 (26·2 to 101)	67·1 (36·1 to 123)	68·2 (37·5 to 121)	66·9 (38·8 to 118)	57·6 (32·3 to 98·6)	32·5 (17·7 to 60·8)	21·5 (10·1 to 42·9)	17·9 (9·5 to 32·8)	12 (6·4 to 22)	8·8 (5 to 15·6)	5·5 (3·1 to 10·1)	4·8 (2·8 to 8·1)	2·7 (1·6 to 4·8)	1·8 (1·1 to 2·8)	0·6 (0·3 to 1·1)	470
Syphilis	1·1 (0·5 to 2)	2·5 (1·5 to 3·9)	2 (1·3 to 3·1)	1·1 (0·7 to 1·7)	1·9 (1·2 to 2·9)	1 (0·7 to 1·6)	0·3 (0·2 to 0·4)	0·5 (0·3 to 0·7)	0·4 (0·3 to 0·6)	0·5 (0·3 to 0·7)	0·3 (0·2 to 0·4)	0·5 (0·4 to 0·8)	0·3 (0·2 to 0·4)	0·4 (0·2 to 0·5)	0·1 (0·1 to 0·1)	16·1
Chlamydial infection	4·3 (1·9 to 8·2)	5·5 (2·6 to 9·7)	6·7 (3·9 to 11)	7·4 (4·6 to 11·7)	5·8 (3·6 to 9·4)	1·7 (1·1 to 2·4)	0·8 (0·5 to 1·1)	0·9 (0·5 to 1·5)	0·4 (0·2 to 0·7)	0·4 (0·3 to 0·7)	0·3 (0·2 to 0·4)	0·4 (0·3 to 0·7)	0·2 (0·1 to 0·3)	0·2 (0·1 to 0·2)	0 (0 to 0·1)	39·7
Gonococcal infection	2·2 (1·4 to 3·2)	4·9 (3·1 to 7·1)	5·9 (3·8 to 8·8)	8·6 (5·6 to 13·5)	8·1 (5 to 12·7)	3·5 (2·1 to 5·6)	1·4 (0·8 to 2·1)	2·4 (1·4 to 3·8)	2·1 (1·2 to 3·5)	1·5 (0·9 to 2·4)	1·2 (0·6 to 1·5)	1 (0·6 to 1·5)	0·5 (0·3 to 0·8)	0·4 (0·2 to 0·6)	0·1 (0 to 0·1)	46·8
Trichomoniasis	21·6 (6·8 to 49·8)	22·6 (7 to 53·4)	19·8 (6·4 to 47·4)	16·7 (5·5 to 40·6)	14·8 (4·8 to 34·2)	11·3 (3·7 to 26·2)	7·8 (2·5 to 19·1)	4·8 (1·7 to 11·4)	3 (1·1 to 7)	2 (0·7 to 4·4)	1·2 (0·4 to 2·5)	0·7 (0·3 to 1·5)	0·4 (0·1 to 0·8)	0·1 (0 to 0·3)	0 (0 to 0·1)	141
Genital herpes	14·5 (4·8 to 35·8)	16 (5·3 to 38·6)	15·3 (4·8 to 37·9)	13·4 (4·2 to 32·5)	12·4 (3·9 to 29·7)	10·8 (3·3 to 26·7)	9·2 (2·8 to 22·6)	7·3 (2·2 to 17·7)	5·1 (1·5 to 12·5)	3·6 (1·1 to 8·8)	2·4 (0·7 to 5·9)	1·7 (0·5 to 4·2)	1·1 (0·3 to 2·8)	0·6 (0·2 to 1·4)	0·3 (0·1 to 0·8)	128
Other sexually transmitted infections	9·8 (5 to 16·9)	15·6 (9·5 to 24·4)	18·3 (11·6 to 28·5)	19·8 (12·4 to 31)	14·5 (8·4 to 24·8)	4·2 (2·3 to 6·7)	2 (1 to 3·5)	2 (1·2 to 3·3)	0·9 (0·5 to 1·6)	0·8 (0·4 to 1·2)	0·4 (0·2 to 0·6)	0·4 (0·2 to 0·6)	0·3 (0·2 to 0·4)	0·2 (0·1 to 0·3)	0 (0 to 0)	98·1
Respiratory infections and tuberculosis	312 (222 to 429)	281 (195 to 385)	228 (159 to 321)	208 (144 to 287)	205 (144 to 286)	216 (152 to 297)	236 (168 to 330)	220 (158 to 301)	235 (165 to 326)	308 (213 to 435)	354 (249 to 479)	375 (265 to 499)	377 (275 to 497)	298 (203 to 391)	239 (155 to 321)	5,990
Tuberculosis	26·5 (13·9 to 44·8)	19·1 (8·8 to 34·5)	13·5 (6 to 24·1)	11·3 (5·5 to 19·4)	6·9 (3 to 13·3)	9·8 (5·4 to 16)	6·8 (4·3 to 10)	6·4 (3·8 to 9·6)	4·9 (3·2 to 7·2)	3·5 (2·4 to 4·9)	2·8 (2 to 4)	2·2 (1·6 to 3·1)	1·9 (1·3 to 2·6)	0·9 (0·6 to 1·2)	0·4 (0·3 to 0·5)	149
Latent tuberculosis infection	0 (0 to 0)	0 (0 to 0)	0 (0 to 0)	0 (0 to 0)	0 (0 to 0)	0 (0 to 0)	0 (0 to 0)	0 (0 to 0)	0 (0 to 0)	0 (0 to 0)	0 (0 to 0)	0 (0 to 0)	0 (0 to 0)	0 (0 to 0)	0 (0 to 0)	0
Drug-susceptible tuberculosis	25·9 (13·1 to 44·3)	18·7 (8·4 to 34·2)	13·2 (5·9 to 23·8)	11 (5·2 to 19·1)	6·7 (2·8 to 13·1)	9·5 (5·1 to 15·8)	6·6 (4·2 to 9·8)	6·2 (3·6 to 9·3)	4·8 (3 to 6·9)	3·4 (2·3 to 4·7)	2·7 (1·9 to 3·9)	2·1 (1·5 to 3)	1·8 (1·2 to 2·6)	0·8 (0·6 to 1·2)	0·4 (0·3 to 0·5)	146
Multidrug-resistant tuberculosis without extensive drug resistar	0·5 (0·1 to 1·9)	0·4 (0·1 to 1·3)	0·3 (0 to 1)	0·3 (0 to 1)	0·2 (0 to 0·6)	0·3 (0 to 0·9)	0·2 (0 to 0·6)	0·2 (0 to 0·6)	0·1 (0 to 0·5)	0·1 (0 to 0·3)	0·1 (0 to 0·3)	0·1 (0 to 0·2)	0·1 (0 to 0·2)	0 (0 to 0·1)	0 (0 to 0)	3·4
Extensively drug-resistant tuberculosis	0·1 (0 to 0·2)	0 (0 to 0·2)	0 (0 to 0·1)	0 (0 to 0·1)	0 (0 to 0·1)	0 (0 to 0·1)	0 (0 to 0·1)	0 (0 to 0·1)	0 (0 to 0·1)	0 (0 to 0·1)	0 (0 to 0)	0 (0 to 0)	0 (0 to 0)	0 (0 to 0)	0 (0 to 0)	0·5
Lower respiratory infections	154 (94·4 to 241)	152 (91·5 to 229)	116 (73·9 to 179)	112 (68·8 to 171)	121 (78·1 to 181)	140 (86·7 to 206)	174 (111 to 259)	170 (112 to 246)	200 (132 to 292)	284 (192 to 410)	338 (235 to 460)	362 (253 to 487)	369 (266 to 486)	293 (199 to 386)	236 (152 to 317)	4,350
Upper respiratory infections	102 (54·6 to 171)	87·3 (46·2 to 150)	77·3 (38·4 to 131)	64·5 (33·5 to 115)	57·2 (29·9 to 99·1)	48 (25·6 to 80·6)	40·3 (21·4 to 66·8)	32·6 (17·4 to 55·3)	23·7 (12·4 to 41)	16·6 (8·9 to 28·4)	10·8 (5·5 to 18·5)	7·8 (4·2 to 12·9)	5 (2·7 to 8·4)	2·9 (1·6 to 5)	1·9 (1 to 3·2)	1,110
Otitis media	29·7 (13·3 to 57·9)	23 (8·9 to 45·7)	21·1 (9·5 to 41·3)	20·1 (8·9 to 38·2)	20 (9·6 to 37·9)	18·6 (8·1 to 34·7)	15·3 (7·5 to 28·2)	11·2 (4·9 to 21·2)	6·4 (3 to 11·9)	4 (1·6 to 8)	2·8 (1·3 to 5·2)	2·3 (1 to 4·3)	1·7 (0·7 to 3·1)	1 (0·5 to 1·9)	0·6 (0·3 to 1·2)	378
Enteric infections	105 (70·5 to 148)	106 (72·2 to 149)	110 (75·8 to 153)	99·4 (68·3 to 139)	111 (75·9 to 155)	105 (72·9 to 145)	109 (75·7 to 149)	116 (81·1 to 156)	118 (85·7 to 158)	122 (87·3 to 164)	108 (77·6 to 146)	98·3 (70·7 to 132)	76·4 (55·9 to 101)	46·9 (34·6 to 61·5)	30 (22·2 to 39·2)	2,410
Diarrheal diseases	104 (70·4 to 148)	106 (72 to 149)	110 (75·4 to 153)	99·2 (68 to 139)	111 (75·9 to 155)	105 (72·9 to 145)	109 (75·6 to 149)	116 (81 to 156)	118 (85·6 to 158)	122 (87·1 to 163)	108 (77·6 to 146)	98·3 (70·6 to 132)	76·3 (55·8 to 101)	46·8 (34·5 to 61·5)	30 (22·2 to 39·1)	2,400
Typhoid and paratyphoid	0 (0 to 0)	0 (0 to 0)	0 (0 to 0)	0 (0 to 0)	0 (0 to 0)	0 (0 to 0)	0 (0 to 0)	0 (0 to 0)	0 (0 to 0)	0 (0 to 0)	0 (0 to 0)	0 (0 to 0)	0 (0 to 0)	0 (0 to 0)	0 (0 to 0)	0·2
Typhoid fever	0 (0 to 0)	0 (0 to 0)	0 (0 to 0)	0 (0 to 0)	0 (0 to 0)	0 (0 to 0)	0 (0 to 0)	0 (0 to 0)	0 (0 to 0)	0 (0 to 0)	0 (0 to 0)	0 (0 to 0)	0 (0 to 0)	0 (0 to 0)	0 (0 to 0)	0·1
Paratyphoid fever	0 (0 to 0)	0 (0 to 0)	0 (0 to 0)	0 (0 to 0)	0 (0 to 0)	0 (0 to 0)	0 (0 to 0)	0 (0 to 0)	0 (0 to 0)	0 (0 to 0)	0 (0 to 0)	0 (0 to 0)	0 (0 to 0)	0 (0 to 0)	0 (0 to 0)	0·1
Invasive Non-typhoidal Salmonella (iNTS)	0 (0 to 0·1)	0 (0 to 0)	0 (0 to 0)	0 (0 to 0)	0 (0 to 0)	0 (0 to 0)	0 (0 to 0)	0 (0 to 0)	0 (0 to 0)	0 (0 to 0)	0 (0 to 0)	0 (0 to 0)	0 (0 to 0)	0 (0 to 0)	0 (0 to 0)	0·7
Other intestinal infectious diseases	0·2 (0 to 0·5)	0·4 (0·1 to 1·2)	0·2 (0 to 0·6)	0·2 (0 to 0·7)	0·2 (0 to 0·4)	0·1 (0 to 0·4)	0·1 (0 to 0·3)	0·1 (0 to 0·3)	0·1 (0 to 0·4)	0·1 (0 to 0·3)	0·1 (0 to 0·2)	0·1 (0 to 0·2)	0·1 (0 to 0·3)	0·1 (0 to 0·2)	0·1 (0 to 0·2)	4·9
Neglected tropical diseases and malaria	130 (70 to 214)	105 (54·5 to 175)	102 (54·4 to 168)	82·7 (43·8 to 137)	74·1 (39·5 to 129)	74·4 (38·8 to 128)	76 (39·2 to 126)	71·1 (36·1 to 124)	64·6 (32·3 to 113)	59·9 (29·1 to 103)	50·4 (23·5 to 92·8)	41·3 (18·2 to 78·4)	29·4 (13·3 to 57·1)	16·8 (7·6 to 32·6)	11·2 (4·9 to 20·3)	1,500
Malaria	0 (0 to 0)	0 (0 to 0)	0 (0 to 0)	0 (0 to 0)	0 (0 to 0)	0 (0 to 0)	0 (0 to 0)	0 (0 to 0)	0 (0 to 0)	0 (0 to 0)	0 (0 to 0)	0 (0 to 0)	0 (0 to 0)	0 (0 to 0)	0 (0 to 0)	0
Chagas disease	0 (0 to 0)	0 (0 to 0)	0 (0 to 0)	0 (0 to 0)	0 (0 to 0)	0 (0 to 0)	0 (0 to 0)	0 (0 to 0)	0 (0 to 0)	0 (0 to 0)	0 (0 to 0)	0 (0 to 0)	0 (0 to 0)	0 (0 to 0)	0 (0 to 0)	0
Leishmaniasis	0 (0 to 0)	0 (0 to 0)	0 (0 to 0)	0 (0 to 0)	0 (0 to 0)	0 (0 to 0)	0 (0 to 0)	0 (0 to 0)	0 (0 to 0)	0 (0 to 0)	0 (0 to 0)	0 (0 to 0)	0 (0 to 0)	0 (0 to 0)	0 (0 to 0)	0
Visceral leishmaniasis	0 (0 to 0)	0 (0 to 0)	0 (0 to 0)	0 (0 to 0)	0 (0 to 0)	0 (0 to 0)	0 (0 to 0)	0 (0 to 0)	0 (0 to 0)	0 (0 to 0)	0 (0 to 0)	0 (0 to 0)	0 (0 to 0)	0 (0 to 0)	0 (0 to 0)	0
Cutaneous and mucocutaneous leishmaniasis	0 (0 to 0)	0 (0 to 0)	0 (0 to 0)	0 (0 to 0)	0 (0 to 0)	0 (0 to 0)	0 (0 to 0)	0 (0 to 0)	0 (0 to 0)	0 (0 to 0)	0 (0 to 0)	0 (0 to 0)	0 (0 to 0)	0 (0 to 0)	0 (0 to 0)	0
African trypanosomiasis	0 (0 to 0)	0 (0 to 0)	0 (0 to 0)	0 (0 to 0)	0 (0 to 0)	0 (0 to 0)	0 (0 to 0)	0 (0 to 0)	0 (0 to 0)	0 (0 to 0)	0 (0 to 0)	0 (0 to 0)	0 (0 to 0)	0 (0 to 0)	0 (0 to 0)	0
Schistosomiasis	0 (0 to 0)	0 (0 to 0)	0 (0 to 0)	0 (0 to 0)	0 (0 to 0)	0 (0 to 0)	0 (0 to 0)	0 (0 to 0)	0 (0 to 0)	0 (0 to 0)	0 (0 to 0)	0 (0 to 0)	0 (0 to 0)	0 (0 to 0)	0 (0 to 0)	0
Cysticercosis	36·2 (8·8 to 84·2)	36·2 (10·9 to 83·4)	35·9 (11·2 to 82·1)	35·8 (11·7 to 81)	39·5 (13 to 89·5)	42·3 (13·3 to 91·2)	49 (17·6 to 94·9)	56·5 (23·5 to 110)	56 (24·3 to 105)	53·5 (22·5 to 97·3)	44·7 (17·9 to 86·3)	37·2 (14·4 to 75·1)	26·2 (10·5 to 53·4)	14·4 (5·8 to 30·3)	8·5 (3·1 to 17·3)	602
Cystic echinococcosis	0 (0 to 0)	0 (0 to 0)	0 (0 to 0)	0 (0 to 0)	0 (0 to 0)	0 (0 to 0)	0 (0 to 0)	0 (0 to 0)	0 (0 to 0)	0 (0 to 0)	0 (0 to 0)	0 (0 to 0)	0 (0 to 0)	0 (0 to 0)	0 (0 to 0)	0·2
Lymphatic filariasis	0 (0 to 0)	0 (0 to 0)	0 (0 to 0)	0 (0 to 0)	0 (0 to 0)	0 (0 to 0)	0 (0 to 0)	0 (0 to 0)	0 (0 to 0)	0 (0 to 0)	0 (0 to 0)	0 (0 to 0)	0 (0 to 0)	0 (0 to 0)	0 (0 to 0)	0
Onchocerciasis	0 (0 to 0)	0 (0 to 0)	0 (0 to 0)	0 (0 to 0)	0 (0 to 0)	0 (0 to 0)	0 (0 to 0)	0 (0 to 0)	0 (0 to 0)	0 (0 to 0)	0 (0 to 0)	0 (0 to 0)	0 (0 to 0)	0 (0 to 0)	0 (0 to 0)	0
Trachoma	0 (0 to 0)	0 (0 to 0)	0 (0 to 0)	0 (0 to 0)	0 (0 to 0)	0 (0 to 0)	0 (0 to 0)	0 (0 to 0)	0 (0 to 0)	0 (0 to 0)	0 (0 to 0)	0 (0 to 0)	0 (0 to 0)	0 (0 to 0)	0 (0 to 0)	0
Dengue	13·1 (2·9 to 30·4)	8·9 (0·6 to 22·7)	7·6 (0·4 to 18·8)	6·1 (0·2 to 15·6)	6·1 (1 to 16)	5·3 (0·5 to 13·7)	4·6 (0·3 to 11·4)	4·2 (0·3 to 10·8)	3·2 (0·3 to 8·3)	2·5 (0·3 to 6·6)	2·6 (0·6 to 5·8)	1·8 (0·4 to 4·2)	1·6 (0·4 to 3·3)	1·2 (0·2 to 2·9)	1·5 (0·2 to 3·8)	129
Yellow fever	0 (0 to 0)	0 (0 to 0)	0 (0 to 0)	0 (0 to 0)	0 (0 to 0)	0 (0 to 0)	0 (0 to 0)	0 (0 to 0)	0 (0 to 0)	0 (0 to 0)	0 (0 to 0)	0 (0 to 0)	0 (0 to 0)	0 (0 to 0)	0 (0 to 0)	0
Rabies	0 (0 to 0)	0 (0 to 0)	0 (0 to 0)	0 (0 to 0)	0 (0 to 0)	0 (0 to 0)	0 (0 to 0)	0 (0 to 0)	0 (0 to 0)	0 (0 to 0)	0 (0 to 0)	0 (0 to 0)	0 (0 to 0)	0 (0 to 0)	0 (0 to 0)	0·1
Intestinal nematode infections	60·9 (29·3 to 106)	41·5 (19·4 to 76·2)	36·7 (18·5 to 62·3)	18·3 (9·2 to 31·4)	9·3 (4·6 to 16·7)	8·2 (4 to 14·6)	7 (3·5 to 12·4)	5·2 (2·6 to 9·1)	3·2 (1·5 to 5·7)	2·3 (1·1 to 4)	1·6 (0·8 to 2·9)	1·1 (0·6 to 1·9)	0·7 (0·3 to 1·2)	0·3 (0·1 to 0·5)	0·1 (0·1 to 0·2)	445
Ascariasis	1·4 (0·4 to 3·2)	1·3 (0·3 to 2·8)	1·1 (0·3 to 2·5)	0·9 (0·3 to 2)	0·8 (0·2 to 1·8)	0·6 (0·2 to 1·4)	0·6 (0·1 to 1·2)	0·5 (0·1 to 1·1)	0·3 (0·1 to 0·7)	0·3 (0·1 to 0·6)	0·2 (0 to 0·4)	0·1 (0 to 0·3)	0·1 (0 to 0·2)	0 (0 to 0·1)	0 (0 to 0·1)	19·6
Trichuriasis	25 (11·2 to 46·4)	16·8 (7·5 to 31·7)	12 (5·3 to 22·8)	8·4 (3·7 to 15·5)	6·8 (2·9 to 12·9)	5·3 (2·3 to 10·1)	4·3 (1·8 to 8·1)	3 (1·3 to 5·8)	1·8 (0·8 to 3·5)	1·2 (0·5 to 2·3)	0·8 (0·3 to 1·6)	0·5 (0·2 to 1)	0·3 (0·1 to 0·5)	0·1 (0 to 0·2)	0 (0 to 0·1)	236
Hookworm disease	34·5 (11·8 to 65·4)	23·5 (8 to 50·3)	23·6 (9·8 to 41·6)	9·1 (3·8 to 16·7)	1·8 (0·7 to 3·7)	2·3 (0·9 to 4·5)	2·2 (0·9 to 4·2)	1·7 (0·6 to 3·2)	1 (0·4 to 1·9)	0·8 (0·3 to 1·7)	0·6 (0·2 to 1·3)	0·5 (0·2 to 1)	0·3 (0·1 to 0·6)	0·1 (0·1 to 0·3)	0·1 (0 to 0·1)	189
Food-borne trematodiases	0 (0 to 0)	0 (0 to 0)	0 (0 to 0)	0 (0 to 0)	0 (0 to 0)	0 (0 to 0)	0 (0 to 0)	0 (0 to 0)	0 (0 to 0)	0 (0 to 0)	0 (0 to 0)	0 (0 to 0)	0 (0 to 0)	0 (0 to 0)	0 (0 to 0)	0
Leprosy	0 (0 to 0·1)	0·1 (0 to 0·1)	0·1 (0 to 0·1)	0·1 (0 to 0·1)	0·1 (0 to 0·1)	0·1 (0 to 0·2)	0·1 (0 to 0·2)	0·1 (0 to 0·2)	0·1 (0 to 0·1)	0·1 (0 to 0·1)	0·1 (0 to 0·1)	0 (0 to 0·1)	0 (0 to 0·1)	0 (0 to 0)	0 (0 to 0)	1·1
Ebola	0 (0 to 0)	0 (0 to 0)	0 (0 to 0)	0 (0 to 0)	0 (0 to 0)	0 (0 to 0)	0 (0 to 0)	0 (0 to 0)	0 (0 to 0)	0 (0 to 0)	0 (0 to 0)	0 (0 to 0)	0 (0 to 0)	0 (0 to 0)	0 (0 to 0)	0
Zika virus	0·1 (0 to 0·5)	0·1 (0 to 0·5)	0·1 (0 to 0·4)	0·1 (0 to 0·4)	0·1 (0 to 0·3)	0 (0 to 0·2)	0 (0 to 0·1)	0 (0 to 0·1)	0 (0 to 0·1)	0 (0 to 0)	0 (0 to 0)	0 (0 to 0)	0 (0 to 0)	0 (0 to 0)	0 (0 to 0)	1
Guinea worm disease	0 (0 to 0)	0 (0 to 0)	0 (0 to 0)	0 (0 to 0)	0 (0 to 0)	0 (0 to 0)	0 (0 to 0)	0 (0 to 0)	0 (0 to 0)	0 (0 to 0)	0 (0 to 0)	0 (0 to 0)	0 (0 to 0)	0 (0 to 0)	0 (0 to 0)	0
Other neglected tropical diseases	19·7 (5·8 to 41·4)	18·5 (5·3 to 42·2)	21·1 (6·6 to 42)	22·3 (8·1 to 42·9)	18·9 (5·7 to 37·9)	18·4 (6·9 to 35·6)	15·3 (5·1 to 30·3)	5·1 (1·5 to 11)	2·1 (0·6 to 5·1)	1·6 (0·4 to 3·6)	1·4 (0·5 to 3·1)	1 (0·3 to 2·4)	0·9 (0·3 to 2)	0·9 (0·4 to 1·6)	1·1 (0·3 to 2·1)	317
Other infectious diseases	121 (86·7 to 165)	122 (82·5 to 172)	111 (75·2 to 157)	105 (70·7 to 145)	107 (70·1 to 153)	88·4 (63·2 to 120)	89·4 (58·9 to 126)	70·1 (51·6 to 90·4)	52·9 (37·9 to 70·3)	39·9 (29·2 to 52·2)	27·1 (20·8 to 34·5)	22 (16·5 to 28·3)	18·7 (13·9 to 24·6)	8·3 (6 to 10·7)	5·4 (3·8 to 7·3)	1,780
Meningitis	31·5 (19 to 47·3)	29·8 (19·3 to 43·4)	25·7 (16·4 to 39·1)	20·2 (12·7 to 29·3)	21·7 (13·7 to 31·2)	19·2 (13·4 to 26·3)	13·7 (9·8 to 18·7)	17·5 (12·4 to 23·8)	14·7 (10·1 to 20·6)	11·5 (7·8 to 16·5)	11 (7·3 to 15·5)	8·2 (5·3 to 11·4)	5·8 (3·7 to 8·4)	1·6 (1·1 to 2·1)	0·8 (0·6 to 1)	457
Encephalitis	34·5 (20·2 to 60·1)	33·4 (20·8 to 51·7)	29·3 (19 to 43·8)	25·9 (16·8 to 40·2)	32·3 (17·7 to 53·4)	18 (11·8 to 27·2)	25·3 (14·1 to 40·4)	23·5 (13·1 to 35·8)	16 (9·1 to 24·5)	11·5 (6·7 to 18·7)	5·4 (3·5 to 8·2)	5·1 (2·8 to 8·7)	3·4 (1·8 to 5·9)	1·4 (0·9 to 2·1)	0·6 (0·3 to 1·1)	559
Diphtheria	0 (0 to 0)	0 (0 to 0)	0 (0 to 0)	0 (0 to 0)	0 (0 to 0)	0 (0 to 0)	0 (0 to 0)	0 (0 to 0)	0 (0 to 0)	0 (0 to 0)	0 (0 to 0)	0 (0 to 0)	0 (0 to 0)	0 (0 to 0)	0 (0 to 0)	0·2
Tetanus	0·2 (0·1 to 0·6)	0·2 (0·1 to 0·6)	0 (0 to 0·1)	0·2 (0·1 to 0·7)	0·1 (0 to 0·3)	1·4 (0·4 to 3·5)	0·9 (0·3 to 2·1)	0·1 (0 to 0·4)	0·1 (0 to 0·2)	0·1 (0 to 0·1)	0 (0 to 0·1)	0 (0 to 0)	0 (0 to 0)	0 (0 to 0)	0 (0 to 0)	4·1
Measles	0 (0 to 0)	0 (0 to 0)	0 (0 to 0)	0 (0 to 0)	0 (0 to 0)	0 (0 to 0)	0 (0 to 0)	0 (0 to 0)	0 (0 to 0)	0 (0 to 0)	0 (0 to 0)	0 (0 to 0)	0 (0 to 0)	0 (0 to 0)	0 (0 to 0)	0
Varicella and herpes zoster	2·4 (0·6 to 5·3)	2·4 (0·6 to 5·2)	2·3 (0·7 to 5)	2·2 (0·6 to 4·5)	2·6 (1 to 5·4)	2·8 (1·2 to 5·5)	3·1 (1·3 to 5·9)	3·1 (1·2 to 5·8)	2·6 (1·1 to 4·9)	2·3 (1·2 to 3·9)	1·8 (0·9 to 3)	2·1 (0·9 to 3·4)	1·8 (0·8 to 2·9)	1·1 (0·4 to 1·8)	0·9 (0·3 to 1·5)	40·3
Acute hepatitis	10·7 (6·7 to 15·9)	7·1 (4·5 to 10·5)	4·7 (2·8 to 7·5)	6 (3·9 to 8·6)	4·6 (3 to 6·4)	3·4 (2·1 to 4·9)	3·2 (2·1 to 4·7)	4·1 (2·6 to 5·8)	1·4 (1 to 2)	1·3 (0·8 to 2)	1·1 (0·7 to 1·5)	0·5 (0·3 to 0·7)	0·7 (0·4 to 1)	0·2 (0·1 to 0·3)	0·1 (0 to 0·1)	91·9
Acute hepatitis A	6·8 (3·3 to 11·3)	4·4 (2·1 to 7·2)	3·1 (1·3 to 5·6)	2·4 (1 to 4·2)	1·8 (0·7 to 3·3)	1·2 (0·4 to 2·4)	0·9 (0·3 to 1·8)	1·2 (0·4 to 3·2)	0·7 (0·1 to 1·3)	0·2 (0·1 to 0·4)	0·4 (0·1 to 0·9)	0·2 (0·1 to 0·4)	0·4 (0·1 to 0·7)	0 (0 to 0·1)	0 (0 to 0)	55·2
Acute hepatitis B	2·9 (1·2 to 5·3)	1·7 (0·8 to 2·9)	0·9 (0·5 to 1·5)	2·9 (1·8 to 4·5)	2 (1·2 to 3·1)	1·7 (1 to 2·6)	1·6 (0·9 to 2·5)	2·5 (0·9 to 4·2)	0·4 (0·2 to 1·1)	0·7 (0·3 to 1·2)	0·5 (0·1 to 0·9)	0·2 (0 to 0·4)	0·2 (0 to 0·5)	0·1 (0 to 0·2)	0 (0 to 0·1)	25·5
Acute hepatitis C	0·2 (0·1 to 0·5)	0·5 (0·1 to 1·1)	0·3 (0·1 to 0·6)	0·3 (0·1 to 0·7)	0·5 (0·1 to 1)	0·3 (0·1 to 0·5)	0·4 (0·2 to 0·8)	0·2 (0·1 to 0·8)	0·2 (0·1 to 0·4)	0·3 (0·1 to 0·5)	0·1 (0 to 0·3)	0·1 (0 to 0·1)	0 (0 to 0·1)	0 (0 to 0)	0 (0 to 0)	4·8
Acute hepatitis E	0·7 (0·4 to 1·4)	0·6 (0·2 to 1·1)	0·4 (0·2 to 0·8)	0·3 (0·2 to 0·6)	0·3 (0·2 to 0·5)	0·2 (0·1 to 0·4)	0·2 (0·1 to 0·4)	0·1 (0·1 to 0·2)	0·1 (0·1 to 0·2)	0·2 (0·1 to 0·3)	0·1 (0 to 0·1)	0·1 (0 to 0·1)	0·1 (0 to 0·2)	0 (0 to 0·1)	0 (0 to 0·1)	6·5
Other unspecified infectious diseases	41·7 (19·3 to 72·6)	49·4 (19·7 to 88·2)	48·7 (22·8 to 83·6)	50·8 (23·4 to 83·2)	45·5 (22·9 to 74·3)	43·5 (23·9 to 68·2)	43·2 (21·3 to 70·9)	21·8 (13·8 to 31·8)	18·1 (10·4 to 26·7)	13·3 (8·3 to 19·6)	7·8 (5·2 to 11·3)	6·1 (3·9 to 9·9)	7 (4·4 to 11·1)	4 (2·6 to 5·9)	3 (1·8 to 4·5)	623
Maternal and neonatal disorders	997 (778 to 1,250)	934 (724 to 1,190)	664 (518 to 841)	448 (339 to 574)	331 (246 to 432)	252 (184 to 339)	199 (143 to 268)	146 (104 to 200)	89·7 (63·8 to 124)	49·1 (33·6 to 70·6)	21·3 (13·6 to 31·3)	7·2 (4 to 12)	2·3 (1·3 to 3·7)	0·5 (0·3 to 0·9)	0·2 (0·1 to 0·3)	23,200
Maternal disorders	429 (293 to 604)	450 (300 to 649)	254 (173 to 363)	109 (76·2 to 156)	31·8 (21·6 to 45·4)	4·4 (3·2 to 6)	0 (0 to 0)	0 (0 to 0)	0 (0 to 0)	0 (0 to 0)	0 (0 to 0)	0 (0 to 0)	0 (0 to 0)	0 (0 to 0)	0 (0 to 0)	1,830
Maternal hemorrhage	47·5 (27·7 to 73·5)	49·5 (29·2 to 79·5)	31 (18·6 to 48·7)	11·6 (6·5 to 18·3)	2 (1 to 3·2)	0 (0 to 0·1)	0 (0 to 0)	0 (0 to 0)	0 (0 to 0)	0 (0 to 0)	0 (0 to 0)	0 (0 to 0)	0 (0 to 0)	0 (0 to 0)	0 (0 to 0)	202
Maternal sepsis and other maternal infections	11·7 (6·4 to 18·8)	11 (5·9 to 18·3)	5·6 (3·1 to 9·6)	2 (0·9 to 3·4)	0·7 (0·4 to 1·2)	0·1 (0 to 0·2)	0 (0 to 0)	0 (0 to 0)	0 (0 to 0)	0 (0 to 0)	0 (0 to 0)	0 (0 to 0)	0 (0 to 0)	0 (0 to 0)	0 (0 to 0)	50·3
Maternal hypertensive disorders	111 (67·5 to 171)	99·3 (59·5 to 150)	44·8 (26·8 to 69·3)	13·2 (8 to 19·8)	2·1 (1·3 to 3·4)	0 (0 to 0)	0 (0 to 0)	0 (0 to 0)	0 (0 to 0)	0 (0 to 0)	0 (0 to 0)	0 (0 to 0)	0 (0 to 0)	0 (0 to 0)	0 (0 to 0)	426
Maternal obstructed labor and uterine rupture	4 (2·3 to 6·5)	6·7 (3·4 to 12·1)	6·2 (3·2 to 11·3)	2·1 (1·1 to 4)	0·5 (0·2 to 1)	0 (0 to 0)	0 (0 to 0)	0 (0 to 0)	0 (0 to 0)	0 (0 to 0)	0 (0 to 0)	0 (0 to 0)	0 (0 to 0)	0 (0 to 0)	0 (0 to 0)	25·8
Maternal abortion and miscarriage	32 (14·8 to 58·8)	30·9 (14 to 62·5)	19·1 (9 to 34·6)	8·2 (3·8 to 15·7)	3·2 (1·5 to 5·7)	0 (0 to 0·1)	0 (0 to 0)	0 (0 to 0)	0 (0 to 0)	0 (0 to 0)	0 (0 to 0)	0 (0 to 0)	0 (0 to 0)	0 (0 to 0)	0 (0 to 0)	134
Ectopic pregnancy	17 (9·4 to 28·6)	14·5 (7·8 to 24·4)	3·9 (2·1 to 6·6)	1·5 (0·8 to 2·5)	0·2 (0·1 to 0·4)	0 (0 to 0)	0 (0 to 0)	0 (0 to 0)	0 (0 to 0)	0 (0 to 0)	0 (0 to 0)	0 (0 to 0)	0 (0 to 0)	0 (0 to 0)	0 (0 to 0)	56·6
Indirect maternal deaths	2·5 (1·3 to 4·8)	3·2 (1·7 to 5·9)	1·6 (0·8 to 2·8)	0·4 (0·2 to 0·8)	0·1 (0 to 0·2)	0 (0 to 0)	–	–	–	–	–	–	–	–	–	NA
Late maternal deaths	38·2 (20·3 to 66·8)	39·7 (20·9 to 67·5)	21·8 (12 to 37·4)	9·2 (5·1 to 15·9)	2·8 (1·6 to 4·4)	0·4 (0·2 to 0·6)	–	–	–	–	–	–	–	–	–	NA
Maternal deaths aggravated by HIV/AIDS	1·8 (0·7 to 3·7)	2·5 (1·1 to 4·5)	1·4 (0·7 to 2·6)	0·6 (0·3 to 1)	0·2 (0·1 to 0·3)	0 (0 to 0)	–	–	–	–	–	–	–	–	–	NA
Neonatal disorders	567 (412 to 758)	484 (356 to 642)	410 (295 to 544)	339 (244 to 450)	299 (214 to 400)	248 (180 to 335)	199 (143 to 268)	146 (104 to 200)	89·7 (63·8 to 124)	49·1 (33·6 to 70·6)	21·3 (13·6 to 31·3)	7·2 (4 to 12)	2·3 (1·3 to 3·7)	0·5 (0·3 to 0·9)	0·2 (0·1 to 0·3)	21,400
Neonatal preterm birth	200 (140 to 270)	170 (119 to 236)	142 (95·3 to 195)	116 (81·7 to 159)	99·5 (68 to 138)	79·5 (54·5 to 111)	60·2 (41·8 to 83)	41·2 (27·4 to 57·5)	23·9 (17·7 to 31·1)	11·4 (8·4 to 14·9)	4·4 (3·1 to 6·1)	1·1 (0·6 to 1·9)	0·5 (0·2 to 1)	0·3 (0·1 to 0·5)	0·1 (0 to 0·3)	11,400
Neonatal encephalopathy due to birth asphyxia and trauma	49·9 (25·1 to 83·8)	42·7 (21·9 to 71·7)	36·7 (18·6 to 63·3)	30·8 (15·3 to 49·7)	27·3 (14·3 to 45·6)	22·7 (10·8 to 36·5)	19 (9·4 to 31·9)	14·5 (6·8 to 25·2)	8·3 (5·2 to 12·4)	3·2 (1·9 to 5)	0·8 (0·4 to 1·4)	0·1 (0·1 to 0·3)	0 (0 to 0·1)	0 (0 to 0)	0 (0 to 0)	3,000
Neonatal sepsis and other neonatal infections	296 (183 to 445)	252 (165 to 376)	215 (137 to 323)	179 (113 to 266)	160 (102 to 237)	135 (83·3 to 199)	111 (67·9 to 163)	83·4 (51·1 to 128)	53·3 (32·2 to 83·4)	32 (19 to 51·4)	15 (8·1 to 24·5)	5·5 (2·6 to 10·1)	1·7 (0·8 to 3)	0·3 (0·1 to 0·5)	0 (0 to 0)	5,410
Hemolytic disease and other neonatal jaundice	21·4 (15·5 to 27·9)	18·5 (13·3 to 24·1)	15·9 (11·4 to 20·9)	13·4 (9·7 to 17·9)	12·1 (8·8 to 16·1)	10·4 (7·5 to 13·8)	8·7 (6·2 to 11·5)	6·6 (4·8 to 8·8)	4·2 (3 to 5·5)	2·5 (1·8 to 3·2)	1·1 (0·8 to 1·5)	0·4 (0·3 to 0·6)	0·1 (0·1 to 0·1)	0 (0 to 0)	0 (0 to 0)	262
Other neonatal disorders	0 (0 to 0)	0 (0 to 0)	0 (0 to 0)	0 (0 to 0)	0 (0 to 0)	0 (0 to 0)	0 (0 to 0)	0 (0 to 0)	0 (0 to 0)	0 (0 to 0)	0 (0 to 0)	0 (0 to 0)	0 (0 to 0)	0 (0 to 0)	0 (0 to 0)	1,350
Nutritional deficiencies	511 (191 to 998)	455 (162 to 976)	524 (200 to 998)	557 (249 to 1,020)	480 (195 to 904)	466 (203 to 844)	396 (172 to 727)	251 (110 to 477)	122 (63·3 to 229)	105 (58·6 to 188)	85·4 (49·1 to 150)	77·9 (44·9 to 130)	75·4 (45·6 to 125)	50·6 (31·4 to 75·9)	33·2 (22·1 to 46·2)	6,780
Protein-energy malnutrition	35 (22·4 to 50·8)	32·4 (21·2 to 48)	33·2 (21·9 to 47·3)	31·9 (22 to 44·3)	30·3 (19 to 44·5)	28·4 (18·5 to 40)	34 (23·4 to 46·5)	42·1 (29·7 to 57·3)	33·9 (23·9 to 46·7)	38·8 (27·1 to 53·8)	36 (24·7 to 51·6)	35·3 (23·7 to 49·4)	37·4 (25 to 54·1)	26·9 (17·2 to 38·5)	24·6 (15·6 to 35·4)	823
Iodine deficiency	13·8 (4·6 to 27)	12·7 (4·9 to 23·6)	11·1 (4·4 to 21·5)	9·4 (3·5 to 18·4)	8·8 (3·3 to 16·4)	7·4 (2·8 to 14·6)	6·2 (2·4 to 11·6)	4·7 (1·7 to 9·2)	3·2 (1·1 to 6)	2·2 (0·8 to 4·4)	1·5 (0·5 to 2·4)	1 (0·6 to 1·6)	0·7 (0·4 to 1·1)	0·4 (0·2 to 0·7)	0·2 (0·1 to 0·4)	118
Vitamin A deficiency	3·8 (1·7 to 7·1)	2·8 (1·3 to 5·1)	2·2 (1 to 4)	1·6 (0·7 to 2·9)	1·3 (0·6 to 2·4)	1·1 (0·5 to 2)	0·9 (0·4 to 1·7)	0·7 (0·3 to 1·4)	0·5 (0·2 to 1)	0·4 (0·2 to 0·8)	0·3 (0·2 to 0·6)	0·2 (0·1 to 0·4)	0·1 (0·1 to 0·2)	0·1 (0 to 0·1)	0 (0 to 0·1)	71·8
Dietary iron deficiency	441 (129 to 936)	392 (105 to 908)	470 (157 to 940)	506 (202 to 967)	423 (135 to 842)	416 (155 to 791)	345 (127 to 680)	199 (60 to 425)	76·5 (20·4 to 185)	56·9 (15·4 to 131)	42·9 (10·9 to 107)	37·2 (8·7 to 88·2)	32·3 (8 to 77·2)	20·2 (5·9 to 46)	6·6 (1·5 to 16·2)	5,610
Other nutritional deficiencies	17·9 (10·1 to 28·3)	14·7 (8·4 to 22·5)	6·9 (3·6 to 11·4)	7·8 (4·4 to 12·5)	16·8 (9·3 to 26·8)	12·5 (7·2 to 19·2)	9·6 (5·5 to 14·2)	4·5 (2·5 to 7)	7·6 (4·2 to 11·8)	6·3 (3·5 to 10·2)	4·7 (2·4 to 7·7)	4·1 (2 to 7·1)	4·8 (1·8 to 8·8)	3 (0·9 to 5·2)	1·7 (0·5 to 3·2)	157
Non-communicable diseases	15,300 (11,900 to 19,300)	16,700 (13,300 to 20,900)	17,200 (13,700 to 21,600)	19,000 (15,300 to 23,000)	22,300 (18,200 to 27,000)	23,600 (19,400 to 28,400)	24,900 (20,500 to 29,800)	26,300 (21,900 to 31,100)	23,600 (20,100 to 27,600)	22,000 (18,700 to 25,500)	18,800 (16,100 to 21,700)	16,400 (14,200 to 18,700)	12,900 (11,200 to 14,700)	8,090 (7,160 to 9,090)	5,640 (5,170 to 6,120)	309,000
Neoplasms	1,310 (928 to 1,820)	2,200 (1,590 to 2,970)	2,850 (2,020 to 3,900)	4,100 (3,070 to 5,420)	5,310 (3,940 to 6,810)	5,630 (4,230 to 7,280)	5,830 (4,500 to 7,470)	5,950 (4,660 to 7,510)	4,930 (3,970 to 6,060)	3,950 (3,140 to 4,850)	2,810 (2,250 to 3,410)	1,940 (1,560 to 2,350)	1,230 (945 to 1,520)	562 (418 to 696)	305 (209 to 382)	51,000
Lip and oral cavity cancer	13·4 (7·6 to 21·3)	9 (5·1 to 13·9)	6·4 (3·8 to 10·3)	14·9 (9·3 to 22·7)	22·5 (13·8 to 34·5)	27 (17·7 to 39·8)	26·7 (17·9 to 38·9)	31·8 (20·3 to 46·5)	29·1 (19·1 to 42·1)	24·1 (15·9 to 34·4)	18·7 (12·6 to 27·1)	12·8 (8·2 to 18·6)	8·3 (5·4 to 12·2)	5·3 (3·3 to 7·4)	3·2 (1·9 to 4·4)	270
Nasopharynx cancer	19·2 (10·7 to 31·6)	19·3 (10·8 to 31·2)	12·8 (7 to 20·4)	13·7 (8 to 22·4)	16·4 (10·2 to 24·6)	14·5 (9 to 22)	13·8 (8·4 to 20·6)	13·4 (7·8 to 21·8)	12·5 (7·5 to 19·4)	6·3 (4 to 9·3)	4·1 (2·5 to 6·6)	3 (1·8 to 4·9)	3·1 (1·8 to 5)	2·2 (1·2 to 3·8)	0·8 (0·5 to 1·4)	192
Other pharynx cancer	5·4 (2·8 to 9·3)	5·7 (3·1 to 9·6)	5·4 (2·8 to 9·8)	5·4 (3 to 9)	15·1 (8·4 to 23·8)	15·3 (9·2 to 23·6)	12·9 (7·7 to 20·1)	16 (9·7 to 24)	9·2 (5·8 to 13·9)	6·9 (4·4 to 10·3)	4·3 (2·7 to 6·5)	3·9 (2·3 to 6·3)	2·9 (1·8 to 4·2)	1·6 (0·9 to 2·7)	1·5 (0·9 to 2·6)	117
Esophageal cancer	8·5 (4·8 to 13·8)	11 (5·7 to 19)	10 (5·6 to 16·5)	14·3 (8·2 to 23·6)	27·7 (16·2 to 42·2)	41 (25·6 to 59·8)	59·2 (35·8 to 91·2)	69·4 (43·7 to 103)	67 (42·3 to 97·7)	58·6 (37·6 to 86·6)	49·8 (30·9 to 76·2)	27·6 (16·9 to 41·9)	18·7 (12·1 to 26·7)	7·3 (4·4 to 10·9)	2·9 (1·6 to 4·6)	474
Stomach cancer	37·9 (22·9 to 59·2)	57·1 (34·1 to 87·7)	59·1 (35·4 to 91·8)	79·3 (53·5 to 118)	133 (88 to 192)	168 (115 to 239)	235 (161 to 337)	265 (184 to 363)	235 (171 to 313)	210 (155 to 284)	204 (154 to 271)	163 (116 to 218)	120 (86·1 to 162)	51·6 (33·9 to 71·2)	26·6 (16·9 to 38·7)	2,070
Colon and rectum cancer	76·1 (45·7 to 120)	130 (79·5 to 204)	133 (80·6 to 202)	243 (155 to 350)	348 (235 to 505)	519 (340 to 750)	557 (373 to 791)	679 (475 to 959)	623 (442 to 851)	537 (381 to 727)	456 (337 to 606)	345 (246 to 463)	244 (168 to 343)	118 (78·6 to 165)	52·6 (30·5 to 74·5)	5,130
Liver cancer	16·7 (8·7 to 28·1)	24·7 (13·2 to 43·1)	27·8 (14·7 to 48·4)	45·7 (26·5 to 74·1)	60·7 (35·2 to 100)	85·4 (51·2 to 130)	88·4 (53·2 to 133)	82·4 (53·1 to 126)	78·6 (51·1 to 122)	63·2 (39·7 to 94·9)	55·1 (36·2 to 81)	45·7 (30·7 to 66·1)	23·1 (14·4 to 33·7)	10 (6·6 to 14)	3·4 (1·9 to 5·1)	743
Liver cancer due to hepatitis B	7·7 (3·5 to 14·6)	11·8 (5·5 to 21·6)	12·6 (5·9 to 24·3)	18·9 (8·8 to 34)	21·7 (10·5 to 39·6)	26·4 (12·2 to 48·1)	24·3 (11·8 to 44)	19·3 (9·2 to 34·9)	14·5 (6·8 to 25·9)	9·6 (4·2 to 18·8)	7·1 (3 to 13)	5 (2·1 to 9·5)	2·3 (1·1 to 4·2)	0·9 (0·4 to 1·7)	0·3 (0·1 to 0·6)	189
Liver cancer due to hepatitis C	0·8 (0·2 to 1·8)	1·8 (0·7 to 3·9)	2·9 (0·9 to 6·5)	6·4 (2·7 to 12·5)	11·3 (4·7 to 21·6)	20·2 (9·6 to 36·2)	24·8 (10·6 to 45·1)	25·3 (12·2 to 46·6)	25·7 (13·6 to 42·3)	22·5 (12·3 to 38·4)	21·7 (11·3 to 36·2)	20·6 (11·9 to 32·9)	11·7 (6·9 to 18·2)	5·5 (3·2 to 8·5)	2 (1 to 3·2)	204
Liver cancer due to alcohol use	1·4 (0·5 to 3)	3 (1·2 to 5·9)	4·5 (1·7 to 9·5)	8·6 (3·7 to 16·7)	12·7 (5·3 to 24·2)	19 (8·4 to 35·7)	20·1 (9·1 to 37·2)	20·4 (9·9 to 37·5)	21·7 (11 to 37·1)	17·1 (8·5 to 29·9)	12·8 (6·6 to 21·9)	8·7 (4·4 to 15·1)	3·7 (1·9 to 6·6)	1·3 (0·6 to 2·4)	0·4 (0·1 to 0·8)	156
Liver cancer due to NASH	2·6 (1·1 to 5)	3·2 (1·3 to 6·5)	3·2 (1·3 to 6·3)	5·1 (2·3 to 10·5)	7·1 (3 to 14·1)	10·1 (4·5 to 18·9)	10·5 (4·7 to 19·2)	10·1 (5 to 19)	10·3 (5·2 to 19·3)	9·2 (4·6 to 16·4)	8 (4·8 to 15·4)	8 (4·1 to 14·3)	3·9 (1·9 to 6·8)	1·6 (0·8 to 3)	0·5 (0·2 to 1·1)	97·9
Liver cancer due to other causes	4·2 (1·9 to 7·9)	5 (2·1 to 9·7)	4·7 (2 to 9·4)	6·7 (3·1 to 12·6)	7·9 (3·7 to 15·2)	9·8 (4·6 to 18·5)	8·8 (4 to 16·1)	7·3 (3·4 to 13·7)	6·3 (2·9 to 11·4)	4·8 (2·1 to 9·2)	4·2 (2 to 8·2)	3·3 (1·5 to 6·1)	1·6 (0·8 to 2·9)	0·6 (0·3 to 1·2)	0·2 (0·1 to 0·4)	97
Gallbladder and biliary tract cancer	3·8 (2·1 to 6·3)	6·4 (3·7 to 10·9)	11·7 (6·2 to 19·7)	26·5 (16 to 42·2)	47·8 (28·5 to 74)	72·9 (45·2 to 105)	93·6 (60·8 to 139)	107 (70·2 to 154)	87·8 (58 to 127)	69·2 (44·9 to 102)	50·5 (33·6 to 76·9)	29 (17·5 to 48·4)	13·8 (8 to 25·5)	7·4 (4·5 to 12·5)	3·6 (2·1 to 6·2)	632
Pancreatic cancer	7·7 (4·3 to 12·9)	11·8 (6·8 to 19·4)	26·8 (16·3 to 41·2)	62·7 (41·2 to 92·3)	92 (58·1 to 137)	148 (96·7 to 215)	182 (119 to 276)	234 (153 to 339)	228 (156 to 316)	203 (143 to 285)	142 (101 to 196)	101 (67·9 to 145)	65·1 (43·4 to 94·8)	25·1 (16 to 36)	15·2 (9·8 to 21·6)	1,550
Larynx cancer	1·7 (0·9 to 2·7)	2·2 (1 to 4·1)	1·2 (0·6 to 2·2)	3·1 (1·6 to 5·3)	5·5 (3·2 to 8·7)	4·3 (2·6 to 9·3)	6·8 (4·2 to 12·1)	11·6 (7·3 to 17·8)	5·9 (3·5 to 11·4)	6·9 (4·4 to 10·7)	5·7 (3·7 to 8·4)	4·3 (2·7 to 6·3)	2·4 (1·5 to 3·7)	1·2 (0·7 to 1·9)	0·4 (0·2 to 0·6)	64·4
Tracheal, bronchus, and lung cancer	49·9 (27·5 to 81·9)	76·1 (42·8 to 122)	82·6 (47·5 to 132)	144 (90·9 to 227)	242 (157 to 365)	317 (213 to 469)	388 (262 to 579)	438 (304 to 619)	375 (255 to 526)	369 (260 to 512)	236 (169 to 327)	161 (110 to 226)	88·3 (58·6 to 129)	28 (17·4 to 41·4)	15·8 (9·2 to 23·2)	3,060
Malignant skin melanoma	4·1 (2·2 to 7·6)	7·3 (3·8 to 12·6)	9 (4·6 to 17·7)	4·7 (2·2 to 13·7)	8 (4·2 to 17·8)	8·3 (4·6 to 16·7)	9·2 (5·3 to 16·5)	10·6 (6·5 to 17·5)	11·5 (6·7 to 17·9)	13·2 (6 to 20·2)	7·5 (4 to 10·8)	6·8 (3·5 to 10·2)	4·8 (2·8 to 7·1)	2·5 (1·4 to 3·9)	1·8 (1 to 2·9)	120
Non-melanoma skin cancer	0·9 (0·6 to 1·2)	0·7 (0·5 to 1)	0·7 (0·4 to 1·1)	2·8 (1·8 to 4·5)	1·7 (1·1 to 2·7)	4·3 (2·7 to 6·5)	6·2 (3·6 to 9·9)	4·3 (2·7 to 6·3)	5 (3·2 to 7·3)	7·2 (4·6 to 10·5)	4·9 (3·4 to 6·7)	4·5 (3·2 to 6)	4·6 (3·2 to 6·3)	4·3 (2·8 to 5·9)	2·9 (1·8 to 4·3)	56
Non-melanoma skin cancer (squamous-cell carcinoma)	0·9 (0·6 to 1·2)	0·7 (0·5 to 1)	0·7 (0·4 to 1·1)	2·8 (1·7 to 4·5)	1·7 (1·1 to 2·7)	4·3 (2·7 to 6·5)	6·2 (3·6 to 9·9)	4·3 (2·7 to 6·3)	5 (3·2 to 7·3)	7·2 (4·5 to 10·5)	4·9 (3·4 to 6·7)	4·5 (3·2 to 6)	4·6 (3·2 to 6·3)	4·3 (2·8 to 5·9)	2·9 (1·8 to 4·3)	56
Non-melanoma skin cancer (basal-cell carcinoma)	0 (0 to 0)	0 (0 to 0)	0 (0 to 0)	0 (0 to 0)	0 (0 to 0)	0 (0 to 0)	0 (0 to 0)	0 (0 to 0)	0 (0 to 0)	0 (0 to 0)	0 (0 to 0)	0 (0 to 0)	0 (0 to 0)	0 (0 to 0)	0 (0 to 0)	0
Breast cancer	202 (126 to 296)	640 (401 to 931)	1,010 (659 to 1,480)	1,690 (1,160 to 2,340)	2,020 (1,400 to 2,780)	1,930 (1,380 to 2,590)	1,730 (1,230 to 2,330)	1,430 (1,040 to 1,920)	1,040 (756 to 1,370)	810 (584 to 1,080)	531 (387 to 702)	358 (261 to 478)	239 (168 to 322)	118 (80·7 to 162)	82·2 (46·4 to 127)	13,900
Cervical cancer	208 (119 to 343)	494 (295 to 767)	759 (443 to 1,150)	917 (591 to 1,360)	1,090 (714 to 1,580)	946 (628 to 1,380)	902 (589 to 1,280)	828 (549 to 1,170)	601 (405 to 850)	394 (271 to 537)	243 (175 to 331)	158 (109 to 218)	83 (56·7 to 116)	36·6 (23 to 53·8)	24·6 (14·9 to 36)	7,770
Uterine cancer	15·1 (7·7 to 27)	17·7 (10 to 29)	31·8 (18·4 to 52·3)	62·1 (38·2 to 95)	116 (73·2 to 178)	162 (104 to 241)	292 (187 to 427)	514 (329 to 752)	479 (318 to 683)	347 (235 to 482)	206 (141 to 293)	124 (82·3 to 178)	64·4 (43·7 to 89·6)	29·8 (19·8 to 41·5)	13·5 (8·6 to 19·5)	2,490
Ovarian cancer	63·6 (33·8 to 104)	93·4 (49·6 to 145)	122 (66·4 to 189)	140 (87·8 to 217)	244 (138 to 373)	302 (169 to 452)	332 (177 to 489)	308 (176 to 444)	257 (133 to 364)	175 (99·9 to 240)	109 (61·4 to 158)	64·7 (38·7 to 96·9)	44·9 (24·4 to 66·4)	20·9 (11·4 to 31·5)	8·1 (3·6 to 12·9)	2,390
Prostate cancer	0 (0 to 0)	0 (0 to 0)	0 (0 to 0)	0 (0 to 0)	0 (0 to 0)	0 (0 to 0)	0 (0 to 0)	0 (0 to 0)	0 (0 to 0)	0 (0 to 0)	0 (0 to 0)	0 (0 to 0)	0 (0 to 0)	0 (0 to 0)	0 (0 to 0)	0
Testicular cancer	–	–	–	–	–	–	–	–	–	–	–	–	–	–	–	NA
Kidney cancer	20·8 (10·8 to 36·2)	25·8 (13·2 to 46·3)	27·8 (15·2 to 45·1)	31·3 (17·7 to 53)	44·1 (24·7 to 73)	50·3 (30·6 to 78·8)	51·1 (30·5 to 82·9)	46·2 (28 to 71·3)	38·7 (24·5 to 59·5)	28·7 (17·7 to 43)	16·9 (10·3 to 26·3)	12·1 (7·2 to 19·1)	6·7 (3·9 to 10·9)	3·2 (1·9 to 5·1)	1·4 (0·8 to 2·1)	457
Bladder cancer	2·8 (1·5 to 4·8)	5 (2·9 to 8·2)	4 (2·1 to 7)	15·4 (9·7 to 23·6)	31·1 (19·5 to 46·1)	49 (32·6 to 71·9)	45·1 (29·4 to 68)	49·4 (32·9 to 70·4)	60·7 (40·9 to 84·5)	62·4 (43·5 to 88)	58·7 (41·1 to 83·3)	48·7 (33·1 to 69·7)	39·1 (26 to 56·6)	19·6 (12·7 to 28·2)	10·4 (6·7 to 14·7)	503
Brain and central nervous system cancer	39·1 (15·8 to 69·4)	40·5 (16 to 70·7)	40·3 (17·9 to 67·7)	41·3 (20·2 to 67·3)	47·7 (23·5 to 81·4)	55 (25·2 to 96·4)	59·7 (25·3 to 101)	57 (22·3 to 96·7)	41·6 (18·4 to 68·8)	29·5 (11·8 to 51·2)	20·7 (7·9 to 36·5)	11·9 (4·2 to 23·3)	5·9 (2·3 to 11·2)	2·8 (1 to 4·9)	1 (0·4 to 1·9)	724
Thyroid cancer	11·4 (4·4 to 24)	13·8 (5·9 to 29·1)	14 (7·1 to 24)	14 (7·3 to 23·6)	27·9 (16·4 to 46·1)	46·6 (28·2 to 73·6)	39 (24·2 to 57·5)	42·3 (26·5 to 62·9)	42·4 (27·3 to 62)	40·2 (25·9 to 58·3)	25·7 (16·7 to 39·1)	16·3 (10·3 to 24)	10·3 (6·1 to 15·6)	3·9 (2·2 to 6)	2·2 (1·3 to 3·4)	370
Mesothelioma	1·6 (0·8 to 3)	1·2 (0·5 to 3·4)	3·9 (2·2 to 7·2)	4 (2·3 to 7·5)	3·6 (2 to 8·4)	3·8 (1·9 to 10·1)	6·5 (4 to 10·2)	2·3 (1·5 to 3·9)	2·5 (1·5 to 3·5)	3 (1·9 to 4·3)	1·5 (0·8 to 2·2)	2·1 (0·8 to 3·2)	0·5 (0·3 to 0·7)	0·5 (0·2 to 0·7)	0·2 (0·1 to 0·3)	38·7
Hodgkin lymphoma	15·6 (7·2 to 28·1)	13·4 (5·4 to 25·5)	9 (3·8 to 17·2)	8·2 (3·5 to 14·9)	3·2 (1·6 to 6)	2·8 (1·4 to 5·4)	3·9 (2·4 to 6)	4·7 (2·6 to 7·2)	4·6 (2·4 to 7·3)	2·3 (1·4 to 3·6)	2·4 (1·5 to 3·8)	1·7 (1 to 2·7)	0·9 (0·5 to 1·4)	0·2 (0·1 to 0·4)	0·1 (0 to 0·2)	102
Non-Hodgkin lymphoma	176 (111 to 265)	165 (108 to 243)	166 (103 to 247)	172 (110 to 247)	237 (151 to 346)	238 (157 to 346)	218 (147 to 329)	210 (142 to 310)	168 (117 to 246)	113 (77·1 to 165)	89·3 (63·5 to 125)	52·1 (35 to 75·1)	26·1 (17·4 to 37·7)	9·7 (6·1 to 13·8)	4·5 (2·8 to 6·6)	2,310
Multiple myeloma	2·6 (1·4 to 4·4)	7·2 (3·5 to 12·7)	10·5 (5·7 to 18)	32 (17·8 to 50·8)	68·5 (34·2 to 105)	89·6 (54·2 to 136)	139 (80·2 to 206)	162 (98·4 to 236)	142 (85·3 to 201)	121 (76·9 to 176)	95·1 (56·9 to 136)	52·8 (32·3 to 79·2)	29·5 (16·7 to 47·9)	12·3 (6·8 to 19·2)	6·5 (3·1 to 10·9)	971
Leukemia	184 (108 to 301)	207 (118 to 312)	173 (100 to 269)	173 (107 to 260)	191 (120 to 288)	158 (99·7 to 230)	159 (100 to 235)	168 (103 to 252)	119 (74·6 to 178)	107 (68·3 to 159)	70·1 (44·5 to 105)	46·9 (28·4 to 72·4)	26·5 (15·9 to 41·4)	12·5 (7·3 to 18·7)	7·8 (4·5 to 12)	2,410
Acute lymphoid leukemia	18·9 (8·3 to 35·6)	19·6 (8·7 to 36·7)	16·8 (6·1 to 34·2)	18·2 (7·6 to 37·4)	12·9 (5·7 to 25)	6·9 (3·1 to 13·3)	8·5 (3·8 to 15·8)	5·1 (2·2 to 10)	5·1 (2·3 to 9·7)	2·3 (1 to 5·1)	1·2 (0·4 to 4·7)	0·7 (0·2 to 3·3)	0·2 (0·1 to 0·9)	0·2 (0·1 to 0·8)	1 (0·1 to 2·3)	399
Chronic lymphoid leukemia	1·1 (0·6 to 1·8)	2·7 (1·2 to 5·1)	0·9 (0·4 to 1·8)	1·3 (0·5 to 2·4)	3·6 (1·6 to 6·9)	6·8 (3·3 to 12·1)	11·9 (5·2 to 22·1)	16·4 (7·6 to 31·7)	20·7 (9·9 to 39·2)	18·8 (8·2 to 34·7)	9·3 (4·2 to 17·4)	6·3 (2·9 to 12·2)	2·1 (0·9 to 4·2)	1·7 (0·7 to 3·3)	0 (0 to 0·1)	105
Acute myeloid leukemia	39·6 (17·8 to 76·2)	24·1 (10·9 to 45·9)	34·3 (14·8 to 65)	37·2 (17·4 to 65·9)	44·1 (22·5 to 77·4)	39·6 (19·4 to 71·4)	41·8 (20·6 to 71·5)	40·4 (19·5 to 70·3)	23 (11·1 to 42·6)	26·2 (12 to 47)	14·1 (6·7 to 26·5)	9 (4·2 to 16·3)	3·6 (1·7 to 7)	0·6 (0·2 to 2)	1·8 (0·4 to 4·6)	556
Chronic myeloid leukemia	21·3 (8·6 to 44·1)	19 (7·4 to 40·4)	2·8 (1·1 to 6)	10·5 (4·5 to 20·1)	19·2 (9·1 to 36)	13·1 (6 to 23·5)	11·7 (5·4 to 22·3)	12·6 (6 to 23·3)	8 (3·7 to 15·6)	12·4 (5·7 to 22·2)	5·2 (2·2 to 10)	2·9 (1·3 to 5·7)	0·5 (0·2 to 1)	2·9 (1·2 to 5·5)	0 (0 to 0·1)	187
Other leukemia	104 (49·4 to 181)	141 (72·2 to 218)	118 (61·1 to 187)	106 (61·1 to 165)	111 (62·1 to 171)	91·2 (50·7 to 140)	84·7 (48·2 to 129)	93·5 (51·4 to 149)	62·4 (35·7 to 97·8)	47·5 (27·1 to 77·7)	40·3 (23·3 to 64·2)	27·9 (16·2 to 44·9)	20·1 (11·4 to 31)	7·1 (3·9 to 11·4)	4·9 (2·4 to 8·1)	1,170
Other malignant neoplasms	109 (74·6 to 156)	93·8 (61·6 to 138)	87·8 (55·6 to 133)	120 (78·9 to 176)	145 (95·1 to 214)	155 (102 to 225)	150 (101 to 210)	154 (105 to 211)	142 (98·1 to 196)	110 (74·7 to 158)	81·2 (57 to 111)	63·6 (44·5 to 87·2)	42·4 (27·9 to 62·3)	22·3 (13·8 to 34)	10·5 (6·2 to 16·7)	1,900
Other neoplasms	11 (4·7 to 17·5)	17·3 (5·1 to 28·1)	9·9 (3·4 to 15·7)	20·2 (9 to 29·8)	18·5 (8·8 to 26·8)	19·1 (10·6 to 28·9)	22 (11·5 to 32·7)	15·1 (10·4 to 20·5)	20·9 (13·3 to 28·3)	28·4 (15·5 to 40·2)	21·7 (14 to 29·3)	18·1 (10·6 to 25·5)	8·5 (5·7 to 11·6)	5·3 (2·9 to 7·7)	1·2 (0·8 to 1·8)	246
Myelodysplastic, myeloproliferative, and other hematopoietic n	11 (4·7 to 17·5)	17·3 (5·1 to 28·1)	9·9 (3·4 to 15·7)	20·2 (9 to 29·8)	18·5 (8·8 to 26·8)	19·1 (10·6 to 28·9)	22 (11·5 to 32·7)	15·1 (10·4 to 20·5)	20·9 (13·3 to 28·3)	28·4 (15·5 to 40·2)	21·7 (14 to 29·3)	18·1 (10·6 to 25·5)	8·5 (5·7 to 11·6)	5·3 (2·9 to 7·7)	1·2 (0·8 to 1·8)	246
Benign and in situ intestinal neoplasms	0 (0 to 0)	0 (0 to 0)	0 (0 to 0)	0 (0 to 0)	0 (0 to 0)	0 (0 to 0)	0 (0 to 0)	0 (0 to 0)	0 (0 to 0)	0 (0 to 0)	0 (0 to 0)	0 (0 to 0)	0 (0 to 0)	0 (0 to 0)	0 (0 to 0)	0
Benign and in situ cervical and uterine neoplasms	0 (0 to 0)	0 (0 to 0)	0 (0 to 0)	0 (0 to 0)	0 (0 to 0)	0 (0 to 0)	0 (0 to 0)	0 (0 to 0)	0 (0 to 0)	0 (0 to 0)	0 (0 to 0)	0 (0 to 0)	0 (0 to 0)	0 (0 to 0)	0 (0 to 0)	0
Other benign and in situ neoplasms	0 (0 to 0)	0 (0 to 0)	0 (0 to 0)	0 (0 to 0)	0 (0 to 0)	0 (0 to 0)	0 (0 to 0)	0 (0 to 0)	0 (0 to 0)	0 (0 to 0)	0 (0 to 0)	0 (0 to 0)	0 (0 to 0)	0 (0 to 0)	0 (0 to 0)	0
Cardiovascular diseases	989 (724 to 1,330)	1,200 (881 to 1,580)	1,490 (1,090 to 2,020)	2,060 (1,550 to 2,700)	3,090 (2,350 to 3,990)	3,590 (2,740 to 4,650)	4,300 (3,300 to 5,580)	5,450 (4,280 to 6,790)	5,600 (4,500 to 6,840)	6,300 (5,200 to 7,650)	6,140 (5,080 to 7,320)	5,900 (4,860 to 6,970)	4,950 (3,940 to 5,940)	3,250 (2,380 to 3,860)	2,390 (1,680 to 2,830)	58,200
Rheumatic heart disease	222 (153 to 310)	177 (123 to 245)	147 (99·4 to 204)	137 (94·6 to 194)	108 (74·9 to 150)	80·4 (56·2 to 112)	56·4 (39·4 to 77·7)	41 (29·4 to 55·2)	29·4 (21·2 to 40)	25·4 (18·8 to 34)	20·2 (14·5 to 27)	15·7 (11·6 to 21)	11 (7·9 to 14·5)	6·9 (4·9 to 9·2)	5 (3·3 to 6·6)	1,550

Ischemic heart disease	0 (0 to 0)	0 (0 to 0)	0 (0 to 0)	0 (0 to 0)	0 (0 to 0)	0 (0 to 0)	11·6 (8·1 to 16·3)	34·6 (22·5 to 52·2)	93·8 (61·2 to 140)	235 (155 to 343)	326 (223 to 463)	501 (355 to 671)	819 (590 to 1,100)	1,010 (731 to 1,320)	1,330 (1,020 to 1,740)	1,660 (1,280 to 2,100)	1,720 (1,290 to 2,220)	1,730 (1,370 to 2,130)	1,770 (1,380 to 2,230)	1,700 (1,370 to 2,070)	1,520 (1,180 to 1,860)	1,060 (795 to 1,300)	836 (578 to 1,040)	16,300
Stroke	49·1 (29·7 to 74·5)	7·4 (4·2 to 11·6)	9·4 (4 to 18·8)	15 (9·4 to 21·8)	37·5 (26 to 51·8)	64·4 (45·6 to 88·1)	130 (90·6 to 179)	213 (145 to 303)	350 (237 to 506)	439 (297 to 608)	616 (422 to 884)	850 (583 to 1,200)	1,350 (962 to 1,830)	1,660 (1,190 to 2,250)	1,910 (1,380 to 2,600)	2,630 (1,950 to 3,410)	2,660 (2,080 to 3,370)	3,300 (2,580 to 4,200)	3,050 (2,460 to 3,750)	2,900 (2,310 to 3,560)	2,290 (1,770 to 2,840)	1,340 (971 to 1,640)	913 (641 to 1,120)	26,800
Ischemic stroke	0·6 (0·3 to 2·1)	0·4 (0·2 to 0·7)	0·3 (0·2 to 0·5)	4·2 (2·9 to 5·8)	13·6 (8·6 to 20·8)	16·7 (9·7 to 27)	35·9 (25·1 to 50·4)	58·5 (40 to 80·7)	70·5 (46·7 to 100)	83·1 (57·1 to 120)	95·1 (65·6 to 130)	127 (88·3 to 173)	184 (130 to 247)	210 (154 to 284)	258 (185 to 344)	571 (424 to 763)	679 (502 to 893)	1,830 (1,370 to 2,360)	1,880 (1,500 to 2,330)	2,320 (1,850 to 2,880)	1,880 (1,440 to 2,340)	1,090 (791 to 1,360)	772 (541 to 951)	12,200
Intracerebral hemorrhage	0·2 (0·1 to 0·7)	0·2 (0·1 to 0·4)	1·9 (0·6 to 4·3)	1·7 (1 to 2·7)	6 (4 to 8·6)	22·8 (15·4 to 33·8)	59·6 (39·7 to 85·9)	85·7 (54·4 to 129)	162 (104 to 243)	203 (134 to 297)	320 (204 to 475)	460 (304 to 668)	856 (582 to 1,200)	1,060 (732 to 1,510)	1,250 (892 to 1,730)	1,750 (1,240 to 2,300)	1,720 (1,300 to 2,230)	1,300 (957 to 1,760)	1,050 (783 to 1,380)	493 (349 to 659)	358 (257 to 469)	211 (141 to 280)	121 (83·9 to 150)	11,500
Subarachnoid hemorrhage	48·3 (29·2 to 73·4)	6·7 (3·8 to 10·7)	7·2 (2·8 to 15)	9 (4·8 to 14·6)	17·8 (10·9 to 26·9)	24·9 (14·9 to 38·4)	34·8 (20·2 to 61)	68·9 (39·2 to 110)	117 (68·7 to 186)	152 (91·5 to 234)	201 (125 to 322)	264 (163 to 397)	311 (199 to 461)	387 (255 to 569)	408 (268 to 593)	310 (208 to 447)	267 (184 to 370)	166 (116 to 229)	117 (82·4 to 159)	81·3 (57·4 to 113)	49·8 (34·7 to 67·1)	33·2 (21·9 to 46·8)	19·5 (12·6 to 27·2)	3,100
Hypertensive heart disease	0 (0 to 0)	0 (0 to 0)	0 (0 to 0)	0 (0 to 0)	0 (0 to 0)	0 (0 to 0)	8·5 (5·3 to 13·4)	22·2 (13·1 to 38·4)	42·3 (24 to 69)	86·2 (51·5 to 132)	125 (74·7 to 209)	181 (116 to 293)	360 (229 to 532)	437 (296 to 667)	551 (368 to 820)	647 (449 to 979)	737 (509 to 1,010)	743 (538 to 1,060)	785 (539 to 1,090)	740 (536 to 1,010)	596 (440 to 817)	431 (291 to 589)	327 (218 to 462)	6,820
Non-rheumatic valvular heart disease	0 (0 to 0)	0 (0 to 0)	0 (0 to 0)	0 (0 to 0)	0 (0 to 0)	0 (0 to 0)	13·1 (8·7 to 18·2)	8·4 (5·4 to 13·1)	15·9 (9·3 to 25·4)	5·8 (3·5 to 8·9)	6 (3·6 to 9·3)	9·2 (5·6 to 14·1)	12·4 (7·9 to 18·7)	8·3 (5·2 to 13·4)	9·9 (6·5 to 14·5)	11·8 (8 to 17)	13·1 (9 to 18·5)	15·9 (11·1 to 22·3)	10·8 (7·5 to 15·2)	8·4 (5·7 to 11·7)	8·1 (5·5 to 11·1)	3·9 (2·6 to 5·4)	4·2 (2·7 to 5·8)	165
Non-rheumatic calcific aortic valve disease	0 (0 to 0)	0 (0 to 0)	0 (0 to 0)	0 (0 to 0)	0 (0 to 0)	0 (0 to 0)	7·8 (4·4 to 12·5)	6·5 (3·8 to 9·9)	6·3 (3 to 11·6)	2·9 (1·1 to 5·1)	3·9 (1·6 to 6·7)	2·9 (1·4 to 5·2)	5·3 (2·9 to 9)	4·7 (2·2 to 8·2)	5·1 (3 to 8·1)	6·3 (3·8 to 9·9)	6·1 (3·2 to 11·2)	7·4 (3·5 to 14·4)	6·3 (3·7 to 10·4)	5·1 (3 to 8·2)	6·2 (4 to 8·9)	2·6 (1·6 to 3·9)	2·8 (1·6 to 4·4)	88·2
Non-rheumatic degenerative mitral valve disease	0 (0 to 0)	0 (0 to 0)	0 (0 to 0)	0 (0 to 0)	0 (0 to 0)	0 (0 to 0)	5·1 (2·6 to 10)	1·8 (0·8 to 5)	9·3 (4·9 to 15·1)	2·6 (1·1 to 5·6)	2 (0·6 to 5·3)	6·2 (3·5 to 9·8)	7 (3·8 to 11·5)	3·6 (1·6 to 6·5)	4·8 (2·7 to 7·8)	5·5 (3·1 to 8·5)	6·9 (3·3 to 11·1)	8·4 (3·2 to 14·3)	4·5 (2 to 7·2)	3·2 (1·5 to 5·4)	1·9 (0·9 to 3·2)	1·3 (0·6 to 2)	1·4 (0·5 to 2·3)	75·4
Other non-rheumatic valve diseases	0 (0 to 0)	0 (0 to 0)	0 (0 to 0)	0 (0 to 0)	0 (0 to 0)	0 (0 to 0)	0·3 (0·1 to 0·6)	0·2 (0·1 to 0·3)	0·3 (0·1 to 0·6)	0·2 (0·1 to 0·6)	0·1 (0 to 0·2)	0·1 (0 to 0·2)	0·1 (0 to 0·2)	0·1 (0 to 0·2)	0·1 (0 to 0·1)	0 (0 to 0·1)	0 (0 to 0·1)	0·1 (0 to 0·2)	0 (0 to 0·1)	0 (0 to 0·1)	0 (0 to 0)	0 (0 to 0)	0 (0 to 0)	1·6
Cardiomyopathy and myocarditis	11·2 (4·5 to 17·7)	5 (3·2 to 7·6)	11·9 (5·2 to 23·9)	16·4 (9·3 to 29)	11·6 (7·4 to 18·1)	21·1 (13·9 to 31·6)	24·9 (16·3 to 36)	40·7 (25·2 to 63·1)	65·6 (37·6 to 105)	87·2 (46·2 to 134)	87·7 (46 to 138)	80·7 (50·2 to 123)	88·2 (55·2 to 131)	93·4 (59·1 to 138)	108 (70·6 to 162)	108 (73·1 to 155)	88·2 (59·6 to 128)	73·9 (50·4 to 105)	62·4 (41·8 to 91·5)	47·1 (29·7 to 72·2)	44·3 (27·8 to 68·7)	26·8 (16 to 44·8)	17·2 (10·2 to 30·6)	1,220
Myocarditis	3 (0·5 to 8·2)	1·4 (0·5 to 3·5)	3·4 (1·5 to 6·2)	4·1 (2 to 6·7)	4·4 (2·8 to 6·8)	4·8 (2·5 to 7·9)	2·1 (0·9 to 3·8)	3·1 (1·4 to 6)	8·1 (3·1 to 15·7)	8·1 (3·8 to 13·7)	3·9 (1·9 to 7·7)	2·1 (1·1 to 4)	5·9 (2·5 to 11·5)	4·3 (2 to 7·9)	3 (1·6 to 5·4)	2·6 (1·3 to 4·8)	1·1 (0·6 to 2)	2·2 (1·1 to 3·8)	2·1 (1 to 3·8)	2·4 (1·1 to 4·9)	3·2 (1·4 to 6·2)	2·4 (1·1 to 4·5)	1·9 (0·9 to 3·3)	79·6
Alcoholic cardiomyopathy	0 (0 to 0)	0 (0 to 0)	0 (0 to 0)	0 (0 to 0)	0 (0 to 0)	0 (0 to 0)	0·7 (0·4 to 1·2)	0·8 (0·5 to 1·3)	1 (0·6 to 1·6)	0·6 (0·4 to 1·1)	0·5 (0·3 to 1)	0·4 (0·3 to 0·8)	0·6 (0·2 to 2·8)	0·7 (0·2 to 3·2)	0·5 (0·2 to 2·3)	0·5 (0·2 to 2)	0·6 (0·2 to 1·9)	0·3 (0·1 to 0·7)	0·6 (0·2 to 1·2)	0·2 (0·1 to 0·6)	0·2 (0·1 to 0·4)	0·1 (0 to 0·2)	0·1 (0·1 to 0·2)	8·4
Other cardiomyopathy	8·2 (3·1 to 14·9)	3·6 (1·3 to 6·2)	8·6 (3·2 to 21)	12·3 (6·5 to 23·6)	7·2 (3·7 to 12·7)	16·3 (10 to 26)	22·1 (13·9 to 32·8)	36·8 (22·9 to 57·2)	56·5 (30·5 to 95)	78·5 (37·9 to 125)	83·2 (40·7 to 133)	78·1 (47·8 to 120)	81·7 (50·6 to 124)	88·4 (55·8 to 131)	105 (67·5 to 158)	104 (71 to 152)	86·6 (58·1 to 127)	71·4 (48·5 to 102)	59·7 (39·6 to 87·2)	44·5 (28 to 68·4)	40·9 (25·9 to 63·8)	24·2 (14·2 to 40·6)	15·1 (8·7 to 28)	1,130
Atrial fibrillation and flutter	0 (0 to 0)	0 (0 to 0)	0 (0 to 0)	0 (0 to 0)	0 (0 to 0)	0 (0 to 0)	0 (0 to 0)	0 (0 to 0)	0 (0 to 0)	1 (0·7 to 1·8)	3·1 (1·9 to 4·6)	19·6 (11·4 to 27·9)	38·4 (19 to 53·6)	45·8 (26·3 to 63·1)	68·7 (45·9 to 92·8)	89 (64·9 to 117)	112 (81·2 to 145)	154 (114 to 198)	167 (127 to 212)	219 (167 to 273)	241 (173 to 298)	223 (150 to 277)	161 (103 to 202)	1,540
Aortic aneurysm	--	--	--	--	--	--	0·9 (0·7 to 1·1)	3·8 (2·3 to 6·2)	4·4 (2·5 to 7·1)	6 (3·3 to 9·8)	11·3 (6·7 to 17·8)	27·5 (17·9 to 41·1)	29·8 (19·6 to 42·4)	36·2 (24·4 to 52·4)	40·2 (27·3 to 55·7)	45·5 (31·2 to 64·5)	52·7 (35·3 to 74·4)	41·7 (27·6 to 58·9)	48·5 (32·6 to 68·2)	43·5 (29·9 to 59·9)	38·6 (26·7 to 53·8)	20·1 (13·2 to 27·3)	12·9 (8·3 to 17·8)	NA
Endocarditis	0·2 (0 to 1·1)	0·4 (0·1 to 1·7)	3·5 (1·4 to 7·8)	2·5 (1·1 to 6·8)	2 (0·9 to 6)	9·6 (3·7 to 16·8)	22·4 (7·9 to 39·1)	51·4 (18·8 to 91·9)	67 (23·4 to 127)	27·3 (14·6 to 46)	56·8 (22·4 to 101)	54·8 (19·5 to 97·9)	57·4 (20 to 101)	52·7 (17·5 to 90·5)	41·6 (19·3 to 68·6)	51·5 (20·2 to 84·7)	25·5 (14·4 to 39·4)	23·2 (13·9 to 35·5)	22 (11·4 to 33·4)	27 (13·3 to 43·6)	19·7 (9·9 to 32)	13·3 (6·6 to 20·9)	12·1 (5 to 19·6)	644
Other cardiovascular and circulatory diseases	15 (9·5 to 23·2)	4·2 (2·4 to 7·5)	5·1 (2·1 to 12·9)	8 (4·2 to 14·6)	20·2 (13 to 29·7)	24·4 (15·8 to 35·3)	47·9 (32 to 69·1)	75·7 (50·8 to 110)	129 (84·8 to 189)	132 (86·9 to 191)	116 (75·1 to 171)	199 (134 to 279)	222 (154 to 308)	162 (114 to 225)	151 (109 to 205)	144 (107 to 188)	122 (82 to 172)	146 (107 to 197)	146 (111 to 186)	136 (100 to 181)	123 (86·3 to 165)	79·9 (54 to 106)	58·3 (36·8 to 82·2)	2,270
Chronic respiratory diseases	0 (0 to 0)	0 (0 to 0)	0·1 (0·1 to 0·1)	447 (248 to 779)	474 (278 to 790)	444 (276 to 681)	408 (260 to 631)	417 (275 to 600)	434 (301 to 604)	403 (283 to 551)	359 (262 to 483)	336 (252 to 451)	356 (267 to 495)	357 (262 to 544)	362 (267 to 583)	399 (288 to 662)	377 (269 to 613)	381 (280 to 582)	332 (242 to 511)	304 (224 to 450)	278 (209 to 373)	180 (134 to 240)	133 (96·3 to 174)	7,180
Chronic obstructive pulmonary disease	0 (0 to 0)	0 (0 to 0)	0·1 (0·1 to 0·1)	15·8 (7·8 to 26·6)	9·9 (3·5 to 18·3)	17·5 (7·6 to 29·5)	31·3 (17·5 to 47·9)	47·1 (27·9 to 70·6)	72 (47·9 to 103)	77 (48·7 to 108)	92·4 (64 to 126)	103 (72 to 140)	131 (91·4 to 188)	151 (107 to 253)	175 (123 to 300)	235 (162 to 424)	248 (172 to 435)	256 (180 to 408)	230 (163 to 380)	225 (163 to 343)	223 (165 to 302)	149 (111 to 201)	111 (80·5 to 147)	2,600
Pneumoconiosis	0 (0 to 0)	0 (0 to 0)	0 (0 to 0)	0 (0 to 0)	0 (0 to 0)	0 (0 to 0)	0·1 (0 to 0·2)	0·1 (0 to 0·1)	0·1 (0 to 0·1)	0·1 (0 to 0·1)	0·1 (0 to 0·1)	0·1 (0·1 to 0·2)	0·1 (0·1 to 0·2)	0·2 (0·1 to 0·2)	0·1 (0·1 to 0·2)	0·1 (0·1 to 0·2)	0·2 (0·1 to 0·3)	0·2 (0·1 to 0·3)	0·3 (0·2 to 0·5)	0·3 (0·2 to 0·4)	0·3 (0·2 to 0·4)	0·1 (0·1 to 0·2)	0·1 (0·1 to 0·2)	2·4
Silicosis	0 (0 to 0)	0 (0 to 0)	0 (0 to 0)	0 (0 to 0)	0 (0 to 0)	0 (0 to 0)	0 (0 to 0)	0 (0 to 0)	0 (0 to 0)	0 (0 to 0)	0 (0 to 0)	0 (0 to 0)	0 (0 to 0)	0 (0 to 0)	0 (0 to 0)	0 (0 to 0)	0 (0 to 0·1)	0 (0 to 0)	0 (0 to 0·1)	0 (0 to 0·1)	0 (0 to 0·1)	0 (0 to 0)	0 (0 to 0)	0·2
Asbestosis	0 (0 to 0)	0 (0 to 0)	0 (0 to 0)	0 (0 to 0)	0 (0 to 0)	0 (0 to 0)	0 (0 to 0)	0 (0 to 0·1)	0 (0 to 0·1)	0 (0 to 0·1)	0 (0 to 0)	0 (0 to 0·1)	0 (0 to 0·1)	0 (0 to 0·1)	0·1 (0 to 0·1)	0 (0 to 0·1)	0 (0 to 0·1)	0 (0 to 0·1)	0·1 (0 to 0·2)	0 (0 to 0·1)	0·1 (0 to 0·1)	0 (0 to 0)	0 (0 to 0)	0·6
Coal workers pneumoconiosis	0 (0 to 0)	0 (0 to 0)	0 (0 to 0)	0 (0 to 0)	0 (0 to 0)	0 (0 to 0)	0 (0 to 0)	0 (0 to 0)	0 (0 to 0)	0 (0 to 0)	0 (0 to 0)	0 (0 to 0)	0 (0 to 0)	0 (0 to 0)	0 (0 to 0)	0 (0 to 0)	0 (0 to 0)	0 (0 to 0)	0 (0 to 0)	0 (0 to 0)	0 (0 to 0)	0 (0 to 0)	0 (0 to 0)	0·1
Other pneumoconiosis	0 (0 to 0)	0 (0 to 0)	0 (0 to 0)	0 (0 to 0)	0 (0 to 0)	0 (0 to 0)	0 (0 to 0·1)	0 (0 to 0·1)	0 (0 to 0)	0 (0 to 0)	0 (0 to 0·1)	0·1 (0 to 0·1)	0·1 (0 to 0·1)	0·1 (0 to 0·2)	0·1 (0 to 0·1)	0·1 (0 to 0·2)	0·1 (0·1 to 0·2)	0·1 (0·1 to 0·2)	0·2 (0·1 to 0·3)	0·2 (0·1 to 0·3)	0·2 (0·1 to 0·3)	0·1 (0 to 0·2)	0·1 (0 to 0·1)	1·5
Asthma	0 (0 to 0)	0 (0 to 0)	0 (0 to 0)	394 (199 to 709)	437 (247 to 754)	386 (219 to 627)	362 (218 to 589)	341 (200 to 524)	309 (191 to 468)	279 (183 to 400)	229 (151 to 329)	189 (130 to 267)	176 (121 to 248)	161 (113 to 234)	136 (92·7 to 199)	126 (87·1 to 193)	100 (69·9 to 154)	90 (62·4 to 133)	71·4 (49·2 to 106)	58·3 (39 to 84·4)	39·7 (26·8 to 55·8)	21·7 (14·3 to 30·6)	15·4 (10·1 to 22·6)	3,920
Interstitial lung disease and pulmonary sarcoidosis	0 (0 to 0)	0 (0 to 0)	0 (0 to 0)	4·9 (1·1 to 14·6)	2 (0·9 to 4·4)	2·4 (1·2 to 4·6)	2·6 (1·3 to 5·2)	7·5 (3·2 to 14·4)	13·1 (5·4 to 24·8)	13·9 (7·2 to 24·2)	13·6 (7·3 to 22·3)	19·7 (8·3 to 34·6)	28·8 (12·8 to 50·1)	28·9 (14·4 to 47·4)	30·2 (15·9 to 50·7)	33 (16·6 to 55)	25·1 (14·2 to 42·7)	28·8 (15·8 to 46·7)	25·5 (12·6 to 42·4)	14·6 (8·5 to 24·9)	10 (5·4 to 18·5)	6·4 (3·5 to 11·7)	3·8 (2 to 7)	314
Other chronic respiratory diseases	0 (0 to 0)	0 (0 to 0)	0 (0 to 0)	32·3 (17·9 to 57·8)	25·2 (12·8 to 40·3)	38·6 (21·5 to 60·1)	12 (6·3 to 19·3)	20·9 (10·9 to 36·1)	39·2 (23·9 to 63·1)	33·3 (20·5 to 52·2)	24·7 (15·7 to 40·6)	24·4 (14·1 to 48·8)	21·1 (12·1 to 43·5)	15·9 (8·2 to 40·7)	20·6 (8·1 to 70·6)	4·6 (1·9 to 13·7)	3 (1·5 to 6·2)	5·6 (2·6 to 10·6)	5 (2 to 9·8)	5·7 (2 to 11·3)	4·6 (1·4 to 9·5)	2·8 (0·8 to 6·1)	2·7 (0·7 to 5·9)	343
Digestive diseases	39·8 (26·3 to 57·5)	29·3 (16·8 to 48·6)	51·8 (27 to 93·1)	123 (83·5 to 172)	131 (96·9 to 175)	150 (116 to 197)	234 (176 to 307)	440 (321 to 598)	579 (415 to 793)	618 (440 to 859)	561 (404 to 769)	584 (427 to 769)	678 (517 to 878)	689 (516 to 895)	725 (549 to 953)	677 (514 to 860)	548 (426 to 684)	507 (389 to 643)	442 (334 to 564)	370 (282 to 469)	268 (210 to 330)	175 (132 to 215)	98·4 (71·9 to 121)	8,720
Cirrhosis and other chronic liver diseases	0 (0 to 0)	0 (0 to 0)	0·1 (0·1 to 0·2)	15·3 (8·5 to 25·1)	19·8 (11·8 to 32·5)	33·8 (19·1 to 53·5)	59·9 (37·7 to 91·7)	90·4 (56·3 to 139)	107 (61·5 to 171)	120 (74·6 to 175)	94·4 (59·3 to 138)	128 (86·5 to 184)	153 (103 to 216)	155 (106 to 220)	191 (132 to 271)	166 (116 to 223)	136 (95·4 to 183)	128 (90·1 to 177)	101 (69·4 to 142)	72·3 (49·6 to 99·6)	47·6 (34 to 62·6)	26·1 (17·9 to 34·5)	13·3 (8·8 to 17·7)	1,860
Cirrhosis and other chronic liver diseases due to hepatitis B	0 (0 to 0)	0 (0 to 0)	0 (0 to 0)	0 (0 to 0)	0 (0 to 0·1)	0·1 (0 to 0·3)	0·3 (0·1 to 0·8)	1·8 (0·6 to 3·9)	4 (1·2 to 9)	6·8 (3·1 to 12·6)	6·7 (2·8 to 12·9)	9·6 (4·5 to 17·5)	11·1 (5 to 21·1)	10·4 (4·8 to 20·5)	10·9 (4·9 to 20·5)	8·2 (3·5 to 16·1)	5·9 (2·7 to 11)	5·3 (2·4 to 10·1)	4·2 (1·9 to 8)	3 (1·4 to 5·5)	2 (1 to 3·3)	1·1 (0·5 to 2)	0·5 (0·2 to 1·1)	91·8
Cirrhosis and other chronic liver diseases due to hepatitis C	0 (0 to 0)	0 (0 to 0)	0 (0 to 0)	0·1 (0 to 0·1)	0·2 (0·1 to 0·4)	0·6 (0·2 to 1·2)	1·6 (0·6 to 3·5)	8·4 (3·3 to 16·4)	18·9 (7·8 to 39·4)	26·9 (13·5 to 46)	22·8 (10·9 to 40·9)	30·6 (15·6 to 51·6)	34·2 (17·1 to 58·7)	32·1 (16·4 to 56·5)	36·2 (19·1 to 62·7)	28·9 (12·6 to 53)	22·3 (11·6 to 38·6)	20 (10·8 to 33·6)	14·8 (7·3 to 26·8)	10·6 (4·9 to 19·1)	7·2 (3·6 to 11·7)	4·1 (2 to 7·1)	2·1 (0·9 to 4)	322
Cirrhosis and other chronic liver diseases due to alcohol use	0 (0 to 0)	0 (0 to 0)	0 (0 to 0)	0 (0 to 0)	0 (0 to 0)	0 (0 to 0)	0·2 (0 to 0·5)	4·1 (1·5 to 8·6)	11 (4·2 to 23·2)	23 (10·8 to 39·4)	25·8 (12·3 to 44·8)	40·5 (21·8 to 67·3)	51·9 (26·5 to 84·5)	55·8 (28·9 to 88·7)	72·2 (39·4 to 115)	61·8 (35·1 to 98)	46·6 (25·8 to 74·2)	39·3 (21·8 to 62·7)	26·5 (14·2 to 42·4)	17 (8·9 to 29·3)	10·7 (5·9 to 17·1)	5·6 (3 to 9·6)	2·7 (1·2 to 5)	495
Cirrhosis and other chronic liver diseases due to NAFLD	0 (0 to 0)	0 (0 to 0)	0 (0 to 0)	0 (0 to 0)	0 (0 to 0)	0 (0 to 0)	2·2 (0·9 to 4·6)	8·6 (3·4 to 16·7)	17·6 (6·6 to 34·3)	23·2 (11 to 41·1)	18·4 (8·2 to 35·5)	26 (12·7 to 47·3)	33·1 (15·7 to 59·3)	34·2 (16·2 to 63·6)	40·9 (19·8 to 72·4)	36·2 (16·5 to 64·6)	32 (17·3 to 51)	32·4 (18·6 to 53·5)	27·1 (13·5 to 45·9)	20·2 (10·2 to 32·8)	13·5 (7·8 to 20·8)	7·5 (4·1 to 11·7)	3·8 (1·8 to 6·4)	377
Cirrhosis and other chronic liver diseases due to other causes	0 (0 to 0)	0 (0 to 0)	0·1 (0·1 to 0·2)	15·3 (8·4 to 25)	19·6 (11·6 to 32·1)	33·1 (18·7 to 52·3)	55·6 (35 to 84·8)	67·5 (39·7 to 104)	55·4 (28·7 to 99·1)	40·2 (20·7 to 71·8)	20·6 (9·4 to 38·5)	21·1 (9·9 to 37·3)	22·6 (10·6 to 42·1)	22·8 (10·2 to 42·2)	31·1 (14·6 to 57·4)	30·8 (15·3 to 54·7)	28·7 (14·1 to 49·3)	31·4 (16·2 to 52)	28·3 (15 to 48·2)	21·6 (11·8 to 35·8)	14·3 (8·7 to 21·5)	7·9 (4·3 to 12)	4 (1·9 to 6·5)	572
Upper digestive system diseases	0 (0 to 0)	0 (0 to 0)	0 (0 to 0)	10·2 (5·2 to 17·6)	14·2 (6·7 to 25·5)	23·4 (14 to 37·4)	75 (41·3 to 126)	189 (108 to 329)	262 (145 to 446)	285 (161 to 493)	268 (150 to 445)	238 (136 to 387)	267 (164 to 427)	273 (171 to 424)	273 (176 to 419)	241 (159 to 376)	189 (130 to 282)	166 (118 to 233)	140 (100 to 192)	107 (74·7 to 146)	73·9 (53·8 to 99·6)	51·7 (37·4 to 69·7)	30·4 (21·3 to 39·9)	3,180
Peptic ulcer disease	0 (0 to 0)	0 (0 to 0)	0 (0 to 0)	0·4 (0·1 to 0·9)	2·1 (1·3 to 3·5)	4·3 (2·8 to 6·5)	5·9 (4 to 8·5)	17·8 (11·8 to 25·9)	14 (8·3 to 22·5)	27·1 (17·7 to 40·4)	29·5 (19·7 to 42)	25·8 (17·4 to 36·4)	44·2 (30·3 to 61·9)	56·3 (39·9 to 79)	65·4 (45·9 to 90·8)	66·2 (45·1 to 93)	56·3 (39·9 to 76·9)	61·7 (43·4 to 85·9)	65·9 (44 to 94·3)	54·2 (35·1 to 77·8)	35·1 (23·9 to 47·6)	26·4 (17·1 to 37·1)	16·9 (10·7 to 23·2)	676
Gastritis and duodenitis	0 (0 to 0)	0 (0 to 0)	0 (0 to 0)	9·8 (4·9 to 17·4)	11·8 (4·5 to 23·6)	11·2 (4·3 to 22·1)	15·2 (5·6 to 30·9)	20·1 (8·1 to 37·2)	33·7 (16·7 to 55·5)	37·8 (18·8 to 64·1)	35·3 (15·6 to 64·4)	29·5 (14·9 to 56)	44·5 (22·1 to 83·7)	51·9 (26·5 to 89·8)	58·8 (33·6 to 100)	46·9 (25·1 to 79·5)	37·5 (22·1 to 59·2)	31·5 (19·3 to 47·9)	23·2 (14·7 to 34·9)	14·8 (9·1 to 22)	13 (8·4 to 18·7)	11·5 (6·8 to 16·4)	5·7 (3·2 to 8·4)	544
Gastroesophageal reflux disease	0 (0 to 0)	0 (0 to 0)	0 (0 to 0)	0 (0 to 0)	0·2 (0·1 to 0·6)	7·9 (3·4 to 15·2)	53·9 (25·4 to 98·4)	151 (74·5 to 290)	214 (103 to 389)	220 (104 to 423)	203 (97·2 to 376)	182 (85·8 to 329)	178 (88·2 to 312)	165 (80·3 to 296)	149 (69·5 to 279)	128 (62·7 to 234)	95·1 (47·7 to 173)	72·5 (36·7 to 129)	51·2 (25·4 to 94·1)	38·2 (18·7 to 68)	25·8 (12·9 to 46·2)	13·8 (6·9 to 24·5)	7·8 (3·7 to 14·1)	1,960
Appendicitis	0 (0 to 0)	0 (0 to 0)	0 (0 to 0)	4·2 (2·1 to 8)	17·2 (10·6 to 27)	21·3 (11·6 to 34)	18·1 (9·3 to 30·3)	21·4 (10·5 to 35·9)	14·4 (5·9 to 26)	10·5 (4·5 to 19)	16·8 (8·8 to 26·7)	12·2 (7·2 to 18·8)	10·8 (6·3 to 17·6)	14·6 (8·2 to 24·1)	16·2 (8·8 to 26·4)	9·6 (5·8 to 14·5)	8·6 (5·3 to 13)	6·4 (3·8 to 10)	4·7 (2·7 to 7·8)	3·7 (2·1 to 6·1)	3·3 (1·9 to 5·2)	2·6 (1·5 to 4·1)	1·2 (0·7 to 1·9)	218
Paralytic ileus and intestinal obstruction	18·9 (10·3 to 30·3)	24·2 (13·6 to 39·9)	46·9 (24 to 85·7)	30·7 (16·7 to 50·2)	17·5 (10 to 26·9)	20 (9·4 to 34·3)	14·2 (6·3 to 25·9)	18·1 (7·4 to 34·2)	20·3 (10·6 to 35·4)	11 (5·1 to 21·5)	16·7 (8·7 to 30·7)	18·8 (9·6 to 34·2)	23·9 (12·3 to 44·3)	37·1 (22 to 59·1)	37·9 (22·9 to 61·8)	52 (33 to 79)	47 (28·7 to 76)	53·1 (31·6 to 83·3)	55·2 (33·5 to 88·8)	55·3 (33·8 to 90·8)	39·9 (22·9 to 69·7)	31·3 (19 to 54·9)	20·4 (11·4 to 33·7)	711
Inguinal, femoral, and abdominal hernia	20·8 (11·3 to 35·5)	5 (1·7 to 12·1)	4·8 (2·3 to 8·8)	29·3 (15·4 to 51·9)	39 (19·7 to 67·2)	25·9 (11·3 to 46·9)	19·9 (8·3 to 35·8)	22·3 (10 to 40·8)	25·6 (11·6 to 45·2)	29·2 (14·1 to 51·3)	29·8 (14 to 52)	34 (19·5 to 54)	37 (21·3 to 58·7)	34·6 (20·6 to 53·5)	31·9 (19·4 to 49·7)	27·9 (17·4 to 41·3)	15·2 (9·8 to 23·3)	11·3 (7·1 to 16·7)	8·6 (5·5 to 13·3)	6·2 (4 to 10)	5 (3·4 to 8·3)	3·8 (2·4 to 6·1)	2 (1·2 to 3·3)	469
Inflammatory bowel disease	0 (0 to 0)	0 (0 to 0)	0 (0 to 0)	0·6 (0·3 to 2·4)	0·4 (0·2 to 1·2)	0·4 (0·3 to 0·7)	2 (1·3 to 3)	10·2 (5·6 to 15·4)	16·1 (9·7 to 25·6)	14·9 (10·1 to 21·2)	10 (5·5 to 15·9)	17·3 (11·1 to 25·5)	14·4 (9·5 to 21)	9·9 (5·8 to 15·3)	10·7 (6·5 to 16·9)	9·2 (5·6 to 15)	8 (5·2 to 11·7)	6·2 (4·1 to 9·2)	5·6 (3·8 to 8)	3·2 (2·2 to 4·6)	2·5 (1·6 to 3·6)	2·1 (1·4 to 2·9)	0·5 (0·4 to 0·8)	144
Vascular intestinal disorders	0 (0 to 0)	0 (0 to 0)	0 (0 to 0)	0·2 (0·1 to 0·4)	0·3 (0·2 to 0·5)	0·2 (0·1 to 0·4)	0·3 (0·1 to 0·4)	0·7 (0·4 to 1·2)	4·1 (2·1 to 7·2)	1·5 (0·8 to 2·4)	3·8 (2 to 6·3)	5·2 (3 to 8·4)	11·5 (6·8 to 18·2)	9·2 (5·6 to 14·6)	10·4 (6·4 to 15·6)	17 (10·9 to 25·3)	15·2 (9·9 to 22·4)	20·1 (12·7 to 30·2)	20·9 (13·3 to 30·6)	17·6 (11 to 25·6)	13·8 (8·6 to 20·1)	7 (4·5 to 10)	3·2 (2 to 4·7)	162
Gallbladder and biliary diseases	0 (0 to 0)	0 (0 to 0)	0 (0 to 0)	3·3 (1·8 to 5·8)	8·8 (2·9 to 17·8)	14·9 (5·4 to 29·5)	26·2 (12·7 to 47·7)	47·5 (26·2 to 78·3)	71·1 (41·3 to 113)	82·9 (51·2 to 127)	86·3 (53 to 133)	90·1 (55·8 to 134)	104 (67 to 154)	96·9 (60·6 to 145)	91·5 (59·5 to 134)	84·1 (57·1 to 118)	67·2 (45·2 to 94·3)	51·7 (35·7 to 72·7)	41·2 (27·6 to 57·3)	32·5 (22·2 to 45·5)	23·5 (16·3 to 32·6)	13·9 (9·5 to 19·3)	7·4 (5 to 10·5)	1,050
Pancreatitis	0 (0 to 0)	0 (0 to 0)	0 (0 to 0)	0·3 (0·1 to 1·1)	0·5 (0·3 to 0·8)	0·8 (0·5 to 1·3)	4 (2·2 to 6·3)	18·2 (9·4 to 29·3)	23·2 (11·2 to 39·1)	27·3 (12·1 to 46·9)	8·3 (5 to 12·9)	17·3 (10·1 to 26·9)	17·9 (10·6 to 27·9)	14·8 (8·9 to 22·9)	19·2 (12·1 to 28·9)	18·3 (11·6 to 26·4)	14·4 (9·7 to 21)	12·1 (7·8 to 17·8)	11·4 (7·7 to 16·2)	11·6 (7 to 17·2)	6·7 (4·4 to 9·5)	3·1 (2 to 4·3)	1·6 (1·1 to 2·3)	231
Other digestive diseases	0 (0 to 0)	0 (0 to 0)	0 (0 to 0)	29 (15·9 to 46)	13·6 (8·3 to 20·9)	9·8 (6 to 15·3)	14 (5·6 to 23·1)	21·6 (7·3 to 37·7)	35·5 (11·9 to 65·1)	36·2 (11·9 to 62·1)	27·4 (9·3 to 48·8)	24·1 (12 to 39·3)	38·9 (15·7 to 65·2)	43·9 (20·2 to 72·2)	42·9 (22·4 to 69)	51·6 (28 to 80)	47·5 (26·2 to 73·1)	52·2 (26·7 to 81·7)	53 (25·8 to 83·3)	60·4 (27·8 to 95)	51·6 (25·8 to 75·7)	33·7 (16 to 49·4)	18·4 (9·1 to 27)	705
Neurological disorders	0·4 (0·1 to 0·9)	1·4 (0·5 to 2·8)	51·2 (23·5 to 90·8)	167 (73·4 to 315)	361 (147 to 662)	1,010 (270 to 2,160)	1,380 (395 to 2,940)	1,620 (491 to 3,320)	1,650 (524 to 3,350)	1,520 (496 to 2,990)	1,360 (460 to 2,710)	1,230 (439 to 2,430)	1,160 (477 to 2,260)	1,020 (462 to 1,870)	877 (440 to 1,530)	834 (476 to 1,360)	754 (459 to 1,240)	834 (511 to 1,450)	956 (576 to 1,790)	1,380 (753 to 2,780)	1,500 (752 to 3,070)	1,260 (586 to 2,560)	1,110 (488 to 2,250)	22,000
Alzheimer's disease and other dementias	0 (0 to 0)	0 (0 to 0)	0 (0 to 0)	0 (0 to 0)	0 (0 to 0)	0 (0 to 0)	0 (0 to 0)	0 (0 to 0)	0 (0 to 0)	0 (0 to 0)	0 (0 to 0)	6·2 (1·4 to 19·4)	34·4 (10·1 to 93·7)	66 (23·5 to 170)	113 (43 to 284)	195 (78·4 to 468)	288 (114 to 695)	443 (191 to 1,030)	608 (260 to 1,400)	1,060 (457 to 2,470)	1,270 (521 to 2,830)	1,140 (466 to 2,440)	1,050 (422 to 2,200)	6,280
Parkinson's disease	0 (0 to 0)	0 (0 to 0)	0 (0 to 0)	0 (0 to 0)	0 (0 to 0)	0 (0 to 0)	0 (0 to 0)	0·4 (0·3 to 0·6)	0·6 (0·3 to 1·1)	1·3 (0·7 to 2·3)	2·2 (1·3 to 3·6)	6·9 (5 to 9·5)	15·3 (11·1 to 21·4)	21·1 (15·4 to 28·9)	31 (21·7 to 42)	57·5 (44·2 to 72·6)	77·2 (60·1 to 97·5)	124 (97·6 to 151)	174 (142 to 215)	192 (151 to 239)	152 (118 to 190)	76·3 (56·9 to 94·4)	37·3 (27·4 to 45·7)	969
Idiopathic epilepsy	0·3 (0·1 to 0·8)	1 (0·2 to 2·4)	19·9 (5·4 to 43·4)	111 (41·9 to 223)	168 (58·7 to 343)	211 (77·5 to 421)	252 (103 to 494)	300 (136 to 547)	287 (138 to 522)	256 (125 to 454)	205 (101 to 354)	169 (82·1 to 293)	164 (84·9 to 280)	134 (66·4 to 229)	110 (47·7 to 208)	104 (44·8 to 195)	83·5 (34·9 to 155)	73·8 (30·9 to 135)	59·1 (25·2 to 107)	49·2 (20·8 to 90)	34·5 (13·4 to 64·6)	19·3 (7·7 to 37)	11·3 (4·6 to 21·7)	2,820
Multiple sclerosis	0 (0 to 0)	0 (0 to 0)	0 (0 to 0)	0 (0 to 0)	0 (0 to 0)	0·2 (0·1 to 0·3)	1 (0·6 to 1·5)	5 (3 to 7·6)	8·1 (5·3 to 12·4)	11·3 (7·4 to 16)	17·9 (11 to 25·8)	16·5 (10·8 to 22·5)	16·4 (11·4 to 23·2)	16·3 (10·3 to 22·1)	12·2 (8·8 to 17)	9·7 (6·9 to 13·5)	5·8 (4 to 7·6)	3·7 (2·7 to 5·3)	2·2 (1·6 to 2·9)	1·6 (1 to 2·3)	1·1 (0·6 to 1·7)	0·6 (0·3 to 0·8)	0·3 (0·2 to 0·4)	130
Motor neuron disease	0 (0 to 0·1)	0·4 (0·2 to 0·6)	7·6 (2·9 to 16)	12 (6·7 to 19·6)	3·2 (1·8 to 5·3)	0·6 (0·4 to 0·8)	0·6 (0·4 to 0·9)	0·7 (0·5 to 1·1)	0·8 (0·6 to 1·2)	0·8 (0·6 to 1·2)	1 (0·7 to 1·4)	6·7 (4·2 to 10·3)	12·5 (8·1 to 18·7)	19·8 (12·3 to 29·5)	14 (9·1 to 20·3)	20 (13·6 to 28·5)	14·2 (9·5 to 20·1)	10·9 (7·1 to 15·8)	9·2 (6·3 to 13·1)	2·7 (1·8 to 4)	0·1 (0·1 to 0·2)	0·1 (0 to 0·1)	0 (0 to 0·1)	138
Headache disorders	0 (0 to 0)	0 (0 to 0)	0 (0 to 0)	0 (0 to 0)	136 (2·1 to 356)	714 (32·1 to 1,820)	1,060 (118 to 2,650)	1,240 (164 to 2,920)	1,270 (186 to 2,990)	1,170 (196 to 2,640)	1,070 (197 to 2,420)	969 (206 to 2,130)	865 (202 to 1,950)	706 (176 to 1,530)	549 (147 to 1,160)	405 (119 to 859)	257 (82·7 to 531)	157 (52·1 to 333)	90·2 (27·9 to 185)	57·3 (18·7 to 119)	35·2 (12 to 71·5)	16 (5·3 to 33·1)	6·6 (1·6 to 15·2)	10,800
Migraine	0 (0 to 0)	0 (0 to 0)	0 (0 to 0)	0 (0 to 0)	126 (1·5 to 351)	676 (20·7 to 1,790)	993 (79·1 to 2,600)	1,160 (112 to 2,850)	1,180 (125 to 2,800)	1,080 (130 to 2,570)	984 (144 to 2,300)	887 (152 to 2,050)	786 (145 to 1,800)	634 (123 to 1,410)	490 (106 to 1,090)	356 (85 to 788)	220 (58·3 to 490)	132 (38·6 to 291)	75 (20·5 to 162)	47·8 (13 to 106)	29·1 (8·4 to 64·5)	13 (3·6 to 27·7)	5·1 (1·1 to 11·8)	9,870
Tension-type headache	0 (0 to 0)	0 (0 to 0)	0 (0 to 0)	0 (0 to 0)	9·8 (0·3 to 62)	38·4 (2 to 246)	65·3 (11·3 to 284)	86·4 (18·6 to 349)	93·8 (21·8 to 346)	89 (20·7 to 302)	87·8 (23·2 to 273)	81·3 (24·4 to 227)	79·1 (25·9 to 208)	72·2 (22·5 to 196)	58·4 (18·6 to 149)	49·6 (15 to 137)	37·2 (10·7 to 113)	25·6 (6·5 to 87·7)	15·2 (3·5 to 52·3)	9·5 (2·2 to 35)	6·1 (1·4 to 21·8)	3 (0·6 to 10·7)	1·5 (0·1 to 7·3)	909
Other neurological disorders	0 (0 to 0)	0 (0 to 0)	23·7 (11·4 to 40·5)	44·3 (19·4 to 82·4)	53·9 (18·3 to 112)	82·9 (28·4 to 171)	72 (22·7 to 156)	71·4 (34·9 to 126)	79·4 (45·1 to 124)	78·9 (47·2 to 122)	60·3 (36·7 to 91·2)	54·5 (33·9 to 83·1)	57·1 (36·5 to 83·1)	58·8 (39·4 to 80·9)	48 (33 to 65·6)	42·7 (31 to 58·5)	28·6 (20·9 to 37·9)	21·6 (15·8 to 28)	12·9 (9·5 to 16·8)	9·2 (6·8 to 12·4)	7·3 (5·1 to 10·7)	4·1 (2·9 to 5·6)	2·2 (1·6 to 3)	914
Mental disorders	0·2 (0·1 to 0·3)	0·5 (0·3 to 0·8)	8·1 (4·5 to 12·9)	53·6 (32·7 to 81·3)	380 (245 to 566)	1,380 (907 to 2,000)	2,400 (1,650 to 3,350)	3,040 (2,040 to 4,220)	3,160 (2,190 to 4,360)	2,880 (1,980 to 3,950)	2,630 (1,820 to 3,540)	2,290 (1,610 to 3,140)	2,150 (1,520 to 2,920)	1,880 (1,360 to 2,530)	1,630 (1,150 to 2,210)	1,300 (902 to 1,770)	889 (612 to 1,190)	626 (440 to 826)	407 (282 to 552)	288 (197 to 388)	198 (137 to 265)	110 (73·1 to 152)	64·3 (40·8 to 94·3)	27,800
Schizophrenia	0 (0 to 0)	0 (0 to 0)	0 (0 to 0)	0 (0 to 0)	0 (0 to 0)	6 (2·5 to 11·8)	49·3 (17·1 to 97·3)	189 (100 to 314)	330 (184 to 513)	372 (227 to 551)	358 (219 to 535)	308 (194 to 428)	278 (182 to 382)	228 (150 to 313)	179 (119 to 249)	125 (80·9 to 181)	76·8 (47·4 to 109)	46·7 (28·6 to 68·6)	25·3 (15·1 to 39·3)	14 (7·8 to 21·7)	6·9 (3·3 to 11·1)	2·6 (1·2 to 4·5)	1 (0·3 to 2)	2,600
Depressive disorders	0 (0 to 0)	0 (0 to 0)	0 (0 to 0)	0·2 (0·1 to 0·5)	28·9 (9·3 to 61·4)	328 (168 to 590)	855 (485 to 1,380)	1,140 (650 to 1,810)	1,110 (637 to 1,740)	998 (587 to 1,590)	956 (581 to 1,460)	876 (501 to 1,380)	870 (554 to 1,300)	818 (524 to 1,220)	753 (458 to 1,130)	635 (378 to 965)	444 (283 to 647)	321 (203 to 453)	214 (129 to 322)	158 (93 to 241)	117 (74·6 to 171)	69·5 (41·8 to 107)	43·2 (23·3 to 71·7)	10,700
Major depressive disorder	0 (0 to 0)	0 (0 to 0)	0 (0 to 0)	0·2 (0·1 to 0·4)	26 (7·3 to 57·3)	305 (144 to 566)	783 (429 to 1,300)	1,010 (548 to 1,650)	938 (517 to 1,490)	826 (453 to 1,420)	792 (456 to 1,240)	728 (396 to 1,180)	730 (434 to 1,130)	693 (424 to 1,040)	646 (378 to 1,000)	549 (309 to 855)	384 (236 to 567)	279 (173 to 408)	187 (109 to 294)	140 (79·6 to 218)	105 (64·6 to 158)	62·9 (36·2 to 101)	39·2 (20·3 to 66·9)	9,220
Dysthymia	0 (0 to 0)	0 (0 to 0)	0 (0 to 0)	0 (0 to 0·1)	2·9 (0 to 7·7)	23 (8·3 to 45·5)	71·7 (33·9 to 128)	137 (69·4 to 238)	170 (89·5 to 296)	172 (97·1 to 286)	164 (90·7 to 277)	148 (78·3 to 253)	140 (78·3 to 245)	125 (73·1 to 207)	107 (62·1 to 173)	86·8 (45·9 to 146)	60 (31·6 to 102)	41·9 (23·2 to 68·8)	26·7 (15·6 to 44·1)	18·2 (9·5 to 30·6)	12 (6·4 to 20·4)	6·7 (3·6 to 10·9)	4 (2 to 7)	1,520
Bipolar disorder	0 (0 to 0)	0 (0 to 0)	0 (0 to 0)	0 (0 to 0)	0 (0 to 0)	88·2 (36·1 to 161)	344 (159 to 603)	469 (238 to 789)	457 (241 to 760)	385 (205 to 622)	330 (185 to 518)	281 (153 to 460)	258 (147 to 436)	219 (123 to 364)	177 (100 to 292)	132 (72·5 to 211)	82·7 (44·7 to 135)	51·4 (26·7 to 84·1)	28·3 (14·2 to 46·9)	15·6 (8 to 26)	8·3 (4 to 14·1)	3·7 (1·7 to 6·4)	1·7 (0·8 to 3)	3,330
Anxiety disorders	0 (0 to 0)	0 (0 to 0)	0 (0 to 0)	10·3 (2·8 to 22·5)	170 (80·6 to 296)	477 (259 to 789)	687 (414 to 1,060)	822 (470 to 1,350)	834 (462 to 1,370)	756 (433 to 1,200)	679 (405 to 1,060)	588 (334 to 955)	541 (311 to 890)	462 (275 to 727)	384 (231 to 582)	299 (175 to 463)	207 (121 to 328)	149 (87·3 to 239)	98·3 (57·9 to 156)	68·2 (40·7 to 106)	42·8 (24·7 to 67·8)	20·8 (11·9 to 32·5)	10·2 (5·4 to 16·6)	7,300
Eating disorders	0 (0 to 0)	0 (0 to 0)	0 (0 to 0)	0 (0 to 0)	1·9 (0·7 to 4)	32·1 (14·9 to 56·3)	128 (64·5 to 229)	224 (113 to 380)	205 (113 to 339)	143 (76·6 to 235)	93 (50·5 to 153)	49·7 (26·6 to 84·6)	26·5 (12·7 to 46·4)	0 (0 to 0)	0 (0 to 0)	0 (0 to 0)	0 (0 to 0)	0 (0 to 0)	0 (0 to 0)	0 (0 to 0)	0 (0 to 0)	0 (0 to 0)	0 (0 to 0)	903
Anorexia nervosa	0 (0 to 0)	0 (0 to 0)	0 (0 to 0)	0 (0 to 0)	1·8 (0·6 to 3·8)	17·4 (6·6 to 32·5)	51 (23·2 to 93·8)	56·4 (27 to 103)	42·8 (20·4 to 76·3)	24·6 (10·8 to 45·6)	15·8 (6·2 to 30·3)	10·5 (3·9 to 20·4)	7·5 (2·5 to 15·1)	0 (0 to 0)	0 (0 to 0)	0 (0 to 0)	0 (0 to 0)	0 (0 to 0)	0 (0 to 0)	0 (0 to 0)	0 (0 to 0)	0 (0 to 0)	0 (0 to 0)	228
Bulimia nervosa	0 (0 to 0)	0 (0 to 0)	0 (0 to 0)	0 (0 to 0)	0·2 (0·1 to 0·4)	14·7 (3·8 to 30·7)	76·9 (31·4 to 156)	168 (75·1 to 309)	162 (78·5 to 283)	119 (59 to 203)	77·2 (38·4 to 134)	39·2 (18·2 to 70·3)	19 (7·7 to 36·1)	0 (0 to 0)	0 (0 to 0)	0 (0 to 0)	0 (0 to 0)	0 (0 to 0)	0 (0 to 0)	0 (0 to 0)	0 (0 to 0)	0 (0 to 0)	0 (0 to 0)	675
Autism spectrum disorders	0·1 (0·1 to 0·2)	0·3 (0·2 to 0·5)	5·3 (2·9 to 8·6)	23·7 (13·4 to 37·1)	31·9 (17·5 to 52·4)	33·6 (19·1 to 51·7)	34·6 (20·1 to 54·1)	37·3 (20·3 to 62·2)	34·8 (19·8 to 54·4)	29·9 (16·6 to 46·4)	25·6 (14·1 to 41)	21 (11·9 to 32·9)	19 (10·9 to 30·2)	16·4 (8·7 to 25·5)	13·9 (7·9 to 22·9)	11·1 (6 to 17·3)	7·9 (4·2 to 12·5)	5·8 (3·2 to 9·2)	4 (2·1 to 6·5)	2·8 (1·5 to 4·6)	1·9 (1 to 3)	1 (0·6 to 1·6)	0·6 (0·3 to 0·9)	362
Attention-deficit/hyperactivity disorder	0 (0 to 0)	0 (0 to 0)	0 (0 to 0)	3·2 (1·4 to 6·3)	38·1 (19·8 to 69·1)	55·6 (30 to 98·8)	48·5 (25·9 to 87·5)	38·7 (20·4 to 70·5)	28·6 (14·8 to 51·3)	20 (9·4 to 35)	14·4 (7 to 25·8)	10·1 (4·6 to 18·3)	7·7 (3·6 to 14·3)	5·6 (2·4 to 10·4)	3·5 (1·3 to 6·7)	1·8 (0·7 to 3·8)	0·7 (0·2 to 1·6)	0·3 (0 to 0·7)	0·1 (0 to 0·2)	0 (0 to 0)	0 (0 to 0)	0 (0 to 0)	0 (0 to 0)	277
Conduct disorder	0 (0 to 0)	0 (0 to 0)	0 (0 to 0)	0 (0 to 0)	85·6 (40·3 to 158)	333 (168 to 583)	200 (99·1 to 351)	0 (0 to 0)	0 (0 to 0)	0 (0 to 0)	0 (0 to 0)	0 (0 to 0)	0 (0 to 0)	0 (0 to 0)	0 (0 to 0)	0 (0 to 0)	0 (0 to 0)	0 (0 to 0)	0 (0 to 0)	0 (0 to 0)	0 (0 to 0)	0 (0 to 0)	0 (0 to 0)	619
Idiopathic developmental intellectual disability	0 (0 to 0·1)	0·2 (0 to 0·3)	2·8 (0·5 to 5·5)	16·2 (4·9 to 30·5)	24·6 (9·7 to 44·9)	25·2 (9·1 to 46·4)	25·6 (8·8 to 47·9)	26·8 (8·9 to 50·4)	25·1 (8·3 to 47)	21·2 (7·6 to 39·3)	18 (6·3 to 33·8)	14·7 (5·1 to 28·2)	13·1 (4·3 to 24·4)	11 (3·5 to 21)	8·9 (2·8 to 17·1)	6·8 (2·2 to 12·7)	4·4 (1·3 to 8·5)	2·8 (0·9 to 5·4)	1·7 (0·5 to 3·4)	1·3 (0·5 to 2·5)	0·8 (0·3 to 1·6)	0·4 (0·1 to 0·7)	0·2 (0 to 0·3)	252
Other mental disorders	0 (0 to 0)	0 (0 to 0)	0 (0 to 0)	0 (0 to 0)	0 (0 to 0)	4·6 (2·2 to 7·9)	32·4 (13·2 to 61·4)	90 (43·6 to 153)	136 (73·4 to 223)	153 (91·4 to 245)	154 (91·4 to 242)	139 (83·1 to 218)	134 (81·3 to 210)	121 (73·2 to 186)	107 (64·9 to 163)	89 (53 to 136)	65·2 (40·8 to 101)	49·9 (30·6 to 76·1)	35·6 (22·5 to 52·9)	27·6 (17·6 to 40·7)	20 (12·9 to 28·8)	11·8 (7·4 to 17·5)	7·4 (4·7 to 10·9)	1,380
Substance use disorders	0 (0 to 0)	0 (0 to 0)	0 (0 to 0)	0·1 (0 to 0·1)	0·1 (0·1 to 0·2)	14·8 (7·7 to 25·3)	245 (152 to 370)	486 (310 to 691)	488 (316 to 693)	378 (235 to 561)	276 (178 to 406)	195 (125 to 288)	158 (104 to 225)	120 (72·4 to 178)	93·2 (59·9 to 135)	66·2 (41·6 to 97·7)	43 (27·3 to 64·2)	30·3 (19·5 to 43·5)	21·2 (14·4 to 30·2)	15·9 (10·7 to 23·3)	11·7 (7·7 to 16·8)	7·5 (5 to 11)	6·3 (3·8 to 9·9)	2,660
Alcohol use disorders	0 (0 to 0)	0 (0 to 0)	0 (0 to 0)	0·1 (0 to 0·1)	0·1 (0·1 to 0·2)	6·1 (2·2 to 13·1)	68·4 (32·9 to 125)	196 (95·1 to 355)	222 (117 to 371)	194 (97·9 to 331)	151 (85·8 to 245)	108 (55·2 to 184)	85·7 (48·3 to 135)	62·5 (35·2 to 105)	47·9 (27 to 75·5)	33·8 (19 to 55·5)	21·1 (11·5 to 35)	15·3 (8·4 to 23·9)	10·6 (5·8 to 16·7)	8·3 (4·4 to 13·7)	6·7 (3·8 to 10·7)	4·8 (2·8 to 7·9)	4·2 (2·2 to 7·5)	1,250
Drug use disorders	0 (0 to 0)	0 (0 to 0)	0 (0 to 0)	0 (0 to 0)	0 (0 to 0)	8·7 (3·7 to 16·7)	177 (105 to 279)	290 (181 to 432)	266 (166 to 387)	184 (110 to 280)	125 (72·9 to 191)	87·1 (51 to 135)	72·3 (44·5 to 110)	57 (31 to 88·6)	45·3 (24·5 to 72·8)	32·4 (17 to 52·2)	21·9 (11·2 to 34·5)	15·1 (7·9 to 24·3)	10·6 (6 to 16·3)	7·6 (4·5 to 11·4)	5 (2·9 to 7·6)	2·8 (1·6 to 4·4)	2 (1·2 to 3·2)	1,410
Opioid use disorders	0 (0 to 0)	[illegible]	[illegible]	[illegible]	[illegible]	[illegible]	[illegible]	[illegible]	[illegible]	[illegible]	[illegible]	[illegible]	[illegible]	[illegible]	[illegible]	[illegible]	[illegible]	[illegible]	[illegible]	[illegible]	[illegible]	[illegible]	[illegible]	[illegible]
Cocaine use disorders	0 (0 to 0)	[illegible]	[illegible]	[illegible]	[illegible]	[illegible]	[illegible]	[illegible]	[illegible]	[illegible]	[illegible]	[illegible]	[illegible]	[illegible]	[illegible]	[illegible]	[illegible]	[illegible]	[illegible]	[illegible]	[illegible]	[illegible]	[illegible]	[illegible]
Amphetamine use disorders	0 (0 to 0)	[illegible]	[illegible]	[illegible]	[illegible]	[illegible]	[illegible]	[illegible]	[illegible]	[illegible]	[illegible]	[illegible]	[illegible]	[illegible]	[illegible]	[illegible]	[illegible]	[illegible]	[illegible]	[illegible]	[illegible]	[illegible]	[illegible]	[illegible]
Cannabis use disorders	0 (0 to 0)	[illegible]	[illegible]	[illegible]	[illegible]	[illegible]	[illegible]	[illegible]	[illegible]	[illegible]	[illegible]	[illegible]	[illegible]	[illegible]	[illegible]	[illegible]	[illegible]	[illegible]	[illegible]	[illegible]	[illegible]	[illegible]	[illegible]	[illegible]
Other drug use disorders	0 (0 to 0)	[illegible]	[illegible]	[illegible]	[illegible]	[illegible]	[illegible]	[illegible]	[illegible]	[illegible]	[illegible]	[illegible]	[illegible]	[illegible]	[illegible]	[illegible]	[illegible]	[illegible]	[illegible]	[illegible]	[illegible]	[illegible]	[illegible]	[illegible]
Diabetes and kidney diseases	0·1 (0·1 to 0·2)	[illegible]	[illegible]	[illegible]	[illegible]	[illegible]	[illegible]	[illegible]	[illegible]	[illegible]	[illegible]	[illegible]	[illegible]	[illegible]	[illegible]	[illegible]	[illegible]	[illegible]	[illegible]	[illegible]	[illegible]	[illegible]	[illegible]	[illegible]
Diabetes mellitus	0·1 (0·1 to 0·2)	[illegible]	[illegible]	[illegible]	[illegible]	[illegible]	[illegible]	[illegible]	[illegible]	[illegible]	[illegible]	[illegible]	[illegible]	[illegible]	[illegible]	[illegible]	[illegible]	[illegible]	[illegible]	[illegible]	[illegible]	[illegible]	[illegible]	[illegible]
Diabetes mellitus type 1	0·1 (0·1 to 0·2)	[illegible]	[illegible]	[illegible]	[illegible]	[illegible]	[illegible]	[illegible]	[illegible]	[illegible]	[illegible]	[illegible]	[illegible]	[illegible]	[illegible]	[illegible]	[illegible]	[illegible]	[illegible]	[illegible]	[illegible]	[illegible]	[illegible]	[illegible]
Diabetes mellitus type 2	0 (0 to 0)	[illegible]	[illegible]	[illegible]	[illegible]	[illegible]	[illegible]	[illegible]	[illegible]	[illegible]	[illegible]	[illegible]	[illegible]	[illegible]	[illegible]	[illegible]	[illegible]	[illegible]	[illegible]	[illegible]	[illegible]	[illegible]	[illegible]	[illegible]
Chronic kidney disease	0 (0 to 0)	[illegible]	[illegible]	[illegible]	[illegible]	[illegible]	[illegible]	[illegible]	[illegible]	[illegible]	[illegible]	[illegible]	[illegible]	[illegible]	[illegible]	[illegible]	[illegible]	[illegible]	[illegible]	[illegible]	[illegible]	[illegible]	[illegible]	[illegible]
Chronic kidney disease due to diabetes mellitus type 1	0 (0 to 0)	[illegible]	[illegible]	[illegible]	[illegible]	[illegible]	[illegible]	[illegible]	[illegible]	[illegible]	[illegible]	[illegible]	[illegible]	[illegible]	[illegible]	[illegible]	[illegible]	[illegible]	[illegible]	[illegible]	[illegible]	[illegible]	[illegible]	[illegible]
Chronic kidney disease due to diabetes mellitus type 2	0 (0 to 0)	[illegible]	[illegible]	[illegible]	[illegible]	[illegible]	[illegible]	[illegible]	[illegible]	[illegible]	[illegible]	[illegible]	[illegible]	[illegible]	[illegible]	[illegible]	[illegible]	[illegible]	[illegible]	[illegible]	[illegible]	[illegible]	[illegible]	[illegible]
Chronic kidney disease due to hypertension	0 (0 to 0)	[illegible]	[illegible]	[illegible]	[illegible]	[illegible]	[illegible]	[illegible]	[illegible]	[illegible]	[illegible]	[illegible]	[illegible]	[illegible]	[illegible]	[illegible]	[illegible]	[illegible]	[illegible]	[illegible]	[illegible]	[illegible]	[illegible]	[illegible]
Chronic kidney disease due to glomerulonephritis	0 (0 to 0)	[illegible]	[illegible]	[illegible]	[illegible]	[illegible]	[illegible]	[illegible]	[illegible]	[illegible]	[illegible]	[illegible]	[illegible]	[illegible]	[illegible]	[illegible]	[illegible]	[illegible]	[illegible]	[illegible]	[illegible]	[illegible]	[illegible]	[illegible]
Chronic kidney disease due to other and unspecified causes	0 (0 to 0)	[illegible]	[illegible]	[illegible]	[illegible]	[illegible]	[illegible]	[illegible]	[illegible]	[illegible]	[illegible]	[illegible]	[illegible]	[illegible]	[illegible]	[illegible]	[illegible]	[illegible]	[illegible]	[illegible]	[illegible]	[illegible]	[illegible]	[illegible]
Acute glomerulonephritis	0 (0 to 0)	[illegible]	[illegible]	[illegible]	[illegible]	[illegible]	[illegible]	[illegible]	[illegible]	[illegible]	[illegible]	[illegible]	[illegible]	[illegible]	[illegible]	[illegible]	[illegible]	[illegible]	[illegible]	[illegible]	[illegible]	[illegible]	[illegible]	[illegible]
Skin and subcutaneous diseases	0 (0 to 0)	[illegible]	[illegible]	[illegible]	[illegible]	[illegible]	[illegible]	[illegible]	[illegible]	[illegible]	[illegible]	[illegible]	[illegible]	[illegible]	[illegible]	[illegible]	[illegible]	[illegible]	[illegible]	[illegible]	[illegible]	[illegible]	[illegible]	[illegible]
Dermatitis	0 (0 to 0)	[illegible]	[illegible]	[illegible]	[illegible]	[illegible]	[illegible]	[illegible]	[illegible]	[illegible]	[illegible]	[illegible]	[illegible]	[illegible]	[illegible]	[illegible]	[illegible]	[illegible]	[illegible]	[illegible]	[illegible]	[illegible]	[illegible]	[illegible]
Atopic dermatitis	0 (0 to 0)	[illegible]	[illegible]	[illegible]	[illegible]	[illegible]	[illegible]	[illegible]	[illegible]	[illegible]	[illegible]	[illegible]	[illegible]	[illegible]	[illegible]	[illegible]	[illegible]	[illegible]	[illegible]	[illegible]	[illegible]	[illegible]	[illegible]	[illegible]
Contact dermatitis	0 (0 to 0)	[illegible]	[illegible]	[illegible]	[illegible]	[illegible]	[illegible]	[illegible]	[illegible]	[illegible]	[illegible]	[illegible]	[illegible]	[illegible]	[illegible]	[illegible]	[illegible]	[illegible]	[illegible]	[illegible]	[illegible]	[illegible]	[illegible]	[illegible]
Seborrhoeic dermatitis	0 (0 to 0)	[illegible]	[illegible]	[illegible]	[illegible]	[illegible]	[illegible]	[illegible]	[illegible]	[illegible]	[illegible]	[illegible]	[illegible]	[illegible]	[illegible]	[illegible]	[illegible]	[illegible]	[illegible]	[illegible]	[illegible]	[illegible]	[illegible]	[illegible]
Psoriasis	0 (0 to 0)	[illegible]	[illegible]	[illegible]	[illegible]	[illegible]	[illegible]	[illegible]	[illegible]	[illegible]	[illegible]	[illegible]	[illegible]	[illegible]	[illegible]	[illegible]	[illegible]	[illegible]	[illegible]	[illegible]	[illegible]	[illegible]	[illegible]	[illegible]
Bacterial skin diseases	0 (0 to 0)	[illegible]	[illegible]	[illegible]	[illegible]	[illegible]	[illegible]	[illegible]	[illegible]	[illegible]	[illegible]	[illegible]	[illegible]	[illegible]	[illegible]	[illegible]	[illegible]	[illegible]	[illegible]	[illegible]	[illegible]	[illegible]	[illegible]	[illegible]
Cellulitis	0 (0 to 0)	[illegible]	[illegible]	[illegible]	[illegible]	[illegible]	[illegible]	[illegible]	[illegible]	[illegible]	[illegible]	[illegible]	[illegible]	[illegible]	[illegible]	[illegible]	[illegible]	[illegible]	[illegible]	[illegible]	[illegible]	[illegible]	[illegible]	[illegible]
Pyoderma	0 (0 to 0)	[illegible]	[illegible]	[illegible]	[illegible]	[illegible]	[illegible]	[illegible]	[illegible]	[illegible]	[illegible]	[illegible]	[illegible]	[illegible]	[illegible]	[illegible]	[illegible]	[illegible]	[illegible]	[illegible]	[illegible]	[illegible]	[illegible]	[illegible]
Scabies	0 (0 to 0)	[illegible]	[illegible]	[illegible]	[illegible]	[illegible]	[illegible]	[illegible]	[illegible]	[illegible]	[illegible]	[illegible]	[illegible]	[illegible]	[illegible]	[illegible]	[illegible]	[illegible]	[illegible]	[illegible]	[illegible]	[illegible]	[illegible]	[illegible]
Fungal skin diseases	0 (0 to 0)	[illegible]	[illegible]	[illegible]	[illegible]	[illegible]	[illegible]	[illegible]	[illegible]	[illegible]	[illegible]	[illegible]	[illegible]	[illegible]	[illegible]	[illegible]	[illegible]	[illegible]	[illegible]	[illegible]	[illegible]	[illegible]	[illegible]	[illegible]
Viral skin diseases	0 (0 to 0)	[illegible]	[illegible]	[illegible]	[illegible]	[illegible]	[illegible]	[illegible]	[illegible]	[illegible]	[illegible]	[illegible]	[illegible]	[illegible]	[illegible]	[illegible]	[illegible]	[illegible]	[illegible]	[illegible]	[illegible]	[illegible]	[illegible]	[illegible]
Acne vulgaris	0 (0 to 0)	[illegible]	[illegible]	[illegible]	[illegible]	[illegible]	[illegible]	[illegible]	[illegible]	[illegible]	[illegible]	[illegible]	[illegible]	[illegible]	[illegible]	[illegible]	[illegible]	[illegible]	[illegible]	[illegible]	[illegible]	[illegible]	[illegible]	[illegible]
Alopecia areata	0 (0 to 0)	[illegible]	[illegible]	[illegible]	[illegible]	[illegible]	[illegible]	[illegible]	[illegible]	[illegible]	[illegible]	[illegible]	[illegible]	[illegible]	[illegible]	[illegible]	[illegible]	[illegible]	[illegible]	[illegible]	[illegible]	[illegible]	[illegible]	[illegible]
Pruritus	0 (0 to 0)	[illegible]	[illegible]	[illegible]	[illegible]	[illegible]	[illegible]	[illegible]	[illegible]	[illegible]	[illegible]	[illegible]	[illegible]	[illegible]	[illegible]	[illegible]	[illegible]	[illegible]	[illegible]	[illegible]	[illegible]	[illegible]	[illegible]	[illegible]
Urticaria	0 (0 to 0)	[illegible]	[illegible]	[illegible]	[illegible]	[illegible]	[illegible]	[illegible]	[illegible]	[illegible]	[illegible]	[illegible]	[illegible]	[illegible]	[illegible]	[illegible]	[illegible]	[illegible]	[illegible]	[illegible]	[illegible]	[illegible]	[illegible]	[illegible]
Decubitus ulcer	0 (0 to 0)	[illegible]	[illegible]	[illegible]	[illegible]	[illegible]	[illegible]	[illegible]	[illegible]	[illegible]	[illegible]	[illegible]	[illegible]	[illegible]	[illegible]	[illegible]	[illegible]	[illegible]	[illegible]	[illegible]	[illegible]	[illegible]	[illegible]	[illegible]
Other skin and subcutaneous diseases	0 (0 to 0)	[illegible]	[illegible]	[illegible]	[illegible]	[illegible]	[illegible]	[illegible]	[illegible]	[illegible]	[illegible]	[illegible]	[illegible]	[illegible]	[illegible]	[illegible]	[illegible]	[illegible]	[illegible]	[illegible]	[illegible]	[illegible]	[illegible]	[illegible]
Sense organ diseases	0 (0 to 0·1)	[illegible]	[illegible]	[illegible]	[illegible]	[illegible]	[illegible]	[illegible]	[illegible]	[illegible]	[illegible]	[illegible]	[illegible]	[illegible]	[illegible]	[illegible]	[illegible]	[illegible]	[illegible]	[illegible]	[illegible]	[illegible]	[illegible]	[illegible]
Blindness and vision loss	0 (0 to 0)	[illegible]	[illegible]	[illegible]	[illegible]	[illegible]	[illegible]	[illegible]	[illegible]	[illegible]	[illegible]	[illegible]	[illegible]	[illegible]	[illegible]	[illegible]	[illegible]	[illegible]	[illegible]	[illegible]	[illegible]	[illegible]	[illegible]	[illegible]
Glaucoma	0 (0 to 0)	[illegible]	[illegible]	[illegible]	[illegible]	[illegible]	[illegible]	[illegible]	[illegible]	[illegible]	[illegible]	[illegible]	[illegible]	[illegible]	[illegible]	[illegible]	[illegible]	[illegible]	[illegible]	[illegible]	[illegible]	[illegible]	[illegible]	[illegible]
Cataract	0 (0 to 0)	[illegible]	[illegible]	[illegible]	[illegible]	[illegible]	[illegible]	[illegible]	[illegible]	[illegible]	[illegible]	[illegible]	[illegible]	[illegible]	[illegible]	[illegible]	[illegible]	[illegible]	[illegible]	[illegible]	[illegible]	[illegible]	[illegible]	[illegible]
Age-related macular degeneration	0 (0 to 0)	[illegible]	[illegible]	[illegible]	[illegible]	[illegible]	[illegible]	[illegible]	[illegible]	[illegible]	[illegible]	[illegible]	[illegible]	[illegible]	[illegible]	[illegible]	[illegible]	[illegible]	[illegible]	[illegible]	[illegible]	[illegible]	[illegible]	[illegible]
Refraction disorders	0 (0 to 0)	[illegible]	[illegible]	[illegible]	[illegible]	[illegible]	[illegible]	[illegible]	[illegible]	[illegible]	[illegible]	[illegible]	[illegible]	[illegible]	[illegible]	[illegible]	[illegible]	[illegible]	[illegible]	[illegible]	[illegible]	[illegible]	[illegible]	[illegible]
Near vision loss	0 (0 to 0)	[illegible]	[illegible]	[illegible]	[illegible]	[illegible]	[illegible]	[illegible]	[illegible]	[illegible]	[illegible]	[illegible]	[illegible]	[illegible]	[illegible]	[illegible]	[illegible]	[illegible]	[illegible]	[illegible]	[illegible]	[illegible]	[illegible]	[illegible]
Other vision loss	0 (0 to 0)	[illegible]	[illegible]	[illegible]	[illegible]	[illegible]	[illegible]	[illegible]	[illegible]	[illegible]	[illegible]	[illegible]	[illegible]	[illegible]	[illegible]	[illegible]	[illegible]	[illegible]	[illegible]	[illegible]	[illegible]	[illegible]	[illegible]	[illegible]
Age-related and other hearing loss	0 (0 to 0)	[illegible]	[illegible]	[illegible]	[illegible]	[illegible]	[illegible]	[illegible]	[illegible]	[illegible]	[illegible]	[illegible]	[illegible]	[illegible]	[illegible]	[illegible]	[illegible]	[illegible]	[illegible]	[illegible]	[illegible]	[illegible]	[illegible]	[illegible]
Other sense organ diseases	0 (0 to 0·1)	[illegible]	[illegible]	[illegible]	[illegible]	[illegible]	[illegible]	[illegible]	[illegible]	[illegible]	[illegible]	[illegible]	[illegible]	[illegible]	[illegible]	[illegible]	[illegible]	[illegible]	[illegible]	[illegible]	[illegible]	[illegible]	[illegible]	[illegible]
Musculoskeletal disorders	0 (0 to 0)	[illegible]	[illegible]	[illegible]	[illegible]	[illegible]	[illegible]	[illegible]	[illegible]	[illegible]	[illegible]	[illegible]	[illegible]	[illegible]	[illegible]	[illegible]	[illegible]	[illegible]	[illegible]	[illegible]	[illegible]	[illegible]	[illegible]	[illegible]
Rheumatoid arthritis	0 (0 to 0)	[illegible]	[illegible]	[illegible]	[illegible]	[illegible]	[illegible]	[illegible]	[illegible]	[illegible]	[illegible]	[illegible]	[illegible]	[illegible]	[illegible]	[illegible]	[illegible]	[illegible]	[illegible]	[illegible]	[illegible]	[illegible]	[illegible]	[illegible]
Osteoarthritis	0 (0 to 0)	[illegible]	[illegible]	[illegible]	[illegible]	[illegible]	[illegible]	[illegible]	[illegible]	[illegible]	[illegible]	[illegible]	[illegible]	[illegible]	[illegible]	[illegible]	[illegible]	[illegible]	[illegible]	[illegible]	[illegible]	[illegible]	[illegible]	[illegible]
Osteoarthritis hip	0 (0 to 0)	[illegible]	[illegible]	[illegible]	[illegible]	[illegible]	[illegible]	[illegible]	[illegible]	[illegible]	[illegible]	[illegible]	[illegible]	[illegible]	[illegible]	[illegible]	[illegible]	[illegible]	[illegible]	[illegible]	[illegible]	[illegible]	[illegible]	[illegible]
Osteoarthritis knee	0 (0 to 0)	[illegible]	[illegible]	[illegible]	[illegible]	[illegible]	[illegible]	[illegible]	[illegible]	[illegible]	[illegible]	[illegible]	[illegible]	[illegible]	[illegible]	[illegible]	[illegible]	[illegible]	[illegible]	[illegible]	[illegible]	[illegible]	[illegible]	[illegible]
Osteoarthritis hand	0 (0 to 0)	[illegible]	[illegible]	[illegible]	[illegible]	[illegible]	[illegible]	[illegible]	[illegible]	[illegible]	[illegible]	[illegible]	[illegible]	[illegible]	[illegible]	[illegible]	[illegible]	[illegible]	[illegible]	[illegible]	[illegible]	[illegible]	[illegible]	[illegible]
Osteoarthritis other	0 (0 to 0)	[illegible]	[illegible]	[illegible]	[illegible]	[illegible]	[illegible]	[illegible]	[illegible]	[illegible]	[illegible]	[illegible]	[illegible]	[illegible]	[illegible]	[illegible]	[illegible]	[illegible]	[illegible]	[illegible]	[illegible]	[illegible]	[illegible]	[illegible]
Low back pain	0 (0 to 0)	[illegible]	[illegible]	[illegible]	[illegible]	[illegible]	[illegible]	[illegible]	[illegible]	[illegible]	[illegible]	[illegible]	[illegible]	[illegible]	[illegible]	[illegible]	[illegible]	[illegible]	[illegible]	[illegible]	[illegible]	[illegible]	[illegible]	[illegible]
Neck pain	0 (0 to 0)	[illegible]	[illegible]	[illegible]	[illegible]	[illegible]	[illegible]	[illegible]	[illegible]	[illegible]	[illegible]	[illegible]	[illegible]	[illegible]	[illegible]	[illegible]	[illegible]	[illegible]	[illegible]	[illegible]	[illegible]	[illegible]	[illegible]	[illegible]
Gout	0 (0 to 0)	[illegible]	[illegible]	[illegible]	[illegible]	[illegible]	[illegible]	[illegible]	[illegible]	[illegible]	[illegible]	[illegible]	[illegible]	[illegible]	[illegible]	[illegible]	[illegible]	[illegible]	[illegible]	[illegible]	[illegible]	[illegible]	[illegible]	[illegible]
Other musculoskeletal disorders	0 (0 to 0)	[illegible]	[illegible]	[illegible]	[illegible]	[illegible]	[illegible]	[illegible]	[illegible]	[illegible]	[illegible]	[illegible]	[illegible]	[illegible]	[illegible]	[illegible]	[illegible]	[illegible]	[illegible]	[illegible]	[illegible]	[illegible]	[illegible]	[illegible]
Other non-communicable diseases	1,350 (721 to 2,080)	[illegible]	[illegible]	[illegible]	[illegible]	[illegible]	[illegible]	[illegible]	[illegible]	[illegible]	[illegible]	[illegible]	[illegible]	[illegible]	[illegible]	[illegible]	[illegible]	[illegible]	[illegible]	[illegible]	[illegible]	[illegible]	[illegible]	[illegible]
Congenital birth defects	1,330 (700 to 2,040)	[illegible]	[illegible]	[illegible]	[illegible]	[illegible]	[illegible]	[illegible]	[illegible]	[illegible]	[illegible]	[illegible]	[illegible]	[illegible]	[illegible]	[illegible]	[illegible]	[illegible]	[illegible]	[illegible]	[illegible]	[illegible]	[illegible]	[illegible]
Neural tube defects	77·3 (39·2 to 140)	[illegible]	[illegible]	[illegible]	[illegible]	[illegible]	[illegible]	[illegible]	[illegible]	[illegible]	[illegible]	[illegible]	[illegible]	[illegible]	[illegible]	[illegible]	[illegible]	[illegible]	[illegible]	[illegible]	[illegible]	[illegible]	[illegible]	[illegible]
Congenital heart anomalies	574 (275 to 946)	[illegible]	[illegible]	[illegible]	[illegible]	[illegible]	[illegible]	[illegible]	[illegible]	[illegible]	[illegible]	[illegible]	[illegible]	[illegible]	[illegible]	[illegible]	[illegible]	[illegible]	[illegible]	[illegible]	[illegible]	[illegible]	[illegible]	[illegible]
Orofacial clefts	1·4 (0·2 to 3·9)	[illegible]	[illegible]	[illegible]	[illegible]	[illegible]	[illegible]	[illegible]	[illegible]	[illegible]	[illegible]	[illegible]	[illegible]	[illegible]	[illegible]	[illegible]	[illegible]	[illegible]	[illegible]	[illegible]	[illegible]	[illegible]	[illegible]	[illegible]
Down syndrome	25·7 (13·4 to 46·9)	[illegible]	[illegible]	[illegible]	[illegible]	[illegible]	[illegible]	[illegible]	[illegible]	[illegible]	[illegible]	[illegible]	[illegible]	[illegible]	[illegible]	[illegible]	[illegible]	[illegible]	[illegible]	[illegible]	[illegible]	[illegible]	[illegible]	[illegible]
Turner syndrome	0 (0 to 0)	[illegible]	[illegible]	[illegible]	[illegible]	[illegible]	[illegible]	[illegible]	[illegible]	[illegible]	[illegible]	[illegible]	[illegible]	[illegible]	[illegible]	[illegible]	[illegible]	[illegible]	[illegible]	[illegible]	[illegible]	[illegible]	[illegible]	[illegible]
Klinefelter syndrome	0 (0 to 0)	[illegible]	[illegible]	[illegible]	[illegible]	[illegible]	[illegible]	[illegible]	[illegible]	[illegible]	[illegible]	[illegible]	[illegible]	[illegible]	[illegible]	[illegible]	[illegible]	[illegible]	[illegible]	[illegible]	[illegible]	[illegible]	[illegible]	[illegible]
Other chromosomal abnormalities	106 (14·2 to 201)	[illegible]	[illegible]	[illegible]	[illegible]	[illegible]	[illegible]	[illegible]	[illegible]	[illegible]	[illegible]	[illegible]	[illegible]	[illegible]	[illegible]	[illegible]	[illegible]	[illegible]	[illegible]	[illegible]	[illegible]	[illegible]	[illegible]	[illegible]
Congenital musculoskeletal and limb anomalies	25·2 (12·1 to 46·2)	[illegible]	[illegible]	[illegible]	[illegible]	[illegible]	[illegible]	[illegible]	[illegible]	[illegible]	[illegible]	[illegible]	[illegible]	[illegible]	[illegible]	[illegible]	[illegible]	[illegible]	[illegible]	[illegible]	[illegible]	[illegible]	[illegible]	[illegible]
Urogenital congenital anomalies	16·4 (5·6 to 32·1)	[illegible]	[illegible]	[illegible]	[illegible]	[illegible]	[illegible]	[illegible]	[illegible]	[illegible]	[illegible]	[illegible]	[illegible]	[illegible]	[illegible]	[illegible]	[illegible]	[illegible]	[illegible]	[illegible]	[illegible]	[illegible]	[illegible]	[illegible]
Digestive congenital anomalies	168 (52·9 to 300)	[illegible]	[illegible]	[illegible]	[illegible]	[illegible]	[illegible]	[illegible]	[illegible]	[illegible]	[illegible]	[illegible]	[illegible]	[illegible]	[illegible]	[illegible]	[illegible]	[illegible]	[illegible]	[illegible]	[illegible]	[illegible]	[illegible]	[illegible]
Other congenital birth defects	333 (155 to 587)	[illegible]	[illegible]	[illegible]	[illegible]	[illegible]	[illegible]	[illegible]	[illegible]	[illegible]	[illegible]	[illegible]	[illegible]	[illegible]	[illegible]	[illegible]	[illegible]	[illegible]	[illegible]	[illegible]	[illegible]	[illegible]	[illegible]	[illegible]
Urinary diseases and male infertility	0·1 (0 to 0·5)	[illegible]	[illegible]	[illegible]	[illegible]	[illegible]	[illegible]	[illegible]	[illegible]	[illegible]	[illegible]	[illegible]	[illegible]	[illegible]	[illegible]	[illegible]	[illegible]	[illegible]	[illegible]	[illegible]	[illegible]	[illegible]	[illegible]	[illegible]
Urolithiasis	0 (0 to 0)	[illegible]	[illegible]	[illegible]	[illegible]	[illegible]	[illegible]	[illegible]	[illegible]	[illegible]	[illegible]	[illegible]	[illegible]	[illegible]	[illegible]	[illegible]	[illegible]	[illegible]	[illegible]	[illegible]	[illegible]	[illegible]	[illegible]	[illegible]

Benign prostatic hyperplasia	0 (0 to 0)	0 (0 to 0)	0 (0 to 0)	0 (0 to 0)	0 (0 to 0)	0 (0 to 0)	0 (0 to 0)	0 (0 to 0)	0 (0 to 0)	0 (0 to 0)	0 (0 to 0)	0 (0 to 0)	0 (0 to 0)	0 (0 to 0)	0 (0 to 0)	0 (0 to 0)	0 (0 to 0)	0 (0 to 0)	0 (0 to 0)	0 (0 to 0)	0 (0 to 0)	0 (0 to 0)	0 (0 to 0)	0
Male infertility	--	--	--	--	--	--	--	--	--	--	--	--	--	--	--	--	--	--	--	--	--	--	--	NA
Other urinary diseases	0 (0 to 0·1)	0·6 (0·2 to 1·3)	0·3 (0·2 to 0·6)	3·6 (2·2 to 5·6)	3·2 (2 to 5)	4·1 (2·6 to 6·4)	4·1 (2·4 to 6·5)	3·4 (2·1 to 5)	4·7 (2·9 to 7·2)	6·1 (3·8 to 9·4)	5 (3·1 to 7·7)	5 (3 to 7·9)	5·4 (3·4 to 8·1)	3 (1·8 to 4·8)	3·5 (2·1 to 5·3)	2·9 (1·8 to 4·7)	1·2 (0·7 to 1·8)	0·8 (0·5 to 1·2)	0·5 (0·3 to 0·8)	1 (0·3 to 2)	1·6 (0·2 to 4·1)	0·8 (0·1 to 1·8)	0·1 (0 to 0·2)	61·2
Gynecological diseases	0 (0 to 0)	0 (0 to 0)	0 (0 to 0)	0 (0 to 0)	0 (0 to 0)	159 (80·1 to 283)	447 (248 to 756)	1,100 (692 to 1,700)	1,600 (1,050 to 2,350)	1,720 (1,140 to 2,470)	1,660 (1,120 to 2,390)	1,480 (977 to 2,120)	1,180 (785 to 1,680)	765 (472 to 1,130)	448 (274 to 645)	280 (164 to 432)	192 (116 to 283)	131 (81·8 to 189)	81·7 (50·6 to 120)	58·2 (35·7 to 88·8)	40·8 (26 to 59·7)	23·7 (14·9 to 35·6)	13·7 (7·7 to 22·9)	11,400
Uterine fibroids	0 (0 to 0)	0 (0 to 0)	0 (0 to 0)	0 (0 to 0)	0 (0 to 0)	2·5 (0·8 to 5·5)	7·6 (3·6 to 15)	24·2 (12·6 to 46·3)	55·9 (27·4 to 108)	101 (49·5 to 193)	152 (77·3 to 285)	167 (90·8 to 288)	145 (78·2 to 251)	97·1 (52·1 to 167)	62·6 (33·1 to 108)	31·5 (15·1 to 61·3)	16·9 (7·9 to 32·7)	9·8 (4·8 to 18·7)	4·8 (2·3 to 9·5)	2·9 (1·4 to 5·4)	1·4 (0·7 to 2·5)	0·7 (0·4 to 1·4)	0·3 (0·2 to 0·5)	883
Polycystic ovarian syndrome	0 (0 to 0)	0 (0 to 0)	0 (0 to 0)	0 (0 to 0)	0 (0 to 0)	5·6 (2 to 12·4)	27·9 (11·8 to 58)	35·9 (14·6 to 73)	33·8 (14·2 to 69·6)	28·7 (11·8 to 59·5)	24·8 (10·2 to 51·9)	20·7 (8·4 to 42·8)	17·1 (6·9 to 35·1)	4·1 (1·6 to 8·8)	0 (0 to 0)	0 (0 to 0)	0 (0 to 0)	0 (0 to 0)	0 (0 to 0)	0 (0 to 0)	0 (0 to 0)	0 (0 to 0)	0 (0 to 0)	199
Female infertility	0 (0 to 0)	0 (0 to 0)	0 (0 to 0)	0 (0 to 0)	0 (0 to 0)	0 (0 to 0)	0 (0 to 0)	44·2 (10·3 to 117)	63·1 (16·1 to 165)	68·8 (17 to 189)	65·4 (10 to 187)	54·7 (8·5 to 157)	1·3 (0·1 to 10·8)	0 (0 to 0)	0 (0 to 0)	0 (0 to 0)	0 (0 to 0)	0 (0 to 0)	0 (0 to 0)	0 (0 to 0)	0 (0 to 0)	0 (0 to 0)	0 (0 to 0)	298
Endometriosis	0 (0 to 0)	0 (0 to 0)	0 (0 to 0)	0 (0 to 0)	0 (0 to 0)	0·1 (0 to 0·1)	37·9 (15 to 80·2)	168 (78·6 to 327)	195 (93·9 to 351)	144 (68·1 to 262)	112 (55·5 to 201)	95·7 (48 to 167)	80·6 (42·3 to 136)	36·8 (19·1 to 63·6)	0 (0 to 0)	0 (0 to 0)	0 (0 to 0)	0 (0 to 0)	0 (0 to 0)	0 (0 to 0)	0 (0 to 0)	0 (0 to 0)	0 (0 to 0)	871
Genital prolapse	0 (0 to 0)	0 (0 to 0)	0 (0 to 0)	0 (0 to 0)	0 (0 to 0)	0 (0 to 0)	0·3 (0·1 to 0·5)	1·7 (0·8 to 3·5)	3·6 (1·4 to 7·5)	6·4 (2·6 to 13)	11·7 (5·5 to 23·2)	16·5 (7·2 to 34)	22·2 (10·8 to 43·3)	25·6 (12·7 to 46·6)	28·6 (14·9 to 49·8)	20·5 (10·5 to 38)	18·6 (10 to 32·1)	14·1 (8·1 to 24·2)	8·7 (4·5 to 16·1)	7·4 (4 to 12·6)	5 (2·7 to 9)	3 (1·5 to 5·5)	2·1 (1·2 to 3·7)	196
Premenstrual syndrome	0 (0 to 0)	0 (0 to 0)	0 (0 to 0)	0 (0 to 0)	0 (0 to 0)	146 (69·8 to 268)	357 (175 to 633)	501 (253 to 894)	502 (288 to 818)	452 (240 to 747)	444 (257 to 699)	384 (214 to 624)	258 (146 to 436)	84·4 (42·1 to 153)	0 (0 to 0)	0 (0 to 0)	0 (0 to 0)	0 (0 to 0)	0 (0 to 0)	0 (0 to 0)	0 (0 to 0)	0 (0 to 0)	0 (0 to 0)	3,130
Other gynecological diseases	0 (0 to 0)	0 (0 to 0)	0 (0 to 0)	0 (0 to 0)	0 (0 to 0)	4·9 (1·1 to 12)	16·8 (6·6 to 33·9)	325 (180 to 534)	750 (417 to 1,190)	919 (542 to 1,400)	849 (504 to 1,340)	737 (404 to 1,170)	659 (374 to 1,040)	517 (247 to 826)	357 (205 to 546)	228 (122 to 366)	156 (90 to 241)	107 (62 to 161)	68·2 (39·3 to 103)	47·9 (28·2 to 77)	34·4 (20·5 to 51·7)	20 (11·7 to 31·7)	11·3 (5·6 to 20·4)	5,810
Hemoglobinopathies and hemolytic anemias	8·8 (5·4 to 13·7)	7·6 (4·8 to 11·3)	35·9 (21·9 to 55·1)	117 (73·5 to 173)	153 (97·7 to 230)	135 (85·3 to 202)	244 (160 to 347)	358 (217 to 570)	345 (200 to 598)	311 (195 to 479)	213 (130 to 321)	169 (107 to 244)	162 (105 to 232)	167 (112 to 239)	139 (92·6 to 191)	105 (67·8 to 155)	59·8 (37·8 to 88·4)	45·1 (28·9 to 66·1)	29·7 (19·2 to 46·2)	25·3 (16·4 to 37·3)	22·2 (14·5 to 33)	13·4 (8·7 to 19·5)	5·8 (3·7 to 8·6)	2,870
Thalassemias	0 (0 to 0)	0 (0 to 0)	0 (0 to 0)	0·3 (0·1 to 0·7)	0·2 (0·1 to 0·4)	0·1 (0 to 0·2)	0·1 (0 to 0·2)	0·1 (0 to 0·2)	0·1 (0 to 0·2)	0 (0 to 0·1)	0 (0 to 0·1)	0 (0 to 0)	0 (0 to 0·1)	0 (0 to 0·1)	0 (0 to 0·1)	0 (0 to 0)	0 (0 to 0)	0 (0 to 0)	0 (0 to 0)	0 (0 to 0)	0 (0 to 0)	0 (0 to 0)	0 (0 to 0)	1·1
Thalassemias trait	0 (0 to 0)	0·1 (0 to 0·2)	0·9 (0·4 to 1·7)	4·1 (1·7 to 7·4)	4·7 (1·4 to 10·9)	2·7 (0·4 to 6·8)	12 (5·7 to 22·2)	16 (7·1 to 27·9)	16·4 (7·6 to 28·5)	13·5 (5·6 to 25·1)	11·9 (5·8 to 20·9)	9·9 (4·8 to 16·8)	1·8 (0·5 to 3·7)	3·2 (1·2 to 6·1)	2·8 (1·1 to 5·5)	2 (0·6 to 3·9)	1 (0·2 to 2·3)	0·8 (0·2 to 1·8)	0·7 (0·2 to 1·5)	0·6 (0·2 to 1·3)	0·5 (0·2 to 1)	0·3 (0·1 to 0·5)	0·1 (0 to 0·2)	106
Sickle cell disorders	8·7 (5·2 to 13·6)	4·8 (2·6 to 8·2)	22·3 (11·8 to 39·3)	55·2 (31·6 to 92)	88·4 (60·6 to 122)	98·4 (62·1 to 146)	158 (99·4 to 233)	202 (122 to 310)	182 (106 to 291)	153 (86·6 to 247)	112 (59·4 to 190)	77·7 (45·6 to 125)	89 (53·9 to 143)	84·7 (48·3 to 136)	51·6 (30·3 to 82·1)	17·1 (8·8 to 30·5)	3·3 (1·4 to 6·5)	0·5 (0·2 to 1)	0·1 (0 to 0·1)	0 (0 to 0)	0 (0 to 0)	0 (0 to 0)	0 (0 to 0)	1,410
Sickle cell trait	0·1 (0 to 0·2)	2·2 (0·9 to 4)	8·3 (4·1 to 14·1)	37·7 (18·5 to 64·1)	42·7 (14·8 to 87·5)	24·5 (6·1 to 56·5)	21·4 (9 to 38·8)	103 (22·7 to 269)	99·8 (17 to 305)	101 (25·3 to 210)	14·3 (6·1 to 25·5)	26·8 (13·3 to 46·2)	29·3 (12·4 to 51·4)	29 (13·5 to 48·3)	25·6 (12·2 to 43·7)	17·3 (7 to 31·1)	9·2 (3 to 18·6)	7·6 (2·8 to 14·9)	6·2 (2·3 to 11·8)	5·6 (2·2 to 10·7)	4·8 (2·1 to 8·8)	2·5 (1·1 to 4·4)	0·9 (0·4 to 1·7)	619
G6PD deficiency	0 (0 to 0)	0 (0 to 0·1)	0 (0 to 0·1)	0·8 (0·4 to 1·4)	1·3 (0·5 to 2·4)	1·2 (0·5 to 2·3)	22·3 (9·1 to 42·2)	10·6 (4·5 to 19·8)	16·6 (6·9 to 34·1)	16·5 (6·4 to 33·4)	36·6 (17·1 to 63·9)	16·6 (8·6 to 28)	10·5 (4·3 to 20·2)	12·7 (4·8 to 25·7)	13·9 (5·8 to 25)	11·2 (5·2 to 20)	6·4 (2·8 to 11·5)	2·8 (1·3 to 4·9)	1·1 (0·5 to 2)	0·5 (0·2 to 0·9)	0·2 (0·1 to 0·4)	0·1 (0 to 0·1)	0 (0 to 0)	182
G6PD trait	0 (0 to 0)	0 (0 to 0)	0 (0 to 0)	0 (0 to 0)	0 (0 to 0)	0 (0 to 0)	3·5 (1·4 to 6·7)	0·1 (0 to 0·2)	0·1 (0 to 0·2)	0·1 (0 to 0·2)	3·4 (1·4 to 6·3)	3·2 (1·3 to 5·8)	0·8 (0·1 to 2)	0 (0 to 0)	0 (0 to 0)	0 (0 to 0)	0 (0 to 0)	0 (0 to 0)	0 (0 to 0)	0 (0 to 0)	0 (0 to 0)	0 (0 to 0)	0 (0 to 0)	11·2
Other hemoglobinopathies and hemolytic anemias	0 (0 to 0)	0·5 (0·2 to 0·9)	4·3 (1·6 to 8)	18·4 (7·2 to 35)	15·4 (4 to 36·2)	8·6 (1·9 to 21·3)	27·2 (8·5 to 58·4)	26·4 (8·5 to 54·6)	30·5 (12·3 to 59·7)	27·9 (9·9 to 56·9)	35·3 (15·2 to 63·4)	34·6 (16·3 to 61·6)	30·9 (14·1 to 55·8)	37·3 (20·2 to 58·6)	44·6 (27·2 to 66·8)	57·8 (35·2 to 89·1)	39·8 (25·3 to 60·2)	33·3 (21·9 to 48·3)	21·6 (14·3 to 32·6)	18·6 (12·4 to 27·7)	16·6 (10·7 to 24·7)	10·6 (6·9 to 15·3)	4·8 (3·1 to 6·9)	545
Endocrine, metabolic, blood, and immune disorders	18·1 (10·7 to 28·6)	25·3 (14·6 to 39·7)	84·1 (42 to 180)	118 (78·4 to 194)	92·4 (59·4 to 142)	92·3 (61·4 to 135)	135 (83·7 to 207)	173 (115 to 249)	239 (159 to 346)	288 (191 to 420)	320 (209 to 459)	380 (250 to 540)	443 (286 to 622)	463 (304 to 696)	491 (324 to 730)	440 (288 to 679)	360 (243 to 562)	281 (187 to 426)	240 (159 to 345)	171 (118 to 245)	133 (89·7 to 187)	70 (46·5 to 97·3)	46 (27·9 to 68)	5,100
Oral disorders	0 (0 to 0)	0 (0 to 0)	0 (0 to 0)	46·9 (26 to 77·3)	63·5 (33·1 to 109)	73·3 (38·7 to 124)	113 (61·1 to 190)	196 (101 to 354)	280 (142 to 537)	323 (161 to 608)	344 (167 to 655)	348 (170 to 633)	397 (211 to 690)	451 (254 to 764)	516 (300 to 816)	545 (338 to 843)	481 (298 to 739)	411 (257 to 626)	308 (194 to 458)	233 (147 to 346)	161 (100 to 234)	88·5 (54·8 to 128)	51·7 (32·6 to 75·4)	5,430
Caries of deciduous teeth	0 (0 to 0)	0 (0 to 0)	0 (0 to 0)	14·8 (5·6 to 30·1)	15·4 (6·1 to 33·4)	0·9 (0·1 to 2·5)	0 (0 to 0)	0 (0 to 0)	0 (0 to 0)	0 (0 to 0)	0 (0 to 0)	0 (0 to 0)	0 (0 to 0)	0 (0 to 0)	0 (0 to 0)	0 (0 to 0)	0 (0 to 0)	0 (0 to 0)	0 (0 to 0)	0 (0 to 0)	0 (0 to 0)	0 (0 to 0)	0 (0 to 0)	31·1
Caries of permanent teeth	0 (0 to 0)	0 (0 to 0)	0 (0 to 0)	0 (0 to 0)	15·6 (5·5 to 34·7)	34·2 (13·6 to 73·7)	38·5 (15·2 to 83)	48·6 (20·3 to 100)	47·8 (18·3 to 94·5)	40·1 (16·2 to 83·3)	35·7 (14·5 to 72·6)	29·1 (12·1 to 58·6)	26·3 (11 to 55·5)	23·5 (9·5 to 50·3)	19·4 (7·2 to 43·3)	14·6 (5·2 to 32·3)	9·4 (3·6 to 20)	6·6 (2·6 to 14)	4·5 (1·8 to 8·9)	3·3 (1·3 to 7)	2·1 (0·8 to 4·3)	1 (0·4 to 2·3)	0·5 (0·2 to 1·2)	401
Periodontal diseases	0 (0 to 0)	0 (0 to 0)	0 (0 to 0)	0 (0 to 0)	0 (0 to 0)	0 (0 to 0)	13·7 (4 to 35·3)	56·7 (17·2 to 143)	125 (40·1 to 292)	173 (59·5 to 387)	198 (71·2 to 441)	199 (72·6 to 433)	202 (76·9 to 428)	182 (71·4 to 377)	150 (58·9 to 307)	111 (43·6 to 228)	71·4 (27·8 to 147)	48·8 (19·1 to 98·8)	32·5 (13 to 66·3)	23·6 (9 to 48·1)	16·2 (6·2 to 33·4)	8·9 (3·2 to 18·7)	5 (1·8 to 10·6)	1,620
Other oral disorders	0 (0 to 0)	0 (0 to 0)	0 (0 to 0)	32·1 (18·5 to 51·3)	32·5 (18·3 to 50·2)	38·1 (21·4 to 60·1)	60·5 (35·4 to 94·5)	81·1 (48·7 to 127)	86·9 (52·8 to 131)	81·4 (49·2 to 123)	73·3 (44·7 to 113)	64·8 (38·6 to 98·5)	62·8 (37·5 to 94·4)	57 (34·5 to 85·3)	49·6 (30·3 to 74·1)	41 (25·2 to 62·5)	29·8 (18·3 to 45·3)	20·6 (12·6 to 31·3)	11·8 (7·3 to 17·8)	7·1 (4·2 to 10·8)	3·9 (2·4 to 5·8)	1·9 (1·2 to 2·8)	1·1 (0·7 to 1·8)	837
Sudden infant death syndrome	--	10·4 (5·8 to 17·9)	27·9 (11·8 to 53·7)	--	--	--	--	--	--	--	--	--	--	--	--	--	--	--	--	--	--	--	--	NA
Injuries	96·4 (65 to 138)	33·4 (22·2 to 48·1)	94·4 (57·7 to 150)	305 (228 to 400)	497 (409 to 589)	627 (518 to 754)	1,100 (883 to 1,320)	1,640 (1,290 to 2,050)	1,850 (1,420 to 2,390)	1,660 (1,280 to 2,140)	1,340 (1,030 to 1,700)	1,200 (954 to 1,490)	1,190 (941 to 1,470)	981 (779 to 1,230)	865 (686 to 1,070)	786 (627 to 965)	594 (479 to 722)	506 (410 to 619)	424 (342 to 516)	395 (317 to 477)	357 (287 to 431)	242 (197 to 288)	181 (145 to 216)	17,000
Transport injuries	7·1 (4·7 to 10·4)	2·5 (1·7 to 3·7)	9·5 (5·1 to 16·1)	54·3 (34·8 to 83·9)	133 (93·8 to 181)	148 (107 to 200)	282 (202 to 380)	330 (233 to 470)	387 (272 to 561)	280 (197 to 396)	207 (152 to 279)	194 (148 to 256)	237 (173 to 309)	188 (143 to 238)	173 (134 to 217)	172 (134 to 213)	129 (102 to 160)	94·4 (74 to 117)	76·7 (60 to 95·3)	61·1 (48·2 to 76·4)	50·4 (38·6 to 64·4)	31·9 (24·7 to 39·4)	25 (18·9 to 31)	3,270
Road injuries	6·6 (4·3 to 9·7)	1·5 (0·9 to 2·3)	8·1 (4·1 to 14·2)	49·8 (30·9 to 77·5)	124 (85·6 to 170)	137 (98·3 to 186)	262 (185 to 355)	305 (215 to 432)	360 (252 to 528)	259 (182 to 367)	193 (140 to 260)	179 (136 to 236)	222 (163 to 290)	176 (135 to 224)	162 (123 to 203)	160 (125 to 200)	121 (95·2 to 150)	88·3 (69·3 to 109)	70·9 (55·2 to 88·1)	56·3 (44·3 to 70·4)	46·1 (35·4 to 59)	28·3 (21·9 to 35·3)	22·2 (16·7 to 27·8)	3,040
Pedestrian road injuries	4·1 (2·7 to 6)	0·7 (0·5 to 1·1)	2 (0·9 to 3·7)	15·6 (9·1 to 25·6)	35·4 (23·8 to 50·2)	46·8 (30·3 to 67·5)	34·4 (22·2 to 50·4)	50·5 (36·1 to 69)	53·2 (35·7 to 78·8)	54·6 (38·4 to 78·3)	46·6 (33·5 to 63·5)	43·5 (32·4 to 58·3)	59·2 (42·7 to 79·3)	50·8 (37·7 to 66·6)	49·3 (37·2 to 63·9)	59·9 (46 to 76·7)	45·3 (34·5 to 57·1)	32 (24·9 to 40·7)	30·2 (22·5 to 39·3)	24·7 (18·7 to 31·8)	21·8 (16·2 to 28·7)	12·7 (9·6 to 16·3)	10·7 (7·9 to 13·5)	784
Cyclist road injuries	0 (0 to 0)	0 (0 to 0)	0 (0 to 0)	2·8 (1·7 to 4·6)	7 (4·4 to 10·4)	11·2 (7·7 to 15·9)	16·6 (11·4 to 22·9)	23·1 (16·3 to 31·7)	31·1 (22·4 to 44)	27·2 (19·9 to 36·3)	26·4 (18·7 to 35·8)	26·1 (18·9 to 35·5)	32·9 (23·6 to 43·8)	27·3 (20·2 to 36·1)	24·9 (18·1 to 33·2)	22·9 (16·8 to 29·9)	18 (13·4 to 23·9)	14·1 (10·3 to 18·4)	8·8 (6·2 to 11·7)	7 (5 to 9·2)	5·3 (3·8 to 7)	3·2 (2·3 to 4·2)	2 (1·4 to 2·6)	338
Motorcyclist road injuries	0 (0 to 0)	0·1 (0 to 0·1)	0·4 (0·2 to 1)	2 (1·2 to 3·2)	4·9 (2·8 to 8·2)	8·6 (6 to 11·9)	23·2 (13·6 to 36·9)	27·8 (19·9 to 38·2)	31·8 (20·2 to 49·1)	26·3 (16·6 to 40·5)	16·1 (11 to 22·9)	14·2 (10·1 to 20)	17 (13 to 21·8)	11·5 (8·3 to 15·4)	11·1 (7·9 to 14·7)	9·3 (6·8 to 12·3)	7·8 (5·8 to 10·2)	6 (4·3 to 8)	4·8 (3·5 to 6·3)	4·4 (3·2 to 5·8)	3·3 (2·4 to 4·5)	2·2 (1·6 to 3)	1·8 (1·3 to 2·4)	235
Motor vehicle road injuries	2·4 (1·6 to 3·6)	0·6 (0·4 to 1)	5·5 (2·9 to 9·4)	27·6 (17·1 to 43·8)	75 (50·9 to 104)	67·7 (44 to 95·9)	183 (124 to 259)	197 (133 to 288)	235 (154 to 363)	144 (92 to 213)	97·2 (67·4 to 138)	88·7 (64·2 to 125)	105 (73·5 to 142)	80·4 (59·3 to 105)	70·5 (52·5 to 91)	63·9 (49·2 to 80·9)	46·7 (36·2 to 58·7)	33·7 (26·1 to 42·4)	25 (18·6 to 32·6)	18·8 (14·6 to 23·7)	14·6 (10·9 to 18·9)	9·6 (7·2 to 12·4)	7·5 (5·4 to 9·6)	1,600
Other road injuries	0 (0 to 0)	0 (0 to 0)	0·2 (0·1 to 0·4)	1·8 (0·9 to 3)	1·8 (1·1 to 2·9)	2·3 (1·5 to 3·3)	4·7 (3 to 7·3)	7 (4·5 to 11·1)	8·7 (5·5 to 14)	7·2 (4·9 to 10·6)	6·5 (4·6 to 9·1)	6·5 (4·5 to 9·2)	7·9 (5·6 to 10·8)	6·4 (4·7 to 8·8)	5·8 (4·2 to 8)	4·5 (3·1 to 6·1)	3·5 (2·5 to 4·8)	2·6 (1·8 to 3·5)	2·1 (1·5 to 2·8)	1·5 (1·1 to 2·1)	1 (0·7 to 1·4)	0·6 (0·4 to 0·8)	0·3 (0·2 to 0·4)	83
Other transport injuries	0·5 (0·3 to 0·8)	1·1 (0·6 to 1·6)	1·4 (0·7 to 2·4)	4·5 (2·9 to 6·9)	9·1 (5·7 to 13·6)	11 (7·4 to 15·7)	20·4 (12·4 to 33·6)	24·1 (15·1 to 37·9)	26·7 (16·7 to 41·7)	20·8 (13·7 to 31·5)	14·3 (9·7 to 21·2)	14·7 (10 to 21·1)	15·1 (10·5 to 22·9)	12·1 (8·4 to 16·7)	11·8 (8·3 to 16·3)	11·4 (8·2 to 15·8)	7·9 (5·6 to 10·8)	6·1 (4·5 to 8·1)	5·8 (4 to 8·1)	4·7 (3·4 to 6·6)	4·3 (3 to 6·1)	3·7 (2·6 to 5·1)	2·8 (1·9 to 3·8)	234
Unintentional injuries	76·1 (49·1 to 109)	26·4 (16·6 to 39·4)	73·3 (44·4 to 118)	187 (134 to 244)	278 (214 to 355)	328 (250 to 426)	404 (299 to 534)	568 (432 to 732)	631 (483 to 830)	624 (480 to 806)	599 (452 to 793)	556 (416 to 728)	577 (437 to 747)	541 (402 to 715)	506 (380 to 658)	485 (371 to 627)	382 (293 to 485)	350 (276 to 440)	311 (246 to 389)	312 (248 to 383)	293 (233 to 356)	203 (164 to 243)	151 (120 to 181)	8,460
Falls	2·2 (1·5 to 3·1)	2 (1·2 to 3·1)	4·3 (2·6 to 6·8)	25·1 (16·9 to 34·5)	45·1 (33·3 to 61·5)	58 (41·7 to 79·7)	72 (51·3 to 101)	98·2 (70·3 to 136)	117 (84·2 to 159)	114 (84·6 to 153)	111 (79·5 to 151)	106 (74·7 to 146)	121 (88·6 to 162)	126 (92·2 to 170)	137 (101 to 182)	150 (114 to 193)	136 (105 to 173)	139 (108 to 173)	140 (111 to 175)	167 (134 to 204)	177 (142 to 216)	134 (106 to 162)	104 (82·8 to 125)	2,280
Drowning	0·2 (0·1 to 0·2)	0·1 (0·1 to 0·2)	1·4 (0·7 to 2·5)	13·3 (8 to 20·2)	13·2 (9·2 to 19)	13·8 (10·1 to 18·7)	13·8 (10·3 to 18·3)	12·7 (9·2 to 17·4)	13 (9·4 to 18)	14·3 (10·5 to 19·4)	11·6 (8·5 to 15·7)	10·1 (7·4 to 13·7)	8·9 (6·7 to 11·6)	6 (4·6 to 7·8)	6·1 (4·7 to 7·8)	4·6 (3·6 to 5·8)	3·6 (2·8 to 4·6)	3·4 (2·6 to 4·3)	2·4 (1·8 to 3·2)	1·5 (1·1 to 1·8)	1 (0·8 to 1·3)	0·7 (0·5 to 0·8)	0·4 (0·3 to 0·5)	156
Fire, heat, and hot substances	0·5 (0·3 to 0·7)	1 (0·7 to 1·5)	6·9 (3·8 to 11·8)	29·8 (19·7 to 42·1)	49·9 (36·1 to 66·4)	64 (43 to 92·9)	86·4 (58·3 to 127)	111 (75·7 to 158)	129 (88·6 to 183)	126 (85·9 to 182)	127 (87·6 to 182)	108 (75·3 to 155)	113 (80·1 to 159)	102 (70·2 to 145)	93·4 (65·3 to 132)	76·9 (53 to 111)	55·1 (38·5 to 78·3)	45·8 (32·7 to 64)	32·2 (22·9 to 45·1)	27·4 (20·4 to 36·4)	21·7 (16·5 to 28·6)	12·4 (9·4 to 16·2)	8·7 (6·6 to 11·1)	1,430
Poisonings	0·5 (0·3 to 0·7)	0·3 (0·2 to 0·5)	0·8 (0·4 to 1·4)	6·2 (3·8 to 9·8)	9·4 (6·2 to 14·5)	9·6 (5·6 to 15·3)	13·8 (9·1 to 20·2)	17·6 (11·7 to 24·7)	16 (11 to 22·8)	14·1 (9·6 to 20·5)	12·6 (8·3 to 18·3)	10·6 (7·3 to 14·8)	9 (5·9 to 13)	7·4 (4·9 to 10·9)	6·2 (3·9 to 9·1)	5·3 (3·5 to 7·9)	5·4 (3·8 to 7·5)	3·7 (2·5 to 5·1)	2·5 (1·7 to 3·6)	1·9 (1·3 to 2·6)	1·4 (0·9 to 1·9)	0·7 (0·5 to 1)	0·5 (0·3 to 0·6)	155
Poisoning by carbon monoxide	0·1 (0·1 to 0·2)	0·1 (0·1 to 0·1)	0·4 (0·2 to 0·7)	2 (1·1 to 3·2)	2·8 (1·7 to 4·4)	2·6 (1·4 to 4·3)	4 (2·6 to 6·2)	5·6 (3·5 to 8·2)	4·5 (2·9 to 6·6)	4·4 (2·8 to 6·6)	3 (1·9 to 4·7)	2·6 (1·6 to 3·9)	2·4 (1·5 to 3·6)	2 (1·2 to 3·1)	1·7 (1·1 to 2·7)	1·4 (0·8 to 2·2)	1·4 (0·9 to 2·1)	0·9 (0·6 to 1·4)	0·5 (0·3 to 0·8)	0·5 (0·3 to 0·8)	0·3 (0·2 to 0·5)	0·2 (0·1 to 0·3)	0·1 (0·1 to 0·2)	43·6
Poisoning by other means	0·4 (0·2 to 0·5)	0·2 (0·1 to 0·3)	0·4 (0·2 to 0·7)	4·3 (2·6 to 6·7)	6·6 (4·2 to 10·5)	7 (3·9 to 11·7)	9·7 (6·2 to 14·7)	12 (7·7 to 17·1)	11·4 (7·5 to 16·9)	9·7 (6·2 to 14·2)	9·5 (6·2 to 14·2)	8 (5·3 to 11·5)	6·6 (4·2 to 9·6)	5·4 (3·4 to 8·1)	4·5 (2·8 to 6·8)	4 (2·6 to 5·9)	4 (2·7 to 5·7)	2·8 (1·9 to 3·9)	2 (1·4 to 2·9)	1·4 (0·9 to 2)	1 (0·7 to 1·4)	0·5 (0·4 to 0·8)	0·4 (0·2 to 0·5)	112
Exposure to mechanical forces	1 (0·6 to 1·5)	1·5 (1 to 2·2)	3·9 (2·6 to 5·8)	22·1 (14·5 to 31·2)	44·8 (30·8 to 62·7)	57·6 (40·3 to 82·5)	87·3 (62·7 to 119)	144 (105 to 188)	156 (112 to 209)	161 (118 to 213)	159 (116 to 213)	140 (105 to 184)	130 (95·9 to 174)	114 (84·5 to 154)	92·4 (65·9 to 128)	83·6 (60·7 to 116)	61·3 (43·8 to 84·2)	47·6 (33·1 to 66·3)	34 (23·8 to 47·3)	26·1 (18·1 to 36·6)	19·3 (13·5 to 27)	11·9 (8·4 to 16·4)	7·9 (5·6 to 10·8)	1,610
Unintentional firearm injuries	0·2 (0·1 to 0·3)	0·2 (0·1 to 0·3)	0·7 (0·4 to 1·1)	5 (2·7 to 7·7)	10·8 (6 to 15·9)	15·3 (10·9 to 20·8)	34·5 (25·1 to 45·7)	72·6 (48·8 to 102)	69·1 (44·6 to 100)	79·4 (52·6 to 117)	78·5 (52·8 to 114)	63·2 (44·5 to 88·3)	52·5 (36·4 to 73·8)	35·7 (26·1 to 47·5)	22·7 (16·9 to 29·5)	21·4 (16·2 to 27·1)	13·7 (10·4 to 17·6)	11·2 (8·4 to 14·3)	6·6 (4·8 to 8·7)	4·8 (3·5 to 6·6)	3·6 (2·6 to 4·9)	2·3 (1·6 to 3·2)	1·7 (1·2 to 2·3)	606
Other exposure to mechanical forces	0·8 (0·5 to 1·3)	1·4 (0·9 to 2)	3·3 (2·1 to 4·9)	17 (10·7 to 25·7)	34 (21·6 to 50·5)	42·3 (26·9 to 65·5)	52·8 (33·7 to 81·1)	71 (47·5 to 105)	86·6 (59·9 to 126)	81·3 (55 to 118)	80 (53·8 to 118)	76·8 (53·7 to 110)	77·8 (52·7 to 111)	78·8 (54·8 to 112)	69·7 (47·6 to 100)	62·2 (42·2 to 90)	47·6 (32·7 to 67·8)	36·5 (24·7 to 52)	27·4 (18·7 to 39)	21·3 (14·3 to 30)	15·7 (10·7 to 22·3)	9·6 (6·7 to 13·3)	6·2 (4·4 to 8·6)	1,000
Adverse effects of medical treatment	14·4 (3·4 to 25·1)	7 (2·7 to 11·8)	26 (12 to 52·9)	25·3 (14·1 to 42·8)	24·9 (7·8 to 41)	21·7 (6·6 to 35·5)	17·8 (7·9 to 29·3)	37·2 (16·6 to 61·9)	37·2 (18·5 to 61·3)	37·5 (19·9 to 59·9)	35·6 (15·8 to 58·5)	49·4 (18·2 to 83)	65·9 (22 to 109)	68·9 (21·1 to 115)	60·9 (21·5 to 99·1)	62·9 (23·6 to 99·7)	41 (18·8 to 63·1)	42·2 (18·5 to 63·8)	43·4 (17·6 to 64·6)	43 (15·3 to 64·1)	37·6 (14·7 to 56·6)	20·5 (9·7 to 30·4)	13·9 (7·1 to 20·6)	834
Animal contact	0 (0 to 0)	0 (0 to 0·1)	0·5 (0·3 to 0·8)	3·5 (2·5 to 4·9)	5·3 (3·3 to 8·5)	7·9 (5·1 to 12·1)	9·2 (5·9 to 13·4)	11 (7·1 to 15·8)	11·3 (7·3 to 16·2)	10·3 (6·8 to 14·7)	9·5 (6·2 to 13·8)	8·8 (5·9 to 12·5)	9·3 (6·1 to 13·2)	8·5 (5·8 to 12)	8·3 (5·8 to 11·8)	7·1 (4·9 to 10·2)	5·6 (3·9 to 8·1)	5·6 (4·1 to 7·4)	4·4 (3·1 to 5·9)	2·9 (2 to 4)	2·3 (1·6 to 3·1)	1·5 (1·1 to 2)	1·1 (0·8 to 1·4)	134
Venomous animal contact	0 (0 to 0)	0 (0 to 0)	0·3 (0·2 to 0·4)	1·9 (1·2 to 2·9)	3·7 (2·1 to 6·3)	5·8 (3·4 to 9·2)	6·8 (4·2 to 10·3)	8·2 (5 to 12·4)	8·4 (5·1 to 12·7)	7·6 (4·8 to 11·3)	7 (4·3 to 10·5)	6·3 (3·9 to 9·1)	6 (3·7 to 8·9)	5·7 (3·7 to 8·3)	5·2 (3·4 to 7·6)	4·5 (2·9 to 6·7)	3·5 (2·2 to 5·1)	2·8 (1·8 to 4)	2·2 (1·5 to 3)	1·8 (1·2 to 2·4)	1·4 (1 to 1·9)	0·9 (0·7 to 1·2)	0·6 (0·5 to 0·9)	90·4
Non-venomous animal contact	0 (0 to 0)	0 (0 to 0)	0·2 (0·1 to 0·4)	1·6 (1·1 to 2·2)	1·6 (1 to 2·7)	2·2 (1·3 to 3·5)	2·4 (1·6 to 3·7)	2·8 (1·8 to 4·3)	2·9 (1·9 to 4·5)	2·7 (1·8 to 4·2)	2·5 (1·6 to 4·1)	2·5 (1·6 to 3·9)	3·2 (2·2 to 4·7)	2·8 (1·8 to 4·1)	3·1 (2·1 to 4·5)	2·6 (1·7 to 3·8)	2·2 (1·4 to 3·1)	2·8 (2 to 3·8)	2·2 (1·5 to 3·1)	1·1 (0·8 to 1·7)	0·9 (0·6 to 1·3)	0·6 (0·4 to 0·8)	0·4 (0·3 to 0·6)	43·4
Foreign body	50·6 (32·8 to 73·2)	12·2 (7·5 to 19·3)	22 (11·3 to 39·1)	24·8 (16·5 to 35·7)	23·1 (17·4 to 29·7)	22·4 (16·9 to 29)	17·8 (12·8 to 24·6)	24·7 (17·7 to 33·8)	28·6 (21·2 to 38·2)	28·3 (20·8 to 37·2)	24 (17·5 to 32·1)	21 (15·1 to 28)	20·2 (15·2 to 26·4)	18·2 (13·5 to 23·7)	16·8 (12·9 to 21·4)	15·3 (11·8 to 19·3)	12·5 (9·9 to 15·6)	11·4 (9·1 to 14·2)	9·9 (7·7 to 12·7)	8·8 (6·9 to 10·8)	5·8 (4·7 to 7·1)	3·9 (3·1 to 4·7)	3·2 (2·5 to 3·9)	426
Pulmonary aspiration and foreign body in airway	50·2 (32·4 to 72·8)	12·1 (7·5 to 19·2)	20·9 (10·2 to 38·4)	18·9 (11·6 to 28·5)	15·2 (11·4 to 20·2)	13·6 (10·3 to 17·9)	7·8 (6 to 10)	11·8 (8·7 to 15·6)	15·1 (11·3 to 20·6)	15·6 (11·3 to 21·3)	12·6 (9·3 to 17·2)	10·5 (7·8 to 14)	10·3 (7·9 to 13·4)	9·3 (6·8 to 12·7)	9·1 (6·7 to 12·3)	8·6 (6·3 to 11·4)	7·3 (5·5 to 9·4)	7·2 (5·5 to 9·5)	6·6 (4·7 to 9)	5·9 (4·4 to 7·8)	3·6 (2·8 to 4·7)	2·6 (1·8 to 3·3)	2·2 (1·6 to 2·8)	277
Foreign body in eyes	0 (0 to 0)	0 (0 to 0)	0·2 (0·1 to 0·5)	1·1 (0·3 to 2·5)	2 (0·6 to 4·5)	2·7 (0·8 to 6·1)	3·8 (1·2 to 8·6)	5·2 (1·7 to 11·7)	5·8 (2·1 to 12·4)	5·6 (2 to 11·4)	5·3 (2·1 to 10·7)	4·8 (2·1 to 9·7)	4·6 (2·2 to 8·6)	3·8 (2 to 6·9)	3·2 (1·8 to 5·2)	2·7 (1·6 to 4·2)	2·1 (1·3 to 3·1)	1·6 (1·1 to 2·4)	1·2 (0·8 to 1·8)	1 (0·6 to 1·4)	0·7 (0·5 to 1)	0·4 (0·3 to 0·6)	0·2 (0·2 to 0·3)	58
Foreign body in other body part	0·4 (0·1 to 0·7)	0·1 (0 to 0·1)	0·8 (0·5 to 1·4)	4·8 (3·1 to 7)	5·9 (4 to 8·3)	6·1 (4·2 to 8·6)	6·2 (4·2 to 8·8)	7·7 (5·2 to 10·7)	7·7 (5·4 to 10·5)	7·2 (5·2 to 9·8)	6·2 (4·4 to 8·7)	5·6 (4·1 to 7·9)	5·4 (3·8 to 7·4)	5 (3·5 to 6·9)	4·5 (3·2 to 6·1)	4 (2·9 to 5·5)	3·1 (2·2 to 4·2)	2·5 (1·8 to 3·4)	2·1 (1·6 to 2·9)	1·9 (1·4 to 2·5)	1·4 (1 to 1·9)	0·9 (0·7 to 1·3)	0·7 (0·5 to 1)	90·3
Environmental heat and cold exposure	0 (0 to 0)	0·1 (0 to 0·1)	1·2 (0·8 to 1·7)	8·1 (5·2 to 12·1)	15·8 (10·4 to 23·4)	21·3 (13·8 to 31·2)	27·4 (18·1 to 39·4)	34·5 (23 to 48·7)	37·6 (25·6 to 54·2)	36·7 (25 to 52·7)	34·7 (23·5 to 50)	32 (21·9 to 44·9)	32·4 (22·2 to 45·8)	31·4 (21·9 to 43·7)	30·1 (20·9 to 41·8)	27·7 (18·9 to 38·7)	22·6 (15·6 to 30·7)	19·2 (13·4 to 26·3)	15·2 (10·6 to 20·6)	13·3 (9·4 to 18·1)	10·6 (7·4 to 14·2)	6·8 (4·8 to 9·2)	4·7 (3·3 to 6·5)	463
Exposure to forces of nature	0 (0 to 0)	0 (0 to 0)	0 (0 to 0)	0 (0 to 0)	0 (0 to 0)	0·2 (0·1 to 0·3)	0·5 (0·3 to 0·8)	0·8 (0·5 to 1·3)	0·9 (0·6 to 1·5)	1·2 (0·8 to 1·8)	1·7 (1·1 to 2·5)	1·9 (1·3 to 2·9)	1·6 (1 to 2·4)	1 (0·7 to 1·5)	0·9 (0·6 to 1·4)	0·9 (0·6 to 1·4)	0·6 (0·4 to 0·9)	0·5 (0·3 to 0·7)	0·3 (0·2 to 0·5)	0·2 (0·2 to 0·4)	0·2 (0·1 to 0·2)	0·1 (0·1 to 0·1)	0·1 (0 to 0·1)	13·7
Other unintentional injuries	6·7 (3·8 to 10·6)	2·1 (1·3 to 3·4)	6·3 (4 to 9·2)	28·5 (18·3 to 41·1)	46·7 (31 to 67·2)	51·6 (35 to 75·6)	58·1 (38 to 88·7)	76 (51·7 to 113)	84·9 (58·9 to 122)	82 (57·4 to 118)	72·9 (49·9 to 103)	68·6 (47·8 to 98·7)	65·5 (45 to 92·8)	56·6 (37·3 to 82·8)	53·4 (34·7 to 76·5)	50·6 (34·4 to 71·7)	39·1 (27·3 to 55·1)	32·1 (22·4 to 44·2)	26·4 (19 to 35·9)	20·5 (14·6 to 27·8)	16 (11·6 to 21·6)	10·2 (7·5 to 13·8)	6·7 (4·9 to 8·8)	961
Self-harm and interpersonal violence	13·3 (7·2 to 21·9)	4·5 (2·5 to 7·3)	11·7 (5·8 to 21·1)	64·2 (39·5 to 105)	85·5 (61·9 to 112)	151 (115 to 199)	414 (316 to 542)	745 (540 to 1,020)	836 (562 to 1,170)	755 (517 to 1,090)	530 (366 to 746)	453 (325 to 619)	372 (264 to 506)	252 (184 to 333)	186 (138 to 245)	129 (99·6 to 167)	81·8 (64·6 to 102)	61 (48·4 to 76)	36·9 (29·8 to 45·1)	21·6 (17·8 to 26·4)	13·9 (11·3 to 16·9)	7 (5·5 to 8·6)	5·4 (4·2 to 6·6)	5,230
Self-harm	0 (0 to 0)	0 (0 to 0)	0 (0 to 0)	0 (0 to 0)	0 (0 to 0)	25·7 (16·3 to 39·1)	83·1 (55·8 to 119)	138 (91·8 to 199)	155 (97·9 to 233)	129 (85·1 to 189)	91·5 (60·3 to 134)	82·2 (55·8 to 117)	67·2 (45·8 to 95·3)	50·7 (35·6 to 70·6)	35·5 (25·7 to 47·9)	26·5 (19·2 to 35·3)	18·7 (13·7 to 25·8)	15·8 (11·3 to 21·9)	10·1 (7·4 to 13·5)	6·8 (5·1 to 8·8)	4·4 (3·2 to 5·8)	1·8 (1·4 to 2·3)	1·8 (1·2 to 2·4)	943
Self-harm by firearm	0 (0 to 0)	0 (0 to 0)	0 (0 to 0)	0 (0 to 0)	0 (0 to 0)	0·9 (0·5 to 1·6)	6·1 (4 to 9)	19·9 (13·1 to 29·5)	14·6 (8·9 to 22·4)	23·2 (15·1 to 34·4)	16·7 (9·1 to 28·2)	13 (7·8 to 21·1)	9·6 (6·5 to 13·7)	8·4 (5·7 to 11·9)	5·6 (4 to 7·6)	1·7 (1·2 to 2·2)	1·5 (1 to 2)	1·6 (1·1 to 2·1)	1·3 (1 to 1·8)	1·2 (0·9 to 1·6)	0·9 (0·6 to 1·2)	0·2 (0·1 to 0·2)	0·3 (0·2 to 0·3)	127
Self-harm by other specified means	0 (0 to 0)	0 (0 to 0)	0 (0 to 0)	0 (0 to 0)	0 (0 to 0)	24·8 (15·7 to 37·7)	77 (51·6 to 111)	118 (78·3 to 170)	140 (88·5 to 211)	106 (70·1 to 155)	74·8 (49·5 to 109)	69·2 (47·5 to 98·2)	57·7 (39·1 to 81·6)	42·4 (29·9 to 59)	29·9 (21·6 to 40·2)	24·8 (17·9 to 33·2)	17·2 (12·6 to 23·7)	14·2 (10·1 to 19·8)	8·8 (6·4 to 11·8)	5·6 (4·2 to 7·2)	3·5 (2·6 to 4·6)	1·6 (1·2 to 2)	1·6 (1·1 to 2)	817
Interpersonal violence	13·3 (7·2 to 21·9)	4·5 (2·5 to 7·3)	11·5 (5·7 to 20·8)	62·5 (38·4 to 103)	84·7 (61·1 to 111)	125 (92·6 to 166)	330 (241 to 446)	606 (421 to 842)	679 (440 to 962)	621 (413 to 908)	435 (291 to 633)	367 (254 to 523)	303 (205 to 426)	199 (142 to 273)	149 (106 to 205)	102 (75·9 to 134)	62·5 (47·6 to 80)	44·8 (34·1 to 58·1)	26·6 (20·9 to 33·1)	14·6 (11·6 to 18·5)	9·4 (7·5 to 11·8)	5·1 (3·9 to 6·5)	3·6 (2·7 to 4·5)	4,260
Physical violence by firearm	1·6 (0·9 to 2·7)	0·2 (0·1 to 0·3)	1·4 (0·7 to 2·5)	19·6 (11·8 to 32·5)	30·7 (21·2 to 41·6)	42·7 (29·7 to 59·7)	139 (95·6 to 194)	274 (181 to 396)	277 (173 to 406)	290 (184 to 438)	251 (154 to 382)	185 (120 to 275)	166 (106 to 242)	83 (55·2 to 120)	47·1 (31 to 67)	30·5 (20·9 to 43)	15·9 (11·1 to 21·5)	5·6 (4 to 7·5)	2·1 (1·6 to 2·7)	0·7 (0·5 to 0·9)	0·8 (0·6 to 1·1)	0·2 (0·1 to 0·2)	0·1 (0·1 to 0·1)	1,860
Physical violence by sharp object	1·3 (0·7 to 2·1)	0·1 (0 to 0·1)	1·2 (0·6 to 2)	14·3 (8·9 to 23·4)	20·8 (14·5 to 27·8)	31·7 (22·2 to 44)	104 (72·8 to 147)	187 (125 to 269)	252 (156 to 370)	187 (119 to 283)	98·1 (62·3 to 146)	105 (70·6 to 153)	67·9 (45·7 to 97·3)	44·9 (31·9 to 62·2)	52·8 (35·8 to 74·4)	29·2 (21·2 to 39·5)	16·1 (11·9 to 21)	16·3 (11·9 to 21·8)	9 (6·8 to 11·5)	4·8 (3·6 to 6·4)	1·9 (1·4 to 2·6)	1·8 (1·4 to 2·3)	2·1 (1·6 to 2·8)	1,250
Sexual violence	0 (0 to 0)	0 (0 to 0)	0 (0 to 0)	0·7 (0·4 to 1·1)	6·5 (3·8 to 10·2)	15·8 (9 to 24·9)	40·4 (22·1 to 65·1)	60·9 (35·4 to 93·4)	55·5 (31·4 to 89·6)	46·4 (25·5 to 77·4)	37·4 (20·8 to 59·5)	27 (15·4 to 44)	20·7 (12·2 to 34·7)	13·8 (7·9 to 23·2)	9·1 (5·2 to 14·6)	6·8 (4·2 to 10·4)	4·9 (3 to 7·4)	3·7 (2·3 to 5·5)	2·6 (1·6 to 3·8)	2 (1·3 to 2·9)	1·2 (0·7 to 2)	0·3 (0·2 to 0·4)	0 (0 to 0·1)	356
Physical violence by other means	10·4 (5·7 to 17·1)	4·2 (2·3 to 6·9)	8·9 (4·3 to 16·3)	28 (17 to 46·6)	26·7 (19 to 35·8)	34·8 (25·2 to 47·7)	47·1 (34 to 64·6)	84·2 (58·7 to 118)	94·5 (61·7 to 134)	97 (64·6 to 140)	48 (33·7 to 66·1)	50 (35·4 to 69)	48·5 (34·3 to 66·5)	57·2 (41·4 to 78·2)	40·1 (28·7 to 54·2)	35·3 (26·7 to 46·3)	25·6 (19·5 to 32·7)	19·3 (14·4 to 25·1)	12·9 (10 to 16·3)	7·2 (5·4 to 9·4)	5·4 (4·1 to 6·9)	2·8 (2·1 to 3·7)	1·3 (0·9 to 1·8)	789
Conflict and terrorism	0 (0 to 0)	0 (0 to 0)	0 (0 to 0)	0 (0 to 0)	0 (0 to 0)	0 (0 to 0)	0 (0 to 0)	0·3 (0·1 to 0·6)	1·7 (0·9 to 3·2)	4·7 (2·6 to 9·1)	3 (1·7 to 5·7)	3·3 (1·8 to 5·9)	2·2 (1·2 to 3·9)	1·6 (0·9 to 2·8)	1·1 (0·7 to 1·9)	0·8 (0·5 to 1·4)	0·5 (0·3 to 0·8)	0·3 (0·2 to 0·5)	0·2 (0·1 to 0·3)	0·2 (0·1 to 0·2)	0·1 (0·1 to 0·1)	0·1 (0 to 0·1)	0 (0 to 0)	20·1

SR Table 2b: Saint Lucia DALYS by cause and age group for females with 95% uncertainty intervals, 2019

Cause	Early Neonatal	Late Neonatal	Post Neonatal	1 to 4	5 to 9	10 to 14	15 to 19	20 to 24	25 to 29	30 to 34	35 to 39	40 to 44	45 to 49	50 to 54	55 to 59	60 to 64	65 to 69	70 to 74	75 to 79	80 to 84	85 to 89	90 to 94	95 plus	Total
All causes	605 (422 to 850)	202 (142 to 282)	141 (105 to 187)	329 (254 to 423)	329 (263 to 415)	445 (347 to 568)	696 (549 to 880)	888 (706 to 1,100)	1,040 (839 to 1,290)	1,120 (905 to 1,370)	1,350 (1,100 to 1,650)	1,620 (1,340 to 1,940)	1,980 (1,650 to 2,330)	2,120 (1,780 to 2,490)	2,090 (1,760 to 2,450)	1,970 (1,690 to 2,290)	1,740 (1,500 to 2,000)	1,660 (1,450 to 1,890)	1,450 (1,270 to 1,650)	1,300 (1,150 to 1,440)	1,030 (923 to 1,130)	592 (545 to 640)	182 (171 to 193)	24,900
Communicable, maternal, neonatal, and nutritional diseases	522 (361 to 730)	159 (111 to 221)	72·8 (52·5 to 96·3)	119 (90·1 to 153)	95·7 (66·8 to 139)	83·2 (59·3 to 116)	105 (76·1 to 144)	123 (96·8 to 163)	137 (108 to 174)	139 (109 to 178)	147 (114 to 191)	153 (117 to 200)	144 (110 to 190)	132 (100 to 172)	111 (83·6 to 148)	90·4 (70·9 to 116)	72·3 (56·9 to 90·6)	74·3 (58·8 to 93·9)	70·8 (55·4 to 89)	71 (56·5 to 88·4)	69·4 (55·5 to 85·5)	45·2 (34·8 to 55·8)	16·8 (12·8 to 21)	2,750
HIV/AIDS and sexually transmitted infections	0·9 (0·5 to 1·5)	0·3 (0·1 to 0·6)	1·1 (1 to 1·2)	1·2 (1·1 to 1·2)	1·3 (1·2 to 1·4)	1·1 (1 to 1·2)	2·9 (2·6 to 3·5)	12·8 (11·3 to 14·8)	19·5 (16·9 to 23·1)	24·5 (21·6 to 28·4)	22·5 (19·6 to 26·4)	28·1 (25·1 to 32·5)	21·8 (19·3 to 25·4)	14·1 (12·5 to 16·4)	8·2 (7 to 9·9)	5·2 (4·4 to 6·3)	2·9 (2·5 to 3·7)	1·4 (1·1 to 2)	0·8 (0·6 to 1·1)	0·5 (0·4 to 0·7)	0·3 (0·3 to 0·4)	0·1 (0·1 to 0·2)	0 (0 to 0)	172
HIV/AIDS	0 (0 to 0)	0 (0 to 0)	1 (0·9 to 1·1)	1·1 (1·1 to 1·2)	1·3 (1·2 to 1·4)	0·9 (0·9 to 1)	2·1 (1·9 to 2·2)	10·6 (9·6 to 11·9)	16·2 (14·5 to 18·7)	20·3 (18·5 to 23)	17·7 (16·1 to 19·6)	22·8 (21·1 to 24·8)	17·1 (15·7 to 18·5)	11·3 (10·5 to 12·2)	6·4 (5·8 to 7·1)	4·1 (3·7 to 4·6)	2·2 (1·9 to 2·5)	0·9 (0·7 to 1)	0·4 (0·3 to 0·5)	0·2 (0·2 to 0·3)	0·1 (0·1 to 0·2)	0 (0 to 0)	0 (0 to 0)	137
HIV/AIDS - Drug-susceptible Tuberculosis	0 (0 to 0)	0 (0 to 0)	0·1 (0·1 to 0·1)	0·1 (0·1 to 0·2)	0·1 (0·1 to 0·2)	0·1 (0·1 to 0·1)	0·2 (0·1 to 0·3)	1·1 (0·8 to 1·6)	1·6 (1·1 to 2·3)	2·2 (1·4 to 3·2)	1·8 (1·2 to 2·5)	2·2 (1·5 to 3·3)	1·8 (1·2 to 2·6)	1 (0·7 to 1·5)	0·6 (0·4 to 0·8)	0·3 (0·2 to 0·5)	0·2 (0·1 to 0·3)	0·1 (0 to 0·1)	0 (0 to 0)	0 (0 to 0)	0 (0 to 0)	0 (0 to 0)	0 (0 to 0)	13·5
HIV/AIDS - Multidrug-resistant Tuberculosis without extensive dru	0 (0 to 0)	0 (0 to 0)	0 (0 to 0)	0 (0 to 0)	0 (0 to 0)	0 (0 to 0)	0 (0 to 0)	0 (0 to 0·1)	0 (0 to 0·1)	0 (0 to 0·1)	0 (0 to 0·1)	0 (0 to 0·1)	0 (0 to 0·1)	0 (0 to 0)	0 (0 to 0)	0 (0 to 0)	0 (0 to 0)	0 (0 to 0)	0 (0 to 0)	0 (0 to 0)	0 (0 to 0)	0 (0 to 0)	0 (0 to 0)	0·2
HIV/AIDS - Extensively drug-resistant Tuberculosis	0 (0 to 0)	0 (0 to 0)	0 (0 to 0)	0 (0 to 0)	0 (0 to 0)	0 (0 to 0)	0 (0 to 0)	0 (0 to 0)	0 (0 to 0)	0 (0 to 0)	0 (0 to 0)	0 (0 to 0)	0 (0 to 0)	0 (0 to 0)	0 (0 to 0)	0 (0 to 0)	0 (0 to 0)	0 (0 to 0)	0 (0 to 0)	0 (0 to 0)	0 (0 to 0)	0 (0 to 0)	0 (0 to 0)	0
HIV/AIDS resulting in other diseases	0 (0 to 0)	0 (0 to 0)	0·9 (0·8 to 1)	1 (0·9 to 1·1)	1·2 (1·1 to 1·3)	0·8 (0·8 to 0·9)	1·9 (1·7 to 2)	9·5 (8·3 to 10·8)	14·6 (12·7 to 17·2)	18·1 (16 to 20·7)	15·9 (14·1 to 17·8)	20·6 (18·6 to 22·6)	15·3 (13·7 to 16·8)	10·3 (9·3 to 11·3)	5·8 (5·2 to 6·5)	3·8 (3·3 to 4·3)	2 (1·7 to 2·3)	0·8 (0·6 to 1)	0·4 (0·3 to 0·5)	0·2 (0·2 to 0·3)	0·1 (0·1 to 0·2)	0 (0 to 0)	0 (0 to 0)	123
Sexually transmitted infections excluding HIV	0·9 (0·5 to 1·5)	0·3 (0·1 to 0·6)	0·1 (0 to 0·2)	0 (0 to 0)	0 (0 to 0)	0·2 (0·1 to 0·3)	0·9 (0·6 to 1·4)	2·1 (1·3 to 3·6)	3·3 (1·9 to 5·9)	4·2 (2·4 to 7)	4·8 (3 to 7·9)	5·3 (3·3 to 9·1)	4·7 (2·8 to 7·8)	2·7 (1·5 to 4·8)	1·8 (0·9 to 3·3)	1·1 (0·6 to 2·1)	0·8 (0·4 to 1·5)	0·6 (0·3 to 1)	0·4 (0·2 to 0·7)	0·3 (0·2 to 0·5)	0·2 (0·1 to 0·3)	0·1 (0·1 to 0·1)	0 (0 to 0)	34·7
Syphilis	0·9 (0·5 to 1·5)	0·3 (0·1 to 0·6)	0·1 (0 to 0·2)	0 (0 to 0)	0 (0 to 0)	0·1 (0 to 0·1)	0·1 (0·1 to 0·2)	0·1 (0·1 to 0·2)	0·2 (0·1 to 0·3)	0·3 (0·2 to 0·4)	0·2 (0·1 to 0·3)	0·1 (0·1 to 0·2)	0·2 (0·1 to 0·3)	0·1 (0·1 to 0·2)	0 (0 to 0)	0 (0 to 0)	0 (0 to 0)	0 (0 to 0)	0 (0 to 0)	0 (0 to 0)	0 (0 to 0)	0 (0 to 0)	0 (0 to 0)	2·9
Chlamydial infection	0 (0 to 0)	0 (0 to 0)	0 (0 to 0)	0 (0 to 0)	0 (0 to 0)	0 (0 to 0)	0·1 (0·1 to 0·1)	0·3 (0·2 to 0·4)	0·3 (0·2 to 0·6)	0·4 (0·2 to 0·6)	0·5 (0·3 to 0·8)	0·6 (0·4 to 1)	0·5 (0·3 to 0·8)	0·2 (0·1 to 0·2)	0·1 (0·1 to 0·1)	0 (0 to 0·1)	0 (0 to 0)	0 (0 to 0)	0 (0 to 0)	0 (0 to 0)	0 (0 to 0)	0 (0 to 0)	0 (0 to 0)	3·1
Gonococcal infection	0 (0 to 0)	[illegible]	[illegible]	[illegible]	[illegible]	[illegible]	[illegible]	[illegible]	[illegible]	[illegible]	[illegible]	[illegible]	[illegible]	[illegible]	[illegible]	[illegible]	[illegible]	[illegible]	[illegible]	[illegible]	[illegible]	[illegible]	0 (0 to 0)	4·6
Trichomoniasis	0 (0 to 0)	[illegible]	[illegible]	[illegible]	[illegible]	[illegible]	[illegible]	[illegible]	[illegible]	[illegible]	[illegible]	[illegible]	[illegible]	[illegible]	[illegible]	[illegible]	[illegible]	[illegible]	[illegible]	[illegible]	[illegible]	[illegible]	0 (0 to 0)	9·3
Genital herpes	0 (0 to 0)	[illegible]	[illegible]	[illegible]	[illegible]	[illegible]	[illegible]	[illegible]	[illegible]	[illegible]	[illegible]	[illegible]	[illegible]	[illegible]	[illegible]	[illegible]	[illegible]	[illegible]	[illegible]	[illegible]	[illegible]	[illegible]	0 (0 to 0)	7·2
Other sexually transmitted infections	0 (0 to 0)	[illegible]	[illegible]	[illegible]	[illegible]	[illegible]	[illegible]	[illegible]	[illegible]	[illegible]	[illegible]	[illegible]	[illegible]	[illegible]	[illegible]	[illegible]	[illegible]	[illegible]	[illegible]	[illegible]	[illegible]	[illegible]	0 (0 to 0)	7·7
Respiratory infections and tuberculosis	12·6 (7·6 to 19·4)	[illegible]	[illegible]	[illegible]	[illegible]	[illegible]	[illegible]	[illegible]	[illegible]	[illegible]	[illegible]	[illegible]	[illegible]	[illegible]	[illegible]	[illegible]	[illegible]	[illegible]	[illegible]	[illegible]	[illegible]	[illegible]	13·4 (9·8 to 17)	577
Tuberculosis	0 (0 to 0)	[illegible]	[illegible]	[illegible]	[illegible]	[illegible]	[illegible]	[illegible]	[illegible]	[illegible]	[illegible]	[illegible]	[illegible]	[illegible]	[illegible]	[illegible]	[illegible]	[illegible]	[illegible]	[illegible]	[illegible]	[illegible]	0·1 (0·1 to 0·1)	41·1
Latent tuberculosis infection	0 (0 to 0)	[illegible]	[illegible]	[illegible]	[illegible]	[illegible]	[illegible]	[illegible]	[illegible]	[illegible]	[illegible]	[illegible]	[illegible]	[illegible]	[illegible]	[illegible]	[illegible]	[illegible]	[illegible]	[illegible]	[illegible]	[illegible]	0 (0 to 0)	0
Drug-susceptible tuberculosis	0 (0 to 0)	[illegible]	[illegible]	[illegible]	[illegible]	[illegible]	[illegible]	[illegible]	[illegible]	[illegible]	[illegible]	[illegible]	[illegible]	[illegible]	[illegible]	[illegible]	[illegible]	[illegible]	[illegible]	[illegible]	[illegible]	[illegible]	0·1 (0·1 to 0·1)	40·7
Multidrug-resistant tuberculosis without extensive drug resistanc	0 (0 to 0)	[illegible]	[illegible]	[illegible]	[illegible]	[illegible]	[illegible]	[illegible]	[illegible]	[illegible]	[illegible]	[illegible]	[illegible]	[illegible]	[illegible]	[illegible]	[illegible]	[illegible]	[illegible]	[illegible]	[illegible]	[illegible]	0 (0 to 0)	0·4
Extensively drug-resistant tuberculosis	0 (0 to 0)	[illegible]	[illegible]	[illegible]	[illegible]	[illegible]	[illegible]	[illegible]	[illegible]	[illegible]	[illegible]	[illegible]	[illegible]	[illegible]	[illegible]	[illegible]	[illegible]	[illegible]	[illegible]	[illegible]	[illegible]	[illegible]	0 (0 to 0)	0·1
Lower respiratory infections	12·6 (7·6 to 19·4)	[illegible]	[illegible]	[illegible]	[illegible]	[illegible]	[illegible]	[illegible]	[illegible]	[illegible]	[illegible]	[illegible]	[illegible]	[illegible]	[illegible]	[illegible]	[illegible]	[illegible]	[illegible]	[illegible]	[illegible]	[illegible]	13·2 (9·7 to 16·9)	449
Upper respiratory infections	0 (0 to 0)	[illegible]	[illegible]	[illegible]	[illegible]	[illegible]	[illegible]	[illegible]	[illegible]	[illegible]	[illegible]	[illegible]	[illegible]	[illegible]	[illegible]	[illegible]	[illegible]	[illegible]	[illegible]	[illegible]	[illegible]	[illegible]	0 (0 to 0·1)	66·5
Otitis media	0 (0 to 0)	[illegible]	[illegible]	[illegible]	[illegible]	[illegible]	[illegible]	[illegible]	[illegible]	[illegible]	[illegible]	[illegible]	[illegible]	[illegible]	[illegible]	[illegible]	[illegible]	[illegible]	[illegible]	[illegible]	[illegible]	[illegible]	0 (0 to 0)	20·7
Enteric infections	1·3 (0·7 to 2·1)	[illegible]	[illegible]	[illegible]	[illegible]	[illegible]	[illegible]	[illegible]	[illegible]	[illegible]	[illegible]	[illegible]	[illegible]	[illegible]	[illegible]	[illegible]	[illegible]	[illegible]	[illegible]	[illegible]	[illegible]	[illegible]	1·1 (0·8 to 1·5)	224
Diarrheal diseases	1·3 (0·7 to 2·1)	[illegible]	[illegible]	[illegible]	[illegible]	[illegible]	[illegible]	[illegible]	[illegible]	[illegible]	[illegible]	[illegible]	[illegible]	[illegible]	[illegible]	[illegible]	[illegible]	[illegible]	[illegible]	[illegible]	[illegible]	[illegible]	1·1 (0·8 to 1·5)	223
Typhoid and paratyphoid	0 (0 to 0)	[illegible]	[illegible]	[illegible]	[illegible]	[illegible]	[illegible]	[illegible]	[illegible]	[illegible]	[illegible]	[illegible]	[illegible]	[illegible]	[illegible]	[illegible]	[illegible]	[illegible]	[illegible]	[illegible]	[illegible]	[illegible]	0 (0 to 0)	0·1
Typhoid fever	0 (0 to 0)	[illegible]	[illegible]	[illegible]	[illegible]	[illegible]	[illegible]	[illegible]	[illegible]	[illegible]	[illegible]	[illegible]	[illegible]	[illegible]	[illegible]	[illegible]	[illegible]	[illegible]	[illegible]	[illegible]	[illegible]	[illegible]	0 (0 to 0)	0
Paratyphoid fever	0 (0 to 0)	[illegible]	[illegible]	[illegible]	[illegible]	[illegible]	[illegible]	[illegible]	[illegible]	[illegible]	[illegible]	[illegible]	[illegible]	[illegible]	[illegible]	[illegible]	[illegible]	[illegible]	[illegible]	[illegible]	[illegible]	[illegible]	0 (0 to 0)	0
Invasive Non-typhoidal Salmonella (iNTS)	0 (0 to 0)	[illegible]	[illegible]	[illegible]	[illegible]	[illegible]	[illegible]	[illegible]	[illegible]	[illegible]	[illegible]	[illegible]	[illegible]	[illegible]	[illegible]	[illegible]	[illegible]	[illegible]	[illegible]	[illegible]	[illegible]	[illegible]	0 (0 to 0)	0·2
Other intestinal infectious diseases	0 (0 to 0)	[illegible]	[illegible]	[illegible]	[illegible]	[illegible]	[illegible]	[illegible]	[illegible]	[illegible]	[illegible]	[illegible]	[illegible]	[illegible]	[illegible]	[illegible]	[illegible]	[illegible]	[illegible]	[illegible]	[illegible]	[illegible]	0 (0 to 0)	0·4
Neglected tropical diseases and malaria	0·2 (0·1 to 0·3)	[illegible]	[illegible]	[illegible]	[illegible]	[illegible]	[illegible]	[illegible]	[illegible]	[illegible]	[illegible]	[illegible]	[illegible]	[illegible]	[illegible]	[illegible]	[illegible]	[illegible]	[illegible]	[illegible]	[illegible]	[illegible]	0·4 (0·2 to 0·6)	124
Malaria	0 (0 to 0)	[illegible]	[illegible]	[illegible]	[illegible]	[illegible]	[illegible]	[illegible]	[illegible]	[illegible]	[illegible]	[illegible]	[illegible]	[illegible]	[illegible]	[illegible]	[illegible]	[illegible]	[illegible]	[illegible]	[illegible]	[illegible]	0 (0 to 0)	0
Chagas disease	0 (0 to 0)	[illegible]	[illegible]	[illegible]	[illegible]	[illegible]	[illegible]	[illegible]	[illegible]	[illegible]	[illegible]	[illegible]	[illegible]	[illegible]	[illegible]	[illegible]	[illegible]	[illegible]	[illegible]	[illegible]	[illegible]	[illegible]	0 (0 to 0)	0
Leishmaniasis	0 (0 to 0)	[illegible]	[illegible]	[illegible]	[illegible]	[illegible]	[illegible]	[illegible]	[illegible]	[illegible]	[illegible]	[illegible]	[illegible]	[illegible]	[illegible]	[illegible]	[illegible]	[illegible]	[illegible]	[illegible]	[illegible]	[illegible]	0 (0 to 0)	0
Visceral leishmaniasis	0 (0 to 0)	[illegible]	[illegible]	[illegible]	[illegible]	[illegible]	[illegible]	[illegible]	[illegible]	[illegible]	[illegible]	[illegible]	[illegible]	[illegible]	[illegible]	[illegible]	[illegible]	[illegible]	[illegible]	[illegible]	[illegible]	[illegible]	0 (0 to 0)	0
Cutaneous and mucocutaneous leishmaniasis	0 (0 to 0)	[illegible]	[illegible]	[illegible]	[illegible]	[illegible]	[illegible]	[illegible]	[illegible]	[illegible]	[illegible]	[illegible]	[illegible]	[illegible]	[illegible]	[illegible]	[illegible]	[illegible]	[illegible]	[illegible]	[illegible]	[illegible]	0 (0 to 0)	0
African trypanosomiasis	0 (0 to 0)	[illegible]	[illegible]	[illegible]	[illegible]	[illegible]	[illegible]	[illegible]	[illegible]	[illegible]	[illegible]	[illegible]	[illegible]	[illegible]	[illegible]	[illegible]	[illegible]	[illegible]	[illegible]	[illegible]	[illegible]	[illegible]	0 (0 to 0)	0
Schistosomiasis	0 (0 to 0)	[illegible]	[illegible]	[illegible]	[illegible]	[illegible]	[illegible]	[illegible]	[illegible]	[illegible]	[illegible]	[illegible]	[illegible]	[illegible]	[illegible]	[illegible]	[illegible]	[illegible]	[illegible]	[illegible]	[illegible]	[illegible]	0 (0 to 0)	0·5
Cysticercosis	0 (0 to 0)	[illegible]	[illegible]	[illegible]	[illegible]	[illegible]	[illegible]	[illegible]	[illegible]	[illegible]	[illegible]	[illegible]	[illegible]	[illegible]	[illegible]	[illegible]	[illegible]	[illegible]	[illegible]	[illegible]	[illegible]	[illegible]	0·2 (0·1 to 0·4)	61·8
Cystic echinococcosis	0 (0 to 0)	[illegible]	[illegible]	[illegible]	[illegible]	[illegible]	[illegible]	[illegible]	[illegible]	[illegible]	[illegible]	[illegible]	[illegible]	[illegible]	[illegible]	[illegible]	[illegible]	[illegible]	[illegible]	[illegible]	[illegible]	[illegible]	0 (0 to 0)	0
Lymphatic filariasis	0 (0 to 0)	[illegible]	[illegible]	[illegible]	[illegible]	[illegible]	[illegible]	[illegible]	[illegible]	[illegible]	[illegible]	[illegible]	[illegible]	[illegible]	[illegible]	[illegible]	[illegible]	[illegible]	[illegible]	[illegible]	[illegible]	[illegible]	0 (0 to 0)	0
Onchocerciasis	0 (0 to 0)	[illegible]	[illegible]	[illegible]	[illegible]	[illegible]	[illegible]	[illegible]	[illegible]	[illegible]	[illegible]	[illegible]	[illegible]	[illegible]	[illegible]	[illegible]	[illegible]	[illegible]	[illegible]	[illegible]	[illegible]	[illegible]	0 (0 to 0)	0
Trachoma	0 (0 to 0)	[illegible]	[illegible]	[illegible]	[illegible]	[illegible]	[illegible]	[illegible]	[illegible]	[illegible]	[illegible]	[illegible]	[illegible]	[illegible]	[illegible]	[illegible]	[illegible]	[illegible]	[illegible]	[illegible]	[illegible]	[illegible]	0 (0 to 0)	0
Dengue	0 (0 to 0)	[illegible]	[illegible]	[illegible]	[illegible]	[illegible]	[illegible]	[illegible]	[illegible]	[illegible]	[illegible]	[illegible]	[illegible]	[illegible]	[illegible]	[illegible]	[illegible]	[illegible]	[illegible]	[illegible]	[illegible]	[illegible]	0 (0 to 0·1)	22·5
Yellow fever	0 (0 to 0)	[illegible]	[illegible]	[illegible]	[illegible]	[illegible]	[illegible]	[illegible]	[illegible]	[illegible]	[illegible]	[illegible]	[illegible]	[illegible]	[illegible]	[illegible]	[illegible]	[illegible]	[illegible]	[illegible]	[illegible]	[illegible]	0 (0 to 0)	0
Rabies	0 (0 to 0)	[illegible]	[illegible]	[illegible]	[illegible]	[illegible]	[illegible]	[illegible]	[illegible]	[illegible]	[illegible]	[illegible]	[illegible]	[illegible]	[illegible]	[illegible]	[illegible]	[illegible]	[illegible]	[illegible]	[illegible]	[illegible]	0 (0 to 0)	0·1
Intestinal nematode infections	0 (0 to 0)	[illegible]	[illegible]	[illegible]	[illegible]	[illegible]	[illegible]	[illegible]	[illegible]	[illegible]	[illegible]	[illegible]	[illegible]	[illegible]	[illegible]	[illegible]	[illegible]	[illegible]	[illegible]	[illegible]	[illegible]	[illegible]	0 (0 to 0)	9·8
Ascariasis	0 (0 to 0)	[illegible]	[illegible]	[illegible]	[illegible]	[illegible]	[illegible]	[illegible]	[illegible]	[illegible]	[illegible]	[illegible]	[illegible]	[illegible]	[illegible]	[illegible]	[illegible]	[illegible]	[illegible]	[illegible]	[illegible]	[illegible]	0 (0 to 0)	1·6
Trichuriasis	0 (0 to 0)	[illegible]	[illegible]	[illegible]	[illegible]	[illegible]	[illegible]	[illegible]	[illegible]	[illegible]	[illegible]	[illegible]	[illegible]	[illegible]	[illegible]	[illegible]	[illegible]	[illegible]	[illegible]	[illegible]	[illegible]	[illegible]	0 (0 to 0)	0·8
Hookworm disease	0 (0 to 0)	[illegible]	[illegible]	[illegible]	[illegible]	[illegible]	[illegible]	[illegible]	[illegible]	[illegible]	[illegible]	[illegible]	[illegible]	[illegible]	[illegible]	[illegible]	[illegible]	[illegible]	[illegible]	[illegible]	[illegible]	[illegible]	0 (0 to 0)	7·4
Food-borne trematodiases	0 (0 to 0)	[illegible]	[illegible]	[illegible]	[illegible]	[illegible]	[illegible]	[illegible]	[illegible]	[illegible]	[illegible]	[illegible]	[illegible]	[illegible]	[illegible]	[illegible]	[illegible]	[illegible]	[illegible]	[illegible]	[illegible]	[illegible]	0 (0 to 0)	0
Leprosy	0 (0 to 0)	[illegible]	[illegible]	[illegible]	[illegible]	[illegible]	[illegible]	[illegible]	[illegible]	[illegible]	[illegible]	[illegible]	[illegible]	[illegible]	[illegible]	[illegible]	[illegible]	[illegible]	[illegible]	[illegible]	[illegible]	[illegible]	0 (0 to 0)	0·3
Ebola	0 (0 to 0)	[illegible]	[illegible]	[illegible]	[illegible]	[illegible]	[illegible]	[illegible]	[illegible]	[illegible]	[illegible]	[illegible]	[illegible]	[illegible]	[illegible]	[illegible]	[illegible]	[illegible]	[illegible]	[illegible]	[illegible]	[illegible]	0 (0 to 0)	0
Zika virus	0 (0 to 0)	[illegible]	[illegible]	[illegible]	[illegible]	[illegible]	[illegible]	[illegible]	[illegible]	[illegible]	[illegible]	[illegible]	[illegible]	[illegible]	[illegible]	[illegible]	[illegible]	[illegible]	[illegible]	[illegible]	[illegible]	[illegible]	0 (0 to 0)	0·9
Guinea worm disease	0 (0 to 0)	[illegible]	[illegible]	[illegible]	[illegible]	[illegible]	[illegible]	[illegible]	[illegible]	[illegible]	[illegible]	[illegible]	[illegible]	[illegible]	[illegible]	[illegible]	[illegible]	[illegible]	[illegible]	[illegible]	[illegible]	[illegible]	0 (0 to 0)	0
Other neglected tropical diseases	0·2 (0·1 to 0·3)	[illegible]	[illegible]	[illegible]	[illegible]	[illegible]	[illegible]	[illegible]	[illegible]	[illegible]	[illegible]	[illegible]	[illegible]	[illegible]	[illegible]	[illegible]	[illegible]	[illegible]	[illegible]	[illegible]	[illegible]	[illegible]	0·1 (0 to 0·2)	28·2
Other infectious diseases	1·4 (0·9 to 2·2)	[illegible]	[illegible]	[illegible]	[illegible]	[illegible]	[illegible]	[illegible]	[illegible]	[illegible]	[illegible]	[illegible]	[illegible]	[illegible]	[illegible]	[illegible]	[illegible]	[illegible]	[illegible]	[illegible]	[illegible]	[illegible]	0·2 (0·1 to 0·2)	108
Meningitis	0·9 (0·6 to 1·4)	[illegible]	[illegible]	[illegible]	[illegible]	[illegible]	[illegible]	[illegible]	[illegible]	[illegible]	[illegible]	[illegible]	[illegible]	[illegible]	[illegible]	[illegible]	[illegible]	[illegible]	[illegible]	[illegible]	[illegible]	[illegible]	0 (0 to 0)	28·3
Encephalitis	0·4 (0·2 to 0·6)	[illegible]	[illegible]	[illegible]	[illegible]	[illegible]	[illegible]	[illegible]	[illegible]	[illegible]	[illegible]	[illegible]	[illegible]	[illegible]	[illegible]	[illegible]	[illegible]	[illegible]	[illegible]	[illegible]	[illegible]	[illegible]	0 (0 to 0)	34·6
Diphtheria	0 (0 to 0)	[illegible]	[illegible]	[illegible]	[illegible]	[illegible]	[illegible]	[illegible]	[illegible]	[illegible]	[illegible]	[illegible]	[illegible]	[illegible]	[illegible]	[illegible]	[illegible]	[illegible]	[illegible]	[illegible]	[illegible]	[illegible]	0 (0 to 0)	0
Tetanus	0 (0 to 0·1)	[illegible]	[illegible]	[illegible]	[illegible]	[illegible]	[illegible]	[illegible]	[illegible]	[illegible]	[illegible]	[illegible]	[illegible]	[illegible]	[illegible]	[illegible]	[illegible]	[illegible]	[illegible]	[illegible]	[illegible]	[illegible]	0 (0 to 0)	1·5
Measles	0 (0 to 0)	[illegible]	[illegible]	[illegible]	[illegible]	[illegible]	[illegible]	[illegible]	[illegible]	[illegible]	[illegible]	[illegible]	[illegible]	[illegible]	[illegible]	[illegible]	[illegible]	[illegible]	[illegible]	[illegible]	[illegible]	[illegible]	0 (0 to 0)	0
Varicella and herpes zoster	0 (0 to 0)	[illegible]	[illegible]	[illegible]	[illegible]	[illegible]	[illegible]	[illegible]	[illegible]	[illegible]	[illegible]	[illegible]	[illegible]	[illegible]	[illegible]	[illegible]	[illegible]	[illegible]	[illegible]	[illegible]	[illegible]	[illegible]	0 (0 to 0)	3·8
Acute hepatitis	0 (0 to 0)	[illegible]	[illegible]	[illegible]	[illegible]	[illegible]	[illegible]	[illegible]	[illegible]	[illegible]	[illegible]	[illegible]	[illegible]	[illegible]	[illegible]	[illegible]	[illegible]	[illegible]	[illegible]	[illegible]	[illegible]	[illegible]	0 (0 to 0)	4·5
Acute hepatitis A	0 (0 to 0)	[illegible]	[illegible]	[illegible]	[illegible]	[illegible]	[illegible]	[illegible]	[illegible]	[illegible]	[illegible]	[illegible]	[illegible]	[illegible]	[illegible]	[illegible]	[illegible]	[illegible]	[illegible]	[illegible]	[illegible]	[illegible]	0 (0 to 0)	2·8
Acute hepatitis B	0 (0 to 0)	[illegible]	[illegible]	[illegible]	[illegible]	[illegible]	[illegible]	[illegible]	[illegible]	[illegible]	[illegible]	[illegible]	[illegible]	[illegible]	[illegible]	[illegible]	[illegible]	[illegible]	[illegible]	[illegible]	[illegible]	[illegible]	0 (0 to 0)	1·1
Acute hepatitis C	0 (0 to 0)	[illegible]	[illegible]	[illegible]	[illegible]	[illegible]	[illegible]	[illegible]	[illegible]	[illegible]	[illegible]	[illegible]	[illegible]	[illegible]	[illegible]	[illegible]	[illegible]	[illegible]	[illegible]	[illegible]	[illegible]	[illegible]	0 (0 to 0)	0·1
Acute hepatitis E	0 (0 to 0)	[illegible]	[illegible]	[illegible]	[illegible]	[illegible]	[illegible]	[illegible]	[illegible]	[illegible]	[illegible]	[illegible]	[illegible]	[illegible]	[illegible]	[illegible]	[illegible]	[illegible]	[illegible]	[illegible]	[illegible]	[illegible]	0 (0 to 0)	0·4
Other unspecified infectious diseases	0·1 (0·1 to 0·2)	[illegible]	[illegible]	[illegible]	[illegible]	[illegible]	[illegible]	[illegible]	[illegible]	[illegible]	[illegible]	[illegible]	[illegible]	[illegible]	[illegible]	[illegible]	[illegible]	[illegible]	[illegible]	[illegible]	[illegible]	[illegible]	0·1 (0 to 0·2)	34·4
Maternal and neonatal disorders	505 (350 to 706)	[illegible]	[illegible]	[illegible]	[illegible]	[illegible]	[illegible]	[illegible]	[illegible]	[illegible]	[illegible]	[illegible]	[illegible]	[illegible]	[illegible]	[illegible]	[illegible]	[illegible]	[illegible]	[illegible]	[illegible]	[illegible]	0 (0 to 0)	1,060
Maternal disorders	0 (0 to 0)	[illegible]	[illegible]	[illegible]	[illegible]	[illegible]	[illegible]	[illegible]	[illegible]	[illegible]	[illegible]	[illegible]	[illegible]	[illegible]	[illegible]	[illegible]	[illegible]	[illegible]	[illegible]	[illegible]	[illegible]	[illegible]	0 (0 to 0)	102
Maternal hemorrhage	0 (0 to 0)	[illegible]	[illegible]	[illegible]	[illegible]	[illegible]	[illegible]	[illegible]	[illegible]	[illegible]	[illegible]	[illegible]	[illegible]	[illegible]	[illegible]	[illegible]	[illegible]	[illegible]	[illegible]	[illegible]	[illegible]	[illegible]	0 (0 to 0)	15·6
Maternal sepsis and other maternal infections	0 (0 to 0)	[illegible]	[illegible]	[illegible]	[illegible]	[illegible]	[illegible]	[illegible]	[illegible]	[illegible]	[illegible]	[illegible]	[illegible]	[illegible]	[illegible]	[illegible]	[illegible]	[illegible]	[illegible]	[illegible]	[illegible]	[illegible]	0 (0 to 0)	3
Maternal hypertensive disorders	0 (0 to 0)	[illegible]	[illegible]	[illegible]	[illegible]	[illegible]	[illegible]	[illegible]	[illegible]	[illegible]	[illegible]	[illegible]	[illegible]	[illegible]	[illegible]	[illegible]	[illegible]	[illegible]	[illegible]	[illegible]	[illegible]	[illegible]	0 (0 to 0)	10·1
Maternal obstructed labor and uterine rupture	0 (0 to 0)	[illegible]	[illegible]	[illegible]	[illegible]	[illegible]	[illegible]	[illegible]	[illegible]	[illegible]	[illegible]	[illegible]	[illegible]	[illegible]	[illegible]	[illegible]	[illegible]	[illegible]	[illegible]	[illegible]	[illegible]	[illegible]	0 (0 to 0)	7
Maternal abortion and miscarriage	0 (0 to 0)	[illegible]	[illegible]	[illegible]	[illegible]	[illegible]	[illegible]	[illegible]	[illegible]	[illegible]	[illegible]	[illegible]	[illegible]	[illegible]	[illegible]	[illegible]	[illegible]	[illegible]	[illegible]	[illegible]	[illegible]	[illegible]	0 (0 to 0)	3·7
Ectopic pregnancy	0 (0 to 0)	[illegible]	[illegible]	[illegible]	[illegible]	[illegible]	[illegible]	[illegible]	[illegible]	[illegible]	[illegible]	[illegible]	[illegible]	[illegible]	[illegible]	[illegible]	[illegible]	[illegible]	[illegible]	[illegible]	[illegible]	[illegible]	0 (0 to 0)	0·9
Indirect maternal deaths	--	[illegible]	[illegible]	[illegible]	[illegible]	[illegible]	[illegible]	[illegible]	[illegible]	[illegible]	[illegible]	[illegible]	[illegible]	[illegible]	[illegible]	[illegible]	[illegible]	[illegible]	[illegible]	[illegible]	[illegible]	[illegible]	--	NA
Late maternal deaths	--	[illegible]	[illegible]	[illegible]	[illegible]	[illegible]	[illegible]	[illegible]	[illegible]	[illegible]	[illegible]	[illegible]	[illegible]	[illegible]	[illegible]	[illegible]	[illegible]	[illegible]	[illegible]	[illegible]	[illegible]	[illegible]	--	NA
Maternal deaths aggravated by HIV/AIDS	--	[illegible]	[illegible]	[illegible]	[illegible]	[illegible]	[illegible]	[illegible]	[illegible]	[illegible]	[illegible]	[illegible]	[illegible]	[illegible]	[illegible]	[illegible]	[illegible]	[illegible]	[illegible]	[illegible]	[illegible]	[illegible]	--	NA
Neonatal disorders	505 (350 to 706)	[illegible]	[illegible]	[illegible]	[illegible]	[illegible]	[illegible]	[illegible]	[illegible]	[illegible]	[illegible]	[illegible]	[illegible]	[illegible]	[illegible]	[illegible]	[illegible]	[illegible]	[illegible]	[illegible]	[illegible]	[illegible]	0 (0 to 0)	963
Neonatal preterm birth	291 (200 to 413)	[illegible]	[illegible]	[illegible]	[illegible]	[illegible]	[illegible]	[illegible]	[illegible]	[illegible]	[illegible]	[illegible]	[illegible]	[illegible]	[illegible]	[illegible]	[illegible]	[illegible]	[illegible]	[illegible]	[illegible]	[illegible]	0 (0 to 0)	479
Neonatal encephalopathy due to birth asphyxia and trauma	113 (73 to 164)	[illegible]	[illegible]	[illegible]	[illegible]	[illegible]	[illegible]	[illegible]	[illegible]	[illegible]	[illegible]	[illegible]	[illegible]	[illegible]	[illegible]	[illegible]	[illegible]	[illegible]	[illegible]	[illegible]	[illegible]	[illegible]	0 (0 to 0)	166
Neonatal sepsis and other neonatal infections	49 (29·5 to 80·4)	[illegible]	[illegible]	[illegible]	[illegible]	[illegible]	[illegible]	[illegible]	[illegible]	[illegible]	[illegible]	[illegible]	[illegible]	[illegible]	[illegible]	[illegible]	[illegible]	[illegible]	[illegible]	[illegible]	[illegible]	[illegible]	0 (0 to 0)	223
Hemolytic disease and other neonatal jaundice	0·1 (0·1 to 0·2)	[illegible]	[illegible]	[illegible]	[illegible]	[illegible]	[illegible]	[illegible]	[illegible]	[illegible]	[illegible]	[illegible]	[illegible]	[illegible]	[illegible]	[illegible]	[illegible]	[illegible]	[illegible]	[illegible]	[illegible]	[illegible]	0 (0 to 0)	13·4
Other neonatal disorders	52·1 (29·9 to 84·8)	[illegible]	[illegible]	[illegible]	[illegible]	[illegible]	[illegible]	[illegible]	[illegible]	[illegible]	[illegible]	[illegible]	[illegible]	[illegible]	[illegible]	[illegible]	[illegible]	[illegible]	[illegible]	[illegible]	[illegible]	[illegible]	0 (0 to 0)	82
Nutritional deficiencies	0 (0 to 0)	[illegible]	[illegible]	[illegible]	[illegible]	[illegible]	[illegible]	[illegible]	[illegible]	[illegible]	[illegible]	[illegible]	[illegible]	[illegible]	[illegible]	[illegible]	[illegible]	[illegible]	[illegible]	[illegible]	[illegible]	[illegible]	1·7 (1·2 to 2·4)	482
Protein-energy malnutrition	0 (0 to 0)	[illegible]	[illegible]	[illegible]	[illegible]	[illegible]	[illegible]	[illegible]	[illegible]	[illegible]	[illegible]	[illegible]	[illegible]	[illegible]	[illegible]	[illegible]	[illegible]	[illegible]	[illegible]	[illegible]	[illegible]	[illegible]	1·4 (0·9 to 1·9)	70·3
Iodine deficiency	0 (0 to 0)	[illegible]	[illegible]	[illegible]	[illegible]	[illegible]	[illegible]	[illegible]	[illegible]	[illegible]	[illegible]	[illegible]	[illegible]	[illegible]	[illegible]	[illegible]	[illegible]	[illegible]	[illegible]	[illegible]	[illegible]	[illegible]	0 (0 to 0)	3
Vitamin A deficiency	0 (0 to 0)	[illegible]	[illegible]	[illegible]	[illegible]	[illegible]	[illegible]	[illegible]	[illegible]	[illegible]	[illegible]	[illegible]	[illegible]	[illegible]	[illegible]	[illegible]	[illegible]	[illegible]	[illegible]	[illegible]	[illegible]	[illegible]	0 (0 to 0)	4·2
Dietary iron deficiency	0 (0 to 0)	[illegible]	[illegible]	[illegible]	[illegible]	[illegible]	[illegible]	[illegible]	[illegible]	[illegible]	[illegible]	[illegible]	[illegible]	[illegible]	[illegible]	[illegible]	[illegible]	[illegible]	[illegible]	[illegible]	[illegible]	[illegible]	0·1 (0 to 0·3)	384
Other nutritional deficiencies	0 (0 to 0)	[illegible]	[illegible]	[illegible]	[illegible]	[illegible]	[illegible]	[illegible]	[illegible]	[illegible]	[illegible]	[illegible]	[illegible]	[illegible]	[illegible]	[illegible]	[illegible]	[illegible]	[illegible]	[illegible]	[illegible]	[illegible]	0·2 (0·1 to 0·4)	21·1
Non-communicable diseases	77·4 (50 to 118)	[illegible]	[illegible]	[illegible]	[illegible]	[illegible]	[illegible]	[illegible]	[illegible]	[illegible]	[illegible]	[illegible]	[illegible]	[illegible]	[illegible]	[illegible]	[illegible]	[illegible]	[illegible]	[illegible]	[illegible]	[illegible]	160 (149 to 171)	20,900
Neoplasms	0·7 (0·4 to 1)	[illegible]	[illegible]	[illegible]	[illegible]	[illegible]	[illegible]	[illegible]	[illegible]	[illegible]	[illegible]	[illegible]	[illegible]	[illegible]	[illegible]	[illegible]	[illegible]	[illegible]	[illegible]	[illegible]	[illegible]	[illegible]	10·6 (8·7 to 12·1)	3,170
Lip and oral cavity cancer	0 (0 to 0)	[illegible]	[illegible]	[illegible]	[illegible]	[illegible]	[illegible]	[illegible]	[illegible]	[illegible]	[illegible]	[illegible]	[illegible]	[illegible]	[illegible]	[illegible]	[illegible]	[illegible]	[illegible]	[illegible]	[illegible]	[illegible]	0·2 (0·2 to 0·3)	28·2
Nasopharynx cancer	0 (0 to 0)	[illegible]	[illegible]	[illegible]	[illegible]	[illegible]	[illegible]	[illegible]	[illegible]	[illegible]	[illegible]	[illegible]	[illegible]	[illegible]	[illegible]	[illegible]	[illegible]	[illegible]	[illegible]	[illegible]	[illegible]	[illegible]	0 (0 to 0·1)	11·3
Other pharynx cancer	0 (0 to 0)	[illegible]	[illegible]	[illegible]	[illegible]	[illegible]	[illegible]	[illegible]	[illegible]	[illegible]	[illegible]	[illegible]	[illegible]	[illegible]	[illegible]	[illegible]	[illegible]	[illegible]	[illegible]	[illegible]	[illegible]	[illegible]	0 (0 to 0)	6·5
Esophageal cancer	0 (0 to 0)	[illegible]	[illegible]	[illegible]	[illegible]	[illegible]	[illegible]	[illegible]	[illegible]	[illegible]	[illegible]	[illegible]	[illegible]	[illegible]	[illegible]	[illegible]	[illegible]	[illegible]	[illegible]	[illegible]	[illegible]	[illegible]	0·2 (0·1 to 0·2)	31·4
Stomach cancer	0 (0 to 0)	[illegible]	[illegible]	[illegible]	[illegible]	[illegible]	[illegible]	[illegible]	[illegible]	[illegible]	[illegible]	[illegible]	[illegible]	[illegible]	[illegible]	[illegible]	[illegible]	[illegible]	[illegible]	[illegible]	[illegible]	[illegible]	1·1 (0·9 to 1·3)	181
Colon and rectum cancer	0 (0 to 0)	[illegible]	[illegible]	[illegible]	[illegible]	[illegible]	[illegible]	[illegible]	[illegible]	[illegible]	[illegible]	[illegible]	[illegible]	[illegible]	[illegible]	[illegible]	[illegible]	[illegible]	[illegible]	[illegible]	[illegible]	[illegible]	1·6 (1·3 to 2)	247
Liver cancer	0 (0 to 0)	[illegible]	[illegible]	[illegible]	[illegible]	[illegible]	[illegible]	[illegible]	[illegible]	[illegible]	[illegible]	[illegible]	[illegible]	[illegible]	[illegible]	[illegible]	[illegible]	[illegible]	[illegible]	[illegible]	[illegible]	[illegible]	0·2 (0·1 to 0·2)	36·3
Liver cancer due to hepatitis B	0 (0 to 0)	[illegible]	[illegible]	[illegible]	[illegible]	[illegible]	[illegible]	[illegible]	[illegible]	[illegible]	[illegible]	[illegible]	[illegible]	[illegible]	[illegible]	[illegible]	[illegible]	[illegible]	[illegible]	[illegible]	[illegible]	[illegible]	0 (0 to 0)	8·2
Liver cancer due to hepatitis C	0 (0 to 0)	[illegible]	[illegible]	[illegible]	[illegible]	[illegible]	[illegible]	[illegible]	[illegible]	[illegible]	[illegible]	[illegible]	[illegible]	[illegible]	[illegible]	[illegible]	[illegible]	[illegible]	[illegible]	[illegible]	[illegible]	[illegible]	0·1 (0 to 0·1)	10·1
Liver cancer due to alcohol use	0 (0 to 0)	[illegible]	[illegible]	[illegible]	[illegible]	[illegible]	[illegible]	[illegible]	[illegible]	[illegible]	[illegible]	[illegible]	[illegible]	[illegible]	[illegible]	[illegible]	[illegible]	[illegible]	[illegible]	[illegible]	[illegible]	[illegible]	0 (0 to 0)	9·3
Liver cancer due to NASH	0 (0 to 0)	[illegible]	[illegible]	[illegible]	[illegible]	[illegible]	[illegible]	[illegible]	[illegible]	[illegible]	[illegible]	[illegible]	[illegible]	[illegible]	[illegible]	[illegible]	[illegible]	[illegible]	[illegible]	[illegible]	[illegible]	[illegible]	0 (0 to 0)	4·6
Liver cancer due to other causes	0 (0 to 0)	[illegible]	[illegible]	[illegible]	[illegible]	[illegible]	[illegible]	[illegible]	[illegible]	[illegible]	[illegible]	[illegible]	[illegible]	[illegible]	[illegible]	[illegible]	[illegible]	[illegible]	[illegible]	[illegible]	[illegible]	[illegible]	0 (0 to 0)	4·1
Gallbladder and biliary tract cancer	0 (0 to 0)	[illegible]	[illegible]	[illegible]	[illegible]	[illegible]	[illegible]	[illegible]	[illegible]	[illegible]	[illegible]	[illegible]	[illegible]	[illegible]	[illegible]	[illegible]	[illegible]	[illegible]	[illegible]	[illegible]	[illegible]	[illegible]	0·1 (0·1 to 0·2)	24·7
Pancreatic cancer	0 (0 to 0)	[illegible]	[illegible]	[illegible]	[illegible]	[illegible]	[illegible]	[illegible]	[illegible]	[illegible]	[illegible]	[illegible]	[illegible]	[illegible]	[illegible]	[illegible]	[illegible]	[illegible]	[illegible]	[illegible]	[illegible]	[illegible]	0·8 (0·6 to 1)	171
Larynx cancer	0 (0 to 0)	[illegible]	[illegible]	[illegible]	[illegible]	[illegible]	[illegible]	[illegible]	[illegible]	[illegible]	[illegible]	[illegible]	[illegible]	[illegible]	[illegible]	[illegible]	[illegible]	[illegible]	[illegible]	[illegible]	[illegible]	[illegible]	0 (0 to 0)	8·5
Tracheal, bronchus, and lung cancer	0 (0 to 0)	[illegible]	[illegible]	[illegible]	[illegible]	[illegible]	[illegible]	[illegible]	[illegible]	[illegible]	[illegible]	[illegible]	[illegible]	[illegible]	[illegible]	[illegible]	[illegible]	[illegible]	[illegible]	[illegible]	[illegible]	[illegible]	0·6 (0·4 to 0·8)	203
Malignant skin melanoma	0 (0 to 0)	[illegible]	[illegible]	[illegible]	[illegible]	[illegible]	[illegible]	[illegible]	[illegible]	[illegible]	[illegible]	[illegible]	[illegible]	[illegible]	[illegible]	[illegible]	[illegible]	[illegible]	[illegible]	[illegible]	[illegible]	[illegible]	0·1 (0·1 to 0·2)	18·9
Non-melanoma skin cancer	0 (0 to 0)	[illegible]	[illegible]	[illegible]	[illegible]	[illegible]	[illegible]	[illegible]	[illegible]	[illegible]	[illegible]	[illegible]	[illegible]	[illegible]	[illegible]	[illegible]	[illegible]	[illegible]	[illegible]	[illegible]	[illegible]	[illegible]	0·2 (0·1 to 0·3)	5·5
Non-melanoma skin cancer (squamous-cell carcinoma)	0 (0 to 0)	[illegible]	[illegible]	[illegible]	[illegible]	[illegible]	[illegible]	[illegible]	[illegible]	[illegible]	[illegible]	[illegible]	[illegible]	[illegible]	[illegible]	[illegible]	[illegible]	[illegible]	[illegible]	[illegible]	[illegible]	[illegible]	0·2 (0·1 to 0·3)	5·5
Non-melanoma skin cancer (basal-cell carcinoma)	0 (0 to 0)	[illegible]	[illegible]	[illegible]	[illegible]	[illegible]	[illegible]	[illegible]	[illegible]	[illegible]	[illegible]	[illegible]	[illegible]	[illegible]	[illegible]	[illegible]	[illegible]	[illegible]	[illegible]	[illegible]	[illegible]	[illegible]	0 (0 to 0)	0
Breast cancer	0 (0 to 0)	[illegible]	[illegible]	[illegible]	[illegible]	[illegible]	[illegible]	[illegible]	[illegible]	[illegible]	[illegible]	[illegible]	[illegible]	[illegible]	[illegible]	[illegible]	[illegible]	[illegible]	[illegible]	[illegible]	[illegible]	[illegible]	2·2 (1·6 to 2·7)	722
Cervical cancer	0 (0 to 0)	[illegible]	[illegible]	[illegible]	[illegible]	[illegible]	[illegible]	[illegible]	[illegible]	[illegible]	[illegible]	[illegible]	[illegible]	[illegible]	[illegible]	[illegible]	[illegible]	[illegible]	[illegible]	[illegible]	[illegible]	[illegible]	0·8 (0·5 to 1·1)	473
Uterine cancer	0 (0 to 0)	[illegible]	[illegible]	[illegible]	[illegible]	[illegible]	[illegible]	[illegible]	[illegible]	[illegible]	[illegible]	[illegible]	[illegible]	[illegible]	[illegible]	[illegible]	[illegible]	[illegible]	[illegible]	[illegible]	[illegible]	[illegible]	0·4 (0·3 to 0·5)	132
Ovarian cancer	0 (0 to 0)	[illegible]	[illegible]	[illegible]	[illegible]	[illegible]	[illegible]	[illegible]	[illegible]	[illegible]	[illegible]	[illegible]	[illegible]	[illegible]	[illegible]	[illegible]	[illegible]	[illegible]	[illegible]	[illegible]	[illegible]	[illegible]	0·3 (0·1 to 0·4)	198
Prostate cancer	0 (0 to 0)	[illegible]	[illegible]	[illegible]	[illegible]	[illegible]	[illegible]	[illegible]	[illegible]	[illegible]	[illegible]	[illegible]	[illegible]	[illegible]	[illegible]	[illegible]	[illegible]	[illegible]	[illegible]	[illegible]	[illegible]	[illegible]	0 (0 to 0)	0
Testicular cancer	--	[illegible]	[illegible]	[illegible]	[illegible]	[illegible]	[illegible]	[illegible]	[illegible]	[illegible]	[illegible]	[illegible]	[illegible]	[illegible]	[illegible]	[illegible]	[illegible]	[illegible]	[illegible]	[illegible]	[illegible]	[illegible]	--	NA
Kidney cancer	0 (0 to 0)	[illegible]	[illegible]	[illegible]	[illegible]	[illegible]	[illegible]	[illegible]	[illegible]	[illegible]	[illegible]	[illegible]	[illegible]	[illegible]	[illegible]	[illegible]	[illegible]	[illegible]	[illegible]	[illegible]	[illegible]	[illegible]	0·1 (0 to 0·1)	29·4
Bladder cancer	0 (0 to 0)	[illegible]	[illegible]	[illegible]	[illegible]	[illegible]	[illegible]	[illegible]	[illegible]	[illegible]	[illegible]	[illegible]	[illegible]	[illegible]	[illegible]	[illegible]	[illegible]	[illegible]	[illegible]	[illegible]	[illegible]	[illegible]	0·5 (0·4 to 0·7)	48
Brain and central nervous system cancer	0·1 (0 to 0·3)	[illegible]	[illegible]	[illegible]	[illegible]	[illegible]	[illegible]	[illegible]	[illegible]	[illegible]	[illegible]	[illegible]	[illegible]	[illegible]	[illegible]	[illegible]	[illegible]	[illegible]	[illegible]	[illegible]	[illegible]	[illegible]	0 (0 to 0·1)	52·9
Thyroid cancer	0 (0 to 0)	[illegible]	[illegible]	[illegible]	[illegible]	[illegible]	[illegible]	[illegible]	[illegible]	[illegible]	[illegible]	[illegible]	[illegible]	[illegible]	[illegible]	[illegible]	[illegible]	[illegible]	[illegible]	[illegible]	[illegible]	[illegible]	0·1 (0·1 to 0·1)	29·7
Mesothelioma	0 (0 to 0)	[illegible]	[illegible]	[illegible]	[illegible]	[illegible]	[illegible]	[illegible]	[illegible]	[illegible]	[illegible]	[illegible]	[illegible]	[illegible]	[illegible]	[illegible]	[illegible]	[illegible]	[illegible]	[illegible]	[illegible]	[illegible]	0 (0 to 0)	6·2
Hodgkin lymphoma	0 (0 to 0)	[illegible]	[illegible]	[illegible]	[illegible]	[illegible]	[illegible]	[illegible]	[illegible]	[illegible]	[illegible]	[illegible]	[illegible]	[illegible]	[illegible]	[illegible]	[illegible]	[illegible]	[illegible]	[illegible]	[illegible]	[illegible]	0 (0 to 0)	7·2
Non-Hodgkin lymphoma	0 (0 to 0)	[illegible]	[illegible]	[illegible]	[illegible]	[illegible]	[illegible]	[illegible]	[illegible]	[illegible]	[illegible]	[illegible]	[illegible]	[illegible]	[illegible]	[illegible]	[illegible]	[illegible]	[illegible]	[illegible]	[illegible]	[illegible]	0·2 (0·2 to 0·3)	130
Multiple myeloma	0 (0 to 0)	[illegible]	[illegible]	[illegible]	[illegible]	[illegible]	[illegible]	[illegible]	[illegible]	[illegible]	[illegible]	[illegible]	[illegible]	[illegible]	[illegible]	[illegible]	[illegible]	[illegible]	[illegible]	[illegible]	[illegible]	[illegible]	0·2 (0·1 to 0·3)	91·6
Leukemia	0·2 (0·1 to 0·3)	[illegible]	[illegible]	[illegible]	[illegible]	[illegible]	[illegible]	[illegible]	[illegible]	[illegible]	[illegible]	[illegible]	[illegible]	[illegible]	[illegible]	[illegible]	[illegible]	[illegible]	[illegible]	[illegible]	[illegible]	[illegible]	0·2 (0·2 to 0·3)	124
Acute lymphoid leukemia	0 (0 to 0·1)	[illegible]	[illegible]	[illegible]	[illegible]	[illegible]	[illegible]	[illegible]	[illegible]	[illegible]	[illegible]	[illegible]	[illegible]	[illegible]	[illegible]	[illegible]	[illegible]	[illegible]	[illegible]	[illegible]	[illegible]	[illegible]	0 (0 to 0)	17·9
Chronic lymphoid leukemia	0 (0 to 0)	[illegible]	[illegible]	[illegible]	[illegible]	[illegible]	[illegible]	[illegible]	[illegible]	[illegible]	[illegible]	[illegible]	[illegible]	[illegible]	[illegible]	[illegible]	[illegible]	[illegible]	[illegible]	[illegible]	[illegible]	[illegible]	0 (0 to 0·1)	9·1
Acute myeloid leukemia	0 (0 to 0·1)	[illegible]	[illegible]	[illegible]	[illegible]	[illegible]	[illegible]	[illegible]	[illegible]	[illegible]	[illegible]	[illegible]	[illegible]	[illegible]	[illegible]	[illegible]	[illegible]	[illegible]	[illegible]	[illegible]	[illegible]	[illegible]	0 (0 to 0)	38

Chronic myeloid leukemia	0 (0 to 0)	0 (0 to 0)	0 (0 to 0)	0·2 (0·1 to 0·3)	0·1 (0·1 to 0·3)	0·1 (0·1 to 0·2)	0·2 (0·1 to 0·3)	0·5 (0·2 to 1)	0·7 (0·3 to 1·4)	0·8 (0·4 to 1·4)	0·8 (0·4 to 1·5)	1 (0·5 to 1·8)	1·1 (0·6 to 2)	1·4 (0·8 to 2·3)	1·3 (0·7 to 2·2)	1·2 (0·7 to 2)	0·8 (0·4 to 1·4)	0·8 (0·4 to 1·4)	0·6 (0·3 to 1)	0·5 (0·3 to 0·8)	0·3 (0·2 to 0·5)	0·1 (0·1 to 0·2)	0 (0 to 0·1)	12·7
Other leukemia	0·1 (0·1 to 0·2)	0 (0 to 0·1)	0·3 (0·1 to 0·5)	1·2 (0·6 to 2·1)	1·5 (0·8 to 2·6)	1·4 (0·8 to 2·3)	1·4 (0·8 to 2·1)	2 (1·2 to 2·9)	2·5 (1·5 to 3·8)	2·3 (1·6 to 3·4)	2·9 (1·8 to 4·2)	4·2 (2·9 to 6)	4·6 (3·1 to 6·4)	3·6 (2·5 to 4·8)	3·6 (2·4 to 5·1)	3·1 (2·2 to 4·3)	2·8 (2 to 3·9)	2·9 (2 to 3·9)	2·1 (1·4 to 3)	1·8 (1·2 to 2·5)	1·4 (1 to 1·9)	0·6 (0·5 to 0·8)	0·1 (0·1 to 0·2)	46·5
Other malignant neoplasms	0·3 (0·2 to 0·4)	0·3 (0·2 to 0·5)	0·8 (0·5 to 1·3)	11·3 (6·5 to 18·5)	6·1 (4·6 to 8·1)	4·9 (3·8 to 6·3)	4·9 (3·7 to 6·3)	5 (3·8 to 6·5)	5·4 (4·1 to 7·2)	6 (4·5 to 7·9)	7·1 (5·5 to 9·2)	9·5 (7·1 to 12·4)	12 (9·2 to 15·7)	12·4 (9·4 to 16·2)	11·3 (8·6 to 14·8)	9·7 (7·5 to 12·8)	8·6 (6·6 to 11·2)	7·2 (5·6 to 9·4)	5·6 (4·3 to 7·5)	4·3 (3·3 to 5·6)	3·5 (2·7 to 4·5)	1·7 (1·3 to 2·1)	0·4 (0·3 to 0·5)	138
Other neoplasms	0 (0 to 0)	0·1 (0 to 0·1)	0·1 (0 to 0·2)	0·6 (0·3 to 1·1)	0·1 (0·1 to 0·2)	0·2 (0·1 to 0·4)	0·2 (0·1 to 0·4)	0·2 (0·2 to 0·3)	0·4 (0·3 to 0·7)	0·5 (0·3 to 0·7)	0·3 (0·2 to 0·5)	1 (0·6 to 1·4)	0·9 (0·6 to 1·3)	1·1 (0·7 to 1·6)	1·2 (0·8 to 1·7)	1·3 (0·9 to 1·8)	1·4 (1 to 1·9)	1·6 (1·1 to 2·1)	1·4 (1·1 to 2)	1·1 (0·8 to 1·5)	0·7 (0·5 to 1)	0·3 (0·2 to 0·5)	0·1 (0 to 0·1)	14·9
Myelodysplastic, myeloproliferative, and other hematopoietic neo	0 (0 to 0)	0·1 (0 to 0·1)	0·1 (0 to 0·2)	0·6 (0·3 to 1·1)	0·1 (0·1 to 0·2)	0·2 (0·1 to 0·4)	0·2 (0·1 to 0·4)	0·2 (0·2 to 0·3)	0·4 (0·3 to 0·7)	0·5 (0·3 to 0·7)	0·3 (0·2 to 0·5)	1 (0·6 to 1·4)	0·9 (0·6 to 1·3)	1·1 (0·7 to 1·6)	1·2 (0·8 to 1·7)	1·3 (0·9 to 1·8)	1·4 (1 to 1·9)	1·6 (1·1 to 2·1)	1·4 (1·1 to 2)	1·1 (0·8 to 1·5)	0·7 (0·5 to 1)	0·3 (0·2 to 0·5)	0·1 (0 to 0·1)	14·9
Benign and in situ intestinal neoplasms	0 (0 to 0)	0 (0 to 0)	0 (0 to 0)	0 (0 to 0)	0 (0 to 0)	0 (0 to 0)	0 (0 to 0)	0 (0 to 0)	0 (0 to 0)	0 (0 to 0)	0 (0 to 0)	0 (0 to 0)	0 (0 to 0)	0 (0 to 0)	0 (0 to 0)	0 (0 to 0)	0 (0 to 0)	0 (0 to 0)	0 (0 to 0)	0 (0 to 0)	0 (0 to 0)	0 (0 to 0)	0 (0 to 0)	0
Benign and in situ cervical and uterine neoplasms	0 (0 to 0)	0 (0 to 0)	0 (0 to 0)	0 (0 to 0)	0 (0 to 0)	0 (0 to 0)	0 (0 to 0)	0 (0 to 0)	0 (0 to 0)	0 (0 to 0)	0 (0 to 0)	0 (0 to 0)	0 (0 to 0)	0 (0 to 0)	0 (0 to 0)	0 (0 to 0)	0 (0 to 0)	0 (0 to 0)	0 (0 to 0)	0 (0 to 0)	0 (0 to 0)	0 (0 to 0)	0 (0 to 0)	0
Other benign and in situ neoplasms	0 (0 to 0)	0 (0 to 0)	0 (0 to 0)	0 (0 to 0)	0 (0 to 0)	0 (0 to 0)	0 (0 to 0)	0 (0 to 0)	0 (0 to 0)	0 (0 to 0)	0 (0 to 0)	0 (0 to 0)	0 (0 to 0)	0 (0 to 0)	0 (0 to 0)	0 (0 to 0)	0 (0 to 0)	0 (0 to 0)	0 (0 to 0)	0 (0 to 0)	0 (0 to 0)	0 (0 to 0)	0 (0 to 0)	0
Cardiovascular diseases	2·2 (1·4 to 3·5)	0·8 (0·5 to 1·2)	1·7 (1·1 to 2·6)	3·6 (2·5 to 5)	5·4 (4·3 to 6·7)	9·1 (7 to 11·7)	19·2 (15 to 24·1)	26·7 (21·1 to 33·7)	37·6 (29·1 to 47·4)	50·3 (40·6 to 62·6)	77·1 (61 to 96·3)	130 (105 to 161)	213 (173 to 266)	270 (217 to 332)	306 (248 to 374)	363 (302 to 439)	366 (308 to 423)	429 (365 to 500)	424 (366 to 490)	429 (366 to 489)	373 (311 to 430)	238 (196 to 268)	79·6 (65·9 to 88·4)	3,850
Rheumatic heart disease	0 (0 to 0)	0 (0 to 0)	0 (0 to 0)	0·3 (0·2 to 0·5)	1·5 (1 to 2·3)	3·5 (2·2 to 5·4)	6·1 (4 to 9·2)	7·3 (4·8 to 10·6)	9·4 (6·7 to 13·3)	10 (7·3 to 13·2)	11·6 (8·4 to 15·5)	13·6 (9·6 to 18·4)	14·4 (10·1 to 19·5)	12·4 (8·5 to 17·4)	9·4 (6·5 to 12·9)	6·1 (4·4 to 8·1)	4·5 (3·3 to 5·9)	3·5 (2·6 to 4·8)	2·7 (1·9 to 3·6)	2·1 (1·5 to 2·8)	1·6 (1·1 to 2·1)	1 (0·7 to 1·3)	0·4 (0·3 to 0·5)	121
Ischemic heart disease	0 (0 to 0)	0 (0 to 0)	0 (0 to 0)	0 (0 to 0)	0 (0 to 0)	0 (0 to 0)	0·7 (0·5 to 0·9)	1·3 (0·9 to 1·8)	2·3 (1·5 to 3·2)	4·8 (3·4 to 6·5)	9·1 (6·6 to 12·3)	19·6 (14·8 to 25·6)	40 (30·6 to 51·1)	59·2 (45·1 to 75·6)	70·8 (53·3 to 90·4)	95·3 (75·7 to 120)	101 (81·9 to 123)	115 (89·3 to 144)	111 (88·4 to 136)	112 (91·4 to 134)	102 (79·3 to 123)	68·3 (53·3 to 82·1)	26·6 (21·2 to 31·3)	939
Stroke	0·9 (0·5 to 1·6)	0·1 (0·1 to 0·2)	0·2 (0·1 to 0·4)	0·5 (0·4 to 0·8)	1·2 (0·9 to 1·7)	2·1 (1·4 to 2·8)	5 (3·6 to 6·7)	7·3 (5·2 to 9·9)	11 (8 to 15)	16·9 (12·4 to 22·2)	28·1 (20·5 to 37·2)	49·2 (36 to 64·6)	83 (61·2 to 110)	109 (81·5 to 145)	122 (92·7 to 159)	147 (113 to 186)	142 (109 to 177)	185 (151 to 221)	188 (156 to 224)	190 (155 to 227)	151 (122 to 179)	86·9 (70·5 to 102)	25·9 (20·5 to 31)	1,550
Ischemic stroke	0·3 (0·1 to 0·4)	0 (0 to 0)	0 (0 to 0)	0·2 (0·1 to 0·3)	0·6 (0·3 to 1)	0·9 (0·5 to 1·6)	1·8 (1·1 to 2·5)	2·5 (1·7 to 3·7)	3·2 (2 to 4·7)	4·7 (3·2 to 6·4)	6·9 (4·9 to 9·3)	11·7 (8·8 to 15·3)	19·1 (14·2 to 24·8)	27·8 (21·3 to 35·9)	35·2 (27·1 to 45·2)	58·3 (45·1 to 73·8)	62·3 (48·5 to 77·3)	117 (94·9 to 141)	129 (107 to 154)	155 (127 to 185)	126 (101 to 150)	72·6 (58·5 to 85·9)	22 (17·4 to 26·5)	857
Intracerebral hemorrhage	0·2 (0·1 to 0·4)	0 (0 to 0·1)	0·1 (0 to 0·2)	0·1 (0·1 to 0·2)	0·3 (0·2 to 0·4)	0·6 (0·4 to 0·7)	1·8 (1·2 to 2·5)	2·8 (1·8 to 4)	4·3 (2·9 to 6)	6·7 (4·6 to 9·2)	11·9 (8·2 to 16·7)	22·5 (15·5 to 31·2)	43·3 (30·2 to 59·9)	58·7 (42·9 to 79·5)	64·2 (47·5 to 84·8)	73 (54·1 to 94·9)	66·5 (49·7 to 83·5)	58 (46·4 to 70·8)	51 (41·6 to 61·4)	29·8 (23·7 to 36·1)	21·8 (17·5 to 26·1)	12·4 (9·9 to 14·8)	3·4 (2·6 to 4·1)	533
Subarachnoid hemorrhage	0·4 (0·2 to 0·8)	0·1 (0 to 0·1)	0·1 (0 to 0·2)	0·2 (0·1 to 0·4)	0·3 (0·2 to 0·5)	0·6 (0·4 to 0·9)	1·5 (0·9 to 2·2)	2 (1·2 to 3·1)	3·4 (2·2 to 5·2)	5·5 (3·6 to 8·1)	9·2 (6·3 to 13·4)	14·9 (9·7 to 21·5)	20·6 (13·9 to 30·2)	23·1 (14·9 to 33·9)	22·8 (15·1 to 33·3)	15·9 (10·4 to 22·8)	13·5 (8·8 to 19·9)	9·5 (6·6 to 13)	7·7 (5·4 to 10·7)	5 (3·5 to 7)	3·3 (2·3 to 4·6)	1·8 (1·2 to 2·5)	0·5 (0·3 to 0·7)	162
Hypertensive heart disease	0 (0 to 0)	0 (0 to 0)	0 (0 to 0)	0 (0 to 0)	0 (0 to 0)	0 (0 to 0)	0·3 (0·2 to 0·5)	0·8 (0·5 to 1·2)	1·2 (0·8 to 1·9)	2·9 (1·8 to 4·4)	6 (3·8 to 8·7)	10·8 (7·3 to 15·1)	24·4 (16·8 to 34·2)	33·3 (22·9 to 46·4)	39·4 (26·9 to 55·3)	44·7 (30·5 to 61·2)	46·7 (33 to 64·2)	49·5 (34·7 to 69)	47·4 (33·4 to 67·4)	48·9 (35·1 to 65)	44·8 (31·2 to 59·7)	30·4 (20·5 to 40·4)	10·3 (7·2 to 13·7)	442
Non-rheumatic valvular heart disease	0 (0 to 0)	0 (0 to 0)	0 (0 to 0)	0 (0 to 0)	0 (0 to 0)	0 (0 to 0)	0·5 (0·3 to 0·6)	0·4 (0·3 to 0·6)	0·5 (0·3 to 0·7)	0·7 (0·4 to 1)	0·9 (0·6 to 1·3)	1·3 (0·9 to 1·9)	2·3 (1·5 to 3·3)	2·2 (1·4 to 3·1)	2·3 (1·5 to 3·3)	2·6 (1·8 to 3·6)	2·2 (1·6 to 3·1)	2·1 (1·5 to 3)	1·9 (1·3 to 2·6)	1·7 (1·2 to 2·3)	1·5 (1 to 2·1)	0·9 (0·6 to 1·2)	0·2 (0·1 to 0·3)	24·2
Non-rheumatic calcific aortic valve disease	0 (0 to 0)	0 (0 to 0)	0 (0 to 0)	0 (0 to 0)	0 (0 to 0)	0 (0 to 0)	0·2 (0·1 to 0·3)	0·2 (0·1 to 0·3)	0·2 (0·1 to 0·3)	0·2 (0·1 to 0·4)	0·3 (0·2 to 0·5)	0·5 (0·3 to 0·9)	1 (0·6 to 1·6)	1·1 (0·6 to 1·7)	1·2 (0·7 to 1·9)	1·6 (1 to 2·4)	1·5 (1 to 2·2)	1·5 (1 to 2·2)	1·4 (1 to 2)	1·3 (0·9 to 1·8)	1·3 (0·9 to 1·8)	0·7 (0·5 to 1)	0·2 (0·1 to 0·2)	14·4
Non-rheumatic degenerative mitral valve disease	0 (0 to 0)	0 (0 to 0)	0 (0 to 0)	0 (0 to 0)	0 (0 to 0)	0 (0 to 0)	0·3 (0·2 to 0·4)	0·2 (0·1 to 0·3)	0·3 (0·2 to 0·5)	0·4 (0·3 to 0·6)	0·5 (0·3 to 0·8)	0·8 (0·5 to 1·2)	1·2 (0·7 to 1·9)	1·1 (0·6 to 1·8)	1·1 (0·6 to 1·7)	1 (0·6 to 1·6)	0·7 (0·4 to 1·2)	0·6 (0·3 to 0·9)	0·4 (0·2 to 0·7)	0·4 (0·2 to 0·6)	0·2 (0·1 to 0·4)	0·2 (0·1 to 0·3)	0 (0 to 0·1)	9·5
Other non-rheumatic valve diseases	0 (0 to 0)	0 (0 to 0)	0 (0 to 0)	0 (0 to 0)	0 (0 to 0)	0 (0 to 0)	0 (0 to 0)	0 (0 to 0)	0 (0 to 0)	0 (0 to 0·1)	0 (0 to 0·1)	0 (0 to 0·1)	0·1 (0 to 0·1)	0 (0 to 0·1)	0 (0 to 0·1)	0 (0 to 0·1)	0 (0 to 0·1)	0 (0 to 0·1)	0 (0 to 0)	0 (0 to 0)	0 (0 to 0)	0 (0 to 0)	0 (0 to 0)	0·4
Cardiomyopathy and myocarditis	0·5 (0·3 to 0·8)	0·3 (0·2 to 0·5)	0·8 (0·5 to 1·3)	1·6 (1·1 to 2·3)	1 (0·7 to 1·4)	1·4 (1 to 2)	1·8 (1·2 to 2·5)	2·7 (1·8 to 3·8)	3·3 (2·1 to 5·1)	4·6 (3 to 6·7)	5·9 (4 to 8·4)	8·3 (5·5 to 11·9)	13·5 (9·1 to 19·1)	15·9 (10·6 to 23)	16·3 (11 to 23)	16·3 (11·5 to 22·4)	15·3 (10·7 to 21·5)	13·8 (9·3 to 19·9)	12·5 (8·4 to 17·7)	10·9 (7·2 to 15·7)	11·3 (7·3 to 16·6)	6·6 (4 to 9·8)	2 (1·1 to 2·9)	167
Myocarditis	0·1 (0·1 to 0·2)	0·1 (0·1 to 0·2)	0·3 (0·2 to 0·6)	0·8 (0·5 to 1·2)	0·7 (0·5 to 0·9)	0·6 (0·4 to 0·9)	0·7 (0·4 to 1)	0·8 (0·5 to 1·2)	1 (0·5 to 1·5)	1·6 (1 to 2·5)	1·4 (0·9 to 2·1)	1·1 (0·7 to 1·7)	2·7 (1·6 to 4·3)	2·3 (1·2 to 3·9)	1·6 (1 to 2·5)	1·5 (0·9 to 2·2)	0·8 (0·5 to 1·3)	1·6 (0·9 to 2·9)	1·6 (0·8 to 2·8)	2 (1 to 3·5)	2·4 (1·4 to 3·7)	1·6 (1 to 2·5)	0·6 (0·4 to 0·9)	28·1
Alcoholic cardiomyopathy	0 (0 to 0)	0 (0 to 0)	0 (0 to 0)	0 (0 to 0)	0 (0 to 0)	0 (0 to 0)	0·1 (0 to 0·2)	0·2 (0·1 to 0·4)	0·4 (0·2 to 0·8)	0·3 (0·1 to 0·5)	0·4 (0·2 to 0·7)	0·5 (0·2 to 0·9)	2·5 (1·2 to 4·1)	3 (1·2 to 5·4)	2·1 (1 to 3·6)	2 (0·9 to 3·5)	2 (0·9 to 3·5)	0·3 (0·1 to 0·6)	0·4 (0·2 to 0·8)	0·4 (0·1 to 0·9)	0·1 (0 to 0·3)	0·1 (0 to 0·1)	0 (0 to 0)	14·9
Other cardiomyopathy	0·4 (0·2 to 0·6)	0·2 (0·1 to 0·4)	0·5 (0·3 to 0·8)	0·8 (0·4 to 1·4)	0·4 (0·2 to 0·6)	0·8 (0·4 to 1·3)	1·1 (0·6 to 1·7)	1·6 (1 to 2·5)	2 (1·1 to 3·3)	2·7 (1·5 to 4·4)	4·1 (2·4 to 6·4)	6·6 (4·1 to 9·9)	8·2 (4·8 to 13)	10·6 (6·9 to 15·9)	12·5 (7·9 to 18·7)	12·8 (8·4 to 19)	12·5 (7·8 to 18·3)	11·8 (7·9 to 17)	10·5 (7 to 14·9)	8·6 (5·6 to 12·4)	8·8 (5·1 to 14)	4·8 (2·6 to 8·1)	1·4 (0·7 to 2·2)	124
Atrial fibrillation and flutter	0 (0 to 0)	0 (0 to 0)	0 (0 to 0)	0 (0 to 0)	0 (0 to 0)	0 (0 to 0)	0 (0 to 0)	0 (0 to 0)	0 (0 to 0)	0·1 (0 to 0·1)	0·2 (0·1 to 0·3)	0·9 (0·6 to 1·4)	1·9 (1·3 to 2·6)	2·8 (2 to 3·9)	4·4 (3·3 to 6)	6·2 (4·6 to 8·2)	8 (6·2 to 10·4)	11·8 (8·9 to 15·7)	13·9 (10·7 to 17·8)	19 (15·1 to 24)	20·9 (16·1 to 27·1)	17·5 (13·2 to 22·7)	5·6 (4·1 to 7·4)	113
Aortic aneurysm	--	--	--	--	--	--	0·1 (0·1 to 0·1)	0·3 (0·2 to 0·4)	0·7 (0·4 to 1)	0·7 (0·4 to 1·2)	1·6 (1 to 2·3)	2·6 (1·7 to 3·6)	4·5 (3·1 to 6·2)	6·1 (4·3 to 8·3)	8·6 (6 to 11·7)	10·4 (7·4 to 14)	13·7 (9·8 to 18·6)	14·4 (9·9 to 19·8)	13·6 (9·4 to 19)	12·4 (8·7 to 17·2)	9·3 (6·5 to 12·9)	5·2 (3·7 to 6·9)	1·4 (1 to 1·8)	NA
Endocarditis	0·1 (0 to 0·1)	0·1 (0 to 0·2)	0·2 (0·1 to 0·4)	0·3 (0·2 to 0·6)	0·4 (0·2 to 0·6)	0·4 (0·3 to 0·7)	1·2 (0·8 to 1·8)	2·4 (1·4 to 3·9)	2·7 (1·6 to 4·4)	2·5 (1·4 to 4)	4·1 (2·5 to 6·5)	4·6 (2·8 to 7·1)	6·2 (3·7 to 9·9)	5 (3 to 8)	5·3 (3·3 to 8·3)	5·3 (3·2 to 8·3)	3·9 (2·5 to 6·1)	3·8 (2·5 to 6)	3·1 (2 to 4·9)	3·7 (2·3 to 5·9)	2·9 (1·8 to 4·7)	1·8 (1·1 to 2·8)	0·6 (0·4 to 0·9)	60·7
Other cardiovascular and circulatory diseases	0·7 (0·4 to 1·2)	0·3 (0·2 to 0·5)	0·5 (0·2 to 0·8)	0·8 (0·5 to 1·3)	1·2 (0·8 to 1·7)	1·7 (1·1 to 2·5)	3·5 (2·3 to 4·9)	4·4 (3 to 6·2)	6·5 (4·3 to 9·1)	7·2 (5·1 to 9·8)	9·7 (6·9 to 13·2)	18·4 (13·2 to 25·5)	21·7 (15·8 to 29·4)	20·9 (15·5 to 27·7)	22·8 (17·4 to 29·3)	23·4 (17·7 to 29·9)	21·3 (16·3 to 27·1)	22·6 (17·7 to 27·9)	19·9 (15·7 to 24·7)	18·6 (14 to 23·9)	19·1 (14·4 to 24·1)	12·7 (9·8 to 15·6)	4·1 (2·8 to 5·5)	262
Chronic respiratory diseases	0 (0 to 0)	0 (0 to 0)	0 (0 to 0)	25·6 (15·5 to 41·5)	24 (14·9 to 38·8)	23·3 (14·3 to 35·8)	23·3 (14·6 to 36·4)	23·8 (15·7 to 34·3)	25·2 (17·5 to 35·5)	25·4 (18·4 to 33·9)	28·9 (22·1 to 37·5)	32·5 (25·4 to 41·4)	40·6 (31·6 to 51·6)	46·4 (36·6 to 57·6)	47·4 (37·5 to 58·6)	46·5 (35·7 to 58·4)	44·1 (33·7 to 55·7)	42·6 (32·8 to 53·5)	36·5 (27·9 to 46·3)	32·5 (24·6 to 41)	28·1 (21·5 to 35·2)	16·6 (12·4 to 20·6)	5·3 (3·8 to 6·7)	619
Chronic obstructive pulmonary disease	0 (0 to 0)	0 (0 to 0)	0 (0 to 0)	0·5 (0·3 to 0·9)	0·5 (0·2 to 1)	0·9 (0·4 to 1·5)	1·8 (1·1 to 2·8)	2·9 (1·8 to 4·2)	3·9 (2·5 to 5·5)	4·7 (3·2 to 6·3)	6·5 (4·5 to 8·8)	9·5 (7 to 12·4)	15·3 (11·1 to 20·2)	22·4 (16·8 to 28·8)	26 (19·8 to 33·3)	31 (23·2 to 40·1)	30·8 (23·3 to 39·8)	30·5 (23·2 to 39·3)	27 (20·4 to 34·9)	25·4 (19·1 to 32·6)	22·7 (17·3 to 28·7)	13·6 (10·1 to 17·3)	4·5 (3·2 to 5·6)	280
Pneumoconiosis	0 (0 to 0)	0 (0 to 0)	0 (0 to 0)	0 (0 to 0)	0 (0 to 0)	0 (0 to 0)	0 (0 to 0)	0 (0 to 0)	0 (0 to 0)	0 (0 to 0)	0 (0 to 0·1)	0 (0 to 0·1)	0 (0 to 0·2)	0·1 (0 to 0·3)	0·1 (0 to 0·1)	0 (0 to 0·1)	0·1 (0 to 0·1)	0 (0 to 0·1)	0·1 (0·1 to 0·2)	0·1 (0 to 0·1)	0·1 (0 to 0·2)	0 (0 to 0)	0 (0 to 0)	1
Silicosis	0 (0 to 0)	0 (0 to 0)	0 (0 to 0)	0 (0 to 0)	0 (0 to 0)	0 (0 to 0)	0 (0 to 0)	0 (0 to 0)	0 (0 to 0)	0 (0 to 0)	0 (0 to 0)	0 (0 to 0)	0 (0 to 0)	0 (0 to 0·1)	0 (0 to 0)	0 (0 to 0)	0 (0 to 0)	0 (0 to 0)	0 (0 to 0)	0 (0 to 0)	0 (0 to 0)	0 (0 to 0)	0 (0 to 0)	0·2
Asbestosis	0 (0 to 0)	0 (0 to 0)	0 (0 to 0)	0 (0 to 0)	0 (0 to 0)	0 (0 to 0)	0 (0 to 0)	0 (0 to 0)	0 (0 to 0)	0 (0 to 0)	0 (0 to 0)	0 (0 to 0)	0 (0 to 0)	0 (0 to 0·1)	0 (0 to 0·1)	0 (0 to 0)	0 (0 to 0)	0 (0 to 0)	0 (0 to 0·1)	0 (0 to 0)	0 (0 to 0·1)	0 (0 to 0)	0 (0 to 0)	0·3
Coal workers pneumoconiosis	0 (0 to 0)	0 (0 to 0)	0 (0 to 0)	0 (0 to 0)	0 (0 to 0)	0 (0 to 0)	0 (0 to 0)	0 (0 to 0)	0 (0 to 0)	0 (0 to 0)	0 (0 to 0)	0 (0 to 0)	0 (0 to 0)	0 (0 to 0)	0 (0 to 0)	0 (0 to 0)	0 (0 to 0)	0 (0 to 0)	0 (0 to 0)	0 (0 to 0)	0 (0 to 0)	0 (0 to 0)	0 (0 to 0)	0·1
Other pneumoconiosis	0 (0 to 0)	0 (0 to 0)	0 (0 to 0)	0 (0 to 0)	0 (0 to 0)	0 (0 to 0)	0 (0 to 0·1)	0 (0 to 0)	0 (0 to 0)	0 (0 to 0)	0 (0 to 0)	0 (0 to 0·1)	0 (0 to 0·1)	0·1 (0 to 0·2)	0 (0 to 0)	0 (0 to 0)	0 (0 to 0)	0 (0 to 0·1)	0 (0 to 0·1)	0 (0 to 0·1)	0 (0 to 0·1)	0 (0 to 0)	0 (0 to 0)	0·4
Asthma	0 (0 to 0)	0 (0 to 0)	0 (0 to 0)	21·3 (11·5 to 36·8)	21·7 (12·6 to 36·1)	20·3 (11·6 to 32·1)	20·2 (11·9 to 33·3)	18·8 (11·1 to 29·2)	17·8 (10·9 to 27·3)	17·2 (11·4 to 25·1)	18·1 (12·4 to 25·1)	18·1 (12·8 to 24·5)	19·4 (14·1 to 25·9)	18·3 (13·1 to 23·8)	14·4 (10·4 to 19·2)	11·1 (7·9 to 15)	9·2 (6·5 to 12·3)	7·6 (5·4 to 10·3)	5·6 (4 to 7·8)	4·1 (2·8 to 5·8)	3 (2·1 to 4·2)	1·7 (1·2 to 2·4)	0·6 (0·4 to 0·8)	268
Interstitial lung disease and pulmonary sarcoidosis	0 (0 to 0)	0 (0 to 0)	0 (0 to 0)	1·1 (0·5 to 1·9)	0·4 (0·2 to 0·6)	0·3 (0·2 to 0·5)	0·4 (0·2 to 0·7)	0·6 (0·4 to 1·1)	1·1 (0·6 to 1·7)	1·8 (1 to 2·9)	2·2 (1·3 to 3·4)	2·3 (1·3 to 3·7)	3·6 (2·2 to 5·5)	3·5 (2·1 to 5·4)	3·9 (2·4 to 6·3)	3·8 (2·1 to 5·8)	3·8 (2·2 to 6)	4·1 (2·5 to 6·5)	3·5 (2·1 to 5·6)	2·7 (1·6 to 4·3)	2·1 (1·1 to 3·5)	1·2 (0·6 to 2)	0·3 (0·1 to 0·5)	42·8
Other chronic respiratory diseases	0 (0 to 0)	0 (0 to 0)	0 (0 to 0)	2·8 (1·4 to 4·9)	1·4 (0·8 to 2·2)	1·8 (1 to 2·8)	0·8 (0·5 to 1·2)	1·5 (1 to 2·2)	2·4 (1·6 to 3·6)	1·7 (1·2 to 2·5)	2 (1·3 to 2·9)	2·5 (1·5 to 3·8)	2·2 (1·4 to 3·4)	2 (1·2 to 2·9)	3·1 (1·7 to 4·8)	0·5 (0·3 to 0·9)	0·3 (0·1 to 0·4)	0·3 (0·2 to 0·6)	0·2 (0·1 to 0·4)	0·3 (0·1 to 0·5)	0·2 (0·1 to 0·4)	0·1 (0 to 0·2)	0 (0 to 0·1)	26
Digestive diseases	1 (0·7 to 1·5)	0·9 (0·5 to 1·6)	2·4 (1·4 to 4)	9·5 (6·6 to 13·3)	7·1 (5·4 to 9·4)	7·7 (5·9 to 9·7)	11·8 (8·8 to 15·7)	20·5 (14·9 to 29)	27·8 (19·7 to 39)	33·5 (24·5 to 45·7)	42·7 (32·1 to 57·5)	54·6 (41·4 to 69·8)	69·6 (54 to 88·7)	76·6 (59·3 to 97·6)	73·3 (58·7 to 93)	68·2 (54·5 to 84·8)	58·7 (46·7 to 72·4)	54·3 (41·6 to 68·1)	41·8 (32·4 to 53)	35·9 (27·7 to 44·4)	27·7 (21·5 to 34·3)	15·1 (11·8 to 18·8)	4 (3·2 to 4·8)	745
Cirrhosis and other chronic liver diseases	0 (0 to 0)	0 (0 to 0)	0 (0 to 0)	1·3 (0·7 to 2·1)	1·2 (0·7 to 1·7)	1·6 (1·1 to 2·2)	2·4 (1·6 to 3·3)	4 (2·9 to 5·5)	5·3 (3·6 to 7·5)	8 (5·6 to 11·2)	11·7 (8·7 to 16·3)	19·7 (14·3 to 26·5)	28·6 (20·8 to 38)	34·7 (25·2 to 46·6)	35 (25·6 to 46·3)	33·3 (25 to 43·4)	29·1 (22·1 to 36·6)	25 (18·5 to 32·7)	17 (12·2 to 23·1)	13·2 (9·5 to 17·5)	8·9 (6·6 to 11·8)	4·4 (3·2 to 5·8)	1·1 (0·8 to 1·5)	286
Cirrhosis and other chronic liver diseases due to hepatitis B	0 (0 to 0)	0 (0 to 0)	0 (0 to 0)	0 (0 to 0)	0 (0 to 0)	0 (0 to 0)	0 (0 to 0)	0·1 (0 to 0·2)	0·2 (0·1 to 0·4)	0·5 (0·2 to 0·8)	0·9 (0·4 to 1·6)	1·5 (0·8 to 2·6)	2·1 (1 to 3·7)	2·3 (1·1 to 4·3)	2 (0·9 to 3·5)	1·6 (0·8 to 3·1)	1·3 (0·6 to 2·3)	1 (0·5 to 1·9)	0·7 (0·3 to 1·4)	0·6 (0·2 to 1)	0·4 (0·2 to 0·6)	0·2 (0·1 to 0·3)	0 (0 to 0·1)	15·3
Cirrhosis and other chronic liver diseases due to hepatitis C	0 (0 to 0)	0 (0 to 0)	0 (0 to 0)	0 (0 to 0)	0 (0 to 0)	0 (0 to 0·1)	0·1 (0 to 0·1)	0·4 (0·2 to 0·7)	0·9 (0·4 to 1·7)	1·8 (0·9 to 2·9)	2·8 (1·5 to 4·8)	4·5 (2·4 to 7·4)	6·6 (3·3 to 10·1)	6·8 (3·6 to 11·9)	6·3 (3·3 to 10·8)	5·6 (2·6 to 9·8)	4·5 (2·5 to 7·7)	3·7 (2·1 to 6)	2·4 (1·3 to 4·1)	1·9 (1 to 3·1)	1·3 (0·7 to 2·1)	0·7 (0·4 to 1·2)	0·2 (0·1 to 0·3)	50·1
Cirrhosis and other chronic liver diseases due to alcohol use	0 (0 to 0)	0 (0 to 0)	0 (0 to 0)	0 (0 to 0)	0 (0 to 0)	0 (0 to 0)	0 (0 to 0)	0·2 (0·1 to 0·4)	0·6 (0·3 to 1·2)	1·7 (0·9 to 2·7)	3·6 (1·8 to 5·7)	6·6 (3·8 to 10·1)	10·3 (5·5 to 15·8)	13·2 (7·5 to 20·1)	14 (8 to 20·9)	13·1 (7·9 to 19·4)	10·6 (6·3 to 16)	8·2 (4·6 to 12·5)	4·8 (2·7 to 7·6)	3·3 (1·7 to 5·5)	2·1 (1·3 to 3·4)	1 (0·6 to 1·7)	0·3 (0·1 to 0·5)	93·6
Cirrhosis and other chronic liver diseases due to NAFLD	0 (0 to 0)	0 (0 to 0)	0 (0 to 0)	0 (0 to 0)	0 (0 to 0)	0 (0 to 0)	0 (0 to 0·2)	0·4 (0·2 to 0·7)	0·9 (0·4 to 1·7)	1·4 (0·8 to 2·5)	2·4 (1·1 to 4·3)	3·9 (2 to 6·7)	6·8 (2·8 to 10·9)	7·5 (3·6 to 13·2)	7·3 (3·6 to 12·2)	6·2 (3·4 to 12·4)	6·8 (3·9 to 10·6)	6·2 (3·6 to 9·7)	4·5 (2·4 to 7·2)	3·6 (2 to 5·8)	2·5 (1·5 to 3·8)	1·2 (0·7 to 1·9)	0·3 (0·2 to 0·5)	62·3
Cirrhosis and other chronic liver diseases due to other causes	0 (0 to 0)	0 (0 to 0)	0 (0 to 0)	1·3 (0·7 to 2·1)	1·1 (0·7 to 1·7)	1·6 (1 to 2·2)	2·2 (1·5 to 3·1)	3 (2 to 4·1)	2·7 (1·5 to 4·1)	2·6 (1·4 to 4·3)	2·5 (1·3 to 4·4)	3·1 (1·5 to 5·6)	4 (2 to 7·3)	4·9 (2·5 to 8·6)	5·5 (2·7 to 9·5)	5·9 (3 to 10·4)	5·9 (2·8 to 10·1)	5·9 (3 to 9·8)	4·6 (2·5 to 7·7)	3·8 (2·1 to 6·3)	2·6 (1·6 to 4)	1·3 (0·8 to 2)	0·3 (0·2 to 0·5)	64·9
Upper digestive system diseases	0 (0 to 0)	0 (0 to 0)	0 (0 to 0)	0·5 (0·3 to 0·9)	0·7 (0·3 to 1·3)	1·2 (0·7 to 2)	4 (2·2 to 7)	9·4 (5·1 to 17·2)	13·3 (7 to 23·2)	14·8 (7·7 to 26·2)	16·6 (9 to 28·4)	17·5 (9·4 to 28·4)	19·7 (11·5 to 32)	19·8 (11·6 to 31·8)	18·1 (11 to 29·1)	15·4 (9·6 to 24·2)	11·9 (7·9 to 18·7)	10·7 (7·4 to 15·4)	8·3 (5·6 to 11·9)	6·8 (4·7 to 9·5)	4·8 (3·5 to 6·3)	2·5 (1·9 to 3·3)	0·7 (0·5 to 0·9)	196
Peptic ulcer disease	0 (0 to 0)	0 (0 to 0)	0 (0 to 0)	0·1 (0·1 to 0·2)	0·1 (0·1 to 0·1)	0·2 (0·1 to 0·2)	0·2 (0·1 to 0·3)	0·3 (0·2 to 0·5)	0·4 (0·3 to 0·6)	0·7 (0·5 to 1)	1 (0·7 to 1·4)	1·4 (1 to 2)	2 (1·4 to 2·8)	2·3 (1·7 to 3·1)	2·5 (1·6 to 3·5)	2·8 (2·1 to 3·6)	2·6 (1·9 to 3·4)	3·1 (2·3 to 4·1)	2·8 (1·8 to 4)	2·9 (1·9 to 4·2)	2·4 (1·7 to 3·2)	1·5 (1·1 to 2)	0·4 (0·3 to 0·6)	29·8
Gastritis and duodenitis	0 (0 to 0)	0 (0 to 0)	0 (0 to 0)	0·4 (0·2 to 0·8)	0·6 (0·3 to 1·2)	0·6 (0·2 to 1·2)	0·9 (0·4 to 1·8)	1·1 (0·5 to 2·1)	1·5 (0·7 to 2·6)	1·8 (0·8 to 3·3)	2·4 (1·2 to 4)	2·3 (1·2 to 4·5)	3·6 (1·8 to 7)	4 (2·2 to 7)	4 (2·2 to 7·1)	3·3 (1·8 to 5·5)	2·5 (1·5 to 4)	2·1 (1·3 to 3·4)	1·4 (0·9 to 2·2)	1 (0·6 to 1·5)	0·8 (0·5 to 1·1)	0·4 (0·3 to 0·6)	0·1 (0·1 to 0·2)	34·9
Gastroesophageal reflux disease	0 (0 to 0)	0 (0 to 0)	0 (0 to 0)	0 (0 to 0)	0 (0 to 0)	0·4 (0·2 to 0·8)	3 (1·4 to 5·5)	8 (4 to 15·5)	11·3 (5·5 to 20·9)	12·2 (5·7 to 23·6)	13·3 (6·2 to 24·4)	13·3 (6·4 to 23·9)	14·1 (7 to 24·9)	13·4 (6·5 to 23·8)	11·5 (5·5 to 21·1)	9·3 (4·5 to 16·8)	6·8 (3·4 to 12·5)	5·5 (2·7 to 9·7)	4·1 (2 to 7·3)	2·8 (1·4 to 5)	1·6 (0·8 to 2·8)	0·6 (0·3 to 1·1)	0·2 (0·1 to 0·3)	131
Appendicitis	0 (0 to 0)	0 (0 to 0)	0 (0 to 0)	0·6 (0·3 to 1·2)	0·9 (0·6 to 1·2)	1·1 (0·7 to 1·8)	1·2 (0·6 to 1·9)	1·2 (0·7 to 1·9)	1 (0·5 to 1·6)	0·9 (0·5 to 1·5)	1·4 (0·8 to 2·1)	1·3 (0·8 to 2·1)	1·5 (0·9 to 2·4)	1·2 (0·7 to 2)	1·2 (0·7 to 2·1)	0·9 (0·5 to 1·4)	0·9 (0·6 to 1·4)	0·9 (0·5 to 1·4)	0·6 (0·3 to 1)	0·5 (0·3 to 0·8)	0·5 (0·3 to 0·8)	0·3 (0·2 to 0·5)	0·1 (0 to 0·1)	18·2
Paralytic ileus and intestinal obstruction	0·7 (0·4 to 1·1)	0·8 (0·5 to 1·4)	2·2 (1·2 to 3·7)	1·7 (1 to 2·9)	0·7 (0·4 to 1·1)	0·7 (0·4 to 1)	0·5 (0·3 to 0·8)	0·6 (0·3 to 0·9)	0·8 (0·4 to 1·2)	0·8 (0·5 to 1·3)	1·5 (0·9 to 2·3)	1·8 (1·1 to 2·8)	2·6 (1·6 to 4·1)	3·1 (1·8 to 4·7)	2·8 (1·8 to 4·1)	3·1 (1·9 to 4·6)	3·3 (2·2 to 4·9)	4 (2·5 to 5·9)	4·1 (2·6 to 6·2)	4·2 (2·8 to 6·4)	3·5 (2·3 to 5·1)	2·1 (1·3 to 3·1)	0·6 (0·4 to 0·8)	46·3
Inguinal, femoral, and abdominal hernia	0·3 (0·2 to 0·5)	0·1 (0 to 0·2)	0·2 (0·1 to 0·4)	1·5 (0·8 to 2·5)	1·9 (1 to 3·3)	1·3 (0·6 to 2·4)	1·1 (0·5 to 1·9)	1 (0·4 to 1·9)	1·2 (0·5 to 2·2)	1·5 (0·7 to 2·7)	1·8 (0·9 to 3·3)	2·1 (1·1 to 3·7)	2·5 (1·3 to 4·2)	2·4 (1·4 to 3·9)	2 (1·2 to 3·3)	1·6 (0·9 to 2·5)	1 (0·6 to 1·5)	0·8 (0·5 to 1·2)	0·6 (0·4 to 0·9)	0·5 (0·3 to 0·8)	0·4 (0·3 to 0·6)	0·3 (0·2 to 0·4)	0·1 (0 to 0·1)	26·3
Inflammatory bowel disease	0 (0 to 0)	0 (0 to 0)	0 (0 to 0)	0·8 (0·4 to 1·4)	0·2 (0·1 to 0·4)	0·1 (0·1 to 0·2)	0·3 (0·2 to 0·5)	0·7 (0·4 to 1)	1·2 (0·7 to 1·7)	1·4 (0·9 to 2)	1·6 (1·1 to 2·4)	2·8 (1·8 to 4·1)	2·4 (1·5 to 3·5)	2·1 (1·5 to 3)	2 (1·4 to 3)	1·9 (1·3 to 2·8)	1·2 (0·8 to 1·8)	1·5 (1 to 2·2)	1·2 (0·8 to 1·8)	0·8 (0·6 to 1·2)	0·7 (0·5 to 1·1)	0·6 (0·4 to 0·8)	0·1 (0·1 to 0·1)	23·7
Vascular intestinal disorders	0 (0 to 0)	0 (0 to 0)	0 (0 to 0)	0 (0 to 0)	0·1 (0 to 0·1)	0 (0 to 0)	0 (0 to 0·1)	0·1 (0·1 to 0·2)	0·2 (0·1 to 0·4)	0·2 (0·1 to 0·3)	0·4 (0·3 to 0·7)	0·8 (0·5 to 1·3)	1·5 (0·9 to 2·3)	1·5 (1 to 2·3)	1·7 (1·1 to 2·5)	2·1 (1·4 to 3·1)	2·3 (1·6 to 3·3)	2·6 (1·8 to 3·9)	2·4 (1·6 to 3·6)	2·3 (1·5 to 3·4)	2 (1·3 to 3)	1 (0·6 to 1·4)	0·2 (0·2 to 0·3)	21·7
Gallbladder and biliary diseases	0 (0 to 0)	0 (0 to 0)	0 (0 to 0)	0·2 (0·1 to 0·3)	0·5 (0·1 to 0·9)	0·8 (0·3 to 1·6)	1·4 (0·6 to 2·6)	2·4 (1·2 to 3·9)	3·2 (1·8 to 5·3)	4·1 (2·4 to 6·5)	5·3 (3·2 to 8·1)	6·3 (3·8 to 9·8)	7·3 (4·6 to 10·8)	7·4 (4·6 to 11·2)	6·4 (4·2 to 9·5)	5·3 (3·5 to 7·8)	4 (2·7 to 5·7)	3·4 (2·4 to 4·7)	2·6 (1·8 to 3·6)	2·1 (1·5 to 2·9)	1·4 (1 to 1·8)	0·8 (0·5 to 1)	0·2 (0·1 to 0·3)	64·9
Pancreatitis	0 (0 to 0)	0 (0 to 0)	0 (0 to 0)	0 (0 to 0)	0 (0 to 0)	0 (0 to 0·1)	0·1 (0 to 0·1)	0·2 (0·1 to 0·3)	0·2 (0·1 to 0·3)	0·2 (0·2 to 0·4)	0·2 (0·2 to 0·4)	0·4 (0·2 to 0·6)	0·5 (0·3 to 0·7)	0·5 (0·3 to 0·7)	0·5 (0·3 to 0·7)	0·5 (0·3 to 0·7)	0·5 (0·3 to 0·6)	0·5 (0·3 to 0·7)	0·4 (0·3 to 0·6)	0·4 (0·2 to 0·5)	0·3 (0·2 to 0·4)	0·1 (0·1 to 0·2)	0 (0 to 0·1)	5·5
Other digestive diseases	0 (0 to 0)	0 (0 to 0)	0 (0 to 0)	2·8 (1·5 to 4·6)	1 (0·6 to 1·5)	0·7 (0·4 to 1·1)	0·8 (0·4 to 1·3)	0·9 (0·5 to 1·4)	1·4 (0·7 to 2·3)	1·6 (0·7 to 2·5)	1·6 (0·8 to 2·6)	2·4 (1·2 to 3·9)	3 (1·5 to 5)	3·9 (1·9 to 6·2)	3·8 (1·9 to 6·2)	4·4 (2·1 to 6·6)	4·4 (2·2 to 6·8)	4·8 (2·4 to 7·5)	4·5 (2·4 to 6·9)	5·1 (2·7 to 7·9)	5·1 (2·7 to 7·8)	3 (1·6 to 4·5)	0·9 (0·5 to 1·2)	55·9
Neurological disorders	0 (0 to 0)	0·2 (0·1 to 0·3)	2·4 (1·4 to 3·9)	12·2 (7 to 19·5)	20·7 (9·7 to 35·7)	57·4 (18·1 to 118)	81·8 (28·1 to 167)	91·1 (31·3 to 185)	92·9 (32·2 to 180)	89·1 (33·2 to 172)	95·6 (37 to 181)	98·2 (39·7 to 184)	102 (47·3 to 189)	89·1 (42·7 to 158)	74·2 (38·8 to 126)	61·9 (36 to 98)	56·4 (34·9 to 88·6)	62·8 (40·4 to 102)	73 (45·8 to 129)	96·1 (56·4 to 187)	93·1 (49·5 to 185)	59·7 (29·1 to 124)	19·5 (9·1 to 42·7)	1,430
Alzheimer's disease and other dementias	0 (0 to 0)	0 (0 to 0)	0 (0 to 0)	0 (0 to 0)	0 (0 to 0)	0 (0 to 0)	0 (0 to 0)	0 (0 to 0)	0 (0 to 0)	0 (0 to 0)	0 (0 to 0)	0·4 (0·1 to 1·5)	2 (0·8 to 7·6)	4·6 (1·7 to 11·8)	8·2 (3·2 to 20·2)	11·7 (5·2 to 26·2)	20·6 (7·7 to 44·8)	29·3 (13·3 to 68·5)	43 (19·5 to 98·5)	70·3 (31·5 to 161)	74·8 (31·5 to 168)	51·6 (21 to 116)	17·8 (7·3 to 41·3)	334
Parkinson's disease	0 (0 to 0)	0 (0 to 0)	0 (0 to 0)	0 (0 to 0)	0 (0 to 0)	0 (0 to 0)	0 (0 to 0)	0 (0 to 0)	0 (0 to 0·1)	0·1 (0·1 to 0·2)	0·2 (0·1 to 0·3)	0·5 (0·4 to 0·7)	1·1 (0·8 to 1·6)	1·9 (1·4 to 2·5)	2·6 (1·9 to 3·4)	4·2 (3·3 to 5·4)	6·1 (4·9 to 7·5)	10·5 (8·6 to 12·6)	14·4 (11·8 to 17·3)	15·6 (12·5 to 18·8)	12·5 (10·1 to 14·9)	5·7 (4·7 to 6·8)	1·2 (1 to 1·4)	76·5
Idiopathic epilepsy	0 (0 to 0)	0 (0 to 0·1)	1·2 (0·6 to 2)	7·5 (4 to 12·5)	10·3 (4·6 to 19·4)	14 (6·1 to 26·1)	18·3 (8·6 to 33·1)	21·3 (10·4 to 35·9)	21·4 (10·9 to 35·9)	20·1 (11 to 32·3)	20 (10·9 to 32)	19·7 (11·3 to 31·6)	20·6 (11·1 to 32·3)	16·4 (8·5 to 27·3)	13 (6·3 to 23)	10·7 (4·8 to 19·3)	8·6 (3·7 to 15·6)	7·7 (3·3 to 14·3)	6·2 (2·6 to 11·2)	4·7 (2 to 8·7)	3 (1·3 to 5·4)	1·4 (0·6 to 2·4)	0·3 (0·1 to 0·6)	246
Multiple sclerosis	0 (0 to 0)	0 (0 to 0)	0 (0 to 0)	0 (0 to 0)	0 (0 to 0)	0 (0 to 0)	0·1 (0 to 0·2)	0·3 (0·2 to 0·5)	0·5 (0·3 to 0·7)	0·6 (0·4 to 1)	1·2 (0·8 to 1·8)	1·3 (0·9 to 1·9)	1·6 (1·1 to 2·4)	1·3 (0·9 to 1·8)	1·1 (0·7 to 1·6)	0·8 (0·5 to 1·1)	0·4 (0·3 to 0·6)	0·3 (0·2 to 0·4)	0·2 (0·1 to 0·2)	0·1 (0 to 0·1)	0·1 (0 to 0·1)	0 (0 to 0)	0 (0 to 0)	9·9
Motor neuron disease	0 (0 to 0)	0·1 (0·1 to 0·2)	0·3 (0·1 to 0·6)	1 (0·5 to 1·7)	0·2 (0·1 to 0·2)	0·2 (0·2 to 0·4)	0·1 (0·1 to 0·1)	0·2 (0·1 to 0·2)	0·2 (0·2 to 0·3)	0·3 (0·2 to 0·4)	0·5 (0·3 to 0·8)	1·1 (0·7 to 1·7)	2·1 (1·4 to 3)	2·6 (1·8 to 3·8)	2·6 (1·8 to 3·8)	2·4 (1·7 to 3·3)	2 (1·3 to 2·7)	1·5 (1 to 2·1)	0·9 (0·6 to 1·3)	0·4 (0·3 to 0·6)	0·2 (0·1 to 0·2)	0·1 (0 to 0·1)	0 (0 to 0)	18·9
Headache disorders	0 (0 to 0)	0 (0 to 0)	0 (0 to 0)	0 (0 to 0)	6·8 (0·1 to 17·6)	37·8 (1·9 to 97·3)	58·3 (6·2 to 145)	65·4 (8·3 to 154)	67·3 (9·8 to 154)	64·7 (10·8 to 143)	69·9 (12·2 to 157)	70·7 (14·1 to 157)	68·4 (16·6 to 154)	57·3 (14·9 to 122)	42·5 (11·7 to 92·7)	29·2 (8·8 to 61·5)	18·3 (5·9 to 38)	11·9 (3·9 to 24·7)	7·1 (2·3 to 14·8)	4·3 (1·3 to 8·9)	2·2 (0·7 to 4·4)	0·7 (0·2 to 1·5)	0·1 (0 to 0·3)	683
Migraine	0 (0 to 0)	0 (0 to 0)	0 (0 to 0)	0 (0 to 0)	6·3 (0·1 to 16·6)	35·8 (1·2 to 95·2)	54·7 (4·3 to 142)	60·9 (6·1 to 149)	62·3 (6·5 to 147)	59·8 (7·2 to 140)	64·2 (9 to 150)	64·7 (10·5 to 150)	62·1 (12 to 145)	51·4 (10·5 to 115)	38 (8·2 to 85·9)	25·6 (6·2 to 57·4)	15·7 (4·2 to 34·8)	9·9 (2·8 to 22·2)	5·9 (1·6 to 12·8)	3·5 (1 to 7·9)	1·8 (0·5 to 3·9)	0·6 (0·2 to 1·3)	0·1 (0 to 0·2)	623
Tension-type headache	0 (0 to 0)	0 (0 to 0)	0 (0 to 0)	0 (0 to 0)	0·5 (0 to 3·1)	2 (0·1 to 13)	3·6 (0·7 to 15·2)	4·5 (0·9 to 17·9)	5 (1·1 to 18·4)	4·9 (1·2 to 16·9)	5·7 (1·5 to 18·1)	5·9 (1·7 to 16·8)	6·3 (2 to 16·2)	5·9 (1·9 to 16·3)	4·5 (1·4 to 11·4)	3·6 (1·1 to 10·1)	2·7 (0·8 to 8)	2 (0·5 to 6·7)	1·2 (0·3 to 4·3)	0·7 (0·2 to 2·5)	0·4 (0·1 to 1·3)	0·1 (0 to 0·5)	0 (0 to 0·1)	59·5
Other neurological disorders	0 (0 to 0)	0 (0 to 0)	0·9 (0·5 to 1·5)	3·7 (2 to 5·9)	3·4 (1·5 to 5·5)	5·4 (2·1 to 10·4)	5·1 (2 to 9·9)	3·9 (1·8 to 7·3)	3·5 (1·8 to 5·8)	3·3 (1·9 to 5·2)	3·8 (2·1 to 5·9)	4·4 (2·6 to 6·7)	5·7 (3·6 to 8·3)	4·7 (3·1 to 6·8)	4·2 (2·8 to 5·9)	2·9 (2 to 4)	2·1 (1·6 to 2·8)	1·6 (1·1 to 2)	1·2 (0·9 to 1·6)	0·8 (0·6 to 1·1)	0·4 (0·3 to 0·6)	0·2 (0·1 to 0·3)	0·1 (0 to 0·1)	61·2
Mental disorders	0 (0 to 0)	0 (0 to 0)	0·4 (0·2 to 0·6)	2·6 (1·6 to 3·8)	19·7 (12·4 to 29·1)	75·5 (50 to 109)	138 (94·1 to 193)	167 (114 to 233)	174 (119 to 240)	163 (115 to 225)	176 (125 to 240)	171 (122 to 236)	174 (125 to 234)	156 (114 to 210)	128 (90·7 to 172)	95·9 (66·9 to 130)	65·1 (46·1 to 86·5)	48·8 (34·8 to 64·3)	33·5 (23·3 to 45·1)	22·2 (15·3 to 30·2)	12·4 (8·8 to 16·7)	5·2 (3·5 to 7·3)	1·3 (0·8 to 1·9)	1,830
Schizophrenia	0 (0 to 0)	0 (0 to 0)	0 (0 to 0)	0 (0 to 0)	0 (0 to 0)	0·3 (0·1 to 0·6)	2·7 (0·9 to 5·2)	10·1 (5·5 to 16·7)	17·8 (9·7 to 27·4)	20·7 (12·1 to 30·5)	23·9 (14·6 to 34·9)	22·8 (14·6 to 32·3)	22·3 (14·6 to 30·6)	19 (12·2 to 26·4)	14·1 (9·3 to 19·5)	9·3 (6 to 13·2)	5·5 (3·6 to 8)	3·6 (2·2 to 5·3)	2·1 (1·2 to 3·1)	1·1 (0·6 to 1·7)	0·4 (0·2 to 0·7)	0·1 (0·1 to 0·2)	0 (0 to 0)	176
Depressive disorders	0 (0 to 0)	0 (0 to 0)	0 (0 to 0)	0 (0 to 0)	1·5 (0·5 to 3·1)	18 (8·8 to 31·7)	49·7 (28·9 to 82)	63·5 (36·8 to 102)	59·8 (34·9 to 92·5)	55·4 (32·8 to 88·2)	64·3 (38·6 to 95·9)	66 (39·8 to 100)	69 (44·6 to 102)	66·5 (42·7 to 98·4)	58·1 (35·2 to 85·2)	42·8 (27·8 to 67·7)	32·1 (19·9 to 45)	23·5 (16·1 to 35·4)	17·6 (10·9 to 26·7)	12·2 (7·6 to 18)	7·3 (4·7 to 10·8)	3·3 (2 to 4·9)	0·8 (0·5 to 1·4)	712
Major depressive disorder	0 (0 to 0)	0 (0 to 0)	0 (0 to 0)	0 (0 to 0)	1·3 (0·3 to 2·9)	16·8 (7·8 to 30·9)	45·8 (25·5 to 75·9)	56·3 (31 to 92·7)	50·8 (28·5 to 81·2)	45·9 (25 to 75·8)	51·8 (30·3 to 81·1)	53·6 (30·8 to 87)	58 (36·4 to 87·8)	56·4 (35 to 84·3)	49·8 (29 to 74·3)	39·5 (22·4 to 59·6)	27·8 (17·1 to 40·1)	21·6 (13·5 to 31·7)	15·4 (9·1 to 24·3)	10·9 (6·4 to 16·8)	6·6 (4 to 9·9)	3 (1·8 to 4·6)	0·8 (0·4 to 1·3)	612
Dysthymia	0 (0 to 0)	0 (0 to 0)	0 (0 to 0)	0 (0 to 0)	0·1 (0 to 0·4)	1·2 (0·4 to 2·4)	3·9 (1·8 to 7)	7·2 (3·7 to 12·7)	9·1 (4·9 to 15·5)	9·5 (5·3 to 16)	11·1 (6 to 17·4)	10·1 (5·9 to 18·5)	11·1 (6·1 to 19·6)	10·1 (5·7 to 16·8)	8·3 (4·8 to 13·3)	6·3 (3·4 to 10·6)	4·3 (2·3 to 7·3)	3·1 (1·8 to 5·2)	2·1 (1·2 to 3·5)	1·3 (0·7 to 2·2)	0·7 (0·4 to 1·2)	0·3 (0·2 to 0·5)	0 (0 to 0·1)	100
Bipolar disorder	0 (0 to 0)	0 (0 to 0)	0 (0 to 0)	0 (0 to 0)	0 (0 to 0)	4·6 (1·8 to 8·6)	18·7 (8·9 to 32·6)	24·4 (12·9 to 40·8)	24·4 (13·2 to 41·4)	21·5 (11·7 to 36·1)	21·6 (11·8 to 36)	20·5 (11·3 to 33·2)	20·4 (11·4 to 33·3)	17·8 (9·7 to 29·7)	13·7 (7·7 to 22·5)	9·5 (5·3 to 15·5)	5·9 (3·1 to 9·6)	3·9 (2·1 to 6·3)	2·2 (1·1 to 3·7)	1·1 (0·6 to 1·9)	0·5 (0·2 to 0·8)	0·2 (0·1 to 0·3)	0 (0 to 0·1)	211
Anxiety disorders	0 (0 to 0)	0 (0 to 0)	0 (0 to 0)	0·5 (0·2 to 1·2)	9·1 (4·5 to 15·9)	26·8 (14·5 to 44·3)	40 (24 to 61·1)	45·6 (25·8 to 74·8)	46·8 (26·6 to 76·1)	44·3 (26·2 to 71·2)	47·2 (28·1 to 73·3)	45·7 (27·2 to 73·5)	45·8 (26·6 to 73·8)	40·3 (24·2 to 63·4)	32·2 (20·3 to 48·3)	23·5 (14·4 to 36·6)	16·1 (9·5 to 25·4)	12·2 (7·2 to 19·4)	8·4 (5·1 to 13·4)	5·4 (3·2 to 8·8)	2·8 (1·6 to 4·5)	1 (0·6 to 1·6)	0·2 (0·1 to 0·3)	494
Eating disorders	0 (0 to 0)	0 (0 to 0)	0 (0 to 0)	0 (0 to 0)	0·1 (0 to 0·2)	1·9 (0·9 to 3·3)	7·9 (4·1 to 14·1)	13·5 (7·1 to 22·8)	12·5 (6·8 to 20·7)	9·1 (5·1 to 15·1)	7 (3·7 to 11·7)	4·2 (2·2 to 7)	2·5 (1·2 to 4·1)	0 (0 to 0)	0 (0 to 0)	0 (0 to 0)	0 (0 to 0)	0 (0 to 0)	0 (0 to 0)	0 (0 to 0)	0 (0 to 0)	0 (0 to 0)	0 (0 to 0)	58·7
Anorexia nervosa	0 (0 to 0)	0 (0 to 0)	0 (0 to 0)	0 (0 to 0)	0·1 (0 to 0·2)	1 (0·4 to 1·9)	3·1 (1·4 to 5·7)	3·3 (1·5 to 6)	2·6 (1·3 to 4·6)	1·6 (0·7 to 3·1)	1·2 (0·5 to 2·3)	0·9 (0·3 to 1·7)	0·7 (0·2 to 1·3)	0 (0 to 0)	0 (0 to 0)	0 (0 to 0)	0 (0 to 0)	0 (0 to 0)	0 (0 to 0)	0 (0 to 0)	0 (0 to 0)	0 (0 to 0)	0 (0 to 0)	14·5
Bulimia nervosa	0 (0 to 0)	0 (0 to 0)	0 (0 to 0)	0 (0 to 0)	0 (0 to 0)	0·9 (0·2 to 1·9)	4·8 (2 to 9·8)	10·1 (4·8 to 18)	9·9 (4·8 to 17·3)	7·5 (3·8 to 12·7)	5·8 (2·8 to 10·2)	3·4 (1·6 to 6)	1·8 (0·8 to 3·2)	0 (0 to 0)	0 (0 to 0)	0 (0 to 0)	0 (0 to 0)	0 (0 to 0)	0 (0 to 0)	0 (0 to 0)	0 (0 to 0)	0 (0 to 0)	0 (0 to 0)	44·2
Autism spectrum disorders	0 (0 to 0)	0 (0 to 0)	0·3 (0·1 to 0·4)	1·2 (0·6 to 1·9)	1·6 (0·9 to 2·6)	1·8 (1 to 2·8)	1·9 (1·1 to 3·1)	1·9 (1·1 to 3)	1·8 (1·1 to 3)	1·6 (1 to 2·6)	1·7 (0·9 to 2·6)	1·6 (0·9 to 2·5)	1·5 (0·9 to 2·4)	1·3 (0·8 to 2·1)	1·1 (0·6 to 1·8)	0·8 (0·4 to 1·3)	0·6 (0·3 to 0·9)	0·4 (0·2 to 0·7)	0·3 (0·2 to 0·5)	0·2 (0·1 to 0·3)	0·1 (0·1 to 0·2)	0 (0 to 0·1)	0 (0 to 0)	21·8
Attention-deficit/hyperactivity disorder	0 (0 to 0)	0 (0 to 0)	0 (0 to 0)	0·2 (0·1 to 0·3)	1·9 (1 to 3·5)	3 (1·6 to 5·2)	2·7 (1·4 to 4·6)	2 (1·1 to 3·7)	1·5 (0·8 to 2·6)	1·1 (0·6 to 2)	0·9 (0·4 to 1·7)	0·7 (0·3 to 1·4)	0·6 (0·3 to 1·1)	0·5 (0·2 to 0·9)	0·3 (0·1 to 0·5)	0·1 (0 to 0·3)	0·1 (0 to 0·1)	0 (0 to 0·1)	0 (0 to 0)	0 (0 to 0)	0 (0 to 0)	0 (0 to 0)	0 (0 to 0)	15·6
Conduct disorder	0 (0 to 0)	0 (0 to 0)	0 (0 to 0)	0 (0 to 0)	4·3 (2 to 8)	17·6 (8·9 to 30·6)	11 (5·5 to 19·1)	0 (0 to 0)	0 (0 to 0)	0 (0 to 0)	0 (0 to 0)	0 (0 to 0)	0 (0 to 0)	0 (0 to 0)	0 (0 to 0)	0 (0 to 0)	0 (0 to 0)	0 (0 to 0)	0 (0 to 0)	0 (0 to 0)	0 (0 to 0)	0 (0 to 0)	0 (0 to 0)	33
Idiopathic developmental intellectual disability	0 (0 to 0)	0 (0 to 0)	0·1 (0 to 0·2)	0·7 (0·2 to 1·3)	1·1 (0·4 to 2)	1·2 (0·4 to 2·2)	1·2 (0·5 to 2·4)	1·3 (0·4 to 2·4)	1·2 (0·4 to 2·2)	1·1 (0·4 to 2)	1·1 (0·4 to 2)	1 (0·3 to 1·9)	0·9 (0·3 to 1·8)	0·8 (0·3 to 1·5)	0·6 (0·2 to 1·2)	0·4 (0·1 to 0·8)	0·3 (0·1 to 0·6)	0·2 (0·1 to 0·4)	0·1 (0 to 0·2)	0·1 (0 to 0·2)	0 (0 to 0·1)	0 (0 to 0)	0 (0 to 0)	13·4
Other mental disorders	0 (0 to 0)	0 (0 to 0)	0 (0 to 0)	0 (0 to 0)	0 (0 to 0)	0·2 (0·1 to 0·4)	1·8 (0·6 to 3·5)	4·7 (2·2 to 8·3)	7·2 (3·8 to 12)	8·5 (4·9 to 13·3)	10·1 (6·1 to 16)	10·1 (6·2 to 15·7)	10·7 (6·2 to 16·5)	9·8 (6 to 15)	8·3 (5 to 12·5)	6·4 (3·8 to 9·9)	4·7 (2·9 to 7·1)	3·8 (2·3 to 5·7)	2·8 (1·8 to 4·2)	2 (1·3 to 3)	1·2 (0·8 to 1·8)	0·5 (0·3 to 0·8)	0·1 (0·1 to 0·2)	93
Substance use disorders	0 (0 to 0)	0 (0 to 0)	0 (0 to 0)	0 (0 to 0)	0 (0 to 0)	0·9 (0·5 to 1·6)	15·1 (9·8 to 21·4)	29·8 (19·7 to 43·5)	31·1 (20·9 to 43·7)	28·6 (19·4 to 40·2)	31·3 (22·4 to 42)	30·7 (22·1 to 41·4)	30·5 (22·3 to 40·8)	22·1 (15·9 to 30)	16·6 (12·3 to 22)	11·3 (8·4 to 15·2)	6·9 (5 to 9·1)	4·8 (3·6 to 6·4)	3·3 (2·4 to 4·4)	2·6 (1·9 to 3·5)	2 (1·4 to 2·7)	1·3 (0·9 to 1·8)	0·4 (0·3 to 0·6)	269
Alcohol use disorders	0 (0 to 0)	0 (0 to 0)	0 (0 to 0)	0 (0 to 0)	0 (0 to 0)	0·4 (0·1 to 0·7)	5·4 (3·2 to 8·5)	14 (8 to 23·4)	16·3 (9·7 to 25·2)	18·1 (11·4 to 27·2)	22·4 (15·7 to 31·2)	24 (16·8 to 33·4)	24·5 (17·3 to 34·1)	17·3 (12·2 to 24·1)	13 (9·4 to 17·8)	9 (6·6 to 12·1)	5·4 (3·8 to 7·3)	3·8 (2·8 to 5·1)	2·6 (1·9 to 3·5)	2·1 (1·5 to 3)	1·7 (1·2 to 2·4)	1·2 (0·8 to 1·6)	0·4 (0·3 to 0·6)	182
Drug use disorders	0 (0 to 0)	0 (0 to 0)	0 (0 to 0)	0 (0 to 0)	0 (0 to 0)	0·6 (0·2 to 1·1)	10·6 (5·7 to 15·2)	15·8 (9·9 to 23·6)	14·8 (9·4 to 21·7)	10·6 (6·3 to 15·9)	8·9 (5·2 to 13·3)	6·7 (4 to 10·4)	6 (3·6 to 9)	4·8 (2·6 to 7·5)	3·6 (2 to 5·7)	2·2 (1·2 to 4)	1·4 (0·8 to 2·4)	0·9 (0·5 to 1·7)	0·6 (0·3 to 1·1)	0·4 (0·2 to 0·7)	0·2 (0·1 to 0·4)	0·1 (0·1 to 0·2)	0 (0 to 0·1)	87·5
Opioid use disorders	0 (0 to 0)	0 (0 to 0)	0 (0 to 0)	0 (0 to 0)	0 (0 to 0)	0 (0 to 0)	1·5 (0·5 to 3·2)	7 (3·1 to 13)	8·6 (4·7 to 14·4)	7 (3·3 to 11·6)	5·8 (3 to 9·8)	4·4 (2 to 7·5)	4 (1·9 to 6·7)	3·5 (1·5 to 6·1)	2·7 (1·2 to 4·8)	1·8 (0·7 to 3·4)	1·1 (0·5 to 2)	0·7 (0·3 to 1·4)	0·5 (0·2 to 0·9)	0·3 (0·1 to 0·6)	0·2 (0·1 to 0·3)	0·1 (0 to 0·1)	0 (0 to 0)	49·2
Cocaine use disorders	0 (0 to 0)	0 (0 to 0)	0 (0 to 0)	0 (0 to 0)	0 (0 to 0)	0 (0 to 0·1)	1·5 (0·5 to 3)	1·8 (0·7 to 3·3)	1·4 (0·6 to 2·5)	1 (0·5 to 1·8)	1 (0·5 to 1·7)	0·8 (0·4 to 1·4)	0·7 (0·4 to 0·9)	0·4 (0·2 to 0·6)	0·3 (0·2 to 0·5)	0·2 (0·1 to 0·3)	0·1 (0·1 to 0·2)	0·1 (0·1 to 0·1)	0·1 (0 to 0·1)	0·1 (0 to 0·1)	0 (0 to 0)	0 (0 to 0)	0 (0 to 0)	9·3
Amphetamine use disorders	0 (0 to 0)	0 (0 to 0)	0 (0 to 0)	0 (0 to 0)	0 (0 to 0)	0 (0 to 0)	0·3 (0·1 to 0·5)	1·2 (0·3 to 2·5)	1·1 (0·3 to 2·4)	0·5 (0·1 to 1·2)	0·3 (0·2 to 0·6)	0·2 (0·1 to 0·3)	0·1 (0·1 to 0·2)	0·1 (0 to 0·1)	0·1 (0 to 0·1)	0 (0 to 0·1)	0 (0 to 0)	0 (0 to 0)	0 (0 to 0)	0 (0 to 0)	0 (0 to 0)	0 (0 to 0)	0 (0 to 0)	4
Cannabis use disorders	0 (0 to 0)	0 (0 to 0)	0 (0 to 0)	0 (0 to 0)	0 (0 to 0)	0·5 (0·2 to 1)	5·3 (2·6 to 9·3)	3·9 (2 to 6·9)	2 (0·9 to 3·6)	1·1 (0·4 to 2·1)	0·7 (0·3 to 1·4)	0·6 (0·2 to 1·2)	0·5 (0·2 to 1·1)	0·3 (0·1 to 0·7)	0·2 (0·1 to 0·3)	0·1 (0 to 0·1)	0 (0 to 0·1)	0 (0 to 0)	0 (0 to 0)	0 (0 to 0)	0 (0 to 0)	0 (0 to 0)	0 (0 to 0)	15·3
Other drug use disorders	0 (0 to 0)	0 (0 to 0)	0 (0 to 0)	0 (0 to 0)	0 (0 to 0)	0 (0 to 0)	1·1 (0·4 to 2·3)	1·9 (0·9 to 3·3)	1·7 (0·8 to 3)	1 (0·5 to 1·8)	0·9 (0·5 to 1·5)	0·7 (0·4 to 1·3)	0·7 (0·4 to 1·1)	0·5 (0·3 to 0·8)	0·3 (0·2 to 0·5)	0·2 (0·1 to 0·3)	0·1 (0·1 to 0·2)	0·1 (0·1 to 0·2)	0·1 (0·1 to 0·1)	0·1 (0·1 to 0·1)	0·1 (0 to 0·1)	0 (0 to 0)	0 (0 to 0)	9·6
Diabetes and kidney diseases	0 (0 to 0)	0 (0 to 0)	1·2 (0·7 to 1·9)	4·4 (3 to 6·3)	5·9 (4·5 to 7·9)	8 (6 to 10·9)	23·1 (18·1 to 28·9)	39·8 (31·1 to 50·3)	60·4 (47·2 to 77·9)	78·7 (61·2 to 98)	113 (87 to 139)	171 (134 to 212)	271 (210 to 339)	360 (282 to 450)	426 (332 to 523)	443 (352 to 535)	414 (329 to 500)	364 (296 to 442)	305 (251 to 363)	246 (202 to 292)	164 (134 to 196)	83·4 (67·5 to 99)	20·7 (16·8 to 24·7)	3,600
Diabetes mellitus	0 (0 to 0)	0 (0 to 0)	0·4 (0·2 to 0·8)	0·9 (0·5 to 1·5)	1·2 (0·7 to 1·7)	1·9 (1·1 to 2·6)	10·4 (7·8 to 13·4)	20·2 (14·9 to 26·6)	32 (22·9 to 43)	43·6 (31·6 to 57·6)	69·7 (51·4 to 92·6)	112 (82·7 to 148)	189 (139 to 249)	266 (198 to 353)	331 (251 to 423)	348 (267 to 431)	331 (251 to 411)	290 (228 to 362)	242 (193 to 296)	195 (156 to 240)	126 (99·8 to 156)	62 (49·5 to 75·9)	14·4 (11·2 to 18)	2,690
Diabetes mellitus type 1	0 (0 to 0)	0 (0 to 0)	0·4 (0·2 to 0·8)	0·9 (0·5 to 1·5)	1·2 (0·7 to 1·7)	1·9 (1·1 to 2·6)	2·3 (1·5 to 3·5)	3·9 (2·5 to 5·8)	5·2 (3·2 to 8)	4·6 (2·9 to 7·1)	7 (4·4 to 10·8)	10·8 (6·8 to 17·5)	4·4 (2·8 to 6·5)	5·5 (3·5 to 8·1)	6 (3·8 to 9·4)	5·1 (3·3 to 8·1)	4·8 (3 to 7·6)	3·7 (2·4 to 5·8)	3·1 (1·9 to 5)	1·6 (1 to 2·7)	1·1 (0·7 to 2)	0·6 (0·4 to 1·1)	0·2 (0·1 to 0·3)	74·3
Diabetes mellitus type 2	0 (0 to 0)	0 (0 to 0)	0 (0 to 0)	0 (0 to 0)	0 (0 to 0)	0 (0 to 0)	8·1 (5·7 to 10·7)	16·3 (11·7 to 22·4)	26·8 (18·3 to 37·2)	39·1 (27·8 to 52·4)	62·7 (45 to 84·7)	101 (73·7 to 134)	184 (136 to 243)	260 (193 to 346)	325 (246 to 415)	343 (263 to 425)	326 (248 to 406)	286 (226 to 357)	239 (190 to 293)	193 (154 to 238)	125 (99 to 155)	61·4 (49 to 75·1)	14·2 (11 to 17·7)	2,610
Chronic kidney disease	0 (0 to 0)	0 (0 to 0)	0·8 (0·5 to 1·3)	3·5 (2·3 to 5·1)	4·7 (3·4 to 6·5)	6·1 (4·2 to 8·8)	12·7 (9 to 17·1)	19·6 (14·1 to 27)	28·4 (19·9 to 38·3)	35 (24·5 to 46·1)	43·1 (30·6 to 59)	59 (43·3 to 80·1)	82·3 (58·7 to 109)	94·5 (68·7 to 127)	94·7 (69·5 to 123)	94·5 (70·8 to 124)	83 (60·3 to 111)	73·7 (55·3 to 97·1)	62·9 (47 to 82·5)	50·7 (38·2 to 64·7)	38 (28·9 to 48·4)	21·4 (15·8 to 27)	6·3 (4·6 to 8·2)	915
Chronic kidney disease due to diabetes mellitus type 1	0 (0 to 0)	0 (0 to 0)	0 (0 to 0)	0 (0 to 0·1)	0·1 (0 to 0·2)	0·2 (0·1 to 0·4)	0·8 (0·2 to 1)	1·4 (0·4 to 2)	2·8 (1·1 to 5·4)	5·1 (2·6 to 8·7)	7·3 (3·6 to 12·4)	8·8 (4·9 to 17·1)	11·9 (5·3 to 21·2)	10·1 (4 to 19)	6·9 (2·2 to 14·7)	5·2 (1·7 to 12)	3·8 (1·4 to 8·3)	2·4 (0·9 to 4·9)	1·3 (0·5 to 3)	0·6 (0·2 to 1·2)	0·2 (0 to 0·4)	0 (0 to 0·1)	0 (0 to 0)	69·2
Chronic kidney disease due to diabetes mellitus type 2	0 (0 to 0)	0 (0 to 0)	0 (0 to 0)	0 (0 to 0)	0 (0 to 0)	0 (0 to 0)	0·2 (0·1 to 0·5)	0·5 (0·2 to 1)	1·2 (0·5 to 2·4)	2·2 (0·9 to 4·2)	3·8 (1·7 to 6·9)	7·3 (3·7 to 12·6)	14·7 (8 to 24)	22·8 (13 to 35·2)	27·4 (15·9 to 40·9)	29·6 (17·8 to 44·3)	26·7 (16·9 to 38·8)	23·9 (15·1 to 34·2)	20 (12·7 to 28·5)	15·6 (9·7 to 21·6)	11·1 (7·2 to 15·6)	6 (3·7 to 9·1)	1·7 (1·2 to 2·7)	215
Chronic kidney disease due to hypertension	0 (0 to 0)	0 (0 to 0)	0 (0 to 0)	0 (0 to 0)	0 (0 to 0)	0 (0 to 0)	0·9 (0·3 to 1·8)	1·5 (0·6 to 3·2)	2·4 (1·1 to 4·4)	3·3 (1·7 to 5·6)	4·5 (2·5 to 7·1)	6·2 (4·1 to 10·4)	9·5 (6·3 to 16·7)	14·4 (8·8 to 22·7)	16·3 (10·5 to 25·5)	18·1 (12·4 to 29·5)	19·3 (12·3 to 29·2)	19·3 (12·6 to 28·1)	18·1 (11·9 to 26·5)	16·5 (10·7 to 22·4)	13·4 (9·4 to 18·6)	8·3 (5·8 to 11·5)	2·6 (1·7 to 3·7)	179
Chronic kidney disease due to glomerulonephritis	0 (0 to 0)	0 (0 to 0)	0·2 (0·1 to 0·4)	1·1 (0·6 to 1·8)	1·7 (0·9 to 2·6)	2·4 (1·4 to 3·8)	4·8 (2·9 to 7·2)	7·1 (4·5 to 10·9)	10 (6·3 to 14·9)	11·3 (6·6 to 17)	13·3 (7·9 to 20·4)	16·9 (9·3 to 25·4)	20·8 (12·3 to 32·4)	21·2 (11·6 to 35)	18·9 (9·8 to 33·5)	16·4 (8·5 to 28)	12·3 (6·1 to 21·4)	9·1 (4·8 to 16)	6·7 (3·3 to 12·3)	4·6 (2·1 to 8·5)	2·8 (1·3 to 5·3)	1·4 (0·6 to 2·5)	0·4 (0·1 to 0·8)	183
Chronic kidney disease due to other and unspecified causes	0 (0 to 0)	0 (0 to 0)	0·6 (0·3 to 1)	2·4 (1·5 to 3·6)	3 (1·9 to 4·3)	3·5 (2·1 to 5·7)	6·3 (3·9 to 9·2)	9·4 (6 to 13·6)	12·1 (7·8 to 17·5)	13·2 (8·3 to 19·1)	14·3 (8·7 to 21·5)	17·9 (11 to 28·2)	24·1 (14·6 to 37·3)	26 (16·4 to 41·1)	24·6 (15·4 to 38·8)	23·3 (14·2 to 36·9)	20·5 (12·3 to 31·9)	19 (12 to 28·3)	16·7 (10·2 to 24·8)	14 (9 to 20·4)	10·5 (6·9 to 15·3)	5·6 (3·7 to 8·2)	1·6 (0·9 to 2·5)	269
Acute glomerulonephritis	0 (0 to 0)	0 (0 to 0)	0 (0 to 0)	0 (0 to 0)	0 (0 to 0·1)	0 (0 to 0)	0 (0 to 0)	0 (0 to 0)	0 (0 to 0)	0 (0 to 0)	0 (0 to 0)	0 (0 to 0)	0 (0 to 0)	0 (0 to 0)	0 (0 to 0)	0 (0 to 0)	0 (0 to 0)	0 (0 to 0)	0 (0 to 0)	0 (0 to 0)	0 (0 to 0)	0 (0 to 0)	0 (0 to 0)	0·2
Skin and subcutaneous diseases	0 (0 to 0)	0 (0 to 0)	2·7 (1·8 to 3·9)	26·7 (16·9 to 39·1)	36·4 (22·9 to 53·7)	40·5 (25·7 to 59·9)	47·3 (29·9 to 70·7)	33 (21·3 to 49·2)	28·6 (18·7 to 41·4)	26·6 (17·8 to 39)	28·4 (19·1 to 41·5)	30·6 (21·2 to 45)	32·4 (22·3 to 47·9)	35·2 (25 to 51·1)	34·7 (24·1 to 49·7)	30·8 (21·1 to 44·2)	26·6 (18·6 to 38·7)	26·8 (18·7 to 39·3)	27·8 (19·8 to 42·4)	24·9 (16·9 to 38·6)	22·4 (14·4 to 34·6)	13·8 (8 to 19·5)	4·6 (2·4 to 6·3)	581
Dermatitis	0 (0 to 0)	0 (0 to 0)	0 (0 to 0)	9·5 (5·1 to 16·5)	12·6 (6·8 to 21·6)	9·8 (5·3 to 16·7)	8·1 (4·4 to 13·4)	6·9 (3·9 to 11·1)	6·1 (3·5 to 10)	5·5 (3·1 to 8·8)	5·5 (3·1 to 8·9)	5·2 (3 to 8·5)	5·4 (3 to 8·8)	5·5 (2·9 to 9)	5 (2·7 to 8·3)	4·2 (2·3 to 6·7)	3·3 (1·8 to 5·5)	2·8 (1·6 to 4·5)	2·1 (1·2 to 3·3)	1·4 (0·8 to 2·2)	0·8 (0·5 to 1·2)	0·3 (0·2 to 0·5)	0·1 (0 to 0·1)	100
Atopic dermatitis	0 (0 to 0)	0 (0 to 0)	0 (0 to 0)	9·4 (5 to 16·3)	12·3 (6·6 to 21·4)	8·9 (4·7 to 15·4)	6·3 (3·2 to 11·2)	4·7 (2·4 to 8·3)	3·7 (1·8 to 6·5)	2·9 (1·4 to 5·3)	2·8 (1·4 to 5·2)	2·6 (1·4 to 4·6)	2·7 (1·4 to 4·6)	2·5 (1·3 to 4·4)	2·3 (1·1 to 4)	1·8 (1 to 3·2)	1·4 (0·7 to 2·3)	1·1 (0·6 to 1·9)	0·9 (0·4 to 1·5)	0·6 (0·3 to 1·1)	0·4 (0·2 to 0·6)	0·2 (0·1 to 0·3)	0 (0 to 0·1)	67·7
Contact dermatitis	0 (0 to 0)	0 (0 to 0)	0 (0 to 0)	0 (0 to 0)	0·1 (0·1 to 0·3)	0·8 (0·3 to 1·6)	1·5 (0·6 to 2·9)	2 (0·8 to 3·7)	2·2 (1 to 4·3)	2·3 (1 to 4·5)	2·4 (1 to 4·8)	2·3 (1 to 4·5)	2·5 (1·1 to 5)	2·7 (1·2 to 5·2)	2·5 (1 to 5)	2·2 (0·9 to 4)	1·8 (0·8 to 3·5)	1·5 (0·7 to 2·8)	1·1 (0·5 to 2·1)	0·7 (0·3 to 1·3)	0·4 (0·2 to 0·7)	0·1 (0·1 to 0·2)	0 (0 to 0·1)	29·2
Seborrhoeic dermatitis	0 (0 to 0)	0 (0 to 0)	0 (0 to 0)	0·1 (0 to 0·1)	0·1 (0·1 to 0·2)	0·1 (0·1 to 0·3)	0·2 (0·1 to 0·3)	0·2 (0·1 to 0·4)	0·2 (0·1 to 0·4)	0·3 (0·1 to 0·4)	0·3 (0·1 to 0·5)	0·2 (0·1 to 0·4)	0·2 (0·1 to 0·4)	0·2 (0·1 to 0·4)	0·2 (0·1 to 0·3)	0·2 (0·1 to 0·3)	0·2 (0·1 to 0·3)	0·2 (0·1 to 0·3)	0·1 (0·1 to 0·2)	0·1 (0 to 0·2)	0·1 (0 to 0·1)	0 (0 to 0)	0 (0 to 0)	3·1
Psoriasis	0 (0 to 0)	0 (0 to 0)	0 (0 to 0)	0·3 (0·2 to 0·5)	0·9 (0·4 to 1·8)	1·4 (0·7 to 2·3)	1·7 (0·7 to 3)	1·9 (0·9 to 3·1)	2·1 (1 to 3·4)	2·2 (1·1 to 3·6)	2·6 (1·4 to 4·1)	2·8 (1·6 to 4·3)	3·1 (1·8 to 4·9)	3·2 (1·9 to 4·8)	2·9 (1·7 to 4·3)	2·4 (1·4 to 3·5)	1·7 (1·1 to 2·6)	1·3 (0·8 to 2)	0·8 (0·5 to 1·2)	0·5 (0·3 to 0·7)	0·2 (0·1 to 0·3)	0·1 (0 to 0·1)	0 (0 to 0)	31·9
Bacterial skin diseases	0 (0 to 0)	0 (0 to 0)	1·2 (0·5 to 2·1)	1·4 (0·7 to 2·5)	1·2 (0·6 to 2)	0·8 (0·5 to 1·4)	0·7 (0·4 to 1·1)	1·1 (0·6 to 1·9)	1·4 (0·8 to 2·7)	2·5 (1·4 to 5·2)	2·5 (1·3 to 4·7)	4·5 (2·4 to 7·9)	4·3 (2·2 to 7·9)	5·7 (2·8 to 9·9)	6 (2·8 to 11·3)	6·1 (3 to 11)	5·2 (2·4 to 9·9)	4 (1·8 to 7·4)	4·6 (1·8 to 8·3)	4·1 (1·4 to 8)	3·8 (1·4 to 6·7)	2·4 (0·7 to 4·2)	0·8 (0·2 to 1·4)	64·4
Cellulitis	0 (0 to 0)	0 (0 to 0)	0·5 (0·2 to 1·1)	0·6 (0·2 to 1·2)	0·7 (0·3 to 1·3)	0·3 (0·1 to 0·6)	0·2 (0·1 to 0·3)	0·4 (0·2 to 0·7)	0·7 (0·4 to 1·7)	0·9 (0·3 to 2·3)	0·9 (0·4 to 2·1)	1·5 (0·6 to 3·2)	1·4 (0·6 to 3)	2·3 (1 to 5·1)	2·6 (1·1 to 6·3)	2·3 (1 to 5·1)	2·2 (0·9 to 5)	1·4 (0·5 to 3·1)	1·5 (0·6 to 3·5)	1·2 (0·4 to 2·6)	1 (0·3 to 2·1)	0·5 (0·2 to 1·1)	0·2 (0 to 0·4)	23·4
Pyoderma	0 (0 to 0)	0 (0 to 0)	0·7 (0·3 to 1·4)	0·8 (0·4 to 1·5)	0·5 (0·3 to 0·9)	0·5 (0·3 to 0·9)	0·5 (0·3 to 0·8)	0·7 (0·4 to 1·2)	0·7 (0·4 to 1·3)	1·7 (0·9 to 3·2)	1·6 (0·8 to 2·8)	3 (1·4 to 5·3)	2·9 (1·5 to 5·3)	3·4 (1·6 to 6)	3·5 (1·3 to 6·2)	3·8 (1·8 to 7)	2·9 (1·2 to 5·4)	2·6 (1·1 to 4·7)	3 (1·1 to 5·4)	2·9 (0·9 to 5·6)	2·8 (0·9 to 5)	1·8 (0·5 to 3·4)	0·6 (0·2 to 1·1)	41
Scabies	0 (0 to 0)	0 (0 to 0)	0·6 (0·3 to 1)	4·1 (2 to 7·2)	5·9 (2·9 to 10·3)	6·7 (3·2 to 11·8)	7·6 (3·8 to 13·2)	7·2 (3·6 to 12·6)	5·2 (2·6 to 9·2)	3·8 (1·8 to 6·7)	3·8 (1·9 to 6·5)	4·1 (2·1 to 7·1)	4·3 (2·2 to 7·5)	3·8 (2 to 6·6)	3 (1·6 to 5·3)	2·2 (1·1 to 4)	1·6 (0·8 to 2·9)	1·3 (0·7 to 2·3)	1·1 (0·6 to 1·9)	0·9 (0·4 to 1·6)	0·6 (0·3 to 1)	0·3 (0·1 to 0·5)	0 (0 to 0·1)	68·1
Fungal skin diseases	0 (0 to 0)	0 (0 to 0)	0·1 (0 to 0·2)	0·8 (0·3 to 1·6)	1·3 (0·5 to 2·8)	1·7 (0·6 to 3·7)	1·9 (0·7 to 4·3)	2·1 (0·8 to 4·5)	2·2 (0·8 to 4·9)	2·1 (0·8 to 4·8)	2·3 (0·9 to 5·1)	2·3 (0·9 to 5)	2·5 (1 to 5·4)	2·6 (1 to 5·7)	2·7 (1·1 to 5·9)	2·5 (1 to 5·7)	2·6 (1 to 5·4)	3 (1·2 to 6·4)	2·8 (1·1 to 6)	2·6 (1 to 5·5)	1·9 (0·8 to 4·1)	0·9 (0·4 to 2)	0·3 (0·1 to 0·5)	41·4
Viral skin diseases	0 (0 to 0)	0 (0 to 0)	0 (0 to 0)	4·6 (2·8 to 6·8)	7·5 (4·7 to 11·6)	4·3 (2·6 to 6·5)	2·8 (1·7 to 4·4)	2·3 (1·4 to 3·6)	1·8 (1·1 to 2·8)	1·4 (0·8 to 2·1)	1·3 (0·8 to 2·1)	1·3 (0·8 to 2)	1·2 (0·7 to 1·9)	1·1 (0·6 to 1·8)	0·9 (0·5 to 1·5)	0·8 (0·4 to 1·2)	0·6 (0·3 to 0·9)	0·4 (0·3 to 0·7)	0·3 (0·2 to 0·4)	0·2 (0·1 to 0·2)	0·1 (0 to 0·1)	0 (0 to 0)	0 (0 to 0)	32·7
Acne vulgaris	0 (0 to 0)	0 (0 to 0)	0 (0 to 0)	0 (0 to 0)	0·4 (0·2 to 0·7)	10·2 (5·8 to 16·6)	18·3 (10·3 to 30·2)	4·9 (2·7 to 8)	2·4 (1·2 to 3·9)	1·6 (0·8 to 2·7)	1·3 (0·6 to 2·2)	0·9 (0·4 to 1·6)	0·7 (0·4 to 1·1)	0·4 (0·2 to 0·7)	0·3 (0·1 to 0·4)	0·2 (0·1 to 0·3)	0·1 (0·1 to 0·2)	0·1 (0 to 0·1)	0 (0 to 0·1)	0 (0 to 0)	0 (0 to 0)	0 (0 to 0)	0 (0 to 0)	41·7
Alopecia areata	0 (0 to 0)	0 (0 to 0)	0 (0 to 0)	0·1 (0 to 0·2)	0·2 (0·1 to 0·3)	0·2 (0·1 to 0·4)	0·5 (0·2 to 0·8)	0·8 (0·4 to 1·2)	1·1 (0·6 to 1·8)	1·1 (0·6 to 1·7)	1·1 (0·6 to 1·6)	0·8 (0·5 to 1·4)	0·8 (0·4 to 1·2)	0·6 (0·3 to 1)	0·5 (0·3 to 0·8)	0·4 (0·2 to 0·6)	0·3 (0·2 to 0·4)	0·2 (0·1 to 0·3)	0·1 (0·1 to 0·2)	0·1 (0 to 0·1)	0 (0 to 0·1)	0 (0 to 0)	0 (0 to 0)	8·8
Pruritus	0 (0 to 0)	0 (0 to 0)	0 (0 to 0)	0·2 (0·1 to 0·4)	0·3 (0·1 to 0·6)	0·3 (0·1 to 0·6)	0·4 (0·2 to 0·8)	0·5 (0·2 to 1)	0·6 (0·3 to 1·1)	0·6 (0·3 to 1·2)	0·7 (0·3 to 1·3)	0·7 (0·3 to 1·3)	0·7 (0·3 to 1·4)	0·7 (0·3 to 1·5)	0·7 (0·3 to 1·4)	0·7 (0·3 to 1·4)	0·6 (0·3 to 1·2)	0·6 (0·2 to 1·1)	0·4 (0·2 to 0·9)	0·3 (0·1 to 0·6)	0·2 (0·1 to 0·3)	0·1 (0 to 0·1)	0 (0 to 0)	9·3
Urticaria	0 (0 to 0)	0 (0 to 0)	0·7 (0·4 to 1·1)	4·7 (2·8 to 7·4)	4·5 (2·5 to 7·4)	3·5 (1·9 to 6)	3·2 (1·7 to 5·6)	3·2 (1·6 to 5·3)	3 (1·5 to 4·9)	2·8 (1·5 to 4·7)	3 (1·7 to 5)	2·8 (1·5 to 4·5)	2·6 (1·4 to 4·3)	2·4 (1·3 to 3·8)	2 (1 to 3·4)	1·6 (0·8 to 2·6)	1·1 (0·6 to 1·9)	0·9 (0·5 to 1·5)	0·7 (0·4 to 1·1)	0·4 (0·2 to 0·7)	0·2 (0·1 to 0·3)	0·1 (0 to 0·1)	0 (0 to 0)	43·2
Decubitus ulcer	0 (0 to 0)	0 (0 to 0)	0 (0 to 0)	0·2 (0 to 0·5)	0·5 (0·1 to 0·9)	0·3 (0·1 to 0·6)	0·3 (0·1 to 0·6)	0·1 (0 to 0·1)	0·6 (0·2 to 1·2)	0·3 (0·1 to 0·6)	1·2 (0·5 to 2·5)	1·4 (0·6 to 2·9)	2·4 (1·1 to 4·9)	4·2 (2 to 8·7)	5·4 (2·8 to 11)	5·1 (2·7 to 9·4)	5·6 (3·1 to 10·4)	8·5 (4·9 to 15)	11·9 (7 to 21·7)	12·4 (7·4 to 22)	13·7 (7·4 to 23·7)	9·2 (4·8 to 13·9)	3·2 (1·6 to 4·6)	86·3
Other skin and subcutaneous diseases	0 (0 to 0)	0 (0 to 0)	0·2 (0·1 to 0·3)	0·9 (0·4 to 1·7)	1·1 (0·5 to 2)	1·4 (0·6 to 2·5)	1·8 (0·9 to 3·3)	2·1 (1·1 to 3·7)	2·2 (1·1 to 4·2)	2·8 (1·4 to 5·1)	3·2 (1·6 to 5·7)	3·9 (2 to 7)	4·4 (2·3 to 8·2)	5 (2·6 to 9·1)	5·2 (2·7 to 9·9)	4·8 (2·5 to 8·7)	3·9 (2 to 7)	3·6 (1·8 to 6·4)	3 (1·6 to 5·1)	2·1 (1·1 to 3·6)	1·1 (0·6 to 1·8)	0·5 (0·3 to 0·9)	0·1 (0·1 to 0·2)	53·1
Sense organ diseases	0 (0 to 0)	0 (0 to 0)	0·2 (0·1 to 0·4)	2·7 (1·7 to 3·9)	6·3 (3·8 to 9·3)	9 (5·8 to 13)	12·1 (7·7 to 17·9)	15·5 (9·7 to 23)	19·2 (12·2 to 28·9)	22·4 (14·2 to 34)	28 (17·6 to 42·2)	34·2 (21·6 to 52)	50·2 (31·1 to 80·1)	65·7 (42·1 to 104)	76·2 (48·4 to 119)	78·6 (50·9 to 119)	75 (49·6 to 111)	78·6 (52·4 to 114)	75·8 (51·9 to 106)	67·4 (46·6 to 92·3)	46·6 (32·8 to 62·9)	22·7 (16·1 to 30)	6·2 (4·5 to 8·2)	793
Blindness and vision loss	0 (0 to 0)	0 (0 to 0)	0 (0 to 0·1)	0·7 (0·4 to 1·2)	1·9 (1·1 to 3·1)	2·7 (1·6 to 4·5)	3·6 (2·1 to 5·7)	4·4 (2·6 to 6·8)	4·7 (2·8 to 7·1)	4·9 (3 to 7·5)	6·4 (4 to 9·8)	8·2 (5·2 to 12·8)	11·5 (7·2 to 18)	14·8 (9·2 to 23·9)	17·7 (11·2 to 28)	19·5 (12·8 to 30·3)	20·4 (13·4 to 30·2)	23·2 (15·3 to 33·1)	23·4 (16 to 32·8)	21·4 (14·6 to 29·2)	15·3 (10·6 to 20·8)	8 (5·6 to 10·8)	2·5 (1·7 to 3·4)	215
Glaucoma	0 (0 to 0)	0 (0 to 0)	0 (0 to 0)	0 (0 to 0)	0 (0 to 0)	0 (0 to 0)	0 (0 to 0)	0 (0 to 0)	0 (0 to 0)	0 (0 to 0)	0 (0 to 0)	0 (0 to 0)	0 (0 to 0·1)	0·2 (0 to 0·4)	0·5 (0·2 to 0·9)	0·8 (0·4 to 1·4)	1·1 (0·6 to 1·9)	1·7 (1 to 2·7)	2·2 (1·3 to 3·4)	2·3 (1·4 to 3·7)	1·8 (1·1 to 2·8)	0·9 (0·5 to 1·4)	0·3 (0·2 to 0·4)	11·8
Cataract	0 (0 to 0)	0 (0 to 0)	0 (0 to 0)	0 (0 to 0)	0 (0 to 0)	0 (0 to 0)	0 (0 to 0)	0 (0 to 0)	0·1 (0 to 0·2)	0·2 (0·1 to 0·5)	0·5 (0·2 to 0·9)	0·8 (0·4 to 1·4)	1·3 (0·7 to 2·2)	1·7 (0·9 to 2·8)	2·1 (1·2 to 3·3)	2·7 (1·6 to 4·1)	3·5 (2·1 to 5·3)	5·2 (3·4 to 7·8)	6·9 (4·6 to 10)	7·2 (4·8 to 10·1)	5·6 (3·8 to 7·9)	2·8 (1·8 to 4)	0·8 (0·5 to 1·3)	41·5
Age-related macular degeneration	0 (0 to 0)	0 (0 to 0)	0 (0 to 0)	0 (0 to 0)	0 (0 to 0)	0 (0 to 0)	0 (0 to 0)	0 (0 to 0)	0 (0 to 0)	0 (0 to 0)	0 (0 to 0)	0 (0 to 0)	0 (0 to 0)	0 (0 to 0·1)	0·1 (0 to 0·2)	0·2 (0·1 to 0·4)	0·2 (0·1 to 0·4)	0·3 (0·1 to 0·4)	0·3 (0·1 to 0·5)	0·3 (0·1 to 0·4)	0·2 (0·1 to 0·3)	0·1 (0 to 0·2)	0 (0 to 0)	1·7
Refraction disorders	0 (0 to 0)	0 (0 to 0)	0 (0 to 0·1)	0·6 (0·3 to 1)	1·6 (0·9 to 2·6)	2·4 (1·4 to 3·9)	3·1 (1·8 to 5·1)	3·7 (2·1 to 5·9)	3·8 (2·2 to 5·8)	3·5 (2 to 5·5)	3·9 (2·3 to 6·1)	4·1 (2·5 to 6·5)	4·7 (2·8 to 7·1)	4·6 (2·7 to 7·2)	4·4 (2·7 to 6·9)	3·9 (2·3 to 6)	3·3 (1·9 to 5)	3·3 (2 to 4·9)	3 (1·8 to 4·4)	2·2 (1·4 to 3·4)	1·2 (0·8 to 1·8)	0·4 (0·3 to 0·6)	0·1 (0 to 0·1)	57·9
Near vision loss	0 (0 to 0)	0 (0 to 0)	0 (0 to 0)	0 (0 to 0)	0 (0 to 0)	0 (0 to 0)	0 (0 to 0·1)	0 (0 to 0·2)	0·1 (0·1 to 0·4)	0·4 (0·2 to 0·9)	0·9 (0·3 to 2·1)	1·8 (0·7 to 4)	4·2 (1·3 to 7·2)	6·1 (2 to 10·4)	6·2 (2·5 to 13)	5·3 (2·7 to 13·8)	6 (2·5 to 12·6)	6 (2·5 to 12·1)	5·3 (2·2 to 10·9)	4·8 (1·8 to 8·4)	2·7 (1·1 to 5·2)	1·2 (0·5 to 2·4)	0·3 (0·1 to 0·7)	50·3
Other vision loss	0 (0 to 0)	0 (0 to 0)	0 (0 to 0)	0·1 (0·1 to 0·2)	0·3 (0·1 to 0·5)	0·4 (0·1 to 0·7)	0·5 (0·1 to 0·9)	0·5 (0·2 to 1)	0·6 (0·2 to 1·2)	0·7 (0·3 to 1·3)	1·1 (0·5 to 1·8)	1·5 (0·8 to 2·6)	2·2 (1·2 to 3·6)	3·3 (2 to 5·3)	4·4 (2·6 to 6·8)	5·4 (3·4 to 7·9)	6·2 (4 to 9)	6·7 (4·3 to 9·8)	5·8 (3·7 to 8·6)	5·2 (3·3 to 7·7)	3·9 (2·5 to 5·8)	2·5 (1·7 to 3·7)	0·9 (0·6 to 1·4)	52·3
Age-related and other hearing loss	0 (0 to 0)	0 (0 to 0)	0·1 (0 to 0·2)	1·4 (0·7 to 2·2)	3·7 (2 to 5·9)	5·5 (3·2 to 8·6)	7·5 (4·4 to 11·4)	10·1 (6 to 15·2)	13·2 (8·1 to 20·1)	15·8 (9·4 to 24·8)	19·4 (11·8 to 30·2)	23·7 (14·4 to 38·5)	36 (21·6 to 58·6)	47·4 (29·3 to 76·4)	54·9 (33·6 to 87·3)	55·2 (34·1 to 85·6)	50·8 (32 to 78·1)	51·6 (33·3 to 76·6)	49·1 (32·5 to 70·3)	43·6 (29·4 to 60·8)	30·1 (20·4 to 41·1)	14·3 (10 to 19·6)	3·6 (2·5 to 4·9)	537
Other sense organ diseases	0 (0 to 0)	0 (0 to 0)	0·1 (0·1 to 0·2)	0·6 (0·3 to 1)	0·7 (0·3 to 1·2)	0·7 (0·3 to 1·4)	0·9 (0·4 to 1·9)	1·1 (0·4 to 2·1)	1·3 (0·6 to 2·6)	1·7 (0·7 to 3·4)	2·1 (1 to 4)	2·2 (1 to 4·3)	2·7 (1·2 to 5·2)	3·6 (1·6 to 7)	3·6 (1·6 to 7)	3·9 (1·8 to 7·3)	3·8 (1·8 to 7·2)	3·8 (1·8 to 7)	3·3 (1·6 to 6)	2·4 (1·2 to 4·5)	1·2 (0·6 to 2·2)	0·4 (0·2 to 0·8)	0·1 (0·1 to 0·3)	40·5
Musculoskeletal disorders	0 (0 to 0)	0 (0 to 0)	0 (0 to 0)	0 (0 to 0)	3·8 (2·1 to 6·4)	20·1 (12·4 to 31)	46·8 (30·6 to 68·9)	72·1 (48·2 to 102)	102 (67·5 to 140)	121 (80·1 to 166)	152 (105 to 214)	175 (120 to 241)	207 (145 to 288)	216 (153 to 303)	204 (140 to 282)	173 (118 to 244)	132 (91·9 to 187)	110 (75·7 to 152)	82·3 (56·1 to 116)	58·3 (41·1 to 81)	35·6 (25·5 to 48·4)	14·4 (10·3 to 19·5)	3·4 (2·4 to 4·7)	1,930
Rheumatoid arthritis	0 (0 to 0)	0 (0 to 0)	0 (0 to 0)	0 (0 to 0)	0·1 (0·1 to 0·2)	0·2 (0·1 to 0·4)	0·8 (0·4 to 1·2)	1·6 (0·6 to 2·8)	2·3 (1·2 to 3·9)	3·1 (1·8 to 4·9)	4·3 (2·5 to 6·5)	5·4 (3·4 to 8·1)	6·6 (4·3 to 9·5)	6·9 (4·5 to 9·9)	6·7 (4·7 to 9·5)	5·4 (3·7 to 7·5)	4·2 (3 to 5·9)	3·3 (2·3 to 4·6)	2·9 (2·1 to 4)	2·5 (1·8 to 3·4)	3·1 (1·9 to 4·5)	1·5 (0·9 to 2·4)	0·4 (0·2 to 0·6)	61·5
Osteoarthritis	0 (0 to 0)	0 (0 to 0)	0 (0 to 0)	0 (0 to 0)	0 (0 to 0)	0 (0 to 0)	0 (0 to 0)	0 (0 to 0)	0 (0 to 0)	0·5 (0·2 to 1·1)	3·7 (1·7 to 7·6)	10·2 (5 to 21·1)	21·3 (10·4 to 42·8)	32·2 (15·7 to 64·8)	38·4 (19 to 76·9)	38·3 (19·1 to 76·7)	32·5 (16·4 to 64·6)	28·8 (14·5 to 57·3)	22·5 (11·2 to 45·3)	16·1 (8·1 to 32·3)	9·1 (4·7 to 18·2)	3·7 (1·9 to 7·3)	0·9 (0·5 to 1·7)	258
Osteoarthritis hip	0 (0 to 0)	0 (0 to 0)	0 (0 to 0)	0 (0 to 0)	0 (0 to 0)	0 (0 to 0)	0 (0 to 0)	0 (0 to 0)	0 (0 to 0)	0·2 (0 to 0·4)	0·3 (0·1 to 0·6)	0·4 (0·1 to 1)	0·8 (0·3 to 1·7)	1·1 (0·4 to 2·6)	1·5 (0·6 to 3·3)	1·6 (0·7 to 3·3)	1·4 (0·6 to 3·1)	1·3 (0·6 to 2·8)	1·1 (0·5 to 2·3)	0·8 (0·4 to 1·6)	0·4 (0·2 to 0·9)	0·2 (0·1 to 0·4)	0 (0 to 0·1)	11·1
Osteoarthritis knee	0 (0 to 0)	0 (0 to 0)	0 (0 to 0)	0 (0 to 0)	0 (0 to 0)	0 (0 to 0)	0 (0 to 0)	0 (0 to 0)	0 (0 to 0)	0·3 (0·1 to 0·7)	2·8 (1·2 to 5·8)	7·8 (3·7 to 16·1)	16·1 (7·6 to 32·9)	24·1 (11·6 to 50)	28·4 (13·5 to 57·1)	28 (13·5 to 55·3)	23·4 (11·5 to 48)	20·5 (10·1 to 41·7)	15·6 (7·6 to 31·6)	10·8 (5·3 to 21·7)	5·8 (2·9 to 11·6)	2·2 (1·1 to 4·5)	0·5 (0·2 to 1)	186
Osteoarthritis hand	0 (0 to 0)	0 (0 to 0)	0 (0 to 0)	0 (0 to 0)	0 (0 to 0)	0 (0 to 0)	0 (0 to 0)	0 (0 to 0)	0 (0 to 0)	0·1 (0 to 0·2)	0·4 (0·1 to 1·1)	1·3 (0·5 to 2·9)	2·8 (1·1 to 6·1)	4·3 (1·9 to 9·2)	5·3 (2·5 to 11·4)	5·2 (2·5 to 11·1)	4·5 (2·1 to 9·3)	4 (1·9 to 8·1)	3·3 (1·6 to 6·8)	2·5 (1·2 to 5·1)	1·6 (0·8 to 3·2)	0·7 (0·4 to 1·5)	0·2 (0·1 to 0·4)	36·1

Osteoarthritis other	0 (0 to 0)	0 (0 to 0)	0 (0 to 0)	0 (0 to 0)	0 (0 to 0)	0 (0 to 0)	0 (0 to 0)	0 (0 to 0)	0 (0 to 0)	0 (0 to 0·1)	0·2 (0·1 to 0·6)	0·7 (0·3 to 1·7)	1·6 (0·6 to 3·5)	2·5 (1·1 to 5·6)	3·2 (1·5 to 7·1)	3·4 (1·6 to 7·4)	3·1 (1·5 to 6·5)	3 (1·5 to 6)	2·5 (1·3 to 5·2)	2 (1·1 to 4·1)	1·3 (0·7 to 2·6)	0·6 (0·3 to 1·2)	0·2 (0·1 to 0·3)	24·6
Low back pain	0 (0 to 0)	0 (0 to 0)	0 (0 to 0)	0 (0 to 0)	3·2 (1·5 to 5·8)	17·4 (10·1 to 28·4)	34·2 (20·2 to 54·9)	43·7 (25·6 to 69·6)	52·8 (30·3 to 80·6)	58 (33·7 to 89·5)	72·9 (43·8 to 114)	80·1 (47·9 to 126)	90·9 (53·5 to 144)	93·1 (56·9 to 145)	85·3 (52·7 to 129)	71·4 (44·9 to 107)	53·9 (33·9 to 82·4)	45·7 (28·6 to 67·9)	36·1 (22·5 to 54)	27 (16·8 to 40·1)	14·3 (8·8 to 21·1)	5·3 (3·3 to 7·8)	1·2 (0·7 to 1·8)	887
Neck pain	0 (0 to 0)	0 (0 to 0)	0 (0 to 0)	0 (0 to 0)	0·1 (0 to 0·4)	1 (0·4 to 1·9)	2·5 (1 to 4·8)	4·2 (1·8 to 8·2)	6·6 (3 to 12·8)	9·9 (4·7 to 18·9)	14·6 (7·4 to 25·5)	17·9 (9 to 31·7)	21 (10·4 to 37·8)	18·6 (9·4 to 35)	16 (7·7 to 29·2)	12·6 (6·4 to 21·9)	9 (4·6 to 15·4)	7·2 (3·8 to 12·3)	5·1 (2·5 to 9·7)	3·2 (1·6 to 5·9)	1·7 (0·9 to 3)	0·7 (0·4 to 1·2)	0·2 (0·1 to 0·3)	152
Gout	0 (0 to 0)	0 (0 to 0)	0 (0 to 0)	0 (0 to 0)	0 (0 to 0)	0 (0 to 0)	0 (0 to 0)	0·1 (0 to 0·1)	0·2 (0·1 to 0·3)	0·2 (0 to 0·6)	0·4 (0·1 to 0·8)	0·5 (0·1 to 1)	0·6 (0·2 to 1·2)	0·7 (0·2 to 1·4)	0·7 (0·3 to 1·3)	0·6 (0·3 to 1·1)	0·5 (0·2 to 0·9)	0·5 (0·2 to 0·8)	0·4 (0·2 to 0·7)	0·3 (0·1 to 0·6)	0·2 (0·1 to 0·4)	0·1 (0 to 0·2)	0 (0 to 0·1)	5·9
Other musculoskeletal disorders	0 (0 to 0)	0 (0 to 0)	0 (0 to 0)	0 (0 to 0)	0·4 (0·2 to 0·6)	1·4 (0·8 to 2·5)	9·3 (6 to 14·5)	22·6 (15 to 32·1)	39·9 (26·6 to 56·9)	49·5 (32·8 to 71·6)	56·4 (37·4 to 81)	60·9 (40·1 to 89·7)	66·6 (44·2 to 95·5)	64·3 (41·3 to 95)	56·7 (36·4 to 83·6)	44·6 (27·6 to 66·6)	31·9 (19·8 to 47·3)	24·2 (13·9 to 37·9)	15·3 (9·1 to 23·8)	9·2 (4·7 to 15·3)	7·2 (4·5 to 10·6)	3·1 (1·8 to 4·6)	0·7 (0·4 to 1·2)	564
Other non-communicable diseases	73·4 (46·9 to 113)	38·2 (24·5 to 56·2)	47·1 (31·8 to 68·1)	55·6 (39·3 to 77)	36·7 (29 to 46·3)	41·1 (31·3 to 53·5)	65·9 (48·7 to 92·2)	107 (75·3 to 151)	145 (102 to 202)	156 (110 to 218)	180 (130 to 246)	188 (133 to 258)	188 (133 to 253)	164 (117 to 221)	135 (95·9 to 182)	110 (79·9 to 150)	86·4 (62·4 to 117)	74·1 (54·4 to 98·9)	58·1 (42·8 to 77·4)	43·1 (32·6 to 56·5)	31·2 (24·6 to 39·3)	15·6 (12·3 to 19·9)	4·2 (3·3 to 5·5)	2,040
Congenital birth defects	71·5 (45·5 to 111)	32·8 (20·3 to 48·7)	30·3 (19 to 46·3)	32 (20·9 to 47·1)	14·8 (11·4 to 19·1)	12·5 (9·3 to 16·7)	11·1 (8·3 to 14·5)	9·8 (7·2 to 12·8)	9·5 (7 to 12·8)	7·7 (5·6 to 10·1)	7·9 (6 to 10·3)	7·6 (5·8 to 10)	7·5 (5·5 to 9·8)	6·5 (4·9 to 8·5)	5 (3·6 to 6·6)	3 (2·2 to 4)	2 (1·4 to 2·7)	0·8 (0·5 to 1·3)	0·5 (0·3 to 0·8)	0·3 (0·2 to 0·5)	0·2 (0·1 to 0·2)	0·1 (0 to 0·1)	0 (0 to 0)	273
Neural tube defects	12·1 (6·6 to 20·7)	3·8 (1·8 to 7·1)	3·1 (1·2 to 6·6)	3·4 (1·5 to 6·8)	0·7 (0·5 to 1·2)	1·1 (0·7 to 1·8)	0·9 (0·6 to 1·4)	0·4 (0·3 to 0·6)	0·6 (0·4 to 0·9)	0·5 (0·3 to 0·8)	0·4 (0·3 to 0·6)	0·4 (0·3 to 0·6)	0·2 (0·1 to 0·3)	0·1 (0·1 to 0·2)	0·2 (0·1 to 0·3)	0·1 (0·1 to 0·1)	0·1 (0·1 to 0·1)	0 (0 to 0)	0 (0 to 0)	0 (0 to 0)	0 (0 to 0)	0 (0 to 0)	0 (0 to 0)	28·1
Congenital heart anomalies	19·3 (9·8 to 32)	12·4 (6·8 to 20·1)	12·6 (6·9 to 20·8)	12·1 (6·8 to 20·1)	4·3 (2·8 to 6·3)	2·8 (1·7 to 4·2)	2·4 (1·6 to 3·7)	2·3 (1·4 to 3·8)	2·5 (1·6 to 3·8)	1·5 (1 to 2·3)	1·9 (1·2 to 2·8)	1·9 (1·3 to 2·8)	1·7 (1·1 to 2·5)	1·4 (0·9 to 2)	0·9 (0·5 to 1·3)	0·4 (0·3 to 0·7)	0·3 (0·2 to 0·4)	0·1 (0 to 0·1)	0 (0 to 0·1)	0 (0 to 0·1)	0 (0 to 0)	0 (0 to 0)	0 (0 to 0)	80·8
Orofacial clefts	0 (0 to 0)	0 (0 to 0)	0·1 (0 to 0·1)	0·1 (0 to 0·3)	0·1 (0 to 0·3)	0·1 (0 to 0·3)	0·2 (0 to 0·3)	0·2 (0 to 0·3)	0·2 (0 to 0·3)	0·1 (0 to 0·3)	0·1 (0 to 0·3)	0·1 (0 to 0·3)	0·1 (0 to 0·3)	0·1 (0 to 0·3)	0·1 (0 to 0·2)	0·1 (0 to 0·2)	0·1 (0 to 0·1)	0 (0 to 0·1)	0 (0 to 0·1)	0 (0 to 0·1)	0 (0 to 0)	0 (0 to 0)	0 (0 to 0)	2
Down syndrome	0·5 (0·2 to 0·9)	0·3 (0·2 to 0·5)	0·7 (0·4 to 1·3)	1·5 (0·7 to 2·7)	0·5 (0·3 to 0·8)	0·4 (0·3 to 0·7)	0·4 (0·3 to 0·6)	0·5 (0·3 to 0·8)	0·5 (0·3 to 0·7)	0·5 (0·3 to 0·7)	0·6 (0·4 to 1)	0·7 (0·4 to 1)	0·9 (0·5 to 1·4)	1 (0·6 to 1·6)	0·8 (0·4 to 1·3)	0·3 (0·1 to 0·5)	0·1 (0 to 0·1)	0 (0 to 0)	0 (0 to 0)	0 (0 to 0)	0 (0 to 0)	0 (0 to 0)	0 (0 to 0)	10·2
Turner syndrome	0 (0 to 0)	0 (0 to 0)	0 (0 to 0)	0 (0 to 0)	0 (0 to 0)	0 (0 to 0)	0 (0 to 0)	0 (0 to 0)	0 (0 to 0)	0 (0 to 0)	0 (0 to 0)	0 (0 to 0)	0 (0 to 0)	0 (0 to 0)	0 (0 to 0)	0 (0 to 0)	0 (0 to 0)	0 (0 to 0)	0 (0 to 0)	0 (0 to 0)	0 (0 to 0)	0 (0 to 0)	0 (0 to 0)	0·2
Klinefelter syndrome	0 (0 to 0)	0 (0 to 0)	0 (0 to 0)	0 (0 to 0)	0 (0 to 0)	0 (0 to 0)	0 (0 to 0)	0 (0 to 0)	0 (0 to 0)	0 (0 to 0)	0 (0 to 0)	0 (0 to 0)	0 (0 to 0)	0 (0 to 0)	0 (0 to 0)	0 (0 to 0)	0 (0 to 0)	0 (0 to 0)	0 (0 to 0)	0 (0 to 0)	0 (0 to 0)	0 (0 to 0)	0 (0 to 0)	0
Other chromosomal abnormalities	3·1 (1·4 to 5·8)	1·9 (0·9 to 3·4)	2·2 (1·2 to 3·9)	1·2 (0·7 to 1·9)	0·6 (0·4 to 0·9)	0·5 (0·3 to 0·8)	0·5 (0·4 to 0·8)	0·5 (0·4 to 0·8)	0·5 (0·3 to 0·7)	0·3 (0·2 to 0·5)	0·2 (0·1 to 0·3)	0·2 (0·1 to 0·3)	0·1 (0·1 to 0·2)	0·1 (0·1 to 0·2)	0·1 (0 to 0·1)	0 (0 to 0)	0 (0 to 0)	0 (0 to 0)	0 (0 to 0)	0 (0 to 0)	0 (0 to 0)	0 (0 to 0)	0 (0 to 0)	12·2
Congenital musculoskeletal and limb anomalies	0·4 (0·2 to 0·8)	0·2 (0·1 to 0·3)	1·2 (0·7 to 2)	2·9 (1·7 to 4·7)	3·5 (2 to 5·4)	3·8 (2·2 to 5·9)	3·8 (2·1 to 6·1)	3 (1·8 to 4·7)	2·8 (1·6 to 4·4)	2·5 (1·4 to 3·9)	2·6 (1·5 to 4·1)	2·5 (1·4 to 3·9)	2·6 (1·4 to 4·1)	2·4 (1·4 to 3·6)	2 (1·1 to 3·1)	1·5 (0·8 to 2·3)	0·9 (0·5 to 1·4)	0·6 (0·3 to 0·9)	0·3 (0·2 to 0·6)	0·2 (0·1 to 0·3)	0·1 (0 to 0·1)	0 (0 to 0)	0 (0 to 0)	39·9
Urogenital congenital anomalies	2·9 (1·3 to 5·7)	1·4 (0·6 to 2·5)	0·6 (0·3 to 1·1)	0·6 (0·4 to 0·9)	0·4 (0·2 to 0·6)	0·5 (0·3 to 0·8)	0·3 (0·2 to 0·5)	0·2 (0·1 to 0·4)	0·3 (0·1 to 0·5)	0·1 (0·1 to 0·2)	0·1 (0·1 to 0·2)	0·1 (0·1 to 0·1)	0·2 (0·1 to 0·4)	0·1 (0 to 0·1)	0 (0 to 0)	0 (0 to 0·1)	0 (0 to 0·1)	0 (0 to 0)	0 (0 to 0)	0 (0 to 0)	0 (0 to 0)	0 (0 to 0)	0 (0 to 0)	8
Digestive congenital anomalies	12·2 (6·2 to 20·7)	6·5 (3·3 to 11)	3·7 (1·8 to 7·3)	2·5 (1·3 to 4·8)	0·6 (0·3 to 0·9)	0·3 (0·2 to 0·4)	0·1 (0·1 to 0·2)	0·1 (0·1 to 0·2)	0·1 (0·1 to 0·1)	0·1 (0·1 to 0·1)	0·1 (0 to 0·1)	0·1 (0 to 0·1)	0·1 (0·1 to 0·2)	0·1 (0 to 0·1)	0·1 (0 to 0·1)	0·1 (0 to 0·1)	0 (0 to 0)	0 (0 to 0)	0 (0 to 0)	0 (0 to 0)	0 (0 to 0)	0 (0 to 0)	0 (0 to 0)	26·5
Other congenital birth defects	20·9 (11·8 to 33·7)	6·4 (3·6 to 10·7)	6·1 (3·4 to 10·5)	7·8 (4·2 to 13·2)	4·2 (2·7 to 6·2)	3 (1·9 to 4·4)	2·4 (1·6 to 3·5)	2·4 (1·6 to 3·7)	2·2 (1·4 to 3·3)	1·9 (1·2 to 2·9)	1·8 (1·1 to 2·7)	1·6 (1 to 2·4)	1·6 (0·9 to 2·4)	1·2 (0·8 to 1·9)	1 (0·6 to 1·5)	0·5 (0·3 to 0·9)	0·4 (0·3 to 0·7)	0·1 (0 to 0·4)	0·1 (0 to 0·3)	0·1 (0 to 0·2)	0 (0 to 0·1)	0 (0 to 0)	0 (0 to 0)	65·7
Urinary diseases and male infertility	0·1 (0 to 0·1)	0·2 (0·1 to 0·3)	0·4 (0·2 to 0·6)	0·8 (0·5 to 1·2)	0·7 (0·5 to 1)	1 (0·7 to 1·4)	1·4 (1 to 2)	1·9 (1·4 to 2·7)	2·7 (1·9 to 3·7)	3·4 (2·4 to 4·7)	4·2 (3·1 to 5·8)	5·5 (3·9 to 7·2)	6·2 (4·4 to 8·4)	6·8 (4·7 to 9·5)	6·5 (4·4 to 9·1)	5·7 (4 to 7·7)	5·4 (3·8 to 7·5)	5·6 (3·9 to 7·8)	5·4 (3·7 to 7·7)	5·8 (3·9 to 8·2)	5·8 (3·7 to 8·4)	3·5 (2·3 to 4·9)	1·1 (0·7 to 1·6)	80·2
Urolithiasis	0 (0 to 0)	0 (0 to 0)	0 (0 to 0)	0 (0 to 0)	0 (0 to 0)	0·1 (0 to 0·1)	0 (0 to 0·1)	0·2 (0·1 to 0·2)	0·3 (0·2 to 0·4)	0·7 (0·4 to 1·1)	1 (0·6 to 1·6)	1 (0·6 to 1·6)	1·7 (0·9 to 2·7)	1·4 (0·8 to 2·2)	1·6 (0·9 to 2·5)	1·2 (0·7 to 1·9)	1 (0·6 to 1·6)	0·6 (0·4 to 1)	0·4 (0·2 to 0·7)	0·3 (0·1 to 0·5)	0·3 (0·1 to 0·5)	0·1 (0 to 0·2)	0 (0 to 0·1)	11·9
Benign prostatic hyperplasia	0 (0 to 0)	0 (0 to 0)	0 (0 to 0)	0 (0 to 0)	0 (0 to 0)	0 (0 to 0)	0 (0 to 0)	0 (0 to 0)	0 (0 to 0)	0 (0 to 0)	0 (0 to 0)	0 (0 to 0)	0 (0 to 0)	0 (0 to 0)	0 (0 to 0)	0 (0 to 0)	0 (0 to 0)	0 (0 to 0)	0 (0 to 0)	0 (0 to 0)	0 (0 to 0)	0 (0 to 0)	0 (0 to 0)	0
Male infertility	--	--	--	--	--	--	--	--	--	--	--	--	--	--	--	--	--	--	--	--	--	--	--	NA
Other urinary diseases	0 (0 to 0·1)	0·1 (0·1 to 0·2)	0 (0 to 0·1)	0·2 (0·2 to 0·4)	0·2 (0·1 to 0·2)	0·3 (0·2 to 0·4)	0·2 (0·2 to 0·4)	0·3 (0·2 to 0·4)	0·2 (0·2 to 0·4)	0·4 (0·2 to 0·5)	0·3 (0·2 to 0·5)	0·4 (0·3 to 0·7)	0·5 (0·3 to 0·7)	0·5 (0·3 to 0·7)	0·4 (0·2 to 0·6)	0·3 (0·2 to 0·5)	0·2 (0·1 to 0·3)	0·1 (0·1 to 0·2)	0·1 (0·1 to 0·2)	0·1 (0·1 to 0·2)	0·1 (0 to 0·1)	0 (0 to 0·1)	0 (0 to 0)	5·1
Gynecological diseases	0 (0 to 0)	0 (0 to 0)	0 (0 to 0)	0 (0 to 0)	0 (0 to 0)	8·5 (4·2 to 14·8)	24·4 (13·7 to 40·5)	57·1 (36·5 to 87·9)	83·6 (54·2 to 123)	93·4 (60·7 to 135)	106 (71·7 to 154)	104 (68·8 to 152)	90·5 (58·5 to 128)	60·1 (36 to 90·3)	33·1 (20·3 to 48·5)	19·9 (11·5 to 30·8)	13 (7·8 to 19·6)	9·5 (5·9 to 13·7)	6·3 (4 to 9·2)	4·1 (2·5 to 6·2)	2·4 (1·5 to 3·5)	1 (0·6 to 1·6)	0·3 (0·1 to 0·4)	717
Uterine fibroids	0 (0 to 0)	0 (0 to 0)	0 (0 to 0)	0 (0 to 0)	0 (0 to 0)	0·1 (0 to 0·3)	0·3 (0·1 to 0·7)	0·9 (0·4 to 2)	2·4 (1 to 4·9)	4·8 (2·1 to 9·8)	8·6 (3·8 to 16·6)	9·8 (4·8 to 18·2)	9·2 (4·6 to 17·4)	6·4 (3 to 12)	3·9 (1·8 to 7·4)	2·1 (1 to 4·3)	1·1 (0·5 to 2·2)	0·6 (0·3 to 1·3)	0·3 (0·2 to 0·7)	0·2 (0·1 to 0·3)	0·1 (0 to 0·1)	0 (0 to 0)	0 (0 to 0)	51
Polycystic ovarian syndrome	0 (0 to 0)	0 (0 to 0)	0 (0 to 0)	0 (0 to 0)	0 (0 to 0)	0·3 (0·1 to 0·6)	1·5 (0·6 to 3)	1·8 (0·7 to 3·8)	1·7 (0·7 to 3·6)	1·5 (0·6 to 3·2)	1·6 (0·7 to 3·2)	1·5 (0·6 to 3)	1·3 (0·5 to 2·7)	0·3 (0·1 to 0·7)	0 (0 to 0)	0 (0 to 0)	0 (0 to 0)	0 (0 to 0)	0 (0 to 0)	0 (0 to 0)	0 (0 to 0)	0 (0 to 0)	0 (0 to 0)	11·5
Female infertility	0 (0 to 0)	0 (0 to 0)	0 (0 to 0)	0 (0 to 0)	0 (0 to 0)	0 (0 to 0)	0 (0 to 0)	1·9 (0·4 to 5·4)	2·9 (0·7 to 7·7)	3·3 (0·7 to 9·7)	3·7 (0·4 to 10·7)	3·2 (0·4 to 9)	0·1 (0 to 0·4)	0 (0 to 0)	0 (0 to 0)	0 (0 to 0)	0 (0 to 0)	0 (0 to 0)	0 (0 to 0)	0 (0 to 0)	0 (0 to 0)	0 (0 to 0)	0 (0 to 0)	15·1
Endometriosis	0 (0 to 0)	0 (0 to 0)	0 (0 to 0)	0 (0 to 0)	0 (0 to 0)	0 (0 to 0)	1·9 (0·8 to 4)	8·4 (4 to 15·4)	9·7 (4·8 to 16·9)	7·4 (3·6 to 13·3)	6·8 (3·5 to 11·9)	6·5 (3·2 to 11·5)	6 (3·3 to 10·2)	2·8 (1·4 to 4·8)	0 (0 to 0)	0 (0 to 0)	0 (0 to 0)	0 (0 to 0)	0 (0 to 0)	0 (0 to 0)	0 (0 to 0)	0 (0 to 0)	0 (0 to 0)	49·6
Genital prolapse	0 (0 to 0)	0 (0 to 0)	0 (0 to 0)	0 (0 to 0)	0 (0 to 0)	0 (0 to 0)	0 (0 to 0)	0·1 (0 to 0·1)	0·2 (0·1 to 0·4)	0·3 (0·1 to 0·7)	0·6 (0·3 to 1·4)	1 (0·4 to 2·1)	1·4 (0·6 to 2·9)	1·6 (0·6 to 3·1)	1·5 (0·6 to 2·9)	1·2 (0·5 to 2·4)	0·9 (0·4 to 1·8)	0·7 (0·3 to 1·4)	0·5 (0·2 to 1)	0·4 (0·2 to 0·8)	0·2 (0·1 to 0·5)	0·1 (0 to 0·2)	0 (0 to 0·1)	10·7
Premenstrual syndrome	0 (0 to 0)	0 (0 to 0)	0 (0 to 0)	0 (0 to 0)	0 (0 to 0)	7·7 (3·7 to 13·8)	19·6 (9·8 to 34·5)	26·5 (13·5 to 47·5)	26·7 (15·3 to 43·6)	25·2 (13 to 41·2)	29·2 (16·4 to 46·3)	28·1 (16·1 to 45·4)	20·5 (11·2 to 34)	6·8 (3·3 to 12·2)	0 (0 to 0)	0 (0 to 0)	0 (0 to 0)	0 (0 to 0)	0 (0 to 0)	0 (0 to 0)	0 (0 to 0)	0 (0 to 0)	0 (0 to 0)	190
Other gynecological diseases	0 (0 to 0)	0 (0 to 0)	0 (0 to 0)	0 (0 to 0)	0 (0 to 0)	0·4 (0·1 to 0·8)	1 (0·4 to 2)	17·5 (9·8 to 27·5)	40 (22·1 to 63·3)	50·9 (30·1 to 78)	55·4 (32·2 to 87·3)	54·1 (29·9 to 85·7)	52·1 (29·3 to 81)	42·2 (20·6 to 68·1)	27·7 (16·4 to 41·7)	16·5 (8·8 to 26·2)	11 (6·2 to 17)	8·1 (4·9 to 12·1)	5·4 (3·2 to 8·4)	3·6 (2 to 5·6)	2·1 (1·2 to 3·2)	0·9 (0·5 to 1·4)	0·2 (0·1 to 0·4)	389
Hemoglobinopathies and hemolytic anemias	0·4 (0·2 to 0·6)	0·3 (0·2 to 0·6)	1·4 (0·9 to 2)	7·8 (4·6 to 12·3)	10·5 (6·9 to 14·7)	8·8 (5·8 to 12·7)	13·1 (8·9 to 18·5)	16·8 (9·8 to 27·7)	18·1 (10 to 30·1)	13·7 (8·3 to 22·6)	13 (8·6 to 18·7)	12·7 (8·4 to 18·1)	12·5 (8·3 to 17·4)	11·3 (7·7 to 15·9)	8·4 (5·6 to 12·2)	6·9 (4·4 to 10·3)	4·1 (2·7 to 6·1)	3·4 (2·2 to 5)	2·7 (1·8 to 3·9)	2·1 (1·4 to 3)	2·4 (1·7 to 3·4)	1·3 (0·9 to 1·9)	0·3 (0·2 to 0·4)	172
Thalassemias	0 (0 to 0)	0 (0 to 0)	0 (0 to 0)	0 (0 to 0·1)	0 (0 to 0·1)	0 (0 to 0)	0 (0 to 0)	0 (0 to 0)	0 (0 to 0)	0 (0 to 0)	0 (0 to 0)	0 (0 to 0)	0 (0 to 0)	0 (0 to 0)	0 (0 to 0)	0 (0 to 0)	0 (0 to 0)	0 (0 to 0)	0 (0 to 0)	0 (0 to 0)	0 (0 to 0)	0 (0 to 0)	0 (0 to 0)	0·1
Thalassemias trait	0 (0 to 0)	0 (0 to 0)	0 (0 to 0·1)	0·1 (0 to 0·3)	0·2 (0·1 to 0·5)	0·2 (0 to 0·3)	0·6 (0·3 to 1·1)	0·8 (0·4 to 1·3)	0·8 (0·3 to 1·3)	0·7 (0·3 to 1·3)	0·7 (0·3 to 1·2)	0·6 (0·3 to 1·2)	0·2 (0·1 to 0·4)	0·2 (0·1 to 0·4)	0·2 (0·1 to 0·3)	0·1 (0 to 0·2)	0·1 (0 to 0·1)	0·1 (0 to 0·1)	0 (0 to 0·1)	0 (0 to 0·1)	0 (0 to 0·1)	0 (0 to 0)	0 (0 to 0)	5·5
Sickle cell disorders	0·4 (0·2 to 0·6)	0·2 (0·1 to 0·4)	0·8 (0·5 to 1·4)	5·4 (2·8 to 9·5)	7 (4·7 to 10)	6·3 (3·9 to 9·4)	8·3 (5·4 to 12·2)	8·2 (5·4 to 12·2)	8·6 (5·4 to 12·7)	6·2 (3·8 to 10)	6·9 (4·2 to 10·7)	6·4 (4 to 9·7)	6·7 (4·2 to 10·1)	4·9 (2·8 to 7·6)	2·2 (1·3 to 3·6)	0·7 (0·3 to 1·2)	0·1 (0 to 0·2)	0 (0 to 0)	0 (0 to 0)	0 (0 to 0)	0 (0 to 0)	0 (0 to 0)	0 (0 to 0)	79·2
Sickle cell trait	0 (0 to 0)	0·1 (0 to 0·2)	0·3 (0·1 to 0·5)	1·4 (0·6 to 2·6)	2·1 (0·8 to 4·1)	1·5 (0·6 to 2·9)	0·9 (0·3 to 1·8)	6 (1·1 to 15·1)	6·6 (1 to 16·4)	4·7 (0·9 to 11·5)	0·9 (0·4 to 1·6)	1·7 (0·8 to 2·9)	2·1 (0·9 to 3·6)	2·1 (0·9 to 3·5)	1·8 (0·8 to 3·1)	1·1 (0·5 to 2)	0·6 (0·2 to 1·2)	0·5 (0·2 to 1·1)	0·4 (0·2 to 0·8)	0·4 (0·1 to 0·7)	0·3 (0·1 to 0·5)	0·1 (0 to 0·2)	0 (0 to 0)	35·6
G6PD deficiency	0 (0 to 0)	0 (0 to 0)	0 (0 to 0)	0 (0 to 0·1)	0·1 (0 to 0·1)	0 (0 to 0·1)	1·3 (0·5 to 2·3)	0·3 (0·1 to 0·5)	0·5 (0·2 to 0·9)	0·4 (0·2 to 0·9)	1·8 (0·9 to 3·1)	1 (0·5 to 1·6)	0·6 (0·2 to 1·1)	0·6 (0·2 to 1·2)	0·5 (0·2 to 1)	0·4 (0·2 to 0·7)	0·2 (0·1 to 0·4)	0·1 (0 to 0·2)	0·1 (0 to 0·1)	0 (0 to 0)	0 (0 to 0)	0 (0 to 0)	0 (0 to 0)	7·9
G6PD trait	0 (0 to 0)	0 (0 to 0)	0 (0 to 0)	0 (0 to 0)	0 (0 to 0)	0 (0 to 0)	0·1 (0·1 to 0·3)	0 (0 to 0)	0 (0 to 0)	0 (0 to 0)	0·2 (0·1 to 0·3)	0·2 (0·1 to 0·3)	0 (0 to 0)	0 (0 to 0)	0 (0 to 0)	0 (0 to 0)	0 (0 to 0)	0 (0 to 0)	0 (0 to 0)	0 (0 to 0)	0 (0 to 0)	0 (0 to 0)	0 (0 to 0)	0·5
Other hemoglobinopathies and hemolytic anemias	0 (0 to 0)	0 (0 to 0)	0·2 (0·1 to 0·4)	0·8 (0·3 to 1·6)	1 (0·3 to 2·2)	0·8 (0·2 to 1·7)	1·8 (0·6 to 3·5)	1·5 (0·6 to 3·1)	1·7 (0·8 to 3·2)	1·7 (0·7 to 3·2)	2·6 (1·3 to 4·6)	2·9 (1·4 to 5)	2·9 (1·6 to 4·9)	3·6 (2·1 to 5·4)	3·8 (2·4 to 5·7)	4·6 (2·9 to 7·1)	3·1 (2·1 to 4·6)	2·7 (1·8 to 3·9)	2·1 (1·5 to 3·1)	1·6 (1·1 to 2·4)	2·1 (1·4 to 3·1)	1·2 (0·8 to 1·8)	0·3 (0·2 to 0·4)	43·2
Endocrine, metabolic, blood, and immune disorders	1·4 (0·8 to 2·6)	1·8 (1 to 3·3)	8·4 (4·8 to 14·6)	12·6 (7·9 to 21·2)	7·5 (5·2 to 11·2)	6·5 (4·3 to 9·7)	9·7 (6·3 to 14·8)	11·3 (7·8 to 16·9)	15·9 (10·8 to 23·1)	19·6 (13·5 to 28·5)	26·1 (17·9 to 36·7)	32·5 (21·6 to 46·5)	40·1 (27·2 to 57·3)	43·1 (29·1 to 63)	42·7 (28·7 to 62·4)	36 (23·8 to 53·7)	28·4 (18·7 to 44·1)	24·5 (16·6 to 35·9)	19·3 (12·7 to 28·4)	13·8 (9·2 to 20·3)	10·8 (7·4 to 16·2)	5·7 (3·9 to 8·7)	1·6 (1 to 2·6)	419
Oral disorders	0 (0 to 0)	0 (0 to 0)	0 (0 to 0)	2·4 (1·3 to 3·8)	3·2 (1·7 to 5·4)	3·8 (2 to 6·6)	6·2 (3·4 to 10·8)	10·4 (5·5 to 19·2)	15·1 (7·7 to 28·3)	18·1 (8·8 to 33·5)	22·6 (10·7 to 41·7)	25·5 (12·2 to 45·9)	31·3 (16·8 to 54·9)	36·2 (20 to 61·6)	39·1 (22·9 to 62·6)	38·6 (23·6 to 60·6)	33·5 (20·6 to 51·4)	30·4 (18·9 to 45·9)	23·8 (15·1 to 35·4)	17 (10·8 to 24·8)	9·6 (6·1 to 14)	4 (2·5 to 5·9)	1 (0·6 to 1·4)	372
Caries of deciduous teeth	0 (0 to 0)	0 (0 to 0)	0 (0 to 0)	0·7 (0·3 to 1·5)	0·8 (0·3 to 1·7)	0 (0 to 0·1)	0 (0 to 0)	0 (0 to 0)	0 (0 to 0)	0 (0 to 0)	0 (0 to 0)	0 (0 to 0)	0 (0 to 0)	0 (0 to 0)	0 (0 to 0)	0 (0 to 0)	0 (0 to 0)	0 (0 to 0)	0 (0 to 0)	0 (0 to 0)	0 (0 to 0)	0 (0 to 0)	0 (0 to 0)	1·6
Caries of permanent teeth	0 (0 to 0)	0 (0 to 0)	0 (0 to 0)	0 (0 to 0)	0·8 (0·3 to 1·7)	1·8 (0·7 to 3·9)	2·1 (0·8 to 4·6)	2·5 (1·1 to 5·3)	2·5 (1 to 5·1)	2·2 (0·9 to 4·5)	2·3 (0·9 to 4·5)	2·1 (0·9 to 4·4)	2 (0·8 to 4·3)	1·9 (0·7 to 3·9)	1·4 (0·5 to 3·3)	1 (0·4 to 2·3)	0·7 (0·2 to 1·4)	0·5 (0·2 to 1)	0·3 (0·1 to 0·7)	0·2 (0·1 to 0·5)	0·1 (0 to 0·3)	0 (0 to 0·1)	0 (0 to 0)	24·5
Periodontal diseases	0 (0 to 0)	0 (0 to 0)	0 (0 to 0)	0 (0 to 0)	0 (0 to 0)	0 (0 to 0)	0·8 (0·2 to 2·1)	3·1 (0·9 to 7·9)	6·9 (2·2 to 16·3)	9·9 (3·4 to 22·6)	13·3 (4·7 to 30·2)	14·9 (5·5 to 32)	16·3 (6·3 to 34·4)	15·1 (6 to 31·9)	11·8 (4·7 to 24·8)	8·3 (3·2 to 16·8)	5·3 (2 to 10·9)	3·8 (1·5 to 7·7)	2·6 (1 to 5·3)	1·8 (0·7 to 3·6)	1 (0·4 to 2·1)	0·4 (0·2 to 0·9)	0·1 (0 to 0·2)	116
Other oral disorders	0 (0 to 0)	0 (0 to 0)	0 (0 to 0)	1·6 (0·9 to 2·5)	1·6 (1 to 2·6)	2 (1·2 to 3·2)	3·3 (1·9 to 5·2)	4·3 (2·6 to 6·6)	4·6 (2·8 to 7)	4·5 (2·7 to 6·8)	4·8 (2·9 to 7·2)	4·7 (2·8 to 7·1)	5 (3 to 7·8)	4·7 (2·8 to 7·2)	3·9 (2·4 to 5·8)	3 (1·8 to 4·5)	2·1 (1·3 to 3·2)	1·6 (0·9 to 2·4)	0·9 (0·6 to 1·4)	0·5 (0·3 to 0·8)	0·2 (0·1 to 0·4)	0·1 (0·1 to 0·1)	0 (0 to 0)	53·5
Sudden infant death syndrome	--	3 (1·6 to 4·9)	6·7 (3·2 to 12·1)	--	--	--	--	--	--	--	--	--	--	--	--	--	--	--	--	--	--	--	--	NA
Injuries	5·2 (3·3 to 7·5)	2·4 (1·6 to 3·6)	7·7 (5·2 to 11·4)	42·9 (30 to 59·4)	45·3 (38·5 to 53·6)	50·4 (42·6 to 59·3)	85 (71·2 to 100)	106 (87·4 to 128)	109 (90·2 to 129)	97·2 (80·6 to 118)	97·9 (80·9 to 118)	98·8 (81·3 to 119)	97·6 (80·6 to 117)	83·2 (68·1 to 101)	69·1 (55·8 to 84·8)	56·3 (46 to 69·5)	45·1 (37 to 54·5)	40·1 (33·2 to 48·4)	34·1 (28·2 to 41)	30·5 (25·4 to 36·4)	26·1 (21·5 to 30·9)	16·1 (13·4 to 19·2)	5·4 (4·5 to 6·2)	1,250
Transport injuries	0·3 (0·2 to 0·5)	0·2 (0·1 to 0·3)	0·8 (0·5 to 1·3)	9·2 (5·7 to 14·6)	14·4 (10·4 to 20)	15·4 (11·5 to 20·6)	29·4 (21·2 to 39·1)	33 (23·8 to 44·1)	31·9 (23·8 to 41·7)	25·8 (19·7 to 33·2)	27·4 (21·2 to 35)	28·5 (21·9 to 36·4)	28·4 (21·7 to 36·2)	23·5 (18·5 to 29)	19·2 (15·3 to 24)	15·1 (12·1 to 18·5)	12 (9·6 to 14·6)	9·3 (7·5 to 11·3)	6·8 (5·4 to 8·2)	4·8 (3·8 to 5·9)	3 (2·4 to 3·7)	1·4 (1·1 to 1·8)	0·4 (0·3 to 0·5)	340
Road injuries	0·2 (0·2 to 0·4)	0·1 (0·1 to 0·2)	0·7 (0·4 to 1·1)	8·1 (4·9 to 13·2)	13·2 (9·4 to 18·4)	13·8 (10·1 to 18·7)	27·3 (19·6 to 36·1)	30·4 (21·6 to 40·6)	29·3 (21·6 to 38·5)	23·5 (17·8 to 30·3)	25·2 (19·5 to 32·3)	25·4 (19·3 to 32·4)	26·2 (20·1 to 33·6)	21·4 (16·6 to 26·7)	17·4 (13·8 to 21·9)	13·9 (11·1 to 17·1)	11·3 (9 to 13·7)	8·7 (7 to 10·6)	6·2 (4·9 to 7·4)	4·4 (3·5 to 5·4)	2·7 (2·1 to 3·4)	1·3 (1 to 1·6)	0·4 (0·3 to 0·4)	311
Pedestrian road injuries	0·1 (0 to 0·1)	0 (0 to 0·1)	0·1 (0·1 to 0·2)	2·6 (1·5 to 4·3)	3·8 (2·6 to 5·5)	3·3 (2·3 to 4·5)	3 (2·2 to 4·1)	3·3 (2·3 to 4·3)	3·4 (2·5 to 4·3)	3·5 (2·6 to 4·5)	4·1 (3·1 to 5·2)	4·4 (3·2 to 5·7)	5 (3·7 to 6·4)	4·7 (3·6 to 6)	4 (3 to 5)	3·7 (2·9 to 4·6)	3·4 (2·6 to 4·2)	2·8 (2·2 to 3·6)	2·2 (1·7 to 2·7)	1·7 (1·3 to 2·1)	1·1 (0·8 to 1·4)	0·5 (0·4 to 0·6)	0·1 (0·1 to 0·2)	60·7
Cyclist road injuries	0 (0 to 0)	0 (0 to 0)	0 (0 to 0)	0·4 (0·2 to 0·8)	0·8 (0·4 to 1·2)	1·5 (0·9 to 2·5)	2 (1·3 to 3·2)	2·2 (1·5 to 3·3)	2·4 (1·7 to 3·4)	2·3 (1·7 to 3·1)	2·7 (2 to 3·7)	2·8 (2 to 3·7)	3 (2·2 to 4·1)	2·9 (2·1 to 3·8)	2·3 (1·6 to 3·1)	1·8 (1·3 to 2·4)	1·3 (1 to 1·8)	1 (0·7 to 1·4)	0·8 (0·5 to 1)	0·5 (0·4 to 0·7)	0·3 (0·2 to 0·4)	0·1 (0·1 to 0·2)	0 (0 to 0)	31·3
Motorcyclist road injuries	0 (0 to 0)	0 (0 to 0)	0 (0 to 0·1)	0·3 (0·2 to 0·4)	0·6 (0·4 to 0·8)	0·7 (0·5 to 0·9)	3 (2·1 to 4)	3·6 (2·5 to 4·9)	3·4 (2·4 to 4·5)	2·3 (1·7 to 2·9)	2·5 (1·9 to 3·2)	2·1 (1·6 to 2·7)	2·2 (1·7 to 2·8)	1·5 (1·2 to 2)	1·3 (1 to 1·8)	1 (0·8 to 1·3)	0·8 (0·6 to 1·1)	0·6 (0·4 to 0·8)	0·5 (0·3 to 0·7)	0·4 (0·3 to 0·5)	0·2 (0·2 to 0·3)	0·1 (0·1 to 0·2)	0 (0 to 0)	27·2
Motor vehicle road injuries	0·1 (0·1 to 0·2)	0·1 (0 to 0·1)	0·5 (0·3 to 0·8)	4·5 (2·7 to 7·4)	7·4 (5·2 to 10·5)	7·9 (5·7 to 11)	18·7 (13·2 to 25·3)	20·6 (14·3 to 27·8)	19 (13·7 to 25·6)	14·5 (10·5 to 19·5)	15 (11·2 to 19·6)	15·2 (11·1 to 19·9)	15·1 (11·2 to 20·1)	11·4 (8·7 to 14·7)	9·1 (7·1 to 11·6)	6·9 (5·4 to 8·6)	5·3 (4·2 to 6·7)	4 (3·1 to 4·9)	2·5 (2 to 3·1)	1·6 (1·3 to 2)	1 (0·8 to 1·2)	0·5 (0·4 to 0·6)	0·1 (0·1 to 0·2)	181
Other road injuries	0 (0 to 0)	0 (0 to 0)	0 (0 to 0)	0·4 (0·1 to 0·8)	0·6 (0·3 to 1)	0·4 (0·2 to 0·6)	0·6 (0·3 to 1)	0·7 (0·4 to 1·2)	1·1 (0·7 to 1·9)	1 (0·6 to 1·7)	1 (0·6 to 1·5)	0·9 (0·6 to 1·4)	0·9 (0·6 to 1·4)	0·9 (0·6 to 1·2)	0·7 (0·5 to 1)	0·6 (0·4 to 0·9)	0·5 (0·3 to 0·7)	0·3 (0·2 to 0·4)	0·2 (0·2 to 0·3)	0·2 (0·1 to 0·2)	0·1 (0·1 to 0·1)	0 (0 to 0·1)	0 (0 to 0)	11·1
Other transport injuries	0·1 (0 to 0·1)	0·1 (0 to 0·1)	0·2 (0·1 to 0·3)	1·1 (0·6 to 1·8)	1·3 (0·8 to 2)	1·6 (1 to 2·5)	2·1 (1·3 to 3·4)	2·6 (1·6 to 4·1)	2·6 (1·7 to 3·9)	2·3 (1·5 to 3·4)	2·2 (1·4 to 3·3)	3·1 (2 to 4·7)	2·1 (1·4 to 3·2)	2·1 (1·4 to 3·1)	1·8 (1·2 to 2·5)	1·2 (0·8 to 1·6)	0·7 (0·5 to 1)	0·6 (0·4 to 0·8)	0·6 (0·4 to 0·9)	0·4 (0·3 to 0·5)	0·3 (0·2 to 0·3)	0·2 (0·1 to 0·2)	0·1 (0 to 0·1)	29
Unintentional injuries	2·9 (1·8 to 4·4)	1·9 (1·2 to 2·9)	5·9 (3·9 to 8·9)	28·5 (19·1 to 40·4)	26 (20·6 to 32·8)	27 (21·3 to 33·5)	28·3 (21·9 to 36·3)	32·7 (25·2 to 42·1)	35·8 (27·9 to 45·8)	36 (28 to 46·4)	39·9 (31 to 51)	42·4 (33·1 to 54)	45·6 (35·2 to 57·5)	43·9 (33·8 to 57·1)	39·3 (30·3 to 50·5)	33·9 (26·3 to 43·8)	27·7 (21·6 to 35·1)	27 (21·5 to 33·3)	24·7 (20·2 to 30·6)	24·3 (20·1 to 29)	22·2 (18 to 26·6)	14·4 (11·9 to 17·2)	4·8 (4 to 5·6)	615
Falls	0 (0 to 0)	0 (0 to 0)	0·1 (0·1 to 0·2)	0·9 (0·6 to 1·2)	1·5 (1 to 2·1)	2·3 (1·5 to 3·2)	3·2 (2·1 to 4·6)	4 (2·7 to 5·8)	4·7 (3·2 to 6·7)	5·1 (3·6 to 7·2)	6·2 (4·3 to 8·7)	6·9 (4·9 to 9·5)	8·3 (6 to 11·5)	9·4 (6·7 to 12·7)	9·5 (6·9 to 12·8)	9·3 (7 to 12·3)	8·5 (6·4 to 10·9)	9·6 (7·5 to 12·1)	10 (8 to 12·6)	12 (9·6 to 14·5)	12·2 (9·7 to 14·8)	7·7 (6·3 to 9·3)	2·5 (2·1 to 3)	134
Drowning	0·2 (0·1 to 0·3)	0·1 (0·1 to 0·2)	0·6 (0·3 to 0·9)	9·6 (6 to 14)	8 (5·8 to 10·9)	7·7 (5·5 to 10·3)	5 (3·5 to 6·6)	3·1 (2·2 to 4·2)	3·1 (2·2 to 4·4)	2·6 (1·9 to 3·5)	2·6 (1·8 to 3·5)	2·5 (1·8 to 3·3)	2·4 (1·8 to 3·2)	1·7 (1·3 to 2·3)	1·2 (0·9 to 1·6)	0·9 (0·7 to 1·2)	0·7 (0·5 to 0·9)	0·5 (0·4 to 0·6)	0·4 (0·3 to 0·6)	0·3 (0·2 to 0·4)	0·3 (0·2 to 0·4)	0·3 (0·2 to 0·3)	0·1 (0·1 to 0·1)	53·8
Fire, heat, and hot substances	0·1 (0·1 to 0·2)	0·1 (0·1 to 0·2)	0·6 (0·4 to 0·8)	4·6 (3 to 6·7)	4·2 (3·2 to 5·2)	4·4 (3·2 to 5·8)	6·1 (4·3 to 8·5)	7·5 (5·4 to 10·1)	8·8 (6·5 to 11·7)	9 (6·6 to 12·4)	10·1 (7·5 to 13·6)	10·1 (7·3 to 13·3)	10 (7·3 to 13·8)	9·5 (6·8 to 12·8)	8 (5·8 to 11)	6·3 (4·5 to 8·7)	4·4 (3·2 to 6·1)	3·8 (2·7 to 5·1)	2·8 (2 to 3·8)	2·1 (1·6 to 2·8)	1·8 (1·4 to 2·2)	1·1 (0·8 to 1·3)	0·4 (0·3 to 0·4)	116
Poisonings	0 (0 to 0)	0 (0 to 0·1)	0·1 (0 to 0·1)	0·7 (0·4 to 1)	0·4 (0·3 to 0·6)	0·5 (0·3 to 0·7)	0·6 (0·4 to 0·9)	0·7 (0·4 to 1)	0·7 (0·5 to 1)	0·6 (0·4 to 0·9)	0·7 (0·4 to 1)	0·7 (0·4 to 0·9)	0·6 (0·4 to 0·9)	0·6 (0·4 to 0·8)	0·5 (0·3 to 0·7)	0·4 (0·2 to 0·5)	0·3 (0·2 to 0·4)	0·2 (0·2 to 0·3)	0·2 (0·1 to 0·3)	0·1 (0·1 to 0·2)	0·1 (0·1 to 0·1)	0 (0 to 0·1)	0 (0 to 0)	8·6
Poisoning by carbon monoxide	0 (0 to 0)	0 (0 to 0)	0 (0 to 0)	0·1 (0 to 0·1)	0·1 (0·1 to 0·2)	0·1 (0·1 to 0·2)	0·2 (0·1 to 0·3)	0·2 (0·1 to 0·3)	0·2 (0·1 to 0·3)	0·2 (0·1 to 0·3)	0·2 (0·1 to 0·3)	0·2 (0·1 to 0·3)	0·2 (0·1 to 0·3)	0·2 (0·1 to 0·2)	0·1 (0·1 to 0·2)	0·1 (0·1 to 0·2)	0·1 (0·1 to 0·1)	0·1 (0 to 0·1)	0 (0 to 0·1)	0 (0 to 0)	0 (0 to 0)	0 (0 to 0)	0 (0 to 0)	2·3
Poisoning by other means	0 (0 to 0)	0 (0 to 0·1)	0·1 (0 to 0·1)	0·6 (0·4 to 0·9)	0·3 (0·2 to 0·5)	0·3 (0·2 to 0·5)	0·4 (0·3 to 0·7)	0·5 (0·3 to 0·7)	0·5 (0·3 to 0·7)	0·4 (0·3 to 0·7)	0·5 (0·3 to 0·7)	0·5 (0·3 to 0·7)	0·5 (0·3 to 0·7)	0·4 (0·3 to 0·6)	0·3 (0·2 to 0·5)	0·3 (0·2 to 0·4)	0·2 (0·1 to 0·3)	0·2 (0·1 to 0·2)	0·1 (0·1 to 0·2)	0·1 (0·1 to 0·2)	0·1 (0·1 to 0·1)	0 (0 to 0)	0 (0 to 0)	6·3
Exposure to mechanical forces	0·2 (0·1 to 0·2)	0·2 (0·1 to 0·3)	0·5 (0·3 to 0·6)	4·6 (3 to 6·6)	5·7 (4·2 to 7·5)	5·7 (4·4 to 7·4)	6·4 (4·7 to 8·5)	7·9 (6 to 10·3)	8·1 (6·2 to 10·6)	7·8 (5·9 to 10·2)	8·7 (6·5 to 11·6)	9·2 (7 to 12)	9·1 (6·7 to 12·3)	8·6 (6·4 to 11·6)	7·1 (5·2 to 9·8)	5·8 (4·2 to 8)	4·4 (3·1 to 6)	3·9 (2·8 to 5·3)	3·3 (2·4 to 4·4)	2·8 (2·1 to 3·6)	2·2 (1·7 to 2·8)	1·6 (1·3 to 2)	0·6 (0·5 to 0·8)	114
Unintentional firearm injuries	0·1 (0 to 0·1)	0 (0 to 0)	0·1 (0 to 0·1)	0·6 (0·3 to 1)	0·9 (0·5 to 1·5)	1·7 (1·1 to 2·5)	2·5 (1·8 to 3·4)	3·4 (2·5 to 4·6)	2·9 (2 to 3·8)	2·8 (2·1 to 3·6)	2·8 (2·1 to 3·7)	2·7 (2 to 3·6)	2·2 (1·7 to 2·9)	1·6 (1·2 to 2·1)	1·3 (1 to 1·7)	0·9 (0·7 to 1·2)	0·6 (0·5 to 0·8)	0·7 (0·5 to 0·9)	0·6 (0·4 to 0·7)	0·7 (0·5 to 0·8)	0·7 (0·5 to 0·9)	0·7 (0·5 to 0·8)	0·3 (0·2 to 0·3)	30·8
Other exposure to mechanical forces	0·1 (0·1 to 0·2)	0·1 (0·1 to 0·2)	0·4 (0·3 to 0·5)	4 (2·7 to 5·8)	4·7 (3·5 to 6·2)	4 (2·9 to 5·5)	3·8 (2·6 to 5·6)	4·6 (3·2 to 6·5)	5·3 (3·7 to 7·4)	5 (3·5 to 7·2)	5·9 (4 to 8·3)	6·4 (4·6 to 8·9)	6·8 (4·8 to 9·6)	7 (5·1 to 9·7)	5·8 (4·1 to 8·2)	4·8 (3·4 to 6·8)	3·8 (2·6 to 5·2)	3·2 (2·3 to 4·5)	2·7 (2 to 3·7)	2·1 (1·5 to 2·8)	1·5 (1·1 to 2)	0·9 (0·7 to 1·2)	0·3 (0·3 to 0·4)	83·4
Adverse effects of medical treatment	0·6 (0·3 to 1)	0·4 (0·2 to 0·6)	1 (0·5 to 1·6)	1·7 (0·9 to 2·8)	0·9 (0·6 to 1·4)	0·8 (0·5 to 1·2)	1 (0·7 to 1·5)	2·2 (1·4 to 3·4)	2·4 (1·5 to 3·6)	3 (2 to 4·5)	2·8 (1·9 to 4·1)	3·7 (2·4 to 5·3)	5 (3·2 to 7·4)	4·2 (2·7 to 6·2)	4·2 (2·8 to 6·3)	3·7 (2·5 to 5·4)	3·2 (2·2 to 4·5)	3·2 (2·1 to 4·4)	3·1 (2·2 to 4·3)	2·6 (1·8 to 3·7)	2·5 (1·6 to 3·5)	1·4 (0·9 to 2)	0·4 (0·2 to 0·6)	54·2
Animal contact	0 (0 to 0)	0 (0 to 0)	0 (0 to 0·1)	0·2 (0·1 to 0·4)	0·3 (0·2 to 0·5)	0·4 (0·3 to 0·6)	0·5 (0·3 to 0·7)	0·5 (0·4 to 0·8)	0·6 (0·4 to 0·8)	0·5 (0·4 to 0·8)	0·6 (0·4 to 0·9)	0·6 (0·4 to 0·9)	0·7 (0·5 to 0·9)	0·6 (0·4 to 0·9)	0·6 (0·4 to 0·8)	0·5 (0·4 to 0·7)	0·4 (0·3 to 0·5)	0·4 (0·3 to 0·5)	0·4 (0·3 to 0·5)	0·4 (0·3 to 0·6)	0·5 (0·3 to 0·7)	0·5 (0·4 to 0·8)	0·2 (0·1 to 0·3)	9·6
Venomous animal contact	0 (0 to 0)	0 (0 to 0)	0 (0 to 0)	0·1 (0·1 to 0·2)	0·2 (0·1 to 0·3)	0·3 (0·2 to 0·4)	0·3 (0·2 to 0·5)	0·4 (0·2 to 0·6)	0·4 (0·3 to 0·6)	0·4 (0·2 to 0·6)	0·4 (0·3 to 0·6)	0·4 (0·3 to 0·6)	0·4 (0·3 to 0·6)	0·4 (0·3 to 0·6)	0·4 (0·2 to 0·5)	0·3 (0·2 to 0·5)	0·2 (0·2 to 0·3)	0·2 (0·2 to 0·3)	0·2 (0·1 to 0·3)	0·2 (0·2 to 0·4)	0·3 (0·2 to 0·4)	0·3 (0·2 to 0·5)	0·1 (0·1 to 0·2)	6·2
Non-venomous animal contact	0 (0 to 0)	0 (0 to 0)	0 (0 to 0)	0·1 (0·1 to 0·2)	0·1 (0·1 to 0·2)	0·1 (0·1 to 0·2)	0·1 (0·1 to 0·2)	0·1 (0·1 to 0·2)	0·2 (0·1 to 0·2)	0·1 (0·1 to 0·2)	0·2 (0·1 to 0·3)	0·2 (0·1 to 0·3)	0·3 (0·2 to 0·4)	0·2 (0·2 to 0·3)	0·2 (0·2 to 0·3)	0·2 (0·1 to 0·3)	0·2 (0·1 to 0·2)	0·2 (0·1 to 0·2)	0·2 (0·1 to 0·2)	0·2 (0·1 to 0·3)	0·2 (0·1 to 0·3)	0·2 (0·1 to 0·3)	0·1 (0 to 0·1)	3·4
Foreign body	1·7 (1 to 2·6)	0·9 (0·5 to 1·5)	2·9 (1·7 to 4·7)	4·4 (2·8 to 6·5)	2·1 (1·7 to 2·7)	1·6 (1·3 to 2·1)	1·1 (0·8 to 1·5)	1·4 (1·1 to 1·9)	1·6 (1·2 to 2·1)	1·5 (1·1 to 2)	1·7 (1·3 to 2·2)	1·9 (1·5 to 2·5)	2·1 (1·6 to 2·7)	2·1 (1·6 to 2·7)	1·9 (1·5 to 2·4)	1·8 (1·3 to 2·2)	1·6 (1·3 to 2)	1·8 (1·4 to 2·2)	1·7 (1·3 to 2·2)	1·7 (1·3 to 2·1)	1·2 (0·9 to 1·5)	0·9 (0·7 to 1·1)	0·3 (0·3 to 0·4)	40
Pulmonary aspiration and foreign body in airway	1·7 (1 to 2·6)	0·9 (0·5 to 1·5)	2·8 (1·6 to 4·6)	4·2 (2·6 to 6·2)	1·8 (1·4 to 2·3)	1·2 (0·9 to 1·6)	0·6 (0·5 to 0·7)	0·8 (0·6 to 1·1)	0·9 (0·7 to 1·2)	0·9 (0·6 to 1·1)	1 (0·8 to 1·3)	1·2 (0·9 to 1·6)	1·4 (1·1 to 1·8)	1·4 (1 to 1·9)	1·3 (1 to 1·7)	1·3 (0·9 to 1·7)	1·3 (1 to 1·6)	1·4 (1·1 to 1·9)	1·4 (1 to 1·9)	1·4 (1·1 to 1·9)	1 (0·8 to 1·3)	0·8 (0·6 to 1)	0·3 (0·2 to 0·4)	31
Foreign body in eyes	0 (0 to 0)	0 (0 to 0)	0 (0 to 0)	0·1 (0 to 0·1)	0·1 (0 to 0·2)	0·1 (0 to 0·3)	0·2 (0·1 to 0·5)	0·3 (0·1 to 0·6)	0·3 (0·1 to 0·7)	0·3 (0·1 to 0·6)	0·3 (0·1 to 0·7)	0·4 (0·2 to 0·7)	0·4 (0·2 to 0·7)	0·3 (0·2 to 0·6)	0·2 (0·1 to 0·4)	0·2 (0·1 to 0·3)	0·1 (0·1 to 0·2)	0·1 (0·1 to 0·2)	0·1 (0·1 to 0·1)	0·1 (0 to 0·1)	0 (0 to 0·1)	0 (0 to 0)	0 (0 to 0)	3·7
Foreign body in other body part	0 (0 to 0)	0 (0 to 0)	0 (0 to 0)	0·2 (0·1 to 0·3)	0·2 (0·2 to 0·3)	0·3 (0·2 to 0·4)	0·3 (0·2 to 0·4)	0·3 (0·2 to 0·5)	0·3 (0·2 to 0·5)	0·3 (0·2 to 0·5)	0·4 (0·3 to 0·5)	0·4 (0·3 to 0·5)	0·4 (0·3 to 0·5)	0·4 (0·3 to 0·5)	0·3 (0·2 to 0·4)	0·3 (0·2 to 0·4)	0·2 (0·2 to 0·3)	0·2 (0·2 to 0·3)	0·2 (0·1 to 0·3)	0·2 (0·1 to 0·2)	0·1 (0·1 to 0·2)	0·1 (0·1 to 0·2)	0 (0 to 0·1)	5·3
Environmental heat and cold exposure	0 (0 to 0)	0 (0 to 0)	0·1 (0 to 0·1)	0·4 (0·3 to 0·6)	0·8 (0·5 to 1·2)	1·1 (0·7 to 1·6)	1·4 (1 to 2·1)	1·8 (1·2 to 2·5)	1·9 (1·3 to 2·8)	2 (1·4 to 2·8)	2·2 (1·5 to 3·1)	2·3 (1·6 to 3·2)	2·5 (1·8 to 3·5)	2·5 (1·7 to 3·5)	2·3 (1·6 to 3·2)	2 (1·4 to 2·8)	1·6 (1·2 to 2·2)	1·5 (1 to 2)	1·2 (0·9 to 1·7)	1·1 (0·8 to 1·4)	0·8 (0·6 to 1)	0·6 (0·4 to 0·8)	0·2 (0·2 to 0·3)	30·4
Exposure to forces of nature	0 (0 to 0)	0 (0 to 0)	0 (0 to 0)	0 (0 to 0)	0 (0 to 0)	0·1 (0·1 to 0·2)	0·2 (0·1 to 0·3)	0·2 (0·1 to 0·4)	0·2 (0·1 to 0·3)	0·2 (0·1 to 0·3)	0·2 (0·2 to 0·4)	0·3 (0·2 to 0·5)	0·3 (0·2 to 0·5)	0·3 (0·2 to 0·5)	0·2 (0·1 to 0·3)	0·2 (0·1 to 0·3)	0·1 (0·1 to 0·2)	0·1 (0·1 to 0·2)	0·1 (0·1 to 0·1)	0·1 (0 to 0·1)	0 (0 to 0)	0 (0 to 0)	0 (0 to 0)	2·9
Other unintentional injuries	0·1 (0 to 0·1)	0 (0 to 0·1)	0·2 (0·1 to 0·3)	1·4 (1 to 2)	2·1 (1·5 to 3)	2·5 (1·7 to 3·5)	2·9 (2 to 4·2)	3·4 (2·3 to 5)	3·7 (2·5 to 5·3)	3·6 (2·5 to 5·4)	4·1 (2·7 to 5·9)	4·3 (2·9 to 6·2)	4·5 (3·1 to 6·5)	4·3 (2·9 to 6·2)	3·8 (2·5 to 5·5)	3·1 (2·1 to 4·5)	2·4 (1·6 to 3·4)	2 (1·3 to 2·8)	1·6 (1·1 to 2·2)	1·1 (0·8 to 1·6)	0·7 (0·5 to 1)	0·3 (0·2 to 0·4)	0·1 (0·1 to 0·1)	52·1
Self-harm and interpersonal violence	2 (1·1 to 3·1)	0·3 (0·2 to 0·6)	1 (0·6 to 1·6)	5·3 (3 to 9)	4·8 (3·6 to 6·6)	8 (6·2 to 10·2)	27·3 (20·8 to 34·9)	40·2 (30·6 to 51·8)	40·9 (30·9 to 53·3)	35·4 (26·3 to 46·1)	30·6 (23·1 to 40·9)	27·9 (21 to 36·9)	23·7 (18·2 to 30·9)	15·8 (12·1 to 20)	10·6 (8·4 to 13·6)	7·3 (5·9 to 9)	5·5 (4·4 to 6·7)	3·9 (3·2 to 4·8)	2·6 (2·1 to 3·1)	1·5 (1·2 to 1·8)	0·9 (0·8 to 1·1)	0·3 (0·3 to 0·4)	0·1 (0·1 to 0·1)	296
Self-harm	0 (0 to 0)	0 (0 to 0)	0 (0 to 0)	0 (0 to 0)	0 (0 to 0)	1·2 (0·7 to 1·7)	6 (4·1 to 8·4)	6·8 (4·8 to 9·4)	6·5 (4·4 to 9·3)	6·2 (4·4 to 8·6)	5·8 (4·2 to 7·9)	5·8 (4·2 to 8)	5·9 (4·2 to 8·2)	4·8 (3·5 to 6·5)	3·7 (2·7 to 5·1)	2·7 (2 to 3·6)	2 (1·5 to 2·7)	1·4 (1 to 1·9)	0·8 (0·6 to 1·1)	0·5 (0·4 to 0·6)	0·3 (0·2 to 0·4)	0·1 (0·1 to 0·1)	0 (0 to 0)	60·6
Self-harm by firearm	0 (0 to 0)	0 (0 to 0)	0 (0 to 0)	0 (0 to 0)	0 (0 to 0)	0 (0 to 0·1)	0·2 (0·1 to 0·2)	0·2 (0·2 to 0·3)	0·2 (0·1 to 0·2)	0·2 (0·2 to 0·3)	0·2 (0·1 to 0·2)	0·1 (0·1 to 0·2)	0·1 (0·1 to 0·2)	0·1 (0·1 to 0·2)	0·1 (0·1 to 0·1)	0 (0 to 0·1)	0 (0 to 0·1)	0 (0 to 0·1)	0 (0 to 0)	0 (0 to 0)	0 (0 to 0)	0 (0 to 0)	0 (0 to 0)	1·7
Self-harm by other specified means	0 (0 to 0)	0 (0 to 0)	0 (0 to 0)	0 (0 to 0)	0 (0 to 0)	1·1 (0·7 to 1·7)	5·8 (4 to 8·2)	6·6 (4·7 to 9·2)	6·4 (4·3 to 9·1)	5·9 (4·2 to 8·2)	5·6 (4 to 7·7)	5·7 (4·1 to 7·9)	5·8 (4·1 to 8)	4·7 (3·4 to 6·4)	3·6 (2·6 to 4·9)	2·7 (2 to 3·6)	2 (1·4 to 2·6)	1·4 (1 to 1·8)	0·8 (0·6 to 1·1)	0·5 (0·4 to 0·6)	0·3 (0·2 to 0·4)	0·1 (0·1 to 0·1)	0 (0 to 0)	58·9
Interpersonal violence	2 (1·1 to 3·1)	0·3 (0·2 to 0·6)	1 (0·6 to 1·6)	5·2 (2·9 to 8·9)	4·8 (3·5 to 6·6)	6·8 (5·1 to 8·9)	21·3 (15·7 to 28·1)	33·2 (24·4 to 43·9)	34·3 (25·1 to 46)	29·1 (20·9 to 39·3)	24·7 (18·1 to 33·7)	22 (16·2 to 30)	17·7 (12·9 to 23·9)	10·9 (8 to 14·4)	6·9 (5·3 to 9)	4·6 (3·5 to 5·9)	3·4 (2·6 to 4·4)	2·5 (2 to 3·1)	1·8 (1·4 to 2·1)	1 (0·8 to 1·2)	0·6 (0·5 to 0·8)	0·2 (0·2 to 0·3)	0·1 (0 to 0·1)	234
Physical violence by firearm	0·1 (0 to 0·1)	0 (0 to 0)	0 (0 to 0·1)	0·6 (0·3 to 1)	0·7 (0·5 to 1)	1·5 (1·1 to 2·1)	8·1 (5·5 to 11)	12 (8·3 to 16·5)	11·5 (8 to 15·8)	9·2 (6·2 to 12·8)	7·6 (5·2 to 10·7)	5·5 (3·8 to 7·8)	5 (3·4 to 7)	2·4 (1·7 to 3·4)	1·4 (1 to 2)	0·7 (0·5 to 1)	0·4 (0·3 to 0·5)	0·2 (0·2 to 0·3)	0·1 (0·1 to 0·1)	0 (0 to 0·1)	0 (0 to 0·1)	0 (0 to 0)	0 (0 to 0)	67·1
Physical violence by sharp object	0·1 (0 to 0·1)	0 (0 to 0)	0 (0 to 0·1)	0·3 (0·2 to 0·5)	0·4 (0·3 to 0·5)	0·8 (0·6 to 1·1)	4·9 (3·5 to 6·7)	9·3 (6·5 to 12·6)	10·3 (7·2 to 14·1)	8·3 (5·7 to 11·6)	7·5 (5·2 to 10·4)	6·6 (4·6 to 9·2)	4·1 (2·9 to 5·7)	2·2 (1·6 to 2·9)	1·3 (1 to 1·7)	0·7 (0·6 to 0·9)	0·5 (0·4 to 0·6)	0·4 (0·3 to 0·5)	0·2 (0·2 to 0·3)	0·2 (0·1 to 0·2)	0·1 (0·1 to 0·1)	0 (0 to 0·1)	0 (0 to 0)	58·1
Sexual violence	0 (0 to 0)	0 (0 to 0)	0 (0 to 0)	0 (0 to 0·1)	0·3 (0·2 to 0·5)	0·8 (0·5 to 1·3)	2·2 (1·2 to 3·6)	3·2 (1·9 to 4·9)	3 (1·7 to 4·8)	2·6 (1·4 to 4·3)	2·5 (1·4 to 3·9)	2 (1·1 to 3·2)	1·6 (1 to 2·8)	1·1 (0·6 to 1·9)	0·7 (0·4 to 1·1)	0·5 (0·3 to 0·8)	0·4 (0·2 to 0·5)	0·3 (0·2 to 0·4)	0·2 (0·1 to 0·3)	0·2 (0·1 to 0·2)	0·1 (0 to 0·1)	0 (0 to 0)	0 (0 to 0)	21·6
Physical violence by other means	1·8 (1 to 2·9)	0·3 (0·2 to 0·5)	0·9 (0·5 to 1·5)	4·3 (2·4 to 7·4)	3·3 (2·4 to 4·6)	3·6 (2·6 to 4·9)	6·1 (4·3 to 8·3)	8·7 (6·2 to 11·7)	9·6 (6·9 to 13·1)	9 (6·3 to 12·4)	7·3 (5·2 to 9·9)	8 (5·8 to 10·9)	7 (5·1 to 9·6)	5·2 (3·7 to 6·9)	3·5 (2·6 to 4·6)	2·6 (2 to 3·5)	2·2 (1·6 to 2·9)	1·6 (1·2 to 2)	1·2 (0·9 to 1·5)	0·6 (0·5 to 0·8)	0·4 (0·3 to 0·5)	0·2 (0·1 to 0·2)	0 (0 to 0·1)	87·4
Conflict and terrorism	0 (0 to 0)	0 (0 to 0)	0 (0 to 0)	0 (0 to 0)	0 (0 to 0)	0 (0 to 0)	0 (0 to 0)	0·1 (0 to 0·2)	0 (0 to 0·1)	0·1 (0 to 0·1)	0 (0 to 0·1)	0 (0 to 0·1)	0 (0 to 0)	0 (0 to 0)	0 (0 to 0)	0 (0 to 0)	0 (0 to 0)	0 (0 to 0)	0 (0 to 0)	0 (0 to 0)	0 (0 to 0)	0 (0 to 0)	0 (0 to 0)	0·4

SR Table 2b: Saint Vincent and the Grenadines DALYS by cause and age group for females with 95% uncertainty intervals, 2019

Cause	Early Neonatal	Late Neonatal	Post Neonatal	1 to 4	5 to 9	10 to 14	15 to 19	20 to 24	25 to 29	30 to 34	35 to 39	40 to 44	45 to 49	50 to 54	55 to 59	60 to 64	65 to 69	70 to 74	75 to 79	80 to 84	85 to 89	90 to 94	95 plus	Total
All causes	359 (236 to 508)	215 (144 to 304)	257 (178 to 360)	221 (171 to 280)	234 (182 to 297)	380 (309 to 472)	505 (407 to 635)	635 (524 to 770)	717 (587 to 861)	793 (656 to 951)	917 (766 to 1,100)	1,070 (901 to 1,260)	1,210 (1,030 to 1,410)	1,350 (1,160 to 1,550)	1,360 (1,160 to 1,570)	1,440 (1,250 to 1,640)	1,230 (1,080 to 1,380)	1,180 (1,050 to 1,310)	1,060 (952 to 1,170)	1,050 (959 to 1,140)	627 (577 to 678)	251 (234 to 268)	75·6 (71·7 to 79·6)	17,100
Communicable, maternal, neonatal, and nutritional diseases	311 (202 to 444)	174 (115 to 244)	144 (101 to 200)	88·3 (64·6 to 118)	72·6 (47·5 to 104)	68·6 (50·6 to 96·8)	74·8 (57 to 98·9)	98·2 (80·6 to 124)	125 (107 to 147)	138 (120 to 161)	140 (121 to 170)	117 (94·7 to 143)	107 (87·9 to 135)	91·1 (73·1 to 116)	76·1 (59 to 98·5)	68·6 (55 to 85·6)	53·4 (42·9 to 65·5)	53·3 (42·1 to 67·1)	54·7 (43·8 to 67·1)	61·8 (49·9 to 76·7)	45 (36·6 to 53·9)	19·9 (15·9 to 24·5)	6·5 (5 to 8)	2,190
HIV/AIDS and sexually transmitted infections	0 (0 to 0)	0 (0 to 0)	4·8 (4·5 to 5·2)	2·8 (2·6 to 3·1)	2·8 (2·6 to 3)	5 (4·7 to 5·4)	8·8 (8·1 to 9·5)	24·3 (22·2 to 26·8)	51·6 (47·2 to 56·7)	65·8 (60·4 to 71·2)	64·1 (59·4 to 69·7)	40·8 (37·5 to 44·9)	38·5 (35·6 to 41·7)	22·1 (20·3 to 24·2)	11·6 (10·5 to 12·7)	8·5 (7·7 to 9·4)	4·7 (4·2 to 5·2)	1·5 (1·3 to 1·8)	0·9 (0·8 to 1·1)	0·6 (0·5 to 0·7)	0·3 (0·3 to 0·4)	0·1 (0 to 0·1)	0 (0 to 0)	359
HIV/AIDS	0 (0 to 0)	0 (0 to 0)	4·8 (4·4 to 5·1)	2·8 (2·6 to 3·1)	2·8 (2·6 to 3)	4·9 (4·6 to 5·3)	8·3 (7·7 to 8·9)	23·2 (21·2 to 25·5)	49·9 (45·8 to 54·9)	63·7 (58·6 to 68·8)	61·7 (57·3 to 66·8)	38·3 (35·4 to 41·8)	36·6 (33·8 to 39·4)	20·9 (19·3 to 22·7)	10·7 (9·8 to 11·6)	7·9 (7·2 to 8·5)	4·3 (3·9 to 4·7)	1·3 (1·1 to 1·4)	0·7 (0·7 to 0·8)	0·5 (0·4 to 0·5)	0·3 (0·2 to 0·3)	0 (0 to 0)	0 (0 to 0)	343
HIV/AIDS - Drug-susceptible Tuberculosis	0 (0 to 0)	0 (0 to 0)	0·2 (0·1 to 0·4)	0·1 (0·1 to 0·2)	0·1 (0·1 to 0·2)	0·2 (0·1 to 0·3)	0·4 (0·3 to 0·6)	1·3 (0·9 to 1·8)	2·4 (1·6 to 3·6)	3 (2 to 4·5)	3 (2 to 4·5)	2 (1·3 to 3)	1·9 (1·2 to 2·8)	1·1 (0·7 to 1·7)	0·5 (0·4 to 0·8)	0·4 (0·3 to 0·6)	0·2 (0·1 to 0·3)	0·1 (0 to 0·1)	0 (0 to 0·1)	0 (0 to 0)	0 (0 to 0)	0 (0 to 0)	0 (0 to 0)	17·1
HIV/AIDS - Multidrug-resistant Tuberculosis without extensive dru	0 (0 to 0)	0 (0 to 0)	0 (0 to 0)	0 (0 to 0)	0 (0 to 0)	0 (0 to 0)	0 (0 to 0)	0 (0 to 0·1)	0 (0 to 0·1)	0 (0 to 0·2)	0 (0 to 0·2)	0 (0 to 0·1)	0 (0 to 0·1)	0 (0 to 0·1)	0 (0 to 0)	0 (0 to 0)	0 (0 to 0)	0 (0 to 0)	0 (0 to 0)	0 (0 to 0)	0 (0 to 0)	0 (0 to 0)	0 (0 to 0)	0·2
HIV/AIDS - Extensively drug-resistant Tuberculosis	0 (0 to 0)	0 (0 to 0)	0 (0 to 0)	0 (0 to 0)	0 (0 to 0)	0 (0 to 0)	0 (0 to 0)	0 (0 to 0)	0 (0 to 0)	0 (0 to 0)	0 (0 to 0)	0 (0 to 0)	0 (0 to 0)	0 (0 to 0)	0 (0 to 0)	0 (0 to 0)	0 (0 to 0)	0 (0 to 0)	0 (0 to 0)	0 (0 to 0)	0 (0 to 0)	0 (0 to 0)	0 (0 to 0)	0
HIV/AIDS resulting in other diseases	0 (0 to 0)	0 (0 to 0)	4·5 (4·2 to 4·9)	2·7 (2·5 to 2·9)	2·6 (2·4 to 2·8)	4·7 (4·3 to 5·1)	7·9 (7·2 to 8·6)	21·9 (19·9 to 24·2)	47·5 (43·3 to 52·4)	60·6 (55·5 to 65·7)	58·6 (53·9 to 63·7)	36·2 (33·3 to 40·1)	34·7 (31·8 to 37·6)	19·7 (18·1 to 21·4)	10·1 (9·2 to 11)	7·5 (6·8 to 8·1)	4 (3·6 to 4·5)	1·2 (1·1 to 1·4)	0·7 (0·6 to 0·8)	0·4 (0·4 to 0·5)	0·3 (0·2 to 0·3)	0 (0 to 0)	0 (0 to 0)	326
Sexually transmitted infections excluding HIV	0 (0 to 0)	0 (0 to 0)	0 (0 to 0)	0 (0 to 0)	0 (0 to 0)	0·1 (0·1 to 0·1)	0·4 (0·3 to 0·7)	1·1 (0·6 to 2)	1·7 (0·9 to 3·1)	2·1 (1·1 to 3·9)	2·4 (1·3 to 4·4)	2·5 (1·4 to 4·7)	1·9 (1 to 3·5)	1·2 (0·6 to 2·4)	0·9 (0·4 to 1·8)	0·6 (0·3 to 1·3)	0·4 (0·2 to 0·8)	0·3 (0·1 to 0·5)	0·2 (0·1 to 0·4)	0·1 (0·1 to 0·2)	0·1 (0 to 0·1)	0 (0 to 0)	0 (0 to 0)	16·1
Syphilis	0 (0 to 0)	0 (0 to 0)	0 (0 to 0)	0 (0 to 0)	0 (0 to 0)	0 (0 to 0)	0 (0 to 0)	0 (0 to 0·1)	0 (0 to 0·1)	0·1 (0 to 0·1)	0·1 (0 to 0·1)	0 (0 to 0)	0 (0 to 0·1)	0 (0 to 0)	0 (0 to 0)	0 (0 to 0)	0 (0 to 0)	0 (0 to 0)	0 (0 to 0)	0 (0 to 0)	0 (0 to 0)	0 (0 to 0)	0 (0 to 0)	0·5
Chlamydial infection	0 (0 to 0)	0 (0 to 0)	0 (0 to 0)	0 (0 to 0)	0 (0 to 0)	0 (0 to 0)	0 (0 to 0·1)	0·1 (0·1 to 0·2)	0·2 (0·1 to 0·3)	0·2 (0·1 to 0·3)	0·2 (0·1 to 0·4)	0·3 (0·2 to 0·5)	0·2 (0·1 to 0·3)	0·1 (0 to 0·1)	0 (0 to 0)	0 (0 to 0)	0 (0 to 0)	0 (0 to 0)	0 (0 to 0)	0 (0 to 0)	0 (0 to 0)	0 (0 to 0)	0 (0 to 0)	1·3
Gonococcal infection	0 (0 to 0)	0 (0 to 0)	0 (0 to 0)	0 (0 to 0)	0 (0 to 0)	0 (0 to 0)	0 (0 to 0·1)	0·1 (0·1 to 0·1)	0·1 (0·1 to 0·2)	0·2 (0·1 to 0·2)	0·2 (0·1 to 0·2)	0·2 (0·2 to 0·3)	0·2 (0·1 to 0·3)	0·1 (0·1 to 0·1)	0 (0 to 0·1)	0 (0 to 0)	0 (0 to 0)	0 (0 to 0)	0 (0 to 0)	0 (0 to 0)	0 (0 to 0)	0 (0 to 0)	0 (0 to 0)	1·3
Trichomoniasis	0 (0 to 0)	0 (0 to 0)	0 (0 to 0)	0 (0 to 0)	0 (0 to 0)	0 (0 to 0)	0·1 (0 to 0·2)	0·4 (0·1 to 0·9)	0·7 (0·2 to 1·5)	0·8 (0·2 to 1·8)	0·8 (0·3 to 1·8)	0·7 (0·2 to 1·8)	0·6 (0·2 to 1·4)	0·5 (0·2 to 1·2)	0·4 (0·1 to 0·9)	0·2 (0·1 to 0·5)	0·1 (0 to 0·3)	0·1 (0 to 0·2)	0·1 (0 to 0·1)	0 (0 to 0·1)	0 (0 to 0)	0 (0 to 0)	0 (0 to 0)	5·4
Genital herpes	0 (0 to 0)	[illegible]	[illegible]	[illegible]	[illegible]	[illegible]	[illegible]	[illegible]	[illegible]	[illegible]	[illegible]	[illegible]	[illegible]	[illegible]	[illegible]	[illegible]	[illegible]	[illegible]	[illegible]	[illegible]	[illegible]	[illegible]	[illegible]	4·2
Other sexually transmitted infections	0 (0 to 0)	[illegible]	[illegible]	[illegible]	[illegible]	[illegible]	[illegible]	[illegible]	[illegible]	[illegible]	[illegible]	[illegible]	[illegible]	[illegible]	[illegible]	[illegible]	[illegible]	[illegible]	[illegible]	[illegible]	[illegible]	[illegible]	[illegible]	3·4
Respiratory infections and tuberculosis	6·9 (4·2 to 11·3)	[illegible]	[illegible]	[illegible]	[illegible]	[illegible]	[illegible]	[illegible]	[illegible]	[illegible]	[illegible]	[illegible]	[illegible]	[illegible]	[illegible]	[illegible]	[illegible]	[illegible]	[illegible]	[illegible]	[illegible]	[illegible]	[illegible]	430
Tuberculosis	0 (0 to 0)	[illegible]	[illegible]	[illegible]	[illegible]	[illegible]	[illegible]	[illegible]	[illegible]	[illegible]	[illegible]	[illegible]	[illegible]	[illegible]	[illegible]	[illegible]	[illegible]	[illegible]	[illegible]	[illegible]	[illegible]	[illegible]	[illegible]	19·7
Latent tuberculosis infection	0 (0 to 0)	[illegible]	[illegible]	[illegible]	[illegible]	[illegible]	[illegible]	[illegible]	[illegible]	[illegible]	[illegible]	[illegible]	[illegible]	[illegible]	[illegible]	[illegible]	[illegible]	[illegible]	[illegible]	[illegible]	[illegible]	[illegible]	[illegible]	0
Drug-susceptible tuberculosis	0 (0 to 0)	[illegible]	[illegible]	[illegible]	[illegible]	[illegible]	[illegible]	[illegible]	[illegible]	[illegible]	[illegible]	[illegible]	[illegible]	[illegible]	[illegible]	[illegible]	[illegible]	[illegible]	[illegible]	[illegible]	[illegible]	[illegible]	[illegible]	19·5
Multidrug-resistant tuberculosis without extensive drug resistanc	0 (0 to 0)	[illegible]	[illegible]	[illegible]	[illegible]	[illegible]	[illegible]	[illegible]	[illegible]	[illegible]	[illegible]	[illegible]	[illegible]	[illegible]	[illegible]	[illegible]	[illegible]	[illegible]	[illegible]	[illegible]	[illegible]	[illegible]	[illegible]	0·2
Extensively drug-resistant tuberculosis	0 (0 to 0)	[illegible]	[illegible]	[illegible]	[illegible]	[illegible]	[illegible]	[illegible]	[illegible]	[illegible]	[illegible]	[illegible]	[illegible]	[illegible]	[illegible]	[illegible]	[illegible]	[illegible]	[illegible]	[illegible]	[illegible]	[illegible]	[illegible]	0
Lower respiratory infections	6·9 (4·2 to 11·3)	[illegible]	[illegible]	[illegible]	[illegible]	[illegible]	[illegible]	[illegible]	[illegible]	[illegible]	[illegible]	[illegible]	[illegible]	[illegible]	[illegible]	[illegible]	[illegible]	[illegible]	[illegible]	[illegible]	[illegible]	[illegible]	[illegible]	351
Upper respiratory infections	0 (0 to 0)	[illegible]	[illegible]	[illegible]	[illegible]	[illegible]	[illegible]	[illegible]	[illegible]	[illegible]	[illegible]	[illegible]	[illegible]	[illegible]	[illegible]	[illegible]	[illegible]	[illegible]	[illegible]	[illegible]	[illegible]	[illegible]	[illegible]	45·5
Otitis media	0 (0 to 0)	[illegible]	[illegible]	[illegible]	[illegible]	[illegible]	[illegible]	[illegible]	[illegible]	[illegible]	[illegible]	[illegible]	[illegible]	[illegible]	[illegible]	[illegible]	[illegible]	[illegible]	[illegible]	[illegible]	[illegible]	[illegible]	[illegible]	13·9
Enteric infections	1 (0·6 to 1·6)	[illegible]	[illegible]	[illegible]	[illegible]	[illegible]	[illegible]	[illegible]	[illegible]	[illegible]	[illegible]	[illegible]	[illegible]	[illegible]	[illegible]	[illegible]	[illegible]	[illegible]	[illegible]	[illegible]	[illegible]	[illegible]	[illegible]	159
Diarrheal diseases	1 (0·6 to 1·6)	[illegible]	[illegible]	[illegible]	[illegible]	[illegible]	[illegible]	[illegible]	[illegible]	[illegible]	[illegible]	[illegible]	[illegible]	[illegible]	[illegible]	[illegible]	[illegible]	[illegible]	[illegible]	[illegible]	[illegible]	[illegible]	[illegible]	158
Typhoid and paratyphoid	0 (0 to 0)	[illegible]	[illegible]	[illegible]	[illegible]	[illegible]	[illegible]	[illegible]	[illegible]	[illegible]	[illegible]	[illegible]	[illegible]	[illegible]	[illegible]	[illegible]	[illegible]	[illegible]	[illegible]	[illegible]	[illegible]	[illegible]	[illegible]	0·1
Typhoid fever	0 (0 to 0)	[illegible]	[illegible]	[illegible]	[illegible]	[illegible]	[illegible]	[illegible]	[illegible]	[illegible]	[illegible]	[illegible]	[illegible]	[illegible]	[illegible]	[illegible]	[illegible]	[illegible]	[illegible]	[illegible]	[illegible]	[illegible]	[illegible]	0
Paratyphoid fever	0 (0 to 0)	[illegible]	[illegible]	[illegible]	[illegible]	[illegible]	[illegible]	[illegible]	[illegible]	[illegible]	[illegible]	[illegible]	[illegible]	[illegible]	[illegible]	[illegible]	[illegible]	[illegible]	[illegible]	[illegible]	[illegible]	[illegible]	[illegible]	0·1
Invasive Non-typhoidal Salmonella (iNTS)	0 (0 to 0)	[illegible]	[illegible]	[illegible]	[illegible]	[illegible]	[illegible]	[illegible]	[illegible]	[illegible]	[illegible]	[illegible]	[illegible]	[illegible]	[illegible]	[illegible]	[illegible]	[illegible]	[illegible]	[illegible]	[illegible]	[illegible]	[illegible]	0·4
Other intestinal infectious diseases	0 (0 to 0)	[illegible]	[illegible]	[illegible]	[illegible]	[illegible]	[illegible]	[illegible]	[illegible]	[illegible]	[illegible]	[illegible]	[illegible]	[illegible]	[illegible]	[illegible]	[illegible]	[illegible]	[illegible]	[illegible]	[illegible]	[illegible]	[illegible]	0·3
Neglected tropical diseases and malaria	0·1 (0 to 0·2)	[illegible]	[illegible]	[illegible]	[illegible]	[illegible]	[illegible]	[illegible]	[illegible]	[illegible]	[illegible]	[illegible]	[illegible]	[illegible]	[illegible]	[illegible]	[illegible]	[illegible]	[illegible]	[illegible]	[illegible]	[illegible]	[illegible]	67·7
Malaria	0 (0 to 0)	[illegible]	[illegible]	[illegible]	[illegible]	[illegible]	[illegible]	[illegible]	[illegible]	[illegible]	[illegible]	[illegible]	[illegible]	[illegible]	[illegible]	[illegible]	[illegible]	[illegible]	[illegible]	[illegible]	[illegible]	[illegible]	[illegible]	0
Chagas disease	0 (0 to 0)	[illegible]	[illegible]	[illegible]	[illegible]	[illegible]	[illegible]	[illegible]	[illegible]	[illegible]	[illegible]	[illegible]	[illegible]	[illegible]	[illegible]	[illegible]	[illegible]	[illegible]	[illegible]	[illegible]	[illegible]	[illegible]	[illegible]	0
Leishmaniasis	0 (0 to 0)	[illegible]	[illegible]	[illegible]	[illegible]	[illegible]	[illegible]	[illegible]	[illegible]	[illegible]	[illegible]	[illegible]	[illegible]	[illegible]	[illegible]	[illegible]	[illegible]	[illegible]	[illegible]	[illegible]	[illegible]	[illegible]	[illegible]	0
Visceral leishmaniasis	0 (0 to 0)	[illegible]	[illegible]	[illegible]	[illegible]	[illegible]	[illegible]	[illegible]	[illegible]	[illegible]	[illegible]	[illegible]	[illegible]	[illegible]	[illegible]	[illegible]	[illegible]	[illegible]	[illegible]	[illegible]	[illegible]	[illegible]	[illegible]	0
Cutaneous and mucocutaneous leishmaniasis	0 (0 to 0)	[illegible]	[illegible]	[illegible]	[illegible]	[illegible]	[illegible]	[illegible]	[illegible]	[illegible]	[illegible]	[illegible]	[illegible]	[illegible]	[illegible]	[illegible]	[illegible]	[illegible]	[illegible]	[illegible]	[illegible]	[illegible]	[illegible]	0
African trypanosomiasis	0 (0 to 0)	[illegible]	[illegible]	[illegible]	[illegible]	[illegible]	[illegible]	[illegible]	[illegible]	[illegible]	[illegible]	[illegible]	[illegible]	[illegible]	[illegible]	[illegible]	[illegible]	[illegible]	[illegible]	[illegible]	[illegible]	[illegible]	[illegible]	0
Schistosomiasis	0 (0 to 0)	[illegible]	[illegible]	[illegible]	[illegible]	[illegible]	[illegible]	[illegible]	[illegible]	[illegible]	[illegible]	[illegible]	[illegible]	[illegible]	[illegible]	[illegible]	[illegible]	[illegible]	[illegible]	[illegible]	[illegible]	[illegible]	[illegible]	0
Cysticercosis	0 (0 to 0)	[illegible]	[illegible]	[illegible]	[illegible]	[illegible]	[illegible]	[illegible]	[illegible]	[illegible]	[illegible]	[illegible]	[illegible]	[illegible]	[illegible]	[illegible]	[illegible]	[illegible]	[illegible]	[illegible]	[illegible]	[illegible]	[illegible]	34·1
Cystic echinococcosis	0 (0 to 0)	[illegible]	[illegible]	[illegible]	[illegible]	[illegible]	[illegible]	[illegible]	[illegible]	[illegible]	[illegible]	[illegible]	[illegible]	[illegible]	[illegible]	[illegible]	[illegible]	[illegible]	[illegible]	[illegible]	[illegible]	[illegible]	[illegible]	0
Lymphatic filariasis	0 (0 to 0)	[illegible]	[illegible]	[illegible]	[illegible]	[illegible]	[illegible]	[illegible]	[illegible]	[illegible]	[illegible]	[illegible]	[illegible]	[illegible]	[illegible]	[illegible]	[illegible]	[illegible]	[illegible]	[illegible]	[illegible]	[illegible]	[illegible]	0
Onchocerciasis	0 (0 to 0)	[illegible]	[illegible]	[illegible]	[illegible]	[illegible]	[illegible]	[illegible]	[illegible]	[illegible]	[illegible]	[illegible]	[illegible]	[illegible]	[illegible]	[illegible]	[illegible]	[illegible]	[illegible]	[illegible]	[illegible]	[illegible]	[illegible]	0
Trachoma	0 (0 to 0)	[illegible]	[illegible]	[illegible]	[illegible]	[illegible]	[illegible]	[illegible]	[illegible]	[illegible]	[illegible]	[illegible]	[illegible]	[illegible]	[illegible]	[illegible]	[illegible]	[illegible]	[illegible]	[illegible]	[illegible]	[illegible]	[illegible]	0
Dengue	0 (0 to 0)	[illegible]	[illegible]	[illegible]	[illegible]	[illegible]	[illegible]	[illegible]	[illegible]	[illegible]	[illegible]	[illegible]	[illegible]	[illegible]	[illegible]	[illegible]	[illegible]	[illegible]	[illegible]	[illegible]	[illegible]	[illegible]	[illegible]	6·3
Yellow fever	0 (0 to 0)	[illegible]	[illegible]	[illegible]	[illegible]	[illegible]	[illegible]	[illegible]	[illegible]	[illegible]	[illegible]	[illegible]	[illegible]	[illegible]	[illegible]	[illegible]	[illegible]	[illegible]	[illegible]	[illegible]	[illegible]	[illegible]	[illegible]	0
Rabies	0 (0 to 0)	[illegible]	[illegible]	[illegible]	[illegible]	[illegible]	[illegible]	[illegible]	[illegible]	[illegible]	[illegible]	[illegible]	[illegible]	[illegible]	[illegible]	[illegible]	[illegible]	[illegible]	[illegible]	[illegible]	[illegible]	[illegible]	[illegible]	0·2
Intestinal nematode infections	0 (0 to 0)	[illegible]	[illegible]	[illegible]	[illegible]	[illegible]	[illegible]	[illegible]	[illegible]	[illegible]	[illegible]	[illegible]	[illegible]	[illegible]	[illegible]	[illegible]	[illegible]	[illegible]	[illegible]	[illegible]	[illegible]	[illegible]	[illegible]	6·8
Ascariasis	0 (0 to 0)	[illegible]	[illegible]	[illegible]	[illegible]	[illegible]	[illegible]	[illegible]	[illegible]	[illegible]	[illegible]	[illegible]	[illegible]	[illegible]	[illegible]	[illegible]	[illegible]	[illegible]	[illegible]	[illegible]	[illegible]	[illegible]	[illegible]	0·5
Trichuriasis	0 (0 to 0)	[illegible]	[illegible]	[illegible]	[illegible]	[illegible]	[illegible]	[illegible]	[illegible]	[illegible]	[illegible]	[illegible]	[illegible]	[illegible]	[illegible]	[illegible]	[illegible]	[illegible]	[illegible]	[illegible]	[illegible]	[illegible]	[illegible]	0
Hookworm disease	0 (0 to 0)	[illegible]	[illegible]	[illegible]	[illegible]	[illegible]	[illegible]	[illegible]	[illegible]	[illegible]	[illegible]	[illegible]	[illegible]	[illegible]	[illegible]	[illegible]	[illegible]	[illegible]	[illegible]	[illegible]	[illegible]	[illegible]	[illegible]	6·2
Food-borne trematodiases	0 (0 to 0)	[illegible]	[illegible]	[illegible]	[illegible]	[illegible]	[illegible]	[illegible]	[illegible]	[illegible]	[illegible]	[illegible]	[illegible]	[illegible]	[illegible]	[illegible]	[illegible]	[illegible]	[illegible]	[illegible]	[illegible]	[illegible]	[illegible]	0
Leprosy	0 (0 to 0)	[illegible]	[illegible]	[illegible]	[illegible]	[illegible]	[illegible]	[illegible]	[illegible]	[illegible]	[illegible]	[illegible]	[illegible]	[illegible]	[illegible]	[illegible]	[illegible]	[illegible]	[illegible]	[illegible]	[illegible]	[illegible]	[illegible]	0·2
Ebola	0 (0 to 0)	[illegible]	[illegible]	[illegible]	[illegible]	[illegible]	[illegible]	[illegible]	[illegible]	[illegible]	[illegible]	[illegible]	[illegible]	[illegible]	[illegible]	[illegible]	[illegible]	[illegible]	[illegible]	[illegible]	[illegible]	[illegible]	[illegible]	0
Zika virus	0 (0 to 0)	[illegible]	[illegible]	[illegible]	[illegible]	[illegible]	[illegible]	[illegible]	[illegible]	[illegible]	[illegible]	[illegible]	[illegible]	[illegible]	[illegible]	[illegible]	[illegible]	[illegible]	[illegible]	[illegible]	[illegible]	[illegible]	[illegible]	1
Guinea worm disease	0 (0 to 0)	[illegible]	[illegible]	[illegible]	[illegible]	[illegible]	[illegible]	[illegible]	[illegible]	[illegible]	[illegible]	[illegible]	[illegible]	[illegible]	[illegible]	[illegible]	[illegible]	[illegible]	[illegible]	[illegible]	[illegible]	[illegible]	[illegible]	0
Other neglected tropical diseases	0·1 (0 to 0·2)	[illegible]	[illegible]	[illegible]	[illegible]	[illegible]	[illegible]	[illegible]	[illegible]	[illegible]	[illegible]	[illegible]	[illegible]	[illegible]	[illegible]	[illegible]	[illegible]	[illegible]	[illegible]	[illegible]	[illegible]	[illegible]	[illegible]	19·2
Other infectious diseases	0·8 (0·5 to 1·2)	[illegible]	[illegible]	[illegible]	[illegible]	[illegible]	[illegible]	[illegible]	[illegible]	[illegible]	[illegible]	[illegible]	[illegible]	[illegible]	[illegible]	[illegible]	[illegible]	[illegible]	[illegible]	[illegible]	[illegible]	[illegible]	[illegible]	74·6
Meningitis	0·5 (0·3 to 0·9)	[illegible]	[illegible]	[illegible]	[illegible]	[illegible]	[illegible]	[illegible]	[illegible]	[illegible]	[illegible]	[illegible]	[illegible]	[illegible]	[illegible]	[illegible]	[illegible]	[illegible]	[illegible]	[illegible]	[illegible]	[illegible]	[illegible]	24
Encephalitis	0·1 (0·1 to 0·2)	[illegible]	[illegible]	[illegible]	[illegible]	[illegible]	[illegible]	[illegible]	[illegible]	[illegible]	[illegible]	[illegible]	[illegible]	[illegible]	[illegible]	[illegible]	[illegible]	[illegible]	[illegible]	[illegible]	[illegible]	[illegible]	[illegible]	12·4
Diphtheria	0 (0 to 0)	[illegible]	[illegible]	[illegible]	[illegible]	[illegible]	[illegible]	[illegible]	[illegible]	[illegible]	[illegible]	[illegible]	[illegible]	[illegible]	[illegible]	[illegible]	[illegible]	[illegible]	[illegible]	[illegible]	[illegible]	[illegible]	[illegible]	0·7
Tetanus	0 (0 to 0)	[illegible]	[illegible]	[illegible]	[illegible]	[illegible]	[illegible]	[illegible]	[illegible]	[illegible]	[illegible]	[illegible]	[illegible]	[illegible]	[illegible]	[illegible]	[illegible]	[illegible]	[illegible]	[illegible]	[illegible]	[illegible]	[illegible]	0
Measles	0 (0 to 0)	[illegible]	[illegible]	[illegible]	[illegible]	[illegible]	[illegible]	[illegible]	[illegible]	[illegible]	[illegible]	[illegible]	[illegible]	[illegible]	[illegible]	[illegible]	[illegible]	[illegible]	[illegible]	[illegible]	[illegible]	[illegible]	[illegible]	0
Varicella and herpes zoster	0 (0 to 0)	[illegible]	[illegible]	[illegible]	[illegible]	[illegible]	[illegible]	[illegible]	[illegible]	[illegible]	[illegible]	[illegible]	[illegible]	[illegible]	[illegible]	[illegible]	[illegible]	[illegible]	[illegible]	[illegible]	[illegible]	[illegible]	[illegible]	3·2
Acute hepatitis	0 (0 to 0)	[illegible]	[illegible]	[illegible]	[illegible]	[illegible]	[illegible]	[illegible]	[illegible]	[illegible]	[illegible]	[illegible]	[illegible]	[illegible]	[illegible]	[illegible]	[illegible]	[illegible]	[illegible]	[illegible]	[illegible]	[illegible]	[illegible]	3·4
Acute hepatitis A	0 (0 to 0)	[illegible]	[illegible]	[illegible]	[illegible]	[illegible]	[illegible]	[illegible]	[illegible]	[illegible]	[illegible]	[illegible]	[illegible]	[illegible]	[illegible]	[illegible]	[illegible]	[illegible]	[illegible]	[illegible]	[illegible]	[illegible]	[illegible]	2·1
Acute hepatitis B	0 (0 to 0)	[illegible]	[illegible]	[illegible]	[illegible]	[illegible]	[illegible]	[illegible]	[illegible]	[illegible]	[illegible]	[illegible]	[illegible]	[illegible]	[illegible]	[illegible]	[illegible]	[illegible]	[illegible]	[illegible]	[illegible]	[illegible]	[illegible]	0·9
Acute hepatitis C	0 (0 to 0)	[illegible]	[illegible]	[illegible]	[illegible]	[illegible]	[illegible]	[illegible]	[illegible]	[illegible]	[illegible]	[illegible]	[illegible]	[illegible]	[illegible]	[illegible]	[illegible]	[illegible]	[illegible]	[illegible]	[illegible]	[illegible]	[illegible]	0·1
Acute hepatitis E	0 (0 to 0)	[illegible]	[illegible]	[illegible]	[illegible]	[illegible]	[illegible]	[illegible]	[illegible]	[illegible]	[illegible]	[illegible]	[illegible]	[illegible]	[illegible]	[illegible]	[illegible]	[illegible]	[illegible]	[illegible]	[illegible]	[illegible]	[illegible]	0·3
Other unspecified infectious diseases	0·2 (0·1 to 0·3)	[illegible]	[illegible]	[illegible]	[illegible]	[illegible]	[illegible]	[illegible]	[illegible]	[illegible]	[illegible]	[illegible]	[illegible]	[illegible]	[illegible]	[illegible]	[illegible]	[illegible]	[illegible]	[illegible]	[illegible]	[illegible]	[illegible]	30·4
Maternal and neonatal disorders	302 (196 to 432)	[illegible]	[illegible]	[illegible]	[illegible]	[illegible]	[illegible]	[illegible]	[illegible]	[illegible]	[illegible]	[illegible]	[illegible]	[illegible]	[illegible]	[illegible]	[illegible]	[illegible]	[illegible]	[illegible]	[illegible]	[illegible]	[illegible]	736
Maternal disorders	0 (0 to 0)	[illegible]	[illegible]	[illegible]	[illegible]	[illegible]	[illegible]	[illegible]	[illegible]	[illegible]	[illegible]	[illegible]	[illegible]	[illegible]	[illegible]	[illegible]	[illegible]	[illegible]	[illegible]	[illegible]	[illegible]	[illegible]	[illegible]	81·9
Maternal hemorrhage	0 (0 to 0)	[illegible]	[illegible]	[illegible]	[illegible]	[illegible]	[illegible]	[illegible]	[illegible]	[illegible]	[illegible]	[illegible]	[illegible]	[illegible]	[illegible]	[illegible]	[illegible]	[illegible]	[illegible]	[illegible]	[illegible]	[illegible]	[illegible]	7
Maternal sepsis and other maternal infections	0 (0 to 0)	[illegible]	[illegible]	[illegible]	[illegible]	[illegible]	[illegible]	[illegible]	[illegible]	[illegible]	[illegible]	[illegible]	[illegible]	[illegible]	[illegible]	[illegible]	[illegible]	[illegible]	[illegible]	[illegible]	[illegible]	[illegible]	[illegible]	1·6
Maternal hypertensive disorders	0 (0 to 0)	[illegible]	[illegible]	[illegible]	[illegible]	[illegible]	[illegible]	[illegible]	[illegible]	[illegible]	[illegible]	[illegible]	[illegible]	[illegible]	[illegible]	[illegible]	[illegible]	[illegible]	[illegible]	[illegible]	[illegible]	[illegible]	[illegible]	21·2
Maternal obstructed labor and uterine rupture	0 (0 to 0)	[illegible]	[illegible]	[illegible]	[illegible]	[illegible]	[illegible]	[illegible]	[illegible]	[illegible]	[illegible]	[illegible]	[illegible]	[illegible]	[illegible]	[illegible]	[illegible]	[illegible]	[illegible]	[illegible]	[illegible]	[illegible]	[illegible]	3·6
Maternal abortion and miscarriage	0 (0 to 0)	[illegible]	[illegible]	[illegible]	[illegible]	[illegible]	[illegible]	[illegible]	[illegible]	[illegible]	[illegible]	[illegible]	[illegible]	[illegible]	[illegible]	[illegible]	[illegible]	[illegible]	[illegible]	[illegible]	[illegible]	[illegible]	[illegible]	4·7
Ectopic pregnancy	0 (0 to 0)	[illegible]	[illegible]	[illegible]	[illegible]	[illegible]	[illegible]	[illegible]	[illegible]	[illegible]	[illegible]	[illegible]	[illegible]	[illegible]	[illegible]	[illegible]	[illegible]	[illegible]	[illegible]	[illegible]	[illegible]	[illegible]	[illegible]	8
Indirect maternal deaths	--	[illegible]	[illegible]	[illegible]	[illegible]	[illegible]	[illegible]	[illegible]	[illegible]	[illegible]	[illegible]	[illegible]	[illegible]	[illegible]	[illegible]	[illegible]	[illegible]	[illegible]	[illegible]	[illegible]	[illegible]	[illegible]	[illegible]	NA
Late maternal deaths	--	[illegible]	[illegible]	[illegible]	[illegible]	[illegible]	[illegible]	[illegible]	[illegible]	[illegible]	[illegible]	[illegible]	[illegible]	[illegible]	[illegible]	[illegible]	[illegible]	[illegible]	[illegible]	[illegible]	[illegible]	[illegible]	[illegible]	NA
Maternal deaths aggravated by HIV/AIDS	--	[illegible]	[illegible]	[illegible]	[illegible]	[illegible]	[illegible]	[illegible]	[illegible]	[illegible]	[illegible]	[illegible]	[illegible]	[illegible]	[illegible]	[illegible]	[illegible]	[illegible]	[illegible]	[illegible]	[illegible]	[illegible]	[illegible]	NA
Neonatal disorders	302 (196 to 432)	[illegible]	[illegible]	[illegible]	[illegible]	[illegible]	[illegible]	[illegible]	[illegible]	[illegible]	[illegible]	[illegible]	[illegible]	[illegible]	[illegible]	[illegible]	[illegible]	[illegible]	[illegible]	[illegible]	[illegible]	[illegible]	[illegible]	654
Neonatal preterm birth	159 (100 to 232)	[illegible]	[illegible]	[illegible]	[illegible]	[illegible]	[illegible]	[illegible]	[illegible]	[illegible]	[illegible]	[illegible]	[illegible]	[illegible]	[illegible]	[illegible]	[illegible]	[illegible]	[illegible]	[illegible]	[illegible]	[illegible]	[illegible]	302
Neonatal encephalopathy due to birth asphyxia and trauma	52·4 (32·4 to 80·8)	[illegible]	[illegible]	[illegible]	[illegible]	[illegible]	[illegible]	[illegible]	[illegible]	[illegible]	[illegible]	[illegible]	[illegible]	[illegible]	[illegible]	[illegible]	[illegible]	[illegible]	[illegible]	[illegible]	[illegible]	[illegible]	[illegible]	82·8
Neonatal sepsis and other neonatal infections	58·9 (35 to 90·6)	[illegible]	[illegible]	[illegible]	[illegible]	[illegible]	[illegible]	[illegible]	[illegible]	[illegible]	[illegible]	[illegible]	[illegible]	[illegible]	[illegible]	[illegible]	[illegible]	[illegible]	[illegible]	[illegible]	[illegible]	[illegible]	[illegible]	197
Hemolytic disease and other neonatal jaundice	2·5 (1·3 to 4·3)	[illegible]	[illegible]	[illegible]	[illegible]	[illegible]	[illegible]	[illegible]	[illegible]	[illegible]	[illegible]	[illegible]	[illegible]	[illegible]	[illegible]	[illegible]	[illegible]	[illegible]	[illegible]	[illegible]	[illegible]	[illegible]	[illegible]	13·5
Other neonatal disorders	29·6 (16·5 to 48)	[illegible]	[illegible]	[illegible]	[illegible]	[illegible]	[illegible]	[illegible]	[illegible]	[illegible]	[illegible]	[illegible]	[illegible]	[illegible]	[illegible]	[illegible]	[illegible]	[illegible]	[illegible]	[illegible]	[illegible]	[illegible]	[illegible]	58·6
Nutritional deficiencies	0 (0 to 0)	[illegible]	[illegible]	[illegible]	[illegible]	[illegible]	[illegible]	[illegible]	[illegible]	[illegible]	[illegible]	[illegible]	[illegible]	[illegible]	[illegible]	[illegible]	[illegible]	[illegible]	[illegible]	[illegible]	[illegible]	[illegible]	[illegible]	362
Protein-energy malnutrition	0 (0 to 0)	[illegible]	[illegible]	[illegible]	[illegible]	[illegible]	[illegible]	[illegible]	[illegible]	[illegible]	[illegible]	[illegible]	[illegible]	[illegible]	[illegible]	[illegible]	[illegible]	[illegible]	[illegible]	[illegible]	[illegible]	[illegible]	[illegible]	80·6
Iodine deficiency	0 (0 to 0)	[illegible]	[illegible]	[illegible]	[illegible]	[illegible]	[illegible]	[illegible]	[illegible]	[illegible]	[illegible]	[illegible]	[illegible]	[illegible]	[illegible]	[illegible]	[illegible]	[illegible]	[illegible]	[illegible]	[illegible]	[illegible]	[illegible]	3·6
Vitamin A deficiency	0 (0 to 0)	[illegible]	[illegible]	[illegible]	[illegible]	[illegible]	[illegible]	[illegible]	[illegible]	[illegible]	[illegible]	[illegible]	[illegible]	[illegible]	[illegible]	[illegible]	[illegible]	[illegible]	[illegible]	[illegible]	[illegible]	[illegible]	[illegible]	3·5
Dietary iron deficiency	0 (0 to 0)	[illegible]	[illegible]	[illegible]	[illegible]	[illegible]	[illegible]	[illegible]	[illegible]	[illegible]	[illegible]	[illegible]	[illegible]	[illegible]	[illegible]	[illegible]	[illegible]	[illegible]	[illegible]	[illegible]	[illegible]	[illegible]	[illegible]	265
Other nutritional deficiencies	0 (0 to 0)	[illegible]	[illegible]	[illegible]	[illegible]	[illegible]	[illegible]	[illegible]	[illegible]	[illegible]	[illegible]	[illegible]	[illegible]	[illegible]	[illegible]	[illegible]	[illegible]	[illegible]	[illegible]	[illegible]	[illegible]	[illegible]	[illegible]	8·8
Non-communicable diseases	45·2 (26·7 to 74·8)	[illegible]	[illegible]	[illegible]	[illegible]	[illegible]	[illegible]	[illegible]	[illegible]	[illegible]	[illegible]	[illegible]	[illegible]	[illegible]	[illegible]	[illegible]	[illegible]	[illegible]	[illegible]	[illegible]	[illegible]	[illegible]	[illegible]	14,100
Neoplasms	0·4 (0·3 to 0·7)	[illegible]	[illegible]	[illegible]	[illegible]	[illegible]	[illegible]	[illegible]	[illegible]	[illegible]	[illegible]	[illegible]	[illegible]	[illegible]	[illegible]	[illegible]	[illegible]	[illegible]	[illegible]	[illegible]	[illegible]	[illegible]	[illegible]	2,350
Lip and oral cavity cancer	0 (0 to 0)	[illegible]	[illegible]	[illegible]	[illegible]	[illegible]	[illegible]	[illegible]	[illegible]	[illegible]	[illegible]	[illegible]	[illegible]	[illegible]	[illegible]	[illegible]	[illegible]	[illegible]	[illegible]	[illegible]	[illegible]	[illegible]	[illegible]	23·5
Nasopharynx cancer	0 (0 to 0)	[illegible]	[illegible]	[illegible]	[illegible]	[illegible]	[illegible]	[illegible]	[illegible]	[illegible]	[illegible]	[illegible]	[illegible]	[illegible]	[illegible]	[illegible]	[illegible]	[illegible]	[illegible]	[illegible]	[illegible]	[illegible]	[illegible]	8·5
Other pharynx cancer	0 (0 to 0)	[illegible]	[illegible]	[illegible]	[illegible]	[illegible]	[illegible]	[illegible]	[illegible]	[illegible]	[illegible]	[illegible]	[illegible]	[illegible]	[illegible]	[illegible]	[illegible]	[illegible]	[illegible]	[illegible]	[illegible]	[illegible]	[illegible]	8·4
Esophageal cancer	0 (0 to 0)	[illegible]	[illegible]	[illegible]	[illegible]	[illegible]	[illegible]	[illegible]	[illegible]	[illegible]	[illegible]	[illegible]	[illegible]	[illegible]	[illegible]	[illegible]	[illegible]	[illegible]	[illegible]	[illegible]	[illegible]	[illegible]	[illegible]	9·3
Stomach cancer	0 (0 to 0)	[illegible]	[illegible]	[illegible]	[illegible]	[illegible]	[illegible]	[illegible]	[illegible]	[illegible]	[illegible]	[illegible]	[illegible]	[illegible]	[illegible]	[illegible]	[illegible]	[illegible]	[illegible]	[illegible]	[illegible]	[illegible]	[illegible]	119
Colon and rectum cancer	0 (0 to 0)	[illegible]	[illegible]	[illegible]	[illegible]	[illegible]	[illegible]	[illegible]	[illegible]	[illegible]	[illegible]	[illegible]	[illegible]	[illegible]	[illegible]	[illegible]	[illegible]	[illegible]	[illegible]	[illegible]	[illegible]	[illegible]	[illegible]	186
Liver cancer	0 (0 to 0)	[illegible]	[illegible]	[illegible]	[illegible]	[illegible]	[illegible]	[illegible]	[illegible]	[illegible]	[illegible]	[illegible]	[illegible]	[illegible]	[illegible]	[illegible]	[illegible]	[illegible]	[illegible]	[illegible]	[illegible]	[illegible]	[illegible]	29·5
Liver cancer due to hepatitis B	0 (0 to 0)	[illegible]	[illegible]	[illegible]	[illegible]	[illegible]	[illegible]	[illegible]	[illegible]	[illegible]	[illegible]	[illegible]	[illegible]	[illegible]	[illegible]	[illegible]	[illegible]	[illegible]	[illegible]	[illegible]	[illegible]	[illegible]	[illegible]	6·7
Liver cancer due to hepatitis C	0 (0 to 0)	[illegible]	[illegible]	[illegible]	[illegible]	[illegible]	[illegible]	[illegible]	[illegible]	[illegible]	[illegible]	[illegible]	[illegible]	[illegible]	[illegible]	[illegible]	[illegible]	[illegible]	[illegible]	[illegible]	[illegible]	[illegible]	[illegible]	8·3
Liver cancer due to alcohol use	0 (0 to 0)	[illegible]	[illegible]	[illegible]	[illegible]	[illegible]	[illegible]	[illegible]	[illegible]	[illegible]	[illegible]	[illegible]	[illegible]	[illegible]	[illegible]	[illegible]	[illegible]	[illegible]	[illegible]	[illegible]	[illegible]	[illegible]	[illegible]	7·2
Liver cancer due to NASH	0 (0 to 0)	[illegible]	[illegible]	[illegible]	[illegible]	[illegible]	[illegible]	[illegible]	[illegible]	[illegible]	[illegible]	[illegible]	[illegible]	[illegible]	[illegible]	[illegible]	[illegible]	[illegible]	[illegible]	[illegible]	[illegible]	[illegible]	[illegible]	3·8
Liver cancer due to other causes	0 (0 to 0)	[illegible]	[illegible]	[illegible]	[illegible]	[illegible]	[illegible]	[illegible]	[illegible]	[illegible]	[illegible]	[illegible]	[illegible]	[illegible]	[illegible]	[illegible]	[illegible]	[illegible]	[illegible]	[illegible]	[illegible]	[illegible]	[illegible]	3·5
Gallbladder and biliary tract cancer	0 (0 to 0)	[illegible]	[illegible]	[illegible]	[illegible]	[illegible]	[illegible]	[illegible]	[illegible]	[illegible]	[illegible]	[illegible]	[illegible]	[illegible]	[illegible]	[illegible]	[illegible]	[illegible]	[illegible]	[illegible]	[illegible]	[illegible]	[illegible]	16
Pancreatic cancer	0 (0 to 0)	[illegible]	[illegible]	[illegible]	[illegible]	[illegible]	[illegible]	[illegible]	[illegible]	[illegible]	[illegible]	[illegible]	[illegible]	[illegible]	[illegible]	[illegible]	[illegible]	[illegible]	[illegible]	[illegible]	[illegible]	[illegible]	[illegible]	84·1
Larynx cancer	0 (0 to 0)	[illegible]	[illegible]	[illegible]	[illegible]	[illegible]	[illegible]	[illegible]	[illegible]	[illegible]	[illegible]	[illegible]	[illegible]	[illegible]	[illegible]	[illegible]	[illegible]	[illegible]	[illegible]	[illegible]	[illegible]	[illegible]	[illegible]	4·5
Tracheal, bronchus, and lung cancer	0 (0 to 0)	[illegible]	[illegible]	[illegible]	[illegible]	[illegible]	[illegible]	[illegible]	[illegible]	[illegible]	[illegible]	[illegible]	[illegible]	[illegible]	[illegible]	[illegible]	[illegible]	[illegible]	[illegible]	[illegible]	[illegible]	[illegible]	[illegible]	119
Malignant skin melanoma	0 (0 to 0)	[illegible]	[illegible]	[illegible]	[illegible]	[illegible]	[illegible]	[illegible]	[illegible]	[illegible]	[illegible]	[illegible]	[illegible]	[illegible]	[illegible]	[illegible]	[illegible]	[illegible]	[illegible]	[illegible]	[illegible]	[illegible]	[illegible]	16·8
Non-melanoma skin cancer	0 (0 to 0)	[illegible]	[illegible]	[illegible]	[illegible]	[illegible]	[illegible]	[illegible]	[illegible]	[illegible]	[illegible]	[illegible]	[illegible]	[illegible]	[illegible]	[illegible]	[illegible]	[illegible]	[illegible]	[illegible]	[illegible]	[illegible]	[illegible]	7·8
Non-melanoma skin cancer (squamous-cell carcinoma)	0 (0 to 0)	[illegible]	[illegible]	[illegible]	[illegible]	[illegible]	[illegible]	[illegible]	[illegible]	[illegible]	[illegible]	[illegible]	[illegible]	[illegible]	[illegible]	[illegible]	[illegible]	[illegible]	[illegible]	[illegible]	[illegible]	[illegible]	[illegible]	7·8
Non-melanoma skin cancer (basal-cell carcinoma)	0 (0 to 0)	[illegible]	[illegible]	[illegible]	[illegible]	[illegible]	[illegible]	[illegible]	[illegible]	[illegible]	[illegible]	[illegible]	[illegible]	[illegible]	[illegible]	[illegible]	[illegible]	[illegible]	[illegible]	[illegible]	[illegible]	[illegible]	[illegible]	0
Breast cancer	0 (0 to 0)	[illegible]	[illegible]	[illegible]	[illegible]	[illegible]	[illegible]	[illegible]	[illegible]	[illegible]	[illegible]	[illegible]	[illegible]	[illegible]	[illegible]	[illegible]	[illegible]	[illegible]	[illegible]	[illegible]	[illegible]	[illegible]	[illegible]	583
Cervical cancer	0 (0 to 0)	[illegible]	[illegible]	[illegible]	[illegible]	[illegible]	[illegible]	[illegible]	[illegible]	[illegible]	[illegible]	[illegible]	[illegible]	[illegible]	[illegible]	[illegible]	[illegible]	[illegible]	[illegible]	[illegible]	[illegible]	[illegible]	[illegible]	420
Uterine cancer	0 (0 to 0)	[illegible]	[illegible]	[illegible]	[illegible]	[illegible]	[illegible]	[illegible]	[illegible]	[illegible]	[illegible]	[illegible]	[illegible]	[illegible]	[illegible]	[illegible]	[illegible]	[illegible]	[illegible]	[illegible]	[illegible]	[illegible]	[illegible]	135
Ovarian cancer	0 (0 to 0)	[illegible]	[illegible]	[illegible]	[illegible]	[illegible]	[illegible]	[illegible]	[illegible]	[illegible]	[illegible]	[illegible]	[illegible]	[illegible]	[illegible]	[illegible]	[illegible]	[illegible]	[illegible]	[illegible]	[illegible]	[illegible]	[illegible]	116
Prostate cancer	0 (0 to 0)	[illegible]	[illegible]	[illegible]	[illegible]	[illegible]	[illegible]	[illegible]	[illegible]	[illegible]	[illegible]	[illegible]	[illegible]	[illegible]	[illegible]	[illegible]	[illegible]	[illegible]	[illegible]	[illegible]	[illegible]	[illegible]	[illegible]	0
Testicular cancer	--	[illegible]	[illegible]	[illegible]	[illegible]	[illegible]	[illegible]	[illegible]	[illegible]	[illegible]	[illegible]	[illegible]	[illegible]	[illegible]	[illegible]	[illegible]	[illegible]	[illegible]	[illegible]	[illegible]	[illegible]	[illegible]	[illegible]	NA
Kidney cancer	0 (0 to 0)	[illegible]	[illegible]	[illegible]	[illegible]	[illegible]	[illegible]	[illegible]	[illegible]	[illegible]	[illegible]	[illegible]	[illegible]	[illegible]	[illegible]	[illegible]	[illegible]	[illegible]	[illegible]	[illegible]	[illegible]	[illegible]	[illegible]	34·7
Bladder cancer	0 (0 to 0)	[illegible]	[illegible]	[illegible]	[illegible]	[illegible]	[illegible]	[illegible]	[illegible]	[illegible]	[illegible]	[illegible]	[illegible]	[illegible]	[illegible]	[illegible]	[illegible]	[illegible]	[illegible]	[illegible]	[illegible]	[illegible]	[illegible]	18·8
Brain and central nervous system cancer	0·1 (0 to 0·1)	[illegible]	[illegible]	[illegible]	[illegible]	[illegible]	[illegible]	[illegible]	[illegible]	[illegible]	[illegible]	[illegible]	[illegible]	[illegible]	[illegible]	[illegible]	[illegible]	[illegible]	[illegible]	[illegible]	[illegible]	[illegible]	[illegible]	34
Thyroid cancer	0 (0 to 0)	[illegible]	[illegible]	[illegible]	[illegible]	[illegible]	[illegible]	[illegible]	[illegible]	[illegible]	[illegible]	[illegible]	[illegible]	[illegible]	[illegible]	[illegible]	[illegible]	[illegible]	[illegible]	[illegible]	[illegible]	[illegible]	[illegible]	22·4
Mesothelioma	0 (0 to 0)	[illegible]	[illegible]	[illegible]	[illegible]	[illegible]	[illegible]	[illegible]	[illegible]	[illegible]	[illegible]	[illegible]	[illegible]	[illegible]	[illegible]	[illegible]	[illegible]	[illegible]	[illegible]	[illegible]	[illegible]	[illegible]	[illegible]	2·2
Hodgkin lymphoma	0 (0 to 0)	[illegible]	[illegible]	[illegible]	[illegible]	[illegible]	[illegible]	[illegible]	[illegible]	[illegible]	[illegible]	[illegible]	[illegible]	[illegible]	[illegible]	[illegible]	[illegible]	[illegible]	[illegible]	[illegible]	[illegible]	[illegible]	[illegible]	8
Non-Hodgkin lymphoma	0 (0 to 0)	[illegible]	[illegible]	[illegible]	[illegible]	[illegible]	[illegible]	[illegible]	[illegible]	[illegible]	[illegible]	[illegible]	[illegible]	[illegible]	[illegible]	[illegible]	[illegible]	[illegible]	[illegible]	[illegible]	[illegible]	[illegible]	[illegible]	107
Multiple myeloma	0 (0 to 0)	[illegible]	[illegible]	[illegible]	[illegible]	[illegible]	[illegible]	[illegible]	[illegible]	[illegible]	[illegible]	[illegible]	[illegible]	[illegible]	[illegible]	[illegible]	[illegible]	[illegible]	[illegible]	[illegible]	[illegible]	[illegible]	[illegible]	26
Leukemia	0·1 (0·1 to 0·2)	[illegible]	[illegible]	[illegible]	[illegible]	[illegible]	[illegible]	[illegible]	[illegible]	[illegible]	[illegible]	[illegible]	[illegible]	[illegible]	[illegible]	[illegible]	[illegible]	[illegible]	[illegible]	[illegible]	[illegible]	[illegible]	[illegible]	97·9
Acute lymphoid leukemia	0 (0 to 0·1)	[illegible]	[illegible]	[illegible]	[illegible]	[illegible]	[illegible]	[illegible]	[illegible]	[illegible]	[illegible]	[illegible]	[illegible]	[illegible]	[illegible]	[illegible]	[illegible]	[illegible]	[illegible]	[illegible]	[illegible]	[illegible]	[illegible]	20·7
Chronic lymphoid leukemia	0 (0 to 0)	[illegible]	[illegible]	[illegible]	[illegible]	[illegible]	[illegible]	[illegible]	[illegible]	[illegible]	[illegible]	[illegible]	[illegible]	[illegible]	[illegible]	[illegible]	[illegible]	[illegible]	[illegible]	[illegible]	[illegible]	[illegible]	[illegible]	8
Acute myeloid leukemia	0 (0 to 0)	[illegible]	[illegible]	[illegible]	[illegible]	[illegible]	[illegible]	[illegible]	[illegible]	[illegible]	[illegible]	[illegible]	[illegible]	[illegible]	[illegible]	[illegible]	[illegible]	[illegible]	[illegible]	[illegible]	[illegible]	[illegible]	[illegible]	21·5

Chronic myeloid leukemia	0 (0 to 0)	0 (0 to 0)	0 (0 to 0)	0·1 (0 to 0·1)	0·1 (0 to 0·1)	0·2 (0·1 to 0·3)	0·1 (0·1 to 0·2)	0·5 (0·2 to 0·9)	0·6 (0·2 to 1)	0·6 (0·3 to 1·1)	0·6 (0·3 to 1·1)	0·7 (0·4 to 1·2)	0·7 (0·4 to 1·2)	0·9 (0·5 to 1·5)	0·9 (0·5 to 1·5)	0·8 (0·5 to 1·3)	0·6 (0·3 to 0·9)	0·5 (0·3 to 0·9)	0·4 (0·2 to 0·7)	0·4 (0·2 to 0·6)	0·1 (0·1 to 0·2)	0 (0 to 0·1)	0 (0 to 0)	8·8
Other leukemia	0·1 (0 to 0·1)	0 (0 to 0·1)	0·6 (0·3 to 1)	0·6 (0·3 to 1)	0·7 (0·4 to 1·2)	2·2 (1·2 to 3·5)	1·3 (0·8 to 2)	2·1 (1·4 to 3·1)	2·5 (1·6 to 3·7)	2·2 (1·5 to 3·2)	2·6 (1·7 to 3·8)	3·2 (2·3 to 4·5)	3·6 (2·5 to 5)	2·8 (2 to 3·7)	2·8 (2 to 3·8)	2·7 (2 to 3·5)	2·2 (1·6 to 3)	2·3 (1·6 to 3·1)	1·7 (1·2 to 2·4)	1·6 (1·1 to 2·3)	0·8 (0·6 to 1·1)	0·3 (0·2 to 0·3)	0·1 (0 to 0·1)	38·9
Other malignant neoplasms	0·2 (0·1 to 0·3)	0·2 (0·1 to 0·3)	0·9 (0·6 to 1·5)	3·3 (2·1 to 5·1)	2·8 (2·2 to 3·7)	6·1 (4·8 to 7·8)	4·7 (3·5 to 6)	4·5 (3·6 to 5·8)	4·2 (3·2 to 5·4)	4·2 (3·2 to 5·5)	5 (3·9 to 6·3)	6·8 (5·2 to 8·8)	8 (6·2 to 10)	9·4 (7·5 to 12)	8·4 (6·4 to 10·9)	8·4 (6·6 to 10·6)	6·8 (5·4 to 8·5)	6 (4·8 to 7·4)	4·7 (3·8 to 5·8)	4·2 (3·4 to 5·1)	2·4 (1·8 to 2·9)	0·8 (0·6 to 1)	0·2 (0·1 to 0·2)	102
Other neoplasms	0 (0 to 0)	0·1 (0 to 0·2)	0·2 (0·1 to 0·5)	0·5 (0·2 to 0·9)	0·1 (0·1 to 0·2)	0·3 (0·2 to 0·5)	0·3 (0·2 to 0·4)	0·3 (0·2 to 0·4)	0·4 (0·3 to 0·7)	0·4 (0·3 to 0·6)	0·3 (0·2 to 0·4)	0·9 (0·6 to 1·2)	0·8 (0·5 to 1·1)	1 (0·7 to 1·5)	1 (0·7 to 1·5)	1·2 (0·9 to 1·7)	1·2 (0·9 to 1·7)	1·4 (1 to 2)	1·4 (1 to 1·9)	1·1 (0·8 to 1·6)	0·5 (0·4 to 0·7)	0·2 (0·1 to 0·2)	0 (0 to 0)	13·7
Myelodysplastic, myeloproliferative, and other hematopoietic neo	0 (0 to 0)	0·1 (0 to 0·2)	0·2 (0·1 to 0·5)	0·5 (0·2 to 0·9)	0·1 (0·1 to 0·2)	0·3 (0·2 to 0·5)	0·3 (0·2 to 0·4)	0·3 (0·2 to 0·4)	0·4 (0·3 to 0·7)	0·4 (0·3 to 0·6)	0·3 (0·2 to 0·4)	0·9 (0·6 to 1·2)	0·8 (0·5 to 1·1)	1 (0·7 to 1·5)	1 (0·7 to 1·5)	1·2 (0·9 to 1·7)	1·2 (0·9 to 1·7)	1·4 (1 to 2)	1·4 (1 to 1·9)	1·1 (0·8 to 1·6)	0·5 (0·4 to 0·7)	0·2 (0·1 to 0·2)	0 (0 to 0)	13·7
Benign and in situ intestinal neoplasms	0 (0 to 0)	0 (0 to 0)	0 (0 to 0)	0 (0 to 0)	0 (0 to 0)	0 (0 to 0)	0 (0 to 0)	0 (0 to 0)	0 (0 to 0)	0 (0 to 0)	0 (0 to 0)	0 (0 to 0)	0 (0 to 0)	0 (0 to 0)	0 (0 to 0)	0 (0 to 0)	0 (0 to 0)	0 (0 to 0)	0 (0 to 0)	0 (0 to 0)	0 (0 to 0)	0 (0 to 0)	0 (0 to 0)	0
Benign and in situ cervical and uterine neoplasms	0 (0 to 0)	0 (0 to 0)	0 (0 to 0)	0 (0 to 0)	0 (0 to 0)	0 (0 to 0)	0 (0 to 0)	0 (0 to 0)	0 (0 to 0)	0 (0 to 0)	0 (0 to 0)	0 (0 to 0)	0 (0 to 0)	0 (0 to 0)	0 (0 to 0)	0 (0 to 0)	0 (0 to 0)	0 (0 to 0)	0 (0 to 0)	0 (0 to 0)	0 (0 to 0)	0 (0 to 0)	0 (0 to 0)	0
Other benign and in situ neoplasms	0 (0 to 0)	0 (0 to 0)	0 (0 to 0)	0 (0 to 0)	0 (0 to 0)	0 (0 to 0)	0 (0 to 0)	0 (0 to 0)	0 (0 to 0)	0 (0 to 0)	0 (0 to 0)	0 (0 to 0)	0 (0 to 0)	0 (0 to 0)	0 (0 to 0)	0 (0 to 0)	0 (0 to 0)	0 (0 to 0)	0 (0 to 0)	0 (0 to 0)	0 (0 to 0)	0 (0 to 0)	0 (0 to 0)	0
Cardiovascular diseases	1·4 (0·9 to 2·3)	0·9 (0·6 to 1·5)	4·2 (2·5 to 6·4)	2·4 (1·7 to 3·4)	3·8 (3·1 to 4·7)	11 (8·8 to 13·6)	17·6 (14·2 to 21·2)	25·7 (20·1 to 31·8)	32·6 (25·4 to 41·9)	43·7 (34 to 54·8)	62·9 (49·6 to 78·4)	106 (84 to 132)	161 (129 to 197)	208 (169 to 251)	231 (192 to 276)	313 (264 to 370)	307 (268 to 351)	375 (332 to 426)	377 (332 to 420)	429 (382 to 477)	275 (238 to 305)	116 (99·2 to 129)	36·6 (30·3 to 40·2)	3,140
Rheumatic heart disease	0 (0 to 0)	0 (0 to 0)	0 (0 to 0)	0·2 (0·1 to 0·3)	1·1 (0·7 to 1·7)	3·3 (2·3 to 4·8)	4·2 (2·9 to 6·2)	4·8 (3·3 to 6·8)	5·7 (4 to 8)	5·8 (4·2 to 7·8)	6·1 (4·2 to 8·3)	6·7 (4·7 to 9·3)	6·1 (4·2 to 8·4)	5·2 (3·8 to 7·3)	4 (2·8 to 5·3)	2·8 (2 to 3·8)	1·9 (1·4 to 2·5)	1·5 (1·1 to 2)	1·1 (0·8 to 1·5)	1 (0·7 to 1·2)	0·5 (0·4 to 0·7)	0·2 (0·2 to 0·3)	0·1 (0·1 to 0·1)	62·3
Ischemic heart disease	0 (0 to 0)	0 (0 to 0)	0 (0 to 0)	0 (0 to 0)	0 (0 to 0)	0 (0 to 0)	1·7 (1·3 to 2·3)	3·2 (2·1 to 4·5)	4·9 (3·5 to 6·5)	9 (6·4 to 12·4)	14·6 (10·2 to 20·5)	29 (21·1 to 38·7)	50·3 (37·7 to 65·5)	68·6 (54·1 to 85)	81 (62·6 to 102)	124 (97·1 to 154)	127 (107 to 150)	158 (131 to 189)	165 (138 to 193)	182 (153 to 211)	128 (107 to 147)	56·3 (46·6 to 65·4)	18·3 (15·2 to 20·8)	1,220
Stroke	0·7 (0·4 to 1·3)	0·2 (0·1 to 0·3)	0·9 (0·4 to 1·6)	0·5 (0·3 to 0·7)	1 (0·7 to 1·4)	2·9 (2·1 to 3·7)	5·2 (3·7 to 6·7)	7·7 (5·9 to 9·8)	10·5 (7·5 to 14·5)	15·1 (10·6 to 20·8)	22·1 (17 to 27·9)	37·3 (28·9 to 48·3)	57 (43·7 to 71·7)	76·6 (56·6 to 101)	81·6 (62·6 to 103)	106 (84·5 to 133)	102 (79·3 to 130)	129 (104 to 157)	127 (106 to 150)	150 (125 to 176)	81·5 (66·8 to 95·3)	29 (23·9 to 34·1)	8·4 (6·5 to 10·3)	1,050
Ischemic stroke	0·2 (0·1 to 0·4)	0 (0 to 0)	0·1 (0 to 0·1)	0·2 (0·1 to 0·2)	0·5 (0·3 to 0·7)	0·8 (0·5 to 1·2)	1·5 (1·1 to 2)	2·2 (1·7 to 2·9)	2·6 (1·7 to 3·6)	3·8 (2·6 to 5·4)	5 (3·7 to 6·5)	8·6 (6·5 to 11)	12·8 (10 to 16·3)	18·7 (13·9 to 24·6)	23·7 (18·5 to 29·4)	41·7 (33·4 to 51·5)	43·9 (32·2 to 57·8)	81·1 (65·4 to 98·8)	85·1 (71·2 to 100)	120 (100 to 140)	66·5 (55·1 to 77·7)	23·8 (19·5 to 28)	7·1 (5·4 to 8·8)	550
Intracerebral hemorrhage	0·2 (0·1 to 0·4)	0·1 (0 to 0·2)	0·5 (0·2 to 1)	0·1 (0·1 to 0·2)	0·3 (0·2 to 0·4)	1·1 (0·7 to 1·5)	2·3 (1·6 to 3·1)	3·6 (2·6 to 4·7)	5·1 (3·4 to 7·5)	7·5 (5 to 10·7)	11·4 (8·4 to 14·9)	20 (14·9 to 26·8)	34 (25·5 to 44·3)	46·5 (33·5 to 63)	47·4 (35·6 to 60·4)	56·9 (44·3 to 71·7)	52 (37·9 to 67·6)	43·1 (34·5 to 52·9)	38·4 (31·6 to 46)	27·1 (22·3 to 32·3)	13·7 (10·7 to 16·9)	4·8 (3·9 to 5·7)	1·2 (0·9 to 1·6)	417
Subarachnoid hemorrhage	0·3 (0·1 to 0·5)	0·1 (0 to 0·2)	0·3 (0·1 to 0·7)	0·2 (0·1 to 0·3)	0·3 (0·2 to 0·4)	1 (0·6 to 1·4)	1·4 (0·9 to 2·1)	1·9 (1·2 to 2·8)	2·8 (1·7 to 4·2)	3·8 (2·4 to 5·8)	5·7 (3·8 to 8·3)	8·8 (5·9 to 12·2)	10·1 (6·7 to 14·2)	11·4 (7·8 to 16·3)	10·5 (7·2 to 15)	7·9 (5·2 to 11·4)	6·4 (4·3 to 9)	4·5 (3·2 to 6·2)	3·6 (2·5 to 5)	2·6 (1·8 to 3·6)	1·2 (0·9 to 1·7)	0·5 (0·3 to 0·6)	0·1 (0·1 to 0·2)	85·6
Hypertensive heart disease	0 (0 to 0)	0 (0 to 0)	0 (0 to 0)	0 (0 to 0)	0 (0 to 0)	0 (0 to 0)	0·7 (0·4 to 1)	1·6 (0·9 to 2·3)	2 (1·2 to 3)	4·2 (2·5 to 6)	7·6 (4·5 to 11·9)	13·1 (8·4 to 19·3)	23·9 (15·2 to 33·4)	32·7 (22·6 to 44·9)	36·6 (25 to 50·3)	46·3 (31 to 64)	43·6 (31·3 to 59)	51·1 (37·1 to 68·7)	49·1 (35·1 to 67·3)	55·4 (39·1 to 73·7)	34·9 (24·7 to 45·5)	16 (11 to 20·8)	5·5 (3·6 to 7·1)	424
Non-rheumatic valvular heart disease	0 (0 to 0)	0 (0 to 0)	0 (0 to 0)	0 (0 to 0)	0 (0 to 0)	0 (0 to 0)	0·6 (0·4 to 0·8)	0·5 (0·3 to 0·8)	0·6 (0·4 to 0·8)	0·7 (0·4 to 1)	0·8 (0·5 to 1·1)	1·1 (0·7 to 1·6)	1·5 (1 to 2·3)	1·4 (1 to 2)	1·5 (1 to 2·1)	1·8 (1·2 to 2·5)	1·4 (1·1 to 2)	1·4 (1 to 2)	1·3 (0·9 to 1·8)	1·3 (0·9 to 1·7)	0·8 (0·6 to 1·1)	0·3 (0·2 to 0·4)	0·1 (0 to 0·1)	17
Non-rheumatic calcific aortic valve disease	0 (0 to 0)	0 (0 to 0)	0 (0 to 0)	0 (0 to 0)	0 (0 to 0)	0 (0 to 0)	0·2 (0·1 to 0·3)	0·3 (0·2 to 0·4)	0·2 (0·1 to 0·3)	0·2 (0·1 to 0·4)	0·3 (0·1 to 0·5)	0·4 (0·2 to 0·7)	0·6 (0·3 to 1·1)	0·6 (0·4 to 1·1)	0·7 (0·4 to 1·1)	1·1 (0·7 to 1·6)	0·9 (0·6 to 1·3)	1 (0·6 to 1·4)	0·9 (0·6 to 1·3)	1 (0·7 to 1·3)	0·6 (0·4 to 0·9)	0·2 (0·2 to 0·3)	0 (0 to 0·1)	9·3
Non-rheumatic degenerative mitral valve disease	0 (0 to 0)	0 (0 to 0)	0 (0 to 0)	0 (0 to 0)	0 (0 to 0)	0 (0 to 0)	0·3 (0·2 to 0·5)	0·2 (0·1 to 0·4)	0·3 (0·2 to 0·4)	0·3 (0·2 to 0·5)	0·4 (0·2 to 0·6)	0·6 (0·3 to 0·9)	0·7 (0·4 to 1·2)	0·7 (0·4 to 1·1)	0·6 (0·4 to 1)	0·6 (0·4 to 1)	0·4 (0·3 to 0·7)	0·3 (0·2 to 0·5)	0·3 (0·1 to 0·4)	0·2 (0·1 to 0·4)	0·1 (0·1 to 0·2)	0·1 (0 to 0·1)	0 (0 to 0)	6·2
Other non-rheumatic valve diseases	0 (0 to 0)	0 (0 to 0)	0 (0 to 0)	0 (0 to 0)	0 (0 to 0)	0 (0 to 0)	0·1 (0·1 to 0·2)	0·1 (0 to 0·1)	0·1 (0 to 0·2)	0·1 (0·1 to 0·3)	0·1 (0 to 0·2)	0·1 (0 to 0·2)	0·2 (0·1 to 0·4)	0·1 (0 to 0·3)	0·1 (0·1 to 0·3)	0·1 (0 to 0·1)	0·1 (0 to 0·2)	0·1 (0 to 0·2)	0·1 (0 to 0·2)	0·1 (0 to 0·2)	0 (0 to 0·1)	0 (0 to 0)	0 (0 to 0)	1·5
Cardiomyopathy and myocarditis	0·2 (0·1 to 0·3)	0·2 (0·1 to 0·4)	1·5 (0·8 to 2·4)	0·7 (0·4 to 1·1)	0·4 (0·3 to 0·6)	1·2 (0·8 to 1·8)	0·9 (0·6 to 1·3)	1·4 (0·9 to 2)	1·4 (0·9 to 2·1)	1·7 (1 to 2·6)	2 (1·2 to 3)	2·6 (1·7 to 3·9)	3·4 (2·2 to 5·1)	4·1 (2·7 to 5·9)	4·1 (2·7 to 5·9)	4·6 (3 to 6·8)	3·8 (2·6 to 5·4)	3·7 (2·5 to 5·1)	3·4 (2·3 to 4·8)	3·4 (2·2 to 4·9)	2·5 (1·6 to 3·6)	0·9 (0·6 to 1·4)	0·3 (0·2 to 0·4)	48·4
Myocarditis	0 (0 to 0·1)	0·1 (0 to 0·1)	0·4 (0·2 to 0·8)	0·3 (0·1 to 0·5)	0·2 (0·1 to 0·3)	0·4 (0·3 to 0·7)	0·2 (0·2 to 0·4)	0·3 (0·2 to 0·5)	0·3 (0·2 to 0·5)	0·4 (0·2 to 0·7)	0·3 (0·2 to 0·5)	0·2 (0·1 to 0·4)	0·5 (0·3 to 0·9)	0·4 (0·2 to 0·7)	0·3 (0·2 to 0·4)	0·3 (0·2 to 0·4)	0·1 (0·1 to 0·2)	0·3 (0·1 to 0·5)	0·3 (0·1 to 0·5)	0·4 (0·2 to 0·7)	0·4 (0·2 to 0·6)	0·2 (0·1 to 0·3)	0·1 (0 to 0·1)	6·5
Alcoholic cardiomyopathy	0 (0 to 0)	0 (0 to 0)	0 (0 to 0)	0 (0 to 0)	0 (0 to 0)	0 (0 to 0)	0 (0 to 0·1)	0·1 (0 to 0·1)	0·1 (0 to 0·2)	0·1 (0 to 0·2)	0·1 (0 to 0·2)	0·1 (0 to 0·2)	0·5 (0·2 to 0·9)	0·5 (0·2 to 1)	0·4 (0·2 to 0·6)	0·4 (0·1 to 0·6)	0·3 (0·1 to 0·6)	0·1 (0 to 0·1)	0·1 (0 to 0·1)	0·1 (0 to 0·2)	0 (0 to 0)	0 (0 to 0)	0 (0 to 0)	2·8
Other cardiomyopathy	0·2 (0·1 to 0·3)	0·2 (0·1 to 0·3)	1·1 (0·5 to 1·8)	0·4 (0·2 to 0·7)	0·2 (0·1 to 0·3)	0·8 (0·4 to 1·4)	0·6 (0·4 to 1)	1 (0·6 to 1·6)	1 (0·5 to 1·6)	1·2 (0·7 to 1·8)	1·6 (0·9 to 2·5)	2·2 (1·4 to 3·4)	2·4 (1·5 to 3·7)	3·2 (2 to 4·6)	3·4 (2·2 to 5)	4 (2·5 to 5·9)	3·4 (2·2 to 4·9)	3·3 (2·2 to 4·7)	3 (2 to 4·3)	2·9 (1·8 to 4·3)	2·1 (1·3 to 3·2)	0·8 (0·5 to 1·2)	0·2 (0·1 to 0·3)	39·2
Atrial fibrillation and flutter	0 (0 to 0)	0 (0 to 0)	0 (0 to 0)	0 (0 to 0)	0 (0 to 0)	0 (0 to 0)	0 (0 to 0)	0 (0 to 0)	0 (0 to 0)	0·1 (0 to 0·1)	0·1 (0·1 to 0·2)	0·7 (0·5 to 1)	1·1 (0·7 to 1·6)	1·6 (1·2 to 2·3)	2·6 (1·9 to 3·6)	4 (3 to 5·3)	4·9 (3·8 to 6·5)	7·2 (5·7 to 9·5)	8·6 (6·8 to 10·8)	12·9 (10·4 to 16·3)	10·4 (8·3 to 13·8)	5·8 (4·5 to 7·7)	1·8 (1·3 to 2·4)	61·8
Aortic aneurysm	--	--	--	--	--	--	0·1 (0 to 0·1)	0·1 (0·1 to 0·2)	0·3 (0·2 to 0·4)	0·2 (0·1 to 0·4)	0·5 (0·3 to 0·7)	0·7 (0·5 to 1·1)	1·1 (0·7 to 1·5)	1·5 (1 to 2)	2 (1·4 to 2·7)	2·7 (1·9 to 3·7)	3·2 (2·3 to 4·3)	3·7 (2·7 to 5·1)	3·5 (2·5 to 4·8)	3·5 (2·4 to 4·9)	1·9 (1·4 to 2·5)	0·7 (0·5 to 0·9)	0·2 (0·1 to 0·2)	NA
Endocarditis	0 (0 to 0)	0 (0 to 0·1)	0·3 (0·1 to 0·5)	0·1 (0·1 to 0·2)	0·1 (0·1 to 0·2)	0·3 (0·2 to 0·4)	0·5 (0·3 to 0·7)	0·9 (0·5 to 1·5)	0·9 (0·5 to 1·4)	0·7 (0·4 to 1·1)	1 (0·6 to 1·8)	1·1 (0·6 to 1·7)	1·2 (0·7 to 1·8)	1 (0·6 to 1·6)	1 (0·6 to 1·6)	1·1 (0·7 to 1·8)	0·8 (0·5 to 1·2)	0·8 (0·5 to 1·2)	0·6 (0·4 to 1)	0·8 (0·5 to 1·3)	0·5 (0·3 to 0·7)	0·2 (0·1 to 0·3)	0·1 (0 to 0·1)	13·9
Other cardiovascular and circulatory diseases	0·5 (0·3 to 0·7)	0·4 (0·3 to 0·7)	1·5 (0·8 to 2·4)	0·9 (0·5 to 1·3)	1·2 (0·8 to 1·7)	3·4 (2·4 to 4·5)	3·7 (2·6 to 5·2)	5·5 (3·6 to 8)	6·4 (4·4 to 9)	6·4 (4·6 to 8·6)	8·1 (6·1 to 10·7)	13·4 (9·6 to 17·9)	14·7 (10·7 to 18·8)	14·2 (10·4 to 19·1)	15·5 (11·5 to 20·4)	17·3 (13·8 to 21·4)	15·2 (11·5 to 19·3)	16·1 (12·1 to 21)	14·5 (10·6 to 19·9)	15·6 (11·6 to 20·7)	11·8 (8·8 to 14·9)	5 (3·8 to 6·3)	1·5 (1·1 to 1·9)	193
Chronic respiratory diseases	0 (0 to 0)	0 (0 to 0)	0 (0 to 0)	17 (9·1 to 30·2)	17·6 (10·4 to 29·8)	16·7 (10·4 to 26·3)	14·5 (9 to 23)	13·2 (8·8 to 19·4)	12·3 (8·4 to 17·6)	12 (8·4 to 16·7)	12·6 (9·1 to 16·9)	13·1 (9·9 to 16·7)	13·9 (10·7 to 17·6)	15·7 (12·2 to 19·6)	15·9 (12·6 to 19·9)	17 (13·1 to 21·6)	15·2 (11·6 to 19·6)	14·5 (11·1 to 18·5)	13 (9·8 to 16·9)	12·4 (9·2 to 16)	7·6 (5·7 to 9·7)	3 (2·2 to 3·8)	0·9 (0·7 to 1·2)	258
Chronic obstructive pulmonary disease	0 (0 to 0)	0 (0 to 0)	0 (0 to 0)	0·2 (0·1 to 0·3)	0·3 (0·1 to 0·7)	0·6 (0·2 to 1)	0·9 (0·5 to 1·5)	1·3 (0·7 to 2)	1·5 (0·9 to 2·3)	1·8 (1·1 to 2·7)	2·3 (1·5 to 3·3)	3 (2·1 to 4·1)	3·9 (2·7 to 5·2)	5·6 (4 to 7·3)	6·7 (4·8 to 8·7)	9·2 (6·8 to 12·1)	8·7 (6·3 to 11·6)	8·9 (6·5 to 11·5)	8·5 (6·3 to 11·2)	9·1 (6·5 to 11·9)	6 (4·4 to 7·7)	2·4 (1·8 to 3·1)	0·7 (0·5 to 0·9)	81·6
Pneumoconiosis	0 (0 to 0)	0 (0 to 0)	0 (0 to 0)	0 (0 to 0)	0 (0 to 0)	0 (0 to 0)	0 (0 to 0)	0 (0 to 0)	0 (0 to 0)	0 (0 to 0)	0 (0 to 0)	0 (0 to 0)	0 (0 to 0)	0·1 (0 to 0·1)	0 (0 to 0)	0 (0 to 0)	0 (0 to 0)	0 (0 to 0)	0 (0 to 0·1)	0 (0 to 0)	0 (0 to 0)	0 (0 to 0)	0 (0 to 0)	0·3
Silicosis	0 (0 to 0)	0 (0 to 0)	0 (0 to 0)	0 (0 to 0)	0 (0 to 0)	0 (0 to 0)	0 (0 to 0)	0 (0 to 0)	0 (0 to 0)	0 (0 to 0)	0 (0 to 0)	0 (0 to 0)	0 (0 to 0)	0 (0 to 0)	0 (0 to 0)	0 (0 to 0)	0 (0 to 0)	0 (0 to 0)	0 (0 to 0)	0 (0 to 0)	0 (0 to 0)	0 (0 to 0)	0 (0 to 0)	0
Asbestosis	0 (0 to 0)	0 (0 to 0)	0 (0 to 0)	0 (0 to 0)	0 (0 to 0)	0 (0 to 0)	0 (0 to 0)	0 (0 to 0)	0 (0 to 0)	0 (0 to 0)	0 (0 to 0)	0 (0 to 0)	0 (0 to 0)	0 (0 to 0)	0 (0 to 0)	0 (0 to 0)	0 (0 to 0)	0 (0 to 0)	0 (0 to 0)	0 (0 to 0)	0 (0 to 0)	0 (0 to 0)	0 (0 to 0)	0·1
Coal workers pneumoconiosis	0 (0 to 0)	0 (0 to 0)	0 (0 to 0)	0 (0 to 0)	0 (0 to 0)	0 (0 to 0)	0 (0 to 0)	0 (0 to 0)	0 (0 to 0)	0 (0 to 0)	0 (0 to 0)	0 (0 to 0)	0 (0 to 0)	0 (0 to 0)	0 (0 to 0)	0 (0 to 0)	0 (0 to 0)	0 (0 to 0)	0 (0 to 0)	0 (0 to 0)	0 (0 to 0)	0 (0 to 0)	0 (0 to 0)	0
Other pneumoconiosis	0 (0 to 0)	0 (0 to 0)	0 (0 to 0)	0 (0 to 0)	0 (0 to 0)	0 (0 to 0)	0 (0 to 0)	0 (0 to 0)	0 (0 to 0)	0 (0 to 0)	0 (0 to 0)	0 (0 to 0)	0 (0 to 0)	0 (0 to 0)	0 (0 to 0)	0 (0 to 0)	0 (0 to 0)	0 (0 to 0)	0 (0 to 0)	0 (0 to 0)	0 (0 to 0)	0 (0 to 0)	0 (0 to 0)	0·1
Asthma	0 (0 to 0)	0 (0 to 0)	0 (0 to 0)	16 (8·2 to 29)	16·6 (9·6 to 28·6)	15 (8·9 to 24·8)	13 (7·6 to 21·7)	11·2 (6·7 to 17·1)	9·8 (6 to 14·8)	9·2 (6 to 13·6)	9·1 (6·2 to 12·9)	8·8 (6·2 to 12·1)	8·6 (6·1 to 11·5)	8·6 (6·3 to 11·1)	7·2 (5·3 to 9·4)	6·2 (4·4 to 8·3)	5·1 (3·6 to 6·8)	4·1 (2·9 to 5·5)	3·1 (2·2 to 4·4)	2·2 (1·5 to 3·3)	1·1 (0·8 to 1·5)	0·4 (0·3 to 0·6)	0·1 (0·1 to 0·2)	155
Interstitial lung disease and pulmonary sarcoidosis	0 (0 to 0)	0 (0 to 0)	0 (0 to 0)	0·3 (0·1 to 0·5)	0·1 (0·1 to 0·2)	0·1 (0·1 to 0·2)	0·1 (0·1 to 0·2)	0·2 (0·1 to 0·3)	0·3 (0·2 to 0·5)	0·4 (0·2 to 0·7)	0·6 (0·3 to 1)	0·7 (0·4 to 1)	0·9 (0·5 to 1·4)	1·1 (0·6 to 1·6)	1·3 (0·8 to 2)	1·5 (0·8 to 2·3)	1·3 (0·8 to 2)	1·5 (0·8 to 2·3)	1·2 (0·7 to 1·9)	0·9 (0·5 to 1·6)	0·5 (0·2 to 0·8)	0·2 (0·1 to 0·3)	0 (0 to 0·1)	13·2
Other chronic respiratory diseases	0 (0 to 0)	0 (0 to 0)	0 (0 to 0)	0·5 (0·3 to 0·9)	0·6 (0·2 to 1·1)	0·9 (0·4 to 1·6)	0·4 (0·2 to 0·6)	0·6 (0·3 to 0·9)	0·7 (0·5 to 1·1)	0·6 (0·4 to 0·8)	0·6 (0·4 to 0·8)	0·6 (0·4 to 0·9)	0·5 (0·3 to 0·7)	0·5 (0·3 to 0·7)	0·7 (0·4 to 1·2)	0·1 (0·1 to 0·3)	0·1 (0 to 0·1)	0·1 (0 to 0·2)	0·1 (0 to 0·1)	0·1 (0 to 0·1)	0 (0 to 0·1)	0 (0 to 0)	0 (0 to 0)	7·7
Digestive diseases	0·4 (0·3 to 0·7)	0·7 (0·4 to 1·2)	3·5 (1·9 to 5·9)	5 (3·4 to 6·8)	4·5 (3·1 to 6·2)	6·8 (5·4 to 8·6)	8·2 (6·3 to 10·6)	13·8 (10·3 to 19)	17 (12·4 to 23·9)	20·6 (14·9 to 28·1)	24·9 (18·4 to 33·4)	30·6 (23·7 to 39·2)	36·2 (28 to 46·2)	42·2 (32·8 to 54)	41·2 (32·2 to 51·3)	42·9 (33·8 to 53·8)	33·9 (27·8 to 41·2)	31·3 (24·5 to 38·5)	25·1 (19·7 to 31·2)	22·5 (18·1 to 27·7)	12·3 (9·8 to 15·2)	4·5 (3·7 to 5·4)	1·2 (0·9 to 1·4)	429
Cirrhosis and other chronic liver diseases	0 (0 to 0)	0 (0 to 0)	0 (0 to 0)	0·3 (0·2 to 0·4)	0·3 (0·2 to 0·5)	1 (0·6 to 1·5)	1 (0·6 to 1·5)	1·7 (1·1 to 2·3)	1·9 (1·3 to 2·8)	2·9 (1·9 to 4·1)	4·3 (2·9 to 6)	6·5 (4·5 to 8·7)	9·4 (6·5 to 13)	12·6 (8·8 to 17·5)	12·6 (9 to 16·9)	13·4 (9·7 to 18·2)	10·6 (7·2 to 15·3)	8·9 (6·5 to 11·7)	6 (4·4 to 8·1)	4·7 (3·4 to 6·2)	2 (1·5 to 2·7)	0·6 (0·5 to 0·8)	0·2 (0·1 to 0·2)	101
Cirrhosis and other chronic liver diseases due to hepatitis B	0 (0 to 0)	0 (0 to 0)	0 (0 to 0)	0 (0 to 0)	0 (0 to 0)	0 (0 to 0)	0 (0 to 0)	0 (0 to 0·1)	0·1 (0 to 0·2)	0·2 (0·1 to 0·3)	0·3 (0·1 to 0·5)	0·5 (0·2 to 0·9)	0·7 (0·3 to 1·2)	0·8 (0·4 to 1·5)	0·7 (0·3 to 1·2)	0·6 (0·3 to 1·2)	0·4 (0·2 to 0·9)	0·3 (0·2 to 0·6)	0·2 (0·1 to 0·5)	0·2 (0·1 to 0·3)	0·1 (0 to 0·1)	0 (0 to 0)	0 (0 to 0)	5·1
Cirrhosis and other chronic liver diseases due to hepatitis C	0 (0 to 0)	0 (0 to 0)	0 (0 to 0)	0 (0 to 0)	0 (0 to 0)	0 (0 to 0)	0 (0 to 0·1)	0·2 (0·1 to 0·3)	0·3 (0·1 to 0·6)	0·6 (0·3 to 1·1)	1 (0·5 to 1·7)	1·5 (0·8 to 2·5)	2 (1 to 3·5)	2·5 (1·3 to 4·3)	2·3 (1·2 to 4·2)	2·3 (1·1 to 4·2)	1·7 (0·9 to 3)	1·4 (0·8 to 2·3)	0·9 (0·5 to 1·5)	0·7 (0·3 to 1·1)	0·3 (0·2 to 0·5)	0·1 (0·1 to 0·2)	0 (0 to 0)	17·9
Cirrhosis and other chronic liver diseases due to alcohol use	0 (0 to 0)	0 (0 to 0)	0 (0 to 0)	0 (0 to 0)	0 (0 to 0)	0 (0 to 0)	0 (0 to 0)	0·1 (0 to 0·2)	0·2 (0·1 to 0·4)	0·6 (0·3 to 1)	1·2 (0·6 to 2·1)	2·1 (1·2 to 3·5)	3·4 (1·8 to 5·4)	4·7 (2·6 to 7·6)	4·9 (2·8 to 7·6)	5·2 (3 to 7·9)	3·8 (2 to 6·1)	2·8 (1·6 to 4·3)	1·6 (0·9 to 2·6)	1·1 (0·6 to 1·9)	0·5 (0·3 to 0·7)	0·1 (0·1 to 0·2)	0 (0 to 0·1)	32·6
Cirrhosis and other chronic liver diseases due to NAFLD	0 (0 to 0)	0 (0 to 0)	0 (0 to 0)	0 (0 to 0)	0 (0 to 0)	0 (0 to 0)	0 (0 to 0·1)	0·2 (0·1 to 0·3)	0·3 (0·1 to 0·6)	0·6 (0·3 to 1)	0·8 (0·4 to 1·6)	1·3 (0·7 to 2·3)	2 (1 to 3·7)	2·8 (1·3 to 5)	2·7 (1·3 to 4·7)	2·9 (1·4 to 5·2)	2·5 (1·3 to 4·2)	2·3 (1·3 to 3·5)	1·6 (0·9 to 2·6)	1·3 (0·7 to 2)	0·6 (0·3 to 0·9)	0·2 (0·1 to 0·3)	0 (0 to 0·1)	22·2
Cirrhosis and other chronic liver diseases due to other causes	0 (0 to 0)	0 (0 to 0)	0 (0 to 0)	0·3 (0·2 to 0·4)	0·3 (0·2 to 0·5)	1 (0·6 to 1·5)	0·9 (0·6 to 1·4)	1·2 (0·8 to 1·8)	1 (0·6 to 1·6)	0·9 (0·5 to 1·7)	0·9 (0·4 to 1·6)	1 (0·5 to 1·8)	1·3 (0·6 to 2·4)	1·8 (0·8 to 3·1)	2 (1 to 3·5)	2·4 (1·2 to 4·1)	2·2 (1 to 3·9)	2·1 (1·1 to 3·5)	1·6 (0·9 to 2·8)	1·4 (0·7 to 2·3)	0·6 (0·4 to 0·9)	0·2 (0·1 to 0·3)	0 (0 to 0·1)	23·4
Upper digestive system diseases	0 (0 to 0)	0 (0 to 0)	0 (0 to 0)	0·4 (0·2 to 0·7)	0·6 (0·3 to 1·1)	1·1 (0·7 to 1·8)	2·8 (1·6 to 4·6)	6·2 (3·5 to 10·8)	8·1 (4·5 to 14)	9·4 (5·1 to 16·2)	10·4 (5·9 to 17·4)	10·8 (6·2 to 17·6)	11·8 (7·4 to 18·4)	12·4 (8 to 19·1)	12·4 (8 to 19·5)	11·8 (8 to 17·6)	9·1 (6·3 to 13·3)	7·9 (5·8 to 10·8)	6·3 (4·5 to 8·6)	5·4 (4 to 7·3)	2·9 (2·1 to 3·8)	1·1 (0·8 to 1·4)	0·3 (0·2 to 0·4)	131
Peptic ulcer disease	0 (0 to 0)	0 (0 to 0)	0 (0 to 0)	0·1 (0 to 0·1)	0 (0 to 0·1)	0·3 (0·2 to 0·4)	0·2 (0·1 to 0·2)	0·3 (0·2 to 0·5)	0·4 (0·2 to 0·6)	0·7 (0·5 to 0·9)	0·9 (0·6 to 1·2)	1·2 (0·8 to 1·7)	1·8 (1·1 to 2·6)	2·2 (1·5 to 3)	2·3 (1·6 to 3·2)	2·9 (2·1 to 3·9)	2·5 (1·8 to 3·3)	2·7 (1·9 to 3·6)	2·5 (1·7 to 3·6)	2·6 (1·8 to 3·6)	1·4 (1 to 2)	0·6 (0·4 to 0·7)	0·2 (0·1 to 0·2)	25·6
Gastritis and duodenitis	0 (0 to 0)	0 (0 to 0)	0 (0 to 0)	0·4 (0·2 to 0·7)	0·5 (0·2 to 1·1)	0·5 (0·2 to 1·1)	0·7 (0·3 to 1·3)	0·9 (0·5 to 1·6)	1·1 (0·6 to 1·8)	1·3 (0·7 to 2·3)	1·7 (0·9 to 2·9)	1·6 (0·9 to 2·9)	2·4 (1·4 to 4·4)	2·9 (1·7 to 4·8)	3·1 (1·8 to 5·1)	2·9 (1·8 to 4·5)	2·3 (1·5 to 3·4)	2 (1·3 to 2·9)	1·5 (1 to 2·2)	1·1 (0·7 to 1·7)	0·7 (0·5 to 1)	0·3 (0·2 to 0·4)	0·1 (0 to 0·1)	28
Gastroesophageal reflux disease	0 (0 to 0)	0 (0 to 0)	0 (0 to 0)	0 (0 to 0)	0 (0 to 0)	0·3 (0·1 to 0·6)	2 (1 to 3·7)	5 (2·4 to 9·6)	6·7 (3·2 to 12·3)	7·4 (3·4 to 14·3)	7·8 (3·7 to 14·4)	7·9 (3·8 to 14·4)	7·6 (3·8 to 13·2)	7·4 (3·6 to 13·3)	6·9 (3·3 to 12·8)	6 (3 to 11)	4·3 (2·2 to 7·8)	3·1 (1·6 to 5·5)	2·3 (1·1 to 4·1)	1·6 (0·8 to 2·9)	0·8 (0·4 to 1·4)	0·3 (0·1 to 0·5)	0·1 (0 to 0·1)	77·5
Appendicitis	0 (0 to 0)	0 (0 to 0)	0 (0 to 0)	0·2 (0·1 to 0·3)	0·3 (0·2 to 0·5)	0·9 (0·5 to 1·3)	0·7 (0·3 to 1·1)	0·6 (0·3 to 1·1)	0·5 (0·2 to 0·8)	0·4 (0·2 to 0·7)	0·6 (0·3 to 1)	0·5 (0·3 to 0·8)	0·6 (0·4 to 0·9)	0·5 (0·3 to 0·8)	0·5 (0·3 to 0·8)	0·4 (0·2 to 0·6)	0·4 (0·2 to 0·5)	0·3 (0·2 to 0·5)	0·2 (0·1 to 0·4)	0·2 (0·1 to 0·3)	0·1 (0·1 to 0·2)	0·1 (0 to 0·1)	0 (0 to 0)	8
Paralytic ileus and intestinal obstruction	0·3 (0·2 to 0·4)	0·6 (0·3 to 1)	3 (1·6 to 5·1)	1 (0·5 to 1·6)	0·5 (0·3 to 0·7)	0·8 (0·5 to 1·2)	0·5 (0·3 to 0·8)	0·5 (0·3 to 0·7)	0·6 (0·3 to 0·9)	0·6 (0·3 to 1)	1·2 (0·7 to 1·8)	1·5 (0·9 to 2·3)	2 (1·2 to 3)	2·7 (1·7 to 4·1)	2·4 (1·6 to 3·6)	3·1 (2 to 4·8)	2·9 (2 to 4·2)	3·4 (2·2 to 5)	3·3 (2·2 to 4·7)	3·4 (2·3 to 4·8)	2 (1·4 to 2·8)	0·8 (0·5 to 1·1)	0·2 (0·1 to 0·3)	37·1
Inguinal, femoral, and abdominal hernia	0·2 (0·1 to 0·3)	0·1 (0 to 0·2)	0·5 (0·3 to 1)	1·3 (0·7 to 2·2)	1·6 (0·8 to 2·8)	1·2 (0·5 to 2)	0·9 (0·4 to 1·5)	0·8 (0·4 to 1·4)	0·9 (0·4 to 1·5)	1·1 (0·6 to 1·9)	1·3 (0·7 to 2·1)	1·6 (1 to 2·5)	1·8 (1·1 to 2·8)	2 (1·3 to 2·9)	1·8 (1·2 to 2·6)	1·8 (1·2 to 2·5)	1·2 (0·8 to 1·6)	1·1 (0·7 to 1·5)	0·9 (0·6 to 1·3)	0·9 (0·6 to 1·3)	0·5 (0·3 to 0·8)	0·2 (0·1 to 0·3)	0 (0 to 0·1)	23·9
Inflammatory bowel disease	0 (0 to 0)	0 (0 to 0)	0 (0 to 0)	0·4 (0·2 to 0·6)	0·1 (0·1 to 0·2)	0·2 (0·1 to 0·3)	0·3 (0·2 to 0·5)	0·6 (0·4 to 0·9)	0·9 (0·6 to 1·4)	1·1 (0·7 to 1·6)	1·3 (0·8 to 1·8)	2·1 (1·3 to 3)	1·8 (1·1 to 2·6)	1·6 (1·1 to 2·4)	1·6 (1·1 to 2·4)	1·7 (1·1 to 2·5)	1 (0·7 to 1·3)	1·2 (0·8 to 1·7)	1 (0·7 to 1·4)	0·7 (0·4 to 1)	0·4 (0·3 to 0·6)	0·2 (0·1 to 0·3)	0 (0 to 0)	18·1
Vascular intestinal disorders	0 (0 to 0)	0 (0 to 0)	0 (0 to 0)	0 (0 to 0)	0 (0 to 0)	0 (0 to 0)	0 (0 to 0)	0 (0 to 0)	0 (0 to 0)	0 (0 to 0·1)	0·1 (0 to 0·1)	0·1 (0·1 to 0·2)	0·2 (0·1 to 0·2)	0·2 (0·1 to 0·3)	0·3 (0·2 to 0·4)	0·4 (0·3 to 0·6)	0·5 (0·4 to 0·6)	0·7 (0·4 to 0·9)	0·7 (0·5 to 0·9)	0·6 (0·4 to 0·9)	0·4 (0·2 to 0·5)	0·1 (0·1 to 0·2)	0 (0 to 0)	4·2
Gallbladder and biliary diseases	0 (0 to 0)	0 (0 to 0)	0 (0 to 0)	0·1 (0·1 to 0·3)	0·4 (0·1 to 0·7)	0·6 (0·2 to 1·2)	1·1 (0·5 to 1·8)	1·8 (1 to 2·7)	2·3 (1·4 to 3·6)	3 (1·9 to 4·5)	3·8 (2·4 to 5·8)	4·7 (3 to 6·7)	5·2 (3·5 to 7·4)	5·6 (3·8 to 8·1)	5·3 (3·7 to 7·4)	5·1 (3·6 to 7·1)	3·8 (2·8 to 5)	3·1 (2·2 to 4·2)	2·5 (1·8 to 3·4)	2·1 (1·5 to 2·9)	1 (0·7 to 1·4)	0·4 (0·3 to 0·5)	0·1 (0·1 to 0·1)	51·9
Pancreatitis	0 (0 to 0)	0 (0 to 0)	0 (0 to 0)	0 (0 to 0·1)	0 (0 to 0·1)	0·1 (0·1 to 0·2)	0·3 (0·2 to 0·4)	0·9 (0·6 to 1·4)	0·8 (0·5 to 1·2)	0·9 (0·5 to 1·4)	0·8 (0·5 to 1·2)	1·3 (0·8 to 1·9)	1·5 (0·9 to 2·3)	1·6 (1 to 2·3)	1·5 (1 to 2·1)	1·6 (1 to 2·3)	1·3 (1 to 1·7)	1·3 (0·8 to 1·8)	1 (0·7 to 1·4)	0·8 (0·5 to 1·2)	0·4 (0·3 to 0·6)	0·1 (0·1 to 0·2)	0 (0 to 0)	16·2
Other digestive diseases	0 (0 to 0)	0 (0 to 0)	0 (0 to 0)	1·2 (0·7 to 1·9)	0·6 (0·3 to 0·8)	0·9 (0·5 to 1·3)	0·7 (0·4 to 1)	0·7 (0·4 to 1·2)	1 (0·5 to 1·6)	1·1 (0·6 to 1·7)	1·1 (0·6 to 1·9)	1·6 (0·9 to 2·5)	2 (1·2 to 3·2)	2·9 (1·6 to 4·7)	2·8 (1·6 to 4·5)	3·6 (2 to 5·7)	3·2 (2 to 4·6)	3·4 (1·9 to 5·3)	3·2 (1·8 to 4·9)	3·7 (2·1 to 5·6)	2·5 (1·5 to 3·5)	0·9 (0·6 to 1·4)	0·3 (0·2 to 0·4)	37·5
Neurological disorders	0 (0 to 0)	0·1 (0 to 0·1)	2·7 (1·6 to 4·2)	7·3 (3·7 to 12·9)	14·7 (6·3 to 26·5)	42·9 (14·7 to 87·6)	54·6 (17·7 to 114)	56·8 (21·1 to 113)	54 (18·8 to 107)	52·7 (19·6 to 102)	55·2 (20·6 to 107)	56·6 (22·8 to 108)	52·9 (24·2 to 100)	47·4 (22·7 to 86·6)	42·3 (22·1 to 72·4)	39·4 (23·2 to 64·2)	34·2 (21·4 to 55·6)	37·2 (23·8 to 62·9)	43 (26·7 to 78·4)	61·8 (34·7 to 123)	47·3 (24·2 to 96·4)	22·6 (10·9 to 47·5)	8 (3·7 to 17·6)	834
Alzheimer's disease and other dementias	0 (0 to 0)	0 (0 to 0)	0 (0 to 0)	0 (0 to 0)	0 (0 to 0)	0 (0 to 0)	0 (0 to 0)	0 (0 to 0)	0 (0 to 0)	0 (0 to 0)	0 (0 to 0)	0·3 (0·1 to 0·9)	1·4 (0·4 to 4)	2·9 (1 to 7·2)	5·1 (2 to 12·2)	8·8 (3·6 to 20·5)	12·4 (5·1 to 30)	19 (8·2 to 43·4)	26 (11·4 to 61·6)	45·6 (19·3 to 106)	38·9 (15·8 to 88·3)	20 (8·2 to 45·1)	7·4 (3·1 to 17·1)	188
Parkinson's disease	0 (0 to 0)	0 (0 to 0)	0 (0 to 0)	0 (0 to 0)	0 (0 to 0)	0 (0 to 0)	0 (0 to 0)	0 (0 to 0)	0 (0 to 0)	0·1 (0 to 0·1)	0·1 (0·1 to 0·2)	0·3 (0·2 to 0·4)	0·6 (0·4 to 0·8)	1 (0·8 to 1·3)	1·4 (1·1 to 1·9)	2·6 (2 to 3·2)	3·5 (2·8 to 4·3)	6 (5 to 7·1)	8·7 (7·3 to 10·3)	10·6 (8·8 to 12·5)	5·9 (4·8 to 6·9)	1·8 (1·4 to 2·1)	0·4 (0·3 to 0·4)	43
Idiopathic epilepsy	0 (0 to 0)	0 (0 to 0·1)	1·2 (0·6 to 2)	4·6 (2·1 to 8·7)	6·8 (2·5 to 13·4)	10·6 (5·1 to 19·7)	11·6 (5·5 to 21·3)	12·9 (7 to 22·5)	11·6 (6·1 to 20)	10·8 (5·7 to 18·4)	10·3 (5·5 to 17·5)	9·9 (5·4 to 16·1)	9·1 (5 to 15·2)	7·6 (4 to 12·8)	6·3 (2·9 to 10·9)	5·7 (2·5 to 10·3)	4·4 (1·9 to 8)	3·7 (1·6 to 6·7)	3 (1·3 to 5·5)	2·4 (1 to 4·4)	1·2 (0·5 to 2·3)	0·4 (0·2 to 0·8)	0·1 (0 to 0·2)	134
Multiple sclerosis	0 (0 to 0)	0 (0 to 0)	0 (0 to 0)	0 (0 to 0)	0 (0 to 0)	0 (0 to 0·1)	0·1 (0 to 0·2)	0·3 (0·2 to 0·4)	0·4 (0·3 to 0·6)	0·5 (0·3 to 0·8)	1 (0·7 to 1·4)	1·1 (0·8 to 1·6)	1·2 (0·8 to 1·7)	1·1 (0·7 to 1·5)	0·9 (0·6 to 1·3)	0·7 (0·5 to 1·1)	0·3 (0·2 to 0·5)	0·3 (0·2 to 0·4)	0·1 (0·1 to 0·2)	0·1 (0·1 to 0·1)	0 (0 to 0·1)	0 (0 to 0)	0 (0 to 0)	8·3
Motor neuron disease	0 (0 to 0)	0 (0 to 0·1)	0·3 (0·1 to 0·5)	0·2 (0·1 to 0·3)	0 (0 to 0·1)	0·1 (0·1 to 0·2)	0 (0 to 0·1)	0·1 (0·1 to 0·1)	0·1 (0·1 to 0·1)	0·1 (0·1 to 0·1)	0·2 (0·1 to 0·2)	0·3 (0·2 to 0·4)	0·5 (0·4 to 0·7)	0·7 (0·5 to 1)	0·7 (0·5 to 0·9)	0·7 (0·5 to 0·9)	0·5 (0·4 to 0·7)	0·4 (0·3 to 0·6)	0·3 (0·2 to 0·4)	0·1 (0·1 to 0·2)	0 (0 to 0·1)	0 (0 to 0)	0 (0 to 0)	5·4
Headache disorders	0 (0 to 0)	0 (0 to 0)	0 (0 to 0)	0 (0 to 0)	5·4 (0·1 to 14)	27·8 (1·2 to 69·8)	39·2 (4·2 to 97·7)	40·8 (5·5 to 96·2)	39·6 (6·1 to 91·8)	39·2 (6·5 to 87·6)	41·3 (7·5 to 91·6)	42·1 (8·4 to 93·7)	37 (8·9 to 82·7)	31·5 (8·2 to 67·3)	25·5 (6·9 to 53·6)	19 (5·7 to 41·1)	11·6 (3·8 to 23·8)	6·8 (2·3 to 14·3)	4 (1·3 to 8·2)	2·5 (0·8 to 5·2)	1·1 (0·4 to 2·2)	0·3 (0·1 to 0·6)	0·1 (0 to 0·1)	415
Migraine	0 (0 to 0)	0 (0 to 0)	0 (0 to 0)	0 (0 to 0)	5 (0·1 to 13·6)	26·3 (0·8 to 68·9)	36·8 (2·9 to 96·3)	38 (3·7 to 93·7)	36·7 (4·2 to 87·4)	36·2 (4·6 to 84·2)	38 (5·3 to 88)	38·5 (6·3 to 88·7)	33·6 (6·2 to 77·3)	28·2 (5·6 to 64·5)	22·8 (4·9 to 50·6)	16·7 (3·9 to 37·8)	10 (2·6 to 21·8)	5·7 (1·6 to 12·4)	3·3 (0·9 to 7·4)	2 (0·6 to 4·5)	0·9 (0·3 to 1·9)	0·2 (0·1 to 0·5)	0 (0 to 0·1)	379
Tension-type headache	0 (0 to 0)	0 (0 to 0)	0 (0 to 0)	0 (0 to 0)	0·4 (0 to 2·5)	1·5 (0·1 to 9·6)	2·4 (0·5 to 10·4)	2·8 (0·6 to 11)	2·9 (0·7 to 10·8)	3 (0·7 to 10·2)	3·4 (0·9 to 10·8)	3·5 (1 to 10·1)	3·4 (1·1 to 8·9)	3·2 (1 to 8·8)	2·7 (0·9 to 6·8)	2·3 (0·7 to 6·5)	1·7 (0·5 to 5·2)	1·1 (0·3 to 3·8)	0·7 (0·1 to 2·4)	0·4 (0·1 to 1·5)	0·2 (0 to 0·7)	0·1 (0 to 0·2)	0 (0 to 0·1)	35·7
Other neurological disorders	0 (0 to 0)	0 (0 to 0)	1·2 (0·7 to 2)	2·5 (1·3 to 4)	2·4 (1 to 4·7)	4·3 (2 to 8·1)	3·6 (1·6 to 6·8)	2·7 (1·3 to 4·7)	2·2 (1·2 to 3·6)	2·1 (1·2 to 3·3)	2·3 (1·4 to 3·6)	2·6 (1·7 to 4)	3 (2 to 4·3)	2·7 (1·9 to 3·8)	2·4 (1·6 to 3·3)	2 (1·4 to 2·6)	1·3 (1 to 1·8)	1 (0·7 to 1·2)	0·8 (0·6 to 1)	0·6 (0·4 to 0·8)	0·2 (0·2 to 0·3)	0·1 (0·1 to 0·1)	0 (0 to 0)	40
Mental disorders	0 (0 to 0)	0 (0 to 0)	0·3 (0·2 to 0·5)	2·2 (1·3 to 3·3)	15·8 (10·2 to 23·3)	55·7 (36·7 to 80·5)	91·4 (61·5 to 128)	102 (69·7 to 142)	101 (68 to 140)	98·5 (68·4 to 136)	103 (73·8 to 139)	102 (71·9 to 140)	93·5 (67·3 to 128)	85·5 (61·4 to 116)	76·7 (54·4 to 104)	62 (43·5 to 84·6)	41·1 (29·1 to 55·4)	27·8 (19·5 to 37)	18·7 (13 to 25·1)	12·7 (8·7 to 17·3)	6·2 (4·3 to 8·3)	2·1 (1·4 to 2·9)	0·6 (0·4 to 0·8)	1,100
Schizophrenia	0 (0 to 0)	0 (0 to 0)	0 (0 to 0)	0 (0 to 0)	0 (0 to 0)	0·2 (0·1 to 0·5)	1·8 (0·7 to 3·6)	6·1 (3·1 to 10·3)	10·2 (5·8 to 15·8)	12·3 (7·3 to 18·5)	13·7 (8·3 to 20·1)	13·4 (8·6 to 18·7)	11·9 (7·8 to 16·9)	10·2 (6·5 to 14·1)	8·3 (5·4 to 11·6)	6 (3·9 to 8·4)	3·5 (2·2 to 5)	2 (1·3 to 3)	1·1 (0·6 to 1·6)	0·6 (0·3 to 1)	0·2 (0·1 to 0·4)	0·1 (0 to 0·1)	0 (0 to 0)	102
Depressive disorders	0 (0 to 0)	0 (0 to 0)	0 (0 to 0)	0 (0 to 0)	1·2 (0·4 to 2·5)	13·2 (6·6 to 24·6)	32·6 (18·8 to 52·1)	38·6 (22·1 to 60·2)	35·2 (20·6 to 54·6)	34 (20 to 54)	37·3 (23·4 to 56·4)	38·7 (23·7 to 59·2)	37·5 (24·4 to 56·6)	36·8 (23·8 to 54·4)	35 (21·8 to 52)	29·8 (17·9 to 44·7)	20·3 (13 to 29·3)	14·1 (9·2 to 20·2)	9·8 (6 to 14·5)	7 (4·1 to 10·6)	3·6 (2·3 to 5·3)	1·3 (0·8 to 2)	0·4 (0·2 to 0·6)	426
Major depressive disorder	0 (0 to 0)	0 (0 to 0)	0 (0 to 0)	0 (0 to 0)	1·1 (0·3 to 2·3)	12·2 (6 to 23·2)	30 (16·8 to 49·1)	34·1 (18·5 to 56·2)	29·9 (16·9 to 47·5)	28·2 (15·4 to 46·9)	31 (18·3 to 48·9)	32·3 (18·6 to 51·8)	31·5 (19·8 to 47·7)	31·2 (19·3 to 46·9)	30 (17·8 to 45·8)	25·7 (14·5 to 39·6)	17·6 (10·9 to 25·8)	12·3 (7·8 to 18)	8·6 (5·1 to 13·2)	6·2 (3·5 to 9·8)	3·3 (2 to 4·8)	1·2 (0·7 to 1·8)	0·3 (0·2 to 0·6)	367
Dysthymia	0 (0 to 0)	0 (0 to 0)	0 (0 to 0)	0 (0 to 0)	0·1 (0 to 0·3)	0·9 (0·3 to 1·9)	2·6 (1·2 to 4·7)	4·4 (2·3 to 7·8)	5·3 (2·8 to 9·1)	5·8 (3·2 to 9·4)	6·3 (3·5 to 10·4)	6·4 (3·4 to 11·3)	6 (3·3 to 10·5)	5·5 (3·2 to 9·3)	5 (2·9 to 8·2)	4·1 (2·2 to 6·7)	2·7 (1·4 to 4·6)	1·8 (1 to 3)	1·2 (0·7 to 2)	0·8 (0·4 to 1·3)	0·4 (0·2 to 0·6)	0·1 (0·1 to 0·2)	0 (0 to 0·1)	59·6
Bipolar disorder	0 (0 to 0)	0 (0 to 0)	0 (0 to 0)	0 (0 to 0)	0 (0 to 0)	3·4 (1·3 to 6·2)	12·2 (5·8 to 21·7)	14·7 (7·5 to 24·7)	13·7 (7 to 22·9)	12·4 (6·6 to 20·8)	12·2 (6·5 to 19·9)	11·8 (6·5 to 19·6)	10·7 (5·9 to 18·1)	9·5 (5·4 to 15·7)	8 (4·4 to 13·2)	6 (3·3 to 9·7)	3·7 (2 to 6)	2·2 (1·2 to 3·5)	1·2 (0·6 to 2)	0·7 (0·3 to 1·1)	0·2 (0·1 to 0·4)	0·1 (0 to 0·1)	0 (0 to 0)	123
Anxiety disorders	0 (0 to 0)	0 (0 to 0)	0 (0 to 0)	0·5 (0·1 to 0·9)	7·3 (3·5 to 12·4)	19·9 (10·6 to 33·8)	26·9 (15·9 to 41·6)	28·5 (16 to 46·1)	27·6 (15·1 to 45·1)	27 (15·9 to 42)	28 (16·4 to 42·8)	27·5 (15·6 to 43·4)	24·8 (14·2 to 39·6)	22·3 (13·2 to 33·8)	19·3 (12·1 to 28·9)	15·2 (9·2 to 23·5)	10·2 (6 to 16·3)	7 (4·1 to 11)	4·7 (2·9 to 7·4)	3·1 (1·9 to 4·9)	1·4 (0·9 to 2·2)	0·4 (0·2 to 0·7)	0·1 (0·1 to 0·2)	302
Eating disorders	0 (0 to 0)	0 (0 to 0)	0 (0 to 0)	0 (0 to 0)	0·1 (0 to 0·2)	1·4 (0·6 to 2·4)	5·2 (2·7 to 9·2)	8·2 (4·4 to 14·4)	7·2 (3·8 to 11·8)	5·4 (3 to 8·7)	4 (2·2 to 6·5)	2·5 (1·3 to 4·2)	1·3 (0·7 to 2·3)	0 (0 to 0)	0 (0 to 0)	0 (0 to 0)	0 (0 to 0)	0 (0 to 0)	0 (0 to 0)	0 (0 to 0)	0 (0 to 0)	0 (0 to 0)	0 (0 to 0)	35·2
Anorexia nervosa	0 (0 to 0)	0 (0 to 0)	0 (0 to 0)	0 (0 to 0)	0·1 (0 to 0·2)	0·7 (0·3 to 1·3)	2·1 (1 to 3·8)	2·1 (1 to 3·9)	1·5 (0·7 to 2·7)	0·9 (0·4 to 1·8)	0·7 (0·3 to 1·3)	0·5 (0·2 to 1)	0·4 (0·1 to 0·7)	0 (0 to 0)	0 (0 to 0)	0 (0 to 0)	0 (0 to 0)	0 (0 to 0)	0 (0 to 0)	0 (0 to 0)	0 (0 to 0)	0 (0 to 0)	0 (0 to 0)	9
Bulimia nervosa	0 (0 to 0)	0 (0 to 0)	0 (0 to 0)	0 (0 to 0)	0 (0 to 0)	0·6 (0·2 to 1·3)	3·2 (1·3 to 6·4)	6·2 (2·8 to 11·8)	5·7 (2·7 to 10·2)	4·5 (2·3 to 7·4)	3·3 (1·6 to 5·5)	1·9 (1 to 3·5)	0·9 (0·4 to 1·8)	0 (0 to 0)	0 (0 to 0)	0 (0 to 0)	0 (0 to 0)	0 (0 to 0)	0 (0 to 0)	0 (0 to 0)	0 (0 to 0)	0 (0 to 0)	0 (0 to 0)	26·3
Autism spectrum disorders	0 (0 to 0)	0 (0 to 0)	0·2 (0·1 to 0·4)	1 (0·6 to 1·6)	1·3 (0·7 to 2·1)	1·3 (0·8 to 2·2)	1·3 (0·7 to 2)	1·2 (0·7 to 1·9)	1·1 (0·6 to 1·7)	1 (0·5 to 1·6)	1 (0·5 to 1·5)	0·9 (0·5 to 1·5)	0·8 (0·4 to 1·3)	0·7 (0·4 to 1·1)	0·6 (0·4 to 1)	0·5 (0·3 to 0·8)	0·3 (0·2 to 0·6)	0·2 (0·1 to 0·4)	0·2 (0·1 to 0·3)	0·1 (0·1 to 0·2)	0·1 (0 to 0·1)	0 (0 to 0)	0 (0 to 0)	13·9
Attention-deficit/hyperactivity disorder	0 (0 to 0)	0 (0 to 0)	0 (0 to 0)	0·1 (0·1 to 0·3)	1·5 (0·8 to 2·8)	2·2 (1·2 to 3·9)	1·8 (1 to 3·2)	1·3 (0·7 to 2·3)	0·9 (0·5 to 1·6)	0·7 (0·3 to 1·2)	0·5 (0·3 to 1)	0·4 (0·2 to 0·8)	0·3 (0·2 to 0·6)	0·2 (0·1 to 0·5)	0·2 (0·1 to 0·3)	0·1 (0 to 0·2)	0 (0 to 0·1)	0 (0 to 0)	0 (0 to 0)	0 (0 to 0)	0 (0 to 0)	0 (0 to 0)	0 (0 to 0)	10·4
Conduct disorder	0 (0 to 0)	0 (0 to 0)	0 (0 to 0)	0 (0 to 0)	3·5 (1·6 to 6·6)	13·1 (6·6 to 23·1)	7·4 (3·6 to 12·9)	0 (0 to 0)	0 (0 to 0)	0 (0 to 0)	0 (0 to 0)	0 (0 to 0)	0 (0 to 0)	0 (0 to 0)	0 (0 to 0)	0 (0 to 0)	0 (0 to 0)	0 (0 to 0)	0 (0 to 0)	0 (0 to 0)	0 (0 to 0)	0 (0 to 0)	0 (0 to 0)	24
Idiopathic developmental intellectual disability	0 (0 to 0)	0 (0 to 0)	0·1 (0 to 0·2)	0·6 (0·2 to 1·1)	0·9 (0·3 to 1·6)	0·9 (0·3 to 1·6)	0·8 (0·3 to 1·6)	0·8 (0·3 to 1·5)	0·7 (0·2 to 1·3)	0·6 (0·2 to 1·2)	0·6 (0·2 to 1·1)	0·6 (0·2 to 1)	0·5 (0·2 to 0·9)	0·4 (0·1 to 0·8)	0·4 (0·1 to 0·7)	0·3 (0·1 to 0·5)	0·2 (0·1 to 0·3)	0·1 (0 to 0·2)	0·1 (0 to 0·1)	0·1 (0 to 0·1)	0 (0 to 0)	0 (0 to 0)	0 (0 to 0)	8·5
Other mental disorders	0 (0 to 0)	0 (0 to 0)	0 (0 to 0)	0 (0 to 0)	0 (0 to 0)	0·2 (0·1 to 0·3)	1·2 (0·5 to 2·3)	2·9 (1·4 to 5·2)	4·2 (2·3 to 6·8)	5·2 (3 to 8·1)	6 (3·4 to 9·3)	6·1 (3·5 to 9·4)	5·7 (3·5 to 8·8)	5·4 (3·3 to 8·3)	5 (3 to 7·4)	4·2 (2·5 to 6·3)	2·9 (1·8 to 4·4)	2·1 (1·3 to 3·2)	1·6 (1 to 2·4)	1·2 (0·7 to 1·7)	0·6 (0·4 to 0·9)	0·2 (0·1 to 0·3)	0·1 (0 to 0·1)	54·7
Substance use disorders	0 (0 to 0)	0 (0 to 0)	0 (0 to 0)	0 (0 to 0)	0 (0 to 0)	0·5 (0·2 to 0·8)	8 (5 to 12)	16·3 (10·3 to 23·6)	16·4 (10·8 to 23·6)	14·8 (9·4 to 21·5)	14·3 (10·1 to 19·6)	12·9 (8·9 to 17·7)	11 (7·6 to 15·1)	8·5 (5·9 to 11·7)	6·6 (4·6 to 8·9)	4·9 (3·5 to 6·6)	2·9 (2·1 to 4)	1·9 (1·4 to 2·6)	1·4 (0·9 to 1·9)	1·1 (0·8 to 1·6)	0·7 (0·4 to 0·9)	0·3 (0·2 to 0·5)	0·1 (0·1 to 0·2)	122
Alcohol use disorders	0 (0 to 0)	0 (0 to 0)	0 (0 to 0)	0 (0 to 0)	0 (0 to 0)	0·3 (0·1 to 0·5)	3·4 (1·8 to 5·5)	8·4 (4·7 to 14·1)	9·2 (5·3 to 14·4)	9·4 (5·7 to 15·1)	10 (6·9 to 14·6)	9·6 (6·3 to 13·7)	8·3 (5·6 to 11·7)	6·3 (4·2 to 8·9)	4·8 (3·2 to 6·6)	3·6 (2·4 to 5·1)	2 (1·4 to 2·9)	1·4 (0·9 to 2)	1 (0·7 to 1·4)	0·9 (0·6 to 1·3)	0·6 (0·4 to 0·8)	0·3 (0·2 to 0·4)	0·1 (0·1 to 0·2)	79·5
Drug use disorders	0 (0 to 0)	0 (0 to 0)	0 (0 to 0)	0 (0 to 0)	0 (0 to 0)	0·2 (0·1 to 0·4)	4·6 (2·6 to 7·3)	7·9 (4·8 to 11·9)	7·2 (4·3 to 10·8)	5·4 (3 to 8·3)	4·2 (2·5 to 6·5)	3·2 (1·8 to 5·2)	2·7 (1·5 to 4)	2·3 (1·3 to 3·7)	1·8 (1 to 2·9)	1·3 (0·7 to 2·1)	0·8 (0·4 to 1·4)	0·5 (0·3 to 0·9)	0·3 (0·2 to 0·6)	0·2 (0·1 to 0·4)	0·1 (0·1 to 0·2)	0 (0 to 0·1)	0 (0 to 0)	43
Opioid use disorders	0 (0 to 0)	0 (0 to 0)	0 (0 to 0)	0 (0 to 0)	0 (0 to 0)	0 (0 to 0)	0·9 (0·3 to 1·8)	3·7 (1·6 to 6·7)	4·2 (2·1 to 7·1)	3·6 (1·6 to 6·1)	2·9 (1·4 to 5)	2·2 (1 to 3·9)	1·8 (0·8 to 3·1)	1·7 (0·7 to 2·9)	1·4 (0·6 to 2·4)	1 (0·4 to 1·8)	0·6 (0·3 to 1·1)	0·4 (0·1 to 0·7)	0·2 (0·1 to 0·4)	0·2 (0 to 0·3)	0·1 (0 to 0·1)	0 (0 to 0)	0 (0 to 0)	24·9
Cocaine use disorders	0 (0 to 0)	0 (0 to 0)	0 (0 to 0)	0 (0 to 0)	0 (0 to 0)	0 (0 to 0·1)	0·9 (0·3 to 2)	0·9 (0·3 to 1·8)	0·6 (0·2 to 1·2)	0·4 (0·1 to 0·9)	0·3 (0·1 to 0·8)	0·2 (0 to 0·6)	0·2 (0·1 to 0·3)	0·2 (0·1 to 0·3)	0·1 (0·1 to 0·2)	0·1 (0·1 to 0·2)	0·1 (0 to 0·1)	0 (0 to 0·1)	0 (0 to 0·1)	0 (0 to 0)	0 (0 to 0)	0 (0 to 0)	0 (0 to 0)	4·3
Amphetamine use disorders	0 (0 to 0)	0 (0 to 0)	0 (0 to 0)	0 (0 to 0)	0 (0 to 0)	0 (0 to 0)	0·2 (0·1 to 0·3)	0·7 (0·2 to 1·5)	0·6 (0·2 to 1·3)	0·3 (0 to 0·8)	0·2 (0·1 to 0·3)	0·1 (0·1 to 0·2)	0·1 (0 to 0·1)	0 (0 to 0·1)	0 (0 to 0·1)	0 (0 to 0)	0 (0 to 0)	0 (0 to 0)	0 (0 to 0)	0 (0 to 0)	0 (0 to 0)	0 (0 to 0)	0 (0 to 0)	2·3
Cannabis use disorders	0 (0 to 0)	0 (0 to 0)	0 (0 to 0)	0 (0 to 0)	0 (0 to 0)	0·2 (0·1 to 0·4)	1·8 (0·8 to 3·4)	1·4 (0·6 to 2·6)	0·8 (0·3 to 1·5)	0·5 (0·2 to 0·9)	0·3 (0·1 to 0·7)	0·3 (0·1 to 0·5)	0·2 (0·1 to 0·4)	0·1 (0·1 to 0·3)	0·1 (0 to 0·2)	0 (0 to 0·1)	0 (0 to 0)	0 (0 to 0)	0 (0 to 0)	0 (0 to 0)	0 (0 to 0)	0 (0 to 0)	0 (0 to 0)	5·8
Other drug use disorders	0 (0 to 0)	0 (0 to 0)	0 (0 to 0)	0 (0 to 0)	0 (0 to 0)	0 (0 to 0)	0·8 (0·3 to 1·5)	1·1 (0·5 to 2)	1 (0·5 to 1·6)	0·6 (0·3 to 1·1)	0·5 (0·3 to 0·9)	0·4 (0·2 to 0·7)	0·4 (0·2 to 0·6)	0·3 (0·1 to 0·4)	0·2 (0·1 to 0·3)	0·1 (0·1 to 0·2)	0·1 (0·1 to 0·1)	0·1 (0·1 to 0·1)	0·1 (0·1 to 0·1)	0·1 (0 to 0·1)	0 (0 to 0)	0 (0 to 0)	0 (0 to 0)	5·7
Diabetes and kidney diseases	0 (0 to 0)	0 (0 to 0)	2·5 (1·5 to 4·1)	3 (1·9 to 4·3)	4·4 (3·2 to 6)	9·9 (7·5 to 12·8)	21·1 (16·9 to 25·8)	34·8 (27·3 to 42·5)	45·5 (35·6 to 56·4)	57·3 (45 to 70·8)	77·4 (61·7 to 94·4)	115 (92·9 to 139)	167 (136 to 202)	240 (195 to 291)	281 (228 to 340)	323 (266 to 387)	292 (246 to 347)	257 (216 to 301)	219 (184 to 258)	190 (157 to 223)	93 (77·2 to 109)	32·1 (26·3 to 37·7)	8 (6·5 to 9·4)	2,470
Diabetes mellitus	0 (0 to 0)	0 (0 to 0)	0·9 (0·4 to 1·7)	0·6 (0·3 to 1)	0·8 (0·5 to 1·1)	2·6 (1·4 to 3·9)	9·2 (7·1 to 11·8)	17 (13 to 21·8)	22·9 (17·1 to 29·2)	30·3 (22·6 to 39·3)	45·4 (34·8 to 57·7)	73·8 (57·3 to 93·7)	115 (90·5 to 145)	176 (136 to 221)	218 (170 to 271)	254 (203 to 313)	235 (191 to 286)	206 (168 to 247)	176 (145 to 213)	152 (123 to 185)	71 (57 to 87·3)	23·6 (18·6 to 28·6)	5·6 (4·5 to 6·8)	1,840
Diabetes mellitus type 1	0 (0 to 0)	0 (0 to 0)	0·9 (0·4 to 1·7)	0·6 (0·3 to 1)	0·8 (0·5 to 1·1)	2·6 (1·4 to 3·9)	2·5 (1·6 to 3·9)	4·5 (2·9 to 6·8)	5·2 (3·3 to 8)	4·5 (2·6 to 7·4)	6·6 (4·2 to 10·4)	10·2 (6·5 to 16)	3·5 (2·2 to 5·4)	4·8 (3 to 7·8)	5·4 (3·4 to 8·8)	5 (3·1 to 8·3)	4·7 (3 to 7·6)	3·5 (2·1 to 6)	3·1 (1·9 to 5·3)	1·7 (1 to 3·2)	0·8 (0·5 to 1·4)	0·3 (0·2 to 0·5)	0·1 (0·1 to 0·2)	71·3
Diabetes mellitus type 2	0 (0 to 0)	0 (0 to 0)	0 (0 to 0)	0 (0 to 0)	0 (0 to 0)	0 (0 to 0)	6·7 (4·9 to 8·8)	12·5 (9 to 16·5)	17·6 (12·6 to 23·4)	25·8 (18·8 to 34·4)	38·8 (29 to 50·4)	63·6 (48·1 to 81·5)	112 (87·6 to 141)	171 (133 to 215)	213 (165 to 265)	249 (199 to 307)	230 (186 to 280)	202 (164 to 242)	173 (142 to 209)	150 (122 to 183)	70·2 (56·3 to 86·4)	23·3 (18·3 to 28·2)	5·5 (4·4 to 6·7)	1,760
Chronic kidney disease	0 (0 to 0)	0 (0 to 0)	1·6 (1 to 2·6)	2·4 (1·6 to 3·5)	3·5 (2·4 to 5)	7·2 (5·1 to 9·7)	11·7 (8·6 to 15·5)	17·6 (12·6 to 23)	22·5 (16·2 to 30·4)	26·9 (19·6 to 35·7)	31·9 (22·5 to 43)	40·8 (30·9 to 53·2)	51·6 (38·5 to 69·2)	63·8 (47·8 to 81·6)	62·9 (46·5 to 82·2)	69·1 (52·6 to 89·5)	57·4 (43·7 to 74·3)	51·4 (39·4 to 65·4)	43·2 (33·8 to 54·7)	37·4 (28·4 to 48·1)	21·9 (17·4 to 26·9)	8·5 (6·7 to 10·5)	2·4 (1·8 to 3)	636
Chronic kidney disease due to diabetes mellitus type 1	0 (0 to 0)	0 (0 to 0)	0 (0 to 0)	0 (0 to 0·1)	0·1 (0 to 0·2)	0·2 (0·1 to 0·4)	0·5 (0·2 to 1)	1·1 (0·5 to 2·3)	2·7 (1·1 to 4·9)	4·4 (2·2 to 7·3)	5·9 (3 to 9·7)	7·4 (4 to 12)	7·9 (3·7 to 13·9)	7·4 (3·1 to 14)	4·9 (1·6 to 10·5)	4 (1·3 to 8·8)	2·6 (1 to 5·7)	1·8 (0·7 to 3·6)	1 (0·4 to 2·2)	0·4 (0·1 to 1)	0·1 (0 to 0·2)	0 (0 to 0)	0 (0 to 0)	52·6
Chronic kidney disease due to diabetes mellitus type 2	0 (0 to 0)	0 (0 to 0)	0 (0 to 0)	0 (0 to 0)	0 (0 to 0)	0 (0 to 0)	0·2 (0·1 to 0·5)	0·5 (0·2 to 1·1)	1·1 (0·4 to 2·1)	1·8 (0·8 to 3·4)	2·9 (1·3 to 5·3)	5·2 (2·5 to 9)	9·5 (5·2 to 15·1)	15·9 (9·2 to 24)	18·8 (11·2 to 27·5)	22·3 (13·5 to 32·1)	19 (12·7 to 27·1)	17·1 (11·4 to 23·8)	14·2 (9·5 to 19·7)	11·9 (8 to 16·8)	6·8 (4·7 to 9·5)	2·5 (1·7 to 3·6)	0·7 (0·4 to 1)	151
Chronic kidney disease due to hypertension	0 (0 to 0)	0 (0 to 0)	0 (0 to 0)	0 (0 to 0)	0 (0 to 0)	0 (0 to 0)	0·8 (0·3 to 1·5)	1·3 (0·5 to 2·4)	1·8 (0·9 to 3·2)	2·4 (1·2 to 4)	3·2 (1·7 to 5·1)	4·6 (2·8 to 7)	6·5 (4·1 to 9·9)	9·5 (5·9 to 14·6)	11 (6·9 to 16·8)	14·3 (9·3 to 20·7)	13·5 (8·6 to 19·6)	13·4 (9·1 to 19·3)	12·4 (8·5 to 18·1)	11·9 (8 to 16·7)	7·7 (5·5 to 10·4)	3·2 (2·3 to 4·3)	0·9 (0·7 to 1·3)	118
Chronic kidney disease due to glomerulonephritis	0 (0 to 0)	0 (0 to 0)	0·5 (0·2 to 0·9)	0·7 (0·4 to 1·2)	1·2 (0·7 to 1·8)	2·9 (1·8 to 4·4)	4·4 (2·6 to 6·4)	6·5 (4 to 9·8)	7·8 (4·8 to 11·8)	8·5 (5·1 to 12·7)	9·6 (5·8 to 14·8)	11·6 (6·6 to 18)	12·9 (7·5 to 19·6)	14·2 (7·8 to 22·8)	12·5 (6·7 to 21·2)	11·9 (5·9 to 21)	8·4 (4·1 to 14·8)	6·4 (3·2 to 10·9)	4·7 (2·2 to 8·4)	3·5 (1·6 to 6·3)	1·6 (0·7 to 2·8)	0·5 (0·3 to 1)	0·1 (0·1 to 0·3)	130
Chronic kidney disease due to other and unspecified causes	0 (0 to 0)	0 (0 to 0)	1·2 (0·7 to 1·9)	1·6 (1 to 2·5)	2·2 (1·4 to 3·4)	4·1 (2·5 to 6·2)	5·8 (3·8 to 8·3)	8·1 (5·2 to 11·8)	9·2 (5·9 to 13·2)	9·8 (6·2 to 14·3)	10·3 (6·4 to 15·6)	12·1 (7·4 to 18·4)	14·7 (9·2 to 22·3)	16·9 (10·4 to 25·3)	15·7 (9·7 to 25)	16·4 (10 to 25·6)	13·8 (8·3 to 20·8)	12·8 (8·2 to 18·4)	11 (6·8 to 16·5)	9·7 (6·1 to 14·6)	5·7 (3·7 to 8·3)	2·2 (1·5 to 3·1)	0·6 (0·4 to 0·9)	184
Acute glomerulonephritis	0 (0 to 0)	0 (0 to 0)	0 (0 to 0)	0 (0 to 0)	0·1 (0·1 to 0·3)	0·2 (0·1 to 0·4)	0·1 (0 to 0·2)	0·2 (0·1 to 0·4)	0·1 (0·1 to 0·3)	0·2 (0·1 to 0·3)	0·1 (0·1 to 0·3)	0·1 (0 to 0·2)	0·1 (0 to 0·2)	0 (0 to 0·1)	0·1 (0 to 0·3)	0·1 (0 to 0·2)	0 (0 to 0·1)	0·1 (0 to 0·2)	0 (0 to 0·1)	0·1 (0 to 0·1)	0 (0 to 0·1)	0 (0 to 0·1)	0 (0 to 0)	1·8
Skin and subcutaneous diseases	0 (0 to 0)	0 (0 to 0)	3 (1·8 to 4·6)	21·9 (13·9 to 31·8)	28·7 (17·9 to 43)	29·7 (19·1 to 43·9)	31·3 (20·2 to 46·9)	20·5 (13·2 to 30·7)	16·8 (10·9 to 24·6)	15·9 (10·5 to 23·1)	16·7 (11·1 to 23·8)	18 (12·4 to 25·9)	17·2 (11·6 to 24·5)	19·3 (13·6 to 27·6)	20·1 (13·9 to 29·9)	19·7 (14 to 28·4)	16·2 (11·7 to 23·6)	15·9 (11·3 to 22·7)	16·4 (11·2 to 24·5)	16·3 (10·9 to 24·6)	11·1 (7·1 to 16·9)	4·7 (3·1 to 6·9)	1·5 (1 to 2·3)	361
Dermatitis	0 (0 to 0)	0 (0 to 0)	0 (0 to 0)	7·9 (4·2 to 13·4)	10 (5·4 to 17·6)	7·2 (4 to 12·2)	5·4 (3 to 8·9)	4·3 (2·4 to 7)	3·6 (2 to 6)	3·3 (1·8 to 5·3)	3·2 (1·8 to 5·2)	3·1 (1·8 to 5)	2·9 (1·6 to 4·8)	3 (1·7 to 5)	3 (1·7 to 4·9)	2·7 (1·5 to 4·4)	2·1 (1·2 to 3·4)	1·6 (0·9 to 2·5)	1·2 (0·7 to 1·9)	0·8 (0·5 to 1·3)	0·4 (0·2 to 0·6)	0·1 (0·1 to 0·2)	0 (0 to 0·1)	66·1
Atopic dermatitis	0 (0 to 0)	0 (0 to 0)	0 (0 to 0)	7·9 (4·2 to 13·4)	9·8 (5·3 to 17·3)	6·6 (3·5 to 11·4)	4·3 (2·2 to 7·5)	2·9 (1·5 to 5·2)	2·1 (1·1 to 3·9)	1·8 (0·9 to 3·2)	1·7 (0·8 to 3)	1·6 (0·8 to 2·8)	1·4 (0·8 to 2·6)	1·4 (0·7 to 2·4)	1·4 (0·7 to 2·4)	1·2 (0·6 to 2·1)	0·9 (0·4 to 1·5)	0·6 (0·3 to 1·1)	0·5 (0·3 to 0·8)	0·4 (0·2 to 0·7)	0·2 (0·1 to 0·3)	0·1 (0 to 0·1)	0 (0 to 0)	46·7
Contact dermatitis	0 (0 to 0)	0 (0 to 0)	0 (0 to 0)	0 (0 to 0)	0·1 (0 to 0·2)	0·6 (0·2 to 1·1)	1 (0·4 to 2)	1·2 (0·5 to 2·4)	1·3 (0·6 to 2·5)	1·4 (0·6 to 2·7)	1·4 (0·6 to 2·9)	1·4 (0·6 to 2·7)	1·4 (0·6 to 2·6)	1·5 (0·6 to 2·8)	1·5 (0·6 to 3)	1·4 (0·6 to 2·6)	1·1 (0·5 to 2·2)	0·9 (0·4 to 1·7)	0·6 (0·3 to 1·1)	0·4 (0·2 to 0·7)	0·2 (0·1 to 0·3)	0·1 (0 to 0·1)	0 (0 to 0)	17·5
Seborrhoeic dermatitis	0 (0 to 0)	0 (0 to 0)	0 (0 to 0)	0·1 (0 to 0·1)	0·1 (0 to 0·1)	0·1 (0 to 0·2)	0·1 (0·1 to 0·2)	0·1 (0·1 to 0·2)	0·1 (0·1 to 0·3)	0·2 (0·1 to 0·3)	0·2 (0·1 to 0·3)	0·1 (0·1 to 0·3)	0·1 (0·1 to 0·2)	0·1 (0·1 to 0·2)	0·1 (0 to 0·2)	0·1 (0·1 to 0·2)	0·1 (0·1 to 0·2)	0·1 (0 to 0·2)	0·1 (0 to 0·1)	0·1 (0 to 0·1)	0 (0 to 0)	0 (0 to 0)	0 (0 to 0)	1·9
Psoriasis	0 (0 to 0)	0 (0 to 0)	0 (0 to 0)	0·3 (0·2 to 0·4)	0·8 (0·3 to 1·5)	1 (0·5 to 1·8)	1·2 (0·6 to 2)	1·2 (0·6 to 2)	1·2 (0·6 to 2)	1·4 (0·7 to 2·2)	1·6 (0·9 to 2·4)	1·7 (0·9 to 2·7)	1·7 (1 to 2·6)	1·8 (1·1 to 2·8)	1·8 (1·1 to 2·7)	1·6 (0·9 to 2·4)	1·1 (0·7 to 1·7)	0·8 (0·5 to 1·2)	0·5 (0·3 to 0·7)	0·3 (0·2 to 0·4)	0·1 (0·1 to 0·2)	0 (0 to 0)	0 (0 to 0)	20·1
Bacterial skin diseases	0 (0 to 0)	0 (0 to 0)	1·7 (0·7 to 3·1)	0·9 (0·5 to 1·4)	0·8 (0·5 to 1·3)	0·7 (0·3 to 1·1)	0·5 (0·3 to 0·9)	0·9 (0·4 to 1·4)	0·8 (0·4 to 1·4)	1·6 (0·8 to 2·7)	1·4 (0·7 to 2·4)	2·7 (1·2 to 4·7)	2·2 (1 to 3·6)	3·1 (1·4 to 5·2)	3·1 (1·4 to 5·8)	3·6 (1·8 to 6·8)	2·8 (1·3 to 5·1)	2·4 (1·1 to 4·4)	2·6 (1·1 to 4·9)	2·8 (1 to 5·2)	1·8 (0·7 to 3·4)	0·8 (0·3 to 1·4)	0·2 (0·1 to 0·4)	37·5
Cellulitis	0 (0 to 0)	0 (0 to 0)	0·5 (0·1 to 1·2)	0·3 (0·1 to 0·6)	0·4 (0·2 to 0·7)	0·2 (0·1 to 0·4)	0·1 (0 to 0·2)	0·2 (0·1 to 0·4)	0·3 (0·1 to 0·6)	0·3 (0·1 to 0·7)	0·4 (0·1 to 0·8)	0·6 (0·3 to 1·3)	0·4 (0·2 to 0·9)	0·9 (0·4 to 2)	0·9 (0·4 to 2·4)	0·9 (0·4 to 2·3)	0·8 (0·4 to 1·9)	0·6 (0·2 to 1·3)	0·6 (0·2 to 1·4)	0·5 (0·2 to 1·2)	0·3 (0·1 to 0·7)	0·1 (0 to 0·2)	0 (0 to 0·1)	9·5
Pyoderma	0 (0 to 0)	0 (0 to 0)	1·2 (0·4 to 2·4)	0·6 (0·3 to 1)	0·4 (0·2 to 0·8)	0·5 (0·2 to 0·8)	0·4 (0·2 to 0·7)	0·7 (0·3 to 1·1)	0·5 (0·3 to 0·9)	1·2 (0·6 to 2·1)	1·1 (0·5 to 1·8)	2·1 (0·9 to 3·6)	1·7 (0·7 to 2·9)	2·2 (0·9 to 3·8)	2·2 (0·8 to 3·9)	2·6 (1·3 to 4·6)	1·9 (0·8 to 3·5)	1·8 (0·7 to 3·3)	2 (0·8 to 3·8)	2·3 (0·8 to 4·2)	1·5 (0·6 to 2·9)	0·7 (0·2 to 1·2)	0·2 (0·1 to 0·4)	28
Scabies	0 (0 to 0)	0 (0 to 0)	0·5 (0·3 to 0·9)	3·4 (1·7 to 6)	4·7 (2·3 to 8·2)	4·9 (2·4 to 8·5)	5·2 (2·6 to 9·1)	4·5 (2·2 to 8)	3·1 (1·6 to 5·4)	2·3 (1·1 to 3·9)	2·3 (1·1 to 3·9)	2·5 (1·3 to 4·2)	2·3 (1·2 to 4)	2·1 (1·1 to 3·6)	1·8 (0·9 to 3·2)	1·4 (0·7 to 2·5)	1 (0·5 to 1·8)	0·8 (0·4 to 1·3)	0·6 (0·3 to 1·1)	0·5 (0·3 to 0·9)	0·3 (0·1 to 0·5)	0·1 (0·1 to 0·2)	0 (0 to 0·1)	44·2
Fungal skin diseases	0 (0 to 0)	0 (0 to 0)	0·1 (0 to 0·2)	0·6 (0·2 to 1·4)	1·1 (0·4 to 2·3)	1·2 (0·5 to 2·8)	1·3 (0·5 to 3)	1·3 (0·5 to 2·9)	1·3 (0·5 to 2·8)	1·3 (0·5 to 2·9)	1·4 (0·5 to 3)	1·4 (0·5 to 3)	1·3 (0·5 to 2·9)	1·4 (0·6 to 3·1)	1·6 (0·7 to 3·6)	1·7 (0·6 to 3·6)	1·6 (0·6 to 3·5)	1·7 (0·7 to 3·6)	1·6 (0·6 to 3·4)	1·5 (0·6 to 3·2)	1 (0·4 to 2)	0·4 (0·2 to 0·8)	0·1 (0 to 0·2)	25
Viral skin diseases	0 (0 to 0)	0 (0 to 0)	0 (0 to 0)	3·7 (2·3 to 5·6)	6 (3·8 to 9·2)	3·1 (2 to 4·8)	1·9 (1·2 to 3)	1·5 (0·9 to 2·4)	1 (0·6 to 1·7)	0·8 (0·5 to 1·3)	0·8 (0·4 to 1·2)	0·8 (0·4 to 1·2)	0·7 (0·4 to 1)	0·6 (0·4 to 1)	0·6 (0·3 to 0·9)	0·5 (0·3 to 0·8)	0·4 (0·2 to 0·6)	0·2 (0·1 to 0·4)	0·1 (0·1 to 0·2)	0·1 (0·1 to 0·1)	0 (0 to 0·1)	0 (0 to 0)	0 (0 to 0)	22·9
Acne vulgaris	0 (0 to 0)	0 (0 to 0)	0 (0 to 0)	0 (0 to 0)	0·3 (0·2 to 0·5)	7·2 (4·1 to 11·7)	11·6 (6·7 to 19)	2·8 (1·5 to 4·7)	1·3 (0·7 to 2·2)	0·9 (0·4 to 1·5)	0·7 (0·3 to 1·2)	0·5 (0·2 to 0·9)	0·3 (0·2 to 0·5)	0·2 (0·1 to 0·4)	0·2 (0·1 to 0·3)	0·1 (0·1 to 0·2)	0·1 (0 to 0·1)	0 (0 to 0·1)	0 (0 to 0)	0 (0 to 0)	0 (0 to 0)	0 (0 to 0)	0 (0 to 0)	26·3
Alopecia areata	0 (0 to 0)	0 (0 to 0)	0 (0 to 0)	0·1 (0 to 0·2)	0·1 (0·1 to 0·3)	0·2 (0·1 to 0·3)	0·3 (0·2 to 0·5)	0·5 (0·3 to 0·8)	0·6 (0·4 to 1)	0·7 (0·4 to 1)	0·6 (0·4 to 1)	0·5 (0·3 to 0·8)	0·4 (0·2 to 0·7)	0·3 (0·2 to 0·6)	0·3 (0·2 to 0·5)	0·3 (0·1 to 0·4)	0·2 (0·1 to 0·3)	0·1 (0·1 to 0·2)	0·1 (0 to 0·1)	0 (0 to 0·1)	0 (0 to 0)	0 (0 to 0)	0 (0 to 0)	5·3
Pruritus	0 (0 to 0)	0 (0 to 0)	0 (0 to 0)	0·2 (0·1 to 0·3)	0·2 (0·1 to 0·5)	0·2 (0·1 to 0·5)	0·3 (0·1 to 0·5)	0·3 (0·1 to 0·6)	0·3 (0·1 to 0·7)	0·4 (0·2 to 0·7)	0·4 (0·2 to 0·8)	0·4 (0·2 to 0·8)	0·4 (0·2 to 0·8)	0·4 (0·2 to 0·8)	0·4 (0·2 to 0·9)	0·4 (0·2 to 0·9)	0·4 (0·2 to 0·8)	0·3 (0·1 to 0·6)	0·2 (0·1 to 0·5)	0·2 (0·1 to 0·4)	0·1 (0 to 0·2)	0 (0 to 0·1)	0 (0 to 0)	5·6
Urticaria	0 (0 to 0)	0 (0 to 0)	0·6 (0·3 to 0·9)	3·9 (2·3 to 6·2)	3·6 (2 to 5·8)	2·5 (1·3 to 4·3)	2·2 (1·1 to 3·8)	2 (1 to 3·3)	1·7 (0·9 to 2·9)	1·7 (0·9 to 2·8)	1·8 (1 to 3)	1·6 (0·9 to 2·8)	1·4 (0·8 to 2·4)	1·3 (0·7 to 2·1)	1·2 (0·6 to 2)	1 (0·5 to 1·7)	0·7 (0·4 to 1·2)	0·5 (0·3 to 0·9)	0·4 (0·2 to 0·6)	0·2 (0·1 to 0·4)	0·1 (0·1 to 0·2)	0 (0 to 0)	0 (0 to 0)	28·6
Decubitus ulcer	0 (0 to 0)	0 (0 to 0)	0 (0 to 0)	0·1 (0 to 0·2)	0·2 (0·1 to 0·4)	0·3 (0·1 to 0·6)	0·3 (0·1 to 0·5)	0·1 (0 to 0·1)	0·4 (0·1 to 0·8)	0·2 (0·1 to 0·4)	0·7 (0·3 to 1·4)	0·9 (0·4 to 1·7)	1·4 (0·6 to 2·4)	2·6 (1·3 to 4·5)	3·4 (1·7 to 6·1)	3·6 (2 to 6·5)	3·5 (2 to 6·4)	5·5 (3·2 to 9·4)	7·6 (4·6 to 13)	8·8 (5 to 14·3)	6·9 (3·9 to 11·2)	3·1 (1·8 to 4·7)	1·1 (0·6 to 1·6)	50·6
Other skin and subcutaneous diseases	0 (0 to 0)	0 (0 to 0)	0·1 (0·1 to 0·2)	0·8 (0·4 to 1·4)	0·8 (0·4 to 1·5)	1 (0·5 to 1·8)	1·2 (0·6 to 2·3)	1·2 (0·6 to 2·3)	1·3 (0·6 to 2·3)	1·4 (0·7 to 2·7)	1·7 (0·8 to 3·1)	1·9 (0·9 to 3·5)	2·1 (1 to 3·9)	2·4 (1·1 to 4·3)	2·7 (1·3 to 5)	2·8 (1·3 to 5·1)	2·3 (1·1 to 4·2)	1·9 (0·9 to 3·5)	1·5 (0·7 to 2·7)	1 (0·5 to 1·8)	0·5 (0·2 to 0·8)	0·1 (0·1 to 0·3)	0 (0 to 0·1)	28·7
Sense organ diseases	0 (0 to 0)	0 (0 to 0)	0·2 (0·1 to 0·3)	2·2 (1·4 to 3·4)	5·1 (3·2 to 7·5)	6·8 (4·3 to 9·9)	8·2 (5·1 to 12)	9·8 (6 to 14·6)	11·5 (7·3 to 17·3)	13·7 (8·6 to 20·5)	16·7 (10·4 to 25·1)	20·6 (13·1 to 31·6)	27·3 (17·4 to 43·3)	36·3 (23·1 to 57·5)	45·9 (29·3 to 71·1)	51·5 (33 to 77·7)	48·3 (31·8 to 71·3)	45·8 (30·7 to 65·7)	43·6 (29·6 to 61·6)	39·6 (27·5 to 54·3)	23·8 (16·6 to 31·8)	9·5 (6·7 to 12·7)	2·8 (2 to 3·7)	469
Blindness and vision loss	0 (0 to 0)	0 (0 to 0)	0 (0 to 0·1)	0·6 (0·4 to 1·1)	1·6 (0·9 to 2·5)	2 (1·2 to 3·3)	2·5 (1·5 to 3·8)	2·8 (1·7 to 4·3)	2·8 (1·7 to 4·2)	3·1 (1·9 to 4·7)	3·8 (2·4 to 5·6)	5 (3·1 to 7·6)	6·2 (3·9 to 9·6)	8·1 (5·1 to 13·1)	10·6 (6·7 to 17)	12·8 (8·1 to 19·6)	13·2 (8·6 to 19·5)	13·6 (9·1 to 19·7)	13·6 (9·3 to 19·3)	12·8 (8·8 to 17·8)	8 (5·6 to 10·9)	3·4 (2·4 to 4·6)	1·1 (0·8 to 1·6)	128
Glaucoma	0 (0 to 0)	0 (0 to 0)	0 (0 to 0)	0 (0 to 0)	0 (0 to 0)	0 (0 to 0)	0 (0 to 0)	0 (0 to 0)	0 (0 to 0)	0 (0 to 0)	0 (0 to 0)	0 (0 to 0)	0 (0 to 0)	0·1 (0 to 0·2)	0·3 (0·1 to 0·5)	0·5 (0·3 to 0·9)	0·7 (0·4 to 1·2)	1 (0·6 to 1·6)	1·3 (0·8 to 2)	1·4 (0·8 to 2·2)	1 (0·6 to 1·5)	0·4 (0·2 to 0·6)	0·1 (0·1 to 0·2)	6·8
Cataract	0 (0 to 0)	0 (0 to 0)	0 (0 to 0)	0 (0 to 0)	0 (0 to 0)	0 (0 to 0)	0 (0 to 0)	0 (0 to 0)	0·1 (0 to 0·1)	0·2 (0 to 0·3)	0·3 (0·1 to 0·6)	0·5 (0·2 to 0·9)	0·7 (0·3 to 1·2)	0·9 (0·5 to 1·5)	1·3 (0·7 to 2)	1·7 (1 to 2·7)	2·3 (1·4 to 3·4)	3·1 (2 to 4·7)	4 (2·6 to 5·8)	4·3 (2·9 to 6·1)	3 (2 to 4·1)	1·2 (0·8 to 1·7)	0·4 (0·2 to 0·6)	23·9
Age-related macular degeneration	0 (0 to 0)	0 (0 to 0)	0 (0 to 0)	0 (0 to 0)	0 (0 to 0)	0 (0 to 0)	0 (0 to 0)	0 (0 to 0)	0 (0 to 0)	0 (0 to 0)	0 (0 to 0)	0 (0 to 0)	0 (0 to 0)	0 (0 to 0·1)	0·1 (0 to 0·1)	0·1 (0 to 0·2)	0·1 (0·1 to 0·3)	0·1 (0·1 to 0·3)	0·2 (0·1 to 0·3)	0·1 (0·1 to 0·2)	0·1 (0 to 0·1)	0 (0 to 0·1)	0 (0 to 0)	1
Refraction disorders	0 (0 to 0)	0 (0 to 0)	0 (0 to 0)	0·5 (0·3 to 0·9)	1·3 (0·7 to 2·1)	1·7 (1 to 2·8)	2·1 (1·2 to 3·4)	2·3 (1·4 to 3·7)	2·2 (1·3 to 3·4)	2·1 (1·2 to 3·4)	2·3 (1·4 to 3·5)	2·4 (1·4 to 3·8)	2·5 (1·5 to 3·8)	2·5 (1·5 to 3·9)	2·6 (1·6 to 4·1)	2·5 (1·5 to 3·9)	2·1 (1·2 to 3·1)	1·9 (1·1 to 2·8)	1·7 (1·1 to 2·6)	1·3 (0·8 to 2)	0·6 (0·4 to 0·9)	0·2 (0·1 to 0·3)	0 (0 to 0·1)	35
Near vision loss	0 (0 to 0)	0 (0 to 0)	0 (0 to 0)	0 (0 to 0)	0 (0 to 0)	0 (0 to 0)	0 (0 to 0·1)	0·1 (0 to 0·1)	0·1 (0 to 0·2)	0·3 (0·1 to 0·6)	0·5 (0·2 to 1·2)	1·1 (0·4 to 2·3)	1·8 (0·7 to 3·8)	2·8 (1·1 to 5·9)	3·8 (1·5 to 8)	4·4 (1·9 to 9·4)	4 (1·6 to 8)	3·6 (1·5 to 7·2)	3·1 (1·3 to 6·4)	2·5 (1·1 to 5·1)	1·4 (0·6 to 2·7)	0·5 (0·2 to 1)	0·1 (0·1 to 0·3)	30·1
Other vision loss	0 (0 to 0)	0 (0 to 0)	0 (0 to 0)	0·1 (0·1 to 0·2)	0·2 (0·1 to 0·5)	0·3 (0·1 to 0·6)	0·4 (0·1 to 0·7)	0·4 (0·2 to 0·8)	0·4 (0·2 to 0·8)	0·5 (0·2 to 0·9)	0·7 (0·3 to 1·1)	0·9 (0·5 to 1·5)	1·2 (0·7 to 1·9)	1·8 (1 to 2·8)	2·6 (1·6 to 3·9)	3·5 (2·2 to 5·1)	4 (2·5 to 5·7)	3·9 (2·5 to 5·7)	3·4 (2·2 to 5)	3·1 (2 to 4·6)	2 (1·3 to 3·1)	1·1 (0·7 to 1·6)	0·4 (0·3 to 0·6)	30·9
Age-related and other hearing loss	0 (0 to 0)	0 (0 to 0)	0·1 (0 to 0·1)	1·1 (0·6 to 1·9)	3 (1·7 to 4·8)	4·2 (2·5 to 6·4)	5·1 (3 to 7·7)	6·3 (3·8 to 9·7)	7·9 (4·8 to 12)	9·7 (5·8 to 15)	11·6 (6·8 to 18·1)	14·3 (8·4 to 22·6)	19·6 (11·8 to 31·3)	26·3 (16·1 to 42)	33·2 (20·4 to 52·3)	36·2 (22·1 to 56·4)	32·6 (20·6 to 49·9)	30·1 (19·3 to 44)	28·1 (18·5 to 40·4)	25·4 (16·8 to 35·5)	15·2 (10·2 to 20·6)	5·9 (4·1 to 8)	1·6 (1·1 to 2·1)	317
Other sense organ diseases	0 (0 to 0)	0 (0 to 0)	0·1 (0 to 0·2)	0·5 (0·2 to 0·8)	0·5 (0·3 to 1)	0·5 (0·2 to 1)	0·6 (0·3 to 1·1)	0·7 (0·3 to 1·3)	0·8 (0·3 to 1·5)	1 (0·4 to 2)	1·3 (0·6 to 2·4)	1·4 (0·6 to 2·5)	1·5 (0·7 to 2·7)	2 (0·9 to 3·7)	2·1 (1 to 4·2)	2·5 (1·2 to 4·8)	2·5 (1·1 to 4·5)	2·1 (1 to 4)	1·9 (0·9 to 3·4)	1·4 (0·7 to 2·6)	0·6 (0·3 to 1·1)	0·2 (0·1 to 0·3)	0·1 (0 to 0·1)	24·2
Musculoskeletal disorders	0 (0 to 0)	0 (0 to 0)	0 (0 to 0)	0 (0 to 0)	2·9 (1·6 to 5·1)	15 (9·4 to 22·8)	31·3 (20·9 to 46)	45·4 (30·7 to 64·4)	59·7 (40·2 to 82·6)	72·8 (48·4 to 98·7)	88·5 (61·7 to 122)	102 (70·9 to 140)	109 (75·7 to 154)	116 (80·4 to 164)	120 (82·5 to 166)	110 (75·2 to 156)	82·1 (56·9 to 116)	62·2 (43·4 to 86·4)	45·8 (30·6 to 64·9)	33·3 (23·1 to 46·3)	17·6 (12·5 to 23·9)	5·6 (4 to 7·7)	1·4 (1 to 2)	1,120
Rheumatoid arthritis	0 (0 to 0)	0 (0 to 0)	0 (0 to 0)	0 (0 to 0)	0 (0 to 0·1)	0·1 (0·1 to 0·2)	0·4 (0·2 to 0·7)	0·8 (0·4 to 1·4)	1·1 (0·5 to 1·8)	1·5 (0·8 to 2·4)	1·9 (1·1 to 2·9)	2·2 (1·3 to 3·4)	2·3 (1·4 to 3·5)	2·4 (1·5 to 3·6)	2·5 (1·6 to 3·6)	2·2 (1·4 to 3·1)	1·6 (1·1 to 2·3)	1·2 (0·8 to 1·7)	1 (0·7 to 1·4)	0·8 (0·6 to 1·2)	0·8 (0·5 to 1·2)	0·3 (0·2 to 0·4)	0·1 (0 to 0·1)	23·4
Osteoarthritis	0 (0 to 0)	0 (0 to 0)	0 (0 to 0)	0 (0 to 0)	0 (0 to 0)	0 (0 to 0)	0 (0 to 0)	0 (0 to 0)	0 (0 to 0)	0·3 (0·1 to 0·7)	2·2 (1 to 4·6)	6·1 (3 to 12·4)	11·4 (5·7 to 23·5)	17·6 (8·5 to 35·4)	22·9 (11·3 to 46·4)	24·7 (12·5 to 49·7)	20·4 (10·3 to 41·6)	16·4 (8·2 to 33·7)	12·6 (6·3 to 25·4)	9·2 (4·7 to 18·6)	4·5 (2·3 to 9·1)	1·5 (0·8 to 3)	0·4 (0·2 to 0·8)	150
Osteoarthritis hip	0 (0 to 0)	0 (0 to 0)	0 (0 to 0)	0 (0 to 0)	0 (0 to 0)	0 (0 to 0)	0 (0 to 0)	0 (0 to 0)	0 (0 to 0)	0·1 (0 to 0·2)	0·2 (0 to 0·4)	0·2 (0·1 to 0·6)	0·4 (0·1 to 0·9)	0·6 (0·3 to 1·4)	0·9 (0·4 to 1·9)	1 (0·4 to 2·1)	0·9 (0·4 to 1·9)	0·8 (0·3 to 1·6)	0·6 (0·3 to 1·3)	0·4 (0·2 to 0·9)	0·2 (0·1 to 0·5)	0·1 (0 to 0·2)	0 (0 to 0)	6·4
Osteoarthritis knee	0 (0 to 0)	0 (0 to 0)	0 (0 to 0)	0 (0 to 0)	0 (0 to 0)	0 (0 to 0)	0 (0 to 0)	0 (0 to 0)	0 (0 to 0)	0·2 (0·1 to 0·4)	1·6 (0·7 to 3·5)	4·7 (2·1 to 9·6)	8·7 (4·1 to 17·8)	13·2 (6·3 to 26·7)	16·9 (8·1 to 34·2)	18·1 (9 to 35·9)	14·8 (7·4 to 29·5)	11·7 (5·7 to 23·6)	8·7 (4·3 to 17·7)	6·2 (3 to 12·5)	2·9 (1·4 to 5·9)	0·9 (0·4 to 1·8)	0·2 (0·1 to 0·4)	109
Osteoarthritis hand	0 (0 to 0)	0 (0 to 0)	0 (0 to 0)	0 (0 to 0)	0 (0 to 0)	0 (0 to 0)	0 (0 to 0)	0 (0 to 0)	0 (0 to 0)	0 (0 to 0·1)	0·3 (0·1 to 0·7)	0·8 (0·3 to 1·7)	1·5 (0·6 to 3·3)	2·4 (1·1 to 5·3)	3·2 (1·4 to 6·9)	3·4 (1·6 to 7·3)	2·8 (1·4 to 5·8)	2·3 (1·1 to 4·8)	1·8 (0·9 to 3·7)	1·4 (0·7 to 2·9)	0·8 (0·4 to 1·6)	0·3 (0·1 to 0·6)	0·1 (0 to 0·2)	21·1

Osteoarthritis other	0 (0 to 0)	0 (0 to 0)	0 (0 to 0)	0 (0 to 0)	0 (0 to 0)	0 (0 to 0)	0 (0 to 0)	0 (0 to 0)	0 (0 to 0)	0 (0 to 0)	0·1 (0 to 0·3)	0·4 (0·2 to 1)	0·9 (0·3 to 1·9)	1·4 (0·6 to 3·1)	1·9 (0·9 to 4·1)	2·2 (1 to 4·6)	2 (0·9 to 4·1)	1·7 (0·8 to 3·5)	1·4 (0·7 to 2·9)	1·2 (0·6 to 2·4)	0·6 (0·3 to 1·3)	0·2 (0·1 to 0·5)	0·1 (0 to 0·1)	14·2
Low back pain	0 (0 to 0)	0 (0 to 0)	0 (0 to 0)	0 (0 to 0)	2·5 (1·2 to 4·7)	12·5 (7·4 to 20·1)	22·4 (13·3 to 36·1)	26·6 (15·5 to 42·2)	30·3 (17·7 to 48)	34·5 (19·9 to 53·4)	42·1 (25·2 to 65·9)	46·4 (27·9 to 70·4)	47·7 (28·4 to 75·2)	49·7 (30·1 to 77·9)	49·9 (31·3 to 76)	45·1 (29·3 to 67·7)	33·5 (21·2 to 51·2)	25·8 (16·2 to 39)	20 (12·4 to 30·2)	15·3 (9·7 to 23)	7 (4·4 to 10·3)	2·1 (1·3 to 3·1)	0·5 (0·3 to 0·8)	514
Neck pain	0 (0 to 0)	0 (0 to 0)	0 (0 to 0)	0 (0 to 0)	0·1 (0 to 0·3)	0·7 (0·3 to 1·5)	1·7 (0·7 to 3·2)	2·6 (1·1 to 5)	3·9 (1·8 to 7·4)	6 (2·8 to 11·5)	8·6 (4·4 to 15·1)	10·6 (5·4 to 18·8)	11·3 (5·5 to 20·4)	10·2 (5·2 to 19·2)	9·6 (4·6 to 16·9)	8·2 (4 to 14·4)	5·7 (2·9 to 9·8)	4·2 (2·2 to 7·2)	2·9 (1·4 to 5·4)	1·9 (0·9 to 3·4)	0·9 (0·4 to 1·5)	0·3 (0·1 to 0·5)	0·1 (0 to 0·1)	89·5
Gout	0 (0 to 0)	0 (0 to 0)	0 (0 to 0)	0 (0 to 0)	0 (0 to 0)	0 (0 to 0)	0 (0 to 0)	0 (0 to 0·1)	0·1 (0 to 0·2)	0·1 (0 to 0·4)	0·2 (0 to 0·5)	0·3 (0·1 to 0·6)	0·3 (0·1 to 0·7)	0·4 (0·1 to 0·7)	0·4 (0·2 to 0·8)	0·4 (0·1 to 0·7)	0·3 (0·1 to 0·6)	0·3 (0·1 to 0·5)	0·2 (0·1 to 0·4)	0·2 (0·1 to 0·3)	0·1 (0 to 0·2)	0 (0 to 0·1)	0 (0 to 0)	3·4
Other musculoskeletal disorders	0 (0 to 0)	0 (0 to 0)	0 (0 to 0)	0 (0 to 0)	0·2 (0·1 to 0·4)	1·6 (0·9 to 2·7)	6·8 (4·3 to 10·3)	15·4 (10·1 to 21·7)	24·3 (16·6 to 34·5)	30·4 (20·1 to 42·6)	33·5 (22·3 to 48·7)	36·4 (23·4 to 53·2)	36·2 (24·4 to 52·5)	35·9 (23·4 to 53·2)	34·3 (22·4 to 50·4)	29·5 (18·2 to 45·3)	20·4 (12·7 to 30·6)	14·3 (8·6 to 22·2)	9·1 (5·5 to 13·5)	5·9 (3·2 to 9·1)	4·3 (2·7 to 6·1)	1·4 (0·9 to 2·1)	0·4 (0·2 to 0·6)	340
Other non-communicable diseases	42·8 (24·8 to 71·5)	36·3 (23·5 to 54·3)	79·9 (50·5 to 121)	37·4 (26·8 to 53)	26·8 (20·6 to 34·1)	41·5 (32·5 to 51·9)	57·4 (42·1 to 79·2)	80 (58 to 111)	97·5 (72·5 to 131)	104 (75·6 to 142)	115 (84·2 to 155)	121 (87·9 to 162)	111 (81·5 to 147)	101 (73·5 to 135)	89·2 (66·1 to 118)	80 (59·1 to 106)	61·3 (46·2 to 81·2)	50·2 (37·9 to 65·6)	40·2 (31·3 to 51·1)	33·6 (26·5 to 42·4)	20·1 (15·9 to 25)	7·5 (6 to 9·3)	2·1 (1·6 to 2·6)	1,440
Congenital birth defects	41·4 (23·7 to 69·9)	32·5 (20·8 to 49·5)	53·3 (31·7 to 81·5)	19·7 (13·1 to 30·3)	10·2 (7·6 to 13·6)	12·5 (9·2 to 16·4)	8·8 (6·7 to 11·5)	7·4 (5·6 to 9·4)	6·4 (4·8 to 8·4)	5 (3·6 to 6·5)	4·9 (3·8 to 6·4)	4·7 (3·6 to 6)	4·2 (3·2 to 5·5)	3·8 (3 to 4·9)	3 (2·2 to 4)	2 (1·4 to 2·6)	1·2 (0·9 to 1·6)	0·5 (0·3 to 0·7)	0·3 (0·2 to 0·4)	0·2 (0·1 to 0·3)	0·1 (0 to 0·1)	0 (0 to 0)	0 (0 to 0)	222
Neural tube defects	7·5 (3·6 to 14)	4·4 (2·1 to 8·4)	7·3 (3 to 15)	3·8 (1·6 to 7·3)	0·6 (0·4 to 0·9)	1·4 (0·9 to 2·3)	0·8 (0·5 to 1·2)	0·3 (0·2 to 0·4)	0·5 (0·3 to 0·7)	0·4 (0·3 to 0·7)	0·3 (0·2 to 0·5)	0·3 (0·2 to 0·4)	0·1 (0·1 to 0·2)	0·1 (0·1 to 0·1)	0·1 (0·1 to 0·2)	0·1 (0 to 0·1)	0·1 (0 to 0·1)	0 (0 to 0)	0 (0 to 0)	0 (0 to 0)	0 (0 to 0)	0 (0 to 0)	0 (0 to 0)	28·1
Congenital heart anomalies	12·9 (6·3 to 24·5)	14·2 (8·4 to 23·2)	25·2 (14·4 to 41·2)	6·8 (3·6 to 14·1)	2·8 (1·7 to 4·5)	3·5 (1·9 to 5·3)	2·4 (1·5 to 3·4)	2·2 (1·5 to 3·2)	2·1 (1·3 to 3·2)	1·2 (0·7 to 1·9)	1·5 (1 to 2·1)	1·4 (1 to 2)	1·3 (0·9 to 1·9)	1·2 (0·8 to 1·7)	0·7 (0·5 to 1)	0·4 (0·3 to 0·6)	0·2 (0·1 to 0·3)	0 (0 to 0·1)	0 (0 to 0)	0 (0 to 0)	0 (0 to 0)	0 (0 to 0)	0 (0 to 0)	80·2
Orofacial clefts	0·2 (0·1 to 0·6)	0·1 (0 to 0·2)	0·4 (0·1 to 1·1)	0·2 (0·1 to 0·3)	0·1 (0 to 0·2)	0·1 (0 to 0·2)	0·1 (0 to 0·2)	0·1 (0 to 0·2)	0·1 (0 to 0·2)	0·1 (0 to 0·2)	0·1 (0 to 0·2)	0·1 (0 to 0·2)	0·1 (0 to 0·2)	0·1 (0 to 0·2)	0·1 (0 to 0·2)	0·1 (0 to 0·1)	0 (0 to 0·1)	0 (0 to 0·1)	0 (0 to 0)	0 (0 to 0)	0 (0 to 0)	0 (0 to 0)	0 (0 to 0)	2
Down syndrome	0·2 (0·1 to 0·4)	0·1 (0 to 0·2)	0·3 (0·1 to 0·4)	0·2 (0·1 to 0·4)	0·2 (0·1 to 0·3)	0·2 (0·1 to 0·3)	0·2 (0·1 to 0·3)	0·2 (0·1 to 0·3)	0·2 (0·1 to 0·2)	0·1 (0·1 to 0·2)	0·1 (0·1 to 0·2)	0·1 (0·1 to 0·2)	0·1 (0·1 to 0·2)	0·1 (0·1 to 0·2)	0·1 (0·1 to 0·2)	0 (0 to 0·1)	0 (0 to 0)	0 (0 to 0)	0 (0 to 0)	0 (0 to 0)	0 (0 to 0)	0 (0 to 0)	0 (0 to 0)	2·5
Turner syndrome	0 (0 to 0)	0 (0 to 0)	0 (0 to 0)	0 (0 to 0)	0 (0 to 0)	0 (0 to 0)	0 (0 to 0)	0 (0 to 0)	0 (0 to 0)	0 (0 to 0)	0 (0 to 0)	0 (0 to 0)	0 (0 to 0)	0 (0 to 0)	0 (0 to 0)	0 (0 to 0)	0 (0 to 0)	0 (0 to 0)	0 (0 to 0)	0 (0 to 0)	0 (0 to 0)	0 (0 to 0)	0 (0 to 0)	0·1
Klinefelter syndrome	0 (0 to 0)	0 (0 to 0)	0 (0 to 0)	0 (0 to 0)	0 (0 to 0)	0 (0 to 0)	0 (0 to 0)	0 (0 to 0)	0 (0 to 0)	0 (0 to 0)	0 (0 to 0)	0 (0 to 0)	0 (0 to 0)	0 (0 to 0)	0 (0 to 0)	0 (0 to 0)	0 (0 to 0)	0 (0 to 0)	0 (0 to 0)	0 (0 to 0)	0 (0 to 0)	0 (0 to 0)	0 (0 to 0)	0
Other chromosomal abnormalities	0·6 (0·3 to 1·2)	0·5 (0·3 to 1)	1·1 (0·6 to 1·8)	0·5 (0·3 to 0·7)	0·3 (0·2 to 0·4)	0·3 (0·2 to 0·4)	0·2 (0·2 to 0·3)	0·2 (0·2 to 0·3)	0·2 (0·1 to 0·2)	0·1 (0·1 to 0·2)	0·1 (0·1 to 0·1)	0·1 (0 to 0·1)	0 (0 to 0·1)	0 (0 to 0)	0 (0 to 0)	0 (0 to 0)	0 (0 to 0)	0 (0 to 0)	0 (0 to 0)	0 (0 to 0)	0 (0 to 0)	0 (0 to 0)	0 (0 to 0)	4·3
Congenital musculoskeletal and limb anomalies	1·9 (0·8 to 3·6)	1·2 (0·6 to 2·2)	3 (1·7 to 4·8)	3·1 (1·8 to 4·8)	3·4 (2 to 5·3)	3·4 (2 to 5·3)	3 (1·7 to 4·4)	2·1 (1·3 to 3·2)	1·8 (1·1 to 2·7)	1·5 (0·9 to 2·4)	1·6 (0·9 to 2·5)	1·6 (1 to 2·4)	1·5 (0·9 to 2·2)	1·3 (0·8 to 2·1)	1·2 (0·7 to 1·8)	0·9 (0·5 to 1·5)	0·6 (0·4 to 0·9)	0·3 (0·2 to 0·5)	0·2 (0·1 to 0·3)	0·1 (0 to 0·1)	0 (0 to 0·1)	0 (0 to 0)	0 (0 to 0)	33·6
Urogenital congenital anomalies	0·7 (0·3 to 1·8)	0·5 (0·3 to 1)	0·4 (0·2 to 0·7)	0·3 (0·2 to 0·4)	0·2 (0·1 to 0·3)	0·3 (0·2 to 0·4)	0·2 (0·1 to 0·2)	0·1 (0·1 to 0·2)	0·1 (0·1 to 0·2)	0·1 (0 to 0·1)	0·1 (0 to 0·1)	0 (0 to 0·1)	0·1 (0 to 0·1)	0 (0 to 0)	0 (0 to 0)	0 (0 to 0)	0 (0 to 0)	0 (0 to 0)	0 (0 to 0)	0 (0 to 0)	0 (0 to 0)	0 (0 to 0)	0 (0 to 0)	3·1
Digestive congenital anomalies	5·5 (2·6 to 11·3)	5·3 (2·7 to 9·3)	5·4 (2·5 to 9·9)	1 (0·5 to 2·3)	0·3 (0·2 to 0·5)	0·2 (0·1 to 0·3)	0·1 (0·1 to 0·1)	0·1 (0 to 0·1)	0 (0 to 0·1)	0 (0 to 0·1)	0 (0 to 0·1)	0 (0 to 0·1)	0·1 (0 to 0·1)	0 (0 to 0)	0·1 (0 to 0·1)	0 (0 to 0·1)	0 (0 to 0)	0 (0 to 0)	0 (0 to 0)	0 (0 to 0)	0 (0 to 0)	0 (0 to 0)	0 (0 to 0)	18·2
Other congenital birth defects	11·7 (6 to 21·3)	6·2 (3·4 to 10·7)	10·4 (5·5 to 17·5)	3·8 (2 to 6·8)	2·4 (1·5 to 3·6)	3·1 (2 to 4·6)	1·9 (1·3 to 2·8)	2 (1·3 to 2·9)	1·5 (0·9 to 2·2)	1·3 (0·8 to 1·9)	1·2 (0·8 to 1·7)	1 (0·6 to 1·5)	1 (0·6 to 1·5)	0·9 (0·6 to 1·4)	0·7 (0·4 to 1)	0·4 (0·2 to 0·6)	0·3 (0·2 to 0·5)	0·1 (0 to 0·2)	0·1 (0 to 0·2)	0 (0 to 0·1)	0 (0 to 0·1)	0 (0 to 0)	0 (0 to 0)	49·9
Urinary diseases and male infertility	0·1 (0 to 0·1)	0·3 (0·1 to 0·4)	1·1 (0·6 to 1·9)	0·8 (0·5 to 1·1)	0·6 (0·4 to 0·9)	1·3 (1 to 1·8)	1·5 (1·1 to 2)	2·3 (1·6 to 3·2)	2·8 (2 to 3·9)	3·5 (2·5 to 5)	4·3 (3·1 to 5·8)	5·6 (4 to 7·4)	5·8 (4·3 to 7·9)	6·9 (4·9 to 9·4)	6·8 (4·8 to 9·4)	6·8 (4·9 to 9·3)	6·1 (4·1 to 8·5)	6·6 (4·7 to 8·9)	6·8 (4·6 to 9·5)	8·1 (5·5 to 11·5)	5·4 (3·5 to 7·8)	2·2 (1·5 to 3·1)	0·6 (0·4 to 0·9)	86·3
Urolithiasis	0 (0 to 0)	0 (0 to 0)	0 (0 to 0)	0 (0 to 0)	0 (0 to 0)	0 (0 to 0)	0 (0 to 0)	0 (0 to 0·1)	0·1 (0 to 0·1)	0·1 (0·1 to 0·2)	0·2 (0·1 to 0·3)	0·2 (0·1 to 0·3)	0·2 (0·1 to 0·4)	0·2 (0 to 0·4)	0·2 (0·1 to 0·4)	0·1 (0·1 to 0·3)	0·1 (0 to 0·1)	0 (0 to 0·1)	0 (0 to 0)	0 (0 to 0)	0 (0 to 0)	0 (0 to 0)	0 (0 to 0)	1·6
Benign prostatic hyperplasia	0 (0 to 0)	0 (0 to 0)	0 (0 to 0)	0 (0 to 0)	0 (0 to 0)	0 (0 to 0)	0 (0 to 0)	0 (0 to 0)	0 (0 to 0)	0 (0 to 0)	0 (0 to 0)	0 (0 to 0)	0 (0 to 0)	0 (0 to 0)	0 (0 to 0)	0 (0 to 0)	0 (0 to 0)	0 (0 to 0)	0 (0 to 0)	0 (0 to 0)	0 (0 to 0)	0 (0 to 0)	0 (0 to 0)	0
Male infertility	--	--	--	--	--	--	--	--	--	--	--	--	--	--	--	--	--	--	--	--	--	--	--	NA
Other urinary diseases	0 (0 to 0)	0·1 (0 to 0·1)	0 (0 to 0·1)	0·2 (0·1 to 0·3)	0·1 (0·1 to 0·2)	0·2 (0·1 to 0·3)	0·2 (0·1 to 0·2)	0·1 (0·1 to 0·2)	0·1 (0·1 to 0·2)	0·2 (0·1 to 0·3)	0·2 (0·1 to 0·3)	0·2 (0·1 to 0·3)	0·2 (0·1 to 0·3)	0·2 (0·1 to 0·2)	0·2 (0·1 to 0·2)	0·1 (0·1 to 0·2)	0·1 (0 to 0·1)	0 (0 to 0·1)	0 (0 to 0)	0 (0 to 0)	0 (0 to 0)	0 (0 to 0)	0 (0 to 0)	2·4
Gynecological diseases	0 (0 to 0)	0 (0 to 0)	0 (0 to 0)	0 (0 to 0)	0 (0 to 0)	6·3 (3·1 to 11)	20·6 (11·9 to 33·3)	35·7 (22·6 to 54·7)	49·2 (32·5 to 72·1)	57 (37·3 to 82·1)	63·4 (42·4 to 91·1)	62·7 (41·3 to 90)	49·6 (32·4 to 70·3)	33·5 (20·4 to 49·6)	20·2 (12·6 to 29·2)	13·2 (7·6 to 20·2)	8·4 (5·2 to 12·6)	5·6 (3·5 to 8·2)	3·7 (2·3 to 5·5)	2·5 (1·5 to 3·8)	1·2 (0·8 to 1·8)	0·4 (0·3 to 0·6)	0·1 (0·1 to 0·2)	433
Uterine fibroids	0 (0 to 0)	0 (0 to 0)	0 (0 to 0)	0 (0 to 0)	0 (0 to 0)	0·1 (0 to 0·2)	0·2 (0·1 to 0·5)	0·6 (0·3 to 1·2)	1·4 (0·6 to 3)	3 (1·4 to 6)	5·2 (2·4 to 10·2)	6 (3 to 11·3)	5·2 (2·6 to 9·4)	3·6 (1·7 to 6·8)	2·4 (1·1 to 4·4)	1·4 (0·6 to 2·7)	0·7 (0·3 to 1·4)	0·4 (0·2 to 0·7)	0·2 (0·1 to 0·4)	0·1 (0 to 0·2)	0 (0 to 0·1)	0 (0 to 0)	0 (0 to 0)	30·5
Polycystic ovarian syndrome	0 (0 to 0)	0 (0 to 0)	0 (0 to 0)	0 (0 to 0)	0 (0 to 0)	0·2 (0·1 to 0·5)	1 (0·4 to 2·1)	1·2 (0·5 to 2·4)	1 (0·4 to 2·2)	1 (0·4 to 2)	0·9 (0·4 to 1·9)	0·9 (0·4 to 1·8)	0·7 (0·3 to 1·4)	0·2 (0·1 to 0·4)	0 (0 to 0)	0 (0 to 0)	0 (0 to 0)	0 (0 to 0)	0 (0 to 0)	0 (0 to 0)	0 (0 to 0)	0 (0 to 0)	0 (0 to 0)	7·1
Female infertility	0 (0 to 0)	0 (0 to 0)	0 (0 to 0)	0 (0 to 0)	0 (0 to 0)	0 (0 to 0)	0 (0 to 0)	1·2 (0·2 to 3·3)	1·6 (0·4 to 4·4)	2 (0·4 to 5·8)	2·2 (0·3 to 6·6)	1·9 (0·2 to 5·5)	0 (0 to 0·2)	0 (0 to 0)	0 (0 to 0)	0 (0 to 0)	0 (0 to 0)	0 (0 to 0)	0 (0 to 0)	0 (0 to 0)	0 (0 to 0)	0 (0 to 0)	0 (0 to 0)	8·9
Endometriosis	0 (0 to 0)	0 (0 to 0)	0 (0 to 0)	0 (0 to 0)	0 (0 to 0)	0 (0 to 0)	1·3 (0·5 to 2·7)	5·3 (2·5 to 9·9)	5·8 (2·8 to 10·5)	4·6 (2·3 to 8·4)	4·1 (2·2 to 7·4)	4 (2 to 7)	3·3 (1·8 to 5·4)	1·6 (0·8 to 2·7)	0 (0 to 0)	0 (0 to 0)	0 (0 to 0)	0 (0 to 0)	0 (0 to 0)	0 (0 to 0)	0 (0 to 0)	0 (0 to 0)	0 (0 to 0)	30·2
Genital prolapse	0 (0 to 0)	0 (0 to 0)	0 (0 to 0)	0 (0 to 0)	0 (0 to 0)	0 (0 to 0)	0 (0 to 0)	0 (0 to 0·1)	0·1 (0 to 0·2)	0·2 (0·1 to 0·5)	0·5 (0·2 to 0·9)	0·7 (0·3 to 1·6)	0·9 (0·4 to 1·9)	1·1 (0·4 to 2·1)	1·1 (0·5 to 2·1)	1 (0·4 to 1·9)	0·7 (0·3 to 1·4)	0·5 (0·3 to 1)	0·4 (0·2 to 0·7)	0·3 (0·1 to 0·5)	0·1 (0·1 to 0·3)	0·1 (0 to 0·1)	0 (0 to 0)	7·7
Premenstrual syndrome	0 (0 to 0)	0 (0 to 0)	0 (0 to 0)	0 (0 to 0)	0 (0 to 0)	5·7 (2·7 to 10·2)	13 (6·5 to 23·2)	16·2 (8·2 to 29)	15·4 (8·5 to 25·3)	15 (7·7 to 24·6)	17 (9·7 to 26·9)	16·6 (9·4 to 26·4)	11 (6·1 to 18·7)	3·7 (1·8 to 6·8)	0 (0 to 0)	0 (0 to 0)	0 (0 to 0)	0 (0 to 0)	0 (0 to 0)	0 (0 to 0)	0 (0 to 0)	0 (0 to 0)	0 (0 to 0)	114
Other gynecological diseases	0 (0 to 0)	0 (0 to 0)	0 (0 to 0)	0 (0 to 0)	0 (0 to 0)	0·3 (0·1 to 0·7)	5 (2 to 9·6)	11·2 (6·3 to 18)	23·8 (13·7 to 37·4)	31·2 (18·8 to 47·4)	33·5 (20·1 to 52·7)	32·6 (18·5 to 52)	28·5 (16·2 to 44·2)	23·4 (11·6 to 37·4)	16·7 (9·9 to 25·3)	10·8 (5·7 to 17·1)	7 (4 to 10·9)	4·7 (2·8 to 7)	3·1 (1·9 to 4·8)	2·1 (1·2 to 3·3)	1 (0·6 to 1·6)	0·4 (0·2 to 0·6)	0·1 (0 to 0·2)	235
Hemoglobinopathies and hemolytic anemias	0·2 (0·1 to 0·4)	0·3 (0·2 to 0·5)	2·2 (1·4 to 3·5)	5·3 (3·2 to 8·3)	7·3 (4·8 to 10·3)	10·6 (7·1 to 15·1)	13·5 (8·3 to 21·5)	17·2 (10·4 to 26)	16·3 (10 to 23·6)	11·7 (6 to 19·2)	9·3 (6·3 to 13·6)	9 (6·1 to 12·3)	8·1 (5·3 to 11·3)	7·8 (5·5 to 10·9)	5·8 (4 to 8·2)	5·4 (3·5 to 7·9)	3·1 (2 to 4·5)	2·5 (1·7 to 3·7)	2 (1·4 to 3)	1·7 (1·2 to 2·3)	1·4 (1 to 2)	0·5 (0·4 to 0·7)	0·1 (0·1 to 0·2)	141
Thalassemias	0 (0 to 0)	0 (0 to 0)	0 (0 to 0)	0 (0 to 0·1)	0 (0 to 0)	0 (0 to 0)	0 (0 to 0)	0 (0 to 0)	0 (0 to 0)	0 (0 to 0)	0 (0 to 0)	0 (0 to 0)	0 (0 to 0)	0 (0 to 0)	0 (0 to 0)	0 (0 to 0)	0 (0 to 0)	0 (0 to 0)	0 (0 to 0)	0 (0 to 0)	0 (0 to 0)	0 (0 to 0)	0 (0 to 0)	0·1
Thalassemias trait	0 (0 to 0)	0 (0 to 0)	0 (0 to 0·1)	0·1 (0 to 0·3)	0·2 (0·1 to 0·4)	0·1 (0 to 0·3)	0·5 (0·2 to 0·9)	0·5 (0·3 to 0·9)	0·5 (0·2 to 0·8)	0·5 (0·2 to 0·8)	0·4 (0·2 to 0·8)	0·4 (0·2 to 0·7)	0·1 (0 to 0·2)	0·1 (0 to 0·2)	0·1 (0 to 0·2)	0·1 (0 to 0·2)	0 (0 to 0·1)	0 (0 to 0·1)	0 (0 to 0·1)	0 (0 to 0)	0 (0 to 0)	0 (0 to 0)	0 (0 to 0)	3·9
Sickle cell disorders	0·2 (0·1 to 0·4)	0·2 (0·1 to 0·4)	1·7 (1 to 3)	3·1 (1·7 to 5·2)	4·3 (3 to 6·2)	8·5 (5·3 to 12·6)	8 (5 to 11·6)	8·3 (5·3 to 12·2)	7·4 (4·7 to 11·3)	5·1 (3 to 7·9)	5·1 (3·1 to 8)	4·5 (2·8 to 6·8)	4·3 (2·5 to 6·4)	3·2 (1·9 to 5·2)	1·4 (0·8 to 2·3)	0·5 (0·2 to 0·8)	0·1 (0 to 0·2)	0 (0 to 0)	0 (0 to 0)	0 (0 to 0)	0 (0 to 0)	0 (0 to 0)	0 (0 to 0)	65·9
Sickle cell trait	0 (0 to 0)	0·1 (0 to 0·1)	0·3 (0·1 to 0·5)	1·3 (0·6 to 2·3)	1·8 (0·7 to 3·3)	1·2 (0·5 to 2·5)	3·9 (0·8 to 10·2)	7·2 (2·1 to 13·7)	7 (1·8 to 12·7)	4·7 (0·7 to 10·5)	0·3 (0·1 to 0·7)	1·1 (0·6 to 1·8)	1·2 (0·6 to 2·2)	1·3 (0·6 to 2·2)	1·1 (0·6 to 1·9)	0·8 (0·4 to 1·4)	0·5 (0·2 to 0·8)	0·4 (0·1 to 0·7)	0·3 (0·1 to 0·5)	0·2 (0·1 to 0·4)	0·1 (0·1 to 0·2)	0 (0 to 0·1)	0 (0 to 0)	35
G6PD deficiency	0 (0 to 0)	0 (0 to 0)	0 (0 to 0)	0 (0 to 0)	0 (0 to 0·1)	0·1 (0 to 0·1)	0·1 (0 to 0·2)	0·2 (0·1 to 0·5)	0·3 (0·1 to 0·7)	0·3 (0·1 to 0·7)	1·5 (0·8 to 2·5)	0·8 (0·4 to 1·2)	0·5 (0·2 to 0·9)	0·5 (0·2 to 1)	0·4 (0·2 to 0·7)	0·3 (0·1 to 0·6)	0·2 (0·1 to 0·3)	0·1 (0 to 0·1)	0 (0 to 0·1)	0 (0 to 0)	0 (0 to 0)	0 (0 to 0)	0 (0 to 0)	5·4
G6PD trait	0 (0 to 0)	0 (0 to 0)	0 (0 to 0)	0 (0 to 0)	0 (0 to 0)	0 (0 to 0)	0 (0 to 0)	0 (0 to 0)	0 (0 to 0)	0 (0 to 0)	0·1 (0 to 0·2)	0·1 (0 to 0·2)	0 (0 to 0)	0 (0 to 0)	0 (0 to 0)	0 (0 to 0)	0 (0 to 0)	0 (0 to 0)	0 (0 to 0)	0 (0 to 0)	0 (0 to 0)	0 (0 to 0)	0 (0 to 0)	0·2
Other hemoglobinopathies and hemolytic anemias	0 (0 to 0)	0 (0 to 0)	0·2 (0·1 to 0·4)	0·7 (0·2 to 1·4)	0·9 (0·3 to 1·8)	0·7 (0·2 to 1·4)	1 (0·3 to 2)	1 (0·4 to 2)	1·1 (0·5 to 2·1)	1·2 (0·5 to 2)	1·8 (0·9 to 3·1)	2·1 (1·2 to 3·4)	2 (1·1 to 3·3)	2·6 (1·6 to 3·9)	2·8 (1·8 to 4)	3·7 (2·3 to 5·6)	2·4 (1·6 to 3·5)	2 (1·4 to 3)	1·6 (1·1 to 2·5)	1·4 (1 to 1·9)	1·3 (0·9 to 1·8)	0·5 (0·3 to 0·7)	0·1 (0·1 to 0·1)	30·9
Endocrine, metabolic, blood, and immune disorders	1·1 (0·6 to 2·1)	2·5 (1·4 to 4·2)	19·8 (11·3 to 31·9)	9·8 (5·9 to 17)	6 (4·1 to 8·6)	7·8 (5·4 to 11)	8·6 (6 to 12·2)	10·6 (7·7 to 14·8)	13·6 (9·5 to 19)	16 (11·2 to 22·4)	19·7 (13·9 to 26·8)	23·6 (16·5 to 32·9)	26·2 (18·3 to 36·8)	28·6 (20·3 to 39·7)	29·8 (20·8 to 42·5)	27·5 (18·6 to 39·7)	20·9 (14·8 to 31·1)	17·5 (12·3 to 25·1)	14 (9·6 to 19·4)	11·3 (7·8 to 16·5)	7·1 (4·8 to 10·5)	2·7 (1·8 to 3·9)	0·8 (0·5 to 1·1)	325
Oral disorders	0 (0 to 0)	0 (0 to 0)	0 (0 to 0)	2 (1·1 to 3·3)	2·7 (1·4 to 4·7)	3·1 (1·6 to 5·4)	4·4 (2·4 to 7·6)	6·7 (3·5 to 12·4)	9·1 (4·6 to 17·2)	11·2 (5·5 to 20·7)	13·6 (6·6 to 25·5)	15·4 (7·6 to 28)	17·1 (9 to 30·3)	20·1 (11·3 to 34·6)	23·7 (13·9 to 37·6)	25·3 (15·4 to 39·9)	21·4 (13·1 to 33)	17·5 (10·9 to 26·8)	13·5 (8·5 to 20·1)	9·9 (6·2 to 14·4)	4·8 (3 to 7)	1·6 (1 to 2·4)	0·4 (0·3 to 0·6)	224
Caries of deciduous teeth	0 (0 to 0)	0 (0 to 0)	0 (0 to 0)	0·7 (0·3 to 1·3)	0·7 (0·3 to 1·4)	0 (0 to 0·1)	0 (0 to 0)	0 (0 to 0)	0 (0 to 0)	0 (0 to 0)	0 (0 to 0)	0 (0 to 0)	0 (0 to 0)	0 (0 to 0)	0 (0 to 0)	0 (0 to 0)	0 (0 to 0)	0 (0 to 0)	0 (0 to 0)	0 (0 to 0)	0 (0 to 0)	0 (0 to 0)	0 (0 to 0)	1·4
Caries of permanent teeth	0 (0 to 0)	0 (0 to 0)	0 (0 to 0)	0 (0 to 0)	0·7 (0·3 to 1·6)	1·6 (0·6 to 3·3)	1·6 (0·7 to 3·5)	1·8 (0·7 to 3·7)	1·7 (0·7 to 3·5)	1·6 (0·7 to 3·2)	1·6 (0·7 to 3·4)	1·5 (0·6 to 3·1)	1·4 (0·6 to 2·8)	1·3 (0·5 to 2·7)	1·1 (0·4 to 2·5)	0·9 (0·3 to 1·9)	0·6 (0·2 to 1·2)	0·4 (0·2 to 0·8)	0·3 (0·1 to 0·5)	0·2 (0·1 to 0·4)	0·1 (0 to 0·2)	0 (0 to 0·1)	0 (0 to 0)	18·4
Periodontal diseases	0 (0 to 0)	0 (0 to 0)	0 (0 to 0)	0 (0 to 0)	0 (0 to 0)	0 (0 to 0)	0·5 (0·2 to 1·4)	2 (0·6 to 4·8)	4·1 (1·3 to 9·6)	6 (2 to 13·3)	7·8 (2·7 to 17·6)	8·8 (3·1 to 19·3)	8·8 (3·3 to 18·6)	8·2 (3·2 to 17·4)	7·1 (2·8 to 14·8)	5·4 (2·1 to 11)	3·3 (1·3 to 6·8)	2·2 (0·9 to 4·4)	1·5 (0·6 to 3)	1 (0·4 to 2·1)	0·5 (0·2 to 1·1)	0·2 (0·1 to 0·4)	0 (0 to 0·1)	67·4
Other oral disorders	0 (0 to 0)	0 (0 to 0)	0 (0 to 0)	1·4 (0·8 to 2·1)	1·3 (0·8 to 2·1)	1·5 (0·9 to 2·3)	2·2 (1·3 to 3·5)	2·7 (1·6 to 4·1)	2·7 (1·6 to 4·2)	2·7 (1·6 to 4·1)	2·8 (1·7 to 4·2)	2·8 (1·7 to 4·2)	2·7 (1·6 to 4·1)	2·5 (1·5 to 3·8)	2·3 (1·4 to 3·5)	1·9 (1·2 to 2·9)	1·4 (0·8 to 2·1)	0·9 (0·5 to 1·4)	0·5 (0·3 to 0·8)	0·3 (0·2 to 0·5)	0·1 (0·1 to 0·2)	0 (0 to 0·1)	0 (0 to 0)	32·9
Sudden infant death syndrome	--	0·7 (0·4 to 1·2)	3·4 (1·6 to 6·4)	--	--	--	--	--	--	--	--	--	--	--	--	--	--	--	--	--	--	--	--	NA
Injuries	3 (1·8 to 4·6)	2·1 (1·4 to 3·3)	13·6 (8·3 to 20·2)	23·1 (16 to 32·2)	24·6 (20·6 to 29·2)	50·8 (42·7 to 59·1)	65·5 (56·1 to 76·2)	86·2 (71·8 to 102)	80·3 (65·6 to 96·7)	70·2 (57·1 to 84·8)	63·9 (52·3 to 77·7)	60·7 (49·9 to 72·8)	53·4 (44·2 to 64·4)	46·1 (37·8 to 55·8)	39·4 (32·5 to 47·9)	34·9 (28·5 to 42·2)	26·5 (21·7 to 32)	22·4 (18·6 to 27)	19·3 (16·1 to 23·3)	18·8 (15·7 to 22·1)	12·4 (10·6 to 14·4)	5·7 (4·8 to 6·7)	1·9 (1·6 to 2·2)	825
Transport injuries	0·1 (0·1 to 0·1)	0·1 (0·1 to 0·2)	0·8 (0·5 to 1·3)	2·6 (1·5 to 4·2)	4·3 (3 to 6·1)	10·3 (7·2 to 14·1)	14 (9·8 to 18·7)	16·2 (11·5 to 22·3)	13·8 (10·2 to 18·5)	10·8 (8 to 14·1)	10·8 (8·3 to 13·9)	11 (8·5 to 13·9)	10·2 (7·9 to 12·7)	8·8 (7·1 to 10·7)	7·5 (6·1 to 9·2)	6·5 (5·2 to 7·9)	4·9 (3·9 to 6)	3·6 (2·9 to 4·5)	2·7 (2·2 to 3·3)	2 (1·6 to 2·5)	1 (0·8 to 1·2)	0·3 (0·3 to 0·4)	0·1 (0·1 to 0·1)	143
Road injuries	0·1 (0 to 0·1)	0·1 (0 to 0·1)	0·7 (0·4 to 1·1)	2·3 (1·3 to 3·8)	3·9 (2·8 to 5·5)	9·3 (6·4 to 12·8)	13·1 (9·1 to 17·5)	15 (10·5 to 20·6)	12·8 (9·5 to 17)	9·9 (7·3 to 13)	10 (7·7 to 12·8)	9·9 (7·7 to 12·5)	9·4 (7·3 to 11·8)	8·1 (6·5 to 9·8)	6·9 (5·6 to 8·4)	6 (4·8 to 7·3)	4·6 (3·7 to 5·7)	3·4 (2·7 to 4·2)	2·5 (2 to 3)	1·9 (1·5 to 2·3)	0·9 (0·7 to 1·1)	0·3 (0·2 to 0·4)	0·1 (0·1 to 0·1)	131
Pedestrian road injuries	0 (0 to 0)	0 (0 to 0·1)	0·2 (0·1 to 0·3)	1 (0·5 to 1·6)	1·5 (1 to 2·2)	3·4 (2·1 to 5·2)	2·2 (1·5 to 2·9)	2·5 (1·5 to 3·8)	2·2 (1·6 to 2·9)	2·2 (1·5 to 3)	2·3 (1·6 to 3·3)	2·3 (1·7 to 3)	2·4 (1·7 to 3·3)	2·3 (1·7 to 3)	2 (1·6 to 2·5)	1·9 (1·5 to 2·5)	1·7 (1·2 to 2·1)	1·3 (1 to 1·6)	1 (0·8 to 1·3)	0·8 (0·7 to 1·1)	0·4 (0·3 to 0·5)	0·1 (0·1 to 0·2)	0 (0 to 0)	33·7
Cyclist road injuries	0 (0 to 0)	0 (0 to 0)	0 (0 to 0)	0·1 (0·1 to 0·2)	0·3 (0·2 to 0·4)	0·9 (0·6 to 1·4)	1·1 (0·7 to 1·6)	1·1 (0·8 to 1·6)	1·1 (0·8 to 1·6)	1·1 (0·8 to 1·4)	1·2 (0·9 to 1·6)	1·2 (0·9 to 1·6)	1·2 (0·9 to 1·6)	1·2 (0·9 to 1·6)	1·1 (0·8 to 1·4)	0·9 (0·6 to 1·2)	0·7 (0·5 to 0·9)	0·5 (0·3 to 0·6)	0·3 (0·2 to 0·5)	0·2 (0·2 to 0·3)	0·1 (0·1 to 0·2)	0 (0 to 0·1)	0 (0 to 0)	14·5
Motorcyclist road injuries	0 (0 to 0)	0 (0 to 0)	0 (0 to 0·1)	0·1 (0 to 0·1)	0·2 (0·1 to 0·2)	0·4 (0·3 to 0·6)	1·2 (0·9 to 1·6)	1·5 (1·1 to 2·1)	1·3 (1 to 1·8)	0·9 (0·7 to 1·2)	1 (0·7 to 1·3)	0·8 (0·6 to 1·1)	0·8 (0·6 to 1)	0·6 (0·5 to 0·9)	0·6 (0·5 to 0·8)	0·5 (0·4 to 0·7)	0·4 (0·3 to 0·5)	0·3 (0·2 to 0·4)	0·2 (0·2 to 0·3)	0·2 (0·1 to 0·3)	0·1 (0·1 to 0·1)	0 (0 to 0·1)	0 (0 to 0)	11·4
Motor vehicle road injuries	0 (0 to 0·1)	0 (0 to 0)	0·5 (0·3 to 0·7)	1·1 (0·6 to 1·9)	1·9 (1·3 to 2·8)	4·4 (2·8 to 6·5)	8·5 (5·8 to 11·7)	9·6 (6·4 to 13·6)	7·8 (5·6 to 10·7)	5·4 (3·8 to 7·4)	5·3 (3·8 to 7·2)	5·2 (3·9 to 6·7)	4·7 (3·5 to 6·2)	3·6 (2·8 to 4·7)	3 (2·3 to 3·7)	2·4 (1·9 to 3)	1·7 (1·3 to 2·2)	1·2 (1 to 1·5)	0·8 (0·6 to 1)	0·5 (0·4 to 0·7)	0·2 (0·2 to 0·3)	0·1 (0·1 to 0·1)	0 (0 to 0)	68·1
Other road injuries	0 (0 to 0)	0 (0 to 0)	0 (0 to 0)	0·1 (0 to 0·1)	0·1 (0·1 to 0·2)	0·2 (0·1 to 0·2)	0·2 (0·1 to 0·3)	0·3 (0·2 to 0·4)	0·4 (0·3 to 0·5)	0·3 (0·2 to 0·5)	0·3 (0·2 to 0·4)	0·3 (0·2 to 0·4)	0·3 (0·2 to 0·4)	0·3 (0·2 to 0·4)	0·3 (0·2 to 0·3)	0·2 (0·2 to 0·3)	0·2 (0·1 to 0·2)	0·1 (0·1 to 0·1)	0·1 (0·1 to 0·1)	0·1 (0 to 0·1)	0 (0 to 0)	0 (0 to 0)	0 (0 to 0)	3·6
Other transport injuries	0 (0 to 0)	0 (0 to 0·1)	0·1 (0·1 to 0·2)	0·3 (0·2 to 0·4)	0·4 (0·3 to 0·5)	1 (0·7 to 1·3)	0·9 (0·7 to 1·3)	1·2 (0·7 to 1·9)	1·1 (0·6 to 1·6)	0·9 (0·6 to 1·3)	0·8 (0·6 to 1·1)	1·1 (0·8 to 1·4)	0·7 (0·5 to 0·9)	0·7 (0·5 to 1·1)	0·6 (0·5 to 0·8)	0·5 (0·4 to 0·6)	0·3 (0·2 to 0·4)	0·2 (0·2 to 0·3)	0·2 (0·2 to 0·3)	0·1 (0·1 to 0·2)	0·1 (0·1 to 0·1)	0 (0 to 0)	0 (0 to 0)	11·4
Unintentional injuries	1·3 (0·8 to 2)	1·6 (1 to 2·5)	9·9 (5·9 to 15·3)	16·6 (11 to 23·6)	16·5 (13 to 20·8)	29·1 (22·9 to 36·1)	22·7 (18·5 to 27·7)	25·1 (19·6 to 31·2)	25·3 (19·9 to 32·1)	24·7 (19·4 to 31·5)	26·3 (20·4 to 33·5)	27·2 (21·1 to 34·7)	26·3 (20·6 to 33·3)	25·9 (20·1 to 33)	24·4 (19 to 31·3)	22·8 (17·9 to 28·8)	17·6 (13·9 to 22·1)	16 (13 to 19·8)	14·7 (12 to 18)	15·6 (13 to 18·7)	10·9 (9·2 to 12·7)	5·2 (4·3 to 6·2)	1·8 (1·5 to 2·1)	408
Falls	0 (0 to 0)	0 (0 to 0)	0·2 (0·1 to 0·3)	0·5 (0·3 to 0·7)	1 (0·7 to 1·6)	1·7 (1·2 to 2·4)	2·1 (1·5 to 3·1)	2·6 (1·8 to 3·6)	2·8 (2 to 4)	3·1 (2·2 to 4·4)	3·7 (2·6 to 5·1)	4·1 (2·9 to 5·8)	4·5 (3·3 to 6·2)	5·2 (3·7 to 7)	5·6 (4·1 to 7·5)	6 (4·4 to 7·9)	5·2 (3·9 to 6·7)	5·3 (4·2 to 6·7)	5·6 (4·5 to 6·8)	7·1 (5·9 to 8·7)	5·4 (4·4 to 6·6)	2·5 (2 to 3)	0·8 (0·6 to 1)	75·1
Drowning	0·1 (0·1 to 0·2)	0·1 (0·1 to 0·2)	1·3 (0·7 to 2·1)	5·5 (3·4 to 8·3)	4·8 (3·3 to 6·5)	11·1 (8·1 to 14·5)	4·9 (3·5 to 6·7)	3·1 (2·1 to 4·4)	2·8 (2 to 3·9)	2·2 (1·5 to 3·1)	2 (1·4 to 2·8)	1·8 (1·3 to 2·5)	1·7 (1·2 to 2·2)	1·2 (0·9 to 1·7)	0·9 (0·7 to 1·1)	0·7 (0·5 to 0·9)	0·5 (0·4 to 0·7)	0·3 (0·3 to 0·5)	0·3 (0·2 to 0·4)	0·2 (0·2 to 0·3)	0·2 (0·1 to 0·2)	0·1 (0·1 to 0·1)	0 (0 to 0)	46·1
Fire, heat, and hot substances	0·1 (0·1 to 0·1)	0·2 (0·1 to 0·3)	1·7 (1 to 2·6)	3·8 (2·4 to 5·7)	3·6 (2·8 to 4·5)	5·4 (4·2 to 6·8)	6 (4·5 to 7·7)	7·3 (5·5 to 9·3)	8 (6·1 to 10·4)	8 (6 to 10·5)	8·5 (6·5 to 10·9)	8·4 (6·3 to 11)	7·5 (5·6 to 9·8)	7·2 (5·5 to 9·5)	6·4 (4·9 to 8·4)	5·5 (4·2 to 7·4)	3·8 (2·9 to 5)	3·1 (2·4 to 4)	2·4 (1·8 to 3·1)	2·1 (1·6 to 2·6)	1·4 (1·1 to 1·7)	0·6 (0·5 to 0·7)	0·2 (0·2 to 0·2)	101
Poisonings	0 (0 to 0)	0 (0 to 0·1)	0·1 (0·1 to 0·2)	0·3 (0·2 to 0·5)	0·3 (0·2 to 0·4)	0·4 (0·2 to 0·5)	0·4 (0·3 to 0·6)	0·4 (0·3 to 0·6)	0·4 (0·3 to 0·6)	0·4 (0·3 to 0·6)	0·4 (0·3 to 0·6)	0·4 (0·3 to 0·5)	0·3 (0·2 to 0·5)	0·3 (0·2 to 0·4)	0·3 (0·2 to 0·4)	0·2 (0·1 to 0·3)	0·2 (0·1 to 0·3)	0·1 (0·1 to 0·2)	0·1 (0·1 to 0·1)	0·1 (0·1 to 0·1)	0 (0 to 0·1)	0 (0 to 0)	0 (0 to 0)	5·2
Poisoning by carbon monoxide	0 (0 to 0)	0 (0 to 0)	0 (0 to 0)	0·1 (0·1 to 0·2)	0·1 (0·1 to 0·1)	0·1 (0·1 to 0·2)	0·1 (0·1 to 0·2)	0·2 (0·1 to 0·3)	0·2 (0·1 to 0·2)	0·2 (0·1 to 0·2)	0·1 (0·1 to 0·2)	0·2 (0·1 to 0·2)	0·1 (0·1 to 0·2)	0·1 (0·1 to 0·2)	0·1 (0·1 to 0·1)	0·1 (0·1 to 0·1)	0·1 (0·1 to 0·1)	0·1 (0 to 0·1)	0 (0 to 0·1)	0 (0 to 0)	0 (0 to 0)	0 (0 to 0)	0 (0 to 0)	2
Poisoning by other means	0 (0 to 0)	0 (0 to 0·1)	0·1 (0·1 to 0·2)	0·2 (0·1 to 0·3)	0·2 (0·1 to 0·3)	0·2 (0·1 to 0·4)	0·3 (0·2 to 0·4)	0·3 (0·2 to 0·4)	0·3 (0·2 to 0·4)	0·2 (0·1 to 0·4)	0·3 (0·2 to 0·4)	0·2 (0·1 to 0·3)	0·2 (0·1 to 0·3)	0·2 (0·1 to 0·3)	0·2 (0·1 to 0·3)	0·1 (0·1 to 0·2)	0·1 (0·1 to 0·2)	0·1 (0 to 0·1)	0·1 (0 to 0·1)	0·1 (0 to 0·1)	0 (0 to 0)	0 (0 to 0)	0 (0 to 0)	3·3
Exposure to mechanical forces	0·1 (0 to 0·1)	0·1 (0·1 to 0·2)	0·6 (0·3 to 0·8)	2·2 (1·4 to 3·2)	3 (2·3 to 3·9)	5 (3·8 to 6·4)	4·4 (3·4 to 5·6)	5·4 (4·1 to 7)	5 (3·8 to 6·4)	4·7 (3·6 to 6·2)	5·1 (3·8 to 6·7)	5·3 (4 to 6·9)	4·8 (3·5 to 6·4)	4·7 (3·5 to 6·3)	4·1 (3 to 5·6)	3·6 (2·6 to 4·9)	2·6 (1·9 to 3·6)	2·2 (1·6 to 2·9)	1·8 (1·3 to 2·4)	1·6 (1·2 to 2·1)	1 (0·8 to 1·3)	0·5 (0·4 to 0·7)	0·2 (0·2 to 0·3)	67·9
Unintentional firearm injuries	0 (0 to 0)	0 (0 to 0)	0·1 (0 to 0·1)	0·2 (0·1 to 0·3)	0·3 (0·2 to 0·5)	1·3 (0·7 to 2)	1·5 (1 to 2·1)	2·1 (1·4 to 2·9)	1·5 (1·1 to 2·1)	1·5 (1·1 to 2)	1·4 (1 to 1·8)	1·3 (0·9 to 1·6)	1 (0·7 to 1·3)	0·7 (0·5 to 0·9)	0·6 (0·5 to 0·8)	0·5 (0·4 to 0·6)	0·3 (0·2 to 0·4)	0·3 (0·2 to 0·4)	0·3 (0·2 to 0·3)	0·3 (0·2 to 0·4)	0·2 (0·2 to 0·3)	0·2 (0·1 to 0·2)	0·1 (0·1 to 0·1)	15·7
Other exposure to mechanical forces	0·1 (0 to 0·1)	0·1 (0·1 to 0·2)	0·5 (0·3 to 0·7)	2 (1·3 to 2·9)	2·6 (2 to 3·5)	3·8 (2·8 to 5)	2·9 (2 to 3·9)	3·3 (2·4 to 4·6)	3·5 (2·5 to 4·8)	3·3 (2·3 to 4·5)	3·7 (2·6 to 5·1)	4 (3 to 5·5)	3·8 (2·8 to 5·3)	4 (2·9 to 5·4)	3·5 (2·5 to 4·8)	3·1 (2·2 to 4·3)	2·3 (1·6 to 3·2)	1·8 (1·3 to 2·6)	1·5 (1·1 to 2)	1·3 (0·9 to 1·7)	0·8 (0·6 to 1)	0·4 (0·3 to 0·5)	0·1 (0·1 to 0·2)	52·3
Adverse effects of medical treatment	0·3 (0·2 to 0·4)	0·3 (0·1 to 0·4)	0·9 (0·5 to 1·6)	0·7 (0·4 to 1·2)	0·4 (0·3 to 0·6)	0·5 (0·3 to 0·8)	0·6 (0·4 to 0·9)	1·2 (0·7 to 1·8)	1·1 (0·6 to 1·7)	1·2 (0·7 to 1·9)	1·2 (0·8 to 1·7)	1·6 (1 to 2·4)	1·8 (1·2 to 2·7)	1·8 (1·2 to 2·5)	1·7 (1·1 to 2·5)	1·8 (1·2 to 2·5)	1·4 (0·9 to 1·9)	1·4 (1 to 2)	1·4 (1 to 1·9)	1·3 (0·9 to 1·8)	0·9 (0·6 to 1·2)	0·3 (0·2 to 0·5)	0·1 (0·1 to 0·1)	23·6
Animal contact	0 (0 to 0)	0 (0 to 0)	0·2 (0·1 to 0·3)	0·3 (0·2 to 0·6)	0·4 (0·2 to 0·5)	0·5 (0·4 to 0·7)	0·4 (0·3 to 0·6)	0·5 (0·3 to 0·7)	0·6 (0·4 to 0·8)	0·5 (0·4 to 0·7)	0·5 (0·4 to 0·7)	0·6 (0·4 to 0·7)	0·6 (0·4 to 0·8)	0·5 (0·4 to 0·7)	0·6 (0·4 to 0·8)	0·5 (0·4 to 0·7)	0·4 (0·3 to 0·5)	0·4 (0·3 to 0·6)	0·5 (0·3 to 0·7)	0·8 (0·5 to 1·2)	0·7 (0·5 to 1·1)	0·6 (0·4 to 0·8)	0·2 (0·1 to 0·3)	10·3
Venomous animal contact	0 (0 to 0)	0 (0 to 0)	0 (0 to 0·1)	0·1 (0·1 to 0·2)	0·1 (0·1 to 0·2)	0·3 (0·2 to 0·4)	0·2 (0·1 to 0·4)	0·3 (0·2 to 0·4)	0·3 (0·2 to 0·5)	0·3 (0·2 to 0·4)	0·3 (0·2 to 0·4)	0·3 (0·2 to 0·4)	0·3 (0·2 to 0·4)	0·2 (0·1 to 0·3)	0·3 (0·2 to 0·4)	0·2 (0·2 to 0·3)	0·2 (0·1 to 0·3)	0·2 (0·1 to 0·3)	0·2 (0·1 to 0·3)	0·3 (0·2 to 0·5)	0·3 (0·2 to 0·5)	0·3 (0·2 to 0·4)	0·1 (0·1 to 0·2)	5
Non-venomous animal contact	0 (0 to 0)	0 (0 to 0)	0·1 (0·1 to 0·2)	0·2 (0·1 to 0·4)	0·2 (0·1 to 0·3)	0·2 (0·1 to 0·4)	0·2 (0·1 to 0·3)	0·2 (0·1 to 0·3)	0·2 (0·1 to 0·3)	0·2 (0·1 to 0·3)	0·2 (0·2 to 0·4)	0·2 (0·2 to 0·4)	0·3 (0·2 to 0·4)	0·3 (0·2 to 0·4)	0·3 (0·2 to 0·4)	0·3 (0·2 to 0·4)	0·2 (0·1 to 0·3)	0·2 (0·2 to 0·3)	0·3 (0·2 to 0·4)	0·5 (0·3 to 0·8)	0·4 (0·2 to 0·6)	0·3 (0·2 to 0·4)	0·1 (0·1 to 0·2)	5·3
Foreign body	0·7 (0·4 to 1·1)	0·7 (0·4 to 1·2)	4·7 (2·6 to 7·6)	1·9 (1·2 to 2·8)	1·1 (0·8 to 1·3)	1·6 (1·2 to 2)	0·8 (0·6 to 1)	1 (0·8 to 1·3)	1 (0·8 to 1·3)	0·9 (0·7 to 1·2)	1 (0·8 to 1·3)	1·1 (0·8 to 1·4)	1·1 (0·9 to 1·4)	1·1 (0·9 to 1·4)	1 (0·8 to 1·3)	1·1 (0·8 to 1·4)	0·9 (0·8 to 1·2)	1 (0·8 to 1·3)	1 (0·8 to 1·3)	1·1 (0·8 to 1·4)	0·5 (0·4 to 0·7)	0·3 (0·2 to 0·3)	0·1 (0·1 to 0·1)	25·8
Pulmonary aspiration and foreign body in airway	0·7 (0·4 to 1·1)	0·7 (0·4 to 1·2)	4·6 (2·5 to 7·5)	1·8 (1·1 to 2·6)	0·8 (0·6 to 1)	1·3 (1 to 1·6)	0·4 (0·3 to 0·5)	0·6 (0·4 to 0·8)	0·6 (0·5 to 0·8)	0·5 (0·4 to 0·7)	0·6 (0·4 to 0·8)	0·7 (0·5 to 0·9)	0·7 (0·5 to 0·9)	0·8 (0·5 to 1)	0·7 (0·5 to 0·9)	0·8 (0·5 to 1·1)	0·7 (0·5 to 0·9)	0·8 (0·6 to 1·1)	0·8 (0·6 to 1·1)	1 (0·7 to 1·2)	0·5 (0·4 to 0·6)	0·2 (0·2 to 0·3)	0·1 (0·1 to 0·1)	20·3
Foreign body in eyes	0 (0 to 0)	0 (0 to 0)	0 (0 to 0)	0 (0 to 0·1)	0·1 (0 to 0·2)	0·1 (0 to 0·2)	0·1 (0 to 0·3)	0·2 (0·1 to 0·4)	0·2 (0·1 to 0·4)	0·2 (0·1 to 0·4)	0·2 (0·1 to 0·4)	0·2 (0·1 to 0·4)	0·2 (0·1 to 0·4)	0·2 (0·1 to 0·3)	0·1 (0·1 to 0·2)	0·1 (0·1 to 0·2)	0·1 (0·1 to 0·1)	0·1 (0 to 0·1)	0·1 (0 to 0·1)	0 (0 to 0·1)	0 (0 to 0)	0 (0 to 0)	0 (0 to 0)	2·3
Foreign body in other body part	0 (0 to 0)	0 (0 to 0)	0 (0 to 0)	0·1 (0·1 to 0·2)	0·2 (0·1 to 0·2)	0·2 (0·1 to 0·3)	0·2 (0·1 to 0·3)	0·2 (0·2 to 0·3)	0·2 (0·1 to 0·3)	0·2 (0·1 to 0·3)	0·2 (0·1 to 0·3)	0·2 (0·2 to 0·3)	0·2 (0·2 to 0·3)	0·2 (0·2 to 0·3)	0·2 (0·1 to 0·3)	0·2 (0·1 to 0·2)	0·1 (0·1 to 0·2)	0·1 (0·1 to 0·2)	0·1 (0·1 to 0·2)	0·1 (0·1 to 0·2)	0·1 (0 to 0·1)	0 (0 to 0·1)	0 (0 to 0)	3·2
Environmental heat and cold exposure	0 (0 to 0)	0 (0 to 0)	0·1 (0·1 to 0·1)	0·4 (0·2 to 0·5)	0·7 (0·5 to 1)	0·9 (0·6 to 1·4)	1·1 (0·8 to 1·6)	1·3 (0·9 to 1·8)	1·3 (0·9 to 1·9)	1·4 (1 to 2)	1·5 (1·1 to 2·1)	1·6 (1·1 to 2·2)	1·6 (1·1 to 2·1)	1·6 (1·1 to 2·2)	1·6 (1·1 to 2·2)	1·5 (1·1 to 2·1)	1·2 (0·8 to 1·6)	1 (0·7 to 1·3)	0·8 (0·6 to 1·1)	0·7 (0·5 to 0·9)	0·4 (0·3 to 0·6)	0·2 (0·2 to 0·3)	0·1 (0·1 to 0·1)	21
Exposure to forces of nature	0 (0 to 0)	0 (0 to 0)	0 (0 to 0)	0 (0 to 0)	0·1 (0 to 0·1)	0·1 (0 to 0·1)	0·2 (0·1 to 0·3)	0·2 (0·1 to 0·3)	0·2 (0·1 to 0·3)	0·2 (0·1 to 0·3)	0·2 (0·1 to 0·3)	0·2 (0·1 to 0·3)	0·2 (0·1 to 0·2)	0·1 (0·1 to 0·2)	0·1 (0·1 to 0·2)	0·1 (0·1 to 0·2)	0·1 (0·1 to 0·1)	0·1 (0 to 0·1)	0·1 (0 to 0·1)	0 (0 to 0)	0 (0 to 0)	0 (0 to 0)	0 (0 to 0)	2
Other unintentional injuries	0 (0 to 0·1)	0 (0 to 0·1)	0·2 (0·1 to 0·3)	0·8 (0·5 to 1·1)	1·3 (0·9 to 1·9)	1·8 (1·3 to 2·5)	1·8 (1·2 to 2·6)	2 (1·4 to 2·9)	2 (1·4 to 2·9)	2 (1·4 to 2·9)	2·2 (1·5 to 3·1)	2·3 (1·6 to 3·3)	2·2 (1·6 to 3·2)	2·2 (1·5 to 3·1)	2·1 (1·4 to 3)	1·8 (1·3 to 2·6)	1·4 (0·9 to 1·9)	1·1 (0·7 to 1·5)	0·8 (0·6 to 1·2)	0·6 (0·4 to 0·9)	0·3 (0·2 to 0·4)	0·1 (0·1 to 0·2)	0 (0 to 0)	29·3
Self-harm and interpersonal violence	1·6 (0·8 to 2·7)	0·5 (0·2 to 0·8)	2·8 (1·6 to 4·8)	4 (2·3 to 6·8)	3·8 (2·8 to 5·2)	11·5 (8·8 to 14·8)	28·8 (22·4 to 36·8)	45 (34·2 to 57·7)	41·2 (30·4 to 53·7)	34·7 (26 to 45·9)	26·8 (20·1 to 35·4)	22·4 (17 to 29·8)	17 (12·9 to 22·4)	11·4 (8·8 to 15)	7·4 (5·9 to 9·3)	5·6 (4·5 to 6·8)	4 (3·2 to 4·8)	2·8 (2·3 to 3·4)	1·9 (1·6 to 2·3)	1·1 (0·9 to 1·4)	0·5 (0·4 to 0·6)	0·2 (0·1 to 0·2)	0 (0 to 0)	275
Self-harm	0 (0 to 0)	0 (0 to 0)	0 (0 to 0)	0 (0 to 0)	0 (0 to 0)	1 (0·6 to 1·5)	3·7 (2·6 to 5·2)	4·7 (3·3 to 6·5)	4·7 (3·2 to 6·7)	4·5 (3·2 to 6·4)	3·7 (2·6 to 5)	3·1 (2·3 to 4·3)	2·8 (2 to 3·7)	2·3 (1·7 to 3·1)	1·7 (1·3 to 2·2)	1·4 (1·1 to 1·9)	1 (0·7 to 1·3)	0·7 (0·5 to 0·9)	0·4 (0·3 to 0·6)	0·3 (0·2 to 0·3)	0·1 (0·1 to 0·2)	0 (0 to 0)	0 (0 to 0)	36·1
Self-harm by firearm	0 (0 to 0)	0 (0 to 0)	0 (0 to 0)	0 (0 to 0)	0 (0 to 0)	0 (0 to 0)	0·1 (0·1 to 0·1)	0·1 (0·1 to 0·2)	0·1 (0·1 to 0·2)	0·2 (0·1 to 0·2)	0·1 (0·1 to 0·1)	0·1 (0 to 0·1)	0·1 (0 to 0·1)	0·1 (0 to 0·1)	0 (0 to 0·1)	0 (0 to 0)	0 (0 to 0)	0 (0 to 0)	0 (0 to 0)	0 (0 to 0)	0 (0 to 0)	0 (0 to 0)	0 (0 to 0)	1
Self-harm by other specified means	0 (0 to 0)	0 (0 to 0)	0 (0 to 0)	0 (0 to 0)	0 (0 to 0)	1 (0·6 to 1·5)	3·6 (2·5 to 5)	4·5 (3·2 to 6·3)	4·6 (3·1 to 6·6)	4·3 (3·1 to 6·2)	3·6 (2·6 to 4·9)	3·1 (2·3 to 4·2)	2·7 (2 to 3·6)	2·2 (1·7 to 3)	1·7 (1·2 to 2·2)	1·4 (1 to 1·9)	1 (0·7 to 1·3)	0·7 (0·5 to 0·9)	0·4 (0·3 to 0·5)	0·3 (0·2 to 0·3)	0·1 (0·1 to 0·1)	0 (0 to 0)	0 (0 to 0)	35·1
Interpersonal violence	1·6 (0·8 to 2·7)	0·5 (0·2 to 0·8)	2·8 (1·6 to 4·8)	3·9 (2·3 to 6·7)	3·8 (2·7 to 5·2)	10·4 (7·7 to 13·6)	25·1 (18·9 to 32·9)	40·3 (29·7 to 52·6)	36·4 (26·2 to 48·5)	30·1 (22 to 40·4)	23·1 (16·8 to 31)	19·3 (14·3 to 26·2)	14·2 (10·4 to 19·1)	9·1 (6·7 to 12·4)	5·7 (4·3 to 7·4)	4·2 (3·2 to 5·2)	3 (2·3 to 3·8)	2·1 (1·6 to 2·7)	1·5 (1·2 to 1·9)	0·8 (0·7 to 1·1)	0·4 (0·3 to 0·5)	0·1 (0·1 to 0·2)	0 (0 to 0)	238
Physical violence by firearm	0 (0 to 0·1)	0 (0 to 0)	0·1 (0 to 0·2)	0·4 (0·2 to 0·8)	0·5 (0·3 to 0·7)	2·1 (1·5 to 2·8)	7·8 (5·3 to 10·9)	11·5 (8·3 to 15·4)	9·4 (6·5 to 12·7)	7·3 (5·1 to 10·2)	5·4 (3·7 to 7·3)	3·6 (2·5 to 5·1)	3·2 (2·2 to 4·5)	1·7 (1·1 to 2·5)	1 (0·7 to 1·3)	0·5 (0·4 to 0·7)	0·3 (0·2 to 0·4)	0·2 (0·1 to 0·2)	0·1 (0·1 to 0·1)	0 (0 to 0)	0 (0 to 0)	0 (0 to 0)	0 (0 to 0)	54·9
Physical violence by sharp object	0·1 (0·1 to 0·2)	0 (0 to 0)	0·2 (0·1 to 0·3)	0·3 (0·2 to 0·6)	0·5 (0·4 to 0·6)	2 (1·5 to 2·7)	9 (6·5 to 12·4)	17·1 (12·4 to 23)	16·2 (11·4 to 21·9)	12·9 (9·1 to 17·9)	10·3 (7·3 to 13·9)	8·4 (5·7 to 12)	4·9 (3·5 to 6·9)	2·7 (2 to 3·8)	1·6 (1·1 to 2·1)	0·9 (0·7 to 1·2)	0·6 (0·5 to 0·8)	0·4 (0·3 to 0·6)	0·2 (0·2 to 0·3)	0·2 (0·1 to 0·2)	0·1 (0·1 to 0·1)	0 (0 to 0)	0 (0 to 0)	88·8
Sexual violence	0 (0 to 0)	0 (0 to 0)	0 (0 to 0)	0 (0 to 0)	0·3 (0·2 to 0·4)	0·6 (0·4 to 1)	1·5 (0·8 to 2·4)	2 (1·2 to 3·1)	1·7 (1 to 2·8)	1·6 (0·9 to 2·6)	1·4 (0·8 to 2·3)	1·2 (0·7 to 1·9)	0·9 (0·5 to 1·5)	0·6 (0·4 to 1)	0·4 (0·2 to 0·7)	0·3 (0·2 to 0·5)	0·2 (0·1 to 0·3)	0·2 (0·1 to 0·2)	0·1 (0·1 to 0·2)	0·1 (0·1 to 0·1)	0 (0 to 0·1)	0 (0 to 0)	0 (0 to 0)	13·2
Physical violence by other means	1·4 (0·7 to 2·5)	0·4 (0·2 to 0·7)	2·5 (1·4 to 4·4)	3·1 (1·8 to 5·5)	2·5 (1·8 to 3·6)	5·7 (4·1 to 7·6)	6·8 (5 to 9·2)	9·7 (6·6 to 13·8)	9·1 (6·5 to 12·2)	8·3 (5·5 to 11·6)	6 (4·3 to 7·9)	6·1 (4·4 to 8·4)	5·2 (3·6 to 7·3)	4·1 (3 to 5·7)	2·7 (2 to 3·6)	2·4 (1·8 to 3)	1·8 (1·4 to 2·4)	1·3 (1 to 1·8)	1·1 (0·8 to 1·4)	0·6 (0·4 to 0·7)	0·3 (0·2 to 0·3)	0·1 (0·1 to 0·1)	0 (0 to 0)	81·4
Conflict and terrorism	0 (0 to 0)	0 (0 to 0)	0 (0 to 0)	0 (0 to 0)	0 (0 to 0)	0 (0 to 0)	0 (0 to 0)	0 (0 to 0)	0 (0 to 0)	0 (0 to 0)	0 (0 to 0)	0 (0 to 0)	0 (0 to 0)	0 (0 to 0)	0 (0 to 0)	0 (0 to 0)	0 (0 to 0)	0 (0 to 0)	0 (0 to 0)	0 (0 to 0)	0 (0 to 0)	0 (0 to 0)	0 (0 to 0)	0

SR Table 2b: Suriname DALYS by cause and age group for females with 95% uncertainty intervals, 2019

Cause	Early Neonatal	Late Neonatal	Post Neonatal	1 to 4	5 to 9	10 to 14	15 to 19	20 to 24	25 to 29	30 to 34	35 to 39	40 to 44	45 to 49	50 to 54	55 to 59	60 to 64	65 to 69	70 to 74	75 to 79	80 to 84	85 to 89	90 to 94	95 plus	Total
All causes	4,340 (3,120 to 5,930)	1,500 (1,080 to 2,040)	2,580 (1,930 to 3,430)	1,600 (1,290 to 1,950)	1,690 (1,350 to 2,120)	2,230 (1,780 to 2,760)	3,400 (2,830 to 4,140)	3,670 (3,050 to 4,450)	4,240 (3,520 to 5,060)	4,780 (3,960 to 5,710)	5,070 (4,260 to 6,000)	5,060 (4,240 to 5,990)	5,870 (4,950 to 6,910)	6,880 (5,860 to 8,040)	7,510 (6,440 to 8,690)	6,900 (5,980 to 7,930)	5,780 (5,050 to 6,560)	5,410 (4,790 to 6,090)	4,850 (4,310 to 5,420)	3,710 (3,330 to 4,110)	2,130 (1,910 to 2,360)	1,000 (910 to 1,100)	472 (437 to 507)	90,700
Communicable, maternal, neonatal, and nutritional diseases	3,730 (2,640 to 5,100)	1,180 (831 to 1,610)	1,470 (1,040 to 2,010)	684 (521 to 886)	551 (385 to 778)	442 (328 to 610)	552 (429 to 727)	662 (549 to 828)	803 (679 to 954)	958 (826 to 1,120)	816 (688 to 974)	696 (581 to 858)	595 (490 to 747)	540 (426 to 685)	477 (374 to 614)	368 (291 to 472)	285 (224 to 357)	277 (216 to 356)	278 (212 to 365)	234 (176 to 303)	154 (115 to 203)	79·1 (57 to 104)	39·6 (27·8 to 54·3)	15,900
HIV/AIDS and sexually transmitted infections	140 (39·2 to 319)	40·3 (11·3 to 93)	117 (76·5 to 189)	34·9 (31·7 to 38·9)	29·4 (27 to 32)	21·5 (20 to 23·3)	63 (57·5 to 69·2)	187 (170 to 207)	302 (277 to 333)	448 (414 to 489)	345 (318 to 376)	276 (255 to 301)	202 (185 to 221)	127 (117 to 139)	81·6 (74·8 to 89·4)	44·1 (40·2 to 48·9)	26·6 (24·2 to 29·4)	9·9 (8·6 to 11·7)	5·3 (4·5 to 6·3)	2·9 (2·5 to 3·5)	1·5 (1·3 to 1·8)	0·3 (0·3 to 0·4)	0·1 (0·1 to 0·2)	2,510
HIV/AIDS	0 (0 to 0)	0 (0 to 0)	61·6 (57·3 to 66·2)	32·6 (30·2 to 35·4)	28·3 (26·2 to 30·4)	20·9 (19·3 to 22·5)	60·1 (55·1 to 65·8)	181 (165 to 200)	292 (269 to 321)	436 (404 to 474)	332 (305 to 361)	263 (244 to 284)	192 (177 to 205)	120 (111 to 129)	76·7 (70·6 to 82·6)	41·1 (37·8 to 44·4)	24·6 (22·7 to 26·6)	8·5 (7·7 to 9·4)	4·3 (3·9 to 4·8)	2·3 (2·1 to 2·6)	1·2 (1·1 to 1·4)	0·2 (0·2 to 0·3)	0·1 (0·1 to 0·1)	2,180
HIV/AIDS - Drug-susceptible Tuberculosis	0 (0 to 0)	0 (0 to 0)	3·6 (2·3 to 5·6)	1·8 (1·2 to 2·8)	1·6 (1 to 2·5)	1·3 (0·8 to 2)	3·7 (2·5 to 5·6)	10·8 (7·3 to 16)	17·1 (11·2 to 25·6)	26·3 (16·9 to 39·9)	20·4 (13 to 30·8)	15·8 (10·1 to 23·8)	11·5 (7·3 to 17·8)	7·3 (4·7 to 11·1)	4·8 (3·2 to 7)	2·5 (1·7 to 3·8)	1·5 (1 to 2·2)	0·5 (0·4 to 0·8)	0·3 (0·2 to 0·4)	0·2 (0·1 to 0·2)	0·1 (0·1 to 0·1)	0 (0 to 0)	0 (0 to 0)	131
HIV/AIDS - Multidrug-resistant Tuberculosis without extensive dru	0 (0 to 0)	0 (0 to 0)	0 (0 to 0·2)	0 (0 to 0·1)	0 (0 to 0·1)	0 (0 to 0·1)	0 (0 to 0·1)	0·1 (0 to 0·4)	0·2 (0 to 0·6)	0·3 (0 to 1)	0·2 (0 to 0·8)	0·2 (0 to 0·6)	0·1 (0 to 0·4)	0·1 (0 to 0·3)	0 (0 to 0·2)	0 (0 to 0·1)	0 (0 to 0·1)	0 (0 to 0)	0 (0 to 0)	0 (0 to 0)	0 (0 to 0)	0 (0 to 0)	0 (0 to 0)	1·3
HIV/AIDS - Extensively drug-resistant Tuberculosis	0 (0 to 0)	0 (0 to 0)	0 (0 to 0)	0 (0 to 0)	0 (0 to 0)	0 (0 to 0)	0 (0 to 0)	0 (0 to 0·1)	0 (0 to 0·1)	0 (0 to 0·2)	0 (0 to 0·1)	0 (0 to 0·1)	0 (0 to 0·1)	0 (0 to 0)	0 (0 to 0)	0 (0 to 0)	0 (0 to 0)	0 (0 to 0)	0 (0 to 0)	0 (0 to 0)	0 (0 to 0)	0 (0 to 0)	0 (0 to 0)	0·2
HIV/AIDS resulting in other diseases	0 (0 to 0)	0 (0 to 0)	57·9 (53 to 62·7)	30·8 (28·2 to 33·7)	26·6 (24·3 to 28·9)	19·6 (18 to 21·4)	56·3 (51 to 61·7)	170 (154 to 190)	275 (250 to 306)	409 (375 to 448)	312 (284 to 343)	247 (227 to 270)	180 (165 to 195)	113 (103 to 122)	71·9 (65·4 to 78)	38·5 (35·2 to 42)	23 (21·1 to 25·2)	8 (7·1 to 8·8)	4 (3·6 to 4·5)	2·2 (1·9 to 2·4)	1·1 (1 to 1·3)	0·2 (0·2 to 0·3)	0·1 (0·1 to 0·1)	2,050
Sexually transmitted infections excluding HIV	140 (39·2 to 319)	40·3 (11·3 to 92·9)	55·8 (15·4 to 126)	2·3 (0·6 to 5·7)	1·1 (0·3 to 2·7)	0·6 (0·3 to 1·2)	3 (1·6 to 5·1)	6·1 (3·3 to 10·8)	9·7 (5·1 to 16·9)	12·6 (6·7 to 22·3)	13·1 (7·1 to 23)	12·5 (6·7 to 22·6)	10·5 (5·6 to 18·8)	7 (3·4 to 13·7)	4·9 (2·3 to 9·7)	3 (1·4 to 6·2)	2 (0·9 to 4)	1·4 (0·7 to 2·8)	1 (0·5 to 1·9)	0·6 (0·3 to 1·1)	0·3 (0·1 to 0·5)	0·1 (0·1 to 0·2)	0 (0 to 0·1)	328
Syphilis	140 (39·2 to 319)	40·3 (11·3 to 92·9)	55·8 (15·4 to 126)	2·3 (0·6 to 5·7)	1·1 (0·3 to 2·7)	0·2 (0·1 to 0·5)	0·3 (0·1 to 0·7)	0·1 (0·1 to 0·2)	0·3 (0·1 to 0·7)	0·5 (0·2 to 1·1)	0·4 (0·2 to 0·8)	0·1 (0·1 to 0·3)	0·2 (0·1 to 0·4)	0·2 (0·1 to 0·3)	0·1 (0 to 0·1)	0·1 (0 to 0·1)	0·1 (0 to 0·1)	0·1 (0 to 0·1)	0 (0 to 0·1)	0 (0 to 0·1)	0 (0 to 0)	0 (0 to 0)	0 (0 to 0)	242
Chlamydial infection	0 (0 to 0)	0 (0 to 0)	0 (0 to 0)	0 (0 to 0)	0 (0 to 0)	0·1 (0 to 0·1)	0·3 (0·2 to 0·5)	0·7 (0·4 to 1·2)	0·9 (0·4 to 1·6)	1·1 (0·6 to 1·9)	1·4 (0·7 to 2·2)	1·4 (0·8 to 2·4)	1 (0·5 to 1·7)	0·3 (0·2 to 0·5)	0·2 (0·1 to 0·4)	0·1 (0 to 0·2)	0 (0 to 0·1)	0 (0 to 0·1)	0 (0 to 0·1)	0 (0 to 0·1)	0 (0 to 0)	0 (0 to 0)	0 (0 to 0)	7·6
Gonococcal infection	0 (0 to 0)	0 (0 to 0)	0 (0 to 0)	0 (0 to 0)	0 (0 to 0)	0·1 (0 to 0·2)	0·5 (0·2 to 1)	0·7 (0·3 to 1·3)	0·9 (0·4 to 1·7)	1·1 (0·6 to 2·1)	1·2 (0·6 to 2·3)	1·3 (0·6 to 2·5)	1·1 (0·5 to 2·1)	0·6 (0·2 to 1·2)	0·4 (0·1 to 0·8)	0·2 (0·1 to 0·6)	0·2 (0·1 to 0·5)	0·2 (0·1 to 0·4)	0·1 (0 to 0·3)	0·1 (0 to 0·1)	0 (0 to 0·1)	0 (0 to 0)	0 (0 to 0)	8·5
Trichomoniasis	0 (0 to 0)	0 (0 to 0)	0 (0 to 0)	0 (0 to 0)	0 (0 to 0)	0 (0 to 0·1)	0·4 (0·1 to 1·1)	2 (0·6 to 4·7)	3·6 (1·2 to 8·3)	4·3 (1·3 to 10)	3·9 (1·3 to 9·1)	3·5 (1·2 to 8·6)	3·4 (1·1 to 8·1)	2·8 (0·9 to 6·5)	1·9 (0·6 to 4·5)	1·1 (0·4 to 2·5)	0·7 (0·2 to 1·6)	0·4 (0·2 to 1)	0·3 (0·1 to 0·6)	0·1 (0 to 0·3)	0·1 (0 to 0·1)	0 (0 to 0)	0 (0 to 0)	28·5
Genital herpes	0 (0 to 0)	0 ([illegible])	0 ([illegible])	0 ([illegible])	0 ([illegible])	0·1 ([illegible])	0·6 ([illegible])	1·3 ([illegible])	1·9 ([illegible])	2·4 ([illegible])	2·5 ([illegible])	2·4 ([illegible])	2·4 ([illegible])	2·3 ([illegible])	1·9 ([illegible])	1·4 ([illegible])	0·9 ([illegible])	0·7 ([illegible])	0·4 ([illegible])	0·3 ([illegible])	0·1 ([illegible])	0·1 ([illegible])	0 ([illegible])	21·6
Other sexually transmitted infections	0 (0 to 0)	[illegible]	[illegible]	[illegible]	[illegible]	[illegible]	[illegible]	[illegible]	[illegible]	[illegible]	[illegible]	[illegible]	[illegible]	[illegible]	[illegible]	[illegible]	[illegible]	[illegible]	[illegible]	[illegible]	[illegible]	[illegible]	[illegible]	19·3
Respiratory infections and tuberculosis	80·6 (42·6 to 140)	[illegible]	[illegible]	[illegible]	[illegible]	[illegible]	[illegible]	[illegible]	[illegible]	[illegible]	[illegible]	[illegible]	[illegible]	[illegible]	[illegible]	[illegible]	[illegible]	[illegible]	[illegible]	[illegible]	[illegible]	[illegible]	[illegible]	2,230
Tuberculosis	0 (0 to 0)	[illegible]	[illegible]	[illegible]	[illegible]	[illegible]	[illegible]	[illegible]	[illegible]	[illegible]	[illegible]	[illegible]	[illegible]	[illegible]	[illegible]	[illegible]	[illegible]	[illegible]	[illegible]	[illegible]	[illegible]	[illegible]	[illegible]	133
Latent tuberculosis infection	0 (0 to 0)	[illegible]	[illegible]	[illegible]	[illegible]	[illegible]	[illegible]	[illegible]	[illegible]	[illegible]	[illegible]	[illegible]	[illegible]	[illegible]	[illegible]	[illegible]	[illegible]	[illegible]	[illegible]	[illegible]	[illegible]	[illegible]	[illegible]	0
Drug-susceptible tuberculosis	0 (0 to 0)	[illegible]	[illegible]	[illegible]	[illegible]	[illegible]	[illegible]	[illegible]	[illegible]	[illegible]	[illegible]	[illegible]	[illegible]	[illegible]	[illegible]	[illegible]	[illegible]	[illegible]	[illegible]	[illegible]	[illegible]	[illegible]	[illegible]	132
Multidrug-resistant tuberculosis without extensive drug resistanc	0 (0 to 0)	[illegible]	[illegible]	[illegible]	[illegible]	[illegible]	[illegible]	[illegible]	[illegible]	[illegible]	[illegible]	[illegible]	[illegible]	[illegible]	[illegible]	[illegible]	[illegible]	[illegible]	[illegible]	[illegible]	[illegible]	[illegible]	[illegible]	1
Extensively drug-resistant tuberculosis	0 (0 to 0)	[illegible]	[illegible]	[illegible]	[illegible]	[illegible]	[illegible]	[illegible]	[illegible]	[illegible]	[illegible]	[illegible]	[illegible]	[illegible]	[illegible]	[illegible]	[illegible]	[illegible]	[illegible]	[illegible]	[illegible]	[illegible]	[illegible]	0·2
Lower respiratory infections	80·6 (42·5 to 140)	[illegible]	[illegible]	[illegible]	[illegible]	[illegible]	[illegible]	[illegible]	[illegible]	[illegible]	[illegible]	[illegible]	[illegible]	[illegible]	[illegible]	[illegible]	[illegible]	[illegible]	[illegible]	[illegible]	[illegible]	[illegible]	[illegible]	1,790
Upper respiratory infections	0 (0 to 0·1)	[illegible]	[illegible]	[illegible]	[illegible]	[illegible]	[illegible]	[illegible]	[illegible]	[illegible]	[illegible]	[illegible]	[illegible]	[illegible]	[illegible]	[illegible]	[illegible]	[illegible]	[illegible]	[illegible]	[illegible]	[illegible]	[illegible]	230
Otitis media	0 (0 to 0)	[illegible]	[illegible]	[illegible]	[illegible]	[illegible]	[illegible]	[illegible]	[illegible]	[illegible]	[illegible]	[illegible]	[illegible]	[illegible]	[illegible]	[illegible]	[illegible]	[illegible]	[illegible]	[illegible]	[illegible]	[illegible]	[illegible]	72·2
Enteric infections	22·1 (9·4 to 43·6)	[illegible]	[illegible]	[illegible]	[illegible]	[illegible]	[illegible]	[illegible]	[illegible]	[illegible]	[illegible]	[illegible]	[illegible]	[illegible]	[illegible]	[illegible]	[illegible]	[illegible]	[illegible]	[illegible]	[illegible]	[illegible]	[illegible]	1,270
Diarrheal diseases	22·1 (9·4 to 43·6)	[illegible]	[illegible]	[illegible]	[illegible]	[illegible]	[illegible]	[illegible]	[illegible]	[illegible]	[illegible]	[illegible]	[illegible]	[illegible]	[illegible]	[illegible]	[illegible]	[illegible]	[illegible]	[illegible]	[illegible]	[illegible]	[illegible]	1,260
Typhoid and paratyphoid	0 (0 to 0)	[illegible]	[illegible]	[illegible]	[illegible]	[illegible]	[illegible]	[illegible]	[illegible]	[illegible]	[illegible]	[illegible]	[illegible]	[illegible]	[illegible]	[illegible]	[illegible]	[illegible]	[illegible]	[illegible]	[illegible]	[illegible]	[illegible]	2·2
Typhoid fever	0 (0 to 0)	[illegible]	[illegible]	[illegible]	[illegible]	[illegible]	[illegible]	[illegible]	[illegible]	[illegible]	[illegible]	[illegible]	[illegible]	[illegible]	[illegible]	[illegible]	[illegible]	[illegible]	[illegible]	[illegible]	[illegible]	[illegible]	[illegible]	1·9
Paratyphoid fever	0 (0 to 0)	[illegible]	[illegible]	[illegible]	[illegible]	[illegible]	[illegible]	[illegible]	[illegible]	[illegible]	[illegible]	[illegible]	[illegible]	[illegible]	[illegible]	[illegible]	[illegible]	[illegible]	[illegible]	[illegible]	[illegible]	[illegible]	[illegible]	0·3
Invasive Non-typhoidal Salmonella (iNTS)	0 (0 to 0)	[illegible]	[illegible]	[illegible]	[illegible]	[illegible]	[illegible]	[illegible]	[illegible]	[illegible]	[illegible]	[illegible]	[illegible]	[illegible]	[illegible]	[illegible]	[illegible]	[illegible]	[illegible]	[illegible]	[illegible]	[illegible]	[illegible]	3
Other intestinal infectious diseases	0 (0 to 0)	[illegible]	[illegible]	[illegible]	[illegible]	[illegible]	[illegible]	[illegible]	[illegible]	[illegible]	[illegible]	[illegible]	[illegible]	[illegible]	[illegible]	[illegible]	[illegible]	[illegible]	[illegible]	[illegible]	[illegible]	[illegible]	[illegible]	1·8
Neglected tropical diseases and malaria	0·8 (0·2 to 2)	[illegible]	[illegible]	[illegible]	[illegible]	[illegible]	[illegible]	[illegible]	[illegible]	[illegible]	[illegible]	[illegible]	[illegible]	[illegible]	[illegible]	[illegible]	[illegible]	[illegible]	[illegible]	[illegible]	[illegible]	[illegible]	[illegible]	925
Malaria	0 (0 to 0)	[illegible]	[illegible]	[illegible]	[illegible]	[illegible]	[illegible]	[illegible]	[illegible]	[illegible]	[illegible]	[illegible]	[illegible]	[illegible]	[illegible]	[illegible]	[illegible]	[illegible]	[illegible]	[illegible]	[illegible]	[illegible]	[illegible]	21·5
Chagas disease	0 (0 to 0)	[illegible]	[illegible]	[illegible]	[illegible]	[illegible]	[illegible]	[illegible]	[illegible]	[illegible]	[illegible]	[illegible]	[illegible]	[illegible]	[illegible]	[illegible]	[illegible]	[illegible]	[illegible]	[illegible]	[illegible]	[illegible]	[illegible]	1·8
Leishmaniasis	0 (0 to 0)	[illegible]	[illegible]	[illegible]	[illegible]	[illegible]	[illegible]	[illegible]	[illegible]	[illegible]	[illegible]	[illegible]	[illegible]	[illegible]	[illegible]	[illegible]	[illegible]	[illegible]	[illegible]	[illegible]	[illegible]	[illegible]	[illegible]	11·5
Visceral leishmaniasis	0 (0 to 0)	[illegible]	[illegible]	[illegible]	[illegible]	[illegible]	[illegible]	[illegible]	[illegible]	[illegible]	[illegible]	[illegible]	[illegible]	[illegible]	[illegible]	[illegible]	[illegible]	[illegible]	[illegible]	[illegible]	[illegible]	[illegible]	[illegible]	2·6
Cutaneous and mucocutaneous leishmaniasis	0 (0 to 0)	[illegible]	[illegible]	[illegible]	[illegible]	[illegible]	[illegible]	[illegible]	[illegible]	[illegible]	[illegible]	[illegible]	[illegible]	[illegible]	[illegible]	[illegible]	[illegible]	[illegible]	[illegible]	[illegible]	[illegible]	[illegible]	[illegible]	9
African trypanosomiasis	0 (0 to 0)	[illegible]	[illegible]	[illegible]	[illegible]	[illegible]	[illegible]	[illegible]	[illegible]	[illegible]	[illegible]	[illegible]	[illegible]	[illegible]	[illegible]	[illegible]	[illegible]	[illegible]	[illegible]	[illegible]	[illegible]	[illegible]	[illegible]	0
Schistosomiasis	0 (0 to 0)	[illegible]	[illegible]	[illegible]	[illegible]	[illegible]	[illegible]	[illegible]	[illegible]	[illegible]	[illegible]	[illegible]	[illegible]	[illegible]	[illegible]	[illegible]	[illegible]	[illegible]	[illegible]	[illegible]	[illegible]	[illegible]	[illegible]	350
Cysticercosis	0 (0 to 0)	[illegible]	[illegible]	[illegible]	[illegible]	[illegible]	[illegible]	[illegible]	[illegible]	[illegible]	[illegible]	[illegible]	[illegible]	[illegible]	[illegible]	[illegible]	[illegible]	[illegible]	[illegible]	[illegible]	[illegible]	[illegible]	[illegible]	180
Cystic echinococcosis	0 (0 to 0)	[illegible]	[illegible]	[illegible]	[illegible]	[illegible]	[illegible]	[illegible]	[illegible]	[illegible]	[illegible]	[illegible]	[illegible]	[illegible]	[illegible]	[illegible]	[illegible]	[illegible]	[illegible]	[illegible]	[illegible]	[illegible]	[illegible]	0·2
Lymphatic filariasis	0 (0 to 0)	[illegible]	[illegible]	[illegible]	[illegible]	[illegible]	[illegible]	[illegible]	[illegible]	[illegible]	[illegible]	[illegible]	[illegible]	[illegible]	[illegible]	[illegible]	[illegible]	[illegible]	[illegible]	[illegible]	[illegible]	[illegible]	[illegible]	0
Onchocerciasis	0 (0 to 0)	[illegible]	[illegible]	[illegible]	[illegible]	[illegible]	[illegible]	[illegible]	[illegible]	[illegible]	[illegible]	[illegible]	[illegible]	[illegible]	[illegible]	[illegible]	[illegible]	[illegible]	[illegible]	[illegible]	[illegible]	[illegible]	[illegible]	0
Trachoma	0 (0 to 0)	[illegible]	[illegible]	[illegible]	[illegible]	[illegible]	[illegible]	[illegible]	[illegible]	[illegible]	[illegible]	[illegible]	[illegible]	[illegible]	[illegible]	[illegible]	[illegible]	[illegible]	[illegible]	[illegible]	[illegible]	[illegible]	[illegible]	0
Dengue	0 (0 to 0)	[illegible]	[illegible]	[illegible]	[illegible]	[illegible]	[illegible]	[illegible]	[illegible]	[illegible]	[illegible]	[illegible]	[illegible]	[illegible]	[illegible]	[illegible]	[illegible]	[illegible]	[illegible]	[illegible]	[illegible]	[illegible]	[illegible]	220
Yellow fever	0 (0 to 0)	[illegible]	[illegible]	[illegible]	[illegible]	[illegible]	[illegible]	[illegible]	[illegible]	[illegible]	[illegible]	[illegible]	[illegible]	[illegible]	[illegible]	[illegible]	[illegible]	[illegible]	[illegible]	[illegible]	[illegible]	[illegible]	[illegible]	1·1
Rabies	0 (0 to 0)	[illegible]	[illegible]	[illegible]	[illegible]	[illegible]	[illegible]	[illegible]	[illegible]	[illegible]	[illegible]	[illegible]	[illegible]	[illegible]	[illegible]	[illegible]	[illegible]	[illegible]	[illegible]	[illegible]	[illegible]	[illegible]	[illegible]	1·6
Intestinal nematode infections	0 (0 to 0)	[illegible]	[illegible]	[illegible]	[illegible]	[illegible]	[illegible]	[illegible]	[illegible]	[illegible]	[illegible]	[illegible]	[illegible]	[illegible]	[illegible]	[illegible]	[illegible]	[illegible]	[illegible]	[illegible]	[illegible]	[illegible]	[illegible]	25·2
Ascariasis	0 (0 to 0)	[illegible]	[illegible]	[illegible]	[illegible]	[illegible]	[illegible]	[illegible]	[illegible]	[illegible]	[illegible]	[illegible]	[illegible]	[illegible]	[illegible]	[illegible]	[illegible]	[illegible]	[illegible]	[illegible]	[illegible]	[illegible]	[illegible]	1·4
Trichuriasis	0 (0 to 0)	[illegible]	[illegible]	[illegible]	[illegible]	[illegible]	[illegible]	[illegible]	[illegible]	[illegible]	[illegible]	[illegible]	[illegible]	[illegible]	[illegible]	[illegible]	[illegible]	[illegible]	[illegible]	[illegible]	[illegible]	[illegible]	[illegible]	0
Hookworm disease	0 (0 to 0)	[illegible]	[illegible]	[illegible]	[illegible]	[illegible]	[illegible]	[illegible]	[illegible]	[illegible]	[illegible]	[illegible]	[illegible]	[illegible]	[illegible]	[illegible]	[illegible]	[illegible]	[illegible]	[illegible]	[illegible]	[illegible]	[illegible]	23·8
Food-borne trematodiases	0 (0 to 0)	[illegible]	[illegible]	[illegible]	[illegible]	[illegible]	[illegible]	[illegible]	[illegible]	[illegible]	[illegible]	[illegible]	[illegible]	[illegible]	[illegible]	[illegible]	[illegible]	[illegible]	[illegible]	[illegible]	[illegible]	[illegible]	[illegible]	0
Leprosy	0 (0 to 0)	[illegible]	[illegible]	[illegible]	[illegible]	[illegible]	[illegible]	[illegible]	[illegible]	[illegible]	[illegible]	[illegible]	[illegible]	[illegible]	[illegible]	[illegible]	[illegible]	[illegible]	[illegible]	[illegible]	[illegible]	[illegible]	[illegible]	1·4
Ebola	0 (0 to 0)	[illegible]	[illegible]	[illegible]	[illegible]	[illegible]	[illegible]	[illegible]	[illegible]	[illegible]	[illegible]	[illegible]	[illegible]	[illegible]	[illegible]	[illegible]	[illegible]	[illegible]	[illegible]	[illegible]	[illegible]	[illegible]	[illegible]	0
Zika virus	0 (0 to 0)	[illegible]	[illegible]	[illegible]	[illegible]	[illegible]	[illegible]	[illegible]	[illegible]	[illegible]	[illegible]	[illegible]	[illegible]	[illegible]	[illegible]	[illegible]	[illegible]	[illegible]	[illegible]	[illegible]	[illegible]	[illegible]	[illegible]	1·1
Guinea worm disease	0 (0 to 0)	[illegible]	[illegible]	[illegible]	[illegible]	[illegible]	[illegible]	[illegible]	[illegible]	[illegible]	[illegible]	[illegible]	[illegible]	[illegible]	[illegible]	[illegible]	[illegible]	[illegible]	[illegible]	[illegible]	[illegible]	[illegible]	[illegible]	0
Other neglected tropical diseases	0·8 (0·2 to 2)	[illegible]	[illegible]	[illegible]	[illegible]	[illegible]	[illegible]	[illegible]	[illegible]	[illegible]	[illegible]	[illegible]	[illegible]	[illegible]	[illegible]	[illegible]	[illegible]	[illegible]	[illegible]	[illegible]	[illegible]	[illegible]	[illegible]	109
Other infectious diseases	12·7 (6·6 to 22·1)	[illegible]	[illegible]	[illegible]	[illegible]	[illegible]	[illegible]	[illegible]	[illegible]	[illegible]	[illegible]	[illegible]	[illegible]	[illegible]	[illegible]	[illegible]	[illegible]	[illegible]	[illegible]	[illegible]	[illegible]	[illegible]	[illegible]	672
Meningitis	7·4 (2·9 to 14·4)	[illegible]	[illegible]	[illegible]	[illegible]	[illegible]	[illegible]	[illegible]	[illegible]	[illegible]	[illegible]	[illegible]	[illegible]	[illegible]	[illegible]	[illegible]	[illegible]	[illegible]	[illegible]	[illegible]	[illegible]	[illegible]	[illegible]	169
Encephalitis	2·3 (0·9 to 5·4)	[illegible]	[illegible]	[illegible]	[illegible]	[illegible]	[illegible]	[illegible]	[illegible]	[illegible]	[illegible]	[illegible]	[illegible]	[illegible]	[illegible]	[illegible]	[illegible]	[illegible]	[illegible]	[illegible]	[illegible]	[illegible]	[illegible]	151
Diphtheria	0 (0 to 0)	[illegible]	[illegible]	[illegible]	[illegible]	[illegible]	[illegible]	[illegible]	[illegible]	[illegible]	[illegible]	[illegible]	[illegible]	[illegible]	[illegible]	[illegible]	[illegible]	[illegible]	[illegible]	[illegible]	[illegible]	[illegible]	[illegible]	0·3
Tetanus	0·2 (0·1 to 0·6)	[illegible]	[illegible]	[illegible]	[illegible]	[illegible]	[illegible]	[illegible]	[illegible]	[illegible]	[illegible]	[illegible]	[illegible]	[illegible]	[illegible]	[illegible]	[illegible]	[illegible]	[illegible]	[illegible]	[illegible]	[illegible]	[illegible]	9·1
Measles	0 (0 to 0)	[illegible]	[illegible]	[illegible]	[illegible]	[illegible]	[illegible]	[illegible]	[illegible]	[illegible]	[illegible]	[illegible]	[illegible]	[illegible]	[illegible]	[illegible]	[illegible]	[illegible]	[illegible]	[illegible]	[illegible]	[illegible]	[illegible]	0
Varicella and herpes zoster	0·2 (0 to 0·5)	[illegible]	[illegible]	[illegible]	[illegible]	[illegible]	[illegible]	[illegible]	[illegible]	[illegible]	[illegible]	[illegible]	[illegible]	[illegible]	[illegible]	[illegible]	[illegible]	[illegible]	[illegible]	[illegible]	[illegible]	[illegible]	[illegible]	27·3
Acute hepatitis	0 (0 to 0)	[illegible]	[illegible]	[illegible]	[illegible]	[illegible]	[illegible]	[illegible]	[illegible]	[illegible]	[illegible]	[illegible]	[illegible]	[illegible]	[illegible]	[illegible]	[illegible]	[illegible]	[illegible]	[illegible]	[illegible]	[illegible]	[illegible]	23·7
Acute hepatitis A	0 (0 to 0)	[illegible]	[illegible]	[illegible]	[illegible]	[illegible]	[illegible]	[illegible]	[illegible]	[illegible]	[illegible]	[illegible]	[illegible]	[illegible]	[illegible]	[illegible]	[illegible]	[illegible]	[illegible]	[illegible]	[illegible]	[illegible]	[illegible]	11·2
Acute hepatitis B	0 (0 to 0)	[illegible]	[illegible]	[illegible]	[illegible]	[illegible]	[illegible]	[illegible]	[illegible]	[illegible]	[illegible]	[illegible]	[illegible]	[illegible]	[illegible]	[illegible]	[illegible]	[illegible]	[illegible]	[illegible]	[illegible]	[illegible]	[illegible]	9·5
Acute hepatitis C	0 (0 to 0)	[illegible]	[illegible]	[illegible]	[illegible]	[illegible]	[illegible]	[illegible]	[illegible]	[illegible]	[illegible]	[illegible]	[illegible]	[illegible]	[illegible]	[illegible]	[illegible]	[illegible]	[illegible]	[illegible]	[illegible]	[illegible]	[illegible]	1·2
Acute hepatitis E	0 (0 to 0)	[illegible]	[illegible]	[illegible]	[illegible]	[illegible]	[illegible]	[illegible]	[illegible]	[illegible]	[illegible]	[illegible]	[illegible]	[illegible]	[illegible]	[illegible]	[illegible]	[illegible]	[illegible]	[illegible]	[illegible]	[illegible]	[illegible]	1·9
Other unspecified infectious diseases	2·5 (0·9 to 6·8)	[illegible]	[illegible]	[illegible]	[illegible]	[illegible]	[illegible]	[illegible]	[illegible]	[illegible]	[illegible]	[illegible]	[illegible]	[illegible]	[illegible]	[illegible]	[illegible]	[illegible]	[illegible]	[illegible]	[illegible]	[illegible]	[illegible]	208
Maternal and neonatal disorders	3,480 (2,460 to 4,750)	[illegible]	[illegible]	[illegible]	[illegible]	[illegible]	[illegible]	[illegible]	[illegible]	[illegible]	[illegible]	[illegible]	[illegible]	[illegible]	[illegible]	[illegible]	[illegible]	[illegible]	[illegible]	[illegible]	[illegible]	[illegible]	[illegible]	6,590
Maternal disorders	0 (0 to 0)	[illegible]	[illegible]	[illegible]	[illegible]	[illegible]	[illegible]	[illegible]	[illegible]	[illegible]	[illegible]	[illegible]	[illegible]	[illegible]	[illegible]	[illegible]	[illegible]	[illegible]	[illegible]	[illegible]	[illegible]	[illegible]	[illegible]	695
Maternal hemorrhage	0 (0 to 0)	[illegible]	[illegible]	[illegible]	[illegible]	[illegible]	[illegible]	[illegible]	[illegible]	[illegible]	[illegible]	[illegible]	[illegible]	[illegible]	[illegible]	[illegible]	[illegible]	[illegible]	[illegible]	[illegible]	[illegible]	[illegible]	[illegible]	129
Maternal sepsis and other maternal infections	0 (0 to 0)	[illegible]	[illegible]	[illegible]	[illegible]	[illegible]	[illegible]	[illegible]	[illegible]	[illegible]	[illegible]	[illegible]	[illegible]	[illegible]	[illegible]	[illegible]	[illegible]	[illegible]	[illegible]	[illegible]	[illegible]	[illegible]	[illegible]	29·3
Maternal hypertensive disorders	0 (0 to 0)	[illegible]	[illegible]	[illegible]	[illegible]	[illegible]	[illegible]	[illegible]	[illegible]	[illegible]	[illegible]	[illegible]	[illegible]	[illegible]	[illegible]	[illegible]	[illegible]	[illegible]	[illegible]	[illegible]	[illegible]	[illegible]	[illegible]	137
Maternal obstructed labor and uterine rupture	0 (0 to 0)	[illegible]	[illegible]	[illegible]	[illegible]	[illegible]	[illegible]	[illegible]	[illegible]	[illegible]	[illegible]	[illegible]	[illegible]	[illegible]	[illegible]	[illegible]	[illegible]	[illegible]	[illegible]	[illegible]	[illegible]	[illegible]	[illegible]	19·5
Maternal abortion and miscarriage	0 (0 to 0)	[illegible]	[illegible]	[illegible]	[illegible]	[illegible]	[illegible]	[illegible]	[illegible]	[illegible]	[illegible]	[illegible]	[illegible]	[illegible]	[illegible]	[illegible]	[illegible]	[illegible]	[illegible]	[illegible]	[illegible]	[illegible]	[illegible]	22·5
Ectopic pregnancy	0 (0 to 0)	[illegible]	[illegible]	[illegible]	[illegible]	[illegible]	[illegible]	[illegible]	[illegible]	[illegible]	[illegible]	[illegible]	[illegible]	[illegible]	[illegible]	[illegible]	[illegible]	[illegible]	[illegible]	[illegible]	[illegible]	[illegible]	[illegible]	54·1
Indirect maternal deaths	--	[illegible]	[illegible]	[illegible]	[illegible]	[illegible]	[illegible]	[illegible]	[illegible]	[illegible]	[illegible]	[illegible]	[illegible]	[illegible]	[illegible]	[illegible]	[illegible]	[illegible]	[illegible]	[illegible]	[illegible]	[illegible]	[illegible]	NA
Late maternal deaths	--	[illegible]	[illegible]	[illegible]	[illegible]	[illegible]	[illegible]	[illegible]	[illegible]	[illegible]	[illegible]	[illegible]	[illegible]	[illegible]	[illegible]	[illegible]	[illegible]	[illegible]	[illegible]	[illegible]	[illegible]	[illegible]	[illegible]	NA
Maternal deaths aggravated by HIV/AIDS	--	[illegible]	[illegible]	[illegible]	[illegible]	[illegible]	[illegible]	[illegible]	[illegible]	[illegible]	[illegible]	[illegible]	[illegible]	[illegible]	[illegible]	[illegible]	[illegible]	[illegible]	[illegible]	[illegible]	[illegible]	[illegible]	[illegible]	NA
Neonatal disorders	3,480 (2,460 to 4,750)	[illegible]	[illegible]	[illegible]	[illegible]	[illegible]	[illegible]	[illegible]	[illegible]	[illegible]	[illegible]	[illegible]	[illegible]	[illegible]	[illegible]	[illegible]	[illegible]	[illegible]	[illegible]	[illegible]	[illegible]	[illegible]	[illegible]	5,900
Neonatal preterm birth	1,820 (1,180 to 2,670)	[illegible]	[illegible]	[illegible]	[illegible]	[illegible]	[illegible]	[illegible]	[illegible]	[illegible]	[illegible]	[illegible]	[illegible]	[illegible]	[illegible]	[illegible]	[illegible]	[illegible]	[illegible]	[illegible]	[illegible]	[illegible]	[illegible]	2,750
Neonatal encephalopathy due to birth asphyxia and trauma	890 (511 to 1,400)	[illegible]	[illegible]	[illegible]	[illegible]	[illegible]	[illegible]	[illegible]	[illegible]	[illegible]	[illegible]	[illegible]	[illegible]	[illegible]	[illegible]	[illegible]	[illegible]	[illegible]	[illegible]	[illegible]	[illegible]	[illegible]	[illegible]	1,200
Neonatal sepsis and other neonatal infections	492 (199 to 934)	[illegible]	[illegible]	[illegible]	[illegible]	[illegible]	[illegible]	[illegible]	[illegible]	[illegible]	[illegible]	[illegible]	[illegible]	[illegible]	[illegible]	[illegible]	[illegible]	[illegible]	[illegible]	[illegible]	[illegible]	[illegible]	[illegible]	1,450
Hemolytic disease and other neonatal jaundice	22·5 (10·4 to 41·1)	[illegible]	[illegible]	[illegible]	[illegible]	[illegible]	[illegible]	[illegible]	[illegible]	[illegible]	[illegible]	[illegible]	[illegible]	[illegible]	[illegible]	[illegible]	[illegible]	[illegible]	[illegible]	[illegible]	[illegible]	[illegible]	[illegible]	72·6
Other neonatal disorders	251 (128 to 449)	[illegible]	[illegible]	[illegible]	[illegible]	[illegible]	[illegible]	[illegible]	[illegible]	[illegible]	[illegible]	[illegible]	[illegible]	[illegible]	[illegible]	[illegible]	[illegible]	[illegible]	[illegible]	[illegible]	[illegible]	[illegible]	[illegible]	425
Nutritional deficiencies	0·2 (0·1 to 0·3)	[illegible]	[illegible]	[illegible]	[illegible]	[illegible]	[illegible]	[illegible]	[illegible]	[illegible]	[illegible]	[illegible]	[illegible]	[illegible]	[illegible]	[illegible]	[illegible]	[illegible]	[illegible]	[illegible]	[illegible]	[illegible]	[illegible]	1,680
Protein-energy malnutrition	0·2 (0·1 to 0·2)	[illegible]	[illegible]	[illegible]	[illegible]	[illegible]	[illegible]	[illegible]	[illegible]	[illegible]	[illegible]	[illegible]	[illegible]	[illegible]	[illegible]	[illegible]	[illegible]	[illegible]	[illegible]	[illegible]	[illegible]	[illegible]	[illegible]	262
Iodine deficiency	0 (0 to 0)	[illegible]	[illegible]	[illegible]	[illegible]	[illegible]	[illegible]	[illegible]	[illegible]	[illegible]	[illegible]	[illegible]	[illegible]	[illegible]	[illegible]	[illegible]	[illegible]	[illegible]	[illegible]	[illegible]	[illegible]	[illegible]	[illegible]	16·5
Vitamin A deficiency	0 (0 to 0)	[illegible]	[illegible]	[illegible]	[illegible]	[illegible]	[illegible]	[illegible]	[illegible]	[illegible]	[illegible]	[illegible]	[illegible]	[illegible]	[illegible]	[illegible]	[illegible]	[illegible]	[illegible]	[illegible]	[illegible]	[illegible]	[illegible]	24·8
Dietary iron deficiency	0 (0 to 0·1)	[illegible]	[illegible]	[illegible]	[illegible]	[illegible]	[illegible]	[illegible]	[illegible]	[illegible]	[illegible]	[illegible]	[illegible]	[illegible]	[illegible]	[illegible]	[illegible]	[illegible]	[illegible]	[illegible]	[illegible]	[illegible]	[illegible]	1,350
Other nutritional deficiencies	0 (0 to 0)	[illegible]	[illegible]	[illegible]	[illegible]	[illegible]	[illegible]	[illegible]	[illegible]	[illegible]	[illegible]	[illegible]	[illegible]	[illegible]	[illegible]	[illegible]	[illegible]	[illegible]	[illegible]	[illegible]	[illegible]	[illegible]	[illegible]	31
Non-communicable diseases	554 (318 to 878)	[illegible]	[illegible]	[illegible]	[illegible]	[illegible]	[illegible]	[illegible]	[illegible]	[illegible]	[illegible]	[illegible]	[illegible]	[illegible]	[illegible]	[illegible]	[illegible]	[illegible]	[illegible]	[illegible]	[illegible]	[illegible]	[illegible]	68,100
Neoplasms	6·6 (2·7 to 15·3)	[illegible]	[illegible]	[illegible]	[illegible]	[illegible]	[illegible]	[illegible]	[illegible]	[illegible]	[illegible]	[illegible]	[illegible]	[illegible]	[illegible]	[illegible]	[illegible]	[illegible]	[illegible]	[illegible]	[illegible]	[illegible]	[illegible]	9,580
Lip and oral cavity cancer	0 (0 to 0)	[illegible]	[illegible]	[illegible]	[illegible]	[illegible]	[illegible]	[illegible]	[illegible]	[illegible]	[illegible]	[illegible]	[illegible]	[illegible]	[illegible]	[illegible]	[illegible]	[illegible]	[illegible]	[illegible]	[illegible]	[illegible]	[illegible]	66·8
Nasopharynx cancer	0 (0 to 0)	[illegible]	[illegible]	[illegible]	[illegible]	[illegible]	[illegible]	[illegible]	[illegible]	[illegible]	[illegible]	[illegible]	[illegible]	[illegible]	[illegible]	[illegible]	[illegible]	[illegible]	[illegible]	[illegible]	[illegible]	[illegible]	[illegible]	57·6
Other pharynx cancer	0 (0 to 0)	[illegible]	[illegible]	[illegible]	[illegible]	[illegible]	[illegible]	[illegible]	[illegible]	[illegible]	[illegible]	[illegible]	[illegible]	[illegible]	[illegible]	[illegible]	[illegible]	[illegible]	[illegible]	[illegible]	[illegible]	[illegible]	[illegible]	22·8
Esophageal cancer	0 (0 to 0)	[illegible]	[illegible]	[illegible]	[illegible]	[illegible]	[illegible]	[illegible]	[illegible]	[illegible]	[illegible]	[illegible]	[illegible]	[illegible]	[illegible]	[illegible]	[illegible]	[illegible]	[illegible]	[illegible]	[illegible]	[illegible]	[illegible]	56·3
Stomach cancer	0 (0 to 0)	[illegible]	[illegible]	[illegible]	[illegible]	[illegible]	[illegible]	[illegible]	[illegible]	[illegible]	[illegible]	[illegible]	[illegible]	[illegible]	[illegible]	[illegible]	[illegible]	[illegible]	[illegible]	[illegible]	[illegible]	[illegible]	[illegible]	390
Colon and rectum cancer	0 (0 to 0)	[illegible]	[illegible]	[illegible]	[illegible]	[illegible]	[illegible]	[illegible]	[illegible]	[illegible]	[illegible]	[illegible]	[illegible]	[illegible]	[illegible]	[illegible]	[illegible]	[illegible]	[illegible]	[illegible]	[illegible]	[illegible]	[illegible]	973
Liver cancer	0·1 (0 to 0·3)	[illegible]	[illegible]	[illegible]	[illegible]	[illegible]	[illegible]	[illegible]	[illegible]	[illegible]	[illegible]	[illegible]	[illegible]	[illegible]	[illegible]	[illegible]	[illegible]	[illegible]	[illegible]	[illegible]	[illegible]	[illegible]	[illegible]	145
Liver cancer due to hepatitis B	0 (0 to 0)	[illegible]	[illegible]	[illegible]	[illegible]	[illegible]	[illegible]	[illegible]	[illegible]	[illegible]	[illegible]	[illegible]	[illegible]	[illegible]	[illegible]	[illegible]	[illegible]	[illegible]	[illegible]	[illegible]	[illegible]	[illegible]	[illegible]	34
Liver cancer due to hepatitis C	0 (0 to 0)	[illegible]	[illegible]	[illegible]	[illegible]	[illegible]	[illegible]	[illegible]	[illegible]	[illegible]	[illegible]	[illegible]	[illegible]	[illegible]	[illegible]	[illegible]	[illegible]	[illegible]	[illegible]	[illegible]	[illegible]	[illegible]	[illegible]	40·4
Liver cancer due to alcohol use	0 (0 to 0)	[illegible]	[illegible]	[illegible]	[illegible]	[illegible]	[illegible]	[illegible]	[illegible]	[illegible]	[illegible]	[illegible]	[illegible]	[illegible]	[illegible]	[illegible]	[illegible]	[illegible]	[illegible]	[illegible]	[illegible]	[illegible]	[illegible]	33·2
Liver cancer due to NASH	0 (0 to 0)	[illegible]	[illegible]	[illegible]	[illegible]	[illegible]	[illegible]	[illegible]	[illegible]	[illegible]	[illegible]	[illegible]	[illegible]	[illegible]	[illegible]	[illegible]	[illegible]	[illegible]	[illegible]	[illegible]	[illegible]	[illegible]	[illegible]	18·9
Liver cancer due to other causes	0·1 (0 to 0·3)	[illegible]	[illegible]	[illegible]	[illegible]	[illegible]	[illegible]	[illegible]	[illegible]	[illegible]	[illegible]	[illegible]	[illegible]	[illegible]	[illegible]	[illegible]	[illegible]	[illegible]	[illegible]	[illegible]	[illegible]	[illegible]	[illegible]	18·1
Gallbladder and biliary tract cancer	0 (0 to 0)	[illegible]	[illegible]	[illegible]	[illegible]	[illegible]	[illegible]	[illegible]	[illegible]	[illegible]	[illegible]	[illegible]	[illegible]	[illegible]	[illegible]	[illegible]	[illegible]	[illegible]	[illegible]	[illegible]	[illegible]	[illegible]	[illegible]	92·5
Pancreatic cancer	0 (0 to 0)	[illegible]	[illegible]	[illegible]	[illegible]	[illegible]	[illegible]	[illegible]	[illegible]	[illegible]	[illegible]	[illegible]	[illegible]	[illegible]	[illegible]	[illegible]	[illegible]	[illegible]	[illegible]	[illegible]	[illegible]	[illegible]	[illegible]	441
Larynx cancer	0 (0 to 0)	[illegible]	[illegible]	[illegible]	[illegible]	[illegible]	[illegible]	[illegible]	[illegible]	[illegible]	[illegible]	[illegible]	[illegible]	[illegible]	[illegible]	[illegible]	[illegible]	[illegible]	[illegible]	[illegible]	[illegible]	[illegible]	[illegible]	19·1
Tracheal, bronchus, and lung cancer	0 (0 to 0)	[illegible]	[illegible]	[illegible]	[illegible]	[illegible]	[illegible]	[illegible]	[illegible]	[illegible]	[illegible]	[illegible]	[illegible]	[illegible]	[illegible]	[illegible]	[illegible]	[illegible]	[illegible]	[illegible]	[illegible]	[illegible]	[illegible]	801
Malignant skin melanoma	0 (0 to 0)	[illegible]	[illegible]	[illegible]	[illegible]	[illegible]	[illegible]	[illegible]	[illegible]	[illegible]	[illegible]	[illegible]	[illegible]	[illegible]	[illegible]	[illegible]	[illegible]	[illegible]	[illegible]	[illegible]	[illegible]	[illegible]	[illegible]	33·5
Non-melanoma skin cancer	0 (0 to 0)	[illegible]	[illegible]	[illegible]	[illegible]	[illegible]	[illegible]	[illegible]	[illegible]	[illegible]	[illegible]	[illegible]	[illegible]	[illegible]	[illegible]	[illegible]	[illegible]	[illegible]	[illegible]	[illegible]	[illegible]	[illegible]	[illegible]	22·1
Non-melanoma skin cancer (squamous-cell carcinoma)	0 (0 to 0)	[illegible]	[illegible]	[illegible]	[illegible]	[illegible]	[illegible]	[illegible]	[illegible]	[illegible]	[illegible]	[illegible]	[illegible]	[illegible]	[illegible]	[illegible]	[illegible]	[illegible]	[illegible]	[illegible]	[illegible]	[illegible]	[illegible]	22·1
Non-melanoma skin cancer (basal-cell carcinoma)	0 (0 to 0)	[illegible]	[illegible]	[illegible]	[illegible]	[illegible]	[illegible]	[illegible]	[illegible]	[illegible]	[illegible]	[illegible]	[illegible]	[illegible]	[illegible]	[illegible]	[illegible]	[illegible]	[illegible]	[illegible]	[illegible]	[illegible]	[illegible]	0
Breast cancer	0 (0 to 0)	[illegible]	[illegible]	[illegible]	[illegible]	[illegible]	[illegible]	[illegible]	[illegible]	[illegible]	[illegible]	[illegible]	[illegible]	[illegible]	[illegible]	[illegible]	[illegible]	[illegible]	[illegible]	[illegible]	[illegible]	[illegible]	[illegible]	1,830
Cervical cancer	0 (0 to 0)	[illegible]	[illegible]	[illegible]	[illegible]	[illegible]	[illegible]	[illegible]	[illegible]	[illegible]	[illegible]	[illegible]	[illegible]	[illegible]	[illegible]	[illegible]	[illegible]	[illegible]	[illegible]	[illegible]	[illegible]	[illegible]	[illegible]	1,760
Uterine cancer	0 (0 to 0)	[illegible]	[illegible]	[illegible]	[illegible]	[illegible]	[illegible]	[illegible]	[illegible]	[illegible]	[illegible]	[illegible]	[illegible]	[illegible]	[illegible]	[illegible]	[illegible]	[illegible]	[illegible]	[illegible]	[illegible]	[illegible]	[illegible]	226
Ovarian cancer	0 (0 to 0)	[illegible]	[illegible]	[illegible]	[illegible]	[illegible]	[illegible]	[illegible]	[illegible]	[illegible]	[illegible]	[illegible]	[illegible]	[illegible]	[illegible]	[illegible]	[illegible]	[illegible]	[illegible]	[illegible]	[illegible]	[illegible]	[illegible]	581
Prostate cancer	0 (0 to 0)	[illegible]	[illegible]	[illegible]	[illegible]	[illegible]	[illegible]	[illegible]	[illegible]	[illegible]	[illegible]	[illegible]	[illegible]	[illegible]	[illegible]	[illegible]	[illegible]	[illegible]	[illegible]	[illegible]	[illegible]	[illegible]	[illegible]	0
Testicular cancer	--	[illegible]	[illegible]	[illegible]	[illegible]	[illegible]	[illegible]	[illegible]	[illegible]	[illegible]	[illegible]	[illegible]	[illegible]	[illegible]	[illegible]	[illegible]	[illegible]	[illegible]	[illegible]	[illegible]	[illegible]	[illegible]	[illegible]	NA
Kidney cancer	0·2 (0 to 0·5)	[illegible]	[illegible]	[illegible]	[illegible]	[illegible]	[illegible]	[illegible]	[illegible]	[illegible]	[illegible]	[illegible]	[illegible]	[illegible]	[illegible]	[illegible]	[illegible]	[illegible]	[illegible]	[illegible]	[illegible]	[illegible]	[illegible]	115
Bladder cancer	0 (0 to 0)	[illegible]	[illegible]	[illegible]	[illegible]	[illegible]	[illegible]	[illegible]	[illegible]	[illegible]	[illegible]	[illegible]	[illegible]	[illegible]	[illegible]	[illegible]	[illegible]	[illegible]	[illegible]	[illegible]	[illegible]	[illegible]	[illegible]	66·6
Brain and central nervous system cancer	2·7 (0·5 to 10·3)	[illegible]	[illegible]	[illegible]	[illegible]	[illegible]	[illegible]	[illegible]	[illegible]	[illegible]	[illegible]	[illegible]	[illegible]	[illegible]	[illegible]	[illegible]	[illegible]	[illegible]	[illegible]	[illegible]	[illegible]	[illegible]	[illegible]	422
Thyroid cancer	0 (0 to 0)	[illegible]	[illegible]	[illegible]	[illegible]	[illegible]	[illegible]	[illegible]	[illegible]	[illegible]	[illegible]	[illegible]	[illegible]	[illegible]	[illegible]	[illegible]	[illegible]	[illegible]	[illegible]	[illegible]	[illegible]	[illegible]	[illegible]	69·5
Mesothelioma	0 (0 to 0)	[illegible]	[illegible]	[illegible]	[illegible]	[illegible]	[illegible]	[illegible]	[illegible]	[illegible]	[illegible]	[illegible]	[illegible]	[illegible]	[illegible]	[illegible]	[illegible]	[illegible]	[illegible]	[illegible]	[illegible]	[illegible]	[illegible]	19
Hodgkin lymphoma	0 (0 to 0)	[illegible]	[illegible]	[illegible]	[illegible]	[illegible]	[illegible]	[illegible]	[illegible]	[illegible]	[illegible]	[illegible]	[illegible]	[illegible]	[illegible]	[illegible]	[illegible]	[illegible]	[illegible]	[illegible]	[illegible]	[illegible]	[illegible]	41·7
Non-Hodgkin lymphoma	0 (0 to 0)	[illegible]	[illegible]	[illegible]	[illegible]	[illegible]	[illegible]	[illegible]	[illegible]	[illegible]	[illegible]	[illegible]	[illegible]	[illegible]	[illegible]	[illegible]	[illegible]	[illegible]	[illegible]	[illegible]	[illegible]	[illegible]	[illegible]	327
Multiple myeloma	0 (0 to 0)	[illegible]	[illegible]	[illegible]	[illegible]	[illegible]	[illegible]	[illegible]	[illegible]	[illegible]	[illegible]	[illegible]	[illegible]	[illegible]	[illegible]	[illegible]	[illegible]	[illegible]	[illegible]	[illegible]	[illegible]	[illegible]	[illegible]	98·4
Leukemia	1·4 (0·2 to 4·5)	[illegible]	[illegible]	[illegible]	[illegible]	[illegible]	[illegible]	[illegible]	[illegible]	[illegible]	[illegible]	[illegible]	[illegible]	[illegible]	[illegible]	[illegible]	[illegible]	[illegible]	[illegible]	[illegible]	[illegible]	[illegible]	[illegible]	394
Acute lymphoid leukemia	0·2 (0 to 0·6)	[illegible]	[illegible]	[illegible]	[illegible]	[illegible]	[illegible]	[illegible]	[illegible]	[illegible]	[illegible]	[illegible]	[illegible]	[illegible]	[illegible]	[illegible]	[illegible]	[illegible]	[illegible]	[illegible]	[illegible]	[illegible]	[illegible]	63·7
Chronic lymphoid leukemia	0 (0 to 0)	[illegible]	[illegible]	[illegible]	[illegible]	[illegible]	[illegible]	[illegible]	[illegible]	[illegible]	[illegible]	[illegible]	[illegible]	[illegible]	[illegible]	[illegible]	[illegible]	[illegible]	[illegible]	[illegible]	[illegible]	[illegible]	[illegible]	15·9
Acute myeloid leukemia	0·2 (0 to 0·6)	[illegible]	[illegible]	[illegible]	[illegible]	[illegible]	[illegible]	[illegible]	[illegible]	[illegible]	[illegible]	[illegible]	[illegible]	[illegible]	[illegible]	[illegible]	[illegible]	[illegible]	[illegible]	[illegible]	[illegible]	[illegible]	[illegible]	83·3

Chronic myeloid leukemia	0 (0 to 0·1)	0 (0 to 0·1)	0·1 (0 to 0·1)	0·4 (0·1 to 1·1)	0·4 (0·2 to 0·9)	0·6 (0·3 to 1·3)	0·9 (0·3 to 1·8)	1·5 (0·6 to 2·8)	2·1 (0·9 to 4)	2·2 (1 to 4·2)	2·3 (1 to 4·3)	1·9 (0·8 to 3·7)	1·9 (0·9 to 3·6)	3 (1·5 to 5·5)	3·2 (1·6 to 5·6)	3 (1·5 to 5·4)	1·8 (0·9 to 3·2)	1·6 (0·8 to 3)	1·3 (0·6 to 2·3)	0·8 (0·4 to 1·5)	0·3 (0·1 to 0·5)	0·1 (0 to 0·2)	0 (0 to 0·1)	29·4
Other leukemia	1·1 (0·2 to 3·3)	0·4 (0·1 to 1·2)	5·6 (2·5 to 11·3)	5·7 (2 to 12·2)	8·3 (3·5 to 15·9)	11·4 (5·5 to 20)	12·2 (5·8 to 22·4)	10·6 (5·5 to 17·6)	13·2 (7 to 22·4)	11·7 (6·2 to 19·4)	13·8 (7·1 to 23·1)	12·8 (7·7 to 20)	14·2 (8·1 to 22·4)	12·9 (7·5 to 20·4)	15·9 (9 to 26)	13·4 (7·6 to 22·3)	10·6 (6·2 to 16·5)	10·5 (6 to 16·1)	8·1 (4·7 to 12·6)	5·4 (3·1 to 8·7)	2·7 (1·4 to 4·4)	0·9 (0·5 to 1·5)	0·3 (0·2 to 0·5)	202
Other malignant neoplasms	2·1 (0·8 to 4·3)	2·1 (0·6 to 5·7)	9 (4·8 to 15·9)	21·1 (13 to 32·4)	21·1 (15·4 to 28·4)	27·1 (19·7 to 37·4)	25·5 (16·8 to 36·9)	18·6 (13·7 to 25·4)	22·6 (16·1 to 31·4)	21·7 (15·2 to 30·2)	24·4 (16·4 to 34·4)	27·1 (18·6 to 38·2)	32 (22·1 to 44·8)	39·4 (28·1 to 54·2)	41·1 (29 to 57·6)	35·9 (24·9 to 50·2)	29 (20·4 to 39·6)	25·3 (17·6 to 34·1)	19·2 (13·1 to 27)	12·6 (8·5 to 17·7)	7·1 (4·5 to 10·4)	2·6 (1·6 to 3·9)	0·9 (0·5 to 1·4)	467
Other neoplasms	0·1 (0 to 0·2)	0·3 (0·1 to 0·6)	1·3 (0·5 to 2·6)	2·1 (1·1 to 4)	0·5 (0·3 to 0·8)	1·1 (0·5 to 2·4)	1·1 (0·5 to 2)	0·8 (0·5 to 1·3)	1·6 (0·8 to 3·2)	1·6 (0·8 to 2·9)	1 (0·5 to 1·6)	2·3 (1·3 to 3·6)	2 (1·2 to 3·3)	2·8 (1·8 to 4·2)	3·3 (2·1 to 5·1)	3·8 (2·6 to 5·3)	3·6 (2·5 to 5·2)	4·2 (2·6 to 6·3)	3·9 (2·4 to 6·2)	2·5 (1·5 to 3·8)	1·1 (0·6 to 1·8)	0·4 (0·2 to 0·7)	0·1 (0·1 to 0·2)	41·5
Myelodysplastic, myeloproliferative, and other hematopoietic neo	0·1 (0 to 0·2)	0·3 (0·1 to 0·6)	1·3 (0·5 to 2·6)	2·1 (1·1 to 4)	0·5 (0·3 to 0·8)	1·1 (0·5 to 2·4)	1·1 (0·5 to 2)	0·8 (0·5 to 1·3)	1·6 (0·8 to 3·2)	1·6 (0·8 to 2·9)	1 (0·5 to 1·6)	2·3 (1·3 to 3·6)	2 (1·2 to 3·3)	2·8 (1·8 to 4·2)	3·3 (2·1 to 5·1)	3·8 (2·6 to 5·3)	3·6 (2·5 to 5·2)	4·2 (2·6 to 6·3)	3·9 (2·4 to 6·2)	2·5 (1·5 to 3·8)	1·1 (0·6 to 1·8)	0·4 (0·2 to 0·7)	0·1 (0·1 to 0·2)	41·5
Benign and in situ intestinal neoplasms	0 (0 to 0)	0 (0 to 0)	0 (0 to 0)	0 (0 to 0)	0 (0 to 0)	0 (0 to 0)	0 (0 to 0)	0 (0 to 0)	0 (0 to 0)	0 (0 to 0)	0 (0 to 0)	0 (0 to 0)	0 (0 to 0)	0 (0 to 0)	0 (0 to 0)	0 (0 to 0)	0 (0 to 0)	0 (0 to 0)	0 (0 to 0)	0 (0 to 0)	0 (0 to 0)	0 (0 to 0)	0 (0 to 0)	0
Benign and in situ cervical and uterine neoplasms	0 (0 to 0)	0 (0 to 0)	0 (0 to 0)	0 (0 to 0)	0 (0 to 0)	0 (0 to 0)	0 (0 to 0)	0 (0 to 0)	0 (0 to 0)	0 (0 to 0)	0 (0 to 0)	0 (0 to 0)	0 (0 to 0)	0 (0 to 0)	0 (0 to 0)	0 (0 to 0)	0 (0 to 0)	0 (0 to 0)	0 (0 to 0)	0 (0 to 0)	0 (0 to 0)	0 (0 to 0)	0 (0 to 0)	0
Other benign and in situ neoplasms	0 (0 to 0)	0 (0 to 0)	0 (0 to 0)	0 (0 to 0)	0 (0 to 0)	0 (0 to 0)	0 (0 to 0)	0 (0 to 0)	0 (0 to 0)	0 (0 to 0)	0 (0 to 0)	0 (0 to 0)	0 (0 to 0)	0 (0 to 0)	0 (0 to 0)	0 (0 to 0)	0 (0 to 0)	0 (0 to 0)	0 (0 to 0)	0 (0 to 0)	0 (0 to 0)	0 (0 to 0)	0 (0 to 0)	0
Cardiovascular diseases	18·5 (8 to 35·6)	6·8 (3·1 to 13·3)	38·7 (18·6 to 70·8)	15·8 (9 to 26·1)	27·3 (19·5 to 36·7)	52·2 (35·7 to 72·3)	112 (76·5 to 156)	115 (81·6 to 154)	169 (116 to 224)	235 (167 to 319)	355 (250 to 478)	453 (329 to 591)	762 (582 to 978)	1,120 (877 to 1,410)	1,450 (1,130 to 1,790)	1,610 (1,270 to 1,960)	1,530 (1,250 to 1,820)	1,610 (1,340 to 1,890)	1,640 (1,360 to 1,940)	1,330 (1,090 to 1,570)	729 (570 to 882)	358 (270 to 429)	176 (127 to 211)	13,900
Rheumatic heart disease	0 (0 to 0)	0 (0 to 0)	0 (0 to 0)	1·2 (0·6 to 2)	6·5 (3·9 to 10·3)	15·1 (8·9 to 23·3)	22·2 (14·1 to 33·5)	20·9 (13·4 to 30)	25·5 (16·8 to 35·1)	26·9 (17·6 to 37·4)	28 (18·6 to 40·3)	25·2 (17·6 to 35·4)	24·3 (16·6 to 33·9)	22·3 (15·6 to 30·3)	18·3 (12·8 to 25·6)	11·2 (7·8 to 15·8)	7·5 (5·1 to 10·2)	5·5 (3·8 to 7·5)	4·3 (3 to 5·8)	2·7 (1·9 to 3·9)	1·4 (0·9 to 1·9)	0·6 (0·4 to 0·9)	0·3 (0·2 to 0·5)	270
Ischemic heart disease	0 (0 to 0)	0 (0 to 0)	0 (0 to 0)	0 (0 to 0)	0 (0 to 0)	0 (0 to 0)	11·9 (6·4 to 19·4)	14·7 (8·1 to 23·6)	24·6 (15 to 38·1)	47·4 (28·9 to 73·1)	87·1 (53·5 to 134)	128 (82·9 to 190)	249 (168 to 350)	397 (278 to 542)	539 (382 to 728)	648 (474 to 850)	593 (443 to 762)	616 (459 to 786)	588 (439 to 753)	496 (369 to 633)	264 (191 to 346)	150 (105 to 196)	77 (53·2 to 99·3)	4,930
Stroke	9·7 (3·4 to 20·4)	1·6 (0·6 to 3·4)	8·3 (2·8 to 20)	3·6 (1·9 to 6·6)	8·8 (5·7 to 12·8)	17·1 (11 to 25·5)	42·9 (26·8 to 62·7)	44·6 (30 to 64·2)	67·2 (44·8 to 96·7)	99·2 (65·3 to 141)	157 (102 to 221)	197 (136 to 276)	335 (242 to 451)	497 (358 to 670)	630 (456 to 852)	669 (494 to 878)	676 (518 to 863)	714 (535 to 909)	767 (592 to 967)	583 (446 to 740)	298 (214 to 389)	125 (88·9 to 164)	58·3 (39·3 to 77·7)	6,010
Ischemic stroke	2·2 (0·7 to 4·7)	0·1 (0 to 0·2)	0·3 (0·1 to 0·8)	1 (0·7 to 1·4)	2·9 (1·6 to 4·6)	4·1 (2·5 to 6·4)	8·7 (5·5 to 13·3)	10·4 (6·7 to 15·3)	12·6 (7·9 to 18·3)	19 (12·1 to 28·2)	26·3 (17·9 to 37·9)	35·5 (24·3 to 50·8)	56·4 (40·2 to 80·7)	88·2 (61·5 to 122)	122 (82·5 to 176)	192 (132 to 268)	211 (149 to 293)	373 (270 to 488)	430 (318 to 569)	414 (310 to 526)	219 (157 to 289)	92·7 (64·6 to 124)	44·8 (29·9 to 59·6)	2,370
Intracerebral hemorrhage	3·9 (1·2 to 8·7)	0·8 (0·3 to 2)	5·1 (1·4 to 13·2)	1·2 (0·5 to 2·8)	3·3 (1·9 to 5·4)	7·2 (4 to 11·7)	23·3 (13·7 to 36·7)	23·8 (14·5 to 36·2)	36·9 (22·9 to 55·8)	56·3 (33 to 85·5)	94·7 (56·9 to 142)	120 (76·1 to 174)	223 (153 to 308)	341 (239 to 469)	436 (305 to 610)	430 (301 to 591)	424 (305 to 566)	315 (216 to 432)	316 (222 to 431)	157 (107 to 225)	72·9 (47·2 to 105)	29·7 (19·5 to 42)	12·5 (7·9 to 18)	3,130
Subarachnoid hemorrhage	3·6 (1 to 8·5)	0·6 (0·2 to 1·5)	2·9 (0·8 to 7·8)	1·4 (0·5 to 2·7)	2·6 (1·3 to 4·6)	5·7 (2·7 to 10·8)	10·8 (5·2 to 18·5)	10·4 (5·4 to 17·5)	17·7 (10 to 29·8)	23·9 (13·2 to 38·6)	35·8 (18·9 to 59·7)	42·4 (24·2 to 65·9)	55·7 (33·5 to 85·9)	67·7 (42·2 to 103)	71·9 (43·8 to 112)	46·4 (26·9 to 71·6)	40·8 (24·8 to 63·4)	25·6 (15·9 to 37·8)	21·9 (12·8 to 35·2)	11·9 (6·7 to 18·9)	5·7 (3·4 to 9·3)	2·4 (1·4 to 3·9)	1 (0·6 to 1·6)	509
Hypertensive heart disease	0 (0 to 0)	0 (0 to 0)	0 (0 to 0)	0 (0 to 0)	0 (0 to 0)	0 (0 to 0)	3·6 (1·7 to 6·3)	5·2 (2·7 to 8·9)	8·6 (4·4 to 14·5)	17·7 (9 to 29·2)	32·2 (15·9 to 53·6)	39·9 (22 to 63·7)	75·7 (45·3 to 116)	115 (70·7 to 180)	147 (88·1 to 226)	158 (86·7 to 247)	138 (85·6 to 213)	159 (98 to 247)	168 (106 to 253)	146 (88·1 to 218)	90·7 (55·7 to 131)	41·7 (24·9 to 61·1)	22·1 (12·5 to 33·6)	1,370
Non-rheumatic valvular heart disease	0 (0 to 0)	0 (0 to 0)	0 (0 to 0)	0 (0 to 0)	0 (0 to 0)	0 (0 to 0)	6·1 (3 to 10·3)	3·7 (1·9 to 6·7)	4·6 (2·4 to 7·9)	5 (2·6 to 8·4)	5·8 (3·2 to 10)	6·5 (3·8 to 10·4)	9·8 (5·6 to 16·3)	10·2 (6·5 to 15·2)	12 (7·2 to 18·1)	12·5 (7·7 to 18·5)	10·1 (6·5 to 15·2)	9·1 (5·9 to 13·2)	8·3 (5·4 to 11·9)	6·3 (3·6 to 10·3)	3·8 (2 to 6·5)	1·7 (0·9 to 3)	0·6 (0·2 to 1)	116
Non-rheumatic calcific aortic valve disease	0 (0 to 0)	0 (0 to 0)	0 (0 to 0)	0 (0 to 0)	0 (0 to 0)	0 (0 to 0)	1·4 (0·4 to 3·1)	1·3 (0·5 to 2·5)	1·1 (0·5 to 2·3)	1 (0·4 to 2)	1·3 (0·4 to 2·8)	1·6 (0·7 to 3·2)	2·5 (1·1 to 4·8)	3·1 (1·6 to 5·5)	3·9 (2 to 6·8)	5·2 (3 to 8·7)	4·8 (2·8 to 7·7)	5 (3 to 7·8)	5 (2·9 to 7·5)	3·8 (2·2 to 6·7)	2·6 (1·3 to 4·7)	1·1 (0·5 to 2)	0·3 (0·1 to 0·6)	45
Non-rheumatic degenerative mitral valve disease	0 (0 to 0)	0 (0 to 0)	0 (0 to 0)	0 (0 to 0)	0 (0 to 0)	0 (0 to 0)	4·6 (2·2 to 8·2)	2·3 (1 to 4·3)	3·4 (1·7 to 5·8)	3·8 (1·9 to 6·5)	4·4 (2·3 to 7·5)	4·8 (2·6 to 7·9)	7 (3·8 to 11·7)	7 (4·2 to 10·7)	7·9 (4·6 to 12·2)	7·2 (4·2 to 11·1)	5·1 (2·9 to 8·2)	4 (2·4 to 6·5)	3·2 (1·8 to 5·1)	2·3 (1·1 to 4·2)	1·1 (0·5 to 2·4)	0·6 (0·3 to 1·2)	0·2 (0·1 to 0·5)	68·8
[truncated: 2,482,101 more chars]
