# Supplementary material for: Global burden of 369 diseases and injuries in 204 countries and territories, 1990–2019: a systematic analysis for the Global Burden of Disease Study 2019
Source: Lancet. 2020 Oct 17;396(10258):1204–22. doi: 10.1016/S0140-6736(20)30925-9 (PMC7567026; doi:10.1016/S0140-6736(20)30925-9)
Supplement: Supplementary appendix 2 [file mmc2d.pdf]

THE LANCET

Supplementary appendix 2d (Part 4 of 5)

This appendix formed part of the original submission and has been peer reviewed. We post it as supplied by the authors.

Supplement to: GBD 2019 Diseases and Injuries Collaborators. Global burden of 369 diseases and injuries in 204 countries and territories, 1990–2019: a systematic analysis for the Global Burden of Disease Study 2019. *Lancet* 2020; **396:** 1204–22.

Appendix 2D: supplementary results appendix to "Global burden of 369 diseases and injuries in 204 countries and territories, 1990–2019: a systematic analysis for the Global Burden of Disease Study 2019"

This supplement provides additional figures and more detailed results for "Global burden of 369 diseases and injuries in 204 countries and territories, 1990–2019: a systematic analysis for the Global Burden of Disease Study 2019."

List of supplementary results appendix figures and tables:

Appendix 2A

Figure S1: Leading GBD Level 3 causes of age-standardised YLDs rates by location for a) females and b) males, 2019. 1

Figure S2: Leading GBD Level 3 causes of age-standardised prevalence rates by location for a) females and b) males, 2019. 3

Figure S3: Trends of age-standardised YLD rates per 100,000 for the top eight Level 3 causes of non-fatal burden in 2019 by SDI quintile for both sexes, from 1990 to 2019. 5

Figure S4: Death counts due to fatal discontinuities by type, 1980–2019. 6

Figure S5: Leading level 3 causes of all-age DALY counts by location for females in 1990 (A) males in 1990 (B) females in 2019 (C) males in 2019 (D) 10

Figure S6: Leading 20 GBD cause hierarchy Level 3 causes of YLLs by SDI grouping and sex for 1990, 2010, and 2019, with percentage change in number of YLLs, and all-age and age-standardised rates. 14

Figure S7: Global distribution of total YLDs by 23 GBD age groups for Level 2 causes of non-fatal burden in 1990 and 2019 for a) females and b) males. 24

Figure S8: Global leading 30 level 3 causes of DALYs for 1990, 2010, and 2019, with percentage change in number of DALYs, and age-standardised rates, by sex and SDI quintile 26

Table S1: All-age DALYs counts and age standardised DALY rates per 100,000 individuals for all causes and level 1 cause groups by location for 1990, 2010, 2019 , both sexes combined. 36

Appendix 2B

Table S2a: Global DALYS by cause and age group for males with 95% uncertainty intervals, by location for 2019. 4734

Appendix 2C

Table S2b: Global DALYS by cause and age group for females with 95% uncertainty intervals, by location for 2019. 6708

Appendix 2D

Table S2c: Global DALYS by cause and age group for both sexes combined with 95% uncertainty intervals, by location for 2019.   8680

Appendix 2E

Table S3: Global deaths and YLL numbers, age-standardised rates per 100,000, and percentage change between 2010 and 2019 for males for all GBD causes and Levels 1 through 4 of the cause hierarchy 10630

Table S4: Global deaths by cause and age group for females (1990, 2010, 2019) 10635

Table S5: Global deaths by cause and age group for males (1990, 2010, 2019) 10642

Table S6: Global deaths by cause and age group for both sexes combined (1990, 2010, 2019) 10648

Table S7: Global YLLs by cause and age group for females. 10654

Table S8: Global YLLs by cause and age group for males. 10656

Table S9: Global YLLs by cause and age group for both sexes combined 10658

Table S10: Deaths due to fatal discontinuities by country, all ages, for both sexes combined 10660

Table S11: Diarrhoeal diseases and lower respiratory infection aetiologies deaths, mortality rates, and annual percentage change from 1990 to 2019. 10676

Table S12: Deaths by location, cause, and age group for males. 10677

Table S13: Deaths by location, cause, and age group for females. 13007

Table S14: Deaths by location, cause, and age group for both sexes combined. 15354

Table S15: Distribution of percentage change in age-standardised deaths for Level 1 causes by location from 1990-2010 and 2010-2019, both sexes. 17543

Table S16: Global deaths and YLL numbers, age-standardised rates per 100,000, and percentage change between 2010 and 2019 for females for all GBD causes and Levels 1 through 4 of the cause hierarchy. 17561

Table S17: Global prevalence, incidence and YLDs for 2019, percentage change of YLDs counts, and percentage change of YLD rates between 2010 and 2019 by cause and by age 17566

Table S18: Prevalence, incidence, and YLDs for 2019, percentage change of YLD counts, and percentage change of age-standardised YLD rates between 2010 and 2019 by cause and GBD location. 17707

SR Table 2c: Global DALYS by cause and age group for both sexes with 95% uncertainty intervals, 2019

Cause	Early Neonatal	Late Neonatal	Post Neonatal	1 to 4	5 to 9
All causes	170,000,000	43,300,000	122,000,000	132,000,000	59,800,000
Communicable, maternal, neonatal, and nutritional diseases	153,000,000	36,500,000	93,100,000	95,700,000	29,300,000
HIV/AIDS and sexually transmitted infections	4,250,000	1,160,000	3,100,000	2,660,000	1,070,000
HIV/AIDS	158	558	1,430,000	2,590,000	1,040,000
[illegible]	5	50·4	356,000	682,000	278,000
[illegible]	0·3	2·4	43,300	79,500	29,400
[illegible]	0	0	1,210	1,440	655
HIV/AIDS resulting in other diseases	153	505	1,030,000	1,820,000	729,000
Sexually transmitted infections excluding HIV	4,250,000	1,160,000	1,670,000	74,300	34,100
Syphilis	4,250,000	1,160,000	1,670,000	74,300	34,100
Chlamydial infection	0	0	0	0	0
Gonococcal infection	0	0	0	0	0
Trichomoniasis	0	0	0	0	0
Genital herpes	0	0	0	0	0
Other sexually transmitted infections	0	0	0	0	0
Respiratory infections and tuberculosis	10,300,000	6,950,000	29,600,000	17,900,000	4,160,000
Tuberculosis	0·9	33·6	2,240,000	2,310,000	848,000
Latent tuberculosis infection	0	0	0	0	0
Drug-susceptible tuberculosis	0·9	32·1	2,040,000	2,130,000	780,000
[illegible]	0	1·4	190,000	175,000	64,600
Extensively drug-resistant tuberculosis	0	0·1	9,080	6,590	3,200
Lower respiratory infections	10,300,000	6,940,000	27,000,000	14,500,000	2,300,000
Upper respiratory infections	3,720	14,200	272,000	750,000	631,000
Otitis media	15,400	3,520	35,700	304,000	381,000
[illegible]	[illegible]	[illegible]	[illegible]	[illegible]	[illegible]

Diabetes mellitus type 1	3,280	8,510	120,000	108,000	126,000	153,000	190,000	275,000	326,000	341,000	406,000	537,000	272,000	321,000	352,000	297,000	277,000	193,000	140,000	71,400	40,100	17,200	5,270	4,680,000
Diabetes mellitus type 2	0	0	0	0	0	0	206,000	396,000	720,000	1,290,000	1,910,000	2,930,000	4,810,000	6,800,000	8,180,000	9,380,000	9,230,000	7,680,000	5,790,000	3,990,000	2,020,000	763,000	207,000	68,300,000
Chronic kidney disease	3·3	63	332,000	533,000	472,000	482,000	884,000	1,190,000	1,370,000	1,670,000	1,920,000	2,290,000	2,720,000	3,450,000	3,870,000	4,140,000	4,140,000	3,720,000	3,150,000	2,500,000	1,640,000	776,000	285,000	41,600,000
[illegible]	0	0	2,260	7,670	11,900	16,500	42,000	86,900	149,000	253,000	342,000	425,000	451,000	433,000	332,000	256,000	191,000	122,000	64,800	27,200	7,540	1,070	97·5	3,230,000
[illegible]	0	0	0	0	0	0	19,800	39,900	62,800	110,000	176,000	310,000	545,000	924,000	1,220,000	1,380,000	1,410,000	1,250,000	1,010,000	738,000	428,000	181,000	62,600	[illegible]
[illegible]	0	0	0	0	0	0	78,900	134,000	182,000	242,000	303,000	399,000	528,000	751,000	938,000	1,120,000	1,200,000	1,130,000	1,020,000	871,000	625,000	320,000	122,000	[illegible]
[illegible]	1·1	20	94,800	182,000	162,000	184,000	331,000	411,000	435,000	467,000	493,000	517,000	500,000	547,000	553,000	523,000	451,000	363,000	291,000	211,000	119,000	50,000	15,800	[illegible]
[illegible]	2·2	43	235,000	344,000	298,000	281,000	412,000	516,000	538,000	598,000	606,000	639,000	698,000	791,000	825,000	862,000	894,000	853,000	764,000	657,000	460,000	224,000	84,100	11,600,000
Acute glomerulonephritis	0	0·5	8,810	14,200	18,500	13,200	8,280	10,200	12,100	15,100	14,600	17,000	19,200	21,800	20,200	22,700	26,400	21,900	18,500	14,000	7,830	2,320	574	307,000
Skin and subcutaneous diseases	177	2,280	417,000	3,320,000	4,140,000	4,410,000	4,660,000	3,080,000	2,750,000	2,650,000	2,320,000	2,110,000	2,100,000	2,060,000	1,910,000	1,750,000	1,610,000	1,290,000	959,000	690,000	406,000	184,000	63,400	42,900,000
Dermatitis	0	0	0	1,020,000	1,280,000	993,000	787,000	685,000	689,000	688,000	582,000	511,000	502,000	497,000	446,000	394,000	351,000	261,000	178,000	116,000	58,600	22,500	6,540	10,100,000
Atopic dermatitis	0	0	0	1,000,000	1,240,000	889,000	631,000	503,000	469,000	442,000	361,000	314,000	306,000	292,000	256,000	218,000	188,000	140,000	100,000	69,800	37,700	15,200	4,500	7,480,000
Contact dermatitis	0	0	0	0	19,400	79,300	133,000	162,000	197,000	220,000	198,000	178,000	179,000	189,000	176,000	161,000	146,000	107,000	66,900	39,100	17,400	6,000	1,720	2,280,000
Seborrhoeic dermatitis	0	0	0	14,800	18,500	24,900	23,100	20,600	23,600	25,400	22,300	19,300	17,600	16,400	14,300	14,000	16,300	14,200	10,500	7,010	3,490	1,270	313	308,000
Psoriasis	0	0	0	46,200	109,000	142,000	160,000	177,000	214,000	252,000	268,000	283,000	312,000	327,000	309,000	277,000	239,000	176,000	110,000	64,800	28,800	9,230	2,090	3,510,000
Bacterial skin diseases	27·1	276	207,000	153,000	58,900	58,100	75,400	68,000	60,500	87,000	90,700	97,200	118,000	134,000	161,000	166,000	161,000	146,000	135,000	98,900	67,700	36,800	14,700	2,200,000
Cellulitis	4	42·5	16,100	23,600	10,600	10,800	20,600	22,800	18,000	26,100	26,700	29,400	36,100	38,600	46,100	48,600	44,200	40,200	33,600	26,800	17,900	10,000	4,200	551,000
Pyoderma	23·1	234	191,000	129,000	48,200	47,300	54,800	45,200	42,500	60,900	64,000	67,800	81,400	95,600	115,000	117,000	117,000	106,000	101,000	72,100	49,800	26,800	10,500	1,640,000
Scabies	53·2	697	61,800	415,000	510,000	501,000	527,000	503,000	426,000	341,000	273,000	254,000	238,000	209,000	160,000	127,000	104,000	72,200	51,600	36,100	19,000	6,780	1,690	4,840,000
Fungal skin diseases	20·4	273	28,100	237,000	338,000	287,000	206,000	174,000	178,000	182,000	169,000	158,000	154,000	153,000	149,000	141,000	150,000	151,000	129,000	114,000	78,300	36,600	11,600	3,220,000
Viral skin diseases	0	0	0	660,000	1,070,000	655,000	404,000	290,000	239,000	213,000	179,000	164,000	163,000	158,000	137,000	123,000	106,000	75,400	45,900	26,700	12,700	4,410	1,170	4,730,000
Acne vulgaris	0	0	0	0	82,400	1,200,000	1,940,000	620,000	343,000	249,000	170,000	116,000	82,600	53,400	34,600	24,300	17,800	11,600	6,780	3,720	1,530	418	65·3	4,960,000
Alopecia areata	0	0	0	12,400	16,600	21,000	35,000	53,800	76,000	80,700	67,800	52,900	44,200	36,700	29,900	26,300	20,200	13,500	7,390	3,950	1,630	410	66·3	601,000
Pruritus	1·1	15·2	2,220	26,900	38,900	38,800	42,200	47,300	55,500	63,000	58,700	54,200	54,900	55,600	51,400	50,200	49,400	38,800	26,700	17,700	8,990	3,350	929	786,000
Urticaria	61·9	832	91,700	602,000	511,000	358,000	307,000	280,000	261,000	256,000	227,000	190,000	169,000	152,000	132,000	114,000	95,800	67,000	42,600	25,400	10,900	3,500	873	3,900,000
Decubitus ulcer	0	0	0	1,230	1,430	1,980	3,670	4,280	5,380	8,340	9,200	10,600	13,400	19,900	25,400	31,300	43,800	54,500	63,500	70,800	58,800	37,000	16,800	481,000
Other skin and subcutaneous diseases	13·5	186	25,600	146,000	129,000	155,000	179,000	178,000	199,000	225,000	223,000	223,000	247,000	266,000	274,000	280,000	268,000	224,000	163,000	112,000	58,800	23,500	6,890	3,600,000
Sense organ diseases	848	2,830	58,600	585,000	1,120,000	1,300,000	1,420,000	1,590,000	1,990,000	2,460,000	2,600,000	2,960,000	3,970,000	5,040,000	5,800,000	6,610,000	7,390,000	6,820,000	5,750,000	4,520,000	2,650,000	1,140,000	341,000	66,100,000
Blindness and vision loss	401	1,210	12,600	136,000	267,000	303,000	315,000	333,000	368,000	460,000	609,000	865,000	1,270,000	1,730,000	2,150,000	2,540,000	2,930,000	2,720,000	2,260,000	1,730,000	993,000	427,000	134,000	22,600,000
Glaucoma	0	0	0	0	0	0	0	0	0	0	0	0	2,170	16,400	42,500	75,200	109,000	123,000	126,000	117,000	82,400	39,900	15,100	748,000
Cataract	0	0	0	0	0	0	0	6,370	26,000	54,000	97,200	158,000	249,000	359,000	496,000	683,000	953,000	1,060,000	991,000	805,000	485,000	198,000	58,900	6,680,000
Age-related macular degeneration	0	0	0	0	0	0	0	0	0	0	0	0	2,180	16,800	44,300	76,800	100,000	96,600	82,800	67,800	45,300	22,400	8,700	564,000
Refraction disorders	22·1	236	8,270	116,000	233,000	260,000	263,000	261,000	261,000	269,000	296,000	342,000	435,000	530,000	606,000	644,000	647,000	535,000	403,000	273,000	133,000	43,800	10,300	6,570,000
Near vision loss	0	0	18·7	533	1,670	2,750	3,700	11,000	24,100	70,200	132,000	247,000	416,000	574,000	664,000	702,000	687,000	535,000	384,000	262,000	127,000	45,200	10,900	4,900,000
Other vision loss	379	971	4,330	20,300	32,800	40,600	48,100	54,300	57,000	67,300	84,100	118,000	170,000	238,000	294,000	364,000	431,000	373,000	272,000	204,000	120,000	77,000	29,800	3,100,000
Age-related and other hearing loss	246	784	29,700	367,000	767,000	919,000	1,020,000	1,160,000	1,500,000	1,830,000	1,810,000	1,920,000	2,490,000	3,040,000	3,390,000	3,750,000	4,120,000	3,790,000	3,250,000	2,620,000	1,580,000	682,000	197,000	40,200,000
Other sense organ diseases	201	844	16,200	81,100	87,500	82,300	90,700	95,800	120,000	170,000	182,000	178,000	201,000	264,000	266,000	310,000	347,000	304,000	248,000	166,000	75,600	27,000	10,300	3,320,000
Musculoskeletal disorders	0	0	0	0	418,000	2,000,000	3,950,000	5,530,000	8,020,000	10,300,000	11,700,000	13,000,000	14,800,000	15,700,000	15,100,000	14,000,000	12,400,000	9,390,000	6,460,000	4,280,000	2,100,000	753,000	202,000	150,000,000
Rheumatoid arthritis	0	0	0	0	4,400	15,000	33,600	59,100	91,200	128,000	157,000	195,000	255,000	312,000	349,000	388,000	405,000	343,000	251,000	165,000	79,000	26,100	6,290	3,260,000
Osteoarthritis	0	0	0	0	0	0	0	0	0	52,700	297,000	767,000	1,520,000	2,320,000	2,730,000	2,860,000	2,710,000	2,160,000	1,570,000	1,090,000	580,000	232,000	67,200	18,900,000
Osteoarthritis hip	0	0	0	0	0	0	0	0	0	18,000	26,900	40,300	64,800	95,600	126,000	148,000	154,000	133,000	99,900	69,800	38,500	16,400	5,000	1,040,000
Osteoarthritis knee	0	0	0	0	0	0	0	0	0	23,300	195,000	519,000	1,030,000	1,540,000	1,710,000	1,730,000	1,610,000	1,250,000	887,000	593,000	298,000	108,000	28,000	11,500,000
Osteoarthritis hand	0	0	0	0	0	0	0	0	0	8,360	54,300	148,000	305,000	480,000	634,000	690,000	655,000	532,000	393,000	288,000	167,000	75,800	24,500	4,450,000
Osteoarthritis other	0	0	0	0	0	0	0	0	0	3,080	21,200	59,600	123,000	199,000	256,000	288,000	290,000	242,000	186,000	137,000	77,100	32,300	9,720	1,920,000
Low back pain	0	0	0	0	354,000	1,600,000	2,630,000	3,040,000	3,700,000	4,300,000	4,960,000	5,460,000	5,960,000	6,160,000	5,820,000	5,360,000	4,740,000	3,670,000	2,650,000	1,910,000	954,000	335,000	85,600	63,700,000
Neck pain	0	0	0	0	38,300	213,000	446,000	681,000	1,120,000	1,740,000	2,070,000	2,340,000	2,700,000	2,550,000	2,200,000	1,870,000	1,570,000	1,130,000	715,000	418,000	197,000	68,700	19,800	22,100,000
Gout	0	0	0	0	0	0	915	7,600	26,700	56,000	84,200	115,000	153,000	181,000	193,000	201,000	203,000	173,000	127,000	86,100	44,600	17,100	5,050	1,620,000
Other musculoskeletal disorders	0	0	0	0	21,800	171,000	846,000	1,740,000	3,080,000	4,000,000	4,110,000	4,130,000	4,250,000	4,170,000	3,800,000	3,340,000	2,750,000	1,920,000	1,150,000	609,000	247,000	73,700	18,300	40,400,000
Other non-communicable diseases	15,500,000	5,510,000	19,200,000	10,700,000	4,830,000	4,090,000	4,680,000	6,460,000	8,150,000	9,170,000	8,990,000	8,640,000	8,110,000	7,480,000	6,710,000	6,270,000	5,750,000	4,600,000	3,430,000	2,430,000	1,340,000	584,000	198,000	153,000,000
Congenital birth defects	15,300,000	5,080,000	15,500,000	7,110,000	2,070,000	1,480,000	1,060,000	858,000	751,000	624,000	549,000	491,000	383,000	342,000	288,000	225,000	160,000	68,000	40,300	23,700	10,800	3,650	924	52,500,000
Neural tube defects	2,940,000	607,000	2,010,000	1,670,000	137,000	93,800	59,200	44,600	34,300	25,800	19,300	15,600	10,500	7,840	5,900	4,520	3,670	1,220	724	429	203	73·1	18·9	7,690,000
Congenital heart anomalies	4,410,000	2,220,000	6,640,000	2,210,000	800,000	564,000	352,000	312,000	257,000	187,000	169,000	146,000	84,500	70,200	59,700	43,900	33,100	6,690	4,310	2,830	1,360	517	154	18,600,000
Orofacial clefts	103,000	37,000	106,000	37,900	27,300	25,200	23,600	22,300	21,700	21,000	18,500	16,300	15,100	13,400	10,900	8,980	7,270	5,080	3,330	2,070	996	351	88·4	528,000
Down syndrome	269,000	118,000	605,000	278,000	71,300	55,500	48,400	46,500	41,900	36,500	33,300	35,500	29,600	34,600	35,600	25,600	11,400	0	0	0	0	0	0	1,780,000
Turner syndrome	9	26·6	409	1,520	1,540	1,300	1,710	1,500	1,340	1,160	918	706	558	271	192	135	93·2	59	34·6	20·2	10	3·7	1	13,500
Klinefelter syndrome	0·8	2·4	36·1	126	109	80·6	63·2	199	179	153	121	95·6	79·9	16·5	11·4	7	4·2	2·3	1·1	0·5	0·2	0	0	1,290
Other chromosomal abnormalities	614,000	262,000	660,000	186,000	71,700	54,200	47,200	37,300	33,900	23,600	20,300	14,600	10,200	7,280	5,590	3,600	2,660	0	0	0	0	0	0	2,050,000
Congenital musculoskeletal and limb a[illegible]	413,000	110,000	474,000	487,000	320,000	280,000	229,000	159,000	150,000	148,000	132,000	120,000	115,000	106,000	89,300	71,700	51,300	29,000	15,300	8,110	3,490	1,150	280	3,510,000
Urogenital congenital anomalies	493,000	107,000	189,000	96,300	49,900	34,200	25,700	21,400	18,000	14,200	10,900	9,710	5,970	4,350	3,050	2,090	1,640	4·3	0·1	0	0	0	0	1,090,000
Digestive congenital anomalies	1,500,000	584,000	1,590,000	409,000	56,600	27,600	12,800	9,580	8,270	6,590	5,820	5,430	4,600	4,730	4,520	4,190	4,000	631	435	301	167	71·9	22·5	4,290,000
Other congenital birth defects	4,530,000	1,040,000	3,270,000	1,740,000	538,000	347,000	255,000	203,000	184,000	160,000	138,000	126,000	107,000	92,600	73,500	60,000	49,200	25,300	16,200	9,910	4,570	1,480	360	13,000,000
Urinary diseases and male infertility	46,400	27,600	186,000	198,000	119,000	103,000	189,000	308,000	307,000	365,000	374,000	398,000	423,000	556,000	702,000	873,000	1,030,000	973,000	812,000	653,000	403,000	209,000	82,400	9,340,000
Urolithiasis	0	0	0	1,810	3,260	4,320	7,780	14,000	22,700	33,200	41,000	46,500	59,400	63,000	64,000	62,200	58,200	44,000	34,800	23,700	12,600	5,740	2,090	604,000
Benign prostatic hyperplasia	0	0	0	0	0	0	0	0	0	0	0	3,630	25,900	77,000	177,000	291,000	399,000	384,000	269,000	158,000	59,400	14,200	2,500	1,860,000
Male infertility	0	0	0	0	0	0	0	49,100	68,600	76,000	64,800	54,900	8,480	0	0	0	0	0	0	0	0	0	0	322,000
Other urinary diseases	33,600	12,500	33,400	79,300	31,900	27,000	44,500	32,600	38,500	61,400	62,800	66,100	87,000	82,600	103,000	113,000	111,000	105,000	81,700	75,800	40,200	19,300	5,950	1,350,000
Gynecological diseases	0	0	0	0	0	471,000	1,240,000	2,680,000	4,100,000	4,730,000	4,430,000	3,880,000	3,020,000	1,940,000	1,120,000	757,000	539,000	355,000	219,000	144,000	74,400	30,300	8,380	29,700,000
Uterine fibroids	0	0	0	0	0	5,730	13,200	29,600	75,200	153,000	227,000	236,000	217,000	155,000	113,000	73,600	33,400	22,900	11,800	8,550	2,650	837	211	1,380,000
Polycystic ovarian syndrome	0	0	0	0	0	14,400	64,400	88,700	91,200	89,200	80,500	72,300	61,600	14,700	0	0	0	0	0	0	0	0	0	577,000
Female infertility	0	0	0	0	0	0	0	85,700	140,000	166,000	141,000	120,000	5,810	0	0	0	0	0	0	0	0	0	0	659,000
Endometriosis	0	0	0	0	0	106	83,800	385,000	469,000	396,000	320,000	275,000	220,000	101,000	0	0	0	0	0	0	0	0	0	2,250,000
Genital prolapse	0	0	0	0	0	5,340	7,280	7,860	10,100	17,200	24,000	30,400	46,100	52,200	57,300	52,900	40,900	34,200	24,000	17,500	9,460	3,950	1,190	442,000
Premenstrual syndrome	0	0	0	0	0	421,000	917,000	1,100,000	1,130,000	1,160,000	1,180,000	1,080,000	737,000	243,000	0	0	0	0	0	0	0	0	0	7,970,000
Other gynecological diseases	0	0	0	0	0	25,000	152,000	980,000	2,180,000	2,750,000	2,460,000	2,070,000	1,740,000	1,370,000	947,000	630,000	465,000	298,000	184,000	118,000	62,300	25,500	6,980	16,500,000
Hemoglobinopathies and hemolytic anemias	61,500	70,800	624,000	2,260,000	1,500,000	967,000	974,000	1,120,000	979,000	834,000	597,000	466,000	382,000	362,000	329,000	332,000	303,000	244,000	184,000	121,000	68,400	26,100	7,850	12,800,000
Thalassemias	24,800	18,200	105,000	198,000	131,000	74,100	63,600	40,100	25,100	22,800	15,800	15,700	14,800	14,200	8,000	3,540	1,130	215	34·1	3·8	0·3	0	0	776,000
Thalassemias trait	1,140	9,300	85,100	309,000	426,000	260,000	223,000	309,000	310,000	295,000	207,000	138,000	122,000	119,000	109,000	86,600	77,400	59,300	43,200	27,400	14,700	4,340	1,010	3,230,000
Sickle cell disorders	31,100	18,900	320,000	1,350,000	394,000	272,000	299,000	247,000	167,000	121,000	77,200	61,400	34,000	19,700	7,440	2,600	1,300	428	89·2	16·6	2·7	0·4	0	3,420,000
Sickle cell trait	1,900	19,300	62,800	234,000	322,000	205,000	161,000	365,000	337,000	233,000	119,000	79,100	58,900	51,100	46,400	38,400	32,000	24,000	17,700	9,560	4,620	1,390	298	2,430,000
G6PD deficiency	988	1,130	7,590	26,500	28,500	38,600	76,700	60,300	51,700	62,000	61,200	50,800	42,100	46,000	38,500	34,600	25,700	14,700	7,560	2,820	796	162	23·7	679,000
G6PD trait	0·3	13·6	4·7	17·6	24·8	17·1	6,530	515	163	1,520	5,590	5,920	3,160	97·9	7·2	5·8	4·9	3·6	2·7	1·8	1	0·4	0·1	23,600
Other hemoglobinopathies and hemolyt[illegible]	1,530	3,930	43,900	145,000	201,000	118,000	145,000	96,000	87,800	98,600	112,000	115,000	108,000	112,000	120,000	166,000	165,000	145,000	115,000	81,000	48,300	20,200	6,520	2,260,000
Endocrine, metabolic, blood, and immune disorders	108,000	94,900	649,000	814,000	775,000	668,000	675,000	681,000	900,000	1,220,000	1,500,000	1,730,000	2,020,000	2,230,000	2,170,000	1,970,000	1,660,000	1,240,000	847,000	531,000	269,000	113,000	41,700	22,900,000
Oral disorders	0	0	0	310,000	363,000	395,000	549,000	813,000	1,110,000	1,400,000	1,550,000	1,670,000	1,870,000	2,050,000	2,100,000	2,110,000	2,050,000	1,730,000	1,320,000	962,000	512,000	201,000	56,800	23,100,000
Caries of deciduous teeth	0	0	0	95,300	98,900	5,360	0	0	0	0	0	0	0	0	0	0	0	0	0	0	0	0	0	200,000
Caries of permanent teeth	0	0	0	0	70,500	183,000	197,000	211,000	204,000	190,000	173,000	150,000	133,000	120,000	102,000	86,200	69,700	49,400	30,800	18,500	8,100	2,620	574	2,000,000
Periodontal diseases	0	0	0	0	0	0	62,200	193,000	385,000	584,000	723,000	818,000	894,000	884,000	757,000	612,000	472,000	313,000	195,000	117,000	55,900	20,200	5,380	7,090,000
Other oral disorders	0	0	0	214,000	194,000	207,000	290,000	342,000	380,000	397,000	363,000	337,000	331,000	306,000	257,000	212,000	171,000	111,000	60,500	31,800	12,800	4,320	1,240	4,220,000
Sudden infant death syndrome	–	230,000	2,200,000	–	–	–	–	–	–	–	–	–	–	–	–	–	–	–	–	–	–	–	–	NA
Injuries	1,090,000	726,000	4,980,000	11,800,000	8,180,000	8,120,000	17,200,000	23,800,000	22,800,000	22,100,000	20,000,000	17,800,000	16,600,000	15,400,000	13,300,000	11,500,000	9,990,000	7,900,000	6,100,000	4,800,000	2,970,000	1,370,000	469,000	249,000,000
Transport injuries	67,400	49,200	662,000	2,790,000	2,660,000	2,330,000	5,800,000	8,110,000	7,650,000	7,410,000	6,760,000	6,080,000	5,760,000	5,370,000	4,520,000	3,750,000	3,040,000	2,110,000	1,370,000	836,000	380,000	122,000	30,600	77,600,000
Road injuries	61,500	45,400	625,000	2,610,000	2,500,000	2,130,000	5,370,000	7,600,000	7,140,000	6,980,000	6,350,000	5,730,000	5,420,000	5,060,000	4,270,000	3,550,000	2,890,000	2,000,000	1,290,000	788,000	355,000	111,000	26,600	72,900,000
Pedestrian road injuries	23,700	16,500	231,000	1,260,000	1,220,000	899,000	1,370,000	1,890,000	1,870,000	1,990,000	1,850,000	1,700,000	1,700,000	1,710,000	1,450,000	1,320,000	1,110,000	812,000	557,000	352,000	159,000	45,300	10,500	23,500,000
Cyclist road injuries	0	0	0	101,000	139,000	203,000	339,000	399,000	444,000	523,000	528,000	564,000	612,000	612,000	532,000	478,000	412,000	284,000	175,000	102,000	45,200	14,400	2,860	6,510,000
Motorcyclist road injuries	5,930	4,350	73,300	195,000	180,000	250,000	1,410,000	2,160,000	1,900,000	1,720,000	1,530,000	1,360,000	1,250,000	1,100,000	906,000	677,000	536,000	341,000	202,000	121,000	52,600	16,900	4,020	16,000,000
Motor vehicle road injuries	31,100	23,800	312,000	1,030,000	927,000	751,000	2,190,000	3,080,000	2,850,000	2,680,000	2,370,000	2,040,000	1,790,000	1,570,000	1,320,000	1,040,000	793,000	537,000	337,000	200,000	93,200	32,900	8,720	26,000,000
Other road injuries	768	669	9,000	28,800	31,100	32,400	59,600	80,400	74,000	66,100	65,200	63,100	67,800	65,800	56,100	41,800	37,000	27,000	19,400	12,100	5,500	1,730	472	846,000
Other transport injuries	5,820	3,820	37,500	177,000	156,000	193,000	425,000	506,000	510,000	432,000	411,000	359,000	336,000	309,000	253,000	197,000	156,000	108,000	75,100	48,200	24,700	10,800	4,020	4,740,000
Unintentional injuries	636,000	540,000	3,720,000	7,860,000	4,720,000	4,180,000	4,820,000	5,890,000	6,030,000	6,510,000	6,450,000	6,320,000	6,340,000	6,280,000	5,920,000	5,680,000	5,380,000	4,700,000	4,030,000	3,540,000	2,390,000	1,200,000	427,000	104,000,000
Falls	108,000	42,400	450,000	855,000	733,000	799,000	1,050,000	1,450,000	1,710,000	2,050,000	2,240,000	2,380,000	2,550,000	2,770,000	2,820,000	2,940,000	3,020,000	2,920,000	2,710,000	2,580,000	1,850,000	966,000	348,000	39,400,000
Drowning	34,100	16,000	340,000	2,760,000	1,680,000	1,240,000	1,070,000	956,000	752,000	717,000	618,000	531,000	470,000	433,000	341,000	286,000	249,000	194,000	144,000	107,000	54,600	16,200	3,860	13,000,000
Fire, heat, and hot substances	26,600	22,700	297,000	883,000	329,000	265,000	415,000	547,000	547,000	598,000	548,000	475,000	462,000	427,000	383,000	338,000	283,000	217,000	160,000	128,000	70,900	28,200	8,850	7,460,000
Poisonings	25,900	22,700	256,000	618,000	203,000	205,000	218,000	274,000	279,000	273,000	251,000	241,000	229,000	219,000	185,000	158,000	127,000	93,600	62,100	42,600	20,600	5,520	1,250	4,010,000
Poisoning by carbon monoxide	5,270	4,300	42,700	134,000	74,100	81,400	121,000	153,000	155,000	161,000	146,000	139,000	128,000	117,000	100,000	87,300	67,700	49,100	33,600	23,500	11,400	2,930	536	1,840,000
Poisoning by other means	20,600	18,400	213,000	484,000	129,000	124,000	96,400	121,000	124,000	112,000	105,000	103,000	101,000	102,000	85,200	70,300	59,000	44,500	28,600	19,100	9,190	2,590	713	2,170,000
Exposure to mechanical forces	82,100	178,000	326,000	491,000	393,000	363,000	613,000	836,000	897,000	948,000	945,000	895,000	901,000	840,000	750,000	646,000	538,000	401,000	282,000	196,000	107,000	44,400	13,900	11,700,000
Unintentional firearm injuries	11,100	22,700	53,500	84,700	49,000	50,600	122,000	161,000	147,000	125,000	107,000	93,400	82,900	64,700	52,000	42,100	32,400	23,200	16,900	11,200	5,950	2,660	947	1,360,000
Other exposure to mechanical forces	71,100	155,000	272,000	406,000	344,000	312,000	491,000	675,000	750,000	822,000	837,000	802,000	818,000	775,000	698,000	604,000	506,000	377,000	265,000	185,000	101,000	41,700	12,900	10,300,000
Adverse effects of medical treatment	70,400	41,900	384,000	452,000	144,000	102,000	108,000	184,000	173,000	162,000	165,000	181,000	186,000	180,000	207,000	256,000	254,000	206,000	162,000	118,000	65,600	27,900	8,540	3,840,000
Animal contact	28,300	20,500	208,000	596,000	499,000	455,000	324,000	334,000	326,000	316,000	286,000	307,000	308,000	280,000	235,000	202,000	193,000	131,000	91,700	50,700	20,300	6,730	1,850	5,220,000
Venomous animal contact	23,300	16,100	155,000	397,000	402,000	387,000	267,000	277,000	270,000	260,000	229,000	250,000	246,000	215,000	175,000	150,000	148,000	98,300	68,600	35,900	12,600	3,730	908	4,090,000
Non-venomous animal contact	4,930	4,410	53,100	199,000	96,600	68,300	56,700	56,900	55,700	56,100	56,300	57,300	62,100	64,800	59,600	51,900	44,900	33,000	23,100	14,800	7,660	3,000	945	1,130,000
Foreign body	211,000	175,000	1,250,000	630,000	267,000	246,000	230,000	290,000	294,000	308,000	302,000	300,000	293,000	288,000	270,000	248,000	220,000	183,000	152,000	130,000	93,700	53,400	24,100	6,460,000
Pulmonary aspiration and foreign body[illegible]	205,000	172,000	1,210,000	504,000	186,000	157,000	127,000	177,000	174,000	181,000	175,000	175,000	169,000	171,000	167,000	155,000	138,000	119,000	105,000	97,000	75,800	45,600	21,300	4,710,000
Foreign body in eyes	44·5	133	2,260	12,200	19,700	25,400	32,400	40,300	46,900	52,100	50,700	51,100	52,500	48,800	39,300	34,200	29,800	21,500	14,800	9,570	4,620	1,660	484	590,000
Foreign body in other body part	5,440	2,560	34,100	113,000	61,400	63,400	69,900	72,800	72,700	74,900	75,400	73,600	71,600	68,800	63,400	59,100	52,000	42,400	32,500	23,000	13,200	6,140	2,260	1,150,000
Environmental heat and cold exposure	11,900	7,410	59,800	94,400	68,500	68,900	107,000	135,000	149,000	185,000	197,000	207,000	210,000	217,000	205,000	182,000	141,000	106,000	82,300	61,900	35,200	15,500	4,950	2,550,000
Exposure to forces of nature	1,620	1,350	8,690	37,300	40,300	61,000	79,000	93,700	90,100	95,100	91,100	83,800	69,800	56,600	45,300	37,100	27,600	18,500	11,600	6,840	3,110	1,050	263	961,000
Other unintentional injuries	37,300	12,300	140,000	448,000	368,000	373,000	598,000	790,000	823,000	859,000	800,000	716,000	662,000	566,000	482,000	393,000	321,000	227,000	165,000	119,000	69,200	31,800	10,800	9,010,000
Self-harm and interpersonal violence	388,000	136,000	600,000	1,150,000	798,000	1,620,000	6,580,000	9,770,000	9,160,000	8,150,000	6,790,000	5,420,000	4,540,000	3,750,000	2,880,000	2,090,000	1,570,000	1,090,000	706,000	423,000	198,000	55,500	12,200	67,900,000
Self-harm	0	0	0	0	0	642,000	3,130,000	4,690,000	4,580,000	4,130,000	3,490,000	2,870,000	2,520,000	2,210,000	1,790,000	1,330,000	1,030,000	732,000	486,000	311,000	146,000	38,400	7,320	34,300,000
Self-harm by firearm	0	0	0	0	0	50,000	180,000	285,000	268,000	240,000	214,000	193,000	187,000	168,000	152,000	114,000	83,900	57,300	37,000	22,700	11,200	3,510	727	2,270,000
Self-harm by other specified means	0	0	0	0	0	592,000	2,950,000	4,400,000	4,310,000	3,890,000	3,280,000	2,680,000	2,340,000	2,040,000	1,640,000	1,220,000	942,000	675,000	449,000	289,000	135,000	34,900	6,590	31,900,000
Interpersonal violence	112,000	33,000	275,000	672,000	476,000	666,000	2,910,000	4,340,000	3,960,000	3,440,000	2,740,000	2,090,000	1,600,000	1,210,000	863,000	605,000	426,000	273,000	163,000	74,900	33,400	11,400	3,590	27,000,000
Physical violence by firearm	17,700	5,380	45,500	136,000	85,400	169,000	1,400,000	2,080,000	1,730,000	1,350,000	1,030,000	716,000	469,000	331,000	210,000	119,000	66,500	36,400	20,100	6,870	2,860	1,070	538	10,000,000
Physical violence by sharp object	9,880	4,410	28,400	124,000	62,900	94,200	562,000	901,000	883,000	787,000	607,000	452,000	344,000	256,000	174,000	126,000	83,500	50,400	29,000	13,300	5,910	2,190	817	5,600,000
Sexual violence	0	0	0	5,330	41,200	88,700	190,000	277,000	305,000	287,000	229,000	191,000	165,000	116,000	63,800	43,500	35,100	23,400	14,700	8,990	3,470	552	29·8	2,090,000
Physical violence by other means	84,500	23,200	201,000	406,000	287,000	315,000	761,000	1,080,000	1,040,000	1,020,000	872,000	725,000	621,000	505,000	416,000	316,000	241,000	163,000	98,900	45,700	21,200	7,610	2,210	9,250,000
Conflict and terrorism	276,000	103,000	322,000	457,000	309,000	296,000	484,000	648,000	543,000	506,000	511,000	425,000	389,000	314,000	216,000	148,000	113,000	80,000	55,600	36,000	18,600	5,510	1,210	6,260,000

SR Table 2c: Lao People's Democratic Republic DALYS by cause and age group for both sexes with 95% uncertainty intervals, 2019

Cause	Early Neonatal	Late Neonatal	Post Neonatal	1 to 4	5 to 9	10 to 14	15 to 19	20 to 24	25 to 29	30 to 34	35 to 39	40 to 44	45 to 49	50 to 54	55 to 59	60 to 64	65 to 69	70 to 74	75 to 79	80 to 84	85 to 89	90 to 94	95 plus	Total
All causes	249,000 (199,000 to 303,000)	56,500 (36,100 to 83,800)	254,000 (187,000 to 331,000)	92,300 (64,900 to 126,000)	64,600 (52,000 to 78,200)	63,100 (51,100 to 76,800)	92,600 (75,300 to 111,000)	113,000 (91,300 to 135,000)	118,000 (93,100 to 144,000)	121,000 (90,700 to 151,000)	116,000 (88,500 to 146,000)	119,000 (93,100 to 150,000)	125,000 (101,000 to 155,000)	136,000 (111,000 to 167,000)	141,000 (115,000 to 171,000)	138,000 (114,000 to 163,000)	119,000 (101,000 to 138,000)	93,200 (81,200 to 105,000)	74,200 (66,600 to 81,200)	49,600 (45,200 to 52,900)	23,900 (21,800 to 25,400)	8,250 (7,500 to 8,810)	2,160 (1,990 to 2,300)	2,370,000
Communicable, maternal, neonatal, and nutritional diseases	217,000 (172,000 to 267,000)	47,200 (30,000 to 69,900)	194,000 (136,000 to 258,000)	59,300 (39,600 to 85,100)	25,500 (19,300 to 32,800)	16,200 (12,700 to 20,400)	17,200 (13,300 to 21,900)	21,600 (16,500 to 27,200)	24,100 (17,600 to 36,700)	23,700 (15,800 to 44,300)	21,500 (14,200 to 39,900)	18,800 (12,900 to 34,300)	17,100 (12,100 to 28,900)	16,000 (11,600 to 23,700)	14,600 (11,000 to 20,100)	14,600 (10,800 to 19,800)	13,200 (10,000 to 17,300)	10,800 (8,340 to 13,500)	9,210 (7,370 to 11,300)	6,810 (5,430 to 8,280)	3,600 (2,880 to 4,380)	1,320 (1,040 to 1,630)	373 (289 to 468)	794,000
HIV/AIDS and sexually transmitted infections	1,660 (470 to 3,960)	487 (131 to 1,170)	1,290 (212 to 5,490)	2,520 (27·6 to 19,500)	365 (28·2 to 1,990)	299 (35·2 to 1,960)	658 (115 to 3,440)	1,120 (242 to 5,840)	2,380 (335 to 14,700)	3,500 (382 to 22,300)	3,290 (338 to 21,400)	2,610 (258 to 17,400)	1,860 (186 to 12,000)	1,120 (115 to 7,730)	654 (77·7 to 4,640)	521 (61·6 to 4,150)	413 (50 to 3,450)	201 (26·5 to 1,750)	69·2 (13·9 to 428)	19·3 (5·1 to 96·6)	7·6 (2·3 to 35·2)	2·1 (0·7 to 9·1)	0·5 (0·2 to 1·6)	25,000
HIV/AIDS	0·1 (0 to 0·3)	0·2 (0 to 1·1)	646 (3·9 to 4,790)	2,500 (16·2 to 19,500)	352 (19·5 to 1,990)	280 (19·1 to 1,950)	593 (60·1 to 3,380)	999 (131 to 5,750)	2,220 (187 to 14,500)	3,330 (234 to 22,100)	3,140 (200 to 21,200)	2,490 (147 to 17,300)	1,770 (107 to 11,900)	1,070 (66·1 to 7,660)	614 (41·7 to 4,610)	493 (36·9 to 4,120)	394 (31·2 to 3,440)	188 (14·8 to 1,740)	60·2 (7·1 to 416)	15 (1·9 to 92·4)	5·5 (0·8 to 32·9)	1·4 (0·2 to 8·4)	0·3 (0·1 to 1·5)	21,200
HIV/AIDS - Drug-susceptible Tuberculosis	0 (0 to 0)	0·1 (0 to 0·1)	200 (3·5 to 1,290)	800 (14·3 to 5,060)	125 (16·9 to 543)	96·5 (17 to 428)	233 (40·9 to 1,020)	410 (92·6 to 1,450)	797 (142 to 3,080)	1,120 (177 to 4,850)	1,050 (159 to 4,820)	839 (114 to 4,310)	594 (85·3 to 3,140)	362 (49·8 to 1,940)	201 (34·9 to 1,150)	156 (30·6 to 944)	115 (27·5 to 690)	52·8 (12 to 330)	19·9 (6·1 to 93·3)	5·3 (1·6 to 23·2)	2 (0·6 to 7·6)	0·5 (0·2 to 1·8)	0·1 (0 to 0·3)	7,190
HIV/AIDS - Multidrug-resistant Tuberculosis without exten	0 (0 to 0)	0 (0 to 0)	2·9 (0 to 25·8)	11·4 (0 to 101)	1·6 (0 to 10·5)	1·2 (0 to 8)	2·8 (0·1 to 17·6)	4·7 (0·2 to 29·8)	9·2 (0·2 to 61·3)	13·1 (0·3 to 87·7)	12 (0·2 to 81·6)	9·8 (0·2 to 63·6)	7·2 (0·1 to 49·3)	4·6 (0·1 to 31·1)	2·5 (0·1 to 17·8)	1·9 (0 to 13·3)	1·3 (0 to 9·7)	0·6 (0 to 4·3)	0·2 (0 to 1·6)	0·1 (0 to 0·4)	0 (0 to 0·1)	0 (0 to 0)	0 (0 to 0)	86·9
HIV/AIDS - Extensively drug-resistant Tuberculosis	0 (0 to 0)	0 (0 to 0)	0·5 (0 to 5·2)	2·1 (0 to 20·1)	0·3 (0 to 2·1)	0·2 (0 to 1·6)	0·5 (0 to 3·3)	0·8 (0 to 5·2)	1·6 (0 to 11·3)	2·4 (0 to 15·6)	2·1 (0 to 14·9)	1·8 (0 to 12·1)	1·3 (0 to 9·5)	0·8 (0 to 5·5)	0·4 (0 to 3·1)	0·3 (0 to 2·5)	0·2 (0 to 1·9)	0·1 (0 to 0·8)	0 (0 to 0·3)	0 (0 to 0·1)	0 (0 to 0)	0 (0 to 0)	0 (0 to 0)	15·7
HIV/AIDS resulting in other diseases	0·1 (0 to 0·3)	0·2 (0 to 1)	442 (0·1 to 3,690)	1,680 (0·4 to 15,200)	225 (0·1 to 1,520)	182 (0·2 to 1,500)	357 (12·9 to 2,580)	583 (18·8 to 4,110)	1,410 (20·1 to 11,500)	2,190 (22·9 to 18,100)	2,080 (18·3 to 17,200)	1,640 (13·8 to 13,500)	1,160 (8·2 to 9,350)	699 (5·3 to 5,590)	410 (1 to 3,520)	334 (0·7 to 3,030)	278 (0·4 to 2,740)	134 (0·2 to 1,410)	40 (0·1 to 318)	9·7 (0 to 75·2)	3·5 (0 to 27·2)	0·9 (0 to 6·8)	0·2 (0 to 1·2)	13,900
Sexually transmitted infections excluding HIV	1,660 (470 to 3,960)	487 (131 to 1,170)	644 (171 to 1,540)	18 (4·3 to 43·6)	12·7 (3·5 to 31)	20 (11·4 to 33·8)	65 (34·7 to 111)	125 (71·7 to 202)	161 (92·7 to 263)	167 (90·4 to 279)	146 (84·7 to 253)	115 (61·6 to 200)	91·1 (49·9 to 163)	56·6 (29·4 to 107)	40·5 (21·1 to 74·2)	28·1 (13·6 to 53·8)	19 (9·6 to 35·4)	13·4 (6·8 to 26)	9 (4·6 to 16·4)	4·3 (2·3 to 7·5)	2·1 (1·1 to 3·8)	0·7 (0·3 to 1·3)	0·2 (0·1 to 0·4)	3,890
Syphilis	1,660 (470 to 3,960)	487 (131 to 1,170)	644 (171 to 1,540)	18 (4·3 to 43·6)	12·7 (3·5 to 31)	5 (2·5 to 10·6)	4·4 (2·8 to 8·1)	3·4 (2·1 to 5·9)	5·5 (2·8 to 10·5)	5·5 (2·6 to 10·1)	9·5 (3·4 to 25·3)	2·6 ([illegible])	4·3 ([illegible])	4 ([illegible])	3·4 ([illegible])	5·1 ([illegible])	3·5 ([illegible])	4 ([illegible])	1·5 ([illegible])	0·7 ([illegible])	0·3 ([illegible])	0·1 ([illegible])	0 ([illegible])	[illegible]
Chlamydial infection	0 (0 to 0)	0 (0 to 0)	0 (0 to 0)	0 (0 to 0)	0 (0 to 0)	0·7 (0·4 to 1·3)	5·8 (3·3 to 9·5)	21 (8·8 to 38·5)	31·3 (14·6 to 57·2)	29·3 (12·3 to 54·2)	23·7 (11 to 43)	[illegible]	[illegible]	[illegible]	[illegible]	[illegible]	[illegible]	[illegible]	[illegible]	[illegible]	[illegible]	[illegible]	[illegible]	[illegible]
Gonococcal infection	0 (0 to 0)	0 (0 to 0)	0 (0 to 0)	0 (0 to 0)	0 (0 to 0)	3·6 (1·6 to 7·7)	16·4 (5 to 35·5)	25·6 (10·4 to 48·7)	19·9 (7·5 to 39·8)	13·7 (5·5 to 27·3)	12·1 (4·9 to 22·7)	[illegible]	[illegible]	[illegible]	[illegible]	[illegible]	[illegible]	[illegible]	[illegible]	[illegible]	[illegible]	[illegible]	[illegible]	[illegible]
Trichomoniasis	0 (0 to 0)	0 (0 to 0)	0 (0 to 0)	0 (0 to 0)	0 (0 to 0)	[illegible]	[illegible]	[illegible]	[illegible]	[illegible]	[illegible]	[illegible]	[illegible]	[illegible]	[illegible]	[illegible]	[illegible]	[illegible]	[illegible]	[illegible]	[illegible]	[illegible]	[illegible]	[illegible]
Genital herpes	0 (0 to 0)	0 (0 to 0)	0 (0 to 0)	0 (0 to 0)	0 (0 to 0)	[illegible]	[illegible]	[illegible]	[illegible]	[illegible]	[illegible]	[illegible]	[illegible]	[illegible]	[illegible]	[illegible]	[illegible]	[illegible]	[illegible]	[illegible]	[illegible]	[illegible]	[illegible]	[illegible]
Other sexually transmitted infections	0 (0 to 0)	0 (0 to 0)	0 (0 to 0)	0 (0 to 0)	0 (0 to 0)	[illegible]	[illegible]	[illegible]	[illegible]	[illegible]	[illegible]	[illegible]	[illegible]	[illegible]	[illegible]	[illegible]	[illegible]	[illegible]	[illegible]	[illegible]	[illegible]	[illegible]	[illegible]	[illegible]
Respiratory infections and tuberculosis	13,600 (7,650 to 21,900)	9,190 (4,950 to 15,300)	104,000 (65,300 to 150,000)	15,600 (9,140 to 23,800)	6,240 (4,660 to 8,190)	[illegible]	[illegible]	[illegible]	[illegible]	[illegible]	[illegible]	[illegible]	[illegible]	[illegible]	[illegible]	[illegible]	[illegible]	[illegible]	[illegible]	[illegible]	[illegible]	[illegible]	[illegible]	[illegible]

Non-rheumatic degenerative mitral valve disease	0 (0 to 0)	0 (0 to 0)	0 (0 to 0)	0 (0 to 0)	0 (0 to 0)	0 (0 to 0)	6·7 (2·5 to 14·1)	9·5 (3·2 to 21·2)	7 (2·5 to 15·5)	10·3 (4 to 21·7)	9 (3·3 to 19·9)	7·3 (3·1 to 15·9)	13·6 (6·7 to 27·9)	13·4 (7 to 27)	20·2 (11·2 to 36·5)	17·7 (10·3 to 30·5)	17·5 (10·6 to 29·2)	16·2 (10·3 to 25·6)	14·1 (8·8 to 22·5)	10·5 (6·5 to 16·8)	4·6 (2·7 to 7·2)	1·9 (1·1 to 3·1)	0·5 (0·3 to 0·8)	180
Other non-rheumatic valve diseases	0 (0 to 0)	0 (0 to 0)	0 (0 to 0)	0 (0 to 0)	0 (0 to 0)	0 (0 to 0)	3·6 (1·1 to 8·2)	5 (1·7 to 11·4)	3 (0·9 to 7·1)	3·5 (0·9 to 8·5)	5·2 (1·3 to 13)	5 (1·3 to 11·8)	4 (0·8 to 9·8)	4 (0·9 to 9·9)	3·9 (0·7 to 10)	4·8 (0·8 to 12·9)	3·3 (0·5 to 8·9)	2·5 (0·4 to 6·8)	2·6 (0·5 to 6·9)	1·5 (0·3 to 4·3)	0·7 (0·1 to 1·8)	0·3 (0 to 0·9)	0·1 (0 to 0·3)	52·9
Cardiomyopathy and myocarditis	35·2 (9·5 to 111)	46·4 (15·3 to 111)	662 (180 to 1,750)	113 (44·1 to 262)	76·8 (33·6 to 183)	96·9 (41·5 to 197)	144 (77·4 to 258)	174 (88·4 to 311)	230 (116 to 398)	274 (138 to 480)	304 (151 to 563)	318 (162 to 570)	339 (169 to 607)	359 (184 to 671)	412 (216 to 754)	375 (205 to 657)	313 (187 to 515)	281 (176 to 428)	246 (166 to 362)	206 (122 to 324)	111 (57·7 to 185)	49·8 (22·9 to 84·1)	15·8 (6·8 to 30·3)	5,180
Myocarditis	9·6 (2 to 29·3)	14·6 (3·6 to 36·7)	273 (61·2 to 846)	52·7 (16·3 to 120)	40·4 (16 to 97·9)	31·4 (12·1 to 70·7)	35·2 (16 to 71·1)	38 (17·4 to 73·7)	39·8 (18·2 to 79·4)	34·9 (15·6 to 66·6)	32·6 (14·6 to 64·8)	33·7 (14·3 to 70·4)	32·1 (13·1 to 71·2)	33·3 (12·6 to 76·8)	31·4 (12·7 to 76)	28·8 (10·5 to 72·7)	21·2 (9 to 42·2)	17·9 (8·2 to 35·5)	19 (8·8 to 38·6)	17·3 (6·1 to 41·1)	11·1 (2·8 to 27·6)	5·6 (1·1 to 14·1)	2·1 (0·3 to 5·9)	856
Alcoholic cardiomyopathy	0 (0 to 0)	0 (0 to 0)	0 (0 to 0)	0 (0 to 0)	0 (0 to 0)	0 (0 to 0)	6·4 (2·8 to 13·3)	8·7 (3·3 to 19·4)	13 (4·3 to 32·2)	25·3 (8·8 to 53·4)	38 (14·2 to 80·2)	42·5 (15·7 to 93·7)	39·9 (15·4 to 87·6)	31·4 (11·3 to 72·9)	35 (14·2 to 75·6)	19·4 (7·7 to 42·1)	11·6 (4·8 to 24·3)	8·4 (3·4 to 18·2)	5·2 (2·3 to 11·9)	2·1 (0·9 to 4·5)	1·3 (0·5 to 2·8)	1·2 (0·5 to 2·6)	0·5 (0·2 to 1·1)	290
Other cardiomyopathy	25·7 (6·7 to 82·5)	31·8 (10·7 to 78·9)	389 (101 to 1,080)	60·8 (21·2 to 149)	36·4 (13·2 to 84·9)	65·5 (26·8 to 134)	102 (52·1 to 186)	127 (63·4 to 231)	177 (89 to 302)	214 (110 to 372)	233 (117 to 430)	242 (124 to 444)	267 (128 to 474)	294 (151 to 557)	346 (180 to 629)	327 (180 to 566)	280 (167 to 465)	255 (158 to 392)	222 (149 to 328)	187 (113 to 294)	98·8 (53·1 to 163)	43 (20·8 to 73·4)	13·2 (5·8 to 23·8)	4,040
Atrial fibrillation and flutter	0 (0 to 0)	0 (0 to 0)	0 (0 to 0)	0 (0 to 0)	0 (0 to 0)	0 (0 to 0)	0 (0 to 0)	0 (0 to 0)	0 (0 to 0)	8·4 (4·6 to 13·3)	28 (13 to 52)	76 (44·3 to 117)	136 (85·2 to 204)	213 (135 to 319)	325 (212 to 471)	421 (288 to 607)	482 (332 to 677)	480 (339 to 670)	488 (363 to 654)	441 (342 to 568)	261 (204 to 329)	115 (89 to 141)	29·1 (21·1 to 38·6)	3,500
Aortic aneurysm	--	--	--	--	--	--	27·4 (13 to 48·3)	26·6 (13·5 to 46·3)	33·9 (17·8 to 58·5)	46·2 (23·9 to 78·6)	59·7 (31·9 to 98·5)	65·9 (37·3 to 106)	72·6 (41·5 to 114)	94·6 (56·1 to 144)	131 (83·4 to 198)	155 (105 to 218)	156 (107 to 214)	140 (96 to 192)	118 (85·3 to 157)	85·3 (62·1 to 111)	38·2 (27·3 to 50·6)	11·5 (8 to 15·5)	1·9 (1·1 to 2·8)	NA
Endocarditis	1·4 (0·4 to 3·5)	5·8 (2 to 13·1)	171 (53·1 to 419)	21·7 (8·8 to 45·3)	22·1 (10·3 to 40·3)	21·3 (10 to 39·8)	111 (56 to 195)	151 (79·8 to 256)	230 (126 to 394)	200 (103 to 343)	183 (95·1 to 317)	194 (96·7 to 351)	138 (70·3 to 243)	124 (67·6 to 222)	113 (61·9 to 199)	81·8 (46·5 to 138)	78·1 (47·3 to 132)	53·7 (32·5 to 87·6)	38·3 (23·4 to 63·7)	23·3 (14 to 37·8)	12·6 (7·3 to 20·4)	4·1 (2·4 to 7·2)	0·8 (0·5 to 1·4)	1,980
Other cardiovascular and circulatory diseases	16·8 (6·2 to 39·8)	27·9 (11·2 to 57·6)	457 (172 to 894)	135 (69·9 to 242)	173 (105 to 265)	122 (66·7 to 198)	190 (121 to 282)	268 (166 to 399)	267 (168 to 403)	280 (176 to 413)	250 (153 to 377)	327 (216 to 485)	292 (196 to 435)	306 (201 to 464)	312 (212 to 476)	294 (202 to 438)	251 (178 to 366)	194 (139 to 276)	167 (123 to 235)	116 (87·3 to 162)	54·1 (39·8 to 74·2)	20·2 (14·4 to 28·7)	5·7 (4 to 7·7)	4,530
Chronic respiratory diseases	0 (0 to 0)	0 (0 to 0)	1·6 (1·2 to 2)	3,320 (2,020 to 5,550)	2,260 (1,610 to 3,190)	1,990 (1,470 to 2,580)	2,140 (1,590 to 2,820)	2,570 (1,930 to 3,290)	2,830 (2,120 to 3,630)	3,030 (2,210 to 3,980)	3,160 (2,330 to 4,160)	3,570 (2,660 to 4,710)	4,600 (3,480 to 6,000)	5,950 (4,580 to 7,790)	7,560 (5,710 to 9,860)	9,250 (6,820 to 12,500)	9,270 (6,840 to 12,500)	8,320 (6,400 to 10,600)	6,900 (5,290 to 8,810)	4,660 (3,580 to 6,020)	2,240 (1,740 to 2,860)	723 (548 to 967)	180 (138 to 243)	84,500
Chronic obstructive pulmonary disease	0 (0 to 0)	0 (0 to 0)	1·6 (1·2 to 2)	183 (91·8 to 367)	192 (127 to 280)	[illegible]	[illegible]	[illegible]	[illegible]	[illegible]	[illegible]	[illegible]	[illegible]	[illegible]	[illegible]	[illegible]	[illegible]	[illegible]	[illegible]	[illegible]	[illegible]	[illegible]	[illegible]	[illegible]
Pneumoconiosis	0 (0 to 0)	0 (0 to 0)	0 (0 to 0)	0 (0 to 0)	0 (0 to 0)	[illegible]	[illegible]	[illegible]	[illegible]	[illegible]	[illegible]	[illegible]	[illegible]	[illegible]	[illegible]	[illegible]	[illegible]	[illegible]	[illegible]	[illegible]	[illegible]	[illegible]	[illegible]	[illegible]
Silicosis	0 (0 to 0)	0 (0 to 0)	0 (0 to 0)	0 (0 to 0)	0 (0 to 0)	[illegible]	[illegible]	[illegible]	[illegible]	[illegible]	[illegible]	[illegible]	[illegible]	[illegible]	[illegible]	[illegible]	[illegible]	[illegible]	[illegible]	[illegible]	[illegible]	[illegible]	[illegible]	[illegible]
Asbestosis	0 (0 to 0)	0 (0 to 0)	0 (0 to 0)	0 (0 to 0)	0 (0 to 0)	[illegible]	[illegible]	[illegible]	[illegible]	[illegible]	[illegible]	[illegible]	[illegible]	[illegible]	[illegible]	[illegible]	[illegible]	[illegible]	[illegible]	[illegible]	[illegible]	[illegible]	[illegible]	[illegible]
Coal workers pneumoconiosis	0 (0 to 0)	0 (0 to 0)	0 (0 to 0)	0 (0 to 0)	0 (0 to 0)	[illegible]	[illegible]	[illegible]	[illegible]	[illegible]	[illegible]	[illegible]	[illegible]	[illegible]	[illegible]	[illegible]	[illegible]	[illegible]	[illegible]	[illegible]	[illegible]	[illegible]	[illegible]	[illegible]
Other pneumoconiosis	0 (0 to 0)	0 (0 to 0)	0 (0 to 0)	0 (0 to 0)	0 (0 to 0)	[illegible]	[illegible]	[illegible]	[illegible]	[illegible]	[illegible]	[illegible]	[illegible]	[illegible]	[illegible]	[illegible]	[illegible]	[illegible]	[illegible]	[illegible]	[illegible]	[illegible]	[illegible]	[illegible]
Asthma	0 (0 to 0)	0 (0 to 0)	0 (0 to 0)	2,900 (1,740 to 4,900)	1,810 (1,250 to 2,670)	[illegible]	[illegible]	[illegible]	[illegible]	[illegible]	[illegible]	[illegible]	[illegible]	[illegible]	[illegible]	[illegible]	[illegible]	[illegible]	[illegible]	[illegible]	[illegible]	[illegible]	[illegible]	[illegible]
Interstitial lung disease and pulmonary sarcoidosis	0 (0 to 0)	0 (0 to 0)	[illegible]	[illegible]	[illegible]	[illegible]	[illegible]	[illegible]	[illegible]	[illegible]	[illegible]	[illegible]	[illegible]	[illegible]	[illegible]	[illegible]	[illegible]	[illegible]	[illegible]	[illegible]	[illegible]	[illegible]	[illegible]	[illegible]
Other chronic respiratory diseases	0 (0 to 0)	0 (0 to 0)	[illegible]	[illegible]	[illegible]	[illegible]	[illegible]	[illegible]	[illegible]	[illegible]	[illegible]	[illegible]	[illegible]	[illegible]	[illegible]	[illegible]	[illegible]	[illegible]	[illegible]	[illegible]	[illegible]	[illegible]	[illegible]	[illegible]
Digestive diseases	57·4 (32·5 to 92·9)	102 (54 to 180)	[illegible]	[illegible]	[illegible]	[illegible]	[illegible]	[illegible]	[illegible]	[illegible]	[illegible]	[illegible]	[illegible]	[illegible]	[illegible]	[illegible]	[illegible]	[illegible]	[illegible]	[illegible]	[illegible]	[illegible]	[illegible]	[illegible]
Cirrhosis and other chronic liver diseases	0 (0 to 0)	0 (0 to 0)	[illegible]	[illegible]	[illegible]	[illegible]	[illegible]	[illegible]	[illegible]	[illegible]	[illegible]	[illegible]	[illegible]	[illegible]	[illegible]	[illegible]	[illegible]	[illegible]	[illegible]	[illegible]	[illegible]	[illegible]	[illegible]	[illegible]
Cirrhosis and other chronic liver diseases due to hepatitis	0 (0 to 0)	0 (0 to 0)	[illegible]	[illegible]	[illegible]	[illegible]	[illegible]	[illegible]	[illegible]	[illegible]	[illegible]	[illegible]	[illegible]	[illegible]	[illegible]	[illegible]	[illegible]	[illegible]	[illegible]	[illegible]	[illegible]	[illegible]	[illegible]	[illegible]
Cirrhosis and other chronic liver diseases due to hepatitis	0 (0 to 0)	0 (0 to 0)	[illegible]	[illegible]	[illegible]	[illegible]	[illegible]	[illegible]	[illegible]	[illegible]	[illegible]	[illegible]	[illegible]	[illegible]	[illegible]	[illegible]	[illegible]	[illegible]	[illegible]	[illegible]	[illegible]	[illegible]	[illegible]	[illegible]
Cirrhosis and other chronic liver diseases due to alcohol u	0 (0 to 0)	0 (0 to 0)	[illegible]	[illegible]	[illegible]	[illegible]	[illegible]	[illegible]	[illegible]	[illegible]	[illegible]	[illegible]	[illegible]	[illegible]	[illegible]	[illegible]	[illegible]	[illegible]	[illegible]	[illegible]	[illegible]	[illegible]	[illegible]	[illegible]
Cirrhosis and other chronic liver diseases due to NAFLD	0 (0 to 0)	0 (0 to 0)	[illegible]	[illegible]	[illegible]	[illegible]	[illegible]	[illegible]	[illegible]	[illegible]	[illegible]	[illegible]	[illegible]	[illegible]	[illegible]	[illegible]	[illegible]	[illegible]	[illegible]	[illegible]	[illegible]	[illegible]	[illegible]	[illegible]
Cirrhosis and other chronic liver diseases due to other cau	0 (0 to 0)	0 (0 to 0)	[illegible]	[illegible]	[illegible]	[illegible]	[illegible]	[illegible]	[illegible]	[illegible]	[illegible]	[illegible]	[illegible]	[illegible]	[illegible]	[illegible]	[illegible]	[illegible]	[illegible]	[illegible]	[illegible]	[illegible]	[illegible]	[illegible]
Upper digestive system diseases	0 (0 to 0)	0 (0 to 0)	[illegible]	[illegible]	[illegible]	[illegible]	[illegible]	[illegible]	[illegible]	[illegible]	[illegible]	[illegible]	[illegible]	[illegible]	[illegible]	[illegible]	[illegible]	[illegible]	[illegible]	[illegible]	[illegible]	[illegible]	[illegible]	[illegible]
Peptic ulcer disease	0 (0 to 0)	0 (0 to 0)	[illegible]	[illegible]	[illegible]	[illegible]	[illegible]	[illegible]	[illegible]	[illegible]	[illegible]	[illegible]	[illegible]	[illegible]	[illegible]	[illegible]	[illegible]	[illegible]	[illegible]	[illegible]	[illegible]	[illegible]	[illegible]	[illegible]
Gastritis and duodenitis	0 (0 to 0)	0 (0 to 0)	[illegible]	[illegible]	[illegible]	[illegible]	[illegible]	[illegible]	[illegible]	[illegible]	[illegible]	[illegible]	[illegible]	[illegible]	[illegible]	[illegible]	[illegible]	[illegible]	[illegible]	[illegible]	[illegible]	[illegible]	[illegible]	[illegible]
Gastroesophageal reflux disease	0 (0 to 0)	0 (0 to 0)	[illegible]	[illegible]	[illegible]	[illegible]	[illegible]	[illegible]	[illegible]	[illegible]	[illegible]	[illegible]	[illegible]	[illegible]	[illegible]	[illegible]	[illegible]	[illegible]	[illegible]	[illegible]	[illegible]	[illegible]	[illegible]	[illegible]
Appendicitis	0 (0 to 0)	0 (0 to 0)	[illegible]	[illegible]	[illegible]	[illegible]	[illegible]	[illegible]	[illegible]	[illegible]	[illegible]	[illegible]	[illegible]	[illegible]	[illegible]	[illegible]	[illegible]	[illegible]	[illegible]	[illegible]	[illegible]	[illegible]	[illegible]	[illegible]
Paralytic ileus and intestinal obstruction	41·5 (24·1 to 67·9)	90·5 (48·3 to 158)	[illegible]	[illegible]	[illegible]	[illegible]	[illegible]	[illegible]	[illegible]	[illegible]	[illegible]	[illegible]	[illegible]	[illegible]	[illegible]	[illegible]	[illegible]	[illegible]	[illegible]	[illegible]	[illegible]	[illegible]	[illegible]	[illegible]
Inguinal, femoral, and abdominal hernia	15·8 (6·4 to 32·5)	11·9 (3·7 to 29·8)	[illegible]	[illegible]	[illegible]	[illegible]	[illegible]	[illegible]	[illegible]	[illegible]	[illegible]	[illegible]	[illegible]	[illegible]	[illegible]	[illegible]	[illegible]	[illegible]	[illegible]	[illegible]	[illegible]	[illegible]	[illegible]	[illegible]
Inflammatory bowel disease	0 (0 to 0)	0 (0 to 0)	[illegible]	[illegible]	[illegible]	[illegible]	[illegible]	[illegible]	[illegible]	[illegible]	[illegible]	[illegible]	[illegible]	[illegible]	[illegible]	[illegible]	[illegible]	[illegible]	[illegible]	[illegible]	[illegible]	[illegible]	[illegible]	[illegible]
Vascular intestinal disorders	0 (0 to 0)	0 (0 to 0)	[illegible]	[illegible]	[illegible]	[illegible]	[illegible]	[illegible]	[illegible]	[illegible]	[illegible]	[illegible]	[illegible]	[illegible]	[illegible]	[illegible]	[illegible]	[illegible]	[illegible]	[illegible]	[illegible]	[illegible]	[illegible]	[illegible]
Gallbladder and biliary diseases	0 (0 to 0)	0 (0 to 0)	[illegible]	[illegible]	[illegible]	[illegible]	[illegible]	[illegible]	[illegible]	[illegible]	[illegible]	[illegible]	[illegible]	[illegible]	[illegible]	[illegible]	[illegible]	[illegible]	[illegible]	[illegible]	[illegible]	[illegible]	[illegible]	[illegible]
Pancreatitis	0 (0 to 0)	0 (0 to 0)	[illegible]	[illegible]	[illegible]	[illegible]	[illegible]	[illegible]	[illegible]	[illegible]	[illegible]	[illegible]	[illegible]	[illegible]	[illegible]	[illegible]	[illegible]	[illegible]	[illegible]	[illegible]	[illegible]	[illegible]	[illegible]	[illegible]
Other digestive diseases	0 (0 to 0)	0 (0 to 0)	[illegible]	[illegible]	[illegible]	[illegible]	[illegible]	[illegible]	[illegible]	[illegible]	[illegible]	[illegible]	[illegible]	[illegible]	[illegible]	[illegible]	[illegible]	[illegible]	[illegible]	[illegible]	[illegible]	[illegible]	[illegible]	[illegible]
Neurological disorders	2·4 (0·5 to 5·2)	6·8 (1·5 to 14·5)	[illegible]	[illegible]	[illegible]	[illegible]	[illegible]	[illegible]	[illegible]	[illegible]	[illegible]	[illegible]	[illegible]	[illegible]	[illegible]	[illegible]	[illegible]	[illegible]	[illegible]	[illegible]	[illegible]	[illegible]	[illegible]	[illegible]
Alzheimer's disease and other dementias	0 (0 to 0)	0 (0 to 0)	[illegible]	[illegible]	[illegible]	[illegible]	[illegible]	[illegible]	[illegible]	[illegible]	[illegible]	[illegible]	[illegible]	[illegible]	[illegible]	[illegible]	[illegible]	[illegible]	[illegible]	[illegible]	[illegible]	[illegible]	[illegible]	[illegible]
Parkinson's disease	0 (0 to 0)	0 (0 to 0)	[illegible]	[illegible]	[illegible]	[illegible]	[illegible]	[illegible]	[illegible]	[illegible]	[illegible]	[illegible]	[illegible]	[illegible]	[illegible]	[illegible]	[illegible]	[illegible]	[illegible]	[illegible]	[illegible]	[illegible]	[illegible]	[illegible]
Idiopathic epilepsy	2·3 (0·4 to 5·1)	6·7 (1·4 to 14·4)	[illegible]	[illegible]	[illegible]	[illegible]	[illegible]	[illegible]	[illegible]	[illegible]	[illegible]	[illegible]	[illegible]	[illegible]	[illegible]	[illegible]	[illegible]	[illegible]	[illegible]	[illegible]	[illegible]	[illegible]	[illegible]	[illegible]
Multiple sclerosis	0 (0 to 0)	0 (0 to 0)	[illegible]	[illegible]	[illegible]	[illegible]	[illegible]	[illegible]	[illegible]	[illegible]	[illegible]	[illegible]	[illegible]	[illegible]	[illegible]	[illegible]	[illegible]	[illegible]	[illegible]	[illegible]	[illegible]	[illegible]	[illegible]	[illegible]
Motor neuron disease	0·1 (0 to 0·1)	0·1 (0 to 0·1)	[illegible]	[illegible]	[illegible]	[illegible]	[illegible]	[illegible]	[illegible]	[illegible]	[illegible]	[illegible]	[illegible]	[illegible]	[illegible]	[illegible]	[illegible]	[illegible]	[illegible]	[illegible]	[illegible]	[illegible]	[illegible]	[illegible]
Headache disorders	0 (0 to 0)	0 (0 to 0)	[illegible]	[illegible]	[illegible]	[illegible]	[illegible]	[illegible]	[illegible]	[illegible]	[illegible]	[illegible]	[illegible]	[illegible]	[illegible]	[illegible]	[illegible]	[illegible]	[illegible]	[illegible]	[illegible]	[illegible]	[illegible]	[illegible]
Migraine	0 (0 to 0)	0 (0 to 0)	[illegible]	[illegible]	[illegible]	[illegible]	[illegible]	[illegible]	[illegible]	[illegible]	[illegible]	[illegible]	[illegible]	[illegible]	[illegible]	[illegible]	[illegible]	[illegible]	[illegible]	[illegible]	[illegible]	[illegible]	[illegible]	[illegible]
Tension-type headache	0 (0 to 0)	0 (0 to 0)	[illegible]	[illegible]	[illegible]	[illegible]	[illegible]	[illegible]	[illegible]	[illegible]	[illegible]	[illegible]	[illegible]	[illegible]	[illegible]	[illegible]	[illegible]	[illegible]	[illegible]	[illegible]	[illegible]	[illegible]	[illegible]	[illegible]
Other neurological disorders	0 (0 to 0)	0 (0 to 0)	[illegible]	[illegible]	[illegible]	[illegible]	[illegible]	[illegible]	[illegible]	[illegible]	[illegible]	[illegible]	[illegible]	[illegible]	[illegible]	[illegible]	[illegible]	[illegible]	[illegible]	[illegible]	[illegible]	[illegible]	[illegible]	[illegible]
Mental disorders	2·5 (1·5 to 3·8)	7·5 (4·6 to 11·5)	[illegible]	[illegible]	[illegible]	[illegible]	[illegible]	[illegible]	[illegible]	[illegible]	[illegible]	[illegible]	[illegible]	[illegible]	[illegible]	[illegible]	[illegible]	[illegible]	[illegible]	[illegible]	[illegible]	[illegible]	[illegible]	[illegible]
Schizophrenia	0 (0 to 0)	0 (0 to 0)	[illegible]	[illegible]	[illegible]	[illegible]	[illegible]	[illegible]	[illegible]	[illegible]	[illegible]	[illegible]	[illegible]	[illegible]	[illegible]	[illegible]	[illegible]	[illegible]	[illegible]	[illegible]	[illegible]	[illegible]	[illegible]	[illegible]
Depressive disorders	0 (0 to 0)	0 (0 to 0)	[illegible]	[illegible]	[illegible]	[illegible]	[illegible]	[illegible]	[illegible]	[illegible]	[illegible]	[illegible]	[illegible]	[illegible]	[illegible]	[illegible]	[illegible]	[illegible]	[illegible]	[illegible]	[illegible]	[illegible]	[illegible]	[illegible]
Major depressive disorder	0 (0 to 0)	0 (0 to 0)	[illegible]	[illegible]	[illegible]	[illegible]	[illegible]	[illegible]	[illegible]	[illegible]	[illegible]	[illegible]	[illegible]	[illegible]	[illegible]	[illegible]	[illegible]	[illegible]	[illegible]	[illegible]	[illegible]	[illegible]	[illegible]	[illegible]
Dysthymia	0 (0 to 0)	0 (0 to 0)	[illegible]	[illegible]	[illegible]	[illegible]	[illegible]	[illegible]	[illegible]	[illegible]	[illegible]	[illegible]	[illegible]	[illegible]	[illegible]	[illegible]	[illegible]	[illegible]	[illegible]	[illegible]	[illegible]	[illegible]	[illegible]	[illegible]
Bipolar disorder	0 (0 to 0)	0 (0 to 0)	[illegible]	[illegible]	[illegible]	[illegible]	[illegible]	[illegible]	[illegible]	[illegible]	[illegible]	[illegible]	[illegible]	[illegible]	[illegible]	[illegible]	[illegible]	[illegible]	[illegible]	[illegible]	[illegible]	[illegible]	[illegible]	[illegible]
Anxiety disorders	0 (0 to 0)	0 (0 to 0)	[illegible]	[illegible]	[illegible]	[illegible]	[illegible]	[illegible]	[illegible]	[illegible]	[illegible]	[illegible]	[illegible]	[illegible]	[illegible]	[illegible]	[illegible]	[illegible]	[illegible]	[illegible]	[illegible]	[illegible]	[illegible]	[illegible]
Eating disorders	0 (0 to 0)	0 (0 to 0)	[illegible]	[illegible]	[illegible]	[illegible]	[illegible]	[illegible]	[illegible]	[illegible]	[illegible]	[illegible]	[illegible]	[illegible]	[illegible]	[illegible]	[illegible]	[illegible]	[illegible]	[illegible]	[illegible]	[illegible]	[illegible]	[illegible]
Anorexia nervosa	0 (0 to 0)	0 (0 to 0)	[illegible]	[illegible]	[illegible]	[illegible]	[illegible]	[illegible]	[illegible]	[illegible]	[illegible]	[illegible]	[illegible]	[illegible]	[illegible]	[illegible]	[illegible]	[illegible]	[illegible]	[illegible]	[illegible]	[illegible]	[illegible]	[illegible]
Bulimia nervosa	0 (0 to 0)	0 (0 to 0)	[illegible]	[illegible]	[illegible]	[illegible]	[illegible]	[illegible]	[illegible]	[illegible]	[illegible]	[illegible]	[illegible]	[illegible]	[illegible]	[illegible]	[illegible]	[illegible]	[illegible]	[illegible]	[illegible]	[illegible]	[illegible]	[illegible]
Autism spectrum disorders	2 (1·3 to 3)	5·8 (3·8 to 8·7)	[illegible]	[illegible]	[illegible]	[illegible]	[illegible]	[illegible]	[illegible]	[illegible]	[illegible]	[illegible]	[illegible]	[illegible]	[illegible]	[illegible]	[illegible]	[illegible]	[illegible]	[illegible]	[illegible]	[illegible]	[illegible]	[illegible]
Attention-deficit/hyperactivity disorder	0 (0 to 0)	0 (0 to 0)	[illegible]	[illegible]	[illegible]	[illegible]	[illegible]	[illegible]	[illegible]	[illegible]	[illegible]	[illegible]	[illegible]	[illegible]	[illegible]	[illegible]	[illegible]	[illegible]	[illegible]	[illegible]	[illegible]	[illegible]	[illegible]	[illegible]
Conduct disorder	0 (0 to 0)	0 (0 to 0)	[illegible]	[illegible]	[illegible]	[illegible]	[illegible]	[illegible]	[illegible]	[illegible]	[illegible]	[illegible]	[illegible]	[illegible]	[illegible]	[illegible]	[illegible]	[illegible]	[illegible]	[illegible]	[illegible]	[illegible]	[illegible]	[illegible]
Idiopathic developmental intellectual disability	0·5 (0·1 to 1·3)	1·6 (0·3 to 4·2)	[illegible]	[illegible]	[illegible]	[illegible]	[illegible]	[illegible]	[illegible]	[illegible]	[illegible]	[illegible]	[illegible]	[illegible]	[illegible]	[illegible]	[illegible]	[illegible]	[illegible]	[illegible]	[illegible]	[illegible]	[illegible]	[illegible]
Other mental disorders	0 (0 to 0)	0 (0 to 0)	[illegible]	[illegible]	[illegible]	[illegible]	[illegible]	[illegible]	[illegible]	[illegible]	[illegible]	[illegible]	[illegible]	[illegible]	[illegible]	[illegible]	[illegible]	[illegible]	[illegible]	[illegible]	[illegible]	[illegible]	[illegible]	[illegible]
Substance use disorders	0 (0 to 0)	0 (0 to 0·1)	[illegible]	[illegible]	[illegible]	[illegible]	[illegible]	[illegible]	[illegible]	[illegible]	[illegible]	[illegible]	[illegible]	[illegible]	[illegible]	[illegible]	[illegible]	[illegible]	[illegible]	[illegible]	[illegible]	[illegible]	[illegible]	[illegible]
Alcohol use disorders	0 (0 to 0)	0 (0 to 0·1)	[illegible]	[illegible]	[illegible]	[illegible]	[illegible]	[illegible]	[illegible]	[illegible]	[illegible]	[illegible]	[illegible]	[illegible]	[illegible]	[illegible]	[illegible]	[illegible]	[illegible]	[illegible]	[illegible]	[illegible]	[illegible]	[illegible]
Drug use disorders	0 (0 to 0)	0 (0 to 0)	[illegible]	[illegible]	[illegible]	[illegible]	[illegible]	[illegible]	[illegible]	[illegible]	[illegible]	[illegible]	[illegible]	[illegible]	[illegible]	[illegible]	[illegible]	[illegible]	[illegible]	[illegible]	[illegible]	[illegible]	[illegible]	[illegible]
Opioid use disorders	0 (0 to 0)	0 (0 to 0)	[illegible]	[illegible]	[illegible]	[illegible]	[illegible]	[illegible]	[illegible]	[illegible]	[illegible]	[illegible]	[illegible]	[illegible]	[illegible]	[illegible]	[illegible]	[illegible]	[illegible]	[illegible]	[illegible]	[illegible]	[illegible]	[illegible]
Cocaine use disorders	0 (0 to 0)	0 (0 to 0)	[illegible]	[illegible]	[illegible]	[illegible]	[illegible]	[illegible]	[illegible]	[illegible]	[illegible]	[illegible]	[illegible]	[illegible]	[illegible]	[illegible]	[illegible]	[illegible]	[illegible]	[illegible]	[illegible]	[illegible]	[illegible]	[illegible]
Amphetamine use disorders	0 (0 to 0)	0 (0 to 0)	[illegible]	[illegible]	[illegible]	[illegible]	[illegible]	[illegible]	[illegible]	[illegible]	[illegible]	[illegible]	[illegible]	[illegible]	[illegible]	[illegible]	[illegible]	[illegible]	[illegible]	[illegible]	[illegible]	[illegible]	[illegible]	[illegible]
Cannabis use disorders	0 (0 to 0)	0 (0 to 0)	[illegible]	[illegible]	[illegible]	[illegible]	[illegible]	[illegible]	[illegible]	[illegible]	[illegible]	[illegible]	[illegible]	[illegible]	[illegible]	[illegible]	[illegible]	[illegible]	[illegible]	[illegible]	[illegible]	[illegible]	[illegible]	[illegible]
Other drug use disorders	0 (0 to 0)	0 (0 to 0)	[illegible]	[illegible]	[illegible]	[illegible]	[illegible]	[illegible]	[illegible]	[illegible]	[illegible]	[illegible]	[illegible]	[illegible]	[illegible]	[illegible]	[illegible]	[illegible]	[illegible]	[illegible]	[illegible]	[illegible]	[illegible]	[illegible]
Diabetes and kidney diseases	3·7 (2·2 to 6·2)	12·2 (5·1 to 23·9)	[illegible]	[illegible]	[illegible]	[illegible]	[illegible]	[illegible]	[illegible]	[illegible]	[illegible]	[illegible]	[illegible]	[illegible]	[illegible]	[illegible]	[illegible]	[illegible]	[illegible]	[illegible]	[illegible]	[illegible]	[illegible]	[illegible]
Diabetes mellitus	3·7 (2·2 to 6·2)	12 (5 to 23·7)	[illegible]	[illegible]	[illegible]	[illegible]	[illegible]	[illegible]	[illegible]	[illegible]	[illegible]	[illegible]	[illegible]	[illegible]	[illegible]	[illegible]	[illegible]	[illegible]	[illegible]	[illegible]	[illegible]	[illegible]	[illegible]	[illegible]
Diabetes mellitus type 1	3·7 (2·2 to 6·2)	12 (5 to 23·7)	[illegible]	[illegible]	[illegible]	[illegible]	[illegible]	[illegible]	[illegible]	[illegible]	[illegible]	[illegible]	[illegible]	[illegible]	[illegible]	[illegible]	[illegible]	[illegible]	[illegible]	[illegible]	[illegible]	[illegible]	[illegible]	[illegible]
Diabetes mellitus type 2	0 (0 to 0)	0 (0 to 0)	[illegible]	[illegible]	[illegible]	[illegible]	[illegible]	[illegible]	[illegible]	[illegible]	[illegible]	[illegible]	[illegible]	[illegible]	[illegible]	[illegible]	[illegible]	[illegible]	[illegible]	[illegible]	[illegible]	[illegible]	[illegible]	[illegible]
Chronic kidney disease	0 (0 to 0)	0·1 (0·1 to 0·2)	[illegible]	[illegible]	[illegible]	[illegible]	[illegible]	[illegible]	[illegible]	[illegible]	[illegible]	[illegible]	[illegible]	[illegible]	[illegible]	[illegible]	[illegible]	[illegible]	[illegible]	[illegible]	[illegible]	[illegible]	[illegible]	[illegible]
Chronic kidney disease due to diabetes mellitus type 1	0 (0 to 0)	0 (0 to 0)	[illegible]	[illegible]	[illegible]	[illegible]	[illegible]	[illegible]	[illegible]	[illegible]	[illegible]	[illegible]	[illegible]	[illegible]	[illegible]	[illegible]	[illegible]	[illegible]	[illegible]	[illegible]	[illegible]	[illegible]	[illegible]	[illegible]
Chronic kidney disease due to diabetes mellitus type 2	0 (0 to 0)	0 (0 to 0)	[illegible]	[illegible]	[illegible]	[illegible]	[illegible]	[illegible]	[illegible]	[illegible]	[illegible]	[illegible]	[illegible]	[illegible]	[illegible]	[illegible]	[illegible]	[illegible]	[illegible]	[illegible]	[illegible]	[illegible]	[illegible]	[illegible]
Chronic kidney disease due to hypertension	0 (0 to 0)	0 (0 to 0)	[illegible]	[illegible]	[illegible]	[illegible]	[illegible]	[illegible]	[illegible]	[illegible]	[illegible]	[illegible]	[illegible]	[illegible]	[illegible]	[illegible]	[illegible]	[illegible]	[illegible]	[illegible]	[illegible]	[illegible]	[illegible]	[illegible]
Chronic kidney disease due to glomerulonephritis	0 (0 to 0)	0 (0 to 0·1)	[illegible]	[illegible]	[illegible]	[illegible]	[illegible]	[illegible]	[illegible]	[illegible]	[illegible]	[illegible]	[illegible]	[illegible]	[illegible]	[illegible]	[illegible]	[illegible]	[illegible]	[illegible]	[illegible]	[illegible]	[illegible]	[illegible]
Chronic kidney disease due to other and unspecified caus	0 (0 to 0)	0·1 (0·1 to 0·2)	[illegible]	[illegible]	[illegible]	[illegible]	[illegible]	[illegible]	[illegible]	[illegible]	[illegible]	[illegible]	[illegible]	[illegible]	[illegible]	[illegible]	[illegible]	[illegible]	[illegible]	[illegible]	[illegible]	[illegible]	[illegible]	[illegible]
Acute glomerulonephritis	0 (0 to 0)	0 (0 to 0)	[illegible]	[illegible]	[illegible]	[illegible]	[illegible]	[illegible]	[illegible]	[illegible]	[illegible]	[illegible]	[illegible]	[illegible]	[illegible]	[illegible]	[illegible]	[illegible]	[illegible]	[illegible]	[illegible]	[illegible]	[illegible]	[illegible]
Skin and subcutaneous diseases	0·3 (0·2 to 0·5)	3·6 (1·9 to 6)	[illegible]	[illegible]	[illegible]	[illegible]	[illegible]	[illegible]	[illegible]	[illegible]	[illegible]	[illegible]	[illegible]	[illegible]	[illegible]	[illegible]	[illegible]	[illegible]	[illegible]	[illegible]	[illegible]	[illegible]	[illegible]	[illegible]
Dermatitis	0 (0 to 0)	0 (0 to 0)	[illegible]	[illegible]	[illegible]	[illegible]	[illegible]	[illegible]	[illegible]	[illegible]	[illegible]	[illegible]	[illegible]	[illegible]	[illegible]	[illegible]	[illegible]	[illegible]	[illegible]	[illegible]	[illegible]	[illegible]	[illegible]	[illegible]
Atopic dermatitis	0 (0 to 0)	0 (0 to 0)	[illegible]	[illegible]	[illegible]	[illegible]	[illegible]	[illegible]	[illegible]	[illegible]	[illegible]	[illegible]	[illegible]	[illegible]	[illegible]	[illegible]	[illegible]	[illegible]	[illegible]	[illegible]	[illegible]	[illegible]	[illegible]	[illegible]
Contact dermatitis	0 (0 to 0)	0 (0 to 0)	[illegible]	[illegible]	[illegible]	[illegible]	[illegible]	[illegible]	[illegible]	[illegible]	[illegible]	[illegible]	[illegible]	[illegible]	[illegible]	[illegible]	[illegible]	[illegible]	[illegible]	[illegible]	[illegible]	[illegible]	[illegible]	[illegible]
Seborrhoeic dermatitis	0 (0 to 0)	0 (0 to 0)	[illegible]	[illegible]	[illegible]	[illegible]	[illegible]	[illegible]	[illegible]	[illegible]	[illegible]	[illegible]	[illegible]	[illegible]	[illegible]	[illegible]	[illegible]	[illegible]	[illegible]	[illegible]	[illegible]	[illegible]	[illegible]	[illegible]
Psoriasis	0 (0 to 0)	0 (0 to 0)	[illegible]	[illegible]	[illegible]	[illegible]	[illegible]	[illegible]	[illegible]	[illegible]	[illegible]	[illegible]	[illegible]	[illegible]	[illegible]	[illegible]	[illegible]	[illegible]	[illegible]	[illegible]	[illegible]	[illegible]	[illegible]	[illegible]
Bacterial skin diseases	0 (0 to 0·1)	0·3 (0·2 to 0·6)	[illegible]	[illegible]	[illegible]	[illegible]	[illegible]	[illegible]	[illegible]	[illegible]	[illegible]	[illegible]	[illegible]	[illegible]	[illegible]	[illegible]	[illegible]	[illegible]	[illegible]	[illegible]	[illegible]	[illegible]	[illegible]	[illegible]
Cellulitis	0 (0 to 0)	0·1 (0·1 to 0·1)	[illegible]	[illegible]	[illegible]	[illegible]	[illegible]	[illegible]	[illegible]	[illegible]	[illegible]	[illegible]	[illegible]	[illegible]	[illegible]	[illegible]	[illegible]	[illegible]	[illegible]	[illegible]	[illegible]	[illegible]	[illegible]	[illegible]
Pyoderma	0 (0 to 0·1)	0·3 (0·1 to 0·5)	[illegible]	[illegible]	[illegible]	[illegible]	[illegible]	[illegible]	[illegible]	[illegible]	[illegible]	[illegible]	[illegible]	[illegible]	[illegible]	[illegible]	[illegible]	[illegible]	[illegible]	[illegible]	[illegible]	[illegible]	[illegible]	[illegible]
Scabies	0·2 (0·1 to 0·3)	2·3 (1·1 to 3·9)	[illegible]	[illegible]	[illegible]	[illegible]	[illegible]	[illegible]	[illegible]	[illegible]	[illegible]	[illegible]	[illegible]	[illegible]	[illegible]	[illegible]	[illegible]	[illegible]	[illegible]	[illegible]	[illegible]	[illegible]	[illegible]	[illegible]
Fungal skin diseases	0 (0 to 0)	0·2 (0·1 to 0·5)	[illegible]	[illegible]	[illegible]	[illegible]	[illegible]	[illegible]	[illegible]	[illegible]	[illegible]	[illegible]	[illegible]	[illegible]	[illegible]	[illegible]	[illegible]	[illegible]	[illegible]	[illegible]	[illegible]	[illegible]	[illegible]	[illegible]
Viral skin diseases	0 (0 to 0)	0 (0 to 0)	[illegible]	[illegible]	[illegible]	[illegible]	[illegible]	[illegible]	[illegible]	[illegible]	[illegible]	[illegible]	[illegible]	[illegible]	[illegible]	[illegible]	[illegible]	[illegible]	[illegible]	[illegible]	[illegible]	[illegible]	[illegible]	[illegible]
Acne vulgaris	0 (0 to 0)	0 (0 to 0)	[illegible]	[illegible]	[illegible]	[illegible]	[illegible]	[illegible]	[illegible]	[illegible]	[illegible]	[illegible]	[illegible]	[illegible]	[illegible]	[illegible]	[illegible]	[illegible]	[illegible]	[illegible]	[illegible]	[illegible]	[illegible]	[illegible]
Alopecia areata	0 (0 to 0)	0 (0 to 0)	[illegible]	[illegible]	[illegible]	[illegible]	[illegible]	[illegible]	[illegible]	[illegible]	[illegible]	[illegible]	[illegible]	[illegible]	[illegible]	[illegible]	[illegible]	[illegible]	[illegible]	[illegible]	[illegible]	[illegible]	[illegible]	[illegible]
Pruritus	0 (0 to 0)	0 (0 to 0)	[illegible]	[illegible]	[illegible]	[illegible]	[illegible]	[illegible]	[illegible]	[illegible]	[illegible]	[illegible]	[illegible]	[illegible]	[illegible]	[illegible]	[illegible]	[illegible]	[illegible]	[illegible]	[illegible]	[illegible]	[illegible]	[illegible]
Urticaria	0 (0 to 0)	0·4 (0·1 to 0·7)	[illegible]	[illegible]	[illegible]	[illegible]	[illegible]	[illegible]	[illegible]	[illegible]	[illegible]	[illegible]	[illegible]	[illegible]	[illegible]	[illegible]	[illegible]	[illegible]	[illegible]	[illegible]	[illegible]	[illegible]	[illegible]	[illegible]
Decubitus ulcer	0 (0 to 0)	0 (0 to 0)	[illegible]	[illegible]	[illegible]	[illegible]	[illegible]	[illegible]	[illegible]	[illegible]	[illegible]	[illegible]	[illegible]	[illegible]	[illegible]	[illegible]	[illegible]	[illegible]	[illegible]	[illegible]	[illegible]	[illegible]	[illegible]	[illegible]
Other skin and subcutaneous diseases	0 (0 to 0·1)	0·3 (0·2 to 0·6)	[illegible]	[illegible]	[illegible]	[illegible]	[illegible]	[illegible]	[illegible]	[illegible]	[illegible]	[illegible]	[illegible]	[illegible]	[illegible]	[illegible]	[illegible]	[illegible]	[illegible]	[illegible]	[illegible]	[illegible]	[illegible]	[illegible]
Sense organ diseases	1·1 (0·6 to 1·9)	3·7 (2·1 to 6·1)	[illegible]	[illegible]	[illegible]	[illegible]	[illegible]	[illegible]	[illegible]	[illegible]	[illegible]	[illegible]	[illegible]	[illegible]	[illegible]	[illegible]	[illegible]	[illegible]	[illegible]	[illegible]	[illegible]	[illegible]	[illegible]	[illegible]
Blindness and vision loss	0·2 (0·1 to 0·4)	0·6 (0·3 to 1·3)	[illegible]	[illegible]	[illegible]	[illegible]	[illegible]	[illegible]	[illegible]	[illegible]	[illegible]	[illegible]	[illegible]	[illegible]	[illegible]	[illegible]	[illegible]	[illegible]	[illegible]	[illegible]	[illegible]	[illegible]	[illegible]	[illegible]
Glaucoma	0 (0 to 0)	0 (0 to 0)	[illegible]	[illegible]	[illegible]	[illegible]	[illegible]	[illegible]	[illegible]	[illegible]	[illegible]	[illegible]	[illegible]	[illegible]	[illegible]	[illegible]	[illegible]	[illegible]	[illegible]	[illegible]	[illegible]	[illegible]	[illegible]	[illegible]
Cataract	0 (0 to 0)	0 (0 to 0)	[illegible]	[illegible]	[illegible]	[illegible]	[illegible]	[illegible]	[illegible]	[illegible]	[illegible]	[illegible]	[illegible]	[illegible]	[illegible]	[illegible]	[illegible]	[illegible]	[illegible]	[illegible]	[illegible]	[illegible]	[illegible]	[illegible]
Age-related macular degeneration	0 (0 to 0)	0 (0 to 0)	[illegible]	[illegible]	[illegible]	[illegible]	[illegible]	[illegible]	[illegible]	[illegible]	[illegible]	[illegible]	[illegible]	[illegible]	[illegible]	[illegible]	[illegible]	[illegible]	[illegible]	[illegible]	[illegible]	[illegible]	[illegible]	[illegible]
Refraction disorders	0 (0 to 0)	0·1 (0 to 0·3)	[illegible]	[illegible]	[illegible]	[illegible]	[illegible]	[illegible]	[illegible]	[illegible]	[illegible]	[illegible]	[illegible]	[illegible]	[illegible]	[illegible]	[illegible]	[illegible]	[illegible]	[illegible]	[illegible]	[illegible]	[illegible]	[illegible]
Near vision loss	0 (0 to 0)	0 (0 to 0)	[illegible]	[illegible]	[illegible]	[illegible]	[illegible]	[illegible]	[illegible]	[illegible]	[illegible]	[illegible]	[illegible]	[illegible]	[illegible]	[illegible]	[illegible]	[illegible]	[illegible]	[illegible]	[illegible]	[illegible]	[illegible]	[illegible]
Other vision loss	0·2 (0·1 to 0·4)	0·5 (0·2 to 1)	[illegible]	[illegible]	[illegible]	[illegible]	[illegible]	[illegible]	[illegible]	[illegible]	[illegible]	[illegible]	[illegible]	[illegible]	[illegible]	[illegible]	[illegible]	[illegible]	[illegible]	[illegible]	[illegible]	[illegible]	[illegible]	[illegible]
Age-related and other hearing loss	0·7 (0·3 to 1·4)	2·1 (0·9 to 4)	[illegible]	[illegible]	[illegible]	[illegible]	[illegible]	[illegible]	[illegible]	[illegible]	[illegible]	[illegible]	[illegible]	[illegible]	[illegible]	[illegible]	[illegible]	[illegible]	[illegible]	[illegible]	[illegible]	[illegible]	[illegible]	[illegible]
Other sense organ diseases	0·2 (0·1 to 0·5)	1 (0·5 to 1·8)	[illegible]	[illegible]	[illegible]	[illegible]	[illegible]	[illegible]	[illegible]	[illegible]	[illegible]	[illegible]	[illegible]	[illegible]	[illegible]	[illegible]	[illegible]	[illegible]	[illegible]	[illegible]	[illegible]	[illegible]	[illegible]	[illegible]
Musculoskeletal disorders	0 (0 to 0)	0 (0 to 0)	[illegible]	[illegible]	[illegible]	[illegible]	[illegible]	[illegible]	[illegible]	[illegible]	[illegible]	[illegible]	[illegible]	[illegible]	[illegible]	[illegible]	[illegible]	[illegible]	[illegible]	[illegible]	[illegible]	[illegible]	[illegible]	[illegible]
Rheumatoid arthritis	0 (0 to 0)	0 (0 to 0)	[illegible]	[illegible]	[illegible]	[illegible]	[illegible]	[illegible]	[illegible]	[illegible]	[illegible]	[illegible]	[illegible]	[illegible]	[illegible]	[illegible]	[illegible]	[illegible]	[illegible]	[illegible]	[illegible]	[illegible]	[illegible]	[illegible]
Osteoarthritis	0 (0 to 0)	0 (0 to 0)	[illegible]	[illegible]	[illegible]	[illegible]	[illegible]	[illegible]	[illegible]	[illegible]	[illegible]	[illegible]	[illegible]	[illegible]	[illegible]	[illegible]	[illegible]	[illegible]	[illegible]	[illegible]	[illegible]	[illegible]	[illegible]	[illegible]
Osteoarthritis hip	0 (0 to 0)	0 (0 to 0)	[illegible]	[illegible]	[illegible]	[illegible]	[illegible]	[illegible]	[illegible]	[illegible]	[illegible]	[illegible]	[illegible]	[illegible]	[illegible]	[illegible]	[illegible]	[illegible]	[illegible]	[illegible]	[illegible]	[illegible]	[illegible]	[illegible]
Osteoarthritis knee	0 (0 to 0)	0 (0 to 0)	[illegible]	[illegible]	[illegible]	[illegible]	[illegible]	[illegible]	[illegible]	[illegible]	[illegible]	[illegible]	[illegible]	[illegible]	[illegible]	[illegible]	[illegible]	[illegible]	[illegible]	[illegible]	[illegible]	[illegible]	[illegible]	[illegible]
Osteoarthritis hand	0 (0 to 0)	0 (0 to 0)	[illegible]	[illegible]	[illegible]	[illegible]	[illegible]	[illegible]	[illegible]	[illegible]	[illegible]	[illegible]	[illegible]	[illegible]	[illegible]	[illegible]	[illegible]	[illegible]	[illegible]	[illegible]	[illegible]	[illegible]	[illegible]	[illegible]
Osteoarthritis other	0 (0 to 0)	0 (0 to 0)	[illegible]	[illegible]	[illegible]	[illegible]	[illegible]	[illegible]	[illegible]	[illegible]	[illegible]	[illegible]	[illegible]	[illegible]	[illegible]	[illegible]	[illegible]	[illegible]	[illegible]	[illegible]	[illegible]	[illegible]	[illegible]	[illegible]
Low back pain	0 (0 to 0)	0 (0 to 0)	[illegible]	[illegible]	[illegible]	[illegible]	[illegible]	[illegible]	[illegible]	[illegible]	[illegible]	[illegible]	[illegible]	[illegible]	[illegible]	[illegible]	[illegible]	[illegible]	[illegible]	[illegible]	[illegible]	[illegible]	[illegible]	[illegible]
Neck pain	0 (0 to 0)	0 (0 to 0)	[illegible]	[illegible]	[illegible]	[illegible]	[illegible]	[illegible]	[illegible]	[illegible]	[illegible]	[illegible]	[illegible]	[illegible]	[illegible]	[illegible]	[illegible]	[illegible]	[illegible]	[illegible]	[illegible]	[illegible]	[illegible]	[illegible]
Gout	0 (0 to 0)	0 (0 to 0)	[illegible]	[illegible]	[illegible]	[illegible]	[illegible]	[illegible]	[illegible]	[illegible]	[illegible]	[illegible]	[illegible]	[illegible]	[illegible]	[illegible]	[illegible]	[illegible]	[illegible]	[illegible]	[illegible]	[illegible]	[illegible]	[illegible]
Other musculoskeletal disorders	0 (0 to 0)	0 (0 to 0)	[illegible]	[illegible]	[illegible]	[illegible]	[illegible]	[illegible]	[illegible]	[illegible]	[illegible]	[illegible]	[illegible]	[illegible]	[illegible]	[illegible]	[illegible]	[illegible]	[illegible]	[illegible]	[illegible]	[illegible]	[illegible]	[illegible]
Other non-communicable diseases	30,800 (15,800 to 50,900)	8,620 (4,790 to 13,900)	[illegible]	[illegible]	[illegible]	[illegible]	[illegible]	[illegible]	[illegible]	[illegible]	[illegible]	[illegible]	[illegible]	[illegible]	[illegible]	[illegible]	[illegible]	[illegible]	[illegible]	[illegible]	[illegible]	[illegible]	[illegible]	[illegible]
Congenital birth defects	30,600 (15,700 to 50,700)	7,780 (4,060 to 13,000)	[illegible]	[illegible]	[illegible]	[illegible]	[illegible]	[illegible]	[illegible]	[illegible]	[illegible]	[illegible]	[illegible]	[illegible]	[illegible]	[illegible]	[illegible]	[illegible]	[illegible]	[illegible]	[illegible]	[illegible]	[illegible]	[illegible]
Neural tube defects	6,210 (1,980 to 13,500)	619 (156 to 1,600)	[illegible]	[illegible]	[illegible]	[illegible]	[illegible]	[illegible]	[illegible]	[illegible]	[illegible]	[illegible]	[illegible]	[illegible]	[illegible]	[illegible]	[illegible]	[illegible]	[illegible]	[illegible]	[illegible]	[illegible]	[illegible]	[illegible]
Congenital heart anomalies	10,600 (4,500 to 18,800)	4,360 (2,290 to 7,280)	[illegible]	[illegible]	[illegible]	[illegible]	[illegible]	[illegible]	[illegible]	[illegible]	[illegible]	[illegible]	[illegible]	[illegible]	[illegible]	[illegible]	[illegible]	[illegible]	[illegible]	[illegible]	[illegible]	[illegible]	[illegible]	[illegible]
Orofacial clefts	160 (50·3 to 401)	60·1 (18·8 to 145)	[illegible]	[illegible]	[illegible]	[illegible]	[illegible]	[illegible]	[illegible]	[illegible]	[illegible]	[illegible]	[illegible]	[illegible]	[illegible]	[illegible]	[illegible]	[illegible]	[illegible]	[illegible]	[illegible]	[illegible]	[illegible]	[illegible]
Down syndrome	162 (68·8 to 351)	64 (26·5 to 128)	[illegible]	[illegible]	[illegible]	[illegible]	[illegible]	[illegible]	[illegible]	[illegible]	[illegible]	[illegible]	[illegible]	[illegible]	[illegible]	[illegible]	[illegible]	[illegible]	[illegible]	[illegible]	[illegible]	[illegible]	[illegible]	[illegible]
Turner syndrome	0 (0 to 0)	0 (0 to 0·1)	[illegible]	[illegible]	[illegible]	[illegible]	[illegible]	[illegible]	[illegible]	[illegible]	[illegible]	[illegible]	[illegible]	[illegible]	[illegible]	[illegible]	[illegible]	[illegible]	[illegible]	[illegible]	[illegible]	[illegible]	[illegible]	[illegible]
Klinefelter syndrome	0 (0 to 0)	0 (0 to 0)	[illegible]	[illegible]	[illegible]	[illegible]	[illegible]	[illegible]	[illegible]	[illegible]	[illegible]	[illegible]	[illegible]	[illegible]	[illegible]	[illegible]	[illegible]	[illegible]	[illegible]	[illegible]	[illegible]	[illegible]	[illegible]	[illegible]
Other chromosomal abnormalities	408 (147 to 1,070)	153 (65·4 to 335)	[illegible]	[illegible]	[illegible]	[illegible]	[illegible]	[illegible]	[illegible]	[illegible]	[illegible]	[illegible]	[illegible]	[illegible]	[illegible]	[illegible]	[illegible]	[illegible]	[illegible]	[illegible]	[illegible]	[illegible]	[illegible]	[illegible]
Congenital musculoskeletal and limb anomalies	413 (171 to 877)	90·7 (52·4 to 157)	[illegible]	[illegible]	[illegible]	[illegible]	[illegible]	[illegible]	[illegible]	[illegible]	[illegible]	[illegible]	[illegible]	[illegible]	[illegible]	[illegible]	[illegible]	[illegible]	[illegible]	[illegible]	[illegible]	[illegible]	[illegible]	[illegible]
Urogenital congenital anomalies	540 (186 to 1,200)	65 (24·7 to 139)	[illegible]	[illegible]	[illegible]	[illegible]	[illegible]	[illegible]	[illegible]	[illegible]	[illegible]	[illegible]	[illegible]	[illegible]	[illegible]	[illegible]	[illegible]	[illegible]	[illegible]	[illegible]	[illegible]	[illegible]	[illegible]	[illegible]
Digestive congenital anomalies	3,110 (1,070 to 6,310)	913 (332 to 1,780)	[illegible]	[illegible]	[illegible]	[illegible]	[illegible]	[illegible]	[illegible]	[illegible]	[illegible]	[illegible]	[illegible]	[illegible]	[illegible]	[illegible]	[illegible]	[illegible]	[illegible]	[illegible]	[illegible]	[illegible]	[illegible]	[illegible]
Other congenital birth defects	9,050 (3,120 to 19,400)	1,460 (556 to 3,080)	[illegible]	[illegible]	[illegible]	[illegible]	[illegible]	[illegible]	[illegible]	[illegible]	[illegible]	[illegible]	[illegible]	[illegible]	[illegible]	[illegible]	[illegible]	[illegible]	[illegible]	[illegible]	[illegible]	[illegible]	[illegible]	[illegible]
Urinary diseases and male infertility	1·4 (0·7 to 2·7)	5·5 (2·6 to 11·2)	[illegible]	[illegible]	[illegible]	[illegible]	[illegible]	[illegible]	[illegible]	[illegible]	[illegible]	[illegible]	[illegible]	[illegible]	[illegible]	[illegible]	[illegible]	[illegible]	[illegible]	[illegible]	[illegible]	[illegible]	[illegible]	[illegible]
Urolithiasis	0 (0 to 0)	0 (0 to 0)	[illegible]	[illegible]	[illegible]	[illegible]	[illegible]	[illegible]	[illegible]	[illegible]	[illegible]	[illegible]	[illegible]	[illegible]	[illegible]	[illegible]	[illegible]	[illegible]	[illegible]	[illegible]	[illegible]	[illegible]	[illegible]	[illegible]
Benign prostatic hyperplasia	0 (0 to 0)	0 (0 to 0)	[illegible]	[illegible]	[illegible]	[illegible]	[illegible]	[illegible]	[illegible]	[illegible]	[illegible]	[illegible]	[illegible]	[illegible]	[illegible]	[illegible]	[illegible]	[illegible]	[illegible]	[illegible]	[illegible]	[illegible]	[illegible]	[illegible]
Male infertility	0 (0 to 0)	0 (0 to 0)	[illegible]	[illegible]	[illegible]	[illegible]	[illegible]	[illegible]	[illegible]	[illegible]	[illegible]	[illegible]	[illegible]	[illegible]	[illegible]	[illegible]	[illegible]	[illegible]	[illegible]	[illegible]	[illegible]	[illegible]	[illegible]	[illegible]
Other urinary diseases	0·4 (0·2 to 0·9)	2 (0·7 to 4·2)	[illegible]	[illegible]	[illegible]	[illegible]	[illegible]	[illegible]	[illegible]	[illegible]	[illegible]	[illegible]	[illegible]	[illegible]	[illegible]	[illegible]	[illegible]	[illegible]	[illegible]	[illegible]	[illegible]	[illegible]	[illegible]	[illegible]
Gynecological diseases	0 (0 to 0)	0 (0 to 0)	[illegible]	[illegible]	[illegible]	[illegible]	[illegible]	[illegible]	[illegible]	[illegible]	[illegible]	[illegible]	[illegible]	[illegible]	[illegible]	[illegible]	[illegible]	[illegible]	[illegible]	[illegible]	[illegible]	[illegible]	[illegible]	[illegible]
Uterine fibroids	0 (0 to 0)	0 (0 to 0)	[illegible]	[illegible]	[illegible]	[illegible]	[illegible]	[illegible]	[illegible]	[illegible]	[illegible]	[illegible]	[illegible]	[illegible]	[illegible]	[illegible]	[illegible]	[illegible]	[illegible]	[illegible]	[illegible]	[illegible]	[illegible]	[illegible]
Polycystic ovarian syndrome	0 (0 to 0)	0 (0 to 0)	[illegible]	[illegible]	[illegible]	[illegible]	[illegible]	[illegible]	[illegible]	[illegible]	[illegible]	[illegible]	[illegible]	[illegible]	[illegible]	[illegible]	[illegible]	[illegible]	[illegible]	[illegible]	[illegible]	[illegible]	[illegible]	[illegible]
Female infertility	0 (0 to 0)	0 (0 to 0)	[illegible]	[illegible]	[illegible]	[illegible]	[illegible]	[illegible]	[illegible]	[illegible]	[illegible]	[illegible]	[illegible]	[illegible]	[illegible]	[illegible]	[illegible]	[illegible]	[illegible]	[illegible]	[illegible]	[illegible]	[illegible]	[illegible]
Endometriosis	0 (0 to 0)	0 (0 to 0)	[illegible]	[illegible]	[illegible]	[illegible]	[illegible]	[illegible]	[illegible]	[illegible]	[illegible]	[illegible]	[illegible]	[illegible]	[illegible]	[illegible]	[illegible]	[illegible]	[illegible]	[illegible]	[illegible]	[illegible]	[illegible]	[illegible]
Genital prolapse	0 (0 to 0)	0 (0 to 0)	[illegible]	[illegible]	[illegible]	[illegible]	[illegible]	[illegible]	[illegible]	[illegible]	[illegible]	[illegible]	[illegible]	[illegible]	[illegible]	[illegible]	[illegible]	[illegible]	[illegible]	[illegible]	[illegible]	[illegible]	[illegible]	[illegible]
Premenstrual syndrome	0 (0 to 0)	0 (0 to 0)	[illegible]	[illegible]	[illegible]	[illegible]	[illegible]	[illegible]	[illegible]	[illegible]	[illegible]	[illegible]	[illegible]	[illegible]	[illegible]	[illegible]	[illegible]	[illegible]	[illegible]	[illegible]	[illegible]	[illegible]	[illegible]	[illegible]
Other gynecological diseases	0 (0 to 0)	0 (0 to 0)	[illegible]	[illegible]	[illegible]	[illegible]	[illegible]	[illegible]	[illegible]	[illegible]	[illegible]	[illegible]	[illegible]	[illegible]	[illegible]	[illegible]	[illegible]	[illegible]	[illegible]	[illegible]	[illegible]	[illegible]	[illegible]	[illegible]
Hemoglobinopathies and hemolytic anemias	35·6 (23 to 52·1)	68·5 (45·4 to 96)	[illegible]	[illegible]	[illegible]	[illegible]	[illegible]	[illegible]	[illegible]	[illegible]	[illegible]	[illegible]	[illegible]	[illegible]	[illegible]	[illegible]	[illegible]	[illegible]	[illegible]	[illegible]	[illegible]	[illegible]	[illegible]	[illegible]
Thalassemias	31·8 (18·8 to 48·3)	20 (12 to 32·1)	[illegible]	[illegible]	[illegible]	[illegible]	[illegible]	[illegible]	[illegible]	[illegible]	[illegible]	[illegible]	[illegible]	[illegible]	[illegible]	[illegible]	[illegible]	[illegible]	[illegible]	[illegible]	[illegible]	[illegible]	[illegible]	[illegible]
Thalassemias trait	3·2 (1·6 to 5·8)	38·6 (23·2 to 57·6)	[illegible]	[illegible]	[illegible]	[illegible]	[illegible]	[illegible]	[illegible]	[illegible]	[illegible]	[illegible]	[illegible]	[illegible]	[illegible]	[illegible]	[illegible]	[illegible]	[illegible]	[illegible]	[illegible]	[illegible]	[illegible]	[illegible]
Sickle cell disorders	0 (0 to 0·1)	0 (0 to 0)	[illegible]	[illegible]	[illegible]	[illegible]	[illegible]	[illegible]	[illegible]	[illegible]	[illegible]	[illegible]	[illegible]	[illegible]	[illegible]	[illegible]	[illegible]	[illegible]	[illegible]	[illegible]	[illegible]	[illegible]	[illegible]	[illegible]
Sickle cell trait	0·2 (0·1 to 0·3)	2 (1·1 to 3·2)	[illegible]	[illegible]	[illegible]	[illegible]	[illegible]	[illegible]	[illegible]	[illegible]	[illegible]	[illegible]	[illegible]	[illegible]	[illegible]	[illegible]	[illegible]	[illegible]	[illegible]	[illegible]	[illegible]	[illegible]	[illegible]	[illegible]

G6PD deficiency	0·1 (0 to 0·2)	0·7 (0·3 to 1·1)	1·7 (0·7 to 3·1)	3·1 (0·9 to 7·1)	6·5 (2·7 to 11·9)	16·1 (6·9 to 31·4)	51·6 (24·3 to 93·6)	84·4 (41·2 to 146)	103 (55·1 to 168)	94·7 (50·4 to 149)	73·7 (38 to 121)	94·4 (60·4 to 137)	73·5 (45·4 to 114)	66·2 (38·8 to 105)	61·1 (33·9 to 98)	44·1 (23·9 to 70·3)	25·5 (12·8 to 41·5)	12·4 (5·9 to 20·7)	5·3 (2·5 to 8·6)	1·7 (0·8 to 3)	0·4 (0·2 to 0·7)	0·1 (0 to 0·1)	0 (0 to 0)	821
G6PD trait	0 (0 to 0)	0 (0 to 0)	0 (0 to 0)	0 (0 to 0)	0 (0 to 0)	0 (0 to 0)	0·3 (0·2 to 0·5)	0·1 (0·1 to 0·2)	0·1 (0·1 to 0·2)	0·1 (0·1 to 0·2)	0·2 (0·1 to 0·3)	6·8 (3·1 to 12·4)	6·7 (3·3 to 11·4)	0·1 (0·1 to 0·2)	0 (0 to 0)	0 (0 to 0)	0 (0 to 0)	0 (0 to 0)	0 (0 to 0)	0 (0 to 0)	0 (0 to 0)	0 (0 to 0)	0 (0 to 0)	14·6
Other hemoglobinopathies and hemolytic anemias	0·3 (0·2 to 0·6)	7·2 (4·2 to 10·9)	47·7 (24·8 to 82·3)	108 (49·8 to 198)	146 (60·4 to 283)	83·8 (35·6 to 156)	111 (58·8 to 192)	97·6 (54 to 165)	110 (58·5 to 182)	112 (63 to 189)	113 (65·3 to 184)	145 (89·4 to 223)	148 (96·3 to 222)	137 (84·5 to 209)	146 (92·2 to 219)	175 (112 to 252)	149 (99·6 to 205)	120 (84·1 to 163)	86·4 (60·9 to 119)	57·5 (40·6 to 78·3)	28·6 (20·9 to 38)	9·2 (6·7 to 12·1)	2·5 (1·7 to 3·4)	2,140
Endocrine, metabolic, blood, and immune disorders	93 (44·1 to 160)	77·5 (42·4 to 133)	1,170 (469 to 2,300)	394 (245 to 582)	393 (251 to 598)	329 (222 to 463)	398 (277 to 548)	452 (303 to 646)	668 (435 to 986)	923 (592 to 1,450)	1,100 (685 to 1,670)	1,190 (731 to 1,820)	1,150 (708 to 1,700)	1,090 (682 to 1,630)	947 (598 to 1,450)	670 (436 to 1,030)	455 (311 to 667)	277 (193 to 380)	186 (134 to 253)	107 (74 to 150)	49·2 (32·8 to 71·2)	18·2 (11·1 to 27·8)	5·5 (3·1 to 8·7)	12,100
Oral disorders	0 (0 to 0)	0 (0 to 0)	0 (0 to 0)	383 (216 to 637)	435 (236 to 748)	481 (254 to 829)	573 (313 to 956)	693 (398 to 1,120)	765 (425 to 1,210)	766 (432 to 1,230)	709 (401 to 1,150)	669 (381 to 1,130)	684 (400 to 1,150)	735 (428 to 1,200)	777 (454 to 1,250)	742 (435 to 1,200)	651 (386 to 1,040)	486 (294 to 768)	374 (229 to 566)	227 (143 to 340)	102 (61·9 to 154)	34·3 (20·8 to 51·7)	8·8 (5·3 to 13·4)	10,300
Caries of deciduous teeth	0 (0 to 0)	0 (0 to 0)	0 (0 to 0)	125 (45·6 to 262)	115 (44·3 to 247)	5·9 (1·1 to 16)	0 (0 to 0)	0 (0 to 0)	0 (0 to 0)	0 (0 to 0)	0 (0 to 0)	0 (0 to 0)	0 (0 to 0)	0 (0 to 0)	0 (0 to 0)	0 (0 to 0)	0 (0 to 0)	0 (0 to 0)	0 (0 to 0)	0 (0 to 0)	0 (0 to 0)	0 (0 to 0)	0 (0 to 0)	245
Caries of permanent teeth	0 (0 to 0)	0 (0 to 0)	0 (0 to 0)	0 (0 to 0)	103 (38·5 to 218)	248 (104 to 499)	234 (95·6 to 470)	234 (97·9 to 475)	214 (85·5 to 441)	178 (72·6 to 372)	145 (59·5 to 309)	110 (45 to 232)	85·1 (34·2 to 183)	70·1 (28·2 to 144)	57·2 (22·7 to 128)	43·6 (15·7 to 95·1)	30·9 (12 to 63·7)	19·2 (7·8 to 39·8)	12·1 (4·9 to 24)	6·2 (2·5 to 12·8)	2·3 (0·9 to 4·8)	0·6 (0·2 to 1·4)	0·1 (0 to 0·3)	1,790
Periodontal diseases	0 (0 to 0)	0 (0 to 0)	0 (0 to 0)	0 (0 to 0)	0 (0 to 0)	0 (0 to 0)	8·8 (2·2 to 23·4)	24·6 (7·2 to 64·7)	53 (15·2 to 133)	86 (26·4 to 231)	115 (36·5 to 299)	147 (45·4 to 377)	177 (57 to 436)	201 (67·7 to 462)	205 (69 to 490)	177 (60·6 to 413)	135 (47·6 to 300)	87·8 (32·1 to 196)	60·3 (23·3 to 134)	33·9 (12·8 to 75·3)	14·5 (5·1 to 31·6)	4·6 (1·6 to 9·8)	1·1 (0·3 to 2·3)	1,530
Other oral disorders	0 (0 to 0)	0 (0 to 0)	0 (0 to 0)	259 (154 to 397)	217 (126 to 336)	227 (133 to 348)	330 (200 to 503)	391 (242 to 602)	416 (251 to 628)	394 (231 to 590)	331 (198 to 503)	277 (170 to 426)	239 (147 to 364)	204 (123 to 302)	165 (101 to 247)	121 (73·7 to 183)	83·3 (50·5 to 126)	46·6 (28·1 to 69·9)	25·4 (15·8 to 37·7)	11·2 (7 to 16·7)	3·7 (2·3 to 5·6)	1·1 (0·6 to 1·6)	0·3 (0·2 to 0·4)	3,740
Sudden infant death syndrome	--	685 (277 to 1,420)	5,960 (1,280 to 14,400)	--	--	--	--	--	--	--	--	--	--	--	--	--	--	--	--	--	--	--	--	NA
Injuries	540 (355 to 783)	376 (194 to 814)	8,210 (4,800 to 12,500)	9,090 (5,290 to 13,800)	11,500 (8,360 to 14,700)	10,700 (8,360 to 13,000)	24,500 (19,100 to 29,100)	31,600 (23,500 to 38,300)	25,500 (18,300 to 31,700)	20,800 (13,500 to 26,000)	16,300 (10,700 to 21,100)	13,900 (9,550 to 18,100)	11,600 (8,870 to 15,100)	9,850 (7,680 to 12,500)	8,150 (6,410 to 10,100)	6,150 (4,920 to 7,500)	4,270 (3,460 to 5,150)	2,840 (2,350 to 3,340)	2,020 (1,700 to 2,370)	1,220 (1,030 to 1,430)	592 (489 to 706)	205 (169 to 247)	56·3 (45·1 to 70)	220,000
Transport injuries	84·6 (36·5 to 175)	56·2 (24·9 to 116)	1,430 (671 to 2,470)	1,640 (908 to 2,750)	3,470 (2,200 to 4,960)	3,240 (2,230 to 4,460)	9,540 (6,580 to 12,800)	12,100 (8,130 to 16,300)	8,990 (5,880 to 12,400)	6,830 (4,180 to 9,390)	5,180 (3,450 to 7,160)	4,520 (3,030 to 6,180)	3,990 (2,860 to 5,430)	3,530 (2,600 to 4,600)	2,940 (2,190 to 3,880)	2,150 (1,640 to 2,760)	1,480 (1,110 to 1,910)	953 (731 to 1,210)	615 (478 to 769)	319 (254 to 390)	126 (98·5 to 154)	34·6 (27·4 to 42·8)	9 (7 to 11·2)	73,200
Road injuries	76·3 (32·4 to 162)	52·1 (23·2 to 107)	1,310 (601 to 2,250)	1,500 (815 to 2,540)	3,240 (2,020 to 4,660)	3,030 (2,070 to 4,150)	8,970 (6,210 to 12,000)	11,400 (7,540 to 15,400)	8,400 (5,500 to 11,600)	6,390 (3,900 to 8,780)	4,860 (3,210 to 6,670)	4,240 (2,810 to 5,780)	3,760 (2,700 to 5,110)	3,320 (2,440 to 4,350)	2,780 (2,070 to 3,660)	2,020 (1,550 to 2,600)	1,400 (1,050 to 1,790)	902 (694 to 1,140)	580 (453 to 727)	300 (238 to 368)	117 (91·6 to 145)	31·7 (24·8 to 39·1)	8·1 (6·2 to 10·1)	68,700
Pedestrian road injuries	32·7 (12·7 to 69·6)	17·5 (7 to 37)	522 (225 to 1,030)	616 (313 to 1,150)	1,390 (813 to 2,230)	1,120 (667 to 1,730)	1,430 (816 to 2,380)	1,770 (961 to 2,930)	1,310 (740 to 2,130)	1,270 (717 to 2,080)	1,000 (586 to 1,600)	872 (505 to 1,460)	903 (541 to 1,460)	919 (586 to 1,410)	783 (500 to 1,180)	721 (491 to 1,030)	507 (344 to 743)	358 (246 to 497)	282 (205 to 380)	142 (104 to 185)	52·8 (39·6 to 69·5)	12·2 (9·3 to 15·6)	3·4 (2·5 to 4·5)	16,000
Cyclist road injuries	0 (0 to 0)	0 (0 to 0)	0 (0 to 0)	125 (54·2 to 246)	308 (155 to 541)	305 (167 to 524)	420 (250 to 703)	488 (297 to 778)	404 (257 to 633)	467 (278 to 725)	364 (229 to 530)	318 (211 to 463)	393 (256 to 599)	361 (243 to 544)	313 (207 to 470)	249 (165 to 358)	173 (118 to 250)	102 (67·8 to 146)	37·7 (27·4 to 49·4)	21·5 (15·6 to 28·1)	10·2 (7·4 to 13·4)	3·2 (2·3 to 4·3)	0·6 (0·4 to 0·8)	4,860
Motorcyclist road injuries	20·2 (6·6 to 56·9)	10·2 (3·6 to 24·5)	371 (117 to 800)	310 (133 to 619)	617 (315 to 1,070)	772 (418 to 1,250)	4,100 (2,510 to 6,010)	5,450 (3,390 to 7,900)	3,860 (2,430 to 5,790)	2,510 (1,530 to 3,710)	1,880 (1,150 to 2,640)	1,620 (1,050 to 2,290)	1,300 (886 to 1,840)	1,060 (744 to 1,470)	879 (620 to 1,200)	501 (367 to 659)	333 (244 to 435)	193 (140 to 254)	111 (79·9 to 145)	60·4 (43 to 81·2)	25·3 (18·3 to 33·7)	7·9 (5·7 to 10·6)	2 (1·4 to 2·6)	26,000
Motor vehicle road injuries	22 (7·8 to 48·8)	23·8 (9·6 to 48·9)	389 (143 to 772)	421 (192 to 821)	890 (460 to 1,520)	795 (459 to 1,300)	2,890 (1,610 to 4,550)	3,520 (1,990 to 5,630)	2,710 (1,560 to 4,220)	2,040 (1,120 to 3,200)	1,530 (844 to 2,410)	1,360 (805 to 2,120)	1,100 (690 to 1,660)	915 (599 to 1,350)	756 (519 to 1,130)	525 (363 to 759)	364 (243 to 541)	237 (164 to 339)	138 (96·6 to 201)	69·8 (51·1 to 96)	26·4 (19·1 to 35)	7·8 (5·8 to 10·4)	1·9 (1·4 to 2·6)	20,700
Other road injuries	1·5 (0·4 to 4·3)	0·6 (0·2 to 1·9)	23·4 (7·8 to 53·2)	25·2 (9·2 to 54·2)	42·7 (18·5 to 86)	40·5 (19·2 to 78·4)	131 (59·7 to 267)	163 (79·8 to 307)	122 (60·7 to 222)	106 (52·3 to 192)	78·2 (39·2 to 148)	70·3 (36·9 to 123)	61·1 (32·7 to 104)	60·8 (32 to 112)	44·3 (25·9 to 73·5)	27·8 (15·3 to 47·8)	19·5 (10·6 to 34·8)	12·1 (6·9 to 21·1)	11·4 (6·7 to 19)	6·2 (3·7 to 10·1)	2·3 (1·4 to 3·6)	0·6 (0·4 to 0·8)	0·2 (0·1 to 0·3)	1,050
Other transport injuries	8·3 (2·8 to 19)	4·1 (1·6 to 9)	120 (49 to 245)	141 (69·5 to 266)	224 (121 to 389)	212 (125 to 351)	572 (293 to 1,030)	741 (394 to 1,270)	585 (314 to 1,000)	447 (223 to 786)	324 (176 to 561)	276 (159 to 440)	235 (133 to 390)	210 (126 to 344)	165 (99·9 to 263)	125 (78·5 to 191)	84·8 (53·4 to 134)	50·7 (33 to 76·1)	34·8 (22·5 to 52·8)	19·1 (12·9 to 28)	8·5 (5·8 to 12·4)	2·9 (2·1 to 4·1)	0·9 (0·6 to 1·3)	4,590
Unintentional injuries	324 (214 to 487)	288 (136 to 676)	6,420 (3,700 to 10,300)	6,990 (4,070 to 10,800)	7,480 (5,250 to 9,880)	6,110 (4,610 to 7,730)	7,570 (5,650 to 9,950)	8,440 (6,210 to 10,800)	7,200 (5,300 to 9,240)	6,320 (4,240 to 8,100)	5,350 (3,740 to 6,980)	4,740 (3,440 to 6,170)	4,210 (3,170 to 5,420)	3,810 (2,950 to 4,900)	3,350 (2,640 to 4,250)	2,810 (2,220 to 3,480)	2,090 (1,680 to 2,570)	1,500 (1,210 to 1,820)	1,190 (987 to 1,430)	812 (661 to 978)	430 (351 to 527)	162 (129 to 201)	44·7 (35·1 to 57·7)	87,600
Falls	52·8 (21·9 to 99·6)	30·7 (14·4 to 57·1)	720 (290 to 1,490)	904 (465 to 1,620)	1,010 (648 to 1,440)	943 (672 to 1,280)	1,490 (1,040 to 2,060)	1,790 (1,240 to 2,460)	1,810 (1,290 to 2,430)	1,680 (1,170 to 2,240)	1,630 (1,150 to 2,160)	1,590 (1,130 to 2,120)	1,450 (1,060 to 1,930)	1,540 (1,130 to 2,020)	1,450 (1,110 to 1,890)	1,390 (1,050 to 1,800)	1,080 (833 to 1,360)	865 (667 to 1,090)	750 (594 to 958)	555 (433 to 706)	309 (241 to 396)	123 (95·1 to 158)	34·2 (25·3 to 45·6)	23,200
Drowning	50·7 (25·8 to 93·8)	23·3 (10·5 to 48·4)	1,040 (468 to 2,050)	3,780 (1,830 to 6,750)	4,010 (2,400 to 6,070)	2,890 (1,900 to 4,210)	2,810 (1,620 to 4,420)	2,360 (1,380 to 3,770)	1,610 (965 to 2,630)	1,300 (702 to 2,060)	1,000 (556 to 1,660)	840 (490 to 1,350)	711 (410 to 1,160)	551 (349 to 855)	442 (265 to 677)	314 (194 to 470)	232 (152 to 341)	140 (91 to 211)	95·9 (64·9 to 137)	46·2 (31 to 67·4)	20·3 (13·8 to 29·5)	4·5 (2·9 to 6·4)	1·1 (0·7 to 1·6)	24,300
Fire, heat, and hot substances	12·2 (5·4 to 22·5)	11·5 (5·3 to 20·3)	524 (150 to 943)	527 (246 to 927)	411 (239 to 603)	374 (248 to 522)	558 (362 to 812)	725 (447 to 1,130)	712 (449 to 1,100)	648 (432 to 963)	526 (348 to 744)	452 (315 to 632)	384 (273 to 531)	322 (231 to 436)	263 (193 to 353)	200 (142 to 268)	150 (106 to 206)	86·9 (61·6 to 116)	66·2 (46·8 to 88·5)	43·5 (31·2 to 58·2)	23·7 (17·1 to 33·2)	7·8 (5·4 to 11)	2 (1·3 to 3)	7,030
Poisonings	8·5 (3·3 to 18·3)	9·2 (1·8 to 33·2)	127 (39·4 to 288)	246 (96·9 to 472)	145 (71·7 to 264)	120 (66·2 to 202)	173 (92·1 to 297)	214 (116 to 358)	187 (102 to 305)	136 (71·6 to 220)	115 (62·2 to 197)	101 (56·8 to 165)	79·7 (44·1 to 136)	57·2 (34·2 to 92)	42·5 (25·1 to 71·9)	33·3 (19·6 to 54·8)	20·4 (12·2 to 32·4)	10·7 (6·7 to 16·2)	6·7 (4·2 to 10·4)	5·8 (3·6 to 9·2)	2·9 (1·8 to 4·5)	0·9 (0·6 to 1·3)	0·3 (0·1 to 0·5)	1,840
Poisoning by carbon monoxide	2·7 (0·6 to 7·3)	0·7 (0·1 to 2·3)	7·1 (1·3 to 23·7)	20·6 (5·4 to 55·6)	17·7 (6·7 to 37·2)	14·4 (6·3 to 30·4)	45 (18·8 to 94·8)	50·9 (23·4 to 96·6)	38·2 (16·3 to 73·8)	33·3 (12·9 to 67·6)	24·1 (10·8 to 47·6)	27·3 (12·9 to 51·9)	18 (7·7 to 37)	11·2 (5·3 to 21·8)	8·4 (3·8 to 16·4)	5·2 (2·4 to 9·8)	4·2 (2 to 7·8)	2·6 (1·3 to 4·5)	1·7 (0·8 to 3·2)	1·2 (0·6 to 2·5)	0·7 (0·3 to 1·4)	0·2 (0·1 to 0·3)	0·1 (0 to 0·2)	336
Poisoning by other means	5·8 (1·8 to 13)	8·4 (1·6 to 31·5)	120 (34·8 to 276)	225 (86·4 to 441)	127 (58·6 to 228)	106 (57·7 to 175)	128 (64·2 to 230)	163 (81·5 to 285)	149 (76·4 to 255)	103 (53·1 to 179)	90·6 (47·4 to 155)	73·6 (40 to 125)	61·7 (33·7 to 108)	46 (27·6 to 75·8)	34·2 (20 to 57·7)	28·1 (16·2 to 46·8)	16·2 (9·3 to 26·4)	8·1 (4·9 to 12·5)	5 (3 to 7·9)	4·6 (2·7 to 7·3)	2·2 (1·3 to 3·4)	0·7 (0·4 to 1·1)	0·2 (0·1 to 0·4)	1,510
Exposure to mechanical forces	14·9 (1·7 to 63·3)	63 (1·4 to 439)	291 (32·2 to 1,460)	260 (135 to 540)	362 (224 to 543)	344 (227 to 510)	562 (373 to 842)	787 (507 to 1,150)	749 (486 to 1,070)	701 (463 to 997)	609 (414 to 871)	525 (362 to 739)	486 (352 to 677)	421 (300 to 574)	373 (261 to 510)	272 (199 to 361)	186 (137 to 251)	121 (90·7 to 164)	78·2 (57·4 to 104)	43·2 (31·5 to 58·1)	19·1 (14·3 to 25·5)	6·4 (4·8 to 8·6)	1·8 (1·3 to 2·4)	7,270
Unintentional firearm injuries	2·7 (0·1 to 15·1)	12 (0·1 to 72·1)	48·7 (3 to 314)	26·8 (12·6 to 60·4)	31·3 (17·7 to 60·4)	36·4 (22·2 to 62·6)	72·4 (37·9 to 157)	115 (53·1 to 265)	92·8 (49·2 to 173)	91·6 (51·7 to 173)	85·4 (48·3 to 161)	70·1 (43·3 to 119)	60·6 (37·1 to 100)	48·7 (31·8 to 76·1)	37·9 (25·7 to 56·2)	26·5 (18·4 to 37·8)	17·9 (12·5 to 25·3)	11·3 (7·7 to 17·1)	6·4 (4·6 to 8·9)	3·7 (2·6 to 5·2)	1·7 (1·2 to 2·4)	0·6 (0·4 to 0·9)	0·2 (0·1 to 0·3)	901
Other exposure to mechanical forces	12·2 (1·1 to 52·1)	50·9 (0·9 to 364)	242 (22·9 to 1,350)	233 (114 to 492)	330 (198 to 506)	308 (200 to 466)	489 (317 to 756)	671 (434 to 998)	656 (416 to 969)	609 (400 to 886)	524 (355 to 758)	454 (309 to 654)	425 (299 to 599)	372 (262 to 514)	335 (232 to 462)	245 (178 to 330)	169 (123 to 230)	110 (81 to 151)	71·8 (52·5 to 96·4)	39·5 (28·5 to 54)	17·4 (12·8 to 23·5)	5·8 (4·3 to 7·8)	1·6 (1·2 to 2·1)	6,370
Adverse effects of medical treatment	69·5 (36·7 to 117)	40·5 (18·7 to 81·5)	512 (164 to 987)	169 (84·1 to 301)	156 (86·6 to 248)	118 (72·2 to 180)	122 (73·3 to 188)	180 (102 to 277)	146 (81·4 to 247)	146 (70·3 to 253)	128 (64·8 to 216)	125 (64·8 to 217)	136 (68·4 to 250)	111 (56·8 to 206)	128 (64·8 to 245)	121 (62·8 to 224)	95·4 (51·4 to 173)	64·1 (37·5 to 113)	48·4 (28·7 to 82·7)	30·9 (18·6 to 52·1)	14·4 (8·8 to 23·9)	5·1 (3·1 to 8·2)	1·4 (0·9 to 2·1)	2,670
Animal contact	6·5 (3·3 to 13)	9·7 (3·8 to 20·8)	236 (76·2 to 492)	287 (113 to 593)	466 (185 to 928)	307 (126 to 597)	264 (125 to 518)	272 (120 to 516)	238 (116 to 434)	192 (94·1 to 340)	197 (99·7 to 373)	165 (81·1 to 301)	168 (82·3 to 333)	179 (90·3 to 332)	147 (76·4 to 274)	113 (58·8 to 207)	80·2 (41·6 to 144)	54·7 (28·3 to 95·1)	36 (19·5 to 63·9)	20·9 (11·6 to 35)	9 (5·1 to 15·2)	2·8 (1·7 to 4·6)	0·9 (0·5 to 1·4)	3,450
Venomous animal contact	3·2 (1·2 to 6·7)	2·8 (0·6 to 8·2)	110 (26 to 276)	165 (55·1 to 362)	290 (93·2 to 637)	196 (70·8 to 416)	169 (62·8 to 363)	179 (65·8 to 392)	134 (53·6 to 288)	110 (42·1 to 216)	116 (47·1 to 246)	98·1 (36·1 to 198)	84·1 (29·9 to 183)	85 (33·4 to 180)	72·9 (27·7 to 154)	51·2 (20·7 to 107)	37·6 (13·6 to 75·1)	22·5 (8·8 to 45·2)	14·9 (6·2 to 31·2)	8·9 (3·5 to 17·5)	3·5 (1·4 to 7·1)	1·1 (0·5 to 2·2)	0·4 (0·2 to 0·6)	1,960
Non-venomous animal contact	3·4 (1·5 to 7·7)	6·9 (2·6 to 15·8)	126 (37·6 to 263)	122 (40 to 282)	175 (63·3 to 394)	110 (41·8 to 262)	94·7 (40·5 to 205)	92·6 (43·9 to 194)	103 (48 to 218)	81·9 (41·3 to 161)	81·2 (40·4 to 163)	67·1 (34·6 to 134)	83·6 (40·3 to 174)	94·1 (47·7 to 186)	74·2 (36·4 to 154)	61·8 (31 to 119)	42·7 (21·6 to 80)	32·2 (16·9 to 59·6)	21·1 (11 to 40·2)	12 (6·4 to 22·4)	5·5 (3·1 to 10·1)	1·7 (1 to 3)	0·5 (0·3 to 0·9)	1,490
Foreign body	89·3 (45·5 to 175)	89·7 (43·5 to 164)	2,650 (1,180 to 4,910)	356 (190 to 605)	269 (167 to 423)	226 (144 to 327)	188 (126 to 261)	270 (171 to 395)	238 (158 to 336)	196 (131 to 275)	154 (103 to 220)	141 (98·3 to 199)	120 (86·1 to 170)	109 (79 to 149)	98·8 (72·7 to 137)	83·7 (60·4 to 115)	60·8 (45·5 to 80·8)	48·3 (35·9 to 66·8)	40·3 (30 to 51·5)	26·2 (20·1 to 34·1)	13·6 (10 to 18·1)	5·3 (4 to 7)	1·6 (1·2 to 2)	5,480
Pulmonary aspiration and foreign body in airway	88·3 (44·8 to 173)	89·2 (43 to 162)	2,600 (1,170 to 4,800)	327 (164 to 567)	236 (139 to 382)	192 (116 to 294)	145 (90·1 to 215)	218 (124 to 340)	180 (104 to 272)	137 (72 to 209)	99·3 (54·4 to 162)	91·6 (55 to 146)	75·9 (47·3 to 124)	69 (42·8 to 105)	63·3 (41·3 to 98·1)	54·3 (33·7 to 84·7)	39·6 (25·9 to 59·7)	33·7 (22·7 to 52·4)	29·5 (20·5 to 40·5)	19·6 (14 to 27·2)	10·4 (7·1 to 14·5)	4·2 (2·9 to 5·8)	1·2 (0·9 to 1·7)	4,800
Foreign body in eyes	0 (0 to 0·1)	0·1 (0 to 0·2)	1·4 (0·4 to 3·1)	7 (2 to 16)	9·7 (2·7 to 22·6)	11·7 (3·6 to 27·4)	15·8 (5 to 36·6)	21·2 (6·5 to 47)	24·9 (8·5 to 52·8)	24·7 (8·8 to 51·9)	21·2 (7·8 to 44·8)	17·7 (7·4 to 35·4)	14·7 (6·9 to 27·7)	11·6 (6 to 20·8)	8·9 (5·1 to 15)	6·7 (3·9 to 10·8)	4·8 (3 to 7·3)	3·1 (2 to 4·6)	2·1 (1·4 to 3·1)	1·2 (0·8 to 1·8)	0·5 (0·3 to 0·7)	0·2 (0·1 to 0·2)	0 (0 to 0·1)	209
Foreign body in other body part	0·9 (0·1 to 3)	0·4 (0·2 to 1)	57·3 (9·3 to 187)	21·8 (11·9 to 41·3)	22·7 (14·5 to 36·5)	23·2 (15·8 to 33·6)	27 (19·2 to 37·5)	30·4 (21·9 to 41·7)	33 (24 to 44·2)	34·7 (24·9 to 46·4)	33·5 (24·2 to 44)	31·2 (22·7 to 41·4)	29·8 (22·3 to 39·3)	28·6 (21·1 to 38·1)	26·6 (19·8 to 35·3)	22·7 (16·5 to 30·1)	16·4 (11·9 to 21·8)	11·6 (8·4 to 15·2)	8·7 (6·4 to 11·3)	5·4 (3·9 to 7·2)	2·7 (2 to 3·6)	1 (0·7 to 1·4)	0·3 (0·2 to 0·4)	470
Environmental heat and cold exposure	1·6 (0·6 to 3·6)	1·9 (0·4 to 5·3)	56·1 (12·5 to 161)	33·2 (16 to 63·2)	38·8 (23·4 to 65·7)	38·3 (26 to 55·4)	68 (41·7 to 110)	94·8 (54·2 to 162)	83 (49·3 to 137)	81·9 (48·4 to 141)	69·2 (41·6 to 109)	62·2 (37·7 to 106)	56·4 (34·9 to 91·3)	52 (31·3 to 82·1)	41·9 (26·2 to 67·6)	37·9 (21·3 to 68)	24·4 (14·5 to 41·1)	18·8 (10·8 to 30·2)	13·4 (7·6 to 22·1)	8·7 (5 to 14·6)	4·4 (2·6 to 6·9)	1·6 (0·9 to 2·5)	0·4 (0·2 to 0·6)	889
Exposure to forces of nature	1 (0·9 to 1·1)	2·7 (2·5 to 3)	22·3 (20·2 to 24·5)	108 (97·9 to 119)	104 (93·7 to 115)	129 (114 to 145)	127 (113 to 143)	120 (107 to 136)	112 (98·5 to 126)	93·5 (81·2 to 107)	75·3 (64·6 to 88·4)	56·9 (49·1 to 66·5)	46·3 (40·4 to 53·9)	38·8 (33·7 to 45·1)	30·2 (26·1 to 35)	22·7 (19·6 to 26·3)	14·5 (12·4 to 17)	9·9 (8·6 to 11·5)	6·2 (5·4 to 7·4)	3·5 (3 to 4·1)	1·4 (1·1 to 1·6)	0·4 (0·3 to 0·4)	0·1 (0·1 to 0·1)	1,130
Other unintentional injuries	17·4 (7·2 to 35·3)	6·3 (2·6 to 15·1)	241 (94·2 to 481)	315 (143 to 719)	510 (254 to 1,090)	611 (307 to 1,310)	1,210 (572 to 2,530)	1,620 (842 to 2,970)	1,310 (723 to 2,360)	1,150 (621 to 2,110)	842 (449 to 1,590)	687 (393 to 1,280)	571 (332 to 1,010)	426 (262 to 722)	338 (208 to 572)	224 (146 to 365)	146 (96 to 225)	79·8 (54·3 to 118)	53 (36·9 to 76·7)	28·1 (20·3 to 38·8)	12·6 (9·1 to 17·4)	4·2 (3·1 to 5·7)	1·1 (0·8 to 1·4)	10,400
Self-harm and interpersonal violence	131 (55·8 to 251)	31·3 (13·9 to 60·8)	369 (147 to 744)	464 (204 to 871)	576 (356 to 922)	1,330 (911 to 1,870)	7,380 (4,940 to 10,300)	11,000 (7,300 to 15,100)	9,290 (6,010 to 12,700)	7,660 (4,500 to 10,700)	5,730 (3,360 to 8,310)	4,620 (2,820 to 6,810)	3,430 (2,300 to 4,820)	2,510 (1,800 to 3,470)	1,850 (1,320 to 2,550)	1,200 (882 to 1,650)	697 (521 to 908)	389 (303 to 506)	215 (166 to 277)	92·1 (73·7 to 117)	36·4 (28·7 to 46·5)	9·3 (7·3 to 11·8)	2·5 (2 to 3·3)	59,000
Self-harm	0 (0 to 0)	0 (0 to 0)	0 (0 to 0)	0 (0 to 0)	0 (0 to 0)	449 (265 to 688)	3,420 (2,180 to 5,000)	4,890 (2,980 to 7,020)	3,850 (2,380 to 5,530)	3,080 (1,720 to 4,520)	2,130 (1,220 to 3,200)	1,640 (991 to 2,470)	1,240 (788 to 1,920)	914 (603 to 1,360)	696 (474 to 998)	471 (318 to 698)	294 (204 to 425)	178 (122 to 263)	94·6 (64·9 to 134)	47 (33·8 to 67·3)	19·4 (13·5 to 28·4)	4·6 (3·2 to 6·9)	1·2 (0·8 to 1·8)	23,400
Self-harm by firearm	0 (0 to 0)	0 (0 to 0)	0 (0 to 0)	0 (0 to 0)	0 (0 to 0)	6·2 (3 to 11·1)	48·7 (18·6 to 110)	97·4 (38·3 to 227)	78·6 (32 to 179)	60·7 (25·3 to 130)	40·1 (16 to 95·5)	36·4 (15·8 to 86·4)	34·7 (15·5 to 82·7)	18·4 (8·3 to 44·4)	16·9 (8 to 43·4)	8·4 (4·2 to 20·9)	4·5 (2·4 to 10·1)	3·5 (1·8 to 7·8)	1·8 (1·1 to 3·5)	1·6 (0·8 to 3·6)	0·7 (0·3 to 1·5)	0·2 (0·1 to 0·5)	0·1 (0 to 0·1)	459
Self-harm by other specified means	0 (0 to 0)	0 (0 to 0)	0 (0 to 0)	0 (0 to 0)	0 (0 to 0)	443 (261 to 680)	3,370 (2,160 to 4,910)	4,790 (2,930 to 6,870)	3,770 (2,330 to 5,440)	3,020 (1,680 to 4,440)	2,090 (1,200 to 3,150)	1,610 (958 to 2,400)	1,210 (768 to 1,860)	895 (592 to 1,330)	679 (462 to 975)	462 (313 to 683)	290 (201 to 419)	174 (120 to 257)	92·8 (63·7 to 132)	45·4 (32·7 to 64·5)	18·7 (13·1 to 27)	4·3 (3 to 6·4)	1·2 (0·8 to 1·7)	23,000
Interpersonal violence	131 (55·8 to 251)	31·3 (13·9 to 60·8)	367 (146 to 742)	457 (198 to 862)	566 (348 to 912)	865 (548 to 1,310)	3,920 (2,280 to 6,300)	6,100 (3,440 to 9,610)	5,390 (3,010 to 8,510)	4,480 (2,400 to 7,090)	3,530 (1,860 to 5,680)	2,920 (1,590 to 4,770)	2,140 (1,340 to 3,210)	1,560 (1,010 to 2,350)	1,130 (728 to 1,720)	712 (491 to 1,030)	396 (277 to 565)	208 (152 to 298)	119 (85·1 to 165)	44·3 (33·9 to 56·1)	16·7 (12·6 to 21·5)	4·7 (3·4 to 6)	1·3 (0·9 to 1·8)	35,100
Physical violence by firearm	6·2 (1·9 to 15·2)	3·7 (1·1 to 9·1)	34·2 (9·4 to 87·9)	61·4 (20·4 to 128)	67·2 (30·5 to 131)	105 (40 to 210)	998 (416 to 1,990)	1,780 (723 to 3,690)	1,440 (584 to 2,950)	1,390 (538 to 2,680)	1,120 (430 to 2,240)	932 (398 to 1,810)	536 (239 to 1,010)	411 (197 to 762)	274 (130 to 511)	159 (75·8 to 288)	59·1 (29·5 to 104)	24·9 (11·6 to 46·9)	13 (6·2 to 25·2)	3·5 (1·9 to 6)	1·3 (0·7 to 2·1)	0·3 (0·2 to 0·5)	0·2 (0·1 to 0·3)	9,410
Physical violence by sharp object	24·7 (8·7 to 54)	3·3 (1 to 8·8)	41·8 (14·1 to 97·8)	116 (42·9 to 263)	146 (71·6 to 262)	249 (119 to 442)	1,860 (817 to 3,430)	3,090 (1,440 to 5,350)	2,830 (1,330 to 4,770)	2,120 (818 to 3,670)	1,590 (647 to 2,840)	1,260 (520 to 2,300)	1,010 (448 to 1,710)	652 (306 to 1,120)	465 (228 to 807)	281 (154 to 453)	159 (85·4 to 274)	79·6 (47·2 to 125)	40·1 (25·4 to 62·6)	14·4 (9·9 to 19·9)	5·6 (4 to 7·7)	1·6 (1·2 to 2·2)	0·5 (0·3 to 0·8)	16,000
Sexual violence	0 (0 to 0)	0 (0 to 0)	0 (0 to 0)	6 (3·5 to 9·9)	46·7 (27·6 to 73·6)	109 (63 to 172)	223 (127 to 353)	241 (140 to 364)	198 (114 to 322)	166 (93·7 to 268)	131 (73·2 to 210)	102 (58·5 to 166)	80·7 (47·1 to 134)	53·9 (30·6 to 89·4)	29·4 (16·8 to 48)	18·1 (11 to 27·5)	12·4 (7·7 to 18·8)	7·4 (4·7 to 11)	4·9 (3·2 to 7·1)	2·7 (1·7 to 4)	0·9 (0·6 to 1·5)	0·1 (0·1 to 0·2)	0 (0 to 0)	1,430
Physical violence by other means	99·9 (41·4 to 192)	24·3 (10·5 to 48·3)	291 (113 to 595)	274 (115 to 534)	306 (171 to 533)	402 (225 to 683)	840 (451 to 1,480)	998 (539 to 1,780)	918 (509 to 1,560)	813 (468 to 1,350)	696 (398 to 1,090)	621 (380 to 1,010)	514 (342 to 767)	438 (305 to 622)	365 (248 to 537)	255 (179 to 356)	166 (120 to 231)	96·7 (69·9 to 131)	61 (43·2 to 82·8)	23·7 (17·1 to 31·2)	8·9 (6·4 to 11·9)	2·6 (1·8 to 3·5)	0·7 (0·5 to 0·9)	8,220
Conflict and terrorism	0 (0 to 0)	0 (0 to 0)	0 (0 to 0)	0·2 (0·1 to 0·4)	1·5 (0·9 to 2·6)	4·3 (2·4 to 7·6)	14·2 (8·2 to 24·5)	13·4 (7·7 to 22·3)	20·8 (12·3 to 34·3)	67·8 (33·2 to 127)	45·9 (24·4 to 83·3)	42·7 (21·9 to 80·1)	38·9 (19·4 to 72·9)	35·4 (17·7 to 69·1)	21·5 (11 to 41)	11·9 (6·2 to 22·7)	5·5 (2·9 to 11)	2·6 (1·4 to 4·9)	1·4 (0·7 to 2·6)	0·7 (0·4 to 1·2)	0·3 (0·1 to 0·5)	0·1 (0 to 0·1)	0 (0 to 0)	329

SR Table 2c: United States of America DALYS by cause and age group for both sexes with 95% uncertainty intervals, 2019

Cause	Early Neonatal	Late Neonatal	Post Neonatal	1 to 4	5 to 9	10 to 14
All causes	991,000 (914,000 to 1,080,000)	247,000 (229,000 to 268,000)	679,000 (636,000 to 726,000)	749,000 (618,000 to 912,000)	863,000 (668,000 to 1,100,000)	1,430,000 (1,100,000 to 1,840,000)
Communicable, maternal, neonatal, and nutritional diseases	758,000 (689,000 to 829,000)	145,000 (131,000 to 159,000)	155,000 (139,000 to 173,000)	151,000 (121,000 to 190,000)	145,000 (110,000 to 197,000)	131,000 (100,000 to 171,000)
HIV/AIDS and sexually transmitted infections	89·2 (77·7 to 103)	39·5 (32·9 to 46·9)	1,310 (1,270 to 1,340)	1,090 (1,060 to 1,120)	653 (632 to 675)	922 (847 to 1,030)
HIV/AIDS	0·1 (0·1 to 0·1)	0·3 (0·2 to 0·4)	1,270 (1,240 to 1,310)	1,080 (1,050 to 1,110)	650 (630 to 673)	692 (670 to 715)
HIV/AIDS - Drug-susceptible Tuberculosis	0 (0 to 0)	0 (0 to 0)	25·2 (15·8 to 38·8)	21 (13·2 to 32·3)	12·3 (7·7 to 18·9)	13·1 (8·2 to 20·2)
HIV/AIDS - Multidrug-resistant Tuberculosis wit[illegible]	0 (0 to 0)	0 (0 to 0)	0·9 (0·2 to 2·5)	0·8 (0·2 to 2·1)	0·4 (0·1 to 1·2)	0·5 (0·1 to 1·3)
HIV/AIDS - Extensively drug-resistant Tubercul[illegible]	0 (0 to 0)	0 (0 to 0)	0·2 (0·1 to 0·7)	0·2 (0·1 to 0·6)	0·1 (0 to 0·3)	0·1 (0 to 0·3)
HIV/AIDS resulting in other diseases	0·1 (0·1 to 0·1)	0·3 (0·2 to 0·4)	1,240 (1,200 to 1,290)	1,060 (1,030 to 1,090)	638 (617 to 661)	678 (655 to 703)
Sexually transmitted infections excluding HIV	89·1 (77·6 to 102)	39·2 (32·6 to 46·7)	34·3 (27·5 to 42·8)	5·2 (4·1 to 6·8)	2·2 (1·8 to 2·6)	230 (165 to 337)
Syphilis	89·1 (77·6 to 102)	39·2 (32·6 to 46·7)	34·3 (27·5 to 42·8)	5·2 (4·1 to 6·8)	2·2 (1·8 to 2·6)	51·4 (48·3 to 55·9)
Chlamydial infection	0 (0 to 0)	0 (0 to 0)	0 (0 to 0)	0 (0 to 0)	0 (0 to 0)	23 (18·1 to 30·3)
Gonococcal infection	0 (0 to 0)	0 (0 to 0)	0 (0 to 0)	0 (0 to 0)	0 (0 to 0)	30·3 (23·1 to 44·4)
Trichomoniasis	0 (0 to 0)	0 (0 to 0)	0 (0 to 0)	0 (0 to 0)	0 (0 to 0)	9·2 (2·5 to 24)
Genital herpes	0 (0 to 0)	0 (0 to 0)	0 (0 to 0)	0 (0 to 0)	0 (0 to 0)	60·5 (20·1 to 143)
Other sexually transmitted infections	0 (0 to 0)	0 (0 to 0)	0 (0 to 0)	0 (0 to 0)	0 (0 to 0)	55·1 (35·5 to 83·8)
Respiratory infections and tuberculosis	2,320 (2,070 to 2,600)	4,280 (3,810 to 4,780)	36,600 (32,300 to 41,900)	46,300 (32,200 to 66,800)	41,500 (26,300 to 64,500)	35,800 (22,100 to 56,700)
Tuberculosis	0 (0 to 0)	0·1 (0 to 0·1)	98·3 (87·9 to 109)	146 (128 to 169)	66·6 (58·8 to 77)	89·5 (68·2 to 124)
Latent tuberculosis infection	0 (0 to 0)	0 (0 to 0)	0 (0 to 0)	0 (0 to 0)	0 (0 to 0)	0 (0 to 0)
Drug-susceptible tuberculosis	0 (0 to 0)	0·1 (0 to 0·1)	94·9 (84·2 to 106)	142 (123 to 164)	64·5 (56·3 to 74·6)	87·1 (65·7 to 122)
Multidrug-resistant tuberculosis without extensiv[illegible]	0 (0 to 0)	0 (0 to 0)	2·7 (0·8 to 6·5)	3·8 (1·2 to 9·3)	1·7 (0·6 to 4·2)	2 (0·6 to 4·5)
[illegible]	[illegible]	[illegible]	[illegible]	[illegible]	[illegible]	[illegible]

[illegible]

Multiple sclerosis	0 (0 to 0)	0 (0 to 0)	0 (0 to 0)	0 (0 to 0)	41·2 (28·2 to 55·7)	205 (142 to 285)	898 (642 to 1,220)	2,670 (1,910 to 3,590)	6,120 (4,270 to 8,160)	10,100 (7,520 to 13,100)	13,700 (10,600 to 17,300)	16,400 (13,200 to 20,700)	21,000 (17,000 to 26,900)	25,500 (20,900 to 31,700)	29,800 (22,900 to 35,400)	29,300 (21,000 to 33,900)	23,500 (15,800 to 27,400)	16,300 (10,800 to 19,200)	8,870 (5,820 to 10,600)	4,290 (2,860 to 5,190)	1,900 (1,290 to 2,340)	709 (521 to 879)	207 (152 to 264)	211,000
Motor neuron disease	102 (90·9 to 114)	375 (330 to 418)	6,760 (6,050 to 7,540)	2,100 (1,910 to 2,300)	776 (705 to 848)	622 (552 to 695)	621 (554 to 697)	640 (570 to 710)	981 (890 to 1,080)	1,690 (1,560 to 1,860)	3,120 (2,930 to 3,340)	5,940 (5,520 to 6,450)	10,200 (9,590 to 10,800)	16,500 (15,600 to 17,500)	26,300 (25,000 to 27,600)	34,900 (33,400 to 36,600)	37,800 (36,300 to 39,300)	34,300 (32,500 to 35,900)	23,500 (21,600 to 25,200)	11,900 (10,400 to 13,000)	4,370 (3,670 to 4,860)	1,160 (937 to 1,320)	246 (186 to 291)	225,000
Headache disorders	0 (0 to 0)	0 (0 to 0)	0 (0 to 0)	0 (0 to 0)	16,600 (395 to 44,800)	97,200 (5,060 to 231,000)	168,000 (16,400 to 404,000)	200,000 (22,600 to 465,000)	231,000 (30,800 to 521,000)	233,000 (40,700 to 517,000)	237,000 (47,500 to 513,000)	220,000 (52,900 to 474,000)	210,000 (55,000 to 459,000)	188,000 (48,900 to 405,000)	172,000 (49,000 to 370,000)	139,000 (42,600 to 294,000)	97,900 (31,600 to 198,000)	59,900 (20,100 to 122,000)	32,100 (9,870 to 65,600)	17,200 (5,660 to 35,700)	9,540 (3,200 to 20,000)	4,060 (1,260 to 8,620)	1,170 (256 to 2,690)	2,330,000
Migraine	0 (0 to 0)	0 (0 to 0)	0 (0 to 0)	0 (0 to 0)	14,600 (264 to 39,400)	88,900 (3,420 to 220,000)	155,000 (11,100 to 389,000)	185,000 (15,600 to 446,000)	211,000 (20,200 to 492,000)	212,000 (26,600 to 484,000)	213,000 (33,600 to 487,000)	197,000 (37,100 to 443,000)	187,000 (38,400 to 416,000)	165,000 (34,900 to 372,000)	150,000 (33,900 to 333,000)	118,000 (30,100 to 259,000)	82,100 (22,400 to 176,000)	49,300 (14,500 to 107,000)	26,200 (7,130 to 55,100)	14,000 (3,780 to 30,500)	7,690 (2,280 to 16,700)	3,230 (853 to 7,040)	883 (168 to 2,060)	2,080,000
Tension-type headache	0 (0 to 0)	0 (0 to 0)	0 (0 to 0)	0 (0 to 0)	2,050 (47·3 to 13,000)	8,370 (614 to 43,500)	13,400 (2,170 to 59,300)	15,800 (3,030 to 64,400)	19,300 (3,800 to 76,900)	21,400 (4,820 to 75,600)	24,500 (6,200 to 82,200)	23,200 (6,430 to 70,500)	23,000 (7,170 to 66,800)	22,900 (6,530 to 65,500)	22,000 (6,640 to 64,500)	20,200 (5,760 to 63,800)	15,800 (4,460 to 52,800)	10,600 (2,760 to 37,000)	5,890 (1,360 to 22,200)	3,190 (740 to 12,300)	1,840 (415 to 7,100)	833 (152 to 3,350)	286 (24·2 to 1,440)	254,000
Other neurological disorders	0 (0 to 0)	0 (0 to 0)	4,380 (3,730 to 5,260)	6,560 (4,720 to 9,150)	7,270 (4,550 to 11,100)	10,900 (7,020 to 16,600)	12,900 (9,840 to 17,200)	11,800 (9,770 to 14,500)	9,700 (8,040 to 11,700)	8,880 (7,440 to 10,500)	10,700 (9,180 to 12,500)	12,400 (10,800 to 14,300)	17,300 (15,200 to 19,600)	19,000 (17,100 to 21,100)	23,100 (21,100 to 25,300)	23,700 (21,900 to 25,900)	23,000 (21,300 to 24,800)	21,300 (19,800 to 23,100)	17,200 (15,800 to 18,600)	11,900 (10,700 to 13,000)	6,840 (5,910 to 7,580)	3,040 (2,540 to 3,400)	1,150 (908 to 1,410)	263,000
Mental disorders	98·7 (65 to 141)	296 (195 to 422)	4,810 (3,180 to 6,860)	24,500 (16,500 to 34,300)	90,800 (62,300 to 129,000)	329,000 (227,000 to 461,000)	585,000 (413,000 to 805,000)	660,000 (469,000 to 885,000)	735,000 (533,000 to 979,000)	711,000 (515,000 to 945,000)	681,000 (503,000 to 881,000)	592,000 (441,000 to 765,000)	575,000 (430,000 to 736,000)	527,000 (393,000 to 680,000)	511,000 (383,000 to 662,000)	428,000 (320,000 to 549,000)	327,000 (246,000 to 415,000)	231,000 (174,000 to 292,000)	142,000 (106,000 to 179,000)	82,200 (61,600 to 104,000)	47,400 (36,000 to 60,000)	23,200 (17,000 to 29,900)	8,570 (6,030 to 11,300)	7,310,000
Schizophrenia	0 (0 to 0)	0 (0 to 0)	0 (0 to 0)	0 (0 to 0)	0 (0 to 0)	1,350 (659 to 2,320)	13,300 (8,330 to 19,700)	56,100 (36,500 to 82,200)	101,000 (68,000 to 140,000)	118,000 (81,700 to 158,000)	122,000 (84,500 to 161,000)	109,000 (78,100 to 140,000)	107,000 (76,600 to 135,000)	98,100 (71,200 to 125,000)	91,200 (65,800 to 116,000)	71,100 (51,400 to 89,700)	48,400 (35,300 to 61,700)	29,500 (21,400 to 38,100)	15,100 (11,000 to 19,500)	7,060 (5,130 to 9,190)	3,160 (2,290 to 4,150)	1,170 (816 to 1,560)	322 (207 to 447)	993,000
Depressive disorders	0 (0 to 0)	0 (0 to 0)	0 (0 to 0)	36·9 (15·9 to 76·6)	3,840 (1,910 to 6,740)	101,000 (61,900 to 159,000)	265,000 (172,000 to 392,000)	296,000 (189,000 to 440,000)	286,000 (185,000 to 408,000)	252,000 (162,000 to 367,000)	232,000 (158,000 to 319,000)	199,000 (135,000 to 281,000)	193,000 (133,000 to 267,000)	179,000 (124,000 to 247,000)	173,000 (121,000 to 242,000)	147,000 (101,000 to 206,000)	116,000 (80,600 to 159,000)	85,400 (60,200 to 115,000)	54,700 (37,400 to 75,400)	33,500 (22,500 to 46,200)	20,700 (14,400 to 27,800)	10,900 (7,430 to 15,200)	4,350 (2,690 to 6,440)	2,650,000
Major depressive disorder	0 (0 to 0)	0 (0 to 0)	0 (0 to 0)	29·3 (10·7 to 68·8)	3,190 (1,360 to 5,940)	95,400 (57,000 to 151,000)	248,000 (157,000 to 368,000)	267,000 (165,000 to 410,000)	248,000 (157,000 to 360,000)	210,000 (130,000 to 317,000)	188,000 (125,000 to 261,000)	157,000 (104,000 to 226,000)	152,000 (102,000 to 212,000)	142,000 (96,800 to 195,000)	139,000 (94,700 to 197,000)	120,000 (79,100 to 171,000)	96,700 (65,200 to 133,000)	71,800 (50,000 to 98,300)	46,500 (30,500 to 65,200)	28,600 (18,700 to 40,400)	17,800 (12,200 to 24,400)	9,380 (6,170 to 13,300)	3,720 (2,220 to 5,680)	2,240,000
Dysthymia	0 (0 to 0)	0 (0 to 0)	0 (0 to 0)	7·7 (2 to 18·3)	648 (297 to 1,210)	5,700 (3,000 to 9,580)	17,200 (9,900 to 27,600)	28,700 (17,200 to 45,400)	38,600 (22,900 to 59,100)	42,000 (26,000 to 63,500)	44,300 (28,600 to 67,100)	41,800 (26,800 to 63,100)	41,300 (26,200 to 63,100)	37,400 (23,800 to 57,300)	34,400 (22,100 to 52,700)	26,900 (16,400 to 40,600)	19,600 (11,800 to 30,200)	13,600 (8,390 to 20,500)	8,250 (5,340 to 12,200)	4,820 (2,970 to 7,300)	2,930 (1,760 to 4,300)	1,560 (986 to 2,300)	632 (367 to 1,040)	410,000
Bipolar disorder	0 (0 to 0)	0 (0 to 0)	0 (0 to 0)	0 (0 to 0)	0 (0 to 0)	15,200 (9,410 to 22,600)	48,900 (30,400 to 73,300)	44,400 (27,600 to 66,800)	39,900 (25,000 to 60,300)	36,700 (23,400 to 55,100)	36,200 (22,900 to 54,000)	34,000 (21,800 to 49,600)	35,200 (22,400 to 51,300)	34,300 (22,000 to 50,600)	34,800 (22,500 to 52,300)	30,300 (19,700 to 44,100)	22,800 (14,700 to 33,200)	15,200 (9,790 to 22,100)	8,490 (5,500 to 12,100)	4,290 (2,760 to 6,110)	2,120 (1,350 to 3,070)	887 (560 to 1,280)	281 (173 to 432)	444,000
Anxiety disorders	0 (0 to 0)	0 (0 to 0)	0 (0 to 0)	1,530 (853 to 2,490)	23,300 (13,900 to 36,700)	69,700 (43,900 to 103,000)	114,000 (76,400 to 164,000)	150,000 (95,200 to 221,000)	180,000 (115,000 to 265,000)	181,000 (119,000 to 257,000)	180,000 (120,000 to 253,000)	162,000 (109,000 to 230,000)	160,000 (106,000 to 230,000)	149,000 (102,000 to 210,000)	144,000 (99,600 to 197,000)	119,000 (78,500 to 168,000)	90,900 (59,500 to 129,000)	64,500 (42,800 to 91,700)	39,800 (27,200 to 56,100)	22,900 (15,400 to 32,300)	12,600 (8,380 to 17,900)	5,690 (3,730 to 8,090)	1,840 (1,170 to 2,650)	1,870,000
Eating disorders	0 (0 to 0)	0 (0 to 0)	0 (0 to 0)	0 (0 to 0)	554 (206 to 1,120)	10,800 (5,720 to 17,800)	37,000 (19,700 to 65,300)	49,100 (27,700 to 80,400)	51,300 (30,600 to 80,300)	45,900 (27,400 to 69,200)	35,100 (20,600 to 54,000)	19,800 (11,700 to 30,900)	11,800 (7,070 to 18,500)	0 (0 to 0)	0 (0 to 0)	0 (0 to 0)	0 (0 to 0)	0 (0 to 0)	0 (0 to 0)	0 (0 to 0)	0 (0 to 0)	0 (0 to 0)	0 (0 to 0)	261,000
Anorexia nervosa	0 (0 to 0)	0 (0 to 0)	0 (0 to 0)	0 (0 to 0)	478 (144 to 1,040)	4,910 (2,650 to 7,940)	14,700 (7,950 to 25,100)	16,600 (9,190 to 28,000)	15,000 (8,800 to 24,300)	10,600 (6,260 to 17,000)	7,690 (4,450 to 12,400)	5,320 (3,200 to 8,470)	3,960 (2,480 to 6,140)	0 (0 to 0)	0 (0 to 0)	0 (0 to 0)	0 (0 to 0)	0 (0 to 0)	0 (0 to 0)	0 (0 to 0)	0 (0 to 0)	0 (0 to 0)	0 (0 to 0)	79,200
Bulimia nervosa	0 (0 to 0)	0 (0 to 0)	0 (0 to 0)	0 (0 to 0)	76·6 (22·5 to 178)	5,860 (2,340 to 11,700)	22,400 (9,770 to 46,700)	32,500 (15,200 to 59,600)	36,200 (19,000 to 61,100)	35,400 (19,400 to 56,800)	27,400 (15,100 to 44,100)	14,400 (7,910 to 24,500)	7,870 (4,150 to 13,600)	0 (0 to 0)	0 (0 to 0)	0 (0 to 0)	0 (0 to 0)	0 (0 to 0)	0 (0 to 0)	0 (0 to 0)	0 (0 to 0)	0 (0 to 0)	0 (0 to 0)	182,000
Autism spectrum disorders	91·7 (59·8 to 131)	273 (177 to 389)	4,370 (2,840 to 6,250)	18,800 (12,400 to 26,900)	23,700 (15,400 to 34,200)	24,800 (16,100 to 36,000)	24,000 (15,700 to 34,300)	22,700 (14,900 to 32,400)	22,900 (15,200 to 32,700)	21,100 (13,900 to 30,000)	19,800 (13,100 to 28,300)	17,300 (11,400 to 24,900)	17,100 (11,300 to 24,300)	16,400 (10,800 to 23,200)	16,400 (11,000 to 23,400)	14,400 (9,570 to 20,300)	11,400 (7,580 to 16,100)	8,340 (5,740 to 11,900)	5,270 (3,610 to 7,430)	3,110 (2,140 to 4,360)	1,740 (1,200 to 2,450)	800 (549 to 1,130)	274 (190 to 386)	295,000
Attention-deficit/hyperactivity disorder	0 (0 to 0)	0 (0 to 0)	0 (0 to 0)	997 (515 to 1,780)	10,700 (5,580 to 19,000)	15,300 (8,130 to 27,200)	11,700 (6,290 to 20,900)	7,740 (4,110 to 13,700)	5,700 (3,020 to 10,100)	4,100 (2,110 to 7,180)	3,100 (1,500 to 5,420)	2,220 (1,100 to 4,010)	1,820 (884 to 3,240)	1,390 (659 to 2,480)	983 (468 to 1,750)	515 (244 to 960)	213 (84·1 to 442)	73·4 (18·9 to 178)	17·4 (2·9 to 50·9)	0·9 (0·1 to 3·4)	0 (0 to 0·1)	0 (0 to 0)	0 (0 to 0)	66,500
Conduct disorder	0 (0 to 0)	0 (0 to 0)	0 (0 to 0)	0 (0 to 0)	23,700 (12,300 to 42,100)	83,200 (45,100 to 138,000)	53,600 (28,500 to 87,900)	0 (0 to 0)	0 (0 to 0)	0 (0 to 0)	0 (0 to 0)	0 (0 to 0)	0 (0 to 0)	0 (0 to 0)	0 (0 to 0)	0 (0 to 0)	0 (0 to 0)	0 (0 to 0)	0 (0 to 0)	0 (0 to 0)	0 (0 to 0)	0 (0 to 0)	0 (0 to 0)	161,000
Idiopathic developmental intellectual disability	7 (0·4 to 19·5)	22·4 (1·1 to 59·8)	438 (21·5 to 1,060)	3,130 (687 to 6,110)	5,120 (1,770 to 9,210)	5,540 (1,960 to 9,890)	5,430 (1,940 to 9,700)	5,260 (1,880 to 9,280)	5,290 (1,880 to 9,360)	4,860 (1,710 to 8,640)	4,580 (1,610 to 8,060)	4,040 (1,560 to 7,080)	4,090 (1,590 to 7,030)	3,970 (1,610 to 6,780)	3,940 (1,630 to 6,720)	3,310 (1,390 to 5,630)	2,460 (1,000 to 4,230)	1,680 (723 to 2,900)	1,020 (455 to 1,740)	615 (296 to 1,010)	328 (162 to 537)	139 (71·5 to 223)	46·2 (23·6 to 74·9)	65,300
Other mental disorders	0 (0 to 0)	0 (0 to 0)	0 (0 to 0)	0 (0 to 0)	0 (0 to 0)	1,780 (978 to 2,900)	12,000 (6,680 to 18,900)	28,600 (16,000 to 44,500)	42,900 (25,100 to 65,500)	47,000 (28,900 to 71,100)	48,400 (30,500 to 73,400)	44,500 (28,200 to 66,700)	45,700 (29,000 to 68,500)	45,100 (28,900 to 67,100)	46,500 (30,100 to 68,500)	41,900 (27,200 to 61,100)	34,100 (22,200 to 49,400)	25,800 (17,200 to 37,200)	17,100 (11,500 to 24,500)	10,800 (7,280 to 15,300)	6,730 (4,500 to 9,520)	3,570 (2,390 to 5,010)	1,450 (968 to 2,050)	504,000
Substance use disorders	0·3 (0·1 to 0·6)	1 (0·4 to 1·9)	17·9 (7·5 to 34·7)	171 (85·8 to 288)	252 (131 to 424)	5,920 (3,600 to 9,350)	285,000 (212,000 to 369,000)	1,000,000 (766,000 to 1,270,000)	1,320,000 (1,050,000 to 1,620,000)	1,120,000 (923,000 to 1,320,000)	885,000 (753,000 to 1,030,000)	668,000 (580,000 to 766,000)	620,000 (545,000 to 710,000)	524,000 (459,000 to 601,000)	425,000 (370,000 to 486,000)	253,000 (215,000 to 295,000)	139,000 (116,000 to 164,000)	73,300 (59,100 to 90,000)	37,700 (30,400 to 46,000)	20,600 (16,300 to 25,400)	12,300 (9,730 to 15,500)	6,650 (4,990 to 8,600)	3,050 (2,220 to 4,140)	7,400,000
[illegible]																								

SR Table 2c: Latin America and Caribbean DALYS by cause and age group for both sexes with 95% uncertainty intervals, 2019

Cause	Early Neonatal	Late Neonatal	Post Neonatal	1 to 4	5 to 9	10 to 14	15 to 19	20 to 24	25 to 29	30 to 34	35 to 39	40 to 44	45 to 49	50 to 54	55 to 59	60 to 64	65 to 69	70 to 74	75 to 79	80 to 84	85 to 89	90 to 94	95 plus	Total
All causes	6,310,000 (5.020.000 to 7.820.000)	1,960,000 (1.580.000 to 2.410.000)	5,680,000 (4.490.000 to 7.150.000)	3,950,000 (3.290.000 to 4.660.000)	3,100,000 (2.540.000 to 3.760.000)	3,860,000 (3.110.000 to 4.780.000)	6,650,000 (5.650.000 to 7.870.000)	8,660,000 (7.540.000 to 10.000.000)	9,040,000 (7.820.000 to 10.500.000)	9,300,000 (8.020.000 to 10.800.000)	9,830,000 (8.530.000 to 11.500.000)	10,100,000 (8.790.000 to 11.700.000)	10,500,000 (9.200.000 to 12.100.000)	11,400,000 (10.100.000 to 13.100.000)	12,000,000 (10.700.000 to 13.600.000)	11,900,000 (10.600.000 to 13.400.000)	11,000,000 (9.910.000 to 12.300.000)	9,760,000 (8.860.000 to 10.800.000)	7,990,000 (7.310.000 to 8.780.000)	6,150,000 (5.650.000 to 6.700.000)	3,920,000 (3.630.000 to 4.230.000)	1,910,000 (1.780.000 to 2.050.000)	788,000 (742.000 to 837.000)	166.000.000
Communicable, maternal, neonatal, and nutritional diseases	5,020,000 (3.950.000 to 6.270.000)	1,460,000 (1.160.000 to 1.810.000)	3,150,000 (2.480.000 to 3.940.000)	1,670,000 (1.400.000 to 1.970.000)	876,000 (701.000 to 1.090.000)	632,000 (509.000 to 780.000)	698,000 (582.000 to 839.000)	835,000 (721.000 to 972.000)	973,000 (856.000 to 1.110.000)	1,040,000 (922.000 to 1.190.000)	1,090,000 (969.000 to 1.240.000)	1,000,000 (894.000 to 1.130.000)	896,000 (799.000 to 1.010.000)	808,000 (717.000 to 913.000)	756,000 (677.000 to 849.000)	679,000 (614.000 to 756.000)	616,000 (559.000 to 676.000)	573,000 (522.000 to 633.000)	523,000 (475.000 to 570.000)	454,000 (398.000 to 498.000)	343,000 (288.000 to 379.000)	186,000 (151.000 to 210.000)	85,000 (64.400 to 97.000)	24.400.000
HIV/AIDS and sexually transmitted infections	99,400 (41.700 to 196.000)	31,400 (12.700 to 61.700)	82,300 (55.800 to 125.000)	48,600 (33.900 to 126.000)	24,800 (20.800 to 30.000)	23,500 (20.400 to 26.800)	50,400 (42.300 to 60.400)	128,000 (117.000 to 144.000)	252,000 (228.000 to 288.000)	324,000 (294.000 to 368.000)	347,000 (319.000 to 386.000)	306,000 (285.000 to 337.000)	250,000 (233.000 to 270.000)	166,000 (155.000 to 179.000)	115,000 (107.000 to 125.000)	68,400 (63.100 to 75.500)	42,800 (38.900 to 48.200)	13,600 (11.600 to 16.600)	6,250 (5.250 to 7.650)	2,340 (1.890 to 3.090)	1,110 (879 to 1.480)	388 (300 to 528)	161 (132 to 206)	2.380.000
HIV/AIDS	3·9 (2·6 to 6·2)	13·1 (8·7 to 20·8)	40,200 (36.000 to 57.900)	47,000 (32.400 to 122.000)	24,000 (20.100 to 29.000)	22,300 (19.300 to 25.600)	45,900 (38.300 to 55.400)	118,000 (109.000 to 131.000)	236,000 (215.000 to 268.000)	305,000 (280.000 to 345.000)	328,000 (304.000 to 360.000)	288,000 (270.000 to 314.000)	236,000 (222.000 to 254.000)	157,000 (148.000 to 166.000)	108,000 (102.000 to 115.000)	63,600 (59.200 to 69.200)	39,100 (36.100 to 43.100)	11,000 (9.580 to 13.000)	4,550 (3.980 to 5.240)	1,260 (1.130 to 1.400)	495 (431 to 564)	160 (135 to 189)	84·3 (73·9 to 95·2)	2.070.000
HIV/AIDS - Drug-susceptible Tuberculosis	0·1 (0 to 0·1)	0·6 (0·4 to 0·9)	4,410 (2.750 to 6.680)	4,280 (2.650 to 6.570)	2,500 (1.610 to 3.770)	2,170 (1.400 to 3.240)	4,340 (2.850 to 6.440)	12,100 (8.030 to 17.700)	23,900 (15.700 to 35.000)	30,200 (19.800 to 44.400)	32,500 (21.200 to 47.900)	28,600 (18.700 to 42.600)	23,200 (15.100 to 34.400)	15,300 (10.000 to 22.700)	10,500 (6.870 to 15.400)	6,140 (4.100 to 8.980)	3,760 (2.500 to 5.500)	1,010 (707 to 1.430)	420 (297 to 592)	119 (82·3 to 170)	44·9 (31·7 to 63·3)	14·4 (10·4 to 19·9)	7·4 (5·2 to 10·4)	205.000
HIV/AIDS - Multidrug-resistant Tuberculosis wit	0 (0 to 0)	0 (0 to 0·1)	348 (110 to 873)	295 (98·3 to 680)	156 (54·3 to 370)	131 (43·9 to 323)	288 (101 to 690)	952 (345 to 2.230)	1,890 (685 to 4.400)	2,300 (816 to 5.540)	2,350 (794 to 5.850)	1,970 (658 to 4.800)	1,570 (510 to 3.830)	1,000 (328 to 2.470)	702 (223 to 1.730)	417 (138 to 1.010)	269 (90·9 to 650)	62·2 (22·3 to 146)	25·9 (9·5 to 59·8)	8·3 (3 to 19·6)	3·2 (1·3 to 7·2)	1·2 (0·5 to 2·5)	0·7 (0·3 to 1·4)	14.700
HIV/AIDS - Extensively drug-resistant Tuberculo	0 (0 to 0)	0 (0 to 0)	57·5 (17·5 to 140)	48·6 (15·4 to 110)	25·5 (8·6 to 58·6)	21·5 (6·8 to 50·9)	46·8 (15·5 to 113)	154 (52 to 351)	306 (103 to 701)	374 (125 to 850)	382 (123 to 902)	322 (102 to 770)	255 (80·2 to 612)	163 (51·2 to 390)	114 (34·6 to 273)	66·9 (21 to 160)	43 (13·7 to 103)	9·5 (3·2 to 22·4)	4 (1·4 to 9·3)	1·3 (0·4 to 3·1)	0·5 (0·2 to 1·1)	0·2 (0·1 to 0·4)	0·1 (0 to 0·2)	2.390
HIV/AIDS resulting in other diseases	3·9 (2·5 to 6·1)	12·5 (8·1 to 20·1)	35,400 (30.300 to 53.600)	42,400 (28.000 to 117.000)	21,300 (17.400 to 26.300)	20,000 (16.800 to 23.400)	41,200 (33.300 to 51.000)	105,000 (93.500 to 119.000)	210,000 (186.000 to 242.000)	272,000 (243.000 to 312.000)	293,000 (263.000 to 328.000)	257,000 (235.000 to 285.000)	210,000 (193.000 to 231.000)	140,000 (128.000 to 151.000)	96,600 (88.100 to 104.000)	56,900 (51.800 to 63.200)	35,000 (31.400 to 39.500)	9,930 (8.470 to 12.000)	4,100 (3.500 to 4.790)	1,130 (996 to 1.280)	447 (381 to 517)	144 (119 to 173)	76·2 (65 to 87)	1.850.000
Sexually transmitted infections excluding HIV	99,400 (41.700 to 196.000)	31,300 (12.700 to 61.700)	42,100 (17.800 to 83.000)	1,630 (641 to 3.350)	768 (289 to 1.540)	1,190 (914 to 1.590)	4,490 (3.090 to 6.640)	9,960 (6.240 to 16.500)	15,300 (8.870 to 26.500)	18,600 (10.600 to 32.700)	19,000 (10.700 to 33.200)	17,600 (9.860 to 32.500)	14,200 (7.980 to 25.600)	9,290 (4.880 to 18.000)	6,700 (3.530 to 12.800)	4,800 (2.600 to 9.220)	3,640 (2.080 to 6.670)	2,580 (1.550 to 4.640)	1,690 (1.040 to 2.980)	1,080 (699 to 1.810)	612 (417 to 978)	228 (151 to 363)	76·8 (52·2 to 120)	306.000
Syphilis	99,400 (41.700 to 196.000)	31,300 (12.700 to 61.700)	42,100 (17.800 to 83.000)	1,630 (641 to 3.350)	768 (289 to 1.540)	370 (303 to 457)	463 (373 to 590)	430 (308 to 637)	726 (563 to 985)	873 (711 to 1.110)	919 (720 to 1.250)	615 (483 to 778)	695 (572 to 867)	648 (509 to 833)	512 (395 to 671)	511 (396 to 662)	455 (356 to 592)	381 (302 to 467)	221 (176 to 271)	175 (144 to 208)	96·4 (78·4 to 117)	45·8 (36·6 to 55·4)	16·8 (13 to 20·8)	183.000
Chlamydial infection	0 (0 to 0)	0 (0 to 0)	0 (0 to 0)	0 (0 to 0)	0 (0 to 0)	81 (59·8 to 110)	442 (308 to 641)	1,330 (922 to 1.920)	1,890 (1.190 to 2.890)	2,090 (1.290 to 3.330)	2,210 (1.410 to 3.340)	2,070 (1.340 to 3.100)	1,380 (922 to 2.120)	481 (345 to 667)	249 (200 to 314)	151 (133 to 174)	86·4 (76·5 to 97)	88·1 (77·4 to 101)	68·3 (59 to 77·6)	61·8 (51·3 to 70·6)	39·2 (31·9 to 45·7)	12 (9·1 to 14·3)	4·9 (3·6 to 6·2)	12.700
Gonococcal infection	0 (0 to 0)	0 (0 to 0)	0 (0 to 0)	0 (0 to 0)	0 (0 to 0)	151 (107 to 222)	832 (615 to 1.150)	1,390 (1.040 to 1.840)	1,250 (974 to 1.600)	1,240 (1.000 to 1.540)	1,210 (999 to 1.510)	1,390 (1.180 to 1.680)	1,250 (1.080 to 1.460)	634 (548 to 743)	441 (389 to 504)	396 (352 to 454)	482 (427 to 540)	322 (284 to 364)	256 (222 to 289)	147 (123 to 168)	106 (86·1 to 123)	31·1 (23·6 to 36·9)	8·8 (6·4 to 11·1)	11.500
Trichomoniasis	0 (0 to 0)	0 (0 to 0)	0 (0 to 0)	0 (0 to 0)	0 (0 to 0)	26·3 (7·5 to 68)	507 (158 to 1.220)	2,660 (931 to 6.220)	5,290 (1.770 to 12.300)	6,090 (2.020 to 14.200)	5,790 (1.910 to 13.300)	5,170 (1.770 to 12.100)	4,320 (1.390 to 9.840)	3,240 (1.020 to 7.590)	2,120 (690 to 4.920)	1,230 (418 to 2.910)	813 (288 to 1.870)	524 (196 to 1.200)	308 (120 to 690)	166 (63·7 to 372)	70·4 (27·6 to 157)	20·8 (7·7 to 45·8)	3·6 (1·2 to 8·6)	38.300
Genital herpes	0 (0 to 0)	0 (0 to 0)	0 (0 to 0)	0 (0 to 0)	0 (0 to 0)	202 (66·4 to 487)	905 (305 to 2.200)	1,990 (665 to 4.840)	3,130 (1.070 to 7.530)	3,990 (1.340 to 9.710)	4,350 (1.400 to 10.600)	4,130 (1.330 to 10.300)	3,710 (1.170 to 9.340)	3,310 (1.030 to 8.090)	2,780 (853 to 6.740)	2,150 (652 to 5.240)	1,570 (470 to 3.840)	1,090 (328 to 2.690)	697 (211 to 1.710)	423 (126 to 1.040)	215 (65·2 to 522)	85·7 (25·9 to 208)	28·3 (8·6 to 69·1)	34.700
[illegible]	[illegible]	[illegible]	[illegible]	[illegible]	[illegible]	[illegible]	[illegible]	[illegible]	[illegible]	[illegible]	[illegible]	[illegible]	[illegible]	[illegible]	[illegible]	[illegible]	[illegible]	[illegible]	[illegible]	[illegible]	[illegible]	[illegible]	[illegible]	[illegible]

Multiple sclerosis	0 (0 to 0)	0 (0 to 0)	0 (0 to 0)	0 (0 to 0)	[illegible]
Motor neuron disease	63·4 (49·5 to 80)	89·1 (62·1 to 131)	3,250 (2,340 to 4,290)	1,770 (1,300 to 2,340)	[illegible]
Headache disorders	0 (0 to 0)	0 (0 to 0)	0 (0 to 0)	0 (0 to 0)	[illegible]
Migraine	0 (0 to 0)	0 (0 to 0)	0 (0 to 0)	0 (0 to 0)	[illegible]
Tension-type headache	0 (0 to 0)	0 (0 to 0)	0 (0 to 0)	0 (0 to 0)	[illegible]
Other neurological disorders	0 (0 to 0)	0 (0 to 0)	16,400 (12,400 to 21,200)	28,500 (20,000 to 38,700)	[illegible]
Mental disorders	143 (94·6 to 207)	429 (281 to 621)	7,220 (4,710 to 10,500)	40,300 (26,900 to 56,600)	[illegible]
Schizophrenia	0 (0 to 0)	0 (0 to 0)	0 (0 to 0)	0 (0 to 0)	[illegible]
Depressive disorders	0 (0 to 0)	0 (0 to 0)	0 (0 to 0)	116 (48·4 to 255)	[illegible]
Major depressive disorder	0 (0 to 0)	0 (0 to 0)	0 (0 to 0)	99·8 (35·9 to 242)	[illegible]
Dysthymia	0 (0 to 0)	0 (0 to 0)	0 (0 to 0)	16·6 (3·9 to 41·6)	[illegible]
Bipolar disorder	0 (0 to 0)	0 (0 to 0)	0 (0 to 0)	0 (0 to 0)	[illegible]
Anxiety disorders	0 (0 to 0)	0 (0 to 0)	0 (0 to 0)	5,150 (2,970 to 8,160)	[illegible]
Eating disorders	0 (0 to 0)	0 (0 to 0)	0 (0 to 0)	0 (0 to 0)	[illegible]
Anorexia nervosa	0 (0 to 0)	0 (0 to 0)	0 (0 to 0)	0 (0 to 0)	[illegible]
Bulimia nervosa	0 (0 to 0)	0 (0 to 0)	0 (0 to 0)	0 (0 to 0)	[illegible]
Autism spectrum disorders	128 (83·8 to 185)	378 (248 to 547)	6,090 (3,980 to 8,890)	26,000 (16,900 to 37,600)	[illegible]
Attention-deficit/hyperactivity disorder	0 (0 to 0)	0 (0 to 0)	0 (0 to 0)	1,880 (1,010 to 3,400)	[illegible]
Conduct disorder	0 (0 to 0)	0 (0 to 0)	0 (0 to 0)	0 (0 to 0)	[illegible]
Idiopathic developmental intellectual disability	15 (1·4 to 40)	50·3 (3·9 to 128)	1,130 (107 to 2,550)	7,240 (1,950 to 13,900)	[illegible]
Other mental disorders	0 (0 to 0)	0 (0 to 0)	0 (0 to 0)	0 (0 to 0)	[illegible]
Substance use disorders	0·1 (0·1 to 0·2)	0·3 (0·2 to 0·6)	6·3 (3·2 to 10·9)	39·8 (21·8 to 64·1)	[illegible]
Alcohol use disorders	0·1 (0·1 to 0·2)	0·3 (0·2 to 0·6)	6·3 (3·2 to 10·9)	39·8 (21·8 to 64·1)	[illegible]
Drug use disorders	0 (0 to 0)	0 (0 to 0)	0 (0 to 0)	0 (0 to 0)	[illegible]
Opioid use disorders	0 (0 to 0)	0 (0 to 0)	0 (0 to 0)	0 (0 to 0)	[illegible]
Cocaine use disorders	0 (0 to 0)	0 (0 to 0)	0 (0 to 0)	0 (0 to 0)	[illegible]
Amphetamine use disorders	0 (0 to 0)	0 (0 to 0)	0 (0 to 0)	0 (0 to 0)	[illegible]
Cannabis use disorders	0 (0 to 0)	0 (0 to 0)	0 (0 to 0)	0 (0 to 0)	[illegible]
Other drug use disorders	0 (0 to 0)	0 (0 to 0)	0 (0 to 0)	0 (0 to 0)	[illegible]
Diabetes and kidney diseases	119 (89·7 to 167)	365 (268 to 521)	34,800 (26,000 to 45,400)	41,900 (32,900 to 51,500)	[illegible]
Diabetes mellitus	118 (89·4 to 167)	359 (262 to 516)	7,770 (5,190 to 10,800)	6,780 (5,030 to 8,950)	[illegible]
Diabetes mellitus type 1	118 (89·4 to 167)	359 (262 to 516)	7,770 (5,190 to 10,800)	6,780 (5,030 to 8,950)	[illegible]
Diabetes mellitus type 2	0 (0 to 0)	0 (0 to 0)	0 (0 to 0)	0 (0 to 0)	[illegible]
Chronic kidney disease	0·3 (0·2 to 0·6)	5·9 (3·1 to 10·2)	26,700 (19,900 to 34,600)	34,400 (26,900 to 42,900)	[illegible]
Chronic kidney disease due to diabetes mellitu[illegible]	0 (0 to 0)	0 (0 to 0)	199 (101 to 374)	597 (364 to 932)	[illegible]
Chronic kidney disease due to diabetes mellitu[illegible]	0 (0 to 0)	0 (0 to 0)	0 (0 to 0)	0 (0 to 0)	[illegible]
Chronic kidney disease due to hypertension	0 (0 to 0)	0 (0 to 0)	0 (0 to 0)	0 (0 to 0)	[illegible]
Chronic kidney disease due to glomerulonephri[illegible]	0·1 (0 to 0·2)	1·9 (1 to 3·4)	8,160 (4,450 to 13,400)	11,500 (7,060 to 16,900)	[illegible]
Chronic kidney disease due to other and unspe[illegible]	0·2 (0·1 to 0·4)	3·9 (2·1 to 6·8)	18,400 (12,600 to 25,200)	22,300 (16,500 to 29,300)	[illegible]
Acute glomerulonephritis	0 (0 to 0)	0·1 (0 to 0·1)	300 (218 to 400)	647 (472 to 894)	[illegible]
Skin and subcutaneous diseases	14·1 (8·2 to 23·1)	184 (108 to 302)	30,500 (21,600 to 42,600)	260,000 (165,000 to 388,000)	[illegible]
Dermatitis	0 (0 to 0)	0 (0 to 0)	0 (0 to 0)	85,900 (45,900 to 144,000)	[illegible]
Atopic dermatitis	0 (0 to 0)	0 (0 to 0)	0 (0 to 0)	85,100 (45,500 to 143,000)	[illegible]
Contact dermatitis	0 (0 to 0)	0 (0 to 0)	0 (0 to 0)	0 (0 to 0)	[illegible]
Seborrhoeic dermatitis	0 (0 to 0)	0 (0 to 0)	0 (0 to 0)	800 (433 to 1,310)	[illegible]
Psoriasis	0 (0 to 0)	0 (0 to 0)	0 (0 to 0)	3,900 (2,690 to 5,330)	[illegible]
Bacterial skin diseases	1·7 (0·8 to 3·1)	17·2 (8·8 to 32)	13,200 (8,600 to 21,200)	9,900 (6,940 to 15,000)	[illegible]
Cellulitis	0·4 (0·3 to 0·7)	4·8 (3·1 to 7·1)	2,820 (1,560 to 4,630)	2,820 (1,920 to 4,030)	[illegible]
Pyoderma	1·2 (0·5 to 2·5)	12·4 (5 to 25·7)	10,400 (6,500 to 17,800)	7,090 (4,710 to 10,900)	[illegible]
Scabies	6·3 (3·2 to 11)	83·4 (42·7 to 143)	7,430 (3,870 to 12,900)	49,500 (25,200 to 87,700)	[illegible]
Fungal skin diseases	0·9 (0·3 to 1·8)	11·3 (4·3 to 23·6)	1,040 (390 to 2,220)	7,750 (2,890 to 17,000)	[illegible]
Viral skin diseases	0 (0 to 0)	0 (0 to 0)	0 (0 to 0)	45,900 (29,100 to 69,600)	[illegible]
Acne vulgaris	0 (0 to 0)	0 (0 to 0)	0 (0 to 0)	0 (0 to 0)	[illegible]
Alopecia areata	0 (0 to 0)	0 (0 to 0)	0 (0 to 0)	897 (562 to 1,360)	[illegible]
Pruritus	0·1 (0 to 0·1)	1 (0·4 to 2·1)	149 (64·1 to 291)	1,750 (756 to 3,520)	[illegible]
Urticaria	4·3 (2·5 to 6·8)	58·9 (35 to 92·7)	6,550 (4,120 to 10,100)	43,400 (26,700 to 67,800)	[illegible]
Decubitus ulcer	0 (0 to 0)	0 (0 to 0)	0 (0 to 0)	366 (212 to 764)	[illegible]
Other skin and subcutaneous diseases	0·9 (0·4 to 1·7)	12 (5·7 to 22·5)	2,180 (1,330 to 3,690)	10,600 (5,370 to 19,000)	[illegible]
Sense organ diseases	40·3 (23 to 66·4)	142 (84·8 to 225)	3,040 (1,970 to 4,440)	31,900 (20,900 to 45,600)	[illegible]
Blindness and vision loss	20·2 (9·5 to 38)	62·9 (30·9 to 117)	937 (514 to 1,610)	12,000 (7,180 to 19,100)	[illegible]
Glaucoma	0 (0 to 0)	0 (0 to 0)	0 (0 to 0)	0 (0 to 0)	[illegible]
Cataract	0 (0 to 0)	0 (0 to 0)	0 (0 to 0)	0 (0 to 0)	[illegible]
Age-related macular degeneration	0 (0 to 0)	0 (0 to 0)	0 (0 to 0)	0 (0 to 0)	[illegible]
Refraction disorders	1·3 (0·5 to 2·6)	14·7 (6·6 to 28·6)	670 (349 to 1,160)	10,300 (5,950 to 16,700)	[illegible]
Near vision loss	0 (0 to 0)	0 (0 to 0)	0·8 (0·3 to 2)	24·1 (7·8 to 58·9)	[illegible]
Other vision loss	18·9 (8·9 to 35·5)	48·2 (23·1 to 88·9)	267 (139 to 457)	1,650 (985 to 2,620)	[illegible]
Age-related and other hearing loss	5·8 (2·2 to 13·7)	18·4 (8·1 to 39·2)	932 (517 to 1,490)	14,100 (7,900 to 22,200)	[illegible]
Other sense organ diseases	14·2 (6·7 to 26·3)	60·5 (31·1 to 104)	1,170 (642 to 1,910)	5,870 (3,170 to 9,770)	[illegible]
Musculoskeletal disorders	0 (0 to 0)	0 (0 to 0)	0 (0 to 0)	0 (0 to 0)	[illegible]
Rheumatoid arthritis	0 (0 to 0)	0 (0 to 0)	0 (0 to 0)	0 (0 to 0)	[illegible]
Osteoarthritis	0 (0 to 0)	0 (0 to 0)	0 (0 to 0)	0 (0 to 0)	[illegible]
Osteoarthritis hip	0 (0 to 0)	0 (0 to 0)	0 (0 to 0)	0 (0 to 0)	[illegible]
Osteoarthritis knee	0 (0 to 0)	0 (0 to 0)	0 (0 to 0)	0 (0 to 0)	[illegible]
Osteoarthritis hand	0 (0 to 0)	0 (0 to 0)	0 (0 to 0)	0 (0 to 0)	[illegible]
Osteoarthritis other	0 (0 to 0)	0 (0 to 0)	0 (0 to 0)	0 (0 to 0)	[illegible]
Low back pain	0 (0 to 0)	0 (0 to 0)	0 (0 to 0)	0 (0 to 0)	[illegible]
Neck pain	0 (0 to 0)	0 (0 to 0)	0 (0 to 0)	0 (0 to 0)	[illegible]
Gout	0 (0 to 0)	0 (0 to 0)	0 (0 to 0)	0 (0 to 0)	[illegible]
Other musculoskeletal disorders	0 (0 to 0)	0 (0 to 0)	0 (0 to 0)	0 (0 to 0)	[illegible]
Other non-communicable diseases	1,210,000 (916,000 to 1,580,000)	447,000 (353,000 to 571,000)	1,700,000 (1,300,000 to 2,210,000)	540,000 (429,000 to 667,000)	[illegible]
Congenital birth defects	1,200,000 (905,000 to 1,570,000)	424,000 (332,000 to 544,000)	1,490,000 (1,130,000 to 1,940,000)	403,000 (304,000 to 512,000)	[illegible]
Neural tube defects	153,000 (111,000 to 211,000)	25,900 (17,100 to 40,800)	84,300 (58,300 to 119,000)	71,500 (49,800 to 106,000)	[illegible]
Congenital heart anomalies	402,000 (287,000 to 539,000)	203,000 (151,000 to 264,000)	727,000 (518,000 to 976,000)	146,000 (102,000 to 195,000)	[illegible]
Orofacial clefts	2,910 (2,110 to 3,970)	1,080 (806 to 1,470)	5,180 (3,750 to 7,030)	1,630 (1,150 to 2,260)	[illegible]
Down syndrome	20,300 (15,500 to 26,400)	9,440 (7,280 to 12,100)	90,100 (66,600 to 123,000)	25,200 (19,500 to 32,400)	[illegible]
Turner syndrome	0·8 (0·3 to 1·5)	2·3 (0·8 to 4·4)	35·1 (12·8 to 71·3)	134 (48·9 to 257)	[illegible]
Klinefelter syndrome	0·1 (0 to 0·2)	0·2 (0·1 to 0·5)	3·7 (1·6 to 7·2)	12·5 (5·3 to 24·2)	[illegible]
Other chromosomal abnormalities	47,900 (27,700 to 63,800)	16,200 (10,700 to 21,600)	70,500 (37,700 to 92,800)	13,600 (8,800 to 17,600)	[illegible]
Congenital musculoskeletal and limb anomalie[illegible]	23,200 (17,200 to 30,900)	5,900 (4,550 to 7,730)	32,200 (23,200 to 44,000)	40,000 (24,600 to 59,600)	[illegible]
Urogenital congenital anomalies	54,100 (37,800 to 90,600)	18,000 (13,000 to 29,800)	17,700 (11,900 to 30,900)	6,090 (4,170 to 8,900)	[illegible]
Digestive congenital anomalies	161,000 (114,000 to 225,000)	75,100 (54,800 to 98,900)	184,000 (125,000 to 248,000)	22,800 (14,900 to 30,700)	[illegible]
Other congenital birth defects	333,000 (238,000 to 441,000)	68,800 (50,100 to 89,700)	280,000 (199,000 to 372,000)	76,200 (49,300 to 102,000)	[illegible]
Urinary diseases and male infertility	797 (515 to 1,060)	975 (709 to 1,320)	23,700 (17,500 to 30,500)	13,100 (10,300 to 16,400)	[illegible]
Urolithiasis	0 (0 to 0)	0 (0 to 0)	0 (0 to 0)	154 (95 to 282)	[illegible]
Benign prostatic hyperplasia	0 (0 to 0)	0 (0 to 0)	0 (0 to 0)	0 (0 to 0)	[illegible]
Male infertility	0 (0 to 0)	0 (0 to 0)	0 (0 to 0)	0 (0 to 0)	[illegible]
Other urinary diseases	281 (176 to 375)	330 (223 to 497)	1,030 (709 to 1,540)	1,880 (1,390 to 2,500)	[illegible]
Gynecological diseases	0 (0 to 0)	0 (0 to 0)	0 (0 to 0)	0 (0 to 0)	[illegible]
Uterine fibroids	0 (0 to 0)	0 (0 to 0)	0 (0 to 0)	0 (0 to 0)	[illegible]
Polycystic ovarian syndrome	0 (0 to 0)	0 (0 to 0)	0 (0 to 0)	0 (0 to 0)	[illegible]
Female infertility	0 (0 to 0)	0 (0 to 0)	0 (0 to 0)	0 (0 to 0)	[illegible]
Endometriosis	0 (0 to 0)	0 (0 to 0)	0 (0 to 0)	0 (0 to 0)	[illegible]
Genital prolapse	0 (0 to 0)	0 (0 to 0)	0 (0 to 0)	0 (0 to 0)	[illegible]
Premenstrual syndrome	0 (0 to 0)	0 (0 to 0)	0 (0 to 0)	0 (0 to 0)	[illegible]
Other gynecological diseases	0 (0 to 0)	0 (0 to 0)	0 (0 to 0)	0 (0 to 0)	[illegible]
Hemoglobinopathies and hemolytic anemias	1,070 (833 to 1,380)	1,410 (1,080 to 1,830)	15,000 (11,500 to 19,000)	31,000 (22,800 to 40,100)	[illegible]
Thalassemias	65·8 (33·5 to 108)	59·5 (29·5 to 97·1)	1,470 (832 to 2,250)	2,110 (1,290 to 3,120)	[illegible]
Thalassemias trait	9·9 (5·5 to 17·1)	73·5 (45·7 to 116)	563 (353 to 861)	1,910 (1,170 to 3,020)	[illegible]
Sickle cell disorders	727 (527 to 978)	621 (412 to 931)	8,340 (5,550 to 11,900)	14,600 (8,300 to 22,100)	[illegible]
Sickle cell trait	32·6 (18·8 to 54·1)	284 (175 to 432)	853 (544 to 1,270)	2,980 (1,850 to 4,510)	[illegible]
G6PD deficiency	51 (29·2 to 77·6)	100 (69 to 142)	1,300 (839 to 1,910)	3,110 (2,030 to 4,530)	[illegible]
G6PD trait	0 (0 to 0)	1·1 (0·4 to 2·7)	0·2 (0·1 to 0·3)	0·6 (0·3 to 0·9)	[illegible]
Other hemoglobinopathies and hemolytic anem[illegible]	183 (116 to 266)	272 (187 to 395)	2,480 (1,760 to 3,440)	6,300 (4,260 to 9,160)	[illegible]
Endocrine, metabolic, blood, and immune disorders	9,430 (6,970 to 12,900)	8,180 (6,210 to 11,300)	82,400 (62,200 to 111,000)	70,600 (54,900 to 90,800)	[illegible]
Oral disorders	0 (0 to 0)	0 (0 to 0)	0 (0 to 0)	22,900 (12,800 to 36,800)	[illegible]
Caries of deciduous teeth	0 (0 to 0)	0 (0 to 0)	0 (0 to 0)	7,280 (2,840 to 14,800)	[illegible]
Caries of permanent teeth	0 (0 to 0)	0 (0 to 0)	0 (0 to 0)	0 (0 to 0)	[illegible]
Periodontal diseases	0 (0 to 0)	0 (0 to 0)	0 (0 to 0)	0 (0 to 0)	[illegible]
Other oral disorders	0 (0 to 0)	0 (0 to 0)	0 (0 to 0)	15,600 (9,320 to 23,400)	[illegible]
Sudden infant death syndrome	–	13,200 (10,100 to 17,100)	84,400 (59,800 to 118,000)	–	[illegible]
Injuries	50,900 (39,400 to 66,100)	35,100 (25,700 to 59,400)	458,000 (349,000 to 591,000)	623,000 (496,000 to 769,000)	[illegible]
Transport injuries	4,030 (2,950 to 5,450)	2,850 (2,140 to 3,780)	42,500 (32,000 to 54,900)	143,000 (110,000 to 182,000)	[illegible]
Road injuries	3,580 (2,620 to 4,840)	2,450 (1,860 to 3,220)	39,900 (30,000 to 51,600)	136,000 (105,000 to 174,000)	[illegible]
Pedestrian road injuries	1,690 (1,230 to 2,300)	1,040 (769 to 1,380)	13,500 (9,750 to 17,800)	74,700 (56,700 to 95,700)	[illegible]
Cyclist road injuries	0 (0 to 0)	0 (0 to 0)	0 (0 to 0)	6,030 (4,450 to 8,300)	[illegible]
Motorcyclist road injuries	436 (303 to 645)	330 (232 to 482)	4,370 (3,240 to 5,780)	8,640 (6,460 to 11,500)	[illegible]
Motor vehicle road injuries	1,290 (930 to 1,780)	985 (742 to 1,320)	20,800 (15,800 to 26,900)	43,800 (33,800 to 57,300)	[illegible]
Other road injuries	167 (124 to 226)	101 (76·1 to 135)	1,160 (846 to 1,580)	2,980 (2,240 to 3,840)	[illegible]
Other transport injuries	447 (300 to 682)	399 (272 to 607)	2,620 (1,980 to 3,400)	6,720 (5,220 to 8,620)	[illegible]
Unintentional injuries	29,700 (22,300 to 39,400)	28,300 (20,500 to 53,300)	376,000 (281,000 to 491,000)	411,000 (321,000 to 509,000)	[illegible]
Falls	1,590 (1,180 to 2,100)	863 (678 to 1,110)	16,000 (12,500 to 20,600)	33,000 (25,900 to 41,000)	[illegible]
Drowning	1,670 (1,220 to 2,280)	895 (644 to 1,210)	15,400 (10,500 to 21,900)	142,000 (103,000 to 185,000)	[illegible]
Fire, heat, and hot substances	715 (535 to 967)	771 (544 to 1,050)	13,600 (9,610 to 18,400)	44,000 (30,900 to 57,900)	[illegible]
Poisonings	1,040 (696 to 1,600)	1,020 (653 to 1,890)	5,060 (3,530 to 7,350)	17,200 (12,400 to 23,400)	[illegible]
Poisoning by carbon monoxide	98·1 (60·4 to 166)	110 (60·6 to 242)	795 (545 to 1,170)	3,170 (2,230 to 4,550)	[illegible]
Poisoning by other means	943 (613 to 1,460)	914 (581 to 1,670)	4,270 (2,910 to 6,340)	14,000 (9,940 to 19,500)	[illegible]
Exposure to mechanical forces	2,250 (629 to 8,030)	4,260 (694 to 28,800)	9,050 (6,290 to 15,400)	25,200 (19,200 to 32,500)	[illegible]
Unintentional firearm injuries	1,040 (193 to 4,390)	1,700 (110 to 14,700)	2,270 (912 to 6,990)	5,400 (3,060 to 9,590)	[illegible]
Other exposure to mechanical forces	1,210 (400 to 4,460)	2,560 (507 to 16,600)	6,780 (5,200 to 10,100)	19,800 (15,200 to 25,200)	[illegible]
Adverse effects of medical treatment	4,440 (3,240 to 5,930)	3,170 (2,340 to 4,250)	22,900 (16,900 to 31,000)	15,700 (11,700 to 21,300)	[illegible]
Animal contact	278 (202 to 383)	319 (248 to 402)	3,000 (2,170 to 4,090)	8,880 (7,070 to 11,000)	[illegible]
Venomous animal contact	194 (146 to 265)	251 (190 to 322)	2,170 (1,540 to 3,000)	6,680 (5,310 to 8,350)	[illegible]
Non-venomous animal contact	83·2 (53 to 126)	67·9 (51·7 to 88)	823 (595 to 1,100)	2,210 (1,680 to 2,850)	[illegible]
Foreign body	15,300 (11,400 to 20,200)	15,800 (12,500 to 20,000)	281,000 (208,000 to 372,000)	99,400 (76,000 to 128,000)	[illegible]
Pulmonary aspiration and foreign body in airw[illegible]	15,200 (11,400 to 20,100)	15,700 (12,400 to 19,900)	280,000 (207,000 to 370,000)	95,300 (73,000 to 124,000)	[illegible]
Foreign body in eyes	3·7 (1 to 8·2)	11·1 (3·1 to 24·4)	191 (54·4 to 415)	1,030 (315 to 2,320)	[illegible]
Foreign body in other body part	103 (59·4 to 171)	99·5 (64 to 162)	1,120 (741 to 1,700)	3,040 (2,090 to 4,340)	[illegible]
Environmental heat and cold exposure	310 (121 to 634)	255 (137 to 487)	1,200 (808 to 1,800)	3,120 (2,150 to 4,520)	[illegible]
Exposure to forces of nature	12·1 (11 to 13·3)	33·7 (30·6 to 37)	279 (254 to 307)	1,410 (1,270 to 1,570)	[illegible]
Other unintentional injuries	2,050 (1,420 to 2,960)	876 (621 to 1,270)	8,030 (6,190 to 10,200)	20,900 (16,300 to 26,600)	[illegible]
Self-harm and interpersonal violence	17,200 (13,400 to 22,300)	3,930 (3,020 to 5,090)	39,700 (30,300 to 51,400)	69,600 (54,900 to 87,500)	[illegible]
Self-harm	0 (0 to 0)	0 (0 to 0)	0 (0 to 0)	0 (0 to 0)	[illegible]
Self-harm by firearm	0 (0 to 0)	0 (0 to 0)	0 (0 to 0)	0 (0 to 0)	[illegible]
Self-harm by other specified means	0 (0 to 0)	0 (0 to 0)	0 (0 to 0)	0 (0 to 0)	[illegible]
Interpersonal violence	17,200 (13,400 to 22,300)	3,930 (3,020 to 5,090)	39,400 (30,100 to 51,100)	68,900 (54,200 to 86,500)	[illegible]
Physical violence by firearm	3,090 (2,380 to 3,990)	941 (723 to 1,220)	11,500 (8,590 to 15,100)	20,700 (16,200 to 26,700)	[illegible]
Physical violence by sharp object	2,070 (1,590 to 2,690)	338 (256 to 450)	3,200 (2,410 to 4,160)	5,220 (4,080 to 6,750)	[illegible]
Sexual violence	0 (0 to 0)	0 (0 to 0)	0 (0 to 0)	275 (160 to 440)	[illegible]
Physical violence by other means	12,000 (9,380 to 15,800)	2,650 (2,040 to 3,500)	24,800 (18,900 to 32,000)	42,700 (32,900 to 54,500)	[illegible]
Conflict and terrorism	0 (0 to 0)	0 (0 to 0)	0 (0 to 0)	16·3 (9·4 to 30·1)	[illegible]

SR Table 2c: Caribbean DALYS by cause and age group for both sexes with 95%uncertainty intervals, 2019

Cause	Early Neonatal	Late Neonatal	Post Neonatal	1 to 4	5 to 9	10 to 14	15 to 19	20 to 24	25 to 29	30 to 34	35 to 39	40 to 44	45 to 49	50 to 54	55 to 59	60 to 64	65 to 69	70 to 74	75 to 79	80 to 84	85 to 89	90 to 94	95 plus	Total
All causes	1,030,000 (792,000 to 1,300,000)	364,000 (285,000 to 458,000)	788,000 (596,000 to 1,030,000)	780,000 (660,000 to 912,000)	388,000 (324,000 to 457,000)	369,000 (302,000 to 445,000)	520,000 (437,000 to 622,000)	664,000 (564,000 to 775,000)	740,000 (634,000 to 861,000)	803,000 (690,000 to 930,000)	817,000 (703,000 to 945,000)	862,000 (743,000 to 996,000)	968,000 (829,000 to 1,120,000)	1,100,000 (948,000 to 1,280,000)	1,160,000 (999,000 to 1,340,000)	1,110,000 (974,000 to 1,270,000)	1,060,000 (931,000 to 1,200,000)	987,000 (874,000 to 1,110,000)	800,000 (714,000 to 892,000)	612,000 (551,000 to 673,000)	392,000 (356,000 to 429,000)	197,000 (181,000 to 215,000)	92,900 (86,300 to 99,500)	16,600,000
Communicable, maternal, neonatal, and nutritional diseases	862,000 (653,000 to 1,110,000)	277,000 (212,000 to 353,000)	538,000 (394,000 to 720,000)	388,000 (308,000 to 491,000)	122,000 (97,700 to 150,000)	77,700 (62,800 to 97,200)	88,700 (71,600 to 110,000)	106,000 (89,500 to 124,000)	130,000 (109,000 to 154,000)	152,000 (129,000 to 182,000)	154,000 (130,000 to 184,000)	142,000 (122,000 to 166,000)	122,000 (106,000 to 140,000)	101,000 (88,300 to 115,000)	81,300 (71,000 to 93,200)	65,300 (56,900 to 74,500)	56,100 (48,500 to 64,400)	52,800 (45,300 to 60,800)	47,000 (40,800 to 54,600)	39,600 (34,200 to 45,000)	28,800 (24,300 to 32,900)	15,900 (12,900 to 18,300)	8,250 (6,440 to 9,780)	3,660,000
HIV/AIDS and sexually transmitted infections	48,800 (16,900 to 101,000)	15,400 (5,450 to 32,600)	25,600 (13,400 to 46,400)	18,300 (13,100 to 25,700)	9,970 (6,800 to 13,700)	9,190 (6,530 to 12,300)	17,400 (10,300 to 26,700)	21,500 (15,900 to 30,300)	40,500 (29,100 to 58,600)	61,100 (46,400 to 83,300)	72,400 (56,100 to 94,600)	68,200 (56,600 to 83,000)	59,500 (50,800 to 70,300)	40,900 (35,600 to 46,900)	23,800 (20,500 to 27,400)	12,200 (10,300 to 14,400)	6,620 (5,500 to 7,800)	2,580 (2,060 to 3,160)	1,190 (941 to 1,470)	344 (271 to 433)	157 (124 to 203)	51·9 (39·4 to 69·1)	17·7 (13·4 to 24·4)	556,000
HIV/AIDS	0·8 (0·5 to 1·2)	2·7 (1·8 to 4)	7,540 (5,780 to 10,100)	17,600 (12,500 to 24,800)	9,620 (6,430 to 13,400)	9,010 (6,360 to 12,100)	16,800 (9,800 to 26,100)	20,400 (14,900 to 29,200)	39,100 (27,900 to 57,000)	59,400 (44,700 to 81,600)	70,700 (54,500 to 92,700)	66,600 (55,100 to 81,600)	58,200 (49,600 to 68,900)	40,000 (34,700 to 46,200)	23,100 (19,900 to 26,800)	11,800 (9,930 to 13,800)	6,250 (5,210 to 7,480)	2,340 (1,850 to 2,900)	1,040 (809 to 1,320)	247 (193 to 311)	97·5 (76·4 to 122)	27·3 (21·2 to 34·4)	8·6 (6·9 to 10·7)	460,000
HIV/AIDS - Drug-susceptible Tuberculosis	0 (0 to 0)	0·2 (0·1 to 0·2)	525 (320 to 826)	1,530 (926 to 2,420)	895 (551 to 1,410)	758 (469 to 1,190)	1,220 (754 to 1,910)	1,500 (972 to 2,280)	2,730 (1,770 to 4,200)	4,180 (2,710 to 6,390)	5,260 (3,390 to 8,150)	5,410 (3,410 to 8,370)	4,790 (3,070 to 7,470)	3,450 (2,180 to 5,360)	1,970 (1,260 to 3,030)	1,010 (655 to 1,560)	485 (317 to 740)	206 (137 to 309)	87·3 (58·9 to 131)	20·2 (13·9 to 29·1)	8·3 (5·8 to 11·7)	2·3 (1·6 to 3·2)	0·6 (0·4 to 0·8)	36,000
HIV/AIDS - Multidrug-resistant Tuberculosis without exten	0 (0 to 0)	0 (0 to 0)	6·7 (1·2 to 22·3)	18·2 (2·4 to 68·5)	10·4 (1·4 to 38·6)	8·8 (1·2 to 32·3)	14·5 (2·2 to 51·8)	19·6 (4·2 to 63·2)	35·8 (7·8 to 116)	54·3 (11·8 to 170)	66·5 (14·1 to 214)	68·1 (14·2 to 227)	61·8 (13·1 to 204)	45 (9·4 to 149)	25·9 (5·5 to 84·9)	13·5 (2·9 to 44)	6·7 (1·6 to 20·9)	2·6 (0·6 to 8·5)	1·1 (0·2 to 3·5)	0·3 (0·1 to 0·8)	0·1 (0 to 0·4)	0 (0 to 0·1)	0 (0 to 0)	460
HIV/AIDS - Extensively drug-resistant Tuberculosis	0 (0 to 0)	0 (0 to 0)	1·1 (0·2 to 3·8)	3 (0·4 to 11·5)	1·7 (0·2 to 6·4)	1·4 (0·2 to 5·4)	2·4 (0·3 to 8·5)	3·2 (0·6 to 10·2)	5·8 (1·2 to 18·8)	8·9 (1·8 to 27·3)	10·9 (2·1 to 34·3)	11·2 (2·2 to 35·7)	10·2 (2 to 31·8)	7·4 (1·4 to 23·5)	4·2 (0·8 to 13·4)	2·2 (0·4 to 7)	1·1 (0·2 to 3·2)	0·4 (0·1 to 1·4)	0·2 (0 to 0·6)	0 (0 to 0·1)	0 (0 to 0·1)	0 (0 to 0)	0 (0 to 0)	75·4
HIV/AIDS resulting in other diseases	0·8 (0·5 to 1·2)	2·5 (1·6 to 3·8)	7,010 (5,190 to 9,550)	16,000 (10,800 to 23,500)	8,710 (5,620 to 12,500)	8,240 (5,640 to 11,300)	15,500 (8,550 to 24,700)	18,900 (13,400 to 27,600)	36,300 (25,300 to 54,300)	55,100 (40,500 to 78,100)	65,300 (49,100 to 87,600)	61,200 (49,500 to 75,900)	53,300 (44,500 to 64,500)	36,500 (30,700 to 42,100)	21,100 (17,600 to 24,800)	10,700 (8,730 to 12,900)	5,760 (4,680 to 7,010)	2,130 (1,620 to 2,710)	955 (720 to 1,230)	226 (173 to 289)	89·1 (68·2 to 113)	25 (18·7 to 32)	8 (6·3 to 10·1)	423,000
Sexually transmitted infections excluding HIV	48,800 (16,900 to 101,000)	15,400 (5,450 to 32,600)	18,100 (6,250 to 37,300)	709 (250 to 1,490)	352 (122 to 724)	180 (109 to 284)	586 (385 to 871)	1,070 (709 to 1,580)	1,410 (895 to 2,240)	1,730 (1,080 to 2,710)	1,690 (1,030 to 2,710)	1,540 (976 to 2,580)	1,360 (837 to 2,250)	897 (530 to 1,530)	650 (382 to 1,120)	432 (248 to 752)	365 (215 to 624)	247 (150 to 419)	150 (88·6 to 260)	97·2 (58·6 to 167)	59·7 (37·6 to 98·6)	24·6 (15·1 to 39·6)	9·1 (5·3 to 15·8)	95,900
Syphilis	48,800 (16,900 to 101,000)	15,400 (5,450 to 32,600)	18,100 (6,250 to 37,300)	709 (250 to 1,490)	352 (122 to 724)	71·4 (38·4 to 128)	108 (60·2 to 199)	96·7 (48·9 to 201)	155 (86·8 to 289)	188 (113 to 330)	220 (106 to 531)	99·7 (56·5 to 183)	126 (73·1 to 230)	124 (65·9 to 239)	111 (56·2 to 216)	89·9 (45·1 to 185)	95 (45·7 to 195)	57·5 (31·9 to 109)	24 (14·6 to 40·8)	18·8 (12·2 to 32·2)	10·2 (6·3 to 17·5)	4·8 (3·2 to 7·4)	1·8 (1·1 to 2·9)	85,000
Chlamydial infection	0 (0 to 0)	0 (0 to 0)	0 (0 to 0)	0 (0 to 0)	0 (0 to 0)	9·1 (5·3 to 16)	51·9 (34·2 to 78·8)	163 (107 to 240)	196 (117 to 305)	201 (120 to 311)	208 (133 to 315)	207 (138 to 311)	154 (101 to 232)	57·3 (36·7 to 86·5)	28·3 (19·8 to 42·8)	13·6 (8·4 to 24·2)	7·2 (4·6 to 12·2)	7·2 (4·5 to 11·5)	5 (3·3 to 7·7)	3·5 (2·4 to 5)	3 (1·8 to 5·2)	1·3 (0·8 to 2·1)	0·5 (0·3 to 0·9)	1,320
Gonococcal infection	0 (0 to 0)	0 (0 to 0)	0 (0 to 0)	0 (0 to 0)	0 (0 to 0)	23·5 (12·3 to 42·2)	134 (77·1 to 221)	227 (141 to 346)	174 (111 to 274)	200 (131 to 327)	185 (122 to 292)	218 (149 to 358)	190 (127 to 301)	98 (65·4 to 151)	58·3 (39·5 to 96·4)	40·9 (25·6 to 72·2)	48·6 (31 to 76·6)	29·7 (19·5 to 45·8)	20·2 (13·6 to 30·1)	10 (7·2 to 14·3)	9 (5·5 to 15)	3·5 (2·1 to 5·5)	0·9 (0·5 to 1·5)	1,670
Trichomoniasis	0 (0 to 0)	0 (0 to 0)	0 (0 to 0)	0 (0 to 0)	0 (0 to 0)	2·2 (0·5 to 5·6)	36·9 (11·6 to 89·8)	169 (60 to 399)	324 (110 to 737)	370 (121 to 881)	328 (108 to 762)	290 (99·2 to 695)	271 (88 to 631)	216 (68·6 to 508)	147 (49·5 to 354)	83·5 (29 to 199)	58·5 (20·5 to 135)	39·3 (14·5 to 89·8)	23·3 (8·8 to 53·2)	12·2 (4·7 to 27·1)	5·1 (2 to 11·1)	1·6 (0·6 to 3·5)	0·3 (0·1 to 0·7)	2,380
Genital herpes	0 (0 to 0)	0 (0 to 0)	0 (0 to 0)	0 (0 to 0)	0 (0 to 0)	15·3 (5·1 to 36·5)	69·6 (21·7 to 166)	147 (48·5 to 360)	234 (80 to 571)	293 (98·3 to 706)	293 (97·5 to 725)	283 (90 to 706)	287 (89·3 to 724)	266 (81·2 to 658)	225 (68 to 546)	164 (49·2 to 399)	127 (37·8 to 310)	93·2 (28·2 to 230)	61·4 (18·7 to 150)	37·1 (11·1 to 90·9)	19·3 (5·8 to 46·5)	8·1 (2·4 to 19·4)	3·1 (0·9 to 7·4)	2,630
Other sexually transmitted infections	0 (0 to 0)	0 (0 to 0)	0 (0 to 0)	0 (0 to 0)	0 (0 to 0)	58·8 (33·6 to 98·7)	185 (118 to 284)	272 (184 to 385)	326 (222 to 467)	474 (324 to 664)	453 (299 to 661)	443 (301 to 662)	330 (220 to 507)	135 (90·6 to 199)	80·9 (55·1 to 120)	40·1 (25·1 to 72·2)	28·9 (17·5 to 48·5)	19·8 (12·8 to 30·8)	16·2 (9·8 to 26·8)	15·6 (8·7 to 29·9)	13·1 (6·7 to 27·2)	5·3 (2·7 to 11·4)	2·5 (1·1 to 6·1)	2,900
Respiratory infections and tuberculosis	52,500 (31,900 to 81,800)	45,100 (26,900 to 68,500)	151,000 (101,000 to 221,000)	103,000 (71,500 to 142,000)	24,400 (18,400 to 30,800)	14,300 (11,200 to 18,100)	13,600 (10,700 to 17,200)	20,600 (16,400 to 25,300)	24,200 (19,400 to 29,500)	26,300 (21,500 to 32,400)	23,900 (18,900 to 30,000)	23,400 (18,400 to 29,300)	22,800 (18,200 to 28,300)	24,600 (19,900 to 30,400)	26,000 (21,200 to 31,700)	27,500 (22,400 to 32,700)	28,100 (23,500 to 33,500)	31,600 (26,000 to 37,500)	31,400 (26,400 to 37,300)	28,700 (24,000 to 33,400)	22,000 (18,000 to 25,600)	12,800 (10,100 to 15,000)	6,930 (5,250 to 8,350)	784,000
Tuberculosis	0 (0 to 0)	0·1 (0 to 0·2)	6,050 (3,550 to 9,410)	9,250 (6,150 to 14,100)	2,890 (2,070 to 4,080)	2,230 (1,640 to 3,120)	3,400 (2,420 to 4,700)	8,920 (6,680 to 11,800)	11,500 (8,660 to 15,200)	12,400 (9,330 to 16,700)	10,300 (7,670 to 14,500)	9,220 (6,470 to 12,500)	7,010 (5,060 to 9,550)	6,470 (4,760 to 8,860)	5,240 (3,800 to 7,210)	4,670 (3,540 to 6,340)	3,390 (2,570 to 4,520)	2,680 (2,000 to 3,500)	1,790 (1,420 to 2,320)	1,160 (881 to 1,490)	655 (502 to 831)	204 (154 to 255)	55·5 (41·2 to 69·9)	110,000
Latent tuberculosis infection	0 (0 to 0)	0 (0 to 0)	0 (0 to 0)	0 (0 to 0)	0 (0 to 0)	0 (0 to 0)	0 (0 to 0)	0 (0 to 0)	0 (0 to 0)	0 (0 to 0)	0 (0 to 0)	0 (0 to 0)	0 (0 to 0)	0 (0 to 0)	0 (0 to 0)	0 (0 to 0)	0 (0 to 0)	0 (0 to 0)	0 (0 to 0)	0 (0 to 0)	0 (0 to 0)	0 (0 to 0)	0 (0 to 0)	0
Drug-susceptible tuberculosis	0 (0 to 0)	0·1 (0 to 0·2)	5,980 (3,520 to 9,340)	9,150 (6,070 to 13,900)	2,870 (2,020 to 4,040)	2,210 (1,620 to 3,070)	3,360 (2,400 to 4,660)	8,820 (6,540 to 11,700)	11,400 (8,520 to 15,100)	12,200 (9,240 to 16,500)	10,200 (7,530 to 14,300)	9,120 (6,410 to 12,400)	6,920 (5,000 to 9,440)	6,390 (4,700 to 8,790)	5,170 (3,730 to 7,110)	4,610 (3,500 to 6,250)	3,350 (2,530 to 4,440)	2,640 (1,970 to 3,440)	1,770 (1,390 to 2,290)	1,140 (868 to 1,470)	644 (492 to 823)	200 (151 to 252)	54·4 (40·1 to 68·6)	108,000
Multidrug-resistant tuberculosis without extensive drug re	0 (0 to 0)	0 (0 to 0)	58·5 (7·6 to 227)	85·4 (11·5 to 330)	25·4 (4·7 to 87·2)	20·8 (3·8 to 75)	34·4 (6·4 to 115)	86·6 (17·8 to 309)	110 (23·6 to 368)	120 (24·8 to 410)	100 (21·3 to 335)	91·3 (20·3 to 296)	72·9 (17·5 to 225)	70 (17·5 to 205)	58·8 (14·4 to 177)	52·6 (13·2 to 164)	39·4 (10 to 115)	32·2 (8·5 to 93·6)	22·5 (6·3 to 65·5)	14·9 (4·2 to 42·6)	8·7 (2·4 to 25·9)	3 (0·9 to 8·7)	1 (0·3 to 2·5)	1,110
Extensively drug-resistant tuberculosis	0 (0 to 0)	0 (0 to 0)	9·7 (1·2 to 37·6)	14·1 (1·8 to 58·1)	3·9 (0·6 to 15·7)	3·3 (0·6 to 11·8)	5·7 (1 to 19·1)	13·8 (2·5 to 46·3)	17·4 (3·4 to 58·3)	19·2 (3·6 to 65·9)	16·3 (3·2 to 55·2)	14·8 (3 to 47·9)	11·9 (2·6 to 39·1)	11·4 (2·6 to 34·4)	9·6 (2·2 to 27·6)	8·6 (2 to 26·8)	6·5 (1·5 to 19·7)	5·2 (1·4 to 15·4)	3·7 (0·9 to 10·3)	2·4 (0·6 to 7)	1·4 (0·4 to 4·3)	0·5 (0·1 to 1·4)	0·2 (0 to 0·4)	180
Lower respiratory infections	52,300 (31,800 to 81,600)	45,000 (26,800 to 68,200)	144,000 (94,900 to 210,000)	86,600 (57,100 to 124,000)	15,000 (10,600 to 20,200)	7,000 (5,240 to 9,240)	5,500 (4,020 to 7,300)	7,200 (5,510 to 9,290)	8,610 (6,570 to 11,100)	10,300 (7,870 to 13,100)	10,300 (7,900 to 13,400)	11,300 (8,690 to 14,400)	13,000 (10,200 to 16,500)	15,700 (12,400 to 19,400)	18,700 (15,200 to 23,100)	21,300 (17,100 to 25,800)	23,600 (19,400 to 28,600)	28,100 (23,000 to 33,900)	29,100 (24,200 to 34,900)	27,200 (22,600 to 31,700)	21,200 (17,300 to 24,700)	12,500 (9,830 to 14,700)	6,840 (5,170 to 8,260)	620,000
[illegible]	[illegible]	[illegible]	[illegible]	[illegible]	[illegible]	[illegible]	[illegible]	[illegible]	[illegible]	[illegible]	[illegible]	[illegible]	[illegible]	[illegible]	[illegible]	[illegible]	[illegible]	[illegible]	[illegible]	[illegible]	[illegible]	[illegible]	[illegible]	[illegible]

Other chronic respiratory diseases	0 (0 to 0)	0 (0 to 0)	0 (0 to 0)	6,460 (2,220 to 15,600)	1,350 (844 to 2,300)
Digestive diseases	2,330 (1,210 to 3,960)	2,190 (1,030 to 4,130)	16,400 (6,490 to 34,300)	25,100 (11,000 to 45,900)	11,700 (8,090 to 15,800)
Cirrhosis and other chronic liver diseases	0 (0 to 0)	0 (0 to 0·1)	4·7 (2·4 to 8)	5,530 (1,850 to 11,300)	2,570 (1,470 to 4,000)
Cirrhosis and other chronic liver diseases due to hepatitis	0 (0 to 0)	0 (0 to 0)	0 (0 to 0)	11 (1·7 to 31·4)	13·1 (3·4 to 29·9)
Cirrhosis and other chronic liver diseases due to hepatitis	0 (0 to 0)	0 (0 to 0)	0 (0 to 0)	30·2 (7 to 77·9)	38·5 (13·8 to 84·4)
Cirrhosis and other chronic liver diseases due to alcohol	0 (0 to 0)	0 (0 to 0)	0 (0 to 0)	0 (0 to 0)	0 (0 to 0)
Cirrhosis and other chronic liver diseases due to NAFLD	0 (0 to 0)	0 (0 to 0)	0 (0 to 0)	0 (0 to 0)	0 (0 to 0)
Cirrhosis and other chronic liver diseases due to other ca	0 (0 to 0)	0 (0 to 0·1)	4·7 (2·4 to 8)	5,490 (1,840 to 11,200)	2,520 (1,440 to 3,890)
Upper digestive system diseases	0 (0 to 0)	0 (0 to 0)	0 (0 to 0)	2,170 (840 to 4,220)	1,020 (620 to 1,530)
Peptic ulcer disease	0 (0 to 0)	0 (0 to 0)	0 (0 to 0)	1,460 (431 to 3,050)	474 (240 to 795)
Gastritis and duodenitis	0 (0 to 0)	0 (0 to 0)	0 (0 to 0)	712 (308 to 1,410)	535 (278 to 889)
Gastroesophageal reflux disease	0 (0 to 0)	0 (0 to 0)	0 (0 to 0)	0 (0 to 0)	9·2 (3·5 to 19·1)
Appendicitis	0 (0 to 0)	0 (0 to 0)	0 (0 to 0)	4,280 (1,510 to 9,200)	2,160 (1,250 to 3,380)
Paralytic ileus and intestinal obstruction	1,280 (678 to 2,110)	1,630 (784 to 3,130)	12,200 (5,240 to 25,500)	3,400 (1,350 to 8,340)	1,130 (669 to 1,880)
Inguinal, femoral, and abdominal hernia	1,050 (425 to 2,070)	568 (181 to 1,290)	4,180 (915 to 11,800)	3,540 (1,820 to 6,360)	3,160 (1,860 to 4,890)
Inflammatory bowel disease	0 (0 to 0)	0 (0 to 0)	0 (0 to 0)	975 (176 to 2,740)	185 (79·1 to 362)
Vascular intestinal disorders	0 (0 to 0)	0 (0 to 0)	0 (0 to 0)	85·2 (25·6 to 200)	60·2 (29·3 to 122)
Gallbladder and biliary diseases	0 (0 to 0)	0 (0 to 0)	0 (0 to 0)	328 (134 to 692)	285 (171 to 440)
Pancreatitis	0 (0 to 0)	0 (0 to 0)	0 (0 to 0)	249 (70 to 564)	39·4 (21·6 to 69·8)

[illegible]

Exposure to forces of nature	1·6 (1·5 to 1·8)	4·6 (4·2 to 5)	37·9 (34·4 to 41·7)	205 (181 to 245)	1,090 (751 to 1,600)	8,370 (5,170 to 13,700)	9,790 (6,050 to 15,600)	14,900 (9,090 to 23,500)	9,050 (5,530 to 14,900)	8,610 (5,220 to 13,600)	8,380 (5,000 to 12,900)	6,780 (4,140 to 10,700)
Other unintentional injuries	1,080 (588 to 1,870)	409 (213 to 757)	1,450 (807 to 2,440)	4,600 (2,720 to 8,180)	4,320 (2,810 to 7,210)	4,210 (2,950 to 6,640)	5,260 (3,810 to 7,650)	6,750 (4,810 to 9,180)	7,440 (5,450 to 9,760)	7,320 (5,420 to 9,580)	6,370 (4,720 to 8,380)	5,840 (4,420 to 7,490)
Self-harm and interpersonal violence	2,780 (1,660 to 4,500)	753 (432 to 1,260)	3,320 (1,920 to 5,430)	9,780 (5,390 to 16,200)	7,990 (4,120 to 12,600)	14,500 (8,710 to 20,800)	66,300 (45,500 to 86,300)	111,000 (81,600 to 138,000)	107,000 (80,500 to 132,000)	91,700 (70,000 to 114,000)	70,300 (52,200 to 88,500)	54,300 (39,400 to 70,000)
Self-harm	0 (0 to 0)	0 (0 to 0)	0 (0 to 0)	0 (0 to 0)	0 (0 to 0)	2,890 (2,110 to 3,910)	14,800 (11,300 to 19,100)	22,600 (17,900 to 28,700)	22,400 (18,500 to 27,800)	22,400 (18,500 to 27,900)	18,900 (15,400 to 23,200)	17,000 (13,800 to 20,700)
Self-harm by firearm	0 (0 to 0)	0 (0 to 0)	0 (0 to 0)	0 (0 to 0)	0 (0 to 0)	80 (50·2 to 137)	1,040 (704 to 1,660)	1,860 (1,220 to 3,070)	1,780 (1,200 to 2,900)	1,700 (1,170 to 2,670)	1,270 (866 to 1,940)	1,110 (724 to 1,700)
Self-harm by other specified means	0 (0 to 0)	0 (0 to 0)	0 (0 to 0)	0 (0 to 0)	0 (0 to 0)	2,810 (2,060 to 3,810)	13,700 (10,500 to 17,800)	20,700 (16,400 to 26,200)	20,600 (17,000 to 25,500)	20,700 (17,100 to 25,400)	17,600 (14,300 to 21,600)	15,900 (12,900 to 19,300)
Interpersonal violence	2,780 (1,660 to 4,500)	753 (432 to 1,260)	3,310 (1,920 to 5,420)	9,720 (5,340 to 16,100)	7,940 (4,080 to 12,500)	11,600 (6,020 to 17,700)	51,400 (30,300 to 70,600)	88,100 (60,100 to 114,000)	84,000 (58,000 to 109,000)	68,800 (47,500 to 90,300)	51,000 (33,200 to 68,300)	37,100 (23,300 to 51,800)
Physical violence by firearm	193 (97·4 to 363)	60·4 (28·2 to 124)	266 (132 to 523)	971 (480 to 2,050)	1,260 (680 to 2,360)	3,020 (1,580 to 5,240)	27,900 (15,800 to 40,700)	53,300 (35,800 to 71,500)	48,700 (33,700 to 64,900)	36,900 (25,900 to 49,700)	25,300 (17,400 to 35,800)	16,200 (10,600 to 22,900)
Physical violence by sharp object	212 (95·3 to 406)	40·6 (14·5 to 101)	139 (75·1 to 298)	632 (323 to 1,610)	770 (398 to 1,960)	1,590 (865 to 3,100)	13,100 (7,500 to 24,000)	21,000 (13,500 to 36,000)	20,700 (13,200 to 34,600)	18,500 (11,800 to 31,100)	13,900 (8,500 to 23,200)	10,800 (6,650 to 18,100)
Sexual violence	0 (0 to 0)	0 (0 to 0)	0 (0 to 0)	29·6 (17·7 to 46·9)	236 (143 to 370)	512 (301 to 804)	1,160 (660 to 1,850)	1,540 (895 to 2,330)	1,420 (803 to 2,280)	1,260 (710 to 2,070)	1,010 (564 to 1,630)	758 (435 to 1,230)
Physical violence by other means	2,380 (1,380 to 3,950)	652 (364 to 1,110)	2,900 (1,660 to 4,870)	8,090 (4,250 to 14,000)	5,670 (2,550 to 9,370)	6,430 (2,670 to 11,300)	9,220 (3,750 to 16,600)	12,300 (5,760 to 21,100)	13,200 (6,030 to 22,300)	12,200 (5,710 to 20,600)	10,700 (4,510 to 18,600)	9,390 (4,010 to 17,200)
Conflict and terrorism	0 (0 to 0)	0 (0 to 0)	0 (0 to 0)	2·8 (1·6 to 4·9)	3·5 (2 to 6·4)	12·2 (7·2 to 20·1)	67·4 (35·2 to 120)	53·2 (28·6 to 91·6)	124 (70·5 to 207)	197 (107 to 331)	179 (102 to 301)	170 (102 to 278)

Exposure to forces of nature	4,980 (3,130 to 7,790)	3,770 (2,350 to 5,860)	2,950 (1,900 to 4,500)	2,350 (1,510 to 3,680)	1,730 (1,110 to 2,570)	1,100 (693 to 1,660)	626 (398 to 935)	319 (202 to 497)	114 (73 to 178)	34 (21·4 to 55·3)	8·1 (4·6 to 16·5)	85,300
Other unintentional injuries	5,360 (4,030 to 7,130)	4,490 (3,430 to 5,820)	3,690 (2,730 to 4,870)	2,620 (1,910 to 3,480)	2,060 (1,520 to 2,800)	1,560 (1,120 to 2,130)	1,120 (807 to 1,530)	708 (504 to 973)	405 (290 to 556)	184 (132 to 254)	79·7 (57·7 to 108)	77,300
Self-harm and interpersonal violence	43,200 (32,900 to 54,700)	33,200 (26,100 to 41,800)	24,400 (19,800 to 30,400)	16,200 (13,200 to 19,400)	11,000 (9,190 to 12,900)	7,740 (6,620 to 9,100)	4,910 (4,180 to 5,710)	2,720 (2,320 to 3,140)	1,390 (1,170 to 1,630)	418 (351 to 481)	146 (121 to 169)	685,000
Self-harm	16,400 (13,300 to 20,100)	14,900 (12,100 to 17,900)	12,200 (9,990 to 14,700)	8,700 (7,210 to 10,500)	6,350 (5,290 to 7,610)	4,940 (4,110 to 5,960)	3,330 (2,760 to 4,000)	2,030 (1,660 to 2,430)	1,040 (847 to 1,260)	291 (232 to 342)	86·3 (66·8 to 104)	191,000
Self-harm by firearm	878 (604 to 1,350)	787 (549 to 1,160)	544 (383 to 787)	423 (289 to 609)	270 (193 to 381)	204 (152 to 281)	124 (93·1 to 164)	85·3 (65·4 to 112)	45·9 (35·3 to 59·2)	8·1 (6 to 10·8)	3·4 (2·4 to 4·6)	12,200
Self-harm by other specified means	15,500 (12,600 to 19,100)	14,100 (11,500 to 17,000)	11,700 (9,570 to 14,100)	8,280 (6,880 to 10,000)	6,080 (5,060 to 7,320)	4,740 (3,930 to 5,730)	3,210 (2,640 to 3,850)	1,950 (1,580 to 2,340)	993 (805 to 1,200)	282 (225 to 335)	82·9 (64·1 to 99·7)	179,000
Interpersonal violence	26,600 (17,600 to 36,700)	18,000 (11,800 to 24,800)	12,000 (8,130 to 16,800)	7,440 (5,100 to 9,860)	4,590 (3,250 to 6,010)	2,770 (2,070 to 3,530)	1,560 (1,210 to 1,940)	684 (548 to 839)	343 (277 to 419)	127 (98·1 to 159)	59·1 (46·2 to 73·9)	491,000
Physical violence by firearm	10,700 (7,230 to 15,500)	6,460 (4,460 to 9,150)	3,910 (2,730 to 5,600)	2,270 (1,650 to 3,130)	1,200 (895 to 1,650)	608 (451 to 821)	245 (190 to 322)	97·6 (77·5 to 127)	51 (40·6 to 63·8)	15·8 (12·2 to 20·1)	8·5 (6·7 to 10·9)	240,000
Physical violence by sharp object	7,680 (5,120 to 12,400)	4,970 (3,390 to 8,140)	2,990 (2,090 to 4,450)	1,690 (1,180 to 2,640)	971 (729 to 1,440)	678 (523 to 912)	368 (284 to 482)	180 (140 to 230)	89·3 (68·7 to 114)	43·3 (33·2 to 56·3)	21·2 (15·9 to 27·5)	121,000
Sexual violence	605 (358 to 1,010)	433 (248 to 695)	289 (171 to 463)	205 (128 to 305)	161 (101 to 239)	121 (76·6 to 179)	82·9 (53·9 to 121)	53·6 (35·1 to 78·5)	24·8 (14·7 to 38·4)	4·5 (2·8 to 7)	0·3 (0·2 to 0·6)	9,910
Physical violence by other means	7,660 (3,580 to 13,100)	6,130 (3,170 to 10,300)	4,840 (2,580 to 7,990)	3,280 (1,820 to 4,950)	2,260 (1,350 to 3,400)	1,360 (898 to 1,930)	866 (611 to 1,150)	353 (263 to 450)	178 (135 to 224)	62·9 (47·9 to 80·5)	29 (21·9 to 37)	120,000
Conflict and terrorism	196 (123 to 317)	190 (121 to 296)	138 (89·9 to 208)	81·8 (53·7 to 123)	50·7 (33·6 to 76·7)	24·2 (16 to 36·5)	12·6 (8·2 to 19·2)	6·7 (4·2 to 10)	3·4 (2·2 to 5)	1 (0·6 to 1·5)	0·3 (0·2 to 0·5)	1,510

SR Table 2c: Antigua and Barbuda DALYS by cause and age group for both sexes with 95% uncertainty intervals, 2019

Cause	Early Neonatal	Late Neonatal	Post Neonatal	1 to 4	5 to 9	10 to 14	15 to 19	20 to 24	25 to 29	30 to 34	35 to 39	40 to 44	45 to 49	50 to 54	55 to 59	60 to 64	65 to 69	70 to 74	75 to 79	80 to 84	85 to 89	90 to 94	95 plus	Total
All causes	315 (224 to 435)	157 (112 to 215)	227 (172 to 297)	387 (286 to 520)	337 (269 to 428)	426 (331 to 543)	691 (559 to 858)	978 (818 to 1,160)	1,080 (905 to 1,290)	1,090 (906 to 1,320)	1,250 (1,050 to 1,490)	1,380 (1,160 to 1,650)	1,660 (1,390 to 1,960)	2,050 (1,730 to 2,400)	2,270 (1,930 to 2,630)	2,300 (1,990 to 2,640)	2,000 (1,750 to 2,270)	1,830 (1,610 to 2,060)	1,500 (1,340 to 1,670)	1,180 (1,080 to 1,290)	705 (657 to 753)	318 (302 to 335)	108 (103 to 113)	24,200
Communicable, maternal, neonatal, and nutritional diseases	262 (186 to 362)	119 (83·7 to 165)	112 (82·4 to 148)	129 (95·1 to 172)	86·8 (61·2 to 125)	66·9 (48·4 to 93·4)	78·2 (59 to 103)	97·1 (79·2 to 122)	117 (97·3 to 145)	133 (112 to 161)	143 (122 to 172)	137 (116 to 171)	131 (111 to 160)	126 (105 to 156)	117 (96·8 to 140)	103 (84 to 130)	86 (70·1 to 105)	84·7 (70·4 to 103)	81·7 (63·5 to 104)	77·1 (63·1 to 93·1)	56 (42·5 to 70·5)	28·3 (21·2 to 36·9)	11·3 (8·3 to 14·8)	2,380
HIV/AIDS and sexually transmitted infections	0·2 (0·1 to 0·3)	0·1 (0 to 0·2)	2·9 (2·7 to 3)	4 (3·8 to 4·2)	3·2 (3 to 3·4)	2·3 (2·1 to 2·4)	5·7 (5·2 to 6·4)	20·6 (18·8 to 22·8)	39·6 (36·5 to 43·9)	56·1 (51·8 to 61·1)	61·5 (57·5 to 66·6)	54·8 (51·2 to 59)	47·6 (44·3 to 51·2)	34·4 (32·1 to 36·7)	25·1 (23·3 to 27·2)	14·2 (13 to 15·4)	9·3 (8·5 to 10·2)	2·6 (2·2 to 3)	1·4 (1·2 to 1·6)	0·7 (0·6 to 0·8)	0·3 (0·3 to 0·4)	0·1 (0·1 to 0·1)	0 (0 to 0)	387
HIV/AIDS	0 (0 to 0)	0 (0 to 0)	2·8 (2·7 to 3)	4 (3·8 to 4·2)	3·2 (3 to 3·4)	2·1 (2 to 2·2)	5 (4·7 to 5·4)	19·1 (17·6 to 20·9)	37·5 (34·6 to 41·4)	53·5 (49·5 to 58·2)	58·8 (55 to 63)	52·1 (48·9 to 55·8)	45·3 (42·4 to 48·3)	32·8 (30·7 to 34·8)	24 (22 to 25·8)	13·4 (12·3 to 14·5)	8·8 (8·1 to 9·5)	2·2 (2 to 2·5)	1·2 (1·1 to 1·3)	0·5 (0·5 to 0·5)	0·2 (0·2 to 0·3)	0·1 (0·1 to 0·1)	0 (0 to 0)	367
HIV/AIDS - Drug-susceptible Tuberculosis	0 (0 to 0)	0 (0 to 0)	0 (0 to 0·1)	0·1 (0 to 0·1)	0 (0 to 0·1)	0 (0 to 0)	0·1 (0·1 to 0·1)	0·4 (0·3 to 0·5)	0·7 (0·5 to 1·1)	0·9 (0·6 to 1·3)	0·9 (0·6 to 1·4)	0·8 (0·6 to 1·2)	0·7 (0·5 to 1)	0·5 (0·3 to 0·7)	0·4 (0·3 to 0·5)	0·2 (0·2 to 0·3)	0·2 (0·1 to 0·2)	0 (0 to 0·1)	0 (0 to 0)	0 (0 to 0)	0 (0 to 0)	0 (0 to 0)	0 (0 to 0)	6
HIV/AIDS - Multidrug-resistant Tuberculosis without extensive dru	0 (0 to 0)	0 (0 to 0)	0 (0 to 0)	0 (0 to 0)	0 (0 to 0)	0 (0 to 0)	0 (0 to 0)	0 (0 to 0)	0 (0 to 0)	0 (0 to 0·1)	0 (0 to 0·1)	0 (0 to 0·1)	0 (0 to 0)	0 (0 to 0)	0 (0 to 0)	0 (0 to 0)	0 (0 to 0)	0 (0 to 0)	0 (0 to 0)	0 (0 to 0)	0 (0 to 0)	0 (0 to 0)	0 (0 to 0)	0·1
HIV/AIDS - Extensively drug-resistant Tuberculosis	0 (0 to 0)	0 (0 to 0)	0 (0 to 0)	0 (0 to 0)	0 (0 to 0)	0 (0 to 0)	0 (0 to 0)	0 (0 to 0)	0 (0 to 0)	0 (0 to 0)	0 (0 to 0)	0 (0 to 0)	0 (0 to 0)	0 (0 to 0)	0 (0 to 0)	0 (0 to 0)	0 (0 to 0)	0 (0 to 0)	0 (0 to 0)	0 (0 to 0)	0 (0 to 0)	0 (0 to 0)	0 (0 to 0)	0
HIV/AIDS resulting in other diseases	0 (0 to 0)	0 (0 to 0)	2·8 (2·6 to 2·9)	3·9 (3·7 to 4·2)	3·1 (3 to 3·3)	2·1 (2 to 2·2)	4·9 (4·6 to 5·3)	18·7 (17·2 to 20·5)	36·8 (34 to 40·5)	52·6 (48·8 to 57·3)	57·8 (54 to 62·1)	51·2 (48 to 54·8)	44·6 (41·7 to 47·6)	32·3 (30·2 to 34·2)	23·6 (21·9 to 25·3)	13·2 (12·1 to 14·3)	8·6 (7·9 to 9·3)	2·2 (1·9 to 2·4)	1·1 (1 to 1·3)	0·5 (0·5 to 0·5)	0·2 (0·2 to 0·3)	0·1 (0·1 to 0·1)	0 (0 to 0)	360
Sexually transmitted infections excluding HIV	0·2 (0·1 to 0·3)	0·1 (0 to 0·2)	0·1 (0 to 0·2)	0 (0 to 0)	0 (0 to 0)	0·1 (0·1 to 0·2)	0·7 (0·4 to 1·2)	1·5 (0·9 to 2·4)	2·1 (1·2 to 3·5)	2·6 (1·4 to 4·4)	2·7 (1·5 to 4·7)	2·7 (1·4 to 5)	2·3 (1·2 to 4·2)	1·6 (0·9 to 3·1)	1·2 (0·6 to 2·2)	0·8 (0·4 to 1·5)	0·6 (0·3 to 1)	0·4 (0·2 to 0·7)	0·2 (0·1 to 0·4)	0·2 (0·1 to 0·3)	0·1 (0 to 0·1)	0 (0 to 0)	0 (0 to 0)	20
Syphilis	0·2 (0·1 to 0·3)	0·1 (0 to 0·2)	0·1 (0 to 0·2)	0 (0 to 0)	0 (0 to 0)	0 (0 to 0)	0·1 (0 to 0·1)	0·1 (0 to 0·2)	0·1 (0·1 to 0·2)	0·1 (0·1 to 0·2)	0·1 (0·1 to 0·2)	0·1 (0 to 0·1)	0·1 (0·1 to 0·2)	0·1 (0·1 to 0·2)	0·1 (0·1 to 0·2)	0·1 (0·1 to 0·2)	0·1 (0·1 to 0·2)	0·1 (0 to 0·1)	0 (0 to 0·1)	0 (0 to 0)	0 (0 to 0)	0 (0 to 0)	0 (0 to 0)	1·7
Chlamydial infection	0 (0 to 0)	0 (0 to 0)	0 (0 to 0)	0 (0 to 0)	0 (0 to 0)	0 (0 to 0)	0·1 (0 to 0·1)	0·2 (0·1 to 0·4)	0·3 (0·1 to 0·6)	0·3 (0·2 to 0·6)	0·4 (0·2 to 0·6)	0·4 (0·2 to 0·6)	0·3 (0·1 to 0·4)	0·1 (0 to 0·2)	0·1 (0 to 0·1)	0 (0 to 0·1)	0 (0 to 0)	0 (0 to 0)	0 (0 to 0)	0 (0 to 0)	0 (0 to 0)	0 (0 to 0)	0 (0 to 0)	2·1
Gonococcal infection	0 (0 to 0)	0 (0 to 0)	0 (0 to 0)	0 (0 to 0)	0 (0 to 0)	0 (0 to 0)	0·2 (0·1 to 0·3)	0·2 (0·1 to 0·4)	0·2 (0·1 to 0·3)	0·2 (0·1 to 0·2)	0·2 (0·1 to 0·2)	0·2 (0·1 to 0·3)	0·2 (0·1 to 0·2)	0·1 (0·1 to 0·1)	0·1 (0 to 0·1)	0 (0 to 0·1)	0 (0 to 0)	0 (0 to 0)	0 (0 to 0)	0 (0 to 0)	0 (0 to 0)	0 (0 to 0)	0 (0 to 0)	1·6
Trichomoniasis	0 (0 to 0)	0 (0 to 0)	0 (0 to 0)	0 (0 to 0)	0 (0 to 0)	0 (0 to 0)	0·1 (0 to 0·1)	0·3 (0·1 to 0·7)	0·6 (0·2 to 1·3)	0·7 (0·2 to 1·7)	0·7 (0·2 to 1·7)	0·7 (0·2 to 1·7)	0·6 (0·1 to 1·5)	0·5 (0·2 to 1·2)	0·3 (0·1 to 0·8)	0·2 (0·1 to 0·4)	0·1 (0 to 0·3)	0·1 (0 to 0·2)	0 (0 to 0·1)	0 (0 to 0)	0 (0 to 0)	0 (0 to 0)	0 (0 to 0)	5
Genital herpes	0 (0 to 0)	0 (0 to 0)	0 (0 to 0)	0 (0 to 0)	0 (0 to 0)	0 (0 to 0·1)	0·1 (0 to 0·3)	0·3 (0·1 to 0·7)	0·5 (0·1 to 1·1)	0·6 (0·2 to 1·5)	0·6 (0·2 to 1·6)	0·7 (0·2 to 1·7)	0·7 (0·2 to 1·6)	0·6 (0·2 to 1·5)	0·5 (0·2 to 1·2)	0·4 (0·1 to 0·9)	0·3 (0·1 to 0·6)	0·2 (0·1 to 0·4)	0·1 (0 to 0·3)	0·1 (0 to 0·2)	0 (0 to 0·1)	0 (0 to 0)	0 (0 to 0)	5·6
Other sexually transmitted infections	0 (0 to 0)	0 (0 to 0)	0 (0 to 0)	0 (0 to 0)	0 (0 to 0)	0 (0 to 0·1)	0·2 (0·1 to 0·4)	0·3 (0·2 to 0·5)	0·4 (0·3 to 0·7)	0·6 (0·4 to 1)	0·7 (0·4 to 1·1)	0·7 (0·4 to 1·2)	0·5 (0·3 to 0·8)	0·2 (0·1 to 0·3)	0·1 (0·1 to 0·2)	0 (0 to 0·1)	0 (0 to 0)	0 (0 to 0)	0 (0 to 0)	0 (0 to 0)	0 (0 to 0)	0 (0 to 0)	0 (0 to 0)	4
Respiratory infections and tuberculosis	8·9 (4·9 to 14·4)	12·1 (6·6 to 19·5)	38 (25·8 to 55·4)	38·2 (25·4 to 56·7)	15·2 (11·2 to 20·9)	11·9 (8·3 to 16·8)	13·7 (9·7 to 18·9)	18·6 (14·1 to 24·2)	20·1 (15·2 to 26·5)	18·8 (14·3 to 24·3)	20·8 (15·3 to 27·3)	21·2 (15·7 to 27·7)	25·6 (19·2 to 33·7)	33·1 (24 to 45·2)	40·5 (31·1 to 51·1)	49·2 (34·6 to 68·5)	46·9 (33·8 to 63·3)	55·8 (44·3 to 70·7)	59·1 (42·5 to 79·5)	60·6 (47·2 to 75·8)	45·8 (33·1 to 59)	23·6 (17·1 to 31·3)	9·5 (6·7 to 12·8)	687
Tuberculosis	0 (0 to 0)	0 (0 to 0)	0·1 (0·1 to 0·2)	0·5 (0·3 to 0·9)	0·7 (0·3 to 1·3)	0·4 (0·1 to 0·7)	0·4 (0·3 to 0·5)	1·8 (1·1 to 2·7)	2·4 (1·4 to 3·8)	2·1 (1·3 to 3·4)	1·8 (1·1 to 2·7)	1·6 (1 to 2·4)	1·5 (0·9 to 2·2)	1·6 (1·1 to 2·3)	1·4 (1 to 2)	1·2 (0·9 to 1·8)	0·8 (0·6 to 1·1)	0·8 (0·6 to 1·1)	0·6 (0·5 to 0·8)	0·5 (0·3 to 0·6)	0·3 (0·2 to 0·3)	0·1 (0·1 to 0·1)	0 (0 to 0)	20·8
Latent tuberculosis infection	0 (0 to 0)	0 (0 to 0)	0 (0 to 0)	0 (0 to 0)	0 (0 to 0)	0 (0 to 0)	0 (0 to 0)	0 (0 to 0)	0 (0 to 0)	0 (0 to 0)	0 (0 to 0)	0 (0 to 0)	0 (0 to 0)	0 (0 to 0)	0 (0 to 0)	0 (0 to 0)	0 (0 to 0)	0 (0 to 0)	0 (0 to 0)	0 (0 to 0)	0 (0 to 0)	0 (0 to 0)	0 (0 to 0)	0
Drug-susceptible tuberculosis	0 (0 to 0)	0 (0 to 0)	0·1 (0·1 to 0·2)	0·5 (0·3 to 0·9)	0·7 (0·3 to 1·3)	0·4 (0·1 to 0·7)	0·4 (0·3 to 0·5)	1·7 (1 to 2·7)	2·4 (1·4 to 3·8)	2·1 (1·3 to 3·3)	1·8 (1·1 to 2·7)	1·6 (1 to 2·4)	1·4 (0·9 to 2·2)	1·6 (1 to 2·3)	1·4 (1 to 2)	1·2 (0·9 to 1·7)	0·8 (0·6 to 1·1)	0·8 (0·6 to 1·1)	0·6 (0·5 to 0·8)	0·5 (0·3 to 0·6)	0·3 (0·2 to 0·3)	0·1 (0·1 to 0·1)	0 (0 to 0)	20·6
Multidrug-resistant tuberculosis without extensive drug resistanc	0 (0 to 0)	0 (0 to 0)	0 (0 to 0)	0 (0 to 0)	0 (0 to 0)	0 (0 to 0)	0 (0 to 0)	0 (0 to 0·1)	0 (0 to 0·1)	0 (0 to 0·1)	0 (0 to 0·1)	0 (0 to 0·1)	0 (0 to 0·1)	0 (0 to 0·1)	0 (0 to 0·1)	0 (0 to 0)	0 (0 to 0)	0 (0 to 0)	0 (0 to 0)	0 (0 to 0)	0 (0 to 0)	0 (0 to 0)	0 (0 to 0)	0·2
Extensively drug-resistant tuberculosis	0 (0 to 0)	0 (0 to 0)	0 (0 to 0)	0 (0 to 0)	0 (0 to 0)	0 (0 to 0)	0 (0 to 0)	0 (0 to 0)	0 (0 to 0)	0 (0 to 0)	0 (0 to 0)	0 (0 to 0)	0 (0 to 0)	0 (0 to 0)	0 (0 to 0)	0 (0 to 0)	0 (0 to 0)	0 (0 to 0)	0 (0 to 0)	0 (0 to 0)	0 (0 to 0)	0 (0 to 0)	0 (0 to 0)	0
Lower respiratory infections	8·9 (4·9 to 14·4)	12 (6·5 to 19·4)	36·5 (24·3 to 54)	30·4 (18·3 to 48·3)	6·1 (4·5 to 7·9)	3·9 (3 to 5)	5·5 (4·1 to 7)	9·3 (7 to 12·3)	10·8 (7·7 to 15·1)	10·2 (7·3 to 14)	12·8 (8·8 to 17·9)	13·5 (9·3 to 18·6)	18·4 (12·6 to 25·6)	26·3 (17·4 to 37·8)	34·9 (25·8 to 45·6)	44·9 (30·7 to 63·8)	43·9 (30·9 to 60·5)	53·6 (41·9 to 68·8)	57·6 (41·1 to 77·9)	59·6 (46·2 to 74·7)	45·3 (32·6 to 58·4)	23·4 (16·9 to 31)	9·4 (6·6 to 12·7)	577
Upper respiratory infections	0 (0 to 0)	0·1 (0 to 0·1)	1·2 (0·7 to 2)	5·2 (3 to 8·2)	5·8 (3·2 to 10)	5·7 (3 to 9·8)	5·9 (3·1 to 10·6)	6 (3·2 to 10·1)	5·5 (3 to 9·2)	5·3 (2·8 to 9)	5·1 (2·6 to 8·7)	4·9 (2·6 to 8·5)	4·5 (2·4 to 7·8)	4 (2·2 to 6·6)	3·2 (1·7 to 5·3)	2·4 (1·3 to 4)	1·8 (0·9 to 3)	1·2 (0·6 to 2)	0·7 (0·4 to 1·2)	0·4 (0·2 to 0·7)	0·2 (0·1 to 0·3)	0·1 (0 to 0·1)	0 (0 to 0)	68·8
Otitis media	0 (0 to 0)	0 (0 to 0)	0·2 (0·1 to 0·4)	2 (1·1 to 3·5)	2·7 (1·4 to 4·5)	1·9 (1 to 3·4)	1·9 (1 to 3·3)	1·6 (0·7 to 3·1)	1·3 (0·6 to 2·4)	1·1 (0·5 to 2·2)	1·1 (0·5 to 2·1)	1·2 (0·5 to 2·2)	1·3 (0·6 to 2·3)	1·2 (0·6 to 2·2)	1 (0·5 to 1·8)	0·7 (0·3 to 1·3)	0·4 (0·2 to 0·7)	0·2 (0·1 to 0·5)	0·1 (0·1 to 0·3)	0·1 (0 to 0·2)	0·1 (0 to 0·1)	0 (0 to 0)	0 (0 to 0)	20·1
Enteric infections	0·5 (0·3 to 0·9)	2·1 (0·9 to 4·2)	14·5 (9·3 to 21·9)	15·7 (10·4 to 22·3)	10·1 (6·2 to 15·6)	9·7 (5·8 to 15·5)	7·5 (4·7 to 11·2)	6·5 (4·2 to 9·7)	7·2 (4·9 to 10·2)	7·8 (5·2 to 10·9)	8·6 (6 to 12)	9·4 (6·5 to 13·1)	10·3 (7·2 to 14·3)	11 (8 to 15)	10·6 (7·6 to 14·2)	11 (8·1 to 14·5)	11·9 (8·8 to 15·6)	12 (9 to 15·7)	10·5 (7·7 to 13·8)	7·8 (6 to 9·9)	4·3 (3·2 to 5·5)	1·8 (1·4 to 2·3)	0·6 (0·5 to 0·8)	191
Diarrheal diseases	0·5 (0·3 to 0·9)	2·1 (0·9 to 4·2)	14·5 (9·2 to 21·9)	15·6 (10·3 to 22·1)	10 (6·2 to 15·5)	9·7 (5·7 to 15·5)	7·4 (4·7 to 11·2)	6·5 (4·2 to 9·7)	7·2 (4·8 to 10·2)	7·7 (5·2 to 10·8)	8·6 (6 to 11·9)	9·3 (6·5 to 13·1)	10·3 (7·2 to 14·3)	11 (8 to 15)	10·6 (7·6 to 14·2)	11 (8·1 to 14·5)	11·8 (8·8 to 15·5)	12 (9 to 15·7)	10·5 (7·7 to 13·8)	7·8 (6 to 9·8)	4·3 (3·2 to 5·5)	1·8 (1·4 to 2·3)	0·6 (0·5 to 0·8)	191
Typhoid and paratyphoid	0 (0 to 0)	0 (0 to 0)	0 (0 to 0)	0 (0 to 0)	0 (0 to 0)	0 (0 to 0)	0 (0 to 0)	0 (0 to 0)	0 (0 to 0)	0 (0 to 0)	0 (0 to 0)	0 (0 to 0)	0 (0 to 0)	0 (0 to 0)	0 (0 to 0)	0 (0 to 0)	0 (0 to 0)	0 (0 to 0)	0 (0 to 0)	0 (0 to 0)	0 (0 to 0)	0 (0 to 0)	0 (0 to 0)	0·1
Typhoid fever	0 (0 to 0)	0 (0 to 0)	0 (0 to 0)	0 (0 to 0)	0 (0 to 0)	0 (0 to 0)	0 (0 to 0)	0 (0 to 0)	0 (0 to 0)	0 (0 to 0)	0 (0 to 0)	0 (0 to 0)	0 (0 to 0)	0 (0 to 0)	0 (0 to 0)	0 (0 to 0)	0 (0 to 0)	0 (0 to 0)	0 (0 to 0)	0 (0 to 0)	0 (0 to 0)	0 (0 to 0)	0 (0 to 0)	0
Paratyphoid fever	0 (0 to 0)	0 (0 to 0)	0 (0 to 0)	0 (0 to 0)	0 (0 to 0)	0 (0 to 0)	0 (0 to 0)	0 (0 to 0)	0 (0 to 0)	0 (0 to 0)	0 (0 to 0)	0 (0 to 0)	0 (0 to 0)	0 (0 to 0)	0 (0 to 0)	0 (0 to 0)	0 (0 to 0)	0 (0 to 0)	0 (0 to 0)	0 (0 to 0)	0 (0 to 0)	0 (0 to 0)	0 (0 to 0)	0
Invasive Non-typhoidal Salmonella (iNTS)	0 (0 to 0)	0 (0 to 0)	0 (0 to 0)	0 (0 to 0·1)	0 (0 to 0)	0 (0 to 0)	0 (0 to 0)	0 (0 to 0)	0 (0 to 0)	0 (0 to 0)	0 (0 to 0)	0 (0 to 0)	0 (0 to 0)	0 (0 to 0)	0 (0 to 0)	0 (0 to 0)	0 (0 to 0)	0 (0 to 0)	0 (0 to 0)	0 (0 to 0)	0 (0 to 0)	0 (0 to 0)	0 (0 to 0)	0·1
Other intestinal infectious diseases	0 (0 to 0)	0 (0 to 0)	0 (0 to 0·1)	0·1 (0 to 0·2)	0 (0 to 0·1)	0 (0 to 0·1)	0 (0 to 0)	0 (0 to 0)	0 (0 to 0)	0 (0 to 0)	0 (0 to 0)	0 (0 to 0)	0 (0 to 0)	0 (0 to 0)	0 (0 to 0)	0 (0 to 0)	0 (0 to 0)	0 (0 to 0)	0 (0 to 0)	0 (0 to 0)	0 (0 to 0)	0 (0 to 0)	0 (0 to 0)	0·3
Neglected tropical diseases and malaria	0 (0 to 0)	0·1 (0·1 to 0·2)	1 (0·6 to 1·6)	3·8 (2 to 6·7)	5·7 (3 to 11·6)	6·3 (2·4 to 14·1)	6·8 (2·8 to 15·6)	9·5 (3·6 to 19·6)	9·5 (3·2 to 20·4)	9·2 (3·4 to 19·1)	6·5 (2·7 to 14·6)	6·2 (2·4 to 13·8)	5·7 (2·4 to 12·4)	5·5 (2·3 to 11·2)	4·5 (2 to 9·3)	3·2 (1·4 to 6·6)	2·1 (0·9 to 4·4)	1·5 (0·6 to 3·1)	1 (0·4 to 2·1)	0·6 (0·2 to 1·2)	0·3 (0·2 to 0·7)	0·1 (0·1 to 0·3)	0·1 (0 to 0·1)	89·8
Malaria	0 (0 to 0)	0 (0 to 0)	0 (0 to 0)	0 (0 to 0)	0 (0 to 0)	0 (0 to 0)	0 (0 to 0)	0 (0 to 0)	0 (0 to 0)	0 (0 to 0)	0 (0 to 0)	0 (0 to 0)	0 (0 to 0)	0 (0 to 0)	0 (0 to 0)	0 (0 to 0)	0 (0 to 0)	0 (0 to 0)	0 (0 to 0)	0 (0 to 0)	0 (0 to 0)	0 (0 to 0)	0 (0 to 0)	0
Chagas disease	0 (0 to 0)	0 (0 to 0)	0 (0 to 0)	0 (0 to 0)	0 (0 to 0)	0 (0 to 0)	0 (0 to 0)	0 (0 to 0)	0 (0 to 0)	0 (0 to 0)	0 (0 to 0)	0 (0 to 0)	0 (0 to 0)	0 (0 to 0)	0 (0 to 0)	0 (0 to 0)	0 (0 to 0)	0 (0 to 0)	0 (0 to 0)	0 (0 to 0)	0 (0 to 0)	0 (0 to 0)	0 (0 to 0)	0
Leishmaniasis	0 (0 to 0)	0 (0 to 0)	0 (0 to 0)	0 (0 to 0)	0 (0 to 0)	0 (0 to 0)	0 (0 to 0)	0 (0 to 0)	0 (0 to 0)	0 (0 to 0)	0 (0 to 0)	0 (0 to 0)	0 (0 to 0)	0 (0 to 0)	0 (0 to 0)	0 (0 to 0)	0 (0 to 0)	0 (0 to 0)	0 (0 to 0)	0 (0 to 0)	0 (0 to 0)	0 (0 to 0)	0 (0 to 0)	0
Visceral leishmaniasis	0 (0 to 0)	0 (0 to 0)	0 (0 to 0)	0 (0 to 0)	0 (0 to 0)	0 (0 to 0)	0 (0 to 0)	0 (0 to 0)	0 (0 to 0)	0 (0 to 0)	0 (0 to 0)	0 (0 to 0)	0 (0 to 0)	0 (0 to 0)	0 (0 to 0)	0 (0 to 0)	0 (0 to 0)	0 (0 to 0)	0 (0 to 0)	0 (0 to 0)	0 (0 to 0)	0 (0 to 0)	0 (0 to 0)	0
Cutaneous and mucocutaneous leishmaniasis	0 (0 to 0)	0 (0 to 0)	0 (0 to 0)	0 (0 to 0)	0 (0 to 0)	0 (0 to 0)	0 (0 to 0)	0 (0 to 0)	0 (0 to 0)	0 (0 to 0)	0 (0 to 0)	0 (0 to 0)	0 (0 to 0)	0 (0 to 0)	0 (0 to 0)	0 (0 to 0)	0 (0 to 0)	0 (0 to 0)	0 (0 to 0)	0 (0 to 0)	0 (0 to 0)	0 (0 to 0)	0 (0 to 0)	0
African trypanosomiasis	0 (0 to 0)	0 (0 to 0)	0 (0 to 0)	0 (0 to 0)	0 (0 to 0)	0 (0 to 0)	0 (0 to 0)	0 (0 to 0)	0 (0 to 0)	0 (0 to 0)	0 (0 to 0)	0 (0 to 0)	0 (0 to 0)	0 (0 to 0)	0 (0 to 0)	0 (0 to 0)	0 (0 to 0)	0 (0 to 0)	0 (0 to 0)	0 (0 to 0)	0 (0 to 0)	0 (0 to 0)	0 (0 to 0)	0
Schistosomiasis	0 (0 to 0)	0 (0 to 0)	0 (0 to 0)	0·2 (0 to 0·7)	2 (0·3 to 5·9)	3·9 (0·6 to 11·3)	4·3 (1 to 12·5)	7·2 (1·6 to 16·7)	7·6 (1·8 to 17·8)	7·2 (2·1 to 16·7)	4·3 (1·1 to 11·6)	4·2 (0·9 to 11·5)	4 (0·9 to 10)	3·8 (0·9 to 9·1)	3·1 (0·8 to 7·7)	2·3 (0·6 to 5·7)	1·5 (0·4 to 3·8)	1·1 (0·3 to 2·7)	0·7 (0·2 to 1·8)	0·4 (0·1 to 1)	0·2 (0·1 to 0·5)	0·1 (0 to 0·2)	0 (0 to 0·1)	58·2
Cysticercosis	0 (0 to 0)	0 (0 to 0)	0 (0 to 0)	0 (0 to 0)	0 (0 to 0)	0 (0 to 0·1)	0 (0 to 0·1)	0·1 (0 to 0·3)	0·1 (0 to 0·3)	0·1 (0 to 0·3)	0·1 (0 to 0·3)	0·1 (0 to 0·3)	0·1 (0 to 0·3)	0·1 (0 to 0·3)	0·1 (0 to 0·3)	0·1 (0 to 0·3)	0·1 (0 to 0·2)	0 (0 to 0·1)	0 (0 to 0·1)	0 (0 to 0)	0 (0 to 0)	0 (0 to 0)	0 (0 to 0)	1·1
Cystic echinococcosis	0 (0 to 0)	0 (0 to 0)	0 (0 to 0)	0 (0 to 0)	0 (0 to 0)	0 (0 to 0)	0 (0 to 0)	0 (0 to 0)	0 (0 to 0)	0 (0 to 0)	0 (0 to 0)	0 (0 to 0)	0 (0 to 0)	0 (0 to 0)	0 (0 to 0)	0 (0 to 0)	0 (0 to 0)	0 (0 to 0)	0 (0 to 0)	0 (0 to 0)	0 (0 to 0)	0 (0 to 0)	0 (0 to 0)	0
Lymphatic filariasis	0 (0 to 0)	0 (0 to 0)	0 (0 to 0)	0 (0 to 0)	0 (0 to 0)	0 (0 to 0)	0 (0 to 0)	0 (0 to 0)	0 (0 to 0)	0 (0 to 0)	0 (0 to 0)	0 (0 to 0)	0 (0 to 0)	0 (0 to 0)	0 (0 to 0)	0 (0 to 0)	0 (0 to 0)	0 (0 to 0)	0 (0 to 0)	0 (0 to 0)	0 (0 to 0)	0 (0 to 0)	0 (0 to 0)	0
Onchocerciasis	0 (0 to 0)	0 (0 to 0)	0 (0 to 0)	0 (0 to 0)	0 (0 to 0)	0 (0 to 0)	0 (0 to 0)	0 (0 to 0)	0 (0 to 0)	0 (0 to 0)	0 (0 to 0)	0 (0 to 0)	0 (0 to 0)	0 (0 to 0)	0 (0 to 0)	0 (0 to 0)	0 (0 to 0)	0 (0 to 0)	0 (0 to 0)	0 (0 to 0)	0 (0 to 0)	0 (0 to 0)	0 (0 to 0)	0
Trachoma	0 (0 to 0)	0 (0 to 0)	0 (0 to 0)	0 (0 to 0)	0 (0 to 0)	0 (0 to 0)	0 (0 to 0)	0 (0 to 0)	0 (0 to 0)	0 (0 to 0)	0 (0 to 0)	0 (0 to 0)	0 (0 to 0)	0 (0 to 0)	0 (0 to 0)	0 (0 to 0)	0 (0 to 0)	0 (0 to 0)	0 (0 to 0)	0 (0 to 0)	0 (0 to 0)	0 (0 to 0)	0 (0 to 0)	0
Dengue	0 (0 to 0)	0 (0 to 0)	0·3 (0·1 to 0·7)	0·6 (0·1 to 1·9)	0·6 (0·2 to 1·5)	0·6 (0·2 to 1·3)	0·8 (0·3 to 1·6)	0·8 (0·3 to 1·6)	0·7 (0·3 to 1·3)	0·6 (0·2 to 1·3)	0·6 (0·2 to 1·1)	0·5 (0·2 to 1)	0·5 (0·2 to 1)	0·5 (0·2 to 1)	0·4 (0·1 to 0·8)	0·4 (0·2 to 0·8)	0·3 (0·1 to 0·6)	0·2 (0·1 to 0·4)	0·1 (0 to 0·3)	0·1 (0 to 0·2)	0·1 (0 to 0·1)	0 (0 to 0·1)	0 (0 to 0)	8·8
Yellow fever	0 (0 to 0)	0 (0 to 0)	0 (0 to 0)	0 (0 to 0)	0 (0 to 0)	0 (0 to 0)	0 (0 to 0)	0 (0 to 0)	0 (0 to 0)	0 (0 to 0)	0 (0 to 0)	0 (0 to 0)	0 (0 to 0)	0 (0 to 0)	0 (0 to 0)	0 (0 to 0)	0 (0 to 0)	0 (0 to 0)	0 (0 to 0)	0 (0 to 0)	0 (0 to 0)	0 (0 to 0)	0 (0 to 0)	0
Rabies	0 (0 to 0)	0 (0 to 0)	0 (0 to 0)	0 (0 to 0·1)	0 (0 to 0·2)	0 (0 to 0·1)	0 (0 to 0)	0 (0 to 0)	0 (0 to 0)	0 (0 to 0)	0 (0 to 0)	0 (0 to 0)	0 (0 to 0)	0 (0 to 0)	0 (0 to 0)	0 (0 to 0)	0 (0 to 0)	0 (0 to 0)	0 (0 to 0)	0 (0 to 0)	0 (0 to 0)	0 (0 to 0)	0 (0 to 0)	0·2
Intestinal nematode infections	0 (0 to 0)	0 (0 to 0)	0 (0 to 0)	0·1 (0 to 0·2)	0·1 (0·1 to 0·3)	0·1 (0 to 0·1)	0·6 (0·2 to 1)	0·5 (0·2 to 1)	0·3 (0·1 to 0·7)	0·3 (0·1 to 0·6)	0·4 (0·2 to 0·7)	0·2 (0·1 to 0·3)	0 (0 to 0·1)	0 (0 to 0·1)	0 (0 to 0·1)	0 (0 to 0)	0 (0 to 0)	0 (0 to 0)	0 (0 to 0)	0 (0 to 0)	0 (0 to 0)	0 (0 to 0)	0 (0 to 0)	2·7
Ascariasis	0 (0 to 0)	0 (0 to 0)	0 (0 to 0)	0 (0 to 0·2)	0 (0 to 0·1)	0 (0 to 0)	0 (0 to 0)	0 (0 to 0)	0 (0 to 0)	0 (0 to 0)	0 (0 to 0)	0 (0 to 0)	0 (0 to 0)	0 (0 to 0)	0 (0 to 0)	0 (0 to 0)	0 (0 to 0)	0 (0 to 0)	0 (0 to 0)	0 (0 to 0)	0 (0 to 0)	0 (0 to 0)	0 (0 to 0)	0·1
Trichuriasis	0 (0 to 0)	0 (0 to 0)	0 (0 to 0)	0 (0 to 0)	0 (0 to 0)	0 (0 to 0)	0 (0 to 0)	0 (0 to 0)	0 (0 to 0)	0 (0 to 0)	0 (0 to 0)	0 (0 to 0)	0 (0 to 0)	0 (0 to 0)	0 (0 to 0)	0 (0 to 0)	0 (0 to 0)	0 (0 to 0)	0 (0 to 0)	0 (0 to 0)	0 (0 to 0)	0 (0 to 0)	0 (0 to 0)	0
Hookworm disease	0 (0 to 0)	0 (0 to 0)	0 (0 to 0)	0 (0 to 0·1)	0·1 (0 to 0·2)	0·1 (0 to 0·1)	0·6 (0·2 to 1)	0·5 (0·2 to 1)	0·3 (0·1 to 0·7)	0·3 (0·1 to 0·6)	0·4 (0·2 to 0·7)	0·2 (0·1 to 0·3)	0 (0 to 0·1)	0 (0 to 0·1)	0 (0 to 0·1)	0 (0 to 0)	0 (0 to 0)	0 (0 to 0)	0 (0 to 0)	0 (0 to 0)	0 (0 to 0)	0 (0 to 0)	0 (0 to 0)	2·6
Food-borne trematodiases	0 (0 to 0)	0 (0 to 0)	0 (0 to 0)	0 (0 to 0)	0 (0 to 0)	0 (0 to 0)	0 (0 to 0)	0 (0 to 0)	0 (0 to 0)	0 (0 to 0)	0 (0 to 0)	0 (0 to 0)	0 (0 to 0)	0 (0 to 0)	0 (0 to 0)	0 (0 to 0)	0 (0 to 0)	0 (0 to 0)	0 (0 to 0)	0 (0 to 0)	0 (0 to 0)	0 (0 to 0)	0 (0 to 0)	0
Leprosy	0 (0 to 0)	0 (0 to 0)	0 (0 to 0)	0 (0 to 0)	0 (0 to 0)	0 (0 to 0)	0 (0 to 0)	0 (0 to 0)	0 (0 to 0)	0 (0 to 0)	0 (0 to 0)	0 (0 to 0)	0 (0 to 0)	0 (0 to 0)	0 (0 to 0)	0 (0 to 0)	0 (0 to 0)	0 (0 to 0)	0 (0 to 0)	0 (0 to 0)	0 (0 to 0)	0 (0 to 0)	0 (0 to 0)	0
Ebola	0 (0 to 0)	0 (0 to 0)	0 (0 to 0)	0 (0 to 0)	0 (0 to 0)	0 (0 to 0)	0 (0 to 0)	0 (0 to 0)	0 (0 to 0)	0 (0 to 0)	0 (0 to 0)	0 (0 to 0)	0 (0 to 0)	0 (0 to 0)	0 (0 to 0)	0 (0 to 0)	0 (0 to 0)	0 (0 to 0)	0 (0 to 0)	0 (0 to 0)	0 (0 to 0)	0 (0 to 0)	0 (0 to 0)	0
Zika virus	0 (0 to 0)	0 (0 to 0)	0 (0 to 0·2)	0·2 (0 to 0·8)	0·2 (0 to 0·9)	0·2 (0 to 0·9)	0·3 (0 to 1·2)	0·3 (0 to 1·3)	0·3 (0 to 1·3)	0·3 (0 to 1·3)	0·3 (0 to 1·1)	0·2 (0 to 0·9)	0·1 (0 to 0·6)	0·1 (0 to 0·5)	0·1 (0 to 0·4)	0·1 (0 to 0·2)	0 (0 to 0·1)	0 (0 to 0·1)	0 (0 to 0)	0 (0 to 0)	0 (0 to 0)	0 (0 to 0)	0 (0 to 0)	2·8
Guinea worm disease	0 (0 to 0)	0 (0 to 0)	0 (0 to 0)	0 (0 to 0)	0 (0 to 0)	0 (0 to 0)	0 (0 to 0)	0 (0 to 0)	0 (0 to 0)	0 (0 to 0)	0 (0 to 0)	0 (0 to 0)	0 (0 to 0)	0 (0 to 0)	0 (0 to 0)	0 (0 to 0)	0 (0 to 0)	0 (0 to 0)	0 (0 to 0)	0 (0 to 0)	0 (0 to 0)	0 (0 to 0)	0 (0 to 0)	0
Other neglected tropical diseases	0 (0 to 0)	0·1 (0·1 to 0·2)	0·7 (0·3 to 1·1)	2·7 (1·3 to 5)	2·9 (1·3 to 5·5)	1·5 (0·6 to 2·9)	0·8 (0·3 to 1·5)	0·6 (0·3 to 1·2)	0·5 (0·2 to 1·1)	0·7 (0·3 to 1·2)	0·9 (0·4 to 1·6)	1 (0·4 to 2)	0·9 (0·4 to 1·8)	0·9 (0·4 to 1·7)	0·8 (0·4 to 1·4)	0·3 (0·2 to 0·6)	0·2 (0·1 to 0·3)	0·2 (0·1 to 0·3)	0·1 (0 to 0·2)	0·1 (0 to 0·1)	0·1 (0 to 0·1)	0 (0 to 0)	0 (0 to 0)	15·9
Other infectious diseases	0·9 (0·5 to 1·4)	2·8 (1·5 to 4·8)	8·7 (6 to 12·2)	11·8 (7·7 to 17·6)	6·8 (5·1 to 8·9)	5·1 (3·8 to 6·5)	5·7 (4·5 to 7·2)	6·2 (4·9 to 7·7)	6·1 (4·8 to 7·8)	5·7 (4·5 to 7·1)	6·3 (5 to 8)	6·9 (5·5 to 8·3)	7·2 (5·6 to 9·5)	7·4 (5·8 to 9·9)	6·6 (5·3 to 9·1)	6·2 (4·6 to 7·9)	4·3 (3·2 to 5·5)	3·4 (2·6 to 4·4)	2·2 (1·7 to 2·9)	1·5 (1·1 to 2)	1 (0·7 to 1·4)	0·3 (0·2 to 0·5)	0·1 (0·1 to 0·2)	113
Meningitis	0·6 (0·3 to 1·1)	2·3 (1·1 to 4·2)	4·9 (3·1 to 7·6)	5·8 (3·3 to 9·6)	2·3 (1·6 to 3·3)	1·5 (1·2 to 2·1)	1·8 (1·3 to 2·4)	1·8 (1·4 to 2·4)	1·7 (1·3 to 2·2)	1·5 (1·1 to 1·9)	1·6 (1·2 to 2·1)	1·5 (1·1 to 1·9)	1·8 (1·4 to 2·2)	2·1 (1·6 to 2·7)	2 (1·5 to 2·6)	2 (1·5 to 2·5)	1·4 (1 to 1·8)	1·1 (0·8 to 1·4)	0·8 (0·6 to 1·1)	0·6 (0·4 to 0·7)	0·3 (0·2 to 0·4)	0·1 (0·1 to 0·1)	0 (0 to 0)	39·3
Encephalitis	0·1 (0·1 to 0·2)	0·2 (0·1 to 0·4)	1·8 (1 to 3)	2·8 (1·4 to 5)	1·4 (0·9 to 1·9)	1·1 (0·8 to 1·6)	1·2 (0·9 to 1·7)	1·3 (0·9 to 1·7)	1 (0·7 to 1·4)	0·9 (0·7 to 1·2)	0·8 (0·6 to 1·2)	0·9 (0·7 to 1·2)	0·9 (0·6 to 1·2)	0·8 (0·6 to 1)	0·8 (0·6 to 1)	0·7 (0·5 to 0·9)	0·4 (0·3 to 0·6)	0·3 (0·2 to 0·5)	0·2 (0·1 to 0·3)	0·1 (0·1 to 0·2)	0·1 (0 to 0·1)	0 (0 to 0)	0 (0 to 0)	17·7
Diphtheria	0 (0 to 0)	0 (0 to 0)	0 (0 to 0)	0 (0 to 0)	0 (0 to 0)	0 (0 to 0)	0 (0 to 0)	0 (0 to 0)	0 (0 to 0)	0 (0 to 0)	0 (0 to 0)	0 (0 to 0)	0 (0 to 0)	0 (0 to 0)	0 (0 to 0)	0 (0 to 0)	0 (0 to 0)	0 (0 to 0)	0 (0 to 0)	0 (0 to 0)	0 (0 to 0)	0 (0 to 0)	0 (0 to 0)	0
Tetanus	0 (0 to 0)	0 (0 to 0)	0 (0 to 0)	0 (0 to 0·1)	0 (0 to 0·2)	0 (0 to 0·2)	0 (0 to 0·2)	0·1 (0 to 0·2)	0·1 (0 to 0·2)	0·1 (0 to 0·2)	0·1 (0 to 0·3)	0·1 (0 to 0·4)	0·1 (0 to 0·6)	0·2 (0·1 to 0·8)	0·2 (0·1 to 0·8)	0·2 (0·1 to 0·7)	0·1 (0 to 0·5)	0·1 (0 to 0·5)	0·1 (0 to 0·3)	0 (0 to 0·1)	0 (0 to 0)	0 (0 to 0)	0 (0 to 0)	1·6
Measles	0 (0 to 0)	0 (0 to 0)	0 (0 to 0)	0 (0 to 0)	0 (0 to 0)	0 (0 to 0)	0 (0 to 0)	0 (0 to 0)	0 (0 to 0)	0 (0 to 0)	0 (0 to 0)	0 (0 to 0)	0 (0 to 0)	0 (0 to 0)	0 (0 to 0)	0 (0 to 0)	0 (0 to 0)	0 (0 to 0)	0 (0 to 0)	0 (0 to 0)	0 (0 to 0)	0 (0 to 0)	0 (0 to 0)	0
Varicella and herpes zoster	0 (0 to 0)	0·1 (0 to 0·1)	0·3 (0·2 to 0·6)	0·3 (0·1 to 0·6)	0·3 (0·2 to 0·5)	0·2 (0·1 to 0·3)	0·2 (0·1 to 0·3)	0·3 (0·2 to 0·4)	0·3 (0·2 to 0·4)	0·3 (0·2 to 0·5)	0·3 (0·2 to 0·4)	0·4 (0·2 to 0·6)	0·3 (0·2 to 0·5)	0·4 (0·2 to 0·5)	0·3 (0·2 to 0·5)	0·3 (0·2 to 0·5)	0·3 (0·2 to 0·4)	0·2 (0·2 to 0·4)	0·2 (0·1 to 0·3)	0·1 (0·1 to 0·2)	0·1 (0·1 to 0·2)	0·1 (0 to 0·1)	0 (0 to 0)	5·1
Acute hepatitis	0 (0 to 0)	0 (0 to 0)	0·1 (0 to 0·1)	0·2 (0·1 to 0·3)	0·4 (0·3 to 0·7)	0·6 (0·4 to 0·9)	0·6 (0·4 to 0·9)	0·6 (0·4 to 0·9)	0·6 (0·4 to 0·8)	0·5 (0·3 to 0·7)	0·4 (0·3 to 0·6)	0·4 (0·3 to 0·6)	0·4 (0·3 to 0·5)	0·4 (0·3 to 0·5)	0·3 (0·2 to 0·4)	0·2 (0·1 to 0·3)	0·1 (0·1 to 0·2)	0·1 (0·1 to 0·2)	0 (0 to 0·1)	0 (0 to 0·1)	0 (0 to 0)	0 (0 to 0)	0 (0 to 0)	6·1
Acute hepatitis A	0 (0 to 0)	0 (0 to 0)	0 (0 to 0)	0·1 (0·1 to 0·2)	0·4 (0·2 to 0·6)	0·5 (0·3 to 0·7)	0·5 (0·3 to 0·7)	0·4 (0·3 to 0·6)	0·3 (0·2 to 0·5)	0·3 (0·2 to 0·4)	0·2 (0·1 to 0·3)	0·2 (0·1 to 0·3)	0·1 (0·1 to 0·2)	0·1 (0 to 0·2)	0·1 (0 to 0·1)	0 (0 to 0·1)	0 (0 to 0)	0 (0 to 0)	0 (0 to 0)	0 (0 to 0)	0 (0 to 0)	0 (0 to 0)	0 (0 to 0)	3·2
Acute hepatitis B	0 (0 to 0)	0 (0 to 0)	0 (0 to 0·1)	0 (0 to 0·1)	0 (0 to 0·1)	0·1 (0 to 0·1)	0·1 (0 to 0·1)	0·1 (0·1 to 0·2)	0·2 (0·1 to 0·3)	0·2 (0·1 to 0·3)	0·2 (0·1 to 0·3)	0·2 (0·1 to 0·3)	0·2 (0·1 to 0·3)	0·2 (0·1 to 0·3)	0·2 (0·1 to 0·3)	0·1 (0·1 to 0·2)	0·1 (0·1 to 0·1)	0·1 (0·1 to 0·1)	0 (0 to 0·1)	0 (0 to 0·1)	0 (0 to 0)	0 (0 to 0)	0 (0 to 0)	2
Acute hepatitis C	0 (0 to 0)	0 (0 to 0)	0 (0 to 0)	0 (0 to 0)	0 (0 to 0)	0 (0 to 0)	0 (0 to 0)	0 (0 to 0)	0 (0 to 0)	0 (0 to 0)	0 (0 to 0)	0 (0 to 0)	0 (0 to 0)	0 (0 to 0)	0 (0 to 0)	0 (0 to 0)	0 (0 to 0)	0 (0 to 0)	0 (0 to 0)	0 (0 to 0)	0 (0 to 0)	0 (0 to 0)	0 (0 to 0)	0·2
Acute hepatitis E	0 (0 to 0)	0 (0 to 0)	0 (0 to 0)	0 (0 to 0)	0 (0 to 0·1)	0 (0 to 0·1)	0·1 (0 to 0·1)	0·1 (0 to 0·1)	0·1 (0 to 0·1)	0 (0 to 0·1)	0 (0 to 0·1)	0 (0 to 0)	0 (0 to 0)	0 (0 to 0)	0 (0 to 0)	0 (0 to 0)	0 (0 to 0)	0 (0 to 0)	0 (0 to 0)	0 (0 to 0)	0 (0 to 0)	0 (0 to 0)	0 (0 to 0)	0·6
Other unspecified infectious diseases	0·2 (0·1 to 0·3)	0·3 (0·2 to 0·4)	1·3 (0·8 to 2)	2·4 (1·4 to 3·9)	2·3 (1·1 to 4·1)	1·5 (0·8 to 2·5)	1·9 (1·1 to 2·8)	2·1 (1·3 to 3·1)	2·5 (1·6 to 3·8)	2·5 (1·6 to 3·6)	3 (2 to 4·5)	3·2 (2·1 to 4·7)	3·8 (2·5 to 5·5)	3·8 (2·5 to 5·5)	3·4 (2·1 to 5)	2·8 (1·6 to 4·2)	2 (1·1 to 2·9)	1·5 (0·8 to 2·3)	0·9 (0·5 to 1·4)	0·6 (0·3 to 1·1)	0·5 (0·2 to 0·8)	0·2 (0·1 to 0·3)	0 (0 to 0·1)	42·7
Maternal and neonatal disorders	251 (177 to 348)	101 (69·9 to 142)	30·3 (20·5 to 42·3)	18·9 (14·4 to 23·9)	13·7 (10·9 to 18)	15·1 (11·4 to 19·7)	20·4 (15·7 to 25·5)	22·3 (17·3 to 27)	21·3 (16·7 to 26·1)	20·2 (15·9 to 24·8)	17·9 (14·2 to 22·5)	15·1 (11·8 to 18·8)	12·6 (9·7 to 16·2)	10·5 (7·8 to 13·5)	7·7 (5·9 to 9·9)	4·8 (3·5 to 6·2)	2·6 (2 to 3·4)	1·1 (0·9 to 1·5)	0·4 (0·3 to 0·5)	0·1 (0·1 to 0·1)	0 (0 to 0)	0 (0 to 0)	0 (0 to 0)	588
Maternal disorders	0 (0 to 0)	0 (0 to 0)	0 (0 to 0)	0 (0 to 0)	0 (0 to 0)	0·2 (0·1 to 0·2)	4·5 (3·4 to 6)	6·3 (4·7 to 8·3)	6·1 (4·5 to 8)	5·8 (4·3 to 7·6)	4·3 (3·2 to 5·6)	2·2 (1·6 to 2·9)	0·6 (0·4 to 0·9)	0·1 (0·1 to 0·1)	0 (0 to 0)	0 (0 to 0)	0 (0 to 0)	0 (0 to 0)	0 (0 to 0)	0 (0 to 0)	0 (0 to 0)	0 (0 to 0)	0 (0 to 0)	30
Maternal hemorrhage	0 (0 to 0)	0 (0 to 0)	0 (0 to 0)	0 (0 to 0)	0 (0 to 0)	0 (0 to 0)	0·6 (0·4 to 1)	1 (0·6 to 1·6)	1 (0·6 to 1·7)	1 (0·6 to 1·5)	0·6 (0·4 to 1)	0·3 (0·2 to 0·4)	0·1 (0 to 0·1)	0 (0 to 0)	0 (0 to 0)	0 (0 to 0)	0 (0 to 0)	0 (0 to 0)	0 (0 to 0)	0 (0 to 0)	0 (0 to 0)	0 (0 to 0)	0 (0 to 0)	4·5
Maternal sepsis and other maternal infections	0 (0 to 0)	0 (0 to 0)	0 (0 to 0)	0 (0 to 0)	0 (0 to 0)	0 (0 to 0)	0·6 (0·3 to 0·9)	0·9 (0·5 to 1·3)	0·9 (0·5 to 1·3)	0·7 (0·4 to 1·1)	0·5 (0·3 to 0·8)	0·2 (0·1 to 0·4)	0·1 (0 to 0·1)	0 (0 to 0)	0 (0 to 0)	0 (0 to 0)	0 (0 to 0)	0 (0 to 0)	0 (0 to 0)	0 (0 to 0)	0 (0 to 0)	0 (0 to 0)	0 (0 to 0)	3·9
Maternal hypertensive disorders	0 (0 to 0)	0 (0 to 0)	0 (0 to 0)	0 (0 to 0)	0 (0 to 0)	0 (0 to 0·1)	1 (0·7 to 1·4)	1·1 (0·8 to 1·5)	1·1 (0·7 to 1·5)	0·9 (0·6 to 1·3)	0·6 (0·4 to 0·9)	0·3 (0·2 to 0·4)	0·1 (0 to 0·1)	0 (0 to 0)	0 (0 to 0)	0 (0 to 0)	0 (0 to 0)	0 (0 to 0)	0 (0 to 0)	0 (0 to 0)	0 (0 to 0)	0 (0 to 0)	0 (0 to 0)	5·2
Maternal obstructed labor and uterine rupture	0 (0 to 0)	0 (0 to 0)	0 (0 to 0)	0 (0 to 0)	0 (0 to 0)	0 (0 to 0)	0·2 (0·1 to 0·2)	0·2 (0·1 to 0·3)	0·2 (0·1 to 0·3)	0·2 (0·1 to 0·3)	0·1 (0·1 to 0·3)	0·1 (0 to 0·1)	0 (0 to 0)	0 (0 to 0)	0 (0 to 0)	0 (0 to 0)	0 (0 to 0)	0 (0 to 0)	0 (0 to 0)	0 (0 to 0)	0 (0 to 0)	0 (0 to 0)	0 (0 to 0)	1
Maternal abortion and miscarriage	0 (0 to 0)	0 (0 to 0)	0 (0 to 0)	0 (0 to 0)	0 (0 to 0)	0 (0 to 0)	0·4 (0·2 to 0·6)	0·5 (0·2 to 0·8)	0·4 (0·2 to 0·7)	0·3 (0·2 to 0·6)	0·3 (0·1 to 0·5)	0·2 (0·1 to 0·3)	0·1 (0 to 0·2)	0 (0 to 0)	0 (0 to 0)	0 (0 to 0)	0 (0 to 0)	0 (0 to 0)	0 (0 to 0)	0 (0 to 0)	0 (0 to 0)	0 (0 to 0)	0 (0 to 0)	2·2
Ectopic pregnancy	0 (0 to 0)	0 (0 to 0)	0 (0 to 0)	0 (0 to 0)	0 (0 to 0)	0 (0 to 0)	0·3 (0·2 to 0·5)	0·6 (0·3 to 0·9)	0·5 (0·3 to 0·9)	0·6 (0·3 to 0·9)	0·4 (0·2 to 0·6)	0·2 (0·1 to 0·3)	0·1 (0 to 0·2)	0 (0 to 0)	0 (0 to 0)	0 (0 to 0)	0 (0 to 0)	0 (0 to 0)	0 (0 to 0)	0 (0 to 0)	0 (0 to 0)	0 (0 to 0)	0 (0 to 0)	2·6
Indirect maternal deaths	--	--	--	--	--	0 (0 to 0)	0·1 (0·1 to 0·2)	0·2 (0·1 to 0·3)	0·1 (0·1 to 0·2)	0·1 (0·1 to 0·2)	0·1 (0 to 0·1)	0 (0 to 0)	0 (0 to 0)	0 (0 to 0)	--	--	--	--	--	--	--	--	--	NA
Late maternal deaths	--	--	--	--	--	0 (0 to 0)	0·4 (0·2 to 0·6)	0·5 (0·3 to 0·8)	0·5 (0·3 to 0·8)	0·4 (0·2 to 0·7)	0·3 (0·2 to 0·6)	0·2 (0·1 to 0·3)	0·1 (0 to 0·1)	0 (0 to 0)	--	--	--	--	--	--	--	--	--	NA
Maternal deaths aggravated by HIV/AIDS	--	--	--	--	--	0 (0 to 0)	0 (0 to 0)	0 (0 to 0)	0 (0 to 0)	0 (0 to 0)	0 (0 to 0)	0 (0 to 0)	0 (0 to 0)	0 (0 to 0)	--	--	--	--	--	--	--	--	--	NA
Neonatal disorders	251 (177 to 348)	101 (69·9 to 142)	30·3 (20·5 to 42·3)	18·9 (14·4 to 23·9)	13·7 (10·9 to 18)	14·9 (11·2 to 19·5)	15·9 (11·8 to 20·9)	16 (11·7 to 20·6)	15·2 (11·2 to 19·5)	14·5 (10·9 to 18·9)	13·6 (10·1 to 17·5)	12·9 (9·6 to 16·7)	12 (9·2 to 15·4)	10·4 (7·8 to 13·4)	7·7 (5·8 to 9·9)	4·8 (3·5 to 6·2)	2·6 (2 to 3·4)	1·1 (0·9 to 1·5)	0·4 (0·3 to 0·5)	0·1 (0·1 to 0·1)	0 (0 to 0)	0 (0 to 0)	0 (0 to 0)	558
Neonatal preterm birth	148 (101 to 204)	50·5 (33 to 72·3)	16·6 (10·8 to 23·8)	8·4 (6·4 to 11)	9·1 (6·6 to 12·2)	9·9 (7·1 to 13·1)	10·4 (7·5 to 13·8)	10·4 (7·4 to 13·3)	9·9 (7·2 to 13·1)	9·4 (6·8 to 12·6)	8·8 (6·4 to 11·6)	8·3 (6·1 to 10·7)	7·6 (5·6 to 10)	6·5 (4·8 to 8·6)	4·6 (3·4 to 6·1)	2·7 (2 to 3·6)	1·4 (1·1 to 1·9)	0·6 (0·5 to 0·8)	0·2 (0·2 to 0·3)	0 (0 to 0·1)	0 (0 to 0)	0 (0 to 0)	0 (0 to 0)	323
Neonatal encephalopathy due to birth asphyxia and trauma	42·2 (26·5 to 63·8)	10 (5·7 to 15·7)	3·4 (2·1 to 5·2)	2·7 (1·7 to 4)	1·7 (1 to 2·7)	1·9 (1·1 to 2·9)	2 (1·2 to 3·1)	2·1 (1·3 to 3·2)	2 (1·1 to 3·1)	1·9 (1·1 to 3)	1·8 (1·2 to 2·8)	1·8 (1·2 to 2·8)	1·7 (1 to 2·5)	1·5 (0·9 to 2·3)	1·2 (0·7 to 1·9)	0·8 (0·5 to 1·2)	0·4 (0·3 to 0·6)	0·1 (0·1 to 0·2)	0 (0 to 0·1)	0 (0 to 0)	0 (0 to 0)	0 (0 to 0)	0 (0 to 0)	79·3
Neonatal sepsis and other neonatal infections	20·4 (12·1 to 32·1)	19·3 (11·3 to 30·5)	4·5 (2·5 to 7·3)	2·2 (1·3 to 3·3)	2·6 (1·5 to 4·1)	2·9 (1·6 to 4·6)	3·1 (1·8 to 4·9)	3·2 (1·8 to 5·2)	3 (1·7 to 4·7)	2·8 (1·6 to 4·6)	2·7 (1·5 to 4·3)	2·6 (1·4 to 4·1)	2·4 (1·3 to 3·9)	2·2 (1·2 to 3·5)	1·7 (0·9 to 2·6)	1·1 (0·6 to 1·8)	0·7 (0·4 to 1·1)	0·3 (0·2 to 0·6)	0·1 (0·1 to 0·2)	0 (0 to 0)	0 (0 to 0)	0 (0 to 0)	0 (0 to 0)	77·9
Hemolytic disease and other neonatal jaundice	3·1 (1·7 to 5·6)	2 (0·9 to 3·8)	0·4 (0·2 to 0·6)	0·3 (0·2 to 0·6)	0·3 (0·2 to 0·3)	0·3 (0·2 to 0·4)	0·3 (0·2 to 0·4)	0·3 (0·2 to 0·4)	0·3 (0·2 to 0·4)	0·3 (0·2 to 0·4)	0·3 (0·2 to 0·4)	0·3 (0·2 to 0·4)	0·3 (0·2 to 0·3)	0·2 (0·2 to 0·3)	0·2 (0·1 to 0·2)	0·1 (0·1 to 0·2)	0·1 (0·1 to 0·1)	0 (0 to 0·1)	0 (0 to 0)	0 (0 to 0)	0 (0 to 0)	0 (0 to 0)	0 (0 to 0)	9·2
Other neonatal disorders	37·5 (22·9 to 58·6)	19·5 (11·9 to 29·4)	5·5 (3·4 to 8·4)	5·2 (4 to 6·7)	0 (0 to 0)	0 (0 to 0)	0 (0 to 0)	0 (0 to 0)	0 (0 to 0)	0 (0 to 0)	0 (0 to 0)	0 (0 to 0)	0 (0 to 0)	0 (0 to 0)	0 (0 to 0)	0 (0 to 0)	0 (0 to 0)	0 (0 to 0)	0 (0 to 0)	0 (0 to 0)	0 (0 to 0)	0 (0 to 0)	0 (0 to 0)	67·6
Nutritional deficiencies	0 (0 to 0·1)	0·9 (0·5 to 1·5)	16·1 (10·7 to 23·7)	36·4 (20·6 to 59·9)	31·8 (14·2 to 59·1)	16·6 (7 to 30·9)	18·4 (8·6 to 33·3)	13·5 (6·7 to 24·7)	13·5 (6·8 to 25·1)	15·1 (7·1 to 27·7)	21·3 (10·2 to 38·6)	24·4 (11·9 to 44·3)	22·2 (11 to 38·6)	24·3 (13·2 to 41·9)	21·4 (12 to 34)	14·9 (8·9 to 24·2)	9·1 (6·2 to 13·7)	8·2 (5·6 to 11·7)	7 (5·1 to 9·4)	5·8 (4·2 to 8·2)	4·2 (2·9 to 5·7)	2·3 (1·5 to 3·2)	1 (0·7 to 1·4)	328
Protein-energy malnutrition	0 (0 to 0)	0·1 (0 to 0·2)	9·9 (6 to 15·8)	11·1 (6·2 to 18·6)	2·5 (1·7 to 3·5)	1·4 (1 to 2)	1·8 (1·3 to 2·4)	2·2 (1·6 to 3)	2·3 (1·7 to 3·2)	2·3 (1·6 to 3·2)	2·9 (2·1 to 4)	3·1 (2·2 to 3·9)	3·6 (2·6 to 4·7)	4·4 (3·3 to 5·8)	4·8 (3·5 to 6·4)	5·4 (4·1 to 7)	4·8 (3·6 to 6·2)	5·3 (3·7 to 7·3)	4·9 (3·7 to 6·4)	4·5 (3·1 to 6·3)	3·2 (2·2 to 4·6)	1·9 (1·2 to 2·7)	0·9 (0·6 to 1·3)	83·4
Iodine deficiency	0 (0 to 0)	0 (0 to 0)	0 (0 to 0)	0 (0 to 0)	0·1 (0 to 0·2)	0·2 (0·1 to 0·3)	0·2 (0·1 to 0·5)	0·3 (0·1 to 0·5)	0·3 (0·1 to 0·6)	0·3 (0·1 to 0·6)	0·3 (0·2 to 0·6)	0·3 (0·1 to 0·6)	0·3 (0·2 to 0·5)	0·3 (0·1 to 0·5)	0·2 (0·1 to 0·4)	0·2 (0·1 to 0·3)	0·1 (0 to 0·2)	0·1 (0 to 0·1)	0 (0 to 0·1)	0 (0 to 0)	0 (0 to 0)	0 (0 to 0)	0 (0 to 0)	3·4
Vitamin A deficiency	0 (0 to 0)	0 (0 to 0)	0·1 (0 to 0·2)	0·7 (0·3 to 1·2)	0·8 (0·4 to 1·3)	0·5 (0·2 to 0·9)	0·3 (0·1 to 0·5)	0·2 (0·1 to 0·4)	0·2 (0·1 to 0·4)	0·2 (0·1 to 0·3)	0·2 (0·1 to 0·4)	0·1 (0·1 to 0·3)	0·1 (0·1 to 0·2)	0·1 (0·1 to 0·2)	0·1 (0 to 0·1)	0·1 (0 to 0·1)	0 (0 to 0·1)	0 (0 to 0·1)	0 (0 to 0)	0 (0 to 0)	0 (0 to 0)	0 (0 to 0)	0 (0 to 0)	3·8
Dietary iron deficiency	0 (0 to 0)	0·8 (0·4 to 1·3)	6 (2·9 to 10·8)	24·5 (10·6 to 46·8)	28·3 (11·1 to 55·2)	14·2 (5·1 to 28·2)	15·6 (5·9 to 30·5)	10·1 (3·7 to 20·9)	9·8 (3·6 to 20·5)	11·6 (4·1 to 23·9)	17·3 (6·4 to 34·3)	20·3 (8·2 to 39·8)	17·5 (6·9 to 34)	18·6 (8 to 35·6)	15·4 (6·5 to 27·5)	8·7 (3·5 to 17·5)	3·5 (1·2 to 7·6)	2·5 (0·8 to 5·4)	1·7 (0·5 to 3·7)	1·1 (0·4 to 2·4)	0·8 (0·3 to 1·7)	0·4 (0·1 to 0·7)	0·1 (0 to 0·1)	229
Other nutritional deficiencies	0 (0 to 0)	0 (0 to 0)	0·1 (0·1 to 0·1)	0·1 (0·1 to 0·2)	0·2 (0·1 to 0·4)	0·2 (0·1 to 0·4)	0·5 (0·3 to 0·7)	0·6 (0·4 to 0·9)	0·8 (0·5 to 1·3)	0·6 (0·4 to 0·9)	0·6 (0·4 to 1)	0·7 (0·4 to 0·9)	0·6 (0·4 to 0·9)	0·9 (0·6 to 1·3)	0·8 (0·5 to 1·1)	0·6 (0·4 to 0·9)	0·6 (0·4 to 0·8)	0·4 (0·3 to 0·6)	0·3 (0·2 to 0·4)	0·2 (0·1 to 0·3)	0·1 (0·1 to 0·2)	0·1 (0 to 0·1)	0 (0 to 0)	8·9
Non-communicable diseases	51 (33·8 to 73·9)	34·9 (23·9 to 48·8)	102 (74·1 to 136)	194 (142 to 262)	196 (151 to 251)	298 (223 to 392)	473 (359 to 615)	610 (477 to 766)	703 (556 to 874)	776 (617 to 969)	923 (756 to 1,130)	1,090 (901 to 1,320)	1,380 (1,150 to 1,640)	1,770 (1,490 to 2,070)	2,020 (1,720 to 2,350)	2,090 (1,800 to 2,410)	1,840 (1,610 to 2,090)	1,680 (1,490 to 1,890)	1,370 (1,230 to 1,530)	1,070 (972 to 1,160)	627 (582 to 672)	279 (262 to 296)	92·5 (87·1 to 97·9)	19,700
Neoplasms	0·7 (0·3 to 1·2)	0·9 (0·4 to 2·4)	3·3 (2·2 to 4·9)	26·4 (16·3 to 40·7)	21·7 (16·9 to 27·5)	17·3 (13·8 to 21·4)	23·3 (18·7 to 29)	32·2 (26·8 to 39·4)	39·1 (33·2 to 46·8)	52 (43 to 62)	86·4 (71·7 to 104)	132 (111 to 158)	209 (170 to 255)	320 (261 to 397)	418 (349 to 508)	456 (376 to 544)	393 (335 to 459)	365 (309 to 427)	294 (252 to 342)	210 (176 to 244)	103 (85·6 to 122)	38·4 (30·1 to 45·3)	10·1 (7·8 to 12·8)	3,250
Lip and oral cavity cancer	0 (0 to 0)	0 (0 to 0)	0 (0 to 0)	0 (0 to 0)	0 (0 to 0·1)	0·1 (0 to 0·1)	0·1 (0·1 to 0·2)	0·2 (0·1 to 0·3)	0·3 (0·2 to 0·4)	0·4 (0·3 to 0·6)	0·8 (0·5 to 1·2)	1·4 (0·9 to 2·1)	2·9 (1·9 to 4·3)	5·3 (3·7 to 7·4)	6·9 (4·9 to 9·5)	7·2 (5·1 to 9·7)	5·1 (3·6 to 6·9)	4·3 (3·1 to 5·8)	3·2 (2·5 to 4·2)	2·2 (1·6 to 2·9)	0·8 (0·6 to 1·1)	0·3 (0·2 to 0·4)	0·1 (0·1 to 0·1)	41·7
Nasopharynx cancer	0 (0 to 0)	0 (0 to 0)	0 (0 to 0)	0 (0 to 0)	0·1 (0 to 0·1)	0·1 (0·1 to 0·2)	0·2 (0·1 to 0·3)	0·3 (0·1 to 0·4)	0·3 (0·1 to 0·4)	0·2 (0·1 to 0·3)	0·3 (0·2 to 0·5)	0·5 (0·3 to 0·8)	0·9 (0·6 to 1·2)	1·4 (1 to 2)	1·9 (1·3 to 2·6)	1·6 (1 to 2·3)	1·1 (0·7 to 1·6)	0·9 (0·6 to 1·3)	0·5 (0·3 to 0·7)	0·3 (0·2 to 0·5)	0·2 (0·1 to 0·2)	0·1 (0 to 0·1)	0 (0 to 0)	10·7
Other pharynx cancer	0 (0 to 0)	0 (0 to 0)	0 (0 to 0)	0 (0 to 0)	0 (0 to 0)	0 (0 to 0)	0 (0 to 0)	0·1 (0 to 0·2)	0·1 (0·1 to 0·2)	0·2 (0·1 to 0·3)	0·5 (0·3 to 0·6)	1 (0·5 to 1·2)	2 (1·4 to 3·1)	3·8 (2·5 to 5·4)	5·2 (3·4 to 7·2)	5·2 (3·2 to 8)	3·4 (2·2 to 5·1)	2·5 (1·7 to 3·8)	1·6 (1 to 2·3)	1 (0·6 to 1·5)	0·4 (0·3 to 0·6)	0·2 (0·1 to 0·2)	0·1 (0 to 0·1)	27·1
Esophageal cancer	0 (0 to 0)	0 (0 to 0)	0 (0 to 0)	0 (0 to 0)	0 (0 to 0)	0 (0 to 0)	0 (0 to 0·1)	0·1 (0·1 to 0·2)	0·2 (0·1 to 0·3)	0·3 (0·2 to 0·5)	0·9 (0·6 to 1·4)	2·1 (1·3 to 3·1)	4·7 (3·4 to 6·4)	8·9 (6·5 to 12·1)	11·8 (8·1 to 16·1)	12·8 (9 to 18)	9·6 (7·1 to 12·9)	8·1 (5·8 to 11·4)	5·7 (4 to 8)	3·7 (2·5 to 5·1)	1·7 (1·2 to 2·3)	0·6 (0·4 to 0·8)	0·1 (0·1 to 0·2)	71·4
Stomach cancer	0 (0 to 0)	0 (0 to 0)	0 (0 to 0)	0 (0 to 0)	0 (0 to 0)	0·1 (0 to 0·1)	0·4 (0·3 to 0·5)	0·9 (0·6 to 1·3)	2 (1·4 to 2·7)	3·2 (2·1 to 4·5)	5·5 (3·7 to 7·9)	8·2 (6·2 to 10·8)	12·9 (9·8 to 16·9)	22·3 (17·2 to 30·5)	30·8 (21·6 to 40·8)	36·4 (27·8 to 46·3)	30·6 (24·5 to 37·6)	28·3 (21·3 to 36·5)	22·3 (17·3 to 27·8)	15·9 (11·9 to 20·5)	7·5 (5·6 to 9·9)	2·7 (2 to 3·4)	0·7 (0·5 to 0·9)	231
Colon and rectum cancer	0 (0 to 0)	0 (0 to 0)	0 (0 to 0)	0 (0 to 0)	0·2 (0·1 to 0·2)	0·2 (0·1 to 0·2)	0·7 (0·5 to 1)	1·5 (1·1 to 2·1)	2·4 (1·8 to 3·2)	4 (2·9 to 5·2)	6·7 (5 to 9)	10 (7·5 to 13·3)	17·1 (12·5 to 23)	30 (22·2 to 39·9)	42·3 (31·5 to 54·9)	49·4 (38 to 63·1)	43·9 (34·2 to 55·4)	40·9 (32 to 51·5)	33·5 (26·9 to 41·3)	25 (19·8 to 31·3)	12·6 (9·6 to 16)	4·9 (3·6 to 6·3)	1·3 (0·9 to 1·7)	327
Liver cancer	0 (0 to 0)	0 (0 to 0)	0·1 (0 to 0·1)	0·2 (0·2 to 0·4)	0·2 (0·2 to 0·3)	0·3 (0·2 to 0·4)	0·2 (0·1 to 0·3)	0·5 (0·3 to 0·8)	0·8 (0·5 to 1·2)	0·8 (0·5 to 1·3)	1·6 (1·1 to 2·5)	2·4 (1·6 to 3·4)	3·9 (2·5 to 5·7)	6·7 (4·5 to 9·4)	9·8 (6·9 to 13·8)	10·7 (7·8 to 14·4)	8·9 (6·5 to 11·9)	8·1 (6·1 to 10·8)	5·8 (4·5 to 7·4)	3·6 (2·9 to 4·6)	1·2 (0·9 to 1·6)	0·4 (0·3 to 0·5)	0·1 (0·1 to 0·1)	66·6
Liver cancer due to hepatitis B	0 (0 to 0)	0 (0 to 0)	0 (0 to 0)	0 (0 to 0)	0 (0 to 0)	0 (0 to 0·1)	0·1 (0 to 0·2)	0·3 (0·2 to 0·5)	0·5 (0·3 to 0·8)	0·5 (0·3 to 0·8)	0·9 (0·5 to 1·5)	1·2 (0·6 to 1·9)	1·7 (0·9 to 2·8)	2·6 (1·4 to 4·3)	3·5 (1·9 to 5·7)	3·2 (1·7 to 5·5)	2·1 (1·1 to 3·5)	1·6 (0·7 to 2·8)	1 (0·5 to 1·7)	0·6 (0·3 to 1)	0·2 (0·1 to 0·3)	0 (0 to 0·1)	0 (0 to 0)	19·9
Liver cancer due to hepatitis C	0 (0 to 0)	0 (0 to 0)	0 (0 to 0)	0 (0 to 0)	0 (0 to 0)	0 (0 to 0)	0 (0 to 0)	0 (0 to 0)	0 (0 to 0·1)	0 (0 to 0·1)	0·1 (0 to 0·2)	0·2 (0·1 to 0·4)	0·5 (0·2 to 0·9)	1 (0·5 to 1·8)	1·8 (0·8 to 3·2)	2 (0·9 to 3·6)	1·9 (1·1 to 3)	1·9 (1·1 to 3·3)	1·6 (0·9 to 2·7)	1·2 (0·7 to 1·9)	0·5 (0·3 to 0·7)	0·2 (0·1 to 0·3)	0·1 (0 to 0·1)	13
Liver cancer due to alcohol use	0 (0 to 0)	0 (0 to 0)	0 (0 to 0)	0 (0 to 0)	0 (0 to 0)	0 (0 to 0)	0 (0 to 0)	0 (0 to 0·1)	0·1 (0 to 0·2)	0·1 (0·1 to 0·3)	0·4 (0·1 to 0·7)	0·6 (0·3 to 1·1)	1·2 (0·5 to 2·1)	2 (1 to 3·7)	3·4 (1·8 to 5·5)	4·1 (2·2 to 6·4)	3·7 (2·2 to 5·6)	3·4 (2·1 to 5·2)	2·2 (1·4 to 3·2)	1·2 (0·7 to 1·8)	0·3 (0·2 to 0·5)	0·1 (0 to 0·1)	0 (0 to 0)	23·1
Liver cancer due to NASH	0 (0 to 0)	0 (0 to 0)	0 (0 to 0)	0 (0 to 0)	0 (0 to 0)	0 (0 to 0)	0 (0 to 0)	0·1 (0 to 0·1)	0·1 (0 to 0·1)	0·1 (0 to 0·1)	0·1 (0 to 0·2)	0·2 (0·1 to 0·3)	0·3 (0·1 to 0·5)	0·5 (0·2 to 0·9)	0·7 (0·4 to 1·4)	0·8 (0·4 to 1·5)	0·8 (0·4 to 1·4)	0·8 (0·4 to 1·3)	0·7 (0·4 to 1·1)	0·5 (0·3 to 0·8)	0·2 (0·1 to 0·3)	0·1 (0 to 0·1)	0 (0 to 0)	5·8
Liver cancer due to other causes	0 (0 to 0)	0 (0 to 0)	0·1 (0 to 0·1)	0·2 (0·2 to 0·4)	0·2 (0·2 to 0·3)	0·3 (0·2 to 0·3)	0·1 (0 to 0·1)	0·1 (0·1 to 0·2)	0·1 (0·1 to 0·2)	0·1 (0 to 0·2)	0·1 (0·1 to 0·3)	0·2 (0·1 to 0·3)	0·3 (0·2 to 0·5)	0·5 (0·2 to 0·9)	0·6 (0·3 to 1·1)	0·5 (0·3 to 1)	0·4 (0·2 to 0·7)	0·4 (0·2 to 0·7)	0·3 (0·2 to 0·5)	0·2 (0·1 to 0·3)	0·1 (0 to 0·1)	0 (0 to 0)	0 (0 to 0)	4·8
Gallbladder and biliary tract cancer	0 (0 to 0)	0 (0 to 0)	0 (0 to 0)	0 (0 to 0)	0 (0 to 0)	0 (0 to 0)	0 (0 to 0)	0·1 (0·1 to 0·2)	0·1 (0·1 to 0·2)	0·2 (0·1 to 0·3)	0·5 (0·3 to 0·8)	0·9 (0·6 to 1·6)	1·6 (1·1 to 2·9)	3·1 (2·1 to 5)	4·5 (3 to 7·4)	5·1 (3·5 to 8·4)	4·5 (3·1 to 7·3)	4 (2·8 to 6·5)	3 (2·1 to 5)	1·9 (1·3 to 3)	0·8 (0·6 to 1·4)	0·3 (0·2 to 0·5)	0·1 (0 to 0·1)	30·6
Pancreatic cancer	0 (0 to 0)	0 (0 to 0)	0 (0 to 0)	0 (0 to 0)	0 (0 to 0)	0 (0 to 0)	0·2 (0·1 to 0·3)	0·3 (0·2 to 0·4)	0·5 (0·3 to 0·7)	1·1 (0·8 to 1·6)	2·3 (1·6 to 3·2)	3·8 (2·9 to 5·9)	8·4 (5·9 to 10·4)	14 (10·3 to 18·9)	18 (13·6 to 27·6)	20·1 (16·2 to 33)	17·8 (13·7 to 26·7)	16·9 (13·5 to 23·8)	12·5 (9 to 17)	8·1 (5·8 to 11·6)	3·8 (2·9 to 4·9)	1·3 (0·9 to 1·8)	0·3 (0·2 to 0·4)	138
Larynx cancer	0 (0 to 0)	0 (0 to 0)	0 (0 to 0)	0 (0 to 0)	0 (0 to 0)	0 (0 to 0)	0 (0 to 0)	0·1 (0·1 to 0·2)	0·1 (0·1 to 0·2)	0·1 (0·1 to 0·2)	0·4 (0·3 to 0·7)	0·9 (0·5 to 1·6)	2·3 (1·4 to 3·6)	5 (3·3 to 7·2)	6·9 (4·7 to 9·3)	7·8 (5·4 to 11·2)	5·6 (3·8 to 7·7)	4·4 (3·1 to 6·2)	2·9 (2·1 to 4·1)	1·9 (1·3 to 2·6)	0·7 (0·4 to 1)	0·2 (0·1 to 0·3)	0 (0 to 0·1)	39·5
Tracheal, bronchus, and lung cancer	0 (0 to 0)	0 (0 to 0)	0 (0 to 0)	0 (0 to 0)	0 (0 to 0)	0 (0 to 0·1)	0·3 (0·2 to 0·5)	0·5 (0·4 to 0·8)	0·8 (0·5 to 1)	1·5 (1 to 2·1)	3·2 (2·2 to 4·5)	6·1 (4·4 to 8·2)	12·9 (8·8 to 17·6)	24 (17·1 to 32·8)	34·7 (25·8 to 44·8)	40·3 (31 to 51·1)	31·9 (25·2 to 39·3)	27·3 (21·7 to 33·7)	18·2 (14·5 to 22·6)	11·4 (8·8 to 14·9)	4·8 (3·6 to 6·2)	1·4 (1·1 to 1·8)	0·4 (0·3 to 0·5)	220
Malignant skin melanoma	0 (0 to 0)	0 (0 to 0)	0 (0 to 0·1)	0·2 (0 to 0·5)	0·1 (0 to 0·1)	0·1 (0 to 0·1)	0·2 (0·1 to 0·3)	0·5 (0·3 to 0·7)	0·7 (0·5 to 1)	0·8 (0·6 to 1·1)	1·6 (1·1 to 2·3)	1·6 (1·1 to 2·4)	2·1 (1·4 to 3)	2·9 (2 to 4·1)	3·3 (2·2 to 4·8)	3 (2 to 4·2)	2·4 (1·6 to 3·3)	2·1 (1·4 to 3)	1·5 (1 to 2)	1·2 (0·8 to 1·7)	0·7 (0·5 to 0·9)	0·2 (0·2 to 0·4)	0·1 (0·1 to 0·1)	25·1
Non-melanoma skin cancer	0 (0 to 0)	0 (0 to 0)	0 (0 to 0)	0 (0 to 0)	0 (0 to 0)	0 (0 to 0)	0 (0 to 0)	0·1 (0·1 to 0·2)	0·3 (0·2 to 0·4)	0·3 (0·2 to 0·5)	0·4 (0·3 to 0·6)	0·7 (0·4 to 0·9)	0·8 (0·5 to 1·2)	1·5 (0·9 to 2·1)	1·8 (1·1 to 2·6)	2·1 (1·2 to 3)	1·9 (1·2 to 2·6)	1·8 (1·1 to 2·4)	1·7 (1 to 2·3)	1·6 (0·9 to 2·2)	1 (0·5 to 1·4)	0·6 (0·3 to 0·9)	0·3 (0·1 to 0·4)	17·2
Non-melanoma skin cancer (squamous-cell carcinoma)	0 (0 to 0)	0 (0 to 0)	0 (0 to 0)	0 (0 to 0)	0 (0 to 0)	0 (0 to 0)	0 (0 to 0)	0·1 (0·1 to 0·2)	0·3 (0·2 to 0·4)	0·3 (0·2 to 0·5)	0·4 (0·3 to 0·6)	0·7 (0·4 to 0·9)	0·8 (0·5 to 1·2)	1·5 (0·9 to 2·1)	1·8 (1·1 to 2·6)	2·1 (1·2 to 3)	1·9 (1·2 to 2·6)	1·8 (1·1 to 2·4)	1·7 (1 to 2·3)	1·6 (0·9 to 2·2)	1 (0·5 to 1·4)	0·6 (0·3 to 0·9)	0·3 (0·1 to 0·4)	17·2
Non-melanoma skin cancer (basal-cell carcinoma)	0 (0 to 0)	0 (0 to 0)	0 (0 to 0)	0 (0 to 0)	0 (0 to 0)	0 (0 to 0)	0 (0 to 0)	0 (0 to 0)	0 (0 to 0)	0 (0 to 0)	0 (0 to 0)	0 (0 to 0)	0 (0 to 0)	0 (0 to 0)	0 (0 to 0)	0 (0 to 0)	0 (0 to 0)	0 (0 to 0)	0 (0 to 0)	0 (0 to 0)	0 (0 to 0)	0 (0 to 0)	0 (0 to 0)	0
Breast cancer	0 (0 to 0)	0 (0 to 0)	0 (0 to 0)	0 (0 to 0)	0 (0 to 0)	0 (0 to 0)	0·4 (0·3 to 0·7)	0·7 (0·5 to 1)	2·6 (1·9 to 3·7)	9·5 (6·8 to 13·2)	19·6 (14·3 to 26·4)	35·4 (25·6 to 46·9)	53·3 (39·3 to 71·3)	69·6 (51·1 to 91·2)	77·5 (56·9 to 101)	58·7 (43·2 to 76·9)	47·2 (35·2 to 61·4)	34·3 (26·1 to 43·3)	23·3 (17·8 to 30)	15·1 (11·1 to 20·1)	9·3 (6·8 to 12·4)	3·6 (2·6 to 4·8)	1·1 (0·7 to 1·7)	461
Cervical cancer	0 (0 to 0)	0 (0 to 0)	0 (0 to 0)	0 (0 to 0)	0 (0 to 0)	0 (0 to 0)	0·3 (0·2 to 0·5)	1 (0·6 to 1·6)	2·7 (1·6 to 4·1)	6 (3·7 to 9)	11·6 (7·3 to 16·6)	17 (11·5 to 24)	22·4 (15·3 to 31·8)	24·7 (16·6 to 35·7)	25·7 (17·9 to 35·3)	20·2 (13·8 to 27·7)	15·5 (10·9 to 20·8)	12 (8·5 to 16·4)	7·6 (5·4 to 10)	4·4 (3 to 6)	2 (1·4 to 2·7)	0·8 (0·5 to 1·1)	0·2 (0·2 to 0·3)	174
Uterine cancer	0 (0 to 0)	0 (0 to 0)	0 (0 to 0)	0 (0 to 0)	0 (0 to 0)	0 (0 to 0)	0 (0 to 0)	0·2 (0·1 to 0·3)	0·4 (0·2 to 0·7)	0·9 (0·5 to 1·5)	1·7 (1·1 to 2·5)	3 (1·9 to 4·5)	5·4 (3·5 to 7·8)	7·8 (5 to 11·4)	10·1 (6·6 to 14·9)	12·4 (8·2 to 18)	10·9 (7·5 to 15·7)	8·7 (6 to 12·5)	5·3 (3·6 to 7·5)	3·1 (2·1 to 4·3)	1·4 (1 to 1·9)	0·5 (0·4 to 0·7)	0·1 (0·1 to 0·2)	72
Ovarian cancer	0 (0 to 0)	0 (0 to 0)	0 (0 to 0)	0 (0 to 0)	0·1 (0·1 to 0·2)	0·3 (0·1 to 0·4)	0·8 (0·4 to 1·3)	1 (0·6 to 1·6)	1 (0·7 to 1·5)	1·7 (1·2 to 2·5)	3·1 (2·1 to 4·4)	6·1 (4 to 8·8)	10·1 (6·2 to 14·4)	13·6 (8·2 to 19·6)	15·6 (9 to 22·2)	13·7 (7·8 to 19)	11·7 (6·3 to 16·6)	8·7 (4·6 to 12)	5·4 (2·6 to 7·6)	3·1 (1·5 to 4·5)	1·6 (0·8 to 2·3)	0·6 (0·3 to 0·8)	0·1 (0 to 0·2)	98·3
Prostate cancer	0 (0 to 0)	0 (0 to 0)	0 (0 to 0)	0 (0 to 0)	0 (0 to 0)	0 (0 to 0)	0 (0 to 0)	0·2 (0·2 to 0·3)	0·5 (0·4 to 0·7)	0·5 (0·4 to 0·7)	0·7 (0·5 to 1)	1·4 (0·9 to 2·2)	4·9 (3·1 to 7·6)	15·7 (10·4 to 23·3)	33·9 (21·5 to 51·1)	69·3 (47·7 to 98·4)	75·4 (53·1 to 107)	94·7 (67·1 to 130)	99·9 (71·9 to 132)	80·2 (54·9 to 106)	40·6 (28·4 to 53·1)	15·4 (10·3 to 20·6)	4 (2·4 to 5·9)	537
Testicular cancer	0 (0 to 0·2)	0 (0 to 0·1)	0 (0 to 0·1)	0·1 (0 to 0·3)	0·1 (0·1 to 0·2)	0·1 (0·1 to 0·2)	0·4 (0·2 to 0·7)	0·8 (0·5 to 1·3)	1 (0·6 to 1·6)	0·5 (0·3 to 0·8)	0·5 (0·3 to 0·8)	0·3 (0·2 to 0·5)	0·2 (0·1 to 0·3)	0·2 (0·1 to 0·4)	0·2 (0·1 to 0·3)	0·2 (0·1 to 0·3)	0·1 (0·1 to 0·3)	0·1 (0·1 to 0·2)	0·1 (0·1 to 0·2)	0·1 (0 to 0·1)	0 (0 to 0·1)	0 (0 to 0)	0 (0 to 0)	5·3
Kidney cancer	0·1 (0 to 0·5)	0·1 (0 to 0·4)	0·2 (0·1 to 0·3)	1·6 (0·9 to 2·7)	1·1 (0·7 to 1·5)	0·4 (0·2 to 0·6)	0·6 (0·3 to 0·9)	0·9 (0·6 to 1·4)	1·1 (0·7 to 1·7)	1·1 (0·6 to 1·7)	1·3 (0·8 to 2·1)	2·1 (1·3 to 3·2)	3·5 (2·4 to 4·9)	5·8 (4·1 to 8·3)	7·7 (5 to 11·2)	7·9 (5·3 to 11·4)	6·1 (4·3 to 8·4)	5 (3·3 to 7·3)	3·1 (2·2 to 4·4)	1·9 (1·3 to 2·8)	0·7 (0·4 to 1)	0·2 (0·2 to 0·4)	0·1 (0 to 0·1)	52·6
Bladder cancer	0 (0 to 0)	0 (0 to 0)	0 (0 to 0)	0 (0 to 0)	0 (0 to 0)	0 (0 to 0)	0·1 (0 to 0·1)	0·1 (0·1 to 0·1)	0·1 (0·1 to 0·2)	0·3 (0·2 to 0·4)	0·4 (0·3 to 0·7)	0·8 (0·6 to 1·2)	1·5 (1 to 2)	3·4 (2·6 to 4·6)	5·5 (3·7 to 7·5)	7·3 (5·1 to 10·2)	7·3 (5·4 to 9·7)	7·8 (5·6 to 10·4)	6·9 (4·8 to 9·3)	5·2 (3·6 to 7)	2·7 (1·9 to 3·8)	1·1 (0·8 to 1·5)	0·3 (0·2 to 0·4)	50·9
Brain and central nervous system cancer	0·3 (0 to 1·4)	0·2 (0 to 1·2)	0·6 (0·3 to 1·2)	4·2 (1·6 to 8·2)	4·1 (1·8 to 6·4)	3·2 (1·5 to 5)	2·4 (1·1 to 3·8)	2·7 (1·1 to 4·2)	2·9 (1·2 to 4·4)	3 (1·3 to 4·5)	4·1 (1·8 to 6)	4·8 (2·1 to 7·2)	5·8 (2·5 to 9)	7·8 (3·4 to 12·3)	9 (3·8 to 13·9)	7·8 (3·2 to 11·9)	5·9 (2·5 to 8·9)	4·2 (1·7 to 6·5)	2·5 (1 to 4)	1·2 (0·5 to 2)	0·5 (0·2 to 0·9)	0·2 (0·1 to 0·3)	0 (0 to 0·1)	77·6
Thyroid cancer	0 (0 to 0)	0 (0 to 0)	0 (0 to 0)	0 (0 to 0)	0·1 (0 to 0·1)	0·1 (0 to 0·1)	0·1 (0·1 to 0·2)	0·2 (0·1 to 0·3)	0·3 (0·2 to 0·5)	0·3 (0·2 to 0·5)	0·6 (0·4 to 0·9)	0·9 (0·5 to 1·3)	1·4 (0·9 to 2)	2·4 (1·6 to 3·4)	2·9 (2 to 4)	2·8 (1·9 to 3·8)	2·5 (1·8 to 3·4)	2·3 (1·6 to 3·1)	1·5 (1·1 to 2·1)	1 (0·7 to 1·4)	0·4 (0·3 to 0·6)	0·2 (0·1 to 0·2)	0 (0 to 0·1)	19·9
Mesothelioma	0 (0 to 0)	0 (0 to 0)	0 (0 to 0)	0 (0 to 0)	0 (0 to 0)	0 (0 to 0)	0 (0 to 0)	0·1 (0 to 0·1)	0·1 (0 to 0·1)	0·1 (0 to 0·1)	0·1 (0 to 0·2)	0·2 (0·1 to 0·2)	0·2 (0·2 to 0·3)	0·4 (0·3 to 0·5)	0·5 (0·4 to 0·7)	0·2 (0·2 to 0·3)	0·2 (0·1 to 0·2)	0·2 (0·1 to 0·2)	0·1 (0·1 to 0·2)	0·1 (0·1 to 0·1)	0 (0 to 0·1)	0 (0 to 0)	0 (0 to 0)	2·5
Hodgkin lymphoma	0 (0 to 0)	0 (0 to 0)	0 (0 to 0)	0 (0 to 0·1)	0·1 (0 to 0·1)	0·1 (0 to 0·1)	0·2 (0·2 to 0·3)	0·5 (0·3 to 0·7)	0·5 (0·3 to 0·7)	0·3 (0·2 to 0·5)	0·3 (0·2 to 0·5)	0·3 (0·2 to 0·4)	0·2 (0·2 to 0·4)	0·3 (0·2 to 0·4)	0·3 (0·2 to 0·5)	0·3 (0·2 to 0·4)	0·3 (0·2 to 0·3)	0·2 (0·1 to 0·3)	0·1 (0·1 to 0·2)	0·1 (0·1 to 0·1)	0 (0 to 0)	0 (0 to 0)	0 (0 to 0)	4·2
Non-Hodgkin lymphoma	0 (0 to 0)	0 (0 to 0)	0 (0 to 0)	2·3 (1·4 to 3·7)	2·4 (1·5 to 3·6)	2 (1·4 to 2·8)	3·1 (2·2 to 4·3)	5 (3·4 to 7·1)	5·3 (3·9 to 7·2)	4·6 (3·1 to 6·7)	5·4 (3·9 to 7·3)	5·2 (3·8 to 7·2)	7·2 (5·2 to 9·7)	9·9 (7·2 to 13·5)	12·3 (9·1 to 16·3)	12·4 (9·1 to 16·1)	9·8 (6·8 to 13·6)	8·4 (5·7 to 11·5)	5·6 (4 to 7·6)	3·2 (2·3 to 4·5)	1·2 (0·8 to 1·6)	0·4 (0·3 to 0·5)	0·1 (0·1 to 0·1)	106
Multiple myeloma	0 (0 to 0)	0 (0 to 0)	0 (0 to 0)	0 (0 to 0)	0 (0 to 0)	0 (0 to 0)	0 (0 to 0)	0·2 (0·1 to 0·3)	0·2 (0·2 to 0·3)	0·3 (0·2 to 0·4)	0·8 (0·6 to 1·1)	1·8 (1·4 to 2·5)	3·8 (2·8 to 5·2)	7·5 (5·7 to 10)	11 (8·3 to 14·6)	12·6 (9·3 to 16·4)	11·2 (8·7 to 14·2)	9·7 (7·5 to 12·3)	6·7 (5·2 to 8·3)	3·9 (2·9 to 4·9)	1·5 (1·1 to 1·9)	0·4 (0·3 to 0·5)	0·1 (0·1 to 0·1)	71·7
Leukemia	0·2 (0 to 0·6)	0·3 (0 to 1·3)	1 (0·6 to 1·5)	7·9 (4·5 to 13·2)	7·5 (5·1 to 10·8)	6·3 (4·5 to 8·6)	7·3 (5·2 to 9·9)	7·8 (5·4 to 10·9)	6·7 (4·9 to 8·9)	5 (3·7 to 6·5)	5·9 (4·4 to 7·9)	6·1 (4·5 to 8·2)	7·5 (5·5 to 9·9)	9·2 (6·7 to 12·2)	10·8 (7·9 to 14·4)	10·9 (7·9 to 14·6)	9·1 (6·6 to 11·9)	8·3 (6·3 to 10·9)	6 (4·5 to 8)	4·1 (3 to 5·5)	2 (1·4 to 2·8)	0·7 (0·5 to 1)	0·2 (0·1 to 0·2)	131
Acute lymphoid leukemia	0 (0 to 0·1)	0 (0 to 0·1)	0·2 (0·1 to 0·4)	3 (1·6 to 5·3)	3·2 (2 to 4·9)	2·2 (1·3 to 3·2)	2·3 (1·5 to 3·5)	1·4 (0·8 to 2·2)	0·7 (0·5 to 1·1)	0·5 (0·3 to 0·7)	0·4 (0·3 to 0·7)	0·4 (0·3 to 0·7)	0·4 (0·2 to 0·7)	0·6 (0·3 to 0·9)	0·6 (0·3 to 0·9)	0·5 (0·3 to 0·7)	0·4 (0·2 to 0·6)	0·3 (0·2 to 0·4)	0·2 (0·1 to 0·4)	0·1 (0·1 to 0·2)	0 (0 to 0·1)	0 (0 to 0)	0 (0 to 0)	17·6
Chronic lymphoid leukemia	0 (0 to 0)	0 (0 to 0)	0 (0 to 0)	0 (0 to 0)	0 (0 to 0)	0 (0 to 0)	0 (0 to 0)	0·1 (0·1 to 0·1)	0·1 (0 to 0·1)	0 (0 to 0·1)	0 (0 to 0·1)	0·1 (0 to 0·1)	0·1 (0 to 0·1)	0·2 (0·1 to 0·3)	0·3 (0·2 to 0·4)	0·3 (0·2 to 0·5)	0·3 (0·2 to 0·5)	0·3 (0·2 to 0·4)	0·2 (0·1 to 0·3)	0·1 (0·1 to 0·3)	0·1 (0 to 0·1)	0 (0 to 0)	0 (0 to 0)	2·2
Acute myeloid leukemia	0·1 (0 to 0·2)	0·1 (0 to 0·6)	0·3 (0·2 to 0·5)	2·5 (1·3 to 4·5)	2·1 (1·2 to 3·5)	2·2 (1·4 to 3·3)	2·7 (1·7 to 4·1)	2·8 (1·8 to 4·1)	2·7 (1·8 to 3·8)	1·7 (1·1 to 2·5)	2·1 (1·5 to 3·1)	1·8 (1·2 to 2·6)	2·5 (1·6 to 3·5)	2·9 (1·9 to 4·2)	3·1 (2 to 4·5)	2·9 (1·8 to 4·3)	2·2 (1·4 to 3·2)	1·7 (1·1 to 2·6)	1·1 (0·7 to 1·6)	0·6 (0·4 to 0·9)	0·2 (0·1 to 0·3)	0·1 (0 to 0·1)	0 (0 to 0)	38·4

Chronic myeloid leukemia	0 (0 to 0)	0 (0 to 0)	0 (0 to 0·1)	0·3 (0·1 to 0·5)	0·1 (0·1 to 0·2)	[illegible]	[illegible]	[illegible]	[illegible]	[illegible]	[illegible]	[illegible]	[illegible]	[illegible]	[illegible]	[illegible]	[illegible]	[illegible]	[illegible]	[illegible]	[illegible]	[illegible]	12·8
Other leukemia	0·1 (0 to 0·4)	0·1 (0 to 0·7)	0·4 (0·3 to 0·7)	2·1 (1·1 to 3·6)	2·1 (1·2 to 3·5)	[illegible]	[illegible]	[illegible]	[illegible]	[illegible]	[illegible]	[illegible]	[illegible]	[illegible]	[illegible]	[illegible]	[illegible]	[illegible]	[illegible]	[illegible]	[illegible]	[illegible]	59·6
Other malignant neoplasms	0·2 (0·1 to 0·4)	0·3 (0·1 to 1)	1·3 (0·8 to 2)	9·6 (5·6 to 15·4)	5·6 (4 to 7·8)	[illegible]	[illegible]	[illegible]	[illegible]	[illegible]	[illegible]	[illegible]	[illegible]	[illegible]	[illegible]	[illegible]	[illegible]	[illegible]	[illegible]	[illegible]	[illegible]	[illegible]	134
Other neoplasms	0 (0 to 0)	0 (0 to 0)	0·1 (0 to 0·1)	0·3 (0·1 to 0·4)	0·2 (0·1 to 0·2)	[illegible]	[illegible]	[illegible]	[illegible]	[illegible]	[illegible]	[illegible]	[illegible]	[illegible]	[illegible]	[illegible]	[illegible]	[illegible]	[illegible]	[illegible]	[illegible]	[illegible]	8·2
Myelodysplastic, myeloproliferative, and other hematopoietic neo	0 (0 to 0)	0 (0 to 0)	0·1 (0 to 0·1)	0·3 (0·1 to 0·4)	0·2 (0·1 to 0·2)	[illegible]	[illegible]	[illegible]	[illegible]	[illegible]	[illegible]	[illegible]	[illegible]	[illegible]	[illegible]	[illegible]	[illegible]	[illegible]	[illegible]	[illegible]	[illegible]	[illegible]	8·2
Benign and in situ intestinal neoplasms	0 (0 to 0)	0 (0 to 0)	0 (0 to 0)	0 (0 to 0)	0 (0 to 0)	[illegible]	[illegible]	[illegible]	[illegible]	[illegible]	[illegible]	[illegible]	[illegible]	[illegible]	[illegible]	[illegible]	[illegible]	[illegible]	[illegible]	[illegible]	[illegible]	[illegible]	0
Benign and in situ cervical and uterine neoplasms	0 (0 to 0)	0 (0 to 0)	0 (0 to 0)	0 (0 to 0)	0 (0 to 0)	[illegible]	[illegible]	[illegible]	[illegible]	[illegible]	[illegible]	[illegible]	[illegible]	[illegible]	[illegible]	[illegible]	[illegible]	[illegible]	[illegible]	[illegible]	[illegible]	[illegible]	0
Other benign and in situ neoplasms	0 (0 to 0)	0 (0 to 0)	0 (0 to 0)	0 (0 to 0)	0 (0 to 0)	[illegible]	[illegible]	[illegible]	[illegible]	[illegible]	[illegible]	[illegible]	[illegible]	[illegible]	[illegible]	[illegible]	[illegible]	[illegible]	[illegible]	[illegible]	[illegible]	[illegible]	0
Cardiovascular diseases	1·1 (0·6 to 1·8)	0·7 (0·4 to 1·1)	2·9 (1·7 to 4·4)	4·4 (2·8 to 7)	5·3 (4·1 to 6·7)	[illegible]	[illegible]	[illegible]	[illegible]	[illegible]	[illegible]	[illegible]	[illegible]	[illegible]	[illegible]	[illegible]	[illegible]	[illegible]	[illegible]	[illegible]	[illegible]	[illegible]	4,030
Rheumatic heart disease	0 (0 to 0)	0 (0 to 0)	0 (0 to 0)	0·3 (0·2 to 0·5)	1·2 (0·7 to 2·1)	[illegible]	[illegible]	[illegible]	[illegible]	[illegible]	[illegible]	[illegible]	[illegible]	[illegible]	[illegible]	[illegible]	[illegible]	[illegible]	[illegible]	[illegible]	[illegible]	[illegible]	72·2
Ischemic heart disease	0 (0 to 0)	0 (0 to 0)	0 (0 to 0)	0 (0 to 0)	0 (0 to 0)	[illegible]	[illegible]	[illegible]	[illegible]	[illegible]	[illegible]	[illegible]	[illegible]	[illegible]	[illegible]	[illegible]	[illegible]	[illegible]	[illegible]	[illegible]	[illegible]	[illegible]	1,460
Stroke	0·4 (0·2 to 0·7)	0·1 (0·1 to 0·2)	0·4 (0·2 to 0·8)	0·7 (0·5 to 1·1)	1·2 (1 to 1·6)	[illegible]	[illegible]	[illegible]	[illegible]	[illegible]	[illegible]	[illegible]	[illegible]	[illegible]	[illegible]	[illegible]	[illegible]	[illegible]	[illegible]	[illegible]	[illegible]	[illegible]	1,310
Ischemic stroke	0·1 (0 to 0·1)	0 (0 to 0)	0 (0 to 0)	0·2 (0·2 to 0·3)	0·6 (0·4 to 0·8)	[illegible]	[illegible]	[illegible]	[illegible]	[illegible]	[illegible]	[illegible]	[illegible]	[illegible]	[illegible]	[illegible]	[illegible]	[illegible]	[illegible]	[illegible]	[illegible]	[illegible]	519
Intracerebral hemorrhage	0·1 (0 to 0·2)	0 (0 to 0·1)	0·2 (0·1 to 0·4)	0·2 (0·1 to 0·3)	0·3 (0·2 to 0·5)	[illegible]	[illegible]	[illegible]	[illegible]	[illegible]	[illegible]	[illegible]	[illegible]	[illegible]	[illegible]	[illegible]	[illegible]	[illegible]	[illegible]	[illegible]	[illegible]	[illegible]	651
Subarachnoid hemorrhage	0·2 (0·1 to 0·4)	0·1 (0 to 0·1)	0·2 (0·1 to 0·4)	0·3 (0·2 to 0·6)	0·4 (0·3 to 0·5)	[illegible]	[illegible]	[illegible]	[illegible]	[illegible]	[illegible]	[illegible]	[illegible]	[illegible]	[illegible]	[illegible]	[illegible]	[illegible]	[illegible]	[illegible]	[illegible]	[illegible]	143
Hypertensive heart disease	0 (0 to 0)	0 (0 to 0)	0 (0 to 0)	0 (0 to 0)	0 (0 to 0)	[illegible]	[illegible]	[illegible]	[illegible]	[illegible]	[illegible]	[illegible]	[illegible]	[illegible]	[illegible]	[illegible]	[illegible]	[illegible]	[illegible]	[illegible]	[illegible]	[illegible]	526
Non-rheumatic valvular heart disease	0 (0 to 0)	0 (0 to 0)	0 (0 to 0)	0 (0 to 0)	0 (0 to 0)	[illegible]	[illegible]	[illegible]	[illegible]	[illegible]	[illegible]	[illegible]	[illegible]	[illegible]	[illegible]	[illegible]	[illegible]	[illegible]	[illegible]	[illegible]	[illegible]	[illegible]	36·3
Non-rheumatic calcific aortic valve disease	0 (0 to 0)	0 (0 to 0)	0 (0 to 0)	0 (0 to 0)	0 (0 to 0)	[illegible]	[illegible]	[illegible]	[illegible]	[illegible]	[illegible]	[illegible]	[illegible]	[illegible]	[illegible]	[illegible]	[illegible]	[illegible]	[illegible]	[illegible]	[illegible]	[illegible]	17·5
Non-rheumatic degenerative mitral valve disease	0 (0 to 0)	0 (0 to 0)	0 (0 to 0)	0 (0 to 0)	0 (0 to 0)	[illegible]	[illegible]	[illegible]	[illegible]	[illegible]	[illegible]	[illegible]	[illegible]	[illegible]	[illegible]	[illegible]	[illegible]	[illegible]	[illegible]	[illegible]	[illegible]	[illegible]	14·9
Other non-rheumatic valve diseases	0 (0 to 0)	0 (0 to 0)	0 (0 to 0)	0 (0 to 0)	0 (0 to 0)	[illegible]	[illegible]	[illegible]	[illegible]	[illegible]	[illegible]	[illegible]	[illegible]	[illegible]	[illegible]	[illegible]	[illegible]	[illegible]	[illegible]	[illegible]	[illegible]	[illegible]	3·8
Cardiomyopathy and myocarditis	0·3 (0·1 to 0·4)	0·2 (0·1 to 0·4)	1·3 (0·8 to 2·1)	1·5 (0·8 to 2·6)	0·8 (0·6 to 1)	[illegible]	[illegible]	[illegible]	[illegible]	[illegible]	[illegible]	[illegible]	[illegible]	[illegible]	[illegible]	[illegible]	[illegible]	[illegible]	[illegible]	[illegible]	[illegible]	[illegible]	146
Myocarditis	0·1 (0 to 0·2)	0·1 (0·1 to 0·2)	0·6 (0·4 to 1)	0·8 (0·4 to 1·4)	0·4 (0·3 to 0·5)	[illegible]	[illegible]	[illegible]	[illegible]	[illegible]	[illegible]	[illegible]	[illegible]	[illegible]	[illegible]	[illegible]	[illegible]	[illegible]	[illegible]	[illegible]	[illegible]	[illegible]	23·1
Alcoholic cardiomyopathy	0 (0 to 0)	[illegible]	[illegible]	[illegible]	[illegible]	[illegible]	[illegible]	[illegible]	[illegible]	[illegible]	[illegible]	[illegible]	[illegible]	[illegible]	[illegible]	[illegible]	[illegible]	[illegible]	[illegible]	[illegible]	[illegible]	[illegible]	15·8
Other cardiomyopathy	0·2 (0·1 to 0·3)	[illegible]	[illegible]	[illegible]	[illegible]	[illegible]	[illegible]	[illegible]	[illegible]	[illegible]	[illegible]	[illegible]	[illegible]	[illegible]	[illegible]	[illegible]	[illegible]	[illegible]	[illegible]	[illegible]	[illegible]	[illegible]	107
Atrial fibrillation and flutter	0 (0 to 0)	[illegible]	[illegible]	[illegible]	[illegible]	[illegible]	[illegible]	[illegible]	[illegible]	[illegible]	[illegible]	[illegible]	[illegible]	[illegible]	[illegible]	[illegible]	[illegible]	[illegible]	[illegible]	[illegible]	[illegible]	[illegible]	99·6
Aortic aneurysm	--	[illegible]	[illegible]	[illegible]	[illegible]	[illegible]	[illegible]	[illegible]	[illegible]	[illegible]	[illegible]	[illegible]	[illegible]	[illegible]	[illegible]	[illegible]	[illegible]	[illegible]	[illegible]	[illegible]	[illegible]	[illegible]	NA
Endocarditis	0 (0 to 0·1)	[illegible]	[illegible]	[illegible]	[illegible]	[illegible]	[illegible]	[illegible]	[illegible]	[illegible]	[illegible]	[illegible]	[illegible]	[illegible]	[illegible]	[illegible]	[illegible]	[illegible]	[illegible]	[illegible]	[illegible]	[illegible]	33·3
Other cardiovascular and circulatory diseases	0·4 (0·2 to 0·6)	[illegible]	[illegible]	[illegible]	[illegible]	[illegible]	[illegible]	[illegible]	[illegible]	[illegible]	[illegible]	[illegible]	[illegible]	[illegible]	[illegible]	[illegible]	[illegible]	[illegible]	[illegible]	[illegible]	[illegible]	[illegible]	262
Chronic respiratory diseases	0 (0 to 0)	[illegible]	[illegible]	[illegible]	[illegible]	[illegible]	[illegible]	[illegible]	[illegible]	[illegible]	[illegible]	[illegible]	[illegible]	[illegible]	[illegible]	[illegible]	[illegible]	[illegible]	[illegible]	[illegible]	[illegible]	[illegible]	435
Chronic obstructive pulmonary disease	0 (0 to 0)	[illegible]	[illegible]	[illegible]	[illegible]	[illegible]	[illegible]	[illegible]	[illegible]	[illegible]	[illegible]	[illegible]	[illegible]	[illegible]	[illegible]	[illegible]	[illegible]	[illegible]	[illegible]	[illegible]	[illegible]	[illegible]	170
Pneumoconiosis	0 (0 to 0)	[illegible]	[illegible]	[illegible]	[illegible]	[illegible]	[illegible]	[illegible]	[illegible]	[illegible]	[illegible]	[illegible]	[illegible]	[illegible]	[illegible]	[illegible]	[illegible]	[illegible]	[illegible]	[illegible]	[illegible]	[illegible]	1·5
Silicosis	0 (0 to 0)	[illegible]	[illegible]	[illegible]	[illegible]	[illegible]	[illegible]	[illegible]	[illegible]	[illegible]	[illegible]	[illegible]	[illegible]	[illegible]	[illegible]	[illegible]	[illegible]	[illegible]	[illegible]	[illegible]	[illegible]	[illegible]	0
Asbestosis	0 (0 to 0)	[illegible]	[illegible]	[illegible]	[illegible]	[illegible]	[illegible]	[illegible]	[illegible]	[illegible]	[illegible]	[illegible]	[illegible]	[illegible]	[illegible]	[illegible]	[illegible]	[illegible]	[illegible]	[illegible]	[illegible]	[illegible]	0·4
Coal workers pneumoconiosis	0 (0 to 0)	[illegible]	[illegible]	[illegible]	[illegible]	[illegible]	[illegible]	[illegible]	[illegible]	[illegible]	[illegible]	[illegible]	[illegible]	[illegible]	[illegible]	[illegible]	[illegible]	[illegible]	[illegible]	[illegible]	[illegible]	[illegible]	0
Other pneumoconiosis	0 (0 to 0)	[illegible]	[illegible]	[illegible]	[illegible]	[illegible]	[illegible]	[illegible]	[illegible]	[illegible]	[illegible]	[illegible]	[illegible]	[illegible]	[illegible]	[illegible]	[illegible]	[illegible]	[illegible]	[illegible]	[illegible]	[illegible]	1·1
Asthma	0 (0 to 0)	[illegible]	[illegible]	[illegible]	[illegible]	[illegible]	[illegible]	[illegible]	[illegible]	[illegible]	[illegible]	[illegible]	[illegible]	[illegible]	[illegible]	[illegible]	[illegible]	[illegible]	[illegible]	[illegible]	[illegible]	[illegible]	216
Interstitial lung disease and pulmonary sarcoidosis	0 (0 to 0)	[illegible]	[illegible]	[illegible]	[illegible]	[illegible]	[illegible]	[illegible]	[illegible]	[illegible]	[illegible]	[illegible]	[illegible]	[illegible]	[illegible]	[illegible]	[illegible]	[illegible]	[illegible]	[illegible]	[illegible]	[illegible]	16·1
Other chronic respiratory diseases	0 (0 to 0)	[illegible]	[illegible]	[illegible]	[illegible]	[illegible]	[illegible]	[illegible]	[illegible]	[illegible]	[illegible]	[illegible]	[illegible]	[illegible]	[illegible]	[illegible]	[illegible]	[illegible]	[illegible]	[illegible]	[illegible]	[illegible]	31·1
Digestive diseases	0·6 (0·4 to 0·8)	[illegible]	[illegible]	[illegible]	[illegible]	[illegible]	[illegible]	[illegible]	[illegible]	[illegible]	[illegible]	[illegible]	[illegible]	[illegible]	[illegible]	[illegible]	[illegible]	[illegible]	[illegible]	[illegible]	[illegible]	[illegible]	845
Cirrhosis and other chronic liver diseases	0 (0 to 0)	[illegible]	[illegible]	[illegible]	[illegible]	[illegible]	[illegible]	[illegible]	[illegible]	[illegible]	[illegible]	[illegible]	[illegible]	[illegible]	[illegible]	[illegible]	[illegible]	[illegible]	[illegible]	[illegible]	[illegible]	[illegible]	349
Cirrhosis and other chronic liver diseases due to hepatitis B	0 (0 to 0)	[illegible]	[illegible]	[illegible]	[illegible]	[illegible]	[illegible]	[illegible]	[illegible]	[illegible]	[illegible]	[illegible]	[illegible]	[illegible]	[illegible]	[illegible]	[illegible]	[illegible]	[illegible]	[illegible]	[illegible]	[illegible]	23·3
Cirrhosis and other chronic liver diseases due to hepatitis C	0 (0 to 0)	[illegible]	[illegible]	[illegible]	[illegible]	[illegible]	[illegible]	[illegible]	[illegible]	[illegible]	[illegible]	[illegible]	[illegible]	[illegible]	[illegible]	[illegible]	[illegible]	[illegible]	[illegible]	[illegible]	[illegible]	[illegible]	66·7
Cirrhosis and other chronic liver diseases due to alcohol use	0 (0 to 0)	[illegible]	[illegible]	[illegible]	[illegible]	[illegible]	[illegible]	[illegible]	[illegible]	[illegible]	[illegible]	[illegible]	[illegible]	[illegible]	[illegible]	[illegible]	[illegible]	[illegible]	[illegible]	[illegible]	[illegible]	[illegible]	148
Cirrhosis and other chronic liver diseases due to NAFLD	0 (0 to 0)	[illegible]	[illegible]	[illegible]	[illegible]	[illegible]	[illegible]	[illegible]	[illegible]	[illegible]	[illegible]	[illegible]	[illegible]	[illegible]	[illegible]	[illegible]	[illegible]	[illegible]	[illegible]	[illegible]	[illegible]	[illegible]	57·8
Cirrhosis and other chronic liver diseases due to other causes	0 (0 to 0)	[illegible]	[illegible]	[illegible]	[illegible]	[illegible]	[illegible]	[illegible]	[illegible]	[illegible]	[illegible]	[illegible]	[illegible]	[illegible]	[illegible]	[illegible]	[illegible]	[illegible]	[illegible]	[illegible]	[illegible]	[illegible]	53·2
Upper digestive system diseases	0 (0 to 0)	[illegible]	[illegible]	[illegible]	[illegible]	[illegible]	[illegible]	[illegible]	[illegible]	[illegible]	[illegible]	[illegible]	[illegible]	[illegible]	[illegible]	[illegible]	[illegible]	[illegible]	[illegible]	[illegible]	[illegible]	[illegible]	203
Peptic ulcer disease	0 (0 to 0)	[illegible]	[illegible]	[illegible]	[illegible]	[illegible]	[illegible]	[illegible]	[illegible]	[illegible]	[illegible]	[illegible]	[illegible]	[illegible]	[illegible]	[illegible]	[illegible]	[illegible]	[illegible]	[illegible]	[illegible]	[illegible]	33·8
Gastritis and duodenitis	0 (0 to 0)	[illegible]	[illegible]	[illegible]	[illegible]	[illegible]	[illegible]	[illegible]	[illegible]	[illegible]	[illegible]	[illegible]	[illegible]	[illegible]	[illegible]	[illegible]	[illegible]	[illegible]	[illegible]	[illegible]	[illegible]	[illegible]	42·7
Gastroesophageal reflux disease	0 (0 to 0)	[illegible]	[illegible]	[illegible]	[illegible]	[illegible]	[illegible]	[illegible]	[illegible]	[illegible]	[illegible]	[illegible]	[illegible]	[illegible]	[illegible]	[illegible]	[illegible]	[illegible]	[illegible]	[illegible]	[illegible]	[illegible]	127
Appendicitis	0 (0 to 0)	[illegible]	[illegible]	[illegible]	[illegible]	[illegible]	[illegible]	[illegible]	[illegible]	[illegible]	[illegible]	[illegible]	[illegible]	[illegible]	[illegible]	[illegible]	[illegible]	[illegible]	[illegible]	[illegible]	[illegible]	[illegible]	15·4
Paralytic ileus and intestinal obstruction	0·4 (0·3 to 0·7)	[illegible]	[illegible]	[illegible]	[illegible]	[illegible]	[illegible]	[illegible]	[illegible]	[illegible]	[illegible]	[illegible]	[illegible]	[illegible]	[illegible]	[illegible]	[illegible]	[illegible]	[illegible]	[illegible]	[illegible]	[illegible]	59·2
Inguinal, femoral, and abdominal hernia	0·1 (0·1 to 0·2)	[illegible]	[illegible]	[illegible]	[illegible]	[illegible]	[illegible]	[illegible]	[illegible]	[illegible]	[illegible]	[illegible]	[illegible]	[illegible]	[illegible]	[illegible]	[illegible]	[illegible]	[illegible]	[illegible]	[illegible]	[illegible]	55·1
Inflammatory bowel disease	0 (0 to 0)	[illegible]	[illegible]	[illegible]	[illegible]	[illegible]	[illegible]	[illegible]	[illegible]	[illegible]	[illegible]	[illegible]	[illegible]	[illegible]	[illegible]	[illegible]	[illegible]	[illegible]	[illegible]	[illegible]	[illegible]	[illegible]	21·9
Vascular intestinal disorders	0 (0 to 0)	[illegible]	[illegible]	[illegible]	[illegible]	[illegible]	[illegible]	[illegible]	[illegible]	[illegible]	[illegible]	[illegible]	[illegible]	[illegible]	[illegible]	[illegible]	[illegible]	[illegible]	[illegible]	[illegible]	[illegible]	[illegible]	8·9
Gallbladder and biliary diseases	0 (0 to 0)	[illegible]	[illegible]	[illegible]	[illegible]	[illegible]	[illegible]	[illegible]	[illegible]	[illegible]	[illegible]	[illegible]	[illegible]	[illegible]	[illegible]	[illegible]	[illegible]	[illegible]	[illegible]	[illegible]	[illegible]	[illegible]	58·1
Pancreatitis	0 (0 to 0)	[illegible]	[illegible]	[illegible]	[illegible]	[illegible]	[illegible]	[illegible]	[illegible]	[illegible]	[illegible]	[illegible]	[illegible]	[illegible]	[illegible]	[illegible]	[illegible]	[illegible]	[illegible]	[illegible]	[illegible]	[illegible]	28
Other digestive diseases	0 (0 to 0)	[illegible]	[illegible]	[illegible]	[illegible]	[illegible]	[illegible]	[illegible]	[illegible]	[illegible]	[illegible]	[illegible]	[illegible]	[illegible]	[illegible]	[illegible]	[illegible]	[illegible]	[illegible]	[illegible]	[illegible]	[illegible]	45·7
Neurological disorders	0 (0 to 0)	[illegible]	[illegible]	[illegible]	[illegible]	[illegible]	[illegible]	[illegible]	[illegible]	[illegible]	[illegible]	[illegible]	[illegible]	[illegible]	[illegible]	[illegible]	[illegible]	[illegible]	[illegible]	[illegible]	[illegible]	[illegible]	1,310
Alzheimer's disease and other dementias	0 (0 to 0)	[illegible]	[illegible]	[illegible]	[illegible]	[illegible]	[illegible]	[illegible]	[illegible]	[illegible]	[illegible]	[illegible]	[illegible]	[illegible]	[illegible]	[illegible]	[illegible]	[illegible]	[illegible]	[illegible]	[illegible]	[illegible]	265
Parkinson's disease	0 (0 to 0)	[illegible]	[illegible]	[illegible]	[illegible]	[illegible]	[illegible]	[illegible]	[illegible]	[illegible]	[illegible]	[illegible]	[illegible]	[illegible]	[illegible]	[illegible]	[illegible]	[illegible]	[illegible]	[illegible]	[illegible]	[illegible]	88·3
Idiopathic epilepsy	0 (0 to 0)	[illegible]	[illegible]	[illegible]	[illegible]	[illegible]	[illegible]	[illegible]	[illegible]	[illegible]	[illegible]	[illegible]	[illegible]	[illegible]	[illegible]	[illegible]	[illegible]	[illegible]	[illegible]	[illegible]	[illegible]	[illegible]	311
Multiple sclerosis	0 (0 to 0)	[illegible]	[illegible]	[illegible]	[illegible]	[illegible]	[illegible]	[illegible]	[illegible]	[illegible]	[illegible]	[illegible]	[illegible]	[illegible]	[illegible]	[illegible]	[illegible]	[illegible]	[illegible]	[illegible]	[illegible]	[illegible]	18·3
Motor neuron disease	0 (0 to 0)	[illegible]	[illegible]	[illegible]	[illegible]	[illegible]	[illegible]	[illegible]	[illegible]	[illegible]	[illegible]	[illegible]	[illegible]	[illegible]	[illegible]	[illegible]	[illegible]	[illegible]	[illegible]	[illegible]	[illegible]	[illegible]	12
Headache disorders	0 (0 to 0)	[illegible]	[illegible]	[illegible]	[illegible]	[illegible]	[illegible]	[illegible]	[illegible]	[illegible]	[illegible]	[illegible]	[illegible]	[illegible]	[illegible]	[illegible]	[illegible]	[illegible]	[illegible]	[illegible]	[illegible]	[illegible]	539
Migraine	0 (0 to 0)	[illegible]	[illegible]	[illegible]	[illegible]	[illegible]	[illegible]	[illegible]	[illegible]	[illegible]	[illegible]	[illegible]	[illegible]	[illegible]	[illegible]	[illegible]	[illegible]	[illegible]	[illegible]	[illegible]	[illegible]	[illegible]	487
Tension-type headache	0 (0 to 0)	[illegible]	[illegible]	[illegible]	[illegible]	[illegible]	[illegible]	[illegible]	[illegible]	[illegible]	[illegible]	[illegible]	[illegible]	[illegible]	[illegible]	[illegible]	[illegible]	[illegible]	[illegible]	[illegible]	[illegible]	[illegible]	52·3
Other neurological disorders	0 (0 to 0)	[illegible]	[illegible]	[illegible]	[illegible]	[illegible]	[illegible]	[illegible]	[illegible]	[illegible]	[illegible]	[illegible]	[illegible]	[illegible]	[illegible]	[illegible]	[illegible]	[illegible]	[illegible]	[illegible]	[illegible]	[illegible]	76
Mental disorders	0 (0 to 0)	[illegible]	[illegible]	[illegible]	[illegible]	[illegible]	[illegible]	[illegible]	[illegible]	[illegible]	[illegible]	[illegible]	[illegible]	[illegible]	[illegible]	[illegible]	[illegible]	[illegible]	[illegible]	[illegible]	[illegible]	[illegible]	1,620
Schizophrenia	0 (0 to 0)	[illegible]	[illegible]	[illegible]	[illegible]	[illegible]	[illegible]	[illegible]	[illegible]	[illegible]	[illegible]	[illegible]	[illegible]	[illegible]	[illegible]	[illegible]	[illegible]	[illegible]	[illegible]	[illegible]	[illegible]	[illegible]	193
Depressive disorders	0 (0 to 0)	[illegible]	[illegible]	[illegible]	[illegible]	[illegible]	[illegible]	[illegible]	[illegible]	[illegible]	[illegible]	[illegible]	[illegible]	[illegible]	[illegible]	[illegible]	[illegible]	[illegible]	[illegible]	[illegible]	[illegible]	[illegible]	527
Major depressive disorder	0 (0 to 0)	[illegible]	[illegible]	[illegible]	[illegible]	[illegible]	[illegible]	[illegible]	[illegible]	[illegible]	[illegible]	[illegible]	[illegible]	[illegible]	[illegible]	[illegible]	[illegible]	[illegible]	[illegible]	[illegible]	[illegible]	[illegible]	435
Dysthymia	0 (0 to 0)	[illegible]	[illegible]	[illegible]	[illegible]	[illegible]	[illegible]	[illegible]	[illegible]	[illegible]	[illegible]	[illegible]	[illegible]	[illegible]	[illegible]	[illegible]	[illegible]	[illegible]	[illegible]	[illegible]	[illegible]	[illegible]	92·2
Bipolar disorder	0 (0 to 0)	[illegible]	[illegible]	[illegible]	[illegible]	[illegible]	[illegible]	[illegible]	[illegible]	[illegible]	[illegible]	[illegible]	[illegible]	[illegible]	[illegible]	[illegible]	[illegible]	[illegible]	[illegible]	[illegible]	[illegible]	[illegible]	204
Anxiety disorders	0 (0 to 0)	[illegible]	[illegible]	[illegible]	[illegible]	[illegible]	[illegible]	[illegible]	[illegible]	[illegible]	[illegible]	[illegible]	[illegible]	[illegible]	[illegible]	[illegible]	[illegible]	[illegible]	[illegible]	[illegible]	[illegible]	[illegible]	401
Eating disorders	0 (0 to 0)	[illegible]	[illegible]	[illegible]	[illegible]	[illegible]	[illegible]	[illegible]	[illegible]	[illegible]	[illegible]	[illegible]	[illegible]	[illegible]	[illegible]	[illegible]	[illegible]	[illegible]	[illegible]	[illegible]	[illegible]	[illegible]	53·1
Anorexia nervosa	0 (0 to 0)	[illegible]	[illegible]	[illegible]	[illegible]	[illegible]	[illegible]	[illegible]	[illegible]	[illegible]	[illegible]	[illegible]	[illegible]	[illegible]	[illegible]	[illegible]	[illegible]	[illegible]	[illegible]	[illegible]	[illegible]	[illegible]	12·1
Bulimia nervosa	0 (0 to 0)	[illegible]	[illegible]	[illegible]	[illegible]	[illegible]	[illegible]	[illegible]	[illegible]	[illegible]	[illegible]	[illegible]	[illegible]	[illegible]	[illegible]	[illegible]	[illegible]	[illegible]	[illegible]	[illegible]	[illegible]	[illegible]	41
Autism spectrum disorders	0 (0 to 0)	[illegible]	[illegible]	[illegible]	[illegible]	[illegible]	[illegible]	[illegible]	[illegible]	[illegible]	[illegible]	[illegible]	[illegible]	[illegible]	[illegible]	[illegible]	[illegible]	[illegible]	[illegible]	[illegible]	[illegible]	[illegible]	45·1
Attention-deficit/hyperactivity disorder	0 (0 to 0)	[illegible]	[illegible]	[illegible]	[illegible]	[illegible]	[illegible]	[illegible]	[illegible]	[illegible]	[illegible]	[illegible]	[illegible]	[illegible]	[illegible]	[illegible]	[illegible]	[illegible]	[illegible]	[illegible]	[illegible]	[illegible]	30·3
Conduct disorder	0 (0 to 0)	[illegible]	[illegible]	[illegible]	[illegible]	[illegible]	[illegible]	[illegible]	[illegible]	[illegible]	[illegible]	[illegible]	[illegible]	[illegible]	[illegible]	[illegible]	[illegible]	[illegible]	[illegible]	[illegible]	[illegible]	[illegible]	48·5
Idiopathic developmental intellectual disability	0 (0 to 0)	[illegible]	[illegible]	[illegible]	[illegible]	[illegible]	[illegible]	[illegible]	[illegible]	[illegible]	[illegible]	[illegible]	[illegible]	[illegible]	[illegible]	[illegible]	[illegible]	[illegible]	[illegible]	[illegible]	[illegible]	[illegible]	9·2
Other mental disorders	0 (0 to 0)	[illegible]	[illegible]	[illegible]	[illegible]	[illegible]	[illegible]	[illegible]	[illegible]	[illegible]	[illegible]	[illegible]	[illegible]	[illegible]	[illegible]	[illegible]	[illegible]	[illegible]	[illegible]	[illegible]	[illegible]	[illegible]	112
Substance use disorders	0 (0 to 0)	[illegible]	[illegible]	[illegible]	[illegible]	[illegible]	[illegible]	[illegible]	[illegible]	[illegible]	[illegible]	[illegible]	[illegible]	[illegible]	[illegible]	[illegible]	[illegible]	[illegible]	[illegible]	[illegible]	[illegible]	[illegible]	545
Alcohol use disorders	0 (0 to 0)	[illegible]	[illegible]	[illegible]	[illegible]	[illegible]	[illegible]	[illegible]	[illegible]	[illegible]	[illegible]	[illegible]	[illegible]	[illegible]	[illegible]	[illegible]	[illegible]	[illegible]	[illegible]	[illegible]	[illegible]	[illegible]	428
Drug use disorders	0 (0 to 0)	[illegible]	[illegible]	[illegible]	[illegible]	[illegible]	[illegible]	[illegible]	[illegible]	[illegible]	[illegible]	[illegible]	[illegible]	[illegible]	[illegible]	[illegible]	[illegible]	[illegible]	[illegible]	[illegible]	[illegible]	[illegible]	117
Opioid use disorders	0 (0 to 0)	[illegible]	[illegible]	[illegible]	[illegible]	[illegible]	[illegible]	[illegible]	[illegible]	[illegible]	[illegible]	[illegible]	[illegible]	[illegible]	[illegible]	[illegible]	[illegible]	[illegible]	[illegible]	[illegible]	[illegible]	[illegible]	52·9
Cocaine use disorders	0 (0 to 0)	[illegible]	[illegible]	[illegible]	[illegible]	[illegible]	[illegible]	[illegible]	[illegible]	[illegible]	[illegible]	[illegible]	[illegible]	[illegible]	[illegible]	[illegible]	[illegible]	[illegible]	[illegible]	[illegible]	[illegible]	[illegible]	24·1
Amphetamine use disorders	0 (0 to 0)	[illegible]	[illegible]	[illegible]	[illegible]	[illegible]	[illegible]	[illegible]	[illegible]	[illegible]	[illegible]	[illegible]	[illegible]	[illegible]	[illegible]	[illegible]	[illegible]	[illegible]	[illegible]	[illegible]	[illegible]	[illegible]	7·5
Cannabis use disorders	0 (0 to 0)	[illegible]	[illegible]	[illegible]	[illegible]	[illegible]	[illegible]	[illegible]	[illegible]	[illegible]	[illegible]	[illegible]	[illegible]	[illegible]	[illegible]	[illegible]	[illegible]	[illegible]	[illegible]	[illegible]	[illegible]	[illegible]	14
Other drug use disorders	0 (0 to 0)	[illegible]	[illegible]	[illegible]	[illegible]	[illegible]	[illegible]	[illegible]	[illegible]	[illegible]	[illegible]	[illegible]	[illegible]	[illegible]	[illegible]	[illegible]	[illegible]	[illegible]	[illegible]	[illegible]	[illegible]	[illegible]	17·9
Diabetes and kidney diseases	0 (0 to 0)	[illegible]	[illegible]	[illegible]	[illegible]	[illegible]	[illegible]	[illegible]	[illegible]	[illegible]	[illegible]	[illegible]	[illegible]	[illegible]	[illegible]	[illegible]	[illegible]	[illegible]	[illegible]	[illegible]	[illegible]	[illegible]	3,090
Diabetes mellitus	0 (0 to 0)	[illegible]	[illegible]	[illegible]	[illegible]	[illegible]	[illegible]	[illegible]	[illegible]	[illegible]	[illegible]	[illegible]	[illegible]	[illegible]	[illegible]	[illegible]	[illegible]	[illegible]	[illegible]	[illegible]	[illegible]	[illegible]	2,060
Diabetes mellitus type 1	0 (0 to 0)	[illegible]	[illegible]	[illegible]	[illegible]	[illegible]	[illegible]	[illegible]	[illegible]	[illegible]	[illegible]	[illegible]	[illegible]	[illegible]	[illegible]	[illegible]	[illegible]	[illegible]	[illegible]	[illegible]	[illegible]	[illegible]	107
Diabetes mellitus type 2	0 (0 to 0)	[illegible]	[illegible]	[illegible]	[illegible]	[illegible]	[illegible]	[illegible]	[illegible]	[illegible]	[illegible]	[illegible]	[illegible]	[illegible]	[illegible]	[illegible]	[illegible]	[illegible]	[illegible]	[illegible]	[illegible]	[illegible]	1,950
Chronic kidney disease	0 (0 to 0)	[illegible]	[illegible]	[illegible]	[illegible]	[illegible]	[illegible]	[illegible]	[illegible]	[illegible]	[illegible]	[illegible]	[illegible]	[illegible]	[illegible]	[illegible]	[illegible]	[illegible]	[illegible]	[illegible]	[illegible]	[illegible]	1,030
Chronic kidney disease due to diabetes mellitus type 1	0 (0 to 0)	[illegible]	[illegible]	[illegible]	[illegible]	[illegible]	[illegible]	[illegible]	[illegible]	[illegible]	[illegible]	[illegible]	[illegible]	[illegible]	[illegible]	[illegible]	[illegible]	[illegible]	[illegible]	[illegible]	[illegible]	[illegible]	73·2
Chronic kidney disease due to diabetes mellitus type 2	0 (0 to 0)	[illegible]	[illegible]	[illegible]	[illegible]	[illegible]	[illegible]	[illegible]	[illegible]	[illegible]	[illegible]	[illegible]	[illegible]	[illegible]	[illegible]	[illegible]	[illegible]	[illegible]	[illegible]	[illegible]	[illegible]	[illegible]	252
Chronic kidney disease due to hypertension	0 (0 to 0)	[illegible]	[illegible]	[illegible]	[illegible]	[illegible]	[illegible]	[illegible]	[illegible]	[illegible]	[illegible]	[illegible]	[illegible]	[illegible]	[illegible]	[illegible]	[illegible]	[illegible]	[illegible]	[illegible]	[illegible]	[illegible]	215
Chronic kidney disease due to glomerulonephritis	0 (0 to 0)	[illegible]	[illegible]	[illegible]	[illegible]	[illegible]	[illegible]	[illegible]	[illegible]	[illegible]	[illegible]	[illegible]	[illegible]	[illegible]	[illegible]	[illegible]	[illegible]	[illegible]	[illegible]	[illegible]	[illegible]	[illegible]	219
Chronic kidney disease due to other and unspecified causes	0 (0 to 0)	[illegible]	[illegible]	[illegible]	[illegible]	[illegible]	[illegible]	[illegible]	[illegible]	[illegible]	[illegible]	[illegible]	[illegible]	[illegible]	[illegible]	[illegible]	[illegible]	[illegible]	[illegible]	[illegible]	[illegible]	[illegible]	276
Acute glomerulonephritis	0 (0 to 0)	[illegible]	[illegible]	[illegible]	[illegible]	[illegible]	[illegible]	[illegible]	[illegible]	[illegible]	[illegible]	[illegible]	[illegible]	[illegible]	[illegible]	[illegible]	[illegible]	[illegible]	[illegible]	[illegible]	[illegible]	[illegible]	0·2
Skin and subcutaneous diseases	0 (0 to 0)	[illegible]	[illegible]	[illegible]	[illegible]	[illegible]	[illegible]	[illegible]	[illegible]	[illegible]	[illegible]	[illegible]	[illegible]	[illegible]	[illegible]	[illegible]	[illegible]	[illegible]	[illegible]	[illegible]	[illegible]	[illegible]	557
Dermatitis	0 (0 to 0)	[illegible]	[illegible]	[illegible]	[illegible]	[illegible]	[illegible]	[illegible]	[illegible]	[illegible]	[illegible]	[illegible]	[illegible]	[illegible]	[illegible]	[illegible]	[illegible]	[illegible]	[illegible]	[illegible]	[illegible]	[illegible]	93·6
Atopic dermatitis	0 (0 to 0)	[illegible]	[illegible]	[illegible]	[illegible]	[illegible]	[illegible]	[illegible]	[illegible]	[illegible]	[illegible]	[illegible]	[illegible]	[illegible]	[illegible]	[illegible]	[illegible]	[illegible]	[illegible]	[illegible]	[illegible]	[illegible]	63·2
Contact dermatitis	0 (0 to 0)	[illegible]	[illegible]	[illegible]	[illegible]	[illegible]	[illegible]	[illegible]	[illegible]	[illegible]	[illegible]	[illegible]	[illegible]	[illegible]	[illegible]	[illegible]	[illegible]	[illegible]	[illegible]	[illegible]	[illegible]	[illegible]	27·2
Seborrhoeic dermatitis	0 (0 to 0)	[illegible]	[illegible]	[illegible]	[illegible]	[illegible]	[illegible]	[illegible]	[illegible]	[illegible]	[illegible]	[illegible]	[illegible]	[illegible]	[illegible]	[illegible]	[illegible]	[illegible]	[illegible]	[illegible]	[illegible]	[illegible]	3·2
Psoriasis	0 (0 to 0)	[illegible]	[illegible]	[illegible]	[illegible]	[illegible]	[illegible]	[illegible]	[illegible]	[illegible]	[illegible]	[illegible]	[illegible]	[illegible]	[illegible]	[illegible]	[illegible]	[illegible]	[illegible]	[illegible]	[illegible]	[illegible]	29·7
Bacterial skin diseases	0 (0 to 0)	[illegible]	[illegible]	[illegible]	[illegible]	[illegible]	[illegible]	[illegible]	[illegible]	[illegible]	[illegible]	[illegible]	[illegible]	[illegible]	[illegible]	[illegible]	[illegible]	[illegible]	[illegible]	[illegible]	[illegible]	[illegible]	42·9
Cellulitis	0 (0 to 0)	[illegible]	[illegible]	[illegible]	[illegible]	[illegible]	[illegible]	[illegible]	[illegible]	[illegible]	[illegible]	[illegible]	[illegible]	[illegible]	[illegible]	[illegible]	[illegible]	[illegible]	[illegible]	[illegible]	[illegible]	[illegible]	12·6
Pyoderma	0 (0 to 0)	[illegible]	[illegible]	[illegible]	[illegible]	[illegible]	[illegible]	[illegible]	[illegible]	[illegible]	[illegible]	[illegible]	[illegible]	[illegible]	[illegible]	[illegible]	[illegible]	[illegible]	[illegible]	[illegible]	[illegible]	[illegible]	30·2
Scabies	0 (0 to 0)	[illegible]	[illegible]	[illegible]	[illegible]	[illegible]	[illegible]	[illegible]	[illegible]	[illegible]	[illegible]	[illegible]	[illegible]	[illegible]	[illegible]	[illegible]	[illegible]	[illegible]	[illegible]	[illegible]	[illegible]	[illegible]	69·2
Fungal skin diseases	0 (0 to 0)	[illegible]	[illegible]	[illegible]	[illegible]	[illegible]	[illegible]	[illegible]	[illegible]	[illegible]	[illegible]	[illegible]	[illegible]	[illegible]	[illegible]	[illegible]	[illegible]	[illegible]	[illegible]	[illegible]	[illegible]	[illegible]	40·5
Viral skin diseases	0 (0 to 0)	[illegible]	[illegible]	[illegible]	[illegible]	[illegible]	[illegible]	[illegible]	[illegible]	[illegible]	[illegible]	[illegible]	[illegible]	[illegible]	[illegible]	[illegible]	[illegible]	[illegible]	[illegible]	[illegible]	[illegible]	[illegible]	34·6
Acne vulgaris	0 (0 to 0)	[illegible]	[illegible]	[illegible]	[illegible]	[illegible]	[illegible]	[illegible]	[illegible]	[illegible]	[illegible]	[illegible]	[illegible]	[illegible]	[illegible]	[illegible]	[illegible]	[illegible]	[illegible]	[illegible]	[illegible]	[illegible]	45·2
Alopecia areata	0 (0 to 0)	[illegible]	[illegible]	[illegible]	[illegible]	[illegible]	[illegible]	[illegible]	[illegible]	[illegible]	[illegible]	[illegible]	[illegible]	[illegible]	[illegible]	[illegible]	[illegible]	[illegible]	[illegible]	[illegible]	[illegible]	[illegible]	6·9
Pruritus	0 (0 to 0)	[illegible]	[illegible]	[illegible]	[illegible]	[illegible]	[illegible]	[illegible]	[illegible]	[illegible]	[illegible]	[illegible]	[illegible]	[illegible]	[illegible]	[illegible]	[illegible]	[illegible]	[illegible]	[illegible]	[illegible]	[illegible]	8·3
Urticaria	0 (0 to 0)	[illegible]	[illegible]	[illegible]	[illegible]	[illegible]	[illegible]	[illegible]	[illegible]	[illegible]	[illegible]	[illegible]	[illegible]	[illegible]	[illegible]	[illegible]	[illegible]	[illegible]	[illegible]	[illegible]	[illegible]	[illegible]	38·7
Decubitus ulcer	0 (0 to 0)	[illegible]	[illegible]	[illegible]	[illegible]	[illegible]	[illegible]	[illegible]	[illegible]	[illegible]	[illegible]	[illegible]	[illegible]	[illegible]	[illegible]	[illegible]	[illegible]	[illegible]	[illegible]	[illegible]	[illegible]	[illegible]	92·1
Other skin and subcutaneous diseases	0 (0 to 0)	[illegible]	[illegible]	[illegible]	[illegible]	[illegible]	[illegible]	[illegible]	[illegible]	[illegible]	[illegible]	[illegible]	[illegible]	[illegible]	[illegible]	[illegible]	[illegible]	[illegible]	[illegible]	[illegible]	[illegible]	[illegible]	55·2
Sense organ diseases	0 (0 to 0)	[illegible]	[illegible]	[illegible]	[illegible]	[illegible]	[illegible]	[illegible]	[illegible]	[illegible]	[illegible]	[illegible]	[illegible]	[illegible]	[illegible]	[illegible]	[illegible]	[illegible]	[illegible]	[illegible]	[illegible]	[illegible]	721
Blindness and vision loss	0 (0 to 0)	[illegible]	[illegible]	[illegible]	[illegible]	[illegible]	[illegible]	[illegible]	[illegible]	[illegible]	[illegible]	[illegible]	[illegible]	[illegible]	[illegible]	[illegible]	[illegible]	[illegible]	[illegible]	[illegible]	[illegible]	[illegible]	188
Glaucoma	0 (0 to 0)	[illegible]	[illegible]	[illegible]	[illegible]	[illegible]	[illegible]	[illegible]	[illegible]	[illegible]	[illegible]	[illegible]	[illegible]	[illegible]	[illegible]	[illegible]	[illegible]	[illegible]	[illegible]	[illegible]	[illegible]	[illegible]	9·5
Cataract	0 (0 to 0)	[illegible]	[illegible]	[illegible]	[illegible]	[illegible]	[illegible]	[illegible]	[illegible]	[illegible]	[illegible]	[illegible]	[illegible]	[illegible]	[illegible]	[illegible]	[illegible]	[illegible]	[illegible]	[illegible]	[illegible]	[illegible]	30·2
Age-related macular degeneration	0 (0 to 0)	[illegible]	[illegible]	[illegible]	[illegible]	[illegible]	[illegible]	[illegible]	[illegible]	[illegible]	[illegible]	[illegible]	[illegible]	[illegible]	[illegible]	[illegible]	[illegible]	[illegible]	[illegible]	[illegible]	[illegible]	[illegible]	1·3
Refraction disorders	0 (0 to 0)	[illegible]	[illegible]	[illegible]	[illegible]	[illegible]	[illegible]	[illegible]	[illegible]	[illegible]	[illegible]	[illegible]	[illegible]	[illegible]	[illegible]	[illegible]	[illegible]	[illegible]	[illegible]	[illegible]	[illegible]	[illegible]	51·2
Near vision loss	0 (0 to 0)	[illegible]	[illegible]	[illegible]	[illegible]	[illegible]	[illegible]	[illegible]	[illegible]	[illegible]	[illegible]	[illegible]	[illegible]	[illegible]	[illegible]	[illegible]	[illegible]	[illegible]	[illegible]	[illegible]	[illegible]	[illegible]	48·3
Other vision loss	0 (0 to 0)	[illegible]	[illegible]	[illegible]	[illegible]	[illegible]	[illegible]	[illegible]	[illegible]	[illegible]	[illegible]	[illegible]	[illegible]	[illegible]	[illegible]	[illegible]	[illegible]	[illegible]	[illegible]	[illegible]	[illegible]	[illegible]	47·6
Age-related and other hearing loss	0 (0 to 0)	[illegible]	[illegible]	[illegible]	[illegible]	[illegible]	[illegible]	[illegible]	[illegible]	[illegible]	[illegible]	[illegible]	[illegible]	[illegible]	[illegible]	[illegible]	[illegible]	[illegible]	[illegible]	[illegible]	[illegible]	[illegible]	497
Other sense organ diseases	0 (0 to 0)	[illegible]	[illegible]	[illegible]	[illegible]	[illegible]	[illegible]	[illegible]	[illegible]	[illegible]	[illegible]	[illegible]	[illegible]	[illegible]	[illegible]	[illegible]	[illegible]	[illegible]	[illegible]	[illegible]	[illegible]	[illegible]	36·1
Musculoskeletal disorders	0 (0 to 0)	[illegible]	[illegible]	[illegible]	[illegible]	[illegible]	[illegible]	[illegible]	[illegible]	[illegible]	[illegible]	[illegible]	[illegible]	[illegible]	[illegible]	[illegible]	[illegible]	[illegible]	[illegible]	[illegible]	[illegible]	[illegible]	1,630
Rheumatoid arthritis	0 (0 to 0)	[illegible]	[illegible]	[illegible]	[illegible]	[illegible]	[illegible]	[illegible]	[illegible]	[illegible]	[illegible]	[illegible]	[illegible]	[illegible]	[illegible]	[illegible]	[illegible]	[illegible]	[illegible]	[illegible]	[illegible]	[illegible]	38·1
Osteoarthritis	0 (0 to 0)	[illegible]	[illegible]	[illegible]	[illegible]	[illegible]	[illegible]	[illegible]	[illegible]	[illegible]	[illegible]	[illegible]	[illegible]	[illegible]	[illegible]	[illegible]	[illegible]	[illegible]	[illegible]	[illegible]	[illegible]	[illegible]	215
Osteoarthritis hip	0 (0 to 0)	[illegible]	[illegible]	[illegible]	[illegible]	[illegible]	[illegible]	[illegible]	[illegible]	[illegible]	[illegible]	[illegible]	[illegible]	[illegible]	[illegible]	[illegible]	[illegible]	[illegible]	[illegible]	[illegible]	[illegible]	[illegible]	10·9
Osteoarthritis knee	0 (0 to 0)	[illegible]	[illegible]	[illegible]	[illegible]	[illegible]	[illegible]	[illegible]	[illegible]	[illegible]	[illegible]	[illegible]	[illegible]	[illegible]	[illegible]	[illegible]	[illegible]	[illegible]	[illegible]	[illegible]	[illegible]	[illegible]	154
Osteoarthritis hand	0 (0 to 0)	[illegible]	[illegible]	[illegible]	[illegible]	[illegible]	[illegible]	[illegible]	[illegible]	[illegible]	[illegible]	[illegible]	[illegible]	[illegible]	[illegible]	[illegible]	[illegible]	[illegible]	[illegible]	[illegible]	[illegible]	[illegible]	25·5

Osteoarthritis other	0 (0 to 0)	0 (0 to 0)	0 (0 to 0)	0 (0 to 0)	0 (0 to 0)	0 (0 to 0)	0 (0 to 0)	0 (0 to 0)	0 (0 to 0)	0 (0 to 0·1)	0·3 (0·1 to 0·6)	0·8 (0·3 to 2)	1·8 (0·8 to 4)	2·9 (1·4 to 6)	3·7 (1·6 to 7·8)	3·8 (1·8 to 8·1)	3·5 (1·7 to 7·2)	2·9 (1·4 to 6)	2·2 (1·1 to 4·5)	1·4 (0·7 to 3)	0·8 (0·4 to 1·7)	0·3 (0·2 to 0·7)	0·1 (0·1 to 0·2)	24·7
Low back pain	0 (0 to 0)	0 (0 to 0)	0 (0 to 0)	0 (0 to 0)	2·8 (1·3 to 5·1)	14·9 (8·9 to 23·7)	29·5 (17·5 to 47)	38·2 (22·7 to 60·7)	45·5 (26·3 to 70·3)	53·2 (30 to 82·6)	61·3 (37 to 94·6)	68·1 (40·7 to 104)	73·4 (44·4 to 116)	77·3 (46·4 to 123)	71·2 (44 to 107)	59·3 (37 to 87·9)	46·7 (29·5 to 72·3)	35·3 (22 to 53·5)	24·1 (15 to 36·2)	14·9 (9·5 to 22·4)	7·3 (4·4 to 10·8)	2·5 (1·6 to 3·7)	0·7 (0·4 to 1)	726
Neck pain	0 (0 to 0)	0 (0 to 0)	0 (0 to 0)	0 (0 to 0)	0·2 (0 to 0·4)	1 (0·4 to 2·1)	2·5 (1·1 to 4·6)	4·2 (1·8 to 8·1)	6·6 (3·1 to 12·7)	10·3 (4·9 to 19·9)	13·9 (7·1 to 24)	17·3 (8·8 to 31·1)	19·5 (9·8 to 34·8)	17·8 (9 to 33·8)	15·2 (7·3 to 27·4)	11·8 (6·1 to 20·7)	8·6 (4·6 to 14·4)	6·2 (3·3 to 10·7)	3·9 (2 to 7·1)	2 (1 to 3·6)	1 (0·5 to 1·7)	0·4 (0·2 to 0·6)	0·1 (0·1 to 0·2)	142
Gout	0 (0 to 0)	0 (0 to 0)	0 (0 to 0)	0 (0 to 0)	0 (0 to 0)	0 (0 to 0)	0 (0 to 0)	0·1 (0 to 0·2)	0·2 (0·1 to 0·5)	0·4 (0·2 to 0·8)	0·6 (0·2 to 1)	0·8 (0·4 to 1·4)	0·9 (0·4 to 1·5)	1 (0·5 to 1·8)	1 (0·5 to 1·7)	0·8 (0·4 to 1·5)	0·8 (0·4 to 1·3)	0·6 (0·3 to 1·1)	0·5 (0·3 to 0·8)	0·3 (0·1 to 0·6)	0·2 (0·1 to 0·3)	0·1 (0 to 0·1)	0 (0 to 0)	8·2
Other musculoskeletal disorders	0 (0 to 0)	0 (0 to 0)	0 (0 to 0)	0 (0 to 0)	0·2 (0·1 to 0·3)	0·8 (0·4 to 1·5)	7·5 (4·4 to 11·9)	18·2 (11·7 to 26·3)	33·2 (21 to 50·1)	44·5 (28·5 to 66·7)	48·9 (31·2 to 72·3)	53·9 (33·6 to 81)	57·4 (37·7 to 86)	58·5 (37·2 to 87·7)	52·9 (33·3 to 78)	42 (25·2 to 64·7)	31·3 (19·6 to 46·6)	21·7 (12·6 to 33·8)	12·3 (7·3 to 18·9)	6 (3 to 9·9)	4·2 (2·6 to 6)	1·5 (0·9 to 2·2)	0·4 (0·2 to 0·6)	495
Other non-communicable diseases	48·6 (31·8 to 70·5)	32·3 (21·7 to 45·8)	79·8 (57 to 109)	66·9 (44·2 to 99·9)	34·3 (27·9 to 42·9)	34·1 (26·6 to 42·9)	51·4 (39·8 to 67·9)	76·8 (57·8 to 105)	96·4 (70·6 to 132)	109 (78·6 to 151)	122 (88·3 to 169)	128 (91·9 to 178)	128 (92·4 to 172)	126 (92·7 to 167)	117 (87·7 to 153)	105 (79·3 to 137)	87·2 (65·6 to 114)	73·4 (55·9 to 94·5)	55 (44·1 to 69)	37·3 (30·2 to 45·7)	23 (18·9 to 27·8)	9·8 (8 to 11·8)	3·1 (2·5 to 3·9)	1,640
Congenital birth defects	46·5 (30 to 68·3)	29·1 (19·1 to 41·6)	52·7 (36·6 to 75·2)	35·9 (22·2 to 55·3)	13·3 (10·5 to 16·8)	11·4 (9 to 14·5)	11·8 (9·3 to 15)	10·8 (8·5 to 13·6)	9·2 (7·4 to 11·2)	7·1 (5·6 to 9)	7 (5·5 to 8·7)	6·2 (4·8 to 8)	6·1 (4·8 to 7·8)	6·1 (4·8 to 7·8)	5·2 (4 to 6·6)	3·2 (2·5 to 4·1)	2·1 (1·6 to 2·7)	0·7 (0·4 to 1)	0·4 (0·2 to 0·6)	0·2 (0·1 to 0·3)	0·1 (0·1 to 0·1)	0 (0 to 0·1)	0 (0 to 0)	265
Neural tube defects	2·8 (1·5 to 4·6)	1·2 (0·7 to 2·1)	3·1 (1·6 to 5·8)	2·7 (1·4 to 5)	0·4 (0·3 to 0·5)	0·5 (0·3 to 0·6)	0·5 (0·4 to 0·7)	0·3 (0·2 to 0·5)	0·3 (0·2 to 0·4)	0·3 (0·2 to 0·3)	0·2 (0·1 to 0·3)	0·2 (0·1 to 0·2)	0·1 (0·1 to 0·2)	0·1 (0·1 to 0·1)	0·1 (0·1 to 0·1)	0·1 (0 to 0·1)	0 (0 to 0·1)	0 (0 to 0)	0 (0 to 0)	0 (0 to 0)	0 (0 to 0)	0 (0 to 0)	0 (0 to 0)	13
Congenital heart anomalies	20·1 (11·5 to 32·3)	15·7 (10·2 to 23)	27·2 (17·9 to 40·5)	17·2 (9·1 to 29·4)	4·7 (3·4 to 6·3)	3·3 (2·4 to 4·4)	3·5 (2·6 to 5)	3·3 (2·5 to 4·4)	2·8 (2·1 to 3·7)	1·9 (1·4 to 2·5)	2 (1·5 to 2·6)	1·7 (1·2 to 2·2)	1·6 (1·2 to 2·1)	1·5 (1 to 2)	1·1 (0·8 to 1·5)	0·7 (0·5 to 0·9)	0·5 (0·3 to 0·6)	0·1 (0 to 0·1)	0 (0 to 0·1)	0 (0 to 0)	0 (0 to 0)	0 (0 to 0)	0 (0 to 0)	109
Orofacial clefts	0·3 (0·1 to 0·7)	0·4 (0·1 to 0·9)	0·5 (0·2 to 1·5)	0·2 (0·1 to 0·4)	0·2 (0·1 to 0·3)	0·2 (0·1 to 0·4)	0·2 (0·1 to 0·4)	0·2 (0·1 to 0·4)	0·2 (0·1 to 0·4)	0·2 (0·1 to 0·3)	0·2 (0·1 to 0·4)	0·2 (0·1 to 0·3)	0·2 (0·1 to 0·3)	0·2 (0·1 to 0·3)	0·1 (0·1 to 0·3)	0·1 (0 to 0·2)	0·1 (0 to 0·1)	0·1 (0 to 0·1)	0 (0 to 0·1)	0 (0 to 0)	0 (0 to 0)	0 (0 to 0)	0 (0 to 0)	3·8
Down syndrome	0·5 (0·3 to 1)	0·4 (0·2 to 0·7)	1·8 (1 to 3)	1·7 (1 to 3)	0·6 (0·4 to 0·8)	0·6 (0·4 to 0·8)	0·8 (0·5 to 1·2)	0·9 (0·6 to 1·2)	0·7 (0·5 to 1·1)	0·7 (0·4 to 1)	0·7 (0·5 to 1)	0·7 (0·4 to 0·9)	0·8 (0·6 to 1·1)	1 (0·6 to 1·4)	1·1 (0·7 to 1·6)	0·5 (0·4 to 0·8)	0·2 (0·1 to 0·3)	0 (0 to 0)	0 (0 to 0)	0 (0 to 0)	0 (0 to 0)	0 (0 to 0)	0 (0 to 0)	13·7
Turner syndrome	0 (0 to 0)	0 (0 to 0)	0 (0 to 0)	0 (0 to 0)	0 (0 to 0)	0 (0 to 0)	0 (0 to 0)	0 (0 to 0)	0 (0 to 0)	0 (0 to 0)	0 (0 to 0)	0 (0 to 0)	0 (0 to 0)	0 (0 to 0)	0 (0 to 0)	0 (0 to 0)	0 (0 to 0)	0 (0 to 0)	0 (0 to 0)	0 (0 to 0)	0 (0 to 0)	0 (0 to 0)	0 (0 to 0)	0·1
Klinefelter syndrome	0 (0 to 0)	0 (0 to 0)	0 (0 to 0)	0 (0 to 0)	0 (0 to 0)	0 (0 to 0)	0 (0 to 0)	0 (0 to 0)	0 (0 to 0)	0 (0 to 0)	0 (0 to 0)	0 (0 to 0)	0 (0 to 0)	0 (0 to 0)	0 (0 to 0)	0 (0 to 0)	0 (0 to 0)	0 (0 to 0)	0 (0 to 0)	0 (0 to 0)	0 (0 to 0)	0 (0 to 0)	0 (0 to 0)	0
Other chromosomal abnormalities	3·8 (2·3 to 6·5)	2·6 (1·4 to 4·3)	5·4 (3·3 to 8·9)	2 (1·1 to 3·3)	0·8 (0·6 to 1·1)	0·7 (0·5 to 1)	0·9 (0·6 to 1·2)	0·7 (0·5 to 0·9)	0·6 (0·4 to 0·8)	0·4 (0·3 to 0·5)	0·3 (0·2 to 0·4)	0·2 (0·1 to 0·3)	0·1 (0·1 to 0·2)	0·1 (0·1 to 0·2)	0·1 (0 to 0·1)	0 (0 to 0)	0 (0 to 0)	0 (0 to 0)	0 (0 to 0)	0 (0 to 0)	0 (0 to 0)	0 (0 to 0)	0 (0 to 0)	18·8
Congenital musculoskeletal and limb anomalies	2·1 (1·1 to 4)	1·1 (0·5 to 2)	2·6 (1·6 to 4·2)	3·4 (2·2 to 4·9)	3·4 (2·1 to 5)	3·5 (2·1 to 5·4)	3·3 (2 to 5)	2·7 (1·7 to 3·9)	2·5 (1·6 to 3·6)	2·2 (1·4 to 3·4)	2·2 (1·4 to 3·3)	2·1 (1·3 to 3·2)	2·1 (1·3 to 3·1)	2 (1·3 to 3)	1·7 (1·1 to 2·5)	1·2 (0·8 to 1·9)	0·8 (0·5 to 1·2)	0·5 (0·3 to 0·7)	0·2 (0·1 to 0·4)	0·1 (0·1 to 0·2)	0 (0 to 0·1)	0 (0 to 0)	0 (0 to 0)	39·8
Urogenital congenital anomalies	0·5 (0·3 to 1)	0·2 (0·1 to 0·4)	0·3 (0·2 to 0·5)	0·4 (0·2 to 0·6)	0·3 (0·2 to 0·4)	0·2 (0·1 to 0·3)	0·2 (0·1 to 0·3)	0·2 (0·1 to 0·3)	0·1 (0·1 to 0·2)	0·1 (0·1 to 0·2)	0·1 (0·1 to 0·1)	0·1 (0·1 to 0·1)	0·1 (0 to 0·1)	0 (0 to 0·1)	0 (0 to 0)	0 (0 to 0)	0 (0 to 0)	0 (0 to 0)	0 (0 to 0)	0 (0 to 0)	0 (0 to 0)	0 (0 to 0)	0 (0 to 0)	2·9
Digestive congenital anomalies	4·2 (2·4 to 7·1)	3 (1·8 to 4·7)	3·3 (1·9 to 5·5)	1·4 (0·8 to 2·6)	0·3 (0·2 to 0·5)	0·2 (0·1 to 0·2)	0·1 (0·1 to 0·2)	0·1 (0·1 to 0·1)	0·1 (0·1 to 0·1)	0·1 (0 to 0·1)	0 (0 to 0·1)	0 (0 to 0·1)	0 (0 to 0·1)	0 (0 to 0·1)	0 (0 to 0·1)	0 (0 to 0·1)	0 (0 to 0)	0 (0 to 0)	0 (0 to 0)	0 (0 to 0)	0 (0 to 0)	0 (0 to 0)	0 (0 to 0)	13·1
Other congenital birth defects	12·1 (7·3 to 18·4)	4·5 (2·7 to 7)	8·4 (5·1 to 12·6)	6·8 (3·8 to 11·3)	2·7 (2 to 3·8)	2·2 (1·6 to 3)	2·2 (1·6 to 3·1)	2·5 (1·7 to 3·5)	1·7 (1·2 to 2·5)	1·4 (1 to 2)	1·2 (0·8 to 1·8)	1·1 (0·7 to 1·7)	1·1 (0·7 to 1·7)	1·1 (0·7 to 1·7)	0·9 (0·6 to 1·4)	0·5 (0·3 to 0·9)	0·4 (0·3 to 0·7)	0·1 (0 to 0·3)	0·1 (0 to 0·2)	0 (0 to 0·1)	0 (0 to 0·1)	0 (0 to 0)	0 (0 to 0)	51
Urinary diseases and male infertility	0·1 (0 to 0·1)	0·2 (0·1 to 0·3)	0·8 (0·5 to 1·3)	1 (0·6 to 1·4)	0·6 (0·4 to 0·7)	0·7 (0·5 to 1)	1·1 (0·9 to 1·5)	2·5 (1·7 to 3·7)	2·9 (2 to 4·5)	3·5 (2·5 to 5·5)	4·5 (3·2 to 6·2)	4·9 (3·6 to 6·8)	5·6 (4 to 7·5)	8·3 (6·2 to 10·6)	12 (8·8 to 15·9)	15·2 (11·2 to 20·1)	15·4 (11·5 to 20·2)	15·7 (11·5 to 20·8)	13·5 (10·1 to 17·7)	10·8 (8·1 to 13·8)	6·6 (4·7 to 8·6)	2·9 (2 to 3·8)	1 (0·6 to 1·4)	130
Urolithiasis	0 (0 to 0)	0 (0 to 0)	0 (0 to 0)	0 (0 to 0)	0 (0 to 0)	0 (0 to 0·1)	0 (0 to 0·1)	0·1 (0·1 to 0·2)	0·2 (0·1 to 0·3)	0·3 (0·2 to 0·5)	0·5 (0·3 to 0·8)	0·5 (0·3 to 0·9)	0·8 (0·4 to 1·3)	0·8 (0·5 to 1·3)	1 (0·6 to 1·5)	0·8 (0·5 to 1·2)	0·6 (0·4 to 0·9)	0·4 (0·2 to 0·5)	0·2 (0·1 to 0·3)	0·1 (0·1 to 0·2)	0·1 (0 to 0·1)	0 (0 to 0)	0 (0 to 0)	6·4
Benign prostatic hyperplasia	0 (0 to 0)	0 (0 to 0)	0 (0 to 0)	0 (0 to 0)	0 (0 to 0)	0 (0 to 0)	0 (0 to 0)	0 (0 to 0)	0 (0 to 0)	0 (0 to 0)	0 (0 to 0)	0 (0 to 0·1)	0·2 (0·1 to 0·5)	1 (0·5 to 1·8)	2·9 (1·4 to 5·1)	4·9 (2·5 to 8·3)	6 (3·4 to 9·7)	5·6 (3·1 to 9·2)	3·7 (2·1 to 6)	1·7 (1 to 2·7)	0·6 (0·3 to 1)	0·2 (0·1 to 0·3)	0 (0 to 0)	26·9
Male infertility	0 (0 to 0)	0 (0 to 0)	0 (0 to 0)	0 (0 to 0)	0 (0 to 0)	0 (0 to 0)	0 (0 to 0)	0·7 (0·2 to 1·7)	0·8 (0·2 to 2)	0·8 (0·2 to 2·3)	0·8 (0·2 to 2)	0·8 (0·2 to 2)	0·1 (0 to 0·3)	0 (0 to 0)	0 (0 to 0)	0 (0 to 0)	0 (0 to 0)	0 (0 to 0)	0 (0 to 0)	0 (0 to 0)	0 (0 to 0)	0 (0 to 0)	0 (0 to 0)	4
Other urinary diseases	0 (0 to 0)	0·1 (0 to 0·2)	0·1 (0 to 0·1)	0·2 (0·1 to 0·3)	0·2 (0·1 to 0·2)	0·2 (0·1 to 0·3)	0·2 (0·2 to 0·3)	0·4 (0·2 to 0·6)	0·3 (0·2 to 0·4)	0·4 (0·3 to 0·6)	0·6 (0·4 to 0·9)	0·5 (0·3 to 0·7)	0·7 (0·4 to 1·1)	0·8 (0·5 to 1·2)	0·9 (0·5 to 1·4)	1·3 (0·6 to 2·2)	1 (0·5 to 1·7)	0·8 (0·4 to 1·5)	0·9 (0·4 to 1·5)	0·8 (0·3 to 1·4)	0·4 (0·1 to 0·7)	0·2 (0·1 to 0·3)	0·1 (0 to 0·1)	10·9
Gynecological diseases	0 (0 to 0)	0 (0 to 0)	0 (0 to 0)	0 (0 to 0)	0 (0 to 0)	4·4 (2·1 to 7·5)	12·4 (7 to 21·2)	28·2 (17·8 to 44·3)	41·9 (27·2 to 61·5)	51·6 (33·5 to 74·6)	55·4 (36·9 to 80·2)	56·3 (36·9 to 83)	47·2 (30·7 to 67·4)	31·7 (19·4 to 48·2)	17·6 (10·6 to 25·5)	10·3 (6 to 15·6)	7 (4·2 to 10·2)	4·6 (2·9 to 6·6)	2·8 (1·8 to 4·1)	1·6 (1 to 2·4)	0·9 (0·6 to 1·4)	0·4 (0·2 to 0·6)	0·1 (0·1 to 0·2)	374
Uterine fibroids	0 (0 to 0)	0 (0 to 0)	0 (0 to 0)	0 (0 to 0)	0 (0 to 0)	0·1 (0 to 0·2)	0·2 (0·1 to 0·4)	0·5 (0·2 to 1·1)	1·3 (0·5 to 2·6)	2·8 (1·3 to 5·6)	4·7 (2·3 to 9·2)	5·5 (2·8 to 10·1)	5·2 (2·7 to 9·3)	3·6 (1·8 to 6·5)	2·3 (1·2 to 4·1)	1·2 (0·6 to 2·2)	0·7 (0·3 to 1·3)	0·4 (0·2 to 0·7)	0·2 (0·1 to 0·3)	0·1 (0·1 to 0·2)	0 (0 to 0·1)	0 (0 to 0)	0 (0 to 0)	28·7
Polycystic ovarian syndrome	0 (0 to 0)	0 (0 to 0)	0 (0 to 0)	0 (0 to 0)	0 (0 to 0)	0·2 (0·1 to 0·4)	0·8 (0·3 to 1·6)	1 (0·4 to 2)	0·9 (0·4 to 2)	0·9 (0·4 to 1·9)	0·9 (0·4 to 1·8)	0·8 (0·4 to 1·7)	0·7 (0·3 to 1·4)	0·2 (0·1 to 0·4)	0 (0 to 0)	0 (0 to 0)	0 (0 to 0)	0 (0 to 0)	0 (0 to 0)	0 (0 to 0)	0 (0 to 0)	0 (0 to 0)	0 (0 to 0)	6·4
Female infertility	0 (0 to 0)	0 (0 to 0)	0 (0 to 0)	0 (0 to 0)	0 (0 to 0)	0 (0 to 0)	0 (0 to 0)	1 (0·2 to 2·7)	1·5 (0·4 to 3·9)	1·8 (0·4 to 5·5)	1·9 (0·3 to 5·6)	1·8 (0·2 to 5)	0 (0 to 0·2)	0 (0 to 0)	0 (0 to 0)	0 (0 to 0)	0 (0 to 0)	0 (0 to 0)	0 (0 to 0)	0 (0 to 0)	0 (0 to 0)	0 (0 to 0)	0 (0 to 0)	8
Endometriosis	0 (0 to 0)	0 (0 to 0)	0 (0 to 0)	0 (0 to 0)	0 (0 to 0)	0 (0 to 0)	1 (0·4 to 2)	4·2 (1·9 to 8·1)	4·9 (2·4 to 8·9)	4 (1·9 to 7·4)	3·5 (1·8 to 6·3)	3·4 (1·7 to 6)	3 (1·6 to 4·9)	1·4 (0·7 to 2·5)	0 (0 to 0)	0 (0 to 0)	0 (0 to 0)	0 (0 to 0)	0 (0 to 0)	0 (0 to 0)	0 (0 to 0)	0 (0 to 0)	0 (0 to 0)	25·4
Genital prolapse	0 (0 to 0)	0 (0 to 0)	0 (0 to 0)	0 (0 to 0)	0 (0 to 0)	0 (0 to 0)	0 (0 to 0)	0 (0 to 0·1)	0·1 (0 to 0·2)	0·2 (0·1 to 0·4)	0·4 (0·1 to 0·8)	0·6 (0·2 to 1·2)	0·8 (0·3 to 1·6)	0·9 (0·4 to 1·8)	0·8 (0·4 to 1·6)	0·6 (0·3 to 1·3)	0·5 (0·2 to 1)	0·4 (0·2 to 0·7)	0·2 (0·1 to 0·5)	0·2 (0·1 to 0·3)	0·1 (0 to 0·2)	0 (0 to 0·1)	0 (0 to 0)	5·8
Premenstrual syndrome	0 (0 to 0)	0 (0 to 0)	0 (0 to 0)	0 (0 to 0)	0 (0 to 0)	4 (1·9 to 7·1)	9·9 (4·9 to 17·8)	13·4 (6·5 to 23·2)	13·5 (7·8 to 21·7)	14 (7·1 to 22·9)	15·3 (8·6 to 24·4)	15·2 (8·5 to 24·7)	10·6 (5·8 to 17·9)	3·5 (1·7 to 6·5)	0 (0 to 0)	0 (0 to 0)	0 (0 to 0)	0 (0 to 0)	0 (0 to 0)	0 (0 to 0)	0 (0 to 0)	0 (0 to 0)	0 (0 to 0)	99·3
Other gynecological diseases	0 (0 to 0)	0 (0 to 0)	0 (0 to 0)	0 (0 to 0)	0 (0 to 0)	0·2 (0 to 0·4)	0·5 (0·2 to 1·1)	8·1 (4·5 to 13·3)	19·7 (11 to 31·6)	27·9 (16·5 to 43·5)	28·8 (16·6 to 46·1)	29 (16·2 to 46·1)	26·9 (14·9 to 42·1)	22·1 (10·4 to 35·7)	14·5 (8·5 to 21·8)	8·5 (4·5 to 13·6)	5·8 (3·2 to 9)	3·8 (2·4 to 5·8)	2·3 (1·4 to 3·6)	1·4 (0·8 to 2·1)	0·8 (0·5 to 1·2)	0·3 (0·2 to 0·5)	0·1 (0 to 0·2)	201
Hemoglobinopathies and hemolytic anemias	0·2 (0·1 to 0·3)	0·4 (0·2 to 0·5)	2·3 (1·5 to 3·3)	9·2 (5·9 to 13·7)	9·4 (6·8 to 12·7)	7·2 (5·3 to 9·5)	10·8 (8·3 to 14·1)	14 (9·4 to 20·1)	12·5 (8·3 to 18·1)	8·9 (6·1 to 13·9)	8·7 (6·4 to 11·5)	8·1 (5·9 to 10·8)	7·9 (5·7 to 10·4)	8·3 (6·2 to 11)	7·5 (5·6 to 9·8)	6·3 (4·8 to 8·6)	4 (3 to 5·5)	3·4 (2·6 to 4·3)	2·5 (2 to 3·3)	1·8 (1·4 to 2·3)	1·7 (1·3 to 2·1)	0·8 (0·6 to 1)	0·2 (0·2 to 0·3)	136
Thalassemias	0 (0 to 0)	0 (0 to 0)	0 (0 to 0)	0·1 (0 to 0·2)	0 (0 to 0·1)	0 (0 to 0)	0 (0 to 0)	0 (0 to 0)	0 (0 to 0)	0 (0 to 0)	0 (0 to 0)	0 (0 to 0)	0 (0 to 0)	0 (0 to 0)	0 (0 to 0)	0 (0 to 0)	0 (0 to 0)	0 (0 to 0)	0 (0 to 0)	0 (0 to 0)	0 (0 to 0)	0 (0 to 0)	0 (0 to 0)	0·1
Thalassemias trait	0 (0 to 0)	0 (0 to 0)	0 (0 to 0·1)	0·2 (0·1 to 0·3)	0·2 (0·1 to 0·4)	0·1 (0 to 0·2)	0·3 (0·2 to 0·5)	0·4 (0·2 to 0·6)	0·4 (0·2 to 0·6)	0·4 (0·2 to 0·6)	0·3 (0·2 to 0·6)	0·2 (0·1 to 0·4)	0·1 (0 to 0·2)	0·1 (0 to 0·2)	0·1 (0 to 0·2)	0·1 (0 to 0·1)	0 (0 to 0·1)	0 (0 to 0·1)	0 (0 to 0·1)	0 (0 to 0)	0 (0 to 0)	0 (0 to 0)	0 (0 to 0)	3
Sickle cell disorders	0·2 (0·1 to 0·3)	0·2 (0·1 to 0·4)	1·7 (1 to 2·6)	6·1 (3·5 to 10·1)	5·7 (4·1 to 7·4)	5·1 (3·8 to 6·7)	7·6 (5·6 to 10·2)	7·6 (5·4 to 10·6)	5·6 (3·8 to 7·5)	3·9 (2·6 to 5·5)	4·2 (2·7 to 6)	3·7 (2·5 to 5·2)	3·7 (2·5 to 5·4)	3·3 (2·1 to 5·1)	1·9 (1·1 to 3)	0·6 (0·3 to 1·1)	0·1 (0 to 0·2)	0 (0 to 0)	0 (0 to 0)	0 (0 to 0)	0 (0 to 0)	0 (0 to 0)	0 (0 to 0)	61·1
Sickle cell trait	0 (0 to 0)	0·1 (0 to 0·2)	0·3 (0·2 to 0·6)	1·5 (0·7 to 2·5)	1·9 (0·8 to 3·6)	1 (0·4 to 1·9)	0·4 (0·1 to 0·8)	3·2 (0·5 to 7·5)	3·6 (0·7 to 8)	2·1 (0·4 to 6)	0·5 (0·2 to 1)	0·9 (0·4 to 1·7)	1 (0·4 to 1·7)	1 (0·5 to 1·8)	0·9 (0·5 to 1·6)	0·6 (0·3 to 1·1)	0·3 (0·1 to 0·6)	0·3 (0·1 to 0·5)	0·2 (0·1 to 0·4)	0·2 (0·1 to 0·3)	0·1 (0·1 to 0·2)	0 (0 to 0·1)	0 (0 to 0)	20·3
G6PD deficiency	0 (0 to 0)	0 (0 to 0)	0 (0 to 0·1)	0·4 (0·2 to 0·9)	0·5 (0·2 to 1)	0·4 (0·2 to 0·7)	1·4 (0·7 to 2·1)	1·9 (0·8 to 3·3)	2 (1 to 3·3)	1·5 (0·7 to 2·4)	2 (1·1 to 3·1)	1·5 (0·8 to 2·3)	1·4 (0·8 to 2·2)	1·6 (1 to 2·4)	1·8 (1·1 to 2·6)	1·6 (1 to 2·2)	0·8 (0·5 to 1·1)	0·5 (0·3 to 0·7)	0·2 (0·1 to 0·4)	0·1 (0·1 to 0·2)	0 (0 to 0·1)	0 (0 to 0)	0 (0 to 0)	19·7
G6PD trait	0 (0 to 0)	0 (0 to 0)	0 (0 to 0)	0 (0 to 0)	0 (0 to 0)	0 (0 to 0)	0·1 (0 to 0·1)	0 (0 to 0)	0 (0 to 0)	0 (0 to 0)	0·1 (0 to 0·1)	0·1 (0 to 0·2)	0 (0 to 0)	0 (0 to 0)	0 (0 to 0)	0 (0 to 0)	0 (0 to 0)	0 (0 to 0)	0 (0 to 0)	0 (0 to 0)	0 (0 to 0)	0 (0 to 0)	0 (0 to 0)	0·2
Other hemoglobinopathies and hemolytic anemias	0 (0 to 0)	0 (0 to 0)	0·2 (0·1 to 0·4)	0·9 (0·4 to 1·7)	1·1 (0·4 to 2·1)	0·6 (0·2 to 1·1)	1·1 (0·5 to 2·2)	0·9 (0·4 to 1·7)	0·9 (0·5 to 1·6)	1 (0·5 to 1·8)	1·5 (0·8 to 2·6)	1·7 (0·9 to 2·9)	1·7 (0·9 to 2·6)	2·2 (1·3 to 3·4)	2·8 (1·9 to 4)	3·5 (2·5 to 5)	2·8 (2 to 3·8)	2·6 (1·9 to 3·4)	2 (1·6 to 2·7)	1·5 (1·2 to 2)	1·5 (1·2 to 2)	0·7 (0·6 to 0·9)	0·2 (0·2 to 0·3)	31·6
Endocrine, metabolic, blood, and immune disorders	1·8 (0·9 to 3·3)	2·3 (1·3 to 3·8)	20·9 (12·4 to 34·1)	18·4 (11 to 29·1)	7·9 (5·7 to 10·6)	6·6 (4·8 to 9·2)	9 (6·7 to 12·4)	10·7 (7·8 to 14·8)	14·7 (10·9 to 21·2)	18·4 (13·4 to 25·9)	23·6 (16·7 to 32·9)	27·1 (19 to 38·6)	32·1 (22·8 to 46·1)	38·7 (27·2 to 54·7)	41 (29·6 to 56·3)	37·2 (26·2 to 51·2)	29·7 (20·9 to 42)	25·3 (18·8 to 34·5)	19·1 (14·1 to 25·6)	13 (9·7 to 17·3)	8·6 (6·4 to 11·3)	3·8 (2·8 to 5·1)	1·2 (0·9 to 1·8)	411
Oral disorders	0 (0 to 0)	0 (0 to 0)	0 (0 to 0)	2·5 (1·4 to 4·1)	3·2 (1·8 to 5·6)	3·8 (2 to 6·6)	6·2 (3·4 to 10·8)	10·6 (5·5 to 19·9)	15·3 (7·7 to 28·8)	19·6 (9·4 to 37·1)	22·6 (10·4 to 41·8)	25·8 (12·3 to 47)	29 (14·7 to 52·2)	32·5 (17·4 to 57·1)	33·5 (18·8 to 54)	32·2 (19·1 to 51·7)	29 (17·3 to 45·1)	23·7 (14·7 to 36·9)	16·7 (10·4 to 24·7)	10 (6·3 to 14·8)	5·2 (3·2 to 7·6)	2 (1·2 to 2·9)	0·6 (0·3 to 0·9)	324
Caries of deciduous teeth	0 (0 to 0)	0 (0 to 0)	0 (0 to 0)	0·8 (0·3 to 1·7)	0·8 (0·3 to 1·8)	0·1 (0 to 0·1)	0 (0 to 0)	0 (0 to 0)	0 (0 to 0)	0 (0 to 0)	0 (0 to 0)	0 (0 to 0)	0 (0 to 0)	0 (0 to 0)	0 (0 to 0)	0 (0 to 0)	0 (0 to 0)	0 (0 to 0)	0 (0 to 0)	0 (0 to 0)	0 (0 to 0)	0 (0 to 0)	0 (0 to 0)	1·7
Caries of permanent teeth	0 (0 to 0)	0 (0 to 0)	0 (0 to 0)	0 (0 to 0)	0·7 (0·3 to 1·6)	1·8 (0·7 to 3·9)	2·1 (0·8 to 4·4)	2·5 (1·1 to 5·2)	2·4 (1 to 4·9)	2·2 (0·9 to 4·7)	2·2 (0·9 to 4·3)	2 (0·8 to 4·1)	1·9 (0·8 to 4)	1·8 (0·7 to 3·6)	1·4 (0·5 to 3·1)	1 (0·3 to 2)	0·6 (0·2 to 1·3)	0·4 (0·2 to 0·9)	0·3 (0·1 to 0·5)	0·1 (0·1 to 0·3)	0·1 (0 to 0·1)	0 (0 to 0·1)	0 (0 to 0)	23·6
Periodontal diseases	0 (0 to 0)	0 (0 to 0)	0 (0 to 0)	0 (0 to 0)	0 (0 to 0)	0 (0 to 0)	0·9 (0·3 to 2·3)	3·6 (1·1 to 9·1)	7·6 (2·4 to 18)	11·3 (3·9 to 25·9)	13·9 (5 to 30·9)	15·9 (5·8 to 33·7)	16·8 (6·5 to 34·9)	16·1 (6·3 to 33·7)	12·9 (5·1 to 27)	9·1 (3·6 to 18·5)	6 (2·3 to 12·3)	4 (1·5 to 8)	2·4 (1 to 4·9)	1·4 (0·5 to 2·7)	0·7 (0·3 to 1·4)	0·2 (0·1 to 0·5)	0·1 (0 to 0·1)	123
Other oral disorders	0 (0 to 0)	0 (0 to 0)	0 (0 to 0)	1·7 (1 to 2·6)	1·6 (1 to 2·5)	2 (1·2 to 3·1)	3·2 (1·9 to 5)	4·1 (2·5 to 6·3)	4·4 (2·6 to 6·7)	4·5 (2·7 to 6·9)	4·5 (2·7 to 6·8)	4·5 (2·7 to 6·9)	4·5 (2·8 to 6·8)	4·3 (2·6 to 6·5)	3·6 (2·1 to 5·4)	2·8 (1·7 to 4·1)	2 (1·2 to 3·1)	1·3 (0·8 to 2)	0·7 (0·4 to 1·1)	0·3 (0·2 to 0·5)	0·1 (0·1 to 0·2)	0 (0 to 0·1)	0 (0 to 0)	50·3
Sudden infant death syndrome	--	0·4 (0·3 to 0·7)	3 (1·8 to 4·9)	--	--	--	--	--	--	--	--	--	--	--	--	--	--	--	--	--	--	--	--	NA
Injuries	2·4 (1·6 to 3·4)	2·3 (1·5 to 3·4)	14·3 (9·5 to 20·9)	64·2 (41·7 to 97·2)	55 (47·2 to 64·1)	60·9 (51·5 to 71·1)	140 (121 to 162)	271 (237 to 310)	259 (228 to 292)	186 (160 to 214)	183 (154 to 216)	152 (127 to 182)	147 (121 to 177)	146 (120 to 177)	130 (107 to 156)	105 (86·9 to 128)	74·8 (61·5 to 90·6)	59·8 (49·9 to 71·3)	45·7 (38 to 54·4)	34·7 (29·3 to 41)	21·7 (18·3 to 25·1)	11 (9·4 to 12·7)	4·2 (3·5 to 4·8)	2,170
Transport injuries	0·1 (0·1 to 0·2)	0·1 (0·1 to 0·2)	0·9 (0·6 to 1·4)	7·8 (4·5 to 12·7)	9·7 (6·8 to 12·8)	11·7 (8·8 to 15)	31·9 (23·8 to 41·2)	60 (45·4 to 76·9)	54·8 (40·6 to 69·9)	38·4 (29·7 to 47·8)	38·4 (29·5 to 48·8)	32·5 (25·4 to 39·6)	33 (26 to 40·7)	33 (25·9 to 41·1)	29·7 (23·2 to 36·4)	23·2 (18·4 to 28·9)	15·9 (12·7 to 19·5)	11·4 (9·3 to 13·9)	7·8 (6·3 to 9·3)	4·6 (3·7 to 5·6)	2·1 (1·7 to 2·6)	0·8 (0·6 to 0·9)	0·2 (0·2 to 0·3)	448
Road injuries	0·1 (0·1 to 0·1)	0·1 (0 to 0·1)	0·7 (0·5 to 1·1)	6·8 (3·9 to 11·1)	8·7 (6 to 11·6)	10·3 (7·7 to 13·4)	29·2 (21·6 to 37·9)	55 (41·4 to 70·9)	50·2 (36·9 to 64·2)	34·9 (26·8 to 44)	35 (26·5 to 44·9)	29·3 (22·9 to 36)	30·3 (23·7 to 37·6)	30·2 (23·7 to 37·8)	26·7 (20·8 to 33·1)	21·3 (17 to 26·6)	14·9 (11·8 to 18·2)	10·4 (8·4 to 12·7)	7·1 (5·8 to 8·6)	4·2 (3·4 to 5·2)	2 (1·6 to 2·4)	0·7 (0·6 to 0·9)	0·2 (0·2 to 0·3)	408
Pedestrian road injuries	0 (0 to 0)	0 (0 to 0)	0·1 (0·1 to 0·2)	2 (1·1 to 3·5)	2·4 (1·6 to 3·3)	2·1 (1·5 to 2·8)	2·3 (1·6 to 3)	3·2 (2·4 to 4·2)	3·6 (2·7 to 4·6)	3·3 (2·5 to 4·2)	3·8 (2·9 to 4·8)	3·7 (2·9 to 4·7)	4·3 (3·3 to 5·3)	4·8 (3·7 to 6·1)	4·8 (3·6 to 6)	4·3 (3·3 to 5·3)	3·4 (2·6 to 4·2)	2·7 (2·2 to 3·4)	2·1 (1·7 to 2·6)	1·4 (1·1 to 1·8)	0·7 (0·5 to 0·8)	0·2 (0·2 to 0·3)	0·1 (0·1 to 0·1)	55·3
Cyclist road injuries	0 (0 to 0)	0 (0 to 0)	0 (0 to 0)	0·5 (0·2 to 0·8)	0·8 (0·5 to 1·1)	1·8 (1·2 to 2·6)	3·2 (2·1 to 4·9)	4·4 (3·1 to 6·2)	4·3 (3·1 to 5·7)	3·9 (2·9 to 5·3)	4·3 (3·2 to 5·6)	4·2 (3·1 to 5·5)	4·5 (3·2 to 5·9)	4·7 (3·5 to 6·1)	4·1 (3 to 5·4)	3·3 (2·5 to 4·4)	2·4 (1·8 to 3·2)	1·7 (1·2 to 2·2)	1·1 (0·8 to 1·4)	0·6 (0·4 to 0·8)	0·3 (0·2 to 0·4)	0·1 (0·1 to 0·1)	0 (0 to 0)	50
Motorcyclist road injuries	0 (0 to 0)	0 (0 to 0)	0·1 (0·1 to 0·2)	0·7 (0·3 to 1·2)	1 (0·5 to 1·6)	1·2 (0·7 to 1·8)	7·5 (4·3 to 11·4)	17·5 (10·8 to 26·2)	16·6 (10·8 to 23·8)	10·1 (6·7 to 14·2)	9·6 (6·4 to 13·6)	6·6 (4·6 to 9)	6·5 (4·6 to 8·9)	5·7 (4 to 7·7)	4·8 (3·4 to 6·4)	3·1 (2·3 to 4·2)	2·1 (1·6 to 2·7)	1·2 (0·9 to 1·6)	0·8 (0·6 to 1·1)	0·5 (0·4 to 0·6)	0·2 (0·2 to 0·3)	0·1 (0·1 to 0·1)	0 (0 to 0)	95·8
Motor vehicle road injuries	0 (0 to 0·1)	0 (0 to 0·1)	0·5 (0·3 to 0·7)	3·4 (1·8 to 5·6)	4·4 (2·8 to 6)	5 (3·6 to 6·7)	15·6 (10·5 to 21·9)	29 (20·3 to 39·6)	24·9 (17·2 to 33·9)	16·7 (12·2 to 22·6)	16·4 (11·6 to 22·1)	13·8 (10·3 to 17·9)	14 (10·5 to 18·3)	13·9 (10·5 to 17·9)	12·1 (9 to 15·6)	9·9 (7·5 to 12·8)	6·3 (4·9 to 8)	4·4 (3·4 to 5·5)	2·8 (2·2 to 3·5)	1·6 (1·2 to 2)	0·7 (0·5 to 0·9)	0·3 (0·2 to 0·3)	0·1 (0·1 to 0·1)	196
Other road injuries	0 (0 to 0)	0 (0 to 0)	0 (0 to 0)	0·2 (0·1 to 0·5)	0·2 (0·1 to 0·4)	0·3 (0·2 to 0·4)	0·5 (0·3 to 0·8)	0·9 (0·6 to 1·4)	0·9 (0·6 to 1·3)	0·9 (0·6 to 1·2)	0·9 (0·7 to 1·3)	1 (0·7 to 1·3)	1·1 (0·8 to 1·4)	1·1 (0·8 to 1·5)	1 (0·7 to 1·3)	0·8 (0·6 to 1)	0·6 (0·5 to 0·8)	0·4 (0·3 to 0·5)	0·3 (0·2 to 0·4)	0·2 (0·1 to 0·2)	0·1 (0·1 to 0·1)	0 (0 to 0)	0 (0 to 0)	11·4
Other transport injuries	0 (0 to 0)	0 (0 to 0·1)	0·2 (0·1 to 0·3)	1·1 (0·6 to 1·8)	1 (0·7 to 1·4)	1·4 (1 to 1·9)	2·8 (1·9 to 3·9)	5 (3·2 to 7·2)	4·6 (3 to 6·7)	3·5 (2·5 to 5)	3·4 (2·4 to 4·7)	3·2 (2·4 to 4·1)	2·7 (2 to 3·6)	2·8 (2·1 to 3·7)	3 (2·2 to 3·9)	1·9 (1·3 to 2·5)	1·1 (0·8 to 1·4)	1 (0·8 to 1·2)	0·6 (0·5 to 0·9)	0·3 (0·2 to 0·4)	0·2 (0·1 to 0·2)	0·1 (0·1 to 0·1)	0 (0 to 0)	39·9
Unintentional injuries	1·7 (1·1 to 2·5)	2 (1·3 to 3)	12·4 (8·1 to 18·5)	52·8 (34 to 81·1)	42·7 (36·3 to 50·2)	44·2 (37·4 to 52·6)	76·1 (62·6 to 91·1)	132 (108 to 162)	127 (107 to 151)	97·5 (80·4 to 119)	100 (80·6 to 123)	90·3 (73·3 to 113)	90 (70·5 to 112)	93·5 (74·7 to 117)	84·9 (67·7 to 106)	71·3 (57·9 to 89·5)	52·4 (42·2 to 65·7)	43·9 (35·7 to 53·6)	35·1 (28·7 to 42·6)	28·5 (23·9 to 33·8)	18·9 (15·9 to 21·9)	10 (8·5 to 11·6)	3·8 (3·2 to 4·4)	1,310
Falls	0 (0 to 0)	0 (0 to 0)	0·3 (0·2 to 0·5)	1·4 (1 to 2·1)	2 (1·5 to 2·7)	3·3 (2·4 to 4·5)	5·6 (4·1 to 7·8)	8·5 (6·3 to 11·3)	9·2 (6·9 to 12·2)	9·4 (6·9 to 12·8)	10·8 (8·1 to 14·4)	11·4 (8·6 to 15·4)	12·7 (9·5 to 16·8)	14·6 (10·9 to 18·9)	14·3 (10·9 to 18·3)	13·7 (10·7 to 17·4)	11·6 (9·1 to 14·7)	11·8 (9·6 to 14·6)	11·2 (9 to 13·5)	11·8 (9·6 to 14·4)	8·9 (7·1 to 10·6)	4·7 (3·8 to 5·7)	1·7 (1·4 to 2·1)	179
Drowning	0·2 (0·1 to 0·2)	0·1 (0·1 to 0·2)	1·5 (1 to 2·2)	19·1 (10·6 to 31·4)	16·8 (13·3 to 20·7)	14·8 (11·7 to 18·2)	21·5 (16·1 to 28·4)	30 (21·6 to 41)	24·9 (18·3 to 32·2)	14·3 (11·3 to 18·3)	13·9 (10·5 to 18)	9·7 (7·2 to 12·8)	8·4 (6·1 to 11·6)	8·3 (6·4 to 10·7)	6·8 (5·2 to 8·8)	5·1 (4 to 6·5)	2·8 (2·2 to 3·5)	1·7 (1·3 to 2·1)	1·1 (0·8 to 1·5)	0·6 (0·4 to 0·8)	0·3 (0·3 to 0·4)	0·2 (0·1 to 0·2)	0·1 (0·1 to 0·1)	202
Fire, heat, and hot substances	0·1 (0·1 to 0·1)	0·2 (0·1 to 0·3)	1·6 (1·1 to 2·4)	9·3 (5·8 to 14·5)	5·4 (4·4 to 6·6)	5 (3·9 to 6·5)	6·9 (5·3 to 9·1)	9·4 (7·3 to 12·2)	10·4 (8·1 to 13·4)	10·7 (8·1 to 14·4)	11·3 (8·4 to 15·1)	11 (8·2 to 14·9)	11·2 (8·4 to 15·2)	11·4 (8·4 to 15·4)	10·3 (7·8 to 13·7)	8·7 (6·8 to 11·3)	6·1 (4·7 to 8·1)	5·1 (4 to 6·6)	3·8 (2·9 to 4·9)	2·8 (2·3 to 3·5)	1·8 (1·5 to 2·2)	0·9 (0·7 to 1·1)	0·3 (0·3 to 0·4)	144
Poisonings	0 (0 to 0)	0·1 (0 to 0·1)	0·3 (0·2 to 0·5)	2·2 (1·2 to 3·8)	0·6 (0·5 to 0·9)	0·6 (0·4 to 0·8)	0·9 (0·7 to 1·2)	1·2 (0·9 to 1·5)	1·3 (0·9 to 1·6)	1·1 (0·8 to 1·4)	1·1 (0·8 to 1·4)	1 (0·7 to 1·2)	0·9 (0·7 to 1·2)	0·9 (0·6 to 1·1)	0·8 (0·6 to 1)	0·6 (0·4 to 0·8)	0·5 (0·4 to 0·6)	0·4 (0·3 to 0·5)	0·3 (0·2 to 0·4)	0·2 (0·2 to 0·3)	0·1 (0·1 to 0·1)	0 (0 to 0·1)	0 (0 to 0)	15·1
Poisoning by carbon monoxide	0 (0 to 0)	0 (0 to 0)	0·1 (0 to 0·1)	0·9 (0·4 to 1·5)	0·3 (0·2 to 0·4)	0·2 (0·1 to 0·3)	0·4 (0·3 to 0·5)	0·6 (0·4 to 0·8)	0·7 (0·5 to 1)	0·6 (0·4 to 0·8)	0·6 (0·4 to 0·7)	0·4 (0·3 to 0·6)	0·4 (0·3 to 0·6)	0·3 (0·2 to 0·5)	0·4 (0·3 to 0·5)	0·3 (0·2 to 0·4)	0·3 (0·2 to 0·3)	0·2 (0·1 to 0·2)	0·1 (0·1 to 0·2)	0·1 (0·1 to 0·1)	0 (0 to 0·1)	0 (0 to 0)	0 (0 to 0)	6·8
Poisoning by other means	0 (0 to 0)	0·1 (0 to 0·1)	0·3 (0·2 to 0·4)	1·4 (0·7 to 2·5)	0·4 (0·3 to 0·6)	0·4 (0·2 to 0·6)	0·5 (0·4 to 0·8)	0·5 (0·4 to 0·8)	0·6 (0·4 to 0·8)	0·5 (0·4 to 0·7)	0·6 (0·4 to 0·8)	0·5 (0·3 to 0·7)	0·5 (0·3 to 0·7)	0·5 (0·4 to 0·7)	0·4 (0·3 to 0·6)	0·3 (0·2 to 0·4)	0·2 (0·2 to 0·3)	0·2 (0·2 to 0·3)	0·1 (0·1 to 0·2)	0·1 (0·1 to 0·2)	0·1 (0·1 to 0·1)	0 (0 to 0)	0 (0 to 0)	8·3
Exposure to mechanical forces	0·1 (0·1 to 0·2)	0·3 (0·2 to 0·4)	1·1 (0·7 to 1·6)	8·7 (5·4 to 13·7)	9·4 (7·3 to 11·8)	11 (8·5 to 13·8)	28 (20·7 to 37·4)	61·7 (43·9 to 84)	58·8 (43·1 to 78·4)	43·1 (32·3 to 57)	43 (31·9 to 57·6)	38·4 (29·9 to 50·4)	37 (27·3 to 48·6)	37·9 (28·3 to 50·4)	33·1 (24·7 to 43·7)	25·8 (18·9 to 34·2)	17·5 (13 to 23·8)	12·8 (9·3 to 17·3)	8·7 (6·3 to 11·8)	5·3 (4 to 7·1)	3 (2·3 to 3·9)	1·5 (1·2 to 1·9)	0·6 (0·5 to 0·8)	487
Unintentional firearm injuries	0 (0 to 0·1)	0·1 (0 to 0·1)	0·3 (0·2 to 0·4)	1·5 (0·8 to 2·7)	1·8 (1·2 to 2·8)	3·6 (2·3 to 5·1)	16·6 (11·2 to 23·4)	42·3 (28·1 to 60·8)	37·1 (25·4 to 51·6)	22·3 (15·2 to 31·8)	20·7 (14·4 to 29)	15·7 (11·5 to 20·9)	13·3 (9·2 to 18·3)	12·3 (8·7 to 17·2)	10·2 (7·3 to 14·3)	7·1 (5·1 to 9·7)	4 (3 to 5·2)	2·8 (2·1 to 3·6)	1·7 (1·3 to 2·2)	1·3 (1 to 1·6)	0·9 (0·7 to 1·1)	0·5 (0·4 to 0·7)	0·3 (0·2 to 0·3)	216
Other exposure to mechanical forces	0·1 (0·1 to 0·1)	0·2 (0·1 to 0·3)	0·8 (0·6 to 1·2)	7·2 (4·5 to 11·4)	7·5 (5·8 to 9·6)	7·4 (5·4 to 9·9)	11·4 (8 to 16·1)	19·4 (13·6 to 26·7)	21·8 (15·2 to 30·7)	20·8 (14·3 to 29)	22·4 (15·6 to 31·8)	22·7 (15·9 to 32·4)	23·7 (16·4 to 33·9)	25·5 (18·1 to 35·9)	22·9 (16·2 to 32)	18·6 (13·1 to 26·2)	13·5 (9·5 to 19)	10·1 (7 to 14·1)	7 (4·9 to 9·7)	4·1 (2·9 to 5·7)	2·1 (1·5 to 2·9)	0·9 (0·7 to 1·3)	0·3 (0·3 to 0·5)	270
Adverse effects of medical treatment	0·4 (0·3 to 0·7)	0·4 (0·3 to 0·7)	1·4 (0·8 to 2·4)	1·6 (0·9 to 2·6)	0·8 (0·6 to 1·1)	0·7 (0·5 to 1)	1 (0·7 to 1·4)	2·2 (1·5 to 3)	2·1 (1·5 to 3)	1·8 (1·3 to 2·5)	2·1 (1·5 to 2·9)	2·3 (1·6 to 3·1)	2·8 (2 to 3·9)	3·6 (2·6 to 5)	4·2 (3 to 5·8)	4·5 (3·2 to 6·3)	3·8 (2·7 to 5)	3·7 (2·7 to 4·9)	3·4 (2·5 to 4·5)	2·6 (1·9 to 3·4)	1·7 (1·2 to 2·2)	0·7 (0·5 to 1)	0·2 (0·2 to 0·3)	48
Animal contact	0 (0 to 0)	0 (0 to 0)	0·1 (0·1 to 0·2)	0·6 (0·4 to 1)	0·5 (0·3 to 0·7)	0·6 (0·4 to 0·8)	0·7 (0·5 to 1)	0·9 (0·6 to 1·2)	1·1 (0·8 to 1·4)	0·8 (0·6 to 1·1)	1 (0·7 to 1·3)	1 (0·7 to 1·3)	1·1 (0·8 to 1·5)	1·2 (0·9 to 1·6)	1·3 (1 to 1·7)	1·2 (0·9 to 1·6)	0·9 (0·7 to 1·2)	0·9 (0·7 to 1·2)	0·8 (0·7 to 1·1)	0·9 (0·6 to 1·2)	0·8 (0·5 to 1·1)	0·7 (0·5 to 0·9)	0·3 (0·2 to 0·5)	17·5
Venomous animal contact	0 (0 to 0)	0 (0 to 0)	0·1 (0 to 0·1)	0·3 (0·2 to 0·5)	0·3 (0·2 to 0·4)	0·4 (0·3 to 0·5)	0·5 (0·3 to 0·7)	0·6 (0·4 to 0·8)	0·7 (0·5 to 0·9)	0·6 (0·4 to 0·8)	0·7 (0·5 to 0·9)	0·6 (0·4 to 0·9)	0·7 (0·5 to 0·9)	0·7 (0·5 to 0·9)	0·7 (0·5 to 0·9)	0·7 (0·5 to 0·9)	0·5 (0·4 to 0·7)	0·5 (0·3 to 0·7)	0·5 (0·4 to 0·6)	0·5 (0·3 to 0·8)	0·5 (0·3 to 0·7)	0·5 (0·3 to 0·6)	0·2 (0·1 to 0·3)	10·6
Non-venomous animal contact	0 (0 to 0)	0 (0 to 0)	0·1 (0 to 0·1)	0·3 (0·1 to 0·5)	0·2 (0·1 to 0·3)	0·2 (0·1 to 0·3)	0·3 (0·2 to 0·4)	0·3 (0·2 to 0·5)	0·4 (0·2 to 0·6)	0·3 (0·2 to 0·4)	0·3 (0·2 to 0·5)	0·4 (0·3 to 0·5)	0·5 (0·3 to 0·7)	0·5 (0·4 to 0·7)	0·6 (0·4 to 0·8)	0·5 (0·4 to 0·7)	0·4 (0·3 to 0·6)	0·5 (0·3 to 0·6)	0·4 (0·2 to 0·5)	0·4 (0·2 to 0·6)	0·3 (0·2 to 0·4)	0·2 (0·1 to 0·3)	0·1 (0·1 to 0·2)	7
Foreign body	0·8 (0·4 to 1·3)	0·8 (0·4 to 1·3)	5·2 (2·9 to 8·9)	5·9 (3·5 to 9·7)	2·3 (1·8 to 2·8)	2·2 (1·8 to 2·7)	1·7 (1·3 to 2·2)	2·7 (2·1 to 3·5)	2·9 (2·2 to 3·7)	2·2 (1·8 to 3)	2·4 (1·9 to 3·2)	2·3 (1·8 to 3)	2·5 (2 to 3·2)	2·9 (2·2 to 3·8)	2·8 (2·3 to 3·5)	2·8 (2·3 to 3·4)	2·4 (1·9 to 2·8)	2·2 (1·8 to 2·6)	2 (1·6 to 2·4)	1·7 (1·4 to 2·1)	0·8 (0·6 to 1·1)	0·5 (0·3 to 0·6)	0·2 (0·1 to 0·3)	52·3
Pulmonary aspiration and foreign body in airway	0·7 (0·4 to 1·3)	0·8 (0·4 to 1·3)	5·1 (2·9 to 8·8)	5·5 (3·1 to 9·1)	1·7 (1·4 to 2·1)	1·5 (1·2 to 1·9)	1 (0·7 to 1·2)	1·7 (1·3 to 2·2)	1·8 (1·4 to 2·3)	1·2 (1 to 1·6)	1·4 (1·1 to 1·8)	1·2 (0·9 to 1·6)	1·4 (1·1 to 1·7)	1·8 (1·3 to 2·5)	1·8 (1·4 to 2·4)	2 (1·5 to 2·4)	1·7 (1·3 to 2·1)	1·6 (1·3 to 2)	1·5 (1·1 to 2)	1·3 (1 to 1·7)	0·6 (0·4 to 0·8)	0·3 (0·2 to 0·5)	0·2 (0·1 to 0·2)	38
Foreign body in eyes	0 (0 to 0)	0 (0 to 0)	0 (0 to 0)	0·1 (0 to 0·2)	0·2 (0 to 0·4)	0·2 (0·1 to 0·5)	0·3 (0·1 to 0·7)	0·4 (0·1 to 0·9)	0·4 (0·2 to 0·9)	0·5 (0·2 to 1)	0·5 (0·2 to 1)	0·5 (0·2 to 1)	0·5 (0·2 to 0·9)	0·4 (0·2 to 0·8)	0·3 (0·2 to 0·5)	0·3 (0·2 to 0·4)	0·2 (0·1 to 0·3)	0·1 (0·1 to 0·2)	0·1 (0·1 to 0·2)	0·1 (0 to 0·1)	0 (0 to 0)	0 (0 to 0)	0 (0 to 0)	5·1
Foreign body in other body part	0 (0 to 0)	0 (0 to 0)	0·1 (0·1 to 0·2)	0·4 (0·2 to 0·6)	0·4 (0·3 to 0·6)	0·5 (0·3 to 0·6)	0·4 (0·3 to 0·6)	0·6 (0·4 to 0·8)	0·7 (0·5 to 1)	0·5 (0·4 to 0·7)	0·5 (0·4 to 0·7)	0·6 (0·4 to 0·8)	0·7 (0·5 to 0·9)	0·7 (0·5 to 0·9)	0·7 (0·5 to 0·9)	0·6 (0·4 to 0·8)	0·5 (0·4 to 0·6)	0·4 (0·3 to 0·6)	0·4 (0·3 to 0·5)	0·3 (0·2 to 0·4)	0·2 (0·1 to 0·2)	0·1 (0·1 to 0·1)	0 (0 to 0·1)	9·2
Environmental heat and cold exposure	0 (0 to 0)	0 (0 to 0)	0·1 (0·1 to 0·2)	0·5 (0·4 to 0·8)	0·9 (0·6 to 1·3)	1·3 (0·9 to 1·9)	2 (1·3 to 2·8)	2·7 (1·8 to 3·8)	3 (2·1 to 4·3)	3·3 (2·3 to 4·7)	3·5 (2·4 to 4·9)	3·7 (2·5 to 5·2)	3·9 (2·7 to 5·5)	4 (2·8 to 5·5)	3·6 (2·6 to 5)	3·1 (2·2 to 4·1)	2·5 (1·8 to 3·4)	2 (1·5 to 2·7)	1·5 (1·1 to 2)	1·1 (0·8 to 1·5)	0·8 (0·5 to 1)	0·5 (0·4 to 0·7)	0·2 (0·2 to 0·3)	44·3
Exposure to forces of nature	0 (0 to 0)	0 (0 to 0)	0 (0 to 0)	0 (0 to 0)	0 (0 to 0)	0 (0 to 0)	0 (0 to 0)	0·2 (0·1 to 0·3)	0·2 (0·1 to 0·3)	0·3 (0·2 to 0·5)	0·4 (0·2 to 0·5)	0·4 (0·3 to 0·6)	0·4 (0·3 to 0·7)	0·5 (0·3 to 0·8)	0·4 (0·3 to 0·7)	0·4 (0·2 to 0·6)	0·4 (0·2 to 0·6)	0·4 (0·2 to 0·6)	0·3 (0·2 to 0·5)	0·2 (0·1 to 0·3)	0·1 (0·1 to 0·2)	0·1 (0 to 0·1)	0 (0 to 0)	4·7
Other unintentional injuries	0·1 (0·1 to 0·1)	0·1 (0·1 to 0·1)	0·6 (0·5 to 0·8)	3·5 (2·4 to 4·9)	4 (3 to 5·2)	4·7 (3·6 to 6)	7·7 (5·8 to 10·4)	12·8 (9·5 to 16·9)	13·2 (10 to 17)	10·3 (7·8 to 13·2)	10·6 (8·2 to 13·7)	9·2 (6·9 to 12·1)	9 (6·9 to 11·8)	8·3 (6·2 to 11)	7·2 (5·4 to 9·4)	5·5 (4·1 to 7·3)	4 (2·9 to 5·3)	2·9 (2·1 to 3·9)	2 (1·5 to 2·8)	1·2 (0·9 to 1·6)	0·6 (0·5 to 0·8)	0·2 (0·2 to 0·3)	0·1 (0·1 to 0·1)	118
Self-harm and interpersonal violence	0·5 (0·3 to 0·8)	0·1 (0·1 to 0·2)	0·9 (0·6 to 1·5)	3·5 (1·9 to 5·9)	2·6 (2 to 3·5)	5 (4·1 to 6·2)	31·9 (24 to 41·3)	78·2 (60·6 to 97·3)	77·1 (61·1 to 95·2)	50·1 (40·1 to 61·6)	44·5 (35·5 to 55·8)	29·6 (23·1 to 37·1)	23·8 (19·1 to 29·6)	19·9 (15·5 to 25·1)	15·3 (12 to 19·6)	10·9 (8·6 to 13·4)	6·4 (5·2 to 7·8)	4·4 (3·6 to 5·4)	2·8 (2·3 to 3·4)	1·6 (1·3 to 1·9)	0·7 (0·6 to 0·8)	0·2 (0·2 to 0·3)	0·1 (0·1 to 0·1)	410
Self-harm	0 (0 to 0)	0 (0 to 0)	0 (0 to 0)	0 (0 to 0)	0 (0 to 0)	0·6 (0·4 to 0·9)	6·8 (4·7 to 9·5)	19 (12·8 to 26·8)	24·2 (16·7 to 34)	17·7 (12·5 to 24·2)	17·3 (11·9 to 24·4)	10·9 (7·8 to 14·9)	8·4 (6·1 to 11·5)	7·5 (5·6 to 10·2)	5·9 (4·3 to 7·9)	4·4 (3·3 to 5·7)	2·6 (1·9 to 3·4)	1·9 (1·5 to 2·5)	1·2 (0·9 to 1·6)	0·8 (0·6 to 1·1)	0·4 (0·3 to 0·5)	0·1 (0·1 to 0·1)	0 (0 to 0)	130
Self-harm by firearm	0 (0 to 0)	0 (0 to 0)	0 (0 to 0)	0 (0 to 0)	0 (0 to 0)	0 (0 to 0)	0·2 (0·1 to 0·3)	0·5 (0·3 to 0·8)	0·5 (0·3 to 0·8)	0·4 (0·2 to 0·6)	0·3 (0·2 to 0·5)	0·2 (0·1 to 0·3)	0·2 (0·1 to 0·3)	0·2 (0·2 to 0·3)	0·2 (0·1 to 0·2)	0·1 (0·1 to 0·2)	0·1 (0·1 to 0·1)	0·1 (0 to 0·1)	0 (0 to 0·1)	0 (0 to 0)	0 (0 to 0)	0 (0 to 0)	0 (0 to 0)	3·1
Self-harm by other specified means	0 (0 to 0)	0 (0 to 0)	0 (0 to 0)	0 (0 to 0)	0 (0 to 0)	0·6 (0·4 to 0·8)	6·6 (4·5 to 9·2)	18·5 (12·5 to 25·9)	23·7 (16·4 to 33·4)	17·3 (12·2 to 23·8)	17 (11·6 to 24)	10·7 (7·7 to 14·6)	8·2 (6 to 11·2)	7·3 (5·4 to 9·9)	5·7 (4·1 to 7·7)	4·3 (3·2 to 5·6)	2·5 (1·9 to 3·3)	1·9 (1·4 to 2·4)	1·2 (0·9 to 1·6)	0·8 (0·6 to 1·1)	0·4 (0·3 to 0·4)	0·1 (0·1 to 0·1)	0 (0 to 0)	127
Interpersonal violence	0·5 (0·3 to 0·8)	0·1 (0·1 to 0·2)	0·9 (0·6 to 1·5)	3·5 (1·9 to 5·8)	2·6 (1·9 to 3·4)	4·3 (3·4 to 5·6)	25·1 (17·8 to 33·7)	59 (44·4 to 76·1)	52·7 (39·6 to 69·5)	32·4 (25 to 41·8)	27·2 (20·8 to 34·8)	18·6 (13·6 to 24·9)	15·3 (12·1 to 19·4)	12·4 (9·3 to 16·1)	9·4 (7·2 to 12·2)	6·4 (5 to 8·1)	3·8 (3 to 4·9)	2·5 (2 to 3)	1·5 (1·2 to 1·9)	0·7 (0·6 to 0·8)	0·3 (0·3 to 0·4)	0·1 (0·1 to 0·1)	0 (0 to 0·1)	279
Physical violence by firearm	0 (0 to 0)	0 (0 to 0)	0 (0 to 0·1)	0·2 (0·1 to 0·4)	0·3 (0·2 to 0·3)	0·7 (0·5 to 0·9)	11·6 (8 to 16·2)	30 (22·1 to 39·4)	25·1 (18·2 to 33·6)	13·3 (10 to 17·5)	9·7 (7·1 to 12·7)	5·3 (3·7 to 7·3)	3·9 (2·9 to 5·2)	2·5 (1·8 to 3·5)	1·6 (1·2 to 2·2)	0·9 (0·7 to 1·2)	0·4 (0·3 to 0·6)	0·2 (0·2 to 0·3)	0·1 (0·1 to 0·1)	0 (0 to 0)	0 (0 to 0)	0 (0 to 0)	0 (0 to 0)	106
Physical violence by sharp object	0 (0 to 0)	0 (0 to 0)	0 (0 to 0)	0·1 (0·1 to 0·2)	0·2 (0·1 to 0·2)	0·3 (0·2 to 0·4)	3·9 (2·7 to 5·4)	10·6 (7·8 to 13·7)	9·5 (7 to 12·5)	5·9 (4·5 to 7·7)	4·9 (3·7 to 6·3)	3·1 (2·3 to 4·1)	2·4 (1·8 to 3)	1·8 (1·4 to 2·4)	1·2 (0·9 to 1·5)	0·8 (0·6 to 1)	0·5 (0·4 to 0·6)	0·3 (0·3 to 0·4)	0·2 (0·1 to 0·2)	0·1 (0·1 to 0·1)	0·1 (0 to 0·1)	0 (0 to 0)	0 (0 to 0)	45·9
Sexual violence	0 (0 to 0)	0 (0 to 0)	0 (0 to 0)	0 (0 to 0·1)	0·3 (0·2 to 0·4)	0·7 (0·4 to 1·1)	1·7 (1 to 2·8)	2·5 (1·4 to 3·7)	2·2 (1·3 to 3·6)	2·1 (1·2 to 3·4)	1·8 (1 to 3)	1·6 (0·9 to 2·5)	1·2 (0·7 to 2·1)	0·9 (0·5 to 1·5)	0·6 (0·3 to 0·9)	0·4 (0·3 to 0·6)	0·3 (0·2 to 0·5)	0·2 (0·1 to 0·3)	0·1 (0·1 to 0·2)	0·1 (0·1 to 0·1)	0 (0 to 0·1)	0 (0 to 0)	0 (0 to 0)	16·9
Physical violence by other means	0·5 (0·3 to 0·8)	0·1 (0·1 to 0·2)	0·9 (0·5 to 1·4)	3·1 (1·6 to 5·2)	1·9 (1·3 to 2·6)	2·6 (2 to 3·4)	7·9 (5·5 to 10·6)	15·9 (11·8 to 20·5)	15·9 (12 to 20·9)	11 (8·6 to 14·3)	10·8 (8·1 to 14)	8·6 (6·2 to 11·6)	7·8 (6·1 to 10)	7·1 (5·2 to 9·5)	5·9 (4·5 to 7·8)	4·3 (3·2 to 5·5)	2·6 (2 to 3·5)	1·7 (1·4 to 2·1)	1·1 (0·8 to 1·4)	0·5 (0·4 to 0·6)	0·2 (0·2 to 0·3)	0·1 (0·1 to 0·1)	0 (0 to 0)	111
Conflict and terrorism	0 (0 to 0)	0 (0 to 0)	0 (0 to 0)	0 (0 to 0)	0 (0 to 0)	0 (0 to 0)	0 (0 to 0)	0 (0 to 0)	0 (0 to 0)	0 (0 to 0)	0 (0 to 0)	0 (0 to 0)	0 (0 to 0)	0 (0 to 0)	0 (0 to 0)	0 (0 to 0)	0 (0 to 0)	0 (0 to 0)	0·1 (0 to 0·1)	0·1 (0·1 to 0·1)	0 (0 to 0)	0 (0 to 0)	0 (0 to 0)	0·1

SR Table 2c: Bahamas DALYS by cause and age group for both sexes with 95% uncertainty intervals, 2019

Cause	Early Neonatal	Late Neonatal	Post Neonatal	1 to 4	5 to 9	10 to 14	15 to 19	20 to 24	25 to 29	30 to 34	35 to 39	40 to 44	45 to 49	50 to 54	55 to 59	60 to 64	65 to 69	70 to 74	75 to 79	80 to 84	85 to 89	90 to 94	95 plus	Total
All causes	1,480 (1,180 to 1,850)	700 (424 to 1,140)	1,280 (1,030 to 1,590)	1,510 (1,230 to 1,870)	1,850 (1,510 to 2,240)	2,640 (2,150 to 3,260)	4,220 (3,530 to 5,040)	6,140 (5,270 to 7,140)	7,310 (6,310 to 8,430)	7,770 (6,680 to 8,980)	8,700 (7,450 to 10,200)	9,460 (8,080 to 11,200)	10,400 (8,680 to 12,300)	11,000 (9,230 to 13,100)	10,800 (9,000 to 12,800)	9,860 (8,310 to 11,600)	8,000 (6,850 to 9,340)	6,150 (5,310 to 7,140)	5,150 (4,480 to 5,930)	3,780 (3,330 to 4,300)	2,080 (1,850 to 2,340)	998 (905 to 1,100)	455 (421 to 493)	122,000
Communicable, maternal, neonatal, and nutritional diseases	1,240 (968 to 1,570)	531 (315 to 901)	585 (455 to 728)	441 (343 to 575)	448 (320 to 628)	393 (296 to 525)	451 (359 to 575)	648 (551 to 773)	977 (867 to 1,110)	1,250 (1,130 to 1,400)	1,450 (1,330 to 1,610)	1,520 (1,390 to 1,690)	1,340 (1,220 to 1,500)	1,010 (893 to 1,160)	726 (629 to 848)	511 (428 to 607)	372 (304 to 457)	268 (211 to 339)	241 (187 to 310)	201 (162 to 245)	131 (103 to 163)	75·1 (58·1 to 92·4)	36 (25·4 to 46·4)	14,900
HIV/AIDS and sexually transmitted infections	0·3 (0·2 to 0·4)	0·1 (0·1 to 0·3)	35·6 (33·7 to 37·3)	30·8 (29·3 to 32·6)	31·4 (29·7 to 33·1)	30·9 (29·2 to 32·7)	69·3 (63·8 to 77·1)	237 (219 to 259)	526 (493 to 572)	792 (748 to 850)	980 (922 to 1,050)	1,050 (986 to 1,110)	889 (835 to 945)	559 (525 to 592)	309 (290 to 328)	149 (139 to 159)	82·9 (77·2 to 88)	18·8 (16·8 to 20·6)	10·1 (9 to 11·1)	4·8 (4·4 to 5·3)	2·1 (1·9 to 2·3)	0·5 (0·4 to 0·6)	0·3 (0·2 to 0·3)	5,810
HIV/AIDS	0 (0 to 0)	0 (0 to 0)	35·4 (33·6 to 37·2)	30·8 (29·3 to 32·6)	31·4 (29·7 to 33·1)	30·2 (28·5 to 32)	66 (61·2 to 72·4)	230 (213 to 250)	516 (485 to 560)	780 (737 to 836)	968 (911 to 1,030)	1,040 (975 to 1,100)	879 (824 to 933)	552 (520 to 585)	304 (285 to 323)	146 (136 to 155)	81 (75·7 to 85·7)	17·7 (15·9 to 19·1)	9·3 (8·4 to 10)	4·4 (4 to 4·7)	1·9 (1·7 to 2)	0·4 (0·4 to 0·5)	0·2 (0·2 to 0·3)	5,720
HIV/AIDS - Drug-susceptible Tuberculosis	0 (0 to 0)	0 (0 to 0)	1·1 (0·6 to 1·6)	0·9 (0·6 to 1·4)	1 (0·6 to 1·5)	0·9 (0·6 to 1·4)	1·9 (1·3 to 2·8)	6·7 (4·4 to 9·8)	15·6 (10·2 to 23·2)	24 (15·3 to 35·5)	30·8 (19·6 to 45·7)	33·4 (21·3 to 50·2)	26·3 (16·5 to 39·2)	16·6 (10·6 to 24·8)	9·2 (5·9 to 13·5)	4·5 (3 to 6·6)	2·5 (1·6 to 3·6)	0·5 (0·4 to 0·8)	0·3 (0·2 to 0·4)	0·1 (0·1 to 0·2)	0·1 (0 to 0·1)	0 (0 to 0)	0 (0 to 0)	176
HIV/AIDS - Multidrug-resistant Tuberculosis without extensive d	0 (0 to 0)	0 (0 to 0)	0 (0 to 0·2)	0 (0 to 0·2)	0 (0 to 0·2)	0 (0 to 0·2)	0·1 (0 to 0·3)	0·3 (0 to 1·1)	0·6 (0·1 to 2·4)	0·9 (0·1 to 3·8)	1·2 (0·1 to 4·8)	1·3 (0·2 to 5·2)	1 (0·1 to 4·2)	0·7 (0·1 to 2·6)	0·4 (0 to 1·4)	0·2 (0 to 0·7)	0·1 (0 to 0·4)	0 (0 to 0·1)	0 (0 to 0)	0 (0 to 0)	0 (0 to 0)	0 (0 to 0)	0 (0 to 0)	6·8
HIV/AIDS - Extensively drug-resistant Tuberculosis	0 (0 to 0)	0 (0 to 0)	0 (0 to 0)	0 (0 to 0)	0 (0 to 0)	0 (0 to 0)	0 (0 to 0)	0 (0 to 0·2)	0·1 (0 to 0·4)	0·2 (0 to 0·6)	0·2 (0 to 0·8)	0·2 (0 to 0·9)	0·2 (0 to 0·7)	0·1 (0 to 0·4)	0·1 (0 to 0·2)	0 (0 to 0·1)	0 (0 to 0·1)	0 (0 to 0)	0 (0 to 0)	0 (0 to 0)	0 (0 to 0)	0 (0 to 0)	0 (0 to 0)	1·1
HIV/AIDS resulting in other diseases	0 (0 to 0)	0 (0 to 0)	34·3 (32·3 to 36·3)	29·8 (28·2 to 31·7)	30·3 (28·7 to 32·1)	29·2 (27·5 to 31)	64 (59 to 70·6)	223 (206 to 243)	500 (468 to 543)	755 (708 to 813)	935 (878 to 1,000)	1,000 (938 to 1,070)	852 (797 to 907)	535 (500 to 569)	295 (275 to 314)	141 (131 to 150)	78·5 (72·9 to 82·9)	17·1 (15·4 to 18·4)	9 (8·2 to 9·7)	4·2 (3·9 to 4·6)	1·8 (1·6 to 1·9)	0·4 (0·4 to 0·5)	0·2 (0·2 to 0·2)	5,540
Sexually transmitted infections excluding HIV	0·3 (0·2 to 0·4)	0·1 (0·1 to 0·3)	0·1 (0·1 to 0·3)	0 (0 to 0)	0 (0 to 0)	0·6 (0·4 to 1·1)	3·3 (2 to 5·4)	6·8 (4·1 to 10·9)	9·7 (5·6 to 16·4)	11·8 (6·7 to 19·8)	12·3 (7·3 to 20·8)	12·2 (7 to 21·6)	10·3 (5·8 to 18·2)	6·5 (3·5 to 12·4)	4·5 (2·2 to 8·8)	2·8 (1·4 to 5·8)	1·8 (0·9 to 3·7)	1·1 (0·6 to 2·3)	0·7 (0·4 to 1·5)	0·4 (0·2 to 0·8)	0·2 (0·1 to 0·4)	0·1 (0 to 0·2)	0 (0 to 0·1)	86
Syphilis	0·3 (0·2 to 0·4)	0·1 (0·1 to 0·3)	0·1 (0·1 to 0·3)	0 (0 to 0)	0 (0 to 0)	0·1 (0·1 to 0·2)	0·3 (0·2 to 0·4)	0·3 (0·2 to 0·5)	0·5 (0·3 to 0·7)	0·6 (0·4 to 0·9)	0·6 (0·4 to 0·8)	0·3 (0·2 to 0·4)	0·4 (0·3 to 0·6)	0·3 (0·2 to 0·4)	0·2 (0·1 to 0·3)	0·2 (0·1 to 0·2)	0·1 (0·1 to 0·2)	0·1 (0·1 to 0·1)	0·1 (0 to 0·1)	0 (0 to 0·1)	0 (0 to 0)	0 (0 to 0)	0 (0 to 0)	4·6
Chlamydial infection	0 (0 to 0)	0 (0 to 0)	0 (0 to 0)	0 (0 to 0)	0 (0 to 0)	0 (0 to 0·1)	0·3 (0·2 to 0·5)	1·1 (0·6 to 1·8)	1·5 (0·8 to 2·5)	1·5 (0·7 to 2·6)	1·7 (1 to 2·6)	1·7 (1 to 2·7)	1·2 (0·7 to 1·9)	0·4 (0·2 to 0·8)	0·2 (0·1 to 0·3)	0·1 (0 to 0·1)	0 (0 to 0·1)	0 (0 to 0)	0 (0 to 0)	0 (0 to 0)	0 (0 to 0)	0 (0 to 0)	0 (0 to 0)	9·8
Gonococcal infection	0 (0 to 0)	0 (0 to 0)	0 (0 to 0)	0 (0 to 0)	0 (0 to 0)	0·1 (0·1 to 0·2)	0·8 (0·3 to 1·5)	1·2 (0·6 to 2)	0·9 (0·5 to 1·6)	1·1 (0·7 to 1·6)	1·1 (0·8 to 1·6)	1·3 (0·9 to 1·8)	1·1 (0·7 to 1·6)	0·6 (0·4 to 0·9)	0·3 (0·2 to 0·4)	0·2 (0·1 to 0·3)	0·2 (0·1 to 0·3)	0·1 (0·1 to 0·2)	0·1 (0 to 0·1)	0 (0 to 0·1)	0 (0 to 0)	0 (0 to 0)	0 (0 to 0)	9·1
Trichomoniasis	0 (0 to 0)	0 (0 to 0)	0 (0 to 0)	0 (0 to 0)	0 (0 to 0)	0 (0 to 0·1)	0·3 (0·1 to 0·7)	1·4 (0·5 to 3·2)	2·7 (0·9 to 5·9)	3 (1 to 7·1)	2·9 (1 to 6·8)	2·8 (0·9 to 6·9)	2·5 (0·8 to 5·8)	2 (0·6 to 4·7)	1·3 (0·5 to 3·2)	0·8 (0·3 to 1·8)	0·5 (0·2 to 1)	0·3 (0·1 to 0·6)	0·2 (0·1 to 0·3)	0·1 (0 to 0·2)	0 (0 to 0·1)	0 (0 to 0)	0 (0 to 0)	20·7
Genital herpes	0 (0 to 0)	0 (0 to 0)	0 (0 to 0)	0 (0 to 0)	0 (0 to 0)	0·1 (0 to 0·3)	0·6 (0·2 to 1·5)	1·3 (0·4 to 3·1)	2 (0·7 to 4·9)	2·5 (0·8 to 6·2)	2·7 (0·9 to 6·7)	2·8 (0·9 to 7)	2·8 (0·9 to 7)	2·5 (0·7 to 6·1)	2 (0·6 to 5)	1·5 (0·5 to 3·7)	1 (0·3 to 2·4)	0·6 (0·2 to 1·5)	0·4 (0·1 to 1)	0·2 (0·1 to 0·6)	0·1 (0 to 0·3)	0 (0 to 0·1)	0 (0 to 0)	23·1
Other sexually transmitted infections	0 (0 to 0)	0 (0 to 0)	0 (0 to 0)	0 (0 to 0)	0 (0 to 0)	0·2 (0·1 to 0·4)	1·1 (0·6 to 1·8)	1·6 (1 to 2·5)	2·1 (1·3 to 3·3)	3·1 (1·9 to 4·7)	3·3 (2·2 to 4·8)	3·4 (2·1 to 5·3)	2·3 (1·4 to 3·8)	0·8 (0·5 to 1·2)	0·4 (0·3 to 0·6)	0·2 (0·1 to 0·2)	0·1 (0·1 to 0·1)	0·1 (0 to 0·1)	0 (0 to 0·1)	0 (0 to 0)	0 (0 to 0)	0 (0 to 0)	0 (0 to 0)	18·7
Respiratory infections and tuberculosis	41·8 (28·5 to 60·7)	51·7 (28·1 to 86)	210 (142 to 295)	110 (77·3 to 152)	69·4 (49·4 to 96)	64·4 (44·5 to 90·6)	77·9 (56·1 to 108)	114 (84·4 to 154)	140 (104 to 183)	155 (116 to 206)	167 (121 to 227)	170 (121 to 233)	180 (130 to 247)	197 (140 to 267)	192 (138 to 254)	194 (138 to 265)	176 (123 to 247)	164 (117 to 225)	163 (117 to 226)	149 (116 to 190)	103 (77·3 to 133)	62·3 (46·6 to 77·1)	30·2 (20·2 to 40)	2,980
Tuberculosis	0 (0 to 0)	0 (0 to 0)	2·1 (1·4 to 3)	2·9 (1·8 to 4·2)	3·1 (1·7 to 5)	3 (2 to 4·3)	7·8 (5·2 to 11·4)	24·1 (16·2 to 35·3)	32 (21·9 to 43·9)	33·6 (23·9 to 45·8)	31 (21·4 to 44)	29·3 (20 to 41·5)	28·5 (18·9 to 40·9)	28 (18·8 to 40·4)	22·8 (15·2 to 32)	19·1 (13 to 27)	13·4 (9·3 to 19·1)	10·1 (7·3 to 13·6)	7·7 (5·4 to 10·2)	4·7 (3·3 to 6·4)	2·5 (1·7 to 3·5)	0·8 (0·6 to 1·1)	0·3 (0·2 to 0·3)	307
Latent tuberculosis infection	0 (0 to 0)	0 (0 to 0)	0 (0 to 0)	0 (0 to 0)	0 (0 to 0)	0 (0 to 0)	0 (0 to 0)	0 (0 to 0)	0 (0 to 0)	0 (0 to 0)	0 (0 to 0)	0 (0 to 0)	0 (0 to 0)	0 (0 to 0)	0 (0 to 0)	0 (0 to 0)	0 (0 to 0)	0 (0 to 0)	0 (0 to 0)	0 (0 to 0)	0 (0 to 0)	0 (0 to 0)	0 (0 to 0)	0
Drug-susceptible tuberculosis	0 (0 to 0)	0 (0 to 0)	2 (1·3 to 2·9)	2·8 (1·7 to 4·1)	3 (1·6 to 4·9)	2·9 (1·9 to 4·3)	7·5 (4·9 to 11·1)	23·2 (15·5 to 34·1)	30·9 (20·7 to 42·5)	32·5 (22·9 to 44·3)	30 (20·3 to 42·6)	28·3 (19 to 40)	27·5 (18·4 to 39·8)	27 (18 to 39·5)	22 (14·7 to 31·2)	18·4 (12·3 to 26·1)	12·9 (8·8 to 18·5)	9·7 (6·7 to 13·2)	7·4 (5·2 to 9·9)	4·5 (3·2 to 6·3)	2·4 (1·6 to 3·3)	0·8 (0·5 to 1·1)	0·2 (0·2 to 0·3)	296
Multidrug-resistant tuberculosis without extensive drug resista	0 (0 to 0)	0 (0 to 0)	0·1 (0 to 0·3)	0·1 (0 to 0·3)	0·1 (0 to 0·3)	0·1 (0 to 0·3)	0·3 (0 to 1)	0·7 (0·1 to 2·8)	0·9 (0·1 to 3·7)	1 (0·1 to 3·8)	0·9 (0·1 to 3·5)	0·9 (0·1 to 3·4)	0·9 (0·1 to 3·7)	0·9 (0·1 to 3·3)	0·7 (0·1 to 2·9)	0·6 (0·1 to 2·3)	0·4 (0 to 1·8)	0·3 (0 to 1·2)	0·2 (0 to 1)	0·2 (0 to 0·6)	0·1 (0 to 0·3)	0 (0 to 0·1)	0 (0 to 0)	9·3
Extensively drug-resistant tuberculosis	0 (0 to 0)	0 (0 to 0)	0 (0 to 0)	0 (0 to 0)	0 (0 to 0)	0 (0 to 0)	0 (0 to 0·2)	0·1 (0 to 0·4)	0·1 (0 to 0·6)	0·2 (0 to 0·6)	0·1 (0 to 0·6)	0·1 (0 to 0·5)	0·1 (0 to 0·5)	0·1 (0 to 0·6)	0·1 (0 to 0·5)	0·1 (0 to 0·4)	0·1 (0 to 0·3)	0·1 (0 to 0·2)	0 (0 to 0·2)	0 (0 to 0·1)	0 (0 to 0·1)	0 (0 to 0)	0 (0 to 0)	1·5
Lower respiratory infections	41·8 (28·4 to 60·6)	51·4 (27·8 to 85·8)	202 (134 to 286)	76·2 (47·5 to 115)	26·1 (18·3 to 35·8)	22·7 (15·7 to 32·1)	35·1 (22·6 to 52·9)	58 (36·8 to 86·4)	77·7 (50·3 to 115)	94·7 (62·7 to 137)	109 (70·6 to 162)	116 (71·2 to 169)	127 (81·4 to 187)	148 (97·7 to 215)	152 (104 to 211)	163 (110 to 231)	155 (102 to 223)	149 (102 to 209)	152 (107 to 214)	143 (110 to 182)	99·2 (74·4 to 129)	61·1 (45·5 to 76)	29·8 (19·8 to 39·5)	2,290
Upper respiratory infections	0·1 (0 to 0·1)	0·3 (0·2 to 0·4)	5·9 (3·7 to 9·1)	22·2 (13 to 36·4)	27·9 (15·4 to 47)	29·2 (15·3 to 50·3)	27 (14·1 to 47·8)	25·7 (13·7 to 44·7)	25 (14·1 to 41·5)	22·6 (12·3 to 37·5)	22·4 (11·8 to 37·5)	20·6 (11·3 to 37)	19·2 (10·4 to 32·6)	16·1 (8·8 to 27·7)	13·2 (7·2 to 21·7)	9·8 (5·3 to 16·3)	6·6 (3·6 to 11)	4·2 (2·3 to 6·9)	2·7 (1·5 to 4·4)	1·6 (0·9 to 2·6)	0·7 (0·4 to 1·1)	0·3 (0·2 to 0·5)	0·1 (0·1 to 0·2)	303
Otitis media	0 (0 to 0)	0 (0 to 0·1)	1 (0·5 to 1·8)	8·6 (4·6 to 15)	12·4 (6·9 to 20·4)	9·5 (5 to 16·7)	8 (4·1 to 13·7)	6·5 (3 to 12·9)	5·5 (2·6 to 10·5)	4·5 (2 to 8·7)	4·5 (2·2 to 8·4)	4·8 (2·2 to 8·9)	5·1 (2·6 to 9·1)	4·7 (2·2 to 8·5)	3·8 (1·9 to 6·6)	2·5 (1·1 to 4·9)	1·4 (0·7 to 2·6)	0·7 (0·3 to 1·5)	0·5 (0·2 to 0·9)	0·3 (0·1 to 0·6)	0·2 (0·1 to 0·3)	0·1 (0 to 0·1)	0 (0 to 0·1)	84·6
Enteric infections	1·6 (1 to 2·3)	6·1 (3·3 to 10·7)	53·9 (33·4 to 83)	51·2 (34·9 to 74·7)	45·9 (28·2 to 73)	46·7 (27·2 to 73·7)	31·5 (20·1 to 46·4)	27·2 (17·9 to 39·7)	31·1 (20·7 to 43·8)	32·6 (22·5 to 44·5)	35·6 (25·1 to 49·2)	38·5 (27·3 to 52)	41 (29·3 to 55·5)	40·7 (28·9 to 55·4)	38·1 (27·6 to 51·6)	36·9 (26·9 to 49)	34·4 (25·2 to 45·7)	30·1 (22·1 to 40)	26·8 (19·4 to 35·7)	19·5 (14·3 to 25·7)	10·1 (7·5 to 13·2)	4·6 (3·4 to 6·1)	2 (1·5 to 2·6)	686
Diarrheal diseases	1·6 (1 to 2·3)	6·1 (3·3 to 10·7)	53·6 (33·3 to 82·8)	50·6 (34·3 to 73·9)	45·1 (27·4 to 72·3)	45·9 (26·5 to 73·2)	31·2 (19·9 to 46·1)	26·9 (17·6 to 39·4)	30·7 (20·5 to 43·3)	32·2 (22·2 to 44·2)	35·4 (24·9 to 49)	38·2 (27 to 51·5)	40·8 (29·1 to 55·4)	40·5 (28·6 to 55·2)	37·8 (27·4 to 51·4)	36·6 (26·6 to 48·7)	34·2 (25 to 45·4)	30 (21·9 to 39·9)	26·7 (19·3 to 35·5)	19·5 (14·3 to 25·6)	10 (7·5 to 13·1)	4·6 (3·4 to 6·1)	2 (1·5 to 2·6)	680
Typhoid and paratyphoid	0 (0 to 0)	0 (0 to 0)	0 (0 to 0)	0 (0 to 0)	0 (0 to 0)	0 (0 to 0)	0 (0 to 0)	0 (0 to 0)	0 (0 to 0)	0 (0 to 0)	0 (0 to 0)	0 (0 to 0)	0 (0 to 0)	0 (0 to 0)	0 (0 to 0)	0 (0 to 0)	0 (0 to 0)	0 (0 to 0)	0 (0 to 0)	0 (0 to 0)	0 (0 to 0)	0 (0 to 0)	0 (0 to 0)	0·1
Typhoid fever	0 (0 to 0)	0 (0 to 0)	0 (0 to 0)	0 (0 to 0)	0 (0 to 0)	0 (0 to 0)	0 (0 to 0)	0 (0 to 0)	0 (0 to 0)	0 (0 to 0)	0 (0 to 0)	0 (0 to 0)	0 (0 to 0)	0 (0 to 0)	0 (0 to 0)	0 (0 to 0)	0 (0 to 0)	0 (0 to 0)	0 (0 to 0)	0 (0 to 0)	0 (0 to 0)	0 (0 to 0)	0 (0 to 0)	0
Paratyphoid fever	0 (0 to 0)	0 (0 to 0)	0 (0 to 0)	0 (0 to 0)	0 (0 to 0)	0 (0 to 0)	0 (0 to 0)	0 (0 to 0)	0 (0 to 0)	0 (0 to 0)	0 (0 to 0)	0 (0 to 0)	0 (0 to 0)	0 (0 to 0)	0 (0 to 0)	0 (0 to 0)	0 (0 to 0)	0 (0 to 0)	0 (0 to 0)	0 (0 to 0)	0 (0 to 0)	0 (0 to 0)	0 (0 to 0)	0
Invasive Non-typhoidal Salmonella (iNTS)	0 (0 to 0)	0 (0 to 0)	0·2 (0·1 to 0·4)	0·5 (0·2 to 1·1)	0·7 (0·3 to 1·3)	0·6 (0·3 to 1·2)	0·1 (0·1 to 0·3)	0·2 (0·1 to 0·3)	0·4 (0·1 to 0·8)	0·2 (0·1 to 0·4)	0·2 (0·1 to 0·3)	0·3 (0·1 to 0·6)	0·1 (0 to 0·3)	0·2 (0·1 to 0·3)	0·2 (0·1 to 0·4)	0·3 (0·1 to 0·5)	0·2 (0·1 to 0·4)	0·1 (0 to 0·1)	0·1 (0 to 0·2)	0 (0 to 0·1)	0 (0 to 0)	0 (0 to 0·1)	0 (0 to 0)	4·6
Other intestinal infectious diseases	0 (0 to 0)	0 (0 to 0)	0·1 (0 to 0·4)	0·2 (0·1 to 0·5)	0·1 (0 to 0·3)	0·1 (0 to 0·3)	0·1 (0 to 0·2)	0·1 (0 to 0·3)	0·1 (0 to 0·2)	0·1 (0 to 0·3)	0·1 (0 to 0·2)	0·1 (0 to 0·2)	0 (0 to 0·1)	0 (0 to 0·1)	0 (0 to 0·1)	0 (0 to 0·1)	0 (0 to 0·1)	0 (0 to 0)	0 (0 to 0)	0 (0 to 0)	0 (0 to 0)	0 (0 to 0)	0 (0 to 0)	1·4
Neglected tropical diseases and malaria	0 (0 to 0·1)	0·4 (0·2 to 0·6)	3·8 (2·2 to 6·1)	11·3 (5·9 to 19·6)	17·9 (8·6 to 31·9)	12 (6 to 21·8)	10·3 (5·8 to 17)	12·5 (7 to 20·2)	14·4 (8·2 to 24)	14·8 (7·6 to 24·7)	16·9 (9·5 to 28·9)	18·9 (10·2 to 33·8)	19·5 (9·6 to 37·3)	21·3 (10·6 to 38·1)	22·3 (11·1 to 41·6)	20·2 (9·2 to 37·3)	16·5 (7·2 to 31·4)	13 (5·7 to 24·1)	10·3 (4·3 to 19)	6·7 (2·9 to 12·5)	3·2 (1·4 to 6·1)	1·4 (0·6 to 2·6)	0·6 (0·3 to 1·2)	268
Malaria	0 (0 to 0)	0 (0 to 0)	0 (0 to 0)	0 (0 to 0)	0 (0 to 0)	0 (0 to 0)	0 (0 to 0)	0 (0 to 0)	0 (0 to 0)	0 (0 to 0)	0 (0 to 0)	0 (0 to 0)	0 (0 to 0)	0 (0 to 0)	0 (0 to 0)	0 (0 to 0)	0 (0 to 0)	0 (0 to 0)	0 (0 to 0)	0 (0 to 0)	0 (0 to 0)	0 (0 to 0)	0 (0 to 0)	0
Chagas disease	0 (0 to 0)	0 (0 to 0)	0 (0 to 0)	0 (0 to 0)	0 (0 to 0)	0 (0 to 0)	0 (0 to 0)	0 (0 to 0)	0 (0 to 0)	0 (0 to 0)	0 (0 to 0)	0 (0 to 0)	0 (0 to 0)	0 (0 to 0)	0 (0 to 0)	0 (0 to 0)	0 (0 to 0)	0 (0 to 0)	0 (0 to 0)	0 (0 to 0)	0 (0 to 0)	0 (0 to 0)	0 (0 to 0)	0·1
Leishmaniasis	0 (0 to 0)	0 (0 to 0)	0 (0 to 0)	0 (0 to 0)	0 (0 to 0)	0 (0 to 0)	0 (0 to 0)	0 (0 to 0)	0 (0 to 0)	0 (0 to 0)	0 (0 to 0)	0 (0 to 0)	0 (0 to 0)	0 (0 to 0)	0 (0 to 0)	0 (0 to 0)	0 (0 to 0)	0 (0 to 0)	0 (0 to 0)	0 (0 to 0)	0 (0 to 0)	0 (0 to 0)	0 (0 to 0)	0
Visceral leishmaniasis	0 (0 to 0)	0 (0 to 0)	0 (0 to 0)	0 (0 to 0)	0 (0 to 0)	0 (0 to 0)	0 (0 to 0)	0 (0 to 0)	0 (0 to 0)	0 (0 to 0)	0 (0 to 0)	0 (0 to 0)	0 (0 to 0)	0 (0 to 0)	0 (0 to 0)	0 (0 to 0)	0 (0 to 0)	0 (0 to 0)	0 (0 to 0)	0 (0 to 0)	0 (0 to 0)	0 (0 to 0)	0 (0 to 0)	0
Cutaneous and mucocutaneous leishmaniasis	0 (0 to 0)	0 (0 to 0)	0 (0 to 0)	0 (0 to 0)	0 (0 to 0)	0 (0 to 0)	0 (0 to 0)	0 (0 to 0)	0 (0 to 0)	0 (0 to 0)	0 (0 to 0)	0 (0 to 0)	0 (0 to 0)	0 (0 to 0)	0 (0 to 0)	0 (0 to 0)	0 (0 to 0)	0 (0 to 0)	0 (0 to 0)	0 (0 to 0)	0 (0 to 0)	0 (0 to 0)	0 (0 to 0)	0
African trypanosomiasis	0 (0 to 0)	0 (0 to 0)	0 (0 to 0)	0 (0 to 0)	0 (0 to 0)	0 (0 to 0)	0 (0 to 0)	0 (0 to 0)	0 (0 to 0)	0 (0 to 0)	0 (0 to 0)	0 (0 to 0)	0 (0 to 0)	0 (0 to 0)	0 (0 to 0)	0 (0 to 0)	0 (0 to 0)	0 (0 to 0)	0 (0 to 0)	0 (0 to 0)	0 (0 to 0)	0 (0 to 0)	0 (0 to 0)	0
Schistosomiasis	0 (0 to 0)	0 (0 to 0)	0 (0 to 0)	0 (0 to 0)	0 (0 to 0)	0 (0 to 0)	0 (0 to 0)	0 (0 to 0)	0 (0 to 0)	0 (0 to 0)	0 (0 to 0)	0 (0 to 0)	0 (0 to 0)	0 (0 to 0)	0 (0 to 0)	0 (0 to 0)	0 (0 to 0)	0 (0 to 0)	0 (0 to 0)	0 (0 to 0)	0 (0 to 0)	0 (0 to 0)	0 (0 to 0)	0
Cysticercosis	0 (0 to 0)	0 (0 to 0)	0 (0 to 0)	0 (0 to 0)	0 (0 to 0)	0·2 (0 to 0·4)	1·1 (0·2 to 2·9)	3·6 (0·9 to 9)	6·4 (2 to 13·7)	7·5 (2·4 to 16·6)	8·8 (2·8 to 20·1)	10·7 (3·4 to 24·6)	12·9 (4·4 to 30·2)	14·8 (5·2 to 30·9)	16·9 (6·4 to 35·6)	17·3 (6·3 to 34·1)	14·9 (5·7 to 29·6)	11·8 (4·7 to 22·8)	9·4 (3·5 to 18)	6·1 (2·4 to 11·9)	2·8 (1·1 to 5·7)	1·2 (0·4 to 2·4)	0·5 (0·2 to 1·1)	147
Cystic echinococcosis	0 (0 to 0)	0 (0 to 0)	0 (0 to 0)	0 (0 to 0)	0 (0 to 0)	0 (0 to 0)	0 (0 to 0)	0 (0 to 0)	0 (0 to 0)	0 (0 to 0)	0 (0 to 0)	0 (0 to 0)	0 (0 to 0)	0 (0 to 0)	0 (0 to 0)	0 (0 to 0)	0 (0 to 0)	0 (0 to 0)	0 (0 to 0)	0 (0 to 0)	0 (0 to 0)	0 (0 to 0)	0 (0 to 0)	0·1
Lymphatic filariasis	0 (0 to 0)	0 (0 to 0)	0 (0 to 0)	0 (0 to 0)	0 (0 to 0)	0 (0 to 0)	0 (0 to 0)	0 (0 to 0)	0 (0 to 0)	0 (0 to 0)	0 (0 to 0)	0 (0 to 0)	0 (0 to 0)	0 (0 to 0)	0 (0 to 0)	0 (0 to 0)	0 (0 to 0)	0 (0 to 0)	0 (0 to 0)	0 (0 to 0)	0 (0 to 0)	0 (0 to 0)	0 (0 to 0)	0
Onchocerciasis	0 (0 to 0)	0 (0 to 0)	0 (0 to 0)	0 (0 to 0)	0 (0 to 0)	0 (0 to 0)	0 (0 to 0)	0 (0 to 0)	0 (0 to 0)	0 (0 to 0)	0 (0 to 0)	0 (0 to 0)	0 (0 to 0)	0 (0 to 0)	0 (0 to 0)	0 (0 to 0)	0 (0 to 0)	0 (0 to 0)	0 (0 to 0)	0 (0 to 0)	0 (0 to 0)	0 (0 to 0)	0 (0 to 0)	0
Trachoma	0 (0 to 0)	0 (0 to 0)	0 (0 to 0)	0 (0 to 0)	0 (0 to 0)	0 (0 to 0)	0 (0 to 0)	0 (0 to 0)	0 (0 to 0)	0 (0 to 0)	0 (0 to 0)	0 (0 to 0)	0 (0 to 0)	0 (0 to 0)	0 (0 to 0)	0 (0 to 0)	0 (0 to 0)	0 (0 to 0)	0 (0 to 0)	0 (0 to 0)	0 (0 to 0)	0 (0 to 0)	0 (0 to 0)	0
Dengue	0 (0 to 0)	0 (0 to 0·1)	1·2 (0·4 to 2·7)	1·5 (0·5 to 2·9)	3·1 (1·1 to 6·1)	3 (1 to 5·8)	3·1 (1·2 to 5·8)	2·8 (1 to 5·5)	2·9 (1·3 to 5·3)	2·5 (0·9 to 4·6)	2·5 (0·9 to 4·5)	2·3 (1 to 4·3)	2 (0·7 to 3·8)	2 (0·7 to 4)	1·6 (0·5 to 3·1)	1·4 (0·5 to 2·7)	0·9 (0·3 to 1·8)	0·6 (0·2 to 1·2)	0·4 (0·1 to 0·9)	0·3 (0·1 to 0·6)	0·2 (0·1 to 0·3)	0·1 (0 to 0·2)	0·1 (0 to 0·2)	34·3
Yellow fever	0 (0 to 0)	0 (0 to 0)	0 (0 to 0)	0 (0 to 0)	0 (0 to 0)	0 (0 to 0)	0 (0 to 0)	0 (0 to 0)	0 (0 to 0)	0 (0 to 0)	0 (0 to 0)	0 (0 to 0)	0 (0 to 0)	0 (0 to 0)	0 (0 to 0)	0 (0 to 0)	0 (0 to 0)	0 (0 to 0)	0 (0 to 0)	0 (0 to 0)	0 (0 to 0)	0 (0 to 0)	0 (0 to 0)	0
Rabies	0 (0 to 0)	0 (0 to 0)	0 (0 to 0)	0 (0 to 0)	0 (0 to 0·1)	0 (0 to 0)	0 (0 to 0)	0 (0 to 0)	0 (0 to 0)	0 (0 to 0)	0 (0 to 0)	0 (0 to 0)	0 (0 to 0)	0 (0 to 0)	0 (0 to 0)	0 (0 to 0)	0 (0 to 0)	0 (0 to 0)	0 (0 to 0)	0 (0 to 0)	0 (0 to 0)	0 (0 to 0)	0 (0 to 0)	0·1
Intestinal nematode infections	0 (0 to 0)	0 (0 to 0)	0 (0 to 0·1)	0·3 (0·1 to 0·5)	0·6 (0·2 to 1·3)	0·3 (0·1 to 0·8)	2 (0·8 to 4)	2·5 (0·8 to 4·9)	1·8 (0·7 to 3·6)	1·4 (0·4 to 2·9)	1·7 (0·8 to 3·1)	1·1 (0·4 to 2)	0·1 (0 to 0·2)	0·2 (0·1 to 0·3)	0·2 (0·1 to 0·3)	0·1 (0·1 to 0·2)	0·1 (0 to 0·1)	0·1 (0 to 0·1)	0 (0 to 0·1)	0 (0 to 0·1)	0 (0 to 0)	0 (0 to 0)	0 (0 to 0)	12·5
Ascariasis	0 (0 to 0)	0 (0 to 0)	0 (0 to 0·1)	0·1 (0 to 0·3)	0·1 (0 to 0·3)	0 (0 to 0·1)	0 (0 to 0·1)	0 (0 to 0·1)	0 (0 to 0·1)	0 (0 to 0·1)	0 (0 to 0·1)	0 (0 to 0·1)	0 (0 to 0·1)	0 (0 to 0·1)	0 (0 to 0)	0 (0 to 0·1)	0 (0 to 0)	0 (0 to 0)	0 (0 to 0)	0 (0 to 0)	0 (0 to 0)	0 (0 to 0)	0 (0 to 0)	0·4
Trichuriasis	0 (0 to 0)	0 (0 to 0)	0 (0 to 0)	0 (0 to 0)	0 (0 to 0)	0 (0 to 0)	0 (0 to 0)	0 (0 to 0)	0 (0 to 0)	0 (0 to 0)	0 (0 to 0)	0 (0 to 0)	0 (0 to 0)	0 (0 to 0)	0 (0 to 0)	0 (0 to 0)	0 (0 to 0)	0 (0 to 0)	0 (0 to 0)	0 (0 to 0)	0 (0 to 0)	0 (0 to 0)	0 (0 to 0)	0
Hookworm disease	0 (0 to 0)	0 (0 to 0)	0 (0 to 0)	0·2 (0 to 0·4)	0·5 (0·2 to 1·2)	0·3 (0·1 to 0·8)	2 (0·7 to 4)	2·5 (0·7 to 4·9)	1·8 (0·6 to 3·5)	1·4 (0·4 to 2·9)	1·7 (0·8 to 3·1)	1·1 (0·4 to 2)	0·1 (0 to 0·2)	0·2 (0·1 to 0·3)	0·1 (0·1 to 0·3)	0·1 (0 to 0·2)	0·1 (0 to 0·1)	0 (0 to 0·1)	0 (0 to 0·1)	0 (0 to 0)	0 (0 to 0)	0 (0 to 0)	0 (0 to 0)	12·1
Food-borne trematodiases	0 (0 to 0)	0 (0 to 0)	0 (0 to 0)	0 (0 to 0)	0 (0 to 0)	0 (0 to 0)	0 (0 to 0)	0 (0 to 0)	0 (0 to 0)	0 (0 to 0)	0 (0 to 0)	0 (0 to 0)	0 (0 to 0)	0 (0 to 0)	0 (0 to 0)	0 (0 to 0)	0 (0 to 0)	0 (0 to 0)	0 (0 to 0)	0 (0 to 0)	0 (0 to 0)	0 (0 to 0)	0 (0 to 0)	0
Leprosy	0 (0 to 0)	0 (0 to 0)	0 (0 to 0)	0 (0 to 0)	0 (0 to 0)	0 (0 to 0)	0 (0 to 0)	0 (0 to 0)	0 (0 to 0)	0 (0 to 0)	0 (0 to 0·1)	0 (0 to 0·1)	0·1 (0 to 0·1)	0·1 (0 to 0·1)	0·1 (0 to 0·1)	0·1 (0 to 0·1)	0 (0 to 0·1)	0 (0 to 0)	0 (0 to 0)	0 (0 to 0)	0 (0 to 0)	0 (0 to 0)	0 (0 to 0)	0·5
Ebola	0 (0 to 0)	0 (0 to 0)	0 (0 to 0)	0 (0 to 0)	0 (0 to 0)	0 (0 to 0)	0 (0 to 0)	0 (0 to 0)	0 (0 to 0)	0 (0 to 0)	0 (0 to 0)	0 (0 to 0)	0 (0 to 0)	0 (0 to 0)	0 (0 to 0)	0 (0 to 0)	0 (0 to 0)	0 (0 to 0)	0 (0 to 0)	0 (0 to 0)	0 (0 to 0)	0 (0 to 0)	0 (0 to 0)	0
Zika virus	0 (0 to 0)	0 (0 to 0)	0 (0 to 0·2)	0·1 (0 to 0·7)	0·2 (0 to 0·8)	0·2 (0 to 0·8)	0·2 (0 to 1)	0·2 (0 to 1)	0·2 (0 to 1)	0·2 (0 to 1·1)	0·2 (0 to 1)	0·2 (0 to 0·8)	0·1 (0 to 0·6)	0·1 (0 to 0·5)	0·1 (0 to 0·3)	0 (0 to 0·2)	0 (0 to 0·1)	0 (0 to 0·1)	0 (0 to 0)	0 (0 to 0)	0 (0 to 0)	0 (0 to 0)	0 (0 to 0)	2·1
Guinea worm disease	0 (0 to 0)	0 (0 to 0)	0 (0 to 0)	0 (0 to 0)	0 (0 to 0)	0 (0 to 0)	0 (0 to 0)	0 (0 to 0)	0 (0 to 0)	0 (0 to 0)	0 (0 to 0)	0 (0 to 0)	0 (0 to 0)	0 (0 to 0)	0 (0 to 0)	0 (0 to 0)	0 (0 to 0)	0 (0 to 0)	0 (0 to 0)	0 (0 to 0)	0 (0 to 0)	0 (0 to 0)	0 (0 to 0)	0
Other neglected tropical diseases	0 (0 to 0·1)	0·3 (0·2 to 0·5)	2·5 (1·3 to 4·3)	9·5 (4·3 to 17·4)	14 (5·8 to 27·1)	8·4 (3·1 to 17·1)	3·9 (1·6 to 7·8)	3·3 (1·3 to 6·5)	3 (1·2 to 5·8)	3·2 (1·2 to 6·1)	3·7 (1·6 to 7·2)	4·6 (2·1 to 8·4)	4·2 (2 to 7·7)	4 (1·8 to 7·2)	3·5 (1·6 to 6·5)	1·3 (0·6 to 2·5)	0·6 (0·3 to 1·1)	0·5 (0·3 to 0·9)	0·4 (0·2 to 0·7)	0·2 (0·1 to 0·4)	0·2 (0·1 to 0·3)	0·1 (0 to 0·1)	0 (0 to 0·1)	71·6
Other infectious diseases	3·1 (2·2 to 4·3)	8 (4·2 to 14·1)	37 (26·7 to 51·6)	32·5 (22·8 to 46·5)	29·7 (21·7 to 39·7)	27·1 (20·6 to 35·3)	29·5 (23·2 to 37·2)	30·3 (23·9 to 38·8)	32 (24·5 to 40·8)	33·3 (25·3 to 42·6)	35·8 (27·3 to 46·9)	36·1 (27·6 to 47·8)	36·2 (26·9 to 48·4)	32·5 (24·2 to 42·8)	27·8 (20·6 to 36·3)	21·7 (16·5 to 27·9)	14·4 (11 to 18·2)	10 (7·7 to 12·5)	6·6 (5·2 to 8·2)	4·4 (3·3 to 5·5)	2·8 (2 to 3·6)	1 (0·7 to 1·2)	0·4 (0·3 to 0·6)	492
Meningitis	1·6 (1·1 to 2·4)	5·3 (2·6 to 9·8)	16·7 (10·2 to 26·2)	9·6 (5·8 to 14·9)	7·2 (5·3 to 9·3)	6·4 (4·7 to 8·8)	7·9 (5·5 to 10·9)	7·9 (5·4 to 11·3)	8·2 (5·6 to 11·5)	9·1 (6·3 to 12·5)	9·1 (6·2 to 13)	8·4 (5·8 to 11·7)	8·6 (5·8 to 12·2)	8·2 (5·6 to 11·7)	6·7 (4·8 to 9·2)	5·7 (4 to 7·8)	3·8 (2·7 to 5·1)	2·5 (1·8 to 3·4)	1·9 (1·4 to 2·6)	1·2 (0·9 to 1·6)	0·6 (0·4 to 0·8)	0·2 (0·1 to 0·2)	0·1 (0 to 0·1)	137
Encephalitis	0·6 (0·4 to 1)	1·2 (0·6 to 2·2)	12·1 (7·7 to 18·4)	11·7 (7·1 to 20·2)	8·6 (5·9 to 12·8)	8·8 (6·2 to 12·7)	9·6 (6·7 to 13·4)	9·8 (6·8 to 14)	8·5 (6 to 12·1)	8·2 (5·7 to 11·6)	7·5 (5·3 to 10·5)	7·5 (5·4 to 10·4)	6·4 (4·5 to 9·2)	4·7 (3·3 to 6·5)	4·2 (3 to 5·8)	3·4 (2·5 to 4·8)	2·2 (1·6 to 3)	1·4 (1 to 2)	0·8 (0·6 to 1·1)	0·5 (0·3 to 0·7)	0·2 (0·1 to 0·3)	0·1 (0 to 0·1)	0 (0 to 0·1)	118
Diphtheria	0 (0 to 0)	0 (0 to 0)	0 (0 to 0)	0 (0 to 0)	0 (0 to 0)	0 (0 to 0)	0 (0 to 0)	0 (0 to 0)	0 (0 to 0)	0 (0 to 0)	0 (0 to 0)	0 (0 to 0)	0 (0 to 0)	0 (0 to 0)	0 (0 to 0)	0 (0 to 0)	0 (0 to 0)	0 (0 to 0)	0 (0 to 0)	0 (0 to 0)	0 (0 to 0)	0 (0 to 0)	0 (0 to 0)	0
Tetanus	0 (0 to 0)	0 (0 to 0·1)	0 (0 to 0·1)	0 (0 to 0)	0·1 (0 to 0·1)	0 (0 to 0·1)	0·1 (0 to 0·1)	0·1 (0 to 0·2)	0·1 (0 to 0·2)	0·1 (0 to 0·3)	0·2 (0·1 to 0·4)	0·2 (0·1 to 0·4)	0·2 (0·1 to 0·5)	0·3 (0·1 to 0·7)	0·3 (0·1 to 0·6)	0·2 (0·1 to 0·5)	0·2 (0·1 to 0·4)	0·1 (0 to 0·3)	0·1 (0 to 0·2)	0·1 (0 to 0·1)	0 (0 to 0·1)	0 (0 to 0)	0 (0 to 0)	2·4
Measles	0 (0 to 0)	0 (0 to 0)	0 (0 to 0)	0 (0 to 0)	0 (0 to 0)	0 (0 to 0)	0 (0 to 0)	0 (0 to 0)	0 (0 to 0)	0 (0 to 0)	0 (0 to 0)	0 (0 to 0)	0 (0 to 0)	0 (0 to 0)	0 (0 to 0)	0 (0 to 0)	0 (0 to 0)	0 (0 to 0)	0 (0 to 0)	0 (0 to 0)	0 (0 to 0)	0 (0 to 0)	0 (0 to 0)	0
Varicella and herpes zoster	0·2 (0·1 to 0·6)	0·4 (0·2 to 1·1)	1·1 (0·5 to 2·5)	0·6 (0·3 to 1·2)	0·8 (0·4 to 1·5)	1·1 (0·5 to 2)	0·8 (0·4 to 1·4)	1·1 (0·5 to 1·8)	1·2 (0·6 to 2)	1·3 (0·7 to 2·2)	1·8 (1 to 3·1)	1·5 (0·8 to 2·4)	1·9 (1 to 3·2)	1·7 (1 to 2·7)	1·4 (0·8 to 2·4)	1·4 (0·8 to 2·4)	0·9 (0·5 to 1·6)	0·9 (0·5 to 1·5)	0·6 (0·4 to 1·1)	0·4 (0·2 to 0·7)	0·2 (0·1 to 0·3)	0·1 (0·1 to 0·2)	0 (0 to 0·1)	21·5
Acute hepatitis	0 (0 to 0)	0 (0 to 0)	0·3 (0·1 to 0·5)	0·6 (0·3 to 0·9)	2 (1·3 to 3·2)	2·9 (1·9 to 4·3)	3 (1·9 to 4·2)	3·1 (2·1 to 4·3)	3 (2·2 to 4·2)	2·6 (1·8 to 3·6)	2·4 (1·7 to 3·4)	2·4 (1·8 to 3·2)	1·9 (1·4 to 2·7)	1·6 (1·2 to 2·2)	1·2 (0·9 to 1·7)	0·8 (0·6 to 1·1)	0·5 (0·4 to 0·6)	0·3 (0·2 to 0·4)	0·2 (0·1 to 0·3)	0·1 (0·1 to 0·2)	0·1 (0 to 0·1)	0 (0 to 0)	0 (0 to 0)	29·2
Acute hepatitis A	0 (0 to 0)	0 (0 to 0)	0·1 (0 to 0·1)	0·5 (0·2 to 0·7)	1·7 (1 to 2·8)	2·5 (1·5 to 3·7)	2·4 (1·5 to 3·5)	2·1 (1·3 to 3)	1·6 (0·9 to 2·5)	1·4 (0·9 to 2·3)	1 (0·6 to 1·6)	1 (0·6 to 1·6)	0·8 (0·4 to 1·2)	0·6 (0·3 to 0·9)	0·4 (0·2 to 0·7)	0·2 (0·1 to 0·4)	0·1 (0·1 to 0·2)	0·1 (0 to 0·1)	0 (0 to 0·1)	0 (0 to 0)	0 (0 to 0)	0 (0 to 0)	0 (0 to 0)	16·4
Acute hepatitis B	0 (0 to 0)	0 (0 to 0)	0·2 (0·1 to 0·4)	0·1 (0 to 0·1)	0·2 (0·1 to 0·3)	0·2 (0·1 to 0·4)	0·3 (0·1 to 0·6)	0·7 (0·4 to 1·2)	1·1 (0·7 to 1·8)	0·9 (0·5 to 1·4)	1·1 (0·7 to 1·7)	1·1 (0·7 to 1·6)	0·8 (0·5 to 1·3)	0·9 (0·6 to 1·2)	0·7 (0·4 to 1)	0·4 (0·3 to 0·7)	0·3 (0·2 to 0·4)	0·2 (0·1 to 0·3)	0·1 (0 to 0·1)	0·1 (0 to 0·1)	0 (0 to 0·1)	0 (0 to 0)	0 (0 to 0)	9·3
Acute hepatitis C	0 (0 to 0)	0 (0 to 0)	0 (0 to 0)	0·1 (0 to 0·1)	0 (0 to 0·1)	0 (0 to 0·1)	0 (0 to 0·1)	0·1 (0 to 0·2)	0·1 (0 to 0·2)	0·1 (0 to 0·2)	0·1 (0 to 0·2)	0·1 (0 to 0·2)	0·2 (0·1 to 0·3)	0·1 (0·1 to 0·3)	0·1 (0 to 0·2)	0·1 (0 to 0·1)	0·1 (0 to 0·1)	0 (0 to 0·1)	0 (0 to 0)	0 (0 to 0)	0 (0 to 0)	0 (0 to 0)	0 (0 to 0)	1·2
Acute hepatitis E	0 (0 to 0)	0 (0 to 0)	0 (0 to 0·1)	0 (0 to 0)	0·1 (0·1 to 0·2)	0·2 (0·1 to 0·5)	0·3 (0·1 to 0·5)	0·2 (0·1 to 0·5)	0·3 (0·1 to 0·5)	0·2 (0·1 to 0·4)	0·2 (0·1 to 0·3)	0·2 (0·1 to 0·3)	0·2 (0·1 to 0·3)	0·1 (0 to 0·1)	0·1 (0 to 0·1)	0·1 (0·1 to 0·2)	0 (0 to 0·1)	0 (0 to 0·1)	0 (0 to 0·1)	0 (0 to 0)	0 (0 to 0)	0 (0 to 0)	0 (0 to 0)	2·3
Other unspecified infectious diseases	0·6 (0·4 to 1·1)	1 (0·6 to 1·7)	5·4 (3·6 to 7·8)	8·6 (5 to 13·9)	10·9 (5·5 to 19·8)	7·9 (4·3 to 13·8)	8·1 (5·2 to 12·4)	8·4 (4·8 to 13)	10·9 (6·2 to 16·9)	12 (6·7 to 18·5)	14·8 (8·8 to 22)	16·1 (9·6 to 24·1)	17·2 (10·3 to 26·2)	15·9 (9·9 to 23)	13·9 (8·4 to 19·9)	10·1 (5·9 to 14·4)	6·8 (4 to 9·7)	4·7 (2·9 to 6·5)	2·9 (1·8 to 4)	2·1 (1·2 to 3)	1·7 (1 to 2·4)	0·6 (0·4 to 0·8)	0·3 (0·2 to 0·4)	181
Maternal and neonatal disorders	1,190 (925 to 1,500)	461 (275 to 774)	188 (126 to 268)	94·4 (73·3 to 119)	88·8 (67 to 114)	104 (77 to 133)	123 (96·9 to 152)	136 (109 to 167)	139 (112 to 171)	129 (104 to 159)	110 (88·5 to 135)	81·6 (63·7 to 102)	63·1 (47·9 to 80·7)	47·9 (35·4 to 61·6)	35·1 (25·7 to 45·4)	22·4 (16·4 to 29·1)	11·4 (8·4 to 14·9)	5·1 (3·6 to 7)	2·2 (1·5 to 3)	0·6 (0·4 to 0·8)	0·1 (0·1 to 0·2)	0 (0 to 0)	0 (0 to 0)	3,040
Maternal disorders	0 (0 to 0)	0 (0 to 0)	0 (0 to 0)	0 (0 to 0)	0 (0 to 0)	0·8 (0·6 to 1·1)	27·1 (20·4 to 34·8)	45·3 (32·8 to 60·1)	51·4 (36·2 to 69·7)	50·7 (36·7 to 70·3)	38·2 (27·7 to 50·8)	15·5 (11·5 to 20·7)	3·8 (2·8 to 5·2)	0·5 (0·4 to 0·6)	0 (0 to 0)	0 (0 to 0)	0 (0 to 0)	0 (0 to 0)	0 (0 to 0)	0 (0 to 0)	0 (0 to 0)	0 (0 to 0)	0 (0 to 0)	233
Maternal hemorrhage	0 (0 to 0)	0 (0 to 0)	0 (0 to 0)	0 (0 to 0)	0 (0 to 0)	0 (0 to 0)	1·8 (1·1 to 2·9)	3·4 (1·9 to 5·7)	4·7 (2·8 to 7·3)	4·2 (2·3 to 6·5)	3·3 (2 to 5·2)	1·3 (0·7 to 2)	0·1 (0·1 to 0·2)	0 (0 to 0)	0 (0 to 0)	0 (0 to 0)	0 (0 to 0)	0 (0 to 0)	0 (0 to 0)	0 (0 to 0)	0 (0 to 0)	0 (0 to 0)	0 (0 to 0)	18·8
Maternal sepsis and other maternal infections	0 (0 to 0)	0 (0 to 0)	0 (0 to 0)	0 (0 to 0)	0 (0 to 0)	0 (0 to 0)	1·1 (0·6 to 1·8)	2·1 (1·2 to 3·2)	2·3 (1·3 to 3·8)	2 (1·1 to 3·3)	1·4 (0·8 to 2·4)	0·5 (0·3 to 0·9)	0·1 (0·1 to 0·2)	0 (0 to 0)	0 (0 to 0)	0 (0 to 0)	0 (0 to 0)	0 (0 to 0)	0 (0 to 0)	0 (0 to 0)	0 (0 to 0)	0 (0 to 0)	0 (0 to 0)	9·7
Maternal hypertensive disorders	0 (0 to 0)	0 (0 to 0)	0 (0 to 0)	0 (0 to 0)	0 (0 to 0)	0·2 (0·1 to 0·2)	4·8 (3·3 to 6·8)	6 (4 to 8·5)	6·8 (4·5 to 9·9)	6 (4 to 8·7)	4·2 (2·8 to 6·1)	1·6 (1 to 2·3)	0·3 (0·2 to 0·5)	0 (0 to 0)	0 (0 to 0)	0 (0 to 0)	0 (0 to 0)	0 (0 to 0)	0 (0 to 0)	0 (0 to 0)	0 (0 to 0)	0 (0 to 0)	0 (0 to 0)	29·8
Maternal obstructed labor and uterine rupture	0 (0 to 0)	0 (0 to 0)	0 (0 to 0)	0 (0 to 0)	0 (0 to 0)	0 (0 to 0)	0·6 (0·3 to 1)	1 (0·5 to 1·7)	1·2 (0·6 to 2·2)	1·2 (0·7 to 2·2)	1·1 (0·6 to 2)	0·4 (0·2 to 0·8)	0·1 (0 to 0·2)	0 (0 to 0)	0 (0 to 0)	0 (0 to 0)	0 (0 to 0)	0 (0 to 0)	0 (0 to 0)	0 (0 to 0)	0 (0 to 0)	0 (0 to 0)	0 (0 to 0)	5·7
Maternal abortion and miscarriage	0 (0 to 0)	0 (0 to 0)	0 (0 to 0)	0 (0 to 0)	0 (0 to 0)	0·1 (0 to 0·1)	1·9 (1 to 3·4)	3·2 (1·7 to 5·7)	3·3 (1·7 to 5·8)	3·1 (1·6 to 5·5)	2·4 (1·2 to 4·5)	1·2 (0·6 to 2·1)	0·4 (0·2 to 0·8)	0 (0 to 0)	0 (0 to 0)	0 (0 to 0)	0 (0 to 0)	0 (0 to 0)	0 (0 to 0)	0 (0 to 0)	0 (0 to 0)	0 (0 to 0)	0 (0 to 0)	15·7
Ectopic pregnancy	0 (0 to 0)	0 (0 to 0)	0 (0 to 0)	0 (0 to 0)	0 (0 to 0)	0 (0 to 0)	1·8 (1 to 2·9)	4·6 (2·6 to 7·2)	5·6 (3·2 to 9·1)	5·9 (3·5 to 9·3)	4 (2·4 to 6·4)	1·3 (0·8 to 2·1)	0·4 (0·2 to 0·7)	0 (0 to 0)	0 (0 to 0)	0 (0 to 0)	0 (0 to 0)	0 (0 to 0)	0 (0 to 0)	0 (0 to 0)	0 (0 to 0)	0 (0 to 0)	0 (0 to 0)	23·7
Indirect maternal deaths	--	--	--	--	--	0·1 (0·1 to 0·2)	4 (2·4 to 6·3)	6·7 (3·8 to 10·6)	6·6 (3·8 to 10·9)	5·9 (3·4 to 9·7)	3·7 (2·1 to 6·1)	1·2 (0·7 to 1·9)	0·2 (0·1 to 0·3)	0 (0 to 0)	--	--	--	--	--	--	--	--	--	NA
Late maternal deaths	--	--	--	--	--	0·1 (0 to 0·1)	2·2 (1·3 to 3·3)	3·5 (2 to 5·4)	3·6 (2 to 5·7)	3·5 (2 to 5·6)	2·6 (1·5 to 4·3)	1 (0·6 to 1·7)	0·3 (0·2 to 0·4)	0 (0 to 0)	--	--	--	--	--	--	--	--	--	NA
Maternal deaths aggravated by HIV/AIDS	--	--	--	--	--	0 (0 to 0)	0·1 (0 to 0·1)	0·3 (0·1 to 0·5)	0·4 (0·2 to 0·8)	0·5 (0·3 to 1)	0·4 (0·2 to 0·6)	0·2 (0·1 to 0·2)	0 (0 to 0·1)	0 (0 to 0)	--	--	--	--	--	--	--	--	--	NA
Neonatal disorders	1,190 (925 to 1,500)	461 (275 to 774)	188 (126 to 268)	94·4 (73·3 to 119)	88·8 (67 to 114)	104 (76·2 to 132)	95·9 (71·4 to 124)	91·1 (67·6 to 117)	87·6 (65·6 to 112)	78·2 (58·7 to 101)	72·2 (53·1 to 92·3)	66·1 (48·8 to 85·7)	59·2 (43·9 to 76·9)	47·5 (34·9 to 61·2)	35·1 (25·7 to 45·4)	22·4 (16·4 to 29·1)	11·4 (8·4 to 14·9)	5·1 (3·6 to 7)	2·2 (1·5 to 3)	0·6 (0·4 to 0·8)	0·1 (0·1 to 0·2)	0 (0 to 0)	0 (0 to 0)	2,810
Neonatal preterm birth	438 (312 to 590)	122 (67·5 to 211)	58·2 (35·7 to 88·4)	33·2 (24·8 to 43·3)	46·1 (33·7 to 59·5)	53·3 (38·9 to 70·3)	48·7 (35·9 to 63·7)	44·9 (32·2 to 59·5)	42·8 (31·6 to 56·1)	37·2 (26·7 to 48·7)	33·7 (24·4 to 44·3)	29·9 (21·8 to 39·6)	25·9 (18·9 to 34·4)	20 (14·5 to 26·2)	13·8 (10·1 to 18)	8·1 (5·9 to 10·6)	3·9 (3 to 4·9)	1·6 (1·2 to 2·1)	0·7 (0·5 to 1)	0·2 (0·1 to 0·3)	0·1 (0 to 0·1)	0 (0 to 0)	0 (0 to 0)	1,060
Neonatal encephalopathy due to birth asphyxia and trauma	297 (197 to 428)	56·3 (28·3 to 99·9)	27·1 (14·8 to 43·5)	11·5 (8 to 16·1)	9·7 (5·4 to 14·8)	11·4 (6·6 to 17·5)	10·7 (6·2 to 16·4)	10·4 (6·1 to 16)	9·9 (5·8 to 15·1)	9·1 (5·2 to 14·2)	8·4 (4·9 to 13·4)	7·9 (4·5 to 12)	7·2 (4·1 to 11·2)	5·8 (3·2 to 9)	4·6 (2·6 to 7·3)	3·2 (1·9 to 4·8)	1·5 (0·9 to 2·1)	0·5 (0·3 to 0·7)	0·1 (0·1 to 0·2)	0 (0 to 0)	0 (0 to 0)	0 (0 to 0)	0 (0 to 0)	492
Neonatal sepsis and other neonatal infections	205 (136 to 288)	177 (103 to 297)	60·1 (35·8 to 95)	19·5 (12·4 to 28·7)	28 (17 to 42·8)	33 (20·6 to 49·5)	30·9 (18·8 to 46·6)	30·2 (18 to 44·8)	29·4 (17·8 to 43·8)	26·9 (16·4 to 40·7)	25·4 (15·1 to 37·9)	23·8 (14·7 to 35·7)	22 (13·4 to 32·7)	18·2 (10·8 to 27·4)	14 (8·5 to 20·9)	9·4 (5·6 to 14·5)	5·2 (3 to 8)	2·6 (1·4 to 4·1)	1·2 (0·6 to 2)	0·3 (0·2 to 0·6)	0·1 (0 to 0·1)	0 (0 to 0)	0 (0 to 0)	762
Hemolytic disease and other neonatal jaundice	1·6 (0·8 to 2·6)	1·1 (0·6 to 1·7)	0·9 (0·6 to 1·3)	3·5 (2·2 to 5)	5 (3·2 to 7·3)	5·9 (4 to 8·5)	5·6 (3·8 to 7·7)	5·6 (4·1 to 7·2)	5·4 (4 to 7)	5 (3·7 to 6·4)	4·7 (3·5 to 6)	4·5 (3·3 to 5·8)	4·1 (3·1 to 5·3)	3·5 (2·6 to 4·4)	2·6 (2 to 3·4)	1·7 (1·3 to 2·2)	0·9 (0·7 to 1·2)	0·5 (0·3 to 0·6)	0·2 (0·1 to 0·3)	0·1 (0 to 0·1)	0 (0 to 0)	0 (0 to 0)	0 (0 to 0)	62·4
Other neonatal disorders	253 (170 to 349)	104 (59·1 to 176)	41·7 (24·7 to 63)	26·7 (21 to 33·5)	0 (0 to 0)	0 (0 to 0)	0 (0 to 0)	0 (0 to 0)	0 (0 to 0)	0 (0 to 0)	0 (0 to 0)	0 (0 to 0)	0 (0 to 0)	0 (0 to 0)	0 (0 to 0)	0 (0 to 0)	0 (0 to 0)	0 (0 to 0)	0 (0 to 0)	0 (0 to 0)	0 (0 to 0)	0 (0 to 0)	0 (0 to 0)	425
Nutritional deficiencies	0·2 (0·1 to 0·3)	3·5 (1·9 to 5·7)	55·7 (37·6 to 78·8)	111 (59·4 to 191)	165 (77·4 to 308)	107 (49 to 200)	110 (54·7 to 195)	91 (43·2 to 166)	94·1 (50·9 to 159)	90·7 (44 to 160)	108 (59·6 to 185)	124 (65·6 to 207)	115 (60·4 to 193)	116 (62·5 to 190)	102 (54·8 to 170)	67 (39·6 to 111)	36 (22·5 to 55·5)	26·6 (17·6 to 40·7)	21·4 (14·7 to 30·7)	15·6 (10·3 to 22·8)	10·1 (6·9 to 14·9)	5·3 (3·7 to 7·4)	2·5 (1·8 to 3·5)	1,580
Protein-energy malnutrition	0·1 (0·1 to 0·2)	0·4 (0·3 to 0·6)	31 (20·4 to 44·2)	16·6 (9·7 to 27)	17·3 (10·8 to 26·1)	15·6 (9·4 to 24·5)	16·2 (9·9 to 24·6)	17·6 (11·2 to 26·4)	18·9 (11·9 to 27·4)	18·6 (12·4 to 27·1)	20·6 (13·9 to 28·4)	19·9 (13·7 to 28·2)	21 (14·4 to 29·1)	20·8 (14·2 to 28·6)	19·6 (13·7 to 26·8)	17·5 (12·7 to 23·9)	13·8 (9·8 to 18·2)	11·6 (8·1 to 15·7)	10·3 (7·8 to 13)	7·7 (5·4 to 10·4)	5 (3·5 to 6·9)	3 (2·1 to 4)	1·8 (1·2 to 2·5)	325
Iodine deficiency	0 (0 to 0)	0 (0 to 0)	0 (0 to 0)	0 (0 to 0·1)	0·3 (0·1 to 0·5)	0·7 (0·3 to 1·4)	0·9 (0·4 to 1·8)	1·1 (0·4 to 2·1)	1·2 (0·5 to 2·3)	1·2 (0·6 to 2·2)	1·2 (0·6 to 2·2)	1·2 (0·6 to 2·1)	1·2 (0·6 to 2)	1 (0·5 to 1·7)	0·8 (0·4 to 1·4)	0·6 (0·3 to 1)	0·4 (0·2 to 0·6)	0·2 (0·1 to 0·4)	0·1 (0·1 to 0·2)	0·1 (0 to 0·1)	0 (0 to 0·1)	0 (0 to 0)	0 (0 to 0)	12·5
Vitamin A deficiency	0 (0 to 0)	0·1 (0 to 0·1)	0·4 (0·2 to 0·6)	2·4 (1·4 to 4)	3·3 (1·8 to 5·8)	2·5 (1·2 to 4·4)	1·3 (0·5 to 2·5)	1 (0·4 to 2)	0·9 (0·5 to 1·6)	0·8 (0·4 to 1·3)	0·7 (0·4 to 1·1)	0·6 (0·3 to 1·1)	0·6 (0·3 to 0·9)	0·5 (0·2 to 0·8)	0·4 (0·2 to 0·6)	0·3 (0·1 to 0·5)	0·2 (0·1 to 0·3)	0·1 (0·1 to 0·2)	0·1 (0 to 0·2)	0·1 (0 to 0·1)	0 (0 to 0)	0 (0 to 0)	0 (0 to 0)	16·2
Dietary iron deficiency	0 (0 to 0·1)	3 (1·6 to 5·1)	22·9 (10·2 to 41·6)	90·6 (39·6 to 171)	140 (56·8 to 282)	84·5 (30·9 to 177)	84·1 (31·7 to 169)	62·9 (20·2 to 133)	62·6 (22·8 to 121)	62·8 (20·2 to 128)	78·5 (33·1 to 153)	93·9 (39·6 to 174)	85·2 (35·3 to 164)	85·1 (35·7 to 155)	73·8 (31·3 to 138)	43 (17·7 to 84·2)	17 (6·1 to 36)	11·4 (4·4 to 24·5)	8·5 (3·1 to 17·7)	6 (2·2 to 12)	4 (1·5 to 7·9)	1·8 (0·7 to 3·4)	0·5 (0·2 to 1)	1,120
Other nutritional deficiencies	0 (0 to 0)	0 (0 to 0)	1·4 (0·7 to 2·7)	1 (0·4 to 2)	3·4 (1·9 to 5·4)	3·8 (2·2 to 6·2)	7·2 (4·3 to 11·1)	8·4 (5·1 to 12·6)	10·4 (6·3 to 16·1)	7·3 (4·6 to 10·7)	7·5 (4·7 to 11)	7·9 (5·2 to 11·1)	7·1 (4·7 to 10·1)	8·6 (5·6 to 12)	7·1 (4·7 to 10)	5·6 (3·7 to 8·1)	4·7 (3·1 to 6·6)	3·2 (2·1 to 4·7)	2·4 (1·6 to 3·4)	1·8 (1 to 2·8)	1 (0·6 to 1·6)	0·6 (0·3 to 1)	0·2 (0·1 to 0·4)	101
Non-communicable diseases	218 (154 to 325)	152 (88·4 to 253)	557 (419 to 718)	669 (504 to 861)	902 (682 to 1,160)	1,540 (1,150 to 2,030)	2,240 (1,720 to 2,890)	2,860 (2,240 to 3,560)	3,570 (2,830 to 4,390)	4,210 (3,440 to 5,110)	5,240 (4,310 to 6,310)	6,320 (5,200 to 7,670)	7,700 (6,290 to 9,350)	8,930 (7,370 to 10,800)	9,230 (7,640 to 11,000)	8,760 (7,350 to 10,400)	7,240 (6,180 to 8,520)	5,620 (4,840 to 6,550)	4,710 (4,090 to 5,420)	3,440 (3,030 to 3,910)	1,870 (1,650 to 2,100)	882 (798 to 976)	400 (368 to 435)	87,300
Neoplasms	1·9 (1·3 to 2·6)	2·3 (1·3 to 4)	15·2 (10·8 to 20·7)	64·3 (43·7 to 93·7)	83·8 (65·7 to 104)	93·7 (73·7 to 117)	141 (110 to 175)	206 (164 to 257)	309 (240 to 397)	505 (391 to 649)	787 (599 to 1,020)	1,070 (820 to 1,380)	1,490 (1,160 to 1,920)	1,880 (1,490 to 2,390)	2,080 (1,650 to 2,630)	2,030 (1,670 to 2,500)	1,670 (1,360 to 2,060)	1,280 (1,050 to 1,550)	994 (819 to 1,200)	637 (530 to 765)	296 (243 to 359)	116 (92 to 141)	34·5 (24·6 to 42·8)	15,800
Lip and oral cavity cancer	0 (0 to 0)	0 (0 to 0)	0 (0 to 0)	0 (0 to 0)	0·3 (0·2 to 0·5)	0·5 (0·3 to 0·8)	1·2 (0·9 to 1·5)	2·1 (1·5 to 2·8)	3·3 (2·5 to 4·4)	5·9 (4·2 to 8)	10·9 (7·5 to 14·8)	20·6 (14·2 to 29·2)	33·6 (24 to 46·7)	48·3 (34·1 to 66·6)	52·5 (37·9 to 70·1)	46 (33·5 to 61·6)	33·4 (25·6 to 42·9)	22·3 (17 to 29)	16·5 (12·7 to 21·7)	9·7 (7·5 to 12·3)	3·9 (3·1 to 4·9)	1·6 (1·3 to 2)	0·6 (0·4 to 0·7)	313
Nasopharynx cancer	0 (0 to 0)	0 (0 to 0)	0 (0 to 0)	0 (0 to 0)	0·4 (0·3 to 0·5)	0·7 (0·5 to 0·9)	1·3 (0·9 to 1·8)	1·8 (1·3 to 2·5)	2·3 (1·6 to 3·2)	2·4 (1·6 to 3·3)	3·1 (2·1 to 4·6)	5·6 (3·7 to 8·2)	7·9 (5·6 to 11)	10·5 (7·3 to 14·5)	11 (8 to 14·9)	7·9 (5·4 to 10·8)	5·4 (3·8 to 7·2)	3·5 (2·5 to 4·7)	1·8 (1·3 to 2·5)	1·1 (0·8 to 1·5)	0·8 (0·6 to 1·1)	0·3 (0·2 to 0·4)	0·1 (0·1 to 0·1)	67·8
Other pharynx cancer	0 (0 to 0)	0 (0 to 0)	0 (0 to 0)	0 (0 to 0)	0 (0 to 0)	0 (0 to 0)	0 (0 to 0)	1·1 (0·7 to 1·7)	1·4 (0·9 to 2)	2·3 (1·6 to 3·1)	4·8 (3 to 7·1)	10·8 (7 to 16·3)	22·4 (15·4 to 32·6)	30·3 (21·1 to 42·5)	34·1 (24·5 to 47)	28·7 (19·7 to 40)	19·4 (13·1 to 27·4)	11·8 (8·1 to 16·4)	7 (4·9 to 10)	3·9 (2·8 to 5·4)	1·8 (1·3 to 2·4)	0·7 (0·5 to 0·9)	0·2 (0·2 to 0·3)	181
Esophageal cancer	0 (0 to 0)	0 (0 to 0)	0 (0 to 0)	0 (0 to 0)	0 (0 to 0)	0 (0 to 0)	0 (0 to 0)	1·4 (1 to 1·9)	2·4 (1·6 to 3·4)	5·2 (3·3 to 7·6)	14·5 (9·6 to 21)	34·6 (23·8 to 49·3)	61·5 (44·2 to 85·6)	85·6 (61·4 to 117)	98·1 (70·2 to 138)	83·4 (58·3 to 115)	65 (46 to 90)	43·8 (30·3 to 59·9)	28·4 (20 to 39·8)	15·7 (11·5 to 21·6)	7·1 (5·3 to 9·3)	2·5 (1·9 to 3·1)	0·6 (0·4 to 0·8)	550
Stomach cancer	0 (0 to 0)	0 (0 to 0)	0 (0 to 0)	0 (0 to 0)	0 (0 to 0)	0 (0 to 0)	2·1 (1·6 to 2·6)	5·1 (3·7 to 6·7)	12·8 (8·2 to 18·3)	25·8 (18 to 35·8)	40·3 (26·5 to 57·9)	59·7 (41·4 to 82)	77·1 (56·1 to 101)	106 (79·3 to 138)	118 (85·4 to 157)	121 (87·9 to 162)	101 (74 to 135)	76·4 (55·9 to 103)	60·6 (44·5 to 80·9)	37·7 (27·9 to 49·5)	17·7 (13·6 to 22·7)	6·8 (5·2 to 8·4)	2 (1·4 to 2·5)	870
Colon and rectum cancer	0 (0 to 0)	0 (0 to 0)	0 (0 to 0)	0 (0 to 0)	0·9 (0·6 to 1·2)	1·1 (0·8 to 1·5)	5·7 (4·2 to 7·4)	12·8 (9·3 to 17)	25·1 (17·3 to 36·2)	49·2 (36·3 to 67·6)	73·5 (53·6 to 102)	100 (74·2 to 134)	137 (101 to 181)	199 (148 to 264)	231 (174 to 303)	236 (183 to 300)	210 (157 to 269)	160 (123 to 205)	129 (100 to 165)	81·8 (65·3 to 99·8)	37·3 (29·2 to 46·9)	15·2 (11·7 to 18·6)	4·7 (3·3 to 5·9)	1,710
Liver cancer	0·1 (0 to 0·1)	0·1 (0 to 0·1)	0·3 (0·2 to 0·5)	0·9 (0·6 to 1·3)	1·1 (0·8 to 1·4)	1·8 (1·4 to 2·2)	1·4 (1 to 1·9)	3·4 (2·4 to 4·8)	6·2 (4·2 to 9·1)	8·1 (5·8 to 11·5)	14·6 (10·3 to 20·2)	22·2 (15·6 to 29·9)	30·5 (21·5 to 42·2)	42·5 (30 to 60·4)	51·6 (37·7 to 69·1)	47·9 (35·7 to 63·1)	38·7 (28·8 to 51·8)	28·6 (21·1 to 37·7)	20·8 (16 to 26·8)	11·9 (8·9 to 15·3)	3·7 (2·8 to 4·7)	1·1 (0·8 to 1·5)	0·2 (0·1 to 0·2)	338
Liver cancer due to hepatitis B	0 (0 to 0)	0 (0 to 0)	0 (0 to 0)	0 (0 to 0)	0 (0 to 0)	0·4 (0·3 to 0·6)	0·6 (0·4 to 1)	1·9 (1·2 to 2·7)	3·8 (2·3 to 5·9)	4·9 (3·2 to 7·2)	8·2 (4·7 to 12·3)	11·3 (6·7 to 17·1)	13·7 (7·8 to 21·6)	16·6 (9·1 to 27·3)	18·3 (10·4 to 28·9)	14·6 (7·6 to 23·9)	9·4 (4·9 to 15·7)	5·7 (2·8 to 10·2)	3·7 (2 to 6·3)	1·8 (0·8 to 3·1)	0·5 (0·3 to 0·9)	0·1 (0·1 to 0·3)	0 (0 to 0)	116
Liver cancer due to hepatitis C	0 (0 to 0)	0 (0 to 0)	0 (0 to 0)	0 (0 to 0)	0 (0 to 0)	0 (0 to 0)	0 (0 to 0)	0·1 (0 to 0·1)	0·2 (0·1 to 0·4)	0·3 (0·2 to 0·6)	0·9 (0·3 to 1·7)	1·8 (0·8 to 3·3)	3·4 (1·6 to 6·2)	6·2 (3·2 to 10·8)	8·9 (4·4 to 16·2)	9·3 (4·6 to 16·5)	8·2 (4·4 to 12·9)	6·8 (3·8 to 10·4)	5·8 (3·2 to 9·2)	4·1 (2·5 to 6·3)	1·5 (0·9 to 2·1)	0·5 (0·3 to 0·8)	0·1 (0·1 to 0·1)	58·2
Liver cancer due to alcohol use	0 (0 to 0)	0 (0 to 0)	0 (0 to 0)	0 (0 to 0)	0 (0 to 0)	0 (0 to 0)	0 (0 to 0·1)	0·3 (0·1 to 0·5)	0·8 (0·3 to 1·6)	1·4 (0·7 to 2·5)	3·3 (1·5 to 6·1)	5·8 (3 to 10·1)	9 (4·3 to 15·7)	13·6 (6·8 to 23·8)	17·3 (9·1 to 28·4)	17·5 (9·9 to 27·3)	15·8 (9·6 to 24)	11·8 (7 to 18·7)	7·6 (4·8 to 11·2)	3·6 (2·2 to 5·6)	1 (0·6 to 1·5)	0·3 (0·1 to 0·4)	0 (0 to 0·1)	109
Liver cancer due to NASH	0 (0 to 0)	0 (0 to 0)	0 (0 to 0)	0 (0 to 0)	0 (0 to 0)	0 (0 to 0)	0·3 (0·2 to 0·5)	0·5 (0·3 to 0·9)	0·6 (0·3 to 1·1)	0·6 (0·3 to 1·1)	1 (0·5 to 1·8)	1·5 (0·7 to 2·6)	2·2 (1·1 to 3·9)	3·3 (1·8 to 5·7)	4 (2·1 to 7·1)	4 (2·1 to 6·8)	3·5 (1·8 to 6·1)	2·9 (1·6 to 5·1)	2·6 (1·5 to 4·4)	1·7 (0·9 to 2·8)	0·5 (0·3 to 0·8)	0·2 (0·1 to 0·3)	0 (0 to 0·1)	29·4
Liver cancer due to other causes	0·1 (0 to 0·1)	0·1 (0 to 0·1)	0·3 (0·2 to 0·5)	0·9 (0·6 to 1·3)	1·1 (0·8 to 1·4)	1·3 (1 to 1·7)	0·4 (0·2 to 0·7)	0·7 (0·4 to 1·1)	0·8 (0·4 to 1·4)	0·9 (0·4 to 1·5)	1·3 (0·7 to 2·4)	1·7 (0·9 to 3·1)	2·2 (1·1 to 4)	2·8 (1·4 to 5·1)	3 (1·5 to 5·2)	2·5 (1·3 to 4·5)	1·9 (1 to 3·1)	1·4 (0·7 to 2·3)	1·1 (0·5 to 1·8)	0·6 (0·3 to 1·1)	0·2 (0·1 to 0·3)	0·1 (0 to 0·1)	0 (0 to 0)	25·4
Gallbladder and biliary tract cancer	0 (0 to 0)	0 (0 to 0)	0 (0 to 0)	0 (0 to 0)	0 (0 to 0)	0 (0 to 0)	0 (0 to 0)	0·6 (0·4 to 0·8)	1·3 (1 to 1·8)	2·4 (1·8 to 3·4)	5·2 (3·7 to 7·7)	9·2 (6·5 to 14·1)	13·9 (9·8 to 21·5)	21·3 (14·9 to 32·7)	26 (18·6 to 40·7)	26·7 (19·4 to 42·8)	22 (15·8 to 34·7)	16·6 (12·1 to 26·9)	12·3 (9·1 to 19·6)	7·3 (5·3 to 11·9)	3 (2·2 to 4·9)	1·1 (0·8 to 1·8)	0·3 (0·2 to 0·5)	169
Pancreatic cancer	0 (0 to 0)	0 (0 to 0)	0 (0 to 0)	0 (0 to 0)	0 (0 to 0)	0 (0 to 0)	0·8 (0·6 to 1)	1·2 (0·9 to 1·6)	2·8 (1·9 to 4)	8 (5·3 to 11·3)	14·8 (9·6 to 21·7)	27·9 (19 to 39·3)	42·5 (30·9 to 56·4)	58·1 (43·6 to 76·4)	69·1 (49·7 to 93·1)	68·7 (49·3 to 93·2)	56·6 (40·6 to 75·8)	42·8 (30·4 to 58·7)	29·9 (21·2 to 40·8)	17·9 (12·9 to 24·1)	7·8 (5·9 to 10)	2·8 (2·1 to 3·4)	0·8 (0·5 to 1)	452
Larynx cancer	0 (0 to 0)	0 (0 to 0)	0 (0 to 0)	0 (0 to 0)	0 (0 to 0)	0 (0 to 0)	0 (0 to 0)	1 (0·7 to 1·4)	1·1 (0·9 to 1·4)	1·9 (1·4 to 2·5)	5·3 (3·7 to 7·3)	12·9 (8·9 to 18)	26·3 (17·7 to 39·5)	42·6 (30·4 to 58·3)	48·7 (35·9 to 66·5)	45·8 (34·1 to 59·8)	34·4 (25·4 to 45·5)	21·5 (16 to 28·2)	13·9 (10·1 to 18·5)	7·1 (5·4 to 9·4)	2·9 (2·1 to 3·8)	0·9 (0·7 to 1·2)	0·2 (0·1 to 0·3)	266
Tracheal, bronchus, and lung cancer	0 (0 to 0)	0 (0 to 0)	0 (0 to 0)	0 (0 to 0)	0 (0 to 0)	0·5 (0·3 to 0·9)	3·5 (2·6 to 4·5)	5·2 (3·7 to 7·1)	8·5 (5·8 to 12)	20·6 (14·8 to 28·4)	42 (28·7 to 59·2)	79·1 (57·1 to 106)	140 (94·8 to 195)	203 (141 to 283)	252 (183 to 343)	245 (179 to 324)	191 (145 to 252)	130 (99·2 to 166)	86·5 (67·2 to 110)	46·4 (35·4 to 60·4)	19·7 (15·1 to 24·9)	6·5 (5 to 7·9)	1·7 (1·2 to 2·2)	1,480
Malignant skin melanoma	0 (0 to 0)	0 (0 to 0)	0·2 (0 to 0·7)	0·7 (0·1 to 2·9)	0·3 (0·2 to 0·5)	0·4 (0·3 to 0·7)	1 (0·7 to 1·5)	2·7 (1·8 to 3·9)	4·8 (3 to 6·9)	6·7 (4·3 to 9·7)	12 (7·9 to 17·6)	12·4 (8·3 to 18·3)	13·3 (9 to 19·6)	15·2 (10·3 to 22·2)	14·8 (10·1 to 22·7)	11·2 (8 to 16·5)	8·5 (5·9 to 11·8)	6·3 (4·5 to 9·4)	4·4 (3·2 to 6·1)	3·1 (2·2 to 4·6)	1·6 (1·1 to 2·2)	0·6 (0·4 to 0·8)	0·3 (0·2 to 0·4)	121
Non-melanoma skin cancer	0 (0 to 0)	0 (0 to 0)	0 (0 to 0)	0 (0 to 0)	0 (0 to 0)	0 (0 to 0)	0 (0 to 0)	0·6 (0·4 to 0·8)	1·5 (1 to 2·1)	2·1 (1·4 to 3)	2·7 (1·8 to 3·9)	4·1 (2·9 to 5·8)	4·4 (3·1 to 6·3)	6·3 (4·4 to 8·8)	6·5 (4·7 to 8·9)	6·5 (4·8 to 8·6)	5·6 (4·2 to 7·3)	4·4 (3·2 to 5·8)	4 (3·1 to 5·2)	3·3 (2·5 to 4·3)	2·1 (1·6 to 2·7)	1·3 (1 to 1·7)	0·6 (0·4 to 0·8)	56
Non-melanoma skin cancer (squamous-cell carcinoma)	0 (0 to 0)	0 (0 to 0)	0 (0 to 0)	0 (0 to 0)	0 (0 to 0)	0 (0 to 0)	0 (0 to 0)	0·6 (0·4 to 0·8)	1·5 (1 to 2·1)	2·1 (1·4 to 3)	2·7 (1·8 to 3·9)	4·1 (2·9 to 5·8)	4·4 (3·1 to 6·3)	6·3 (4·4 to 8·8)	6·5 (4·7 to 8·9)	6·5 (4·8 to 8·6)	5·6 (4·2 to 7·3)	4·4 (3·2 to 5·8)	4 (3·1 to 5·2)	3·3 (2·5 to 4·3)	2·1 (1·6 to 2·7)	1·3 (1 to 1·7)	0·6 (0·4 to 0·8)	56
Non-melanoma skin cancer (basal-cell carcinoma)	0 (0 to 0)	0 (0 to 0)	0 (0 to 0)	0 (0 to 0)	0 (0 to 0)	0 (0 to 0)	0 (0 to 0)	0 (0 to 0)	0 (0 to 0)	0 (0 to 0)	0 (0 to 0)	0 (0 to 0)	0 (0 to 0)	0 (0 to 0)	0 (0 to 0)	0 (0 to 0)	0 (0 to 0)	0 (0 to 0)	0 (0 to 0)	0 (0 to 0)	0 (0 to 0)	0 (0 to 0)	0 (0 to 0)	0
Breast cancer	0 (0 to 0)	0 (0 to 0)	0 (0 to 0)	0 (0 to 0)	0 (0 to 0)	0 (0 to 0)	3·3 (2·5 to 4·3)	7·2 (5 to 10·2)	31·6 (20·9 to 47·3)	112 (76 to 160)	203 (143 to 274)	271 (189 to 376)	365 (260 to 491)	378 (271 to 510)	350 (246 to 483)	287 (218 to 378)	193 (143 to 253)	128 (95·1 to 170)	91·7 (67·7 to 120)	58·9 (43·9 to 76·2)	28·2 (21·2 to 36·2)	11 (8·2 to 13·8)	4·5 (3 to 5·8)	2,520
Cervical cancer	0 (0 to 0)	0 (0 to 0)	0 (0 to 0)	0 (0 to 0)	0 (0 to 0)	0 (0 to 0)	2 (1·4 to 2·9)	8·2 (5·2 to 12·7)	24·6 (15 to 38·7)	52·2 (33·1 to 79·4)	89·7 (60·2 to 129)	92·4 (62·8 to 135)	116 (80·7 to 168)	119 (82 to 167)	103 (73·8 to 141)	75·3 (54·5 to 102)	53·2 (37·6 to 72·5)	33·6 (24·8 to 45)	22·4 (17 to 29)	13·6 (9·6 to 18·7)	5 (3·5 to 6·6)	1·9 (1·3 to 2·7)	0·7 (0·4 to 1·1)	813
Uterine cancer	0 (0 to 0)	0 (0 to 0)	0 (0 to 0)	0 (0 to 0)	0 (0 to 0)	0 (0 to 0)	0 (0 to 0)	1·8 (1·3 to 2·4)	4·4 (2·8 to 6·5)	9·2 (6 to 13·3)	15·7 (9·9 to 23·4)	20·4 (13·4 to 30·2)	31·2 (20·8 to 44·8)	38·1 (25·9 to 54·2)	42·2 (30·3 to 58·6)	50 (35·9 to 67·9)	40·4 (29·4 to 54)	26·9 (19·9 to 35·7)	16·8 (12·2 to 22·3)	10 (7·1 to 13·5)	3·6 (2·6 to 4·7)	1·4 (1 to 1·8)	0·5 (0·3 to 0·7)	313
Ovarian cancer	0 (0 to 0)	0 (0 to 0)	0 (0 to 0)	0 (0 to 0)	1·4 (0·6 to 2·4)	2·5 (1·3 to 4·2)	7·1 (3·8 to 10·7)	10·3 (5·8 to 16·2)	10·7 (6·1 to 16·4)	17·5 (9·7 to 27·4)	29 (16·9 to 43·4)	44·1 (27·5 to 62·2)	60·7 (38·4 to 86)	69·6 (46·3 to 98·4)	71·4 (47·9 to 101)	61·2 (39·7 to 85·5)	45·5 (30·1 to 63·2)	28·2 (19·1 to 38·7)	18·4 (12·3 to 25·2)	10·8 (7·2 to 14·6)	4·4 (3 to 5·7)	1·6 (1·1 to 2·1)	0·4 (0·2 to 0·5)	495
Prostate cancer	0 (0 to 0)	0 (0 to 0)	0 (0 to 0)	0 (0 to 0)	0 (0 to 0)	0 (0 to 0)	0 (0 to 0)	1·1 (0·6 to 1·4)	2·9 (1·5 to 3·9)	3·8 (1·9 to 5·1)	4·9 (2·8 to 6·8)	11·2 (6·9 to 15·9)	31·1 (19·9 to 46·7)	78·3 (52·6 to 109)	146 (97·8 to 208)	252 (180 to 365)	285 (200 to 406)	312 (222 to 433)	296 (213 to 417)	219 (158 to 311)	113 (82·8 to 168)	46·6 (33·8 to 69·3)	12·7 (8·5 to 19·3)	1,820
Testicular cancer	0 (0 to 0·1)	0 (0 to 0·1)	0 (0 to 0·1)	0·1 (0·1 to 0·3)	0·1 (0·1 to 0·2)	0·1 (0·1 to 0·2)	0·4 (0·2 to 0·6)	0·7 (0·4 to 1·1)	1 (0·6 to 1·6)	0·6 (0·4 to 0·9)	0·5 (0·3 to 0·8)	0·4 (0·2 to 0·6)	0·2 (0·1 to 0·4)	0·2 (0·1 to 0·3)	0·1 (0·1 to 0·2)	0·1 (0·1 to 0·2)	0·1 (0·1 to 0·1)	0·1 (0 to 0·1)	0·1 (0 to 0·1)	0 (0 to 0)	0 (0 to 0)	0 (0 to 0)	0 (0 to 0)	4·9
Kidney cancer	0·1 (0·1 to 0·2)	0·1 (0 to 0·1)	0·9 (0·6 to 1·4)	4·7 (2·9 to 7·3)	4·4 (3·2 to 5·9)	2·4 (1·8 to 3·4)	3·6 (2·4 to 5)	5·8 (4 to 8)	8·1 (5·5 to 11·3)	9·8 (6 to 14·8)	11·6 (7·4 to 16·9)	17·7 (11·6 to 25·4)	24·7 (17·8 to 34)	33 (24·1 to 43·5)	36·2 (25·3 to 50·9)	32·2 (23·2 to 42·8)	24·4 (17·9 to 32·6)	16·2 (11·8 to 21·8)	10·1 (7·5 to 13·3)	5·4 (3·9 to 7·2)	1·9 (1·3 to 2·5)	0·7 (0·5 to 1)	0·2 (0·1 to 0·3)	254
Bladder cancer	0 (0 to 0)	0 (0 to 0)	0 (0 to 0)	0 (0 to 0)	0 (0 to 0)	0 (0 to 0)	0·2 (0·2 to 0·3)	0·4 (0·3 to 0·5)	0·5 (0·4 to 0·7)	1·6 (1·1 to 2·4)	2·6 (1·7 to 3·7)	4·6 (3·1 to 6·5)	7·2 (5·1 to 9·6)	13·1 (9·6 to 17·7)	17·9 (12·6 to 24·5)	21·1 (15·5 to 28·1)	20·2 (14·7 to 27)	17·3 (12·4 to 23·8)	14·8 (10·7 to 20)	10 (7·2 to 13·4)	5·1 (3·8 to 6·7)	2·1 (1·6 to 2·8)	0·7 (0·4 to 0·9)	139
Brain and central nervous system cancer	0·4 (0·1 to 0·6)	0·4 (0·2 to 0·8)	2·8 (1·2 to 4·6)	10·4 (4·7 to 16·9)	17·3 (9·2 to 25·3)	16 (7·9 to 23·9)	12·3 (5·4 to 18·8)	14·1 (5·6 to 22·5)	17·9 (7 to 28·7)	20·7 (8·5 to 31·4)	27·2 (12 to 41·4)	29·4 (13·5 to 44·2)	29·7 (14·1 to 43·8)	33·3 (15·6 to 48·4)	32·9 (15·8 to 48·8)	25·4 (12·1 to 37)	18·2 (8·9 to 26·3)	10·5 (4·9 to 14·8)	6·4 (2·9 to 9·2)	3 (1·4 to 4·4)	1·1 (0·5 to 1·7)	0·4 (0·2 to 0·5)	0·1 (0 to 0·1)	330
Thyroid cancer	0 (0 to 0)	0 (0 to 0)	0 (0 to 0)	0 (0 to 0)	0·3 (0·2 to 0·3)	0·3 (0·2 to 0·4)	0·7 (0·5 to 0·9)	1·1 (0·8 to 1·5)	2 (1·4 to 2·7)	2·5 (1·8 to 3·3)	4·3 (3 to 6)	5·2 (3·7 to 7·2)	7·6 (5·4 to 10·5)	10·5 (7·5 to 14·3)	10·8 (7·9 to 14·5)	9·2 (7 to 11·9)	7·4 (5·6 to 9·8)	5·5 (4·2 to 7)	3·8 (2·9 to 4·8)	2·3 (1·8 to 3)	0·9 (0·7 to 1·1)	0·3 (0·3 to 0·4)	0·1 (0·1 to 0·1)	74·5
Mesothelioma	0 (0 to 0)	0 (0 to 0)	0 (0 to 0)	0 (0 to 0)	0 (0 to 0)	0 (0 to 0)	0 (0 to 0)	0·9 (0·6 to 1·2)	1·2 (0·8 to 1·7)	1·3 (0·9 to 1·7)	2·4 (1·6 to 3·5)	2·6 (1·9 to 3·5)	3·8 (2·7 to 5·3)	5 (3·7 to 6·8)	5·5 (4 to 7·4)	2·2 (1·6 to 2·9)	1·5 (1·2 to 2·1)	1·4 (1 to 1·8)	1·1 (0·8 to 1·5)	0·6 (0·5 to 0·8)	0·3 (0·2 to 0·4)	0·1 (0·1 to 0·1)	0 (0 to 0)	30
Hodgkin lymphoma	0 (0 to 0)	0 (0 to 0)	0 (0 to 0)	0·2 (0·1 to 0·2)	0·3 (0·2 to 0·4)	0·7 (0·5 to 0·9)	4 (2·7 to 5·7)	7·3 (4·9 to 10·4)	7·4 (4·8 to 10·5)	6·3 (4·2 to 8·9)	5·7 (3·8 to 8)	5·3 (3·6 to 7·3)	4 (2·7 to 5·7)	3·9 (2·7 to 5·3)	3·5 (2·5 to 5)	3 (2·2 to 4)	2·3 (1·7 to 3·3)	1·4 (1 to 2)	1·2 (0·8 to 1·6)	0·6 (0·4 to 0·9)	0·3 (0·2 to 0·4)	0·1 (0 to 0·1)	0 (0 to 0)	57·3
Non-Hodgkin lymphoma	0 (0 to 0)	0 (0 to 0)	0 (0 to 0)	5·9 (3·6 to 8·9)	9·1 (6 to 13·7)	10·9 (7·2 to 16)	18·9 (12·8 to 26·4)	30·3 (21 to 43·1)	38·1 (27·5 to 51·7)	42·2 (29·9 to 58·9)	45·5 (33·4 to 61·2)	43·6 (31·2 to 58·3)	49·5 (36·5 to 67·1)	54·5 (40·6 to 72·4)	55·9 (41·3 to 73·5)	49·4 (37·1 to 65·4)	37·7 (27·3 to 51·6)	26·4 (18·9 to 36·3)	17·8 (13·1 to 23·9)	9·3 (6·9 to 12·7)	3·2 (2·4 to 4·2)	1·1 (0·8 to 1·3)	0·3 (0·2 to 0·4)	550
Multiple myeloma	0 (0 to 0)	0 (0 to 0)	0 (0 to 0)	0 (0 to 0)	0 (0 to 0)	0 (0 to 0)	0 (0 to 0)	1·6 (1 to 2·1)	2·5 (1·7 to 3·4)	4·2 (2·9 to 5·8)	10·5 (7·2 to 14·8)	22·7 (15·9 to 30·6)	40·1 (28·2 to 55)	64·5 (46·9 to 87·5)	79·8 (58 to 107)	81·2 (61·4 to 108)	67·1 (51·5 to 87·9)	49·5 (37·9 to 65·1)	34·2 (26·9 to 43·7)	18·4 (13·8 to 24·2)	6·7 (5·1 to 8·8)	1·9 (1·4 to 2·6)	0·5 (0·3 to 0·6)	486
Leukemia	0·5 (0·3 to 0·7)	0·6 (0·3 to 1)	4·2 (2·8 to 6·2)	16·4 (10·3 to 25·2)	26·4 (19·5 to 35·6)	29·2 (20·9 to 40·4)	35·9 (26·5 to 47·1)	38·6 (27·9 to 52·1)	40·6 (29·6 to 54·1)	36·4 (26·4 to 49·4)	40·4 (28·6 to 54·4)	38·9 (28·5 to 51·9)	39·7 (29·4 to 53)	39·2 (29·7 to 51·1)	38·8 (28·8 to 50·7)	34·3 (26·5 to 43·7)	27·5 (21·7 to 35·2)	20·7 (15·8 to 26·3)	14·9 (11·8 to 18·5)	9·3 (7·3 to 11·9)	4·2 (3·3 to 5·2)	1·5 (1·2 to 1·9)	0·5 (0·3 to 0·6)	539
Acute lymphoid leukemia	0·1 (0·1 to 0·2)	0·1 (0 to 0·1)	1·2 (0·7 to 1·9)	7·8 (4·7 to 12·3)	14·1 (9·7 to 20·2)	12·3 (8·2 to 18·2)	14·2 (9·6 to 20·1)	9·4 (6·1 to 14·1)	5·9 (3·8 to 9)	4·7 (3·1 to 6·9)	4·1 (2·6 to 6·3)	3·8 (2·5 to 5·7)	2·7 (1·8 to 4)	2·5 (1·6 to 3·7)	2·2 (1·5 to 3·2)	1·4 (1 to 2·1)	1·4 (1 to 2)	0·9 (0·6 to 1·3)	0·6 (0·4 to 0·8)	0·4 (0·2 to 0·5)	0·1 (0·1 to 0·2)	0·1 (0 to 0·1)	0 (0 to 0)	90·1
Chronic lymphoid leukemia	0 (0 to 0)	0 (0 to 0)	0 (0 to 0)	0 (0 to 0)	0 (0 to 0)	0 (0 to 0)	0 (0 to 0)	1 (0·6 to 1·5)	0·9 (0·6 to 1·3)	0·8 (0·5 to 1·2)	1·6 (1 to 2·4)	2·3 (1·5 to 3·2)	1·6 (1 to 2·3)	4·7 (3·2 to 6·6)	6·7 (4·6 to 9·3)	7·5 (5·5 to 10·2)	7·1 (5·2 to 9·4)	5·2 (3·7 to 6·9)	4·1 (3 to 5·5)	2·7 (1·9 to 3·6)	1·2 (0·9 to 1·6)	0·5 (0·4 to 0·6)	0·1 (0·1 to 0·2)	47·9
Acute myeloid leukemia	0·1 (0·1 to 0·2)	0·4 (0·2 to 0·7)	1·7 (1 to 2·7)	6·6 (3·9 to 10·6)	8·9 (5·9 to 13)	12·7 (8·6 to 18·8)	16·4 (11·7 to 23)	17·4 (11·9 to 23·7)	20·6 (14·6 to 28·1)	16·7 (11·5 to 23·2)	19·1 (12·6 to 26·6)	16·1 (11·3 to 22·2)	18·7 (13·2 to 25·8)	16·2 (11·6 to 21·8)	14·4 (10·2 to 19·7)	11·5 (8·4 to 15·6)	8 (5·8 to 10·8)	5·6 (4 to 7·5)	3·5 (2·5 to 4·8)	1·9 (1·4 to 2·7)	0·7 (0·5 to 1)	0·2 (0·1 to 0·3)	0·1 (0 to 0·1)	218
Chronic myeloid leukemia	0 (0 to 0)	0 (0 to 0)	0·2 (0·1 to 0·3)	0·5 (0·3 to 0·9)	0·4 (0·3 to 0·7)	0·5 (0·3 to 0·7)	1 (0·6 to 1·5)	3·4 (2·1 to 5·3)	5·4 (3·5 to 7·9)	6 (3·9 to 8·7)	6·7 (4·3 to 9·8)	5·6 (3·6 to 8·1)	5·7 (3·7 to 8·3)	6·3 (4·3 to 8·8)	6·1 (4·2 to 8·5)	5·6 (3·8 to 7·8)	4·1 (2·8 to 5·7)	2·9 (2 to 4)	2·2 (1·5 to 3)	1·3 (0·9 to 1·9)	0·5 (0·4 to 0·7)	0·2 (0·1 to 0·3)	0·1 (0 to 0·1)	64·6
Other leukemia	0·2 (0·1 to 0·3)	0·1 (0·1 to 0·2)	1·1 (0·7 to 1·7)	1·6 (1 to 2·7)	2·9 (1·8 to 4·5)	3·8 (2·3 to 6)	4·3 (2·7 to 6·4)	7·3 (4·9 to 10·8)	7·7 (5·2 to 11)	8·2 (5·5 to 11·8)	8·9 (6·1 to 12·5)	11·1 (7·7 to 15·7)	11 (7·5 to 15·6)	9·5 (6·8 to 13)	9·5 (6·5 to 12·9)	8·2 (6 to 10·9)	6·9 (5·2 to 9·2)	6·1 (4·4 to 8·2)	4·5 (3·2 to 6)	3 (2·2 to 4)	1·6 (1·2 to 2·1)	0·6 (0·4 to 0·8)	0·2 (0·1 to 0·2)	119
Other malignant neoplasms	0·8 (0·5 to 1·4)	0·9 (0·5 to 1·6)	6·1 (4·3 to 8·4)	22·9 (15·1 to 34·5)	20·3 (16 to 26·7)	24·8 (19·4 to 32·1)	33·1 (25·7 to 42·3)	35·8 (28·1 to 46·3)	38·3 (29·4 to 50·2)	41·2 (31·3 to 54·6)	46·6 (35 to 61·1)	53·9 (40·2 to 71)	60·4 (44·8 to 78·7)	68·7 (52·6 to 91·4)	67·7 (51·2 to 87·5)	57·7 (44·7 to 73·4)	44·1 (34·5 to 55)	30·9 (24·5 to 38·3)	22·7 (18·1 to 28·4)	14·8 (11·5 to 18·6)	7·6 (5·8 to 9·7)	3·1 (2·4 to 3·9)	0·9 (0·6 to 1·2)	703

Other neoplasms	0·1 (0·1 to 0·2)	0·2 (0·1 to 0·4)	0·7 (0·3 to 1·2)	2 (1·1 to 3·3)	1·5 (0·9 to 2·3)	1·8 (1·1 to 2·5)	2·1 (1·2 to 3·2)	1·7 (1·1 to 2·5)	3·4 (2 to 5·1)	3 (1·9 to 4·5)	3·5 (2·1 to 5·4)	5 (3·3 to 7·1)	4·9 (3·3 to 7·4)	7·5 (4·9 to 11)	8·5 (5·7 to 12·7)	9·5 (6·5 to 14·6)	8·5 (6·3 to 12·6)	8·1 (5·7 to 12·8)	6·8 (5 to 10·3)	4·3 (3·1 to 6·5)	2 (1·4 to 3·1)	0·7 (0·5 to 1·1)	0·2 (0·1 to 0·2)	85·8
Myelodysplastic, myeloproliferative, and other hematopoietic r	0·1 (0·1 to 0·2)	0·2 (0·1 to 0·4)	0·7 (0·3 to 1·2)	2 (1·1 to 3·3)	1·5 (0·9 to 2·3)	1·8 (1·1 to 2·5)	2·1 (1·2 to 3·2)	1·7 (1·1 to 2·5)	3·4 (2 to 5·1)	3 (1·9 to 4·5)	3·5 (2·1 to 5·4)	5 (3·3 to 7·1)	4·9 (3·3 to 7·4)	7·5 (4·9 to 11)	8·5 (5·7 to 12·7)	9·5 (6·5 to 14·6)	8·5 (6·3 to 12·6)	8·1 (5·7 to 12·8)	6·8 (5 to 10·3)	4·3 (3·1 to 6·5)	2 (1·4 to 3·1)	0·7 (0·5 to 1·1)	0·2 (0·1 to 0·2)	85·8
Benign and in situ intestinal neoplasms	0 (0 to 0)	0 (0 to 0)	0 (0 to 0)	0 (0 to 0)	0 (0 to 0)	0 (0 to 0)	0 (0 to 0)	0 (0 to 0)	0 (0 to 0)	0 (0 to 0)	0 (0 to 0)	0 (0 to 0)	0 (0 to 0)	0 (0 to 0)	0 (0 to 0)	0 (0 to 0)	0 (0 to 0)	0 (0 to 0)	0 (0 to 0)	0 (0 to 0)	0 (0 to 0)	0 (0 to 0)	0 (0 to 0)	0
Benign and in situ cervical and uterine neoplasms	0 (0 to 0)	0 (0 to 0)	0 (0 to 0)	0 (0 to 0)	0 (0 to 0)	0 (0 to 0)	0 (0 to 0)	0 (0 to 0)	0 (0 to 0)	0 (0 to 0)	0 (0 to 0)	0 (0 to 0)	0 (0 to 0)	0 (0 to 0)	0 (0 to 0)	0 (0 to 0)	0 (0 to 0)	0 (0 to 0)	0 (0 to 0)	0 (0 to 0)	0 (0 to 0)	0 (0 to 0)	0 (0 to 0)	0
Other benign and in situ neoplasms	0 (0 to 0)	0 (0 to 0)	0 (0 to 0)	0 (0 to 0)	0 (0 to 0)	0 (0 to 0)	0 (0 to 0)	0 (0 to 0)	0 (0 to 0)	0 (0 to 0)	0 (0 to 0)	0 (0 to 0)	0 (0 to 0)	0 (0 to 0)	0 (0 to 0)	0 (0 to 0)	0 (0 to 0)	0 (0 to 0)	0 (0 to 0)	0 (0 to 0)	0 (0 to 0)	0 (0 to 0)	0 (0 to 0)	0
Cardiovascular diseases	5·4 (3·7 to 7·7)	3·2 (1·8 to 5·6)	17·1 (11·6 to 24·9)	13·1 (9·2 to 17·8)	23·9 (18·8 to 30·1)	46 (35 to 59·1)	118 (92·9 to 149)	196 (152 to 254)	307 (238 to 397)	473 (365 to 611)	784 (608 to 1,010)	1,190 (915 to 1,550)	1,720 (1,330 to 2,250)	2,240 (1,770 to 2,850)	2,500 (2,010 to 3,130)	2,530 (2,060 to 3,110)	2,240 (1,870 to 2,710)	1,820 (1,530 to 2,160)	1,650 (1,400 to 1,940)	1,310 (1,110 to 1,520)	753 (630 to 873)	365 (296 to 422)	165 (119 to 197)	20,500
Rheumatic heart disease	0 (0 to 0)	0 (0 to 0)	0 (0 to 0)	0·9 (0·4 to 1·6)	5·6 (3·2 to 9·3)	14·2 (8·3 to 23·6)	22·7 (14·7 to 33·9)	27·2 (18 to 39·6)	31·8 (21·8 to 44·8)	33·1 (23·3 to 46·2)	35·6 (25·4 to 49·1)	34·8 (24·7 to 48·7)	30·5 (21·7 to 42·8)	25·1 (17·9 to 34·7)	19 (13·5 to 25·9)	11·8 (8·6 to 16)	7·5 (5·5 to 10·1)	4·5 (3·3 to 6)	3·3 (2·5 to 4·3)	1·9 (1·4 to 2·5)	0·9 (0·7 to 1·2)	0·4 (0·3 to 0·6)	0·2 (0·1 to 0·3)	311
Ischemic heart disease	0 (0 to 0)	0 (0 to 0)	0 (0 to 0)	0 (0 to 0)	0 (0 to 0)	0 (0 to 0)	9·4 (6·7 to 12·9)	25·9 (17·9 to 36·7)	56·1 (38·7 to 78·2)	110 (78·2 to 151)	209 (152 to 281)	376 (275 to 514)	598 (443 to 811)	852 (653 to 1,100)	992 (771 to 1,270)	1,040 (815 to 1,310)	905 (740 to 1,110)	700 (575 to 850)	583 (472 to 707)	461 (374 to 547)	275 (222 to 332)	134 (108 to 161)	61·7 (43·6 to 76·2)	7,390
Stroke	1·2 (0·6 to 2·1)	0·3 (0·2 to 0·6)	1·6 (0·8 to 2·9)	1·8 (1·3 to 2·4)	5·1 (3·8 to 6·7)	9·5 (7 to 12·7)	26·7 (19 to 36·8)	49·9 (33·7 to 72·6)	71·5 (47·8 to 105)	117 (79·4 to 165)	194 (132 to 273)	296 (201 to 416)	415 (296 to 568)	566 (401 to 791)	594 (436 to 789)	597 (462 to 760)	570 (426 to 742)	487 (377 to 619)	490 (400 to 600)	364 (282 to 453)	194 (152 to 235)	83·6 (64·7 to 102)	33·9 (23·4 to 43·8)	5,170
Ischemic stroke	0·2 (0·1 to 0·4)	0 (0 to 0·1)	0·1 (0·1 to 0·2)	0·9 (0·7 to 1·3)	2·6 (1·7 to 3·9)	4·2 (2·6 to 6·3)	6·8 (4·8 to 9·5)	10·2 (7·4 to 13·9)	13·7 (9·9 to 18·7)	20·6 (14·3 to 28)	30·4 (21·8 to 41·3)	45·9 (33·9 to 61)	66·3 (50 to 86·8)	99·6 (73 to 132)	122 (91·9 to 160)	180 (138 to 231)	184 (137 to 235)	245 (190 to 311)	268 (217 to 325)	262 (203 to 327)	146 (114 to 177)	63·4 (48·5 to 78·4)	26·6 (18·2 to 34·2)	1,800
Intracerebral hemorrhage	0·3 (0·2 to 0·6)	0·1 (0 to 0·2)	0·8 (0·4 to 1·6)	0·3 (0·2 to 0·5)	1·3 (0·9 to 1·8)	2·6 (1·8 to 3·5)	11·4 (7·5 to 16·7)	24·4 (15·2 to 36·7)	37 (23·1 to 57·3)	66 (42·1 to 98·4)	115 (74·7 to 169)	185 (121 to 267)	280 (193 to 389)	389 (270 to 561)	399 (288 to 541)	371 (280 to 482)	350 (256 to 463)	221 (166 to 284)	206 (164 to 259)	91·7 (69·6 to 117)	43·9 (33·4 to 55·2)	18·4 (14 to 23)	6·5 (4·4 to 8·5)	2,820
Subarachnoid hemorrhage	0·7 (0·3 to 1·2)	0·2 (0·1 to 0·4)	0·7 (0·3 to 1·3)	0·5 (0·3 to 0·8)	1·2 (0·8 to 1·8)	2·8 (1·8 to 4·1)	8·5 (5·3 to 13·2)	15·2 (9·2 to 24·8)	20·7 (11·9 to 33·2)	29·9 (18·2 to 46·2)	48·6 (30·7 to 72·3)	65·1 (42·1 to 96·4)	68·8 (45·8 to 101)	76·8 (51·4 to 111)	73 (48·5 to 106)	45·7 (31·6 to 62·8)	36·8 (25·2 to 51·2)	20·9 (14·5 to 28·8)	16·7 (12·3 to 22·6)	10·1 (7·2 to 13·9)	4·5 (3·3 to 6·1)	1·9 (1·3 to 2·5)	0·8 (0·5 to 1·2)	550
Hypertensive heart disease	0 (0 to 0)	0 (0 to 0)	0 (0 to 0)	0 (0 to 0)	0 (0 to 0)	0 (0 to 0)	6·8 (4·2 to 10·2)	16·2 (10·4 to 25)	29·1 (17·2 to 44·9)	65·5 (41·1 to 96·1)	133 (87·3 to 191)	221 (148 to 311)	368 (251 to 519)	474 (322 to 653)	552 (391 to 752)	548 (395 to 748)	476 (347 to 644)	401 (299 to 524)	362 (269 to 477)	311 (234 to 402)	170 (129 to 222)	86·9 (63·8 to 114)	40·5 (26·6 to 53·1)	4,260
Non-rheumatic valvular heart disease	0 (0 to 0)	0 (0 to 0)	0 (0 to 0)	0 (0 to 0)	0 (0 to 0)	0 (0 to 0)	3·7 (2·5 to 5·2)	4·1 (2·8 to 5·9)	6·4 (4·2 to 9·2)	8·2 (5·6 to 11·7)	10·2 (6·5 to 15·1)	12·8 (8·8 to 18·1)	15·3 (10·5 to 21·6)	14·5 (10·3 to 20)	15·8 (11·5 to 21·2)	15·1 (10·9 to 20)	12·6 (9·6 to 16·4)	8·8 (6·7 to 11·5)	7·4 (5·7 to 9·6)	5·5 (4·2 to 7·1)	3 (2·2 to 3·8)	1·3 (1 to 1·7)	0·5 (0·3 to 0·6)	145
Non-rheumatic calcific aortic valve disease	0 (0 to 0)	0 (0 to 0)	0 (0 to 0)	0 (0 to 0)	0 (0 to 0)	0 (0 to 0)	1·8 (1·1 to 2·7)	2·1 (1·4 to 3·3)	3·3 (2·1 to 5·2)	3·3 (2·1 to 5·2)	4·8 (2·8 to 7·7)	6·5 (4·1 to 9·8)	8·3 (5·5 to 12·2)	8·1 (5·5 to 11·5)	9·3 (6·5 to 13·2)	9·7 (6·8 to 13·3)	8·9 (6·5 to 11·9)	6·5 (4·8 to 8·5)	5·6 (4·2 to 7·4)	4·3 (3·2 to 5·6)	2·4 (1·8 to 3·1)	1 (0·7 to 1·4)	0·3 (0·2 to 0·5)	86·1
Non-rheumatic degenerative mitral valve disease	0 (0 to 0)	0 (0 to 0)	0 (0 to 0)	0 (0 to 0)	0 (0 to 0)	0 (0 to 0)	1·5 (0·9 to 2·4)	1·3 (0·8 to 2)	2·6 (1·6 to 3·9)	3·5 (2·2 to 5·2)	4·6 (2·7 to 7·2)	5·6 (3·6 to 8·5)	6 (3·7 to 9)	5·6 (3·6 to 8·3)	5·8 (3·8 to 8·4)	4·9 (3·2 to 7·3)	3·4 (2·2 to 5)	2 (1·3 to 3)	1·5 (1 to 2·2)	1 (0·6 to 1·4)	0·5 (0·3 to 0·7)	0·3 (0·2 to 0·4)	0·1 (0 to 0·2)	50·1
Other non-rheumatic valve diseases	0 (0 to 0)	0 (0 to 0)	0 (0 to 0)	0 (0 to 0)	0 (0 to 0)	0 (0 to 0)	0·4 (0·2 to 0·9)	0·7 (0·3 to 1·2)	0·5 (0·2 to 1)	1·4 (0·7 to 2·4)	0·9 (0·4 to 1·6)	0·7 (0·3 to 1·2)	1 (0·4 to 1·9)	0·9 (0·4 to 1·6)	0·6 (0·3 to 1·2)	0·4 (0·2 to 0·7)	0·4 (0·2 to 0·6)	0·4 (0·2 to 0·7)	0·3 (0·1 to 0·5)	0·3 (0·1 to 0·5)	0·1 (0·1 to 0·2)	0·1 (0 to 0·1)	0 (0 to 0·1)	8·9
Cardiomyopathy and myocarditis	1·3 (0·8 to 2)	1·2 (0·6 to 2·2)	7·5 (5 to 11·1)	5·3 (3·4 to 7·8)	5·3 (4 to 6·8)	8·6 (6·4 to 11·3)	18·5 (13·2 to 25·4)	28·2 (18·9 to 38·8)	47·8 (31·3 to 65·5)	64·1 (41·9 to 88·9)	96·1 (63 to 138)	115 (79·2 to 159)	134 (88·8 to 190)	137 (93·6 to 189)	135 (95·7 to 188)	111 (77·9 to 152)	83·3 (60·8 to 111)	55·1 (40·3 to 72·9)	42·4 (31·3 to 56·1)	28·9 (21·1 to 37·9)	18·5 (13·3 to 24·8)	7·8 (5·5 to 10·6)	3·4 (2·2 to 5)	1,160
Myocarditis	0·1 (0 to 0·2)	0·2 (0·1 to 0·3)	0·9 (0·5 to 1·6)	0·8 (0·4 to 1·4)	0·9 (0·6 to 1·4)	0·9 (0·5 to 1·3)	1·6 (0·9 to 2·7)	1·7 (1 to 2·9)	2·7 (1·4 to 4·7)	3·6 (1·8 to 6·4)	3·8 (1·9 to 6·7)	3·6 (2 to 6·1)	3·2 (1·8 to 5·5)	3·3 (1·8 to 5·8)	2·4 (1·3 to 4·3)	1·8 (1 to 3·1)	1·4 (0·8 to 2·4)	1 (0·6 to 1·6)	0·9 (0·5 to 1·5)	0·8 (0·4 to 1·3)	0·6 (0·3 to 1·1)	0·3 (0·2 to 0·5)	0·2 (0·1 to 0·4)	36·8
Alcoholic cardiomyopathy	0 (0 to 0)	0 (0 to 0)	0 (0 to 0)	0 (0 to 0)	0 (0 to 0)	0 (0 to 0)	1·2 (0·7 to 1·9)	2·3 (1·3 to 4)	6·5 (3·2 to 11·7)	14·7 (7·2 to 25)	22·1 (11·7 to 38·1)	32·3 (18·8 to 52·4)	47·7 (28·5 to 73·8)	49·5 (30·5 to 73·5)	45 (27·2 to 70·2)	33 (19·6 to 50·5)	24·2 (14·9 to 36)	11·1 (6·2 to 17·7)	7 (3·5 to 11·4)	1·6 (0·7 to 2·9)	0·6 (0·3 to 1·2)	0·2 (0·1 to 0·4)	0·1 (0 to 0·1)	299
Other cardiomyopathy	1·2 (0·7 to 1·8)	1·1 (0·6 to 1·9)	6·6 (4·3 to 9·8)	4·5 (2·8 to 6·8)	4·4 (3·1 to 5·7)	7·7 (5·6 to 10·3)	15·7 (11·1 to 21·6)	24·2 (16·2 to 33·6)	38·6 (25 to 54·3)	45·8 (29·5 to 65·4)	70·2 (44 to 102)	79·3 (53·9 to 113)	82·9 (52·8 to 121)	84·6 (56·3 to 122)	88·1 (61·5 to 123)	75·9 (52·3 to 106)	57·7 (40·9 to 79·1)	43 (31·1 to 57·6)	34·6 (25·4 to 46·3)	26·5 (19·4 to 35·4)	17·3 (12·5 to 23)	7·3 (5·1 to 9·9)	3·1 (2 to 4·7)	820
Atrial fibrillation and flutter	0 (0 to 0)	0 (0 to 0)	0 (0 to 0)	0 (0 to 0)	0 (0 to 0)	0 (0 to 0)	0 (0 to 0)	0 (0 to 0)	0 (0 to 0)	1·4 (0·9 to 2)	1·7 (1·1 to 2·4)	7·3 (5·3 to 10·1)	11·8 (8·4 to 16·7)	17·5 (12·9 to 23·3)	26·3 (19·8 to 34·7)	33·6 (25·7 to 44·8)	37·4 (29 to 48·2)	41·8 (33·2 to 52·5)	47·6 (37·6 to 59·4)	52 (41·5 to 64·9)	38·7 (30·7 to 49·1)	25·4 (19·6 to 32·7)	11·8 (8·3 to 15·7)	354
Aortic aneurysm	--	--	--	--	--	--	1·1 (0·7 to 1·6)	1·5 (1 to 2·4)	3·7 (2·3 to 5·5)	4·9 (3·4 to 7·1)	9·8 (6·5 to 14·1)	13·2 (8·9 to 18·9)	16·8 (11·3 to 25)	23·8 (15·7 to 34·2)	34·5 (23 to 48·6)	41·4 (29·2 to 57)	41·9 (29·3 to 58·5)	34·5 (23·4 to 48·2)	29·8 (21·1 to 41·3)	20·5 (14·1 to 28·8)	10 (7·1 to 13·5)	3·8 (2·7 to 5)	1·4 (0·9 to 1·9)	NA
Endocarditis	0·1 (0·1 to 0·3)	0·2 (0·1 to 0·4)	1·2 (0·7 to 2)	1·1 (0·6 to 1·7)	1·1 (0·7 to 1·8)	2·1 (1·3 to 3·2)	6 (3·7 to 9·5)	11·2 (6·9 to 16·7)	14·4 (8·2 to 23)	17·3 (9·7 to 28·5)	23·4 (13·3 to 36·3)	26·2 (15·8 to 40)	23·8 (14·3 to 38·3)	22·2 (13·8 to 33·2)	18·9 (11·9 to 27·9)	15·9 (10·3 to 23·7)	10·6 (7·1 to 15·7)	7·7 (5 to 11·2)	5·4 (3·8 to 7·9)	4·8 (3·1 to 7·2)	2·5 (1·5 to 3·9)	1·1 (0·7 to 1·7)	0·6 (0·3 to 1·3)	218
Other cardiovascular and circulatory diseases	2·7 (1·6 to 4·3)	1·5 (0·7 to 2·7)	6·8 (4 to 10·7)	4·1 (2·7 to 6·1)	6·8 (4·5 to 9·8)	11·7 (7·9 to 16·7)	23·1 (16·2 to 31·6)	31·4 (22·7 to 42·5)	46 (32·1 to 61·7)	51·9 (37 to 71·8)	71·8 (50·6 to 98·1)	89·2 (65·2 to 119)	98·4 (72·8 to 130)	100 (72·2 to 136)	101 (76 to 130)	94·8 (72·2 to 121)	77·8 (60·4 to 97·1)	61·8 (48·4 to 77·2)	56·3 (43·3 to 71·2)	43·3 (32·9 to 54·4)	28·1 (20·9 to 35·6)	13·2 (9·6 to 16·8)	6 (3·9 to 7·8)	1,030
Chronic respiratory diseases	0 (0 to 0)	0 (0 to 0)	0 (0 to 0)	126 (69·4 to 219)	136 (80·5 to 224)	126 (75·9 to 201)	101 (64·9 to 153)	95·7 (65·9 to 133)	101 (74·2 to 138)	101 (75 to 130)	108 (83·2 to 138)	113 (88·4 to 141)	129 (102 to 161)	155 (124 to 193)	172 (136 to 214)	173 (138 to 218)	157 (124 to 198)	137 (108 to 171)	120 (96 to 148)	83·4 (67·2 to 103)	45·6 (36·9 to 56·1)	22·5 (17·8 to 27·4)	9·7 (7·2 to 12·1)	2,210
Chronic obstructive pulmonary disease	0 (0 to 0)	0 (0 to 0)	0 (0 to 0)	1·3 (0·9 to 1·8)	2·2 (1·2 to 3·5)	3·9 (2·1 to 6·5)	7·1 (4·5 to 10·1)	11·3 (7·7 to 15·9)	15·1 (10·8 to 20·3)	18·1 (13·1 to 23·7)	23·7 (17·4 to 30·9)	31·8 (23·9 to 40·9)	46·8 (35·9 to 59·9)	69·3 (54·3 to 89)	90·4 (68·1 to 116)	110 (86·5 to 142)	108 (84·7 to 137)	97·8 (76·2 to 124)	89·9 (71·5 to 112)	64·7 (52·1 to 80·9)	36·6 (29·4 to 45·4)	18·3 (14·5 to 22·5)	7·7 (5·7 to 9·6)	854
Pneumoconiosis	0 (0 to 0)	0 (0 to 0)	0 (0 to 0)	0 (0 to 0)	0 (0 to 0)	0 (0 to 0)	0·5 (0·3 to 0·7)	0·1 (0·1 to 0·2)	0·4 (0·2 to 0·7)	0·1 (0·1 to 0·2)	0·3 (0·2 to 0·4)	0·2 (0·1 to 0·3)	0·5 (0·3 to 0·7)	0·6 (0·3 to 0·9)	0·4 (0·2 to 0·7)	0·5 (0·3 to 0·7)	0·6 (0·4 to 1)	0·4 (0·2 to 0·5)	0·3 (0·2 to 0·5)	0·3 (0·2 to 0·4)	0·2 (0·1 to 0·3)	0·1 (0·1 to 0·1)	0 (0 to 0)	5·5
Silicosis	0 (0 to 0)	0 (0 to 0)	0 (0 to 0)	0 (0 to 0)	0 (0 to 0)	0 (0 to 0)	0·2 (0·1 to 0·3)	0·1 (0 to 0·1)	0·4 (0·2 to 0·7)	0·1 (0 to 0·1)	0·2 (0·1 to 0·3)	0·1 (0 to 0·1)	0·4 (0·2 to 0·6)	0·3 (0·2 to 0·5)	0·2 (0·1 to 0·4)	0·3 (0·2 to 0·6)	0·4 (0·2 to 0·6)	0·2 (0·1 to 0·3)	0·2 (0·1 to 0·3)	0·2 (0·1 to 0·3)	0·1 (0·1 to 0·2)	0 (0 to 0·1)	0 (0 to 0)	3·3
Asbestosis	0 (0 to 0)	0 (0 to 0)	0 (0 to 0)	0 (0 to 0)	0 (0 to 0)	0 (0 to 0)	0·2 (0·1 to 0·4)	0 (0 to 0·1)	0 (0 to 0)	0 (0 to 0)	0 (0 to 0·1)	0 (0 to 0·1)	0 (0 to 0·1)	0·1 (0 to 0·2)	0·1 (0 to 0·2)	0·1 (0 to 0·1)	0·1 (0 to 0·2)	0·1 (0 to 0·1)	0·1 (0 to 0·1)	0 (0 to 0·1)	0 (0 to 0·1)	0 (0 to 0)	0 (0 to 0)	0·9
Coal workers pneumoconiosis	0 (0 to 0)	0 (0 to 0)	0 (0 to 0)	0 (0 to 0)	0 (0 to 0)	0 (0 to 0)	0 (0 to 0)	0 (0 to 0)	0 (0 to 0)	0 (0 to 0)	0 (0 to 0)	0 (0 to 0)	0 (0 to 0)	0 (0 to 0·1)	0 (0 to 0)	0 (0 to 0·1)	0 (0 to 0)	0 (0 to 0)	0 (0 to 0)	0 (0 to 0)	0 (0 to 0)	0 (0 to 0)	0 (0 to 0)	0·1
Other pneumoconiosis	0 (0 to 0)	0 (0 to 0)	0 (0 to 0)	0 (0 to 0)	0 (0 to 0)	0 (0 to 0)	0·1 (0 to 0·2)	0 (0 to 0·1)	0 (0 to 0)	0 (0 to 0)	0 (0 to 0·1)	0·1 (0·1 to 0·2)	0·1 (0 to 0·1)	0·1 (0·1 to 0·2)	0·1 (0 to 0·2)	0·1 (0 to 0·1)	0·2 (0·1 to 0·3)	0·1 (0 to 0·1)	0·1 (0 to 0·1)	0·1 (0 to 0·1)	0 (0 to 0·1)	0 (0 to 0)	0 (0 to 0)	1·1
Asthma	0 (0 to 0)	0 (0 to 0)	0 (0 to 0)	112 (56·4 to 203)	128 (72·4 to 216)	113 (63·7 to 187)	84·9 (50·8 to 136)	71·1 (43·7 to 107)	65·7 (42·3 to 98·8)	62·1 (41·8 to 87·5)	58·8 (41·2 to 82·7)	53·5 (38·2 to 73·3)	52·6 (38·2 to 72·3)	52·3 (37·6 to 69·7)	43 (31·3 to 58·2)	37 (27·1 to 50·3)	27·4 (20 to 36·8)	19·1 (13·5 to 26·1)	13·2 (9·6 to 18)	8·2 (5·8 to 11·3)	3·6 (2·5 to 5)	1·7 (1·2 to 2·4)	0·8 (0·5 to 1·2)	1,010
Interstitial lung disease and pulmonary sarcoidosis	0 (0 to 0)	0 (0 to 0)	0 (0 to 0)	2·5 (1·5 to 4·2)	0·8 (0·5 to 1·3)	0·9 (0·5 to 1·4)	2·3 (1·3 to 3·8)	3·3 (1·7 to 5·1)	3·8 (2·2 to 6·2)	7·6 (4·3 to 12·5)	9·7 (5·4 to 15·5)	11·3 (6·7 to 17·7)	13·6 (8·7 to 20·6)	17·1 (10·7 to 25·7)	18·4 (12·2 to 26·1)	20 (13·1 to 29)	17·9 (12 to 25·8)	16·7 (11 to 24·1)	13·9 (9·4 to 20·1)	8·5 (5·5 to 12·5)	4·3 (2·7 to 6·2)	2 (1·3 to 3)	1 (0·5 to 1·6)	176
Other chronic respiratory diseases	0 (0 to 0)	0 (0 to 0)	0 (0 to 0)	10·4 (6·5 to 16·3)	5·6 (3·7 to 7·8)	8·4 (5·5 to 12)	6·1 (4·1 to 8·5)	9·8 (7·2 to 13·3)	15·9 (11 to 22·4)	13·2 (9·1 to 18·5)	15·5 (10·6 to 22)	15·9 (10·9 to 23)	15·1 (10·3 to 21·5)	16·2 (10·5 to 23·7)	19·4 (12·6 to 29·3)	4·9 (3 to 7·8)	3·2 (1·9 to 4·9)	2·9 (1·8 to 4·4)	2·2 (1·3 to 3·3)	1·8 (1 to 2·9)	0·9 (0·5 to 1·5)	0·4 (0·2 to 0·6)	0·2 (0·1 to 0·4)	168
Digestive diseases	2·9 (2 to 4·1)	3·6 (1·9 to 6·3)	25·9 (15·6 to 41·5)	40·5 (28·2 to 56·6)	39·7 (29·4 to 53·7)	43·6 (33·6 to 56·2)	70·1 (53·3 to 90·8)	124 (91·9 to 163)	182 (136 to 239)	247 (186 to 319)	343 (269 to 442)	425 (324 to 545)	512 (394 to 671)	601 (465 to 770)	529 (421 to 663)	467 (369 to 588)	341 (270 to 422)	232 (185 to 285)	178 (140 to 224)	113 (92·8 to 136)	53·9 (41·8 to 67)	24·6 (18·2 to 30·5)	9·3 (6·6 to 11·8)	4,610
Cirrhosis and other chronic liver diseases	0 (0 to 0)	0 (0 to 0)	0 (0 to 0)	4·2 (2·5 to 6·7)	4·5 (3·2 to 6)	6·7 (4·7 to 9·1)	14·1 (9·4 to 20·2)	26 (18·1 to 36·7)	44·8 (30·6 to 62·9)	80 (55·9 to 108)	138 (102 to 184)	196 (141 to 263)	250 (180 to 340)	325 (243 to 443)	269 (200 to 354)	230 (175 to 297)	156 (120 to 201)	91·9 (70·8 to 118)	61·5 (46·1 to 80·6)	34·8 (27·3 to 43·4)	13·7 (10·2 to 17·8)	5·5 (3·8 to 7·1)	1·9 (1·2 to 2·6)	1,950
Cirrhosis and other chronic liver diseases due to hepatitis B	0 (0 to 0)	0 (0 to 0)	0 (0 to 0)	0 (0 to 0)	0 (0 to 0)	0 (0 to 0·1)	0·1 (0 to 0·3)	0·9 (0·4 to 1·9)	2·7 (0·9 to 5·8)	6·4 (3·1 to 11·5)	13 (5·8 to 24·4)	19 (9·4 to 34·3)	23·5 (11·3 to 45)	28 (13 to 55·3)	19·3 (8·8 to 35·2)	14·2 (6·2 to 27·6)	8·6 (4·1 to 15·7)	4·8 (2·2 to 8·9)	3·3 (1·6 to 5·7)	1·8 (0·8 to 3·1)	0·7 (0·4 to 1·2)	0·3 (0·1 to 0·5)	0·1 (0 to 0·2)	147
Cirrhosis and other chronic liver diseases due to hepatitis C	0 (0 to 0)	0 (0 to 0)	0 (0 to 0)	0 (0 to 0·1)	0·1 (0 to 0·1)	0·2 (0·1 to 0·3)	0·6 (0·2 to 1·3)	3·5 (1·6 to 6·5)	10·2 (4·9 to 18·3)	20·4 (11·1 to 32·4)	35·2 (18·3 to 61·2)	47·7 (25·4 to 79·6)	56·6 (30·8 to 94·1)	68·2 (36·4 to 116)	52·1 (27·2 to 91·3)	41·6 (19·6 to 76·9)	27·2 (15 to 44·5)	15·5 (8·8 to 25·8)	9·9 (5·3 to 17·5)	5·6 (3·1 to 9·1)	2·3 (1·3 to 3·6)	0·9 (0·5 to 1·5)	0·3 (0·1 to 0·6)	398
Cirrhosis and other chronic liver diseases due to alcohol use	0 (0 to 0)	0 (0 to 0)	0 (0 to 0)	0 (0 to 0)	0 (0 to 0)	0 (0 to 0)	0·1 (0 to 0·2)	2·2 (0·9 to 4·6)	7·8 (3·5 to 14·9)	22·9 (13 to 36·4)	52·2 (29·2 to 82·2)	83·3 (50·4 to 131)	112 (67·6 to 169)	152 (91·1 to 230)	130 (80 to 185)	110 (71 to 157)	70·3 (43·5 to 98·8)	37·8 (23·3 to 55·1)	22·1 (12·9 to 33·2)	11 (6·6 to 16·3)	4·1 (2·5 to 6·3)	1·5 (0·8 to 2·5)	0·5 (0·2 to 0·9)	820
Cirrhosis and other chronic liver diseases due to NAFLD	0 (0 to 0)	0 (0 to 0)	0 (0 to 0)	0 (0 to 0)	0 (0 to 0)	0 (0 to 0)	0·7 (0·3 to 1·4)	2·8 (1·3 to 5·3)	7·2 (3·2 to 13·2)	12·9 (6·3 to 22)	19·9 (9·1 to 37·8)	27·1 (12·8 to 51·8)	36·6 (17·3 to 66·3)	48 (22·7 to 85·6)	39·7 (18·7 to 72·5)	35·7 (16·5 to 64·3)	27·5 (16 to 43·9)	18·2 (10·5 to 28·4)	13·6 (7·2 to 22·1)	8·3 (4·6 to 13)	3·4 (2 to 5·1)	1·4 (0·8 to 2·2)	0·5 (0·2 to 0·9)	303
Cirrhosis and other chronic liver diseases due to other causes	0 (0 to 0)	0 (0 to 0)	0 (0 to 0)	4·1 (2·5 to 6·7)	4·4 (3·1 to 6)	6·5 (4·5 to 8·8)	12·7 (8·2 to 17·7)	16·6 (10·8 to 24·6)	16·9 (9·1 to 28·4)	17·4 (8·7 to 29·1)	18 (8·4 to 32·7)	18·6 (8·9 to 34·6)	21·8 (10·4 to 38·8)	28·9 (14 to 52·3)	28 (13·7 to 51·1)	27·8 (14·2 to 47·9)	22·2 (10·8 to 38·9)	15·6 (8·4 to 25·4)	12·6 (6·8 to 20·6)	8·1 (4·4 to 12·9)	3·3 (1·9 to 4·9)	1·4 (0·7 to 2·2)	0·5 (0·2 to 0·8)	285
Upper digestive system diseases	0 (0 to 0)	0 (0 to 0)	0 (0 to 0)	2·1 (1·2 to 3·4)	3 (1·6 to 5·2)	5·6 (3·5 to 9·3)	18·7 (10·9 to 30·8)	44·2 (25·8 to 76·7)	63·8 (37·3 to 107)	74·6 (44·8 to 125)	83·6 (51·2 to 132)	88·9 (55·5 to 136)	101 (66 to 152)	105 (70·1 to 154)	96·5 (65·4 to 141)	82·9 (58·9 to 120)	62 (44·1 to 86·6)	44·1 (32·4 to 60·1)	34·2 (25·4 to 46·4)	21·3 (16·1 to 28·6)	10·5 (7·7 to 14·1)	4·9 (3·5 to 6·5)	1·7 (1·2 to 2·3)	948
Peptic ulcer disease	0 (0 to 0)	0 (0 to 0)	0 (0 to 0)	0·4 (0·2 to 0·7)	0·4 (0·2 to 0·5)	0·9 (0·6 to 1·2)	2·1 (1·4 to 3·2)	5·3 (3·5 to 7·7)	8 (5·1 to 11·9)	12·3 (8·2 to 18·3)	17·5 (12·1 to 24·6)	20·3 (13·4 to 30·1)	25·4 (16·7 to 37·8)	32·9 (22 to 47·9)	30·9 (21 to 43·8)	30·2 (21·4 to 41·6)	25·2 (17·3 to 34·5)	19·2 (13·3 to 26·4)	16·8 (11·8 to 23·2)	11 (8·2 to 14·3)	5·6 (3·9 to 7·8)	2·7 (1·8 to 3·9)	1 (0·6 to 1·4)	268
Gastritis and duodenitis	0 (0 to 0)	0 (0 to 0)	0 (0 to 0)	1·7 (0·9 to 3)	2·6 (1·1 to 4·7)	2·6 (1·2 to 5)	3·3 (1·6 to 6·1)	4·8 (2·7 to 8·1)	6·8 (3·8 to 11·1)	8·9 (5 to 14·6)	12 (7 to 19·2)	13·1 (7·6 to 22·4)	19 (11·3 to 31·9)	19·1 (11·4 to 30·8)	19·2 (11·9 to 30·3)	15·9 (10·1 to 24·2)	11·6 (7·6 to 17)	8·1 (5·3 to 11·6)	5·7 (3·7 to 8·1)	3·2 (2·2 to 4·6)	1·7 (1·1 to 2·4)	0·8 (0·6 to 1·2)	0·3 (0·2 to 0·4)	160
Gastroesophageal reflux disease	0 (0 to 0)	0 (0 to 0)	0 (0 to 0)	0 (0 to 0)	0·1 (0 to 0·1)	2·2 (0·9 to 4)	13·2 (6·5 to 24·6)	34·1 (16·7 to 66·3)	49·1 (24·2 to 90·7)	53·3 (25·1 to 100)	54·1 (25·4 to 99·5)	55·5 (26·5 to 101)	56·4 (27·7 to 98·9)	52·9 (25·8 to 96·2)	46·3 (22 to 88)	36·8 (17·8 to 68·4)	25·2 (12·8 to 45·4)	16·8 (8·5 to 29·7)	11·8 (6 to 21·1)	7·1 (3·5 to 12·5)	3·3 (1·6 to 5·9)	1·3 (0·7 to 2·3)	0·5 (0·2 to 0·9)	520
Appendicitis	0 (0 to 0)	0 (0 to 0)	0 (0 to 0)	1·9 (1·1 to 3·3)	2·8 (1·9 to 4·1)	4·5 (2·7 to 6·7)	5·8 (3·7 to 8·8)	6·3 (3·9 to 9·4)	5·8 (3·5 to 8·7)	5·5 (3·5 to 8·1)	7·3 (4·6 to 10·9)	6 (3·9 to 9)	6 (3·9 to 9·1)	5·7 (3·6 to 8·6)	4·8 (3 to 7·3)	3·6 (2·2 to 5·3)	3·4 (2·3 to 4·9)	2·4 (1·6 to 3·4)	1·5 (0·9 to 2·3)	1 (0·6 to 1·4)	0·6 (0·4 to 0·8)	0·3 (0·2 to 0·4)	0·1 (0 to 0·1)	74·9
Paralytic ileus and intestinal obstruction	2·2 (1·6 to 3)	3·1 (1·7 to 5·4)	22·5 (13·5 to 35·6)	8·7 (4·9 to 14)	5 (3·4 to 7)	5·1 (3·4 to 7·1)	5·3 (3·3 to 8·2)	6·6 (3·7 to 10·2)	8·4 (4·4 to 13·3)	9·6 (5·3 to 14·9)	16·3 (9·9 to 25·3)	18·3 (11·2 to 28·4)	22·2 (13·7 to 33·9)	28·1 (17·7 to 41·7)	27·6 (18·5 to 40·4)	28·5 (18·9 to 40·4)	25·7 (18·1 to 35·8)	22·9 (16·1 to 32)	21·4 (15·1 to 29·6)	16·3 (11·8 to 22·4)	8·5 (5·9 to 12)	4·1 (2·7 to 6)	2 (1·2 to 3·2)	318
Inguinal, femoral, and abdominal hernia	0·7 (0·4 to 1·2)	0·5 (0·2 to 0·9)	3·4 (1·8 to 6·2)	12 (7·1 to 18·6)	16·9 (9·2 to 28)	13·1 (7 to 22)	10·2 (5·3 to 16·8)	10·7 (5·8 to 17·6)	12·9 (7·5 to 20·8)	14·4 (8·4 to 23·2)	17·7 (10·1 to 28·5)	22 (13·7 to 34·9)	25·7 (15·9 to 39·8)	27·9 (18·1 to 42)	25·7 (17·2 to 38)	21·6 (14·6 to 31·6)	14·6 (10·6 to 20·3)	9·5 (7·1 to 12·7)	6·8 (5·2 to 8·8)	4 (3·1 to 5·2)	1·9 (1·4 to 2·6)	0·9 (0·6 to 1·2)	0·3 (0·2 to 0·4)	273
Inflammatory bowel disease	0 (0 to 0)	0 (0 to 0)	0 (0 to 0)	1·3 (0·7 to 2·2)	0·5 (0·4 to 0·8)	0·4 (0·3 to 0·6)	1·5 (1 to 2·2)	2·8 (2 to 4·1)	4·8 (3·3 to 7·1)	6·4 (4·3 to 9·1)	7·2 (5 to 10)	8·7 (6 to 12·3)	8 (5·7 to 11·1)	7·7 (5·5 to 10·5)	7·7 (5·5 to 10·7)	6·5 (4·7 to 8·7)	4·3 (3·1 to 5·8)	3·8 (2·8 to 5)	3 (2·1 to 4·3)	1·8 (1·3 to 2·4)	0·9 (0·6 to 1·2)	0·5 (0·4 to 0·7)	0·1 (0·1 to 0·2)	78
Vascular intestinal disorders	0 (0 to 0)	0 (0 to 0)	0 (0 to 0)	0·3 (0·2 to 0·5)	0·5 (0·3 to 0·7)	0·3 (0·2 to 0·5)	0·7 (0·4 to 1·1)	1·1 (0·7 to 1·7)	2·3 (1·4 to 3·5)	3·2 (1·9 to 4·9)	4·5 (2·8 to 7)	5·6 (3·5 to 8·7)	9·6 (5·7 to 15·1)	12 (7·8 to 17·9)	12·8 (8·4 to 18·8)	15 (10 to 21·4)	13·7 (9·4 to 18·9)	11 (7·6 to 15·4)	10·1 (6·7 to 14·3)	7·1 (4·9 to 9·7)	3·5 (2·4 to 4·9)	1·6 (1·1 to 2·1)	0·5 (0·3 to 0·7)	115
Gallbladder and biliary diseases	0 (0 to 0)	0 (0 to 0)	0 (0 to 0)	0·7 (0·4 to 1·1)	1·5 (0·7 to 2·9)	3 (1·4 to 5·1)	5·2 (3 to 8·5)	9·2 (5·8 to 13·9)	13·3 (8·4 to 19·9)	18·1 (11·9 to 26·2)	22 (14·5 to 31·7)	27·1 (18·3 to 38)	30·7 (20·7 to 43·4)	35·1 (24·5 to 49·2)	35 (24·9 to 48·2)	31·3 (22·4 to 42·6)	25 (18·2 to 33·9)	18·8 (13·4 to 25·2)	15·4 (11 to 21·5)	10·1 (7·2 to 13·7)	4·6 (3·3 to 6·5)	2·3 (1·6 to 3·1)	0·9 (0·6 to 1·3)	309
Pancreatitis	0 (0 to 0)	0 (0 to 0)	0 (0 to 0)	0·5 (0·3 to 0·8)	0·3 (0·2 to 0·4)	0·9 (0·6 to 1·4)	3·8 (2·5 to 5·6)	9·7 (6·3 to 14·8)	16·7 (10·7 to 25)	23·8 (15·5 to 35·6)	32·4 (21·2 to 47·7)	37·3 (23·7 to 54·8)	37 (24·4 to 54·5)	31·2 (20·8 to 44·4)	27 (18·3 to 39·2)	22·4 (15·1 to 31·2)	15·9 (11·2 to 21·4)	11 (8 to 14·9)	8·4 (6 to 11·5)	5 (3·6 to 6·6)	2·2 (1·5 to 3)	0·9 (0·6 to 1·2)	0·3 (0·2 to 0·5)	287
Other digestive diseases	0 (0 to 0)	0 (0 to 0)	0 (0 to 0)	8·8 (5·3 to 13·7)	4·7 (3 to 6·4)	3·9 (2·6 to 5·6)	4·8 (2·8 to 7·4)	7 (3·7 to 10·6)	9·6 (5 to 15·3)	11·5 (6·1 to 18·1)	13·5 (7·3 to 20·9)	15·6 (9·2 to 25·2)	21 (12 to 33·8)	23·5 (13·6 to 35·9)	23·3 (13·6 to 34·9)	25·5 (15·4 to 37·5)	20·9 (13·3 to 29·5)	16·9 (10·4 to 23·9)	15·6 (9·8 to 22·5)	12·1 (7·2 to 16·8)	7·5 (4·6 to 10·6)	3·7 (2·2 to 5·3)	1·5 (0·7 to 2·3)	251
Neurological disorders	0·1 (0 to 0·1)	0·3 (0·1 to 0·6)	16·3 (9·9 to 24)	40·5 (18·7 to 70·7)	85·9 (36·5 to 153)	253 (84·5 to 517)	324 (124 to 655)	349 (135 to 678)	356 (147 to 665)	336 (139 to 628)	343 (155 to 623)	349 (159 to 639)	342 (170 to 595)	306 (164 to 515)	269 (154 to 430)	238 (149 to 368)	208 (137 to 332)	206 (136 to 357)	233 (149 to 421)	270 (154 to 533)	197 (101 to 400)	120 (57·1 to 256)	82·9 (35·6 to 171)	4,920
Alzheimer's disease and other dementias	0 (0 to 0)	0 (0 to 0)	0 (0 to 0)	0 (0 to 0)	0 (0 to 0)	0 (0 to 0)	0 (0 to 0)	0 (0 to 0)	0 (0 to 0)	0 (0 to 0)	0 (0 to 0)	1·9 (0·4 to 6·2)	10 (2·9 to 27·4)	19·3 (7 to 47·1)	33·2 (13 to 76·7)	53·4 (21·7 to 126)	73 (28·4 to 182)	99·3 (43·1 to 237)	132 (57·7 to 321)	194 (80·1 to 463)	159 (63·5 to 365)	106 (42 to 246)	78·4 (31·3 to 168)	959
Parkinson's disease	0 (0 to 0)	0 (0 to 0)	0 (0 to 0)	0 (0 to 0)	0 (0 to 0)	0 (0 to 0)	0 (0 to 0)	0·2 (0·1 to 0·3)	0·5 (0·3 to 0·7)	0·5 (0·3 to 0·8)	1·2 (0·8 to 1·7)	3·2 (2·4 to 4·2)	6·6 (5 to 8·8)	11·1 (8·6 to 14·3)	16·2 (12·7 to 20·3)	24·6 (19·9 to 30·2)	34·1 (28·5 to 41·5)	46·3 (38·4 to 55·6)	61·6 (51·1 to 72·8)	54·2 (44·6 to 65·5)	28 (23·3 to 32·9)	10·4 (8·3 to 12·3)	3·1 (2·2 to 3·8)	302
Idiopathic epilepsy	0·1 (0 to 0·1)	0·2 (0 to 0·4)	5·4 (2·6 to 9·5)	24·9 (9·7 to 46·5)	40·8 (13·9 to 82·7)	59 (21·5 to 120)	73·8 (32·5 to 133)	86·7 (42·4 to 150)	88·6 (45·2 to 146)	79·8 (43 to 132)	78·6 (44·1 to 122)	71·7 (39·1 to 113)	68 (37·2 to 109)	55·3 (26·8 to 92·5)	46·1 (21·8 to 79·5)	36·9 (15·7 to 63·7)	26·7 (10·5 to 47·2)	18·9 (7·3 to 34·2)	14·6 (5·4 to 26·3)	9·3 (3·5 to 16·8)	4·5 (1·6 to 8·5)	2 (0·7 to 3·6)	0·8 (0·3 to 1·5)	893
Multiple sclerosis	0 (0 to 0)	0 (0 to 0)	0 (0 to 0)	0 (0 to 0)	0·1 (0 to 0·1)	0·1 (0·1 to 0·3)	0·8 (0·4 to 1·4)	2·6 (1·5 to 3·7)	3·6 (2·3 to 5)	4·8 (3·3 to 6·4)	8·5 (5·6 to 11·3)	9·2 (6·5 to 12·9)	9·3 (6·5 to 12·7)	7·9 (5·6 to 10·7)	7·3 (5·3 to 10)	5·2 (3·8 to 7·1)	2·6 (1·9 to 3·4)	1·5 (1·1 to 2)	0·8 (0·6 to 1·1)	0·3 (0·3 to 0·5)	0·1 (0·1 to 0·2)	0·1 (0 to 0·1)	0 (0 to 0)	64·9
Motor neuron disease	0 (0 to 0)	0·1 (0·1 to 0·3)	2·2 (1·2 to 3·5)	1·3 (0·8 to 2)	0·3 (0·2 to 0·4)	0·6 (0·4 to 0·9)	0·9 (0·7 to 1·3)	1·3 (1 to 1·8)	1·3 (1 to 1·8)	1·9 (1·3 to 2·5)	4 (2·8 to 5·6)	5·8 (4 to 8·1)	6·9 (4·8 to 9·5)	9·2 (6·5 to 12·7)	9·2 (6·4 to 12·6)	8·2 (5·9 to 10·8)	6·5 (4·7 to 8·8)	3·7 (2·7 to 4·9)	2 (1·4 to 2·6)	0·7 (0·5 to 1)	0·2 (0·2 to 0·3)	0·1 (0 to 0·1)	0 (0 to 0)	66·4
Headache disorders	0 (0 to 0)	0 (0 to 0)	0 (0 to 0)	0 (0 to 0)	27·7 (0·5 to 70·7)	162 (7·3 to 407)	213 (22 to 527)	229 (31·4 to 535)	237 (38·4 to 541)	227 (41·3 to 510)	227 (45 to 500)	232 (52·7 to 514)	215 (55·8 to 474)	178 (46·9 to 385)	135 (38·5 to 278)	93·2 (29·4 to 197)	54·9 (18·3 to 112)	30·1 (10·1 to 62·5)	17·3 (5·7 to 35·8)	8·9 (2·9 to 18·5)	3·8 (1·3 to 7·8)	1·3 (0·4 to 2·8)	0·4 (0·1 to 0·9)	2,290
Migraine	0 (0 to 0)	0 (0 to 0)	0 (0 to 0)	0 (0 to 0)	25·4 (0·3 to 68·5)	152 (4·9 to 401)	199 (15·1 to 517)	212 (21·9 to 517)	217 (26·2 to 513)	207 (29·3 to 487)	206 (30·3 to 474)	210 (38·1 to 486)	192 (38·1 to 439)	157 (32·2 to 349)	119 (26·5 to 258)	80·7 (20·4 to 179)	46·2 (13 to 99·9)	24·7 (7·3 to 54·7)	14·1 (4 to 31)	7·3 (2·1 to 16·3)	3·1 (0·9 to 6·9)	1·1 (0·3 to 2·3)	0·3 (0·1 to 0·7)	2,070
Tension-type headache	0 (0 to 0)	0 (0 to 0)	0 (0 to 0)	0 (0 to 0)	2·3 (0·1 to 15)	9·9 (0·7 to 69·2)	14·7 (2·6 to 70·3)	17·8 (3·6 to 74·6)	19·9 (4·1 to 79·5)	19·8 (4·6 to 74·4)	21·4 (5·2 to 73·1)	22·3 (6·4 to 71·3)	22·3 (6·5 to 65·1)	20·6 (6·1 to 60·1)	15·8 (4·7 to 43·7)	12·6 (3·9 to 37·5)	8·7 (2·5 to 28·4)	5·3 (1·4 to 18·8)	3·1 (0·7 to 11·5)	1·6 (0·4 to 5·8)	0·7 (0·2 to 2·6)	0·3 (0·1 to 1)	0·1 (0 to 0·5)	219
Other neurological disorders	0 (0 to 0)	0 (0 to 0)	8·8 (5·4 to 13·5)	14·4 (7·8 to 23·4)	17·1 (7·6 to 31)	31·6 (14·9 to 56·9)	34·8 (20 to 55·6)	28·6 (16·7 to 43·3)	24·8 (15·5 to 36·5)	22·1 (13·8 to 32)	23·1 (15 to 33·8)	24·9 (16·5 to 35·9)	26·5 (16·9 to 37·9)	24·9 (17·1 to 34·7)	21·8 (15·1 to 29·8)	16·5 (12 to 22·1)	10·6 (7·7 to 14·1)	6·5 (4·8 to 8·7)	4·7 (3·5 to 6·2)	2·8 (2 to 3·7)	1 (0·7 to 1·4)	0·4 (0·3 to 0·5)	0·2 (0·1 to 0·2)	346
Mental disorders	0·1 (0 to 0·1)	0·2 (0·1 to 0·2)	2·8 (1·7 to 4·1)	17·1 (11·3 to 24·5)	115 (77·1 to 167)	399 (270 to 571)	562 (386 to 783)	621 (428 to 867)	666 (462 to 922)	647 (455 to 885)	652 (470 to 874)	646 (468 to 882)	626 (453 to 844)	551 (398 to 744)	458 (332 to 614)	338 (240 to 454)	214 (152 to 283)	133 (95·6 to 175)	85·9 (60·5 to 114)	48·9 (33·8 to 65·9)	22·8 (15·9 to 30·3)	9·5 (6·4 to 13)	4 (2·6 to 5·7)	6,820
Schizophrenia	0 (0 to 0)	0 (0 to 0)	0 (0 to 0)	0 (0 to 0)	0 (0 to 0)	1·8 (0·8 to 3·5)	13·8 (6 to 26)	48·6 (25·9 to 79·7)	84·6 (49 to 129)	100 (62·4 to 146)	107 (66·4 to 155)	104 (68·6 to 143)	97·8 (65·5 to 133)	82 (55 to 111)	61·6 (40·8 to 83·7)	40·3 (27·2 to 56·1)	22·6 (15 to 31·2)	12·2 (8 to 17)	6·7 (4·2 to 9·7)	3·1 (1·9 to 4·5)	1·1 (0·6 to 1·7)	0·3 (0·2 to 0·5)	0·1 (0 to 0·2)	788
Depressive disorders	0 (0 to 0)	0 (0 to 0)	0 (0 to 0)	0 (0 to 0·1)	5·7 (2·2 to 11·1)	67·2 (34·3 to 119)	159 (92·1 to 254)	198 (115 to 312)	195 (116 to 298)	187 (112 to 290)	200 (125 to 294)	212 (132 to 328)	218 (141 to 318)	206 (136 to 304)	183 (116 to 274)	143 (87·5 to 211)	93·5 (59·5 to 134)	60·5 (38·8 to 86·1)	40·8 (25·2 to 60)	24·7 (14·6 to 37·7)	12·5 (8 to 18)	5·7 (3·5 to 8·4)	2·5 (1·4 to 4·1)	2,210
Major depressive disorder	0 (0 to 0)	0 (0 to 0)	0 (0 to 0)	0 (0 to 0·1)	5 (1·7 to 10·3)	61·1 (29·5 to 112)	142 (79 to 235)	168 (89·7 to 274)	158 (88·3 to 252)	147 (82·7 to 240)	159 (96·2 to 242)	170 (101 to 268)	176 (108 to 266)	169 (108 to 249)	152 (89·1 to 232)	120 (68·7 to 181)	78·7 (49 to 114)	51·3 (32·3 to 74·3)	34·9 (20·6 to 53·8)	21·4 (12·2 to 33·7)	11·1 (6·9 to 16·3)	5·1 (3 to 7·7)	2·3 (1·2 to 3·8)	1,830
Dysthymia	0 (0 to 0)	0 (0 to 0)	0 (0 to 0)	0 (0 to 0)	0·7 (0·1 to 1·7)	6·1 (2·5 to 12)	16·9 (8·4 to 29·4)	29·5 (14·6 to 51·6)	37·6 (19·6 to 67·6)	39·5 (21·8 to 64·3)	41·2 (22·9 to 68·1)	42·3 (22·3 to 72·6)	41·5 (22·7 to 70·2)	37 (21·8 to 61·4)	30·9 (18·3 to 50·6)	23·2 (12·5 to 38·9)	14·8 (7·8 to 24·9)	9·2 (5·3 to 14·9)	5·8 (3·4 to 9·4)	3·2 (1·8 to 5·5)	1·5 (0·8 to 2·5)	0·6 (0·3 to 1)	0·3 (0·1 to 0·4)	382
Bipolar disorder	0 (0 to 0)	0 (0 to 0)	0 (0 to 0)	0 (0 to 0)	0 (0 to 0)	22·5 (9·2 to 41·6)	77·8 (37 to 139)	96·7 (50·2 to 162)	95·9 (49·6 to 159)	84·6 (46·4 to 139)	81 (43·8 to 131)	80·8 (44 to 127)	78·1 (44·2 to 128)	67·7 (37·2 to 111)	52·9 (29·6 to 85·3)	36·1 (20·4 to 58·3)	20·8 (11·6 to 32·7)	11·4 (6·2 to 18·5)	6·2 (3·4 to 10·2)	2·8 (1·5 to 4·7)	1 (0·5 to 1·8)	0·3 (0·2 to 0·6)	0·1 (0·1 to 0·2)	817
Anxiety disorders	0 (0 to 0)	0 (0 to 0)	0 (0 to 0)	2·2 (0·8 to 4·3)	38·8 (20·2 to 66·7)	118 (65·7 to 192)	149 (88·9 to 232)	161 (91·2 to 260)	164 (92·5 to 268)	158 (93 to 249)	157 (93·4 to 236)	156 (86·2 to 249)	151 (87·4 to 242)	133 (78 to 201)	109 (69·2 to 161)	80·5 (49·7 to 123)	51·4 (30·6 to 79·9)	32·2 (19·6 to 51·3)	20·8 (13 to 32·6)	11·4 (6·9 to 17·4)	4·8 (2·9 to 7·3)	1·8 (1 to 2·8)	0·6 (0·3 to 1)	1,700
Eating disorders	0 (0 to 0)	0 (0 to 0)	0 (0 to 0)	0 (0 to 0)	0·5 (0·2 to 1)	9·8 (4·7 to 17·1)	32·8 (17·1 to 58·4)	52·2 (28·3 to 88·7)	51·6 (28·8 to 84·1)	42·7 (24·1 to 68·6)	31·1 (16·9 to 50·5)	17·9 (9·5 to 29·8)	9·3 (5 to 15·4)	0 (0 to 0)	0 (0 to 0)	0 (0 to 0)	0 (0 to 0)	0 (0 to 0)	0 (0 to 0)	0 (0 to 0)	0 (0 to 0)	0 (0 to 0)	0 (0 to 0)	248
Anorexia nervosa	0 (0 to 0)	0 (0 to 0)	0 (0 to 0)	0 (0 to 0)	0·4 (0·1 to 1)	4·6 (2·1 to 8·3)	12·2 (5·9 to 22·1)	12·7 (6·3 to 22·9)	10·1 (5 to 17·3)	6·4 (3·1 to 11·5)	4·5 (2·2 to 8·1)	3·4 (1·5 to 5·7)	2·5 (1·2 to 4·6)	0 (0 to 0)	0 (0 to 0)	0 (0 to 0)	0 (0 to 0)	0 (0 to 0)	0 (0 to 0)	0 (0 to 0)	0 (0 to 0)	0 (0 to 0)	0 (0 to 0)	56·9
Bulimia nervosa	0 (0 to 0)	0 (0 to 0)	0 (0 to 0)	0 (0 to 0)	0·1 (0 to 0·1)	5·2 (1·8 to 11)	20·7 (9 to 41·1)	39·5 (18·1 to 72·7)	41·5 (20·8 to 71·8)	36·3 (19·2 to 60·7)	26·6 (13·9 to 45·2)	14·5 (7·2 to 25·1)	6·8 (3·1 to 12·3)	0 (0 to 0)	0 (0 to 0)	0 (0 to 0)	0 (0 to 0)	0 (0 to 0)	0 (0 to 0)	0 (0 to 0)	0 (0 to 0)	0 (0 to 0)	0 (0 to 0)	191
Autism spectrum disorders	0·1 (0 to 0·1)	0·2 (0·1 to 0·2)	2·5 (1·6 to 3·8)	11·5 (7·2 to 17)	16·8 (10·5 to 25·7)	19·3 (12·5 to 28·5)	17·9 (11·8 to 26·9)	17·5 (11·3 to 26·3)	16·6 (10·3 to 24·4)	14·7 (9·2 to 22·1)	13·7 (8·7 to 20·4)	12·9 (8·2 to 19·5)	12·1 (7·6 to 17·9)	10·4 (6·5 to 15·7)	8·4 (5·4 to 12·6)	6·2 (3·9 to 9·2)	4 (2·5 to 6)	2·5 (1·6 to 3·8)	1·6 (1·1 to 2·5)	1 (0·6 to 1·4)	0·4 (0·3 to 0·6)	0·2 (0·1 to 0·2)	0·1 (0 to 0·1)	190
Attention-deficit/hyperactivity disorder	0 (0 to 0)	0 (0 to 0)	0 (0 to 0)	1·4 (0·7 to 2·7)	18 (9·8 to 32·1)	28·8 (15·5 to 50·9)	22·8 (12·6 to 40·1)	17·1 (9·4 to 30·2)	12·8 (6·9 to 22·4)	9·5 (5 to 16·6)	7·4 (3·7 to 12·7)	6 (3 to 10·5)	4·8 (2·5 to 8·3)	3·4 (1·7 to 6·1)	2 (0·9 to 3·6)	1 (0·4 to 1·8)	0·4 (0·2 to 0·7)	0·1 (0 to 0·3)	0 (0 to 0·1)	0 (0 to 0)	0 (0 to 0)	0 (0 to 0)	0 (0 to 0)	135
Conduct disorder	0 (0 to 0)	0 (0 to 0)	0 (0 to 0)	0 (0 to 0)	31·8 (16·2 to 56·6)	126 (69·5 to 208)	74·8 (39·4 to 125)	0 (0 to 0)	0 (0 to 0)	0 (0 to 0)	0 (0 to 0)	0 (0 to 0)	0 (0 to 0)	0 (0 to 0)	0 (0 to 0)	0 (0 to 0)	0 (0 to 0)	0 (0 to 0)	0 (0 to 0)	0 (0 to 0)	0 (0 to 0)	0 (0 to 0)	0 (0 to 0)	232
Idiopathic developmental intellectual disability	0 (0 to 0)	0 (0 to 0)	0·3 (0 to 0·7)	1·8 (0·2 to 4)	3·5 (1 to 6·8)	4 (1 to 7·7)	3·7 (0·9 to 7·1)	3·6 (0·9 to 7·1)	3·4 (0·9 to 6·6)	3 (0·8 to 5·8)	2·8 (0·7 to 5·3)	2·5 (0·6 to 5)	2·3 (0·6 to 4·4)	1·9 (0·5 to 3·7)	1·5 (0·4 to 2·8)	1 (0·2 to 2)	0·6 (0·1 to 1·2)	0·3 (0·1 to 0·7)	0·2 (0 to 0·4)	0·1 (0 to 0·2)	0 (0 to 0·1)	0 (0 to 0)	0 (0 to 0)	36·4
Other mental disorders	0 (0 to 0)	0 (0 to 0)	0 (0 to 0)	0 (0 to 0)	0 (0 to 0)	1·7 (0·8 to 2·8)	10·8 (5·1 to 18·8)	26·6 (13·5 to 44·7)	40·8 (23·6 to 64·2)	47·7 (27·9 to 74)	51·9 (30·9 to 80·8)	52·9 (32·1 to 81·5)	52·6 (32·2 to 80·4)	47·4 (28·8 to 73·4)	39·9 (24·5 to 61·1)	30·4 (18·6 to 45·8)	20·4 (12·8 to 30·9)	13·5 (8·7 to 19·9)	9·5 (6·2 to 13·7)	5·9 (3·8 to 8·5)	2·9 (1·8 to 4·1)	1·2 (0·8 to 1·8)	0·5 (0·3 to 0·8)	457
Substance use disorders	0 (0 to 0)	0 (0 to 0)	0 (0 to 0)	0 (0 to 0)	0 (0 to 0)	5 (2·7 to 8·3)	85·3 (56·3 to 120)	185 (125 to 253)	227 (158 to 306)	233 (168 to 308)	246 (183 to 326)	245 (185 to 321)	230 (172 to 305)	193 (146 to 251)	154 (118 to 200)	98·5 (74·2 to 126)	56 (42·6 to 72·7)	28·1 (21·4 to 36·5)	15·6 (11·9 to 20)	8·3 (6·2 to 10·8)	4 (3 to 5·5)	2 (1·4 to 2·9)	1·1 (0·7 to 1·7)	2,020
Alcohol use disorders	0 (0 to 0)	0 (0 to 0)	0 (0 to 0)	0 (0 to 0)	0 (0 to 0)	2·1 (0·9 to 4·2)	30·7 (18·1 to 47·6)	89 (53·1 to 141)	131 (85·9 to 189)	160 (109 to 221)	186 (135 to 254)	198 (143 to 266)	192 (140 to 262)	165 (122 to 219)	135 (101 to 178)	86·8 (64·3 to 112)	49·4 (36·8 to 64·5)	24·3 (18·4 to 31·7)	13·2 (9·8 to 16·9)	6·8 (5 to 9·2)	3·3 (2·4 to 4·7)	1·8 (1·2 to 2·6)	1 (0·6 to 1·6)	1,480
Drug use disorders	0 (0 to 0)	0 (0 to 0)	0 (0 to 0)	0 (0 to 0)	0 (0 to 0)	2·8 (1·4 to 5)	54·6 (33·7 to 81·4)	95·9 (64·4 to 135)	95·2 (65·2 to 133)	72·9 (50·8 to 103)	60·2 (42·1 to 82·9)	47·5 (32·4 to 64·7)	38·1 (26·8 to 52·4)	27·3 (18·1 to 38·4)	19·2 (12·5 to 27·3)	11·7 (7·4 to 17·5)	6·7 (4·1 to 9·8)	3·8 (2·2 to 5·8)	2·4 (1·5 to 3·6)	1·4 (0·9 to 2·1)	0·7 (0·4 to 1)	0·3 (0·2 to 0·4)	0·1 (0·1 to 0·2)	541
Opioid use disorders	0 (0 to 0)	0 (0 to 0)	0 (0 to 0)	0 (0 to 0)	0 (0 to 0)	0 (0 to 0)	9·3 (4·4 to 16)	40·4 (21·4 to 65·7)	49·3 (28·9 to 76·7)	40·7 (25·1 to 64)	33·4 (20·7 to 52)	26·6 (15·7 to 40·1)	21·8 (12·8 to 33·2)	17·3 (9·3 to 27·3)	13 (7 to 20·5)	8·4 (4·5 to 13·6)	4·8 (2·6 to 7·6)	2·6 (1·3 to 4·4)	1·7 (0·9 to 2·7)	1 (0·5 to 1·6)	0·4 (0·2 to 0·7)	0·2 (0·1 to 0·3)	0·1 (0·1 to 0·2)	271
Cocaine use disorders	0 (0 to 0)	0 (0 to 0)	0 (0 to 0)	0 (0 to 0)	0 (0 to 0)	0·3 (0·2 to 0·6)	12 (5·3 to 21·8)	14·4 (7·7 to 23·6)	12·9 (8 to 19·5)	10·5 (6·3 to 15·7)	9·6 (6·2 to 13·9)	7·3 (4·5 to 11·1)	5·5 (3·3 to 8·1)	3·1 (1·7 to 4·8)	2·1 (1·2 to 3·4)	1·2 (0·6 to 2·1)	0·7 (0·4 to 1·3)	0·4 (0·2 to 0·8)	0·3 (0·1 to 0·5)	0·1 (0·1 to 0·3)	0·1 (0 to 0·1)	0 (0 to 0)	0 (0 to 0)	80·6
Amphetamine use disorders	0 (0 to 0)	0 (0 to 0)	0 (0 to 0)	0 (0 to 0)	0 (0 to 0)	0 (0 to 0)	1·7 (0·5 to 3·6)	7·7 (3·2 to 14·2)	7·8 (3·5 to 14)	4·6 (2·3 to 8·4)	3·1 (1·7 to 5·1)	2·1 (1·4 to 3·1)	1·4 (0·9 to 2)	0·9 (0·6 to 1·4)	0·6 (0·4 to 0·9)	0·3 (0·2 to 0·5)	0·2 (0·1 to 0·3)	0·1 (0 to 0·1)	0 (0 to 0·1)	0 (0 to 0)	0 (0 to 0)	0 (0 to 0)	0 (0 to 0)	30·5
Cannabis use disorders	0 (0 to 0)	0 (0 to 0)	0 (0 to 0)	0 (0 to 0)	0 (0 to 0)	2·3 (0·9 to 4·4)	22·1 (10·9 to 40·1)	17·9 (8·1 to 32·4)	10·3 (4·7 to 19·6)	6·1 (2·8 to 11)	4·3 (1·9 to 8·1)	3·5 (1·5 to 6·5)	3 (1·3 to 5·4)	2 (1 to 3·7)	1·2 (0·5 to 2·2)	0·6 (0·2 to 1·1)	0·3 (0·1 to 0·5)	0·1 (0 to 0·2)	0 (0 to 0·1)	0 (0 to 0)	0 (0 to 0)	0 (0 to 0)	0 (0 to 0)	73·8
Other drug use disorders	0 (0 to 0)	0 (0 to 0)	0 (0 to 0)	0 (0 to 0)	0 (0 to 0)	0·2 (0·1 to 0·4)	9·5 (4·8 to 15·9)	15·4 (9·3 to 23·9)	14·9 (9·5 to 21·8)	11·1 (7 to 16·2)	9·8 (6·5 to 13·8)	8 (5·3 to 11·5)	6·6 (4·4 to 9·3)	3·9 (2·5 to 5·7)	2·3 (1·4 to 3·4)	1·2 (0·7 to 1·8)	0·8 (0·5 to 1·2)	0·5 (0·3 to 0·8)	0·4 (0·3 to 0·6)	0·3 (0·2 to 0·4)	0·1 (0·1 to 0·2)	0·1 (0 to 0·1)	0 (0 to 0)	85
Diabetes and kidney diseases	0 (0 to 0·1)	0·1 (0·1 to 0·3)	11·3 (7·3 to 16·7)	14·8 (10·3 to 20·1)	24·2 (17·7 to 32·5)	34·1 (24·9 to 46·4)	99·1 (75·9 to 125)	174 (137 to 219)	271 (208 to 343)	354 (279 to 443)	492 (395 to 621)	663 (516 to 824)	923 (727 to 1,150)	1,220 (951 to 1,560)	1,330 (1,050 to 1,640)	1,350 (1,080 to 1,660)	1,160 (951 to 1,410)	860 (701 to 1,040)	697 (577 to 826)	453 (374 to 545)	212 (174 to 252)	92·4 (76·1 to 110)	34·7 (26·6 to 41·5)	10,500
Diabetes mellitus	0 (0 to 0·1)	0·1 (0·1 to 0·3)	2·7 (1·4 to 5·1)	2·3 (1·4 to 3·7)	3·1 (2·1 to 4·4)	6·2 (4·5 to 8·7)	34·1 (25·7 to 42·8)	70·9 (54·8 to 92·4)	119 (89·5 to 153)	170 (128 to 220)	258 (193 to 335)	374 (284 to 487)	573 (428 to 746)	818 (621 to 1,050)	890 (680 to 1,130)	934 (724 to 1,180)	812 (641 to 1,020)	602 (473 to 756)	489 (392 to 600)	312 (250 to 386)	138 (110 to 168)	58·2 (46·2 to 70·7)	21 (16 to 26·2)	6,690
Diabetes mellitus type 1	0 (0 to 0·1)	0·1 (0·1 to 0·3)	2·7 (1·4 to 5·1)	2·3 (1·4 to 3·7)	3·1 (2·1 to 4·4)	6·2 (4·5 to 8·7)	12·5 (9 to 16·8)	25 (17·9 to 33·9)	40 (28·3 to 54·5)	33·4 (22·1 to 49·6)	49·5 (34·9 to 71)	64·9 (44·1 to 95·6)	24·3 (16·7 to 35·3)	30·9 (20·5 to 45·5)	33·3 (23 to 49·4)	28 (19·4 to 41·7)	24·3 (16·5 to 36)	14·5 (10·1 to 21·4)	11·8 (8 to 18)	5·3 (3·7 to 8·1)	2·4 (1·7 to 3·5)	1·1 (0·8 to 1·6)	0·5 (0·3 to 0·7)	416
Diabetes mellitus type 2	0 (0 to 0)	0 (0 to 0)	0 (0 to 0)	0 (0 to 0)	0 (0 to 0)	0 (0 to 0)	21·6 (14·9 to 29)	45·9 (32·1 to 64)	79·1 (55·2 to 107)	137 (101 to 182)	209 (151 to 282)	310 (226 to 411)	548 (408 to 718)	787 (602 to 1,020)	857 (654 to 1,090)	906 (701 to 1,150)	788 (621 to 994)	587 (462 to 736)	478 (382 to 585)	307 (247 to 380)	135 (108 to 165)	57·1 (45·2 to 69·5)	20·5 (15·5 to 25·7)	6,270
Chronic kidney disease	0 (0 to 0)	0 (0 to 0)	8·6 (5·4 to 12·9)	12·1 (8·2 to 16·7)	20·4 (14·3 to 28·1)	27·4 (18·9 to 39·3)	64·3 (45·8 to 87·6)	103 (75·2 to 138)	151 (108 to 206)	183 (133 to 246)	233 (167 to 313)	288 (209 to 384)	350 (253 to 472)	401 (291 to 548)	440 (334 to 577)	418 (315 to 541)	351 (276 to 445)	258 (197 to 328)	207 (159 to 259)	141 (109 to 182)	73·9 (57·7 to 92·5)	34·1 (26·4 to 42·4)	13·7 (9·8 to 17·6)	3,780
Chronic kidney disease due to diabetes mellitus type 1	0 (0 to 0)	0 (0 to 0)	0·1 (0 to 0·1)	0·3 (0·1 to 0·6)	0·6 (0·2 to 1·3)	0·9 (0·3 to 2)	2·5 (1 to 5)	5·4 (2·3 to 10·9)	15·2 (6·3 to 29·3)	28·2 (13·5 to 49·7)	42·5 (21·1 to 71·2)	51·1 (24·4 to 91·7)	51·8 (25 to 90·6)	44 (16·8 to 84·5)	33·1 (11·1 to 70·8)	23·3 (8·3 to 49·8)	15·4 (6·2 to 32·9)	8·5 (3·2 to 17·3)	4·5 (1·7 to 9·9)	1·5 (0·6 to 3·5)	0·3 (0·1 to 0·7)	0 (0 to 0·1)	0 (0 to 0)	329
Chronic kidney disease due to diabetes mellitus type 2	0 (0 to 0)	0 (0 to 0)	0 (0 to 0)	0 (0 to 0)	0 (0 to 0)	0 (0 to 0)	1 (0·3 to 2·3)	2·5 (0·9 to 5·4)	6 (2·5 to 12·8)	11·1 (4·7 to 21·9)	19·7 (9·1 to 37·2)	34·6 (16·3 to 60)	61·6 (33·1 to 102)	95·1 (53·6 to 150)	125 (71·7 to 184)	129 (78·4 to 188)	111 (72·7 to 158)	82·5 (53·3 to 115)	65·2 (42·5 to 91·1)	43·2 (27·5 to 60·9)	21·3 (13·7 to 30·1)	9·2 (5·6 to 13·3)	3·5 (1·9 to 5·3)	821
Chronic kidney disease due to hypertension	0 (0 to 0)	0 (0 to 0)	0 (0 to 0)	0 (0 to 0)	0 (0 to 0)	0 (0 to 0)	4·7 (2·2 to 9)	8·1 (3·6 to 14·3)	13·2 (6·8 to 21·7)	17·5 (9·8 to 28·8)	23·9 (13·9 to 37·3)	34·1 (21·6 to 52·4)	48·4 (31·1 to 72·5)	64·6 (39·4 to 97·7)	82·6 (54·3 to 119)	91·3 (58·8 to 136)	86·4 (58·4 to 124)	69 (46·6 to 98·1)	61·2 (43 to 85·3)	46·1 (32·3 to 63·4)	26·9 (19·2 to 36·2)	13·5 (9·8 to 18·1)	5·6 (3·6 to 7·8)	697
Chronic kidney disease due to glomerulonephritis	0 (0 to 0)	0 (0 to 0)	2·4 (1·2 to 4·5)	3·9 (2·2 to 6·2)	7·3 (4·4 to 11·1)	11 (6·5 to 16·6)	26·4 (15·9 to 39·7)	41·6 (25·6 to 61·7)	57·9 (35·1 to 88·8)	63·7 (39·4 to 93·2)	76·6 (45·8 to 115)	88 (51·6 to 132)	95 (58·5 to 144)	98·3 (57·1 to 157)	98 (55 to 160)	80·6 (43·7 to 130)	58 (29·3 to 98·4)	36·4 (19·9 to 63)	25·2 (12·7 to 43·4)	14·4 (6·6 to 26)	5·9 (2·7 to 10·5)	2·3 (1·1 to 4·2)	0·8 (0·3 to 1·7)	894
Chronic kidney disease due to other and unspecified causes	0 (0 to 0)	0 (0 to 0)	6·1 (3·6 to 9·4)	8 (5·3 to 11·6)	12·5 (8·2 to 18·5)	15·6 (9·7 to 24·3)	29·8 (19·3 to 43·9)	45·2 (28·4 to 66·4)	58·5 (36 to 86·6)	62·5 (38·3 to 94)	70·2 (41·6 to 108)	80 (45·9 to 124)	92·9 (55·5 to 141)	99·1 (61·1 to 155)	102 (62 to 159)	94·3 (56·8 to 149)	80 (48·2 to 123)	61·6 (38·6 to 92·6)	50·8 (30·8 to 74·9)	35·9 (23 to 54·8)	19·6 (12·8 to 28·2)	9 (6·2 to 12·9)	3·8 (2·4 to 5·6)	1,040
Acute glomerulonephritis	0 (0 to 0)	0 (0 to 0)	0 (0 to 0·1)	0·4 (0·1 to 0·9)	0·8 (0·4 to 1·4)	0·5 (0·2 to 1·1)	0·7 (0·4 to 1·2)	0·6 (0·3 to 1·2)	0·7 (0·3 to 1·2)	0·6 (0·3 to 1·2)	0·8 (0·4 to 1·4)	0·9 (0·4 to 1·7)	1·2 (0·6 to 2·1)	0·5 (0·3 to 1)	0·9 (0·4 to 1·7)	0·6 (0·3 to 1·1)	0·3 (0·2 to 0·7)	0·5 (0·2 to 1)	0·3 (0·1 to 0·6)	0·2 (0·1 to 0·4)	0·1 (0·1 to 0·2)	0·1 (0 to 0·1)	0 (0 to 0)	10·7
Skin and subcutaneous diseases	0 (0 to 0)	0·1 (0 to 0·1)	19·2 (12·3 to 27·5)	119 (76·8 to 177)	175 (109 to 258)	210 (135 to 311)	222 (144 to 333)	149 (97·8 to 220)	132 (88·9 to 193)	122 (81·7 to 174)	125 (84·5 to 176)	134 (93·6 to 189)	136 (94·2 to 192)	146 (103 to 208)	143 (99·8 to 201)	129 (93·4 to 183)	106 (75·9 to 150)	87·9 (61·8 to 125)	86·5 (62·2 to 128)	68·9 (49 to 105)	42·2 (30 to 68)	21·7 (15·1 to 36·4)	12·2 (7·5 to 25·7)	2,390
Dermatitis	0 (0 to 0)	0 (0 to 0)	0 (0 to 0)	40·9 (22·4 to 69·6)	57·5 (30·6 to 98·9)	47·1 (25·8 to 79·7)	33·6 (18·8 to 55·2)	28·1 (15·9 to 45·2)	25·4 (14·6 to 40·8)	23 (12·9 to 36·9)	21·7 (12·3 to 34·4)	21 (12·3 to 34·1)	21 (11·9 to 33·8)	20·4 (10·9 to 33·3)	19 (10·6 to 31·6)	15·8 (8·6 to 25·3)	11·7 (6·5 to 18·6)	8·2 (4·7 to 13·2)	5·7 (3·3 to 9·1)	3·4 (2 to 5·3)	1·5 (0·9 to 2·5)	0·6 (0·4 to 0·9)	0·2 (0·1 to 0·4)	406
Atopic dermatitis	0 (0 to 0)	0 (0 to 0)	0 (0 to 0)	40·5 (22·2 to 69·2)	56·3 (29·6 to 97·6)	42·4 (22·3 to 73·5)	26·1 (13·7 to 45·4)	18·9 (9·8 to 32·6)	14·8 (7·4 to 25·8)	12·1 (6·1 to 21·1)	11 (5·6 to 19·3)	10·5 (5·6 to 18·2)	10·2 (5·3 to 17·9)	9·4 (4·8 to 16·4)	8·6 (4·3 to 15·6)	6·8 (3·6 to 12·1)	4·8 (2·5 to 8·1)	3·2 (1·7 to 5·4)	2·4 (1·2 to 4)	1·5 (0·8 to 2·6)	0·7 (0·4 to 1·3)	0·3 (0·2 to 0·5)	0·1 (0·1 to 0·2)	281
Contact dermatitis	0 (0 to 0)	0 (0 to 0)	0 (0 to 0)	0 (0 to 0)	0·7 (0·3 to 1·5)	3·9 (1·6 to 7·9)	6·6 (2·7 to 12)	8·2 (3·4 to 15·6)	9·5 (4·4 to 18)	9·7 (4·2 to 18·3)	9·6 (4 to 19·5)	9·4 (4·4 to 19)	9·8 (4·5 to 19·3)	10·2 (4·5 to 19)	9·7 (4·3 to 19)	8·2 (3·5 to 15·1)	6·3 (3 to 12)	4·5 (2·1 to 8·5)	3 (1·4 to 5·7)	1·6 (0·8 to 3)	0·7 (0·3 to 1·3)	0·3 (0·1 to 0·5)	0·1 (0 to 0·2)	112
Seborrhoeic dermatitis	0 (0 to 0)	0 (0 to 0)	0 (0 to 0)	0·4 (0·2 to 0·7)	0·5 (0·2 to 0·9)	0·8 (0·4 to 1·4)	0·9 (0·4 to 1·6)	1 (0·5 to 1·7)	1·1 (0·6 to 2)	1·2 (0·6 to 2)	1·1 (0·6 to 1·9)	1·1 (0·5 to 1·8)	1 (0·5 to 1·6)	0·9 (0·4 to 1·5)	0·8 (0·4 to 1·4)	0·7 (0·3 to 1·2)	0·6 (0·3 to 1·1)	0·5 (0·3 to 0·8)	0·4 (0·2 to 0·6)	0·3 (0·1 to 0·4)	0·1 (0·1 to 0·2)	0 (0 to 0·1)	0 (0 to 0)	13·2
Psoriasis	0 (0 to 0)	0 (0 to 0)	0 (0 to 0)	1·5 (0·9 to 2·2)	4·5 (2·2 to 7·1)	6·7 (3·7 to 10·5)	7·1 (3·8 to 11·3)	7·6 (4·3 to 12·1)	8·6 (4·9 to 13·2)	9·1 (5·5 to 14·1)	10·1 (6·4 to 14·9)	10·8 (6·6 to 15·2)	11·8 (7·3 to 17·3)	11·6 (7·5 to 16·8)	10·9 (7 to 15·8)	8·8 (5·8 to 12·6)	6·1 (3·9 to 8·6)	3·8 (2·4 to 5·4)	2·2 (1·4 to 3·2)	1·1 (0·7 to 1·6)	0·4 (0·2 to 0·6)	0·1 (0·1 to 0·2)	0 (0 to 0·1)	123
Bacterial skin diseases	0 (0 to 0)	0 (0 to 0)	7·2 (3·3 to 12·5)	4·1 (2·3 to 6·2)	4·3 (2·7 to 6·7)	3·9 (2·3 to 6)	3·3 (2 to 5·2)	4·4 (2·5 to 7·1)	4·6 (2·6 to 7·9)	7·3 (4 to 12·3)	6 (3·2 to 10·5)	9·8 (5·1 to 17·5)	8·8 (4·7 to 15·6)	9·8 (5·3 to 16·8)	9·7 (4·9 to 17)	9·2 (4·8 to 15·7)	6·5 (3·3 to 11·6)	4·5 (2·3 to 8·9)	4·3 (2·1 to 8·1)	3·4 (1·7 to 6·5)	1·9 (0·9 to 4)	0·9 (0·4 to 2)	0·6 (0·2 to 1·4)	114
Cellulitis	0 (0 to 0)	0 (0 to 0)	1·8 (0·6 to 3·8)	1·1 (0·5 to 1·9)	1·5 (0·7 to 2·4)	1 (0·6 to 1·7)	0·7 (0·4 to 1·3)	1 (0·5 to 1·8)	1·3 (0·7 to 2·6)	1·6 (0·8 to 2·9)	1·4 (0·7 to 2·7)	1·9 (1 to 3·9)	1·7 (0·9 to 3·5)	2·3 (1·1 to 4·7)	2·1 (1·1 to 4·3)	2 (0·9 to 4·2)	1·6 (0·7 to 3·2)	0·9 (0·4 to 2)	0·8 (0·3 to 1·8)	0·5 (0·2 to 1·3)	0·3 (0·1 to 0·7)	0·1 (0 to 0·4)	0·1 (0 to 0·4)	25·7
Pyoderma	0 (0 to 0)	0 (0 to 0)	5·4 (2·5 to 9·4)	3 (1·6 to 4·9)	2·8 (1·6 to 4·8)	2·9 (1·6 to 4·7)	2·5 (1·4 to 4·2)	3·4 (1·9 to 5·7)	3·3 (1·8 to 5·8)	5·8 (3·1 to 10·1)	4·6 (2·4 to 8)	7·9 (3·9 to 14·2)	7·1 (3·6 to 12·6)	7·5 (3·9 to 13)	7·5 (3·6 to 13·6)	7·2 (3·7 to 12·1)	4·9 (2·4 to 9)	3·6 (1·7 to 7·1)	3·5 (1·7 to 6·5)	2·8 (1·3 to 5·5)	1·6 (0·7 to 3·4)	0·8 (0·4 to 1·7)	0·5 (0·2 to 1)	88·8
Scabies	0 (0 to 0)	0 (0 to 0·1)	2·7 (1·4 to 4·6)	19·6 (9·8 to 34·2)	30·3 (14·7 to 53·6)	35·4 (17·4 to 61·5)	35·3 (17·9 to 61·2)	31·9 (16 to 56·7)	23·5 (11·9 to 41·3)	16·8 (7·7 to 30·3)	16 (7·9 to 27·3)	17·6 (8·9 to 30)	17·8 (9·2 to 30·4)	15·4 (7·8 to 25·9)	12·1 (6·2 to 21·1)	8·6 (4·4 to 15)	5·7 (2·9 to 10)	3·9 (2 to 6·8)	3·1 (1·6 to 5·4)	2·1 (1·1 to 3·7)	1·2 (0·6 to 2)	0·5 (0·3 to 0·9)	0·2 (0·1 to 0·4)	300
Fungal skin diseases	0 (0 to 0)	0 (0 to 0)	0·5 (0·2 to 1)	3·8 (1·4 to 8·2)	7·2 (2·7 to 15·6)	9·5 (3·5 to 21·1)	9·3 (3·3 to 20·9)	9·9 (3·8 to 21·6)	10·4 (3·9 to 23·2)	10·2 (3·8 to 23·3)	10·3 (4 to 22·5)	10·4 (4·1 to 22·4)	10·7 (4·2 to 23·7)	11 (4·3 to 24)	11·6 (4·6 to 25·3)	10·6 (4·1 to 22·9)	9·9 (4 to 21·4)	9·6 (3·7 to 20·5)	8·5 (3·2 to 17·8)	6·7 (2·6 to 14·5)	4·1 (1·6 to 8·6)	2 (0·8 to 4·1)	0·9 (0·4 to 1·8)	167
Viral skin diseases	0 (0 to 0)	0 (0 to 0)	0 (0 to 0)	21·4 (13·4 to 32·7)	39 (24·5 to 60·7)	23·2 (14·7 to 35·9)	13·4 (8·2 to 20·9)	10·7 (6·7 to 16·6)	8 (4·9 to 12·1)	6·2 (3·7 to 9·7)	5·7 (3·4 to 8·7)	5·5 (3·4 to 8·5)	5·1 (3·1 to 8·2)	4·6 (2·7 to 7·1)	3·8 (2·3 to 5·9)	3·1 (1·9 to 4·7)	2·1 (1·3 to 3·3)	1·3 (0·8 to 2)	0·8 (0·5 to 1·2)	0·4 (0·2 to 0·6)	0·2 (0·1 to 0·2)	0·1 (0 to 0·1)	0 (0 to 0)	155
Acne vulgaris	0 (0 to 0)	0 (0 to 0)	0 (0 to 0)	0 (0 to 0)	2·1 (1·2 to 3·5)	54·8 (31·3 to 91·3)	90·3 (51·6 to 147)	23·3 (13·1 to 37·2)	10·8 (5·9 to 17·6)	7·2 (4 to 12·1)	5·4 (2·8 to 9·2)	3·9 (2·2 to 6·4)	2·8 (1·6 to 4·5)	1·8 (1 to 2·9)	1·1 (0·6 to 1·8)	0·7 (0·4 to 1·1)	0·4 (0·2 to 0·7)	0·2 (0·1 to 0·4)	0·2 (0·1 to 0·2)	0·1 (0 to 0·1)	0 (0 to 0)	0 (0 to 0)	0 (0 to 0)	205
Alopecia areata	0 (0 to 0)	0 (0 to 0)	0 (0 to 0)	0·4 (0·2 to 0·7)	0·7 (0·3 to 1·2)	1 (0·5 to 1·8)	1·7 (1 to 2·8)	2·7 (1·6 to 4·4)	3·7 (2·3 to 6)	3·7 (2·3 to 5·9)	3·4 (2 to 5·2)	2·8 (1·7 to 4·5)	2·4 (1·4 to 3·7)	1·9 (1·1 to 3·1)	1·6 (0·9 to 2·4)	1·2 (0·7 to 2)	0·8 (0·5 to 1·2)	0·5 (0·3 to 0·8)	0·3 (0·2 to 0·5)	0·1 (0·1 to 0·2)	0·1 (0 to 0·1)	0 (0 to 0)	0 (0 to 0)	29·1
Pruritus	0 (0 to 0)	0 (0 to 0)	0·1 (0 to 0·1)	0·8 (0·3 to 1·6)	1·4 (0·6 to 2·8)	1·6 (0·7 to 3·2)	1·7 (0·7 to 3·4)	2·1 (0·9 to 4·2)	2·4 (1·1 to 4·7)	2·6 (1·1 to 5)	2·6 (1·1 to 5·1)	2·7 (1·2 to 5·1)	2·7 (1·2 to 5·4)	2·7 (1·2 to 5·5)	2·7 (1·1 to 5·5)	2·6 (1·1 to 5·2)	2·1 (0·9 to 4·4)	1·6 (0·7 to 3·2)	1·2 (0·5 to 2·3)	0·7 (0·3 to 1·4)	0·3 (0·1 to 0·7)	0·1 (0·1 to 0·2)	0·1 (0 to 0·1)	34·8
Urticaria	0 (0 to 0)	0 (0 to 0)	2·8 (1·7 to 4·3)	19·6 (11·8 to 30·5)	19·9 (11·6 to 32·2)	16 (8·6 to 26·7)	13·1 (7·1 to 22·2)	12·2 (6·7 to 20·1)	11·5 (6·2 to 18·7)	11·2 (6·3 to 18·1)	11·2 (6·4 to 17·9)	10·4 (5·9 to 16·1)	9·4 (5·1 to 15·4)	8·3 (4·8 to 13·5)	7·2 (3·9 to 11·8)	5·5 (3 to 9·2)	3·8 (2·1 to 6·4)	2·5 (1·4 to 4)	1·7 (0·9 to 2·7)	0·9 (0·5 to 1·5)	0·4 (0·2 to 0·6)	0·1 (0·1 to 0·2)	0·1 (0 to 0·1)	168
Decubitus ulcer	0 (0 to 0)	0 (0 to 0)	0 (0 to 0)	1·5 (0·5 to 3)	2·7 (1·3 to 4·4)	3·4 (1·5 to 5·7)	4·2 (1·7 to 6·5)	4·6 (1·6 to 8·5)	11 (5·3 to 19)	8·4 (3·8 to 14·5)	15·1 (8·2 to 26·4)	19·1 (10·1 to 35·1)	22·8 (13 to 40·3)	33·8 (19·8 to 58)	39·1 (23·9 to 64·3)	41 (25·7 to 69·5)	38·6 (25·1 to 65·7)	39 (25·1 to 67)	48·3 (31·8 to 83·8)	43·8 (29·1 to 77·4)	29·5 (19·5 to 53·6)	16 (10·3 to 28·9)	9·7 (5·4 to 22·6)	432
Other skin and subcutaneous diseases	0 (0 to 0)	0 (0 to 0)	6 (2·9 to 10·6)	5·9 (2·9 to 10·4)	5·7 (3 to 10·1)	7·7 (3·8 to 14)	9·3 (5 to 16·5)	11·1 (6·4 to 19)	12·2 (6·5 to 20·5)	16·5 (9·6 to 27·5)	17·1 (9·7 to 28·1)	20 (11·3 to 32·4)	21·2 (11·5 to 36·1)	25 (14·2 to 42·8)	23·9 (13·2 to 40·3)	22·3 (12 to 38·4)	18·3 (10·1 to 30·9)	12·7 (6·8 to 22·1)	10·2 (5·5 to 16·8)	6·1 (3·4 to 10·4)	2·7 (1·5 to 4·5)	1·2 (0·7 to 2)	0·4 (0·2 to 0·7)	255
Sense organ diseases	0 (0 to 0)	0 (0 to 0·1)	1 (0·6 to 1·5)	11·7 (7·5 to 17·2)	29·7 (19 to 43·8)	43·8 (27·9 to 62·7)	50·8 (32·5 to 73·8)	64·5 (40·6 to 95·1)	82·8 (51·6 to 125)	99·4 (61·8 to 153)	118 (72·5 to 182)	149 (92·4 to 231)	209 (131 to 334)	266 (170 to 422)	309 (198 to 480)	312 (200 to 472)	277 (182 to 408)	239 (160 to 346)	218 (151 to 308)	165 (116 to 230)	94·4 (66·6 to 128)	45 (32 to 60·2)	20·3 (14·5 to 26·8)	2,810
Blindness and vision loss	0 (0 to 0)	0 (0 to 0)	0·2 (0·1 to 0·3)	3·2 (1·8 to 5·3)	8·4 (5 to 13·6)	12·1 (7·3 to 19·1)	13·6 (8·2 to 21·1)	16·1 (9·4 to 24·9)	18·1 (11·3 to 26·8)	20·2 (12·6 to 30·7)	25·5 (16·4 to 38·6)	35 (22·2 to 53·3)	47·7 (30 to 75·3)	60·4 (38·2 to 94·6)	73 (46·9 to 115)	78·8 (51·6 to 121)	76·4 (49·8 to 113)	70·7 (47·4 to 101)	66·7 (45·8 to 94·8)	52 (35·5 to 71·8)	30·6 (21·3 to 41·6)	15·5 (10·8 to 21·2)	7·9 (5·5 to 11)	732
Glaucoma	0 (0 to 0)	0 (0 to 0)	0 (0 to 0)	0 (0 to 0)	0 (0 to 0)	0 (0 to 0)	0 (0 to 0)	0 (0 to 0)	0 (0 to 0)	0 (0 to 0)	0 (0 to 0)	0 (0 to 0)	0·1 (0 to 0·2)	0·8 (0·3 to 1·6)	2·2 (1 to 3·8)	3·6 (2 to 6)	4·6 (2·7 to 7·2)	5·4 (3·3 to 8·2)	6·5 (3·9 to 10·2)	5·9 (3·6 to 8·9)	3·9 (2·4 to 6)	2 (1·2 to 3)	1 (0·5 to 1·6)	36
Cataract	0 (0 to 0)	0 (0 to 0)	0 (0 to 0)	0 (0 to 0)	0 (0 to 0)	0 (0 to 0)	0 (0 to 0)	0·1 (0 to 0·1)	0·4 (0·2 to 0·7)	0·9 (0·5 to 1·6)	1·8 (0·9 to 3·1)	3·1 (1·6 to 5·1)	4·9 (2·8 to 8·1)	6·4 (3·7 to 10·1)	8·1 (4·8 to 12·4)	9·8 (6 to 15·1)	11·9 (7·3 to 17·7)	14·5 (9·6 to 21·3)	17·9 (11·7 to 26·2)	16·2 (10·8 to 22·8)	10·4 (7 to 14·5)	5·2 (3·5 to 7·2)	2·6 (1·6 to 3·8)	114
Age-related macular degeneration	0 (0 to 0)	0 (0 to 0)	0 (0 to 0)	0 (0 to 0)	0 (0 to 0)	0 (0 to 0)	0 (0 to 0)	0 (0 to 0)	0 (0 to 0)	0 (0 to 0)	0 (0 to 0)	0 (0 to 0)	0 (0 to 0·1)	0·3 (0·1 to 0·5)	0·5 (0·3 to 0·9)	0·7 (0·3 to 1·3)	0·8 (0·4 to 1·3)	0·8 (0·4 to 1·3)	0·8 (0·4 to 1·3)	0·6 (0·3 to 1)	0·4 (0·2 to 0·6)	0·2 (0·1 to 0·3)	0·1 (0 to 0·1)	5·1
Refraction disorders	0 (0 to 0)	0 (0 to 0)	0·1 (0 to 0·2)	2·7 (1·5 to 4·6)	7·3 (4·2 to 12·3)	10·4 (6 to 16·7)	11·7 (6·9 to 18·9)	13·5 (7·6 to 21·7)	14·3 (8·6 to 22·2)	14·1 (8·3 to 21·4)	15·1 (9·2 to 22·8)	16·6 (10·1 to 25·7)	17·9 (11 to 27·3)	17·4 (10·3 to 26·9)	16·7 (10·3 to 25·8)	14·4 (8·9 to 22·1)	11·4 (7 to 17·2)	9·4 (5·8 to 14·4)	8·2 (5·2 to 12·4)	5·3 (3·2 to 7·9)	2·4 (1·5 to 3·5)	0·8 (0·5 to 1·2)	0·3 (0·2 to 0·5)	210
Near vision loss	0 (0 to 0)	0 (0 to 0)	0 (0 to 0)	0 (0 to 0)	0·1 (0 to 0·1)	0·1 (0 to 0·3)	0·1 (0 to 0·4)	0·4 (0·1 to 0·8)	0·8 (0·3 to 1·8)	2 (0·8 to 4·4)	3·9 (1·4 to 9)	8 (3·2 to 17)	14 (5·7 to 29·7)	20·5 (8·4 to 42·7)	25·9 (10·6 to 55)	27·1 (11·4 to 56·2)	23·6 (10 to 47·4)	19·1 (8 to 37·9)	15·8 (6·5 to 32·1)	10·8 (4·6 to 21·5)	5·5 (2·4 to 10·9)	2·4 (1 to 4·7)	1 (0·4 to 2·2)	181
Other vision loss	0 (0 to 0)	0 (0 to 0)	0·1 (0 to 0·1)	0·5 (0·2 to 0·8)	1·1 (0·5 to 1·9)	1·6 (0·7 to 2·8)	1·8 (0·8 to 3·3)	2·2 (1 to 4)	2·6 (1·3 to 4·5)	3·3 (1·6 to 5·5)	4·7 (2·6 to 7·9)	7·3 (4·1 to 12)	10·7 (6 to 16·8)	15·1 (9·3 to 22·8)	19·6 (12·2 to 29·2)	23·2 (15·2 to 33·7)	24·1 (15·6 to 34·5)	21·5 (14·1 to 30·5)	17·5 (11·1 to 25·5)	13·3 (8·7 to 19·2)	8 (5·1 to 12)	5 (3·4 to 7·1)	3 (1·9 to 4·4)	186
Age-related and other hearing loss	0 (0 to 0)	0 (0 to 0)	0·4 (0·2 to 0·6)	6 (3·3 to 9·7)	18 (10·3 to 28·1)	27·9 (16·8 to 40·8)	33 (19·9 to 48·9)	43·7 (26·1 to 65·9)	59·1 (35·8 to 91·2)	71·9 (42·8 to 114)	83·6 (49·6 to 133)	105 (61·6 to 168)	151 (89·6 to 247)	192 (118 to 309)	222 (138 to 352)	218 (134 to 343)	187 (117 to 285)	158 (101 to 234)	142 (94·9 to 207)	108 (72·4 to 151)	61·4 (42·2 to 84)	28·6 (20 to 38·9)	11·9 (8·4 to 15·9)	1,930
Other sense organ diseases	0 (0 to 0)	0 (0 to 0)	0·5 (0·3 to 0·8)	2·6 (1·3 to 4·5)	3·4 (1·6 to 5·7)	3·8 (1·9 to 6·5)	4·1 (2 to 7·5)	4·6 (2 to 8·6)	5·6 (2·5 to 10·9)	7·3 (3·2 to 14·1)	8·6 (4 to 15·9)	9·2 (4·2 to 17·6)	10·5 (4·7 to 19·8)	13·5 (6·3 to 26)	13·7 (6·4 to 26·6)	14·8 (7 to 27·5)	13·6 (6·3 to 25·2)	11 (5·1 to 20·2)	9·3 (4·3 to 17·1)	5·8 (2·9 to 10·8)	2·4 (1·1 to 4·3)	0·9 (0·4 to 1·6)	0·5 (0·2 to 0·9)	146
Musculoskeletal disorders	0 (0 to 0)	0 (0 to 0)	0 (0 to 0)	0 (0 to 0)	15·9 (8·4 to 27·3)	89·2 (56 to 139)	192 (129 to 274)	294 (198 to 407)	418 (284 to 579)	506 (338 to 688)	591 (415 to 801)	676 (472 to 916)	761 (534 to 1,060)	782 (545 to 1,100)	751 (515 to 1,040)	633 (437 to 897)	455 (317 to 638)	315 (216 to 442)	221 (151 to 316)	132 (90·7 to 187)	67·3 (48 to 91·8)	26 (18·7 to 36)	10·4 (7·4 to 14·5)	6,940
Rheumatoid arthritis	0 (0 to 0)	0 (0 to 0)	0 (0 to 0)	0 (0 to 0)	0·3 (0·1 to 0·4)	0·9 (0·5 to 1·5)	2·5 (1·4 to 4)	4·9 (2·4 to 8·3)	7·3 (4 to 11·6)	9·7 (5·5 to 14·8)	12·7 (7·8 to 18·9)	15·2 (9·6 to 22)	17·4 (11·3 to 25·3)	18 (12·1 to 25·6)	17·7 (11·9 to 24·8)	14·6 (10·2 to 20·3)	10·9 (7·7 to 14·6)	7·3 (5·1 to 10·1)	5·9 (4·3 to 8·2)	3·9 (2·8 to 5·4)	3·8 (2·6 to 5·4)	1·6 (1·1 to 2·4)	0·6 (0·4 to 1·1)	155
Osteoarthritis	0 (0 to 0)	0 (0 to 0)	0 (0 to 0)	0 (0 to 0)	0 (0 to 0)	0 (0 to 0)	0 (0 to 0)	0 (0 to 0)	0 (0 to 0)	2·3 (1 to 5)	14·6 (7·1 to 30·1)	40·8 (20·2 to 84·2)	80·4 (39·4 to 163)	119 (57 to 242)	143 (69·2 to 290)	141 (69·9 to 280)	112 (55·8 to 225)	82·8 (41·1 to 170)	61·6 (30·4 to 122)	38·2 (19·4 to 76·5)	18 (9·3 to 36·1)	7·4 (3·8 to 14·7)	3 (1·5 to 5·9)	863
Osteoarthritis hip	0 (0 to 0)	0 (0 to 0)	0 (0 to 0)	0 (0 to 0)	0 (0 to 0)	0 (0 to 0)	0 (0 to 0)	0 (0 to 0)	0 (0 to 0)	0·8 (0·3 to 1·7)	1·2 (0·4 to 2·8)	2·1 (0·8 to 4·8)	3·5 (1·5 to 7·9)	5·3 (2·3 to 11·8)	6·8 (3 to 14·7)	7·3 (3·2 to 15·6)	6·1 (2·7 to 12·8)	4·7 (2·1 to 9·7)	3·5 (1·7 to 7·5)	2·2 (1·1 to 4·5)	1·1 (0·5 to 2·2)	0·4 (0·2 to 0·9)	0·2 (0·1 to 0·4)	45·3
Osteoarthritis knee	0 (0 to 0)	0 (0 to 0)	0 (0 to 0)	0 (0 to 0)	0 (0 to 0)	0 (0 to 0)	0 (0 to 0)	0 (0 to 0)	0 (0 to 0)	1·2 (0·4 to 2·8)	10·8 (5 to 22·2)	30·8 (14·7 to 63·7)	60·4 (28·7 to 124)	88·1 (41·4 to 180)	104 (49·3 to 217)	102 (48·8 to 206)	79·7 (38·6 to 163)	58·2 (28·5 to 120)	42·3 (20·7 to 86·2)	25·4 (12·6 to 51·9)	11·4 (5·6 to 23·1)	4·4 (2·2 to 8·8)	1·6 (0·8 to 3·2)	620
Osteoarthritis hand	0 (0 to 0)	0 (0 to 0)	0 (0 to 0)	0 (0 to 0)	0 (0 to 0)	0 (0 to 0)	0 (0 to 0)	0 (0 to 0)	0 (0 to 0)	0·2 (0 to 0·6)	1·5 (0·4 to 3·8)	4·3 (1·6 to 9·5)	8·8 (3·5 to 18·8)	13·3 (6 to 28·3)	16·3 (7·8 to 36·4)	16·1 (7·5 to 34)	12·7 (6 to 27·4)	9·7 (4·8 to 19·6)	7·5 (3·6 to 15·7)	5 (2·5 to 10·4)	2·6 (1·3 to 5·4)	1·2 (0·6 to 2·6)	0·6 (0·3 to 1·2)	99·9
Osteoarthritis other	0 (0 to 0)	0 (0 to 0)	0 (0 to 0)	0 (0 to 0)	0 (0 to 0)	0 (0 to 0)	0 (0 to 0)	0 (0 to 0)	0 (0 to 0)	0·1 (0 to 0·4)	1·1 (0·3 to 2·6)	3·7 (1·4 to 8·4)	7·7 (3·3 to 17)	12 (5·3 to 25·5)	15·3 (6·9 to 33·6)	15·8 (7·3 to 34)	13·1 (6·3 to 27·6)	10·2 (5 to 21·4)	8·2 (4 to 17)	5·6 (2·8 to 11·5)	2·9 (1·5 to 6)	1·3 (0·7 to 2·6)	0·6 (0·3 to 1·2)	97·5
Low back pain	0 (0 to 0)	0 (0 to 0)	0 (0 to 0)	0 (0 to 0)	13·4 (6·1 to 24·4)	76·4 (44·5 to 124)	133 (77·5 to 211)	166 (99·5 to 256)	201 (117 to 316)	221 (123 to 338)	256 (155 to 386)	284 (173 to 438)	307 (188 to 482)	309 (188 to 485)	288 (181 to 435)	240 (153 to 362)	173 (110 to 266)	122 (76·8 to 180)	89·5 (56·2 to 136)	56·7 (35·3 to 84·3)	25·4 (15·4 to 36·9)	9·4 (6 to 14)	3·7 (2·3 to 5·6)	2,970
Neck pain	0 (0 to 0)	0 (0 to 0)	0 (0 to 0)	0 (0 to 0)	0·8 (0·2 to 1·9)	5·2 (2·2 to 10·5)	11·3 (5 to 21·4)	17·9 (8·1 to 35·3)	28·9 (13·6 to 57·2)	43·3 (20·8 to 81·6)	58·5 (29·7 to 102)	72 (36·5 to 128)	81·4 (40·8 to 145)	71·1 (36·4 to 133)	61·7 (29·3 to 108)	47·8 (24·2 to 83)	31·9 (16·8 to 53·3)	21·2 (11·3 to 36·4)	14·2 (7·1 to 25·7)	7·7 (3·8 to 13·8)	3·5 (1·9 to 6)	1·4 (0·7 to 2·4)	0·6 (0·3 to 1·1)	580
Gout	0 (0 to 0)	0 (0 to 0)	0 (0 to 0)	0 (0 to 0)	0 (0 to 0)	0 (0 to 0)	0 (0 to 0·1)	0·4 (0·2 to 0·7)	1 (0·3 to 2·2)	1·7 (0·7 to 3·4)	2·5 (1·1 to 4·4)	3·2 (1·5 to 5·7)	3·9 (1·8 to 6·6)	4·1 (1·9 to 7·6)	4 (1·9 to 6·9)	3·5 (1·7 to 6·4)	2·8 (1·3 to 4·8)	2·2 (1·1 to 3·6)	1·8 (0·9 to 3)	1·2 (0·6 to 2)	0·6 (0·3 to 1·1)	0·3 (0·2 to 0·5)	0·1 (0·1 to 0·2)	33·3
Other musculoskeletal disorders	0 (0 to 0)	0 (0 to 0)	0 (0 to 0)	0 (0 to 0)	1·5 (1 to 2·3)	6·7 (3·8 to 11·8)	45·7 (28·8 to 69·2)	105 (71·9 to 147)	180 (120 to 259)	227 (150 to 328)	246 (166 to 343)	261 (170 to 381)	271 (184 to 393)	262 (173 to 388)	237 (153 to 346)	187 (117 to 290)	125 (78·4 to 185)	79·5 (47·6 to 123)	48·1 (28·9 to 74·7)	24·5 (13 to 40·2)	16 (10·4 to 22·7)	5·9 (3·4 to 9)	2·4 (1·2 to 4·2)	2,330
Other non-communicable diseases	208 (146 to 316)	142 (82·1 to 238)	448 (328 to 584)	222 (165 to 301)	172 (135 to 216)	194 (152 to 243)	275 (213 to 353)	399 (303 to 533)	525 (397 to 697)	589 (438 to 770)	651 (495 to 847)	657 (493 to 877)	628 (464 to 820)	585 (445 to 760)	528 (397 to 682)	465 (356 to 604)	357 (274 to 457)	269 (209 to 345)	213 (167 to 271)	146 (115 to 182)	83·2 (66·9 to 103)	37·5 (29·7 to 45·7)	16·3 (12 to 20·9)	7,810

Congenital birth defects	194 (133 to 301)	117 (67·7 to 196)	245 (172 to 334)	100 (70·4 to 141)	61·6 (46·1 to 81·5)	62 (47·4 to 79·2)	62·4 (47·2 to 79·9)	55·6 (41·8 to 70·8)	52·8 (38·4 to 67·9)	43·1 (31·8 to 54·9)	40·7 (30 to 52·6)	33·4 (25·2 to 43·1)	29·5 (22·5 to 37·5)	26·1 (20·1 to 33·5)	20·2 (15·2 to 25·8)	12·1 (9·3 to 15·7)	7·1 (5·4 to 9·1)	2·2 (1·4 to 3·3)	1·3 (0·8 to 2)	0·7 (0·4 to 1·1)	0·3 (0·2 to 0·4)	0·1 (0·1 to 0·2)	0 (0 to 0·1)	1,170
Neural tube defects	11·5 (5·3 to 20·4)	4·2 (1·8 to 8·7)	9·6 (4·8 to 17·3)	6 (3·2 to 10·1)	1·9 (1·3 to 2·5)	2·6 (1·8 to 3·7)	2·8 (1·9 to 4)	2·1 (1·4 to 3·2)	2·3 (1·5 to 3·6)	1·6 (1 to 2·3)	0·9 (0·6 to 1·2)	0·9 (0·6 to 1·3)	0·6 (0·4 to 0·8)	0·4 (0·3 to 0·5)	0·3 (0·2 to 0·4)	0·2 (0·1 to 0·3)	0·1 (0·1 to 0·1)	0 (0 to 0·1)	0 (0 to 0)	0 (0 to 0)	0 (0 to 0)	0 (0 to 0)	0 (0 to 0)	48
Congenital heart anomalies	73 (42·2 to 130)	55·7 (31·3 to 94·6)	114 (75·3 to 166)	38·4 (23·9 to 62·8)	17·6 (11·5 to 26·5)	15·4 (10·9 to 21·8)	16·9 (11·9 to 23·4)	16·1 (10·5 to 22·6)	16·3 (10·1 to 23·1)	12·6 (8·1 to 18)	12·4 (7·9 to 17·6)	9 (6·2 to 12·4)	6·9 (4·8 to 9·6)	5·4 (3·7 to 7·4)	3·2 (2·2 to 4·5)	2 (1·4 to 2·9)	1·3 (0·9 to 1·7)	0·2 (0·1 to 0·4)	0·1 (0 to 0·2)	0·1 (0 to 0·2)	0 (0 to 0·1)	0 (0 to 0)	0 (0 to 0)	417
Orofacial clefts	0 (0 to 0·1)	0 (0 to 0)	0·3 (0·1 to 0·6)	0·8 (0·4 to 1·4)	0·9 (0·4 to 1·5)	1 (0·4 to 1·8)	0·9 (0·4 to 1·6)	0·9 (0·4 to 1·7)	0·9 (0·4 to 1·6)	0·8 (0·4 to 1·5)	0·8 (0·3 to 1·5)	0·8 (0·3 to 1·5)	0·8 (0·3 to 1·4)	0·7 (0·3 to 1·2)	0·6 (0·2 to 1)	0·4 (0·2 to 0·8)	0·3 (0·1 to 0·5)	0·2 (0·1 to 0·4)	0·1 (0·1 to 0·2)	0·1 (0 to 0·1)	0 (0 to 0·1)	0 (0 to 0)	0 (0 to 0)	11·3
Down syndrome	3 (1·6 to 4·9)	2·4 (1·2 to 4·3)	12 (7 to 18·7)	7·5 (4·5 to 11·9)	3·3 (2·1 to 4·9)	4 (2·8 to 5·6)	5·2 (3·5 to 7·4)	5·5 (3·5 to 8)	5·4 (3·5 to 7·9)	5·7 (3·5 to 8·8)	6·6 (4·1 to 9·6)	6·1 (3·9 to 9·1)	6·3 (4·2 to 9·1)	6·5 (4·3 to 9·2)	5·8 (3·8 to 8·2)	2·5 (1·7 to 3·6)	1 (0·6 to 1·4)	0 (0 to 0)	0 (0 to 0)	0 (0 to 0)	0 (0 to 0)	0 (0 to 0)	0 (0 to 0)	88·7
Turner syndrome	0 (0 to 0)	0 (0 to 0)	0 (0 to 0)	0 (0 to 0·1)	0 (0 to 0·1)	0 (0 to 0·1)	0·1 (0 to 0·1)	0·1 (0 to 0·1)	0·1 (0 to 0·1)	0 (0 to 0·1)	0 (0 to 0·1)	0 (0 to 0)	0 (0 to 0)	0 (0 to 0)	0 (0 to 0)	0 (0 to 0)	0 (0 to 0)	0 (0 to 0)	0 (0 to 0)	0 (0 to 0)	0 (0 to 0)	0 (0 to 0)	0 (0 to 0)	0·5
Klinefelter syndrome	0 (0 to 0)	0 (0 to 0)	0 (0 to 0)	0 (0 to 0)	0 (0 to 0)	0 (0 to 0)	0 (0 to 0)	0 (0 to 0)	0 (0 to 0)	0 (0 to 0)	0 (0 to 0)	0 (0 to 0)	0 (0 to 0)	0 (0 to 0)	0 (0 to 0)	0 (0 to 0)	0 (0 to 0)	0 (0 to 0)	0 (0 to 0)	0 (0 to 0)	0 (0 to 0)	0 (0 to 0)	0 (0 to 0)	0
Other chromosomal abnormalities	29·2 (17 to 49·7)	20 (10·3 to 36·5)	38·5 (24·3 to 59·8)	9·2 (5·8 to 14·3)	5·4 (3·7 to 7·9)	5·5 (3·8 to 7·7)	6·6 (4·4 to 9·7)	5·1 (3·4 to 7·6)	5·5 (3·3 to 8·4)	3 (1·9 to 4·6)	2·6 (1·6 to 4·1)	1·3 (0·8 to 1·8)	0·8 (0·5 to 1·3)	0·7 (0·4 to 1)	0·4 (0·2 to 0·6)	0·2 (0·1 to 0·3)	0·1 (0·1 to 0·1)	0 (0 to 0)	0 (0 to 0)	0 (0 to 0)	0 (0 to 0)	0 (0 to 0)	0 (0 to 0)	134
Congenital musculoskeletal and limb anomalies	7·1 (4 to 12·2)	3·7 (1·9 to 6·7)	11·1 (7 to 16·9)	14 (8·4 to 21·1)	17·3 (10·6 to 26·1)	19·8 (11·5 to 30·4)	16·9 (10·3 to 25·6)	11·7 (7·5 to 17·4)	10·6 (6·6 to 15·9)	9·2 (5·4 to 14)	9·1 (5·5 to 13·8)	8·9 (5·4 to 13·3)	8·6 (5·4 to 12·9)	7·7 (4·8 to 11·7)	6·5 (4 to 10·1)	4·8 (2·9 to 7·1)	2·9 (1·8 to 4·3)	1·4 (0·8 to 2·2)	0·8 (0·4 to 1·3)	0·4 (0·2 to 0·6)	0·1 (0·1 to 0·2)	0 (0 to 0·1)	0 (0 to 0)	173
Urogenital congenital anomalies	4·1 (2 to 12)	1·9 (0·9 to 3·6)	2·8 (1·6 to 4·8)	1·9 (1·2 to 2·8)	1·4 (0·9 to 2·1)	1·2 (0·8 to 1·8)	0·9 (0·6 to 1·3)	0·8 (0·5 to 1·2)	0·7 (0·4 to 1)	0·7 (0·4 to 1)	0·4 (0·3 to 0·6)	0·3 (0·2 to 0·5)	0·3 (0·2 to 0·5)	0·2 (0·1 to 0·2)	0·2 (0·1 to 0·3)	0·1 (0·1 to 0·2)	0·1 (0 to 0·1)	0 (0 to 0)	0 (0 to 0)	0 (0 to 0)	0 (0 to 0)	0 (0 to 0)	0 (0 to 0)	18·1
Digestive congenital anomalies	15·2 (8·8 to 27·2)	9·9 (5 to 17·9)	14·7 (8·9 to 23·5)	3·7 (2·3 to 6)	1·4 (0·8 to 2·2)	0·8 (0·5 to 1·1)	0·6 (0·4 to 0·9)	0·4 (0·2 to 0·5)	0·4 (0·3 to 0·6)	0·3 (0·2 to 0·4)	0·2 (0·2 to 0·3)	0·2 (0·1 to 0·3)	0·2 (0·1 to 0·3)	0·2 (0·1 to 0·3)	0·1 (0·1 to 0·2)	0·1 (0·1 to 0·2)	0·1 (0·1 to 0·1)	0 (0 to 0)	0 (0 to 0)	0 (0 to 0)	0 (0 to 0)	0 (0 to 0)	0 (0 to 0)	48·4
Other congenital birth defects	50·9 (31·8 to 78·7)	19·5 (10·1 to 34·9)	41·8 (27·1 to 61·6)	18·8 (12 to 29·1)	12·4 (8·3 to 18)	11·6 (8·4 to 16)	11·5 (8·1 to 16·1)	13 (8·3 to 18·2)	10·6 (6·7 to 15·3)	9·1 (5·8 to 12·9)	7·6 (5 to 11)	5·9 (3·9 to 8·6)	5 (3·3 to 7·6)	4·5 (3 to 6·9)	3·1 (2 to 4·8)	1·7 (1·1 to 3)	1·3 (0·8 to 2·2)	0·4 (0·1 to 0·9)	0·2 (0·1 to 0·7)	0·2 (0 to 0·4)	0·1 (0 to 0·2)	0 (0 to 0·1)	0 (0 to 0)	229
Urinary diseases and male infertility	0·4 (0·2 to 0·6)	1·2 (0·6 to 2·1)	6·3 (4 to 9·5)	3·6 (2·4 to 5)	3 (2·2 to 4·1)	4·7 (3·5 to 6·3)	8·3 (6·2 to 10·9)	17·2 (12·2 to 23·6)	22·3 (15·8 to 30)	30·2 (21·4 to 42·4)	40 (28·4 to 56)	41·4 (29·5 to 55·9)	43·6 (30·1 to 59·4)	54·1 (39 to 73·9)	65·7 (47·8 to 88·3)	73·9 (53·9 to 95·4)	69·7 (52·2 to 89·7)	61·4 (46·3 to 78·9)	55·8 (40·4 to 72·5)	42·4 (30·6 to 56·6)	25·2 (17·7 to 33·2)	11·6 (8·2 to 15·5)	4·8 (3·1 to 6·5)	687
Urolithiasis	0 (0 to 0)	0 (0 to 0)	0 (0 to 0)	0 (0 to 0·1)	0·1 (0 to 0·1)	0·1 (0·1 to 0·2)	0·2 (0·1 to 0·4)	0·5 (0·3 to 0·8)	0·9 (0·5 to 1·3)	1·7 (1 to 2·8)	2·7 (1·6 to 4·3)	2·4 (1·4 to 4)	3·9 (2·4 to 6·2)	3·5 (2·1 to 5·6)	3·8 (2·2 to 5·8)	2·9 (1·8 to 4·3)	1·9 (1·2 to 2·9)	1·1 (0·7 to 1·7)	0·7 (0·4 to 1·1)	0·3 (0·2 to 0·4)	0·1 (0·1 to 0·2)	0·1 (0 to 0·1)	0 (0 to 0)	26·9
Benign prostatic hyperplasia	0 (0 to 0)	0 (0 to 0)	0 (0 to 0)	0 (0 to 0)	0 (0 to 0)	0 (0 to 0)	0 (0 to 0)	0 (0 to 0)	0 (0 to 0)	0 (0 to 0)	0 (0 to 0)	0·1 (0 to 0·4)	1 (0·4 to 2·1)	4 (1·9 to 7)	11·6 (5·7 to 20·3)	18·6 (9·6 to 31·7)	20·6 (11·6 to 32·9)	17·3 (9·5 to 28·1)	11·9 (6·6 to 19·2)	6·2 (3·5 to 10)	2·2 (1·2 to 3·5)	0·6 (0·3 to 1)	0·1 (0·1 to 0·2)	94·3
Male infertility	0 (0 to 0)	0 (0 to 0)	0 (0 to 0)	0 (0 to 0)	0 (0 to 0)	0 (0 to 0)	0 (0 to 0)	2·8 (0·8 to 7·3)	3·5 (1 to 8·9)	3·6 (1 to 9·8)	3·5 (1 to 9·1)	3·2 (0·9 to 8·1)	0·5 (0·1 to 1·3)	0 (0 to 0)	0 (0 to 0)	0 (0 to 0)	0 (0 to 0)	0 (0 to 0)	0 (0 to 0)	0 (0 to 0)	0 (0 to 0)	0 (0 to 0)	0 (0 to 0)	17
Other urinary diseases	0·2 (0·1 to 0·3)	0·7 (0·3 to 1·3)	0·6 (0·3 to 1·1)	0·8 (0·5 to 1·1)	0·7 (0·4 to 1)	1·4 (0·9 to 2·1)	1·6 (1 to 2·4)	3·2 (1·8 to 5·2)	1·8 (1·1 to 2·8)	2·8 (1·7 to 4·6)	4·3 (2·3 to 7·1)	4·1 (2·4 to 6·4)	4·9 (2·8 to 7·8)	5·1 (2·9 to 8·3)	4·6 (2·7 to 7·2)	5·4 (3·1 to 8·6)	4·2 (2·2 to 6·9)	2·6 (1·3 to 4·5)	2·6 (1·3 to 4·5)	1·8 (0·9 to 3·3)	0·9 (0·4 to 1·6)	0·5 (0·2 to 0·9)	0·1 (0·1 to 0·3)	54·8
Gynecological diseases	0 (0 to 0)	0 (0 to 0)	0 (0 to 0)	0 (0 to 0)	0 (0 to 0)	22·5 (11·3 to 39·6)	57·1 (32·2 to 96·3)	126 (78·5 to 192)	190 (125 to 279)	218 (144 to 313)	235 (158 to 336)	235 (155 to 344)	195 (128 to 277)	128 (77·3 to 190)	72·7 (44·7 to 105)	43·9 (25·9 to 67·1)	27 (16·4 to 39·9)	16·9 (10·9 to 24·7)	11 (6·9 to 16·5)	6·3 (3·8 to 9·6)	3·1 (1·9 to 4·6)	1·4 (0·9 to 2·2)	0·7 (0·4 to 1·1)	1,590
Uterine fibroids	0 (0 to 0)	0 (0 to 0)	0 (0 to 0)	0 (0 to 0)	0 (0 to 0)	0·4 (0·1 to 0·8)	0·9 (0·4 to 1·9)	2·2 (0·9 to 4·6)	5·7 (2·4 to 12·2)	11·7 (5·3 to 24·2)	20·4 (10 to 38·7)	23·1 (11·6 to 43·8)	21·2 (10·8 to 38·6)	14·1 (6·8 to 26·2)	8·9 (4·3 to 16·9)	4·8 (2·2 to 9·3)	2·4 (1·1 to 4·5)	1·2 (0·5 to 2·3)	0·6 (0·3 to 1·2)	0·3 (0·1 to 0·5)	0·1 (0 to 0·2)	0 (0 to 0·1)	0 (0 to 0)	118
Polycystic ovarian syndrome	0 (0 to 0)	0 (0 to 0)	0 (0 to 0)	0 (0 to 0)	0 (0 to 0)	1 (0·4 to 2·1)	4·2 (1·7 to 8·6)	5 (2 to 10·3)	4·8 (2 to 10·1)	4·4 (1·8 to 9·1)	4·2 (1·7 to 8·5)	4 (1·6 to 8·2)	3·3 (1·4 to 6·7)	0·8 (0·3 to 1·7)	0 (0 to 0)	0 (0 to 0)	0 (0 to 0)	0 (0 to 0)	0 (0 to 0)	0 (0 to 0)	0 (0 to 0)	0 (0 to 0)	0 (0 to 0)	31·6
Female infertility	0 (0 to 0)	0 (0 to 0)	0 (0 to 0)	0 (0 to 0)	0 (0 to 0)	0 (0 to 0)	0 (0 to 0)	4·2 (0·8 to 11·6)	6·4 (1·5 to 17·3)	7·7 (1·6 to 22·8)	8·2 (1·1 to 25)	7·3 (0·9 to 20·8)	0·1 (0 to 1·1)	0 (0 to 0)	0 (0 to 0)	0 (0 to 0)	0 (0 to 0)	0 (0 to 0)	0 (0 to 0)	0 (0 to 0)	0 (0 to 0)	0 (0 to 0)	0 (0 to 0)	33·9
Endometriosis	0 (0 to 0)	0 (0 to 0)	0 (0 to 0)	0 (0 to 0)	0 (0 to 0)	0 (0 to 0)	4·3 (1·7 to 9·3)	17·3 (8 to 31·6)	20·7 (10·2 to 37·4)	16 (8 to 28·5)	13·9 (7 to 24·9)	13·4 (6·9 to 22·9)	11·8 (6·3 to 19·6)	5·4 (2·7 to 9·1)	0 (0 to 0)	0 (0 to 0)	0 (0 to 0)	0 (0 to 0)	0 (0 to 0)	0 (0 to 0)	0 (0 to 0)	0 (0 to 0)	0 (0 to 0)	103
Genital prolapse	0 (0 to 0)	0 (0 to 0)	0 (0 to 0)	0 (0 to 0)	0 (0 to 0)	0 (0 to 0)	0 (0 to 0)	0·1 (0 to 0·3)	0·4 (0·1 to 0·9)	0·8 (0·3 to 1·7)	1·5 (0·6 to 3·1)	2·3 (0·9 to 5)	3·1 (1·4 to 6·4)	3·4 (1·5 to 6·8)	3·3 (1·4 to 6·5)	2·7 (1·2 to 5·5)	1·9 (0·9 to 3·8)	1·3 (0·6 to 2·6)	1 (0·4 to 1·9)	0·6 (0·3 to 1·1)	0·3 (0·1 to 0·6)	0·2 (0·1 to 0·3)	0·1 (0 to 0·2)	23
Premenstrual syndrome	0 (0 to 0)	0 (0 to 0)	0 (0 to 0)	0 (0 to 0)	0 (0 to 0)	20·2 (9·5 to 37·3)	45·3 (23 to 81·4)	58·7 (28·7 to 104)	59·8 (34·8 to 97·6)	57·7 (30·2 to 93·4)	62·1 (34·4 to 98·3)	61·5 (33·9 to 99)	42·8 (24·4 to 72·8)	14 (7·1 to 25·4)	0 (0 to 0)	0 (0 to 0)	0 (0 to 0)	0 (0 to 0)	0 (0 to 0)	0 (0 to 0)	0 (0 to 0)	0 (0 to 0)	0 (0 to 0)	422
Other gynecological diseases	0 (0 to 0)	0 (0 to 0)	0 (0 to 0)	0 (0 to 0)	0 (0 to 0)	0·9 (0·3 to 2·2)	2·5 (1·2 to 4·2)	38·4 (21·5 to 62)	92·1 (51·6 to 144)	119 (71·2 to 185)	125 (74·4 to 194)	124 (70·5 to 195)	113 (64·5 to 175)	89·8 (44·6 to 144)	60·5 (36·2 to 92)	36·4 (19·6 to 57·8)	22·8 (13·1 to 35·3)	14·4 (8·9 to 21·8)	9·5 (5·8 to 14·5)	5·4 (3·1 to 8·4)	2·7 (1·6 to 4·1)	1·2 (0·7 to 2)	0·6 (0·3 to 1)	857
Hemoglobinopathies and hemolytic anemias	0·7 (0·5 to 1)	1·4 (0·9 to 2·1)	10 (6·8 to 14·3)	26·5 (17·5 to 37·8)	41·8 (28 to 60·1)	37 (26·2 to 50·1)	54·6 (39·5 to 72·7)	71·7 (49·1 to 100)	65·5 (43·6 to 97·6)	58·7 (39·7 to 84·5)	52·4 (37·5 to 70·5)	47·1 (33·9 to 65·1)	39·5 (28·1 to 54·3)	35·5 (25·9 to 48·1)	29 (20·7 to 39·1)	22·9 (16 to 31·5)	13·2 (9·3 to 18·4)	9·3 (6·7 to 12·6)	7·2 (5·2 to 9·7)	4·9 (3·5 to 6·7)	4 (3 to 5·4)	1·9 (1·4 to 2·4)	0·7 (0·5 to 0·9)	636
Thalassemias	0 (0 to 0)	0 (0 to 0)	0 (0 to 0)	0·1 (0 to 0·2)	0 (0 to 0·1)	0 (0 to 0)	0 (0 to 0)	0 (0 to 0)	0 (0 to 0)	0 (0 to 0)	0 (0 to 0)	0 (0 to 0)	0 (0 to 0)	0 (0 to 0)	0 (0 to 0)	0 (0 to 0)	0 (0 to 0)	0 (0 to 0)	0 (0 to 0)	0 (0 to 0)	0 (0 to 0)	0 (0 to 0)	0 (0 to 0)	0·2
Thalassemias trait	0 (0 to 0)	0 (0 to 0)	0·2 (0·1 to 0·3)	0·7 (0·3 to 1·3)	1·1 (0·4 to 2·2)	0·7 (0·2 to 1·5)	1·6 (0·7 to 2·6)	1·6 (0·7 to 3·1)	1·8 (0·9 to 3·3)	1·6 (0·7 to 3)	1·6 (0·8 to 2·7)	1·4 (0·6 to 2·6)	0·4 (0·2 to 0·8)	0·5 (0·2 to 1·1)	0·5 (0·2 to 0·9)	0·3 (0·1 to 0·6)	0·2 (0·1 to 0·4)	0·1 (0 to 0·3)	0·1 (0 to 0·2)	0·1 (0 to 0·1)	0 (0 to 0·1)	0 (0 to 0)	0 (0 to 0)	14·7
Sickle cell disorders	0·6 (0·4 to 0·9)	0·8 (0·4 to 1·3)	7·2 (4·7 to 11)	14·3 (9 to 21·4)	22 (16 to 29·7)	24·6 (17·4 to 33·6)	38·6 (27·4 to 53)	41·9 (29 to 59·7)	39·7 (25·9 to 57·2)	31·3 (20·9 to 43·8)	30·5 (19·7 to 44·5)	23·5 (15·4 to 34·4)	18·3 (11·5 to 26·9)	12·7 (7·9 to 19·7)	5·8 (3·5 to 9·3)	1·7 (0·9 to 2·9)	0·3 (0·1 to 0·6)	0 (0 to 0·1)	0 (0 to 0)	0 (0 to 0)	0 (0 to 0)	0 (0 to 0)	0 (0 to 0)	314
Sickle cell trait	0 (0 to 0·1)	0·5 (0·3 to 0·9)	1·7 (0·9 to 3)	7·5 (3·9 to 12·9)	12·6 (5·6 to 23·1)	7·6 (3·2 to 14·3)	4·5 (2·1 to 8·2)	19·2 (4·8 to 39·8)	13 (2·6 to 37·3)	14·4 (3·6 to 32·4)	2·8 (1·1 to 5·3)	5·3 (2·5 to 8·6)	6 (2·8 to 10·8)	6 (2·9 to 10·3)	5·5 (2·7 to 9·3)	3·7 (1·8 to 6·3)	1·9 (0·7 to 3·6)	1·4 (0·6 to 2·6)	1·2 (0·5 to 2·1)	0·9 (0·4 to 1·4)	0·5 (0·3 to 0·9)	0·2 (0·1 to 0·3)	0·1 (0 to 0·1)	116
G6PD deficiency	0 (0 to 0)	0 (0 to 0·1)	0·1 (0 to 0·1)	0·6 (0·3 to 1·1)	0·8 (0·4 to 1·6)	0·9 (0·4 to 1·7)	3·8 (2 to 6·3)	4·2 (1·6 to 8·4)	6 (2·6 to 10·9)	6·3 (2·5 to 11·9)	10·2 (5·7 to 16·4)	8·6 (4·5 to 14)	6·9 (3·5 to 11·6)	6·9 (3·7 to 10·9)	6·5 (3·9 to 9·8)	4·7 (2·7 to 7·2)	2·4 (1·3 to 3·7)	1·2 (0·7 to 1·9)	0·6 (0·3 to 0·9)	0·2 (0·1 to 0·3)	0·1 (0 to 0·2)	0 (0 to 0)	0 (0 to 0)	71·2
G6PD trait	0 (0 to 0)	0 (0 to 0)	0 (0 to 0)	0 (0 to 0)	0 (0 to 0)	0 (0 to 0)	0·3 (0·1 to 0·7)	0 (0 to 0)	0 (0 to 0)	0 (0 to 0)	0·3 (0·1 to 0·7)	0·4 (0·1 to 0·7)	0·1 (0 to 0·1)	0 (0 to 0)	0 (0 to 0)	0 (0 to 0)	0 (0 to 0)	0 (0 to 0)	0 (0 to 0)	0 (0 to 0)	0 (0 to 0)	0 (0 to 0)	0 (0 to 0)	1·1
Other hemoglobinopathies and hemolytic anemias	0 (0 to 0)	0·1 (0·1 to 0·2)	0·8 (0·4 to 1·5)	3·3 (1·5 to 6·3)	5·2 (2·1 to 10·4)	3·2 (1·2 to 6·6)	5·7 (2·4 to 10·9)	4·7 (1·9 to 8·9)	5 (2·4 to 8·9)	5·1 (2·3 to 9·1)	7 (3·7 to 12·4)	7·9 (4·4 to 13)	7·8 (4·5 to 12·7)	9·4 (5·6 to 14·2)	10·6 (6·9 to 15·6)	12·5 (8·3 to 18·3)	8·4 (5·9 to 11·9)	6·5 (4·6 to 8·8)	5·3 (3·9 to 7)	3·8 (2·8 to 5)	3·4 (2·5 to 4·4)	1·6 (1·2 to 2·1)	0·6 (0·4 to 0·8)	118
Endocrine, metabolic, blood, and immune disorders	12·3 (7·4 to 23·7)	15·5 (7·8 to 30)	143 (93·8 to 224)	80·5 (50·9 to 132)	50·6 (36 to 72·9)	48·2 (35·2 to 69·5)	64·6 (47·4 to 86·5)	82·2 (59·1 to 108)	126 (92 to 171)	157 (114 to 216)	188 (137 to 255)	193 (141 to 267)	200 (146 to 276)	213 (156 to 286)	207 (148 to 285)	183 (132 to 254)	135 (98·4 to 192)	99·3 (72·7 to 139)	77·6 (56·6 to 111)	54·4 (38·8 to 75·1)	32·8 (23·9 to 47·7)	15·1 (10·8 to 22·5)	7 (4·4 to 11·3)	2,380
Oral disorders	0 (0 to 0)	0 (0 to 0)	0 (0 to 0)	10·7 (6·1 to 17·7)	15·2 (8·2 to 25·7)	19·4 (10·2 to 33·3)	28 (15·1 to 48·7)	46 (24·3 to 85·5)	67·8 (33·2 to 130)	82·4 (39·4 to 155)	95·4 (44·7 to 181)	108 (51·7 to 195)	121 (62 to 217)	129 (69·5 to 227)	134 (76·1 to 216)	129 (77·2 to 206)	105 (63·1 to 163)	79·9 (49·3 to 125)	60·1 (37·4 to 89·6)	37·4 (23·4 to 55·9)	17·8 (11 to 26·3)	7·4 (4·6 to 10·9)	3·1 (1·9 to 4·6)	1,290
Caries of deciduous teeth	0 (0 to 0)	0 (0 to 0)	0 (0 to 0)	3·5 (1·4 to 7·2)	3·9 (1·6 to 8·7)	0·3 (0 to 0·7)	0 (0 to 0)	0 (0 to 0)	0 (0 to 0)	0 (0 to 0)	0 (0 to 0)	0 (0 to 0)	0 (0 to 0)	0 (0 to 0)	0 (0 to 0)	0 (0 to 0)	0 (0 to 0)	0 (0 to 0)	0 (0 to 0)	0 (0 to 0)	0 (0 to 0)	0 (0 to 0)	0 (0 to 0)	7·7
Caries of permanent teeth	0 (0 to 0)	0 (0 to 0)	0 (0 to 0)	0 (0 to 0)	3·5 (1·2 to 7·5)	9·1 (3·6 to 20)	9·4 (3·7 to 20·9)	11 (4·6 to 23)	10·9 (4·3 to 22·3)	9·5 (3·8 to 19·7)	9·1 (3·7 to 17·8)	8·4 (3·5 to 17·3)	7·9 (3·3 to 17)	7 (2·8 to 14·2)	5·5 (2·1 to 12·1)	3·8 (1·3 to 8·5)	2·3 (0·9 to 4·9)	1·4 (0·6 to 3)	0·9 (0·4 to 1·8)	0·5 (0·2 to 1·1)	0·2 (0·1 to 0·5)	0·1 (0 to 0·2)	0 (0 to 0·1)	101
Periodontal diseases	0 (0 to 0)	0 (0 to 0)	0 (0 to 0)	0 (0 to 0)	0 (0 to 0)	0 (0 to 0)	4·2 (1·2 to 10·9)	15·7 (4·8 to 39·3)	34 (10·7 to 82·2)	47·9 (16·7 to 111)	58·8 (21 to 130)	66·7 (24·3 to 143)	70·2 (27 to 145)	64·3 (25·2 to 134)	52·4 (20·7 to 112)	36·7 (14·6 to 74·5)	22·3 (8·8 to 44·9)	13·6 (5·3 to 27·8)	8·9 (3·5 to 17·6)	5·2 (2 to 10·3)	2·4 (0·9 to 4·9)	1 (0·4 to 2)	0·4 (0·1 to 0·8)	505
Other oral disorders	0 (0 to 0)	0 (0 to 0)	0 (0 to 0)	7·2 (4·4 to 11·1)	7·8 (4·6 to 12·1)	10·1 (6 to 15·9)	14·3 (8·7 to 22·2)	17·7 (10·7 to 27·4)	19·4 (11·8 to 28·6)	19·1 (11·5 to 28·8)	18·9 (11·3 to 28·9)	19 (11·4 to 28·7)	19 (11·8 to 28·7)	17·4 (10·8 to 25·9)	14·7 (8·9 to 21·7)	11·2 (6·7 to 16·6)	7·5 (4·6 to 11·3)	4·6 (2·8 to 6·9)	2·6 (1·6 to 3·8)	1·3 (0·8 to 1·9)	0·5 (0·3 to 0·7)	0·2 (0·1 to 0·3)	0·1 (0 to 0·1)	212
Sudden infant death syndrome	--	6·7 (3·5 to 11·6)	43·3 (26·1 to 67·6)	--	--	--	--	--	--	--	--	--	--	--	--	--	--	--	--	--	--	--	--	NA
Injuries	20·3 (14·9 to 27·5)	17·5 (11·6 to 26·3)	143 (109 to 188)	404 (340 to 483)	495 (453 to 547)	712 (643 to 793)	1,520 (1,340 to 1,770)	2,640 (2,280 to 3,090)	2,760 (2,430 to 3,210)	2,310 (1,980 to 2,680)	2,000 (1,690 to 2,380)	1,630 (1,370 to 1,950)	1,330 (1,110 to 1,580)	1,060 (891 to 1,260)	812 (691 to 953)	583 (501 to 675)	383 (327 to 444)	264 (227 to 307)	202 (174 to 235)	144 (124 to 167)	82·3 (69·7 to 96·1)	41·3 (34·4 to 47·9)	18·8 (15 to 22·1)	19,600
Transport injuries	1 (0·8 to 1·3)	0·9 (0·5 to 1·4)	10 (7·6 to 13·3)	60·4 (49·6 to 75·5)	101 (85·3 to 119)	156 (134 to 184)	430 (351 to 531)	695 (570 to 846)	688 (556 to 819)	601 (498 to 729)	545 (441 to 675)	472 (390 to 572)	425 (350 to 514)	345 (291 to 408)	261 (218 to 312)	175 (150 to 205)	110 (94 to 129)	67·9 (57·5 to 79·8)	45·7 (38·5 to 53·2)	25·1 (20·8 to 29·5)	11 (9·2 to 13·1)	4·1 (3·4 to 4·9)	1·6 (1·3 to 2)	5,230
Road injuries	0·5 (0·3 to 0·7)	0·4 (0·2 to 0·7)	5·3 (3·5 to 7·7)	25·2 (16·4 to 39·1)	53 (37·9 to 71·3)	80·6 (59·7 to 105)	263 (190 to 352)	450 (329 to 588)	459 (339 to 588)	389 (298 to 499)	354 (263 to 467)	284 (212 to 373)	268 (203 to 351)	215 (166 to 274)	167 (129 to 211)	120 (97 to 148)	79·1 (63·3 to 96·3)	47·4 (37·8 to 58·3)	33 (26·5 to 39·9)	19 (15·2 to 23·3)	8·7 (7 to 10·6)	3·3 (2·6 to 4)	1·3 (1 to 1·6)	3,330
Pedestrian road injuries	0·2 (0·1 to 0·3)	0·1 (0·1 to 0·2)	1·3 (0·9 to 2)	10·6 (6·7 to 16·7)	20·9 (14·5 to 28·3)	25·2 (17·7 to 34·9)	31·9 (22·5 to 43·7)	46·5 (32·8 to 64·5)	56 (41 to 72)	58·9 (45·1 to 76·3)	61·5 (45·1 to 82·5)	57·1 (41·9 to 75·9)	58 (42·5 to 77·1)	51·8 (38·9 to 67·4)	44 (32·6 to 57·5)	34 (26·8 to 42·8)	24·8 (19·6 to 30·9)	16·1 (12·7 to 20·1)	12·5 (10 to 15·3)	7·7 (6·1 to 9·3)	3·5 (2·8 to 4·4)	1·2 (1 to 1·5)	0·4 (0·3 to 0·5)	624
Cyclist road injuries	0 (0 to 0)	0 (0 to 0)	0 (0 to 0)	0·7 (0·4 to 1·1)	2·3 (1·5 to 3·5)	7·4 (4·8 to 10·8)	15·2 (9·2 to 23·2)	19·8 (13·5 to 28·2)	21·5 (15·2 to 29·4)	21·4 (15·1 to 29)	21·9 (15·4 to 29·5)	20·9 (15·2 to 28)	20·5 (15 to 28)	19 (13·8 to 24·9)	15·6 (11·3 to 20·6)	11·8 (8·7 to 15·7)	8 (5·9 to 10·5)	4·9 (3·5 to 6·5)	3·3 (2·4 to 4·4)	2 (1·4 to 2·7)	0·9 (0·6 to 1·3)	0·4 (0·3 to 0·5)	0·2 (0·1 to 0·2)	218
Motorcyclist road injuries	0 (0 to 0·1)	0 (0 to 0·1)	0·4 (0·3 to 0·6)	1·4 (0·9 to 2·1)	3·2 (2·3 to 4·2)	6·1 (4·1 to 9)	48·6 (33·5 to 67·8)	108 (70·8 to 155)	122 (89·4 to 156)	95·4 (73·7 to 122)	83 (61·4 to 110)	58·5 (44·4 to 75·6)	51·3 (38·9 to 65·6)	37·4 (29·2 to 47)	27·9 (21·5 to 35·3)	17·4 (13·3 to 22·3)	11·1 (8·4 to 14·4)	6·2 (4·5 to 8·4)	4·3 (3·1 to 5·8)	2·7 (1·9 to 3·7)	1·3 (0·9 to 1·8)	0·5 (0·4 to 0·7)	0·2 (0·2 to 0·3)	688
Motor vehicle road injuries	0·3 (0·2 to 0·4)	0·2 (0·1 to 0·4)	3·5 (2·2 to 5)	12·2 (7·4 to 19)	25·8 (18·4 to 35·4)	40·9 (29·4 to 54·3)	166 (119 to 226)	273 (198 to 367)	256 (186 to 330)	210 (156 to 271)	184 (132 to 247)	144 (103 to 194)	134 (97·7 to 178)	103 (78 to 135)	76·4 (58·5 to 98·7)	54·4 (42·7 to 68·3)	33·3 (26·7 to 40·9)	18·9 (14·9 to 23·4)	12·1 (9·7 to 14·8)	6·2 (5 to 7·6)	2·7 (2·2 to 3·3)	1·1 (0·8 to 1·3)	0·4 (0·3 to 0·6)	1,760
Other road injuries	0 (0 to 0)	0 (0 to 0)	0·1 (0 to 0·1)	0·4 (0·2 to 0·7)	0·8 (0·4 to 1·3)	0·9 (0·6 to 1·3)	1·7 (1·1 to 2·5)	2·5 (1·8 to 3·6)	3·5 (2·3 to 5)	3·6 (2·4 to 5·2)	3·8 (2·6 to 5)	3·8 (2·7 to 5·2)	4 (2·8 to 5·5)	3·8 (2·7 to 5·2)	3·4 (2·4 to 4·6)	2·7 (1·9 to 3·6)	1·9 (1·3 to 2·6)	1·2 (0·8 to 1·6)	0·9 (0·6 to 1·2)	0·5 (0·4 to 0·7)	0·2 (0·2 to 0·3)	0·1 (0·1 to 0·1)	0 (0 to 0)	39·7
Other transport injuries	0·5 (0·4 to 0·7)	0·5 (0·3 to 0·7)	4·7 (3·9 to 6)	35·2 (31·3 to 39·7)	47·7 (43·1 to 52·7)	75·5 (68·2 to 84)	167 (149 to 187)	246 (218 to 282)	229 (203 to 263)	212 (185 to 244)	191 (167 to 221)	188 (166 to 215)	158 (140 to 178)	129 (115 to 145)	93·4 (82·8 to 106)	55·2 (49·6 to 62·3)	31·4 (28·3 to 34·7)	20·5 (18·4 to 22·8)	12·7 (11·2 to 14·6)	6 (5·4 to 6·7)	2·3 (2·1 to 2·6)	0·8 (0·7 to 0·9)	0·3 (0·3 to 0·4)	1,910
Unintentional injuries	12·1 (9 to 15·7)	14·8 (10 to 21·9)	119 (89·4 to 160)	317 (267 to 384)	366 (329 to 411)	489 (439 to 548)	549 (479 to 631)	640 (551 to 753)	675 (582 to 791)	640 (547 to 752)	606 (517 to 706)	565 (482 to 660)	514 (435 to 611)	467 (393 to 549)	387 (328 to 458)	309 (262 to 366)	218 (185 to 258)	166 (141 to 194)	138 (118 to 161)	111 (94·8 to 129)	67·5 (56·8 to 79)	35·9 (29·8 to 41·8)	16·7 (13·3 to 19·7)	7,420
Falls	0·2 (0·1 to 0·2)	0·2 (0·1 to 0·3)	2·4 (1·6 to 3·5)	5·8 (3·9 to 8·2)	9·8 (7·2 to 13·3)	18·6 (13·6 to 24·8)	30·5 (22·9 to 39·9)	45 (35 to 58·2)	54·2 (42·4 to 69·3)	57·6 (44·8 to 73·8)	65·7 (51 to 83·6)	69·1 (53·6 to 88·7)	73·5 (56·4 to 94·3)	76·9 (59·5 to 97)	71·6 (55·7 to 90·9)	65·1 (51·1 to 81·3)	53·1 (42·2 to 66·4)	50·2 (40·3 to 61·4)	50·7 (41·3 to 61·8)	52·1 (42·7 to 62·8)	37·2 (30·4 to 45·1)	21·1 (16·9 to 25·1)	9·7 (7·6 to 11·7)	920
Drowning	0·9 (0·6 to 1·3)	0·7 (0·4 to 1·2)	10·5 (6·5 to 15·6)	60·8 (38·8 to 89·6)	75·3 (55·1 to 97·9)	94·8 (69·6 to 126)	132 (92·1 to 182)	146 (101 to 207)	166 (122 to 226)	155 (115 to 206)	132 (98·9 to 174)	106 (77·9 to 142)	76·7 (55 to 106)	59 (43 to 80·7)	41·3 (31·2 to 54·2)	25·6 (19·5 to 33·3)	14·3 (10·8 to 18·3)	7 (5·4 to 8·8)	4·5 (3·5 to 5·9)	1·9 (1·5 to 2·4)	1·1 (0·8 to 1·5)	0·7 (0·5 to 0·8)	0·3 (0·2 to 0·4)	1,310
Fire, heat, and hot substances	0·4 (0·3 to 0·6)	0·8 (0·5 to 1·4)	10·1 (6·6 to 14·9)	28 (17·6 to 42·4)	26·6 (21 to 33·4)	31·3 (23·9 to 39·8)	42·1 (32·1 to 54·9)	58·6 (44·8 to 76·7)	70·9 (53·8 to 92)	77·1 (58·7 to 101)	76·8 (59·1 to 101)	71·3 (53·9 to 92·4)	67 (50·3 to 89·1)	59·8 (44·6 to 79·1)	50·6 (38·4 to 66·9)	39·8 (30 to 52·7)	26·4 (20·2 to 35·3)	19·1 (14·7 to 25)	14·4 (11·1 to 18·9)	9·9 (7·7 to 12·7)	6 (4·6 to 7·6)	3 (2·4 to 3·7)	1·4 (1 to 1·7)	792
Poisonings	0·1 (0 to 0·1)	0·2 (0·1 to 0·3)	0·8 (0·5 to 1·2)	2·8 (1·7 to 4·2)	2 (1·4 to 2·9)	2·5 (1·6 to 3·6)	3·4 (2·4 to 4·6)	4·9 (3·6 to 6·3)	6·3 (4·7 to 8·5)	6·3 (4·6 to 8·2)	5·8 (4·3 to 7·8)	4·4 (3·3 to 5·8)	3·8 (2·8 to 5)	3 (2·2 to 4)	2·4 (1·7 to 3·2)	1·7 (1·2 to 2·3)	1·2 (0·8 to 1·6)	0·8 (0·6 to 1)	0·6 (0·4 to 0·8)	0·4 (0·3 to 0·5)	0·2 (0·1 to 0·2)	0·1 (0·1 to 0·1)	0 (0 to 0)	53·4
Poisoning by carbon monoxide	0 (0 to 0)	0 (0 to 0·1)	0·2 (0·1 to 0·4)	1·2 (0·6 to 2·1)	0·7 (0·5 to 1·1)	0·8 (0·5 to 1·1)	1·2 (0·8 to 1·8)	2·4 (1·6 to 3·5)	3·7 (2·5 to 5·3)	3·8 (2·5 to 5·3)	3·3 (2·2 to 4·7)	2·2 (1·5 to 3)	1·7 (1·1 to 2·4)	1·2 (0·8 to 1·7)	0·9 (0·7 to 1·3)	0·6 (0·5 to 0·9)	0·4 (0·3 to 0·6)	0·2 (0·2 to 0·3)	0·2 (0·1 to 0·2)	0·1 (0·1 to 0·1)	0 (0 to 0·1)	0 (0 to 0)	0 (0 to 0)	25
Poisoning by other means	0 (0 to 0·1)	0·1 (0·1 to 0·2)	0·6 (0·4 to 0·9)	1·6 (1 to 2·3)	1·3 (0·8 to 1·9)	1·7 (1 to 2·7)	2·2 (1·4 to 3·1)	2·4 (1·7 to 3·5)	2·6 (1·9 to 3·7)	2·5 (1·8 to 3·5)	2·5 (1·7 to 3·4)	2·2 (1·5 to 3·1)	2·1 (1·4 to 2·9)	1·9 (1·3 to 2·6)	1·5 (1 to 2·1)	1·1 (0·7 to 1·6)	0·8 (0·5 to 1·1)	0·5 (0·4 to 0·7)	0·4 (0·3 to 0·6)	0·3 (0·2 to 0·4)	0·1 (0·1 to 0·2)	0·1 (0 to 0·1)	0 (0 to 0)	28·4
Exposure to mechanical forces	0·1 (0·1 to 0·1)	0·2 (0·1 to 0·3)	1·2 (0·8 to 1·6)	6·2 (4·2 to 8·7)	12·5 (8·8 to 17·4)	19·9 (13·7 to 28·8)	31·8 (23·2 to 42·8)	47·5 (35·7 to 64·2)	51·5 (37·9 to 68·4)	50·5 (37·3 to 67·6)	50·7 (37·5 to 68·4)	50·3 (37 to 69·4)	48·3 (34·4 to 66·6)	44·6 (32·1 to 62·6)	38·5 (27·6 to 54·4)	29·9 (20·5 to 42)	20·5 (14·1 to 29·1)	13·9 (9·6 to 19·8)	10 (6·9 to 14·1)	6·5 (4·6 to 9·1)	3·4 (2·4 to 4·7)	1·7 (1·2 to 2·2)	0·8 (0·6 to 1·1)	540
Unintentional firearm injuries	0 (0 to 0)	0 (0 to 0)	0·1 (0·1 to 0·2)	0·6 (0·3 to 0·9)	1 (0·6 to 1·6)	1·9 (1·3 to 2·8)	4·3 (3 to 6)	7·7 (5·3 to 11·2)	7·4 (5 to 10·4)	7 (5 to 9·6)	6·8 (5 to 9·1)	6·6 (4·8 to 8·7)	6·2 (4·5 to 8·2)	5·5 (4 to 7·3)	4·7 (3·4 to 6·3)	3·6 (2·6 to 4·9)	2·5 (1·8 to 3·4)	1·6 (1·2 to 2·2)	1·2 (0·8 to 1·6)	0·8 (0·5 to 1·1)	0·4 (0·3 to 0·6)	0·2 (0·2 to 0·3)	0·1 (0·1 to 0·2)	70·2
Other exposure to mechanical forces	0·1 (0·1 to 0·1)	0·2 (0·1 to 0·3)	1·1 (0·7 to 1·5)	5·6 (3·8 to 8)	11·5 (7·9 to 16·2)	18 (12·2 to 26·4)	27·5 (19·8 to 37·9)	39·8 (29·6 to 54·7)	44·1 (31·9 to 59·7)	43·5 (31·8 to 59·6)	43·9 (31·8 to 59·9)	43·7 (31·7 to 61·7)	42·1 (29·8 to 58·7)	39·2 (27·8 to 55·3)	33·7 (23·8 to 48)	26·2 (18 to 37·5)	18 (12·2 to 25·8)	12·2 (8·3 to 17·5)	8·9 (6 to 12·6)	5·8 (4 to 8·1)	3 (2·1 to 4·1)	1·4 (1·1 to 1·9)	0·7 (0·5 to 0·9)	470
Adverse effects of medical treatment	2·4 (1·6 to 3·5)	2·4 (1·3 to 4·1)	9·2 (5·6 to 14·4)	6 (3·8 to 9)	4·7 (3·3 to 6·5)	5·1 (3·6 to 7·1)	7·4 (4·9 to 10·7)	15·9 (10·7 to 22·2)	18·4 (12·1 to 26·4)	18·1 (12·1 to 25·6)	19·8 (13·1 to 28·2)	21 (14·2 to 29·9)	21·9 (14·9 to 30·5)	24·4 (17 to 33·9)	23·8 (16·8 to 33·2)	22·9 (16·1 to 31·5)	17·8 (13·1 to 24·2)	14·3 (10·5 to 19·4)	13·2 (9·6 to 17·4)	9·5 (7·1 to 12·5)	5·6 (4·1 to 7·4)	2·6 (1·9 to 3·4)	1·3 (0·8 to 2)	287
Animal contact	0 (0 to 0)	0·1 (0 to 0·1)	0·4 (0·3 to 0·6)	1·3 (0·8 to 1·8)	1·6 (1·2 to 2·3)	2·5 (1·8 to 3·5)	3 (2·1 to 4·2)	3·6 (2·5 to 4·7)	5 (3·6 to 6·5)	4 (2·8 to 5·2)	4·9 (3·5 to 6·5)	4·8 (3·5 to 6·4)	5·1 (3·6 to 6·7)	4·8 (3·5 to 6·2)	4·3 (3·1 to 5·7)	3·8 (2·8 to 5)	2·9 (2·1 to 3·8)	2·2 (1·6 to 2·9)	1·8 (1·3 to 2·3)	1·4 (1·1 to 1·9)	0·9 (0·7 to 1·3)	0·8 (0·5 to 1)	0·5 (0·3 to 0·7)	59·5
Venomous animal contact	0 (0 to 0)	0 (0 to 0·1)	0·2 (0·1 to 0·3)	0·8 (0·5 to 1·1)	1 (0·7 to 1·5)	1·8 (1·2 to 2·5)	2·1 (1·4 to 3)	2·4 (1·6 to 3·4)	3·4 (2·3 to 4·6)	2·6 (1·8 to 3·5)	3·5 (2·4 to 4·8)	3·1 (2·2 to 4·3)	3·4 (2·4 to 4·5)	3·1 (2·2 to 4·2)	2·8 (1·9 to 3·7)	2·4 (1·7 to 3·3)	1·7 (1·2 to 2·4)	1·2 (0·8 to 1·6)	1 (0·7 to 1·4)	0·7 (0·5 to 0·9)	0·4 (0·3 to 0·6)	0·4 (0·2 to 0·5)	0·2 (0·1 to 0·3)	38·4
Non-venomous animal contact	0 (0 to 0)	0 (0 to 0)	0·2 (0·1 to 0·3)	0·5 (0·3 to 0·8)	0·6 (0·4 to 0·9)	0·7 (0·5 to 1·1)	0·9 (0·6 to 1·3)	1·2 (0·8 to 1·6)	1·6 (1 to 2·4)	1·4 (0·9 to 2)	1·4 (0·9 to 2)	1·6 (1·1 to 2·4)	1·7 (1·2 to 2·5)	1·6 (1·1 to 2·3)	1·6 (1·1 to 2·2)	1·4 (0·9 to 1·9)	1·1 (0·8 to 1·6)	1 (0·7 to 1·3)	0·8 (0·5 to 1·1)	0·7 (0·5 to 1)	0·5 (0·3 to 0·7)	0·4 (0·2 to 0·6)	0·3 (0·1 to 0·4)	21·1
Foreign body	6·3 (4·3 to 8·8)	6 (3·2 to 10·2)	49·2 (29·7 to 76·2)	27·6 (18 to 39·9)	15·9 (12·5 to 19·8)	19·6 (14·8 to 25·7)	14·6 (11 to 19)	23·7 (17·7 to 31·5)	28·9 (22·1 to 37·4)	26·4 (19·8 to 34·7)	27 (20·7 to 35·5)	25·9 (19·7 to 33·1)	24·6 (19·1 to 32·2)	24·7 (18·4 to 33)	21·1 (16·3 to 27)	18·9 (14·2 to 24·5)	15·8 (12 to 20·2)	12·4 (9·4 to 15·8)	11·2 (9·1 to 14)	9 (6·8 to 11·5)	4 (3 to 5)	2·3 (1·7 to 2·9)	1·2 (0·8 to 1·6)	416
Pulmonary aspiration and foreign body in airway	6·3 (4·3 to 8·8)	6 (3·2 to 10·1)	48·8 (29·4 to 75·7)	26·1 (16·8 to 38·2)	13·6 (10·5 to 17·2)	16·5 (12 to 22)	11·4 (8·4 to 15·4)	19·6 (14·3 to 26·8)	24 (18 to 31·8)	22·1 (16·1 to 29·4)	22·6 (17 to 30·2)	21·1 (15·6 to 28·3)	19·7 (14·8 to 26·5)	20·5 (14·6 to 28·2)	17·4 (12·9 to 22·8)	15·9 (11·6 to 21·3)	13·5 (9·9 to 17·8)	10·7 (8 to 14)	9·8 (7·9 to 12·5)	7·9 (5·9 to 10·4)	3·4 (2·5 to 4·4)	2 (1·4 to 2·5)	1·1 (0·7 to 1·4)	360
Foreign body in eyes	0 (0 to 0)	0 (0 to 0)	0·1 (0 to 0·2)	0·4 (0·1 to 0·9)	0·7 (0·2 to 1·8)	1·1 (0·3 to 2·6)	1·4 (0·4 to 3·3)	1·8 (0·6 to 3·8)	2 (0·7 to 4·2)	2 (0·7 to 4·1)	2 (0·7 to 4·2)	2·1 (0·9 to 4·2)	2 (0·9 to 3·9)	1·7 (0·9 to 3)	1·3 (0·8 to 2·2)	1 (0·6 to 1·6)	0·7 (0·4 to 1·1)	0·5 (0·3 to 0·7)	0·4 (0·2 to 0·5)	0·2 (0·2 to 0·3)	0·1 (0·1 to 0·2)	0 (0 to 0·1)	0 (0 to 0)	21·6
Foreign body in other body part	0 (0 to 0)	0 (0 to 0)	0·3 (0·2 to 0·5)	1·1 (0·7 to 1·6)	1·6 (1·1 to 2·2)	2 (1·4 to 2·8)	1·8 (1·3 to 2·5)	2·3 (1·6 to 3·2)	2·9 (2 to 4·2)	2·4 (1·7 to 3·2)	2·3 (1·7 to 3·2)	2·7 (2 to 3·6)	2·9 (2 to 3·9)	2·5 (1·8 to 3·4)	2·4 (1·7 to 3·3)	1·9 (1·4 to 2·6)	1·5 (1·1 to 2·1)	1·1 (0·8 to 1·6)	1 (0·8 to 1·3)	0·8 (0·6 to 1·1)	0·4 (0·3 to 0·6)	0·3 (0·2 to 0·4)	0·1 (0·1 to 0·2)	34·5
Environmental heat and cold exposure	0 (0 to 0)	0·1 (0 to 0·1)	0·4 (0·3 to 0·5)	1·8 (1·2 to 2·5)	3·6 (2·5 to 5·3)	5·7 (3·8 to 8·1)	8·3 (5·9 to 11·3)	11·7 (8·6 to 15·7)	13·3 (9·7 to 17·7)	13·9 (10·2 to 18·6)	14·2 (10·3 to 19·2)	14·5 (10·5 to 19·6)	14·9 (10·7 to 20)	14·1 (10·2 to 19·1)	13 (9·6 to 17·1)	10·5 (7·5 to 14)	7·6 (5·5 to 10·1)	5·9 (4·4 to 7·7)	4·7 (3·5 to 6)	3·5 (2·6 to 4·4)	2 (1·5 to 2·5)	1·1 (0·9 to 1·4)	0·6 (0·4 to 0·7)	165
Exposure to forces of nature	1·5 (1·3 to 1·6)	4·1 (3·7 to 4·5)	33·7 (30·6 to 37·1)	171 (155 to 188)	203 (185 to 224)	273 (248 to 301)	253 (230 to 279)	251 (228 to 276)	223 (203 to 246)	195 (178 to 215)	174 (158 to 192)	167 (151 to 184)	150 (136 to 165)	133 (120 to 146)	101 (91·9 to 111)	76·5 (69·5 to 84·4)	48·1 (43·8 to 53·1)	33·5 (30·4 to 37)	22·3 (20·3 to 24·7)	13·4 (12·2 to 14·8)	5·7 (5·2 to 6·3)	1·9 (1·8 to 2·1)	0·6 (0·6 to 0·7)	2,540
Other unintentional injuries	0·2 (0·1 to 0·4)	0·2 (0·1 to 0·3)	1·5 (1·1 to 2·1)	5·9 (4·1 to 8·3)	10·7 (7·8 to 14·8)	15·5 (11·4 to 21·3)	22·8 (16·8 to 30·5)	32·2 (24·1 to 43)	36·8 (28·1 to 47·5)	35·3 (26·2 to 45·5)	34·6 (26·3 to 45·1)	31·1 (23·3 to 40·4)	28·3 (20·6 to 38·2)	23·3 (16·7 to 31·5)	19·3 (13·9 to 26·6)	14·4 (10 to 19·9)	9·9 (7 to 14)	6·6 (4·7 to 9·2)	5 (3·5 to 6·9)	3 (2·1 to 4·2)	1·5 (1·1 to 2·1)	0·6 (0·4 to 0·9)	0·3 (0·2 to 0·4)	339
Self-harm and interpersonal violence	7·2 (4·3 to 12)	1·8 (0·9 to 3·5)	14 (8·4 to 23·3)	26·5 (16 to 44·1)	28·2 (20·7 to 39·1)	66·8 (50 to 90·5)	545 (421 to 710)	1,300 (1,040 to 1,620)	1,390 (1,140 to 1,710)	1,070 (839 to 1,340)	854 (663 to 1,110)	590 (423 to 787)	387 (279 to 524)	249 (181 to 338)	165 (119 to 221)	98·5 (75·3 to 127)	55 (42·6 to 71·8)	30 (23·6 to 38·7)	18·2 (14·5 to 22·9)	8·3 (6·7 to 10·1)	3·8 (3 to 4·6)	1·3 (1·1 to 1·6)	0·5 (0·4 to 0·7)	6,920
Self-harm	0 (0 to 0)	0 (0 to 0)	0 (0 to 0)	0 (0 to 0)	0 (0 to 0)	3·5 (2·3 to 5·2)	33·4 (23·3 to 46·3)	64·9 (46·4 to 89·3)	78·5 (56·7 to 108)	78·1 (56·8 to 104)	72·9 (51·5 to 99·7)	58·3 (41·3 to 82·2)	46 (32·8 to 62·5)	37·5 (26·7 to 52·4)	27·2 (19·6 to 37·3)	18·7 (14 to 25·1)	11·7 (8·8 to 15·3)	7·4 (5·5 to 9·7)	4·7 (3·6 to 6·2)	2·6 (2 to 3·4)	1·2 (0·9 to 1·6)	0·4 (0·3 to 0·5)	0·1 (0·1 to 0·1)	547
Self-harm by firearm	0 (0 to 0)	0 (0 to 0)	0 (0 to 0)	0 (0 to 0)	0 (0 to 0)	0·2 (0·1 to 0·3)	2·7 (1·5 to 4·5)	5·5 (3 to 9·4)	6 (3·3 to 10·2)	5·6 (3·3 to 8·9)	4·8 (2·8 to 8)	4·2 (2·5 to 6·7)	3·1 (1·8 to 5)	2·8 (1·7 to 4·5)	1·8 (1·1 to 2·8)	1·3 (0·8 to 2)	0·8 (0·5 to 1·1)	0·5 (0·3 to 0·7)	0·3 (0·2 to 0·4)	0·2 (0·1 to 0·2)	0·1 (0 to 0·1)	0 (0 to 0)	0 (0 to 0)	39·8
Self-harm by other specified means	0 (0 to 0)	0 (0 to 0)	0 (0 to 0)	0 (0 to 0)	0 (0 to 0)	3·3 (2·2 to 4·9)	30·6 (21·4 to 42·7)	59·5 (42·8 to 80·9)	72·4 (52·4 to 101)	72·5 (53 to 96·5)	68·1 (47·4 to 94·4)	54·1 (38·6 to 76·4)	42·9 (30·6 to 58·1)	34·6 (24·6 to 48·6)	25·5 (18·3 to 34·9)	17·4 (12·9 to 23·4)	10·9 (8·2 to 14·4)	6·9 (5·2 to 9·1)	4·5 (3·4 to 5·8)	2·5 (1·8 to 3·2)	1·1 (0·9 to 1·5)	0·4 (0·3 to 0·4)	0·1 (0·1 to 0·1)	507
Interpersonal violence	7·2 (4·3 to 12)	1·8 (0·9 to 3·5)	14 (8·4 to 23·3)	26·1 (15·7 to 43·6)	28 (20·5 to 38·9)	63·1 (46·4 to 86)	509 (388 to 669)	1,230 (976 to 1,540)	1,300 (1,070 to 1,610)	989 (770 to 1,240)	777 (594 to 1,020)	527 (375 to 714)	338 (234 to 467)	210 (147 to 291)	136 (94 to 188)	79·4 (58·4 to 107)	42·9 (32 to 57·8)	22·5 (16·8 to 30·2)	13·3 (10·1 to 17·3)	5·7 (4·5 to 7·1)	2·5 (2 to 3·2)	0·9 (0·7 to 1·2)	0·4 (0·3 to 0·6)	6,330
Physical violence by firearm	0·7 (0·3 to 1·3)	0·3 (0·1 to 0·5)	2·8 (1·6 to 4·6)	6·6 (3·9 to 11·2)	10·5 (7·2 to 15·2)	32·9 (23·2 to 46·2)	374 (278 to 498)	983 (772 to 1,230)	1,030 (836 to 1,280)	741 (571 to 933)	562 (418 to 736)	351 (242 to 480)	209 (140 to 295)	119 (82 to 170)	74·7 (49·7 to 107)	39·8 (28 to 55·2)	18·5 (13·1 to 26·2)	8·1 (5·8 to 11·7)	3·4 (2·3 to 4·9)	1·2 (0·9 to 1·5)	0·5 (0·4 to 0·7)	0·2 (0·1 to 0·2)	0·1 (0·1 to 0·1)	4,570
Physical violence by sharp object	0·6 (0·3 to 1)	0·1 (0·1 to 0·2)	0·8 (0·5 to 1·4)	2·5 (1·6 to 4·1)	3·3 (2·4 to 4·4)	8·3 (6·1 to 11·3)	92·6 (64·1 to 130)	178 (127 to 236)	195 (141 to 268)	176 (124 to 238)	147 (100 to 207)	114 (75·7 to 162)	76·3 (49·4 to 110)	47·5 (33·6 to 64·9)	27·4 (19·8 to 36·8)	16·1 (12·1 to 21·1)	8·5 (6·4 to 11·1)	5·1 (3·9 to 6·8)	3 (2·2 to 4)	1·5 (1·1 to 1·9)	0·6 (0·5 to 0·8)	0·3 (0·2 to 0·4)	0·1 (0·1 to 0·2)	1,100
Sexual violence	0 (0 to 0)	0 (0 to 0)	0 (0 to 0)	0·1 (0·1 to 0·2)	1·3 (0·8 to 2·1)	3·5 (2·1 to 5·5)	7·8 (4·3 to 12·4)	10·7 (6·1 to 16·3)	9·9 (5·6 to 15·9)	8·7 (4·8 to 14·4)	7·8 (4·4 to 12·4)	6·5 (3·7 to 10·5)	5·2 (3 to 8·8)	3·6 (2·1 to 5·9)	2·4 (1·4 to 3·8)	1·7 (1·1 to 2·6)	1·1 (0·7 to 1·7)	0·7 (0·5 to 1·1)	0·5 (0·3 to 0·8)	0·3 (0·2 to 0·5)	0·1 (0·1 to 0·2)	0 (0 to 0)	0 (0 to 0)	72·2
Physical violence by other means	5·9 (3·5 to 9·9)	1·5 (0·7 to 2·7)	10·3 (6·2 to 17·3)	16·9 (9·9 to 28·5)	12·9 (9·3 to 18)	18·3 (13·3 to 25)	35 (25·5 to 46·4)	59·4 (44·1 to 77·8)	67·6 (51 to 88·2)	63·7 (46·1 to 85·1)	60·4 (43·8 to 81·7)	55·7 (38·8 to 76·3)	47·8 (33·2 to 66)	39·9 (29·1 to 53·6)	31·3 (22·6 to 42·6)	21·9 (16·3 to 29)	14·8 (11 to 19·7)	8·6 (6·4 to 11·2)	6·3 (4·8 to 8·4)	2·7 (2·1 to 3·5)	1·2 (1 to 1·6)	0·4 (0·3 to 0·6)	0·2 (0·2 to 0·3)	583
Conflict and terrorism	0 (0 to 0)	0 (0 to 0)	0 (0 to 0)	0 (0 to 0)	0 (0 to 0)	0 (0 to 0)	0 (0 to 0)	0 (0 to 0·1)	0·1 (0 to 0·1)	0·1 (0 to 0·1)	0·1 (0 to 0·1)	1·8 (1·1 to 2·9)	1·6 (1·1 to 2·5)	0·5 (0·3 to 0·8)	1·2 (0·8 to 1·7)	0·2 (0·2 to 0·3)	0·4 (0·3 to 0·6)	0 (0 to 0)	0·2 (0·1 to 0·3)	0 (0 to 0)	0 (0 to 0)	0 (0 to 0)	0 (0 to 0)	6·3

SR Table 2c: Barbados DALYS by cause and age group for both sexes with 95% uncertainty intervals, 2019

Cause	Early Neonatal	Late Neonatal	Post Neonatal	1 to 4	5 to 9	10 to 14	15 to 19	20 to 24	25 to 29	30 to 34	35 to 39	40 to 44
All causes	1,670 (1,150 to 2,390)	507 (352 to 720)	671 (489 to 918)	689 (546 to 857)	1,010 (788 to 1,300)	1,340 (1,050 to 1,700)	2,210 (1,820 to 2,700)	2,550 (2,130 to 3,070)	3,110 (2,620 to 3,740)	3,470 (2,910 to 4,120)	4,010 (3,340 to 4,760)	4,480 (3,730 to 5,330)
Communicable, maternal, neonatal, and nutritional diseases	1,370 (947 to 1,940)	376 (258 to 531)	303 (220 to 415)	237 (184 to 300)	347 (245 to 484)	276 (206 to 361)	262 (212 to 327)	301 (255 to 359)	389 (329 to 456)	411 (359 to 475)	473 (412 to 550)	435 (378 to 507)
HIV/AIDS and sexually transmitted infections	1·3 (0·8 to 2·1)	0·4 (0·2 to 0·8)	8 (7·5 to 8·5)	5·1 (4·8 to 5·3)	5·1 (4·8 to 5·4)	9 (8·5 to 9·6)	16·6 (15 to 18·5)	67·6 (62·5 to 74·4)	137 (127 to 150)	154 (143 to 168)	194 (181 to 209)	164 (153 to 177)
HIV/AIDS	0 (0 to 0)	0 (0 to 0)	7·7 (7·2 to 8·1)	5 (4·8 to 5·3)	5·1 (4·8 to 5·4)	8·5 (8·1 to 9·1)	14·1 (13·2 to 15·2)	62·9 (58·6 to 68·3)	130 (121 to 142)	146 (136 to 157)	185 (174 to 198)	155 (145 to 164)
HIV/AIDS - Drug-susceptible Tuberculosis	0 (0 to 0)	0 (0 to 0)	0·1 (0·1 to 0·2)	0·1 (0·1 to 0·1)	0·1 (0·1 to 0·2)	0·1 (0·1 to 0·2)	0·3 (0·2 to 0·4)	1·1 (0·7 to 1·6)	2·3 (1·6 to 3·4)	2·7 (1·8 to 4)	3·2 (2·2 to 4·8)	2·9 (1·9 to 4·2)
HIV/AIDS - Multidrug-resistant Tuberculosis without extensive d	0 (0 to 0)	0 (0 to 0)	0 (0 to 0)	0 (0 to 0)	0 (0 to 0)	0 (0 to 0)	0 (0 to 0)	0 (0 to 0)	0 (0 to 0·1)	0 (0 to 0·1)	0 (0 to 0·1)	0 (0 to 0·1)
HIV/AIDS - Extensively drug-resistant Tuberculosis	0 (0 to 0)	0 (0 to 0)	0 (0 to 0)	0 (0 to 0)	0 (0 to 0)	0 (0 to 0)	0 (0 to 0)	0 (0 to 0)	0 (0 to 0)	0 (0 to 0)	0 (0 to 0)	0 (0 to 0)
HIV/AIDS resulting in other diseases	0 (0 to 0)	0 (0 to 0)	7·5 (7·1 to 8)	5 (4·7 to 5·2)	5 (4·7 to 5·3)	8·4 (8 to 8·9)	13·9 (13 to 14·9)	61·8 (57·4 to 67)	128 (119 to 140)	143 (133 to 154)	181 (170 to 194)	152 (143 to 162)
Sexually transmitted infections excluding HIV	1·3 (0·8 to 2·1)	0·4 (0·2 to 0·8)	0·3 (0·1 to 0·6)	0 (0 to 0)	0 (0 to 0)	0·4 (0·3 to 0·7)	2·5 (1·5 to 3·8)	4·6 (2·8 to 7·3)	6·7 (3·9 to 11·2)	8·2 (4·8 to 13·4)	9·4 (5·5 to 15·9)	9·2 (5·6 to 15·8)
Syphilis	1·3 (0·8 to 2·1)	0·4 (0·2 to 0·8)	0·3 (0·1 to 0·6)	0 (0 to 0)	0 (0 to 0)	0·1 (0·1 to 0·1)	0·2 (0·1 to 0·3)	0·2 (0·1 to 0·2)	0·3 (0·2 to 0·4)	0·4 (0·2 to 0·6)	0·4 (0·3 to 0·6)	0·2 (0·2 to 0·3)
Chlamydial infection	0 (0 to 0)	0 (0 to 0)	0 (0 to 0)	0 (0 to 0)	0 (0 to 0)	0 (0 to 0)	0·3 (0·1 to 0·6)	0·9 (0·5 to 1·5)	1·3 (0·7 to 2·1)	1·3 (0·7 to 2·3)	1·5 (0·8 to 2·5)	1·5 (0·9 to 2·3)
Gonococcal infection	0 (0 to 0)	0 (0 to 0)	0 (0 to 0)	0 (0 to 0)	0 (0 to 0)	0·1 (0 to 0·2)	0·6 (0·3 to 1·1)	0·8 (0·4 to 1·5)	0·6 (0·3 to 1)	0·6 (0·4 to 1)	0·8 (0·5 to 1·1)	1 (0·7 to 1·3)
Trichomoniasis	0 (0 to 0)	0 (0 to 0)	0 (0 to 0)	0 (0 to 0)	0 (0 to 0)	0 (0 to 0)	0·2 (0·1 to 0·5)	0·8 (0·3 to 2)	1·7 (0·6 to 4·1)	2 (0·6 to 4·7)	2·1 (0·7 to 5·1)	2 (0·7 to 4·8)
Genital herpes	0 (0 to 0)	0 (0 to 0)	0 (0 to 0)	0 (0 to 0)	0 (0 to 0)	0·1 (0 to 0·2)	0·4 (0·1 to 0·9)	0·8 (0·2 to 1·9)	1·3 (0·4 to 3·3)	1·6 (0·6 to 3·9)	2 (0·7 to 4·8)	2 (0·6 to 5·1)
Other sexually transmitted infections	0 (0 to 0)	0 (0 to 0)	0 (0 to 0)	0 (0 to 0)	0 (0 to 0)	0·2 (0·1 to 0·3)	0·9 (0·5 to 1·5)	1·2 (0·7 to 1·8)	1·5 (0·9 to 2·4)	2·3 (1·4 to 3·4)	2·6 (1·7 to 3·9)	2·6 (1·6 to 4)
Respiratory infections and tuberculosis	41·5 (24·8 to 65·9)	31·6 (18·7 to 49·5)	96·6 (59·6 to 146)	50·4 (35·8 to 69)	40·1 (27·8 to 57·3)	35·5 (24·6 to 50·8)	43·4 (30·3 to 60)	45·7 (32·5 to 61·7)	53·8 (39·1 to 71·3)	61·6 (45·7 to 81·1)	66 (48·5 to 87·5)	70·7 (51·7 to 95·7)
Tuberculosis	0 (0 to 0)	0 (0 to 0)	0·3 (0·2 to 0·5)	0·6 (0·3 to 1·1)	1 (0·3 to 1·9)	0·7 (0·5 to 0·9)	1·2 (0·9 to 1·8)	3·5 (2·1 to 5·3)	5·4 (3·4 to 8·2)	5·5 (3·5 to 8·1)	5·1 (3·4 to 7·4)	4·8 (3·1 to 6·7)
Latent tuberculosis infection	0 (0 to 0)	0 (0 to 0)	0 (0 to 0)	0 (0 to 0)	0 (0 to 0)	0 (0 to 0)	0 (0 to 0)	0 (0 to 0)	0 (0 to 0)	0 (0 to 0)	0 (0 to 0)	0 (0 to 0)
Drug-susceptible tuberculosis	0 (0 to 0)	0 (0 to 0)	0·3 (0·2 to 0·5)	0·6 (0·3 to 1·1)	1 (0·3 to 1·9)	0·7 (0·5 to 0·9)	1·2 (0·8 to 1·7)	3·4 (2·1 to 5·2)	5·4 (3·4 to 8·1)	5·5 (3·5 to 8·1)	5·1 (3·4 to 7·3)	4·8 (3·1 to 6·7)
Multidrug-resistant tuberculosis without extensive drug resista	0 (0 to 0)	0 (0 to 0)	0 (0 to 0)	0 (0 to 0)	0 (0 to 0)	0 (0 to 0)	0 (0 to 0)	0 (0 to 0·1)	0 (0 to 0·1)	0 (0 to 0·1)	0 (0 to 0·2)	0 (0 to 0·1)
Extensively drug-resistant tuberculosis	0 (0 to 0)	0 (0 to 0)	0 (0 to 0)	0 (0 to 0)	0 (0 to 0)	0 (0 to 0)	0 (0 to 0)	0 (0 to 0)	0 (0 to 0)	0 (0 to 0)	0 (0 to 0)	0 (0 to 0)
Lower respiratory infections	41·5 (24·8 to 65·9)	31·4 (18·5 to 49·3)	92 (54·7 to 141)	29·5 (18·9 to 44·2)	14 (9·9 to 19·3)	11·9 (8·2 to 16·5)	19·1 (12·5 to 27·7)	21·9 (14·2 to 33·1)	28·2 (18·5 to 42·2)	37·7 (24·8 to 55)	41·4 (27·7 to 60·1)	47·3 (30·6 to 69)
Upper respiratory infections	0 (0 to 0·1)	0·2 (0·1 to 0·3)	3·6 (2·2 to 5·8)	14·4 (8·2 to 23·6)	17 (9·3 to 28·4)	16·9 (9 to 28·7)	17·3 (9·1 to 30·1)	15·7 (8·5 to 26·9)	16 (8·9 to 26·5)	14·9 (8·2 to 24·9)	15·7 (8 to 26·3)	14·6 (7·8 to 25·6)
Otitis media	0 (0 to 0)	0 (0 to 0)	0·6 (0·3 to 1·2)	5·8 (3·1 to 10·1)	8·1 (4·4 to 13·5)	6 (3·2 to 10·6)	5·8 (3·1 to 10·1)	4·7 (2 to 9·2)	4·2 (2 to 7·7)	3·5 (1·5 to 6·6)	3·8 (1·8 to 7·1)	4 (1·9 to 7·3)
Enteric infections	1·8 (1 to 3)	4·6 (2·7 to 7·5)	33·9 (21·9 to 51·5)	36 (24 to 52·7)	33·4 (20·6 to 53·7)	32·3 (19·1 to 51·6)	24·4 (15·4 to 36·7)	18·9 (11·7 to 28·7)	22·8 (15·1 to 32·8)	24·9 (16·8 to 34·8)	29·3 (20 to 40·6)	31·5 (22·4 to 42·9)
Diarrheal diseases	1·8 (1 to 3)	4·6 (2·7 to 7·5)	33·8 (21·7 to 51·3)	35·8 (23·8 to 52·5)	33·2 (20·4 to 53·6)	32·1 (18·9 to 51·4)	24·2 (15·3 to 36·5)	18·8 (11·7 to 28·5)	22·7 (15·1 to 32·7)	24·7 (16·7 to 34·6)	29·2 (20 to 40·5)	31·4 (22·3 to 42·8)
Typhoid and paratyphoid	0 (0 to 0)	0 (0 to 0)	0 (0 to 0)	0 (0 to 0)	0 (0 to 0)	0 (0 to 0·1)	0·1 (0 to 0·2)	0·1 (0 to 0·1)	0 (0 to 0·1)	0·1 (0 to 0·2)	0 (0 to 0·1)	0 (0 to 0·1)
Typhoid fever	0 (0 to 0)	0 (0 to 0)	0 (0 to 0)	0 (0 to 0)	0 (0 to 0)	0 (0 to 0·1)	0·1 (0 to 0·2)	0 (0 to 0·1)	0 (0 to 0·1)	0·1 (0 to 0·2)	0 (0 to 0·1)	0 (0 to 0·1)
Paratyphoid fever	0 (0 to 0)	0 (0 to 0)	0 (0 to 0)	0 (0 to 0)	0 (0 to 0)	0 (0 to 0)	0 (0 to 0)	0 (0 to 0)	0 (0 to 0)	0 (0 to 0)	0 (0 to 0)	0 (0 to 0)
Invasive Non-typhoidal Salmonella (iNTS)	0 (0 to 0)	0 (0 to 0)	0 (0 to 0·1)	0 (0 to 0·1)	0·1 (0 to 0·2)	0·1 (0 to 0·2)	0 (0 to 0)	0 (0 to 0)	0 (0 to 0·1)	0 (0 to 0·1)	0 (0 to 0·1)	0 (0 to 0)
Other intestinal infectious diseases	0 (0 to 0)	0 (0 to 0)	0·1 (0 to 0·2)	0·1 (0 to 0·2)	0·1 (0 to 0·2)	0·1 (0 to 0·2)	0·1 (0 to 0·1)	0·1 (0 to 0·1)	0 (0 to 0·1)	0·1 (0 to 0·2)	0 (0 to 0·1)	0 (0 to 0·1)
Neglected tropical diseases and malaria	0·1 (0 to 0·1)	0·4 (0·2 to 0·8)	6·1 (2·6 to 12·6)	6·9 (3·8 to 11·4)	21·7 (12·9 to 34·6)	13·1 (7·4 to 20·8)	10·6 (6 to 17·8)	10·2 (5·9 to 16·3)	11·8 (6·7 to 19·4)	11·8 (6·8 to 19)	13·6 (8·1 to 21·7)	14·1 (8·1 to 23·1)
Malaria	0 (0 to 0)	0 (0 to 0)	0 (0 to 0)	0 (0 to 0)	0 (0 to 0)	0 (0 to 0)	0 (0 to 0)	0 (0 to 0)	0 (0 to 0)	0 (0 to 0)	0 (0 to 0)	0 (0 to 0)
Chagas disease	0 (0 to 0)	0 (0 to 0)	0 (0 to 0)	0 (0 to 0)	0 (0 to 0)	0 (0 to 0)	0 (0 to 0)	0 (0 to 0)	0 (0 to 0)	0 (0 to 0)	0 (0 to 0)	0 (0 to 0)
Leishmaniasis	0 (0 to 0)	0 (0 to 0)	0 (0 to 0)	0 (0 to 0)	0 (0 to 0)	0 (0 to 0)	0 (0 to 0)	0 (0 to 0)	0 (0 to 0)	0 (0 to 0)	0 (0 to 0)	0 (0 to 0)
Visceral leishmaniasis	0 (0 to 0)	0 (0 to 0)	0 (0 to 0)	0 (0 to 0)	0 (0 to 0)	0 (0 to 0)	0 (0 to 0)	0 (0 to 0)	0 (0 to 0)	0 (0 to 0)	0 (0 to 0)	0 (0 to 0)
Cutaneous and mucocutaneous leishmaniasis	0 (0 to 0)	0 (0 to 0)	0 (0 to 0)	0 (0 to 0)	0 (0 to 0)	0 (0 to 0)	0 (0 to 0)	0 (0 to 0)	0 (0 to 0)	0 (0 to 0)	0 (0 to 0)	0 (0 to 0)
African trypanosomiasis	0 (0 to 0)	0 (0 to 0)	0 (0 to 0)	0 (0 to 0)	0 (0 to 0)	0 (0 to 0)	0 (0 to 0)	0 (0 to 0)	0 (0 to 0)	0 (0 to 0)	0 (0 to 0)	0 (0 to 0)
Schistosomiasis	0 (0 to 0)	0 (0 to 0)	0 (0 to 0)	0 (0 to 0)	0 (0 to 0)	0 (0 to 0)	0 (0 to 0)	0 (0 to 0)	0 (0 to 0)	0 (0 to 0)	0 (0 to 0)	0 (0 to 0)
Cysticercosis	0 (0 to 0)	0 (0 to 0)	0 (0 to 0)	0 (0 to 0)	0 (0 to 0)	0·1 (0 to 0·2)	0·7 (0·1 to 1·9)	2·2 (0·5 to 5·8)	4 (1·2 to 9·3)	4·5 (1·4 to 10·4)	5·5 (1·7 to 12·6)	6·5 (2·1 to 14·3)
Cystic echinococcosis	0 (0 to 0)	0 (0 to 0)	0 (0 to 0)	0 (0 to 0)	0 (0 to 0)	0 (0 to 0)	0 (0 to 0)	0 (0 to 0)	0 (0 to 0)	0 (0 to 0)	0 (0 to 0)	0 (0 to 0)
Lymphatic filariasis	0 (0 to 0)	0 (0 to 0)	0 (0 to 0)	0 (0 to 0)	0 (0 to 0)	0 (0 to 0)	0 (0 to 0)	0 (0 to 0)	0 (0 to 0)	0 (0 to 0)	0 (0 to 0)	0 (0 to 0)
Onchocerciasis	0 (0 to 0)	0 (0 to 0)	0 (0 to 0)	0 (0 to 0)	0 (0 to 0)	0 (0 to 0)	0 (0 to 0)	0 (0 to 0)	0 (0 to 0)	0 (0 to 0)	0 (0 to 0)	0 (0 to 0)
Trachoma	0 (0 to 0)	0 (0 to 0)	0 (0 to 0)	0 (0 to 0)	0 (0 to 0)	0 (0 to 0)	0 (0 to 0)	0 (0 to 0)	0 (0 to 0)	0 (0 to 0)	0 (0 to 0)	0 (0 to 0)
Dengue	0 (0 to 0)	0·2 (0·1 to 0·6)	5 (1·5 to 11·5)	3·6 (1·4 to 6·8)	7·8 (2·8 to 14·7)	5·8 (2·3 to 10·8)	7·5 (3·4 to 14·4)	5·3 (2·5 to 9·9)	5·6 (2·5 to 9·9)	5·2 (2·5 to 9·1)	5·3 (2·5 to 9·5)	5·5 (2·7 to 10)
Yellow fever	0 (0 to 0)	0 (0 to 0)	0 (0 to 0)	0 (0 to 0)	0 (0 to 0)	0 (0 to 0)	0 (0 to 0)	0 (0 to 0)	0 (0 to 0)	0 (0 to 0)	0 (0 to 0)	0 (0 to 0)
Rabies	0 (0 to 0)	0 (0 to 0)	0 (0 to 0)	0 (0 to 0)	0·1 (0 to 0·2)	0 (0 to 0·1)	0 (0 to 0·1)	0 (0 to 0)	0 (0 to 0)	0 (0 to 0)	0 (0 to 0)	0 (0 to 0)
Intestinal nematode infections	0 (0 to 0)	0 (0 to 0)	0 (0 to 0)	0·1 (0 to 0·3)	0·5 (0·2 to 1)	0·3 (0·1 to 0·6)	0·9 (0·3 to 2)	1·2 (0·3 to 2·7)	1 (0·3 to 2·2)	0·7 (0·2 to 1·8)	1 (0·4 to 2·1)	0·3 (0·1 to 0·7)
Ascariasis	0 (0 to 0)	0 (0 to 0)	0 (0 to 0)	0 (0 to 0·1)	0 (0 to 0·1)	0 (0 to 0·1)	0 (0 to 0·1)	0 (0 to 0)	0 (0 to 0)	0 (0 to 0)	0 (0 to 0)	0 (0 to 0)
Trichuriasis	0 (0 to 0)	0 (0 to 0)	0 (0 to 0)	0 (0 to 0)	0 (0 to 0)	0 (0 to 0)	0 (0 to 0)	0 (0 to 0)	0 (0 to 0)	0 (0 to 0)	0 (0 to 0)	0 (0 to 0)
Hookworm disease	0 (0 to 0)	0 (0 to 0)	0 (0 to 0)	0·1 (0 to 0·2)	0·4 (0·2 to 0·9)	0·3 (0·1 to 0·6)	0·9 (0·3 to 2)	1·2 (0·3 to 2·7)	1 (0·3 to 2·2)	0·7 (0·2 to 1·8)	1 (0·4 to 2·1)	0·3 (0·1 to 0·7)
Food-borne trematodiases	0 (0 to 0)	0 (0 to 0)	0 (0 to 0)	0 (0 to 0)	0 (0 to 0)	0 (0 to 0)	0 (0 to 0)	0 (0 to 0)	0 (0 to 0)	0 (0 to 0)	0 (0 to 0)	0 (0 to 0)
Leprosy	0 (0 to 0)	0 (0 to 0)	0 (0 to 0)	0 (0 to 0)	0 (0 to 0)	0 (0 to 0)	0 (0 to 0)	0 (0 to 0)	0 (0 to 0)	0 (0 to 0)	0 (0 to 0)	0 (0 to 0)
Ebola	0 (0 to 0)	0 (0 to 0)	0 (0 to 0)	0 (0 to 0)	0 (0 to 0)	0 (0 to 0)	0 (0 to 0)	0 (0 to 0)	0 (0 to 0)	0 (0 to 0)	0 (0 to 0)	0 (0 to 0)
Zika virus	0 (0 to 0)	0 (0 to 0·1)	0·3 (0 to 0·9)	0·1 (0 to 0·7)	0·2 (0 to 0·9)	0·2 (0 to 1)	0·3 (0 to 1·3)	0·3 (0 to 1·4)	0·3 (0 to 1·4)	0·3 (0 to 1·4)	0·2 (0 to 1·3)	0·2 (0 to 1)
Guinea worm disease	0 (0 to 0)	0 (0 to 0)	0 (0 to 0)	0 (0 to 0)	0 (0 to 0)	0 (0 to 0)	0 (0 to 0)	0 (0 to 0)	0 (0 to 0)	0 (0 to 0)	0 (0 to 0)	0 (0 to 0)
Other neglected tropical diseases	0 (0 to 0·1)	0·2 (0·1 to 0·3)	0·8 (0·4 to 1·6)	3 (1·2 to 6)	13·2 (7 to 22·8)	6·6 (2·8 to 12·5)	1·2 (0·4 to 2·3)	1·1 (0·4 to 2·3)	1 (0·3 to 2·3)	1·2 (0·4 to 2·5)	1·5 (0·5 to 3·1)	1·6 (0·6 to 3·3)
Other infectious diseases	4·9 (3·1 to 7·4)	7·9 (5·1 to 11·9)	29·7 (18·5 to 43·9)	21·3 (14·4 to 31·3)	25·2 (19 to 33·6)	20·2 (15·5 to 26·3)	24·1 (18·7 to 30·7)	21·2 (16·6 to 27)	23·5 (17·9 to 30·7)	26·8 (20·2 to 35·8)	28 (21·4 to 36·3)	29·3 (21·8 to 38·9)
Meningitis	2·7 (1·5 to 4·2)	5·1 (3 to 8)	12·4 (6·6 to 20·1)	5·7 (3·6 to 8·8)	5·3 (4 to 6·8)	4·7 (3·5 to 6·4)	6·5 (4·6 to 8·9)	4·6 (3·4 to 6·3)	4·8 (3·5 to 6·5)	5·8 (4·2 to 7·9)	5·6 (4·2 to 7·6)	5·5 (4·1 to 7·4)
Encephalitis	0·9 (0·5 to 1·5)	1·3 (0·7 to 2·1)	10·8 (5·8 to 18·9)	9·4 (5·3 to 18·1)	7·5 (4·8 to 12·7)	6·6 (4·6 to 9·2)	8·6 (5·9 to 12)	7·1 (4·9 to 10)	6·2 (4·2 to 9)	6·2 (4·4 to 8·7)	5·9 (4·2 to 8·4)	6 (4·3 to 8·5)
Diphtheria	0 (0 to 0)	0 (0 to 0)	0 (0 to 0)	0 (0 to 0)	0 (0 to 0)	0 (0 to 0)	0 (0 to 0)	0 (0 to 0)	0 (0 to 0)	0 (0 to 0)	0 (0 to 0)	0 (0 to 0)
Tetanus	0·1 (0·1 to 0·3)	0·2 (0·1 to 0·4)	0·2 (0·1 to 0·5)	0·1 (0 to 0·2)	0·2 (0·1 to 0·7)	0·2 (0·1 to 0·6)	0·3 (0·1 to 0·8)	0·3 (0·1 to 0·9)	0·3 (0·1 to 0·9)	0·5 (0·2 to 1·3)	0·7 (0·3 to 1·6)	0·8 (0·3 to 1·9)
Measles	0 (0 to 0)	0 (0 to 0)	0 (0 to 0)	0 (0 to 0)	0 (0 to 0)	0 (0 to 0)	0 (0 to 0)	0 (0 to 0)	0 (0 to 0)	0 (0 to 0)	0 (0 to 0)	0 (0 to 0)
Varicella and herpes zoster	0·1 (0·1 to 0·3)	0·3 (0·1 to 0·6)	0·8 (0·4 to 1·6)	0·4 (0·2 to 0·7)	0·6 (0·4 to 1·1)	0·7 (0·4 to 1·1)	0·6 (0·4 to 1)	0·8 (0·4 to 1·2)	0·9 (0·5 to 1·4)	0·9 (0·5 to 1·4)	1·1 (0·7 to 1·8)	1·2 (0·7 to 1·8)
Acute hepatitis	0 (0 to 0)	0 (0 to 0)	0·1 (0·1 to 0·3)	0·3 (0·2 to 0·6)	1·2 (0·7 to 1·9)	1·7 (1·1 to 2·5)	1·8 (1·2 to 2·7)	1·7 (1·2 to 2·4)	1·6 (1·1 to 2·3)	1·4 (1 to 2)	1·4 (1 to 1·9)	1·4 (1 to 1·8)
Acute hepatitis A	0 (0 to 0)	0 (0 to 0)	0 (0 to 0·1)	0·3 (0·1 to 0·5)	1·1 (0·6 to 1·7)	1·4 (0·9 to 2·2)	1·5 (1 to 2·3)	1·2 (0·8 to 1·9)	1 (0·6 to 1·5)	0·9 (0·5 to 1·4)	0·7 (0·4 to 1·1)	0·7 (0·4 to 1)
Acute hepatitis B	0 (0 to 0)	0 (0 to 0)	0·1 (0 to 0·2)	0 (0 to 0·1)	0·1 (0 to 0·1)	0·1 (0 to 0·2)	0·2 (0·1 to 0·3)	0·3 (0·2 to 0·5)	0·5 (0·3 to 0·7)	0·4 (0·3 to 0·6)	0·5 (0·3 to 0·8)	0·5 (0·3 to 0·8)
Acute hepatitis C	0 (0 to 0)	0 (0 to 0)	0 (0 to 0)	0 (0 to 0·1)	0 (0 to 0)	0 (0 to 0)	0 (0 to 0)	0 (0 to 0)	0 (0 to 0)	0 (0 to 0)	0 (0 to 0)	0 (0 to 0·1)
Acute hepatitis E	0 (0 to 0)	0 (0 to 0)	0 (0 to 0)	0 (0 to 0)	0·1 (0 to 0·1)	0·1 (0 to 0·3)	0·1 (0 to 0·3)	0·1 (0 to 0·3)	0·1 (0·1 to 0·2)	0·1 (0 to 0·2)	0·1 (0·1 to 0·2)	0·1 (0 to 0·1)
Other unspecified infectious diseases	1 (0·5 to 1·7)	1·1 (0·6 to 1·8)	4·5 (2·5 to 7·3)	4·3 (2·6 to 7·2)	10·2 (6 to 16·7)	6·3 (3·6 to 10·3)	6·2 (3·8 to 9·6)	6·7 (4·2 to 10·3)	9·6 (5·5 to 15)	12 (6·7 to 19·3)	13·3 (7·8 to 20)	14·5 (8·6 to 21·8)
Maternal and neonatal disorders	1,320 (911 to 1,870)	330 (225 to 469)	105 (70·1 to 153)	81·6 (60·7 to 105)	80·3 (58·3 to 106)	92·2 (67·6 to 122)	112 (86 to 144)	112 (87·7 to 142)	111 (85·2 to 140)	103 (79·6 to 131)	102 (79 to 129)	85·4 (65·3 to 109)
Maternal disorders	0 (0 to 0)	0 (0 to 0)	0 (0 to 0)	0 (0 to 0)	0 (0 to 0)	0·5 (0·4 to 0·6)	16·1 (12·4 to 20·6)	23·2 (17·4 to 30·7)	19·8 (14·8 to 26·3)	19·1 (14·6 to 25)	17·3 (13·1 to 21·8)	7·5 (5·8 to 9·7)
Maternal hemorrhage	0 (0 to 0)	0 (0 to 0)	0 (0 to 0)	0 (0 to 0)	0 (0 to 0)	0 (0 to 0·1)	1·9 (1·2 to 2·9)	3·3 (1·9 to 5)	3·3 (2 to 5)	3·2 (2 to 4·8)	2·9 (1·8 to 4·4)	1·1 (0·7 to 1·7)
Maternal sepsis and other maternal infections	0 (0 to 0)	0 (0 to 0)	0 (0 to 0)	0 (0 to 0)	0 (0 to 0)	0 (0 to 0·1)	1·8 (1·1 to 2·7)	2·7 (1·6 to 4)	2·4 (1·3 to 3·6)	2·1 (1·2 to 3·3)	1·8 (1·1 to 2·8)	0·7 (0·4 to 1·1)
Maternal hypertensive disorders	0 (0 to 0)	0 (0 to 0)	0 (0 to 0)	0 (0 to 0)	0 (0 to 0)	0·1 (0·1 to 0·1)	2·8 (2 to 3·8)	3 (2 to 4·1)	2·7 (1·8 to 3·8)	2·3 (1·6 to 3·3)	1·9 (1·3 to 2·6)	0·8 (0·5 to 1·2)
Maternal obstructed labor and uterine rupture	0 (0 to 0)	0 (0 to 0)	0 (0 to 0)	0 (0 to 0)	0 (0 to 0)	0 (0 to 0)	0·4 (0·2 to 0·7)	0·6 (0·3 to 1)	0·5 (0·3 to 0·9)	0·6 (0·3 to 1)	0·6 (0·3 to 1)	0·2 (0·1 to 0·4)
Maternal abortion and miscarriage	0 (0 to 0)	0 (0 to 0)	0 (0 to 0)	0 (0 to 0)	0 (0 to 0)	0 (0 to 0·1)	1·1 (0·6 to 1·8)	1·6 (0·9 to 2·6)	1·2 (0·6 to 2)	1 (0·5 to 1·7)	0·9 (0·5 to 1·7)	0·5 (0·3 to 0·8)
Ectopic pregnancy	0 (0 to 0)	0 (0 to 0)	0 (0 to 0)	0 (0 to 0)	0 (0 to 0)	0 (0 to 0)	1·3 (0·8 to 2)	2·6 (1·6 to 4·2)	2·3 (1·4 to 3·6)	2·4 (1·4 to 3·6)	2 (1·2 to 3)	0·7 (0·4 to 1·2)
Indirect maternal deaths	--	--	--	--	--	0 (0 to 0·1)	1·3 (0·8 to 1·9)	1·8 (1 to 2·8)	1·3 (0·7 to 2)	1·1 (0·6 to 1·8)	0·9 (0·5 to 1·4)	0·3 (0·2 to 0·5)
Late maternal deaths	--	--	--	--	--	0·1 (0 to 0·1)	1·6 (1 to 2·5)	2·2 (1·3 to 3·4)	1·7 (1 to 2·8)	1·6 (0·9 to 2·7)	1·5 (0·9 to 2·5)	0·7 (0·4 to 1·1)
Maternal deaths aggravated by HIV/AIDS	--	--	--	--	--	0 (0 to 0)	0 (0 to 0)	0·1 (0 to 0·1)	0·1 (0 to 0·1)	0·1 (0 to 0·1)	0·1 (0 to 0·1)	0 (0 to 0)
Neonatal disorders	1,320 (911 to 1,870)	330 (225 to 469)	105 (70·1 to 153)	81·6 (60·7 to 105)	80·3 (58·3 to 106)	91·7 (67·1 to 121)	96·3 (70·3 to 127)	88·9 (65·8 to 116)	90·7 (67 to 118)	83·6 (62·7 to 110)	84·5 (62·5 to 112)	77·9 (58·2 to 102)
Neonatal preterm birth	515 (339 to 755)	98·8 (62·8 to 144)	36·5 (23·9 to 54·4)	21·5 (15·7 to 28·4)	29·4 (20·9 to 38·5)	33·3 (24·1 to 44·8)	34·2 (25·2 to 45)	30·9 (22·2 to 41·7)	31·4 (22·8 to 42·2)	28·5 (20·8 to 38·3)	28·3 (20·8 to 37·6)	25·5 (18·6 to 34·3)
Neonatal encephalopathy due to birth asphyxia and trauma	409 (253 to 607)	56·3 (31·9 to 86·4)	19·8 (10·8 to 32·1)	7·8 (5·3 to 11·2)	7·3 (4·3 to 11·3)	8·3 (4·8 to 13·1)	8·8 (5 to 13·8)	8·2 (4·9 to 12·8)	8·3 (4·9 to 12·9)	7·7 (4·3 to 12·1)	7·9 (4·5 to 12·1)	7·3 (4·2 to 10·9)
Neonatal sepsis and other neonatal infections	205 (121 to 325)	125 (80·1 to 188)	31 (20·3 to 46·4)	27·4 (17·9 to 39)	39·4 (25·8 to 55·7)	45·3 (29·2 to 64·7)	48·1 (30·9 to 70·7)	45 (28·9 to 65·1)	46·2 (30·7 to 68·2)	42·8 (27·8 to 61·8)	43·6 (28 to 62·8)	40·8 (27·3 to 58·6)
Hemolytic disease and other neonatal jaundice	8·4 (3·9 to 15)	2·2 (1·3 to 3·8)	1 (0·6 to 1·8)	3 (1·8 to 4·5)	4·2 (2·4 to 6·4)	4·8 (2·8 to 7·3)	5·2 (3 to 8·1)	4·8 (2·8 to 7·6)	4·8 (2·8 to 7·5)	4·5 (2·5 to 7·1)	4·7 (2·6 to 7·2)	4·3 (2·5 to 6·7)
Other neonatal disorders	178 (106 to 278)	46·7 (28·3 to 72·6)	16·2 (11·4 to 23·2)	21·8 (16·3 to 28·1)	0 (0 to 0)	0 (0 to 0)	0 (0 to 0)	0 (0 to 0)	0 (0 to 0)	0 (0 to 0)	0 (0 to 0)	0 (0 to 0)
Nutritional deficiencies	0·1 (0·1 to 0·1)	1·7 (0·8 to 3)	24·6 (16·2 to 36·4)	36 (17·5 to 68)	141 (75·5 to 241)	73·6 (35·6 to 134)	30·9 (13·7 to 58·8)	25·3 (11·8 to 51·4)	29·6 (13 to 59·4)	29·3 (12·4 to 57·9)	40·8 (18·7 to 75·3)	39·7 (18·6 to 77·8)
Protein-energy malnutrition	0·1 (0·1 to 0·1)	0·3 (0·2 to 0·4)	16·9 (10·8 to 26·4)	7·7 (4·6 to 12)	4·4 (2·9 to 6·4)	3·4 (2·1 to 5·1)	3·7 (2·3 to 5·8)	3·7 (2·3 to 5·5)	4·3 (2·6 to 6·4)	4·6 (3·1 to 6·5)	5·5 (3·8 to 7·8)	5·3 (3·4 to 7·5)
Iodine deficiency	0 (0 to 0)	0 (0 to 0)	0 (0 to 0)	0·1 (0 to 0·1)	0·5 (0·2 to 1)	1·3 (0·4 to 2·4)	1·8 (0·7 to 3·3)	1·9 (0·8 to 3·5)	2·2 (0·9 to 4)	2·2 (0·9 to 4·1)	2·4 (1 to 4·3)	2·2 (1 to 4·1)
Vitamin A deficiency	0 (0 to 0)	0 (0 to 0·1)	0·2 (0·1 to 0·4)	1·2 (0·6 to 2·2)	2 (1 to 3·5)	1·4 (0·6 to 2·6)	0·6 (0·2 to 1·1)	0·4 (0·2 to 0·8)	0·4 (0·2 to 0·7)	0·3 (0·1 to 0·6)	0·3 (0·1 to 0·5)	0·2 (0·1 to 0·4)
Dietary iron deficiency	0 (0 to 0)	1·3 (0·5 to 2·6)	5·8 (1·7 to 13·1)	25·9 (8·5 to 55·8)	133 (67·9 to 232)	66·6 (28·4 to 126)	22·6 (6·4 to 48·3)	17 (4·3 to 40·5)	20·1 (5 to 49·2)	20 (4·7 to 48·4)	30·2 (9·4 to 63·3)	29·2 (9·4 to 65·1)
Other nutritional deficiencies	0 (0 to 0)	0 (0 to 0)	1·6 (0·7 to 3·3)	1 (0·4 to 2·1)	1 (0·6 to 1·7)	0·9 (0·5 to 1·5)	2·2 (1·4 to 3·4)	2·2 (1·4 to 3·2)	2·6 (1·6 to 4)	2·2 (1·4 to 3·2)	2·4 (1·5 to 3·5)	2·8 (1·8 to 4)
Non-communicable diseases	294 (186 to 441)	126 (83·5 to 183)	334 (233 to 466)	388 (293 to 497)	561 (435 to 716)	897 (679 to 1,170)	1,430 (1,110 to 1,830)	1,600 (1,250 to 2,020)	2,010 (1,590 to 2,510)	2,360 (1,900 to 2,890)	2,970 (2,420 to 3,630)	3,540 (2,920 to 4,270)
Neoplasms	2·6 (1·7 to 3·9)	2·2 (1·5 to 3·3)	12·1 (7·8 to 17·9)	42·8 (29·2 to 60·6)	67·2 (53·6 to 83·9)	69·4 (56·1 to 85·9)	112 (89·2 to 143)	120 (96·1 to 156)	160 (128 to 205)	258 (205 to 328)	421 (336 to 534)	602 (475 to 762)
Lip and oral cavity cancer	0 (0 to 0)	0 (0 to 0)	0 (0 to 0)	0 (0 to 0)	0·2 (0·1 to 0·2)	0·2 (0·1 to 0·3)	0·5 (0·4 to 0·7)	0·6 (0·5 to 0·9)	1 (0·7 to 1·3)	2 (1·4 to 2·6)	3·3 (2·4 to 4·6)	6·8 (4·8 to 9·4)
Nasopharynx cancer	0 (0 to 0)	0 (0 to 0)	0 (0 to 0)	0 (0 to 0)	0·2 (0·2 to 0·3)	0·4 (0·3 to 0·6)	1 (0·7 to 1·4)	1 (0·7 to 1·4)	1·2 (0·8 to 1·7)	1·3 (0·9 to 1·9)	1·7 (1·1 to 2·4)	3·2 (2·1 to 4·5)
Other pharynx cancer	0 (0 to 0)	0 (0 to 0)	0 (0 to 0)	0 (0 to 0)	0 (0 to 0)	0 (0 to 0)	0 (0 to 0)	0·4 (0·3 to 0·6)	0·5 (0·3 to 0·7)	1 (0·7 to 1·3)	1·8 (1·3 to 2·8)	4·4 (2·9 to 6·6)
Esophageal cancer	0 (0 to 0)	0 (0 to 0)	0 (0 to 0)	0 (0 to 0)	0 (0 to 0)	0 (0 to 0)	0 (0 to 0)	0·6 (0·5 to 0·8)	1·1 (0·8 to 1·6)	2·5 (1·7 to 3·7)	6·4 (4·5 to 9·2)	16·1 (11 to 22·6)
Stomach cancer	0 (0 to 0)	0 (0 to 0)	0 (0 to 0)	0 (0 to 0)	0 (0 to 0)	0 (0 to 0)	1·5 (1·2 to 2)	2·6 (2 to 3·5)	6 (4·1 to 8·8)	13·6 (9·6 to 19)	20·7 (14·6 to 29)	33·4 (24·3 to 45·5)
Colon and rectum cancer	0 (0 to 0)	0 (0 to 0)	0 (0 to 0)	0 (0 to 0)	0·6 (0·4 to 0·8)	0·8 (0·6 to 1·1)	4·3 (3·4 to 5·7)	6·6 (4·8 to 9·1)	11·9 (8·3 to 16·8)	25·9 (18·6 to 34)	38·5 (28·2 to 51·1)	58·5 (44·4 to 75·9)
Liver cancer	0·1 (0 to 0·2)	0·1 (0 to 0·1)	0·8 (0·5 to 1·2)	2·4 (1·4 to 3·5)	2·3 (1·3 to 4)	2·1 (1·4 to 3·2)	0·7 (0·5 to 0·9)	1·2 (0·8 to 1·7)	2 (1·4 to 2·9)	2·9 (2 to 4)	5·1 (3·6 to 7)	8·2 (6 to 11·5)
Liver cancer due to hepatitis B	0 (0 to 0)	0 (0 to 0)	0 (0 to 0)	0 (0 to 0)	0 (0 to 0)	0·2 (0·1 to 0·3)	0·3 (0·2 to 0·4)	0·6 (0·4 to 0·9)	1·2 (0·7 to 1·9)	1·7 (1·1 to 2·5)	2·6 (1·5 to 4·1)	3·8 (2·2 to 5·9)
Liver cancer due to hepatitis C	0 (0 to 0)	0 (0 to 0)	0 (0 to 0)	0 (0 to 0)	0 (0 to 0)	0 (0 to 0)	0 (0 to 0)	0 (0 to 0)	0·1 (0 to 0·1)	0·1 (0·1 to 0·2)	0·3 (0·1 to 0·6)	0·7 (0·3 to 1·4)
Liver cancer due to alcohol use	0 (0 to 0)	0 (0 to 0)	0 (0 to 0)	0 (0 to 0)	0 (0 to 0)	0 (0 to 0)	0 (0 to 0)	0·1 (0 to 0·2)	0·3 (0·1 to 0·6)	0·6 (0·3 to 1)	1·2 (0·6 to 2·3)	2·4 (1·2 to 4)
Liver cancer due to NASH	0 (0 to 0)	0 (0 to 0)	0 (0 to 0)	0 (0 to 0)	0 (0 to 0)	0 (0 to 0)	0·2 (0·1 to 0·3)	0·2 (0·1 to 0·3)	0·2 (0·1 to 0·4)	0·2 (0·1 to 0·4)	0·4 (0·2 to 0·7)	0·6 (0·3 to 1·1)
Liver cancer due to other causes	0·1 (0 to 0·2)	0·1 (0 to 0·1)	0·8 (0·5 to 1·2)	2·4 (1·4 to 3·5)	2·3 (1·3 to 4)	1·9 (1·3 to 3)	0·2 (0·1 to 0·3)	0·3 (0·1 to 0·4)	0·3 (0·1 to 0·5)	0·3 (0·2 to 0·5)	0·5 (0·2 to 0·9)	0·7 (0·3 to 1·2)
Gallbladder and biliary tract cancer	0 (0 to 0)	0 (0 to 0)	0 (0 to 0)	0 (0 to 0)	0 (0 to 0)	0 (0 to 0)	0 (0 to 0)	0·2 (0·1 to 0·4)	0·4 (0·3 to 0·7)	0·9 (0·6 to 1·5)	2 (1·4 to 3·5)	3·8 (2·6 to 6·7)
Pancreatic cancer	0 (0 to 0)	0 (0 to 0)	0 (0 to 0)	0 (0 to 0)	0 (0 to 0)	0 (0 to 0)	0·8 (0·7 to 1·1)	0·9 (0·7 to 1·2)	1·9 (1·4 to 2·8)	6·2 (4·1 to 9·2)	10·8 (7·3 to 15·7)	21·9 (15·6 to 30·4)
Larynx cancer	0 (0 to 0)	0 (0 to 0)	0 (0 to 0)	0 (0 to 0)	0 (0 to 0)	0 (0 to 0)	0 (0 to 0)	0·3 (0·2 to 0·4)	0·3 (0·2 to 0·4)	0·6 (0·4 to 0·7)	1·3 (1 to 1·8)	3·4 (2·4 to 4·6)
Tracheal, bronchus, and lung cancer	0 (0 to 0)	0 (0 to 0)	0 (0 to 0)	0 (0 to 0)	0 (0 to 0)	0·2 (0·1 to 0·3)	1·4 (1·1 to 1·8)	1·4 (1 to 2)	2·3 (1·6 to 3·4)	6·3 (4·5 to 8·6)	12·3 (9 to 16·6)	24·9 (18·5 to 32·6)
Malignant skin melanoma	0 (0 to 0)	0 (0 to 0)	0·1 (0 to 0·2)	0·2 (0·1 to 0·7)	0·2 (0·1 to 0·3)	0·1 (0·1 to 0·2)	0·4 (0·3 to 0·5)	0·7 (0·5 to 1)	1·2 (0·8 to 1·7)	1·8 (1·2 to 2·5)	3·1 (2·1 to 4·3)	3·3 (2·2 to 4·7)
Non-melanoma skin cancer	0 (0 to 0)	0 (0 to 0)	0 (0 to 0)	0 (0 to 0)	0 (0 to 0)	0 (0 to 0)	0 (0 to 0)	0·2 (0·2 to 0·3)	0·4 (0·3 to 0·5)	0·5 (0·4 to 0·7)	0·8 (0·5 to 1·1)	1·2 (0·9 to 1·7)
Non-melanoma skin cancer (squamous-cell carcinoma)	0 (0 to 0)	0 (0 to 0)	0 (0 to 0)	0 (0 to 0)	0 (0 to 0)	0 (0 to 0)	0 (0 to 0)	0·2 (0·2 to 0·3)	0·4 (0·3 to 0·5)	0·5 (0·4 to 0·7)	0·8 (0·5 to 1·1)	1·2 (0·9 to 1·7)
Non-melanoma skin cancer (basal-cell carcinoma)	0 (0 to 0)	0 (0 to 0)	0 (0 to 0)	0 (0 to 0)	0 (0 to 0)	0 (0 to 0)	0 (0 to 0)	0 (0 to 0)	0 (0 to 0)	0 (0 to 0)	0 (0 to 0)	0 (0 to 0)
Breast cancer	0 (0 to 0)	0 (0 to 0)	0 (0 to 0)	0 (0 to 0)	0 (0 to 0)	0 (0 to 0)	2 (1·6 to 2·6)	3·8 (2·7 to 5·2)	12·8 (8·7 to 18·8)	43 (30·7 to 60·3)	104 (76·9 to 135)	146 (105 to 195)
Cervical cancer	0 (0 to 0)	0 (0 to 0)	0 (0 to 0)	0 (0 to 0)	0 (0 to 0)	0 (0 to 0)	1·6 (1·1 to 2·3)	5·6 (3·6 to 8·5)	13·9 (8·8 to 21·4)	28·6 (19·3 to 40·5)	56·4 (39·7 to 78·1)	71·2 (51·3 to 98·2)
Uterine cancer	0 (0 to 0)	0 (0 to 0)	0 (0 to 0)	0 (0 to 0)	0 (0 to 0)	0 (0 to 0)	0 (0 to 0)	1·3 (0·9 to 1·7)	2·4 (1·7 to 3·5)	4·9 (3·4 to 7)	10·5 (6·9 to 15·8)	15·5 (10·3 to 22·5)
Ovarian cancer	0 (0 to 0)	0 (0 to 0)	0 (0 to 0)	0 (0 to 0)	0·7 (0·3 to 1·1)	0·7 (0·3 to 1·3)	3·3 (1·4 to 4·9)	4·5 (1·9 to 6·9)	4·4 (2·3 to 6·8)	7 (3·5 to 10·7)	13·8 (6·9 to 19·9)	22·2 (11·7 to 31·4)
Prostate cancer	0 (0 to 0)	0 (0 to 0)	0 (0 to 0)	0 (0 to 0)	0 (0 to 0)	0 (0 to 0)	0 (0 to 0)	0·5 (0·4 to 0·7)	1·4 (0·9 to 1·8)	2·1 (1·3 to 2·8)	2·4 (1·5 to 3·2)	5·7 (3·7 to 8·1)
Testicular cancer	0 (0 to 0·6)	0 (0 to 0·1)	0·1 (0 to 0·2)	0·2 (0 to 0·5)	0·1 (0·1 to 0·2)	0·2 (0·1 to 0·3)	0·6 (0·4 to 1)	0·9 (0·5 to 1·3)	1·5 (0·9 to 2·3)	1 (0·6 to 1·6)	0·9 (0·6 to 1·3)	0·7 (0·4 to 1)
Kidney cancer	0·2 (0·1 to 0·3)	0·1 (0 to 0·1)	0·7 (0·4 to 1·2)	2·9 (1·7 to 4·5)	3·4 (2·5 to 4·5)	1·9 (1·3 to 2·7)	3·1 (2·2 to 4·4)	3·7 (2·5 to 5·2)	5 (3·4 to 7·3)	6·6 (4·2 to 9·8)	7·4 (4·9 to 10·7)	12·2 (8·2 to 16·8)
Bladder cancer	0 (0 to 0)	0 (0 to 0)	0 (0 to 0)	0 (0 to 0)	0 (0 to 0)	0 (0 to 0)	0·2 (0·1 to 0·2)	0·2 (0·2 to 0·3)	0·3 (0·2 to 0·4)	1 (0·7 to 1·5)	1·6 (1·1 to 2·3)	3·1 (2·1 to 4·3)
Brain and central nervous system cancer	0·4 (0·1 to 0·8)	0·4 (0·1 to 0·7)	2 (0·6 to 3·6)	6·1 (2 to 10·2)	11·8 (4·9 to 18·7)	10·4 (4 to 16·2)	9·3 (3·1 to 15·2)	8·3 (3 to 13·8)	9·8 (3·2 to 15·9)	12·4 (4·2 to 19·3)	16·1 (5·6 to 25·5)	18·7 (6·6 to 29)
Thyroid cancer	0 (0 to 0)	0 (0 to 0)	0 (0 to 0)	0 (0 to 0)	0·2 (0·1 to 0·2)	0·1 (0·1 to 0·2)	0·4 (0·3 to 0·6)	0·5 (0·4 to 0·7)	0·8 (0·6 to 1·1)	1·1 (0·9 to 1·5)	2·1 (1·5 to 2·9)	2·7 (2 to 3·9)
Mesothelioma	0 (0 to 0)	0 (0 to 0)	0 (0 to 0)	0 (0 to 0)	0 (0 to 0)	0 (0 to 0)	0 (0 to 0)	0·5 (0·3 to 0·7)	0·5 (0·3 to 0·7)	0·5 (0·4 to 0·7)	1·1 (0·8 to 1·6)	1·2 (0·8 to 1·7)
Hodgkin lymphoma	0 (0 to 0)	0 (0 to 0)	0 (0 to 0)	0·1 (0·1 to 0·1)	0·2 (0·1 to 0·2)	0·4 (0·3 to 0·5)	2·4 (1·7 to 3·4)	3·4 (2·3 to 4·9)	3·7 (2·6 to 5·4)	3·4 (2·3 to 4·8)	3 (2·1 to 4·2)	2·9 (2·1 to 4)
Non-Hodgkin lymphoma	0 (0 to 0)	0 (0 to 0)	0 (0 to 0)	4·2 (2·7 to 6·4)	8·3 (5·5 to 12·1)	10·4 (7·1 to 15)	19·2 (13·1 to 27)	21·9 (15 to 31·7)	25·1 (19 to 34·3)	29·9 (21 to 41·5)	32 (24·2 to 42·9)	33·4 (24·5 to 45·5)
Multiple myeloma	0 (0 to 0)	0 (0 to 0)	0 (0 to 0)	0 (0 to 0)	0 (0 to 0)	0 (0 to 0)	0 (0 to 0)	0·8 (0·6 to 1·2)	1·4 (1 to 1·8)	2·5 (1·8 to 3·3)	6·3 (4·5 to 8·6)	14·3 (10·4 to 19·5)
Leukemia	0·9 (0·6 to 1·2)	0·7 (0·4 to 1)	3·5 (2·2 to 5·5)	11·3 (7·2 to 17·2)	22·4 (16·6 to 29·9)	24·4 (17·7 to 32·5)	33·3 (25·2 to 44·6)	26·1 (19·5 to 35·7)	25·2 (18·4 to 33·9)	24·5 (18·6 to 32·5)	27·2 (20 to 36·6)	28·7 (21·4 to 38)
Acute lymphoid leukemia	0·1 (0 to 0·4)	0·1 (0 to 0·2)	0·6 (0·4 to 1·1)	3·7 (2·2 to 6)	8·2 (5 to 12·3)	6 (3·7 to 9·4)	8·3 (5·3 to 12·5)	3·9 (2·3 to 6·2)	2·1 (1·3 to 3·2)	1·8 (1·1 to 2·8)	1·5 (0·9 to 2·3)	1·6 (1 to 2·4)
Chronic lymphoid leukemia	0 (0 to 0)	0 (0 to 0)	0 (0 to 0)	0 (0 to 0)	0 (0 to 0)	0 (0 to 0)	0 (0 to 0)	0·4 (0·2 to 0·6)	0·4 (0·2 to 0·7)	0·4 (0·2 to 0·6)	0·5 (0·3 to 0·8)	0·9 (0·5 to 1·4)
Acute myeloid leukemia	0·2 (0·1 to 0·3)	0·4 (0·3 to 0·7)	1·5 (0·9 to 2·4)	5·5 (3·3 to 8·6)	9·4 (6·2 to 13·4)	12·1 (8·3 to 16·9)	17·2 (12·4 to 23·8)	12·9 (9 to 17·8)	13·6 (9·7 to 19)	11·7 (8·3 to 16·2)	14 (9·9 to 19·4)	12·6 (8·8 to 17·4)
Chronic myeloid leukemia	0 (0 to 0)	0 (0 to 0)	0·1 (0 to 0·2)	0·3 (0·1 to 0·5)	0·3 (0·2 to 0·5)	0·4 (0·2 to 0·6)	0·9 (0·4 to 1·5)	1·7 (0·9 to 2·9)	2·5 (1·4 to 4·3)	2·9 (1·7 to 4·7)	3·1 (1·9 to 4·8)	2·7 (1·6 to 4·3)
Other leukemia	0·5 (0·3 to 0·7)	0·2 (0·1 to 0·3)	1·3 (0·8 to 2·1)	1·8 (1 to 3)	4·5 (2·7 to 7)	5·9 (3·7 to 9)	6·9 (4·6 to 10·1)	7·3 (5 to 10·7)	6·5 (4·4 to 9·4)	7·7 (5·3 to 10·8)	8·1 (5·6 to 11·4)	10·9 (7·8 to 15)
Other malignant neoplasms	1 (0·6 to 1·4)	0·9 (0·6 to 1·3)	4·7 (3 to 7)	14·8 (9·6 to 21·4)	16·1 (12·5 to 20·9)	16·4 (12·9 to 20·6)	25·3 (19·4 to 32·9)	20·3 (15·5 to 26·9)	20·6 (16 to 27)	23·5 (18·4 to 30·8)	27·2 (21 to 35·4)	32·5 (24·4 to 43·6)

Cause	45 to 49	50 to 54	55 to 59	60 to 64	65 to 69	70 to 74	75 to 79	80 to 84	85 to 89	90 to 94	95 plus	Total
All causes	5,810 (4,820 to 6,870)	6,930 (5,760 to 8,210)	8,820 (7,380 to 10,400)	10,400 (8,800 to 12,100)	9,840 (8,430 to 11,300)	9,930 (8,560 to 11,300)	8,150 (7,120 to 9,190)	6,530 (5,820 to 7,240)	4,690 (4,250 to 5,120)	2,270 (2,090 to 2,430)	563 (528 to 594)	99,600
Communicable, maternal, neonatal, and nutritional diseases	487 (425 to 560)	427 (356 to 506)	453 (375 to 538)	479 (390 to 579)	443 (352 to 546)	467 (367 to 600)	450 (348 to 571)	435 (351 to 523)	379 (295 to 471)	217 (166 to 268)	59·4 (44·5 to 75·3)	9,470
HIV/AIDS and sexually transmitted infections	189 (176 to 204)	112 (103 to 122)	89 (81·7 to 97·5)	59·8 (54·1 to 66·4)	38·3 (34·5 to 43)	14·7 (12·6 to 17·5)	7·4 (6·2 to 8·9)	4·2 (3·6 to 5·1)	2·4 (2 to 2·8)	0·8 (0·6 to 1)	0·2 (0·2 to 0·3)	1,280
HIV/AIDS	181 (169 to 193)	106 (98·8 to 114)	84·2 (78 to 90·8)	56·1 (51·4 to 61)	35·6 (32·5 to 38·9)	12·7 (11 to 14·4)	6·1 (5·2 to 7)	3·4 (2·9 to 3·9)	1·8 (1·6 to 2·1)	0·6 (0·5 to 0·7)	0·2 (0·2 to 0·2)	1,210
HIV/AIDS - Drug-susceptible Tuberculosis	3·1 (2·1 to 4·6)	1·9 (1·3 to 2·8)	1·7 (1·1 to 2·4)	1·1 (0·8 to 1·7)	0·7 (0·5 to 1·1)	0·3 (0·2 to 0·4)	0·1 (0·1 to 0·2)	0·1 (0·1 to 0·1)	0 (0 to 0·1)	0 (0 to 0)	0 (0 to 0)	22
HIV/AIDS - Multidrug-resistant Tuberculosis without extensive d	0 (0 to 0·1)	0 (0 to 0·1)	0 (0 to 0·1)	0 (0 to 0)	0 (0 to 0)	0 (0 to 0)	0 (0 to 0)	0 (0 to 0)	0 (0 to 0)	0 (0 to 0)	0 (0 to 0)	0·2
HIV/AIDS - Extensively drug-resistant Tuberculosis	0 (0 to 0)	0 (0 to 0)	0 (0 to 0)	0 (0 to 0)	0 (0 to 0)	0 (0 to 0)	0 (0 to 0)	0 (0 to 0)	0 (0 to 0)	0 (0 to 0)	0 (0 to 0)	0
HIV/AIDS resulting in other diseases	177 (166 to 190)	104 (96·6 to 112)	82·6 (76·3 to 89·1)	54·9 (50·2 to 59·9)	34·8 (31·7 to 38·3)	12·4 (10·7 to 14·1)	5·9 (5·1 to 6·8)	3·3 (2·9 to 3·8)	1·8 (1·5 to 2·1)	0·6 (0·5 to 0·7)	0·2 (0·2 to 0·2)	1,180
Sexually transmitted infections excluding HIV	8·7 (5 to 14·9)	6 (3·3 to 11·4)	4·8 (2·5 to 9·3)	3·7 (1·9 to 7·2)	2·8 (1·5 to 5·4)	2·1 (1·1 to 3·9)	1·3 (0·7 to 2·4)	0·8 (0·5 to 1·5)	0·5 (0·3 to 0·9)	0·2 (0·1 to 0·4)	0 (0 to 0·1)	74·1
Syphilis	0·3 (0·2 to 0·5)	0·3 (0·2 to 0·4)	0·3 (0·2 to 0·5)	0·3 (0·2 to 0·5)	0·3 (0·2 to 0·5)	0·2 (0·2 to 0·4)	0·1 (0·1 to 0·2)	0·1 (0·1 to 0·2)	0·1 (0·1 to 0·1)	0 (0 to 0·1)	0 (0 to 0)	5·9
Chlamydial infection	1·2 (0·7 to 1·9)	0·5 (0·2 to 0·8)	0·2 (0·2 to 0·3)	0·1 (0·1 to 0·2)	0·1 (0 to 0·1)	0·1 (0 to 0·1)	0·1 (0 to 0·1)	0 (0 to 0·1)	0 (0 to 0·1)	0 (0 to 0)	0 (0 to 0)	9
Gonococcal infection	0·9 (0·6 to 1·3)	0·5 (0·4 to 0·8)	0·4 (0·2 to 0·5)	0·3 (0·2 to 0·5)	0·3 (0·2 to 0·5)	0·2 (0·1 to 0·4)	0·2 (0·1 to 0·3)	0·1 (0·1 to 0·2)	0·1 (0·1 to 0·1)	0 (0 to 0·1)	0 (0 to 0)	7·5
Trichomoniasis	2·1 (0·7 to 4·8)	1·8 (0·6 to 4·1)	1·4 (0·5 to 3·3)	0·9 (0·3 to 2·1)	0·6 (0·2 to 1·4)	0·4 (0·1 to 0·9)	0·2 (0·1 to 0·5)	0·1 (0 to 0·3)	0·1 (0 to 0·1)	0 (0 to 0)	0 (0 to 0)	16·4
Genital herpes	2·2 (0·7 to 5·6)	2·1 (0·7 to 5·5)	2 (0·6 to 5·2)	1·8 (0·5 to 4·4)	1·3 (0·4 to 3·3)	1 (0·3 to 2·4)	0·6 (0·2 to 1·5)	0·4 (0·1 to 0·9)	0·2 (0·1 to 0·5)	0·1 (0 to 0·2)	0 (0 to 0)	19·8
Other sexually transmitted infections	2 (1·3 to 3·3)	0·8 (0·5 to 1·2)	0·5 (0·4 to 0·7)	0·3 (0·2 to 0·4)	0·2 (0·1 to 0·3)	0·1 (0·1 to 0·2)	0·1 (0·1 to 0·1)	0·1 (0 to 0·1)	0·1 (0 to 0·1)	0 (0 to 0)	0 (0 to 0)	15·4
Respiratory infections and tuberculosis	90 (65·5 to 120)	108 (74·3 to 150)	146 (108 to 194)	208 (143 to 287)	225 (154 to 308)	288 (200 to 411)	322 (227 to 438)	340 (261 to 423)	314 (235 to 401)	187 (140 to 237)	51·6 (37·7 to 66·5)	2,920
Tuberculosis	5·2 (3·5 to 7·4)	5·3 (3·5 to 7·5)	5·2 (3·6 to 7·4)	5·5 (3·8 to 7·7)	4·4 (3 to 6·3)	4·2 (3 to 5·6)	3·1 (2·2 to 4·3)	2·4 (1·7 to 3·2)	1·6 (1·1 to 2·2)	0·6 (0·4 to 0·8)	0·1 (0·1 to 0·1)	65·6
Latent tuberculosis infection	0 (0 to 0)	0 (0 to 0)	0 (0 to 0)	0 (0 to 0)	0 (0 to 0)	0 (0 to 0)	0 (0 to 0)	0 (0 to 0)	0 (0 to 0)	0 (0 to 0)	0 (0 to 0)	0
Drug-susceptible tuberculosis	5·1 (3·4 to 7·4)	5·3 (3·5 to 7·5)	5·2 (3·6 to 7·3)	5·5 (3·7 to 7·6)	4·3 (3 to 6·3)	4·1 (3 to 5·5)	3·1 (2·2 to 4·2)	2·4 (1·7 to 3·2)	1·6 (1·1 to 2·2)	0·6 (0·4 to 0·8)	0·1 (0·1 to 0·1)	65·1
Multidrug-resistant tuberculosis without extensive drug resista	0 (0 to 0·2)	0 (0 to 0·2)	0 (0 to 0·2)	0 (0 to 0·2)	0 (0 to 0·2)	0 (0 to 0·2)	0 (0 to 0·1)	0 (0 to 0·1)	0 (0 to 0·1)	0 (0 to 0)	0 (0 to 0)	0·4
Extensively drug-resistant tuberculosis	0 (0 to 0)	0 (0 to 0)	0 (0 to 0)	0 (0 to 0)	0 (0 to 0)	0 (0 to 0)	0 (0 to 0)	0 (0 to 0)	0 (0 to 0)	0 (0 to 0)	0 (0 to 0)	0·1
Lower respiratory infections	65·5 (43·5 to 95·5)	83·8 (53·8 to 123)	124 (87·3 to 170)	188 (124 to 269)	209 (138 to 293)	276 (188 to 400)	314 (218 to 431)	335 (256 to 418)	311 (232 to 398)	186 (139 to 235)	51·3 (37·4 to 66·2)	2,560
Upper respiratory infections	14·7 (8 to 25·7)	13·8 (7·7 to 23·7)	13·1 (7·3 to 21·9)	11·5 (6·1 to 19·2)	8·9 (4·8 to 14·8)	6·5 (3·5 to 11·1)	4 (2·1 to 6·5)	2·5 (1·3 to 4·2)	1·3 (0·7 to 2·2)	0·6 (0·3 to 0·9)	0·1 (0·1 to 0·2)	223
Otitis media	4·6 (2·3 to 8·3)	4·7 (2·1 to 8·8)	4·3 (2·1 to 7·8)	3·5 (1·6 to 6·7)	2·1 (1 to 3·9)	1·3 (0·6 to 2·7)	0·9 (0·4 to 1·6)	0·6 (0·3 to 1·2)	0·4 (0·2 to 0·7)	0·2 (0·1 to 0·3)	0 (0 to 0·1)	69·4
Enteric infections	37·5 (26·1 to 52·7)	40·9 (28·3 to 56·5)	45·1 (31·9 to 63·3)	53 (38·5 to 71·2)	57·6 (41·7 to 77·7)	60·8 (43·7 to 80·7)	51·3 (38·1 to 68)	40·9 (30·1 to 53·3)	25·4 (18·6 to 32·6)	11·5 (8·6 to 14·8)	2·7 (2 to 3·5)	721
Diarrheal diseases	37·4 (26 to 52·6)	40·8 (28·2 to 56·4)	45 (31·7 to 63·2)	52·8 (38·2 to 70·9)	57·4 (41·6 to 77·6)	60·7 (43·6 to 80·6)	51·2 (37·9 to 67·8)	40·8 (30 to 53·2)	25·4 (18·5 to 32·6)	11·5 (8·6 to 14·7)	2·7 (2 to 3·4)	718
Typhoid and paratyphoid	0 (0 to 0·1)	0 (0 to 0·1)	0·1 (0 to 0·2)	0·1 (0 to 0·2)	0·1 (0 to 0·2)	0 (0 to 0·1)	0 (0 to 0·1)	0 (0 to 0·1)	0 (0 to 0·1)	0 (0 to 0)	0 (0 to 0)	0·8
Typhoid fever	0 (0 to 0·1)	0 (0 to 0·1)	0 (0 to 0·1)	0 (0 to 0·1)	0·1 (0 to 0·2)	0 (0 to 0·1)	0 (0 to 0·1)	0 (0 to 0·1)	0 (0 to 0·1)	0 (0 to 0)	0 (0 to 0)	0·7
Paratyphoid fever	0 (0 to 0)	0 (0 to 0)	0 (0 to 0·1)	0 (0 to 0·1)	0 (0 to 0)	0 (0 to 0)	0 (0 to 0)	0 (0 to 0)	0 (0 to 0)	0 (0 to 0)	0 (0 to 0)	0·1
Invasive Non-typhoidal Salmonella (iNTS)	0 (0 to 0·1)	0 (0 to 0·1)	0 (0 to 0·1)	0·2 (0 to 0·3)	0·1 (0 to 0·1)	0 (0 to 0·1)	0·1 (0 to 0·1)	0 (0 to 0)	0 (0 to 0)	0 (0 to 0)	0 (0 to 0)	1
Other intestinal infectious diseases	0 (0 to 0·1)	0 (0 to 0·1)	0 (0 to 0·1)	0 (0 to 0·1)	0 (0 to 0·1)	0 (0 to 0·1)	0 (0 to 0·1)	0 (0 to 0·1)	0 (0 to 0·1)	0 (0 to 0)	0 (0 to 0)	1
Neglected tropical diseases and malaria	15·5 (8·7 to 26·7)	18·6 (9·4 to 32·9)	22·1 (12 to 37·4)	27·5 (14·5 to 48·7)	26 (13·1 to 46·3)	24·9 (12·6 to 41·5)	18·9 (8·8 to 33·9)	13·7 (6·7 to 24·3)	8·3 (4·2 to 14·8)	3·6 (1·9 to 6·7)	0·9 (0·4 to 1·6)	300
Malaria	0 (0 to 0)	0 (0 to 0)	0 (0 to 0)	0 (0 to 0)	0 (0 to 0)	0 (0 to 0)	0 (0 to 0)	0 (0 to 0)	0 (0 to 0)	0 (0 to 0)	0 (0 to 0)	0
Chagas disease	0 (0 to 0)	0 (0 to 0)	0 (0 to 0)	0 (0 to 0)	0 (0 to 0)	0 (0 to 0)	0 (0 to 0)	0 (0 to 0)	0 (0 to 0)	0 (0 to 0)	0 (0 to 0)	0
Leishmaniasis	0 (0 to 0)	0 (0 to 0)	0 (0 to 0)	0 (0 to 0)	0 (0 to 0)	0 (0 to 0)	0 (0 to 0)	0 (0 to 0)	0 (0 to 0)	0 (0 to 0)	0 (0 to 0)	0
Visceral leishmaniasis	0 (0 to 0)	0 (0 to 0)	0 (0 to 0)	0 (0 to 0)	0 (0 to 0)	0 (0 to 0)	0 (0 to 0)	0 (0 to 0)	0 (0 to 0)	0 (0 to 0)	0 (0 to 0)	0
Cutaneous and mucocutaneous leishmaniasis	0 (0 to 0)	0 (0 to 0)	0 (0 to 0)	0 (0 to 0)	0 (0 to 0)	0 (0 to 0)	0 (0 to 0)	0 (0 to 0)	0 (0 to 0)	0 (0 to 0)	0 (0 to 0)	0
African trypanosomiasis	0 (0 to 0)	0 (0 to 0)	0 (0 to 0)	0 (0 to 0)	0 (0 to 0)	0 (0 to 0)	0 (0 to 0)	0 (0 to 0)	0 (0 to 0)	0 (0 to 0)	0 (0 to 0)	0
Schistosomiasis	0 (0 to 0)	0 (0 to 0)	0 (0 to 0)	0 (0 to 0)	0 (0 to 0)	0 (0 to 0)	0 (0 to 0)	0 (0 to 0)	0 (0 to 0)	0 (0 to 0)	0 (0 to 0)	0
Cysticercosis	8·5 (2·6 to 18·9)	10·7 (3·9 to 23·7)	14·7 (5·5 to 29·7)	18·8 (7 to 38·4)	19·8 (8·3 to 39·6)	19·2 (7·9 to 36·3)	14·8 (5·8 to 29·9)	10·9 (4·4 to 21·5)	6·3 (2·4 to 12·7)	2·6 (1·1 to 5·5)	0·6 (0·2 to 1·2)	150
Cystic echinococcosis	0 (0 to 0)	0 (0 to 0)	0 (0 to 0)	0 (0 to 0)	0 (0 to 0)	0 (0 to 0)	0 (0 to 0)	0 (0 to 0)	0 (0 to 0)	0 (0 to 0)	0 (0 to 0)	0
Lymphatic filariasis	0 (0 to 0)	0 (0 to 0)	0 (0 to 0)	0 (0 to 0)	0 (0 to 0)	0 (0 to 0)	0 (0 to 0)	0 (0 to 0)	0 (0 to 0)	0 (0 to 0)	0 (0 to 0)	0
Onchocerciasis	0 (0 to 0)	0 (0 to 0)	0 (0 to 0)	0 (0 to 0)	0 (0 to 0)	0 (0 to 0)	0 (0 to 0)	0 (0 to 0)	0 (0 to 0)	0 (0 to 0)	0 (0 to 0)	0
Trachoma	0 (0 to 0)	0 (0 to 0)	0 (0 to 0)	0 (0 to 0)	0 (0 to 0)	0 (0 to 0)	0 (0 to 0)	0 (0 to 0)	0 (0 to 0)	0 (0 to 0)	0 (0 to 0)	0
Dengue	5·2 (2·6 to 9·3)	6·2 (2·6 to 13·1)	5·5 (2·5 to 11·1)	7·5 (3·3 to 15·7)	5·3 (2·3 to 10·6)	4·6 (2·3 to 8·4)	3·3 (1·5 to 6·1)	2·3 (1·2 to 3·9)	1·6 (0·8 to 2·7)	0·8 (0·4 to 1·4)	0·2 (0·1 to 0·4)	99·3
Yellow fever	0 (0 to 0)	0 (0 to 0)	0 (0 to 0)	0 (0 to 0)	0 (0 to 0)	0 (0 to 0)	0 (0 to 0)	0 (0 to 0)	0 (0 to 0)	0 (0 to 0)	0 (0 to 0)	0
Rabies	0 (0 to 0·1)	0 (0 to 0·1)	0 (0 to 0)	0 (0 to 0·1)	0 (0 to 0·1)	0 (0 to 0)	0 (0 to 0)	0 (0 to 0)	0 (0 to 0)	0 (0 to 0)	0 (0 to 0)	0·3
Intestinal nematode infections	0·1 (0 to 0·2)	0·1 (0·1 to 0·3)	0·2 (0·1 to 0·3)	0·1 (0·1 to 0·3)	0·1 (0 to 0·2)	0·1 (0 to 0·2)	0·1 (0 to 0·2)	0·1 (0 to 0·1)	0 (0 to 0·1)	0 (0 to 0)	0 (0 to 0)	7·1
Ascariasis	0 (0 to 0)	0 (0 to 0)	0 (0 to 0)	0 (0 to 0·1)	0 (0 to 0)	0 (0 to 0·1)	0 (0 to 0)	0 (0 to 0)	0 (0 to 0)	0 (0 to 0)	0 (0 to 0)	0·3
Trichuriasis	0 (0 to 0)	0 (0 to 0)	0 (0 to 0)	0 (0 to 0)	0 (0 to 0)	0 (0 to 0)	0 (0 to 0)	0 (0 to 0)	0 (0 to 0)	0 (0 to 0)	0 (0 to 0)	0
Hookworm disease	0·1 (0 to 0·2)	0·1 (0 to 0·3)	0·1 (0·1 to 0·3)	0·1 (0 to 0·2)	0·1 (0 to 0·2)	0·1 (0 to 0·2)	0·1 (0 to 0·1)	0 (0 to 0·1)	0 (0 to 0·1)	0 (0 to 0)	0 (0 to 0)	6·8
Food-borne trematodiases	0 (0 to 0)	0 (0 to 0)	0 (0 to 0)	0 (0 to 0)	0 (0 to 0)	0 (0 to 0)	0 (0 to 0)	0 (0 to 0)	0 (0 to 0)	0 (0 to 0)	0 (0 to 0)	0
Leprosy	0 (0 to 0)	0 (0 to 0·1)	0 (0 to 0·1)	0 (0 to 0·1)	0 (0 to 0·1)	0 (0 to 0·1)	0 (0 to 0)	0 (0 to 0)	0 (0 to 0)	0 (0 to 0)	0 (0 to 0)	0·4
Ebola	0 (0 to 0)	0 (0 to 0)	0 (0 to 0)	0 (0 to 0)	0 (0 to 0)	0 (0 to 0)	0 (0 to 0)	0 (0 to 0)	0 (0 to 0)	0 (0 to 0)	0 (0 to 0)	0
Zika virus	0·1 (0 to 0·7)	0·1 (0 to 0·6)	0·1 (0 to 0·4)	0·1 (0 to 0·3)	0 (0 to 0·2)	0 (0 to 0·1)	0 (0 to 0)	0 (0 to 0)	0 (0 to 0)	0 (0 to 0)	0 (0 to 0)	2·8
Guinea worm disease	0 (0 to 0)	0 (0 to 0)	0 (0 to 0)	0 (0 to 0)	0 (0 to 0)	0 (0 to 0)	0 (0 to 0)	0 (0 to 0)	0 (0 to 0)	0 (0 to 0)	0 (0 to 0)	0
Other neglected tropical diseases	1·5 (0·6 to 3)	1·4 (0·5 to 3)	1·6 (0·7 to 3·4)	0·9 (0·4 to 1·7)	0·7 (0·3 to 1·3)	0·9 (0·5 to 1·6)	0·7 (0·4 to 1·2)	0·4 (0·2 to 0·7)	0·4 (0·2 to 0·6)	0·2 (0·1 to 0·3)	0·1 (0 to 0·1)	40·1
Other infectious diseases	35·7 (25·1 to 47·3)	34·5 (25·1 to 46·2)	38·6 (27·7 to 51·8)	40·1 (29·3 to 51·8)	31 (22·5 to 40·9)	27·5 (20·1 to 35·7)	17·4 (12·8 to 22·6)	12·1 (8·7 to 15·9)	10·2 (6·6 to 13·7)	3·5 (2·2 to 4·7)	0·8 (0·5 to 1·1)	513
Meningitis	6·8 (5 to 9·2)	7·1 (5·2 to 9·8)	7·9 (5·6 to 10·4)	8·8 (6·3 to 11·8)	6·7 (4·9 to 8·8)	5·8 (4·3 to 7·8)	4·3 (3·2 to 5·6)	2·9 (2·1 to 4)	2 (1·5 to 2·8)	0·6 (0·4 to 0·7)	0·1 (0·1 to 0·1)	122
Encephalitis	6·3 (4·3 to 8·8)	5·1 (3·5 to 7·3)	6 (4·2 to 8·8)	6·2 (4·3 to 9)	4·5 (3·2 to 6·7)	3·8 (2·7 to 5·6)	2·1 (1·4 to 2·9)	1·4 (0·9 to 2)	0·7 (0·5 to 1)	0·3 (0·2 to 0·4)	0·1 (0 to 0·1)	113
Diphtheria	0 (0 to 0)	0 (0 to 0)	0 (0 to 0)	0 (0 to 0)	0 (0 to 0)	0 (0 to 0)	0 (0 to 0)	0 (0 to 0)	0 (0 to 0)	0 (0 to 0)	0 (0 to 0)	0
Tetanus	1·1 (0·4 to 3)	1·7 (0·7 to 4·2)	2·1 (0·7 to 5·4)	2 (0·8 to 4·8)	1·8 (0·7 to 4·3)	1·8 (0·7 to 4·3)	1·4 (0·6 to 3)	0·8 (0·3 to 1·6)	0·5 (0·2 to 1·1)	0·2 (0·1 to 0·4)	0 (0 to 0·1)	17·3
Measles	0 (0 to 0)	0 (0 to 0)	0 (0 to 0)	0 (0 to 0)	0 (0 to 0)	0 (0 to 0)	0 (0 to 0)	0 (0 to 0)	0 (0 to 0)	0 (0 to 0)	0 (0 to 0)	0
Varicella and herpes zoster	1·3 (0·8 to 2)	1·3 (0·8 to 2)	1·3 (0·7 to 2·1)	1·7 (1 to 2·6)	1·3 (0·7 to 2·1)	1·4 (0·9 to 2·1)	1 (0·6 to 1·6)	0·8 (0·5 to 1·3)	0·6 (0·4 to 1)	0·3 (0·2 to 0·5)	0·2 (0·1 to 0·3)	19·4
Acute hepatitis	1·3 (0·9 to 1·7)	1·2 (0·9 to 1·6)	1·1 (0·8 to 1·5)	0·9 (0·7 to 1·3)	0·7 (0·5 to 0·8)	0·5 (0·4 to 0·7)	0·3 (0·2 to 0·4)	0·2 (0·1 to 0·3)	0·2 (0·1 to 0·2)	0·1 (0 to 0·1)	0 (0 to 0)	19·1
Acute hepatitis A	0·6 (0·4 to 0·9)	0·5 (0·3 to 0·8)	0·4 (0·2 to 0·7)	0·3 (0·1 to 0·6)	0·2 (0·1 to 0·3)	0·1 (0 to 0·2)	0·1 (0 to 0·2)	0·1 (0 to 0·1)	0 (0 to 0·1)	0 (0 to 0)	0 (0 to 0)	11·3
Acute hepatitis B	0·5 (0·3 to 0·8)	0·6 (0·4 to 0·8)	0·6 (0·3 to 0·8)	0·5 (0·3 to 0·7)	0·4 (0·2 to 0·5)	0·3 (0·2 to 0·5)	0·1 (0·1 to 0·2)	0·1 (0 to 0·2)	0·1 (0 to 0·1)	0 (0 to 0)	0 (0 to 0)	5·8
Acute hepatitis C	0 (0 to 0·1)	0·1 (0 to 0·1)	0·1 (0 to 0·1)	0·1 (0 to 0·1)	0·1 (0 to 0·1)	0 (0 to 0·1)	0 (0 to 0)	0 (0 to 0)	0 (0 to 0)	0 (0 to 0)	0 (0 to 0)	0·6
Acute hepatitis E	0·1 (0·1 to 0·2)	0·1 (0 to 0·1)	0·1 (0 to 0·1)	0·1 (0 to 0·1)	0·1 (0 to 0·1)	0·1 (0 to 0·1)	0 (0 to 0·1)	0 (0 to 0)	0 (0 to 0·1)	0 (0 to 0)	0 (0 to 0)	1·4
Other unspecified infectious diseases	19 (10·3 to 29·4)	18·1 (10·2 to 28)	20·2 (11·5 to 30·9)	20·4 (11·1 to 29·7)	16·1 (8·8 to 23·4)	14·2 (7·6 to 20·5)	8·3 (4·2 to 12·2)	6 (3 to 9·3)	6·2 (2·9 to 9·6)	2·1 (0·9 to 3·3)	0·5 (0·2 to 0·7)	221
Maternal and neonatal disorders	81·6 (59·8 to 106)	73·4 (54·8 to 97·4)	66 (49·3 to 85·2)	52·3 (38·7 to 69·8)	32·7 (23·8 to 44)	18·2 (12·8 to 25·4)	7·1 (4·7 to 10)	2 (1·2 to 3)	0·4 (0·2 to 0·6)	0 (0 to 0·1)	0 (0 to 0)	2,960
Maternal disorders	2·1 (1·4 to 3)	0·3 (0·2 to 0·4)	0 (0 to 0)	0 (0 to 0)	0 (0 to 0)	0 (0 to 0)	0 (0 to 0)	0 (0 to 0)	0 (0 to 0)	0 (0 to 0)	0 (0 to 0)	106
Maternal hemorrhage	0·2 (0·1 to 0·3)	0 (0 to 0)	0 (0 to 0)	0 (0 to 0)	0 (0 to 0)	0 (0 to 0)	0 (0 to 0)	0 (0 to 0)	0 (0 to 0)	0 (0 to 0)	0 (0 to 0)	15·9
Maternal sepsis and other maternal infections	0·2 (0·1 to 0·3)	0 (0 to 0)	0 (0 to 0)	0 (0 to 0)	0 (0 to 0)	0 (0 to 0)	0 (0 to 0)	0 (0 to 0)	0 (0 to 0)	0 (0 to 0)	0 (0 to 0)	11·7
Maternal hypertensive disorders	0·2 (0·1 to 0·2)	0 (0 to 0)	0 (0 to 0)	0 (0 to 0)	0 (0 to 0)	0 (0 to 0)	0 (0 to 0)	0 (0 to 0)	0 (0 to 0)	0 (0 to 0)	0 (0 to 0)	13·8
Maternal obstructed labor and uterine rupture	0·1 (0 to 0·1)	0 (0 to 0)	0 (0 to 0)	0 (0 to 0)	0 (0 to 0)	0 (0 to 0)	0 (0 to 0)	0 (0 to 0)	0 (0 to 0)	0 (0 to 0)	0 (0 to 0)	3
Maternal abortion and miscarriage	0·2 (0·1 to 0·4)	0 (0 to 0)	0 (0 to 0)	0 (0 to 0)	0 (0 to 0)	0 (0 to 0)	0 (0 to 0)	0 (0 to 0)	0 (0 to 0)	0 (0 to 0)	0 (0 to 0)	6·5
Ectopic pregnancy	0·3 (0·2 to 0·5)	0 (0 to 0)	0 (0 to 0)	0 (0 to 0)	0 (0 to 0)	0 (0 to 0)	0 (0 to 0)	0 (0 to 0)	0 (0 to 0)	0 (0 to 0)	0 (0 to 0)	11·6
Indirect maternal deaths	0·1 (0 to 0·1)	0 (0 to 0)	--	--	--	--	--	--	--	--	--	NA
Late maternal deaths	0·2 (0·1 to 0·3)	0 (0 to 0)	--	--	--	--	--	--	--	--	--	NA
Maternal deaths aggravated by HIV/AIDS	0 (0 to 0)	0 (0 to 0)	--	--	--	--	--	--	--	--	--	NA
Neonatal disorders	79·5 (57·8 to 105)	73 (54·6 to 97·1)	66 (49·3 to 85·2)	52·3 (38·7 to 69·8)	32·7 (23·8 to 44)	18·2 (12·8 to 25·4)	7·1 (4·7 to 10)	2 (1·2 to 3)	0·4 (0·2 to 0·6)	0 (0 to 0·1)	0 (0 to 0)	2,860
Neonatal preterm birth	25·3 (18·3 to 34·2)	22·1 (16 to 30)	18·7 (13·3 to 25)	13·4 (9·7 to 17·5)	7·7 (5·7 to 9·9)	3·7 (2·8 to 4·7)	1·2 (0·9 to 1·6)	0·2 (0·1 to 0·3)	0·1 (0 to 0·1)	0 (0 to 0)	0 (0 to 0)	1,010
Neonatal encephalopathy due to birth asphyxia and trauma	7·5 (4·2 to 11·2)	7 (4 to 10·7)	6·5 (3·7 to 10·5)	5·5 (3·2 to 8·7)	3 (1·7 to 4·7)	1·2 (0·7 to 1·8)	0·3 (0·2 to 0·5)	0 (0 to 0·1)	0 (0 to 0)	0 (0 to 0)	0 (0 to 0)	588
Neonatal sepsis and other neonatal infections	42·2 (26·8 to 61·9)	39·6 (26·1 to 58·3)	36·6 (24·1 to 52·2)	30·1 (19·6 to 44·8)	19·8 (12·6 to 29·5)	12 (7·5 to 18·3)	5 (2·9 to 7·8)	1·6 (0·8 to 2·6)	0·3 (0·1 to 0·5)	0 (0 to 0)	0 (0 to 0)	928
Hemolytic disease and other neonatal jaundice	4·5 (2·7 to 6·9)	4·3 (2·9 to 6·3)	4·1 (2·9 to 5·6)	3·4 (2·4 to 4·6)	2·2 (1·6 to 2·9)	1·3 (1 to 1·7)	0·5 (0·4 to 0·7)	0·2 (0·1 to 0·2)	0 (0 to 0)	0 (0 to 0)	0 (0 to 0)	72·6
Other neonatal disorders	0 (0 to 0)	0 (0 to 0)	0 (0 to 0)	0 (0 to 0)	0 (0 to 0)	0 (0 to 0)	0 (0 to 0)	0 (0 to 0)	0 (0 to 0)	0 (0 to 0)	0 (0 to 0)	263
Nutritional deficiencies	37·1 (19·1 to 68·5)	40·2 (21·3 to 76·1)	45·6 (23·7 to 86·4)	37·9 (21·9 to 63)	32·3 (19·9 to 52·4)	32·2 (20·7 to 50·3)	26·3 (18·4 to 38·6)	22·2 (14·8 to 32·9)	18·5 (12·5 to 26·6)	10 (6·8 to 13·6)	3·2 (2·2 to 4·4)	778
Protein-energy malnutrition	6·7 (4·4 to 9·5)	7·5 (5·1 to 10·4)	9·3 (6·5 to 12·9)	11·1 (7·5 to 15·2)	10·7 (7·4 to 15)	12·5 (8·4 to 17·9)	11·7 (8·6 to 15·2)	10·1 (6·8 to 14·6)	8·9 (5·8 to 12·8)	5·6 (3·5 to 7·9)	2 (1·3 to 2·9)	156
Iodine deficiency	2·3 (1 to 4·3)	2·2 (1 to 3·9)	2·1 (0·9 to 3·8)	1·7 (0·8 to 3·1)	1·3 (0·6 to 2·2)	0·9 (0·4 to 1·6)	0·5 (0·3 to 0·9)	0·3 (0·2 to 0·5)	0·2 (0·1 to 0·3)	0·1 (0 to 0·1)	0 (0 to 0)	26·1
Vitamin A deficiency	0·2 (0·1 to 0·4)	0·2 (0·1 to 0·3)	0·2 (0·1 to 0·3)	0·1 (0·1 to 0·3)	0·1 (0·1 to 0·2)	0·1 (0 to 0·2)	0·1 (0 to 0·1)	0 (0 to 0·1)	0 (0 to 0)	0 (0 to 0)	0 (0 to 0)	8
Dietary iron deficiency	24·7 (7·6 to 56·3)	26 (8·2 to 60·9)	29·5 (9·2 to 66·9)	19·4 (5·7 to 43·4)	14·6 (4·4 to 34·4)	12·8 (4·2 to 28·9)	9·2 (3 to 20·7)	7 (2·3 to 15·1)	5·5 (1·9 to 12·5)	2 (0·7 to 4·2)	0·5 (0·1 to 1)	523
Other nutritional deficiencies	3·2 (2·1 to 4·8)	4·3 (2·8 to 6·2)	4·5 (2·9 to 6·5)	5·5 (3·3 to 8·1)	5·6 (3·4 to 8·7)	5·9 (3·4 to 9·2)	4·8 (2·8 to 7)	4·8 (2·5 to 7·4)	3·9 (2·2 to 6·4)	2·4 (1·2 to 4)	0·6 (0·3 to 1·1)	64·7
Non-communicable diseases	4,810 (3,970 to 5,760)	6,050 (5,010 to 7,190)	7,890 (6,570 to 9,280)	9,480 (8,010 to 11,000)	9,060 (7,750 to 10,400)	9,160 (7,910 to 10,500)	7,470 (6,520 to 8,440)	5,920 (5,260 to 6,570)	4,180 (3,790 to 4,560)	1,980 (1,820 to 2,140)	485 (454 to 517)	83,000
Neoplasms	949 (734 to 1,200)	1,390 (1,090 to 1,740)	1,990 (1,550 to 2,460)	2,590 (2,080 to 3,120)	2,440 (2,000 to 2,910)	2,440 (2,030 to 2,880)	1,880 (1,570 to 2,250)	1,290 (1,080 to 1,490)	781 (641 to 912)	310 (249 to 359)	53 (41·1 to 61·8)	18,000
Lip and oral cavity cancer	14·2 (10·1 to 19·7)	21·3 (15·3 to 28·5)	33·4 (24·8 to 45·1)	36·8 (26·6 to 48)	31·5 (24 to 39·8)	28·4 (21·5 to 36·6)	20·7 (16·1 to 27)	13·8 (10·5 to 17·4)	7·2 (5·7 to 8·9)	3 (2·4 to 3·6)	0·6 (0·5 to 0·8)	225
Nasopharynx cancer	5·7 (3·9 to 8)	8·1 (5·7 to 11·5)	12 (8·8 to 16·4)	11 (7·7 to 15·1)	8·8 (6·3 to 11·8)	7·5 (5·3 to 10)	4 (2·8 to 5·6)	2·5 (1·8 to 3·4)	2·4 (1·7 to 3·3)	0·9 (0·7 to 1·2)	0·1 (0·1 to 0·2)	73·1
Other pharynx cancer	11·8 (7·8 to 17)	17·1 (12 to 23·9)	27·5 (19·2 to 38·1)	29·2 (19·7 to 41·2)	23 (15·9 to 31·6)	18·6 (12·7 to 26)	11·1 (7·7 to 15·5)	6·8 (5 to 9·2)	3·9 (2·8 to 5·2)	1·5 (1·1 to 1·9)	0·3 (0·2 to 0·4)	159
Esophageal cancer	36·4 (24·7 to 51·2)	53·4 (39·6 to 71·8)	81·7 (57·5 to 114)	94·1 (66·2 to 129)	79·3 (56·8 to 106)	71·8 (50·1 to 96·9)	49·8 (35·3 to 69)	32 (23·5 to 42·8)	19·4 (14·5 to 25·1)	7 (5·3 to 8·7)	1·1 (0·7 to 1·3)	553
Stomach cancer	52·8 (36·4 to 72·2)	76·7 (57·2 to 100)	111 (79·8 to 144)	153 (111 to 201)	149 (110 to 196)	148 (111 to 193)	114 (83·8 to 150)	80·2 (60 to 105)	50·8 (38 to 63·8)	19·9 (15·5 to 23·9)	3·5 (2·6 to 4·2)	1,040
Colon and rectum cancer	101 (74·5 to 133)	159 (122 to 207)	262 (193 to 340)	366 (276 to 462)	343 (263 to 430)	348 (273 to 430)	285 (223 to 358)	202 (159 to 247)	121 (94·7 to 151)	46·8 (36·4 to 55·5)	8·7 (6·7 to 10·3)	2,390
Liver cancer	14 (9·9 to 19·2)	21·6 (15·4 to 29·5)	36·1 (26·5 to 48·1)	44·6 (33·4 to 57·3)	44·5 (33·4 to 57·6)	44·5 (33·9 to 58·2)	31·9 (24·1 to 41·8)	19 (14·5 to 24·2)	7·8 (5·9 to 10·1)	2·6 (1·9 to 3·3)	0·4 (0·3 to 0·5)	
Liver cancer due to hepatitis B	5·7 (3·1 to 9·1)	7·6 (4·3 to 12·4)	11·7 (6·5 to 19)	12·2 (6·6 to 20·1)	9·7 (5 to 16·3)	8 (3·8 to 13·6)	5 (2·7 to 8·5)	2·6 (1·2 to 4·7)	1 (0·5 to 1·8)	0·3 (0·1 to 0·5)	0 (0 to 0·1)	74·2
Liver cancer due to hepatitis C	1·6 (0·7 to 3)	3·2 (1·7 to 5·5)	6·2 (2·8 to 11·3)	8·6 (4·2 to 15)	9 (5 to 14·7)	10·1 (5·7 to 16·7)	8·6 (4·7 to 14·1)	6·4 (3·6 to 9·8)	3·1 (2 to 4·6)	1·2 (0·7 to 1·7)	0·2 (0·1 to 0·3)	59·5
Liver cancer due to alcohol use	4·5 (2·2 to 7·7)	7·4 (3·7 to 12·3)	13·2 (7 to 20·6)	17·6 (10·1 to 26·9)	19·7 (12·4 to 29)	19·7 (12·1 to 29·1)	12·6 (7·8 to 18·5)	6·4 (3·8 to 9·6)	2·2 (1·4 to 3·5)	0·6 (0·3 to 0·9)	0·1 (0 to 0·1)	109
Liver cancer due to NASH	1·1 (0·5 to 2)	1·8 (1 to 3·2)	3 (1·6 to 5·3)	3·9 (2 to 6·6)	4·1 (2·2 to 7·3)	4·7 (2·7 to 8·1)	4·1 (2·4 to 6·9)	2·8 (1·5 to 4·4)	1·1 (0·6 to 1·8)	0·4 (0·2 to 0·7)	0·1 (0 to 0·1)	28·9
Liver cancer due to other causes	1 (0·5 to 1·8)	1·5 (0·7 to 2·6)	2·1 (1 to 3·8)	2·3 (1·2 to 4·1)	2 (1·1 to 3·5)	2 (1 to 3·4)	1·5 (0·8 to 2·7)	0·9 (0·5 to 1·6)	0·4 (0·2 to 0·6)	0·1 (0·1 to 0·2)	0 (0 to 0)	23·7
Gallbladder and biliary tract cancer	7·1 (4·9 to 12·3)	12·5 (8·3 to 21·4)	19·9 (13·4 to 34·5)	27·4 (19·1 to 48)	25·6 (18 to 45)	25·9 (18·2 to 45·3)	19 (13·7 to 33·5)	12·1 (8·9 to 20·7)	6·5 (4·8 to 11·2)	2·4 (1·7 to 4·2)	0·4 (0·3 to 0·8)	166
Pancreatic cancer	41·2 (28·7 to 55·8)	62·3 (46·4 to 81·7)	103 (73·2 to 135)	141 (102 to 189)	128 (93·8 to 170)	128 (93·3 to 167)	89·4 (63·4 to 120)	58 (42·2 to 77·3)	32·6 (24·2 to 41·1)	11·9 (9·2 to 14·5)	2·1 (1·6 to 2·6)	840
Larynx cancer	8·9 (5·9 to 13)	15 (11·1 to 20·2)	24·7 (18 to 33·3)	29·8 (22·6 to 38·9)	26·4 (20·5 to 33·7)	22·6 (17 to 29·1)	14·2 (10·7 to 18·9)	8·6 (6·7 to 11)	4·2 (3·2 to 5·4)	1·4 (1·1 to 1·8)	0·2 (0·1 to 0·2)	162
Tracheal, bronchus, and lung cancer	54·8 (39·2 to 74·1)	87·7 (61 to 125)	141 (101 to 190)	182 (132 to 238)	172 (128 to 223)	157 (119 to 199)	101 (78·5 to 129)	58·4 (44·6 to 73·8)	32·5 (25 to 41·3)	10·9 (8·6 to 13)	1·8 (1·3 to 2·1)	1,050
Malignant skin melanoma	4·4 (2·9 to 6·3)	5·4 (3·6 to 7·7)	7·3 (4·9 to 10·5)	7·3 (5 to 10·2)	6·4 (4·5 to 8·7)	6·3 (4·4 to 8·8)	4·3 (3 to 5·9)	3·5 (2·4 to 4·7)	2·3 (1·6 to 3·1)	0·9 (0·6 to 1·1)	0·2 (0·2 to 0·3)	59·4
Non-melanoma skin cancer	1·4 (1 to 2)	2·4 (1·8 to 3·3)	3·4 (2·4 to 4·7)	4·7 (3·5 to 6·1)	4·8 (3·6 to 6·4)	5 (3·8 to 6·5)	4·7 (3·5 to 6·1)	4·1 (3·1 to 5·1)	3·4 (2·6 to 4·3)	2·3 (1·7 to 2·9)	0·7 (0·5 to 1)	40
Non-melanoma skin cancer (squamous-cell carcinoma)	1·4 (1 to 2)	2·4 (1·8 to 3·3)	3·4 (2·4 to 4·7)	4·7 (3·5 to 6·1)	4·8 (3·6 to 6·4)	5 (3·8 to 6·5)	4·7 (3·5 to 6·1)	4 (3·1 to 5·1)	3·4 (2·6 to 4·3)	2·3 (1·7 to 2·9)	0·7 (0·5 to 1)	39·9
Non-melanoma skin cancer (basal-cell carcinoma)	0 (0 to 0)	0 (0 to 0)	0 (0 to 0)	0 (0 to 0)	0 (0 to 0)	0 (0 to 0)	0 (0 to 0)	0 (0 to 0)	0 (0 to 0)	0 (0 to 0)	0 (0 to 0)	0
Breast cancer	206 (148 to 276)	306 (213 to 408)	346 (246 to 466)	366 (265 to 474)	281 (211 to 357)	235 (176 to 300)	163 (120 to 217)	108 (80·6 to 141)	71·7 (54·2 to 91·7)	28·6 (22 to 35·3)	7·6 (5·7 to 9·3)	2,430
Cervical cancer	90·3 (63·1 to 128)	105 (73·7 to 141)	112 (79·7 to 151)	120 (87·4 to 163)	90·2 (66·2 to 125)	77·1 (56·7 to 102)	51·4 (37·7 to 67·9)	33·1 (23·9 to 44·1)	17·5 (12·9 to 23·2)	6·9 (4·9 to 9·4)	1·6 (1·1 to 2·3)	882
Uterine cancer	27·1 (18·5 to 37·9)	40 (27·1 to 57·8)	54·3 (37·5 to 74·3)	86·7 (62·2 to 116)	76·5 (55·9 to 103)	70·1 (52·1 to 93·4)	42·8 (31·5 to 57·9)	25·2 (18 to 33·7)	13·2 (9·8 to 16·8)	5 (3·7 to 6·5)	1·1 (0·8 to 1·5)	477
Ovarian cancer	36·4 (17·5 to 52·4)	48·1 (24·3 to 67·3)	59·4 (30·6 to 82·3)	66·3 (33·8 to 93·1)	56·5 (28·5 to 78·1)	45·4 (22·9 to 62·9)	28·5 (13·4 to 39·6)	17·3 (8·1 to 23·3)	10 (4·4 to 13·5)	3·9 (1·6 to 5)	0·6 (0·2 to 0·8)	429
Prostate cancer	20 (12·7 to 28·9)	55·4 (36·8 to 79)	147 (98·8 to 206)	333 (225 to 456)	437 (298 to 583)	569 (394 to 759)	541 (390 to 717)	417 (298 to 545)	267 (195 to 346)	114 (79·5 to 146)	15·2 (10·2 to 20·8)	2,930
Testicular cancer	0·5 (0·3 to 0·7)	0·4 (0·2 to 0·6)	0·3 (0·2 to 0·5)	0·4 (0·2 to 0·5)	0·3 (0·2 to 0·5)	0·3 (0·2 to 0·5)	0·3 (0·2 to 0·4)	0·2 (0·1 to 0·3)	0·1 (0·1 to 0·2)	0·1 (0 to 0·1)	0 (0 to 0)	9·1
Kidney cancer	20·6 (13·8 to 28·9)	29·8 (22 to 39·1)	44·5 (31·2 to 60·3)	52·2 (37·1 to 71)	44·7 (32·1 to 59·5)	39·4 (28·5 to 53·1)	25·1 (18·3 to 33·8)	14·8 (10·6 to 20)	6·6 (4·8 to 9·1)	2·6 (1·9 to 3·5)	0·5 (0·3 to 0·6)	328
Bladder cancer	5·8 (3·9 to 8·2)	11·7 (8·6 to 15·6)	21·4 (15 to 28·2)	33·3 (24·7 to 43·8)	36·5 (27·1 to 47·8)	41·9 (30·7 to 55·6)	35·5 (26·1 to 46·6)	26·8 (19·9 to 35·8)	18 (13·3 to 23·5)	7·6 (5·8 to 9·4)	1·4 (1 to 1·8)	246
Brain and central nervous system cancer	22·5 (7·9 to 35)	27·7 (10·1 to 42·3)	36·4 (13 to 54·3)	36·9 (13·3 to 55·3)	29·8 (10·9 to 44·5)	22·9 (8·1 to 33·9)	13·6 (4·8 to 20·7)	6·7 (2·3 to 10·3)	3·4 (1·1 to 5·5)	1·1 (0·4 to 1·6)	0·2 (0·1 to 0·3)	307
Thyroid cancer	4·7 (3·3 to 6·5)	7·4 (5·2 to 10·4)	9·9 (7·1 to 13·3)	11·4 (8·6 to 14·8)	10·4 (7·7 to 13·8)	10·3 (8 to 13·1)	7 (5·4 to 8·8)	4·7 (3·6 to 5·9)	2·4 (1·8 to 3)	0·9 (0·7 to 1·2)	0·2 (0·1 to 0·2)	77·4
Mesothelioma	2·1 (1·5 to 2·9)	3 (2·2 to 4)	4·3 (3·2 to 5·7)	2·2 (1·6 to 3)	1·8 (1·4 to 2·3)	2·2 (1·7 to 2·9)	1·6 (1·2 to 2·1)	1·1 (0·9 to 1·4)	0·6 (0·4 to 0·8)	0·2 (0·1 to 0·3)	0 (0 to 0)	23
Hodgkin lymphoma	2·6 (1·8 to 3·8)	2·8 (2 to 4)	3·4 (2·4 to 4·7)	3·6 (2·6 to 5)	3·3 (2·5 to 4·6)	2·6 (1·9 to 3·6)	2 (1·5 to 2·7)	1·2 (0·8 to 1·6)	0·6 (0·4 to 0·8)	0·2 (0·1 to 0·2)	0 (0 to 0)	41·8
Non-Hodgkin lymphoma	46·4 (33·9 to 62·2)	56·8 (41·8 to 74·8)	78·6 (57·3 to 102)	94·4 (71·4 to 122)	80·6 (59·2 to 106)	74·2 (54·3 to 99·5)	51·1 (37·3 to 68·8)	29·4 (21·4 to 38·7)	14 (10·4 to 18·3)	4·7 (3·7 to 5·7)	0·8 (0·6 to 1)	715
Multiple myeloma	30 (21·3 to 41·1)	54·5 (40·4 to 73·1)	89 (65·4 to 118)	117 (86·5 to 156)	114 (86·5 to 149)	108 (81·8 to 139)	72·6 (55·7 to 92·8)	41·6 (32·2 to 52·7)	20·6 (15·7 to 25·9)	6·2 (4·6 to 7·6)	1 (0·7 to 1·3)	680
Leukemia	35·2 (26·1 to 47·1)	39·4 (29·9 to 52·3)	51·4 (38·3 to 65·6)	60·5 (46·4 to 76·3)	55·4 (43·1 to 69·3)	55·2 (42·6 to 70·4)	39·7 (30·9 to 49·3)	27·3 (21·5 to 34)	16·3 (12·6 to 20·1)	6 (4·8 to 7·2)	1·1 (0·8 to 1·3)	616
Acute lymphoid leukemia	1·4 (0·9 to 2·1)	1·7 (1·1 to 2·6)	2 (1·3 to 2·9)	1·8 (1·1 to 2·8)	1·7 (1·1 to 2·5)	1·4 (0·9 to 2·1)	1 (0·6 to 1·7)	0·7 (0·4 to 1·1)	0·3 (0·2 to 0·4)	0·1 (0·1 to 0·2)	0 (0 to 0)	50·1
Chronic lymphoid leukemia	0·8 (0·5 to 1·2)	2·3 (1·5 to 3·5)	4·8 (3·1 to 7·1)	7 (4·4 to 10)	7·9 (5·3 to 11·4)	7·6 (4·9 to 11·1)	5·8 (3·8 to 8·3)	4 (2·7 to 5·7)	2·5 (1·6 to 3·7)	1 (0·7 to 1·4)	0·2 (0·1 to 0·2)	46·4
Acute myeloid leukemia	16·7 (11·6 to 22·8)	17·9 (13 to 24)	21·1 (15·1 to 28)	23·5 (17·2 to 30·8)	18·5 (13·7 to 24)	16·8 (12·1 to 22·3)	10·6 (7·7 to 14·3)	6·3 (4·6 to 8·6)	2·9 (2 to 4)	0·9 (0·6 to 1·2)	0·2 (0·1 to 0·3)	247
Chronic myeloid leukemia	3·3 (2 to 5·1)	4·3 (2·7 to 6·6)	5·7 (3·5 to 8·8)	7·1 (4·3 to 10·5)	6·2 (3·9 to 9·2)	5·7 (3·5 to 8·4)	4·4 (2·8 to 6·6)	3 (1·9 to 4·2)	1·5 (1 to 2·2)	0·6 (0·4 to 0·9)	0·1 (0·1 to 0·2)	56·8
Other leukemia	13 (9·1 to 18·3)	13·1 (9·2 to 17·9)	17·8 (12·6 to 23·8)	21·2 (15·3 to 28·8)	21·1 (15·5 to 27·3)	23·8 (17·8 to 31·5)	17·8 (13·3 to 23·1)	13·3 (9·8 to 17·6)	9 (6·8 to 11·5)	3·4 (2·6 to 4·2)	0·6 (0·4 to 0·7)	216
Other malignant neoplasms	43·2 (32·3 to 57·2)	55·6 (41·7 to 73·1)	69·3 (52·8 to 90·7)	76·9 (59·7 to 98·6)	67·3 (52·4 to 86·7)	62·7 (49·3 to 80)	45·5 (36·3 to 58·2)	31·7 (25·4 to 39·5)	21·8 (17·2 to 27)	8·6 (6·7 to 10·5)	1·6 (1·2 to 2)	687

Other neoplasms	0·1 (0 to 0·1)	0·1 (0 to 0·2)	0·3 (0·1 to 0·6)	0·7 (0·4 to 1·1)	0·5 (0·3 to 0·7)	0·6 (0·4 to 0·9)	0·8 (0·5 to 1·2)	0·6 (0·4 to 0·8)	1 (0·7 to 1·4)	0·9 (0·6 to 1·4)	1 (0·7 to 1·4)	1·8 (1·2 to 2·5)	2 (1·4 to 2·9)	3·2 (2·2 to 4·7)	4·8 (3·4 to 6·9)	6·8 (5 to 9·9)	7·2 (5·4 to 10)	8·9 (6·6 to 13·1)	7·5 (5·6 to 10·6)	5·2 (3·8 to 7·1)	3·1 (2·3 to 4·3)	1·2 (0·8 to 1·6)	0·2 (0·1 to 0·2)	58·5
Myelodysplastic, myeloproliferative, and other hematopoietic n	0·1 (0 to 0·1)	0·1 (0 to 0·2)	0·3 (0·1 to 0·6)	0·7 (0·4 to 1·1)	0·5 (0·3 to 0·7)	0·6 (0·4 to 0·9)	0·8 (0·5 to 1·2)	0·6 (0·4 to 0·8)	1 (0·7 to 1·4)	0·9 (0·6 to 1·4)	1 (0·7 to 1·4)	1·8 (1·2 to 2·5)	2 (1·4 to 2·9)	3·2 (2·2 to 4·7)	4·8 (3·4 to 6·9)	6·8 (5 to 9·9)	7·2 (5·4 to 10)	8·9 (6·6 to 13·1)	7·5 (5·6 to 10·6)	5·2 (3·8 to 7·1)	3·1 (2·3 to 4·3)	1·2 (0·8 to 1·6)	0·2 (0·1 to 0·2)	58·5
Benign and in situ intestinal neoplasms	0 (0 to 0)	0 (0 to 0)	0 (0 to 0)	0 (0 to 0)	0 (0 to 0)	0 (0 to 0)	0 (0 to 0)	0 (0 to 0)	0 (0 to 0)	0 (0 to 0)	0 (0 to 0)	0 (0 to 0)	0 (0 to 0)	0 (0 to 0)	0 (0 to 0)	0 (0 to 0)	0 (0 to 0)	0 (0 to 0)	0 (0 to 0)	0 (0 to 0)	0 (0 to 0)	0 (0 to 0)	0 (0 to 0)	0
Benign and in situ cervical and uterine neoplasms	0 (0 to 0)	0 (0 to 0)	0 (0 to 0)	0 (0 to 0)	0 (0 to 0)	0 (0 to 0)	0 (0 to 0)	0 (0 to 0)	0 (0 to 0)	0 (0 to 0)	0 (0 to 0)	0 (0 to 0)	0 (0 to 0)	0 (0 to 0)	0 (0 to 0)	0 (0 to 0)	0 (0 to 0)	0 (0 to 0)	0 (0 to 0)	0 (0 to 0)	0 (0 to 0)	0 (0 to 0)	0 (0 to 0)	0
Other benign and in situ neoplasms	0 (0 to 0)	0 (0 to 0)	0 (0 to 0)	0 (0 to 0)	0 (0 to 0)	0 (0 to 0)	0 (0 to 0)	0 (0 to 0)	0 (0 to 0)	0 (0 to 0)	0 (0 to 0)	0 (0 to 0)	0 (0 to 0)	0 (0 to 0)	0 (0 to 0)	0 (0 to 0)	0 (0 to 0)	0 (0 to 0)	0 (0 to 0)	0 (0 to 0)	0 (0 to 0)	0 (0 to 0)	0 (0 to 0)	0
Cardiovascular diseases	5·1 (3 to 7·8)	1·9 (1·2 to 2·8)	7·6 (4·7 to 12)	5·2 (3·8 to 6·9)	12·7 (9·8 to 16·4)	24·2 (18·2 to 31·2)	62·8 (49·6 to 78·8)	76·2 (59·4 to 97·5)	106 (83·4 to 137)	164 (128 to 207)	242 (186 to 309)	390 (307 to 491)	674 (531 to 842)	936 (735 to 1,170)	1,420 (1,120 to 1,790)	1,900 (1,530 to 2,320)	2,060 (1,710 to 2,460)	2,330 (1,950 to 2,740)	2,040 (1,730 to 2,370)	1,770 (1,500 to 2,060)	1,400 (1,180 to 1,600)	718 (586 to 812)	195 (153 to 221)	16,600
Rheumatic heart disease	0 (0 to 0)	0 (0 to 0)	0 (0 to 0)	0·6 (0·3 to 1)	3·6 (2·1 to 5·9)	8·7 (5·2 to 13·9)	15·3 (10·3 to 22·9)	16 (10·3 to 23·4)	18·4 (12·6 to 26·7)	19·7 (13·5 to 27·6)	22·4 (15·9 to 30·8)	22·9 (16·1 to 31·1)	23·6 (16·5 to 32·2)	22·3 (16·1 to 30)	21·5 (15·6 to 28·7)	17·4 (12·6 to 22·8)	12·9 (9·6 to 16·6)	10 (7·4 to 12·9)	7·2 (5·4 to 9·5)	4·6 (3·4 to 6)	3 (2·3 to 4)	1·5 (1·1 to 1·9)	0·5 (0·3 to 0·6)	252
Ischemic heart disease	0 (0 to 0)	0 (0 to 0)	0 (0 to 0)	0 (0 to 0)	0 (0 to 0)	0 (0 to 0)	4 (2·9 to 5·6)	7·6 (5·3 to 10·9)	14·8 (10·3 to 20·5)	33 (24·2 to 44·9)	56·5 (40·9 to 75·5)	104 (78·8 to 137)	217 (167 to 284)	340 (260 to 433)	540 (416 to 675)	761 (609 to 931)	827 (683 to 991)	892 (743 to 1,050)	745 (622 to 871)	634 (527 to 743)	523 (437 to 615)	271 (218 to 317)	72·6 (56·2 to 84·8)	6,040
Stroke	1·5 (0·6 to 2·8)	0·2 (0·1 to 0·4)	0·9 (0·4 to 1·7)	1·1 (0·8 to 1·4)	3·2 (2·2 to 4·5)	5·9 (4·2 to 7·8)	16·7 (11·7 to 22·6)	22·3 (15·5 to 31·4)	30 (21·2 to 42·2)	50·9 (35·6 to 69·9)	79·7 (54·3 to 111)	129 (89·8 to 177)	234 (167 to 310)	311 (225 to 425)	473 (335 to 651)	622 (473 to 801)	703 (529 to 923)	861 (658 to 1,100)	784 (613 to 997)	673 (514 to 846)	508 (406 to 608)	243 (185 to 295)	64·7 (48·1 to 79·6)	5,820
Ischemic stroke	0·3 (0·1 to 0·6)	0 (0 to 0)	0·1 (0·1 to 0·1)	0·7 (0·5 to 0·9)	1·8 (1·1 to 2·9)	2·8 (1·7 to 4·2)	4·9 (3·4 to 6·8)	6·1 (4·3 to 8·2)	7·9 (5·5 to 10·9)	12 (8·6 to 16·1)	17·7 (12·7 to 23·6)	27·8 (20·1 to 37·4)	49·3 (37·1 to 63·5)	76·1 (56·9 to 101)	123 (90·2 to 163)	233 (178 to 298)	284 (209 to 369)	505 (384 to 644)	488 (379 to 617)	527 (406 to 654)	414 (331 to 497)	203 (154 to 246)	55·7 (41·5 to 68·7)	3,040
Intracerebral hemorrhage	0·3 (0·1 to 0·6)	0·1 (0 to 0·1)	0·3 (0·2 to 0·7)	0·1 (0·1 to 0·2)	0·6 (0·4 to 0·9)	1·3 (0·9 to 1·8)	5·5 (3·7 to 7·8)	7·8 (5 to 11·3)	11·2 (7·4 to 16·6)	21·7 (14·1 to 31·4)	34·7 (22·4 to 51·3)	61·4 (39·7 to 87·7)	130 (88·8 to 178)	172 (120 to 242)	269 (187 to 379)	322 (237 to 439)	356 (262 to 474)	308 (226 to 404)	261 (195 to 342)	124 (90·6 to 165)	80·8 (63 to 99·7)	34·9 (26·2 to 43·3)	7·7 (5·8 to 9·5)	2,210
Subarachnoid hemorrhage	0·9 (0·4 to 1·6)	0·2 (0·1 to 0·3)	0·5 (0·2 to 0·9)	0·3 (0·1 to 0·4)	0·8 (0·5 to 1·2)	1·8 (1·1 to 2·6)	6·2 (3·9 to 9·4)	8·4 (5·1 to 13·4)	10·9 (6·6 to 17·1)	17·1 (11 to 25·3)	27·3 (16·8 to 39·9)	39·9 (26·3 to 58·8)	55·1 (37·6 to 79·8)	62·9 (42·1 to 89·9)	81 (52·9 to 116)	66·1 (45·2 to 93·2)	63 (42·9 to 89·8)	47·4 (33 to 65·2)	34·7 (24·2 to 47)	22 (15·5 to 29·8)	13·5 (9·8 to 18·1)	5·9 (4·2 to 8·1)	1·4 (1 to 1·9)	567
Hypertensive heart disease	0 (0 to 0)	0 (0 to 0)	0 (0 to 0)	0 (0 to 0)	0 (0 to 0)	0 (0 to 0)	0·9 (0·5 to 1·3)	1·6 (1 to 2·5)	2·5 (1·5 to 4)	6·5 (4·2 to 9·8)	12·7 (8·2 to 18·5)	22·8 (15·6 to 33)	48·9 (31·3 to 72·6)	73·5 (49·8 to 105)	113 (76·1 to 161)	152 (104 to 215)	159 (112 to 219)	177 (127 to 246)	161 (114 to 218)	146 (107 to 192)	104 (75·8 to 139)	57·7 (40·9 to 75·9)	16·8 (11·3 to 22·7)	1,260
Non-rheumatic valvular heart disease	0 (0 to 0)	0 (0 to 0)	0 (0 to 0)	0 (0 to 0)	0 (0 to 0)	0 (0 to 0)	1·9 (1·3 to 2·6)	1·6 (1·1 to 2·2)	2·4 (1·6 to 3·5)	3·4 (2·3 to 4·8)	3·8 (2·6 to 5·3)	5·4 (3·7 to 7·7)	8·3 (5·7 to 11·8)	9·1 (6·2 to 12·7)	14 (9·8 to 18·5)	17·1 (12·6 to 22·8)	17 (12·5 to 22·5)	16·1 (12·1 to 20·7)	13 (10 to 16·7)	10·8 (8·2 to 13·9)	7·6 (5·9 to 9·8)	3·6 (2·7 to 4·5)	0·7 (0·5 to 0·9)	136
Non-rheumatic calcific aortic valve disease	0 (0 to 0)	0 (0 to 0)	0 (0 to 0)	0 (0 to 0)	0 (0 to 0)	0 (0 to 0)	1·2 (0·8 to 1·8)	1·1 (0·8 to 1·6)	1·6 (1 to 2·5)	2 (1·3 to 2·8)	2·4 (1·6 to 3·5)	3·7 (2·4 to 5·4)	5·8 (3·9 to 8·6)	6·5 (4·4 to 9·3)	10·4 (7·1 to 13·9)	13·5 (9·7 to 18)	14 (10·3 to 18·6)	13·4 (10·1 to 17·4)	11·2 (8·7 to 14·4)	9·5 (7·1 to 12·2)	6·8 (5·2 to 8·8)	3·1 (2·4 to 3·9)	0·6 (0·4 to 0·8)	107
Non-rheumatic degenerative mitral valve disease	0 (0 to 0)	0 (0 to 0)	0 (0 to 0)	0 (0 to 0)	0 (0 to 0)	0 (0 to 0)	0·4 (0·2 to 0·6)	0·3 (0·2 to 0·5)	0·6 (0·4 to 1·1)	1·1 (0·7 to 1·6)	1·1 (0·6 to 1·7)	1·5 (0·9 to 2·4)	1·9 (1·1 to 3·1)	2·1 (1·2 to 3·2)	3·1 (1·8 to 4·8)	3·2 (1·9 to 5)	2·5 (1·5 to 3·9)	2·1 (1·3 to 3·2)	1·5 (0·9 to 2·2)	1 (0·6 to 1·5)	0·7 (0·4 to 1)	0·3 (0·2 to 0·5)	0·1 (0 to 0·1)	23·4
Other non-rheumatic valve diseases	0 (0 to 0)	0 (0 to 0)	0 (0 to 0)	0 (0 to 0)	0 (0 to 0)	0 (0 to 0)	0·3 (0·1 to 0·5)	0·2 (0·1 to 0·3)	0·2 (0·1 to 0·3)	0·4 (0·2 to 0·7)	0·3 (0·2 to 0·6)	0·3 (0·1 to 0·5)	0·6 (0·2 to 1·1)	0·5 (0·2 to 1)	0·6 (0·2 to 1·1)	0·4 (0·2 to 0·7)	0·4 (0·2 to 0·8)	0·5 (0·2 to 1·1)	0·3 (0·2 to 0·7)	0·3 (0·2 to 0·6)	0·2 (0·1 to 0·3)	0·1 (0·1 to 0·2)	0 (0 to 0·1)	5·6
Cardiomyopathy and myocarditis	1 (0·5 to 1·6)	0·6 (0·4 to 1)	2·9 (1·7 to 4·6)	1·6 (1·1 to 2·4)	2 (1·5 to 2·7)	3·2 (2·3 to 4·4)	8 (5·8 to 10·8)	9·2 (6·4 to 12·7)	14·2 (9·9 to 19·9)	21 (14·7 to 29·3)	28·1 (19·2 to 38·5)	37·2 (26·1 to 52·2)	58·3 (40·3 to 81·1)	71·8 (49·6 to 96·5)	93 (66·1 to 128)	103 (73·7 to 137)	90·4 (65·6 to 118)	81·4 (61·7 to 109)	60·4 (44·8 to 78·7)	43·9 (32·8 to 58·6)	35 (25·7 to 46·1)	16·1 (11·5 to 21·3)	4 (2·7 to 5·4)	786
Myocarditis	0·1 (0·1 to 0·2)	0·1 (0·1 to 0·2)	0·6 (0·3 to 1·1)	0·4 (0·2 to 0·7)	0·6 (0·4 to 0·9)	0·6 (0·4 to 0·8)	1·2 (0·7 to 1·9)	1 (0·6 to 1·7)	1·4 (0·8 to 2·3)	2 (1·1 to 3·2)	1·9 (1·1 to 3·1)	1·9 (1 to 3·2)	2·2 (1·2 to 3·6)	2·7 (1·5 to 4·4)	2·6 (1·4 to 4·2)	2·8 (1·5 to 4·7)	2·4 (1·4 to 4·1)	2·4 (1·3 to 4)	2·1 (1·2 to 3·5)	2 (1·1 to 3·8)	2 (1 to 3·7)	1·1 (0·7 to 1·8)	0·4 (0·3 to 0·7)	34·6
Alcoholic cardiomyopathy	0 (0 to 0)	0 (0 to 0)	0 (0 to 0)	0 (0 to 0)	0 (0 to 0)	0 (0 to 0)	0·7 (0·4 to 1·1)	1 (0·6 to 1·6)	2·6 (1·4 to 4·4)	6·1 (3·5 to 9·6)	8·1 (4·5 to 12·9)	13 (8·1 to 19·8)	26·5 (16·5 to 39·4)	31·9 (20 to 46·2)	38 (23·2 to 56·6)	37·6 (22·8 to 55·1)	34·1 (22·3 to 49·6)	20·1 (11·4 to 31·9)	12·6 (7·2 to 19·9)	3·5 (1·8 to 6·2)	1·7 (0·8 to 3·3)	0·6 (0·3 to 1·1)	0·1 (0·1 to 0·2)	238
Other cardiomyopathy	0·9 (0·4 to 1·4)	0·5 (0·3 to 0·8)	2·3 (1·3 to 3·7)	1·2 (0·7 to 1·8)	1·4 (1 to 2)	2·7 (1·8 to 3·7)	6·2 (4·3 to 8·6)	7·1 (4·9 to 10)	10·2 (6·9 to 14·4)	13 (8·4 to 19·1)	18 (12·1 to 25·3)	22·3 (15·1 to 31·9)	29·5 (18·8 to 43·6)	37·3 (24·5 to 52·6)	52·4 (35·7 to 72·7)	62·7 (43·4 to 88·4)	53·9 (38·2 to 72·4)	58·9 (42·6 to 80·2)	45·7 (33·4 to 60·7)	38·3 (28·5 to 51·8)	31·3 (22·8 to 41·3)	14·3 (10·1 to 19·3)	3·4 (2·3 to 4·8)	513
Atrial fibrillation and flutter	0 (0 to 0)	0 (0 to 0)	0 (0 to 0)	0 (0 to 0)	0 (0 to 0)	0 (0 to 0)	0 (0 to 0)	0 (0 to 0)	0 (0 to 0)	0·4 (0·3 to 0·6)	0·7 (0·4 to 1)	2·7 (1·8 to 3·9)	5·7 (3·9 to 8)	10 (7 to 13·9)	19·9 (14·5 to 26·4)	32·4 (24·1 to 43)	42·1 (32 to 55)	59·9 (46·2 to 77·4)	65·5 (51·6 to 83·5)	78·4 (63·4 to 96·8)	75 (58·4 to 94·2)	49·8 (39·8 to 64)	13·3 (10·1 to 16·9)	456
Aortic aneurysm	--	--	--	--	--	--	0·5 (0·3 to 0·7)	0·4 (0·3 to 0·6)	0·9 (0·6 to 1·5)	1·5 (1 to 2·2)	2·5 (1·6 to 3·8)	3·8 (2·6 to 5·3)	6·2 (4·2 to 8·8)	9·9 (6·8 to 14·3)	20·6 (14·5 to 28·2)	31·7 (21·5 to 44·3)	37 (25 to 52·4)	41·3 (28·8 to 57·7)	33·7 (23·4 to 47·1)	25·7 (18 to 35·3)	16·6 (12 to 22·6)	6·8 (4·9 to 8·9)	1·3 (1 to 1·7)	NA
Endocarditis	0·2 (0·1 to 0·3)	0·2 (0·1 to 0·3)	0·7 (0·4 to 1·3)	0·5 (0·3 to 0·8)	0·7 (0·4 to 1)	1·2 (0·7 to 1·8)	3·9 (2·4 to 6)	5·5 (3·4 to 8·2)	6·6 (4 to 10·2)	9·3 (5·5 to 15)	11·2 (7 to 17·6)	14·1 (8·7 to 22·7)	16·6 (10 to 24·9)	17·5 (11·1 to 26·4)	20·2 (13 to 30·1)	21·5 (13·8 to 31·9)	17·3 (11·6 to 25·5)	16·3 (10·6 to 23·5)	11 (7·9 to 15)	10 (6·6 to 14·4)	7 (4·6 to 10·2)	3·1 (2 to 4·6)	0·9 (0·6 to 1·3)	195
Other cardiovascular and circulatory diseases	2·4 (1·3 to 4)	0·8 (0·5 to 1·3)	3·1 (1·6 to 5)	1·5 (1 to 2·1)	3·3 (2·2 to 4·6)	5·3 (3·6 to 7·6)	11·7 (8·6 to 16)	12·1 (8·9 to 16·1)	16·3 (11·6 to 21·9)	18·4 (13·6 to 24·4)	24·5 (18·1 to 32·2)	44·1 (31·7 to 61)	48·3 (36·1 to 63·1)	54·4 (42·1 to 70·1)	76·4 (58·5 to 96·3)	94·4 (71·8 to 119)	90·1 (71·5 to 111)	95·9 (74·9 to 121)	84·7 (66·4 to 105)	72·6 (57 to 91·1)	61·9 (46·6 to 77·6)	29·5 (22·7 to 36·6)	7·7 (5·7 to 9·8)	860
Chronic respiratory diseases	0 (0 to 0)	0 (0 to 0)	0 (0 to 0)	82·2 (43·5 to 146)	86·3 (50·6 to 144)	76 (46·1 to 120)	68·6 (42·8 to 106)	58·7 (37·6 to 87·2)	62·4 (42 to 91·8)	64 (44·6 to 88·8)	69·4 (50·8 to 94·9)	71·2 (54·8 to 92)	89·4 (70·3 to 112)	112 (87·6 to 139)	149 (117 to 182)	191 (152 to 231)	190 (152 to 228)	208 (168 to 252)	172 (139 to 212)	140 (113 to 168)	105 (85·3 to 124)	50·8 (41·2 to 60·2)	12·5 (10 to 14·8)	2,060
Chronic obstructive pulmonary disease	0 (0 to 0)	0 (0 to 0)	0 (0 to 0)	0·5 (0·4 to 0·7)	1·2 (0·7 to 2)	2·1 (1 to 3·5)	3·7 (2·2 to 5·5)	5 (3·2 to 7·3)	6·8 (4·4 to 9·6)	8·3 (5·4 to 11·6)	11·1 (7·7 to 14·9)	14·4 (10·5 to 18·6)	22·7 (17·1 to 29·3)	35·5 (27·4 to 44·4)	59 (45·6 to 74·7)	95·7 (73 to 119)	105 (82·9 to 130)	127 (98·9 to 158)	116 (89·6 to 145)	99·7 (77·8 to 124)	79·4 (62·9 to 96·2)	38·9 (31·1 to 46·9)	9·5 (7·5 to 11·4)	842
Pneumoconiosis	0 (0 to 0)	0 (0 to 0)	0 (0 to 0)	0 (0 to 0)	0 (0 to 0)	0 (0 to 0)	0 (0 to 0·1)	0 (0 to 0)	0 (0 to 0)	0 (0 to 0)	0 (0 to 0)	0 (0 to 0)	0·1 (0 to 0·1)	0·1 (0·1 to 0·1)	0·1 (0·1 to 0·1)	0·1 (0·1 to 0·2)	0·2 (0·1 to 0·3)	0·2 (0·1 to 0·2)	0·2 (0·1 to 0·2)	0·1 (0·1 to 0·2)	0·1 (0·1 to 0·2)	0·1 (0 to 0·1)	0 (0 to 0)	1·4
Silicosis	0 (0 to 0)	0 (0 to 0)	0 (0 to 0)	0 (0 to 0)	0 (0 to 0)	0 (0 to 0)	0 (0 to 0)	0 (0 to 0)	0 (0 to 0)	0 (0 to 0)	0 (0 to 0)	0 (0 to 0)	0 (0 to 0)	0 (0 to 0)	0 (0 to 0)	0 (0 to 0·1)	0 (0 to 0·1)	0 (0 to 0·1)	0 (0 to 0·1)	0 (0 to 0·1)	0 (0 to 0)	0 (0 to 0)	0 (0 to 0)	0·3
Asbestosis	0 (0 to 0)	0 (0 to 0)	0 (0 to 0)	0 (0 to 0)	0 (0 to 0)	0 (0 to 0)	0 (0 to 0)	0 (0 to 0)	0 (0 to 0)	0 (0 to 0)	0 (0 to 0)	0 (0 to 0)	0 (0 to 0)	0 (0 to 0)	0 (0 to 0)	0 (0 to 0)	0 (0 to 0)	0 (0 to 0)	0 (0 to 0)	0 (0 to 0)	0 (0 to 0)	0 (0 to 0)	0 (0 to 0)	0·2
Coal workers pneumoconiosis	0 (0 to 0)	0 (0 to 0)	0 (0 to 0)	0 (0 to 0)	0 (0 to 0)	0 (0 to 0)	0 (0 to 0)	0 (0 to 0)	0 (0 to 0)	0 (0 to 0)	0 (0 to 0)	0 (0 to 0)	0 (0 to 0)	0 (0 to 0)	0 (0 to 0)	0 (0 to 0)	0 (0 to 0)	0 (0 to 0)	0 (0 to 0)	0 (0 to 0)	0 (0 to 0)	0 (0 to 0)	0 (0 to 0)	0·1
Other pneumoconiosis	0 (0 to 0)	0 (0 to 0)	0 (0 to 0)	0 (0 to 0)	0 (0 to 0)	0 (0 to 0)	0 (0 to 0)	0 (0 to 0)	0 (0 to 0)	0 (0 to 0)	0 (0 to 0)	0 (0 to 0)	0 (0 to 0)	0 (0 to 0·1)	0·1 (0 to 0·1)	0 (0 to 0·1)	0·1 (0·1 to 0·2)	0·1 (0·1 to 0·2)	0·1 (0·1 to 0·1)	0·1 (0·1 to 0·1)	0·1 (0 to 0·1)	0 (0 to 0)	0 (0 to 0)	0·8
Asthma	0 (0 to 0)	0 (0 to 0)	0 (0 to 0)	77·7 (39·4 to 141)	82·4 (47·2 to 139)	70 (40·4 to 115)	61·4 (35·9 to 99·4)	49·3 (29·2 to 77·5)	49·5 (30·2 to 77·5)	48·8 (31·1 to 73)	50 (33·5 to 73·1)	47·4 (33·3 to 65·9)	54·4 (38·8 to 74·4)	60·5 (43·3 to 81·2)	66·8 (47·6 to 90·1)	73·7 (53·7 to 96·4)	62·8 (45·6 to 82)	55·5 (40·6 to 73)	36·7 (27·2 to 48·6)	26·6 (19·1 to 35·5)	15·7 (11·3 to 20·9)	7·7 (5·8 to 10)	2 (1·4 to 2·6)	999
Interstitial lung disease and pulmonary sarcoidosis	0 (0 to 0)	0 (0 to 0)	0 (0 to 0)	1 (0·5 to 1·7)	0·4 (0·2 to 0·6)	0·4 (0·2 to 0·6)	0·9 (0·5 to 1·6)	1·1 (0·6 to 1·8)	1·4 (0·9 to 2·1)	2·5 (1·6 to 3·9)	3·5 (2·2 to 5·4)	4·3 (2·7 to 6·2)	6·3 (4·2 to 9)	8·8 (5·8 to 12·5)	13·3 (8·6 to 18·8)	17·4 (11·7 to 25)	18·1 (12·1 to 26)	21·6 (14 to 30·6)	16·8 (11·4 to 24·3)	11·3 (7·2 to 17·1)	7·5 (4·6 to 12·2)	3·4 (2 to 5·3)	0·8 (0·4 to 1·2)	141
Other chronic respiratory diseases	0 (0 to 0)	0 (0 to 0)	0 (0 to 0)	3 (1·9 to 4·4)	2·3 (1·4 to 3·5)	3·5 (1·9 to 5·3)	2·5 (1·6 to 3·6)	3·3 (2·3 to 4·6)	4·6 (3·2 to 6·4)	4·3 (3 to 6)	4·8 (3·4 to 6·6)	5 (3·5 to 6·9)	5·9 (4 to 8·4)	6·8 (4·5 to 10·1)	9·9 (6·4 to 14·3)	3·8 (2·2 to 6·1)	3 (1·7 to 4·9)	3·4 (2·1 to 5·4)	2·6 (1·6 to 4)	2·4 (1·3 to 3·9)	1·8 (0·9 to 3·2)	0·7 (0·4 to 1·2)	0·2 (0·1 to 0·3)	73·9
Digestive diseases	2·7 (1·7 to 4)	2·1 (1·3 to 3·3)	11·7 (6·9 to 19·7)	18·3 (13 to 25)	20·7 (14·5 to 28·4)	22 (16·2 to 28·7)	38·4 (29·1 to 49·7)	56·5 (41·3 to 77·9)	83 (60·3 to 114)	115 (87·1 to 151)	151 (113 to 194)	192 (150 to 243)	270 (211 to 340)	318 (245 to 406)	393 (302 to 499)	437 (342 to 546)	364 (290 to 450)	332 (262 to 406)	233 (185 to 288)	181 (147 to 216)	118 (95·1 to 143)	52·1 (40·6 to 62)	11·1 (8·6 to 13)	3,420
Cirrhosis and other chronic liver diseases	0 (0 to 0)	0 (0 to 0)	0 (0 to 0)	1·1 (0·7 to 1·7)	1·6 (1·1 to 2·3)	2·6 (1·6 to 3·7)	5·7 (3·7 to 8)	7·4 (5·2 to 10·4)	12·8 (8·8 to 18·4)	26·7 (18·5 to 36·6)	41·5 (29·8 to 56·4)	66·8 (47·8 to 90·3)	112 (81·7 to 151)	138 (99·9 to 183)	178 (129 to 240)	193 (141 to 252)	151 (113 to 194)	119 (89·7 to 152)	67·5 (50·6 to 86·9)	44·8 (34·7 to 56·4)	24·1 (18·4 to 31·2)	9 (6·7 to 11·6)	1·7 (1·2 to 2·2)	1,200
Cirrhosis and other chronic liver diseases due to hepatitis B	0 (0 to 0)	0 (0 to 0)	0 (0 to 0)	0 (0 to 0)	0 (0 to 0)	0 (0 to 0)	0·1 (0 to 0·2)	0·3 (0·1 to 0·6)	0·8 (0·3 to 1·8)	2·3 (1·1 to 4)	4 (1·8 to 7·3)	6·6 (3·3 to 12)	10·8 (5·3 to 19·7)	12·1 (5·6 to 22·3)	13·2 (6 to 24)	12·2 (5·7 to 22·7)	8·6 (4·1 to 15·9)	6·5 (3 to 12·3)	3·8 (1·8 to 6·7)	2·5 (1·2 to 4·4)	1·3 (0·7 to 2·2)	0·5 (0·2 to 0·9)	0·1 (0 to 0·2)	85·7
Cirrhosis and other chronic liver diseases due to hepatitis C	0 (0 to 0)	0 (0 to 0)	0 (0 to 0)	0 (0 to 0)	0 (0 to 0)	0·1 (0 to 0·1)	0·3 (0·1 to 0·6)	1 (0·5 to 1·8)	2·9 (1·5 to 5·1)	6·6 (3·8 to 10·5)	9·9 (5·1 to 17·2)	15·3 (8·1 to 25·8)	24 (12·7 to 40·2)	27·3 (14·1 to 46·2)	32·6 (16·6 to 56·8)	33·1 (15·8 to 61·3)	25 (13·9 to 44·2)	19·1 (10·5 to 31·8)	10·5 (5·5 to 17·8)	6·9 (3·7 to 11·7)	3·8 (2·2 to 6·3)	1·5 (0·8 to 2·5)	0·3 (0·1 to 0·5)	220
Cirrhosis and other chronic liver diseases due to alcohol use	0 (0 to 0)	0 (0 to 0)	0 (0 to 0)	0 (0 to 0)	0 (0 to 0)	0 (0 to 0)	0 (0 to 0·1)	0·7 (0·3 to 1·4)	2·5 (1·1 to 4·8)	8·5 (4·8 to 13·7)	16·7 (9 to 26·1)	29·9 (18·5 to 45)	52·8 (31·3 to 79·2)	67 (40·5 to 99·7)	90·8 (56·5 to 129)	98·1 (62·5 to 141)	72·4 (47·3 to 102)	52·7 (32·6 to 76·3)	26·7 (16·6 to 39·3)	16 (9·8 to 24)	8 (4·9 to 11·9)	2·8 (1·6 to 4·3)	0·5 (0·2 to 0·8)	546
Cirrhosis and other chronic liver diseases due to NAFLD	0 (0 to 0)	0 (0 to 0)	0 (0 to 0)	0 (0 to 0)	0 (0 to 0)	0 (0 to 0)	0·3 (0·1 to 0·6)	0·8 (0·4 to 1·5)	2·1 (0·9 to 4·1)	4·2 (2·2 to 7·1)	5·9 (2·7 to 11·2)	9·2 (4·4 to 16·5)	16 (7 to 29·8)	20·1 (9 to 37·6)	25·3 (12 to 46·4)	29 (13·8 to 53·2)	25·6 (14·7 to 40·9)	22·6 (12·9 to 35·4)	14·3 (7·5 to 22·8)	10·3 (5·5 to 15·9)	5·8 (3·4 to 8·9)	2·2 (1·2 to 3·5)	0·4 (0·2 to 0·8)	194
Cirrhosis and other chronic liver diseases due to other causes	0 (0 to 0)	0 (0 to 0)	0 (0 to 0)	1·1 (0·7 to 1·6)	1·6 (1·1 to 2·2)	2·5 (1·6 to 3·5)	5 (3·2 to 7·1)	4·6 (3 to 6·9)	4·4 (2·4 to 7·4)	5·1 (2·6 to 9·6)	5 (2·3 to 8·8)	5·9 (2·7 to 11·1)	8·8 (4·3 to 16·9)	11·2 (5·6 to 20·1)	16·2 (8·4 to 30·1)	20·8 (10·9 to 36·8)	19 (9·5 to 33·4)	17·8 (9·3 to 30)	12·3 (6·5 to 20·5)	9·1 (4·9 to 14·9)	5·2 (3 to 8)	2 (1·1 to 3·1)	0·4 (0·2 to 0·7)	158
Upper digestive system diseases	0 (0 to 0)	0 (0 to 0)	0 (0 to 0)	1 (0·5 to 1·7)	1·7 (0·8 to 2·9)	3·2 (1·9 to 5·1)	11·7 (6·7 to 19·4)	25·3 (14·1 to 46·1)	38·2 (21·4 to 65·7)	45·5 (25·5 to 78·1)	52·4 (29·8 to 86·8)	56 (33·2 to 91·1)	68·4 (42·9 to 107)	76 (48·8 to 116)	86·3 (57·5 to 131)	91·2 (62·6 to 134)	76·9 (54·6 to 110)	69·6 (50·7 to 95·7)	51·5 (37·7 to 69·8)	39·2 (29·2 to 51·8)	25·5 (19 to 33)	11·5 (8·7 to 14·8)	2·4 (1·8 to 3·1)	834
Peptic ulcer disease	0 (0 to 0)	0 (0 to 0)	0 (0 to 0)	0·2 (0·1 to 0·3)	0·2 (0·1 to 0·3)	0·5 (0·3 to 0·8)	1·3 (0·8 to 1·9)	2·1 (1·4 to 3)	3·1 (2 to 4·5)	5·5 (3·7 to 8)	7·1 (4·8 to 9·8)	9 (6 to 12·7)	13·4 (9 to 19·3)	18·1 (12 to 25·5)	25·3 (16·8 to 35·5)	33·1 (23·5 to 44·8)	30·8 (21·7 to 41·9)	33·2 (23·5 to 45·4)	28 (19·6 to 39)	23·7 (17·6 to 31·1)	16·5 (11·5 to 22·2)	7·8 (5·7 to 10·3)	1·7 (1·2 to 2·2)	261
Gastritis and duodenitis	0 (0 to 0)	0 (0 to 0)	0 (0 to 0)	0·9 (0·4 to 1·5)	1·4 (0·6 to 2·7)	1·4 (0·6 to 2·7)	1·8 (0·8 to 3·5)	2·2 (1·1 to 3·9)	3·1 (1·5 to 5·4)	4·3 (2·2 to 7·5)	6 (3·2 to 10·2)	7·2 (3·8 to 13·4)	10·6 (5·7 to 19·3)	11·9 (6·5 to 20·5)	14·4 (8·2 to 24·3)	14·2 (8·3 to 22·8)	11·5 (7 to 18·3)	9·4 (5·9 to 14·3)	5·8 (3·9 to 8·4)	3·8 (2·5 to 5·4)	2·6 (1·7 to 3·6)	1·2 (0·8 to 1·7)	0·2 (0·2 to 0·3)	114
Gastroesophageal reflux disease	0 (0 to 0)	0 (0 to 0)	0 (0 to 0)	0 (0 to 0)	0 (0 to 0·1)	1·3 (0·5 to 2·4)	8·6 (4·3 to 16)	21 (10·4 to 41·4)	32 (15·4 to 58·9)	35·7 (16·8 to 67·2)	39·3 (18·6 to 72·3)	39·7 (18·9 to 72·5)	44·4 (21·8 to 78·3)	46 (22·3 to 83·8)	46·7 (22·2 to 88·2)	44 (21·4 to 81·6)	34·6 (17·5 to 63·1)	27·1 (13·7 to 47·9)	17·7 (9 to 31·6)	11·7 (5·8 to 20·9)	6·5 (3·2 to 11·4)	2·5 (1·3 to 4·4)	0·5 (0·2 to 0·9)	459
Appendicitis	0 (0 to 0)	0 (0 to 0)	0 (0 to 0)	0·6 (0·4 to 1)	1·3 (0·9 to 1·9)	2·3 (1·4 to 3·8)	3·3 (1·9 to 4·9)	2·9 (1·7 to 4·5)	2·6 (1·5 to 4·1)	2·6 (1·6 to 4·1)	3·1 (1·9 to 5)	2·8 (1·7 to 4·3)	3·2 (2 to 5)	3·2 (2 to 5·1)	3·7 (2·3 to 5·8)	3·6 (2·3 to 5·7)	3·8 (2·5 to 5·8)	3·6 (2·4 to 5·8)	2·1 (1·3 to 3·4)	1·7 (1·1 to 2·5)	1·3 (0·8 to 2)	0·6 (0·4 to 0·9)	0·1 (0·1 to 0·2)	48·5
Paralytic ileus and intestinal obstruction	2 (1·3 to 3)	1·9 (1·2 to 3)	10·4 (6·1 to 17·5)	4·2 (2·5 to 6·4)	3 (2·1 to 4·2)	3 (2 to 4·1)	4 (2·7 to 6)	4 (2·6 to 5·8)	5 (3·2 to 7·5)	6·2 (4·1 to 9)	10·4 (6·8 to 15·2)	12·4 (8·3 to 17·9)	17·4 (11·4 to 25·5)	23·8 (15·3 to 34·5)	31·8 (20·5 to 46·7)	41·9 (28 to 59·7)	42 (29·1 to 58·5)	51 (35·6 to 69·4)	43·8 (30·7 to 59·5)	39·7 (29·4 to 53·2)	27·6 (20·3 to 36·6)	12·8 (9·3 to 16·6)	3 (2·2 to 4)	401
Inguinal, femoral, and abdominal hernia	0·6 (0·3 to 1·1)	0·2 (0·1 to 0·4)	1·3 (0·7 to 2·1)	7·2 (4·2 to 11·6)	9·5 (5·3 to 16·3)	6·8 (3·6 to 11·6)	5·8 (3·1 to 9·7)	5·5 (2·9 to 9·4)	7 (3·9 to 11·7)	8·3 (4·8 to 14)	11·1 (6·2 to 18·3)	13·5 (8 to 21·5)	17·6 (10·4 to 28)	20·4 (12·3 to 31·4)	22·9 (14·8 to 35·1)	23·1 (15·1 to 33·2)	17·8 (12·3 to 25·3)	14·4 (10·6 to 19)	9·7 (7·2 to 12·4)	7·1 (5·5 to 8·9)	4·7 (3·4 to 6·3)	2·1 (1·5 to 2·8)	0·4 (0·3 to 0·5)	217
Inflammatory bowel disease	0 (0 to 0)	0 (0 to 0)	0 (0 to 0)	0·3 (0·2 to 0·6)	0·2 (0·2 to 0·4)	0·2 (0·2 to 0·3)	0·9 (0·6 to 1·2)	1·6 (1·1 to 2·3)	2·6 (1·8 to 4)	3·9 (2·6 to 5·5)	5·3 (3·5 to 7·8)	6·7 (4·5 to 9·4)	7 (4·9 to 9·9)	7·6 (5·2 to 10·8)	9·1 (6·4 to 12·3)	9·5 (6·8 to 13)	6·8 (4·9 to 9·2)	7·1 (5·2 to 9·4)	4·9 (3·6 to 6·6)	3·2 (2·4 to 4·2)	2·1 (1·5 to 2·9)	1·2 (0·8 to 1·6)	0·2 (0·1 to 0·2)	80·7
Vascular intestinal disorders	0 (0 to 0)	0 (0 to 0)	0 (0 to 0)	0·1 (0 to 0·1)	0·1 (0·1 to 0·2)	0·1 (0 to 0·1)	0·2 (0·1 to 0·3)	0·2 (0·2 to 0·4)	0·4 (0·3 to 0·7)	0·7 (0·4 to 1)	0·9 (0·5 to 1·4)	1·3 (0·8 to 2)	2·6 (1·5 to 3·9)	3·4 (2·2 to 5)	5·1 (3·3 to 7·5)	7·7 (4·8 to 11·2)	8 (5·3 to 11·2)	8·9 (6 to 12·8)	7·4 (5 to 10·6)	6·3 (4·3 to 8·9)	4·3 (2·9 to 6·2)	1·8 (1·2 to 2·5)	0·3 (0·2 to 0·5)	59·8
Gallbladder and biliary diseases	0 (0 to 0)	0 (0 to 0)	0 (0 to 0)	0·3 (0·2 to 0·5)	0·8 (0·3 to 1·6)	1·5 (0·7 to 2·7)	2·7 (1·4 to 4·7)	4·1 (2·3 to 6·5)	5·9 (3·4 to 9·5)	8 (4·7 to 12·6)	10·6 (6·4 to 15·9)	12·6 (7·8 to 19)	15·8 (10·3 to 23·2)	18·3 (12·2 to 26·8)	21 (14·3 to 29·4)	22·3 (15·5 to 31·1)	19 (13·5 to 26)	17·6 (12·7 to 24·3)	13·2 (9·2 to 18)	10·2 (7·1 to 14·1)	6·2 (4·3 to 8·5)	2·9 (2 to 4·1)	0·6 (0·4 to 0·9)	194
Pancreatitis	0 (0 to 0)	0 (0 to 0)	0 (0 to 0)	0·1 (0·1 to 0·2)	0·1 (0·1 to 0·1)	0·3 (0·2 to 0·5)	1·4 (1 to 2·1)	2·7 (1·8 to 3·9)	4·6 (2·8 to 7)	7·7 (4·8 to 11·5)	9·6 (6·2 to 14)	12·4 (8·2 to 17·7)	14 (8·9 to 20·4)	12·9 (8·4 to 18·5)	15·9 (10·5 to 23·1)	17 (11·4 to 23·8)	13·2 (9·2 to 18·5)	12·5 (8·7 to 17·2)	8·8 (6·3 to 12)	6·2 (4·3 to 8·2)	3·6 (2·6 to 5)	1·3 (0·9 to 1·8)	0·3 (0·2 to 0·4)	145
Other digestive diseases	0 (0 to 0)	0 (0 to 0)	0 (0 to 0)	3·3 (1·8 to 5·2)	2·3 (1·4 to 3·3)	2 (1·2 to 2·8)	2·8 (1·4 to 4·2)	3 (1·5 to 4·6)	3·8 (2 to 6·2)	5·1 (2·6 to 8·1)	5·8 (2·9 to 9)	7·5 (3·9 to 11·7)	11·2 (5·8 to 17·8)	14·2 (7·5 to 21·7)	19·6 (10·4 to 30·8)	27·8 (14·4 to 41·5)	25·6 (13·6 to 37·9)	28·2 (14·7 to 41·1)	24·2 (12·7 to 35·3)	22·7 (12·1 to 32·4)	18·8 (10·1 to 26·6)	8·8 (4·8 to 12·5)	2 (1 to 2·8)	239
Neurological disorders	0 (0 to 0·1)	0·4 (0·2 to 0·7)	13·3 (8·3 to 20·6)	26·5 (13 to 49·1)	53·6 (23·6 to 95)	149 (48·3 to 309)	217 (82·9 to 428)	211 (82·4 to 416)	227 (90·3 to 432)	224 (90·5 to 413)	244 (103 to 442)	248 (116 to 452)	275 (143 to 480)	273 (148 to 459)	290 (173 to 462)	311 (202 to 470)	307 (203 to 481)	366 (236 to 619)	387 (248 to 703)	462 (269 to 916)	410 (218 to 834)	237 (115 to 490)	72·8 (33 to 155)	5,010
Alzheimer's disease and other dementias	0 (0 to 0)	0 (0 to 0)	0 (0 to 0)	0 (0 to 0)	0 (0 to 0)	0 (0 to 0)	0 (0 to 0)	0 (0 to 0)	0 (0 to 0)	0 (0 to 0)	0 (0 to 0)	1·3 (0·3 to 4·3)	8 (2·5 to 22·2)	17·4 (6·5 to 44·7)	36·4 (14·2 to 89)	69·7 (27·3 to 166)	109 (43·5 to 266)	178 (73·8 to 426)	220 (90·9 to 528)	328 (137 to 785)	325 (131 to 751)	206 (81·5 to 461)	67·3 (27·2 to 150)	1,570
Parkinson's disease	0 (0 to 0)	0 (0 to 0)	0 (0 to 0)	0 (0 to 0)	0 (0 to 0)	0 (0 to 0)	0 (0 to 0)	0·1 (0·1 to 0·1)	0·2 (0·1 to 0·3)	0·3 (0·2 to 0·5)	0·6 (0·4 to 0·9)	1·6 (1·1 to 2·2)	3·7 (2·8 to 5)	6·9 (5·1 to 9·3)	12·8 (9·7 to 16·6)	25·2 (20·2 to 30·9)	39·8 (32·6 to 48·1)	71·3 (59·6 to 84·8)	94·9 (78·8 to 111)	90·8 (75·3 to 106)	61·8 (51·1 to 72·1)	22·8 (18·7 to 26·6)	3·8 (3 to 4·4)	437
Idiopathic epilepsy	0 (0 to 0·1)	0·1 (0 to 0·3)	3·8 (1·9 to 7)	15·9 (6 to 32·1)	25·3 (9·1 to 50·5)	34·9 (13·6 to 66·6)	49·9 (24·5 to 87)	50·1 (25·7 to 84·6)	52·5 (26·7 to 86·5)	50·6 (27·6 to 81·9)	50·8 (28·5 to 82·9)	47·9 (26·1 to 77·6)	52·7 (30·9 to 84·7)	46·8 (25·5 to 79)	48·2 (25·9 to 83·7)	46·8 (23·6 to 81·7)	38·7 (17·7 to 68·2)	33·1 (14·7 to 58·3)	24·6 (11·2 to 43·4)	17·6 (7·7 to 31·3)	10·6 (4·7 to 18·6)	4·6 (2 to 8·2)	0·9 (0·4 to 1·7)	706
Multiple sclerosis	0 (0 to 0)	0 (0 to 0)	0 (0 to 0)	0 (0 to 0)	0 (0 to 0·1)	0·1 (0 to 0·3)	0·7 (0·3 to 1·4)	1·9 (1·1 to 2·9)	2·7 (1·7 to 3·8)	3·8 (2·7 to 5·1)	7·5 (5·1 to 10·1)	8·3 (5·8 to 11·4)	9·9 (6·8 to 13·5)	9·5 (6·6 to 12·8)	11 (7·4 to 14·8)	10·1 (6·7 to 13·3)	5·4 (3·9 to 7·2)	4·2 (2·9 to 5·6)	2·1 (1·5 to 2·8)	0·9 (0·7 to 1·3)	0·5 (0·4 to 0·7)	0·2 (0·1 to 0·2)	0 (0 to 0·1)	78·8
Motor neuron disease	0 (0 to 0)	0·3 (0·2 to 0·5)	2·9 (1·6 to 5)	1·4 (0·8 to 2·1)	0·4 (0·2 to 0·5)	0·7 (0·5 to 1·1)	1·2 (0·8 to 1·6)	1·2 (0·9 to 1·7)	1·2 (0·9 to 1·6)	1·9 (1·3 to 2·6)	3·9 (2·8 to 5·3)	6·2 (4·3 to 8·5)	9·6 (6·8 to 13·3)	14·2 (9·9 to 19·4)	19·5 (13·6 to 26·3)	23·2 (16·8 to 30·6)	20·7 (14·5 to 28)	15·9 (11·5 to 21·5)	8·7 (6 to 11·7)	3·5 (2·4 to 4·7)	1·3 (0·9 to 1·8)	0·4 (0·3 to 0·5)	0·1 (0·1 to 0·1)	138
Headache disorders	0 (0 to 0)	0 (0 to 0)	0 (0 to 0)	0 (0 to 0)	16·9 (0·3 to 44·8)	93·3 (4·3 to 235)	138 (15 to 342)	141 (19·8 to 332)	154 (24·4 to 353)	152 (28 to 339)	166 (32·5 to 359)	166 (37·3 to 363)	170 (43·6 to 372)	156 (41·4 to 335)	137 (38·3 to 288)	111 (35·6 to 228)	75·1 (25 to 154)	48·1 (16·2 to 102)	25·9 (8·3 to 53·1)	14·7 (4·8 to 30·6)	7·5 (2·5 to 15·4)	2·5 (0·8 to 5·3)	0·4 (0·1 to 0·9)	1,770
Migraine	0 (0 to 0)	0 (0 to 0)	0 (0 to 0)	0 (0 to 0)	15·5 (0·2 to 43·5)	87·5 (2·9 to 230)	129 (10·4 to 334)	130 (13·4 to 317)	141 (16·6 to 338)	139 (19 to 321)	150 (23 to 346)	150 (27 to 346)	152 (30·2 to 341)	138 (29 to 306)	121 (27·4 to 269)	96·1 (24·3 to 210)	63·3 (16·7 to 135)	39·6 (11·2 to 85·3)	21·1 (5·9 to 46·6)	12·1 (3·4 to 26·7)	6·1 (1·8 to 13·4)	2 (0·6 to 4·5)	0·3 (0·1 to 0·7)	1,590
Tension-type headache	0 (0 to 0)	0 (0 to 0)	0 (0 to 0)	0 (0 to 0)	1·4 (0 to 9)	5·8 (0·3 to 41·5)	9·5 (1·6 to 45·2)	10·8 (2·3 to 46·7)	12·9 (2·8 to 51·7)	13·3 (3 to 48·4)	15·6 (3·9 to 53·8)	16 (4·6 to 50·6)	17·7 (5·8 to 49·5)	17·9 (5·4 to 53·3)	16·1 (5 to 44·3)	15 (4·7 to 43·5)	11·8 (3·4 to 38)	8·5 (2·1 to 29·7)	4·7 (1·1 to 17·7)	2·6 (0·6 to 9·8)	1·4 (0·3 to 5)	0·5 (0·1 to 1·9)	0·1 (0 to 0·5)	182
Other neurological disorders	0 (0 to 0)	0 (0 to 0)	6·6 (3·9 to 10·8)	9·2 (4·9 to 15·7)	11 (5·5 to 19·4)	20·1 (10·4 to 34·1)	27·2 (17 to 41·1)	17·4 (10·5 to 26·5)	15·8 (10·2 to 22·2)	15·2 (9·6 to 21·8)	15·5 (9·9 to 22·3)	17·2 (11·3 to 25·1)	21·3 (14·4 to 30·2)	22·4 (15·9 to 30·6)	25·7 (17·9 to 35·9)	24·7 (17·2 to 34·1)	18·7 (13·6 to 25·5)	15·3 (11·1 to 21)	10·4 (7·4 to 14·3)	6·8 (4·7 to 9·4)	3·3 (2·2 to 4·6)	1·3 (1 to 1·8)	0·3 (0·2 to 0·4)	305
Mental disorders	0 (0 to 0·1)	0·1 (0·1 to 0·2)	2·1 (1·3 to 3)	12 (7·8 to 17·2)	71·4 (48·2 to 104)	233 (157 to 330)	368 (253 to 516)	386 (268 to 528)	437 (303 to 592)	435 (310 to 599)	478 (346 to 637)	469 (336 to 638)	503 (369 to 680)	490 (358 to 662)	475 (342 to 633)	415 (298 to 556)	301 (214 to 400)	220 (156 to 289)	133 (93·8 to 176)	83·4 (57·1 to 112)	46·4 (32·6 to 62·1)	18·8 (13 to 25·7)	4·1 (2·6 to 5·9)	5,580
Schizophrenia	0 (0 to 0)	0 (0 to 0)	0 (0 to 0)	0 (0 to 0)	0 (0 to 0)	1·1 (0·5 to 2·1)	9 (4 to 15·7)	29·8 (15·9 to 48·9)	54·8 (31·7 to 81·8)	67·2 (41·3 to 97·9)	77·6 (49·3 to 112)	74·8 (49·3 to 103)	76·9 (51·5 to 103)	70·3 (48·3 to 95·9)	61·4 (41·7 to 84·6)	47·4 (31·6 to 65·9)	30·4 (20·3 to 41·8)	19 (12·3 to 26·9)	9·6 (6·1 to 13·6)	4·8 (2·9 to 7)	2 (1·1 to 3)	0·6 (0·3 to 0·9)	0·1 (0 to 0·1)	637
Depressive disorders	0 (0 to 0)	0 (0 to 0)	0 (0 to 0)	0 (0 to 0·1)	3·5 (1·4 to 6·8)	39·5 (20·1 to 70·6)	108 (63·5 to 170)	130 (73·9 to 202)	134 (80·3 to 205)	129 (78·2 to 199)	150 (95·7 to 219)	157 (99·8 to 237)	179 (120 to 263)	188 (124 to 275)	195 (124 to 286)	180 (113 to 270)	136 (86·6 to 192)	103 (66·9 to 146)	65·6 (40·8 to 97·1)	43·6 (26·3 to 64·7)	26·3 (16·8 to 37·8)	11·4 (7·1 to 17·3)	2·6 (1·5 to 4·3)	1,980
Major depressive disorder	0 (0 to 0)	0 (0 to 0)	0 (0 to 0)	0 (0 to 0·1)	3·1 (1·1 to 6·4)	36 (17 to 66·4)	96·7 (55·3 to 155)	112 (61 to 182)	109 (62·4 to 173)	103 (58·9 to 167)	120 (74·3 to 180)	127 (75·9 to 196)	147 (93 to 223)	155 (98·9 to 229)	163 (99·8 to 247)	153 (91·5 to 234)	115 (72·3 to 165)	88·6 (56·2 to 127)	56·8 (33·7 to 86·4)	38·2 (22·6 to 58·4)	23·4 (14·8 to 34·7)	10·2 (6·1 to 15·9)	2·4 (1·2 to 4)	1,660
Dysthymia	0 (0 to 0)	0 (0 to 0)	0 (0 to 0)	0 (0 to 0)	0·4 (0·1 to 1·1)	3·5 (1·4 to 6·9)	11 (5·3 to 19·2)	18·2 (9·4 to 31·8)	24·5 (12·8 to 42·3)	26·4 (15·2 to 44·4)	29·9 (17·1 to 49·3)	30·2 (16·4 to 52)	32·9 (17·9 to 56·8)	32·4 (18·9 to 53·8)	31·3 (18·3 to 50·9)	27·7 (15 to 46·9)	20·3 (10·8 to 33·8)	14·7 (8·4 to 24·3)	8·8 (5·1 to 14·4)	5·3 (3 to 9)	2·9 (1·6 to 4·9)	1·2 (0·7 to 2)	0·3 (0·1 to 0·5)	322
Bipolar disorder	0 (0 to 0)	0 (0 to 0)	0 (0 to 0)	0 (0 to 0)	0 (0 to 0)	13·1 (5·4 to 24·4)	50·8 (24·7 to 91·3)	60·5 (30·9 to 100)	64·5 (34·4 to 106)	59·3 (31·9 to 99·1)	62·1 (33·8 to 99)	60·8 (33·4 to 99·5)	64·7 (37 to 106)	61·4 (33·7 to 100)	56 (31·5 to 92·6)	45·2 (24·7 to 72·4)	30·1 (16·6 to 48)	19·2 (10·5 to 30·7)	9·7 (5·4 to 15·6)	4·8 (2·5 to 7·8)	2·1 (1·1 to 3·5)	0·7 (0·3 to 1·1)	0·1 (0·1 to 0·2)	665
Anxiety disorders	0 (0 to 0)	0 (0 to 0)	0 (0 to 0)	1·4 (0·5 to 2·8)	23·4 (11·9 to 40·3)	68 (37·7 to 112)	96·5 (58 to 149)	98·4 (56·1 to 160)	107 (61·7 to 177)	105 (62·5 to 167)	114 (68·6 to 175)	112 (63·8 to 176)	119 (70 to 189)	115 (69·3 to 174)	110 (69·8 to 163)	95·2 (58·1 to 145)	69·8 (42·1 to 109)	51·1 (31·4 to 79·6)	31 (19·4 to 49·1)	18·7 (11·3 to 28·8)	9·4 (5·6 to 14·6)	3·3 (1·8 to 5·2)	0·6 (0·3 to 1)	1,350
Eating disorders	0 (0 to 0)	0 (0 to 0)	0 (0 to 0)	0 (0 to 0)	0·3 (0·1 to 0·6)	4·9 (2·4 to 8·5)	18 (9·6 to 31)	26·6 (14·1 to 44·7)	27·6 (15 to 45·6)	23·3 (12·9 to 38)	18·5 (10 to 30·5)	10·3 (5·5 to 17·3)	5·8 (3·1 to 9·9)	0 (0 to 0)	0 (0 to 0)	0 (0 to 0)	0 (0 to 0)	0 (0 to 0)	0 (0 to 0)	0 (0 to 0)	0 (0 to 0)	0 (0 to 0)	0 (0 to 0)	135
Anorexia nervosa	0 (0 to 0)	0 (0 to 0)	0 (0 to 0)	0 (0 to 0)	0·2 (0·1 to 0·5)	2·3 (1·1 to 4·3)	6·8 (3·3 to 12·1)	6·6 (3·2 to 11·8)	5·4 (2·8 to 9·2)	3·5 (1·6 to 6)	2·7 (1·2 to 4·9)	2 (0·9 to 3·6)	1·6 (0·6 to 2·9)	0 (0 to 0)	0 (0 to 0)	0 (0 to 0)	0 (0 to 0)	0 (0 to 0)	0 (0 to 0)	0 (0 to 0)	0 (0 to 0)	0 (0 to 0)	0 (0 to 0)	31·2
Bulimia nervosa	0 (0 to 0)	0 (0 to 0)	0 (0 to 0)	0 (0 to 0)	0 (0 to 0·1)	2·6 (0·8 to 5·4)	11·2 (5 to 22·6)	20 (9·2 to 36·3)	22·2 (11·2 to 38·4)	19·8 (10·5 to 32·9)	15·8 (8·3 to 26·8)	8·3 (4·1 to 14·9)	4·2 (2 to 7·8)	0 (0 to 0)	0 (0 to 0)	0 (0 to 0)	0 (0 to 0)	0 (0 to 0)	0 (0 to 0)	0 (0 to 0)	0 (0 to 0)	0 (0 to 0)	0 (0 to 0)	104
Autism spectrum disorders	0 (0 to 0·1)	0·1 (0·1 to 0·2)	1·8 (1·1 to 2·6)	7·8 (4·8 to 11·4)	10·5 (6·6 to 15·7)	11·4 (7·2 to 17)	11·9 (7·5 to 17·8)	10·9 (7 to 16·4)	11 (7 to 16·4)	10·1 (6·3 to 15)	10·2 (6·4 to 15·5)	9·5 (6 to 14·1)	9·7 (6·1 to 14·4)	9·2 (5·7 to 13·8)	8·7 (5·5 to 13·1)	7·6 (4·8 to 11·1)	5·7 (3·6 to 8·5)	4·2 (2·6 to 6·4)	2·6 (1·6 to 3·8)	1·6 (1 to 2·5)	0·9 (0·5 to 1·3)	0·3 (0·2 to 0·5)	0·1 (0 to 0·1)	146
Attention-deficit/hyperactivity disorder	0 (0 to 0)	0 (0 to 0)	0 (0 to 0)	1 (0·5 to 1·8)	11·2 (6·2 to 20·1)	17 (9·3 to 29·5)	15 (8·2 to 26·8)	10·6 (5·8 to 18·6)	8·4 (4·6 to 14·9)	6·4 (3·4 to 11)	5·4 (2·8 to 9·6)	4·3 (2·1 to 7·5)	3·7 (1·8 to 6·5)	2·9 (1·4 to 5·1)	2 (0·9 to 3·7)	1·1 (0·5 to 2·1)	0·5 (0·2 to 1)	0·2 (0·1 to 0·4)	0 (0 to 0·1)	0 (0 to 0)	0 (0 to 0)	0 (0 to 0)	0 (0 to 0)	89·8
Conduct disorder	0 (0 to 0)	0 (0 to 0)	0 (0 to 0)	0 (0 to 0)	19·7 (10 to 35·1)	74 (40·1 to 122)	49·1 (25·9 to 81·1)	0 (0 to 0)	0 (0 to 0)	0 (0 to 0)	0 (0 to 0)	0 (0 to 0)	0 (0 to 0)	0 (0 to 0)	0 (0 to 0)	0 (0 to 0)	0 (0 to 0)	0 (0 to 0)	0 (0 to 0)	0 (0 to 0)	0 (0 to 0)	0 (0 to 0)	0 (0 to 0)	143
Idiopathic developmental intellectual disability	0 (0 to 0)	0 (0 to 0)	0·3 (0 to 0·7)	1·8 (0·4 to 3·6)	2·8 (0·8 to 5·2)	2·9 (0·8 to 5·7)	3 (0·8 to 5·7)	2·7 (0·7 to 5·2)	2·7 (0·7 to 5·3)	2·5 (0·6 to 4·8)	2·4 (0·6 to 4·9)	2·2 (0·6 to 4·5)	2·2 (0·6 to 4·4)	2·1 (0·5 to 4·1)	1·9 (0·4 to 3·9)	1·5 (0·3 to 3·2)	1·1 (0·2 to 2·1)	0·7 (0·1 to 1·4)	0·4 (0·1 to 0·8)	0·2 (0·1 to 0·5)	0·1 (0 to 0·2)	0 (0 to 0·1)	0 (0 to 0)	33·4
Other mental disorders	0 (0 to 0)	0 (0 to 0)	0 (0 to 0)	0 (0 to 0)	0 (0 to 0)	1 (0·5 to 1·7)	7 (3·1 to 12)	16·5 (8·4 to 28·5)	26·5 (15·1 to 42·1)	32 (18·9 to 49·8)	37·9 (23 to 58·2)	38·1 (23·7 to 59·4)	41·5 (25·1 to 64·2)	41·2 (25·6 to 63·1)	40·3 (25·1 to 61·3)	36·3 (23·1 to 54·9)	28 (18 to 42·1)	21·8 (14·1 to 32·9)	14·4 (9·5 to 21·1)	9·7 (6·3 to 14·1)	5·7 (3·8 to 8·2)	2·4 (1·6 to 3·5)	0·5 (0·4 to 0·8)	401
Substance use disorders	0 (0 to 0)	0 (0 to 0)	0 (0 to 0)	0 (0 to 0)	0 (0 to 0)	3 (1·6 to 4·9)	47·4 (30·8 to 66·8)	93·9 (62·3 to 133)	112 (77 to 153)	105 (74·5 to 146)	103 (75·6 to 136)	96·8 (70·7 to 128)	104 (77·4 to 138)	93·1 (69·5 to 125)	92 (68·5 to 120)	73·4 (55·1 to 96·7)	48 (36 to 60·9)	29·4 (22·2 to 38·3)	15·8 (11·8 to 20·3)	9·2 (6·8 to 12·3)	5·6 (4 to 7·6)	2·9 (2 to 4·1)	0·9 (0·6 to 1·4)	1,030
Alcohol use disorders	0 (0 to 0)	0 (0 to 0)	0 (0 to 0)	0 (0 to 0)	0 (0 to 0)	1·2 (0·5 to 2·3)	15 (8·3 to 24·7)	38·5 (21·2 to 62·8)	53·7 (32·9 to 81·7)	61·1 (39·5 to 93·7)	66·7 (47·2 to 92·9)	68 (48·5 to 95·4)	76·9 (55·2 to 106)	70·7 (50·9 to 98·1)	73 (53 to 98·2)	59·3 (43·1 to 80·9)	39·1 (28·5 to 51·5)	23·5 (17 to 31·5)	12·2 (8·9 to 16·3)	6·9 (4·9 to 9·6)	4·3 (2·9 to 6·1)	2·3 (1·5 to 3·5)	0·7 (0·4 to 1·2)	673
Drug use disorders	0 (0 to 0)	0 (0 to 0)	0 (0 to 0)	0 (0 to 0)	0 (0 to 0)	1·8 (0·9 to 3·2)	32·3 (20·8 to 47·1)	55·4 (36·3 to 80·4)	57·8 (37·6 to 82·2)	43·7 (28·4 to 63·2)	36·4 (24·4 to 51·5)	28·8 (19·1 to 41·1)	26·8 (17·7 to 39·2)	22·4 (14·1 to 33·3)	19 (11·7 to 28·5)	14 (8·3 to 21·3)	9 (5·4 to 13·8)	6 (3·5 to 9·1)	3·6 (2·2 to 5·4)	2·3 (1·4 to 3·4)	1·3 (0·8 to 2)	0·6 (0·4 to 0·9)	0·2 (0·1 to 0·2)	361
Opioid use disorders	0 (0 to 0)	0 (0 to 0)	0 (0 to 0)	0 (0 to 0)	0 (0 to 0)	0 (0 to 0)	6·8 (3·1 to 11·7)	27·3 (14·5 to 46·4)	35 (20·1 to 56)	28·6 (16·3 to 45·9)	24·5 (14·5 to 37·4)	19·5 (11 to 30·1)	18·4 (10·6 to 29·7)	16·5 (8·9 to 27)	14·6 (7·9 to 23·1)	11·2 (5·8 to 18·1)	7·2 (3·8 to 11·7)	4·7 (2·3 to 7·7)	2·7 (1·4 to 4·6)	1·8 (0·9 to 2·8)	1 (0·5 to 1·6)	0·5 (0·2 to 0·8)	0·1 (0·1 to 0·2)	220
Cocaine use disorders	0 (0 to 0)	0 (0 to 0)	0 (0 to 0)	0 (0 to 0)	0 (0 to 0)	0·1 (0 to 0·1)	3·3 (1·4 to 6·3)	4 (2·1 to 6·7)	3·7 (2 to 5·8)	3·3 (2·1 to 5)	3·2 (2 to 4·8)	2·5 (1·6 to 3·9)	2·3 (1·5 to 3·4)	1·3 (0·9 to 1·8)	1·2 (0·8 to 1·8)	0·8 (0·5 to 1·1)	0·5 (0·4 to 0·8)	0·3 (0·2 to 0·5)	0·2 (0·1 to 0·3)	0·1 (0·1 to 0·1)	0·1 (0 to 0·1)	0 (0 to 0)	0 (0 to 0)	27
Amphetamine use disorders	0 (0 to 0)	0 (0 to 0)	0 (0 to 0)	0 (0 to 0)	0 (0 to 0)	0 (0 to 0)	1·1 (0·4 to 2·3)	4·5 (1·8 to 8·3)	4·7 (2·1 to 8·6)	2·5 (1·1 to 4·7)	1·6 (0·8 to 3)	1 (0·6 to 1·6)	0·8 (0·5 to 1·2)	0·6 (0·4 to 1)	0·5 (0·3 to 0·8)	0·3 (0·2 to 0·5)	0·2 (0·1 to 0·3)	0·1 (0 to 0·2)	0 (0 to 0·1)	0 (0 to 0)	0 (0 to 0)	0 (0 to 0)	0 (0 to 0)	17·9
Cannabis use disorders	0 (0 to 0)	0 (0 to 0)	0 (0 to 0)	0 (0 to 0)	0 (0 to 0)	1·7 (0·7 to 3·1)	18·3 (9·9 to 30·7)	14·3 (7·4 to 24·2)	9 (4·2 to 15·9)	5·4 (2·6 to 9·5)	4 (1·9 to 6·9)	3·2 (1·5 to 5·4)	2·8 (1·3 to 5)	2·1 (1 to 3·9)	1·5 (0·7 to 2·6)	0·8 (0·4 to 1·6)	0·4 (0·2 to 0·8)	0·2 (0·1 to 0·4)	0·1 (0 to 0·2)	0 (0 to 0·1)	0 (0 to 0)	0 (0 to 0)	0 (0 to 0)	63·9
Other drug use disorders	0 (0 to 0)	0 (0 to 0)	0 (0 to 0)	0 (0 to 0)	0 (0 to 0)	0 (0 to 0·1)	2·9 (1·4 to 5·1)	5·4 (2·9 to 8·9)	5·5 (3·2 to 8·7)	3·8 (2·3 to 5·9)	3·2 (2 to 4·8)	2·6 (1·7 to 3·9)	2·6 (1·7 to 3·7)	1·8 (1·1 to 2·7)	1·3 (0·8 to 1·8)	0·8 (0·6 to 1·1)	0·6 (0·4 to 0·8)	0·7 (0·5 to 0·9)	0·5 (0·4 to 0·7)	0·4 (0·3 to 0·5)	0·2 (0·1 to 0·3)	0·1 (0·1 to 0·1)	0 (0 to 0)	32·3
Diabetes and kidney diseases	0 (0 to 0·1)	0·1 (0·1 to 0·2)	5·9 (3·6 to 9·2)	6·6 (4·5 to 9·3)	12·9 (9·4 to 17·5)	19 (14 to 26·3)	62·6 (49·5 to 78·3)	90·2 (71·6 to 115)	137 (107 to 173)	193 (156 to 238)	274 (222 to 332)	387 (302 to 479)	634 (502 to 783)	942 (741 to 1,170)	1,330 (1,070 to 1,620)	1,680 (1,370 to 2,030)	1,670 (1,370 to 2,000)	1,680 (1,390 to 1,990)	1,370 (1,140 to 1,620)	1,020 (855 to 1,200)	627 (529 to 732)	278 (232 to 323)	57·8 (45·9 to 68·3)	12,500
Diabetes mellitus	0 (0 to 0·1)	0·1 (0·1 to 0·2)	2 (0·9 to 3·8)	1·4 (0·9 to 2·2)	2·3 (1·6 to 3·2)	4·7 (2·9 to 6·7)	27·7 (21·1 to 35·3)	45·4 (35·8 to 57·4)	72·6 (56·1 to 94·6)	108 (83·9 to 140)	166 (129 to 209)	250 (194 to 316)	437 (334 to 560)	689 (524 to 866)	992 (768 to 1,230)	1,260 (1,010 to 1,560)	1,290 (1,030 to 1,570)	1,300 (1,050 to 1,560)	1,080 (884 to 1,290)	794 (650 to 952)	478 (397 to 572)	207 (169 to 245)	40·8 (32 to 49·1)	9,240
Diabetes mellitus type 1	0 (0 to 0·1)	0·1 (0·1 to 0·2)	2 (0·9 to 3·8)	1·4 (0·9 to 2·2)	2·3 (1·6 to 3·2)	4·7 (2·9 to 6·7)	10·1 (7·3 to 13·9)	15·3 (11·2 to 21·1)	23·5 (17 to 32)	20·5 (14·2 to 29·4)	29·4 (20·2 to 41·5)	41·5 (29·3 to 59·9)	17·3 (12 to 24·5)	24·5 (16·5 to 34·9)	36·3 (23·9 to 54·1)	38·4 (25·8 to 56·4)	38·6 (25·2 to 56·1)	30·3 (20 to 44·3)	24·7 (15·7 to 39·2)	11·8 (7·7 to 19·3)	7·1 (4·7 to 12·5)	3·5 (2·2 to 5·7)	0·8 (0·5 to 1·4)	384
Diabetes mellitus type 2	0 (0 to 0)	0 (0 to 0)	0 (0 to 0)	0 (0 to 0)	0 (0 to 0)	0 (0 to 0)	17·6 (12·4 to 23·6)	30·1 (21·6 to 40)	49·1 (35·1 to 68)	87·4 (65 to 116)	137 (102 to 179)	208 (159 to 268)	420 (319 to 540)	664 (502 to 839)	956 (740 to 1,180)	1,220 (979 to 1,510)	1,250 (999 to 1,530)	1,270 (1,020 to 1,530)	1,050 (863 to 1,270)	782 (640 to 938)	471 (391 to 563)	203 (165 to 240)	40 (31·4 to 48·3)	8,860
Chronic kidney disease	0 (0 to 0)	0 (0 to 0)	4 (2·4 to 6·5)	5·2 (3·4 to 7·6)	10·5 (7·3 to 15)	14·3 (9·8 to 20·7)	34·8 (24·6 to 48·3)	44·8 (31·6 to 61·4)	64·3 (45·3 to 88·5)	84·9 (60·6 to 113)	108 (80·5 to 146)	137 (98·9 to 187)	197 (146 to 266)	253 (184 to 342)	341 (258 to 451)	425 (317 to 550)	389 (289 to 517)	376 (293 to 483)	293 (225 to 374)	225 (174 to 279)	149 (118 to 186)	71·1 (54 to 87·9)	16·9 (12·8 to 21·4)	3,250
Chronic kidney disease due to diabetes mellitus type 1	0 (0 to 0)	0 (0 to 0)	0 (0 to 0·1)	0·1 (0 to 0·3)	0·3 (0·1 to 0·7)	0·5 (0·2 to 1·1)	1·4 (0·6 to 2·7)	2·2 (0·9 to 4·6)	5·9 (2·6 to 11·2)	12·2 (6 to 21·5)	18·2 (9·2 to 30)	22·4 (11·6 to 37·1)	27·5 (12·7 to 48)	26·8 (10·9 to 52·1)	25·3 (8·4 to 56·4)	23·7 (8·4 to 52·4)	16·7 (6·3 to 38·8)	12·2 (4·7 to 25·9)	6·2 (2·4 to 13·9)	2·4 (0·9 to 5·7)	0·6 (0·2 to 1·4)	0·1 (0 to 0·2)	0 (0 to 0)	205
Chronic kidney disease due to diabetes mellitus type 2	0 (0 to 0)	0 (0 to 0)	0 (0 to 0)	0 (0 to 0)	0 (0 to 0)	0 (0 to 0)	0·6 (0·2 to 1·3)	1·1 (0·4 to 2·4)	2·5 (1·1 to 5·3)	5·2 (2·4 to 9·7)	9·2 (4·3 to 17·1)	16·8 (8·2 to 29·6)	35·2 (20·3 to 56·8)	61·1 (36·7 to 91·5)	98·9 (58·7 to 147)	133 (83·2 to 192)	126 (81·7 to 185)	122 (82·9 to 168)	94 (60·6 to 130)	70 (46·9 to 97·5)	43·9 (28·4 to 61·6)	19·9 (12·3 to 28·8)	4·5 (2·5 to 6·6)	844
Chronic kidney disease due to hypertension	0 (0 to 0)	0 (0 to 0)	0 (0 to 0)	0 (0 to 0)	0 (0 to 0)	0 (0 to 0)	2·7 (1·1 to 5·3)	4·1 (1·8 to 7·6)	6·5 (3·1 to 11·3)	9 (4·8 to 15·1)	12·4 (7·2 to 19·7)	17·4 (10·5 to 26·9)	28·5 (17·9 to 42·6)	41·5 (25·9 to 64·1)	64 (41·4 to 94·3)	92·4 (61·3 to 135)	94·9 (63 to 140)	100 (68·3 to 140)	86·1 (59·1 to 121)	72·9 (51·4 to 99·2)	53·6 (38·7 to 72·8)	27·9 (19·8 to 37)	6·8 (4·8 to 9·2)	721
Chronic kidney disease due to glomerulonephritis	0 (0 to 0)	0 (0 to 0)	1·1 (0·5 to 2·1)	1·7 (0·9 to 2·7)	3·8 (2·3 to 5·9)	5·7 (3·5 to 8·9)	14·1 (8·7 to 21·2)	17·2 (10·5 to 25·2)	23·6 (14·9 to 35·8)	28·8 (18 to 42·1)	34·6 (21·7 to 50·6)	40·7 (24·2 to 60·4)	52·4 (31·7 to 80·5)	60·3 (35·7 to 93·5)	74·2 (40·4 to 121)	80·8 (42·8 to 135)	62·9 (30·1 to 113)	52 (26·8 to 86·5)	34·9 (17·3 to 62·1)	22·6 (11·1 to 41·6)	11·8 (5·4 to 20·6)	4·8 (2·2 to 8·3)	1 (0·4 to 2·1)	629
Chronic kidney disease due to other and unspecified causes	0 (0 to 0)	0 (0 to 0)	2·8 (1·5 to 4·8)	3·4 (2·1 to 5·2)	6·5 (4·2 to 9·7)	8·2 (4·9 to 12·7)	16·1 (9·8 to 23·6)	20·1 (12·2 to 29·3)	25·7 (16 to 36·9)	29·7 (18·3 to 44·8)	33·4 (20·2 to 51)	39·6 (23·7 to 59·2)	53·7 (32·1 to 82·3)	63·5 (38·9 to 97·7)	79 (48·7 to 124)	94·3 (55·5 to 152)	89·2 (54·3 to 140)	89·8 (56·9 to 135)	72 (45·4 to 108)	57·4 (35·4 to 85)	39·2 (26 to 56·2)	18·4 (12·1 to 26·2)	4·7 (3 to 6·8)	847
Acute glomerulonephritis	0 (0 to 0)	0 (0 to 0)	0 (0 to 0)	0 (0 to 0)	0 (0 to 0)	0 (0 to 0)	0 (0 to 0)	0 (0 to 0)	0 (0 to 0)	0 (0 to 0)	0 (0 to 0)	0 (0 to 0)	0 (0 to 0)	0 (0 to 0)	0 (0 to 0)	0 (0 to 0)	0 (0 to 0)	0 (0 to 0·1)	0 (0 to 0)	0 (0 to 0)	0 (0 to 0)	0 (0 to 0)	0 (0 to 0)	0·3
Skin and subcutaneous diseases	0 (0 to 0)	0·1 (0 to 0·1)	19·7 (12 to 31)	80·9 (51·5 to 118)	111 (70·9 to 164)	123 (79·6 to 182)	144 (94·6 to 214)	94·4 (63·1 to 136)	92·3 (63·5 to 132)	90 (62·6 to 126)	98·9 (69·9 to 141)	109 (76·7 to 152)	126 (90·2 to 174)	156 (112 to 218)	190 (137 to 264)	216 (157 to 308)	216 (154 to 333)	232 (166 to 370)	234 (167 to 384)	205 (145 to 331)	171 (119 to 271)	91·7 (62·3 to 141)	27·1 (16·9 to 43·8)	2,830
Dermatitis	0 (0 to 0)	0 (0 to 0)	0 (0 to 0)	27·2 (14·6 to 47·6)	35·3 (18·6 to 59·8)	27·3 (15 to 46·5)	21·8 (12 to 35·2)	17·3 (9·8 to 27·6)	16·7 (9·4 to 26·9)	15·4 (8·9 to 24·5)	15·7 (8·9 to 25)	15 (8·7 to 24)	16·6 (9·1 to 27·1)	17·8 (9·8 to 29·1)	19·2 (10·7 to 32·1)	18·9 (10·5 to 30·1)	16 (9·1 to 25·7)	13·2 (7·5 to 21·2)	8·7 (5 to 13·8)	5·6 (3·3 to 8·9)	3 (1·8 to 4·8)	1·2 (0·7 to 1·8)	0·2 (0·1 to 0·4)	312
Atopic dermatitis	0 (0 to 0)	0 (0 to 0)	0 (0 to 0)	27 (14·5 to 47·2)	34·6 (18·2 to 59)	24·6 (13·2 to 42·4)	16·9 (9 to 29)	11·6 (5·9 to 20·3)	9·7 (5 to 17·6)	8·1 (4·2 to 14·7)	8 (4 to 13·7)	7·5 (3·9 to 13)	8·1 (4·2 to 14)	8·2 (4·3 to 14·2)	8·7 (4·5 to 15·1)	8·2 (4·1 to 14·2)	6·5 (3·4 to 11)	5·2 (2·8 to 8·7)	3·6 (1·9 to 6·1)	2·5 (1·3 to 4·3)	1·4 (0·8 to 2·5)	0·6 (0·3 to 1)	0·1 (0·1 to 0·2)	201
Contact dermatitis	0 (0 to 0)	0 (0 to 0)	0 (0 to 0)	0 (0 to 0)	0·4 (0·2 to 0·9)	2·3 (0·9 to 4·5)	4·3 (1·9 to 8)	5 (2·1 to 9·3)	6·2 (2·8 to 11·6)	6·5 (2·9 to 12)	7 (3 to 13·3)	6·8 (3 to 13·4)	7·7 (3·4 to 15)	8·8 (3·8 to 16·6)	9·8 (4·2 to 19·8)	9·9 (4·2 to 18·2)	8·7 (4 to 16·6)	7·2 (3·3 to 13·5)	4·5 (2·2 to 8·4)	2·7 (1·2 to 5·1)	1·4 (0·6 to 2·6)	0·5 (0·2 to 0·9)	0·1 (0 to 0·2)	99·8
Seborrhoeic dermatitis	0 (0 to 0)	0 (0 to 0)	0 (0 to 0)	0·2 (0·1 to 0·4)	0·3 (0·1 to 0·6)	0·4 (0·2 to 0·8)	0·6 (0·3 to 1)	0·6 (0·3 to 1)	0·7 (0·4 to 1·3)	0·8 (0·4 to 1·4)	0·8 (0·4 to 1·4)	0·8 (0·4 to 1·3)	0·8 (0·4 to 1·3)	0·8 (0·4 to 1·3)	0·8 (0·4 to 1·4)	0·8 (0·4 to 1·4)	0·9 (0·4 to 1·4)	0·8 (0·4 to 1·4)	0·6 (0·3 to 1)	0·4 (0·2 to 0·7)	0·2 (0·1 to 0·4)	0·1 (0 to 0·2)	0 (0 to 0)	11·4
Psoriasis	0 (0 to 0)	0 (0 to 0)	0 (0 to 0)	1 (0·6 to 1·4)	2·7 (1·3 to 4·4)	3·8 (2·1 to 6)	4·5 (2·6 to 7)	4·6 (2·7 to 6·9)	5·5 (3·2 to 8·2)	6 (3·6 to 9)	7·1 (4·3 to 10·6)	7·7 (4·8 to 11·3)	9·1 (5·6 to 13·4)	9·9 (6·3 to 14·6)	10·7 (7 to 15·5)	10·2 (6·9 to 14·6)	8 (5·1 to 11·3)	5·9 (3·7 to 8·3)	3·3 (2·1 to 4·7)	1·7 (1·1 to 2·6)	0·7 (0·4 to 1·2)	0·2 (0·1 to 0·4)	0 (0 to 0·1)	103
Bacterial skin diseases	0 (0 to 0)	0 (0 to 0)	14·7 (7·4 to 25·9)	5·2 (3 to 8·4)	6·5 (4 to 10·6)	5·5 (3 to 8·6)	7·2 (4·1 to 12·2)	7·9 (4·5 to 13·8)	10·2 (5·2 to 18·4)	15·8 (8·7 to 26·7)	14·9 (7·9 to 26·7)	22·8 (11·7 to 39·4)	25·4 (12·9 to 46·2)	33·3 (16·3 to 59·4)	42·8 (21·5 to 80)	48·6 (25·4 to 87)	46·3 (23·7 to 95)	42·9 (22·6 to 88·3)	42·1 (22·3 to 89·8)	36·1 (18·5 to 76)	26·7 (13·6 to 54·6)	14·4 (7·6 to 27·5)	3·9 (1·9 to 6·9)	473
Cellulitis	0 (0 to 0)	0 (0 to 0)	4·8 (1·5 to 10·2)	1·7 (0·7 to 3·1)	2·7 (1·1 to 4·5)	2·1 (0·8 to 4·1)	2·8 (1·1 to 5·7)	2·5 (1 to 5·4)	4 (1·7 to 8·2)	5 (2·1 to 9·8)	4·5 (1·9 to 9·7)	5·7 (2·5 to 12·4)	7 (2·9 to 15·6)	9·5 (4·3 to 19·2)	11·4 (5·1 to 24·5)	12·5 (5·2 to 25·8)	12 (5·5 to 29·8)	10·4 (4·3 to 28·8)	8·5 (3·6 to 24·9)	7 (3 to 19·1)	4·9 (2 to 14·7)	2·5 (1·1 to 7·6)	0·7 (0·3 to 1·7)	122
Pyoderma	0 (0 to 0)	0 (0 to 0)	9·9 (4·9 to 18)	3·5 (1·9 to 6)	3·7 (2·2 to 6·7)	3·3 (1·7 to 5·6)	4·4 (2·4 to 7·7)	5·4 (3·1 to 9·3)	6·2 (2·9 to 11·3)	10·9 (5·8 to 18·4)	10·3 (5·1 to 18·7)	17·1 (8·3 to 29·8)	18·4 (8·7 to 33·3)	23·8 (10·9 to 42·7)	31·4 (14·8 to 55·2)	36·2 (17·9 to 63·7)	34·2 (16·8 to 65·7)	32·5 (16·3 to 65·4)	33·6 (17·6 to 69·1)	29·1 (15·1 to 57·9)	21·8 (10·7 to 44·1)	11·9 (6·1 to 22)	3·3 (1·5 to 5·7)	351
Scabies	0 (0 to 0)	0 (0 to 0)	1·8 (1 to 3·2)	13 (6·6 to 23·1)	18·5 (8·9 to 32·9)	20·5 (10·3 to 35·6)	22·8 (11·7 to 39)	19·4 (9·6 to 33·9)	15·2 (7·5 to 26·8)	11·2 (5·5 to 20·1)	11·6 (5·8 to 20·1)	12·6 (6·5 to 21·6)	13·9 (7·2 to 23·8)	13·3 (6·8 to 22·4)	12·2 (6·2 to 21·1)	10·2 (5·3 to 17·9)	7·8 (4 to 13·8)	6·3 (3·2 to 11·1)	4·7 (2·4 to 8·1)	3·5 (1·8 to 6·2)	2·3 (1·2 to 3·9)	1 (0·5 to 1·8)	0·2 (0·1 to 0·4)	222
Fungal skin diseases	0 (0 to 0)	0 (0 to 0)	0·3 (0·1 to 0·7)	2·5 (0·9 to 5·3)	4·3 (1·6 to 9·3)	5·4 (2 to 12)	6 (2·1 to 13·7)	6 (2·2 to 12·6)	6·7 (2·6 to 14·9)	6·8 (2·6 to 15·4)	7·4 (2·9 to 16·2)	7·4 (2·9 to 16·2)	8·3 (3·3 to 18·5)	9·5 (3·8 to 21·1)	11·6 (4·6 to 25·7)	12·6 (4·8 to 27·5)	13·6 (5·5 to 29·2)	15·4 (6 to 33·1)	12·8 (4·9 to 26·8)	11·1 (4·3 to 23·7)	8·1 (3·2 to 16·9)	3·8 (1·6 to 8)	0·9 (0·4 to 1·8)	161
Viral skin diseases	0 (0 to 0)	0 (0 to 0)	0 (0 to 0)	14·3 (9·1 to 21·7)	24 (15 to 36·9)	13·5 (8·5 to 20·3)	8·8 (5·5 to 13·5)	6·6 (4·1 to 10)	5·2 (3·2 to 8·1)	4·2 (2·5 to 6·4)	4·2 (2·5 to 6·4)	3·9 (2·4 to 6·1)	4 (2·5 to 6·2)	4 (2·4 to 6·2)	3·8 (2·2 to 6)	3·7 (2·2 to 5·8)	2·9 (1·8 to 4·5)	2·1 (1·3 to 3·3)	1·2 (0·7 to 1·8)	0·6 (0·4 to 1)	0·3 (0·2 to 0·5)	0·1 (0·1 to 0·2)	0 (0 to 0)	107
Acne vulgaris	0 (0 to 0)	0 (0 to 0)	0 (0 to 0)	0 (0 to 0)	1·2 (0·6 to 2)	29·5 (16·6 to 48·4)	52·5 (29·6 to 85·6)	12·4 (7·1 to 20·1)	6·2 (3·4 to 10·1)	4·3 (2·3 to 7·2)	3·5 (1·8 to 6)	2·5 (1·3 to 4)	2 (1·1 to 3·2)	1·4 (0·8 to 2·2)	1 (0·6 to 1·6)	0·7 (0·4 to 1·2)	0·5 (0·3 to 0·8)	0·3 (0·2 to 0·6)	0·2 (0·1 to 0·3)	0·1 (0·1 to 0·2)	0 (0 to 0·1)	0 (0 to 0)	0 (0 to 0)	118
Alopecia areata	0 (0 to 0)	0 (0 to 0)	0 (0 to 0)	0·3 (0·1 to 0·5)	0·4 (0·2 to 0·7)	0·6 (0·3 to 1)	1·1 (0·6 to 1·8)	1·7 (1 to 2·7)	2·4 (1·4 to 3·8)	2·5 (1·5 to 3·9)	2·5 (1·5 to 3·9)	2 (1·2 to 3·2)	1·9 (1·1 to 3)	1·7 (1 to 2·7)	1·6 (0·9 to 2·5)	1·5 (0·9 to 2·4)	1·1 (0·6 to 1·7)	0·8 (0·4 to 1·3)	0·4 (0·2 to 0·7)	0·2 (0·1 to 0·4)	0·1 (0·1 to 0·2)	0 (0 to 0)	0 (0 to 0)	22·7
Pruritus	0 (0 to 0)	0 (0 to 0)	0 (0 to 0·1)	0·5 (0·2 to 1·1)	0·8 (0·3 to 1·6)	0·9 (0·4 to 1·8)	1·1 (0·5 to 2·1)	1·3 (0·5 to 2·5)	1·5 (0·7 to 3)	1·7 (0·7 to 3·2)	1·9 (0·8 to 3·6)	1·8 (0·8 to 3·5)	2·1 (0·9 to 4·1)	2·3 (1 to 4·7)	2·7 (1·1 to 5·5)	3 (1·3 to 6·2)	2·9 (1·3 to 5·6)	2·5 (1·1 to 4·9)	1·7 (0·7 to 3·4)	1·1 (0·5 to 2·3)	0·6 (0·3 to 1·3)	0·2 (0·1 to 0·5)	0·1 (0 to 0·1)	30·8
Urticaria	0 (0 to 0)	0 (0 to 0)	1·9 (1·1 to 3)	13 (7·8 to 20·5)	12·2 (7 to 20)	9·3 (5·1 to 15·6)	8·5 (4·7 to 13·8)	7·6 (4·1 to 12·5)	7·5 (4 to 12·1)	7·5 (4 to 12·3)	8·1 (4·7 to 12·7)	7·5 (4·3 to 11·8)	7·4 (4·2 to 12·3)	7·3 (4·3 to 11·9)	7·3 (4 to 11·7)	6·6 (3·6 to 10·7)	5·1 (2·8 to 8·3)	4·1 (2·3 to 6·6)	2·6 (1·4 to 4·1)	1·5 (0·9 to 2·4)	0·8 (0·4 to 1·2)	0·3 (0·1 to 0·4)	0·1 (0 to 0·1)	126
Decubitus ulcer	0 (0 to 0)	0 (0 to 0)	0 (0 to 0)	0·8 (0·2 to 1·8)	1·9 (0·9 to 3·5)	2·4 (0·8 to 4)	3·8 (1·4 to 6·7)	4·2 (1·3 to 10·1)	8·8 (3·8 to 16·3)	6·8 (2·6 to 14)	12·7 (6·6 to 24·7)	15·5 (7·8 to 30·2)	22·3 (12 to 40·5)	38·8 (23·2 to 67·9)	57·3 (33·2 to 104)	78·4 (47·5 to 141)	91·1 (56·1 to 180)	121 (77·5 to 229)	144 (95·5 to 268)	135 (88·1 to 240)	124 (81 to 210)	68·5 (44·2 to 113)	21·3 (12·8 to 34·8)	959
Other skin and subcutaneous diseases	0 (0 to 0)	0 (0 to 0)	0·9 (0·5 to 1·6)	3·1 (1·5 to 5·9)	3·2 (1·6 to 5·9)	4·2 (2 to 8)	5·4 (2·6 to 10)	5·5 (2·8 to 10·1)	6·4 (3·1 to 11·6)	7·9 (4 to 14·5)	9·3 (4·6 to 17)	10·6 (5·5 to 18·6)	13·2 (6·8 to 24·1)	16·5 (8·3 to 29·2)	19·9 (9·9 to 36·3)	22·1 (11·2 to 40·4)	20·1 (10·3 to 36·2)	17·6 (8·7 to 31·9)	12·7 (6·6 to 22·3)	8·3 (4·3 to 14·5)	4·3 (2·3 to 7·4)	1·8 (1 to 3)	0·3 (0·2 to 0·6)	193
Sense organ diseases	0 (0 to 0)	0 (0 to 0)	0·6 (0·4 to 1)	7 (4·3 to 10)	15·8 (9·7 to 23·5)	22 (13·8 to 32·8)	28·7 (18·2 to 42·2)	34·4 (21·1 to 50·5)	48 (30 to 73·5)	60·1 (36·8 to 93)	77·2 (47·4 to 121)	96 (59·1 to 151)	151 (91·8 to 244)	213 (132 to 343)	288 (178 to 458)	342 (215 to 535)	344 (220 to 521)	343 (224 to 499)	287 (194 to 412)	236 (161 to 328)	160 (111 to 218)	74·3 (52·7 to 99·6)	17·4 (12·6 to 23·5)	2,840
Blindness and vision loss	0 (0 to 0)	0 (0 to 0)	0·1 (0 to 0·1)	1·3 (0·7 to 2·1)	2·9 (1·7 to 4·6)	3·8 (2·2 to 6·2)	4·7 (2·8 to 7·3)	5·2 (3 to 8)	6·2 (3·7 to 9·5)	7·3 (4·3 to 11·6)	10·4 (6·2 to 16·5)	14·6 (8·5 to 24·2)	23·3 (13·2 to 40)	33·8 (19·2 to 59·1)	48·4 (27·7 to 85·3)	61·5 (35·8 to 104)	66·3 (39·5 to 108)	69·5 (42·5 to 109)	59·2 (37·6 to 91·8)	49·4 (32·5 to 74·7)	34·3 (22·9 to 49·1)	17·2 (11·7 to 24·2)	4·9 (3·3 to 6·9)	524
Glaucoma	0 (0 to 0)	0 (0 to 0)	0 (0 to 0)	0 (0 to 0)	0 (0 to 0)	0 (0 to 0)	0 (0 to 0)	0 (0 to 0)	0 (0 to 0)	0 (0 to 0)	0 (0 to 0)	0 (0 to 0)	0 (0 to 0·1)	0·3 (0·1 to 0·4)	0·8 (0·4 to 1·6)	1·8 (0·9 to 3·1)	2·7 (1·5 to 4·3)	3·8 (2·3 to 6)	4·3 (2·7 to 6·9)	4·4 (2·7 to 6·5)	3·5 (2·1 to 5·4)	1·8 (1·1 to 2·8)	0·5 (0·3 to 0·9)	24
Cataract	0 (0 to 0)	0 (0 to 0)	0 (0 to 0)	0 (0 to 0)	0 (0 to 0)	0 (0 to 0)	0 (0 to 0)	0 (0 to 0)	0·1 (0 to 0·2)	0·2 (0·1 to 0·4)	0·5 (0·3 to 0·9)	0·9 (0·4 to 1·5)	1·6 (0·8 to 2·7)	2·4 (1·2 to 3·8)	3·5 (2 to 5·8)	5·1 (3 to 8·1)	7·2 (4·3 to 10·8)	10·6 (6·6 to 15·5)	12·5 (8·2 to 18·2)	12·5 (8·2 to 17·7)	9·7 (6·3 to 13·5)	4·9 (3·2 to 6·9)	1·4 (0·9 to 2·1)	73·1
Age-related macular degeneration	0 (0 to 0)	0 (0 to 0)	0 (0 to 0)	0 (0 to 0)	0 (0 to 0)	0 (0 to 0)	0 (0 to 0)	0 (0 to 0)	0 (0 to 0)	0 (0 to 0)	0 (0 to 0)	0 (0 to 0)	0 (0 to 0)	0·1 (0 to 0·1)	0·2 (0·1 to 0·3)	0·3 (0·2 to 0·5)	0·4 (0·2 to 0·6)	0·5 (0·3 to 0·7)	0·4 (0·2 to 0·7)	0·4 (0·2 to 0·6)	0·2 (0·1 to 0·4)	0·1 (0·1 to 0·2)	0 (0 to 0·1)	2·6
Refraction disorders	0 (0 to 0)	0 (0 to 0)	0 (0 to 0·1)	1·1 (0·6 to 1·8)	2·5 (1·4 to 4·1)	3·3 (1·9 to 5·5)	4·1 (2·4 to 6·6)	4·4 (2·5 to 7)	5 (2·9 to 7·6)	5 (2·9 to 7·9)	5·8 (3·4 to 8·9)	6·2 (3·6 to 9·7)	7·3 (4·4 to 11·1)	7·8 (4·7 to 12·1)	8·6 (5·3 to 13·5)	8·6 (5·2 to 13·3)	7·7 (4·5 to 11·7)	7·5 (4·5 to 11·4)	6·2 (3·7 to 9·1)	4·4 (2·7 to 6·7)	2·4 (1·5 to 3·6)	0·9 (0·5 to 1·3)	0·2 (0·1 to 0·3)	98·9
Near vision loss	0 (0 to 0)	0 (0 to 0)	0 (0 to 0)	0 (0 to 0)	0 (0 to 0·1)	0·1 (0 to 0·1)	0·1 (0 to 0·2)	0·2 (0·1 to 0·6)	0·5 (0·2 to 1·2)	1·4 (0·5 to 3)	2·9 (1 to 6·3)	5·9 (2·3 to 12·6)	11·4 (4·6 to 23·9)	18·5 (7·3 to 38·7)	27·2 (10·3 to 56·3)	33·6 (14 to 69·8)	33·5 (13·6 to 66·9)	31·8 (13·3 to 63·9)	24·4 (10·3 to 49·7)	18·3 (7·8 to 36·5)	11·3 (4·9 to 22·2)	4·9 (2 to 9·9)	1·1 (0·4 to 2·4)	227
Other vision loss	0 (0 to 0)	0 (0 to 0)	0 (0 to 0)	0·2 (0·1 to 0·4)	0·4 (0·1 to 0·8)	0·5 (0·2 to 0·9)	0·5 (0·2 to 1)	0·5 (0·2 to 1·1)	0·6 (0·3 to 1·2)	0·8 (0·4 to 1·4)	1·1 (0·6 to 2)	1·7 (0·9 to 2·8)	2·8 (1·4 to 4·8)	4·9 (2·8 to 7·6)	8·1 (4·7 to 12·2)	12 (7·6 to 18)	14·9 (9·7 to 21·4)	15·5 (10 to 22·3)	11·4 (7·2 to 16·7)	9·5 (6·3 to 13·8)	7 (4·5 to 10·6)	4·6 (3·1 to 6·6)	1·6 (1 to 2·4)	98·6
Age-related and other hearing loss	0 (0 to 0)	0 (0 to 0)	0·2 (0·1 to 0·4)	4 (2·2 to 6·3)	10·8 (6·1 to 17·2)	16 (9·3 to 24·1)	21·3 (12·6 to 31·8)	26·4 (15·9 to 41)	38·1 (23·2 to 58·9)	47·9 (29 to 74·8)	60·6 (35·8 to 97·5)	74·8 (43·8 to 120)	119 (69·9 to 196)	168 (102 to 271)	225 (138 to 360)	263 (162 to 416)	259 (163 to 398)	256 (163 to 376)	213 (143 to 311)	178 (118 to 247)	121 (82 to 166)	55·3 (38·3 to 75·3)	12 (8·5 to 16·3)	2,170
Other sense organ diseases	0 (0 to 0)	0 (0 to 0)	0·3 (0·2 to 0·6)	1·7 (0·8 to 2·8)	2 (1 to 3·4)	2·2 (1·1 to 3·9)	2·7 (1·2 to 4·8)	2·8 (1·2 to 5·3)	3·6 (1·6 to 7·1)	4·9 (2·1 to 9·7)	6·2 (2·9 to 11·4)	6·6 (3 to 12·7)	8·3 (3·7 to 15·6)	11·7 (5·6 to 22·4)	13·8 (6·4 to 27·1)	17·6 (8·3 to 32·9)	18·5 (8·5 to 33·9)	17·4 (8·1 to 31·7)	14 (6·6 to 24·8)	9·5 (4·7 to 18·1)	4·7 (2·3 to 8·5)	1·7 (0·8 to 3·1)	0·5 (0·2 to 0·9)	151
Musculoskeletal disorders	0 (0 to 0)	0 (0 to 0)	0 (0 to 0)	0 (0 to 0)	10·1 (5·5 to 16·8)	52·3 (33·2 to 80·8)	126 (84·5 to 181)	181 (122 to 254)	267 (180 to 376)	331 (220 to 456)	424 (297 to 578)	484 (342 to 660)	602 (425 to 838)	685 (483 to 957)	765 (527 to 1,060)	766 (527 to 1,080)	631 (436 to 882)	515 (357 to 718)	341 (231 to 481)	227 (160 to 318)	144 (105 to 196)	55·9 (41·5 to 75·8)	12·1 (8·9 to 16·6)	6,620
Rheumatoid arthritis	0 (0 to 0)	0 (0 to 0)	0 (0 to 0)	0 (0 to 0)	0·2 (0·1 to 0·4)	0·6 (0·3 to 1)	1·8 (1·1 to 2·8)	3·3 (1·7 to 5·5)	5·2 (2·9 to 8·4)	7·2 (4 to 11·1)	10·4 (6·5 to 15·7)	13 (7·9 to 19·2)	16·9 (11 to 24·2)	20 (13·5 to 28·1)	23·5 (16·5 to 32·5)	23·6 (16·7 to 32·2)	20·5 (14·5 to 27·5)	17·1 (12·2 to 23·6)	13·9 (10·1 to 19·3)	10·5 (7·6 to 14·4)	12·6 (8·6 to 18·2)	5·5 (3·8 to 8·3)	1·3 (0·8 to 2)	207
Osteoarthritis	0 (0 to 0)	0 (0 to 0)	0 (0 to 0)	0 (0 to 0)	0 (0 to 0)	0 (0 to 0)	0 (0 to 0)	0 (0 to 0)	0 (0 to 0)	1·6 (0·7 to 3·3)	10·7 (5·1 to 22·2)	29·3 (14·5 to 59·1)	63·4 (31·1 to 129)	103 (50·2 to 210)	145 (70·4 to 288)	169 (84·7 to 334)	154 (76·5 to 303)	134 (66·2 to 271)	92·8 (45·9 to 186)	63·2 (31·9 to 128)	35·8 (18·1 to 72·3)	14·3 (7·3 to 28·7)	3 (1·6 to 6)	1,020
Osteoarthritis hip	0 (0 to 0)	0 (0 to 0)	0 (0 to 0)	0 (0 to 0)	0 (0 to 0)	0 (0 to 0)	0 (0 to 0)	0 (0 to 0)	0 (0 to 0)	0·5 (0·2 to 1·2)	0·9 (0·3 to 2·1)	1·5 (0·6 to 3·4)	2·8 (1·2 to 6·3)	4·6 (1·9 to 10)	6·9 (3 to 15·3)	8·7 (3·8 to 18·9)	8·5 (3·8 to 17·7)	7·7 (3·6 to 15·7)	5·4 (2·5 to 11·2)	3·7 (1·7 to 7·8)	2·1 (1 to 4·4)	0·9 (0·4 to 1·8)	0·2 (0·1 to 0·4)	54·4
Osteoarthritis knee	0 (0 to 0)	0 (0 to 0)	0 (0 to 0)	0 (0 to 0)	0 (0 to 0)	0 (0 to 0)	0 (0 to 0)	0 (0 to 0)	0 (0 to 0)	0·8 (0·3 to 1·7)	7·9 (3·6 to 16·2)	22·1 (10·4 to 46·7)	47·6 (22·7 to 98·7)	76·8 (36 to 159)	106 (50·7 to 218)	122 (59·3 to 246)	110 (53·8 to 222)	94 (46·3 to 191)	63·8 (31·2 to 130)	42·1 (20·6 to 85)	22·7 (11·2 to 45·8)	8·5 (4·2 to 17·3)	1·7 (0·8 to 3·3)	725
Osteoarthritis hand	0 (0 to 0)	0 (0 to 0)	0 (0 to 0)	0 (0 to 0)	0 (0 to 0)	0 (0 to 0)	0 (0 to 0)	0 (0 to 0)	0 (0 to 0)	0·1 (0 to 0·4)	1·1 (0·3 to 2·7)	3 (1·1 to 6·9)	6·9 (2·9 to 15·1)	11·5 (5·1 to 25·4)	16·5 (7·8 to 36·9)	19·1 (9·1 to 41·2)	17·5 (8·3 to 36·6)	15·5 (7·2 to 32·3)	11·3 (5·5 to 23·3)	8·2 (4·1 to 17·1)	5·2 (2·5 to 10·6)	2·4 (1·2 to 4·8)	0·6 (0·3 to 1·2)	119
Osteoarthritis other	0 (0 to 0)	0 (0 to 0)	0 (0 to 0)	0 (0 to 0)	0 (0 to 0)	0 (0 to 0)	0 (0 to 0)	0 (0 to 0)	0 (0 to 0)	0·1 (0 to 0·3)	0·8 (0·2 to 2)	2·6 (1 to 6)	6·1 (2·6 to 13·8)	10·4 (4·6 to 22·3)	15·4 (7 to 33·9)	18·9 (9 to 39·9)	18 (8·4 to 37·3)	16·4 (8·1 to 33·5)	12·3 (6·1 to 24·8)	9·2 (4·7 to 19)	5·8 (3 to 11·7)	2·6 (1·3 to 5·2)	0·6 (0·3 to 1·2)	119
Low back pain	0 (0 to 0)	0 (0 to 0)	0 (0 to 0)	0 (0 to 0)	8·3 (4 to 15·1)	44·5 (26·6 to 72)	86·7 (50·7 to 136)	103 (60·8 to 165)	133 (77·1 to 208)	151 (83·9 to 233)	189 (111 to 287)	206 (122 to 318)	245 (149 to 384)	272 (164 to 424)	294 (182 to 443)	289 (180 to 427)	237 (149 to 365)	196 (124 to 293)	135 (83·1 to 200)	95·3 (59·4 to 142)	50·8 (31·2 to 73·8)	18·3 (11·5 to 27·1)	3·8 (2·3 to 5·7)	2,760
Neck pain	0 (0 to 0)	0 (0 to 0)	0 (0 to 0)	0 (0 to 0)	0·5 (0·1 to 1·2)	3 (1·4 to 5·9)	7·4 (3·1 to 13·5)	11 (4·9 to 21·4)	18·9 (8·4 to 37·6)	29·1 (14 to 55·6)	42·5 (22·1 to 73·2)	51·8 (26·3 to 93·1)	64·2 (31·7 to 117)	61·8 (31 to 115)	62·3 (30·4 to 111)	57 (29·4 to 98·5)	43·7 (23·3 to 74)	34·1 (18·2 to 58·1)	21·3 (10·6 to 39·1)	12·8 (6·4 to 23·2)	6·8 (3·5 to 11·7)	2·7 (1·4 to 4·6)	0·6 (0·3 to 1·1)	531
Gout	0 (0 to 0)	0 (0 to 0)	0 (0 to 0)	0 (0 to 0)	0 (0 to 0)	0 (0 to 0)	0 (0 to 0·1)	0·2 (0·1 to 0·4)	0·7 (0·2 to 1·3)	1·2 (0·4 to 2·3)	1·8 (0·8 to 3·3)	2·4 (1 to 4·2)	3·1 (1·5 to 5·4)	3·5 (1·6 to 6·5)	4 (1·7 to 7·1)	4·2 (2 to 7·5)	3·9 (1·9 to 6·6)	3·5 (1·8 to 5·7)	2·6 (1·4 to 4·3)	2 (1 to 3·4)	1·3 (0·7 to 2·1)	0·6 (0·3 to 1)	0·1 (0·1 to 0·2)	35·2
Other musculoskeletal disorders	0 (0 to 0)	0 (0 to 0)	0 (0 to 0)	0 (0 to 0)	1·1 (0·7 to 1·7)	4·3 (2·4 to 7)	30·3 (19·7 to 44·7)	62·7 (43·2 to 87·8)	109 (72·3 to 158)	141 (94 to 204)	170 (114 to 244)	181 (118 to 264)	209 (139 to 303)	225 (145 to 331)	237 (155 to 345)	224 (139 to 335)	172 (109 to 257)	131 (79·9 to 201)	75·4 (47·6 to 112)	43·6 (24·5 to 70)	36·6 (25·4 to 50·2)	14·4 (9·4 to 20·9)	3·3 (1·9 to 5·3)	2,070
Other non-communicable diseases	284 (178 to 426)	119 (78·1 to 174)	261 (178 to 372)	107 (80·2 to 140)	98·7 (77·7 to 125)	104 (79·9 to 132)	155 (121 to 202)	198 (150 to 268)	275 (205 to 379)	325 (240 to 441)	388 (285 to 523)	399 (291 to 543)	433 (317 to 574)	447 (329 to 592)	497 (377 to 648)	544 (416 to 702)	489 (377 to 627)	474 (366 to 592)	376 (296 to 459)	290 (225 to 357)	210 (168 to 256)	94 (73·7 to 115)	21·6 (16·5 to 26·2)	6,590

Congenital birth defects	274 (172 to 414)	106 (68·2 to 158)	176 (116 to 256)	59·4 (41·5 to 81·9)	40·3 (31·6 to 51·8)	37·9 (28·6 to 47·9)	44·1 (33·5 to 55·8)	32·7 (25·3 to 42·6)	30·8 (23·6 to 39·6)	26·8 (20·5 to 34·2)	25·8 (20·1 to 33·1)	22·3 (17·3 to 28·6)	23·1 (17·5 to 29·1)	22·6 (17·5 to 29·1)	22·1 (16·9 to 28)	16·2 (12·4 to 21)	11·1 (8·6 to 14·2)	3·9 (2·5 to 5·6)	2·2 (1·4 to 3·2)	1·3 (0·8 to 2)	0·6 (0·4 to 1)	0·2 (0·1 to 0·4)	0 (0 to 0·1)	980
Neural tube defects	8·1 (4·2 to 14·8)	2·4 (1·2 to 4·1)	4·3 (2 to 8·4)	2·2 (1·2 to 3·9)	0·9 (0·6 to 1·2)	1·2 (0·8 to 1·7)	1·2 (0·9 to 1·7)	0·7 (0·5 to 1)	0·8 (0·5 to 1)	0·6 (0·4 to 0·8)	0·5 (0·4 to 0·7)	0·4 (0·3 to 0·6)	0·4 (0·3 to 0·5)	0·3 (0·2 to 0·4)	0·3 (0·2 to 0·4)	0·2 (0·2 to 0·3)	0·2 (0·1 to 0·2)	0·1 (0 to 0·1)	0 (0 to 0·1)	0 (0 to 0)	0 (0 to 0)	0 (0 to 0)	0 (0 to 0)	25
Congenital heart anomalies	110 (62·6 to 176)	52·3 (32 to 79·4)	89 (54·8 to 135)	24·3 (14·3 to 38·5)	14·2 (9·7 to 19·9)	11·8 (7·5 to 16·5)	15·1 (10·4 to 20·8)	10·4 (7·2 to 14·7)	9·8 (7 to 13·5)	8·3 (5·7 to 11·7)	7·9 (5·5 to 10·9)	6·3 (4·5 to 8·7)	5·6 (3·9 to 7·7)	5 (3·5 to 7)	4 (2·7 to 5·5)	3·2 (2·2 to 4·3)	2·3 (1·7 to 3·1)	0·3 (0·1 to 0·6)	0·2 (0·1 to 0·4)	0·1 (0 to 0·3)	0·1 (0 to 0·1)	0 (0 to 0·1)	0 (0 to 0)	381
Orofacial clefts	0·2 (0 to 1)	0 (0 to 0·2)	0·3 (0·1 to 0·5)	0·5 (0·2 to 0·9)	0·5 (0·2 to 0·9)	0·6 (0·2 to 1)	0·6 (0·2 to 1)	0·5 (0·2 to 1)	0·6 (0·2 to 1·1)	0·5 (0·2 to 1)	0·6 (0·2 to 1)	0·5 (0·2 to 1)	0·6 (0·2 to 1·1)	0·6 (0·2 to 1)	0·6 (0·2 to 1)	0·5 (0·2 to 0·9)	0·4 (0·2 to 0·7)	0·3 (0·1 to 0·5)	0·2 (0·1 to 0·4)	0·1 (0·1 to 0·2)	0·1 (0 to 0·1)	0 (0 to 0·1)	0 (0 to 0)	8·7
Down syndrome	4·1 (2·3 to 6·8)	2·1 (1·1 to 3·5)	8·6 (4·6 to 14·1)	4·5 (2·5 to 7)	2·2 (1·4 to 3·4)	2·4 (1·7 to 3·6)	3·7 (2·4 to 5·5)	3·2 (2·1 to 4·8)	3 (2 to 4·6)	3·5 (2·3 to 5·1)	4 (2·6 to 5·9)	3·9 (2·6 to 5·6)	4·8 (3·1 to 6·8)	5·5 (3·6 to 7·9)	6·6 (4·3 to 9·3)	3·6 (2·4 to 5·2)	1·6 (1 to 2·3)	0 (0 to 0)	0 (0 to 0)	0 (0 to 0)	0 (0 to 0)	0 (0 to 0)	0 (0 to 0)	67·4
Turner syndrome	0 (0 to 0)	0 (0 to 0)	0 (0 to 0)	0 (0 to 0)	0 (0 to 0)	0 (0 to 0)	0 (0 to 0·1)	0 (0 to 0·1)	0 (0 to 0·1)	0 (0 to 0)	0 (0 to 0)	0 (0 to 0)	0 (0 to 0)	0 (0 to 0)	0 (0 to 0)	0 (0 to 0)	0 (0 to 0)	0 (0 to 0)	0 (0 to 0)	0 (0 to 0)	0 (0 to 0)	0 (0 to 0)	0 (0 to 0)	0·3
Klinefelter syndrome	0 (0 to 0)	0 (0 to 0)	0 (0 to 0)	0 (0 to 0)	0 (0 to 0)	0 (0 to 0)	0 (0 to 0)	0 (0 to 0)	0 (0 to 0)	0 (0 to 0)	0 (0 to 0)	0 (0 to 0)	0 (0 to 0)	0 (0 to 0)	0 (0 to 0)	0 (0 to 0)	0 (0 to 0)	0 (0 to 0)	0 (0 to 0)	0 (0 to 0)	0 (0 to 0)	0 (0 to 0)	0 (0 to 0)	0
Other chromosomal abnormalities	27·3 (15·4 to 44)	11·6 (7 to 19)	19·6 (11·6 to 31·3)	4·4 (2·9 to 6·8)	2·9 (2 to 4)	2·7 (1·8 to 3·8)	3·8 (2·5 to 5·5)	2·4 (1·6 to 3·5)	2·6 (1·7 to 3·8)	1·5 (1 to 2·2)	1·3 (0·8 to 1·9)	0·7 (0·5 to 1)	0·6 (0·4 to 0·9)	0·5 (0·3 to 0·7)	0·4 (0·2 to 0·5)	0·2 (0·1 to 0·3)	0·1 (0·1 to 0·2)	0 (0 to 0)	0 (0 to 0)	0 (0 to 0)	0 (0 to 0)	0 (0 to 0)	0 (0 to 0)	82·4
Congenital musculoskeletal and limb anomalies	9·1 (5 to 15·7)	2·8 (1·6 to 4·5)	7·2 (4·5 to 10·7)	8 (4·9 to 12·2)	9·3 (5·8 to 14·2)	10 (6·1 to 15·2)	9·6 (5·7 to 14·7)	6·9 (4·2 to 10·5)	6·9 (4·4 to 10·2)	6·1 (3·6 to 9·1)	6·6 (4 to 9·9)	6·2 (3·8 to 9·2)	6·6 (4·1 to 10)	6·7 (4·2 to 10·2)	6·7 (4·3 to 9·9)	5·7 (3·5 to 8·6)	4·1 (2·5 to 6·1)	2·5 (1·4 to 3·9)	1·3 (0·7 to 2·1)	0·7 (0·4 to 1·1)	0·3 (0·2 to 0·5)	0·1 (0·1 to 0·2)	0 (0 to 0)	123
Urogenital congenital anomalies	17·4 (9·3 to 29·4)	6·4 (3·4 to 11·2)	4·9 (2·8 to 8·2)	2 (1·3 to 2·9)	1·5 (1 to 2·2)	1·8 (1·1 to 2·8)	1·4 (0·8 to 2·4)	0·9 (0·5 to 1·5)	0·9 (0·5 to 1·5)	0·6 (0·4 to 1)	0·5 (0·3 to 0·9)	0·3 (0·2 to 0·4)	0·7 (0·4 to 1·1)	0·3 (0·2 to 0·4)	0·3 (0·2 to 0·4)	0·3 (0·2 to 0·5)	0·4 (0·2 to 0·6)	0 (0 to 0)	0 (0 to 0)	0 (0 to 0)	0 (0 to 0)	0 (0 to 0)	0 (0 to 0)	40·6
Digestive congenital anomalies	38·6 (22·3 to 60·3)	15·5 (9·2 to 25)	17·5 (10·3 to 27·6)	3·9 (2·5 to 6·2)	1·5 (1 to 2·3)	0·8 (0·6 to 1·2)	0·6 (0·4 to 0·9)	0·3 (0·2 to 0·4)	0·3 (0·2 to 0·4)	0·2 (0·2 to 0·3)	0·2 (0·1 to 0·2)	0·2 (0·1 to 0·2)	0·2 (0·2 to 0·3)	0·2 (0·1 to 0·3)	0·2 (0·1 to 0·3)	0·3 (0·2 to 0·4)	0·2 (0·1 to 0·3)	0 (0 to 0)	0 (0 to 0)	0 (0 to 0)	0 (0 to 0)	0 (0 to 0)	0 (0 to 0)	80·8
Other congenital birth defects	59·4 (34·1 to 94·4)	12·9 (7·8 to 20·1)	24·7 (14·4 to 39)	9·6 (5·9 to 14·5)	7·3 (5 to 10·3)	6·6 (4·6 to 9·3)	8·1 (5·3 to 11·2)	7·3 (4·8 to 10·6)	5·8 (3·8 to 8·9)	5·4 (3·4 to 8·1)	4·2 (2·8 to 6·4)	3·6 (2·4 to 5·7)	3·6 (2·3 to 5·7)	3·7 (2·4 to 5·8)	3·2 (2 to 5·3)	2·2 (1·3 to 3·9)	1·9 (1·2 to 3·4)	0·6 (0·2 to 1·6)	0·4 (0·1 to 1·1)	0·3 (0·1 to 0·7)	0·2 (0 to 0·4)	0·1 (0 to 0·2)	0 (0 to 0)	171
Urinary diseases and male infertility	0·9 (0·4 to 1·4)	1·4 (0·8 to 2·2)	5·9 (3·4 to 9·5)	2·6 (1·8 to 3·6)	2·4 (1·8 to 3·1)	3·6 (2·7 to 4·7)	7 (5·4 to 9·1)	11·8 (8·4 to 15·8)	14·5 (10·5 to 19·9)	20·8 (14·8 to 28·1)	28·8 (20·1 to 38·8)	32 (22·9 to 43·3)	40·6 (28·3 to 55·2)	57·2 (40·9 to 78·7)	90·8 (63·8 to 119)	131 (93·3 to 171)	137 (100 to 178)	165 (114 to 216)	152 (104 to 201)	135 (86·4 to 182)	104 (67·7 to 136)	47·9 (31 to 64)	10·7 (7·1 to 14·3)	1,200
Urolithiasis	0 (0 to 0)	0 (0 to 0)	0 (0 to 0)	0 (0 to 0)	0 (0 to 0·1)	0·1 (0·1 to 0·2)	0·1 (0·1 to 0·2)	0·3 (0·2 to 0·5)	0·5 (0·3 to 0·9)	1 (0·6 to 1·6)	1·8 (1·1 to 3)	1·8 (1 to 2·9)	3·4 (2 to 5·3)	3·5 (2·1 to 5·3)	5 (3 to 7·5)	5 (3 to 7·4)	3·8 (2·2 to 5·6)	3 (1·8 to 4·7)	2·1 (1·1 to 3·5)	1 (0·6 to 1·6)	0·6 (0·3 to 1)	0·2 (0·1 to 0·4)	0 (0 to 0·1)	33·5
Benign prostatic hyperplasia	0 (0 to 0)	0 (0 to 0)	0 (0 to 0)	0 (0 to 0)	0 (0 to 0)	0 (0 to 0)	0 (0 to 0)	0 (0 to 0)	0 (0 to 0)	0 (0 to 0)	0 (0 to 0)	0·1 (0 to 0·2)	0·8 (0·3 to 1·7)	3·5 (1·7 to 6·3)	11·9 (5·8 to 20·5)	22·9 (11·9 to 39·5)	29·3 (16·4 to 46·4)	29 (15·8 to 47·2)	18·6 (10·2 to 30)	10·5 (5·9 to 17·2)	4·5 (2·5 to 7·3)	1·3 (0·7 to 2·1)	0·2 (0·1 to 0·2)	133
Male infertility	0 (0 to 0)	0 (0 to 0)	0 (0 to 0)	0 (0 to 0)	0 (0 to 0)	0 (0 to 0)	0 (0 to 0)	1·7 (0·5 to 4·6)	2·3 (0·6 to 5·6)	2·4 (0·7 to 6·5)	2·5 (0·7 to 6·4)	2·3 (0·7 to 6)	0·4 (0·1 to 1·1)	0 (0 to 0)	0 (0 to 0)	0 (0 to 0)	0 (0 to 0)	0 (0 to 0)	0 (0 to 0)	0 (0 to 0)	0 (0 to 0)	0 (0 to 0)	0 (0 to 0)	11·7
Other urinary diseases	0·2 (0·1 to 0·3)	0·5 (0·2 to 0·8)	0·2 (0·1 to 0·4)	0·4 (0·3 to 0·7)	0·4 (0·3 to 0·6)	0·6 (0·4 to 0·9)	0·9 (0·6 to 1·4)	1·2 (0·6 to 2)	0·8 (0·5 to 1·3)	1·4 (0·9 to 2·1)	2·2 (1·2 to 3·5)	2 (1·2 to 3·1)	3·2 (1·7 to 5·2)	3·1 (1·8 to 5·4)	4·1 (2·1 to 6·8)	6·2 (2·9 to 10·8)	5·9 (2·6 to 10·2)	5·1 (2·2 to 9·5)	5·1 (1·9 to 9·2)	4·3 (1·6 to 8·1)	2·6 (1 to 5)	1·5 (0·6 to 2·9)	0·2 (0·1 to 0·4)	52
Gynecological diseases	0 (0 to 0)	0 (0 to 0)	0 (0 to 0)	0 (0 to 0)	0 (0 to 0)	12·6 (6 to 22·2)	35·6 (19·8 to 59·8)	73·3 (45·6 to 114)	120 (76·7 to 178)	141 (90·9 to 203)	167 (111 to 242)	166 (108 to 241)	153 (99·2 to 219)	111 (65·4 to 167)	73·1 (45 to 106)	51·6 (29·6 to 79·5)	36·3 (21·9 to 53·8)	26·6 (16·6 to 38·5)	16·3 (10·3 to 24·2)	10·2 (6·1 to 15·4)	6 (3·8 to 8·9)	2·6 (1·6 to 4)	0·7 (0·4 to 1·1)	1,200
Uterine fibroids	0 (0 to 0)	0 (0 to 0)	0 (0 to 0)	0 (0 to 0)	0 (0 to 0)	0·2 (0·1 to 0·5)	0·6 (0·3 to 1·2)	1·4 (0·6 to 2·8)	3·7 (1·5 to 7·6)	7·7 (3·5 to 16·1)	14·5 (7 to 28)	16·5 (8·2 to 29·9)	16·8 (8·8 to 30·4)	12·5 (6·2 to 23·7)	9·2 (4·5 to 17·1)	5·8 (2·6 to 10·9)	3·3 (1·5 to 6·3)	1·9 (0·9 to 3·7)	1 (0·5 to 1·9)	0·5 (0·3 to 0·9)	0·2 (0·1 to 0·4)	0·1 (0·1 to 0·2)	0 (0 to 0)	95·9
Polycystic ovarian syndrome	0 (0 to 0)	0 (0 to 0)	0 (0 to 0)	0 (0 to 0)	0 (0 to 0)	0·5 (0·2 to 1·1)	2·6 (1·1 to 5·3)	3 (1·2 to 6·3)	3 (1·3 to 6·3)	2·8 (1·2 to 5·9)	2·9 (1·2 to 6)	2·8 (1·1 to 5·5)	2·6 (1·1 to 5·2)	0·7 (0·3 to 1·5)	0 (0 to 0)	0 (0 to 0)	0 (0 to 0)	0 (0 to 0)	0 (0 to 0)	0 (0 to 0)	0 (0 to 0)	0 (0 to 0)	0 (0 to 0)	21
Female infertility	0 (0 to 0)	0 (0 to 0)	0 (0 to 0)	0 (0 to 0)	0 (0 to 0)	0 (0 to 0)	0 (0 to 0)	2·5 (0·5 to 7)	4·2 (1 to 11·2)	5 (1·1 to 14·7)	5·8 (0·8 to 17·3)	5·2 (0·6 to 14·6)	0·1 (0 to 0·8)	0 (0 to 0)	0 (0 to 0)	0 (0 to 0)	0 (0 to 0)	0 (0 to 0)	0 (0 to 0)	0 (0 to 0)	0 (0 to 0)	0 (0 to 0)	0 (0 to 0)	22·8
Endometriosis	0 (0 to 0)	0 (0 to 0)	0 (0 to 0)	0 (0 to 0)	0 (0 to 0)	0 (0 to 0)	2·7 (1·1 to 5·8)	10·9 (5·3 to 19·8)	13·7 (6·6 to 25·2)	10·9 (5·3 to 19·8)	10·3 (5·5 to 18·2)	9·8 (5 to 17)	9·5 (5·1 to 15·9)	4·9 (2·4 to 8·2)	0 (0 to 0)	0 (0 to 0)	0 (0 to 0)	0 (0 to 0)	0 (0 to 0)	0 (0 to 0)	0 (0 to 0)	0 (0 to 0)	0 (0 to 0)	72·6
Genital prolapse	0 (0 to 0)	0 (0 to 0)	0 (0 to 0)	0 (0 to 0)	0 (0 to 0)	0 (0 to 0)	0 (0 to 0)	0·1 (0 to 0·2)	0·3 (0·1 to 0·5)	0·5 (0·2 to 1·1)	1·1 (0·4 to 2·2)	1·6 (0·6 to 3·6)	2·5 (1·1 to 5)	3·1 (1·3 to 6)	3·5 (1·6 to 7)	3·4 (1·6 to 6·5)	2·9 (1·5 to 5·3)	2·3 (1·2 to 4·3)	1·6 (0·8 to 2·9)	1·1 (0·5 to 2)	0·7 (0·3 to 1·2)	0·3 (0·2 to 0·6)	0·1 (0 to 0·2)	25
Premenstrual syndrome	0 (0 to 0)	0 (0 to 0)	0 (0 to 0)	0 (0 to 0)	0 (0 to 0)	11·4 (5·1 to 20·6)	28·6 (14·1 to 51·7)	35·6 (17·6 to 63·6)	39·2 (22·1 to 64·7)	38·3 (19·7 to 63·3)	45·8 (26·2 to 72·5)	44·4 (25 to 70·2)	34·2 (18·7 to 56·7)	12·3 (6 to 22·3)	0 (0 to 0)	0 (0 to 0)	0 (0 to 0)	0 (0 to 0)	0 (0 to 0)	0 (0 to 0)	0 (0 to 0)	0 (0 to 0)	0 (0 to 0)	290
Other gynecological diseases	0 (0 to 0)	0 (0 to 0)	0 (0 to 0)	0 (0 to 0)	0 (0 to 0)	0·5 (0·1 to 1)	1·1 (0·5 to 1·9)	19·8 (11·4 to 32·2)	56·1 (31·1 to 90·9)	76·1 (44·4 to 118)	87·1 (51·6 to 137)	85·8 (48·6 to 137)	87·8 (50·1 to 137)	77·7 (37·1 to 124)	60·4 (35·4 to 91·2)	42·5 (22·7 to 68·1)	30·2 (17·6 to 46·5)	22·4 (13·5 to 33·5)	13·7 (8·2 to 21·2)	8·6 (4·8 to 13·6)	5·1 (3·1 to 7·8)	2·2 (1·2 to 3·5)	0·6 (0·3 to 1)	678
Hemoglobinopathies and hemolytic anemias	0·6 (0·4 to 0·9)	0·7 (0·4 to 1)	4 (2·5 to 6·2)	9 (5·7 to 13·8)	21·9 (15·5 to 30·7)	18 (12·5 to 24·9)	23·9 (17·4 to 31·7)	22·7 (15·5 to 33)	21·8 (14·5 to 33·6)	20·5 (14·1 to 30·2)	19·9 (13·7 to 28·1)	17·8 (12·4 to 24·8)	17·1 (11·8 to 23·9)	17·1 (12 to 24)	18 (12·7 to 24·7)	17·6 (12·5 to 25·2)	13·4 (9·3 to 18·5)	12·6 (9·1 to 17)	9·6 (7 to 12·8)	7·2 (5·2 to 9·4)	7·9 (5·9 to 10·3)	3·7 (2·8 to 4·9)	0·9 (0·6 to 1·2)	306
Thalassemias	0 (0 to 0)	0 (0 to 0)	0 (0 to 0)	0·1 (0 to 0·1)	0 (0 to 0·1)	0 (0 to 0)	0 (0 to 0)	0 (0 to 0)	0 (0 to 0)	0 (0 to 0)	0 (0 to 0)	0 (0 to 0)	0 (0 to 0)	0 (0 to 0)	0 (0 to 0)	0 (0 to 0)	0 (0 to 0)	0 (0 to 0)	0 (0 to 0)	0 (0 to 0)	0 (0 to 0)	0 (0 to 0)	0 (0 to 0)	0·2
Thalassemias trait	0 (0 to 0)	0 (0 to 0)	0·1 (0 to 0·1)	0·3 (0·1 to 0·6)	0·7 (0·3 to 1·2)	0·4 (0·2 to 0·9)	0·7 (0·3 to 1·2)	0·7 (0·3 to 1·4)	0·8 (0·3 to 1·5)	0·7 (0·3 to 1·4)	0·8 (0·3 to 1·4)	0·4 (0·1 to 1)	0·2 (0·1 to 0·5)	0·2 (0·1 to 0·6)	0·3 (0·1 to 0·6)	0·2 (0·1 to 0·5)	0·2 (0·1 to 0·4)	0·2 (0 to 0·4)	0·1 (0 to 0·3)	0·1 (0 to 0·2)	0·1 (0 to 0·2)	0 (0 to 0·1)	0 (0 to 0)	7·2
Sickle cell disorders	0·6 (0·4 to 0·9)	0·4 (0·3 to 0·7)	3·1 (1·8 to 5·2)	5 (3·2 to 7·8)	9·5 (6·8 to 13)	10·6 (7·2 to 15)	17·9 (13 to 24·6)	14·5 (9·9 to 20·7)	12·1 (8 to 17·6)	9·9 (6·6 to 14·2)	10·1 (6·3 to 15·4)	8·5 (5·2 to 12·7)	8·2 (5·2 to 12·3)	6·7 (4 to 10·5)	4 (2·2 to 6·3)	1·6 (0·8 to 2·9)	0·3 (0·1 to 0·6)	0·1 (0 to 0·1)	0 (0 to 0)	0 (0 to 0)	0 (0 to 0)	0 (0 to 0)	0 (0 to 0)	123
Sickle cell trait	0 (0 to 0)	0·2 (0·1 to 0·3)	0·5 (0·2 to 1·1)	2·3 (0·8 to 4·8)	6·1 (3·1 to 10·2)	3·7 (1·7 to 6·7)	1 (0·3 to 2·1)	3·6 (0·6 to 10·6)	3·9 (0·6 to 13·3)	4·5 (1·1 to 11·5)	1·4 (0·5 to 2·7)	1·9 (0·7 to 3·8)	1·9 (0·6 to 4·2)	2·1 (0·7 to 4·4)	2·5 (0·9 to 4·9)	1·8 (0·6 to 3·7)	1·5 (0·5 to 3·2)	1·5 (0·6 to 3)	1·2 (0·5 to 2·3)	1 (0·4 to 1·8)	0·8 (0·4 to 1·4)	0·3 (0·1 to 0·5)	0·1 (0 to 0·1)	43·6
G6PD deficiency	0 (0 to 0)	0 (0 to 0·1)	0 (0 to 0·1)	0·4 (0·2 to 0·8)	0·7 (0·3 to 1·4)	0·7 (0·3 to 1·4)	2·4 (1·1 to 4·1)	2·4 (0·9 to 4·5)	3·2 (1·5 to 5·8)	3·5 (1·6 to 6·3)	4·5 (2·4 to 7·1)	3·8 (2 to 6·2)	3·7 (1·8 to 6)	3·8 (2·1 to 5·9)	4·8 (2·9 to 7·3)	4·5 (2·6 to 6·8)	2·6 (1·5 to 3·9)	1·8 (1 to 2·8)	0·9 (0·4 to 1·4)	0·4 (0·2 to 0·6)	0·2 (0·1 to 0·4)	0·1 (0 to 0·1)	0 (0 to 0)	44·6
G6PD trait	0 (0 to 0)	0 (0 to 0)	0 (0 to 0)	0 (0 to 0)	0 (0 to 0)	0 (0 to 0)	0·2 (0 to 0·3)	0 (0 to 0)	0 (0 to 0)	0 (0 to 0)	0·2 (0·1 to 0·4)	0·2 (0 to 0·4)	0 (0 to 0)	0 (0 to 0)	0 (0 to 0)	0 (0 to 0)	0 (0 to 0)	0 (0 to 0)	0 (0 to 0)	0 (0 to 0)	0 (0 to 0)	0 (0 to 0)	0 (0 to 0)	0·5
Other hemoglobinopathies and hemolytic anemias	0 (0 to 0)	0 (0 to 0·1)	0·2 (0·1 to 0·5)	1 (0·3 to 2·1)	4·9 (2·5 to 8·4)	2·5 (1·1 to 4·8)	1·7 (0·6 to 3·6)	1·4 (0·6 to 2·8)	1·8 (0·8 to 3·6)	1·8 (0·8 to 3·6)	3 (1·5 to 5·4)	3 (1·6 to 5·2)	3 (1·7 to 5·1)	4·2 (2·5 to 6·4)	6·5 (4·3 to 9·6)	9·6 (6·4 to 14·3)	8·8 (6 to 12·2)	9·1 (6·4 to 12·1)	7·4 (5·3 to 9·8)	5·7 (4·2 to 7·3)	6·8 (5·1 to 8·9)	3·4 (2·5 to 4·4)	0·8 (0·5 to 1)	86·5
Endocrine, metabolic, blood, and immune disorders	7·7 (4·5 to 12·7)	7·3 (4·2 to 12)	56·9 (32 to 96·6)	28·4 (17·9 to 50·8)	24·6 (17·1 to 36·9)	20·4 (14·2 to 29·5)	26·3 (18·6 to 36·3)	29·5 (21·1 to 42·8)	44·4 (32 to 62·9)	60·8 (43·7 to 87)	77·2 (56·2 to 109)	84 (59·6 to 120)	104 (72·5 to 146)	126 (86·3 to 180)	154 (107 to 223)	169 (118 to 244)	142 (101 to 209)	133 (94·3 to 190)	102 (71·8 to 142)	72·5 (53·4 to 101)	55·5 (40·8 to 76·3)	24·6 (18 to 34·4)	6·1 (4·3 to 8·6)	1,550
Oral disorders	0 (0 to 0)	0 (0 to 0)	0 (0 to 0)	7·2 (4 to 11·7)	9·4 (5·1 to 16·3)	11·4 (6·1 to 20·1)	18·1 (9·9 to 31)	27·9 (14·7 to 50·9)	43·5 (21·9 to 84)	54·4 (26·3 to 103)	68·7 (32·4 to 128)	76·7 (36·3 to 139)	95·4 (48·9 to 170)	113 (61·6 to 199)	139 (79 to 221)	159 (96·1 to 254)	149 (89·1 to 232)	134 (82·9 to 205)	93·7 (58·9 to 138)	63·9 (40·3 to 94·3)	36·4 (22·2 to 53·9)	14·9 (9·2 to 21·7)	3·2 (2 to 4·8)	1,320
Caries of deciduous teeth	0 (0 to 0)	0 (0 to 0)	0 (0 to 0)	2·4 (0·9 to 4·8)	2·5 (1 to 5·3)	0·2 (0 to 0·4)	0 (0 to 0)	0 (0 to 0)	0 (0 to 0)	0 (0 to 0)	0 (0 to 0)	0 (0 to 0)	0 (0 to 0)	0 (0 to 0)	0 (0 to 0)	0 (0 to 0)	0 (0 to 0)	0 (0 to 0)	0 (0 to 0)	0 (0 to 0)	0 (0 to 0)	0 (0 to 0)	0 (0 to 0)	5
Caries of permanent teeth	0 (0 to 0)	0 (0 to 0)	0 (0 to 0)	0 (0 to 0)	2·2 (0·8 to 4·8)	5·4 (2·2 to 12·1)	6·3 (2·5 to 13·4)	6·9 (2·9 to 14·5)	7·1 (2·9 to 14·5)	6·4 (2·6 to 13·1)	6·8 (2·8 to 13·6)	6·2 (2·6 to 12·6)	6·4 (2·6 to 13·3)	6·3 (2·5 to 13·1)	5·8 (2·2 to 13·1)	4·7 (1·7 to 10·3)	3·2 (1·2 to 6·9)	2·4 (0·9 to 4·9)	1·5 (0·6 to 2·9)	0·9 (0·4 to 2)	0·5 (0·2 to 1)	0·2 (0·1 to 0·4)	0 (0 to 0·1)	79·2
Periodontal diseases	0 (0 to 0)	0 (0 to 0)	0 (0 to 0)	0 (0 to 0)	0 (0 to 0)	0 (0 to 0)	2·5 (0·7 to 6·7)	9·1 (2·8 to 22·8)	21·2 (6·7 to 51)	30·9 (10·6 to 68·8)	41·4 (14·7 to 93·3)	46·6 (17·2 to 100)	54·2 (20·7 to 114)	54·8 (21·4 to 115)	51·8 (20·5 to 108)	42·9 (17 to 86·6)	29·7 (11·6 to 60·9)	21·1 (8·2 to 42·4)	13 (5·2 to 25·6)	8·3 (3·2 to 16·5)	4·6 (1·8 to 9·5)	1·8 (0·7 to 3·8)	0·4 (0·1 to 0·8)	434
Other oral disorders	0 (0 to 0)	0 (0 to 0)	0 (0 to 0)	4·8 (2·8 to 7·5)	4·8 (2·8 to 7·5)	5·8 (3·4 to 9·1)	9·3 (5·7 to 14·4)	10·9 (6·6 to 16·7)	12·6 (7·6 to 19)	12·8 (7·7 to 19·2)	13·7 (8·3 to 20·8)	13·6 (8·1 to 20·4)	15 (9 to 22·7)	15·1 (9·2 to 23)	14·8 (9 to 22·3)	13·3 (8·2 to 20)	10·3 (6·2 to 15·3)	7·3 (4·5 to 11·1)	3·9 (2·4 to 5·8)	2·1 (1·3 to 3·1)	0·9 (0·6 to 1·4)	0·3 (0·2 to 0·5)	0·1 (0 to 0·1)	171
Sudden infant death syndrome	--	3·2 (1·9 to 5·1)	17·8 (9·9 to 29·2)	--	--	--	--	--	--	--	--	--	--	--	--	--	--	--	--	--	--	--	--	NA
Injuries	13·3 (8·6 to 19·6)	5·8 (3·8 to 8·4)	34·4 (22·8 to 51·2)	64·4 (46·9 to 86·1)	106 (87·9 to 128)	167 (138 to 198)	521 (430 to 633)	644 (551 to 773)	717 (618 to 853)	693 (594 to 815)	563 (470 to 672)	502 (413 to 612)	508 (409 to 614)	454 (367 to 547)	475 (387 to 582)	442 (361 to 530)	342 (280 to 410)	297 (248 to 356)	224 (188 to 266)	178 (150 to 211)	133 (112 to 154)	66·8 (56·1 to 77·3)	18·2 (15·2 to 21·3)	7,170
Transport injuries	0·6 (0·4 to 1)	0·4 (0·2 to 0·6)	3 (1·8 to 4·6)	10·1 (6·6 to 15·1)	25·5 (18·2 to 33·8)	41·1 (29·4 to 53·9)	137 (97·4 to 187)	162 (116 to 213)	176 (130 to 228)	170 (126 to 219)	144 (108 to 188)	133 (99·4 to 168)	140 (104 to 180)	127 (98 to 159)	134 (103 to 168)	120 (93·2 to 151)	89·7 (69·9 to 111)	70·3 (55·8 to 85·9)	46·9 (37·1 to 57·6)	29·4 (23·5 to 35·6)	16·4 (13 to 20·1)	6·3 (5 to 7·7)	1·4 (1·1 to 1·6)	1,780
Road injuries	0·6 (0·3 to 0·9)	0·3 (0·2 to 0·5)	2·7 (1·6 to 4·2)	9·3 (6 to 14·2)	24·1 (16·8 to 32·4)	38·8 (27·4 to 51)	133 (92·5 to 182)	156 (112 to 206)	170 (125 to 220)	164 (121 to 211)	138 (104 to 181)	127 (94·7 to 161)	134 (100 to 173)	121 (93·7 to 153)	128 (97·6 to 161)	115 (89·6 to 146)	86·6 (67·1 to 107)	67·4 (53·2 to 82·4)	45 (35·4 to 55·5)	28·4 (22·6 to 34·5)	15·8 (12·5 to 19·4)	6 (4·8 to 7·4)	1·3 (1 to 1·6)	1,710
Pedestrian road injuries	0·2 (0·1 to 0·3)	0·1 (0·1 to 0·1)	0·5 (0·3 to 0·8)	2·8 (1·8 to 4·5)	7·3 (5 to 10·1)	9·4 (6·6 to 12·9)	12·5 (8·8 to 17·3)	12·6 (9 to 16·9)	15·3 (11·4 to 20·5)	17·8 (13·2 to 23·3)	17·7 (13·2 to 23·5)	18·1 (13·3 to 23·1)	21·2 (15·5 to 27·9)	21·4 (16·3 to 28)	24·9 (18·7 to 32·6)	24·6 (18·8 to 31·5)	20·8 (15·9 to 26·2)	18·3 (14·2 to 23·1)	13·9 (10·7 to 17·7)	9·6 (7·5 to 11·8)	5·5 (4·3 to 6·9)	2 (1·6 to 2·4)	0·4 (0·3 to 0·5)	277
Cyclist road injuries	0 (0 to 0)	0 (0 to 0)	0 (0 to 0)	0·5 (0·3 to 0·7)	1·6 (1 to 2·5)	4·7 (3 to 7)	10 (6·6 to 14·6)	9·9 (7 to 14)	11·6 (8·3 to 15·5)	12·5 (9·1 to 16·8)	13·2 (9·3 to 17·8)	13·3 (9·8 to 17·8)	15·1 (11 to 20·1)	15·6 (11·3 to 20·4)	15·9 (11·5 to 21·3)	14·7 (10·5 to 19·5)	11·6 (8·3 to 15·1)	8·7 (6·3 to 11·4)	5·5 (4 to 7·2)	3·5 (2·5 to 4·7)	1·9 (1·3 to 2·6)	0·8 (0·5 to 1·1)	0·2 (0·1 to 0·2)	171
Motorcyclist road injuries	0 (0 to 0·1)	0 (0 to 0·1)	0·2 (0·1 to 0·3)	0·5 (0·3 to 0·9)	1·3 (0·8 to 2·2)	2·5 (1·4 to 4·2)	20·9 (11·7 to 33·6)	30·7 (17·7 to 48·2)	37·2 (23·4 to 57·4)	34·2 (22·1 to 51·7)	25·7 (17·1 to 37·7)	20·3 (14 to 28·9)	21·1 (14·9 to 29·1)	17·3 (12·6 to 23)	17·7 (12·5 to 24·1)	13·4 (9·8 to 18)	9·9 (7·3 to 13·1)	6·7 (4·9 to 9)	4·3 (3·2 to 5·8)	2·9 (2·1 to 4)	1·7 (1·2 to 2·4)	0·7 (0·5 to 1)	0·2 (0·1 to 0·2)	270
Motor vehicle road injuries	0·3 (0·2 to 0·5)	0·2 (0·1 to 0·3)	2 (1·2 to 3·1)	5·2 (3·3 to 7·8)	13·6 (9·1 to 18·5)	21·7 (14·3 to 29·5)	88·5 (59·6 to 123)	102 (69·9 to 140)	105 (73·9 to 143)	97·4 (65·7 to 131)	79·3 (56·6 to 108)	72·7 (51·7 to 94·8)	74·3 (51·6 to 99)	64·1 (47 to 84·3)	66·8 (48·3 to 88·1)	59·7 (44·2 to 78·1)	41·8 (30·8 to 53)	31·8 (23·8 to 39·9)	20 (15·1 to 25·3)	11·4 (8·7 to 14·1)	6·2 (4·8 to 7·8)	2·4 (1·9 to 2·9)	0·5 (0·4 to 0·6)	966
Other road injuries	0 (0 to 0)	0 (0 to 0)	0 (0 to 0·1)	0·2 (0·1 to 0·4)	0·3 (0·2 to 0·5)	0·4 (0·3 to 0·6)	0·8 (0·6 to 1·1)	1·1 (0·8 to 1·6)	1·5 (1·1 to 2)	1·7 (1·2 to 2·4)	2·1 (1·5 to 2·9)	2·4 (1·6 to 3·2)	2·8 (2 to 3·9)	3·1 (2·1 to 4·2)	3·2 (2·2 to 4·4)	3·1 (2·2 to 4·2)	2·5 (1·8 to 3·5)	1·9 (1·3 to 2·6)	1·3 (0·9 to 1·8)	0·8 (0·6 to 1·1)	0·4 (0·3 to 0·6)	0·2 (0·1 to 0·2)	0 (0 to 0)	30·1
Other transport injuries	0·1 (0 to 0·1)	0·1 (0 to 0·1)	0·3 (0·2 to 0·5)	0·8 (0·5 to 1·1)	1·4 (1 to 1·8)	2·4 (1·6 to 3·3)	4·6 (3 to 6·8)	5·4 (3·5 to 7·8)	6 (4 to 8·7)	6·4 (4·3 to 9·4)	5·6 (3·7 to 8·1)	5·8 (4 to 8·2)	5·4 (3·7 to 7·7)	5·1 (3·7 to 6·9)	5·9 (4·3 to 8·2)	4·5 (3·3 to 6)	3·1 (2·3 to 3·9)	3 (2·2 to 3·9)	1·9 (1·4 to 2·5)	1 (0·8 to 1·3)	0·6 (0·5 to 0·8)	0·3 (0·2 to 0·4)	0·1 (0·1 to 0·1)	69·7
Unintentional injuries	6·7 (4·3 to 9·8)	4·4 (2·8 to 6·3)	25·6 (16·6 to 39·9)	45·7 (32·9 to 62·2)	69·2 (54·6 to 85·6)	97·7 (77·7 to 121)	166 (133 to 208)	163 (130 to 206)	186 (150 to 231)	195 (156 to 240)	188 (148 to 239)	186 (147 to 234)	205 (159 to 261)	207 (161 to 264)	224 (176 to 285)	225 (178 to 282)	187 (148 to 237)	178 (146 to 218)	148 (122 to 178)	132 (111 to 157)	107 (89·7 to 125)	57·6 (47·9 to 66·6)	16·3 (13·6 to 19·1)	3,020
Falls	0·1 (0·1 to 0·2)	0·1 (0·1 to 0·1)	0·9 (0·6 to 1·3)	2·4 (1·7 to 3·4)	5·4 (3·8 to 7·7)	9·8 (6·9 to 13·8)	16·6 (12·1 to 22·4)	19·1 (14 to 26·2)	24 (17·7 to 32·9)	27·3 (20·4 to 37·2)	31·8 (23·4 to 43·2)	34·5 (25·5 to 46·4)	41·9 (31·3 to 55·7)	47·8 (36 to 63·4)	54·9 (41·6 to 72·2)	61 (47·1 to 78)	57 (44·6 to 71·5)	65·6 (52·7 to 79·8)	63 (51·7 to 77·4)	69·6 (57·4 to 84)	64·1 (52·4 to 76·7)	36·1 (29·5 to 42·6)	10·6 (8·7 to 12·6)	744
Drowning	0·7 (0·4 to 1·1)	0·3 (0·2 to 0·5)	3·7 (2·2 to 6)	17·3 (10·8 to 26·1)	27·7 (19·8 to 36·7)	39 (28 to 51·6)	71 (50·6 to 95·8)	52·3 (35·3 to 73·7)	53·8 (38·8 to 73·9)	50·7 (37·3 to 65·9)	37·7 (27·4 to 50·4)	31·9 (23·7 to 42·3)	29·4 (21·2 to 39)	23·8 (17·1 to 32·1)	23·5 (16·9 to 32)	19·2 (14·4 to 25·1)	12·4 (9·6 to 16·1)	8·1 (6·3 to 10·2)	5·3 (4·1 to 6·7)	2·5 (2 to 3·2)	1·8 (1·4 to 2·3)	1 (0·8 to 1·3)	0·3 (0·2 to 0·4)	513
Fire, heat, and hot substances	0·2 (0·2 to 0·4)	0·3 (0·2 to 0·4)	2·8 (1·8 to 4·4)	6·9 (4·5 to 10)	9·7 (7·4 to 12·5)	12·2 (8·8 to 16·5)	17·4 (12·6 to 23·3)	19·1 (13·7 to 26·2)	22·7 (16·7 to 30·9)	26·3 (18·8 to 35·6)	28 (19·9 to 39·2)	27·5 (19·7 to 37·8)	30·3 (21·9 to 42·2)	30·3 (21·4 to 42·1)	31·5 (22·6 to 44·5)	30·2 (21·8 to 41·7)	22·9 (16·5 to 31·6)	19·9 (14·7 to 27)	14·2 (10·6 to 18·8)	10·5 (8·2 to 13·9)	7·9 (6·3 to 10)	4 (3·2 to 4·8)	1 (0·8 to 1·3)	376
Poisonings	0·1 (0 to 0·1)	0·1 (0·1 to 0·2)	0·4 (0·3 to 0·7)	1·3 (0·8 to 2)	1·2 (0·9 to 1·8)	1·5 (1 to 2·2)	2·2 (1·6 to 3)	2·5 (1·8 to 3·3)	3·1 (2·3 to 4·2)	3·4 (2·5 to 4·3)	3 (2·2 to 4·1)	2·6 (1·8 to 3·4)	2·6 (1·9 to 3·5)	2·4 (1·7 to 3·2)	2·4 (1·7 to 3·2)	2·1 (1·5 to 2·9)	1·7 (1·2 to 2·3)	1·4 (1 to 1·8)	0·9 (0·7 to 1·2)	0·7 (0·5 to 0·9)	0·4 (0·3 to 0·5)	0·2 (0·1 to 0·2)	0 (0 to 0)	36·3
Poisoning by carbon monoxide	0 (0 to 0)	0 (0 to 0)	0 (0 to 0)	0·1 (0·1 to 0·2)	0·2 (0·1 to 0·3)	0·3 (0·2 to 0·4)	0·4 (0·3 to 0·6)	0·5 (0·3 to 0·7)	0·6 (0·4 to 0·9)	0·7 (0·4 to 1)	0·6 (0·4 to 0·9)	0·5 (0·3 to 0·7)	0·5 (0·3 to 0·7)	0·4 (0·2 to 0·6)	0·4 (0·3 to 0·6)	0·4 (0·2 to 0·6)	0·3 (0·2 to 0·5)	0·2 (0·2 to 0·4)	0·2 (0·1 to 0·2)	0·1 (0·1 to 0·2)	0·1 (0 to 0·1)	0 (0 to 0)	0 (0 to 0)	6·4
Poisoning by other means	0·1 (0 to 0·1)	0·1 (0·1 to 0·2)	0·4 (0·3 to 0·7)	1·2 (0·7 to 1·8)	1 (0·7 to 1·5)	1·3 (0·8 to 1·9)	1·8 (1·2 to 2·5)	2 (1·4 to 2·7)	2·5 (1·9 to 3·4)	2·7 (2 to 3·5)	2·5 (1·8 to 3·3)	2·1 (1·5 to 2·8)	2·2 (1·6 to 2·9)	2 (1·4 to 2·7)	2 (1·4 to 2·7)	1·8 (1·2 to 2·4)	1·4 (1 to 1·9)	1·1 (0·8 to 1·5)	0·8 (0·6 to 1)	0·6 (0·4 to 0·7)	0·3 (0·3 to 0·4)	0·1 (0·1 to 0·2)	0 (0 to 0)	29·9
Exposure to mechanical forces	0·1 (0·1 to 0·2)	0·2 (0·1 to 0·3)	1 (0·6 to 1·3)	4·3 (3 to 6·1)	8·9 (6·2 to 12·4)	13·5 (9·6 to 19·4)	27·7 (20·5 to 37·2)	34 (25·7 to 45·3)	38·9 (29·2 to 51·9)	41·1 (31·1 to 54·5)	40·5 (30·2 to 54·8)	41·1 (30·2 to 55·2)	44·4 (32·5 to 61·7)	44·6 (31·5 to 62·4)	46·3 (33·3 to 64·3)	42·6 (30·4 to 59·2)	33·1 (23·2 to 46·4)	26·4 (18·5 to 36·9)	17·6 (12·3 to 24·8)	12·3 (8·7 to 17·2)	7·7 (5·5 to 10·5)	3·9 (2·8 to 5·2)	1·1 (0·8 to 1·4)	531
Unintentional firearm injuries	0 (0 to 0·1)	0 (0 to 0)	0·2 (0·1 to 0·2)	0·5 (0·3 to 0·8)	1 (0·7 to 1·5)	2·6 (1·8 to 3·8)	12·1 (8·2 to 17·8)	16·2 (11·4 to 23·6)	17·1 (11·9 to 23·8)	16·8 (12 to 22·8)	13·3 (9·6 to 17·5)	12·2 (8·8 to 16·3)	11·4 (8·4 to 14·9)	9·7 (7·3 to 12·7)	9·8 (7·5 to 12·6)	7·9 (6 to 9·9)	5·5 (4·1 to 7·1)	4·1 (3·1 to 5·2)	2·5 (1·9 to 3·3)	1·9 (1·4 to 2·5)	1·4 (1 to 1·7)	0·8 (0·6 to 1)	0·3 (0·2 to 0·4)	147
Other exposure to mechanical forces	0·1 (0·1 to 0·2)	0·2 (0·1 to 0·2)	0·8 (0·5 to 1·1)	3·8 (2·6 to 5·4)	7·9 (5·4 to 11·2)	10·9 (7·5 to 16·1)	15·6 (10·6 to 22·8)	17·8 (12·3 to 25·8)	21·8 (15·2 to 31·4)	24·4 (16·9 to 35·3)	27·2 (18·2 to 39·8)	28·9 (20 to 41·7)	33 (22·6 to 48·3)	34·9 (23·8 to 50·6)	36·6 (25·1 to 52·3)	34·7 (23·8 to 49·9)	27·6 (18·3 to 39·9)	22·3 (15·1 to 31·9)	15·1 (10·3 to 21·7)	10·4 (7·1 to 14·7)	6·4 (4·5 to 8·9)	3·1 (2·2 to 4·2)	0·8 (0·6 to 1·1)	384
Adverse effects of medical treatment	2·4 (1·5 to 3·6)	1·6 (1 to 2·5)	5·2 (3 to 8·6)	3·1 (2 to 5)	2·5 (1·8 to 3·7)	2·5 (1·7 to 3·5)	4·4 (3·1 to 6·2)	7·5 (5·2 to 10·9)	8·4 (5·9 to 11·9)	8·8 (6·3 to 12·2)	9·7 (6·9 to 13·4)	10·9 (7·6 to 15·4)	13·8 (9·4 to 19·3)	16·6 (11·6 to 22·8)	21·3 (15·1 to 29·1)	26·6 (18·6 to 36·9)	23·8 (17·3 to 32·1)	25·5 (19 to 34·4)	23·2 (17·4 to 31)	18·4 (14·1 to 23·9)	14·3 (10·8 to 18·8)	6·9 (5·1 to 8·7)	1·7 (1·2 to 2·3)	259
Animal contact	0 (0 to 0)	0 (0 to 0)	0·1 (0·1 to 0·1)	0·5 (0·3 to 0·7)	0·8 (0·5 to 1·3)	1·2 (0·8 to 1·8)	1·6 (1·1 to 2·3)	1·7 (1·1 to 2·4)	2 (1·3 to 2·8)	1·9 (1·3 to 2·7)	2·2 (1·5 to 3·2)	2·2 (1·5 to 3·2)	2·6 (1·8 to 3·6)	2·7 (1·8 to 3·9)	2·9 (2·1 to 4·1)	2·9 (2 to 4·1)	2·5 (1·8 to 3·4)	2·1 (1·5 to 2·9)	1·6 (1·1 to 2·1)	1·2 (0·9 to 1·7)	1 (0·7 to 1·3)	0·7 (0·5 to 0·9)	0·2 (0·2 to 0·3)	34·6
Venomous animal contact	0 (0 to 0)	0 (0 to 0)	0 (0 to 0·1)	0·3 (0·2 to 0·4)	0·6 (0·3 to 0·9)	0·9 (0·5 to 1·4)	1·2 (0·7 to 1·8)	1·2 (0·8 to 1·8)	1·4 (0·9 to 2·1)	1·4 (0·9 to 2)	1·6 (1·1 to 2·3)	1·6 (1 to 2·2)	1·8 (1·2 to 2·5)	1·8 (1·2 to 2·5)	1·9 (1·3 to 2·7)	1·8 (1·2 to 2·6)	1·5 (1 to 2·1)	1·2 (0·8 to 1·7)	0·9 (0·7 to 1·2)	0·7 (0·5 to 1)	0·6 (0·4 to 0·8)	0·4 (0·3 to 0·6)	0·1 (0·1 to 0·2)	22·9
Non-venomous animal contact	0 (0 to 0)	0 (0 to 0)	0 (0 to 0·1)	0·2 (0·1 to 0·3)	0·2 (0·2 to 0·4)	0·3 (0·2 to 0·6)	0·4 (0·3 to 0·7)	0·5 (0·3 to 0·7)	0·6 (0·4 to 0·8)	0·5 (0·4 to 0·8)	0·6 (0·4 to 1)	0·7 (0·4 to 1·1)	0·8 (0·6 to 1·3)	0·9 (0·6 to 1·4)	1 (0·7 to 1·6)	1·1 (0·7 to 1·6)	1 (0·7 to 1·4)	0·9 (0·6 to 1·3)	0·6 (0·4 to 0·9)	0·5 (0·4 to 0·7)	0·4 (0·3 to 0·5)	0·2 (0·2 to 0·3)	0·1 (0·1 to 0·1)	11·7
Foreign body	2·8 (1·7 to 4·2)	1·6 (1 to 2·4)	10·5 (6 to 17·7)	5·3 (3·6 to 7·5)	4·2 (3·3 to 5·4)	5·6 (4·2 to 7·3)	5·1 (3·8 to 6·7)	5·7 (4·3 to 7·5)	7 (5·3 to 9·1)	7·1 (5·3 to 9·2)	6·9 (5·1 to 9·1)	7·2 (5·5 to 9·3)	8·2 (6·3 to 10·5)	8·6 (6·5 to 11·3)	9·6 (7·5 to 12·1)	10·7 (8·3 to 13·6)	9·7 (7·6 to 12·4)	9·6 (7·4 to 12)	8·1 (6·6 to 9·9)	6·9 (5·3 to 8·8)	4 (3·2 to 4·8)	2·2 (1·7 to 2·6)	0·6 (0·5 to 0·8)	147
Pulmonary aspiration and foreign body in airway	2·7 (1·7 to 4·2)	1·6 (0·9 to 2·4)	10·3 (5·9 to 17·4)	4·4 (2·8 to 6·5)	2·9 (2·2 to 3·8)	3·9 (2·8 to 5·1)	3 (2·3 to 4)	3·4 (2·5 to 4·5)	4·1 (3·1 to 5·5)	4·4 (3·3 to 5·8)	4 (2·9 to 5·3)	4 (3·1 to 5·3)	4·7 (3·5 to 6)	5·2 (3·8 to 7·1)	6·1 (4·4 to 8·1)	7·3 (5·2 to 9·8)	6·8 (5·1 to 9·2)	7·1 (5·2 to 9·2)	6·2 (4·9 to 7·8)	5·4 (4 to 7)	3 (2·3 to 3·8)	1·7 (1·3 to 2·1)	0·5 (0·4 to 0·6)	103
Foreign body in eyes	0 (0 to 0)	0 (0 to 0)	0 (0 to 0·1)	0·3 (0·1 to 0·6)	0·5 (0·1 to 1·1)	0·7 (0·2 to 1·5)	0·9 (0·3 to 2·1)	1·1 (0·3 to 2·4)	1·3 (0·4 to 2·7)	1·3 (0·5 to 2·8)	1·4 (0·5 to 3)	1·5 (0·6 to 3)	1·6 (0·7 to 3)	1·5 (0·8 to 2·7)	1·3 (0·8 to 2·2)	1·2 (0·7 to 1·9)	1 (0·6 to 1·5)	0·8 (0·5 to 1·2)	0·5 (0·4 to 0·8)	0·4 (0·2 to 0·6)	0·2 (0·1 to 0·3)	0·1 (0·1 to 0·1)	0 (0 to 0)	17·6
Foreign body in other body part	0 (0 to 0)	0 (0 to 0)	0·2 (0·1 to 0·3)	0·6 (0·4 to 0·9)	0·9 (0·6 to 1·3)	1·1 (0·7 to 1·6)	1·1 (0·8 to 1·6)	1·2 (0·9 to 1·7)	1·5 (1·1 to 2·2)	1·4 (1 to 2)	1·5 (1·1 to 2·1)	1·7 (1·2 to 2·2)	2 (1·4 to 2·6)	1·9 (1·4 to 2·6)	2·2 (1·6 to 2·9)	2·2 (1·6 to 2·9)	1·9 (1·4 to 2·6)	1·7 (1·2 to 2·3)	1·4 (1 to 1·8)	1·2 (0·8 to 1·6)	0·7 (0·5 to 1)	0·4 (0·3 to 0·6)	0·1 (0·1 to 0·2)	26·8
Environmental heat and cold exposure	0 (0 to 0)	0 (0 to 0)	0·2 (0·1 to 0·2)	1 (0·7 to 1·5)	2·1 (1·4 to 3·2)	3·2 (2·1 to 4·7)	4·7 (3·1 to 6·8)	5·6 (3·8 to 8·1)	6·9 (4·7 to 9·8)	7·6 (5·2 to 10·9)	8·7 (5·8 to 12·3)	9·1 (6·2 to 12·9)	10·5 (7·3 to 14·8)	11·1 (7·7 to 15·5)	11·7 (8·3 to 16·2)	11·5 (8 to 16)	9·7 (6·7 to 13·3)	8·3 (5·8 to 11·3)	5·9 (4·2 to 8)	4·4 (3·1 to 5·9)	2·7 (2 to 3·7)	1·3 (1 to 1·8)	0·4 (0·3 to 0·5)	127
Exposure to forces of nature	0 (0 to 0)	0 (0 to 0)	0 (0 to 0)	0 (0 to 0)	0 (0 to 0)	0 (0 to 0)	0 (0 to 0·1)	0 (0 to 0)	0·1 (0 to 0·1)	0 (0 to 0·1)	0 (0 to 0·1)	0 (0 to 0·1)	0·1 (0 to 0·1)	0·1 (0 to 0·1)	0·1 (0 to 0·1)	0 (0 to 0·1)	0 (0 to 0·1)	0 (0 to 0)	0 (0 to 0)	0 (0 to 0)	0 (0 to 0)	0 (0 to 0)	0 (0 to 0)	0·6
Other unintentional injuries	0·2 (0·1 to 0·4)	0·1 (0·1 to 0·2)	0·9 (0·6 to 1·2)	3·5 (2·4 to 5·1)	6·6 (4·6 to 9·4)	9·2 (6·7 to 12·8)	14·9 (11·1 to 20·2)	15·9 (11·7 to 21·9)	19·1 (14·1 to 25·6)	20·3 (15 to 27·3)	19·8 (14·5 to 27·1)	19·2 (13·9 to 26·1)	20·8 (14·9 to 28·3)	19·4 (13·5 to 27·3)	19·9 (13·9 to 28·1)	18·1 (12·6 to 25·8)	14·3 (9·9 to 20·4)	11·4 (7·9 to 16·2)	7·9 (5·3 to 11·1)	5·3 (3·6 to 7·5)	3·1 (2·2 to 4·3)	1·3 (0·9 to 1·8)	0·3 (0·2 to 0·4)	251
Self-harm and interpersonal violence	6 (3·5 to 9·8)	1 (0·6 to 1·7)	5·7 (3·2 to 9·6)	8·6 (5·1 to 13·5)	11·7 (8·7 to 15·9)	27·8 (20·9 to 36·4)	218 (164 to 291)	319 (250 to 408)	355 (281 to 445)	328 (261 to 410)	231 (175 to 296)	184 (140 to 243)	164 (123 to 215)	120 (89·7 to 155)	116 (88·2 to 150)	97·1 (76·4 to 122)	65·4 (52 to 80·6)	48·4 (39·2 to 59·1)	29·8 (24 to 36·7)	16·5 (13·5 to 20·1)	9·2 (7·6 to 11)	3 (2·4 to 3·5)	0·6 (0·5 to 0·7)	2,370
Self-harm	0 (0 to 0)	0 (0 to 0)	0 (0 to 0)	0 (0 to 0)	0 (0 to 0)	3·6 (2·4 to 5·2)	32 (22·9 to 44·2)	45·5 (32·4 to 61·7)	58 (42·5 to 76·7)	64·2 (46·7 to 85·3)	53·8 (39·2 to 71·9)	46·7 (33·6 to 64·5)	47·7 (33·9 to 65·5)	40·3 (28·7 to 54)	42·5 (30·8 to 56·3)	39·2 (28·9 to 52·3)	27·9 (21·3 to 36)	23·1 (17·6 to 30·1)	14·8 (11·3 to 19·4)	9·5 (7·2 to 12·3)	5·3 (4 to 6·7)	1·6 (1·2 to 2)	0·2 (0·2 to 0·3)	556
Self-harm by firearm	0 (0 to 0)	0 (0 to 0)	0 (0 to 0)	0 (0 to 0)	0 (0 to 0)	0·1 (0·1 to 0·2)	2·6 (1·6 to 4)	2·9 (1·7 to 4·7)	3·3 (1·9 to 5·1)	3·6 (2·2 to 5·7)	2·6 (1·6 to 4·1)	2·3 (1·4 to 3·6)	2·4 (1·4 to 3·7)	2·2 (1·4 to 3·4)	2·1 (1·3 to 3·1)	1·8 (1·1 to 2·9)	1·3 (0·8 to 1·9)	1·1 (0·7 to 1·6)	0·7 (0·5 to 1)	0·5 (0·4 to 0·7)	0·3 (0·2 to 0·4)	0 (0 to 0·1)	0 (0 to 0)	29·9
Self-harm by other specified means	0 (0 to 0)	0 (0 to 0)	0 (0 to 0)	0 (0 to 0)	0 (0 to 0)	3·4 (2·3 to 5)	29·5 (20·9 to 40·7)	42·6 (30·4 to 57·9)	54·8 (40·2 to 73)	60·5 (44·1 to 80·6)	51·2 (37·2 to 68·7)	44·3 (31·7 to 61)	45·3 (32·3 to 62·3)	38 (27 to 51·1)	40·5 (29·1 to 53·6)	37·4 (27·5 to 49·8)	26·7 (20·3 to 34·5)	22 (16·7 to 28·6)	14·1 (10·8 to 18·6)	9 (6·8 to 11·7)	5 (3·8 to 6·4)	1·5 (1·2 to 1·9)	0·2 (0·2 to 0·3)	526
Interpersonal violence	6 (3·5 to 9·8)	1 (0·6 to 1·7)	5·7 (3·2 to 9·5)	8·4 (4·9 to 13·2)	11·6 (8·5 to 15·7)	24·2 (17·8 to 32·2)	186 (135 to 254)	274 (207 to 356)	296 (226 to 380)	264 (203 to 343)	177 (130 to 236)	135 (97·5 to 186)	114 (81·4 to 156)	79 (57·2 to 108)	72·5 (52·8 to 99·2)	56·9 (41·7 to 74·9)	36·9 (28 to 48·9)	25·2 (19·2 to 32·5)	15 (11·4 to 19)	6·9 (5·4 to 8·7)	3·8 (3 to 4·7)	1·4 (1·1 to 1·8)	0·4 (0·3 to 0·5)	1,800
Physical violence by firearm	0·1 (0·1 to 0·2)	0 (0 to 0)	0·2 (0·1 to 0·4)	0·6 (0·3 to 0·9)	1·2 (0·9 to 1·8)	4·7 (3·4 to 6·5)	83·2 (58·9 to 114)	129 (97·3 to 167)	141 (108 to 182)	121 (92·4 to 158)	67·2 (48·8 to 89·6)	44·7 (31·4 to 62·4)	34·6 (23·7 to 48·5)	20·1 (14 to 28·3)	17·4 (12·1 to 24·8)	11·9 (8 to 16·3)	6·1 (4·3 to 8·4)	3·3 (2·4 to 4·5)	1·2 (0·9 to 1·7)	0·5 (0·4 to 0·6)	0·3 (0·2 to 0·3)	0·1 (0·1 to 0·1)	0 (0 to 0)	689
Physical violence by sharp object	0·3 (0·2 to 0·6)	0 (0 to 0·1)	0·3 (0·2 to 0·5)	0·8 (0·5 to 1·2)	1·5 (1·1 to 2)	4·1 (3·1 to 5·5)	62·4 (44·4 to 87·4)	94·3 (70·5 to 126)	99·3 (74·7 to 128)	88·6 (67·3 to 117)	63·4 (46 to 85·7)	48·2 (33·9 to 67·1)	37·6 (26·4 to 51·9)	24·1 (17·2 to 33·2)	19·1 (14 to 26·1)	14 (10·3 to 18·4)	8·2 (6·2 to 10·5)	6 (4·6 to 7·6)	3·1 (2·4 to 4)	1·7 (1·3 to 2·1)	0·9 (0·7 to 1·1)	0·4 (0·3 to 0·6)	0·1 (0·1 to 0·1)	579
Sexual violence	0 (0 to 0)	0 (0 to 0)	0 (0 to 0)	0·1 (0·1 to 0·1)	0·8 (0·5 to 1·3)	2 (1·2 to 3·2)	5 (2·8 to 8)	6·5 (3·8 to 10)	6·4 (3·6 to 10·3)	5·8 (3·2 to 9·6)	5·7 (3·2 to 9)	4·6 (2·6 to 7·5)	4·1 (2·4 to 6·9)	3·2 (1·8 to 5·2)	2·4 (1·4 to 3·9)	2 (1·3 to 3·1)	1·6 (1 to 2·3)	1·2 (0·7 to 1·8)	0·8 (0·5 to 1·1)	0·5 (0·3 to 0·8)	0·3 (0·2 to 0·4)	0 (0 to 0·1)	0 (0 to 0)	53·1
Physical violence by other means	5·6 (3·2 to 9·1)	0·9 (0·5 to 1·6)	5·2 (2·9 to 8·7)	7 (3·9 to 11·1)	8·1 (5·8 to 11·2)	13·2 (9·5 to 18·1)	35·3 (25·9 to 48·3)	44 (32·9 to 57·9)	49·3 (37 to 64·5)	47·7 (36·4 to 63·1)	41·1 (30 to 54·3)	37·6 (27·2 to 51·7)	37·9 (27·2 to 52·1)	31·7 (22·9 to 43·2)	33·5 (24·3 to 45·9)	29 (21·1 to 38·4)	21·1 (15·7 to 28·2)	14·7 (10·9 to 19·2)	9·8 (7·2 to 12·8)	4·2 (3·2 to 5·5)	2·4 (1·9 to 3)	0·8 (0·6 to 1)	0·2 (0·2 to 0·3)	480
Conflict and terrorism	0 (0 to 0)	0 (0 to 0)	0 (0 to 0)	0 (0 to 0)	0 (0 to 0)	0 (0 to 0)	0 (0 to 0)	0 (0 to 0)	0·1 (0 to 0·2)	0·1 (0·1 to 0·2)	0·1 (0·1 to 0·2)	1·9 (1·2 to 3·2)	1·8 (1·2 to 3)	0·4 (0·3 to 0·7)	1·2 (0·8 to 2)	0·9 (0·6 to 1·3)	0·5 (0·3 to 0·7)	0 (0 to 0·1)	0 (0 to 0)	0·1 (0·1 to 0·2)	0 (0 to 0)	0 (0 to 0)	0 (0 to 0)	7·4

SR Table 2c: Belize DALYS by cause and age group for both sexes with 95% uncertainty intervals, 2019

Cause	Early Neonatal	Late Neonatal	Post Neonatal	1 to 4	5 to 9	10 to 14	15 to 19	20 to 24	25 to 29	30 to 34	35 to 39	40 to 44	45 to 49	50 to 54	55 to 59	60 to 64	65 to 69	70 to 74	75 to 79	80 to 84	85 to 89	90 to 94	95 plus	Total
All causes	4,280 (3,500 to 5,230)	1,800 (1,430 to 2,220)	2,830 (2,180 to 3,760)	2,620 (2,160 to 3,150)	2,620 (2,060 to 3,270)	3,390 (2,710 to 4,180)	6,220 (5,320 to 7,340)	7,550 (6,570 to 8,670)	8,330 (7,310 to 9,460)	8,190 (7,170 to 9,310)	7,300 (6,340 to 8,410)	6,960 (6,030 to 8,020)	7,250 (6,280 to 8,320)	7,100 (6,170 to 8,120)	7,200 (6,250 to 8,190)	6,230 (5,460 to 7,060)	4,990 (4,410 to 5,640)	4,010 (3,560 to 4,490)	3,060 (2,730 to 3,400)	2,460 (2,220 to 2,710)	1,540 (1,400 to 1,680)	784 (722 to 846)	307 (288 to 327)	107,000
Communicable, maternal, neonatal, and nutritional diseases	3,640 (2,930 to 4,450)	1,420 (1,110 to 1,750)	1,540 (1,180 to 2,020)	1,010 (802 to 1,260)	797 (563 to 1,110)	630 (475 to 841)	732 (595 to 920)	935 (805 to 1,110)	1,340 (1,200 to 1,500)	1,440 (1,300 to 1,590)	1,310 (1,180 to 1,460)	1,120 (1,000 to 1,270)	907 (806 to 1,030)	669 (581 to 784)	539 (460 to 627)	407 (337 to 494)	300 (243 to 367)	258 (207 to 325)	223 (176 to 277)	222 (181 to 263)	162 (129 to 197)	95·8 (72·9 to 118)	41·7 (30·1 to 52·4)	19,700
HIV/AIDS and sexually transmitted infections	0·6 (0·4 to 1)	0·2 (0·1 to 0·4)	59·6 (56·3 to 63·2)	54·8 (51·4 to 58)	42·1 (38 to 47·1)	29·1 (26·7 to 32·3)	83·4 (77·7 to 90)	294 (272 to 322)	692 (646 to 753)	804 (752 to 864)	778 (728 to 829)	622 (583 to 668)	454 (426 to 483)	250 (234 to 266)	139 (130 to 147)	66·2 (61·6 to 71·2)	33·2 (30·7 to 36)	9·3 (8·3 to 10·6)	4·2 (3·7 to 4·8)	2·2 (2 to 2·6)	1·1 (1 to 1·2)	0·3 (0·3 to 0·4)	0·1 (0·1 to 0·2)	4,420
HIV/AIDS	0 (0 to 0)	0 (0 to 0·1)	59·5 (56·2 to 63)	54·8 (51·4 to 58)	42·1 (38 to 47·1)	28·3 (26 to 31·3)	79·1 (74·1 to 84·7)	286 (265 to 313)	681 (636 to 739)	792 (743 to 852)	767 (717 to 820)	612 (573 to 658)	447 (419 to 475)	246 (230 to 262)	136 (128 to 144)	64·5 (60 to 69·2)	32·1 (29·7 to 34·5)	8·7 (7·8 to 9·6)	3·8 (3·4 to 4·3)	2 (1·8 to 2·2)	0·9 (0·9 to 1·1)	0·3 (0·2 to 0·3)	0·1 (0·1 to 0·1)	4,340
HIV/AIDS - Drug-susceptible Tuberculosis	0 (0 to 0)	0 (0 to 0)	4·2 (2·6 to 6·4)	3·9 (2·4 to 5·9)	3·1 (2 to 4·6)	2·1 (1·4 to 3·1)	5·9 (3·9 to 8·7)	21·5 (14·2 to 31·9)	50 (32·2 to 75)	58·3 (37·4 to 87·9)	56·3 (36·1 to 85·1)	45 (28·1 to 68·3)	32·7 (20·8 to 49·3)	18 (11·6 to 27·1)	10 (6·3 to 14·7)	4·8 (3·2 to 7·1)	2·4 (1·6 to 3·5)	0·7 (0·5 to 0·9)	0·3 (0·2 to 0·4)	0·1 (0·1 to 0·2)	0·1 (0 to 0·1)	0 (0 to 0)	0 (0 to 0)	319
HIV/AIDS - Multidrug-resistant Tuberculosis without extensive dru	0 (0 to 0)	0 (0 to 0)	0·1 (0 to 0·2)	0·1 (0 to 0·2)	0 (0 to 0·2)	0 (0 to 0·1)	0·1 (0 to 0·3)	0·3 (0 to 1·1)	0·6 (0·1 to 2·5)	0·7 (0·1 to 3)	0·7 (0·1 to 2·9)	0·6 (0 to 2·4)	0·4 (0 to 1·7)	0·2 (0 to 1)	0·1 (0 to 0·5)	0·1 (0 to 0·3)	0 (0 to 0·1)	0 (0 to 0)	0 (0 to 0)	0 (0 to 0)	0 (0 to 0)	0 (0 to 0)	0 (0 to 0)	3·9
HIV/AIDS - Extensively drug-resistant Tuberculosis	0 (0 to 0)	0 (0 to 0)	0 (0 to 0)	0 (0 to 0)	0 (0 to 0)	0 (0 to 0)	0 (0 to 0·1)	0 (0 to 0·2)	0·1 (0 to 0·4)	0·1 (0 to 0·5)	0·1 (0 to 0·5)	0·1 (0 to 0·4)	0·1 (0 to 0·3)	0 (0 to 0·2)	0 (0 to 0·1)	0 (0 to 0)	0 (0 to 0)	0 (0 to 0)	0 (0 to 0)	0 (0 to 0)	0 (0 to 0)	0 (0 to 0)	0 (0 to 0)	0·6
HIV/AIDS resulting in other diseases	0 (0 to 0)	0 (0 to 0·1)	55·2 (51·1 to 59·3)	50·8 (47 to 54·5)	38·9 (34·6 to 44·3)	26·1 (23·5 to 29·3)	73·1 (67·1 to 79)	265 (242 to 293)	631 (575 to 693)	733 (674 to 795)	710 (657 to 767)	566 (522 to 614)	414 (382 to 445)	227 (211 to 245)	126 (116 to 136)	59·6 (54·5 to 64·6)	29·7 (26·9 to 32·2)	8 (7·1 to 8·9)	3·5 (3·1 to 4)	1·8 (1·6 to 2)	0·9 (0·8 to 1)	0·3 (0·2 to 0·3)	0·1 (0·1 to 0·1)	4,020
Sexually transmitted infections excluding HIV	0·6 (0·4 to 0·9)	0·2 (0·1 to 0·4)	0·1 (0 to 0·2)	0 (0 to 0)	0 (0 to 0)	0·8 (0·5 to 1·4)	4·3 (2·4 to 7·2)	8 (4·7 to 13·2)	10·6 (5·9 to 17·8)	12·1 (6·8 to 20·7)	11·1 (6·2 to 19·7)	9·9 (5·4 to 18·2)	7·1 (3·9 to 12·8)	4·3 (2·1 to 8·3)	2·8 (1·3 to 5·7)	1·7 (0·7 to 3·5)	1·1 (0·5 to 2·2)	0·7 (0·3 to 1·4)	0·4 (0·2 to 0·8)	0·2 (0·1 to 0·5)	0·1 (0·1 to 0·3)	0·1 (0 to 0·1)	0 (0 to 0)	76·1
Syphilis	0·6 (0·4 to 0·9)	0·2 (0·1 to 0·4)	0·1 (0 to 0·2)	0 (0 to 0)	0 (0 to 0)	0·1 (0·1 to 0·1)	0·2 (0·1 to 0·3)	0·2 (0·1 to 0·3)	0·3 (0·2 to 0·5)	0·4 (0·3 to 0·6)	0·2 (0·2 to 0·3)	0·1 (0·1 to 0·2)	0·2 (0·1 to 0·3)	0·1 (0·1 to 0·2)	0·1 (0·1 to 0·1)	0·1 (0 to 0·1)	0·1 (0 to 0·1)	0 (0 to 0·1)	0 (0 to 0)	0 (0 to 0)	0 (0 to 0)	0 (0 to 0)	0 (0 to 0)	3·1
Chlamydial infection	0 (0 to 0)	0 (0 to 0)	0 (0 to 0)	0 (0 to 0)	0 (0 to 0)	0·1 (0 to 0·1)	0·5 (0·3 to 0·7)	1·2 (0·6 to 2·2)	1·7 (0·9 to 3)	1·7 (0·9 to 2·9)	1·6 (0·8 to 2·6)	1·4 (0·7 to 2·3)	0·8 (0·4 to 1·4)	0·3 (0·1 to 0·5)	0·1 (0 to 0·2)	0 (0 to 0)	0 (0 to 0)	0 (0 to 0)	0 (0 to 0)	0 (0 to 0)	0 (0 to 0)	0 (0 to 0)	0 (0 to 0)	9·3
Gonococcal infection	0 (0 to 0)	0 (0 to 0)	0 (0 to 0)	0 (0 to 0)	0 (0 to 0)	0·1 (0·1 to 0·3)	0·9 (0·3 to 2)	1·2 (0·6 to 2·4)	0·9 (0·5 to 1·6)	0·9 (0·5 to 1·3)	0·7 (0·5 to 0·9)	0·7 (0·5 to 1)	0·6 (0·4 to 0·8)	0·3 (0·2 to 0·4)	0·1 (0·1 to 0·2)	0·1 (0 to 0·1)	0·1 (0 to 0·1)	0 (0 to 0·1)	0 (0 to 0)	0 (0 to 0)	0 (0 to 0)	0 (0 to 0)	0 (0 to 0)	6·7
Trichomoniasis	0 (0 to 0)	0 (0 to 0)	0 (0 to 0)	0 (0 to 0)	0 (0 to 0)	0 (0 to 0·1)	0·4 (0·1 to 1·1)	1·8 (0·6 to 4·3)	3 (1 to 7)	3·2 (1·1 to 7·6)	2·9 (0·9 to 6·8)	2·5 (0·8 to 5·8)	2 (0·7 to 4·7)	1·4 (0·4 to 3·2)	0·9 (0·3 to 2)	0·5 (0·2 to 1·1)	0·3 (0·1 to 0·6)	0·2 (0·1 to 0·3)	0·1 (0 to 0·2)	0 (0 to 0·1)	0 (0 to 0)	0 (0 to 0)	0 (0 to 0)	19·2
Genital herpes	0 (0 to 0)	0 (0 to 0)	0 (0 to 0)	0 (0 to 0)	0 (0 to 0)	0·2 (0·1 to 0·5)	0·9 (0·3 to 2)	1·6 (0·5 to 4·1)	2·3 (0·8 to 5·5)	2·7 (0·9 to 6·6)	2·7 (0·9 to 6·6)	2·4 (0·8 to 6·1)	2·1 (0·7 to 5·1)	1·7 (0·5 to 4·2)	1·4 (0·4 to 3·3)	1 (0·3 to 2·4)	0·6 (0·2 to 1·6)	0·4 (0·1 to 1)	0·2 (0·1 to 0·6)	0·2 (0 to 0·4)	0·1 (0 to 0·2)	0 (0 to 0·1)	0 (0 to 0)	20·5
Other sexually transmitted infections	0 (0 to 0)	0 (0 to 0)	0 (0 to 0)	0 (0 to 0)	0 (0 to 0)	0·3 (0·1 to 0·6)	1·4 (0·7 to 2·6)	1·9 (1·1 to 3·1)	2·4 (1·4 to 3·8)	3·2 (2 to 4·9)	3·1 (1·8 to 4·7)	2·8 (1·6 to 4·5)	1·4 (0·9 to 2·3)	0·5 (0·3 to 0·8)	0·2 (0·1 to 0·4)	0·1 (0 to 0·1)	0 (0 to 0)	0 (0 to 0)	0 (0 to 0)	0 (0 to 0)	0 (0 to 0)	0 (0 to 0)	0 (0 to 0)	17·4
Respiratory infections and tuberculosis	151 (103 to 211)	176 (122 to 246)	549 (369 to 784)	270 (200 to 351)	136 (103 to 181)	114 (84·9 to 153)	163 (123 to 211)	207 (162 to 264)	239 (190 to 305)	256 (201 to 324)	207 (160 to 265)	201 (154 to 262)	203 (156 to 262)	203 (153 to 265)	215 (168 to 268)	204 (153 to 266)	170 (122 to 222)	170 (129 to 225)	159 (118 to 206)	172 (134 to 207)	129 (99·4 to 159)	78·8 (57·4 to 98·5)	34·6 (24·1 to 44·4)	4,410
Tuberculosis	0 (0 to 0)	0 (0 to 0)	11·2 (7·6 to 16·3)	12·6 (9·1 to 17·2)	9 (5·9 to 12·6)	8·9 (6·5 to 12·1)	28 (19·7 to 38·9)	61·1 (42·2 to 84·9)	78·7 (57·5 to 106)	84 (61·7 to 111)	61·4 (44·8 to 83·8)	57·1 (41·2 to 77·8)	51·5 (36·9 to 69·7)	46·9 (33·4 to 66·4)	42 (28·8 to 57·3)	33·1 (23·8 to 45)	22·4 (15·9 to 30·1)	16·9 (12·5 to 22·3)	11·4 (8·4 to 15·4)	8·7 (6·3 to 11·4)	4·7 (3·3 to 6·3)	1·7 (1·1 to 2·3)	0·5 (0·4 to 0·6)	652
Latent tuberculosis infection	0 (0 to 0)	0 (0 to 0)	0 (0 to 0)	0 (0 to 0)	0 (0 to 0)	0 (0 to 0)	0 (0 to 0)	0 (0 to 0)	0 (0 to 0)	0 (0 to 0)	0 (0 to 0)	0 (0 to 0)	0 (0 to 0)	0 (0 to 0)	0 (0 to 0)	0 (0 to 0)	0 (0 to 0)	0 (0 to 0)	0 (0 to 0)	0 (0 to 0)	0 (0 to 0)	0 (0 to 0)	0 (0 to 0)	0
Drug-susceptible tuberculosis	0 (0 to 0)	0 (0 to 0)	11 (7·6 to 16)	12·4 (9 to 16·9)	8·9 (5·9 to 12·5)	8·8 (6·4 to 11·9)	27·7 (19·5 to 38·7)	60·5 (41·7 to 84·2)	77·8 (56·5 to 104)	83·1 (60·8 to 110)	60·8 (44·4 to 83·4)	56·5 (40·6 to 76·7)	50·9 (36·2 to 69·4)	46·4 (32·6 to 65·8)	41·5 (28·5 to 57)	32·7 (23·4 to 44·6)	22·1 (15·8 to 29·8)	16·7 (12·3 to 21·8)	11·3 (8·3 to 15·2)	8·6 (6·2 to 11·3)	4·6 (3·3 to 6·2)	1·7 (1·1 to 2·2)	0·5 (0·4 to 0·6)	645
Multidrug-resistant tuberculosis without extensive drug resistanc	0 (0 to 0)	0 (0 to 0)	0·1 (0 to 0·5)	0·1 (0 to 0·5)	0·1 (0 to 0·3)	0·1 (0 to 0·3)	0·3 (0 to 1·2)	0·6 (0·1 to 2·2)	0·7 (0·1 to 3·1)	0·8 (0·1 to 3·3)	0·6 (0·1 to 2·3)	0·5 (0·1 to 2·3)	0·5 (0 to 2·1)	0·5 (0 to 1·9)	0·4 (0 to 1·8)	0·3 (0 to 1·4)	0·2 (0 to 1)	0·2 (0 to 0·7)	0·1 (0 to 0·5)	0·1 (0 to 0·4)	0 (0 to 0·2)	0 (0 to 0·1)	0 (0 to 0)	6·3
Extensively drug-resistant tuberculosis	0 (0 to 0)	0 (0 to 0)	0 (0 to 0·1)	0 (0 to 0·1)	0 (0 to 0)	0 (0 to 0·1)	0 (0 to 0·2)	0·1 (0 to 0·4)	0·1 (0 to 0·5)	0·1 (0 to 0·5)	0·1 (0 to 0·4)	0·1 (0 to 0·4)	0·1 (0 to 0·4)	0·1 (0 to 0·3)	0·1 (0 to 0·3)	0·1 (0 to 0·2)	0 (0 to 0·2)	0 (0 to 0·1)	0 (0 to 0·1)	0 (0 to 0·1)	0 (0 to 0)	0 (0 to 0)	0 (0 to 0)	1
Lower respiratory infections	151 (103 to 211)	176 (121 to 245)	525 (347 to 758)	203 (140 to 282)	62·9 (45·1 to 84)	48·5 (35·1 to 66·3)	81·2 (55·4 to 114)	100 (67·4 to 146)	123 (85·7 to 173)	141 (95·8 to 201)	118 (81·3 to 167)	120 (81·1 to 173)	132 (91·9 to 183)	140 (95·8 to 196)	161 (119 to 210)	162 (113 to 223)	141 (96·7 to 192)	150 (108 to 204)	146 (105 to 193)	162 (127 to 198)	124 (94·5 to 153)	76·8 (55·6 to 96·4)	34 (23·5 to 43·8)	3,280
Upper respiratory infections	0·1 (0·1 to 0·1)	0·5 (0·3 to 0·9)	10·9 (6·9 to 16·5)	39 (23·5 to 62·3)	42·3 (23·6 to 70·6)	40·5 (20·9 to 69·4)	39·2 (20·9 to 68·9)	33·4 (18·3 to 57·7)	28·4 (15·7 to 47)	24·2 (13·2 to 40·9)	21·5 (11 to 36·1)	18 (9·7 to 31·1)	14·7 (7·8 to 25·4)	11·3 (6·1 to 19·2)	9 (4·9 to 14·9)	6·4 (3·5 to 10·4)	4·5 (2·4 to 7·6)	2·8 (1·6 to 4·7)	1·7 (0·9 to 2·7)	1 (0·5 to 1·7)	0·5 (0·3 to 0·9)	0·2 (0·1 to 0·4)	0·1 (0 to 0·1)	350
Otitis media	0 (0 to 0)	0 (0 to 0·1)	1·7 (0·9 to 3·1)	15·6 (8·3 to 27·2)	21·7 (12 to 36)	16·3 (8·9 to 28·3)	14·8 (7·9 to 24·9)	11·8 (5·3 to 23·8)	8·9 (4·3 to 16·7)	6·7 (2·9 to 12·7)	6·1 (2·9 to 11·3)	5·8 (2·8 to 11·2)	5·3 (2·6 to 9·5)	4·5 (2·1 to 8·2)	3·5 (1·7 to 6·1)	2·3 (1·1 to 4·3)	1·3 (0·6 to 2·4)	0·7 (0·3 to 1·4)	0·4 (0·2 to 0·8)	0·3 (0·1 to 0·6)	0·2 (0·1 to 0·3)	0·1 (0 to 0·2)	0 (0 to 0·1)	128
Enteric infections	12·8 (8·5 to 18·7)	42 (24·6 to 66·7)	268 (159 to 436)	130 (95·7 to 172)	73·8 (48·5 to 108)	78·6 (47·8 to 122)	56·4 (37·8 to 79·3)	43·9 (29·9 to 62·3)	45·9 (33 to 61·9)	45·4 (32·8 to 61)	43·3 (31·6 to 57·9)	42·3 (31·3 to 56·4)	42·2 (31·2 to 55·8)	39·1 (29·2 to 51·2)	37·6 (28·5 to 48·3)	35·1 (25·9 to 46·3)	33·5 (24·6 to 44·5)	30·9 (22·9 to 41·4)	25·8 (19·3 to 33·6)	21·4 (16 to 27·4)	12·7 (9·4 to 16·6)	6·3 (4·7 to 8·3)	2·5 (1·8 to 3·3)	1,170
Diarrheal diseases	12·8 (8·5 to 18·7)	42 (24·6 to 66·7)	268 (158 to 434)	129 (94·6 to 170)	73·1 (47·7 to 107)	77·9 (47·1 to 121)	56 (37·5 to 79)	43·5 (29·6 to 61·8)	45·6 (32·6 to 61·6)	45 (32·3 to 60·5)	43·1 (31·4 to 57·7)	42·1 (31·1 to 56·2)	42·1 (31·2 to 55·7)	39 (29·1 to 51·1)	37·4 (28·4 to 48·2)	35 (25·7 to 46·2)	33·4 (24·5 to 44·4)	30·9 (22·9 to 41·3)	25·8 (19·2 to 33·6)	21·4 (16 to 27·3)	12·7 (9·3 to 16·6)	6·3 (4·7 to 8·3)	2·5 (1·8 to 3·3)	1,160
Typhoid and paratyphoid	0 (0 to 0)	0 (0 to 0)	0 (0 to 0·1)	0 (0 to 0·1)	0 (0 to 0)	0 (0 to 0·1)	0 (0 to 0)	0·1 (0 to 0·1)	0 (0 to 0·1)	0 (0 to 0·1)	0 (0 to 0·1)	0 (0 to 0·1)	0 (0 to 0)	0 (0 to 0·1)	0 (0 to 0·1)	0 (0 to 0)	0 (0 to 0)	0 (0 to 0)	0 (0 to 0)	0 (0 to 0)	0 (0 to 0)	0 (0 to 0)	0 (0 to 0)	0·4
Typhoid fever	0 (0 to 0)	0 (0 to 0)	0 (0 to 0·1)	0 (0 to 0·1)	0 (0 to 0)	0 (0 to 0·1)	0 (0 to 0)	0 (0 to 0·1)	0 (0 to 0·1)	0 (0 to 0·1)	0 (0 to 0·1)	0 (0 to 0·1)	0 (0 to 0)	0 (0 to 0·1)	0 (0 to 0)	0 (0 to 0)	0 (0 to 0)	0 (0 to 0)	0 (0 to 0)	0 (0 to 0)	0 (0 to 0)	0 (0 to 0)	0 (0 to 0)	0·3
Paratyphoid fever	0 (0 to 0)	0 (0 to 0)	0 (0 to 0)	0 (0 to 0·1)	0 (0 to 0)	0 (0 to 0)	0 (0 to 0)	0 (0 to 0)	0 (0 to 0)	0 (0 to 0)	0 (0 to 0)	0 (0 to 0)	0 (0 to 0)	0 (0 to 0)	0 (0 to 0)	0 (0 to 0)	0 (0 to 0)	0 (0 to 0)	0 (0 to 0)	0 (0 to 0)	0 (0 to 0)	0 (0 to 0)	0 (0 to 0)	0·1
Invasive Non-typhoidal Salmonella (iNTS)	0 (0 to 0)	0 (0 to 0)	0·2 (0·1 to 0·5)	0·5 (0·2 to 1)	0·5 (0·2 to 0·9)	0·5 (0·2 to 0·9)	0·1 (0·1 to 0·2)	0·1 (0·1 to 0·2)	0·2 (0·1 to 0·4)	0·1 (0 to 0·2)	0·1 (0 to 0·1)	0·1 (0 to 0·2)	0·1 (0 to 0·1)	0·1 (0 to 0·1)	0·1 (0 to 0·1)	0·1 (0 to 0·2)	0·1 (0 to 0·1)	0 (0 to 0·1)	0 (0 to 0·1)	0 (0 to 0)	0 (0 to 0)	0 (0 to 0)	0 (0 to 0)	2·8
Other intestinal infectious diseases	0 (0 to 0)	0 (0 to 0)	0·4 (0 to 1·2)	0·5 (0·1 to 1·2)	0·3 (0·1 to 0·7)	0·2 (0·1 to 0·6)	0·2 (0 to 0·5)	0·2 (0 to 0·5)	0·1 (0 to 0·2)	0·2 (0 to 0·5)	0·1 (0 to 0·2)	0·1 (0 to 0·2)	0 (0 to 0·1)	0 (0 to 0·1)	0 (0 to 0·1)	0 (0 to 0·1)	0 (0 to 0)	0 (0 to 0)	0 (0 to 0)	0 (0 to 0)	0 (0 to 0)	0 (0 to 0)	0 (0 to 0)	2·4
Neglected tropical diseases and malaria	0·1 (0·1 to 0·2)	0·8 (0·5 to 1·2)	8·5 (5 to 13·1)	26·4 (14·9 to 41·7)	40·2 (21·6 to 67·4)	30·3 (15·6 to 56)	30·9 (15·7 to 55·2)	30·9 (15·4 to 57·1)	32·9 (16·4 to 57·4)	31·4 (16 to 53)	28·9 (14·4 to 49·3)	27·6 (14·9 to 46·5)	24·8 (13 to 41)	23·1 (12·5 to 37·7)	21·8 (12·1 to 35·5)	17·8 (9·4 to 29·1)	14·3 (7·4 to 23·8)	11 (5·9 to 18·4)	7·9 (4·1 to 13·6)	5·7 (2·8 to 9·6)	4 (2·1 to 6·7)	1·8 (1 to 2·9)	0·6 (0·3 to 1)	421
Malaria	0 (0 to 0)	0 (0 to 0)	0 (0 to 0·1)	0·3 (0 to 0·7)	0·4 (0 to 1·1)	0·1 (0 to 0·3)	0·2 (0 to 0·5)	0·1 (0 to 0·4)	0·1 (0 to 0·3)	0·1 (0 to 0·2)	0·1 (0 to 0·2)	0·1 (0 to 0·2)	0·1 (0 to 0·1)	0 (0 to 0·1)	0 (0 to 0)	0 (0 to 0)	0 (0 to 0)	0 (0 to 0)	0 (0 to 0)	0 (0 to 0)	0 (0 to 0)	0 (0 to 0)	0 (0 to 0)	1·7
Chagas disease	0 (0 to 0)	0 (0 to 0)	0 (0 to 0)	0 (0 to 0)	0 (0 to 0)	0 (0 to 0)	0 (0 to 0·1)	0 (0 to 0·1)	0 (0 to 0·1)	0 (0 to 0·1)	0·1 (0 to 0·1)	0·1 (0 to 0·2)	0·1 (0·1 to 0·2)	0·1 (0·1 to 0·2)	0·2 (0·1 to 0·4)	0·3 (0·1 to 0·5)	0·3 (0·1 to 0·5)	0·2 (0·1 to 0·4)	0·1 (0·1 to 0·2)	0·1 (0·1 to 0·2)	0·8 (0·4 to 2·3)	0·3 (0·1 to 0·8)	0·1 (0 to 0·2)	2·8
Leishmaniasis	0 (0 to 0)	0 (0 to 0)	0 (0 to 0)	0 (0 to 0)	0 (0 to 0)	0 (0 to 0)	0 (0 to 0)	0 (0 to 0)	0 (0 to 0)	0 (0 to 0)	0 (0 to 0)	0 (0 to 0)	0 (0 to 0)	0 (0 to 0)	0 (0 to 0)	0 (0 to 0)	0 (0 to 0)	0 (0 to 0)	0 (0 to 0)	0 (0 to 0)	0 (0 to 0)	0 (0 to 0)	0 (0 to 0)	0
Visceral leishmaniasis	0 (0 to 0)	0 (0 to 0)	0 (0 to 0)	0 (0 to 0)	0 (0 to 0)	0 (0 to 0)	0 (0 to 0)	0 (0 to 0)	0 (0 to 0)	0 (0 to 0)	0 (0 to 0)	0 (0 to 0)	0 (0 to 0)	0 (0 to 0)	0 (0 to 0)	0 (0 to 0)	0 (0 to 0)	0 (0 to 0)	0 (0 to 0)	0 (0 to 0)	0 (0 to 0)	0 (0 to 0)	0 (0 to 0)	0
Cutaneous and mucocutaneous leishmaniasis	0 (0 to 0)	0 (0 to 0)	0 (0 to 0)	0 (0 to 0)	0 (0 to 0)	0 (0 to 0)	0 (0 to 0)	0 (0 to 0)	0 (0 to 0)	0 (0 to 0)	0 (0 to 0)	0 (0 to 0)	0 (0 to 0)	0 (0 to 0)	0 (0 to 0)	0 (0 to 0)	0 (0 to 0)	0 (0 to 0)	0 (0 to 0)	0 (0 to 0)	0 (0 to 0)	0 (0 to 0)	0 (0 to 0)	0
African trypanosomiasis	0 (0 to 0)	0 (0 to 0)	0 (0 to 0)	0 (0 to 0)	0 (0 to 0)	0 (0 to 0)	0 (0 to 0)	0 (0 to 0)	0 (0 to 0)	0 (0 to 0)	0 (0 to 0)	0 (0 to 0)	0 (0 to 0)	0 (0 to 0)	0 (0 to 0)	0 (0 to 0)	0 (0 to 0)	0 (0 to 0)	0 (0 to 0)	0 (0 to 0)	0 (0 to 0)	0 (0 to 0)	0 (0 to 0)	0
Schistosomiasis	0 (0 to 0)	0 (0 to 0)	0 (0 to 0)	0 (0 to 0)	0 (0 to 0)	0 (0 to 0)	0 (0 to 0)	0 (0 to 0)	0 (0 to 0)	0 (0 to 0)	0 (0 to 0)	0 (0 to 0)	0 (0 to 0)	0 (0 to 0)	0 (0 to 0)	0 (0 to 0)	0 (0 to 0)	0 (0 to 0)	0 (0 to 0)	0 (0 to 0)	0 (0 to 0)	0 (0 to 0)	0 (0 to 0)	0
Cysticercosis	0 (0 to 0)	0 (0 to 0)	0 (0 to 0)	0·1 (0 to 0·2)	0·4 (0 to 1·3)	1 (0·1 to 2·5)	3·9 (0·5 to 8·6)	9·4 (2·8 to 19·9)	14·6 (5·2 to 29·1)	15·6 (5·4 to 29·9)	14·9 (5·4 to 28·9)	14·6 (5·4 to 29·7)	14·9 (6 to 29·3)	14·5 (5·8 to 26·6)	15·1 (6·4 to 29)	13·8 (6·2 to 25·2)	11·9 (5·3 to 20·5)	9·1 (4·3 to 16·3)	6·7 (3 to 12·2)	4·8 (2 to 8·8)	2·7 (1·1 to 4·8)	1·2 (0·5 to 2·3)	0·4 (0·2 to 0·8)	170
Cystic echinococcosis	0 (0 to 0)	[illegible]	[illegible]	[illegible]	[illegible]	[illegible]	[illegible]	[illegible]	[illegible]	[illegible]	[illegible]	[illegible]	[illegible]	[illegible]	[illegible]	[illegible]	[illegible]	[illegible]	[illegible]	[illegible]	[illegible]	[illegible]	0 (0 to 0)	0·1
Lymphatic filariasis	0 (0 to 0)	[illegible]	[illegible]	[illegible]	[illegible]	[illegible]	[illegible]	[illegible]	[illegible]	[illegible]	[illegible]	[illegible]	[illegible]	[illegible]	[illegible]	[illegible]	[illegible]	[illegible]	[illegible]	[illegible]	[illegible]	[illegible]	0 (0 to 0)	0
Onchocerciasis	0 (0 to 0)	[illegible]	[illegible]	[illegible]	[illegible]	[illegible]	[illegible]	[illegible]	[illegible]	[illegible]	[illegible]	[illegible]	[illegible]	[illegible]	[illegible]	[illegible]	[illegible]	[illegible]	[illegible]	[illegible]	[illegible]	[illegible]	0 (0 to 0)	0
Trachoma	0 (0 to 0)	[illegible]	[illegible]	[illegible]	[illegible]	[illegible]	[illegible]	[illegible]	[illegible]	[illegible]	[illegible]	[illegible]	[illegible]	[illegible]	[illegible]	[illegible]	[illegible]	[illegible]	[illegible]	[illegible]	[illegible]	[illegible]	0 (0 to 0)	0
Dengue	0 (0 to 0)	[illegible]	[illegible]	[illegible]	[illegible]	[illegible]	[illegible]	[illegible]	[illegible]	[illegible]	[illegible]	[illegible]	[illegible]	[illegible]	[illegible]	[illegible]	[illegible]	[illegible]	[illegible]	[illegible]	[illegible]	[illegible]	0·1 (0 to 0·2)	104
Yellow fever	0 (0 to 0)	[illegible]	[illegible]	[illegible]	[illegible]	[illegible]	[illegible]	[illegible]	[illegible]	[illegible]	[illegible]	[illegible]	[illegible]	[illegible]	[illegible]	[illegible]	[illegible]	[illegible]	[illegible]	[illegible]	[illegible]	[illegible]	0 (0 to 0)	0
Rabies	0 (0 to 0)	[illegible]	[illegible]	[illegible]	[illegible]	[illegible]	[illegible]	[illegible]	[illegible]	[illegible]	[illegible]	[illegible]	[illegible]	[illegible]	[illegible]	[illegible]	[illegible]	[illegible]	[illegible]	[illegible]	[illegible]	[illegible]	0 (0 to 0)	1
Intestinal nematode infections	0 (0 to 0)	[illegible]	[illegible]	[illegible]	[illegible]	[illegible]	[illegible]	[illegible]	[illegible]	[illegible]	[illegible]	[illegible]	[illegible]	[illegible]	[illegible]	[illegible]	[illegible]	[illegible]	[illegible]	[illegible]	[illegible]	[illegible]	0 (0 to 0)	31·2
Ascariasis	0 (0 to 0)	[illegible]	[illegible]	[illegible]	[illegible]	[illegible]	[illegible]	[illegible]	[illegible]	[illegible]	[illegible]	[illegible]	[illegible]	[illegible]	[illegible]	[illegible]	[illegible]	[illegible]	[illegible]	[illegible]	[illegible]	[illegible]	0 (0 to 0)	5·9
Trichuriasis	0 (0 to 0)	[illegible]	[illegible]	[illegible]	[illegible]	[illegible]	[illegible]	[illegible]	[illegible]	[illegible]	[illegible]	[illegible]	[illegible]	[illegible]	[illegible]	[illegible]	[illegible]	[illegible]	[illegible]	[illegible]	[illegible]	[illegible]	0 (0 to 0)	1
Hookworm disease	0 (0 to 0)	[illegible]	[illegible]	[illegible]	[illegible]	[illegible]	[illegible]	[illegible]	[illegible]	[illegible]	[illegible]	[illegible]	[illegible]	[illegible]	[illegible]	[illegible]	[illegible]	[illegible]	[illegible]	[illegible]	[illegible]	[illegible]	0 (0 to 0)	24·3
Food-borne trematodiases	0 (0 to 0)	[illegible]	[illegible]	[illegible]	[illegible]	[illegible]	[illegible]	[illegible]	[illegible]	[illegible]	[illegible]	[illegible]	[illegible]	[illegible]	[illegible]	[illegible]	[illegible]	[illegible]	[illegible]	[illegible]	[illegible]	[illegible]	0 (0 to 0)	0
Leprosy	0 (0 to 0)	[illegible]	[illegible]	[illegible]	[illegible]	[illegible]	[illegible]	[illegible]	[illegible]	[illegible]	[illegible]	[illegible]	[illegible]	[illegible]	[illegible]	[illegible]	[illegible]	[illegible]	[illegible]	[illegible]	[illegible]	[illegible]	0 (0 to 0)	1·1
Ebola	0 (0 to 0)	[illegible]	[illegible]	[illegible]	[illegible]	[illegible]	[illegible]	[illegible]	[illegible]	[illegible]	[illegible]	[illegible]	[illegible]	[illegible]	[illegible]	[illegible]	[illegible]	[illegible]	[illegible]	[illegible]	[illegible]	[illegible]	0 (0 to 0)	0
Zika virus	0 (0 to 0)	[illegible]	[illegible]	[illegible]	[illegible]	[illegible]	[illegible]	[illegible]	[illegible]	[illegible]	[illegible]	[illegible]	[illegible]	[illegible]	[illegible]	[illegible]	[illegible]	[illegible]	[illegible]	[illegible]	[illegible]	[illegible]	0 (0 to 0)	2·9
Guinea worm disease	0 (0 to 0)	[illegible]	[illegible]	[illegible]	[illegible]	[illegible]	[illegible]	[illegible]	[illegible]	[illegible]	[illegible]	[illegible]	[illegible]	[illegible]	[illegible]	[illegible]	[illegible]	[illegible]	[illegible]	[illegible]	[illegible]	[illegible]	0 (0 to 0)	0
Other neglected tropical diseases	0·1 (0·1 to 0·2)	[illegible]	[illegible]	[illegible]	[illegible]	[illegible]	[illegible]	[illegible]	[illegible]	[illegible]	[illegible]	[illegible]	[illegible]	[illegible]	[illegible]	[illegible]	[illegible]	[illegible]	[illegible]	[illegible]	[illegible]	[illegible]	0 (0 to 0)	107
Other infectious diseases	7·7 (5·6 to 10·3)	[illegible]	[illegible]	[illegible]	[illegible]	[illegible]	[illegible]	[illegible]	[illegible]	[illegible]	[illegible]	[illegible]	[illegible]	[illegible]	[illegible]	[illegible]	[illegible]	[illegible]	[illegible]	[illegible]	[illegible]	[illegible]	0·2 (0·2 to 0·3)	543
Meningitis	4·6 (3·1 to 6·6)	[illegible]	[illegible]	[illegible]	[illegible]	[illegible]	[illegible]	[illegible]	[illegible]	[illegible]	[illegible]	[illegible]	[illegible]	[illegible]	[illegible]	[illegible]	[illegible]	[illegible]	[illegible]	[illegible]	[illegible]	[illegible]	0 (0 to 0)	160
Encephalitis	1·5 (0·9 to 2·2)	[illegible]	[illegible]	[illegible]	[illegible]	[illegible]	[illegible]	[illegible]	[illegible]	[illegible]	[illegible]	[illegible]	[illegible]	[illegible]	[illegible]	[illegible]	[illegible]	[illegible]	[illegible]	[illegible]	[illegible]	[illegible]	0 (0 to 0)	150
Diphtheria	0 (0 to 0)	[illegible]	[illegible]	[illegible]	[illegible]	[illegible]	[illegible]	[illegible]	[illegible]	[illegible]	[illegible]	[illegible]	[illegible]	[illegible]	[illegible]	[illegible]	[illegible]	[illegible]	[illegible]	[illegible]	[illegible]	[illegible]	0 (0 to 0)	0·3
Tetanus	0·1 (0 to 0·1)	[illegible]	[illegible]	[illegible]	[illegible]	[illegible]	[illegible]	[illegible]	[illegible]	[illegible]	[illegible]	[illegible]	[illegible]	[illegible]	[illegible]	[illegible]	[illegible]	[illegible]	[illegible]	[illegible]	[illegible]	[illegible]	0 (0 to 0)	4·8
Measles	0 (0 to 0)	[illegible]	[illegible]	[illegible]	[illegible]	[illegible]	[illegible]	[illegible]	[illegible]	[illegible]	[illegible]	[illegible]	[illegible]	[illegible]	[illegible]	[illegible]	[illegible]	[illegible]	[illegible]	[illegible]	[illegible]	[illegible]	0 (0 to 0)	0
Varicella and herpes zoster	0·6 (0·2 to 1·2)	[illegible]	[illegible]	[illegible]	[illegible]	[illegible]	[illegible]	[illegible]	[illegible]	[illegible]	[illegible]	[illegible]	[illegible]	[illegible]	[illegible]	[illegible]	[illegible]	[illegible]	[illegible]	[illegible]	[illegible]	[illegible]	0 (0 to 0·1)	26
Acute hepatitis	0 (0 to 0)	[illegible]	[illegible]	[illegible]	[illegible]	[illegible]	[illegible]	[illegible]	[illegible]	[illegible]	[illegible]	[illegible]	[illegible]	[illegible]	[illegible]	[illegible]	[illegible]	[illegible]	[illegible]	[illegible]	[illegible]	[illegible]	0 (0 to 0)	38·2
Acute hepatitis A	0 (0 to 0)	[illegible]	[illegible]	[illegible]	[illegible]	[illegible]	[illegible]	[illegible]	[illegible]	[illegible]	[illegible]	[illegible]	[illegible]	[illegible]	[illegible]	[illegible]	[illegible]	[illegible]	[illegible]	[illegible]	[illegible]	[illegible]	0 (0 to 0)	20·7
Acute hepatitis B	0 (0 to 0)	[illegible]	[illegible]	[illegible]	[illegible]	[illegible]	[illegible]	[illegible]	[illegible]	[illegible]	[illegible]	[illegible]	[illegible]	[illegible]	[illegible]	[illegible]	[illegible]	[illegible]	[illegible]	[illegible]	[illegible]	[illegible]	0 (0 to 0)	11·7
Acute hepatitis C	0 (0 to 0)	[illegible]	[illegible]	[illegible]	[illegible]	[illegible]	[illegible]	[illegible]	[illegible]	[illegible]	[illegible]	[illegible]	[illegible]	[illegible]	[illegible]	[illegible]	[illegible]	[illegible]	[illegible]	[illegible]	[illegible]	[illegible]	0 (0 to 0)	2·5
Acute hepatitis E	0 (0 to 0)	[illegible]	[illegible]	[illegible]	[illegible]	[illegible]	[illegible]	[illegible]	[illegible]	[illegible]	[illegible]	[illegible]	[illegible]	[illegible]	[illegible]	[illegible]	[illegible]	[illegible]	[illegible]	[illegible]	[illegible]	[illegible]	0 (0 to 0)	3·3
Other unspecified infectious diseases	0·9 (0·6 to 1·3)	[illegible]	[illegible]	[illegible]	[illegible]	[illegible]	[illegible]	[illegible]	[illegible]	[illegible]	[illegible]	[illegible]	[illegible]	[illegible]	[illegible]	[illegible]	[illegible]	[illegible]	[illegible]	[illegible]	[illegible]	[illegible]	0·1 (0·1 to 0·2)	151
Maternal and neonatal disorders	3,470 (2,790 to 4,230)	[illegible]	[illegible]	[illegible]	[illegible]	[illegible]	[illegible]	[illegible]	[illegible]	[illegible]	[illegible]	[illegible]	[illegible]	[illegible]	[illegible]	[illegible]	[illegible]	[illegible]	[illegible]	[illegible]	[illegible]	[illegible]	0 (0 to 0)	6,620
Maternal disorders	0 (0 to 0)	[illegible]	[illegible]	[illegible]	[illegible]	[illegible]	[illegible]	[illegible]	[illegible]	[illegible]	[illegible]	[illegible]	[illegible]	[illegible]	[illegible]	[illegible]	[illegible]	[illegible]	[illegible]	[illegible]	[illegible]	[illegible]	0 (0 to 0)	373
Maternal hemorrhage	0 (0 to 0)	[illegible]	[illegible]	[illegible]	[illegible]	[illegible]	[illegible]	[illegible]	[illegible]	[illegible]	[illegible]	[illegible]	[illegible]	[illegible]	[illegible]	[illegible]	[illegible]	[illegible]	[illegible]	[illegible]	[illegible]	[illegible]	0 (0 to 0)	72·4
Maternal sepsis and other maternal infections	0 (0 to 0)	[illegible]	[illegible]	[illegible]	[illegible]	[illegible]	[illegible]	[illegible]	[illegible]	[illegible]	[illegible]	[illegible]	[illegible]	[illegible]	[illegible]	[illegible]	[illegible]	[illegible]	[illegible]	[illegible]	[illegible]	[illegible]	0 (0 to 0)	34·8
Maternal hypertensive disorders	0 (0 to 0)	[illegible]	[illegible]	[illegible]	[illegible]	[illegible]	[illegible]	[illegible]	[illegible]	[illegible]	[illegible]	[illegible]	[illegible]	[illegible]	[illegible]	[illegible]	[illegible]	[illegible]	[illegible]	[illegible]	[illegible]	[illegible]	0 (0 to 0)	72·4
Maternal obstructed labor and uterine rupture	0 (0 to 0)	[illegible]	[illegible]	[illegible]	[illegible]	[illegible]	[illegible]	[illegible]	[illegible]	[illegible]	[illegible]	[illegible]	[illegible]	[illegible]	[illegible]	[illegible]	[illegible]	[illegible]	[illegible]	[illegible]	[illegible]	[illegible]	0 (0 to 0)	13·4
Maternal abortion and miscarriage	0 (0 to 0)	[illegible]	[illegible]	[illegible]	[illegible]	[illegible]	[illegible]	[illegible]	[illegible]	[illegible]	[illegible]	[illegible]	[illegible]	[illegible]	[illegible]	[illegible]	[illegible]	[illegible]	[illegible]	[illegible]	[illegible]	[illegible]	0 (0 to 0)	24·4
Ectopic pregnancy	0 (0 to 0)	[illegible]	[illegible]	[illegible]	[illegible]	[illegible]	[illegible]	[illegible]	[illegible]	[illegible]	[illegible]	[illegible]	[illegible]	[illegible]	[illegible]	[illegible]	[illegible]	[illegible]	[illegible]	[illegible]	[illegible]	[illegible]	0 (0 to 0)	8·9
Indirect maternal deaths	--	[illegible]	[illegible]	[illegible]	[illegible]	[illegible]	[illegible]	[illegible]	[illegible]	[illegible]	[illegible]	[illegible]	[illegible]	[illegible]	[illegible]	[illegible]	[illegible]	[illegible]	[illegible]	[illegible]	[illegible]	[illegible]	--	NA
Late maternal deaths	--	[illegible]	[illegible]	[illegible]	[illegible]	[illegible]	[illegible]	[illegible]	[illegible]	[illegible]	[illegible]	[illegible]	[illegible]	[illegible]	[illegible]	[illegible]	[illegible]	[illegible]	[illegible]	[illegible]	[illegible]	[illegible]	--	NA
Maternal deaths aggravated by HIV/AIDS	--	[illegible]	[illegible]	[illegible]	[illegible]	[illegible]	[illegible]	[illegible]	[illegible]	[illegible]	[illegible]	[illegible]	[illegible]	[illegible]	[illegible]	[illegible]	[illegible]	[illegible]	[illegible]	[illegible]	[illegible]	[illegible]	--	NA
Neonatal disorders	3,470 (2,790 to 4,230)	[illegible]	[illegible]	[illegible]	[illegible]	[illegible]	[illegible]	[illegible]	[illegible]	[illegible]	[illegible]	[illegible]	[illegible]	[illegible]	[illegible]	[illegible]	[illegible]	[illegible]	[illegible]	[illegible]	[illegible]	[illegible]	0 (0 to 0)	6,250
Neonatal preterm birth	1,670 (1,280 to 2,130)	[illegible]	[illegible]	[illegible]	[illegible]	[illegible]	[illegible]	[illegible]	[illegible]	[illegible]	[illegible]	[illegible]	[illegible]	[illegible]	[illegible]	[illegible]	[illegible]	[illegible]	[illegible]	[illegible]	[illegible]	[illegible]	0 (0 to 0)	2,790
Neonatal encephalopathy due to birth asphyxia and trauma	957 (693 to 1,240)	[illegible]	[illegible]	[illegible]	[illegible]	[illegible]	[illegible]	[illegible]	[illegible]	[illegible]	[illegible]	[illegible]	[illegible]	[illegible]	[illegible]	[illegible]	[illegible]	[illegible]	[illegible]	[illegible]	[illegible]	[illegible]	0 (0 to 0)	1,360
Neonatal sepsis and other neonatal infections	409 (282 to 583)	[illegible]	[illegible]	[illegible]	[illegible]	[illegible]	[illegible]	[illegible]	[illegible]	[illegible]	[illegible]	[illegible]	[illegible]	[illegible]	[illegible]	[illegible]	[illegible]	[illegible]	[illegible]	[illegible]	[illegible]	[illegible]	0 (0 to 0)	1,280
Hemolytic disease and other neonatal jaundice	66·9 (39·8 to 106)	[illegible]	[illegible]	[illegible]	[illegible]	[illegible]	[illegible]	[illegible]	[illegible]	[illegible]	[illegible]	[illegible]	[illegible]	[illegible]	[illegible]	[illegible]	[illegible]	[illegible]	[illegible]	[illegible]	[illegible]	[illegible]	0 (0 to 0)	180
Other neonatal disorders	366 (241 to 534)	[illegible]	[illegible]	[illegible]	[illegible]	[illegible]	[illegible]	[illegible]	[illegible]	[illegible]	[illegible]	[illegible]	[illegible]	[illegible]	[illegible]	[illegible]	[illegible]	[illegible]	[illegible]	[illegible]	[illegible]	[illegible]	0 (0 to 0)	645
Nutritional deficiencies	0·3 (0·2 to 0·5)	[illegible]	[illegible]	[illegible]	[illegible]	[illegible]	[illegible]	[illegible]	[illegible]	[illegible]	[illegible]	[illegible]	[illegible]	[illegible]	[illegible]	[illegible]	[illegible]	[illegible]	[illegible]	[illegible]	[illegible]	[illegible]	3·6 (2·6 to 5)	2,150
Protein-energy malnutrition	0·2 (0·1 to 0·3)	[illegible]	[illegible]	[illegible]	[illegible]	[illegible]	[illegible]	[illegible]	[illegible]	[illegible]	[illegible]	[illegible]	[illegible]	[illegible]	[illegible]	[illegible]	[illegible]	[illegible]	[illegible]	[illegible]	[illegible]	[illegible]	2·9 (1·9 to 4·1)	524
Iodine deficiency	0 (0 to 0)	[illegible]	[illegible]	[illegible]	[illegible]	[illegible]	[illegible]	[illegible]	[illegible]	[illegible]	[illegible]	[illegible]	[illegible]	[illegible]	[illegible]	[illegible]	[illegible]	[illegible]	[illegible]	[illegible]	[illegible]	[illegible]	0 (0 to 0)	9
Vitamin A deficiency	0 (0 to 0·1)	[illegible]	[illegible]	[illegible]	[illegible]	[illegible]	[illegible]	[illegible]	[illegible]	[illegible]	[illegible]	[illegible]	[illegible]	[illegible]	[illegible]	[illegible]	[illegible]	[illegible]	[illegible]	[illegible]	[illegible]	[illegible]	0 (0 to 0)	34·8
Dietary iron deficiency	0·1 (0 to 0·2)	[illegible]	[illegible]	[illegible]	[illegible]	[illegible]	[illegible]	[illegible]	[illegible]	[illegible]	[illegible]	[illegible]	[illegible]	[illegible]	[illegible]	[illegible]	[illegible]	[illegible]	[illegible]	[illegible]	[illegible]	[illegible]	0·5 (0·2 to 1)	1,540
Other nutritional deficiencies	0 (0 to 0)	[illegible]	[illegible]	[illegible]	[illegible]	[illegible]	[illegible]	[illegible]	[illegible]	[illegible]	[illegible]	[illegible]	[illegible]	[illegible]	[illegible]	[illegible]	[illegible]	[illegible]	[illegible]	[illegible]	[illegible]	[illegible]	0·2 (0·1 to 0·4)	45·7
Non-communicable diseases	583 (411 to 809)	[illegible]	[illegible]	[illegible]	[illegible]	[illegible]	[illegible]	[illegible]	[illegible]	[illegible]	[illegible]	[illegible]	[illegible]	[illegible]	[illegible]	[illegible]	[illegible]	[illegible]	[illegible]	[illegible]	[illegible]	[illegible]	252 (232 to 272)	67,600
Neoplasms	4·5 (3·1 to 6·2)	[illegible]	[illegible]	[illegible]	[illegible]	[illegible]	[illegible]	[illegible]	[illegible]	[illegible]	[illegible]	[illegible]	[illegible]	[illegible]	[illegible]	[illegible]	[illegible]	[illegible]	[illegible]	[illegible]	[illegible]	[illegible]	18·2 (13·1 to 22·1)	8,650
Lip and oral cavity cancer	0 (0 to 0)	[illegible]	[illegible]	[illegible]	[illegible]	[illegible]	[illegible]	[illegible]	[illegible]	[illegible]	[illegible]	[illegible]	[illegible]	[illegible]	[illegible]	[illegible]	[illegible]	[illegible]	[illegible]	[illegible]	[illegible]	[illegible]	0·2 (0·2 to 0·3)	106
Nasopharynx cancer	0 (0 to 0)	[illegible]	[illegible]	[illegible]	[illegible]	[illegible]	[illegible]	[illegible]	[illegible]	[illegible]	[illegible]	[illegible]	[illegible]	[illegible]	[illegible]	[illegible]	[illegible]	[illegible]	[illegible]	[illegible]	[illegible]	[illegible]	0 (0 to 0·1)	36·5
Other pharynx cancer	0 (0 to 0)	[illegible]	[illegible]	[illegible]	[illegible]	[illegible]	[illegible]	[illegible]	[illegible]	[illegible]	[illegible]	[illegible]	[illegible]	[illegible]	[illegible]	[illegible]	[illegible]	[illegible]	[illegible]	[illegible]	[illegible]	[illegible]	0·1 (0·1 to 0·1)	52·1
Esophageal cancer	0 (0 to 0)	[illegible]	[illegible]	[illegible]	[illegible]	[illegible]	[illegible]	[illegible]	[illegible]	[illegible]	[illegible]	[illegible]	[illegible]	[illegible]	[illegible]	[illegible]	[illegible]	[illegible]	[illegible]	[illegible]	[illegible]	[illegible]	0·2 (0·2 to 0·3)	184
Stomach cancer	0 (0 to 0)	[illegible]	[illegible]	[illegible]	[illegible]	[illegible]	[illegible]	[illegible]	[illegible]	[illegible]	[illegible]	[illegible]	[illegible]	[illegible]	[illegible]	[illegible]	[illegible]	[illegible]	[illegible]	[illegible]	[illegible]	[illegible]	1·7 (1·2 to 2)	720
Colon and rectum cancer	0 (0 to 0)	[illegible]	[illegible]	[illegible]	[illegible]	[illegible]	[illegible]	[illegible]	[illegible]	[illegible]	[illegible]	[illegible]	[illegible]	[illegible]	[illegible]	[illegible]	[illegible]	[illegible]	[illegible]	[illegible]	[illegible]	[illegible]	2 (1·5 to 2·5)	716
Liver cancer	0·1 (0·1 to 0·2)	[illegible]	[illegible]	[illegible]	[illegible]	[illegible]	[illegible]	[illegible]	[illegible]	[illegible]	[illegible]	[illegible]	[illegible]	[illegible]	[illegible]	[illegible]	[illegible]	[illegible]	[illegible]	[illegible]	[illegible]	[illegible]	0·2 (0·2 to 0·3)	234
Liver cancer due to hepatitis B	0 (0 to 0)	[illegible]	[illegible]	[illegible]	[illegible]	[illegible]	[illegible]	[illegible]	[illegible]	[illegible]	[illegible]	[illegible]	[illegible]	[illegible]	[illegible]	[illegible]	[illegible]	[illegible]	[illegible]	[illegible]	[illegible]	[illegible]	0 (0 to 0·1)	77·9
Liver cancer due to hepatitis C	0 (0 to 0)	[illegible]	[illegible]	[illegible]	[illegible]	[illegible]	[illegible]	[illegible]	[illegible]	[illegible]	[illegible]	[illegible]	[illegible]	[illegible]	[illegible]	[illegible]	[illegible]	[illegible]	[illegible]	[illegible]	[illegible]	[illegible]	0·1 (0·1 to 0·2)	39·9
Liver cancer due to alcohol use	0 (0 to 0)	[illegible]	[illegible]	[illegible]	[illegible]	[illegible]	[illegible]	[illegible]	[illegible]	[illegible]	[illegible]	[illegible]	[illegible]	[illegible]	[illegible]	[illegible]	[illegible]	[illegible]	[illegible]	[illegible]	[illegible]	[illegible]	0 (0 to 0·1)	73·6
Liver cancer due to NASH	0 (0 to 0)	[illegible]	[illegible]	[illegible]	[illegible]	[illegible]	[illegible]	[illegible]	[illegible]	[illegible]	[illegible]	[illegible]	[illegible]	[illegible]	[illegible]	[illegible]	[illegible]	[illegible]	[illegible]	[illegible]	[illegible]	[illegible]	0 (0 to 0·1)	20·8
Liver cancer due to other causes	0·1 (0·1 to 0·2)	[illegible]	[illegible]	[illegible]	[illegible]	[illegible]	[illegible]	[illegible]	[illegible]	[illegible]	[illegible]	[illegible]	[illegible]	[illegible]	[illegible]	[illegible]	[illegible]	[illegible]	[illegible]	[illegible]	[illegible]	[illegible]	0 (0 to 0)	22·1
Gallbladder and biliary tract cancer	0 (0 to 0)	[illegible]	[illegible]	[illegible]	[illegible]	[illegible]	[illegible]	[illegible]	[illegible]	[illegible]	[illegible]	[illegible]	[illegible]	[illegible]	[illegible]	[illegible]	[illegible]	[illegible]	[illegible]	[illegible]	[illegible]	[illegible]	0·2 (0·1 to 0·3)	96·6
Pancreatic cancer	0 (0 to 0)	[illegible]	[illegible]	[illegible]	[illegible]	[illegible]	[illegible]	[illegible]	[illegible]	[illegible]	[illegible]	[illegible]	[illegible]	[illegible]	[illegible]	[illegible]	[illegible]	[illegible]	[illegible]	[illegible]	[illegible]	[illegible]	0·9 (0·6 to 1·2)	436
Larynx cancer	0 (0 to 0)	[illegible]	[illegible]	[illegible]	[illegible]	[illegible]	[illegible]	[illegible]	[illegible]	[illegible]	[illegible]	[illegible]	[illegible]	[illegible]	[illegible]	[illegible]	[illegible]	[illegible]	[illegible]	[illegible]	[illegible]	[illegible]	0·1 (0·1 to 0·1)	129
Tracheal, bronchus, and lung cancer	0 (0 to 0)	[illegible]	[illegible]	[illegible]	[illegible]	[illegible]	[illegible]	[illegible]	[illegible]	[illegible]	[illegible]	[illegible]	[illegible]	[illegible]	[illegible]	[illegible]	[illegible]	[illegible]	[illegible]	[illegible]	[illegible]	[illegible]	1·2 (0·9 to 1·4)	979
Malignant skin melanoma	0 (0 to 0)	[illegible]	[illegible]	[illegible]	[illegible]	[illegible]	[illegible]	[illegible]	[illegible]	[illegible]	[illegible]	[illegible]	[illegible]	[illegible]	[illegible]	[illegible]	[illegible]	[illegible]	[illegible]	[illegible]	[illegible]	[illegible]	0·1 (0·1 to 0·1)	40·6
Non-melanoma skin cancer	0 (0 to 0)	[illegible]	[illegible]	[illegible]	[illegible]	[illegible]	[illegible]	[illegible]	[illegible]	[illegible]	[illegible]	[illegible]	[illegible]	[illegible]	[illegible]	[illegible]	[illegible]	[illegible]	[illegible]	[illegible]	[illegible]	[illegible]	0·7 (0·5 to 1)	56·2
Non-melanoma skin cancer (squamous-cell carcinoma)	0 (0 to 0)	[illegible]	[illegible]	[illegible]	[illegible]	[illegible]	[illegible]	[illegible]	[illegible]	[illegible]	[illegible]	[illegible]	[illegible]	[illegible]	[illegible]	[illegible]	[illegible]	[illegible]	[illegible]	[illegible]	[illegible]	[illegible]	0·7 (0·5 to 1)	56·2
Non-melanoma skin cancer (basal-cell carcinoma)	0 (0 to 0)	[illegible]	[illegible]	[illegible]	[illegible]	[illegible]	[illegible]	[illegible]	[illegible]	[illegible]	[illegible]	[illegible]	[illegible]	[illegible]	[illegible]	[illegible]	[illegible]	[illegible]	[illegible]	[illegible]	[illegible]	[illegible]	0 (0 to 0)	0
Breast cancer	0 (0 to 0)	[illegible]	[illegible]	[illegible]	[illegible]	[illegible]	[illegible]	[illegible]	[illegible]	[illegible]	[illegible]	[illegible]	[illegible]	[illegible]	[illegible]	[illegible]	[illegible]	[illegible]	[illegible]	[illegible]	[illegible]	[illegible]	1·1 (0·7 to 1·4)	612
Cervical cancer	0 (0 to 0)	[illegible]	[illegible]	[illegible]	[illegible]	[illegible]	[illegible]	[illegible]	[illegible]	[illegible]	[illegible]	[illegible]	[illegible]	[illegible]	[illegible]	[illegible]	[illegible]	[illegible]	[illegible]	[illegible]	[illegible]	[illegible]	0·8 (0·6 to 1·2)	1,010
Uterine cancer	0 (0 to 0)	[illegible]	[illegible]	[illegible]	[illegible]	[illegible]	[illegible]	[illegible]	[illegible]	[illegible]	[illegible]	[illegible]	[illegible]	[illegible]	[illegible]	[illegible]	[illegible]	[illegible]	[illegible]	[illegible]	[illegible]	[illegible]	0·3 (0·2 to 0·4)	189
Ovarian cancer	0 (0 to 0)	[illegible]	[illegible]	[illegible]	[illegible]	[illegible]	[illegible]	[illegible]	[illegible]	[illegible]	[illegible]	[illegible]	[illegible]	[illegible]	[illegible]	[illegible]	[illegible]	[illegible]	[illegible]	[illegible]	[illegible]	[illegible]	0·1 (0·1 to 0·1)	140
Prostate cancer	0 (0 to 0)	[illegible]	[illegible]	[illegible]	[illegible]	[illegible]	[illegible]	[illegible]	[illegible]	[illegible]	[illegible]	[illegible]	[illegible]	[illegible]	[illegible]	[illegible]	[illegible]	[illegible]	[illegible]	[illegible]	[illegible]	[illegible]	6·2 (4 to 8·8)	803
Testicular cancer	0·1 (0 to 1·7)	[illegible]	[illegible]	[illegible]	[illegible]	[illegible]	[illegible]	[illegible]	[illegible]	[illegible]	[illegible]	[illegible]	[illegible]	[illegible]	[illegible]	[illegible]	[illegible]	[illegible]	[illegible]	[illegible]	[illegible]	[illegible]	0 (0 to 0)	23·4
Kidney cancer	0·3 (0·2 to 0·4)	[illegible]	[illegible]	[illegible]	[illegible]	[illegible]	[illegible]	[illegible]	[illegible]	[illegible]	[illegible]	[illegible]	[illegible]	[illegible]	[illegible]	[illegible]	[illegible]	[illegible]	[illegible]	[illegible]	[illegible]	[illegible]	0·1 (0·1 to 0·2)	187
Bladder cancer	0 (0 to 0)	[illegible]	[illegible]	[illegible]	[illegible]	[illegible]	[illegible]	[illegible]	[illegible]	[illegible]	[illegible]	[illegible]	[illegible]	[illegible]	[illegible]	[illegible]	[illegible]	[illegible]	[illegible]	[illegible]	[illegible]	[illegible]	0·6 (0·4 to 0·8)	114
Brain and central nervous system cancer	1 (0·4 to 1·5)	[illegible]	[illegible]	[illegible]	[illegible]	[illegible]	[illegible]	[illegible]	[illegible]	[illegible]	[illegible]	[illegible]	[illegible]	[illegible]	[illegible]	[illegible]	[illegible]	[illegible]	[illegible]	[illegible]	[illegible]	[illegible]	0·1 (0 to 0·1)	294
Thyroid cancer	0 (0 to 0)	[illegible]	[illegible]	[illegible]	[illegible]	[illegible]	[illegible]	[illegible]	[illegible]	[illegible]	[illegible]	[illegible]	[illegible]	[illegible]	[illegible]	[illegible]	[illegible]	[illegible]	[illegible]	[illegible]	[illegible]	[illegible]	0 (0 to 0·1)	32·4
Mesothelioma	0 (0 to 0)	[illegible]	[illegible]	[illegible]	[illegible]	[illegible]	[illegible]	[illegible]	[illegible]	[illegible]	[illegible]	[illegible]	[illegible]	[illegible]	[illegible]	[illegible]	[illegible]	[illegible]	[illegible]	[illegible]	[illegible]	[illegible]	0 (0 to 0)	46·7
Hodgkin lymphoma	0 (0 to 0)	[illegible]	[illegible]	[illegible]	[illegible]	[illegible]	[illegible]	[illegible]	[illegible]	[illegible]	[illegible]	[illegible]	[illegible]	[illegible]	[illegible]	[illegible]	[illegible]	[illegible]	[illegible]	[illegible]	[illegible]	[illegible]	0 (0 to 0)	84·6
Non-Hodgkin lymphoma	0 (0 to 0)	[illegible]	[illegible]	[illegible]	[illegible]	[illegible]	[illegible]	[illegible]	[illegible]	[illegible]	[illegible]	[illegible]	[illegible]	[illegible]	[illegible]	[illegible]	[illegible]	[illegible]	[illegible]	[illegible]	[illegible]	[illegible]	0·1 (0·1 to 0·1)	247
Multiple myeloma	0 (0 to 0)	[illegible]	[illegible]	[illegible]	[illegible]	[illegible]	[illegible]	[illegible]	[illegible]	[illegible]	[illegible]	[illegible]	[illegible]	[illegible]	[illegible]	[illegible]	[illegible]	[illegible]	[illegible]	[illegible]	[illegible]	[illegible]	0·1 (0 to 0·1)	65·2
Leukemia	1·3 (1 to 1·8)	[illegible]	[illegible]	[illegible]	[illegible]	[illegible]	[illegible]	[illegible]	[illegible]	[illegible]	[illegible]	[illegible]	[illegible]	[illegible]	[illegible]	[illegible]	[illegible]	[illegible]	[illegible]	[illegible]	[illegible]	[illegible]	0·3 (0·2 to 0·4)	503
Acute lymphoid leukemia	0·2 (0·1 to 0·3)	[illegible]	[illegible]	[illegible]	[illegible]	[illegible]	[illegible]	[illegible]	[illegible]	[illegible]	[illegible]	[illegible]	[illegible]	[illegible]	[illegible]	[illegible]	[illegible]	[illegible]	[illegible]	[illegible]	[illegible]	[illegible]	0 (0 to 0)	126
Chronic lymphoid leukemia	0 (0 to 0)	[illegible]	[illegible]	[illegible]	[illegible]	[illegible]	[illegible]	[illegible]	[illegible]	[illegible]	[illegible]	[illegible]	[illegible]	[illegible]	[illegible]	[illegible]	[illegible]	[illegible]	[illegible]	[illegible]	[illegible]	[illegible]	0 (0 to 0)	17
Acute myeloid leukemia	0·2 (0 to 0·5)	[illegible]	[illegible]	[illegible]	[illegible]	[illegible]	[illegible]	[illegible]	[illegible]	[illegible]	[illegible]	[illegible]	[illegible]	[illegible]	[illegible]	[illegible]	[illegible]	[illegible]	[illegible]	[illegible]	[illegible]	[illegible]	0 (0 to 0)	48·2

Chronic myeloid leukemia	0 (0 to 0)	0 (0 to 0)	0·2 (0·1 to 0·4)	0·5 (0·3 to 0·8)	0·4 (0·3 to 0·6)	0·4 (0·3 to 0·6)	0·8 (0·5 to 1·3)	1·5 (0·8 to 2·6)	2·5 (1·4 to 4·2)	2·2 (1·3 to 3·5)	2 (1·2 to 2·9)	1·2 (0·7 to 1·9)	1·4 (0·8 to 2·1)	1·5 (1 to 2·3)	1·5 (0·9 to 2·2)	1·3 (0·8 to 2)	0·9 (0·6 to 1·5)	0·6 (0·4 to 0·9)	0·4 (0·2 to 0·6)	0·2 (0·2 to 0·4)	0·1 (0·1 to 0·2)	0 (0 to 0·1)	0 (0 to 0)	19·9
Other leukemia	1 (0·6 to 1·4)	0·8 (0·3 to 1·2)	5 (3·2 to 7·6)	10·5 (6·8 to 15·5)	14 (9·5 to 20·3)	16·6 (11·6 to 23·1)	22·5 (16·1 to 30·1)	24·9 (18·4 to 32·6)	26·2 (19·9 to 33·6)	24·2 (17·9 to 31·2)	19·1 (14·4 to 24·9)	18·7 (14·3 to 23·6)	21 (16·2 to 26·6)	18·6 (14·8 to 23·3)	18·1 (14 to 22·7)	15·1 (12·1 to 18·8)	12 (9·4 to 15)	9·6 (7·5 to 11·9)	6·6 (5·2 to 8·2)	4·4 (3·5 to 5·5)	2·3 (1·8 to 2·9)	0·9 (0·7 to 1·1)	0·2 (0·2 to 0·3)	292
Other malignant neoplasms	1·5 (1·1 to 2·1)	1·7 (1·2 to 2·3)	9·4 (6·3 to 13·3)	34 (25 to 45·7)	24·6 (19·8 to 30·5)	27·4 (22·2 to 33)	42·4 (34·1 to 51·8)	36·2 (28·9 to 45)	32·2 (26·4 to 39·6)	30·2 (24·5 to 37·1)	26·2 (21 to 32·2)	27·9 (22·3 to 34·8)	32·3 (25·7 to 40·3)	34·3 (27·6 to 42·3)	34·6 (27·4 to 42·9)	27·8 (22·1 to 34·6)	21·8 (17·6 to 26·8)	16·4 (13·2 to 20·1)	11·6 (9·5 to 14)	7·9 (6·4 to 9·5)	4·1 (3·2 to 5·4)	1·7 (1·3 to 2·3)	0·5 (0·3 to 0·6)	487
Other neoplasms	0·1 (0·1 to 0·2)	0·2 (0·1 to 0·4)	0·6 (0·3 to 1·2)	1·9 (1·2 to 2·9)	1·3 (0·8 to 1·9)	1·3 (0·8 to 1·9)	1·5 (1 to 2·2)	1·1 (0·7 to 1·5)	1·8 (1·1 to 2·5)	1·5 (1 to 2·1)	1·3 (0·8 to 1·9)	1·8 (1·2 to 2·5)	1·8 (1·3 to 2·5)	2·5 (1·8 to 3·7)	2·9 (2·1 to 4·3)	3·1 (2·3 to 4·8)	2·8 (2·1 to 4·1)	2·7 (2 to 4·2)	2 (1·5 to 3·3)	1·4 (1 to 2·2)	0·7 (0·5 to 1·2)	0·3 (0·2 to 0·4)	0·1 (0 to 0·1)	34·7
Myelodysplastic, myeloproliferative, and other hematopoietic neo	0·1 (0·1 to 0·2)	0·2 (0·1 to 0·4)	0·6 (0·3 to 1·2)	1·9 (1·2 to 2·9)	1·3 (0·8 to 1·9)	1·3 (0·8 to 1·9)	1·5 (1 to 2·2)	1·1 (0·7 to 1·5)	1·8 (1·1 to 2·5)	1·5 (1 to 2·1)	1·3 (0·8 to 1·9)	1·8 (1·2 to 2·5)	1·8 (1·3 to 2·5)	2·5 (1·8 to 3·7)	2·9 (2·1 to 4·3)	3·1 (2·3 to 4·8)	2·8 (2·1 to 4·1)	2·7 (2 to 4·2)	2 (1·5 to 3·3)	1·4 (1 to 2·2)	0·7 (0·5 to 1·2)	0·3 (0·2 to 0·4)	0·1 (0 to 0·1)	34·7
Benign and in situ intestinal neoplasms	0 (0 to 0)	0 (0 to 0)	0 (0 to 0)	0 (0 to 0)	0 (0 to 0)	0 (0 to 0)	0 (0 to 0)	0 (0 to 0)	0 (0 to 0)	0 (0 to 0)	0 (0 to 0)	0 (0 to 0)	0 (0 to 0)	0 (0 to 0)	0 (0 to 0)	0 (0 to 0)	0 (0 to 0)	0 (0 to 0)	0 (0 to 0)	0 (0 to 0)	0 (0 to 0)	0 (0 to 0)	0 (0 to 0)	0
Benign and in situ cervical and uterine neoplasms	0 (0 to 0)	0 (0 to 0)	0 (0 to 0)	0 (0 to 0)	0 (0 to 0)	0 (0 to 0)	0 (0 to 0)	0 (0 to 0)	0 (0 to 0)	0 (0 to 0)	0 (0 to 0)	0 (0 to 0)	0 (0 to 0)	0 (0 to 0)	0 (0 to 0)	0 (0 to 0)	0 (0 to 0)	0 (0 to 0)	0 (0 to 0)	0 (0 to 0)	0 (0 to 0)	0 (0 to 0)	0 (0 to 0)	0
Other benign and in situ neoplasms	0 (0 to 0)	0 (0 to 0)	0 (0 to 0)	0 (0 to 0)	0 (0 to 0)	0 (0 to 0)	0 (0 to 0)	0 (0 to 0)	0 (0 to 0)	0 (0 to 0)	0 (0 to 0)	0 (0 to 0)	0 (0 to 0)	0 (0 to 0)	0 (0 to 0)	0 (0 to 0)	0 (0 to 0)	0 (0 to 0)	0 (0 to 0)	0 (0 to 0)	0 (0 to 0)	0 (0 to 0)	0 (0 to 0)	0
Cardiovascular diseases	14 (9·5 to 19·3)	7·3 (4·9 to 10·2)	31 (19·8 to 46)	23·8 (18·4 to 30·9)	36·1 (28 to 45·7)	62·5 (48·3 to 79·9)	168 (136 to 204)	204 (162 to 254)	267 (213 to 329)	371 (295 to 455)	405 (322 to 506)	568 (471 to 676)	854 (714 to 1,010)	1,030 (854 to 1,230)	1,300 (1,100 to 1,550)	1,290 (1,120 to 1,500)	1,150 (982 to 1,320)	987 (866 to 1,140)	841 (747 to 939)	703 (601 to 788)	474 (389 to 541)	248 (193 to 282)	99·7 (74·9 to 115)	11,100
Rheumatic heart disease	0 (0 to 0)	0 (0 to 0)	0 (0 to 0)	1·8 (1·1 to 2·9)	9 (5·5 to 14·6)	21·1 (12·9 to 33·8)	37·9 (25·9 to 55·4)	36·9 (25·7 to 52·1)	39·2 (27·2 to 53·2)	38·8 (28 to 51·4)	31·4 (22·4 to 41·8)	28·7 (20·7 to 38)	26·1 (18·8 to 35·3)	20·2 (14·6 to 27)	15·7 (11·5 to 21)	9·1 (6·9 to 11·8)	5·7 (4·3 to 7·3)	3·5 (2·6 to 4·5)	2·4 (1·8 to 3)	1·4 (1·1 to 1·8)	0·8 (0·6 to 1·1)	0·4 (0·3 to 0·5)	0·2 (0·1 to 0·2)	330
Ischemic heart disease	0 (0 to 0)	0 (0 to 0)	0 (0 to 0)	0 (0 to 0)	0 (0 to 0)	0 (0 to 0)	16·8 (12·8 to 21·7)	31·7 (22·8 to 41·7)	54·1 (39·8 to 70·4)	101 (74·9 to 132)	129 (96·1 to 168)	203 (160 to 257)	337 (265 to 426)	449 (361 to 547)	606 (498 to 745)	604 (515 to 700)	548 (472 to 643)	454 (384 to 524)	378 (325 to 434)	313 (266 to 362)	215 (176 to 246)	112 (86·9 to 131)	48·4 (36·4 to 57·1)	4,600
Stroke	4·4 (2·3 to 7·2)	1·1 (0·6 to 1·7)	4·1 (2 to 7·3)	3·7 (2·8 to 4·7)	8·4 (6 to 11·1)	14·1 (10·6 to 18·2)	43·4 (31·5 to 60·2)	57·5 (40·2 to 80·2)	72·9 (51 to 103)	109 (74·4 to 150)	118 (83·7 to 166)	168 (129 to 214)	253 (195 to 315)	304 (233 to 382)	383 (305 to 475)	393 (316 to 481)	338 (257 to 438)	309 (242 to 386)	269 (224 to 322)	219 (174 to 263)	139 (108 to 166)	67·8 (51·1 to 82·1)	24·3 (17·5 to 30·1)	3,300
Ischemic stroke	0·7 (0·4 to 1·2)	0·1 (0·1 to 0·2)	0·3 (0·2 to 0·4)	1·7 (1·2 to 2·3)	4·1 (2·5 to 6·1)	6 (3·8 to 9)	10·4 (7·4 to 14·2)	12 (8·7 to 16·1)	14·6 (10·1 to 19·9)	20·1 (14·5 to 27·1)	21·9 (15·9 to 29·4)	29·4 (22·3 to 37·8)	42·2 (32·8 to 52·7)	56·3 (43·8 to 70·8)	76 (59·9 to 96·5)	112 (89·1 to 138)	108 (83·8 to 139)	143 (111 to 180)	134 (110 to 162)	149 (118 to 180)	99 (77·4 to 118)	50·2 (37·6 to 60·5)	18·7 (13·5 to 23·2)	1,110
Intracerebral hemorrhage	1·3 (0·6 to 2·1)	0·3 (0·2 to 0·5)	2·2 (1 to 4·1)	0·7 (0·5 to 1)	2·3 (1·6 to 3·2)	4·1 (2·9 to 5·4)	20·2 (13·6 to 29·2)	28·7 (18·8 to 41·6)	38·7 (25·7 to 58·8)	63·1 (40·8 to 90·1)	68·7 (45·6 to 101)	103 (76·3 to 137)	169 (128 to 212)	205 (153 to 262)	263 (205 to 328)	253 (200 to 318)	209 (155 to 275)	152 (115 to 195)	125 (99·4 to 153)	63·9 (48 to 80·9)	35·9 (27·4 to 45·3)	15·9 (11·9 to 19·8)	5·1 (3·7 to 6·5)	1,830
Subarachnoid hemorrhage	2·4 (1·3 to 4)	0·7 (0·4 to 1·1)	1·6 (0·7 to 3)	1·3 (0·8 to 1·8)	2 (1·3 to 3)	4 (2·7 to 5·7)	12·9 (8·2 to 19·2)	16·7 (10·1 to 25·9)	19·6 (12·1 to 29·7)	25·3 (15·7 to 38·1)	27·3 (17·3 to 39·9)	35·2 (23·9 to 48·1)	41·7 (29·4 to 56·6)	42·6 (30·4 to 59·4)	43·8 (32·3 to 60·2)	27·6 (20 to 37·1)	21·3 (14·9 to 29·9)	13·5 (9·7 to 18·8)	10·2 (7·5 to 13·5)	6·6 (4·8 to 9)	3·6 (2·6 to 5)	1·8 (1·2 to 2·4)	0·5 (0·4 to 0·8)	362
Hypertensive heart disease	0 (0 to 0)	0 (0 to 0)	0 (0 to 0)	0 (0 to 0)	0 (0 to 0)	0 (0 to 0)	4·2 (2·3 to 6·2)	7·4 (4·3 to 11·2)	11·4 (6·6 to 17·6)	23·5 (14 to 34·1)	32·5 (21 to 47·2)	51·6 (34·3 to 71·5)	90·4 (60·9 to 127)	108 (75 to 150)	127 (87 to 175)	132 (95·6 to 176)	121 (90 to 162)	109 (79·4 to 143)	94·9 (69·7 to 124)	81·5 (61·7 to 107)	54·1 (40·1 to 69·6)	29·3 (20·4 to 39·1)	12·4 (8·4 to 16·6)	1,090
Non-rheumatic valvular heart disease	0 (0 to 0)	0 (0 to 0)	0 (0 to 0)	0 (0 to 0)	0 (0 to 0)	0 (0 to 0)	5·2 (3·8 to 6·8)	4·6 (3·2 to 6·3)	6·1 (4·1 to 8·7)	7·4 (5·2 to 10·1)	6·2 (4·2 to 8·7)	7·5 (5·1 to 10·8)	9·2 (6·5 to 12·4)	8·4 (5·9 to 11·2)	10·2 (7·3 to 14)	9·1 (6·7 to 11·9)	7·7 (5·8 to 10·2)	5·6 (4·3 to 7·1)	4·1 (3·1 to 5·3)	3·5 (2·7 to 4·5)	2·1 (1·6 to 2·6)	1 (0·7 to 1·2)	0·3 (0·2 to 0·4)	98·1
Non-rheumatic calcific aortic valve disease	0 (0 to 0)	0 (0 to 0)	0 (0 to 0)	0 (0 to 0)	0 (0 to 0)	0 (0 to 0)	1·7 (1 to 2·7)	1·5 (0·9 to 2·4)	1·7 (0·9 to 3·1)	1·6 (0·9 to 2·8)	1·5 (0·8 to 2·6)	2·1 (1·2 to 3·5)	2·8 (1·6 to 4·5)	2·6 (1·6 to 4·1)	3·5 (2·2 to 5·3)	3·5 (2·3 to 5)	3·5 (2·4 to 5)	2·7 (1·8 to 3·7)	2·1 (1·6 to 2·8)	2 (1·4 to 2·6)	1·2 (0·8 to 1·6)	0·5 (0·3 to 0·7)	0·2 (0·1 to 0·2)	34·7
Non-rheumatic degenerative mitral valve disease	0 (0 to 0)	0 (0 to 0)	0 (0 to 0)	0 (0 to 0)	0 (0 to 0)	0 (0 to 0)	2·9 (1·9 to 4)	2·6 (1·7 to 3·6)	4 (2·5 to 5·8)	4·9 (3·2 to 7·2)	4·2 (2·7 to 6·1)	5·1 (3·3 to 7·5)	5·8 (3·8 to 8·2)	5·2 (3·6 to 7·2)	6·3 (4·3 to 8·9)	5·3 (3·8 to 7·3)	4 (2·8 to 5·5)	2·6 (1·8 to 3·5)	1·8 (1·2 to 2·5)	1·3 (0·9 to 1·9)	0·8 (0·5 to 1·1)	0·4 (0·3 to 0·6)	0·1 (0·1 to 0·2)	57·2
Other non-rheumatic valve diseases	0 (0 to 0)	0 (0 to 0)	0 (0 to 0)	0 (0 to 0)	0 (0 to 0)	0 (0 to 0)	0·6 (0·3 to 1·1)	0·5 (0·2 to 0·9)	0·4 (0·2 to 0·8)	0·8 (0·4 to 1·4)	0·4 (0·2 to 0·8)	0·3 (0·1 to 0·6)	0·7 (0·3 to 1·3)	0·5 (0·2 to 1)	0·5 (0·2 to 0·9)	0·3 (0·1 to 0·5)	0·3 (0·1 to 0·5)	0·3 (0·2 to 0·5)	0·2 (0·1 to 0·4)	0·2 (0·1 to 0·4)	0·1 (0·1 to 0·2)	0·1 (0 to 0·1)	0 (0 to 0)	6·2
Cardiomyopathy and myocarditis	3·4 (2·2 to 4·8)	2·8 (2 to 3·9)	14·8 (9·5 to 22·1)	9·7 (6·7 to 13·4)	7 (5·5 to 8·7)	9·8 (7·5 to 12·2)	22 (16·2 to 28·6)	24·7 (17·3 to 33)	30·7 (20·8 to 41·6)	38·4 (25·2 to 52·4)	38·7 (26·3 to 55·3)	45·4 (32 to 61·3)	58·9 (39·8 to 79·3)	58·8 (41·1 to 79·6)	61·6 (43·6 to 81·2)	51·1 (37·5 to 67·2)	38·5 (28·5 to 49·9)	28·1 (21·1 to 36·5)	20·7 (15·4 to 27)	15·6 (11·2 to 20·1)	11·5 (8·3 to 15·4)	5·4 (3·8 to 7·2)	1·9 (1·3 to 2·7)	599
Myocarditis	0·4 (0·2 to 0·8)	0·6 (0·3 to 0·9)	3 (1·5 to 5·4)	2·3 (1·3 to 3·7)	2 (1·2 to 2·9)	1·7 (1·1 to 2·5)	3·4 (2 to 5·4)	2·8 (1·7 to 4·4)	3·4 (2 to 5·5)	4·6 (2·9 to 7)	3·2 (1·9 to 5·1)	3·1 (1·7 to 5)	3·5 (1·9 to 5·6)	3·3 (1·9 to 5·3)	2·4 (1·4 to 4)	1·9 (1·1 to 3)	1·5 (0·8 to 2·5)	1 (0·6 to 1·6)	0·9 (0·5 to 1·4)	0·7 (0·4 to 1·3)	0·7 (0·3 to 1·3)	0·4 (0·2 to 0·7)	0·2 (0·1 to 0·4)	47·1
Alcoholic cardiomyopathy	0 (0 to 0)	0 (0 to 0)	0 (0 to 0)	0 (0 to 0)	0 (0 to 0)	0 (0 to 0)	0·5 (0·3 to 0·8)	0·8 (0·4 to 1·3)	1·5 (0·8 to 2·6)	2·8 (1·3 to 5·2)	3·2 (1·6 to 5·7)	4·8 (2·5 to 8·1)	8·9 (4·7 to 13·8)	9 (5·1 to 14·1)	8·4 (4·8 to 13·3)	6·2 (3·6 to 10·1)	4·9 (2·8 to 7·7)	2 (1 to 3·6)	1·2 (0·6 to 2·2)	0·3 (0·2 to 0·5)	0·1 (0·1 to 0·2)	0 (0 to 0·1)	0 (0 to 0)	54·9
Other cardiomyopathy	2·9 (1·9 to 4·2)	2·3 (1·5 to 3·2)	11·7 (7·4 to 18·2)	7·4 (5 to 10·8)	4·9 (3·5 to 6·5)	8·1 (5·9 to 10·5)	18 (12·7 to 24·1)	21 (14 to 28·2)	25·8 (16·7 to 35·6)	31 (19·9 to 43·4)	32·2 (21·2 to 46)	37·5 (26·4 to 51·5)	46·5 (30·8 to 63·5)	46·5 (32·9 to 63·5)	50·8 (35·9 to 67·5)	43 (31·5 to 56·2)	32·2 (23·6 to 41·6)	25·1 (18·9 to 32)	18·6 (13·9 to 24·4)	14·5 (10·6 to 18·7)	10·7 (7·7 to 14·4)	4·9 (3·4 to 6·8)	1·7 (1·1 to 2·3)	497
Atrial fibrillation and flutter	0 (0 to 0)	0 (0 to 0)	0 (0 to 0)	0 (0 to 0)	0 (0 to 0)	0 (0 to 0)	0 (0 to 0)	0 (0 to 0)	0 (0 to 0)	1 (0·6 to 1·4)	1 (0·6 to 1·5)	3·8 (2·6 to 5·3)	6·7 (4·7 to 9·1)	9·5 (6·8 to 13)	15·1 (11·4 to 20·4)	18·6 (14·3 to 24·6)	20·8 (16 to 27·2)	23·7 (18·2 to 30·7)	24·7 (19·6 to 31·2)	28·7 (22·9 to 35·4)	24·2 (19·1 to 30·5)	16·9 (13 to 21·6)	6·5 (4·7 to 8·6)	201
Aortic aneurysm	--	--	--	--	--	--	0·8 (0·6 to 1·2)	0·8 (0·5 to 1·2)	1·7 (1·2 to 2·3)	2 (1·4 to 2·9)	2·7 (1·8 to 3·9)	3·5 (2·4 to 4·9)	4·9 (3·3 to 6·7)	6·3 (4·6 to 8·7)	10 (7·1 to 14)	11·7 (8·3 to 15·8)	11·9 (8·2 to 16·3)	10·6 (7·5 to 14·5)	8·6 (6 to 12)	6·5 (4·4 to 9)	3·6 (2·5 to 4·8)	1·5 (1 to 2)	0·4 (0·3 to 0·6)	NA
Endocarditis	0·2 (0·1 to 0·3)	0·3 (0·2 to 0·4)	1·1 (0·6 to 1·8)	0·9 (0·5 to 1·4)	0·7 (0·4 to 1·1)	1·1 (0·7 to 1·8)	3·1 (2 to 4·7)	4·3 (2·6 to 6·4)	4·7 (2·8 to 7·5)	5·3 (3 to 8·2)	4·8 (3·1 to 7·2)	5·4 (3·5 to 8)	5·3 (3·3 to 8·1)	4·5 (2·9 to 6·5)	4·2 (2·8 to 6)	3·3 (2·2 to 5·1)	2·3 (1·5 to 3·4)	1·7 (1·1 to 2·6)	1·1 (0·8 to 1·7)	1 (0·7 to 1·6)	0·6 (0·4 to 1)	0·3 (0·2 to 0·5)	0·1 (0·1 to 0·2)	56·2
Other cardiovascular and circulatory diseases	6 (3·7 to 9)	3·2 (1·9 to 4·7)	11·1 (6·2 to 18)	7·7 (5·4 to 10·7)	11 (7·1 to 15·9)	16·4 (10·9 to 23·4)	34·3 (25·7 to 45·3)	36·1 (26·6 to 47·3)	46·2 (34·1 to 61·2)	45·5 (32 to 60·4)	40·1 (28·2 to 54)	51·5 (38·1 to 67·4)	61·5 (48·2 to 77·1)	59·4 (46·5 to 73·8)	64·8 (51·5 to 79·7)	58·7 (46·7 to 72·7)	46·7 (37·6 to 57·6)	38 (30·6 to 46·6)	32·5 (25·6 to 39·7)	27·7 (21·8 to 33·9)	21·1 (15·9 to 26·2)	11 (7·9 to 14·2)	4·3 (3 to 5·5)	735
Chronic respiratory diseases	0 (0 to 0)	0 (0 to 0)	0 (0 to 0)	240 (152 to 383)	213 (132 to 338)	189 (121 to 285)	178 (125 to 256)	155 (115 to 209)	158 (121 to 203)	154 (122 to 193)	138 (110 to 172)	135 (111 to 163)	158 (129 to 192)	186 (154 to 225)	222 (179 to 272)	231 (188 to 283)	204 (169 to 249)	192 (156 to 234)	146 (121 to 177)	121 (97·5 to 146)	64·7 (52 to 78·7)	31·3 (24·4 to 37·8)	11·8 (8·6 to 14·3)	3,130
Chronic obstructive pulmonary disease	0 (0 to 0)	0 (0 to 0)	0 (0 to 0)	6·2 (4·4 to 8·4)	5 (3·1 to 7·4)	7·5 (4·6 to 11)	17·8 (12·8 to 23·8)	24·9 (18·3 to 32·5)	31·6 (23·4 to 42·3)	34 (25·5 to 44·6)	37·6 (28·1 to 47·9)	45·7 (35·6 to 57)	70 (54·5 to 89)	99·1 (79·6 to 122)	137 (108 to 172)	163 (131 to 198)	150 (122 to 185)	146 (118 to 180)	114 (92·7 to 140)	97·4 (78·3 to 118)	53·2 (42·7 to 65·1)	25·9 (20·2 to 31·3)	9·9 (7·3 to 12·2)	1,280
Pneumoconiosis	0 (0 to 0)	0 (0 to 0)	0 (0 to 0)	0 (0 to 0)	0 (0 to 0)	0 (0 to 0)	2·9 (1·8 to 4·3)	0·5 (0·3 to 1)	2 (1·1 to 3·3)	0·3 (0·2 to 0·5)	0·7 (0·4 to 1·1)	0·4 (0·3 to 0·6)	1 (0·6 to 1·5)	1 (0·6 to 1·5)	0·9 (0·5 to 1·5)	1 (0·6 to 1·7)	1·3 (0·8 to 2)	0·7 (0·4 to 1)	0·5 (0·3 to 0·8)	0·6 (0·4 to 0·9)	0·4 (0·3 to 0·6)	0·2 (0·1 to 0·3)	0 (0 to 0)	14·4
Silicosis	0 (0 to 0)	0 (0 to 0)	0 (0 to 0)	0 (0 to 0)	0 (0 to 0)	0 (0 to 0)	0·1 (0 to 0·2)	0 (0 to 0·1)	0·8 (0·4 to 1·5)	0 (0 to 0)	0 (0 to 0)	0 (0 to 0)	0·1 (0·1 to 0·3)	0 (0 to 0·1)	0 (0 to 0·1)	0 (0 to 0)	0 (0 to 0·1)	0 (0 to 0)	0 (0 to 0·1)	0 (0 to 0·1)	0 (0 to 0·1)	0 (0 to 0)	0 (0 to 0)	1·4
Asbestosis	0 (0 to 0)	0 (0 to 0)	0 (0 to 0)	0 (0 to 0)	0 (0 to 0)	0 (0 to 0)	0·3 (0·1 to 0·7)	0 (0 to 0·1)	0·1 (0 to 0·1)	0 (0 to 0)	0 (0 to 0)	0 (0 to 0)	0 (0 to 0·1)	0 (0 to 0·1)	0·1 (0 to 0·1)	0 (0 to 0)	0 (0 to 0)	0 (0 to 0)	0 (0 to 0)	0 (0 to 0)	0 (0 to 0·1)	0 (0 to 0)	0 (0 to 0)	0·7
Coal workers pneumoconiosis	0 (0 to 0)	0 (0 to 0)	0 (0 to 0)	0 (0 to 0)	0 (0 to 0)	0 (0 to 0)	2·2 (1·3 to 3·4)	0·4 (0·2 to 0·7)	0·9 (0·4 to 1·6)	0·3 (0·2 to 0·4)	0·6 (0·4 to 1)	0·3 (0·1 to 0·4)	0·7 (0·4 to 1·1)	0·8 (0·5 to 1·3)	0·7 (0·4 to 1·2)	0·9 (0·5 to 1·6)	1·1 (0·7 to 1·8)	0·6 (0·4 to 0·9)	0·4 (0·2 to 0·6)	0·5 (0·3 to 0·8)	0·3 (0·2 to 0·4)	0·2 (0·1 to 0·3)	0 (0 to 0)	10·8
Other pneumoconiosis	0 (0 to 0)	0 (0 to 0)	0 (0 to 0)	0 (0 to 0)	0 (0 to 0)	0 (0 to 0)	0·3 (0·2 to 0·6)	0·1 (0 to 0·2)	0·2 (0·1 to 0·3)	0 (0 to 0·1)	0 (0 to 0·1)	0·1 (0·1 to 0·2)	0·1 (0·1 to 0·2)	0·1 (0 to 0·1)	0·1 (0 to 0·2)	0 (0 to 0·1)	0·1 (0·1 to 0·2)	0·1 (0 to 0·1)	0·1 (0 to 0·1)	0·1 (0 to 0·1)	0·1 (0 to 0·1)	0 (0 to 0)	0 (0 to 0)	1·5
Asthma	0 (0 to 0)	0 (0 to 0)	0 (0 to 0)	190 (102 to 331)	194 (114 to 319)	164 (97·6 to 261)	136 (85·7 to 211)	103 (67 to 150)	91·6 (62·8 to 129)	81·4 (57·3 to 113)	63·8 (45·5 to 87·7)	51·2 (37·6 to 68·2)	48·4 (36·1 to 63·7)	43·9 (33·3 to 55·7)	37·1 (26·9 to 50·1)	30·1 (22·5 to 40·7)	21 (15·8 to 27·7)	15·1 (10·9 to 20·8)	9·4 (6·8 to 12·2)	7 (4·9 to 9·8)	3·3 (2·3 to 4·5)	1·7 (1·2 to 2·3)	0·7 (0·4 to 0·9)	1,290
Interstitial lung disease and pulmonary sarcoidosis	0 (0 to 0)	0 (0 to 0)	0 (0 to 0)	21·5 (13·6 to 32·4)	4·2 (2·8 to 6·2)	4·5 (2·8 to 6·5)	12·1 (7·1 to 18·4)	15·2 (8·4 to 24·2)	15 (8·2 to 24·4)	25·6 (14·7 to 39)	24·4 (15·8 to 35·7)	28 (18·6 to 39)	28·7 (19·1 to 41·5)	33·3 (22 to 46·9)	38 (24·3 to 55·4)	34·5 (22·7 to 50·6)	30·1 (19·7 to 42·4)	28·8 (18·4 to 39·3)	21·1 (14 to 29·9)	14·7 (9·3 to 21·7)	7·3 (4·7 to 10·6)	3·3 (2·1 to 4·8)	1·1 (0·6 to 1·7)	391
Other chronic respiratory diseases	0 (0 to 0)	0 (0 to 0)	0 (0 to 0)	22·8 (14·8 to 33·6)	9·9 (6·6 to 13·9)	13·2 (9·1 to 18·4)	9·2 (6·4 to 13·3)	12·1 (8·6 to 16·6)	17·8 (12·6 to 24·9)	13·2 (9·4 to 18·2)	11·5 (8·3 to 16)	9·8 (7 to 13·6)	9·6 (6·8 to 13·3)	8·7 (6 to 12·5)	9·4 (6·5 to 13·4)	2·4 (1·5 to 3·8)	1·6 (1 to 2·5)	1·4 (0·9 to 2·2)	0·9 (0·6 to 1·6)	0·9 (0·5 to 1·4)	0·5 (0·3 to 0·8)	0·2 (0·1 to 0·4)	0·1 (0 to 0·2)	155
Digestive diseases	9·3 (6·5 to 12·8)	10·1 (6·2 to 15·5)	60·6 (34·9 to 99·1)	87·2 (63·2 to 118)	68·4 (50·8 to 90·2)	68·1 (53·3 to 86·4)	119 (94 to 150)	166 (128 to 216)	222 (171 to 287)	286 (218 to 365)	312 (250 to 387)	360 (297 to 439)	457 (372 to 551)	480 (401 to 574)	481 (405 to 567)	367 (309 to 438)	254 (215 to 298)	183 (151 to 220)	119 (95·6 to 146)	86·9 (71·1 to 101)	47·1 (35·4 to 58·5)	21·6 (16·8 to 26·1)	7·5 (5·5 to 9·3)	4,270
Cirrhosis and other chronic liver diseases	0 (0 to 0)	0 (0 to 0)	0 (0 to 0·1)	13·5 (8·5 to 20·4)	11 (8 to 14·7)	14·1 (10·3 to 18·1)	34·2 (23·6 to 46·3)	46 (33·8 to 61·3)	73·8 (52·6 to 101)	119 (86·6 to 158)	147 (111 to 188)	196 (155 to 243)	283 (225 to 348)	309 (250 to 379)	315 (258 to 383)	228 (186 to 279)	147 (122 to 178)	97·2 (76·7 to 119)	55 (42·2 to 69)	35·7 (28 to 43·7)	16·4 (11·7 to 21·2)	6·5 (4·8 to 8·2)	2·2 (1·5 to 2·8)	2,150
Cirrhosis and other chronic liver diseases due to hepatitis B	0 (0 to 0)	0 (0 to 0)	0 (0 to 0)	0 (0 to 0)	0 (0 to 0·1)	0·1 (0 to 0·2)	0·4 (0·1 to 0·9)	1·7 (0·7 to 3·4)	4·7 (1·6 to 9·2)	9·9 (4·9 to 16·8)	14·6 (6·5 to 27·3)	20 (10 to 35)	27·9 (14·4 to 52)	27·8 (13·8 to 51·2)	23·9 (11·3 to 44·3)	14·8 (7 to 27·6)	8·6 (4·3 to 15·8)	5·4 (2·5 to 9·9)	3·1 (1·5 to 5·3)	2·1 (1 to 3·5)	0·9 (0·5 to 1·5)	0·3 (0·2 to 0·6)	0·1 (0 to 0·2)	166
Cirrhosis and other chronic liver diseases due to hepatitis C	0 (0 to 0)	0 (0 to 0)	0 (0 to 0)	0·1 (0 to 0·2)	0·2 (0·1 to 0·3)	0·4 (0·2 to 0·8)	1·6 (0·6 to 3·3)	6·4 (3 to 11·4)	16·8 (8·1 to 30)	30·3 (17·3 to 46·8)	37·5 (20·1 to 62·8)	48·2 (26·5 to 78·5)	64·9 (36·4 to 103)	66·1 (35·5 to 109)	62·5 (35·1 to 104)	42·4 (20·7 to 73·7)	26·3 (15·1 to 43·1)	16·8 (9·8 to 26·4)	9·1 (5 to 14·9)	6 (3·4 to 9·9)	2·8 (1·6 to 4·5)	1·1 (0·6 to 1·9)	0·4 (0·2 to 0·7)	440
Cirrhosis and other chronic liver diseases due to alcohol use	0 (0 to 0)	0 (0 to 0)	0 (0 to 0)	0 (0 to 0)	0 (0 to 0)	0 (0 to 0)	0·2 (0 to 0·5)	3·9 (1·6 to 7·9)	12·5 (5·5 to 22·6)	33·1 (18·9 to 52·4)	55·2 (30·3 to 83·4)	82 (50·9 to 116)	123 (77·2 to 174)	140 (90·9 to 194)	150 (99·3 to 207)	108 (70·1 to 148)	65·8 (43·2 to 89·8)	40·2 (25 to 57)	19·9 (12·4 to 28·9)	12 (7 to 18·2)	5 (3·1 to 7·6)	1·8 (1·1 to 2·9)	0·6 (0·2 to 1)	853
Cirrhosis and other chronic liver diseases due to NAFLD	0 (0 to 0)	0 (0 to 0)	0 (0 to 0)	0 (0 to 0)	0 (0 to 0)	0 (0 to 0)	1·6 (0·7 to 3·1)	4·9 (2·3 to 8·8)	11·6 (5·3 to 21·4)	19 (9·8 to 32·2)	20·6 (9·8 to 37·6)	27 (12·7 to 48·3)	41·5 (20 to 76)	46·8 (22·5 to 83·4)	46·1 (22·7 to 80·4)	35·3 (17·1 to 61·8)	25·7 (14·9 to 40·3)	18·7 (11·2 to 28·3)	11·9 (6·3 to 18·9)	8·1 (4·6 to 12·4)	3·9 (2·3 to 6)	1·6 (0·9 to 2·5)	0·6 (0·3 to 0·9)	325
Cirrhosis and other chronic liver diseases due to other causes	0 (0 to 0)	0 (0 to 0)	0 (0 to 0·1)	13·4 (8·4 to 20·3)	10·8 (7·8 to 14·5)	13·6 (10 to 17·5)	30·5 (20·7 to 41)	29·1 (19·2 to 41·4)	28·3 (15·9 to 43·7)	26·6 (14·2 to 43·8)	18·9 (9·3 to 33·4)	18·8 (9·3 to 33·2)	25·3 (12·9 to 45·2)	28·7 (14·6 to 49·2)	32·5 (16·6 to 55·6)	27·5 (15·3 to 44·8)	20·8 (10·6 to 36·2)	16 (8·3 to 26·2)	11 (5·9 to 17·5)	7·6 (4·3 to 11·8)	3·7 (2·2 to 5·5)	1·6 (0·9 to 2·4)	0·5 (0·2 to 0·9)	365
Upper digestive system diseases	0 (0 to 0)	0 (0 to 0)	0 (0 to 0)	4·5 (2·7 to 7·3)	5·8 (3 to 9·9)	9·2 (5·3 to 14·8)	29 (16·8 to 47)	57·6 (33·9 to 101)	73·6 (42·6 to 125)	81 (48 to 137)	78·9 (46·4 to 128)	73·8 (45·5 to 117)	75·4 (48·3 to 116)	71·1 (46·8 to 105)	67 (46·4 to 97·9)	53·7 (37·6 to 77·3)	39·9 (28·8 to 56·1)	29·6 (21·3 to 40)	20·6 (15 to 27·4)	15·2 (11·6 to 20)	8·6 (6·2 to 11·3)	4 (3 to 5·3)	1·4 (1 to 1·8)	800
Peptic ulcer disease	0 (0 to 0)	0 (0 to 0)	0 (0 to 0)	0·7 (0·4 to 1·1)	0·5 (0·3 to 0·8)	1·2 (0·9 to 1·7)	3 (2 to 4·3)	5·4 (3·6 to 7·8)	7 (4·6 to 10·5)	10·2 (6·5 to 15·1)	10·3 (7·2 to 14·6)	11·1 (7·7 to 15·7)	12·9 (8·7 to 18·2)	16·3 (11·4 to 21·9)	17·9 (12·6 to 23·6)	15·9 (11·7 to 20·6)	12·7 (9·2 to 17)	10·7 (7·6 to 14·4)	8·4 (5·9 to 11·6)	7 (5·2 to 9·1)	4 (2·7 to 5·5)	1·9 (1·3 to 2·6)	0·7 (0·5 to 1)	158
Gastritis and duodenitis	0 (0 to 0)	0 (0 to 0)	0 (0 to 0)	3·8 (2·1 to 6·4)	5·2 (2·4 to 9·3)	4·9 (2·1 to 9·4)	6·8 (3·4 to 12)	8·4 (5·1 to 13·9)	10·4 (6 to 16·7)	13·2 (7·6 to 21)	15·2 (8·5 to 24·8)	14·5 (8·1 to 25·1)	18·7 (11·3 to 30·9)	17·5 (10·6 to 27·6)	17·7 (11·3 to 26·9)	13·7 (9 to 20)	10·1 (7 to 14)	7·3 (5 to 10·2)	4·8 (3·4 to 6·7)	3·3 (2·3 to 4·6)	2 (1·3 to 2·7)	1 (0·7 to 1·4)	0·3 (0·2 to 0·4)	179
Gastroesophageal reflux disease	0 (0 to 0)	0 (0 to 0)	0 (0 to 0)	0 (0 to 0)	0·1 (0 to 0·2)	3 (1·4 to 5·8)	19·3 (9·5 to 36·4)	43·8 (21·7 to 86·3)	56·2 (27·6 to 104)	57·6 (27 to 109)	53·4 (25·3 to 97·6)	48·3 (23·1 to 87·9)	43·9 (21·6 to 77·6)	37·3 (18·3 to 68·4)	31·5 (14·7 to 59·4)	24·1 (11·6 to 44·8)	17·1 (8·6 to 31·3)	11·5 (5·9 to 20·2)	7·4 (3·8 to 13·4)	4·9 (2·4 to 8·7)	2·6 (1·3 to 4·7)	1·1 (0·6 to 2)	0·4 (0·2 to 0·6)	463
Appendicitis	0 (0 to 0)	0 (0 to 0)	0 (0 to 0)	4·9 (2·8 to 8·4)	5·5 (3·8 to 7·7)	7·1 (4·5 to 10·4)	9·9 (6·6 to 14·4)	8·8 (5·6 to 13)	7·3 (4·6 to 10·9)	6·6 (4·3 to 9·6)	6·2 (4·1 to 9·1)	4·7 (3·2 to 6·8)	4·9 (3·3 to 7)	4·3 (2·8 to 6·2)	4 (2·6 to 5·9)	2·6 (1·8 to 3·7)	2·4 (1·6 to 3·4)	1·8 (1·2 to 2·6)	1·1 (0·7 to 1·6)	0·8 (0·5 to 1·1)	0·6 (0·4 to 0·8)	0·3 (0·2 to 0·4)	0·1 (0·1 to 0·1)	83·9
Paralytic ileus and intestinal obstruction	6·4 (4·5 to 8·8)	8·5 (5·2 to 13·3)	52·1 (29·2 to 85·3)	19·7 (12·4 to 30·3)	8·6 (5·7 to 12·1)	7·9 (5·5 to 10·7)	8·5 (5·4 to 12·6)	8·7 (5·3 to 12·9)	9·5 (5·8 to 14·2)	9·6 (5·8 to 14·6)	12·7 (8·7 to 17·9)	12·8 (9 to 17·5)	15·6 (10·8 to 21·9)	18·6 (13 to 25·6)	18·7 (13·4 to 26·3)	18 (12·8 to 25·2)	16·1 (11·5 to 23)	15·9 (10·9 to 22·8)	13·5 (9·1 to 20)	12·1 (8·4 to 17·5)	7·6 (5·1 to 11·2)	3·8 (2·6 to 5·6)	1·4 (0·9 to 2·3)	306
Inguinal, femoral, and abdominal hernia	2·9 (1·8 to 4·3)	1·6 (0·8 to 2·7)	8·4 (4·4 to 15)	21·2 (13·2 to 31·8)	26·2 (14·2 to 43·6)	18·4 (10·4 to 30·5)	15·2 (8·7 to 24·6)	13·6 (7·9 to 21·7)	14·6 (8·5 to 23)	15·4 (9 to 24·3)	16·4 (9·9 to 26)	17·7 (11·1 to 27·1)	19 (11·7 to 29·7)	18·8 (12 to 27·8)	17·6 (11·8 to 25·4)	14 (9·7 to 19·7)	9·4 (6·7 to 13)	6·5 (4·8 to 8·5)	4·2 (3·2 to 5·5)	3·1 (2·4 to 4)	1·8 (1·3 to 2·4)	0·8 (0·6 to 1·1)	0·3 (0·2 to 0·4)	267
Inflammatory bowel disease	0 (0 to 0)	0 (0 to 0)	0 (0 to 0)	3·9 (2·3 to 6·2)	1·1 (0·7 to 1·6)	0·7 (0·5 to 1)	3 (1·9 to 4·3)	4·3 (2·9 to 6·2)	6·7 (4·4 to 10·2)	8·3 (5·6 to 12·4)	6·7 (4·7 to 9·2)	7·2 (5·3 to 9·8)	6·9 (5 to 9·6)	6 (4·4 to 8·2)	6·4 (4·6 to 8·6)	4·6 (3·3 to 6·3)	3·2 (2·4 to 4·5)	3·1 (2·3 to 4·4)	2·1 (1·5 to 3·1)	1·6 (1·1 to 2·2)	0·8 (0·6 to 1·2)	0·5 (0·3 to 0·6)	0·1 (0·1 to 0·2)	77·4
Vascular intestinal disorders	0 (0 to 0)	0 (0 to 0)	0 (0 to 0)	0·3 (0·2 to 0·4)	0·4 (0·2 to 0·5)	0·2 (0·1 to 0·3)	0·5 (0·3 to 0·7)	0·6 (0·4 to 0·8)	1 (0·6 to 1·5)	1·2 (0·8 to 1·8)	1·3 (0·8 to 1·8)	1·5 (1 to 2·1)	2·5 (1·7 to 3·6)	2·9 (2 to 4·1)	3·4 (2·3 to 4·7)	3·6 (2·6 to 4·9)	3·3 (2·3 to 4·4)	3 (2·1 to 4·1)	2·5 (1·7 to 3·6)	2·1 (1·5 to 3)	1·2 (0·8 to 1·7)	0·6 (0·4 to 0·7)	0·2 (0·1 to 0·2)	32·1
Gallbladder and biliary diseases	0 (0 to 0)	0 (0 to 0)	0 (0 to 0)	1·3 (0·8 to 2·1)	2·5 (1·1 to 4·4)	4·4 (2·2 to 7·7)	8·4 (5 to 13·3)	12·3 (7·9 to 18·1)	16·2 (10·2 to 23·4)	20·7 (13·5 to 29·5)	20·4 (13·4 to 29·2)	22·8 (15·2 to 31·8)	24·7 (16·9 to 33·9)	25·5 (18·2 to 34·8)	25·3 (18·1 to 33)	20·8 (15·4 to 27·3)	16·2 (12·1 to 21·4)	12·8 (9·3 to 17)	9·5 (6·8 to 12·7)	7·4 (5·3 to 9·8)	4 (2·8 to 5·4)	2 (1·4 to 2·7)	0·7 (0·5 to 1)	258
Pancreatitis	0 (0 to 0)	0 (0 to 0)	0 (0 to 0)	0·6 (0·4 to 0·9)	0·2 (0·1 to 0·4)	0·9 (0·6 to 1·2)	3·6 (2·3 to 5·3)	6·4 (4·1 to 9·2)	10·5 (6·7 to 16)	14·1 (9 to 20·8)	14·4 (9·8 to 21·8)	14·8 (9·8 to 21·5)	14 (9·7 to 20·6)	11·3 (7·8 to 15·6)	10·5 (7·4 to 14·6)	7·9 (5·5 to 11)	5·2 (3·9 to 7)	3·9 (2·8 to 5·2)	2·7 (1·9 to 3·6)	1·9 (1·4 to 2·5)	0·9 (0·6 to 1·2)	0·4 (0·3 to 0·5)	0·1 (0·1 to 0·2)	124
Other digestive diseases	0 (0 to 0)	0 (0 to 0)	0 (0 to 0)	17·1 (10·8 to 25·6)	7·1 (4·8 to 10)	5·2 (3·5 to 7·2)	6·8 (3·8 to 10·1)	7·4 (4·3 to 11·2)	9·2 (4·9 to 14·1)	10·2 (5·3 to 15)	8·2 (4·9 to 12·2)	8·9 (5·6 to 12·9)	11·6 (7·3 to 17·2)	12·9 (7·8 to 18·5)	13·4 (8·4 to 19·3)	13·3 (8·5 to 18·8)	10·6 (6·9 to 14·9)	9·3 (5·9 to 13)	7·9 (4·8 to 11·5)	7·1 (4·3 to 10)	5·3 (3·2 to 7·5)	2·8 (1·6 to 3·9)	1 (0·6 to 1·5)	175
Neurological disorders	0·1 (0 to 0·3)	0·4 (0·2 to 0·9)	28·5 (17·9 to 43·3)	69·9 (35·9 to 120)	131 (55·4 to 238)	351 (115 to 706)	476 (176 to 930)	444 (180 to 831)	405 (170 to 747)	362 (156 to 675)	323 (142 to 596)	288 (130 to 526)	258 (133 to 457)	208 (112 to 354)	177 (104 to 281)	150 (95·5 to 231)	137 (88·4 to 223)	142 (90·7 to 247)	147 (92·6 to 266)	190 (108 to 375)	157 (80·3 to 318)	101 (48·4 to 212)	48·8 (21·5 to 102)	4,600
Alzheimer's disease and other dementias	0 (0 to 0)	0 (0 to 0)	0 (0 to 0)	0 (0 to 0)	0 (0 to 0)	0 (0 to 0)	0 (0 to 0)	0 (0 to 0)	0 (0 to 0)	0 (0 to 0)	0 (0 to 0)	1·7 (0·4 to 5·3)	8·7 (2·5 to 24·3)	15·1 (5·3 to 38·3)	24·8 (9·4 to 60·3)	37·4 (15·2 to 88·5)	53 (21 to 129)	73·6 (31·1 to 172)	87·7 (36·8 to 209)	138 (56·9 to 322)	129 (52·3 to 288)	90·3 (36·6 to 202)	45·8 (18·8 to 99·8)	706
Parkinson's disease	0 (0 to 0)	0 (0 to 0)	0 (0 to 0)	0 (0 to 0)	0 (0 to 0)	0 (0 to 0)	0 (0 to 0)	0·3 (0·2 to 0·4)	0·5 (0·3 to 0·7)	0·6 (0·4 to 0·9)	1 (0·7 to 1·4)	2·4 (1·8 to 3·1)	4·9 (3·8 to 6·2)	7·7 (6·1 to 9·7)	11·2 (9 to 13·8)	15·9 (13·2 to 18·8)	21·6 (18·2 to 25·6)	30·7 (26·2 to 35·9)	37·5 (32 to 43·3)	37·4 (31·2 to 43·6)	20·8 (17·2 to 24·1)	7·9 (6·2 to 9·4)	2·1 (1·5 to 2·5)	202
Idiopathic epilepsy	0·1 (0 to 0·3)	0·3 (0·1 to 0·8)	11·8 (6·5 to 19·2)	46·6 (21·7 to 84)	65·1 (23·8 to 127)	85·8 (34 to 165)	119 (58·8 to 204)	117 (66·3 to 199)	109 (63·5 to 175)	95·4 (56·3 to 150)	76 (44·8 to 118)	61·7 (36·2 to 99·5)	56·8 (34 to 89·2)	41·5 (24·5 to 66·6)	33·5 (18·9 to 54·7)	24·4 (12·5 to 41·1)	17·5 (8·3 to 30·2)	12·4 (5·5 to 21·9)	8·6 (3·8 to 15·2)	6·1 (2·6 to 10·9)	3·4 (1·4 to 6·4)	1·6 (0·7 to 3)	0·5 (0·2 to 1)	994
Multiple sclerosis	0 (0 to 0)	0 (0 to 0)	0 (0 to 0)	0 (0 to 0)	0 (0 to 0·1)	0·1 (0·1 to 0·2)	0·6 (0·3 to 0·9)	1·5 (1 to 2·2)	2 (1·5 to 2·8)	2·5 (1·9 to 3·8)	3·5 (2·6 to 5·2)	3·6 (2·7 to 5·5)	3·7 (2·8 to 5·3)	3 (2·3 to 4·4)	2·7 (2·1 to 4)	1·8 (1·4 to 2·6)	0·9 (0·7 to 1·4)	0·6 (0·4 to 0·8)	0·3 (0·2 to 0·4)	0·2 (0·1 to 0·2)	0·1 (0·1 to 0·1)	0 (0 to 0)	0 (0 to 0)	27·2
Motor neuron disease	0 (0 to 0)	0·1 (0·1 to 0·2)	2·8 (1·6 to 4·6)	1·3 (0·9 to 1·9)	0·3 (0·2 to 0·4)	0·5 (0·4 to 0·6)	1·2 (0·8 to 1·6)	1·1 (0·8 to 1·5)	1 (0·7 to 1·3)	1·3 (0·9 to 1·7)	2·1 (1·5 to 2·9)	2·7 (2 to 3·8)	3 (2·2 to 4)	3·7 (2·7 to 4·9)	3·9 (2·9 to 5)	3·1 (2·4 to 4)	2·4 (1·8 to 3·1)	1·3 (1 to 1·7)	0·6 (0·4 to 0·8)	0·3 (0·2 to 0·3)	0·1 (0·1 to 0·1)	0 (0 to 0)	0 (0 to 0)	32·7
Headache disorders	0 (0 to 0)	0 (0 to 0)	0 (0 to 0)	0 (0 to 0)	42·2 (0·7 to 111)	225 (10·4 to 577)	311 (31·9 to 769)	295 (40·2 to 693)	270 (40·7 to 611)	244 (45·6 to 547)	225 (42·8 to 493)	201 (46·4 to 443)	166 (42·8 to 366)	124 (34·3 to 262)	89·6 (25·8 to 190)	59·6 (18·9 to 124)	36·3 (12·2 to 73·7)	19·7 (6·7 to 40·7)	10·3 (3·3 to 21·6)	5·9 (2 to 12·3)	3 (1 to 6·2)	1·1 (0·3 to 2·2)	0·3 (0·1 to 0·6)	2,330
Migraine	0 (0 to 0)	0 (0 to 0)	0 (0 to 0)	0 (0 to 0)	38·7 (0·5 to 104)	212 (6·5 to 571)	290 (22·5 to 757)	273 (28·1 to 664)	247 (28·1 to 587)	223 (28·2 to 524)	203 (31·5 to 474)	182 (33·2 to 420)	149 (29 to 340)	110 (22·9 to 241)	78·9 (17·8 to 175)	51·4 (13·1 to 113)	30·5 (8·6 to 66)	16·2 (4·9 to 34·3)	8·3 (2·4 to 18·7)	4·8 (1·4 to 10·7)	2·4 (0·7 to 5·3)	0·9 (0·3 to 1·8)	0·2 (0 to 0·5)	2,120
Tension-type headache	0 (0 to 0)	0 (0 to 0)	0 (0 to 0)	0 (0 to 0)	3·5 (0·1 to 23·4)	13·8 (0·8 to 99·6)	21·3 (3·7 to 109)	22·6 (4·6 to 102)	22·7 (4·9 to 94·6)	21·2 (5 to 79·5)	21·2 (5·2 to 74·4)	19·3 (5·5 to 62)	17·3 (5·8 to 50·9)	14·4 (4·1 to 43·3)	10·7 (3·3 to 29·4)	8·1 (2·5 to 23·3)	5·8 (1·6 to 18·3)	3·5 (0·9 to 12·6)	1·9 (0·4 to 7)	1·1 (0·2 to 3·9)	0·5 (0·1 to 2)	0·2 (0 to 0·8)	0·1 (0 to 0·3)	209
Other neurological disorders	0 (0 to 0)	0 (0 to 0)	13·9 (8·5 to 21·4)	22 (12·4 to 35·2)	23·3 (10·1 to 44·1)	39·2 (17·3 to 71·6)	44·3 (23·9 to 73·7)	29 (16·8 to 48·3)	21·9 (13·2 to 34·7)	18 (11·1 to 26·6)	15·6 (9·6 to 23·2)	15 (9·7 to 22·5)	15·2 (10·2 to 22·6)	13·1 (9·2 to 17·7)	11·7 (8·5 to 16·3)	8·2 (6 to 10·9)	5·2 (3·9 to 6·8)	3·4 (2·6 to 4·4)	2·2 (1·6 to 2·8)	1·4 (1·1 to 1·8)	0·6 (0·5 to 0·8)	0·3 (0·2 to 0·3)	0·1 (0·1 to 0·1)	304
Mental disorders	0·1 (0·1 to 0·2)	0·3 (0·2 to 0·5)	5·7 (3·6 to 8·3)	32·3 (20·8 to 46·6)	179 (121 to 260)	560 (378 to 797)	829 (574 to 1,150)	812 (556 to 1,110)	766 (529 to 1,040)	698 (498 to 940)	648 (463 to 861)	571 (411 to 764)	495 (358 to 659)	397 (287 to 525)	318 (229 to 426)	226 (162 to 301)	148 (107 to 197)	92·6 (67 to 122)	54·7 (38·6 to 73·6)	34·4 (23·9 to 46·5)	18·6 (13·1 to 25)	8·2 (5·6 to 11·1)	2·7 (1·7 to 3·9)	6,900
Schizophrenia	0 (0 to 0)	0 (0 to 0)	0 (0 to 0)	0 (0 to 0)	0 (0 to 0)	2·6 (1·1 to 4·9)	19·8 (8·9 to 36)	59·7 (33·2 to 95·9)	92·9 (53·9 to 139)	103 (64·6 to 150)	100 (63·5 to 145)	86·8 (58·3 to 120)	72·8 (49·3 to 98·9)	54·8 (37·8 to 73·8)	39·8 (27·4 to 53·8)	25·1 (16·6 to 34·4)	14·3 (9·7 to 20)	7·7 (4·8 to 10·8)	3·8 (2·4 to 5·4)	1·8 (1·1 to 2·7)	0·7 (0·4 to 1·1)	0·2 (0·1 to 0·3)	0 (0 to 0·1)	686
Depressive disorders	0 (0 to 0)	0 (0 to 0)	0 (0 to 0)	0·1 (0 to 0·2)	8·6 (3·3 to 17·4)	96·9 (49·9 to 171)	255 (149 to 404)	294 (178 to 461)	253 (154 to 382)	224 (137 to 346)	219 (140 to 320)	205 (129 to 314)	186 (121 to 276)	158 (105 to 230)	134 (85·5 to 198)	100 (62·9 to 146)	68·2 (44·9 to 98·4)	44·3 (28·8 to 62·5)	27·3 (17·4 to 40·8)	18·3 (11·3 to 27·6)	10·7 (6·8 to 15·5)	5·1 (3·2 to 7·5)	1·8 (1 to 2·8)	2,310
Major depressive disorder	0 (0 to 0)	0 (0 to 0)	0 (0 to 0)	0·1 (0 to 0·2)	7·5 (2·5 to 15·9)	88·4 (42·9 to 164)	230 (131 to 374)	256 (140 to 413)	210 (125 to 325)	181 (105 to 294)	179 (108 to 266)	168 (99·6 to 263)	154 (95 to 230)	132 (85·1 to 193)	113 (68·8 to 169)	85 (51·3 to 126)	58·2 (37 to 84·7)	38·1 (24·5 to 54·8)	23·7 (14·7 to 36·6)	16·1 (9·6 to 25·1)	9·5 (6 to 14)	4·5 (2·7 to 6·9)	1·6 (0·9 to 2·6)	1,960
Dysthymia	0 (0 to 0)	0 (0 to 0)	0 (0 to 0)	0 (0 to 0)	1·1 (0·2 to 2·6)	8·5 (3·6 to 16·4)	24·7 (12·3 to 43)	38 (18·9 to 66·9)	43·1 (22·1 to 74)	42·6 (23·8 to 70·4)	40·6 (23·7 to 67·8)	36·7 (19·8 to 63·3)	32·2 (17·9 to 55·6)	26·1 (15·3 to 43·8)	21 (12·2 to 34·3)	15 (8·1 to 25·4)	10 (5·3 to 16·9)	6·2 (3·5 to 10·2)	3·6 (2·2 to 5·8)	2·2 (1·2 to 3·7)	1·2 (0·6 to 2)	0·5 (0·3 to 0·8)	0·2 (0·1 to 0·3)	354
Bipolar disorder	0 (0 to 0)	0 (0 to 0)	0 (0 to 0)	0 (0 to 0)	0 (0 to 0)	31·4 (13·4 to 56·9)	114 (53·2 to 196)	124 (65·6 to 210)	110 (56·7 to 184)	92 (50·2 to 152)	81 (43·9 to 131)	71·6 (40·1 to 114)	62 (35 to 103)	48·3 (27·2 to 80·6)	36·7 (21·2 to 60·7)	24·1 (13·7 to 39·2)	14·5 (8 to 23·3)	8 (4·5 to 13)	4 (2·2 to 6·5)	2 (1·1 to 3·2)	0·8 (0·4 to 1·4)	0·3 (0·1 to 0·5)	0·1 (0 to 0·1)	824
Anxiety disorders	0 (0 to 0)	0 (0 to 0)	0 (0 to 0)	3·5 (1·3 to 6·8)	57·2 (29·7 to 96·7)	161 (89·1 to 261)	212 (129 to 327)	201 (116 to 328)	183 (104 to 305)	165 (97·8 to 263)	149 (88·6 to 229)	130 (74·5 to 209)	112 (63·9 to 175)	89·2 (54 to 134)	70·4 (44·5 to 104)	50 (29·8 to 78·5)	33·1 (19·9 to 52·9)	20·6 (12·9 to 33)	12 (7·5 to 19)	7·2 (4·3 to 11·1)	3·5 (2·1 to 5·5)	1·4 (0·8 to 2·2)	0·4 (0·2 to 0·6)	1,660
Eating disorders	0 (0 to 0)	0 (0 to 0)	0 (0 to 0)	0 (0 to 0)	0·6 (0·2 to 1·2)	10·2 (4·9 to 17·7)	34·3 (18·1 to 60)	45·8 (24·3 to 77·7)	39·1 (21·9 to 62·8)	30·1 (16·1 to 49·1)	20·3 (11 to 34·2)	9·9 (5·1 to 17)	4·4 (2·2 to 7·3)	0 (0 to 0)	0 (0 to 0)	0 (0 to 0)	0 (0 to 0)	0 (0 to 0)	0 (0 to 0)	0 (0 to 0)	0 (0 to 0)	0 (0 to 0)	0 (0 to 0)	195
Anorexia nervosa	0 (0 to 0)	0 (0 to 0)	0 (0 to 0)	0 (0 to 0)	0·5 (0·2 to 1·2)	5 (2·1 to 9·1)	13·3 (6·6 to 23·6)	11·8 (5·6 to 21·2)	7·9 (4 to 14·5)	4·5 (2·1 to 8·1)	3 (1·3 to 5·6)	2 (0·8 to 3·8)	1·3 (0·5 to 2·4)	0 (0 to 0)	0 (0 to 0)	0 (0 to 0)	0 (0 to 0)	0 (0 to 0)	0 (0 to 0)	0 (0 to 0)	0 (0 to 0)	0 (0 to 0)	0 (0 to 0)	49·3
Bulimia nervosa	0 (0 to 0)	0 (0 to 0)	0 (0 to 0)	0 (0 to 0)	0·1 (0 to 0·2)	5·2 (1·7 to 10·7)	21 (9·1 to 42·8)	34 (15·4 to 63·9)	31·2 (15·9 to 53·5)	25·7 (13·1 to 43·3)	17·3 (8·7 to 30·2)	7·9 (3·6 to 14·4)	3·1 (1·3 to 5·7)	0 (0 to 0)	0 (0 to 0)	0 (0 to 0)	0 (0 to 0)	0 (0 to 0)	0 (0 to 0)	0 (0 to 0)	0 (0 to 0)	0 (0 to 0)	0 (0 to 0)	145
Autism spectrum disorders	0·1 (0·1 to 0·1)	0·3 (0·2 to 0·4)	4·6 (2·9 to 6·9)	19·6 (12·6 to 29·4)	25·7 (16·7 to 39·6)	26·9 (17·2 to 40·2)	25·9 (16 to 39·1)	22·1 (13·7 to 33·1)	18·9 (12·1 to 27·9)	15·9 (10·1 to 23·6)	13·4 (8·4 to 20)	11·2 (7 to 16·6)	9·4 (5·8 to 14·1)	7·4 (4·7 to 11·2)	5·9 (3·7 to 8·9)	4·2 (2·6 to 6·3)	2·9 (1·8 to 4·3)	1·9 (1·1 to 2·8)	1·2 (0·7 to 1·8)	0·7 (0·5 to 1·1)	0·4 (0·2 to 0·5)	0·2 (0·1 to 0·2)	0 (0 to 0·1)	219
Attention-deficit/hyperactivity disorder	0 (0 to 0)	0 (0 to 0)	0 (0 to 0)	2·5 (1·2 to 4·7)	27·7 (15 to 49·2)	40·7 (22·2 to 72·6)	33·3 (18·2 to 59·4)	21·8 (11·9 to 37·8)	14·8 (7·9 to 25·8)	10·3 (5·3 to 17·4)	7·4 (3·8 to 12·6)	5·2 (2·7 to 9·2)	3·7 (1·9 to 6·5)	2·4 (1·2 to 4·4)	1·4 (0·6 to 2·6)	0·7 (0·3 to 1·2)	0·3 (0·1 to 0·5)	0·1 (0 to 0·2)	0 (0 to 0·1)	0 (0 to 0)	0 (0 to 0)	0 (0 to 0)	0 (0 to 0)	172
Conduct disorder	0 (0 to 0)	0 (0 to 0)	0 (0 to 0)	0 (0 to 0)	49 (25·2 to 86)	177 (97·3 to 292)	109 (57·9 to 182)	0 (0 to 0)	0 (0 to 0)	0 (0 to 0)	0 (0 to 0)	0 (0 to 0)	0 (0 to 0)	0 (0 to 0)	0 (0 to 0)	0 (0 to 0)	0 (0 to 0)	0 (0 to 0)	0 (0 to 0)	0 (0 to 0)	0 (0 to 0)	0 (0 to 0)	0 (0 to 0)	335
Idiopathic developmental intellectual disability	0 (0 to 0)	0·1 (0 to 0·1)	1·1 (0·1 to 2·4)	6·6 (1·8 to 12·6)	10·4 (3·8 to 18·7)	11 (4 to 20·6)	10·6 (3·5 to 19·5)	9·1 (3·3 to 16·6)	7·6 (2·8 to 13·7)	6·4 (2·3 to 11·5)	5·3 (2 to 9·7)	4·3 (1·6 to 7·9)	3·5 (1·3 to 6·3)	2·7 (1 to 4·8)	2 (0·7 to 3·6)	1·3 (0·4 to 2·5)	0·8 (0·3 to 1·6)	0·5 (0·2 to 0·9)	0·3 (0·1 to 0·5)	0·2 (0·1 to 0·3)	0·1 (0 to 0·2)	0 (0 to 0·1)	0 (0 to 0)	83·9
Other mental disorders	0 (0 to 0)	0 (0 to 0)	0 (0 to 0)	0 (0 to 0)	0 (0 to 0)	2·4 (1·1 to 4)	15·6 (7·4 to 26·8)	34·3 (17·2 to 57·6)	46·9 (26 to 74·3)	51·5 (30·9 to 79·9)	51·4 (31·3 to 79·5)	46·5 (28·6 to 71·9)	41 (25·1 to 62·7)	33·7 (20·4 to 51)	27·6 (17·2 to 41·6)	20·2 (12·6 to 30·5)	14·2 (9·1 to 21·2)	9·6 (6·2 to 14·1)	6·2 (4·1 to 9)	4·2 (2·7 to 6·1)	2·3 (1·5 to 3·3)	1·1 (0·7 to 1·6)	0·4 (0·2 to 0·5)	409
Substance use disorders	0 (0 to 0)	0 (0 to 0)	0 (0 to 0)	0 (0 to 0·1)	0 (0 to 0·1)	6·7 (3·6 to 10·8)	110 (72·3 to 157)	192 (130 to 266)	201 (142 to 271)	190 (137 to 255)	173 (129 to 226)	154 (115 to 203)	139 (105 to 185)	108 (81·9 to 140)	92·3 (69·6 to 119)	56·3 (42·6 to 74)	32·4 (24·5 to 41·4)	16 (12·1 to 20·9)	8·2 (6·2 to 10·3)	4·7 (3·5 to 6·1)	2·3 (1·7 to 3·2)	1·2 (0·8 to 1·7)	0·5 (0·3 to 0·8)	1,490
Alcohol use disorders	0 (0 to 0)	0 (0 to 0)	0 (0 to 0)	0 (0 to 0·1)	0 (0 to 0·1)	2·9 (1·2 to 5·6)	38·1 (22 to 59·9)	94·3 (56·3 to 150)	119 (78·8 to 173)	133 (92·1 to 188)	133 (95·9 to 178)	126 (91·4 to 170)	118 (87·2 to 159)	93·7 (69·8 to 124)	82·3 (60·7 to 108)	50·5 (37·6 to 66·8)	28·9 (21·5 to 37·3)	13·9 (10·3 to 18·6)	6·9 (5·1 to 9)	3·8 (2·7 to 5·2)	1·9 (1·3 to 2·7)	1 (0·6 to 1·4)	0·4 (0·3 to 0·7)	1,050
Drug use disorders	0 (0 to 0)	0 (0 to 0)	0 (0 to 0)	0 (0 to 0)	0 (0 to 0)	3·8 (1·9 to 6·4)	71·8 (45·4 to 108)	97·3 (63·4 to 138)	81·4 (54 to 115)	57 (38·2 to 80·3)	40·1 (27·4 to 56·2)	28·2 (19 to 39·5)	20·9 (14·3 to 29·2)	14·2 (9·4 to 19·9)	9·9 (6·3 to 14·4)	5·8 (3·6 to 8·6)	3·5 (2·1 to 5·3)	2 (1·3 to 3·1)	1·2 (0·8 to 1·9)	0·8 (0·5 to 1·2)	0·4 (0·3 to 0·6)	0·2 (0·1 to 0·3)	0·1 (0·1 to 0·1)	439
Opioid use disorders	0 (0 to 0)	0 (0 to 0)	0 (0 to 0)	0 (0 to 0)	0 (0 to 0)	0 (0 to 0)	8·7 (3·5 to 16·1)	31·4 (16·2 to 55·6)	34·2 (19·2 to 55·1)	27·3 (14·9 to 42·8)	20·4 (11·4 to 32·9)	14·6 (7·9 to 23·4)	10·8 (6·2 to 17·3)	8·1 (4·3 to 12·9)	5·9 (3 to 9·5)	3·7 (1·9 to 6·1)	2·2 (1·1 to 3·7)	1·2 (0·6 to 2·1)	0·7 (0·4 to 1·2)	0·5 (0·2 to 0·8)	0·3 (0·1 to 0·4)	0·1 (0·1 to 0·2)	0·1 (0 to 0·1)	170
Cocaine use disorders	0 (0 to 0)	0 (0 to 0)	0 (0 to 0)	0 (0 to 0)	0 (0 to 0)	0·5 (0·2 to 0·8)	17·7 (8·3 to 32·3)	17·9 (9·9 to 29·4)	13·6 (8 to 20·9)	10·3 (6·2 to 16·1)	7·5 (4·5 to 11·4)	5·1 (3·1 to 7·9)	3·8 (2·3 to 6)	2·1 (1·2 to 3·3)	1·5 (0·8 to 2·5)	0·8 (0·4 to 1·4)	0·5 (0·2 to 0·9)	0·3 (0·1 to 0·5)	0·2 (0·1 to 0·3)	0·1 (0 to 0·2)	0·1 (0 to 0·1)	0 (0 to 0)	0 (0 to 0)	81·8
Amphetamine use disorders	0 (0 to 0)	0 (0 to 0)	0 (0 to 0)	0 (0 to 0)	0 (0 to 0)	0 (0 to 0)	2·4 (0·8 to 5·3)	9·5 (3·7 to 18)	8·4 (3·7 to 15·5)	4·1 (1·6 to 7·8)	2 (0·9 to 3·9)	1·1 (0·7 to 1·8)	0·7 (0·4 to 1·1)	0·5 (0·3 to 0·8)	0·3 (0·2 to 0·5)	0·2 (0·1 to 0·3)	0·1 (0·1 to 0·2)	0 (0 to 0·1)	0 (0 to 0)	0 (0 to 0)	0 (0 to 0)	0 (0 to 0)	0 (0 to 0)	29·4
Cannabis use disorders	0 (0 to 0)	0 (0 to 0)	0 (0 to 0)	0 (0 to 0)	0 (0 to 0)	3 (1·3 to 5·5)	30·1 (16 to 50·9)	21·4 (11 to 36·4)	11·5 (5·2 to 20·7)	6·5 (3 to 12·2)	4·3 (2 to 7·8)	3·1 (1·4 to 5·7)	2·3 (1 to 4·2)	1·5 (0·6 to 2·8)	0·9 (0·4 to 1·6)	0·4 (0·2 to 0·7)	0·2 (0·1 to 0·3)	0·1 (0 to 0·1)	0 (0 to 0·1)	0 (0 to 0)	0 (0 to 0)	0 (0 to 0)	0 (0 to 0)	85·3
Other drug use disorders	0 (0 to 0)	0 (0 to 0)	0 (0 to 0)	0 (0 to 0)	0 (0 to 0)	0·3 (0·1 to 0·6)	12·9 (6·2 to 23·1)	17·2 (9·5 to 27·8)	13·7 (7·9 to 20·9)	8·8 (5·2 to 14)	5·9 (3·5 to 9·1)	4·2 (2·5 to 6·6)	3·3 (2·1 to 5)	2·1 (1·3 to 3·2)	1·3 (0·8 to 2·1)	0·8 (0·4 to 1·2)	0·5 (0·3 to 0·8)	0·4 (0·3 to 0·6)	0·3 (0·2 to 0·4)	0·2 (0·1 to 0·3)	0·1 (0·1 to 0·2)	0·1 (0 to 0·1)	0 (0 to 0)	72·2
Diabetes and kidney diseases	0·1 (0·1 to 0·2)	0·3 (0·2 to 0·6)	31·6 (20·2 to 47)	37·5 (27·3 to 48·9)	51·4 (38·4 to 67·5)	61·6 (47 to 79·6)	193 (152 to 237)	255 (207 to 310)	363 (294 to 443)	443 (359 to 538)	476 (390 to 576)	584 (475 to 699)	845 (704 to 1,000)	1,120 (931 to 1,330)	1,280 (1,070 to 1,500)	1,160 (977 to 1,360)	969 (821 to 1,130)	753 (645 to 879)	524 (451 to 604)	366 (311 to 421)	188 (156 to 218)	83·9 (68 to 98·5)	28·9 (22·4 to 34·7)	9,810
Diabetes mellitus	0·1 (0·1 to 0·2)	0·3 (0·2 to 0·6)	7 (3·5 to 12·8)	6·1 (3·9 to 8·8)	6·3 (4·2 to 8·9)	11·1 (7·6 to 15·2)	59·7 (47·9 to 74·1)	95·2 (75·7 to 118)	144 (113 to 180)	192 (151 to 238)	229 (180 to 286)	313 (250 to 388)	499 (404 to 620)	704 (575 to 867)	829 (677 to 998)	769 (636 to 924)	664 (546 to 792)	523 (435 to 628)	357 (299 to 423)	238 (199 to 280)	119 (96·9 to 142)	49·9 (40·1 to 60·2)	16·5 (12·8 to 20)	5,830
Diabetes mellitus type 1	0·1 (0·1 to 0·2)	0·3 (0·2 to 0·6)	7 (3·5 to 12·8)	6·1 (3·9 to 8·8)	6·3 (4·2 to 8·9)	11·1 (7·6 to 15·2)	21·5 (15·5 to 28·3)	33·7 (25·1 to 45·2)	50·7 (37·5 to 67·8)	38 (26 to 55·6)	41·1 (29·2 to 59)	51·6 (37·2 to 72·9)	18·2 (12·9 to 27·1)	21·9 (15·2 to 34·4)	25·3 (17·4 to 40·6)	18·7 (12·6 to 29)	15·7 (11 to 25·6)	9·6 (6·7 to 15·5)	6·9 (4·7 to 11·3)	3·3 (2·3 to 5)	1·7 (1·2 to 2·6)	0·8 (0·6 to 1·2)	0·3 (0·2 to 0·5)	390
Diabetes mellitus type 2	0 (0 to 0)	0 (0 to 0)	0 (0 to 0)	0 (0 to 0)	0 (0 to 0)	0 (0 to 0)	38·2 (27·7 to 49·6)	61·6 (45·5 to 80·9)	93·5 (69·3 to 124)	154 (119 to 196)	188 (145 to 241)	261 (205 to 332)	480 (388 to 598)	682 (557 to 842)	804 (656 to 971)	750 (620 to 901)	648 (530 to 775)	513 (428 to 619)	350 (293 to 415)	235 (196 to 276)	117 (95·4 to 140)	49·1 (39·4 to 59·4)	16·1 (12·6 to 19·6)	5,440
Chronic kidney disease	0 (0 to 0)	0 (0 to 0)	24·5 (15·2 to 38·2)	31 (22 to 41·7)	42·9 (30·7 to 58·5)	49·4 (35·3 to 67·8)	131 (96·3 to 173)	158 (117 to 205)	217 (162 to 285)	249 (186 to 322)	246 (188 to 323)	270 (208 to 346)	345 (275 to 431)	411 (316 to 528)	452 (356 to 566)	389 (299 to 490)	305 (243 to 373)	230 (190 to 277)	167 (136 to 202)	128 (102 to 157)	68·7 (53·8 to 84·2)	33·9 (24·7 to 41·8)	12·4 (9·1 to 15·9)	3,960
Chronic kidney disease due to diabetes mellitus type 1	0 (0 to 0)	0 (0 to 0)	0·2 (0·1 to 0·4)	0·6 (0·2 to 1·4)	1·1 (0·5 to 2·6)	1·5 (0·7 to 3·3)	5·3 (2·2 to 10·4)	9·1 (3·7 to 18·2)	24·1 (10·5 to 47·1)	41·6 (20·6 to 70·2)	45·3 (23·9 to 76·5)	49·2 (26·1 to 81·5)	53·6 (26·3 to 88·8)	48 (19·8 to 90·7)	36 (12·1 to 75·3)	22·7 (7·9 to 49·5)	13·7 (5·3 to 30·6)	7·8 (3·2 to 15·8)	3·7 (1·4 to 8)	1·4 (0·5 to 3·3)	0·3 (0·1 to 0·7)	0 (0 to 0·1)	0 (0 to 0)	365
Chronic kidney disease due to diabetes mellitus type 2	0 (0 to 0)	0 (0 to 0)	0 (0 to 0)	0 (0 to 0)	0 (0 to 0)	0 (0 to 0)	2·2 (0·8 to 5·1)	4·1 (1·6 to 8·8)	9·3 (3·7 to 19·5)	15·9 (6·7 to 30)	21·2 (10 to 38·7)	33·3 (16·7 to 57·8)	61·6 (34·4 to 97·5)	98·7 (58·3 to 147)	129 (77·1 to 183)	121 (71·5 to 171)	97·3 (63·5 to 135)	73·8 (51·4 to 101)	53·2 (35·6 to 71·1)	39·9 (27 to 56·3)	20·3 (13·5 to 28·6)	9·5 (5·7 to 13·9)	3·4 (1·9 to 4·9)	794
Chronic kidney disease due to hypertension	0 (0 to 0)	0 (0 to 0)	0 (0 to 0)	0 (0 to 0)	0 (0 to 0)	0 (0 to 0)	8·2 (3·7 to 15·2)	11·8 (5·6 to 20·4)	18 (9·6 to 30·2)	22·5 (12·7 to 35·6)	25 (15·1 to 38·3)	31·6 (20·3 to 47·2)	46·4 (30·9 to 66·4)	64·6 (41·2 to 96·3)	83·7 (54·3 to 120)	84·5 (53·6 to 123)	74·7 (50·5 to 105)	61·4 (44 to 82·9)	49·4 (34·9 to 67·2)	41·8 (29·9 to 56)	24·8 (17·8 to 32·6)	13·3 (9·1 to 17·7)	5·1 (3·5 to 7·2)	667
Chronic kidney disease due to glomerulonephritis	0 (0 to 0)	0 (0 to 0)	7 (3·4 to 13)	9·9 (5·7 to 15·7)	15·5 (8·9 to 24)	20 (12·2 to 29·6)	54·2 (33·6 to 81)	63·8 (38·6 to 93·2)	83 (53·5 to 122)	85·5 (52·8 to 127)	78·6 (51·2 to 114)	81·3 (48·7 to 122)	93·2 (57·6 to 134)	101 (56·6 to 160)	102 (56·4 to 165)	76·1 (39·8 to 125)	51·1 (26·1 to 88·6)	32·9 (17·6 to 53·4)	20·8 (10·5 to 35·8)	13·6 (6·3 to 25)	5·6 (2·5 to 9·7)	2·3 (1·1 to 4·2)	0·8 (0·3 to 1·6)	998
Chronic kidney disease due to other and unspecified causes	0 (0 to 0)	0 (0 to 0)	17·4 (10·2 to 27·3)	20·5 (13·3 to 28·5)	26·2 (17·4 to 38·5)	27·8 (17·3 to 42·4)	61 (39 to 87)	69·1 (43·4 to 97·5)	82·7 (53·6 to 117)	83·2 (53·1 to 126)	75·6 (46·6 to 112)	75 (45·4 to 112)	90·2 (55 to 135)	99·2 (60·9 to 155)	101 (60·9 to 163)	84·8 (51 to 135)	68·1 (40·5 to 103)	53·7 (33·9 to 79·7)	39·8 (24·7 to 60·9)	31 (19·5 to 47·1)	17·7 (11·6 to 25·3)	8·7 (5·9 to 12·5)	3·2 (2 to 4·7)	1,140
Acute glomerulonephritis	0 (0 to 0)	0 (0 to 0)	0 (0 to 0·1)	0·3 (0·1 to 0·7)	2·2 (0·9 to 4·3)	1·1 (0·5 to 2·3)	1·8 (0·9 to 3·4)	1·6 (0·6 to 3·2)	1·5 (0·6 to 3·3)	1·8 (0·8 to 3·6)	1 (0·5 to 1·8)	0·8 (0·4 to 1·4)	0·9 (0·5 to 1·4)	0·3 (0·2 to 0·5)	0·9 (0·4 to 1·7)	0·4 (0·2 to 0·9)	0·2 (0·1 to 0·4)	0·4 (0·2 to 0·8)	0·2 (0·1 to 0·3)	0·2 (0·1 to 0·3)	0·1 (0·1 to 0·2)	0·1 (0·1 to 0·2)	0 (0 to 0)	15·9
Skin and subcutaneous diseases	0 (0 to 0)	0·1 (0·1 to 0·2)	21·7 (14·6 to 30·3)	197 (124 to 296)	263 (162 to 388)	283 (179 to 423)	300 (194 to 453)	179 (114 to 267)	138 (90 to 205)	117 (75 to 171)	106 (68·9 to 156)	96·7 (64·3 to 140)	88·4 (59·6 to 129)	81 (54·6 to 117)	74·1 (50·4 to 107)	61·6 (43·1 to 88·9)	49·7 (34·7 to 73·5)	39·6 (26·3 to 57·9)	30·6 (21·1 to 44·7)	23·9 (16·5 to 35·1)	15·7 (11 to 23·5)	8·2 (5·7 to 12·7)	3·2 (2·2 to 5·2)	2,180
Dermatitis	0 (0 to 0)	0 (0 to 0)	0 (0 to 0)	69·4 (38·2 to 117)	87·8 (46·5 to 148)	65·7 (36 to 109)	49·1 (28·1 to 81)	36·1 (20·1 to 57·7)	29·2 (17·1 to 47·2)	24·9 (14·1 to 40·7)	21·4 (11·8 to 34·2)	18·2 (10·6 to 29·5)	16·2 (8·9 to 26·2)	14·4 (8 to 23·6)	12·9 (7 to 20·5)	10·2 (5·7 to 16·4)	7·9 (4·4 to 12·8)	5·6 (3·2 to 8·8)	3·6 (2·1 to 5·7)	2·3 (1·4 to 3·7)	1·2 (0·7 to 2)	0·5 (0·3 to 0·8)	0·2 (0·1 to 0·3)	477
Atopic dermatitis	0 (0 to 0)	0 (0 to 0)	0 (0 to 0)	68·8 (37·7 to 116)	86 (45·2 to 146)	59·2 (31·6 to 101)	38·2 (20 to 65·3)	24·3 (12·4 to 40·9)	17 (8·7 to 29·9)	13·2 (6·5 to 23·3)	10·9 (5·5 to 19·2)	9·1 (4·8 to 16·1)	7·9 (4·2 to 13·6)	6·6 (3·5 to 11·5)	5·8 (2·9 to 10)	4·4 (2·3 to 7·5)	3·2 (1·7 to 5·5)	2·2 (1·2 to 3·7)	1·5 (0·8 to 2·4)	1 (0·6 to 1·8)	0·6 (0·3 to 1)	0·3 (0·1 to 0·4)	0·1 (0 to 0·1)	360
Contact dermatitis	0 (0 to 0)	0 (0 to 0)	0 (0 to 0)	0 (0 to 0)	1·1 (0·4 to 2·4)	5·5 (2·1 to 10·7)	9·7 (4 to 17·9)	10·5 (4·6 to 19·9)	10·9 (5·1 to 20·1)	10·5 (4·4 to 19·8)	9·5 (4 to 18·6)	8·2 (3·8 to 16·6)	7·6 (3·4 to 14·4)	7·2 (3·2 to 13·6)	6·6 (2·9 to 12·8)	5·4 (2·3 to 9·9)	4·3 (2 to 8·3)	3 (1·5 to 5·6)	1·9 (0·9 to 3·4)	1·1 (0·5 to 2·1)	0·6 (0·2 to 1)	0·2 (0·1 to 0·4)	0·1 (0 to 0·1)	104
Seborrhoeic dermatitis	0 (0 to 0)	0 (0 to 0)	0 (0 to 0)	0·6 (0·3 to 1·1)	0·7 (0·3 to 1·3)	1·1 (0·5 to 1·9)	1·3 (0·7 to 2·4)	1·2 (0·6 to 2·2)	1·3 (0·7 to 2·2)	1·3 (0·6 to 2·1)	1·1 (0·6 to 2)	0·9 (0·5 to 1·6)	0·8 (0·4 to 1·3)	0·6 (0·3 to 1·1)	0·5 (0·3 to 0·9)	0·4 (0·2 to 0·7)	0·4 (0·2 to 0·7)	0·3 (0·2 to 0·6)	0·2 (0·1 to 0·4)	0·2 (0·1 to 0·3)	0·1 (0·1 to 0·2)	0 (0 to 0·1)	0 (0 to 0)	13·2
Psoriasis	0 (0 to 0)	0 (0 to 0)	0 (0 to 0)	2·9 (1·9 to 4·2)	7·9 (4·1 to 13)	11·1 (6·2 to 17·4)	12·1 (7·2 to 18·4)	11·6 (6·6 to 17·4)	11·6 (7 to 17·5)	11·7 (7·1 to 17·3)	11·7 (7·4 to 17·6)	11·2 (7·1 to 16)	10·7 (6·8 to 15·3)	9·8 (6·4 to 14·3)	8·7 (5·9 to 12·2)	6·9 (4·5 to 9·7)	4·9 (3·4 to 6·8)	3·1 (2 to 4·5)	1·7 (1·1 to 2·5)	0·9 (0·6 to 1·3)	0·4 (0·2 to 0·6)	0·1 (0·1 to 0·2)	0 (0 to 0·1)	139
Bacterial skin diseases	0 (0 to 0)	0 (0 to 0)	8·4 (4 to 14·2)	5·3 (3·4 to 7·9)	5·2 (3·3 to 7·8)	4·5 (2·9 to 6·8)	4·6 (2·9 to 6·9)	4·5 (2·8 to 7·1)	5·4 (3·4 to 8·3)	6·1 (3·8 to 9·1)	5·4 (3·3 to 8·6)	6·8 (4·1 to 10·8)	6·9 (4·5 to 11·3)	7·5 (4·4 to 11·7)	7·5 (4·4 to 12·2)	6·8 (3·8 to 10·9)	5·3 (2·7 to 9·2)	4·3 (2·2 to 7·9)	3·8 (1·9 to 7·4)	3·3 (1·6 to 6·3)	2·2 (1·1 to 4·6)	1·2 (0·6 to 2·5)	0·5 (0·2 to 1·1)	105
Cellulitis	0 (0 to 0)	0 (0 to 0)	2·3 (0·9 to 4·6)	1·6 (0·8 to 2·6)	2 (1·1 to 3·2)	1·4 (0·9 to 2·3)	1·4 (0·8 to 2·6)	1·3 (0·7 to 2·5)	1·8 (1 to 3·5)	1·8 (0·9 to 3·2)	1·5 (0·8 to 2·8)	1·7 (0·9 to 3·2)	1·7 (0·9 to 3·3)	1·9 (1·1 to 3·6)	1·8 (1 to 3·6)	1·6 (0·8 to 3)	1·2 (0·6 to 2·3)	0·9 (0·4 to 1·9)	0·7 (0·3 to 1·5)	0·5 (0·2 to 1·2)	0·3 (0·1 to 0·8)	0·2 (0·1 to 0·4)	0·1 (0 to 0·2)	27·8
Pyoderma	0 (0 to 0)	0 (0 to 0)	6·1 (2·8 to 10·3)	3·7 (2·2 to 6)	3·2 (1·9 to 5·3)	3·1 (1·8 to 5·2)	3·1 (1·9 to 5·2)	3·2 (1·9 to 5·1)	3·5 (2 to 5·5)	4·3 (2·6 to 6·8)	3·9 (2·2 to 6)	5·1 (2·9 to 8·1)	5·2 (3 to 8·2)	5·5 (3·2 to 8·9)	5·7 (2·9 to 8·9)	5·2 (2·8 to 8·4)	4 (2 to 6·8)	3·4 (1·6 to 6·3)	3·1 (1·5 to 6·2)	2·7 (1·3 to 5·2)	1·9 (0·9 to 3·8)	1·1 (0·5 to 2·1)	0·4 (0·2 to 0·9)	77·6
Scabies	0 (0 to 0)	0·1 (0 to 0·1)	5 (2·6 to 8·7)	33·8 (17 to 60·1)	47 (22·9 to 82·9)	50·7 (24·9 to 88·8)	52·5 (26·9 to 89·8)	41·9 (21 to 73)	27·4 (13·8 to 47·2)	18·4 (9 to 33·4)	16·1 (8 to 27·6)	15·7 (8·1 to 26·8)	14 (7·1 to 24·1)	11 (5·6 to 18·6)	8·4 (4·3 to 14·9)	5·7 (3 to 10·1)	4 (2 to 6·9)	2·7 (1·4 to 4·8)	2 (1 to 3·4)	1·5 (0·8 to 2·7)	0·9 (0·5 to 1·6)	0·5 (0·2 to 0·8)	0·2 (0·1 to 0·3)	359
Fungal skin diseases	0 (0 to 0)	0 (0 to 0)	0·8 (0·3 to 1·9)	6·4 (2·4 to 14)	10·9 (4·1 to 24)	13·1 (4·9 to 29)	13·6 (4·8 to 30·2)	12·6 (4·8 to 27·7)	11·8 (4·6 to 26·9)	11 (4·2 to 24·7)	10·1 (3·9 to 22·2)	9·1 (3·5 to 19·8)	8·3 (3·2 to 18)	7·7 (3·1 to 16·8)	7·9 (3·1 to 17·1)	7 (2·6 to 15·2)	6·8 (2·7 to 14·5)	6·6 (2·6 to 14·2)	5·4 (2·1 to 11·3)	4·7 (1·8 to 9·9)	3·3 (1·3 to 6·9)	1·7 (0·7 to 3·5)	0·6 (0·2 to 1·3)	159
Viral skin diseases	0 (0 to 0)	0 (0 to 0)	0 (0 to 0)	36·3 (23 to 55·5)	59·7 (36·7 to 91·1)	32·5 (20·3 to 49)	19·6 (12·2 to 29·8)	13·8 (8·4 to 21)	9·2 (5·6 to 14·1)	6·7 (4·1 to 10·3)	5·7 (3·4 to 8·7)	4·8 (2·9 to 7·5)	4 (2·3 to 6·2)	3·2 (1·9 to 5)	2·6 (1·6 to 4)	2 (1·3 to 3·1)	1·5 (0·9 to 2·3)	0·9 (0·6 to 1·4)	0·5 (0·3 to 0·8)	0·3 (0·2 to 0·4)	0·1 (0·1 to 0·2)	0 (0 to 0·1)	0 (0 to 0)	203
Acne vulgaris	0 (0 to 0)	0 (0 to 0)	0 (0 to 0)	0 (0 to 0)	2·9 (1·6 to 4·7)	69·1 (39·1 to 112)	112 (62·9 to 186)	24·5 (13·6 to 40·3)	10·4 (5·7 to 17·4)	6·6 (3·7 to 11·1)	4·5 (2·4 to 7·4)	2·9 (1·7 to 4·7)	1·8 (1 to 3)	1 (0·6 to 1·7)	0·6 (0·4 to 1)	0·4 (0·2 to 0·6)	0·2 (0·1 to 0·4)	0·1 (0·1 to 0·2)	0·1 (0 to 0·1)	0 (0 to 0·1)	0 (0 to 0)	0 (0 to 0)	0 (0 to 0)	237
Alopecia areata	0 (0 to 0)	0 (0 to 0)	0 (0 to 0)	0·7 (0·3 to 1·2)	1 (0·5 to 1·8)	1·4 (0·8 to 2·5)	2·5 (1·4 to 4·1)	3·5 (2 to 5·7)	4·3 (2·5 to 6·8)	4 (2·4 to 6·3)	3·4 (2 to 5·3)	2·5 (1·5 to 3·8)	1·8 (1 to 2·8)	1·3 (0·8 to 2·1)	1 (0·6 to 1·6)	0·8 (0·5 to 1·2)	0·5 (0·3 to 0·8)	0·3 (0·2 to 0·5)	0·2 (0·1 to 0·3)	0·1 (0·1 to 0·1)	0 (0 to 0·1)	0 (0 to 0)	0 (0 to 0)	29·4
Pruritus	0 (0 to 0)	0 (0 to 0)	0·1 (0 to 0·2)	1·3 (0·6 to 2·6)	1·9 (0·8 to 3·9)	2 (0·9 to 4·1)	2·4 (1 to 4·8)	2·6 (1·1 to 5)	2·6 (1·1 to 5·2)	2·6 (1·1 to 5·2)	2·5 (1·1 to 4·9)	2·2 (0·9 to 4·2)	2 (0·9 to 3·9)	1·8 (0·8 to 3·6)	1·7 (0·7 to 3·4)	1·6 (0·7 to 3·1)	1·4 (0·6 to 2·7)	1 (0·4 to 2)	0·7 (0·3 to 1·3)	0·5 (0·2 to 0·9)	0·2 (0·1 to 0·5)	0·1 (0 to 0·2)	0 (0 to 0·1)	31·4
Urticaria	0 (0 to 0)	0 (0 to 0·1)	5 (3 to 7·8)	33·2 (20·1 to 51·4)	30·5 (17·3 to 50·1)	22·3 (12·4 to 37·4)	19·1 (10·4 to 31·8)	15·8 (8·6 to 26·3)	13·2 (7·2 to 21·2)	12 (6·6 to 19·7)	11 (6·4 to 18)	9 (5·2 to 14·4)	7·3 (4·1 to 12·3)	5·8 (3·3 to 9·3)	4·8 (2·8 to 7·7)	3·6 (1·9 to 5·8)	2·5 (1·4 to 4·1)	1·7 (0·9 to 2·8)	1 (0·6 to 1·7)	0·6 (0·4 to 1)	0·3 (0·2 to 0·5)	0·1 (0·1 to 0·2)	0 (0 to 0·1)	199
Decubitus ulcer	0 (0 to 0)	0 (0 to 0)	0 (0 to 0)	0·3 (0·1 to 0·6)	0·5 (0·3 to 0·9)	0·6 (0·3 to 1)	0·8 (0·4 to 1·4)	1 (0·3 to 2·2)	1·8 (0·7 to 3·3)	1·1 (0·5 to 2·1)	1·8 (0·9 to 3·6)	1·9 (0·9 to 4)	2·5 (1·4 to 5·2)	3·9 (2·3 to 7·2)	4·6 (2·8 to 8·3)	4·7 (2·9 to 8·5)	4·9 (3 to 9·2)	5·6 (3·6 to 10·2)	6·5 (4·3 to 11·1)	6·3 (4·3 to 11·1)	5·2 (3·5 to 9·4)	3·2 (2·1 to 5·5)	1·4 (0·9 to 2·8)	58·6
Other skin and subcutaneous diseases	0 (0 to 0)	0 (0 to 0)	2·4 (1·3 to 4)	7·8 (3·8 to 14·2)	7·8 (3·7 to 14·4)	9·8 (4·5 to 18·9)	11·8 (5·8 to 22·4)	11 (5·6 to 20·7)	11 (5·3 to 20·3)	12·2 (6·2 to 22·3)	12·4 (6·3 to 22)	12·6 (6·5 to 22·9)	12·8 (6·5 to 23·3)	13·4 (7 to 23·5)	13·4 (6·9 to 24·2)	12 (6·1 to 21·3)	9·9 (5 to 17·3)	7·5 (3·8 to 13·5)	5·3 (2·7 to 9·1)	3·5 (1·8 to 6·1)	1·7 (0·9 to 3)	0·8 (0·4 to 1·4)	0·2 (0·1 to 0·4)	179
Sense organ diseases	0 (0 to 0)	0·1 (0 to 0·1)	2 (1·2 to 3)	21·6 (13·7 to 30·8)	48·5 (30·1 to 71·6)	65·7 (41·5 to 95·3)	78·8 (49·5 to 116)	86·6 (53·8 to 126)	98·6 (62·6 to 149)	112 (69·9 to 169)	121 (75·5 to 183)	135 (85·5 to 205)	171 (107 to 272)	200 (128 to 315)	225 (144 to 347)	219 (142 to 332)	202 (134 to 303)	177 (118 to 255)	147 (100 to 207)	122 (84·6 to 168)	80·2 (56·4 to 107)	40·9 (29 to 54·3)	14·9 (10·7 to 19·9)	2,370
Blindness and vision loss	0 (0 to 0)	0 (0 to 0)	0·3 (0·2 to 0·6)	5·8 (3·2 to 9·7)	13·7 (7·9 to 21·5)	18 (10·7 to 28·5)	21·1 (12·7 to 32·1)	22·1 (13·4 to 33·7)	22·1 (13·6 to 32·9)	23·3 (14·3 to 34·8)	27·2 (17·4 to 40·2)	33 (20·7 to 50·1)	40·4 (25·4 to 63·6)	46·7 (29·3 to 73·7)	54·7 (34·7 to 87·6)	57 (36·5 to 88·1)	57·7 (37·7 to 85·2)	54·5 (36·5 to 77·8)	47·2 (32·5 to 66·9)	40·5 (27·8 to 55·5)	27·5 (19 to 37)	15 (10·4 to 20·5)	6·2 (4·3 to 8·5)	634
Glaucoma	0 (0 to 0)	0 (0 to 0)	0 (0 to 0)	0 (0 to 0)	0 (0 to 0)	0 (0 to 0)	0 (0 to 0)	0 (0 to 0)	0 (0 to 0)	0 (0 to 0)	0 (0 to 0)	0 (0 to 0)	0·1 (0 to 0·2)	0·7 (0·2 to 1·3)	1·8 (0·9 to 3·1)	2·8 (1·5 to 4·5)	3·7 (2·2 to 5·9)	4·5 (2·8 to 7)	4·9 (2·9 to 7·8)	4·9 (3 to 7·4)	3·7 (2·3 to 5·7)	2 (1·2 to 3)	0·8 (0·5 to 1·3)	29·9
Cataract	0 (0 to 0)	0 (0 to 0)	0 (0 to 0)	0 (0 to 0)	0 (0 to 0)	0 (0 to 0)	0 (0 to 0)	0·1 (0 to 0·2)	0·5 (0·2 to 1)	1·1 (0·4 to 2·2)	2·1 (1 to 3·5)	3·2 (1·6 to 5·4)	4·5 (2·4 to 7·3)	5·2 (2·9 to 8·4)	6·2 (3·7 to 9·6)	7·2 (4·5 to 10·7)	9·1 (5·8 to 13·8)	11·4 (7·3 to 16·4)	12·7 (8·5 to 18·4)	12·6 (8·4 to 17·7)	9·4 (6·4 to 13·4)	5 (3·3 to 7·1)	2 (1·2 to 3)	92·4
Age-related macular degeneration	0 (0 to 0)	0 (0 to 0)	0 (0 to 0)	0 (0 to 0)	0 (0 to 0)	0 (0 to 0)	0 (0 to 0)	0 (0 to 0)	0 (0 to 0)	0 (0 to 0)	0 (0 to 0)	0 (0 to 0)	0 (0 to 0·1)	0·2 (0·1 to 0·3)	0·4 (0·2 to 0·6)	0·5 (0·2 to 0·9)	0·5 (0·3 to 0·9)	0·5 (0·3 to 0·9)	0·5 (0·3 to 0·8)	0·4 (0·2 to 0·7)	0·3 (0·2 to 0·4)	0·1 (0·1 to 0·2)	0·1 (0 to 0·1)	3·6
Refraction disorders	0 (0 to 0)	0 (0 to 0)	0·2 (0·1 to 0·4)	4·8 (2·5 to 8·2)	11·6 (6·6 to 18·6)	15·1 (8·8 to 25)	17·7 (10·3 to 27·8)	18·1 (10·3 to 28·6)	17 (10·1 to 25·5)	15·7 (9·2 to 24·3)	15·4 (9·4 to 23·5)	14·9 (9 to 23·3)	14·4 (8·8 to 21·8)	12·7 (7·5 to 19·2)	11·7 (7·1 to 18·1)	9·7 (6 to 14·8)	8 (4·8 to 12·2)	6·7 (4·2 to 10·4)	5·4 (3·4 to 8·2)	3·8 (2·3 to 5·8)	2 (1·3 to 3)	0·7 (0·5 to 1·1)	0·2 (0·1 to 0·3)	206
Near vision loss	0 (0 to 0)	0 (0 to 0)	0 (0 to 0)	0 (0 to 0·1)	0·1 (0 to 0·2)	0·1 (0 to 0·4)	0·2 (0·1 to 0·5)	0·5 (0·2 to 1·2)	1 (0·3 to 2·2)	2·3 (0·9 to 5·2)	4·3 (1·5 to 9·4)	7·8 (3·1 to 16·7)	12·2 (4·9 to 25·9)	16·2 (6·5 to 34)	20 (7·9 to 43·4)	20·1 (8·3 to 42·2)	18·2 (7·5 to 36·8)	14·9 (6·1 to 29·4)	11·2 (4·7 to 23·4)	8·4 (3·6 to 16·9)	5 (2·1 to 9·8)	2·4 (1 to 4·7)	0·8 (0·3 to 1·8)	146
Other vision loss	0 (0 to 0)	0 (0 to 0)	0·1 (0·1 to 0·2)	0·9 (0·5 to 1·6)	2 (0·9 to 3·6)	2·7 (1·2 to 5)	3·2 (1·4 to 5·6)	3·4 (1·6 to 5·9)	3·6 (1·8 to 6·4)	4·2 (2·1 to 7·2)	5·3 (2·9 to 8·7)	7·2 (4·2 to 11·3)	9·2 (5 to 14·6)	11·8 (7·5 to 17·9)	14·7 (9·2 to 22·2)	16·7 (10·8 to 24·3)	18·1 (11·6 to 25·7)	16·5 (10·8 to 24)	12·4 (7·8 to 18·3)	10·3 (6·6 to 14·6)	7·1 (4·6 to 10·7)	4·7 (3·2 to 6·8)	2·3 (1·4 to 3·4)	156
Age-related and other hearing loss	0 (0 to 0)	0 (0 to 0)	0·8 (0·4 to 1·4)	11·5 (6 to 19·2)	29·9 (16·6 to 48·1)	42·6 (24·7 to 63·5)	51·8 (30·3 to 76·7)	58·8 (34·5 to 88·8)	70·3 (43·8 to 109)	80·5 (48·8 to 126)	85·2 (51·4 to 135)	94·1 (54·3 to 150)	123 (72·7 to 199)	144 (87·7 to 230)	161 (99·2 to 255)	153 (94·1 to 239)	136 (86·8 to 206)	115 (75·2 to 170)	93·9 (63·2 to 135)	77·8 (52·5 to 108)	50·8 (34·5 to 69·2)	25·2 (17·5 to 34·1)	8·4 (6 to 11·3)	1,610
Other sense organ diseases	0 (0 to 0)	0 (0 to 0·1)	0·9 (0·4 to 1·4)	4·3 (2·2 to 7·3)	5 (2·4 to 8·6)	5·1 (2·5 to 9·2)	5·8 (2·8 to 10·8)	5·7 (2·5 to 10·3)	6·2 (2·8 to 11·8)	7·7 (3·4 to 14·8)	8·3 (3·7 to 15·7)	7·7 (3·5 to 14·9)	8 (3·5 to 14·7)	9·3 (4·4 to 18·2)	9 (4·2 to 17·6)	9·3 (4·4 to 17·4)	8·9 (4·1 to 16·4)	7·2 (3·5 to 13·5)	5·6 (2·6 to 10·1)	3·8 (1·9 to 7·1)	1·8 (0·9 to 3·3)	0·7 (0·4 to 1·3)	0·3 (0·2 to 0·6)	121
Musculoskeletal disorders	0 (0 to 0)	0 (0 to 0)	0 (0 to 0)	0 (0 to 0)	24·4 (13 to 42·5)	125 (77·8 to 192)	271 (178 to 400)	354 (236 to 506)	451 (298 to 635)	510 (337 to 697)	539 (371 to 742)	553 (382 to 765)	561 (390 to 792)	526 (366 to 756)	487 (333 to 676)	395 (271 to 563)	294 (204 to 416)	204 (138 to 287)	131 (87·8 to 188)	86·3 (59·2 to 123)	48·6 (34·3 to 68)	19·8 (14 to 27·8)	6·4 (4·5 to 9·1)	5,590
Rheumatoid arthritis	0 (0 to 0)	0 (0 to 0)	0 (0 to 0)	0 (0 to 0)	0·5 (0·3 to 0·9)	1·3 (0·7 to 2·1)	4 (2·3 to 6·2)	6·6 (3·2 to 11·1)	8·6 (4·7 to 14)	10·9 (6·6 to 16·7)	12·8 (8 to 19·3)	14 (8·8 to 20·5)	14·7 (9·8 to 20·8)	13·9 (9·6 to 19·6)	13·2 (9·4 to 18)	10 (7 to 13·7)	7·3 (5·2 to 9·7)	4·8 (3·5 to 6·5)	3·4 (2·5 to 4·5)	2·4 (1·8 to 3·2)	2·8 (1·9 to 3·8)	1·3 (0·9 to 1·9)	0·4 (0·3 to 0·6)	133
Osteoarthritis	0 (0 to 0)	0 (0 to 0)	0 (0 to 0)	0 (0 to 0)	0 (0 to 0)	0 (0 to 0)	0 (0 to 0)	0 (0 to 0)	0 (0 to 0)	2·6 (1·1 to 5·3)	14·7 (7·1 to 30·3)	36·1 (17·7 to 72·9)	63·4 (31·2 to 128)	84·6 (40·9 to 169)	98·2 (47·5 to 195)	93 (45·9 to 184)	76·4 (38·4 to 152)	56·9 (28·4 to 115)	38·5 (19·2 to 77·7)	26·4 (13·3 to 52·5)	14·5 (7·4 to 28·6)	6·3 (3·2 to 12·5)	2 (1·1 to 4)	613
Osteoarthritis hip	0 (0 to 0)	0 (0 to 0)	0 (0 to 0)	0 (0 to 0)	0 (0 to 0)	0 (0 to 0)	0 (0 to 0)	0 (0 to 0)	0 (0 to 0)	0·9 (0·3 to 2)	1·3 (0·5 to 2·9)	1·9 (0·7 to 4·1)	2·9 (1·2 to 6·5)	3·9 (1·7 to 8·9)	4·9 (2·2 to 10·5)	5 (2·2 to 10·7)	4·4 (2 to 9·7)	3·4 (1·6 to 7·2)	2·4 (1·1 to 4·9)	1·6 (0·8 to 3·3)	0·9 (0·4 to 1·9)	0·4 (0·2 to 0·8)	0·1 (0·1 to 0·3)	33·9
Osteoarthritis knee	0 (0 to 0)	0 (0 to 0)	0 (0 to 0)	0 (0 to 0)	0 (0 to 0)	0 (0 to 0)	0 (0 to 0)	0 (0 to 0)	0 (0 to 0)	1·3 (0·5 to 2·8)	10·9 (4·9 to 23·1)	27·3 (12·8 to 56·3)	47·6 (22·9 to 97·3)	62·8 (29·3 to 129)	71·7 (33·9 to 144)	67 (32·5 to 136)	54·4 (26·4 to 111)	39·9 (19·6 to 82)	26·4 (13·1 to 53·4)	17·5 (8·6 to 35·4)	9·2 (4·5 to 18·4)	3·7 (1·8 to 7·5)	1·1 (0·6 to 2·2)	441
Osteoarthritis hand	0 (0 to 0)	0 (0 to 0)	0 (0 to 0)	0 (0 to 0)	0 (0 to 0)	0 (0 to 0)	0 (0 to 0)	0 (0 to 0)	0 (0 to 0)	0·2 (0 to 0·6)	1·4 (0·4 to 3·7)	3·7 (1·4 to 8·6)	6·8 (2·8 to 14·8)	9·2 (4 to 20)	10·8 (4·9 to 23·7)	10·2 (4·8 to 21·6)	8·4 (4·1 to 17·7)	6·3 (3 to 12·9)	4·4 (2·2 to 9)	3·3 (1·6 to 6·8)	2 (1 to 4·2)	1 (0·5 to 2)	0·4 (0·2 to 0·8)	68·1

Osteoarthritis other	0 (0 to 0)	0 (0 to 0)	0 (0 to 0)	0 (0 to 0)	0 (0 to 0)	0 (0 to 0)	0 (0 to 0)	0 (0 to 0)	0 (0 to 0)	0·2 (0 to 0·4)	1·1 (0·4 to 2·7)	3·3 (1·2 to 7·5)	6·2 (2·5 to 13·6)	8·7 (3·9 to 18·4)	10·8 (4·8 to 23·4)	10·7 (5 to 22·5)	9·2 (4·3 to 19·6)	7·2 (3·5 to 14·9)	5·3 (2·7 to 11·2)	4 (2 to 8·2)	2·4 (1·2 to 4·8)	1·2 (0·6 to 2·4)	0·4 (0·2 to 0·8)	70·6
Low back pain	0 (0 to 0)	0 (0 to 0)	0 (0 to 0)	0 (0 to 0)	21·3 (10·5 to 39·1)	111 (65·9 to 177)	200 (121 to 312)	221 (132 to 353)	239 (137 to 369)	248 (142 to 381)	262 (158 to 399)	256 (156 to 389)	247 (151 to 386)	224 (138 to 349)	200 (124 to 298)	160 (101 to 236)	119 (75·8 to 182)	84·1 (52 to 126)	56 (34·7 to 84·9)	39·5 (25·2 to 59·2)	20·6 (12·6 to 29·8)	8·1 (5·1 to 12)	2·5 (1·6 to 3·8)	2,720
Neck pain	0 (0 to 0)	0 (0 to 0)	0 (0 to 0)	0 (0 to 0)	1·1 (0·3 to 2·8)	7·3 (3 to 15·1)	16·4 (7·1 to 31·3)	22·9 (10·1 to 45·2)	33·2 (15·8 to 65·7)	46·9 (22·5 to 89·1)	57·8 (29·4 to 99·1)	62·8 (32·2 to 113)	63·1 (31·9 to 111)	49·9 (25·5 to 92)	41·8 (20·3 to 74·6)	31·1 (15·7 to 55·2)	21·5 (11·4 to 35·8)	14·4 (7·6 to 24·9)	8·8 (4·5 to 16·1)	5·3 (2·6 to 9·3)	2·8 (1·4 to 4·7)	1·2 (0·6 to 2)	0·4 (0·2 to 0·8)	489
Gout	0 (0 to 0)	0 (0 to 0)	0 (0 to 0)	0 (0 to 0)	0 (0 to 0)	0 (0 to 0)	0·1 (0 to 0·2)	0·5 (0·2 to 0·9)	1·2 (0·4 to 2·5)	2 (0·7 to 4)	2·6 (1·2 to 4·7)	2·9 (1·4 to 5·3)	3·1 (1·6 to 5·3)	3 (1·4 to 5·4)	2·9 (1·4 to 5·1)	2·4 (1·1 to 4·4)	2 (1·1 to 3·4)	1·6 (0·8 to 2·7)	1·2 (0·6 to 2)	0·9 (0·4 to 1·6)	0·6 (0·3 to 1)	0·3 (0·2 to 0·5)	0·1 (0·1 to 0·2)	27·5
Other musculoskeletal disorders	0 (0 to 0)	0 (0 to 0)	0 (0 to 0)	0 (0 to 0)	1·4 (0·9 to 2·1)	5·5 (3·1 to 10·4)	50·5 (30·8 to 80·9)	103 (68·2 to 149)	169 (111 to 253)	200 (129 to 292)	189 (122 to 271)	181 (109 to 273)	171 (108 to 251)	150 (92·5 to 230)	131 (80·3 to 197)	98·4 (57·8 to 155)	67·2 (40·4 to 105)	42·3 (23·6 to 70·7)	23 (12·7 to 37·3)	11·9 (4·8 to 21·2)	7·4 (4 to 11·4)	2·6 (1·4 to 4·5)	0·8 (0·4 to 1·5)	1,600
Other non-communicable diseases	555 (387 to 781)	324 (237 to 419)	829 (595 to 1,150)	349 (275 to 441)	233 (178 to 299)	236 (183 to 301)	380 (290 to 512)	446 (328 to 605)	523 (389 to 716)	542 (398 to 745)	523 (382 to 717)	472 (345 to 645)	423 (310 to 569)	359 (265 to 475)	316 (238 to 418)	268 (202 to 353)	211 (158 to 276)	164 (124 to 212)	117 (90·8 to 150)	86 (66·9 to 108)	53·7 (41·9 to 66·4)	25·3 (19·7 to 31·2)	9·1 (6·8 to 11·5)	7,440
Congenital birth defects	536 (369 to 763)	278 (199 to 367)	523 (359 to 742)	192 (146 to 249)	95·5 (74 to 120)	85·7 (67 to 108)	91 (69·9 to 113)	66·2 (50·9 to 82·4)	56 (42·7 to 70)	43·2 (32·8 to 55·1)	32·8 (25·7 to 40·8)	25·1 (19·4 to 31·7)	21·5 (17 to 27·1)	17·5 (13·8 to 21·9)	13·6 (10·6 to 16·9)	7·7 (5·7 to 10)	4·5 (3·4 to 5·9)	1·5 (0·9 to 2·4)	0·8 (0·5 to 1·3)	0·5 (0·3 to 0·8)	0·2 (0·1 to 0·4)	0·1 (0·1 to 0·2)	0 (0 to 0·1)	2,090
Neural tube defects	42·3 (23·3 to 68·8)	14·6 (8·3 to 23·5)	31·6 (17 to 55·7)	21·2 (12·3 to 34·1)	3·4 (2·5 to 4·6)	4·5 (3·3 to 5·9)	5 (3·5 to 6·9)	2·6 (1·8 to 3·7)	2·7 (1·8 to 3·9)	1·8 (1·2 to 2·5)	0·9 (0·6 to 1·1)	0·7 (0·5 to 1)	0·4 (0·3 to 0·6)	0·2 (0·2 to 0·3)	0·2 (0·1 to 0·3)	0·1 (0·1 to 0·2)	0·1 (0·1 to 0·1)	0 (0 to 0)	0 (0 to 0)	0 (0 to 0)	0 (0 to 0)	0 (0 to 0)	0 (0 to 0)	132
Congenital heart anomalies	183 (112 to 268)	126 (84·7 to 172)	239 (155 to 347)	64·5 (44 to 92·9)	25·7 (18·3 to 35·4)	19·8 (14·3 to 27·4)	24·4 (16·7 to 32·7)	17·6 (12·3 to 24·6)	16·2 (11 to 21·8)	11·5 (7·5 to 16·2)	8·4 (5·9 to 11·6)	5·5 (3·9 to 7·6)	4·2 (3 to 5·7)	3 (2·1 to 4·1)	1·8 (1·2 to 2·5)	1·1 (0·7 to 1·5)	0·7 (0·5 to 1)	0·1 (0 to 0·2)	0·1 (0 to 0·1)	0 (0 to 0·1)	0 (0 to 0·1)	0 (0 to 0)	0 (0 to 0)	752
Orofacial clefts	0·9 (0·4 to 1·7)	0·4 (0·2 to 0·8)	1·7 (0·8 to 3·4)	1·4 (0·7 to 2·5)	1·3 (0·6 to 2·5)	1·3 (0·6 to 2·4)	1·3 (0·6 to 2·5)	1·1 (0·5 to 2·1)	1 (0·4 to 1·8)	0·9 (0·4 to 1·6)	0·8 (0·3 to 1·4)	0·7 (0·3 to 1·2)	0·6 (0·3 to 1·1)	0·5 (0·2 to 0·9)	0·4 (0·2 to 0·7)	0·3 (0·1 to 0·5)	0·2 (0·1 to 0·4)	0·1 (0·1 to 0·2)	0·1 (0 to 0·2)	0·1 (0 to 0·1)	0 (0 to 0·1)	0 (0 to 0)	0 (0 to 0)	15·2
Down syndrome	10·2 (5·3 to 15·7)	7 (4 to 10·7)	34 (18·5 to 54)	16·8 (10·3 to 25·1)	6 (4·1 to 8·3)	6·4 (4·3 to 9·1)	9·3 (6·2 to 13·3)	7·8 (5·3 to 10·9)	7 (4·6 to 10·6)	6·7 (4·1 to 10)	5·9 (4 to 8·2)	4·9 (3·5 to 6·8)	4·9 (3·5 to 6·8)	4·7 (3·2 to 6·4)	4·2 (3 to 5·7)	1·7 (1·1 to 2·4)	0·7 (0·4 to 1)	0 (0 to 0)	0 (0 to 0)	0 (0 to 0)	0 (0 to 0)	0 (0 to 0)	0 (0 to 0)	138
Turner syndrome	0 (0 to 0)	0 (0 to 0)	0 (0 to 0)	0·1 (0 to 0·1)	0·1 (0 to 0·1)	0·1 (0 to 0·1)	0·1 (0 to 0·2)	0·1 (0 to 0·2)	0·1 (0 to 0·1)	0 (0 to 0·1)	0 (0 to 0·1)	0 (0 to 0)	0 (0 to 0)	0 (0 to 0)	0 (0 to 0)	0 (0 to 0)	0 (0 to 0)	0 (0 to 0)	0 (0 to 0)	0 (0 to 0)	0 (0 to 0)	0 (0 to 0)	0 (0 to 0)	0·6
Klinefelter syndrome	0 (0 to 0)	0 (0 to 0)	0 (0 to 0)	0 (0 to 0)	0 (0 to 0)	0 (0 to 0)	0 (0 to 0)	0 (0 to 0)	0 (0 to 0)	0 (0 to 0)	0 (0 to 0)	0 (0 to 0)	0 (0 to 0)	0 (0 to 0)	0 (0 to 0)	0 (0 to 0)	0 (0 to 0)	0 (0 to 0)	0 (0 to 0)	0 (0 to 0)	0 (0 to 0)	0 (0 to 0)	0 (0 to 0)	0·1
Other chromosomal abnormalities	19·6 (11·7 to 29·9)	11·4 (6·8 to 18·2)	23 (13·3 to 37·1)	7·2 (5 to 10)	4·4 (3·1 to 6·1)	4 (2·8 to 5·6)	4·4 (3·1 to 6·1)	3 (2·1 to 4·1)	2·4 (1·6 to 3·3)	1·3 (0·9 to 1·8)	0·8 (0·6 to 1·2)	0·5 (0·3 to 0·7)	0·3 (0·2 to 0·4)	0·2 (0·1 to 0·3)	0·1 (0·1 to 0·2)	0·1 (0 to 0·1)	0 (0 to 0)	0 (0 to 0)	0 (0 to 0)	0 (0 to 0)	0 (0 to 0)	0 (0 to 0)	0 (0 to 0)	82·7
Congenital musculoskeletal and limb anomalies	16·2 (9·9 to 24·9)	7·3 (4·6 to 11)	23·6 (15·4 to 35)	26·7 (16·3 to 41·9)	26·6 (15·6 to 40·8)	27·6 (15·7 to 42·2)	23·8 (14·1 to 35·6)	14·4 (9·3 to 21·2)	11·8 (7·5 to 17·2)	9·4 (5·5 to 14·4)	8·6 (5·2 to 13)	7·4 (4·4 to 11·3)	6·3 (4 to 9·6)	5·2 (3·2 to 7·7)	4·2 (2·6 to 6·3)	2·9 (1·8 to 4·3)	1·8 (1·1 to 2·6)	0·9 (0·5 to 1·3)	0·4 (0·2 to 0·7)	0·2 (0·1 to 0·3)	0·1 (0·1 to 0·2)	0 (0 to 0·1)	0 (0 to 0)	226
Urogenital congenital anomalies	16·4 (9·3 to 29·5)	8·1 (5 to 12·9)	9·2 (5·3 to 15·3)	4 (2·6 to 6·1)	2·6 (1·7 to 3·9)	2·3 (1·5 to 3·4)	1·5 (1 to 2·3)	1·2 (0·8 to 1·8)	1 (0·6 to 1·5)	0·8 (0·5 to 1·2)	0·4 (0·3 to 0·6)	0·3 (0·2 to 0·4)	0·3 (0·2 to 0·5)	0·1 (0·1 to 0·2)	0·2 (0·1 to 0·3)	0·1 (0·1 to 0·1)	0 (0 to 0·1)	0 (0 to 0)	0 (0 to 0)	0 (0 to 0)	0 (0 to 0)	0 (0 to 0)	0 (0 to 0)	48·6
Digestive congenital anomalies	93·6 (57·5 to 144)	54·2 (34·8 to 81·6)	64·8 (38 to 104)	12·9 (8·4 to 20·1)	3·8 (2·5 to 5·8)	1·9 (1·3 to 2·7)	1·5 (1 to 2·2)	0·6 (0·4 to 0·9)	0·6 (0·4 to 0·9)	0·4 (0·3 to 0·5)	0·2 (0·2 to 0·3)	0·2 (0·1 to 0·3)	0·2 (0·2 to 0·3)	0·2 (0·1 to 0·2)	0·1 (0·1 to 0·2)	0·1 (0·1 to 0·2)	0·1 (0·1 to 0·1)	0 (0 to 0)	0 (0 to 0)	0 (0 to 0)	0 (0 to 0)	0 (0 to 0)	0 (0 to 0)	235
Other congenital birth defects	154 (98·6 to 220)	48·9 (31 to 68·9)	96·8 (59·2 to 147)	37·1 (25·7 to 51·5)	21·6 (15·2 to 29·4)	17·8 (12·7 to 24·7)	19·7 (13·7 to 27·7)	17·8 (11·9 to 24·9)	13·2 (8·6 to 19)	10·4 (6·8 to 14·9)	6·8 (4·5 to 10·3)	4·9 (3·2 to 7·8)	4·1 (2·7 to 6·7)	3·4 (2·2 to 5·6)	2·4 (1·5 to 4)	1·3 (0·8 to 2·5)	1 (0·6 to 1·9)	0·4 (0·1 to 0·9)	0·2 (0·1 to 0·6)	0·2 (0·1 to 0·4)	0·1 (0 to 0·2)	0 (0 to 0·1)	0 (0 to 0)	462
Urinary diseases and male infertility	1·2 (0·7 to 1·8)	3·1 (1·9 to 4·5)	14 (8·5 to 21·3)	6·9 (4·9 to 9·2)	4·7 (3·4 to 6·2)	6·4 (4·8 to 8·5)	12 (9·5 to 15·2)	19·4 (14·6 to 25·4)	23·7 (18 to 31·3)	30·1 (22·7 to 39·3)	30·5 (22·2 to 39·9)	29·6 (22·2 to 38·2)	32·8 (24·4 to 42·7)	38·8 (29·4 to 49·7)	48·7 (36·2 to 62)	51·8 (39 to 66·5)	48·1 (36·2 to 62·3)	44·2 (33·1 to 56·9)	35·2 (26 to 45·5)	29·1 (21·2 to 37·9)	18·8 (13·8 to 24·9)	9 (6·2 to 11·9)	3·3 (2·2 to 4·5)	542
Urolithiasis	0 (0 to 0)	0 (0 to 0)	0 (0 to 0)	0·1 (0·1 to 0·2)	0·1 (0·1 to 0·2)	0·2 (0·1 to 0·4)	0·3 (0·2 to 0·6)	0·7 (0·5 to 1·2)	1·2 (0·8 to 1·8)	2·5 (1·5 to 4)	3 (1·8 to 4·6)	2·5 (1·5 to 3·8)	4·1 (2·5 to 6·2)	3·5 (2·2 to 5·2)	4 (2·5 to 6)	2·9 (1·9 to 4·1)	1·8 (1·2 to 2·6)	1·1 (0·7 to 1·6)	0·7 (0·4 to 1·1)	0·3 (0·2 to 0·5)	0·2 (0·1 to 0·3)	0·1 (0 to 0·1)	0 (0 to 0)	29·3
Benign prostatic hyperplasia	0 (0 to 0)	0 (0 to 0)	0 (0 to 0)	0 (0 to 0)	0 (0 to 0)	0 (0 to 0)	0 (0 to 0)	0 (0 to 0)	0 (0 to 0)	0 (0 to 0)	0 (0 to 0)	0·1 (0 to 0·3)	0·8 (0·3 to 1·7)	3 (1·4 to 5·3)	8·7 (4·3 to 14·8)	13·6 (7 to 23·4)	16·2 (9·2 to 25·9)	14·5 (7·8 to 24)	9·6 (5·3 to 15·9)	5·3 (3 to 8·6)	2·1 (1·1 to 3·4)	0·7 (0·3 to 1·1)	0·1 (0·1 to 0·2)	74·7
Male infertility	0 (0 to 0)	0 (0 to 0)	0 (0 to 0)	0 (0 to 0)	0 (0 to 0)	0 (0 to 0)	0 (0 to 0)	2·5 (0·7 to 6·6)	2·7 (0·7 to 6·7)	2·5 (0·7 to 6·8)	2·1 (0·6 to 5·4)	1·7 (0·5 to 4·5)	0·2 (0 to 0·7)	0 (0 to 0)	0 (0 to 0)	0 (0 to 0)	0 (0 to 0)	0 (0 to 0)	0 (0 to 0)	0 (0 to 0)	0 (0 to 0)	0 (0 to 0)	0 (0 to 0)	11·7
Other urinary diseases	0·6 (0·4 to 0·9)	2·1 (1·3 to 3·2)	2 (0·9 to 3·6)	1·6 (1 to 2·4)	1·3 (0·8 to 1·9)	2·3 (1·5 to 3·3)	3·2 (2·2 to 4·5)	5·3 (3·1 to 7·7)	2·8 (1·6 to 4·5)	4·2 (2·5 to 6·3)	5·2 (2·9 to 8·2)	4·5 (2·8 to 6·7)	5·3 (3·1 to 8·2)	5·2 (3·3 to 8·1)	5·3 (3·1 to 8)	6·1 (3·3 to 9·3)	4·7 (2·5 to 7·8)	3·2 (1·6 to 5·6)	2·8 (1·3 to 4·8)	2·5 (1·2 to 4·6)	1·3 (0·6 to 2·5)	0·7 (0·3 to 1·2)	0·2 (0·1 to 0·4)	72·5
Gynecological diseases	0 (0 to 0)	0 (0 to 0)	0 (0 to 0)	0 (0 to 0)	0 (0 to 0)	31·4 (16·2 to 53·9)	102 (58·3 to 168)	160 (103 to 244)	213 (139 to 311)	230 (151 to 333)	229 (154 to 331)	199 (129 to 287)	150 (96·8 to 212)	87·5 (53 to 131)	46·1 (28·3 to 67·1)	26·4 (15·5 to 41)	16·4 (10·1 to 24·1)	9·9 (6·2 to 14·6)	5·7 (3·6 to 8·3)	3·6 (2·2 to 5·5)	2·2 (1·4 to 3·3)	1·1 (0·6 to 1·6)	0·4 (0·2 to 0·6)	1,510
Uterine fibroids	0 (0 to 0)	0 (0 to 0)	0 (0 to 0)	0 (0 to 0)	0 (0 to 0)	0·5 (0·2 to 1·1)	1·1 (0·4 to 2·5)	2·6 (1 to 5·7)	6·1 (2·4 to 12·9)	12·1 (5·2 to 25·3)	18·4 (8·1 to 37·2)	18·3 (8·4 to 34·9)	14·4 (6·6 to 27·5)	9·1 (4·2 to 17·2)	5·3 (2·4 to 10·2)	2·8 (1·2 to 5·6)	1·4 (0·6 to 2·6)	0·6 (0·3 to 1·3)	0·3 (0·1 to 0·6)	0·1 (0·1 to 0·3)	0·1 (0 to 0·1)	0 (0 to 0)	0 (0 to 0)	93·2
Polycystic ovarian syndrome	0 (0 to 0)	0 (0 to 0)	0 (0 to 0)	0 (0 to 0)	0 (0 to 0)	1·3 (0·5 to 2·9)	5·5 (2·3 to 11·4)	6·3 (2·6 to 12·9)	5·3 (2·2 to 10·9)	4·5 (1·9 to 9·3)	3·9 (1·6 to 7·8)	3·3 (1·4 to 6·7)	2·3 (0·9 to 4·7)	0·5 (0·2 to 1·1)	0 (0 to 0)	0 (0 to 0)	0 (0 to 0)	0 (0 to 0)	0 (0 to 0)	0 (0 to 0)	0 (0 to 0)	0 (0 to 0)	0 (0 to 0)	33
Female infertility	0 (0 to 0)	0 (0 to 0)	0 (0 to 0)	0 (0 to 0)	0 (0 to 0)	0 (0 to 0)	0 (0 to 0)	3·1 (0·4 to 9·4)	4 (0·7 to 11·4)	4·1 (0·5 to 13·1)	3·6 (0·1 to 12·1)	2·8 (0·1 to 9·8)	0 (0 to 0·1)	0 (0 to 0)	0 (0 to 0)	0 (0 to 0)	0 (0 to 0)	0 (0 to 0)	0 (0 to 0)	0 (0 to 0)	0 (0 to 0)	0 (0 to 0)	0 (0 to 0)	17·8
Endometriosis	0 (0 to 0)	0 (0 to 0)	0 (0 to 0)	0 (0 to 0)	0 (0 to 0)	0 (0 to 0)	7 (2·8 to 14·9)	25·8 (12·5 to 47)	27 (13·3 to 47·7)	19·8 (9·6 to 35·3)	15·7 (8 to 28)	13·4 (7 to 23·1)	10·4 (5·6 to 17)	4·3 (2·1 to 7·3)	0 (0 to 0)	0 (0 to 0)	0 (0 to 0)	0 (0 to 0)	0 (0 to 0)	0 (0 to 0)	0 (0 to 0)	0 (0 to 0)	0 (0 to 0)	124
Genital prolapse	0 (0 to 0)	0 (0 to 0)	0 (0 to 0)	0 (0 to 0)	0 (0 to 0)	0 (0 to 0)	0 (0 to 0·1)	0·2 (0·1 to 0·4)	0·5 (0·2 to 1·1)	1 (0·4 to 2)	1·8 (0·8 to 3·6)	2·4 (1·1 to 5·2)	3·1 (1·5 to 6·2)	2·9 (1·4 to 5·6)	2·7 (1·3 to 5·1)	2 (1 to 3·9)	1·4 (0·7 to 2·8)	1 (0·5 to 1·8)	0·6 (0·3 to 1·1)	0·4 (0·2 to 0·8)	0·3 (0·1 to 0·5)	0·1 (0·1 to 0·3)	0·1 (0 to 0·1)	20·5
Premenstrual syndrome	0 (0 to 0)	0 (0 to 0)	0 (0 to 0)	0 (0 to 0)	0 (0 to 0)	28·1 (13·2 to 50·2)	64·7 (32·3 to 116)	71·4 (35·7 to 126)	66 (37·7 to 108)	60·5 (30·7 to 99·3)	60·8 (33·8 to 97·5)	53·4 (30·2 to 85·1)	32·9 (18·2 to 55·2)	9·6 (5 to 17·7)	0 (0 to 0)	0 (0 to 0)	0 (0 to 0)	0 (0 to 0)	0 (0 to 0)	0 (0 to 0)	0 (0 to 0)	0 (0 to 0)	0 (0 to 0)	447
Other gynecological diseases	0 (0 to 0)	0 (0 to 0)	0 (0 to 0)	0 (0 to 0)	0 (0 to 0)	1·6 (0·5 to 3·3)	23·8 (8·6 to 47·4)	50·4 (28·4 to 81·8)	104 (58·4 to 165)	128 (76·4 to 198)	125 (74·3 to 192)	105 (60·1 to 166)	86·4 (49·7 to 133)	61·1 (30·5 to 97·9)	38 (22·3 to 58·1)	21·6 (11·6 to 35)	13·6 (7·9 to 21·2)	8·3 (5 to 12·6)	4·8 (2·9 to 7·2)	3·1 (1·8 to 4·8)	1·9 (1·1 to 2·9)	0·9 (0·5 to 1·4)	0·3 (0·2 to 0·6)	778
Hemoglobinopathies and hemolytic anemias	1·1 (0·8 to 1·5)	2·3 (1·6 to 3·3)	14·1 (9·5 to 19·9)	39·3 (25·6 to 56·4)	54·7 (35·7 to 78·6)	39·3 (26·9 to 56·2)	74·7 (48·2 to 117)	81·5 (49·2 to 123)	70·7 (41·7 to 107)	50·4 (27·7 to 79·9)	40 (25·2 to 63·6)	26·7 (18·6 to 36·9)	21·6 (15·2 to 29·3)	18·8 (13·1 to 26·2)	15·3 (10·6 to 21·8)	11·9 (7·8 to 17·4)	6·5 (4·2 to 9·7)	4·8 (3·1 to 7)	3·4 (2·3 to 4·9)	2·6 (1·7 to 3·9)	2·3 (1·6 to 3·2)	1·2 (0·8 to 1·7)	0·4 (0·3 to 0·6)	583
Thalassemias	0 (0 to 0)	0 (0 to 0)	0 (0 to 0)	0·1 (0 to 0·2)	0 (0 to 0·1)	0 (0 to 0)	0 (0 to 0)	0 (0 to 0)	0 (0 to 0)	0 (0 to 0)	0 (0 to 0)	0 (0 to 0)	0 (0 to 0)	0 (0 to 0)	0 (0 to 0)	0 (0 to 0)	0 (0 to 0)	0 (0 to 0)	0 (0 to 0)	0 (0 to 0)	0 (0 to 0)	0 (0 to 0)	0 (0 to 0)	0·2
Thalassemias trait	0 (0 to 0)	0 (0 to 0·1)	0·3 (0·2 to 0·6)	1·4 (0·6 to 2·4)	2·1 (1 to 3·6)	1·2 (0·5 to 2·5)	2·7 (1·2 to 5)	2·5 (1·3 to 4·3)	2·3 (1·2 to 3·8)	2·1 (1 to 3·5)	1·7 (0·8 to 2·9)	1·4 (0·6 to 2·4)	0·3 (0·1 to 0·6)	0·5 (0·2 to 0·8)	0·4 (0·2 to 0·8)	0·3 (0·1 to 0·5)	0·2 (0·1 to 0·3)	0·1 (0 to 0·2)	0·1 (0 to 0·2)	0·1 (0 to 0·1)	0 (0 to 0·1)	0 (0 to 0)	0 (0 to 0)	19·6
Sickle cell disorders	1 (0·7 to 1·4)	1 (0·7 to 1·5)	8·4 (5·2 to 13)	16·4 (11·1 to 23·6)	20·5 (15·6 to 26·9)	18·7 (13·7 to 25·7)	30·8 (22·4 to 41·4)	25·2 (18·1 to 34·5)	22·1 (15·3 to 31·1)	15·9 (10·8 to 22·8)	10·8 (7·2 to 15·8)	7·8 (5·3 to 10·8)	6·7 (4·5 to 9·6)	4·5 (2·9 to 6·8)	2 (1·2 to 3·1)	0·5 (0·3 to 0·9)	0·1 (0 to 0·2)	0 (0 to 0)	0 (0 to 0)	0 (0 to 0)	0 (0 to 0)	0 (0 to 0)	0 (0 to 0)	192
Sickle cell trait	0·1 (0 to 0·2)	1 (0·5 to 1·7)	3·4 (1·7 to 5·7)	13·9 (7·2 to 24·4)	21·1 (10·3 to 35·2)	12·5 (5·8 to 22·4)	30·6 (7·5 to 69·3)	43·9 (17·2 to 77)	36·1 (11·8 to 66·4)	22·2 (4·5 to 46·7)	15·6 (1·1 to 40·4)	5·4 (2·7 to 9·4)	5·1 (2·6 to 8·7)	4·7 (2·4 to 7·9)	4·1 (2 to 6·7)	2·7 (1·4 to 4·6)	1·5 (0·7 to 2·7)	1·1 (0·5 to 2)	0·8 (0·4 to 1·4)	0·7 (0·3 to 1·1)	0·5 (0·3 to 0·8)	0·2 (0·1 to 0·3)	0 (0 to 0·1)	227
G6PD deficiency	0 (0 to 0)	0·1 (0 to 0·1)	0·1 (0 to 0·2)	0·6 (0·3 to 1·1)	1 (0·4 to 1·6)	1 (0·5 to 1·9)	2·3 (1 to 4·4)	3·5 (1·4 to 6·5)	4·1 (1·9 to 7·4)	4·1 (1·9 to 7·1)	5·3 (2·2 to 10·9)	4·4 (2·6 to 6·6)	3·1 (1·7 to 4·5)	2·6 (1·5 to 3·8)	2·5 (1·5 to 3·5)	1·6 (1 to 2·3)	0·8 (0·5 to 1·2)	0·5 (0·3 to 0·7)	0·2 (0·1 to 0·3)	0·1 (0 to 0·1)	0 (0 to 0·1)	0 (0 to 0)	0 (0 to 0)	37·8
G6PD trait	0 (0 to 0)	0 (0 to 0)	0 (0 to 0)	0 (0 to 0)	0 (0 to 0)	0 (0 to 0)	0 (0 to 0)	0 (0 to 0)	0 (0 to 0)	0 (0 to 0)	0·3 (0 to 0·6)	0·3 (0·1 to 0·6)	0·1 (0 to 0·2)	0 (0 to 0)	0 (0 to 0)	0 (0 to 0)	0 (0 to 0)	0 (0 to 0)	0 (0 to 0)	0 (0 to 0)	0 (0 to 0)	0 (0 to 0)	0 (0 to 0)	0·8
Other hemoglobinopathies and hemolytic anemias	0 (0 to 0)	0·2 (0·1 to 0·4)	1·9 (0·9 to 3·4)	6·9 (3·2 to 12·8)	10 (4·2 to 18·7)	5·8 (2·5 to 10·9)	8·2 (3·4 to 16·5)	6·4 (2·8 to 12·4)	6·1 (2·8 to 11·7)	6·2 (3 to 10·9)	6·4 (2·9 to 13)	7·4 (3·7 to 12·6)	6·3 (3·3 to 10·4)	6·5 (3·7 to 10·4)	6·3 (3·9 to 9·7)	6·8 (4·2 to 10·3)	3·9 (2·5 to 5·9)	3·1 (2·1 to 4·5)	2·2 (1·6 to 3·2)	1·8 (1·2 to 2·6)	1·8 (1·2 to 2·4)	1 (0·7 to 1·4)	0·4 (0·2 to 0·6)	106
Endocrine, metabolic, blood, and immune disorders	15·8 (10·7 to 25·1)	18·3 (11·9 to 28·2)	160 (101 to 247)	92·1 (66·9 to 137)	54·8 (38·6 to 78)	45·4 (33·8 to 63·7)	59·9 (44·7 to 83·3)	61·4 (45·2 to 84·1)	85·2 (62·5 to 112)	102 (77·4 to 135)	99 (72·5 to 132)	99·5 (70·7 to 137)	104 (75·1 to 143)	104 (75·5 to 147)	98 (71·9 to 136)	80·9 (56·2 to 117)	59·2 (41·8 to 88)	45·1 (31·4 to 66)	31·9 (22·5 to 45·8)	22·8 (16·3 to 32·4)	15·1 (10·7 to 22·7)	7·3 (5·2 to 10·9)	2·6 (1·7 to 4·4)	1,460
Oral disorders	0 (0 to 0)	0 (0 to 0)	0 (0 to 0)	18·3 (10·2 to 30·5)	23·7 (12·8 to 40·5)	28 (15 to 48·5)	40·6 (21·4 to 71·3)	57·1 (29·6 to 106)	74·5 (37·4 to 144)	85·6 (40·5 to 157)	91·8 (43·3 to 172)	92·4 (45·3 to 170)	93·8 (49 to 169)	92·2 (50·9 to 160)	94·4 (53·9 to 150)	88·9 (53·7 to 141)	75·9 (46 to 119)	58·3 (36·5 to 88·5)	39·9 (24·8 to 60)	27·4 (17·2 to 40·6)	15·1 (9·4 to 22·2)	6·7 (4·2 to 9·9)	2·3 (1·4 to 3·3)	1,110
Caries of deciduous teeth	0 (0 to 0)	0 (0 to 0)	0 (0 to 0)	6·1 (2·3 to 12·2)	6·1 (2·6 to 13·5)	0·4 (0·1 to 1)	0 (0 to 0)	0 (0 to 0)	0 (0 to 0)	0 (0 to 0)	0 (0 to 0)	0 (0 to 0)	0 (0 to 0)	0 (0 to 0)	0 (0 to 0)	0 (0 to 0)	0 (0 to 0)	0 (0 to 0)	0 (0 to 0)	0 (0 to 0)	0 (0 to 0)	0 (0 to 0)	0 (0 to 0)	12·6
Caries of permanent teeth	0 (0 to 0)	0 (0 to 0)	0 (0 to 0)	0 (0 to 0)	5·7 (2 to 12·6)	13·5 (5·5 to 29·5)	14·4 (5·7 to 31)	14·5 (6·1 to 30·5)	12·7 (4·9 to 26·1)	10·5 (4·2 to 22·3)	9·4 (3·8 to 19)	7·7 (3·2 to 15·6)	6·5 (2·6 to 13·8)	5·2 (2 to 10·9)	4 (1·5 to 8·8)	2·7 (1 to 5·8)	1·7 (0·6 to 3·5)	1·1 (0·4 to 2·2)	0·6 (0·3 to 1·3)	0·4 (0·2 to 0·8)	0·2 (0·1 to 0·4)	0·1 (0 to 0·2)	0 (0 to 0·1)	111
Periodontal diseases	0 (0 to 0)	0 (0 to 0)	0 (0 to 0)	0 (0 to 0)	0 (0 to 0)	0 (0 to 0)	5·3 (1·5 to 13·7)	17·6 (5·3 to 44·1)	34·7 (10·9 to 84·7)	47·1 (16 to 106)	53·8 (19·1 to 122)	54·6 (19·8 to 119)	51·7 (19·7 to 110)	43·1 (17 to 91)	33·8 (13·4 to 72·3)	22·6 (9·1 to 46·6)	14·1 (5·5 to 29·2)	8·6 (3·3 to 17·8)	5·2 (2·1 to 10·2)	3·4 (1·3 to 6·7)	1·8 (0·7 to 3·8)	0·8 (0·3 to 1·6)	0·2 (0·1 to 0·5)	398
Other oral disorders	0 (0 to 0)	0 (0 to 0)	0 (0 to 0)	12·2 (7·1 to 18·5)	11·9 (6·9 to 18·5)	14·1 (8·2 to 22·2)	20·9 (12·8 to 32·7)	22·7 (14 to 34·7)	22·2 (13·2 to 33·5)	20·6 (12·4 to 30·9)	18·7 (11·3 to 28·6)	16·5 (10 to 25)	14·8 (9 to 22·1)	12·2 (7·4 to 18·4)	9·9 (6 to 14·8)	7·3 (4·4 to 11)	5·1 (3 to 7·6)	3·1 (1·9 to 4·7)	1·6 (1 to 2·4)	0·9 (0·5 to 1·3)	0·4 (0·2 to 0·5)	0·1 (0·1 to 0·2)	0 (0 to 0·1)	215
Sudden infant death syndrome	--	22·6 (15·3 to 32·6)	118 (69·3 to 185)	--	--	--	--	--	--	--	--	--	--	--	--	--	--	--	--	--	--	--	--	NA
Injuries	60·1 (45·1 to 80·1)	42 (31·2 to 54·4)	250 (172 to 354)	439 (340 to 558)	462 (398 to 535)	645 (571 to 717)	2,200 (1,970 to 2,460)	3,110 (2,770 to 3,480)	3,110 (2,790 to 3,460)	2,550 (2,270 to 2,830)	1,780 (1,550 to 2,020)	1,340 (1,150 to 1,560)	1,080 (906 to 1,260)	778 (655 to 916)	620 (526 to 719)	424 (360 to 494)	279 (239 to 324)	192 (164 to 223)	133 (113 to 153)	104 (89·8 to 120)	65·4 (54·8 to 76·4)	34·8 (29·2 to 40·5)	13·9 (11·4 to 16·3)	19,700
Transport injuries	2·9 (2·1 to 3·8)	2·5 (1·8 to 3·4)	20·8 (14·1 to 29·6)	82·3 (58·7 to 112)	131 (99·2 to 166)	177 (142 to 216)	615 (484 to 767)	825 (649 to 1,010)	832 (677 to 1,010)	667 (527 to 805)	475 (373 to 584)	389 (308 to 481)	331 (262 to 406)	239 (191 to 291)	192 (153 to 234)	127 (104 to 152)	80·3 (67 to 95)	49·8 (41·1 to 59·3)	30·7 (25·3 to 36·3)	19·5 (15·8 to 23·7)	9·7 (7·7 to 11·7)	3·9 (3·1 to 4·7)	1·3 (1·1 to 1·6)	5,300
Road injuries	2·1 (1·6 to 2·9)	1·6 (1·1 to 2·3)	16·4 (10·8 to 23·6)	72·1 (50·8 to 99·7)	118 (86·8 to 152)	156 (120 to 195)	563 (435 to 708)	757 (588 to 935)	764 (619 to 934)	604 (479 to 737)	433 (338 to 539)	351 (278 to 435)	303 (241 to 372)	218 (173 to 267)	173 (137 to 210)	117 (95·7 to 141)	74·8 (62·1 to 89·4)	45·2 (36·9 to 54·4)	28·2 (23·2 to 33·5)	18·2 (14·6 to 22·1)	9 (7·2 to 10·9)	3·5 (2·8 to 4·3)	1·2 (0·9 to 1·4)	4,830
Pedestrian road injuries	0·8 (0·6 to 1·1)	0·5 (0·3 to 0·7)	3·8 (2·5 to 5·5)	27 (18·9 to 37)	42·6 (30·1 to 56·6)	42·1 (30·3 to 53·5)	60·8 (36·7 to 92·9)	65·5 (45·3 to 90·4)	73·9 (50·1 to 105)	74·5 (57·4 to 93·8)	59·8 (44·8 to 78·6)	55·4 (41·9 to 71·1)	53·4 (39·5 to 70)	43·3 (34·4 to 53·3)	38·7 (30·5 to 48·2)	28·6 (23·1 to 34·8)	20·4 (16·8 to 24·5)	13·8 (11·3 to 16·8)	9·7 (8 to 11·7)	6·9 (5·6 to 8·5)	3·5 (2·8 to 4·3)	1·3 (1 to 1·6)	0·4 (0·3 to 0·5)	727
Cyclist road injuries	0 (0 to 0)	0 (0 to 0)	0 (0 to 0)	4·3 (2·4 to 7·1)	9·1 (5·8 to 13·8)	27·9 (17·9 to 41·9)	68·9 (45·2 to 101)	72·4 (47·3 to 103)	66·7 (46·1 to 91·5)	60·7 (41·7 to 84·3)	48·3 (34·9 to 64·6)	40·1 (29·4 to 53·6)	35·1 (26 to 46·1)	29·3 (21·7 to 38)	23·7 (17 to 31·5)	17·1 (12·8 to 22·7)	11·6 (8·8 to 15)	6·8 (5·1 to 9)	4·1 (3 to 5·3)	2·5 (1·9 to 3·4)	1·2 (0·9 to 1·6)	0·5 (0·4 to 0·7)	0·2 (0·1 to 0·2)	530
Motorcyclist road injuries	0·2 (0·1 to 0·2)	0·2 (0·1 to 0·3)	1·5 (1 to 2·2)	4 (2·7 to 5·6)	7·5 (4·3 to 12·1)	11·4 (7·4 to 17·2)	120 (86·2 to 158)	208 (159 to 259)	209 (165 to 259)	161 (120 to 215)	101 (75·5 to 132)	68·1 (52·2 to 88·1)	54·5 (41·2 to 70·3)	35·1 (28 to 43·3)	26·7 (20·9 to 32·5)	15 (11·9 to 18·6)	9·4 (7·1 to 11·9)	5·1 (3·8 to 6·7)	3·1 (2·3 to 4·2)	2 (1·5 to 2·8)	1·1 (0·8 to 1·4)	0·5 (0·3 to 0·6)	0·2 (0·1 to 0·2)	1,040
Motor vehicle road injuries	1·1 (0·8 to 1·5)	0·9 (0·6 to 1·3)	10·8 (7·1 to 15·8)	35·7 (24·9 to 49·5)	57·2 (40·9 to 76·2)	72·9 (53·6 to 95·7)	309 (236 to 393)	405 (312 to 499)	408 (323 to 499)	301 (227 to 388)	219 (163 to 281)	183 (141 to 235)	156 (115 to 202)	107 (83·7 to 133)	80·4 (62·4 to 99·7)	54·5 (43·5 to 66·5)	31·8 (25·9 to 38·2)	18·4 (15 to 22·5)	10·6 (8·6 to 12·7)	6·2 (5 to 7·6)	3 (2·4 to 3·6)	1·2 (1 to 1·4)	0·4 (0·3 to 0·5)	2,470
Other road injuries	0·1 (0 to 0·1)	0 (0 to 0·1)	0·3 (0·1 to 0·5)	1·1 (0·6 to 2·1)	1·8 (1·1 to 2·8)	1·9 (1·3 to 2·8)	4·5 (3 to 6·6)	6·5 (4·3 to 9·6)	7·1 (5 to 10·4)	6·5 (4·5 to 9·2)	5·1 (3·7 to 7·1)	4·5 (3·2 to 5·9)	4·5 (3·3 to 5·9)	3·6 (2·6 to 4·8)	3 (2·2 to 4)	2·2 (1·6 to 2·8)	1·6 (1·2 to 2·1)	1 (0·7 to 1·3)	0·7 (0·5 to 0·9)	0·4 (0·3 to 0·6)	0·2 (0·2 to 0·3)	0·1 (0·1 to 0·1)	0 (0 to 0)	56·7
Other transport injuries	0·7 (0·5 to 1·1)	0·9 (0·5 to 1·3)	4·4 (2·7 to 6·8)	10·2 (6·3 to 15·9)	12·7 (8·9 to 17·9)	21·2 (14·9 to 28·2)	51·7 (32·6 to 76·9)	68 (44·2 to 97)	67·3 (45·1 to 98·5)	62·9 (42·1 to 87·9)	42·6 (27·4 to 62·5)	37·6 (25 to 52·7)	27·3 (17·9 to 39·2)	20·9 (14·5 to 29·4)	19·7 (13·5 to 28·4)	10·1 (7·5 to 13·6)	5·5 (4·2 to 7·2)	4·6 (3·5 to 5·9)	2·5 (1·8 to 3·3)	1·4 (1 to 1·7)	0·7 (0·5 to 0·9)	0·3 (0·3 to 0·4)	0·1 (0·1 to 0·2)	473
Unintentional injuries	36·5 (27·7 to 48·3)	34·3 (25·3 to 44·6)	197 (130 to 289)	301 (226 to 399)	282 (229 to 339)	357 (297 to 418)	617 (513 to 744)	681 (558 to 825)	663 (554 to 788)	597 (494 to 715)	480 (405 to 574)	412 (338 to 501)	355 (289 to 432)	301 (248 to 371)	262 (220 to 313)	198 (165 to 241)	143 (119 to 171)	110 (92·3 to 131)	84·1 (70·4 to 99·9)	74 (63 to 86·3)	50·9 (42·4 to 59·7)	29·3 (24·6 to 34·3)	12 (9·8 to 14·1)	6,280
Falls	0·5 (0·4 to 0·6)	0·4 (0·3 to 0·6)	5·4 (3·6 to 8·1)	11·9 (8·8 to 16·3)	16·6 (12·4 to 22·1)	29·3 (21·9 to 38·4)	51·7 (40·1 to 66·7)	60·9 (47·3 to 77·4)	63·7 (49·8 to 81·1)	64·4 (50·1 to 82·1)	62·7 (49·9 to 80·5)	59·6 (46·5 to 76·1)	58·7 (46·1 to 74·7)	56·4 (43·8 to 71·3)	53·1 (42·2 to 65·8)	45·4 (36·4 to 56·6)	36·8 (29·7 to 44·7)	34 (27·6 to 41)	29·9 (24·6 to 36)	33·1 (27·4 to 39·3)	26·3 (21·4 to 31·5)	15·7 (12·9 to 18·7)	6·4 (5 to 7·5)	823
Drowning	2·5 (1·8 to 3·5)	1·8 (1·2 to 2·6)	20·8 (13·1 to 32·5)	117 (80·3 to 166)	118 (85 to 154)	141 (109 to 175)	243 (180 to 321)	213 (150 to 289)	190 (138 to 256)	154 (113 to 200)	103 (76·7 to 134)	75·5 (55·6 to 99·5)	53 (38·5 to 71·6)	37·8 (27·3 to 51·1)	28·1 (20·6 to 36·7)	16·2 (12·4 to 21)	8·8 (6·9 to 11·2)	4·4 (3·6 to 5·4)	2·6 (2 to 3·3)	1·3 (1 to 1·6)	0·8 (0·6 to 1)	0·5 (0·4 to 0·6)	0·2 (0·2 to 0·3)	1,530
Fire, heat, and hot substances	0·5 (0·4 to 0·7)	0·9 (0·7 to 1·3)	9·6 (6·4 to 14·3)	26·9 (18·5 to 38·1)	27·5 (21·5 to 35)	35·4 (26·2 to 46·9)	47·9 (35·6 to 63·4)	51·3 (38·9 to 66·8)	54·6 (41·9 to 70)	55·5 (41·9 to 72·7)	47·4 (35·6 to 62·5)	41·5 (30·5 to 54·4)	37 (27·6 to 49·1)	30·2 (22·6 to 40·1)	25 (18·8 to 33·1)	18·2 (13·7 to 24·5)	11·9 (8·9 to 16·2)	8·2 (6·2 to 10·9)	5·3 (3·9 to 7·1)	3·9 (3 to 5·1)	2·5 (1·9 to 3·2)	1·2 (1 to 1·6)	0·5 (0·4 to 0·6)	543
Poisonings	0·5 (0·3 to 0·7)	0·9 (0·6 to 1·3)	4 (2·5 to 6·5)	13·2 (8·4 to 19·9)	5·7 (4·2 to 7·5)	5·4 (3·9 to 7·2)	8·9 (6·5 to 11·9)	11·1 (8·1 to 14·8)	13·8 (10·1 to 18·9)	13·1 (9·4 to 17·3)	9·1 (6·6 to 12·3)	6·2 (4·5 to 8·3)	5 (3·6 to 6·6)	3·6 (2·6 to 4·7)	2·8 (2·1 to 3·7)	1·9 (1·4 to 2·4)	1·3 (1 to 1·7)	0·8 (0·6 to 1·1)	0·6 (0·4 to 0·7)	0·4 (0·3 to 0·5)	0·2 (0·2 to 0·3)	0·1 (0·1 to 0·1)	0 (0 to 0)	109
Poisoning by carbon monoxide	0·3 (0·2 to 0·4)	0·5 (0·2 to 0·8)	1 (0·5 to 1·9)	6·4 (3·6 to 10·9)	2·7 (1·8 to 4)	2·4 (1·5 to 3·4)	5 (3·2 to 7·3)	6·3 (4 to 9·6)	7·8 (4·9 to 12·1)	7·7 (5 to 11·3)	5·3 (3·2 to 8)	3·5 (2·1 to 5·1)	2·6 (1·6 to 3·9)	1·6 (1 to 2·4)	1·5 (1 to 2·2)	0·9 (0·6 to 1·3)	0·7 (0·5 to 0·9)	0·4 (0·3 to 0·6)	0·3 (0·2 to 0·4)	0·2 (0·2 to 0·3)	0·1 (0·1 to 0·2)	0 (0 to 0·1)	0 (0 to 0)	57·2
Poisoning by other means	0·2 (0·1 to 0·2)	0·4 (0·3 to 0·7)	3 (1·9 to 4·8)	6·8 (4·3 to 10·2)	3 (2·1 to 4)	3 (2·1 to 4·4)	3·9 (2·8 to 5·3)	4·8 (3·5 to 6·3)	6·1 (4·5 to 7·9)	5·4 (4 to 6·9)	3·8 (2·9 to 4·9)	2·8 (2 to 3·5)	2·4 (1·7 to 3·1)	2 (1·5 to 2·6)	1·4 (1 to 1·8)	1 (0·7 to 1·3)	0·6 (0·4 to 0·8)	0·4 (0·3 to 0·6)	0·3 (0·2 to 0·4)	0·2 (0·1 to 0·2)	0·1 (0·1 to 0·1)	0·1 (0 to 0·1)	0 (0 to 0)	51·4
Exposure to mechanical forces	1·1 (0·7 to 1·6)	1·8 (1·2 to 2·5)	7·6 (5·2 to 11·3)	31·6 (22·4 to 44·2)	43·8 (31·8 to 57·5)	58·6 (44·2 to 76·6)	151 (113 to 196)	199 (151 to 258)	184 (141 to 236)	171 (132 to 218)	141 (111 to 179)	124 (95·9 to 161)	102 (78·6 to 131)	82·7 (63·7 to 107)	70·4 (54·1 to 90·5)	48 (36·3 to 62·9)	31·9 (23·3 to 43·7)	21·5 (15·7 to 29·2)	14 (10 to 19·1)	9·7 (7·1 to 12·9)	5·6 (4·1 to 7·5)	3 (2·2 to 3·8)	1·2 (1 to 1·6)	1,510
Unintentional firearm injuries	0·5 (0·3 to 0·8)	0·5 (0·3 to 0·8)	2·5 (1·5 to 4·1)	7·3 (4·3 to 11·6)	11·3 (7·3 to 16·8)	22·3 (15·1 to 33)	97·8 (68·1 to 131)	137 (98·4 to 183)	117 (84 to 159)	101 (72·7 to 136)	75·1 (54·9 to 99·4)	61·5 (44·8 to 84·3)	44 (32·2 to 58·1)	30·7 (23·5 to 40)	24·4 (18·5 to 31·8)	13·9 (10·9 to 17·3)	7·9 (6·2 to 10)	4·9 (3·8 to 6·2)	2·9 (2·3 to 3·7)	2·2 (1·8 to 2·7)	1·5 (1·2 to 1·9)	1 (0·8 to 1·3)	0·5 (0·4 to 0·6)	768
Other exposure to mechanical forces	0·6 (0·4 to 0·8)	1·3 (0·9 to 1·8)	5·1 (3·5 to 7·2)	24·2 (17 to 34·3)	32·5 (23·5 to 43·1)	36·3 (26·4 to 49·3)	53·6 (37·6 to 74·6)	62 (43·3 to 86·4)	67·4 (47·8 to 94·4)	70·2 (49·3 to 97·2)	66·1 (48·3 to 91·1)	62·7 (44·7 to 87·8)	57·8 (41·1 to 82)	52 (37·1 to 70·9)	46 (32·9 to 63·1)	34·1 (24·3 to 47·5)	24 (16·7 to 33·8)	16·6 (11·6 to 23·4)	11·1 (7·6 to 15·5)	7·5 (5·2 to 10·4)	4·1 (2·8 to 5·7)	2 (1·4 to 2·7)	0·7 (0·5 to 1)	738
Adverse effects of medical treatment	12·8 (8·4 to 18)	12·2 (8·3 to 17)	40·9 (24·3 to 63·7)	22·6 (14·6 to 34·4)	14·7 (10·5 to 20)	14·8 (10·5 to 20·9)	21·3 (14·9 to 29·8)	38·4 (26·5 to 53·6)	39·8 (27·6 to 55·2)	35·5 (24·4 to 47·7)	31 (22·4 to 42·4)	30 (22·1 to 40·6)	31·1 (22·4 to 41·9)	32·9 (23·9 to 44·9)	32·3 (23·8 to 43·3)	29·4 (21·2 to 40·9)	22·9 (16·8 to 30·7)	18·9 (14·3 to 25·1)	15·6 (11·7 to 21·4)	12·8 (9·7 to 17)	8·1 (6·1 to 10·7)	4 (3 to 5·3)	1·4 (0·9 to 2·1)	523
Animal contact	0·3 (0·2 to 0·4)	0·7 (0·4 to 1·1)	3·1 (2 to 4·8)	8·3 (5·6 to 11·7)	7 (5·2 to 9·5)	9·4 (7 to 12·6)	12·2 (8·8 to 16·6)	11·6 (8·5 to 15·2)	16·7 (12·4 to 22·2)	11·5 (8·6 to 15·1)	12·4 (9·2 to 16·6)	10·8 (8 to 14·4)	11·4 (8·2 to 15·2)	9·8 (7·3 to 13)	9·5 (7 to 12·4)	8·2 (6·1 to 10·8)	6·4 (4·6 to 8·7)	4·9 (3·7 to 6·5)	3·7 (2·8 to 4·9)	2·9 (2·2 to 3·9)	2·1 (1·6 to 2·8)	1·9 (1·3 to 2·6)	1 (0·6 to 1·4)	166
Venomous animal contact	0·2 (0·1 to 0·2)	0·5 (0·3 to 0·9)	1·9 (1 to 3)	5·7 (3·7 to 8·5)	4·5 (3 to 6·3)	6·9 (4·8 to 9·4)	8·4 (5·5 to 12·1)	7·6 (5·2 to 10·1)	11·6 (7·9 to 16·1)	7·6 (5·4 to 10·1)	9·2 (6·5 to 12·3)	7·1 (5 to 9·8)	7·5 (5·1 to 10·5)	6·3 (4·5 to 8·8)	6·3 (4·5 to 8·4)	5·5 (3·9 to 7·6)	3·9 (2·6 to 5·6)	2·8 (1·9 to 4)	2·5 (1·8 to 3·4)	2 (1·4 to 2·7)	1·5 (1·1 to 2·1)	1·5 (1 to 2·1)	0·8 (0·5 to 1·1)	112
Non-venomous animal contact	0·1 (0·1 to 0·2)	0·2 (0·1 to 0·4)	1·3 (0·6 to 2·3)	2·6 (1·4 to 4·5)	2·5 (1·5 to 4·1)	2·6 (1·5 to 4·1)	3·8 (2·4 to 5·9)	4 (2·5 to 6·1)	5·2 (2·8 to 8·2)	4 (2·5 to 5·7)	3·2 (2·1 to 4·9)	3·6 (2·3 to 5·7)	3·9 (2·4 to 5·8)	3·5 (2·3 to 5)	3·2 (2·2 to 4·6)	2·7 (1·7 to 4·2)	2·5 (1·6 to 3·8)	2·1 (1·4 to 3·1)	1·2 (0·8 to 1·8)	1 (0·6 to 1·4)	0·6 (0·4 to 0·9)	0·4 (0·3 to 0·7)	0·2 (0·1 to 0·4)	54·4
Foreign body	17·6 (12·6 to 24·4)	15·1 (10·4 to 21·5)	102 (61·9 to 163)	54·9 (38·7 to 75·7)	25·8 (20·2 to 32)	30·3 (23·4 to 37·5)	25·1 (19·7 to 32·1)	30 (23 to 38·5)	33 (25·5 to 41·4)	28·5 (21·7 to 36·5)	22·3 (17 to 28·3)	20·1 (15·6 to 25·7)	18·6 (14·6 to 23·5)	17·2 (12·5 to 22·6)	15·2 (11·7 to 19·3)	12·5 (9·5 to 16·4)	9·8 (7·7 to 12·5)	7·8 (6·1 to 9·6)	6·2 (5·2 to 7·3)	5·5 (4·2 to 6·9)	2·7 (2 to 3·4)	1·6 (1·2 to 2)	0·8 (0·6 to 1)	502
Pulmonary aspiration and foreign body in airway	17·6 (12·6 to 24·3)	15·1 (10·3 to 21·4)	101 (61·4 to 162)	52·6 (36·5 to 73·4)	22·5 (17·1 to 28·3)	26 (19·8 to 33·3)	20·4 (15·6 to 26·2)	25 (18·5 to 33)	28 (21 to 36·7)	24·1 (17·6 to 31·8)	18·1 (13·6 to 23·7)	16·2 (12·1 to 21·1)	15·1 (11·5 to 19·4)	14·4 (10·1 to 19·7)	12·8 (9·4 to 16·7)	10·6 (7·8 to 14·5)	8·4 (6·4 to 11)	6·7 (5·2 to 8·6)	5·5 (4·5 to 6·6)	4·9 (3·6 to 6·3)	2·4 (1·7 to 3)	1·4 (1 to 1·8)	0·7 (0·5 to 0·9)	450
Foreign body in eyes	0 (0 to 0)	0 (0 to 0)	0·1 (0 to 0·3)	0·7 (0·2 to 1·5)	1·1 (0·3 to 2·7)	1·6 (0·5 to 3·7)	2 (0·7 to 4·7)	2·3 (0·7 to 4·9)	2·3 (0·8 to 4·8)	2·1 (0·8 to 4·5)	2 (0·8 to 4·2)	1·8 (0·8 to 3·6)	1·6 (0·7 to 3)	1·2 (0·6 to 2·2)	0·9 (0·5 to 1·5)	0·7 (0·4 to 1·1)	0·5 (0·3 to 0·8)	0·4 (0·2 to 0·5)	0·2 (0·2 to 0·4)	0·2 (0·1 to 0·2)	0·1 (0·1 to 0·1)	0 (0 to 0·1)	0 (0 to 0)	21·9
Foreign body in other body part	0 (0 to 0)	0 (0 to 0·1)	0·4 (0·3 to 0·7)	1·6 (1·1 to 2·4)	2·2 (1·5 to 3·1)	2·7 (1·8 to 3·8)	2·7 (2 to 3·9)	2·7 (2 to 3·7)	2·8 (2 to 3·7)	2·4 (1·7 to 3·2)	2·1 (1·6 to 2·9)	2·1 (1·5 to 2·8)	2 (1·4 to 2·6)	1·6 (1·2 to 2·2)	1·5 (1·1 to 2)	1·1 (0·8 to 1·5)	0·9 (0·7 to 1·2)	0·7 (0·5 to 0·9)	0·5 (0·4 to 0·6)	0·4 (0·3 to 0·5)	0·2 (0·2 to 0·3)	0·1 (0·1 to 0·2)	0·1 (0 to 0·1)	30·8
Environmental heat and cold exposure	0 (0 to 0)	0·1 (0 to 0·1)	0·5 (0·3 to 0·7)	2·7 (1·8 to 4)	5·6 (3·7 to 8·2)	8·2 (5·4 to 11·8)	11·5 (7·9 to 16)	12·9 (8·9 to 17·8)	13·3 (9·3 to 18·1)	13·1 (9·2 to 18·4)	12·4 (8·7 to 17·1)	11·5 (8·1 to 16)	10·7 (7·7 to 14·7)	9·4 (6·7 to 12·7)	8·3 (5·9 to 11·3)	6·5 (4·6 to 8·9)	4·9 (3·5 to 6·7)	3·7 (2·6 to 4·9)	2·6 (1·8 to 3·4)	2 (1·4 to 2·6)	1·2 (0·9 to 1·6)	0·7 (0·5 to 0·9)	0·3 (0·2 to 0·4)	142
Exposure to forces of nature	0 (0 to 0)	0 (0 to 0)	0 (0 to 0)	0 (0 to 0)	0 (0 to 0)	0·2 (0·1 to 0·3)	0·7 (0·4 to 1)	2·3 (1·5 to 3·8)	2·7 (1·6 to 4·1)	3·3 (2 to 5·4)	1·8 (1·1 to 2·9)	1·7 (1·1 to 2·6)	1·8 (1·1 to 2·8)	1·4 (0·9 to 2·2)	1·1 (0·6 to 1·6)	0·7 (0·5 to 1·1)	0·5 (0·3 to 0·8)	0·4 (0·2 to 0·6)	0·2 (0·1 to 0·3)	0·1 (0·1 to 0·2)	0·1 (0 to 0·1)	0 (0 to 0)	0 (0 to 0)	19
Other unintentional injuries	0·6 (0·4 to 1)	0·4 (0·2 to 0·6)	3·2 (2·2 to 4·5)	11·5 (8·4 to 15·6)	17·8 (12·9 to 24·2)	25 (18·9 to 33·1)	43·3 (32·3 to 57·4)	49·7 (37·5 to 65·1)	51·6 (39 to 67·2)	46·9 (35·3 to 61·1)	37·6 (29·2 to 48·4)	31·1 (23·5 to 40·7)	26·1 (19·7 to 34·5)	19·4 (14·1 to 26·1)	16 (11·9 to 21·9)	11·2 (8·1 to 15·1)	7·8 (5·6 to 10·7)	5·2 (3·7 to 7·3)	3·5 (2·4 to 4·9)	2·4 (1·6 to 3·3)	1·3 (0·9 to 1·8)	0·6 (0·4 to 0·8)	0·2 (0·2 to 0·3)	412
Self-harm and interpersonal violence	20·7 (12·9 to 32·5)	5·2 (3·2 to 8·2)	32·7 (19·9 to 52·8)	55·8 (35·3 to 88·4)	48·8 (35·3 to 65·7)	110 (85·6 to 139)	973 (797 to 1,160)	1,610 (1,370 to 1,880)	1,620 (1,390 to 1,870)	1,280 (1,090 to 1,500)	827 (693 to 978)	544 (433 to 668)	389 (308 to 480)	238 (188 to 300)	166 (130 to 209)	98·8 (79·2 to 123)	55·7 (45 to 67·9)	32·7 (26·7 to 39·8)	17·8 (14·7 to 21·4)	10·7 (8·9 to 12·7)	4·8 (4 to 5·7)	1·6 (1·3 to 1·9)	0·6 (0·4 to 0·7)	8,140
Self-harm	0 (0 to 0)	0 (0 to 0)	0 (0 to 0)	0 (0 to 0)	0 (0 to 0)	13·4 (9·4 to 19)	129 (98 to 174)	205 (153 to 268)	224 (165 to 293)	215 (160 to 287)	149 (112 to 193)	108 (80·7 to 139)	93·7 (69·6 to 120)	68·7 (52·3 to 89·7)	56 (42·1 to 72·1)	37·7 (28·9 to 49)	23 (18 to 29·1)	14·9 (11·5 to 19·1)	8·5 (6·5 to 10·9)	6 (4·6 to 7·7)	2·7 (2·1 to 3·5)	0·8 (0·6 to 1)	0·2 (0·1 to 0·2)	1,360
Self-harm by firearm	0 (0 to 0)	0 (0 to 0)	0 (0 to 0)	0 (0 to 0)	0 (0 to 0)	0·7 (0·4 to 1·3)	17·8 (11·1 to 28·5)	27·5 (16·2 to 43·6)	27·2 (16·7 to 45·8)	25·4 (15·8 to 40·8)	15·5 (9·6 to 23·9)	12·2 (7·2 to 19)	9·1 (5·4 to 13·8)	7·3 (4·4 to 10·8)	5·3 (3·3 to 8·1)	3·5 (2·2 to 5·3)	1·9 (1·2 to 2·8)	1·2 (0·7 to 1·8)	0·6 (0·4 to 1)	0·5 (0·3 to 0·7)	0·2 (0·1 to 0·3)	0 (0 to 0)	0 (0 to 0)	156
Self-harm by other specified means	0 (0 to 0)	0 (0 to 0)	0 (0 to 0)	0 (0 to 0)	0 (0 to 0)	12·6 (8·9 to 17·9)	111 (83·3 to 148)	177 (130 to 236)	197 (145 to 259)	190 (139 to 252)	134 (99·9 to 173)	96 (71·6 to 124)	84·7 (62·5 to 108)	61·4 (46·9 to 80)	50·8 (38·1 to 65·8)	34·2 (26·1 to 44·2)	21·1 (16·3 to 26·7)	13·7 (10·5 to 17·4)	7·9 (6·1 to 10·2)	5·5 (4·2 to 7·2)	2·5 (1·9 to 3·2)	0·8 (0·6 to 1)	0·2 (0·1 to 0·2)	1,200
Interpersonal violence	20·7 (12·9 to 32·5)	5·2 (3·2 to 8·2)	32·6 (19·8 to 52·6)	55·3 (34·8 to 87·9)	48·4 (35 to 65·3)	96·5 (73 to 125)	843 (672 to 1,030)	1,400 (1,180 to 1,650)	1,390 (1,170 to 1,610)	1,060 (872 to 1,250)	671 (543 to 812)	431 (336 to 543)	288 (220 to 366)	166 (122 to 216)	108 (78·7 to 145)	59·4 (44·3 to 79·3)	30·8 (23·2 to 39·6)	16·3 (12·3 to 20·9)	8·7 (6·6 to 11·1)	4·1 (3·3 to 5)	2 (1·6 to 2·4)	0·8 (0·6 to 1)	0·3 (0·2 to 0·4)	6,730
Physical violence by firearm	0·9 (0·5 to 1·5)	0·4 (0·2 to 0·6)	3·3 (1·8 to 5·8)	7·7 (4·7 to 12·5)	11·2 (7·8 to 16·1)	35·5 (25·8 to 46·8)	526 (412 to 651)	920 (766 to 1,090)	884 (741 to 1,040)	635 (521 to 760)	380 (291 to 479)	215 (165 to 278)	134 (98·4 to 176)	68·1 (49·4 to 90·7)	40·5 (28·4 to 55·6)	19·6 (14 to 27)	8·4 (6·1 to 11·2)	3·6 (2·5 to 5)	1·3 (0·9 to 1·8)	0·5 (0·4 to 0·6)	0·2 (0·2 to 0·3)	0·1 (0·1 to 0·1)	0 (0 to 0)	3,890
Physical violence by sharp object	1·5 (0·8 to 2·7)	0·3 (0·2 to 0·6)	2 (1·1 to 3·2)	5·4 (3·6 to 8·2)	6·4 (4·7 to 8·5)	16·1 (12·2 to 21)	219 (171 to 275)	339 (277 to 402)	351 (293 to 416)	292 (240 to 347)	192 (146 to 248)	136 (104 to 171)	86·7 (65·3 to 112)	48·7 (35·6 to 63·2)	28·7 (20·8 to 38·3)	15·5 (11·6 to 20·4)	7·4 (5·6 to 9·4)	4·4 (3·4 to 5·8)	2·2 (1·7 to 2·9)	1·2 (0·9 to 1·5)	0·6 (0·4 to 0·7)	0·3 (0·2 to 0·4)	0·1 (0·1 to 0·2)	1,760
Sexual violence	0 (0 to 0)	0 (0 to 0)	0 (0 to 0)	0·2 (0·1 to 0·4)	2 (1·2 to 3·2)	4·9 (2·9 to 7·8)	11·4 (6·3 to 18·1)	13·8 (7·9 to 21)	11·3 (6·4 to 18·2)	9·4 (5·2 to 15·5)	7·7 (4·3 to 12·2)	5·6 (3·2 to 9·1)	4 (2·4 to 6·8)	2·5 (1·4 to 4·1)	1·6 (0·9 to 2·6)	1·1 (0·7 to 1·7)	0·8 (0·5 to 1·1)	0·5 (0·3 to 0·7)	0·3 (0·2 to 0·5)	0·2 (0·1 to 0·3)	0·1 (0·1 to 0·2)	0 (0 to 0)	0 (0 to 0)	77·5
Physical violence by other means	18·3 (11·3 to 28·9)	4·5 (2·8 to 7·1)	27·3 (16·5 to 43·9)	42 (26·2 to 67·1)	28·7 (19·9 to 39·6)	40·1 (29·9 to 52·3)	86·8 (62·5 to 115)	129 (99·5 to 167)	140 (110 to 180)	122 (92·6 to 159)	91·1 (72·7 to 114)	74·5 (55 to 97)	63·3 (47·1 to 83·8)	46·3 (34·4 to 60·4)	37 (27 to 49·7)	23·3 (17·4 to 31·1)	14·3 (10·7 to 18·3)	7·8 (5·8 to 10·1)	4·9 (3·6 to 6·3)	2·2 (1·7 to 2·8)	1·1 (0·9 to 1·4)	0·4 (0·3 to 0·5)	0·2 (0·1 to 0·2)	1,000
Conflict and terrorism	0 (0 to 0)	0 (0 to 0)	0 (0 to 0)	0 (0 to 0)	0 (0 to 0)	0 (0 to 0)	0 (0 to 0)	0 (0 to 0)	4·2 (2·4 to 7·2)	8·8 (5·4 to 14·1)	6·1 (3·6 to 9·8)	4·3 (2·6 to 6·7)	7·6 (4·8 to 12)	4·1 (2·6 to 6·2)	1·9 (1·3 to 2·9)	1·6 (1 to 2·4)	1·9 (1·2 to 2·9)	1·5 (1 to 2·2)	0·6 (0·4 to 0·9)	0·6 (0·4 to 0·8)	0·1 (0·1 to 0·2)	0 (0 to 0)	0 (0 to 0)	43·2

SR Table 2c: Cuba DALYS by cause and age group for both sexes with 95% uncertainty intervals, 2019

Cause	Early Neonatal	Late Neonatal	Post Neonatal	1 to 4	5 to 9	10 to 14
All causes	13,600 (10,800 to 16,800)	7,360 (5,880 to 8,990)	16,600 (13,900 to 19,700)	24,300 (19,200 to 30,600)	31,700 (24,100 to 41,400)	38,400 (29,700 to 48,700)
Communicable, maternal, neonatal, and nutritional diseases	10,500 (6,690 to 13,300)	4,890 (3,690 to 6,130)	7,140 (5,640 to 8,770)	7,310 (5,470 to 9,800)	7,920 (5,220 to 11,800)	5,290 (3,910 to 7,280)
HIV/AIDS and sexually transmitted infections	0·4 (0·2 to 0·6)	0·2 (0·1 to 0·4)	94·3 (88·4 to 101)	55·3 (48·7 to 69·8)	88·5 (79·6 to 102)	94·5 (84 to 114)
HIV/AIDS	0 (0 to 0·1)	0·1 (0 to 0·3)	94·3 (88·3 to 101)	55·3 (48·7 to 69·8)	88·5 (79·5 to 102)	83·7 (75·4 to 101)
HIV/AIDS - Drug-susceptible Tuberculosis	0 (0 to 0)	0 (0 to 0)	1·2 (0·8 to 1·9)	0·8 (0·5 to 1·1)	1·2 (0·8 to 1·8)	1·1 (0·7 to 1·6)
HIV/AIDS - Multidrug-resistant Tuberculosis without exten	0 (0 to 0)	0 (0 to 0)	0·1 (0 to 0·2)	0 (0 to 0·1)	0·1 (0 to 0·2)	0·1 (0 to 0·2)
HIV/AIDS - Extensively drug-resistant Tuberculosis	0 (0 to 0)	0 (0 to 0)	0 (0 to 0)	0 (0 to 0)	0 (0 to 0)	0 (0 to 0)
HIV/AIDS resulting in other diseases	0 (0 to 0·1)	0·1 (0 to 0·3)	92·9 (87 to 99·4)	54·5 (48 to 68·9)	87·2 (78·4 to 101)	82·5 (74·4 to 99·9)
Sexually transmitted infections excluding HIV	0·4 (0·2 to 0·5)	0·1 (0·1 to 0·2)	0·1 (0 to 0·2)	0 (0 to 0)	0 (0 to 0)	10·8 (6·5 to 18·6)
Syphilis	0·4 (0·2 to 0·5)	0·1 (0·1 to 0·2)	0·1 (0 to 0·2)	0 (0 to 0)	0 (0 to 0)	1·5 (1·2 to 1·8)
Chlamydial infection	0 (0 to 0)	0 (0 to 0)	0 (0 to 0)	0 (0 to 0)	0 (0 to 0)	0·7 (0·5 to 1)
Gonococcal infection	0 (0 to 0)	0 (0 to 0)	0 (0 to 0)	0 (0 to 0)	0 (0 to 0)	2 (0·9 to 4·1)
Trichomoniasis	0 (0 to 0)	0 (0 to 0)	0 (0 to 0)	0 (0 to 0)	0 (0 to 0)	0·3 (0 to 1)
Genital herpes	0 (0 to 0)	0 (0 to 0)	0 (0 to 0)	0 (0 to 0)	0 (0 to 0)	2·5 (0·7 to 6·1)
Other sexually transmitted infections	0 (0 to 0)	0 (0 to 0)	0 (0 to 0)	0 (0 to 0)	0 (0 to 0)	3·9 (1·9 to 7·7)
Respiratory infections and tuberculosis	624 (380 to 879)	613 (415 to 830)	2,250 (1,560 to 3,040)	1,740 (1,280 to 2,300)	1,300 (856 to 1,930)	996 (649 to 1,510)
Tuberculosis	0 (0 to 0)	0 (0 to 0)	1·9 (1·4 to 2·5)	12·8 (8·1 to 20·5)	20·8 (7·2 to 43·2)	7·2 (4·2 to 11·6)
Latent tuberculosis infection	0 (0 to 0)	0 (0 to 0)	0 (0 to 0)	0 (0 to 0)	0 (0 to 0)	0 (0 to 0)
Drug-susceptible tuberculosis	0 (0 to 0)	0 (0 to 0)	1·8 (1·3 to 2·4)	12·4 (7·9 to 19·7)	20·3 (6·8 to 42·9)	7 (4 to 11·4)
Multidrug-resistant tuberculosis without extensive drug res	0 (0 to 0)	0 (0 to 0)	0·1 (0 to 0·2)	0·3 (0·1 to 1·1)	0·5 (0·1 to 1·5)	0·2 (0 to 0·6)
Extensively drug-resistant tuberculosis	0 (0 to 0)	0 (0 to 0)	0 (0 to 0)	0 (0 to 0·1)	0 (0 to 0·1)	0 (0 to 0·1)
Lower respiratory infections	622 (378 to 878)	605 (409 to 822)	2,090 (1,420 to 2,890)	906 (647 to 1,200)	268 (193 to 353)	220 (155 to 300)
Upper respiratory infections	1·1 (0·6 to 1·8)	6·5 (3·6 to 11)	135 (80·4 to 216)	583 (338 to 940)	670 (370 to 1,150)	551 (284 to 952)
Otitis media	0·3 (0 to 1·1)	1·1 (0·4 to 3·1)	24·4 (12·3 to 43·8)	234 (128 to 411)	338 (183 to 554)	219 (117 to 377)
Enteric infections	11·3 (7·6 to 16·2)	98·8 (67·4 to 140)	920 (623 to 1,320)	1,060 (678 to 1,570)	1,000 (597 to 1,570)	882 (506 to 1,380)
Diarrheal diseases	11·3 (7·6 to 16·2)	98·8 (67·3 to 140)	915 (620 to 1,310)	1,050 (671 to 1,560)	998 (593 to 1,570)	877 (502 to 1,370)
Typhoid and paratyphoid	0 (0 to 0)	0 (0 to 0)	0 (0 to 0)	0·1 (0 to 0·1)	0·1 (0 to 0·2)	0·1 (0 to 0·1)
Typhoid fever	0 (0 to 0)	0 (0 to 0)	0 (0 to 0)	0 (0 to 0·1)	0 (0 to 0·1)	0 (0 to 0)
Paratyphoid fever	0 (0 to 0)	0 (0 to 0)	0 (0 to 0)	0 (0 to 0·1)	0·1 (0 to 0·1)	0 (0 to 0·1)
Invasive Non-typhoidal Salmonella (iNTS)	0 (0 to 0)	0·1 (0 to 0·2)	2·2 (0·1 to 6)	4·8 (0·6 to 10·1)	2·6 (0·7 to 4·7)	2·1 (0·6 to 4)
Other intestinal infectious diseases	0 (0 to 0)	0 (0 to 0)	2·4 (0·3 to 6·2)	4·1 (1·4 to 8·7)	2·8 (0·9 to 6·3)	2·5 (0·8 to 5·7)
Neglected tropical diseases and malaria	0·3 (0·1 to 0·7)	6·9 (3·6 to 11·7)	48·4 (22·4 to 87·8)	230 (110 to 411)	419 (194 to 746)	218 (102 to 395)
Malaria	0 (0 to 0)	0 (0 to 0)	0 (0 to 0)	0 (0 to 0)	0 (0 to 0)	0 (0 to 0)
Chagas disease	0 (0 to 0)	0 (0 to 0)	0 (0 to 0)	0 (0 to 0)	0 (0 to 0)	0 (0 to 0)
Leishmaniasis	0 (0 to 0)	0 (0 to 0)	0 (0 to 0)	0 (0 to 0)	0 (0 to 0)	0 (0 to 0)
Visceral leishmaniasis	0 (0 to 0)	0 (0 to 0)	0 (0 to 0)	0 (0 to 0)	0 (0 to 0)	0 (0 to 0)
Cutaneous and mucocutaneous leishmaniasis	0 (0 to 0)	0 (0 to 0)	0 (0 to 0)	0 (0 to 0)	0 (0 to 0)	0 (0 to 0)
African trypanosomiasis	0 (0 to 0)	0 (0 to 0)	0 (0 to 0)	0 (0 to 0)	0 (0 to 0)	0 (0 to 0)
Schistosomiasis	0 (0 to 0)	0 (0 to 0)	0 (0 to 0)	0 (0 to 0)	0 (0 to 0)	0 (0 to 0)
Cysticercosis	0 (0 to 0)	0 (0 to 0)	0 (0 to 0)	0 (0 to 0)	0 (0 to 0)	0 (0 to 0)
Cystic echinococcosis	0 (0 to 0)	0 (0 to 0)	0 (0 to 0)	0 (0 to 0)	0 (0 to 0·1)	0 (0 to 0·1)
Lymphatic filariasis	0 (0 to 0)	0 (0 to 0)	0 (0 to 0)	0 (0 to 0)	0 (0 to 0)	0 (0 to 0)
Onchocerciasis	0 (0 to 0)	0 (0 to 0)	0 (0 to 0)	0 (0 to 0)	0 (0 to 0)	0 (0 to 0)
Trachoma	0 (0 to 0)	0 (0 to 0)	0 (0 to 0)	0 (0 to 0)	0 (0 to 0)	0 (0 to 0)
Dengue	0 (0 to 0)	0 (0 to 0·1)	5·1 (0·1 to 15·8)	19·8 (0·3 to 62·2)	48·3 (1·2 to 143)	46·9 (2·8 to 132)
Yellow fever	0 (0 to 0)	0 (0 to 0)	0 (0 to 0)	0 (0 to 0)	0 (0 to 0)	0 (0 to 0)
Rabies	0 (0 to 0)	0 (0 to 0)	0·1 (0 to 0·3)	2·6 (0·8 to 6·9)	1·1 (0·3 to 2·8)	0·1 (0 to 0·2)
Intestinal nematode infections	0 (0 to 0)	0 (0 to 0)	1·6 (0·6 to 2·8)	37·9 (19 to 65·9)	110 (56·3 to 194)	73·4 (37·8 to 129)
Ascariasis	0 (0 to 0)	0 (0 to 0)	1 (0·2 to 2·3)	23 (10·7 to 42·9)	62·8 (28·2 to 117)	35·4 (15·5 to 66·7)
Trichuriasis	0 (0 to 0)	0 (0 to 0)	0·1 (0 to 0·1)	2 (0·7 to 4·3)	7·6 (3 to 15·9)	5 (1·7 to 11)
Hookworm disease	0 (0 to 0)	0 (0 to 0)	0·4 (0·2 to 0·8)	12·8 (5·8 to 24·1)	40·1 (19·1 to 70·4)	33·1 (15·5 to 61·7)
Food-borne trematodiases	0 (0 to 0)	0 (0 to 0)	0 (0 to 0)	0 (0 to 0)	0 (0 to 0)	0 (0 to 0)
Leprosy	0 (0 to 0)	0 (0 to 0)	0 (0 to 0)	0 (0 to 0)	0 (0 to 0)	0 (0 to 0)
Ebola	0 (0 to 0)	0 (0 to 0)	0 (0 to 0)	0 (0 to 0)	0 (0 to 0)	0 (0 to 0)
Zika virus	0 (0 to 0)	0 (0 to 0)	0 (0 to 0·2)	0·2 (0 to 0·7)	0·2 (0 to 1)	0·2 (0 to 1)
Guinea worm disease	0 (0 to 0)	0 (0 to 0)	0 (0 to 0)	0 (0 to 0)	0 (0 to 0)	0 (0 to 0)
Other neglected tropical diseases	0·3 (0·1 to 0·7)	6·9 (3·6 to 11·7)	41·6 (18·7 to 79·5)	170 (70 to 334)	259 (93·2 to 534)	97·2 (32·8 to 214)
Other infectious diseases	55·2 (29·8 to 80)	186 (122 to 260)	911 (614 to 1,240)	805 (620 to 1,020)	649 (495 to 860)	467 (377 to 596)
Meningitis	20 (11·6 to 29·4)	118 (75·3 to 171)	493 (311 to 731)	328 (233 to 437)	183 (144 to 231)	146 (113 to 187)
Encephalitis	6·1 (3 to 16·4)	21·1 (13·1 to 33·3)	207 (133 to 314)	244 (163 to 361)	166 (118 to 235)	139 (102 to 190)
Diphtheria	0 (0 to 0)	0 (0 to 0)	0 (0 to 0)	0 (0 to 0·1)	0·1 (0·1 to 0·3)	0·1 (0 to 0·2)
Tetanus	0 (0 to 0)	0 (0 to 0)	0 (0 to 0)	0·2 (0 to 0·9)	0·4 (0 to 1·4)	0·3 (0 to 1·4)
Measles	0 (0 to 0)	0 (0 to 0)	0·1 (0 to 0·3)	0·2 (0 to 0·5)	0·2 (0·1 to 0·8)	0·2 (0 to 0·8)
Varicella and herpes zoster	4·8 (1·3 to 9·6)	11·7 (4·4 to 21)	40·4 (17·5 to 67·8)	19·9 (10·2 to 32·3)	35·1 (17 to 56·8)	20·9 (11·3 to 35·2)
Acute hepatitis	0 (0 to 0)	0 (0 to 0·1)	4 (2 to 7·1)	14·7 (8·2 to 24·1)	46·6 (28·7 to 74·6)	51 (31·6 to 78·4)
Acute hepatitis A	0 (0 to 0)	0 (0 to 0)	1 (0·4 to 2·2)	11·8 (5·9 to 20·7)	40·5 (23·6 to 66·9)	45·6 (27·9 to 71·5)
Acute hepatitis B	0 (0 to 0)	0 (0 to 0)	2·3 (0·9 to 4·5)	0·8 (0·3 to 2)	1·8 (0·7 to 3·5)	0·8 (0·3 to 2·1)
Acute hepatitis C	0 (0 to 0)	0 (0 to 0·1)	0·7 (0·3 to 1·2)	1·7 (0·7 to 3·1)	1·4 (0·5 to 2·7)	0·9 (0·4 to 1·7)
Acute hepatitis E	0 (0 to 0)	0 (0 to 0)	0·1 (0 to 0·2)	0·4 (0·2 to 0·6)	2·9 (1·4 to 5·3)	3·7 (0·7 to 8·7)
Other unspecified infectious diseases	24·2 (8 to 42·9)	35·4 (13·6 to 64·6)	153 (69·5 to 247)	184 (107 to 305)	215 (103 to 395)	110 (61 to 193)
Maternal and neonatal disorders	9,780 (6,250 to 12,500)	3,910 (2,940 to 4,930)	2,320 (1,660 to 3,150)	1,630 (1,220 to 2,150)	1,490 (1,100 to 1,930)	1,390 (999 to 1,840)
Maternal disorders	0 (0 to 0)	0 (0 to 0)	0 (0 to 0)	0 (0 to 0)	0 (0 to 0)	15·2 (12 to 18·6)
Maternal hemorrhage	0 (0 to 0)	0 (0 to 0)	0 (0 to 0)	0 (0 to 0)	0 (0 to 0)	0·8 (0·5 to 1·3)
Maternal sepsis and other maternal infections	0 (0 to 0)	0 (0 to 0)	0 (0 to 0)	0 (0 to 0)	0 (0 to 0)	0·6 (0·3 to 1·1)
Maternal hypertensive disorders	0 (0 to 0)	0 (0 to 0)	0 (0 to 0)	0 (0 to 0)	0 (0 to 0)	3·4 (2·4 to 4·6)
Maternal obstructed labor and uterine rupture	0 (0 to 0)	0 (0 to 0)	0 (0 to 0)	0 (0 to 0)	0 (0 to 0)	0·3 (0·1 to 0·5)
Maternal abortion and miscarriage	0 (0 to 0)	0 (0 to 0)	0 (0 to 0)	0 (0 to 0)	0 (0 to 0)	1·3 (0·8 to 2)
Ectopic pregnancy	0 (0 to 0)	0 (0 to 0)	0 (0 to 0)	0 (0 to 0)	0 (0 to 0)	0·2 (0·1 to 0·4)
Indirect maternal deaths	--	--	--	--	--	2·9 (1·9 to 4·2)
Late maternal deaths	--	--	--	--	--	2·2 (1·3 to 3·5)
Maternal deaths aggravated by HIV/AIDS	--	--	--	--	--	0 (0 to 0)
Neonatal disorders	9,780 (6,250 to 12,500)	3,910 (2,940 to 4,930)	2,320 (1,660 to 3,150)	1,630 (1,220 to 2,150)	1,490 (1,100 to 1,930)	1,370 (983 to 1,830)
Neonatal preterm birth	3,000 (1,850 to 4,150)	796 (531 to 1,120)	769 (538 to 1,050)	587 (420 to 787)	786 (567 to 1,040)	720 (508 to 958)
Neonatal encephalopathy due to birth asphyxia and traum	2,350 (1,470 to 3,200)	569 (350 to 873)	518 (320 to 786)	233 (159 to 330)	198 (117 to 300)	186 (110 to 282)
Neonatal sepsis and other neonatal infections	1,410 (875 to 2,000)	1,120 (795 to 1,490)	253 (177 to 337)	344 (200 to 529)	482 (296 to 733)	446 (276 to 694)
Hemolytic disease and other neonatal jaundice	131 (73·7 to 211)	33·6 (21·5 to 49·6)	24·2 (11·6 to 45·8)	23·9 (17·3 to 34·3)	23·6 (17·6 to 30·7)	21·9 (16·3 to 28·4)
Other neonatal disorders	2,880 (1,730 to 4,020)	1,400 (993 to 1,840)	753 (514 to 1,080)	446 (334 to 582)	0 (0 to 0)	0 (0 to 0)
Nutritional deficiencies	1·8 (0·8 to 3·7)	71·4 (36·1 to 124)	598 (344 to 1,010)	1,790 (764 to 3,530)	2,970 (1,180 to 5,800)	1,250 (555 to 2,530)
Protein-energy malnutrition	1·1 (0·6 to 1·7)	3·8 (2·3 to 6)	180 (127 to 243)	56·5 (33·5 to 87·1)	217 (114 to 361)	157 (81·1 to 263)
Iodine deficiency	0 (0 to 0)	0 (0 to 0)	0 (0 to 0)	2·1 (0·8 to 4)	15·6 (6·9 to 29·2)	33·7 (15·1 to 61·2)
Vitamin A deficiency	0·2 (0 to 0·5)	2·2 (0·8 to 4·8)	6·2 (2·2 to 13·7)	28 (9·5 to 62·9)	37·8 (11·9 to 90·4)	14·8 (3·5 to 39·2)
Dietary iron deficiency	0·5 (0 to 2·2)	65·4 (31·9 to 115)	406 (179 to 784)	1,700 (682 to 3,420)	2,640 (932 to 5,400)	992 (336 to 2,240)
Other nutritional deficiencies	0 (0 to 0)	0 (0 to 0)	5·2 (3·3 to 8)	8·7 (4·4 to 15·5)	56 (31·5 to 90·1)	47 (26·2 to 76·9)
Non-communicable diseases	2,990 (1,940 to 6,580)	2,360 (1,750 to 3,400)	8,350 (6,700 to 10,400)	14,400 (11,000 to 18,700)	19,800 (14,800 to 25,800)	27,400 (20,400 to 36,500)
Neoplasms	31·2 (16·4 to 51·1)	48·5 (29·3 to 65·5)	352 (228 to 475)	1,750 (1,250 to 2,250)	2,260 (1,770 to 2,740)	2,140 (1,670 to 2,660)
Lip and oral cavity cancer	0 (0 to 0)	0 (0 to 0)	0 (0 to 0)	0 (0 to 0)	6·9 (4·9 to 10·1)	7·8 (5·2 to 11·3)
Nasopharynx cancer	0 (0 to 0)	0 (0 to 0)	0 (0 to 0)	0 (0 to 0)	8·3 (5·6 to 13·5)	12·7 (8·2 to 19·1)
Other pharynx cancer	0 (0 to 0)	0 (0 to 0)	0 (0 to 0)	0 (0 to 0)	0 (0 to 0)	0 (0 to 0)
Esophageal cancer	0 (0 to 0)	0 (0 to 0)	0 (0 to 0)	0 (0 to 0)	0 (0 to 0)	0 (0 to 0)
Stomach cancer	0 (0 to 0)	0 (0 to 0)	0 (0 to 0)	0 (0 to 0)	0 (0 to 0)	0 (0 to 0)
Colon and rectum cancer	0 (0 to 0)	0 (0 to 0)	0 (0 to 0)	0 (0 to 0)	13·8 (9·4 to 18·7)	16·6 (12 to 22·2)
Liver cancer	0·3 (0·2 to 0·4)	0·7 (0·3 to 1·5)	6 (4 to 8·2)	21·7 (15·7 to 28·3)	22·9 (17·9 to 28·9)	25·5 (19·2 to 33·7)
Liver cancer due to hepatitis B	0 (0 to 0)	0 (0 to 0)	0 (0 to 0)	0 (0 to 0)	0 (0 to 0)	3·8 (1·9 to 6·8)
Liver cancer due to hepatitis C	0 (0 to 0)	0 (0 to 0)	0 (0 to 0)	0 (0 to 0)	0 (0 to 0)	0·1 (0 to 0·2)
Liver cancer due to alcohol use	0 (0 to 0)	0 (0 to 0)	0 (0 to 0)	0 (0 to 0)	0 (0 to 0)	0 (0 to 0)
Liver cancer due to NASH	0 (0 to 0)	0 (0 to 0)	0 (0 to 0)	0 (0 to 0)	0 (0 to 0)	0 (0 to 0)
Liver cancer due to other causes	0·3 (0·2 to 0·4)	0·7 (0·3 to 1·5)	6 (4 to 8·2)	21·7 (15·7 to 28·3)	22·9 (17·9 to 28·9)	21·7 (16·6 to 28·8)
Gallbladder and biliary tract cancer	0 (0 to 0)	0 (0 to 0)	0 (0 to 0)	0 (0 to 0)	0 (0 to 0)	0 (0 to 0)
Pancreatic cancer	0 (0 to 0)	0 (0 to 0)	0 (0 to 0)	0 (0 to 0)	0 (0 to 0)	0 (0 to 0)
Larynx cancer	0 (0 to 0)	0 (0 to 0)	0 (0 to 0)	0 (0 to 0)	0 (0 to 0)	0 (0 to 0)
Tracheal, bronchus, and lung cancer	0 (0 to 0)	0 (0 to 0)	0 (0 to 0)	0 (0 to 0)	0 (0 to 0)	11·8 (8·1 to 16·9)
Malignant skin melanoma	0·1 (0 to 0·2)	0·1 (0·1 to 0·3)	2·1 (0·8 to 5·9)	9·3 (3·8 to 26·2)	6 (4·8 to 7·7)	6·7 (4·6 to 10·2)
Non-melanoma skin cancer	0 (0 to 0)	0 (0 to 0)	0 (0 to 0)	0 (0 to 0)	0 (0 to 0)	0 (0 to 0)
Non-melanoma skin cancer (squamous-cell carcinoma)	0 (0 to 0)	0 (0 to 0)	0 (0 to 0)	0 (0 to 0)	0 (0 to 0)	0 (0 to 0)
Non-melanoma skin cancer (basal-cell carcinoma)	0 (0 to 0)	0 (0 to 0)	0 (0 to 0)	0 (0 to 0)	0 (0 to 0)	0 (0 to 0)
Breast cancer	0 (0 to 0)	0 (0 to 0)	0 (0 to 0)	0 (0 to 0)	0 (0 to 0)	0 (0 to 0)
Cervical cancer	0 (0 to 0)	0 (0 to 0)	0 (0 to 0)	0 (0 to 0)	0 (0 to 0)	0 (0 to 0)
Uterine cancer	0 (0 to 0)	0 (0 to 0)	0 (0 to 0)	0 (0 to 0)	0 (0 to 0)	0 (0 to 0)
Ovarian cancer	0 (0 to 0)	0 (0 to 0)	0 (0 to 0)	0 (0 to 0)	9·7 (5·3 to 15·1)	13·7 (8·2 to 21·1)
Prostate cancer	0 (0 to 0)	0 (0 to 0)	0 (0 to 0)	0 (0 to 0)	0 (0 to 0)	0 (0 to 0)
Testicular cancer	1·1 (0 to 12·5)	0·7 (0·1 to 7·2)	3·7 (0·7 to 16·1)	12 (3 to 38·3)	10·2 (6·4 to 15·4)	10·7 (5·6 to 18·3)
Kidney cancer	1·3 (0·7 to 1·9)	1·8 (1·2 to 2·6)	19·9 (13·1 to 29·5)	106 (69·6 to 150)	97·1 (61·3 to 150)	38·9 (22·3 to 62)
Bladder cancer	0 (0 to 0)	0 (0 to 0)	0 (0 to 0)	0 (0 to 0)	0 (0 to 0)	0 (0 to 0)
Brain and central nervous system cancer	9·2 (2·1 to 14·7)	13 (2·9 to 22·6)	99·7 (27·3 to 167)	401 (116 to 623)	590 (236 to 875)	500 (201 to 750)
Thyroid cancer	0 (0 to 0)	0 (0 to 0)	0 (0 to 0)	0 (0 to 0)	7·7 (5·5 to 11·9)	6·3 (4·8 to 8·7)
Mesothelioma	0 (0 to 0)	0 (0 to 0)	0 (0 to 0)	0 (0 to 0)	0 (0 to 0)	0 (0 to 0)
Hodgkin lymphoma	0 (0 to 0)	0 (0 to 0)	0 (0 to 0)	9·2 (4·3 to 14·9)	13·2 (6·5 to 22·2)	25 (11·9 to 42·6)
Non-Hodgkin lymphoma	0 (0 to 0)	0 (0 to 0)	0 (0 to 0)	175 (124 to 235)	241 (177 to 325)	238 (172 to 328)
Multiple myeloma	0 (0 to 0)	0 (0 to 0)	0 (0 to 0)	0 (0 to 0)	0 (0 to 0)	0 (0 to 0)
Leukemia	7·7 (4·3 to 11·5)	15·2 (10·5 to 20·5)	109 (71·4 to 150)	517 (366 to 708)	747 (547 to 983)	674 (479 to 909)
Acute lymphoid leukemia	1·1 (0·5 to 2·4)	1 (0·5 to 2·5)	35·8 (20·9 to 53·7)	320 (212 to 453)	521 (360 to 713)	369 (245 to 534)
Chronic lymphoid leukemia	0 (0 to 0)	0 (0 to 0)	0 (0 to 0)	0 (0 to 0)	0 (0 to 0)	0 (0 to 0)
Acute myeloid leukemia	3·2 (1·2 to 5·3)	9·5 (5·5 to 13·6)	43 (25·3 to 66·7)	155 (101 to 231)	174 (105 to 269)	237 (149 to 345)
Chronic myeloid leukemia	0·2 (0·1 to 0·4)	0·1 (0 to 0·4)	3·1 (1·4 to 6·2)	17·1 (10 to 27)	11·2 (6·3 to 17·4)	11·7 (7 to 18·2)
Other leukemia	3·2 (1·7 to 5)	4·5 (2·5 to 7·7)	26·7 (15·8 to 40·7)	24·8 (15·8 to 37·9)	40·9 (22 to 71·5)	56·4 (31·5 to 91·5)
Other malignant neoplasms	11·3 (5·2 to 28·1)	15·8 (10·1 to 23·6)	111 (76·5 to 151)	492 (359 to 643)	466 (369 to 577)	535 (410 to 684)
Other neoplasms	0·2 (0·1 to 0·8)	1·1 (0·2 to 2)	1·2 (0·9 to 2·5)	12·2 (8 to 18)	22·1 (10·9 to 34·9)	13·1 (8·7 to 19·2)
Myelodysplastic, myeloproliferative, and other hematopoie	0·2 (0·1 to 0·8)	1·1 (0·2 to 2)	1·2 (0·9 to 2·5)	12·2 (8 to 18)	22·1 (10·9 to 34·9)	13·1 (8·7 to 19·2)
Benign and in situ intestinal neoplasms	0 (0 to 0)	0 (0 to 0)	0 (0 to 0)	0 (0 to 0)	0 (0 to 0)	0 (0 to 0)
Benign and in situ cervical and uterine neoplasms	0 (0 to 0)	0 (0 to 0)	0 (0 to 0)	0 (0 to 0)	0 (0 to 0)	0 (0 to 0)
Other benign and in situ neoplasms	0 (0 to 0)	0 (0 to 0)	0 (0 to 0)	0 (0 to 0)	0 (0 to 0)	0 (0 to 0)
Cardiovascular diseases	19·1 (11·2 to 27·6)	31·1 (20·9 to 44·6)	272 (189 to 380)	220 (170 to 274)	389 (291 to 510)	665 (505 to 881)
Rheumatic heart disease	0 (0 to 0)	0 (0 to 0)	0 (0 to 0)	22 (11 to 40·3)	133 (77·8 to 226)	277 (167 to 449)
Ischemic heart disease	0 (0 to 0)	0 (0 to 0)	0 (0 to 0)	0 (0 to 0)	0 (0 to 0)	0 (0 to 0)
Stroke	1·5 (0·5 to 3)	2·6 (1·2 to 5·5)	23·4 (10·8 to 53·8)	35·4 (25·5 to 48·5)	109 (78·4 to 145)	174 (126 to 234)
Ischemic stroke	0·1 (0 to 0·2)	0·3 (0·1 to 0·7)	2·8 (1·7 to 4·7)	22·8 (15·5 to 32·1)	59·4 (39·4 to 86)	75·6 (46·8 to 116)
Intracerebral hemorrhage	0 (0 to 0·1)	0·1 (0 to 0·2)	9·9 (4·2 to 22·7)	4·2 (2·8 to 6·4)	21·1 (13·8 to 30·3)	41·4 (28·6 to 58·9)
Subarachnoid hemorrhage	1·3 (0·4 to 2·9)	2·2 (1 to 4·6)	10·7 (4 to 29·6)	8·4 (4·5 to 16·3)	28·1 (17·4 to 41·8)	57·4 (36·1 to 85·5)
Hypertensive heart disease	0 (0 to 0)	0 (0 to 0)	0 (0 to 0)	0 (0 to 0)	0 (0 to 0)	0 (0 to 0)
Non-rheumatic valvular heart disease	0 (0 to 0)	0 (0 to 0)	0 (0 to 0)	0 (0 to 0)	0 (0 to 0)	0 (0 to 0)
Non-rheumatic calcific aortic valve disease	0 (0 to 0)	0 (0 to 0)	0 (0 to 0)	0 (0 to 0)	0 (0 to 0)	0 (0 to 0)

Cause	15 to 19	20 to 24	25 to 29	30 to 34	35 to 39	40 to 44	45 to 49	50 to 54	55 to 59	60 to 64	65 to 69	70 to 74	75 to 79	80 to 84	85 to 89	90 to 94	95 plus	Total
All causes	63,200 (50,200 to 78,600)	78,700 (63,500 to 96,700)	99,200 (80,500 to 120,000)	118,000 (96,400 to 143,000)	113,000 (93,400 to 136,000)	145,000 (121,000 to 174,000)	241,000 (200,000 to 289,000)	322,000 (267,000 to 383,000)	363,000 (302,000 to 433,000)	313,000 (262,000 to 369,000)	331,000 (279,000 to 388,000)	336,000 (286,000 to 390,000)	305,000 (261,000 to 351,000)	236,000 (205,000 to 268,000)	155,000 (136,000 to 175,000)	86,800 (77,600 to 96,500)	42,300 (38,800 to 45,800)	3,480,000
Communicable, maternal, neonatal, and nutritional diseases	6,320 (4,770 to 8,450)	7,160 (5,600 to 9,150)	8,850 (7,160 to 11,200)	10,900 (8,960 to 13,600)	9,590 (7,880 to 11,900)	10,400 (8,290 to 13,400)	14,400 (11,600 to 18,000)	16,100 (12,900 to 20,000)	16,300 (13,100 to 20,100)	13,500 (10,700 to 16,700)	14,500 (11,200 to 18,200)	17,300 (13,200 to 22,100)	18,900 (14,600 to 24,300)	16,900 (13,800 to 20,300)	12,800 (10,200 to 15,800)	8,240 (6,410 to 10,100)	4,770 (3,550 to 5,950)	250,000
HIV/AIDS and sexually transmitted infections	246 (210 to 298)	1,020 (913 to 1,150)	2,010 (1,810 to 2,270)	3,280 (2,970 to 3,650)	2,810 (2,530 to 3,200)	2,530 (2,270 to 2,860)	3,040 (2,720 to 3,420)	2,120 (1,890 to 2,450)	1,410 (1,210 to 1,690)	690 (566 to 875)	483 (388 to 630)	168 (117 to 250)	87 (60·2 to 131)	37·7 (23·7 to 64·6)	16·8 (9·8 to 29·9)	6·5 (3·6 to 11·9)	2·4 (1·3 to 4·4)	20,300
HIV/AIDS	163 (149 to 182)	846 (773 to 934)	1,740 (1,590 to 1,950)	2,930 (2,700 to 3,240)	2,480 (2,270 to 2,780)	2,160 (1,980 to 2,390)	2,600 (2,370 to 2,850)	1,840 (1,690 to 2,050)	1,210 (1,070 to 1,440)	583 (491 to 748)	399 (331 to 524)	103 (74·3 to 157)	42·4 (32·8 to 59·1)	12·5 (10·3 to 16·1)	3·8 (2·7 to 5·7)	1 (0·4 to 1·9)	0·4 (0·2 to 0·7)	17,400
HIV/AIDS - Drug-susceptible Tuberculosis	2·3 (1·6 to 3·4)	12·2 (8·2 to 17·7)	25·1 (16·9 to 36·6)	42 (28·1 to 61·1)	35·9 (24·3 to 52·2)	31·7 (21·4 to 46)	37·7 (25·6 to 54·7)	27·5 (18·9 to 39·7)	18·9 (13·5 to 26·4)	9·7 (7 to 13·2)	6·8 (5 to 9·2)	2 (1·5 to 2·7)	0·8 (0·6 to 1·1)	0·2 (0·2 to 0·3)	0·1 (0·1 to 0·1)	0 (0 to 0)	0 (0 to 0)	257
HIV/AIDS - Multidrug-resistant Tuberculosis without exten	0·1 (0 to 0·4)	0·7 (0·1 to 2·3)	1·4 (0·2 to 4·7)	2·4 (0·4 to 7·8)	2 (0·3 to 6·6)	1·8 (0·3 to 5·8)	2·1 (0·3 to 6·9)	1·5 (0·2 to 4·9)	1 (0·2 to 3·1)	0·5 (0·1 to 1·5)	0·3 (0·1 to 1)	0·1 (0 to 0·2)	0 (0 to 0·1)	0 (0 to 0)	0 (0 to 0)	0 (0 to 0)	0 (0 to 0)	14·3
HIV/AIDS - Extensively drug-resistant Tuberculosis	0 (0 to 0·1)	0·1 (0 to 0·4)	0·2 (0 to 0·7)	0·4 (0·1 to 1·3)	0·3 (0·1 to 1·1)	0·3 (0 to 0·9)	0·3 (0·1 to 1·1)	0·2 (0 to 0·8)	0·1 (0 to 0·5)	0·1 (0 to 0·2)	0 (0 to 0·1)	0 (0 to 0)	0 (0 to 0)	0 (0 to 0)	0 (0 to 0)	0 (0 to 0)	0 (0 to 0)	2·2
HIV/AIDS resulting in other diseases	161 (147 to 179)	833 (761 to 921)	1,720 (1,570 to 1,930)	2,880 (2,650 to 3,190)	2,450 (2,230 to 2,740)	2,120 (1,950 to 2,340)	2,550 (2,330 to 2,810)	1,810 (1,660 to 2,030)	1,190 (1,050 to 1,420)	572 (482 to 738)	392 (325 to 516)	100 (72·1 to 154)	41·6 (32 to 58·1)	12·2 (10·1 to 15·9)	3·8 (2·6 to 5·6)	1 (0·4 to 1·8)	0·4 (0·2 to 0·7)	17,200
Sexually transmitted infections excluding HIV	83 (52·2 to 125)	172 (110 to 267)	263 (158 to 424)	347 (207 to 562)	327 (202 to 525)	373 (233 to 608)	441 (268 to 739)	279 (148 to 513)	198 (104 to 377)	107 (52·5 to 211)	83·9 (40·1 to 171)	65·6 (32·1 to 129)	44·6 (22·9 to 88·9)	25·3 (12·5 to 50·8)	13 (6·4 to 25·6)	5·5 (2·8 to 10·8)	2 (1 to 4)	2,840
Syphilis	6·8 (4·2 to 10)	4·9 (3 to 7·8)	12·8 (7·7 to 20·5)	19·3 (12·5 to 29·4)	13·9 (9·1 to 19·6)	7·3 (5·1 to 10·6)	16·6 (11·2 to 23·7)	10·1 (7 to 14·3)	5·2 (3·6 to 7·2)	3·9 (2·7 to 5·3)	3·8 (2·7 to 5·2)	3·8 (2·7 to 5·2)	2·5 (1·8 to 3·3)	2 (1·4 to 2·7)	1·1 (0·8 to 1·4)	0·6 (0·5 to 0·9)	0·3 (0·2 to 0·4)	117
Chlamydial infection	8·8 (5·8 to 13·2)	30 (18 to 48)	42·8 (23·5 to 73·6)	46·3 (24·2 to 78·1)	47·1 (28·2 to 72·4)	55·6 (35·2 to 83·8)	56·6 (34·6 to 86·1)	20·7 (11·4 to 34·7)	10·8 (7·8 to 14·8)	3·9 (2·4 to 6)	1·8 (1·1 to 2·8)	2 (1·2 to 3·2)	1·4 (0·9 to 2·1)	0·9 (0·6 to 1·4)	0·4 (0·2 to 0·6)	0·2 (0·1 to 0·3)	0·1 (0·1 to 0·1)	330
Gonococcal infection	19·7 (9·1 to 35·6)	33·7 (19·2 to 54·9)	33·4 (19·5 to 51·3)	38·7 (25·9 to 55·8)	38·9 (26·4 to 53·8)	55·1 (36·4 to 79·3)	67·7 (43·3 to 98·2)	30·4 (19·1 to 46·9)	19·6 (13·1 to 28·4)	9·4 (5·6 to 14·6)	9·3 (5·4 to 14·7)	6·8 (4·1 to 10·9)	5 (3·2 to 7·6)	2 (1·3 to 3·1)	1·1 (0·6 to 1·6)	0·4 (0·3 to 0·7)	0·2 (0·1 to 0·2)	373
Trichomoniasis	6·3 (1·9 to 15·8)	29·8 (9·7 to 71·1)	62·5 (20·9 to 143)	78·4 (25·3 to 189)	65·2 (21·5 to 156)	68·5 (23·4 to 169)	89 (28·7 to 206)	79·4 (24·6 to 190)	54·1 (17·7 to 133)	27 (9·1 to 64·5)	20·2 (7·1 to 47·2)	14·5 (5·2 to 32·7)	9·2 (3·4 to 20·7)	4·8 (1·8 to 10·5)	2 (0·8 to 4·5)	0·7 (0·2 to 1·6)	0·1 (0 to 0·3)	612
Genital herpes	12·8 (3·9 to 32·4)	28·3 (9·2 to 70·1)	49·1 (15·9 to 119)	67 (22·1 to 162)	62·9 (20·6 to 155)	70·7 (22·4 to 174)	97·7 (30·3 to 248)	100 (30·8 to 246)	84·3 (26·5 to 206)	54·3 (16·2 to 134)	44·6 (13·6 to 109)	35 (10·7 to 86·7)	24·5 (7·3 to 60·3)	14·7 (4·4 to 35·9)	7·8 (2·4 to 19)	3·5 (1·1 to 8·3)	1·3 (0·4 to 3·1)	761
Other sexually transmitted infections	28·7 (17·4 to 45)	45·4 (29·4 to 65·8)	62·1 (40·1 to 94·2)	97·6 (62·7 to 146)	98·8 (66 to 144)	116 (76·8 to 173)	113 (73·6 to 172)	38·3 (22·6 to 59·2)	23·6 (14·9 to 35·8)	8·3 (4·9 to 12·8)	4·2 (2·4 to 6·5)	3·5 (2·1 to 5·6)	2 (1·3 to 3)	0·9 (0·5 to 1·3)	0·6 (0·3 to 0·9)	0·2 (0·1 to 0·3)	0·1 (0 to 0·1)	647
Respiratory infections and tuberculosis	1,170 (773 to 1,720)	1,330 (914 to 1,870)	1,520 (1,070 to 2,050)	1,790 (1,270 to 2,490)	1,710 (1,260 to 2,250)	2,330 (1,690 to 3,110)	4,100 (3,010 to 5,500)	5,730 (4,250 to 7,670)	7,480 (5,680 to 9,540)	7,880 (5,820 to 10,200)	9,530 (6,800 to 12,700)	12,800 (9,430 to 17,100)	15,000 (11,200 to 19,900)	14,200 (11,400 to 17,400)	11,200 (8,670 to 14,000)	7,460 (5,760 to 9,240)	4,450 (3,270 to 5,570)	117,000
Tuberculosis	6·8 (4·7 to 9·5)	48·8 (18·1 to 87)	93·4 (43·2 to 159)	112 (57·2 to 186)	92·4 (48·6 to 150)	107 (62·8 to 169)	137 (81·2 to 210)	181 (121 to 261)	164 (109 to 230)	136 (95 to 193)	113 (79·6 to 157)	116 (82·4 to 158)	99·6 (70·3 to 136)	61·8 (43 to 85·7)	37·7 (26·2 to 51·6)	17·5 (12 to 23·8)	6·7 (5 to 8·3)	1,570
Latent tuberculosis infection	0 (0 to 0)	0 (0 to 0)	0 (0 to 0)	0 (0 to 0)	0 (0 to 0)	0 (0 to 0)	0 (0 to 0)	0 (0 to 0)	0 (0 to 0)	0 (0 to 0)	0 (0 to 0)	0 (0 to 0)	0 (0 to 0)	0 (0 to 0)	0 (0 to 0)	0 (0 to 0)	0 (0 to 0)	0
Drug-susceptible tuberculosis	6·5 (4·4 to 9·2)	47·2 (16·8 to 85·2)	90·5 (41·4 to 157)	108 (55·2 to 182)	89·1 (45·7 to 146)	103 (59·4 to 165)	131 (76·4 to 204)	173 (114 to 251)	156 (102 to 223)	129 (89·2 to 183)	107 (73·8 to 150)	110 (77·3 to 150)	94·4 (65·6 to 130)	58·6 (40·5 to 82·5)	35·7 (24·6 to 49·6)	16·6 (11·1 to 22·5)	6·3 (4·5 to 8)	1,500
Multidrug-resistant tuberculosis without extensive drug res	0·3 (0 to 0·8)	1·4 (0·2 to 4·2)	2·7 (0·5 to 7·5)	3·3 (0·6 to 9·9)	3 (0·6 to 9)	3·7 (0·7 to 11·3)	5·2 (0·9 to 16·1)	7·3 (1·2 to 20·9)	7 (1·1 to 21·1)	5·9 (1 to 18·4)	5 (0·8 to 16·2)	5·1 (0·9 to 15·5)	4·5 (0·8 to 14·3)	2·8 (0·5 to 8·4)	1·7 (0·3 to 5·2)	0·8 (0·1 to 2·6)	0·3 (0 to 1)	61
Extensively drug-resistant tuberculosis	0 (0 to 0·1)	0·2 (0 to 0·5)	0·3 (0·1 to 0·9)	0·4 (0·1 to 1·2)	0·4 (0·1 to 1·2)	0·5 (0·1 to 1·5)	0·7 (0·1 to 2·2)	1·1 (0·2 to 3·3)	1·1 (0·2 to 3·5)	0·9 (0·1 to 2·8)	0·8 (0·1 to 2·6)	0·8 (0·1 to 2·6)	0·7 (0·1 to 2·2)	0·4 (0·1 to 1·4)	0·3 (0 to 0·8)	0·1 (0 to 0·4)	0 (0 to 0·2)	8·9
Lower respiratory infections	341 (234 to 492)	475 (315 to 693)	615 (410 to 878)	886 (584 to 1,270)	951 (662 to 1,360)	1,530 (1,020 to 2,140)	3,030 (2,110 to 4,300)	4,630 (3,310 to 6,450)	6,540 (4,770 to 8,590)	7,250 (5,190 to 9,620)	9,020 (6,350 to 12,200)	12,400 (9,060 to 16,700)	14,700 (10,900 to 19,600)	14,000 (11,200 to 17,200)	11,100 (8,580 to 13,900)	7,410 (5,710 to 9,170)	4,430 (3,250 to 5,550)	104,000
Upper respiratory infections	598 (309 to 1,030)	591 (324 to 1,000)	615 (338 to 1,010)	624 (331 to 1,040)	516 (266 to 854)	527 (279 to 942)	678 (361 to 1,180)	662 (363 to 1,100)	556 (299 to 910)	358 (190 to 598)	308 (163 to 518)	239 (132 to 399)	166 (90·8 to 273)	98 (53·8 to 162)	51·7 (28·7 to 84·8)	25·9 (14·9 to 42·9)	10·6 (6 to 18)	8,570
Otitis media	228 (120 to 385)	213 (92·6 to 427)	195 (95·3 to 367)	173 (76·3 to 321)	148 (73 to 275)	171 (78·1 to 319)	248 (123 to 442)	264 (127 to 482)	219 (111 to 394)	131 (62·3 to 262)	89·8 (44·1 to 167)	59·5 (26·2 to 115)	45·2 (22·6 to 80·6)	31·7 (15·2 to 57)	18·1 (8·5 to 32·2)	9·1 (4·1 to 16·2)	3·8 (1·8 to 7)	3,060
Enteric infections	665 (421 to 996)	559 (349 to 845)	731 (492 to 1,050)	912 (618 to 1,300)	897 (624 to 1,260)	1,130 (799 to 1,590)	1,800 (1,280 to 2,480)	2,200 (1,580 to 2,960)	2,180 (1,570 to 2,950)	1,960 (1,440 to 2,590)	2,430 (1,790 to 3,170)	2,730 (2,030 to 3,520)	2,640 (1,990 to 3,430)	1,930 (1,500 to 2,470)	1,110 (847 to 1,410)	538 (408 to 676)	228 (175 to 286)	28,600
Diarrheal diseases	663 (419 to 994)	557 (345 to 843)	729 (491 to 1,050)	910 (616 to 1,290)	896 (623 to 1,260)	1,130 (797 to 1,580)	1,790 (1,280 to 2,480)	2,200 (1,580 to 2,950)	2,170 (1,570 to 2,940)	1,960 (1,440 to 2,590)	2,430 (1,780 to 3,160)	2,730 (2,030 to 3,510)	2,640 (1,990 to 3,430)	1,930 (1,500 to 2,470)	1,110 (846 to 1,410)	538 (408 to 675)	227 (175 to 286)	28,600
Typhoid and paratyphoid	0·1 (0 to 0·1)	0·1 (0 to 0·1)	0·1 (0 to 0·1)	0·1 (0 to 0·1)	0 (0 to 0·1)	0 (0 to 0·1)	0·1 (0 to 0·2)	0·1 (0 to 0·2)	0·1 (0·1 to 0·2)	0·5 (0·1 to 1·6)	2·2 (0·1 to 6·2)	2·4 (0 to 7·4)	2 (0 to 6·6)	1·3 (0 to 4·4)	0·6 (0 to 1·8)	0·1 (0 to 0·2)	0 (0 to 0)	9·9
Typhoid fever	0 (0 to 0)	0 (0 to 0·1)	0 (0 to 0·1)	0 (0 to 0·1)	0 (0 to 0·1)	0 (0 to 0·1)	0·1 (0 to 0·2)	0·1 (0 to 0·1)	0·1 (0 to 0·2)	0·5 (0 to 1·6)	2·2 (0 to 6·2)	2·3 (0 to 7·3)	2 (0 to 6·6)	1·3 (0 to 4·4)	0·6 (0 to 1·7)	0·1 (0 to 0·2)	0 (0 to 0)	9·4
Paratyphoid fever	0 (0 to 0·1)	0 (0 to 0)	0 (0 to 0)	0 (0 to 0)	0 (0 to 0)	0 (0 to 0)	0 (0 to 0)	0 (0 to 0)	0 (0 to 0·1)	0 (0 to 0)	0 (0 to 0·1)	0 (0 to 0)	0 (0 to 0)	0 (0 to 0)	0 (0 to 0)	0 (0 to 0)	0 (0 to 0)	0·5
Invasive Non-typhoidal Salmonella (iNTS)	0·3 (0·1 to 0·6)	0·3 (0·1 to 0·6)	0·4 (0·2 to 0·7)	0·1 (0 to 0·3)	0·3 (0·1 to 0·6)	0·1 (0·1 to 0·4)	0·3 (0·1 to 0·6)	0·1 (0 to 0·6)	0·2 (0·1 to 0·7)	0·2 (0 to 1·3)	0·5 (0·2 to 1·1)	0·2 (0 to 0·7)	0·2 (0 to 1)	0 (0 to 0·3)	0 (0 to 0·2)	0 (0 to 0·2)	0 (0 to 0·1)	15·1
Other intestinal infectious diseases	1·6 (0·4 to 3·5)	1·5 (0·4 to 3·4)	0·9 (0·3 to 2)	2·5 (0·9 to 5·1)	1·2 (0·5 to 2·5)	1·6 (0·7 to 3·4)	1·6 (0·6 to 3·3)	1·8 (0·6 to 3·6)	1·4 (0·4 to 3·1)	1 (0·2 to 2·5)	1·7 (0·5 to 4)	1·2 (0·3 to 2·8)	0·8 (0·2 to 2)	0·8 (0·2 to 2)	1 (0·2 to 2·6)	0·5 (0·1 to 1·2)	0·3 (0·1 to 0·9)	33·1
Neglected tropical diseases and malaria	210 (94·5 to 379)	193 (86·4 to 364)	180 (81·9 to 329)	185 (85·7 to 327)	162 (75·9 to 293)	156 (75·6 to 286)	198 (95·6 to 344)	220 (97·7 to 382)	191 (90·5 to 329)	91·1 (41·1 to 162)	69·1 (28·6 to 135)	59 (24·6 to 111)	47 (19·7 to 92)	31 (12·4 to 59·9)	20·7 (8·4 to 40·4)	13 (4·4 to 26·9)	8·7 (1·7 to 21·3)	2,960
Malaria	0 (0 to 0)	0 (0 to 0)	0 (0 to 0)	0 (0 to 0)	0 (0 to 0)	0 (0 to 0)	0 (0 to 0)	0 (0 to 0)	0 (0 to 0)	0 (0 to 0)	0 (0 to 0)	0 (0 to 0)	0 (0 to 0)	0 (0 to 0)	0 (0 to 0)	0 (0 to 0)	0 (0 to 0)	0
Chagas disease	0 (0 to 0)	0 (0 to 0)	0 (0 to 0)	0 (0 to 0)	0 (0 to 0)	0 (0 to 0)	0 (0 to 0·1)	0 (0 to 0)	0 (0 to 0·1)	0 (0 to 0·1)	0 (0 to 0)	0 (0 to 0)	0 (0 to 0)	0 (0 to 0)	0 (0 to 0)	0 (0 to 0)	0 (0 to 0)	0·2
Leishmaniasis	0 (0 to 0)	0 (0 to 0)	0 (0 to 0)	0 (0 to 0)	0 (0 to 0)	0 (0 to 0)	0 (0 to 0)	0 (0 to 0)	0 (0 to 0)	0 (0 to 0)	0 (0 to 0)	0 (0 to 0)	0 (0 to 0)	0 (0 to 0)	0 (0 to 0)	0 (0 to 0)	0 (0 to 0)	0
Visceral leishmaniasis	0 (0 to 0)	0 (0 to 0)	0 (0 to 0)	0 (0 to 0)	0 (0 to 0)	0 (0 to 0)	0 (0 to 0)	0 (0 to 0)	0 (0 to 0)	0 (0 to 0)	0 (0 to 0)	0 (0 to 0)	0 (0 to 0)	0 (0 to 0)	0 (0 to 0)	0 (0 to 0)	0 (0 to 0)	0
Cutaneous and mucocutaneous leishmaniasis	0 (0 to 0)	0 (0 to 0)	0 (0 to 0)	0 (0 to 0)	0 (0 to 0)	0 (0 to 0)	0 (0 to 0)	0 (0 to 0)	0 (0 to 0)	0 (0 to 0)	0 (0 to 0)	0 (0 to 0)	0 (0 to 0)	0 (0 to 0)	0 (0 to 0)	0 (0 to 0)	0 (0 to 0)	0
African trypanosomiasis	0 (0 to 0)	0 (0 to 0)	0 (0 to 0)	0 (0 to 0)	0 (0 to 0)	0 (0 to 0)	0 (0 to 0)	0 (0 to 0)	0 (0 to 0)	0 (0 to 0)	0 (0 to 0)	0 (0 to 0)	0 (0 to 0)	0 (0 to 0)	0 (0 to 0)	0 (0 to 0)	0 (0 to 0)	0
Schistosomiasis	0 (0 to 0)	0 (0 to 0)	0 (0 to 0)	0 (0 to 0)	0 (0 to 0)	0 (0 to 0)	0 (0 to 0)	0 (0 to 0)	0 (0 to 0)	0 (0 to 0)	0 (0 to 0)	0 (0 to 0)	0 (0 to 0)	0 (0 to 0)	0 (0 to 0)	0 (0 to 0)	0 (0 to 0)	0
Cysticercosis	0 (0 to 0)	0 (0 to 0)	0 (0 to 0)	0 (0 to 0)	0 (0 to 0)	0 (0 to 0)	0 (0 to 0)	0 (0 to 0)	0 (0 to 0)	0 (0 to 0)	0 (0 to 0)	0 (0 to 0)	0 (0 to 0)	0 (0 to 0)	0 (0 to 0)	0 (0 to 0)	0 (0 to 0)	0
Cystic echinococcosis	0·1 (0 to 0·1)	0·1 (0 to 0·1)	0·1 (0 to 0·2)	0·1 (0 to 0·2)	0·1 (0 to 0·2)	0·1 (0 to 0·2)	0·1 (0 to 0·4)	0·2 (0 to 0·4)	0·2 (0 to 0·5)	0·1 (0 to 0·4)	0·1 (0 to 0·4)	0·1 (0 to 0·3)	0·1 (0 to 0·3)	0·1 (0 to 0·2)	0·1 (0 to 0·1)	0 (0 to 0·1)	0 (0 to 0)	1·7
Lymphatic filariasis	0 (0 to 0)	0 (0 to 0)	0 (0 to 0)	0 (0 to 0)	0 (0 to 0)	0 (0 to 0)	0 (0 to 0)	0 (0 to 0)	0 (0 to 0)	0 (0 to 0)	0 (0 to 0)	0 (0 to 0)	0 (0 to 0)	0 (0 to 0)	0 (0 to 0)	0 (0 to 0)	0 (0 to 0)	0
Onchocerciasis	0 (0 to 0)	0 (0 to 0)	0 (0 to 0)	0 (0 to 0)	0 (0 to 0)	0 (0 to 0)	0 (0 to 0)	0 (0 to 0)	0 (0 to 0)	0 (0 to 0)	0 (0 to 0)	0 (0 to 0)	0 (0 to 0)	0 (0 to 0)	0 (0 to 0)	0 (0 to 0)	0 (0 to 0)	0
Trachoma	0 (0 to 0)	0 (0 to 0)	0 (0 to 0)	0 (0 to 0)	0 (0 to 0)	0 (0 to 0)	0 (0 to 0)	0 (0 to 0)	0 (0 to 0)	0 (0 to 0)	0 (0 to 0)	0 (0 to 0)	0 (0 to 0)	0 (0 to 0)	0 (0 to 0)	0 (0 to 0)	0 (0 to 0)	0
Dengue	51 (4·3 to 143)	50·1 (4·2 to 135)	52·4 (5·9 to 136)	49·5 (5·6 to 133)	39·6 (3·3 to 104)	40 (3·3 to 111)	52·8 (6·3 to 143)	56·1 (5·4 to 150)	49·7 (2 to 139)	34·1 (1·3 to 95·7)	29·9 (1 to 86·1)	25·5 (0·9 to 74)	20·6 (0·5 to 62·1)	13·4 (0·4 to 38·4)	9·1 (0·2 to 26·3)	7·3 (0·4 to 20·6)	6·6 (0·2 to 18·9)	708
Yellow fever	0 (0 to 0)	0 (0 to 0)	0 (0 to 0)	0 (0 to 0)	0 (0 to 0)	0 (0 to 0)	0 (0 to 0)	0 (0 to 0)	0 (0 to 0)	0 (0 to 0)	0 (0 to 0)	0 (0 to 0)	0 (0 to 0)	0 (0 to 0)	0 (0 to 0)	0 (0 to 0)	0 (0 to 0)	0
Rabies	0·5 (0·1 to 1·2)	0·5 (0·1 to 1·1)	0·4 (0·1 to 0·9)	0 (0 to 0)	0 (0 to 0)	0 (0 to 0)	4 (1 to 11·1)	1·2 (0·3 to 3·3)	0·2 (0·1 to 0·6)	1 (0·2 to 2·9)	1·8 (0·5 to 4·1)	1·5 (0·4 to 3·9)	0·2 (0·1 to 0·5)	0·2 (0 to 0·7)	0 (0 to 0·1)	0 (0 to 0)	0 (0 to 0)	15·4
Intestinal nematode infections	105 (52·8 to 190)	102 (46·5 to 185)	83·7 (39·8 to 155)	80·7 (39 to 147)	63 (31·1 to 116)	41·7 (21 to 73·1)	48·8 (24·4 to 87·2)	49·2 (24·5 to 89·1)	43·5 (21·8 to 77)	28·8 (14·6 to 49·9)	22·7 (11·6 to 39·1)	19·1 (9·3 to 34)	15·3 (7·8 to 26·6)	9·4 (4·7 to 16·3)	5·1 (2·6 to 8·7)	2·3 (1·1 to 3·8)	0·7 (0·4 to 1·2)	944
Ascariasis	23·3 (9·4 to 44·6)	20·9 (8·6 to 39·5)	21·4 (8·9 to 41·1)	21·5 (8·8 to 40·6)	18·4 (7·5 to 35·4)	18·7 (8 to 35·4)	22·7 (9·7 to 44)	21·9 (9 to 43·1)	18·9 (7·9 to 38·5)	12·6 (5·1 to 24·9)	9·7 (4 to 19·2)	8·2 (3·3 to 15·7)	6·5 (2·8 to 12·7)	3·8 (1·6 to 7·5)	2 (0·8 to 3·8)	0·9 (0·4 to 1·9)	0·3 (0·1 to 0·6)	354
Trichuriasis	4·7 (1·5 to 10)	4·8 (1·6 to 9·8)	4 (1·3 to 8·7)	3·2 (1·4 to 6·5)	2·1 (0·8 to 4·5)	1·9 (0·9 to 3·6)	2·2 (1 to 4·2)	2 (0·9 to 3·8)	1·6 (0·7 to 3)	0·9 (0·4 to 1·8)	0·7 (0·3 to 1·3)	0·5 (0·2 to 0·9)	0·4 (0·2 to 0·7)	0·2 (0·1 to 0·4)	0·1 (0 to 0·2)	0 (0 to 0·1)	0 (0 to 0)	44
Hookworm disease	76·9 (34·8 to 153)	76·4 (30·1 to 148)	58·3 (23·8 to 118)	55·9 (23·1 to 107)	42·5 (19 to 82)	21·2 (10 to 37·9)	23·9 (10·1 to 45·5)	25·2 (10·7 to 48·2)	23·1 (10·7 to 43·1)	15·2 (7 to 28·2)	12·4 (5·5 to 22·3)	10·4 (4·5 to 19·7)	8·5 (3·9 to 15·7)	5·4 (2·4 to 10·1)	3 (1·4 to 5·5)	1·3 (0·6 to 2·3)	0·4 (0·2 to 0·7)	546
Food-borne trematodiases	0 (0 to 0)	0 (0 to 0)	0 (0 to 0)	0 (0 to 0)	0 (0 to 0)	0 (0 to 0)	0 (0 to 0)	0 (0 to 0)	0 (0 to 0)	0 (0 to 0)	0 (0 to 0)	0 (0 to 0)	0 (0 to 0)	0 (0 to 0)	0 (0 to 0)	0 (0 to 0)	0 (0 to 0)	0
Leprosy	0·2 (0·1 to 0·3)	0·3 (0·2 to 0·4)	0·4 (0·3 to 0·7)	0·6 (0·4 to 1)	0·7 (0·4 to 1·1)	1 (0·6 to 1·5)	1·6 (1 to 2·5)	2·1 (1·3 to 3·3)	2·2 (1·4 to 3·4)	1·8 (1·1 to 2·6)	1·8 (1·1 to 2·6)	1·7 (1 to 2·5)	1·4 (0·8 to 2·1)	1 (0·6 to 1·4)	0·6 (0·4 to 0·9)	0·3 (0·1 to 0·6)	0·1 (0·1 to 0·2)	17·7
Ebola	0 (0 to 0)	0 (0 to 0)	0 (0 to 0)	0 (0 to 0)	0 (0 to 0)	0 (0 to 0)	0 (0 to 0)	0 (0 to 0)	0 (0 to 0)	0 (0 to 0)	0 (0 to 0)	0 (0 to 0)	0 (0 to 0)	0 (0 to 0)	0 (0 to 0)	0 (0 to 0)	0 (0 to 0)	0
Zika virus	0·3 (0 to 1·2)	0·3 (0 to 1·2)	0·3 (0 to 1·2)	0·3 (0 to 1·3)	0·3 (0 to 1·1)	0·2 (0 to 0·9)	0·2 (0 to 0·7)	0·2 (0 to 0·6)	0·1 (0 to 0·4)	0·1 (0 to 0·3)	0·1 (0 to 0·2)	0 (0 to 0·1)	0 (0 to 0·1)	0 (0 to 0)	0 (0 to 0)	0 (0 to 0)	0 (0 to 0)	3
Guinea worm disease	0 (0 to 0)	0 (0 to 0)	0 (0 to 0)	0 (0 to 0)	0 (0 to 0)	0 (0 to 0)	0 (0 to 0)	0 (0 to 0)	0 (0 to 0)	0 (0 to 0)	0 (0 to 0)	0 (0 to 0)	0 (0 to 0)	0 (0 to 0)	0 (0 to 0)	0 (0 to 0)	0 (0 to 0)	0
Other neglected tropical diseases	53·4 (19·2 to 114)	40 (10·7 to 97·5)	42·3 (11·9 to 100)	53·3 (18·3 to 122)	58·2 (19·7 to 123)	72·9 (25·1 to 157)	90·9 (32·7 to 187)	111 (41·6 to 217)	95·2 (36·5 to 196)	25·3 (8·8 to 53·8)	12·8 (4·2 to 28·4)	11 (4 to 24·8)	9·4 (3·7 to 19·7)	7 (2·6 to 14·7)	5·9 (2·3 to 12·3)	3·1 (1·3 to 6·1)	1·1 (0·5 to 2·2)	1,270
Other infectious diseases	518 (406 to 656)	472 (370 to 612)	516 (407 to 661)	567 (439 to 728)	501 (390 to 654)	586 (447 to 751)	898 (697 to 1,170)	994 (776 to 1,280)	955 (732 to 1,220)	661 (522 to 825)	553 (440 to 700)	469 (370 to 606)	321 (254 to 414)	193 (149 to 261)	119 (85·4 to 171)	46·2 (33·5 to 68·4)	20·4 (13·9 to 33·9)	11,500
Meningitis	151 (114 to 197)	124 (94 to 164)	130 (99·9 to 165)	150 (116 to 190)	132 (97·8 to 176)	155 (114 to 202)	256 (188 to 345)	311 (226 to 422)	339 (251 to 445)	258 (195 to 342)	227 (173 to 293)	196 (147 to 251)	144 (109 to 188)	83·6 (62·9 to 110)	44·5 (32·6 to 59·3)	15 (11·3 to 19·2)	5·6 (4·4 to 7)	4,010
Encephalitis	147 (109 to 203)	134 (96 to 192)	127 (93·7 to 173)	133 (97·5 to 180)	104 (77 to 140)	117 (87·7 to 160)	158 (117 to 220)	154 (115 to 205)	141 (104 to 191)	98·4 (73·7 to 133)	78·2 (58·1 to 105)	66·6 (49·1 to 91)	37·9 (27·6 to 53·1)	20·9 (14·3 to 31·8)	9·5 (6·4 to 14·6)	4·1 (2·9 to 6)	1·8 (1·3 to 2·7)	2,310
Diphtheria	0·1 (0 to 0·1)	0·1 (0 to 0·1)	0·1 (0 to 0·1)	0 (0 to 0·1)	0 (0 to 0)	0 (0 to 0)	0 (0 to 0·1)	0 (0 to 0)	0 (0 to 0)	0 (0 to 0)	0 (0 to 0)	0 (0 to 0)	0 (0 to 0)	0 (0 to 0)	0 (0 to 0)	0 (0 to 0)	0 (0 to 0)	0·6
Tetanus	0·3 (0 to 2·3)	0·3 (0 to 1·7)	0·3 (0 to 1·4)	0·3 (0 to 1·7)	0·3 (0 to 1·6)	0·4 (0 to 1·8)	0·8 (0·1 to 2·9)	2 (0·2 to 4·6)	2 (0·3 to 5·1)	1·2 (0·2 to 2·8)	1·3 (0·2 to 3·4)	1·2 (0·2 to 3·5)	0·9 (0·2 to 2·6)	0·5 (0·1 to 1·9)	0·2 (0 to 0·9)	0·1 (0 to 0·4)	0 (0 to 0·2)	13·1
Measles	0·1 (0 to 0·3)	0·1 (0 to 0·1)	0·1 (0 to 0·1)	0 (0 to 0·1)	0 (0 to 0)	0 (0 to 0)	0 (0 to 0·1)	0 (0 to 0)	0 (0 to 0)	0 (0 to 0)	0 (0 to 0)	0 (0 to 0)	0 (0 to 0)	0 (0 to 0)	0 (0 to 0)	0 (0 to 0)	0 (0 to 0)	1·2
Varicella and herpes zoster	21·4 (11·6 to 33·8)	24 (14 to 38)	29·4 (17·6 to 45·7)	29·9 (17·1 to 48·5)	25·5 (13·8 to 46·5)	33 (19·3 to 53·3)	48·9 (27·7 to 86·9)	57·5 (34 to 92·3)	50 (26·6 to 84·8)	50·3 (30·3 to 80·9)	43·8 (26·2 to 72·1)	48·4 (30·2 to 75)	39·9 (25·3 to 63)	27·8 (16·6 to 48·1)	15·2 (8·8 to 29·9)	8·8 (5·3 to 16·8)	4·8 (2·5 to 12·3)	691
Acute hepatitis	55 (35 to 83·2)	49·7 (31·8 to 70·7)	46·9 (29·5 to 68·2)	45·6 (29·4 to 66·3)	33·4 (22 to 48·9)	34·2 (22·8 to 48·8)	38·3 (25·6 to 55·9)	35·4 (24·1 to 50·9)	27·9 (19·2 to 39·4)	16·5 (11·7 to 22·4)	11·3 (8·5 to 15)	9·5 (7 to 12·8)	6·4 (4·5 to 8·8)	3·2 (2·2 to 4·5)	2·1 (1·4 to 3·1)	0·9 (0·5 to 1·4)	0·3 (0·2 to 0·6)	533
Acute hepatitis A	48·1 (30 to 73·1)	40·9 (25·1 to 60·3)	34·1 (19·3 to 53·1)	31·4 (17·8 to 49·9)	20·3 (10·6 to 33·1)	19·2 (9·9 to 32)	20·9 (11 to 34·4)	16·5 (7·7 to 29·5)	11·2 (4·7 to 20·4)	5·3 (2·6 to 9·7)	3·4 (2 to 5·3)	2·2 (1 to 3·8)	1·5 (0·7 to 2·5)	0·5 (0·2 to 1·1)	0·2 (0 to 0·9)	0 (0 to 0·2)	0 (0 to 0·1)	355
Acute hepatitis B	1·2 (0·4 to 2·8)	3·3 (1·5 to 5·6)	7 (4 to 11·6)	9 (5·7 to 13·8)	9·4 (6·2 to 13·4)	10·6 (6·9 to 14·6)	12·4 (7·5 to 19·1)	14·3 (8·7 to 20·6)	12·8 (7·7 to 19)	8 (4·5 to 12)	5·4 (3·3 to 8·1)	4·8 (3 to 7·2)	2·8 (1·7 to 4·2)	1·2 (0·7 to 2)	0·6 (0·4 to 1)	0·2 (0·1 to 0·4)	0 (0 to 0·1)	109
Acute hepatitis C	1·2 (0·5 to 2·4)	1·4 (0·6 to 2·7)	1·6 (0·7 to 3·1)	1·4 (0·7 to 2·6)	1·1 (0·5 to 2)	2 (1 to 3·5)	2·4 (1·2 to 5·2)	2·5 (1·1 to 5·6)	2·1 (0·8 to 5·5)	1·7 (0·7 to 4)	1·2 (0·4 to 2·9)	1·2 (0·5 to 2·8)	0·9 (0·4 to 2·1)	0·5 (0·3 to 1)	0·3 (0·1 to 0·5)	0·1 (0·1 to 0·3)	0·1 (0 to 0·1)	26·5
Acute hepatitis E	4·6 (1 to 9·7)	4·2 (0·8 to 9·8)	4·1 (2 to 8·3)	3·7 (1·7 to 7·6)	2·6 (1·2 to 4·8)	2·4 (1·1 to 4·4)	2·6 (1·3 to 4·7)	2·1 (0·9 to 4·2)	1·8 (0·8 to 3·3)	1·5 (0·8 to 2·7)	1·3 (0·6 to 2·3)	1·2 (0·6 to 2·2)	1·2 (0·6 to 2·1)	0·9 (0·5 to 1·6)	1·1 (0·4 to 1·8)	0·5 (0·1 to 0·9)	0·2 (0·1 to 0·4)	42·7
Other unspecified infectious diseases	142 (86·5 to 218)	140 (90·6 to 214)	183 (117 to 271)	208 (135 to 305)	205 (138 to 310)	246 (162 to 368)	396 (256 to 586)	435 (289 to 628)	394 (257 to 568)	236 (153 to 335)	191 (130 to 268)	148 (103 to 233)	92 (63·1 to 147)	57·1 (35·1 to 100)	47·5 (26·8 to 88·1)	17·4 (9·6 to 31·7)	7·8 (4·1 to 14·9)	3,870
Maternal and neonatal disorders	2,000 (1,570 to 2,530)	2,360 (1,890 to 2,910)	2,540 (2,040 to 3,140)	2,560 (2,060 to 3,150)	1,870 (1,460 to 2,290)	1,600 (1,210 to 2,050)	1,900 (1,410 to 2,510)	1,810 (1,340 to 2,370)	1,420 (1,040 to 1,910)	811 (598 to 1,080)	534 (391 to 713)	298 (216 to 413)	132 (92·7 to 182)	37·7 (25·3 to 54·4)	10·5 (6 to 15·7)	3·2 (1·5 to 5·5)	1·1 (0·5 to 2)	40,400
Maternal disorders	472 (368 to 589)	815 (607 to 1,050)	879 (662 to 1,140)	869 (657 to 1,120)	490 (363 to 629)	175 (132 to 226)	43·1 (30·8 to 58·7)	5·3 (4 to 7·2)	0 (0 to 0)	0 (0 to 0)	0 (0 to 0)	0 (0 to 0)	0 (0 to 0)	0 (0 to 0)	0 (0 to 0)	0 (0 to 0)	0 (0 to 0)	3,760
Maternal hemorrhage	54·8 (30·2 to 91·4)	107 (62·5 to 167)	133 (81·1 to 203)	135 (84·2 to 203)	72·5 (43·6 to 109)	22·9 (14 to 34·7)	1·9 (1·1 to 3·3)	0·2 (0·1 to 0·3)	0 (0 to 0)	0 (0 to 0)	0 (0 to 0)	0 (0 to 0)	0 (0 to 0)	0 (0 to 0)	0 (0 to 0)	0 (0 to 0)	0 (0 to 0)	528
Maternal sepsis and other maternal infections	24·9 (13·9 to 42·2)	49·9 (28·6 to 81·4)	53·7 (33·1 to 83·2)	50·2 (29·4 to 77·7)	28·6 (16·8 to 45·9)	9·8 (5·7 to 15·7)	2·7 (1·5 to 4·4)	0·4 (0 to 1·2)	0 (0 to 0)	0 (0 to 0)	0 (0 to 0)	0 (0 to 0)	0 (0 to 0)	0 (0 to 0)	0 (0 to 0)	0 (0 to 0)	0 (0 to 0)	221
Maternal hypertensive disorders	70 (49·1 to 94·8)	85·9 (57·9 to 120)	91·3 (61·7 to 129)	76·5 (51·6 to 107)	37·9 (25·3 to 54·6)	11 (6·7 to 16·6)	1·1 (0·8 to 1·6)	0·1 (0 to 0·1)	0 (0 to 0)	0 (0 to 0)	0 (0 to 0)	0 (0 to 0)	0 (0 to 0)	0 (0 to 0)	0 (0 to 0)	0 (0 to 0)	0 (0 to 0)	377
Maternal obstructed labor and uterine rupture	5·9 (3·2 to 9·4)	11·3 (6·5 to 18)	13·4 (7·9 to 21·5)	13·1 (7·5 to 22)	6·5 (3·6 to 10·7)	1·5 (0·8 to 2·5)	0·6 (0·3 to 1·1)	0 (0 to 0·1)	0 (0 to 0)	0 (0 to 0)	0 (0 to 0)	0 (0 to 0)	0 (0 to 0)	0 (0 to 0)	0 (0 to 0)	0 (0 to 0)	0 (0 to 0)	52·5
Maternal abortion and miscarriage	40·5 (22·1 to 64·9)	63 (34·9 to 103)	56·5 (30·3 to 91·5)	52·1 (26·5 to 89·4)	35 (17·5 to 59·5)	20·6 (11·1 to 35·1)	13·7 (8·1 to 21·1)	0·5 (0·2 to 1)	0 (0 to 0)	0 (0 to 0)	0 (0 to 0)	0 (0 to 0)	0 (0 to 0)	0 (0 to 0)	0 (0 to 0)	0 (0 to 0)	0 (0 to 0)	283
Ectopic pregnancy	14·2 (8·8 to 21·6)	37·1 (22·9 to 56·6)	45·8 (28·1 to 69·8)	51·1 (30·9 to 80)	25·3 (15·2 to 39·6)	7·5 (4·6 to 11·7)	2·1 (1·2 to 3·5)	0 (0 to 0)	0 (0 to 0)	0 (0 to 0)	0 (0 to 0)	0 (0 to 0)	0 (0 to 0)	0 (0 to 0)	0 (0 to 0)	0 (0 to 0)	0 (0 to 0)	183
Indirect maternal deaths	107 (71 to 153)	203 (130 to 294)	217 (136 to 336)	205 (129 to 300)	99·6 (61·3 to 145)	27·8 (17·3 to 42·5)	1·6 (0·8 to 2·6)	0 (0 to 0·1)	--	--	--	--	--	--	--	--	--	NA
Late maternal deaths	68 (43·6 to 101)	113 (68·8 to 174)	112 (68·3 to 174)	115 (70·6 to 177)	73·2 (44·1 to 115)	26·6 (16·4 to 40·8)	6·5 (4·2 to 9·5)	0·7 (0·5 to 1·1)	--	--	--	--	--	--	--	--	--	NA
Maternal deaths aggravated by HIV/AIDS	0 (0 to 0·1)	0·2 (0·1 to 0·4)	0·6 (0·2 to 1·4)	1 (0·4 to 2·2)	0·6 (0·3 to 1·2)	0·2 (0 to 0·4)	0 (0 to 0·1)	0 (0 to 0)	--	--	--	--	--	--	--	--	--	NA
Neonatal disorders	1,530 (1,110 to 2,060)	1,550 (1,130 to 2,050)	1,660 (1,240 to 2,180)	1,690 (1,260 to 2,200)	1,380 (1,010 to 1,800)	1,430 (1,040 to 1,870)	1,850 (1,370 to 2,460)	1,810 (1,330 to 2,370)	1,420 (1,040 to 1,910)	811 (598 to 1,080)	534 (391 to 713)	298 (216 to 413)	132 (92·7 to 182)	37·7 (25·3 to 54·4)	10·5 (6 to 15·7)	3·2 (1·5 to 5·5)	1·1 (0·5 to 2)	36,600
Neonatal preterm birth	798 (569 to 1,090)	797 (564 to 1,070)	853 (606 to 1,130)	862 (617 to 1,150)	697 (499 to 925)	715 (519 to 958)	915 (658 to 1,220)	872 (632 to 1,170)	659 (477 to 876)	357 (264 to 475)	225 (171 to 288)	119 (88·7 to 157)	52·7 (36·8 to 72·7)	13·9 (7·8 to 22·2)	6·2 (2·8 to 10·7)	2·8 (1·2 to 4·9)	1·1 (0·5 to 2)	14,600
Neonatal encephalopathy due to birth asphyxia and traum	208 (124 to 327)	214 (128 to 329)	227 (135 to 348)	233 (138 to 347)	191 (114 to 288)	200 (119 to 304)	264 (163 to 397)	258 (152 to 391)	215 (134 to 321)	130 (83·7 to 194)	80·3 (55·2 to 109)	33·2 (21·6 to 47·5)	9·6 (5·5 to 14·8)	1·4 (0·6 to 2·8)	0·2 (0 to 0·4)	0 (0 to 0)	0 (0 to 0)	6,320
Neonatal sepsis and other neonatal infections	498 (295 to 785)	511 (302 to 777)	550 (332 to 827)	566 (335 to 856)	467 (276 to 703)	485 (286 to 741)	643 (387 to 988)	645 (390 to 970)	520 (294 to 814)	309 (187 to 473)	218 (129 to 347)	139 (80 to 229)	67 (36·1 to 110)	21·3 (12·3 to 35)	3·9 (1·7 to 7·3)	0·4 (0·1 to 0·9)	0 (0 to 0)	9,700
Hemolytic disease and other neonatal jaundice	24·8 (18·4 to 32·3)	25·4 (18·9 to 33·2)	27·3 (20·3 to 35·6)	28·1 (20·9 to 36·7)	23·1 (17 to 29·9)	24·2 (17·8 to 31·2)	32 (23·7 to 41·5)	31·8 (23·5 to 41·4)	25·6 (18·9 to 33·6)	15·2 (11·2 to 19·8)	10·9 (8 to 14)	6·8 (5 to 8·8)	3·2 (2·3 to 4·3)	1 (0·7 to 1·4)	0·2 (0·1 to 0·3)	0 (0 to 0)	0 (0 to 0)	538
Other neonatal disorders	0 (0 to 0)	0 (0 to 0)	0 (0 to 0)	0 (0 to 0)	0 (0 to 0)	0 (0 to 0)	0 (0 to 0)	0 (0 to 0)	0 (0 to 0)	0 (0 to 0)	0 (0 to 0)	0 (0 to 0)	0 (0 to 0)	0 (0 to 0)	0 (0 to 0)	0 (0 to 0)	0 (0 to 0)	5,470
Nutritional deficiencies	1,500 (700 to 2,930)	1,230 (525 to 2,530)	1,370 (644 to 2,750)	1,570 (752 to 3,090)	1,650 (766 to 3,110)	2,020 (945 to 3,920)	2,520 (1,180 to 4,700)	3,060 (1,470 to 5,470)	2,650 (1,310 to 4,910)	1,400 (699 to 2,530)	888 (503 to 1,540)	742 (444 to 1,250)	611 (384 to 1,020)	451 (265 to 757)	336 (193 to 576)	176 (105 to 291)	66·3 (43·5 to 101)	28,900
Protein-energy malnutrition	189 (100 to 311)	203 (113 to 325)	226 (127 to 348)	249 (146 to 381)	218 (129 to 331)	243 (149 to 358)	354 (223 to 523)	392 (249 to 565)	372 (247 to 532)	285 (193 to 392)	286 (197 to 384)	269 (188 to 371)	230 (167 to 305)	162 (117 to 218)	101 (72·9 to 133)	54·9 (39·3 to 73·3)	27·7 (20·3 to 36·8)	4,480
Iodine deficiency	52·6 (22 to 97·2)	61·9 (24·5 to 113)	71·7 (28·7 to 135)	76·4 (32·9 to 143)	63·5 (27·5 to 117)	67·1 (28·4 to 118)	88·5 (39·1 to 160)	90·1 (39·8 to 154)	73·5 (31·1 to 134)	45·7 (18·9 to 83·7)	37·3 (17·9 to 63·9)	28·2 (13·5 to 50)	19·8 (10·7 to 31·3)	12·2 (6·3 to 20·3)	7·2 (3·8 to 11·3)	3·2 (1·6 to 5)	1·1 (0·6 to 1·8)	851
Vitamin A deficiency	0 (0 to 0)	0 (0 to 0)	0 (0 to 0)	0 (0 to 0)	0 (0 to 0)	0 (0 to 0)	0 (0 to 0)	0 (0 to 0)	0 (0 to 0)	0 (0 to 0)	0 (0 to 0)	0 (0 to 0)	0 (0 to 0)	0 (0 to 0)	0 (0 to 0)	0 (0 to 0)	0 (0 to 0)	89·2
Dietary iron deficiency	1,170 (432 to 2,500)	862 (227 to 2,090)	924 (280 to 2,240)	1,140 (396 to 2,590)	1,280 (449 to 2,670)	1,610 (581 to 3,450)	1,970 (674 to 4,060)	2,410 (900 to 4,790)	2,060 (793 to 4,290)	981 (339 to 2,050)	469 (148 to 1,110)	370 (122 to 858)	305 (105 to 693)	235 (72·7 to 527)	203 (71·5 to 431)	104 (38·3 to 217)	30·7 (11·7 to 62·3)	21,900
Other nutritional deficiencies	88·9 (47·6 to 146)	104 (58·4 to 166)	146 (81·8 to 224)	102 (59·7 to 157)	86·8 (50·1 to 133)	96·2 (58·1 to 143)	111 (67·5 to 167)	165 (102 to 242)	139 (87·2 to 211)	90·3 (58·8 to 132)	95·6 (61·7 to 137)	74·6 (49·8 to 108)	56·8 (38·1 to 82·4)	42 (27·9 to 63·1)	24·8 (16·9 to 36·4)	14 (8·6 to 20·9)	6·7 (3·7 to 10·2)	1,560
Non-communicable diseases	43,600 (32,600 to 57,100)	53,400 (40,700 to 67,900)	68,600 (53,200 to 86,800)	84,500 (66,100 to 105,000)	84,500 (67,800 to 105,000)	114,000 (92,800 to 139,000)	199,000 (164,000 to 239,000)	277,000 (229,000 to 329,000)	321,000 (267,000 to 380,000)	281,000 (236,000 to 333,000)	299,000 (252,000 to 350,000)	300,000 (254,000 to 349,000)	268,000 (230,000 to 310,000)	201,000 (175,000 to 230,000)	127,000 (112,000 to 144,000)	69,000 (61,400 to 76,800)	32,400 (29,600 to 35,800)	2,900,000
Neoplasms	2,780 (2,110 to 3,530)	3,270 (2,470 to 4,180)	4,450 (3,450 to 5,590)	6,990 (5,430 to 8,700)	9,210 (7,260 to 11,400)	16,500 (13,000 to 20,400)	36,000 (28,400 to 45,100)	63,300 (49,700 to 78,700)	84,900 (66,700 to 106,000)	79,500 (63,400 to 98,200)	83,800 (67,900 to 102,000)	78,500 (64,300 to 93,900)	64,400 (53,000 to 76,900)	41,100 (33,800 to 48,800)	22,100 (17,900 to 26,200)	9,650 (7,660 to 11,400)	3,310 (2,520 to 3,940)	616,000
Lip and oral cavity cancer	14·9 (10 to 21·3)	21·9 (14·1 to 32·1)	33·5 (21·9 to 49·2)	83·4 (55·9 to 121)	124 (82·6 to 182)	330 (223 to 472)	891 (610 to 1,310)	1,910 (1,370 to 2,640)	2,380 (1,700 to 3,250)	1,930 (1,410 to 2,600)	1,700 (1,250 to 2,240)	1,480 (1,090 to 1,930)	1,120 (850 to 1,420)	720 (547 to 920)	353 (265 to 459)	150 (113 to 191)	62 (44·4 to 77·9)	13,300
Nasopharynx cancer	18·8 (10·1 to 33·6)	22·7 (11·7 to 42·5)	29·3 (16·6 to 48)	37·9 (22 to 60·4)	39·9 (25 to 61·1)	98·2 (60·4 to 147)	215 (148 to 309)	428 (290 to 614)	498 (339 to 747)	335 (230 to 475)	281 (179 to 403)	228 (147 to 339)	138 (86·1 to 208)	75·7 (46·2 to 116)	51·6 (30·7 to 82·7)	24 (14 to 37·7)	9·4 (5·4 to 15·2)	2,550
Other pharynx cancer	0 (0 to 0)	7·7 (5·2 to 11)	9·7 (6·4 to 14·1)	23·3 (13·7 to 37)	43·4 (26·5 to 68·4)	132 (81·4 to 200)	472 (321 to 678)	897 (631 to 1,230)	1,160 (761 to 1,650)	883 (585 to 1,260)	712 (458 to 1,040)	568 (380 to 822)	373 (256 to 532)	186 (127 to 258)	93·9 (63·9 to 133)	40·8 (24·5 to 64·2)	16·3 (9·5 to 26·3)	5,610
Esophageal cancer	0 (0 to 0)	11·5 (7·6 to 16·4)	21·9 (13·2 to 33·9)	44·7 (28·2 to 67·5)	153 (102 to 226)	661 (419 to 987)	1,990 (1,400 to 2,750)	3,680 (2,710 to 4,840)	4,950 (3,570 to 6,720)	3,650 (2,680 to 4,860)	2,990 (2,130 to 4,040)	2,460 (1,810 to 3,290)	1,540 (1,130 to 2,040)	992 (713 to 1,360)	442 (331 to 580)	190 (142 to 242)	50·7 (33·8 to 70·8)	23,800
Stomach cancer	11 (7·2 to 16·6)	30·2 (20 to 43·7)	80 (54·4 to 114)	194 (123 to 284)	292 (195 to 428)	589 (404 to 818)	1,270 (938 to 1,700)	2,520 (1,860 to 3,360)	3,240 (2,460 to 4,170)	3,060 (2,300 to 3,980)	3,270 (2,460 to 4,280)	3,130 (2,430 to 3,940)	2,560 (1,930 to 3,320)	1,580 (1,210 to 2,010)	835 (633 to 1,090)	359 (260 to 469)	117 (85·2 to 150)	23,100
Colon and rectum cancer	69·1 (48·4 to 97·6)	129 (89·6 to 180)	239 (175 to 322)	526 (364 to 711)	781 (566 to 1,060)	1,510 (1,140 to 2,030)	3,390 (2,510 to 4,440)	5,590 (4,120 to 7,460)	7,490 (5,600 to 9,820)	8,240 (6,150 to 10,500)	9,360 (7,180 to 12,000)	9,760 (7,710 to 12,100)	9,260 (7,300 to 11,500)	6,360 (5,030 to 7,890)	3,590 (2,790 to 4,440)	1,640 (1,250 to 2,090)	534 (386 to 675)	68,500
Liver cancer	15·1 (9·6 to 23·1)	24·5 (16 to 36·3)	43·7 (28·6 to 65·3)	62·7 (40 to 93·2)	86·4 (55·4 to 133)	180 (118 to 263)	443 (292 to 649)	871 (596 to 1,270)	1,330 (899 to 1,860)	1,260 (895 to 1,700)	1,470 (1,060 to 1,940)	1,480 (1,110 to 1,930)	1,200 (921 to 1,520)	736 (578 to 927)	329 (245 to 426)	113 (80·6 to 147)	22·9 (16·1 to 31·4)	9,740
Liver cancer due to hepatitis B	6·7 (3·6 to 11·4)	12·8 (7·8 to 19·5)	25·9 (14·6 to 41·2)	36·7 (21 to 58)	46·3 (26·2 to 74·4)	88·4 (47·9 to 148)	192 (99·2 to 327)	335 (185 to 585)	467 (257 to 774)	380 (202 to 644)	354 (179 to 595)	297 (141 to 512)	214 (116 to 366)	115 (57·4 to 195)	47·6 (24·9 to 80·7)	14·8 (7 to 27·6)	2·8 (1·1 to 5·9)	2,640
Liver cancer due to hepatitis C	0·1 (0 to 0·4)	0·5 (0·2 to 1)	1·3 (0·4 to 2·9)	2·8 (1·2 to 5·8)	5·6 (2·1 to 11·7)	15·6 (7 to 29·1)	51 (22·3 to 92·4)	125 (60·6 to 215)	222 (98·5 to 403)	231 (106 to 407)	285 (155 to 456)	320 (175 to 519)	308 (165 to 502)	234 (138 to 351)	124 (77·1 to 184)	49·4 (29·3 to 74·2)	11·2 (6·1 to 17·7)	1,990
Liver cancer due to alcohol use	0·4 (0·1 to 0·8)	1·9 (0·9 to 3·6)	5·4 (2·1 to 10·8)	10·7 (5 to 19·7)	19·3 (8·2 to 37·9)	47·7 (21 to 84·7)	133 (60 to 238)	288 (138 to 482)	467 (252 to 754)	488 (267 to 759)	641 (389 to 950)	657 (402 to 976)	481 (301 to 673)	252 (154 to 368)	96·3 (57·1 to 149)	27·3 (14·9 to 43·6)	4·6 (2 to 8·2)	3,620
Liver cancer due to NASH	3·5 (1·8 to 6·1)	4 (2·1 to 7·1)	4·7 (2·1 to 8·5)	5·4 (2·6 to 9·8)	6·8 (3·1 to 13)	13·4 (6·7 to 24·6)	34·8 (16·3 to 65·2)	68·6 (34·5 to 122)	104 (48·6 to 183)	101 (50·9 to 180)	126 (67·5 to 233)	145 (79·9 to 251)	145 (82·9 to 236)	101 (55·4 to 168)	45·4 (25·5 to 73·6)	16 (8·2 to 28·3)	3·3 (1·4 to 6·2)	927
Liver cancer due to other causes	4·5 (2·3 to 7·7)	5·4 (2·8 to 9·2)	6·4 (2·9 to 11·5)	7·1 (3·3 to 13·3)	8·4 (4 to 15·9)	14·8 (7·3 to 26·2)	33 (16·5 to 58·7)	55·2 (26·1 to 100)	71·2 (34·9 to 133)	59·3 (30·2 to 112)	62·3 (32·4 to 108)	61·2 (30·5 to 104)	53·4 (27·7 to 89·2)	34·2 (18·2 to 60·1)	14·9 (8 to 25·2)	5·1 (2·6 to 9·3)	1 (0·4 to 2)	571
Gallbladder and biliary tract cancer	0 (0 to 0)	5·5 (4·3 to 8·3)	8·4 (5·2 to 17·4)	19·1 (11·4 to 36·7)	40·3 (25·8 to 72·1)	96·7 (61 to 165)	240 (150 to 404)	444 (287 to 777)	653 (432 to 1,140)	611 (405 to 1,090)	650 (441 to 1,170)	672 (462 to 1,130)	538 (381 to 903)	353 (249 to 559)	168 (116 to 275)	69·5 (46·6 to 117)	24·6 (16·5 to 41·6)	4,590
Pancreatic cancer	16·8 (9·7 to 27·3)	29·6 (16·6 to 50)	60·7 (37·5 to 95)	141 (93 to 201)	240 (167 to 335)	594 (428 to 808)	1,500 (1,090 to 2,030)	2,600 (1,880 to 3,450)	3,750 (2,770 to 4,970)	3,410 (2,590 to 4,470)	3,700 (2,690 to 4,870)	3,460 (2,580 to 4,480)	2,800 (2,050 to 3,710)	1,730 (1,250 to 2,310)	781 (569 to 1,030)	340 (248 to 448)	118 (84 to 156)	25,300
Larynx cancer	0 (0 to 0)	14·2 (9·6 to 20)	21·3 (14·5 to 29·8)	40·1 (26 to 59·7)	96·6 (61·3 to 149)	369 (215 to 582)	1,380 (918 to 2,010)	3,210 (2,260 to 4,320)	4,070 (2,900 to 5,510)	3,780 (2,740 to 5,040)	3,510 (2,580 to 4,710)	2,830 (2,110 to 3,730)	1,930 (1,470 to 2,500)	1,060 (817 to 1,380)	452 (335 to 600)	187 (133 to 249)	54·6 (36·6 to 73·6)	23,000
Tracheal, bronchus, and lung cancer	66·8 (45·7 to 95·4)	84·8 (57·2 to 120)	153 (111 to 207)	416 (289 to 588)	880 (631 to 1,200)	2,090 (1,540 to 2,760)	6,010 (4,360 to 8,220)	14,300 (10,700 to 18,700)	22,600 (16,300 to 30,400)	23,200 (17,700 to 29,800)	24,000 (18,800 to 30,200)	20,300 (16,000 to 25,200)	14,600 (11,600 to 18,300)	7,870 (6,220 to 9,900)	4,030 (3,100 to 4,960)	1,530 (1,190 to 1,870)	522 (394 to 659)	143,000
Malignant skin melanoma	13·3 (8·1 to 20)	30·1 (18·1 to 43·7)	53·1 (31·5 to 75·3)	78·2 (46·5 to 110)	110 (74·1 to 160)	146 (93·7 to 209)	263 (167 to 383)	329 (213 to 488)	363 (240 to 543)	253 (169 to 392)	255 (174 to 380)	247 (176 to 364)	188 (131 to 280)	140 (97 to 217)	89 (62·1 to 127)	37·3 (25·5 to 53·8)	20·6 (13·5 to 30·9)	2,640
Non-melanoma skin cancer	0 (0 to 0)	19·8 (13·9 to 27·5)	46·1 (33·8 to 62·8)	74·9 (53·9 to 100)	83·8 (59·2 to 115)	170 (121 to 232)	244 (170 to 346)	506 (354 to 697)	637 (463 to 870)	567 (417 to 741)	704 (524 to 922)	735 (555 to 941)	790 (579 to 1,020)	721 (548 to 913)	564 (424 to 711)	403 (288 to 502)	222 (150 to 287)	6,490
Non-melanoma skin cancer (squamous-cell carcinoma)	0 (0 to 0)	19·8 (13·9 to 27·5)	46·1 (33·7 to 62·8)	74·9 (53·9 to 99·9)	83·7 (59·2 to 115)	170 (121 to 232)	244 (170 to 346)	506 (354 to 697)	637 (463 to 870)	567 (416 to 741)	703 (524 to 922)	735 (555 to 941)	790 (579 to 1,020)	721 (548 to 912)	564 (423 to 711)	403 (288 to 502)	222 (150 to 287)	6,490
Non-melanoma skin cancer (basal-cell carcinoma)	0 (0 to 0)	0 (0 to 0)	0 (0 to 0)	0 (0 to 0)	0 (0 to 0·1)	0 (0 to 0·1)	0·1 (0 to 0·2)	0·1 (0 to 0·2)	0·1 (0 to 0·2)	0·1 (0 to 0·2)	0·1 (0·1 to 0·2)	0·1 (0·1 to 0·3)	0·1 (0 to 0·2)	0·1 (0 to 0·2)	0·1 (0 to 0·1)	0 (0 to 0·1)	0 (0 to 0)	1·1
Breast cancer	16·2 (10·7 to 24·9)	31·2 (21·4 to 45·6)	165 (111 to 236)	754 (514 to 1,060)	1,310 (909 to 1,780)	2,330 (1,700 to 3,160)	4,540 (3,260 to 6,030)	6,390 (4,800 to 8,430)	7,040 (5,140 to 9,310)	5,240 (3,940 to 6,900)	4,930 (3,750 to 6,410)	4,050 (3,090 to 5,110)	3,370 (2,540 to 4,340)	2,340 (1,730 to 3,010)	1,430 (1,070 to 1,870)	634 (461 to 818)	250 (165 to 351)	44,800
Cervical cancer	17 (9·5 to 29)	92·3 (53·3 to 153)	363 (214 to 567)	830 (518 to 1,270)	1,140 (755 to 1,630)	1,730 (1,160 to 2,400)	2,710 (1,910 to 3,700)	3,210 (2,190 to 4,420)	2,970 (2,100 to 4,040)	1,980 (1,390 to 2,700)	1,770 (1,270 to 2,370)	1,470 (1,050 to 1,960)	1,050 (759 to 1,410)	597 (424 to 821)	262 (179 to 361)	121 (79·4 to 168)	40·8 (26·1 to 59·9)	20,300
Uterine cancer	0 (0 to 0)	31·2 (18·7 to 48·2)	87·7 (48·9 to 145)	228 (134 to 369)	325 (207 to 483)	509 (337 to 742)	1,090 (731 to 1,560)	1,650 (1,110 to 2,320)	2,190 (1,530 to 3,190)	2,170 (1,520 to 3,020)	2,290 (1,580 to 3,200)	1,940 (1,390 to 2,620)	1,510 (1,050 to 2,080)	914 (639 to 1,240)	400 (288 to 550)	177 (126 to 233)	61·2 (41·6 to 83·2)	15,600
Ovarian cancer	43·5 (25 to 70·2)	69·8 (40·7 to 106)	91·6 (57 to 140)	190 (114 to 275)	266 (160 to 387)	526 (320 to 744)	1,120 (629 to 1,590)	1,590 (856 to 2,230)	1,660 (927 to 2,320)	1,140 (669 to 1,610)	1,060 (594 to 1,430)	814 (459 to 1,120)	571 (280 to 776)	326 (151 to 465)	150 (78·9 to 220)	54·1 (32·3 to 78)	14·6 (7·5 to 22·3)	9,700
Prostate cancer	0 (0 to 0)	7·3 (4·8 to 10·5)	14·4 (10·6 to 23·3)	22·3 (16·2 to 32)	25·1 (17·8 to 36·8)	79 (50·3 to 119)	380 (246 to 565)	1,410 (846 to 2,050)	3,080 (1,870 to 4,420)	5,000 (2,960 to 6,970)	7,560 (4,430 to 10,600)	10,500 (6,060 to 14,300)	11,100 (6,600 to 14,800)	8,560 (4,920 to 11,400)	5,240 (3,170 to 6,870)	2,410 (1,350 to 3,210)	793 (449 to 1,140)	56,200
Testicular cancer	41·9 (20·5 to 74·7)	66·5 (39·4 to 104)	100 (60·6 to 152)	76·3 (44·2 to 119)	60·8 (34·8 to 96·1)	57·1 (31·6 to 91·9)	59·7 (27·9 to 97·7)	58 (30 to 94·9)	38·2 (18·7 to 63·4)	28·3 (15 to 47·5)	33·6 (15·2 to 60·4)	37·9 (18 to 64·9)	32·2 (15·1 to 56·4)	18·1 (8·8 to 30·7)	12·6 (6·8 to 19·8)	5·5 (2·9 to 9·2)	1·6 (0·9 to 2·5)	767
Kidney cancer	57 (29·7 to 97·8)	80·2 (45·4 to 133)	102 (61·6 to 159)	131 (74·9 to 201)	136 (81·3 to 212)	271 (175 to 406)	586 (379 to 871)	1,050 (734 to 1,480)	1,440 (965 to 2,090)	1,270 (864 to 1,820)	1,190 (810 to 1,660)	1,060 (734 to 1,470)	726 (497 to 1,000)	424 (308 to 571)	167 (120 to 222)	64·6 (42·6 to 91·7)	18·7 (11·6 to 27·4)	9,040
Bladder cancer	6 (4·9 to 7·5)	7 (5 to 10·1)	10·8 (7 to 16·9)	42·2 (24·5 to 66·9)	57·7 (36·1 to 89·9)	134 (86 to 203)	331 (236 to 447)	833 (604 to 1,130)	1,430 (1,040 to 1,910)	1,700 (1,250 to 2,240)	2,270 (1,640 to 3,120)	2,530 (1,910 to 3,280)	2,270 (1,650 to 3,060)	1,580 (1,160 to 2,080)	856 (654 to 1,090)	419 (301 to 547)	144 (101 to 191)	14,600
Brain and central nervous system cancer	350 (125 to 553)	386 (117 to 611)	494 (150 to 776)	629 (199 to 950)	676 (230 to 1,070)	945 (301 to 1,500)	1,520 (479 to 2,400)	2,090 (675 to 3,210)	2,410 (734 to 3,790)	1,720 (527 to 2,720)	1,660 (480 to 2,560)	1,280 (367 to 1,980)	804 (231 to 1,250)	364 (114 to 590)	147 (51·9 to 236)	55·9 (20 to 89·3)	13·7 (5·9 to 22·7)	17,200
Thyroid cancer	10·4 (6·6 to 16·4)	13·4 (8·4 to 21·7)	26·2 (14·3 to 44·1)	40·5 (23·2 to 64·5)	63·3 (40·9 to 95·9)	90·5 (58·9 to 133)	190 (128 to 273)	341 (235 to 480)	378 (261 to 537)	302 (211 to 414)	326 (236 to 439)	321 (237 to 421)	242 (175 to 323)	156 (112 to 210)	75·2 (53·5 to 102)	33·8 (23·6 to 45·8)	10·3 (6·7 to 15·2)	2,630
Mesothelioma	0 (0 to 0)	16·2 (10·6 to 22·4)	15·8 (10·4 to 22·8)	15·2 (10·8 to 20·6)	28·4 (19·7 to 39·1)	31·7 (22·5 to 45·9)	55·9 (39·8 to 79·6)	98·3 (69·4 to 139)	131 (95·1 to 175)	50·8 (38 to 66·9)	37·2 (27·8 to 50·8)	51·1 (37·8 to 67)	35·5 (26·6 to 46)	22·3 (16·8 to 29)	10·2 (7·6 to 13·6)	3·9 (2·8 to 5·1)	1·4 (1 to 1·9)	605
Hodgkin lymphoma	128 (56·2 to 201)	222 (93 to 357)	278 (102 to 418)	260 (98·6 to 396)	191 (76·9 to 293)	227 (86·8 to 335)	278 (104 to 422)	340 (134 to 506)	326 (135 to 486)	256 (103 to 370)	257 (106 to 375)	211 (81·3 to 312)	165 (66·7 to 244)	96·9 (35·6 to 152)	43·7 (17·6 to 67·7)	16·2 (5·4 to 28·9)	5·5 (1·5 to 10)	3,350
Non-Hodgkin lymphoma	339 (248 to 459)	410 (289 to 576)	504 (380 to 670)	612 (444 to 847)	555 (416 to 720)	707 (530 to 931)	1,230 (914 to 1,640)	1,710 (1,300 to 2,180)	2,290 (1,700 to 3,060)	1,930 (1,440 to 2,480)	2,050 (1,500 to 2,640)	1,850 (1,360 to 2,430)	1,320 (998 to 1,720)	658 (511 to 827)	254 (190 to 324)	84·8 (65·1 to 105)	23 (16·5 to 29·2)	17,200
Multiple myeloma	0 (0 to 0)	11·4 (7·2 to 17·1)	16·2 (10·9 to 23·6)	29·2 (19·1 to 42·8)	64·9 (41·6 to 95·9)	177 (124 to 254)	521 (362 to 724)	1,050 (750 to 1,440)	1,610 (1,140 to 2,200)	1,350 (1,010 to 1,850)	1,470 (1,070 to 1,930)	1,240 (933 to 1,620)	964 (731 to 1,280)	491 (359 to 676)	207 (144 to 294)	62·3 (42·5 to 91·4)	16·8 (10·9 to 25·6)	9,290
Leukemia	768 (543 to 1,060)	701 (490 to 967)	715 (513 to 965)	684 (501 to 918)	662 (477 to 892)	742 (544 to 988)	1,280 (934 to 1,700)	1,560 (1,140 to 2,120)	1,820 (1,310 to 2,430)	1,640 (1,210 to 2,190)	1,680 (1,230 to 2,240)	1,480 (1,100 to 1,940)	1,380 (1,050 to 1,770)	849 (625 to 1,120)	419 (295 to 549)	165 (119 to 216)	47·5 (30·1 to 69·3)	18,600
Acute lymphoid leukemia	401 (267 to 571)	271 (180 to 397)	172 (113 to 252)	143 (93·5 to 210)	110 (72·3 to 157)	126 (83·5 to 183)	154 (101 to 225)	174 (115 to 258)	196 (134 to 283)	138 (93·9 to 194)	160 (106 to 231)	126 (85·2 to 176)	111 (68·4 to 158)	69·2 (43·5 to 100)	25·5 (13·8 to 39·2)	13·1 (6·9 to 19·5)	3·8 (1·4 to 7·1)	3,640
Chronic lymphoid leukemia	0 (0 to 0)	17·7 (10·1 to 27·5)	12·1 (7·4 to 18·9)	14·1 (6·5 to 21·4)	18·3 (12 to 26·8)	36·7 (24·1 to 53·5)	43·8 (27·1 to 68·8)	153 (103 to 225)	252 (163 to 381)	275 (184 to 406)	357 (238 to 526)	325 (223 to 457)	324 (227 to 441)	215 (150 to 297)	112 (74·9 to 154)	48·1 (32·2 to 67·8)	10·8 (6·4 to 17·1)	2,210
Acute myeloid leukemia	289 (178 to 425)	288 (193 to 419)	368 (258 to 513)	332 (236 to 453)	335 (232 to 464)	345 (244 to 478)	645 (462 to 853)	686 (478 to 966)	696 (484 to 961)	601 (428 to 834)	518 (360 to 715)	411 (290 to 584)	331 (228 to 476)	178 (123 to 257)	71 (45·3 to 109)	24·6 (16·1 to 36·8)	6·2 (3·4 to 10·5)	6,750
Chronic myeloid leukemia	22·3 (11·5 to 40·7)	59·4 (36·7 to 93·9)	86·7 (55·2 to 129)	112 (74·2 to 166)	115 (75·3 to 168)	108 (69·4 to 158)	222 (145 to 322)	289 (187 to 420)	369 (249 to 517)	368 (245 to 525)	331 (222 to 470)	310 (212 to 427)	286 (204 to 396)	181 (124 to 257)	72·9 (48·3 to 104)	33·1 (22·3 to 46·3)	12·6 (8 to 18·1)	3,020
Other leukemia	56·6 (33·4 to 90·4)	65·3 (42·6 to 97·5)	76·3 (49·1 to 111)	83·2 (55·1 to 122)	84·3 (55·7 to 125)	127 (86·9 to 184)	218 (149 to 311)	254 (174 to 357)	303 (201 to 426)	253 (174 to 363)	310 (214 to 429)	312 (219 to 436)	325 (231 to 434)	206 (143 to 285)	138 (93·1 to 193)	46·3 (31·6 to 64·3)	14 (8·6 to 21·6)	3,030
Other malignant neoplasms	757 (512 to 1,030)	669 (458 to 893)	634 (475 to 807)	690 (510 to 886)	647 (486 to 840)	902 (676 to 1,200)	1,740 (1,290 to 2,290)	2,500 (1,810 to 3,350)	2,850 (2,100 to 3,780)	2,360 (1,700 to 3,070)	2,380 (1,740 to 3,050)	2,130 (1,600 to 2,700)	1,610 (1,200 to 2,080)	1,030 (773 to 1,330)	569 (434 to 735)	248 (181 to 326)	88·8 (63·3 to 117)	23,400
Other neoplasms	24·9 (13·4 to 36·7)	21·5 (11 to 31·2)	30·3 (14·5 to 46·2)	15·5 (9·2 to 24·6)	27·1 (14·3 to 40·9)	39·6 (27·2 to 55·2)	66·3 (45·3 to 93·6)	108 (71·5 to 160)	159 (102 to 231)	171 (112 to 240)	213 (143 to 293)	227 (153 to 315)	190 (132 to 277)	102 (73·5 to 163)	47·2 (32·7 to 83·5)	14·8 (9·2 to 31·9)	2·8 (1·6 to 6·7)	1,510
Myelodysplastic, myeloproliferative, and other hematopoie	24·9 (13·4 to 36·7)	21·5 (11 to 31·2)	30·3 (14·5 to 46·2)	15·5 (9·2 to 24·6)	27·1 (14·3 to 40·9)	39·6 (27·2 to 55·2)	66·3 (45·3 to 93·6)	108 (71·5 to 160)	159 (102 to 231)	171 (112 to 240)	213 (143 to 293)	227 (153 to 315)	190 (132 to 277)	102 (73·5 to 163)	47·2 (32·7 to 83·5)	14·8 (9·2 to 31·9)	2·8 (1·6 to 6·7)	1,510
Benign and in situ intestinal neoplasms	0 (0 to 0)	0 (0 to 0)	0 (0 to 0)	0 (0 to 0)	0 (0 to 0)	0 (0 to 0)	0 (0 to 0)	0 (0 to 0)	0 (0 to 0)	0 (0 to 0)	0 (0 to 0)	0 (0 to 0)	0 (0 to 0)	0 (0 to 0)	0 (0 to 0)	0 (0 to 0)	0 (0 to 0)	0
Benign and in situ cervical and uterine neoplasms	0 (0 to 0)	0 (0 to 0)	0 (0 to 0)	0 (0 to 0)	0 (0 to 0)	0 (0 to 0)	0 (0 to 0)	0 (0 to 0)	0 (0 to 0)	0 (0 to 0)	0 (0 to 0)	0 (0 to 0)	0 (0 to 0)	0 (0 to 0)	0 (0 to 0)	0 (0 to 0)	0 (0 to 0)	0
Other benign and in situ neoplasms	0 (0 to 0)	0 (0 to 0)	0 (0 to 0)	0 (0 to 0)	0 (0 to 0)	0 (0 to 0)	0 (0 to 0)	0 (0 to 0)	0 (0 to 0)	0 (0 to 0)	0 (0 to 0)	0 (0 to 0)	0 (0 to 0)	0 (0 to 0)	0 (0 to 0)	0 (0 to 0)	0 (0 to 0)	0
Cardiovascular diseases	1,500 (1,170 to 1,870)	2,120 (1,680 to 2,660)	3,270 (2,640 to 4,030)	5,500 (4,480 to 6,680)	7,230 (5,810 to 8,930)	13,400 (10,900 to 16,700)	29,300 (23,500 to 36,600)	49,900 (39,100 to 62,200)	67,300 (53,700 to 84,000)	68,500 (55,800 to 84,100)	82,100 (67,600 to 98,900)	94,100 (78,200 to 113,000)	93,900 (78,900 to 111,000)	78,700 (66,000 to 92,300)	53,000 (43,700 to 61,700)	31,300 (25,400 to 36,200)	15,800 (12,500 to 18,200)	698,000
Rheumatic heart disease	482 (308 to 744)	582 (385 to 858)	712 (474 to 1,010)	826 (573 to 1,150)	797 (578 to 1,070)	899 (655 to 1,220)	1,220 (881 to 1,650)	1,240 (901 to 1,690)	1,160 (846 to 1,590)	655 (481 to 888)	521 (390 to 690)	349 (262 to 461)	262 (200 to 337)	140 (106 to 181)	76·5 (56·8 to 97·9)	40·8 (30·2 to 52·1)	19·2 (14·1 to 24·7)	10,400
Ischemic heart disease	121 (85·9 to 158)	336 (236 to 471)	775 (586 to 989)	1,810 (1,400 to 2,270)	2,770 (2,170 to 3,520)	5,720 (4,460 to 7,130)	14,100 (11,000 to 18,100)	25,500 (20,100 to 32,100)	36,000 (28,500 to 45,500)	38,100 (30,600 to 47,100)	45,500 (37,200 to 55,800)	51,400 (42,200 to 62,000)	50,400 (42,000 to 60,200)	42,300 (35,200 to 50,800)	29,000 (24,000 to 34,000)	18,000 (14,500 to 20,900)	9,570 (7,490 to 11,000)	371,000
Stroke	418 (297 to 557)	617 (433 to 823)	888 (639 to 1,170)	1,570 (1,160 to 2,130)	2,130 (1,550 to 2,840)	3,850 (2,890 to 5,080)	8,190 (6,140 to 10,600)	13,800 (10,000 to 18,100)	18,000 (13,400 to 23,400)	18,000 (14,000 to 23,000)	22,100 (16,900 to 28,400)	26,400 (20,500 to 33,400)	27,900 (22,100 to 34,400)	23,400 (18,600 to 28,600)	15,100 (11,800 to 18,300)	7,950 (6,130 to 9,710)	3,520 (2,600 to 4,440)	194,000
Ischemic stroke	134 (89·9 to 190)	177 (121 to 246)	239 (168 to 326)	407 (294 to 550)	489 (356 to 647)	893 (674 to 1,160)	2,110 (1,600 to 2,720)	4,130 (3,070 to 5,340)	6,360 (4,910 to 8,230)	8,290 (6,550 to 10,600)	11,200 (8,700 to 14,400)	16,700 (13,100 to 21,000)	18,700 (14,500 to 23,400)	18,100 (14,400 to 22,200)	12,100 (9,400 to 14,700)	6,580 (5,060 to 8,020)	3,020 (2,230 to 3,810)	110,000
Intracerebral hemorrhage	142 (95·1 to 200)	229 (149 to 323)	357 (240 to 507)	691 (482 to 963)	1,040 (718 to 1,450)	2,070 (1,480 to 2,840)	4,590 (3,340 to 6,150)	7,690 (5,400 to 10,300)	9,470 (6,850 to 12,600)	8,210 (6,080 to 10,700)	9,360 (6,910 to 12,300)	8,570 (6,290 to 11,000)	8,210 (6,360 to 10,300)	4,750 (3,570 to 6,020)	2,700 (2,080 to 3,340)	1,230 (939 to 1,540)	445 (327 to 573)	69,800
Subarachnoid hemorrhage	142 (92·6 to 207)	211 (129 to 316)	292 (183 to 431)	474 (323 to 690)	596 (396 to 838)	887 (609 to 1,230)	1,490 (1,050 to 2,050)	2,020 (1,370 to 2,790)	2,210 (1,540 to 3,130)	1,490 (1,100 to 2,020)	1,540 (1,110 to 2,120)	1,130 (808 to 1,510)	978 (718 to 1,300)	580 (420 to 767)	315 (240 to 412)	147 (107 to 198)	57 (39·6 to 77·7)	14,700
Hypertensive heart disease	11·5 (7·2 to 18·4)	37·8 (22 to 58·1)	86·9 (48·3 to 140)	186 (105 to 276)	307 (182 to 451)	637 (374 to 934)	1,660 (967 to 2,470)	2,840 (1,590 to 4,070)	3,780 (2,080 to 5,340)	3,990 (2,100 to 5,560)	4,770 (2,630 to 6,420)	5,680 (3,040 to 7,720)	5,630 (2,890 to 7,600)	4,570 (2,390 to 5,990)	3,100 (1,590 to 4,010)	1,780 (905 to 2,300)	947 (446 to 1,260)	40,000
Non-rheumatic valvular heart disease	59·5 (42·1 to 81·4)	74·1 (51·4 to 104)	109 (72·5 to 157)	152 (108 to 207)	156 (109 to 210)	259 (185 to 355)	454 (319 to 628)	646 (465 to 893)	852 (598 to 1,160)	767 (555 to 1,030)	829 (623 to 1,090)	717 (542 to 944)	567 (431 to 746)	407 (310 to 531)	217 (164 to 279)	99·1 (73·7 to 128)	31·5 (22·1 to 41·1)	6,390
Non-rheumatic calcific aortic valve disease	31·1 (20 to 46)	49·9 (32·4 to 71·6)	58·2 (35·9 to 91)	68·2 (42·4 to 104)	72·6 (45·9 to 108)	129 (83·5 to 194)	235 (150 to 358)	376 (246 to 571)	510 (329 to 766)	508 (342 to 723)	582 (407 to 799)	539 (390 to 741)	439 (328 to 597)	320 (238 to 422)	169 (126 to 219)	74·6 (54·9 to 98·5)	22·7 (15·2 to 30·8)	4,180

Non-rheumatic degenerative mitral valve disease	0 (0 to 0)	[illegible]	23·4 (14·7 to 35)	8·5 (5·2 to 12·4)	2,170
Other non-rheumatic valve diseases	0 (0 to 0)	[illegible]	1·1 (0·2 to 2·5)	0·3 (0·1 to 0·9)	44·4
Cardiomyopathy and myocarditis	12 (4·1 to 17·8)	[illegible]	324 (230 to 436)	145 (102 to 199)	23,200
Myocarditis	2·3 (0·7 to 4·2)	[illegible]	19 (9 to 40·4)	14 (6·7 to 28·2)	788
Alcoholic cardiomyopathy	0 (0 to 0)	[illegible]	36·1 (18·2 to 61·7)	11·5 (6·4 to 18·9)	13,800
Other cardiomyopathy	9·7 (3·4 to 14·8)	[illegible]	269 (186 to 368)	119 (81·3 to 165)	8,580
Atrial fibrillation and flutter	0 (0 to 0)	[illegible]	1,780 (1,380 to 2,250)	879 (661 to 1,130)	15,300
Aortic aneurysm	--	[illegible]	382 (282 to 501)	130 (88·5 to 171)	NA
Endocarditis	2·7 (0·4 to 5·4)	[illegible]	32 (20·8 to 52·6)	16·3 (9·8 to 28·7)	2,310
Other cardiovascular and circulatory diseases	2·9 (1·1 to 8·1)	[illegible]	374 (279 to 466)	180 (126 to 232)	12,500
Chronic respiratory diseases	0 (0 to 0)	[illegible]	3,080 (2,530 to 3,650)	1,360 (1,080 to 1,620)	131,000
Chronic obstructive pulmonary disease	0 (0 to 0)	[illegible]	2,950 (2,410 to 3,500)	1,300 (1,030 to 1,550)	93,500
Pneumoconiosis	0 (0 to 0)	[illegible]	2·8 (2 to 3·9)	1·1 (0·8 to 1·6)	67·3
Silicosis	0 (0 to 0)	[illegible]	1·4 (0·9 to 2·2)	0·3 (0·2 to 0·5)	34·4
Asbestosis	0 (0 to 0)	[illegible]	0·2 (0·1 to 0·4)	0·1 (0 to 0·2)	6·8
Coal workers pneumoconiosis	0 (0 to 0)	[illegible]	0·1 (0·1 to 0·2)	0 (0 to 0·1)	2·1
Other pneumoconiosis	0 (0 to 0)	[illegible]	1·1 (0·6 to 1·7)	0·7 (0·4 to 1·1)	24
Asthma	0 (0 to 0)	[illegible]	98·1 (71·1 to 128)	44·8 (32·1 to 58·7)	33,000
Interstitial lung disease and pulmonary sarcoidosis	0 (0 to 0)	[illegible]	23·5 (13·7 to 41)	10·2 (5·6 to 18·3)	1,780
Other chronic respiratory diseases	0 (0 to 0)	[illegible]	7·7 (5·5 to 11·2)	4·2 (2·8 to 6·2)	2,110
Digestive diseases	36·9 (26 to 48·9)	[illegible]	1,820 (1,460 to 2,170)	718 (560 to 852)	137,000
Cirrhosis and other chronic liver diseases	0 (0 to 0)	[illegible]	289 (214 to 367)	103 (76 to 129)	56,100
Cirrhosis and other chronic liver diseases due to hepatitis	0 (0 to 0)	[illegible]	14·4 (6·8 to 25·1)	5·1 (1·8 to 10·5)	3,950
Cirrhosis and other chronic liver diseases due to hepatitis	0 (0 to 0)	[illegible]	48·7 (25·8 to 81·8)	17·9 (7·2 to 32·2)	10,700
Cirrhosis and other chronic liver diseases due to alcohol u	0 (0 to 0)	[illegible]	86·4 (51·7 to 131)	29 (13·8 to 48·5)	25,500
Cirrhosis and other chronic liver diseases due to NAFLD	0 (0 to 0)	[illegible]	73 (39·3 to 116)	26·7 (11·6 to 45)	8,960
Cirrhosis and other chronic liver diseases due to other cau	0 (0 to 0)	[illegible]	66·2 (35·4 to 107)	24·1 (11·1 to 41·1)	6,960
Upper digestive system diseases	0 (0 to 0)	[illegible]	499 (394 to 625)	190 (147 to 240)	32,100
Peptic ulcer disease	0 (0 to 0)	[illegible]	280 (212 to 355)	111 (83·3 to 143)	7,920
Gastritis and duodenitis	0 (0 to 0)	[illegible]	104 (71·8 to 141)	35·2 (24 to 47·7)	6,430
Gastroesophageal reflux disease	0 (0 to 0)	[illegible]	114 (58·2 to 202)	43·6 (21 to 77·4)	17,800
Appendicitis	0 (0 to 0)	[illegible]	40·5 (27·6 to 58·4)	14·6 (9·8 to 20·2)	2,790
Paralytic ileus and intestinal obstruction	25·1 (18·3 to 35·2)	[illegible]	290 (180 to 393)	130 (87·3 to 170)	8,970
Inguinal, femoral, and abdominal hernia	11·8 (3·2 to 21·9)	[illegible]	133 (93·5 to 175)	54·9 (38·7 to 72)	10,100
Inflammatory bowel disease	0 (0 to 0)	[illegible]	20·7 (14·9 to 28·2)	6·1 (4·3 to 8·4)	1,680
Vascular intestinal disorders	0 (0 to 0)	[illegible]	220 (156 to 299)	85·5 (58·4 to 115)	6,970
Gallbladder and biliary diseases	0 (0 to 0)	[illegible]	178 (131 to 236)	71·4 (53·2 to 93·5)	8,860
Pancreatitis	0 (0 to 0)	[illegible]	41 (28·9 to 60·2)	14·6 (9·9 to 25·5)	5,920
Other digestive diseases	0 (0 to 0)	[illegible]	112 (67·8 to 154)	48·1 (28·9 to 66·4)	3,760
Neurological disorders	1 (0·2 to 2·3)	[illegible]	8,950 (4,450 to 18,700)	5,410 (2,520 to 11,700)	167,000
Alzheimer's disease and other dementias	0 (0 to 0)	[illegible]	7,900 (3,380 to 17,800)	5,070 (2,140 to 11,400)	58,300
Parkinson's disease	0 (0 to 0)	[illegible]	820 (667 to 966)	262 (205 to 313)	14,700
Idiopathic epilepsy	1 (0·2 to 2·3)	[illegible]	80·2 (26·5 to 164)	33·8 (10·6 to 69·4)	13,200
Multiple sclerosis	0 (0 to 0)	[illegible]	5·2 (3·9 to 6·8)	2·2 (1·4 to 2·9)	2,100
Motor neuron disease	0 (0 to 0·1)	[illegible]	8·8 (6·5 to 11·3)	5·8 (4·4 to 7·3)	3,200
Headache disorders	0 (0 to 0)	[illegible]	112 (36·2 to 235)	32·4 (7·2 to 77·7)	67,000
Migraine	0 (0 to 0)	[illegible]	90·1 (25·8 to 196)	24·6 (5·3 to 55·9)	60,100
Tension-type headache	0 (0 to 0)	[illegible]	22·3 (4·3 to 84·6)	7·8 (0·6 to 41·2)	6,930
Other neurological disorders	0 (0 to 0)	[illegible]	23·6 (17·8 to 30·2)	9·8 (7·3 to 12·4)	9,060
Mental disorders	1·7 (1·1 to 2·4)	[illegible]	1,110 (737 to 1,550)	449 (277 to 668)	234,000
Schizophrenia	0 (0 to 0)	[illegible]	21·9 (10·8 to 35·7)	5·6 (2·4 to 10·2)	24,500
Depressive disorders	0 (0 to 0)	[illegible]	782 (475 to 1,190)	332 (180 to 533)	97,300
Major depressive disorder	0 (0 to 0)	[illegible]	728 (432 to 1,120)	310 (162 to 509)	84,900
Dysthymia	0 (0 to 0)	[illegible]	53·6 (29·4 to 89·5)	21·8 (11·4 to 38·1)	12,400
Bipolar disorder	0 (0 to 0)	[illegible]	30·4 (16 to 51·4)	10·1 (5 to 17·7)	26,000
Anxiety disorders	0 (0 to 0)	[illegible]	144 (81·1 to 223)	47·7 (25·5 to 77·3)	50,500
Eating disorders	0 (0 to 0)	[illegible]	0 (0 to 0)	0 (0 to 0)	4,000
Anorexia nervosa	0 (0 to 0)	[illegible]	0 (0 to 0)	0 (0 to 0)	935
Bulimia nervosa	0 (0 to 0)	[illegible]	0 (0 to 0)	0 (0 to 0)	3,060
Autism spectrum disorders	1·4 (0·9 to 2)	[illegible]	16·2 (10·3 to 24·5)	6 (3·8 to 9·1)	5,670
Attention-deficit/hyperactivity disorder	0 (0 to 0)	[illegible]	0 (0 to 0)	0 (0 to 0)	3,340
Conduct disorder	0 (0 to 0)	[illegible]	0 (0 to 0)	0 (0 to 0)	4,870
Idiopathic developmental intellectual disability	0·3 (0 to 0·7)	[illegible]	2·6 (0·8 to 5·2)	0·8 (0·2 to 1·6)	1,920
Other mental disorders	0 (0 to 0)	[illegible]	111 (72 to 160)	46·5 (30·7 to 67)	15,700
Substance use disorders	0 (0 to 0)	[illegible]	161 (108 to 233)	93·6 (61·1 to 140)	49,900
Alcohol use disorders	0 (0 to 0)	[illegible]	129 (82·5 to 194)	76·8 (46 to 122)	38,000
Drug use disorders	0 (0 to 0)	[illegible]	32·6 (21·4 to 47·2)	16·8 (10·9 to 24·9)	11,900
Opioid use disorders	0 (0 to 0)	[illegible]	22·9 (13·1 to 36·4)	12·6 (7 to 20·4)	6,200
Cocaine use disorders	0 (0 to 0)	[illegible]	2·9 (1·8 to 4·3)	1 (0·6 to 1·6)	1,790
Amphetamine use disorders	0 (0 to 0)	[illegible]	0·4 (0·2 to 0·9)	0·2 (0·1 to 0·5)	668
Cannabis use disorders	0 (0 to 0)	[illegible]	0·2 (0·1 to 0·3)	0 (0 to 0·1)	1,530
Other drug use disorders	0 (0 to 0)	[illegible]	6·1 (4·6 to 7·7)	2·9 (2·2 to 3·8)	1,690
Diabetes and kidney diseases	0·7 (0·1 to 1)	[illegible]	4,320 (3,510 to 5,260)	1,680 (1,360 to 2,060)	243,000
Diabetes mellitus	0·7 (0·1 to 1)	[illegible]	2,820 (2,140 to 3,640)	1,050 (795 to 1,360)	163,000
Diabetes mellitus type 1	0·7 (0·1 to 1)	[illegible]	22·8 (14·9 to 37·4)	9·3 (6 to 15)	3,640
Diabetes mellitus type 2	0 (0 to 0)	[illegible]	2,800 (2,130 to 3,600)	1,040 (786 to 1,350)	160,000
Chronic kidney disease	0 (0 to 0)	[illegible]	1,500 (1,170 to 1,880)	629 (493 to 772)	79,300
Chronic kidney disease due to diabetes mellitus type 1	0 (0 to 0)	[illegible]	1·1 (0·3 to 2·8)	0·1 (0 to 0·3)	4,410
Chronic kidney disease due to diabetes mellitus type 2	0 (0 to 0)	[illegible]	339 (210 to 499)	132 (72·3 to 200)	18,800
Chronic kidney disease due to hypertension	0 (0 to 0)	[illegible]	589 (422 to 780)	252 (182 to 327)	18,000
Chronic kidney disease due to glomerulonephritis	0 (0 to 0)	[illegible]	95·1 (43·9 to 168)	34·6 (14 to 69·2)	15,900
Chronic kidney disease due to other and unspecified caus	0 (0 to 0)	[illegible]	479 (327 to 660)	210 (139 to 298)	22,300
Acute glomerulonephritis	0 (0 to 0)	[illegible]	0·4 (0·2 to 0·6)	0 (0 to 0·1)	28·7
Skin and subcutaneous diseases	0·1 (0·1 to 0·2)	[illegible]	714 (475 to 1,160)	327 (217 to 586)	60,400
Dermatitis	0 (0 to 0)	[illegible]	52·6 (30·9 to 83)	20·6 (11·9 to 33·1)	11,800
Atopic dermatitis	0 (0 to 0)	[illegible]	26 (14·2 to 44·1)	10·2 (5·5 to 17·1)	7,530
Contact dermatitis	0 (0 to 0)	[illegible]	22·5 (10·3 to 41·3)	9 (3·8 to 17·9)	3,800
Seborrhoeic dermatitis	0 (0 to 0)	[illegible]	4 (2 to 6·9)	1·4 (0·7 to 2·3)	434
Psoriasis	0 (0 to 0)	[illegible]	12·2 (7 to 18)	3·8 (2·1 to 5·9)	4,570
Bacterial skin diseases	0 (0 to 0)	[illegible]	161 (87·4 to 446)	90·7 (46·4 to 264)	5,940
Cellulitis	0 (0 to 0)	[illegible]	48·3 (20·5 to 182)	29·4 (12·4 to 105)	2,150
Pyoderma	0 (0 to 0)	[illegible]	113 (63·3 to 290)	61·3 (31·9 to 169)	3,800
Scabies	0·1 (0 to 0·1)	[illegible]	46·8 (24·5 to 80·4)	18·7 (9·4 to 32·4)	8,470
Fungal skin diseases	0 (0 to 0)	[illegible]	173 (70·4 to 360)	76·2 (30·9 to 155)	6,150
Viral skin diseases	0 (0 to 0)	[illegible]	5 (2·9 to 7·8)	1·8 (1 to 2·8)	4,040
Acne vulgaris	0 (0 to 0)	[illegible]	0·6 (0·3 to 0·9)	0·1 (0·1 to 0·2)	4,010
Alopecia areata	0 (0 to 0)	[illegible]	1·2 (0·6 to 2·1)	0·3 (0·1 to 0·5)	856
Pruritus	0 (0 to 0)	[illegible]	10·8 (4·8 to 20·7)	4·4 (1·9 to 8·6)	1,140
Urticaria	0 (0 to 0·1)	[illegible]	11·6 (6·5 to 18·6)	4·3 (2·2 to 7·6)	4,750
Decubitus ulcer	0 (0 to 0)	[illegible]	165 (104 to 312)	81·2 (51·6 to 170)	1,660
Other skin and subcutaneous diseases	0 (0 to 0)	[illegible]	74·3 (39·4 to 127)	25 (13 to 43·3)	7,020
Sense organ diseases	0·4 (0·2 to 0·6)	[illegible]	4,540 (3,250 to 6,000)	1,990 (1,430 to 2,600)	139,000
Blindness and vision loss	0·2 (0·1 to 0·3)	[illegible]	1,860 (1,300 to 2,500)	894 (626 to 1,230)	46,100
Glaucoma	0 (0 to 0)	[illegible]	239 (143 to 362)	112 (64·3 to 181)	3,150
Cataract	0 (0 to 0)	[illegible]	690 (462 to 961)	320 (201 to 475)	10,200
Age-related macular degeneration	0 (0 to 0)	[illegible]	17·3 (10·2 to 27·4)	7·6 (4·4 to 12·1)	368
Refraction disorders	0 (0 to 0)	[illegible]	93 (59·4 to 138)	30·5 (17·3 to 48·2)	9,950
Near vision loss	0 (0 to 0)	[illegible]	233 (97·5 to 458)	96·1 (37·1 to 202)	9,130
Other vision loss	0·2 (0·1 to 0·3)	[illegible]	592 (400 to 839)	328 (206 to 477)	13,200
Age-related and other hearing loss	0 (0 to 0·1)	[illegible]	2,600 (1,830 to 3,490)	1,050 (744 to 1,410)	87,200
Other sense organ diseases	0·1 (0·1 to 0·3)	[illegible]	76·2 (35·9 to 137)	41·8 (19·3 to 74·4)	5,690
Musculoskeletal disorders	0 (0 to 0)	[illegible]	1,850 (1,250 to 2,640)	732 (508 to 1,040)	245,000
Rheumatoid arthritis	0 (0 to 0)	[illegible]	75 (50·3 to 135)	30·4 (19·5 to 57·5)	6,390
Osteoarthritis	0 (0 to 0)	[illegible]	630 (327 to 1,250)	246 (126 to 482)	38,000
Osteoarthritis hip	0 (0 to 0)	[illegible]	37·1 (17·8 to 77·9)	14·7 (6·8 to 30·7)	1,950
Osteoarthritis knee	0 (0 to 0)	[illegible]	373 (183 to 752)	135 (66·6 to 267)	27,000
Osteoarthritis hand	0 (0 to 0)	[illegible]	105 (51·1 to 217)	46·5 (23·2 to 95·7)	4,550
Osteoarthritis other	0 (0 to 0)	[illegible]	116 (58·7 to 233)	49·8 (25·8 to 99)	4,460
Low back pain	0 (0 to 0)	[illegible]	896 (575 to 1,320)	333 (216 to 493)	112,000
Neck pain	0 (0 to 0)	[illegible]	121 (62·5 to 205)	53·3 (28·3 to 94)	20,600
Gout	0 (0 to 0)	[illegible]	26·7 (14·2 to 44·2)	12·8 (6·6 to 21·5)	1,320
Other musculoskeletal disorders	0 (0 to 0)	[illegible]	97·9 (60 to 186)	56·9 (33·7 to 108)	67,200
Other non-communicable diseases	2,900 (1,860 to 6,510)	[illegible]	1,440 (1,090 to 1,900)	569 (417 to 767)	178,000
Congenital birth defects	2,840 (1,820 to 6,450)	[illegible]	13·3 (8·1 to 21·3)	5·1 (3·1 to 8)	26,100
Neural tube defects	74·2 (36·6 to 144)	[illegible]	0·3 (0·2 to 0·5)	0·1 (0·1 to 0·2)	1,450
Congenital heart anomalies	1,080 (577 to 2,800)	[illegible]	1·4 (0·5 to 2·9)	0·6 (0·2 to 1·2)	8,200
Orofacial clefts	0·6 (0·3 to 1·2)	[illegible]	1·4 (0·7 to 2·3)	0·5 (0·3 to 0·9)	312
Down syndrome	31·5 (13·6 to 67·4)	[illegible]	0 (0 to 0)	0 (0 to 0)	2,050
Turner syndrome	0 (0 to 0)	[illegible]	0 (0 to 0)	0 (0 to 0)	9·9
Klinefelter syndrome	0 (0 to 0)	[illegible]	0 (0 to 0)	0 (0 to 0)	1·1
Other chromosomal abnormalities	157 (68·6 to 298)	[illegible]	0 (0 to 0)	0 (0 to 0)	1,120
Congenital musculoskeletal and limb anomalies	67·3 (29·4 to 222)	[illegible]	6·5 (3·6 to 10·8)	2·4 (1·3 to 3·8)	3,940
Urogenital congenital anomalies	70·9 (29·4 to 297)	[illegible]	0 (0 to 0)	0 (0 to 0)	411
Digestive congenital anomalies	651 (341 to 1,490)	[illegible]	0·1 (0 to 0·1)	0 (0 to 0)	2,560
Other congenital birth defects	712 (375 to 1,410)	[illegible]	3·6 (1 to 9·3)	1·4 (0·4 to 3·8)	6,000
Urinary diseases and male infertility	0·4 (0·1 to 1·4)	[illegible]	278 (209 to 360)	97·5 (69·1 to 146)	14,300
Urolithiasis	0 (0 to 0)	[illegible]	10·1 (6·3 to 15·8)	2·5 (1·3 to 4·2)	1,590
Benign prostatic hyperplasia	0 (0 to 0)	[illegible]	59·8 (32·7 to 97·8)	16·2 (8·7 to 26·6)	4,800
Male infertility	0 (0 to 0)	[illegible]	0 (0 to 0)	0 (0 to 0)	461
Other urinary diseases	0·2 (0·1 to 0·8)	[illegible]	19 (8·3 to 50·5)	6·7 (2·9 to 18)	1,250
Gynecological diseases	0 (0 to 0)	[illegible]	112 (69·3 to 168)	47·4 (25·8 to 78·1)	46,100
Uterine fibroids	0 (0 to 0)	[illegible]	2·5 (1·2 to 4·8)	0·7 (0·3 to 1·4)	3,750
Polycystic ovarian syndrome	0 (0 to 0)	[illegible]	0 (0 to 0)	0 (0 to 0)	739
Female infertility	0 (0 to 0)	[illegible]	0 (0 to 0)	0 (0 to 0)	786
Endometriosis	0 (0 to 0)	[illegible]	0 (0 to 0)	0 (0 to 0)	3,290
Genital prolapse	0 (0 to 0)	[illegible]	14·2 (7 to 26·2)	6·5 (3·3 to 11·7)	1,050
Premenstrual syndrome	0 (0 to 0)	[illegible]	0 (0 to 0)	0 (0 to 0)	10,500
Other gynecological diseases	0 (0 to 0)	[illegible]	94·9 (53·2 to 151)	40·2 (19·8 to 71·2)	26,000
Hemoglobinopathies and hemolytic anemias	1·1 [illegible]	[illegible]	51 [illegible]	21 [illegible]	[illegible]
Thalassemias	[illegible]	[illegible]	[illegible]	[illegible]	[illegible]
Thalassemias trait	[illegible]	[illegible]	[illegible]	[illegible]	[illegible]
Sickle cell disorders	[illegible]	[illegible]	[illegible]	[illegible]	[illegible]
Sickle cell trait	[illegible]	[illegible]	[illegible]	[illegible]	[illegible]

G6PD deficiency	0 (0 to 0·1)	0·7 (0·3 to 1·6)	0·7 (0·3 to 1·2)	5·6 (3 to 9·4)	9·9 (4·6 to 16·8)	9·6 (4·5 to 17)	72·7 (30·9 to 135)	47·8 (21·3 to 84·1)	65·1 (33·9 to 104)	79·2 (40·2 to 131)	106 (63·2 to 164)	86·9 (48·7 to 137)	106 (58·2 to 163)	96·7 (58·3 to 148)	88 (51·1 to 137)	47·4 (26·5 to 75)	24·2 (12·5 to 38·7)	14·6 (7·2 to 24·2)	7·5 (3·6 to 12·7)	2·2 (1·1 to 3·5)	1·1 (0·6 to 1·8)	0·3 (0·2 to 0·5)	0·1 (0 to 0·1)	872
G6PD trait	0 (0 to 0)	0 (0 to 0·1)	0 (0 to 0)	0 (0 to 0)	0 (0 to 0)	0 (0 to 0)	6·3 (1·6 to 15·1)	0·1 (0 to 0·3)	0·1 (0 to 0·3)	0·3 (0·1 to 0·4)	6·3 (1·9 to 12·6)	6·6 (1·4 to 14·1)	0·3 (0·1 to 0·6)	0 (0 to 0)	0 (0 to 0)	0 (0 to 0)	0 (0 to 0)	0 (0 to 0)	0 (0 to 0)	0 (0 to 0)	0 (0 to 0)	0 (0 to 0)	0 (0 to 0)	20·1
Other hemoglobinopathies and hemolytic anemias	0 (0 to 0·1)	2·4 (1·1 to 4·2)	14·9 (6·3 to 29·6)	62·6 (25·7 to 124)	97·4 (34·2 to 194)	37·9 (12·5 to 84·6)	83·6 (34·6 to 166)	68·6 (27·8 to 146)	81·7 (38 to 164)	100 (47·1 to 189)	118 (59·2 to 210)	140 (73·6 to 247)	186 (101 to 308)	246 (142 to 393)	266 (167 to 410)	240 (145 to 390)	166 (107 to 250)	150 (103 to 224)	132 (93·1 to 189)	79·7 (52·3 to 120)	73·6 (50·3 to 112)	41·6 (28·1 to 61·3)	18·4 (12·1 to 28·4)	2,410
Endocrine, metabolic, blood, and immune disorders	55·3 (31·2 to 92·9)	93·2 (57·4 to 135)	834 (551 to 1,180)	613 (429 to 839)	525 (365 to 739)	393 (275 to 564)	466 (303 to 694)	490 (317 to 738)	711 (457 to 1,100)	1,000 (634 to 1,560)	1,160 (734 to 1,740)	1,660 (1,040 to 2,520)	2,930 (1,860 to 4,350)	3,770 (2,420 to 5,730)	3,990 (2,540 to 6,100)	3,070 (1,950 to 4,830)	2,860 (1,760 to 4,540)	2,570 (1,590 to 4,030)	2,010 (1,230 to 3,100)	1,230 (752 to 1,930)	664 (413 to 1,120)	307 (196 to 490)	126 (75·5 to 222)	31,500
Oral disorders	0 (0 to 0)	0 (0 to 0)	0 (0 to 0)	279 (156 to 458)	384 (207 to 674)	390 (205 to 693)	636 (346 to 1,110)	1,040 (545 to 1,900)	1,640 (829 to 3,120)	2,240 (1,090 to 4,160)	2,240 (1,060 to 4,220)	2,770 (1,340 to 5,040)	4,420 (2,310 to 7,810)	5,600 (3,050 to 9,550)	6,160 (3,570 to 9,900)	5,320 (3,310 to 7,950)	5,600 (3,600 to 8,210)	5,250 (3,400 to 7,600)	4,100 (2,700 to 5,850)	2,630 (1,760 to 3,700)	1,460 (951 to 2,050)	681 (451 to 959)	273 (177 to 383)	53,100
Caries of deciduous teeth	0 (0 to 0)	0 (0 to 0)	0 (0 to 0)	92·8 (35·1 to 187)	97·7 (40·2 to 218)	4·9 (0·7 to 13·1)	0 (0 to 0)	0 (0 to 0)	0 (0 to 0)	0 (0 to 0)	0 (0 to 0)	0 (0 to 0)	0 (0 to 0)	0 (0 to 0)	0 (0 to 0)	0 (0 to 0)	0 (0 to 0)	0 (0 to 0)	0 (0 to 0)	0 (0 to 0)	0 (0 to 0)	0 (0 to 0)	0 (0 to 0)	195
Caries of permanent teeth	0 (0 to 0)	0 (0 to 0)	0 (0 to 0)	0 (0 to 0)	98·5 (35·4 to 217)	197 (80 to 423)	237 (95·6 to 495)	277 (115 to 561)	298 (120 to 606)	295 (119 to 625)	249 (98 to 510)	252 (107 to 522)	335 (137 to 704)	353 (147 to 730)	307 (121 to 671)	202 (78·6 to 418)	151 (62·7 to 306)	114 (47·6 to 228)	78·4 (33·9 to 154)	46·9 (19·2 to 92·6)	24·4 (9·6 to 49·3)	10·6 (3·9 to 22·5)	3·7 (1·4 to 8·2)	3,530
Periodontal diseases	0 (0 to 0)	0 (0 to 0)	0 (0 to 0)	0 (0 to 0)	0 (0 to 0)	0 (0 to 0)	80·2 (23·4 to 202)	312 (94·5 to 800)	750 (238 to 1,810)	1,210 (410 to 2,740)	1,280 (459 to 2,950)	1,600 (584 to 3,480)	2,370 (909 to 5,030)	2,510 (997 to 5,300)	2,070 (827 to 4,320)	1,250 (500 to 2,610)	948 (369 to 1,940)	714 (284 to 1,430)	503 (198 to 997)	313 (122 to 612)	174 (66·3 to 343)	80 (30·3 to 162)	30·6 (11·4 to 62·2)	16,200
Other oral disorders	0 (0 to 0)	0 (0 to 0)	0 (0 to 0)	187 (110 to 282)	188 (108 to 289)	188 (109 to 289)	319 (193 to 492)	404 (250 to 622)	479 (287 to 719)	526 (314 to 792)	443 (264 to 673)	483 (292 to 721)	679 (411 to 1,010)	714 (443 to 1,070)	615 (369 to 907)	409 (242 to 610)	351 (213 to 528)	267 (162 to 401)	161 (97·7 to 242)	82·1 (50·9 to 124)	36·3 (22·4 to 53·5)	14·9 (9·3 to 21·8)	6·1 (3·8 to 9·2)	6,550
Sudden infant death syndrome	--	40·8 (26·7 to 58)	301 (182 to 458)	--	--	--	--	--	--	--	--	--	--	--	--	--	--	--	--	--	--	--	--	NA
Injuries	165 (96·1 to 240)	119 (86 to 158)	1,140 (831 to 1,550)	2,580 (2,080 to 3,120)	3,950 (3,310 to 4,670)	5,630 (4,710 to 6,650)	13,300 (11,200 to 15,700)	18,200 (15,400 to 21,400)	21,700 (18,500 to 25,200)	23,100 (19,500 to 27,100)	19,200 (15,900 to 23,000)	21,000 (17,400 to 25,300)	27,800 (22,500 to 34,000)	29,200 (23,800 to 35,200)	26,200 (21,400 to 32,100)	18,400 (15,000 to 22,400)	17,500 (14,300 to 21,200)	18,000 (15,000 to 21,600)	18,000 (14,900 to 21,400)	17,500 (14,400 to 20,900)	14,600 (12,000 to 17,300)	9,630 (7,860 to 11,400)	5,070 (4,120 to 5,950)	332,000
Transport injuries	4·5 (2·8 to 6·1)	4·4 (3·1 to 6)	84·9 (59 to 116)	503 (371 to 646)	1,010 (788 to 1,260)	1,450 (1,130 to 1,790)	3,410 (2,560 to 4,380)	4,650 (3,630 to 5,860)	5,760 (4,570 to 7,150)	5,680 (4,520 to 6,940)	4,650 (3,650 to 5,730)	5,110 (4,080 to 6,400)	6,400 (5,040 to 8,010)	6,520 (5,110 to 8,150)	5,780 (4,590 to 7,100)	3,640 (2,910 to 4,450)	3,130 (2,500 to 3,800)	2,510 (2,030 to 3,070)	1,880 (1,530 to 2,270)	1,140 (902 to 1,400)	595 (472 to 725)	265 (208 to 327)	110 (85·8 to 137)	64,300
Road injuries	4·2 (2·6 to 5·7)	3·7 (2·5 to 5·1)	76·4 (51·1 to 106)	458 (328 to 600)	931 (717 to 1,170)	1,340 (1,030 to 1,670)	3,220 (2,400 to 4,160)	4,370 (3,390 to 5,560)	5,430 (4,280 to 6,770)	5,320 (4,230 to 6,540)	4,360 (3,420 to 5,390)	4,730 (3,780 to 5,940)	5,970 (4,700 to 7,490)	6,070 (4,760 to 7,590)	5,330 (4,250 to 6,610)	3,420 (2,730 to 4,190)	2,960 (2,360 to 3,600)	2,350 (1,880 to 2,880)	1,780 (1,430 to 2,150)	1,080 (859 to 1,330)	563 (447 to 688)	249 (196 to 308)	102 (80·1 to 128)	60,100
Pedestrian road injuries	1·2 (0·8 to 1·7)	0·6 (0·4 to 0·9)	12·7 (8·1 to 18·1)	192 (132 to 263)	446 (331 to 583)	525 (383 to 683)	612 (444 to 803)	647 (492 to 819)	795 (627 to 992)	840 (649 to 1,060)	776 (599 to 975)	964 (753 to 1,220)	1,380 (1,060 to 1,790)	1,500 (1,160 to 1,940)	1,510 (1,170 to 1,930)	1,050 (826 to 1,330)	1,040 (818 to 1,300)	938 (739 to 1,180)	784 (617 to 970)	517 (406 to 643)	277 (216 to 341)	119 (92·4 to 146)	49·5 (38·8 to 60·7)	15,000
Cyclist road injuries	0 (0 to 0)	0 (0 to 0)	0 (0 to 0)	45·7 (27·3 to 71·3)	109 (71·2 to 159)	316 (219 to 436)	683 (454 to 1,020)	766 (539 to 1,060)	836 (597 to 1,150)	875 (619 to 1,180)	803 (578 to 1,090)	892 (661 to 1,170)	1,170 (874 to 1,530)	1,330 (970 to 1,720)	1,230 (902 to 1,630)	826 (625 to 1,060)	695 (518 to 892)	541 (403 to 720)	374 (283 to 497)	207 (153 to 271)	101 (71·2 to 135)	44·5 (30·9 to 60·4)	17·4 (12·1 to 23·8)	11,900
Motorcyclist road injuries	0·1 (0·1 to 0·2)	0·2 (0·1 to 0·2)	6·6 (4·3 to 8·9)	25·1 (18·1 to 33·2)	51·8 (36·7 to 70·5)	68·6 (52·5 to 86·7)	455 (336 to 595)	918 (711 to 1,160)	1,290 (1,010 to 1,620)	1,300 (1,030 to 1,630)	943 (733 to 1,180)	820 (647 to 1,040)	1,050 (819 to 1,320)	982 (775 to 1,240)	835 (649 to 1,050)	478 (368 to 609)	397 (301 to 518)	288 (210 to 383)	219 (157 to 296)	143 (100 to 198)	83 (57·7 to 116)	41·3 (28·5 to 57·6)	18 (12·3 to 25·2)	10,400
Motor vehicle road injuries	2·4 (1·4 to 3·2)	2·7 (1·8 to 3·9)	50·7 (33·5 to 70·3)	150 (105 to 200)	263 (188 to 348)	354 (247 to 474)	1,320 (929 to 1,780)	1,780 (1,260 to 2,330)	2,180 (1,620 to 2,810)	1,970 (1,400 to 2,550)	1,540 (1,120 to 1,970)	1,710 (1,280 to 2,230)	1,880 (1,400 to 2,450)	1,790 (1,330 to 2,330)	1,350 (1,020 to 1,720)	823 (614 to 1,040)	600 (451 to 754)	408 (310 to 505)	268 (208 to 334)	146 (111 to 186)	71·8 (54·1 to 93·1)	32·6 (24·6 to 42·1)	13·4 (10·1 to 17·3)	18,700
Other road injuries	0·4 (0·2 to 0·8)	0·2 (0·1 to 0·3)	6·4 (3·3 to 11·7)	44·9 (21·5 to 77·6)	60·4 (33·6 to 100)	74·1 (44·2 to 115)	143 (86 to 234)	264 (163 to 423)	331 (206 to 526)	325 (210 to 508)	300 (199 to 442)	345 (225 to 502)	479 (329 to 711)	467 (319 to 669)	408 (280 to 571)	238 (169 to 320)	232 (165 to 313)	173 (125 to 238)	131 (96·5 to 174)	67·5 (49·4 to 89·2)	30·8 (22·3 to 41·3)	12 (8·5 to 16·1)	4·2 (2·9 to 5·8)	4,140
Other transport injuries	0·3 (0·2 to 0·5)	0·7 (0·5 to 1·1)	8·5 (5·8 to 12·2)	44·7 (30·7 to 63)	76·1 (53·5 to 104)	115 (82·8 to 153)	193 (135 to 270)	275 (190 to 386)	331 (234 to 454)	368 (259 to 508)	292 (205 to 407)	377 (268 to 511)	432 (312 to 585)	453 (327 to 607)	446 (323 to 597)	216 (164 to 281)	166 (127 to 213)	164 (121 to 211)	108 (80·9 to 139)	60·3 (45·4 to 78·8)	31·8 (24 to 41·2)	15·4 (11·5 to 20·1)	7·4 (5·6 to 9·7)	4,180
Unintentional injuries	50·2 (31·3 to 69·1)	95·4 (68·2 to 131)	951 (674 to 1,330)	1,870 (1,460 to 2,350)	2,710 (2,170 to 3,350)	3,440 (2,720 to 4,290)	5,180 (4,100 to 6,530)	5,770 (4,520 to 7,290)	6,860 (5,450 to 8,800)	7,660 (5,990 to 10,000)	6,690 (5,130 to 8,890)	7,870 (6,050 to 10,400)	11,200 (8,660 to 14,700)	13,100 (10,000 to 17,000)	12,400 (9,640 to 16,100)	9,630 (7,500 to 12,400)	10,300 (8,160 to 12,900)	12,100 (9,790 to 14,600)	13,600 (11,200 to 16,400)	14,900 (12,100 to 17,900)	13,200 (10,800 to 15,700)	9,130 (7,450 to 10,900)	4,880 (3,960 to 5,740)	174,000
Falls	1·9 (1·1 to 2·6)	2·1 (1·4 to 2·9)	65·5 (45·8 to 91·4)	204 (154 to 266)	355 (264 to 470)	528 (401 to 696)	935 (702 to 1,230)	1,180 (882 to 1,550)	1,460 (1,100 to 1,980)	1,720 (1,310 to 2,270)	1,730 (1,310 to 2,300)	2,200 (1,680 to 2,890)	3,630 (2,820 to 4,640)	4,810 (3,790 to 6,030)	5,110 (4,000 to 6,430)	4,590 (3,650 to 5,650)	5,830 (4,690 to 7,220)	8,150 (6,660 to 9,950)	10,500 (8,500 to 12,600)	12,600 (10,200 to 15,200)	11,800 (9,610 to 14,000)	8,190 (6,610 to 9,810)	4,350 (3,510 to 5,140)	90,000
Drowning	1·6 (1 to 2·3)	1·5 (1 to 2·2)	43·6 (29·1 to 63·8)	488 (339 to 654)	800 (589 to 1,040)	1,020 (767 to 1,320)	1,360 (996 to 1,810)	1,070 (760 to 1,450)	1,140 (862 to 1,470)	1,030 (796 to 1,320)	727 (555 to 938)	864 (667 to 1,120)	838 (622 to 1,090)	891 (654 to 1,160)	617 (465 to 806)	405 (310 to 522)	283 (220 to 364)	196 (152 to 247)	144 (114 to 180)	64·5 (50·5 to 80·1)	41·9 (32·5 to 52·8)	32·4 (25·7 to 39·5)	20·8 (15·4 to 25·6)	12,100
Fire, heat, and hot substances	0·5 (0·3 to 0·7)	1·1 (0·8 to 1·5)	28·1 (20·2 to 38)	157 (112 to 207)	303 (211 to 435)	362 (241 to 546)	550 (359 to 816)	655 (424 to 998)	816 (535 to 1,220)	1,040 (679 to 1,590)	924 (593 to 1,420)	997 (634 to 1,510)	1,420 (901 to 2,180)	1,520 (984 to 2,310)	1,390 (908 to 2,150)	948 (618 to 1,450)	814 (533 to 1,240)	694 (458 to 1,030)	544 (364 to 803)	371 (253 to 528)	232 (166 to 327)	129 (95·4 to 175)	65·3 (49·6 to 84·3)	14,000
Poisonings	0·3 (0·2 to 0·4)	1·4 (0·9 to 1·9)	11·8 (7·6 to 16·5)	65·5 (42 to 99)	52·7 (33·4 to 79)	60·2 (39·2 to 88·5)	84·5 (56·6 to 121)	96·3 (66·6 to 133)	110 (78 to 152)	112 (78·7 to 157)	92·1 (62·8 to 131)	95·8 (67·4 to 134)	130 (91 to 181)	139 (97·1 to 188)	122 (85·9 to 170)	80·1 (56·2 to 111)	72·5 (51·3 to 97·9)	59·6 (42·9 to 80·7)	45·3 (32·6 to 59·5)	30·4 (22·1 to 39·8)	16·5 (12 to 21·9)	7·6 (5·6 to 10·1)	2·9 (2·1 to 3·8)	1,490
Poisoning by carbon monoxide	0 (0 to 0·1)	0 (0 to 0)	0·9 (0·5 to 1·4)	9·1 (5 to 15·9)	13·1 (8·3 to 20·2)	14·6 (9·7 to 21·9)	23·1 (15·4 to 32·7)	29·4 (20·1 to 39·7)	35·5 (25·3 to 49·3)	31·6 (21·7 to 44·7)	25·3 (17·1 to 35·1)	25·2 (17·3 to 35·6)	33 (22·3 to 46·4)	31·4 (21·3 to 45)	33·1 (22·5 to 47·3)	20·2 (13·9 to 28)	19·6 (13·9 to 27·1)	15 (10·4 to 20·5)	12·4 (8·8 to 16·9)	9·2 (6·7 to 12·1)	4·6 (3·2 to 6·2)	1·9 (1·4 to 2·6)	0·7 (0·5 to 1)	389
Poisoning by other means	0·2 (0·1 to 0·4)	1·4 (0·9 to 1·9)	11 (7·1 to 15·3)	56·5 (35·9 to 86·9)	39·6 (23·6 to 61·6)	45·7 (28 to 71·4)	61·4 (38·3 to 91·4)	66·9 (42·1 to 96·4)	74·8 (48·7 to 109)	79·9 (52·5 to 118)	66·8 (43·8 to 101)	70·7 (47·7 to 102)	97·3 (64·8 to 140)	107 (71·9 to 150)	88·9 (58·6 to 129)	59·8 (40·4 to 85·1)	52·8 (35·5 to 74·1)	44·6 (31·1 to 62·9)	32·8 (22·7 to 45·1)	21·2 (14·5 to 29·1)	11·9 (8·3 to 16·3)	5·7 (4 to 7·8)	2·2 (1·6 to 3)	1,100
Exposure to mechanical forces	1·1 (0·7 to 1·5)	3·2 (2·2 to 4·3)	33·6 (23·4 to 47·6)	235 (171 to 322)	425 (300 to 591)	522 (364 to 754)	835 (589 to 1,160)	1,070 (765 to 1,500)	1,250 (889 to 1,770)	1,460 (1,020 to 2,080)	1,310 (908 to 1,890)	1,530 (1,070 to 2,230)	2,160 (1,520 to 3,120)	2,430 (1,710 to 3,490)	2,210 (1,560 to 3,170)	1,500 (1,040 to 2,160)	1,320 (888 to 1,900)	1,130 (765 to 1,620)	855 (588 to 1,220)	576 (393 to 813)	359 (250 to 500)	213 (154 to 285)	114 (85·8 to 151)	21,500
Unintentional firearm injuries	0·2 (0·1 to 0·3)	0·2 (0·1 to 0·4)	4 (2·6 to 6·4)	21·7 (13·8 to 32·8)	35·6 (23·3 to 54)	53·4 (36·6 to 76)	113 (78·3 to 155)	144 (102 to 200)	153 (109 to 208)	181 (129 to 250)	164 (115 to 224)	195 (135 to 268)	277 (193 to 377)	311 (220 to 417)	282 (198 to 376)	192 (135 to 260)	168 (118 to 228)	145 (102 to 196)	112 (81·3 to 152)	86·9 (64·1 to 115)	64·2 (48·5 to 83·4)	53·9 (40·7 to 68·6)	37 (27·7 to 47·9)	2,790
Other exposure to mechanical forces	0·9 (0·6 to 1·3)	3 (2 to 4)	29·6 (20·3 to 42)	213 (154 to 293)	390 (275 to 550)	468 (324 to 702)	722 (504 to 1,030)	925 (648 to 1,320)	1,090 (770 to 1,580)	1,270 (878 to 1,870)	1,140 (780 to 1,690)	1,330 (926 to 1,980)	1,880 (1,300 to 2,770)	2,120 (1,480 to 3,070)	1,930 (1,330 to 2,790)	1,310 (898 to 1,910)	1,150 (764 to 1,680)	981 (657 to 1,430)	744 (500 to 1,080)	489 (325 to 703)	294 (197 to 420)	159 (110 to 220)	77·4 (55·1 to 107)	18,700
Adverse effects of medical treatment	14 (8·2 to 22·6)	20·8 (14·3 to 29·5)	57·1 (37·4 to 91·5)	56 (36·7 to 83·1)	28·1 (16·8 to 50·3)	21 (12·9 to 38·2)	31·1 (20·4 to 51·9)	60·5 (40·1 to 99·8)	80·4 (54·4 to 122)	103 (70·8 to 145)	86·9 (62·8 to 126)	108 (76·7 to 156)	182 (128 to 255)	231 (163 to 326)	248 (170 to 352)	217 (155 to 301)	199 (142 to 283)	228 (170 to 305)	218 (161 to 303)	139 (100 to 219)	94·2 (67·5 to 152)	46·7 (31·9 to 80·1)	24·3 (16 to 42)	2,490
Animal contact	0·1 (0·1 to 0·2)	0·3 (0·2 to 0·4)	3·7 (2·6 to 5·3)	29 (19·9 to 41·1)	52·9 (34·4 to 78·9)	64·7 (41·2 to 95·9)	91·2 (60·9 to 128)	103 (69·4 to 144)	132 (94·1 to 184)	134 (93·2 to 189)	130 (91·6 to 181)	151 (108 to 208)	235 (172 to 317)	264 (191 to 359)	254 (186 to 336)	197 (144 to 264)	197 (143 to 262)	192 (146 to 248)	184 (140 to 237)	184 (132 to 248)	161 (114 to 223)	170 (120 to 237)	114 (76·2 to 165)	3,040
Venomous animal contact	0·1 (0·1 to 0·1)	0·1 (0·1 to 0·2)	2·1 (1·4 to 3·3)	16·3 (10·2 to 24·9)	33·5 (20 to 53·9)	44 (26·5 to 66·3)	59·9 (37·2 to 87·3)	70·2 (44·5 to 103)	80 (52 to 116)	84·4 (55·3 to 124)	75·6 (48·7 to 111)	82·7 (55·7 to 118)	125 (85·5 to 176)	132 (87·5 to 186)	121 (82·1 to 171)	90·9 (63 to 128)	85·1 (59·7 to 116)	89·7 (65·4 to 118)	103 (76·8 to 134)	125 (85·2 to 177)	124 (83·7 to 177)	143 (99·2 to 203)	97·7 (64·4 to 140)	1,790
Non-venomous animal contact	0·1 (0 to 0·1)	0·2 (0·1 to 0·2)	1·6 (1 to 2·4)	12·7 (8·9 to 18·1)	19·4 (12·5 to 30·5)	20·6 (12·7 to 33·5)	31·3 (19·8 to 47·1)	33·3 (21·7 to 49·3)	52·4 (37·1 to 74·1)	49·9 (32·7 to 73·6)	54·1 (37·2 to 77)	68·2 (46·8 to 95·4)	109 (74·9 to 151)	132 (92·6 to 186)	133 (93·5 to 177)	106 (74·1 to 149)	111 (75·6 to 156)	102 (72·2 to 137)	81·3 (56·2 to 112)	59 (41·1 to 81·8)	37·6 (25·3 to 53·8)	26·2 (16·7 to 39·6)	16·3 (9·9 to 25·5)	1,260
Foreign body	24·8 (14·8 to 34·8)	59·2 (39·3 to 87)	635 (417 to 948)	332 (237 to 437)	174 (134 to 224)	188 (142 to 244)	166 (122 to 223)	200 (148 to 274)	248 (187 to 335)	264 (202 to 345)	235 (177 to 313)	298 (227 to 380)	445 (352 to 567)	573 (439 to 729)	559 (440 to 705)	429 (330 to 550)	443 (348 to 560)	449 (346 to 564)	422 (342 to 511)	371 (290 to 466)	228 (180 to 282)	162 (132 to 193)	97·7 (74·6 to 118)	7,010
Pulmonary aspiration and foreign body in airway	24·6 (14·7 to 34·6)	58·8 (38·8 to 86·5)	627 (408 to 937)	291 (205 to 386)	116 (89·2 to 147)	125 (92·3 to 164)	85·5 (64·7 to 110)	102 (75 to 134)	134 (102 to 174)	141 (107 to 184)	126 (95·1 to 162)	166 (127 to 215)	252 (192 to 326)	378 (269 to 509)	384 (285 to 502)	304 (224 to 406)	313 (234 to 418)	331 (243 to 431)	305 (239 to 383)	266 (196 to 347)	154 (114 to 198)	101 (73·6 to 127)	58·8 (41·2 to 76·3)	4,840
Foreign body in eyes	0 (0 to 0·1)	0·1 (0 to 0·2)	1·8 (0·5 to 3·9)	10·2 (3·1 to 23·2)	18 (5·2 to 42·3)	21·1 (6·4 to 49·5)	31·2 (9·9 to 73·2)	40·7 (13 to 89·4)	49·5 (17·4 to 105)	54·9 (19·2 to 116)	47·8 (18 to 100)	53·4 (22·8 to 108)	72·3 (33·5 to 139)	69·7 (35·9 to 126)	55·8 (32 to 92·4)	37·7 (22·4 to 59·7)	33·8 (20·8 to 51·8)	29·2 (18·6 to 43·4)	22·8 (14·8 to 33·8)	15·1 (9·9 to 22)	8·6 (5·8 to 12·4)	4·2 (2·8 to 6)	1·7 (1·2 to 2·4)	680
Foreign body in other body part	0·2 (0·1 to 0·3)	0·4 (0·2 to 0·6)	6·9 (4·3 to 10·5)	30·3 (20·4 to 44·7)	40·5 (26·8 to 58·2)	41·9 (28·5 to 59)	49·6 (34·5 to 70)	57·4 (40·4 to 80·3)	64·6 (46·2 to 89·6)	68·5 (48·3 to 95·5)	61·8 (43·6 to 85·6)	78·2 (56·5 to 106)	121 (87·7 to 165)	125 (89 to 171)	120 (84·9 to 163)	87·9 (63 to 122)	96·9 (67·8 to 133)	89·3 (65·3 to 118)	93·6 (65·2 to 133)	90 (61 to 127)	65·5 (46 to 92·8)	57·8 (39 to 79·6)	37·1 (24·8 to 52·6)	1,480
Environmental heat and cold exposure	0·2 (0·1 to 0·2)	1·2 (0·8 to 1·9)	5·4 (3·5 to 8)	35·8 (23·3 to 53·5)	71·1 (44·6 to 107)	90·2 (58·2 to 132)	140 (91·7 to 205)	183 (123 to 262)	232 (157 to 335)	276 (186 to 396)	248 (167 to 358)	287 (196 to 412)	419 (285 to 594)	466 (319 to 655)	432 (297 to 603)	315 (217 to 437)	295 (205 to 408)	272 (191 to 374)	224 (158 to 305)	167 (120 to 222)	113 (84·7 to 151)	81·6 (61·5 to 105)	53·1 (39·1 to 72)	4,410
Exposure to forces of nature	0·1 (0·1 to 0·1)	0·3 (0·3 to 0·4)	2·9 (2·6 to 3·1)	15·3 (13·9 to 16·8)	15·9 (14·4 to 17·5)	18·8 (16·9 to 20·7)	22·3 (20·1 to 24·6)	21·7 (19·6 to 24·2)	24·9 (22·4 to 27·6)	21·3 (19·1 to 23·7)	17·2 (15·3 to 19·5)	21·2 (18·9 to 23·8)	25·1 (22·3 to 28·5)	25·4 (22·5 to 28·7)	18·1 (15·9 to 20·6)	13·7 (12·2 to 15·6)	11·2 (9·9 to 12·6)	9·1 (8·1 to 10·3)	6·5 (5·8 to 7·3)	4 (3·6 to 4·5)	2·1 (1·8 to 2·3)	0·9 (0·8 to 1)	0·3 (0·3 to 0·3)	298
Other unintentional injuries	5·6 (3·2 to 8·8)	4·2 (2·7 to 6·5)	64 (47 to 86·3)	253 (185 to 344)	427 (307 to 585)	561 (427 to 744)	963 (723 to 1,270)	1,130 (846 to 1,500)	1,370 (1,050 to 1,770)	1,500 (1,150 to 1,950)	1,190 (908 to 1,560)	1,320 (993 to 1,720)	1,730 (1,280 to 2,270)	1,700 (1,240 to 2,310)	1,460 (1,050 to 1,990)	926 (655 to 1,300)	821 (589 to 1,140)	674 (473 to 954)	521 (366 to 731)	329 (228 to 462)	190 (132 to 268)	90·8 (62·5 to 128)	38·3 (26·7 to 53·8)	17,300
Self-harm and interpersonal violence	110 (59·3 to 178)	19 (11·7 to 29·6)	109 (66·5 to 165)	211 (146 to 327)	233 (183 to 296)	734 (577 to 907)	4,700 (3,690 to 5,900)	7,760 (6,190 to 9,600)	9,120 (7,490 to 11,100)	9,730 (7,890 to 11,900)	7,840 (6,240 to 9,610)	7,990 (6,280 to 10,200)	10,100 (7,790 to 13,100)	9,580 (7,240 to 12,200)	8,000 (6,130 to 10,200)	5,140 (4,010 to 6,550)	4,040 (3,200 to 5,080)	3,440 (2,740 to 4,310)	2,540 (2,000 to 3,190)	1,530 (1,200 to 1,910)	783 (634 to 968)	239 (191 to 286)	76·9 (62·4 to 91·4)	94,100
Self-harm	0 (0 to 0)	0 (0 to 0)	0 (0 to 0)	0 (0 to 0)	0 (0 to 0)	297 (209 to 409)	2,130 (1,600 to 2,760)	3,360 (2,520 to 4,400)	4,070 (3,180 to 5,150)	4,810 (3,820 to 6,040)	4,230 (3,260 to 5,350)	4,780 (3,610 to 6,350)	6,600 (4,900 to 8,760)	6,950 (5,090 to 9,130)	6,070 (4,510 to 7,940)	4,110 (3,130 to 5,360)	3,280 (2,530 to 4,190)	2,870 (2,230 to 3,670)	2,160 (1,660 to 2,770)	1,360 (1,040 to 1,720)	699 (552 to 883)	204 (159 to 249)	59·7 (46·7 to 72·7)	58,000
Self-harm by firearm	0 (0 to 0)	0 (0 to 0)	0 (0 to 0)	0 (0 to 0)	0 (0 to 0)	4·7 (3·1 to 7)	151 (69·5 to 243)	133 (62·2 to 219)	123 (58·5 to 197)	113 (65·7 to 169)	80·5 (47·4 to 123)	83·5 (49·9 to 126)	85·6 (54·3 to 127)	105 (66·6 to 164)	84·9 (55·4 to 126)	57·5 (38·5 to 89)	43·3 (30·2 to 63·6)	46·7 (33·5 to 70·6)	33·5 (25 to 46·6)	25·5 (19 to 36·5)	15·2 (10·9 to 20·9)	1·9 (1·4 to 2·5)	0·7 (0·6 to 1)	1,190
Self-harm by other specified means	0 (0 to 0)	0 (0 to 0)	0 (0 to 0)	0 (0 to 0)	0 (0 to 0)	292 (206 to 404)	1,980 (1,490 to 2,560)	3,230 (2,420 to 4,230)	3,940 (3,080 to 5,010)	4,700 (3,710 to 5,910)	4,140 (3,190 to 5,250)	4,700 (3,550 to 6,240)	6,520 (4,850 to 8,650)	6,850 (5,020 to 9,020)	5,990 (4,450 to 7,840)	4,050 (3,080 to 5,270)	3,240 (2,490 to 4,140)	2,830 (2,190 to 3,630)	2,120 (1,630 to 2,730)	1,330 (1,020 to 1,690)	684 (540 to 867)	202 (157 to 247)	59 (46 to 71·9)	56,900
Interpersonal violence	110 (59·3 to 178)	19 (11·7 to 29·6)	108 (65·5 to 164)	203 (138 to 318)	227 (178 to 291)	432 (329 to 553)	2,560 (1,850 to 3,390)	4,390 (3,390 to 5,640)	5,050 (3,920 to 6,520)	4,920 (3,770 to 6,310)	3,610 (2,760 to 4,630)	3,180 (2,370 to 4,180)	3,490 (2,640 to 4,650)	2,570 (1,990 to 3,340)	1,880 (1,410 to 2,460)	1,010 (766 to 1,290)	746 (578 to 951)	555 (438 to 691)	379 (298 to 487)	168 (132 to 207)	83·4 (66·3 to 104)	34·8 (26·7 to 44·7)	17·1 (13·1 to 22)	35,700
Physical violence by firearm	0·3 (0·2 to 0·5)	0·1 (0·1 to 0·2)	3 (1·8 to 4·7)	10·5 (7·4 to 14·8)	22·1 (16·6 to 28·8)	42·9 (31·5 to 56·9)	246 (160 to 364)	343 (229 to 488)	286 (194 to 411)	267 (182 to 368)	160 (111 to 225)	129 (89·6 to 181)	125 (88·3 to 176)	98·2 (72·4 to 135)	90·6 (65·9 to 126)	50·2 (36·4 to 65·8)	34·4 (26·2 to 44·6)	24·4 (18·6 to 30·6)	16·1 (12 to 21·1)	7·8 (5·8 to 10)	4·3 (3·2 to 5·5)	2 (1·5 to 2·6)	0·9 (0·6 to 1·2)	1,960
Physical violence by sharp object	5·6 (2·8 to 10)	2·2 (1·1 to 4)	7·3 (4·8 to 10·5)	47·5 (32·6 to 73)	59·3 (44·8 to 78·2)	149 (108 to 199)	1,820 (1,260 to 2,480)	3,270 (2,480 to 4,250)	3,850 (2,890 to 5,080)	3,670 (2,730 to 4,770)	2,670 (1,980 to 3,490)	2,240 (1,620 to 3,010)	2,300 (1,670 to 3,140)	1,470 (1,090 to 1,960)	860 (632 to 1,150)	413 (310 to 539)	257 (194 to 334)	182 (139 to 233)	102 (78·2 to 133)	50·1 (38 to 64·8)	25 (18·6 to 32·5)	13·3 (10·1 to 17·5)	6·3 (4·6 to 8·5)	23,500
Sexual violence	0 (0 to 0)	0 (0 to 0)	0 (0 to 0)	3·6 (2·1 to 5·8)	32·1 (19·5 to 50·3)	65·6 (38·3 to 103)	172 (95·5 to 273)	241 (139 to 368)	241 (136 to 387)	236 (131 to 388)	180 (101 to 287)	163 (93·6 to 264)	184 (108 to 310)	149 (84·2 to 242)	100 (58·7 to 160)	62·6 (38·7 to 94·2)	53·1 (33 to 78·3)	43·6 (27·1 to 64·9)	32·2 (20·7 to 47)	21 (13·6 to 30·1)	10·1 (6 to 15·8)	2 (1·2 to 3·1)	0·2 (0·1 to 0·3)	1,990
Physical violence by other means	104 (56·5 to 168)	16·8 (10·3 to 26·4)	97·3 (58·7 to 147)	141 (94·3 to 225)	114 (86·4 to 151)	175 (128 to 231)	319 (233 to 429)	535 (382 to 737)	665 (479 to 913)	745 (525 to 1,010)	598 (425 to 812)	651 (486 to 862)	884 (655 to 1,170)	854 (653 to 1,120)	831 (618 to 1,100)	479 (357 to 629)	402 (304 to 527)	305 (235 to 388)	229 (172 to 304)	88·6 (67·3 to 113)	44 (33·7 to 55·1)	17·5 (13·1 to 23)	9·8 (7·3 to 12·5)	8,300
Conflict and terrorism	0 (0 to 0)	0 (0 to 0)	0 (0 to 0)	0 (0 to 0)	0 (0 to 0)	0 (0 to 0)	0 (0 to 0·1)	0·1 (0 to 0·1)	0·3 (0·2 to 0·6)	0·8 (0·5 to 1·5)	5·6 (3·3 to 10·3)	16·4 (10·2 to 26·9)	40·1 (25·3 to 65·4)	57·8 (36·7 to 93·3)	45 (29·6 to 67·7)	22·4 (15 to 33·8)	13·7 (9 to 20)	7·2 (4·7 to 10·8)	3 (2 to 4·5)	1·3 (0·8 to 1·9)	0·6 (0·4 to 0·8)	0·2 (0·1 to 0·3)	0·1 (0 to 0·1)	214

SR Table 2c: Dominica DALYS by cause and age group for both sexes with 95% uncertainty intervals, 2019

Cause	Early Neonatal	Late Neonatal	Post Neonatal	1 to 4	5 to 9	10 to 14	15 to 19	20 to 24	25 to 29	30 to 34	35 to 39	40 to 44	45 to 49	50 to 54	55 to 59	60 to 64	65 to 69	70 to 74	75 to 79	80 to 84	85 to 89	90 to 94	95 plus	Total
All causes	923 (645 to 1,290)	263 (185 to 367)	481 (349 to 659)	388 (304 to 487)	311 (256 to 381)	516 (420 to 629)	782 (643 to 944)	[illegible]	[illegible]	[illegible]	[illegible]	[illegible]	[illegible]	[illegible]	[illegible]	[illegible]	[illegible]	[illegible]	[illegible]	[illegible]	[illegible]	[illegible]	129 (115 to 142)	25,000
Communicable, maternal, neonatal, and nutritional diseases	806 (561 to 1,120)	207 (146 to 293)	257 (179 to 357)	135 (105 to 174)	75·7 (57·7 to 99·8)	80·2 (63·6 to 101)	91·3 (74 to 113)	[illegible]	[illegible]	[illegible]	[illegible]	[illegible]	[illegible]	[illegible]	[illegible]	[illegible]	[illegible]	[illegible]	[illegible]	[illegible]	[illegible]	[illegible]	10·7 (8 to 13·7)	3,120
HIV/AIDS and sexually transmitted infections	30·4 (8·4 to 69)	9·7 (2·7 to 22)	22 (10·2 to 43·8)	4·7 (4·2 to 5·5)	2·1 (1·9 to 2·4)	3·6 (3·3 to 3·9)	4·5 (4·1 to 5·2)	[illegible]	[illegible]	[illegible]	[illegible]	[illegible]	[illegible]	[illegible]	[illegible]	[illegible]	[illegible]	[illegible]	[illegible]	[illegible]	[illegible]	[illegible]	0 (0 to 0)	335
HIV/AIDS	0 (0 to 0)	0 (0 to 0)	5·9 (5·6 to 6·2)	4·1 (3·8 to 4·3)	1·8 (1·7 to 2)	3·3 (3·1 to 3·5)	3·7 (3·5 to 4)	[illegible]	[illegible]	[illegible]	[illegible]	[illegible]	[illegible]	[illegible]	[illegible]	[illegible]	[illegible]	[illegible]	[illegible]	[illegible]	[illegible]	[illegible]	0 (0 to 0)	261
HIV/AIDS - Drug-susceptible Tuberculosis	0 (0 to 0)	0 (0 to 0)	0·8 (0·5 to 1·2)	0·5 (0·3 to 0·8)	0·2 (0·1 to 0·3)	0·4 (0·3 to 0·6)	0·5 (0·3 to 0·7)	[illegible]	[illegible]	[illegible]	[illegible]	[illegible]	[illegible]	[illegible]	[illegible]	[illegible]	[illegible]	[illegible]	[illegible]	[illegible]	[illegible]	[illegible]	0 (0 to 0)	32·8
HIV/AIDS - Multidrug-resistant Tuberculosis without extensive dru	0 (0 to 0)	0 (0 to 0)	0 (0 to 0·1)	0 (0 to 0)	0 (0 to 0)	0 (0 to 0)	0 (0 to 0)	[illegible]	[illegible]	[illegible]	[illegible]	[illegible]	[illegible]	[illegible]	[illegible]	[illegible]	[illegible]	[illegible]	[illegible]	[illegible]	[illegible]	[illegible]	0 (0 to 0)	0·5
HIV/AIDS - Extensively drug-resistant Tuberculosis	0 (0 to 0)	0 (0 to 0)	0 (0 to 0)	0 (0 to 0)	0 (0 to 0)	0 (0 to 0)	0 (0 to 0)	[illegible]	[illegible]	[illegible]	[illegible]	[illegible]	[illegible]	[illegible]	[illegible]	[illegible]	[illegible]	[illegible]	[illegible]	[illegible]	[illegible]	[illegible]	0 (0 to 0)	0·1
HIV/AIDS resulting in other diseases	0 (0 to 0)	0 (0 to 0)	5·1 (4·6 to 5·6)	3·5 (3·1 to 3·9)	1·6 (1·5 to 1·8)	2·9 (2·6 to 3·2)	3·3 (2·9 to 3·5)	[illegible]	[illegible]	[illegible]	[illegible]	[illegible]	[illegible]	[illegible]	[illegible]	[illegible]	[illegible]	[illegible]	[illegible]	[illegible]	[illegible]	[illegible]	0 (0 to 0)	227
Sexually transmitted infections excluding HIV	30·4 (8·4 to 69)	9·7 (2·7 to 22)	16·1 (4·3 to 37·9)	0·6 (0·2 to 1·4)	0·3 (0·1 to 0·6)	0·2 (0·1 to 0·4)	0·8 (0·5 to 1·4)	[illegible]	[illegible]	[illegible]	[illegible]	[illegible]	[illegible]	[illegible]	[illegible]	[illegible]	[illegible]	[illegible]	[illegible]	[illegible]	[illegible]	[illegible]	0 (0 to 0)	74
Syphilis	30·4 (8·4 to 69)	9·7 (2·7 to 22)	16·1 (4·3 to 37·9)	0·6 (0·2 to 1·4)	0·3 (0·1 to 0·6)	0·1 (0 to 0·2)	0·2 (0·1 to 0·5)	[illegible]	[illegible]	[illegible]	[illegible]	[illegible]	[illegible]	[illegible]	[illegible]	[illegible]	[illegible]	[illegible]	[illegible]	[illegible]	[illegible]	[illegible]	0 (0 to 0)	60·4
Chlamydial infection	0 (0 to 0)	0 (0 to 0)	0 (0 to 0)	0 (0 to 0)	0 (0 to 0)	0 (0 to 0)	0·1 (0 to 0·1)	[illegible]	[illegible]	[illegible]	[illegible]	[illegible]	[illegible]	[illegible]	[illegible]	[illegible]	[illegible]	[illegible]	[illegible]	[illegible]	[illegible]	[illegible]	0 (0 to 0)	1·4
Gonococcal infection	0 (0 to 0)	0 (0 to 0)	0 (0 to 0)	0 (0 to 0)	0 (0 to 0)	0 (0 to 0·1)	0·2 (0·1 to 0·3)	[illegible]	[illegible]	[illegible]	[illegible]	[illegible]	[illegible]	[illegible]	[illegible]	[illegible]	[illegible]	[illegible]	[illegible]	[illegible]	[illegible]	[illegible]	0 (0 to 0)	1·3
Trichomoniasis	0 (0 to 0)	0 (0 to 0)	0 (0 to 0)	0 (0 to 0)	0 (0 to 0)	0 (0 to 0)	0·1 (0 to 0·1)	[illegible]	[illegible]	[illegible]	[illegible]	[illegible]	[illegible]	[illegible]	[illegible]	[illegible]	[illegible]	[illegible]	[illegible]	[illegible]	[illegible]	[illegible]	0 (0 to 0)	3·4
Genital herpes	0 (0 to 0)	0 (0 to 0)	0 (0 to 0)	0 (0 to 0)	0 (0 to 0)	0 (0 to 0·1)	0·1 (0 to 0·3)	[illegible]	[illegible]	[illegible]	[illegible]	[illegible]	[illegible]	[illegible]	[illegible]	[illegible]	[illegible]	[illegible]	[illegible]	[illegible]	[illegible]	[illegible]	0 (0 to 0)	4·1
Other sexually transmitted infections	0 (0 to 0)	0 (0 to 0)	0 (0 to 0)	0 (0 to 0)	0 (0 to 0)	0·1 (0 to 0·2)	0·2 (0·1 to 0·4)	[illegible]	[illegible]	[illegible]	[illegible]	[illegible]	[illegible]	[illegible]	[illegible]	[illegible]	[illegible]	[illegible]	[illegible]	[illegible]	[illegible]	[illegible]	0 (0 to 0)	3·4
Respiratory infections and tuberculosis	21·4 (12 to 34·6)	16·1 (9·5 to 26·7)	78·1 (47·7 to 122)	36·5 (25·4 to 53·6)	14·2 (10·5 to 19·4)	16 (11·8 to 21·7)	17·7 (13·1 to 23·5)	[illegible]	[illegible]	[illegible]	[illegible]	[illegible]	[illegible]	[illegible]	[illegible]	[illegible]	[illegible]	[illegible]	[illegible]	[illegible]	[illegible]	[illegible]	8·7 (6·2 to 11·6)	778
Tuberculosis	0 (0 to 0)	0 (0 to 0)	1·5 (0·8 to 2·4)	1·7 (1·1 to 2·5)	1·1 (0·7 to 1·7)	1·6 (1·1 to 2·3)	3·4 (2·1 to 5·2)	[illegible]	[illegible]	[illegible]	[illegible]	[illegible]	[illegible]	[illegible]	[illegible]	[illegible]	[illegible]	[illegible]	[illegible]	[illegible]	[illegible]	[illegible]	0·1 (0·1 to 0·2)	101
Latent tuberculosis infection	0 (0 to 0)	0 (0 to 0)	0 (0 to 0)	0 (0 to 0)	0 (0 to 0)	0 (0 to 0)	0 (0 to 0)	[illegible]	[illegible]	[illegible]	[illegible]	[illegible]	[illegible]	[illegible]	[illegible]	[illegible]	[illegible]	[illegible]	[illegible]	[illegible]	[illegible]	[illegible]	0 (0 to 0)	0
Drug-susceptible tuberculosis	0 (0 to 0)	0 (0 to 0)	1·5 (0·8 to 2·3)	1·7 (1·1 to 2·5)	1·1 (0·7 to 1·7)	1·6 (1·1 to 2·3)	3·3 (2·1 to 5·1)	[illegible]	[illegible]	[illegible]	[illegible]	[illegible]	[illegible]	[illegible]	[illegible]	[illegible]	[illegible]	[illegible]	[illegible]	[illegible]	[illegible]	[illegible]	0·1 (0·1 to 0·2)	99·2
Multidrug-resistant tuberculosis without extensive drug resistanc	0 (0 to 0)	0 (0 to 0)	0 (0 to 0·1)	0 (0 to 0·1)	0 (0 to 0·1)	0 (0 to 0·1)	0 (0 to 0·2)	[illegible]	[illegible]	[illegible]	[illegible]	[illegible]	[illegible]	[illegible]	[illegible]	[illegible]	[illegible]	[illegible]	[illegible]	[illegible]	[illegible]	[illegible]	0 (0 to 0)	1·2
Extensively drug-resistant tuberculosis	0 (0 to 0)	0 (0 to 0)	0 (0 to 0)	0 (0 to 0)	0 (0 to 0)	0 (0 to 0)	0 (0 to 0)	[illegible]	[illegible]	[illegible]	[illegible]	[illegible]	[illegible]	[illegible]	[illegible]	[illegible]	[illegible]	[illegible]	[illegible]	[illegible]	[illegible]	[illegible]	0 (0 to 0)	0·2
Lower respiratory infections	21·4 (12 to 34·6)	16 (9·4 to 26·5)	74·8 (44·5 to 117)	28·2 (17·6 to 43·4)	5·5 (4 to 7·2)	7·3 (5·1 to 10·1)	7·3 (5·1 to 10·2)	[illegible]	[illegible]	[illegible]	[illegible]	[illegible]	[illegible]	[illegible]	[illegible]	[illegible]	[illegible]	[illegible]	[illegible]	[illegible]	[illegible]	[illegible]	8·5 (6 to 11·4)	603
Upper respiratory infections	0 (0 to 0)	0·1 (0 to 0·1)	1·7 (1 to 2·9)	4·9 (3 to 7·7)	5·2 (2·9 to 8·7)	5·2 (2·8 to 8·8)	5·2 (2·8 to 8·9)	[illegible]	[illegible]	[illegible]	[illegible]	[illegible]	[illegible]	[illegible]	[illegible]	[illegible]	[illegible]	[illegible]	[illegible]	[illegible]	[illegible]	[illegible]	0 (0 to 0·1)	56·4
Otitis media	0 (0 to 0)	0 (0 to 0)	0·2 (0·1 to 0·3)	1·7 (0·9 to 3)	2·5 (1·4 to 4·2)	1·9 (1·1 to 3·3)	1·8 (1 to 3·1)	[illegible]	[illegible]	[illegible]	[illegible]	[illegible]	[illegible]	[illegible]	[illegible]	[illegible]	[illegible]	[illegible]	[illegible]	[illegible]	[illegible]	[illegible]	0 (0 to 0)	18·6
Enteric infections	1 (0·5 to 1·7)	2·5 (1·3 to 4·5)	22·4 (12 to 37·4)	14 (9·2 to 20)	8·7 (5·5 to 13·4)	9 (5·5 to 13·8)	6·6 (4·3 to 9·7)	[illegible]	[illegible]	[illegible]	[illegible]	[illegible]	[illegible]	[illegible]	[illegible]	[illegible]	[illegible]	[illegible]	[illegible]	[illegible]	[illegible]	[illegible]	0·6 (0·4 to 1)	170
Diarrheal diseases	1 (0·5 to 1·7)	2·5 (1·3 to 4·5)	22·3 (11·9 to 37·4)	13·8 (9 to 19·8)	8·5 (5·3 to 13·3)	8·8 (5·4 to 13·6)	6·5 (4·2 to 9·6)	[illegible]	[illegible]	[illegible]	[illegible]	[illegible]	[illegible]	[illegible]	[illegible]	[illegible]	[illegible]	[illegible]	[illegible]	[illegible]	[illegible]	[illegible]	0·6 (0·4 to 1)	168
Typhoid and paratyphoid	0 (0 to 0)	0 (0 to 0)	0 (0 to 0)	0·1 (0 to 0·2)	0 (0 to 0·1)	0·1 (0 to 0·1)	0 (0 to 0·1)	[illegible]	[illegible]	[illegible]	[illegible]	[illegible]	[illegible]	[illegible]	[illegible]	[illegible]	[illegible]	[illegible]	[illegible]	[illegible]	[illegible]	[illegible]	0 (0 to 0)	0·5
Typhoid fever	0 (0 to 0)	[illegible]	[illegible]	[illegible]	[illegible]	[illegible]	[illegible]	[illegible]	[illegible]	[illegible]	[illegible]	[illegible]	[illegible]	[illegible]	[illegible]	[illegible]	[illegible]	[illegible]	[illegible]	[illegible]	[illegible]	[illegible]	0 (0 to 0)	0·4
Paratyphoid fever	0 (0 to 0)	[illegible]	[illegible]	[illegible]	[illegible]	[illegible]	[illegible]	[illegible]	[illegible]	[illegible]	[illegible]	[illegible]	[illegible]	[illegible]	[illegible]	[illegible]	[illegible]	[illegible]	[illegible]	[illegible]	[illegible]	[illegible]	0 (0 to 0)	0·1
Invasive Non-typhoidal Salmonella (iNTS)	0 (0 to 0)	[illegible]	[illegible]	[illegible]	[illegible]	[illegible]	[illegible]	[illegible]	[illegible]	[illegible]	[illegible]	[illegible]	[illegible]	[illegible]	[illegible]	[illegible]	[illegible]	[illegible]	[illegible]	[illegible]	[illegible]	[illegible]	0 (0 to 0)	0·8
Other intestinal infectious diseases	0 (0 to 0)	[illegible]	[illegible]	[illegible]	[illegible]	[illegible]	[illegible]	[illegible]	[illegible]	[illegible]	[illegible]	[illegible]	[illegible]	[illegible]	[illegible]	[illegible]	[illegible]	[illegible]	[illegible]	[illegible]	[illegible]	[illegible]	0 (0 to 0)	0·4
Neglected tropical diseases and malaria	0·1 (0 to 0·1)	[illegible]	[illegible]	[illegible]	[illegible]	[illegible]	[illegible]	[illegible]	[illegible]	[illegible]	[illegible]	[illegible]	[illegible]	[illegible]	[illegible]	[illegible]	[illegible]	[illegible]	[illegible]	[illegible]	[illegible]	[illegible]	0·2 (0·1 to 0·4)	78·6
Malaria	0 (0 to 0)	[illegible]	[illegible]	[illegible]	[illegible]	[illegible]	[illegible]	[illegible]	[illegible]	[illegible]	[illegible]	[illegible]	[illegible]	[illegible]	[illegible]	[illegible]	[illegible]	[illegible]	[illegible]	[illegible]	[illegible]	[illegible]	0 (0 to 0)	0
Chagas disease	0 (0 to 0)	[illegible]	[illegible]	[illegible]	[illegible]	[illegible]	[illegible]	[illegible]	[illegible]	[illegible]	[illegible]	[illegible]	[illegible]	[illegible]	[illegible]	[illegible]	[illegible]	[illegible]	[illegible]	[illegible]	[illegible]	[illegible]	0 (0 to 0)	0
Leishmaniasis	0 (0 to 0)	[illegible]	[illegible]	[illegible]	[illegible]	[illegible]	[illegible]	[illegible]	[illegible]	[illegible]	[illegible]	[illegible]	[illegible]	[illegible]	[illegible]	[illegible]	[illegible]	[illegible]	[illegible]	[illegible]	[illegible]	[illegible]	0 (0 to 0)	0
Visceral leishmaniasis	0 (0 to 0)	[illegible]	[illegible]	[illegible]	[illegible]	[illegible]	[illegible]	[illegible]	[illegible]	[illegible]	[illegible]	[illegible]	[illegible]	[illegible]	[illegible]	[illegible]	[illegible]	[illegible]	[illegible]	[illegible]	[illegible]	[illegible]	0 (0 to 0)	0
Cutaneous and mucocutaneous leishmaniasis	0 (0 to 0)	[illegible]	[illegible]	[illegible]	[illegible]	[illegible]	[illegible]	[illegible]	[illegible]	[illegible]	[illegible]	[illegible]	[illegible]	[illegible]	[illegible]	[illegible]	[illegible]	[illegible]	[illegible]	[illegible]	[illegible]	[illegible]	0 (0 to 0)	0
African trypanosomiasis	0 (0 to 0)	[illegible]	[illegible]	[illegible]	[illegible]	[illegible]	[illegible]	[illegible]	[illegible]	[illegible]	[illegible]	[illegible]	[illegible]	[illegible]	[illegible]	[illegible]	[illegible]	[illegible]	[illegible]	[illegible]	[illegible]	[illegible]	0 (0 to 0)	0
Schistosomiasis	0 (0 to 0)	[illegible]	[illegible]	[illegible]	[illegible]	[illegible]	[illegible]	[illegible]	[illegible]	[illegible]	[illegible]	[illegible]	[illegible]	[illegible]	[illegible]	[illegible]	[illegible]	[illegible]	[illegible]	[illegible]	[illegible]	[illegible]	0 (0 to 0)	0
Cysticercosis	0 (0 to 0)	[illegible]	[illegible]	[illegible]	[illegible]	[illegible]	[illegible]	[illegible]	[illegible]	[illegible]	[illegible]	[illegible]	[illegible]	[illegible]	[illegible]	[illegible]	[illegible]	[illegible]	[illegible]	[illegible]	[illegible]	[illegible]	0·1 (0·1 to 0·3)	44·8
Cystic echinococcosis	0 (0 to 0)	[illegible]	[illegible]	[illegible]	[illegible]	[illegible]	[illegible]	[illegible]	[illegible]	[illegible]	[illegible]	[illegible]	[illegible]	[illegible]	[illegible]	[illegible]	[illegible]	[illegible]	[illegible]	[illegible]	[illegible]	[illegible]	0 (0 to 0)	0
Lymphatic filariasis	0 (0 to 0)	[illegible]	[illegible]	[illegible]	[illegible]	[illegible]	[illegible]	[illegible]	[illegible]	[illegible]	[illegible]	[illegible]	[illegible]	[illegible]	[illegible]	[illegible]	[illegible]	[illegible]	[illegible]	[illegible]	[illegible]	[illegible]	0 (0 to 0)	0
Onchocerciasis	0 (0 to 0)	[illegible]	[illegible]	[illegible]	[illegible]	[illegible]	[illegible]	[illegible]	[illegible]	[illegible]	[illegible]	[illegible]	[illegible]	[illegible]	[illegible]	[illegible]	[illegible]	[illegible]	[illegible]	[illegible]	[illegible]	[illegible]	0 (0 to 0)	0
Trachoma	0 (0 to 0)	[illegible]	[illegible]	[illegible]	[illegible]	[illegible]	[illegible]	[illegible]	[illegible]	[illegible]	[illegible]	[illegible]	[illegible]	[illegible]	[illegible]	[illegible]	[illegible]	[illegible]	[illegible]	[illegible]	[illegible]	[illegible]	0 (0 to 0)	0
Dengue	0 (0 to 0)	[illegible]	[illegible]	[illegible]	[illegible]	[illegible]	[illegible]	[illegible]	[illegible]	[illegible]	[illegible]	[illegible]	[illegible]	[illegible]	[illegible]	[illegible]	[illegible]	[illegible]	[illegible]	[illegible]	[illegible]	[illegible]	0 (0 to 0)	9·5
Yellow fever	0 (0 to 0)	[illegible]	[illegible]	[illegible]	[illegible]	[illegible]	[illegible]	[illegible]	[illegible]	[illegible]	[illegible]	[illegible]	[illegible]	[illegible]	[illegible]	[illegible]	[illegible]	[illegible]	[illegible]	[illegible]	[illegible]	[illegible]	0 (0 to 0)	0
Rabies	0 (0 to 0)	[illegible]	[illegible]	[illegible]	[illegible]	[illegible]	[illegible]	[illegible]	[illegible]	[illegible]	[illegible]	[illegible]	[illegible]	[illegible]	[illegible]	[illegible]	[illegible]	[illegible]	[illegible]	[illegible]	[illegible]	[illegible]	0 (0 to 0)	0·2
Intestinal nematode infections	0 (0 to 0)	[illegible]	[illegible]	[illegible]	[illegible]	[illegible]	[illegible]	[illegible]	[illegible]	[illegible]	[illegible]	[illegible]	[illegible]	[illegible]	[illegible]	[illegible]	[illegible]	[illegible]	[illegible]	[illegible]	[illegible]	[illegible]	0 (0 to 0)	2·8
Ascariasis	0 (0 to 0)	[illegible]	[illegible]	[illegible]	[illegible]	[illegible]	[illegible]	[illegible]	[illegible]	[illegible]	[illegible]	[illegible]	[illegible]	[illegible]	[illegible]	[illegible]	[illegible]	[illegible]	[illegible]	[illegible]	[illegible]	[illegible]	0 (0 to 0)	0·1
Trichuriasis	0 (0 to 0)	[illegible]	[illegible]	[illegible]	[illegible]	[illegible]	[illegible]	[illegible]	[illegible]	[illegible]	[illegible]	[illegible]	[illegible]	[illegible]	[illegible]	[illegible]	[illegible]	[illegible]	[illegible]	[illegible]	[illegible]	[illegible]	0 (0 to 0)	0
Hookworm disease	0 (0 to 0)	[illegible]	[illegible]	[illegible]	[illegible]	[illegible]	[illegible]	[illegible]	[illegible]	[illegible]	[illegible]	[illegible]	[illegible]	[illegible]	[illegible]	[illegible]	[illegible]	[illegible]	[illegible]	[illegible]	[illegible]	[illegible]	0 (0 to 0)	2·6
Food-borne trematodiases	0 (0 to 0)	[illegible]	[illegible]	[illegible]	[illegible]	[illegible]	[illegible]	[illegible]	[illegible]	[illegible]	[illegible]	[illegible]	[illegible]	[illegible]	[illegible]	[illegible]	[illegible]	[illegible]	[illegible]	[illegible]	[illegible]	[illegible]	0 (0 to 0)	0
Leprosy	0 (0 to 0)	[illegible]	[illegible]	[illegible]	[illegible]	[illegible]	[illegible]	[illegible]	[illegible]	[illegible]	[illegible]	[illegible]	[illegible]	[illegible]	[illegible]	[illegible]	[illegible]	[illegible]	[illegible]	[illegible]	[illegible]	[illegible]	0 (0 to 0)	0·5
Ebola	0 (0 to 0)	[illegible]	[illegible]	[illegible]	[illegible]	[illegible]	[illegible]	[illegible]	[illegible]	[illegible]	[illegible]	[illegible]	[illegible]	[illegible]	[illegible]	[illegible]	[illegible]	[illegible]	[illegible]	[illegible]	[illegible]	[illegible]	0 (0 to 0)	0
Zika virus	0 (0 to 0)	[illegible]	[illegible]	[illegible]	[illegible]	[illegible]	[illegible]	[illegible]	[illegible]	[illegible]	[illegible]	[illegible]	[illegible]	[illegible]	[illegible]	[illegible]	[illegible]	[illegible]	[illegible]	[illegible]	[illegible]	[illegible]	0 (0 to 0)	3·8
Guinea worm disease	0 (0 to 0)	[illegible]	[illegible]	[illegible]	[illegible]	[illegible]	[illegible]	[illegible]	[illegible]	[illegible]	[illegible]	[illegible]	[illegible]	[illegible]	[illegible]	[illegible]	[illegible]	[illegible]	[illegible]	[illegible]	[illegible]	[illegible]	0 (0 to 0)	0
Other neglected tropical diseases	0·1 (0 to 0·1)	[illegible]	[illegible]	[illegible]	[illegible]	[illegible]	[illegible]	[illegible]	[illegible]	[illegible]	[illegible]	[illegible]	[illegible]	[illegible]	[illegible]	[illegible]	[illegible]	[illegible]	[illegible]	[illegible]	[illegible]	[illegible]	0 (0 to 0·1)	16·9
Other infectious diseases	2·2 (1·3 to 3·5)	[illegible]	[illegible]	[illegible]	[illegible]	[illegible]	[illegible]	[illegible]	[illegible]	[illegible]	[illegible]	[illegible]	[illegible]	[illegible]	[illegible]	[illegible]	[illegible]	[illegible]	[illegible]	[illegible]	[illegible]	[illegible]	0·2 (0·1 to 0·4)	173
Meningitis	1·4 (0·7 to 2·5)	[illegible]	[illegible]	[illegible]	[illegible]	[illegible]	[illegible]	[illegible]	[illegible]	[illegible]	[illegible]	[illegible]	[illegible]	[illegible]	[illegible]	[illegible]	[illegible]	[illegible]	[illegible]	[illegible]	[illegible]	[illegible]	0 (0 to 0)	44·5
Encephalitis	0·3 (0·2 to 0·6)	[illegible]	[illegible]	[illegible]	[illegible]	[illegible]	[illegible]	[illegible]	[illegible]	[illegible]	[illegible]	[illegible]	[illegible]	[illegible]	[illegible]	[illegible]	[illegible]	[illegible]	[illegible]	[illegible]	[illegible]	[illegible]	0 (0 to 0)	36·1
Diphtheria	0 (0 to 0)	[illegible]	[illegible]	[illegible]	[illegible]	[illegible]	[illegible]	[illegible]	[illegible]	[illegible]	[illegible]	[illegible]	[illegible]	[illegible]	[illegible]	[illegible]	[illegible]	[illegible]	[illegible]	[illegible]	[illegible]	[illegible]	0 (0 to 0)	0·1
Tetanus	0·1 (0 to 0·1)	[illegible]	[illegible]	[illegible]	[illegible]	[illegible]	[illegible]	[illegible]	[illegible]	[illegible]	[illegible]	[illegible]	[illegible]	[illegible]	[illegible]	[illegible]	[illegible]	[illegible]	[illegible]	[illegible]	[illegible]	[illegible]	0 (0 to 0)	4·5
Measles	0 (0 to 0)	[illegible]	[illegible]	[illegible]	[illegible]	[illegible]	[illegible]	[illegible]	[illegible]	[illegible]	[illegible]	[illegible]	[illegible]	[illegible]	[illegible]	[illegible]	[illegible]	[illegible]	[illegible]	[illegible]	[illegible]	[illegible]	0 (0 to 0)	0
Varicella and herpes zoster	0 (0 to 0·1)	[illegible]	[illegible]	[illegible]	[illegible]	[illegible]	[illegible]	[illegible]	[illegible]	[illegible]	[illegible]	[illegible]	[illegible]	[illegible]	[illegible]	[illegible]	[illegible]	[illegible]	[illegible]	[illegible]	[illegible]	[illegible]	0·1 (0 to 0·3)	5·9
Acute hepatitis	0 (0 to 0)	[illegible]	[illegible]	[illegible]	[illegible]	[illegible]	[illegible]	[illegible]	[illegible]	[illegible]	[illegible]	[illegible]	[illegible]	[illegible]	[illegible]	[illegible]	[illegible]	[illegible]	[illegible]	[illegible]	[illegible]	[illegible]	0 (0 to 0)	5·4
Acute hepatitis A	0 (0 to 0)	[illegible]	[illegible]	[illegible]	[illegible]	[illegible]	[illegible]	[illegible]	[illegible]	[illegible]	[illegible]	[illegible]	[illegible]	[illegible]	[illegible]	[illegible]	[illegible]	[illegible]	[illegible]	[illegible]	[illegible]	[illegible]	0 (0 to 0)	2·9
Acute hepatitis B	0 (0 to 0)	[illegible]	[illegible]	[illegible]	[illegible]	[illegible]	[illegible]	[illegible]	[illegible]	[illegible]	[illegible]	[illegible]	[illegible]	[illegible]	[illegible]	[illegible]	[illegible]	[illegible]	[illegible]	[illegible]	[illegible]	[illegible]	0 (0 to 0)	1·9
Acute hepatitis C	0 (0 to 0)	[illegible]	[illegible]	[illegible]	[illegible]	[illegible]	[illegible]	[illegible]	[illegible]	[illegible]	[illegible]	[illegible]	[illegible]	[illegible]	[illegible]	[illegible]	[illegible]	[illegible]	[illegible]	[illegible]	[illegible]	[illegible]	0 (0 to 0)	0·1
Acute hepatitis E	0 (0 to 0)	[illegible]	[illegible]	[illegible]	[illegible]	[illegible]	[illegible]	[illegible]	[illegible]	[illegible]	[illegible]	[illegible]	[illegible]	[illegible]	[illegible]	[illegible]	[illegible]	[illegible]	[illegible]	[illegible]	[illegible]	[illegible]	0 (0 to 0)	0·5
Other unspecified infectious diseases	0·3 (0·1 to 0·7)	[illegible]	[illegible]	[illegible]	[illegible]	[illegible]	[illegible]	[illegible]	[illegible]	[illegible]	[illegible]	[illegible]	[illegible]	[illegible]	[illegible]	[illegible]	[illegible]	[illegible]	[illegible]	[illegible]	[illegible]	[illegible]	0·1 (0 to 0·2)	46·5
Maternal and neonatal disorders	751 (526 to 1,060)	[illegible]	[illegible]	[illegible]	[illegible]	[illegible]	[illegible]	[illegible]	[illegible]	[illegible]	[illegible]	[illegible]	[illegible]	[illegible]	[illegible]	[illegible]	[illegible]	[illegible]	[illegible]	[illegible]	[illegible]	[illegible]	0 (0 to 0)	1,300
Maternal disorders	0 (0 to 0)	[illegible]	[illegible]	[illegible]	[illegible]	[illegible]	[illegible]	[illegible]	[illegible]	[illegible]	[illegible]	[illegible]	[illegible]	[illegible]	[illegible]	[illegible]	[illegible]	[illegible]	[illegible]	[illegible]	[illegible]	[illegible]	0 (0 to 0)	57·4
Maternal hemorrhage	0 (0 to 0)	[illegible]	[illegible]	[illegible]	[illegible]	[illegible]	[illegible]	[illegible]	[illegible]	[illegible]	[illegible]	[illegible]	[illegible]	[illegible]	[illegible]	[illegible]	[illegible]	[illegible]	[illegible]	[illegible]	[illegible]	[illegible]	0 (0 to 0)	16·7
Maternal sepsis and other maternal infections	0 (0 to 0)	[illegible]	[illegible]	[illegible]	[illegible]	[illegible]	[illegible]	[illegible]	[illegible]	[illegible]	[illegible]	[illegible]	[illegible]	[illegible]	[illegible]	[illegible]	[illegible]	[illegible]	[illegible]	[illegible]	[illegible]	[illegible]	0 (0 to 0)	10·3
Maternal hypertensive disorders	0 (0 to 0)	[illegible]	[illegible]	[illegible]	[illegible]	[illegible]	[illegible]	[illegible]	[illegible]	[illegible]	[illegible]	[illegible]	[illegible]	[illegible]	[illegible]	[illegible]	[illegible]	[illegible]	[illegible]	[illegible]	[illegible]	[illegible]	0 (0 to 0)	1·7
Maternal obstructed labor and uterine rupture	0 (0 to 0)	[illegible]	[illegible]	[illegible]	[illegible]	[illegible]	[illegible]	[illegible]	[illegible]	[illegible]	[illegible]	[illegible]	[illegible]	[illegible]	[illegible]	[illegible]	[illegible]	[illegible]	[illegible]	[illegible]	[illegible]	[illegible]	0 (0 to 0)	0·9
Maternal abortion and miscarriage	0 (0 to 0)	[illegible]	[illegible]	[illegible]	[illegible]	[illegible]	[illegible]	[illegible]	[illegible]	[illegible]	[illegible]	[illegible]	[illegible]	[illegible]	[illegible]	[illegible]	[illegible]	[illegible]	[illegible]	[illegible]	[illegible]	[illegible]	0 (0 to 0)	8·3
Ectopic pregnancy	0 (0 to 0)	[illegible]	[illegible]	[illegible]	[illegible]	[illegible]	[illegible]	[illegible]	[illegible]	[illegible]	[illegible]	[illegible]	[illegible]	[illegible]	[illegible]	[illegible]	[illegible]	[illegible]	[illegible]	[illegible]	[illegible]	[illegible]	0 (0 to 0)	8·5
Indirect maternal deaths	--	[illegible]	[illegible]	[illegible]	[illegible]	[illegible]	[illegible]	[illegible]	[illegible]	[illegible]	[illegible]	[illegible]	[illegible]	[illegible]	[illegible]	[illegible]	[illegible]	[illegible]	[illegible]	[illegible]	[illegible]	[illegible]	--	NA
Late maternal deaths	--	[illegible]	[illegible]	[illegible]	[illegible]	[illegible]	[illegible]	[illegible]	[illegible]	[illegible]	[illegible]	[illegible]	[illegible]	[illegible]	[illegible]	[illegible]	[illegible]	[illegible]	[illegible]	[illegible]	[illegible]	[illegible]	--	NA
Maternal deaths aggravated by HIV/AIDS	--	[illegible]	[illegible]	[illegible]	[illegible]	[illegible]	[illegible]	[illegible]	[illegible]	[illegible]	[illegible]	[illegible]	[illegible]	[illegible]	[illegible]	[illegible]	[illegible]	[illegible]	[illegible]	[illegible]	[illegible]	[illegible]	--	NA
Neonatal disorders	751 (526 to 1,060)	[illegible]	[illegible]	[illegible]	[illegible]	[illegible]	[illegible]	[illegible]	[illegible]	[illegible]	[illegible]	[illegible]	[illegible]	[illegible]	[illegible]	[illegible]	[illegible]	[illegible]	[illegible]	[illegible]	[illegible]	[illegible]	0 (0 to 0)	1,250
Neonatal preterm birth	239 (150 to 356)	[illegible]	[illegible]	[illegible]	[illegible]	[illegible]	[illegible]	[illegible]	[illegible]	[illegible]	[illegible]	[illegible]	[illegible]	[illegible]	[illegible]	[illegible]	[illegible]	[illegible]	[illegible]	[illegible]	[illegible]	[illegible]	0 (0 to 0)	388
Neonatal encephalopathy due to birth asphyxia and trauma	325 (205 to 482)	[illegible]	[illegible]	[illegible]	[illegible]	[illegible]	[illegible]	[illegible]	[illegible]	[illegible]	[illegible]	[illegible]	[illegible]	[illegible]	[illegible]	[illegible]	[illegible]	[illegible]	[illegible]	[illegible]	[illegible]	[illegible]	0 (0 to 0)	422
Neonatal sepsis and other neonatal infections	102 (59·5 to 165)	[illegible]	[illegible]	[illegible]	[illegible]	[illegible]	[illegible]	[illegible]	[illegible]	[illegible]	[illegible]	[illegible]	[illegible]	[illegible]	[illegible]	[illegible]	[illegible]	[illegible]	[illegible]	[illegible]	[illegible]	[illegible]	0 (0 to 0)	302
Hemolytic disease and other neonatal jaundice	11·7 (6·3 to 19·7)	[illegible]	[illegible]	[illegible]	[illegible]	[illegible]	[illegible]	[illegible]	[illegible]	[illegible]	[illegible]	[illegible]	[illegible]	[illegible]	[illegible]	[illegible]	[illegible]	[illegible]	[illegible]	[illegible]	[illegible]	[illegible]	0 (0 to 0)	25·8
Other neonatal disorders	72·4 (42·8 to 113)	[illegible]	[illegible]	[illegible]	[illegible]	[illegible]	[illegible]	[illegible]	[illegible]	[illegible]	[illegible]	[illegible]	[illegible]	[illegible]	[illegible]	[illegible]	[illegible]	[illegible]	[illegible]	[illegible]	[illegible]	[illegible]	0 (0 to 0)	108
Nutritional deficiencies	0 (0 to 0·1)	[illegible]	[illegible]	[illegible]	[illegible]	[illegible]	[illegible]	[illegible]	[illegible]	[illegible]	[illegible]	[illegible]	[illegible]	[illegible]	[illegible]	[illegible]	[illegible]	[illegible]	[illegible]	[illegible]	[illegible]	[illegible]	0·9 (0·6 to 1·3)	284
Protein-energy malnutrition	0 (0 to 0)	[illegible]	[illegible]	[illegible]	[illegible]	[illegible]	[illegible]	[illegible]	[illegible]	[illegible]	[illegible]	[illegible]	[illegible]	[illegible]	[illegible]	[illegible]	[illegible]	[illegible]	[illegible]	[illegible]	[illegible]	[illegible]	0·6 (0·4 to 1)	65·1
Iodine deficiency	0 (0 to 0)	[illegible]	[illegible]	[illegible]	[illegible]	[illegible]	[illegible]	[illegible]	[illegible]	[illegible]	[illegible]	[illegible]	[illegible]	[illegible]	[illegible]	[illegible]	[illegible]	[illegible]	[illegible]	[illegible]	[illegible]	[illegible]	0 (0 to 0)	4·2
Vitamin A deficiency	0 (0 to 0)	[illegible]	[illegible]	[illegible]	[illegible]	[illegible]	[illegible]	[illegible]	[illegible]	[illegible]	[illegible]	[illegible]	[illegible]	[illegible]	[illegible]	[illegible]	[illegible]	[illegible]	[illegible]	[illegible]	[illegible]	[illegible]	0 (0 to 0)	2·9
Dietary iron deficiency	0 (0 to 0)	[illegible]	[illegible]	[illegible]	[illegible]	[illegible]	[illegible]	[illegible]	[illegible]	[illegible]	[illegible]	[illegible]	[illegible]	[illegible]	[illegible]	[illegible]	[illegible]	[illegible]	[illegible]	[illegible]	[illegible]	[illegible]	0·1 (0 to 0·2)	192
Other nutritional deficiencies	0 (0 to 0)	[illegible]	[illegible]	[illegible]	[illegible]	[illegible]	[illegible]	[illegible]	[illegible]	[illegible]	[illegible]	[illegible]	[illegible]	[illegible]	[illegible]	[illegible]	[illegible]	[illegible]	[illegible]	[illegible]	[illegible]	[illegible]	0·1 (0·1 to 0·2)	19·6
Non-communicable diseases	111 (70·2 to 170)	[illegible]	[illegible]	[illegible]	[illegible]	[illegible]	[illegible]	[illegible]	[illegible]	[illegible]	[illegible]	[illegible]	[illegible]	[illegible]	[illegible]	[illegible]	[illegible]	[illegible]	[illegible]	[illegible]	[illegible]	[illegible]	115 (101 to 126)	19,600
Neoplasms	1·1 (0·7 to 1·9)	[illegible]	[illegible]	[illegible]	[illegible]	[illegible]	[illegible]	[illegible]	[illegible]	[illegible]	[illegible]	[illegible]	[illegible]	[illegible]	[illegible]	[illegible]	[illegible]	[illegible]	[illegible]	[illegible]	[illegible]	[illegible]	12·4 (9·5 to 15·5)	3,980
Lip and oral cavity cancer	0 (0 to 0)	[illegible]	[illegible]	[illegible]	[illegible]	[illegible]	[illegible]	[illegible]	[illegible]	[illegible]	[illegible]	[illegible]	[illegible]	[illegible]	[illegible]	[illegible]	[illegible]	[illegible]	[illegible]	[illegible]	[illegible]	[illegible]	0·2 (0·1 to 0·3)	65·5
Nasopharynx cancer	0 (0 to 0)	[illegible]	[illegible]	[illegible]	[illegible]	[illegible]	[illegible]	[illegible]	[illegible]	[illegible]	[illegible]	[illegible]	[illegible]	[illegible]	[illegible]	[illegible]	[illegible]	[illegible]	[illegible]	[illegible]	[illegible]	[illegible]	0 (0 to 0)	14·2
Other pharynx cancer	0 (0 to 0)	[illegible]	[illegible]	[illegible]	[illegible]	[illegible]	[illegible]	[illegible]	[illegible]	[illegible]	[illegible]	[illegible]	[illegible]	[illegible]	[illegible]	[illegible]	[illegible]	[illegible]	[illegible]	[illegible]	[illegible]	[illegible]	0·1 (0 to 0·1)	44·7
Esophageal cancer	0 (0 to 0)	[illegible]	[illegible]	[illegible]	[illegible]	[illegible]	[illegible]	[illegible]	[illegible]	[illegible]	[illegible]	[illegible]	[illegible]	[illegible]	[illegible]	[illegible]	[illegible]	[illegible]	[illegible]	[illegible]	[illegible]	[illegible]	0·2 (0·1 to 0·3)	106
Stomach cancer	0 (0 to 0)	[illegible]	[illegible]	[illegible]	[illegible]	[illegible]	[illegible]	[illegible]	[illegible]	[illegible]	[illegible]	[illegible]	[illegible]	[illegible]	[illegible]	[illegible]	[illegible]	[illegible]	[illegible]	[illegible]	[illegible]	[illegible]	1·4 (0·9 to 1·9)	388
Colon and rectum cancer	0 (0 to 0)	[illegible]	[illegible]	[illegible]	[illegible]	[illegible]	[illegible]	[illegible]	[illegible]	[illegible]	[illegible]	[illegible]	[illegible]	[illegible]	[illegible]	[illegible]	[illegible]	[illegible]	[illegible]	[illegible]	[illegible]	[illegible]	1·2 (0·8 to 1·7)	277
Liver cancer	0 (0 to 0·1)	[illegible]	[illegible]	[illegible]	[illegible]	[illegible]	[illegible]	[illegible]	[illegible]	[illegible]	[illegible]	[illegible]	[illegible]	[illegible]	[illegible]	[illegible]	[illegible]	[illegible]	[illegible]	[illegible]	[illegible]	[illegible]	0·1 (0·1 to 0·2)	70
Liver cancer due to hepatitis B	0 (0 to 0)	[illegible]	[illegible]	[illegible]	[illegible]	[illegible]	[illegible]	[illegible]	[illegible]	[illegible]	[illegible]	[illegible]	[illegible]	[illegible]	[illegible]	[illegible]	[illegible]	[illegible]	[illegible]	[illegible]	[illegible]	[illegible]	0 (0 to 0)	20·4
Liver cancer due to hepatitis C	0 (0 to 0)	[illegible]	[illegible]	[illegible]	[illegible]	[illegible]	[illegible]	[illegible]	[illegible]	[illegible]	[illegible]	[illegible]	[illegible]	[illegible]	[illegible]	[illegible]	[illegible]	[illegible]	[illegible]	[illegible]	[illegible]	[illegible]	0·1 (0 to 0·1)	13·4
Liver cancer due to alcohol use	0 (0 to 0)	[illegible]	[illegible]	[illegible]	[illegible]	[illegible]	[illegible]	[illegible]	[illegible]	[illegible]	[illegible]	[illegible]	[illegible]	[illegible]	[illegible]	[illegible]	[illegible]	[illegible]	[illegible]	[illegible]	[illegible]	[illegible]	0 (0 to 0)	25
Liver cancer due to NASH	0 (0 to 0)	[illegible]	[illegible]	[illegible]	[illegible]	[illegible]	[illegible]	[illegible]	[illegible]	[illegible]	[illegible]	[illegible]	[illegible]	[illegible]	[illegible]	[illegible]	[illegible]	[illegible]	[illegible]	[illegible]	[illegible]	[illegible]	0 (0 to 0)	6·3
Liver cancer due to other causes	0 (0 to 0·1)	[illegible]	[illegible]	[illegible]	[illegible]	[illegible]	[illegible]	[illegible]	[illegible]	[illegible]	[illegible]	[illegible]	[illegible]	[illegible]	[illegible]	[illegible]	[illegible]	[illegible]	[illegible]	[illegible]	[illegible]	[illegible]	0 (0 to 0)	4·9
Gallbladder and biliary tract cancer	0 (0 to 0)	[illegible]	[illegible]	[illegible]	[illegible]	[illegible]	[illegible]	[illegible]	[illegible]	[illegible]	[illegible]	[illegible]	[illegible]	[illegible]	[illegible]	[illegible]	[illegible]	[illegible]	[illegible]	[illegible]	[illegible]	[illegible]	0·1 (0·1 to 0·2)	39·6
Pancreatic cancer	0 (0 to 0)	[illegible]	[illegible]	[illegible]	[illegible]	[illegible]	[illegible]	[illegible]	[illegible]	[illegible]	[illegible]	[illegible]	[illegible]	[illegible]	[illegible]	[illegible]	[illegible]	[illegible]	[illegible]	[illegible]	[illegible]	[illegible]	0·4 (0·3 to 0·6)	165
Larynx cancer	0 (0 to 0)	[illegible]	[illegible]	[illegible]	[illegible]	[illegible]	[illegible]	[illegible]	[illegible]	[illegible]	[illegible]	[illegible]	[illegible]	[illegible]	[illegible]	[illegible]	[illegible]	[illegible]	[illegible]	[illegible]	[illegible]	[illegible]	0·1 (0 to 0·1)	51·7
Tracheal, bronchus, and lung cancer	0 (0 to 0)	[illegible]	[illegible]	[illegible]	[illegible]	[illegible]	[illegible]	[illegible]	[illegible]	[illegible]	[illegible]	[illegible]	[illegible]	[illegible]	[illegible]	[illegible]	[illegible]	[illegible]	[illegible]	[illegible]	[illegible]	[illegible]	0·6 (0·4 to 0·8)	357
Malignant skin melanoma	0 (0 to 0)	0 (0 to 0)	0 (0 to 0)	[illegible]	[illegible]	[illegible]	[illegible]	[illegible]	[illegible]	[illegible]	[illegible]	[illegible]	[illegible]	[illegible]	[illegible]	[illegible]	[illegible]	[illegible]	[illegible]	[illegible]	[illegible]	[illegible]	0 (0 to 0·1)	11
Non-melanoma skin cancer	0 (0 to 0)	0 (0 to 0)	0 (0 to 0)	[illegible]	[illegible]	[illegible]	[illegible]	[illegible]	[illegible]	[illegible]	[illegible]	[illegible]	[illegible]	[illegible]	[illegible]	[illegible]	[illegible]	[illegible]	[illegible]	[illegible]	[illegible]	0·5 (0·3 to 0·7)	0·2 (0·1 to 0·3)	10·9
Non-melanoma skin cancer (squamous-cell carcinoma)	0 (0 to 0)	0 (0 to 0)	0 (0 to 0)	[illegible]	[illegible]	[illegible]	[illegible]	[illegible]	[illegible]	[illegible]	[illegible]	[illegible]	[illegible]	[illegible]	[illegible]	[illegible]	[illegible]	[illegible]	[illegible]	[illegible]	[illegible]	0·5 (0·3 to 0·7)	0·2 (0·1 to 0·3)	10·9
Non-melanoma skin cancer (basal-cell carcinoma)	0 (0 to 0)	0 (0 to 0)	0 (0 to 0)	[illegible]	[illegible]	[illegible]	[illegible]	[illegible]	[illegible]	[illegible]	[illegible]	[illegible]	[illegible]	[illegible]	[illegible]	[illegible]	[illegible]	[illegible]	[illegible]	[illegible]	[illegible]	0 (0 to 0)	0 (0 to 0)	0
Breast cancer	0 (0 to 0)	0 (0 to 0)	0 (0 to 0)	[illegible]	[illegible]	[illegible]	[illegible]	[illegible]	[illegible]	[illegible]	[illegible]	[illegible]	[illegible]	[illegible]	[illegible]	[illegible]	[illegible]	[illegible]	[illegible]	[illegible]	[illegible]	4·5 (3·1 to 6·4)	1·5 (0·9 to 2·2)	362
Cervical cancer	0 (0 to 0)	0 (0 to 0)	0 (0 to 0)	[illegible]	[illegible]	[illegible]	[illegible]	[illegible]	[illegible]	[illegible]	[illegible]	[illegible]	[illegible]	[illegible]	[illegible]	[illegible]	[illegible]	[illegible]	[illegible]	[illegible]	[illegible]	1·5 (0·9 to 2·3)	0·4 (0·3 to 0·7)	213
Uterine cancer	0 (0 to 0)	0 (0 to 0)	0 (0 to 0)	[illegible]	[illegible]	[illegible]	[illegible]	[illegible]	[illegible]	[illegible]	[illegible]	[illegible]	[illegible]	[illegible]	[illegible]	[illegible]	[illegible]	[illegible]	[illegible]	[illegible]	[illegible]	0·6 (0·4 to 0·8)	0·2 (0·1 to 0·2)	48·8
Ovarian cancer	0 (0 to 0)	0 (0 to 0)	0 (0 to 0)	[illegible]	[illegible]	[illegible]	[illegible]	[illegible]	[illegible]	[illegible]	[illegible]	[illegible]	[illegible]	[illegible]	[illegible]	[illegible]	[illegible]	[illegible]	[illegible]	[illegible]	[illegible]	0·3 (0·2 to 0·5)	0·1 (0 to 0·1)	38·9
Prostate cancer	0 (0 to 0)	0 (0 to 0)	0 (0 to 0)	[illegible]	[illegible]	[illegible]	[illegible]	[illegible]	[illegible]	[illegible]	[illegible]	[illegible]	[illegible]	[illegible]	[illegible]	[illegible]	[illegible]	[illegible]	[illegible]	[illegible]	[illegible]	26·5 (18·3 to 36·6)	4 (2·6 to 6·1)	818
Testicular cancer	0 (0 to 0)	0 (0 to 0)	0 (0 to 0·1)	[illegible]	[illegible]	[illegible]	[illegible]	[illegible]	[illegible]	[illegible]	[illegible]	[illegible]	[illegible]	[illegible]	[illegible]	[illegible]	[illegible]	[illegible]	[illegible]	[illegible]	[illegible]	0 (0 to 0)	0 (0 to 0)	2·9
Kidney cancer	0·1 (0 to 0·2)	0 (0 to 0·1)	0·4 (0·2 to 0·7)	[illegible]	[illegible]	[illegible]	[illegible]	[illegible]	[illegible]	[illegible]	[illegible]	[illegible]	[illegible]	[illegible]	[illegible]	[illegible]	[illegible]	[illegible]	[illegible]	[illegible]	[illegible]	0·3 (0·2 to 0·5)	0·1 (0 to 0·1)	58·9
Bladder cancer	0 (0 to 0)	0 (0 to 0)	0 (0 to 0)	[illegible]	[illegible]	[illegible]	[illegible]	[illegible]	[illegible]	[illegible]	[illegible]	[illegible]	[illegible]	[illegible]	[illegible]	[illegible]	[illegible]	[illegible]	[illegible]	[illegible]	[illegible]	1·8 (1·3 to 2·5)	0·4 (0·3 to 0·6)	64·3
Brain and central nervous system cancer	0·1 (0 to 0·2)	0·1 (0 to 0·2)	0·6 (0·2 to 1·1)	[illegible]	[illegible]	[illegible]	[illegible]	[illegible]	[illegible]	[illegible]	[illegible]	[illegible]	[illegible]	[illegible]	[illegible]	[illegible]	[illegible]	[illegible]	[illegible]	[illegible]	[illegible]	0·1 (0 to 0·2)	0 (0 to 0)	42·4
Thyroid cancer	0 (0 to 0)	0 (0 to 0)	0 (0 to 0)	[illegible]	[illegible]	[illegible]	[illegible]	[illegible]	[illegible]	[illegible]	[illegible]	[illegible]	[illegible]	[illegible]	[illegible]	[illegible]	[illegible]	[illegible]	[illegible]	[illegible]	[illegible]	0·1 (0·1 to 0·2)	0 (0 to 0)	14·9
Mesothelioma	0 (0 to 0)	0 (0 to 0)	0 (0 to 0)	[illegible]	[illegible]	[illegible]	[illegible]	[illegible]	[illegible]	[illegible]	[illegible]	[illegible]	[illegible]	[illegible]	[illegible]	[illegible]	[illegible]	[illegible]	[illegible]	[illegible]	[illegible]	0 (0 to 0·1)	0 (0 to 0)	8·6
Hodgkin lymphoma	0 (0 to 0)	0 (0 to 0)	0 (0 to 0)	[illegible]	[illegible]	[illegible]	[illegible]	[illegible]	[illegible]	[illegible]	[illegible]	[illegible]	[illegible]	[illegible]	[illegible]	[illegible]	[illegible]	[illegible]	[illegible]	[illegible]	[illegible]	0 (0 to 0·1)	0 (0 to 0)	15·8
Non-Hodgkin lymphoma	0 (0 to 0)	0 (0 to 0)	0 (0 to 0)	[illegible]	[illegible]	[illegible]	[illegible]	[illegible]	[illegible]	[illegible]	[illegible]	[illegible]	[illegible]	[illegible]	[illegible]	[illegible]	[illegible]	[illegible]	[illegible]	[illegible]	[illegible]	0·8 (0·6 to 1·2)	0·2 (0·1 to 0·3)	184
Multiple myeloma	0 (0 to 0)	0 (0 to 0)	0 (0 to 0)	[illegible]	[illegible]	[illegible]	[illegible]	[illegible]	[illegible]	[illegible]	[illegible]	[illegible]	[illegible]	[illegible]	[illegible]	[illegible]	[illegible]	[illegible]	[illegible]	[illegible]	[illegible]	0·9 (0·6 to 1·3)	0·2 (0·1 to 0·3)	119
Leukemia	0·4 (0·2 to 0·9)	0·3 (0·1 to 0·8)	2·5 (1·4 to 4·2)	[illegible]	[illegible]	[illegible]	[illegible]	[illegible]	[illegible]	[illegible]	[illegible]	[illegible]	[illegible]	[illegible]	[illegible]	[illegible]	[illegible]	[illegible]	[illegible]	[illegible]	[illegible]	1·1 (0·8 to 1·5)	0·2 (0·2 to 0·4)	173
Acute lymphoid leukemia	[illegible]	[illegible]	[illegible]	[illegible]	[illegible]	[illegible]	[illegible]	[illegible]	[illegible]	[illegible]	[illegible]	[illegible]	[illegible]	[illegible]	[illegible]	[illegible]	[illegible]	[illegible]	[illegible]	[illegible]	[illegible]	[illegible]	[illegible]	51·5
Chronic lymphoid leukemia	[illegible]	[illegible]	[illegible]	[illegible]	[illegible]	[illegible]	[illegible]	[illegible]	[illegible]	[illegible]	[illegible]	[illegible]	[illegible]	[illegible]	[illegible]	[illegible]	[illegible]	[illegible]	[illegible]	[illegible]	[illegible]	[illegible]	[illegible]	[illegible]
Acute myeloid leukemia	[illegible]	[illegible]	[illegible]	[illegible]	[illegible]	[illegible]	[illegible]	[illegible]	[illegible]	[illegible]	[illegible]	[illegible]	[illegible]	[illegible]	[illegible]	[illegible]	[illegible]	[illegible]	[illegible]	[illegible]	[illegible]	[illegible]	[illegible]	35·4

Chronic myeloid leukemia	0 (0 to 0)	0 (0 to 0)	0·1 (0 to 0·2)	0·2 (0·1 to 0·4)	0·1 (0 to 0·2)	0·1 (0·1 to 0·3)	0·2 (0·1 to 0·4)	0·4 (0·2 to 0·8)	0·8 (0·4 to 1·5)	0·7 (0·3 to 1·3)	1 (0·5 to 1·7)	0·6 (0·3 to 1·1)	0·7 (0·4 to 1·4)	1·3 (0·7 to 2·2)	1·4 (0·7 to 2·6)	1·4 (0·7 to 2·6)	1·3 (0·7 to 2·4)	0·9 (0·4 to 1·8)	0·8 (0·4 to 1·5)	0·6 (0·3 to 1·1)	0·2 (0·1 to 0·4)	0·1 (0 to 0·1)	0 (0 to 0)	12·9
Other leukemia	0·2 (0·1 to 0·6)	0·1 (0 to 0·2)	1 (0·5 to 1·9)	1·5 (0·7 to 2·8)	1·4 (0·7 to 2·5)	3 (1·5 to 5·2)	2·4 (1·2 to 4)	3·1 (1·7 to 5·3)	2·9 (1·6 to 4·6)	3 (1·7 to 4·7)	2·9 (1·6 to 4·5)	3·2 (2 to 4·8)	4·2 (2·6 to 6·3)	4·3 (2·6 to 6·6)	4·9 (3·1 to 7·3)	5·3 (3·4 to 8·1)	5·3 (3·5 to 7·7)	5·1 (3·4 to 7·4)	4·9 (3·3 to 6·9)	4 (2·7 to 5·8)	2·1 (1·4 to 3·1)	0·8 (0·5 to 1·1)	0·2 (0·1 to 0·3)	65·7
Other malignant neoplasms	0·5 (0·2 to 0·9)	0·5 (0·2 to 1)	3·5 (2 to 5·8)	14·3 (8·7 to 22·8)	7·6 (5·5 to 11·1)	10·5 (7·6 to 14·1)	10·8 (7·7 to 14·8)	8·9 (6·3 to 12)	8·1 (6 to 10·6)	8·4 (6·1 to 11·2)	8·1 (6 to 10·8)	8·3 (5·9 to 11·4)	11·2 (7·8 to 15·5)	14·5 (10·2 to 19·3)	16·2 (11·6 to 22·2)	16 (11·7 to 21·1)	14·4 (10·4 to 18·9)	11·8 (8·8 to 15·1)	10·4 (7·8 to 13·4)	8·3 (6·4 to 10·7)	4·7 (3·4 to 6·2)	1·8 (1·2 to 2·4)	0·4 (0·3 to 0·6)	199
Other neoplasms	0 (0 to 0·1)	0·1 (0 to 0·1)	0·2 (0·1 to 0·5)	0·6 (0·4 to 1)	0·3 (0·2 to 0·4)	0·5 (0·3 to 0·7)	0·4 (0·3 to 0·6)	0·3 (0·2 to 0·4)	0·5 (0·3 to 0·7)	0·5 (0·3 to 0·7)	0·4 (0·3 to 0·6)	0·6 (0·4 to 0·8)	0·7 (0·5 to 0·9)	1·1 (0·8 to 1·6)	1·4 (1 to 2·1)	1·8 (1·3 to 2·6)	2 (1·5 to 2·8)	2·1 (1·6 to 2·9)	2·2 (1·6 to 3)	1·8 (1·3 to 2·4)	0·8 (0·6 to 1·1)	0·3 (0·2 to 0·4)	0·1 (0 to 0·1)	18·7
Myelodysplastic, myeloproliferative, and other hematopoietic neo	0 (0 to 0·1)	0·1 (0 to 0·1)	0·2 (0·1 to 0·5)	0·6 (0·4 to 1)	0·3 (0·2 to 0·4)	0·5 (0·3 to 0·7)	0·4 (0·3 to 0·6)	0·3 (0·2 to 0·4)	0·5 (0·3 to 0·7)	0·5 (0·3 to 0·7)	0·4 (0·3 to 0·6)	0·6 (0·4 to 0·8)	0·7 (0·5 to 0·9)	1·1 (0·8 to 1·6)	1·4 (1 to 2·1)	1·8 (1·3 to 2·6)	2 (1·5 to 2·8)	2·1 (1·6 to 2·9)	2·2 (1·6 to 3)	1·8 (1·3 to 2·4)	0·8 (0·6 to 1·1)	0·3 (0·2 to 0·4)	0·1 (0 to 0·1)	18·7
Benign and in situ intestinal neoplasms	0 (0 to 0)	0 (0 to 0)	0 (0 to 0)	0 (0 to 0)	0 (0 to 0)	0 (0 to 0)	0 (0 to 0)	0 (0 to 0)	0 (0 to 0)	0 (0 to 0)	0 (0 to 0)	0 (0 to 0)	0 (0 to 0)	0 (0 to 0)	0 (0 to 0)	0 (0 to 0)	0 (0 to 0)	0 (0 to 0)	0 (0 to 0)	0 (0 to 0)	0 (0 to 0)	0 (0 to 0)	0 (0 to 0)	0
Benign and in situ cervical and uterine neoplasms	0 (0 to 0)	0 (0 to 0)	0 (0 to 0)	0 (0 to 0)	0 (0 to 0)	0 (0 to 0)	0 (0 to 0)	0 (0 to 0)	0 (0 to 0)	0 (0 to 0)	0 (0 to 0)	0 (0 to 0)	0 (0 to 0)	0 (0 to 0)	0 (0 to 0)	0 (0 to 0)	0 (0 to 0)	0 (0 to 0)	0 (0 to 0)	0 (0 to 0)	0 (0 to 0)	0 (0 to 0)	0 (0 to 0)	0
Other benign and in situ neoplasms	0 (0 to 0)	0 (0 to 0)	0 (0 to 0)	0 (0 to 0)	0 (0 to 0)	0 (0 to 0)	0 (0 to 0)	0 (0 to 0)	0 (0 to 0)	0 (0 to 0)	0 (0 to 0)	0 (0 to 0)	0 (0 to 0)	0 (0 to 0)	0 (0 to 0)	0 (0 to 0)	0 (0 to 0)	0 (0 to 0)	0 (0 to 0)	0 (0 to 0)	0 (0 to 0)	0 (0 to 0)	0 (0 to 0)	0
Cardiovascular diseases	2·9 (1·5 to 5·1)	1·1 (0·6 to 1·9)	6·6 (3·6 to 11·2)	4·5 (2·9 to 6·9)	4·8 (3·6 to 6·1)	12·7 (9·1 to 17·4)	25·6 (18·9 to 34·6)	31·4 (22·7 to 43·9)	41·8 (30·4 to 57·8)	58·8 (42·4 to 79·4)	83·4 (60·5 to 111)	108 (78·3 to 142)	180 (136 to 236)	298 (231 to 379)	399 (309 to 515)	484 (379 to 609)	525 (422 to 639)	522 (433 to 636)	558 (466 to 665)	545 (463 to 636)	334 (280 to 390)	158 (124 to 186)	53·8 (42·3 to 63·6)	4,440
Rheumatic heart disease	0 (0 to 0)	0 (0 to 0)	0 (0 to 0)	0·3 (0·2 to 0·4)	1·2 (0·7 to 1·8)	3·6 (2·3 to 5·4)	5·9 (4 to 8·4)	5·8 (3·9 to 8·2)	6·4 (4·5 to 9)	7 (4·9 to 9·7)	6·6 (4·7 to 9·3)	6·2 (4·3 to 8·6)	6·7 (4·8 to 9·1)	6·2 (4·5 to 8·4)	5·4 (3·9 to 7·5)	4·2 (3 to 5·8)	3·4 (2·5 to 4·5)	2·4 (1·8 to 3·4)	2·2 (1·6 to 3·1)	1·7 (1·2 to 2·3)	0·9 (0·7 to 1·3)	0·4 (0·3 to 0·6)	0·2 (0·1 to 0·3)	76·8
Ischemic heart disease	0 (0 to 0)	0 (0 to 0)	0 (0 to 0)	0 (0 to 0)	0 (0 to 0)	0 (0 to 0)	1·5 (0·9 to 2·2)	2·7 (1·6 to 4·2)	4·9 (3 to 7·5)	9·3 (6 to 14)	16·6 (11 to 24·7)	25·3 (16·9 to 35·6)	46·9 (32·8 to 65·3)	88·6 (63·2 to 120)	127 (94·3 to 175)	162 (120 to 211)	179 (136 to 228)	179 (139 to 229)	187 (148 to 236)	178 (138 to 220)	115 (89 to 147)	55·8 (42·8 to 70·3)	19·8 (14·4 to 24·9)	1,400
Stroke	0·7 (0·3 to 1·4)	0·1 (0 to 0·2)	0·5 (0·2 to 1·1)	0·4 (0·3 to 0·7)	0·9 (0·6 to 1·2)	2·1 (1·4 to 2·9)	4·8 (3·3 to 6·6)	6·4 (4·3 to 9)	8·3 (5·9 to 11·5)	12·7 (8·9 to 17·8)	18·4 (13·2 to 26·1)	26 (17·9 to 35·3)	44·9 (32·8 to 60·9)	75·1 (54·4 to 98·9)	102 (74·5 to 138)	127 (92·8 to 166)	143 (107 to 183)	153 (118 to 198)	174 (136 to 215)	168 (134 to 208)	94·1 (74 to 119)	40·7 (30·2 to 51)	12·9 (9·4 to 17)	1,220
Ischemic stroke	0·2 (0·1 to 0·3)	0 (0 to 0)	0 (0 to 0·1)	0·2 (0·1 to 0·3)	0·5 (0·3 to 0·8)	0·8 (0·5 to 1·2)	1·4 (0·9 to 1·9)	1·7 (1·2 to 2·3)	2 (1·4 to 2·8)	3 (2·1 to 4·2)	3·9 (2·8 to 5·2)	5·4 (3·9 to 7·3)	9·4 (6·8 to 12·6)	16·6 (12 to 22·3)	24·5 (17·6 to 33·4)	45·4 (32·7 to 61·9)	56·1 (41·3 to 73·7)	84·6 (63·5 to 109)	104 (79·8 to 132)	128 (101 to 160)	74·7 (57·6 to 93·8)	33·1 (24·3 to 41·8)	10·8 (7·9 to 14·3)	606
Intracerebral hemorrhage	0·2 (0·1 to 0·4)	0 (0 to 0·1)	0·3 (0·1 to 0·6)	0·1 (0·1 to 0·2)	0·2 (0·1 to 0·3)	0·6 (0·4 to 0·9)	1·8 (1·1 to 2·7)	2·6 (1·6 to 3·9)	3·5 (2·3 to 5·3)	5·9 (3·8 to 8·7)	9·1 (6 to 13·3)	13·8 (9·2 to 19·5)	26·4 (18·2 to 37·1)	46·4 (32·8 to 63·2)	63 (44·1 to 87·1)	70·3 (49·1 to 94·4)	76·1 (53·9 to 102)	61·6 (44·4 to 84·9)	62·7 (45·1 to 83)	35·4 (25·2 to 48·8)	17·1 (11·8 to 23·2)	6·7 (4·6 to 8·9)	1·8 (1·2 to 2·5)	506
Subarachnoid hemorrhage	0·3 (0·1 to 0·7)	0·1 (0 to 0·1)	0·2 (0·1 to 0·5)	0·2 (0·1 to 0·3)	0·2 (0·1 to 0·3)	0·7 (0·4 to 1·1)	1·6 (0·9 to 2·6)	2·1 (1·2 to 3·4)	2·7 (1·6 to 4·2)	3·8 (2·3 to 5·8)	5·5 (3·5 to 8·4)	6·7 (4·2 to 10·1)	9·2 (5·8 to 13·8)	12·1 (8·2 to 17·6)	14·5 (9·5 to 22·4)	11·1 (7·2 to 17·3)	10·7 (6·8 to 15·6)	7·2 (4·8 to 10·9)	6·6 (4·3 to 9·6)	4·9 (3·3 to 7·5)	2·2 (1·5 to 3·2)	0·9 (0·6 to 1·3)	0·2 (0·1 to 0·3)	104
Hypertensive heart disease	0 (0 to 0)	0 (0 to 0)	0 (0 to 0)	0 (0 to 0)	0 (0 to 0)	0 (0 to 0)	0·9 (0·5 to 1·4)	1·6 (0·9 to 2·6)	2·5 (1·4 to 3·9)	5·6 (3·3 to 8·9)	9·9 (6 to 15·5)	14·9 (9·3 to 22·5)	29·9 (19·6 to 43·6)	51·3 (33 to 75·1)	68·1 (44·9 to 99·3)	84·2 (57·6 to 120)	90·8 (62 to 127)	88·6 (61 to 122)	93·5 (64·9 to 130)	96·1 (69·5 to 130)	55·8 (40·1 to 75)	28 (19·6 to 37·7)	10·6 (7·1 to 14·8)	732
Non-rheumatic valvular heart disease	0 (0 to 0)	0 (0 to 0)	0 (0 to 0)	0 (0 to 0)	0 (0 to 0)	0 (0 to 0)	0·6 (0·3 to 1)	0·5 (0·3 to 1)	0·9 (0·5 to 1·5)	1 (0·6 to 1·6)	1·2 (0·7 to 2)	1·4 (0·8 to 2·1)	[illegible]	[illegible]	[illegible]	[illegible]	[illegible]	[illegible]	[illegible]	[illegible]	[illegible]	0·6 (0·4 to 0·8)	0·1 (0·1 to 0·2)	34·3
Non-rheumatic calcific aortic valve disease	0 (0 to 0)	0 (0 to 0)	0 (0 to 0)	0 (0 to 0)	0 (0 to 0)	0 (0 to 0)	0·3 (0·2 to 0·6)	0·3 (0·1 to 0·5)	0·4 (0·2 to 0·7)	0·4 (0·2 to 0·6)	0·5 (0·2 to 0·8)	0·6 (0·3 to 0·9)	[illegible]	[illegible]	[illegible]	[illegible]	[illegible]	[illegible]	[illegible]	[illegible]	[illegible]	0·4 (0·2 to 0·6)	0·1 (0 to 0·1)	19
Non-rheumatic degenerative mitral valve disease	0 (0 to 0)	[illegible]	[illegible]	[illegible]	[illegible]	[illegible]	[illegible]	[illegible]	[illegible]	[illegible]	[illegible]	[illegible]	[illegible]	[illegible]	[illegible]	[illegible]	[illegible]	[illegible]	[illegible]	[illegible]	[illegible]	0·2 (0·1 to 0·3)	0 (0 to 0)	14·6
Other non-rheumatic valve diseases	0 (0 to 0)	[illegible]	[illegible]	[illegible]	[illegible]	[illegible]	[illegible]	[illegible]	[illegible]	[illegible]	[illegible]	[illegible]	[illegible]	[illegible]	[illegible]	[illegible]	[illegible]	[illegible]	[illegible]	[illegible]	[illegible]	0 (0 to 0)	0 (0 to 0)	0·6
Cardiomyopathy and myocarditis	1·2 (0·6 to 2·2)	[illegible]	[illegible]	[illegible]	[illegible]	[illegible]	[illegible]	[illegible]	[illegible]	[illegible]	[illegible]	[illegible]	[illegible]	[illegible]	[illegible]	[illegible]	[illegible]	[illegible]	[illegible]	[illegible]	[illegible]	7·9 (5·2 to 11·6)	2·3 (1·4 to 3·6)	467
Myocarditis	0·1 (0 to 0·2)	[illegible]	[illegible]	[illegible]	[illegible]	[illegible]	[illegible]	[illegible]	[illegible]	[illegible]	[illegible]	[illegible]	[illegible]	[illegible]	[illegible]	[illegible]	[illegible]	[illegible]	[illegible]	[illegible]	[illegible]	0·5 (0·2 to 1)	0·2 (0·1 to 0·4)	16·2
Alcoholic cardiomyopathy	0 (0 to 0)	[illegible]	[illegible]	[illegible]	[illegible]	[illegible]	[illegible]	[illegible]	[illegible]	[illegible]	[illegible]	[illegible]	[illegible]	[illegible]	[illegible]	[illegible]	[illegible]	[illegible]	[illegible]	[illegible]	[illegible]	0 (0 to 0·1)	0 (0 to 0)	21·4
Other cardiomyopathy	1·1 (0·5 to 2·1)	[illegible]	[illegible]	[illegible]	[illegible]	[illegible]	[illegible]	[illegible]	[illegible]	[illegible]	[illegible]	[illegible]	[illegible]	[illegible]	[illegible]	[illegible]	[illegible]	[illegible]	[illegible]	[illegible]	[illegible]	7·4 (4·9 to 10·8)	2·1 (1·2 to 3·2)	429
Atrial fibrillation and flutter	0 (0 to 0)	[illegible]	[illegible]	[illegible]	[illegible]	[illegible]	[illegible]	[illegible]	[illegible]	[illegible]	[illegible]	[illegible]	[illegible]	[illegible]	[illegible]	[illegible]	[illegible]	[illegible]	[illegible]	[illegible]	[illegible]	11·1 (8·5 to 14·1)	3·4 (2·4 to 4·6)	109
Aortic aneurysm	--	[illegible]	[illegible]	[illegible]	[illegible]	[illegible]	[illegible]	[illegible]	[illegible]	[illegible]	[illegible]	[illegible]	[illegible]	[illegible]	[illegible]	[illegible]	[illegible]	[illegible]	[illegible]	[illegible]	[illegible]	2·1 (1·4 to 2·8)	0·5 (0·3 to 0·9)	NA
Endocarditis	0·1 (0 to 0·1)	[illegible]	[illegible]	[illegible]	[illegible]	[illegible]	[illegible]	[illegible]	[illegible]	[illegible]	[illegible]	[illegible]	[illegible]	[illegible]	[illegible]	[illegible]	[illegible]	[illegible]	[illegible]	[illegible]	[illegible]	0·7 (0·4 to 1·1)	0·3 (0·1 to 0·4)	38·6
Other cardiovascular and circulatory diseases	0·9 (0·4 to 1·8)	[illegible]	[illegible]	[illegible]	[illegible]	[illegible]	[illegible]	[illegible]	[illegible]	[illegible]	[illegible]	[illegible]	[illegible]	[illegible]	[illegible]	[illegible]	[illegible]	[illegible]	[illegible]	[illegible]	[illegible]	9·6 (6·9 to 13)	3·2 (2·2 to 4·5)	263
Chronic respiratory diseases	0 (0 to 0)	[illegible]	[illegible]	[illegible]	[illegible]	[illegible]	[illegible]	[illegible]	[illegible]	[illegible]	[illegible]	[illegible]	[illegible]	[illegible]	[illegible]	[illegible]	[illegible]	[illegible]	[illegible]	[illegible]	[illegible]	12·7 (9·6 to 16·3)	3·4 (2·4 to 4·3)	645
Chronic obstructive pulmonary disease	0 (0 to 0)	[illegible]	[illegible]	[illegible]	[illegible]	[illegible]	[illegible]	[illegible]	[illegible]	[illegible]	[illegible]	[illegible]	[illegible]	[illegible]	[illegible]	[illegible]	[illegible]	[illegible]	[illegible]	[illegible]	[illegible]	10·1 (7·5 to 13·1)	2·7 (1·9 to 3·5)	304
Pneumoconiosis	0 (0 to 0)	[illegible]	[illegible]	[illegible]	[illegible]	[illegible]	[illegible]	[illegible]	[illegible]	[illegible]	[illegible]	[illegible]	[illegible]	[illegible]	[illegible]	[illegible]	[illegible]	[illegible]	[illegible]	[illegible]	[illegible]	0 (0 to 0)	0 (0 to 0)	0·6
Silicosis	0 (0 to 0)	[illegible]	[illegible]	[illegible]	[illegible]	[illegible]	[illegible]	[illegible]	[illegible]	[illegible]	[illegible]	[illegible]	[illegible]	[illegible]	[illegible]	[illegible]	[illegible]	[illegible]	[illegible]	[illegible]	[illegible]	0 (0 to 0)	0 (0 to 0)	0·1
Asbestosis	0 (0 to 0)	[illegible]	[illegible]	[illegible]	[illegible]	[illegible]	[illegible]	[illegible]	[illegible]	[illegible]	[illegible]	[illegible]	[illegible]	[illegible]	[illegible]	[illegible]	[illegible]	[illegible]	[illegible]	[illegible]	[illegible]	0 (0 to 0)	0 (0 to 0)	0·2
Coal workers pneumoconiosis	0 (0 to 0)	[illegible]	[illegible]	[illegible]	[illegible]	[illegible]	[illegible]	[illegible]	[illegible]	[illegible]	[illegible]	[illegible]	[illegible]	[illegible]	[illegible]	[illegible]	[illegible]	[illegible]	[illegible]	[illegible]	[illegible]	0 (0 to 0)	0 (0 to 0)	0
Other pneumoconiosis	0 (0 to 0)	[illegible]	[illegible]	[illegible]	[illegible]	[illegible]	[illegible]	[illegible]	[illegible]	[illegible]	[illegible]	[illegible]	[illegible]	[illegible]	[illegible]	[illegible]	[illegible]	[illegible]	[illegible]	[illegible]	[illegible]	0 (0 to 0)	0 (0 to 0)	0·2
Asthma	0 (0 to 0)	[illegible]	[illegible]	[illegible]	[illegible]	[illegible]	[illegible]	[illegible]	[illegible]	[illegible]	[illegible]	[illegible]	[illegible]	[illegible]	[illegible]	[illegible]	[illegible]	[illegible]	[illegible]	[illegible]	[illegible]	1·8 (1·2 to 2·5)	0·5 (0·3 to 0·8)	280
Interstitial lung disease and pulmonary sarcoidosis	0 (0 to 0)	[illegible]	[illegible]	[illegible]	[illegible]	[illegible]	[illegible]	[illegible]	[illegible]	[illegible]	[illegible]	[illegible]	[illegible]	[illegible]	[illegible]	[illegible]	[illegible]	[illegible]	[illegible]	[illegible]	[illegible]	0·6 (0·3 to 0·9)	0·1 (0·1 to 0·2)	25·8
Other chronic respiratory diseases	0 (0 to 0)	[illegible]	[illegible]	[illegible]	[illegible]	[illegible]	[illegible]	[illegible]	[illegible]	[illegible]	[illegible]	[illegible]	[illegible]	[illegible]	[illegible]	[illegible]	[illegible]	[illegible]	[illegible]	[illegible]	[illegible]	0·2 (0·1 to 0·4)	0·1 (0 to 0·1)	34·2
Digestive diseases	1·2 (0·6 to 2)	[illegible]	[illegible]	[illegible]	[illegible]	[illegible]	[illegible]	[illegible]	[illegible]	[illegible]	[illegible]	[illegible]	[illegible]	[illegible]	[illegible]	[illegible]	[illegible]	[illegible]	[illegible]	[illegible]	[illegible]	9·5 (7 to 12·5)	2·6 (1·7 to 3·6)	796
Cirrhosis and other chronic liver diseases	0 (0 to 0)	[illegible]	[illegible]	[illegible]	[illegible]	[illegible]	[illegible]	[illegible]	[illegible]	[illegible]	[illegible]	[illegible]	[illegible]	[illegible]	[illegible]	[illegible]	[illegible]	[illegible]	[illegible]	[illegible]	[illegible]	1·9 (1·2 to 2·7)	0·5 (0·3 to 0·8)	291
Cirrhosis and other chronic liver diseases due to hepatitis B	0 (0 to 0)	[illegible]	[illegible]	[illegible]	[illegible]	[illegible]	[illegible]	[illegible]	[illegible]	[illegible]	[illegible]	[illegible]	[illegible]	[illegible]	[illegible]	[illegible]	[illegible]	[illegible]	[illegible]	[illegible]	[illegible]	0·1 (0 to 0·2)	0 (0 to 0)	19·7
Cirrhosis and other chronic liver diseases due to hepatitis C	0 (0 to 0)	[illegible]	[illegible]	[illegible]	[illegible]	[illegible]	[illegible]	[illegible]	[illegible]	[illegible]	[illegible]	[illegible]	[illegible]	[illegible]	[illegible]	[illegible]	[illegible]	[illegible]	[illegible]	[illegible]	[illegible]	0·3 (0·1 to 0·6)	0·1 (0 to 0·2)	55·1
Cirrhosis and other chronic liver diseases due to alcohol use	0 (0 to 0)	[illegible]	[illegible]	[illegible]	[illegible]	[illegible]	[illegible]	[illegible]	[illegible]	[illegible]	[illegible]	[illegible]	[illegible]	[illegible]	[illegible]	[illegible]	[illegible]	[illegible]	[illegible]	[illegible]	[illegible]	0·5 (0·3 to 0·8)	0·1 (0 to 0·2)	123
Cirrhosis and other chronic liver diseases due to NAFLD	0 (0 to 0)	[illegible]	[illegible]	[illegible]	[illegible]	[illegible]	[illegible]	[illegible]	[illegible]	[illegible]	[illegible]	[illegible]	[illegible]	[illegible]	[illegible]	[illegible]	[illegible]	[illegible]	[illegible]	[illegible]	[illegible]	0·5 (0·3 to 0·8)	0·1 (0·1 to 0·2)	47·1
Cirrhosis and other chronic liver diseases due to other causes	0 (0 to 0)	[illegible]	[illegible]	[illegible]	[illegible]	[illegible]	[illegible]	[illegible]	[illegible]	[illegible]	[illegible]	[illegible]	[illegible]	[illegible]	[illegible]	[illegible]	[illegible]	[illegible]	[illegible]	[illegible]	[illegible]	0·5 (0·2 to 0·8)	0·1 (0·1 to 0·3)	45·9
Upper digestive system diseases	0 (0 to 0)	[illegible]	[illegible]	[illegible]	[illegible]	[illegible]	[illegible]	[illegible]	[illegible]	[illegible]	[illegible]	[illegible]	[illegible]	[illegible]	[illegible]	[illegible]	[illegible]	[illegible]	[illegible]	[illegible]	[illegible]	1·8 (1·2 to 2·5)	0·5 (0·3 to 0·7)	181
Peptic ulcer disease	0 (0 to 0)	[illegible]	[illegible]	[illegible]	[illegible]	[illegible]	[illegible]	[illegible]	[illegible]	[illegible]	[illegible]	[illegible]	[illegible]	[illegible]	[illegible]	[illegible]	[illegible]	[illegible]	[illegible]	[illegible]	[illegible]	1·1 (0·6 to 1·6)	0·3 (0·2 to 0·5)	50·9
Gastritis and duodenitis	0 (0 to 0)	[illegible]	[illegible]	[illegible]	[illegible]	[illegible]	[illegible]	[illegible]	[illegible]	[illegible]	[illegible]	[illegible]	[illegible]	[illegible]	[illegible]	[illegible]	[illegible]	[illegible]	[illegible]	[illegible]	[illegible]	0·3 (0·2 to 0·5)	0·1 (0 to 0·1)	34·4
Gastroesophageal reflux disease	0 (0 to 0)	[illegible]	[illegible]	[illegible]	[illegible]	[illegible]	[illegible]	[illegible]	[illegible]	[illegible]	[illegible]	[illegible]	[illegible]	[illegible]	[illegible]	[illegible]	[illegible]	[illegible]	[illegible]	[illegible]	[illegible]	0·4 (0·2 to 0·8)	0·1 (0·1 to 0·2)	95·4
Appendicitis	0 (0 to 0)	[illegible]	[illegible]	[illegible]	[illegible]	[illegible]	[illegible]	[illegible]	[illegible]	[illegible]	[illegible]	[illegible]	[illegible]	[illegible]	[illegible]	[illegible]	[illegible]	[illegible]	[illegible]	[illegible]	[illegible]	0·1 (0·1 to 0·2)	0 (0 to 0)	14·7
Paralytic ileus and intestinal obstruction	0·8 (0·4 to 1·5)	[illegible]	[illegible]	[illegible]	[illegible]	[illegible]	[illegible]	[illegible]	[illegible]	[illegible]	[illegible]	[illegible]	[illegible]	[illegible]	[illegible]	[illegible]	[illegible]	[illegible]	[illegible]	[illegible]	[illegible]	2 (1·3 to 2·9)	0·6 (0·3 to 0·9)	83·5
Inguinal, femoral, and abdominal hernia	0·3 (0·1 to 0·6)	[illegible]	[illegible]	[illegible]	[illegible]	[illegible]	[illegible]	[illegible]	[illegible]	[illegible]	[illegible]	[illegible]	[illegible]	[illegible]	[illegible]	[illegible]	[illegible]	[illegible]	[illegible]	[illegible]	[illegible]	0·4 (0·2 to 0·5)	0·1 (0 to 0·2)	49·3
Inflammatory bowel disease	0 (0 to 0)	[illegible]	[illegible]	[illegible]	[illegible]	[illegible]	[illegible]	[illegible]	[illegible]	[illegible]	[illegible]	[illegible]	[illegible]	[illegible]	[illegible]	[illegible]	[illegible]	[illegible]	[illegible]	[illegible]	[illegible]	0·2 (0·1 to 0·3)	0 (0 to 0·1)	20·5
Vascular intestinal disorders	0 (0 to 0)	[illegible]	[illegible]	[illegible]	[illegible]	[illegible]	[illegible]	[illegible]	[illegible]	[illegible]	[illegible]	[illegible]	[illegible]	[illegible]	[illegible]	[illegible]	[illegible]	[illegible]	[illegible]	[illegible]	[illegible]	0·8 (0·5 to 1·2)	0·2 (0·1 to 0·4)	26·8
Gallbladder and biliary diseases	0 (0 to 0)	[illegible]	[illegible]	[illegible]	[illegible]	[illegible]	[illegible]	[illegible]	[illegible]	[illegible]	[illegible]	[illegible]	[illegible]	[illegible]	[illegible]	[illegible]	[illegible]	[illegible]	[illegible]	[illegible]	[illegible]	0·5 (0·4 to 0·7)	0·1 (0·1 to 0·2)	40·5
Pancreatitis	0 (0 to 0)	[illegible]	[illegible]	[illegible]	[illegible]	[illegible]	[illegible]	[illegible]	[illegible]	[illegible]	[illegible]	[illegible]	[illegible]	[illegible]	[illegible]	[illegible]	[illegible]	[illegible]	[illegible]	[illegible]	[illegible]	0·3 (0·2 to 0·5)	0·1 (0 to 0·1)	37
Other digestive diseases	0 (0 to 0)	[illegible]	[illegible]	[illegible]	[illegible]	[illegible]	[illegible]	[illegible]	[illegible]	[illegible]	[illegible]	[illegible]	[illegible]	[illegible]	[illegible]	[illegible]	[illegible]	[illegible]	[illegible]	[illegible]	[illegible]	1·6 (0·9 to 2·5)	0·5 (0·2 to 0·8)	51·5
Neurological disorders	0 (0 to 0)	[illegible]	[illegible]	[illegible]	[illegible]	[illegible]	[illegible]	[illegible]	[illegible]	[illegible]	[illegible]	[illegible]	[illegible]	[illegible]	[illegible]	[illegible]	[illegible]	[illegible]	[illegible]	[illegible]	[illegible]	43·8 (20·6 to 93·9)	14·1 (6·5 to 31)	1,170
Alzheimer's disease and other dementias	0 (0 to 0)	[illegible]	[illegible]	[illegible]	[illegible]	[illegible]	[illegible]	[illegible]	[illegible]	[illegible]	[illegible]	[illegible]	[illegible]	[illegible]	[illegible]	[illegible]	[illegible]	[illegible]	[illegible]	[illegible]	[illegible]	38·1 (14·4 to 88·4)	12·9 (5·2 to 29·8)	295
Parkinson's disease	0 (0 to 0)	[illegible]	[illegible]	[illegible]	[illegible]	[illegible]	[illegible]	[illegible]	[illegible]	[illegible]	[illegible]	[illegible]	[illegible]	[illegible]	[illegible]	[illegible]	[illegible]	[illegible]	[illegible]	[illegible]	[illegible]	4 (3 to 4·9)	0·8 (0·5 to 1)	89·3
Idiopathic epilepsy	0 (0 to 0)	[illegible]	[illegible]	[illegible]	[illegible]	[illegible]	[illegible]	[illegible]	[illegible]	[illegible]	[illegible]	[illegible]	[illegible]	[illegible]	[illegible]	[illegible]	[illegible]	[illegible]	[illegible]	[illegible]	[illegible]	1·1 (0·6 to 1·8)	0·3 (0·1 to 0·4)	289
Multiple sclerosis	0 (0 to 0)	[illegible]	[illegible]	[illegible]	[illegible]	[illegible]	[illegible]	[illegible]	[illegible]	[illegible]	[illegible]	[illegible]	[illegible]	[illegible]	[illegible]	[illegible]	[illegible]	[illegible]	[illegible]	[illegible]	[illegible]	0 (0 to 0)	0 (0 to 0)	5·9
Motor neuron disease	0 (0 to 0)	[illegible]	[illegible]	[illegible]	[illegible]	[illegible]	[illegible]	[illegible]	[illegible]	[illegible]	[illegible]	[illegible]	[illegible]	[illegible]	[illegible]	[illegible]	[illegible]	[illegible]	[illegible]	[illegible]	[illegible]	0 (0 to 0·1)	0 (0 to 0)	19·5
Headache disorders	0 (0 to 0)	[illegible]	[illegible]	[illegible]	[illegible]	[illegible]	[illegible]	[illegible]	[illegible]	[illegible]	[illegible]	[illegible]	[illegible]	[illegible]	[illegible]	[illegible]	[illegible]	[illegible]	[illegible]	[illegible]	[illegible]	0·4 (0·1 to 0·9)	0·1 (0 to 0·2)	395
Migraine	0 (0 to 0)	[illegible]	[illegible]	[illegible]	[illegible]	[illegible]	[illegible]	[illegible]	[illegible]	[illegible]	[illegible]	[illegible]	[illegible]	[illegible]	[illegible]	[illegible]	[illegible]	[illegible]	[illegible]	[illegible]	[illegible]	0·4 (0·1 to 0·7)	0·1 (0 to 0·2)	356
Tension-type headache	0 (0 to 0)	[illegible]	[illegible]	[illegible]	[illegible]	[illegible]	[illegible]	[illegible]	[illegible]	[illegible]	[illegible]	[illegible]	[illegible]	[illegible]	[illegible]	[illegible]	[illegible]	[illegible]	[illegible]	[illegible]	[illegible]	0·1 (0 to 0·3)	0 (0 to 0·1)	38·9
Other neurological disorders	0 (0 to 0)	[illegible]	[illegible]	[illegible]	[illegible]	[illegible]	[illegible]	[illegible]	[illegible]	[illegible]	[illegible]	[illegible]	[illegible]	[illegible]	[illegible]	[illegible]	[illegible]	[illegible]	[illegible]	[illegible]	[illegible]	0·2 (0·1 to 0·3)	0·1 (0 to 0·1)	77·1
Mental disorders	0 (0 to 0)	[illegible]	[illegible]	[illegible]	[illegible]	[illegible]	[illegible]	[illegible]	[illegible]	[illegible]	[illegible]	[illegible]	[illegible]	[illegible]	[illegible]	[illegible]	[illegible]	[illegible]	[illegible]	[illegible]	[illegible]	3·1 (2·1 to 4·3)	0·9 (0·6 to 1·2)	1,210
Schizophrenia	0 (0 to 0)	[illegible]	[illegible]	[illegible]	[illegible]	[illegible]	[illegible]	[illegible]	[illegible]	[illegible]	[illegible]	[illegible]	[illegible]	[illegible]	[illegible]	[illegible]	[illegible]	[illegible]	[illegible]	[illegible]	[illegible]	0·1 (0 to 0·1)	0 (0 to 0)	132
Depressive disorders	0 (0 to 0)	[illegible]	[illegible]	[illegible]	[illegible]	[illegible]	[illegible]	[illegible]	[illegible]	[illegible]	[illegible]	[illegible]	[illegible]	[illegible]	[illegible]	[illegible]	[illegible]	[illegible]	[illegible]	[illegible]	[illegible]	1·9 (1·2 to 2·9)	0·6 (0·3 to 0·9)	408
Major depressive disorder	0 (0 to 0)	[illegible]	[illegible]	[illegible]	[illegible]	[illegible]	[illegible]	[illegible]	[illegible]	[illegible]	[illegible]	[illegible]	[illegible]	[illegible]	[illegible]	[illegible]	[illegible]	[illegible]	[illegible]	[illegible]	[illegible]	1·7 (1 to 2·6)	0·5 (0·3 to 0·8)	340
Dysthymia	0 (0 to 0)	[illegible]	[illegible]	[illegible]	[illegible]	[illegible]	[illegible]	[illegible]	[illegible]	[illegible]	[illegible]	[illegible]	[illegible]	[illegible]	[illegible]	[illegible]	[illegible]	[illegible]	[illegible]	[illegible]	[illegible]	0·2 (0·1 to 0·3)	0·1 (0 to 0·1)	68·1
Bipolar disorder	0 (0 to 0)	[illegible]	[illegible]	[illegible]	[illegible]	[illegible]	[illegible]	[illegible]	[illegible]	[illegible]	[illegible]	[illegible]	[illegible]	[illegible]	[illegible]	[illegible]	[illegible]	[illegible]	[illegible]	[illegible]	[illegible]	0·1 (0·1 to 0·2)	0 (0 to 0)	144
Anxiety disorders	0 (0 to 0)	[illegible]	[illegible]	[illegible]	[illegible]	[illegible]	[illegible]	[illegible]	[illegible]	[illegible]	[illegible]	[illegible]	[illegible]	[illegible]	[illegible]	[illegible]	[illegible]	[illegible]	[illegible]	[illegible]	[illegible]	0·6 (0·3 to 0·9)	0·1 (0·1 to 0·2)	298
Eating disorders	0 (0 to 0)	[illegible]	[illegible]	[illegible]	[illegible]	[illegible]	[illegible]	[illegible]	[illegible]	[illegible]	[illegible]	[illegible]	[illegible]	[illegible]	[illegible]	[illegible]	[illegible]	[illegible]	[illegible]	[illegible]	[illegible]	0 (0 to 0)	0 (0 to 0)	29·6
Anorexia nervosa	0 (0 to 0)	[illegible]	[illegible]	[illegible]	[illegible]	[illegible]	[illegible]	[illegible]	[illegible]	[illegible]	[illegible]	[illegible]	[illegible]	[illegible]	[illegible]	[illegible]	[illegible]	[illegible]	[illegible]	[illegible]	[illegible]	0 (0 to 0)	0 (0 to 0)	7·2
Bulimia nervosa	0 (0 to 0)	[illegible]	[illegible]	[illegible]	[illegible]	[illegible]	[illegible]	[illegible]	[illegible]	[illegible]	[illegible]	[illegible]	[illegible]	[illegible]	[illegible]	[illegible]	[illegible]	[illegible]	[illegible]	[illegible]	[illegible]	0 (0 to 0)	0 (0 to 0)	22·4
Autism spectrum disorders	0 (0 to 0)	[illegible]	[illegible]	[illegible]	[illegible]	[illegible]	[illegible]	[illegible]	[illegible]	[illegible]	[illegible]	[illegible]	[illegible]	[illegible]	[illegible]	[illegible]	[illegible]	[illegible]	[illegible]	[illegible]	[illegible]	0·1 (0 to 0·1)	0 (0 to 0)	35
Attention-deficit/hyperactivity disorder	0 (0 to 0)	[illegible]	[illegible]	[illegible]	[illegible]	[illegible]	[illegible]	[illegible]	[illegible]	[illegible]	[illegible]	[illegible]	[illegible]	[illegible]	[illegible]	[illegible]	[illegible]	[illegible]	[illegible]	[illegible]	[illegible]	0 (0 to 0)	0 (0 to 0)	24·1
Conduct disorder	0 (0 to 0)	[illegible]	[illegible]	[illegible]	[illegible]	[illegible]	[illegible]	[illegible]	[illegible]	[illegible]	[illegible]	[illegible]	[illegible]	[illegible]	[illegible]	[illegible]	[illegible]	[illegible]	[illegible]	[illegible]	[illegible]	0 (0 to 0)	0 (0 to 0)	41·9
Idiopathic developmental intellectual disability	0 (0 to 0)	[illegible]	[illegible]	[illegible]	[illegible]	[illegible]	[illegible]	[illegible]	[illegible]	[illegible]	[illegible]	[illegible]	[illegible]	[illegible]	[illegible]	[illegible]	[illegible]	[illegible]	[illegible]	[illegible]	[illegible]	0 (0 to 0)	0 (0 to 0)	10·6
Other mental disorders	0 (0 to 0)	[illegible]	[illegible]	[illegible]	[illegible]	[illegible]	[illegible]	[illegible]	[illegible]	[illegible]	[illegible]	[illegible]	[illegible]	[illegible]	[illegible]	[illegible]	[illegible]	[illegible]	[illegible]	[illegible]	[illegible]	0·4 (0·3 to 0·6)	0·1 (0·1 to 0·2)	84·3
Substance use disorders	0 (0 to 0)	[illegible]	[illegible]	[illegible]	[illegible]	[illegible]	[illegible]	[illegible]	[illegible]	[illegible]	[illegible]	[illegible]	[illegible]	[illegible]	[illegible]	[illegible]	[illegible]	[illegible]	[illegible]	[illegible]	[illegible]	0·8 (0·6 to 1·1)	0·3 (0·2 to 0·4)	386
Alcohol use disorders	0 (0 to 0)	[illegible]	[illegible]	[illegible]	[illegible]	[illegible]	[illegible]	[illegible]	[illegible]	[illegible]	[illegible]	[illegible]	[illegible]	[illegible]	[illegible]	[illegible]	[illegible]	[illegible]	[illegible]	[illegible]	[illegible]	0·7 (0·5 to 1)	0·2 (0·2 to 0·4)	287
Drug use disorders	0 (0 to 0)	[illegible]	[illegible]	[illegible]	[illegible]	[illegible]	[illegible]	[illegible]	[illegible]	[illegible]	[illegible]	[illegible]	[illegible]	[illegible]	[illegible]	[illegible]	[illegible]	[illegible]	[illegible]	[illegible]	[illegible]	0·1 (0·1 to 0·1)	0 (0 to 0)	98·7
Opioid use disorders	0 (0 to 0)	[illegible]	[illegible]	[illegible]	[illegible]	[illegible]	[illegible]	[illegible]	[illegible]	[illegible]	[illegible]	[illegible]	[illegible]	[illegible]	[illegible]	[illegible]	[illegible]	[illegible]	[illegible]	[illegible]	[illegible]	0·1 (0 to 0·1)	0 (0 to 0)	33·5
Cocaine use disorders	0 (0 to 0)	[illegible]	[illegible]	[illegible]	[illegible]	[illegible]	[illegible]	[illegible]	[illegible]	[illegible]	[illegible]	[illegible]	[illegible]	[illegible]	[illegible]	[illegible]	[illegible]	[illegible]	[illegible]	[illegible]	[illegible]	0 (0 to 0)	0 (0 to 0)	29·1
Amphetamine use disorders	0 (0 to 0)	[illegible]	[illegible]	[illegible]	[illegible]	[illegible]	[illegible]	[illegible]	[illegible]	[illegible]	[illegible]	[illegible]	[illegible]	[illegible]	[illegible]	[illegible]	[illegible]	[illegible]	[illegible]	[illegible]	[illegible]	0 (0 to 0)	0 (0 to 0)	5·2
Cannabis use disorders	0 (0 to 0)	[illegible]	[illegible]	[illegible]	[illegible]	[illegible]	[illegible]	[illegible]	[illegible]	[illegible]	[illegible]	[illegible]	[illegible]	[illegible]	[illegible]	[illegible]	[illegible]	[illegible]	[illegible]	[illegible]	[illegible]	0 (0 to 0)	0 (0 to 0)	13·6
Other drug use disorders	0 (0 to 0)	[illegible]	[illegible]	[illegible]	[illegible]	[illegible]	[illegible]	[illegible]	[illegible]	[illegible]	[illegible]	[illegible]	[illegible]	[illegible]	[illegible]	[illegible]	[illegible]	[illegible]	[illegible]	[illegible]	[illegible]	0 (0 to 0)	0 (0 to 0)	17·4
Diabetes and kidney diseases	0 (0 to 0)	[illegible]	[illegible]	[illegible]	[illegible]	[illegible]	[illegible]	[illegible]	[illegible]	[illegible]	[illegible]	[illegible]	[illegible]	[illegible]	[illegible]	[illegible]	[illegible]	[illegible]	[illegible]	[illegible]	[illegible]	52·2 (41·5 to 64)	14·1 (10·8 to 18)	3,110
Diabetes mellitus	0 (0 to 0)	[illegible]	[illegible]	[illegible]	[illegible]	[illegible]	[illegible]	[illegible]	[illegible]	[illegible]	[illegible]	[illegible]	[illegible]	[illegible]	[illegible]	[illegible]	[illegible]	[illegible]	[illegible]	[illegible]	[illegible]	32·1 (24·5 to 41·8)	8·3 (5·9 to 11·4)	1,990
Diabetes mellitus type 1	0 (0 to 0)	[illegible]	[illegible]	[illegible]	[illegible]	[illegible]	[illegible]	[illegible]	[illegible]	[illegible]	[illegible]	[illegible]	[illegible]	[illegible]	[illegible]	[illegible]	[illegible]	[illegible]	[illegible]	[illegible]	[illegible]	0·5 (0·3 to 0·8)	0·1 (0·1 to 0·2)	83
Diabetes mellitus type 2	0 (0 to 0)	[illegible]	[illegible]	[illegible]	[illegible]	[illegible]	[illegible]	[illegible]	[illegible]	[illegible]	[illegible]	[illegible]	[illegible]	[illegible]	[illegible]	[illegible]	[illegible]	[illegible]	[illegible]	[illegible]	[illegible]	31·6 (24·1 to 41·2)	8·2 (5·8 to 11·2)	1,910
Chronic kidney disease	0 (0 to 0)	[illegible]	[illegible]	[illegible]	[illegible]	[illegible]	[illegible]	[illegible]	[illegible]	[illegible]	[illegible]	[illegible]	[illegible]	[illegible]	[illegible]	[illegible]	[illegible]	[illegible]	[illegible]	[illegible]	[illegible]	20·1 (15 to 25·7)	5·8 (4·2 to 7·9)	1,110
Chronic kidney disease due to diabetes mellitus type 1	0 (0 to 0)	[illegible]	[illegible]	[illegible]	[illegible]	[illegible]	[illegible]	[illegible]	[illegible]	[illegible]	[illegible]	[illegible]	[illegible]	[illegible]	[illegible]	[illegible]	[illegible]	[illegible]	[illegible]	[illegible]	[illegible]	0 (0 to 0·1)	0 (0 to 0)	80·7
Chronic kidney disease due to diabetes mellitus type 2	0 (0 to 0)	[illegible]	[illegible]	[illegible]	[illegible]	[illegible]	[illegible]	[illegible]	[illegible]	[illegible]	[illegible]	[illegible]	[illegible]	[illegible]	[illegible]	[illegible]	[illegible]	[illegible]	[illegible]	[illegible]	[illegible]	5·8 (3·5 to 8·5)	1·6 (0·9 to 2·5)	270
Chronic kidney disease due to hypertension	0 (0 to 0)	[illegible]	[illegible]	[illegible]	[illegible]	[illegible]	[illegible]	[illegible]	[illegible]	[illegible]	[illegible]	[illegible]	[illegible]	[illegible]	[illegible]	[illegible]	[illegible]	[illegible]	[illegible]	[illegible]	[illegible]	8·1 (5·6 to 11)	2·4 (1·6 to 3·5)	232
Chronic kidney disease due to glomerulonephritis	0 (0 to 0)	[illegible]	[illegible]	[illegible]	[illegible]	[illegible]	[illegible]	[illegible]	[illegible]	[illegible]	[illegible]	[illegible]	[illegible]	[illegible]	[illegible]	[illegible]	[illegible]	[illegible]	[illegible]	[illegible]	[illegible]	1·4 (0·6 to 2·6)	0·4 (0·1 to 0·8)	239
Chronic kidney disease due to other and unspecified causes	0 (0 to 0)	[illegible]	[illegible]	[illegible]	[illegible]	[illegible]	[illegible]	[illegible]	[illegible]	[illegible]	[illegible]	[illegible]	[illegible]	[illegible]	[illegible]	[illegible]	[illegible]	[illegible]	[illegible]	[illegible]	[illegible]	4·7 (3·1 to 7)	1·4 (0·8 to 2·2)	292
Acute glomerulonephritis	0 (0 to 0)	[illegible]	[illegible]	[illegible]	[illegible]	[illegible]	[illegible]	[illegible]	[illegible]	[illegible]	[illegible]	[illegible]	[illegible]	[illegible]	[illegible]	[illegible]	[illegible]	[illegible]	[illegible]	[illegible]	[illegible]	0 (0 to 0)	0 (0 to 0)	0·5
Skin and subcutaneous diseases	0 (0 to 0)	[illegible]	[illegible]	[illegible]	[illegible]	[illegible]	[illegible]	[illegible]	[illegible]	[illegible]	[illegible]	[illegible]	[illegible]	[illegible]	[illegible]	[illegible]	[illegible]	[illegible]	[illegible]	[illegible]	[illegible]	10·8 (7·2 to 16·1)	3·8 (2·3 to 6·4)	505
Dermatitis	0 (0 to 0)	[illegible]	[illegible]	[illegible]	[illegible]	[illegible]	[illegible]	[illegible]	[illegible]	[illegible]	[illegible]	[illegible]	[illegible]	[illegible]	[illegible]	[illegible]	[illegible]	[illegible]	[illegible]	[illegible]	[illegible]	0·2 (0·1 to 0·3)	0·1 (0 to 0·1)	74·3
Atopic dermatitis	0 (0 to 0)	[illegible]	[illegible]	[illegible]	[illegible]	[illegible]	[illegible]	[illegible]	[illegible]	[illegible]	[illegible]	[illegible]	[illegible]	[illegible]	[illegible]	[illegible]	[illegible]	[illegible]	[illegible]	[illegible]	[illegible]	0·1 (0·1 to 0·2)	0 (0 to 0)	51
Contact dermatitis	0 (0 to 0)	[illegible]	[illegible]	[illegible]	[illegible]	[illegible]	[illegible]	[illegible]	[illegible]	[illegible]	[illegible]	[illegible]	[illegible]	[illegible]	[illegible]	[illegible]	[illegible]	[illegible]	[illegible]	[illegible]	[illegible]	0·1 (0 to 0·2)	0 (0 to 0)	20·9
Seborrhoeic dermatitis	0 (0 to 0)	[illegible]	[illegible]	[illegible]	[illegible]	[illegible]	[illegible]	[illegible]	[illegible]	[illegible]	[illegible]	[illegible]	[illegible]	[illegible]	[illegible]	[illegible]	[illegible]	[illegible]	[illegible]	[illegible]	[illegible]	0 (0 to 0)	0 (0 to 0)	2·5
Psoriasis	0 (0 to 0)	[illegible]	[illegible]	[illegible]	[illegible]	[illegible]	[illegible]	[illegible]	[illegible]	[illegible]	[illegible]	[illegible]	[illegible]	[illegible]	[illegible]	[illegible]	[illegible]	[illegible]	[illegible]	[illegible]	[illegible]	0 (0 to 0·1)	0 (0 to 0)	22·9
Bacterial skin diseases	0 (0 to 0)	[illegible]	[illegible]	[illegible]	[illegible]	[illegible]	[illegible]	[illegible]	[illegible]	[illegible]	[illegible]	[illegible]	[illegible]	[illegible]	[illegible]	[illegible]	[illegible]	[illegible]	[illegible]	[illegible]	[illegible]	1·8 (0·7 to 3·4)	0·5 (0·2 to 1·3)	70·1
Cellulitis	0 (0 to 0)	[illegible]	[illegible]	[illegible]	[illegible]	[illegible]	[illegible]	[illegible]	[illegible]	[illegible]	[illegible]	[illegible]	[illegible]	[illegible]	[illegible]	[illegible]	[illegible]	[illegible]	[illegible]	[illegible]	[illegible]	0·3 (0·1 to 0·6)	0·1 (0 to 0·2)	16·5
Pyoderma	0 (0 to 0)	[illegible]	[illegible]	[illegible]	[illegible]	[illegible]	[illegible]	[illegible]	[illegible]	[illegible]	[illegible]	[illegible]	[illegible]	[illegible]	[illegible]	[illegible]	[illegible]	[illegible]	[illegible]	[illegible]	[illegible]	1·5 (0·6 to 3)	0·4 (0·1 to 1·1)	53·6
Scabies	0 (0 to 0)	[illegible]	[illegible]	[illegible]	[illegible]	[illegible]	[illegible]	[illegible]	[illegible]	[illegible]	[illegible]	[illegible]	[illegible]	[illegible]	[illegible]	[illegible]	[illegible]	[illegible]	[illegible]	[illegible]	[illegible]	0·2 (0·1 to 0·3)	0 (0 to 0·1)	54·5
Fungal skin diseases	0 (0 to 0)	[illegible]	[illegible]	[illegible]	[illegible]	[illegible]	[illegible]	[illegible]	[illegible]	[illegible]	[illegible]	[illegible]	[illegible]	[illegible]	[illegible]	[illegible]	[illegible]	[illegible]	[illegible]	[illegible]	[illegible]	0·6 (0·3 to 1·3)	0·2 (0·1 to 0·4)	33·9
Viral skin diseases	0 (0 to 0)	[illegible]	[illegible]	[illegible]	[illegible]	[illegible]	[illegible]	[illegible]	[illegible]	[illegible]	[illegible]	[illegible]	[illegible]	[illegible]	[illegible]	[illegible]	[illegible]	[illegible]	[illegible]	[illegible]	[illegible]	0 (0 to 0)	0 (0 to 0)	27·9
Acne vulgaris	0 (0 to 0)	[illegible]	[illegible]	[illegible]	[illegible]	[illegible]	[illegible]	[illegible]	[illegible]	[illegible]	[illegible]	[illegible]	[illegible]	[illegible]	[illegible]	[illegible]	[illegible]	[illegible]	[illegible]	[illegible]	[illegible]	0 (0 to 0)	0 (0 to 0)	32·6
Alopecia areata	0 (0 to 0)	[illegible]	[illegible]	[illegible]	[illegible]	[illegible]	[illegible]	[illegible]	[illegible]	[illegible]	[illegible]	[illegible]	[illegible]	[illegible]	[illegible]	[illegible]	[illegible]	[illegible]	[illegible]	[illegible]	[illegible]	0 (0 to 0)	0 (0 to 0)	5
Pruritus	0 (0 to 0)	[illegible]	[illegible]	[illegible]	[illegible]	[illegible]	[illegible]	[illegible]	[illegible]	[illegible]	[illegible]	[illegible]	[illegible]	[illegible]	[illegible]	[illegible]	[illegible]	[illegible]	[illegible]	[illegible]	[illegible]	0 (0 to 0·1)	0 (0 to 0)	6·3
Urticaria	0 (0 to 0)	[illegible]	[illegible]	[illegible]	[illegible]	[illegible]	[illegible]	[illegible]	[illegible]	[illegible]	[illegible]	[illegible]	[illegible]	[illegible]	[illegible]	[illegible]	[illegible]	[illegible]	[illegible]	[illegible]	[illegible]	0 (0 to 0·1)	0 (0 to 0)	30·2
Decubitus ulcer	0 (0 to 0)	[illegible]	[illegible]	[illegible]	[illegible]	[illegible]	[illegible]	[illegible]	[illegible]	[illegible]	[illegible]	[illegible]	[illegible]	[illegible]	[illegible]	[illegible]	[illegible]	[illegible]	[illegible]	[illegible]	[illegible]	7·6 (4·5 to 11·9)	2·9 (1·6 to 5·1)	106
Other skin and subcutaneous diseases	0 (0 to 0)	[illegible]	[illegible]	[illegible]	[illegible]	[illegible]	[illegible]	[illegible]	[illegible]	[illegible]	[illegible]	[illegible]	[illegible]	[illegible]	[illegible]	[illegible]	[illegible]	[illegible]	[illegible]	[illegible]	[illegible]	0·3 (0·2 to 0·5)	0·1 (0 to 0·1)	40·7
Sense organ diseases	0 (0 to 0)	[illegible]	[illegible]	[illegible]	[illegible]	[illegible]	[illegible]	[illegible]	[illegible]	[illegible]	[illegible]	[illegible]	[illegible]	[illegible]	[illegible]	[illegible]	[illegible]	[illegible]	[illegible]	[illegible]	[illegible]	15·2 (10·8 to 20·1)	4·5 (3·2 to 5·9)	626
Blindness and vision loss	0 (0 to 0)	[illegible]	[illegible]	[illegible]	[illegible]	[illegible]	[illegible]	[illegible]	[illegible]	[illegible]	[illegible]	[illegible]	[illegible]	[illegible]	[illegible]	[illegible]	[illegible]	[illegible]	[illegible]	[illegible]	[illegible]	5·4 (3·8 to 7·3)	1·8 (1·2 to 2·5)	173
Glaucoma	0 (0 to 0)	[illegible]	[illegible]	[illegible]	[illegible]	[illegible]	[illegible]	[illegible]	[illegible]	[illegible]	[illegible]	[illegible]	[illegible]	[illegible]	[illegible]	[illegible]	[illegible]	[illegible]	[illegible]	[illegible]	[illegible]	0·7 (0·4 to 1·1)	0·2 (0·1 to 0·4)	10·9
Cataract	0 (0 to 0)	[illegible]	[illegible]	[illegible]	[illegible]	[illegible]	[illegible]	[illegible]	[illegible]	[illegible]	[illegible]	[illegible]	[illegible]	[illegible]	[illegible]	[illegible]	[illegible]	[illegible]	[illegible]	[illegible]	[illegible]	1·8 (1·2 to 2·6)	0·6 (0·4 to 0·9)	31·4
Age-related macular degeneration	0 (0 to 0)	[illegible]	[illegible]	[illegible]	[illegible]	[illegible]	[illegible]	[illegible]	[illegible]	[illegible]	[illegible]	[illegible]	[illegible]	[illegible]	[illegible]	[illegible]	[illegible]	[illegible]	[illegible]	[illegible]	[illegible]	0·1 (0 to 0·1)	0 (0 to 0)	1·4
Refraction disorders	0 (0 to 0)	[illegible]	[illegible]	[illegible]	[illegible]	[illegible]	[illegible]	[illegible]	[illegible]	[illegible]	[illegible]	[illegible]	[illegible]	[illegible]	[illegible]	[illegible]	[illegible]	[illegible]	[illegible]	[illegible]	[illegible]	0·3 (0·2 to 0·4)	0·1 (0 to 0·1)	40·9
Near vision loss	0 (0 to 0)	[illegible]	[illegible]	[illegible]	[illegible]	[illegible]	[illegible]	[illegible]	[illegible]	[illegible]	[illegible]	[illegible]	[illegible]	[illegible]	[illegible]	[illegible]	[illegible]	[illegible]	[illegible]	[illegible]	[illegible]	0·8 (0·3 to 1·7)	0·2 (0·1 to 0·5)	43
Other vision loss	0 (0 to 0)	[illegible]	[illegible]	[illegible]	[illegible]	[illegible]	[illegible]	[illegible]	[illegible]	[illegible]	[illegible]	[illegible]	[illegible]	[illegible]	[illegible]	[illegible]	[illegible]	[illegible]	[illegible]	[illegible]	[illegible]	1·7 (1·2 to 2·5)	0·7 (0·4 to 1)	45
Age-related and other hearing loss	0 (0 to 0)	[illegible]	[illegible]	[illegible]	[illegible]	[illegible]	[illegible]	[illegible]	[illegible]	[illegible]	[illegible]	[illegible]	[illegible]	[illegible]	[illegible]	[illegible]	[illegible]	[illegible]	[illegible]	[illegible]	[illegible]	9·5 (6·6 to 12·8)	2·6 (1·8 to 3·5)	424
Other sense organ diseases	0 (0 to 0)	[illegible]	[illegible]	[illegible]	[illegible]	[illegible]	[illegible]	[illegible]	[illegible]	[illegible]	[illegible]	[illegible]	[illegible]	[illegible]	[illegible]	[illegible]	[illegible]	[illegible]	[illegible]	[illegible]	[illegible]	0·3 (0·1 to 0·5)	0·1 (0 to 0·2)	29·5
Musculoskeletal disorders	0 (0 to 0)	[illegible]	[illegible]	[illegible]	[illegible]	[illegible]	[illegible]	[illegible]	[illegible]	[illegible]	[illegible]	[illegible]	[illegible]	[illegible]	[illegible]	[illegible]	[illegible]	[illegible]	[illegible]	[illegible]	[illegible]	8·5 (6 to 11·7)	2·2 (1·6 to 3·1)	1,260
Rheumatoid arthritis	0 (0 to 0)	[illegible]	[illegible]	[illegible]	[illegible]	[illegible]	[illegible]	[illegible]	[illegible]	[illegible]	[illegible]	[illegible]	[illegible]	[illegible]	[illegible]	[illegible]	[illegible]	[illegible]	[illegible]	[illegible]	[illegible]	0·9 (0·6 to 1·2)	0·2 (0·1 to 0·3)	29·8
Osteoarthritis	0 (0 to 0)	[illegible]	[illegible]	[illegible]	[illegible]	[illegible]	[illegible]	[illegible]	[illegible]	[illegible]	[illegible]	[illegible]	[illegible]	[illegible]	[illegible]	[illegible]	[illegible]	[illegible]	[illegible]	[illegible]	[illegible]	2·4 (1·2 to 4·6)	0·6 (0·3 to 1·2)	180
Osteoarthritis hip	0 (0 to 0)	[illegible]	[illegible]	[illegible]	[illegible]	[illegible]	[illegible]	[illegible]	[illegible]	[illegible]	[illegible]	[illegible]	[illegible]	[illegible]	[illegible]	[illegible]	[illegible]	[illegible]	[illegible]	[illegible]	[illegible]	0·1 (0·1 to 0·3)	0 (0 to 0·1)	9·3
Osteoarthritis knee	0 (0 to 0)	[illegible]	[illegible]	[illegible]	[illegible]	[illegible]	[illegible]	[illegible]	[illegible]	[illegible]	[illegible]	[illegible]	[illegible]	[illegible]	[illegible]	[illegible]	[illegible]	[illegible]	[illegible]	[illegible]	[illegible]	1·4 (0·7 to 2·8)	0·3 (0·2 to 0·7)	128
Osteoarthritis hand	0 (0 to 0)	[illegible]	[illegible]	[illegible]	[illegible]	[illegible]	[illegible]	[illegible]	[illegible]	[illegible]	[illegible]	[illegible]	[illegible]	[illegible]	[illegible]	[illegible]	[illegible]	[illegible]	[illegible]	[illegible]	[illegible]	0·4 (0·2 to 0·8)	0·1 (0·1 to 0·3)	21·3

Osteoarthritis other	0 (0 to 0)	0 (0 to 0)	0 (0 to 0)	0 (0 to 0)	0 (0 to 0)	0 (0 to 0)	0 (0 to 0)	0 (0 to 0)	0 (0 to 0)	0 (0 to 0·1)	0·2 (0·1 to 0·4)	0·5 (0·2 to 1·2)	1·2 (0·5 to 2·6)	2·2 (1 to 4·7)	2·9 (1·3 to 6·1)	3·2 (1·5 to 7)	3 (1·4 to 6·2)	2·4 (1·2 to 5·2)	2·3 (1·1 to 4·7)	1·9 (1 to 3·9)	1·1 (0·5 to 2·1)	0·4 (0·2 to 0·9)	0·1 (0·1 to 0·2)	21·4
Low back pain	0 (0 to 0)	0 (0 to 0)	0 (0 to 0)	0 (0 to 0)	2·5 (1·1 to 4·3)	13·4 (7·9 to 20·9)	25·2 (14·7 to 39·4)	28·7 (16·9 to 45)	33·9 (19·9 to 52·7)	39·6 (22·7 to 61·2)	41·5 (25·2 to 64·6)	40·3 (24·2 to 62·1)	48·9 (29·4 to 76·9)	57·8 (35·5 to 89·1)	55·6 (34·5 to 82·8)	49·8 (31·3 to 74·7)	40·1 (25·6 to 62·1)	30·2 (18·8 to 44·7)	25·7 (16 to 38·9)	19·9 (12·7 to 29·8)	9·7 (6 to 14·2)	3·2 (2 to 4·7)	0·8 (0·5 to 1·2)	567
Neck pain	0 (0 to 0)	0 (0 to 0)	0 (0 to 0)	0 (0 to 0)	0·1 (0 to 0·3)	0·9 (0·4 to 1·8)	2·1 (0·9 to 3·9)	3 (1·3 to 6·1)	4·8 (2·2 to 9·5)	7·6 (3·6 to 14·6)	9·3 (4·8 to 16·6)	10·1 (5·1 to 17·8)	12·7 (6·4 to 23)	13·1 (6·8 to 24·2)	11·8 (5·7 to 21·1)	9·9 (5 to 17·3)	7·3 (3·9 to 12·2)	5·2 (2·8 to 9)	4 (2 to 7·4)	2·7 (1·3 to 4·9)	1·3 (0·7 to 2·2)	0·5 (0·2 to 0·8)	0·1 (0·1 to 0·2)	107
Gout	0 (0 to 0)	0 (0 to 0)	0 (0 to 0)	0 (0 to 0)	0 (0 to 0)	0 (0 to 0)	0 (0 to 0)	0·1 (0 to 0·1)	0·2 (0·1 to 0·3)	0·3 (0·1 to 0·6)	0·4 (0·2 to 0·7)	0·4 (0·2 to 0·8)	0·6 (0·3 to 1·1)	0·8 (0·4 to 1·4)	0·7 (0·4 to 1·4)	0·7 (0·3 to 1·4)	0·6 (0·3 to 1·1)	0·5 (0·3 to 0·9)	0·5 (0·3 to 0·8)	0·4 (0·2 to 0·7)	0·2 (0·1 to 0·4)	0·1 (0 to 0·2)	0 (0 to 0)	6·6
Other musculoskeletal disorders	0 (0 to 0)	0 (0 to 0)	0 (0 to 0)	0 (0 to 0)	0·1 (0·1 to 0·2)	1·1 (0·6 to 1·9)	6·5 (3·9 to 10·1)	14·4 (9·6 to 20·6)	24·9 (16 to 37·2)	33·7 (22·3 to 49·4)	32·7 (20·9 to 48)	31·1 (19·2 to 46·8)	36·8 (23·8 to 54·5)	41·5 (25·8 to 63·3)	39·3 (24·4 to 59·1)	33·9 (19·7 to 54·3)	25·5 (15·7 to 38·6)	17·4 (10 to 28)	12·5 (7·3 to 19·1)	7·8 (4 to 13)	4·7 (2·9 to 7)	1·5 (0·9 to 2·4)	0·4 (0·2 to 0·7)	366
Other non-communicable diseases	106 (65·9 to 163)	48·5 (31·8 to 71·9)	151 (98·5 to 216)	63·6 (46·1 to 87·7)	32·6 (26·3 to 39·6)	48·5 (38·3 to 60·7)	57·2 (44·4 to 72·8)	67·9 (51·6 to 89·3)	81·3 (61·4 to 109)	90·2 (66·7 to 121)	87·3 (65·8 to 118)	77·2 (56·6 to 105)	83·7 (61·6 to 112)	89·5 (64·7 to 119)	84·5 (62·1 to 113)	81·1 (60·4 to 108)	71·1 (53 to 94·6)	57·8 (43·5 to 75·4)	51·8 (39·4 to 66·6)	41·8 (32·5 to 52·7)	24·9 (19·8 to 31·1)	10·1 (8 to 12·4)	2·7 (2·1 to 3·6)	1,510
Congenital birth defects	102 (63·8 to 158)	44·1 (28·2 to 65·9)	106 (65·8 to 161)	37·7 (26·1 to 53·9)	14·5 (11·2 to 18·3)	18·9 (14 to 25·1)	15·6 (11·1 to 21·5)	11·3 (8·2 to 15·6)	9·5 (6·8 to 13)	7·6 (5·7 to 10·2)	6·5 (4·9 to 8·5)	4·7 (3·5 to 6·1)	4·8 (3·6 to 6·3)	4·9 (3·6 to 6·4)	4·2 (3·1 to 5·5)	2·7 (2 to 3·5)	1·9 (1·4 to 2·5)	0·5 (0·3 to 0·8)	0·4 (0·2 to 0·6)	0·2 (0·1 to 0·4)	0·1 (0·1 to 0·2)	0 (0 to 0·1)	0 (0 to 0)	399
Neural tube defects	8 (3·3 to 16·3)	2·3 (0·9 to 5)	4·8 (1·8 to 10·8)	4 (1·3 to 9·6)	0·5 (0·3 to 0·7)	1 (0·6 to 1·7)	0·8 (0·5 to 1·4)	0·3 (0·2 to 0·5)	0·4 (0·2 to 0·6)	0·3 (0·2 to 0·5)	0·2 (0·1 to 0·3)	0·1 (0·1 to 0·2)	0·1 (0·1 to 0·1)	0·1 (0 to 0·1)	0·1 (0 to 0·1)	0 (0 to 0·1)	0 (0 to 0·1)	0 (0 to 0)	0 (0 to 0)	0 (0 to 0)	0 (0 to 0)	0 (0 to 0)	0 (0 to 0)	23
Congenital heart anomalies	31·9 (18 to 52·9)	18·1 (10·6 to 28·2)	44·8 (25·1 to 74·3)	13·5 (7·8 to 21·7)	3·9 (2·5 to 5·6)	5·3 (3·3 to 8)	4·3 (2·5 to 6·7)	3 (1·9 to 4·7)	2·6 (1·6 to 4)	1·9 (1·2 to 2·9)	1·7 (1·1 to 2·6)	1·2 (0·8 to 1·7)	1·1 (0·7 to 1·6)	0·9 (0·6 to 1·4)	0·6 (0·4 to 0·9)	0·5 (0·3 to 0·7)	0·3 (0·2 to 0·5)	0 (0 to 0·1)	0 (0 to 0·1)	0 (0 to 0)	0 (0 to 0)	0 (0 to 0)	0 (0 to 0)	136
Orofacial clefts	0·4 (0·1 to 1·1)	0·2 (0·1 to 0·6)	0·6 (0·1 to 1·9)	0·2 (0·1 to 0·4)	0·2 (0·1 to 0·3)	0·2 (0·1 to 0·3)	0·2 (0·1 to 0·3)	0·2 (0·1 to 0·3)	0·2 (0·1 to 0·3)	0·1 (0·1 to 0·3)	0·1 (0·1 to 0·2)	0·1 (0 to 0·2)	0·1 (0·1 to 0·2)	0·1 (0·1 to 0·2)	0·1 (0 to 0·2)	0·1 (0 to 0·2)	0·1 (0 to 0·1)	0 (0 to 0·1)	0 (0 to 0·1)	0 (0 to 0·1)	0 (0 to 0)	0 (0 to 0)	0 (0 to 0)	3·2
Down syndrome	1·1 (0·5 to 2·1)	0·7 (0·4 to 1·3)	4 (1·9 to 7·8)	2·4 (1·2 to 4·5)	0·7 (0·4 to 1·1)	1·1 (0·6 to 1·7)	1·1 (0·7 to 1·8)	1 (0·6 to 1·6)	0·8 (0·5 to 1·2)	0·8 (0·5 to 1·4)	0·8 (0·5 to 1·3)	0·7 (0·4 to 1·1)	0·9 (0·5 to 1·4)	0·9 (0·6 to 1·5)	1 (0·6 to 1·6)	0·5 (0·3 to 0·8)	0·2 (0·1 to 0·4)	0 (0 to 0)	0 (0 to 0)	0 (0 to 0)	0 (0 to 0)	0 (0 to 0)	0 (0 to 0)	18·9
Turner syndrome	0 (0 to 0)	0 (0 to 0)	0 (0 to 0)	0 (0 to 0)	0 (0 to 0)	0 (0 to 0)	0 (0 to 0)	0 (0 to 0)	0 (0 to 0)	0 (0 to 0)	0 (0 to 0)	0 (0 to 0)	0 (0 to 0)	0 (0 to 0)	0 (0 to 0)	0 (0 to 0)	0 (0 to 0)	0 (0 to 0)	0 (0 to 0)	0 (0 to 0)	0 (0 to 0)	0 (0 to 0)	0 (0 to 0)	0·1
Klinefelter syndrome	0 (0 to 0)	0 (0 to 0)	0 (0 to 0)	0 (0 to 0)	0 (0 to 0)	0 (0 to 0)	0 (0 to 0)	0 (0 to 0)	0 (0 to 0)	0 (0 to 0)	0 (0 to 0)	0 (0 to 0)	0 (0 to 0)	0 (0 to 0)	0 (0 to 0)	0 (0 to 0)	0 (0 to 0)	0 (0 to 0)	0 (0 to 0)	0 (0 to 0)	0 (0 to 0)	0 (0 to 0)	0 (0 to 0)	0
Other chromosomal abnormalities	11·1 (5·6 to 20·8)	5·7 (2·9 to 9·9)	12·9 (6·6 to 23·4)	2·5 (1·5 to 4·4)	1·1 (0·7 to 1·5)	1·5 (0·8 to 2·5)	1·5 (0·9 to 2·6)	0·9 (0·6 to 1·5)	0·9 (0·5 to 1·4)	0·5 (0·3 to 0·8)	0·4 (0·2 to 0·7)	0·2 (0·1 to 0·3)	0·1 (0·1 to 0·2)	0·1 (0·1 to 0·2)	0·1 (0 to 0·2)	0 (0 to 0·1)	0 (0 to 0)	0 (0 to 0)	0 (0 to 0)	0 (0 to 0)	0 (0 to 0)	0 (0 to 0)	0 (0 to 0)	39·5
Congenital musculoskeletal and limb anomalies	6·3 (3·3 to 11·9)	2·5 (1·3 to 4·5)	7 (3·9 to 12)	4·3 (2·7 to 6·5)	4 (2·5 to 6)	4·3 (2·7 to 6·3)	3·6 (2·3 to 5·4)	2·4 (1·6 to 3·4)	2·2 (1·4 to 3)	1·7 (1 to 2·5)	1·6 (1·1 to 2·4)	1·3 (0·9 to 1·9)	1·4 (0·9 to 2)	1·5 (1 to 2·3)	1·4 (0·9 to 2·1)	1 (0·6 to 1·5)	0·7 (0·5 to 1)	0·3 (0·2 to 0·5)	0·2 (0·1 to 0·3)	0·1 (0·1 to 0·2)	0 (0 to 0·1)	0 (0 to 0)	0 (0 to 0)	47·9
Urogenital congenital anomalies	0·6 (0·3 to 1·1)	0·2 (0·1 to 0·4)	0·4 (0·2 to 0·6)	0·3 (0·2 to 0·5)	0·2 (0·1 to 0·3)	0·2 (0·1 to 0·3)	0·2 (0·1 to 0·2)	0·1 (0·1 to 0·2)	0·1 (0·1 to 0·2)	0·1 (0·1 to 0·1)	0·1 (0 to 0·1)	0 (0 to 0·1)	0 (0 to 0·1)	0 (0 to 0·1)	0 (0 to 0)	0 (0 to 0)	0 (0 to 0)	0 (0 to 0)	0 (0 to 0)	0 (0 to 0)	0 (0 to 0)	0 (0 to 0)	0 (0 to 0)	2·7
Digestive congenital anomalies	11·7 (5·8 to 21·6)	5·5 (3 to 9·2)	9·1 (4·7 to 16·1)	1·8 (1 to 3)	0·4 (0·3 to 0·7)	0·3 (0·2 to 0·5)	0·2 (0·1 to 0·3)	0·1 (0·1 to 0·1)	0·1 (0 to 0·1)	0·1 (0 to 0·1)	0 (0 to 0)	0 (0 to 0)	0 (0 to 0·1)	0 (0 to 0·1)	0 (0 to 0)	0 (0 to 0·1)	0 (0 to 0·1)	0 (0 to 0)	0 (0 to 0)	0 (0 to 0)	0 (0 to 0)	0 (0 to 0)	0 (0 to 0)	29·7
Other congenital birth defects	31 (17·6 to 51·8)	8·8 (5 to 14)	22·8 (12·1 to 38·3)	8·6 (4·8 to 13·8)	3·6 (2·3 to 5·1)	5 (3·1 to 7·3)	3·7 (2·2 to 5·8)	3·3 (2 to 5)	2·3 (1·4 to 3·6)	2 (1·3 to 3·1)	1·5 (0·9 to 2·3)	1·1 (0·7 to 1·6)	1 (0·7 to 1·6)	1·1 (0·7 to 1·8)	0·8 (0·5 to 1·3)	0·5 (0·3 to 0·8)	0·4 (0·3 to 0·7)	0·1 (0 to 0·3)	0·1 (0 to 0·2)	0·1 (0 to 0·2)	0 (0 to 0·1)	0 (0 to 0)	0 (0 to 0)	97·9
Urinary diseases and male infertility	0·1 (0 to 0·2)	0·1 (0·1 to 0·3)	0·9 (0·4 to 1·7)	0·5 (0·3 to 0·7)	0·4 (0·3 to 0·5)	0·6 (0·4 to 0·9)	0·8 (0·6 to 1·1)	1·5 (1 to 2·4)	1·8 (1·1 to 2·9)	2·3 (1·5 to 3·8)	2·6 (1·7 to 3·9)	2·3 (1·6 to 3·4)	2·6 (1·8 to 3·6)	4·2 (2·9 to 6)	6·7 (4·4 to 9·6)	9·4 (6·2 to 13·2)	10·2 (7 to 14·3)	9·7 (6·9 to 13·3)	9·3 (6·5 to 12·9)	7·9 (5·6 to 11·1)	4·3 (3 to 6·2)	1·7 (1·2 to 2·5)	0·4 (0·3 to 0·6)	80·6
Urolithiasis	0 (0 to 0)	0 (0 to 0)	0 (0 to 0)	0 (0 to 0)	0 (0 to 0)	0 (0 to 0)	0 (0 to 0·1)	0·1 (0 to 0·1)	0·1 (0·1 to 0·2)	0·2 (0·1 to 0·4)	0·3 (0·1 to 0·5)	0·3 (0·1 to 0·5)	0·4 (0·2 to 0·7)	0·5 (0·3 to 0·8)	0·6 (0·3 to 0·9)	0·5 (0·2 to 0·7)	0·3 (0·2 to 0·5)	0·2 (0·1 to 0·4)	0·2 (0·1 to 0·3)	0·1 (0 to 0·1)	0 (0 to 0·1)	0 (0 to 0)	0 (0 to 0)	3·8
Benign prostatic hyperplasia	0 (0 to 0)	0 (0 to 0)	0 (0 to 0)	0 (0 to 0)	0 (0 to 0)	0 (0 to 0)	0 (0 to 0)	0 (0 to 0)	0 (0 to 0)	0 (0 to 0)	0 (0 to 0)	0 (0 to 0)	0·2 (0·1 to 0·4)	0·8 (0·4 to 1·5)	2·6 (1·2 to 4·5)	4·7 (2·4 to 8·1)	5·4 (3·1 to 8·5)	4·6 (2·5 to 7·6)	3·8 (2·1 to 6·3)	2·3 (1·3 to 3·7)	0·8 (0·4 to 1·2)	0·2 (0·1 to 0·3)	0 (0 to 0)	25·4
Male infertility	0 (0 to 0)	0 (0 to 0)	0 (0 to 0)	0 (0 to 0)	0 (0 to 0)	0 (0 to 0)	0 (0 to 0)	0·5 (0·1 to 1·3)	0·6 (0·2 to 1·5)	0·7 (0·2 to 1·8)	0·6 (0·2 to 1·5)	0·5 (0·1 to 1·3)	0·1 (0 to 0·2)	0 (0 to 0)	0 (0 to 0)	0 (0 to 0)	0 (0 to 0)	0 (0 to 0)	0 (0 to 0)	0 (0 to 0)	0 (0 to 0)	0 (0 to 0)	0 (0 to 0)	2·9
Other urinary diseases	0 (0 to 0·1)	0·1 (0 to 0·2)	0·2 (0·1 to 0·4)	0·2 (0·1 to 0·3)	0·1 (0·1 to 0·2)	0·2 (0·2 to 0·3)	0·2 (0·1 to 0·3)	0·3 (0·2 to 0·5)	0·2 (0·1 to 0·4)	0·3 (0·2 to 0·5)	0·5 (0·3 to 0·9)	0·4 (0·2 to 0·6)	0·6 (0·3 to 0·9)	0·8 (0·4 to 1·4)	0·9 (0·4 to 1·7)	1·1 (0·5 to 2·1)	1·1 (0·5 to 2·1)	0·9 (0·4 to 1·7)	1 (0·4 to 2)	0·9 (0·4 to 1·9)	0·4 (0·2 to 0·9)	0·2 (0·1 to 0·4)	0 (0 to 0·1)	10·8
Gynecological diseases	0 (0 to 0)	0 (0 to 0)	0 (0 to 0)	0 (0 to 0)	0 (0 to 0)	3·8 (1·9 to 6·7)	10·4 (6 to 17·6)	21 (13·3 to 32·5)	30·3 (19·7 to 44·2)	36·9 (24·3 to 53·3)	35·3 (23·7 to 51·3)	30·6 (19·9 to 43·9)	28·8 (18·8 to 41)	21·8 (13 to 32·8)	12·3 (7·5 to 18·1)	7·8 (4·5 to 12)	5·7 (3·5 to 8·5)	4 (2·5 to 5·7)	2·9 (1·9 to 4·3)	2·1 (1·3 to 3·1)	1·2 (0·8 to 1·8)	0·5 (0·3 to 0·7)	0·2 (0·1 to 0·3)	256
Uterine fibroids	0 (0 to 0)	0 (0 to 0)	0 (0 to 0)	0 (0 to 0)	0 (0 to 0)	0·1 (0 to 0·1)	0·2 (0·1 to 0·3)	0·4 (0·1 to 0·8)	0·9 (0·4 to 1·9)	2 (0·9 to 4·1)	2·9 (1·4 to 5·8)	2·9 (1·4 to 5·2)	3·1 (1·5 to 5·5)	2·3 (1·1 to 4·2)	1·5 (0·7 to 2·7)	0·8 (0·4 to 1·6)	0·5 (0·2 to 0·9)	0·3 (0·1 to 0·5)	0·2 (0·1 to 0·3)	0·1 (0 to 0·2)	0 (0 to 0·1)	0 (0 to 0)	0 (0 to 0)	18
Polycystic ovarian syndrome	0 (0 to 0)	0 (0 to 0)	0 (0 to 0)	0 (0 to 0)	0 (0 to 0)	0·1 (0·1 to 0·3)	0·7 (0·3 to 1·4)	0·7 (0·3 to 1·5)	0·7 (0·3 to 1·4)	0·7 (0·3 to 1·4)	0·6 (0·2 to 1·1)	0·5 (0·2 to 0·9)	0·4 (0·2 to 0·9)	0·1 (0 to 0·3)	0 (0 to 0)	0 (0 to 0)	0 (0 to 0)	0 (0 to 0)	0 (0 to 0)	0 (0 to 0)	0 (0 to 0)	0 (0 to 0)	0 (0 to 0)	4·5
Female infertility	0 (0 to 0)	0 (0 to 0)	0 (0 to 0)	0 (0 to 0)	0 (0 to 0)	0 (0 to 0)	0 (0 to 0)	0·7 (0·1 to 1·9)	1 (0·3 to 2·7)	1·3 (0·3 to 3·7)	1·2 (0·2 to 3·6)	1 (0·1 to 2·7)	0 (0 to 0·2)	0 (0 to 0)	0 (0 to 0)	0 (0 to 0)	0 (0 to 0)	0 (0 to 0)	0 (0 to 0)	0 (0 to 0)	0 (0 to 0)	0 (0 to 0)	0 (0 to 0)	5·2
Endometriosis	0 (0 to 0)	0 (0 to 0)	0 (0 to 0)	0 (0 to 0)	0 (0 to 0)	0 (0 to 0)	0·8 (0·3 to 1·6)	3 (1·4 to 5·5)	3·4 (1·7 to 6·1)	2·9 (1·4 to 5·1)	2·2 (1·1 to 4)	1·9 (0·9 to 3·2)	1·8 (1 to 3)	1 (0·5 to 1·8)	0 (0 to 0)	0 (0 to 0)	0 (0 to 0)	0 (0 to 0)	0 (0 to 0)	0 (0 to 0)	0 (0 to 0)	0 (0 to 0)	0 (0 to 0)	17
Genital prolapse	0 (0 to 0)	0 (0 to 0)	0 (0 to 0)	0 (0 to 0)	0 (0 to 0)	0 (0 to 0)	0·1 (0 to 0·2)	0·1 (0 to 0·3)	0·1 (0·1 to 0·3)	0·2 (0·1 to 0·4)	0·3 (0·1 to 0·6)	0·4 (0·2 to 0·8)	0·6 (0·3 to 1·1)	0·7 (0·3 to 1·3)	0·6 (0·3 to 1·2)	0·5 (0·3 to 1)	0·5 (0·2 to 0·9)	0·4 (0·2 to 0·7)	0·3 (0·1 to 0·6)	0·2 (0·1 to 0·4)	0·1 (0·1 to 0·3)	0·1 (0 to 0·1)	0 (0 to 0)	5·3
Premenstrual syndrome	0 (0 to 0)	0 (0 to 0)	0 (0 to 0)	0 (0 to 0)	0 (0 to 0)	3·4 (1·6 to 6·3)	8·2 (4·1 to 14·9)	9·5 (4·8 to 16·7)	9·5 (5·5 to 15·6)	9·8 (5·1 to 15·8)	9·5 (5·3 to 15·1)	8·1 (4·6 to 13·4)	6·4 (3·7 to 10·7)	2·4 (1·2 to 4·4)	0 (0 to 0)	0 (0 to 0)	0 (0 to 0)	0 (0 to 0)	0 (0 to 0)	0 (0 to 0)	0 (0 to 0)	0 (0 to 0)	0 (0 to 0)	66·8
Other gynecological diseases	0 (0 to 0)	0 (0 to 0)	0 (0 to 0)	0 (0 to 0)	0 (0 to 0)	0·2 (0 to 0·4)	0·6 (0·3 to 1)	6·6 (3·9 to 10·3)	14·6 (8·5 to 22·9)	20·2 (12·2 to 31·3)	18·6 (11·1 to 29·5)	15·9 (8·9 to 25·1)	16·5 (9·4 to 25·2)	15·2 (7·6 to 24·3)	10·2 (5·9 to 15·6)	6·4 (3·4 to 10·4)	4·7 (2·7 to 7·3)	3·3 (2 to 4·9)	2·5 (1·5 to 3·8)	1·8 (1 to 2·8)	1 (0·6 to 1·6)	0·4 (0·2 to 0·7)	0·1 (0·1 to 0·2)	139
Hemoglobinopathies and hemolytic anemias	0·5 (0·3 to 0·7)	0·5 (0·3 to 0·7)	4 (2·5 to 6·5)	8·6 (5·5 to 13)	8·6 (6·1 to 12)	13·7 (9·2 to 19·4)	16·1 (11·4 to 22·3)	16·7 (11·4 to 23·1)	15·6 (10·4 to 22)	12·2 (7·9 to 17·6)	9·2 (6·4 to 12·8)	7·4 (5·2 to 10·3)	7·5 (5·3 to 10·5)	7·2 (5·2 to 10·1)	6·5 (4·7 to 8·7)	6·2 (4·4 to 8·5)	4·5 (3·1 to 6·2)	3·6 (2·6 to 4·8)	3·3 (2·4 to 4·5)	2·8 (2 to 3·7)	2·6 (1·9 to 3·5)	1·1 (0·8 to 1·5)	0·3 (0·2 to 0·4)	159
Thalassemias	0 (0 to 0)	0 (0 to 0)	0 (0 to 0)	0·1 (0 to 0·1)	0 (0 to 0)	0 (0 to 0)	0 (0 to 0)	0 (0 to 0)	0 (0 to 0)	0 (0 to 0)	0 (0 to 0)	0 (0 to 0)	0 (0 to 0)	0 (0 to 0)	0 (0 to 0)	0 (0 to 0)	0 (0 to 0)	0 (0 to 0)	0 (0 to 0)	0 (0 to 0)	0 (0 to 0)	0 (0 to 0)	0 (0 to 0)	0·1
Thalassemias trait	0 (0 to 0)	0 (0 to 0)	0 (0 to 0·1)	0·1 (0 to 0·2)	0·1 (0 to 0·3)	0·1 (0 to 0·2)	0·3 (0·1 to 0·5)	0·3 (0·2 to 0·5)	0·3 (0·2 to 0·5)	0·3 (0·2 to 0·5)	0·3 (0·1 to 0·5)	0·2 (0·1 to 0·4)	0·1 (0 to 0·1)	0·1 (0 to 0·2)	0·1 (0 to 0·2)	0·1 (0 to 0·1)	0 (0 to 0·1)	0 (0 to 0·1)	0 (0 to 0·1)	0 (0 to 0·1)	0 (0 to 0)	0 (0 to 0)	0 (0 to 0)	2·6
Sickle cell disorders	0·4 (0·3 to 0·7)	0·3 (0·2 to 0·6)	3·5 (2 to 5·9)	6·8 (4·1 to 10·8)	6·4 (4·5 to 9·1)	11·8 (7·4 to 17·2)	12·9 (8·6 to 18·9)	10·6 (6·7 to 15·7)	8·3 (5·1 to 12·9)	6·1 (3·7 to 9·1)	4·8 (3 to 7·3)	3·5 (2·1 to 5·6)	3·6 (2·2 to 5·7)	2·3 (1·4 to 3·7)	1·2 (0·7 to 2)	0·5 (0·2 to 0·8)	0·1 (0 to 0·2)	0 (0 to 0)	0 (0 to 0)	0 (0 to 0)	0 (0 to 0)	0 (0 to 0)	0 (0 to 0)	83·3
Sickle cell trait	0 (0 to 0)	0·1 (0 to 0·1)	0·3 (0·2 to 0·5)	0·9 (0·4 to 1·5)	1·1 (0·5 to 2·2)	0·9 (0·3 to 1·7)	0·4 (0·1 to 0·8)	3·5 (0·7 to 7·2)	4·2 (1·2 to 7·8)	3 (0·6 to 6·8)	0·3 (0·1 to 0·7)	0·7 (0·4 to 1·2)	0·8 (0·4 to 1·4)	0·9 (0·5 to 1·6)	0·9 (0·5 to 1·6)	0·7 (0·4 to 1·2)	0·4 (0·2 to 0·8)	0·4 (0·2 to 0·6)	0·3 (0·2 to 0·6)	0·3 (0·1 to 0·5)	0·2 (0·1 to 0·3)	0·1 (0 to 0·1)	0 (0 to 0)	20·5
G6PD deficiency	0 (0 to 0)	0 (0 to 0)	0 (0 to 0·1)	0·3 (0·1 to 0·6)	0·3 (0·1 to 0·7)	0·5 (0·2 to 0·9)	1·4 (0·8 to 2·3)	1·2 (0·5 to 2·4)	1·7 (0·7 to 3)	1·6 (0·7 to 3)	2·2 (1·2 to 3·6)	1·5 (0·8 to 2·4)	1·4 (0·7 to 2·4)	1·8 (1 to 2·9)	1·8 (1 to 2·8)	1·4 (0·8 to 2·1)	0·8 (0·5 to 1·4)	0·5 (0·3 to 0·8)	0·3 (0·1 to 0·5)	0·1 (0·1 to 0·2)	0·1 (0 to 0·1)	0 (0 to 0)	0 (0 to 0)	18·9
G6PD trait	0 (0 to 0)	0 (0 to 0)	0 (0 to 0)	0 (0 to 0)	0 (0 to 0)	0 (0 to 0)	0·1 (0 to 0·1)	0 (0 to 0)	0 (0 to 0)	0 (0 to 0)	0·1 (0 to 0·1)	0·1 (0 to 0·1)	0 (0 to 0)	0 (0 to 0)	0 (0 to 0)	0 (0 to 0)	0 (0 to 0)	0 (0 to 0)	0 (0 to 0)	0 (0 to 0)	0 (0 to 0)	0 (0 to 0)	0 (0 to 0)	0·2
Other hemoglobinopathies and hemolytic anemias	0 (0 to 0)	0 (0 to 0)	0·2 (0·1 to 0·3)	0·5 (0·2 to 0·8)	0·5 (0·2 to 1·1)	0·5 (0·2 to 1)	1·1 (0·5 to 1·8)	1 (0·6 to 1·6)	1·1 (0·6 to 1·9)	1·2 (0·7 to 2)	1·5 (0·9 to 2·3)	1·4 (0·9 to 2·3)	1·6 (1 to 2·5)	2·1 (1·3 to 3·1)	2·6 (1·7 to 3·7)	3·5 (2·5 to 5)	3 (2 to 4·3)	2·7 (1·9 to 3·6)	2·7 (1·9 to 3·7)	2·3 (1·7 to 3·2)	2·3 (1·6 to 3·1)	1 (0·7 to 1·4)	0·3 (0·2 to 0·4)	33·1
Endocrine, metabolic, blood, and immune disorders	2·9 (1·4 to 5·2)	2·7 (1·4 to 4·7)	29·3 (16·1 to 48·8)	14·8 (8·7 to 22·6)	6·4 (4·3 to 9)	8·1 (5·4 to 11·5)	9·1 (6·4 to 12·9)	9·7 (6·8 to 13·6)	13·3 (9·2 to 18·9)	17 (11·7 to 24)	18·8 (12·9 to 26·5)	17·3 (11·9 to 23·9)	21 (14·8 to 28·7)	27·1 (18·7 to 39)	28·4 (19·9 to 39·8)	27·4 (19 to 38·3)	23·4 (16·7 to 33·3)	19·4 (13·6 to 27)	17·8 (12·7 to 24·5)	15·1 (10·7 to 20·3)	9·7 (6·7 to 13·7)	4·2 (2·8 to 5·9)	1·2 (0·8 to 1·8)	344
Oral disorders	0 (0 to 0)	0 (0 to 0)	0 (0 to 0)	2·1 (1·2 to 3·5)	2·8 (1·5 to 4·7)	3·4 (1·8 to 5·9)	5·2 (2·8 to 9)	7·6 (4 to 14·2)	10·8 (5·4 to 20·5)	14·1 (6·7 to 26)	15 (7·2 to 28·2)	14·9 (7·1 to 27·4)	18·9 (9·9 to 34·2)	24·1 (13·1 to 42·1)	26·4 (15·3 to 42·8)	27·6 (16·8 to 44·3)	25·4 (15·3 to 39·8)	20·7 (13 to 31·6)	18 (11·3 to 27·1)	13·7 (8·7 to 20·1)	7 (4·4 to 10·4)	2·6 (1·6 to 3·8)	0·7 (0·4 to 1)	261
Caries of deciduous teeth	0 (0 to 0)	0 (0 to 0)	0 (0 to 0)	0·7 (0·3 to 1·4)	0·7 (0·3 to 1·5)	0 (0 to 0·1)	0 (0 to 0)	0 (0 to 0)	0 (0 to 0)	0 (0 to 0)	0 (0 to 0)	0 (0 to 0)	0 (0 to 0)	0 (0 to 0)	0 (0 to 0)	0 (0 to 0)	0 (0 to 0)	0 (0 to 0)	0 (0 to 0)	0 (0 to 0)	0 (0 to 0)	0 (0 to 0)	0 (0 to 0)	1·5
Caries of permanent teeth	0 (0 to 0)	0 (0 to 0)	0 (0 to 0)	0 (0 to 0)	0·7 (0·2 to 1·5)	1·6 (0·6 to 3·5)	1·8 (0·7 to 3·8)	1·9 (0·8 to 3·9)	1·8 (0·7 to 3·8)	1·7 (0·7 to 3·5)	1·5 (0·6 to 3·2)	1·2 (0·5 to 2·5)	1·3 (0·5 to 2·7)	1·4 (0·6 to 2·8)	1·1 (0·4 to 2·5)	0·9 (0·3 to 1·8)	0·6 (0·2 to 1·2)	0·4 (0·1 to 0·8)	0·3 (0·1 to 0·6)	0·2 (0·1 to 0·4)	0·1 (0 to 0·2)	0 (0 to 0·1)	0 (0 to 0)	18·6
Periodontal diseases	0 (0 to 0)	0 (0 to 0)	0 (0 to 0)	0 (0 to 0)	0 (0 to 0)	0 (0 to 0)	0·7 (0·2 to 1·8)	2·4 (0·7 to 6)	5·1 (1·6 to 12·4)	7·8 (2·7 to 17·8)	8·9 (3·1 to 20·1)	8·9 (3·2 to 19·2)	10·6 (4 to 22·1)	11·5 (4·6 to 23·9)	9·8 (3·9 to 20·2)	7·4 (2·9 to 15·1)	4·9 (1·9 to 9·9)	3·1 (1·2 to 6·2)	2·4 (0·9 to 4·8)	1·7 (0·7 to 3·4)	0·8 (0·3 to 1·7)	0·3 (0·1 to 0·6)	0·1 (0 to 0·2)	86·4
Other oral disorders	0 (0 to 0)	0 (0 to 0)	0 (0 to 0)	1·4 (0·8 to 2·1)	1·4 (0·8 to 2·2)	1·7 (1 to 2·7)	2·7 (1·6 to 4·1)	3 (1·8 to 4·6)	3·2 (1·9 to 4·8)	3·4 (2 to 5)	3 (1·8 to 4·7)	2·6 (1·6 to 4)	3 (1·8 to 4·5)	3·2 (2 to 4·8)	2·8 (1·7 to 4·2)	2·3 (1·4 to 3·5)	1·7 (1·1 to 2·6)	1·1 (0·7 to 1·7)	0·7 (0·5 to 1·1)	0·4 (0·3 to 0·7)	0·2 (0·1 to 0·3)	0·1 (0 to 0·1)	0 (0 to 0)	38
Sudden infant death syndrome	--	1·2 (0·5 to 2·4)	10·1 (3·3 to 21·6)	--	--	--	--	--	--	--	--	--	--	--	--	--	--	--	--	--	--	--	--	NA
Injuries	6·3 (4 to 9·7)	3·6 (2·2 to 5·6)	30·4 (18·6 to 46·7)	61·4 (43·3 to 85·8)	51·3 (42·3 to 61·1)	99·1 (80·7 to 119)	212 (170 to 260)	253 (203 to 313)	260 (209 to 313)	218 (176 to 264)	193 (156 to 237)	134 (108 to 163)	130 (104 to 159)	144 (116 to 178)	127 (103 to 155)	100 (81·3 to 121)	77·6 (63·1 to 93·1)	58 (47·9 to 70)	50·9 (42·7 to 59·9)	44·4 (37·5 to 52·1)	26·1 (21·9 to 30·8)	12 (10 to 14)	3·6 (2·9 to 4·3)	2,290
Transport injuries	0·5 (0·2 to 0·9)	0·3 (0·1 to 0·5)	3 (1·6 to 5·3)	11·5 (6·7 to 17·9)	14·5 (10·2 to 19·7)	32·5 (23·5 to 42·6)	71·4 (50·4 to 98·1)	81·7 (56·7 to 112)	79·8 (54·5 to 106)	66·9 (48·8 to 88·3)	60·7 (44·5 to 80·6)	43·9 (32·5 to 57·9)	42·8 (32·2 to 55·8)	47·8 (35·4 to 63·5)	42·2 (32 to 56·2)	31·5 (24·6 to 40·5)	23·5 (18·1 to 30·2)	15·8 (12·3 to 20·4)	12·5 (9·9 to 15·7)	8·6 (7 to 10·7)	4 (3·1 to 4·9)	1·4 (1·1 to 1·7)	0·4 (0·3 to 0·4)	697
Road injuries	0·4 (0·2 to 0·8)	0·2 (0·1 to 0·4)	2·7 (1·4 to 4·7)	10·7 (6·1 to 16·9)	13·6 (9·6 to 18·6)	30·4 (22·2 to 40·1)	68·3 (47·6 to 94·1)	78·1 (54·7 to 107)	76·3 (52·1 to 102)	63·6 (46·3 to 83·8)	57·9 (42·2 to 76·9)	41·5 (30·9 to 54·5)	40·9 (30·8 to 53·3)	45·6 (33·8 to 60·8)	40 (30·3 to 53·1)	30·1 (23·6 to 39)	22·6 (17·5 to 29·1)	15 (11·7 to 19·6)	11·9 (9·4 to 14·9)	8·2 (6·7 to 10·2)	3·8 (3 to 4·7)	1·3 (1 to 1·6)	0·3 (0·3 to 0·4)	663
Pedestrian road injuries	0·2 (0·1 to 0·4)	0·1 (0 to 0·1)	0·8 (0·4 to 1·6)	5·1 (2·9 to 8·3)	6·1 (4 to 8·8)	11·2 (7·3 to 16·1)	9·1 (5·6 to 14)	8·6 (5·2 to 13·1)	8·7 (5·4 to 13·1)	9·1 (5·9 to 13·3)	9·4 (6·1 to 13·6)	7·8 (5·2 to 11·5)	8·6 (5·8 to 12·6)	10·6 (7·2 to 15·7)	10·5 (7·1 to 15·4)	8·8 (6·2 to 12·5)	7·6 (5·3 to 10·5)	5·7 (4·2 to 7·8)	5·1 (3·8 to 6·9)	3·8 (2·9 to 5)	1·8 (1·4 to 2·4)	0·6 (0·4 to 0·7)	0·1 (0·1 to 0·2)	139
Cyclist road injuries	0 (0 to 0)	0 (0 to 0)	0 (0 to 0)	0·4 (0·2 to 0·8)	0·8 (0·5 to 1·2)	3·4 (2·1 to 5·2)	5·2 (3·4 to 7·8)	4·6 (3·2 to 6·4)	4·6 (3·3 to 6·5)	4·5 (3·3 to 6·3)	4·5 (3·2 to 6·1)	3·7 (2·7 to 4·9)	3·9 (2·8 to 5)	4·7 (3·5 to 6·3)	4·2 (3·1 to 5·6)	3·4 (2·5 to 4·5)	2·5 (1·9 to 3·5)	1·6 (1·2 to 2·2)	1·2 (0·9 to 1·6)	0·8 (0·6 to 1·1)	0·4 (0·3 to 0·5)	0·1 (0·1 to 0·2)	0 (0 to 0)	54·6
Motorcyclist road injuries	0·1 (0 to 0·1)	0 (0 to 0·1)	0·4 (0·2 to 0·8)	0·9 (0·4 to 1·7)	1·4 (0·8 to 2·3)	3·1 (1·7 to 5·4)	20 (12·1 to 32·1)	26·8 (15·9 to 40·6)	28·3 (17·7 to 42·3)	21·6 (14·1 to 30·8)	18·5 (12·3 to 26·2)	11·6 (7·8 to 16·8)	10·4 (7·4 to 14·5)	10·8 (7·5 to 15·4)	8·9 (6 to 12·8)	5·5 (4 to 7·5)	3·7 (2·7 to 5·2)	2 (1·5 to 2·7)	1·5 (1·1 to 2)	1 (0·8 to 1·4)	0·5 (0·3 to 0·7)	0·2 (0·1 to 0·2)	0 (0 to 0·1)	177
Motor vehicle road injuries	0·1 (0·1 to 0·3)	0·1 (0 to 0·2)	1·4 (0·7 to 2·5)	4·1 (2·1 to 6·8)	5·3 (3·4 to 8)	12·4 (8·2 to 17·4)	33·7 (22·2 to 48·9)	37·6 (25·2 to 55·3)	34 (21·6 to 47·6)	27·8 (18·3 to 38·8)	24·9 (16·8 to 35·5)	17·9 (12·1 to 25)	17·3 (12·2 to 23·8)	18·7 (13 to 27)	15·8 (11 to 21·9)	11·8 (8·6 to 16·2)	8·2 (5·9 to 11·5)	5·4 (3·9 to 7·5)	3·8 (2·8 to 5·1)	2·3 (1·8 to 3·1)	1 (0·8 to 1·4)	0·4 (0·3 to 0·5)	0·1 (0·1 to 0·1)	284
Other road injuries	0 (0 to 0)	0 (0 to 0)	0 (0 to 0·1)	0·1 (0·1 to 0·3)	0·1 (0·1 to 0·3)	0·3 (0·2 to 0·4)	0·4 (0·3 to 0·6)	0·6 (0·4 to 0·8)	0·6 (0·4 to 0·9)	0·6 (0·5 to 0·9)	0·6 (0·5 to 0·8)	0·6 (0·4 to 0·8)	0·7 (0·5 to 0·9)	0·8 (0·5 to 1)	0·7 (0·5 to 0·9)	0·6 (0·4 to 0·8)	0·5 (0·3 to 0·6)	0·3 (0·2 to 0·4)	0·3 (0·2 to 0·4)	0·2 (0·1 to 0·2)	0·1 (0·1 to 0·1)	0 (0 to 0)	0 (0 to 0)	8·1
Other transport injuries	0·1 (0 to 0·2)	0·1 (0 to 0·1)	0·4 (0·2 to 0·7)	0·9 (0·5 to 1·5)	0·9 (0·5 to 1·3)	2·1 (1·3 to 3·2)	3·1 (1·8 to 5·1)	3·6 (2·2 to 5·7)	3·5 (2·1 to 5·9)	3·3 (1·9 to 5·1)	2·9 (1·8 to 4·5)	2·4 (1·5 to 3·6)	2 (1·3 to 2·9)	2·2 (1·4 to 3·3)	2·2 (1·4 to 3·4)	1·4 (1 to 2)	0·9 (0·6 to 1·3)	0·8 (0·5 to 1·2)	0·6 (0·4 to 0·8)	0·4 (0·3 to 0·5)	0·2 (0·1 to 0·2)	0·1 (0·1 to 0·1)	0 (0 to 0)	33·9
Unintentional injuries	4·1 (2·5 to 6·3)	3 (1·9 to 4·9)	24·9 (15·1 to 38·8)	45·2 (31·3 to 63·2)	33·4 (26·2 to 41·2)	55·2 (43·9 to 67·6)	73·4 (56·2 to 95·9)	71·1 (55·8 to 89·1)	74·9 (59·3 to 93·6)	69·3 (55·1 to 85·2)	66·8 (53·2 to 82·2)	52·2 (41·5 to 65·1)	54·5 (43·1 to 67·6)	64·6 (51·2 to 79·6)	60·2 (48·2 to 74·1)	52·1 (41·6 to 63·8)	42·3 (34 to 51·5)	34·5 (28·3 to 42)	33·2 (27·6 to 39·3)	32·5 (27·2 to 38·7)	20·7 (17·2 to 24·7)	10·2 (8·4 to 12·1)	3·1 (2·5 to 3·8)	981
Falls	0·1 (0 to 0·1)	0 (0 to 0·1)	0·6 (0·3 to 1·1)	1·5 (0·9 to 2·4)	1·9 (1·4 to 2·6)	3·8 (2·7 to 5·1)	6·3 (4·7 to 8·4)	7 (5·2 to 9·4)	8·1 (6 to 10·8)	8·7 (6·5 to 11·4)	9·5 (7·2 to 12·2)	8·5 (6·5 to 11·1)	10·3 (7·8 to 13·5)	13·9 (10·6 to 18)	13·8 (10·7 to 17·6)	13·1 (10·1 to 16·8)	11·5 (8·8 to 14·5)	10·8 (8·5 to 13·8)	11·5 (9·1 to 14·3)	13·9 (10·9 to 18)	9·9 (7·7 to 12·6)	5·1 (3·9 to 6·5)	1·5 (1·1 to 1·9)	171
Drowning	0·4 (0·2 to 0·6)	0·2 (0·1 to 0·3)	2·9 (1·4 to 5·4)	16 (9 to 26·4)	14·3 (8·9 to 20·7)	24·7 (16·6 to 33·8)	31·9 (18·4 to 48·3)	23·5 (14·8 to 35·4)	21·8 (13·8 to 32·9)	16·4 (10·6 to 23·9)	15·1 (9·5 to 23·1)	9·6 (6·1 to 14·8)	7·8 (4·8 to 11·8)	9·1 (5·9 to 13·6)	7·1 (4·4 to 11·2)	4·7 (3 to 6·9)	3 (2 to 4·5)	1·7 (1·1 to 2·5)	1·2 (0·8 to 1·8)	0·6 (0·4 to 0·9)	0·4 (0·2 to 0·5)	0·2 (0·1 to 0·2)	0·1 (0 to 0·1)	213
Fire, heat, and hot substances	0·2 (0·1 to 0·3)	0·2 (0·1 to 0·3)	2·9 (1·5 to 4·8)	8·1 (4·6 to 12·3)	5 (3·6 to 6·8)	7·2 (5·3 to 9·4)	8·2 (6 to 10·8)	8·8 (6·5 to 11·6)	9·6 (7·3 to 12·4)	10·5 (7·9 to 13·5)	9·6 (7·2 to 12·5)	8·2 (6·2 to 10·7)	8·9 (6·7 to 11·7)	9·5 (7·1 to 12·6)	8·6 (6·5 to 11·6)	7·3 (5·5 to 9·7)	5·5 (4·1 to 7·2)	4·3 (3·2 to 5·8)	3·8 (2·8 to 5)	3·3 (2·4 to 4·5)	2·2 (1·6 to 3)	1·1 (0·7 to 1·5)	0·4 (0·2 to 0·5)	133
Poisonings	0·2 (0·1 to 0·5)	0·4 (0·1 to 0·9)	2·2 (0·8 to 4·4)	6·4 (3 to 12·2)	1·9 (1 to 3·4)	2·2 (1·2 to 4·1)	2·9 (1·6 to 5·6)	3·7 (2 to 7·5)	4·8 (2·6 to 10·3)	4·6 (2·5 to 9·6)	4·1 (2·2 to 9)	2·4 (1·4 to 4·9)	2·1 (1·2 to 4·5)	2·2 (1·2 to 4·5)	1·8 (1·1 to 3·8)	1·4 (0·8 to 2·7)	1 (0·6 to 1·9)	0·8 (0·5 to 1·4)	0·7 (0·4 to 1·2)	0·6 (0·4 to 1)	0·3 (0·2 to 0·5)	0·1 (0·1 to 0·2)	0 (0 to 0)	46·6
Poisoning by carbon monoxide	0 (0 to 0·1)	0 (0 to 0·1)	0·1 (0 to 0·2)	0·5 (0·2 to 1·3)	0·2 (0·1 to 0·3)	0·2 (0·1 to 0·5)	0·5 (0·2 to 1)	0·7 (0·4 to 1·3)	0·7 (0·3 to 1·3)	0·7 (0·3 to 1·2)	0·6 (0·3 to 1·1)	0·4 (0·2 to 0·6)	0·3 (0·1 to 0·5)	0·2 (0·1 to 0·3)	0·3 (0·1 to 0·4)	0·2 (0·1 to 0·4)	0·2 (0·1 to 0·4)	0·1 (0·1 to 0·2)	0·1 (0·1 to 0·3)	0·1 (0 to 0·2)	0 (0 to 0·1)	0 (0 to 0)	0 (0 to 0)	6·2
Poisoning by other means	0·2 (0·1 to 0·4)	0·3 (0·1 to 0·8)	2·1 (0·8 to 4·3)	5·9 (2·8 to 11·6)	1·7 (0·9 to 3·2)	1·9 (1·1 to 3·8)	2·4 (1·2 to 4·8)	3 (1·6 to 6·5)	4·2 (2·2 to 9·4)	3·9 (2 to 8·8)	3·5 (1·9 to 8·1)	2 (1·2 to 4·4)	1·8 (1 to 4·1)	2 (1·1 to 4·3)	1·6 (0·9 to 3·5)	1·1 (0·6 to 2·5)	0·8 (0·5 to 1·6)	0·6 (0·4 to 1·2)	0·5 (0·3 to 1·1)	0·5 (0·3 to 0·9)	0·2 (0·1 to 0·4)	0·1 (0·1 to 0·2)	0 (0 to 0)	40·5
Exposure to mechanical forces	0·1 (0 to 0·4)	0·2 (0 to 1·3)	0·6 (0·2 to 2·3)	2·1 (1·2 to 3·6)	2·7 (1·9 to 3·6)	4·8 (3·5 to 6·7)	9 (6·1 to 13·3)	10·9 (7·8 to 15·3)	11·3 (8·1 to 16)	11 (7·8 to 15·3)	10·4 (7·7 to 14·4)	8·8 (6·4 to 12)	9·4 (6·8 to 12·7)	10·7 (7·9 to 14·5)	9·8 (7·1 to 13·8)	8·1 (5·8 to 11)	5·8 (4·1 to 8)	4 (2·8 to 5·5)	3·3 (2·4 to 4·6)	2·5 (1·8 to 3·5)	1·3 (1 to 1·8)	0·6 (0·4 to 0·8)	0·2 (0·1 to 0·3)	128
Unintentional firearm injuries	0 (0 to 0·1)	0 (0 to 0·2)	0·1 (0 to 0·3)	0·1 (0·1 to 0·3)	0·1 (0·1 to 0·3)	0·2 (0·1 to 0·3)	2·6 (1·4 to 4·8)	3·8 (2 to 6·4)	3·5 (1·8 to 6·2)	2·8 (1·6 to 4·5)	2·4 (1·4 to 4)	1·8 (1·1 to 2·8)	1·6 (1·1 to 2·3)	1·7 (1·1 to 2·5)	1·5 (1 to 2·1)	1·1 (0·8 to 1·5)	0·8 (0·5 to 1)	0·5 (0·4 to 0·6)	0·4 (0·3 to 0·5)	0·3 (0·2 to 0·4)	0·2 (0·1 to 0·2)	0·1 (0·1 to 0·1)	0 (0 to 0·1)	26·2
Other exposure to mechanical forces	0·1 (0 to 0·4)	0·2 (0 to 1·1)	0·6 (0·2 to 2·1)	2 (1·2 to 3·4)	2·5 (1·7 to 3·4)	4·2 (3 to 5·9)	6·3 (4·2 to 9·4)	7·1 (4·8 to 10·5)	7·9 (5·5 to 11·5)	8·2 (5·7 to 11·5)	7·9 (5·7 to 11)	7 (5 to 9·8)	7·8 (5·5 to 10·9)	9 (6·4 to 12·4)	8·3 (5·9 to 12)	7 (4·9 to 9·7)	5·1 (3·5 to 7·1)	3·5 (2·4 to 4·9)	2·9 (2·1 to 4·1)	2·2 (1·6 to 3·1)	1·2 (0·8 to 1·6)	0·5 (0·4 to 0·7)	0·2 (0·1 to 0·2)	102
Adverse effects of medical treatment	1·1 (0·6 to 1·8)	0·8 (0·4 to 1·3)	3·2 (1·8 to 5·5)	1·9 (1·1 to 2·9)	1·1 (0·7 to 1·5)	1·6 (1 to 2·3)	1·8 (1·1 to 2·7)	2·9 (1·8 to 4·2)	3 (2 to 4·4)	2·9 (1·8 to 4·6)	2·9 (1·9 to 4·4)	2·6 (1·7 to 3·8)	3·2 (2·1 to 4·6)	4·3 (2·9 to 6·2)	4·8 (3·2 to 7)	5·2 (3·5 to 7·5)	5·1 (3·5 to 7·2)	4·6 (3·2 to 6·3)	5 (3·6 to 6·9)	4·7 (3·3 to 6·5)	2·8 (2 to 3·9)	1·2 (0·9 to 1·7)	0·3 (0·2 to 0·5)	67·1
Animal contact	0 (0 to 0)	0 (0 to 0)	0·1 (0 to 0·2)	0·3 (0·2 to 0·5)	0·3 (0·2 to 0·5)	0·6 (0·4 to 0·8)	0·6 (0·4 to 0·9)	0·6 (0·4 to 0·9)	0·8 (0·5 to 1)	0·7 (0·5 to 0·9)	0·7 (0·5 to 1)	0·6 (0·4 to 0·9)	0·7 (0·5 to 1)	0·9 (0·6 to 1·2)	0·9 (0·6 to 1·2)	0·8 (0·6 to 1·1)	0·7 (0·5 to 1)	0·6 (0·4 to 0·8)	0·5 (0·3 to 0·8)	0·5 (0·3 to 0·8)	0·4 (0·2 to 0·6)	0·3 (0·1 to 0·5)	0·1 (0 to 0·2)	11·9
Venomous animal contact	0 (0 to 0)	0 (0 to 0)	0·1 (0 to 0·1)	0·2 (0·1 to 0·3)	0·2 (0·1 to 0·3)	0·4 (0·2 to 0·5)	0·4 (0·3 to 0·6)	0·4 (0·3 to 0·6)	0·5 (0·3 to 0·7)	0·4 (0·3 to 0·6)	0·5 (0·3 to 0·7)	0·4 (0·3 to 0·6)	0·4 (0·3 to 0·6)	0·5 (0·4 to 0·7)	0·5 (0·4 to 0·7)	0·5 (0·3 to 0·6)	0·4 (0·3 to 0·5)	0·3 (0·2 to 0·4)	0·3 (0·2 to 0·4)	0·3 (0·2 to 0·4)	0·2 (0·1 to 0·4)	0·2 (0·1 to 0·3)	0·1 (0 to 0·2)	7·2
Non-venomous animal contact	0 (0 to 0)	0 (0 to 0)	0·1 (0 to 0·1)	0·1 (0·1 to 0·3)	0·1 (0·1 to 0·2)	0·2 (0·1 to 0·3)	0·2 (0·1 to 0·3)	0·2 (0·1 to 0·3)	0·3 (0·2 to 0·4)	0·2 (0·1 to 0·3)	0·2 (0·1 to 0·4)	0·2 (0·1 to 0·3)	0·3 (0·2 to 0·4)	0·4 (0·2 to 0·5)	0·4 (0·2 to 0·5)	0·3 (0·2 to 0·5)	0·3 (0·2 to 0·5)	0·3 (0·2 to 0·4)	0·2 (0·1 to 0·4)	0·3 (0·1 to 0·4)	0·2 (0·1 to 0·3)	0·1 (0 to 0·2)	0·1 (0 to 0·1)	4·8
Foreign body	2 (1 to 3·5)	1·1 (0·6 to 2)	11·4 (5·5 to 20·4)	6·1 (3·5 to 9·7)	2·5 (1·6 to 3·6)	4·1 (2·8 to 5·9)	2·4 (1·7 to 3·4)	2·7 (1·8 to 3·7)	3·1 (2·1 to 4·3)	2·7 (1·9 to 3·7)	2·8 (1·9 to 3·9)	2·3 (1·6 to 3·1)	2·4 (1·8 to 3·2)	3·3 (2·3 to 4·4)	3·1 (2·2 to 4·3)	2·9 (2 to 4·1)	2·7 (1·9 to 3·8)	2·3 (1·6 to 3·2)	2·2 (1·6 to 3)	2·1 (1·6 to 3)	1 (0·7 to 1·3)	0·5 (0·3 to 0·6)	0·2 (0·1 to 0·2)	65·7
Pulmonary aspiration and foreign body in airway	1·9 (1 to 3·5)	1·1 (0·6 to 2)	11·3 (5·4 to 20·2)	5·8 (3·2 to 9·3)	2 (1·3 to 3·1)	3·5 (2·2 to 5·2)	1·8 (1·1 to 2·7)	2 (1·3 to 3)	2·3 (1·5 to 3·5)	2 (1·3 to 2·9)	2·1 (1·3 to 3·1)	1·6 (1·1 to 2·3)	1·7 (1·1 to 2·4)	2·5 (1·6 to 3·6)	2·4 (1·6 to 3·6)	2·3 (1·4 to 3·5)	2·1 (1·4 to 3·3)	1·9 (1·2 to 2·8)	1·8 (1·3 to 2·6)	1·8 (1·3 to 2·6)	0·8 (0·5 to 1·1)	0·4 (0·3 to 0·5)	0·1 (0·1 to 0·2)	55·3
Foreign body in eyes	0 (0 to 0)	0 (0 to 0)	0 (0 to 0)	0·1 (0 to 0·2)	0·1 (0 to 0·3)	0·2 (0·1 to 0·5)	0·3 (0·1 to 0·6)	0·3 (0·1 to 0·7)	0·3 (0·1 to 0·7)	0·4 (0·1 to 0·7)	0·3 (0·1 to 0·7)	0·3 (0·1 to 0·6)	0·3 (0·2 to 0·6)	0·3 (0·2 to 0·6)	0·3 (0·2 to 0·4)	0·2 (0·1 to 0·4)	0·2 (0·1 to 0·3)	0·1 (0·1 to 0·2)	0·1 (0·1 to 0·2)	0·1 (0·1 to 0·1)	0 (0 to 0·1)	0 (0 to 0)	0 (0 to 0)	4
Foreign body in other body part	0 (0 to 0)	0 (0 to 0)	0·1 (0 to 0·2)	0·2 (0·2 to 0·3)	0·3 (0·2 to 0·4)	0·4 (0·3 to 0·6)	0·4 (0·3 to 0·5)	0·4 (0·3 to 0·5)	0·4 (0·3 to 0·6)	0·4 (0·3 to 0·5)	0·4 (0·3 to 0·5)	0·4 (0·3 to 0·5)	0·4 (0·3 to 0·6)	0·5 (0·3 to 0·6)	0·5 (0·3 to 0·6)	0·4 (0·3 to 0·5)	0·3 (0·2 to 0·5)	0·3 (0·2 to 0·4)	0·3 (0·2 to 0·4)	0·3 (0·2 to 0·4)	0·1 (0·1 to 0·2)	0·1 (0 to 0·1)	0 (0 to 0)	6·4
Environmental heat and cold exposure	0 (0 to 0)	0 (0 to 0)	0·1 (0·1 to 0·3)	0·4 (0·3 to 0·6)	0·7 (0·5 to 1·1)	1·1 (0·8 to 1·6)	1·8 (1·3 to 2·4)	2·1 (1·5 to 2·8)	2·3 (1·6 to 3·1)	2·5 (1·8 to 3·3)	2·4 (1·7 to 3·2)	2·2 (1·5 to 2·9)	2·5 (1·8 to 3·4)	3 (2·1 to 3·9)	2·9 (2·1 to 3·8)	2·5 (1·8 to 3·3)	2 (1·4 to 2·6)	1·7 (1·2 to 2·2)	1·5 (1·1 to 2)	1·4 (1 to 2)	0·8 (0·6 to 1·1)	0·5 (0·3 to 0·7)	0·2 (0·1 to 0·2)	34·6
Exposure to forces of nature	0 (0 to 0)	0 (0 to 0)	0 (0 to 0)	0·2 (0·2 to 0·4)	0·1 (0·1 to 0·2)	0·2 (0·1 to 0·4)	0·4 (0·3 to 0·6)	0·5 (0·4 to 0·8)	0·6 (0·4 to 1)	0·6 (0·4 to 0·8)	0·7 (0·4 to 1)	0·7 (0·4 to 1)	1 (0·6 to 1·4)	1·3 (0·9 to 2·1)	1·6 (1 to 2·4)	1·8 (1·2 to 2·7)	1·8 (1·1 to 2·7)	1·6 (1 to 2·3)	1·5 (1 to 2·4)	1·4 (0·9 to 2·1)	0·9 (0·6 to 1·3)	0·4 (0·3 to 0·6)	0·2 (0·1 to 0·2)	17·5
Other unintentional injuries	0·2 (0·1 to 0·3)	0·1 (0 to 0·1)	0·7 (0·4 to 1·1)	2·2 (1·3 to 3·7)	2·9 (1·8 to 4·5)	5 (3·2 to 7·8)	8·2 (5 to 13·6)	8·4 (5·4 to 13·4)	9·5 (5·9 to 14·6)	8·8 (5·7 to 13·3)	8·6 (5·5 to 13·6)	6·3 (4·4 to 9·1)	6·3 (4·3 to 9)	6·5 (4·6 to 9)	5·7 (4 to 8·1)	4·3 (3·1 to 6)	3·2 (2·3 to 4·4)	2·2 (1·6 to 3·1)	1·8 (1·3 to 2·5)	1·3 (0·9 to 1·8)	0·7 (0·5 to 0·9)	0·2 (0·2 to 0·3)	0·1 (0 to 0·1)	93·3
Self-harm and interpersonal violence	1·7 (0·8 to 3·1)	0·3 (0·1 to 0·6)	2·5 (1·2 to 4·3)	4·7 (2·5 to 7·9)	3·4 (2·3 to 5·1)	11·4 (7·8 to 15·9)	66·9 (45 to 98·1)	100 (70·4 to 138)	105 (74·5 to 141)	81·4 (57·9 to 113)	65·7 (46·1 to 92·2)	37·8 (26·9 to 51·6)	32·3 (22·7 to 44·6)	31·8 (22·6 to 44·3)	24·4 (17·2 to 33·5)	16·8 (12·2 to 22·2)	11·8 (8·9 to 15·4)	7·7 (5·7 to 10·2)	5·2 (4 to 6·9)	3·4 (2·6 to 4·3)	1·5 (1·1 to 1·9)	0·4 (0·3 to 0·5)	0·1 (0·1 to 0·1)	616
Self-harm	0 (0 to 0)	0 (0 to 0)	0 (0 to 0)	0 (0 to 0)	0 (0 to 0)	1·8 (1·1 to 2·8)	11·9 (7·6 to 18)	19·1 (12·3 to 27·7)	24·7 (16·1 to 34·9)	21·9 (14·2 to 31·3)	18·7 (12 to 27·2)	11·1 (7·5 to 15·8)	9·9 (6·8 to 14·5)	11·5 (7·7 to 16·8)	9·6 (6·5 to 13·8)	7·1 (4·5 to 10·3)	5·3 (3·6 to 7·6)	3·8 (2·5 to 5·4)	2·8 (1·9 to 3·9)	2 (1·4 to 2·9)	0·9 (0·6 to 1·2)	0·2 (0·2 to 0·3)	0 (0 to 0·1)	162
Self-harm by firearm	0 (0 to 0)	0 (0 to 0)	0 (0 to 0)	0 (0 to 0)	0 (0 to 0)	0 (0 to 0·1)	0·5 (0·2 to 1)	0·8 (0·4 to 1·4)	0·8 (0·4 to 1·4)	0·7 (0·4 to 1·2)	0·6 (0·3 to 1·1)	0·4 (0·2 to 0·6)	0·3 (0·2 to 0·6)	0·4 (0·2 to 0·7)	0·3 (0·2 to 0·5)	0·2 (0·1 to 0·4)	0·2 (0·1 to 0·3)	0·1 (0·1 to 0·2)	0·1 (0 to 0·1)	0·1 (0 to 0·1)	0 (0 to 0)	0 (0 to 0)	0 (0 to 0)	5·4
Self-harm by other specified means	0 (0 to 0)	0 (0 to 0)	0 (0 to 0)	0 (0 to 0)	0 (0 to 0)	1·7 (1·1 to 2·7)	11·4 (7·2 to 17·2)	18·3 (11·9 to 26·5)	23·9 (15·5 to 34)	21·2 (13·7 to 30·5)	18·2 (11·5 to 26·5)	10·7 (7·2 to 15·4)	9·6 (6·5 to 14)	11·1 (7·4 to 16)	9·3 (6·2 to 13·4)	6·9 (4·4 to 9·9)	5·2 (3·5 to 7·4)	3·7 (2·4 to 5·3)	2·7 (1·8 to 3·8)	2 (1·3 to 2·8)	0·9 (0·6 to 1·2)	0·2 (0·2 to 0·3)	0 (0 to 0·1)	157
Interpersonal violence	1·7 (0·8 to 3·1)	0·3 (0·1 to 0·6)	2·5 (1·2 to 4·3)	4·6 (2·4 to 7·9)	3·4 (2·2 to 5)	9·6 (6·3 to 13·9)	55 (33·8 to 84·8)	80·9 (53·6 to 116)	80·1 (51·4 to 115)	59·4 (38 to 86·5)	46·6 (29·2 to 70·1)	26·4 (16·6 to 38·2)	22 (14·6 to 32·8)	19·7 (12·6 to 29·8)	14·2 (9·1 to 21·3)	9·1 (6·3 to 12·8)	5·9 (4·1 to 8·3)	3·5 (2·4 to 5·1)	2·4 (1·7 to 3·4)	1·3 (1 to 1·6)	0·6 (0·5 to 0·7)	0·2 (0·1 to 0·2)	0·1 (0 to 0·1)	449
Physical violence by firearm	0 (0 to 0·1)	0 (0 to 0)	0·1 (0 to 0·2)	0·3 (0·1 to 0·7)	0·4 (0·2 to 0·7)	1·9 (0·9 to 3·4)	21·4 (10·7 to 36·6)	32·1 (17·3 to 51·5)	30·7 (16·6 to 50·7)	19·9 (10·1 to 34·1)	14·1 (6·7 to 24·3)	6·7 (3·2 to 11·7)	5·2 (2·7 to 9·3)	4 (1·9 to 7·3)	2·6 (1·3 to 4·7)	1·4 (0·7 to 2·4)	0·8 (0·4 to 1·4)	0·3 (0·2 to 0·6)	0·2 (0·1 to 0·3)	0·1 (0 to 0·1)	0 (0 to 0)	0 (0 to 0)	0 (0 to 0)	142
Physical violence by sharp object	0·1 (0 to 0·3)	0 (0 to 0)	0·2 (0·1 to 0·3)	0·5 (0·2 to 0·9)	0·5 (0·3 to 0·8)	2 (1·1 to 3·4)	22·8 (11·8 to 39·5)	34·7 (19·7 to 56)	34·5 (19·7 to 54·1)	26·9 (15·4 to 43·1)	21·1 (11·5 to 35)	11·8 (6·5 to 18·3)	8·9 (5·3 to 14·1)	7·6 (4·2 to 12·9)	4·7 (2·7 to 7·7)	2·8 (1·8 to 4·5)	1·6 (1 to 2·5)	1 (0·6 to 1·7)	0·6 (0·4 to 1)	0·4 (0·3 to 0·5)	0·2 (0·1 to 0·2)	0·1 (0 to 0·1)	0 (0 to 0)	183
Sexual violence	0 (0 to 0)	0 (0 to 0)	0 (0 to 0)	0 (0 to 0)	0·2 (0·1 to 0·4)	0·6 (0·4 to 0·9)	1·4 (0·8 to 2·3)	1·8 (1 to 2·7)	1·6 (0·9 to 2·6)	1·5 (0·8 to 2·5)	1·2 (0·7 to 2)	0·9 (0·5 to 1·4)	0·8 (0·5 to 1·4)	0·7 (0·4 to 1·1)	0·5 (0·3 to 0·7)	0·4 (0·2 to 0·5)	0·3 (0·2 to 0·4)	0·2 (0·1 to 0·3)	0·1 (0·1 to 0·2)	0·1 (0·1 to 0·2)	0 (0 to 0·1)	0 (0 to 0)	0 (0 to 0)	12·4
Physical violence by other means	1·6 (0·8 to 2·8)	0·3 (0·1 to 0·5)	2·2 (1 to 3·9)	3·8 (1·9 to 6·5)	2·2 (1·4 to 3·5)	5·1 (3·1 to 7·8)	9·4 (5·4 to 15·8)	12·3 (7·3 to 19·9)	13·3 (7·5 to 21)	11·1 (6·6 to 17·9)	10·2 (5·9 to 17·3)	7 (4·2 to 11·2)	7 (4·3 to 10·8)	7·5 (4·6 to 11·7)	6·5 (3·9 to 10·5)	4·5 (2·9 to 6·7)	3·2 (2·1 to 4·7)	1·9 (1·3 to 2·9)	1·5 (1 to 2·2)	0·7 (0·5 to 1)	0·3 (0·3 to 0·4)	0·1 (0·1 to 0·1)	0 (0 to 0)	112
Conflict and terrorism	0 (0 to 0)	0 (0 to 0)	0 (0 to 0)	0 (0 to 0)	0 (0 to 0)	0 (0 to 0)	0 (0 to 0)	0 (0 to 0)	0 (0 to 0)	0·1 (0 to 0·1)	0·4 (0·2 to 0·6)	0·4 (0·2 to 0·6)	0·4 (0·3 to 0·7)	0·6 (0·4 to 0·9)	0·6 (0·4 to 0·9)	0·6 (0·4 to 0·8)	0·6 (0·4 to 0·9)	0·4 (0·2 to 0·5)	0·1 (0 to 0·1)	0 (0 to 0·1)	0 (0 to 0)	0 (0 to 0)	0 (0 to 0)	4·1

SR Table 2c: Dominican Republic DALYS by cause and age group for both sexes with 95% uncertainty intervals, 2019

Cause	Early Neonatal	Late Neonatal	Post Neonatal	1 to 4	5 to 9	10 to 14	15 to 19	20 to 24	25 to 29	30 to 34	35 to 39	40 to 44	45 to 49	50 to 54	55 to 59	60 to 64	65 to 69	70 to 74	75 to 79	80 to 84	85 to 89	90 to 94	95 plus	Total
All causes	276,000 (198,000 to 380,000)	67,900 (48,800 to 92,700)	113,000 (84,300 to 150,000)	93,400 (75,100 to 114,000)	70,100 (58,100 to 84,100)	78,500 (63,700 to 96,700)	120,000 (98,700 to 147,000)	173,000 (141,000 to 206,000)	185,000 (154,000 to 220,000)	191,000 (160,000 to 227,000)	191,000 (158,000 to 228,000)	196,000 (161,000 to 234,000)	203,000 (166,000 to 245,000)	222,000 (178,000 to 272,000)	208,000 (167,000 to 255,000)	215,000 (174,000 to 261,000)	182,000 (150,000 to 220,000)	175,000 (146,000 to 208,000)	125,000 (108,000 to 147,000)	102,000 (90,400 to 117,000)	73,000 (65,000 to 81,600)	29,800 (26,600 to 33,100)	10,100 (9,220 to 11,000)	3,300,000
Communicable, maternal, neonatal, and nutritional diseases	247,000 (173,000 to 336,000)	56,400 (39,500 to 77,500)	68,000 (46,700 to 96,100)	41,900 (31,000 to 55,200)	20,900 (16,500 to 26,400)	15,800 (12,400 to 20,100)	17,900 (14,300 to 21,900)	24,500 (19,700 to 29,800)	27,600 (21,900 to 35,000)	29,700 (23,600 to 37,800)	28,700 (22,700 to 37,000)	26,200 (21,300 to 32,900)	22,800 (18,700 to 27,700)	19,200 (15,800 to 23,700)	14,500 (11,900 to 18,000)	12,000 (9,620 to 15,100)	9,130 (7,320 to 11,300)	8,600 (6,900 to 10,900)	6,370 (5,190 to 7,940)	5,400 (4,320 to 6,670)	4,670 (3,630 to 5,740)	2,060 (1,540 to 2,600)	745 (551 to 950)	710,000
HIV/AIDS and sexually transmitted infections	8,660 (2,700 to 18,700)	2,950 (908 to 6,510)	4,660 (1,760 to 9,960)	862 (367 to 2,000)	437 (291 to 597)	355 (218 to 520)	1,220 (665 to 2,270)	3,010 (1,690 to 5,390)	5,900 (3,140 to 11,500)	8,640 (5,120 to 15,200)	10,600 (6,700 to 16,900)	10,500 (7,590 to 15,100)	9,690 (7,000 to 13,100)	6,910 (5,200 to 8,960)	3,980 (2,970 to 5,330)	2,040 (1,470 to 2,820)	1,040 (733 to 1,480)	542 (360 to 802)	261 (170 to 387)	78·3 (53·4 to 116)	47·5 (32 to 72·4)	17·9 (11·6 to 27·6)	6·2 (3·9 to 10·7)	82,400
HIV/AIDS	0 (0 to 0·1)	0·2 (0·1 to 0·2)	581 (308 to 942)	716 (269 to 1,840)	364 (239 to 507)	290 (163 to 444)	1,040 (505 to 2,100)	2,690 (1,400 to 5,100)	5,500 (2,760 to 11,100)	8,210 (4,730 to 14,900)	10,200 (6,340 to 16,500)	10,200 (7,300 to 14,900)	9,430 (6,780 to 12,800)	6,710 (5,030 to 8,750)	3,840 (2,840 to 5,130)	1,930 (1,390 to 2,720)	947 (651 to 1,390)	479 (306 to 744)	225 (139 to 349)	52·1 (33 to 79·9)	24·9 (15·9 to 38·3)	9 (5·7 to 13·8)	3 (1·8 to 4·7)	63,400
HIV/AIDS - Drug-susceptible Tuberculosis	0 (0 to 0)	0 (0 to 0)	92·2 (57·7 to 146)	112 (70·1 to 172)	55·8 (36·6 to 85·6)	47 (31·1 to 71)	157 (103 to 241)	427 (282 to 651)	779 (506 to 1,180)	1,190 (781 to 1,830)	1,530 (1,000 to 2,360)	1,630 (1,050 to 2,500)	1,580 (1,020 to 2,460)	1,190 (775 to 1,840)	668 (431 to 1,030)	348 (226 to 540)	157 (101 to 238)	81·1 (53·6 to 122)	35·1 (23·9 to 51·8)	8·7 (6 to 12·5)	4·3 (3 to 6·3)	1·3 (0·9 to 1·8)	0·3 (0·2 to 0·4)	10,100
HIV/AIDS - Multidrug-resistant Tuberculosis without exte	0 (0 to 0)	0 (0 to 0)	1·4 (0·1 to 6·8)	1·6 (0·1 to 7·9)	0·8 (0·1 to 3·9)	0·6 (0·1 to 3·2)	2·2 (0·2 to 10·8)	5·9 (0·5 to 27·9)	10·6 (0·9 to 50·2)	16 (1·3 to 78)	20·6 (1·7 to 99·8)	22 (1·8 to 106)	21·6 (1·7 to 106)	16·5 (1·3 to 79·9)	9·3 (0·7 to 43·8)	4·8 (0·4 to 22·8)	2·2 (0·2 to 10·1)	1·1 (0·1 to 5·1)	0·5 (0 to 2·1)	0·1 (0 to 0·5)	0·1 (0 to 0·3)	0 (0 to 0·1)	0 (0 to 0)	138
HIV/AIDS - Extensively drug-resistant Tuberculosis	0 (0 to 0)	0 (0 to 0)	0·2 (0 to 1·1)	0·3 (0 to 1·4)	0·1 (0 to 0·6)	0·1 (0 to 0·5)	0·4 (0 to 1·8)	1 (0·1 to 4·8)	1·7 (0·1 to 8·5)	2·6 (0·2 to 12·9)	3·4 (0·2 to 16·6)	3·6 (0·3 to 17·8)	3·6 (0·3 to 17·4)	2·7 (0·2 to 13·3)	1·5 (0·1 to 7·4)	0·8 (0·1 to 3·9)	0·4 (0 to 1·7)	0·2 (0 to 0·8)	0·1 (0 to 0·3)	0 (0 to 0·1)	0 (0 to 0)	0 (0 to 0)	0 (0 to 0)	22·8
HIV/AIDS resulting in other diseases	0 (0 to 0·1)	0·1 (0·1 to 0·2)	487 (210 to 858)	602 (158 to 1,730)	307 (182 to 450)	242 (116 to 393)	884 (352 to 1,940)	2,260 (978 to 4,700)	4,710 (2,010 to 10,200)	7,000 (3,500 to 13,500)	8,600 (4,820 to 14,900)	8,550 (5,580 to 13,100)	7,820 (5,160 to 11,100)	5,500 (3,710 to 7,600)	3,160 (2,090 to 4,450)	1,580 (994 to 2,390)	787 (481 to 1,220)	397 (219 to 658)	189 (103 to 313)	43·2 (24·4 to 70·4)	20·4 (11·5 to 33·4)	7·7 (4·4 to 12·6)	2·7 (1·5 to 4·4)	53,200
Sexually transmitted infections excluding HIV	8,660 (2,700 to 18,700)	2,950 (908 to 6,510)	4,080 (1,250 to 9,610)	146 (43·4 to 345)	73·6 (23·3 to 161)	64·5 (29·8 to 144)	173 (95 to 295)	325 (186 to 531)	395 (233 to 650)	432 (251 to 707)	421 (233 to 814)	330 (189 to 563)	261 (152 to 470)	196 (103 to 351)	144 (76·7 to 257)	101 (49·6 to 193)	94·1 (47·5 to 186)	62·6 (34·2 to 109)	36·2 (19·6 to 61·6)	26·2 (13·4 to 51·5)	22·6 (10·9 to 45·1)	8·9 (4·1 to 18)	3·2 (1·3 to 7·4)	19,000
Syphilis	8,660 (2,700 to 18,700)	2,950 (908 to 6,510)	4,080 (1,250 to 9,610)	146 (43·4 to 345)	73·6 (23·3 to 161)	28·5 (9·8 to 72·5)	46·9 (15·1 to 124)	50·6 (16 to 146)	73·5 (24·5 to 208)	74·4 (25·4 to 186)	102 (25·8 to 364)	44·1 (14·4 to 116)	44·4 (14·3 to 118)	46·2 (14 to 129)	45·7 (15·3 to 117)	35·5 (11·3 to 95)	39 (12·6 to 103)	23·2 (8·7 to 53·5)	9·4 (4 to 21·7)	7·7 (3·2 to 18·9)	5·2 (2·3 to 11·5)	2·3 (1 to 4·5)	0·8 (0·3 to 1·8)	16,600

Other cardiovascular and circulatory diseases	[illegible]
Chronic respiratory diseases	[illegible]
Chronic obstructive pulmonary disease	[illegible]
Pneumoconiosis	[illegible]
Silicosis	[illegible]
Asbestosis	[illegible]
Coal workers pneumoconiosis	[illegible]
Other pneumoconiosis	[illegible]
Asthma	[illegible]
Interstitial lung disease and pulmonary sarcoidosis	[illegible]
Other chronic respiratory diseases	[illegible]
Digestive diseases	[illegible]
Cirrhosis and other chronic liver diseases	[illegible]
Cirrhosis and other chronic liver diseases due to hepati…	[illegible]
Cirrhosis and other chronic liver diseases due to hepati…	[illegible]
Cirrhosis and other chronic liver diseases due to alcoh…	[illegible]
Cirrhosis and other chronic liver diseases due to NAFL…	[illegible]
Cirrhosis and other chronic liver diseases due to other…	[illegible]
Upper digestive system diseases	[illegible]
Peptic ulcer disease	[illegible]
Gastritis and duodenitis	[illegible]
Gastroesophageal reflux disease	[illegible]
Appendicitis	[illegible]
Paralytic ileus and intestinal obstruction	[illegible]
Inguinal, femoral, and abdominal hernia	[illegible]
Inflammatory bowel disease	[illegible]
Vascular intestinal disorders	[illegible]
Gallbladder and biliary diseases	[illegible]
Pancreatitis	[illegible]
Other digestive diseases	[illegible]
Neurological disorders	[illegible]
Alzheimer's disease and other dementias	[illegible]
Parkinson's disease	[illegible]
Idiopathic epilepsy	[illegible]
Multiple sclerosis	[illegible]
Motor neuron disease	[illegible]
Headache disorders	[illegible]
Migraine	[illegible]
Tension-type headache	[illegible]
Other neurological disorders	[illegible]
Mental disorders	[illegible]
Schizophrenia	[illegible]
Depressive disorders	[illegible]
Major depressive disorder	[illegible]
Dysthymia	[illegible]
Bipolar disorder	[illegible]
Anxiety disorders	[illegible]
Eating disorders	[illegible]
Anorexia nervosa	[illegible]
Bulimia nervosa	[illegible]
Autism spectrum disorders	[illegible]
Attention-deficit/hyperactivity disorder	[illegible]
Conduct disorder	[illegible]
Idiopathic developmental intellectual disability	[illegible]
Other mental disorders	[illegible]
Substance use disorders	[illegible]
Alcohol use disorders	[illegible]
Drug use disorders	[illegible]
Opioid use disorders	[illegible]
Cocaine use disorders	[illegible]
Amphetamine use disorders	[illegible]
Cannabis use disorders	[illegible]
Other drug use disorders	[illegible]
Diabetes and kidney diseases	[illegible]
Diabetes mellitus	[illegible]
Diabetes mellitus type 1	[illegible]
Diabetes mellitus type 2	[illegible]
Chronic kidney disease	[illegible]
Chronic kidney disease due to diabetes mellitus type 1	[illegible]
Chronic kidney disease due to diabetes mellitus type 2	[illegible]
Chronic kidney disease due to hypertension	[illegible]
Chronic kidney disease due to glomerulonephritis	[illegible]
Chronic kidney disease due to other and unspecified cau…	[illegible]
Acute glomerulonephritis	[illegible]
Skin and subcutaneous diseases	[illegible]
Dermatitis	[illegible]
Atopic dermatitis	[illegible]
Contact dermatitis	[illegible]
Seborrhoeic dermatitis	[illegible]
Psoriasis	[illegible]
Bacterial skin diseases	[illegible]
Cellulitis	[illegible]
Pyoderma	[illegible]
Scabies	[illegible]
Fungal skin diseases	[illegible]
Viral skin diseases	[illegible]
Acne vulgaris	[illegible]
Alopecia areata	[illegible]
Pruritus	[illegible]
Urticaria	[illegible]
Decubitus ulcer	[illegible]
Other skin and subcutaneous diseases	[illegible]
Sense organ diseases	[illegible]
Blindness and vision loss	[illegible]
Glaucoma	[illegible]
Cataract	[illegible]
Age-related macular degeneration	[illegible]
Refraction disorders	[illegible]
Near vision loss	[illegible]
Other vision loss	[illegible]
Age-related and other hearing loss	[illegible]
Other sense organ diseases	[illegible]
Musculoskeletal disorders	[illegible]
Rheumatoid arthritis	[illegible]
Osteoarthritis	[illegible]
Osteoarthritis hip	[illegible]
Osteoarthritis knee	[illegible]
Osteoarthritis hand	[illegible]
Osteoarthritis other	[illegible]
Low back pain	[illegible]
Neck pain	[illegible]
Gout	[illegible]
Other musculoskeletal disorders	[illegible]
Other non-communicable diseases	[illegible]
Congenital birth defects	[illegible]
Neural tube defects	[illegible]
Congenital heart anomalies	[illegible]
Orofacial clefts	[illegible]
Down syndrome	[illegible]
Turner syndrome	[illegible]
Klinefelter syndrome	[illegible]
Other chromosomal abnormalities	[illegible]
Congenital musculoskeletal and limb anomalies	[illegible]
Urogenital congenital anomalies	[illegible]
Digestive congenital anomalies	[illegible]
Other congenital birth defects	[illegible]
Urinary diseases and male infertility	[illegible]
Urolithiasis	[illegible]
Benign prostatic hyperplasia	[illegible]
Male infertility	[illegible]
Other urinary diseases	[illegible]
Gynecological diseases	[illegible]
Uterine fibroids	[illegible]
Polycystic ovarian syndrome	[illegible]
Female infertility	[illegible]
Endometriosis	[illegible]
Genital prolapse	[illegible]
Premenstrual syndrome	[illegible]
Other gynecological diseases	[illegible]
Haemoglobinopathies and haemolytic anaemias	[illegible]
Thalassaemias	[illegible]
Thalassaemias trait	[illegible]
Sickle cell disorders	[illegible]
Sickle cell trait	[illegible]
G6PD deficiency	[illegible]
G6PD trait	[illegible]
Other haemoglobinopathies and haemolytic anaemias	[illegible]
Endocrine, metabolic, blood, and immune disorders	[illegible]
Oral disorders	[illegible]
Caries of deciduous teeth	[illegible]
Caries of permanent teeth	[illegible]
Periodontal diseases	[illegible]
Edentulism	[illegible]
Other oral disorders	[illegible]
Sudden infant death syndrome	[illegible]
Injuries	[illegible]
Transport injuries	[illegible]
Road injuries	[illegible]
Pedestrian road injuries	[illegible]
Cyclist road injuries	[illegible]
Motorcyclist road injuries	[illegible]
Motor vehicle road injuries	[illegible]
Other road injuries	[illegible]

Other transport injuries	11·9 (3·8 to 28·6)	7·3 (2·9 to 16·1)	42 (19·2 to 89·6)	94·6 (50·9 to 192)	110 (70·6 to 178)	133 (89·1 to 206)	238 (144 to 390)	424 (248 to 711)	425 (251 to 696)	434 (251 to 728)	360 (217 to 622)	323 (190 to 549)
Unintentional injuries	1,220 (733 to 1,940)	839 (509 to 1,420)	5,730 (3,250 to 9,000)	8,150 (5,420 to 11,800)	6,790 (5,470 to 8,160)	7,210 (5,850 to 8,850)	8,680 (6,480 to 11,100)	12,400 (9,060 to 16,400)	12,600 (9,360 to 15,900)	12,100 (9,160 to 15,500)	10,900 (8,170 to 13,900)	9,870 (7,500 to 12,600)
Falls	32 (13·4 to 68)	13·3 (6·8 to 25·6)	182 (92 to 336)	385 (217 to 601)	468 (341 to 624)	637 (471 to 849)	957 (688 to 1,270)	1,370 (963 to 1,910)	1,500 (1,060 to 2,080)	1,540 (1,120 to 2,100)	1,570 (1,160 to 2,080)	1,550 (1,140 to 2,050)
Drowning	86·5 (41·5 to 177)	36·7 (17·3 to 69)	375 (180 to 732)	1,840 (910 to 3,230)	1,710 (1,040 to 2,680)	1,870 (1,190 to 2,750)	2,110 (1,140 to 3,470)	2,260 (1,200 to 3,670)	1,830 (1,010 to 2,920)	1,590 (835 to 2,630)	1,250 (637 to 1,990)	962 (530 to 1,590)
Fire, heat, and hot substances	43·6 (21·8 to 97·8)	51·3 (25·6 to 108)	531 (238 to 1,070)	1,420 (651 to 2,670)	963 (647 to 1,420)	855 (599 to 1,160)	973 (695 to 1,320)	1,260 (894 to 1,690)	1,350 (989 to 1,820)	1,400 (1,000 to 1,880)	1,300 (951 to 1,770)	1,150 (819 to 1,580)
Poisonings	52·2 (18 to 114)	66·2 (17·9 to 182)	247 (91·1 to 584)	568 (262 to 1,090)	247 (154 to 420)	194 (119 to 302)	180 (115 to 284)	295 (171 to 493)	289 (170 to 446)	263 (149 to 410)	230 (136 to 370)	190 (114 to 294)
Poisoning by carbon monoxide	6·1 (1·1 to 19·5)	4·4 (0·7 to 15·4)	9·7 (2·5 to 28·3)	62·8 (19·8 to 173)	44·9 (23·2 to 85·2)	44·1 (22·1 to 82·6)	59·2 (29·8 to 109)	109 (50·3 to 196)	96·6 (44·5 to 171)	84·8 (35·5 to 157)	69·7 (31·4 to 130)	64·6 (29·6 to 115)
Poisoning by other means	46·1 (14·7 to 104)	61·8 (16·8 to 173)	237 (87·1 to 564)	505 (231 to 996)	202 (123 to 342)	150 (92·2 to 238)	121 (77 to 187)	186 (110 to 304)	192 (115 to 307)	178 (104 to 295)	160 (94·9 to 269)	125 (74·7 to 193)
Exposure to mechanical forces	34·7 (4·7 to 148)	69 (3·3 to 368)	165 (42·7 to 658)	472 (259 to 760)	645 (448 to 895)	766 (547 to 1,030)	1,480 (993 to 2,110)	2,470 (1,570 to 3,830)	2,480 (1,630 to 3,630)	2,420 (1,630 to 3,660)	2,190 (1,510 to 3,120)	2,020 (1,420 to 2,810)
Unintentional firearm injuries	16 (1·3 to 84·4)	32·6 (0·7 to 191)	72·3 (9·3 to 285)	140 (58·2 to 291)	169 (95·2 to 291)	241 (150 to 361)	770 (416 to 1,330)	1,500 (775 to 2,730)	1,350 (692 to 2,370)	1,220 (660 to 2,150)	1,000 (577 to 1,760)	869 (520 to 1,410)
Other exposure to mechanical forces	18·7 (2·1 to 87)	36·5 (1·7 to 218)	92·6 (25·8 to 365)	332 (182 to 539)	476 (314 to 707)	525 (349 to 786)	714 (456 to 1,070)	970 (642 to 1,400)	1,120 (760 to 1,640)	1,200 (809 to 1,780)	1,190 (805 to 1,740)	1,150 (781 to 1,710)
Adverse effects of medical treatment	243 (123 to 398)	160 (81·4 to 283)	552 (262 to 1,000)	416 (237 to 719)	273 (163 to 403)	205 (132 to 308)	220 (131 to 360)	468 (253 to 775)	469 (238 to 789)	492 (255 to 853)	466 (242 to 788)	481 (257 to 852)
Animal contact	0·5 (0·2 to 1·1)	0·5 (0·3 to 1·2)	6·9 (4·4 to 12·6)	38·8 (25·3 to 55·6)	53·3 (33·8 to 83·6)	70·9 (45·7 to 105)	83·9 (53·3 to 121)	96·8 (62·6 to 139)	97·8 (65·4 to 141)	92·5 (61·1 to 132)	89·1 (58·2 to 129)	83·3 (54·4 to 119)
Venomous animal contact	0·2 (0·1 to 0·5)	0·3 (0·1 to 0·7)	4·6 (2·8 to 8·5)	26·2 (16 to 39·5)	37·5 (22·1 to 61·4)	50·9 (31 to 78·8)	61·1 (36·9 to 90·7)	71·5 (44·9 to 106)	73·3 (48·1 to 106)	68·2 (43·7 to 101)	64·6 (41·2 to 94·3)	59 (37·4 to 85·5)
Non-venomous animal contact	0·3 (0·1 to 0·7)	0·2 (0·1 to 0·6)	2·2 (1·3 to 4·1)	12·6 (7·6 to 19·2)	15·8 (9·9 to 26·5)	20 (12·2 to 33·1)	22·8 (14·1 to 36·3)	25·3 (15·6 to 39·1)	24·5 (14·5 to 38·7)	24·3 (14·8 to 38·8)	24·5 (15·2 to 40·2)	24·3 (14·9 to 38·4)
Foreign body	643 (330 to 1,140)	410 (225 to 677)	3,420 (1,560 to 6,120)	2,200 (1,250 to 3,660)	1,290 (854 to 1,890)	1,270 (845 to 1,850)	680 (434 to 988)	1,120 (672 to 1,670)	1,190 (730 to 1,770)	1,090 (658 to 1,590)	987 (608 to 1,500)	969 (589 to 1,460)
Pulmonary aspiration and foreign body in airway	641 (329 to 1,140)	409 (224 to 676)	3,410 (1,550 to 6,100)	2,120 (1,190 to 3,560)	1,190 (772 to 1,790)	1,160 (753 to 1,730)	559 (329 to 861)	973 (552 to 1,510)	1,040 (602 to 1,590)	955 (521 to 1,450)	859 (483 to 1,370)	849 (471 to 1,310)
Foreign body in eyes	0·1 (0 to 0·2)	0·2 (0·1 to 0·5)	3·8 (1 to 8·3)	19·4 (5·9 to 43·9)	28·3 (8·1 to 66·4)	33·5 (10·4 to 78·9)	44·2 (13·9 to 104)	55·9 (17·9 to 123)	60·4 (21·3 to 128)	58·6 (20·9 to 123)	55·2 (21 to 114)	51·7 (22·3 to 103)
Foreign body in other body part	1·5 (0·5 to 4·3)	0·8 (0·5 to 1·4)	11 (6 to 18·2)	58·5 (33·4 to 96)	70·9 (44·1 to 110)	73·5 (51 to 104)	76·9 (54·9 to 102)	88·6 (63·6 to 120)	83·8 (60·7 to 111)	80·2 (58·2 to 108)	73·2 (53·7 to 100)	67·6 (50·3 to 91·4)
Environmental heat and cold exposure	1·4 (0·6 to 3)	1·6 (0·8 to 2·9)	17·4 (10·5 to 28·4)	86·9 (58·3 to 125)	146 (95·7 to 210)	187 (126 to 263)	266 (185 to 365)	345 (231 to 470)	380 (263 to 522)	389 (273 to 540)	370 (261 to 510)	355 (250 to 485)
Exposure to forces of nature	0 (0 to 0)	0 (0 to 0)	0 (0 to 0)	0·1 (0·1 to 0·2)	0·5 (0·3 to 0·9)	3·1 (2 to 5)	20·5 (12·5 to 32·7)	32·2 (20·2 to 49·8)	54·4 (34 to 86)	43·7 (27·4 to 68·5)	36·7 (23·7 to 54·9)	34·5 (22 to 51·2)
Other unintentional injuries	85·3 (39·4 to 158)	29·6 (13·4 to 57·1)	227 (119 to 415)	729 (377 to 1,370)	1,000 (587 to 1,590)	1,150 (682 to 1,820)	1,710 (909 to 2,840)	2,710 (1,300 to 4,570)	2,920 (1,500 to 5,050)	2,740 (1,410 to 4,780)	2,390 (1,270 to 4,000)	2,070 (1,130 to 3,300)
Self-harm and interpersonal violence	817 (385 to 1,570)	155 (74·2 to 305)	1,150 (513 to 2,330)	2,080 (946 to 4,060)	1,690 (1,060 to 2,510)	2,990 (2,040 to 4,380)	14,100 (9,350 to 20,800)	27,100 (17,800 to 37,800)	25,200 (17,800 to 34,500)	20,900 (14,600 to 29,100)	15,400 (10,300 to 22,200)	11,400 (7,370 to 17,000)
Self-harm	0 (0 to 0)	0 (0 to 0)	0 (0 to 0)	0 (0 to 0)	0 (0 to 0)	397 (237 to 609)	2,310 (1,450 to 3,460)	4,580 (2,800 to 6,900)	4,630 (2,870 to 6,940)	4,350 (2,560 to 6,600)	3,240 (1,980 to 5,230)	2,670 (1,610 to 4,390)
Self-harm by firearm	0 (0 to 0)	0 (0 to 0)	0 (0 to 0)	0 (0 to 0)	0 (0 to 0)	26·6 (14·3 to 53·7)	318 (136 to 674)	796 (343 to 1,560)	777 (349 to 1,470)	688 (304 to 1,320)	509 (223 to 997)	426 (190 to 849)
Self-harm by other specified means	0 (0 to 0)	0 (0 to 0)	0 (0 to 0)	0 (0 to 0)	0 (0 to 0)	370 (222 to 571)	2,000 (1,250 to 2,960)	3,780 (2,310 to 5,700)	3,860 (2,410 to 5,740)	3,660 (2,240 to 5,520)	2,730 (1,650 to 4,300)	2,240 (1,380 to 3,590)
Interpersonal violence	817 (385 to 1,570)	155 (74·2 to 305)	1,150 (512 to 2,320)	2,070 (942 to 4,060)	1,680 (1,050 to 2,500)	2,590 (1,670 to 3,970)	11,800 (7,270 to 18,100)	22,500 (14,000 to 32,600)	20,600 (13,400 to 29,900)	16,600 (10,700 to 24,800)	12,100 (7,510 to 18,700)	8,760 (5,360 to 13,600)
Physical violence by firearm	84 (28·4 to 199)	18·2 (5·8 to 44·3)	125 (41·5 to 301)	285 (102 to 618)	368 (192 to 626)	959 (508 to 1,690)	7,700 (4,220 to 12,700)	15,400 (8,870 to 23,500)	14,100 (8,660 to 21,300)	11,000 (6,590 to 16,900)	7,900 (4,670 to 12,600)	5,390 (2,980 to 9,000)
Physical violence by sharp object	68·6 (23·9 to 162)	7 (2·4 to 17·5)	45·8 (18·9 to 96·5)	109 (49·7 to 236)	130 (71·5 to 221)	324 (168 to 551)	2,580 (1,310 to 4,640)	4,970 (2,560 to 8,300)	4,470 (2,510 to 7,640)	3,860 (2,010 to 6,650)	2,820 (1,480 to 4,910)	2,200 (1,230 to 3,910)
Sexual violence	0 (0 to 0)	0 (0 to 0)	0 (0 to 0)	7 (4·1 to 11·1)	51·2 (31·6 to 80)	107 (61·2 to 169)	250 (144 to 399)	343 (200 to 518)	305 (174 to 492)	257 (144 to 417)	210 (119 to 333)	157 (91·3 to 254)
Physical violence by other means	665 (319 to 1,300)	130 (60·6 to 257)	981 (435 to 1,970)	1,670 (719 to 3,380)	1,130 (653 to 1,760)	1,200 (656 to 1,950)	1,290 (759 to 2,170)	1,830 (1,060 to 2,960)	1,700 (1,030 to 2,740)	1,480 (915 to 2,370)	1,220 (748 to 2,020)	1,010 (587 to 1,640)
Conflict and terrorism	0 (0 to 0)	0 (0 to 0)	0 (0 to 0)	0 (0 to 0)	0·1 (0 to 0·1)	0·1 (0 to 0·1)	0·2 (0·1 to 0·3)	0·4 (0·2 to 0·6)	1·1 (0·6 to 1·8)	3·7 (2·2 to 6)	7·5 (4·5 to 12·3)	6 (3·7 to 9·8)

Other transport injuries	245 (153 to 415)	211 (133 to 334)	170 (111 to 275)	128 (83·1 to 204)	83·1 (53·9 to 123)	67·6 (44·7 to 100)	37·8 (26·8 to 53·2)	19·9 (14·6 to 26·2)	11 (8·3 to 14·2)	4·2 (3·1 to 5·4)	1·4 (1·1 to 1·9)	3,580
Unintentional injuries	8,490 (6,500 to 10,900)	7,820 (6,000 to 10,000)	6,310 (4,950 to 8,110)	5,640 (4,400 to 7,270)	4,300 (3,340 to 5,440)	3,740 (3,000 to 4,620)	2,720 (2,220 to 3,360)	2,390 (1,960 to 2,860)	1,910 (1,520 to 2,420)	852 (666 to 1,090)	288 (213 to 399)	141,000
Falls	1,510 (1,140 to 1,980)	1,610 (1,210 to 2,140)	1,410 (1,070 to 1,840)	1,410 (1,050 to 1,860)	1,180 (895 to 1,530)	1,230 (954 to 1,570)	1,030 (811 to 1,280)	1,140 (900 to 1,430)	1,100 (820 to 1,510)	529 (393 to 711)	187 (127 to 286)	22,500
Drowning	685 (380 to 1,140)	555 (314 to 913)	362 (201 to 614)	308 (177 to 509)	187 (105 to 293)	125 (76·6 to 198)	76·8 (47·5 to 123)	43·6 (25 to 66·4)	51·1 (21 to 80)	21·8 (8·9 to 34)	4·8 (2·5 to 7·2)	18,300
Fire, heat, and hot substances	1,030 (756 to 1,400)	916 (668 to 1,240)	747 (540 to 1,030)	621 (458 to 840)	443 (320 to 609)	350 (254 to 479)	231 (166 to 319)	177 (127 to 249)	138 (96·2 to 194)	57 (39·6 to 83·6)	17·4 (11·8 to 28·8)	16,000
Poisonings	160 (96·8 to 270)	121 (75·6 to 193)	82·1 (54·4 to 122)	68·8 (44·5 to 105)	47·1 (32·2 to 67·3)	38·2 (25·8 to 56·2)	27 (18·2 to 39·9)	21·3 (14 to 32·7)	14·7 (8·7 to 22·3)	5 (2·9 to 7·7)	1·1 (0·7 to 1·7)	3,410
Poisoning by carbon monoxide	41·9 (19·9 to 82·2)	25 (13 to 45·7)	19·6 (10·2 to 34·6)	14·3 (7·5 to 24·8)	11·4 (6·7 to 18·7)	9·8 (5·5 to 15·9)	5·3 (2·8 to 9·2)	4 (2·1 to 7·4)	3·2 (1·5 to 6)	1·2 (0·5 to 2·2)	0·2 (0·1 to 0·3)	792
Poisoning by other means	118 (70·7 to 194)	95·7 (58·9 to 151)	62·5 (41·4 to 95)	54·5 (34·7 to 86·6)	35·7 (23·5 to 52·6)	28·4 (19·3 to 42·5)	21·7 (14·3 to 32·7)	17·3 (11·1 to 27)	11·5 (7·1 to 17·6)	3·9 (2·3 to 6)	0·9 (0·6 to 1·4)	2,610
Exposure to mechanical forces	1,730 (1,230 to 2,360)	1,540 (1,110 to 2,120)	1,260 (900 to 1,740)	1,010 (717 to 1,370)	736 (526 to 1,000)	547 (385 to 757)	364 (257 to 506)	247 (175 to 345)	152 (108 to 207)	61·5 (43·8 to 84·8)	21·1 (14·9 to 29·5)	22,900
Unintentional firearm injuries	635 (398 to 980)	517 (341 to 837)	372 (249 to 584)	274 (190 to 405)	173 (120 to 248)	117 (83·8 to 157)	70·9 (51·6 to 94·2)	49·5 (36·4 to 66·8)	35·6 (25·2 to 48·4)	15·3 (11·1 to 20·3)	5·6 (3·9 to 8·1)	9,640
Other exposure to mechanical forces	1,090 (749 to 1,580)	1,030 (699 to 1,460)	889 (606 to 1,280)	731 (499 to 1,050)	563 (382 to 807)	431 (292 to 616)	293 (199 to 422)	198 (134 to 284)	116 (79·5 to 167)	46·3 (31·9 to 65·2)	15·5 (10·6 to 22)	13,200
Adverse effects of medical treatment	487 (269 to 831)	572 (323 to 1,020)	505 (286 to 894)	546 (313 to 918)	434 (252 to 762)	409 (254 to 654)	316 (195 to 493)	252 (162 to 379)	190 (126 to 262)	69·5 (46·8 to 93·8)	19 (12 to 26·5)	8,250
Animal contact	78·5 (51·5 to 113)	73·5 (49·8 to 104)	65·3 (45 to 93·5)	56·4 (38·9 to 79·6)	45·9 (31·6 to 64·2)	36·4 (25·5 to 50·5)	25·3 (17·6 to 35·8)	18·5 (13 to 26)	11·6 (8·3 to 16·2)	5·4 (3·8 to 8·3)	1·7 (1·2 to 3)	1,130
Venomous animal contact	53·3 (34·6 to 76·6)	49·9 (33·1 to 70·7)	43·8 (29·8 to 63·2)	37·8 (25·7 to 52·6)	30·2 (20·7 to 41·4)	23·6 (16·3 to 32)	16 (11·1 to 22·5)	11·8 (8·2 to 16·4)	7·6 (5·4 to 10·9)	3·7 (2·6 to 6)	1·2 (0·8 to 2·1)	796
Non-venomous animal contact	25·2 (15·9 to 38·2)	23·6 (15 to 36·2)	21·5 (13·6 to 32·4)	18·6 (12·2 to 28·4)	15·7 (10 to 24)	12·8 (8·3 to 19·3)	9·3 (5·9 to 13·9)	6·7 (4·4 to 10)	4 (2·6 to 6)	1·7 (1·1 to 2·6)	0·6 (0·4 to 0·9)	336
Foreign body	824 (513 to 1,220)	836 (510 to 1,260)	656 (406 to 979)	681 (428 to 1,050)	542 (347 to 809)	492 (322 to 703)	315 (218 to 434)	251 (178 to 340)	120 (83·8 to 167)	49 (34·8 to 68·8)	17·7 (12·1 to 25·6)	20,100
Pulmonary aspiration and foreign body in airway	718 (410 to 1,100)	744 (423 to 1,170)	583 (342 to 910)	617 (367 to 973)	489 (295 to 760)	434 (276 to 640)	269 (180 to 379)	210 (141 to 295)	95·9 (64·8 to 137)	37·9 (24·5 to 55·8)	14 (9 to 21·6)	18,400
Foreign body in eyes	45·3 (21·6 to 86·3)	36·5 (18·6 to 65·2)	27·8 (16·1 to 46·1)	22 (13·2 to 34·7)	17·6 (10·8 to 27)	13·6 (8·8 to 20·4)	9·7 (6·2 to 14·4)	6·6 (4·3 to 9·8)	3·6 (2·4 to 5·2)	1·3 (0·9 to 1·9)	0·4 (0·3 to 0·6)	596
Foreign body in other body part	60·9 (45·1 to 81·2)	55·4 (40·3 to 73·6)	45·1 (33·5 to 58·7)	41·6 (30·1 to 56·7)	35·6 (25·8 to 48·4)	44·1 (28 to 68·2)	36·6 (23·7 to 55·1)	34·3 (22 to 52·9)	20 (12·4 to 32·4)	9·8 (6 to 15·7)	3·3 (1·9 to 5·5)	1,070
Environmental heat and cold exposure	331 (232 to 449)	308 (218 to 412)	269 (191 to 359)	229 (161 to 310)	186 (131 to 250)	154 (108 to 204)	111 (80·4 to 148)	84 (60·9 to 110)	52 (38·1 to 68·3)	21·8 (16 to 29·3)	7·5 (5·2 to 10·1)	4,300
Exposure to forces of nature	32·9 (20·9 to 50)	27·4 (17·5 to 40·7)	20·9 (13·5 to 31·4)	15·8 (10·5 to 23·7)	12·2 (8·2 to 18·5)	8·8 (6 to 12·9)	5·8 (3·8 to 8·4)	3·6 (2·3 to 5·1)	1·8 (1·2 to 2·7)	0·6 (0·4 to 0·9)	0·2 (0·1 to 0·3)	356
Other unintentional injuries	1,630 (930 to 2,740)	1,260 (794 to 1,860)	928 (596 to 1,360)	698 (478 to 979)	488 (341 to 679)	346 (240 to 491)	226 (160 to 305)	147 (105 to 198)	83·8 (60·5 to 116)	31 (22·5 to 41·7)	10·1 (7·1 to 14·6)	23,600
Self-harm and interpersonal violence	8,220 (5,560 to 12,300)	6,000 (3,950 to 8,990)	3,900 (2,640 to 5,800)	2,820 (1,960 to 4,030)	1,650 (1,180 to 2,360)	1,090 (796 to 1,550)	588 (433 to 781)	323 (248 to 433)	198 (152 to 267)	53·8 (41·4 to 69·8)	15·1 (10·9 to 23·7)	148,000
Self-harm	2,160 (1,380 to 3,420)	1,910 (1,220 to 3,020)	1,420 (906 to 2,250)	1,230 (782 to 1,860)	762 (494 to 1,170)	549 (367 to 831)	299 (200 to 440)	183 (124 to 284)	118 (81·3 to 182)	27·7 (19·1 to 42·2)	6 (3·4 to 14·8)	30,800
Self-harm by firearm	322 (151 to 618)	278 (127 to 555)	178 (83·4 to 337)	150 (67·9 to 282)	85·5 (41·6 to 165)	60·4 (29·7 to 116)	29·9 (14·9 to 57·9)	18·8 (10 to 37)	12·2 (7 to 21·3)	2·4 (1·2 to 4·5)	0·5 (0·2 to 1·5)	4,680
Self-harm by other specified means	1,840 (1,180 to 2,840)	1,630 (1,040 to 2,620)	1,250 (801 to 1,980)	1,080 (680 to 1,640)	677 (435 to 1,030)	489 (329 to 737)	269 (181 to 389)	164 (110 to 253)	106 (71·6 to 165)	25·3 (17·4 to 38·5)	5·5 (3·1 to 13·7)	26,200
Interpersonal violence	6,050 (3,810 to 9,480)	4,080 (2,490 to 6,510)	2,470 (1,550 to 3,920)	1,590 (1,000 to 2,430)	882 (603 to 1,330)	542 (366 to 794)	288 (207 to 420)	139 (107 to 179)	79·1 (61·3 to 102)	25·9 (19·9 to 32·7)	9·1 (6·7 to 12·3)	117,000
Physical violence by firearm	3,680 (2,010 to 6,320)	2,290 (1,200 to 3,870)	1,280 (663 to 2,140)	794 (423 to 1,360)	384 (209 to 678)	190 (102 to 353)	71·3 (39·3 to 119)	28·7 (17·7 to 45·4)	16·7 (11 to 25)	3·3 (2·2 to 5)	1·1 (0·7 to 1·9)	72,000
Physical violence by sharp object	1,400 (794 to 2,450)	957 (532 to 1,650)	600 (336 to 1,060)	362 (223 to 593)	206 (135 to 328)	145 (92·7 to 220)	76·8 (50 to 121)	38·2 (27·1 to 53·6)	22·6 (15·8 to 31)	10·1 (7·2 to 13·5)	4·4 (2·9 to 6·6)	25,400
Sexual violence	114 (67·3 to 191)	77·7 (43·7 to 126)	49·4 (28·9 to 78·4)	35·9 (21·8 to 54·3)	27·2 (16·7 to 40·8)	20 (12·4 to 29·9)	13·4 (8·6 to 19·6)	9 (5·8 to 13·3)	4·1 (2·4 to 6·5)	0·6 (0·4 to 0·9)	0 (0 to 0·1)	2,040
Physical violence by other means	857 (546 to 1,380)	763 (481 to 1,230)	548 (353 to 871)	398 (261 to 584)	265 (185 to 394)	187 (132 to 262)	126 (90·8 to 174)	63 (46·1 to 84·4)	35·6 (26·2 to 47·7)	11·9 (8·7 to 15·7)	3·5 (2·5 to 5)	17,600
Conflict and terrorism	6·1 (3·8 to 9·4)	6 (3·8 to 9·5)	5 (3·1 to 8·1)	2·1 (1·4 to 3·1)	1·3 (0·8 to 2)	1·7 (1·1 to 2·6)	1·1 (0·7 to 1·7)	0·7 (0·4 to 1)	0·7 (0·4 to 1)	0·1 (0·1 to 0·2)	0 (0 to 0·1)	43·7

SR Table 2c: Malaysia DALYS by cause and age group for both sexes with 95% uncertainty intervals, 2019

Cause	Early Neonatal	Late Neonatal	Post Neonatal	1 to 4	5 to 9	10 to 14	15 to 19	20 to 24	25 to 29	30 to 34	35 to 39
All causes	126,000 (101,000 to 153,000)	43,900 (35,600 to 53,200)	89,500 (74,200 to 107,000)	94,400 (68,600 to 126,000)	129,000 (101,000 to 161,000)	174,000 (138,000 to 218,000)	302,000 (245,000 to 370,000)	380,000 (316,000 to 458,000)	425,000 (352,000 to 516,000)	476,000 (398,000 to 571,000)	470,000 (392,000 to 561,000)
Communicable, maternal, neonatal, and nutritional diseases	86,600 (67,300 to 109,000)	29,000 (22,800 to 36,200)	45,100 (35,200 to 55,500)	49,800 (35,500 to 67,100)	44,700 (33,400 to 59,300)	34,100 (26,300 to 43,700)	40,500 (31,000 to 52,400)	46,500 (37,300 to 57,300)	58,100 (47,700 to 71,100)	72,200 (60,400 to 85,900)	73,500 (61,400 to 87,900)
HIV/AIDS and sexually transmitted infections	871 (276 to 2,060)	280 (85·1 to 623)	1,100 (832 to 1,560)	834 (706 to 992)	731 (610 to 863)	821 (653 to 999)	1,160 (952 to 1,390)	2,740 (2,270 to 3,240)	7,160 (5,960 to 8,550)	15,600 (12,700 to 18,900)	19,300 (15,700 to 23,600)
HIV/AIDS	0·5 (0·2 to 0·8)	1·6 (0·8 to 2·4)	787 (653 to 933)	826 (695 to 986)	725 (605 to 856)	760 (589 to 934)	935 (794 to 1,110)	2,270 (1,900 to 2,670)	6,520 (5,400 to 7,830)	14,800 (12,000 to 18,200)	18,700 (15,200 to 22,900)
HIV/AIDS - Drug-susceptible Tuberculosis	0 (0 to 0)	0·1 (0 to 0·1)	139 (88·9 to 206)	146 (97·9 to 214)	117 (79·3 to 171)	114 (78·7 to 165)	165 (111 to 242)	417 (281 to 608)	1,200 (810 to 1,760)	2,700 (1,790 to 4,010)	3,370 (2,240 to 5,040)
HIV/AIDS - Multidrug-resistant Tuberculosis without exte	0 (0 to 0)	0 (0 to 0)	3·1 (0·6 to 9·9)	3·1 (0·6 to 9·7)	2·4 (0·5 to 7·6)	2·3 (0·4 to 7·3)	3·5 (0·7 to 11)	8·6 (1·6 to 27·1)	24·8 (4·7 to 78)	56·5 (10·4 to 179)	70·6 (12·9 to 225)
HIV/AIDS - Extensively drug-resistant Tuberculosis	0 (0 to 0)	0 (0 to 0)	0·6 (0·1 to 1·9)	0·6 (0·1 to 1·8)	0·4 (0·1 to 1·4)	0·4 (0·1 to 1·4)	0·6 (0·1 to 2·1)	1·6 (0·3 to 5·1)	4·6 (0·8 to 14·8)	10·6 (1·8 to 34·3)	13·3 (2·3 to 43·3)
HIV/AIDS resulting in other diseases	0·5 (0·2 to 0·8)	1·5 (0·7 to 2·4)	644 (497 to 802)	676 (531 to 842)	605 (471 to 744)	643 (466 to 827)	766 (603 to 957)	1,840 (1,440 to 2,300)	5,290 (4,070 to 6,670)	12,100 (9,100 to 15,700)	15,300 (11,300 to 19,800)
Sexually transmitted infections excluding HIV	871 (275 to 2,060)	279 (83·3 to 622)	317 (92·6 to 755)	8·4 (2 to 22·8)	5·8 (1·7 to 12·9)	61·8 (37·1 to 103)	222 (114 to 394)	467 (259 to 786)	644 (355 to 1,100)	716 (381 to 1,230)	603 (331 to 1,070)
Syphilis	871 (275 to 2,060)	279 (83·3 to 622)	317 (92·6 to 755)	8·4 (2 to 22·8)	5·8 (1·7 to 12·9)	13·4 (8·7 to 24·9)	11·6 (8·7 to 15·9)	10·7 (7·6 to 15·7)	16·3 (10·8 to 24·8)	16·8 (10·3 to 27)	16 (10·1 to 25·7)
Chlamydial infection	0 (0 to 0)	0 (0 to 0)	0 (0 to 0)	0 (0 to 0)	0 (0 to 0)	2·5 (1·5 to 4·5)	19·4 (10·5 to 33·3)	76·1 (27·7 to 154)	131 (55·7 to 247)	132 (51·1 to 253)	104 (43·2 to 194)
Gonococcal infection	0 (0 to 0)	0 (0 to 0)	0 (0 to 0)	0 (0 to 0)	0 (0 to 0)	12·2 (5·3 to 25·4)	55·6 (14·4 to 124)	91 (33·3 to 191)	70·5 (21·4 to 149)	50·2 (16 to 111)	40·5 (14·5 to 82·6)
Trichomoniasis	0 (0 to 0)	0 (0 to 0)	0 (0 to 0)	0 (0 to 0)	0 (0 to 0)	1·8 (0 to 5·7)	25·3 (7·3 to 62·4)	97·1 (32·6 to 237)	154 (49·9 to 347)	162 (51·6 to 377)	140 (45·9 to 334)
Genital herpes	0 (0 to 0)	0 (0 to 0)	0 (0 to 0)	0 (0 to 0)	0 (0 to 0)	7·5 (2·1 to 18·1)	36·1 (10·5 to 87·1)	80·9 (25·4 to 200)	127 (40·8 to 312)	161 (52·4 to 388)	155 (49·2 to 379)
Other sexually transmitted infections	0 (0 to 0)	0 (0 to 0)	0 (0 to 0)	0 (0 to 0)	0 (0 to 0)	24·4 (12·4 to 48)	73·8 (31 to 143)	111 (57·5 to 189)	145 (77·4 to 247)	194 (97·7 to 314)	149 (82·6 to 241)
Respiratory infections and tuberculosis	2,510 (1,590 to 3,780)	2,060 (1,400 to 3,050)	11,700 (7,660 to 17,200)	5,840 (3,730 to 8,640)	7,770 (5,760 to 10,500)	6,830 (5,120 to 9,080)	9,370 (7,020 to 12,200)	14,200 (11,000 to 18,100)	18,100 (14,000 to 22,900)	23,000 (17,400 to 29,800)	23,800 (17,400 to 31,300)
Tuberculosis	0 (0 to 0)	0 (0 to 0)	370 (236 to 538)	225 (108 to 371)	584 (355 to 917)	550 (349 to 797)	1,070 (726 to 1,540)	3,390 (2,350 to 4,670)	4,910 (3,450 to 6,730)	4,980 (3,550 to 6,730)	4,900 (3,450 to 6,740)
Latent tuberculosis infection	0 (0 to 0)	0 (0 to 0)	0 (0 to 0)	0 (0 to 0)	0 (0 to 0)	0 (0 to 0)	0 (0 to 0)	0 (0 to 0)	0 (0 to 0)	0 (0 to 0)	0 (0 to 0)
Drug-susceptible tuberculosis	0 (0 to 0)	0 (0 to 0)	363 (230 to 529)	221 (105 to 368)	576 (344 to 905)	541 (342 to 788)	1,050 (711 to 1,510)	3,330 (2,290 to 4,570)	4,830 (3,360 to 6,680)	4,890 (3,500 to 6,600)	4,810 (3,370 to 6,640)
Multidrug-resistant tuberculosis without extensive drug r	0 (0 to 0)	0 (0 to 0)	6·6 (1·3 to 22·4)	2·9 (0·6 to 9·6)	7·2 (1·6 to 22·2)	7·8 (1·6 to 24·2)	17·9 (3·6 to 54·3)	49·1 (10·4 to 144)	72·2 (14·8 to 218)	76·4 (15·2 to 236)	76·5 (15·5 to 227)
Extensively drug-resistant tuberculosis	0 (0 to 0)	0 (0 to 0)	1·3 (0·2 to 4·4)	0·5 (0·1 to 1·6)	1·1 (0·2 to 3·6)	1·3 (0·2 to 4·3)	3·3 (0·6 to 11·2)	8·3 (1·6 to 26·4)	12·5 (2·4 to 40·7)	13·6 (2·5 to 45·4)	13·9 (2·6 to 44·7)
Lower respiratory infections	2,510 (1,580 to 3,780)	2,020 (1,370 to 3,020)	10,600 (6,610 to 16,100)	2,110 (953 to 3,990)	3,240 (2,430 to 4,400)	3,280 (2,360 to 4,490)	5,240 (3,720 to 7,270)	7,760 (5,470 to 10,800)	10,300 (7,300 to 14,000)	15,400 (10,600 to 21,100)	16,700 (11,100 to 23,100)
Upper respiratory infections	5·2 (2·9 to 8·8)	33 (18·3 to 55)	632 (363 to 1,040)	2,540 (1,430 to 4,230)	2,690 (1,460 to 4,540)	2,290 (1,200 to 3,880)	2,390 (1,240 to 4,200)	2,450 (1,310 to 4,270)	2,390 (1,330 to 4,010)	2,210 (1,190 to 3,750)	1,830 (927 to 3,080)
Otitis media	0·2 (0·1 to 0·4)	1·8 (0·9 to 3·3)	90·6 (48·5 to 158)	966 (529 to 1,640)	1,250 (682 to 2,120)	709 (365 to 1,290)	675 (349 to 1,200)	626 (274 to 1,220)	535 (244 to 989)	437 (182 to 868)	365 (173 to 680)
Enteric infections	106 (66·1 to 164)	390 (250 to 619)	5,080 (3,360 to 7,520)	9,840 (6,020 to 15,300)	8,150 (4,890 to 12,900)	6,670 (3,580 to 11,200)	5,530 (2,810 to 10,000)	4,270 (2,430 to 7,690)	4,070 (2,670 to 6,420)	3,850 (2,660 to 5,600)	3,390 (2,400 to 4,760)
Diarrheal diseases	106 (66·1 to 164)	384 (244 to 612)	4,960 (3,240 to 7,380)	7,090 (4,570 to 10,700)	3,340 (2,180 to 5,140)	1,910 (1,240 to 2,850)	1,540 (1,020 to 2,230)	1,650 (1,090 to 2,410)	2,290 (1,550 to 3,220)	2,740 (1,910 to 3,850)	2,820 (1,980 to 3,900)
Typhoid and paratyphoid	0 (0 to 0)	0 (0 to 0)	35·8 (11·6 to 87·4)	2,550 (636 to 6,330)	4,570 (1,720 to 9,330)	4,610 (1,710 to 9,130)	3,870 (1,220 to 8,390)	2,530 (864 to 5,700)	1,720 (650 to 3,800)	1,060 (377 to 2,400)	542 (205 to 1,150)
Typhoid fever	0 (0 to 0)	0 (0 to 0)	32·1 (10·1 to 79·2)	2,280 (566 to 5,720)	4,090 (1,540 to 8,280)	4,140 (1,520 to 8,290)	3,500 (1,090 to 7,590)	2,290 (765 to 5,230)	1,550 (598 to 3,430)	959 (340 to 2,180)	490 (183 to 1,030)
Paratyphoid fever	0 (0 to 0)	0 (0 to 0)	3·7 (1 to 10·2)	274 (61·2 to 703)	481 (149 to 1,220)	466 (143 to 1,060)	373 (102 to 899)	242 (70 to 586)	166 (50 to 402)	102 (28·8 to 260)	51·7 (16·4 to 120)
Invasive Non-typhoidal Salmonella (iNTS)	0 (0 to 0)	5·9 (3·1 to 10)	83·6 (43·6 to 144)	186 (62·3 to 422)	218 (88·1 to 443)	140 (50·8 to 294)	105 (34·3 to 242)	81·7 (22·7 to 191)	58·9 (17·1 to 139)	42·2 (11·6 to 99)	28·8 (8·7 to 65·7)
Other intestinal infectious diseases	0 (0 to 0)	0 (0 to 0)	9·5 (1·6 to 24·1)	15·7 (8·6 to 27·1)	9·5 (2·3 to 23·5)	8 (1·2 to 21·1)	6·4 (0·8 to 16·7)	6·4 (0·9 to 16·4)	3·1 (0·6 to 8)	8 (1·9 to 20·1)	3·8 (1 to 9·1)
Neglected tropical diseases and malaria	1·2 (0·4 to 2·4)	82·7 (40 to 176)	894 (438 to 1,900)	2,230 (1,200 to 4,620)	4,950 (3,140 to 7,610)	4,330 (2,730 to 6,530)	5,070 (3,240 to 7,740)	5,490 (3,400 to 8,420)	5,500 (3,460 to 8,480)	4,590 (2,880 to 7,150)	3,650 (2,310 to 5,490)
Malaria	0·1 (0 to 0·2)	1·1 (0·4 to 2·3)	8·1 (2·7 to 17·3)	37·4 (12·2 to 75·7)	51·3 (22·4 to 97·9)	29 (19·6 to 43·2)	98·2 (44·9 to 209)	93·3 (44·2 to 189)	75·5 (40 to 140)	64·9 (35·4 to 114)	51·9 (29·2 to 85·5)
Chagas disease	0 (0 to 0)	0 (0 to 0)	0 (0 to 0)	0 (0 to 0)	0 (0 to 0)	0 (0 to 0)	0 (0 to 0)	0 (0 to 0)	0 (0 to 0)	0 (0 to 0)	0 (0 to 0)
Leishmaniasis	0 (0 to 0)	0 (0 to 0)	0 (0 to 0)	0 (0 to 0)	0 (0 to 0)	0 (0 to 0)	0 (0 to 0)	0 (0 to 0)	0 (0 to 0)	0 (0 to 0)	0 (0 to 0)
Visceral leishmaniasis	0 (0 to 0)	0 (0 to 0)	0 (0 to 0)	0 (0 to 0)	0 (0 to 0)	0 (0 to 0)	0 (0 to 0)	0 (0 to 0)	0 (0 to 0)	0 (0 to 0)	0 (0 to 0)
Cutaneous and mucocutaneous leishmaniasis	0 (0 to 0)	0 (0 to 0)	0 (0 to 0)	0 (0 to 0)	0 (0 to 0)	0 (0 to 0)	0 (0 to 0)	0 (0 to 0)	0 (0 to 0)	0 (0 to 0)	0 (0 to 0)
African trypanosomiasis	0 (0 to 0)	0 (0 to 0)	0 (0 to 0)	0 (0 to 0)	0 (0 to 0)	0 (0 to 0)	0 (0 to 0)	0 (0 to 0)	0 (0 to 0)	0 (0 to 0)	0 (0 to 0)
Schistosomiasis	0 (0 to 0)	0 (0 to 0)	0 (0 to 0)	0 (0 to 0)	0 (0 to 0)	0 (0 to 0)	0 (0 to 0)	0 (0 to 0)	0 (0 to 0)	0 (0 to 0)	0 (0 to 0)
Cysticercosis	0 (0 to 0)	0 (0 to 0)	0 (0 to 0)	0 (0 to 0)	0·1 (0 to 0·3)	0·6 (0·2 to 1·7)	2·2 (0·6 to 5·5)	5·1 (1·3 to 12·4)	8·2 (2·5 to 19·9)	10·1 (3·7 to 22)	11·1 (4·1 to 24·8)
Cystic echinococcosis	0 (0 to 0)	0 (0 to 0)	0 (0 to 0)	0·3 (0 to 0·9)	1·5 (0·1 to 4·3)	2·2 (0·2 to 5·9)	3·1 (0·4 to 8·8)	3·3 (0·4 to 8·5)	3·8 (0·4 to 9·9)	4·2 (0·4 to 11·1)	3·8 (0·2 to 10·6)
Lymphatic filariasis	0 (0 to 0)	0 (0 to 0)	0 (0 to 0)	0 (0 to 0)	819 (419 to 1,570)	850 (444 to 1,560)	939 (462 to 1,740)	1,030 (524 to 1,890)	1,090 (570 to 2,060)	1,070 (546 to 2,040)	919 (472 to 1,710)
Onchocerciasis	0 (0 to 0)	0 (0 to 0)	0 (0 to 0)	0 (0 to 0)	0 (0 to 0)	0 (0 to 0)	0 (0 to 0)	0 (0 to 0)	0 (0 to 0)	0 (0 to 0)	0 (0 to 0)
Trachoma	0 (0 to 0)	0 (0 to 0)	0 (0 to 0)	0 (0 to 0)	0 (0 to 0)	0 (0 to 0)	0 (0 to 0)	0 (0 to 0)	0 (0 to 0)	0 (0 to 0)	0 (0 to 0)
Dengue	0 (0 to 0)	45·4 (10·8 to 135)	603 (198 to 1,580)	749 (229 to 2,970)	1,450 (612 to 3,190)	1,310 (596 to 2,520)	1,300 (616 to 2,530)	1,590 (648 to 3,420)	2,090 (867 to 4,620)	1,700 (707 to 3,400)	1,280 (512 to 2,630)
Yellow fever	0 (0 to 0)	0 (0 to 0)	0 (0 to 0)	0 (0 to 0)	0 (0 to 0)	0 (0 to 0)	0 (0 to 0)	0 (0 to 0)	0 (0 to 0)	0 (0 to 0)	0 (0 to 0)
Rabies	0 (0 to 0)	0 (0 to 0)	0 (0 to 0)	0 (0 to 0)	4·2 (0·1 to 11·2)	4 (0·1 to 11·5)	0 (0 to 0·1)	0 (0 to 0·1)	0 (0 to 0·1)	0 (0 to 0·2)	0 (0 to 0·2)
Intestinal nematode infections	0 (0 to 0)	0 (0 to 0)	24·3 (12·2 to 42·6)	540 (276 to 931)	1,540 (749 to 2,740)	1,630 (794 to 2,970)	2,400 (1,180 to 4,350)	2,540 (1,250 to 4,520)	1,970 (979 to 3,520)	1,480 (753 to 2,630)	1,100 (567 to 1,920)

Cause	40 to 44	45 to 49	50 to 54	55 to 59	60 to 64	65 to 69	70 to 74	75 to 79	80 to 84	85 to 89	90 to 94	95 plus	Total
All causes	459,000 (380,000 to 549,000)	482,000 (394,000 to 579,000)	551,000 (451,000 to 660,000)	637,000 (523,000 to 763,000)	682,000 (569,000 to 808,000)	672,000 (566,000 to 790,000)	613,000 (520,000 to 716,000)	417,000 (358,000 to 481,000)	293,000 (255,000 to 333,000)	139,000 (122,000 to 156,000)	63,800 (57,500 to 70,400)	10,900 (10,100 to 11,800)	7,730,000
Communicable, maternal, neonatal, and nutritional diseases	63,100 (51,900 to 77,400)	56,900 (45,600 to 70,800)	58,500 (44,400 to 73,800)	65,100 (48,000 to 82,900)	73,600 (51,200 to 95,000)	77,700 (52,700 to 100,000)	81,400 (55,500 to 108,000)	62,400 (41,900 to 80,400)	46,200 (32,400 to 58,000)	23,800 (16,900 to 29,500)	11,700 (8,260 to 14,400)	2,040 (1,440 to 2,570)	1,200,000
HIV/AIDS and sexually transmitted infections	14,100 (11,400 to 17,300)	9,120 (7,580 to 11,000)	6,520 (5,480 to 7,800)	4,940 (4,130 to 5,990)	3,210 (2,690 to 3,820)	1,210 (1,010 to 1,420)	173 (128 to 237)	76·1 (50·4 to 121)	21·7 (11·9 to 38·8)	8 (4·6 to 15·1)	2·6 (1·4 to 5·1)	0·5 (0·3 to 0·8)	90,000
HIV/AIDS	13,600 (11,000 to 16,800)	8,730 (7,200 to 10,500)	6,260 (5,270 to 7,510)	4,750 (3,960 to 5,760)	3,080 (2,570 to 3,680)	1,110 (933 to 1,300)	104 (78·6 to 130)	31·5 (23·9 to 39)	4·6 (3·6 to 5·6)	2·3 (1·8 to 2·7)	0·6 (0·5 to 0·8)	0·3 (0·2 to 0·3)	84,100
HIV/AIDS - Drug-susceptible Tuberculosis	2,420 (1,580 to 3,620)	1,530 (1,000 to 2,310)	1,090 (717 to 1,650)	825 (542 to 1,230)	535 (356 to 793)	195 (131 to 287)	19·4 (14·1 to 25·8)	5·9 (4·3 to 8)	0·8 (0·6 to 1·1)	0·4 (0·3 to 0·6)	0·1 (0·1 to 0·2)	0 (0 to 0·1)	15,000
HIV/AIDS - Multidrug-resistant Tuberculosis without exte	51·2 (9·4 to 163)	32·9 (6 to 105)	23·8 (4·3 to 75·8)	18 (3·3 to 57·4)	11·6 (2·1 to 36·8)	4·1 (0·8 to 13)	0·3 (0·1 to 0·9)	0·1 (0 to 0·3)	0 (0 to 0)	0 (0 to 0)	0 (0 to 0)	0 (0 to 0)	317
HIV/AIDS - Extensively drug-resistant Tuberculosis	9·6 (1·7 to 31·3)	6·2 (1·1 to 20·2)	4·5 (0·8 to 14·6)	3·4 (0·6 to 11)	2·2 (0·4 to 7·1)	0·8 (0·1 to 2·5)	0·1 (0 to 0·2)	0 (0 to 0)	0 (0 to 0)	0 (0 to 0)	0 (0 to 0)	0 (0 to 0)	59·4
HIV/AIDS resulting in other diseases	11,100 (8,300 to 14,500)	7,150 (5,550 to 8,930)	5,140 (4,010 to 6,470)	3,910 (3,000 to 5,000)	2,530 (1,980 to 3,160)	910 (720 to 1,110)	84·1 (59·5 to 110)	25·5 (17·9 to 32·5)	3·7 (2·7 to 4·7)	1·8 (1·3 to 2·3)	0·5 (0·4 to 0·7)	0·2 (0·2 to 0·3)	68,700
Sexually transmitted infections excluding HIV	496 (268 to 899)	396 (207 to 723)	261 (132 to 509)	191 (87·3 to 380)	136 (60·2 to 282)	100 (48·5 to 194)	68·9 (35·1 to 129)	44·6 (21·1 to 87·4)	17·2 (7·8 to 34·5)	5·8 (2·4 to 12·6)	2 (0·8 to 4·4)	0·2 (0·1 to 0·6)	5,910
Syphilis	8·2 (5·4 to 12·3)	8·7 (5·3 to 14·7)	6·7 (3·6 to 13·4)	4·7 (2·7 to 9·7)	7 (2·5 to 17·2)	4·9 (2·2 to 10·2)	6·4 (2·2 to 15·3)	2·4 (1·1 to 5·1)	1 (0·5 to 1·8)	0·2 (0·1 to 0·6)	0·1 (0·1 to 0·3)	0 (0 to 0)	1,620
Chlamydial infection	79·8 (34·8 to 142)	53·1 (23·9 to 95·1)	20·6 (5·3 to 44·3)	8·7 (5 to 14·1)	3·3 (1·6 to 7·2)	2·2 (0·9 to 5·2)	2·4 (0·9 to 5·3)	3·1 (1·1 to 7·3)	0·7 (0·2 to 1·5)	0·1 (0 to 0·4)	0 (0 to 0·1)	0 (0 to 0)	639
Gonococcal infection	36·6 (14·8 to 70·5)	34·7 (13·3 to 67·1)	19·4 (10 to 34)	13·8 (6·3 to 26·3)	8·2 (3·4 to 18·1)	11·5 (4·3 to 27·4)	8·8 (3·5 to 18·5)	11 (3·9 to 26·2)	1·6 (0·6 to 3·5)	0·3 (0·1 to 1·1)	0·1 (0 to 0·4)	0 (0 to 0)	466
Trichomoniasis	121 (40·1 to 302)	101 (31·8 to 235)	80·8 (26·2 to 188)	58·4 (18·8 to 143)	39·1 (13·5 to 89·8)	26·2 (9·1 to 62·7)	14·6 (5·4 to 32·6)	7 (2·5 to 15·7)	3·5 (1·3 to 7·7)	1·1 (0·4 to 2·6)	0·3 (0·1 to 0·6)	0 (0 to 0)	1,030
Genital herpes	136 (43·6 to 342)	117 (36·2 to 280)	104 (31·4 to 262)	89·4 (27·3 to 215)	70·6 (21·3 to 175)	50·1 (15·9 to 127)	31·7 (10 to 79·6)	16·7 (5·2 to 41·6)	9·6 (2·8 to 23·7)	3·8 (1·1 to 9·6)	1·4 (0·4 to 3·5)	0·2 (0·1 to 0·5)	1,200
Other sexually transmitted infections	114 (67·3 to 179)	80·9 (42·5 to 136)	29·4 (16·3 to 47·4)	16·5 (10 to 26·6)	7·5 (3·3 to 16·3)	5·4 (2·2 to 12·4)	5 (2 to 10)	4·4 (1·6 to 10·3)	0·7 (0·3 to 1·5)	0·2 (0·1 to 0·6)	0 (0 to 0·2)	0 (0 to 0)	962
Respiratory infections and tuberculosis	23,000 (16,500 to 30,600)	25,300 (18,100 to 34,800)	32,300 (21,300 to 45,000)	41,600 (26,700 to 57,000)	55,100 (34,400 to 74,300)	62,800 (38,500 to 83,900)	69,500 (43,700 to 94,500)	53,900 (33,300 to 71,000)	40,200 (26,400 to 51,800)	20,800 (13,900 to 26,200)	10,200 (6,890 to 12,900)	1,770 (1,170 to 2,270)	562,000
Tuberculosis	4,240 (3,010 to 5,860)	4,320 (3,070 to 6,050)	4,370 (3,020 to 6,170)	4,580 (3,230 to 6,560)	5,610 (4,000 to 7,700)	6,840 (4,920 to 9,300)	6,610 (4,540 to 8,990)	3,920 (2,850 to 5,360)	2,610 (1,850 to 3,570)	1,120 (809 to 1,480)	456 (335 to 595)	81·7 (56·7 to 114)	65,700
Latent tuberculosis infection	0 (0 to 0)	0 (0 to 0)	0 (0 to 0)	0 (0 to 0)	0 (0 to 0)	0 (0 to 0)	0 (0 to 0)	0 (0 to 0)	0 (0 to 0)	0 (0 to 0)	0 (0 to 0)	0 (0 to 0)	0
Drug-susceptible tuberculosis	4,160 (2,930 to 5,730)	4,240 (2,990 to 5,970)	4,280 (2,950 to 6,080)	4,490 (3,170 to 6,400)	5,500 (3,910 to 7,570)	6,700 (4,810 to 9,200)	6,470 (4,460 to 8,800)	3,840 (2,780 to 5,290)	2,560 (1,810 to 3,480)	1,100 (790 to 1,450)	447 (327 to 581)	80 (55·2 to 113)	64,500
Multidrug-resistant tuberculosis without extensive drug r	67·1 (12·6 to 225)	70·9 (13·8 to 231)	74·9 (14·1 to 240)	80·6 (15·6 to 254)	94·4 (18·2 to 298)	118 (22 to 358)	112 (24 to 356)	66·6 (13·1 to 196)	44·8 (8·5 to 150)	19·1 (3·6 to 57·4)	7·8 (1·5 to 23·6)	1·4 (0·3 to 4·4)	1,070
Extensively drug-resistant tuberculosis	12·2 (2·1 to 40·5)	13·1 (2·3 to 45·5)	14 (2·4 to 48·1)	15·3 (2·6 to 51)	17·5 (3·1 to 58·1)	21·8 (4·1 to 70·9)	20·6 (4 to 68·9)	12·2 (2·1 to 39·1)	8·2 (1·5 to 26·8)	3·5 (0·6 to 11·8)	1·4 (0·2 to 4·5)	0·3 (0 to 0·9)	196
Lower respiratory infections	17,000 (10,900 to 23,400)	19,600 (12,600 to 27,900)	26,600 (15,800 to 37,900)	35,900 (20,800 to 50,600)	48,600 (27,900 to 66,800)	55,400 (31,800 to 75,900)	62,600 (37,000 to 86,000)	49,800 (29,200 to 66,200)	37,500 (23,700 to 48,500)	19,600 (12,800 to 24,800)	9,760 (6,450 to 12,400)	1,680 (1,100 to 2,190)	463,000
Upper respiratory infections	1,440 (761 to 2,570)	1,140 (592 to 1,970)	968 (521 to 1,670)	826 (439 to 1,390)	663 (358 to 1,120)	481 (252 to 837)	312 (170 to 530)	166 (89·4 to 273)	93·8 (49·8 to 160)	38·1 (21·4 to 63·2)	15·9 (9·1 to 25·5)	2·6 (1·5 to 4·3)	25,600
Otitis media	323 (143 to 620)	293 (144 to 520)	287 (135 to 516)	243 (129 to 425)	193 (93·8 to 369)	112 (56·5 to 206)	64·7 (31·7 to 124)	34·3 (17·3 to 62·3)	20·8 (9·1 to 39·2)	9·2 (4·2 to 16·2)	3·9 (1·8 to 7·1)	0·7 (0·3 to 1·3)	7,240
Enteric infections	2,980 (2,100 to 4,110)	3,020 (2,120 to 4,200)	3,350 (2,340 to 4,810)	3,740 (2,490 to 5,430)	4,720 (3,150 to 6,790)	5,670 (3,880 to 8,300)	5,740 (3,840 to 8,600)	4,820 (3,160 to 7,440)	3,730 (2,410 to 5,720)	1,890 (1,210 to 2,960)	925 (589 to 1,560)	169 (101 to 288)	92,100
Diarrheal diseases	2,640 (1,830 to 3,680)	2,760 (1,920 to 3,930)	3,130 (2,150 to 4,580)	3,550 (2,290 to 5,230)	4,560 (3,010 to 6,610)	5,550 (3,800 to 8,130)	5,660 (3,790 to 8,480)	4,790 (3,130 to 7,390)	3,700 (2,390 to 5,700)	1,880 (1,200 to 2,950)	922 (587 to 1,550)	168 (101 to 288)	68,200
Typhoid and paratyphoid	307 (119 to 660)	230 (82·6 to 489)	191 (71 to 419)	165 (55·3 to 381)	126 (43·1 to 284)	84·5 (32·6 to 188)	47·4 (17·9 to 104)	22·5 (8·1 to 45·6)	11·4 (3·7 to 23·6)	4·2 (1·7 to 7·9)	1·4 (0·6 to 3·1)	0·3 (0·1 to 0·6)	22,700
Typhoid fever	278 (107 to 600)	209 (75·4 to 451)	173 (63·3 to 386)	151 (51·1 to 351)	115 (38·7 to 266)	77·3 (29·6 to 170)	43·3 (16 to 95·2)	20·6 (7·3 to 42)	10·4 (3·5 to 22·1)	3·8 (1·6 to 7·3)	1·3 (0·5 to 2·8)	0·2 (0·1 to 0·6)	20,400
Paratyphoid fever	28·8 (8·9 to 68·2)	21·3 (5·7 to 53·2)	17·3 (5·3 to 41·5)	14·7 (4·1 to 35)	10·9 (3·1 to 25·7)	7·2 (2·1 to 18·2)	4·1 (1·2 to 9·9)	1·9 (0·6 to 4·4)	0·9 (0·3 to 2·2)	0·3 (0·1 to 0·8)	0·1 (0 to 0·3)	0 (0 to 0·1)	2,270
Invasive Non-typhoidal Salmonella (iNTS)	22 (7·4 to 49·5)	20·1 (6·2 to 44·7)	22·3 (7·1 to 47·9)	27·5 (8·6 to 61·8)	32·2 (10·3 to 68·7)	31·9 (10·4 to 66·4)	25·5 (9 to 51·2)	15·2 (5·2 to 30·9)	8·8 (3 to 18·7)	3·2 (1 to 7)	1·2 (0·2 to 2·9)	0·2 (0 to 0·7)	1,160
Other intestinal infectious diseases	3·8 (1·1 to 8·9)	2·4 (0·6 to 5·9)	2·4 (0·5 to 5·9)	2 (0·4 to 4·9)	1·8 (0·3 to 4·9)	3 (0·6 to 7·7)	2 (0·4 to 5·1)	1·1 (0·2 to 2·7)	0·9 (0·2 to 2·3)	0·8 (0·1 to 2·1)	0·3 (0·1 to 0·9)	0·1 (0 to 0·2)	91
Neglected tropical diseases and malaria	3,110 (1,970 to 4,920)	2,480 (1,530 to 3,860)	2,090 (1,360 to 3,100)	1,890 (1,180 to 2,890)	1,440 (850 to 2,380)	967 (625 to 1,450)	675 (445 to 1,020)	408 (264 to 612)	235 (152 to 364)	110 (67·9 to 202)	50·3 (30·9 to 97·6)	11·7 (6·6 to 29)	50,300
Malaria	44·3 (24·1 to 75·2)	32·3 (18·5 to 62·5)	29·2 (15·1 to 62·2)	27 (13 to 60)	18 (8·9 to 38·9)	13·3 (6·1 to 29·4)	10·1 (3·9 to 24·8)	4 (1·7 to 9·2)	1·8 (0·8 to 3·6)	0·6 (0·3 to 1·1)	0·2 (0·1 to 0·4)	0 (0 to 0·1)	691
Chagas disease	0 (0 to 0)	0 (0 to 0)	0 (0 to 0)	0 (0 to 0)	0 (0 to 0)	0 (0 to 0)	0 (0 to 0)	0 (0 to 0)	0 (0 to 0)	0 (0 to 0)	0 (0 to 0)	0 (0 to 0)	0
Leishmaniasis	0 (0 to 0)	0 (0 to 0)	0 (0 to 0)	0 (0 to 0)	0 (0 to 0)	0 (0 to 0)	0 (0 to 0)	0 (0 to 0)	0 (0 to 0)	0 (0 to 0)	0 (0 to 0)	0 (0 to 0)	0
Visceral leishmaniasis	0 (0 to 0)	0 (0 to 0)	0 (0 to 0)	0 (0 to 0)	0 (0 to 0)	0 (0 to 0)	0 (0 to 0)	0 (0 to 0)	0 (0 to 0)	0 (0 to 0)	0 (0 to 0)	0 (0 to 0)	0
Cutaneous and mucocutaneous leishmaniasis	0 (0 to 0)	0 (0 to 0)	0 (0 to 0)	0 (0 to 0)	0 (0 to 0)	0 (0 to 0)	0 (0 to 0)	0 (0 to 0)	0 (0 to 0)	0 (0 to 0)	0 (0 to 0)	0 (0 to 0)	0
African trypanosomiasis	0 (0 to 0)	0 (0 to 0)	0 (0 to 0)	0 (0 to 0)	0 (0 to 0)	0 (0 to 0)	0 (0 to 0)	0 (0 to 0)	0 (0 to 0)	0 (0 to 0)	0 (0 to 0)	0 (0 to 0)	0
Schistosomiasis	0 (0 to 0)	0 (0 to 0)	0 (0 to 0)	0 (0 to 0)	0 (0 to 0)	0 (0 to 0)	0 (0 to 0)	0 (0 to 0)	0 (0 to 0)	0 (0 to 0)	0 (0 to 0)	0 (0 to 0)	0
Cysticercosis	12·3 (4·1 to 27·4)	14·2 (5 to 32·6)	18·2 (5·9 to 42·9)	24·2 (8·9 to 55·8)	30·1 (10·5 to 69·9)	27·2 (9·4 to 71·8)	22·7 (3·7 to 60·6)	15·1 (2·1 to 38·5)	10·3 (1·8 to 25·2)	4·2 (0·9 to 11)	1·8 (0·4 to 4·8)	0·3 (0·1 to 0·8)	218
Cystic echinococcosis	3·3 (0·2 to 9·2)	3·2 (0·1 to 9·6)	3·6 (0·1 to 10·2)	4·1 (0·1 to 11·5)	3·9 (0·1 to 11·5)	3·3 (0·1 to 9·6)	2·9 (0 to 8·4)	1·7 (0 to 4·7)	1 (0 to 2·8)	0·5 (0 to 1·5)	0·2 (0 to 0·7)	0 (0 to 0·1)	50·2
Lymphatic filariasis	761 (398 to 1,400)	642 (334 to 1,210)	576 (299 to 1,080)	513 (266 to 951)	423 (224 to 759)	317 (171 to 576)	219 (115 to 405)	121 (65·4 to 220)	72·8 (38·8 to 131)	30·6 (16·4 to 55·5)	12·7 (6·8 to 22·9)	2·2 (1·1 to 4·1)	10,400
Onchocerciasis	0 (0 to 0)	0 (0 to 0)	0 (0 to 0)	0 (0 to 0)	0 (0 to 0)	0 (0 to 0)	0 (0 to 0)	0 (0 to 0)	0 (0 to 0)	0 (0 to 0)	0 (0 to 0)	0 (0 to 0)	0
Trachoma	0 (0 to 0)	0 (0 to 0)	0 (0 to 0)	0 (0 to 0)	0 (0 to 0)	0 (0 to 0)	0 (0 to 0)	0 (0 to 0)	0 (0 to 0)	0 (0 to 0)	0 (0 to 0)	0 (0 to 0)	0
Dengue	1,200 (454 to 2,570)	1,010 (387 to 2,170)	814 (327 to 1,690)	769 (305 to 1,550)	658 (241 to 1,560)	395 (172 to 785)	283 (131 to 547)	187 (83·2 to 370)	104 (50·1 to 213)	54·4 (22·6 to 142)	27·8 (12·9 to 76·1)	7·8 (3·4 to 25·6)	17,600
Yellow fever	0 (0 to 0)	0 (0 to 0)	0 (0 to 0)	0 (0 to 0)	0 (0 to 0)	0 (0 to 0)	0 (0 to 0)	0 (0 to 0)	0 (0 to 0)	0 (0 to 0)	0 (0 to 0)	0 (0 to 0)	0
Rabies	0 (0 to 0·2)	0 (0 to 0·2)	0 (0 to 0·1)	0 (0 to 0·1)	0 (0 to 0·1)	0 (0 to 0·1)	0 (0 to 0·1)	0 (0 to 0·1)	0 (0 to 0)	0 (0 to 0)	0 (0 to 0)	0 (0 to 0)	8·5
Intestinal nematode infections	790 (395 to 1,370)	517 (260 to 924)	411 (207 to 732)	336 (168 to 590)	251 (126 to 434)	160 (81·8 to 281)	101 (52·1 to 173)	55·9 (29·9 to 95·1)	29·9 (16·2 to 51·4)	11·2 (5·8 to 18·8)	4·2 (2·3 to 7·1)	0·6 (0·3 to 1)	15,900

Other cardiovascular and circulatory diseases	24·8 (8·4 to 84·8)	43·6 (22·5 to 80·3)	254 (121 to 462)	205 (100 to 364)	578 (363 to 892)	635 (402 to 981)	1,240 (800 to 1,820)	1,650 (1,110 to 2,370)	2,110 (1,450 to 3,000)	2,380 (1,670 to 3,340)	2,180 (1,510 to 3,040)	3,650 (2,460 to 5,360)
Chronic respiratory diseases	0 (0 to 0)	0 (0 to 0)	3·9 (1·8 to 5·8)	4,150 (2,240 to 7,230)	5,860 (3,570 to 9,510)	5,160 (3,390 to 7,790)	4,880 (3,410 to 6,810)	5,950 (4,530 to 7,710)	7,340 (5,700 to 9,250)	8,720 (6,690 to 10,800)	9,120 (7,160 to 11,300)	9,190 (7,450 to 11,200)
Chronic obstructive pulmonary disease	0 (0 to 0)	0 (0 to 0)	3·9 (1·8 to 5·8)	135 (71 to 213)	314 (148 to 531)	527 (289 to 809)	1,150 (772 to 1,580)	1,970 (1,410 to 2,640)	2,830 (2,100 to 3,610)	3,670 (2,770 to 4,610)	4,210 (3,230 to 5,310)	4,550 (3,640 to 5,610)
Pneumoconiosis	0 (0 to 0)	0 (0 to 0)	0 (0 to 0)	0 (0 to 0)	0 (0 to 0)	0 (0 to 0)	0·3 (0·1 to 0·6)	0·8 (0·4 to 1·6)	1·3 (0·6 to 2·4)	2·7 (1·5 to 4·6)	7·3 (3·7 to 12·4)	12·3 (6 to 21·9)
Silicosis	0 (0 to 0)	0 (0 to 0)	0 (0 to 0)	0 (0 to 0)	0 (0 to 0)	0 (0 to 0)	0·1 (0 to 0·1)	0·3 (0·1 to 0·6)	0·6 (0·2 to 1·3)	1·9 (0·9 to 3·4)	5·9 (2·6 to 10·8)	11·4 (5·2 to 20·6)
Asbestosis	0 (0 to 0)	0 (0 to 0)	0 (0 to 0)	0 (0 to 0)	0 (0 to 0)	0 (0 to 0)	0·1 (0 to 0·4)	0·4 (0·1 to 1·1)	0·5 (0·1 to 1·2)	0·5 (0·1 to 1·3)	0·5 (0·2 to 1)	0·4 (0·1 to 0·8)
Coal workers pneumoconiosis	0 (0 to 0)	0 (0 to 0)	0 (0 to 0)	0 (0 to 0)	0 (0 to 0)	0 (0 to 0)	0 (0 to 0)	0 (0 to 0·1)	0·1 (0 to 0·1)	0·1 (0 to 0·2)	0·3 (0·1 to 0·9)	0·2 (0·1 to 0·3)
Other pneumoconiosis	0 (0 to 0)	0 (0 to 0)	0 (0 to 0)	0 (0 to 0)	0 (0 to 0)	0 (0 to 0)	0·1 (0 to 0·1)	0·1 (0 to 0·2)	0·1 (0·1 to 0·3)	0·2 (0·1 to 0·4)	0·5 (0·2 to 1·1)	0·4 (0·2 to 0·7)
Asthma	0 (0 to 0)	0 (0 to 0)	0 (0 to 0)	3,710 (1,890 to 6,810)	4,990 (2,810 to 8,550)	3,860 (2,240 to 6,330)	3,140 (1,920 to 5,050)	2,960 (1,940 to 4,410)	3,370 (2,250 to 4,770)	3,760 (2,540 to 5,110)	3,570 (2,500 to 4,830)	3,320 (2,380 to 4,420)
Interstitial lung disease and pulmonary sarcoidosis	0 (0 to 0)	0 (0 to 0)	0 (0 to 0)	22 (8·9 to 52·1)	20·5 (12·3 to 32·4)	25·4 (16 to 40·4)	49·8 (25·6 to 88·1)	78·8 (46·5 to 127)	165 (92·1 to 273)	174 (93·4 to 300)	249 (137 to 404)	359 (223 to 532)
Other chronic respiratory diseases	0 (0 to 0)	0 (0 to 0)	0 (0 to 0)	283 (141 to 571)	532 (279 to 844)	750 (447 to 1,140)	543 (364 to 798)	951 (659 to 1,330)	975 (695 to 1,310)	1,110 (811 to 1,500)	1,080 (777 to 1,500)	943 (672 to 1,350)
Digestive diseases	173 (118 to 249)	293 (202 to 421)	1,880 (1,100 to 3,030)	1,350 (790 to 2,350)	1,830 (1,350 to 2,420)	1,940 (1,440 to 2,580)	3,430 (2,510 to 4,480)	5,300 (3,960 to 6,950)	8,070 (5,970 to 10,500)	12,800 (9,630 to 16,700)	15,700 (11,500 to 20,500)	18,000 (13,600 to 23,900)
Cirrhosis and other chronic liver diseases	0 (0 to 0)	0 (0 to 0)	2·6 (1·3 to 4·4)	220 (70·5 to 541)	378 (241 to 568)	456 (302 to 647)	952 (625 to 1,410)	1,590 (1,080 to 2,260)	2,600 (1,710 to 3,720)	5,730 (3,760 to 8,090)	8,480 (5,800 to 12,100)	10,700 (7,400 to 15,100)
Cirrhosis and other chronic liver diseases due to hepati	0 (0 to 0)	0 (0 to 0)	0 (0 to 0)	2·4 (0·6 to 7)	10·5 (4·7 to 19·9)	23·6 (11·2 to 42·3)	86·9 (36·6 to 169)	478 (248 to 779)	1,120 (604 to 1,830)	2,720 (1,670 to 4,130)	4,120 (2,590 to 6,380)	5,090 (3,250 to 7,370)
Cirrhosis and other chronic liver diseases due to hepati	0 (0 to 0)	0 (0 to 0)	0 (0 to 0)	1·8 (0·5 to 5·2)	7·8 (3·5 to 15·8)	17·5 (8·1 to 33·1)	63·9 (27·2 to 132)	282 (139 to 503)	630 (299 to 1,130)	1,480 (797 to 2,430)	2,190 (1,160 to 3,620)	2,740 (1,480 to 4,540)
Cirrhosis and other chronic liver diseases due to alcoho	0 (0 to 0)	0 (0 to 0)	0 (0 to 0)	0 (0 to 0)	0 (0 to 0)	0 (0 to 0)	4·1 (1 to 11·6)	60·2 (25·3 to 129)	157 (60·2 to 346)	573 (298 to 987)	1,180 (555 to 2,090)	1,720 (916 to 2,950)
Cirrhosis and other chronic liver diseases due to NAFL[illegible]	0 (0 to 0)	0 (0 to 0)	0 (0 to 0)	0 (0 to 0)	0 (0 to 0)	0 (0 to 0)	34·7 (15·2 to 71·5)	102 (48·2 to 180)	197 (83·8 to 376)	373 (177 to 663)	467 (200 to 913)	584 (261 to 1,090)
Cirrhosis and other chronic liver diseases due to other [illegible]	0 (0 to 0)	0 (0 to 0)	2·6 (1·3 to 4·4)	216 (68·6 to 531)	359 (229 to 543)	414 (273 to 589)	762 (498 to 1,130)	664 (397 to 1,010)	499 (259 to 861)	576 (296 to 1,040)	527 (254 to 968)	535 (267 to 969)
Upper digestive system diseases	0 (0 to 0)	0 (0 to 0)	0 (0 to 0)	233 (106 to 415)	277 (133 to 491)	325 (187 to 542)	830 (500 to 1,320)	1,570 (937 to 2,640)	2,190 (1,290 to 3,580)	2,700 (1,610 to 4,260)	2,670 (1,590 to 4,150)	2,530 (1,620 to 3,780)
Peptic ulcer disease	0 (0 to 0)	0 (0 to 0)	0 (0 to 0)	10·9 (2·2 to 29·2)	23·8 (11·9 to 41·7)	65·3 (36·3 to 110)	177 (111 to 281)	266 (159 to 417)	422 (248 to 672)	664 (373 to 1,100)	694 (424 to 1,080)	754 (463 to 1,220)
Gastritis and duodenitis	0 (0 to 0)	0 (0 to 0)	0 (0 to 0)	222 (96·5 to 408)	249 (108 to 459)	175 (73·5 to 354)	192 (80·7 to 367)	234 (108 to 444)	310 (137 to 572)	431 (182 to 822)	557 (259 to 1,040)	535 (267 to 959)
Gastroesophageal reflux disease	0 (0 to 0)	0 (0 to 0)	0 (0 to 0)	0 (0 to 0)	4·2 (1·6 to 9)	85·1 (35·6 to 164)	462 (222 to 916)	1,070 (530 to 2,090)	1,460 (685 to 2,730)	1,600 (736 to 3,030)	1,420 (661 to 2,690)	1,240 (589 to 2,340)
Appendicitis	0 (0 to 0)	0 (0 to 0)	0 (0 to 0)	48·6 (18·1 to 123)	192 (98·5 to 338)	270 (139 to 457)	332 (169 to 562)	313 (152 to 558)	309 (150 to 541)	194 (100 to 331)	185 (104 to 300)	136 (78·3 to 212)
Paralytic ileus and intestinal obstruction	154 (108 to 218)	280 (194 to 402)	1,790 (1,050 to 2,870)	341 (142 to 756)	365 (226 to 569)	298 (179 to 445)	420 (246 to 654)	383 (226 to 594)	601 (333 to 1,040)	672 (348 to 1,190)	831 (447 to 1,410)	942 (529 to 1,530)
Inguinal, femoral, and abdominal hernia	19·4 (5·6 to 42·1)	13·7 (4·8 to 31·3)	88·6 (34·4 to 199)	253 (102 to 513)	309 (124 to 569)	211 (84·4 to 414)	183 (76 to 362)	220 (89·2 to 406)	305 (139 to 553)	399 (190 to 709)	461 (224 to 800)	545 (285 to 959)
Inflammatory bowel disease	0 (0 to 0)	0 (0 to 0)	0 (0 to 0)	11·1 (2·1 to 33)	13·2 (7 to 23·7)	19·4 (10·9 to 34)	32·8 (20·8 to 48·5)	68·7 (43·8 to 103)	100 (63·6 to 158)	96·6 (64 to 144)	100 (68·4 to 144)	109 (71·8 to 156)
Vascular intestinal disorders	0 (0 to 0)	0 (0 to 0)	0 (0 to 0)	45·1 (10·9 to 127)	29·7 (14·3 to 56·6)	42·9 (18·4 to 90·7)	62·6 (33·6 to 118)	143 (80·8 to 236)	152 (88·5 to 241)	206 (114 to 344)	139 (83·9 to 221)	156 (98·5 to 238)
Gallbladder and biliary diseases	0 (0 to 0)	0 (0 to 0)	0 (0 to 0)	44·6 (24·5 to 78·3)	85·4 (47·9 to 141)	130 (59·3 to 242)	269 (147 to 457)	382 (198 to 633)	623 (356 to 1,000)	1,030 (654 to 1,520)	1,080 (690 to 1,590)	1,230 (797 to 1,820)
Pancreatitis	0 (0 to 0)	0 (0 to 0)	0 (0 to 0)	5·8 (2·6 to 12·5)	57·4 (24·5 to 106)	56 (32·2 to 91·6)	231 (119 to 399)	466 (236 to 832)	924 (494 to 1,680)	1,460 (708 to 2,820)	1,380 (681 to 2,690)	1,380 (742 to 2,520)
Other digestive diseases	0 (0 to 0)	0 (0 to 0)	0 (0 to 0)	145 (71·3 to 278)	125 (79 to 201)	137 (87·7 to 208)	115 (71·7 to 187)	162 (96·9 to 276)	263 (150 to 449)	299 (172 to 503)	345 (193 to 563)	327 (191 to 546)
Neurological disorders	7 (1·6 to 16·3)	21 (4·7 to 48·6)	1,760 (1,120 to 2,630)	3,600 (1,150 to 6,950)	7,380 (3,120 to 13,900)	16,800 (5,260 to 35,400)	23,400 (6,800 to 51,600)	26,800 (7,790 to 56,900)	28,600 (7,950 to 60,700)	28,500 (8,060 to 60,500)	24,800 (7,140 to 52,900)	21,300 (6,870 to 44,300)
Alzheimer's disease and other dementias	0 (0 to 0)	0 (0 to 0)	0 (0 to 0)	0 (0 to 0)	0 (0 to 0)	0 (0 to 0)	0 (0 to 0)	0 (0 to 0)	0 (0 to 0)	0 (0 to 0)	0 (0 to 0)	143 (35·5 to 439)
Parkinson's disease	0 (0 to 0)	0 (0 to 0)	0 (0 to 0)	0 (0 to 0)	0 (0 to 0)	0 (0 to 0)	0 (0 to 0)	5·1 (2·1 to 7·7)	13·7 (6·7 to 25)	50·8 (31·1 to 80·4)	48·6 (24·7 to 85·3)	212 (157 to 282)
Idiopathic epilepsy	6·9 (1·6 to 16·3)	20·8 (4·5 to 48·4)	691 (353 to 1,200)	2,630 (821 to 5,120)	3,840 (1,340 to 7,530)	3,830 (1,430 to 7,540)	3,880 (1,540 to 7,600)	3,920 (1,730 to 7,400)	3,700 (1,640 to 7,060)	3,350 (1,590 to 6,220)	2,810 (1,370 to 4,990)	2,350 (1,070 to 4,220)
Multiple sclerosis	0 (0 to 0)	0 (0 to 0)	0 (0 to 0)	0 (0 to 0)	0·4 (0·2 to 0·7)	1·7 (0·7 to 3·3)	9·3 (5·1 to 16·1)	41·3 (25 to 68·4)	60·7 (41·3 to 87·9)	101 (72·2 to 139)	131 (91·9 to 177)	152 (110 to 206)
Motor neuron disease	0·1 (0 to 0·1)	0·2 (0·1 to 0·2)	3·9 (2·6 to 6·1)	12·5 (7·2 to 21·1)	15·4 (12 to 19·5)	17·3 (13·3 to 22·1)	20·5 (15·9 to 25·9)	22 (16·9 to 27·9)	21·9 (16·7 to 28·1)	23·8 (17·8 to 31·2)	28·2 (19·7 to 39·2)	29·4 (17·2 to 48·3)
Headache disorders	0 (0 to 0)	0 (0 to 0)	0 (0 to 0)	0 (0 to 0)	2,250 (48·4 to 5,890)	11,200 (484 to 29,500)	17,900 (1,790 to 44,700)	21,600 (2,650 to 52,600)	23,900 (3,190 to 55,500)	24,400 (3,750 to 56,000)	21,200 (3,490 to 49,000)	17,800 (3,350 to 40,700)
Migraine	0 (0 to 0)	0 (0 to 0)	0 (0 to 0)	0 (0 to 0)	2,020 (31·3 to 5,460)	10,400 (325 to 28,000)	16,600 (1,170 to 43,700)	20,000 (1,850 to 50,500)	22,100 (2,210 to 53,900)	22,500 (2,560 to 53,600)	19,400 (2,440 to 46,900)	16,300 (2,350 to 38,600)
Tension-type headache	0 (0 to 0)	0 (0 to 0)	0 (0 to 0)	0 (0 to 0)	230 (5·5 to 1,380)	804 (45·5 to 5,380)	1,260 (196 to 6,590)	1,560 (301 to 7,140)	1,820 (367 to 7,640)	1,910 (413 to 7,270)	1,820 (404 to 6,370)	1,540 (404 to 5,230)
Other neurological disorders	0 (0 to 0)	0 (0 to 0)	1,070 (683 to 1,580)	959 (323 to 1,830)	1,270 (456 to 2,480)	1,740 (750 to 3,290)	1,620 (791 to 2,860)	1,160 (655 to 1,930)	803 (453 to 1,360)	643 (355 to 1,070)	641 (370 to 1,050)	635 (377 to 996)
Mental disorders	7·2 (4·5 to 10·8)	21·9 (13·9 to 32)	372 (228 to 543)	1,940 (1,230 to 2,830)	10,200 (6,620 to 14,700)	33,900 (22,900 to 49,700)	52,600 (36,400 to 75,000)	52,600 (36,400 to 72,400)	56,900 (39,900 to 78,000)	57,500 (40,700 to 77,600)	51,400 (36,400 to 68,500)	43,800 (31,500 to 58,800)
Schizophrenia	0 (0 to 0)	0 (0 to 0)	0 (0 to 0)	0 (0 to 0)	0 (0 to 0)	194 (83·4 to 372)	1,610 (778 to 2,890)	5,840 (3,280 to 9,660)	10,000 (5,980 to 14,900)	11,500 (7,080 to 16,600)	10,100 (6,520 to 14,200)	8,080 (5,510 to 11,200)
Depressive disorders	0 (0 to 0)	0 (0 to 0)	0 (0 to 0)	4·6 (1·9 to 9·9)	435 (181 to 856)	10,100 (5,410 to 17,100)	24,000 (14,500 to 37,400)	21,600 (13,000 to 33,600)	19,700 (12,200 to 29,500)	18,600 (11,500 to 28,000)	17,800 (11,500 to 25,600)	16,400 (10,700 to 24,300)
Major depressive disorder	0 (0 to 0)	0 (0 to 0)	0 (0 to 0)	3·7 (1·3 to 8·8)	368 (138 to 781)	9,680 (5,080 to 16,600)	22,600 (13,400 to 35,800)	19,000 (11,000 to 30,400)	15,900 (9,580 to 24,700)	13,800 (8,150 to 22,000)	12,800 (7,960 to 18,700)	11,500 (7,100 to 17,400)
Dysthymia	0 (0 to 0)	0 (0 to 0)	0 (0 to 0)	0·9 (0·2 to 2·3)	66·4 (11·8 to 170)	458 (188 to 882)	1,370 (667 to 2,300)	2,610 (1,280 to 4,660)	3,830 (2,100 to 6,770)	4,830 (2,770 to 7,640)	5,030 (2,900 to 8,090)	4,920 (2,670 to 8,190)
Bipolar disorder	0 (0 to 0)	0 (0 to 0)	0 (0 to 0)	0 (0 to 0)	0 (0 to 0)	528 (179 to 1,060)	2,070 (912 to 3,700)	2,900 (1,340 to 4,960)	3,120 (1,560 to 5,240)	3,010 (1,520 to 4,950)	2,590 (1,370 to 4,360)	2,200 (1,200 to 3,720)
Anxiety disorders	0 (0 to 0)	0 (0 to 0)	0 (0 to 0)	270 (106 to 525)	3,990 (2,040 to 6,690)	10,100 (5,450 to 16,800)	13,700 (8,250 to 21,400)	15,200 (8,710 to 24,700)	15,700 (9,060 to 25,800)	15,400 (9,330 to 23,900)	13,300 (7,930 to 20,500)	11,300 (6,480 to 18,300)
Eating disorders	0 (0 to 0)	0 (0 to 0)	0 (0 to 0)	0 (0 to 0)	38 (13·3 to 78)	549 (274 to 971)	1,660 (886 to 2,800)	2,250 (1,190 to 3,850)	2,300 (1,330 to 3,560)	2,220 (1,200 to 3,550)	1,500 (802 to 2,470)	692 (376 to 1,150)

[illegible]

Other transport injuries	5·3 (1·9 to 12·1)	4·8 (2·4 to 9·2)	82·8 (47·4 to 135)	178 (104 to 293)	516 (323 to 785)	926 (596 to 1,460)	4,780 (2,800 to 7,900)	6,500 (3,980 to 9,920)	5,220 (3,290 to 7,910)	4,770 (2,760 to 7,770)	3,230 (1,980 to 5,220)	2,490 (1,490 to 3,980)	2,190 (1,330 to 3,520)	2,160 (1,360 to 3,380)	1,920 (1,220 to 2,940)	1,810 (1,110 to 2,760)	1,480 (898 to 2,340)	1,140 (679 to 1,770)	674 (405 to 1,060)	346 (224 to 530)	122 (80·4 to 184)	56·5 (36·8 to 83·6)	13·7 (8·2 to 21·7)	40,600
Unintentional injuries	445 (267 to 702)	513 (336 to 752)	4,570 (2,930 to 6,720)	3,880 (2,200 to 6,540)	9,020 (7,030 to 11,200)	11,500 (9,120 to 14,300)	17,800 (13,600 to 23,300)	21,700 (16,900 to 26,900)	22,000 (17,500 to 26,700)	22,100 (17,600 to 27,000)	18,800 (15,100 to 23,300)	15,900 (12,600 to 19,500)	14,400 (11,400 to 18,100)	14,500 (11,300 to 18,000)	14,800 (11,700 to 18,400)	15,000 (11,800 to 18,700)	13,400 (10,700 to 16,900)	11,800 (9,170 to 14,600)	8,100 (6,240 to 10,100)	5,810 (4,450 to 7,240)	3,030 (2,310 to 3,790)	1,490 (1,150 to 1,830)	268 (202 to 342)	251,000
Falls	23·7 (8·4 to 55·8)	15·4 (6·4 to 33·1)	239 (131 to 396)	522 (272 to 978)	1,280 (934 to 1,680)	1,870 (1,390 to 2,460)	3,940 (2,900 to 5,270)	5,360 (4,020 to 7,020)	5,950 (4,540 to 7,790)	6,180 (4,700 to 7,910)	5,860 (4,380 to 7,540)	5,420 (4,060 to 7,050)	5,090 (3,880 to 6,560)	6,020 (4,580 to 7,680)	6,480 (4,920 to 8,240)	7,510 (5,620 to 9,740)	7,070 (5,170 to 9,340)	6,790 (4,840 to 9,040)	4,990 (3,550 to 6,580)	3,860 (2,760 to 5,050)	2,070 (1,480 to 2,690)	1,040 (732 to 1,340)	181 (128 to 244)	87,800
Drowning	27·3 (15·7 to 45·6)	13·1 (7·2 to 22)	318 (171 to 563)	1,420 (568 to 2,980)	4,180 (2,590 to 6,080)	5,220 (3,430 to 7,510)	6,040 (3,500 to 9,750)	5,270 (3,270 to 7,900)	4,270 (2,760 to 6,280)	3,730 (2,490 to 5,450)	2,740 (1,800 to 4,020)	1,970 (1,340 to 2,910)	1,670 (1,090 to 2,490)	1,330 (861 to 1,940)	1,130 (729 to 1,670)	928 (637 to 1,330)	763 (510 to 1,110)	591 (394 to 865)	342 (235 to 478)	169 (114 to 238)	74·9 (49·6 to 108)	25·6 (17·2 to 35)	4·9 (3·2 to 7·3)	42,200
Fire, heat, and hot substances	8·7 (4·5 to 16·5)	9·2 (4·5 to 19·8)	166 (93·1 to 296)	306 (136 to 681)	571 (405 to 807)	732 (531 to 1,000)	1,250 (850 to 1,750)	1,720 (1,100 to 2,490)	1,850 (1,250 to 2,660)	1,960 (1,360 to 2,670)	1,640 (1,180 to 2,190)	1,400 (1,030 to 1,890)	1,240 (911 to 1,650)	1,200 (895 to 1,590)	1,170 (879 to 1,600)	1,130 (798 to 1,580)	1,010 (726 to 1,390)	719 (494 to 1,030)	477 (311 to 690)	310 (206 to 449)	158 (110 to 220)	66·3 (45·5 to 91·1)	12·1 (8·2 to 17·5)	19,100
Poisonings	6·6 (2·5 to 15·4)	3·5 (0·7 to 13)	25·3 (8·9 to 68)	69 (27·3 to 180)	115 (65·4 to 230)	203 (120 to 354)	716 (410 to 1,190)	1,170 (689 to 1,960)	1,360 (843 to 2,150)	1,360 (785 to 2,240)	1,070 (656 to 1,740)	853 (532 to 1,350)	635 (376 to 1,040)	505 (318 to 792)	450 (265 to 710)	421 (250 to 694)	309 (180 to 518)	212 (125 to 340)	118 (67·7 to 190)	79·5 (48·3 to 122)	41·3 (26·1 to 61·5)	15·9 (10 to 24·4)	3·1 (1·8 to 5·4)	9,750
Poisoning by carbon monoxide	0·8 (0·2 to 3·5)	1·1 (0·2 to 4·1)	5·6 (1·8 to 15·8)	26·6 (9·3 to 66·6)	39·7 (20 to 69·5)	42·3 (23 to 77·3)	124 (54·9 to 266)	168 (79·5 to 310)	167 (83·8 to 299)	222 (94·9 to 456)	172 (76 to 335)	181 (82·8 to 347)	118 (52·9 to 225)	75·6 (36·3 to 143)	59·4 (27·4 to 114)	43·2 (20·9 to 86·3)	46·1 (21·8 to 85·8)	38·4 (17·1 to 72·2)	22·8 (8·9 to 45·6)	9·8 (4·5 to 20·1)	6·6 (3·1 to 12·8)	2·1 (1 to 4·1)	0·4 (0·2 to 0·8)	1,570
Poisoning by other means	5·8 (1·9 to 13·7)	2·4 (0·5 to 10·4)	19·7 (6·7 to 60·3)	42·4 (15 to 141)	75·4 (38·4 to 188)	161 (90·4 to 309)	592 (337 to 1,020)	1,000 (586 to 1,670)	1,190 (718 to 1,880)	1,140 (643 to 1,880)	901 (541 to 1,470)	671 (415 to 1,060)	518 (305 to 865)	430 (264 to 690)	390 (230 to 633)	378 (216 to 629)	263 (153 to 440)	173 (103 to 285)	94·9 (53·9 to 155)	69·6 (41·2 to 109)	34·7 (21·6 to 53·8)	13·8 (8·6 to 21·6)	2·7 (1·5 to 4·7)	8,180
Exposure to mechanical forces	15·2 (3·1 to 59·2)	26·9 (3 to 137)	167 (58·6 to 481)	327 (201 to 505)	631 (431 to 892)	803 (566 to 1,110)	1,420 (971 to 2,010)	2,030 (1,370 to 2,900)	2,240 (1,600 to 3,090)	2,440 (1,730 to 3,310)	2,210 (1,610 to 3,020)	1,950 (1,430 to 2,610)	1,930 (1,390 to 2,660)	1,820 (1,320 to 2,490)	1,830 (1,330 to 2,490)	1,580 (1,140 to 2,180)	1,250 (900 to 1,700)	911 (662 to 1,230)	521 (383 to 703)	306 (223 to 412)	134 (97 to 181)	58·7 (43·6 to 78·5)	11 (8·3 to 14·7)	24,600
Unintentional firearm injuries	1 (0·2 to 6)	4·3 (0·4 to 23·4)	18·7 (5·9 to 54·2)	50·8 (28·5 to 86·4)	65·7 (43·7 to 96·3)	84 (57·2 to 122)	123 (81 to 188)	206 (128 to 331)	238 (160 to 370)	272 (182 to 415)	291 (181 to 454)	224 (156 to 318)	213 (147 to 310)	196 (137 to 275)	176 (124 to 247)	136 (98·7 to 181)	107 (77·4 to 143)	72·7 (52·2 to 98·1)	37·8 (26·9 to 51·1)	23·4 (16·7 to 32)	10·6 (7·6 to 14·3)	4·8 (3·4 to 6·4)	0·9 (0·6 to 1·2)	2,560
Other exposure to mechanical forces	14·2 (2·8 to 55·3)	22·6 (2·5 to 111)	149 (50·4 to 436)	276 (165 to 431)	565 (378 to 815)	719 (499 to 1,020)	1,300 (875 to 1,860)	1,820 (1,210 to 2,640)	2,010 (1,400 to 2,820)	2,170 (1,540 to 3,000)	1,920 (1,380 to 2,640)	1,730 (1,260 to 2,320)	1,710 (1,230 to 2,400)	1,630 (1,180 to 2,250)	1,660 (1,180 to 2,280)	1,450 (1,030 to 2,000)	1,140 (815 to 1,570)	839 (607 to 1,140)	483 (350 to 655)	283 (205 to 380)	123 (88·9 to 167)	54 (40·1 to 72·1)	10·1 (7·5 to 13·6)	22,100
Adverse effects of medical treatment	25·4 (8·4 to 65·4)	27·5 (12·8 to 57·3)	233 (142 to 359)	144 (78·1 to 269)	177 (116 to 251)	185 (118 to 264)	261 (161 to 391)	398 (245 to 608)	395 (255 to 575)	426 (257 to 638)	427 (265 to 657)	410 (259 to 609)	487 (312 to 737)	414 (271 to 599)	563 (357 to 814)	614 (412 to 870)	585 (387 to 866)	457 (289 to 652)	277 (181 to 398)	179 (118 to 256)	78·7 (53·5 to 109)	36·8 (24·4 to 50·7)	6·2 (4 to 8·9)	6,810
Animal contact	2·8 (1·7 to 5·1)	2·7 (1·5 to 5·4)	41·6 (24 to 76·7)	94·8 (50·9 to 172)	288 (188 to 432)	281 (179 to 423)	338 (226 to 507)	399 (266 to 593)	399 (273 to 586)	336 (227 to 492)	336 (223 to 483)	274 (184 to 410)	272 (181 to 415)	354 (247 to 516)	376 (246 to 569)	359 (232 to 537)	311 (202 to 466)	312 (176 to 479)	175 (105 to 261)	96·6 (58·9 to 144)	44·6 (28·2 to 65·9)	17·6 (12 to 24·9)	3·7 (2·3 to 5·5)	5,120
Venomous animal contact	2·4 (1·4 to 4·3)	1·6 (0·8 to 3·4)	30·2 (16·2 to 59·6)	59·7 (28·5 to 120)	228 (139 to 351)	215 (131 to 342)	264 (169 to 412)	316 (197 to 494)	297 (191 to 464)	225 (143 to 351)	233 (144 to 355)	192 (118 to 312)	187 (113 to 310)	258 (164 to 397)	289 (182 to 459)	287 (172 to 447)	253 (155 to 389)	254 (136 to 395)	140 (79·7 to 217)	75·7 (42·7 to 119)	33·4 (19·7 to 51·4)	13·2 (8·3 to 19·5)	3 (1·7 to 4·6)	3,860
Non-venomous animal contact	0·5 (0·2 to 0·9)	1·1 (0·6 to 2·3)	11·5 (6·3 to 19·7)	35·1 (20 to 61·1)	60·4 (37·2 to 98·3)	66·3 (39·6 to 104)	73·3 (46·6 to 111)	82·6 (52·9 to 121)	102 (64 to 155)	111 (69·7 to 170)	103 (64·1 to 159)	82·2 (51·9 to 129)	85·8 (54·1 to 131)	96 (63·3 to 140)	86·4 (56·5 to 128)	72·1 (46·4 to 110)	58·8 (36·7 to 89·5)	58·2 (35·2 to 95·6)	35 (21·9 to 56·3)	20·9 (13·2 to 33·5)	11·3 (7·3 to 17·2)	4·4 (2·8 to 6·3)	0·8 (0·5 to 1·1)	1,260
Foreign body	324 (180 to 551)	404 (261 to 610)	3,200 (1,790 to 5,240)	517 (250 to 955)	791 (552 to 1,120)	983 (692 to 1,370)	858 (599 to 1,230)	1,280 (873 to 1,880)	1,430 (1,050 to 2,070)	1,550 (1,100 to 2,070)	1,210 (876 to 1,650)	967 (702 to 1,310)	844 (606 to 1,180)	848 (611 to 1,160)	955 (694 to 1,300)	905 (650 to 1,250)	873 (650 to 1,130)	948 (691 to 1,300)	718 (519 to 959)	510 (372 to 693)	290 (201 to 409)	169 (118 to 231)	34·2 (22·8 to 47·4)	20,600
Pulmonary aspiration and foreign body in airway	324 (180 to 551)	403 (260 to 610)	3,180 (1,780 to 5,200)	445 (193 to 874)	688 (461 to 1,010)	860 (575 to 1,230)	703 (463 to 1,070)	1,070 (691 to 1,660)	1,190 (829 to 1,780)	1,290 (881 to 1,800)	960 (650 to 1,400)	751 (507 to 1,100)	648 (426 to 985)	658 (429 to 965)	776 (530 to 1,110)	749 (501 to 1,090)	745 (528 to 994)	853 (588 to 1,210)	655 (462 to 896)	469 (331 to 646)	266 (178 to 385)	156 (107 to 218)	31·7 (20·8 to 44·5)	17,900
Foreign body in eyes	0·1 (0 to 0·2)	0·3 (0·1 to 0·6)	4·6 (1·2 to 10·1)	22·3 (6·3 to 51·3)	33·9 (9·4 to 78·5)	42·2 (13 to 99·3)	61·1 (19·1 to 142)	89·5 (27·6 to 201)	112 (38·1 to 237)	120 (42·7 to 255)	105 (39·2 to 223)	87·6 (36·6 to 175)	72·5 (33·8 to 137)	60·9 (31·1 to 110)	49·9 (28·4 to 85·2)	41·5 (24·4 to 66·8)	32 (19·6 to 49·1)	22·9 (14·5 to 34·2)	13·4 (8·7 to 19·8)	8·4 (5·5 to 12·4)	3·6 (2·4 to 5·1)	1·5 (1 to 2·1)	0·3 (0·2 to 0·4)	986
Foreign body in other body part	0·5 (0·2 to 1)	0·9 (0·5 to 1·4)	15·6 (8·3 to 28·6)	50 (29·7 to 79·2)	69·4 (47·2 to 98·7)	80·3 (55·9 to 114)	94·6 (65·8 to 131)	115 (79·8 to 159)	130 (93·2 to 179)	145 (103 to 200)	141 (101 to 188)	128 (92·8 to 178)	123 (88 to 167)	130 (94 to 177)	129 (94·6 to 174)	115 (82·3 to 154)	96·1 (69·1 to 128)	72 (51·1 to 96·1)	49·6 (35·8 to 67)	32·5 (23·1 to 43·8)	20·2 (13·9 to 28·5)	10·9 (7·4 to 16·3)	2·2 (1·5 to 3·1)	1,750
Environmental heat and cold exposure	0·5 (0·2 to 0·9)	0·7 (0·4 to 1·1)	14·1 (8 to 25·7)	48·4 (30·5 to 71·5)	98·6 (65·9 to 144)	116 (79·9 to 163)	163 (114 to 225)	206 (146 to 281)	223 (158 to 308)	238 (168 to 326)	204 (144 to 282)	176 (123 to 246)	159 (112 to 215)	152 (108 to 206)	139 (99·3 to 190)	128 (90·5 to 174)	106 (76·3 to 145)	78·9 (56·6 to 107)	46·9 (33·7 to 62·6)	30·4 (22·2 to 41·7)	15·3 (11 to 21)	7 (4·9 to 9·8)	1·2 (0·8 to 1·6)	2,350
Exposure to forces of nature	0 (0 to 0)	0·1 (0·1 to 0·1)	0·9 (0·8 to 1)	4·7 (4·3 to 5·2)	6·2 (5·3 to 7·2)	9·1 (7·6 to 11·4)	16·5 (12·6 to 22)	24 (18 to 32·9)	44·6 (31·8 to 62·3)	48·7 (34·7 to 69·1)	52·5 (36·7 to 78·1)	34·3 (23·7 to 48)	28·7 (20·1 to 39·5)	25·9 (17·9 to 36·4)	21·7 (15·2 to 30·4)	17·2 (12·4 to 23·7)	13·1 (9·4 to 18)	9·4 (6·7 to 13)	5·5 (3·9 to 7·4)	3·2 (2·3 to 4·5)	1·4 (1 to 2)	0·5 (0·4 to 0·8)	0·1 (0·1 to 0·1)	368
Other unintentional injuries	10·8 (5·2 to 30·7)	9·4 (5·7 to 14·6)	160 (105 to 235)	432 (267 to 661)	879 (597 to 1,290)	1,140 (755 to 1,700)	2,830 (1,620 to 4,560)	3,820 (2,260 to 5,810)	3,810 (2,390 to 5,480)	3,790 (2,460 to 5,770)	3,070 (2,060 to 4,600)	2,400 (1,660 to 3,330)	2,090 (1,430 to 3,000)	1,790 (1,240 to 2,500)	1,710 (1,160 to 2,460)	1,360 (950 to 1,920)	1,120 (762 to 1,570)	728 (521 to 1,020)	432 (306 to 594)	260 (191 to 351)	123 (89·3 to 164)	57·3 (42·1 to 75·3)	11 (7·9 to 14·7)	32,000
Self-harm and interpersonal violence	54·7 (26·9 to 105)	14·5 (7·7 to 26·9)	145 (80 to 258)	266 (131 to 510)	549 (409 to 739)	1,440 (1,080 to 1,940)	10,800 (7,410 to 16,800)	17,800 (12,400 to 26,600)	19,000 (13,700 to 27,100)	18,500 (13,400 to 26,500)	13,600 (9,990 to 18,500)	10,300 (7,720 to 13,600)	8,040 (5,920 to 10,800)	6,880 (5,060 to 9,170)	6,180 (4,590 to 8,060)	4,900 (3,620 to 6,550)	3,640 (2,730 to 4,890)	2,670 (2,010 to 3,500)	1,340 (1,000 to 1,750)	696 (531 to 904)	300 (223 to 390)	114 (84·7 to 144)	22·6 (15·4 to 30·3)	127,000
Self-harm	0 (0 to 0)	0 (0 to 0)	0 (0 to 0)	0 (0 to 0)	0 (0 to 0)	326 (180 to 698)	6,160 (3,740 to 11,600)	10,300 (6,350 to 17,300)	11,700 (7,710 to 18,300)	12,100 (7,940 to 18,400)	8,710 (5,940 to 12,600)	6,670 (4,610 to 9,260)	5,270 (3,580 to 7,720)	4,660 (3,080 to 6,650)	4,340 (2,950 to 5,990)	3,560 (2,370 to 5,150)	2,720 (1,850 to 3,860)	2,070 (1,430 to 2,870)	1,020 (705 to 1,410)	546 (385 to 754)	241 (168 to 329)	91·4 (62·5 to 122)	18 (11·3 to 25·6)	80,500
Self-harm by firearm	0 (0 to 0)	0 (0 to 0)	0 (0 to 0)	0 (0 to 0)	0 (0 to 0)	17·6 (8·7 to 34·5)	166 (58·8 to 441)	307 (138 to 636)	346 (147 to 656)	319 (122 to 623)	236 (90·8 to 421)	194 (75·7 to 353)	186 (71·7 to 336)	107 (36·3 to 203)	111 (41 to 201)	48·2 (22·3 to 87·2)	27·2 (14·4 to 46·6)	19·2 (10·9 to 34·1)	8·4 (4·9 to 14·2)	7·2 (4 to 13·1)	2·4 (1·4 to 4·5)	1·6 (0·8 to 3)	0·3 (0·2 to 0·6)	2,100
Self-harm by other specified means	0 (0 to 0)	0 (0 to 0)	0 (0 to 0)	0 (0 to 0)	0 (0 to 0)	308 (169 to 672)	5,990 (3,640 to 11,200)	9,990 (6,150 to 16,900)	11,300 (7,460 to 17,700)	11,800 (7,700 to 17,900)	8,480 (5,810 to 12,300)	6,480 (4,450 to 8,950)	5,090 (3,460 to 7,390)	4,550 (3,010 to 6,510)	4,230 (2,880 to 5,850)	3,510 (2,320 to 5,110)	2,690 (1,830 to 3,830)	2,050 (1,410 to 2,840)	1,010 (699 to 1,400)	539 (379 to 743)	238 (167 to 326)	89·8 (61·2 to 120)	17·7 (11 to 25)	78,400
Interpersonal violence	54·6 (26·8 to 105)	14·4 (7·6 to 26·8)	141 (76·2 to 254)	254 (122 to 490)	525 (387 to 714)	1,090 (809 to 1,430)	4,590 (2,980 to 7,280)	7,400 (4,860 to 11,400)	7,190 (4,740 to 10,900)	6,290 (4,250 to 9,410)	4,730 (3,230 to 6,880)	3,590 (2,590 to 5,090)	2,720 (1,990 to 3,710)	2,180 (1,630 to 2,880)	1,810 (1,330 to 2,430)	1,320 (997 to 1,680)	917 (712 to 1,190)	596 (456 to 770)	317 (239 to 414)	149 (116 to 190)	58·9 (45·2 to 75·9)	22·5 (16·7 to 29·8)	4·5 (3·3 to 6·1)	46,000
Physical violence by firearm	0·9 (0·3 to 1·9)	0·6 (0·2 to 1·4)	3·2 (1·6 to 6·4)	10·9 (5 to 22·5)	15·6 (10·6 to 22·9)	31·7 (18·1 to 59)	743 (289 to 1,540)	1,440 (599 to 2,880)	1,240 (569 to 2,440)	1,070 (483 to 2,020)	772 (380 to 1,420)	575 (279 to 1,110)	332 (162 to 663)	238 (121 to 433)	191 (94·5 to 357)	106 (55 to 192)	53·2 (30·4 to 93·9)	26·8 (15 to 49·5)	12·9 (7·2 to 24·2)	3·7 (2·7 to 5·2)	1·8 (1·3 to 2·5)	0·8 (0·6 to 1·2)	0·3 (0·2 to 0·5)	6,870
Physical violence by sharp object	7·8 (2·8 to 17)	0·8 (0·4 to 1·8)	8·2 (4·8 to 13·5)	30·6 (17·1 to 51·1)	49·9 (33·8 to 72)	94·6 (61·5 to 141)	953 (429 to 1,970)	1,770 (897 to 3,370)	1,830 (965 to 3,460)	1,440 (748 to 2,830)	993 (558 to 1,840)	716 (440 to 1,230)	542 (350 to 894)	384 (259 to 602)	330 (223 to 511)	247 (176 to 355)	160 (115 to 227)	102 (74·5 to 139)	50·1 (35·5 to 67·7)	24·7 (17·8 to 33·4)	10·7 (7·9 to 14·3)	4·5 (3·3 to 6)	1·1 (0·7 to 1·6)	9,750
Sexual violence	0 (0 to 0)	0 (0 to 0)	0 (0 to 0)	19·2 (11·2 to 31·4)	162 (95·7 to 255)	390 (225 to 616)	852 (484 to 1,350)	996 (579 to 1,510)	869 (499 to 1,410)	780 (441 to 1,260)	628 (352 to 1,010)	496 (283 to 805)	392 (229 to 650)	280 (158 to 464)	166 (95·1 to 271)	114 (69·8 to 174)	84·6 (52·7 to 128)	55·5 (34·9 to 82·1)	30·7 (20·2 to 44·7)	18·5 (12 to 27·5)	6·4 (3·9 to 10·1)	1·1 (0·7 to 1·8)	0 (0 to 0·1)	6,340
Physical violence by other means	46 (21·8 to 88·6)	13 (6·7 to 24·6)	130 (67·9 to 237)	193 (77·7 to 401)	298 (199 to 448)	570 (388 to 816)	2,040 (1,200 to 3,540)	3,200 (1,990 to 5,150)	3,260 (2,040 to 5,250)	2,990 (1,910 to 4,600)	2,340 (1,540 to 3,670)	1,800 (1,240 to 2,580)	1,450 (1,030 to 2,050)	1,280 (926 to 1,720)	1,130 (816 to 1,530)	856 (633 to 1,120)	619 (456 to 821)	411 (303 to 547)	224 (165 to 299)	102 (74·6 to 135)	40 (29 to 54·1)	16 (11·4 to 21·8)	3·2 (2·3 to 4·3)	23,000
Conflict and terrorism	0·1 (0·1 to 0·1)	0·1 (0·1 to 0·1)	0·2 (0·2 to 0·2)	1·7 (1·5 to 1·9)	5·2 (3·4 to 8·9)	5·2 (3·1 to 9·2)	8·3 (5·5 to 13·2)	13·1 (9·1 to 19·9)	14·5 (9·9 to 23)	14·3 (9·7 to 21·4)	15·9 (9·5 to 30)	19·4 (7·5 to 61·1)	12·9 (5·5 to 36·6)	14·3 (5·7 to 41·8)	14 (5 to 45)	11·2 (3·6 to 40·5)	6·5 (2·3 to 21·8)	3·4 (1·2 to 10·8)	1·5 (0·7 to 4·4)	0·7 (0·3 to 2)	0·3 (0·1 to 0·7)	0·1 (0·1 to 0·3)	0 (0 to 0·1)	163

SR Table 2c: Grenada DALYS by cause and age group for both sexes with 95% uncertainty intervals, 2019

Cause	Early Neonatal	Late Neonatal	Post Neonatal	1 to 4	5 to 9	10 to 14	15 to 19	20 to 24	25 to 29	30 to 34	35 to 39
All causes	876 (610 to 1,210)	193 (128 to 282)	464 (341 to 632)	462 (358 to 584)	489 (392 to 622)	579 (459 to 732)	975 (809 to 1,170)	1,330 (1,120 to 1,570)	1,390 (1,160 to 1,650)	1,400 (1,190 to 1,640)	1,390 (1,190 to 1,610)
Communicable, maternal, neonatal, and nutritional diseases	766 (534 to 1,060)	150 (96·8 to 220)	242 (172 to 334)	176 (133 to 231)	147 (104 to 214)	98·1 (74·3 to 130)	111 (88·7 to 140)	138 (114 to 169)	159 (134 to 189)	153 (130 to 179)	146 (125 to 172)
HIV/AIDS and sexually transmitted infections	0·1 (0·1 to 0·2)	0 (0 to 0·1)	2·1 (2 to 2·2)	2·2 (2·1 to 2·3)	2·1 (2 to 2·2)	1·8 (1·7 to 2)	3·6 (3·1 to 4·2)	13·1 (11·7 to 15)	36·8 (33·6 to 41·2)	39·4 (36·1 to 44·1)	45·7 (42·3 to 49·7)
HIV/AIDS	0 (0 to 0)	0 (0 to 0)	2 (1·9 to 2·2)	2·2 (2·1 to 2·3)	2·1 (2 to 2·2)	1·7 (1·6 to 1·8)	2·6 (2·5 to 2·8)	11·1 (10·1 to 12·3)	34·1 (31·6 to 37·7)	36·7 (33·8 to 40·6)	43·2 (40·2 to 46·9)
HIV/AIDS - Drug-susceptible Tuberculosis	0 (0 to 0)	0 (0 to 0)	0 (0 to 0·1)	0 (0 to 0·1)	0 (0 to 0·1)	0 (0 to 0)	0·1 (0 to 0·1)	0·3 (0·2 to 0·4)	0·8 (0·6 to 1·1)	0·9 (0·7 to 1·3)	1 (0·6 to 1·4)
HIV/AIDS - Multidrug-resistant Tuberculosis without extensive dru	0 (0 to 0)	0 (0 to 0)	0 (0 to 0)	0 (0 to 0)	0 (0 to 0)	0 (0 to 0)	0 (0 to 0)	0 (0 to 0)	0 (0 to 0·1)	0 (0 to 0·1)	0 (0 to 0·1)
HIV/AIDS - Extensively drug-resistant Tuberculosis	0 (0 to 0)	0 (0 to 0)	0 (0 to 0)	0 (0 to 0)	0 (0 to 0)	0 (0 to 0)	0 (0 to 0)	0 (0 to 0)	0 (0 to 0)	0 (0 to 0)	0 (0 to 0)
HIV/AIDS resulting in other diseases	0 (0 to 0)	0 (0 to 0)	2 (1·9 to 2·1)	2·1 (2 to 2·3)	2 (1·9 to 2·2)	1·6 (1·5 to 1·8)	2·5 (2·4 to 2·7)	10·8 (9·9 to 12·1)	33·3 (30·8 to 36·9)	35·8 (32·8 to 39·5)	42·3 (39·2 to 45·9)
Sexually transmitted infections excluding HIV	0·1 (0·1 to 0·2)	0 (0 to 0·1)	0·1 (0 to 0·1)	0 (0 to 0)	0 (0 to 0)	0·2 (0·1 to 0·3)	1 (0·6 to 1·6)	2 (1·2 to 3·3)	2·7 (1·5 to 4·6)	2·7 (1·5 to 4·7)	2·4 (1·4 to 4·2)
Syphilis	0·1 (0·1 to 0·2)	0 (0 to 0·1)	0·1 (0 to 0·1)	0 (0 to 0)	0 (0 to 0)	0 (0 to 0)	0·1 (0·1 to 0·2)	0·1 (0·1 to 0·2)	0·2 (0·1 to 0·3)	0·1 (0·1 to 0·2)	0·2 (0·1 to 0·2)
Chlamydial infection	0 (0 to 0)	0 (0 to 0)	0 (0 to 0)	0 (0 to 0)	0 (0 to 0)	0 (0 to 0)	0·1 (0·1 to 0·1)	0·3 (0·1 to 0·5)	0·4 (0·2 to 0·8)	0·4 (0·2 to 0·7)	0·3 (0·2 to 0·6)
Gonococcal infection	0 (0 to 0)	0 (0 to 0)	0 (0 to 0)	0 (0 to 0)	0 (0 to 0)	0 (0 to 0·1)	0·2 (0·1 to 0·5)	0·3 (0·2 to 0·6)	0·2 (0·1 to 0·4)	0·2 (0·1 to 0·3)	0·1 (0·1 to 0·2)
Trichomoniasis	0 (0 to 0)	0 (0 to 0)	0 (0 to 0)	0 (0 to 0)	0 (0 to 0)	0 (0 to 0)	0·1 (0 to 0·2)	0·4 (0·1 to 1)	0·7 (0·2 to 1·7)	0·7 (0·2 to 1·7)	0·6 (0·2 to 1·4)
Genital herpes	0 (0 to 0)	0 (0 to 0)	0 (0 to 0)	0 (0 to 0)	0 (0 to 0)	0 (0 to 0·1)	0·2 (0 to 0·4)	0·4 (0·1 to 0·9)	0·6 (0·2 to 1·4)	0·6 (0·2 to 1·5)	0·6 (0·2 to 1·4)
Other sexually transmitted infections	0 (0 to 0)	0 (0 to 0)	0 (0 to 0)	0 (0 to 0)	0 (0 to 0)	0·1 (0 to 0·1)	0·3 (0·2 to 0·6)	0·5 (0·3 to 0·7)	0·6 (0·3 to 0·9)	0·7 (0·4 to 1·1)	0·6 (0·4 to 1)
Respiratory infections and tuberculosis	32·1 (20·1 to 49·5)	19 (11·3 to 29·9)	105 (66·6 to 157)	50·4 (35·8 to 70·8)	24·5 (18·1 to 32·3)	19·2 (14·1 to 26·5)	24·7 (17·8 to 33·7)	33·3 (24·4 to 45·1)	33·2 (24·5 to 44)	35·7 (26·2 to 47·6)	33·9 (24·8 to 45·2)
Tuberculosis	0 (0 to 0)	0 (0 to 0)	0·2 (0·1 to 0·3)	0·5 (0·3 to 0·9)	0·8 (0·3 to 1·4)	0·4 (0·2 to 0·8)	0·6 (0·5 to 0·9)	2·4 (1·5 to 3·6)	3·2 (2 to 4·9)	2·7 (1·8 to 4)	2·2 (1·5 to 3)
Latent tuberculosis infection	0 (0 to 0)	0 (0 to 0)	0 (0 to 0)	0 (0 to 0)	0 (0 to 0)	0 (0 to 0)	0 (0 to 0)	0 (0 to 0)	0 (0 to 0)	0 (0 to 0)	0 (0 to 0)
Drug-susceptible tuberculosis	0 (0 to 0)	0 (0 to 0)	0·2 (0·1 to 0·3)	0·5 (0·3 to 0·9)	0·7 (0·3 to 1·4)	0·4 (0·2 to 0·8)	0·6 (0·5 to 0·9)	2·4 (1·4 to 3·5)	3·1 (2 to 4·8)	2·7 (1·8 to 4)	2·1 (1·5 to 3)
Multidrug-resistant tuberculosis without extensive drug resistanc	0 (0 to 0)	0 (0 to 0)	0 (0 to 0)	0 (0 to 0)	0 (0 to 0)	0 (0 to 0)	0 (0 to 0)	0 (0 to 0·1)	0 (0 to 0·2)	0 (0 to 0·2)	0 (0 to 0·1)
Extensively drug-resistant tuberculosis	0 (0 to 0)	0 (0 to 0)	0 (0 to 0)	0 (0 to 0)	0 (0 to 0)	0 (0 to 0)	0 (0 to 0)	0 (0 to 0)	0 (0 to 0)	0 (0 to 0)	0 (0 to 0)
Lower respiratory infections	32·1 (20·1 to 49·4)	18·9 (11·2 to 29·7)	103 (64·1 to 155)	40 (25·7 to 59·3)	12·4 (8·8 to 17·2)	8·8 (6·3 to 12·1)	13·9 (9·6 to 19·4)	20·4 (13·5 to 29·8)	20·8 (14·1 to 29·7)	25·9 (17·3 to 37)	25·8 (17·8 to 36)
Upper respiratory infections	0 (0 to 0)	0·1 (0·1 to 0·2)	1·8 (1·1 to 2·9)	7·1 (4·1 to 11·3)	7·6 (4·2 to 13)	7·3 (3·9 to 12·4)	7·5 (4 to 13·4)	8 (4·3 to 13·7)	7·2 (3·9 to 11·6)	5·7 (3 to 9·5)	4·7 (2·4 to 7·8)
Otitis media	0 (0 to 0)	0 (0 to 0)	0·3 (0·2 to 0·6)	2·8 (1·6 to 4·9)	3·7 (2 to 6·1)	2·7 (1·4 to 4·7)	2·6 (1·4 to 4·5)	2·5 (1·1 to 4·8)	2 (1 to 3·6)	1·4 (0·6 to 2·7)	1·2 (0·6 to 2·2)
Enteric infections	0·9 (0·5 to 1·4)	1·7 (1 to 2·8)	20·3 (12·6 to 32)	19·5 (13·2 to 28·4)	16·6 (10 to 26·9)	15·9 (9·4 to 25·4)	12·5 (7·9 to 18·1)	11·6 (7·3 to 16·9)	11·9 (7·9 to 16·8)	10·7 (7·4 to 14·7)	9·7 (6·7 to 13·3)
Diarrheal diseases	0·9 (0·5 to 1·4)	1·7 (1 to 2·8)	20·2 (12·5 to 31·9)	19·4 (13·1 to 28·2)	16·6 (9·9 to 26·8)	15·9 (9·4 to 25·4)	12·5 (7·9 to 18)	11·5 (7·3 to 16·8)	11·8 (7·9 to 16·8)	10·6 (7·3 to 14·7)	9·7 (6·7 to 13·3)
Typhoid and paratyphoid	0 (0 to 0)	0 (0 to 0)	0 (0 to 0)	0 (0 to 0)	0 (0 to 0)	0 (0 to 0)	0 (0 to 0)	0 (0 to 0)	0 (0 to 0)	0 (0 to 0)	0 (0 to 0)
Typhoid fever	0 (0 to 0)	0 (0 to 0)	0 (0 to 0)	0 (0 to 0)	0 (0 to 0)	0 (0 to 0)	0 (0 to 0)	0 (0 to 0)	0 (0 to 0)	0 (0 to 0)	0 (0 to 0)
Paratyphoid fever	0 (0 to 0)	0 (0 to 0)	0 (0 to 0)	0 (0 to 0)	0 (0 to 0)	0 (0 to 0)	0 (0 to 0)	0 (0 to 0)	0 (0 to 0)	0 (0 to 0)	0 (0 to 0)
Invasive Non-typhoidal Salmonella (iNTS)	0 (0 to 0)	0 (0 to 0)	0 (0 to 0)	0 (0 to 0·1)	0 (0 to 0)	0 (0 to 0)	0 (0 to 0)	0 (0 to 0)	0 (0 to 0)	0 (0 to 0)	0 (0 to 0)
Other intestinal infectious diseases	0 (0 to 0)	0 (0 to 0)	0·1 (0 to 0·2)	0·1 (0 to 0·2)	0·1 (0 to 0·1)	0 (0 to 0·1)	0 (0 to 0·1)	0 (0 to 0·1)	0 (0 to 0)	0 (0 to 0·1)	0 (0 to 0)
Neglected tropical diseases and malaria	0·1 (0·1 to 0·3)	0·3 (0·2 to 0·5)	2·4 (1·4 to 4·1)	6·7 (3·9 to 10·8)	8 (4·4 to 13·4)	4 (2·3 to 6·7)	4·1 (2·3 to 6·8)	6 (3·4 to 9·8)	6·2 (3·4 to 10·2)	5·6 (3 to 9·1)	5 (2·7 to 8·3)
Malaria	0 (0 to 0)	0 (0 to 0)	0 (0 to 0)	0 (0 to 0)	0 (0 to 0)	0 (0 to 0)	0 (0 to 0)	0 (0 to 0)	0 (0 to 0)	0 (0 to 0)	0 (0 to 0)
Chagas disease	0 (0 to 0)	0 (0 to 0)	0 (0 to 0)	0 (0 to 0)	0 (0 to 0)	0 (0 to 0)	0 (0 to 0)	0 (0 to 0)	0 (0 to 0)	0 (0 to 0)	0 (0 to 0)
Leishmaniasis	0 (0 to 0)	0 (0 to 0)	0 (0 to 0)	0 (0 to 0)	0 (0 to 0)	0 (0 to 0)	0 (0 to 0)	0 (0 to 0)	0 (0 to 0)	0 (0 to 0)	0 (0 to 0)
Visceral leishmaniasis	0 (0 to 0)	0 (0 to 0)	0 (0 to 0)	0 (0 to 0)	0 (0 to 0)	0 (0 to 0)	0 (0 to 0)	0 (0 to 0)	0 (0 to 0)	0 (0 to 0)	0 (0 to 0)
Cutaneous and mucocutaneous leishmaniasis	0 (0 to 0)	0 (0 to 0)	0 (0 to 0)	0 (0 to 0)	0 (0 to 0)	0 (0 to 0)	0 (0 to 0)	0 (0 to 0)	0 (0 to 0)	0 (0 to 0)	0 (0 to 0)
African trypanosomiasis	0 (0 to 0)	0 (0 to 0)	0 (0 to 0)	0 (0 to 0)	0 (0 to 0)	0 (0 to 0)	0 (0 to 0)	0 (0 to 0)	0 (0 to 0)	0 (0 to 0)	0 (0 to 0)
Schistosomiasis	0 (0 to 0)	0 (0 to 0)	0 (0 to 0)	0 (0 to 0)	0 (0 to 0)	0 (0 to 0)	0 (0 to 0)	0 (0 to 0)	0 (0 to 0)	0 (0 to 0)	0 (0 to 0)
Cysticercosis	0 (0 to 0)	0 (0 to 0)	0 (0 to 0)	0 (0 to 0)	0 (0 to 0)	0 (0 to 0·1)	0·4 (0 to 1·1)	1·5 (0·4 to 3·6)	2·6 (0·9 to 5·5)	2·6 (0·9 to 5·7)	2·7 (0·9 to 5·8)
Cystic echinococcosis	0 (0 to 0)	0 (0 to 0)	0 (0 to 0)	0 (0 to 0)	0 (0 to 0)	0 (0 to 0)	0 (0 to 0)	0 (0 to 0)	0 (0 to 0)	0 (0 to 0)	0 (0 to 0)
Lymphatic filariasis	0 (0 to 0)	0 (0 to 0)	0 (0 to 0)	0 (0 to 0)	0 (0 to 0)	0 (0 to 0)	0 (0 to 0)	0 (0 to 0)	0 (0 to 0)	0 (0 to 0)	0 (0 to 0)
Onchocerciasis	0 (0 to 0)	0 (0 to 0)	0 (0 to 0)	0 (0 to 0)	0 (0 to 0)	0 (0 to 0)	0 (0 to 0)	0 (0 to 0)	0 (0 to 0)	0 (0 to 0)	0 (0 to 0)
Trachoma	0 (0 to 0)	0 (0 to 0)	0 (0 to 0)	0 (0 to 0)	0 (0 to 0)	0 (0 to 0)	0 (0 to 0)	0 (0 to 0)	0 (0 to 0)	0 (0 to 0)	0 (0 to 0)
Dengue	0 (0 to 0)	0 (0 to 0)	0·2 (0·1 to 0·4)	0·6 (0·3 to 1·3)	1·3 (0·5 to 3·1)	1·2 (0·5 to 2·4)	1·4 (0·5 to 3·3)	1·7 (0·6 to 4·2)	1·6 (0·5 to 4·1)	1 (0·4 to 2·5)	0·7 (0·3 to 1·4)
Yellow fever	0 (0 to 0)	0 (0 to 0)	0 (0 to 0)	0 (0 to 0)	0 (0 to 0)	0 (0 to 0)	0 (0 to 0)	0 (0 to 0)	0 (0 to 0)	0 (0 to 0)	0 (0 to 0)
Rabies	0 (0 to 0)	0 (0 to 0)	0 (0 to 0)	0 (0 to 0)	0 (0 to 0)	0 (0 to 0)	0 (0 to 0)	0 (0 to 0)	0 (0 to 0)	0 (0 to 0)	0 (0 to 0)
Intestinal nematode infections	0 (0 to 0)	0 (0 to 0)	0 (0 to 0·1)	0·3 (0·1 to 0·5)	0·6 (0·3 to 1)	0·4 (0·2 to 0·7)	0·9 (0·4 to 1·8)	1·1 (0·5 to 2·1)	0·7 (0·3 to 1·4)	0·5 (0·2 to 1)	0·5 (0·2 to 0·9)
Ascariasis	0 (0 to 0)	0 (0 to 0)	0 (0 to 0·1)	0·1 (0 to 0·3)	0·1 (0 to 0·3)	0·1 (0 to 0·2)	0·1 (0 to 0·2)	0·1 (0 to 0·1)	0·1 (0 to 0·1)	0 (0 to 0·1)	0 (0 to 0·1)
Trichuriasis	0 (0 to 0)	0 (0 to 0)	0 (0 to 0)	0 (0 to 0)	0 (0 to 0)	0 (0 to 0)	0 (0 to 0)	0 (0 to 0)	0 (0 to 0)	0 (0 to 0)	0 (0 to 0)
Hookworm disease	0 (0 to 0)	0 (0 to 0)	0 (0 to 0)	0·2 (0·1 to 0·3)	0·4 (0·2 to 0·7)	0·3 (0·1 to 0·6)	0·9 (0·4 to 1·6)	1·1 (0·4 to 2)	0·7 (0·3 to 1·3)	0·4 (0·2 to 0·9)	0·4 (0·2 to 0·8)
Food-borne trematodiases	0 (0 to 0)	0 (0 to 0)	0 (0 to 0)	0 (0 to 0)	0 (0 to 0)	0 (0 to 0)	0 (0 to 0)	0 (0 to 0)	0 (0 to 0)	0 (0 to 0)	0 (0 to 0)
Leprosy	0 (0 to 0)	0 (0 to 0)	0 (0 to 0)	0 (0 to 0)	0 (0 to 0)	0 (0 to 0)	0 (0 to 0)	0 (0 to 0)	0 (0 to 0)	0 (0 to 0)	0 (0 to 0·1)
Ebola	0 (0 to 0)	0 (0 to 0)	0 (0 to 0)	0 (0 to 0)	0 (0 to 0)	0 (0 to 0)	0 (0 to 0)	0 (0 to 0)	0 (0 to 0)	0 (0 to 0)	0 (0 to 0)
Zika virus	0 (0 to 0)	0 (0 to 0·1)	0·4 (0·1 to 1·1)	0·2 (0 to 0·7)	0·2 (0 to 1)	0·2 (0 to 1)	0·3 (0 to 1·3)	0·3 (0 to 1·4)	0·3 (0 to 1·4)	0·3 (0 to 1·4)	0·3 (0 to 1·2)
Guinea worm disease	0 (0 to 0)	0 (0 to 0)	0 (0 to 0)	0 (0 to 0)	0 (0 to 0)	0 (0 to 0)	0 (0 to 0)	0 (0 to 0)	0 (0 to 0)	0 (0 to 0)	0 (0 to 0)
Other neglected tropical diseases	0·1 (0·1 to 0·3)	0·3 (0·2 to 0·5)	1·8 (1 to 3·4)	5·7 (3·1 to 9·6)	5·9 (3 to 10·6)	2·1 (0·9 to 4)	1 (0·5 to 1·9)	1·2 (0·6 to 2·8)	0·9 (0·4 to 1·8)	1·1 (0·5 to 2·1)	0·8 (0·4 to 1·5)
Other infectious diseases	1·1 (0·7 to 1·7)	2 (1·2 to 3)	12 (7·9 to 18)	10·7 (7·1 to 15·4)	8·5 (6·2 to 12)	5·9 (4·5 to 7·9)	7·7 (6·1 to 9·8)	8·3 (6·3 to 11·5)	7·9 (5·7 to 11·1)	7·8 (5·9 to 10·8)	6·9 (5·3 to 9·2)
Meningitis	0·5 (0·3 to 0·8)	1·1 (0·6 to 1·7)	4 (2·3 to 6·3)	2·3 (1·4 to 3·4)	1·4 (1·1 to 1·8)	1·1 (0·9 to 1·4)	1·6 (1·2 to 2)	1·4 (1 to 1·7)	1·1 (0·9 to 1·4)	1·2 (1 to 1·6)	1 (0·8 to 1·3)
Encephalitis	0·2 (0·1 to 0·3)	0·3 (0·1 to 0·6)	3·5 (1·9 to 7·1)	3·2 (1·7 to 6·8)	1·9 (1·2 to 3·2)	1·7 (1·2 to 2·6)	2·2 (1·6 to 3·3)	2·1 (1·5 to 3·5)	1·5 (1·1 to 2·5)	1·4 (1 to 2·1)	1·1 (0·8 to 1·6)
Diphtheria	0 (0 to 0)	0 (0 to 0)	0 (0 to 0)	0 (0 to 0)	0 (0 to 0)	0 (0 to 0)	0 (0 to 0)	0 (0 to 0)	0 (0 to 0)	0 (0 to 0)	0 (0 to 0)
Tetanus	0 (0 to 0)	0 (0 to 0)	0 (0 to 0·1)	0 (0 to 0·1)	0 (0 to 0·3)	0 (0 to 0·2)	0·1 (0 to 0·3)	0·1 (0 to 0·4)	0·1 (0 to 0·3)	0·1 (0·1 to 0·4)	0·2 (0·1 to 0·4)
Measles	0 (0 to 0)	0 (0 to 0)	0 (0 to 0)	0 (0 to 0)	0 (0 to 0)	0 (0 to 0)	0 (0 to 0)	0 (0 to 0)	0 (0 to 0)	0 (0 to 0)	0 (0 to 0)
Varicella and herpes zoster	0·1 (0 to 0·2)	0·2 (0·1 to 0·3)	0·7 (0·4 to 1·3)	0·3 (0·2 to 0·6)	0·3 (0·2 to 0·9)	0·3 (0·2 to 0·6)	0·3 (0·2 to 0·6)	0·3 (0·1 to 0·7)	0·3 (0·2 to 0·7)	0·3 (0·1 to 0·7)	0·3 (0·2 to 0·7)
Acute hepatitis	0 (0 to 0)	0 (0 to 0)	0·1 (0·1 to 0·2)	0·2 (0·1 to 0·3)	0·6 (0·3 to 0·9)	0·7 (0·5 to 1·1)	0·8 (0·5 to 1·2)	0·9 (0·6 to 1·3)	0·7 (0·5 to 1·1)	0·6 (0·4 to 0·8)	0·5 (0·3 to 0·6)
Acute hepatitis A	0 (0 to 0)	0 (0 to 0)	0 (0 to 0)	0·1 (0·1 to 0·2)	0·5 (0·3 to 0·7)	0·6 (0·4 to 0·9)	0·6 (0·4 to 0·9)	0·6 (0·3 to 0·9)	0·4 (0·2 to 0·6)	0·3 (0·2 to 0·5)	0·2 (0·1 to 0·3)
Acute hepatitis B	0 (0 to 0)	0 (0 to 0)	0·1 (0 to 0·2)	0 (0 to 0·1)	0·1 (0 to 0·1)	0·1 (0 to 0·1)	0·1 (0·1 to 0·2)	0·2 (0·1 to 0·3)	0·3 (0·2 to 0·4)	0·2 (0·1 to 0·3)	0·2 (0·2 to 0·3)
Acute hepatitis C	0 (0 to 0)	0 (0 to 0)	0 (0 to 0)	0 (0 to 0)	0 (0 to 0)	0 (0 to 0)	0 (0 to 0)	0 (0 to 0)	0 (0 to 0)	0 (0 to 0)	0 (0 to 0)
Acute hepatitis E	0 (0 to 0)	0 (0 to 0)	0 (0 to 0)	0 (0 to 0)	0 (0 to 0·1)	0·1 (0 to 0·1)	0·1 (0 to 0·2)	0·1 (0 to 0·2)	0·1 (0 to 0·1)	0·1 (0 to 0·1)	0 (0 to 0·1)
Other unspecified infectious diseases	0·4 (0·2 to 0·6)	0·4 (0·2 to 0·8)	3 (1·8 to 4·9)	4·1 (2·5 to 6·3)	4·2 (2·2 to 7·2)	2 (1·1 to 3·3)	2·7 (1·8 to 4)	3·5 (2·3 to 5·7)	4·1 (2·5 to 6·7)	4·1 (2·6 to 6·4)	3·8 (2·5 to 5·6)
Maternal and neonatal disorders	732 (510 to 1,020)	125 (80·9 to 185)	73·4 (46·8 to 110)	34·3 (26·4 to 43·3)	28·3 (20·7 to 36·4)	30·1 (22·5 to 39·2)	40·1 (31·9 to 49·4)	45·8 (36·6 to 56·3)	42·8 (34·1 to 52·6)	35·8 (29 to 43·5)	26·9 (21·6 to 33·1)
Maternal disorders	0 (0 to 0)	0 (0 to 0)	0 (0 to 0)	0 (0 to 0)	0 (0 to 0)	0·2 (0·2 to 0·3)	8·9 (7 to 11·1)	12·4 (9·7 to 15·7)	11·8 (9 to 15·2)	11·3 (8·8 to 13·9)	7·3 (5·9 to 9·1)
Maternal hemorrhage	0 (0 to 0)	0 (0 to 0)	0 (0 to 0)	0 (0 to 0)	0 (0 to 0)	0 (0 to 0)	0·9 (0·6 to 1·4)	1·6 (1 to 2·5)	1·8 (1·1 to 2·8)	1·8 (1·2 to 2·6)	1·2 (0·8 to 1·8)
Maternal sepsis and other maternal infections	0 (0 to 0)	0 (0 to 0)	0 (0 to 0)	0 (0 to 0)	0 (0 to 0)	0 (0 to 0)	0·2 (0·1 to 0·4)	0·4 (0·2 to 0·7)	0·3 (0·2 to 0·6)	0·3 (0·1 to 0·4)	0·1 (0·1 to 0·2)
Maternal hypertensive disorders	0 (0 to 0)	0 (0 to 0)	0 (0 to 0)	0 (0 to 0)	0 (0 to 0)	0·1 (0·1 to 0·1)	4·1 (3·1 to 5·3)	4·7 (3·4 to 6·3)	4·3 (3·1 to 6)	3·7 (2·7 to 5)	2·2 (1·6 to 3)
Maternal obstructed labor and uterine rupture	0 (0 to 0)	0 (0 to 0)	0 (0 to 0)	0 (0 to 0)	0 (0 to 0)	0 (0 to 0)	0·2 (0·1 to 0·3)	0·3 (0·2 to 0·4)	0·3 (0·2 to 0·4)	0·3 (0·2 to 0·5)	0·2 (0·1 to 0·4)
Maternal abortion and miscarriage	0 (0 to 0)	0 (0 to 0)	0 (0 to 0)	0 (0 to 0)	0 (0 to 0)	0 (0 to 0)	0·4 (0·2 to 0·7)	0·7 (0·3 to 1·1)	0·5 (0·3 to 0·9)	0·5 (0·3 to 0·9)	0·4 (0·2 to 0·6)
Ectopic pregnancy	0 (0 to 0)	0 (0 to 0)	0 (0 to 0)	0 (0 to 0)	0 (0 to 0)	0 (0 to 0)	0·3 (0·2 to 0·4)	0·5 (0·3 to 0·8)	0·5 (0·3 to 0·8)	0·5 (0·3 to 0·7)	0·3 (0·2 to 0·4)
Indirect maternal deaths	--	--	--	--	--	0 (0 to 0)	0·4 (0·2 to 0·6)	0·6 (0·3 to 0·9)	0·5 (0·3 to 0·8)	0·5 (0·3 to 0·8)	0·3 (0·2 to 0·4)
Late maternal deaths	--	--	--	--	--	0 (0 to 0)	0·6 (0·4 to 1)	0·8 (0·5 to 1·3)	0·7 (0·4 to 1·2)	0·7 (0·4 to 1·1)	0·4 (0·3 to 0·7)
Maternal deaths aggravated by HIV/AIDS	--	--	--	--	--	0 (0 to 0)	0 (0 to 0)	0 (0 to 0)	0 (0 to 0)	0 (0 to 0)	0 (0 to 0)
Neonatal disorders	732 (510 to 1,020)	125 (80·9 to 185)	73·4 (46·8 to 110)	34·3 (26·4 to 43·3)	28·3 (20·7 to 36·4)	29·9 (22·3 to 38·9)	31·2 (23·4 to 40·4)	33·4 (25·1 to 43)	31 (23·2 to 40·2)	24·5 (18·4 to 31·5)	19·6 (14·6 to 25·5)
Neonatal preterm birth	315 (215 to 456)	37·6 (22·2 to 58·3)	26·5 (16·7 to 40·4)	10·4 (7·7 to 13·6)	12·4 (8·9 to 16·1)	13·1 (9·4 to 17·5)	13·4 (9·9 to 17·9)	14·1 (10·4 to 18·7)	13 (9·5 to 17·4)	10·1 (7·4 to 13·3)	8 (5·8 to 10·6)
Neonatal encephalopathy due to birth asphyxia and trauma	246 (167 to 354)	25·3 (14·3 to 40·1)	16·5 (8·9 to 28·9)	5·4 (3·5 to 7·6)	3·7 (2·2 to 5·9)	3·9 (2·3 to 6·2)	4·2 (2·4 to 6·5)	4·5 (2·6 to 7·1)	4·2 (2·4 to 6·6)	3·4 (1·9 to 5·2)	2·7 (1·6 to 4·2)
Neonatal sepsis and other neonatal infections	91·5 (56·7 to 142)	45·7 (27·7 to 72·2)	20·1 (12·4 to 31·4)	8·3 (5·4 to 12·1)	10·8 (6·9 to 16·2)	11·4 (7·4 to 17·3)	12 (7·7 to 17·3)	13 (8·3 to 19)	12·2 (7·7 to 17·6)	9·7 (6·3 to 14·3)	7·9 (5 to 11·5)
Hemolytic disease and other neonatal jaundice	2·4 (1·4 to 4)	0·7 (0·4 to 1·1)	0·6 (0·3 to 1)	1·2 (0·7 to 1·7)	1·4 (0·8 to 2·1)	1·5 (1 to 2·2)	1·6 (1·1 to 2·3)	1·8 (1·3 to 2·3)	1·7 (1·2 to 2·1)	1·3 (1 to 1·7)	1·1 (0·8 to 1·4)
Other neonatal disorders	76·7 (47·7 to 119)	16·1 (9·7 to 24·6)	9·6 (6·5 to 14·4)	9 (6·8 to 11·4)	0 (0 to 0)	0 (0 to 0)	0 (0 to 0)	0 (0 to 0)	0 (0 to 0)	0 (0 to 0)	0 (0 to 0)
Nutritional deficiencies	0·1 (0 to 0·1)	1·5 (0·8 to 2·3)	26·3 (17·3 to 37·7)	52·7 (29·8 to 83·8)	58·8 (29·5 to 108)	21·1 (9·3 to 41·2)	18·6 (8·7 to 35·7)	20·4 (10·1 to 36·8)	20·7 (10·6 to 37·6)	17·8 (9·1 to 31·3)	17·9 (8·5 to 30·9)
Protein-energy malnutrition	0 (0 to 0·1)	0·1 (0·1 to 0·2)	15 (9·4 to 22·5)	7·8 (4·5 to 12·4)	3 (2 to 4·1)	1·8 (1·2 to 2·6)	2·3 (1·6 to 3·2)	2·8 (1·9 to 4)	2·8 (1·8 to 4·1)	2·8 (2 to 3·8)	2·9 (2·1 to 3·9)
Iodine deficiency	0 (0 to 0)	0 (0 to 0)	0 (0 to 0)	0 (0 to 0)	0·1 (0 to 0·2)	0·3 (0·1 to 0·5)	0·4 (0·2 to 0·8)	0·6 (0·2 to 1)	0·6 (0·2 to 1·1)	0·5 (0·2 to 0·9)	0·4 (0·2 to 0·8)
Vitamin A deficiency	0 (0 to 0)	0·1 (0 to 0·1)	0·3 (0·1 to 0·5)	1·4 (0·7 to 2·4)	1·6 (0·8 to 2·8)	0·8 (0·4 to 1·6)	0·3 (0·1 to 0·7)	0·3 (0·1 to 0·5)	0·3 (0·1 to 0·5)	0·2 (0·1 to 0·3)	0·1 (0·1 to 0·2)
Dietary iron deficiency	0 (0 to 0·1)	1·3 (0·7 to 2)	10·6 (5·2 to 18·4)	43·3 (21·7 to 74·8)	53·8 (25·1 to 102)	17·9 (6·6 to 37·2)	14·7 (5·2 to 31·4)	15·8 (5·8 to 31·6)	15·9 (6·1 to 32·4)	13·6 (5·2 to 26·8)	13·7 (4·5 to 26·3)
Other nutritional deficiencies	0 (0 to 0)	0 (0 to 0)	0·4 (0·2 to 0·7)	0·3 (0·1 to 0·6)	0·3 (0·2 to 0·5)	0·3 (0·2 to 0·5)	0·8 (0·5 to 1·2)	0·9 (0·5 to 1·4)	1·1 (0·7 to 1·8)	0·8 (0·5 to 1·1)	0·7 (0·5 to 1·1)
Non-communicable diseases	102 (68·3 to 151)	39·7 (26·1 to 59·3)	187 (130 to 263)	226 (171 to 296)	269 (209 to 344)	392 (293 to 511)	642 (501 to 813)	848 (672 to 1,050)	941 (743 to 1,150)	980 (804 to 1,170)	1,020 (855 to 1,210)
Neoplasms	0·9 (0·6 to 1·3)	0·7 (0·4 to 1)	5·4 (3·5 to 8·1)	24 (15·9 to 35·1)	29 (23 to 35·5)	27·1 (22·7 to 32·1)	45·6 (38·1 to 52·8)	60 (47·6 to 72·2)	69·9 (54·9 to 87·3)	110 (90·5 to 133)	146 (121 to 174)
Lip and oral cavity cancer	0 (0 to 0)	0 (0 to 0)	0 (0 to 0)	0 (0 to 0)	0·1 (0 to 0·1)	0·1 (0·1 to 0·1)	0·3 (0·2 to 0·4)	0·4 (0·3 to 0·6)	0·5 (0·3 to 0·7)	0·9 (0·6 to 1·3)	1·4 (1 to 2)
Nasopharynx cancer	0 (0 to 0)	0 (0 to 0)	0 (0 to 0)	0 (0 to 0)	0·1 (0·1 to 0·2)	0·2 (0·1 to 0·2)	0·4 (0·3 to 0·6)	0·4 (0·3 to 0·7)	0·4 (0·3 to 0·6)	0·4 (0·3 to 0·6)	0·5 (0·3 to 0·7)
Other pharynx cancer	0 (0 to 0)	0 (0 to 0)	0 (0 to 0)	0 (0 to 0)	0 (0 to 0)	0 (0 to 0)	0 (0 to 0)	0·2 (0·1 to 0·4)	0·2 (0·1 to 0·3)	0·4 (0·2 to 0·5)	0·6 (0·4 to 0·9)
Esophageal cancer	0 (0 to 0)	0 (0 to 0)	0 (0 to 0)	0 (0 to 0)	0 (0 to 0)	0 (0 to 0)	0 (0 to 0)	0·3 (0·2 to 0·5)	0·5 (0·3 to 0·7)	1 (0·7 to 1·4)	2·4 (1·6 to 3·5)
Stomach cancer	0 (0 to 0)	0 (0 to 0)	0 (0 to 0)	0 (0 to 0)	0 (0 to 0)	0 (0 to 0)	0·5 (0·4 to 0·8)	1·2 (0·8 to 1·6)	2·3 (1·5 to 3·3)	4·7 (3·3 to 6·4)	6·4 (4·3 to 9·1)
Colon and rectum cancer	0 (0 to 0)	0 (0 to 0)	0 (0 to 0)	0 (0 to 0)	0·2 (0·2 to 0·4)	0·3 (0·2 to 0·3)	1·4 (1 to 1·8)	2·7 (1·9 to 3·6)	3·9 (2·8 to 5·2)	8·1 (5·8 to 10·8)	10·8 (7·6 to 14·5)
Liver cancer	0 (0 to 0)	0 (0 to 0)	0·1 (0·1 to 0·2)	0·3 (0·2 to 0·5)	0·3 (0·2 to 0·4)	0·4 (0·3 to 0·6)	0·3 (0·2 to 0·5)	0·7 (0·5 to 1)	0·9 (0·6 to 1·4)	1·3 (0·8 to 1·9)	2 (1·3 to 2·9)
Liver cancer due to hepatitis B	0 (0 to 0)	0 (0 to 0)	0 (0 to 0)	0 (0 to 0)	0 (0 to 0)	0·1 (0 to 0·2)	0·1 (0·1 to 0·2)	0·4 (0·2 to 0·5)	0·5 (0·3 to 0·8)	0·7 (0·4 to 1·1)	1 (0·6 to 1·7)
Liver cancer due to hepatitis C	0 (0 to 0)	0 (0 to 0)	0 (0 to 0)	0 (0 to 0)	0 (0 to 0)	0 (0 to 0)	0 (0 to 0)	0 (0 to 0)	0 (0 to 0·1)	0·1 (0 to 0·1)	0·1 (0 to 0·3)
Liver cancer due to alcohol use	0 (0 to 0)	0 (0 to 0)	0 (0 to 0)	0 (0 to 0)	0 (0 to 0)	0 (0 to 0)	0 (0 to 0)	0·1 (0 to 0·1)	0·1 (0 to 0·3)	0·2 (0·1 to 0·4)	0·5 (0·2 to 0·9)
Liver cancer due to NASH	0 (0 to 0)	0 (0 to 0)	0 (0 to 0)	0 (0 to 0)	0 (0 to 0)	0 (0 to 0)	0·1 (0 to 0·1)	0·1 (0·1 to 0·2)	0·1 (0 to 0·2)	0·1 (0 to 0·2)	0·1 (0·1 to 0·2)
Liver cancer due to other causes	0 (0 to 0)	0 (0 to 0)	0·1 (0·1 to 0·2)	0·3 (0·2 to 0·5)	0·3 (0·2 to 0·4)	0·3 (0·3 to 0·4)	0·1 (0·1 to 0·2)	0·2 (0·1 to 0·3)	0·1 (0·1 to 0·2)	0·2 (0·1 to 0·3)	0·2 (0·1 to 0·4)
Gallbladder and biliary tract cancer	0 (0 to 0)	0 (0 to 0)	0 (0 to 0)	0 (0 to 0)	0 (0 to 0)	0 (0 to 0)	0 (0 to 0)	0·1 (0·1 to 0·2)	0·2 (0·1 to 0·4)	0·4 (0·2 to 0·7)	0·7 (0·5 to 1·1)
Pancreatic cancer	0 (0 to 0)	0 (0 to 0)	0 (0 to 0)	0 (0 to 0)	0 (0 to 0)	0 (0 to 0)	0·4 (0·3 to 0·6)	0·5 (0·3 to 0·9)	0·9 (0·5 to 1·5)	2·6 (1·9 to 3·4)	4·2 (3 to 5·7)
Larynx cancer	0 (0 to 0)	0 (0 to 0)	0 (0 to 0)	0 (0 to 0)	0 (0 to 0)	0 (0 to 0)	0 (0 to 0)	0·1 (0·1 to 0·1)	0·1 (0·1 to 0·1)	0·2 (0·1 to 0·2)	0·4 (0·2 to 0·6)
Tracheal, bronchus, and lung cancer	0 (0 to 0)	0 (0 to 0)	0 (0 to 0)	0 (0 to 0)	0 (0 to 0)	0·1 (0·1 to 0·2)	0·8 (0·6 to 1·1)	1 (0·7 to 1·5)	1·3 (0·9 to 1·8)	3·3 (2·1 to 5)	6 (4·2 to 8·6)
Malignant skin melanoma	0 (0 to 0)	0 (0 to 0)	0 (0 to 0·1)	0·2 (0 to 0·4)	0·1 (0 to 0·1)	0·1 (0 to 0·1)	0·2 (0·1 to 0·3)	0·4 (0·3 to 0·8)	0·5 (0·4 to 1)	0·7 (0·5 to 1·2)	1·2 (0·8 to 2·1)
Non-melanoma skin cancer	0 (0 to 0)	0 (0 to 0)	0 (0 to 0)	0 (0 to 0)	0 (0 to 0)	0 (0 to 0)	0 (0 to 0)	0·1 (0·1 to 0·2)	0·2 (0·1 to 0·3)	0·3 (0·2 to 0·5)	0·4 (0·2 to 0·6)
Non-melanoma skin cancer (squamous-cell carcinoma)	0 (0 to 0)	0 (0 to 0)	0 (0 to 0)	0 (0 to 0)	0 (0 to 0)	0 (0 to 0)	0 (0 to 0)	0·1 (0·1 to 0·2)	0·2 (0·1 to 0·3)	0·3 (0·2 to 0·5)	0·4 (0·2 to 0·6)
Non-melanoma skin cancer (basal-cell carcinoma)	0 (0 to 0)	0 (0 to 0)	0 (0 to 0)	0 (0 to 0)	0 (0 to 0)	0 (0 to 0)	0 (0 to 0)	0 (0 to 0)	0 (0 to 0)	0 (0 to 0)	0 (0 to 0)
Breast cancer	0 (0 to 0)	0 (0 to 0)	0 (0 to 0)	0 (0 to 0)	0 (0 to 0)	0 (0 to 0)	0·7 (0·4 to 1)	1·4 (1 to 2)	5 (3·3 to 7)	17·3 (12·1 to 24)	29·5 (21·6 to 39·6)
Cervical cancer	0 (0 to 0)	0 (0 to 0)	0 (0 to 0)	0 (0 to 0)	0 (0 to 0)	0 (0 to 0)	0·8 (0·4 to 1·3)	3 (1·8 to 4·9)	8 (4·9 to 12·8)	17 (10·8 to 25·1)	25·6 (16·8 to 36·3)
Uterine cancer	0 (0 to 0)	0 (0 to 0)	0 (0 to 0)	0 (0 to 0)	0 (0 to 0)	0 (0 to 0)	0 (0 to 0)	0·9 (0·5 to 1·3)	1·8 (1 to 3)	3·7 (2·3 to 5·9)	5·4 (3·4 to 7·9)
Ovarian cancer	0 (0 to 0)	0 (0 to 0)	0 (0 to 0)	0 (0 to 0)	0·3 (0·1 to 0·5)	0·5 (0·3 to 0·7)	1·6 (0·9 to 2·6)	2·5 (1·4 to 3·9)	2·4 (1·4 to 3·6)	3·8 (2·1 to 5·5)	5·3 (3·1 to 7·5)
Prostate cancer	0 (0 to 0)	0 (0 to 0)	0 (0 to 0)	0 (0 to 0)	0 (0 to 0)	0 (0 to 0)	0 (0 to 0)	0·3 (0·2 to 0·5)	0·6 (0·4 to 0·9)	0·8 (0·6 to 1·1)	0·9 (0·6 to 1·2)
Testicular cancer	0 (0 to 0·3)	0 (0 to 0)	0 (0 to 0·2)	0 (0 to 0·1)	0·1 (0 to 0·1)	0·1 (0 to 0·1)	0·2 (0·2 to 0·4)	0·4 (0·3 to 0·7)	0·5 (0·3 to 0·8)	0·3 (0·2 to 0·5)	0·3 (0·2 to 0·4)
Kidney cancer	0 (0 to 0·1)	0 (0 to 0)	0·3 (0·2 to 0·4)	1·3 (0·8 to 2)	1·2 (0·9 to 1·7)	0·5 (0·4 to 0·7)	0·8 (0·6 to 1)	1·2 (0·8 to 1·6)	1·3 (0·9 to 1·9)	1·6 (1·1 to 2·5)	1·6 (1·1 to 2·3)
Bladder cancer	0 (0 to 0)	0 (0 to 0)	0 (0 to 0)	0 (0 to 0)	0 (0 to 0)	0 (0 to 0)	0·1 (0·1 to 0·1)	0·1 (0·1 to 0·2)	0·1 (0·1 to 0·2)	0·5 (0·3 to 0·6)	0·6 (0·4 to 0·9)
Brain and central nervous system cancer	0·1 (0·1 to 0·2)	0·1 (0 to 0·2)	1·1 (0·5 to 1·9)	4 (1·7 to 6·8)	5·5 (2·9 to 8·1)	4·3 (2·1 to 6·5)	3·6 (1·8 to 5·4)	4 (2 to 5·8)	4 (1·9 to 6)	4·8 (2·2 to 7·1)	5·2 (2·4 to 7·5)
Thyroid cancer	0 (0 to 0)	0 (0 to 0)	0 (0 to 0)	0 (0 to 0)	0·1 (0·1 to 0·1)	0·1 (0·1 to 0·1)	0·3 (0·2 to 0·4)	0·3 (0·2 to 0·5)	0·4 (0·3 to 0·7)	0·6 (0·4 to 0·9)	0·9 (0·6 to 1·3)
Mesothelioma	0 (0 to 0)	0 (0 to 0)	0 (0 to 0)	0 (0 to 0)	0 (0 to 0)	0 (0 to 0)	0 (0 to 0)	0·3 (0·2 to 0·6)	0·3 (0·2 to 0·4)	0·3 (0·2 to 0·4)	0·6 (0·4 to 0·8)
Hodgkin lymphoma	0 (0 to 0)	0 (0 to 0)	0 (0 to 0)	0·1 (0 to 0·1)	0·1 (0·1 to 0·2)	0·2 (0·1 to 0·3)	0·9 (0·6 to 1·3)	1·5 (1 to 2·2)	1·3 (0·9 to 1·9)	1·2 (0·8 to 1·6)	0·9 (0·6 to 1·3)
Non-Hodgkin lymphoma	0 (0 to 0)	0 (0 to 0)	0 (0 to 0)	4 (2·4 to 6·3)	5·9 (4 to 8·4)	5·6 (4 to 7·8)	10·8 (8·3 to 13·7)	14·7 (10·4 to 20·3)	14·2 (10·3 to 19·1)	15·9 (11·5 to 21·8)	14·9 (11·1 to 20)
Multiple myeloma	0 (0 to 0)	0 (0 to 0)	0 (0 to 0)	0 (0 to 0)	0 (0 to 0)	0 (0 to 0)	0 (0 to 0)	0·2 (0·1 to 0·3)	0·3 (0·2 to 0·4)	0·5 (0·3 to 0·7)	1 (0·7 to 1·4)
Leukemia	0·3 (0·2 to 0·4)	0·2 (0·1 to 0·3)	1·7 (1 to 2·6)	6·5 (4·1 to 9·9)	8·8 (6·4 to 11·7)	8 (6·1 to 10·3)	11·1 (8·7 to 13·9)	10·1 (7·3 to 13·1)	8·3 (6·2 to 11)	7·5 (5·8 to 9·6)	7·4 (5·5 to 9·7)
Acute lymphoid leukemia	0 (0 to 0·1)	0 (0 to 0·1)	0·3 (0·2 to 0·5)	2·2 (1·2 to 3·7)	3·4 (2 to 5·3)	2·5 (1·6 to 3·8)	3·6 (2·3 to 5·1)	1·9 (1·2 to 2·8)	1 (0·6 to 1·6)	0·7 (0·5 to 1·1)	0·5 (0·3 to 0·8)
Chronic lymphoid leukemia	0 (0 to 0)	0 (0 to 0)	0 (0 to 0)	0 (0 to 0)	0 (0 to 0)	0 (0 to 0)	0 (0 to 0)	0·3 (0·2 to 0·4)	0·2 (0·1 to 0·3)	0·2 (0·1 to 0·2)	0·3 (0·2 to 0·5)
Acute myeloid leukemia	0 (0 to 0·1)	0·1 (0 to 0·2)	0·4 (0·2 to 0·6)	2 (1·2 to 3·5)	2 (1·3 to 3·1)	2·2 (1·5 to 3·3)	3·4 (2·3 to 5)	2·9 (1·9 to 4·3)	2·8 (1·9 to 3·9)	2·2 (1·5 to 3·1)	2·1 (1·4 to 3·1)

Cause	40 to 44	45 to 49	50 to 54	55 to 59	60 to 64	65 to 69	70 to 74	75 to 79	80 to 84	85 to 89	90 to 94	95 plus	Total
All causes	1,620 (1,380 to 1,870)	2,200 (1,910 to 2,540)	2,840 (2,480 to 3,220)	3,140 (2,790 to 3,500)	2,980 (2,710 to 3,290)	2,910 (2,670 to 3,170)	2,730 (2,520 to 2,950)	2,290 (2,130 to 2,470)	1,170 (1,100 to 1,250)	573 (539 to 610)	297 (281 to 315)	116 (110 to 122)	32,400
Communicable, maternal, neonatal, and nutritional diseases	146 (122 to 175)	158 (132 to 188)	170 (141 to 205)	162 (132 to 198)	143 (119 to 169)	139 (111 to 174)	147 (113 to 187)	146 (122 to 177)	89·8 (71·7 to 112)	56·4 (44·8 to 68)	32 (24·8 to 38·5)	15·6 (11·3 to 19·6)	3,690
HIV/AIDS and sexually transmitted infections	41·4 (38·2 to 45·2)	36·8 (34·1 to 39·8)	31·9 (29·4 to 34·7)	19·3 (17·5 to 21·3)	10·2 (9·1 to 11·4)	5·8 (5·1 to 6·6)	2·1 (1·7 to 2·6)	1 (0·8 to 1·2)	0·3 (0·3 to 0·4)	0·1 (0·1 to 0·2)	0 (0 to 0·1)	0 (0 to 0)	296
HIV/AIDS	38·9 (36·3 to 42·1)	34·4 (32·1 to 37)	30·1 (27·9 to 32·3)	18·1 (16·6 to 19·6)	9·4 (8·5 to 10·3)	5·2 (4·6 to 5·8)	1·6 (1·4 to 1·9)	0·7 (0·6 to 0·8)	0·2 (0·2 to 0·2)	0·1 (0·1 to 0·1)	0 (0 to 0)	0 (0 to 0)	274
HIV/AIDS - Drug-susceptible Tuberculosis	0·8 (0·6 to 1·1)	0·7 (0·5 to 1)	0·7 (0·5 to 1)	0·5 (0·3 to 0·7)	0·3 (0·2 to 0·4)	0·1 (0·1 to 0·2)	0 (0 to 0·1)	0 (0 to 0)	0 (0 to 0)	0 (0 to 0)	0 (0 to 0)	0 (0 to 0)	6·4
HIV/AIDS - Multidrug-resistant Tuberculosis without extensive dru	0 (0 to 0·1)	0 (0 to 0·1)	0 (0 to 0·1)	0 (0 to 0)	0 (0 to 0)	0 (0 to 0)	0 (0 to 0)	0 (0 to 0)	0 (0 to 0)	0 (0 to 0)	0 (0 to 0)	0 (0 to 0)	0·1
HIV/AIDS - Extensively drug-resistant Tuberculosis	0 (0 to 0)	0 (0 to 0)	0 (0 to 0)	0 (0 to 0)	0 (0 to 0)	0 (0 to 0)	0 (0 to 0)	0 (0 to 0)	0 (0 to 0)	0 (0 to 0)	0 (0 to 0)	0 (0 to 0)	0
HIV/AIDS resulting in other diseases	38·1 (35·5 to 41·1)	33·7 (31·3 to 36·2)	29·4 (27·2 to 31·5)	17·6 (16 to 19·1)	9·1 (8·2 to 10)	5·1 (4·5 to 5·7)	1·6 (1·3 to 1·9)	0·7 (0·6 to 0·8)	0·2 (0·2 to 0·2)	0·1 (0·1 to 0·1)	0 (0 to 0)	0 (0 to 0)	268
Sexually transmitted infections excluding HIV	2·5 (1·3 to 4·5)	2·4 (1·3 to 4·4)	1·8 (0·9 to 3·5)	1·3 (0·6 to 2·4)	0·8 (0·4 to 1·5)	0·6 (0·3 to 1·1)	0·4 (0·2 to 0·8)	0·3 (0·1 to 0·5)	0·1 (0·1 to 0·2)	0·1 (0 to 0·1)	0 (0 to 0)	0 (0 to 0)	21·4
Syphilis	0·1 (0·1 to 0·1)	0·1 (0·1 to 0·2)	0·2 (0·1 to 0·3)	0·2 (0·1 to 0·3)	0·1 (0·1 to 0·2)	0·1 (0·1 to 0·2)	0·1 (0·1 to 0·2)	0 (0 to 0·1)	0 (0 to 0)	0 (0 to 0)	0 (0 to 0)	0 (0 to 0)	1·9
Chlamydial infection	0·3 (0·2 to 0·6)	0·3 (0·1 to 0·5)	0·1 (0 to 0·2)	0 (0 to 0·1)	0 (0 to 0)	0 (0 to 0)	0 (0 to 0)	0 (0 to 0)	0 (0 to 0)	0 (0 to 0)	0 (0 to 0)	0 (0 to 0)	2·3
Gonococcal infection	0·2 (0·1 to 0·2)	0·1 (0·1 to 0·2)	0·1 (0·1 to 0·1)	0 (0 to 0·1)	0 (0 to 0)	0 (0 to 0·1)	0 (0 to 0)	0 (0 to 0)	0 (0 to 0)	0 (0 to 0)	0 (0 to 0)	0 (0 to 0)	1·7
Trichomoniasis	0·6 (0·2 to 1·6)	0·6 (0·2 to 1·5)	0·5 (0·2 to 1·2)	0·3 (0·1 to 0·8)	0·2 (0·1 to 0·4)	0·1 (0 to 0·3)	0·1 (0 to 0·2)	0·1 (0 to 0·1)	0 (0 to 0)	0 (0 to 0)	0 (0 to 0)	0 (0 to 0)	5·2
Genital herpes	0·6 (0·2 to 1·6)	0·7 (0·2 to 1·8)	0·7 (0·2 to 1·8)	0·6 (0·2 to 1·4)	0·4 (0·1 to 0·9)	0·3 (0·1 to 0·7)	0·2 (0·1 to 0·5)	0·1 (0 to 0·3)	0·1 (0 to 0·1)	0 (0 to 0·1)	0 (0 to 0)	0 (0 to 0)	6
Other sexually transmitted infections	0·7 (0·4 to 1·1)	0·5 (0·3 to 0·9)	0·2 (0·1 to 0·3)	0·1 (0·1 to 0·2)	0 (0 to 0·1)	0 (0 to 0)	0 (0 to 0)	0 (0 to 0)	0 (0 to 0)	0 (0 to 0)	0 (0 to 0)	0 (0 to 0)	4·4
Respiratory infections and tuberculosis	37·8 (27·3 to 50·5)	51 (37·2 to 68·2)	64·3 (47·2 to 86·5)	75·2 (54·8 to 103)	81·1 (62·9 to 102)	91·9 (67·3 to 124)	108 (75·8 to 146)	115 (92·6 to 145)	74·6 (56·5 to 96)	48·7 (38·2 to 59·9)	28 (21 to 34)	14 (9·9 to 17·9)	1,200
Tuberculosis	2·4 (1·6 to 3·4)	2·5 (1·7 to 3·4)	2·4 (1·7 to 3·4)	1·9 (1·4 to 2·7)	1·6 (1·2 to 2·1)	1·5 (1·1 to 2·1)	1·5 (1·1 to 2)	1·1 (0·8 to 1·4)	0·4 (0·3 to 0·5)	0·2 (0·1 to 0·2)	0·1 (0 to 0·1)	0 (0 to 0)	28·6
Latent tuberculosis infection	0 (0 to 0)	0 (0 to 0)	0 (0 to 0)	0 (0 to 0)	0 (0 to 0)	0 (0 to 0)	0 (0 to 0)	0 (0 to 0)	0 (0 to 0)	0 (0 to 0)	0 (0 to 0)	0 (0 to 0)	0
Drug-susceptible tuberculosis	2·4 (1·6 to 3·4)	2·4 (1·7 to 3·3)	2·4 (1·6 to 3·3)	1·9 (1·3 to 2·6)	1·6 (1·1 to 2·1)	1·5 (1·1 to 2·1)	1·5 (1·1 to 2)	1·1 (0·8 to 1·4)	0·4 (0·3 to 0·5)	0·2 (0·1 to 0·2)	0·1 (0 to 0·1)	0 (0 to 0)	28·2
Multidrug-resistant tuberculosis without extensive drug resistanc	0 (0 to 0·2)	0 (0 to 0·2)	0 (0 to 0·2)	0 (0 to 0·1)	0 (0 to 0·1)	0 (0 to 0·1)	0 (0 to 0·1)	0 (0 to 0·1)	0 (0 to 0)	0 (0 to 0)	0 (0 to 0)	0 (0 to 0)	0·4
Extensively drug-resistant tuberculosis	0 (0 to 0)	0 (0 to 0)	0 (0 to 0)	0 (0 to 0)	0 (0 to 0)	0 (0 to 0)	0 (0 to 0)	0 (0 to 0)	0 (0 to 0)	0 (0 to 0)	0 (0 to 0)	0 (0 to 0)	0·1
Lower respiratory infections	29·3 (20 to 42)	42·1 (28·8 to 59·2)	55·5 (39·2 to 77·5)	68·3 (48·8 to 95·7)	76·1 (58·3 to 96·9)	88 (63·4 to 120)	104 (72·8 to 143)	113 (90·5 to 143)	73·7 (55·7 to 95·2)	48·3 (37·9 to 59·6)	27·9 (20·9 to 33·9)	13·9 (9·9 to 17·9)	1,060
Upper respiratory infections	4·7 (2·5 to 8·3)	4·9 (2·6 to 8·4)	4·7 (2·6 to 8)	3·7 (2 to 6·2)	2·6 (1·4 to 4·3)	1·9 (1 to 3·2)	1·4 (0·8 to 2·3)	0·9 (0·5 to 1·6)	0·4 (0·2 to 0·6)	0·2 (0·1 to 0·3)	0·1 (0 to 0·1)	0 (0 to 0)	82·5
Otitis media	1·3 (0·6 to 2·5)	1·6 (0·8 to 2·8)	1·7 (0·8 to 3·1)	1·3 (0·6 to 2·3)	0·8 (0·4 to 1·6)	0·5 (0·2 to 0·9)	0·3 (0·1 to 0·6)	0·2 (0·1 to 0·4)	0·1 (0 to 0·2)	0 (0 to 0·1)	0 (0 to 0)	0 (0 to 0)	27
Enteric infections	10·8 (7·4 to 14·6)	13 (9·2 to 18)	14·3 (10·2 to 20·1)	13·1 (9·2 to 18·3)	12 (8·8 to 16·1)	12·4 (9·1 to 16·4)	12·9 (9·6 to 16·8)	11·8 (8·7 to 15·2)	6·1 (4·6 to 8·1)	2·9 (2·2 to 3·7)	1·4 (1 to 1·8)	0·5 (0·4 to 0·7)	242
Diarrheal diseases	10·8 (7·4 to 14·6)	12·9 (9·2 to 17·9)	14·2 (10·2 to 20·1)	13·1 (9·2 to 18·3)	11·9 (8·8 to 16·1)	12·4 (9·1 to 16·4)	12·9 (9·6 to 16·8)	11·7 (8·7 to 15·1)	6·1 (4·6 to 8·1)	2·9 (2·2 to 3·7)	1·4 (1 to 1·8)	0·5 (0·4 to 0·7)	242
Typhoid and paratyphoid	0 (0 to 0)	0 (0 to 0)	0 (0 to 0)	0 (0 to 0)	0 (0 to 0)	0 (0 to 0)	0 (0 to 0)	0 (0 to 0)	0 (0 to 0)	0 (0 to 0)	0 (0 to 0)	0 (0 to 0)	0·1
Typhoid fever	0 (0 to 0)	0 (0 to 0)	0 (0 to 0)	0 (0 to 0)	0 (0 to 0)	0 (0 to 0)	0 (0 to 0)	0 (0 to 0)	0 (0 to 0)	0 (0 to 0)	0 (0 to 0)	0 (0 to 0)	0
Paratyphoid fever	0 (0 to 0)	0 (0 to 0)	0 (0 to 0)	0 (0 to 0)	0 (0 to 0)	0 (0 to 0)	0 (0 to 0)	0 (0 to 0)	0 (0 to 0)	0 (0 to 0)	0 (0 to 0)	0 (0 to 0)	0
Invasive Non-typhoidal Salmonella (iNTS)	0 (0 to 0)	0 (0 to 0)	0 (0 to 0)	0 (0 to 0)	0 (0 to 0)	0 (0 to 0)	0 (0 to 0)	0 (0 to 0)	0 (0 to 0)	0 (0 to 0)	0 (0 to 0)	0 (0 to 0)	0·1
Other intestinal infectious diseases	0 (0 to 0)	0 (0 to 0)	0 (0 to 0)	0 (0 to 0)	0 (0 to 0)	0 (0 to 0)	0 (0 to 0)	0 (0 to 0)	0 (0 to 0)	0 (0 to 0)	0 (0 to 0)	0 (0 to 0)	0·5
Neglected tropical diseases and malaria	5·7 (3·1 to 9·8)	6·6 (3·6 to 11·6)	7·7 (3·9 to 13·8)	8·3 (4·3 to 13·9)	7·3 (3·8 to 12·8)	6·6 (3·2 to 11·7)	6·4 (3·4 to 11·1)	5·3 (2·6 to 8·9)	2·4 (1·2 to 4·2)	1·1 (0·6 to 1·9)	0·5 (0·3 to 0·9)	0·2 (0·1 to 0·4)	107
Malaria	0 (0 to 0)	0 (0 to 0)	0 (0 to 0)	0 (0 to 0)	0 (0 to 0)	0 (0 to 0)	0 (0 to 0)	0 (0 to 0)	0 (0 to 0)	0 (0 to 0)	0 (0 to 0)	0 (0 to 0)	0
Chagas disease	0 (0 to 0)	0 (0 to 0)	0 (0 to 0)	0 (0 to 0·1)	0 (0 to 0·1)	0 (0 to 0)	0 (0 to 0·1)	0 (0 to 0·1)	0 (0 to 0·1)	0 (0 to 0)	0 (0 to 0)	0 (0 to 0)	0·2
Leishmaniasis	0 (0 to 0)	0 (0 to 0)	0 (0 to 0)	0 (0 to 0)	0 (0 to 0)	0 (0 to 0)	0 (0 to 0)	0 (0 to 0)	0 (0 to 0)	0 (0 to 0)	0 (0 to 0)	0 (0 to 0)	0
Visceral leishmaniasis	0 (0 to 0)	0 (0 to 0)	0 (0 to 0)	0 (0 to 0)	0 (0 to 0)	0 (0 to 0)	0 (0 to 0)	0 (0 to 0)	0 (0 to 0)	0 (0 to 0)	0 (0 to 0)	0 (0 to 0)	0
Cutaneous and mucocutaneous leishmaniasis	0 (0 to 0)	0 (0 to 0)	0 (0 to 0)	0 (0 to 0)	0 (0 to 0)	0 (0 to 0)	0 (0 to 0)	0 (0 to 0)	0 (0 to 0)	0 (0 to 0)	0 (0 to 0)	0 (0 to 0)	0
African trypanosomiasis	0 (0 to 0)	0 (0 to 0)	0 (0 to 0)	0 (0 to 0)	0 (0 to 0)	0 (0 to 0)	0 (0 to 0)	0 (0 to 0)	0 (0 to 0)	0 (0 to 0)	0 (0 to 0)	0 (0 to 0)	0
Schistosomiasis	0 (0 to 0)	0 (0 to 0)	0 (0 to 0)	0 (0 to 0)	0 (0 to 0)	0 (0 to 0)	0 (0 to 0)	0 (0 to 0)	0 (0 to 0)	0 (0 to 0)	0 (0 to 0)	0 (0 to 0)	0
Cysticercosis	3·3 (1·1 to 7)	4·4 (1·6 to 9·3)	5·5 (1·9 to 11·1)	6·1 (2·4 to 11·5)	5·9 (2·5 to 11·1)	5·6 (2·3 to 10·5)	5·4 (2·5 to 10·1)	4·5 (1·8 to 8·1)	2·1 (0·9 to 3·9)	0·9 (0·4 to 1·7)	0·4 (0·2 to 0·8)	0·1 (0·1 to 0·3)	54·1
Cystic echinococcosis	0 (0 to 0)	0 (0 to 0)	0 (0 to 0)	0 (0 to 0)	0 (0 to 0)	0 (0 to 0)	0 (0 to 0)	0 (0 to 0)	0 (0 to 0)	0 (0 to 0)	0 (0 to 0)	0 (0 to 0)	0
Lymphatic filariasis	0 (0 to 0)	0 (0 to 0)	0 (0 to 0)	0 (0 to 0)	0 (0 to 0)	0 (0 to 0)	0 (0 to 0)	0 (0 to 0)	0 (0 to 0)	0 (0 to 0)	0 (0 to 0)	0 (0 to 0)	0
Onchocerciasis	0 (0 to 0)	0 (0 to 0)	0 (0 to 0)	0 (0 to 0)	0 (0 to 0)	0 (0 to 0)	0 (0 to 0)	0 (0 to 0)	0 (0 to 0)	0 (0 to 0)	0 (0 to 0)	0 (0 to 0)	0
Trachoma	0 (0 to 0)	0 (0 to 0)	0 (0 to 0)	0 (0 to 0)	0 (0 to 0)	0 (0 to 0)	0 (0 to 0)	0 (0 to 0)	0 (0 to 0)	0 (0 to 0)	0 (0 to 0)	0 (0 to 0)	0
Dengue	0·7 (0·3 to 1·3)	0·7 (0·3 to 1·3)	0·7 (0·3 to 1·4)	0·8 (0·3 to 1·8)	0·5 (0·2 to 1)	0·3 (0·1 to 0·7)	0·3 (0·1 to 0·5)	0·2 (0 to 0·4)	0·1 (0 to 0·2)	0 (0 to 0·1)	0 (0 to 0·1)	0 (0 to 0·1)	14·2
Yellow fever	0 (0 to 0)	0 (0 to 0)	0 (0 to 0)	0 (0 to 0)	0 (0 to 0)	0 (0 to 0)	0 (0 to 0)	0 (0 to 0)	0 (0 to 0)	0 (0 to 0)	0 (0 to 0)	0 (0 to 0)	0
Rabies	0 (0 to 0)	0 (0 to 0)	0 (0 to 0)	0 (0 to 0)	0 (0 to 0)	0 (0 to 0)	0 (0 to 0)	0 (0 to 0)	0 (0 to 0)	0 (0 to 0)	0 (0 to 0)	0 (0 to 0)	0·1
Intestinal nematode infections	0·5 (0·2 to 0·8)	0·2 (0·1 to 0·3)	0·2 (0·1 to 0·3)	0·1 (0·1 to 0·3)	0·1 (0·1 to 0·2)	0·1 (0 to 0·1)	0·1 (0 to 0·1)	0·1 (0 to 0·1)	0 (0 to 0)	0 (0 to 0)	0 (0 to 0)	0 (0 to 0)	6·3
Ascariasis	0 (0 to 0·1)	0 (0 to 0·1)	0 (0 to 0·1)	0 (0 to 0·1)	0 (0 to 0·1)	0 (0 to 0)	0 (0 to 0·1)	0 (0 to 0)	0 (0 to 0)	0 (0 to 0)	0 (0 to 0)	0 (0 to 0)	0·9
Trichuriasis	0 (0 to 0)	0 (0 to 0)	0 (0 to 0)	0 (0 to 0)	0 (0 to 0)	0 (0 to 0)	0 (0 to 0)	0 (0 to 0)	0 (0 to 0)	0 (0 to 0)	0 (0 to 0)	0 (0 to 0)	0
Hookworm disease	0·4 (0·2 to 0·7)	0·1 (0 to 0·2)	0·1 (0 to 0·2)	0·1 (0 to 0·2)	0·1 (0 to 0·2)	0·1 (0 to 0·1)	0 (0 to 0·1)	0 (0 to 0·1)	0 (0 to 0)	0 (0 to 0)	0 (0 to 0)	0 (0 to 0)	5·3
Food-borne trematodiases	0 (0 to 0)	0 (0 to 0)	0 (0 to 0)	0 (0 to 0)	0 (0 to 0)	0 (0 to 0)	0 (0 to 0)	0 (0 to 0)	0 (0 to 0)	0 (0 to 0)	0 (0 to 0)	0 (0 to 0)	0
Leprosy	0 (0 to 0·1)	0·1 (0 to 0·1)	0·1 (0 to 0·2)	0·1 (0 to 0·1)	0·1 (0 to 0·1)	0·1 (0 to 0·1)	0 (0 to 0·1)	0 (0 to 0·1)	0 (0 to 0)	0 (0 to 0)	0 (0 to 0)	0 (0 to 0)	0·6
Ebola	0 (0 to 0)	0 (0 to 0)	0 (0 to 0)	0 (0 to 0)	0 (0 to 0)	0 (0 to 0)	0 (0 to 0)	0 (0 to 0)	0 (0 to 0)	0 (0 to 0)	0 (0 to 0)	0 (0 to 0)	0
Zika virus	0·2 (0 to 1)	0·1 (0 to 0·7)	0·1 (0 to 0·6)	0·1 (0 to 0·4)	0·1 (0 to 0·2)	0 (0 to 0·1)	0 (0 to 0·1)	0 (0 to 0)	0 (0 to 0)	0 (0 to 0)	0 (0 to 0)	0 (0 to 0)	3·1
Guinea worm disease	0 (0 to 0)	0 (0 to 0)	0 (0 to 0)	0 (0 to 0)	0 (0 to 0)	0 (0 to 0)	0 (0 to 0)	0 (0 to 0)	0 (0 to 0)	0 (0 to 0)	0 (0 to 0)	0 (0 to 0)	0
Other neglected tropical diseases	1 (0·5 to 1·9)	1·1 (0·6 to 2·1)	1·1 (0·5 to 1·9)	1·1 (0·5 to 2)	0·6 (0·3 to 1·2)	0·5 (0·3 to 0·9)	0·6 (0·3 to 1·1)	0·5 (0·3 to 1)	0·2 (0·1 to 0·4)	0·1 (0·1 to 0·2)	0·1 (0 to 0·2)	0·1 (0 to 0·2)	28
Other infectious diseases	7·3 (5·5 to 9·5)	9·6 (7·2 to 12·9)	10·3 (8 to 14·1)	9·7 (7·4 to 13·3)	7·6 (5·8 to 9·9)	5·6 (4·2 to 7·3)	4·5 (3·3 to 5·7)	2·9 (2·1 to 3·6)	1·3 (1 to 1·6)	0·7 (0·5 to 0·9)	0·3 (0·2 to 0·3)	0·1 (0·1 to 0·2)	139
Meningitis	1 (0·8 to 1·3)	1·3 (1 to 1·6)	1·4 (1·1 to 1·8)	1·3 (1 to 1·6)	1·1 (0·9 to 1·4)	0·9 (0·7 to 1·2)	0·7 (0·6 to 0·9)	0·6 (0·4 to 0·7)	0·2 (0·2 to 0·3)	0·1 (0·1 to 0·1)	0 (0 to 0)	0 (0 to 0)	25·4
Encephalitis	1·1 (0·8 to 1·7)	1·2 (0·9 to 1·8)	1·1 (0·8 to 1·7)	1·1 (0·8 to 1·7)	0·8 (0·6 to 1·3)	0·6 (0·5 to 0·9)	0·5 (0·4 to 0·7)	0·3 (0·2 to 0·4)	0·1 (0·1 to 0·2)	0 (0 to 0·1)	0 (0 to 0)	0 (0 to 0)	26·2
Diphtheria	0 (0 to 0)	0 (0 to 0)	0 (0 to 0)	0 (0 to 0)	0 (0 to 0)	0 (0 to 0)	0 (0 to 0)	0 (0 to 0)	0 (0 to 0)	0 (0 to 0)	0 (0 to 0)	0 (0 to 0)	0
Tetanus	0·2 (0·1 to 0·5)	0·3 (0·1 to 0·9)	0·5 (0·2 to 1·4)	0·5 (0·2 to 1·7)	0·4 (0·2 to 1·2)	0·3 (0·1 to 1)	0·2 (0·1 to 0·7)	0·2 (0·1 to 0·5)	0·1 (0 to 0·2)	0 (0 to 0)	0 (0 to 0)	0 (0 to 0)	3·3
Measles	0 (0 to 0)	0 (0 to 0)	0 (0 to 0)	0 (0 to 0)	0 (0 to 0)	0 (0 to 0)	0 (0 to 0)	0 (0 to 0)	0 (0 to 0)	0 (0 to 0)	0 (0 to 0)	0 (0 to 0)	0
Varicella and herpes zoster	0·3 (0·2 to 0·7)	0·4 (0·2 to 0·8)	0·5 (0·3 to 0·8)	0·4 (0·2 to 0·6)	0·3 (0·2 to 0·6)	0·3 (0·2 to 0·5)	0·3 (0·2 to 0·6)	0·3 (0·2 to 0·5)	0·1 (0·1 to 0·3)	0·1 (0 to 0·2)	0 (0 to 0·1)	0 (0 to 0·1)	6·3
Acute hepatitis	0·5 (0·3 to 0·6)	0·5 (0·3 to 0·6)	0·5 (0·4 to 0·6)	0·4 (0·3 to 0·5)	0·3 (0·2 to 0·3)	0·2 (0·1 to 0·2)	0·1 (0·1 to 0·2)	0·1 (0·1 to 0·1)	0 (0 to 0)	0 (0 to 0)	0 (0 to 0)	0 (0 to 0)	7·7
Acute hepatitis A	0·2 (0·1 to 0·3)	0·2 (0·1 to 0·3)	0·1 (0·1 to 0·2)	0·1 (0 to 0·1)	0 (0 to 0·1)	0 (0 to 0·1)	0 (0 to 0)	0 (0 to 0)	0 (0 to 0)	0 (0 to 0)	0 (0 to 0)	0 (0 to 0)	3·9
Acute hepatitis B	0·2 (0·2 to 0·3)	0·2 (0·2 to 0·4)	0·3 (0·2 to 0·4)	0·3 (0·2 to 0·3)	0·2 (0·1 to 0·2)	0·1 (0·1 to 0·2)	0·1 (0·1 to 0·1)	0 (0 to 0·1)	0 (0 to 0)	0 (0 to 0)	0 (0 to 0)	0 (0 to 0)	2·9
Acute hepatitis C	0 (0 to 0)	0 (0 to 0)	0 (0 to 0)	0 (0 to 0)	0 (0 to 0)	0 (0 to 0)	0 (0 to 0)	0 (0 to 0)	0 (0 to 0)	0 (0 to 0)	0 (0 to 0)	0 (0 to 0)	0·2
Acute hepatitis E	0 (0 to 0·1)	0·1 (0 to 0·1)	0 (0 to 0·1)	0 (0 to 0·1)	0·1 (0 to 0·1)	0 (0 to 0)	0 (0 to 0)	0 (0 to 0)	0 (0 to 0)	0 (0 to 0)	0 (0 to 0)	0 (0 to 0)	0·7
Other unspecified infectious diseases	4·2 (2·6 to 6·1)	5·9 (3·7 to 8·8)	6·4 (4·3 to 9·4)	6·1 (4 to 9·1)	4·7 (2·9 to 6·6)	3·3 (1·9 to 4·8)	2·6 (1·4 to 3·7)	1·5 (0·8 to 2·2)	0·7 (0·4 to 1)	0·5 (0·3 to 0·6)	0·2 (0·1 to 0·2)	0·1 (0 to 0·1)	68·2
Maternal and neonatal disorders	22·1 (17 to 27·6)	20·2 (15 to 25·7)	17·8 (13·4 to 23)	12·7 (9·6 to 16·7)	7·5 (5·5 to 9·8)	3·9 (2·9 to 5·4)	1·8 (1·3 to 2·5)	0·7 (0·5 to 1)	0·1 (0·1 to 0·2)	0 (0 to 0)	0 (0 to 0)	0 (0 to 0)	1,300
Maternal disorders	3 (2·4 to 3·8)	0·7 (0·6 to 1)	0·1 (0·1 to 0·1)	0 (0 to 0)	0 (0 to 0)	0 (0 to 0)	0 (0 to 0)	0 (0 to 0)	0 (0 to 0)	0 (0 to 0)	0 (0 to 0)	0 (0 to 0)	55·8
Maternal hemorrhage	0·5 (0·3 to 0·8)	0·1 (0 to 0·1)	0 (0 to 0)	0 (0 to 0)	0 (0 to 0)	0 (0 to 0)	0 (0 to 0)	0 (0 to 0)	0 (0 to 0)	0 (0 to 0)	0 (0 to 0)	0 (0 to 0)	8
Maternal sepsis and other maternal infections	0·1 (0 to 0·1)	0 (0 to 0)	0 (0 to 0)	0 (0 to 0)	0 (0 to 0)	0 (0 to 0)	0 (0 to 0)	0 (0 to 0)	0 (0 to 0)	0 (0 to 0)	0 (0 to 0)	0 (0 to 0)	1·4
Maternal hypertensive disorders	0·8 (0·6 to 1·1)	0·2 (0·1 to 0·3)	0 (0 to 0)	0 (0 to 0)	0 (0 to 0)	0 (0 to 0)	0 (0 to 0)	0 (0 to 0)	0 (0 to 0)	0 (0 to 0)	0 (0 to 0)	0 (0 to 0)	20·2
Maternal obstructed labor and uterine rupture	0·1 (0·1 to 0·2)	0 (0 to 0)	0 (0 to 0)	0 (0 to 0)	0 (0 to 0)	0 (0 to 0)	0 (0 to 0)	0 (0 to 0)	0 (0 to 0)	0 (0 to 0)	0 (0 to 0)	0 (0 to 0)	1·3
Maternal abortion and miscarriage	0·2 (0·1 to 0·3)	0·1 (0 to 0·2)	0 (0 to 0)	0 (0 to 0)	0 (0 to 0)	0 (0 to 0)	0 (0 to 0)	0 (0 to 0)	0 (0 to 0)	0 (0 to 0)	0 (0 to 0)	0 (0 to 0)	2·8
Ectopic pregnancy	0·1 (0·1 to 0·1)	0 (0 to 0·1)	0 (0 to 0)	0 (0 to 0)	0 (0 to 0)	0 (0 to 0)	0 (0 to 0)	0 (0 to 0)	0 (0 to 0)	0 (0 to 0)	0 (0 to 0)	0 (0 to 0)	2·2
Indirect maternal deaths	0·1 (0·1 to 0·1)	0 (0 to 0)	0 (0 to 0)	--	--	--	--	--	--	--	--	--	NA
Late maternal deaths	0·2 (0·1 to 0·3)	0 (0 to 0·1)	0 (0 to 0)	--	--	--	--	--	--	--	--	--	NA
Maternal deaths aggravated by HIV/AIDS	0 (0 to 0)	0 (0 to 0)	0 (0 to 0)	--	--	--	--	--	--	--	--	--	NA
Neonatal disorders	19·1 (14·1 to 24·4)	19·5 (14·4 to 24·9)	17·7 (13·3 to 22·9)	12·7 (9·6 to 16·7)	7·5 (5·5 to 9·8)	3·9 (2·9 to 5·4)	1·8 (1·3 to 2·5)	0·7 (0·5 to 1)	0·1 (0·1 to 0·2)	0 (0 to 0)	0 (0 to 0)	0 (0 to 0)	1,250
Neonatal preterm birth	7·6 (5·5 to 9·9)	7·5 (5·5 to 9·8)	6·5 (4·8 to 8·6)	4·4 (3·2 to 5·8)	2·4 (1·7 to 3·1)	1·1 (0·9 to 1·5)	0·5 (0·4 to 0·7)	0·2 (0·1 to 0·3)	0 (0 to 0·1)	0 (0 to 0)	0 (0 to 0)	0 (0 to 0)	504
Neonatal encephalopathy due to birth asphyxia and trauma	2·7 (1·6 to 4·2)	2·8 (1·7 to 4·3)	2·6 (1·5 to 4)	1·9 (1·1 to 3·1)	1·2 (0·6 to 1·9)	0·6 (0·4 to 0·9)	0·2 (0·1 to 0·3)	0 (0 to 0·1)	0 (0 to 0)	0 (0 to 0)	0 (0 to 0)	0 (0 to 0)	332
Neonatal sepsis and other neonatal infections	7·8 (5 to 11·1)	8·1 (5·1 to 11·9)	7·5 (4·9 to 11)	5·6 (3·6 to 8·4)	3·4 (2·1 to 5·1)	1·9 (1·2 to 3)	1 (0·6 to 1·6)	0·4 (0·2 to 0·6)	0·1 (0 to 0·1)	0 (0 to 0)	0 (0 to 0)	0 (0 to 0)	278
Hemolytic disease and other neonatal jaundice	1·1 (0·8 to 1·4)	1·1 (0·8 to 1·5)	1·1 (0·8 to 1·4)	0·8 (0·6 to 1)	0·5 (0·4 to 0·6)	0·3 (0·2 to 0·4)	0·2 (0·1 to 0·2)	0·1 (0 to 0·1)	0 (0 to 0)	0 (0 to 0)	0 (0 to 0)	0 (0 to 0)	20·3
Other neonatal disorders	0 (0 to 0)	0 (0 to 0)	0 (0 to 0)	0 (0 to 0)	0 (0 to 0)	0 (0 to 0)	0 (0 to 0)	0 (0 to 0)	0 (0 to 0)	0 (0 to 0)	0 (0 to 0)	0 (0 to 0)	111
Nutritional deficiencies	20·9 (10·4 to 36·1)	20·8 (11·1 to 36·9)	23·4 (12·8 to 39)	23·4 (13·2 to 38·7)	17·6 (10·4 to 27·2)	12·2 (8·2 to 18·4)	11·4 (7·8 to 17)	9·5 (6·7 to 13·8)	4·9 (3·4 to 7)	2·8 (1·9 to 4·2)	1·7 (1·1 to 2·3)	0·7 (0·5 to 0·9)	405
Protein-energy malnutrition	3·1 (2·2 to 4)	4·2 (3 to 5·5)	5·1 (3·8 to 6·9)	5·4 (3·9 to 7·2)	5·4 (3·9 to 7·2)	5·6 (4·1 to 7·2)	6 (4·2 to 8·3)	5·4 (3·9 to 7)	2·8 (2 to 3·8)	1·6 (1·1 to 2·2)	1 (0·7 to 1·4)	0·5 (0·4 to 0·7)	87·5
Iodine deficiency	0·4 (0·2 to 0·7)	0·5 (0·2 to 0·8)	0·4 (0·2 to 0·8)	0·3 (0·2 to 0·6)	0·2 (0·1 to 0·4)	0·2 (0·1 to 0·3)	0·1 (0·1 to 0·2)	0·1 (0 to 0·1)	0 (0 to 0·1)	0 (0 to 0)	0 (0 to 0)	0 (0 to 0)	5·2
Vitamin A deficiency	0·1 (0·1 to 0·2)	0·1 (0·1 to 0·2)	0·1 (0·1 to 0·2)	0·1 (0·1 to 0·2)	0·1 (0 to 0·1)	0 (0 to 0·1)	0 (0 to 0·1)	0 (0 to 0·1)	0 (0 to 0)	0 (0 to 0)	0 (0 to 0)	0 (0 to 0)	6·1
Dietary iron deficiency	16·5 (6·6 to 31·4)	15·1 (5·8 to 30·6)	16·4 (6·5 to 31·4)	16·4 (6·7 to 31)	10·9 (4·3 to 20·3)	5·5 (2 to 11·5)	4·4 (1·7 to 9·2)	3·3 (1·2 to 7·1)	1·7 (0·6 to 3·4)	1 (0·4 to 2·1)	0·5 (0·2 to 1)	0·1 (0 to 0·2)	292
Other nutritional deficiencies	0·8 (0·5 to 1·2)	0·9 (0·6 to 1·4)	1·4 (0·9 to 1·9)	1·1 (0·8 to 1·6)	0·9 (0·6 to 1·4)	0·9 (0·6 to 1·4)	0·8 (0·5 to 1·3)	0·7 (0·4 to 1)	0·4 (0·2 to 0·6)	0·2 (0·1 to 0·4)	0·1 (0·1 to 0·2)	0·1 (0 to 0·1)	14
Non-communicable diseases	1,270 (1,070 to 1,480)	1,820 (1,570 to 2,120)	2,450 (2,140 to 2,780)	2,780 (2,480 to 3,110)	2,700 (2,450 to 2,980)	2,660 (2,440 to 2,900)	2,490 (2,290 to 2,690)	2,070 (1,920 to 2,240)	1,050 (975 to 1,120)	498 (465 to 533)	255 (239 to 273)	96·5 (90·2 to 104)	25,800
Neoplasms	201 (165 to 244)	327 (274 to 385)	501 (424 to 579)	620 (532 to 711)	635 (555 to 721)	621 (553 to 699)	593 (518 to 661)	458 (394 to 515)	190 (165 to 213)	73·6 (62·5 to 82·4)	28 (23·2 to 31·9)	7·7 (5·9 to 9)	4,770
Lip and oral cavity cancer	3·1 (2 to 4·6)	6·9 (4·6 to 10·2)	11·1 (7·9 to 15·4)	14·1 (10·1 to 19·3)	12·5 (9·2 to 16·2)	11·4 (8·6 to 15)	8·7 (6·6 to 11·2)	6·2 (4·8 to 8)	2·3 (1·7 to 3)	0·7 (0·5 to 0·9)	0·3 (0·2 to 0·4)	0·1 (0·1 to 0·1)	81·1
Nasopharynx cancer	1 (0·7 to 1·3)	1·8 (1·3 to 2·5)	2·8 (2 to 3·9)	3·4 (2·5 to 4·7)	2·5 (1·8 to 3·4)	2·1 (1·5 to 2·8)	1·5 (1·1 to 2·1)	0·8 (0·6 to 1·1)	0·3 (0·2 to 0·4)	0·2 (0·1 to 0·2)	0·1 (0 to 0·1)	0 (0 to 0)	19
Other pharynx cancer	1·5 (0·9 to 2·1)	4·1 (2·7 to 5·9)	6·3 (4·6 to 8·6)	8·3 (5·7 to 12)	7·3 (5 to 10·2)	6·1 (3·9 to 8·8)	4·3 (2·9 to 6·5)	2·6 (1·7 to 3·8)	1 (0·6 to 1·4)	0·3 (0·3 to 0·4)	0·2 (0·1 to 0·2)	0 (0 to 0·1)	43·5
Esophageal cancer	6·5 (3·8 to 9·5)	16·4 (11·4 to 22·8)	26·7 (20 to 35·4)	34·6 (25·4 to 46·5)	32 (24·2 to 41·1)	28·5 (20·7 to 37·3)	22·8 (16·9 to 30·8)	14·2 (10·1 to 19·3)	4·9 (3·5 to 6·8)	1·6 (1·3 to 2)	0·5 (0·4 to 0·7)	0·1 (0·1 to 0·2)	193
Stomach cancer	9·9 (7·2 to 13·6)	16·7 (11·7 to 22·9)	27·1 (21·1 to 34·3)	33·7 (26·7 to 42·1)	36·2 (29·5 to 43·5)	37·4 (29·2 to 48·2)	33·9 (26·1 to 43)	26·5 (21·3 to 32·9)	11·8 (8·9 to 15·3)	5·1 (4 to 6·2)	2 (1·5 to 2·7)	0·6 (0·4 to 0·8)	256
Colon and rectum cancer	16·1 (11·8 to 21·8)	28·1 (20·8 to 36·7)	46 (35·1 to 59·6)	60·9 (47·8 to 75·8)	68·4 (54·3 to 85)	69·7 (55·7 to 86)	62·3 (51·2 to 75·3)	48·6 (39·9 to 58·5)	23 (18·4 to 28·5)	10·5 (8 to 13·3)	4·3 (3·2 to 5·7)	1·2 (0·8 to 1·6)	467
Liver cancer	3·2 (2 to 4·7)	5·6 (3·6 to 8·1)	9·1 (6·3 to 12·6)	12·8 (9·3 to 17·6)	12·4 (9·2 to 16·2)	12·2 (9·4 to 16·1)	10·9 (8·4 to 14)	8·1 (6·2 to 10·2)	3·2 (2·5 to 4)	0·9 (0·7 to 1·2)	0·3 (0·2 to 0·4)	0·1 (0·1 to 0·1)	85
Liver cancer due to hepatitis B	1·5 (0·8 to 2·5)	2·4 (1·3 to 4)	3·4 (1·8 to 5·5)	4·3 (2·4 to 7·1)	3·6 (1·9 to 5·9)	2·8 (1·5 to 4·7)	2 (1 to 3·5)	1·3 (0·7 to 2·2)	0·4 (0·2 to 0·8)	0·1 (0·1 to 0·2)	0 (0 to 0·1)	0 (0 to 0)	24·7
Liver cancer due to hepatitis C	0·3 (0·1 to 0·5)	0·6 (0·2 to 1·1)	1·3 (0·6 to 2·2)	2·1 (1 to 3·9)	2·3 (1·1 to 4)	2·4 (1·4 to 3·8)	2·5 (1·4 to 3·9)	2·2 (1·2 to 3·4)	1·1 (0·7 to 1·7)	0·4 (0·3 to 0·6)	0·2 (0·1 to 0·2)	0 (0 to 0·1)	15·5
Liver cancer due to alcohol use	0·9 (0·4 to 1·7)	1·8 (0·9 to 3·3)	3·2 (1·6 to 5·3)	4·8 (2·6 to 7·8)	5 (2·9 to 7·5)	5·5 (3·6 to 8)	5 (3·1 to 7·3)	3·3 (2·2 to 4·6)	1·1 (0·6 to 1·5)	0·2 (0·1 to 0·4)	0·1 (0 to 0·1)	0 (0 to 0)	31·9
Liver cancer due to NASH	0·2 (0·1 to 0·4)	0·4 (0·2 to 0·7)	0·6 (0·3 to 1·1)	0·9 (0·4 to 1·5)	0·9 (0·5 to 1·5)	0·9 (0·5 to 1·6)	1 (0·6 to 1·6)	0·9 (0·5 to 1·5)	0·4 (0·2 to 0·7)	0·1 (0·1 to 0·2)	0 (0 to 0·1)	0 (0 to 0)	6·7
Liver cancer due to other causes	0·3 (0·1 to 0·5)	0·4 (0·2 to 0·7)	0·6 (0·3 to 1)	0·7 (0·4 to 1·3)	0·6 (0·3 to 1·1)	0·5 (0·3 to 0·9)	0·5 (0·2 to 0·9)	0·4 (0·2 to 0·7)	0·2 (0·1 to 0·3)	0 (0 to 0·1)	0 (0 to 0)	0 (0 to 0)	6·2
Gallbladder and biliary tract cancer	1·4 (0·9 to 2·1)	2·6 (1·8 to 4·1)	4·8 (3·4 to 7·5)	6·5 (4·7 to 10·6)	7 (5 to 10·9)	6·7 (4·9 to 10·2)	6·2 (4·5 to 9·3)	4·5 (3·4 to 6·8)	1·9 (1·4 to 2·9)	0·7 (0·5 to 1·1)	0·3 (0·2 to 0·4)	0·1 (0 to 0·1)	44
Pancreatic cancer	8·5 (6 to 11·6)	17·1 (11·7 to 23·9)	27·1 (20·6 to 35·1)	36·3 (28 to 46·4)	38·3 (30 to 47·7)	38·3 (28·2 to 51·2)	33·9 (24·5 to 45·2)	24 (17·7 to 31·9)	10·1 (7·1 to 13·9)	3·9 (3 to 5)	1·5 (1 to 2)	0·4 (0·3 to 0·5)	248
Larynx cancer	0·9 (0·5 to 1·5)	2·6 (1·7 to 3·9)	4·6 (3·2 to 6·3)	6 (4·4 to 8·2)	6·2 (4·5 to 8·3)	6 (4·3 to 8)	4·5 (3·4 to 6·1)	3 (2·2 to 4)	1 (0·8 to 1·3)	0·3 (0·2 to 0·4)	0·1 (0·1 to 0·1)	0 (0 to 0)	35·9
Tracheal, bronchus, and lung cancer	12·1 (8·7 to 16·5)	27·7 (20·7 to 36)	47·9 (35·3 to 62·5)	66·8 (48·7 to 87·9)	65·7 (50·4 to 83·7)	61·4 (50·5 to 74·5)	52·1 (42·6 to 62·7)	34·1 (27·6 to 41·5)	11·8 (9·3 to 14·8)	4 (3·1 to 5)	1·3 (1 to 1·7)	0·4 (0·2 to 0·5)	398
Malignant skin melanoma	1·3 (0·8 to 2·2)	1·7 (1·1 to 3)	2·3 (1·5 to 4·2)	2·5 (1·7 to 5)	2 (1·3 to 3·5)	1·8 (1·2 to 3·3)	1·6 (1·1 to 2·9)	1·1 (0·8 to 2·1)	0·6 (0·4 to 1)	0·3 (0·2 to 0·4)	0·1 (0·1 to 0·2)	0 (0 to 0·1)	18·7
Non-melanoma skin cancer	0·6 (0·4 to 0·9)	0·9 (0·6 to 1·3)	1·5 (1 to 2)	1·8 (1·3 to 2·5)	2 (1·5 to 2·6)	2·1 (1·4 to 2·8)	1·9 (1·1 to 2·4)	1·6 (0·9 to 2·1)	0·8 (0·5 to 1)	0·4 (0·2 to 0·4)	0·2 (0·1 to 0·2)	0·1 (0 to 0·1)	15
Non-melanoma skin cancer (squamous-cell carcinoma)	0·6 (0·4 to 0·9)	0·9 (0·6 to 1·3)	1·5 (1 to 2)	1·8 (1·3 to 2·5)	2 (1·5 to 2·6)	2·1 (1·4 to 2·8)	1·9 (1·1 to 2·4)	1·6 (0·9 to 2·1)	0·8 (0·5 to 1)	0·4 (0·2 to 0·4)	0·2 (0·1 to 0·2)	0·1 (0 to 0·1)	15
Non-melanoma skin cancer (basal-cell carcinoma)	0 (0 to 0)	0 (0 to 0)	0 (0 to 0)	0 (0 to 0)	0 (0 to 0)	0 (0 to 0)	0 (0 to 0)	0 (0 to 0)	0 (0 to 0)	0 (0 to 0)	0 (0 to 0)	0 (0 to 0)	0
Breast cancer	39·9 (28·5 to 53·2)	56·1 (42·5 to 72·7)	79·7 (60·4 to 102)	77·5 (58·6 to 99·6)	58·7 (44·3 to 74·7)	44·9 (34·3 to 58·3)	40·1 (30·6 to 51·3)	28·4 (21·3 to 36·1)	14·5 (10·7 to 19·1)	7·9 (5·9 to 10·2)	3·4 (2·5 to 4·6)	1·2 (0·8 to 1·8)	506
Cervical cancer	28·7 (19·5 to 39·6)	35·4 (25 to 48·6)	43·1 (30·1 to 58·6)	40·4 (29·3 to 53·5)	31·1 (22·1 to 41·9)	24·3 (17·3 to 32·1)	20·2 (14·5 to 26·9)	13·4 (9·6 to 17·7)	6·3 (4·4 to 8·7)	2·7 (1·9 to 3·6)	1·2 (0·8 to 1·6)	0·4 (0·3 to 0·5)	302
Uterine cancer	7·2 (4·7 to 10·3)	12·4 (8·4 to 17·2)	18·1 (12·7 to 25·7)	22 (15·2 to 31·4)	25·3 (17·5 to 35·9)	23 (16·1 to 32·9)	21·3 (15 to 29·5)	13·6 (9·3 to 18·8)	6·5 (4·4 to 9)	2·6 (1·8 to 3·6)	1·1 (0·8 to 1·6)	0·3 (0·2 to 0·5)	165
Ovarian cancer	8·1 (5·1 to 11·5)	12·2 (7·8 to 16·6)	18·1 (11·2 to 25·3)	19 (11·6 to 27·3)	15·7 (9·3 to 21·3)	13·5 (7·9 to 18·7)	11·1 (6·6 to 15)	6·8 (4·3 to 9·4)	3·2 (2 to 4·6)	1·5 (0·9 to 2·1)	0·6 (0·4 to 0·9)	0·1 (0·1 to 0·2)	126
Prostate cancer	2·2 (1·3 to 3·2)	8·5 (5·5 to 12·3)	25·9 (17·4 to 36·2)	55·5 (37 to 81·2)	102 (71·4 to 141)	128 (87·1 to 174)	165 (92·5 to 216)	156 (89·1 to 200)	60·8 (38·5 to 77·1)	20·1 (13·9 to 25·8)	6·7 (4·6 to 8·6)	1·4 (0·9 to 2)	735
Testicular cancer	0·4 (0·2 to 0·5)	0·2 (0·1 to 0·3)	0·2 (0·1 to 0·3)	0·1 (0·1 to 0·2)	0·1 (0·1 to 0·2)	0·1 (0·1 to 0·2)	0·1 (0·1 to 0·2)	0·1 (0·1 to 0·2)	0 (0 to 0·1)	0 (0 to 0)	0 (0 to 0)	0 (0 to 0)	3·5
Kidney cancer	2·7 (1·9 to 3·7)	4·7 (3·4 to 6·3)	7·5 (5·9 to 9·5)	9·5 (7·1 to 12·8)	8·9 (6·8 to 11·5)	8·1 (6·2 to 10·4)	6·4 (5 to 8·3)	4 (3 to 5·1)	1·4 (1·1 to 1·9)	0·4 (0·3 to 0·5)	0·1 (0·1 to 0·2)	0 (0 to 0·1)	63·7
Bladder cancer	1·2 (0·8 to 1·8)	2·3 (1·6 to 3·2)	5 (3·7 to 6·5)	7·8 (5·9 to 10·2)	9·8 (7·4 to 12·5)	11·1 (8 to 15·2)	11·5 (8·3 to 15·6)	9·9 (7·1 to 13·3)	4·4 (3·1 to 5·9)	1·9 (1·4 to 2·4)	0·8 (0·6 to 1·1)	0·2 (0·2 to 0·3)	67·4
Brain and central nervous system cancer	5·9 (2·5 to 8·8)	7·5 (3·4 to 11·2)	9·9 (4·9 to 14·6)	11·1 (5·5 to 15·9)	8·8 (4·2 to 12·9)	7·3 (3·1 to 10·4)	5·2 (2 to 7·7)	3 (1·2 to 4·6)	1 (0·4 to 1·6)	0·3 (0·1 to 0·5)	0·1 (0 to 0·2)	0 (0 to 0)	96·9
Thyroid cancer	1·2 (0·8 to 1·6)	2·2 (1·5 to 3)	3·5 (2·6 to 4·8)	4·2 (3·1 to 5·8)	3·7 (2·8 to 4·9)	3·6 (2·6 to 4·7)	3·3 (2·5 to 4·2)	2·2 (1·6 to 3)	1 (0·7 to 1·4)	0·4 (0·3 to 0·5)	0·1 (0·1 to 0·2)	0 (0 to 0·1)	28·1
Mesothelioma	0·6 (0·4 to 0·9)	1 (0·7 to 1·4)	1·5 (1·2 to 2·1)	1·7 (1·3 to 2·3)	0·7 (0·5 to 0·9)	0·5 (0·4 to 0·7)	0·6 (0·4 to 0·9)	0·4 (0·3 to 0·5)	0·2 (0·1 to 0·3)	0·1 (0 to 0·1)	0 (0 to 0)	0 (0 to 0)	8·8
Hodgkin lymphoma	0·9 (0·6 to 1·3)	0·9 (0·6 to 1·2)	1 (0·7 to 1·4)	1 (0·7 to 1·4)	0·9 (0·6 to 1·2)	0·8 (0·6 to 1·1)	0·6 (0·4 to 0·8)	0·4 (0·3 to 0·6)	0·1 (0·1 to 0·2)	0 (0 to 0·1)	0 (0 to 0)	0 (0 to 0)	12·9
Non-Hodgkin lymphoma	15·5 (11·3 to 21·7)	22·4 (16·7 to 28·7)	28·7 (22·7 to 36)	33·4 (26·1 to 42·7)	30·4 (24·4 to 37·3)	28·3 (21·9 to 36·5)	23·5 (18·1 to 30·4)	15·4 (11·5 to 19·8)	5·6 (4·2 to 7·4)	1·8 (1·3 to 2·3)	0·6 (0·5 to 0·7)	0·2 (0·1 to 0·2)	292
Multiple myeloma	2·1 (1·5 to 2·9)	4·7 (3·5 to 6·4)	8·8 (6·6 to 11·9)	12·4 (9·4 to 16·7)	12·8 (9·8 to 17)	12·7 (9·8 to 16·8)	10·8 (8·5 to 13·9)	7·3 (5·7 to 9·3)	2·6 (1·9 to 3·5)	0·9 (0·6 to 1·3)	0·3 (0·2 to 0·4)	0·1 (0 to 0·1)	77·3
Leukemia	7·3 (5·8 to 9·4)	9·4 (7·4 to 11·9)	11·4 (9·2 to 13·9)	12·7 (10·2 to 15·7)	11·9 (9·7 to 14·2)	11·3 (9·5 to 13·5)	10·4 (8·6 to 12·5)	7·6 (6·2 to 9·2)	3·3 (2·7 to 4·1)	1·3 (1·1 to 1·6)	0·5 (0·4 to 0·6)	0·1 (0·1 to 0·2)	157
Acute lymphoid leukemia	0·5 (0·3 to 0·8)	0·5 (0·3 to 0·8)	0·7 (0·4 to 1)	0·6 (0·4 to 0·9)	0·5 (0·3 to 0·7)	0·4 (0·3 to 0·6)	0·3 (0·2 to 0·4)	0·2 (0·1 to 0·3)	0·1 (0 to 0·1)	0 (0 to 0)	0 (0 to 0)	0 (0 to 0)	20·1
Chronic lymphoid leukemia	0·3 (0·2 to 0·5)	0·3 (0·2 to 0·4)	1 (0·7 to 1·6)	1·5 (0·9 to 2·2)	1·7 (1·1 to 2·5)	1·7 (1·2 to 2·5)	1·4 (0·9 to 2·1)	1·1 (0·7 to 1·6)	0·4 (0·3 to 0·7)	0·2 (0·1 to 0·3)	0·1 (0 to 0·1)	0 (0 to 0)	10·6
Acute myeloid leukemia	1·8 (1·2 to 2·5)	2·5 (1·7 to 3·4)	2·7 (1·8 to 3·8)	2·6 (1·6 to 3·7)	2·1 (1·4 to 3)	1·6 (1·1 to 2·3)	1·2 (0·7 to 1·9)	0·7 (0·4 to 1·2)	0·3 (0·2 to 0·5)	0·1 (0 to 0·1)	0 (0 to 0)	0 (0 to 0)	35·7

Chronic myeloid leukemia	0 (0 to 0)	0 (0 to 0)	0·1 (0 to 0·2)	0·3 (0·2 to 0·5)	0·2 (0·2 to 0·4)	0·2 (0·1 to 0·3)	0·4 (0·2 to 0·6)	0·7 (0·4 to 1·2)	0·8 (0·5 to 1·3)	0·8 (0·5 to 1·3)	0·9 (0·6 to 1·3)	0·7 (0·5 to 1·1)	1 (0·6 to 1·4)	1·3 (0·9 to 2)	1·4 (0·9 to 2·1)	1·3 (0·9 to 1·9)	1·2 (0·8 to 1·8)	1 (0·6 to 1·4)	0·7 (0·4 to 1)	0·3 (0·2 to 0·4)	0·1 (0 to 0·1)	0 (0 to 0)	0 (0 to 0)	13·6
Other leukemia	0·2 (0·1 to 0·3)	0·1 (0 to 0·1)	0·9 (0·5 to 1·4)	2 (1·1 to 3·2)	3·2 (1·9 to 4·8)	3 (2·1 to 4·3)	3·8 (2·6 to 5·3)	4·2 (2·9 to 5·8)	3·4 (2·4 to 4·8)	3·5 (2·6 to 4·7)	3·5 (2·5 to 4·7)	4 (3 to 5·2)	5·1 (3·9 to 6·7)	5·7 (4·3 to 7·2)	6·6 (5·1 to 8·6)	6·3 (4·9 to 7·8)	6·4 (5·1 to 7·9)	6·6 (5·2 to 8·1)	5 (3·9 to 6·2)	2·3 (1·8 to 2·9)	1 (0·8 to 1·2)	0·4 (0·3 to 0·5)	0·1 (0·1 to 0·1)	77·2
Other malignant neoplasms	0·3 (0·2 to 0·5)	0·3 (0·2 to 0·4)	1·9 (1·2 to 2·8)	6·6 (4·2 to 9·8)	5·6 (4·3 to 7·2)	6·3 (5 to 7·8)	9·8 (7·7 to 12·3)	10·1 (7·7 to 13·1)	8·4 (6·2 to 11·1)	8·8 (6·9 to 11·3)	8·6 (6·7 to 10·9)	10·1 (7·5 to 12·9)	13·8 (10·7 to 17·8)	18·5 (14·2 to 24·1)	20·4 (15·6 to 26·7)	18·1 (14·3 to 22·7)	16·4 (13·1 to 20·7)	13·8 (11·1 to 17·2)	10·2 (8·3 to 12·7)	4·5 (3·6 to 5·8)	2·2 (1·6 to 2·8)	0·9 (0·6 to 1·2)	0·3 (0·2 to 0·4)	196
Other neoplasms	0·1 (0 to 0·1)	0·1 (0 to 0·1)	0·3 (0·2 to 0·6)	1 (0·6 to 1·6)	0·5 (0·3 to 0·8)	0·5 (0·3 to 0·8)	0·7 (0·4 to 0·9)	0·6 (0·4 to 0·8)	0·9 (0·6 to 1·3)	0·8 (0·6 to 1·2)	0·7 (0·5 to 1)	1·2 (0·8 to 1·7)	1·4 (1 to 1·9)	2·4 (1·7 to 3·4)	3·1 (2·2 to 4·4)	3·4 (2·5 to 4·8)	3·6 (2·6 to 4·8)	4·1 (2·9 to 5·5)	3·5 (2·5 to 4·7)	1·6 (1·2 to 2)	0·7 (0·5 to 0·9)	0·3 (0·2 to 0·4)	0·1 (0 to 0·1)	31·4
Myelodysplastic, myeloproliferative, and other hematopoietic neo	0·1 (0 to 0·1)	0·1 (0 to 0·1)	0·3 (0·2 to 0·6)	1 (0·6 to 1·6)	0·5 (0·3 to 0·8)	0·5 (0·3 to 0·8)	0·7 (0·4 to 0·9)	0·6 (0·4 to 0·8)	0·9 (0·6 to 1·3)	0·8 (0·6 to 1·2)	0·7 (0·5 to 1)	1·2 (0·8 to 1·7)	1·4 (1 to 1·9)	2·4 (1·7 to 3·4)	3·1 (2·2 to 4·4)	3·4 (2·5 to 4·8)	3·6 (2·6 to 4·8)	4·1 (2·9 to 5·5)	3·5 (2·5 to 4·7)	1·6 (1·2 to 2)	0·7 (0·5 to 0·9)	0·3 (0·2 to 0·4)	0·1 (0 to 0·1)	31·4
Benign and in situ intestinal neoplasms	0 (0 to 0)	0 (0 to 0)	0 (0 to 0)	0 (0 to 0)	0 (0 to 0)	0 (0 to 0)	0 (0 to 0)	0 (0 to 0)	0 (0 to 0)	0 (0 to 0)	0 (0 to 0)	0 (0 to 0)	0 (0 to 0)	0 (0 to 0)	0 (0 to 0)	0 (0 to 0)	0 (0 to 0)	0 (0 to 0)	0 (0 to 0)	0 (0 to 0)	0 (0 to 0)	0 (0 to 0)	0 (0 to 0)	0
Benign and in situ cervical and uterine neoplasms	0 (0 to 0)	0 (0 to 0)	0 (0 to 0)	0 (0 to 0)	0 (0 to 0)	0 (0 to 0)	0 (0 to 0)	0 (0 to 0)	0 (0 to 0)	0 (0 to 0)	0 (0 to 0)	0 (0 to 0)	0 (0 to 0)	0 (0 to 0)	0 (0 to 0)	0 (0 to 0)	0 (0 to 0)	0 (0 to 0)	0 (0 to 0)	0 (0 to 0)	0 (0 to 0)	0 (0 to 0)	0 (0 to 0)	0
Other benign and in situ neoplasms	0 (0 to 0)	0 (0 to 0)	0 (0 to 0)	0 (0 to 0)	0 (0 to 0)	0 (0 to 0)	0 (0 to 0)	0 (0 to 0)	0 (0 to 0)	0 (0 to 0)	0 (0 to 0)	0 (0 to 0)	0 (0 to 0)	0 (0 to 0)	0 (0 to 0)	0 (0 to 0)	0 (0 to 0)	0 (0 to 0)	0 (0 to 0)	0 (0 to 0)	0 (0 to 0)	0 (0 to 0)	0 (0 to 0)	0
Cardiovascular diseases	2·8 (1·7 to 4·2)	0·8 (0·5 to 1·3)	6·2 (3·8 to 9·5)	4·5 (3·2 to 6·4)	7·2 (5·8 to 8·9)	11·7 (9·3 to 14·5)	32·7 (26·6 to 39·2)	49·3 (39·3 to 59·6)	58·1 (45·9 to 71·9)	82·8 (66·3 to 101)	112 (90·9 to 139)	175 (143 to 211)	316 (264 to 379)	502 (429 to 579)	679 (594 to 771)	722 (649 to 802)	794 (713 to 876)	775 (706 to 840)	694 (629 to 758)	389 (347 to 425)	196 (170 to 215)	110 (91·7 to 122)	42·4 (33 to 48·1)	5,760
Rheumatic heart disease	0 (0 to 0)	0 (0 to 0)	0 (0 to 0)	0·4 (0·2 to 0·6)	2 (1·3 to 3·1)	4·5 (3 to 6·8)	9·2 (6·5 to 12·9)	11 (7·8 to 15)	11·5 (8·3 to 15·7)	11·7 (8·5 to 15·2)	11·2 (8·3 to 14·5)	11·7 (8·4 to 15·6)	12·9 (9·9 to 16·3)	13·2 (9·6 to 18·3)	11·7 (8·7 to 16)	7·5 (5·6 to 9·9)	5·9 (4·4 to 7·8)	4·2 (3·2 to 5·6)	3·2 (2·5 to 4·1)	1·4 (1 to 1·8)	0·6 (0·5 to 0·8)	0·4 (0·3 to 0·5)	0·2 (0·1 to 0·2)	135
Ischemic heart disease	0 (0 to 0)	0 (0 to 0)	0 (0 to 0)	0 (0 to 0)	0 (0 to 0)	0 (0 to 0)	2·2 (1·6 to 2·8)	5 (3·5 to 6·6)	8·1 (5·8 to 11)	16·7 (12·3 to 22·1)	27·6 (21·3 to 35·6)	51·5 (40·9 to 63·9)	108 (87·3 to 132)	187 (152 to 221)	280 (240 to 325)	303 (267 to 339)	343 (305 to 383)	326 (292 to 362)	276 (247 to 306)	143 (125 to 161)	72·5 (60·6 to 83·5)	41·5 (33·9 to 48·2)	16·6 (12·6 to 19·7)	2,210
Stroke	1·2 (0·7 to 1·9)	0·1 (0·1 to 0·2)	0·8 (0·4 to 1·5)	0·7 (0·5 to 1)	1·7 (1·2 to 2·3)	2·7 (2 to 3·5)	8·3 (6·3 to 10·4)	13·7 (9·6 to 18·4)	16·1 (11·2 to 21·7)	24·9 (17·7 to 33·5)	35·5 (26·1 to 48)	56·5 (40·7 to 74·6)	103 (73·8 to 137)	167 (128 to 210)	214 (164 to 268)	237 (189 to 291)	266 (218 to 317)	268 (228 to 314)	258 (207 to 304)	152 (124 to 180)	72·7 (60·5 to 84·6)	37·3 (30·1 to 44·1)	13·1 (9·9 to 15·8)	1,950
Ischemic stroke	0·3 (0·1 to 0·4)	0 (0 to 0)	0·1 (0 to 0·1)	0·3 (0·2 to 0·5)	0·8 (0·5 to 1·3)	1·2 (0·8 to 1·8)	2·4 (1·7 to 3·2)	3·5 (2·5 to 4·7)	4·2 (3 to 5·6)	5·8 (4·3 to 7·7)	7·5 (5·6 to 10·1)	12 (9·1 to 15·7)	22·1 (16·4 to 29·3)	39·7 (30·8 to 49·9)	57 (43·6 to 71·9)	91 (71·2 to 112)	110 (89·9 to 133)	160 (132 to 188)	164 (131 to 195)	120 (98·9 to 144)	59·7 (49·3 to 69·6)	31·3 (25 to 37·4)	11·2 (8·3 to 13·5)	905
Intracerebral hemorrhage	0·3 (0·1 to 0·5)	0 (0 to 0·1)	0·4 (0·2 to 0·7)	0·1 (0·1 to 0·2)	0·4 (0·3 to 0·6)	0·7 (0·5 to 0·9)	3·1 (2·2 to 4·1)	5·5 (3·7 to 7·8)	6·7 (4·4 to 9·7)	11·7 (7·8 to 16·7)	17·9 (12·4 to 24·8)	30·5 (20·9 to 41·6)	61·8 (43·9 to 83·9)	101 (75·2 to 129)	128 (96·8 to 163)	127 (99·1 to 160)	139 (110 to 172)	97·1 (78·6 to 119)	85·2 (66·6 to 104)	27·9 (22·3 to 34·2)	11·6 (9·5 to 13·9)	5·3 (4·2 to 6·4)	1·7 (1·3 to 2·1)	862
Subarachnoid hemorrhage	0·6 (0·3 to 1·1)	0·1 (0 to 0·1)	0·4 (0·2 to 0·8)	0·3 (0·1 to 0·4)	0·5 (0·3 to 0·7)	0·8 (0·5 to 1·1)	2·8 (1·9 to 3·9)	4·7 (3 to 7)	5·2 (3·2 to 7·7)	7·4 (4·8 to 10·6)	10·1 (6·8 to 14·4)	14 (9·3 to 20)	19·3 (12·7 to 27·8)	26 (18·4 to 35·6)	29·3 (21·1 to 39·9)	18·7 (13·5 to 24·9)	16·9 (12·1 to 22·4)	10·9 (8 to 14·6)	8·3 (6·1 to 11)	3·5 (2·5 to 4·7)	1·4 (1 to 1·9)	0·7 (0·5 to 0·9)	0·2 (0·2 to 0·3)	182
Hypertensive heart disease	0 (0 to 0)	0 (0 to 0)	0 (0 to 0)	0 (0 to 0)	0 (0 to 0)	0 (0 to 0)	0·6 (0·4 to 0·9)	1·4 (0·9 to 2·1)	2 (1·2 to 3)	4·5 (3 to 6·3)	7·7 (4·9 to 10·9)	13·3 (9 to 19)	29 (19·8 to 39·6)	48·3 (34·4 to 65·9)	65·3 (46·7 to 88·7)	68·9 (48·5 to 91·8)	71·4 (50·6 to 95·4)	73·2 (54·2 to 96·2)	66·7 (48·5 to 86·7)	40 (29·5 to 52·2)	20·1 (14·3 to 26·1)	12·9 (8·7 to 16·6)	5·6 (3·6 to 7·5)	531
Non-rheumatic valvular heart disease	0 (0 to 0)	0 (0 to 0)	0 (0 to 0)	0 (0 to 0)	0 (0 to 0)	0 (0 to 0)	1·1 (0·8 to 1·5)	1·3 (0·9 to 1·8)	1·8 (1·1 to 2·6)	2·2 (1·5 to 3·1)	2·4 (1·6 to 3·2)	3·5 (2·4 to 4·9)	5·3 (3·7 to 7·3)	6·3 (4·6 to 8·5)	8·6 (6·2 to 11·4)	8·1 (6 to 10·5)	8·5 (6·6 to 11·1)	6·8 (5 to 8·9)	5·2 (3·9 to 6·7)	2·4 (1·9 to 3·1)	0·9 (0·7 to 1·1)	0·4 (0·3 to 0·5)	0·1 (0·1 to 0·1)	64·7
Non-rheumatic calcific aortic valve disease	0 (0 to 0)	0 (0 to 0)	0 (0 to 0)	0 (0 to 0)	0 (0 to 0)	0 (0 to 0)	0·4 (0·3 to 0·7)	0·5 (0·3 to 0·8)	0·6 (0·3 to 1)	0·6 (0·3 to 1)	0·7 (0·4 to 1·1)	1·1 (0·6 to 1·9)	1·9 (1·1 to 3)	2·4 (1·5 to 3·6)	3·3 (2 to 5·1)	3·4 (2·2 to 4·9)	4·2 (2·9 to 6)	3·6 (2·4 to 5·1)	2·8 (1·9 to 4)	1·5 (1·1 to 2)	0·5 (0·4 to 0·7)	0·2 (0·1 to 0·3)	0·1 (0 to 0·1)	27·7
Non-rheumatic degenerative mitral valve disease	0 (0 to 0)	0 (0 to 0)	0 (0 to 0)	0 (0 to 0)	0 (0 to 0)	0 (0 to 0)	0·6 (0·4 to 0·9)	0·8 (0·5 to 1·1)	1·2 (0·7 to 1·8)	1·6 (1·1 to 2·3)	1·6 (1·1 to 2·3)	2·3 (1·6 to 3·3)	3·3 (2·1 to 4·7)	3·9 (2·6 to 5·5)	5·2 (3·5 to 7·3)	4·6 (3·2 to 6·4)	4·2 (2·8 to 5·8)	3·1 (2 to 4·4)	2·3 (1·5 to 3·3)	0·9 (0·6 to 1·3)	0·3 (0·2 to 0·5)	0·2 (0·1 to 0·2)	0 (0 to 0·1)	36·3
Other non-rheumatic valve diseases	0 (0 to 0)	0 (0 to 0)	0 (0 to 0)	0 (0 to 0)	0 (0 to 0)	0 (0 to 0)	0 (0 to 0·1)	0 (0 to 0)	0 (0 to 0·1)	0·1 (0 to 0·1)	0 (0 to 0·1)	0 (0 to 0·1)	0·1 (0 to 0·1)	0·1 (0 to 0·1)	0·1 (0 to 0·1)	0 (0 to 0·1)	0·1 (0 to 0·1)	0·1 (0 to 0·2)	0·1 (0 to 0·1)	0 (0 to 0·1)	0 (0 to 0)	0 (0 to 0)	0 (0 to 0)	0·7
Cardiomyopathy and myocarditis	0·9 (0·6 to 1·3)	0·4 (0·2 to 0·6)	3·4 (2·1 to 5·2)	2 (1·4 to 3)	1·6 (1·2 to 2)	2 (1·5 to 2·5)	4·6 (3·3 to 6)	6·8 (4·8 to 8·9)	8 (5·4 to 10·7)	10·6 (7·2 to 14·2)	12·9 (8·8 to 17·4)	16·6 (11·7 to 22·1)	27·1 (18·4 to 36·7)	37·1 (26 to 48·7)	41·8 (29·5 to 54·6)	36·6 (26·2 to 46·6)	33·1 (23·3 to 43·2)	27·7 (19·4 to 35·9)	21·3 (15·6 to 27·5)	10·4 (7·8 to 13·5)	6 (4·3 to 8·3)	3·1 (2 to 4·3)	1·1 (0·7 to 1·6)	315
Myocarditis	0·1 (0·1 to 0·2)	0·1 (0 to 0·2)	0·8 (0·4 to 1·4)	0·6 (0·3 to 1)	0·5 (0·3 to 0·8)	0·4 (0·2 to 0·6)	0·9 (0·5 to 1·4)	0·9 (0·5 to 1·6)	1 (0·6 to 1·8)	1·4 (0·8 to 2·3)	1·1 (0·6 to 1·8)	1 (0·6 to 1·7)	1·4 (0·8 to 2·4)	1·8 (1 to 3·1)	1·5 (0·8 to 2·6)	1·2 (0·7 to 2)	1 (0·6 to 1·6)	1 (0·5 to 1·6)	0·9 (0·5 to 1·5)	0·6 (0·3 to 1·1)	0·5 (0·2 to 1)	0·3 (0·2 to 0·6)	0·2 (0·1 to 0·3)	19·3
Alcoholic cardiomyopathy	0 (0 to 0)	0 (0 to 0)	0 (0 to 0)	0 (0 to 0)	0 (0 to 0)	0 (0 to 0)	0·2 (0·1 to 0·4)	0·5 (0·2 to 0·8)	0·9 (0·4 to 1·6)	1·8 (0·9 to 3)	2·4 (1·3 to 3·9)	4·1 (2·2 to 6·6)	9·1 (5 to 14·1)	12·3 (6·8 to 18)	12·8 (7·2 to 19·4)	10·2 (5·7 to 15·1)	9·2 (4·9 to 14·1)	4·8 (2·3 to 8·2)	3 (1·5 to 5·1)	0·4 (0·2 to 0·8)	0·1 (0·1 to 0·2)	0 (0 to 0·1)	0 (0 to 0)	72·1
Other cardiomyopathy	0·8 (0·5 to 1·1)	0·3 (0·2 to 0·5)	2·6 (1·6 to 4)	1·5 (0·9 to 2·3)	1·1 (0·7 to 1·5)	1·6 (1·2 to 2·1)	3·5 (2·5 to 4·8)	5·4 (3·7 to 7·2)	6 (4 to 8·3)	7·3 (4·9 to 10)	9·3 (6·2 to 12·7)	11·5 (7·9 to 15·8)	16·5 (11·1 to 22·6)	23 (16·4 to 30·9)	27·5 (19·5 to 35·7)	25·3 (18·5 to 33·7)	22·9 (16·2 to 30·5)	21·9 (15·6 to 28·4)	17·3 (12·8 to 22·7)	9·3 (6·8 to 12·3)	5·3 (3·8 to 7·5)	2·7 (1·8 to 3·8)	0·9 (0·6 to 1·3)	223
Atrial fibrillation and flutter	0 (0 to 0)	0 (0 to 0)	0 (0 to 0)	0 (0 to 0)	0 (0 to 0)	0 (0 to 0)	0 (0 to 0)	0 (0 to 0)	0 (0 to 0)	0·2 (0·1 to 0·3)	0·2 (0·2 to 0·3)	1 (0·7 to 1·3)	2·1 (1·5 to 3)	3·9 (2·8 to 5·4)	6·6 (5 to 9)	8·4 (6·6 to 11·1)	10·8 (8·5 to 13·9)	14·4 (11·6 to 18·5)	16·4 (13·3 to 20·8)	13 (10·6 to 16·2)	8·4 (6·9 to 11)	6·2 (4·9 to 8·2)	2·4 (1·8 to 3·4)	94
Aortic aneurysm	--	--	--	--	--	--	0·3 (0·2 to 0·4)	0·3 (0·2 to 0·5)	0·6 (0·4 to 0·9)	0·9 (0·6 to 1·3)	1·4 (0·9 to 2)	2·1 (1·5 to 2·9)	3·5 (2·5 to 4·7)	6·3 (4·6 to 8·6)	11·3 (8·4 to 14·6)	14·4 (10·7 to 18·7)	17·5 (12·8 to 22·7)	17·2 (12·5 to 23)	14 (10·2 to 18·6)	6·1 (4·5 to 8·1)	2·3 (1·7 to 3·1)	0·9 (0·7 to 1·2)	0·3 (0·2 to 0·4)	NA
Endocarditis	0·1 (0·1 to 0·2)	0·1 (0·1 to 0·2)	0·7 (0·3 to 1·2)	0·5 (0·3 to 0·7)	0·4 (0·3 to 0·6)	0·6 (0·3 to 0·9)	2·1 (1·3 to 3·1)	3·6 (2·1 to 5·7)	3·5 (2 to 6)	4 (2·3 to 6·5)	4·6 (2·7 to 7·4)	5·3 (3·1 to 8·3)	6·6 (4·1 to 10·2)	7·8 (4·9 to 12)	7·8 (4·9 to 12·9)	6·7 (4·2 to 10·4)	5·4 (3·6 to 8·1)	4·7 (3·4 to 7·4)	3·4 (2·2 to 5·3)	2·3 (1·4 to 3·7)	1·1 (0·7 to 2)	0·6 (0·4 to 1·1)	0·3 (0·2 to 0·5)	72·3
Other cardiovascular and circulatory diseases	0·6 (0·3 to 0·9)	0·2 (0·1 to 0·4)	1·3 (0·7 to 2·2)	0·9 (0·5 to 1·4)	1·4 (1 to 2·1)	2 (1·3 to 2·7)	4·2 (3 to 5·7)	6 (4·5 to 7·9)	6·6 (4·8 to 8·9)	7·2 (5·3 to 9·3)	8·7 (6·4 to 11·4)	13·1 (9·7 to 17·4)	17 (13·1 to 21·4)	21·9 (17·3 to 27·1)	26·5 (21·1 to 32·3)	26 (21·1 to 30·6)	26·1 (20·6 to 32·1)	25·1 (20·5 to 30·2)	22·9 (18·5 to 27·4)	13·1 (10·4 to 16)	7·8 (6·2 to 9·5)	4·4 (3·4 to 5·4)	1·8 (1·2 to 2·3)	245
Chronic respiratory diseases	0 (0 to 0)	0 (0 to 0)	0 (0 to 0)	46·8 (25·8 to 81)	43 (25·4 to 70·5)	35 (21·4 to 54·7)	32·2 (20·6 to 49·8)	33·3 (22·2 to 50)	32 (21·7 to 46·8)	29·1 (21 to 40)	26·5 (19·7 to 35·2)	28·8 (22·4 to 36·6)	39·4 (31·4 to 49·2)	54·3 (44·4 to 65·7)	64·4 (53·8 to 77·1)	66·6 (55·7 to 79·4)	69·7 (58·6 to 82·7)	69·7 (59·5 to 81·6)	59·4 (49·7 to 69·8)	29·1 (24·4 to 34·3)	13·5 (11·1 to 15·8)	6·9 (5·5 to 8·2)	2·7 (2·1 to 3·3)	782
Chronic obstructive pulmonary disease	0 (0 to 0)	0 (0 to 0)	0 (0 to 0)	0·5 (0·3 to 0·8)	0·7 (0·4 to 1)	1·1 (0·6 to 1·6)	2·3 (1·6 to 3·1)	3·9 (2·8 to 5·2)	4·6 (3·2 to 6·1)	5·2 (3·8 to 6·7)	5·9 (4·4 to 7·4)	8·4 (6·4 to 10·5)	15·1 (11·9 to 18·6)	26·1 (21·1 to 31·6)	37·4 (30·1 to 45·3)	47·3 (39 to 56·5)	52·5 (43·7 to 63·2)	54 (44·9 to 64·9)	47·6 (39·1 to 56·5)	23·7 (19·6 to 28·3)	11·2 (9·1 to 13·2)	5·7 (4·6 to 6·9)	2·3 (1·8 to 2·8)	355
Pneumoconiosis	0 (0 to 0)	0 (0 to 0)	0 (0 to 0)	0 (0 to 0)	0 (0 to 0)	0 (0 to 0)	0·1 (0·1 to 0·2)	0 (0 to 0·1)	0·1 (0 to 0·2)	0 (0 to 0·1)	0·1 (0 to 0·1)	0·1 (0 to 0·1)	0·2 (0·1 to 0·3)	0·3 (0·2 to 0·5)	0·3 (0·2 to 0·5)	0·4 (0·3 to 0·7)	0·7 (0·4 to 1)	0·4 (0·3 to 0·6)	0·3 (0·2 to 0·5)	0·2 (0·2 to 0·3)	0·1 (0·1 to 0·1)	0 (0 to 0)	0 (0 to 0)	3·5
Silicosis	0 (0 to 0)	0 (0 to 0)	0 (0 to 0)	0 (0 to 0)	0 (0 to 0)	0 (0 to 0)	0 (0 to 0)	0 (0 to 0)	0 (0 to 0·1)	0 (0 to 0)	0 (0 to 0)	0 (0 to 0)	0 (0 to 0)	0 (0 to 0)	0 (0 to 0)	0 (0 to 0)	0 (0 to 0)	0 (0 to 0)	0 (0 to 0·1)	0 (0 to 0)	0 (0 to 0)	0 (0 to 0)	0 (0 to 0)	0·2
Asbestosis	0 (0 to 0)	0 (0 to 0)	0 (0 to 0)	0 (0 to 0)	0 (0 to 0)	0 (0 to 0)	0 (0 to 0)	0 (0 to 0)	0 (0 to 0)	0 (0 to 0)	0 (0 to 0)	0 (0 to 0)	0 (0 to 0)	0 (0 to 0)	0 (0 to 0)	0 (0 to 0)	0 (0 to 0)	0 (0 to 0)	0 (0 to 0)	0 (0 to 0)	0 (0 to 0)	0 (0 to 0)	0 (0 to 0)	0·1
Coal workers pneumoconiosis	0 (0 to 0)	0 (0 to 0)	0 (0 to 0)	0 (0 to 0)	0 (0 to 0)	0 (0 to 0)	0·1 (0 to 0·1)	0 (0 to 0)	0 (0 to 0·1)	0 (0 to 0)	0·1 (0 to 0·1)	0 (0 to 0·1)	0·2 (0·1 to 0·3)	0·3 (0·1 to 0·4)	0·2 (0·1 to 0·4)	0·4 (0·2 to 0·6)	0·5 (0·3 to 0·8)	0·3 (0·2 to 0·5)	0·2 (0·1 to 0·4)	0·2 (0·1 to 0·2)	0·1 (0 to 0·1)	0 (0 to 0)	0 (0 to 0)	2·7
Other pneumoconiosis	0 (0 to 0)	0 (0 to 0)	0 (0 to 0)	0 (0 to 0)	0 (0 to 0)	0 (0 to 0)	0 (0 to 0)	0 (0 to 0)	0 (0 to 0)	0 (0 to 0)	0 (0 to 0)	0 (0 to 0)	0 (0 to 0)	0 (0 to 0)	0 (0 to 0)	0 (0 to 0)	0·1 (0 to 0·2)	0·1 (0 to 0·1)	0·1 (0 to 0·1)	0 (0 to 0·1)	0 (0 to 0)	0 (0 to 0)	0 (0 to 0)	0·4
Asthma	0 (0 to 0)	0 (0 to 0)	0 (0 to 0)	41·2 (20·7 to 75·6)	40 (22·2 to 67·2)	31·2 (17·7 to 51·1)	26·9 (15·5 to 43·9)	25·1 (14·3 to 41)	22·1 (12·8 to 36·2)	18·7 (11·4 to 28·9)	15·2 (9·8 to 22·9)	14·6 (9·7 to 21·5)	16·4 (11·2 to 23·4)	18·2 (12·9 to 25·3)	15·8 (11·3 to 21·8)	12·7 (9·1 to 17·1)	10·7 (7·8 to 14·5)	9·1 (6·6 to 12·1)	6·5 (4·7 to 8·5)	2·9 (2·1 to 3·9)	1·2 (0·9 to 1·7)	0·6 (0·5 to 0·9)	0·3 (0·2 to 0·4)	329
Interstitial lung disease and pulmonary sarcoidosis	0 (0 to 0)	0 (0 to 0)	0 (0 to 0)	0·7 (0·4 to 1·3)	0·2 (0·1 to 0·4)	0·2 (0·1 to 0·3)	0·4 (0·3 to 0·7)	0·7 (0·4 to 1·1)	0·8 (0·4 to 1·2)	1·3 (0·8 to 2·1)	1·4 (0·9 to 2·2)	1·7 (1·1 to 2·5)	2·3 (1·6 to 3·2)	3·2 (2·2 to 4·5)	3·9 (2·6 to 5·6)	3·9 (2·7 to 5·4)	3·9 (2·6 to 5·5)	4·3 (2·8 to 6·2)	3·6 (2·3 to 5·3)	1·6 (1 to 2·4)	0·8 (0·5 to 1·2)	0·4 (0·2 to 0·6)	0·1 (0·1 to 0·2)	35·5
Other chronic respiratory diseases	0 (0 to 0)	0 (0 to 0)	0 (0 to 0)	4·3 (2·7 to 6·6)	2·1 (1·5 to 2·8)	2·5 (1·8 to 3·4)	2·4 (1·7 to 3·2)	3·6 (2·6 to 5·1)	4·4 (3·2 to 6)	3·9 (2·8 to 5·4)	3·8 (2·7 to 5·2)	4·1 (2·9 to 5·7)	5·4 (3·8 to 7·6)	6·5 (4·4 to 9·3)	7 (4·6 to 10·7)	2·4 (1·3 to 3·9)	2 (1·1 to 3·2)	2 (1·2 to 2·9)	1·4 (0·9 to 2·3)	0·7 (0·4 to 1·1)	0·3 (0·1 to 0·4)	0·1 (0·1 to 0·2)	0 (0 to 0·1)	58·9
Digestive diseases	1·4 (0·9 to 2·1)	0·9 (0·5 to 1·5)	9·3 (5·3 to 15·3)	13·3 (9·3 to 18·5)	11·8 (8·9 to 15·7)	11 (8·5 to 14·3)	19·2 (15·4 to 24·3)	33·6 (25·7 to 44·6)	41·8 (31·7 to 56·1)	52·7 (41·3 to 66·9)	62·3 (50·2 to 77·8)	81·5 (65·3 to 99·8)	122 (99·4 to 149)	161 (132 to 192)	164 (137 to 193)	147 (124 to 171)	124 (104 to 148)	105 (87 to 125)	77·3 (64·2 to 91)	34 (29·2 to 39·3)	14·1 (11·4 to 17·2)	6·5 (5·1 to 7·9)	2·1 (1·6 to 2·5)	1,300
Cirrhosis and other chronic liver diseases	0 (0 to 0)	0 (0 to 0)	0 (0 to 0)	1 (0·6 to 1·6)	1·3 (0·9 to 1·8)	1·5 (1·1 to 2)	3·5 (2·5 to 4·4)	5·6 (4·1 to 7·6)	7·6 (5·4 to 10·4)	14·7 (10·9 to 19·1)	23 (17·5 to 29·9)	35·6 (26·9 to 44·9)	60·3 (46·8 to 76·8)	83·3 (65·7 to 105)	85 (68·3 to 103)	73·6 (59·9 to 88·1)	58·9 (46·7 to 72·5)	42·1 (33·1 to 52·6)	26·5 (20·8 to 32·9)	9·5 (7·7 to 11·7)	3 (2·3 to 3·8)	1·1 (0·9 to 1·4)	0·3 (0·3 to 0·4)	537
Cirrhosis and other chronic liver diseases due to hepatitis B	0 (0 to 0)	0 (0 to 0)	0 (0 to 0)	0 (0 to 0)	0 (0 to 0)	0 (0 to 0)	0 (0 to 0·1)	0·2 (0·1 to 0·4)	0·5 (0·2 to 0·9)	1·2 (0·6 to 2)	2·1 (1 to 4·1)	3·4 (1·7 to 6)	5·6 (2·8 to 10)	7·1 (3·4 to 13·1)	6·1 (2·9 to 10·9)	4·5 (2·1 to 8·5)	3·2 (1·6 to 6)	2·2 (1·1 to 4·2)	1·4 (0·7 to 2·5)	0·5 (0·2 to 0·9)	0·1 (0·1 to 0·2)	0·1 (0 to 0·1)	0 (0 to 0)	38·3
Cirrhosis and other chronic liver diseases due to hepatitis C	0 (0 to 0)	0 (0 to 0)	0 (0 to 0)	0 (0 to 0)	0 (0 to 0)	0 (0 to 0·1)	0·2 (0·1 to 0·3)	0·8 (0·4 to 1·4)	1·7 (0·8 to 3)	3·7 (2 to 5·8)	5·8 (3 to 10·1)	8·5 (4·7 to 14·2)	13·4 (7·4 to 22)	17·3 (9·5 to 28·5)	16·3 (8·8 to 27·3)	13·3 (6·4 to 23·5)	10·2 (5·6 to 16·7)	7·1 (4 to 11·6)	4·3 (2·3 to 7·4)	1·5 (0·8 to 2·5)	0·5 (0·3 to 0·8)	0·2 (0·1 to 0·3)	0·1 (0 to 0·1)	105
Cirrhosis and other chronic liver diseases due to alcohol use	0 (0 to 0)	0 (0 to 0)	0 (0 to 0)	0 (0 to 0)	0 (0 to 0)	0 (0 to 0)	0 (0 to 0·1)	0·5 (0·2 to 1·1)	1·4 (0·7 to 2·7)	4·5 (2·7 to 6·9)	9·2 (5·4 to 13·9)	15·9 (10 to 22·8)	28·2 (17·9 to 40·5)	40·5 (26·1 to 54·9)	43·1 (28·7 to 58·5)	37·3 (25 to 50·3)	28·4 (18·9 to 39·4)	18·7 (12 to 26·6)	10·6 (6·9 to 15)	3·2 (2 to 4·8)	0·9 (0·6 to 1·3)	0·3 (0·2 to 0·5)	0·1 (0 to 0·1)	243
Cirrhosis and other chronic liver diseases due to NAFLD	0 (0 to 0)	0 (0 to 0)	0 (0 to 0)	0 (0 to 0)	0 (0 to 0)	0 (0 to 0)	0·2 (0·1 to 0·3)	0·6 (0·3 to 1·1)	1·2 (0·5 to 2·2)	2·3 (1·2 to 3·8)	3·1 (1·4 to 5·8)	4·7 (2·3 to 8·3)	8·2 (3·7 to 14·7)	11·6 (5·6 to 20·8)	11·6 (5·6 to 20·4)	10·6 (5·2 to 18·7)	9·5 (5·4 to 15)	7·7 (4·5 to 11·6)	5·3 (2·9 to 8·6)	2·1 (1·2 to 3·3)	0·7 (0·4 to 1·1)	0·3 (0·2 to 0·4)	0·1 (0 to 0·2)	79·8
Cirrhosis and other chronic liver diseases due to other causes	0 (0 to 0)	0 (0 to 0)	0 (0 to 0)	1 (0·6 to 1·6)	1·3 (0·9 to 1·8)	1·5 (1 to 1·9)	3·1 (2·2 to 4)	3·5 (2·3 to 4·9)	2·8 (1·6 to 4·2)	3 (1·6 to 5)	2·8 (1·4 to 5)	3·1 (1·5 to 5·7)	4·8 (2·4 to 8·3)	6·8 (3·6 to 11·6)	7·9 (4 to 13·6)	8 (4·3 to 13·7)	7·5 (3·7 to 13)	6·5 (3·3 to 10·8)	4·9 (2·6 to 8)	2·1 (1·1 to 3·4)	0·7 (0·4 to 1·1)	0·3 (0·2 to 0·5)	0·1 (0 to 0·2)	71·6
Upper digestive system diseases	0 (0 to 0)	0 (0 to 0)	0 (0 to 0)	0·7 (0·4 to 1·1)	0·9 (0·5 to 1·6)	1·5 (0·9 to 2·3)	5·3 (3·1 to 8·9)	13·5 (7·7 to 23·8)	18 (10·1 to 30·8)	18·7 (11·1 to 31·7)	18·1 (10·8 to 28·7)	20·5 (13 to 31·9)	27 (18·2 to 40·3)	32·2 (22·1 to 46·7)	30·9 (21·9 to 44·1)	27 (20 to 37)	23·7 (18 to 31)	20·7 (15·6 to 27·1)	16·2 (12·6 to 20·7)	7·1 (5·6 to 9)	2·9 (2·2 to 3·8)	1·4 (1 to 1·7)	0·4 (0·3 to 0·6)	287
Peptic ulcer disease	0 (0 to 0)	0 (0 to 0)	0 (0 to 0)	0·1 (0·1 to 0·2)	0·1 (0·1 to 0·2)	0·3 (0·2 to 0·4)	0·6 (0·4 to 0·8)	1·3 (0·9 to 1·9)	1·5 (1 to 2·2)	2·5 (1·6 to 3·5)	3·1 (2·1 to 4·2)	3·9 (2·5 to 5·5)	5·8 (4 to 8·1)	9·4 (6·5 to 13)	10·2 (7·2 to 14·3)	10·8 (8·1 to 14·2)	10·6 (7·7 to 14·4)	10·1 (7·3 to 13·7)	8·7 (6·5 to 11·2)	4 (3·1 to 5)	1·7 (1·2 to 2·2)	0·8 (0·6 to 1)	0·3 (0·2 to 0·4)	85·6
Gastritis and duodenitis	0 (0 to 0)	0 (0 to 0)	0 (0 to 0)	0·6 (0·3 to 1)	0·8 (0·4 to 1·4)	0·7 (0·3 to 1·3)	1·1 (0·5 to 1·8)	1·7 (0·9 to 2·7)	2·1 (1·2 to 3·3)	2·7 (1·6 to 4·3)	3·2 (2 to 5)	4 (2·5 to 6·1)	6·7 (4·3 to 10·3)	7·4 (4·7 to 11·1)	7·7 (5 to 11·2)	6·5 (4·4 to 9·1)	5·9 (4 to 8·2)	5 (3·4 to 6·9)	3·4 (2·4 to 4·8)	1·3 (0·9 to 1·8)	0·5 (0·4 to 0·7)	0·2 (0·2 to 0·3)	0·1 (0 to 0·1)	61·3
Gastroesophageal reflux disease	0 (0 to 0)	0 (0 to 0)	0 (0 to 0)	0 (0 to 0)	0 (0 to 0)	0·5 (0·2 to 1)	3·7 (1·8 to 6·8)	10·5 (5·2 to 20·6)	14·4 (7 to 26·6)	13·6 (6·4 to 25·4)	11·8 (5·6 to 21·5)	12·6 (6 to 22·9)	14·5 (7·2 to 25·6)	15·4 (7·5 to 27·9)	13·1 (6·2 to 24·7)	9·7 (4·7 to 18·1)	7·2 (3·7 to 13)	5·7 (2·9 to 10)	4·1 (2·1 to 7·4)	1·8 (0·9 to 3·2)	0·8 (0·4 to 1·4)	0·3 (0·2 to 0·6)	0·1 (0·1 to 0·2)	140
Appendicitis	0 (0 to 0)	0 (0 to 0)	0 (0 to 0)	0·4 (0·2 to 0·7)	0·7 (0·5 to 1)	1 (0·6 to 1·5)	1·4 (0·8 to 2·1)	1·4 (0·9 to 2·2)	1·1 (0·6 to 1·7)	1 (0·6 to 1·5)	1·1 (0·7 to 1·6)	0·9 (0·6 to 1·3)	1·1 (0·7 to 1·6)	1·2 (0·8 to 1·7)	1·2 (0·8 to 1·7)	0·9 (0·6 to 1·3)	1 (0·7 to 1·4)	0·9 (0·6 to 1·3)	0·5 (0·4 to 0·8)	0·3 (0·2 to 0·4)	0·2 (0·1 to 0·2)	0·1 (0 to 0·1)	0 (0 to 0)	16·2
Paralytic ileus and intestinal obstruction	1·1 (0·7 to 1·6)	0·8 (0·5 to 1·4)	8·4 (4·7 to 14)	3·1 (1·8 to 5·1)	1·7 (1·2 to 2·4)	1·5 (1·1 to 2)	1·9 (1·4 to 2·6)	2·5 (1·6 to 3·6)	2·5 (1·6 to 3·8)	2·8 (1·8 to 4·2)	4 (2·7 to 5·6)	4·5 (3 to 6·4)	6·8 (4·6 to 9·6)	10·4 (7·1 to 15)	11·7 (8 to 17·3)	11·8 (8·5 to 16·3)	11·3 (7·7 to 15·7)	12·4 (8·7 to 16·6)	11 (7·8 to 14·3)	5·9 (4·4 to 7·7)	2·7 (2 to 3·7)	1·4 (1 to 1·8)	0·5 (0·3 to 0·7)	121
Inguinal, femoral, and abdominal hernia	0·4 (0·2 to 0·6)	0·1 (0 to 0·1)	0·9 (0·5 to 1·6)	3·7 (2·2 to 5·8)	4·7 (2·7 to 7·7)	3·2 (1·7 to 5·5)	2·8 (1·6 to 4·8)	3·1 (1·6 to 5·2)	3·5 (1·9 to 5·7)	3·6 (2 to 5·8)	3·8 (2·3 to 6·1)	4·9 (3 to 7·8)	6·7 (4 to 10·5)	8·2 (5·1 to 12·3)	7·8 (5·1 to 11·6)	6·7 (4·5 to 9·5)	5·4 (3·9 to 7·3)	4·7 (3·5 to 6·1)	3·3 (2·6 to 4·2)	1·3 (1 to 1·6)	0·5 (0·4 to 0·7)	0·2 (0·2 to 0·3)	0·1 (0 to 0·1)	79·5
Inflammatory bowel disease	0 (0 to 0)	0 (0 to 0)	0 (0 to 0)	0·9 (0·5 to 1·5)	0·3 (0·2 to 0·5)	0·2 (0·1 to 0·3)	0·9 (0·6 to 1·2)	1·5 (1·1 to 2·2)	2·1 (1·4 to 3·3)	2·9 (2 to 4·2)	2·7 (1·9 to 3·6)	3·2 (2·3 to 4·3)	3·7 (2·6 to 5·1)	4·2 (3 to 5·8)	4·8 (3·3 to 7)	3·9 (2·8 to 5·4)	3·1 (2·2 to 4·3)	3·6 (2·6 to 4·9)	2·6 (1·9 to 3·6)	1·1 (0·8 to 1·4)	0·5 (0·3 to 0·6)	0·3 (0·2 to 0·4)	0·1 (0 to 0·1)	42·8
Vascular intestinal disorders	0 (0 to 0)	0 (0 to 0)	0 (0 to 0)	0 (0 to 0·1)	0·1 (0 to 0·1)	0 (0 to 0·1)	0·1 (0 to 0·1)	0·1 (0·1 to 0·2)	0·2 (0·1 to 0·2)	0·2 (0·2 to 0·3)	0·3 (0·2 to 0·4)	0·4 (0·3 to 0·6)	0·8 (0·5 to 1·2)	1·2 (0·8 to 1·7)	1·4 (1 to 2)	1·8 (1·3 to 2·5)	2·1 (1·5 to 2·8)	2·3 (1·6 to 3·2)	2 (1·4 to 2·8)	1·1 (0·8 to 1·5)	0·5 (0·4 to 0·7)	0·2 (0·1 to 0·3)	0·1 (0 to 0·1)	14·9
Gallbladder and biliary diseases	0 (0 to 0)	0 (0 to 0)	0 (0 to 0)	0·2 (0·1 to 0·3)	0·4 (0·2 to 0·8)	0·7 (0·3 to 1·2)	1·3 (0·7 to 2·2)	2·4 (1·4 to 3·7)	3·1 (1·8 to 4·7)	3·7 (2·5 to 5·4)	3·9 (2·6 to 5·7)	5 (3·4 to 7·3)	6·6 (4·4 to 9·4)	8·3 (5·8 to 11·6)	8·5 (6·1 to 11·7)	7·6 (5·5 to 10)	6·9 (5·1 to 9·3)	6·5 (4·9 to 8·9)	5·3 (3·8 to 7·1)	2·5 (1·8 to 3·4)	1 (0·8 to 1·4)	0·5 (0·4 to 0·7)	0·2 (0·1 to 0·2)	74·9
Pancreatitis	0 (0 to 0)	0 (0 to 0)	0 (0 to 0)	0·1 (0 to 0·1)	0 (0 to 0·1)	0·1 (0·1 to 0·2)	0·4 (0·3 to 0·6)	1·1 (0·7 to 1·5)	1·4 (0·9 to 1·9)	2 (1·4 to 3)	2·4 (1·6 to 3·3)	2·9 (2 to 4·1)	3·6 (2·5 to 5)	3·7 (2·6 to 5·2)	3·6 (2·5 to 5)	3·1 (2·3 to 4·1)	2·5 (1·8 to 3·3)	2·2 (1·6 to 2·9)	1·6 (1·2 to 2·1)	0·7 (0·5 to 0·9)	0·3 (0·2 to 0·4)	0·1 (0·1 to 0·1)	0 (0 to 0)	31·8
Other digestive diseases	0 (0 to 0)	0 (0 to 0)	0 (0 to 0)	3·1 (1·8 to 4·8)	1·6 (1·1 to 2·2)	1·2 (0·8 to 1·6)	1·6 (1 to 2·2)	2·3 (1·3 to 3·2)	2·4 (1·4 to 3·6)	3 (1·8 to 4·5)	3 (1·7 to 4·5)	3·6 (2·1 to 5·4)	6 (3·5 to 9)	8·1 (4·9 to 11·6)	9·1 (5·5 to 13)	10·3 (6·2 to 14·4)	9·3 (5·7 to 12·9)	9·4 (5·6 to 13·1)	8·2 (5 to 11·5)	4·6 (2·9 to 6·2)	2·5 (1·7 to 3·5)	1·3 (0·8 to 1·8)	0·4 (0·3 to 0·6)	91
Neurological disorders	0 (0 to 0)	0·1 (0 to 0·2)	5·8 (3·6 to 8·8)	12·8 (6·1 to 22·1)	24·6 (11·4 to 43)	63·6 (22·4 to 131)	92·2 (37·2 to 182)	106 (44·5 to 202)	100 (42 to 191)	84·5 (37 to 155)	73·7 (33·8 to 134)	78·2 (37·7 to 140)	88·4 (45·9 to 155)	88·7 (49·3 to 151)	78·3 (48·8 to 124)	65·8 (43·4 to 98·4)	63·4 (43·2 to 98·3)	72·2 (49·1 to 119)	81·3 (54·3 to 139)	66·6 (39 to 127)	45·5 (23·5 to 93·6)	30·3 (14·2 to 65·1)	15·4 (6·7 to 33·8)	1,340
Alzheimer's disease and other dementias	0 (0 to 0)	0 (0 to 0)	0 (0 to 0)	0 (0 to 0)	0 (0 to 0)	0 (0 to 0)	0 (0 to 0)	0 (0 to 0)	0 (0 to 0)	0 (0 to 0)	0 (0 to 0)	0·4 (0·1 to 1·2)	2·6 (0·8 to 7)	5·9 (2·1 to 14·6)	10·4 (4 to 24·8)	15·1 (6 to 36·4)	21·1 (8·4 to 52·5)	32·3 (14 to 75·6)	43·4 (18·8 to 102)	46·9 (19·7 to 110)	37·1 (14·9 to 85·6)	27 (10·7 to 62·1)	14·5 (5·8 to 33)	257
Parkinson's disease	0 (0 to 0)	0 (0 to 0)	0 (0 to 0)	0 (0 to 0)	0 (0 to 0)	0 (0 to 0)	0 (0 to 0)	0 (0 to 0·1)	0·1 (0 to 0·1)	0·1 (0·1 to 0·2)	0·2 (0·1 to 0·3)	0·5 (0·4 to 0·7)	1·3 (1 to 1·7)	2·5 (1·9 to 3·2)	3·8 (3·1 to 4·8)	6 (5·1 to 7·1)	10·1 (8·6 to 11·6)	16·8 (14·7 to 18·9)	22·6 (20·1 to 25·7)	13·5 (11·7 to 15·4)	6 (5·1 to 6·9)	2·3 (1·9 to 2·6)	0·6 (0·5 to 0·7)	86·5
Idiopathic epilepsy	0 (0 to 0)	0·1 (0 to 0·1)	2·2 (1·2 to 3·7)	8·7 (3·8 to 15·6)	12·3 (4·4 to 23·1)	16·1 (5·9 to 29·8)	23·3 (11·1 to 41·6)	28·4 (14·6 to 48)	25·6 (12·8 to 43·2)	21·7 (12 to 34·9)	18·6 (10·6 to 29·6)	18·1 (10 to 29·6)	20·9 (12 to 32·5)	18·8 (10·6 to 30·4)	16·3 (8·9 to 26·2)	12·5 (6·4 to 20·4)	10 (4·9 to 16·8)	8·3 (3·7 to 14·2)	6·5 (3 to 11·3)	2·9 (1·2 to 5·2)	1·2 (0·5 to 2·2)	0·6 (0·2 to 1)	0·2 (0·1 to 0·3)	273
Multiple sclerosis	0 (0 to 0)	0 (0 to 0)	0 (0 to 0)	0 (0 to 0)	0 (0 to 0)	0 (0 to 0)	0·2 (0·1 to 0·3)	0·7 (0·5 to 1)	0·8 (0·6 to 1·2)	1·1 (0·8 to 1·6)	1·7 (1·2 to 2·5)	1·9 (1·4 to 2·9)	2·3 (1·7 to 3·5)	2·3 (1·7 to 3·4)	2·3 (1·8 to 3·7)	1·6 (1·2 to 2·4)	0·9 (0·7 to 1·4)	0·6 (0·5 to 1)	0·3 (0·3 to 0·5)	0·1 (0·1 to 0·2)	0 (0 to 0·1)	0 (0 to 0)	0 (0 to 0)	17
Motor neuron disease	0 (0 to 0)	0 (0 to 0)	0·8 (0·4 to 1·5)	0·3 (0·2 to 0·5)	0 (0 to 0·1)	0·1 (0·1 to 0·1)	0·3 (0·2 to 0·5)	0·4 (0·3 to 0·5)	0·3 (0·2 to 0·4)	0·4 (0·3 to 0·6)	0·8 (0·6 to 1·1)	1·3 (0·9 to 1·8)	1·8 (1·3 to 2·5)	2·8 (2 to 3·7)	3·1 (2·4 to 4·1)	2·8 (2·2 to 3·5)	2·7 (2·1 to 3·5)	1·7 (1·3 to 2·2)	0·8 (0·6 to 1·1)	0·2 (0·1 to 0·2)	0 (0 to 0·1)	0 (0 to 0)	0 (0 to 0)	21
Headache disorders	0 (0 to 0)	0 (0 to 0)	0 (0 to 0)	0 (0 to 0)	7·7 (0·1 to 20·1)	39·9 (1·9 to 100)	59·2 (6 to 148)	69·6 (9·2 to 164)	68·5 (10·9 to 158)	57 (10·5 to 126)	48·6 (9·3 to 107)	51·7 (11·9 to 113)	54·3 (14 to 117)	50·7 (13·9 to 108)	36·8 (10·6 to 77·4)	23·7 (7·7 to 49·1)	15·3 (5·2 to 31)	10 (3·2 to 20·7)	5·9 (1·9 to 12·3)	2·4 (0·8 to 4·9)	0·9 (0·3 to 2)	0·4 (0·1 to 0·8)	0·1 (0 to 0·2)	603
Migraine	0 (0 to 0)	0 (0 to 0)	0 (0 to 0)	0 (0 to 0)	7 (0·1 to 19·3)	37·4 (1·2 to 97·3)	55·1 (4·1 to 145)	64·3 (6·5 to 157)	62·7 (7·3 to 150)	52 (6·9 to 122)	44 (6·7 to 102)	46·7 (8·4 to 107)	48·6 (9·9 to 110)	44·7 (9·3 to 101)	32·4 (7·3 to 71·6)	20·4 (5·4 to 44)	12·9 (3·6 to 27·7)	8·2 (2·4 to 17·7)	4·8 (1·4 to 10·8)	1·9 (0·5 to 4·2)	0·8 (0·2 to 1·7)	0·3 (0·1 to 0·6)	0·1 (0 to 0·2)	544
Tension-type headache	0 (0 to 0)	0 (0 to 0)	0 (0 to 0)	0 (0 to 0)	0·6 (0 to 4·3)	2·5 (0·2 to 17·6)	4·1 (0·7 to 20·1)	5·4 (1·1 to 23·6)	5·8 (1·2 to 23·3)	5 (1·1 to 19)	4·6 (1·1 to 15·9)	5 (1·4 to 16·2)	5·7 (1·8 to 16·8)	6 (1·7 to 18)	4·4 (1·4 to 12·1)	3·3 (1 to 9·5)	2·4 (0·7 to 7·8)	1·8 (0·4 to 6·1)	1·1 (0·2 to 4)	0·4 (0·1 to 1·5)	0·2 (0 to 0·6)	0·1 (0 to 0·3)	0 (0 to 0·1)	58·2
Other neurological disorders	0 (0 to 0)	0 (0 to 0)	2·7 (1·6 to 4·4)	3·8 (1·8 to 6·4)	4·5 (1·9 to 8·2)	7·5 (3·2 to 13·3)	9·2 (4·9 to 15·2)	7·3 (4·1 to 11·5)	5 (2·7 to 8·1)	4·1 (2·4 to 6·5)	3·7 (2·2 to 5·7)	4·3 (2·6 to 6·6)	5·3 (3·3 to 7·9)	5·8 (4 to 8)	5·5 (4 to 7·3)	4·1 (3 to 5·5)	3·2 (2·3 to 4·1)	2·4 (1·8 to 3·2)	1·6 (1·2 to 2·1)	0·6 (0·5 to 0·8)	0·2 (0·1 to 0·2)	0·1 (0·1 to 0·1)	0 (0 to 0)	80·9
Mental disorders	0 (0 to 0)	0·1 (0 to 0·1)	1 (0·6 to 1·5)	5·8 (3·8 to 8·2)	32·4 (21·7 to 46·4)	100 (68·4 to 144)	160 (110 to 221)	195 (134 to 268)	198 (138 to 271)	167 (117 to 225)	144 (105 to 191)	149 (108 to 202)	165 (120 to 223)	164 (120 to 219)	132 (97 to 176)	90·7 (65 to 121)	62·1 (44·9 to 81·1)	45·7 (33 to 60·1)	30·6 (21·9 to 40·6)	13 (9·1 to 17·4)	5·6 (4 to 7·6)	2·6 (1·7 to 3·6)	0·9 (0·6 to 1·3)	1,860
Schizophrenia	0 (0 to 0)	0 (0 to 0)	0 (0 to 0)	0 (0 to 0)	0 (0 to 0)	0·5 (0·2 to 0·9)	3·8 (1·7 to 7)	14·8 (8·1 to 24·4)	24·7 (14·3 to 36·7)	25·2 (15·9 to 36·4)	22·9 (14·6 to 32·6)	23·4 (15·5 to 32·5)	24·9 (16·8 to 33·5)	23·2 (16 to 31·3)	16·9 (11·6 to 23)	10·3 (7 to 14)	6·2 (4·1 to 8·8)	3·9 (2·6 to 5·4)	2·2 (1·4 to 3·1)	0·7 (0·4 to 1·1)	0·2 (0·1 to 0·3)	0·1 (0 to 0·1)	0 (0 to 0)	204
Depressive disorders	0 (0 to 0)	0 (0 to 0)	0 (0 to 0)	0 (0 to 0)	1·6 (0·6 to 2·9)	16·8 (8·9 to 29·1)	47·4 (27·4 to 75·1)	68·2 (40·4 to 105)	63·2 (38·4 to 95·9)	51·5 (31 to 76·7)	46·8 (30·4 to 67·4)	51·6 (32·4 to 78·3)	59·9 (38·5 to 87·6)	63·6 (41·8 to 91·9)	54·2 (34·8 to 78·9)	39·4 (25 to 58)	28·1 (18·4 to 39·7)	21·5 (14·1 to 30·3)	15·1 (9·3 to 22·4)	6·8 (4·2 to 10·2)	3·2 (2·1 to 4·7)	1·6 (1 to 2·4)	0·6 (0·3 to 0·9)	641
Major depressive disorder	0 (0 to 0)	0 (0 to 0)	0 (0 to 0)	0 (0 to 0)	1·4 (0·4 to 2·7)	15·3 (7·6 to 27·5)	42·6 (24 to 69·1)	59·1 (32·9 to 94·9)	52·1 (30·5 to 81·8)	41·5 (23·8 to 64·6)	37·9 (23·3 to 55·1)	42 (24·9 to 65·3)	49·2 (31·1 to 73·9)	52·8 (33·7 to 77·5)	45·6 (27·9 to 67·9)	33·4 (19·9 to 49·4)	23·9 (15·3 to 33·8)	18·5 (11·8 to 26·8)	13 (7·8 to 20·1)	6 (3·4 to 9·2)	2·9 (1·8 to 4·2)	1·4 (0·9 to 2·2)	0·5 (0·3 to 0·9)	539
Dysthymia	0 (0 to 0)	0 (0 to 0)	0 (0 to 0)	0 (0 to 0)	0·2 (0 to 0·5)	1·5 (0·6 to 3)	4·7 (2·4 to 8·2)	9·1 (4·6 to 16·3)	11 (5·8 to 19·5)	10 (5·7 to 16·2)	8·9 (5 to 14·7)	9·5 (5·2 to 16·3)	10·7 (5·8 to 18·4)	10·7 (6·2 to 17·7)	8·6 (5·1 to 14·1)	6 (3·2 to 10·2)	4·2 (2·3 to 7)	3·1 (1·7 to 5)	2 (1·2 to 3·3)	0·8 (0·5 to 1·4)	0·3 (0·2 to 0·6)	0·2 (0·1 to 0·3)	0·1 (0 to 0·1)	102
Bipolar disorder	0 (0 to 0)	0 (0 to 0)	0 (0 to 0)	0 (0 to 0)	0 (0 to 0)	5·6 (2·3 to 10·5)	21·9 (10·4 to 38·2)	30·2 (15·3 to 50·7)	28·9 (14·6 to 47·9)	22·3 (12·1 to 36·2)	18·3 (10 to 29·9)	18·9 (10·6 to 30·5)	20·7 (11·8 to 33·9)	20·1 (11·2 to 33·6)	15·2 (8·7 to 25·3)	9·7 (5·6 to 15·7)	6 (3·4 to 9·6)	3·9 (2·1 to 6·4)	2·2 (1·2 to 3·5)	0·7 (0·4 to 1·2)	0·2 (0·1 to 0·4)	0·1 (0 to 0·1)	0 (0 to 0)	225
Anxiety disorders	0 (0 to 0)	0 (0 to 0)	0 (0 to 0)	0·7 (0·2 to 1·3)	10·7 (5·5 to 18·4)	29·2 (16 to 48·1)	41·4 (25·1 to 63·9)	48·7 (27·4 to 77·9)	47·7 (26·6 to 78)	39·6 (23·7 to 62·1)	33·5 (20·6 to 51·1)	35 (19·7 to 55·9)	38·2 (22·7 to 60·6)	37·9 (23·7 to 58·2)	30 (18·9 to 44·8)	20·7 (12·7 to 32)	14·4 (8·8 to 22·7)	10·7 (6·7 to 16·9)	7·2 (4·5 to 11·2)	3 (1·8 to 4·7)	1·2 (0·7 to 1·9)	0·5 (0·3 to 0·8)	0·1 (0·1 to 0·2)	450
Eating disorders	0 (0 to 0)	0 (0 to 0)	0 (0 to 0)	0 (0 to 0)	0·1 (0 to 0·2)	2 (1 to 3·5)	7·4 (3·8 to 13·2)	12·3 (6·5 to 20·8)	11·5 (6·5 to 18·3)	8·2 (4·5 to 13·4)	5·1 (2·9 to 8·3)	3 (1·7 to 5)	1·8 (0·9 to 3)	0 (0 to 0)	0 (0 to 0)	0 (0 to 0)	0 (0 to 0)	0 (0 to 0)	0 (0 to 0)	0 (0 to 0)	0 (0 to 0)	0 (0 to 0)	0 (0 to 0)	51·5
Anorexia nervosa	0 (0 to 0)	0 (0 to 0)	0 (0 to 0)	0 (0 to 0)	0·1 (0 to 0·2)	1 (0·4 to 1·8)	2·8 (1·3 to 5)	3·1 (1·6 to 5·6)	2·3 (1·1 to 3·9)	1·2 (0·6 to 2·2)	0·7 (0·3 to 1·4)	0·6 (0·2 to 1·1)	0·5 (0·2 to 0·9)	0 (0 to 0)	0 (0 to 0)	0 (0 to 0)	0 (0 to 0)	0 (0 to 0)	0 (0 to 0)	0 (0 to 0)	0 (0 to 0)	0 (0 to 0)	0 (0 to 0)	12·3
Bulimia nervosa	0 (0 to 0)	0 (0 to 0)	0 (0 to 0)	0 (0 to 0)	0 (0 to 0)	1·1 (0·3 to 2·2)	4·6 (2 to 9·5)	9·2 (4·1 to 16·8)	9·2 (4·9 to 15·7)	7 (3·7 to 11·9)	4·4 (2·2 to 7·5)	2·4 (1·2 to 4·2)	1·3 (0·5 to 2·3)	0 (0 to 0)	0 (0 to 0)	0 (0 to 0)	0 (0 to 0)	0 (0 to 0)	0 (0 to 0)	0 (0 to 0)	0 (0 to 0)	0 (0 to 0)	0 (0 to 0)	39·2
Autism spectrum disorders	0 (0 to 0)	0·1 (0 to 0·1)	0·9 (0·5 to 1·2)	3·7 (2·4 to 5·4)	4·7 (3 to 7)	4·9 (3·1 to 7·4)	5·2 (3·3 to 7·7)	5·6 (3·4 to 8·4)	5·1 (3·2 to 7·5)	3·9 (2·5 to 5·8)	3·1 (1·9 to 4·6)	3·1 (1·9 to 4·6)	3·2 (2 to 4·8)	3·2 (2 to 4·7)	2·6 (1·6 to 3·9)	1·8 (1·1 to 2·5)	1·2 (0·8 to 1·8)	0·9 (0·6 to 1·3)	0·6 (0·4 to 0·9)	0·2 (0·1 to 0·3)	0·1 (0·1 to 0·1)	0 (0 to 0)	0 (0 to 0)	54·1
Attention-deficit/hyperactivity disorder	0 (0 to 0)	0 (0 to 0)	0 (0 to 0)	0·5 (0·2 to 0·8)	5·1 (2·7 to 9·2)	7·4 (4 to 12·9)	6·6 (3·6 to 11·7)	5·4 (3 to 9·3)	3·9 (2·1 to 6·6)	2·5 (1·3 to 4·2)	1·7 (0·8 to 2·9)	1·4 (0·7 to 2·5)	1·2 (0·6 to 2·2)	1 (0·5 to 1·8)	0·6 (0·3 to 1·1)	0·3 (0·1 to 0·5)	0·1 (0 to 0·2)	0 (0 to 0·1)	0 (0 to 0)	0 (0 to 0)	0 (0 to 0)	0 (0 to 0)	0 (0 to 0)	37·6
Conduct disorder	0 (0 to 0)	0 (0 to 0)	0 (0 to 0)	0 (0 to 0)	8·9 (4·6 to 15·8)	32 (17·4 to 52·7)	21·5 (11·2 to 36·3)	0 (0 to 0)	0 (0 to 0)	0 (0 to 0)	0 (0 to 0)	0 (0 to 0)	0 (0 to 0)	0 (0 to 0)	0 (0 to 0)	0 (0 to 0)	0 (0 to 0)	0 (0 to 0)	0 (0 to 0)	0 (0 to 0)	0 (0 to 0)	0 (0 to 0)	0 (0 to 0)	62·3
Idiopathic developmental intellectual disability	0 (0 to 0)	0 (0 to 0)	0·1 (0 to 0·3)	0·9 (0·2 to 1·8)	1·4 (0·4 to 2·6)	1·4 (0·4 to 2·7)	1·5 (0·5 to 2·8)	1·6 (0·5 to 3)	1·4 (0·4 to 2·7)	1·1 (0·3 to 2·1)	0·9 (0·3 to 1·6)	0·8 (0·2 to 1·6)	0·9 (0·3 to 1·6)	0·8 (0·2 to 1·5)	0·6 (0·2 to 1·2)	0·4 (0·1 to 0·8)	0·3 (0·1 to 0·5)	0·2 (0 to 0·3)	0·1 (0 to 0·2)	0 (0 to 0·1)	0 (0 to 0)	0 (0 to 0)	0 (0 to 0)	14·4
Other mental disorders	0 (0 to 0)	0 (0 to 0)	0 (0 to 0)	0 (0 to 0)	0 (0 to 0)	0·4 (0·2 to 0·7)	3 (1·3 to 5·3)	8·3 (4·2 to 13·8)	12·1 (6·8 to 19·1)	12·3 (7·2 to 18·9)	11·5 (6·8 to 17·7)	12·2 (7·5 to 18·5)	13·7 (8·4 to 21·1)	14 (8·5 to 21·4)	11·5 (7·2 to 17·4)	8·1 (5 to 12·3)	5·9 (3·8 to 8·7)	4·6 (3 to 6·8)	3·3 (2·2 to 4·8)	1·5 (0·9 to 2·1)	0·6 (0·4 to 0·9)	0·3 (0·2 to 0·4)	0·1 (0·1 to 0·2)	124
Substance use disorders	0 (0 to 0)	0 (0 to 0)	0 (0 to 0)	0 (0 to 0)	0 (0 to 0)	1·2 (0·7 to 2·1)	25·2 (16·8 to 36)	53·1 (36·8 to 73·6)	56·1 (40·2 to 76·2)	49·5 (36·9 to 65·8)	43·8 (34·2 to 56·3)	45·6 (34·8 to 58)	53 (40·5 to 67·7)	49 (38·3 to 63·3)	41·4 (32·6 to 51·8)	25·9 (19·6 to 33·5)	16·6 (13·1 to 20·8)	9·2 (7·2 to 11·7)	4·9 (3·9 to 6·2)	1·6 (1·2 to 2·1)	0·7 (0·5 to 0·9)	0·4 (0·3 to 0·5)	0·2 (0·1 to 0·3)	478
Alcohol use disorders	0 (0 to 0)	0 (0 to 0)	0 (0 to 0)	0 (0 to 0)	0 (0 to 0)	0·5 (0·2 to 1)	6·9 (3·9 to 11)	20·9 (12·2 to 34·5)	26·5 (16·7 to 39·9)	27·4 (18·6 to 39·4)	27·5 (20 to 36·9)	32 (23 to 42·6)	40·4 (29·7 to 54·1)	39·8 (29·9 to 52·7)	35·2 (26·5 to 45·2)	22·6 (16·5 to 29·7)	14·6 (11 to 18·8)	7·8 (5·9 to 10·2)	4 (3·1 to 5·1)	1·2 (0·9 to 1·7)	0·5 (0·4 to 0·7)	0·3 (0·2 to 0·4)	0·2 (0·1 to 0·2)	308
Drug use disorders	0 (0 to 0)	0 (0 to 0)	0 (0 to 0)	0 (0 to 0)	0 (0 to 0)	0·7 (0·4 to 1·3)	18·3 (11·6 to 27·6)	32·1 (21·7 to 45·4)	29·7 (21 to 40·2)	22·1 (16·1 to 29·4)	16·3 (11·8 to 21·3)	13·6 (9·9 to 18·1)	12·6 (9·2 to 16·5)	9·2 (6·6 to 12·4)	6·2 (4·3 to 8·4)	3·3 (2·2 to 4·7)	2 (1·4 to 2·8)	1·4 (0·9 to 2)	1 (0·6 to 1·4)	0·4 (0·3 to 0·6)	0·2 (0·1 to 0·2)	0·1 (0 to 0·1)	0 (0 to 0)	169
Opioid use disorders	0 (0 to 0)	0 (0 to 0)	0 (0 to 0)	0 (0 to 0)	0 (0 to 0)	0 (0 to 0)	2 (0·9 to 3·6)	9·5 (5·3 to 15·8)	11·3 (6·8 to 17·6)	9 (5·5 to 13·5)	6·5 (4·1 to 9·7)	5·4 (3·5 to 8·1)	5 (3 to 7·2)	4·3 (2·5 to 6·7)	3·1 (1·8 to 4·7)	1·8 (0·9 to 2·9)	1·1 (0·6 to 1·8)	0·7 (0·4 to 1·2)	0·5 (0·3 to 0·8)	0·2 (0·1 to 0·4)	0·1 (0 to 0·1)	0 (0 to 0·1)	0 (0 to 0)	60·6
Cocaine use disorders	0 (0 to 0)	0 (0 to 0)	0 (0 to 0)	0 (0 to 0)	0 (0 to 0)	0·1 (0·1 to 0·2)	6 (3·2 to 10)	8·7 (5·5 to 13·2)	7·6 (5·2 to 10·9)	6·5 (4·5 to 9)	5·2 (3·6 to 7·2)	4·4 (2·9 to 6·2)	4·1 (2·7 to 5·7)	2·3 (1·6 to 3·4)	1·6 (1 to 2·3)	0·7 (0·5 to 1·1)	0·4 (0·3 to 0·7)	0·3 (0·2 to 0·5)	0·2 (0·1 to 0·3)	0·1 (0 to 0·1)	0 (0 to 0)	0 (0 to 0)	0 (0 to 0)	48·2
Amphetamine use disorders	0 (0 to 0)	0 (0 to 0)	0 (0 to 0)	0 (0 to 0)	0 (0 to 0)	0 (0 to 0)	0·5 (0·2 to 1)	2·5 (1·2 to 4·5)	2·4 (1·1 to 4·2)	1·4 (0·8 to 2·4)	0·9 (0·5 to 1·4)	0·7 (0·5 to 1)	0·6 (0·4 to 0·8)	0·5 (0·3 to 0·6)	0·3 (0·2 to 0·4)	0·1 (0·1 to 0·2)	0·1 (0·1 to 0·1)	0 (0 to 0)	0 (0 to 0)	0 (0 to 0)	0 (0 to 0)	0 (0 to 0)	0 (0 to 0)	10
Cannabis use disorders	0 (0 to 0)	0 (0 to 0)	0 (0 to 0)	0 (0 to 0)	0 (0 to 0)	0·5 (0·2 to 1·1)	5·5 (2·5 to 10·4)	4·9 (2·3 to 8·7)	2·7 (1·2 to 5·3)	1·4 (0·6 to 2·5)	0·8 (0·4 to 1·6)	0·7 (0·3 to 1·3)	0·7 (0·3 to 1·3)	0·5 (0·2 to 1)	0·3 (0·1 to 0·6)	0·1 (0·1 to 0·2)	0·1 (0 to 0·1)	0 (0 to 0·1)	0 (0 to 0)	0 (0 to 0)	0 (0 to 0)	0 (0 to 0)	0 (0 to 0)	18·2
Other drug use disorders	0 (0 to 0)	0 (0 to 0)	0 (0 to 0)	0 (0 to 0)	0 (0 to 0)	0·1 (0 to 0·2)	4·2 (2·3 to 7·1)	6·6 (4·1 to 10·2)	5·7 (3·6 to 8·5)	3·9 (2·6 to 5·5)	2·9 (1·9 to 4·1)	2·4 (1·6 to 3·4)	2·3 (1·6 to 3·2)	1·6 (1 to 2·2)	1 (0·6 to 1·4)	0·5 (0·3 to 0·8)	0·3 (0·2 to 0·5)	0·3 (0·2 to 0·4)	0·2 (0·2 to 0·3)	0·1 (0·1 to 0·2)	0 (0 to 0·1)	0 (0 to 0)	0 (0 to 0)	32·1
Diabetes and kidney diseases	0 (0 to 0)	0 (0 to 0·1)	5·9 (3·6 to 8·9)	6·6 (4·5 to 9·3)	9·6 (7·2 to 12·6)	11·1 (8·4 to 15)	35·8 (28·5 to 43·5)	59·8 (48 to 73·6)	78·5 (61·5 to 98·9)	101 (80·9 to 124)	122 (98·4 to 149)	174 (140 to 212)	299 (241 to 361)	447 (367 to 535)	536 (445 to 636)	549 (472 to 637)	562 (486 to 652)	501 (434 to 577)	404 (352 to 464)	191 (163 to 216)	80·6 (68·1 to 92·8)	36·2 (30·1 to 42·1)	12 (9·6 to 14·3)	4,220
Diabetes mellitus	0 (0 to 0)	0 (0 to 0·1)	1·4 (0·7 to 2·5)	1·1 (0·7 to 1·8)	1·2 (0·8 to 1·7)	2·1 (1·4 to 2·8)	12 (9·7 to 14·9)	23·6 (18·4 to 29·9)	33·6 (25·8 to 43·9)	46 (35·4 to 58·7)	61·2 (47·9 to 76·1)	97·4 (75·1 to 124)	181 (141 to 230)	295 (228 to 367)	357 (286 to 437)	371 (303 to 446)	383 (319 to 459)	353 (297 to 414)	285 (241 to 335)	134 (112 to 157)	55·6 (45·6 to 66)	24·4 (19·7 to 29·6)	7·5 (5·9 to 9·2)	2,730
Diabetes mellitus type 1	0 (0 to 0)	0 (0 to 0·1)	1·4 (0·7 to 2·5)	1·1 (0·7 to 1·8)	1·2 (0·8 to 1·7)	2·1 (1·4 to 2·8)	3·8 (2·8 to 5)	7 (5·2 to 9·3)	8·8 (6·3 to 11·8)	7·2 (5 to 10·2)	9·1 (6·5 to 13·3)	12·9 (9·2 to 18·3)	5·6 (3·9 to 8·1)	8 (5·5 to 11·7)	9·8 (6·8 to 14)	8·2 (5·6 to 11·9)	8·5 (6 to 12·9)	5·9 (4·2 to 9·3)	4·8 (3·4 to 7·6)	1·5 (1 to 2·4)	0·6 (0·4 to 1)	0·3 (0·2 to 0·4)	0·1 (0·1 to 0·2)	108
Diabetes mellitus type 2	0 (0 to 0)	0 (0 to 0)	0 (0 to 0)	0 (0 to 0)	0 (0 to 0)	0 (0 to 0)	8·2 (6·2 to 10·7)	16·6 (12 to 22·1)	24·8 (17·8 to 34·1)	38·8 (28·9 to 50·8)	52·1 (39·6 to 66)	84·5 (63·3 to 110)	176 (135 to 224)	287 (221 to 357)	347 (277 to 426)	363 (297 to 437)	374 (311 to 449)	347 (292 to 408)	280 (237 to 330)	132 (111 to 155)	55 (45·1 to 65·3)	24·1 (19·4 to 29·3)	7·4 (5·8 to 9·1)	2,620
Chronic kidney disease	0 (0 to 0)	0 (0 to 0)	4·5 (2·7 to 7)	5·5 (3·7 to 7·8)	8·4 (6·1 to 11·3)	9·1 (6·4 to 12·7)	23·7 (17·2 to 30·9)	36·1 (26·8 to 47)	44·8 (32·1 to 59·9)	54·6 (40·6 to 71·3)	61·2 (45·2 to 80·3)	76·5 (57·3 to 98·6)	118 (88·9 to 151)	152 (116 to 194)	179 (138 to 230)	179 (146 to 214)	179 (140 to 223)	149 (119 to 183)	119 (94·5 to 150)	56·7 (45·2 to 69·1)	25 (20 to 30·8)	11·8 (9·1 to 14·8)	4·5 (3·3 to 5·6)	1,500
Chronic kidney disease due to diabetes mellitus type 1	0 (0 to 0)	0 (0 to 0)	0 (0 to 0·1)	0·1 (0 to 0·2)	0·2 (0·1 to 0·5)	0·3 (0·1 to 0·6)	1 (0·4 to 2)	2·1 (0·9 to 4·4)	4·7 (2 to 8·7)	9 (4·6 to 15·1)	11·7 (6·1 to 19·5)	13·9 (7·1 to 22·5)	18·3 (8·3 to 30·9)	17·5 (7·2 to 32·6)	14·4 (4·8 to 30)	10·6 (3·8 to 22·2)	8·3 (3·1 to 18·1)	5·1 (2 to 10·6)	2·7 (1 to 6)	0·7 (0·2 to 1·5)	0·1 (0 to 0·3)	0 (0 to 0)	0 (0 to 0)	121
Chronic kidney disease due to diabetes mellitus type 2	0 (0 to 0)	0 (0 to 0)	0 (0 to 0)	0 (0 to 0)	0 (0 to 0)	0 (0 to 0)	0·4 (0·1 to 1)	1 (0·4 to 2·2)	1·9 (0·8 to 3·8)	3·5 (1·5 to 6·7)	5·4 (2·4 to 10·2)	9·6 (4·7 to 16·7)	21·3 (11·6 to 33·7)	37·1 (21·6 to 55·3)	52·5 (31·4 to 77·3)	56·5 (35·1 to 79·5)	58·1 (37·5 to 81·5)	48·5 (33 to 65·3)	38·7 (26·3 to 53)	18 (12·2 to 24·9)	7·7 (5 to 10·5)	3·5 (2·2 to 4·8)	1·2 (0·7 to 1·8)	365
Chronic kidney disease due to hypertension	0 (0 to 0)	0 (0 to 0)	0 (0 to 0)	0 (0 to 0)	0 (0 to 0)	0 (0 to 0)	1·6 (0·7 to 3)	2·8 (1·4 to 5·3)	4·1 (2 to 6·9)	5·2 (2·8 to 8·2)	6·2 (3·6 to 9·7)	9·1 (5·7 to 13·8)	16·1 (10·5 to 23·7)	24·1 (15·6 to 35·2)	33·1 (21·5 to 49)	38·6 (26 to 53·6)	43·8 (29·5 to 62·2)	39·7 (27·8 to 54·9)	35·2 (25·1 to 49·2)	18·5 (13 to 24·7)	9 (6·7 to 11·9)	4·6 (3·3 to 6·1)	1·8 (1·2 to 2·5)	293
Chronic kidney disease due to glomerulonephritis	0 (0 to 0)	0 (0 to 0)	1·3 (0·6 to 2·4)	1·8 (1 to 2·9)	3·1 (1·8 to 4·7)	3·7 (2·2 to 5·5)	9·8 (6·3 to 14·4)	14·4 (9 to 21·2)	16·6 (10·3 to 24·6)	18·6 (11·7 to 26·7)	19·5 (12·1 to 28·8)	23·1 (13·2 to 34·3)	32 (20·1 to 47·8)	37·2 (21·4 to 56·6)	40·2 (22·8 to 63·1)	35·2 (19·4 to 55·3)	30·4 (15·7 to 52·3)	21·4 (11·3 to 36·4)	14·9 (7·2 to 25·8)	5·9 (2·7 to 10·7)	2 (0·9 to 3·4)	0·8 (0·4 to 1·4)	0·3 (0·1 to 0·6)	332
Chronic kidney disease due to other and unspecified causes	0 (0 to 0)	0 (0 to 0)	3·2 (1·8 to 5·3)	3·7 (2·4 to 5·3)	5·2 (3·5 to 7·4)	5·1 (3·1 to 7·8)	10·9 (6·6 to 15·8)	15·8 (10·2 to 22·4)	17·5 (11·3 to 25·4)	18·3 (11·8 to 27·7)	18·4 (11·2 to 27·7)	20·9 (12·5 to 30·9)	30·3 (18·5 to 46·8)	36·1 (21·9 to 55·6)	39·2 (23 to 62)	37·6 (22 to 59·5)	38·7 (22·7 to 59·5)	34 (20·7 to 51)	27·9 (16·8 to 42·2)	13·6 (8·8 to 20)	6·3 (4 to 8·9)	3 (2 to 4·2)	1·1 (0·7 to 1·7)	387
Acute glomerulonephritis	0 (0 to 0)	0 (0 to 0)	0 (0 to 0)	0 (0 to 0)	0 (0 to 0)	0 (0 to 0)	0 (0 to 0)	0 (0 to 0)	0 (0 to 0)	0 (0 to 0)	0 (0 to 0)	0 (0 to 0)	0 (0 to 0)	0 (0 to 0)	0 (0 to 0)	0 (0 to 0)	0 (0 to 0)	0 (0 to 0)	0 (0 to 0)	0 (0 to 0)	0 (0 to 0)	0 (0 to 0)	0 (0 to 0)	0·1
Skin and subcutaneous diseases	0 (0 to 0)	0 (0 to 0)	9·1 (5·4 to 14·7)	38·5 (24·7 to 56·8)	49·4 (31·3 to 71·9)	52·2 (34 to 77·4)	60·9 (40 to 91·1)	45·8 (29·8 to 67·4)	38·9 (25·8 to 55·5)	31·9 (21·6 to 46)	27·7 (19 to 39·2)	31·3 (22·3 to 44·1)	37·1 (25·9 to 52·2)	46·2 (33·4 to 65·7)	46·1 (32·9 to 65·6)	40·9 (30·2 to 57·5)	38·8 (28·3 to 54·1)	38·8 (28·3 to 53·6)	38·7 (27·9 to 54·7)	22 (15·9 to 31·7)	13 (9·1 to 20·4)	7·6 (5·1 to 11·5)	3·4 (2·2 to 5·9)	718
Dermatitis	0 (0 to 0)	0 (0 to 0)	0 (0 to 0)	13 (7 to 22)	15·9 (8·4 to 27·5)	11·7 (6·5 to 19·7)	9·4 (5·4 to 15·3)	8·6 (4·9 to 13·8)	7·5 (4·3 to 12·1)	5·9 (3·4 to 9·5)	4·7 (2·7 to 7·5)	4·8 (2·8 to 7·8)	5·4 (3 to 8·8)	5·9 (3·3 to 9·8)	5·3 (2·9 to 8·6)	4·1 (2·3 to 6·6)	3·3 (1·9 to 5·4)	2·8 (1·6 to 4·5)	2 (1·1 to 3·2)	0·9 (0·5 to 1·4)	0·4 (0·2 to 0·6)	0·2 (0·1 to 0·2)	0·1 (0 to 0·1)	112
Atopic dermatitis	0 (0 to 0)	0 (0 to 0)	0 (0 to 0)	12·8 (6·9 to 21·8)	15·6 (8·2 to 27)	10·6 (5·7 to 17·9)	7·3 (3·9 to 12·6)	5·8 (3 to 10)	4·4 (2·2 to 7·6)	3·1 (1·6 to 5·5)	2·4 (1·2 to 4·2)	2·4 (1·2 to 4·3)	2·6 (1·4 to 4·5)	2·7 (1·4 to 4·8)	2·4 (1·2 to 4)	1·8 (0·9 to 3·1)	1·4 (0·7 to 2·4)	1·1 (0·6 to 1·8)	0·8 (0·4 to 1·4)	0·4 (0·2 to 0·7)	0·2 (0·1 to 0·3)	0·1 (0 to 0·1)	0 (0 to 0)	77·7
Contact dermatitis	0 (0 to 0)	0 (0 to 0)	0 (0 to 0)	0 (0 to 0)	0·2 (0·1 to 0·4)	1 (0·4 to 1·9)	1·9 (0·8 to 3·5)	2·5 (1·1 to 4·6)	2·8 (1·3 to 5·2)	2·5 (1·1 to 4·7)	2·1 (0·9 to 4)	2·1 (1 to 4·2)	2·5 (1·1 to 4·8)	3 (1·4 to 5·4)	2·7 (1·2 to 5·2)	2·2 (0·9 to 4)	1·8 (0·8 to 3·5)	1·5 (0·7 to 2·9)	1 (0·5 to 2)	0·4 (0·2 to 0·8)	0·2 (0·1 to 0·3)	0·1 (0 to 0·1)	0 (0 to 0)	30·4
Seborrhoeic dermatitis	0 (0 to 0)	0 (0 to 0)	0 (0 to 0)	0·1 (0·1 to 0·2)	0·1 (0·1 to 0·2)	0·2 (0·1 to 0·3)	0·2 (0·1 to 0·4)	0·3 (0·2 to 0·5)	0·3 (0·2 to 0·6)	0·3 (0·2 to 0·5)	0·2 (0·1 to 0·4)	0·2 (0·1 to 0·4)	0·2 (0·1 to 0·4)	0·3 (0·1 to 0·4)	0·2 (0·1 to 0·4)	0·2 (0·1 to 0·3)	0·2 (0·1 to 0·3)	0·2 (0·1 to 0·3)	0·1 (0·1 to 0·2)	0·1 (0 to 0·1)	0 (0 to 0)	0 (0 to 0)	0 (0 to 0)	3·6
Psoriasis	0 (0 to 0)	0 (0 to 0)	0 (0 to 0)	0·5 (0·3 to 0·8)	1·3 (0·7 to 2·1)	1·8 (1 to 2·8)	2·1 (1·2 to 3·2)	2·5 (1·5 to 3·7)	2·7 (1·6 to 4·2)	2·5 (1·5 to 3·9)	2·3 (1·4 to 3·4)	2·6 (1·6 to 3·8)	3·2 (2 to 4·7)	3·6 (2·3 to 5·2)	3·3 (2·1 to 4·7)	2·5 (1·6 to 3·5)	1·8 (1·2 to 2·6)	1·4 (0·9 to 1·9)	0·8 (0·6 to 1·2)	0·3 (0·2 to 0·4)	0·1 (0·1 to 0·2)	0 (0 to 0·1)	0 (0 to 0)	35·4
Bacterial skin diseases	0 (0 to 0)	0 (0 to 0)	6·7 (3·3 to 12·2)	2·4 (1·3 to 4)	2·4 (1·5 to 3·6)	2 (1·2 to 3·2)	2·6 (1·5 to 4·5)	3·4 (1·9 to 6·2)	3·8 (2·2 to 7·5)	5·1 (3 to 9·2)	4·3 (2·5 to 7·8)	6·6 (3·8 to 11·4)	7·8 (4·3 to 14·4)	11·3 (6·2 to 20)	12·3 (6·8 to 22·4)	11·8 (6·7 to 20·4)	11·2 (5·5 to 19·3)	10·1 (5·3 to 17·4)	9·8 (5·2 to 17·7)	5·1 (2·8 to 8·9)	2·8 (1·4 to 5)	1·5 (0·7 to 2·8)	0·7 (0·3 to 1·4)	124
Cellulitis	0 (0 to 0)	0 (0 to 0)	0·6 (0·2 to 1·6)	0·3 (0·2 to 0·5)	0·5 (0·3 to 0·8)	0·3 (0·2 to 0·6)	0·3 (0·2 to 0·9)	0·4 (0·2 to 1·2)	0·5 (0·3 to 1·5)	0·6 (0·3 to 1·6)	0·6 (0·3 to 1·4)	0·7 (0·3 to 2)	1 (0·4 to 2·8)	1·4 (0·7 to 3·7)	1·4 (0·7 to 3·8)	1·3 (0·6 to 3·4)	1·2 (0·6 to 3·4)	1·1 (0·5 to 2·9)	0·8 (0·3 to 2·5)	0·4 (0·2 to 1·1)	0·2 (0·1 to 0·5)	0·1 (0 to 0·2)	0 (0 to 0·1)	13·8
Pyoderma	0 (0 to 0)	0 (0 to 0)	6·1 (2·9 to 11)	2·1 (1·1 to 3·5)	1·9 (1·2 to 3)	1·7 (1 to 2·8)	2·2 (1·2 to 3·9)	3 (1·6 to 5·4)	3·2 (1·7 to 6·6)	4·5 (2·5 to 7·9)	3·8 (2 to 6·4)	5·8 (3·3 to 9·8)	6·8 (3·6 to 12·3)	9·9 (5 to 16·9)	10·9 (5·7 to 19·7)	10·5 (5·5 to 17·8)	9·9 (4·7 to 16·6)	9 (4·3 to 15·3)	9·1 (4·6 to 15·6)	4·7 (2·5 to 8·1)	2·6 (1·3 to 4·6)	1·4 (0·7 to 2·5)	0·6 (0·3 to 1·2)	110
Scabies	0 (0 to 0)	0 (0 to 0)	0·9 (0·5 to 1·5)	6·3 (3·1 to 11·3)	8·4 (4·1 to 14·9)	8·9 (4·3 to 16·1)	10 (5·1 to 16·9)	9·8 (5 to 17·4)	6·9 (3·5 to 12·3)	4·3 (2·1 to 7·8)	3·5 (1·7 to 6)	4 (2·1 to 6·9)	4·6 (2·4 to 7·8)	4·5 (2·3 to 7·6)	3·4 (1·7 to 6·1)	2·3 (1·2 to 4)	1·6 (0·8 to 2·8)	1·3 (0·7 to 2·3)	1·1 (0·6 to 1·9)	0·6 (0·3 to 1)	0·3 (0·1 to 0·5)	0·1 (0·1 to 0·2)	0 (0 to 0·1)	83
Fungal skin diseases	0 (0 to 0)	0 (0 to 0)	0·2 (0·1 to 0·3)	1·2 (0·4 to 2·6)	2 (0·8 to 4·3)	2·4 (0·9 to 5·3)	2·6 (1 to 5·9)	3 (1·2 to 6·8)	3 (1·2 to 6·9)	2·6 (1 to 5·8)	2·2 (0·9 to 4·9)	2·4 (0·9 to 5·1)	2·8 (1·1 to 6)	3·2 (1·3 to 7)	3·3 (1·3 to 7·3)	2·8 (1·1 to 6·1)	2·8 (1·1 to 6·1)	3·2 (1·3 to 7)	3 (1·1 to 6·2)	1·7 (0·7 to 3·6)	0·9 (0·4 to 2)	0·5 (0·2 to 1)	0·2 (0·1 to 0·4)	46
Viral skin diseases	0 (0 to 0)	0 (0 to 0)	0 (0 to 0)	6·8 (4·3 to 10·4)	10·8 (6·7 to 16·6)	5·8 (3·7 to 8·8)	3·8 (2·3 to 6)	3·3 (2 to 5·1)	2·3 (1·5 to 3·6)	1·6 (1 to 2·4)	1·2 (0·8 to 1·9)	1·2 (0·8 to 2)	1·3 (0·8 to 2)	1·3 (0·8 to 2·1)	1·1 (0·7 to 1·7)	0·8 (0·5 to 1·2)	0·6 (0·4 to 0·9)	0·5 (0·3 to 0·7)	0·3 (0·2 to 0·4)	0·1 (0·1 to 0·2)	0 (0 to 0·1)	0 (0 to 0)	0 (0 to 0)	43
Acne vulgaris	0 (0 to 0)	0 (0 to 0)	0 (0 to 0)	0 (0 to 0)	0·6 (0·3 to 0·9)	12·8 (7·3 to 20·8)	22·9 (13·5 to 38·5)	6·3 (3·6 to 10·4)	2·8 (1·6 to 4·6)	1·7 (0·9 to 2·8)	1·1 (0·6 to 1·7)	0·8 (0·4 to 1·3)	0·6 (0·4 to 1)	0·5 (0·3 to 0·7)	0·3 (0·2 to 0·5)	0·2 (0·1 to 0·3)	0·1 (0·1 to 0·2)	0·1 (0 to 0·1)	0 (0 to 0·1)	0 (0 to 0)	0 (0 to 0)	0 (0 to 0)	0 (0 to 0)	50·8
Alopecia areata	0 (0 to 0)	0 (0 to 0)	0 (0 to 0)	0·1 (0·1 to 0·2)	0·2 (0·1 to 0·3)	0·2 (0·1 to 0·4)	0·5 (0·3 to 0·8)	0·8 (0·5 to 1·3)	1·1 (0·7 to 1·7)	0·9 (0·6 to 1·5)	0·7 (0·4 to 1·1)	0·6 (0·4 to 1)	0·6 (0·3 to 0·9)	0·5 (0·3 to 0·9)	0·4 (0·2 to 0·7)	0·3 (0·2 to 0·5)	0·2 (0·1 to 0·4)	0·2 (0·1 to 0·3)	0·1 (0·1 to 0·2)	0 (0 to 0·1)	0 (0 to 0)	0 (0 to 0)	0 (0 to 0)	7·7
Pruritus	0 (0 to 0)	0 (0 to 0)	0 (0 to 0)	0·3 (0·1 to 0·5)	0·4 (0·2 to 0·7)	0·4 (0·2 to 0·7)	0·5 (0·2 to 0·9)	0·6 (0·3 to 1·2)	0·7 (0·3 to 1·3)	0·6 (0·3 to 1·2)	0·6 (0·2 to 1·1)	0·6 (0·3 to 1·1)	0·7 (0·3 to 1·4)	0·8 (0·3 to 1·5)	0·7 (0·3 to 1·5)	0·6 (0·3 to 1·3)	0·6 (0·3 to 1·1)	0·5 (0·2 to 1)	0·4 (0·2 to 0·8)	0·2 (0·1 to 0·4)	0·1 (0 to 0·2)	0 (0 to 0·1)	0 (0 to 0)	9·2
Urticaria	0 (0 to 0)	0 (0 to 0)	0·9 (0·6 to 1·4)	6·2 (3·8 to 9·7)	5·5 (3·2 to 8·9)	4 (2·2 to 6·6)	3·7 (2·1 to 6·1)	3·8 (2 to 6·3)	3·4 (1·9 to 5·4)	2·8 (1·6 to 4·7)	2·4 (1·4 to 3·9)	2·4 (1·3 to 3·7)	2·4 (1·4 to 4)	2·4 (1·3 to 3·9)	2 (1·1 to 3·2)	1·4 (0·8 to 2·4)	1·1 (0·6 to 1·8)	0·8 (0·5 to 1·4)	0·6 (0·3 to 1)	0·2 (0·1 to 0·4)	0·1 (0·1 to 0·1)	0 (0 to 0·1)	0 (0 to 0)	46·1
Decubitus ulcer	0 (0 to 0)	0 (0 to 0)	0 (0 to 0)	0·2 (0·1 to 0·6)	0·4 (0·1 to 0·8)	0·4 (0·2 to 0·7)	0·6 (0·2 to 1·2)	1 (0·3 to 2·9)	1·7 (0·7 to 4·1)	1·1 (0·4 to 2·5)	1·9 (1 to 3·6)	2·1 (1·1 to 4·3)	3·5 (1·9 to 7·3)	6·7 (3·8 to 12·9)	8·5 (5 to 16·2)	9·2 (5·3 to 16·4)	10·9 (6·3 to 18·5)	14·1 (8·6 to 21·8)	17·5 (10·8 to 27·4)	11·6 (7·3 to 18·6)	7·8 (5 to 12·6)	5 (3 to 8·2)	2·3 (1·4 to 4·2)	106
Other skin and subcutaneous diseases	0 (0 to 0)	0 (0 to 0)	0·4 (0·2 to 0·7)	1·5 (0·7 to 2·8)	1·4 (0·7 to 2·6)	1·8 (0·8 to 3·5)	2·3 (1·1 to 4·3)	2·6 (1·3 to 4·9)	3 (1·5 to 5·3)	2·8 (1·4 to 5·3)	2·7 (1·3 to 4·9)	3·2 (1·6 to 5·7)	4·2 (2·1 to 7·9)	5·4 (2·7 to 9·8)	5·5 (2·7 to 9·9)	4·9 (2·4 to 8·7)	4·5 (2·4 to 8)	3·8 (2 to 6·8)	3·1 (1·6 to 5·3)	1·3 (0·7 to 2·3)	0·5 (0·3 to 0·9)	0·2 (0·1 to 0·4)	0·1 (0 to 0·1)	55·3
Sense organ diseases	0 (0 to 0)	0 (0 to 0)	0·4 (0·2 to 0·5)	3·8 (2·4 to 5·6)	8·5 (5·4 to 12·6)	11·3 (7·2 to 16·5)	14·7 (9·4 to 21·3)	20·3 (12·5 to 30)	24·9 (15·6 to 37·8)	25·9 (16·1 to 39·4)	26·2 (16·2 to 40)	34·7 (21·3 to 53·6)	55·7 (35 to 88·7)	80·6 (51·4 to 129)	91 (57·8 to 140)	86·2 (55·3 to 131)	82·8 (54·5 to 123)	84·7 (57·2 to 122)	79·2 (54·7 to 111)	43·8 (30·8 to 59·8)	22·8 (15·9 to 30·6)	11·9 (8·4 to 15·8)	4·4 (3·2 to 5·9)	814
Blindness and vision loss	0 (0 to 0)	0 (0 to 0)	0·1 (0 to 0·1)	1·1 (0·6 to 1·8)	2·4 (1·4 to 3·9)	3·1 (1·9 to 4·9)	3·9 (2·3 to 6)	5·1 (2·9 to 7·8)	5·4 (3·4 to 8)	5·3 (3·2 to 8)	5·7 (3·6 to 8·7)	8·2 (5·1 to 12·6)	12·6 (7·8 to 19·9)	18·2 (11·3 to 29·5)	21·6 (13·4 to 33·6)	21·9 (14·3 to 33·5)	23 (15·2 to 33·6)	25·4 (16·9 to 36·4)	24·8 (17·1 to 35·1)	14·1 (9·7 to 19·4)	7·6 (5·2 to 10·2)	4·2 (3 to 5·8)	1·8 (1·2 to 2·5)	216
Glaucoma	0 (0 to 0)	0 (0 to 0)	0 (0 to 0)	0 (0 to 0)	0 (0 to 0)	0 (0 to 0)	0 (0 to 0)	0 (0 to 0)	0 (0 to 0)	0 (0 to 0)	0 (0 to 0)	0 (0 to 0)	0 (0 to 0·1)	0·2 (0·1 to 0·5)	0·6 (0·3 to 1·1)	1 (0·6 to 1·6)	1·4 (0·8 to 2·2)	2 (1·2 to 3)	2·5 (1·5 to 3·9)	1·6 (1 to 2·5)	1 (0·6 to 1·5)	0·5 (0·3 to 0·8)	0·2 (0·1 to 0·3)	11
Cataract	0 (0 to 0)	0 (0 to 0)	0 (0 to 0)	0 (0 to 0)	0 (0 to 0)	0 (0 to 0)	0 (0 to 0)	0 (0 to 0)	0·1 (0·1 to 0·2)	0·2 (0·1 to 0·4)	0·4 (0·2 to 0·7)	0·7 (0·4 to 1·2)	1·3 (0·7 to 2·1)	1·9 (1 to 3)	2·3 (1·4 to 3·6)	2·7 (1·6 to 4)	3·5 (2·2 to 5·2)	5·2 (3·4 to 7·6)	6·7 (4·4 to 9·7)	4·5 (3 to 6·4)	2·7 (1·8 to 3·7)	1·5 (1 to 2·1)	0·6 (0·4 to 0·9)	34·4
Age-related macular degeneration	0 (0 to 0)	0 (0 to 0)	0 (0 to 0)	0 (0 to 0)	0 (0 to 0)	0 (0 to 0)	0 (0 to 0)	0 (0 to 0)	0 (0 to 0)	0 (0 to 0)	0 (0 to 0)	0 (0 to 0)	0 (0 to 0)	0·1 (0 to 0·1)	0·1 (0·1 to 0·2)	0·2 (0·1 to 0·3)	0·2 (0·1 to 0·4)	0·3 (0·1 to 0·4)	0·3 (0·2 to 0·5)	0·2 (0·1 to 0·2)	0·1 (0 to 0·1)	0 (0 to 0·1)	0 (0 to 0)	1·5
Refraction disorders	0 (0 to 0)	0 (0 to 0)	0 (0 to 0·1)	0·9 (0·5 to 1·5)	2·1 (1·2 to 3·5)	2·6 (1·6 to 4·3)	3·3 (1·9 to 5·3)	4·2 (2·4 to 6·5)	4·3 (2·6 to 6·6)	3·6 (2·1 to 5·7)	3·4 (2 to 5·1)	3·8 (2·2 to 5·9)	4·7 (2·9 to 7·2)	5·1 (3·1 to 7·9)	4·8 (3 to 7·3)	3·8 (2·4 to 5·8)	3·3 (2 to 5)	3·3 (2 to 5)	3 (1·8 to 4·4)	1·4 (0·9 to 2·1)	0·6 (0·4 to 0·9)	0·2 (0·1 to 0·3)	0·1 (0 to 0·1)	58·5
Near vision loss	0 (0 to 0)	0 (0 to 0)	0 (0 to 0)	0 (0 to 0)	0 (0 to 0)	0 (0 to 0·1)	0 (0 to 0·1)	0·1 (0 to 0·3)	0·2 (0·1 to 0·6)	0·5 (0·2 to 1·2)	0·9 (0·3 to 2)	2 (0·8 to 4·2)	4 (1·6 to 8·5)	6·6 (2·6 to 13·6)	8·1 (3·2 to 16·8)	7·9 (3·3 to 16·4)	7·4 (3·1 to 14·8)	7·1 (3 to 13·9)	6 (2·5 to 12·2)	2·9 (1·2 to 5·9)	1·4 (0·6 to 2·6)	0·6 (0·3 to 1·3)	0·2 (0·1 to 0·5)	56·1
Other vision loss	0 (0 to 0)	0 (0 to 0)	0 (0 to 0)	0·2 (0·1 to 0·3)	0·3 (0·2 to 0·6)	0·4 (0·2 to 0·8)	0·6 (0·3 to 1)	0·7 (0·3 to 1·3)	0·8 (0·4 to 1·4)	0·9 (0·4 to 1·5)	1 (0·6 to 1·7)	1·6 (0·9 to 2·6)	2·6 (1·5 to 4·3)	4·3 (2·6 to 6·6)	5·6 (3·5 to 8·3)	6·3 (4·1 to 9·1)	7·1 (4·7 to 10·3)	7·6 (4·9 to 11)	6·4 (4·1 to 9·5)	3·5 (2·3 to 5·1)	1·9 (1·2 to 2·9)	1·3 (0·9 to 1·9)	0·7 (0·4 to 1)	54·1
Age-related and other hearing loss	0 (0 to 0)	0 (0 to 0)	0·1 (0·1 to 0·2)	2 (1·1 to 3·3)	5·2 (2·9 to 8·3)	7·3 (4·3 to 11·1)	9·7 (5·7 to 14·7)	13·9 (8·2 to 21·2)	17·9 (10·9 to 27·5)	18·8 (11·4 to 29·2)	18·7 (11·1 to 29·7)	24·5 (14·6 to 39·2)	40·4 (24·1 to 65·7)	58·6 (35·9 to 94·5)	65·7 (40·5 to 104)	60·5 (37 to 94·9)	56 (35·6 to 84·7)	55·7 (35·7 to 81)	51·2 (34·6 to 74·3)	28·3 (19·1 to 39·2)	14·6 (10 to 19·8)	7·5 (5·2 to 10)	2·6 (1·8 to 3·5)	559
Other sense organ diseases	0 (0 to 0)	0 (0 to 0)	0·2 (0·1 to 0·3)	0·8 (0·4 to 1·3)	0·9 (0·5 to 1·6)	0·9 (0·5 to 1·6)	1·1 (0·5 to 2·1)	1·4 (0·6 to 2·7)	1·6 (0·7 to 3·1)	1·8 (0·8 to 3·6)	1·8 (0·8 to 3·4)	2 (0·9 to 3·9)	2·6 (1·2 to 5·1)	3·8 (1·8 to 7·5)	3·8 (1·8 to 7·3)	3·8 (1·8 to 6·9)	3·8 (1·8 to 7·2)	3·6 (1·7 to 6·8)	3·2 (1·5 to 5·7)	1·5 (0·7 to 2·7)	0·6 (0·3 to 1)	0·2 (0·1 to 0·4)	0·1 (0 to 0·2)	39·6
Musculoskeletal disorders	0 (0 to 0)	0 (0 to 0)	0 (0 to 0)	0 (0 to 0)	4·4 (2·4 to 7·4)	21·9 (13·6 to 34·1)	51·6 (34·2 to 75)	84·2 (55·6 to 121)	114 (74·3 to 164)	119 (78 to 163)	118 (80·8 to 165)	143 (99·8 to 195)	183 (127 to 255)	212 (146 to 301)	198 (133 to 275)	156 (106 to 221)	122 (85·5 to 172)	101 (69·2 to 142)	73·3 (49·6 to 104)	32·8 (22·7 to 46·7)	15·5 (11 to 21·5)	6·7 (4·7 to 9·2)	2·2 (1·6 to 3·1)	1,760
Rheumatoid arthritis	0 (0 to 0)	0 (0 to 0)	0 (0 to 0)	0 (0 to 0)	0 (0 to 0·1)	0·2 (0·1 to 0·3)	0·6 (0·3 to 0·9)	1·2 (0·6 to 1·9)	1·7 (0·9 to 2·8)	1·9 (1·1 to 2·9)	2 (1·1 to 3)	2·4 (1·4 to 3·7)	3 (1·8 to 4·5)	3·4 (2·1 to 5)	3·1 (2 to 4·5)	2·4 (1·6 to 3·4)	2 (1·3 to 2·7)	1·5 (1 to 2·1)	1·2 (0·9 to 1·7)	0·6 (0·4 to 0·8)	0·5 (0·3 to 0·7)	0·2 (0·1 to 0·4)	0·1 (0 to 0·1)	27·8
Osteoarthritis	0 (0 to 0)	0 (0 to 0)	0 (0 to 0)	0 (0 to 0)	0 (0 to 0)	0 (0 to 0)	0 (0 to 0)	0 (0 to 0)	0 (0 to 0)	0·6 (0·2 to 1·2)	3 (1·4 to 6·2)	8·8 (4·3 to 17·5)	19·6 (9·6 to 40)	32·4 (15·7 to 67)	37·9 (18·3 to 76·4)	34·8 (17·4 to 68·7)	30·1 (15·3 to 60·2)	26·6 (13·3 to 53·8)	20·5 (10·3 to 41·2)	9·5 (4·9 to 19·2)	4·2 (2·1 to 8·3)	1·9 (1 to 3·7)	0·6 (0·3 to 1·2)	230
Osteoarthritis hip	0 (0 to 0)	0 (0 to 0)	0 (0 to 0)	0 (0 to 0)	0 (0 to 0)	0 (0 to 0)	0 (0 to 0)	0 (0 to 0)	0 (0 to 0)	0·2 (0·1 to 0·4)	0·2 (0·1 to 0·6)	0·4 (0·2 to 1)	0·8 (0·3 to 1·8)	1·4 (0·6 to 3·1)	1·7 (0·8 to 3·8)	1·7 (0·8 to 3·6)	1·6 (0·7 to 3·3)	1·4 (0·7 to 3)	1·1 (0·5 to 2·4)	0·5 (0·2 to 1)	0·2 (0·1 to 0·4)	0·1 (0 to 0·2)	0 (0 to 0·1)	11·3
Osteoarthritis knee	0 (0 to 0)	0 (0 to 0)	0 (0 to 0)	0 (0 to 0)	0 (0 to 0)	0 (0 to 0)	0 (0 to 0)	0 (0 to 0)	0 (0 to 0)	0·3 (0·1 to 0·7)	2·2 (1 to 4·6)	6·7 (3·1 to 13·4)	14·7 (7 to 30·1)	24 (11·3 to 49·2)	27·6 (13 to 56·1)	25·1 (12·2 to 50·3)	21·5 (10·5 to 44)	18·6 (9·2 to 38)	14·1 (6·9 to 27·8)	6·3 (3·1 to 12·9)	2·6 (1·3 to 5·3)	1·1 (0·5 to 2·2)	0·3 (0·2 to 0·7)	165
Osteoarthritis hand	0 (0 to 0)	0 (0 to 0)	0 (0 to 0)	0 (0 to 0)	0 (0 to 0)	0 (0 to 0)	0 (0 to 0)	0 (0 to 0)	0 (0 to 0)	0·1 (0 to 0·2)	0·3 (0·1 to 0·8)	1 (0·4 to 2·2)	2·2 (0·9 to 4·7)	3·8 (1·7 to 8·4)	4·5 (2·1 to 10)	4·1 (1·9 to 8·7)	3·5 (1·7 to 7·5)	3·2 (1·6 to 6·7)	2·6 (1·3 to 5·3)	1·3 (0·6 to 2·7)	0·7 (0·3 to 1·4)	0·3 (0·2 to 0·7)	0·1 (0·1 to 0·3)	27·7

Osteoarthritis other	0 (0 to 0)	0 (0 to 0)	0 (0 to 0)	0 (0 to 0)	0 (0 to 0)	0 (0 to 0)	0 (0 to 0)	0 (0 to 0)	0 (0 to 0)	0 (0 to 0·1)	0·2 (0·1 to 0·6)	0·8 (0·3 to 1·8)	1·9 (0·8 to 4·3)	3·3 (1·4 to 6·9)	4·1 (1·9 to 8·9)	4 (1·8 to 8·1)	3·6 (1·7 to 7·4)	3·3 (1·6 to 6·8)	2·7 (1·4 to 5·6)	1·4 (0·7 to 2·7)	0·6 (0·3 to 1·3)	0·3 (0·2 to 0·6)	0·1 (0·1 to 0·2)	26·3
Low back pain	0 (0 to 0)	0 (0 to 0)	0 (0 to 0)	0 (0 to 0)	3·8 (1·8 to 6·8)	19·3 (11·3 to 31·4)	37·7 (22·5 to 59·4)	51·9 (30·7 to 83)	60 (35 to 95·3)	57 (32·6 to 88)	56·2 (34·2 to 86·9)	65·3 (39·9 to 100)	79·7 (48·7 to 123)	90·1 (55·1 to 140)	81·1 (50·2 to 121)	63 (39·5 to 94·3)	49·2 (31·5 to 75·3)	41·4 (26·1 to 61·4)	31·5 (19·4 to 47)	15·1 (9·6 to 22·8)	6·3 (3·9 to 9·3)	2·6 (1·6 to 3·9)	0·8 (0·5 to 1·2)	812
Neck pain	0 (0 to 0)	0 (0 to 0)	0 (0 to 0)	0 (0 to 0)	0·2 (0·1 to 0·5)	1·3 (0·6 to 2·6)	3·2 (1·4 to 5·8)	5·5 (2·4 to 11·1)	8·5 (3·9 to 17)	11 (5·3 to 21·2)	12·7 (6·4 to 22)	16·4 (8·3 to 29·5)	20·9 (10·5 to 37·4)	20·5 (10·4 to 38·4)	17·3 (8·3 to 30·6)	12·5 (6·3 to 22·3)	9·1 (4·8 to 15·3)	7·1 (3·8 to 12·3)	4·9 (2·4 to 9·2)	2 (1 to 3·5)	0·8 (0·4 to 1·4)	0·4 (0·2 to 0·6)	0·1 (0·1 to 0·2)	154
Gout	0 (0 to 0)	0 (0 to 0)	0 (0 to 0)	0 (0 to 0)	0 (0 to 0)	0 (0 to 0)	0 (0 to 0)	0·1 (0 to 0·2)	0·3 (0·1 to 0·6)	0·4 (0·1 to 0·9)	0·5 (0·2 to 1)	0·7 (0·3 to 1·3)	1 (0·4 to 1·6)	1·1 (0·5 to 2)	1·1 (0·5 to 1·9)	0·9 (0·4 to 1·6)	0·8 (0·4 to 1·3)	0·7 (0·4 to 1·2)	0·6 (0·3 to 1)	0·3 (0·1 to 0·5)	0·1 (0·1 to 0·2)	0·1 (0 to 0·1)	0 (0 to 0)	8·7
Other musculoskeletal disorders	0 (0 to 0)	0 (0 to 0)	0 (0 to 0)	0 (0 to 0)	0·3 (0·2 to 0·5)	1·2 (0·7 to 2·1)	10·2 (6·4 to 16)	25·5 (16·9 to 36·5)	43·3 (27·8 to 63·9)	48·6 (32·2 to 71·2)	44 (29·1 to 63·6)	49·8 (31·4 to 74·2)	58·9 (38·7 to 87·9)	64·8 (40·9 to 98·4)	57·3 (36·9 to 85·6)	42·6 (25·9 to 67)	31·2 (19·8 to 46·5)	23·6 (13·6 to 37·4)	14·5 (8·4 to 22·6)	5·3 (2·6 to 9)	3·6 (2·3 to 5·2)	1·6 (0·9 to 2·3)	0·5 (0·3 to 0·9)	527
Other non-communicable diseases	97·3 (64·6 to 144)	37·2 (23·8 to 56·3)	144 (97·1 to 206)	69·8 (51·4 to 94·3)	49·2 (39·2 to 62·6)	45·6 (36·3 to 56·9)	72·4 (57·2 to 92·2)	107 (81·4 to 143)	128 (94·9 to 176)	127 (94·4 to 169)	120 (90·3 to 162)	125 (91·8 to 171)	139 (102 to 185)	146 (109 to 193)	135 (103 to 176)	115 (88 to 147)	100 (78·1 to 129)	91·5 (71·3 to 116)	73·9 (58·3 to 92·8)	35·3 (28·2 to 44)	17·9 (14·5 to 21·9)	8·6 (6·9 to 10·4)	3 (2·3 to 3·8)	1,990
Congenital birth defects	94·2 (62·4 to 140)	33·2 (21·3 to 50·7)	98·9 (65·3 to 146)	36·4 (25·8 to 51·8)	19 (15·2 to 23·8)	16·3 (12·9 to 20·4)	18·3 (14·7 to 22·8)	16·3 (12·8 to 20·3)	12·9 (9·9 to 16·4)	10 (8 to 12·5)	8·1 (6·3 to 10·1)	7 (5·5 to 8·7)	7·5 (5·8 to 9·3)	7·6 (5·9 to 9·4)	6·3 (5 to 7·9)	3·6 (2·8 to 4·6)	2·4 (1·9 to 3)	0·8 (0·5 to 1·2)	0·5 (0·3 to 0·7)	0·2 (0·1 to 0·3)	0·1 (0 to 0·1)	0 (0 to 0)	0 (0 to 0)	399
Neural tube defects	7·1 (4·1 to 11·8)	2·2 (1·1 to 3·8)	9·3 (4·5 to 16·7)	6·1 (3 to 11)	0·6 (0·5 to 0·9)	0·8 (0·6 to 1·1)	0·8 (0·6 to 1·1)	0·5 (0·4 to 0·7)	0·5 (0·4 to 0·7)	0·4 (0·3 to 0·5)	0·2 (0·2 to 0·3)	0·2 (0·1 to 0·3)	0·2 (0·1 to 0·2)	0·1 (0·1 to 0·2)	0·1 (0·1 to 0·1)	0·1 (0 to 0·1)	0 (0 to 0)	0 (0 to 0)	0 (0 to 0)	0 (0 to 0)	0 (0 to 0)	0 (0 to 0)	0 (0 to 0)	29·3
Congenital heart anomalies	53·8 (34·5 to 80·8)	20·9 (13 to 33·5)	61·5 (39 to 93·8)	16·8 (10·1 to 26·5)	8·3 (6·1 to 10·9)	6 (4·5 to 7·8)	7·6 (5·7 to 10)	6·9 (4·9 to 9·2)	5·2 (3·8 to 7·3)	4 (2·9 to 5·4)	3·4 (2·5 to 4·5)	2·6 (1·9 to 3·4)	2·6 (1·9 to 3·4)	2·5 (1·9 to 3·3)	1·8 (1·3 to 2·4)	1 (0·8 to 1·3)	0·7 (0·5 to 0·9)	0·1 (0 to 0·1)	0 (0 to 0·1)	0 (0 to 0)	0 (0 to 0)	0 (0 to 0)	0 (0 to 0)	206
Orofacial clefts	0 (0 to 0·1)	0 (0 to 0)	0·1 (0·1 to 0·2)	0·3 (0·1 to 0·4)	0·2 (0·1 to 0·4)	0·2 (0·1 to 0·5)	0·3 (0·1 to 0·5)	0·3 (0·1 to 0·5)	0·3 (0·1 to 0·5)	0·2 (0·1 to 0·4)	0·2 (0·1 to 0·3)	0·2 (0·1 to 0·3)	0·2 (0·1 to 0·4)	0·2 (0·1 to 0·4)	0·2 (0·1 to 0·3)	0·1 (0 to 0·2)	0·1 (0 to 0·2)	0·1 (0 to 0·1)	0 (0 to 0·1)	0 (0 to 0)	0 (0 to 0)	0 (0 to 0)	0 (0 to 0)	3·2
Down syndrome	1·2 (0·7 to 2)	0·5 (0·2 to 0·8)	3·2 (1·6 to 5·6)	1·4 (0·9 to 2·4)	0·7 (0·5 to 1)	0·8 (0·6 to 1·2)	1·2 (0·8 to 1·7)	1·3 (0·8 to 1·8)	1 (0·6 to 1·5)	1 (0·6 to 1·5)	0·8 (0·5 to 1·2)	0·8 (0·5 to 1·2)	1·1 (0·7 to 1·6)	1·2 (0·8 to 1·7)	1·4 (0·9 to 2)	0·6 (0·4 to 0·9)	0·3 (0·2 to 0·5)	0 (0 to 0)	0 (0 to 0)	0 (0 to 0)	0 (0 to 0)	0 (0 to 0)	0 (0 to 0)	18·7
Turner syndrome	0 (0 to 0)	0 (0 to 0)	0 (0 to 0)	0 (0 to 0)	0 (0 to 0)	0 (0 to 0)	0 (0 to 0)	0 (0 to 0)	0 (0 to 0)	0 (0 to 0)	0 (0 to 0)	0 (0 to 0)	0 (0 to 0)	0 (0 to 0)	0 (0 to 0)	0 (0 to 0)	0 (0 to 0)	0 (0 to 0)	0 (0 to 0)	0 (0 to 0)	0 (0 to 0)	0 (0 to 0)	0 (0 to 0)	0·1
Klinefelter syndrome	0 (0 to 0)	0 (0 to 0)	0 (0 to 0)	0 (0 to 0)	0 (0 to 0)	0 (0 to 0)	0 (0 to 0)	0 (0 to 0)	0 (0 to 0)	0 (0 to 0)	0 (0 to 0)	0 (0 to 0)	0 (0 to 0)	0 (0 to 0)	0 (0 to 0)	0 (0 to 0)	0 (0 to 0)	0 (0 to 0)	0 (0 to 0)	0 (0 to 0)	0 (0 to 0)	0 (0 to 0)	0 (0 to 0)	0
Other chromosomal abnormalities	6·5 (3·7 to 10·7)	2·9 (1·5 to 5·4)	7 (4 to 12·7)	1·9 (1·2 to 2·9)	1·1 (0·7 to 1·4)	1 (0·7 to 1·3)	1·2 (0·8 to 1·6)	1 (0·7 to 1·3)	0·8 (0·5 to 1)	0·4 (0·3 to 0·6)	0·3 (0·2 to 0·4)	0·2 (0·1 to 0·2)	0·1 (0·1 to 0·2)	0·1 (0·1 to 0·2)	0·1 (0 to 0·1)	0 (0 to 0)	0 (0 to 0)	0 (0 to 0)	0 (0 to 0)	0 (0 to 0)	0 (0 to 0)	0 (0 to 0)	0 (0 to 0)	24·4
Congenital musculoskeletal and limb anomalies	2·2 (1·2 to 3·8)	0·7 (0·4 to 1·2)	3·3 (2·1 to 4·9)	4·4 (2·6 to 6·7)	4·5 (2·7 to 7·1)	4·7 (2·8 to 7·3)	4·4 (2·7 to 6·7)	3·5 (2·1 to 5·2)	3 (1·9 to 4·5)	2·3 (1·4 to 3·6)	2 (1·2 to 3)	2 (1·2 to 3)	2·2 (1·3 to 3·3)	2·2 (1·4 to 3·3)	1·8 (1·2 to 2·8)	1·2 (0·8 to 1·9)	0·8 (0·5 to 1·2)	0·5 (0·3 to 0·7)	0·3 (0·1 to 0·4)	0·1 (0 to 0·2)	0 (0 to 0·1)	0 (0 to 0)	0 (0 to 0)	46
Urogenital congenital anomalies	1·2 (0·7 to 2·2)	0·4 (0·2 to 0·7)	0·6 (0·4 to 1)	0·5 (0·3 to 0·8)	0·4 (0·2 to 0·6)	0·3 (0·2 to 0·5)	0·3 (0·2 to 0·4)	0·3 (0·2 to 0·4)	0·2 (0·1 to 0·3)	0·1 (0·1 to 0·2)	0·1 (0·1 to 0·1)	0·1 (0·1 to 0·1)	0·1 (0·1 to 0·1)	0·1 (0 to 0·1)	0 (0 to 0·1)	0 (0 to 0)	0 (0 to 0)	0 (0 to 0)	0 (0 to 0)	0 (0 to 0)	0 (0 to 0)	0 (0 to 0)	0 (0 to 0)	4·7
Digestive congenital anomalies	8·6 (4·9 to 15·6)	2·7 (1·5 to 4·6)	5·5 (3·2 to 9·4)	1·2 (0·8 to 2)	0·5 (0·3 to 0·8)	0·2 (0·2 to 0·4)	0·2 (0·1 to 0·3)	0·2 (0·1 to 0·2)	0·1 (0·1 to 0·2)	0·1 (0·1 to 0·1)	0·1 (0 to 0·1)	0·1 (0 to 0·1)	0·1 (0 to 0·1)	0·1 (0 to 0·1)	0 (0 to 0·1)	0 (0 to 0·1)	0 (0 to 0·1)	0 (0 to 0)	0 (0 to 0)	0 (0 to 0)	0 (0 to 0)	0 (0 to 0)	0 (0 to 0)	19·8
Other congenital birth defects	13·6 (8·1 to 21·3)	3 (1·6 to 4·8)	8·4 (4·9 to 13·4)	3·8 (2·4 to 6)	2·7 (1·9 to 3·8)	2·2 (1·5 to 3·1)	2·3 (1·6 to 3·3)	2·5 (1·6 to 3·6)	1·8 (1·2 to 2·7)	1·4 (0·9 to 2·2)	1 (0·7 to 1·8)	0·9 (0·5 to 1·6)	1 (0·6 to 1·7)	1 (0·6 to 1·8)	0·8 (0·5 to 1·5)	0·5 (0·3 to 0·9)	0·4 (0·2 to 0·7)	0·2 (0·1 to 0·4)	0·1 (0 to 0·3)	0·1 (0 to 0·1)	0 (0 to 0·1)	0 (0 to 0)	0 (0 to 0)	47·5
Urinary diseases and male infertility	0·2 (0·1 to 0·3)	0·3 (0·1 to 0·4)	2·1 (1·2 to 3·3)	1·1 (0·7 to 1·6)	0·8 (0·6 to 1·1)	1 (0·8 to 1·4)	1·8 (1·4 to 2·3)	4·1 (3 to 5·9)	4·6 (3·2 to 6·6)	5·7 (4·1 to 7·9)	6·5 (4·7 to 8·5)	7 (5·1 to 9·4)	9·1 (6·6 to 11·8)	13·5 (10 to 17·4)	19·1 (14·1 to 24·3)	22·3 (16·1 to 28·5)	24·2 (17·8 to 31·1)	25·5 (18·2 to 32)	22·3 (16·1 to 28·8)	10·8 (8 to 13·9)	5·2 (3·8 to 6·8)	2·4 (1·7 to 3·2)	0·9 (0·6 to 1·2)	190
Urolithiasis	0 (0 to 0)	0 (0 to 0)	0 (0 to 0)	0 (0 to 0)	0 (0 to 0·1)	0 (0 to 0·1)	0·1 (0 to 0·1)	0·2 (0·1 to 0·3)	0·3 (0·2 to 0·4)	0·5 (0·3 to 0·8)	0·8 (0·5 to 1·2)	0·8 (0·5 to 1·2)	1·6 (1 to 2·5)	1·8 (1·1 to 2·6)	2·4 (1·5 to 3·4)	2 (1·3 to 2·9)	1·7 (1·1 to 2·6)	1·4 (0·9 to 2·1)	0·9 (0·6 to 1·5)	0·2 (0·1 to 0·4)	0·1 (0·1 to 0·1)	0 (0 to 0)	0 (0 to 0)	14·9
Benign prostatic hyperplasia	0 (0 to 0)	0 (0 to 0)	0 (0 to 0)	0 (0 to 0)	0 (0 to 0)	0 (0 to 0)	0 (0 to 0)	0 (0 to 0)	0 (0 to 0)	0 (0 to 0)	0 (0 to 0)	0 (0 to 0·1)	0·3 (0·1 to 0·6)	1·3 (0·6 to 2·3)	3·8 (1·8 to 6·5)	5·7 (2·9 to 9·8)	6·6 (3·7 to 10·4)	6·5 (3·5 to 10·4)	4·5 (2·5 to 7)	1·3 (0·7 to 2·1)	0·3 (0·2 to 0·5)	0·1 (0 to 0·1)	0 (0 to 0)	30·3
Male infertility	0 (0 to 0)	0 (0 to 0)	0 (0 to 0)	0 (0 to 0)	0 (0 to 0)	0 (0 to 0)	0 (0 to 0)	0·9 (0·3 to 2·3)	1·1 (0·3 to 2·7)	1 (0·3 to 2·6)	0·8 (0·2 to 2)	0·8 (0·2 to 2)	0·1 (0 to 0·4)	0 (0 to 0)	0 (0 to 0)	0 (0 to 0)	0 (0 to 0)	0 (0 to 0)	0 (0 to 0)	0 (0 to 0)	0 (0 to 0)	0 (0 to 0)	0 (0 to 0)	4·7
Other urinary diseases	0·1 (0 to 0·2)	0·2 (0·1 to 0·3)	0·3 (0·1 to 0·5)	0·3 (0·2 to 0·4)	0·2 (0·2 to 0·3)	0·4 (0·2 to 0·5)	0·5 (0·3 to 0·7)	0·8 (0·5 to 1·2)	0·4 (0·3 to 0·7)	0·7 (0·4 to 1)	1 (0·6 to 1·5)	1 (0·6 to 1·5)	1·6 (0·9 to 2·5)	1·8 (1·1 to 3)	2·1 (1·1 to 3·2)	2·7 (1·4 to 4·2)	2·8 (1·4 to 4·9)	2·2 (1 to 3·9)	2·1 (0·9 to 3·9)	0·9 (0·4 to 1·7)	0·3 (0·1 to 0·5)	0·1 (0·1 to 0·2)	0 (0 to 0·1)	22·4
Gynecological diseases	0 (0 to 0)	0 (0 to 0)	0 (0 to 0)	0 (0 to 0)	0 (0 to 0)	5·5 (2·7 to 9·5)	15·5 (8·8 to 25·3)	37·7 (24·1 to 58·3)	53·5 (34·6 to 78·9)	52·9 (35·3 to 75·9)	50 (34 to 71·9)	51·5 (33·9 to 74·9)	48·3 (32·3 to 68·3)	35·1 (21·3 to 52·6)	18·8 (11·7 to 27·4)	10·5 (6·1 to 15·9)	7·4 (4·6 to 10·8)	5·6 (3·6 to 8·1)	3·9 (2·5 to 5·8)	1·9 (1·1 to 2·8)	1 (0·6 to 1·4)	0·5 (0·3 to 0·7)	0·2 (0·1 to 0·3)	400
Uterine fibroids	0 (0 to 0)	0 (0 to 0)	0 (0 to 0)	0 (0 to 0)	0 (0 to 0)	0·1 (0 to 0·2)	0·2 (0·1 to 0·5)	0·6 (0·3 to 1·3)	1·5 (0·6 to 3·1)	2·7 (1·2 to 5·4)	3·7 (1·6 to 7·5)	4·7 (2·2 to 8·9)	4·5 (2·1 to 8·4)	3·5 (1·6 to 6·7)	2·1 (1 to 4)	1·1 (0·5 to 2·1)	0·6 (0·2 to 1·1)	0·3 (0·1 to 0·7)	0·2 (0·1 to 0·4)	0·1 (0 to 0·1)	0 (0 to 0·1)	0 (0 to 0)	0 (0 to 0)	25·8
Polycystic ovarian syndrome	0 (0 to 0)	0 (0 to 0)	0 (0 to 0)	0 (0 to 0)	0 (0 to 0)	0·2 (0·1 to 0·4)	0·9 (0·4 to 1·8)	1·1 (0·5 to 2·4)	1·1 (0·4 to 2·1)	0·8 (0·3 to 1·7)	0·7 (0·3 to 1·4)	0·7 (0·3 to 1·3)	0·6 (0·3 to 1·3)	0·2 (0·1 to 0·4)	0 (0 to 0)	0 (0 to 0)	0 (0 to 0)	0 (0 to 0)	0 (0 to 0)	0 (0 to 0)	0 (0 to 0)	0 (0 to 0)	0 (0 to 0)	6·2
Female infertility	0 (0 to 0)	0 (0 to 0)	0 (0 to 0)	0 (0 to 0)	0 (0 to 0)	0 (0 to 0)	0 (0 to 0)	1·3 (0·3 to 3·5)	1·8 (0·4 to 4·8)	1·8 (0·4 to 5·3)	1·6 (0·2 to 4·7)	1·6 (0·2 to 4·4)	0 (0 to 0·2)	0 (0 to 0)	0 (0 to 0)	0 (0 to 0)	0 (0 to 0)	0 (0 to 0)	0 (0 to 0)	0 (0 to 0)	0 (0 to 0)	0 (0 to 0)	0 (0 to 0)	8·2
Endometriosis	0 (0 to 0)	0 (0 to 0)	0 (0 to 0)	0 (0 to 0)	0 (0 to 0)	0 (0 to 0)	1·3 (0·5 to 2·6)	5·7 (2·7 to 10·4)	6·4 (3·1 to 11·4)	4·3 (2·2 to 7·7)	3·2 (1·6 to 5·7)	3·3 (1·7 to 5·9)	3·2 (1·8 to 5·2)	1·7 (0·9 to 2·9)	0 (0 to 0)	0 (0 to 0)	0 (0 to 0)	0 (0 to 0)	0 (0 to 0)	0 (0 to 0)	0 (0 to 0)	0 (0 to 0)	0 (0 to 0)	29
Genital prolapse	0 (0 to 0)	0 (0 to 0)	0 (0 to 0)	0 (0 to 0)	0 (0 to 0)	0 (0 to 0)	0 (0 to 0)	0 (0 to 0·1)	0·1 (0 to 0·3)	0·2 (0·1 to 0·5)	0·4 (0·2 to 0·8)	0·6 (0·3 to 1·3)	1 (0·5 to 1·9)	1·2 (0·5 to 2·2)	1·1 (0·6 to 2·1)	0·8 (0·4 to 1·5)	0·7 (0·4 to 1·3)	0·6 (0·3 to 1)	0·5 (0·2 to 0·8)	0·2 (0·1 to 0·4)	0·1 (0·1 to 0·2)	0·1 (0 to 0·1)	0 (0 to 0)	7·7
Premenstrual syndrome	0 (0 to 0)	0 (0 to 0)	0 (0 to 0)	0 (0 to 0)	0 (0 to 0)	5 (2·3 to 8·9)	12·1 (6 to 21·9)	17·2 (8·8 to 30·5)	16·7 (9·4 to 27·7)	13·7 (7·2 to 22·4)	12·7 (7·2 to 20·7)	13·3 (7·5 to 21·1)	10·6 (5·9 to 17·6)	3·9 (2 to 6·9)	0 (0 to 0)	0 (0 to 0)	0 (0 to 0)	0 (0 to 0)	0 (0 to 0)	0 (0 to 0)	0 (0 to 0)	0 (0 to 0)	0 (0 to 0)	105
Other gynecological diseases	0 (0 to 0)	0 (0 to 0)	0 (0 to 0)	0 (0 to 0)	0 (0 to 0)	0·2 (0·1 to 0·5)	1 (0·6 to 1·7)	11·8 (7·1 to 18·7)	25·9 (14·8 to 40·7)	29·4 (17·8 to 44·1)	27·6 (17·1 to 42·7)	27·4 (15·6 to 43·5)	28·4 (16·4 to 43·4)	24·7 (12·2 to 39·1)	15·6 (9·4 to 23·5)	8·7 (4·7 to 13·6)	6·1 (3·5 to 9·4)	4·7 (2·9 to 6·9)	3·3 (2 to 5)	1·6 (0·9 to 2·4)	0·8 (0·5 to 1·2)	0·4 (0·2 to 0·7)	0·1 (0·1 to 0·3)	218
Hemoglobinopathies and hemolytic anemias	0·4 (0·3 to 0·6)	0·5 (0·3 to 0·8)	4·3 (2·9 to 6·5)	10·9 (7·7 to 15·6)	14·6 (10·5 to 19·8)	10·2 (7·5 to 13·4)	17·8 (13·4 to 23)	20·4 (14·1 to 29·4)	18·6 (11·8 to 27·6)	15·4 (10 to 22·4)	12 (8·5 to 16·5)	10 (7·5 to 13·4)	10 (7·3 to 13·2)	10·3 (7·7 to 13·3)	9·3 (6·9 to 11·9)	7·7 (5·8 to 10·2)	5·5 (4·1 to 7·1)	4·8 (3·7 to 6·3)	3·7 (2·8 to 4·7)	1·7 (1·3 to 2·2)	1·3 (1 to 1·7)	0·6 (0·5 to 0·8)	0·2 (0·1 to 0·3)	190
Thalassemias	0 (0 to 0)	0 (0 to 0)	0 (0 to 0)	0 (0 to 0·1)	0 (0 to 0)	0 (0 to 0)	0 (0 to 0)	0 (0 to 0)	0 (0 to 0)	0 (0 to 0)	0 (0 to 0)	0 (0 to 0)	0 (0 to 0)	0 (0 to 0)	0 (0 to 0)	0 (0 to 0)	0 (0 to 0)	0 (0 to 0)	0 (0 to 0)	0 (0 to 0)	0 (0 to 0)	0 (0 to 0)	0 (0 to 0)	0·1
Thalassemias trait	0 (0 to 0)	0 (0 to 0)	0·1 (0 to 0·1)	0·3 (0·1 to 0·5)	0·4 (0·2 to 0·6)	0·1 (0 to 0·3)	0·4 (0·2 to 0·7)	0·5 (0·2 to 0·9)	0·5 (0·2 to 0·9)	0·4 (0·2 to 0·7)	0·3 (0·1 to 0·6)	0·3 (0·2 to 0·5)	0·1 (0 to 0·2)	0·1 (0 to 0·2)	0·1 (0 to 0·2)	0·1 (0 to 0·2)	0·1 (0 to 0·1)	0·1 (0 to 0·1)	0 (0 to 0·1)	0 (0 to 0)	0 (0 to 0)	0 (0 to 0)	0 (0 to 0)	3·9
Sickle cell disorders	0·4 (0·3 to 0·6)	0·3 (0·2 to 0·4)	3·2 (1·9 to 5·2)	6·1 (3·7 to 9·9)	8·3 (6 to 11·1)	7·5 (5·5 to 10)	14·1 (10·1 to 19)	12·6 (8·7 to 17·2)	9·4 (6·4 to 13·3)	7·2 (4·9 to 10·4)	6·2 (4·1 to 8·8)	4·9 (3·2 to 7)	4·6 (3 to 6·8)	3·8 (2·4 to 5·8)	1·8 (1 to 2·8)	0·5 (0·2 to 0·8)	0·1 (0 to 0·2)	0 (0 to 0)	0 (0 to 0)	0 (0 to 0)	0 (0 to 0)	0 (0 to 0)	0 (0 to 0)	90·9
Sickle cell trait	0 (0 to 0)	0·2 (0·1 to 0·3)	0·6 (0·3 to 1)	2·6 (1·4 to 4·2)	3·5 (1·8 to 6)	1·4 (0·5 to 2·8)	0·5 (0·2 to 1)	4·1 (0·7 to 10·4)	5·4 (1 to 12·2)	4·6 (0·8 to 9·9)	2·3 (0·6 to 5·6)	0·8 (0·4 to 1·4)	1·1 (0·4 to 1·9)	1·2 (0·5 to 2·1)	1·2 (0·6 to 2·1)	0·8 (0·4 to 1·5)	0·5 (0·2 to 1)	0·5 (0·2 to 0·9)	0·4 (0·2 to 0·7)	0·2 (0·1 to 0·4)	0·1 (0·1 to 0·2)	0·1 (0 to 0·1)	0 (0 to 0)	32·3
G6PD deficiency	0 (0 to 0)	0 (0 to 0)	0 (0 to 0·1)	0·3 (0·1 to 0·5)	0·5 (0·2 to 0·8)	0·4 (0·2 to 0·8)	1·7 (0·9 to 2·8)	2 (0·8 to 3·8)	1·9 (0·9 to 3·5)	2 (0·9 to 3·3)	1·8 (0·8 to 2·9)	2·3 (1·3 to 3·6)	2·4 (1·3 to 3·7)	2·6 (1·5 to 3·9)	2·8 (1·8 to 4·1)	2 (1·3 to 2·9)	1·2 (0·7 to 1·8)	0·8 (0·4 to 1·2)	0·4 (0·2 to 0·6)	0·1 (0 to 0·1)	0 (0 to 0)	0 (0 to 0)	0 (0 to 0)	25·2
G6PD trait	0 (0 to 0)	0 (0 to 0)	0 (0 to 0)	0 (0 to 0)	0 (0 to 0)	0 (0 to 0)	0·1 (0 to 0·2)	0 (0 to 0)	0 (0 to 0)	0 (0 to 0)	0 (0 to 0)	0·1 (0 to 0·1)	0 (0 to 0)	0 (0 to 0)	0 (0 to 0)	0 (0 to 0)	0 (0 to 0)	0 (0 to 0)	0 (0 to 0)	0 (0 to 0)	0 (0 to 0)	0 (0 to 0)	0 (0 to 0)	0·2
Other hemoglobinopathies and hemolytic anemias	0 (0 to 0)	0 (0 to 0·1)	0·4 (0·2 to 0·7)	1·6 (0·8 to 2·7)	2 (0·9 to 3·7)	0·7 (0·3 to 1·4)	1·1 (0·5 to 2·2)	1·3 (0·6 to 2·3)	1·4 (0·7 to 2·5)	1·2 (0·7 to 2·1)	1·4 (0·7 to 2·2)	1·7 (0·9 to 2·7)	1·8 (1·1 to 2·9)	2·6 (1·6 to 3·8)	3·5 (2·4 to 4·9)	4·2 (3 to 5·8)	3·5 (2·5 to 4·8)	3·5 (2·6 to 4·6)	2·9 (2·2 to 3·7)	1·4 (1·1 to 1·8)	1·1 (0·9 to 1·5)	0·6 (0·4 to 0·8)	0·2 (0·1 to 0·2)	37·9
Endocrine, metabolic, blood, and immune disorders	2·5 (1·5 to 4·3)	2·4 (1·3 to 4·4)	31·4 (19·2 to 52·3)	17·9 (11·7 to 28·4)	10·4 (7·5 to 14·3)	7·8 (5·6 to 11·1)	11·2 (7·9 to 15·5)	14·8 (10·5 to 21·6)	19 (13·1 to 28·8)	22·8 (16·3 to 32·5)	23·2 (17·6 to 31·5)	25·3 (18·4 to 35·7)	32·7 (23·4 to 44·8)	41·3 (29·8 to 59·8)	42·6 (30·7 to 60·5)	36·3 (26·2 to 50·9)	30 (22·2 to 42·3)	26·6 (19·5 to 36·6)	21·5 (15·5 to 29·2)	10·5 (7·7 to 14·3)	5·9 (4·3 to 8·2)	3 (2·1 to 4·1)	1·1 (0·7 to 1·6)	440
Oral disorders	0 (0 to 0)	0 (0 to 0)	0 (0 to 0)	3·4 (1·9 to 5·6)	4·3 (2·3 to 7·4)	4·9 (2·6 to 8·5)	7·8 (4·3 to 13·6)	13·9 (7·1 to 26)	19·5 (9·6 to 37·8)	20·6 (9·8 to 38·5)	20·6 (9·6 to 39·4)	24·3 (11·6 to 44·8)	31·1 (15·9 to 56·7)	37·7 (20·2 to 66·1)	38·4 (21·9 to 62·7)	34·7 (20·8 to 55·5)	31 (18·7 to 48)	28·2 (17·5 to 42·8)	22 (13·8 to 32·9)	10·1 (6·4 to 15·1)	4·4 (2·8 to 6·5)	2 (1·3 to 2·9)	0·7 (0·4 to 1)	360
Caries of deciduous teeth	0 (0 to 0)	0 (0 to 0)	0 (0 to 0)	1·1 (0·4 to 2·3)	1·1 (0·5 to 2·4)	0·1 (0 to 0·2)	0 (0 to 0)	0 (0 to 0)	0 (0 to 0)	0 (0 to 0)	0 (0 to 0)	0 (0 to 0)	0 (0 to 0)	0 (0 to 0)	0 (0 to 0)	0 (0 to 0)	0 (0 to 0)	0 (0 to 0)	0 (0 to 0)	0 (0 to 0)	0 (0 to 0)	0 (0 to 0)	0 (0 to 0)	2·3
Caries of permanent teeth	0 (0 to 0)	0 (0 to 0)	0 (0 to 0)	0 (0 to 0)	1 (0·3 to 2·2)	2·3 (0·9 to 5·1)	2·7 (1·1 to 5·8)	3·5 (1·5 to 7)	3·2 (1·2 to 6·6)	2·4 (1 to 5)	2 (0·8 to 4·1)	2 (0·8 to 4)	2·1 (0·9 to 4·4)	2·1 (0·8 to 4·4)	1·6 (0·6 to 3·6)	1·1 (0·4 to 2·3)	0·7 (0·3 to 1·4)	0·5 (0·2 to 1·1)	0·3 (0·1 to 0·7)	0·1 (0·1 to 0·3)	0·1 (0 to 0·1)	0 (0 to 0·1)	0 (0 to 0)	27·8
Periodontal diseases	0 (0 to 0)	0 (0 to 0)	0 (0 to 0)	0 (0 to 0)	0 (0 to 0)	0 (0 to 0)	1·1 (0·3 to 2·8)	4·5 (1·3 to 11·5)	9·4 (2·9 to 22·5)	11·7 (4 to 26·5)	12·4 (4·4 to 27·6)	14·8 (5·4 to 31·6)	17·6 (6·7 to 37·5)	18·3 (7·2 to 39)	14·4 (5·7 to 29·9)	9·4 (3·7 to 19)	6·1 (2·4 to 12·5)	4·4 (1·7 to 8·8)	3 (1·2 to 5·9)	1·2 (0·5 to 2·5)	0·5 (0·2 to 1·1)	0·2 (0·1 to 0·5)	0·1 (0 to 0·2)	129
Other oral disorders	0 (0 to 0)	0 (0 to 0)	0 (0 to 0)	2·3 (1·4 to 3·5)	2·2 (1·3 to 3·3)	2·5 (1·5 to 3·9)	4 (2·5 to 6·2)	5·4 (3·3 to 8·2)	5·7 (3·4 to 8·6)	4·8 (2·9 to 7·3)	4·1 (2·5 to 6·2)	4·3 (2·6 to 6·4)	4·9 (2·9 to 7·4)	5 (3·1 to 7·5)	4·1 (2·4 to 6·1)	2·9 (1·8 to 4·4)	2·1 (1·3 to 3·2)	1·5 (0·9 to 2·3)	0·9 (0·6 to 1·4)	0·3 (0·2 to 0·5)	0·1 (0·1 to 0·2)	0 (0 to 0·1)	0 (0 to 0)	57·3
Sudden infant death syndrome	--	0·8 (0·4 to 1·3)	7·1 (4·1 to 11·9)	--	--	--	--	--	--	--	--	--	--	--	--	--	--	--	--	--	--	--	--	NA
Injuries	7 (4·7 to 10·2)	3·1 (2 to 4·8)	35·8 (22·4 to 54·7)	60·1 (41·5 to 87·2)	73·5 (61·8 to 86)	88·9 (76·7 to 101)	221 (195 to 247)	342 (287 to 391)	291 (242 to 339)	266 (230 to 307)	218 (188 to 252)	202 (173 to 235)	221 (187 to 256)	216 (186 to 253)	190 (162 to 220)	141 (122 to 164)	111 (96·9 to 128)	92·2 (80·4 to 106)	73·3 (63·7 to 84·7)	36·7 (31·9 to 42·1)	18·6 (16·2 to 21·2)	10·1 (8·7 to 11·7)	4·1 (3·4 to 4·7)	2,920
Transport injuries	0·3 (0·2 to 0·4)	0·1 (0·1 to 0·2)	1·8 (1·2 to 2·7)	6·9 (4·4 to 10·2)	13·7 (10·1 to 17·8)	17·7 (13·9 to 22)	56·8 (43·2 to 72·2)	87·9 (65·3 to 114)	72·7 (56 to 91·7)	66·5 (52·8 to 83·4)	52·9 (41 to 66·6)	49·9 (39·4 to 62·6)	55·5 (44·2 to 68)	51·9 (41·8 to 63·1)	45·1 (37·1 to 54)	31·4 (26·2 to 36·8)	23·2 (19·3 to 27·5)	16·4 (13·7 to 19·3)	10·9 (9·2 to 12·9)	4 (3·3 to 4·6)	1·4 (1·2 to 1·7)	0·6 (0·5 to 0·7)	0·2 (0·2 to 0·2)	668
Road injuries	0·2 (0·1 to 0·3)	0·1 (0 to 0·1)	1·3 (0·8 to 2·1)	5·7 (3·5 to 8·7)	11·9 (8·5 to 16)	15·1 (11·5 to 19·3)	50·9 (38·5 to 65·5)	78·8 (56·8 to 103)	65·1 (49 to 83·1)	59 (46 to 74·8)	47·1 (35·6 to 59·9)	43·9 (33·9 to 56·1)	49·7 (39·2 to 61·8)	46·5 (37·2 to 57·2)	39·5 (32·2 to 47·6)	28·4 (23·5 to 33·6)	21·2 (17·6 to 25·3)	14·5 (12 to 17·3)	9·8 (8·1 to 11·7)	3·6 (3 to 4·3)	1·3 (1·1 to 1·5)	0·5 (0·4 to 0·6)	0·2 (0·1 to 0·2)	594
Pedestrian road injuries	0·1 (0 to 0·1)	0 (0 to 0)	0·2 (0·1 to 0·4)	1·7 (1 to 2·6)	3·6 (2·5 to 5)	3·3 (2·4 to 4·4)	4·2 (3·1 to 5·4)	5·5 (4·1 to 7)	5·4 (4·1 to 6·8)	5·9 (4·6 to 7·5)	5·5 (4·2 to 6·9)	5·8 (4·5 to 7·3)	7·3 (5·7 to 9·1)	7·7 (6·1 to 9·6)	7·4 (5·9 to 9·1)	5·9 (4·8 to 7)	5·1 (4·2 to 6)	4 (3·2 to 4·8)	3·1 (2·5 to 3·7)	1·2 (1 to 1·5)	0·4 (0·4 to 0·5)	0·2 (0·1 to 0·2)	0·1 (0 to 0·1)	83·5
Cyclist road injuries	0 (0 to 0)	0 (0 to 0)	0 (0 to 0)	0·5 (0·3 to 0·8)	1·1 (0·7 to 1·8)	3 (1·9 to 4·5)	6·7 (4·1 to 10·4)	8·2 (5·5 to 11·8)	7·1 (5·1 to 10)	6·8 (4·8 to 9·5)	5·9 (4·2 to 8·1)	5·9 (4·3 to 7·7)	6·7 (5 to 9)	7·2 (5·4 to 9·3)	6·1 (4·5 to 8)	4·5 (3·4 to 5·8)	3·4 (2·6 to 4·4)	2·2 (1·7 to 2·8)	1·4 (1 to 1·8)	0·5 (0·4 to 0·7)	0·2 (0·1 to 0·2)	0·1 (0·1 to 0·1)	0 (0 to 0)	77·4
Motorcyclist road injuries	0 (0 to 0)	0 (0 to 0)	0·1 (0·1 to 0·2)	0·4 (0·2 to 0·5)	0·9 (0·6 to 1·2)	1·3 (0·9 to 1·6)	9·9 (7·3 to 13·1)	19·3 (13·8 to 25·4)	16·7 (12·5 to 21·6)	14·3 (11 to 18·4)	10·6 (8 to 13·6)	8·7 (6·8 to 11·1)	9·4 (7·5 to 11·6)	8 (6·3 to 10·1)	6·5 (5·2 to 7·9)	3·9 (3·1 to 4·9)	2·7 (2·1 to 3·4)	1·7 (1·2 to 2·2)	1·1 (0·8 to 1·5)	0·5 (0·3 to 0·6)	0·2 (0·1 to 0·2)	0·1 (0·1 to 0·1)	0 (0 to 0)	116
Motor vehicle road injuries	0·1 (0·1 to 0·1)	0 (0 to 0·1)	0·9 (0·6 to 1·4)	3 (1·8 to 4·7)	6 (4·2 to 8·1)	7·4 (5·4 to 9·8)	29·7 (22·4 to 38·6)	45·1 (32 to 59·7)	35·1 (25·9 to 45·5)	31·2 (23·7 to 40·6)	24·3 (17·8 to 31·9)	22·6 (17·1 to 29·9)	25·3 (19·2 to 32·3)	22·4 (17·6 to 28·1)	18·5 (14·6 to 22·7)	13·4 (10·8 to 16·1)	9·5 (7·7 to 11·6)	6·3 (5·1 to 7·6)	4 (3·2 to 4·8)	1·3 (1·1 to 1·5)	0·4 (0·4 to 0·5)	0·2 (0·1 to 0·2)	0·1 (0 to 0·1)	307
Other road injuries	0 (0 to 0)	0 (0 to 0)	0 (0 to 0)	0·2 (0·1 to 0·3)	0·2 (0·1 to 0·4)	0·2 (0·2 to 0·3)	0·5 (0·3 to 0·8)	0·8 (0·6 to 1·1)	0·9 (0·6 to 1·2)	0·8 (0·6 to 1·1)	0·8 (0·6 to 1·1)	0·8 (0·6 to 1·1)	1 (0·7 to 1·3)	1·1 (0·8 to 1·5)	1 (0·7 to 1·3)	0·7 (0·5 to 1)	0·6 (0·4 to 0·7)	0·4 (0·3 to 0·5)	0·3 (0·2 to 0·4)	0·1 (0·1 to 0·1)	0 (0 to 0·1)	0 (0 to 0)	0 (0 to 0)	10·6
Other transport injuries	0·1 (0 to 0·1)	0·1 (0 to 0·1)	0·5 (0·3 to 0·8)	1·2 (0·8 to 1·8)	1·7 (1·2 to 2·3)	2·5 (1·8 to 3·3)	5·9 (4 to 8·2)	9·1 (6·2 to 12·9)	7·6 (5·2 to 10·5)	7·5 (5·1 to 10·3)	5·8 (4 to 8·4)	6 (4·3 to 8)	5·7 (4 to 7·8)	5·4 (4·1 to 7·1)	5·5 (4·2 to 7·2)	3·1 (2·3 to 4)	2 (1·5 to 2·5)	1·9 (1·4 to 2·4)	1·1 (0·8 to 1·5)	0·4 (0·3 to 0·4)	0·1 (0·1 to 0·2)	0·1 (0·1 to 0·1)	0 (0 to 0)	73·3
Unintentional injuries	5·6 (3·6 to 8·2)	2·8 (1·8 to 4·4)	32·1 (19·6 to 49·8)	49·6 (34 to 74)	56·1 (45·3 to 68)	63·6 (52·2 to 75·3)	111 (90·5 to 131)	149 (119 to 181)	131 (104 to 161)	122 (99·9 to 149)	105 (85·5 to 127)	107 (87·4 to 129)	121 (98·7 to 148)	126 (103 to 153)	113 (93·4 to 135)	88·7 (74·4 to 107)	72·6 (61·5 to 86·6)	65·1 (56·1 to 77·2)	55·6 (47·8 to 65·7)	30·3 (26·1 to 35)	16·3 (14·1 to 18·6)	9·2 (7·9 to 10·7)	3·8 (3·1 to 4·3)	1,640
Falls	0·1 (0·1 to 0·1)	0 (0 to 0·1)	1·1 (0·6 to 1·8)	2·2 (1·5 to 3·3)	3·5 (2·7 to 4·7)	5·7 (4·3 to 7·5)	10·7 (8·2 to 14·1)	15·4 (11·7 to 19·7)	15·5 (11·8 to 20·5)	15·7 (12·3 to 20·2)	15·7 (12 to 20)	18·1 (14·1 to 23)	23·5 (18·6 to 29·4)	28·3 (22·3 to 35·7)	27·4 (21·8 to 34·1)	24 (19·5 to 29·2)	22 (18 to 26·8)	24·3 (20·2 to 29)	23·8 (19·6 to 28·8)	15·9 (13·1 to 19)	9·5 (8 to 11·1)	5·4 (4·5 to 6·5)	2·1 (1·7 to 2·5)	310
Drowning	0·4 (0·3 to 0·6)	0·1 (0·1 to 0·2)	3·2 (1·9 to 5·1)	17·7 (11·1 to 27·7)	22·6 (16·8 to 29)	23·5 (18·3 to 28·8)	39·5 (30·5 to 48·2)	40·8 (29·5 to 53)	29 (21·1 to 37·9)	25·3 (19·3 to 32·2)	19·4 (15·1 to 24·7)	16·9 (13·2 to 20·9)	16·3 (12·7 to 20·6)	14·3 (11·1 to 18)	11·3 (9·1 to 13·7)	7·4 (6·1 to 8·9)	5 (4 to 6·2)	2·9 (2·4 to 3·5)	1·8 (1·5 to 2·2)	0·5 (0·4 to 0·6)	0·2 (0·2 to 0·3)	0·2 (0·1 to 0·2)	0·1 (0·1 to 0·1)	299
Fire, heat, and hot substances	0·2 (0·1 to 0·2)	0·1 (0·1 to 0·2)	2·7 (1·6 to 4·1)	7·1 (4·6 to 10·9)	7·1 (5·6 to 8·7)	7·4 (5·7 to 9·5)	10·3 (7·7 to 13·3)	14 (10·6 to 18·1)	14·5 (11 to 18·7)	14·5 (11·2 to 19)	12·7 (9·7 to 16·3)	12·8 (9·7 to 16·8)	14·5 (10·9 to 18·9)	14·7 (11 to 19·3)	12·8 (9·7 to 16·6)	9·7 (7·4 to 12·6)	7·1 (5·5 to 9·2)	6 (4·7 to 7·8)	4·5 (3·6 to 5·8)	2·2 (1·7 to 2·8)	1·1 (0·9 to 1·4)	0·6 (0·5 to 0·7)	0·2 (0·2 to 0·3)	177
Poisonings	0 (0 to 0·1)	0·1 (0 to 0·1)	0·4 (0·2 to 0·6)	1·4 (0·9 to 2·2)	0·8 (0·6 to 1·1)	0·8 (0·5 to 1·1)	1·3 (0·9 to 1·7)	1·7 (1·3 to 2·2)	1·8 (1·3 to 2·2)	1·7 (1·3 to 2·1)	1·4 (1 to 1·8)	1·1 (0·9 to 1·4)	1·1 (0·9 to 1·5)	1·1 (0·8 to 1·4)	1 (0·7 to 1·2)	0·6 (0·5 to 0·8)	0·5 (0·4 to 0·6)	0·4 (0·3 to 0·5)	0·3 (0·2 to 0·4)	0·2 (0·1 to 0·2)	0·1 (0·1 to 0·1)	0 (0 to 0)	0 (0 to 0)	17·6
Poisoning by carbon monoxide	0 (0 to 0)	0 (0 to 0)	0 (0 to 0·1)	0·3 (0·1 to 0·5)	0·2 (0·1 to 0·3)	0·2 (0·1 to 0·3)	0·4 (0·3 to 0·6)	0·7 (0·4 to 1)	0·6 (0·4 to 0·9)	0·6 (0·4 to 0·9)	0·5 (0·3 to 0·7)	0·4 (0·3 to 0·6)	0·3 (0·2 to 0·5)	0·3 (0·2 to 0·4)	0·3 (0·2 to 0·4)	0·2 (0·1 to 0·2)	0·2 (0·1 to 0·2)	0·1 (0·1 to 0·2)	0·1 (0·1 to 0·1)	0 (0 to 0·1)	0 (0 to 0)	0 (0 to 0)	0 (0 to 0)	5·5
Poisoning by other means	0 (0 to 0)	0·1 (0 to 0·1)	0·4 (0·2 to 0·6)	1·1 (0·7 to 1·8)	0·6 (0·4 to 0·8)	0·6 (0·4 to 0·8)	0·8 (0·6 to 1·2)	1 (0·8 to 1·4)	1·1 (0·8 to 1·5)	1 (0·8 to 1·3)	0·9 (0·6 to 1·1)	0·7 (0·5 to 1)	0·8 (0·6 to 1·1)	0·8 (0·6 to 1·1)	0·7 (0·5 to 0·9)	0·5 (0·3 to 0·6)	0·3 (0·2 to 0·4)	0·3 (0·2 to 0·4)	0·2 (0·2 to 0·3)	0·1 (0·1 to 0·1)	0·1 (0 to 0·1)	0 (0 to 0)	0 (0 to 0)	12·1
Exposure to mechanical forces	0·2 (0·1 to 0·3)	0·2 (0·1 to 0·3)	1·3 (0·9 to 1·9)	5·4 (3·8 to 7·7)	8·2 (6·4 to 10·4)	10·3 (8·1 to 13·4)	26·5 (21·2 to 31·9)	44·1 (34·6 to 54·2)	37·9 (29·2 to 48)	35·4 (28·1 to 44·2)	29·7 (23·7 to 37·5)	30·6 (24·2 to 39·3)	33 (25·6 to 42·7)	33·6 (25·9 to 43·8)	28·8 (22·1 to 37·8)	20 (15·2 to 26·7)	14·1 (10·5 to 18·9)	10·7 (7·8 to 14·3)	7·5 (5·5 to 10·1)	3·2 (2·4 to 4·3)	1·5 (1·1 to 1·9)	0·9 (0·7 to 1·1)	0·4 (0·3 to 0·5)	383
Unintentional firearm injuries	0 (0 to 0)	0 (0 to 0)	0·1 (0·1 to 0·2)	0·3 (0·2 to 0·4)	0·5 (0·4 to 0·7)	1·2 (0·9 to 1·7)	8·9 (6·5 to 12)	18·3 (12·8 to 24·9)	12·8 (9·1 to 17·2)	11 (7·7 to 14·8)	8 (5·8 to 10·5)	7·3 (5·2 to 9·7)	6·7 (5 to 9·2)	5·6 (4·2 to 7·2)	4·4 (3·4 to 5·8)	2·7 (2·1 to 3·5)	1·8 (1·3 to 2·3)	1·1 (0·8 to 1·4)	0·7 (0·5 to 0·9)	0·3 (0·2 to 0·4)	0·2 (0·1 to 0·2)	0·1 (0·1 to 0·2)	0·1 (0 to 0·1)	92
Other exposure to mechanical forces	0·2 (0·1 to 0·2)	0·2 (0·1 to 0·3)	1·2 (0·8 to 1·8)	5·2 (3·6 to 7·3)	7·7 (6 to 9·8)	9·1 (7·1 to 12)	17·6 (13·4 to 22·6)	25·8 (19·2 to 33·6)	25 (18·4 to 33·3)	24·4 (18·3 to 31·8)	21·6 (16·5 to 28·4)	23·3 (17·5 to 31·4)	26·3 (19·8 to 35·3)	28 (20·9 to 37·6)	24·3 (18·1 to 33)	17·3 (12·7 to 23·6)	12·3 (9 to 16·8)	9·6 (6·9 to 13)	6·8 (4·9 to 9·3)	2·9 (2·1 to 3·9)	1·3 (1 to 1·7)	0·8 (0·6 to 1)	0·3 (0·3 to 0·4)	291
Adverse effects of medical treatment	1·3 (0·8 to 2)	0·9 (0·6 to 1·5)	4·2 (2·4 to 7·6)	2·5 (1·6 to 4)	1·7 (1·2 to 2·3)	1·6 (1·1 to 2·1)	2·5 (1·8 to 3·4)	5·2 (3·5 to 7·5)	4·9 (3·3 to 7)	4·7 (3·4 to 6·5)	4·4 (3·2 to 5·9)	4·8 (3·6 to 6·5)	6·2 (4·4 to 8·3)	8·4 (6·2 to 11·4)	9·3 (6·8 to 12·8)	9·1 (6·6 to 12·8)	8·5 (6·4 to 11·1)	8·1 (6·3 to 10·5)	7·4 (5·7 to 9·5)	3·5 (2·8 to 4·4)	1·7 (1·3 to 2·2)	0·8 (0·6 to 1·1)	0·3 (0·2 to 0·4)	102
Animal contact	0 (0 to 0)	0 (0 to 0·1)	0·3 (0·2 to 0·4)	0·7 (0·5 to 1·1)	0·8 (0·6 to 1·2)	1·1 (0·8 to 1·5)	1·4 (1 to 1·9)	1·7 (1·2 to 2·2)	2 (1·5 to 2·6)	1·5 (1·1 to 2)	1·7 (1·3 to 2·3)	1·8 (1·3 to 2·4)	2·5 (1·8 to 3·2)	2·6 (2 to 3·4)	2·6 (1·9 to 3·4)	2·4 (1·7 to 3·3)	2·3 (1·6 to 3·2)	2 (1·4 to 2·6)	1·5 (1 to 2)	0·6 (0·5 to 0·8)	0·3 (0·2 to 0·4)	0·3 (0·2 to 0·4)	0·1 (0·1 to 0·2)	30·3
Venomous animal contact	0 (0 to 0)	0 (0 to 0)	0 (0 to 0·1)	0·2 (0·1 to 0·3)	0·3 (0·2 to 0·5)	0·5 (0·3 to 0·7)	0·6 (0·4 to 0·9)	0·7 (0·5 to 1)	0·8 (0·5 to 1·1)	0·6 (0·4 to 0·9)	0·6 (0·4 to 0·9)	0·6 (0·4 to 0·9)	0·7 (0·5 to 1)	0·8 (0·5 to 1·1)	0·7 (0·5 to 1)	0·6 (0·4 to 0·8)	0·4 (0·3 to 0·6)	0·4 (0·3 to 0·5)	0·3 (0·2 to 0·5)	0·2 (0·1 to 0·3)	0·1 (0·1 to 0·2)	0·1 (0·1 to 0·2)	0·1 (0 to 0·1)	9·6
Non-venomous animal contact	0 (0 to 0)	0 (0 to 0)	0·2 (0·1 to 0·4)	0·5 (0·3 to 0·8)	0·5 (0·3 to 0·8)	0·6 (0·4 to 0·9)	0·8 (0·6 to 1·2)	0·9 (0·7 to 1·3)	1·2 (0·8 to 1·8)	0·9 (0·6 to 1·3)	1·1 (0·7 to 1·5)	1·2 (0·8 to 1·7)	1·7 (1·1 to 2·3)	1·8 (1·3 to 2·5)	1·8 (1·3 to 2·5)	1·8 (1·2 to 2·6)	1·8 (1·2 to 2·7)	1·6 (1·1 to 2·2)	1·1 (0·8 to 1·6)	0·4 (0·3 to 0·6)	0·2 (0·1 to 0·2)	0·1 (0·1 to 0·2)	0·1 (0 to 0·1)	20·7
Foreign body	3·1 (2 to 4·5)	1·2 (0·7 to 2)	17·7 (10·1 to 28·5)	8·9 (5·8 to 13·6)	5·2 (4·1 to 6·5)	5·3 (4·2 to 6·5)	4·2 (3·3 to 5·1)	6·1 (4·7 to 7·7)	5·8 (4·5 to 7·2)	5·3 (4·1 to 6·7)	4·7 (3·7 to 6)	5 (4 to 6)	6·1 (4·9 to 7·5)	7 (5·6 to 8·7)	6·5 (5·3 to 7·8)	5·9 (4·7 to 7·2)	5·8 (4·5 to 7·2)	5·2 (4·4 to 6·1)	4·5 (3·6 to 5·5)	2·4 (1·9 to 3)	0·9 (0·7 to 1·1)	0·6 (0·4 to 0·7)	0·3 (0·2 to 0·3)	117
Pulmonary aspiration and foreign body in airway	3·1 (1·9 to 4·5)	1·2 (0·7 to 2)	17·6 (10 to 28·4)	8·5 (5·4 to 13)	4·6 (3·6 to 5·8)	4·6 (3·5 to 5·7)	3·2 (2·5 to 4)	4·9 (3·7 to 6·2)	4·6 (3·5 to 5·9)	4·3 (3·2 to 5·5)	3·8 (3 to 4·9)	4 (3·2 to 4·9)	5 (3·9 to 6·3)	5·8 (4·4 to 7·5)	5·5 (4·4 to 6·8)	5·2 (4·1 to 6·5)	5·2 (4 to 6·6)	4·7 (3·9 to 5·6)	4·1 (3·2 to 5·1)	2·2 (1·7 to 2·8)	0·8 (0·7 to 1·1)	0·5 (0·4 to 0·7)	0·2 (0·2 to 0·3)	104
Foreign body in eyes	0 (0 to 0)	0 (0 to 0)	0 (0 to 0·1)	0·1 (0 to 0·3)	0·2 (0·1 to 0·5)	0·3 (0·1 to 0·7)	0·4 (0·1 to 0·9)	0·6 (0·2 to 1·2)	0·6 (0·2 to 1·3)	0·5 (0·2 to 1·1)	0·4 (0·2 to 0·9)	0·5 (0·2 to 1)	0·5 (0·3 to 1)	0·5 (0·3 to 0·9)	0·4 (0·2 to 0·6)	0·3 (0·2 to 0·4)	0·2 (0·1 to 0·3)	0·2 (0·1 to 0·3)	0·1 (0·1 to 0·2)	0·1 (0 to 0·1)	0 (0 to 0)	0 (0 to 0)	0 (0 to 0)	5·9
Foreign body in other body part	0 (0 to 0)	0 (0 to 0)	0·1 (0 to 0·1)	0·3 (0·2 to 0·4)	0·4 (0·2 to 0·5)	0·4 (0·3 to 0·6)	0·5 (0·4 to 0·7)	0·6 (0·4 to 0·9)	0·6 (0·4 to 0·8)	0·5 (0·4 to 0·7)	0·4 (0·3 to 0·6)	0·5 (0·4 to 0·7)	0·6 (0·4 to 0·8)	0·6 (0·4 to 0·8)	0·6 (0·4 to 0·8)	0·4 (0·3 to 0·6)	0·3 (0·2 to 0·4)	0·3 (0·2 to 0·4)	0·2 (0·2 to 0·3)	0·1 (0·1 to 0·1)	0 (0 to 0·1)	0 (0 to 0)	0 (0 to 0)	7·6
Environmental heat and cold exposure	0 (0 to 0)	0 (0 to 0)	0·1 (0·1 to 0·2)	0·6 (0·4 to 0·9)	1·2 (0·8 to 1·7)	1·7 (1·1 to 2·4)	2·5 (1·7 to 3·5)	3·5 (2·3 to 4·9)	3·8 (2·6 to 5·5)	3·5 (2·5 to 5·1)	3·2 (2·2 to 4·4)	3·5 (2·5 to 4·9)	4·2 (2·9 to 5·7)	4·5 (3·2 to 6·2)	4 (2·8 to 5·5)	3·1 (2·2 to 4·2)	2·4 (1·7 to 3·2)	2 (1·5 to 2·7)	1·6 (1·1 to 2·1)	0·8 (0·6 to 1·1)	0·4 (0·3 to 0·6)	0·3 (0·2 to 0·4)	0·2 (0·1 to 0·2)	47·1
Exposure to forces of nature	0 (0 to 0)	0 (0 to 0)	0 (0 to 0)	0 (0 to 0)	0 (0 to 0)	0 (0 to 0)	0·3 (0·2 to 0·4)	0·3 (0·2 to 0·5)	0·7 (0·4 to 1·1)	0·3 (0·2 to 0·5)	0·3 (0·1 to 0·4)	0·3 (0·2 to 0·5)	0·4 (0·2 to 0·6)	0·3 (0·2 to 0·5)	0·3 (0·2 to 0·4)	0·2 (0·1 to 0·3)	0·1 (0·1 to 0·2)	0·1 (0·1 to 0·2)	0·1 (0 to 0·1)	0 (0 to 0)	0 (0 to 0)	0 (0 to 0)	0 (0 to 0)	3·7
Other unintentional injuries	0·2 (0·1 to 0·4)	0·1 (0 to 0·1)	1 (0·6 to 1·4)	3 (2·1 to 4·2)	4·9 (3·8 to 6·3)	6·2 (4·8 to 8)	11·6 (8·9 to 14·8)	16·4 (12·7 to 20·8)	15·2 (11·5 to 19·6)	14·3 (11·1 to 18·3)	12 (9·4 to 15·1)	11·6 (9·1 to 14·7)	12·7 (9·8 to 16·4)	11·2 (8·6 to 14·4)	9·4 (7·2 to 12·2)	6·4 (4·8 to 8·4)	4·7 (3·6 to 6·3)	3·5 (2·6 to 4·8)	2·5 (1·9 to 3·4)	1 (0·7 to 1·4)	0·4 (0·3 to 0·6)	0·2 (0·1 to 0·3)	0·1 (0 to 0·1)	149
Self-harm and interpersonal violence	1·2 (0·7 to 1·9)	0·2 (0·1 to 0·3)	1·9 (1·1 to 3)	3·6 (2·1 to 5·9)	3·7 (2·8 to 4·8)	7·7 (6·1 to 9·6)	53·7 (41 to 68·2)	104 (80·4 to 132)	87 (65·2 to 111)	77·2 (60·8 to 96·9)	59·9 (47·2 to 74·4)	45·9 (36·7 to 58·3)	44·8 (35·7 to 55·7)	38·1 (31·4 to 46·2)	31·5 (26 to 38)	21·1 (17·5 to 25·4)	15·3 (12·8 to 18)	10·7 (8·9 to 12·7)	6·8 (5·7 to 8)	2·4 (2 to 2·9)	0·9 (0·7 to 1·1)	0·3 (0·2 to 0·4)	0·1 (0·1 to 0·1)	618
Self-harm	0 (0 to 0)	0 (0 to 0)	0 (0 to 0)	0 (0 to 0)	0 (0 to 0)	1·6 (1·1 to 2·2)	14 (10·5 to 18·3)	26·8 (19·4 to 36)	24·5 (17·4 to 32·8)	25·4 (18·7 to 33·8)	20·7 (15·4 to 27·1)	18·1 (13·5 to 24·2)	19·5 (14·7 to 25·4)	18·3 (13·8 to 23·6)	15·8 (12·1 to 19·9)	11·4 (8·8 to 14·6)	8·8 (7 to 11)	6·5 (5·2 to 8·2)	4·1 (3·2 to 5·2)	1·4 (1·1 to 1·8)	0·4 (0·3 to 0·5)	0·1 (0·1 to 0·1)	0 (0 to 0)	218
Self-harm by firearm	0 (0 to 0)	0 (0 to 0)	0 (0 to 0)	0 (0 to 0)	0 (0 to 0)	0 (0 to 0)	0·1 (0·1 to 0·2)	0·3 (0·1 to 0·4)	0·2 (0·1 to 0·4)	0·3 (0·2 to 0·4)	0·2 (0·1 to 0·3)	0·2 (0·1 to 0·3)	0·2 (0·1 to 0·3)	0·2 (0·1 to 0·3)	0·2 (0·1 to 0·2)	0·1 (0·1 to 0·2)	0·1 (0·1 to 0·1)	0·1 (0·1 to 0·1)	0 (0 to 0·1)	0 (0 to 0)	0 (0 to 0)	0 (0 to 0)	0 (0 to 0)	2·3
Self-harm by other specified means	0 (0 to 0)	0 (0 to 0)	0 (0 to 0)	0 (0 to 0)	0 (0 to 0)	1·6 (1·1 to 2·2)	13·9 (10·4 to 18·1)	26·6 (19·2 to 35·6)	24·2 (17·2 to 32·5)	25 (18·5 to 33·5)	20·5 (15·2 to 26·9)	17·9 (13·3 to 23·9)	19·3 (14·5 to 25·1)	18·1 (13·6 to 23·4)	15·7 (11·9 to 19·7)	11·3 (8·7 to 14·4)	8·7 (6·9 to 10·9)	6·5 (5·1 to 8·2)	4·1 (3·2 to 5·2)	1·4 (1·1 to 1·7)	0·4 (0·3 to 0·5)	0·1 (0·1 to 0·1)	0 (0 to 0)	215
Interpersonal violence	1·2 (0·7 to 1·9)	0·2 (0·1 to 0·3)	1·8 (1·1 to 3)	3·5 (2·1 to 5·8)	3·6 (2·7 to 4·8)	6 (4·6 to 7·8)	39·6 (28·3 to 52·5)	77·6 (57·4 to 101)	62·5 (45 to 83·4)	51·3 (38·1 to 66·4)	34·5 (25·7 to 45·3)	26 (19·4 to 34·5)	23·7 (17·9 to 31·5)	17·8 (13·8 to 22·9)	13·1 (10·2 to 17·1)	8 (6·3 to 10·2)	5·4 (4·2 to 6·8)	3·4 (2·6 to 4·3)	2 (1·6 to 2·5)	0·6 (0·5 to 0·7)	0·2 (0·2 to 0·3)	0·1 (0·1 to 0·1)	0 (0 to 0)	382
Physical violence by firearm	0 (0 to 0)	0 (0 to 0)	0 (0 to 0)	0·1 (0·1 to 0·2)	0·2 (0·1 to 0·2)	0·3 (0·2 to 0·5)	6·5 (4·5 to 8·9)	13·4 (9·5 to 17·6)	9·3 (6·4 to 12·5)	6·6 (4·7 to 8·7)	3·5 (2·5 to 4·7)	2 (1·4 to 2·8)	1·5 (1·1 to 2·1)	0·9 (0·7 to 1·2)	0·5 (0·4 to 0·7)	0·3 (0·2 to 0·4)	0·2 (0·1 to 0·2)	0·1 (0·1 to 0·1)	0 (0 to 0·1)	0 (0 to 0)	0 (0 to 0)	0 (0 to 0)	0 (0 to 0)	45·4
Physical violence by sharp object	0 (0 to 0)	0 (0 to 0)	0 (0 to 0)	0·1 (0·1 to 0·2)	0·2 (0·1 to 0·3)	0·4 (0·3 to 0·5)	4·8 (3·3 to 6·6)	10·2 (7·3 to 13·5)	7·8 (5·6 to 10·5)	6·3 (4·7 to 8·3)	3·9 (2·9 to 5·2)	2·6 (2 to 3·5)	2 (1·5 to 2·6)	1·4 (1·1 to 1·8)	0·9 (0·7 to 1·2)	0·6 (0·4 to 0·7)	0·4 (0·3 to 0·5)	0·3 (0·2 to 0·4)	0·2 (0·1 to 0·2)	0·1 (0·1 to 0·1)	0 (0 to 0)	0 (0 to 0)	0 (0 to 0)	42·2
Sexual violence	0 (0 to 0)	0 (0 to 0)	0 (0 to 0)	0 (0 to 0·1)	0·4 (0·2 to 0·6)	0·9 (0·5 to 1·4)	2·1 (1·2 to 3·4)	3·2 (1·9 to 4·9)	2·9 (1·6 to 4·6)	2·2 (1·2 to 3·6)	1·7 (0·9 to 2·6)	1·4 (0·8 to 2·4)	1·3 (0·8 to 2·2)	1 (0·6 to 1·7)	0·7 (0·4 to 1·1)	0·4 (0·3 to 0·7)	0·3 (0·2 to 0·5)	0·3 (0·2 to 0·4)	0·2 (0·1 to 0·3)	0·1 (0·1 to 0·1)	0 (0 to 0·1)	0 (0 to 0)	0 (0 to 0)	19·1
Physical violence by other means	1·2 (0·7 to 1·9)	0·2 (0·1 to 0·2)	1·8 (1 to 3)	3·2 (1·9 to 5·5)	2·9 (2·1 to 3·9)	4·5 (3·2 to 6)	26·2 (18·1 to 35·5)	50·8 (36·8 to 66·9)	42·5 (30·1 to 57·1)	36·2 (26·4 to 47·2)	25·4 (18·3 to 33·7)	19·9 (14·6 to 27·1)	18·9 (13·8 to 25·6)	14·5 (10·9 to 19)	11 (8·3 to 14·6)	6·7 (5·1 to 8·8)	4·5 (3·4 to 5·9)	2·7 (2·1 to 3·6)	1·6 (1·2 to 2·1)	0·4 (0·3 to 0·5)	0·2 (0·1 to 0·2)	0·1 (0 to 0·1)	0 (0 to 0)	275
Conflict and terrorism	0 (0 to 0)	0 (0 to 0)	0 (0 to 0)	0 (0 to 0)	0 (0 to 0)	0 (0 to 0)	0 (0 to 0)	0 (0 to 0)	0 (0 to 0)	0·4 (0·2 to 0·7)	4·7 (2·2 to 8·8)	1·7 (0·8 to 3·2)	1·6 (0·8 to 3)	1·9 (1 to 3·5)	2·6 (1·5 to 4·4)	1·7 (1 to 3)	1·1 (0·6 to 2)	0·8 (0·4 to 1·6)	0·6 (0·3 to 1·3)	0·4 (0·2 to 0·7)	0·2 (0·1 to 0·4)	0·1 (0 to 0·2)	0 (0 to 0·1)	18

SR Table 2c: Guyana DALYS by cause and age group for both sexes with 95% uncertainty intervals, 2019

Cause	Early Neonatal	Late Neonatal	Post Neonatal	1 to 4	5 to 9	10 to 14	15 to 19	20 to 24	25 to 29	30 to 34	35 to 39	40 to 44	45 to 49	50 to 54	55 to 59	60 to 64	65 to 69	70 to 74	75 to 79	80 to 84	85 to 89	90 to 94	95 plus	Total
All causes	16,800 (11,900 to 23,400)	2,310 (1,630 to 3,200)	7,160 (5,260 to 9,740)	5,760 (4,550 to 7,210)	6,010 (5,000 to 7,280)	6,920 (5,600 to 8,510)	11,800 (9,810 to 14,300)	19,600 (16,300 to 23,100)	19,900 (16,600 to 23,300)	17,500 (14,700 to 20,600)	17,900 (14,900 to 21,300)	20,400 (16,800 to 24,400)	22,100 (17,900 to 27,000)	24,500 (19,800 to 30,000)	26,300 (21,200 to 32,200)	24,700 (20,300 to 29,800)	21,200 (17,600 to 25,200)	14,700 (12,300 to 17,400)	10,500 (8,930 to 12,300)	6,870 (5,960 to 7,840)	3,400 (2,990 to 3,830)	1,780 (1,620 to 1,930)	489 (453 to 520)	309,000
Communicable, maternal, neonatal, and nutritional diseases	15,000 (10,500 to 20,600)	1,870 (1,310 to 2,620)	4,430 (3,210 to 6,020)	2,520 (1,990 to 3,150)	2,070 (1,560 to 2,700)	1,580 (1,210 to 2,080)	1,910 (1,500 to 2,410)	3,100 (2,570 to 3,680)	3,740 (3,230 to 4,300)	3,630 (3,200 to 4,110)	3,450 (3,030 to 3,920)	3,240 (2,810 to 3,760)	2,780 (2,360 to 3,310)	2,350 (1,940 to 2,840)	2,010 (1,650 to 2,450)	1,650 (1,350 to 2,000)	1,330 (1,060 to 1,660)	945 (755 to 1,200)	723 (587 to 895)	555 (431 to 713)	324 (249 to 409)	195 (152 to 241)	59·6 (44·7 to 76·6)	59,400
HIV/AIDS and sexually transmitted infections	11·7 (4·8 to 22·6)	1·6 (0·7 to 3·3)	146 (138 to 155)	120 (112 to 127)	96·5 (84·2 to 123)	70·1 (61·5 to 85)	146 (134 to 157)	682 (631 to 738)	1,470 (1,370 to 1,590)	1,760 (1,650 to 1,890)	1,670 (1,580 to 1,780)	1,400 (1,310 to 1,490)	1,050 (987 to 1,120)	636 (595 to 681)	382 (349 to 419)	226 (201 to 260)	138 (118 to 163)	38·1 (26·7 to 52·3)	15·4 (11·6 to 20·6)	6·5 (5 to 8·4)	2·6 (2 to 3·5)	0·8 (0·6 to 1·2)	0·2 (0·2 to 0·3)	10,100
HIV/AIDS	0 (0 to 0)	0·1 (0 to 0·2)	143 (135 to 151)	120 (112 to 127)	96·4 (84·1 to 123)	67·7 (59·2 to 82·4)	134 (126 to 144)	657 (610 to 710)	1,450 (1,350 to 1,560)	1,740 (1,630 to 1,870)	1,640 (1,550 to 1,750)	1,370 (1,290 to 1,460)	1,030 (965 to 1,100)	622 (583 to 666)	372 (341 to 408)	220 (195 to 252)	133 (113 to 158)	35·5 (24·5 to 49)	13·9 (10·3 to 19)	5·6 (4·3 to 7·4)	2·2 (1·6 to 3·1)	0·7 (0·4 to 1)	0·2 (0·1 to 0·3)	9,840
HIV/AIDS - Drug-susceptible Tuberculosis	0 (0 to 0)	0 (0 to 0)	17·8 (10·8 to 27·3)	14·6 (8·9 to 22·2)	11·4 (7·2 to 17)	7·8 (5 to 11·6)	15·9 (9·9 to 24)	80·8 (50·9 to 121)	179 (112 to 270)	215 (134 to 323)	203 (127 to 306)	167 (105 to 254)	124 (77·9 to 188)	74·4 (46·6 to 112)	43·4 (28·2 to 64·8)	25·2 (16·3 to 37)	14·9 (9·7 to 21·7)	3·5 (2·4 to 5)	1·4 (0·9 to 2·1)	0·6 (0·4 to 0·9)	0·2 (0·2 to 0·3)	0·1 (0 to 0·1)	0 (0 to 0)	1,200
HIV/AIDS - Multidrug-resistant Tuberculosis without extensive	0 (0 to 0)	0 (0 to 0)	0·4 (0 to 1·9)	0·3 (0 to 1·6)	0·2 (0 to 1·2)	0·1 (0 to 0·8)	0·3 (0 to 1·7)	1·5 (0·1 to 8·5)	3·3 (0·2 to 18·5)	3·9 (0·3 to 22·1)	3·6 (0·2 to 20·8)	3 (0·2 to 17·3)	2·3 (0·2 to 12·9)	1·4 (0·1 to 7·7)	0·8 (0·1 to 4·5)	0·4 (0 to 2·6)	0·3 (0 to 1·5)	0·1 (0 to 0·3)	0 (0 to 0·1)	0 (0 to 0·1)	0 (0 to 0)	0 (0 to 0)	0 (0 to 0)	22
HIV/AIDS - Extensively drug-resistant Tuberculosis	0 (0 to 0)	0 (0 to 0)	0·1 (0 to 0·3)	0 (0 to 0·3)	0 (0 to 0·2)	0 (0 to 0·1)	0 (0 to 0·3)	0·2 (0 to 1·4)	0·5 (0 to 3·1)	0·6 (0 to 3·7)	0·6 (0 to 3·5)	0·5 (0 to 2·9)	0·4 (0 to 2·1)	0·2 (0 to 1·3)	0·1 (0 to 0·7)	0·1 (0 to 0·4)	0 (0 to 0·2)	0 (0 to 0)	0 (0 to 0)	0 (0 to 0)	0 (0 to 0)	0 (0 to 0)	0 (0 to 0)	3·6
HIV/AIDS resulting in other diseases	0 (0 to 0)	0·1 (0 to 0·2)	125 (114 to 136)	105 (94·2 to 114)	84·8 (70·9 to 111)	59·7 (50·7 to 74·8)	118 (107 to 129)	575 (511 to 639)	1,260 (1,130 to 1,390)	1,520 (1,360 to 1,680)	1,440 (1,290 to 1,570)	1,200 (1,080 to 1,310)	900 (806 to 984)	546 (491 to 596)	327 (290 to 366)	194 (165 to 229)	118 (95·9 to 143)	32 (21·1 to 45·7)	12·5 (8·8 to 17·5)	5 (3·6 to 6·8)	2 (1·4 to 2·8)	0·6 (0·3 to 0·9)	0·2 (0·1 to 0·2)	8,620
Sexually transmitted infections excluding HIV	11·7 (4·8 to 22·6)	1·5 (0·6 to 3·2)	2·9 (1 to 7·2)	0·1 (0 to 0·4)	0·1 (0 to 0·1)	2·5 (1·7 to 3·7)	11·6 (7·4 to 17·4)	25·2 (16·4 to 39·1)	28·2 (18·4 to 43·4)	28·2 (17·9 to 44·1)	28·4 (18·6 to 44·5)	29 (18·8 to 45·1)	23·8 (15·2 to 37·8)	14·3 (8·9 to 24)	9·9 (5·7 to 16·9)	6·4 (3·6 to 11)	4·7 (2·9 to 8)	2·5 (1·5 to 4·2)	1·5 (0·9 to 2·5)	0·9 (0·5 to 1·4)	0·4 (0·2 to 0·6)	0·2 (0·1 to 0·3)	0 (0 to 0·1)	234
Syphilis	11·7 (4·8 to 22·6)	1·5 (0·6 to 3·2)	2·9 (1 to 7·2)	0·1 (0 to 0·4)	0·1 (0 to 0·1)	0·8 (0·5 to 1·3)	[illegible]	[illegible]	[illegible]	[illegible]	[illegible]	[illegible]	[illegible]	[illegible]	[illegible]	[illegible]	[illegible]	[illegible]	[illegible]	[illegible]	[illegible]	[illegible]	[illegible]	[illegible]
Chlamydial infection	0 (0 to 0)	0 (0 to 0)	0 (0 to 0)	0 (0 to 0)	0 (0 to 0)	0·2 (0·1 to 0·2)	[illegible]	[illegible]	[illegible]	[illegible]	[illegible]	[illegible]	[illegible]	[illegible]	[illegible]	[illegible]	[illegible]	[illegible]	[illegible]	[illegible]	[illegible]	[illegible]	[illegible]	[illegible]
Gonococcal infection	0 (0 to 0)	0 (0 to 0)	0 (0 to 0)	0 (0 to 0)	0 (0 to 0)	0·4 (0·2 to 0·7)	[illegible]	[illegible]	[illegible]	[illegible]	[illegible]	[illegible]	[illegible]	[illegible]	[illegible]	[illegible]	[illegible]	[illegible]	[illegible]	[illegible]	[illegible]	[illegible]	[illegible]	[illegible]
Trichomoniasis	0 (0 to 0)	0 (0 to 0)	0 (0 to 0)	0 (0 to 0)	0 (0 to 0)	0 (0 to 0·1)	[illegible]	[illegible]	[illegible]	[illegible]	[illegible]	[illegible]	[illegible]	[illegible]	[illegible]	[illegible]	[illegible]	[illegible]	[illegible]	[illegible]	[illegible]	[illegible]	[illegible]	[illegible]
Genital herpes	0 (0 to 0)	0 (0 to 0)	0 (0 to 0)	0 (0 to 0)	0 (0 to 0)	0·3 (0·1 to 0·7)	[illegible]	[illegible]	[illegible]	[illegible]	[illegible]	[illegible]	[illegible]	[illegible]	[illegible]	[illegible]	[illegible]	[illegible]	[illegible]	[illegible]	[illegible]	[illegible]	[illegible]	[illegible]
Other sexually transmitted infections	0 (0 to 0)	0 (0 to 0)	0 (0 to 0)	0 (0 to 0)	0 (0 to 0)	0·8 (0·5 to 1·3)	[illegible]	[illegible]	[illegible]	[illegible]	[illegible]	[illegible]	[illegible]	[illegible]	[illegible]	[illegible]	[illegible]	[illegible]	[illegible]	[illegible]	[illegible]	[illegible]	[illegible]	[illegible]
Respiratory infections and tuberculosis	490 (261 to 806)	[illegible]	[illegible]	[illegible]	[illegible]	[illegible]	[illegible]	[illegible]	[illegible]	[illegible]	[illegible]	[illegible]	[illegible]	[illegible]	[illegible]	[illegible]	[illegible]	[illegible]	[illegible]	[illegible]	[illegible]	[illegible]	[illegible]	[illegible]
Tuberculosis	0 (0 to 0)	[illegible]	[illegible]	[illegible]	[illegible]	[illegible]	[illegible]	[illegible]	[illegible]	[illegible]	[illegible]	[illegible]	[illegible]	[illegible]	[illegible]	[illegible]	[illegible]	[illegible]	[illegible]	[illegible]	[illegible]	[illegible]	[illegible]	[illegible]
Latent tuberculosis infection	0 (0 to 0)	[illegible]	[illegible]	[illegible]	[illegible]	[illegible]	[illegible]	[illegible]	[illegible]	[illegible]	[illegible]	[illegible]	[illegible]	[illegible]	[illegible]	[illegible]	[illegible]	[illegible]	[illegible]	[illegible]	[illegible]	[illegible]	[illegible]	[illegible]
Drug-susceptible tuberculosis	0 (0 to 0)	[illegible]	[illegible]	[illegible]	[illegible]	[illegible]	[illegible]	[illegible]	[illegible]	[illegible]	[illegible]	[illegible]	[illegible]	[illegible]	[illegible]	[illegible]	[illegible]	[illegible]	[illegible]	[illegible]	[illegible]	[illegible]	[illegible]	[illegible]
Multidrug-resistant tuberculosis without extensive drug resistan	0 (0 to 0)	[illegible]	[illegible]	[illegible]	[illegible]	[illegible]	[illegible]	[illegible]	[illegible]	[illegible]	[illegible]	[illegible]	[illegible]	[illegible]	[illegible]	[illegible]	[illegible]	[illegible]	[illegible]	[illegible]	[illegible]	[illegible]	[illegible]	[illegible]
Extensively drug-resistant tuberculosis	0 (0 to 0)	[illegible]	[illegible]	[illegible]	[illegible]	[illegible]	[illegible]	[illegible]	[illegible]	[illegible]	[illegible]	[illegible]	[illegible]	[illegible]	[illegible]	[illegible]	[illegible]	[illegible]	[illegible]	[illegible]	[illegible]	[illegible]	[illegible]	[illegible]
Lower respiratory infections	490 (261 to 806)	[illegible]	[illegible]	[illegible]	[illegible]	[illegible]	[illegible]	[illegible]	[illegible]	[illegible]	[illegible]	[illegible]	[illegible]	[illegible]	[illegible]	[illegible]	[illegible]	[illegible]	[illegible]	[illegible]	[illegible]	[illegible]	[illegible]	[illegible]
Upper respiratory infections	0·2 (0·1 to 0·3)	[illegible]	[illegible]	[illegible]	[illegible]	[illegible]	[illegible]	[illegible]	[illegible]	[illegible]	[illegible]	[illegible]	[illegible]	[illegible]	[illegible]	[illegible]	[illegible]	[illegible]	[illegible]	[illegible]	[illegible]	[illegible]	[illegible]	[illegible]
Otitis media	0 (0 to 0·1)	[illegible]	[illegible]	[illegible]	[illegible]	[illegible]	[illegible]	[illegible]	[illegible]	[illegible]	[illegible]	[illegible]	[illegible]	[illegible]	[illegible]	[illegible]	[illegible]	[illegible]	[illegible]	[illegible]	[illegible]	[illegible]	[illegible]	[illegible]
Enteric infections	79·2 (39·7 to 138)	[illegible]	[illegible]	[illegible]	[illegible]	[illegible]	[illegible]	[illegible]	[illegible]	[illegible]	[illegible]	[illegible]	[illegible]	[illegible]	[illegible]	[illegible]	[illegible]	[illegible]	[illegible]	[illegible]	[illegible]	[illegible]	[illegible]	[illegible]
Diarrheal diseases	79·2 (39·7 to 138)	[illegible]	[illegible]	[illegible]	[illegible]	[illegible]	[illegible]	[illegible]	[illegible]	[illegible]	[illegible]	[illegible]	[illegible]	[illegible]	[illegible]	[illegible]	[illegible]	[illegible]	[illegible]	[illegible]	[illegible]	[illegible]	[illegible]	[illegible]
Typhoid and paratyphoid	0 (0 to 0)	[illegible]	[illegible]	[illegible]	[illegible]	[illegible]	[illegible]	[illegible]	[illegible]	[illegible]	[illegible]	[illegible]	[illegible]	[illegible]	[illegible]	[illegible]	[illegible]	[illegible]	[illegible]	[illegible]	[illegible]	[illegible]	[illegible]	[illegible]
Typhoid fever	0 (0 to 0)	[illegible]	[illegible]	[illegible]	[illegible]	[illegible]	[illegible]	[illegible]	[illegible]	[illegible]	[illegible]	[illegible]	[illegible]	[illegible]	[illegible]	[illegible]	[illegible]	[illegible]	[illegible]	[illegible]	[illegible]	[illegible]	[illegible]	[illegible]
Paratyphoid fever	0 (0 to 0)	[illegible]	[illegible]	[illegible]	[illegible]	[illegible]	[illegible]	[illegible]	[illegible]	[illegible]	[illegible]	[illegible]	[illegible]	[illegible]	[illegible]	[illegible]	[illegible]	[illegible]	[illegible]	[illegible]	[illegible]	[illegible]	[illegible]	[illegible]
Invasive Non-typhoidal Salmonella (iNTS)	0 (0 to 0)	[illegible]	[illegible]	[illegible]	[illegible]	[illegible]	[illegible]	[illegible]	[illegible]	[illegible]	[illegible]	[illegible]	[illegible]	[illegible]	[illegible]	[illegible]	[illegible]	[illegible]	[illegible]	[illegible]	[illegible]	[illegible]	[illegible]	[illegible]
Other intestinal infectious diseases	0 (0 to 0)	[illegible]	[illegible]	[illegible]	[illegible]	[illegible]	[illegible]	[illegible]	[illegible]	[illegible]	[illegible]	[illegible]	[illegible]	[illegible]	[illegible]	[illegible]	[illegible]	[illegible]	[illegible]	[illegible]	[illegible]	[illegible]	[illegible]	[illegible]
Neglected tropical diseases and malaria	2·1 (0·9 to 4·3)	[illegible]	[illegible]	[illegible]	[illegible]	[illegible]	[illegible]	[illegible]	[illegible]	[illegible]	[illegible]	[illegible]	[illegible]	[illegible]	[illegible]	[illegible]	[illegible]	[illegible]	[illegible]	[illegible]	[illegible]	[illegible]	[illegible]	[illegible]
Malaria	0 (0 to 0·1)	[illegible]	[illegible]	[illegible]	[illegible]	[illegible]	[illegible]	[illegible]	[illegible]	[illegible]	[illegible]	[illegible]	[illegible]	[illegible]	[illegible]	[illegible]	[illegible]	[illegible]	[illegible]	[illegible]	[illegible]	[illegible]	[illegible]	[illegible]
Chagas disease	0 (0 to 0)	[illegible]	[illegible]	[illegible]	[illegible]	[illegible]	[illegible]	[illegible]	[illegible]	[illegible]	[illegible]	[illegible]	[illegible]	[illegible]	[illegible]	[illegible]	[illegible]	[illegible]	[illegible]	[illegible]	[illegible]	[illegible]	[illegible]	[illegible]
Leishmaniasis	0 (0 to 0)	[illegible]	[illegible]	[illegible]	[illegible]	[illegible]	[illegible]	[illegible]	[illegible]	[illegible]	[illegible]	[illegible]	[illegible]	[illegible]	[illegible]	[illegible]	[illegible]	[illegible]	[illegible]	[illegible]	[illegible]	[illegible]	[illegible]	[illegible]
Visceral leishmaniasis	0 (0 to 0)	[illegible]	[illegible]	[illegible]	[illegible]	[illegible]	[illegible]	[illegible]	[illegible]	[illegible]	[illegible]	[illegible]	[illegible]	[illegible]	[illegible]	[illegible]	[illegible]	[illegible]	[illegible]	[illegible]	[illegible]	[illegible]	[illegible]	[illegible]
Cutaneous and mucocutaneous leishmaniasis	0 (0 to 0)	[illegible]	[illegible]	[illegible]	[illegible]	[illegible]	[illegible]	[illegible]	[illegible]	[illegible]	[illegible]	[illegible]	[illegible]	[illegible]	[illegible]	[illegible]	[illegible]	[illegible]	[illegible]	[illegible]	[illegible]	[illegible]	[illegible]	[illegible]
African trypanosomiasis	0 (0 to 0)	[illegible]	[illegible]	[illegible]	[illegible]	[illegible]	[illegible]	[illegible]	[illegible]	[illegible]	[illegible]	[illegible]	[illegible]	[illegible]	[illegible]	[illegible]	[illegible]	[illegible]	[illegible]	[illegible]	[illegible]	[illegible]	[illegible]	[illegible]
Schistosomiasis	0 (0 to 0)	[illegible]	[illegible]	[illegible]	[illegible]	[illegible]	[illegible]	[illegible]	[illegible]	[illegible]	[illegible]	[illegible]	[illegible]	[illegible]	[illegible]	[illegible]	[illegible]	[illegible]	[illegible]	[illegible]	[illegible]	[illegible]	[illegible]	[illegible]
Cysticercosis	0 (0 to 0)	[illegible]	[illegible]	[illegible]	[illegible]	[illegible]	[illegible]	[illegible]	[illegible]	[illegible]	[illegible]	[illegible]	[illegible]	[illegible]	[illegible]	[illegible]	[illegible]	[illegible]	[illegible]	[illegible]	[illegible]	[illegible]	[illegible]	[illegible]
Cystic echinococcosis	0 (0 to 0)	[illegible]	[illegible]	[illegible]	[illegible]	[illegible]	[illegible]	[illegible]	[illegible]	[illegible]	[illegible]	[illegible]	[illegible]	[illegible]	[illegible]	[illegible]	[illegible]	[illegible]	[illegible]	[illegible]	[illegible]	[illegible]	[illegible]	[illegible]
Lymphatic filariasis	0 (0 to 0)	[illegible]	[illegible]	[illegible]	[illegible]	[illegible]	[illegible]	[illegible]	[illegible]	[illegible]	[illegible]	[illegible]	[illegible]	[illegible]	[illegible]	[illegible]	[illegible]	[illegible]	[illegible]	[illegible]	[illegible]	[illegible]	[illegible]	[illegible]
Onchocerciasis	0 (0 to 0)	[illegible]	[illegible]	[illegible]	[illegible]	[illegible]	[illegible]	[illegible]	[illegible]	[illegible]	[illegible]	[illegible]	[illegible]	[illegible]	[illegible]	[illegible]	[illegible]	[illegible]	[illegible]	[illegible]	[illegible]	[illegible]	[illegible]	[illegible]
Trachoma	0 (0 to 0)	[illegible]	[illegible]	[illegible]	[illegible]	[illegible]	[illegible]	[illegible]	[illegible]	[illegible]	[illegible]	[illegible]	[illegible]	[illegible]	[illegible]	[illegible]	[illegible]	[illegible]	[illegible]	[illegible]	[illegible]	[illegible]	[illegible]	[illegible]
Dengue	0 (0 to 0)	[illegible]	[illegible]	[illegible]	[illegible]	[illegible]	[illegible]	[illegible]	[illegible]	[illegible]	[illegible]	[illegible]	[illegible]	[illegible]	[illegible]	[illegible]	[illegible]	[illegible]	[illegible]	[illegible]	[illegible]	[illegible]	[illegible]	[illegible]
Yellow fever	0 (0 to 0)	[illegible]	[illegible]	[illegible]	[illegible]	[illegible]	[illegible]	[illegible]	[illegible]	[illegible]	[illegible]	[illegible]	[illegible]	[illegible]	[illegible]	[illegible]	[illegible]	[illegible]	[illegible]	[illegible]	[illegible]	[illegible]	[illegible]	[illegible]
Rabies	0 (0 to 0)	[illegible]	[illegible]	[illegible]	[illegible]	[illegible]	[illegible]	[illegible]	[illegible]	[illegible]	[illegible]	[illegible]	[illegible]	[illegible]	[illegible]	[illegible]	[illegible]	[illegible]	[illegible]	[illegible]	[illegible]	[illegible]	[illegible]	[illegible]
Intestinal nematode infections	0 (0 to 0)	[illegible]	[illegible]	[illegible]	[illegible]	[illegible]	[illegible]	[illegible]	[illegible]	[illegible]	[illegible]	[illegible]	[illegible]	[illegible]	[illegible]	[illegible]	[illegible]	[illegible]	[illegible]	[illegible]	[illegible]	[illegible]	[illegible]	[illegible]
Ascariasis	0 (0 to 0)	[illegible]	[illegible]	[illegible]	[illegible]	[illegible]	[illegible]	[illegible]	[illegible]	[illegible]	[illegible]	[illegible]	[illegible]	[illegible]	[illegible]	[illegible]	[illegible]	[illegible]	[illegible]	[illegible]	[illegible]	[illegible]	[illegible]	[illegible]
Trichuriasis	0 (0 to 0)	[illegible]	[illegible]	[illegible]	[illegible]	[illegible]	[illegible]	[illegible]	[illegible]	[illegible]	[illegible]	[illegible]	[illegible]	[illegible]	[illegible]	[illegible]	[illegible]	[illegible]	[illegible]	[illegible]	[illegible]	[illegible]	[illegible]	[illegible]
Hookworm disease	0 (0 to 0)	[illegible]	[illegible]	[illegible]	[illegible]	[illegible]	[illegible]	[illegible]	[illegible]	[illegible]	[illegible]	[illegible]	[illegible]	[illegible]	[illegible]	[illegible]	[illegible]	[illegible]	[illegible]	[illegible]	[illegible]	[illegible]	[illegible]	[illegible]
Food-borne trematodiases	0 (0 to 0)	[illegible]	[illegible]	[illegible]	[illegible]	[illegible]	[illegible]	[illegible]	[illegible]	[illegible]	[illegible]	[illegible]	[illegible]	[illegible]	[illegible]	[illegible]	[illegible]	[illegible]	[illegible]	[illegible]	[illegible]	[illegible]	[illegible]	[illegible]
Leprosy	0 (0 to 0)	[illegible]	[illegible]	[illegible]	[illegible]	[illegible]	[illegible]	[illegible]	[illegible]	[illegible]	[illegible]	[illegible]	[illegible]	[illegible]	[illegible]	[illegible]	[illegible]	[illegible]	[illegible]	[illegible]	[illegible]	[illegible]	[illegible]	[illegible]
Ebola	0 (0 to 0)	[illegible]	[illegible]	[illegible]	[illegible]	[illegible]	[illegible]	[illegible]	[illegible]	[illegible]	[illegible]	[illegible]	[illegible]	[illegible]	[illegible]	[illegible]	[illegible]	[illegible]	[illegible]	[illegible]	[illegible]	[illegible]	[illegible]	[illegible]
Zika virus	0 (0 to 0)	[illegible]	[illegible]	[illegible]	[illegible]	[illegible]	[illegible]	[illegible]	[illegible]	[illegible]	[illegible]	[illegible]	[illegible]	[illegible]	[illegible]	[illegible]	[illegible]	[illegible]	[illegible]	[illegible]	[illegible]	[illegible]	[illegible]	[illegible]
Guinea worm disease	0 (0 to 0)	[illegible]	[illegible]	[illegible]	[illegible]	[illegible]	[illegible]	[illegible]	[illegible]	[illegible]	[illegible]	[illegible]	[illegible]	[illegible]	[illegible]	[illegible]	[illegible]	[illegible]	[illegible]	[illegible]	[illegible]	[illegible]	[illegible]	[illegible]
Other neglected tropical diseases	2·1 (0·9 to 4·3)	[illegible]	[illegible]	[illegible]	[illegible]	[illegible]	[illegible]	[illegible]	[illegible]	[illegible]	[illegible]	[illegible]	[illegible]	[illegible]	[illegible]	[illegible]	[illegible]	[illegible]	[illegible]	[illegible]	[illegible]	[illegible]	[illegible]	[illegible]
Other infectious diseases	25 (15·4 to 39·[illegible])	[illegible]	[illegible]	[illegible]	[illegible]	[illegible]	[illegible]	[illegible]	[illegible]	[illegible]	[illegible]	[illegible]	[illegible]	[illegible]	[illegible]	[illegible]	[illegible]	[illegible]	[illegible]	[illegible]	[illegible]	[illegible]	[illegible]	[illegible]
Meningitis	12·9 (6·5 to 22·5)	[illegible]	[illegible]	[illegible]	[illegible]	[illegible]	[illegible]	[illegible]	[illegible]	[illegible]	[illegible]	[illegible]	[illegible]	[illegible]	[illegible]	[illegible]	[illegible]	[illegible]	[illegible]	[illegible]	[illegible]	[illegible]	[illegible]	[illegible]
Encephalitis	6·7 (4 to 10·5)	[illegible]	[illegible]	[illegible]	[illegible]	[illegible]	[illegible]	[illegible]	[illegible]	[illegible]	[illegible]	[illegible]	[illegible]	[illegible]	[illegible]	[illegible]	[illegible]	[illegible]	[illegible]	[illegible]	[illegible]	[illegible]	[illegible]	[illegible]
Diphtheria	0 (0 to 0)	[illegible]	[illegible]	[illegible]	[illegible]	[illegible]	[illegible]	[illegible]	[illegible]	[illegible]	[illegible]	[illegible]	[illegible]	[illegible]	[illegible]	[illegible]	[illegible]	[illegible]	[illegible]	[illegible]	[illegible]	[illegible]	[illegible]	[illegible]
Tetanus	0·3 (0·1 to 0·5)	[illegible]	[illegible]	[illegible]	[illegible]	[illegible]	[illegible]	[illegible]	[illegible]	[illegible]	[illegible]	[illegible]	[illegible]	[illegible]	[illegible]	[illegible]	[illegible]	[illegible]	[illegible]	[illegible]	[illegible]	[illegible]	[illegible]	[illegible]
Measles	0 (0 to 0)	[illegible]	[illegible]	[illegible]	[illegible]	[illegible]	[illegible]	[illegible]	[illegible]	[illegible]	[illegible]	[illegible]	[illegible]	[illegible]	[illegible]	[illegible]	[illegible]	[illegible]	[illegible]	[illegible]	[illegible]	[illegible]	[illegible]	[illegible]
Varicella and herpes zoster	0·4 (0·1 to 0·9)	[illegible]	[illegible]	[illegible]	[illegible]	[illegible]	[illegible]	[illegible]	[illegible]	[illegible]	[illegible]	[illegible]	[illegible]	[illegible]	[illegible]	[illegible]	[illegible]	[illegible]	[illegible]	[illegible]	[illegible]	[illegible]	[illegible]	[illegible]
Acute hepatitis	0 (0 to 0)	[illegible]	[illegible]	[illegible]	[illegible]	[illegible]	[illegible]	[illegible]	[illegible]	[illegible]	[illegible]	[illegible]	[illegible]	[illegible]	[illegible]	[illegible]	[illegible]	[illegible]	[illegible]	[illegible]	[illegible]	[illegible]	[illegible]	[illegible]
Acute hepatitis A	0 (0 to 0)	[illegible]	[illegible]	[illegible]	[illegible]	[illegible]	[illegible]	[illegible]	[illegible]	[illegible]	[illegible]	[illegible]	[illegible]	[illegible]	[illegible]	[illegible]	[illegible]	[illegible]	[illegible]	[illegible]	[illegible]	[illegible]	[illegible]	[illegible]
Acute hepatitis B	0 (0 to 0)	[illegible]	[illegible]	[illegible]	[illegible]	[illegible]	[illegible]	[illegible]	[illegible]	[illegible]	[illegible]	[illegible]	[illegible]	[illegible]	[illegible]	[illegible]	[illegible]	[illegible]	[illegible]	[illegible]	[illegible]	[illegible]	[illegible]	[illegible]
Acute hepatitis C	0 (0 to 0)	[illegible]	[illegible]	[illegible]	[illegible]	[illegible]	[illegible]	[illegible]	[illegible]	[illegible]	[illegible]	[illegible]	[illegible]	[illegible]	[illegible]	[illegible]	[illegible]	[illegible]	[illegible]	[illegible]	[illegible]	[illegible]	[illegible]	[illegible]
Acute hepatitis E	0 (0 to 0)	[illegible]	[illegible]	[illegible]	[illegible]	[illegible]	[illegible]	[illegible]	[illegible]	[illegible]	[illegible]	[illegible]	[illegible]	[illegible]	[illegible]	[illegible]	[illegible]	[illegible]	[illegible]	[illegible]	[illegible]	[illegible]	[illegible]	[illegible]
Other unspecified infectious diseases	4·7 (2·3 to 8·2)	[illegible]	[illegible]	[illegible]	[illegible]	[illegible]	[illegible]	[illegible]	[illegible]	[illegible]	[illegible]	[illegible]	[illegible]	[illegible]	[illegible]	[illegible]	[illegible]	[illegible]	[illegible]	[illegible]	[illegible]	[illegible]	[illegible]	[illegible]
Maternal and neonatal disorders	14,400 (10,100 to 19,[illegible])	[illegible]	[illegible]	[illegible]	[illegible]	[illegible]	[illegible]	[illegible]	[illegible]	[illegible]	[illegible]	[illegible]	[illegible]	[illegible]	[illegible]	[illegible]	[illegible]	[illegible]	[illegible]	[illegible]	[illegible]	[illegible]	[illegible]	[illegible]
Maternal disorders	0 (0 to 0)	[illegible]	[illegible]	[illegible]	[illegible]	[illegible]	[illegible]	[illegible]	[illegible]	[illegible]	[illegible]	[illegible]	[illegible]	[illegible]	[illegible]	[illegible]	[illegible]	[illegible]	[illegible]	[illegible]	[illegible]	[illegible]	[illegible]	[illegible]
Maternal hemorrhage	0 (0 to 0)	[illegible]	[illegible]	[illegible]	[illegible]	[illegible]	[illegible]	[illegible]	[illegible]	[illegible]	[illegible]	[illegible]	[illegible]	[illegible]	[illegible]	[illegible]	[illegible]	[illegible]	[illegible]	[illegible]	[illegible]	[illegible]	[illegible]	[illegible]
Maternal sepsis and other maternal infections	0 (0 to 0)	[illegible]	[illegible]	[illegible]	[illegible]	[illegible]	[illegible]	[illegible]	[illegible]	[illegible]	[illegible]	[illegible]	[illegible]	[illegible]	[illegible]	[illegible]	[illegible]	[illegible]	[illegible]	[illegible]	[illegible]	[illegible]	[illegible]	[illegible]
Maternal hypertensive disorders	0 (0 to 0)	[illegible]	[illegible]	[illegible]	[illegible]	[illegible]	[illegible]	[illegible]	[illegible]	[illegible]	[illegible]	[illegible]	[illegible]	[illegible]	[illegible]	[illegible]	[illegible]	[illegible]	[illegible]	[illegible]	[illegible]	[illegible]	[illegible]	[illegible]
Maternal obstructed labor and uterine rupture	0 (0 to 0)	[illegible]	[illegible]	[illegible]	[illegible]	[illegible]	[illegible]	[illegible]	[illegible]	[illegible]	[illegible]	[illegible]	[illegible]	[illegible]	[illegible]	[illegible]	[illegible]	[illegible]	[illegible]	[illegible]	[illegible]	[illegible]	[illegible]	[illegible]
Maternal abortion and miscarriage	0 (0 to 0)	[illegible]	[illegible]	[illegible]	[illegible]	[illegible]	[illegible]	[illegible]	[illegible]	[illegible]	[illegible]	[illegible]	[illegible]	[illegible]	[illegible]	[illegible]	[illegible]	[illegible]	[illegible]	[illegible]	[illegible]	[illegible]	[illegible]	[illegible]
Ectopic pregnancy	0 (0 to 0)	[illegible]	[illegible]	[illegible]	[illegible]	[illegible]	[illegible]	[illegible]	[illegible]	[illegible]	[illegible]	[illegible]	[illegible]	[illegible]	[illegible]	[illegible]	[illegible]	[illegible]	[illegible]	[illegible]	[illegible]	[illegible]	[illegible]	[illegible]
Indirect maternal deaths	--	[illegible]	[illegible]	[illegible]	[illegible]	[illegible]	[illegible]	[illegible]	[illegible]	[illegible]	[illegible]	[illegible]	[illegible]	[illegible]	[illegible]	[illegible]	[illegible]	[illegible]	[illegible]	[illegible]	[illegible]	[illegible]	[illegible]	[illegible]
Late maternal deaths	--	[illegible]	[illegible]	[illegible]	[illegible]	[illegible]	[illegible]	[illegible]	[illegible]	[illegible]	[illegible]	[illegible]	[illegible]	[illegible]	[illegible]	[illegible]	[illegible]	[illegible]	[illegible]	[illegible]	[illegible]	[illegible]	[illegible]	[illegible]
Maternal deaths aggravated by HIV/AIDS	--	[illegible]	[illegible]	[illegible]	[illegible]	[illegible]	[illegible]	[illegible]	[illegible]	[illegible]	[illegible]	[illegible]	[illegible]	[illegible]	[illegible]	[illegible]	[illegible]	[illegible]	[illegible]	[illegible]	[illegible]	[illegible]	[illegible]	[illegible]
Neonatal disorders	14,400 (10,100 to 19,[illegible])	[illegible]	[illegible]	[illegible]	[illegible]	[illegible]	[illegible]	[illegible]	[illegible]	[illegible]	[illegible]	[illegible]	[illegible]	[illegible]	[illegible]	[illegible]	[illegible]	[illegible]	[illegible]	[illegible]	[illegible]	[illegible]	[illegible]	[illegible]
Neonatal preterm birth	6,880 (4,650 to 9,8[illegible])	[illegible]	[illegible]	[illegible]	[illegible]	[illegible]	[illegible]	[illegible]	[illegible]	[illegible]	[illegible]	[illegible]	[illegible]	[illegible]	[illegible]	[illegible]	[illegible]	[illegible]	[illegible]	[illegible]	[illegible]	[illegible]	[illegible]	[illegible]
Neonatal encephalopathy due to birth asphyxia and trauma	3,500 (2,200 to 5,0[illegible])	[illegible]	[illegible]	[illegible]	[illegible]	[illegible]	[illegible]	[illegible]	[illegible]	[illegible]	[illegible]	[illegible]	[illegible]	[illegible]	[illegible]	[illegible]	[illegible]	[illegible]	[illegible]	[illegible]	[illegible]	[illegible]	[illegible]	[illegible]
Neonatal sepsis and other neonatal infections	1,990 (1,220 to 3,0[illegible])	[illegible]	[illegible]	[illegible]	[illegible]	[illegible]	[illegible]	[illegible]	[illegible]	[illegible]	[illegible]	[illegible]	[illegible]	[illegible]	[illegible]	[illegible]	[illegible]	[illegible]	[illegible]	[illegible]	[illegible]	[illegible]	[illegible]	[illegible]
Hemolytic disease and other neonatal jaundice	132 (65·7 to 22[illegible])	[illegible]	[illegible]	[illegible]	[illegible]	[illegible]	[illegible]	[illegible]	[illegible]	[illegible]	[illegible]	[illegible]	[illegible]	[illegible]	[illegible]	[illegible]	[illegible]	[illegible]	[illegible]	[illegible]	[illegible]	[illegible]	[illegible]	[illegible]
Other neonatal disorders	1,870 (1,110 to 2,8[illegible])	[illegible]	[illegible]	[illegible]	[illegible]	[illegible]	[illegible]	[illegible]	[illegible]	[illegible]	[illegible]	[illegible]	[illegible]	[illegible]	[illegible]	[illegible]	[illegible]	[illegible]	[illegible]	[illegible]	[illegible]	[illegible]	[illegible]	[illegible]
Nutritional deficiencies	1·7 (1 to 2·5)	[illegible]	[illegible]	[illegible]	[illegible]	[illegible]	[illegible]	[illegible]	[illegible]	[illegible]	[illegible]	[illegible]	[illegible]	[illegible]	[illegible]	[illegible]	[illegible]	[illegible]	[illegible]	[illegible]	[illegible]	[illegible]	[illegible]	[illegible]
Protein-energy malnutrition	1·4 (0·9 to 2·1)	[illegible]	[illegible]	[illegible]	[illegible]	[illegible]	[illegible]	[illegible]	[illegible]	[illegible]	[illegible]	[illegible]	[illegible]	[illegible]	[illegible]	[illegible]	[illegible]	[illegible]	[illegible]	[illegible]	[illegible]	[illegible]	[illegible]	[illegible]
Iodine deficiency	0 (0 to 0)	[illegible]	[illegible]	[illegible]	[illegible]	[illegible]	[illegible]	[illegible]	[illegible]	[illegible]	[illegible]	[illegible]	[illegible]	[illegible]	[illegible]	[illegible]	[illegible]	[illegible]	[illegible]	[illegible]	[illegible]	[illegible]	[illegible]	[illegible]
Vitamin A deficiency	0·1 (0 to 0·2)	[illegible]	[illegible]	[illegible]	[illegible]	[illegible]	[illegible]	[illegible]	[illegible]	[illegible]	[illegible]	[illegible]	[illegible]	[illegible]	[illegible]	[illegible]	[illegible]	[illegible]	[illegible]	[illegible]	[illegible]	[illegible]	[illegible]	[illegible]
Dietary iron deficiency	0·2 (0 to 0·6)	[illegible]	[illegible]	[illegible]	[illegible]	[illegible]	[illegible]	[illegible]	[illegible]	[illegible]	[illegible]	[illegible]	[illegible]	[illegible]	[illegible]	[illegible]	[illegible]	[illegible]	[illegible]	[illegible]	[illegible]	[illegible]	[illegible]	[illegible]
Other nutritional deficiencies	0 (0 to 0)	[illegible]	[illegible]	[illegible]	[illegible]	[illegible]	[illegible]	[illegible]	[illegible]	[illegible]	[illegible]	[illegible]	[illegible]	[illegible]	[illegible]	[illegible]	[illegible]	[illegible]	[illegible]	[illegible]	[illegible]	[illegible]	[illegible]	[illegible]
Non-communicable diseases	1,660 (1,080 to 2,4[illegible])	[illegible]	[illegible]	[illegible]	[illegible]	[illegible]	[illegible]	[illegible]	[illegible]	[illegible]	[illegible]	[illegible]	[illegible]	[illegible]	[illegible]	[illegible]	[illegible]	[illegible]	[illegible]	[illegible]	[illegible]	[illegible]	[illegible]	[illegible]
Neoplasms	22·5 (7 to 67·8)	[illegible]	[illegible]	[illegible]	[illegible]	[illegible]	[illegible]	[illegible]	[illegible]	[illegible]	[illegible]	[illegible]	[illegible]	[illegible]	[illegible]	[illegible]	[illegible]	[illegible]	[illegible]	[illegible]	[illegible]	[illegible]	[illegible]	[illegible]
Lip and oral cavity cancer	0 (0 to 0)	[illegible]	[illegible]	[illegible]	[illegible]	[illegible]	[illegible]	[illegible]	[illegible]	[illegible]	[illegible]	[illegible]	[illegible]	[illegible]	[illegible]	[illegible]	[illegible]	[illegible]	[illegible]	[illegible]	[illegible]	[illegible]	[illegible]	[illegible]
Nasopharynx cancer	0 (0 to 0)	[illegible]	[illegible]	[illegible]	[illegible]	[illegible]	[illegible]	[illegible]	[illegible]	[illegible]	[illegible]	[illegible]	[illegible]	[illegible]	[illegible]	[illegible]	[illegible]	[illegible]	[illegible]	[illegible]	[illegible]	[illegible]	[illegible]	[illegible]
Other pharynx cancer	0 (0 to 0)	[illegible]	[illegible]	[illegible]	[illegible]	[illegible]	[illegible]	[illegible]	[illegible]	[illegible]	[illegible]	[illegible]	[illegible]	[illegible]	[illegible]	[illegible]	[illegible]	[illegible]	[illegible]	[illegible]	[illegible]	[illegible]	[illegible]	[illegible]
Esophageal cancer	0 (0 to 0)	[illegible]	[illegible]	[illegible]	[illegible]	[illegible]	[illegible]	[illegible]	[illegible]	[illegible]	[illegible]	[illegible]	[illegible]	[illegible]	[illegible]	[illegible]	[illegible]	[illegible]	[illegible]	[illegible]	[illegible]	[illegible]	[illegible]	[illegible]
Stomach cancer	0 (0 to 0)	[illegible]	[illegible]	[illegible]	[illegible]	[illegible]	[illegible]	[illegible]	[illegible]	[illegible]	[illegible]	[illegible]	[illegible]	[illegible]	[illegible]	[illegible]	[illegible]	[illegible]	[illegible]	[illegible]	[illegible]	[illegible]	[illegible]	[illegible]
Colon and rectum cancer	0 (0 to 0)	[illegible]	[illegible]	[illegible]	[illegible]	[illegible]	[illegible]	[illegible]	[illegible]	[illegible]	[illegible]	[illegible]	[illegible]	[illegible]	[illegible]	[illegible]	[illegible]	[illegible]	[illegible]	[illegible]	[illegible]	[illegible]	[illegible]	[illegible]
Liver cancer	0·3 (0·2 to 0·6)	[illegible]	[illegible]	[illegible]	[illegible]	[illegible]	[illegible]	[illegible]	[illegible]	[illegible]	[illegible]	[illegible]	[illegible]	[illegible]	[illegible]	[illegible]	[illegible]	[illegible]	[illegible]	[illegible]	[illegible]	[illegible]	[illegible]	[illegible]
Liver cancer due to hepatitis B	0 (0 to 0)	[illegible]	[illegible]	[illegible]	[illegible]	[illegible]	[illegible]	[illegible]	[illegible]	[illegible]	[illegible]	[illegible]	[illegible]	[illegible]	[illegible]	[illegible]	[illegible]	[illegible]	[illegible]	[illegible]	[illegible]	[illegible]	[illegible]	[illegible]
Liver cancer due to hepatitis C	0 (0 to 0)	[illegible]	[illegible]	[illegible]	[illegible]	[illegible]	[illegible]	[illegible]	[illegible]	[illegible]	[illegible]	[illegible]	[illegible]	[illegible]	[illegible]	[illegible]	[illegible]	[illegible]	[illegible]	[illegible]	[illegible]	[illegible]	[illegible]	[illegible]
Liver cancer due to alcohol use	0 (0 to 0)	[illegible]	[illegible]	[illegible]	[illegible]	[illegible]	[illegible]	[illegible]	[illegible]	[illegible]	[illegible]	[illegible]	[illegible]	[illegible]	[illegible]	[illegible]	[illegible]	[illegible]	[illegible]	[illegible]	[illegible]	[illegible]	[illegible]	[illegible]
Liver cancer due to NASH	0 (0 to 0)	[illegible]	[illegible]	[illegible]	[illegible]	[illegible]	[illegible]	[illegible]	[illegible]	[illegible]	[illegible]	[illegible]	[illegible]	[illegible]	[illegible]	[illegible]	[illegible]	[illegible]	[illegible]	[illegible]	[illegible]	[illegible]	[illegible]	[illegible]
Liver cancer due to other causes	0·3 (0·2 to 0·6)	[illegible]	[illegible]	[illegible]	[illegible]	[illegible]	[illegible]	[illegible]	[illegible]	[illegible]	[illegible]	[illegible]	[illegible]	[illegible]	[illegible]	[illegible]	[illegible]	[illegible]	[illegible]	[illegible]	[illegible]	[illegible]	[illegible]	[illegible]
Gallbladder and biliary tract cancer	0 (0 to 0)	[illegible]	[illegible]	[illegible]	[illegible]	[illegible]	[illegible]	[illegible]	[illegible]	[illegible]	[illegible]	[illegible]	[illegible]	[illegible]	[illegible]	[illegible]	[illegible]	[illegible]	[illegible]	[illegible]	[illegible]	[illegible]	[illegible]	[illegible]
Pancreatic cancer	0 (0 to 0)	[illegible]	[illegible]	[illegible]	[illegible]	[illegible]	[illegible]	[illegible]	[illegible]	[illegible]	[illegible]	[illegible]	[illegible]	[illegible]	[illegible]	[illegible]	[illegible]	[illegible]	[illegible]	[illegible]	[illegible]	[illegible]	[illegible]	[illegible]
Larynx cancer	0 (0 to 0)	[illegible]	[illegible]	[illegible]	[illegible]	[illegible]	[illegible]	[illegible]	[illegible]	[illegible]	[illegible]	[illegible]	[illegible]	[illegible]	[illegible]	[illegible]	[illegible]	[illegible]	[illegible]	[illegible]	[illegible]	[illegible]	[illegible]	[illegible]
Tracheal, bronchus, and lung cancer	0 (0 to 0)	[illegible]	[illegible]	[illegible]	[illegible]	[illegible]	[illegible]	[illegible]	[illegible]	[illegible]	[illegible]	[illegible]	[illegible]	[illegible]	[illegible]	[illegible]	[illegible]	[illegible]	[illegible]	[illegible]	[illegible]	[illegible]	[illegible]	[illegible]
Malignant skin melanoma	0 (0 to 0·1)	[illegible]	[illegible]	[illegible]	[illegible]	[illegible]	[illegible]	[illegible]	[illegible]	[illegible]	[illegible]	[illegible]	[illegible]	[illegible]	[illegible]	[illegible]	[illegible]	[illegible]	[illegible]	[illegible]	[illegible]	[illegible]	[illegible]	[illegible]
Non-melanoma skin cancer	0 (0 to 0)	[illegible]	[illegible]	[illegible]	[illegible]	[illegible]	[illegible]	[illegible]	[illegible]	[illegible]	[illegible]	[illegible]	[illegible]	[illegible]	[illegible]	[illegible]	[illegible]	[illegible]	[illegible]	[illegible]	[illegible]	[illegible]	[illegible]	[illegible]
Non-melanoma skin cancer (squamous-cell carcinoma)	0 (0 to 0)	[illegible]	[illegible]	[illegible]	[illegible]	[illegible]	[illegible]	[illegible]	[illegible]	[illegible]	[illegible]	[illegible]	[illegible]	[illegible]	[illegible]	[illegible]	[illegible]	[illegible]	[illegible]	[illegible]	[illegible]	[illegible]	[illegible]	[illegible]
Non-melanoma skin cancer (basal-cell carcinoma)	0 (0 to 0)	[illegible]	[illegible]	[illegible]	[illegible]	[illegible]	[illegible]	[illegible]	[illegible]	[illegible]	[illegible]	[illegible]	[illegible]	[illegible]	[illegible]	[illegible]	[illegible]	[illegible]	[illegible]	[illegible]	[illegible]	[illegible]	[illegible]	[illegible]
Breast cancer	0 (0 to 0)	[illegible]	[illegible]	[illegible]	[illegible]	[illegible]	[illegible]	[illegible]	[illegible]	[illegible]	[illegible]	[illegible]	[illegible]	[illegible]	[illegible]	[illegible]	[illegible]	[illegible]	[illegible]	[illegible]	[illegible]	[illegible]	[illegible]	[illegible]
Cervical cancer	0 (0 to 0)	[illegible]	[illegible]	[illegible]	[illegible]	[illegible]	[illegible]	[illegible]	[illegible]	[illegible]	[illegible]	[illegible]	[illegible]	[illegible]	[illegible]	[illegible]	[illegible]	[illegible]	[illegible]	[illegible]	[illegible]	[illegible]	[illegible]	[illegible]
Uterine cancer	0 (0 to 0)	[illegible]	[illegible]	[illegible]	[illegible]	[illegible]	[illegible]	[illegible]	[illegible]	[illegible]	[illegible]	[illegible]	[illegible]	[illegible]	[illegible]	[illegible]	[illegible]	[illegible]	[illegible]	[illegible]	[illegible]	[illegible]	[illegible]	[illegible]
Ovarian cancer	0 (0 to 0)	[illegible]	[illegible]	[illegible]	[illegible]	[illegible]	[illegible]	[illegible]	[illegible]	[illegible]	[illegible]	[illegible]	[illegible]	[illegible]	[illegible]	[illegible]	[illegible]	[illegible]	[illegible]	[illegible]	[illegible]	[illegible]	[illegible]	[illegible]
Prostate cancer	0 (0 to 0)	[illegible]	[illegible]	[illegible]	[illegible]	[illegible]	[illegible]	[illegible]	[illegible]	[illegible]	[illegible]	[illegible]	[illegible]	[illegible]	[illegible]	[illegible]	[illegible]	[illegible]	[illegible]	[illegible]	[illegible]	[illegible]	[illegible]	[illegible]
Testicular cancer	0·7 (0 to 7·2)	[illegible]	[illegible]	[illegible]	[illegible]	[illegible]	[illegible]	[illegible]	[illegible]	[illegible]	[illegible]	[illegible]	[illegible]	[illegible]	[illegible]	[illegible]	[illegible]	[illegible]	[illegible]	[illegible]	[illegible]	[illegible]	[illegible]	[illegible]
Kidney cancer	5 (0·1 to 26·4)	[illegible]	[illegible]	[illegible]	[illegible]	[illegible]	[illegible]	[illegible]	[illegible]	[illegible]	[illegible]	[illegible]	[illegible]	[illegible]	[illegible]	[illegible]	[illegible]	[illegible]	[illegible]	[illegible]	[illegible]	[illegible]	[illegible]	[illegible]
Bladder cancer	0 (0 to 0)	[illegible]	[illegible]	[illegible]	[illegible]	[illegible]	[illegible]	[illegible]	[illegible]	[illegible]	[illegible]	[illegible]	[illegible]	[illegible]	[illegible]	[illegible]	[illegible]	[illegible]	[illegible]	[illegible]	[illegible]	[illegible]	[illegible]	[illegible]
Brain and central nervous system cancer	4 (0·2 to 25)	[illegible]	[illegible]	[illegible]	[illegible]	[illegible]	[illegible]	[illegible]	[illegible]	[illegible]	[illegible]	[illegible]	[illegible]	[illegible]	[illegible]	[illegible]	[illegible]	[illegible]	[illegible]	[illegible]	[illegible]	[illegible]	[illegible]	[illegible]
Thyroid cancer	0 (0 to 0)	[illegible]	[illegible]	[illegible]	[illegible]	[illegible]	[illegible]	[illegible]	[illegible]	[illegible]	[illegible]	[illegible]	[illegible]	[illegible]	[illegible]	[illegible]	[illegible]	[illegible]	[illegible]	[illegible]	[illegible]	[illegible]	[illegible]	[illegible]
Mesothelioma	0 (0 to 0)	[illegible]	[illegible]	[illegible]	[illegible]	[illegible]	[illegible]	[illegible]	[illegible]	[illegible]	[illegible]	[illegible]	[illegible]	[illegible]	[illegible]	[illegible]	[illegible]	[illegible]	[illegible]	[illegible]	[illegible]	[illegible]	[illegible]	[illegible]
Hodgkin lymphoma	0 (0 to 0)	[illegible]	[illegible]	[illegible]	[illegible]	[illegible]	[illegible]	[illegible]	[illegible]	[illegible]	[illegible]	[illegible]	[illegible]	[illegible]	[illegible]	[illegible]	[illegible]	[illegible]	[illegible]	[illegible]	[illegible]	[illegible]	[illegible]	[illegible]
Non-Hodgkin lymphoma	0 (0 to 0)	[illegible]	[illegible]	[illegible]	[illegible]	[illegible]	[illegible]	[illegible]	[illegible]	[illegible]	[illegible]	[illegible]	[illegible]	[illegible]	[illegible]	[illegible]	[illegible]	[illegible]	[illegible]	[illegible]	[illegible]	[illegible]	[illegible]	[illegible]
Multiple myeloma	0 (0 to 0)	[illegible]	[illegible]	[illegible]	[illegible]	[illegible]	[illegible]	[illegible]	[illegible]	[illegible]	[illegible]	[illegible]	[illegible]	[illegible]	[illegible]	[illegible]	[illegible]	[illegible]	[illegible]	[illegible]	[illegible]	[illegible]	[illegible]	[illegible]
Leukemia	6·2 (1 to 24)	[illegible]	[illegible]	[illegible]	[illegible]	[illegible]	[illegible]	[illegible]	[illegible]	[illegible]	[illegible]	[illegible]	[illegible]	[illegible]	[illegible]	[illegible]	[illegible]	[illegible]	[illegible]	[illegible]	[illegible]	[illegible]	[illegible]	[illegible]
Acute lymphoid leukemia	2·4 (0·2 to 11·2)	[illegible]	[illegible]	[illegible]	[illegible]	[illegible]	[illegible]	[illegible]	[illegible]	[illegible]	[illegible]	[illegible]	[illegible]	[illegible]	[illegible]	[illegible]	[illegible]	[illegible]	[illegible]	[illegible]	[illegible]	[illegible]	[illegible]	[illegible]
Chronic lymphoid leukemia	0 (0 to 0)	[illegible]	[illegible]	[illegible]	[illegible]	[illegible]	[illegible]	[illegible]	[illegible]	[illegible]	[illegible]	[illegible]	[illegible]	[illegible]	[illegible]	[illegible]	[illegible]	[illegible]	[illegible]	[illegible]	[illegible]	[illegible]	[illegible]	[illegible]
Acute myeloid leukemia	0·8 (0 to 4)	[illegible]	[illegible]	[illegible]	[illegible]	[illegible]	[illegible]	[illegible]	[illegible]	[illegible]	[illegible]	[illegible]	[illegible]	[illegible]	[illegible]	[illegible]	[illegible]	[illegible]	[illegible]	[illegible]	[illegible]	[illegible]	[illegible]	[illegible]
Chronic myeloid leukemia	0 (0 to 0·1)	[illegible]	[illegible]	[illegible]	[illegible]	[illegible]	[illegible]	[illegible]	[illegible]	[illegible]	[illegible]	[illegible]	[illegible]	[illegible]	[illegible]	[illegible]	[illegible]	[illegible]	[illegible]	[illegible]	[illegible]	[illegible]	[illegible]	[illegible]
Other leukemia	3 (0·4 to 11·3)	[illegible]	[illegible]	[illegible]	[illegible]	[illegible]	[illegible]	[illegible]	[illegible]	[illegible]	[illegible]	[illegible]	[illegible]	[illegible]	[illegible]	[illegible]	[illegible]	[illegible]	[illegible]	[illegible]	[illegible]	[illegible]	[illegible]	[illegible]
Other malignant neoplasms	5·9 (2 to 15·1)	[illegible]	[illegible]	[illegible]	[illegible]	[illegible]	[illegible]	[illegible]	[illegible]	[illegible]	[illegible]	[illegible]	[illegible]	[illegible]	[illegible]	[illegible]	[illegible]	[illegible]	[illegible]	[illegible]	[illegible]	[illegible]	[illegible]	[illegible]
Other neoplasms	0·3 (0·1 to 0·5)	[illegible]	[illegible]	[illegible]	[illegible]	[illegible]	[illegible]	[illegible]	[illegible]	[illegible]	[illegible]	[illegible]	[illegible]	[illegible]	[illegible]	[illegible]	[illegible]	[illegible]	[illegible]	[illegible]	[illegible]	[illegible]	[illegible]	[illegible]
Myelodysplastic, myeloproliferative, and other hematopoietic n	0·3 (0·1 to 0·5)	[illegible]	[illegible]	[illegible]	[illegible]	[illegible]	[illegible]	[illegible]	[illegible]	[illegible]	[illegible]	[illegible]	[illegible]	[illegible]	[illegible]	[illegible]	[illegible]	[illegible]	[illegible]	[illegible]	[illegible]	[illegible]	[illegible]	[illegible]
Benign and in situ intestinal neoplasms	0 (0 to 0)	[illegible]	[illegible]	[illegible]	[illegible]	[illegible]	[illegible]	[illegible]	[illegible]	[illegible]	[illegible]	[illegible]	[illegible]	[illegible]	[illegible]	[illegible]	[illegible]	[illegible]	[illegible]	[illegible]	[illegible]	[illegible]	[illegible]	[illegible]
Benign and in situ cervical and uterine neoplasms	0 (0 to 0)	[illegible]	[illegible]	[illegible]	[illegible]	[illegible]	[illegible]	[illegible]	[illegible]	[illegible]	[illegible]	[illegible]	[illegible]	[illegible]	[illegible]	[illegible]	[illegible]	[illegible]	[illegible]	[illegible]	[illegible]	[illegible]	[illegible]	[illegible]
Other benign and in situ neoplasms	0 (0 to 0)	[illegible]	[illegible]	[illegible]	[illegible]	[illegible]	[illegible]	[illegible]	[illegible]	[illegible]	[illegible]	[illegible]	[illegible]	[illegible]	[illegible]	[illegible]	[illegible]	[illegible]	[illegible]	[illegible]	[illegible]	[illegible]	[illegible]	[illegible]
Cardiovascular diseases	75·5 (42·2 to 12[illegible])	[illegible]	[illegible]	[illegible]	[illegible]	[illegible]	[illegible]	[illegible]	[illegible]	[illegible]	[illegible]	[illegible]	[illegible]	[illegible]	[illegible]	[illegible]	[illegible]	[illegible]	[illegible]	[illegible]	[illegible]	[illegible]	[illegible]	[illegible]
Rheumatic heart disease	0 (0 to 0)	[illegible]	[illegible]	[illegible]	[illegible]	[illegible]	[illegible]	[illegible]	[illegible]	[illegible]	[illegible]	[illegible]	[illegible]	[illegible]	[illegible]	[illegible]	[illegible]	[illegible]	[illegible]	[illegible]	[illegible]	[illegible]	[illegible]	[illegible]

Ischemic heart disease	0 (0 to 0)	0 (0 to 0)	0 (0 to 0)	0 (0 to 0)	0 (0 to 0)	[illegible]	[illegible]	[illegible]	[illegible]	[illegible]	[illegible]	[illegible]	[illegible]	[illegible]	[illegible]	[illegible]	[illegible]	[illegible]	[illegible]	[illegible]	[illegible]	[illegible]	103 (81·2 to 122)	24,800
Stroke	34·9 (16·4 to 63·6)	2·8 (1·1 to 5·7)	23·1 (10·5 to 42·5)	14·7 (9·5 to 21·6)	36·6 (26·5 to 47·6)	[illegible]	[illegible]	[illegible]	[illegible]	[illegible]	[illegible]	[illegible]	[illegible]	[illegible]	[illegible]	[illegible]	[illegible]	[illegible]	[illegible]	[illegible]	[illegible]	[illegible]	57·5 (43 to 71·3)	20,400
Ischemic stroke	6·8 (2·9 to 13)	0·2 (0·1 to 0·5)	1·4 (0·7 to 2·6)	4·5 (3·1 to 5·9)	9·5 (6·2 to 13·9)	[illegible]	[illegible]	[illegible]	[illegible]	[illegible]	[illegible]	[illegible]	[illegible]	[illegible]	[illegible]	[illegible]	[illegible]	[illegible]	[illegible]	[illegible]	[illegible]	[illegible]	45·2 (33·7 to 55·8)	7,350
Intracerebral hemorrhage	11·8 (4·7 to 23·6)	0·6 (0·2 to 1·3)	13·3 (5·8 to 25·3)	4·1 (2 to 6·7)	14·7 (10 to 20·5)	[illegible]	[illegible]	[illegible]	[illegible]	[illegible]	[illegible]	[illegible]	[illegible]	[illegible]	[illegible]	[illegible]	[illegible]	[illegible]	[illegible]	[illegible]	[illegible]	[illegible]	11·5 (8·3 to 14·8)	11,400
Subarachnoid hemorrhage	16·3 (7 to 30·9)	2 (0·8 to 4)	8·4 (3·5 to 16·3)	6·2 (3·4 to 10·1)	12·4 (7·9 to 17·8)	[illegible]	[illegible]	[illegible]	[illegible]	[illegible]	[illegible]	[illegible]	[illegible]	[illegible]	[illegible]	[illegible]	[illegible]	[illegible]	[illegible]	[illegible]	[illegible]	[illegible]	0·9 (0·6 to 1·3)	1,640
Hypertensive heart disease	0 (0 to 0)	0 (0 to 0)	0 (0 to 0)	0 (0 to 0)	0 (0 to 0)	[illegible]	[illegible]	[illegible]	[illegible]	[illegible]	[illegible]	[illegible]	[illegible]	[illegible]	[illegible]	[illegible]	[illegible]	[illegible]	[illegible]	[illegible]	[illegible]	[illegible]	35·6 (24·6 to 48·5)	7,260
Non-rheumatic valvular heart disease	0 (0 to 0)	0 (0 to 0)	0 (0 to 0)	0 (0 to 0)	0 (0 to 0)	[illegible]	[illegible]	[illegible]	[illegible]	[illegible]	[illegible]	[illegible]	[illegible]	[illegible]	[illegible]	[illegible]	[illegible]	[illegible]	[illegible]	[illegible]	[illegible]	[illegible]	0·7 (0·5 to 1)	568
Non-rheumatic calcific aortic valve disease	0 (0 to 0)	0 (0 to 0)	0 (0 to 0)	0 (0 to 0)	0 (0 to 0)	[illegible]	[illegible]	[illegible]	[illegible]	[illegible]	[illegible]	[illegible]	[illegible]	[illegible]	[illegible]	[illegible]	[illegible]	[illegible]	[illegible]	[illegible]	[illegible]	[illegible]	0·5 (0·3 to 0·8)	318
Non-rheumatic degenerative mitral valve disease	0 (0 to 0)	0 (0 to 0)	0 (0 to 0)	0 (0 to 0)	0 (0 to 0)	[illegible]	[illegible]	[illegible]	[illegible]	[illegible]	[illegible]	[illegible]	[illegible]	[illegible]	[illegible]	[illegible]	[illegible]	[illegible]	[illegible]	[illegible]	[illegible]	[illegible]	0·1 (0·1 to 0·2)	217
Other non-rheumatic valve diseases	0 (0 to 0)	0 (0 to 0)	0 (0 to 0)	0 (0 to 0)	0 (0 to 0)	[illegible]	[illegible]	[illegible]	[illegible]	[illegible]	[illegible]	[illegible]	[illegible]	[illegible]	[illegible]	[illegible]	[illegible]	[illegible]	[illegible]	[illegible]	[illegible]	[illegible]	0 (0 to 0·1)	33
Cardiomyopathy and myocarditis	16·3 (9·2 to 26·6)	5·7 (3 to 9·8)	56·4 (30·7 to 98·9)	35·2 (22·9 to 51·7)	31·1 (23·2 to 39·7)	[illegible]	[illegible]	[illegible]	[illegible]	[illegible]	[illegible]	[illegible]	[illegible]	[illegible]	[illegible]	[illegible]	[illegible]	[illegible]	[illegible]	[illegible]	[illegible]	[illegible]	3·4 (2·2 to 5)	2,500
Myocarditis	6·2 (2·7 to 11·4)	3·1 (1·6 to 6)	27·6 (13·2 to 51·4)	18·9 (11·1 to 28·9)	18·3 (12·7 to 24·6)	[illegible]	[illegible]	[illegible]	[illegible]	[illegible]	[illegible]	[illegible]	[illegible]	[illegible]	[illegible]	[illegible]	[illegible]	[illegible]	[illegible]	[illegible]	[illegible]	[illegible]	1 (0·6 to 1·6)	517
Alcoholic cardiomyopathy	0 (0 to 0)	0 (0 to 0)	0 (0 to 0)	0 (0 to 0)	0 (0 to 0)	[illegible]	[illegible]	[illegible]	[illegible]	[illegible]	[illegible]	[illegible]	[illegible]	[illegible]	[illegible]	[illegible]	[illegible]	[illegible]	[illegible]	[illegible]	[illegible]	[illegible]	0 (0 to 0·1)	512
Other cardiomyopathy	10·1 (5·1 to 17·6)	2·5 (1·1 to 4·7)	28·7 (13·6 to 53·1)	16·4 (8·6 to 26·8)	12·8 (7·8 to 19)	[illegible]	[illegible]	[illegible]	[illegible]	[illegible]	[illegible]	[illegible]	[illegible]	[illegible]	[illegible]	[illegible]	[illegible]	[illegible]	[illegible]	[illegible]	[illegible]	[illegible]	2·3 (1·3 to 3·7)	1,470
Atrial fibrillation and flutter	0 (0 to 0)	0 (0 to 0)	0 (0 to 0)	0 (0 to 0)	0 (0 to 0)	[illegible]	[illegible]	[illegible]	[illegible]	[illegible]	[illegible]	[illegible]	[illegible]	[illegible]	[illegible]	[illegible]	[illegible]	[illegible]	[illegible]	[illegible]	[illegible]	[illegible]	10 (7·3 to 13)	558
Aortic aneurysm	--	--	--	--	--	[illegible]	[illegible]	[illegible]	[illegible]	[illegible]	[illegible]	[illegible]	[illegible]	[illegible]	[illegible]	[illegible]	[illegible]	[illegible]	[illegible]	[illegible]	[illegible]	[illegible]	1·3 (0·8 to 2)	NA
Endocarditis	1·9 (1 to 3·2)	1·2 (0·5 to 2·2)	10·4 (4·4 to 19·9)	7·4 (3·7 to 13·3)	8·1 (4·3 to 14·5)	[illegible]	[illegible]	[illegible]	[illegible]	[illegible]	[illegible]	[illegible]	[illegible]	[illegible]	[illegible]	[illegible]	[illegible]	[illegible]	[illegible]	[illegible]	[illegible]	[illegible]	0·7 (0·4 to 1·1)	575
Other cardiovascular and circulatory diseases	22·4 (9·4 to 41·4)	4·1 (1·9 to 7·7)	33·3 (15 to 65·9)	17·6 (10·5 to 26·9)	27·2 (18·8 to 36·7)	[illegible]	[illegible]	[illegible]	[illegible]	[illegible]	[illegible]	[illegible]	[illegible]	[illegible]	[illegible]	[illegible]	[illegible]	[illegible]	[illegible]	[illegible]	[illegible]	[illegible]	4·9 (3·4 to 6·2)	1,750
Chronic respiratory diseases	0 (0 to 0)	0 (0 to 0)	0·1 (0 to 0·1)	402 (248 to 656)	360 (228 to 560)	[illegible]	[illegible]	[illegible]	[illegible]	[illegible]	[illegible]	[illegible]	[illegible]	[illegible]	[illegible]	[illegible]	[illegible]	[illegible]	[illegible]	[illegible]	[illegible]	[illegible]	10·7 (8·1 to 13·7)	5,540
Chronic obstructive pulmonary disease	0 (0 to 0)	0 (0 to 0)	0·1 (0 to 0·1)	7·5 (4·8 to 11·2)	7·1 (4·5 to 10·4)	[illegible]	[illegible]	[illegible]	[illegible]	[illegible]	[illegible]	[illegible]	[illegible]	[illegible]	[illegible]	[illegible]	[illegible]	[illegible]	[illegible]	[illegible]	[illegible]	[illegible]	9 (6·7 to 11·5)	2,090
Pneumoconiosis	0 (0 to 0)	[illegible]	[illegible]	[illegible]	[illegible]	[illegible]	[illegible]	[illegible]	[illegible]	[illegible]	[illegible]	[illegible]	[illegible]	[illegible]	[illegible]	[illegible]	[illegible]	[illegible]	[illegible]	[illegible]	[illegible]	[illegible]	0 (0 to 0)	6·6
Silicosis	0 (0 to 0)	[illegible]	[illegible]	[illegible]	[illegible]	[illegible]	[illegible]	[illegible]	[illegible]	[illegible]	[illegible]	[illegible]	[illegible]	[illegible]	[illegible]	[illegible]	[illegible]	[illegible]	[illegible]	[illegible]	[illegible]	[illegible]	0 (0 to 0)	3·3
Asbestosis	0 (0 to 0)	[illegible]	[illegible]	[illegible]	[illegible]	[illegible]	[illegible]	[illegible]	[illegible]	[illegible]	[illegible]	[illegible]	[illegible]	[illegible]	[illegible]	[illegible]	[illegible]	[illegible]	[illegible]	[illegible]	[illegible]	[illegible]	0 (0 to 0)	1·3
Coal workers pneumoconiosis	0 (0 to 0)	0 (0 to 0)	[illegible]	[illegible]	[illegible]	[illegible]	[illegible]	[illegible]	[illegible]	[illegible]	[illegible]	[illegible]	[illegible]	[illegible]	[illegible]	[illegible]	[illegible]	[illegible]	[illegible]	[illegible]	[illegible]	[illegible]	0 (0 to 0)	0·3
Other pneumoconiosis	0 (0 to 0)	0 (0 to 0)	[illegible]	[illegible]	[illegible]	[illegible]	[illegible]	[illegible]	[illegible]	[illegible]	[illegible]	[illegible]	[illegible]	[illegible]	[illegible]	[illegible]	[illegible]	[illegible]	[illegible]	[illegible]	[illegible]	[illegible]	0 (0 to 0)	1·7
Asthma	0 (0 to 0)	0 (0 to 0)	[illegible]	[illegible]	[illegible]	[illegible]	[illegible]	[illegible]	[illegible]	[illegible]	[illegible]	[illegible]	[illegible]	[illegible]	[illegible]	[illegible]	[illegible]	[illegible]	[illegible]	[illegible]	[illegible]	[illegible]	1·3 (0·8 to 2)	2,950
Interstitial lung disease and pulmonary sarcoidosis	0 (0 to 0)	0 (0 to 0)	[illegible]	[illegible]	[illegible]	[illegible]	[illegible]	[illegible]	[illegible]	[illegible]	[illegible]	[illegible]	[illegible]	[illegible]	[illegible]	[illegible]	[illegible]	[illegible]	[illegible]	[illegible]	[illegible]	[illegible]	0·4 (0·2 to 0·7)	239
Other chronic respiratory diseases	0 (0 to 0)	0 (0 to 0)	[illegible]	[illegible]	[illegible]	[illegible]	[illegible]	[illegible]	[illegible]	[illegible]	[illegible]	[illegible]	[illegible]	[illegible]	[illegible]	[illegible]	[illegible]	[illegible]	[illegible]	[illegible]	[illegible]	[illegible]	0·1 (0 to 0·1)	256
Digestive diseases	37·4 (22 to 58·5)	14·7 (8·4 to 25·2)	[illegible]	[illegible]	[illegible]	[illegible]	[illegible]	[illegible]	[illegible]	[illegible]	[illegible]	[illegible]	[illegible]	[illegible]	[illegible]	[illegible]	[illegible]	[illegible]	[illegible]	[illegible]	[illegible]	[illegible]	12·5 (9·7 to 14·9)	15,000
Cirrhosis and other chronic liver diseases	0 (0 to 0)	0 (0 to 0)	[illegible]	[illegible]	[illegible]	[illegible]	[illegible]	[illegible]	[illegible]	[illegible]	[illegible]	[illegible]	[illegible]	[illegible]	[illegible]	[illegible]	[illegible]	[illegible]	[illegible]	[illegible]	[illegible]	[illegible]	3·5 (2·6 to 4·4)	8,420
Cirrhosis and other chronic liver diseases due to hepatitis B	0 (0 to 0)	0 (0 to 0)	[illegible]	[illegible]	[illegible]	[illegible]	[illegible]	[illegible]	[illegible]	[illegible]	[illegible]	[illegible]	[illegible]	[illegible]	[illegible]	[illegible]	[illegible]	[illegible]	[illegible]	[illegible]	[illegible]	[illegible]	0·2 (0·1 to 0·4)	640
Cirrhosis and other chronic liver diseases due to hepatitis C	0 (0 to 0)	0 (0 to 0)	[illegible]	[illegible]	[illegible]	[illegible]	[illegible]	[illegible]	[illegible]	[illegible]	[illegible]	[illegible]	[illegible]	[illegible]	[illegible]	[illegible]	[illegible]	[illegible]	[illegible]	[illegible]	[illegible]	[illegible]	0·6 (0·3 to 1·2)	1,740
Cirrhosis and other chronic liver diseases due to alcohol use	0 (0 to 0)	0 (0 to 0)	[illegible]	[illegible]	[illegible]	[illegible]	[illegible]	[illegible]	[illegible]	[illegible]	[illegible]	[illegible]	[illegible]	[illegible]	[illegible]	[illegible]	[illegible]	[illegible]	[illegible]	[illegible]	[illegible]	[illegible]	1 (0·5 to 1·7)	3,580
Cirrhosis and other chronic liver diseases due to NAFLD	0 (0 to 0)	0 (0 to 0)	[illegible]	[illegible]	[illegible]	[illegible]	[illegible]	[illegible]	[illegible]	[illegible]	[illegible]	[illegible]	[illegible]	[illegible]	[illegible]	[illegible]	[illegible]	[illegible]	[illegible]	[illegible]	[illegible]	[illegible]	0·9 (0·4 to 1·5)	1,210
Cirrhosis and other chronic liver diseases due to other causes	0 (0 to 0)	0 (0 to 0)	[illegible]	[illegible]	[illegible]	[illegible]	[illegible]	[illegible]	[illegible]	[illegible]	[illegible]	[illegible]	[illegible]	[illegible]	[illegible]	[illegible]	[illegible]	[illegible]	[illegible]	[illegible]	[illegible]	[illegible]	0·8 (0·4 to 1·4)	1,240
Upper digestive system diseases	0 (0 to 0)	0 (0 to 0)	[illegible]	[illegible]	[illegible]	[illegible]	[illegible]	[illegible]	[illegible]	[illegible]	[illegible]	[illegible]	[illegible]	[illegible]	[illegible]	[illegible]	[illegible]	[illegible]	[illegible]	[illegible]	[illegible]	[illegible]	3·2 (2·3 to 4·1)	2,480
Peptic ulcer disease	0 (0 to 0)	0 (0 to 0)	[illegible]	[illegible]	[illegible]	[illegible]	[illegible]	[illegible]	[illegible]	[illegible]	[illegible]	[illegible]	[illegible]	[illegible]	[illegible]	[illegible]	[illegible]	[illegible]	[illegible]	[illegible]	[illegible]	[illegible]	2·4 (1·7 to 3·2)	1,140
Gastritis and duodenitis	0 (0 to 0)	0 (0 to 0)	[illegible]	[illegible]	[illegible]	[illegible]	[illegible]	[illegible]	[illegible]	[illegible]	[illegible]	[illegible]	[illegible]	[illegible]	[illegible]	[illegible]	[illegible]	[illegible]	[illegible]	[illegible]	[illegible]	[illegible]	0·4 (0·3 to 0·7)	409
Gastroesophageal reflux disease	0 (0 to 0)	0 (0 to 0)	[illegible]	[illegible]	[illegible]	[illegible]	[illegible]	[illegible]	[illegible]	[illegible]	[illegible]	[illegible]	[illegible]	[illegible]	[illegible]	[illegible]	[illegible]	[illegible]	[illegible]	[illegible]	[illegible]	[illegible]	0·4 (0·2 to 0·6)	925
Appendicitis	0 (0 to 0)	0 (0 to 0)	[illegible]	[illegible]	[illegible]	[illegible]	[illegible]	[illegible]	[illegible]	[illegible]	[illegible]	[illegible]	[illegible]	[illegible]	[illegible]	[illegible]	[illegible]	[illegible]	[illegible]	[illegible]	[illegible]	[illegible]	0·2 (0·1 to 0·3)	408
Paralytic ileus and intestinal obstruction	21·4 (12·4 to 35)	12·1 (7 to 20·3)	[illegible]	[illegible]	[illegible]	[illegible]	[illegible]	[illegible]	[illegible]	[illegible]	[illegible]	[illegible]	[illegible]	[illegible]	[illegible]	[illegible]	[illegible]	[illegible]	[illegible]	[illegible]	[illegible]	[illegible]	1·7 (1·1 to 2·5)	774
Inguinal, femoral, and abdominal hernia	16 (8·9 to 26·3)	2·6 (1·1 to 5·2)	[illegible]	[illegible]	[illegible]	[illegible]	[illegible]	[illegible]	[illegible]	[illegible]	[illegible]	[illegible]	[illegible]	[illegible]	[illegible]	[illegible]	[illegible]	[illegible]	[illegible]	[illegible]	[illegible]	[illegible]	0·5 (0·4 to 0·7)	720
Inflammatory bowel disease	0 (0 to 0)	0 (0 to 0)	[illegible]	[illegible]	[illegible]	[illegible]	[illegible]	[illegible]	[illegible]	[illegible]	[illegible]	[illegible]	[illegible]	[illegible]	[illegible]	[illegible]	[illegible]	[illegible]	[illegible]	[illegible]	[illegible]	[illegible]	0·2 (0·1 to 0·3)	257
Vascular intestinal disorders	0 (0 to 0)	0 (0 to 0)	[illegible]	[illegible]	[illegible]	[illegible]	[illegible]	[illegible]	[illegible]	[illegible]	[illegible]	[illegible]	[illegible]	[illegible]	[illegible]	[illegible]	[illegible]	[illegible]	[illegible]	[illegible]	[illegible]	[illegible]	0·3 (0·2 to 0·5)	150
Gallbladder and biliary diseases	0 (0 to 0)	0 (0 to 0)	[illegible]	[illegible]	[illegible]	[illegible]	[illegible]	[illegible]	[illegible]	[illegible]	[illegible]	[illegible]	[illegible]	[illegible]	[illegible]	[illegible]	[illegible]	[illegible]	[illegible]	[illegible]	[illegible]	[illegible]	0·9 (0·6 to 1·2)	639
Pancreatitis	0 (0 to 0)	0 (0 to 0)	[illegible]	[illegible]	[illegible]	[illegible]	[illegible]	[illegible]	[illegible]	[illegible]	[illegible]	[illegible]	[illegible]	[illegible]	[illegible]	[illegible]	[illegible]	[illegible]	[illegible]	[illegible]	[illegible]	[illegible]	0·3 (0·2 to 0·5)	584
Other digestive diseases	0 (0 to 0)	0 (0 to 0)	[illegible]	[illegible]	[illegible]	[illegible]	[illegible]	[illegible]	[illegible]	[illegible]	[illegible]	[illegible]	[illegible]	[illegible]	[illegible]	[illegible]	[illegible]	[illegible]	[illegible]	[illegible]	[illegible]	[illegible]	1·6 (0·9 to 2·4)	555
Neurological disorders	0·2 (0·1 to 0·5)	0·7 (0·3 to 1·5)	[illegible]	[illegible]	[illegible]	[illegible]	[illegible]	[illegible]	[illegible]	[illegible]	[illegible]	[illegible]	[illegible]	[illegible]	[illegible]	[illegible]	[illegible]	[illegible]	[illegible]	[illegible]	[illegible]	[illegible]	49·1 (21·7 to 109)	9,730
Alzheimer's disease and other dementias	0 (0 to 0)	0 (0 to 0)	[illegible]	[illegible]	[illegible]	[illegible]	[illegible]	[illegible]	[illegible]	[illegible]	[illegible]	[illegible]	[illegible]	[illegible]	[illegible]	[illegible]	[illegible]	[illegible]	[illegible]	[illegible]	[illegible]	[illegible]	45·5 (17·7 to 106)	1,290
Parkinson's disease	0 (0 to 0)	0 (0 to 0)	[illegible]	[illegible]	[illegible]	[illegible]	[illegible]	[illegible]	[illegible]	[illegible]	[illegible]	[illegible]	[illegible]	[illegible]	[illegible]	[illegible]	[illegible]	[illegible]	[illegible]	[illegible]	[illegible]	[illegible]	2·6 (2 to 3·1)	482
Idiopathic epilepsy	0·2 (0 to 0·5)	0·6 (0·1 to 1·4)	[illegible]	[illegible]	[illegible]	[illegible]	[illegible]	[illegible]	[illegible]	[illegible]	[illegible]	[illegible]	[illegible]	[illegible]	[illegible]	[illegible]	[illegible]	[illegible]	[illegible]	[illegible]	[illegible]	[illegible]	0·7 (0·3 to 1·2)	2,630
Multiple sclerosis	0 (0 to 0)	0 (0 to 0)	[illegible]	[illegible]	[illegible]	[illegible]	[illegible]	[illegible]	[illegible]	[illegible]	[illegible]	[illegible]	[illegible]	[illegible]	[illegible]	[illegible]	[illegible]	[illegible]	[illegible]	[illegible]	[illegible]	[illegible]	0 (0 to 0)	50·5
Motor neuron disease	0 (0 to 0)	0·1 (0·1 to 0·2)	[illegible]	[illegible]	[illegible]	[illegible]	[illegible]	[illegible]	[illegible]	[illegible]	[illegible]	[illegible]	[illegible]	[illegible]	[illegible]	[illegible]	[illegible]	[illegible]	[illegible]	[illegible]	[illegible]	[illegible]	0 (0 to 0)	109
Headache disorders	0 (0 to 0)	0 (0 to 0)	[illegible]	[illegible]	[illegible]	[illegible]	[illegible]	[illegible]	[illegible]	[illegible]	[illegible]	[illegible]	[illegible]	[illegible]	[illegible]	[illegible]	[illegible]	[illegible]	[illegible]	[illegible]	[illegible]	[illegible]	0·3 (0·1 to 0·6)	4,370
Migraine	0 (0 to 0)	0 (0 to 0)	[illegible]	[illegible]	[illegible]	[illegible]	[illegible]	[illegible]	[illegible]	[illegible]	[illegible]	[illegible]	[illegible]	[illegible]	[illegible]	[illegible]	[illegible]	[illegible]	[illegible]	[illegible]	[illegible]	[illegible]	0·2 (0 to 0·5)	3,960
Tension-type headache	0 (0 to 0)	0 (0 to 0)	[illegible]	[illegible]	[illegible]	[illegible]	[illegible]	[illegible]	[illegible]	[illegible]	[illegible]	[illegible]	[illegible]	[illegible]	[illegible]	[illegible]	[illegible]	[illegible]	[illegible]	[illegible]	[illegible]	[illegible]	0·1 (0 to 0·3)	403
Other neurological disorders	0 (0 to 0)	0 (0 to 0)	[illegible]	[illegible]	[illegible]	[illegible]	[illegible]	[illegible]	[illegible]	[illegible]	[illegible]	[illegible]	[illegible]	[illegible]	[illegible]	[illegible]	[illegible]	[illegible]	[illegible]	[illegible]	[illegible]	[illegible]	0·1 (0·1 to 0·2)	800
Mental disorders	0·2 (0·1 to 0·3)	0·6 (0·4 to 1)	[illegible]	[illegible]	[illegible]	[illegible]	[illegible]	[illegible]	[illegible]	[illegible]	[illegible]	[illegible]	[illegible]	[illegible]	[illegible]	[illegible]	[illegible]	[illegible]	[illegible]	[illegible]	[illegible]	[illegible]	3·3 (2·1 to 4·8)	15,800
Schizophrenia	0 (0 to 0)	0 (0 to 0)	[illegible]	[illegible]	[illegible]	[illegible]	[illegible]	[illegible]	[illegible]	[illegible]	[illegible]	[illegible]	[illegible]	[illegible]	[illegible]	[illegible]	[illegible]	[illegible]	[illegible]	[illegible]	[illegible]	[illegible]	0 (0 to 0·1)	1,300
Depressive disorders	0 (0 to 0)	0 (0 to 0)	[illegible]	[illegible]	[illegible]	[illegible]	[illegible]	[illegible]	[illegible]	[illegible]	[illegible]	[illegible]	[illegible]	[illegible]	[illegible]	[illegible]	[illegible]	[illegible]	[illegible]	[illegible]	[illegible]	[illegible]	2·3 (1·3 to 3·8)	7,170
Major depressive disorder	0 (0 to 0)	0 (0 to 0)	[illegible]	[illegible]	[illegible]	[illegible]	[illegible]	[illegible]	[illegible]	[illegible]	[illegible]	[illegible]	[illegible]	[illegible]	[illegible]	[illegible]	[illegible]	[illegible]	[illegible]	[illegible]	[illegible]	[illegible]	2·2 (1·1 to 3·5)	6,480
Dysthymia	0 (0 to 0)	0 (0 to 0)	[illegible]	[illegible]	[illegible]	[illegible]	[illegible]	[illegible]	[illegible]	[illegible]	[illegible]	[illegible]	[illegible]	[illegible]	[illegible]	[illegible]	[illegible]	[illegible]	[illegible]	[illegible]	[illegible]	[illegible]	0·2 (0·1 to 0·3)	692
Bipolar disorder	0 (0 to 0)	0 (0 to 0)	[illegible]	[illegible]	[illegible]	[illegible]	[illegible]	[illegible]	[illegible]	[illegible]	[illegible]	[illegible]	[illegible]	[illegible]	[illegible]	[illegible]	[illegible]	[illegible]	[illegible]	[illegible]	[illegible]	[illegible]	0·1 (0 to 0·1)	1,510
Anxiety disorders	0 (0 to 0)	0 (0 to 0)	[illegible]	[illegible]	[illegible]	[illegible]	[illegible]	[illegible]	[illegible]	[illegible]	[illegible]	[illegible]	[illegible]	[illegible]	[illegible]	[illegible]	[illegible]	[illegible]	[illegible]	[illegible]	[illegible]	[illegible]	0·4 (0·2 to 0·6)	3,270
Eating disorders	0 (0 to 0)	0 (0 to 0)	[illegible]	[illegible]	[illegible]	[illegible]	[illegible]	[illegible]	[illegible]	[illegible]	[illegible]	[illegible]	[illegible]	[illegible]	[illegible]	[illegible]	[illegible]	[illegible]	[illegible]	[illegible]	[illegible]	[illegible]	0 (0 to 0)	338
Anorexia nervosa	0 (0 to 0)	0 (0 to 0)	[illegible]	[illegible]	[illegible]	[illegible]	[illegible]	[illegible]	[illegible]	[illegible]	[illegible]	[illegible]	[illegible]	[illegible]	[illegible]	[illegible]	[illegible]	[illegible]	[illegible]	[illegible]	[illegible]	[illegible]	0 (0 to 0)	84·8
Bulimia nervosa	0 (0 to 0)	0 (0 to 0)	[illegible]	[illegible]	[illegible]	[illegible]	[illegible]	[illegible]	[illegible]	[illegible]	[illegible]	[illegible]	[illegible]	[illegible]	[illegible]	[illegible]	[illegible]	[illegible]	[illegible]	[illegible]	[illegible]	[illegible]	0 (0 to 0)	253
Autism spectrum disorders	0·2 (0·1 to 0·3)	0·5 (0·3 to 0·8)	[illegible]	[illegible]	[illegible]	[illegible]	[illegible]	[illegible]	[illegible]	[illegible]	[illegible]	[illegible]	[illegible]	[illegible]	[illegible]	[illegible]	[illegible]	[illegible]	[illegible]	[illegible]	[illegible]	[illegible]	0 (0 to 0·1)	401
Attention-deficit/hyperactivity disorder	0 (0 to 0)	0 (0 to 0)	[illegible]	[illegible]	[illegible]	[illegible]	[illegible]	[illegible]	[illegible]	[illegible]	[illegible]	[illegible]	[illegible]	[illegible]	[illegible]	[illegible]	[illegible]	[illegible]	[illegible]	[illegible]	[illegible]	[illegible]	0 (0 to 0)	301
Conduct disorder	0 (0 to 0)	0 (0 to 0)	[illegible]	[illegible]	[illegible]	[illegible]	[illegible]	[illegible]	[illegible]	[illegible]	[illegible]	[illegible]	[illegible]	[illegible]	[illegible]	[illegible]	[illegible]	[illegible]	[illegible]	[illegible]	[illegible]	[illegible]	0 (0 to 0)	534
Idiopathic developmental intellectual disability	0 (0 to 0·1)	0·1 (0 to 0·3)	[illegible]	[illegible]	[illegible]	[illegible]	[illegible]	[illegible]	[illegible]	[illegible]	[illegible]	[illegible]	[illegible]	[illegible]	[illegible]	[illegible]	[illegible]	[illegible]	[illegible]	[illegible]	[illegible]	[illegible]	0 (0 to 0)	151
Other mental disorders	0 (0 to 0)	0 (0 to 0)	[illegible]	[illegible]	[illegible]	[illegible]	[illegible]	[illegible]	[illegible]	[illegible]	[illegible]	[illegible]	[illegible]	[illegible]	[illegible]	[illegible]	[illegible]	[illegible]	[illegible]	[illegible]	[illegible]	[illegible]	0·4 (0·3 to 0·6)	807
Substance use disorders	0 (0 to 0)	0 (0 to 0)	[illegible]	[illegible]	[illegible]	[illegible]	[illegible]	[illegible]	[illegible]	[illegible]	[illegible]	[illegible]	[illegible]	[illegible]	[illegible]	[illegible]	[illegible]	[illegible]	[illegible]	[illegible]	[illegible]	[illegible]	0·8 (0·5 to 1·1)	3,390
Alcohol use disorders	0 (0 to 0)	0 (0 to 0)	[illegible]	[illegible]	[illegible]	[illegible]	[illegible]	[illegible]	[illegible]	[illegible]	[illegible]	[illegible]	[illegible]	[illegible]	[illegible]	[illegible]	[illegible]	[illegible]	[illegible]	[illegible]	[illegible]	[illegible]	0·7 (0·4 to 1)	2,500
Drug use disorders	0 (0 to 0)	0 (0 to 0)	[illegible]	[illegible]	[illegible]	[illegible]	[illegible]	[illegible]	[illegible]	[illegible]	[illegible]	[illegible]	[illegible]	[illegible]	[illegible]	[illegible]	[illegible]	[illegible]	[illegible]	[illegible]	[illegible]	[illegible]	0·1 (0·1 to 0·2)	890
Opioid use disorders	0 (0 to 0)	0 (0 to 0)	[illegible]	[illegible]	[illegible]	[illegible]	[illegible]	[illegible]	[illegible]	[illegible]	[illegible]	[illegible]	[illegible]	[illegible]	[illegible]	[illegible]	[illegible]	[illegible]	[illegible]	[illegible]	[illegible]	[illegible]	0·1 (0 to 0·1)	440
Cocaine use disorders	0 (0 to 0)	0 (0 to 0)	[illegible]	[illegible]	[illegible]	[illegible]	[illegible]	[illegible]	[illegible]	[illegible]	[illegible]	[illegible]	[illegible]	[illegible]	[illegible]	[illegible]	[illegible]	[illegible]	[illegible]	[illegible]	[illegible]	[illegible]	0 (0 to 0)	148
Amphetamine use disorders	0 (0 to 0)	0 (0 to 0)	[illegible]	[illegible]	[illegible]	[illegible]	[illegible]	[illegible]	[illegible]	[illegible]	[illegible]	[illegible]	[illegible]	[illegible]	[illegible]	[illegible]	[illegible]	[illegible]	[illegible]	[illegible]	[illegible]	[illegible]	0 (0 to 0)	58·7
Cannabis use disorders	0 (0 to 0)	0 (0 to 0)	[illegible]	[illegible]	[illegible]	[illegible]	[illegible]	[illegible]	[illegible]	[illegible]	[illegible]	[illegible]	[illegible]	[illegible]	[illegible]	[illegible]	[illegible]	[illegible]	[illegible]	[illegible]	[illegible]	[illegible]	0 (0 to 0)	104
Other drug use disorders	0 (0 to 0)	0 (0 to 0)	[illegible]	[illegible]	[illegible]	[illegible]	[illegible]	[illegible]	[illegible]	[illegible]	[illegible]	[illegible]	[illegible]	[illegible]	[illegible]	[illegible]	[illegible]	[illegible]	[illegible]	[illegible]	[illegible]	[illegible]	0 (0 to 0)	139
Diabetes and kidney diseases	0·2 (0·1 to 0·5)	0·6 (0·3 to 1·1)	[illegible]	[illegible]	[illegible]	[illegible]	[illegible]	[illegible]	[illegible]	[illegible]	[illegible]	[illegible]	[illegible]	[illegible]	[illegible]	[illegible]	[illegible]	[illegible]	[illegible]	[illegible]	[illegible]	[illegible]	45·1 (36·1 to 54·4)	31,700
Diabetes mellitus	0·2 (0·1 to 0·5)	0·5 (0·3 to 1·1)	[illegible]	[illegible]	[illegible]	[illegible]	[illegible]	[illegible]	[illegible]	[illegible]	[illegible]	[illegible]	[illegible]	[illegible]	[illegible]	[illegible]	[illegible]	[illegible]	[illegible]	[illegible]	[illegible]	[illegible]	30·2 (23·8 to 37·8)	21,900
Diabetes mellitus type 1	0·2 (0·1 to 0·5)	0·5 (0·3 to 1·1)	[illegible]	[illegible]	[illegible]	[illegible]	[illegible]	[illegible]	[illegible]	[illegible]	[illegible]	[illegible]	[illegible]	[illegible]	[illegible]	[illegible]	[illegible]	[illegible]	[illegible]	[illegible]	[illegible]	[illegible]	0·6 (0·4 to 0·9)	1,260
Diabetes mellitus type 2	0 (0 to 0)	0 (0 to 0)	[illegible]	[illegible]	[illegible]	[illegible]	[illegible]	[illegible]	[illegible]	[illegible]	[illegible]	[illegible]	[illegible]	[illegible]	[illegible]	[illegible]	[illegible]	[illegible]	[illegible]	[illegible]	[illegible]	[illegible]	29·7 (23·4 to 37·1)	20,600
Chronic kidney disease	0 (0 to 0)	0 (0 to 0)	[illegible]	[illegible]	[illegible]	[illegible]	[illegible]	[illegible]	[illegible]	[illegible]	[illegible]	[illegible]	[illegible]	[illegible]	[illegible]	[illegible]	[illegible]	[illegible]	[illegible]	[illegible]	[illegible]	[illegible]	14·8 (10·9 to 19·5)	9,820
Chronic kidney disease due to diabetes mellitus type 1	0 (0 to 0)	0 (0 to 0)	[illegible]	[illegible]	[illegible]	[illegible]	[illegible]	[illegible]	[illegible]	[illegible]	[illegible]	[illegible]	[illegible]	[illegible]	[illegible]	[illegible]	[illegible]	[illegible]	[illegible]	[illegible]	[illegible]	[illegible]	0 (0 to 0)	920
Chronic kidney disease due to diabetes mellitus type 2	0 (0 to 0)	0 (0 to 0)	[illegible]	[illegible]	[illegible]	[illegible]	[illegible]	[illegible]	[illegible]	[illegible]	[illegible]	[illegible]	[illegible]	[illegible]	[illegible]	[illegible]	[illegible]	[illegible]	[illegible]	[illegible]	[illegible]	[illegible]	4·2 (2·2 to 6·1)	2,170
Chronic kidney disease due to hypertension	0 (0 to 0)	0 (0 to 0)	[illegible]	[illegible]	[illegible]	[illegible]	[illegible]	[illegible]	[illegible]	[illegible]	[illegible]	[illegible]	[illegible]	[illegible]	[illegible]	[illegible]	[illegible]	[illegible]	[illegible]	[illegible]	[illegible]	[illegible]	6·1 (4·1 to 8·6)	1,700
Chronic kidney disease due to glomerulonephritis	0 (0 to 0)	0 (0 to 0)	[illegible]	[illegible]	[illegible]	[illegible]	[illegible]	[illegible]	[illegible]	[illegible]	[illegible]	[illegible]	[illegible]	[illegible]	[illegible]	[illegible]	[illegible]	[illegible]	[illegible]	[illegible]	[illegible]	[illegible]	0·9 (0·4 to 2)	2,370
Chronic kidney disease due to other and unspecified causes	0 (0 to 0)	0 (0 to 0)	[illegible]	[illegible]	[illegible]	[illegible]	[illegible]	[illegible]	[illegible]	[illegible]	[illegible]	[illegible]	[illegible]	[illegible]	[illegible]	[illegible]	[illegible]	[illegible]	[illegible]	[illegible]	[illegible]	[illegible]	3·7 (2·2 to 5·7)	2,660
Acute glomerulonephritis	0 (0 to 0)	0 (0 to 0)	[illegible]	[illegible]	[illegible]	[illegible]	[illegible]	[illegible]	[illegible]	[illegible]	[illegible]	[illegible]	[illegible]	[illegible]	[illegible]	[illegible]	[illegible]	[illegible]	[illegible]	[illegible]	[illegible]	[illegible]	0 (0 to 0)	13·8
Skin and subcutaneous diseases	0 (0 to 0)	0·2 (0·1 to 0·4)	[illegible]	[illegible]	[illegible]	[illegible]	[illegible]	[illegible]	[illegible]	[illegible]	[illegible]	[illegible]	[illegible]	[illegible]	[illegible]	[illegible]	[illegible]	[illegible]	[illegible]	[illegible]	[illegible]	[illegible]	3·2 (2·1 to 4·8)	3,980
Dermatitis	0 (0 to 0)	0 (0 to 0)	[illegible]	[illegible]	[illegible]	[illegible]	[illegible]	[illegible]	[illegible]	[illegible]	[illegible]	[illegible]	[illegible]	[illegible]	[illegible]	[illegible]	[illegible]	[illegible]	[illegible]	[illegible]	[illegible]	[illegible]	0·2 (0·1 to 0·3)	873
Atopic dermatitis	0 (0 to 0)	0 (0 to 0)	[illegible]	[illegible]	[illegible]	[illegible]	[illegible]	[illegible]	[illegible]	[illegible]	[illegible]	[illegible]	[illegible]	[illegible]	[illegible]	[illegible]	[illegible]	[illegible]	[illegible]	[illegible]	[illegible]	[illegible]	0·1 (0 to 0·1)	644
Contact dermatitis	0 (0 to 0)	0 (0 to 0)	[illegible]	[illegible]	[illegible]	[illegible]	[illegible]	[illegible]	[illegible]	[illegible]	[illegible]	[illegible]	[illegible]	[illegible]	[illegible]	[illegible]	[illegible]	[illegible]	[illegible]	[illegible]	[illegible]	[illegible]	0·1 (0 to 0·1)	203
Seborrhoeic dermatitis	0 (0 to 0)	0 (0 to 0)	[illegible]	[illegible]	[illegible]	[illegible]	[illegible]	[illegible]	[illegible]	[illegible]	[illegible]	[illegible]	[illegible]	[illegible]	[illegible]	[illegible]	[illegible]	[illegible]	[illegible]	[illegible]	[illegible]	[illegible]	0 (0 to 0)	25·2
Psoriasis	0 (0 to 0)	0 (0 to 0)	[illegible]	[illegible]	[illegible]	[illegible]	[illegible]	[illegible]	[illegible]	[illegible]	[illegible]	[illegible]	[illegible]	[illegible]	[illegible]	[illegible]	[illegible]	[illegible]	[illegible]	[illegible]	[illegible]	[illegible]	0 (0 to 0)	240
Bacterial skin diseases	0 (0 to 0)	0 (0 to 0)	[illegible]	[illegible]	[illegible]	[illegible]	[illegible]	[illegible]	[illegible]	[illegible]	[illegible]	[illegible]	[illegible]	[illegible]	[illegible]	[illegible]	[illegible]	[illegible]	[illegible]	[illegible]	[illegible]	[illegible]	0·6 (0·2 to 1·1)	191
Cellulitis	0 (0 to 0)	0 (0 to 0)	[illegible]	[illegible]	[illegible]	[illegible]	[illegible]	[illegible]	[illegible]	[illegible]	[illegible]	[illegible]	[illegible]	[illegible]	[illegible]	[illegible]	[illegible]	[illegible]	[illegible]	[illegible]	[illegible]	[illegible]	0·1 (0 to 0·1)	35·1
Pyoderma	0 (0 to 0)	0 (0 to 0)	[illegible]	[illegible]	[illegible]	[illegible]	[illegible]	[illegible]	[illegible]	[illegible]	[illegible]	[illegible]	[illegible]	[illegible]	[illegible]	[illegible]	[illegible]	[illegible]	[illegible]	[illegible]	[illegible]	[illegible]	0·5 (0·2 to 1·1)	155
Scabies	0 (0 to 0)	0·1 (0·1 to 0·2)	[illegible]	[illegible]	[illegible]	[illegible]	[illegible]	[illegible]	[illegible]	[illegible]	[illegible]	[illegible]	[illegible]	[illegible]	[illegible]	[illegible]	[illegible]	[illegible]	[illegible]	[illegible]	[illegible]	[illegible]	0·2 (0·1 to 0·3)	655
Fungal skin diseases	0 (0 to 0)	0 (0 to 0)	[illegible]	[illegible]	[illegible]	[illegible]	[illegible]	[illegible]	[illegible]	[illegible]	[illegible]	[illegible]	[illegible]	[illegible]	[illegible]	[illegible]	[illegible]	[illegible]	[illegible]	[illegible]	[illegible]	[illegible]	0·6 (0·3 to 1·3)	311
Viral skin diseases	0 (0 to 0)	0 (0 to 0)	[illegible]	[illegible]	[illegible]	[illegible]	[illegible]	[illegible]	[illegible]	[illegible]	[illegible]	[illegible]	[illegible]	[illegible]	[illegible]	[illegible]	[illegible]	[illegible]	[illegible]	[illegible]	[illegible]	[illegible]	0 (0 to 0)	361
Acne vulgaris	0 (0 to 0)	0 (0 to 0)	[illegible]	[illegible]	[illegible]	[illegible]	[illegible]	[illegible]	[illegible]	[illegible]	[illegible]	[illegible]	[illegible]	[illegible]	[illegible]	[illegible]	[illegible]	[illegible]	[illegible]	[illegible]	[illegible]	[illegible]	0 (0 to 0)	401
Alopecia areata	0 (0 to 0)	0 (0 to 0)	[illegible]	[illegible]	[illegible]	[illegible]	[illegible]	[illegible]	[illegible]	[illegible]	[illegible]	[illegible]	[illegible]	[illegible]	[illegible]	[illegible]	[illegible]	[illegible]	[illegible]	[illegible]	[illegible]	[illegible]	0 (0 to 0)	55·8
Pruritus	0 (0 to 0)	0 (0 to 0)	[illegible]	[illegible]	[illegible]	[illegible]	[illegible]	[illegible]	[illegible]	[illegible]	[illegible]	[illegible]	[illegible]	[illegible]	[illegible]	[illegible]	[illegible]	[illegible]	[illegible]	[illegible]	[illegible]	[illegible]	0 (0 to 0·1)	61
Urticaria	0 (0 to 0)	0·1 (0 to 0·1)	[illegible]	[illegible]	[illegible]	[illegible]	[illegible]	[illegible]	[illegible]	[illegible]	[illegible]	[illegible]	[illegible]	[illegible]	[illegible]	[illegible]	[illegible]	[illegible]	[illegible]	[illegible]	[illegible]	[illegible]	0 (0 to 0·1)	366
Decubitus ulcer	0 (0 to 0)	0 (0 to 0)	[illegible]	[illegible]	[illegible]	[illegible]	[illegible]	[illegible]	[illegible]	[illegible]	[illegible]	[illegible]	[illegible]	[illegible]	[illegible]	[illegible]	[illegible]	[illegible]	[illegible]	[illegible]	[illegible]	[illegible]	1·3 (0·8 to 2·2)	103
Other skin and subcutaneous diseases	0 (0 to 0)	0 (0 to 0)	[illegible]	[illegible]	[illegible]	[illegible]	[illegible]	[illegible]	[illegible]	[illegible]	[illegible]	[illegible]	[illegible]	[illegible]	[illegible]	[illegible]	[illegible]	[illegible]	[illegible]	[illegible]	[illegible]	[illegible]	0·2 (0·1 to 0·4)	367
Sense organ diseases	0 (0 to 0·1)	0·1 (0·1 to 0·2)	[illegible]	[illegible]	[illegible]	[illegible]	[illegible]	[illegible]	[illegible]	[illegible]	[illegible]	[illegible]	[illegible]	[illegible]	[illegible]	[illegible]	[illegible]	[illegible]	[illegible]	[illegible]	[illegible]	[illegible]	15·2 (11 to 20·1)	4,920
Blindness and vision loss	0 (0 to 0)	0 (0 to 0·1)	[illegible]	[illegible]	[illegible]	[illegible]	[illegible]	[illegible]	[illegible]	[illegible]	[illegible]	[illegible]	[illegible]	[illegible]	[illegible]	[illegible]	[illegible]	[illegible]	[illegible]	[illegible]	[illegible]	[illegible]	6·4 (4·5 to 8·8)	1,330
Glaucoma	0 (0 to 0)	0 (0 to 0)	[illegible]	[illegible]	[illegible]	[illegible]	[illegible]	[illegible]	[illegible]	[illegible]	[illegible]	[illegible]	[illegible]	[illegible]	[illegible]	[illegible]	[illegible]	[illegible]	[illegible]	[illegible]	[illegible]	[illegible]	0·9 (0·5 to 1·4)	65·8
Cataract	0 (0 to 0)	0 (0 to 0)	[illegible]	[illegible]	[illegible]	[illegible]	[illegible]	[illegible]	[illegible]	[illegible]	[illegible]	[illegible]	[illegible]	[illegible]	[illegible]	[illegible]	[illegible]	[illegible]	[illegible]	[illegible]	[illegible]	[illegible]	2·1 (1·3 to 3·1)	200
Age-related macular degeneration	0 (0 to 0)	0 (0 to 0)	[illegible]	[illegible]	[illegible]	[illegible]	[illegible]	[illegible]	[illegible]	[illegible]	[illegible]	[illegible]	[illegible]	[illegible]	[illegible]	[illegible]	[illegible]	[illegible]	[illegible]	[illegible]	[illegible]	[illegible]	0·1 (0 to 0·1)	8·3
Refraction disorders	0 (0 to 0)	0 (0 to 0)	[illegible]	[illegible]	[illegible]	[illegible]	[illegible]	[illegible]	[illegible]	[illegible]	[illegible]	[illegible]	[illegible]	[illegible]	[illegible]	[illegible]	[illegible]	[illegible]	[illegible]	[illegible]	[illegible]	[illegible]	0·2 (0·1 to 0·3)	398
Near vision loss	0 (0 to 0)	0 (0 to 0)	[illegible]	[illegible]	[illegible]	[illegible]	[illegible]	[illegible]	[illegible]	[illegible]	[illegible]	[illegible]	[illegible]	[illegible]	[illegible]	[illegible]	[illegible]	[illegible]	[illegible]	[illegible]	[illegible]	[illegible]	0·8 (0·3 to 1·7)	318
Other vision loss	0 (0 to 0)	0 (0 to 0·1)	[illegible]	[illegible]	[illegible]	[illegible]	[illegible]	[illegible]	[illegible]	[illegible]	[illegible]	[illegible]	[illegible]	[illegible]	[illegible]	[illegible]	[illegible]	[illegible]	[illegible]	[illegible]	[illegible]	[illegible]	2·4 (1·5 to 3·5)	339
Age-related and other hearing loss	0 (0 to 0)	0 (0 to 0·1)	[illegible]	[illegible]	[illegible]	[illegible]	[illegible]	[illegible]	[illegible]	[illegible]	[illegible]	[illegible]	[illegible]	[illegible]	[illegible]	[illegible]	[illegible]	[illegible]	[illegible]	[illegible]	[illegible]	[illegible]	8·5 (6·1 to 11·5)	3,340
Other sense organ diseases	0 (0 to 0)	0·1 (0 to 0·1)	[illegible]	[illegible]	[illegible]	[illegible]	[illegible]	[illegible]	[illegible]	[illegible]	[illegible]	[illegible]	[illegible]	[illegible]	[illegible]	[illegible]	[illegible]	[illegible]	[illegible]	[illegible]	[illegible]	[illegible]	0·3 (0·2 to 0·6)	248
Musculoskeletal disorders	0 (0 to 0)	0 (0 to 0)	[illegible]	[illegible]	[illegible]	[illegible]	[illegible]	[illegible]	[illegible]	[illegible]	[illegible]	[illegible]	[illegible]	[illegible]	[illegible]	[illegible]	[illegible]	[illegible]	[illegible]	[illegible]	[illegible]	[illegible]	5·9 (4·1 to 8·4)	10,900
Rheumatoid arthritis	0 (0 to 0)	0 (0 to 0)	[illegible]	[illegible]	[illegible]	[illegible]	[illegible]	[illegible]	[illegible]	[illegible]	[illegible]	[illegible]	[illegible]	[illegible]	[illegible]	[illegible]	[illegible]	[illegible]	[illegible]	[illegible]	[illegible]	[illegible]	0·3 (0·2 to 0·4)	180
Osteoarthritis	0 (0 to 0)	0 (0 to 0)	[illegible]	[illegible]	[illegible]	[illegible]	[illegible]	[illegible]	[illegible]	[illegible]	[illegible]	[illegible]	[illegible]	[illegible]	[illegible]	[illegible]	[illegible]	[illegible]	[illegible]	[illegible]	[illegible]	[illegible]	2 (1 to 3·9)	1,300
Osteoarthritis hip	0 (0 to 0)	0 (0 to 0)	[illegible]	[illegible]	[illegible]	[illegible]	[illegible]	[illegible]	[illegible]	[illegible]	[illegible]	[illegible]	[illegible]	[illegible]	[illegible]	[illegible]	[illegible]	[illegible]	[illegible]	[illegible]	[illegible]	[illegible]	0·1 (0·1 to 0·2)	64·2
Osteoarthritis knee	0 (0 to 0)	0 (0 to 0)	[illegible]	[illegible]	[illegible]	[illegible]	[illegible]	[illegible]	[illegible]	[illegible]	[illegible]	[illegible]	[illegible]	[illegible]	[illegible]	[illegible]	[illegible]	[illegible]	[illegible]	[illegible]	[illegible]	[illegible]	1·1 (0·5 to 2·1)	935
Osteoarthritis hand	0 (0 to 0)	0 (0 to 0)	[illegible]	[illegible]	[illegible]	[illegible]	[illegible]	[illegible]	[illegible]	[illegible]	[illegible]	[illegible]	[illegible]	[illegible]	[illegible]	[illegible]	[illegible]	[illegible]	[illegible]	[illegible]	[illegible]	[illegible]	0·4 (0·2 to 0·8)	155
Osteoarthritis other	0 (0 to 0)	0 (0 to 0)	[illegible]	[illegible]	[illegible]	[illegible]	[illegible]	[illegible]	[illegible]	[illegible]	[illegible]	[illegible]	[illegible]	[illegible]	[illegible]	[illegible]	[illegible]	[illegible]	[illegible]	[illegible]	[illegible]	[illegible]	0·4 (0·2 to 0·8)	145
Low back pain	0 (0 to 0)	0 (0 to 0)	[illegible]	[illegible]	[illegible]	[illegible]	[illegible]	[illegible]	[illegible]	[illegible]	[illegible]	[illegible]	[illegible]	[illegible]	[illegible]	[illegible]	[illegible]	[illegible]	[illegible]	[illegible]	[illegible]	[illegible]	2·5 (1·6 to 3·8)	5,310
Neck pain	0 (0 to 0)	0 (0 to 0)	[illegible]	[illegible]	[illegible]	[illegible]	[illegible]	[illegible]	[illegible]	[illegible]	[illegible]	[illegible]	[illegible]	[illegible]	[illegible]	[illegible]	[illegible]	[illegible]	[illegible]	[illegible]	[illegible]	[illegible]	0·4 (0·2 to 0·8)	988
Gout	0 (0 to 0)	0 (0 to 0)	[illegible]	[illegible]	[illegible]	[illegible]	[illegible]	[illegible]	[illegible]	[illegible]	[illegible]	[illegible]	[illegible]	[illegible]	[illegible]	[illegible]	[illegible]	[illegible]	[illegible]	[illegible]	[illegible]	[illegible]	0·1 (0·1 to 0·2)	51·3
Other musculoskeletal disorders	0 (0 to 0)	0 (0 to 0)	[illegible]	[illegible]	[illegible]	[illegible]	[illegible]	[illegible]	[illegible]	[illegible]	[illegible]	[illegible]	[illegible]	[illegible]	[illegible]	[illegible]	[illegible]	[illegible]	[illegible]	[illegible]	[illegible]	[illegible]	0·6 (0·3 to 0·9)	3,090
Other non-communicable diseases	1,520 (973 to 2,290)	352 (212 to 579)	[illegible]	[illegible]	[illegible]	[illegible]	[illegible]	[illegible]	[illegible]	[illegible]	[illegible]	[illegible]	[illegible]	[illegible]	[illegible]	[illegible]	[illegible]	[illegible]	[illegible]	[illegible]	[illegible]	[illegible]	10·5 (8·2 to 12·9)	16,500
Congenital birth defects	1,470 (934 to 2,230)	312 (181 to 528)	[illegible]	[illegible]	[illegible]	[illegible]	[illegible]	[illegible]	[illegible]	[illegible]	[illegible]	[illegible]	[illegible]	[illegible]	[illegible]	[illegible]	[illegible]	[illegible]	[illegible]	[illegible]	[illegible]	[illegible]	0 (0 to 0)	4,540
Neural tube defects	102 (56·6 to 168)	12·8 (6 to 25·6)	[illegible]	[illegible]	[illegible]	[illegible]	[illegible]	[illegible]	[illegible]	[illegible]	[illegible]	[illegible]	[illegible]	[illegible]	[illegible]	[illegible]	[illegible]	[illegible]	[illegible]	[illegible]	[illegible]	[illegible]	0 (0 to 0)	230
Congenital heart anomalies	602 (356 to 942)	159 (83·9 to 298)	[illegible]	[illegible]	[illegible]	[illegible]	[illegible]	[illegible]	[illegible]	[illegible]	[illegible]	[illegible]	[illegible]	[illegible]	[illegible]	[illegible]	[illegible]	[illegible]	[illegible]	[illegible]	[illegible]	[illegible]	0 (0 to 0)	2,000
Orofacial clefts	5·4 (2·5 to 10·4)	1·2 (0·4 to 2·9)	[illegible]	[illegible]	[illegible]	[illegible]	[illegible]	[illegible]	[illegible]	[illegible]	[illegible]	[illegible]	[illegible]	[illegible]	[illegible]	[illegible]	[illegible]	[illegible]	[illegible]	[illegible]	[illegible]	[illegible]	0 (0 to 0)	34·6
Down syndrome	5 (2·7 to 8·6)	1·8 (0·7 to 3·2)	[illegible]	[illegible]	[illegible]	[illegible]	[illegible]	[illegible]	[illegible]	[illegible]	[illegible]	[illegible]	[illegible]	[illegible]	[illegible]	[illegible]	[illegible]	[illegible]	[illegible]	[illegible]	[illegible]	[illegible]	0 (0 to 0)	85·1
Turner syndrome	0 (0 to 0)	0 (0 to 0)	[illegible]	[illegible]	[illegible]	[illegible]	[illegible]	[illegible]	[illegible]	[illegible]	[illegible]	[illegible]	[illegible]	[illegible]	[illegible]	[illegible]	[illegible]	[illegible]	[illegible]	[illegible]	[illegible]	[illegible]	0 (0 to 0)	1·2
Klinefelter syndrome	0 (0 to 0)	0 (0 to 0)	[illegible]	[illegible]	[illegible]	[illegible]	[illegible]	[illegible]	[illegible]	[illegible]	[illegible]	[illegible]	[illegible]	[illegible]	[illegible]	[illegible]	[illegible]	[illegible]	[illegible]	[illegible]	[illegible]	[illegible]	0 (0 to 0)	0·1
Other chromosomal abnormalities	11·1 (5·6 to 18·5)	5·6 (2·2 to 10·1)	[illegible]	[illegible]	[illegible]	[illegible]	[illegible]	[illegible]	[illegible]	[illegible]	[illegible]	[illegible]	[illegible]	[illegible]	[illegible]	[illegible]	[illegible]	[illegible]	[illegible]	[illegible]	[illegible]	[illegible]	0 (0 to 0)	70·5
Congenital musculoskeletal and limb anomalies	23·2 (13·3 to 37·8)	6·2 (3·9 to 9·9)	[illegible]	[illegible]	[illegible]	[illegible]	[illegible]	[illegible]	[illegible]	[illegible]	[illegible]	[illegible]	[illegible]	[illegible]	[illegible]	[illegible]	[illegible]	[illegible]	[illegible]	[illegible]	[illegible]	[illegible]	0 (0 to 0)	423
Urogenital congenital anomalies	67·4 (34·7 to 114)	13·4 (6·1 to 32·2)	[illegible]	[illegible]	[illegible]	[illegible]	[illegible]	[illegible]	[illegible]	[illegible]	[illegible]	[illegible]	[illegible]	[illegible]	[illegible]	[illegible]	[illegible]	[illegible]	[illegible]	[illegible]	[illegible]	[illegible]	0 (0 to 0)	145
Digestive congenital anomalies	269 (159 to 435)	59·3 (30·7 to 113)	[illegible]	[illegible]	[illegible]	[illegible]	[illegible]	[illegible]	[illegible]	[illegible]	[illegible]	[illegible]	[illegible]	[illegible]	[illegible]	[illegible]	[illegible]	[illegible]	[illegible]	[illegible]	[illegible]	[illegible]	0 (0 to 0)	529
Other congenital birth defects	381 (222 to 604)	52·8 (29 to 89·1)	[illegible]	[illegible]	[illegible]	[illegible]	[illegible]	[illegible]	[illegible]	[illegible]	[illegible]	[illegible]	[illegible]	[illegible]	[illegible]	[illegible]	[illegible]	[illegible]	[illegible]	[illegible]	[illegible]	[illegible]	0 (0 to 0)	1,020
Urinary diseases and male infertility	3·1 (1·6 to 4·8)	2·9 (1·4 to 5·3)	[illegible]	[illegible]	[illegible]	[illegible]	[illegible]	[illegible]	[illegible]	[illegible]	[illegible]	[illegible]	[illegible]	[illegible]	[illegible]	[illegible]	[illegible]	[illegible]	[illegible]	[illegible]	[illegible]	[illegible]	3·5 (2·5 to 4·8)	1,150
Urolithiasis	0 (0 to 0)	0 (0 to 0)	[illegible]	[illegible]	[illegible]	[illegible]	[illegible]	[illegible]	[illegible]	[illegible]	[illegible]	[illegible]	[illegible]	[illegible]	[illegible]	[illegible]	[illegible]	[illegible]	[illegible]	[illegible]	[illegible]	[illegible]	0 (0 to 0·1)	84

Benign prostatic hyperplasia	0 (0 to 0)	0 (0 to 0)	0 (0 to 0)	0 (0 to 0)	0 (0 to 0)	0 (0 to 0)	0 (0 to 0)	0 (0 to 0)	0 (0 to 0)	0 (0 to 0)	0 (0 to 0)	0·2 (0 to 0·6)	1·7 (0·6 to 3·6)	6·6 (3·2 to 11·8)	19·8 (9·4 to 34·8)	32·1 (16·6 to 54·8)	38·4 (21·5 to 61·5)	28·9 (16 to 46·3)	18·2 (10·1 to 29·4)	8·6 (4·9 to 13·8)	2·8 (1·6 to 4·6)	0·8 (0·4 to 1·3)	0·1 (0·1 to 0·2)	158
Male infertility	0 (0 to 0)	0 (0 to 0)	0 (0 to 0)	0 (0 to 0)	0 (0 to 0)	0 (0 to 0)	0 (0 to 0)	9·4 (2·5 to 24·9)	10·1 (2·7 to 24·7)	8·5 (2·3 to 22·7)	7·8 (2·2 to 19·9)	7 (2 to 18·3)	1 (0·2 to 2·8)	0 (0 to 0)	0 (0 to 0)	0 (0 to 0)	0 (0 to 0)	0 (0 to 0)	0 (0 to 0)	0 (0 to 0)	0 (0 to 0)	0 (0 to 0)	0 (0 to 0)	43·8
Other urinary diseases	1·4 (0·7 to 2·2)	1·7 (0·8 to 3·2)	2·9 (1·4 to 5·3)	2·6 (1·6 to 3·9)	2·4 (1·5 to 3·8)	4·1 (2·6 to 6·1)	4·2 (2·8 to 6·3)	9·9 (5·8 to 15·4)	4·6 (2·8 to 7·1)	5·9 (3·5 to 9·3)	8·7 (5 to 14)	9 (5·3 to 14·9)	10·3 (5·6 to 17)	11 (5·7 to 18·1)	10·8 (5·6 to 18·6)	12·8 (6·8 to 21·2)	10·4 (5·3 to 18·4)	6 (3 to 10·2)	5 (2·2 to 9·5)	3·1 (1·5 to 5·7)	1·3 (0·6 to 2·5)	0·7 (0·3 to 1·4)	0·2 (0·1 to 0·4)	129
Gynecological diseases	0 (0 to 0)	0 (0 to 0)	0 (0 to 0)	0 (0 to 0)	0 (0 to 0)	49·7 (24·5 to 87·2)	168 (99·8 to 273)	325 (205 to 497)	426 (273 to 629)	402 (263 to 580)	411 (281 to 587)	408 (272 to 591)	307 (203 to 431)	196 (118 to 292)	114 (71·5 to 164)	69·9 (41·7 to 106)	47·2 (29·3 to 69·6)	25·8 (16·4 to 37·3)	15·5 (9·8 to 22·6)	8·7 (5·4 to 13·2)	4·2 (2·6 to 6·1)	1·8 (1·1 to 2·8)	0·4 (0·2 to 0·6)	2,980
Uterine fibroids	0 (0 to 0)	0 (0 to 0)	0 (0 to 0)	0 (0 to 0)	0 (0 to 0)	0·8 (0·3 to 1·8)	2·4 (1·1 to 4·7)	6·2 (2·8 to 12·7)	13·3 (6 to 27)	22·3 (10·7 to 43·7)	37·6 (19·1 to 70·4)	43·4 (23·6 to 77·5)	36·7 (19·9 to 63·1)	23 (11·8 to 40·7)	14·8 (7·6 to 26·2)	7·8 (3·7 to 14·7)	4·2 (2 to 7·9)	1·8 (0·9 to 3·5)	0·9 (0·5 to 1·7)	0·4 (0·2 to 0·8)	0·1 (0·1 to 0·3)	0·1 (0 to 0·1)	0 (0 to 0)	216
Polycystic ovarian syndrome	0 (0 to 0)	0 (0 to 0)	0 (0 to 0)	0 (0 to 0)	0 (0 to 0)	1·6 (0·6 to 3·5)	8 (3·2 to 16·7)	10·4 (4·3 to 21·7)	8·6 (3·4 to 17·5)	6·3 (2·7 to 12·9)	5·7 (2·4 to 11·8)	5·4 (2·3 to 11·1)	4·2 (1·8 to 8·4)	1 (0·4 to 2·1)	0 (0 to 0)	0 (0 to 0)	0 (0 to 0)	0 (0 to 0)	0 (0 to 0)	0 (0 to 0)	0 (0 to 0)	0 (0 to 0)	0 (0 to 0)	51·3
Female infertility	0 (0 to 0)	0 (0 to 0)	0 (0 to 0)	0 (0 to 0)	0 (0 to 0)	0 (0 to 0)	0 (0 to 0)	15 (3·6 to 39·5)	19·8 (5·1 to 54)	19·5 (4·5 to 57·4)	18·8 (3·1 to 55·7)	16·7 (2·8 to 45)	0·3 (0 to 2·6)	0 (0 to 0)	0 (0 to 0)	0 (0 to 0)	0 (0 to 0)	0 (0 to 0)	0 (0 to 0)	0 (0 to 0)	0 (0 to 0)	0 (0 to 0)	0 (0 to 0)	90·1
Endometriosis	0 (0 to 0)	0 (0 to 0)	0 (0 to 0)	0 (0 to 0)	0 (0 to 0)	0 (0 to 0)	11·1 (4·5 to 22)	51·3 (24·3 to 93·5)	52·6 (25 to 95·4)	33·4 (16·2 to 60·1)	27·3 (14·1 to 47·7)	26·7 (13·2 to 46·4)	21 (11 to 34·9)	9·5 (4·7 to 16·1)	0 (0 to 0)	0 (0 to 0)	0 (0 to 0)	0 (0 to 0)	0 (0 to 0)	0 (0 to 0)	0 (0 to 0)	0 (0 to 0)	0 (0 to 0)	233
Genital prolapse	0 (0 to 0)	0 (0 to 0)	0 (0 to 0)	0 (0 to 0)	0 (0 to 0)	0 (0 to 0)	0 (0 to 0·1)	0·4 (0·1 to 0·9)	1 (0·3 to 2·1)	1·6 (0·7 to 3·4)	3 (1·3 to 5·9)	4·5 (1·9 to 9·5)	5·6 (2·6 to 11·7)	6·1 (2·7 to 12·1)	6·4 (3 to 12·2)	5·2 (2·5 to 9·9)	4·2 (2·1 to 7·7)	2·5 (1·3 to 4·6)	1·7 (0·8 to 3)	1 (0·5 to 1·9)	0·5 (0·3 to 1)	0·2 (0·1 to 0·4)	0·1 (0 to 0·1)	44·1
Premenstrual syndrome	0 (0 to 0)	0 (0 to 0)	0 (0 to 0)	0 (0 to 0)	0 (0 to 0)	44·3 (20·3 to 79·9)	104 (53 to 183)	144 (70·3 to 256)	129 (74·8 to 209)	103 (54·7 to 169)	108 (61·4 to 171)	106 (59·6 to 167)	67 (36·8 to 111)	21·7 (10·8 to 38·9)	0 (0 to 0)	0 (0 to 0)	0 (0 to 0)	0 (0 to 0)	0 (0 to 0)	0 (0 to 0)	0 (0 to 0)	0 (0 to 0)	0 (0 to 0)	827
Other gynecological diseases	0 (0 to 0)	0 (0 to 0)	0 (0 to 0)	0 (0 to 0)	0 (0 to 0)	2·9 (1·1 to 6)	42 (19·5 to 71·3)	97·8 (56·9 to 153)	201 (115 to 315)	216 (127 to 327)	211 (127 to 329)	205 (118 to 331)	172 (99·1 to 267)	135 (67 to 219)	93·2 (53·9 to 140)	56·9 (31 to 91)	38·7 (22·7 to 59·8)	21·4 (13 to 32)	12·9 (7·4 to 19·7)	7·3 (4·2 to 11·6)	3·5 (2·1 to 5·4)	1·5 (0·9 to 2·4)	0·3 (0·2 to 0·6)	1,520
Hemoglobinopathies and hemolytic anemias	6 (3·9 to 9·1)	5·2 (3·3 to 7·7)	48·5 (32·1 to 70·9)	106 (73·4 to 149)	175 (131 to 226)	133 (99·5 to 179)	174 (125 to 245)	263 (186 to 349)	217 (152 to 303)	145 (100 to 204)	111 (82·1 to 147)	110 (80·6 to 146)	91·1 (67·5 to 119)	85 (62·8 to 112)	72·8 (55·5 to 96)	62·4 (44·8 to 82·8)	43·2 (31·8 to 58·4)	25·6 (18·6 to 33·5)	14·4 (10·8 to 19·1)	8·1 (6 to 10·9)	6 (4·5 to 7·7)	3 (2·2 to 3·9)	0·7 (0·5 to 1)	1,910
Thalassemias	0 (0 to 0)	0 (0 to 0)	0·1 (0 to 0·2)	0·5 (0·2 to 1·1)	0·3 (0·1 to 0·5)	0·1 (0 to 0·2)	0·1 (0 to 0·1)	0 (0 to 0·1)	0 (0 to 0·1)	0 (0 to 0)	0 (0 to 0)	0 (0 to 0)	0 (0 to 0)	0 (0 to 0)	0 (0 to 0)	0 (0 to 0)	0 (0 to 0)	0 (0 to 0)	0 (0 to 0)	0 (0 to 0)	0 (0 to 0)	0 (0 to 0)	0 (0 to 0)	1·1
Thalassemias trait	0 (0 to 0)	0·1 (0 to 0·2)	0·7 (0·4 to 1·2)	2·4 (1·1 to 4·2)	4·3 (2·1 to 7·4)	2·4 (1 to 4·7)	5 (2·5 to 8·3)	5·3 (2·8 to 8·4)	4·6 (2·5 to 7·5)	3·9 (2 to 6·3)	3·6 (1·9 to 5·9)	3·2 (1·6 to 5·3)	0·8 (0·4 to 1·6)	1·4 (0·7 to 2·3)	1·3 (0·7 to 2·2)	1 (0·5 to 1·7)	0·7 (0·3 to 1·2)	0·4 (0·2 to 0·7)	0·2 (0·1 to 0·4)	0·1 (0·1 to 0·3)	0·1 (0 to 0·1)	0 (0 to 0·1)	0 (0 to 0)	41·7
Sickle cell disorders	5·8 (3·7 to 8·8)	2·5 (1·4 to 4·5)	36·2 (21·5 to 57·7)	65·5 (40·3 to 100)	101 (79 to 132)	89·3 (65·3 to 125)	113 (76·9 to 157)	136 (87·6 to 198)	106 (67·8 to 158)	64·7 (41·4 to 95·4)	55 (34·2 to 82·1)	45·8 (28·7 to 68·8)	34·4 (21·9 to 52·3)	22·6 (12·8 to 36·6)	9·8 (5·2 to 16·5)	2·4 (1·1 to 4·4)	0·4 (0·1 to 0·8)	0 (0 to 0·1)	0 (0 to 0)	0 (0 to 0)	0 (0 to 0)	0 (0 to 0)	0 (0 to 0)	889
Sickle cell trait	0·2 (0·1 to 0·3)	1·9 (1 to 3·2)	7 (3·9 to 11·2)	22·8 (12·9 to 36·3)	40·8 (21·3 to 64·8)	23·1 (11·6 to 41·1)	30·5 (8·6 to 70·4)	83·4 (39·9 to 137)	68·5 (29·8 to 121)	41·7 (13 to 80·5)	4·3 (1·7 to 8·6)	11·3 (6·5 to 17·5)	12·2 (6·7 to 19)	13 (7·6 to 20·6)	12·4 (7 to 19·3)	9·1 (5 to 14·4)	6·8 (3·8 to 10·6)	3·9 (2·3 to 6)	2·2 (1·2 to 3·8)	1·3 (0·7 to 2·2)	0·7 (0·4 to 1·2)	0·3 (0·2 to 0·5)	0 (0 to 0·1)	397
G6PD deficiency	0 (0 to 0)	0·1 (0·1 to 0·3)	0·4 (0·2 to 0·8)	2·8 (1·3 to 5·7)	5·5 (2·5 to 9·9)	5·6 (2·5 to 10·4)	9·8 (4·2 to 17·8)	21·1 (8·8 to 39·6)	22·7 (10·2 to 39·9)	20·3 (9·2 to 35·6)	28·2 (16·9 to 41·5)	25·3 (14 to 39·7)	20·8 (11·6 to 31·3)	19·1 (11 to 29)	17·2 (10·2 to 25·2)	11·8 (7·2 to 17·2)	6 (3·6 to 9·3)	2·8 (1·5 to 4·3)	1·1 (0·6 to 1·7)	0·4 (0·2 to 0·6)	0·2 (0·1 to 0·2)	0 (0 to 0·1)	0 (0 to 0)	221
G6PD trait	0 (0 to 0)	0 (0 to 0)	0 (0 to 0)	0 (0 to 0)	0 (0 to 0)	0 (0 to 0)	0 (0 to 0)	0 (0 to 0)	0 (0 to 0)	0 (0 to 0)	0·8 (0·3 to 1·4)	0·8 (0·4 to 1·4)	0·2 (0·1 to 0·5)	0 (0 to 0)	0 (0 to 0)	0 (0 to 0)	0 (0 to 0)	0 (0 to 0)	0 (0 to 0)	0 (0 to 0)	0 (0 to 0)	0 (0 to 0)	0 (0 to 0)	1·9
Other hemoglobinopathies and hemolytic anemias	0 (0 to 0)	0·5 (0·2 to 0·8)	4·1 (2·1 to 6·9)	12 (6·3 to 20·3)	22·4 (10·5 to 37·1)	12·7 (5·8 to 25·1)	16·5 (8·2 to 28)	17·2 (9·4 to 28·6)	16 (9·1 to 25·9)	14·8 (8·8 to 23·6)	18·9 (11·8 to 28·4)	23·5 (14·9 to 34·9)	22·6 (14·7 to 32·5)	28·8 (19 to 42·2)	32·1 (22·2 to 44·9)	38·1 (26 to 52·8)	29·3 (20·6 to 40·9)	18·5 (13·2 to 25)	10·8 (8·1 to 14·4)	6·2 (4·7 to 8·3)	5 (3·7 to 6·4)	2·6 (2 to 3·4)	0·7 (0·5 to 0·9)	354
Endocrine, metabolic, blood, and immune disorders	45·4 (25·2 to 74·9)	26·4 (14·3 to 52·5)	350 (206 to 589)	204 (129 to 312)	133 (98·2 to 179)	99·9 (74·2 to 137)	117 (85·8 to 159)	164 (118 to 222)	207 (146 to 289)	211 (150 to 293)	231 (164 to 315)	257 (183 to 354)	256 (181 to 356)	271 (190 to 375)	282 (204 to 380)	241 (173 to 335)	189 (140 to 273)	124 (88·6 to 171)	84·6 (59·5 to 115)	50·5 (35·7 to 70·7)	27·6 (20·3 to 39·8)	13·5 (9·7 to 19·9)	3·5 (2·4 to 5·1)	3,590
Oral disorders	0 (0 to 0)	0 (0 to 0)	0 (0 to 0)	34·6 (19·7 to 57·4)	40·4 (21·7 to 69·5)	43 (22·7 to 74)	65·9 (36·4 to 114)	115 (61·2 to 212)	143 (73·1 to 278)	142 (68·1 to 272)	159 (75·1 to 296)	181 (87·7 to 335)	189 (99·5 to 338)	203 (111 to 355)	221 (128 to 351)	220 (134 to 346)	197 (122 to 304)	133 (83·2 to 202)	92·5 (57·9 to 137)	55·3 (35·6 to 81)	25·1 (15·8 to 36·6)	10·3 (6·4 to 14·9)	2·3 (1·4 to 3·4)	2,270
Caries of deciduous teeth	0 (0 to 0)	0 (0 to 0)	0 (0 to 0)	11·5 (4·4 to 23·4)	10·5 (4·3 to 22·8)	0·6 (0·1 to 1·5)	0 (0 to 0)	0 (0 to 0)	0 (0 to 0)	0 (0 to 0)	0 (0 to 0)	0 (0 to 0)	0 (0 to 0)	0 (0 to 0)	0 (0 to 0)	0 (0 to 0)	0 (0 to 0)	0 (0 to 0)	0 (0 to 0)	0 (0 to 0)	0 (0 to 0)	0 (0 to 0)	0 (0 to 0)	22·6
Caries of permanent teeth	0 (0 to 0)	0 (0 to 0)	0 (0 to 0)	0 (0 to 0)	9·6 (3·3 to 21·4)	20·7 (8·3 to 44·5)	23·5 (9·2 to 50·3)	29·6 (12·4 to 60·7)	24·9 (10 to 49·2)	17·8 (7·2 to 37·4)	16·5 (6·8 to 34·5)	15·4 (6·3 to 31·5)	13·3 (5·4 to 27·5)	11·7 (4·7 to 24·3)	9·5 (3·6 to 21·1)	6·7 (2·5 to 14·3)	4·4 (1·7 to 9)	2·4 (1 to 4·9)	1·5 (0·6 to 2·9)	0·8 (0·3 to 1·7)	0·3 (0·1 to 0·7)	0·1 (0 to 0·3)	0 (0 to 0·1)	209
Periodontal diseases	0 (0 to 0)	0 (0 to 0)	0 (0 to 0)	0 (0 to 0)	0 (0 to 0)	0 (0 to 0)	8·4 (2·4 to 22·1)	34·9 (10·5 to 86)	66·2 (20·3 to 155)	77·5 (26·2 to 177)	92·4 (32·8 to 211)	106 (38·7 to 233)	104 (39·3 to 225)	94·4 (37·2 to 207)	78 (31·1 to 162)	54·6 (21·7 to 112)	35·5 (14 to 73·3)	18·9 (7·4 to 38·6)	11·4 (4·5 to 23·2)	6·4 (2·5 to 13·1)	2·8 (1·1 to 5·8)	1·1 (0·4 to 2·4)	0·2 (0·1 to 0·5)	793
Other oral disorders	0 (0 to 0)	0 (0 to 0)	0 (0 to 0)	23·1 (13·7 to 35·4)	20·3 (12 to 31·7)	21·7 (12·8 to 34·1)	33·9 (20·3 to 52·1)	45·8 (27·9 to 70·9)	42·8 (25·5 to 64)	34·2 (20·8 to 51·5)	32·2 (19·4 to 49·1)	32·4 (19·3 to 48·5)	30 (18·3 to 45)	27 (16·4 to 40·2)	23·3 (14 to 34·9)	18 (11 to 26·9)	13·1 (8·1 to 19·7)	7 (4·2 to 10·6)	3·7 (2·3 to 5·6)	1·7 (1 to 2·6)	0·6 (0·4 to 0·9)	0·2 (0·1 to 0·3)	0 (0 to 0·1)	411
Sudden infant death syndrome	–	5·4 (3·1 to 8·7)	56·6 (31·4 to 92)	–	–	–	–	–	–	–	–	–	–	–	–	–	–	–	–	–	–	–	–	NA
Injuries	173 (114 to 250)	47·4 (30·4 to 72·5)	594 (346 to 970)	881 (617 to 1,200)	1,150 (987 to 1,340)	1,540 (1,260 to 1,860)	3,980 (3,130 to 4,930)	7,900 (6,280 to 9,740)	7,080 (5,830 to 8,580)	5,260 (4,230 to 6,440)	4,370 (3,450 to 5,430)	3,980 (3,090 to 5,010)	3,290 (2,540 to 4,250)	2,580 (1,990 to 3,280)	2,020 (1,580 to 2,540)	1,440 (1,150 to 1,750)	982 (807 to 1,180)	616 (512 to 738)	418 (350 to 494)	277 (232 to 323)	155 (129 to 181)	89·2 (75·5 to 103)	28·3 (24·2 to 32)	48,900
Transport injuries	6·5 (4·2 to 9·9)	2·1 (1·3 to 3·4)	33·7 (20·9 to 52)	123 (77·2 to 185)	277 (207 to 363)	367 (268 to 490)	905 (642 to 1,220)	1,800 (1,280 to 2,370)	1,540 (1,140 to 1,990)	1,060 (758 to 1,410)	852 (616 to 1,150)	795 (566 to 1,060)	714 (520 to 953)	590 (423 to 796)	476 (349 to 629)	303 (228 to 395)	190 (142 to 242)	100 (77·2 to 127)	57·7 (44·8 to 71·6)	28·3 (22·8 to 34·1)	11·7 (9·4 to 14·4)	4·6 (3·7 to 5·6)	1·2 (1 to 1·5)	10,200
Road injuries	4·5 (2·8 to 7)	1·2 (0·7 to 2)	24·5 (14·3 to 39·4)	103 (61·2 to 161)	244 (178 to 324)	315 (229 to 425)	809 (560 to 1,100)	1,610 (1,140 to 2,160)	1,370 (1,000 to 1,800)	923 (654 to 1,260)	751 (535 to 1,030)	689 (488 to 935)	637 (457 to 858)	526 (373 to 710)	415 (301 to 555)	272 (203 to 359)	172 (130 to 220)	88 (67·6 to 111)	51·4 (39·9 to 64·1)	25·6 (20·5 to 31·1)	10·6 (8·5 to 13·2)	4·1 (3·3 to 5)	1 (0·8 to 1·3)	9,050
Pedestrian road injuries	2 (1·3 to 3·2)	0·4 (0·2 to 0·6)	6·8 (3·9 to 11·1)	43·5 (25·7 to 67·7)	103 (72·3 to 138)	102 (69·7 to 143)	98·7 (68·6 to 136)	162 (115 to 217)	159 (115 to 210)	135 (95·7 to 185)	124 (86·8 to 171)	132 (91·6 to 183)	134 (94·9 to 186)	126 (87·5 to 174)	112 (77·8 to 151)	78·5 (57·2 to 106)	56·6 (40·9 to 75·5)	32·1 (24 to 42)	20·6 (15·6 to 26·4)	10·9 (8·7 to 13·6)	4·5 (3·6 to 5·7)	1·6 (1·3 to 2)	0·4 (0·3 to 0·5)	1,650
Cyclist road injuries	0 (0 to 0)	0 (0 to 0)	0 (0 to 0)	6·2 (2·9 to 11·7)	17·3 (9·5 to 30)	51·2 (28·6 to 87·8)	95·7 (53·9 to 159)	137 (81 to 220)	114 (71·2 to 172)	89·1 (55·7 to 134)	77 (50·6 to 115)	76 (51·3 to 117)	68·4 (46·8 to 99·9)	64·1 (43·6 to 93·2)	49·6 (34·1 to 72·9)	34·5 (23·8 to 49·5)	23·2 (16·3 to 32)	11·3 (8·2 to 15·5)	6·3 (4·6 to 8·4)	3·2 (2·3 to 4·3)	1·3 (1 to 1·8)	0·5 (0·4 to 0·7)	0·1 (0·1 to 0·2)	927
Motorcyclist road injuries	0·4 (0·2 to 0·6)	0·1 (0·1 to 0·2)	1·9 (1·1 to 3)	4·8 (2·8 to 7·3)	13·5 (9·1 to 18·4)	20·5 (14·2 to 28·4)	146 (97·4 to 205)	392 (269 to 535)	335 (245 to 437)	204 (146 to 274)	166 (118 to 226)	135 (97·1 to 183)	115 (84·5 to 154)	82·9 (60·3 to 111)	60 (44·7 to 79)	32·6 (25·4 to 41·4)	20·5 (15·7 to 26·3)	9·6 (7 to 12·4)	5·8 (4·2 to 7·7)	3·3 (2·3 to 4·4)	1·5 (1 to 2)	0·6 (0·4 to 0·8)	0·2 (0·1 to 0·2)	1,750
Motor vehicle road injuries	2 (1·2 to 3·1)	0·7 (0·4 to 1·2)	15·4 (8·9 to 25·3)	46·9 (27·6 to 76)	107 (78·9 to 143)	139 (97·2 to 190)	463 (318 to 644)	910 (631 to 1,240)	752 (530 to 995)	487 (332 to 679)	377 (259 to 524)	338 (229 to 467)	311 (215 to 430)	246 (169 to 341)	189 (131 to 262)	122 (85·3 to 166)	69 (49·2 to 91·8)	33·4 (24·9 to 43)	17·6 (13·4 to 22·6)	7·7 (6 to 9·4)	3 (2·4 to 3·8)	1·2 (1 to 1·5)	0·3 (0·3 to 0·4)	4,640
Other road injuries	0·1 (0 to 0·2)	0 (0 to 0)	0·4 (0·2 to 0·7)	1·6 (0·7 to 3·1)	3 (1·4 to 5·9)	2·9 (1·8 to 4·8)	5·5 (3·5 to 8·2)	11 (6·7 to 18·2)	11 (7 to 16·9)	8·1 (5·4 to 12)	7·8 (5·2 to 11)	7·7 (5·5 to 10·8)	7·6 (5·4 to 10·2)	6·6 (4·8 to 9)	5·7 (4·1 to 7·5)	4·2 (3 to 5·8)	3·2 (2·3 to 4·2)	1·7 (1·2 to 2·2)	1·1 (0·8 to 1·5)	0·6 (0·4 to 0·8)	0·2 (0·2 to 0·3)	0·1 (0·1 to 0·1)	0 (0 to 0)	90
Other transport injuries	2 (1·3 to 3·1)	0·9 (0·5 to 1·4)	9·2 (5·7 to 14)	20·3 (13·1 to 30·4)	33·2 (24·6 to 43·4)	51·5 (35·3 to 73)	95·5 (61·9 to 140)	184 (114 to 281)	165 (107 to 231)	137 (87·7 to 203)	101 (67·4 to 146)	106 (70·9 to 150)	77·8 (51·4 to 112)	64·6 (44·2 to 92·8)	60·8 (41·8 to 84·8)	31·6 (22·5 to 42·8)	17·4 (12·5 to 23·8)	12·4 (9·1 to 17)	6·3 (4·7 to 8·3)	2·6 (2·1 to 3·2)	1·1 (0·9 to 1·3)	0·5 (0·4 to 0·6)	0·2 (0·1 to 0·2)	1,180
Unintentional injuries	112 (71·4 to 168)	40·6 (25·2 to 63·6)	501 (275 to 854)	656 (440 to 926)	766 (631 to 924)	860 (680 to 1,090)	1,150 (863 to 1,510)	1,780 (1,330 to 2,360)	1,620 (1,260 to 2,030)	1,270 (1,000 to 1,600)	1,150 (902 to 1,420)	1,150 (897 to 1,450)	1,010 (792 to 1,260)	911 (723 to 1,140)	792 (637 to 980)	646 (524 to 787)	494 (405 to 600)	357 (293 to 430)	277 (230 to 330)	209 (175 to 246)	128 (106 to 151)	79·8 (67·2 to 92)	26 (22·1 to 29·5)	16,000
Falls	3·4 (2·2 to 5)	1·1 (0·7 to 1·7)	25·7 (16·4 to 39·9)	47·8 (32·8 to 70·3)	63·1 (50·5 to 79·4)	90·5 (69·5 to 116)	149 (109 to 202)	245 (184 to 322)	239 (184 to 304)	210 (164 to 272)	222 (170 to 282)	241 (185 to 305)	245 (187 to 314)	264 (201 to 340)	248 (190 to 310)	232 (183 to 288)	196 (157 to 240)	169 (136 to 207)	144 (116 to 176)	124 (103 to 148)	83 (67·9 to 99·6)	50·9 (41·8 to 59·3)	16·5 (13·9 to 18·9)	3,310
Drowning	6·6 (4·2 to 9·7)	1·5 (0·9 to 2·2)	38 (23 to 57·9)	205 (120 to 318)	286 (217 to 362)	319 (232 to 425)	419 (277 to 596)	593 (407 to 838)	459 (317 to 631)	333 (240 to 448)	262 (187 to 349)	241 (166 to 332)	169 (115 to 238)	128 (88·9 to 177)	86·5 (62·8 to 119)	51·7 (38 to 67·4)	27·7 (20·4 to 36·8)	12 (9·2 to 15·4)	6·5 (4·9 to 8·5)	2·4 (1·8 to 3·1)	1·2 (0·9 to 1·5)	0·7 (0·5 to 0·9)	0·2 (0·2 to 0·3)	3,650
Fire, heat, and hot substances	2·8 (1·8 to 4)	1·5 (1 to 2·4)	33·5 (20·6 to 51·9)	87·2 (57·2 to 125)	92·2 (73·7 to 113)	90·2 (69·2 to 115)	113 (85·8 to 143)	163 (125 to 213)	161 (127 to 200)	136 (106 to 175)	126 (97·8 to 159)	123 (96·2 to 158)	107 (83·5 to 137)	92·3 (71·9 to 122)	78·4 (61 to 101)	59·6 (46·1 to 77·8)	40·1 (31 to 52·7)	25·7 (19·7 to 33·5)	17·1 (13·3 to 22·3)	10·8 (8·4 to 13·8)	6 (4·7 to 7·5)	3·3 (2·6 to 3·9)	1 (0·8 to 1·2)	1,570
Poisonings	0·7 (0·4 to 1·1)	0·4 (0·2 to 0·7)	4·8 (2·7 to 7·9)	14·9 (9·8 to 21·5)	9 (6·5 to 11·9)	7·7 (5·6 to 10·5)	10·4 (7·5 to 14·1)	16·6 (12·2 to 21·5)	17·4 (12·2 to 23·8)	14·4 (10·3 to 19·6)	12·1 (8·9 to 16·2)	10 (7·4 to 13·2)	7·9 (5·7 to 10·6)	6·2 (4·5 to 8·2)	5·2 (3·9 to 6·7)	3·6 (2·8 to 4·8)	2·7 (2·1 to 3·4)	1·6 (1·2 to 2·1)	1 (0·8 to 1·4)	0·6 (0·5 to 0·8)	0·3 (0·2 to 0·4)	0·1 (0·1 to 0·2)	0 (0 to 0)	148
Poisoning by carbon monoxide	0·3 (0·2 to 0·5)	0·1 (0 to 0·2)	0·6 (0·2 to 1·2)	4·2 (2·2 to 7·5)	2·5 (1·5 to 3·8)	2·3 (1·5 to 3·5)	4 (2·5 to 6·1)	7·4 (4·9 to 10·5)	7·7 (4·3 to 12)	7 (4 to 11·1)	5·5 (3·3 to 8·5)	4·4 (2·8 to 6·6)	3·2 (1·9 to 5·1)	2 (1·2 to 3)	2·1 (1·4 to 3·1)	1·4 (1 to 1·9)	1·1 (0·8 to 1·5)	0·6 (0·4 to 0·8)	0·4 (0·3 to 0·6)	0·2 (0·2 to 0·3)	0·1 (0·1 to 0·1)	0 (0 to 0)	0 (0 to 0)	57·2
Poisoning by other means	0·4 (0·2 to 0·6)	0·3 (0·2 to 0·5)	4·2 (2·4 to 6·9)	10·7 (6·9 to 15·4)	6·4 (4·7 to 8·5)	5·4 (3·7 to 7·5)	6·4 (4·5 to 9·1)	9·2 (6·6 to 12·3)	9·7 (7·2 to 12·7)	7·4 (5·3 to 9·9)	6·6 (4·9 to 8·7)	5·6 (4 to 7·3)	4·8 (3·4 to 6·2)	4·3 (3·1 to 5·6)	3·1 (2·2 to 4·1)	2·2 (1·6 to 3·1)	1·6 (1·1 to 2·1)	1 (0·7 to 1·3)	0·7 (0·5 to 0·9)	0·4 (0·3 to 0·5)	0·2 (0·1 to 0·3)	0·1 (0·1 to 0·1)	0 (0 to 0)	90·6
Exposure to mechanical forces	1·5 (1 to 2·3)	0·9 (0·6 to 1·3)	8·9 (5·9 to 13·2)	38·8 (28·2 to 53)	62·5 (48·1 to 80·3)	73·3 (56·1 to 95·4)	137 (96·8 to 191)	243 (170 to 342)	212 (152 to 286)	169 (124 to 225)	147 (111 to 196)	148 (110 to 190)	125 (93·7 to 165)	110 (82·3 to 147)	92·9 (69·3 to 123)	67·2 (49·4 to 91)	46·6 (33·5 to 63·7)	27·4 (19·6 to 37·6)	17·7 (12·7 to 24·1)	10·7 (7·8 to 14·3)	5·3 (3·9 to 7·1)	2·9 (2·2 to 3·7)	0·9 (0·7 to 1·1)	1,750
Unintentional firearm injuries	0·2 (0·1 to 0·4)	0 (0 to 0·1)	0·7 (0·4 to 1·1)	2·5 (1·6 to 3·7)	4·6 (3·3 to 6·4)	9·8 (7 to 13·5)	35·4 (21·7 to 56·5)	77·9 (45·3 to 127)	57·6 (34·2 to 92)	40·9 (25·8 to 64·6)	31·4 (20·2 to 47)	28·2 (18·6 to 41·6)	20·2 (13·5 to 29·5)	14·3 (10 to 20·4)	11·1 (8·1 to 15·4)	7·2 (5·3 to 9·8)	4·6 (3·4 to 6)	2·6 (2 to 3·5)	1·7 (1·3 to 2·2)	1·2 (0·9 to 1·6)	0·7 (0·5 to 0·9)	0·5 (0·4 to 0·7)	0·2 (0·1 to 0·2)	354
Other exposure to mechanical forces	1·3 (0·8 to 2)	0·8 (0·5 to 1·2)	8·2 (5·5 to 12·2)	36·3 (26·3 to 49·7)	57·8 (44·2 to 74·7)	63·4 (48·5 to 83·7)	101 (72·1 to 141)	165 (114 to 231)	155 (112 to 212)	128 (92·1 to 172)	116 (85·3 to 157)	119 (88·5 to 159)	105 (77·5 to 140)	95·5 (70·8 to 129)	81·8 (60·3 to 110)	60 (43·5 to 82·3)	42 (29·7 to 57·9)	24·7 (17·4 to 34·2)	16 (11·4 to 21·9)	9·5 (6·8 to 12·9)	4·6 (3·3 to 6·2)	2·4 (1·8 to 3·1)	0·7 (0·5 to 0·9)	1,390
Adverse effects of medical treatment	21·8 (13 to 35)	11·3 (6·4 to 18·5)	54 (29·5 to 97·2)	31·8 (18·2 to 54·7)	24·8 (16·9 to 34·7)	21·4 (14·5 to 31·2)	25·9 (17 to 37·8)	61·5 (39·5 to 88·8)	59·5 (39·2 to 91·2)	48·1 (31 to 71·6)	48·8 (31·3 to 71)	54·3 (35·9 to 79·3)	54·6 (35·5 to 83·6)	60·2 (39·7 to 89·9)	60·5 (40·2 to 86·8)	56·1 (38·4 to 81·1)	46·3 (31·3 to 66)	32·4 (22·2 to 44·9)	25·3 (17·7 to 35·4)	16·2 (11·7 to 22·7)	8·8 (6·1 to 12·8)	4·4 (3 to 6·3)	1·2 (0·8 to 1·7)	829
Animal contact	1·7 (1·1 to 2·6)	1·6 (1 to 2·7)	14·5 (9 to 23·1)	31·7 (19·9 to 51·3)	35·1 (24·6 to 50·2)	36·4 (25·6 to 49·5)	37·9 (26·8 to 54·7)	49·3 (33·8 to 68·2)	67·9 (47·3 to 95·4)	40·6 (28·4 to 57·9)	51·4 (34·5 to 73·7)	52·5 (35·1 to 78·9)	55·9 (38·4 to 83·1)	50 (32·9 to 74·2)	49·8 (33·5 to 71·2)	45 (30·8 to 63·7)	35·8 (25·1 to 51·1)	24·9 (18·2 to 34·1)	18·7 (13·7 to 25·9)	13·8 (9·3 to 19·8)	8·9 (5·6 to 13·4)	8·9 (6·2 to 12·3)	3·3 (2·2 to 4·7)	736
Venomous animal contact	1·6 (1 to 2·3)	1·5 (0·9 to 2·5)	12·7 (7·7 to 20·5)	27·3 (16·5 to 44·4)	29·2 (20 to 42·7)	33·4 (23·3 to 46)	32·8 (22·8 to 47·9)	43·5 (29·6 to 61·6)	62·8 (43·6 to 88·6)	36·6 (25·2 to 53)	47·3 (31·5 to 69·5)	47·8 (31·3 to 72·6)	49·7 (33·2 to 75·1)	44 (28·3 to 66·2)	44·3 (29·4 to 64·3)	40·8 (27·4 to 57·9)	32·2 (22 to 46·1)	22 (15·8 to 30·2)	16·9 (12·2 to 23·8)	12·2 (8·1 to 17·7)	8 (5 to 12·2)	8·2 (5·7 to 11·5)	3 (2 to 4·4)	658
Non-venomous animal contact	0·2 (0·1 to 0·3)	0·1 (0·1 to 0·2)	1·8 (0·9 to 3·6)	4·4 (2 to 8·5)	5·9 (3·3 to 9·6)	3 (1·8 to 4·7)	5 (3·1 to 7·7)	5·8 (3·4 to 9·4)	5·2 (3·1 to 8)	3·9 (2·4 to 6·2)	4·1 (2·6 to 6·3)	4·7 (3 to 7·5)	6·3 (3·9 to 10)	6 (4 to 8·8)	5·5 (3·4 to 8·8)	4·2 (2·7 to 6·4)	3·6 (2·4 to 5·5)	2·9 (1·9 to 4·4)	1·8 (1·2 to 2·6)	1·5 (0·9 to 2·5)	0·9 (0·5 to 1·5)	0·7 (0·4 to 1·1)	0·3 (0·1 to 0·5)	77·7
Foreign body	66·9 (40·3 to 106)	20·6 (11 to 34·6)	297 (132 to 566)	132 (82·7 to 196)	84·7 (67·3 to 106)	87·4 (66·8 to 112)	53·9 (40 to 71·8)	91·7 (66·4 to 123)	92·6 (69·3 to 121)	70·7 (53·8 to 90·6)	64·3 (47·9 to 84)	67·9 (49·1 to 90·9)	62 (44·9 to 82·9)	62 (45·1 to 83·6)	54·2 (40·4 to 72·9)	48·6 (35·8 to 63)	40 (29·3 to 54·3)	28 (21·9 to 35·2)	21·2 (15·7 to 28·3)	14·6 (11·1 to 19)	6 (4·4 to 7·9)	3·8 (2·8 to 4·8)	1·3 (1 to 1·6)	1,470
Pulmonary aspiration and foreign body in airway	66·3 (39·9 to 105)	20·3 (10·9 to 34·3)	293 (129 to 561)	122 (73·7 to 185)	72·9 (56·5 to 92·2)	74 (54·9 to 97·8)	43·2 (29·9 to 59·5)	75·5 (52·5 to 104)	75 (52·9 to 100)	59·3 (43·9 to 77·2)	53·2 (37·4 to 72)	54·2 (37·1 to 75·4)	48·5 (33·5 to 68·5)	51·2 (35·9 to 71·1)	44 (31·5 to 59·9)	40·3 (28·3 to 54·4)	32·8 (22·9 to 45)	23·1 (17·6 to 29·9)	17·2 (12·3 to 23·9)	11·6 (8·5 to 15·4)	4·5 (3·2 to 6·1)	2·8 (1·9 to 3·7)	1 (0·7 to 1·2)	1,290
Foreign body in eyes	0 (0 to 0)	0 (0 to 0)	0·2 (0·1 to 0·5)	1·3 (0·4 to 2·9)	2 (0·6 to 4·6)	2·4 (0·8 to 5·7)	3·3 (1·1 to 7·8)	4·6 (1·5 to 10·1)	4·4 (1·6 to 9·3)	3·6 (1·3 to 7·5)	3·5 (1·3 to 7·3)	3·6 (1·6 to 7·2)	3·3 (1·6 to 6·3)	2·7 (1·4 to 4·9)	2·2 (1·3 to 3·6)	1·7 (1 to 2·7)	1·3 (0·8 to 2)	0·8 (0·5 to 1·2)	0·5 (0·3 to 0·8)	0·3 (0·2 to 0·5)	0·1 (0·1 to 0·2)	0·1 (0 to 0·1)	0 (0 to 0)	42
Foreign body in other body part	0·6 (0·3 to 1·2)	0·3 (0·1 to 0·6)	4·3 (1·8 to 10·5)	9 (5 to 16·9)	9·9 (6·7 to 14·6)	11 (6·9 to 17·6)	7·4 (5·2 to 10·3)	11·6 (7·8 to 17)	13·2 (8·8 to 20·3)	7·8 (5·5 to 11)	7·6 (5·4 to 10·7)	10 (6·7 to 14·8)	10·2 (6·9 to 14·9)	8 (5·6 to 11·2)	8 (5·5 to 11·6)	6·6 (4·4 to 10)	5·9 (4 to 8·6)	4·1 (2·7 to 6·3)	3·5 (2·2 to 5·3)	2·7 (1·8 to 4·1)	1·3 (0·8 to 2)	0·9 (0·6 to 1·4)	0·3 (0·2 to 0·5)	144
Environmental heat and cold exposure	0·5 (0·3 to 0·8)	0·3 (0·2 to 0·5)	3·5 (2·3 to 5·1)	10·8 (7·9 to 14·4)	18·7 (13·2 to 25·1)	21·8 (15·6 to 29·7)	33 (24·4 to 44)	50·1 (37·7 to 65·4)	47·4 (35·6 to 62·2)	40·7 (30·7 to 53·3)	38·6 (28·7 to 50·4)	39·9 (29·7 to 52·2)	38·1 (28·5 to 49·2)	35·6 (26·9 to 46·1)	33·6 (25·4 to 43·7)	26·9 (20·1 to 35)	20·8 (15·6 to 26·8)	14·9 (11·4 to 18·9)	11·1 (8·5 to 14·2)	8·2 (6·2 to 10·6)	4·7 (3·6 to 6·1)	3·4 (2·5 to 4·6)	1·2 (0·9 to 1·6)	504
Exposure to forces of nature	0 (0 to 0)	0 (0 to 0)	0 (0 to 0)	0 (0 to 0)	0 (0 to 0)	0·1 (0 to 0·1)	0·8 (0·5 to 1·3)	1 (0·6 to 1·6)	1·6 (0·9 to 2·6)	0·6 (0·4 to 1)	0·6 (0·4 to 0·9)	0·7 (0·4 to 1·1)	0·7 (0·4 to 1·1)	0·5 (0·3 to 0·8)	0·4 (0·2 to 0·6)	0·3 (0·2 to 0·5)	0·2 (0·1 to 0·4)	0·1 (0·1 to 0·2)	0·1 (0 to 0·1)	0 (0 to 0·1)	0 (0 to 0)	0 (0 to 0)	0 (0 to 0)	7·8
Other unintentional injuries	6·5 (4·2 to 9·6)	1·3 (0·9 to 1·9)	20·6 (13·9 to 29·5)	56·2 (39·8 to 76·5)	89·7 (70·7 to 112)	112 (84·4 to 145)	167 (120 to 227)	263 (186 to 369)	258 (186 to 346)	206 (152 to 275)	175 (131 to 230)	175 (129 to 228)	141 (105 to 181)	102 (77·7 to 135)	82·5 (63·2 to 107)	55·1 (42 to 71·1)	38 (29 to 50·1)	21·1 (16 to 28·1)	13·9 (10·5 to 18·3)	7·6 (5·7 to 10·1)	3·4 (2·5 to 4·6)	1·5 (1·1 to 2)	0·4 (0·3 to 0·5)	2,000
Self-harm and interpersonal violence	54·4 (32 to 87·8)	4·8 (2·6 to 8·3)	59·1 (33·1 to 96·1)	101 (59·5 to 161)	111 (80·5 to 153)	310 (231 to 412)	1,930 (1,440 to 2,540)	4,330 (3,270 to 5,540)	3,930 (3,080 to 4,930)	2,930 (2,240 to 3,730)	2,370 (1,700 to 3,140)	2,030 (1,470 to 2,730)	1,570 (1,100 to 2,180)	1,080 (763 to 1,500)	754 (546 to 1,030)	486 (349 to 656)	298 (222 to 390)	159 (119 to 210)	82·9 (62·4 to 109)	39·8 (30·3 to 52)	15·5 (12·1 to 19·6)	4·8 (3·7 to 6·1)	1·1 (0·8 to 1·3)	22,600
Self-harm	0 (0 to 0)	0 (0 to 0)	0 (0 to 0)	0 (0 to 0)	0 (0 to 0)	130 (87·6 to 184)	1,030 (725 to 1,430)	2,150 (1,500 to 2,910)	1,890 (1,340 to 2,520)	1,520 (1,090 to 2,060)	1,330 (885 to 1,820)	1,200 (823 to 1,680)	985 (669 to 1,430)	703 (475 to 1,010)	515 (358 to 724)	356 (245 to 497)	223 (160 to 306)	125 (88·9 to 172)	65·5 (47·6 to 89·6)	33·4 (24·7 to 45·3)	12·8 (9·6 to 16·5)	3·8 (2·8 to 5)	0·8 (0·6 to 1)	12,300
Self-harm by firearm	0 (0 to 0)	0 (0 to 0)	0 (0 to 0)	0 (0 to 0)	0 (0 to 0)	0·7 (0·4 to 1·2)	12·1 (5·6 to 24·3)	28·2 (13 to 57)	23·2 (12·2 to 41·8)	18·8 (10·3 to 35·9)	15·3 (8·5 to 27·6)	15·7 (8·8 to 27·2)	12·1 (6·5 to 21·3)	10·8 (6·4 to 17·8)	7·2 (4 to 12·2)	5·3 (3·2 to 8·3)	3 (1·8 to 4·6)	1·6 (1·1 to 2·6)	0·9 (0·5 to 1·4)	0·4 (0·3 to 0·6)	0·2 (0·1 to 0·2)	0 (0 to 0)	0 (0 to 0)	156
Self-harm by other specified means	0 (0 to 0)	0 (0 to 0)	0 (0 to 0)	0 (0 to 0)	0 (0 to 0)	129 (87·1 to 183)	1,020 (714 to 1,410)	2,120 (1,470 to 2,870)	1,860 (1,320 to 2,490)	1,510 (1,070 to 2,030)	1,310 (874 to 1,800)	1,180 (811 to 1,660)	973 (663 to 1,410)	692 (469 to 993)	508 (354 to 714)	351 (242 to 490)	220 (158 to 302)	123 (87·6 to 170)	64·6 (46·8 to 88·4)	32·9 (24·2 to 44·8)	12·6 (9·5 to 16·3)	3·7 (2·8 to 5)	0·7 (0·5 to 1)	12,100
Interpersonal violence	54·4 (32 to 87·8)	4·8 (2·6 to 8·3)	58·9 (32·9 to 95·9)	100 (58·6 to 159)	111 (79·9 to 152)	176 (122 to 248)	894 (593 to 1,260)	2,170 (1,510 to 2,910)	2,020 (1,440 to 2,720)	1,400 (978 to 1,860)	1,030 (711 to 1,420)	824 (574 to 1,150)	577 (387 to 828)	371 (245 to 524)	235 (161 to 341)	126 (87·7 to 177)	72·7 (52·3 to 98·4)	33·4 (24·7 to 44·2)	17 (12·4 to 22·5)	6·2 (4·8 to 7·7)	2·5 (2 to 3·2)	0·9 (0·7 to 1·2)	0·3 (0·2 to 0·4)	10,300
Physical violence by firearm	1·6 (0·9 to 2·5)	0·3 (0·1 to 0·5)	3·8 (2·1 to 6·5)	9·4 (5·4 to 16)	18·2 (12·7 to 25·8)	47·7 (32·5 to 68·1)	405 (261 to 591)	1,130 (796 to 1,530)	1,050 (752 to 1,400)	698 (496 to 915)	469 (321 to 649)	370 (253 to 521)	253 (166 to 368)	142 (90·3 to 207)	81 (53·6 to 120)	36·5 (24 to 53·1)	16·9 (11·2 to 23·8)	6·2 (4·3 to 8·7)	1·9 (1·3 to 2·7)	0·5 (0·4 to 0·7)	0·2 (0·2 to 0·3)	0·1 (0·1 to 0·1)	0 (0 to 0)	4,740
Physical violence by sharp object	2·5 (1·4 to 4)	0·2 (0·1 to 0·3)	2·3 (1·4 to 3·6)	6·7 (4·2 to 10·2)	10·4 (7·7 to 14·1)	24·1 (16·8 to 33·1)	282 (186 to 406)	656 (445 to 896)	601 (414 to 820)	424 (294 to 577)	319 (216 to 448)	238 (163 to 340)	147 (98·3 to 213)	88·6 (58·3 to 127)	48·8 (34·2 to 71·7)	24·8 (17·7 to 33·9)	12·7 (9·4 to 17)	6·6 (4·8 to 8·7)	3·1 (2·3 to 4)	1·3 (1 to 1·7)	0·5 (0·4 to 0·7)	0·3 (0·2 to 0·3)	0·1 (0·1 to 0·1)	2,900
Sexual violence	0 (0 to 0)	0 (0 to 0)	0 (0 to 0)	0·5 (0·3 to 0·7)	3·5 (2·1 to 5·5)	7·6 (4·4 to 12)	18·4 (10·3 to 29·3)	27·7 (15·9 to 42·3)	22 (12·4 to 35·2)	15·7 (8·7 to 25·9)	13·4 (7·5 to 21·3)	11·1 (6·4 to 18)	8·2 (4·8 to 13·8)	5·7 (3·2 to 9·3)	3·8 (2·3 to 6·2)	2·8 (1·7 to 4·2)	2 (1·2 to 3)	1·2 (0·7 to 1·7)	0·7 (0·5 to 1·1)	0·4 (0·3 to 0·6)	0·2 (0·1 to 0·3)	0 (0 to 0)	0 (0 to 0)	145
Physical violence by other means	50·4 (29·6 to 81·2)	4·3 (2·4 to 7·6)	52·8 (29·5 to 85·8)	83·8 (48·8 to 135)	78·4 (55·6 to 108)	96·6 (66·6 to 137)	188 (123 to 265)	351 (242 to 479)	355 (244 to 496)	259 (181 to 349)	228 (156 to 319)	205 (145 to 288)	170 (115 to 239)	134 (90 to 190)	101 (69·8 to 146)	62·3 (43·8 to 86·9)	41 (29·3 to 55·8)	19·5 (14·2 to 26)	11·3 (8·1 to 15·2)	3·9 (2·9 to 4·9)	1·6 (1·2 to 2)	0·6 (0·4 to 0·7)	0·2 (0·1 to 0·2)	2,500
Conflict and terrorism	0 (0 to 0)	0 (0 to 0)	0 (0 to 0)	0 (0 to 0)	0 (0 to 0)	3·8 (2·1 to 6·7)	4·4 (2·5 to 7·7)	5·4 (3 to 9·2)	5·5 (3·3 to 9)	5·6 (3·5 to 9)	7·7 (4·8 to 11·7)	4·5 (2·9 to 7)	5·4 (3·5 to 8·1)	4·1 (2·7 to 6)	3·7 (2·5 to 5·5)	3·1 (2·1 to 4·5)	2·1 (1·4 to 3)	0·8 (0·6 to 1·2)	0·4 (0·3 to 0·7)	0·2 (0·1 to 0·3)	0·2 (0·1 to 0·3)	0·1 (0 to 0·1)	0 (0 to 0)	57

SR Table 2c: Haiti DALYS by cause and age group for both sexes with 95% uncertainty intervals, 2019

Cause	Early Neonatal	Late Neonatal	Post Neonatal	1 to 4	5 to 9	10 to 14
All causes	613,000 (481,000 to 761,000)	249,000 (196,000 to 308,000)	596,000 (429,000 to 813,000)	597,000 (506,000 to 702,000)	229,000 (189,000 to 266,000)	182,000 (153,000 to 216,000)
Communicable, maternal, neonatal, and nutritional diseases	499,000 (383,000 to 631,000)	186,000 (140,000 to 235,000)	426,000 (296,000 to 594,000)	311,000 (240,000 to 410,000)	76,000 (60,200 to 95,200)	43,500 (34,000 to 56,200)
HIV/AIDS and sexually transmitted infections	38,200 (13,500 to 79,000)	11,900 (4,200 to 25,500)	19,100 (9,860 to 34,100)	16,000 (10,900 to 23,100)	8,550 (5,480 to 12,200)	7,980 (5,400 to 11,000)
HIV/AIDS	0·6 (0·4 to 1)	2·2 (1·3 to 3·3)	5,880 (4,160 to 8,390)	15,400 (10,400 to 22,400)	8,290 (5,240 to 12,000)	7,900 (5,300 to 10,900)
HIV/AIDS - Drug-susceptible Tuberculosis	0 (0 to 0)	0·1 (0·1 to 0·2)	375 (226 to 597)	1,330 (804 to 2,110)	781 (480 to 1,220)	665 (410 to 1,040)
HIV/AIDS - Multidrug-resistant Tuberculosis without exte	0 (0 to 0)	0 (0 to 0)	4·3 (0·3 to 17·2)	15·1 (1·1 to 60·9)	8·7 (0·7 to 34·9)	7·4 (0·6 to 29·6)
HIV/AIDS - Extensively drug-resistant Tuberculosis	0 (0 to 0)	0 (0 to 0)	0·7 (0·1 to 2·9)	2·5 (0·2 to 10·1)	1·4 (0·1 to 5·7)	1·2 (0·1 to 4·8)
HIV/AIDS resulting in other diseases	0·6 (0·4 to 1)	2 (1·2 to 3·2)	5,500 (3,750 to 8,040)	14,100 (8,870 to 21,000)	7,500 (4,500 to 11,200)	7,220 (4,740 to 10,200)
Sexually transmitted infections excluding HIV	38,200 (13,500 to 79,000)	11,800 (4,200 to 25,500)	13,200 (4,470 to 27,000)	533 (186 to 1,140)	264 (92·7 to 543)	81·1 (38·1 to 160)
Syphilis	38,200 (13,500 to 79,000)	11,800 (4,200 to 25,500)	13,200 (4,470 to 27,000)	533 (186 to 1,140)	264 (92·7 to 543)	35·4 (12·6 to 79·2)
Chlamydial infection	0 (0 to 0)	0 (0 to 0)	0 (0 to 0)	0 (0 to 0)	0 (0 to 0)	4·1 (1·8 to 9·1)
Gonococcal infection	0 (0 to 0)	0 (0 to 0)	0 (0 to 0)	0 (0 to 0)	0 (0 to 0)	10·1 (4·5 to 19·3)
Trichomoniasis	0 (0 to 0)	0 (0 to 0)	0 (0 to 0)	0 (0 to 0)	0 (0 to 0)	0·8 (0 to 2·4)
Genital herpes	0 (0 to 0)	0 (0 to 0)	0 (0 to 0)	0 (0 to 0)	0 (0 to 0)	4·7 (1·5 to 11·2)
Other sexually transmitted infections	0 (0 to 0)	0 (0 to 0)	0 (0 to 0)	0 (0 to 0)	0 (0 to 0)	25·9 (11·6 to 49·1)
Respiratory infections and tuberculosis	44,400 (25,700 to 71,800)	39,000 (22,000 to 61,400)	125,000 (80,300 to 190,000)	87,200 (58,500 to 125,000)	15,900 (11,200 to 21,100)	8,050 (5,980 to 10,500)

[illegible]

Other cardiovascular and circulatory diseases	998 (436 to 2,110)	724 (275 to 1,520)	2,960 (794 to 6,850)	3,350 (1,110 to 6,660)	[illegible]	[illegible]	[illegible]	[illegible]	[illegible]	[illegible]	[illegible]	[illegible]	[illegible]	[illegible]	[illegible]	[illegible]	[illegible]	[illegible]	[illegible]	[illegible]	[illegible]	[illegible]	28·3 (17·3 to 56·6)	34,200
Chronic respiratory diseases	0 (0 to 0)	0 (0 to 0)	1·5 (1 to 2·1)	35,100 (14,900 to 55,000)	[illegible]	[illegible]	[illegible]	[illegible]	[illegible]	[illegible]	[illegible]	[illegible]	[illegible]	[illegible]	[illegible]	[illegible]	[illegible]	[illegible]	[illegible]	[illegible]	[illegible]	[illegible]	136 (83·6 to 222)	156,000
Chronic obstructive pulmonary disease	0 (0 to 0)	0 (0 to 0)	1·5 (1 to 2·1)	2,450 (429 to 6,140)	[illegible]	[illegible]	[illegible]	[illegible]	[illegible]	[illegible]	[illegible]	[illegible]	[illegible]	[illegible]	[illegible]	[illegible]	[illegible]	[illegible]	[illegible]	[illegible]	[illegible]	[illegible]	111 (69·6 to 173)	52,100
Pneumoconiosis	0 (0 to 0)	0 (0 to 0)	0 (0 to 0)	0 (0 to 0)	[illegible]	[illegible]	[illegible]	[illegible]	[illegible]	[illegible]	[illegible]	[illegible]	[illegible]	[illegible]	[illegible]	[illegible]	[illegible]	[illegible]	[illegible]	[illegible]	[illegible]	[illegible]	0·2 (0·1 to 0·5)	313
Silicosis	0 (0 to 0)	0 (0 to 0)	0 (0 to 0)	0 (0 to 0)	[illegible]	[illegible]	[illegible]	[illegible]	[illegible]	[illegible]	[illegible]	[illegible]	[illegible]	[illegible]	[illegible]	[illegible]	[illegible]	[illegible]	[illegible]	[illegible]	[illegible]	[illegible]	0 (0 to 0·1)	88·2
Asbestosis	0 (0 to 0)	0 (0 to 0)	0 (0 to 0)	0 (0 to 0)	[illegible]	[illegible]	[illegible]	[illegible]	[illegible]	[illegible]	[illegible]	[illegible]	[illegible]	[illegible]	[illegible]	[illegible]	[illegible]	[illegible]	[illegible]	[illegible]	[illegible]	[illegible]	0 (0 to 0·1)	48
Coal workers pneumoconiosis	0 (0 to 0)	0 (0 to 0)	0 (0 to 0)	0 (0 to 0)	[illegible]	[illegible]	[illegible]	[illegible]	[illegible]	[illegible]	[illegible]	[illegible]	[illegible]	[illegible]	[illegible]	[illegible]	[illegible]	[illegible]	[illegible]	[illegible]	[illegible]	[illegible]	0 (0 to 0·1)	41
Other pneumoconiosis	0 (0 to 0)	0 (0 to 0)	0 (0 to 0)	0 (0 to 0)	[illegible]	[illegible]	[illegible]	[illegible]	[illegible]	[illegible]	[illegible]	[illegible]	[illegible]	[illegible]	[illegible]	[illegible]	[illegible]	[illegible]	[illegible]	[illegible]	[illegible]	[illegible]	0·1 (0 to 0·3)	135
Asthma	0 (0 to 0)	0 (0 to 0)	0 (0 to 0)	26,700 (11,600 to 44,600)	[illegible]	[illegible]	[illegible]	[illegible]	[illegible]	[illegible]	[illegible]	[illegible]	[illegible]	[illegible]	[illegible]	[illegible]	[illegible]	[illegible]	[illegible]	[illegible]	[illegible]	[illegible]	19·2 (8·8 to 48·5)	88,600
Interstitial lung disease and pulmonary sarcoidosis	0 (0 to 0)	0 (0 to 0)	0 (0 to 0)	682 (181 to 2,210)	[illegible]	[illegible]	[illegible]	[illegible]	[illegible]	[illegible]	[illegible]	[illegible]	[illegible]	[illegible]	[illegible]	[illegible]	[illegible]	[illegible]	[illegible]	[illegible]	[illegible]	[illegible]	3·7 (1·6 to 8·2)	4,400
Other chronic respiratory diseases	0 (0 to 0)	0 (0 to 0)	0 (0 to 0)	5,210 (1,180 to 13,800)	[illegible]	[illegible]	[illegible]	[illegible]	[illegible]	[illegible]	[illegible]	[illegible]	[illegible]	[illegible]	[illegible]	[illegible]	[illegible]	[illegible]	[illegible]	[illegible]	[illegible]	[illegible]	1·3 (0·5 to 2·9)	11,000
Digestive diseases	1,530 (558 to 3,040)	1,480 (394 to 3,370)	10,600 (1,590 to 28,400)	16,900 (3,990 to 37,300)	[illegible]	[illegible]	[illegible]	[illegible]	[illegible]	[illegible]	[illegible]	[illegible]	[illegible]	[illegible]	[illegible]	[illegible]	[illegible]	[illegible]	[illegible]	[illegible]	[illegible]	[illegible]	73·4 (48·3 to 119)	191,000
Cirrhosis and other chronic liver diseases	0 (0 to 0)	0 (0 to 0)	2 (1 to 3·5)	3,490 (288 to 9,250)	[illegible]	[illegible]	[illegible]	[illegible]	[illegible]	[illegible]	[illegible]	[illegible]	[illegible]	[illegible]	[illegible]	[illegible]	[illegible]	[illegible]	[illegible]	[illegible]	[illegible]	[illegible]	14 (8·2 to 23·6)	73,300
Cirrhosis and other chronic liver diseases due to hepati	0 (0 to 0)	0 (0 to 0)	0 (0 to 0)	7·6 (0·4 to 26·4)	[illegible]	[illegible]	[illegible]	[illegible]	[illegible]	[illegible]	[illegible]	[illegible]	[illegible]	[illegible]	[illegible]	[illegible]	[illegible]	[illegible]	[illegible]	[illegible]	[illegible]	[illegible]	1·3 (0·4 to 3)	8,840
Cirrhosis and other chronic liver diseases due to hepati	0 (0 to 0)	0 (0 to 0)	0 (0 to 0)	18·3 (1·1 to 59·3)	[illegible]	[illegible]	[illegible]	[illegible]	[illegible]	[illegible]	[illegible]	[illegible]	[illegible]	[illegible]	[illegible]	[illegible]	[illegible]	[illegible]	[illegible]	[illegible]	[illegible]	[illegible]	2·5 (1 to 5·4)	13,400
Cirrhosis and other chronic liver diseases due to alcoho	0 (0 to 0)	0 (0 to 0)	0 (0 to 0)	0 (0 to 0)	[illegible]	[illegible]	[illegible]	[illegible]	[illegible]	[illegible]	[illegible]	[illegible]	[illegible]	[illegible]	[illegible]	[illegible]	[illegible]	[illegible]	[illegible]	[illegible]	[illegible]	[illegible]	3·7 (1·5 to 7·2)	25,600
Cirrhosis and other chronic liver diseases due to NAFL[	0 (0 to 0)	0 (0 to 0)	0 (0 to 0)	0 (0 to 0)	[illegible]	[illegible]	[illegible]	[illegible]	[illegible]	[illegible]	[illegible]	[illegible]	[illegible]	[illegible]	[illegible]	[illegible]	[illegible]	[illegible]	[illegible]	[illegible]	[illegible]	[illegible]	3·2 (1·2 to 6·3)	9,390
Cirrhosis and other chronic liver diseases due to other c	0 (0 to 0)	0 (0 to 0)	2 (1 to 3·5)	3,460 (287 to 9,190)	[illegible]	[illegible]	[illegible]	[illegible]	[illegible]	[illegible]	[illegible]	[illegible]	[illegible]	[illegible]	[illegible]	[illegible]	[illegible]	[illegible]	[illegible]	[illegible]	[illegible]	[illegible]	3·3 (1·3 to 6·7)	16,000
Upper digestive system diseases	0 (0 to 0)	0 (0 to 0)	0 (0 to 0)	1,760 (502 to 3,690)	[illegible]	[illegible]	[illegible]	[illegible]	[illegible]	[illegible]	[illegible]	[illegible]	[illegible]	[illegible]	[illegible]	[illegible]	[illegible]	[illegible]	[illegible]	[illegible]	[illegible]	[illegible]	22·2 (13·7 to 34·5)	38,800
Peptic ulcer disease	0 (0 to 0)	0 (0 to 0)	0 (0 to 0)	1,260 (270 to 2,800)	[illegible]	[illegible]	[illegible]	[illegible]	[illegible]	[illegible]	[illegible]	[illegible]	[illegible]	[illegible]	[illegible]	[illegible]	[illegible]	[illegible]	[illegible]	[illegible]	[illegible]	[illegible]	16·9 (9·8 to 27·4)	18,700
Gastritis and duodenitis	0 (0 to 0)	0 (0 to 0)	0 (0 to 0)	496 (156 to 1,180)	[illegible]	[illegible]	[illegible]	[illegible]	[illegible]	[illegible]	[illegible]	[illegible]	[illegible]	[illegible]	[illegible]	[illegible]	[illegible]	[illegible]	[illegible]	[illegible]	[illegible]	[illegible]	3 (1·4 to 6·2)	7,090
Gastroesophageal reflux disease	0 (0 to 0)	0 (0 to 0)	0 (0 to 0)	0 (0 to 0)	[illegible]	[illegible]	[illegible]	[illegible]	[illegible]	[illegible]	[illegible]	[illegible]	[illegible]	[illegible]	[illegible]	[illegible]	[illegible]	[illegible]	[illegible]	[illegible]	[illegible]	[illegible]	2·3 (1·1 to 4)	13,000
Appendicitis	0 (0 to 0)	0 (0 to 0)	0 (0 to 0)	3,100 (339 to 8,140)	[illegible]	[illegible]	[illegible]	[illegible]	[illegible]	[illegible]	[illegible]	[illegible]	[illegible]	[illegible]	[illegible]	[illegible]	[illegible]	[illegible]	[illegible]	[illegible]	[illegible]	[illegible]	1·2 (0·6 to 2·2)	9,810
Paralytic ileus and intestinal obstruction	771 (251 to 1,550)	1,050 (291 to 2,530)	7,320 (1,140 to 20,900)	2,210 (329 to 7,120)	[illegible]	[illegible]	[illegible]	[illegible]	[illegible]	[illegible]	[illegible]	[illegible]	[illegible]	[illegible]	[illegible]	[illegible]	[illegible]	[illegible]	[illegible]	[illegible]	[illegible]	[illegible]	13·7 (7 to 27)	20,300
Inguinal, femoral, and abdominal hernia	759 (197 to 1,730)	430 (60·8 to 1,100)	3,230 (218 to 10,600)	2,060 (734 to 4,510)	[illegible]	[illegible]	[illegible]	[illegible]	[illegible]	[illegible]	[illegible]	[illegible]	[illegible]	[illegible]	[illegible]	[illegible]	[illegible]	[illegible]	[illegible]	[illegible]	[illegible]	[illegible]	3·7 (1·4 to 7·8)	17,400
Inflammatory bowel disease	0 (0 to 0)	0 (0 to 0)	0 (0 to 0)	803 (65 to 2,520)	[illegible]	[illegible]	[illegible]	[illegible]	[illegible]	[illegible]	[illegible]	[illegible]	[illegible]	[illegible]	[illegible]	[illegible]	[illegible]	[illegible]	[illegible]	[illegible]	[illegible]	[illegible]	1 (0·5 to 2·1)	3,780
Vascular intestinal disorders	0 (0 to 0)	0 (0 to 0)	0 (0 to 0)	55·5 (5·9 to 168)	[illegible]	[illegible]	[illegible]	[illegible]	[illegible]	[illegible]	[illegible]	[illegible]	[illegible]	[illegible]	[illegible]	[illegible]	[illegible]	[illegible]	[illegible]	[illegible]	[illegible]	[illegible]	1·9 (1·1 to 3·7)	1,580
Gallbladder and biliary diseases	0 (0 to 0)	0 (0 to 0)	0 (0 to 0)	233 (50·4 to 591)	[illegible]	[illegible]	[illegible]	[illegible]	[illegible]	[illegible]	[illegible]	[illegible]	[illegible]	[illegible]	[illegible]	[illegible]	[illegible]	[illegible]	[illegible]	[illegible]	[illegible]	[illegible]	5·2 (3·2 to 8)	10,700
Pancreatitis	0 (0 to 0)	0 (0 to 0)	0 (0 to 0)	192 (24·7 to 484)	[illegible]	[illegible]	[illegible]	[illegible]	[illegible]	[illegible]	[illegible]	[illegible]	[illegible]	[illegible]	[illegible]	[illegible]	[illegible]	[illegible]	[illegible]	[illegible]	[illegible]	[illegible]	1·4 (0·8 to 2·7)	5,880
Other digestive diseases	0 (0 to 0)	0 (0 to 0)	0 (0 to 0)	3,060 (335 to 9,610)	[illegible]	[illegible]	[illegible]	[illegible]	[illegible]	[illegible]	[illegible]	[illegible]	[illegible]	[illegible]	[illegible]	[illegible]	[illegible]	[illegible]	[illegible]	[illegible]	[illegible]	[illegible]	9 (4·2 to 18·2)	9,550
Neurological disorders	5·3 (1·4 to 12)	30·5 (11 to 67·9)	3,310 (1,290 to 5,620)	8,870 (3,900 to 13,400)	[illegible]	[illegible]	[illegible]	[illegible]	[illegible]	[illegible]	[illegible]	[illegible]	[illegible]	[illegible]	[illegible]	[illegible]	[illegible]	[illegible]	[illegible]	[illegible]	[illegible]	[illegible]	284 (124 to 653)	141,000
Alzheimer's disease and other dementias	0 (0 to 0)	0 (0 to 0)	0 (0 to 0)	0 (0 to 0)	[illegible]	[illegible]	[illegible]	[illegible]	[illegible]	[illegible]	[illegible]	[illegible]	[illegible]	[illegible]	[illegible]	[illegible]	[illegible]	[illegible]	[illegible]	[illegible]	[illegible]	[illegible]	266 (106 to 638)	13,800
Parkinson's disease	0 (0 to 0)	0 (0 to 0)	0 (0 to 0)	0 (0 to 0)	[illegible]	[illegible]	[illegible]	[illegible]	[illegible]	[illegible]	[illegible]	[illegible]	[illegible]	[illegible]	[illegible]	[illegible]	[illegible]	[illegible]	[illegible]	[illegible]	[illegible]	[illegible]	12·2 (8·8 to 15·7)	5,300
Idiopathic epilepsy	4·9 (0·8 to 11·6)	14 (2·7 to 32·6)	1,590 (449 to 2,890)	6,360 (1,990 to 10,300)	[illegible]	[illegible]	[illegible]	[illegible]	[illegible]	[illegible]	[illegible]	[illegible]	[illegible]	[illegible]	[illegible]	[illegible]	[illegible]	[illegible]	[illegible]	[illegible]	[illegible]	[illegible]	3·6 (1·4 to 6·6)	40,500
Multiple sclerosis	0 (0 to 0)	0 (0 to 0)	0 (0 to 0)	0 (0 to 0)	[illegible]	[illegible]	[illegible]	[illegible]	[illegible]	[illegible]	[illegible]	[illegible]	[illegible]	[illegible]	[illegible]	[illegible]	[illegible]	[illegible]	[illegible]	[illegible]	[illegible]	[illegible]	0·1 (0 to 0·1)	1,280
Motor neuron disease	0·5 (0·2 to 0·9)	16·5 (3·1 to 51·9)	309 (62·1 to 889)	232 (59·5 to 567)	[illegible]	[illegible]	[illegible]	[illegible]	[illegible]	[illegible]	[illegible]	[illegible]	[illegible]	[illegible]	[illegible]	[illegible]	[illegible]	[illegible]	[illegible]	[illegible]	[illegible]	[illegible]	0·1 (0 to 0·1)	1,380
Headache disorders	0 (0 to 0)	0 (0 to 0)	0 (0 to 0)	0 (0 to 0)	[illegible]	[illegible]	[illegible]	[illegible]	[illegible]	[illegible]	[illegible]	[illegible]	[illegible]	[illegible]	[illegible]	[illegible]	[illegible]	[illegible]	[illegible]	[illegible]	[illegible]	[illegible]	1·6 (0·4 to 3·9)	67,100
Migraine	0 (0 to 0)	0 (0 to 0)	0 (0 to 0)	0 (0 to 0)	[illegible]	[illegible]	[illegible]	[illegible]	[illegible]	[illegible]	[illegible]	[illegible]	[illegible]	[illegible]	[illegible]	[illegible]	[illegible]	[illegible]	[illegible]	[illegible]	[illegible]	[illegible]	1·2 (0·3 to 2·9)	61,100
Tension-type headache	0 (0 to 0)	0 (0 to 0)	0 (0 to 0)	0 (0 to 0)	[illegible]	[illegible]	[illegible]	[illegible]	[illegible]	[illegible]	[illegible]	[illegible]	[illegible]	[illegible]	[illegible]	[illegible]	[illegible]	[illegible]	[illegible]	[illegible]	[illegible]	[illegible]	0·4 (0 to 2·2)	5,930
Other neurological disorders	0 (0 to 0)	0 (0 to 0)	1,420 (565 to 2,660)	2,280 (1,110 to 3,760)	[illegible]	[illegible]	[illegible]	[illegible]	[illegible]	[illegible]	[illegible]	[illegible]	[illegible]	[illegible]	[illegible]	[illegible]	[illegible]	[illegible]	[illegible]	[illegible]	[illegible]	[illegible]	0·6 (0·4 to 0·9)	11,400
Mental disorders	6 (3·5 to 9·1)	17·8 (10·3 to 27·2)	303 (179 to 461)	1,680 (1,050 to 2,450)	[illegible]	[illegible]	[illegible]	[illegible]	[illegible]	[illegible]	[illegible]	[illegible]	[illegible]	[illegible]	[illegible]	[illegible]	[illegible]	[illegible]	[illegible]	[illegible]	[illegible]	[illegible]	18·4 (11·6 to 26·7)	206,000
Schizophrenia	0 (0 to 0)	0 (0 to 0)	0 (0 to 0)	0 (0 to 0)	[illegible]	[illegible]	[illegible]	[illegible]	[illegible]	[illegible]	[illegible]	[illegible]	[illegible]	[illegible]	[illegible]	[illegible]	[illegible]	[illegible]	[illegible]	[illegible]	[illegible]	[illegible]	0·2 (0·1 to 0·3)	17,400
Depressive disorders	0 (0 to 0)	0 (0 to 0)	0 (0 to 0)	3·1 (1·3 to 6·9)	[illegible]	[illegible]	[illegible]	[illegible]	[illegible]	[illegible]	[illegible]	[illegible]	[illegible]	[illegible]	[illegible]	[illegible]	[illegible]	[illegible]	[illegible]	[illegible]	[illegible]	[illegible]	12·3 (6·6 to 20)	72,100
Major depressive disorder	0 (0 to 0)	0 (0 to 0)	0 (0 to 0)	2·6 (0·9 to 6·3)	[illegible]	[illegible]	[illegible]	[illegible]	[illegible]	[illegible]	[illegible]	[illegible]	[illegible]	[illegible]	[illegible]	[illegible]	[illegible]	[illegible]	[illegible]	[illegible]	[illegible]	[illegible]	11·1 (5·8 to 18·9)	62,100
Dysthymia	0 (0 to 0)	0 (0 to 0)	0 (0 to 0)	0·5 (0·1 to 1·4)	[illegible]	[illegible]	[illegible]	[illegible]	[illegible]	[illegible]	[illegible]	[illegible]	[illegible]	[illegible]	[illegible]	[illegible]	[illegible]	[illegible]	[illegible]	[illegible]	[illegible]	[illegible]	1·1 (0·6 to 2)	10,000
Bipolar disorder	0 (0 to 0)	0 (0 to 0)	0 (0 to 0)	0 (0 to 0)	[illegible]	[illegible]	[illegible]	[illegible]	[illegible]	[illegible]	[illegible]	[illegible]	[illegible]	[illegible]	[illegible]	[illegible]	[illegible]	[illegible]	[illegible]	[illegible]	[illegible]	[illegible]	0·5 (0·2 to 0·9)	21,700
Anxiety disorders	0 (0 to 0)	0 (0 to 0)	0 (0 to 0)	152 (57·7 to 294)	[illegible]	[illegible]	[illegible]	[illegible]	[illegible]	[illegible]	[illegible]	[illegible]	[illegible]	[illegible]	[illegible]	[illegible]	[illegible]	[illegible]	[illegible]	[illegible]	[illegible]	[illegible]	2·6 (1·4 to 4·1)	52,400
Eating disorders	0 (0 to 0)	0 (0 to 0)	0 (0 to 0)	0 (0 to 0)	[illegible]	[illegible]	[illegible]	[illegible]	[illegible]	[illegible]	[illegible]	[illegible]	[illegible]	[illegible]	[illegible]	[illegible]	[illegible]	[illegible]	[illegible]	[illegible]	[illegible]	[illegible]	0 (0 to 0)	3,580
Anorexia nervosa	0 (0 to 0)	0 (0 to 0)	0 (0 to 0)	0 (0 to 0)	[illegible]	[illegible]	[illegible]	[illegible]	[illegible]	[illegible]	[illegible]	[illegible]	[illegible]	[illegible]	[illegible]	[illegible]	[illegible]	[illegible]	[illegible]	[illegible]	[illegible]	[illegible]	0 (0 to 0)	952
Bulimia nervosa	0 (0 to 0)	0 (0 to 0)	0 (0 to 0)	0 (0 to 0)	[illegible]	[illegible]	[illegible]	[illegible]	[illegible]	[illegible]	[illegible]	[illegible]	[illegible]	[illegible]	[illegible]	[illegible]	[illegible]	[illegible]	[illegible]	[illegible]	[illegible]	[illegible]	0 (0 to 0)	2,620
Autism spectrum disorders	4·2 (2·6 to 6·3)	12·2 (7·7 to 18·3)	193 (123 to 285)	782 (506 to 1,160)	[illegible]	[illegible]	[illegible]	[illegible]	[illegible]	[illegible]	[illegible]	[illegible]	[illegible]	[illegible]	[illegible]	[illegible]	[illegible]	[illegible]	[illegible]	[illegible]	[illegible]	[illegible]	0·3 (0·2 to 0·5)	6,560
Attention-deficit/hyperactivity disorder	0 (0 to 0)	0 (0 to 0)	0 (0 to 0)	98·5 (49·2 to 175)	[illegible]	[illegible]	[illegible]	[illegible]	[illegible]	[illegible]	[illegible]	[illegible]	[illegible]	[illegible]	[illegible]	[illegible]	[illegible]	[illegible]	[illegible]	[illegible]	[illegible]	[illegible]	0 (0 to 0)	5,130
Conduct disorder	0 (0 to 0)	0 (0 to 0)	0 (0 to 0)	0 (0 to 0)	[illegible]	[illegible]	[illegible]	[illegible]	[illegible]	[illegible]	[illegible]	[illegible]	[illegible]	[illegible]	[illegible]	[illegible]	[illegible]	[illegible]	[illegible]	[illegible]	[illegible]	[illegible]	0 (0 to 0)	9,950
Idiopathic developmental intellectual disability	1·8 (0·2 to 4)	5·6 (0·5 to 12·2)	110 (22 to 225)	648 (259 to 1,150)	[illegible]	[illegible]	[illegible]	[illegible]	[illegible]	[illegible]	[illegible]	[illegible]	[illegible]	[illegible]	[illegible]	[illegible]	[illegible]	[illegible]	[illegible]	[illegible]	[illegible]	[illegible]	0·1 (0 to 0·2)	5,700
Other mental disorders	0 (0 to 0)	0 (0 to 0)	0 (0 to 0)	0 (0 to 0)	[illegible]	[illegible]	[illegible]	[illegible]	[illegible]	[illegible]	[illegible]	[illegible]	[illegible]	[illegible]	[illegible]	[illegible]	[illegible]	[illegible]	[illegible]	[illegible]	[illegible]	[illegible]	2·5 (1·6 to 3·5)	11,300
Substance use disorders	0 (0 to 0)	0 (0 to 0)	0·4 (0·2 to 0·6)	2·2 (1·1 to 3·9)	[illegible]	[illegible]	[illegible]	[illegible]	[illegible]	[illegible]	[illegible]	[illegible]	[illegible]	[illegible]	[illegible]	[illegible]	[illegible]	[illegible]	[illegible]	[illegible]	[illegible]	[illegible]	3·7 (2·4 to 5·6)	43,400
Alcohol use disorders	0 (0 to 0)	0 (0 to 0)	0·4 (0·2 to 0·6)	2·2 (1·1 to 3·9)	[illegible]	[illegible]	[illegible]	[illegible]	[illegible]	[illegible]	[illegible]	[illegible]	[illegible]	[illegible]	[illegible]	[illegible]	[illegible]	[illegible]	[illegible]	[illegible]	[illegible]	[illegible]	3·2 (2 to 5·1)	31,100
Drug use disorders	0 (0 to 0)	0 (0 to 0)	0 (0 to 0)	0 (0 to 0)	[illegible]	[illegible]	[illegible]	[illegible]	[illegible]	[illegible]	[illegible]	[illegible]	[illegible]	[illegible]	[illegible]	[illegible]	[illegible]	[illegible]	[illegible]	[illegible]	[illegible]	[illegible]	0·5 (0·3 to 0·8)	12,300
Opioid use disorders	0 (0 to 0)	0 (0 to 0)	0 (0 to 0)	0 (0 to 0)	[illegible]	[illegible]	[illegible]	[illegible]	[illegible]	[illegible]	[illegible]	[illegible]	[illegible]	[illegible]	[illegible]	[illegible]	[illegible]	[illegible]	[illegible]	[illegible]	[illegible]	[illegible]	0·3 (0·1 to 0·5)	3,600
Cocaine use disorders	0 (0 to 0)	0 (0 to 0)	0 (0 to 0)	0 (0 to 0)	[illegible]	[illegible]	[illegible]	[illegible]	[illegible]	[illegible]	[illegible]	[illegible]	[illegible]	[illegible]	[illegible]	[illegible]	[illegible]	[illegible]	[illegible]	[illegible]	[illegible]	[illegible]	0·1 (0 to 0·1)	3,530
Amphetamine use disorders	0 (0 to 0)	0 (0 to 0)	0 (0 to 0)	0 (0 to 0)	[illegible]	[illegible]	[illegible]	[illegible]	[illegible]	[illegible]	[illegible]	[illegible]	[illegible]	[illegible]	[illegible]	[illegible]	[illegible]	[illegible]	[illegible]	[illegible]	[illegible]	[illegible]	0 (0 to 0)	912
Cannabis use disorders	0 (0 to 0)	0 (0 to 0)	0 (0 to 0)	0 (0 to 0)	[illegible]	[illegible]	[illegible]	[illegible]	[illegible]	[illegible]	[illegible]	[illegible]	[illegible]	[illegible]	[illegible]	[illegible]	[illegible]	[illegible]	[illegible]	[illegible]	[illegible]	[illegible]	0 (0 to 0)	1,380
Other drug use disorders	0 (0 to 0)	0 (0 to 0)	0 (0 to 0)	0 (0 to 0)	[illegible]	[illegible]	[illegible]	[illegible]	[illegible]	[illegible]	[illegible]	[illegible]	[illegible]	[illegible]	[illegible]	[illegible]	[illegible]	[illegible]	[illegible]	[illegible]	[illegible]	[illegible]	0·1 (0·1 to 0·2)	2,850
Diabetes and kidney diseases	16·3 (10·1 to 25)	55·1 (27·5 to 97·9)	3,500 (1,150 to 6,500)	5,010 (2,130 to 7,710)	[illegible]	[illegible]	[illegible]	[illegible]	[illegible]	[illegible]	[illegible]	[illegible]	[illegible]	[illegible]	[illegible]	[illegible]	[illegible]	[illegible]	[illegible]	[illegible]	[illegible]	[illegible]	166 (127 to 224)	278,000
Diabetes mellitus	16·3 (10·1 to 25)	54·9 (27·1 to 97·6)	914 (295 to 2,100)	1,150 (455 to 2,480)	[illegible]	[illegible]	[illegible]	[illegible]	[illegible]	[illegible]	[illegible]	[illegible]	[illegible]	[illegible]	[illegible]	[illegible]	[illegible]	[illegible]	[illegible]	[illegible]	[illegible]	[illegible]	108 (79·3 to 150)	184,000
Diabetes mellitus type 1	16·3 (10·1 to 25)	54·9 (27·1 to 97·6)	914 (295 to 2,100)	1,150 (455 to 2,480)	[illegible]	[illegible]	[illegible]	[illegible]	[illegible]	[illegible]	[illegible]	[illegible]	[illegible]	[illegible]	[illegible]	[illegible]	[illegible]	[illegible]	[illegible]	[illegible]	[illegible]	[illegible]	3 (1·7 to 4·9)	20,000
Diabetes mellitus type 2	0 (0 to 0)	0 (0 to 0)	0 (0 to 0)	0 (0 to 0)	[illegible]	[illegible]	[illegible]	[illegible]	[illegible]	[illegible]	[illegible]	[illegible]	[illegible]	[illegible]	[illegible]	[illegible]	[illegible]	[illegible]	[illegible]	[illegible]	[illegible]	[illegible]	105 (77·6 to 146)	164,000
Chronic kidney disease	0 (0 to 0)	0·2 (0·1 to 0·4)	2,580 (682 to 4,900)	3,780 (1,400 to 5,750)	[illegible]	[illegible]	[illegible]	[illegible]	[illegible]	[illegible]	[illegible]	[illegible]	[illegible]	[illegible]	[illegible]	[illegible]	[illegible]	[illegible]	[illegible]	[illegible]	[illegible]	[illegible]	57·8 (39·4 to 92·2)	92,800
Chronic kidney disease due to diabetes mellitus type 1	0 (0 to 0)	0 (0 to 0)	16·4 (4 to 43·4)	40·8 (15·7 to 80·7)	[illegible]	[illegible]	[illegible]	[illegible]	[illegible]	[illegible]	[illegible]	[illegible]	[illegible]	[illegible]	[illegible]	[illegible]	[illegible]	[illegible]	[illegible]	[illegible]	[illegible]	[illegible]	0 (0 to 0)	7,890
Chronic kidney disease due to diabetes mellitus type 2	0 (0 to 0)	0 (0 to 0)	0 (0 to 0)	0 (0 to 0)	[illegible]	[illegible]	[illegible]	[illegible]	[illegible]	[illegible]	[illegible]	[illegible]	[illegible]	[illegible]	[illegible]	[illegible]	[illegible]	[illegible]	[illegible]	[illegible]	[illegible]	[illegible]	14·4 (7·3 to 25·1)	16,400
Chronic kidney disease due to hypertension	0 (0 to 0)	0 (0 to 0)	0 (0 to 0)	0 (0 to 0)	[illegible]	[illegible]	[illegible]	[illegible]	[illegible]	[illegible]	[illegible]	[illegible]	[illegible]	[illegible]	[illegible]	[illegible]	[illegible]	[illegible]	[illegible]	[illegible]	[illegible]	[illegible]	23·7 (14·9 to 40·5)	14,100
Chronic kidney disease due to glomerulonephritis	0 (0 to 0)	0·1 (0 to 0·1)	730 (171 to 1,650)	1,200 (405 to 2,270)	[illegible]	[illegible]	[illegible]	[illegible]	[illegible]	[illegible]	[illegible]	[illegible]	[illegible]	[illegible]	[illegible]	[illegible]	[illegible]	[illegible]	[illegible]	[illegible]	[illegible]	[illegible]	3·5 (1·2 to 8·6)	24,400
Chronic kidney disease due to other and unspecified ca	0 (0 to 0)	0·2 (0·1 to 0·3)	1,830 (492 to 3,640)	2,540 (910 to 4,070)	[illegible]	[illegible]	[illegible]	[illegible]	[illegible]	[illegible]	[illegible]	[illegible]	[illegible]	[illegible]	[illegible]	[illegible]	[illegible]	[illegible]	[illegible]	[illegible]	[illegible]	[illegible]	16·1 (9·9 to 24·7)	30,000
Acute glomerulonephritis	0 (0 to 0)	0 (0 to 0)	8·4 (1·4 to 28·1)	74·2 (7·5 to 246)	[illegible]	[illegible]	[illegible]	[illegible]	[illegible]	[illegible]	[illegible]	[illegible]	[illegible]	[illegible]	[illegible]	[illegible]	[illegible]	[illegible]	[illegible]	[illegible]	[illegible]	[illegible]	0·1 (0 to 0·3)	625
Skin and subcutaneous diseases	0·4 (0·3 to 0·7)	5·7 (3·3 to 9·1)	2,580 (934 to 7,160)	8,610 (5,400 to 12,700)	[illegible]	[illegible]	[illegible]	[illegible]	[illegible]	[illegible]	[illegible]	[illegible]	[illegible]	[illegible]	[illegible]	[illegible]	[illegible]	[illegible]	[illegible]	[illegible]	[illegible]	[illegible]	36·6 (18·7 to 62·4)	74,100
Dermatitis	0 (0 to 0)	0 (0 to 0)	0 (0 to 0)	2,760 (1,490 to 4,670)	[illegible]	[illegible]	[illegible]	[illegible]	[illegible]	[illegible]	[illegible]	[illegible]	[illegible]	[illegible]	[illegible]	[illegible]	[illegible]	[illegible]	[illegible]	[illegible]	[illegible]	[illegible]	1·1 (0·6 to 1·7)	14,900
Atopic dermatitis	0 (0 to 0)	0 (0 to 0)	0 (0 to 0)	2,740 (1,470 to 4,630)	[illegible]	[illegible]	[illegible]	[illegible]	[illegible]	[illegible]	[illegible]	[illegible]	[illegible]	[illegible]	[illegible]	[illegible]	[illegible]	[illegible]	[illegible]	[illegible]	[illegible]	[illegible]	0·5 (0·3 to 0·9)	11,600
Contact dermatitis	0 (0 to 0)	0 (0 to 0)	0 (0 to 0)	0 (0 to 0)	[illegible]	[illegible]	[illegible]	[illegible]	[illegible]	[illegible]	[illegible]	[illegible]	[illegible]	[illegible]	[illegible]	[illegible]	[illegible]	[illegible]	[illegible]	[illegible]	[illegible]	[illegible]	0·5 (0·2 to 0·9)	2,910
Seborrhoeic dermatitis	0 (0 to 0)	0 (0 to 0)	0 (0 to 0)	24·8 (11·6 to 44·8)	[illegible]	[illegible]	[illegible]	[illegible]	[illegible]	[illegible]	[illegible]	[illegible]	[illegible]	[illegible]	[illegible]	[illegible]	[illegible]	[illegible]	[illegible]	[illegible]	[illegible]	[illegible]	0·1 (0 to 0·1)	384
Psoriasis	0 (0 to 0)	0 (0 to 0)	0 (0 to 0)	137 (65·7 to 236)	[illegible]	[illegible]	[illegible]	[illegible]	[illegible]	[illegible]	[illegible]	[illegible]	[illegible]	[illegible]	[illegible]	[illegible]	[illegible]	[illegible]	[illegible]	[illegible]	[illegible]	[illegible]	0·3 (0·1 to 0·4)	4,850
Bacterial skin diseases	0·1 (0 to 0·1)	0·6 (0·3 to 1·1)	1,940 (436 to 6,120)	787 (275 to 2,250)	[illegible]	[illegible]	[illegible]	[illegible]	[illegible]	[illegible]	[illegible]	[illegible]	[illegible]	[illegible]	[illegible]	[illegible]	[illegible]	[illegible]	[illegible]	[illegible]	[illegible]	[illegible]	6·6 (1·4 to 17·1)	7,640
Cellulitis	0 (0 to 0)	0·1 (0·1 to 0·2)	442 (55·9 to 1,700)	191 (44·4 to 779)	[illegible]	[illegible]	[illegible]	[illegible]	[illegible]	[illegible]	[illegible]	[illegible]	[illegible]	[illegible]	[illegible]	[illegible]	[illegible]	[illegible]	[illegible]	[illegible]	[illegible]	[illegible]	1·2 (0·2 to 3·7)	2,050
Pyoderma	0 (0 to 0·1)	0·4 (0·2 to 0·9)	1,500 (302 to 5,280)	596 (162 to 1,810)	[illegible]	[illegible]	[illegible]	[illegible]	[illegible]	[illegible]	[illegible]	[illegible]	[illegible]	[illegible]	[illegible]	[illegible]	[illegible]	[illegible]	[illegible]	[illegible]	[illegible]	[illegible]	5·4 (1·1 to 14·1)	5,580
Scabies	0·2 (0·1 to 0·3)	2·4 (1·2 to 3·9)	210 (110 to 363)	1,360 (690 to 2,390)	[illegible]	[illegible]	[illegible]	[illegible]	[illegible]	[illegible]	[illegible]	[illegible]	[illegible]	[illegible]	[illegible]	[illegible]	[illegible]	[illegible]	[illegible]	[illegible]	[illegible]	[illegible]	1 (0·5 to 1·7)	11,000
Fungal skin diseases	0 (0 to 0·1)	0·4 (0·2 to 0·9)	37·9 (14·3 to 82·5)	277 (103 to 606)	[illegible]	[illegible]	[illegible]	[illegible]	[illegible]	[illegible]	[illegible]	[illegible]	[illegible]	[illegible]	[illegible]	[illegible]	[illegible]	[illegible]	[illegible]	[illegible]	[illegible]	[illegible]	4 (1·6 to 8·1)	4,630
Viral skin diseases	0 (0 to 0)	0 (0 to 0)	0 (0 to 0)	1,450 (927 to 2,220)	[illegible]	[illegible]	[illegible]	[illegible]	[illegible]	[illegible]	[illegible]	[illegible]	[illegible]	[illegible]	[illegible]	[illegible]	[illegible]	[illegible]	[illegible]	[illegible]	[illegible]	[illegible]	0·1 (0·1 to 0·1)	6,590
Acne vulgaris	0 (0 to 0)	0 (0 to 0)	0 (0 to 0)	0 (0 to 0)	[illegible]	[illegible]	[illegible]	[illegible]	[illegible]	[illegible]	[illegible]	[illegible]	[illegible]	[illegible]	[illegible]	[illegible]	[illegible]	[illegible]	[illegible]	[illegible]	[illegible]	[illegible]	0 (0 to 0)	6,820
Alopecia areata	0 (0 to 0)	0 (0 to 0)	0 (0 to 0)	27·8 (13·9 to 46·6)	[illegible]	[illegible]	[illegible]	[illegible]	[illegible]	[illegible]	[illegible]	[illegible]	[illegible]	[illegible]	[illegible]	[illegible]	[illegible]	[illegible]	[illegible]	[illegible]	[illegible]	[illegible]	0 (0 to 0)	869
Pruritus	0 (0 to 0)	0 (0 to 0·1)	4·6 (1·8 to 9·3)	49·8 (22 to 105)	[illegible]	[illegible]	[illegible]	[illegible]	[illegible]	[illegible]	[illegible]	[illegible]	[illegible]	[illegible]	[illegible]	[illegible]	[illegible]	[illegible]	[illegible]	[illegible]	[illegible]	[illegible]	0·2 (0·1 to 0·4)	849
Urticaria	0·1 (0·1 to 0·2)	1·9 (1·1 to 3·2)	211 (128 to 323)	1,320 (795 to 2,020)	[illegible]	[illegible]	[illegible]	[illegible]	[illegible]	[illegible]	[illegible]	[illegible]	[illegible]	[illegible]	[illegible]	[illegible]	[illegible]	[illegible]	[illegible]	[illegible]	[illegible]	[illegible]	0·2 (0·1 to 0·4)	6,320
Decubitus ulcer	0 (0 to 0)	0 (0 to 0)	0 (0 to 0)	109 (14·2 to 430)	[illegible]	[illegible]	[illegible]	[illegible]	[illegible]	[illegible]	[illegible]	[illegible]	[illegible]	[illegible]	[illegible]	[illegible]	[illegible]	[illegible]	[illegible]	[illegible]	[illegible]	[illegible]	21·6 (9·3 to 41·6)	4,480
Other skin and subcutaneous diseases	0 (0 to 0·1)	0·4 (0·2 to 0·8)	172 (41·1 to 911)	337 (161 to 605)	[illegible]	[illegible]	[illegible]	[illegible]	[illegible]	[illegible]	[illegible]	[illegible]	[illegible]	[illegible]	[illegible]	[illegible]	[illegible]	[illegible]	[illegible]	[illegible]	[illegible]	[illegible]	1·5 (0·8 to 2·5)	5,150
Sense organ diseases	0·9 (0·4 to 1·5)	3·3 (1·9 to 5·4)	85·1 (51·5 to 126)	900 (571 to 1,320)	[illegible]	[illegible]	[illegible]	[illegible]	[illegible]	[illegible]	[illegible]	[illegible]	[illegible]	[illegible]	[illegible]	[illegible]	[illegible]	[illegible]	[illegible]	[illegible]	[illegible]	[illegible]	105 (75·1 to 139)	66,400
Blindness and vision loss	0·2 (0 to 0·5)	0·7 (0·2 to 1·6)	13·9 (6·6 to 26·5)	240 (136 to 396)	[illegible]	[illegible]	[illegible]	[illegible]	[illegible]	[illegible]	[illegible]	[illegible]	[illegible]	[illegible]	[illegible]	[illegible]	[illegible]	[illegible]	[illegible]	[illegible]	[illegible]	[illegible]	48·3 (33·6 to 67·8)	19,800
Glaucoma	0 (0 to 0)	0 (0 to 0)	0 (0 to 0)	0 (0 to 0)	[illegible]	[illegible]	[illegible]	[illegible]	[illegible]	[illegible]	[illegible]	[illegible]	[illegible]	[illegible]	[illegible]	[illegible]	[illegible]	[illegible]	[illegible]	[illegible]	[illegible]	[illegible]	6·8 (3·9 to 11)	1,000
Cataract	0 (0 to 0)	0 (0 to 0)	0 (0 to 0)	0 (0 to 0)	[illegible]	[illegible]	[illegible]	[illegible]	[illegible]	[illegible]	[illegible]	[illegible]	[illegible]	[illegible]	[illegible]	[illegible]	[illegible]	[illegible]	[illegible]	[illegible]	[illegible]	[illegible]	16·5 (10·2 to 24·5)	3,110
Age-related macular degeneration	0 (0 to 0)	0 (0 to 0)	0 (0 to 0)	0 (0 to 0)	[illegible]	[illegible]	[illegible]	[illegible]	[illegible]	[illegible]	[illegible]	[illegible]	[illegible]	[illegible]	[illegible]	[illegible]	[illegible]	[illegible]	[illegible]	[illegible]	[illegible]	[illegible]	0·4 (0·2 to 0·6)	107
Refraction disorders	0 (0 to 0)	0·1 (0 to 0·3)	8·6 (3·7 to 16·8)	192 (105 to 329)	[illegible]	[illegible]	[illegible]	[illegible]	[illegible]	[illegible]	[illegible]	[illegible]	[illegible]	[illegible]	[illegible]	[illegible]	[illegible]	[illegible]	[illegible]	[illegible]	[illegible]	[illegible]	1·4 (0·8 to 2·2)	6,060
Near vision loss	0 (0 to 0)	0 (0 to 0)	0 (0 to 0·1)	1 (0·3 to 2·5)	[illegible]	[illegible]	[illegible]	[illegible]	[illegible]	[illegible]	[illegible]	[illegible]	[illegible]	[illegible]	[illegible]	[illegible]	[illegible]	[illegible]	[illegible]	[illegible]	[illegible]	[illegible]	5·7 (2·1 to 12·1)	3,980
Other vision loss	0·2 (0 to 0·5)	0·6 (0·2 to 1·3)	5·2 (2·2 to 10·2)	46·7 (23·8 to 78·7)	[illegible]	[illegible]	[illegible]	[illegible]	[illegible]	[illegible]	[illegible]	[illegible]	[illegible]	[illegible]	[illegible]	[illegible]	[illegible]	[illegible]	[illegible]	[illegible]	[illegible]	[illegible]	17·6 (10·9 to 26·2)	5,550
Age-related and other hearing loss	0·2 (0·1 to 0·5)	0·7 (0·3 to 1·6)	36·4 (17 to 63·4)	496 (258 to 820)	[illegible]	[illegible]	[illegible]	[illegible]	[illegible]	[illegible]	[illegible]	[illegible]	[illegible]	[illegible]	[illegible]	[illegible]	[illegible]	[illegible]	[illegible]	[illegible]	[illegible]	[illegible]	54·9 (38·8 to 73·5)	43,300
Other sense organ diseases	0·4 (0·2 to 0·9)	1·8 (0·9 to 3·3)	34·8 (19 to 58·5)	165 (81·9 to 277)	[illegible]	[illegible]	[illegible]	[illegible]	[illegible]	[illegible]	[illegible]	[illegible]	[illegible]	[illegible]	[illegible]	[illegible]	[illegible]	[illegible]	[illegible]	[illegible]	[illegible]	[illegible]	2·1 (1 to 3·7)	3,280
Musculoskeletal disorders	0 (0 to 0)	0 (0 to 0)	0 (0 to 0)	0 (0 to 0)	[illegible]	[illegible]	[illegible]	[illegible]	[illegible]	[illegible]	[illegible]	[illegible]	[illegible]	[illegible]	[illegible]	[illegible]	[illegible]	[illegible]	[illegible]	[illegible]	[illegible]	[illegible]	37·7 (25·9 to 53·2)	136,000
Rheumatoid arthritis	0 (0 to 0)	0 (0 to 0)	0 (0 to 0)	0 (0 to 0)	[illegible]	[illegible]	[illegible]	[illegible]	[illegible]	[illegible]	[illegible]	[illegible]	[illegible]	[illegible]	[illegible]	[illegible]	[illegible]	[illegible]	[illegible]	[illegible]	[illegible]	[illegible]	2·8 (1·5 to 5)	3,470
Osteoarthritis	0 (0 to 0)	0 (0 to 0)	0 (0 to 0)	0 (0 to 0)	[illegible]	[illegible]	[illegible]	[illegible]	[illegible]	[illegible]	[illegible]	[illegible]	[illegible]	[illegible]	[illegible]	[illegible]	[illegible]	[illegible]	[illegible]	[illegible]	[illegible]	[illegible]	11·8 (6·1 to 23·3)	13,200
Osteoarthritis hip	0 (0 to 0)	0 (0 to 0)	0 (0 to 0)	0 (0 to 0)	[illegible]	[illegible]	[illegible]	[illegible]	[illegible]	[illegible]	[illegible]	[illegible]	[illegible]	[illegible]	[illegible]	[illegible]	[illegible]	[illegible]	[illegible]	[illegible]	[illegible]	[illegible]	0·6 (0·3 to 1·2)	567
Osteoarthritis knee	0 (0 to 0)	0 (0 to 0)	0 (0 to 0)	0 (0 to 0)	[illegible]	[illegible]	[illegible]	[illegible]	[illegible]	[illegible]	[illegible]	[illegible]	[illegible]	[illegible]	[illegible]	[illegible]	[illegible]	[illegible]	[illegible]	[illegible]	[illegible]	[illegible]	6·4 (3·2 to 12·8)	9,420
Osteoarthritis hand	0 (0 to 0)	0 (0 to 0)	0 (0 to 0)	0 (0 to 0)	[illegible]	[illegible]	[illegible]	[illegible]	[illegible]	[illegible]	[illegible]	[illegible]	[illegible]	[illegible]	[illegible]	[illegible]	[illegible]	[illegible]	[illegible]	[illegible]	[illegible]	[illegible]	2·4 (1·1 to 4·8)	1,730
Osteoarthritis other	0 (0 to 0)	0 (0 to 0)	0 (0 to 0)	0 (0 to 0)	[illegible]	[illegible]	[illegible]	[illegible]	[illegible]	[illegible]	[illegible]	[illegible]	[illegible]	[illegible]	[illegible]	[illegible]	[illegible]	[illegible]	[illegible]	[illegible]	[illegible]	[illegible]	2·5 (1·3 to 4·9)	1,470
Low back pain	0 (0 to 0)	0 (0 to 0)	0 (0 to 0)	0 (0 to 0)	[illegible]	[illegible]	[illegible]	[illegible]	[illegible]	[illegible]	[illegible]	[illegible]	[illegible]	[illegible]	[illegible]	[illegible]	[illegible]	[illegible]	[illegible]	[illegible]	[illegible]	[illegible]	16 (10 to 24·1)	74,700
Neck pain	0 (0 to 0)	0 (0 to 0)	0 (0 to 0)	0 (0 to 0)	[illegible]	[illegible]	[illegible]	[illegible]	[illegible]	[illegible]	[illegible]	[illegible]	[illegible]	[illegible]	[illegible]	[illegible]	[illegible]	[illegible]	[illegible]	[illegible]	[illegible]	[illegible]	2·8 (1·5 to 4·9)	13,500
Gout	0 (0 to 0)	0 (0 to 0)	0 (0 to 0)	0 (0 to 0)	[illegible]	[illegible]	[illegible]	[illegible]	[illegible]	[illegible]	[illegible]	[illegible]	[illegible]	[illegible]	[illegible]	[illegible]	[illegible]	[illegible]	[illegible]	[illegible]	[illegible]	[illegible]	0·6 (0·3 to 1·1)	577
Other musculoskeletal disorders	0 (0 to 0)	0 (0 to 0)	0 (0 to 0)	0 (0 to 0)	[illegible]	[illegible]	[illegible]	[illegible]	[illegible]	[illegible]	[illegible]	[illegible]	[illegible]	[illegible]	[illegible]	[illegible]	[illegible]	[illegible]	[illegible]	[illegible]	[illegible]	[illegible]	3·8 (2 to 6·7)	30,900
Other non-communicable diseases	98,800 (58,200 to 160,000)	50,900 (31,900 to 75,700)	95,800 (54,200 to 156,000)	68,700 (46,900 to 104,000)	[illegible]	[illegible]	[illegible]	[illegible]	[illegible]	[illegible]	[illegible]	[illegible]	[illegible]	[illegible]	[illegible]	[illegible]	[illegible]	[illegible]	[illegible]	[illegible]	[illegible]	[illegible]	54·4 (39·5 to 79·9)	516,000
Congenital birth defects	97,500 (57,000 to 159,000)	48,300 (29,600 to 72,400)	74,900 (39,900 to 123,000)	50,500 (28,900 to 88,900)	[illegible]	[illegible]	[illegible]	[illegible]	[illegible]	[illegible]	[illegible]	[illegible]	[illegible]	[illegible]	[illegible]	[illegible]	[illegible]	[illegible]	[illegible]	[illegible]	[illegible]	[illegible]	0·2 (0·1 to 0·4)	316,000
Neural tube defects	18,000 (4,030 to 49,100)	6,800 (1,290 to 19,100)	10,800 (1,550 to 33,900)	10,900 (821 to 38,900)	[illegible]	[illegible]	[illegible]	[illegible]	[illegible]	[illegible]	[illegible]	[illegible]	[illegible]	[illegible]	[illegible]	[illegible]	[illegible]	[illegible]	[illegible]	[illegible]	[illegible]	[illegible]	0 (0 to 0)	50,200
Congenital heart anomalies	34,700 (16,000 to 61,400)	21,600 (9,880 to 36,800)	32,500 (11,200 to 64,200)	19,600 (5,180 to 41,200)	[illegible]	[illegible]	[illegible]	[illegible]	[illegible]	[illegible]	[illegible]	[illegible]	[illegible]	[illegible]	[illegible]	[illegible]	[illegible]	[illegible]	[illegible]	[illegible]	[illegible]	[illegible]	0 (0 to 0)	126,000
Orofacial clefts	225 (42·6 to 787)	77·9 (15·9 to 275)	125 (25·8 to 570)	103 (43 to 234)	[illegible]	[illegible]	[illegible]	[illegible]	[illegible]	[illegible]	[illegible]	[illegible]	[illegible]	[illegible]	[illegible]	[illegible]	[illegible]	[illegible]	[illegible]	[illegible]	[illegible]	[illegible]	0 (0 to 0·1)	847
Down syndrome	1,050 (314 to 2,430)	665 (176 to 1,690)	2,440 (428 to 8,030)	2,550 (457 to 6,960)	[illegible]	[illegible]	[illegible]	[illegible]	[illegible]	[illegible]	[illegible]	[illegible]	[illegible]	[illegible]	[illegible]	[illegible]	[illegible]	[illegible]	[illegible]	[illegible]	[illegible]	[illegible]	0 (0 to 0)	11,200
Turner syndrome	0 (0 to 0·1)	0·1 (0 to 0·2)	1 (0 to 3)	3·5 (1·3 to 6·9)	[illegible]	[illegible]	[illegible]	[illegible]	[illegible]	[illegible]	[illegible]	[illegible]	[illegible]	[illegible]	[illegible]	[illegible]	[illegible]	[illegible]	[illegible]	[illegible]	[illegible]	[illegible]	0 (0 to 0)	21·7
Klinefelter syndrome	0 (0 to 0)	0 (0 to 0)	0·1 (0·1 to 0·3)	0·4 (0·2 to 0·8)	[illegible]	[illegible]	[illegible]	[illegible]	[illegible]	[illegible]	[illegible]	[illegible]	[illegible]	[illegible]	[illegible]	[illegible]	[illegible]	[illegible]	[illegible]	[illegible]	[illegible]	[illegible]	0 (0 to 0)	1·8
Other chromosomal abnormalities	4,530 (1,440 to 10,900)	2,520 (770 to 5,780)	3,570 (656 to 9,660)	1,450 (337 to 3,490)	[illegible]	[illegible]	[illegible]	[illegible]	[illegible]	[illegible]	[illegible]	[illegible]	[illegible]	[illegible]	[illegible]	[illegible]	[illegible]	[illegible]	[illegible]	[illegible]	[illegible]	[illegible]	0 (0 to 0)	13,900
Congenital musculoskeletal and limb anomalies	2,050 (817 to 4,260)	866 (348 to 1,720)	2,040 (1,020 to 3,780)	2,150 (1,210 to 3,500)	[illegible]	[illegible]	[illegible]	[illegible]	[illegible]	[illegible]	[illegible]	[illegible]	[illegible]	[illegible]	[illegible]	[illegible]	[illegible]	[illegible]	[illegible]	[illegible]	[illegible]	[illegible]	0 (0 to 0·1)	12,600
Urogenital congenital anomalies	2,140 (807 to 4,670)	922 (360 to 1,800)	923 (221 to 2,840)	472 (152 to 1,390)	[illegible]	[illegible]	[illegible]	[illegible]	[illegible]	[illegible]	[illegible]	[illegible]	[illegible]	[illegible]	[illegible]	[illegible]	[illegible]	[illegible]	[illegible]	[illegible]	[illegible]	[illegible]	0 (0 to 0)	5,040
Digestive congenital anomalies	13,900 (5,120 to 27,600)	8,120 (2,300 to 16,400)	9,320 (1,270 to 21,800)	3,680 (446 to 9,700)	[illegible]	[illegible]	[illegible]	[illegible]	[illegible]	[illegible]	[illegible]	[illegible]	[illegible]	[illegible]	[illegible]	[illegible]	[illegible]	[illegible]	[illegible]	[illegible]	[illegible]	[illegible]	0 (0 to 0)	36,100
Other congenital birth defects	20,900 (8,360 to 38,400)	6,740 (2,330 to 13,200)	13,100 (2,210 to 34,100)	9,660 (1,490 to 21,300)	[illegible]	[illegible]	[illegible]	[illegible]	[illegible]	[illegible]	[illegible]	[illegible]	[illegible]	[illegible]	[illegible]	[illegible]	[illegible]	[illegible]	[illegible]	[illegible]	[illegible]	[illegible]	0·1 (0 to 0·3)	60,100
Urinary diseases and male infertility	108 (52·4 to 211)	225 (90·8 to 446)	1,520 (356 to 3,300)	1,010 (361 to 1,710)	[illegible]	[illegible]	[illegible]	[illegible]	[illegible]	[illegible]	[illegible]	[illegible]	[illegible]	[illegible]	[illegible]	[illegible]	[illegible]	[illegible]	[illegible]	[illegible]	[illegible]	[illegible]	16·3 (9·2 to 31·6)	15,200
Urolithiasis	0 (0 to 0)	0 (0 to 0)	0 (0 to 0)	41·2 (5·5 to 162)	[illegible]	[illegible]	[illegible]	[illegible]	[illegible]	[illegible]	[illegible]	[illegible]	[illegible]	[illegible]	[illegible]	[illegible]	[illegible]	[illegible]	[illegible]	[illegible]	[illegible]	[illegible]	0·2 (0·1 to 0·7)	1,030
Benign prostatic hyperplasia	0 (0 to 0)	0 (0 to 0)	0 (0 to 0)	0 (0 to 0)	[illegible]	[illegible]	[illegible]	[illegible]	[illegible]	[illegible]	[illegible]	[illegible]	[illegible]	[illegible]	[illegible]	[illegible]	[illegible]	[illegible]	[illegible]	[illegible]	[illegible]	[illegible]	1 (0·5 to 1·6)	1,660
Male infertility	0 (0 to 0)	0 (0 to 0)	0 (0 to 0)	0 (0 to 0)	[illegible]	[illegible]	[illegible]	[illegible]	[illegible]	[illegible]	[illegible]	[illegible]	[illegible]	[illegible]	[illegible]	[illegible]	[illegible]	[illegible]	[illegible]	[illegible]	[illegible]	[illegible]	0 (0 to 0)	549
Other urinary diseases	44·6 (18·2 to 92·4)	131 (48 to 271)	163 (30·1 to 437)	125 (46·4 to 273)	[illegible]	[illegible]	[illegible]	[illegible]	[illegible]	[illegible]	[illegible]	[illegible]	[illegible]	[illegible]	[illegible]	[illegible]	[illegible]	[illegible]	[illegible]	[illegible]	[illegible]	[illegible]	1·1 (0·3 to 2·8)	1,730
Gynecological diseases	0 (0 to 0)	0 (0 to 0)	0 (0 to 0)	0 (0 to 0)	[illegible]	[illegible]	[illegible]	[illegible]	[illegible]	[illegible]	[illegible]	[illegible]	[illegible]	[illegible]	[illegible]	[illegible]	[illegible]	[illegible]	[illegible]	[illegible]	[illegible]	[illegible]	2·4 (1·3 to 3·8)	47,700
Uterine fibroids	0 (0 to 0)	0 (0 to 0)	0 (0 to 0)	0 (0 to 0)	[illegible]	[illegible]	[illegible]	[illegible]	[illegible]	[illegible]	[illegible]	[illegible]	[illegible]	[illegible]	[illegible]	[illegible]	[illegible]	[illegible]	[illegible]	[illegible]	[illegible]	[illegible]	0 (0 to 0·1)	2,850
Polycystic ovarian syndrome	0 (0 to 0)	0 (0 to 0)	0 (0 to 0)	0 (0 to 0)	[illegible]	[illegible]	[illegible]	[illegible]	[illegible]	[illegible]	[illegible]	[illegible]	[illegible]	[illegible]	[illegible]	[illegible]	[illegible]	[illegible]	[illegible]	[illegible]	[illegible]	[illegible]	0 (0 to 0)	529
Female infertility	0 (0 to 0)	0 (0 to 0)	0 (0 to 0)	0 (0 to 0)	[illegible]	[illegible]	[illegible]	[illegible]	[illegible]	[illegible]	[illegible]	[illegible]	[illegible]	[illegible]	[illegible]	[illegible]	[illegible]	[illegible]	[illegible]	[illegible]	[illegible]	[illegible]	0 (0 to 0)	1,210
Endometriosis	0 (0 to 0)	0 (0 to 0)	0 (0 to 0)	0 (0 to 0)	[illegible]	[illegible]	[illegible]	[illegible]	[illegible]	[illegible]	[illegible]	[illegible]	[illegible]	[illegible]	[illegible]	[illegible]	[illegible]	[illegible]	[illegible]	[illegible]	[illegible]	[illegible]	0 (0 to 0)	3,880
Genital prolapse	0 (0 to 0)	0 (0 to 0)	0 (0 to 0)	0 (0 to 0)	[illegible]	[illegible]	[illegible]	[illegible]	[illegible]	[illegible]	[illegible]	[illegible]	[illegible]	[illegible]	[illegible]	[illegible]	[illegible]	[illegible]	[illegible]	[illegible]	[illegible]	[illegible]	0·3 (0·2 to 0·6)	668
Premenstrual syndrome	0 (0 to 0)	0 (0 to 0)	0 (0 to 0)	0 (0 to 0)	[illegible]	[illegible]	[illegible]	[illegible]	[illegible]	[illegible]	[illegible]	[illegible]	[illegible]	[illegible]	[illegible]	[illegible]	[illegible]	[illegible]	[illegible]	[illegible]	[illegible]	[illegible]	0 (0 to 0)	13,800
Other gynecological diseases	0 (0 to 0)	0 (0 to 0)	0 (0 to 0)	0 (0 to 0)	[illegible]	[illegible]	[illegible]	[illegible]	[illegible]	[illegible]	[illegible]	[illegible]	[illegible]	[illegible]	[illegible]	[illegible]	[illegible]	[illegible]	[illegible]	[illegible]	[illegible]	[illegible]	2 (1 to 3·5)	24,700
Hemoglobinopathies and hemolytic anemias	334 (198 to 498)	403 (227 to 671)	3,250 (1,120 to 6,290)	9,280 (3,600 to 16,000)	[illegible]	[illegible]	[illegible]	[illegible]	[illegible]	[illegible]	[illegible]	[illegible]	[illegible]	[illegible]	[illegible]	[illegible]	[illegible]	[illegible]	[illegible]	[illegible]	[illegible]	[illegible]	7 (4·7 to 10·1)	51,600
Thalassemias	0·3 (0·1 to 0·6)	0·3 (0·1 to 0·7)	7·6 (1·6 to 17·1)	49 (10·2 to 107)	[illegible]	[illegible]	[illegible]	[illegible]	[illegible]	[illegible]	[illegible]	[illegible]	[illegible]	[illegible]	[illegible]	[illegible]	[illegible]	[illegible]	[illegible]	[illegible]	[illegible]	[illegible]	0 (0 to 0)	75·9
Thalassemias trait	1 (0·5 to 1·8)	6 (3·5 to 9·1)	58·7 (35·3 to 92·4)	234 (140 to 367)	[illegible]	[illegible]	[illegible]	[illegible]	[illegible]	[illegible]	[illegible]	[illegible]	[illegible]	[illegible]	[illegible]	[illegible]	[illegible]	[illegible]	[illegible]	[illegible]	[illegible]	[illegible]	0·1 (0·1 to 0·2)	2,000
Sickle cell disorders	319 (182 to 483)	294 (126 to 552)	2,720 (629 to 5,720)	7,070 (1,810 to 13,700)	[illegible]	[illegible]	[illegible]	[illegible]	[illegible]	[illegible]	[illegible]	[illegible]	[illegible]	[illegible]	[illegible]	[illegible]	[illegible]	[illegible]	[illegible]	[illegible]	[illegible]	[illegible]	0 (0 to 0)	26,900
Sickle cell trait	11·6 (6·4 to 18·6)	84·2 (50·1 to 131)	267 (164 to 394)	1,070 (682 to 1,610)	[illegible]	[illegible]	[illegible]	[illegible]	[illegible]	[illegible]	[illegible]	[illegible]	[illegible]	[illegible]	[illegible]	[illegible]	[illegible]	[illegible]	[illegible]	[illegible]	[illegible]	[illegible]	0·5 (0·3 to 0·8)	13,200
G6PD deficiency	1·1 (0·5 to 2)	5·7 (2·9 to 9·3)	31·5 (9·1 to 68·1)	271 (59·6 to 586)	[illegible]	[illegible]	[illegible]	[illegible]	[illegible]	[illegible]	[illegible]	[illegible]	[illegible]	[illegible]	[illegible]	[illegible]	[illegible]	[illegible]	[illegible]	[illegible]	[illegible]	[illegible]	0·1 (0 to 0·2)	3,340
G6PD trait	0 (0 to 0)	0 (0 to 0·5)	0 (0 to 0)	0 (0 to 0)	[illegible]	[illegible]	[illegible]	[illegible]	[illegible]	[illegible]	[illegible]	[illegible]	[illegible]	[illegible]	[illegible]	[illegible]	[illegible]	[illegible]	[illegible]	[illegible]	[illegible]	[illegible]	0 (0 to 0)	17·9
Other hemoglobinopathies and hemolytic anemias	1 (0·6 to 1·5)	12·5 (7·7 to 18·9)	163 (96·7 to 252)	586 (350 to 916)	[illegible]	[illegible]	[illegible]	[illegible]	[illegible]	[illegible]	[illegible]	[illegible]	[illegible]	[illegible]	[illegible]	[illegible]	[illegible]	[illegible]	[illegible]	[illegible]	[illegible]	[illegible]	6·2 (4·1 to 9·2)	6,100
Endocrine, metabolic, blood, and immune disorders	865 (450 to 1,480)	983 (463 to 1,810)	8,890 (3,110 to 17,800)	7,090 (3,290 to 12,300)	[illegible]	[illegible]	[illegible]	[illegible]	[illegible]	[illegible]	[illegible]	[illegible]	[illegible]	[illegible]	[illegible]	[illegible]	[illegible]	[illegible]	[illegible]	[illegible]	[illegible]	[illegible]	13·1 (7·4 to 22·7)	47,100
Oral disorders	0 (0 to 0)	0 (0 to 0)	0 (0 to 0)	734 (416 to 1,200)	[illegible]	[illegible]	[illegible]	[illegible]	[illegible]	[illegible]	[illegible]	[illegible]	[illegible]	[illegible]	[illegible]	[illegible]	[illegible]	[illegible]	[illegible]	[illegible]	[illegible]	[illegible]	15·5 (9·7 to 22·4)	30,500
Caries of deciduous teeth	0 (0 to 0)	0 (0 to 0)	0 (0 to 0)	247 (96·7 to 512)	[illegible]	[illegible]	[illegible]	[illegible]	[illegible]	[illegible]	[illegible]	[illegible]	[illegible]	[illegible]	[illegible]	[illegible]	[illegible]	[illegible]	[illegible]	[illegible]	[illegible]	[illegible]	0 (0 to 0)	476
Caries of permanent teeth	0 (0 to 0)	0 (0 to 0)	0 (0 to 0)	0 (0 to 0)	[illegible]	[illegible]	[illegible]	[illegible]	[illegible]	[illegible]	[illegible]	[illegible]	[illegible]	[illegible]	[illegible]	[illegible]	[illegible]	[illegible]	[illegible]	[illegible]	[illegible]	[illegible]	0·2 (0·1 to 0·4)	3,450
Periodontal diseases	0 (0 to 0)	0 (0 to 0)	0 (0 to 0)	0 (0 to 0)	[illegible]	[illegible]	[illegible]	[illegible]	[illegible]	[illegible]	[illegible]	[illegible]	[illegible]	[illegible]	[illegible]	[illegible]	[illegible]	[illegible]	[illegible]	[illegible]	[illegible]	[illegible]	1·4 (0·5 to 3)	9,720
Other oral disorders	0 (0 to 0)	0 (0 to 0)	0 (0 to 0)	487 (296 to 760)	[illegible]	[illegible]	[illegible]	[illegible]	[illegible]	[illegible]	[illegible]	[illegible]	[illegible]	[illegible]	[illegible]	[illegible]	[illegible]	[illegible]	[illegible]	[illegible]	[illegible]	[illegible]	0·3 (0·2 to 0·5)	6,320
Sudden infant death syndrome	--	959 (290 to 2,680)	7,280 (1,020 to 23,200)	--	[illegible]	[illegible]	[illegible]	[illegible]	[illegible]	[illegible]	[illegible]	[illegible]	[illegible]	[illegible]	[illegible]	[illegible]	[illegible]	[illegible]	[illegible]	[illegible]	[illegible]	[illegible]	--	NA
Injuries	8,800 (5,320 to 15,400)	8,320 (3,830 to 30,400)	37,300 (18,400 to 59,500)	106,000 (65,700 to 141,000)	[illegible]	[illegible]	[illegible]	[illegible]	[illegible]	[illegible]	[illegible]	[illegible]	[illegible]	[illegible]	[illegible]	[illegible]	[illegible]	[illegible]	[illegible]	[illegible]	[illegible]	[illegible]	115 (89·2 to 151)	806,000
Transport injuries	836 (352 to 1,730)	589 (273 to 1,150)	2,630 (1,300 to 4,510)	11,100 (6,180 to 19,500)	[illegible]	[illegible]	[illegible]	[illegible]	[illegible]	[illegible]	[illegible]	[illegible]	[illegible]	[illegible]	[illegible]	[illegible]	[illegible]	[illegible]	[illegible]	[illegible]	[illegible]	[illegible]	5·9 (4·3 to 10·7)	101,000
Road injuries	681 (283 to 1,410)	434 (195 to 853)	2,270 (1,110 to 3,950)	10,300 (5,680 to 18,000)	[illegible]	[illegible]	[illegible]	[illegible]	[illegible]	[illegible]	[illegible]	[illegible]	[illegible]	[illegible]	[illegible]	[illegible]	[illegible]	[illegible]	[illegible]	[illegible]	[illegible]	[illegible]	5·2 (3·8 to 9·4)	93,100
Pedestrian road injuries	315 (119 to 688)	181 (68·6 to 395)	729 (300 to 1,480)	4,640 (2,300 to 8,560)	[illegible]	[illegible]	[illegible]	[illegible]	[illegible]	[illegible]	[illegible]	[illegible]	[illegible]	[illegible]	[illegible]	[illegible]	[illegible]	[illegible]	[illegible]	[illegible]	[illegible]	[illegible]	1·9 (1·3 to 3·8)	24,300
Cyclist road injuries	0 (0 to 0)	0 (0 to 0)	0 (0 to 0)	999 (244 to 2,580)	[illegible]	[illegible]	[illegible]	[illegible]	[illegible]	[illegible]	[illegible]	[illegible]	[illegible]	[illegible]	[illegible]	[illegible]	[illegible]	[illegible]	[illegible]	[illegible]	[illegible]	[illegible]	0·7 (0·5 to 0·9)	10,100
Motorcyclist road injuries	102 (24·3 to 273)	84·8 (22·2 to 210)	528 (148 to 1,240)	1,510 (454 to 3,620)	[illegible]	[illegible]	[illegible]	[illegible]	[illegible]	[illegible]	[illegible]	[illegible]	[illegible]	[illegible]	[illegible]	[illegible]	[illegible]	[illegible]	[illegible]	[illegible]	[illegible]	[illegible]	1 (0·7 to 1·4)	22,900
Motor vehicle road injuries	247 (86·4 to 559)	159 (55·4 to 348)	991 (332 to 2,080)	3,080 (987 to 6,850)	[illegible]	[illegible]	[illegible]	[illegible]	[illegible]	[illegible]	[illegible]	[illegible]	[illegible]	[illegible]	[illegible]	[illegible]	[illegible]	[illegible]	[illegible]	[illegible]	[illegible]	[illegible]	1·4 (0·9 to 3·2)	34,900
Other road injuries	16·1 (4·4 to 46·9)	9·1 (2·2 to 27·8)	25·8 (7·2 to 66·4)	70 (21·9 to 180)	[illegible]	[illegible]	[illegible]	[illegible]	[illegible]	[illegible]	[illegible]	[illegible]	[illegible]	[illegible]	[illegible]	[illegible]	[illegible]	[illegible]	[illegible]	[illegible]	[illegible]	[illegible]	0·1 (0·1 to 0·1)	930

Other transport injuries	155 (54·1 to 345)	155 (58·1 to 340)	353 (142 to 704)	806 (360 to 1,760)	618 (312 to 1,440)	577 (311 to 1,390)	662 (347 to 1,490)	779 (410 to 1,800)
Unintentional injuries	6,410 (3,680 to 12,500)	7,230 (2,960 to 29,600)	33,100 (14,800 to 54,600)	88,200 (45,300 to 121,000)	40,900 (29,200 to 51,800)	32,700 (25,600 to 41,600)	29,800 (20,900 to 41,400)	34,700 (24,300 to 49,200)
Falls	92·6 (39·3 to 182)	67·5 (20·3 to 145)	1,080 (181 to 2,780)	3,010 (649 to 6,820)	1,470 (763 to 2,390)	1,330 (864 to 1,880)	1,330 (900 to 1,810)	1,460 (1,000 to 2,070)
Drowning	371 (174 to 716)	245 (102 to 469)	4,260 (1,100 to 8,980)	38,100 (13,300 to 62,000)	19,800 (11,000 to 29,000)	11,300 (7,030 to 16,000)	7,050 (3,600 to 12,100)	4,940 (2,440 to 9,140)
Fire, heat, and hot substances	158 (59·5 to 275)	262 (68 to 494)	3,680 (581 to 7,050)	15,200 (2,960 to 25,900)	5,310 (2,250 to 8,300)	3,060 (1,940 to 4,130)	2,590 (1,690 to 3,530)	2,730 (1,820 to 3,780)
Poisonings	200 (68·8 to 565)	312 (76 to 1,060)	1,160 (380 to 2,800)	5,870 (2,640 to 10,900)	1,150 (559 to 2,580)	487 (250 to 1,210)	402 (207 to 952)	515 (257 to 1,390)
Poisoning by carbon monoxide	34·2 (9 to 93·6)	43·2 (6·7 to 158)	81·5 (12·9 to 265)	913 (255 to 2,100)	261 (86·7 to 701)	158 (62·3 to 481)	195 (80 to 539)	258 (106 to 767)
Poisoning by other means	165 (50·2 to 501)	268 (59·1 to 966)	1,070 (323 to 2,650)	4,960 (2,010 to 9,630)	888 (410 to 1,980)	329 (162 to 804)	207 (102 to 438)	258 (120 to 634)
Exposure to mechanical forces	1,510 (137 to 6,700)	3,240 (54·6 to 24,500)	2,080 (227 to 8,220)	5,070 (1,950 to 9,990)	4,640 (1,790 to 8,220)	3,780 (1,420 to 6,800)	5,550 (1,810 to 12,100)	6,580 (2,300 to 14,400)
Unintentional firearm injuries	824 (52·4 to 4,040)	1,480 (9 to 14,100)	1,250 (58·1 to 5,870)	2,580 (508 to 6,260)	2,310 (584 to 5,440)	2,280 (679 to 5,070)	4,150 (963 to 10,600)	4,960 (1,250 to 13,000)
Other exposure to mechanical forces	684 (21·9 to 3,810)	1,760 (13 to 15,500)	832 (76·8 to 3,570)	2,490 (785 to 5,990)	2,330 (785 to 5,330)	1,490 (636 to 3,180)	1,390 (729 to 3,380)	1,620 (872 to 3,910)
Adverse effects of medical treatment	1,060 (625 to 1,720)	1,020 (505 to 1,730)	3,830 (1,130 to 7,710)	3,290 (1,140 to 5,980)	1,290 (730 to 2,050)	670 (421 to 968)	511 (308 to 756)	845 (498 to 1,320)
Animal contact	12 (4·1 to 22·7)	22·8 (6·7 to 45·4)	161 (36·8 to 328)	637 (163 to 1,300)	292 (148 to 463)	222 (139 to 336)	189 (132 to 266)	186 (126 to 258)
Venomous animal contact	9·8 (3·1 to 20·3)	17·9 (4·7 to 38·1)	106 (24·5 to 233)	436 (111 to 946)	176 (89 to 294)	155 (94 to 238)	131 (88·3 to 195)	131 (89·4 to 184)
Non-venomous animal contact	2·1 (0·5 to 4·7)	4·9 (1 to 11·2)	55·2 (4·7 to 137)	201 (22·7 to 482)	116 (34·4 to 221)	66·6 (29·8 to 122)	57·9 (30·5 to 91·2)	55·5 (29·7 to 88·2)
Foreign body	1,870 (964 to 3,320)	1,530 (805 to 2,480)	15,400 (5,890 to 27,900)	12,500 (6,780 to 19,400)	2,730 (1,720 to 4,120)	1,490 (1,010 to 2,090)	564 (390 to 801)	632 (432 to 906)
Pulmonary aspiration and foreign body in airway	1,860 (959 to 3,310)	1,520 (798 to 2,450)	15,200 (5,850 to 27,700)	12,200 (6,480 to 18,800)	2,540 (1,540 to 3,850)	1,320 (857 to 1,910)	408 (260 to 630)	470 (301 to 737)
Foreign body in eyes	0·1 (0 to 0·2)	0·3 (0·1 to 0·7)	5·3 (1·5 to 11·7)	26·9 (8·1 to 60·9)	40 (11·6 to 93·7)	47·3 (15·2 to 111)	55·8 (18 to 131)	63·4 (20·5 to 138)
Foreign body in other body part	12·7 (3·3 to 35·7)	9·5 (3·1 to 25·5)	150 (27 to 563)	332 (89·2 to 1,140)	158 (89·3 to 316)	123 (82·9 to 190)	101 (73·4 to 136)	98·2 (71·8 to 132)
Environmental heat and cold exposure	204 (41·8 to 506)	172 (69·7 to 386)	416 (150 to 947)	1,170 (543 to 2,350)	940 (460 to 1,860)	673 (386 to 1,180)	788 (448 to 1,700)	961 (535 to 2,130)
Exposure to forces of nature	0 (0 to 0)	0 (0 to 0)	0 (0 to 0)	6·9 (4·1 to 11·4)	824 (503 to 1,290)	7,760 (4,650 to 12,900)	9,130 (5,560 to 14,600)	14,100 (8,480 to 22,300)
Other unintentional injuries	928 (444 to 1,650)	353 (166 to 679)	1,050 (472 to 1,910)	3,250 (1,540 to 6,360)	2,400 (1,110 to 5,290)	1,910 (922 to 4,200)	1,740 (805 to 4,210)	1,750 (897 to 3,750)
Self-harm and interpersonal violence	1,560 (732 to 2,970)	509 (237 to 964)	1,640 (727 to 3,230)	6,480 (2,880 to 12,300)	5,090 (1,640 to 9,350)	8,500 (3,390 to 14,200)	32,300 (14,600 to 49,700)	44,200 (20,200 to 63,800)
Self-harm	0 (0 to 0)	0 (0 to 0)	0 (0 to 0)	0 (0 to 0)	0 (0 to 0)	1,660 (995 to 2,580)	6,750 (4,100 to 10,300)	8,230 (5,040 to 12,600)
Self-harm by firearm	0 (0 to 0)	0 (0 to 0)	0 (0 to 0)	0 (0 to 0)	0 (0 to 0)	35·8 (13·1 to 80·6)	389 (153 to 803)	544 (228 to 1,170)
Self-harm by other specified means	0 (0 to 0)	0 (0 to 0)	0 (0 to 0)	0 (0 to 0)	0 (0 to 0)	1,620 (970 to 2,550)	6,360 (3,840 to 9,830)	7,680 (4,610 to 11,700)
Interpersonal violence	1,560 (731 to 2,970)	509 (237 to 964)	1,640 (724 to 3,230)	6,450 (2,850 to 12,200)	5,060 (1,610 to 9,320)	6,810 (1,900 to 12,400)	25,400 (7,260 to 42,600)	35,900 (11,400 to 55,400)
Physical violence by firearm	81·7 (25·8 to 192)	34·3 (10·6 to 89·3)	81·7 (22·9 to 271)	484 (124 to 1,580)	596 (120 to 1,700)	1,300 (215 to 3,200)	11,600 (1,910 to 22,700)	17,700 (3,120 to 31,300)
Physical violence by sharp object	118 (38·7 to 285)	28·2 (6·1 to 88)	66·6 (21·9 to 214)	382 (119 to 1,280)	450 (125 to 1,570)	854 (224 to 2,350)	6,760 (1,830 to 17,100)	9,380 (2,690 to 23,100)
Sexual violence	0 (0 to 0)	0 (0 to 0)	0 (0 to 0)	14·1 (8·3 to 22·3)	110 (66·1 to 171)	238 (139 to 376)	506 (288 to 807)	629 (363 to 960)
Physical violence by other means	1,360 (624 to 2,640)	447 (206 to 873)	1,490 (644 to 3,020)	5,570 (2,340 to 11,000)	3,910 (1,170 to 7,350)	4,420 (1,080 to 9,020)	6,530 (1,380 to 13,700)	8,180 (1,920 to 17,000)
Conflict and terrorism	0 (0 to 0)	0 (0 to 0)	0 (0 to 0)	2·7 (1·5 to 4·6)	3·2 (1·8 to 5·9)	7·8 (4·5 to 12·9)	60·2 (30·7 to 109)	44·4 (23·5 to 78)

Other transport injuries	760 (398 to 1,880)	733 (384 to 1,760)	591 (317 to 1,470)	542 (297 to 1,380)	355 (203 to 890)	275 (165 to 669)	243 (139 to 654)	155 (94·5 to 388)
Unintentional injuries	29,100 (20,400 to 40,700)	28,100 (20,400 to 37,800)	25,600 (18,300 to 34,200)	21,700 (15,800 to 29,100)	16,300 (11,700 to 22,500)	13,100 (9,370 to 17,900)	10,700 (7,840 to 14,600)	8,690 (6,590 to 11,600)
Falls	1,600 (1,150 to 2,130)	1,750 (1,270 to 2,320)	1,810 (1,300 to 2,400)	1,710 (1,230 to 2,270)	1,530 (1,100 to 2,060)	1,520 (1,090 to 2,070)	1,380 (981 to 1,860)	1,360 (1,000 to 1,810)
Drowning	4,290 (2,260 to 7,550)	3,730 (2,110 to 6,000)	3,110 (1,710 to 5,010)	2,490 (1,400 to 4,120)	1,610 (893 to 2,740)	1,180 (657 to 2,040)	808 (444 to 1,440)	564 (324 to 993)
Fire, heat, and hot substances	3,060 (2,020 to 4,270)	3,080 (2,120 to 4,110)	2,750 (1,930 to 3,660)	2,210 (1,570 to 2,950)	1,660 (1,150 to 2,190)	1,290 (876 to 1,740)	1,030 (702 to 1,360)	798 (527 to 1,090)
Poisonings	638 (305 to 1,880)	638 (302 to 1,980)	532 (255 to 1,610)	386 (186 to 1,150)	273 (127 to 917)	180 (84·9 to 577)	135 (66·6 to 442)	98·2 (47·2 to 344)
Poisoning by carbon monoxide	280 (106 to 988)	288 (112 to 1,100)	243 (90·2 to 896)	175 (64·9 to 646)	105 (37 to 475)	57·7 (19·7 to 236)	54·8 (19·8 to 233)	37·9 (13·6 to 153)
Poisoning by other means	358 (163 to 956)	350 (156 to 991)	289 (140 to 691)	211 (99·1 to 541)	168 (74·6 to 484)	122 (58·5 to 366)	80·1 (38·2 to 244)	60·3 (29·7 to 185)
Exposure to mechanical forces	6,410 (2,490 to 13,800)	6,170 (2,720 to 12,000)	5,300 (2,460 to 10,000)	4,760 (2,160 to 9,420)	3,500 (1,650 to 6,790)	2,840 (1,390 to 5,440)	2,230 (1,120 to 4,390)	1,610 (869 to 2,730)
Unintentional firearm injuries	4,530 (1,180 to 12,100)	4,150 (1,300 to 10,100)	3,340 (1,060 to 8,100)	2,970 (918 to 7,260)	2,000 (651 to 5,090)	1,520 (495 to 3,920)	1,140 (383 to 2,990)	736 (275 to 1,770)
Other exposure to mechanical forces	1,880 (1,070 to 4,170)	2,020 (1,190 to 4,000)	1,960 (1,160 to 4,100)	1,800 (1,080 to 3,740)	1,490 (882 to 2,950)	1,320 (784 to 2,590)	1,090 (669 to 2,060)	873 (556 to 1,480)
Adverse effects of medical treatment	876 (514 to 1,330)	963 (547 to 1,530)	879 (514 to 1,380)	883 (525 to 1,410)	828 (471 to 1,410)	811 (472 to 1,380)	810 (454 to 1,440)	811 (452 to 1,370)
Animal contact	219 (144 to 316)	197 (136 to 277)	201 (134 to 293)	175 (116 to 259)	157 (100 to 251)	136 (89 to 221)	124 (79 to 201)	111 (69·3 to 191)
Venomous animal contact	158 (102 to 231)	141 (95·8 to 197)	146 (95 to 224)	122 (77·7 to 187)	103 (64·2 to 178)	90·6 (57·5 to 161)	81·6 (50·8 to 146)	73·3 (44·3 to 143)
Non-venomous animal contact	60·3 (31·7 to 102)	56 (31 to 88·4)	54·7 (30·7 to 86·8)	53·7 (30·2 to 95·5)	54·4 (29·7 to 96·1)	45·5 (25·9 to 75·8)	42·7 (23·8 to 73)	37·7 (20·5 to 68·1)
Foreign body	656 (465 to 909)	619 (448 to 837)	571 (394 to 790)	524 (379 to 739)	411 (283 to 592)	361 (249 to 519)	302 (210 to 457)	277 (188 to 402)
Pulmonary aspiration and foreign body in airway	486 (321 to 725)	452 (302 to 659)	422 (272 to 634)	394 (259 to 602)	306 (191 to 476)	280 (176 to 434)	236 (148 to 389)	222 (140 to 343)
Foreign body in eyes	68·9 (25·5 to 146)	69·1 (24·6 to 145)	62·3 (24·2 to 129)	52·6 (22·7 to 105)	40·7 (19·6 to 76·8)	30·4 (16·1 to 53·7)	22·9 (13·3 to 37·2)	18·1 (10·8 to 28·4)
Foreign body in other body part	102 (74 to 136)	97·2 (71·4 to 128)	86·6 (63·2 to 115)	77·2 (56·7 to 103)	64·5 (45·6 to 87·8)	51·2 (37·7 to 69·1)	43·1 (30·9 to 58·4)	37·2 (26·1 to 51·3)
Environmental heat and cold exposure	991 (591 to 1,940)	1,070 (638 to 2,000)	946 (577 to 1,790)	859 (505 to 1,700)	750 (436 to 1,480)	670 (369 to 1,320)	631 (329 to 1,270)	526 (278 to 1,070)
Exposure to forces of nature	8,400 (5,030 to 13,900)	8,020 (4,780 to 12,800)	7,820 (4,570 to 12,100)	6,290 (3,760 to 10,000)	4,550 (2,790 to 7,190)	3,410 (2,060 to 5,340)	2,660 (1,660 to 4,130)	2,110 (1,320 to 3,380)
Other unintentional injuries	1,930 (943 to 4,340)	1,900 (986 to 3,980)	1,690 (877 to 3,670)	1,440 (742 to 2,940)	1,070 (561 to 2,300)	743 (408 to 1,480)	588 (325 to 1,190)	423 (242 to 837)
Self-harm and interpersonal violence	41,100 (18,300 to 60,600)	34,800 (15,500 to 51,800)	27,000 (10,500 to 42,500)	20,100 (7,690 to 33,900)	13,300 (4,960 to 22,900)	8,850 (3,500 to 15,200)	5,920 (2,350 to 10,100)	3,890 (1,650 to 6,330)
Self-harm	7,040 (4,330 to 10,500)	6,860 (4,510 to 10,100)	5,820 (3,560 to 8,810)	4,640 (2,840 to 6,880)	3,280 (1,920 to 5,160)	2,470 (1,450 to 3,740)	1,770 (1,060 to 2,710)	1,310 (780 to 1,960)
Self-harm by firearm	458 (179 to 1,040)	465 (224 to 930)	344 (168 to 663)	279 (131 to 557)	184 (79·7 to 368)	150 (67·9 to 313)	92·2 (40·9 to 198)	68·5 (30·3 to 151)
Self-harm by other specified means	6,580 (4,130 to 9,800)	6,390 (4,240 to 9,330)	5,480 (3,380 to 8,250)	4,360 (2,690 to 6,490)	3,100 (1,820 to 4,840)	2,320 (1,340 to 3,500)	1,680 (995 to 2,580)	1,240 (735 to 1,850)
Interpersonal violence	33,900 (10,200 to 53,200)	27,700 (8,400 to 45,200)	21,000 (5,060 to 35,900)	15,300 (3,590 to 29,000)	9,890 (2,100 to 18,500)	6,310 (1,380 to 12,000)	4,110 (860 to 8,240)	2,560 (584 to 4,770)
Physical violence by firearm	15,400 (2,710 to 27,700)	11,000 (1,800 to 21,100)	7,140 (950 to 14,800)	4,270 (538 to 9,690)	2,450 (260 to 5,670)	1,250 (141 to 3,010)	698 (82·3 to 1,810)	362 (48·9 to 841)
Physical violence by sharp object	8,950 (2,550 to 22,800)	7,920 (2,240 to 19,600)	6,080 (1,440 to 15,300)	4,440 (961 to 11,100)	2,590 (522 to 7,100)	1,530 (303 to 4,500)	818 (190 to 2,280)	479 (122 to 1,450)
Sexual violence	572 (319 to 928)	503 (276 to 827)	395 (215 to 636)	260 (148 to 426)	165 (96·5 to 270)	101 (57·5 to 165)	63·9 (37·1 to 103)	47·6 (29 to 71·1)
Physical violence by other means	8,970 (2,150 to 17,700)	8,240 (1,880 to 16,600)	7,400 (1,440 to 15,200)	6,340 (1,150 to 13,800)	4,680 (810 to 9,920)	3,430 (598 to 7,420)	2,530 (439 to 5,380)	1,670 (314 to 3,330)
Conflict and terrorism	105 (57·7 to 179)	160 (84·2 to 279)	130 (70·5 to 226)	107 (58·7 to 181)	86·4 (47·9 to 147)	61·5 (35·1 to 105)	36·6 (20·8 to 62·6)	21 (12 to 35·8)

Other transport injuries	98·8 (64·7 to 215)	70·4 (43·6 to 172)	41·2 (27·2 to 99·5)	19·5 (13·6 to 36·6)	8·2 (5·8 to 14·5)	2·7 (2 to 4·5)	0·7 (0·5 to 1·2)	8,000
Unintentional injuries	6,560 (4,960 to 8,440)	4,670 (3,660 to 5,980)	3,270 (2,540 to 4,190)	2,260 (1,770 to 2,940)	1,130 (852 to 1,470)	424 (321 to 558)	104 (79·6 to 140)	445,000
Falls	1,200 (879 to 1,600)	1,120 (825 to 1,490)	967 (696 to 1,280)	897 (651 to 1,190)	528 (359 to 703)	210 (145 to 290)	52·6 (35·6 to 77·3)	27,500
Drowning	351 (206 to 610)	167 (99·4 to 287)	94·5 (58·6 to 159)	34·9 (22·1 to 57·3)	16·5 (9·8 to 26·8)	5·6 (3·5 to 8·8)	1·3 (0·8 to 2·1)	105,000
Fire, heat, and hot substances	559 (382 to 762)	380 (234 to 530)	249 (155 to 356)	158 (94·9 to 223)	81·6 (49·8 to 118)	28 (17·5 to 39·2)	6·7 (4·2 to 9)	50,400
Poisonings	68·5 (36·1 to 211)	40·3 (21·4 to 114)	26 (13·9 to 72·4)	15·4 (8 to 48·1)	5·7 (3·1 to 15·4)	1·7 (0·9 to 5·5)	0·3 (0·2 to 0·8)	13,100
Poisoning by carbon monoxide	29·5 (12·3 to 104)	17·5 (7 to 57·6)	10·1 (3·8 to 34·8)	6·6 (2·3 to 26·6)	2·1 (0·8 to 8·1)	0·5 (0·2 to 2·3)	0·1 (0 to 0·3)	3,250
Poisoning by other means	39 (19·1 to 117)	22·8 (12·1 to 56·8)	15·9 (7·9 to 41·6)	8·8 (4·4 to 22)	3·5 (1·8 to 8·5)	1·2 (0·6 to 3·5)	0·2 (0·1 to 0·5)	9,880
Exposure to mechanical forces	1,070 (632 to 1,740)	653 (396 to 994)	403 (250 to 592)	229 (146 to 332)	108 (68·5 to 155)	38·2 (23·9 to 54·9)	9·8 (5·6 to 15·4)	67,800
Unintentional firearm injuries	429 (180 to 1,010)	238 (106 to 515)	130 (62·5 to 272)	75·1 (37·7 to 143)	37·9 (18·3 to 70·9)	13·6 (6·6 to 24·1)	3·7 (1·8 to 7)	41,200
Other exposure to mechanical forces	646 (421 to 1,040)	415 (268 to 626)	273 (174 to 406)	154 (102 to 235)	70·4 (46·3 to 104)	24·6 (15·9 to 36·8)	6·2 (3·6 to 10·2)	26,600
Adverse effects of medical treatment	680 (413 to 1,130)	522 (326 to 887)	411 (247 to 695)	262 (156 to 448)	122 (71·1 to 213)	39·2 (23·3 to 71·2)	8·4 (4·9 to 14·8)	21,400
Animal contact	93·7 (58 to 161)	66·5 (41·2 to 109)	47·1 (27·7 to 80)	30·4 (17·8 to 49·3)	14·6 (8·2 to 24·5)	7·3 (3·6 to 14)	2·1 (1 to 4·9)	3,300
Venomous animal contact	59·3 (35·5 to 119)	40 (24·7 to 75·8)	30·7 (17·5 to 56·4)	20 (11·2 to 34·3)	10·2 (5·6 to 18·4)	5·5 (2·5 to 10·9)	1·6 (0·7 to 3·8)	2,250
Non-venomous animal contact	34·4 (17·9 to 61·2)	26·5 (13 to 48·5)	16·4 (8·8 to 27·7)	10·4 (5·6 to 19·3)	4·4 (2·4 to 8·3)	1·8 (1 to 3·6)	0·6 (0·3 to 1·3)	1,060
Foreign body	238 (165 to 347)	174 (120 to 247)	130 (91·7 to 181)	89·3 (63 to 123)	34·2 (23·1 to 48·2)	12·8 (8·9 to 17·5)	3·5 (2·5 to 4·7)	41,100
Pulmonary aspiration and foreign body in airway	193 (126 to 298)	142 (95·2 to 210)	107 (70·4 to 155)	73·8 (49·3 to 105)	27·2 (17·4 to 40·1)	10·1 (6·8 to 14·6)	2·7 (1·8 to 3·8)	38,900
Foreign body in eyes	14·1 (8·7 to 21·5)	9·1 (5·8 to 13·5)	6 (3·9 to 8·9)	3·5 (2·3 to 5·1)	1·5 (1 to 2·1)	0·5 (0·3 to 0·7)	0·1 (0·1 to 0·1)	639
Foreign body in other body part	30·9 (21·9 to 43)	22·5 (15·6 to 34)	17·3 (11·7 to 24·6)	12·1 (7·9 to 18·2)	5·5 (3·6 to 8·4)	2·3 (1·4 to 3·5)	0·6 (0·4 to 1)	1,630
Environmental heat and cold exposure	438 (231 to 886)	378 (175 to 870)	278 (129 to 616)	209 (92·6 to 459)	105 (47·3 to 234)	48·6 (20·6 to 112)	13 (5·4 to 30)	13,200
Exposure to forces of nature	1,550 (983 to 2,350)	981 (605 to 1,490)	546 (343 to 819)	270 (165 to 429)	89·6 (56·2 to 137)	24·1 (15·3 to 36·8)	4·1 (2·6 to 6·2)	78,600
Other unintentional injuries	303 (184 to 541)	180 (112 to 313)	114 (73·4 to 183)	60·5 (40·4 to 89·6)	26·4 (17·9 to 39·2)	8·3 (5·6 to 12·1)	1·8 (1·3 to 2·7)	23,800
Self-harm and interpersonal violence	2,370 (1,110 to 3,840)	1,250 (645 to 1,950)	647 (353 to 993)	270 (164 to 384)	103 (64·3 to 145)	22·7 (14·9 to 30·5)	5·3 (3·1 to 8·1)	260,000
Self-harm	896 (506 to 1,360)	519 (300 to 761)	282 (157 to 415)	143 (82·6 to 215)	55 (32·8 to 81·8)	10·8 (6·5 to 15·9)	1·7 (1 to 2·5)	51,700
Self-harm by firearm	45·1 (19·4 to 98·2)	26·9 (12·1 to 56·3)	14·5 (6·8 to 30·9)	7·9 (3·7 to 15·9)	2·8 (1·5 to 5·2)	0·4 (0·2 to 0·8)	0·1 (0 to 0·2)	3,110
Self-harm by other specified means	850 (480 to 1,280)	492 (277 to 722)	268 (147 to 390)	135 (77·3 to 203)	52·2 (30·5 to 77·1)	10·5 (6·2 to 15·4)	1·6 (0·9 to 2·4)	48,600
Interpersonal violence	1,460 (361 to 2,720)	725 (200 to 1,400)	361 (107 to 672)	126 (51·4 to 201)	47·2 (21·4 to 75·5)	11·7 (6·3 to 17·6)	3·5 (1·5 to 6·3)	207,000
Physical violence by firearm	166 (25·1 to 428)	64·7 (11·5 to 156)	20·7 (4·9 to 55)	6·6 (2 to 15·5)	2·6 (0·9 to 5·9)	0·7 (0·2 to 1·4)	0·2 (0·1 to 0·4)	74,800
Physical violence by sharp object	239 (76·2 to 679)	133 (44·4 to 352)	61·2 (23·6 to 153)	25·4 (12·2 to 53·6)	9·6 (5·3 to 19)	3·7 (2 to 6·6)	0·9 (0·5 to 2·1)	51,300
Sexual violence	35 (21·5 to 52·1)	21·1 (13·2 to 31·5)	13·1 (8·5 to 19·3)	7·4 (4·8 to 10·9)	2·6 (1·6 to 4·1)	0·3 (0·2 to 0·5)	0 (0 to 0)	3,680
Physical violence by other means	1,020 (207 to 2,130)	506 (110 to 1,040)	266 (61·7 to 536)	86·1 (28·3 to 156)	32·3 (12·3 to 57·2)	6·9 (3·6 to 10·9)	2·4 (0·9 to 4·9)	77,100
Conflict and terrorism	11·6 (6·6 to 19·8)	5·7 (3·2 to 9·7)	3 (1·8 to 5·2)	1·5 (0·9 to 2·6)	0·6 (0·3 to 0·9)	0·2 (0·1 to 0·3)	0 (0 to 0·1)	848

SR Table 2c: Jamaica DALYS by cause and age group for both sexes with 95% uncertainty intervals, 2019

Cause	Early Neonatal	Late Neonatal	Post Neonatal	1 to 4	5 to 9	10 to 14	15 to 19	20 to 24	25 to 29	30 to 34	35 to 39	40 to 44	45 to 49	50 to 54	55 to 59	60 to 64	65 to 69	70 to 74	75 to 79	80 to 84	85 to 89	90 to 94	95 plus	Total
All causes	30,900 (21,800 to 42,700)	9,000 (6,400 to 12,400)	6,320 (4,200 to 9,420)	10,800 (8,570 to 13,600)	11,900 (9,370 to 15,000)	15,500 (12,200 to 19,700)	23,400 (18,500 to 29,300)	33,500 (27,500 to 40,400)	39,000 (32,400 to 47,000)	40,800 (34,000 to 49,000)	40,900 (33,900 to 49,200)	41,800 (34,600 to 50,100)	49,100 (40,400 to 59,100)	54,300 (44,600 to 65,100)	59,400 (48,900 to 71,200)	60,700 (50,400 to 72,000)	55,700 (46,900 to 65,500)	54,500 (46,000 to 63,800)	43,300 (36,900 to 50,200)	34,300 (29,700 to 39,200)	24,500 (21,400 to 27,600)	14,200 (12,600 to 15,800)	9,170 (8,420 to 9,940)	763,000
Communicable, maternal, neonatal, and nutritional diseases	27,300 (19,300 to 37,700)	7,350 (5,240 to 10,100)	3,400 (2,270 to 5,200)	4,460 (3,380 to 5,710)	3,480 (2,470 to 4,800)	2,800 (2,130 to 3,620)	3,330 (2,560 to 4,270)	4,430 (3,600 to 5,420)	5,820 (4,950 to 6,880)	6,560 (5,760 to 7,540)	6,810 (6,070 to 7,790)	5,520 (4,840 to 6,410)	4,900 (4,240 to 5,780)	4,060 (3,490 to 4,800)	3,510 (2,950 to 4,230)	2,570 (2,140 to 3,080)	2,120 (1,780 to 2,520)	1,720 (1,350 to 2,130)	1,420 (1,150 to 1,710)	1,230 (962 to 1,540)	1,020 (803 to 1,270)	657 (502 to 800)	473 (348 to 585)	105,000
HIV/AIDS and sexually transmitted infections	4·3 (2·5 to 7·2)	3·1 (1·5 to 5·6)	234 (221 to 247)	294 (279 to 310)	197 (187 to 209)	171 (162 to 181)	363 (334 to 397)	953 (880 to 1,050)	2,290 (2,120 to 2,500)	3,300 (3,100 to 3,550)	3,940 (3,700 to 4,190)	2,980 (2,800 to 3,170)	2,430 (2,280 to 2,590)	1,740 (1,640 to 1,850)	1,210 (1,140 to 1,290)	651 (602 to 702)	561 (521 to 602)	118 (101 to 137)	46 (39 to 54·6)	13·8 (10·8 to 18·4)	6·5 (4·8 to 9·3)	2·7 (1·8 to 4)	1·7 (1·3 to 2·3)	21,500
HIV/AIDS	0 (0 to 0)	0 (0 to 0)	233 (220 to 246)	294 (279 to 310)	197 (187 to 209)	166 (158 to 176)	337 (313 to 364)	900 (836 to 983)	2,210 (2,060 to 2,400)	3,210 (3,020 to 3,450)	3,850 (3,620 to 4,100)	2,900 (2,730 to 3,080)	2,350 (2,210 to 2,500)	1,690 (1,590 to 1,790)	1,180 (1,110 to 1,260)	626 (581 to 671)	544 (506 to 579)	105 (92 to 119)	38·6 (33·5 to 43·7)	7·5 (6·5 to 8·5)	3 (2·4 to 3·7)	0·6 (0·3 to 1)	1 (0·8 to 1·2)	20,900
HIV/AIDS - Drug-susceptible Tuberculosis	0 (0 to 0)	0 (0 to 0)	3 (1·9 to 4·7)	3·9 (2·4 to 6)	2·8 (1·8 to 4·3)	2·3 (1·4 to 3·5)	4·4 (3 to 6·3)	12·4 (8·5 to 18)	30 (20·3 to 43·5)	44·1 (29·4 to 64·7)	53·3 (35·4 to 78·8)	41·3 (27·5 to 60·8)	35·8 (23·8 to 52·6)	26·3 (17·6 to 38·4)	19 (13·1 to 27·4)	11·2 (7·8 to 15·7)	9·2 (6·4 to 13·2)	2·5 (1·8 to 3·2)	0·9 (0·6 to 1·2)	0·2 (0·1 to 0·2)	0·1 (0·1 to 0·1)	0 (0 to 0)	0 (0 to 0)	303
HIV/AIDS - Multidrug-resistant Tuberculosis without extensive d	0 (0 to 0)	0 (0 to 0)	0·1 (0 to 0·5)	0·2 (0 to 0·6)	0·1 (0 to 0·4)	0·1 (0 to 0·4)	0·2 (0 to 0·6)	0·4 (0·1 to 1·7)	1·1 (0·1 to 4)	1·6 (0·2 to 5·8)	1·9 (0·2 to 7)	1·4 (0·2 to 5·4)	1·3 (0·2 to 4·7)	0·9 (0·1 to 3·5)	0·7 (0·1 to 2·4)	0·4 (0 to 1·3)	0·3 (0 to 1·1)	0·1 (0 to 0·2)	0 (0 to 0·1)	0 (0 to 0)	0 (0 to 0)	0 (0 to 0)	0 (0 to 0)	10·7
HIV/AIDS - Extensively drug-resistant Tuberculosis	0 (0 to 0)	0 (0 to 0)	0 (0 to 0·1)	0 (0 to 0·1)	0 (0 to 0·1)	0 (0 to 0·1)	0 (0 to 0·1)	0·1 (0 to 0·3)	0·2 (0 to 0·6)	0·2 (0 to 0·9)	0·3 (0 to 1·1)	0·2 (0 to 0·9)	0·2 (0 to 0·8)	0·2 (0 to 0·6)	0·1 (0 to 0·4)	0·1 (0 to 0·2)	0 (0 to 0·2)	0 (0 to 0)	0 (0 to 0)	0 (0 to 0)	0 (0 to 0)	0 (0 to 0)	0 (0 to 0)	1·7
HIV/AIDS resulting in other diseases	0 (0 to 0)	0 (0 to 0)	230 (217 to 243)	289 (274 to 306)	195 (184 to 206)	164 (155 to 173)	332 (308 to 360)	887 (822 to 969)	2,180 (2,030 to 2,360)	3,170 (2,980 to 3,410)	3,800 (3,570 to 4,040)	2,850 (2,690 to 3,040)	2,320 (2,180 to 2,460)	1,660 (1,560 to 1,760)	1,160 (1,090 to 1,240)	614 (570 to 659)	534 (496 to 568)	102 (89·6 to 117)	37·7 (32·6 to 42·8)	7·3 (6·3 to 8·3)	2·9 (2·3 to 3·6)	0·6 (0·3 to 1)	1 (0·8 to 1·1)	20,500
Sexually transmitted infections excluding HIV	4·3 (2·5 to 7·2)	3·1 (1·5 to 5·6)	0·9 (0·3 to 2·2)	0 (0 to 0)	0 (0 to 0)	4·8 (2·9 to 8·1)	25·8 (14·5 to 44)	53·5 (30·1 to 90·7)	75·9 (40·3 to 134)	89·3 (50·3 to 157)	86·2 (49·9 to 151)	82·5 (48·5 to 142)	74 (43·8 to 124)	45·8 (26·3 to 82·2)	31·3 (16·2 to 60·5)	25·3 (14 to 45·6)	17 (9·7 to 30·3)	12·9 (7·7 to 22·3)	7·5 (4·3 to 13·5)	6·3 (3·9 to 10·5)	3·5 (2·1 to 6·1)	2 (1·2 to 3·3)	0·7 (0·4 to 1·3)	653
Syphilis	4·3 (2·5 to 7·2)	3·1 (1·5 to 5·6)	0·9 (0·3 to 2·2)	0 (0 to 0)	0 (0 to 0)	0·7 (0·5 to 1·1)	1·5 (0·9 to 2·3)	1·3 (0·6 to 2·9)	1·7 (0·9 to 3·2)	3·2 (2 to 4·9)	3·3 (2·2 to 4·8)	2·6 (1·6 to 3·8)	4·9 (2·8 to 7·9)	4·4 (2·3 to 7·8)	3·4 (1·6 to 5·7)	3·3 (1·5 to 5·8)	2·3 (1·3 to 3·8)	2·2 (1·3 to 3·8)	0·9 (0·6 to 1·2)	1 (0·7 to 1·4)	0·5 (0·3 to 0·6)	0·4 (0·3 to 0·6)	0·1 (0·1 to 0·2)	46
Chlamydial infection	0 (0 to 0)	0 (0 to 0)	0 (0 to 0)	0 (0 to 0)	0 (0 to 0)	0·3 (0·2 to 0·4)	2·5 (1·6 to 4)	7·8 (3·8 to 13·6)	11·7 (5·4 to 21·8)	11·8 (6·1 to 20·9)	11·4 (6·7 to 18·2)	11·1 (6·8 to 17·7)	8·6 (5·1 to 13·4)	2·8 (1·5 to 5·1)	1·1 (0·8 to 1·6)	1 (0·6 to 1·6)	0·4 (0·2 to 0·7)	0·4 (0·3 to 0·7)	0·3 (0·2 to 0·4)	0·4 (0·3 to 0·7)	0·2 (0·1 to 0·3)	0·2 (0·1 to 0·2)	0 (0 to 0·1)	72·2
Gonococcal infection	0 (0 to 0)	0 (0 to 0)	0 (0 to 0)	0 (0 to 0)	0 (0 to 0)	0·8 (0·4 to 1·7)	5·7 (2 to 11·9)	7·9 (2·9 to 15·7)	5 (2·1 to 10)	6·5 (3·8 to 10)	6·9 (4·5 to 9·9)	9·5 (6·3 to 14·4)	9·1 (6 to 13·7)	4·3 (2·7 to 6·3)	1·8 (1·1 to 2·7)	2·6 (1·6 to 4·1)	2·3 (1·4 to 3·8)	1·7 (1 to 2·6)	1 (0·6 to 1·6)	1·1 (0·7 to 1·6)	0·5 (0·3 to 0·8)	0·4 (0·2 to 0·6)	0·1 (0 to 0·1)	67·1
Trichomoniasis	0 (0 to 0)	0 (0 to 0)	0 (0 to 0)	0 (0 to 0)	0 (0 to 0)	0·1 (0 to 0·4)	2·4 (0·7 to 5·5)	11·8 (3·9 to 28·1)	22·1 (7 to 51·1)	23·2 (7·2 to 54·8)	20·5 (6·7 to 48·9)	17·3 (5·7 to 41·9)	15·4 (5 to 35·4)	11·7 (3·8 to 27)	8 (2·6 to 19·6)	5 (1·7 to 11·7)	3·1 (1·1 to 7·2)	2 (0·7 to 4·5)	1·2 (0·5 to 2·6)	0·7 (0·3 to 1·5)	0·4 (0·1 to 0·8)	0·1 (0 to 0·3)	0 (0 to 0·1)	145
Genital herpes	0 (0 to 0)	0 (0 to 0)	0 (0 to 0)	0 (0 to 0)	0 (0 to 0)	1·2 (0·4 to 2·8)	5·5 (1·7 to 13·6)	12·9 (4·2 to 31·2)	19·8 (6·6 to 48·6)	22·2 (7·6 to 54·5)	21·5 (6·8 to 53·4)	19·3 (6·2 to 47·5)	18·6 (5·8 to 46·2)	16·6 (5·2 to 40·6)	14·1 (4·3 to 34·6)	11 (3·4 to 27·1)	7·7 (2·3 to 19·1)	5·6 (1·7 to 13·6)	3·5 (1·1 to 8·8)	2·4 (0·8 to 5·8)	1·5 (0·5 to 3·7)	0·8 (0·2 to 1·8)	0·4 (0·1 to 1)	184
Other sexually transmitted infections	0 (0 to 0)	0 (0 to 0)	0 (0 to 0)	0 (0 to 0)	0 (0 to 0)	1·6 (0·8 to 3·2)	8·3 (4·2 to 14·6)	11·8 (6·8 to 19·3)	15·6 (8·8 to 25·5)	22·4 (13·6 to 34·9)	22·6 (14·7 to 33·9)	22·6 (14·6 to 34·4)	17·4 (10·7 to 27·7)	6 (3·6 to 9·1)	2·9 (1·7 to 4·5)	2·4 (1·5 to 3·8)	1·1 (0·7 to 1·8)	1 (0·6 to 1·5)	0·6 (0·4 to 0·8)	0·7 (0·4 to 1·2)	0·4 (0·2 to 0·6)	0·2 (0·1 to 0·3)	0 (0 to 0·1)	138
Respiratory infections and tuberculosis	464 (282 to 717)	400 (254 to 603)	539 (295 to 904)	596 (415 to 820)	517 (361 to 729)	428 (288 to 628)	437 (299 to 647)	566 (391 to 781)	586 (427 to 789)	563 (401 to 775)	513 (370 to 710)	486 (340 to 661)	526 (376 to 732)	563 (394 to 778)	699 (486 to 981)	641 (458 to 878)	619 (433 to 856)	768 (534 to 1,070)	764 (580 to 987)	747 (530 to 993)	659 (487 to 859)	458 (332 to 578)	349 (240 to 442)	12,900
Tuberculosis	0 (0 to 0)	0 (0 to 0)	1·1 (0·7 to 1·8)	6·9 (4·8 to 9·5)	9 (2·8 to 18·4)	4 (2·3 to 6·4)	8·8 (5·8 to 12·6)	49·1 (30·5 to 75·3)	52·7 (33·9 to 79·8)	41·5 (25·6 to 62·2)	39·9 (25·8 to 57)	34·3 (22·2 to 49·8)	35·9 (22·9 to 53·8)	40·9 (26·5 to 59)	35 (23·2 to 50·5)	30·5 (19·7 to 44)	23·8 (16 to 33·6)	22·4 (15·7 to 30·5)	14·2 (9·9 to 19·8)	8·6 (6 to 11·6)	4·7 (3·3 to 6·5)	1·9 (1·3 to 2·6)	1·1 (0·8 to 1·4)	466
Latent tuberculosis infection	0 (0 to 0)	0 (0 to 0)	0 (0 to 0)	0 (0 to 0)	0 (0 to 0)	0 (0 to 0)	0 (0 to 0)	0 (0 to 0)	0 (0 to 0)	0 (0 to 0)	0 (0 to 0)	0 (0 to 0)	0 (0 to 0)	0 (0 to 0)	0 (0 to 0)	0 (0 to 0)	0 (0 to 0)	0 (0 to 0)	0 (0 to 0)	0 (0 to 0)	0 (0 to 0)	0 (0 to 0)	0 (0 to 0)	0
Drug-susceptible tuberculosis	0 (0 to 0)	0 (0 to 0)	1·1 (0·7 to 1·7)	6·7 (4·5 to 9·4)	8·8 (2·6 to 18·2)	3·9 (2·2 to 6·2)	8·5 (5·5 to 12·3)	47·6 (29·1 to 73·3)	51·4 (32·3 to 77·6)	40·5 (24·4 to 61·1)	38·7 (24·7 to 55·7)	33·3 (21·1 to 48·3)	34·7 (21·7 to 52·7)	39·5 (25·4 to 57·4)	33·8 (21·8 to 49·2)	29·5 (19 to 43·1)	22·9 (15·4 to 32·4)	21·6 (14·8 to 29·7)	13·7 (9·5 to 19·3)	8·3 (5·7 to 11·3)	4·6 (3·2 to 6·3)	1·8 (1·2 to 2·5)	1·1 (0·7 to 1·4)	452
Multidrug-resistant tuberculosis without extensive drug resistar	0 (0 to 0)	0 (0 to 0)	0 (0 to 0·1)	0·2 (0 to 0·6)	0·2 (0 to 0·6)	0·1 (0 to 0·3)	0·3 (0 to 0·9)	1·3 (0·2 to 4·5)	1·2 (0·2 to 4·3)	0·9 (0·1 to 3·5)	1 (0·1 to 3·6)	0·9 (0·1 to 3·2)	1 (0·1 to 3·9)	1·2 (0·2 to 4·1)	1 (0·1 to 3·7)	0·9 (0·1 to 3·2)	0·7 (0·1 to 2·4)	0·6 (0·1 to 2·2)	0·4 (0·1 to 1·4)	0·3 (0 to 1)	0·1 (0 to 0·5)	0·1 (0 to 0·2)	0 (0 to 0·1)	12·3
Extensively drug-resistant tuberculosis	0 (0 to 0)	0 (0 to 0)	0 (0 to 0)	0 (0 to 0·1)	0 (0 to 0·1)	0 (0 to 0)	0 (0 to 0·2)	0·2 (0 to 0·7)	0·2 (0 to 0·6)	0·1 (0 to 0·5)	0·2 (0 to 0·6)	0·1 (0 to 0·5)	0·2 (0 to 0·6)	0·2 (0 to 0·7)	0·2 (0 to 0·6)	0·1 (0 to 0·5)	0·1 (0 to 0·4)	0·1 (0 to 0·4)	0·1 (0 to 0·2)	0 (0 to 0·2)	0 (0 to 0·1)	0 (0 to 0)	0 (0 to 0)	1·9
Lower respiratory infections	463 (282 to 716)	397 (251 to 601)	489 (241 to 860)	334 (204 to 507)	184 (132 to 253)	134 (92·8 to 186)	135 (90·1 to 193)	213 (129 to 313)	263 (165 to 406)	301 (186 to 447)	281 (187 to 426)	285 (176 to 430)	333 (212 to 493)	384 (240 to 571)	549 (346 to 809)	523 (346 to 745)	534 (353 to 771)	704 (469 to 1,000)	723 (542 to 950)	720 (501 to 968)	643 (471 to 843)	449 (324 to 570)	344 (235 to 436)	9,380
Upper respiratory infections	0·4 (0·2 to 0·6)	2·2 (1·2 to 3·7)	40·7 (22·9 to 67·4)	179 (100 to 294)	214 (117 to 361)	208 (109 to 356)	213 (111 to 377)	223 (119 to 380)	205 (114 to 346)	172 (93·2 to 294)	149 (74·3 to 251)	125 (67·5 to 220)	114 (61·3 to 200)	98·5 (53·6 to 167)	82·3 (43·5 to 136)	64·7 (34·3 to 111)	47·4 (24·9 to 80·7)	33·5 (18·1 to 56·5)	20·6 (10·6 to 34·7)	13·7 (7·2 to 22·8)	8·3 (4·7 to 14)	4·6 (2·6 to 8)	2·8 (1·5 to 4·7)	2,220
Otitis media	0 (0 to 0·1)	0·2 (0·1 to 0·4)	8·2 (4·1 to 15·2)	75·9 (40·9 to 132)	110 (62·6 to 179)	81 (44·6 to 145)	81·2 (43·2 to 138)	81·1 (34·9 to 160)	65·4 (30·2 to 124)	48·6 (21·1 to 91·7)	43·5 (20·6 to 82·9)	41·6 (18·9 to 77·9)	42·6 (21·1 to 77)	40 (18·7 to 73·3)	32·8 (16·3 to 59·2)	23·7 (10·6 to 45·4)	12·9 (6·9 to 25·9)	8·6 (3·7 to 17·1)	5·7 (2·8 to 10·5)	4·3 (1·9 to 7·8)	3 (1·3 to 5·3)	1·7 (0·8 to 3·1)	1 (0·5 to 1·8)	815
Enteric infections	62 (34·4 to 104)	90·8 (49·8 to 151)	393 (240 to 610)	437 (303 to 617)	334 (210 to 526)	307 (181 to 486)	222 (142 to 320)	211 (139 to 305)	239 (164 to 334)	235 (162 to 323)	230 (165 to 318)	218 (155 to 299)	236 (167 to 323)	233 (165 to 316)	237 (170 to 322)	241 (173 to 318)	257 (189 to 339)	272 (196 to 360)	227 (169 to 296)	191 (144 to 248)	134 (98·2 to 176)	77·4 (57·4 to 99)	47·3 (35·7 to 61·1)	5,130
Diarrheal diseases	62 (34·4 to 104)	90·8 (49·8 to 151)	392 (239 to 609)	434 (300 to 613)	333 (209 to 524)	306 (180 to 484)	222 (142 to 329)	210 (138 to 305)	238 (164 to 334)	234 (161 to 321)	230 (164 to 318)	218 (154 to 299)	236 (167 to 322)	233 (165 to 316)	237 (170 to 321)	241 (173 to 318)	256 (189 to 338)	272 (196 to 359)	227 (169 to 296)	191 (143 to 248)	134 (98·2 to 176)	77·3 (57·3 to 99)	47·2 (35·7 to 61)	5,120
Typhoid and paratyphoid	0 (0 to 0)	0 (0 to 0)	0 (0 to 0)	0 (0 to 0·1)	0 (0 to 0·1)	0 (0 to 0·1)	0 (0 to 0·1)	0 (0 to 0·1)	0 (0 to 0)	0 (0 to 0)	0 (0 to 0)	0 (0 to 0)	0 (0 to 0)	0 (0 to 0)	0 (0 to 0)	0 (0 to 0)	0 (0 to 0)	0 (0 to 0·1)	0 (0 to 0)	0 (0 to 0)	0 (0 to 0)	0 (0 to 0)	0 (0 to 0)	0·5
Typhoid fever	0 (0 to 0)	0 (0 to 0)	0 (0 to 0)	0 (0 to 0)	0 (0 to 0)	0 (0 to 0)	0 (0 to 0)	0 (0 to 0)	0 (0 to 0)	0 (0 to 0)	0 (0 to 0)	0 (0 to 0)	0 (0 to 0)	0 (0 to 0)	0 (0 to 0)	0 (0 to 0)	0 (0 to 0)	0 (0 to 0)	0 (0 to 0)	0 (0 to 0)	0 (0 to 0)	0 (0 to 0)	0 (0 to 0)	0·2
Paratyphoid fever	0 (0 to 0)	0 (0 to 0)	0 (0 to 0)	0 (0 to 0)	0 (0 to 0·1)	0 (0 to 0)	0 (0 to 0)	0 (0 to 0)	0 (0 to 0)	0 (0 to 0)	0 (0 to 0)	0 (0 to 0)	0 (0 to 0)	0 (0 to 0)	0 (0 to 0)	0 (0 to 0)	0 (0 to 0)	0 (0 to 0)	0 (0 to 0)	0 (0 to 0)	0 (0 to 0)	0 (0 to 0)	0 (0 to 0)	0·3
Invasive Non-typhoidal Salmonella (iNTS)	0 (0 to 0)	0 (0 to 0)	0·1 (0 to 0·1)	0·6 (0·1 to 1·3)	0·7 (0·1 to 1·4)	0·3 (0·1 to 0·5)	0·1 (0 to 0·1)	0·1 (0 to 0·1)	0·1 (0 to 0·2)	0 (0 to 0)	0 (0 to 0)	0 (0 to 0)	0 (0 to 0)	0 (0 to 0·1)	0 (0 to 0)	0 (0 to 0)	0 (0 to 0·1)	0 (0 to 0·1)	0 (0 to 0)	0 (0 to 0)	0 (0 to 0)	0 (0 to 0)	0 (0 to 0)	1·9
Other intestinal infectious diseases	0 (0 to 0)	0 (0 to 0)	0·9 (0·1 to 2·5)	1·7 (0·4 to 4·3)	1·1 (0·2 to 2·8)	0·9 (0·2 to 2·2)	0·5 (0·1 to 1·1)	0·6 (0·1 to 1·4)	0·3 (0 to 0·8)	0·8 (0·2 to 2)	0·4 (0·1 to 0·9)	0·4 (0·1 to 1)	0·3 (0·1 to 0·7)	0·3 (0·1 to 0·7)	0·2 (0 to 0·5)	0·2 (0 to 0·5)	0·2 (0 to 0·6)	0·2 (0 to 0·5)	0·1 (0 to 0·3)	0·1 (0 to 0·3)	0·1 (0 to 0·4)	0·1 (0 to 0·2)	0·1 (0 to 0·2)	9·5
Neglected tropical diseases and malaria	0·6 (0·2 to 1)	3·6 (2 to 5·6)	32·2 (18·2 to 52·8)	154 (89·1 to 263)	216 (114 to 366)	155 (84·7 to 259)	156 (88·8 to 248)	216 (123 to 347)	200 (113 to 312)	163 (92·8 to 266)	150 (82·8 to 251)	131 (73·8 to 215)	131 (72·1 to 229)	134 (71·6 to 231)	145 (77·3 to 240)	142 (72·1 to 248)	135 (66·6 to 234)	124 (58·9 to 222)	99 (46·2 to 179)	74·2 (32·7 to 138)	49·3 (23·3 to 94·7)	27 (12·8 to 51·4)	16·5 (7·5 to 30·5)	2,650
Malaria	0 (0 to 0)	0 (0 to 0)	0 (0 to 0)	0 (0 to 0)	0 (0 to 0)	0 (0 to 0)	0 (0 to 0)	0 (0 to 0)	0 (0 to 0)	0 (0 to 0)	0 (0 to 0)	0 (0 to 0)	0 (0 to 0)	0 (0 to 0)	0 (0 to 0)	0 (0 to 0)	0 (0 to 0)	0 (0 to 0)	0 (0 to 0)	0 (0 to 0)	0 (0 to 0)	0 (0 to 0)	0 (0 to 0)	0
Chagas disease	0 (0 to 0)	0 (0 to 0)	0 (0 to 0)	0 (0 to 0)	0 (0 to 0)	0 (0 to 0)	0 (0 to 0)	0 (0 to 0)	0 (0 to 0)	0 (0 to 0)	0 (0 to 0)	0 (0 to 0)	0 (0 to 0)	0 (0 to 0)	0 (0 to 0)	0 (0 to 0)	0 (0 to 0)	0 (0 to 0)	0 (0 to 0)	0 (0 to 0)	0 (0 to 0)	0 (0 to 0)	0 (0 to 0)	0
Leishmaniasis	0 (0 to 0)	0 (0 to 0)	0 (0 to 0)	0 (0 to 0)	0 (0 to 0)	0 (0 to 0)	0 (0 to 0)	0 (0 to 0)	0 (0 to 0)	0 (0 to 0)	0 (0 to 0)	0 (0 to 0)	0 (0 to 0)	0 (0 to 0)	0 (0 to 0)	0 (0 to 0)	0 (0 to 0)	0 (0 to 0)	0 (0 to 0)	0 (0 to 0)	0 (0 to 0)	0 (0 to 0)	0 (0 to 0)	0
Visceral leishmaniasis	0 (0 to 0)	0 (0 to 0)	0 (0 to 0)	0 (0 to 0)	0 (0 to 0)	0 (0 to 0)	0 (0 to 0)	0 (0 to 0)	0 (0 to 0)	0 (0 to 0)	0 (0 to 0)	0 (0 to 0)	0 (0 to 0)	0 (0 to 0)	0 (0 to 0)	0 (0 to 0)	0 (0 to 0)	0 (0 to 0)	0 (0 to 0)	0 (0 to 0)	0 (0 to 0)	0 (0 to 0)	0 (0 to 0)	0
Cutaneous and mucocutaneous leishmaniasis	0 (0 to 0)	0 (0 to 0)	0 (0 to 0)	0 (0 to 0)	0 (0 to 0)	0 (0 to 0)	0 (0 to 0)	0 (0 to 0)	0 (0 to 0)	0 (0 to 0)	0 (0 to 0)	0 (0 to 0)	0 (0 to 0)	0 (0 to 0)	0 (0 to 0)	0 (0 to 0)	0 (0 to 0)	0 (0 to 0)	0 (0 to 0)	0 (0 to 0)	0 (0 to 0)	0 (0 to 0)	0 (0 to 0)	0
African trypanosomiasis	0 (0 to 0)	0 (0 to 0)	0 (0 to 0)	0 (0 to 0)	0 (0 to 0)	0 (0 to 0)	0 (0 to 0)	0 (0 to 0)	0 (0 to 0)	0 (0 to 0)	0 (0 to 0)	0 (0 to 0)	0 (0 to 0)	0 (0 to 0)	0 (0 to 0)	0 (0 to 0)	0 (0 to 0)	0 (0 to 0)	0 (0 to 0)	0 (0 to 0)	0 (0 to 0)	0 (0 to 0)	0 (0 to 0)	0
Schistosomiasis	0 (0 to 0)	0 (0 to 0)	0 (0 to 0)	0 (0 to 0)	0 (0 to 0)	0 (0 to 0)	0 (0 to 0)	0 (0 to 0)	0 (0 to 0)	0 (0 to 0)	0 (0 to 0)	0 (0 to 0)	0 (0 to 0)	0 (0 to 0)	0 (0 to 0)	0 (0 to 0)	0 (0 to 0)	0 (0 to 0)	0 (0 to 0)	0 (0 to 0)	0 (0 to 0)	0 (0 to 0)	0 (0 to 0)	0
Cysticercosis	0 (0 to 0)	0 (0 to 0)	0 (0 to 0)	0 (0 to 0)	0·1 (0 to 0·2)	1·3 (0·3 to 3·6)	9·9 (1 to 27·5)	36·3 (8·4 to 92·6)	58·5 (16·2 to 131)	59·6 (19·2 to 134)	59·7 (20·5 to 140)	63 (21·8 to 140)	73·1 (25·5 to 160)	82·4 (27·4 to 172)	99·5 (38·4 to 190)	114 (47·8 to 215)	114 (47·8 to 215)	109 (42·9 to 203)	86·4 (34·6 to 165)	66·7 (25·3 to 130)	43·5 (17·3 to 89·5)	22·9 (9 to 47·1)	12·8 (4·6 to 25·7)	1,110
Cystic echinococcosis	0 (0 to 0)	0 (0 to 0)	0 (0 to 0)	0 (0 to 0)	0 (0 to 0)	0 (0 to 0)	0 (0 to 0)	0 (0 to 0·1)	0 (0 to 0·1)	0 (0 to 0·1)	0 (0 to 0·1)	0 (0 to 0·1)	0 (0 to 0·1)	0 (0 to 0·1)	0 (0 to 0·1)	0 (0 to 0·1)	0 (0 to 0·1)	0 (0 to 0·1)	0 (0 to 0)	0 (0 to 0)	0 (0 to 0)	0 (0 to 0)	0 (0 to 0)	0·4
Lymphatic filariasis	0 (0 to 0)	0 (0 to 0)	0 (0 to 0)	0 (0 to 0)	0 (0 to 0)	0 (0 to 0)	0 (0 to 0)	0 (0 to 0)	0 (0 to 0)	0 (0 to 0)	0 (0 to 0)	0 (0 to 0)	0 (0 to 0)	0 (0 to 0)	0 (0 to 0)	0 (0 to 0)	0 (0 to 0)	0 (0 to 0)	0 (0 to 0)	0 (0 to 0)	0 (0 to 0)	0 (0 to 0)	0 (0 to 0)	0
Onchocerciasis	0 (0 to 0)	0 (0 to 0)	0 (0 to 0)	0 (0 to 0)	0 (0 to 0)	0 (0 to 0)	0 (0 to 0)	0 (0 to 0)	0 (0 to 0)	0 (0 to 0)	0 (0 to 0)	0 (0 to 0)	0 (0 to 0)	0 (0 to 0)	0 (0 to 0)	0 (0 to 0)	0 (0 to 0)	0 (0 to 0)	0 (0 to 0)	0 (0 to 0)	0 (0 to 0)	0 (0 to 0)	0 (0 to 0)	0
Trachoma	0 (0 to 0)	0 (0 to 0)	0 (0 to 0)	0 (0 to 0)	0 (0 to 0)	0 (0 to 0)	0 (0 to 0)	0 (0 to 0)	0 (0 to 0)	0 (0 to 0)	0 (0 to 0)	0 (0 to 0)	0 (0 to 0)	0 (0 to 0)	0 (0 to 0)	0 (0 to 0)	0 (0 to 0)	0 (0 to 0)	0 (0 to 0)	0 (0 to 0)	0 (0 to 0)	0 (0 to 0)	0 (0 to 0)	0
Dengue	0 (0 to 0)	0·3 (0 to 1·1)	5·7 (1·4 to 11·9)	14·6 (4·5 to 29·4)	19·9 (3·5 to 50·9)	23·2 (4·6 to 55·4)	26·1 (6 to 57·6)	29·1 (7·6 to 61·1)	24·9 (6·9 to 54·5)	18·2 (4·1 to 40·6)	13·9 (2·3 to 32·6)	12 (2·2 to 27·9)	13·9 (3·3 to 29·3)	11·1 (2·4 to 26·2)	9·1 (1·5 to 21·7)	9·4 (2·2 to 21·6)	9·7 (1·9 to 22·9)	6·9 (1·7 to 14·5)	5 (1·5 to 10)	3·1 (1 to 6·7)	2·4 (0·7 to 4·9)	2 (0·6 to 4·4)	2·2 (0·4 to 5·4)	263
Yellow fever	0 (0 to 0)	0 (0 to 0)	0 (0 to 0)	0 (0 to 0)	0 (0 to 0)	0 (0 to 0)	0 (0 to 0)	0 (0 to 0)	0 (0 to 0)	0 (0 to 0)	0 (0 to 0)	0 (0 to 0)	0 (0 to 0)	0 (0 to 0)	0 (0 to 0)	0 (0 to 0)	0 (0 to 0)	0 (0 to 0)	0 (0 to 0)	0 (0 to 0)	0 (0 to 0)	0 (0 to 0)	0 (0 to 0)	0
Rabies	0 (0 to 0)	0 (0 to 0)	0 (0 to 0)	0 (0 to 0·1)	0·1 (0 to 0·2)	0 (0 to 0·1)	0 (0 to 0)	0 (0 to 0)	0 (0 to 0)	0 (0 to 0)	0 (0 to 0)	0 (0 to 0)	0 (0 to 0)	0 (0 to 0)	0 (0 to 0)	0 (0 to 0)	0 (0 to 0)	0 (0 to 0)	0 (0 to 0)	0 (0 to 0)	0 (0 to 0)	0 (0 to 0)	0 (0 to 0)	0·2
Intestinal nematode infections	0 (0 to 0)	0 (0 to 0)	1·1 (0·6 to 2)	29·5 (15·3 to 50·5)	107 (53·8 to 187)	91·2 (45·3 to 164)	93 (47·6 to 161)	125 (62·8 to 219)	90·1 (46·4 to 154)	60·8 (30·6 to 106)	50·7 (26·8 to 84·5)	28·9 (14·6 to 49·2)	18·8 (9·4 to 33·1)	16·2 (8·3 to 28)	13·6 (7·1 to 22·8)	10 (5·2 to 17·5)	6·3 (3·3 to 11·1)	4·6 (2·3 to 7·9)	3·1 (1·6 to 5·3)	2 (1 to 3·3)	1·2 (0·6 to 2·1)	0·5 (0·3 to 0·9)	0·2 (0·1 to 0·4)	754
Ascariasis	0 (0 to 0)	0 (0 to 0)	0·3 (0·1 to 0·6)	4 (1·6 to 7·8)	8·1 (3·4 to 15·4)	4·7 (1·8 to 9·5)	3·2 (1·2 to 6·5)	3 (1·1 to 6·2)	2·8 (1 to 5·6)	2·4 (0·8 to 4·9)	2·1 (0·8 to 4·2)	1·7 (0·6 to 3·5)	1·5 (0·6 to 3)	1·3 (0·5 to 2·7)	1·1 (0·4 to 2·2)	1 (0·4 to 2)	0·6 (0·2 to 1·2)	0·5 (0·2 to 1·1)	0·4 (0·1 to 0·7)	0·2 (0·1 to 0·5)	0·1 (0 to 0·3)	0·1 (0 to 0·2)	0 (0 to 0·1)	39·1
Trichuriasis	0 (0 to 0)	0 (0 to 0)	0·7 (0·3 to 1·3)	19·1 (8·4 to 36·2)	83·3 (37·3 to 156)	73·9 (33·8 to 138)	64·2 (29·2 to 120)	67·8 (30·9 to 126)	50 (22 to 94·5)	32·8 (14·9 to 59·8)	23·7 (10 to 44·1)	16·5 (7·5 to 29·9)	13·8 (6 to 26·6)	11 (4·8 to 19·6)	8·8 (3·9 to 16·3)	6·1 (2·7 to 11·6)	3·7 (1·7 to 7·2)	2·5 (1·1 to 4·6)	1·6 (0·7 to 3)	0·9 (0·4 to 1·8)	0·5 (0·2 to 0·9)	0·2 (0·1 to 0·4)	0·1 (0 to 0·2)	480
Hookworm disease	0 (0 to 0)	0 (0 to 0)	0·2 (0·1 to 0·4)	6·4 (2·9 to 11·7)	15·7 (7·2 to 29·8)	12·6 (5·8 to 23·4)	25·6 (11 to 48·7)	53·9 (19·2 to 107)	37·4 (13·5 to 69·8)	25·5 (9·5 to 55·1)	25·5 (11 to 45·4)	10·7 (5·1 to 19·4)	3·5 (1·5 to 6·8)	3·9 (1·8 to 7·3)	3·8 (1·7 to 6·9)	2·9 (1·3 to 5·4)	1·9 (0·9 to 3·6)	1·6 (0·7 to 3)	1·2 (0·5 to 2·2)	0·8 (0·4 to 1·5)	0·6 (0·3 to 1)	0·3 (0·1 to 0·5)	0·1 (0 to 0·2)	234
Food-borne trematodiases	0 (0 to 0)	0 (0 to 0)	0 (0 to 0)	0 (0 to 0)	0 (0 to 0)	0 (0 to 0)	0 (0 to 0)	0 (0 to 0)	0 (0 to 0)	0 (0 to 0)	0 (0 to 0)	0 (0 to 0)	0 (0 to 0)	0 (0 to 0)	0 (0 to 0)	0 (0 to 0)	0 (0 to 0)	0 (0 to 0)	0 (0 to 0)	0 (0 to 0)	0 (0 to 0)	0 (0 to 0)	0 (0 to 0)	0
Leprosy	0 (0 to 0)	0 (0 to 0)	0 (0 to 0)	0 (0 to 0)	0 (0 to 0)	0 (0 to 0)	0·1 (0 to 0·1)	0·1 (0 to 0·1)	0·1 (0·1 to 0·2)	0·2 (0·1 to 0·3)	0·2 (0·1 to 0·3)	0·2 (0·1 to 0·4)	0·3 (0·2 to 0·4)	0·3 (0·2 to 0·5)	0·3 (0·2 to 0·5)	0·3 (0·2 to 0·5)	0·3 (0·2 to 0·4)	0·2 (0·1 to 0·3)	0·2 (0·1 to 0·3)	0·1 (0·1 to 0·2)	0·1 (0·1 to 0·1)	0 (0 to 0·1)	0 (0 to 0)	3·1
Ebola	0 (0 to 0)	0 (0 to 0)	0 (0 to 0)	0 (0 to 0)	0 (0 to 0)	0 (0 to 0)	0 (0 to 0)	0 (0 to 0)	0 (0 to 0)	0 (0 to 0)	0 (0 to 0)	0 (0 to 0)	0 (0 to 0)	0 (0 to 0)	0 (0 to 0)	0 (0 to 0)	0 (0 to 0)	0 (0 to 0)	0 (0 to 0)	0 (0 to 0)	0 (0 to 0)	0 (0 to 0)	0 (0 to 0)	0
Zika virus	0 (0 to 0)	0 (0 to 0)	0 (0 to 0·2)	0·2 (0 to 1)	0·3 (0 to 1·1)	0·3 (0 to 1·1)	0·3 (0 to 1·2)	0·3 (0 to 1·4)	0·3 (0 to 1·4)	0·3 (0 to 1·5)	0·3 (0 to 1·3)	0·2 (0 to 1·1)	0·2 (0 to 0·7)	0·1 (0 to 0·6)	0·1 (0 to 0·4)	0·1 (0 to 0·3)	0 (0 to 0·2)	0 (0 to 0·1)	0 (0 to 0)	0 (0 to 0)	0 (0 to 0)	0 (0 to 0)	0 (0 to 0)	3·1
Guinea worm disease	0 (0 to 0)	0 (0 to 0)	0 (0 to 0)	0 (0 to 0)	0 (0 to 0)	0 (0 to 0)	0 (0 to 0)	0 (0 to 0)	0 (0 to 0)	0 (0 to 0)	0 (0 to 0)	0 (0 to 0)	0 (0 to 0)	0 (0 to 0)	0 (0 to 0)	0 (0 to 0)	0 (0 to 0)	0 (0 to 0)	0 (0 to 0)	0 (0 to 0)	0 (0 to 0)	0 (0 to 0)	0 (0 to 0)	0
Other neglected tropical diseases	0·6 (0·2 to 1)	3·4 (1·9 to 5·2)	25·3 (12·5 to 42·9)	110 (54·5 to 189)	89·1 (34·4 to 184)	39·1 (13·1 to 79·5)	26·2 (9·9 to 54·5)	25·7 (9·8 to 50·7)	25·7 (9·4 to 50·6)	23·7 (8·7 to 48·7)	25·5 (9·8 to 49·1)	26·5 (11·5 to 48·9)	24·4 (9·5 to 45·2)	24 (10·5 to 43·3)	21·9 (9·4 to 41·3)	8·2 (3·6 to 15·7)	3·9 (1·7 to 7·6)	3·9 (1·9 to 7)	4·3 (1·6 to 7·8)	2·2 (1 to 3·9)	2·2 (1·1 to 3·8)	1·6 (0·7 to 2·7)	1·2 (0·5 to 2·3)	519
Other infectious diseases	85 (54·6 to 127)	101 (64·9 to 152)	206 (117 to 373)	334 (232 to 494)	249 (183 to 340)	210 (157 to 274)	210 (149 to 289)	270 (181 to 383)	288 (188 to 429)	313 (187 to 479)	289 (162 to 456)	270 (153 to 427)	332 (175 to 546)	258 (161 to 384)	241 (148 to 359)	198 (128 to 279)	134 (94 to 180)	104 (74·5 to 138)	63·2 (48·8 to 80·1)	46 (35·7 to 57·5)	35 (27·1 to 43·9)	13·7 (10·5 to 17·2)	8·4 (6·2 to 10·6)	4,260
Meningitis	55·3 (34·2 to 85·7)	74·3 (43·6 to 118)	74·4 (36·4 to 133)	76·7 (48·3 to 114)	55 (42·1 to 70·9)	48·8 (35·2 to 64·8)	47·9 (33·6 to 65·5)	66·3 (46·9 to 91·1)	71·2 (50·3 to 97·1)	73·5 (52·1 to 102)	61·3 (43·6 to 87·4)	50·7 (35·6 to 69·2)	51·8 (36·1 to 68·9)	52·5 (37·8 to 71·9)	47·1 (33·8 to 64·2)	50·2 (36·2 to 69·8)	36·2 (26·1 to 48·4)	29·4 (21·6 to 38·9)	22·8 (16·8 to 30·4)	16·8 (12·1 to 22·1)	11·3 (8·2 to 15·1)	3 (2·2 to 3·9)	2 (1·4 to 2·5)	1,080
Encephalitis	18·8 (9·4 to 30·4)	14 (8·2 to 22·7)	74 (29·9 to 184)	133 (68·2 to 278)	85·9 (54·4 to 154)	70·8 (45·4 to 119)	52·9 (34·9 to 89·1)	63·4 (42·3 to 100)	58·2 (39·4 to 91·2)	60·4 (41·8 to 85·1)	48·2 (34 to 68·4)	48·9 (33·8 to 69·4)	62·9 (41·7 to 92·8)	38·4 (27·3 to 53·5)	45·6 (30 to 64·7)	40·4 (27·6 to 56·6)	29·9 (20·6 to 40·6)	21·3 (14·8 to 29·8)	11·8 (8·3 to 16·6)	9·1 (5·9 to 13·1)	4·9 (3·1 to 7·7)	2 (1·4 to 2·8)	0·8 (0·5 to 1·3)	995
Diphtheria	0 (0 to 0)	0 (0 to 0)	0 (0 to 0)	0·1 (0 to 0·2)	0·1 (0 to 0·4)	0·1 (0 to 0·2)	0 (0 to 0·1)	0 (0 to 0·1)	0 (0 to 0)	0 (0 to 0)	0 (0 to 0)	0 (0 to 0)	0 (0 to 0)	0 (0 to 0)	0 (0 to 0)	0 (0 to 0)	0 (0 to 0)	0 (0 to 0)	0 (0 to 0)	0 (0 to 0)	0 (0 to 0)	0 (0 to 0)	0 (0 to 0)	0·4
Tetanus	0 (0 to 0·1)	0·1 (0 to 0·2)	0·1 (0 to 0·2)	0·2 (0 to 0·5)	0·9 (0·2 to 2·2)	0·6 (0·2 to 1·4)	0·4 (0·1 to 1·2)	0·6 (0·2 to 1·5)	0·5 (0·2 to 1·1)	0·4 (0·1 to 1·1)	0·2 (0 to 1)	0·5 (0·1 to 1·3)	0·9 (0·3 to 2·6)	2 (0·7 to 5·2)	1·5 (0·5 to 3·7)	0·8 (0·3 to 2·8)	0·4 (0·1 to 2·1)	0·4 (0·1 to 2·1)	0·4 (0·1 to 1·2)	0·1 (0 to 0·7)	0 (0 to 0·3)	0 (0 to 0·1)	0 (0 to 0·1)	11
Measles	0 (0 to 0)	0 (0 to 0)	0 (0 to 0)	0 (0 to 0)	0 (0 to 0)	0 (0 to 0)	0 (0 to 0)	0 (0 to 0)	0 (0 to 0)	0 (0 to 0)	0 (0 to 0)	0 (0 to 0)	0 (0 to 0)	0 (0 to 0)	0 (0 to 0)	0 (0 to 0)	0 (0 to 0)	0 (0 to 0)	0 (0 to 0)	0 (0 to 0)	0 (0 to 0)	0 (0 to 0)	0 (0 to 0)	0
Varicella and herpes zoster	0·3 (0·1 to 0·7)	0·6 (0·2 to 1·2)	1·5 (0·7 to 2·9)	1·4 (0·7 to 2·8)	2 (1·2 to 3·4)	2·5 (0·8 to 4·9)	3·2 (1 to 6·6)	4·5 (1·4 to 9·1)	5·1 (2·2 to 9·7)	4·8 (1·9 to 8·9)	4·8 (2·1 to 8·7)	4·3 (1·7 to 8·1)	5·3 (2·4 to 9·8)	5·9 (2·8 to 10·6)	6·4 (3·1 to 11·3)	6·3 (2·8 to 11·2)	5·3 (2·6 to 9·5)	4·3 (2·7 to 7·8)	3·5 (1·9 to 5·8)	3·4 (1·8 to 5·3)	2·7 (1·4 to 4·2)	1·6 (0·8 to 2·4)	1·4 (0·5 to 2·1)	81·7
Acute hepatitis	0 (0 to 0)	0 (0 to 0)	0·6 (0·3 to 1·2)	3·7 (2 to 6·4)	15·7 (9·4 to 24·7)	20·2 (12·5 to 30·2)	20·9 (13·3 to 31)	21·9 (14·2 to 31·7)	20·4 (14·1 to 28·8)	15·7 (10·2 to 21·8)	9·5 (6·5 to 13·8)	10·8 (7·4 to 15·3)	8·8 (5·8 to 13·3)	11·3 (7·9 to 15·7)	8·3 (5·8 to 11·6)	6·8 (4·7 to 9·6)	2·9 (2·1 to 3·9)	2·4 (1·6 to 3·6)	1·6 (1·1 to 2·1)	0·7 (0·5 to 1)	0·9 (0·6 to 1·2)	0·3 (0·2 to 0·4)	0·1 (0·1 to 0·2)	183
Acute hepatitis A	0 (0 to 0)	0 (0 to 0)	0·1 (0 to 0·1)	2·8 (1·2 to 5·3)	12·8 (7 to 21·4)	16·7 (9·7 to 26·3)	16·4 (9·8 to 25·4)	14·6 (8·3 to 22·9)	12·7 (7·5 to 19·9)	10·1 (5·8 to 15·2)	6 (3·2 to 9·8)	4·5 (2·3 to 7·4)	3·6 (1·9 to 6·3)	3·9 (1·8 to 6·7)	3·1 (1·2 to 5·4)	2·2 (0·8 to 4·9)	1 (0·4 to 1·6)	0·3 (0·1 to 0·6)	0·4 (0·1 to 1)	0·2 (0·1 to 0·4)	0·4 (0·1 to 0·7)	0 (0 to 0·1)	0 (0 to 0)	112
Acute hepatitis B	0 (0 to 0)	0 (0 to 0)	0·4 (0·1 to 0·9)	0·4 (0·1 to 0·7)	1·5 (0·8 to 2·8)	1·5 (0·6 to 3)	2·1 (0·9 to 4·1)	5·2 (3·1 to 8·2)	5·5 (3 to 9·4)	3·8 (1·8 to 7·2)	2·4 (1·5 to 3·5)	5·2 (3·3 to 7·8)	3·7 (2·2 to 5·9)	6·2 (3·6 to 9·7)	4 (2·1 to 6·9)	4 (1·7 to 6·4)	1·3 (0·7 to 2·1)	1·4 (0·8 to 2·1)	0·7 (0·3 to 1·2)	0·3 (0·1 to 0·6)	0·3 (0·1 to 0·6)	0·1 (0 to 0·2)	0·1 (0 to 0·1)	50
Acute hepatitis C	0 (0 to 0)	0 (0 to 0)	0·2 (0·1 to 0·3)	0·4 (0·2 to 0·9)	0·3 (0·2 to 0·7)	0·3 (0·1 to 0·6)	0·6 (0·2 to 1·3)	0·4 (0·2 to 0·8)	0·7 (0·3 to 1·2)	0·6 (0·2 to 1·2)	0·4 (0·2 to 0·7)	0·4 (0·2 to 0·9)	0·9 (0·3 to 1·6)	0·8 (0·4 to 1·5)	0·9 (0·4 to 1·5)	0·4 (0·2 to 1·2)	0·4 (0·2 to 0·8)	0·4 (0·2 to 0·8)	0·2 (0·1 to 0·4)	0·1 (0·1 to 0·2)	0·1 (0 to 0·1)	0 (0 to 0·1)	0 (0 to 0)	8·6
Acute hepatitis E	0 (0 to 0)	0 (0 to 0)	0 (0 to 0)	0·1 (0 to 0·2)	1 (0·5 to 1·8)	1·6 (0·4 to 3·5)	1·8 (0·4 to 3·8)	1·6 (0·4 to 3·6)	1·5 (0·2 to 2·9)	1·1 (0·5 to 2·2)	0·8 (0·4 to 1·4)	0·6 (0·3 to 1·1)	0·6 (0·3 to 1)	0·4 (0·2 to 0·7)	0·4 (0·2 to 0·7)	0·2 (0·1 to 0·6)	0·1 (0 to 0·4)	0·1 (0 to 0·3)	0·1 (0 to 0·3)	0 (0 to 0·1)	0 (0 to 0·1)	0 (0 to 0)	0 (0 to 0)	12·9
Other unspecified infectious diseases	10·6 (3·8 to 19·4)	11·6 (6·4 to 19·5)	48·4 (29·8 to 76·5)	111 (66·9 to 169)	88·3 (42·9 to 162)	66·9 (27·8 to 118)	84·3 (35·6 to 149)	113 (42·3 to 209)	132 (47·7 to 253)	158 (47·4 to 309)	165 (53·5 to 315)	155 (55·8 to 290)	202 (65·6 to 391)	148 (53·9 to 262)	132 (54·4 to 233)	93 (40·2 to 155)	59·5 (29·9 to 93·4)	46·2 (23·9 to 71·5)	23·2 (13·4 to 33·6)	16 (10·2 to 22·3)	15·2 (10·7 to 21·1)	6·9 (4·8 to 9·5)	4·1 (2·7 to 5·8)	1,890
Maternal and neonatal disorders	26,700 (18,900 to 36,900)	6,720 (4,750 to 9,260)	1,490 (867 to 2,410)	1,340 (1,010 to 1,720)	960 (717 to 1,260)	1,050 (775 to 1,400)	1,260 (965 to 1,610)	1,560 (1,210 to 1,950)	1,520 (1,200 to 1,910)	1,360 (1,070 to 1,720)	1,010 (784 to 1,270)	742 (564 to 955)	606 (453 to 790)	486 (358 to 641)	376 (283 to 498)	261 (187 to 353)	154 (112 to 210)	79·3 (55·2 to 113)	31·2 (20·3 to 45)	9·7 (5·8 to 15·7)	2·9 (1·7 to 4·6)	0·7 (0·4 to 1·2)	0·2 (0·1 to 0·4)	47,700
Maternal disorders	0 (0 to 0)	0 (0 to 0)	0 (0 to 0)	0 (0 to 0)	0 (0 to 0)	4·8 (3·7 to 6·1)	164 (118 to 216)	384 (270 to 521)	429 (293 to 604)	450 (300 to 649)	254 (173 to 363)	109 (76·2 to 156)	31·8 (21·6 to 45·4)	4·4 (3·2 to 6)	0 (0 to 0)	0 (0 to 0)	0 (0 to 0)	0 (0 to 0)	0 (0 to 0)	0 (0 to 0)	0 (0 to 0)	0 (0 to 0)	0 (0 to 0)	1,830
Maternal hemorrhage	0 (0 to 0)	0 (0 to 0)	0 (0 to 0)	0 (0 to 0)	0 (0 to 0)	0·2 (0·1 to 0·3)	17·9 (10·3 to 29·3)	42·7 (24·4 to 67·5)	47·5 (27·7 to 73·5)	49·5 (29·2 to 79·5)	31 (18·6 to 48·7)	11·6 (6·5 to 18·3)	2 (1 to 3·2)	0 (0 to 0·1)	0 (0 to 0)	0 (0 to 0)	0 (0 to 0)	0 (0 to 0)	0 (0 to 0)	0 (0 to 0)	0 (0 to 0)	0 (0 to 0)	0 (0 to 0)	202
Maternal sepsis and other maternal infections	0 (0 to 0)	0 (0 to 0)	0 (0 to 0)	0 (0 to 0)	0 (0 to 0)	0·2 (0·1 to 0·3)	6·1 (2·8 to 10·8)	13 (6·6 to 22·5)	11·7 (6·4 to 18·8)	11 (5·9 to 18·3)	5·6 (3·1 to 9·6)	2 (0·9 to 3·4)	0·7 (0·4 to 1·2)	0·1 (0 to 0·2)	0 (0 to 0)	0 (0 to 0)	0 (0 to 0)	0 (0 to 0)	0 (0 to 0)	0 (0 to 0)	0 (0 to 0)	0 (0 to 0)	0 (0 to 0)	50·3
Maternal hypertensive disorders	0 (0 to 0)	0 (0 to 0)	0 (0 to 0)	0 (0 to 0)	0 (0 to 0)	1·8 (1·2 to 2·5)	52·7 (34·1 to 76·1)	101 (65·4 to 148)	111 (67·5 to 171)	99·3 (59·5 to 150)	44·8 (26·8 to 69·3)	13·2 (8 to 19·8)	2·1 (1·3 to 3·4)	0 (0 to 0)	0 (0 to 0)	0 (0 to 0)	0 (0 to 0)	0 (0 to 0)	0 (0 to 0)	0 (0 to 0)	0 (0 to 0)	0 (0 to 0)	0 (0 to 0)	426
Maternal obstructed labor and uterine rupture	0 (0 to 0)	0 (0 to 0)	0 (0 to 0)	0 (0 to 0)	0 (0 to 0)	0·1 (0 to 0·2)	2·3 (1·3 to 3·5)	3·7 (2·2 to 6)	4 (2·3 to 6·5)	6·7 (3·4 to 12·1)	6·2 (3·2 to 11·3)	2·1 (1·1 to 4)	0·5 (0·2 to 1·1)	0 (0 to 0)	0 (0 to 0)	0 (0 to 0)	0 (0 to 0)	0 (0 to 0)	0 (0 to 0)	0 (0 to 0)	0 (0 to 0)	0 (0 to 0)	0 (0 to 0)	25·8
Maternal abortion and miscarriage	0 (0 to 0)	0 (0 to 0)	0 (0 to 0)	0 (0 to 0)	0 (0 to 0)	0·2 (0·1 to 0·3)	10·7 (5·5 to 18)	29·9 (14·3 to 52·5)	32 (14·8 to 58·8)	30·9 (14 to 62·5)	19·1 (9 to 34·6)	8·2 (3·8 to 15·7)	3·2 (1·5 to 5·7)	0 (0 to 0·1)	0 (0 to 0)	0 (0 to 0)	0 (0 to 0)	0 (0 to 0)	0 (0 to 0)	0 (0 to 0)	0 (0 to 0)	0 (0 to 0)	0 (0 to 0)	134
Ectopic pregnancy	0 (0 to 0)	0 (0 to 0)	0 (0 to 0)	0 (0 to 0)	0 (0 to 0)	0·1 (0·1 to 0·2)	5·2 (2·9 to 8·2)	14·2 (8 to 23·7)	17 (9·4 to 28·6)	14·5 (7·8 to 24·4)	3·9 (2·1 to 6·6)	1·5 (0·8 to 2·5)	0·2 (0·1 to 0·4)	0 (0 to 0)	0 (0 to 0)	0 (0 to 0)	0 (0 to 0)	0 (0 to 0)	0 (0 to 0)	0 (0 to 0)	0 (0 to 0)	0 (0 to 0)	0 (0 to 0)	56·6
Indirect maternal deaths	--	--	--	--	--	0 (0 to 0·1)	1·2 (0·6 to 2)	2 (1·1 to 3·5)	2·5 (1·3 to 4·8)	3·2 (1·7 to 5·9)	1·6 (0·8 to 2·8)	0·4 (0·2 to 0·8)	0·1 (0 to 0·3)	0 (0 to 0)	--	--	--	--	--	--	--	--	--	NA
Late maternal deaths	--	--	--	--	--	0·4 (0·2 to 0·7)	15 (8·6 to 24·1)	36·2 (19·7 to 58·5)	38·2 (20·3 to 66·8)	39·7 (20·9 to 67·5)	21·8 (12 to 37·4)	9·2 (5·1 to 15·9)	2·8 (1·6 to 4·4)	0·4 (0·2 to 0·6)	--	--	--	--	--	--	--	--	--	NA
Maternal deaths aggravated by HIV/AIDS	--	--	--	--	--	0 (0 to 0)	0·1 (0 to 0·3)	0·8 (0·3 to 1·6)	1·8 (0·7 to 3·7)	2·5 (1·1 to 4·5)	1·4 (0·7 to 2·6)	0·6 (0·3 to 1·1)	0·2 (0·1 to 0·3)	0 (0 to 0)	--	--	--	--	--	--	--	--	--	NA
Neonatal disorders	26,700 (18,900 to 36,900)	6,720 (4,750 to 9,260)	1,490 (867 to 2,410)	1,340 (1,010 to 1,720)	960 (717 to 1,260)	1,050 (770 to 1,390)	1,100 (815 to 1,440)	1,170 (848 to 1,540)	1,090 (815 to 1,420)	912 (689 to 1,180)	761 (567 to 994)	633 (466 to 834)	575 (421 to 755)	481 (354 to 637)	376 (283 to 498)	261 (187 to 353)	154 (112 to 210)	79·3 (55·2 to 113)	31·2 (20·3 to 45)	9·7 (5·8 to 15·7)	2·9 (1·7 to 4·6)	0·7 (0·4 to 1·2)	0·2 (0·1 to 0·4)	45,900
Neonatal preterm birth	16,300 (11,300 to 23,000)	3,290 (2,170 to 4,740)	626 (345 to 1,050)	360 (261 to 459)	377 (268 to 493)	410 (297 to 544)	425 (306 to 567)	447 (321 to 587)	416 (297 to 545)	343 (246 to 457)	280 (200 to 370)	230 (166 to 309)	204 (148 to 268)	165 (118 to 219)	121 (87·6 to 159)	76·4 (53·9 to 102)	42·3 (31·8 to 54·4)	19·1 (14·4 to 24·8)	7 (5 to 9·8)	1·7 (1 to 2·8)	0·8 (0·3 to 1·5)	0·4 (0·2 to 0·7)	0·2 (0·1 to 0·4)	24,100
Neonatal encephalopathy due to birth asphyxia and trauma	5,450 (3,480 to 8,060)	644 (368 to 1,000)	187 (94·7 to 316)	206 (125 to 314)	96·3 (56·9 to 150)	104 (60·5 to 162)	110 (64·1 to 170)	116 (66 to 183)	110 (65·5 to 170)	91·4 (51·3 to 143)	77·2 (44·9 to 122)	65·1 (36·9 to 100)	59·5 (34·9 to 93·1)	50·2 (28·5 to 77·8)	41·1 (22·7 to 65)	29·6 (16·2 to 46·8)	16·3 (10·1 to 24·4)	5·9 (3·5 to 9·2)	1·4 (0·8 to 2·3)	0·2 (0·1 to 0·4)	0 (0 to 0·1)	0 (0 to 0)	0 (0 to 0)	7,460
Neonatal sepsis and other neonatal infections	3,010 (1,830 to 4,750)	2,130 (1,360 to 3,210)	470 (262 to 783)	375 (254 to 527)	451 (292 to 650)	493 (316 to 733)	523 (338 to 772)	562 (358 to 824)	526 (340 to 770)	441 (288 to 649)	373 (245 to 542)	313 (199 to 450)	287 (184 to 418)	245 (156 to 358)	197 (129 to 287)	143 (89·5 to 218)	88·2 (55 to 132)	50·1 (29·1 to 79)	21·2 (11·5 to 34·2)	7·2 (3·8 to 12·7)	1·9 (0·9 to 3·6)	0·3 (0·1 to 0·5)	0 (0 to 0)	10,700
Hemolytic disease and other neonatal jaundice	84·5 (45·7 to 143)	42·6 (24·7 to 68·8)	9·9 (5·5 to 18·3)	30·2 (20·1 to 42·5)	35·9 (23·3 to 49·8)	39·6 (29·4 to 51·1)	42·4 (31·2 to 54·8)	45·6 (33·3 to 58·6)	42·9 (31·6 to 55·7)	36·1 (26·4 to 47)	30·6 (22 to 39·7)	25·9 (19 to 33·7)	24·1 (17·7 to 31·4)	20·8 (15·2 to 27·2)	16·9 (12·4 to 22·2)	12·2 (8·9 to 15·8)	7·5 (5·4 to 9·6)	4·1 (3 to 5·3)	1·7 (1·2 to 2·3)	0·6 (0·4 to 0·8)	0·1 (0·1 to 0·2)	0 (0 to 0)	0 (0 to 0)	554
Other neonatal disorders	1,890 (1,160 to 2,930)	615 (401 to 893)	194 (122 to 301)	373 (281 to 485)	0 (0 to 0)	0 (0 to 0)	0 (0 to 0)	0 (0 to 0)	0 (0 to 0)	0 (0 to 0)	0 (0 to 0)	0 (0 to 0)	0 (0 to 0)	0 (0 to 0)	0 (0 to 0)	0 (0 to 0)	0 (0 to 0)	0 (0 to 0)	0 (0 to 0)	0 (0 to 0)	0 (0 to 0)	0 (0 to 0)	0 (0 to 0)	3,070
Nutritional deficiencies	1·7 (1 to 2·9)	35·7 (20 to 56·2)	508 (322 to 765)	1,300 (710 to 2,140)	1,010 (453 to 1,960)	479 (195 to 918)	682 (308 to 1,290)	655 (299 to 1,210)	692 (315 to 1,240)	619 (286 to 1,190)	672 (316 to 1,170)	695 (355 to 1,190)	643 (314 to 1,100)	650 (349 to 1,080)	600 (330 to 1,030)	433 (253 to 712)	261 (168 to 407)	250 (162 to 370)	187 (125 to 279)	151 (102 to 221)	129 (87·7 to 191)	78·1 (52·5 to 110)	49·9 (35·8 to 67·4)	10,800
Protein-energy malnutrition	1·2 (0·8 to 1·8)	3·7 (2·2 to 5·4)	255 (148 to 410)	187 (113 to 283)	82·7 (54·7 to 115)	52·7 (33·7 to 77·1)	65·2 (43·4 to 92)	66·9 (42·7 to 99·1)	77 (52 to 111)	70·8 (50 to 97·9)	74·8 (52 to 103)	70·3 (49·5 to 96·3)	77·1 (52·2 to 109)	85·4 (58·8 to 117)	97 (67·6 to 133)	115 (77·5 to 159)	104 (73·8 to 142)	124 (81·6 to 177)	98·8 (68·9 to 141)	81 (55 to 114)	68·2 (47·6 to 96·1)	42·3 (28·2 to 58·4)	34·9 (23·2 to 48·7)	1,930
Iodine deficiency	0 (0 to 0)	0 (0 to 0)	0 (0 to 0)	0·7 (0·2 to 1·3)	5·1 (2·1 to 9·6)	12·7 (5·4 to 23·9)	18·6 (7·8 to 34·3)	23·7 (9·1 to 43·9)	24·2 (9·3 to 46)	22 (9·1 to 39·8)	19·1 (8·8 to 34·8)	16·5 (7 to 30·3)	15·6 (6·8 to 27·7)	13·5 (6 to 25·1)	11·2 (4·8 to 20·6)	8·3 (3·7 to 14·7)	5·9 (2·8 to 10)	4·1 (2 to 7·2)	2·6 (1·4 to 4·1)	1·6 (0·8 to 2·5)	1 (0·5 to 1·7)	0·6 (0·3 to 0·9)	0·3 (0·2 to 0·5)	207
Vitamin A deficiency	0·1 (0 to 0·2)	1·2 (0·5 to 2·4)	4·9 (2·7 to 9·2)	28·9 (14·8 to 49·9)	31 (15·7 to 54·5)	19·4 (8·6 to 35·8)	9·7 (3·4 to 19·1)	8·5 (3·9 to 15·4)	7·3 (3·6 to 12·9)	5·5 (2·7 to 9·8)	4·3 (2·2 to 7·5)	3·3 (1·8 to 5·8)	3·1 (1·6 to 5·3)	2·6 (1·4 to 4·4)	2·2 (1·2 to 3·8)	1·6 (0·9 to 2·8)	1·2 (0·6 to 2·1)	1 (0·5 to 1·7)	0·7 (0·4 to 1·2)	0·5 (0·2 to 0·8)	0·3 (0·1 to 0·4)	0·1 (0 to 0·2)	0 (0 to 0·1)	137
Dietary iron deficiency	0·4 (0·1 to 1·3)	30·9 (16·3 to 49·5)	245 (117 to 434)	1,080 (502 to 1,910)	879 (330 to 1,810)	385 (118 to 821)	572 (213 to 1,170)	529 (186 to 1,070)	550 (194 to 1,110)	496 (170 to 1,060)	558 (209 to 1,050)	587 (254 to 1,070)	520 (197 to 982)	521 (236 to 929)	467 (206 to 869)	289 (114 to 566)	127 (46·8 to 268)	98·5 (38·1 to 208)	71·6 (26·3 to 150)	57·5 (20 to 122)	52·1 (19 to 108)	30 (12·2 to 58)	11·9 (4·6 to 22·9)	8,160
Other nutritional deficiencies	0 (0 to 0)	0 (0 to 0)	2·9 (1·8 to 4·2)	8·9 (3·8 to 17·1)	10·4 (6·2 to 17·1)	9·6 (5·4 to 15·7)	16·4 (9·1 to 27·5)	26·5 (15·2 to 40·9)	33·1 (20·3 to 50·5)	25·1 (16 to 36·4)	15·4 (9 to 23·4)	17·8 (11·3 to 26·5)	27 (16·4 to 41)	27·7 (17·5 to 40·5)	22·3 (14·5 to 33)	18·7 (11·3 to 30·6)	22·6 (13·9 to 34·9)	22·8 (13·1 to 38·8)	13·4 (7·5 to 22·2)	10·8 (5·8 to 18·8)	7·4 (3·6 to 12·3)	5·1 (2·2 to 8·4)	2·8 (1 to 4·7)	347
Non-communicable diseases	3,410 (2,050 to 5,050)	1,580 (929 to 2,300)	2,730 (1,620 to 4,410)	5,600 (4,250 to 7,200)	7,230 (5,590 to 9,120)	11,000 (8,410 to 14,600)	16,200 (12,300 to 21,400)	21,600 (16,800 to 27,300)	25,300 (20,100 to 31,700)	27,000 (21,600 to 33,500)	28,100 (22,700 to 34,900)	31,300 (25,400 to 38,000)	39,400 (31,700 to 47,600)	46,100 (37,600 to 55,800)	52,500 (42,900 to 63,200)	55,400 (45,900 to 65,900)	51,700 (43,500 to 61,100)	51,300 (43,200 to 60,100)	40,800 (34,900 to 47,400)	32,300 (28,000 to 36,900)	22,800 (20,000 to 25,800)	13,200 (11,700 to 14,700)	8,430 (7,730 to 9,160)	595,000
Neoplasms	25·7 (17·4 to 40·1)	21·6 (14·7 to 31·1)	74·9 (39·4 to 126)	479 (338 to 665)	780 (632 to 973)	777 (602 to 995)	869 (657 to 1,120)	1,440 (1,100 to 1,860)	2,060 (1,540 to 2,720)	3,220 (2,420 to 4,190)	4,030 (2,920 to 5,410)	5,790 (4,360 to 7,680)	8,180 (6,150 to 10,600)	10,200 (7,700 to 13,200)	12,200 (9,270 to 15,800)	13,600 (10,500 to 17,100)	12,300 (9,830 to 15,500)	11,400 (9,070 to 14,200)	8,110 (6,530 to 9,940)	5,320 (4,220 to 6,550)	3,040 (2,370 to 3,720)	1,360 (990 to 1,690)	664 (474 to 833)	106,000
Lip and oral cavity cancer	0 (0 to 0)	0 (0 to 0)	0 (0 to 0)	0 (0 to 0)	3·3 (1·9 to 5·2)	4·5 (2·9 to 6·8)	7·5 (5 to 10·6)	15·2 (9·7 to 23·1)	17·6 (10·9 to 26·5)	17·6 (11·5 to 25·6)	15·9 (10·5 to 23·3)	36·3 (22·6 to 53·6)	56·3 (36·8 to 82·8)	74·6 (52·7 to 107)	96·3 (66·1 to 135)	105 (73·7 to 144)	92·2 (64 to 128)	71·5 (51·4 to 97·4)	49·8 (36·5 to 65·9)	30·1 (21·7 to 40)	17·4 (12·8 to 23·2)	9·7 (6·7 to 12·7)	5·3 (3·6 to 7)	726
Nasopharynx cancer	0 (0 to 0)	0 (0 to 0)	0 (0 to 0)	0 (0 to 0)	11·1 (6·1 to 18·8)	13·6 (7·3 to 22·5)	19·3 (10·5 to 33·6)	34·4 (17·2 to 63·1)	27·3 (15·4 to 45)	32·7 (18·8 to 51·8)	29·8 (17·2 to 46·1)	32·2 (19·3 to 52)	50·5 (30·5 to 76·7)	48·5 (29·7 to 73·8)	59·4 (34·9 to 89·9)	49·5 (30·2 to 77)	42·7 (26·2 to 65·2)	29·4 (18·4 to 44·2)	12·6 (7·7 to 19·8)	7·3 (4·2 to 11·7)	5·6 (3·2 to 9)	3·3 (1·8 to 5·4)	1·3 (0·8 to 2·2)	511
Other pharynx cancer	0 (0 to 0)	0 (0 to 0)	0 (0 to 0)	0 (0 to 0)	0 (0 to 0)	0 (0 to 0)	0 (0 to 0)	10·9 (5·5 to 19·7)	6·6 (3·5 to 11·1)	9·9 (5·7 to 16·1)	8·8 (4·7 to 15·4)	14·3 (8·5 to 23)	46 (29 to 66·9)	67·1 (41·9 to 101)	80·1 (46·9 to 126)	80·6 (51·8 to 120)	65·3 (39·7 to 99·5)	42·5 (27·6 to 61·9)	23·9 (15·6 to 34·4)	16·7 (9·9 to 26·6)	7·6 (4·8 to 10·9)	3·6 (2 to 5·6)	3·2 (1·8 to 5·5)	487
Esophageal cancer	0 (0 to 0)	0 (0 to 0)	0 (0 to 0)	0 (0 to 0)	0 (0 to 0)	0 (0 to 0)	0 (0 to 0)	4·7 (3 to 7·1)	12·4 (7·1 to 19·9)	22·6 (13·4 to 36·1)	34 (20·7 to 52·3)	45·9 (26·3 to 74·4)	91·8 (56 to 139)	148 (97·1 to 213)	276 (177 to 404)	275 (177 to 396)	305 (203 to 436)	242 (166 to 346)	166 (111 to 240)	94·4 (62·6 to 137)	49·6 (34·3 to 67·5)	18·4 (12·2 to 25·7)	6·5 (4·2 to 9·7)	1,790
Stomach cancer	0 (0 to 0)	0 (0 to 0)	0 (0 to 0)	0 (0 to 0)	0 (0 to 0)	0 (0 to 0)	12·3 (7·7 to 18·3)	25·5 (17·2 to 37·3)	62·8 (39·4 to 94·4)	130 (80·4 to 195)	155 (94·9 to 237)	246 (165 to 359)	377 (247 to 552)	619 (417 to 877)	803 (547 to 1,160)	945 (655 to 1,300)	832 (593 to 1,120)	774 (578 to 1,060)	587 (445 to 775)	382 (295 to 482)	220 (162 to 292)	86·6 (57·9 to 118)	41·5 (26·9 to 59·1)	6,300
Colon and rectum cancer	0 (0 to 0)	0 (0 to 0)	0 (0 to 0)	0 (0 to 0)	8 (5·5 to 11·3)	9·5 (6·8 to 13·4)	31·6 (21·5 to 44·9)	88 (61·4 to 124)	148 (102 to 211)	271 (181 to 378)	294 (192 to 420)	489 (335 to 679)	713 (490 to 1,010)	1,050 (721 to 1,440)	1,360 (973 to 1,830)	1,650 (1,220 to 2,180)	1,510 (1,130 to 1,960)	1,360 (1,020 to 1,760)	981 (758 to 1,250)	633 (482 to 810)	401 (295 to 528)	179 (128 to 234)	83·5 (54·1 to 111)	11,300
Liver cancer	1 (0·4 to 1·9)	0·2 (0·1 to 0·5)	1·4 (0·6 to 2·7)	11·5 (6·7 to 18·8)	9·1 (7 to 11·5)	14·5 (9·9 to 20·5)	6·5 (3·8 to 10·3)	23·7 (14·7 to 35·3)	39·1 (24 to 60·3)	49·3 (29·9 to 76·5)	68·4 (40·6 to 107)	96·6 (61·2 to 146)	130 (82 to 210)	184 (119 to 269)	239 (156 to 352)	252 (174 to 358)	245 (176 to 337)	222 (158 to 301)	164 (121 to 216)	107 (78·4 to 141)	49·9 (35·7 to 65·9)	20 (14·3 to 27)	6·2 (3·9 to 8·8)	1,940
Liver cancer due to hepatitis B	0 (0 to 0)	0 (0 to 0)	0 (0 to 0)	0 (0 to 0)	0 (0 to 0)	2·4 (1·2 to 4·4)	2·9 (1·5 to 5·2)	12 (6·8 to 19·3)	22·8 (12·4 to 38·5)	28 (15·6 to 46·1)	37·3 (19·8 to 62·9)	47·3 (25·1 to 78·4)	56·1 (27·4 to 99·9)	69·2 (35·5 to 117)	83·9 (44·8 to 142)	77·3 (39·1 to 128)	60·9 (30·9 to 102)	46·7 (22 to 80·5)	30·3 (15·7 to 51)	16·8 (7·8 to 29·5)	7·2 (3·6 to 12)	2·7 (1·3 to 4·8)	0·8 (0·3 to 1·6)	605
Liver cancer due to hepatitis C	0 (0 to 0)	0 (0 to 0)	0 (0 to 0)	0 (0 to 0)	0 (0 to 0)	0·1 (0 to 0·2)	0·1 (0 to 0·2)	0·5 (0·2 to 1·1)	1·2 (0·4 to 2·9)	2·5 (1 to 5·4)	4·6 (1·6 to 9·8)	9·4 (4·1 to 17·8)	16·9 (7·2 to 32·6)	30·6 (14·6 to 52)	44·5 (20·3 to 84·3)	49·9 (23·8 to 90·4)	51·6 (27·5 to 88·7)	50·3 (26·7 to 83)	44·7 (23·6 to 73)	36·8 (21·3 to 56·7)	20·2 (12·6 to 31)	9·2 (5·4 to 13·9)	3·1 (1·5 to 5)	376
Liver cancer due to alcohol use	0 (0 to 0)	0 (0 to 0)	0 (0 to 0)	0 (0 to 0)	0 (0 to 0)	0 (0 to 0)	0·2 (0 to 0·3)	1·6 (0·7 to 3·1)	4·4 (1·7 to 9)	7·5 (3·4 to 13·9)	14·2 (6·2 to 28·1)	23·1 (10·5 to 43·8)	35·4 (14·9 to 66·9)	54·3 (26·5 to 97·1)	77·2 (37·6 to 131)	90·8 (48·5 to 149)	100 (58·8 to 156)	94 (54·2 to 150)	61·8 (38·3 to 92·9)	33·5 (20·1 to 49·7)	13·3 (7·6 to 20·3)	4·5 (2·3 to 7·3)	1·1 (0·5 to 2)	617
Liver cancer due to NASH	0 (0 to 0)	0 (0 to 0)	0 (0 to 0)	0 (0 to 0)	0 (0 to 0)	0 (0 to 0)	1·4 (0·7 to 2·5)	3·8 (1·9 to 6·6)	4·2 (1·9 to 7·9)	4·5 (2·1 to 8·6)	5·1 (2·2 to 9·8)	7·5 (3·7 to 14·4)	10·7 (5 to 20·3)	15·5 (7·5 to 28·8)	18·9 (9·4 to 32·9)	20·2 (10·5 to 36·7)	20·9 (11·2 to 38·1)	20·9 (11·3 to 35·8)	19·3 (11 to 33·1)	14·5 (7·8 to 24·4)	6·8 (3·6 to 11·1)	2·7 (1·4 to 4·9)	0·9 (0·3 to 1·7)	178
Liver cancer due to other causes	1 (0·4 to 1·9)	0·2 (0·1 to 0·5)	1·4 (0·6 to 2·7)	11·5 (6·7 to 18·8)	9·1 (7 to 11·5)	12 (8 to 17·2)	2 (1 to 3·5)	5·8 (2·9 to 9·9)	6·4 (3 to 11·4)	6·8 (3·1 to 12·6)	7·1 (3·3 to 13·4)	9·3 (4·6 to 17·2)	11·3 (5·4 to 20·8)	14·2 (6·9 to 26)	14·9 (7·2 to 27·1)	13·4 (6·7 to 24·2)	11·8 (6 to 21)	9·9 (4·7 to 17·4)	8 (4·1 to 14)	5·6 (2·6 to 9·7)	2·5 (1·3 to 4·4)	1 (0·5 to 1·7)	0·3 (0·1 to 0·6)	165
Gallbladder and biliary tract cancer	0 (0 to 0)	0 (0 to 0)	0 (0 to 0)	0 (0 to 0)	0 (0 to 0)	0 (0 to 0)	0 (0 to 0)	4·8 (2·6 to 7)	9·1 (5·5 to 13·6)	14 (9·1 to 20·9)	25·3 (16·3 to 36·9)	44·9 (29·6 to 65·1)	75·3 (49 to 109)	112 (74·7 to 160)	144 (95·7 to 206)	175 (122 to 241)	142 (99·6 to 208)	121 (82 to 191)	86·5 (60·4 to 134)	52·2 (34·8 to 84·9)	26·9 (17·5 to 44·7)	12·4 (7·8 to 21·1)	5·7 (3·4 to 9·8)	1,050
Pancreatic cancer	0 (0 to 0)	0 (0 to 0)	0 (0 to 0)	0 (0 to 0)	0 (0 to 0)	0 (0 to 0)	6·2 (3·4 to 10·6)	4·1 (2·4 to 6·8)	11·9 (6·8 to 19·4)	45·2 (29·4 to 66·2)	61·6 (39·4 to 91·9)	159 (104 to 235)	234 (147 to 346)	357 (233 to 520)	453 (297 to 676)	571 (381 to 811)	481 (326 to 665)	451 (323 to 637)	284 (202 to 396)	182 (124 to 260)	105 (71·9 to 150)	39·7 (25·3 to 56·6)	20·8 (13·9 to 29)	3,460
Larynx cancer	0 (0 to 0)	0 (0 to 0)	0 (0 to 0)	0 (0 to 0)	0 (0 to 0)	0 (0 to 0)	0 (0 to 0)	2·4 (1·7 to 3·2)	3 (2 to 4·4)	6·7 (4·2 to 9·7)	17·2 (9·3 to 29·2)	31·1 (16·6 to 54·5)	61·4 (36·1 to 97·5)	83·8 (53·7 to 124)	127 (81·1 to 187)	157 (103 to 226)	150 (98·3 to 218)	135 (91·4 to 192)	73·3 (50·8 to 105)	37·7 (26·4 to 53·5)	19·5 (12·9 to 27·3)	5·8 (4 to 7·9)	2 (1·3 to 2·9)	912
Tracheal, bronchus, and lung cancer	0 (0 to 0)	0 (0 to 0)	0 (0 to 0)	0 (0 to 0)	0 (0 to 0)	10·8 (6·5 to 17·1)	50·8 (34·8 to 72·5)	66·9 (42·5 to 101)	98·2 (66·3 to 142)	182 (122 to 269)	248 (159 to 376)	466 (307 to 675)	1,000 (688 to 1,390)	1,640 (1,150 to 2,270)	2,180 (1,550 to 2,980)	2,370 (1,760 to 3,110)	1,940 (1,470 to 2,530)	1,850 (1,400 to 2,340)	999 (763 to 1,300)	533 (395 to 700)	233 (169 to 304)	72 (51·1 to 92·9)	32·5 (22·4 to 42·9)	14,000
Malignant skin melanoma	0·1 (0 to 0·2)	0·1 (0 to 0·2)	0·6 (0·2 to 1·6)	3·1 (1·3 to 8·4)	2 (1·7 to 2·5)	2 (1·5 to 2·8)	2·6 (1·7 to 4·1)	8·4 (5 to 13)	7·3 (4 to 15·8)	11·1 (6·2 to 22·5)	17·1 (9·7 to 36)	16·9 (9·3 to 35·7)	27·7 (17 to 47·3)	33·3 (20·5 to 56·8)	35·6 (22·1 to 65·6)	35·1 (22·8 to 54·9)	28·4 (18·8 to 46·8)	31·2 (20·8 to 46·9)	19·9 (13·5 to 31·7)	17·2 (11·4 to 25·2)	10 (6·6 to 14·7)	4·7 (2·9 to 6·6)	3·1 (1·9 to 4·8)	318
Non-melanoma skin cancer	0 (0 to 0)	0 (0 to 0)	0 (0 to 0)	0 (0 to 0)	0 (0 to 0)	0 (0 to 0)	0 (0 to 0)	1·7 (1·3 to 2·2)	3·8 (2·6 to 5·4)	10·6 (4·8 to 16·1)	10·1 (5·9 to 15·3)	12·1 (7·9 to 17·6)	15·4 (10·1 to 22·2)	21·8 (15 to 31·5)	24·9 (17·1 to 35·2)	25·1 (18·3 to 34·2)	25·9 (18·7 to 35·8)	26·3 (19·5 to 34·8)	20·5 (15·1 to 27)	17·1 (12·9 to 22·4)	13·3 (9·8 to 17·2)	10·2 (7·3 to 13)	6·4 (4·3 to 8·8)	245
Non-melanoma skin cancer (squamous-cell carcinoma)	0 (0 to 0)	0 (0 to 0)	0 (0 to 0)	0 (0 to 0)	0 (0 to 0)	0 (0 to 0)	0 (0 to 0)	1·7 (1·3 to 2·2)	3·8 (2·6 to 5·4)	10·6 (4·8 to 16·1)	10·1 (5·9 to 15·3)	12·1 (7·9 to 17·6)	15·4 (10·1 to 22·2)	21·8 (15 to 31·5)	24·9 (17·1 to 35·2)	25·1 (18·3 to 34·1)	25·9 (18·7 to 35·7)	26·3 (19·5 to 34·8)	20·4 (15 to 27)	17·1 (12·9 to 22·4)	13·3 (9·8 to 17·2)	10·2 (7·3 to 13)	6·4 (4·3 to 8·8)	245
Non-melanoma skin cancer (basal-cell carcinoma)	0 (0 to 0)	0 (0 to 0)	0 (0 to 0)	0 (0 to 0)	0 (0 to 0)	0 (0 to 0)	0 (0 to 0)	0 (0 to 0)	0 (0 to 0)	0 (0 to 0)	0 (0 to 0)	0 (0 to 0)	0 (0 to 0)	0 (0 to 0)	0 (0 to 0)	0 (0 to 0)	0 (0 to 0)	0 (0 to 0)	0 (0 to 0)	0 (0 to 0)	0 (0 to 0)	0 (0 to 0)	0 (0 to 0)	0·1
Breast cancer	0 (0 to 0)	0 (0 to 0)	0 (0 to 0)	0 (0 to 0)	0 (0 to 0)	0 (0 to 0)	14·7 (8·9 to 23·5)	49·8 (31·4 to 72·5)	205 (129 to 299)	642 (402 to 933)	1,010 (663 to 1,490)	1,690 (1,170 to 2,340)	2,030 (1,410 to 2,790)	1,950 (1,400 to 2,630)	1,750 (1,250 to 2,360)	1,450 (1,050 to 1,950)	1,060 (766 to 1,390)	835 (606 to 1,110)	549 (406 to 721)	368 (272 to 490)	246 (174 to 332)	120 (83·4 to 165)	82·6 (46·6 to 127)	14,100
Cervical cancer	0 (0 to 0)	0 (0 to 0)	0 (0 to 0)	0 (0 to 0)	0 (0 to 0)	0 (0 to 0)	14·4 (7·9 to 23·8)	72·6 (38 to 121)	208 (119 to 343)	494 (295 to 767)	759 (443 to 1,150)	917 (591 to 1,360)	1,090 (714 to 1,580)	946 (628 to 1,380)	902 (589 to 1,280)	828 (549 to 1,170)	601 (405 to 850)	394 (271 to 537)	243 (175 to 331)	158 (109 to 218)	83 (56·7 to 116)	36·6 (23 to 53·8)	24·6 (14·9 to 36)	7,770
Uterine cancer	0 (0 to 0)	0 (0 to 0)	0 (0 to 0)	0 (0 to 0)	0 (0 to 0)	0 (0 to 0)	0 (0 to 0)	11·5 (6·8 to 17·9)	15·1 (7·7 to 27)	17·7 (10 to 29)	31·8 (18·4 to 52·3)	62·1 (38·2 to 95)	116 (73·2 to 178)	162 (104 to 241)	292 (187 to 427)	514 (329 to 752)	479 (318 to 683)	347 (235 to 482)	206 (141 to 293)	124 (82·3 to 178)	64·4 (43·7 to 89·6)	29·8 (19·8 to 41·5)	13·5 (8·6 to 19·5)	2,490
Ovarian cancer	0 (0 to 0)	0 (0 to 0)	0 (0 to 0)	0 (0 to 0)	6·5 (3·5 to 10·6)	11·9 (6 to 19·3)	31·2 (14·6 to 51·4)	51·9 (27·5 to 86·6)	63·6 (33·8 to 104)	93·4 (49·6 to 145)	122 (66·4 to 189)	140 (87·8 to 217)	244 (138 to 373)	302 (169 to 452)	332 (177 to 489)	308 (176 to 444)	257 (133 to 364)	175 (99·9 to 240)	109 (61·4 to 158)	64·7 (38·7 to 96·9)	44·9 (24·4 to 66·4)	20·9 (11·4 to 31·5)	8·1 (3·6 to 12·9)	2,390
Prostate cancer	0 (0 to 0)	0 (0 to 0)	0 (0 to 0)	0 (0 to 0)	0 (0 to 0)	0 (0 to 0)	0 (0 to 0)	2·5 (1·7 to 3·9)	8·2 (4·7 to 11·6)	5 (3·2 to 10·5)	6·4 (3·9 to 14·1)	26·3 (15·3 to 41·4)	122 (67 to 187)	343 (191 to 525)	823 (396 to 1,260)	1,650 (844 to 2,410)	2,210 (1,060 to 3,210)	2,730 (1,380 to 3,830)	2,460 (1,330 to 3,370)	1,840 (1,050 to 2,520)	1,080 (704 to 1,430)	530 (336 to 723)	246 (150 to 361)	14,100
Testicular cancer	1·3 (0 to 14)	0·3 (0 to 3·9)	0·6 (0·1 to 2·9)	1·8 (0·7 to 4·7)	1·9 (1·2 to 3·3)	1·8 (1 to 3·3)	5·6 (2·8 to 10·1)	12·4 (7 to 19·6)	11·1 (6·6 to 17·5)	8·7 (5·2 to 13·7)	3·6 (2·1 to 6)	8·7 (4·7 to 14·5)	4·2 (2·2 to 7·2)	4 (2·1 to 6·9)	4·1 (2 to 7·1)	4·5 (2·1 to 7·6)	1·1 (0·5 to 2·1)	2·4 (1·2 to 4·5)	3·1 (1·3 to 6)	2 (0·9 to 3·7)	1·2 (0·6 to 2)	0·5 (0·2 to 0·8)	0·2 (0·1 to 0·3)	85·1
Kidney cancer	0·5 (0·3 to 0·7)	0·3 (0·2 to 0·5)	1·1 (0·5 to 1·8)	21·8 (13·3 to 33·3)	22·9 (13·2 to 36·7)	12 (6·3 to 21·9)	16·2 (7·7 to 28·5)	27·5 (14·6 to 47·4)	40·2 (22 to 67·8)	51·8 (28·1 to 89·7)	49·2 (28·1 to 78·8)	59·9 (34·6 to 98)	86·4 (50·1 to 141)	111 (67·1 to 171)	127 (76·2 to 201)	120 (74·1 to 184)	102 (66·1 to 151)	86·4 (54·8 to 126)	56·7 (36 to 84·7)	31 (19·7 to 45·9)	15·8 (9·8 to 23·9)	7·4 (4·7 to 11)	3 (1·8 to 4·5)	1,050
Bladder cancer	0 (0 to 0)	0 (0 to 0)	0 (0 to 0)	0 (0 to 0)	0 (0 to 0)	0 (0 to 0)	1·6 (1·3 to 2)	2·7 (1·8 to 4·1)	4·2 (2·3 to 7)	9·4 (5·4 to 15·2)	13·3 (7·6 to 21·2)	36·6 (23·1 to 56·6)	61·5 (38·8 to 91·1)	111 (73·2 to 160)	143 (92·8 to 213)	195 (129 to 280)	191 (126 to 266)	212 (150 to 305)	151 (106 to 214)	111 (77·3 to 156)	80·2 (54·2 to 113)	38 (25·4 to 53·2)	18·8 (12·4 to 26·9)	1,380
Brain and central nervous system cancer	3 (1·5 to 4·9)	3·1 (1·3 to 5·5)	13·5 (4·8 to 27·2)	82·9 (34·9 to 139)	151 (58·8 to 232)	102 (41·8 to 158)	64·1 (20·2 to 105)	83 (25·2 to 135)	87·3 (30·6 to 137)	82·7 (35·8 to 130)	90·7 (41·7 to 143)	96·1 (46·7 to 147)	108 (56·4 to 166)	142 (63 to 225)	161 (66·9 to 256)	133 (58·1 to 206)	98·3 (45·8 to 147)	70·9 (32 to 106)	42·4 (18·4 to 65·9)	22·4 (9·2 to 36·5)	10·1 (4·7 to 16·3)	4·3 (1·9 to 6·8)	1·5 (0·7 to 2·4)	1,650
Thyroid cancer	0 (0 to 0)	0 (0 to 0)	0 (0 to 0)	0 (0 to 0)	3·4 (2·2 to 5·5)	2·1 (1·6 to 2·8)	7·7 (4·1 to 12·9)	13·3 (6 to 26·9)	17·2 (8·8 to 30·6)	19 (9·9 to 34·6)	18·3 (10·7 to 29·2)	20 (12 to 31)	42 (27·1 to 65·9)	66 (43·2 to 97·4)	57·9 (38·2 to 83·7)	66·2 (45·1 to 92·6)	63·9 (43·5 to 87·1)	56·2 (38·9 to 77·6)	34·1 (23·5 to 48·6)	21·1 (14·5 to 29·3)	12·4 (7·9 to 17·8)	4·9 (3 to 7·1)	2·5 (1·5 to 3·7)	528
Mesothelioma	0 (0 to 0)	0 (0 to 0)	0 (0 to 0)	0 (0 to 0)	0 (0 to 0)	0 (0 to 0)	0 (0 to 0)	2·4 (1·4 to 5·2)	5·1 (3·3 to 7·5)	5·2 (3·5 to 8)	9·8 (6·6 to 14·6)	7·2 (4·8 to 11·5)	10·8 (6·9 to 17·3)	20·6 (13·6 to 30·3)	26·8 (18·7 to 37)	9·2 (6·4 to 13)	11·8 (8·6 to 15·9)	13·3 (9·7 to 17·2)	10·4 (7·7 to 13·6)	6·2 (4·5 to 7·9)	2·4 (1·7 to 3·2)	0·9 (0·6 to 1·2)	0·4 (0·3 to 0·6)	142
Hodgkin lymphoma	0 (0 to 0)	0 (0 to 0)	0 (0 to 0)	1·7 (1·1 to 2·4)	2 (1·6 to 2·5)	4·4 (2·4 to 7·6)	10·9 (6·5 to 17·3)	34·9 (18·3 to 57·9)	32·1 (19·2 to 50·4)	28·9 (16·8 to 45·4)	24·6 (13·7 to 38·8)	20·9 (12·3 to 32·1)	10·4 (6·3 to 16)	12·4 (8·1 to 19·4)	13·9 (9·1 to 20·4)	15·6 (10·2 to 22·2)	12·2 (8·2 to 17·4)	8·2 (5·7 to 11·6)	6·1 (4·3 to 8·3)	3 (2 to 4·3)	1·5 (1 to 2·2)	0·4 (0·2 to 0·7)	0·1 (0·1 to 0·3)	244
Non-Hodgkin lymphoma	0 (0 to 0)	0 (0 to 0)	0 (0 to 0)	36·5 (23 to 55·6)	76·7 (54·9 to 104)	116 (80·5 to 163)	119 (83·9 to 164)	239 (166 to 332)	339 (237 to 467)	386 (269 to 537)	350 (238 to 493)	379 (264 to 525)	536 (375 to 754)	613 (428 to 855)	532 (366 to 734)	508 (365 to 695)	386 (283 to 518)	294 (215 to 387)	177 (133 to 236)	101 (73·1 to 138)	41·3 (29·9 to 56·4)	15·4 (10·5 to 21·2)	7·4 (4·9 to 10)	5,250
Multiple myeloma	0 (0 to 0)	0 (0 to 0)	0 (0 to 0)	0 (0 to 0)	0 (0 to 0)	0 (0 to 0)	0 (0 to 0)	9 (3·4 to 15·2)	9·6 (5·3 to 14·7)	15·3 (8·8 to 23)	22·8 (14 to 35·5)	64·5 (41·2 to 94·4)	164 (88·4 to 237)	276 (152 to 405)	383 (209 to 552)	400 (248 to 556)	376 (229 to 524)	315 (204 to 437)	220 (143 to 300)	111 (76·2 to 155)	60·5 (40·3 to 86·9)	19·9 (13 to 27·9)	7·8 (4·4 to 12·1)	2,450
Leukemia	8·7 (6 to 12·4)	12·4 (8·2 to 17·9)	31·7 (16·3 to 54·4)	173 (110 to 251)	298 (213 to 416)	275 (188 to 388)	244 (166 to 343)	289 (194 to 412)	337 (231 to 480)	330 (218 to 466)	327 (217 to 474)	319 (214 to 453)	349 (237 to 491)	337 (227 to 477)	368 (249 to 515)	358 (244 to 497)	248 (174 to 338)	223 (158 to 308)	150 (108 to 204)	87·6 (59·5 to 122)	49·3 (33·4 to 70·1)	19·4 (12·9 to 26·6)	10·2 (6·3 to 14·9)	4,850
Acute lymphoid leukemia	3·2 (1·5 to 4·8)	6·3 (1·6 to 9·9)	14·4 (6·4 to 26·9)	85 (51·8 to 130)	171 (112 to 246)	108 (65·4 to 169)	102 (63·4 to 154)	34·3 (17·9 to 62·5)	26·9 (13·8 to 46·7)	27 (14·7 to 44·3)	23·5 (11·9 to 41·8)	24·1 (12·3 to 42·8)	17·9 (9·6 to 31·2)	13·3 (7 to 22·2)	15·2 (8·6 to 24·5)	9·1 (5·1 to 15·1)	9·6 (5·5 to 15·3)	6 (3·4 to 10)	2·8 (1·4 to 6·5)	1·7 (0·8 to 4·8)	0·5 (0·2 to 1·6)	0·4 (0·2 to 1·1)	1·1 (0·2 to 2·3)	703
Chronic lymphoid leukemia	0 (0 to 0)	0 (0 to 0)	0 (0 to 0)	0 (0 to 0)	0 (0 to 0)	0 (0 to 0)	0 (0 to 0)	2·3 (1·4 to 3·4)	2 (1·2 to 2·9)	3·8 (1·9 to 6·4)	1·5 (0·8 to 2·5)	1·7 (0·9 to 3·2)	4·1 (2 to 7·4)	11·7 (6·9 to 19·1)	33·2 (17·3 to 53·7)	41·8 (21·7 to 66·4)	35·1 (20·5 to 55·2)	34·3 (19·1 to 57)	16·6 (9·7 to 26·7)	13·4 (7·5 to 21·7)	8 (3·7 to 13)	2·9 (1·6 to 4·6)	0·1 (0 to 0·3)	212
Acute myeloid leukemia	2·2 (1·3 to 3·3)	5·1 (2·9 to 8·6)	11·8 (4·8 to 24)	55·4 (32·9 to 87)	74·5 (43·4 to 123)	99·4 (57·4 to 153)	79·6 (47·8 to 124)	77·2 (45·1 to 124)	88·7 (52·7 to 136)	54·2 (31·3 to 86)	62·2 (33·6 to 102)	67 (38·3 to 111)	73·9 (43·1 to 117)	83·6 (47·9 to 135)	71·7 (43·1 to 113)	66·5 (37·9 to 104)	48·7 (29·1 to 78·5)	42·2 (25·7 to 65·8)	26·5 (15·4 to 42·1)	14·5 (8·3 to 23·7)	6·9 (4 to 11·4)	1·8 (0·9 to 3·8)	2 (0·6 to 4·9)	1,120
Chronic myeloid leukemia	0·2 (0 to 0·4)	0·1 (0 to 0·2)	0·4 (0·1 to 0·9)	3·4 (1·9 to 5·6)	2·9 (1·7 to 4·7)	6·9 (3·7 to 12)	11·4 (6 to 19·8)	36·4 (18·9 to 61·6)	34·6 (18·8 to 59·2)	33·8 (18 to 58)	19·1 (9·4 to 37·1)	21·9 (12·2 to 35·7)	32·2 (18·6 to 53·2)	37·7 (21·3 to 63·1)	41·3 (22·4 to 70·6)	42·2 (22·9 to 69·2)	29·9 (16·7 to 49·4)	24·8 (14·6 to 39)	17·7 (10 to 28·9)	8·4 (4·7 to 13·4)	2·3 (1·2 to 4·2)	3·1 (1·4 to 5·8)	0·1 (0 to 0·2)	411
Other leukemia	3·1 (2 to 4·9)	0·9 (0·3 to 3·5)	5·2 (1·9 to 13·5)	29·2 (17·1 to 46·9)	49·4 (24·7 to 87·4)	60·7 (32·1 to 102)	50·9 (27·1 to 83·6)	139 (84·9 to 211)	185 (107 to 277)	211 (126 to 306)	221 (137 to 324)	204 (129 to 305)	221 (139 to 320)	191 (123 to 278)	207 (130 to 298)	199 (126 to 288)	125 (84·2 to 179)	116 (78·9 to 166)	86·6 (59·3 to 121)	49·7 (33 to 71·3)	31·6 (20·7 to 45·5)	11·1 (7·1 to 16·4)	6·9 (3·7 to 10·6)	2,400
Other malignant neoplasms	10·9 (7·4 to 16·1)	5 (3·1 to 9·3)	23·9 (11·3 to 46·6)	141 (89·5 to 244)	174 (127 to 237)	187 (136 to 251)	195 (141 to 268)	238 (164 to 330)	213 (154 to 289)	202 (146 to 275)	186 (130 to 251)	227 (157 to 323)	287 (196 to 407)	336 (230 to 474)	331 (237 to 453)	325 (237 to 442)	303 (224 to 399)	265 (195 to 358)	176 (134 to 228)	125 (93·2 to 162)	73·6 (51·7 to 103)	36·1 (25·3 to 53·2)	17·1 (11·1 to 24·9)	4,080
Other neoplasms	0·2 (0 to 0·9)	0·1 (0 to 0·6)	2·1 (0·8 to 4·8)	6·3 (3·5 to 11·1)	10·3 (4·7 to 16·7)	8·9 (4·1 to 14)	8·1 (3·2 to 13·5)	5·6 (2·8 to 8·7)	14·1 (8·5 to 22·9)	22·3 (9·7 to 33·9)	20·7 (10·2 to 32·1)	27·3 (15·9 to 38·2)	36·7 (18·7 to 55)	56·9 (24·3 to 90·6)	60·9 (30·4 to 92·9)	52·8 (29·7 to 74·3)	47·1 (33·8 to 65·8)	62·2 (43 to 86·2)	47·3 (33·2 to 66·2)	32·8 (23·4 to 45·1)	17·4 (12·5 to 24)	9·4 (6·2 to 13)	2·8 (1·6 to 4)	552
Myelodysplastic, myeloproliferative, and other hematopoietic n	0·2 (0 to 0·9)	0·1 (0 to 0·6)	2·1 (0·8 to 4·8)	6·3 (3·5 to 11·1)	10·3 (4·7 to 16·7)	8·9 (4·1 to 14)	8·1 (3·2 to 13·5)	5·6 (2·8 to 8·7)	14·1 (8·5 to 22·9)	22·3 (9·7 to 33·9)	20·7 (10·2 to 32·1)	27·3 (15·9 to 38·2)	36·7 (18·7 to 55)	56·9 (24·3 to 90·6)	60·9 (30·4 to 92·9)	52·8 (29·7 to 74·3)	47·1 (33·8 to 65·8)	62·2 (43 to 86·2)	47·3 (33·2 to 66·2)	32·8 (23·4 to 45·1)	17·4 (12·5 to 24)	9·4 (6·2 to 13)	2·8 (1·6 to 4)	552
Benign and in situ intestinal neoplasms	0 (0 to 0)	0 (0 to 0)	0 (0 to 0)	0 (0 to 0)	0 (0 to 0)	0 (0 to 0)	0 (0 to 0)	0 (0 to 0)	0 (0 to 0)	0 (0 to 0)	0 (0 to 0)	0 (0 to 0)	0 (0 to 0)	0 (0 to 0)	0 (0 to 0)	0 (0 to 0)	0 (0 to 0)	0 (0 to 0)	0 (0 to 0)	0 (0 to 0)	0 (0 to 0)	0 (0 to 0)	0 (0 to 0)	0
Benign and in situ cervical and uterine neoplasms	0 (0 to 0)	0 (0 to 0)	0 (0 to 0)	0 (0 to 0)	0 (0 to 0)	0 (0 to 0)	0 (0 to 0)	0 (0 to 0)	0 (0 to 0)	0 (0 to 0)	0 (0 to 0)	0 (0 to 0)	0 (0 to 0)	0 (0 to 0)	0 (0 to 0)	0 (0 to 0)	0 (0 to 0)	0 (0 to 0)	0 (0 to 0)	0 (0 to 0)	0 (0 to 0)	0 (0 to 0)	0 (0 to 0)	0
Other benign and in situ neoplasms	0 (0 to 0)	0 (0 to 0)	0 (0 to 0)	0 (0 to 0)	0 (0 to 0)	0 (0 to 0)	0 (0 to 0)	0 (0 to 0)	0 (0 to 0)	0 (0 to 0)	0 (0 to 0)	0 (0 to 0)	0 (0 to 0)	0 (0 to 0)	0 (0 to 0)	0 (0 to 0)	0 (0 to 0)	0 (0 to 0)	0 (0 to 0)	0 (0 to 0)	0 (0 to 0)	0 (0 to 0)	0 (0 to 0)	0
Cardiovascular diseases	158 (101 to 236)	39·1 (25 to 58·8)	61·2 (31·8 to 107)	103 (70·9 to 144)	251 (193 to 323)	431 (332 to 546)	770 (598 to 961)	1,180 (912 to 1,520)	1,660 (1,260 to 2,180)	2,050 (1,560 to 2,640)	2,810 (2,090 to 3,710)	4,030 (3,040 to 5,320)	6,330 (4,780 to 8,280)	8,290 (6,270 to 10,700)	10,600 (8,090 to 13,500)	12,600 (9,790 to 15,700)	13,100 (10,400 to 16,200)	14,800 (12,100 to 17,800)	12,800 (10,700 to 15,300)	11,000 (9,150 to 13,100)	8,240 (6,650 to 9,820)	4,980 (3,720 to 5,900)	3,450 (2,520 to 4,060)	120,000
Rheumatic heart disease	0 (0 to 0)	0 (0 to 0)	0 (0 to 0)	12·6 (7·9 to 19·3)	80·9 (56·9 to 114)	186 (132 to 254)	254 (178 to 348)	309 (218 to 423)	361 (254 to 504)	287 (203 to 399)	245 (170 to 339)	218 (152 to 302)	176 (123 to 241)	129 (91·1 to 179)	94·3 (67·1 to 128)	72·8 (52·5 to 98·3)	51·7 (37·9 to 69·4)	43·4 (32 to 58·2)	33·1 (24·2 to 43·3)	22·4 (16·5 to 29·8)	15·1 (11·1 to 19·8)	8·8 (6·2 to 11·6)	5·8 (4 to 7·6)	2,610

Ischemic heart disease	0 (0 to 0)	0 (0 to 0)	0 (0 to 0)	0 (0 to 0)	0 (0 to 0)	0 (0 to 0)	40·5 (27·9 to 57·1)	[illegible]
Stroke	108 (64·9 to 167)	17·3 (9·9 to 27·8)	19·4 (8·5 to 37·9)	30·2 (19·9 to 42·4)	82 (56·1 to 113)	124 (87·4 to 167)	237 (164 to 330)	[illegible]
Ischemic stroke	1·4 (0·7 to 3·2)	1 (0·5 to 1·6)	0·7 (0·5 to 1)	9·8 (6·9 to 13·2)	25·6 (16·5 to 38·3)	33·8 (21·4 to 50·5)	59 (41·6 to 80·6)	[illegible]
Intracerebral hemorrhage	14 (6·2 to 29·9)	3 (1·5 to 5·4)	6·9 (2·8 to 14·4)	4·3 (2·5 to 7)	20·2 (12·5 to 31·1)	44·2 (29·4 to 66·4)	108 (70·8 to 157)	[illegible]
Subarachnoid hemorrhage	93 (54·4 to 142)	13·3 (7·2 to 21·7)	11·8 (4·9 to 24·1)	16·1 (8·8 to 25)	36·2 (21·5 to 55·7)	45·7 (27·5 to 72)	70·8 (40·9 to 115)	[illegible]
Hypertensive heart disease	0 (0 to 0)	0 (0 to 0)	0 (0 to 0)	0 (0 to 0)	0 (0 to 0)	0 (0 to 0)	20·4 (13 to 31·1)	[illegible]
Non-rheumatic valvular heart disease	0 (0 to 0)	0 (0 to 0)	0 (0 to 0)	0 (0 to 0)	0 (0 to 0)	0 (0 to 0)	18 (12·5 to 24·5)	[illegible]
Non-rheumatic calcific aortic valve disease	0 (0 to 0)	0 (0 to 0)	0 (0 to 0)	0 (0 to 0)	0 (0 to 0)	0 (0 to 0)	11·5 (7·3 to 17·1)	[illegible]
Non-rheumatic degenerative mitral valve disease	0 (0 to 0)	0 (0 to 0)	0 (0 to 0)	0 (0 to 0)	0 (0 to 0)	0 (0 to 0)	6·1 (3·6 to 11·8)	[illegible]
Other non-rheumatic valve diseases	0 (0 to 0)	0 (0 to 0)	0 (0 to 0)	0 (0 to 0)	0 (0 to 0)	0 (0 to 0)	0·4 (0·2 to 0·8)	[illegible]
Cardiomyopathy and myocarditis	22·6 (13·8 to 32·8)	11·4 (7·6 to 17·1)	24·3 (10·9 to 45·7)	33 (19·7 to 54·1)	34·5 (23·8 to 48·6)	42·8 (29·7 to 60·5)	58·3 (40·3 to 80·6)	[illegible]
Myocarditis	5·7 (1·4 to 10·8)	3·2 (1·5 to 5·8)	5·3 (2·3 to 10·1)	7·2 (3·7 to 12)	7·7 (4·8 to 11·9)	6·9 (4 to 10·5)	6·5 (3·6 to 11)	[illegible]
Alcoholic cardiomyopathy	0 (0 to 0)	0 (0 to 0)	0 (0 to 0)	0 (0 to 0)	0 (0 to 0)	0 (0 to 0)	2·5 (1·5 to 3·9)	[illegible]
Other cardiomyopathy	16·9 (9·2 to 25·9)	8·2 (4·8 to 13·6)	19·1 (7·8 to 39·1)	25·8 (14·9 to 45·7)	26·8 (17·9 to 38·3)	35·9 (24·4 to 52·4)	49·3 (33·6 to 69·1)	[illegible]
Atrial fibrillation and flutter	0 (0 to 0)	0 (0 to 0)	0 (0 to 0)	0 (0 to 0)	0 (0 to 0)	0 (0 to 0)	0 (0 to 0)	[illegible]
Aortic aneurysm	--	--	--	--	--	--	12·5 (8·1 to 18·2)	[illegible]
Endocarditis	1·3 (0·5 to 3·7)	1 (0·2 to 4·4)	7·1 (3·1 to 14·6)	8·6 (4·3 to 18·7)	4·6 (2·1 to 12)	24·8 (10·4 to 41)	46·6 (17·6 to 83·9)	[illegible]
Other cardiovascular and circulatory diseases	25·9 (15·9 to 40·5)	9·4 (5·3 to 16·5)	10·4 (4·9 to 21·6)	18·9 (10·9 to 31·6)	49·2 (32·8 to 72·9)	53·8 (35·1 to 76·3)	81·9 (56·8 to 115)	[illegible]
Chronic respiratory diseases	0 (0 to 0)	0 (0 to 0)	0·2 (0·1 to 0·2)	1,100 (624 to 1,870)	1,130 (688 to 1,830)	985 (629 to 1,500)	801 (525 to 1,220)	[illegible]
Chronic obstructive pulmonary disease	0 (0 to 0)	0 (0 to 0)	0·2 (0·1 to 0·2)	37·7 (25·4 to 54·1)	29·2 (19 to 42·5)	32·7 (18·1 to 52·2)	59·7 (38·3 to 83·2)	[illegible]
Pneumoconiosis	0 (0 to 0)	0 (0 to 0)	0 (0 to 0)	0 (0 to 0)	0 (0 to 0)	0 (0 to 0)	1·1 (0·6 to 1·8)	[illegible]
Silicosis	0 (0 to 0)	0 (0 to 0)	0 (0 to 0)	0 (0 to 0)	0 (0 to 0)	0 (0 to 0)	0·1 (0 to 0·2)	[illegible]
Asbestosis	0 (0 to 0)	0 (0 to 0)	0 (0 to 0)	0 (0 to 0)	0 (0 to 0)	0 (0 to 0)	0·8 (0·4 to 1·3)	[illegible]
Coal workers pneumoconiosis	0 (0 to 0)	0 (0 to 0)	0 (0 to 0)	0 (0 to 0)	0 (0 to 0)	0 (0 to 0)	0 (0 to 0·1)	[illegible]
Other pneumoconiosis	0 (0 to 0)	0 (0 to 0)	0 (0 to 0)	0 (0 to 0)	0 (0 to 0)	0 (0 to 0)	0·2 (0·1 to 0·5)	[illegible]
Asthma	0 (0 to 0)	0 (0 to 0)	0 (0 to 0)	967 (498 to 1,720)	1,060 (623 to 1,750)	877 (537 to 1,380)	695 (425 to 1,120)	[illegible]
Interstitial lung disease and pulmonary sarcoidosis	0 (0 to 0)	0 (0 to 0)	0 (0 to 0)	16·2 (8·6 to 27·5)	3·7 (2 to 6·5)	4·1 (2·3 to 6·9)	5·8 (3 to 10·8)	[illegible]
Other chronic respiratory diseases	0 (0 to 0)	0 (0 to 0)	0 (0 to 0)	82·5 (51 to 132)	40·9 (26·9 to 58·4)	70·7 (44·5 to 100)	39·3 (25·6 to 56·1)	[illegible]
Digestive diseases	69·3 (45·2 to 99·7)	53·9 (31·2 to 88·3)	109 (59·4 to 191)	294 (204 to 407)	324 (236 to 438)	331 (252 to 440)	454 (342 to 600)	[illegible]
Cirrhosis and other chronic liver diseases	0 (0 to 0)	0 (0 to 0)	0·2 (0·1 to 0·3)	27·9 (15·9 to 47·1)	38 (22·4 to 64·6)	55 (31·5 to 91)	91·1 (57·3 to 139)	[illegible]
Cirrhosis and other chronic liver diseases due to hepatitis B	0 (0 to 0)	0 (0 to 0)	0 (0 to 0)	0 (0 to 0·1)	0·1 (0 to 0·3)	0·3 (0·1 to 0·8)	0·8 (0·2 to 1·8)	[illegible]
Cirrhosis and other chronic liver diseases due to hepatitis C	0 (0 to 0)	0 (0 to 0)	0 (0 to 0)	0·1 (0·1 to 0·3)	0·5 (0·2 to 1·1)	1·4 (0·6 to 3)	3·5 (1·3 to 7·5)	[illegible]
Cirrhosis and other chronic liver diseases due to alcohol use	0 (0 to 0)	0 (0 to 0)	0 (0 to 0)	0 (0 to 0)	0 (0 to 0)	0 (0 to 0)	0·4 (0·1 to 1·1)	[illegible]
Cirrhosis and other chronic liver diseases due to NAFLD	0 (0 to 0)	0 (0 to 0)	0 (0 to 0)	0 (0 to 0)	0 (0 to 0)	0 (0 to 0)	4 (1·6 to 8·3)	[illegible]
Cirrhosis and other chronic liver diseases due to other causes	0 (0 to 0)	0 (0 to 0)	0·2 (0·1 to 0·3)	27·7 (15·8 to 46·7)	37·3 (22 to 63·7)	53·3 (30·4 to 88·1)	82·4 (52·4 to 124)	[illegible]
Upper digestive system diseases	0 (0 to 0)	0 (0 to 0)	0 (0 to 0)	16·7 (9 to 27·6)	24·4 (12·5 to 41·6)	41·1 (24·8 to 65·7)	146 (83·7 to 248)	[illegible]
Peptic ulcer disease	0 (0 to 0)	0 (0 to 0)	0 (0 to 0)	1·2 (0·6 to 2·4)	3·4 (2 to 6·1)	6·8 (4·3 to 11·1)	12·8 (8·2 to 19·8)	[illegible]
Gastritis and duodenitis	0 (0 to 0)	0 (0 to 0)	0 (0 to 0)	15·5 (7·9 to 26·4)	20·6 (9·2 to 38·2)	18·7 (7·8 to 36·1)	27·2 (13·1 to 51·3)	[illegible]
Gastroesophageal reflux disease	0 (0 to 0)	0 (0 to 0)	0 (0 to 0)	0 (0 to 0)	0·5 (0·1 to 1·1)	15·5 (7 to 30·2)	106 (50·9 to 198)	[illegible]
Appendicitis	0 (0 to 0)	0 (0 to 0)	0 (0 to 0)	11·9 (6 to 23·7)	30·9 (20 to 49·8)	38·2 (22·8 to 58·7)	38·2 (22·3 to 59·2)	[illegible]
Paralytic ileus and intestinal obstruction	37·9 (22·5 to 58·9)	40·7 (23·5 to 66·2)	93·5 (49·7 to 167)	60·7 (33·6 to 97·7)	43·9 (27·5 to 64·1)	47 (27·5 to 71·8)	32·4 (18 to 51·2)	[illegible]
Inguinal, femoral, and abdominal hernia	31·3 (17·6 to 53·2)	13·2 (6 to 25·7)	15·2 (8 to 26·4)	93·9 (53 to 151)	128 (71·6 to 218)	87·9 (46·3 to 155)	74·7 (39·5 to 127)	[illegible]
Inflammatory bowel disease	0 (0 to 0)	0 (0 to 0)	0 (0 to 0)	6·1 (3·1 to 11)	2·5 (1·4 to 4·4)	2·7 (1·5 to 5·2)	4·3 (2·7 to 7·4)	[illegible]
Vascular intestinal disorders	0 (0 to 0)	0 (0 to 0)	0 (0 to 0)	2 (1·1 to 3·4)	0·8 (0·4 to 1·3)	2·8 (1·4 to 5·6)	0·7 (0·4 to 1·2)	[illegible]
Gallbladder and biliary diseases	0 (0 to 0)	0 (0 to 0)	0 (0 to 0)	4·2 (2·4 to 7·2)	11·2 (4·8 to 20·7)	18·9 (8·4 to 34·6)	32·5 (16·7 to 57·4)	[illegible]
Pancreatitis	0 (0 to 0)	0 (0 to 0)	0 (0 to 0)	3·2 (1·5 to 5·8)	0·8 (0·5 to 1·3)	4·8 (2·5 to 8·2)	5·3 (3·4 to 8·2)	[illegible]
Other digestive diseases	0 (0 to 0)	0 (0 to 0)	0 (0 to 0)	67·8 (40·2 to 106)	43·1 (24·6 to 63·5)	32·1 (17·8 to 48·2)	28·8 (13·3 to 44·6)	[illegible]
Neurological disorders	0·9 (0·4 to 1·9)	2·7 (1 to 5·4)	112 (55·4 to 192)	346 (157 to 640)	690 (294 to 1,240)	1,770 (550 to 3,650)	2,400 (776 to 4,970)	[illegible]
Alzheimer's disease and other dementias	0 (0 to 0)	0 (0 to 0)	0 (0 to 0)	0 (0 to 0)	0 (0 to 0)	0 (0 to 0)	0 (0 to 0)	[illegible]
Parkinson's disease	0 (0 to 0)	0 (0 to 0)	0 (0 to 0)	0 (0 to 0)	0 (0 to 0)	0 (0 to 0)	0 (0 to 0)	[illegible]
Idiopathic epilepsy	0·7 (0·1 to 1·6)	2 (0·4 to 4·8)	39·6 (11·5 to 89·8)	239 (97·5 to 458)	354 (138 to 692)	432 (162 to 853)	523 (225 to 1,000)	[illegible]
Multiple sclerosis	0 (0 to 0)	0 (0 to 0)	0 (0 to 0)	0 (0 to 0)	0·1 (0 to 0·1)	0·3 (0·1 to 0·5)	1·6 (1 to 2·3)	[illegible]
Motor neuron disease	0·3 (0·2 to 0·4)	0·7 (0·4 to 1·1)	28 (12·6 to 54·4)	19·6 (11·9 to 29·9)	3·8 (2·4 to 6)	1·5 (1 to 2·1)	4·8 (3·4 to 6·8)	[illegible]
Headache disorders	0 (0 to 0)	0 (0 to 0)	0 (0 to 0)	0 (0 to 0)	218 (3·6 to 578)	1,150 (52·5 to 2,910)	1,700 (181 to 4,210)	[illegible]
Migraine	0 (0 to 0)	0 (0 to 0)	0 (0 to 0)	0 (0 to 0)	200 (2·6 to 535)	1,080 (35 to 2,890)	1,580 (126 to 4,140)	[illegible]
Tension-type headache	0 (0 to 0)	0 (0 to 0)	0 (0 to 0)	0 (0 to 0)	18·1 (0·5 to 120)	70·4 (3·9 to 512)	116 (19·7 to 573)	[illegible]
Other neurological disorders	0 (0 to 0)	0 (0 to 0)	44·4 (21·7 to 77·3)	88·2 (37·3 to 166)	114 (42·7 to 225)	186 (73·8 to 363)	177 (76·3 to 336)	[illegible]
Mental disorders	0·6 (0·3 to 0·8)	1·6 (1 to 2·4)	27·5 (17·5 to 40·8)	161 (106 to 229)	907 (605 to 1,310)	2,820 (1,890 to 4,020)	4,400 (3,050 to 6,050)	[illegible]
Schizophrenia	0 (0 to 0)	0 (0 to 0)	0 (0 to 0)	0 (0 to 0)	0 (0 to 0)	13·1 (5·6 to 25·5)	110 (49·9 to 203)	[illegible]
Depressive disorders	0 (0 to 0)	0 (0 to 0)	0 (0 to 0)	0·4 (0·2 to 0·8)	44·5 (17·4 to 86·4)	476 (246 to 841)	1,270 (737 to 2,000)	[illegible]
Major depressive disorder	0 (0 to 0)	0 (0 to 0)	0 (0 to 0)	0·3 (0·1 to 0·8)	39 (12·9 to 81·7)	432 (209 to 797)	1,130 (628 to 1,850)	[illegible]
Dysthymia	0 (0 to 0)	0 (0 to 0)	0 (0 to 0)	0·1 (0 to 0·2)	5·5 (1 to 14·2)	43·6 (17·9 to 85·5)	135 (66·7 to 232)	[illegible]
Bipolar disorder	0 (0 to 0)	0 (0 to 0)	0 (0 to 0)	0 (0 to 0)	0 (0 to 0)	164 (69·2 to 292)	641 (299 to 1,130)	[illegible]
Anxiety disorders	0 (0 to 0)	0 (0 to 0)	0 (0 to 0)	17·4 (6·4 to 35·9)	284 (146 to 496)	792 (439 to 1,300)	1,120 (670 to 1,750)	[illegible]
Eating disorders	0 (0 to 0)	0 (0 to 0)	0 (0 to 0)	0 (0 to 0)	3·1 (1·1 to 6·3)	51·2 (24·6 to 89·6)	185 (98·1 to 331)	[illegible]
Anorexia nervosa	0 (0 to 0)	0 (0 to 0)	0 (0 to 0)	0 (0 to 0)	2·7 (0·8 to 6)	25 (10 to 45·5)	71·4 (34·1 to 129)	[illegible]
Bulimia nervosa	0 (0 to 0)	0 (0 to 0)	0 (0 to 0)	0 (0 to 0)	0·4 (0·1 to 0·8)	26·2 (8·3 to 56·4)	113 (47·7 to 228)	[illegible]
Autism spectrum disorders	0·5 (0·3 to 0·7)	1·4 (0·9 to 2)	21·9 (13·9 to 32·4)	97·9 (60·1 to 144)	133 (84 to 199)	140 (89·1 to 209)	146 (95·2 to 218)	[illegible]
Attention-deficit/hyperactivity disorder	0 (0 to 0)	0 (0 to 0)	0 (0 to 0)	12·3 (6·2 to 21·8)	142 (78·5 to 254)	209 (114 to 366)	185 (102 to 323)	[illegible]
Conduct disorder	0 (0 to 0)	0 (0 to 0)	0 (0 to 0)	0 (0 to 0)	251 (128 to 434)	908 (496 to 1,490)	604 (323 to 999)	[illegible]
Idiopathic developmental intellectual disability	0·1 (0 to 0·2)	0·3 (0 to 0·7)	5·5 (0·7 to 11·5)	32·7 (8·6 to 62·8)	50·2 (17·5 to 92·5)	51·8 (17·7 to 96·3)	52·9 (17·2 to 97·7)	[illegible]
Other mental disorders	0 (0 to 0)	0 (0 to 0)	0 (0 to 0)	0 (0 to 0)	0 (0 to 0)	12 (5·6 to 20·4)	86·2 (40·6 to 150)	[illegible]
Substance use disorders	0 (0 to 0)	0 (0 to 0)	0 (0 to 0·1)	0·2 (0·1 to 0·3)	0·2 (0·1 to 0·4)	39·5 (22·3 to 65·2)	710 (438 to 1,040)	[illegible]
Alcohol use disorders	0 (0 to 0)	0 (0 to 0)	0 (0 to 0·1)	0·2 (0·1 to 0·3)	0·2 (0·1 to 0·4)	14·2 (5·8 to 27·4)	168 (83·2 to 287)	[illegible]
Drug use disorders	0 (0 to 0)	0 (0 to 0)	0 (0 to 0)	0 (0 to 0)	0 (0 to 0)	25·3 (13·1 to 44·2)	542 (328 to 838)	[illegible]
Opioid use disorders	0 (0 to 0)	0 (0 to 0)	0 (0 to 0)	0 (0 to 0)	0 (0 to 0)	0 (0 to 0)	49·4 (19·7 to 96·2)	[illegible]
Cocaine use disorders	0 (0 to 0)	0 (0 to 0)	0 (0 to 0)	0 (0 to 0)	0 (0 to 0)	4·2 (2·1 to 7·6)	168 (80·4 to 307)	[illegible]
Amphetamine use disorders	0 (0 to 0)	0 (0 to 0)	0 (0 to 0)	0 (0 to 0)	0 (0 to 0)	0 (0 to 0)	12·5 (3·4 to 25·8)	[illegible]
Cannabis use disorders	0 (0 to 0)	0 (0 to 0)	0 (0 to 0)	0 (0 to 0)	0 (0 to 0)	18·3 (7·3 to 35·3)	195 (102 to 332)	[illegible]
Other drug use disorders	0 (0 to 0)	0 (0 to 0)	0 (0 to 0)	0 (0 to 0)	0 (0 to 0)	2·8 (1·3 to 5·1)	117 (57 to 211)	[illegible]
Diabetes and kidney diseases	0·5 (0·3 to 0·7)	1·4 (0·8 to 2·3)	44·6 (23·1 to 75·2)	104 (71·6 to 142)	161 (118 to 214)	218 (157 to 306)	593 (457 to 766)	[illegible]
Diabetes mellitus	0·5 (0·3 to 0·7)	1·3 (0·8 to 2·3)	10·7 (3·8 to 24·3)	20 (12 to 34·5)	27·7 (18·9 to 38·9)	45·1 (31·8 to 62·2)	225 (169 to 294)	[illegible]
Diabetes mellitus type 1	0·5 (0·3 to 0·7)	1·3 (0·8 to 2·3)	10·7 (3·8 to 24·3)	20 (12 to 34·5)	27·7 (18·9 to 38·9)	45·1 (31·8 to 62·2)	69·1 (49·3 to 93·6)	[illegible]
Diabetes mellitus type 2	0 (0 to 0)	0 (0 to 0)	0 (0 to 0)	0 (0 to 0)	0 (0 to 0)	0 (0 to 0)	156 (109 to 218)	[illegible]
Chronic kidney disease	0 (0 to 0)	0 (0 to 0)	33·9 (17·8 to 56·4)	83·8 (57·2 to 115)	133 (91·2 to 185)	173 (116 to 257)	367 (260 to 526)	[illegible]
Chronic kidney disease due to diabetes mellitus type 1	0 (0 to 0)	0 (0 to 0)	0·3 (0·1 to 0·7)	2 (0·8 to 4·5)	3·7 (1·4 to 9)	5·5 (2·2 to 12·1)	15·3 (6·4 to 32·1)	[illegible]
Chronic kidney disease due to diabetes mellitus type 2	0 (0 to 0)	0 (0 to 0)	0 (0 to 0)	0 (0 to 0)	0 (0 to 0)	0 (0 to 0)	6·2 (2·1 to 14)	[illegible]
Chronic kidney disease due to hypertension	0 (0 to 0)	0 (0 to 0)	0 (0 to 0)	0 (0 to 0)	0 (0 to 0)	0 (0 to 0)	29·6 (13 to 58·2)	[illegible]
Chronic kidney disease due to glomerulonephritis	0 (0 to 0)	0 (0 to 0)	9·5 (4·1 to 18·3)	26·6 (15·2 to 42·2)	47·7 (28·9 to 73·2)	70 (40·1 to 109)	144 (89·3 to 216)	[illegible]
Chronic kidney disease due to other and unspecified causes	0 (0 to 0)	0 (0 to 0)	24 (11·9 to 42·1)	55·2 (37·3 to 78·8)	81·2 (52·5 to 121)	97·3 (58·5 to 156)	173 (109 to 259)	[illegible]
Acute glomerulonephritis	0 (0 to 0)	0 (0 to 0)	0 (0 to 0)	0·1 (0 to 0·1)	0·4 (0·2 to 0·6)	0·4 (0·2 to 0·7)	0·5 (0·3 to 0·9)	[illegible]
Skin and subcutaneous diseases	0 (0 to 0·1)	0·6 (0·4 to 1)	78·3 (39·4 to 150)	974 (609 to 1,440)	1,340 (830 to 2,000)	1,450 (931 to 2,150)	1,670 (1,060 to 2,500)	[illegible]
Dermatitis	0 (0 to 0)	0 (0 to 0)	0 (0 to 0)	344 (186 to 587)	449 (242 to 764)	335 (181 to 566)	268 (151 to 443)	[illegible]
Atopic dermatitis	0 (0 to 0)	0 (0 to 0)	0 (0 to 0)	341 (183 to 580)	439 (238 to 751)	301 (159 to 518)	208 (109 to 360)	[illegible]
Contact dermatitis	0 (0 to 0)	0 (0 to 0)	0 (0 to 0)	0 (0 to 0)	5·7 (2·2 to 11·1)	28·1 (11·2 to 54·1)	53 (21·8 to 101)	[illegible]
Seborrhoeic dermatitis	0 (0 to 0)	0 (0 to 0)	0 (0 to 0)	3 (1·5 to 5·4)	3·7 (1·8 to 6·8)	5·5 (2·6 to 10·1)	7·1 (3·4 to 12·9)	[illegible]
Psoriasis	0 (0 to 0)	0 (0 to 0)	0 (0 to 0)	13·6 (8·6 to 19·8)	37·1 (19·3 to 58·9)	52·6 (28·8 to 83·3)	60·9 (33·6 to 96·7)	[illegible]
Bacterial skin diseases	0 (0 to 0)	0·1 (0 to 0·1)	20·5 (2·9 to 91·7)	18·1 (8·1 to 42·8)	27·6 (15·9 to 51·1)	25·8 (14·5 to 43·9)	22·9 (13·7 to 36·1)	[illegible]
Cellulitis	0 (0 to 0)	0 (0 to 0)	4·7 (0·3 to 39·8)	4·9 (1·8 to 11·1)	5·7 (1·9 to 12·1)	3·8 (2·2 to 7·7)	4 (2·2 to 7·9)	[illegible]
Pyoderma	0 (0 to 0)	0 (0 to 0·1)	15·8 (2·3 to 72·3)	13·2 (5 to 34)	21·8 (12·2 to 42·7)	22 (11·4 to 38·4)	18·9 (10·6 to 31·1)	[illegible]
Scabies	0 (0 to 0)	0·3 (0·1 to 0·4)	23·3 (12·5 to 40·8)	166 (83·9 to 298)	237 (114 to 415)	253 (123 to 440)	282 (145 to 492)	[illegible]
Fungal skin diseases	0 (0 to 0)	0 (0 to 0·1)	4 (1·5 to 8·7)	31·5 (11·6 to 68)	55 (20·7 to 119)	66·6 (24·6 to 149)	74 (27·2 to 165)	[illegible]
Viral skin diseases	0 (0 to 0)	0 (0 to 0)	0 (0 to 0)	180 (113 to 276)	306 (191 to 470)	166 (104 to 250)	108 (66·5 to 166)	[illegible]
Acne vulgaris	0 (0 to 0)	0 (0 to 0)	0 (0 to 0)	0 (0 to 0)	15·2 (8·3 to 24·2)	360 (208 to 591)	639 (370 to 1,040)	[illegible]
Alopecia areata	0 (0 to 0)	0 (0 to 0)	0 (0 to 0)	3·5 (1·6 to 6)	5·3 (2·6 to 9·4)	7·3 (3·9 to 12)	13·6 (7·4 to 22·4)	[illegible]
Pruritus	0 (0 to 0)	0 (0 to 0)	0·5 (0·2 to 1·1)	6·6 (2·8 to 13·1)	10 (4·2 to 20·5)	10·5 (4·3 to 21·2)	13 (5·4 to 25·9)	[illegible]
Urticaria	0 (0 to 0)	0·2 (0·1 to 0·4)	24·1 (14·6 to 37·4)	165 (99·9 to 256)	156 (91·9 to 252)	114 (62·1 to 192)	104 (58·2 to 172)	[illegible]
Decubitus ulcer	0 (0 to 0)	0 (0 to 0)	0 (0 to 0)	7·6 (2·2 to 28·8)	7·9 (4·2 to 21·4)	10·4 (3·4 to 20·9)	13·7 (3·1 to 26·1)	[illegible]
Other skin and subcutaneous diseases	0 (0 to 0)	0 (0 to 0·1)	5·7 (2·6 to 11·7)	38·2 (17·5 to 71·7)	39·6 (19 to 74·3)	50·3 (23·6 to 94·5)	66·9 (33·8 to 126)	[illegible]
Sense organ diseases	0·1 (0 to 0·2)	0·3 (0·2 to 0·6)	9 (5·6 to 13·5)	101 (62·5 to 149)	234 (144 to 347)	315 (202 to 468)	406 (255 to 586)	[illegible]
Blindness and vision loss	0 (0 to 0·1)	0·1 (0 to 0·2)	1·5 (0·7 to 2·7)	27·7 (15·7 to 46·7)	67·2 (39 to 106)	87·4 (51·6 to 139)	110 (66·6 to 171)	[illegible]
Glaucoma	0 (0 to 0)	0 (0 to 0)	0 (0 to 0)	0 (0 to 0)	0 (0 to 0)	0 (0 to 0)	0 (0 to 0)	[illegible]
Cataract	0 (0 to 0)	0 (0 to 0)	0 (0 to 0)	0 (0 to 0)	0 (0 to 0)	0 (0 to 0)	0 (0 to 0)	[illegible]
Age-related macular degeneration	0 (0 to 0)	0 (0 to 0)	0 (0 to 0)	0 (0 to 0)	0 (0 to 0)	0 (0 to 0)	0 (0 to 0)	[illegible]
Refraction disorders	0 (0 to 0)	0 (0 to 0)	1 (0·4 to 1·9)	23·4 (12·8 to 38·9)	58·3 (32·6 to 93·7)	75·4 (43 to 125)	94·4 (54·9 to 151)	[illegible]
Near vision loss	0 (0 to 0)	0 (0 to 0)	0 (0 to 0)	0·1 (0 to 0·3)	0·4 (0·1 to 1·1)	0·7 (0·2 to 2)	1·1 (0·3 to 2·8)	[illegible]
Other vision loss	0 (0 to 0·1)	0·1 (0 to 0·1)	0·5 (0·2 to 1)	4·2 (2·2 to 7·1)	8·5 (3·7 to 15·7)	11·3 (5·5 to 20·4)	14·2 (6·2 to 25·2)	[illegible]
Age-related and other hearing loss	0 (0 to 0)	0·1 (0 to 0·1)	3·3 (1·6 to 5·5)	51·6 (26·2 to 84·1)	141 (78·3 to 221)	201 (116 to 309)	264 (151 to 401)	[illegible]
Other sense organ diseases	0·1 (0 to 0·1)	0·2 (0·1 to 0·4)	4·2 (2·1 to 7·3)	21·4 (11 to 36·7)	25·8 (13·1 to 44·9)	26·4 (12·9 to 46·6)	32·3 (15·6 to 60)	[illegible]
Musculoskeletal disorders	0 (0 to 0)	0 (0 to 0)	0 (0 to 0)	0 (0 to 0)	126 (67·3 to 217)	650 (406 to 987)	1,490 (973 to 2,210)	[illegible]
Rheumatoid arthritis	0 (0 to 0)	0 (0 to 0)	0 (0 to 0)	0 (0 to 0)	2·9 (1·6 to 5·1)	6·4 (3·8 to 10·3)	16 (8·3 to 25·5)	[illegible]
Osteoarthritis	0 (0 to 0)	0 (0 to 0)	0 (0 to 0)	0 (0 to 0)	0 (0 to 0)	0 (0 to 0)	0 (0 to 0)	[illegible]
Osteoarthritis hip	0 (0 to 0)	0 (0 to 0)	0 (0 to 0)	0 (0 to 0)	0 (0 to 0)	0 (0 to 0)	0 (0 to 0)	[illegible]
Osteoarthritis knee	0 (0 to 0)	0 (0 to 0)	0 (0 to 0)	0 (0 to 0)	0 (0 to 0)	0 (0 to 0)	0 (0 to 0)	[illegible]
Osteoarthritis hand	0 (0 to 0)	0 (0 to 0)	0 (0 to 0)	0 (0 to 0)	0 (0 to 0)	0 (0 to 0)	0 (0 to 0)	[illegible]
Osteoarthritis other	0 (0 to 0)	0 (0 to 0)	0 (0 to 0)	0 (0 to 0)	0 (0 to 0)	0 (0 to 0)	0 (0 to 0)	[illegible]
Low back pain	0 (0 to 0)	0 (0 to 0)	0 (0 to 0)	0 (0 to 0)	110 (52·4 to 200)	570 (338 to 911)	1,100 (637 to 1,740)	[illegible]
Neck pain	0 (0 to 0)	0 (0 to 0)	0 (0 to 0)	0 (0 to 0)	5·9 (1·4 to 14·6)	37·2 (15·3 to 74·7)	89·9 (38·4 to 168)	[illegible]
Gout	0 (0 to 0)	0 (0 to 0)	0 (0 to 0)	0 (0 to 0)	0 (0 to 0)	0 (0 to 0)	0·4 (0·1 to 0·9)	[illegible]
Other musculoskeletal disorders	0 (0 to 0)	0 (0 to 0)	0 (0 to 0)	0 (0 to 0)	7·3 (5 to 11·8)	36·3 (19·8 to 61·9)	281 (164 to 436)	[illegible]
Other non-communicable diseases	3,160 (1,770 to 4,730)	1,460 (817 to 2,140)	2,220 (1,200 to 3,730)	1,930 (1,290 to 2,640)	1,280 (1,010 to 1,610)	1,270 (979 to 1,620)	1,670 (1,250 to 2,220)	[illegible]
Congenital birth defects	3,080 (1,700 to 4,660)	1,360 (739 to 2,030)	1,840 (863 to 3,210)	1,250 (645 to 1,850)	592 (382 to 783)	468 (319 to 617)	372 (271 to 485)	[illegible]
Neural tube defects	160 (87 to 272)	52·8 (26·3 to 94·2)	57·8 (23·5 to 117)	37·7 (21·2 to 63·9)	10·5 (7·5 to 14)	15·3 (10·5 to 20·8)	11·2 (8 to 15)	[illegible]
Congenital heart anomalies	1,280 (637 to 2,050)	653 (335 to 1,010)	901 (438 to 1,580)	548 (279 to 870)	244 (137 to 347)	171 (95·9 to 246)	128 (69 to 188)	[illegible]
Orofacial clefts	3·4 (1·1 to 7·4)	1·1 (0·4 to 2·3)	3·3 (1·5 to 6)	6·7 (3·2 to 11·7)	6·7 (3 to 12·2)	6·9 (2·9 to 12·3)	7·1 (2·9 to 13·3)	[illegible]
Down syndrome	40·3 (22·4 to 66·1)	20·8 (11·4 to 32·7)	107 (51·7 to 190)	98·2 (57·1 to 158)	17·4 (11·8 to 25·4)	26 (16·8 to 39·1)	33·4 (21·7 to 48·8)	[illegible]
Turner syndrome	0 (0 to 0)	0 (0 to 0)	0·1 (0 to 0·1)	0·2 (0·1 to 0·4)	0·3 (0·1 to 0·6)	0·3 (0·1 to 0·6)	0·5 (0·2 to 0·8)	[illegible]
Klinefelter syndrome	0 (0 to 0)	0 (0 to 0)	0 (0 to 0)	0 (0 to 0·1)	0 (0 to 0·1)	0 (0 to 0)	0 (0 to 0)	[illegible]
Other chromosomal abnormalities	211 (59·6 to 362)	105 (28·8 to 186)	136 (44·1 to 254)	59·2 (27·4 to 89·1)	24·4 (16·3 to 33·8)	24·4 (14·3 to 35·3)	18 (12·1 to 25·9)	[illegible]
Congenital musculoskeletal and limb anomalies	55·9 (21·3 to 98·9)	19·8 (9·6 to 33·2)	49·5 (30·9 to 75)	97·2 (56·9 to 151)	113 (68·2 to 173)	120 (68·7 to 182)	115 (69·9 to 174)	[illegible]
Urogenital congenital anomalies	108 (46·4 to 285)	42·2 (16·6 to 76·4)	33 (14·2 to 64·4)	24·7 (14 to 37·4)	17·5 (9·9 to 26·7)	13 (7·6 to 19·7)	7·1 (4·5 to 10·7)	[illegible]
Digestive congenital anomalies	486 (218 to 787)	278 (94·7 to 473)	223 (55·6 to 420)	130 (29·4 to 232)	47 (10·8 to 79·2)	18·2 (5·1 to 30)	7·6 (3·1 to 12·1)	[illegible]
Other congenital birth defects	741 (370 to 1,210)	190 (93·7 to 315)	333 (104 to 648)	244 (84·5 to 409)	111 (59·4 to 165)	73·3 (45·2 to 109)	43·7 (28·7 to 70·2)	[illegible]
Urinary diseases and male infertility	6·4 (1·6 to 11)	4·3 (2·4 to 8·5)	15·1 (7·7 to 28·5)	19 (13·1 to 26·4)	17·2 (11·6 to 24·8)	22·2 (15·1 to 32·2)	30·4 (21·4 to 42·2)	[illegible]
Urolithiasis	0 (0 to 0)	0 (0 to 0)	0 (0 to 0)	0·2 (0·1 to 0·4)	0·5 (0·3 to 0·9)	1·6 (1 to 2·5)	1·7 (0·8 to 3)	[illegible]

Benign prostatic hyperplasia	0 (0 to 0)	0 (0 to 0)	0 (0 to 0)	0 (0 to 0)	0 (0 to 0)	0 (0 to 0)	0 (0 to 0)	0 (0 to 0)	0 (0 to 0)	0 (0 to 0)	0 (0 to 0)	0·8 (0 to 2·1)	6·5 (2·4 to 13·9)	27·3 (12·7 to 49·4)	83·4 (39·5 to 147)	140 (72·2 to 241)	173 (97·1 to 277)	173 (96·4 to 280)	111 (61·7 to 179)	61·9 (34·4 to 98·6)	29·5 (15·6 to 48·1)	10·8 (5·9 to 17·8)	4 (2·1 to 6·5)	821
Male infertility	0 (0 to 0)	0 (0 to 0)	0 (0 to 0)	0 (0 to 0)	0 (0 to 0)	0 (0 to 0)	0 (0 to 0)	29·4 (7·8 to 74·9)	34·3 (9·8 to 85·1)	31·8 (8·9 to 86·6)	27·4 (7·7 to 70·3)	23 (6·8 to 59)	3·5 (0·8 to 10·4)	0 (0 to 0)	0 (0 to 0)	0 (0 to 0)	0 (0 to 0)	0 (0 to 0)	0 (0 to 0)	0 (0 to 0)	0 (0 to 0)	0 (0 to 0)	0 (0 to 0)	149
Other urinary diseases	1 (0·3 to 1·9)	1·2 (0·4 to 3·8)	0·6 (0·3 to 1·3)	5·6 (3·7 to 8·3)	5·3 (3·7 to 7·5)	6·6 (4·6 to 9·4)	7·7 (5·2 to 10·6)	6·3 (4·1 to 9·3)	6·9 (4·8 to 10)	9 (5·6 to 13·4)	17·5 (9·6 to 29·6)	17·4 (7·8 to 28·5)	18·8 (10·2 to 28·8)	15·1 (8·7 to 24)	18·2 (9·6 to 30·9)	37·3 (11·4 to 68·6)	29·8 (10·2 to 54·1)	22·7 (7·8 to 43·9)	29·6 (6·8 to 56·9)	25 (4·9 to 50·3)	14·6 (2·9 to 29·2)	14 (1·7 to 27·1)	10 (0·9 to 19·4)	320
Gynecological diseases	0 (0 to 0)	0 (0 to 0)	0 (0 to 0)	0 (0 to 0)	0 (0 to 0)	159 (80·1 to 283)	447 (248 to 756)	1,100 (692 to 1,700)	1,600 (1,050 to 2,350)	1,720 (1,140 to 2,470)	1,660 (1,120 to 2,390)	1,480 (977 to 2,120)	1,180 (785 to 1,680)	765 (472 to 1,130)	448 (274 to 645)	280 (164 to 432)	192 (116 to 283)	131 (81·8 to 189)	81·7 (50·6 to 120)	58·2 (35·7 to 88·8)	40·8 (26 to 59·7)	23·7 (14·9 to 35·6)	13·7 (7·7 to 22·9)	11,400
Uterine fibroids	0 (0 to 0)	0 (0 to 0)	0 (0 to 0)	0 (0 to 0)	0 (0 to 0)	2·5 (0·8 to 5·5)	7·6 (3·6 to 15)	24·2 (12·6 to 46·3)	55·9 (27·4 to 108)	101 (49·5 to 193)	152 (77·3 to 285)	167 (90·8 to 288)	145 (78·2 to 251)	97·1 (52·1 to 167)	62·6 (33·1 to 108)	31·5 (15·1 to 61·3)	16·9 (7·9 to 32·7)	9·8 (4·8 to 18·7)	4·8 (2·3 to 9·5)	2·9 (1·4 to 5·4)	1·4 (0·7 to 2·5)	0·7 (0·4 to 1·4)	0·3 (0·2 to 0·5)	883
Polycystic ovarian syndrome	0 (0 to 0)	0 (0 to 0)	0 (0 to 0)	0 (0 to 0)	0 (0 to 0)	5·6 (2 to 12·4)	27·9 (11·8 to 58)	35·9 (14·6 to 73)	33·8 (14·2 to 69·6)	28·7 (11·8 to 59·5)	24·8 (10·2 to 51·9)	20·7 (8·4 to 42·8)	17·1 (6·9 to 35·1)	4·1 (1·6 to 8·8)	0 (0 to 0)	0 (0 to 0)	0 (0 to 0)	0 (0 to 0)	0 (0 to 0)	0 (0 to 0)	0 (0 to 0)	0 (0 to 0)	0 (0 to 0)	199
Female infertility	0 (0 to 0)	0 (0 to 0)	0 (0 to 0)	0 (0 to 0)	0 (0 to 0)	0 (0 to 0)	0 (0 to 0)	44·2 (10·3 to 117)	63·1 (16·1 to 165)	68·8 (17 to 189)	65·4 (10 to 187)	54·7 (8·5 to 157)	1·3 (0·1 to 10·8)	0 (0 to 0)	0 (0 to 0)	0 (0 to 0)	0 (0 to 0)	0 (0 to 0)	0 (0 to 0)	0 (0 to 0)	0 (0 to 0)	0 (0 to 0)	0 (0 to 0)	298
Endometriosis	0 (0 to 0)	0 (0 to 0)	0 (0 to 0)	0 (0 to 0)	0 (0 to 0)	0·1 (0 to 0·1)	37·9 (15 to 80·2)	168 (78·6 to 327)	195 (93·9 to 351)	144 (68·1 to 262)	112 (55·5 to 201)	95·7 (48 to 167)	80·6 (42·3 to 136)	36·8 (19·1 to 63·6)	0 (0 to 0)	0 (0 to 0)	0 (0 to 0)	0 (0 to 0)	0 (0 to 0)	0 (0 to 0)	0 (0 to 0)	0 (0 to 0)	0 (0 to 0)	871
Genital prolapse	0 (0 to 0)	0 (0 to 0)	0 (0 to 0)	0 (0 to 0)	0 (0 to 0)	0 (0 to 0)	0·3 (0·1 to 0·5)	1·7 (0·8 to 3·5)	3·6 (1·4 to 7·5)	6·4 (2·6 to 13)	11·7 (5·5 to 23·2)	16·5 (7·2 to 34)	22·2 (10·8 to 43·3)	25·6 (12·7 to 46·6)	28·6 (14·9 to 49·8)	20·5 (10·5 to 38)	18·6 (10 to 32·1)	14·1 (8·1 to 24·2)	8·7 (4·5 to 16·1)	7·4 (4 to 12·6)	5 (2·7 to 9)	3 (1·5 to 5·5)	2·1 (1·2 to 3·7)	196
Premenstrual syndrome	0 (0 to 0)	0 (0 to 0)	0 (0 to 0)	0 (0 to 0)	0 (0 to 0)	146 (69·8 to 268)	357 (175 to 633)	501 (253 to 894)	502 (288 to 818)	452 (240 to 747)	444 (257 to 699)	384 (214 to 624)	258 (146 to 436)	84·4 (42·1 to 153)	0 (0 to 0)	0 (0 to 0)	0 (0 to 0)	0 (0 to 0)	0 (0 to 0)	0 (0 to 0)	0 (0 to 0)	0 (0 to 0)	0 (0 to 0)	3,130
Other gynecological diseases	0 (0 to 0)	0 (0 to 0)	0 (0 to 0)	0 (0 to 0)	0 (0 to 0)	4·9 (1·1 to 12)	16·8 (6·6 to 33·9)	325 (180 to 534)	750 (417 to 1,190)	919 (542 to 1,400)	849 (504 to 1,340)	737 (404 to 1,170)	659 (374 to 1,040)	517 (247 to 826)	357 (205 to 546)	228 (122 to 366)	156 (90 to 241)	107 (62 to 161)	68·2 (39·3 to 103)	47·9 (28·2 to 77)	34·4 (20·5 to 51·7)	20 (11·7 to 31·7)	11·3 (5·6 to 20·4)	5,810
Hemoglobinopathies and hemolytic anemias	16·8 (11 to 24·6)	16·9 (11·6 to 23·7)	88·6 (55·4 to 134)	288 (197 to 412)	356 (250 to 491)	306 (219 to 415)	401 (288 to 545)	567 (383 to 820)	522 (344 to 780)	476 (327 to 670)	349 (238 to 495)	290 (205 to 393)	289 (205 to 393)	289 (207 to 388)	259 (188 to 352)	189 (138 to 260)	118 (82·3 to 162)	86·3 (61·5 to 117)	60·5 (43·6 to 83·1)	43·5 (31·4 to 60·5)	38·3 (27·3 to 53·1)	25 (17·8 to 35·1)	13·8 (9·2 to 20·4)	5,090
Thalassemias	0 (0 to 0)	0 (0 to 0)	0·1 (0 to 0·1)	0·8 (0·3 to 1·5)	0·5 (0·2 to 1)	0·2 (0·1 to 0·4)	0·1 (0·1 to 0·3)	0·2 (0·1 to 0·4)	0·1 (0 to 0·3)	0·1 (0 to 0·2)	0 (0 to 0·1)	0 (0 to 0·1)	0 (0 to 0·1)	0 (0 to 0·1)	0 (0 to 0·1)	0 (0 to 0)	0 (0 to 0)	0 (0 to 0)	0 (0 to 0)	0 (0 to 0)	0 (0 to 0)	0 (0 to 0)	0 (0 to 0)	2·3
Thalassemias trait	0 (0 to 0)	0·2 (0·1 to 0·4)	2 (1 to 3·5)	9 (4·3 to 15·5)	10·1 (3·9 to 19·6)	4·7 (1·2 to 10·1)	13 (6·1 to 24·1)	16·9 (7·7 to 29·6)	17·3 (8·3 to 29·8)	14·4 (6·2 to 26·5)	12·7 (6·3 to 22·1)	10·7 (5·2 to 18·4)	2·7 (1 to 5·3)	4·2 (1·8 to 7·7)	4 (1·9 to 7·2)	2·9 (1·2 to 5·5)	1·6 (0·6 to 3·4)	1·4 (0·6 to 2·6)	1·1 (0·4 to 2·1)	0·9 (0·4 to 1·7)	0·8 (0·4 to 1·4)	0·4 (0·2 to 0·7)	0·2 (0·1 to 0·3)	131
Sickle cell disorders	16·2 (10·5 to 24)	10·1 (6 to 16·1)	59·4 (30·8 to 104)	153 (98·2 to 224)	213 (156 to 278)	232 (163 to 323)	277 (193 to 391)	354 (235 to 503)	299 (194 to 439)	258 (167 to 374)	195 (121 to 290)	148 (95·2 to 215)	153 (98·8 to 231)	132 (82·6 to 200)	77·8 (46·2 to 123)	22·8 (11·9 to 39·2)	4·2 (1·8 to 8·2)	0·6 (0·2 to 1·2)	0·1 (0 to 0·2)	0 (0 to 0)	0 (0 to 0)	0 (0 to 0)	0 (0 to 0)	2,610
Sickle cell trait	0·4 (0·1 to 0·9)	5·1 (2·6 to 8·3)	17·7 (9·8 to 28·6)	79·5 (42·6 to 132)	87·8 (38 to 164)	41·3 (13·6 to 84·7)	30·4 (14·1 to 55·3)	110 (28·2 to 279)	108 (22·7 to 319)	108 (31·3 to 220)	20·9 (10 to 37·9)	33·1 (16·5 to 56)	36·8 (16·3 to 62·5)	37·4 (19·3 to 61·1)	35·6 (17·8 to 60·1)	25·2 (12·2 to 44·1)	14·4 (6 to 27·2)	12·3 (5·6 to 21·7)	9·8 (4·2 to 17·8)	8·2 (4 to 14·4)	7·2 (3·8 to 11·8)	3·6 (2 to 6·1)	1·4 (0·7 to 2·3)	834
G6PD deficiency	0 (0 to 0·1)	0·4 (0·2 to 0·7)	0·5 (0·2 to 0·9)	6·6 (3·4 to 11·5)	11·4 (4·9 to 20·8)	12·4 (5·6 to 23·7)	37·3 (19·2 to 62·4)	45 (17·9 to 83·2)	54 (23·9 to 95·5)	54·4 (23·4 to 99·6)	70 (37·7 to 111)	49·3 (24·5 to 79·8)	49·3 (23·2 to 86·5)	58·4 (28·8 to 97·3)	68·7 (38 to 107)	49·3 (29·1 to 77·8)	29·2 (16·3 to 44·2)	14 (7·9 to 21·7)	6·6 (3·5 to 10·3)	2·1 (1·1 to 3·4)	0·9 (0·4 to 1·5)	0·3 (0·2 to 0·6)	0·1 (0 to 0·2)	620
G6PD trait	0 (0 to 0)	0 (0 to 0)	0 (0 to 0)	0 (0 to 0)	0 (0 to 0)	0 (0 to 0)	3·5 (1·4 to 6·7)	0·1 (0 to 0·2)	0·1 (0 to 0·2)	0·1 (0 to 0·2)	3·4 (1·4 to 6·3)	3·2 (1·3 to 5·8)	0·8 (0·1 to 2)	0 (0 to 0)	0 (0 to 0)	0 (0 to 0)	0 (0 to 0)	0 (0 to 0)	0 (0 to 0)	0 (0 to 0)	0 (0 to 0)	0 (0 to 0)	0 (0 to 0)	11·2
Other hemoglobinopathies and hemolytic anemias	0 (0 to 0·1)	1·1 (0·6 to 1·8)	8·9 (4·2 to 16·1)	39·6 (19·1 to 71·1)	32·7 (13 to 66·8)	15·3 (5·2 to 32·5)	39·9 (16·4 to 79·5)	39·9 (17·8 to 75·1)	43·9 (19·9 to 81·3)	41·2 (19 to 76·3)	47·2 (23·6 to 82·1)	46·7 (24·6 to 79·7)	45·6 (24·6 to 77·1)	56·7 (35 to 86·6)	73·2 (48·1 to 106)	88·7 (59·3 to 130)	68·3 (46·4 to 97·4)	58 (40·6 to 79·1)	43 (31·1 to 57·4)	32·3 (23·4 to 43·6)	29·4 (21·3 to 40·2)	20·6 (14·6 to 28·6)	12·1 (8·1 to 18)	884
Endocrine, metabolic, blood, and immune disorders	48·1 (29·5 to 75·2)	47·5 (29·4 to 72·4)	208 (102 to 453)	290 (201 to 450)	191 (135 to 272)	170 (119 to 238)	195 (128 to 284)	252 (173 to 346)	350 (247 to 485)	425 (294 to 597)	468 (320 to 647)	537 (364 to 751)	650 (448 to 907)	694 (476 to 996)	730 (496 to 1,070)	687 (478 to 996)	570 (400 to 838)	487 (339 to 697)	393 (279 to 539)	278 (200 to 386)	198 (140 to 273)	108 (76·8 to 141)	62·8 (41·3 to 88·7)	8,040
Oral disorders	0 (0 to 0)	0 (0 to 0)	0 (0 to 0)	90·7 (50·8 to 151)	122 (65 to 207)	143 (73·6 to 246)	222 (121 to 381)	386 (201 to 708)	546 (273 to 1,040)	620 (302 to 1,160)	650 (309 to 1,240)	657 (319 to 1,200)	742 (385 to 1,330)	816 (451 to 1,430)	896 (512 to 1,430)	927 (565 to 1,470)	839 (512 to 1,320)	730 (457 to 1,120)	526 (330 to 786)	378 (239 to 562)	248 (154 to 360)	132 (82·1 to 193)	73·8 (46·2 to 108)	9,740
Caries of deciduous teeth	0 (0 to 0)	0 (0 to 0)	0 (0 to 0)	30·3 (11·8 to 62·1)	31·5 (12·7 to 69·8)	1·9 (0·3 to 4·9)	0 (0 to 0)	0 (0 to 0)	0 (0 to 0)	0 (0 to 0)	0 (0 to 0)	0 (0 to 0)	0 (0 to 0)	0 (0 to 0)	0 (0 to 0)	0 (0 to 0)	0 (0 to 0)	0 (0 to 0)	0 (0 to 0)	0 (0 to 0)	0 (0 to 0)	0 (0 to 0)	0 (0 to 0)	63·6
Caries of permanent teeth	0 (0 to 0)	0 (0 to 0)	0 (0 to 0)	0 (0 to 0)	29·1 (10·3 to 64·5)	68·5 (27·5 to 148)	78·3 (31·5 to 165)	98·2 (40·5 to 203)	93·7 (37 to 190)	76·5 (31·5 to 158)	66·8 (27·7 to 135)	55 (22·7 to 110)	51·2 (20·8 to 106)	46·6 (18·6 to 97·9)	38·1 (14·5 to 87·2)	27·9 (9·9 to 60·7)	18·3 (6·9 to 38·6)	13·1 (5·1 to 27·6)	8·4 (3·4 to 16·6)	5·6 (2·2 to 11·9)	3·4 (1·3 to 7)	1·6 (0·6 to 3·6)	0·7 (0·2 to 1·7)	781
Periodontal diseases	0 (0 to 0)	0 (0 to 0)	0 (0 to 0)	0 (0 to 0)	0 (0 to 0)	0 (0 to 0)	28·8 (8·2 to 75·5)	119 (35 to 305)	254 (80·4 to 602)	340 (116 to 755)	380 (134 to 871)	388 (141 to 844)	408 (153 to 867)	381 (150 to 801)	318 (125 to 672)	232 (92·8 to 475)	153 (60 to 315)	106 (41 to 215)	66·1 (26·4 to 132)	44·4 (17·1 to 90·4)	28·3 (10·7 to 59·8)	14·7 (5·3 to 30·9)	7·7 (2·8 to 16·2)	3,270
Other oral disorders	0 (0 to 0)	0 (0 to 0)	0 (0 to 0)	60·5 (35·3 to 94·5)	61·3 (35·6 to 92·8)	72·2 (41·9 to 114)	114 (69·1 to 179)	153 (93·9 to 233)	162 (97·7 to 246)	148 (89·4 to 221)	131 (79·4 to 201)	117 (71 to 177)	116 (70·7 to 173)	107 (64·9 to 161)	93·1 (57 to 138)	75·1 (46 to 112)	55·2 (33·1 to 84·4)	38·3 (23·3 to 58·1)	20·9 (13 to 31·7)	11·7 (7·2 to 18)	6 (3·8 to 9·1)	2·8 (1·8 to 4·1)	1·6 (0·9 to 2·5)	1,550
Sudden infant death syndrome	--	23·5 (14 to 37·4)	63 (30·4 to 112)	--	--	--	--	--	--	--	--	--	--	--	--	--	--	--	--	--	--	--	--	NA
Injuries	192 (130 to 274)	70·5 (47·6 to 102)	183 (113 to 284)	710 (533 to 941)	1,170 (974 to 1,400)	1,670 (1,380 to 2,010)	3,870 (3,100 to 4,740)	7,440 (6,140 to 9,010)	7,880 (6,490 to 9,430)	7,210 (5,930 to 8,760)	6,000 (4,800 to 7,430)	5,030 (4,010 to 6,240)	4,890 (3,820 to 6,130)	4,090 (3,260 to 5,120)	3,470 (2,750 to 4,260)	2,660 (2,160 to 3,250)	1,940 (1,580 to 2,360)	1,570 (1,280 to 1,880)	1,100 (902 to 1,320)	843 (688 to 1,000)	629 (511 to 755)	384 (313 to 457)	268 (218 to 317)	63,300
Transport injuries	20·5 (13·7 to 29·7)	8·7 (5·5 to 12·9)	18·6 (10·4 to 30·8)	143 (97·3 to 213)	332 (244 to 432)	401 (299 to 524)	785 (574 to 1,040)	1,280 (960 to 1,690)	1,270 (975 to 1,660)	982 (747 to 1,260)	871 (660 to 1,140)	758 (575 to 971)	852 (639 to 1,100)	780 (599 to 991)	736 (572 to 925)	569 (457 to 692)	413 (331 to 505)	310 (248 to 375)	216 (175 to 262)	145 (117 to 175)	97·2 (78 to 119)	52·9 (42·2 to 64·2)	36·3 (28 to 44·1)	11,100
Road injuries	19 (12·6 to 28)	6·7 (4·1 to 10·2)	15·4 (8·1 to 26·3)	131 (87·3 to 198)	310 (225 to 406)	373 (274 to 493)	739 (532 to 979)	1,210 (904 to 1,590)	1,200 (915 to 1,570)	916 (699 to 1,190)	821 (622 to 1,080)	711 (539 to 915)	801 (601 to 1,040)	736 (563 to 943)	692 (535 to 870)	535 (428 to 654)	390 (312 to 478)	289 (231 to 350)	201 (163 to 245)	135 (108 to 162)	89·8 (71·7 to 111)	47·6 (37·9 to 58)	32·4 (25·1 to 39·6)	10,400
Pedestrian road injuries	10·3 (6·9 to 15)	2·9 (1·8 to 4·5)	4·5 (2·3 to 7·8)	58·5 (36·9 to 92·9)	134 (93·1 to 183)	131 (92·7 to 181)	90·9 (56·7 to 138)	117 (85·3 to 158)	122 (86·1 to 167)	120 (88·9 to 161)	123 (90·1 to 168)	118 (88·3 to 150)	157 (115 to 213)	172 (128 to 225)	171 (129 to 223)	163 (129 to 204)	119 (94 to 149)	101 (79·7 to 124)	76·1 (60·9 to 94)	52·4 (41·4 to 65·7)	37·5 (29·4 to 47·2)	18·9 (14·9 to 23·2)	14 (10·8 to 17·3)	2,110
Cyclist road injuries	0 (0 to 0)	0 (0 to 0)	0 (0 to 0)	9·2 (5·2 to 15·4)	20·4 (12·9 to 31·2)	50·4 (33·2 to 71·8)	81·3 (55·4 to 118)	112 (80·2 to 152)	117 (84·7 to 156)	110 (82·5 to 147)	118 (86·1 to 156)	112 (82·6 to 145)	133 (93·6 to 177)	125 (93 to 162)	110 (82·4 to 145)	88·2 (66·9 to 114)	69·3 (53·2 to 91·1)	45·5 (34 to 59·3)	30·4 (22·4 to 39·2)	20·6 (14·9 to 26·6)	13 (9·5 to 17·3)	6·8 (5 to 9·1)	3·7 (2·6 to 5)	1,380
Motorcyclist road injuries	1·1 (0·7 to 1·7)	0·6 (0·3 to 0·9)	1·3 (0·7 to 2·2)	7·1 (4·7 to 10·7)	18·7 (13·3 to 24·9)	30·7 (22·1 to 42·8)	122 (84·7 to 168)	263 (192 to 353)	269 (198 to 350)	181 (132 to 244)	158 (117 to 214)	121 (90·5 to 157)	121 (88·9 to 165)	104 (79·5 to 131)	88·4 (67·1 to 115)	51·7 (40·2 to 64·7)	36·7 (28·1 to 46·8)	24·8 (18·4 to 32·3)	17 (12·5 to 22·3)	12·5 (9 to 16·7)	8·5 (6·1 to 11·5)	5 (3·6 to 6·7)	3·3 (2·4 to 4·5)	1,650
Motor vehicle road injuries	7·4 (4·9 to 10·9)	3·1 (1·9 to 4·7)	9·3 (4·8 to 15·9)	52·8 (35·3 to 79·6)	134 (96·2 to 172)	155 (109 to 209)	435 (305 to 595)	701 (508 to 953)	673 (497 to 910)	488 (348 to 657)	405 (293 to 563)	343 (245 to 457)	369 (263 to 507)	315 (234 to 416)	303 (224 to 399)	217 (170 to 274)	153 (119 to 191)	109 (85·8 to 133)	71·1 (56·6 to 88·8)	44·7 (35·7 to 55·3)	28·1 (22·3 to 34·7)	15·5 (12·1 to 19·1)	10·7 (8·1 to 13·3)	5,040
Other road injuries	0·2 (0·1 to 0·3)	0·1 (0 to 0·1)	0·3 (0·2 to 0·6)	3·4 (1·9 to 5·5)	3·6 (2·4 to 5·3)	5·2 (3·6 to 6·9)	9·1 (6·3 to 12·6)	14·2 (10·1 to 19·6)	17·5 (12·2 to 23·9)	16·7 (11·8 to 23·1)	16·9 (11·9 to 23)	17·6 (12·4 to 23·9)	20·8 (14·9 to 27·7)	20·1 (14·4 to 27·4)	18·5 (13·1 to 25·5)	15 (10·4 to 20·9)	12·1 (8·5 to 16·5)	9·4 (6·6 to 12·9)	6·6 (4·7 to 9)	4·4 (3·1 to 6)	2·7 (1·9 to 3·7)	1·4 (1 to 1·9)	0·7 (0·5 to 1)	217
Other transport injuries	1·5 (0·9 to 2·3)	2 (1·1 to 3·1)	3·2 (1·9 to 4·9)	11·7 (7·9 to 16·5)	21·8 (14·8 to 30·9)	28·3 (19·8 to 40·8)	46·8 (32·7 to 65·3)	74·8 (52·5 to 107)	76·7 (54·9 to 105)	65·9 (45·7 to 94)	49·9 (35 to 70·2)	47·1 (33·5 to 64·1)	50·8 (34·6 to 73)	43·9 (31·5 to 60·5)	44·7 (32·2 to 61·3)	33·7 (25·1 to 45·1)	23 (17·8 to 29·1)	20·7 (15·6 to 27·6)	14·9 (10·6 to 20·2)	10·6 (7·9 to 13·5)	7·4 (5·6 to 9·4)	5·3 (4·1 to 6·9)	3·8 (2·8 to 5)	688
Unintentional injuries	141 (92·6 to 203)	53·5 (35 to 79·8)	135 (84·6 to 211)	400 (293 to 524)	630 (495 to 793)	836 (647 to 1,070)	1,230 (944 to 1,590)	2,070 (1,620 to 2,610)	2,300 (1,820 to 2,870)	2,240 (1,790 to 2,820)	2,070 (1,610 to 2,600)	1,870 (1,450 to 2,380)	1,910 (1,500 to 2,460)	1,780 (1,380 to 2,310)	1,640 (1,270 to 2,130)	1,420 (1,100 to 1,820)	1,100 (861 to 1,400)	987 (785 to 1,230)	746 (601 to 929)	631 (504 to 761)	493 (397 to 597)	314 (255 to 373)	220 (179 to 262)	25,200
Falls	4 (2·8 to 5·7)	3·7 (2·4 to 5·6)	8·2 (4·9 to 12·7)	53·8 (37·8 to 72·7)	99·8 (75·4 to 131)	149 (111 to 198)	207 (153 to 278)	305 (227 to 404)	352 (268 to 471)	350 (265 to 460)	357 (270 to 469)	339 (255 to 440)	375 (282 to 484)	412 (320 to 534)	408 (315 to 525)	396 (307 to 498)	336 (266 to 417)	342 (276 to 415)	284 (230 to 350)	289 (236 to 346)	259 (211 to 309)	184 (147 to 220)	138 (112 to 163)	5,650
Drowning	0·5 (0·3 to 0·7)	0·3 (0·2 to 0·5)	3·7 (1·9 to 6·6)	34·5 (20·3 to 53)	62·5 (41·3 to 91·3)	64·7 (45·4 to 89·1)	78·1 (51·8 to 109)	99·2 (66·8 to 145)	91 (66·1 to 126)	60·4 (44·6 to 82)	48·1 (35·3 to 65)	41·5 (30·3 to 57·1)	31·3 (22·9 to 41·7)	28·8 (21 to 39·1)	33 (24·4 to 44·7)	22 (16·8 to 28·3)	15·6 (11·8 to 19·9)	12·1 (9·4 to 15·3)	7·2 (5·6 to 9·1)	4·1 (3·2 to 5)	2·7 (2·1 to 3·3)	1·5 (1·2 to 1·8)	1 (0·8 to 1·2)	744
Fire, heat, and hot substances	1 (0·6 to 1·4)	1·6 (1·1 to 2·3)	12 (6·7 to 20·1)	65·1 (43·2 to 93·2)	101 (74·6 to 135)	136 (94·3 to 190)	184 (125 to 260)	242 (171 to 341)	274 (192 to 381)	259 (178 to 369)	251 (175 to 358)	222 (156 to 314)	232 (164 to 323)	212 (147 to 297)	191 (134 to 271)	156 (109 to 220)	112 (79·9 to 161)	95·6 (68·8 to 133)	63·3 (45·9 to 88·2)	50·9 (38·1 to 68)	37·2 (28·3 to 49·6)	20·7 (15·9 to 27)	13·3 (10·2 to 17)	2,930
Poisonings	1·1 (0·7 to 1·7)	0·9 (0·5 to 1·4)	2 (1·1 to 3·4)	12·4 (8 to 19·4)	19 (13·4 to 26·8)	21·1 (14·2 to 30·5)	25·6 (17·6 to 36·3)	34 (23·9 to 46·5)	35·8 (26 to 48·9)	33·5 (24·4 to 45)	26·2 (18·5 to 35·7)	23·5 (16·7 to 31·4)	22·4 (16 to 31)	17·5 (12·4 to 23·9)	14·2 (10 to 19·4)	11·2 (7·9 to 15·2)	9·5 (7 to 12·8)	7·4 (5·4 to 9·9)	4·7 (3·4 to 6·3)	3·3 (2·5 to 4·5)	2·1 (1·5 to 2·9)	1·1 (0·8 to 1·5)	0·7 (0·5 to 0·9)	329
Poisoning by carbon monoxide	0·2 (0·1 to 0·4)	0·1 (0·1 to 0·2)	0·8 (0·4 to 1·4)	3·1 (1·7 to 5·4)	4·9 (3 to 7·1)	5·4 (3·5 to 7·8)	7·1 (4·7 to 10·4)	10·9 (7·4 to 15·3)	11·8 (8·1 to 16·5)	11·5 (7·7 to 17·1)	7·6 (5·1 to 10·8)	5 (3·3 to 7)	4·2 (2·8 to 6·3)	3·1 (2 to 4·5)	2·8 (1·8 to 4·1)	2 (1·3 to 3)	1·9 (1·3 to 2·8)	1·8 (1·2 to 2·5)	0·9 (0·6 to 1·3)	0·8 (0·5 to 1·1)	0·5 (0·3 to 0·7)	0·2 (0·2 to 0·4)	0·2 (0·1 to 0·2)	86·6
Poisoning by other means	0·9 (0·5 to 1·4)	0·7 (0·4 to 1·2)	1·2 (0·7 to 2·1)	9·3 (5·8 to 14·4)	14·1 (9·4 to 20·5)	15·7 (10·2 to 23·6)	18·4 (12 to 27·8)	23·1 (15·2 to 33·2)	24 (16·3 to 34·4)	22·1 (15·3 to 31·2)	18·6 (12·6 to 26·3)	18·5 (12·8 to 25·6)	18·2 (12·6 to 25·4)	14·4 (10·1 to 19·7)	11·4 (7·8 to 15·9)	9·2 (6·3 to 12·7)	7·5 (5·4 to 10·3)	5·7 (3·9 to 7·8)	3·8 (2·7 to 5·2)	2·6 (1·8 to 3·5)	1·7 (1·2 to 2·3)	0·9 (0·6 to 1·2)	0·5 (0·4 to 0·7)	243
Exposure to mechanical forces	2·2 (1·4 to 3·3)	3·1 (2 to 4·6)	8 (5·2 to 11·6)	48·4 (32·4 to 68·2)	103 (70·3 to 145)	163 (116 to 232)	404 (301 to 531)	898 (675 to 1,160)	1,030 (803 to 1,310)	1,040 (832 to 1,310)	928 (732 to 1,150)	818 (630 to 1,040)	792 (591 to 1,040)	669 (507 to 879)	568 (424 to 764)	445 (328 to 604)	316 (229 to 434)	235 (165 to 326)	148 (105 to 210)	98·5 (68·6 to 139)	62·6 (43·6 to 88·2)	34·3 (23·8 to 48·2)	20·1 (14 to 27·6)	8,840
Unintentional firearm injuries	0·6 (0·3 to 1·1)	0·6 (0·4 to 1·1)	1·6 (0·9 to 2·6)	10·4 (6·6 to 15·7)	20·2 (14·1 to 28·9)	43·3 (31·5 to 58·5)	223 (155 to 306)	619 (438 to 848)	683 (511 to 901)	690 (531 to 898)	568 (427 to 733)	465 (343 to 617)	411 (292 to 557)	288 (219 to 369)	215 (164 to 271)	144 (111 to 180)	86·9 (65·7 to 112)	56 (41·4 to 71·9)	30·4 (21·9 to 40·5)	19 (13·6 to 25·5)	11·7 (8·2 to 15·9)	6·3 (4·5 to 8·7)	3·8 (2·7 to 5·3)	4,600
Other exposure to mechanical forces	1·6 (1 to 2·4)	2·5 (1·6 to 3·7)	6·4 (4·1 to 9·4)	37·9 (24 to 55·8)	82·3 (53·4 to 121)	120 (77·9 to 187)	181 (118 to 272)	279 (186 to 411)	343 (236 to 506)	355 (239 to 526)	359 (242 to 538)	353 (242 to 520)	381 (260 to 562)	381 (260 to 565)	353 (241 to 510)	301 (207 to 433)	229 (156 to 331)	179 (121 to 257)	118 (79·9 to 170)	79·5 (54 to 113)	51 (34·4 to 72·6)	28 (19·1 to 39·7)	16·3 (11·3 to 22·7)	4,240
Adverse effects of medical treatment	27·6 (12·7 to 43·5)	14·5 (8·8 to 22·3)	44·9 (24·1 to 81)	49·7 (30·5 to 77·5)	49·8 (27·1 to 71·5)	50 (24·3 to 73·6)	46·3 (23·2 to 68·9)	100 (50·7 to 149)	84·7 (53·6 to 123)	83·4 (50·4 to 120)	86·7 (50 to 130)	84·4 (45·6 to 129)	121 (60·2 to 182)	135 (69·6 to 202)	148 (79·2 to 215)	145 (83·5 to 211)	117 (76 to 166)	133 (79·5 to 185)	118 (72 to 164)	94·8 (55·8 to 128)	67·3 (39·7 to 90·8)	34·8 (21·9 to 46·9)	22 (14·3 to 29·7)	1,860
Animal contact	0·1 (0 to 0·1)	0·2 (0·1 to 0·3)	1·3 (0·8 to 2)	7·6 (5·4 to 10·6)	12·3 (8·1 to 18·8)	18·8 (12·3 to 27·8)	22·5 (14·7 to 32·5)	28·3 (18·3 to 41)	30·8 (20·7 to 42·9)	25·7 (17·3 to 36·4)	23·9 (15·8 to 34·5)	22·2 (14·9 to 31·9)	23·8 (16·2 to 34)	23·1 (15·7 to 33·4)	21·6 (14·8 to 31·3)	18·4 (12·9 to 26·5)	16·2 (11·6 to 22·8)	13 (9·4 to 17·9)	9·4 (6·7 to 12·5)	7 (5 to 9·4)	4·3 (3·1 to 6)	2·6 (1·9 to 3·5)	1·7 (1·2 to 2·3)	335
Venomous animal contact	0 (0 to 0)	0 (0 to 0·1)	0·6 (0·4 to 1)	4·6 (3 to 6·7)	8·6 (5·3 to 13·5)	13·4 (8·2 to 20·5)	16·5 (10·4 to 24·4)	20·8 (13 to 30·6)	21·3 (13·6 to 31)	18·6 (12·1 to 26·9)	17 (11·1 to 24·8)	15·5 (10·2 to 22·3)	15·8 (10·3 to 22·5)	15·2 (10·2 to 22)	13·7 (9·1 to 19·7)	11·4 (7·7 to 16·4)	9·7 (6·9 to 13·6)	7·2 (4·9 to 10)	5·1 (3·5 to 7)	3·7 (2·5 to 5)	2·6 (1·9 to 3·5)	1·6 (1·1 to 2·1)	1 (0·7 to 1·3)	224
Non-venomous animal contact	0 (0 to 0·1)	0·2 (0·1 to 0·3)	0·6 (0·3 to 1·1)	3 (2·1 to 4·2)	3·8 (2·4 to 6·2)	5·4 (3·3 to 8·8)	6·1 (3·8 to 9·7)	7·5 (4·6 to 11·6)	9·5 (6·4 to 13·5)	7·1 (4·4 to 11·3)	6·8 (4·3 to 11·2)	6·7 (4·2 to 10·6)	8·1 (5·3 to 12)	7·9 (5·1 to 11·8)	8 (5·3 to 11·9)	7 (4·6 to 10·2)	6·5 (4·4 to 9·4)	5·9 (4·3 to 8·2)	4·3 (3 to 6)	3·3 (2·3 to 4·6)	1·8 (1·2 to 2·6)	1·1 (0·7 to 1·5)	0·7 (0·5 to 1)	111
Foreign body	95·1 (61·6 to 138)	25·8 (16 to 39·5)	42·9 (22·3 to 77·1)	63·4 (43·3 to 89·4)	61·4 (46·8 to 79·1)	74 (56 to 95·5)	52·9 (38·5 to 72·4)	74·3 (54·4 to 99·7)	85 (64·1 to 112)	75·1 (56 to 98·4)	65·1 (48·1 to 86)	57·6 (42·2 to 76·7)	57·3 (43·3 to 75·3)	52·9 (39·8 to 69·1)	46·9 (36·5 to 59·6)	43·6 (33·5 to 54·6)	33·8 (26·6 to 42·6)	30·2 (24·3 to 36·5)	24·1 (19·1 to 30·2)	18·8 (14·8 to 23·1)	11·3 (9·1 to 13·7)	7 (5·6 to 8·5)	5·3 (4·2 to 6·3)	1,100
Pulmonary aspiration and foreign body in airway	94·5 (61·2 to 138)	25·5 (15·8 to 39·3)	40 (19·7 to 74·1)	48·7 (30·7 to 71·9)	40 (29·6 to 52·5)	49·5 (36·6 to 66·2)	25·1 (19·1 to 32·3)	38·7 (28·6 to 50·7)	48·8 (37·6 to 64·3)	41·7 (31·6 to 55·4)	35 (26·1 to 46·6)	29·8 (22·3 to 39·3)	29·5 (22·1 to 38·9)	27·5 (19·8 to 37·6)	25·1 (18·5 to 33·6)	25·3 (18·5 to 33·5)	19·7 (14·4 to 25·7)	18·8 (14·7 to 23·7)	15·4 (11·4 to 20·6)	12·1 (9·1 to 15·9)	6·8 (5·2 to 8·5)	4·3 (3·1 to 5·5)	3·5 (2·5 to 4·2)	705
Foreign body in eyes	0 (0 to 0)	0 (0 to 0·1)	0·6 (0·2 to 1·3)	3·3 (1 to 7·5)	5·9 (1·7 to 13·8)	8 (2·5 to 18·7)	11·2 (3·5 to 26·2)	15·4 (4·9 to 33·5)	16·6 (5·9 to 35·3)	15·3 (5·4 to 32·2)	13·9 (5·3 to 29)	12·8 (5·5 to 25·7)	12·5 (6 to 23·9)	10·7 (5·6 to 19·3)	8·7 (5 to 14·3)	7·1 (4·1 to 11·2)	5·5 (3·4 to 8·4)	4·3 (2·8 to 6·5)	3 (2 to 4·5)	2·2 (1·4 to 3·2)	1·4 (1 to 2)	0·8 (0·5 to 1·1)	0·4 (0·3 to 0·6)	160
Foreign body in other body part	0·6 (0·3 to 1·1)	0·3 (0·2 to 0·4)	2·3 (1·4 to 3·6)	11·4 (7·6 to 16·5)	15·6 (11·2 to 21·7)	16·4 (11·7 to 22·8)	16·7 (11·7 to 23·4)	20·2 (14·3 to 27·6)	19·6 (14 to 26·9)	18·1 (13·1 to 24·8)	16·2 (11·7 to 22·2)	14·9 (10·7 to 20·4)	15·3 (11 to 20·5)	14·7 (10·7 to 20)	13·1 (9·4 to 17·8)	11·3 (8·1 to 15·4)	8·7 (6·3 to 11·8)	7·1 (5·1 to 9·6)	5·7 (4·2 to 7·5)	4·5 (3·3 to 6)	3·1 (2·2 to 4·2)	1·9 (1·3 to 2·6)	1·4 (1 to 1·9)	239
Environmental heat and cold exposure	0·1 (0·1 to 0·1)	0·2 (0·1 to 0·3)	2 (1·3 to 2·9)	13·9 (9·2 to 20·4)	28·7 (19 to 41·4)	41·7 (27·1 to 60·8)	59·7 (39·9 to 85)	84·5 (57·5 to 117)	92·4 (63·1 to 131)	90·7 (61·5 to 131)	85·4 (58·9 to 121)	82·1 (55·7 to 114)	86 (60·6 to 120)	83·4 (59·1 to 114)	77·6 (54·2 to 107)	67·3 (47·2 to 93·3)	53·8 (37·7 to 73·4)	44·8 (31·7 to 60·5)	32·1 (22·7 to 43·3)	25·2 (17·8 to 33·8)	18·3 (13 to 24·5)	11 (7·9 to 14·9)	7·2 (5 to 9·7)	1,090
Exposure to forces of nature	0 (0 to 0)	0 (0 to 0)	0 (0 to 0)	0 (0 to 0)	0 (0 to 0·1)	0·4 (0·2 to 0·7)	1 (0·6 to 1·6)	1·9 (1·2 to 3)	2·4 (1·5 to 3·8)	2·9 (1·9 to 4·3)	3·9 (2·7 to 5·7)	4·9 (3·4 to 7·1)	4·6 (3·1 to 6·8)	3·4 (2·3 to 4·8)	3·1 (2·1 to 4·4)	2·7 (1·8 to 3·8)	1·9 (1·3 to 2·6)	1·3 (0·9 to 1·8)	0·8 (0·6 to 1·1)	0·5 (0·4 to 0·8)	0·3 (0·2 to 0·5)	0·2 (0·1 to 0·2)	0·1 (0·1 to 0·1)	36·3
Other unintentional injuries	9·5 (5·4 to 15·5)	3·2 (1·8 to 5·2)	10 (6·4 to 14·8)	51·6 (33·2 to 74·3)	93·8 (62·9 to 137)	117 (82·3 to 168)	151 (102 to 218)	205 (144 to 293)	224 (160 to 307)	218 (156 to 307)	191 (137 to 267)	175 (124 to 244)	169 (118 to 237)	147 (98·9 to 214)	134 (88·7 to 192)	116 (78·7 to 165)	88·3 (60·8 to 126)	72·8 (50·7 to 100)	54 (39 to 73·5)	38·4 (27·5 to 53·2)	27·2 (19·7 to 37·5)	16·8 (12·6 to 22·5)	10·9 (8·1 to 14·2)	2,320
Self-harm and interpersonal violence	30·1 (17·2 to 48·6)	8·3 (4·6 to 13·2)	29·8 (14·7 to 54·3)	167 (101 to 274)	210 (148 to 281)	436 (331 to 576)	1,850 (1,380 to 2,440)	4,090 (3,200 to 5,230)	4,310 (3,310 to 5,450)	3,980 (3,000 to 5,190)	3,060 (2,260 to 4,050)	2,410 (1,720 to 3,230)	2,120 (1,480 to 2,920)	1,530 (1,090 to 2,080)	1,090 (775 to 1,480)	672 (507 to 874)	425 (321 to 548)	271 (209 to 342)	142 (111 to 176)	67 (54·9 to 81·9)	38·8 (31·6 to 46·7)	17·2 (13·9 to 20·8)	11·6 (9·2 to 14·2)	27,000
Self-harm	0 (0 to 0)	0 (0 to 0)	0 (0 to 0)	0 (0 to 0)	0 (0 to 0)	57·2 (38·7 to 83·2)	260 (182 to 359)	532 (383 to 739)	591 (419 to 807)	549 (389 to 758)	452 (322 to 620)	382 (269 to 530)	353 (247 to 486)	278 (190 to 386)	215 (147 to 294)	144 (103 to 197)	95 (69·5 to 125)	72·6 (53·5 to 97)	39·4 (29·9 to 51)	21·1 (16·1 to 27·2)	12·7 (9·6 to 16)	5·5 (4·1 to 6·8)	3·8 (2·7 to 4·7)	4,060
Self-harm by firearm	0 (0 to 0)	0 (0 to 0)	0 (0 to 0)	0 (0 to 0)	0 (0 to 0)	2 (1·1 to 3·5)	30·5 (16·8 to 52·2)	105 (62·9 to 164)	120 (74·9 to 189)	134 (85·1 to 204)	90 (56·9 to 138)	75·3 (46·9 to 117)	80 (45·9 to 123)	63·2 (38 to 97·4)	40·4 (24·3 to 63·9)	24·2 (15·2 to 38·4)	13·6 (8·4 to 20·5)	7·3 (4·9 to 10·6)	3·4 (2·5 to 4·6)	2·6 (1·9 to 3·4)	1·8 (1·3 to 2·3)	0·6 (0·4 to 0·8)	0·5 (0·4 to 0·7)	795
Self-harm by other specified means	0 (0 to 0)	0 (0 to 0)	0 (0 to 0)	0 (0 to 0)	0 (0 to 0)	55·2 (37·5 to 80·6)	229 (162 to 316)	427 (301 to 609)	471 (329 to 648)	415 (294 to 572)	362 (258 to 498)	306 (213 to 428)	273 (190 to 384)	215 (145 to 302)	175 (121 to 238)	119 (84·8 to 165)	81·4 (59·3 to 107)	65·3 (48·2 to 88·1)	35·9 (27·3 to 46·9)	18·5 (14 to 24)	11 (8·2 to 13·9)	4·9 (3·6 to 6·1)	3·3 (2·3 to 4·1)	3,270
Interpersonal violence	30·1 (17·2 to 48·6)	8·3 (4·6 to 13·2)	29·4 (14·5 to 53·8)	164 (98·3 to 270)	208 (146 to 279)	377 (273 to 505)	1,590 (1,150 to 2,130)	3,550 (2,700 to 4,610)	3,710 (2,770 to 4,750)	3,420 (2,520 to 4,500)	2,600 (1,870 to 3,510)	2,020 (1,430 to 2,740)	1,760 (1,200 to 2,490)	1,230 (864 to 1,720)	863 (595 to 1,190)	517 (375 to 704)	323 (231 to 432)	196 (144 to 263)	101 (76·4 to 134)	45·2 (35·7 to 57·2)	25·6 (20·2 to 32·4)	11·6 (8·9 to 14·8)	7·7 (5·8 to 9·8)	22,800
Physical violence by firearm	10·5 (6·1 to 16·8)	2·1 (1·2 to 3·4)	10·5 (5 to 19·6)	74·2 (42·8 to 126)	97·6 (66·5 to 133)	202 (143 to 279)	1,110 (787 to 1,520)	2,710 (2,060 to 3,520)	2,820 (2,110 to 3,590)	2,630 (1,930 to 3,440)	1,970 (1,390 to 2,680)	1,440 (1,000 to 2,000)	1,240 (823 to 1,790)	806 (547 to 1,150)	516 (339 to 732)	278 (189 to 392)	157 (107 to 218)	62 (42·7 to 86·8)	24·4 (17 to 34·4)	7·2 (5·5 to 9·5)	2·6 (2 to 3·4)	0·9 (0·7 to 1·2)	1 (0·7 to 1·4)	16,200
Physical violence by sharp object	3 (1·7 to 4·9)	0·4 (0·3 to 0·7)	2·6 (1·4 to 4·5)	25·8 (15·7 to 42·2)	40·3 (28·4 to 53·7)	79·8 (57 to 109)	328 (236 to 449)	571 (422 to 754)	601 (431 to 802)	531 (383 to 722)	427 (305 to 580)	400 (280 to 549)	335 (231 to 468)	259 (184 to 359)	210 (146 to 290)	119 (86·7 to 158)	72·5 (52·9 to 95·2)	73·4 (52·3 to 99·6)	42·6 (31·3 to 57·7)	20·8 (16 to 27·4)	10·8 (8·2 to 14·1)	5·2 (3·9 to 6·7)	4 (3 to 5·3)	4,160
Sexual violence	0 (0 to 0)	0 (0 to 0)	0 (0 to 0)	1·2 (0·7 to 1·9)	10·5 (6·4 to 16·4)	25·1 (14·6 to 39·4)	61·7 (34·4 to 98·2)	91·8 (52·8 to 140)	82·5 (46·5 to 132)	67·6 (37·5 to 111)	54·3 (30·4 to 86·3)	39·8 (22·9 to 64·6)	31·5 (18·5 to 53·2)	22·2 (12·6 to 36·3)	15·2 (8·9 to 24·2)	11·5 (7·1 to 17·2)	8·3 (5·2 to 12·2)	6·2 (3·9 to 9·3)	4·2 (2·7 to 6·1)	3 (1·9 to 4·3)	1·7 (1 to 2·6)	0·4 (0·2 to 0·6)	0 (0 to 0·1)	539
Physical violence by other means	16·6 (9·4 to 27·1)	5·7 (3·1 to 9·3)	16·4 (8·1 to 29·9)	62·6 (37 to 102)	59·6 (42·1 to 81)	70·1 (51·7 to 93·8)	87·1 (65·5 to 115)	174 (132 to 225)	204 (154 to 268)	199 (147 to 265)	144 (107 to 190)	134 (99·1 to 175)	151 (111 to 199)	145 (108 to 191)	122 (90 to 160)	109 (82·6 to 141)	85·7 (64·5 to 111)	54·2 (41 to 70)	30·2 (23·1 to 38)	14·2 (10·6 to 18·4)	10·5 (8 to 13·3)	5·1 (3·8 to 6·5)	2·6 (1·9 to 3·6)	1,900
Conflict and terrorism	0 (0 to 0)	0 (0 to 0)	0 (0 to 0)	0 (0 to 0)	0 (0 to 0)	0 (0 to 0)	0 (0 to 0)	0·4 (0·3 to 0·8)	2·8 (1·6 to 4·9)	7·8 (4·6 to 14·6)	5·5 (3·2 to 9·4)	7·3 (4·3 to 11·7)	8·7 (5·4 to 14·2)	13·2 (8·4 to 20·6)	12·9 (8·6 to 19·4)	11·2 (7·4 to 16·5)	6·7 (4·4 to 9·9)	2 (1·4 to 3)	1 (0·7 to 1·5)	0·6 (0·4 to 0·9)	0·4 (0·3 to 0·6)	0·1 (0·1 to 0·2)	0·1 (0 to 0·1)	80·7

SR Table 2c: Saint Lucia DALYS by cause and age group for both sexes with 95% uncertainty intervals, 2019

Cause	Early Neonatal	Late Neonatal	Post Neonatal	1 to 4	5 to 9	10 to 14	15 to 19	20 to 24	25 to 29	30 to 34	35 to 39	40 to 44	45 to 49	50 to 54	55 to 59	60 to 64	65 to 69	70 to 74	75 to 79	80 to 84	85 to 89	90 to 94	95 plus	Total
All causes	1,360 (928 to 1,950)	307 (212 to 434)	353 (258 to 482)	619 (483 to 793)	681 (547 to 859)	908 (722 to 1,150)	1,590 (1,310 to 1,930)	2,210 (1,880 to 2,600)	2,580 (2,200 to 3,020)	2,610 (2,210 to 3,060)	2,950 (2,490 to 3,480)	3,520 (3,000 to 4,110)	4,280 (3,620 to 5,000)	4,770 (4,040 to 5,550)	4,820 (4,120 to 5,630)	4,640 (3,990 to 5,360)	3,880 (3,370 to 4,460)	4,070 (3,560 to 4,610)	3,200 (2,820 to 3,610)	2,630 (2,340 to 2,910)	1,810 (1,650 to 1,990)	949 (878 to 1,020)	245 (230 to 260)	55,000
Communicable, maternal, neonatal, and nutritional diseases	1,180 (803 to 1,680)	239 (163 to 338)	183 (133 to 252)	221 (168 to 286)	194 (139 to 278)	159 (118 to 212)	183 (141 to 234)	216 (177 to 269)	258 (215 to 311)	270 (230 to 325)	284 (237 to 346)	302 (251 to 371)	295 (242 to 361)	279 (228 to 342)	242 (195 to 304)	207 (168 to 256)	164 (131 to 202)	183 (146 to 231)	157 (130 to 190)	147 (117 to 185)	125 (101 to 155)	72·5 (56·5 to 89·6)	23·1 (18·4 to 28)	5,580
HIV/AIDS and sexually transmitted infections	1·8 (1 to 3·1)	0·4 (0·2 to 0·8)	2·1 (1·9 to 2·4)	1·9 (1·8 to 2)	2·2 (2·1 to 2·3)	2·2 (2 to 2·4)	5·3 (4·6 to 6·3)	23·3 (21 to 26·2)	48·4 (43·9 to 55·6)	64·1 (58·8 to 72·1)	62·5 (57·3 to 68·9)	69·2 (64·3 to 75·9)	51·9 (47·6 to 56·6)	36·4 (33·7 to 40)	23 (20·8 to 25·7)	12·2 (10·9 to 14)	6·7 (5·8 to 7·9)	3 (2·4 to 3·8)	1·5 (1·2 to 2)	0·9 (0·7 to 1·2)	0·5 (0·4 to 0·6)	0·2 (0·1 to 0·2)	0 (0 to 0·1)	420
HIV/AIDS	0 (0 to 0)	0 (0 to 0)	1·8 (1·7 to 1·9)	1·8 (1·8 to 2)	2·2 (2 to 2·3)	1·8 (1·7 to 1·9)	3·6 (3·4 to 3·8)	19·8 (18·1 to 21·9)	43·6 (39·8 to 49·6)	58·4 (54·1 to 65·4)	56·1 (52·1 to 61)	62·6 (58·8 to 66·9)	45·9 (43 to 49)	32·6 (30·6 to 34·7)	20·4 (18·8 to 22)	10·6 (9·7 to 11·6)	5·5 (4·9 to 6·1)	2·1 (1·8 to 2·4)	0·9 (0·8 to 1·1)	0·5 (0·4 to 0·6)	0·2 (0·2 to 0·3)	0·1 (0 to 0·1)	0 (0 to 0)	371
HIV/AIDS - Drug-susceptible Tuberculosis	0 (0 to 0)	0 (0 to 0)	0·2 (0·1 to 0·2)	0·2 (0·1 to 0·3)	0·2 (0·1 to 0·3)	0·2 (0·1 to 0·2)	0·3 (0·2 to 0·5)	2 (1·4 to 2·9)	4·1 (2·8 to 6)	5·8 (3·9 to 8·5)	5·3 (3·5 to 7·7)	6·1 (4 to 9)	4·6 (3 to 6·8)	3·1 (2·1 to 4·6)	1·9 (1·2 to 2·7)	0·9 (0·6 to 1·4)	0·5 (0·3 to 0·7)	0·2 (0·1 to 0·3)	0·1 (0·1 to 0·1)	0 (0 to 0·1)	0 (0 to 0)	0 (0 to 0)	0 (0 to 0)	35·8
HIV/AIDS - Multidrug-resistant Tuberculosis without extensive dru	0 (0 to 0)	0 (0 to 0)	0 (0 to 0)	0 (0 to 0)	0 (0 to 0)	0 (0 to 0)	0 (0 to 0)	0 (0 to 0·1)	0 (0 to 0·2)	0 (0 to 0·3)	0 (0 to 0·2)	0 (0 to 0·3)	0 (0 to 0·2)	0 (0 to 0·1)	0 (0 to 0·1)	0 (0 to 0)	0 (0 to 0)	0 (0 to 0)	0 (0 to 0)	0 (0 to 0)	0 (0 to 0)	0 (0 to 0)	0 (0 to 0)	0·4
HIV/AIDS - Extensively drug-resistant Tuberculosis	0 (0 to 0)	0 (0 to 0)	0 (0 to 0)	0 (0 to 0)	0 (0 to 0)	0 (0 to 0)	0 (0 to 0)	0 (0 to 0)	0 (0 to 0)	0 (0 to 0)	0 (0 to 0)	0 (0 to 0·1)	0 (0 to 0)	0 (0 to 0)	0 (0 to 0)	0 (0 to 0)	0 (0 to 0)	0 (0 to 0)	0 (0 to 0)	0 (0 to 0)	0 (0 to 0)	0 (0 to 0)	0 (0 to 0)	0·1
HIV/AIDS resulting in other diseases	0 (0 to 0)	0 (0 to 0)	1·7 (1·5 to 1·8)	1·6 (1·5 to 1·8)	2 (1·8 to 2·1)	1·7 (1·5 to 1·8)	3·3 (3 to 3·5)	17·8 (15·9 to 19·8)	39·4 (35·3 to 45·5)	52·5 (47·7 to 59·7)	50·8 (46·2 to 56)	56·4 (51·6 to 61·2)	41·2 (37·5 to 44·4)	29·4 (27 to 31·7)	18·5 (16·7 to 20·2)	9·6 (8·5 to 10·7)	5 (4·3 to 5·6)	1·9 (1·6 to 2·2)	0·9 (0·7 to 1)	0·4 (0·4 to 0·5)	0·2 (0·2 to 0·2)	0 (0 to 0·1)	0 (0 to 0)	334
Sexually transmitted infections excluding HIV	1·8 (1 to 3·1)	0·4 (0·2 to 0·8)	0·3 (0·1 to 0·6)	0 (0 to 0·1)	0 (0 to 0)	0·3 (0·2 to 0·5)	1·7 (1·1 to 2·6)	3·4 (2·2 to 5·3)	4·8 (2·9 to 7·7)	5·7 (3·5 to 9·2)	6·4 (4 to 10·2)	6·6 (4·2 to 11)	6 (3·7 to 9·7)	3·8 (2·2 to 6·6)	2·6 (1·4 to 4·7)	1·6 (0·9 to 3)	1·2 (0·7 to 2·2)	0·9 (0·5 to 1·6)	0·6 (0·3 to 1)	0·4 (0·3 to 0·7)	0·3 (0·2 to 0·4)	0·1 (0·1 to 0·2)	0 (0 to 0)	49·1
Syphilis	1·8 (1 to 3·1)	0·4 (0·2 to 0·8)	0·3 (0·1 to 0·6)	0 (0 to 0·1)	0 (0 to 0)	0·1 (0·1 to 0·1)	0·2 (0·1 to 0·3)	0·3 (0·1 to 0·4)	0·4 (0·3 to 0·7)	0·5 (0·3 to 0·7)	0·5 (0·4 to 0·8)	0·3 (0·2 to 0·5)	0·4 (0·3 to 0·7)	0·4 (0·2 to 0·7)	0·3 (0·2 to 0·5)	0·2 (0·1 to 0·4)	0·2 (0·1 to 0·4)	0·2 (0·1 to 0·3)	0·1 (0·1 to 0·1)	0·1 (0·1 to 0·1)	0 (0 to 0·1)	0 (0 to 0)	0 (0 to 0)	6·9
Chlamydial infection	0 (0 to 0)	0 (0 to 0)	0 (0 to 0)	0 (0 to 0)	0 (0 to 0)	0 (0 to 0)	0·2 (0·1 to 0·2)	0·5 (0·3 to 0·8)	0·7 (0·4 to 1·2)	0·7 (0·4 to 1·2)	0·9 (0·5 to 1·4)	0·9 (0·6 to 1·4)	0·7 (0·4 to 1·1)	0·3 (0·1 to 0·4)	0·1 (0·1 to 0·2)	0·1 (0 to 0·1)	0 (0 to 0)	0 (0 to 0)	0 (0 to 0)	0 (0 to 0)	0 (0 to 0)	0 (0 to 0)	0 (0 to 0)	5·2
Gonococcal infection	0 (0 to 0)	0 (0 to 0)	0 (0 to 0)	0 (0 to 0)	0 (0 to 0)	0·1 (0 to 0·1)	0·4 (0·2 to 0·7)	0·6 (0·4 to 1·1)	0·5 (0·3 to 0·8)	0·6 (0·4 to 0·9)	0·7 (0·5 to 0·9)	0·9 (0·6 to 1·3)	0·9 (0·6 to 1·3)	0·4 (0·3 to 0·6)	0·2 (0·2 to 0·3)	0·1 (0·1 to 0·2)	0·2 (0·1 to 0·2)	0·1 (0·1 to 0·2)	0·1 (0·1 to 0·1)	0·1 (0·1 to 0·1)	0 (0 to 0·1)	0 (0 to 0)	0 (0 to 0)	6
Trichomoniasis	0 (0 to 0)	0 (0 to 0)	0 (0 to 0)	0 (0 to 0)	0 (0 to 0)	0 (0 to 0)	0·1 (0 to 0·3)	0·6 (0·2 to 1·5)	1·2 (0·4 to 2·7)	1·3 (0·4 to 3)	1·4 (0·4 to 3·1)	1·3 (0·4 to 3·1)	1·2 (0·4 to 2·9)	1 (0·3 to 2·2)	0·6 (0·2 to 1·5)	0·4 (0·1 to 0·8)	0·2 (0·1 to 0·5)	0·2 (0·1 to 0·4)	0·1 (0 to 0·2)	0·1 (0 to 0·2)	0 (0 to 0·1)	0 (0 to 0)	0 (0 to 0)	9·6
Genital herpes	0 (0 to 0)	0 (0 to 0)	0 (0 to 0)	0 (0 to 0)	0 (0 to 0)	0·1 (0 to 0·1)	0·3 (0·1 to 0·6)	0·6 (0·2 to 1·4)	0·9 (0·3 to 2·2)	1·1 (0·4 to 2·6)	1·3 (0·4 to 3·1)	1·3 (0·4 to 3·2)	1·3 (0·4 to 3·2)	1·2 (0·4 to 3)	1 (0·3 to 2·4)	0·7 (0·2 to 1·8)	0·5 (0·1 to 1·2)	0·4 (0·1 to 0·9)	0·2 (0·1 to 0·6)	0·2 (0 to 0·4)	0·1 (0 to 0·2)	0 (0 to 0·1)	0 (0 to 0)	11
Other sexually transmitted infections	0 (0 to 0)	0 (0 to 0)	0 (0 to 0)	0 (0 to 0)	0 (0 to 0)	0·1 (0·1 to 0·2)	0·5 (0·3 to 0·9)	0·8 (0·5 to 1·2)	1·1 (0·7 to 1·6)	1·5 (1 to 2·2)	1·7 (1·1 to 2·5)	1·9 (1·3 to 2·8)	1·4 (0·9 to 2·2)	0·6 (0·4 to 0·8)	0·3 (0·2 to 0·4)	0·1 (0·1 to 0·2)	0·1 (0·1 to 0·1)	0 (0 to 0·1)	0·1 (0 to 0·1)	0 (0 to 0·1)	0 (0 to 0·1)	0 (0 to 0)	0 (0 to 0)	10·5
Respiratory infections and tuberculosis	31·6 (18·8 to 49·7)	18·6 (11·9 to 28·6)	50·1 (30·8 to 75·7)	50·3 (35·6 to 69·9)	29·2 (21·1 to 40·4)	25·1 (17·6 to 35·6)	30·3 (21·7 to 41·6)	42·2 (31·7 to 55·3)	51·2 (39·3 to 66·4)	53·9 (41·9 to 69·8)	58·1 (44·9 to 76)	68·3 (52·7 to 90·1)	78 (58·5 to 103)	83·4 (61·2 to 111)	83·8 (60·2 to 114)	87·9 (63·9 to 116)	79·2 (56·4 to 107)	103 (74·2 to 141)	95·9 (74 to 122)	99·3 (74·1 to 133)	92·4 (71·7 to 117)	56 (42·8 to 71·6)	18·5 (14·2 to 22·5)	1,390
Tuberculosis	0 (0 to 0)	0 (0 to 0)	0·7 (0·5 to 1·1)	1·4 (0·9 to 2·2)	1·4 (0·8 to 2·1)	1·4 (1·1 to 1·8)	3·1 (2·2 to 4·3)	9·3 (6·2 to 13)	13·3 (9·7 to 17·8)	13·8 (9·8 to 18·2)	13·7 (9·6 to 18·8)	15·1 (10·7 to 20·9)	15·1 (10·5 to 21·2)	14·4 (10·1 to 20·2)	12·6 (8·7 to 17·6)	10·7 (7·7 to 14·7)	7·5 (5·2 to 10·5)	8 (5·8 to 10·7)	5·6 (4 to 7·7)	4·1 (3 to 5·6)	2·6 (1·8 to 3·6)	1 (0·7 to 1·4)	0·2 (0·2 to 0·3)	155
Latent tuberculosis infection	0 (0 to 0)	0 (0 to 0)	0 (0 to 0)	0 (0 to 0)	0 (0 to 0)	0 (0 to 0)	0 (0 to 0)	0 (0 to 0)	0 (0 to 0)	0 (0 to 0)	0 (0 to 0)	0 (0 to 0)	0 (0 to 0)	0 (0 to 0)	0 (0 to 0)	0 (0 to 0)	0 (0 to 0)	0 (0 to 0)	0 (0 to 0)	0 (0 to 0)	0 (0 to 0)	0 (0 to 0)	0 (0 to 0)	0
Drug-susceptible tuberculosis	0 (0 to 0)	0 (0 to 0)	0·7 (0·4 to 1·1)	1·4 (0·9 to 2·1)	1·4 (0·8 to 2·1)	1·4 (1·1 to 1·8)	3 (2·1 to 4·2)	9·2 (6·2 to 12·9)	13·2 (9·7 to 17·6)	13·7 (9·7 to 18·1)	13·5 (9·5 to 18·5)	14·9 (10·6 to 20·4)	14·9 (10·3 to 20·8)	14·2 (10·1 to 20·1)	12·4 (8·5 to 17·3)	10·6 (7·6 to 14·6)	7·4 (5·1 to 10·4)	7·9 (5·8 to 10·5)	5·6 (3·9 to 7·5)	4·1 (2·9 to 5·5)	2·5 (1·8 to 3·5)	1 (0·7 to 1·4)	0·2 (0·2 to 0·3)	153
Multidrug-resistant tuberculosis without extensive drug resistanc	0 (0 to 0)	0 (0 to 0)	0 (0 to 0)	0 (0 to 0·1)	0 (0 to 0·1)	0 (0 to 0·1)	0 (0 to 0·1)	0·1 (0 to 0·3)	0·1 (0 to 0·5)	0·1 (0 to 0·5)	0·1 (0 to 0·5)	0·1 (0 to 0·6)	0·1 (0 to 0·6)	0·1 (0 to 0·6)	0·1 (0 to 0·5)	0·1 (0 to 0·4)	0·1 (0 to 0·3)	0·1 (0 to 0·3)	0·1 (0 to 0·2)	0 (0 to 0·2)	0 (0 to 0·1)	0 (0 to 0)	0 (0 to 0)	1·4
Extensively drug-resistant tuberculosis	0 (0 to 0)	0 (0 to 0)	0 (0 to 0)	0 (0 to 0)	0 (0 to 0)	0 (0 to 0)	0 (0 to 0)	0 (0 to 0·1)	0 (0 to 0·1)	0 (0 to 0·1)	0 (0 to 0·1)	0 (0 to 0·1)	0 (0 to 0·1)	0 (0 to 0·1)	0 (0 to 0·1)	0 (0 to 0·1)	0 (0 to 0·1)	0 (0 to 0)	0 (0 to 0)	0 (0 to 0)	0 (0 to 0)	0 (0 to 0)	0 (0 to 0)	0·2
Lower respiratory infections	31·5 (18·8 to 49·7)	18·5 (11·7 to 28·5)	46·5 (27·2 to 71·7)	35·2 (22·5 to 52)	11·4 (8·1 to 15·6)	8·6 (6·1 to 12·2)	11·3 (7·5 to 16·2)	17·5 (11·6 to 25·9)	23·6 (15·7 to 34)	27·7 (18·9 to 39·4)	31·5 (21·8 to 44·4)	40·8 (26·7 to 59·9)	50·8 (33·7 to 72·8)	58·1 (38·2 to 84·1)	62·5 (40·5 to 91·5)	70·8 (48·2 to 98·5)	67·4 (45·1 to 95·9)	92·1 (63·5 to 128)	88·2 (66·2 to 113)	93·9 (68·6 to 128)	89·1 (68·6 to 113)	54·7 (41·5 to 70·3)	18·2 (14 to 22·3)	1,050
Upper respiratory infections	0 (0 to 0)	0·1 (0·1 to 0·2)	2·5 (1·5 to 3·7)	9·9 (5·7 to 15·8)	11·1 (6·3 to 18·4)	11 (5·9 to 19)	11·8 (6·2 to 20·6)	11·7 (6·5 to 20)	11·2 (6·2 to 18·5)	10 (5·4 to 16·7)	10·3 (5·2 to 17·1)	9·7 (5·3 to 16·9)	9·1 (4·9 to 16)	8·1 (4·5 to 13·5)	6·5 (3·5 to 10·6)	4·8 (2·7 to 8)	3·4 (1·8 to 5·7)	2·6 (1·5 to 4·4)	1·7 (0·9 to 2·7)	1 (0·5 to 1·7)	0·5 (0·3 to 0·8)	0·2 (0·1 to 0·4)	0 (0 to 0·1)	137
Otitis media	0 (0 to 0)	0 (0 to 0)	0·4 (0·2 to 0·8)	3·8 (2 to 6·6)	5·4 (3 to 8·9)	4·1 (2·2 to 7·1)	4·2 (2·2 to 7)	3·7 (1·6 to 7·5)	3·1 (1·5 to 6)	2·5 (1·4 to 4·6)	2·6 (1·3 to 4·9)	2·8 (1·3 to 5)	3 (1·5 to 5·2)	2·9 (1·3 to 5·3)	2·3 (1·1 to 4)	1·5 (0·7 to 2·9)	0·9 (0·4 to 1·6)	0·6 (0·3 to 1·1)	0·4 (0·2 to 0·7)	0·3 (0·1 to 0·5)	0·2 (0·1 to 0·3)	0·1 (0 to 0·1)	0 (0 to 0)	44·8
Enteric infections	2·7 (1·5 to 4·4)	5 (2·8 to 8·2)	29·8 (18·5 to 45·2)	32·7 (22·4 to 45·7)	24·8 (15·4 to 37·9)	25·1 (14·7 to 40·5)	19·9 (12·8 to 29)	17·5 (11·5 to 25)	19·9 (13·4 to 27·9)	20·1 (14·1 to 28)	23·1 (16·3 to 31·2)	25·7 (18·3 to 34·3)	28·6 (20·7 to 38·7)	29·5 (20·8 to 39·8)	27 (19·4 to 36·9)	26·6 (19·4 to 35·6)	26·4 (19·8 to 34·4)	30·1 (22·4 to 39·2)	25·9 (19·7 to 33·1)	21·1 (16·1 to 26·7)	12·8 (9·6 to 16·9)	6·1 (4·5 to 8)	1·6 (1·1 to 2·1)	482
Diarrheal diseases	2·7 (1·5 to 4·4)	5 (2·8 to 8·2)	29·7 (18·5 to 45·2)	32·5 (22·3 to 45·6)	24·7 (15·3 to 37·8)	25 (14·6 to 40·3)	19·9 (12·8 to 28·9)	17·4 (11·5 to 24·9)	19·8 (13·4 to 27·8)	20 (13·9 to 27·9)	23 (16·3 to 31·1)	25·6 (18·3 to 34·3)	28·6 (20·7 to 38·7)	29·4 (20·7 to 39·7)	26·9 (19·4 to 36·8)	26·6 (19·4 to 35·6)	26·4 (19·7 to 34·4)	30·1 (22·4 to 39·1)	25·9 (19·7 to 33·1)	21·1 (16·1 to 26·7)	12·8 (9·5 to 16·8)	6·1 (4·5 to 7·9)	1·6 (1·1 to 2·1)	481
Typhoid and paratyphoid	0 (0 to 0)	0 (0 to 0)	0 (0 to 0)	0 (0 to 0)	0 (0 to 0)	0 (0 to 0)	0 (0 to 0)	0 (0 to 0)	0 (0 to 0)	0 (0 to 0)	0 (0 to 0)	0 (0 to 0)	0 (0 to 0)	0 (0 to 0)	0 (0 to 0·1)	0 (0 to 0)	0 (0 to 0)	0 (0 to 0)	0 (0 to 0)	0 (0 to 0)	0 (0 to 0)	0 (0 to 0)	0 (0 to 0)	0·1
Typhoid fever	0 (0 to 0)	0 (0 to 0)	0 (0 to 0)	0 (0 to 0)	0 (0 to 0)	0 (0 to 0)	0 (0 to 0)	0 (0 to 0)	0 (0 to 0)	0 (0 to 0)	0 (0 to 0)	0 (0 to 0)	0 (0 to 0)	0 (0 to 0)	0 (0 to 0)	0 (0 to 0)	0 (0 to 0)	0 (0 to 0)	0 (0 to 0)	0 (0 to 0)	0 (0 to 0)	0 (0 to 0)	0 (0 to 0)	0
Paratyphoid fever	0 (0 to 0)	0 (0 to 0)	0 (0 to 0)	0 (0 to 0)	0 (0 to 0)	0 (0 to 0)	0 (0 to 0)	0 (0 to 0)	0 (0 to 0)	0 (0 to 0)	0 (0 to 0)	0 (0 to 0)	0 (0 to 0)	0 (0 to 0)	0 (0 to 0·1)	0 (0 to 0)	0 (0 to 0)	0 (0 to 0)	0 (0 to 0)	0 (0 to 0)	0 (0 to 0)	0 (0 to 0)	0 (0 to 0)	0·1
Invasive Non-typhoidal Salmonella (iNTS)	0 (0 to 0)	0 (0 to 0)	0 (0 to 0)	0 (0 to 0·1)	0 (0 to 0·1)	0·1 (0 to 0·1)	0 (0 to 0)	0 (0 to 0)	0 (0 to 0)	0 (0 to 0)	0 (0 to 0)	0 (0 to 0·1)	0 (0 to 0)	0 (0 to 0)	0 (0 to 0)	0 (0 to 0)	0 (0 to 0)	0 (0 to 0)	0 (0 to 0)	0 (0 to 0)	0 (0 to 0)	0 (0 to 0)	0 (0 to 0)	0·4
Other intestinal infectious diseases	0 (0 to 0)	0 (0 to 0)	0·1 (0 to 0·1)	0·1 (0 to 0·3)	0·1 (0 to 0·2)	0·1 (0 to 0·2)	0 (0 to 0·1)	0·1 (0 to 0·1)	0 (0 to 0·1)	0 (0 to 0·1)	0 (0 to 0·1)	0 (0 to 0)	0 (0 to 0·1)	0 (0 to 0·1)	0 (0 to 0)	0 (0 to 0)	0 (0 to 0)	0 (0 to 0)	0 (0 to 0)	0 (0 to 0)	0 (0 to 0)	0 (0 to 0)	0 (0 to 0)	0·8
Neglected tropical diseases and malaria	0·2 (0·1 to 0·3)	0·4 (0·2 to 0·6)	2·8 (1·6 to 4·7)	9·6 (5·8 to 15·3)	12·4 (7 to 20·1)	7·8 (4·7 to 11·9)	7·8 (4·8 to 11·6)	9·9 (6·2 to 15·1)	11·1 (7 to 17·6)	11·4 (6·9 to 18·1)	12·9 (7·7 to 20·6)	14·4 (8·4 to 24·2)	15·6 (8·8 to 27·4)	16·9 (9·1 to 28·3)	16·5 (9·1 to 27·8)	15·3 (7·8 to 25·9)	12·9 (6·2 to 22·7)	12·6 (6·3 to 21·9)	9·8 (4·7 to 17·2)	7·1 (3·4 to 12·2)	4·1 (2·1 to 6·9)	1·8 (1 to 3)	0·5 (0·2 to 0·8)	214
Malaria	0 (0 to 0)	0 (0 to 0)	0 (0 to 0)	0 (0 to 0)	0 (0 to 0)	0 (0 to 0)	0 (0 to 0)	0 (0 to 0)	0 (0 to 0)	0 (0 to 0)	0 (0 to 0)	0 (0 to 0)	0 (0 to 0)	0 (0 to 0)	0 (0 to 0)	0 (0 to 0)	0 (0 to 0)	0 (0 to 0)	0 (0 to 0)	0 (0 to 0)	0 (0 to 0)	0 (0 to 0)	0 (0 to 0)	
Chagas disease	0 (0 to 0)	0 (0 to 0)	0 (0 to 0)	0 (0 to 0)	0 (0 to 0)	0 (0 to 0)	0 (0 to 0)	0 (0 to 0)	0 (0 to 0)	0 (0 to 0)	0 (0 to 0)	0 (0 to 0)	0 (0 to 0)	0 (0 to 0)	0 (0 to 0)	0 (0 to 0)	0 (0 to 0)	0 (0 to 0)	0 (0 to 0)	0 (0 to 0)	0 (0 to 0)	0 (0 to 0)	0 (0 to 0)	0
Leishmaniasis	0 (0 to 0)	0 (0 to 0)	0 (0 to 0)	0 (0 to 0)	0 (0 to 0)	0 (0 to 0)	0 (0 to 0)	0 (0 to 0)	0 (0 to 0)	0 (0 to 0)	0 (0 to 0)	0 (0 to 0)	0 (0 to 0)	0 (0 to 0)	0 (0 to 0)	0 (0 to 0)	0 (0 to 0)	0 (0 to 0)	0 (0 to 0)	0 (0 to 0)	0 (0 to 0)	0 (0 to 0)	0 (0 to 0)	0
Visceral leishmaniasis	0 (0 to 0)	0 (0 to 0)	0 (0 to 0)	0 (0 to 0)	0 (0 to 0)	0 (0 to 0)	0 (0 to 0)	0 (0 to 0)	0 (0 to 0)	0 (0 to 0)	0 (0 to 0)	0 (0 to 0)	0 (0 to 0)	0 (0 to 0)	0 (0 to 0)	0 (0 to 0)	0 (0 to 0)	0 (0 to 0)	0 (0 to 0)	0 (0 to 0)	0 (0 to 0)	0 (0 to 0)	0 (0 to 0)	0
Cutaneous and mucocutaneous leishmaniasis	0 (0 to 0)	0 (0 to 0)	0 (0 to 0)	0 (0 to 0)	0 (0 to 0)	0 (0 to 0)	0 (0 to 0)	0 (0 to 0)	0 (0 to 0)	0 (0 to 0)	0 (0 to 0)	0 (0 to 0)	0 (0 to 0)	0 (0 to 0)	0 (0 to 0)	0 (0 to 0)	0 (0 to 0)	0 (0 to 0)	0 (0 to 0)	0 (0 to 0)	0 (0 to 0)	0 (0 to 0)	0 (0 to 0)	
African trypanosomiasis	0 (0 to 0)	0 (0 to 0)	0 (0 to 0)	0 (0 to 0)	0 (0 to 0)	0 (0 to 0)	0 (0 to 0)	0 (0 to 0)	0 (0 to 0)	0 (0 to 0)	0 (0 to 0)	0 (0 to 0)	0 (0 to 0)	0 (0 to 0)	0 (0 to 0)	0 (0 to 0)	0 (0 to 0)	0 (0 to 0)	0 (0 to 0)	0 (0 to 0)	0 (0 to 0)	0 (0 to 0)	0 (0 to 0)	0
Schistosomiasis	0 (0 to 0)	0 (0 to 0)	0 (0 to 0)	0 (0 to 0)	0 (0 to 0)	0 (0 to 0)	0 (0 to 0·1)	0 (0 to 0·1)	0 (0 to 0·2)	0 (0 to 0·2)	0·1 (0 to 0·3)	0·1 (0 to 0·4)	0·1 (0 to 0·5)	0·2 (0 to 0·7)	0·2 (0 to 0·8)	0·2 (0 to 0·7)	0·1 (0 to 0·4)	0·1 (0 to 0·6)	0·1 (0 to 0·4)	0·1 (0 to 0·2)	0 (0 to 0·1)	0 (0 to 0)	0 (0 to 0)	1·3
Cysticercosis	0 (0 to 0)	0 (0 to 0)	0 (0 to 0)	0 (0 to 0)	0 (0 to 0)	0·1 (0 to 0·2)	0·7 (0·1 to 1·8)	2·5 (0·6 to 6)	4·5 (1·4 to 10·1)	5·2 (1·8 to 11·2)	6·5 (2·3 to 13·7)	7·8 (2·6 to 16·9)	9·5 (3·4 to 20·5)	10·9 (3·7 to 22)	11·7 (4·5 to 21·4)	11·8 (4·7 to 22·2)	10·8 (4·2 to 20·1)	10·4 (4·6 to 19·8)	8·2 (3·2 to 15·5)	6 (2·4 to 11·2)	3·2 (1·4 to 6)	1·3 (0·5 to 2·6)	0·3 (0·1 to 0·5)	111
Cystic echinococcosis	0 (0 to 0)	0 (0 to 0)	0 (0 to 0)	0 (0 to 0)	0 (0 to 0)	0 (0 to 0)	0 (0 to 0)	0 (0 to 0)	0 (0 to 0)	0 (0 to 0)	0 (0 to 0)	0 (0 to 0)	0 (0 to 0)	0 (0 to 0)	0 (0 to 0)	0 (0 to 0)	0 (0 to 0)	0 (0 to 0)	0 (0 to 0)	0 (0 to 0)	0 (0 to 0)	0 (0 to 0)	0 (0 to 0)	
Lymphatic filariasis	0 (0 to 0)	0 (0 to 0)	0 (0 to 0)	0 (0 to 0)	0 (0 to 0)	0 (0 to 0)	0 (0 to 0)	0 (0 to 0)	0 (0 to 0)	0 (0 to 0)	0 (0 to 0)	0 (0 to 0)	0 (0 to 0)	0 (0 to 0)	0 (0 to 0)	0 (0 to 0)	0 (0 to 0)	0 (0 to 0)	0 (0 to 0)	0 (0 to 0)	0 (0 to 0)	0 (0 to 0)	0 (0 to 0)	0
Onchocerciasis	0 (0 to 0)	0 (0 to 0)	0 (0 to 0)	0 (0 to 0)	0 (0 to 0)	0 (0 to 0)	0 (0 to 0)	0 (0 to 0)	0 (0 to 0)	0 (0 to 0)	0 (0 to 0)	0 (0 to 0)	0 (0 to 0)	0 (0 to 0)	0 (0 to 0)	0 (0 to 0)	0 (0 to 0)	0 (0 to 0)	0 (0 to 0)	0 (0 to 0)	0 (0 to 0)	0 (0 to 0)	0 (0 to 0)	0
Trachoma	0 (0 to 0)	0 (0 to 0)	0 (0 to 0)	0 (0 to 0)	0 (0 to 0)	0 (0 to 0)	0 (0 to 0)	0 (0 to 0)	0 (0 to 0)	0 (0 to 0)	0 (0 to 0)	0 (0 to 0)	0 (0 to 0)	0 (0 to 0)	0 (0 to 0)	0 (0 to 0)	0 (0 to 0)	0 (0 to 0)	0 (0 to 0)	0 (0 to 0)	0 (0 to 0)	0 (0 to 0)	0 (0 to 0)	0
Dengue	0 (0 to 0)	0·1 (0 to 0·2)	1·2 (0·4 to 2·8)	2·6 (0·9 to 5·5)	4 (1·5 to 8·2)	2·7 (1·1 to 4·8)	2·8 (1·2 to 5·1)	2·6 (1·2 to 4·6)	2·9 (1·4 to 5)	2·3 (1·1 to 4)	2·4 (1·1 to 4·3)	2·6 (1·2 to 4·5)	2·3 (1·1 to 4)	2·5 (1 to 4·9)	1·8 (0·7 to 3·2)	1·7 (0·8 to 3·1)	1·1 (0·4 to 2·1)	0·9 (0·4 to 1·6)	0·6 (0·2 to 1·2)	0·5 (0·2 to 0·8)	0·3 (0·1 to 0·6)	0·2 (0·1 to 0·3)	0·1 (0 to 0·1)	38·1
Yellow fever	0 (0 to 0)	0 (0 to 0)	0 (0 to 0)	0 (0 to 0)	0 (0 to 0)	0 (0 to 0)	0 (0 to 0)	0 (0 to 0)	0 (0 to 0)	0 (0 to 0)	0 (0 to 0)	0 (0 to 0)	0 (0 to 0)	0 (0 to 0)	0 (0 to 0)	0 (0 to 0)	0 (0 to 0)	0 (0 to 0)	0 (0 to 0)	0 (0 to 0)	0 (0 to 0)	0 (0 to 0)	0 (0 to 0)	0
Rabies	0 (0 to 0)	0 (0 to 0)	0 (0 to 0)	0 (0 to 0·1)	0·1 (0 to 0·2)	0 (0 to 0·1)	0 (0 to 0·1)	0 (0 to 0)	0 (0 to 0)	0 (0 to 0)	0 (0 to 0)	0 (0 to 0)	0 (0 to 0·1)	0 (0 to 0·1)	0 (0 to 0)	0 (0 to 0)	0 (0 to 0)	0 (0 to 0)	0 (0 to 0)	0 (0 to 0)	0 (0 to 0)	0 (0 to 0)	0 (0 to 0)	0·3
Intestinal nematode infections	0 (0 to 0)	0 (0 to 0)	0 (0 to 0·1)	0·4 (0·2 to 0·8)	1·1 (0·6 to 1·8)	1 (0·5 to 1·6)	2 (1 to 3·4)	2·3 (1·1 to 4·1)	1·6 (0·8 to 2·9)	1·3 (0·6 to 2·3)	1·4 (0·7 to 2·3)	0·9 (0·5 to 1·6)	0·5 (0·2 to 0·9)	0·4 (0·2 to 0·8)	0·4 (0·2 to 0·7)	0·3 (0·1 to 0·5)	0·2 (0·1 to 0·3)	0·2 (0·1 to 0·3)	0·1 (0·1 to 0·2)	0·1 (0 to 0·1)	0 (0 to 0·1)	0 (0 to 0)	0 (0 to 0)	14
Ascariasis	0 (0 to 0)	0 (0 to 0)	0 (0 to 0)	0·2 (0·1 to 0·5)	0·4 (0·2 to 0·7)	0·3 (0·1 to 0·5)	0·3 (0·1 to 0·6)	0·3 (0·1 to 0·5)	0·3 (0·1 to 0·5)	0·2 (0·1 to 0·4)	0·2 (0·1 to 0·5)	0·2 (0·1 to 0·4)	0·2 (0·1 to 0·4)	0·2 (0·1 to 0·3)	0·1 (0·1 to 0·3)	0·1 (0 to 0·2)	0·1 (0 to 0·1)	0·1 (0 to 0·1)	0 (0 to 0·1)	0 (0 to 0)	0 (0 to 0)	0 (0 to 0)	0 (0 to 0)	3·2
Trichuriasis	0 (0 to 0)	0 (0 to 0)	0 (0 to 0)	0 (0 to 0·1)	0·1 (0 to 0·3)	0·1 (0·1 to 0·3)	0·2 (0·1 to 0·5)	0·2 (0·1 to 0·5)	0·2 (0·1 to 0·4)	0·1 (0 to 0·3)	0·1 (0 to 0·2)	0·1 (0 to 0·2)	0·1 (0 to 0·2)	0·1 (0 to 0·1)	0 (0 to 0·1)	0 (0 to 0·1)	0 (0 to 0)	0 (0 to 0)	0 (0 to 0)	0 (0 to 0)	0 (0 to 0)	0 (0 to 0)	0 (0 to 0)	1·6
Hookworm disease	0 (0 to 0)	0 (0 to 0)	0 (0 to 0)	0·2 (0·1 to 0·3)	0·5 (0·2 to 1)	0·5 (0·2 to 0·9)	1·4 (0·6 to 2·6)	1·8 (0·8 to 3·4)	1·2 (0·5 to 2·2)	0·9 (0·4 to 1·8)	1 (0·5 to 1·8)	0·6 (0·3 to 1·2)	0·2 (0·1 to 0·4)	0·2 (0·1 to 0·4)	0·2 (0·1 to 0·4)	0·1 (0·1 to 0·3)	0·1 (0 to 0·2)	0·1 (0 to 0·1)	0 (0 to 0·1)	0 (0 to 0·1)	0 (0 to 0)	0 (0 to 0)	0 (0 to 0)	9·2
Food-borne trematodiases	0 (0 to 0)	0 (0 to 0)	0 (0 to 0)	0 (0 to 0)	0 (0 to 0)	0 (0 to 0)	0 (0 to 0)	0 (0 to 0)	0 (0 to 0)	0 (0 to 0)	0 (0 to 0)	0 (0 to 0)	0 (0 to 0)	0 (0 to 0)	0 (0 to 0)	0 (0 to 0)	0 (0 to 0)	0 (0 to 0)	0 (0 to 0)	0 (0 to 0)	0 (0 to 0)	0 (0 to 0)	0 (0 to 0)	0
Leprosy	0 (0 to 0)	0 (0 to 0)	0 (0 to 0)	0 (0 to 0)	0 (0 to 0)	0 (0 to 0)	0 (0 to 0)	0 (0 to 0)	0 (0 to 0·1)	0 (0 to 0·1)	0·1 (0 to 0·1)	0·1 (0 to 0·2)	0·1 (0 to 0·2)	0·1 (0 to 0·2)	0·1 (0 to 0·2)	0·1 (0 to 0·2)	0·1 (0 to 0·1)	0·1 (0 to 0·1)	0·1 (0 to 0·1)	0 (0 to 0·1)	0 (0 to 0)	0 (0 to 0)	0 (0 to 0)	0·9
Ebola	0 (0 to 0)	0 (0 to 0)	0 (0 to 0)	0 (0 to 0)	0 (0 to 0)	0 (0 to 0)	0 (0 to 0)	0 (0 to 0)	0 (0 to 0)	0 (0 to 0)	0 (0 to 0)	0 (0 to 0)	0 (0 to 0)	0 (0 to 0)	0 (0 to 0)	0 (0 to 0)	0 (0 to 0)	0 (0 to 0)	0 (0 to 0)	0 (0 to 0)	0 (0 to 0)	0 (0 to 0)	0 (0 to 0)	0
Zika virus	0 (0 to 0)	0 (0 to 0)	0 (0 to 0·1)	0·2 (0 to 0·8)	0·2 (0 to 1)	0·2 (0 to 1)	0·3 (0 to 1·1)	0·3 (0 to 1·2)	0·3 (0 to 1·2)	0·3 (0 to 1·3)	0·3 (0 to 1·2)	0·2 (0 to 1)	0·2 (0 to 0·7)	0·1 (0 to 0·6)	0·1 (0 to 0·4)	0·1 (0 to 0·3)	0 (0 to 0·1)	0 (0 to 0·1)	0 (0 to 0)	0 (0 to 0)	0 (0 to 0)	0 (0 to 0)	0 (0 to 0)	2·9
Guinea worm disease	0 (0 to 0)	0 (0 to 0)	0 (0 to 0)	0 (0 to 0)	0 (0 to 0)	0 (0 to 0)	0 (0 to 0)	0 (0 to 0)	0 (0 to 0)	0 (0 to 0)	0 (0 to 0)	0 (0 to 0)	0 (0 to 0)	0 (0 to 0)	0 (0 to 0)	0 (0 to 0)	0 (0 to 0)	0 (0 to 0)	0 (0 to 0)	0 (0 to 0)	0 (0 to 0)	0 (0 to 0)	0 (0 to 0)	0
Other neglected tropical diseases	0·2 (0·1 to 0·3)	0·3 (0·2 to 0·5)	1·5 (0·8 to 2·6)	6·3 (3·4 to 11)	7·1 (3·4 to 12·8)	3·9 (1·7 to 7)	2·1 (1 to 3·7)	2·2 (1·1 to 3·8)	1·8 (0·9 to 3)	2·2 (1·1 to 3·9)	2·2 (1·1 to 3·9)	2·7 (1·4 to 4·6)	3 (1·6 to 4·7)	2·6 (1·4 to 4·2)	2·3 (1·2 to 3·9)	1·1 (0·6 to 1·8)	0·7 (0·4 to 1·1)	0·9 (0·5 to 1·5)	0·7 (0·4 to 1·3)	0·4 (0·2 to 0·7)	0·4 (0·2 to 0·6)	0·3 (0·1 to 0·4)	0·1 (0 to 0·2)	45
Other infectious diseases	3·8 (2·4 to 5·6)	4·9 (3 to 7·5)	16·2 (10·5 to 24·1)	22·5 (15·5 to 31·7)	16·1 (12·4 to 21·3)	13·6 (10·6 to 17·4)	15·7 (12·5 to 19·8)	16·8 (13·1 to 21·4)	17·9 (14·9 to 22·9)	18·4 (14·2 to 23·4)	20 (15·3 to 25·4)	22·5 (16·9 to 28·9)	26·3 (19·3 to 35·2)	23·4 (17·2 to 30·3)	18·8 (14·2 to 25)	15·2 (11·3 to 20)	10·4 (7·6 to 13·3)	9·6 (7·4 to 12·5)	5·8 (4·5 to 7·4)	4·1 (3·1 to 5·1)	3·3 (2·3 to 4·4)	1·2 (0·8 to 1·6)	0·3 (0·2 to 0·4)	307
Meningitis	2·2 (1·3 to 3·5)	3·3 (1·9 to 5·3)	6·9 (3·8 to 11·8)	7·1 (4·5 to 10·5)	4·2 (3·2 to 5·5)	3·4 (2·6 to 4·5)	4 (3 to 5·4)	3·8 (2·8 to 5·2)	3·9 (3 to 5·2)	4·1 (3 to 5·3)	4·1 (3·1 to 5·4)	4·5 (3·4 to 6)	5·1 (3·7 to 6·9)	4·8 (3·6 to 6·5)	3·9 (2·9 to 5·3)	3·5 (2·6 to 4·7)	2·3 (1·8 to 3·1)	2·1 (1·6 to 2·8)	1·5 (1·1 to 2)	1 (0·8 to 1·4)	0·7 (0·5 to 1)	0·2 (0·2 to 0·3)	0 (0 to 0·1)	76·8
Encephalitis	0·6 (0·3 to 0·9)	0·6 (0·4 to 1·1)	4·5 (2·5 to 8)	9 (5·1 to 15·2)	5·2 (3·5 to 7·5)	4·7 (3·2 to 7)	4·9 (3·4 to 7)	5·2 (3·5 to 7·8)	4·4 (3·1 to 6·3)	4·2 (3 to 5·8)	3·8 (2·8 to 5·3)	4·3 (3·1 to 5·9)	4·2 (3 to 5·8)	3 (2·1 to 4·2)	2·6 (1·9 to 3·8)	2·1 (1·5 to 2·9)	1·4 (1 to 2·1)	1·2 (0·9 to 1·8)	0·7 (0·5 to 1)	0·4 (0·3 to 0·7)	0·2 (0·2 to 0·4)	0·1 (0 to 0·1)	0 (0 to 0)	67·5
Diphtheria	0 (0 to 0)	0 (0 to 0)	0 (0 to 0)	0 (0 to 0)	0 (0 to 0)	0 (0 to 0)	0 (0 to 0)	0 (0 to 0)	0 (0 to 0)	0 (0 to 0)	0 (0 to 0)	0 (0 to 0)	0 (0 to 0)	0 (0 to 0)	0 (0 to 0)	0 (0 to 0)	0 (0 to 0)	0 (0 to 0)	0 (0 to 0)	0 (0 to 0)	0 (0 to 0)	0 (0 to 0)	0 (0 to 0)	0
Tetanus	0·1 (0 to 0·1)	0 (0 to 0·1)	0 (0 to 0·1)	0 (0 to 0·1)	0·1 (0 to 0·4)	0·1 (0 to 0·3)	0·1 (0 to 0·4)	0·2 (0·1 to 0·6)	0·2 (0·1 to 0·5)	0·3 (0·1 to 0·7)	0·3 (0·1 to 0·8)	0·4 (0·1 to 1·1)	0·6 (0·2 to 1·6)	0·8 (0·3 to 2·5)	0·7 (0·2 to 2·1)	0·5 (0·2 to 1·5)	0·4 (0·1 to 1·2)	0·4 (0·2 to 1·2)	0·3 (0·1 to 0·7)	0·2 (0·1 to 0·4)	0·1 (0 to 0·2)	0 (0 to 0·1)	0 (0 to 0)	5·9
Measles	0 (0 to 0)	0 (0 to 0)	0 (0 to 0)	0 (0 to 0)	0 (0 to 0)	0 (0 to 0)	0 (0 to 0)	0 (0 to 0)	0 (0 to 0)	0 (0 to 0)	0 (0 to 0)	0 (0 to 0)	0 (0 to 0)	0 (0 to 0)	0 (0 to 0)	0 (0 to 0)	0 (0 to 0)	0 (0 to 0)	0 (0 to 0)	0 (0 to 0)	0 (0 to 0)	0 (0 to 0)	0 (0 to 0)	0
Varicella and herpes zoster	0·3 (0·1 to 0·6)	0·3 (0·1 to 0·7)	0·7 (0·3 to 1·5)	0·4 (0·2 to 0·8)	0·6 (0·4 to 1·2)	0·7 (0·3 to 1·3)	0·5 (0·3 to 0·8)	0·6 (0·3 to 1)	0·7 (0·4 to 1·1)	0·8 (0·5 to 1·3)	1·1 (0·8 to 1·9)	1 (0·6 to 1·6)	1·3 (0·7 to 2·1)	1·1 (0·6 to 1·8)	0·9 (0·5 to 1·4)	0·9 (0·6 to 1·5)	0·6 (0·3 to 0·9)	0·8 (0·5 to 1·4)	0·5 (0·3 to 0·9)	0·4 (0·2 to 0·7)	0·2 (0·1 to 0·4)	0·1 (0·1 to 0·2)	0 (0 to 0·1)	14·7
Acute hepatitis	0 (0 to 0)	0 (0 to 0)	0 (0 to 0·1)	0·2 (0·1 to 0·4)	0·8 (0·5 to 1·2)	1·1 (0·7 to 1·6)	1·1 (0·7 to 1·7)	1·1 (0·7 to 1·6)	1 (0·7 to 1·4)	0·8 (0·6 to 1·2)	0·8 (0·5 to 1·1)	0·8 (0·5 to 1·1)	0·7 (0·4 to 0·9)	0·6 (0·4 to 0·8)	0·4 (0·3 to 0·5)	0·3 (0·2 to 0·4)	0·2 (0·1 to 0·2)	0·2 (0·1 to 0·2)	0·1 (0·1 to 0·1)	0 (0 to 0·1)	0 (0 to 0·1)	0 (0 to 0)	0 (0 to 0)	10·1
Acute hepatitis A	0 (0 to 0)	0 (0 to 0)	0 (0 to 0)	0·2 (0·1 to 0·3)	0·7 (0·4 to 1·1)	0·9 (0·5 to 1·4)	0·9 (0·6 to 1·4)	0·8 (0·5 to 1·2)	0·6 (0·3 to 1)	0·5 (0·3 to 0·8)	0·4 (0·2 to 0·7)	0·3 (0·2 to 0·6)	0·3 (0·1 to 0·5)	0·2 (0·1 to 0·3)	0·1 (0 to 0·2)	0·1 (0 to 0·1)	0 (0 to 0)	0 (0 to 0)	0 (0 to 0)	0 (0 to 0)	0 (0 to 0)	0 (0 to 0)	0 (0 to 0)	5·9
Acute hepatitis B	0 (0 to 0)	0 (0 to 0)	0 (0 to 0·1)	0 (0 to 0·1)	0 (0 to 0·1)	0 (0 to 0·1)	0·1 (0 to 0·2)	0·2 (0·1 to 0·3)	0·3 (0·2 to 0·4)	0·3 (0·2 to 0·4)	0·3 (0·2 to 0·4)	0·3 (0·2 to 0·5)	0·3 (0·2 to 0·4)	0·3 (0·2 to 0·4)	0·2 (0·2 to 0·3)	0·1 (0·1 to 0·2)	0·1 (0·1 to 0·1)	0·1 (0·1 to 0·1)	0 (0 to 0·1)	0 (0 to 0)	0 (0 to 0)	0 (0 to 0)	0 (0 to 0)	2·8
Acute hepatitis C	0 (0 to 0)	0 (0 to 0)	0 (0 to 0)	0 (0 to 0)	0 (0 to 0)	0 (0 to 0)	0 (0 to 0)	0 (0 to 0)	0 (0 to 0)	0 (0 to 0)	0 (0 to 0)	0 (0 to 0·1)	0 (0 to 0·1)	0 (0 to 0·1)	0 (0 to 0·1)	0 (0 to 0)	0 (0 to 0)	0 (0 to 0)	0 (0 to 0)	0 (0 to 0)	0 (0 to 0)	0 (0 to 0)	0 (0 to 0)	0·4
Acute hepatitis E	0 (0 to 0)	0 (0 to 0)	0 (0 to 0)	0 (0 to 0·1)	0·1 (0 to 0·1)	0·1 (0 to 0·2)	0·1 (0 to 0·2)	0·1 (0 to 0·2)	0·1 (0·1 to 0·2)	0·1 (0 to 0·2)	0·1 (0 to 0·2)	0·1 (0 to 0·1)	0·1 (0 to 0·1)	0 (0 to 0·1)	0 (0 to 0·1)	0 (0 to 0·1)	0 (0 to 0)	0 (0 to 0)	0 (0 to 0)	0 (0 to 0)	0 (0 to 0)	0 (0 to 0)	0 (0 to 0)	0·9
Other unspecified infectious diseases	0·6 (0·3 to 1)	0·6 (0·4 to 1)	2·8 (1·6 to 4·6)	4·9 (2·9 to 7·7)	5 (2·7 to 9)	3·6 (2·2 to 5·8)	5 (3·2 to 7·3)	5·9 (3·7 to 8·9)	7·6 (4·4 to 11·4)	8·2 (4·8 to 12·3)	9·8 (6·2 to 14·5)	11·4 (7 to 17)	14·6 (8·8 to 21·9)	13·1 (8·1 to 19·4)	10·3 (6·5 to 15)	7·9 (4·8 to 11·6)	5·5 (3·3 to 7·7)	4·9 (2·9 to 7·1)	2·8 (1·6 to 4)	2 (1·1 to 2·8)	2 (1·1 to 2·9)	0·7 (0·3 to 1·1)	0·1 (0·1 to 0·2)	129
Maternal and neonatal disorders	1,140 (777 to 1,610)	208 (142 to 296)	55·8 (37 to 82·5)	45·8 (35·4 to 58·5)	40 (29·7 to 51·5)	45·4 (33·3 to 58·6)	59·4 (46·7 to 75·1)	67·7 (54·3 to 83·7)	69·9 (55·8 to 85·9)	62·9 (50·2 to 76·6)	57·7 (45·7 to 71·4)	47·2 (36·9 to 59·1)	38·8 (29·4 to 50·1)	31·2 (23 to 40·5)	22·7 (16·6 to 29·6)	14·5 (10·6 to 19)	7·8 (5·9 to 10·6)	4·3 (3 to 5·8)	1·7 (1·2 to 2·5)	0·5 (0·3 to 0·8)	0·1 (0·1 to 0·2)	0 (0 to 0)	0 (0 to 0)	2,020
Maternal disorders	0 (0 to 0)	0 (0 to 0)	0 (0 to 0)	0 (0 to 0)	0 (0 to 0)	0·4 (0·3 to 0·5)	11·1 (8·6 to 13·9)	19·4 (15 to 25·1)	22·9 (17·5 to 29·3)	21·3 (16·4 to 27·1)	15·9 (12·3 to 20·2)	8·4 (6·5 to 10·7)	2·3 (1·7 to 3·1)	0·3 (0·2 to 0·3)	0 (0 to 0)	0 (0 to 0)	0 (0 to 0)	0 (0 to 0)	0 (0 to 0)	0 (0 to 0)	0 (0 to 0)	0 (0 to 0)	0 (0 to 0)	102
Maternal hemorrhage	0 (0 to 0)	0 (0 to 0)	0 (0 to 0)	0 (0 to 0)	0 (0 to 0)	0 (0 to 0)	1·4 (0·9 to 2·1)	2·8 (1·7 to 4·2)	3·7 (2·2 to 5·5)	3·5 (2·2 to 5·3)	2·6 (1·6 to 3·9)	1·3 (0·8 to 1·9)	0·2 (0·1 to 0·3)	0 (0 to 0)	0 (0 to 0)	0 (0 to 0)	0 (0 to 0)	0 (0 to 0)	0 (0 to 0)	0 (0 to 0)	0 (0 to 0)	0 (0 to 0)	0 (0 to 0)	15·6
Maternal sepsis and other maternal infections	0 (0 to 0)	0 (0 to 0)	0 (0 to 0)	0 (0 to 0)	0 (0 to 0)	0 (0 to 0)	0·4 (0·2 to 0·7)	0·7 (0·4 to 1·1)	0·7 (0·4 to 1·1)	0·6 (0·3 to 1)	0·4 (0·2 to 0·6)	0·2 (0·1 to 0·3)	0 (0 to 0)	0 (0 to 0)	0 (0 to 0)	0 (0 to 0)	0 (0 to 0)	0 (0 to 0)	0 (0 to 0)	0 (0 to 0)	0 (0 to 0)	0 (0 to 0)	0 (0 to 0)	3
Maternal hypertensive disorders	0 (0 to 0)	0 (0 to 0)	0 (0 to 0)	0 (0 to 0)	0 (0 to 0)	0·1 (0 to 0·1)	1·5 (1 to 2·1)	2 (1·4 to 2·9)	2·5 (1·6 to 3·4)	2 (1·3 to 2·8)	1·3 (0·9 to 1·8)	0·6 (0·4 to 0·9)	0·1 (0·1 to 0·2)	0 (0 to 0)	0 (0 to 0)	0 (0 to 0)	0 (0 to 0)	0 (0 to 0)	0 (0 to 0)	0 (0 to 0)	0 (0 to 0)	0 (0 to 0)	0 (0 to 0)	10·1
Maternal obstructed labor and uterine rupture	0 (0 to 0)	0 (0 to 0)	0 (0 to 0)	0 (0 to 0)	0 (0 to 0)	0 (0 to 0)	0·6 (0·3 to 1·1)	1·2 (0·6 to 2·1)	1·5 (0·8 to 2·7)	1·5 (0·7 to 2·7)	1·3 (0·7 to 2·4)	0·6 (0·3 to 1·1)	0·2 (0·1 to 0·4)	0 (0 to 0)	0 (0 to 0)	0 (0 to 0)	0 (0 to 0)	0 (0 to 0)	0 (0 to 0)	0 (0 to 0)	0 (0 to 0)	0 (0 to 0)	0 (0 to 0)	7
Maternal abortion and miscarriage	0 (0 to 0)	0 (0 to 0)	0 (0 to 0)	0 (0 to 0)	0 (0 to 0)	0 (0 to 0)	0·5 (0·2 to 0·8)	0·8 (0·4 to 1·4)	0·8 (0·4 to 1·4)	0·6 (0·3 to 1·2)	0·5 (0·2 to 0·9)	0·3 (0·2 to 0·6)	0·1 (0·1 to 0·2)	0 (0 to 0)	0 (0 to 0)	0 (0 to 0)	0 (0 to 0)	0 (0 to 0)	0 (0 to 0)	0 (0 to 0)	0 (0 to 0)	0 (0 to 0)	0 (0 to 0)	3·7
Ectopic pregnancy	0 (0 to 0)	0 (0 to 0)	0 (0 to 0)	0 (0 to 0)	0 (0 to 0)	0 (0 to 0)	0·1 (0 to 0·1)	0·2 (0·1 to 0·3)	0·2 (0·1 to 0·3)	0·2 (0·1 to 0·3)	0·1 (0·1 to 0·2)	0·1 (0 to 0·1)	0 (0 to 0)	0 (0 to 0)	0 (0 to 0)	0 (0 to 0)	0 (0 to 0)	0 (0 to 0)	0 (0 to 0)	0 (0 to 0)	0 (0 to 0)	0 (0 to 0)	0 (0 to 0)	0·9
Indirect maternal deaths	--	--	--	--	--	0·1 (0 to 0·1)	[illegible] (0·8 to 2·2)	2·8 (1·5 to 4)	2·8 (1·6 to 4·4)	2·6 (1·2 to 3·5)	1·1 (0·7 to 2·2)	0·5 (0·3 to 0·9)	0·1 (0·1 to 0·2)	0 (0 to 0)	--	--	--	--	--	--	--	--	--	NA
Late maternal deaths	--	--	--	--	--	0 (0 to 0·1)	0·9 (0·5 to 1·3)	1·5 (0·9 to 2·3)	1·7 (1 to 2·8)	1·5 (0·9 to 2·5)	1·1 (0·7 to 1·8)	0·6 (0·3 to 0·9)	0·2 (0·1 to 0·3)	0 (0 to 0)	--	--	--	--	--	--	--	--	--	NA
Maternal deaths aggravated by HIV/AIDS	--	--	--	--	--	0 (0 to 0)	0 (0 to 0)	0 (0 to 0)	0 (0 to 0)	0 (0 to 0)	0 (0 to 0)	0 (0 to 0)	0 (0 to 0)	0 (0 to 0)	--	--	--	--	--	--	--	--	--	NA
Neonatal disorders	1,140 (777 to 1,610)	208 (142 to 296)	55·8 (37 to 82·5)	45·8 (35·4 to 58·5)	40 (29·7 to 51·5)	45 (32·9 to 58·2)	48·3 (36·2 to 63·3)	48·3 (36 to 62·7)	47·1 (34·2 to 61·5)	41·6 (30·9 to 53·7)	41·9 (31 to 53·6)	38·8 (28·8 to 50·7)	36·5 (27·2 to 47·8)	31 (22·7 to 40·2)	22·7 (16·6 to 29·6)	14·5 (10·6 to 19)	7·8 (5·9 to 10·6)	4·3 (3 to 5·8)	1·7 (1·2 to 2·5)	0·5 (0·3 to 0·8)	0·1 (0·1 to 0·2)	0 (0 to 0)	0 (0 to 0)	1,920
Neonatal preterm birth	601 (405 to 859)	84·6 (53·8 to 127)	23·7 (15·3 to 35·5)	15·3 (11·4 to 20)	19·1 (13·6 to 25·4)	21·2 (15·3 to 28·4)	22·6 (16 to 30·2)	22·1 (16·3 to 29·4)	21·3 (15·3 to 28·4)	18·6 (13·5 to 24·9)	18·3 (13·4 to 24·8)	16·7 (12·4 to 22·2)	15·2 (11 to 20·4)	12·4 (8·9 to 16·7)	8·6 (6·2 to 11·5)	5 (3·6 to 6·7)	2·5 (1·8 to 3·3)	1·3 (1 to 1·7)	0·5 (0·3 to 0·7)	0·1 (0·1 to 0·2)	0 (0 to 0·1)	0 (0 to 0)	0 (0 to 0)	930
Neonatal encephalopathy due to birth asphyxia and trauma	271 (171 to 407)	30 (18 to 46·5)	9·4 (5·3 to 15·2)	6·4 (4·2 to 9·3)	4·7 (2·8 to 7·2)	5·3 (3·2 to 8·4)	5·8 (3·3 to 8·7)	5·9 (3·4 to 9·1)	5·7 (3·3 to 9)	5·1 (2·9 to 7·9)	5·2 (3 to 8)	4·9 (2·9 to 7·4)	4·7 (2·7 to 7·4)	4·2 (2·4 to 6·4)	3·1 (1·7 to 4·9)	2·1 (1·2 to 3·3)	1·1 (0·6 to 1·6)	0·4 (0·3 to 0·7)	0·1 (0·1 to 0·2)	0 (0 to 0)	0 (0 to 0)	0 (0 to 0)	0 (0 to 0)	375
Neonatal sepsis and other neonatal infections	100 (59·1 to 165)	57·8 (36·1 to 87·4)	11·6 (7·7 to 17·3)	10·3 (6·7 to 14·9)	14·4 (9·2 to 21·1)	16·3 (10·2 to 24·1)	17·6 (11·2 to 26·4)	17·9 (11·2 to 27·2)	17·7 (10·8 to 26·8)	15·8 (10 to 23·5)	16·2 (10·1 to 23·6)	15·1 (9·4 to 22·8)	14·6 (9·3 to 21·5)	12·8 (8·1 to 18·9)	9·7 (5·9 to 14·5)	6·5 (4·1 to 9·9)	3·8 (2·3 to 6)	2·3 (1·3 to 3·6)	1 (0·5 to 1·7)	0·3 (0·2 to 0·6)	0·1 (0 to 0·1)	0 (0 to 0)	0 (0 to 0)	362
Hemolytic disease and other neonatal jaundice	2·3 (1·1 to 4·1)	0·5 (0·3 to 0·8)	0·4 (0·3 to 0·5)	1·4 (0·9 to 2·1)	1·9 (1·2 to 2·8)	2·1 (1·4 to 3)	2·4 (1·8 to 3·1)	2·4 (1·8 to 3·1)	2·4 (1·8 to 3·1)	2·1 (1·6 to 2·8)	2·2 (1·6 to 2·8)	2·1 (1·5 to 2·7)	2 (1·5 to 2·6)	1·8 (1·3 to 2·3)	1·3 (1 to 1·7)	0·9 (0·7 to 1·1)	0·5 (0·4 to 0·7)	0·3 (0·2 to 0·4)	0·1 (0·1 to 0·2)	0 (0 to 0·1)	0 (0 to 0)	0 (0 to 0)	0 (0 to 0)	29·2
Other neonatal disorders	163 (97·4 to 258)	35·3 (22·6 to 52·3)	10·6 (7·1 to 15·8)	12·3 (9·4 to 15·8)	0 (0 to 0)	0 (0 to 0)	0 (0 to 0)	0 (0 to 0)	0 (0 to 0)	0 (0 to 0)	0 (0 to 0)	0 (0 to 0)	0 (0 to 0)	0 (0 to 0)	0 (0 to 0)	0 (0 to 0)	0 (0 to 0)	0 (0 to 0)	0 (0 to 0)	0 (0 to 0)	0 (0 to 0)	0 (0 to 0)	0 (0 to 0)	221
Nutritional deficiencies	0·1 (0 to 0·1)	1·6 (0·9 to 2·6)	26·4 (17·9 to 38·9)	58·1 (32·9 to 97·5)	68·8 (32·1 to 132)	40·1 (18·3 to 71·4)	44·6 (21 to 78·9)	38·7 (18·8 to 70·6)	39·1 (20·3 to 69)	39·4 (19·1 to 71·3)	50·2 (26·2 to 87·1)	55 (27·6 to 94·5)	55·8 (29·7 to 92·7)	58·5 (33 to 93·7)	50·4 (28·8 to 82·4)	34·9 (20·6 to 55·1)	20·1 (13·1 to 30·4)	20·4 (13·5 to 30·4)	16·5 (11·5 to 23)	13·9 (9·6 to 19·6)	11·6 (8·1 to 16·1)	7·2 (5·2 to 9·5)	2·3 (1·6 to 3·1)	754
Protein-energy malnutrition	0·1 (0 to 0·1)	0·2 (0·1 to 0·3)	15·3 (9·8 to 24)	15·1 (8·2 to 24·4)	5·4 (3·6 to 7·8)	3·5 (2·3 to 4·9)	4 (2·7 to 5·5)	4·9 (3·3 to 6·8)	5·6 (3·7 to 7·8)	5·8 (4·3 to 7·6)	7·2 (5·2 to 9·6)	7·7 (5·5 to 10·3)	9·2 (6·4 to 12·6)	10 (7·1 to 13·8)	9·8 (7 to 13·2)	9·9 (7·3 to 13·5)	8·6 (6·3 to 11·6)	10·8 (7·3 to 15·4)	9·3 (6·6 to 12·8)	8·3 (5·8 to 11·7)	7·3 (5 to 10·3)	5·1 (3·4 to 7·1)	1·8 (1·3 to 2·6)	165
Iodine deficiency	0 (0 to 0)	0 (0 to 0)	0 (0 to 0)	0 (0 to 0)	0·1 (0 to 0·2)	0·3 (0·1 to 0·5)	0·4 (0·2 to 0·7)	0·5 (0·2 to 0·8)	0·5 (0·2 to 0·9)	0·5 (0·3 to 0·9)	0·5 (0·3 to 0·9)	0·5 (0·3 to 0·9)	0·5 (0·3 to 0·8)	0·5 (0·2 to 0·8)	0·4 (0·2 to 0·6)	0·3 (0·1 to 0·4)	0·2 (0·1 to 0·3)	0·1 (0 to 0·2)	0·1 (0 to 0·1)	0 (0 to 0)	0 (0 to 0)	0 (0 to 0)	0 (0 to 0)	5·3
Vitamin A deficiency	0 (0 to 0)	0·1 (0 to 0·1)	0·3 (0·1 to 0·5)	1·4 (0·7 to 2·4)	1·9 (0·9 to 3·4)	1·3 (0·6 to 2·3)	0·5 (0·2 to 1)	0·5 (0·2 to 0·8)	0·4 (0·2 to 0·7)	0·3 (0·2 to 0·6)	0·3 (0·2 to 0·5)	0·3 (0·1 to 0·5)	0·3 (0·1 to 0·4)	0·2 (0·1 to 0·4)	0·2 (0·1 to 0·3)	0·1 (0 to 0·2)	0·1 (0 to 0·2)	0·1 (0 to 0·2)	0·1 (0 to 0·1)	0 (0 to 0·1)	0 (0 to 0)	0 (0 to 0)	0 (0 to 0)	8·3
Dietary iron deficiency	0 (0 to 0)	1·4 (0·7 to 2·2)	10·3 (4·9 to 19)	40·8 (17·5 to 79·6)	60·7 (24·5 to 121)	34·4 (13 to 65·4)	38·3 (15·4 to 72·1)	31·2 (12·1 to 61·9)	30·5 (12·4 to 59·6)	31·1 (11·8 to 62)	40·3 (17·1 to 76·7)	44·3 (17·8 to 82·3)	43·3 (18·6 to 79·9)	44·8 (20·2 to 79·1)	37·7 (16·6 to 69·8)	22·5 (9·2 to 43)	9·3 (3·3 to 19·1)	7·4 (2·5 to 15·1)	5·5 (2·1 to 11·5)	4·1 (1·4 to 9)	3 (1·1 to 5·8)	1·3 (0·5 to 2·4)	0·2 (0·1 to 0·4)	542
Other nutritional deficiencies	0 (0 to 0)	0 (0 to 0)	0·5 (0·3 to 1)	0·8 (0·3 to 1·8)	0·6 (0·4 to 1·1)	0·6 (0·4 to 1)	1·4 (0·9 to 2·1)	1·7 (1 to 2·6)	2·1 (1·4 to 3·2)	1·6 (1·1 to 2·4)	1·9 (1·2 to 2·7)	2·2 (1·4 to 3·2)	2·5 (1·6 to 3·8)	3 (1·9 to 4·2)	2·4 (1·6 to 3·5)	2·2 (1·4 to 3·1)	2 (1·3 to 2·9)	1·8 (1·2 to 3)	1·5 (0·9 to 2·3)	1·4 (0·8 to 2·2)	1·2 (0·6 to 2·2)	0·9 (0·4 to 1·6)	0·2 (0·1 to 0·4)	32·9
Non-communicable diseases	169 (110 to 253)	63 (42·1 to 90·3)	146 (102 to 205)	314 (238 to 405)	380 (298 to 481)	601 (453 to 785)	972 (745 to 1,240)	1,240 (977 to 1,540)	1,510 (1,220 to 1,860)	1,670 (1,360 to 2,040)	2,090 (1,730 to 2,520)	2,670 (2,250 to 3,170)	3,480 (2,940 to 4,100)	4,070 (3,440 to 4,770)	4,260 (3,630 to 4,960)	4,200 (3,600 to 4,830)	3,560 (3,090 to 4,090)	3,740 (3,270 to 4,250)	2,940 (2,590 to 3,320)	2,400 (2,140 to 2,670)	1,640 (1,480 to 1,800)	847 (785 to 915)	214 (200 to 229)	43,200
Neoplasms	1·6 (1 to 2·8)	1·1 (0·7 to 1·7)	4·6 (2·9 to 7)	40·1 (26·1 to 59·8)	43 (35·2 to 52·1)	40·5 (32·5 to 50·5)	57·6 (46·4 to 71·3)	81·9 (66·5 to 101)	116 (94·9 to 141)	167 (137 to 201)	254 (209 to 307)	418 (343 to 511)	635 (512 to 785)	797 (655 to 974)	869 (709 to 1,060)	871 (710 to 1,040)	742 (619 to 886)	794 (660 to 946)	583 (493 to 699)	419 (350 to 497)	264 (220 to 310)	110 (90·1 to 127)	20 (16·5 to 23)	7,330
Lip and oral cavity cancer	0 (0 to 0)	0 (0 to 0)	0 (0 to 0)	0 (0 to 0)	0·1 (0·1 to 0·2)	0·2 (0·1 to 0·3)	0·4 (0·3 to 0·5)	0·7 (0·5 to 0·9)	1·1 (0·8 to 1·4)	1·9 (1·4 to 2·6)	3·5 (2·5 to 4·7)	8·1 (5·8 to 10·8)	15·5 (11·4 to 21·5)	21·8 (16 to 29·4)	23·9 (17·7 to 31·8)	21·8 (15·5 to 29·1)	15·4 (11·8 to 19·9)	15·2 (11·5 to 19·8)	10·2 (7·5 to 13·2)	6·8 (5·3 to 8·6)	3·5 (2·8 to 4·3)	1·6 (1·3 to 1·9)	0·4 (0·3 to 0·4)	152
Nasopharynx cancer	0 (0 to 0)	0 (0 to 0)	0 (0 to 0)	0 (0 to 0)	0·2 (0·2 to 0·3)	0·4 (0·3 to 0·5)	0·7 (0·5 to 1)	0·9 (0·7 to 1·3)	1·1 (0·8 to 1·6)	1·1 (0·8 to 1·5)	1·5 (1 to 2)	3·1 (2·2 to 4·4)	5 (3·5 to 7)	6·7 (4·7 to 9·2)	7 (5 to 9·5)	5·2 (3·7 to 7·1)	3·6 (2·6 to 4·8)	3·3 (2·3 to 4·5)	1·6 (1·2 to 2·2)	1·1 (0·8 to 1·4)	1 (0·7 to 1·4)	0·4 (0·3 to 0·5)	0·1 (0·1 to 0·1)	44
Other pharynx cancer	0 (0 to 0)	0 (0 to 0)	0 (0 to 0)	0 (0 to 0)	0 (0 to 0)	0 (0 to 0)	0 (0 to 0)	0·3 (0·2 to 0·4)	0·4 (0·3 to 0·5)	0·5 (0·4 to 0·7)	1·1 (0·8 to 1·6)	3 (2 to 4·2)	7·3 (5·1 to 10·2)	10 (7·1 to 14)	11·2 (7·9 to 15·3)	9·8 (6·8 to 13·4)	6·5 (4·5 to 8·9)	5·7 (4 to 7·9)	3·1 (2·2 to 4·2)	1·9 (1·4 to 2·6)	1·1 (0·8 to 1·4)	0·4 (0·3 to 0·6)	0·1 (0·1 to 0·2)	62·3
Esophageal cancer	0 (0 to 0)	0 (0 to 0)	0 (0 to 0)	0 (0 to 0)	0 (0 to 0)	0 (0 to 0)	0 (0 to 0)	0·4 (0·3 to 0·6)	0·7 (0·5 to 1)	1·5 (1 to 2·1)	4·1 (2·8 to 5·6)	11·5 (8·2 to 15·5)	25·1 (18·1 to 33·7)	35 (26 to 46·8)	40·1 (28·6 to 54)	37·3 (26·6 to 49·3)	26·2 (18·9 to 34·9)	25·3 (18·1 to 33·3)	15·2 (10·9 to 20·5)	9·8 (7·1 to 13·3)	5·4 (4 to 7)	2·1 (1·6 to 2·6)	0·3 (0·2 to 0·4)	240
Stomach cancer	0 (0 to 0)	0 (0 to 0)	0 (0 to 0)	0 (0 to 0)	0 (0 to 0)	0 (0 to 0)	1 (0·8 to 1·2)	2·2 (1·6 to 2·8)	5·5 (3·9 to 7·9)	10·9 (7·8 to 14·7)	17·4 (12·6 to 23·3)	32·1 (23·6 to 42·5)	47·4 (34·3 to 64·2)	64·7 (47·4 to 84·3)	73·8 (56·1 to 96·7)	77·1 (57·4 to 101)	62·9 (46·8 to 81·4)	68·1 (50·7 to 89·1)	48·5 (38·1 to 60·5)	33·7 (25·6 to 44·1)	21·3 (16·4 to 26·9)	8·9 (7 to 10·8)	1·6 (1·4 to 1·8)	577
Colon and rectum cancer	0 (0 to 0)	0 (0 to 0)	0 (0 to 0)	0 (0 to 0)	0·3 (0·2 to 0·4)	0·3 (0·2 to 0·4)	1·3 (1 to 1·7)	2·9 (2·1 to 3·8)	5·4 (3·9 to 7·5)	10·2 (7·7 to 13·1)	15·7 (11·8 to 20·2)	27·1 (20·9 to 34·7)	42·2 (32·2 to 54·5)	58·1 (44·8 to 74·4)	67·6 (51·6 to 88·5)	72·8 (55·7 to 93·3)	62·6 (47·9 to 81·4)	67·1 (52·5 to 84·2)	50·4 (39·1 to 62·9)	37·1 (29·2 to 46·4)	24·3 (19·3 to 29·2)	11·1 (8·8 to 13·2)	2·1 (1·7 to 2·5)	559
Liver cancer	0 (0 to 0)	0 (0 to 0)	0·1 (0·1 to 0·2)	0·4 (0·3 to 0·7)	0·4 (0·3 to 0·5)	0·5 (0·4 to 0·7)	0·3 (0·2 to 0·4)	0·8 (0·5 to 1)	1·3 (0·9 to 1·9)	1·7 (1·2 to 2·4)	3 (2·2 to 4·1)	5·4 (3·9 to 7·3)	8·7 (6·3 to 11·9)	12 (8·5 to 16·2)	15 (11 to 20)	15·8 (11·9 to 20·7)	14 (10·4 to 18·3)	14·9 (10·9 to 20)	10·2 (7·6 to 13·3)	6·1 (4·8 to 7·9)	2·7 (2 to 3·4)	1 (0·8 to 1·2)	0·2 (0·2 to 0·3)	115
Liver cancer due to hepatitis B	0 (0 to 0)	0 (0 to 0)	0 (0 to 0)	0 (0 to 0)	0 (0 to 0)	0·1 (0·1 to 0·2)	0·1 (0·1 to 0·2)	0·4 (0·3 to 0·6)	0·8 (0·5 to 1·2)	1 (0·6 to 1·5)	1·6 (0·9 to 2·4)	2·6 (1·5 to 3·9)	3·6 (2·1 to 5·7)	4·4 (2·4 to 7·4)	5·1 (2·8 to 7·9)	4·7 (2·4 to 7·5)	3·2 (1·6 to 5·5)	2·9 (1·4 to 5)	1·7 (0·9 to 3)	0·8 (0·4 to 1·6)	0·3 (0·2 to 0·6)	0·1 (0·1 to 0·2)	0 (0 to 0)	33·4
Liver cancer due to hepatitis C	0 (0 to 0)	0 (0 to 0)	0 (0 to 0)	0 (0 to 0)	0 (0 to 0)	0 (0 to 0)	0 (0 to 0)	0 (0 to 0)	0 (0 to 0·1)	0·1 (0 to 0·1)	0·2 (0·1 to 0·4)	0·4 (0·2 to 0·8)	0·9 (0·5 to 1·7)	1·7 (0·8 to 2·9)	2·5 (1·2 to 4·3)	2·8 (1·4 to 5)	2·7 (1·5 to 4·4)	3 (1·7 to 4·9)	2·5 (1·4 to 4·2)	1·9 (1·1 to 2·9)	1 (0·7 to 1·5)	0·4 (0·3 to 0·7)	0·1 (0·1 to 0·2)	20·3
Liver cancer due to alcohol use	0 (0 to 0)	0 (0 to 0)	0 (0 to 0)	0 (0 to 0)	0 (0 to 0)	0 (0 to 0)	0 (0 to 0)	0·1 (0 to 0·1)	0·2 (0·1 to 0·4)	0·4 (0·2 to 0·6)	0·8 (0·4 to 1·4)	2·2 (0·8 to 2·7)	2·9 (1·4 to 4·9)	4·8 (2·2 to 6·8)	6·1 (3 to 9)	6·6 (3·9 to 9·9)	6·6 (4 to 9·7)	7·2 (4·3 to 10·5)	4·4 (2·8 to 6·2)	2·2 (1·4 to 3·3)	0·8 (0·5 to 1·2)	0·2 (0·1 to 0·4)	0 (0 to 0·1)	43·8
Liver cancer due to NASH	0 (0 to 0)	0 (0 to 0)	0 (0 to 0)	0 (0 to 0)	0 (0 to 0)	0·1 (0 to 0·1)	0·1 (0 to 0·1)	0·2 (0·1 to 0·2)	0·2 (0·1 to 0·2)	0·1 (0·1 to 0·2)	0·2 (0·1 to 0·3)	0·4 (0·2 to 0·6)	0·6 (0·3 to 1·1)	0·9 (0·4 to 1·5)	1·1 (0·6 to 1·9)	1·1 (0·6 to 2)	1·1 (0·6 to 1·9)	1·3 (0·7 to 2·2)	1 (0·6 to 1·8)	0·8 (0·4 to 1·3)	0·4 (0·2 to 0·6)	0·1 (0·1 to 0·2)	0 (0 to 0·1)	9·4
Liver cancer due to other causes	0 (0 to 0)	0 (0 to 0)	0·1 (0·1 to 0·2)	0·4 (0·3 to 0·7)	0·4 (0·3 to 0·5)	0·4 (0·3 to 0·6)	0·1 (0 to 0·1)	0·1 (0·1 to 0·2)	0·1 (0·1 to 0·2)	0·1 (0·1 to 0·3)	0·2 (0·1 to 0·3)	0·3 (0·2 to 0·5)	0·5 (0·2 to 0·7)	0·6 (0·3 to 1·1)	0·6 (0·4 to 1·4)	0·6 (0·4 to 1·5)	0·6 (0·4 to 1·3)	0·6 (0·3 to 1)	0·4 (0·2 to 0·8)	0·2 (0·1 to 0·5)	0·1 (0 to 0·2)	0·1 (0 to 0·1)	0 (0 to 0)	7·7
Gallbladder and biliary tract cancer	0 (0 to 0)	0 (0 to 0)	0 (0 to 0)	0 (0 to 0)	0 (0 to 0)	0 (0 to 0)	0 (0 to 0)	0·2 (0·1 to 0·2)	0·3 (0·2 to 0·4)	0·5 (0·4 to 0·8)	1·1 (0·8 to 1·7)	2·3 (1·6 to 3·8)	4 (2·8 to 6·6)	6 (4·2 to 9·8)	7·1 (5 to 11·7)	7·4 (5·4 to 12·4)	6·4 (4·7 to 10·8)	6·5 (4·8 to 11)	4·5 (3·4 to 7·6)	3 (2·3 to 4·9)	1·7 (1·2 to 2·8)	0·7 (0·5 to 1·1)	0·1 (0·1 to 0·2)	51·8
Pancreatic cancer	0 (0 to 0)	0 (0 to 0)	0 (0 to 0)	0 (0 to 0)	0 (0 to 0)	0 (0 to 0)	0·5 (0·4 to 0·6)	0·7 (0·5 to 0·9)	1·6 (1·1 to 2·3)	4·1 (2·7 to 5·9)	7·9 (5·3 to 11)	18·3 (13 to 24·3)	31·6 (23 to 42·8)	42·3 (31·2 to 55·9)	50·6 (37·7 to 66·9)	52·9 (39 to 70·3)	44·4 (32·5 to 59·8)	45·2 (32·4 to 59·4)	30 (21·8 to 39·7)	21·1 (15·6 to 27·7)	12·9 (9·9 to 16·5)	5·2 (4 to 6·4)	1 (0·8 to 1·2)	370
Larynx cancer	0 (0 to 0)	0 (0 to 0)	0 (0 to 0)	0 (0 to 0)	0 (0 to 0)	0 (0 to 0)	0 (0 to 0)	0·4 (0·3 to 0·5)	0·3 (0·3 to 0·4)	0·6 (0·4 to 0·7)	1·5 (1·1 to 2)	4·3 (3·2 to 5·6)	10·5 (7·3 to 14·9)	17 (12·6 to 22·5)	19·5 (14·4 to 25·7)	19·1 (14·6 to 23·9)	14 (10·6 to 18)	13·4 (10·3 to 17·2)	7·9 (6 to 10·3)	4·7 (3·5 to 6)	2·2 (1·6 to 2·8)	0·8 (0·6 to 1)	0·1 (0·1 to 0·1)	116
Tracheal, bronchus, and lung cancer	0 (0 to 0)	0 (0 to 0)	0 (0 to 0)	0 (0 to 0)	0 (0 to 0)	0·2 (0·1 to 0·3)	1·1 (0·8 to 1·3)	1·5 (1·1 to 2)	2·4 (1·7 to 3·3)	5·8 (4·3 to 7·6)	12 (8·8 to 15·9)	27·3 (21 to 35·4)	56·7 (42·5 to 76·2)	82 (61·5 to 109)	96·4 (71·3 to 127)	97·1 (72·4 to 123)	78·1 (59·6 to 99·6)	77·8 (61·5 to 98·7)	47·2 (37·5 to 59·6)	28·8 (22·2 to 36·8)	15·6 (12·1 to 19·6)	5·6 (4·4 to 6·7)	1 (0·8 to 1·2)	636
Malignant skin melanoma	0 (0 to 0)	0 (0 to 0)	0 (0 to 0·1)	0·3 (0·1 to 0·8)	0·1 (0·1 to 0·2)	0·1 (0·1 to 0·2)	0·3 (0·2 to 0·4)	0·8 (0·5 to 1·2)	1·3 (0·9 to 1·8)	1·7 (1·2 to 2·5)	3·3 (2·3 to 4·7)	3·8 (2·7 to 5·3)	4·7 (3·3 to 6·7)	5·2 (3·6 to 7·5)	5·5 (3·5 to 7·2)	4 (2·7 to 5·6)	3·1 (2·2 to 4·6)	3·2 (2·3 to 4·6)	2·1 (1·5 to 3)	1·7 (1·2 to 2·5)	1·2 (0·9 to 1·8)	0·5 (0·4 to 0·7)	0·2 (0·1 to 0·2)	42·5
Non-melanoma skin cancer	0 (0 to 0)	0 (0 to 0)	0 (0 to 0)	0 (0 to 0)	0 (0 to 0)	0 (0 to 0)	0 (0 to 0)	0·1 (0·1 to 0·2)	0·4 (0·2 to 0·5)	0·5 (0·4 to 0·7)	0·6 (0·4 to 0·8)	1·1 (0·8 to 1·5)	1·3 (1 to 1·8)	1·8 (1·4 to 2·4)	1·9 (1·4 to 2·5)	1·9 (1·5 to 2·5)	1·8 (1·3 to 2·3)	1·9 (1·4 to 2·5)	1·7 (1·3 to 2·1)	1·6 (1·2 to 2)	1·3 (1 to 1·6)	0·9 (0·7 to 1·1)	0·3 (0·2 to 0·4)	19·1
Non-melanoma skin cancer (squamous-cell carcinoma)	0 (0 to 0)	0 (0 to 0)	0 (0 to 0)	0 (0 to 0)	0 (0 to 0)	0 (0 to 0)	0 (0 to 0)	0·1 (0·1 to 0·2)	0·4 (0·2 to 0·5)	0·5 (0·4 to 0·7)	0·6 (0·4 to 0·8)	1·1 (0·8 to 1·5)	1·3 (1 to 1·8)	1·8 (1·4 to 2·4)	1·9 (1·4 to 2·5)	1·9 (1·5 to 2·5)	1·8 (1·3 to 2·3)	1·9 (1·4 to 2·5)	1·7 (1·3 to 2·1)	1·6 (1·2 to 2)	1·3 (1 to 1·6)	0·9 (0·7 to 1·1)	0·3 (0·2 to 0·4)	19·1
Non-melanoma skin cancer (basal-cell carcinoma)	0 (0 to 0)	0 (0 to 0)	0 (0 to 0)	0 (0 to 0)	0 (0 to 0)	0 (0 to 0)	0 (0 to 0)	0 (0 to 0)	0 (0 to 0)	0 (0 to 0)	0 (0 to 0)	0 (0 to 0)	0 (0 to 0)	0 (0 to 0)	0 (0 to 0)	0 (0 to 0)	0 (0 to 0)	0 (0 to 0)	0 (0 to 0)	0 (0 to 0)	0 (0 to 0)	0 (0 to 0)	0 (0 to 0)	0
Breast cancer	0 (0 to 0)	0 (0 to 0)	0 (0 to 0)	0 (0 to 0)	0 (0 to 0)	0 (0 to 0)	0·7 (0·5 to 0·8)	1·4 (1 to 2)	6·8 (4·6 to 9·8)	22·1 (16·2 to 29·9)	43·1 (32·4 to 56·2)	76·2 (56·6 to 102)	106 (76·1 to 143)	121 (87 to 163)	105 (76·4 to 139)	77·8 (57·1 to 102)	55·2 (42·4 to 72·8)	43·6 (32·8 to 57·1)	30·7 (22·8 to 40·8)	23·2 (17 to 30·5)	17·5 (13·3 to 22·3)	8·4 (6·5 to 10·4)	2·2 (1·7 to 2·8)	740
Cervical cancer	0 (0 to 0)	0 (0 to 0)	0 (0 to 0)	0 (0 to 0)	0 (0 to 0)	0 (0 to 0)	0·8 (0·6 to 1·1)	3·4 (2·2 to 4·9)	11·3 (7·1 to 16·4)	23·7 (15·9 to 33·7)	42·2 (29·6 to 57·7)	58·8 (43·5 to 77·7)	74·4 (52·3 to 102)	64·4 (47 to 84·7)	56·2 (41·6 to 77·3)	42·4 (29·9 to 56·4)	32·7 (24·3 to 43·8)	25·3 (19·2 to 33·2)	16·1 (12·2 to 21)	11·3 (8·3 to 15·3)	6·7 (4·9 to 9)	3·1 (2·1 to 4·1)	0·8 (0·5 to 1·1)	473
Uterine cancer	0 (0 to 0)	0 (0 to 0)	0 (0 to 0)	0 (0 to 0)	0 (0 to 0)	0 (0 to 0)	0 (0 to 0)	0·5 (0·4 to 0·7)	1·3 (1 to 1·9)	2·6 (1·9 to 3·6)	4·7 (3·2 to 6·7)	8·4 (5·7 to 11·8)	13·8 (9·5 to 19·4)	15·9 (11·2 to 22·2)	16·7 (12 to 23)	19·4 (13·9 to 26·5)	16·3 (12·2 to 21·6)	13·4 (9·8 to 18·1)	8·5 (6·2 to 11·3)	5·7 (4·1 to 7·6)	3·3 (2·5 to 4·2)	1·5 (1·2 to 2)	0·4 (0·3 to 0·5)	132
Ovarian cancer	0 (0 to 0)	0 (0 to 0)	0 (0 to 0)	0 (0 to 0)	0·4 (0·2 to 0·6)	0·6 (0·4 to 1)	1·7 (1 to 2·5)	2·8 (1·8 to 4·2)	3·3 (2·1 to 4·8)	5·5 (3·6 to 8)	9·2 (5·9 to 12·8)	17 (11·1 to 23·7)	26·4 (17 to 36·9)	29 (19 to 40·1)	27·9 (18 to 38·6)	21·9 (13·7 to 31)	18·5 (12 to 26·4)	13·8 (9 to 18·9)	8·9 (6 to 12·3)	5·8 (3·7 to 8·1)	3·7 (2·2 to 5)	1·6 (0·9 to 2·1)	0·3 (0·1 to 0·4)	198
Prostate cancer	0 (0 to 0)	0 (0 to 0)	0 (0 to 0)	0 (0 to 0)	0 (0 to 0)	0 (0 to 0)	0 (0 to 0)	0·5 (0·3 to 0·6)	1·1 (0·7 to 1·5)	1·5 (0·8 to 1·9)	1·8 (1·2 to 2·4)	4·7 (3·3 to 6·5)	15·4 (10·4 to 21·7)	40·4 (28·9 to 56·9)	72 (50·4 to 105)	128 (91·8 to 184)	147 (102 to 215)	218 (157 to 309)	196 (143 to 275)	157 (115 to 214)	104 (77 to 138)	41·8 (30·3 to 53·3)	6·2 (4·2 to 8·3)	1,140
Testicular cancer	0·1 (0 to 1·3)	0 (0 to 0·2)	0·1 (0 to 0·4)	0·3 (0·1 to 0·8)	0·3 (0·2 to 0·5)	0·4 (0·2 to 0·6)	1·3 (0·8 to 2)	2·5 (1·5 to 3·5)	3·8 (2·2 to 5·8)	2·3 (1·4 to 3·5)	2 (1·4 to 2·9)	1·6 (1·1 to 2·5)	1 (0·6 to 1·6)	0·8 (0·5 to 1·1)	0·6 (0·4 to 0·9)	0·5 (0·3 to 0·7)	0·4 (0·2 to 0·6)	0·4 (0·3 to 0·6)	0·4 (0·2 to 0·5)	0·2 (0·1 to 0·3)	0·1 (0·1 to 0·2)	0·1 (0 to 0·1)	0 (0 to 0)	19·3
Kidney cancer	0·1 (0 to 0·1)	0 (0 to 0)	0·2 (0·1 to 0·3)	1·8 (1·1 to 2·8)	1·6 (1·2 to 2·1)	0·8 (0·5 to 1)	1·1 (0·8 to 1·5)	1·6 (1·1 to 2·3)	2·4 (1·6 to 3·3)	2·7 (1·7 to 4·1)	3·1 (2·1 to 4·4)	5·7 (3·9 to 8·2)	9·2 (6·5 to 12·9)	12·2 (8·8 to 16·1)	13·4 (9·5 to 18·3)	12·3 (8·9 to 16·3)	9·3 (6·7 to 12·4)	8·7 (6·3 to 11·6)	5·1 (3·7 to 6·9)	3 (2·2 to 4·1)	1·4 (1 to 1·8)	0·6 (0·4 to 0·8)	0·1 (0·1 to 0·1)	96·4
Bladder cancer	0 (0 to 0)	0 (0 to 0)	0 (0 to 0)	0 (0 to 0)	0 (0 to 0)	0 (0 to 0)	0·1 (0 to 0·1)	0·2 (0·2 to 0·3)	0·3 (0·2 to 0·4)	1 (0·7 to 1·5)	1·6 (1·1 to 2·2)	3·4 (2·4 to 4·6)	6·1 (4·1 to 8·7)	10·6 (7·5 to 14·4)	14·4 (10·6 to 19·2)	17·7 (13·1 to 22·9)	17·1 (12·8 to 22·6)	20·8 (15·1 to 27·4)	16·9 (12·4 to 22·2)	12·9 (9·5 to 17·1)	8·6 (6·5 to 11·1)	4 (3·1 to 5·1)	0·8 (0·6 to 1)	137
Brain and central nervous system cancer	0·2 (0·1 to 0·4)	0·2 (0·1 to 0·3)	0·7 (0·3 to 1·3)	5 (2 to 9)	6·9 (3·3 to 10·3)	5·5 (2·5 to 8·4)	4 (1·7 to 6)	4·6 (1·8 to 6·9)	5·8 (2·1 to 8·9)	6·6 (2·5 to 9·8)	8·5 (3·4 to 12·5)	11 (4·6 to 15·8)	12·6 (5·3 to 18·5)	13·7 (6 to 20·1)	13·2 (5·7 to 19·5)	10·2 (4·5 to 14·4)	7·5 (3·3 to 10·7)	5·9 (2·5 to 8·4)	3·3 (1·4 to 4·8)	1·7 (0·7 to 2·5)	0·9 (0·4 to 1·3)	0·3 (0·1 to 0·4)	0·1 (0 to 0·1)	128
Thyroid cancer	0 (0 to 0)	0 (0 to 0)	0 (0 to 0)	0 (0 to 0)	0·1 (0·1 to 0·2)	0·1 (0·1 to 0·2)	0·3 (0·2 to 0·4)	0·4 (0·3 to 0·5)	0·7 (0·6 to 1)	0·9 (0·7 to 1·2)	1·6 (1·2 to 2·2)	2·5 (1·9 to 3·4)	3·9 (2·8 to 5·4)	5·4 (3·8 to 7·4)	5·5 (4 to 7·3)	4·8 (3·7 to 6·3)	4·2 (3·1 to 5·5)	4 (3·1 to 5·1)	2·7 (2·1 to 3·5)	1·9 (1·5 to 2·5)	1·1 (0·8 to 1·4)	0·5 (0·4 to 0·6)	0·1 (0·1 to 0·1)	40·9
Mesothelioma	0 (0 to 0)	0 (0 to 0)	0 (0 to 0)	0 (0 to 0)	0 (0 to 0)	0 (0 to 0)	0 (0 to 0)	0·4 (0·2 to 0·6)	0·3 (0·2 to 0·5)	0·4 (0·3 to 0·5)	0·8 (0·5 to 1·1)	1 (0·7 to 1·5)	1·5 (1·1 to 2)	1·9 (1·4 to 2·6)	1·9 (1·4 to 2·6)	0·7 (0·5 to 1)	0·5 (0·4 to 0·7)	0·7 (0·5 to 0·9)	0·5 (0·3 to 0·6)	0·3 (0·2 to 0·4)	0·2 (0·1 to 0·2)	0·1 (0 to 0·1)	0 (0 to 0)	11·1
Hodgkin lymphoma	0 (0 to 0)	0 (0 to 0)	0 (0 to 0)	0·1 (0·1 to 0·2)	0·3 (0·2 to 0·4)	0·5 (0·3 to 0·6)	2·1 (1·5 to 3)	4 (2·7 to 5·8)	4·6 (3 to 6·7)	3·9 (2·7 to 5·6)	3·6 (2·5 to 5)	3·9 (2·7 to 5·3)	3·3 (2·2 to 4·5)	3·3 (2·2 to 4·7)	2·8 (1·9 to 4)	2·4 (1·7 to 3·3)	1·8 (1·3 to 2·5)	1·5 (1·1 to 2)	1 (0·8 to 1·4)	0·6 (0·4 to 0·8)	0·3 (0·2 to 0·4)	0·1 (0 to 0·1)	0 (0 to 0)	40·1
Non-Hodgkin lymphoma	0 (0 to 0)	0 (0 to 0)	0 (0 to 0)	4·4 (2·7 to 7·1)	6·3 (4·2 to 9·1)	6·6 (4·4 to 9·6)	10·4 (7 to 14·6)	15·7 (10·6 to 21·8)	19·5 (14·5 to 25·5)	20·7 (14·9 to 27·7)	22·2 (16·1 to 29·3)	26·2 (18·9 to 35)	33·7 (23·4 to 45·9)	35·8 (27·2 to 45·8)	36·2 (25·8 to 48·7)	32·8 (24·9 to 41·1)	25·3 (18·7 to 33·2)	24·5 (19 to 30·8)	15·2 (11·3 to 20·2)	9 (6·6 to 11·9)	4·3 (3·3 to 5·5)	1·6 (1·3 to 1·9)	0·3 (0·2 to 0·4)	351
Multiple myeloma	0 (0 to 0)	0 (0 to 0)	0 (0 to 0)	0 (0 to 0)	0 (0 to 0)	0 (0 to 0)	0 (0 to 0)	0·5 (0·4 to 0·7)	0·8 (0·6 to 1)	1·3 (1 to 1·7)	3·1 (2·3 to 4·2)	7·8 (5·8 to 10·5)	16 (11·6 to 21·8)	25·1 (18·9 to 32·9)	30·6 (22·6 to 40·3)	30·4 (22·5 to 39·4)	27·3 (21·1 to 35·6)	25·7 (20 to 33·1)	17·3 (13·6 to 22·1)	10·3 (7·8 to 13·1)	5·2 (3·9 to 6·5)	1·6 (1·2 to 2)	0·3 (0·2 to 0·3)	203
Leukemia	0·5 (0·3 to 0·7)	0·3 (0·2 to 0·5)	1·4 (0·8 to 2·1)	10·7 (6·8 to 16·2)	14·8 (11 to 19·5)	14 (10 to 18·4)	15·9 (11·8 to 20·9)	16·5 (12·1 to 21·7)	16·8 (12·9 to 21·3)	14·5 (11·1 to 18·5)	16 (12·3 to 21)	19 (14·7 to 24·2)	21·9 (16·9 to 28)	21·3 (16·5 to 27·1)	20·6 (15·9 to 26·2)	18·6 (14·5 to 23·1)	15·4 (12·1 to 19·3)	15·9 (12·6 to 20·2)	10·8 (8·4 to 13·5)	7·6 (6 to 9·4)	4·6 (3·6 to 5·8)	1·9 (1·6 to 2·3)	0·4 (0·3 to 0·4)	279
Acute lymphoid leukemia	0·1 (0 to 0·3)	0·1 (0 to 0·1)	0·4 (0·2 to 0·7)	5·5 (3·4 to 8·6)	8·2 (5·6 to 11·4)	6·3 (4 to 8·9)	7·4 (5 to 10·6)	4·8 (3·1 to 7·4)	2·9 (1·9 to 4·3)	2·2 (1·4 to 3·2)	1·9 (1·2 to 2·8)	2 (1·2 to 2·8)	1·6 (1 to 2·4)	1·5 (1 to 2·2)	1·2 (0·8 to 1·8)	0·8 (0·5 to 1·2)	0·8 (0·5 to 1·1)	0·7 (0·4 to 1)	0·4 (0·3 to 0·6)	0·3 (0·2 to 0·4)	0·1 (0·1 to 0·2)	0·1 (0 to 0·1)	0 (0 to 0)	49·3
Chronic lymphoid leukemia	0 (0 to 0)	0 (0 to 0)	0 (0 to 0)	0 (0 to 0)	0 (0 to 0)	0 (0 to 0)	0 (0 to 0)	0·4 (0·2 to 0·7)	0·5 (0·3 to 0·8)	0·4 (0·2 to 0·6)	0·6 (0·3 to 0·9)	1·1 (0·6 to 1·8)	0·9 (0·6 to 1·6)	2·5 (1·6 to 3·6)	3·5 (2·3 to 5·2)	3·9 (2·6 to 5·5)	3·7 (2·5 to 5·2)	3·7 (2·4 to 5·4)	2·5 (1·7 to 3·7)	1·8 (1·2 to 2·5)	1·1 (0·7 to 1·6)	0·5 (0·3 to 0·7)	0·1 (0 to 0·1)	27·1
Acute myeloid leukemia	0·1 (0 to 0·1)	0·2 (0·1 to 0·3)	0·4 (0·2 to 0·6)	3·1 (1·7 to 5)	3·4 (2·1 to 5·3)	4·2 (2·8 to 5·9)	4·8 (3·2 to 7)	5 (3·4 to 7·2)	6 (4·3 to 8·2)	4·7 (3·3 to 6·3)	5·4 (3·8 to 7·5)	5·1 (3·6 to 6·9)	6·8 (5 to 9·3)	5·9 (4·3 to 7·9)	5 (3·7 to 6·6)	4 (2·8 to 5·4)	2·9 (2·1 to 4)	2·6 (1·9 to 3·5)	1·6 (1·2 to 2·2)	0·9 (0·6 to 1·3)	0·4 (0·3 to 0·6)	0·1 (0·1 to 0·2)	0 (0 to 0)	72·7

Chronic myeloid leukemia	0 (0 to 0)	0 (0 to 0)	0 (0 to 0·1)	0·3 (0·2 to 0·5)	0·2 (0·1 to 0·4)	0·2 (0·1 to 0·3)	0·4 (0·2 to 0·6)	1·2 (0·7 to 1·9)	1·9 (1·1 to 2·9)	1·9 (1·2 to 3)	2·2 (1·3 to 3·3)	2·2 (1·4 to 3·3)	2·5 (1·6 to 3·8)	2·9 (1·9 to 4·3)	2·7 (1·7 to 3·9)	2·6 (1·6 to 3·9)	1·8 (1·2 to 2·7)	1·8 (1·1 to 2·6)	1·3 (0·8 to 2)	0·9 (0·6 to 1·3)	0·5 (0·3 to 0·7)	0·2 (0·1 to 0·3)	0·1 (0 to 0·1)	27·6
Other leukemia	0·3 (0·2 to 0·5)	0·1 (0 to 0·1)	0·5 (0·3 to 0·8)	1·9 (1 to 3·1)	3 (1·8 to 4·6)	3·3 (2·1 to 4·8)	3·3 (2·2 to 4·7)	5 (3·5 to 7)	5·4 (3·8 to 7·3)	5·3 (3·8 to 7·1)	6 (4·3 to 8·1)	8·7 (6·4 to 11·5)	9·9 (7·4 to 13·1)	8·5 (6·4 to 11·2)	8·1 (6 to 10·8)	7·3 (5·5 to 9·6)	6·2 (4·5 to 8·1)	7·2 (5·4 to 9·3)	5 (3·7 to 6·6)	3·7 (2·8 to 4·8)	2·5 (1·9 to 3·3)	1 (0·8 to 1·3)	0·2 (0·1 to 0·2)	103
Other malignant neoplasms	0·6 (0·4 to 0·9)	0·5 (0·3 to 0·7)	2 (1·2 to 3)	16·1 (9·9 to 25)	10·6 (8·4 to 13·2)	9·7 (7·6 to 12·1)	13 (10 to 16·6)	14·4 (11·4 to 17·9)	14·5 (11·7 to 18·1)	15·1 (12·1 to 19·3)	16·6 (13·2 to 21)	21·9 (17·1 to 28)	28 (21·8 to 36·5)	30·6 (23·4 to 39·5)	29·6 (22·7 to 39·2)	25·1 (19·8 to 32·8)	20·3 (16·1 to 26·1)	19 (15·1 to 24·1)	13 (10·4 to 16·9)	9·1 (7·3 to 11·6)	6·5 (5·1 to 8·1)	2·8 (2·2 to 3·4)	0·5 (0·4 to 0·7)	319
Other neoplasms	0·1 (0 to 0·1)	0·1 (0 to 0·2)	0·2 (0·1 to 0·3)	1 (0·6 to 1·6)	0·6 (0·4 to 0·9)	0·7 (0·4 to 0·9)	0·8 (0·4 to 1·1)	0·6 (0·4 to 0·9)	1·2 (0·7 to 1·7)	1 (0·6 to 1·5)	1·2 (0·7 to 1·8)	1·9 (1·3 to 2·6)	2·2 (1·5 to 3·2)	3·2 (2·2 to 4·8)	3·8 (2·5 to 5·6)	4·1 (2·9 to 6·3)	3·9 (2·8 to 5·8)	5 (3·6 to 7·7)	4 (2·8 to 5·8)	2·7 (1·9 to 3·9)	1·6 (1·2 to 2·3)	0·7 (0·4 to 0·9)	0·1 (0·1 to 0·1)	40·4
Myelodysplastic, myeloproliferative, and other hematopoietic neo	0·1 (0 to 0·1)	0·1 (0 to 0·2)	0·2 (0·1 to 0·3)	1 (0·6 to 1·6)	0·6 (0·4 to 0·9)	0·7 (0·4 to 0·9)	0·8 (0·4 to 1·1)	0·6 (0·4 to 0·9)	1·2 (0·7 to 1·7)	1 (0·6 to 1·5)	1·2 (0·7 to 1·8)	1·9 (1·3 to 2·6)	2·2 (1·5 to 3·2)	3·2 (2·2 to 4·8)	3·8 (2·5 to 5·6)	4·1 (2·9 to 6·3)	3·9 (2·8 to 5·8)	5 (3·6 to 7·7)	4 (2·8 to 5·8)	2·7 (1·9 to 3·9)	1·6 (1·2 to 2·3)	0·7 (0·4 to 0·9)	0·1 (0·1 to 0·1)	40·4
Benign and in situ intestinal neoplasms	0 (0 to 0)	0 (0 to 0)	0 (0 to 0)	0 (0 to 0)	0 (0 to 0)	0 (0 to 0)	0 (0 to 0)	0 (0 to 0)	0 (0 to 0)	0 (0 to 0)	0 (0 to 0)	0 (0 to 0)	0 (0 to 0)	0 (0 to 0)	0 (0 to 0)	0 (0 to 0)	0 (0 to 0)	0 (0 to 0)	0 (0 to 0)	0 (0 to 0)	0 (0 to 0)	0 (0 to 0)	0 (0 to 0)	0
Benign and in situ cervical and uterine neoplasms	0 (0 to 0)	0 (0 to 0)	0 (0 to 0)	0 (0 to 0)	0 (0 to 0)	0 (0 to 0)	0 (0 to 0)	0 (0 to 0)	0 (0 to 0)	0 (0 to 0)	0 (0 to 0)	0 (0 to 0)	0 (0 to 0)	0 (0 to 0)	0 (0 to 0)	0 (0 to 0)	0 (0 to 0)	0 (0 to 0)	0 (0 to 0)	0 (0 to 0)	0 (0 to 0)	0 (0 to 0)	0 (0 to 0)	0
Other benign and in situ neoplasms	0 (0 to 0)	0 (0 to 0)	0 (0 to 0)	0 (0 to 0)	0 (0 to 0)	0 (0 to 0)	0 (0 to 0)	0 (0 to 0)	0 (0 to 0)	0 (0 to 0)	0 (0 to 0)	0 (0 to 0)	0 (0 to 0)	0 (0 to 0)	0 (0 to 0)	0 (0 to 0)	0 (0 to 0)	0 (0 to 0)	0 (0 to 0)	0 (0 to 0)	0 (0 to 0)	0 (0 to 0)	0 (0 to 0)	0
Cardiovascular diseases	4·2 (2·6 to 6·7)	1·2 (0·7 to 1·9)	4 (2·5 to 6·4)	6·2 (4·3 to 8·6)	10·3 (8·2 to 12·8)	18 (13·8 to 23)	43·6 (34·3 to 54·2)	66·1 (52·7 to 82)	96·9 (77·3 to 122)	135 (109 to 166)	202 (162 to 248)	354 (286 to 437)	564 (453 to 702)	754 (605 to 923)	884 (727 to 1,080)	997 (828 to 1,190)	907 (761 to 1,060)	1,070 (915 to 1,250)	896 (771 to 1,040)	817 (702 to 932)	600 (509 to 684)	351 (292 to 395)	101 (84·7 to 112)	8,880
Rheumatic heart disease	0 (0 to 0)	0 (0 to 0)	0 (0 to 0)	0·5 (0·3 to 0·8)	2·7 (1·7 to 4·2)	6·3 (4 to 9·7)	11·4 (7·5 to 16·8)	13·7 (9·3 to 19·7)	16·8 (12 to 23·2)	17·2 (12·6 to 23·1)	19·1 (13·9 to 25·7)	21·9 (15·7 to 29·8)	22·4 (15·7 to 30)	19·5 (13·6 to 26·8)	15·1 (10·7 to 20·5)	9·9 (7·2 to 13)	6·8 (5 to 8·9)	5·4 (4 to 7·2)	3·9 (2·8 to 5·1)	2·7 (2 to 3·5)	1·9 (1·4 to 2·5)	1·1 (0·8 to 1·5)	0·4 (0·3 to 0·5)	199
Ischemic heart disease	0 (0 to 0)	0 (0 to 0)	0 (0 to 0)	0 (0 to 0)	0 (0 to 0)	0 (0 to 0)	2 (1·4 to 2·7)	4·9 (3·5 to 6·7)	10·5 (7·3 to 14·2)	20·5 (15·2 to 26·6)	37 (27·7 to 47·9)	77·8 (59·1 to 99·6)	141 (108 to 180)	221 (175 to 278)	263 (207 to 323)	321 (262 to 389)	297 (240 to 359)	331 (275 to 391)	260 (216 to 309)	239 (200 to 279)	173 (139 to 205)	106 (84·6 to 126)	34·1 (27·7 to 39·7)	2,540
Stroke	1·4 (0·7 to 2·4)	0·1 (0·1 to 0·3)	0·4 (0·2 to 0·8)	0·9 (0·7 to 1·2)	2·3 (1·7 to 3·1)	4 (2·9 to 5·2)	10·8 (7·7 to 14·4)	18·4 (12·9 to 25·4)	26·4 (18·7 to 37·1)	40·4 (28·4 to 54·7)	64·1 (45·5 to 86·3)	116 (84·3 to 156)	193 (139 to 261)	262 (191 to 350)	328 (247 to 424)	379 (287 to 483)	341 (258 to 425)	435 (344 to 538)	381 (308 to 463)	342 (276 to 410)	235 (190 to 282)	125 (102 to 146)	32·8 (26·1 to 38·6)	3,340
Ischemic stroke	0·3 (0·1 to 0·5)	0 (0 to 0)	0 (0 to 0·1)	0·5 (0·3 to 0·6)	1·2 (0·7 to 1·8)	1·8 (1·1 to 2·8)	3·3 (2·3 to 4·6)	4·7 (3·3 to 6·3)	6·3 (4·5 to 8·6)	9 (6·6 to 11·7)	13·2 (9·7 to 17·4)	22·7 (17·1 to 30)	37·2 (27·7 to 48·4)	56·6 (42·3 to 74·8)	76·3 (58·3 to 98·1)	126 (95·1 to 161)	127 (97 to 159)	234 (185 to 289)	228 (183 to 276)	264 (213 to 316)	187 (151 to 226)	101 (81·3 to 119)	27·3 (21·8 to 32·4)	1,530
Intracerebral hemorrhage	0·4 (0·2 to 0·6)	0 (0 to 0·1)	0·2 (0·1 to 0·4)	0·2 (0·1 to 0·2)	0·6 (0·4 to 0·8)	1·1 (0·7 to 1·4)	4·2 (2·8 to 6)	8·2 (5·3 to 11·7)	12·4 (8·2 to 18·3)	20·8 (13·5 to 29·6)	34·2 (23·4 to 48·3)	66·1 (45·5 to 91·2)	121 (84·8 to 169)	167 (120 to 225)	211 (156 to 281)	226 (167 to 292)	192 (141 to 246)	184 (140 to 237)	141 (110 to 177)	70·1 (54·7 to 89·2)	42·6 (33·3 to 53·1)	21·6 (17·1 to 26·2)	4·9 (3·9 to 5·8)	1,530
Subarachnoid hemorrhage	0·7 (0·4 to 1·4)	0·1 (0 to 0·2)	0·2 (0·1 to 0·4)	0·3 (0·2 to 0·5)	0·6 (0·4 to 0·8)	1·1 (0·7 to 1·6)	3·3 (2 to 4·9)	5·6 (3·4 to 8·7)	7·6 (4·7 to 12)	10·6 (6·9 to 15·5)	16·7 (11·1 to 24·1)	26·8 (17·7 to 38·3)	34·5 (23·3 to 48·4)	38·3 (25·4 to 55·7)	40·2 (27·1 to 56·8)	27·3 (18·7 to 37·9)	22·2 (15·5 to 31·1)	17 (12·5 to 22·6)	12·5 (9 to 16·7)	8·3 (6·1 to 10·9)	5·1 (3·7 to 6·9)	2·6 (1·9 to 3·4)	0·6 (0·4 to 0·8)	282
Hypertensive heart disease	0 (0 to 0)	0 (0 to 0)	0 (0 to 0)	0 (0 to 0)	0 (0 to 0)	0 (0 to 0)	0·7 (0·5 to 1·1)	1·8 (1·2 to 2·7)	3·2 (1·9 to 4·8)	7·4 (4·8 to 10·7)	14·3 (9·6 to 20·5)	29 (20·1 to 40·4)	58·4 (40·5 to 84)	79·9 (54·6 to 112)	92·7 (65·4 to 128)	100 (69·8 to 139)	92·2 (66·9 to 128)	108 (78·6 to 147)	93·9 (68·6 to 127)	86·5 (63·2 to 114)	68·2 (49·4 to 88·8)	43 (30·5 to 57)	12·9 (8·9 to 17·2)	892
Non-rheumatic valvular heart disease	0 (0 to 0)	0 (0 to 0)	0 (0 to 0)	0 (0 to 0)	0 (0 to 0)	0 (0 to 0)	0·9 (0·7 to 1·2)	1 (0·7 to 1·4)	1·6 (1·1 to 2·3)	2·1 (1·5 to 2·8)	2·4 (1·7 to 3·2)	3·9 (2·7 to 5·3)	5·6 (4 to 7·7)	5·7 (4 to 7·7)	6·4 (4·7 to 8·4)	6·5 (4·7 to 8·6)	5·6 (4·3 to 7·3)	5·6 (4·2 to 7·3)	4·3 (3·2 to 5·5)	3·7 (2·9 to 4·7)	2·7 (2 to 3·4)	1·4 (1·1 to 1·8)	0·3 (0·2 to 0·4)	59·6
Non-rheumatic calcific aortic valve disease	0 (0 to 0)	0 (0 to 0)	0 (0 to 0)	0 (0 to 0)	0 (0 to 0)	0 (0 to 0)	0·5 (0·3 to 0·7)	0·6 (0·4 to 0·9)	1 (0·6 to 1·5)	1·1 (0·7 to 1·5)	1·3 (0·8 to 1·8)	2·3 (1·5 to 3·2)	3·4 (2·3 to 4·9)	3·6 (2·4 to 5)	4·2 (3 to 5·8)	4·5 (3·2 to 6·2)	4·2 (3·1 to 5·6)	4·4 (3·3 to 5·9)	3·5 (2·6 to 4·5)	3·1 (2·4 to 3·9)	2·2 (1·7 to 2·9)	1·2 (0·9 to 1·5)	0·2 (0·2 to 0·3)	41·4
Non-rheumatic degenerative mitral valve disease	0 (0 to 0)	0 (0 to 0)	0 (0 to 0)	0 (0 to 0)	0 (0 to 0)	0 (0 to 0)	0·4 (0·2 to 0·5)	0·3 (0·2 to 0·5)	0·6 (0·4 to 0·9)	0·9 (0·6 to 1·3)	1 (0·7 to 1·5)	1·6 (1 to 2·4)	2·1 (1·4 to 2·9)	2 (1·3 to 3)	2·1 (1·4 to 3·1)	1·9 (1·2 to 2·8)	1·4 (0·9 to 2)	1·1 (0·7 to 1·7)	0·8 (0·5 to 1·1)	0·6 (0·4 to 0·9)	0·4 (0·2 to 0·6)	0·2 (0·2 to 0·4)	0·1 (0 to 0·1)	17·5
Other non-rheumatic valve diseases	0 (0 to 0)	0 (0 to 0)	0 (0 to 0)	0 (0 to 0)	0 (0 to 0)	0 (0 to 0)	[illegible]	[illegible]	[illegible]	[illegible]	[illegible]	[illegible]	[illegible]	[illegible]	[illegible]	[illegible]	[illegible]	[illegible]	[illegible]	[illegible]	[illegible]	[illegible]	[illegible]	0·7
Cardiomyopathy and myocarditis	1·2 (0·7 to 1·8)	0·5 (0·3 to 0·8)	2 (1·2 to 3·1)	2·6 (1·7 to 3·7)	2·1 (1·6 to 2·7)	3 (2·2 to 3·8)	6·8 (4·9 to 9·2)	10·1 (7·3 to 13·2)	15·9 (11·4 to 21·1)	21·5 (15 to 28·7)	29·4 (20·9 to 40·3)	45 (32·7 to 60·5)	65·1 (46·8 to 88·1)	73·8 (52·6 to 101)	72·8 (52·1 to 97·3)	63·2 (46·3 to 83·6)	50·8 (36·8 to 66·4)	48·4 (35·4 to 63·7)	33·6 (24·7 to 43·8)	26·3 (19 to 34·8)	21·5 (15·4 to 28·9)	11 (7·5 to 15)	2·8 (1·8 to 3·8)	609
Myocarditis	0·3 (0·1 to 0·5)	0·2 (0·1 to 0·3)	0·8 (0·4 to 1·3)	1·1 (0·7 to 1·8)	1 (0·7 to 1·3)	1 (0·7 to 1·4)	1·9 (1·1 to 2·8)	2·1 (1·3 to 3)	2·9 (1·8 to 4·6)	3·9 (2·4 to 6)	4 (2·4 to 6·1)	4·5 (2·6 to 7·3)	5·4 (3·2 to 8·3)	5·9 (3·3 to 9·7)	4·2 (2·4 to 7)	3·5 (2 to 5·6)	2·8 (1·6 to 4·4)	3·1 (1·7 to 5·2)	2·6 (1·5 to 4·4)	2·7 (1·5 to 4·5)	2·9 (1·7 to 4·5)	1·9 (1·2 to 2·9)	0·7 (0·4 to 1·1)	59·2
Alcoholic cardiomyopathy	[illegible]	[illegible]	[illegible]	[illegible]	[illegible]	[illegible]	[illegible]	[illegible]	[illegible]	[illegible]	[illegible]	[illegible]	[illegible]	[illegible]	[illegible]	[illegible]	[illegible]	[illegible]	[illegible]	[illegible]	[illegible]	[illegible]	[illegible]	[illegible]
Other cardiomyopathy	[illegible]	[illegible]	[illegible]	[illegible]	[illegible]	[illegible]	[illegible]	[illegible]	[illegible]	[illegible]	[illegible]	[illegible]	[illegible]	[illegible]	[illegible]	[illegible]	[illegible]	[illegible]	[illegible]	[illegible]	[illegible]	[illegible]	[illegible]	[illegible]
Atrial fibrillation and flutter	[illegible]	[illegible]	[illegible]	[illegible]	[illegible]	[illegible]	[illegible]	[illegible]	[illegible]	[illegible]	[illegible]	[illegible]	[illegible]	[illegible]	[illegible]	[illegible]	[illegible]	[illegible]	[illegible]	[illegible]	[illegible]	[illegible]	[illegible]	[illegible]
Aortic aneurysm	[illegible]	[illegible]	[illegible]	[illegible]	[illegible]	[illegible]	[illegible]	[illegible]	[illegible]	[illegible]	[illegible]	[illegible]	[illegible]	[illegible]	[illegible]	[illegible]	[illegible]	[illegible]	[illegible]	[illegible]	[illegible]	[illegible]	[illegible]	[illegible]
Endocarditis	[illegible]	[illegible]	[illegible]	[illegible]	[illegible]	[illegible]	[illegible]	[illegible]	[illegible]	[illegible]	[illegible]	[illegible]	[illegible]	[illegible]	[illegible]	[illegible]	[illegible]	[illegible]	[illegible]	[illegible]	[illegible]	[illegible]	[illegible]	[illegible]
Other cardiovascular and circulatory diseases	[illegible]	[illegible]	[illegible]	[illegible]	[illegible]	[illegible]	[illegible]	[illegible]	[illegible]	[illegible]	[illegible]	[illegible]	[illegible]	[illegible]	[illegible]	[illegible]	[illegible]	[illegible]	[illegible]	[illegible]	[illegible]	[illegible]	[illegible]	[illegible]
Chronic respiratory diseases	[illegible]	[illegible]	[illegible]	[illegible]	[illegible]	[illegible]	[illegible]	[illegible]	[illegible]	[illegible]	[illegible]	[illegible]	[illegible]	[illegible]	[illegible]	[illegible]	[illegible]	[illegible]	[illegible]	[illegible]	[illegible]	[illegible]	[illegible]	[illegible]
Chronic obstructive pulmonary disease	[illegible]	[illegible]	[illegible]	[illegible]	[illegible]	[illegible]	[illegible]	[illegible]	[illegible]	[illegible]	[illegible]	[illegible]	[illegible]	[illegible]	[illegible]	[illegible]	[illegible]	[illegible]	[illegible]	[illegible]	[illegible]	[illegible]	[illegible]	[illegible]
Pneumoconiosis	[illegible]	[illegible]	[illegible]	[illegible]	[illegible]	[illegible]	[illegible]	[illegible]	[illegible]	[illegible]	[illegible]	[illegible]	[illegible]	[illegible]	[illegible]	[illegible]	[illegible]	[illegible]	[illegible]	[illegible]	[illegible]	[illegible]	[illegible]	[illegible]
Silicosis	[illegible]	[illegible]	[illegible]	[illegible]	[illegible]	[illegible]	[illegible]	[illegible]	[illegible]	[illegible]	[illegible]	[illegible]	[illegible]	[illegible]	[illegible]	[illegible]	[illegible]	[illegible]	[illegible]	[illegible]	[illegible]	[illegible]	[illegible]	[illegible]
Asbestosis	[illegible]	[illegible]	[illegible]	[illegible]	[illegible]	[illegible]	[illegible]	[illegible]	[illegible]	[illegible]	[illegible]	[illegible]	[illegible]	[illegible]	[illegible]	[illegible]	[illegible]	[illegible]	[illegible]	[illegible]	[illegible]	[illegible]	[illegible]	[illegible]
Coal workers pneumoconiosis	[illegible]	[illegible]	[illegible]	[illegible]	[illegible]	[illegible]	[illegible]	[illegible]	[illegible]	[illegible]	[illegible]	[illegible]	[illegible]	[illegible]	[illegible]	[illegible]	[illegible]	[illegible]	[illegible]	[illegible]	[illegible]	[illegible]	[illegible]	[illegible]
Other pneumoconiosis	[illegible]	[illegible]	[illegible]	[illegible]	[illegible]	[illegible]	[illegible]	[illegible]	[illegible]	[illegible]	[illegible]	[illegible]	[illegible]	[illegible]	[illegible]	[illegible]	[illegible]	[illegible]	[illegible]	[illegible]	[illegible]	[illegible]	[illegible]	[illegible]
Asthma	[illegible]	[illegible]	[illegible]	[illegible]	[illegible]	[illegible]	[illegible]	[illegible]	[illegible]	[illegible]	[illegible]	[illegible]	[illegible]	[illegible]	[illegible]	[illegible]	[illegible]	[illegible]	[illegible]	[illegible]	[illegible]	[illegible]	[illegible]	[illegible]
Interstitial lung disease and pulmonary sarcoidosis	[illegible]	[illegible]	[illegible]	[illegible]	[illegible]	[illegible]	[illegible]	[illegible]	[illegible]	[illegible]	[illegible]	[illegible]	[illegible]	[illegible]	[illegible]	[illegible]	[illegible]	[illegible]	[illegible]	[illegible]	[illegible]	[illegible]	[illegible]	[illegible]
Other chronic respiratory diseases	[illegible]	[illegible]	[illegible]	[illegible]	[illegible]	[illegible]	[illegible]	[illegible]	[illegible]	[illegible]	[illegible]	[illegible]	[illegible]	[illegible]	[illegible]	[illegible]	[illegible]	[illegible]	[illegible]	[illegible]	[illegible]	[illegible]	[illegible]	[illegible]
Digestive diseases	[illegible]	[illegible]	[illegible]	[illegible]	[illegible]	[illegible]	[illegible]	[illegible]	[illegible]	[illegible]	[illegible]	[illegible]	[illegible]	[illegible]	[illegible]	[illegible]	[illegible]	[illegible]	[illegible]	[illegible]	[illegible]	[illegible]	[illegible]	[illegible]
Cirrhosis and other chronic liver diseases	[illegible]	[illegible]	[illegible]	[illegible]	[illegible]	[illegible]	[illegible]	[illegible]	[illegible]	[illegible]	[illegible]	[illegible]	[illegible]	[illegible]	[illegible]	[illegible]	[illegible]	[illegible]	[illegible]	[illegible]	[illegible]	[illegible]	[illegible]	[illegible]
Cirrhosis and other chronic liver diseases due to hepatitis B	[illegible]	[illegible]	[illegible]	[illegible]	[illegible]	[illegible]	[illegible]	[illegible]	[illegible]	[illegible]	[illegible]	[illegible]	[illegible]	[illegible]	[illegible]	[illegible]	[illegible]	[illegible]	[illegible]	[illegible]	[illegible]	[illegible]	[illegible]	[illegible]
Cirrhosis and other chronic liver diseases due to hepatitis C	[illegible]	[illegible]	[illegible]	[illegible]	[illegible]	[illegible]	[illegible]	[illegible]	[illegible]	[illegible]	[illegible]	[illegible]	[illegible]	[illegible]	[illegible]	[illegible]	[illegible]	[illegible]	[illegible]	[illegible]	[illegible]	[illegible]	[illegible]	[illegible]
Cirrhosis and other chronic liver diseases due to alcohol use	[illegible]	[illegible]	[illegible]	[illegible]	[illegible]	[illegible]	[illegible]	[illegible]	[illegible]	[illegible]	[illegible]	[illegible]	[illegible]	[illegible]	[illegible]	[illegible]	[illegible]	[illegible]	[illegible]	[illegible]	[illegible]	[illegible]	[illegible]	[illegible]
Cirrhosis and other chronic liver diseases due to NAFLD	[illegible]	[illegible]	[illegible]	[illegible]	[illegible]	[illegible]	[illegible]	[illegible]	[illegible]	[illegible]	[illegible]	[illegible]	[illegible]	[illegible]	[illegible]	[illegible]	[illegible]	[illegible]	[illegible]	[illegible]	[illegible]	[illegible]	[illegible]	[illegible]
Cirrhosis and other chronic liver diseases due to other causes	[illegible]	[illegible]	[illegible]	[illegible]	[illegible]	[illegible]	[illegible]	[illegible]	[illegible]	[illegible]	[illegible]	[illegible]	[illegible]	[illegible]	[illegible]	[illegible]	[illegible]	[illegible]	[illegible]	[illegible]	[illegible]	[illegible]	[illegible]	[illegible]
Upper digestive system diseases	[illegible]	[illegible]	[illegible]	[illegible]	[illegible]	[illegible]	[illegible]	[illegible]	[illegible]	[illegible]	[illegible]	[illegible]	[illegible]	[illegible]	[illegible]	[illegible]	[illegible]	[illegible]	[illegible]	[illegible]	[illegible]	[illegible]	[illegible]	[illegible]
Peptic ulcer disease	[illegible]	[illegible]	[illegible]	[illegible]	[illegible]	[illegible]	[illegible]	[illegible]	[illegible]	[illegible]	[illegible]	[illegible]	[illegible]	[illegible]	[illegible]	[illegible]	[illegible]	[illegible]	[illegible]	[illegible]	[illegible]	[illegible]	[illegible]	[illegible]
Gastritis and duodenitis	[illegible]	[illegible]	[illegible]	[illegible]	[illegible]	[illegible]	[illegible]	[illegible]	[illegible]	[illegible]	[illegible]	[illegible]	[illegible]	[illegible]	[illegible]	[illegible]	[illegible]	[illegible]	[illegible]	[illegible]	[illegible]	[illegible]	[illegible]	[illegible]
Gastroesophageal reflux disease	[illegible]	[illegible]	[illegible]	[illegible]	[illegible]	[illegible]	[illegible]	[illegible]	[illegible]	[illegible]	[illegible]	[illegible]	[illegible]	[illegible]	[illegible]	[illegible]	[illegible]	[illegible]	[illegible]	[illegible]	[illegible]	[illegible]	[illegible]	[illegible]
Appendicitis	[illegible]	[illegible]	[illegible]	[illegible]	[illegible]	[illegible]	[illegible]	[illegible]	[illegible]	[illegible]	[illegible]	[illegible]	[illegible]	[illegible]	[illegible]	[illegible]	[illegible]	[illegible]	[illegible]	[illegible]	[illegible]	[illegible]	[illegible]	[illegible]
Paralytic ileus and intestinal obstruction	[illegible]	[illegible]	[illegible]	[illegible]	[illegible]	[illegible]	[illegible]	[illegible]	[illegible]	[illegible]	[illegible]	[illegible]	[illegible]	[illegible]	[illegible]	[illegible]	[illegible]	[illegible]	[illegible]	[illegible]	[illegible]	[illegible]	[illegible]	[illegible]
Inguinal, femoral, and abdominal hernia	[illegible]	[illegible]	[illegible]	[illegible]	[illegible]	[illegible]	[illegible]	[illegible]	[illegible]	[illegible]	[illegible]	[illegible]	[illegible]	[illegible]	[illegible]	[illegible]	[illegible]	[illegible]	[illegible]	[illegible]	[illegible]	[illegible]	[illegible]	[illegible]
Inflammatory bowel disease	[illegible]	[illegible]	[illegible]	[illegible]	[illegible]	[illegible]	[illegible]	[illegible]	[illegible]	[illegible]	[illegible]	[illegible]	[illegible]	[illegible]	[illegible]	[illegible]	[illegible]	[illegible]	[illegible]	[illegible]	[illegible]	[illegible]	[illegible]	[illegible]
Vascular intestinal disorders	[illegible]	[illegible]	[illegible]	[illegible]	[illegible]	[illegible]	[illegible]	[illegible]	[illegible]	[illegible]	[illegible]	[illegible]	[illegible]	[illegible]	[illegible]	[illegible]	[illegible]	[illegible]	[illegible]	[illegible]	[illegible]	[illegible]	[illegible]	[illegible]
Gallbladder and biliary diseases	[illegible]	[illegible]	[illegible]	[illegible]	[illegible]	[illegible]	[illegible]	[illegible]	[illegible]	[illegible]	[illegible]	[illegible]	[illegible]	[illegible]	[illegible]	[illegible]	[illegible]	[illegible]	[illegible]	[illegible]	[illegible]	[illegible]	[illegible]	[illegible]
Pancreatitis	[illegible]	[illegible]	[illegible]	[illegible]	[illegible]	[illegible]	[illegible]	[illegible]	[illegible]	[illegible]	[illegible]	[illegible]	[illegible]	[illegible]	[illegible]	[illegible]	[illegible]	[illegible]	[illegible]	[illegible]	[illegible]	[illegible]	[illegible]	[illegible]
Other digestive diseases	[illegible]	[illegible]	[illegible]	[illegible]	[illegible]	[illegible]	[illegible]	[illegible]	[illegible]	[illegible]	[illegible]	[illegible]	[illegible]	[illegible]	[illegible]	[illegible]	[illegible]	[illegible]	[illegible]	[illegible]	[illegible]	[illegible]	[illegible]	[illegible]
Neurological disorders	[illegible]	[illegible]	[illegible]	[illegible]	[illegible]	[illegible]	[illegible]	[illegible]	[illegible]	[illegible]	[illegible]	[illegible]	[illegible]	[illegible]	[illegible]	[illegible]	[illegible]	[illegible]	[illegible]	[illegible]	[illegible]	[illegible]	[illegible]	[illegible]
Alzheimer's disease and other dementias	[illegible]	[illegible]	[illegible]	[illegible]	[illegible]	[illegible]	[illegible]	[illegible]	[illegible]	[illegible]	[illegible]	[illegible]	[illegible]	[illegible]	[illegible]	[illegible]	[illegible]	[illegible]	[illegible]	[illegible]	[illegible]	[illegible]	[illegible]	[illegible]
Parkinson's disease	[illegible]	[illegible]	[illegible]	[illegible]	[illegible]	[illegible]	[illegible]	[illegible]	[illegible]	[illegible]	[illegible]	[illegible]	[illegible]	[illegible]	[illegible]	[illegible]	[illegible]	[illegible]	[illegible]	[illegible]	[illegible]	[illegible]	[illegible]	[illegible]
Idiopathic epilepsy	[illegible]	[illegible]	[illegible]	[illegible]	[illegible]	[illegible]	[illegible]	[illegible]	[illegible]	[illegible]	[illegible]	[illegible]	[illegible]	[illegible]	[illegible]	[illegible]	[illegible]	[illegible]	[illegible]	[illegible]	[illegible]	[illegible]	[illegible]	[illegible]
Multiple sclerosis	[illegible]	[illegible]	[illegible]	[illegible]	[illegible]	[illegible]	[illegible]	[illegible]	[illegible]	[illegible]	[illegible]	[illegible]	[illegible]	[illegible]	[illegible]	[illegible]	[illegible]	[illegible]	[illegible]	[illegible]	[illegible]	[illegible]	[illegible]	[illegible]
Motor neuron disease	[illegible]	[illegible]	[illegible]	[illegible]	[illegible]	[illegible]	[illegible]	[illegible]	[illegible]	[illegible]	[illegible]	[illegible]	[illegible]	[illegible]	[illegible]	[illegible]	[illegible]	[illegible]	[illegible]	[illegible]	[illegible]	[illegible]	[illegible]	[illegible]
Headache disorders	[illegible]	[illegible]	[illegible]	[illegible]	[illegible]	[illegible]	[illegible]	[illegible]	[illegible]	[illegible]	[illegible]	[illegible]	[illegible]	[illegible]	[illegible]	[illegible]	[illegible]	[illegible]	[illegible]	[illegible]	[illegible]	[illegible]	[illegible]	[illegible]
Migraine	[illegible]	[illegible]	[illegible]	[illegible]	[illegible]	[illegible]	[illegible]	[illegible]	[illegible]	[illegible]	[illegible]	[illegible]	[illegible]	[illegible]	[illegible]	[illegible]	[illegible]	[illegible]	[illegible]	[illegible]	[illegible]	[illegible]	[illegible]	[illegible]
Tension-type headache	[illegible]	[illegible]	[illegible]	[illegible]	[illegible]	[illegible]	[illegible]	[illegible]	[illegible]	[illegible]	[illegible]	[illegible]	[illegible]	[illegible]	[illegible]	[illegible]	[illegible]	[illegible]	[illegible]	[illegible]	[illegible]	[illegible]	[illegible]	[illegible]
Other neurological disorders	[illegible]	[illegible]	[illegible]	[illegible]	[illegible]	[illegible]	[illegible]	[illegible]	[illegible]	[illegible]	[illegible]	[illegible]	[illegible]	[illegible]	[illegible]	[illegible]	[illegible]	[illegible]	[illegible]	[illegible]	[illegible]	[illegible]	[illegible]	[illegible]
Mental disorders	[illegible]	[illegible]	[illegible]	[illegible]	[illegible]	[illegible]	[illegible]	[illegible]	[illegible]	[illegible]	[illegible]	[illegible]	[illegible]	[illegible]	[illegible]	[illegible]	[illegible]	[illegible]	[illegible]	[illegible]	[illegible]	[illegible]	[illegible]	[illegible]
Schizophrenia	[illegible]	[illegible]	[illegible]	[illegible]	[illegible]	[illegible]	[illegible]	[illegible]	[illegible]	[illegible]	[illegible]	[illegible]	[illegible]	[illegible]	[illegible]	[illegible]	[illegible]	[illegible]	[illegible]	[illegible]	[illegible]	[illegible]	[illegible]	[illegible]
Depressive disorders	[illegible]	[illegible]	[illegible]	[illegible]	[illegible]	[illegible]	[illegible]	[illegible]	[illegible]	[illegible]	[illegible]	[illegible]	[illegible]	[illegible]	[illegible]	[illegible]	[illegible]	[illegible]	[illegible]	[illegible]	[illegible]	[illegible]	[illegible]	[illegible]
Major depressive disorder	[illegible]	[illegible]	[illegible]	[illegible]	[illegible]	[illegible]	[illegible]	[illegible]	[illegible]	[illegible]	[illegible]	[illegible]	[illegible]	[illegible]	[illegible]	[illegible]	[illegible]	[illegible]	[illegible]	[illegible]	[illegible]	[illegible]	[illegible]	[illegible]
Dysthymia	[illegible]	[illegible]	[illegible]	[illegible]	[illegible]	[illegible]	[illegible]	[illegible]	[illegible]	[illegible]	[illegible]	[illegible]	[illegible]	[illegible]	[illegible]	[illegible]	[illegible]	[illegible]	[illegible]	[illegible]	[illegible]	[illegible]	[illegible]	[illegible]
Bipolar disorder	[illegible]	[illegible]	[illegible]	[illegible]	[illegible]	[illegible]	[illegible]	[illegible]	[illegible]	[illegible]	[illegible]	[illegible]	[illegible]	[illegible]	[illegible]	[illegible]	[illegible]	[illegible]	[illegible]	[illegible]	[illegible]	[illegible]	[illegible]	[illegible]
Anxiety disorders	[illegible]	[illegible]	[illegible]	[illegible]	[illegible]	[illegible]	[illegible]	[illegible]	[illegible]	[illegible]	[illegible]	[illegible]	[illegible]	[illegible]	[illegible]	[illegible]	[illegible]	[illegible]	[illegible]	[illegible]	[illegible]	[illegible]	[illegible]	[illegible]
Eating disorders	[illegible]	[illegible]	[illegible]	[illegible]	[illegible]	[illegible]	[illegible]	[illegible]	[illegible]	[illegible]	[illegible]	[illegible]	[illegible]	[illegible]	[illegible]	[illegible]	[illegible]	[illegible]	[illegible]	[illegible]	[illegible]	[illegible]	[illegible]	[illegible]
Anorexia nervosa	[illegible]	[illegible]	[illegible]	[illegible]	[illegible]	[illegible]	[illegible]	[illegible]	[illegible]	[illegible]	[illegible]	[illegible]	[illegible]	[illegible]	[illegible]	[illegible]	[illegible]	[illegible]	[illegible]	[illegible]	[illegible]	[illegible]	[illegible]	[illegible]
Bulimia nervosa	[illegible]	[illegible]	[illegible]	[illegible]	[illegible]	[illegible]	[illegible]	[illegible]	[illegible]	[illegible]	[illegible]	[illegible]	[illegible]	[illegible]	[illegible]	[illegible]	[illegible]	[illegible]	[illegible]	[illegible]	[illegible]	[illegible]	[illegible]	[illegible]
Autism spectrum disorders	[illegible]	[illegible]	[illegible]	[illegible]	[illegible]	[illegible]	[illegible]	[illegible]	[illegible]	[illegible]	[illegible]	[illegible]	[illegible]	[illegible]	[illegible]	[illegible]	[illegible]	[illegible]	[illegible]	[illegible]	[illegible]	[illegible]	[illegible]	[illegible]
Attention-deficit/hyperactivity disorder	[illegible]	[illegible]	[illegible]	[illegible]	[illegible]	[illegible]	[illegible]	[illegible]	[illegible]	[illegible]	[illegible]	[illegible]	[illegible]	[illegible]	[illegible]	[illegible]	[illegible]	[illegible]	[illegible]	[illegible]	[illegible]	[illegible]	[illegible]	[illegible]
Conduct disorder	[illegible]	[illegible]	[illegible]	[illegible]	[illegible]	[illegible]	[illegible]	[illegible]	[illegible]	[illegible]	[illegible]	[illegible]	[illegible]	[illegible]	[illegible]	[illegible]	[illegible]	[illegible]	[illegible]	[illegible]	[illegible]	[illegible]	[illegible]	[illegible]
Idiopathic developmental intellectual disability	[illegible]	[illegible]	[illegible]	[illegible]	[illegible]	[illegible]	[illegible]	[illegible]	[illegible]	[illegible]	[illegible]	[illegible]	[illegible]	[illegible]	[illegible]	[illegible]	[illegible]	[illegible]	[illegible]	[illegible]	[illegible]	[illegible]	[illegible]	[illegible]
Other mental disorders	[illegible]	[illegible]	[illegible]	[illegible]	[illegible]	[illegible]	[illegible]	[illegible]	[illegible]	[illegible]	[illegible]	[illegible]	[illegible]	[illegible]	[illegible]	[illegible]	[illegible]	[illegible]	[illegible]	[illegible]	[illegible]	[illegible]	[illegible]	[illegible]
Substance use disorders	[illegible]	[illegible]	[illegible]	[illegible]	[illegible]	[illegible]	[illegible]	[illegible]	[illegible]	[illegible]	[illegible]	[illegible]	[illegible]	[illegible]	[illegible]	[illegible]	[illegible]	[illegible]	[illegible]	[illegible]	[illegible]	[illegible]	[illegible]	[illegible]
Alcohol use disorders	[illegible]	[illegible]	[illegible]	[illegible]	[illegible]	[illegible]	[illegible]	[illegible]	[illegible]	[illegible]	[illegible]	[illegible]	[illegible]	[illegible]	[illegible]	[illegible]	[illegible]	[illegible]	[illegible]	[illegible]	[illegible]	[illegible]	[illegible]	[illegible]
Drug use disorders	[illegible]	[illegible]	[illegible]	[illegible]	[illegible]	[illegible]	[illegible]	[illegible]	[illegible]	[illegible]	[illegible]	[illegible]	[illegible]	[illegible]	[illegible]	[illegible]	[illegible]	[illegible]	[illegible]	[illegible]	[illegible]	[illegible]	[illegible]	[illegible]
Opioid use disorders	[illegible]	[illegible]	[illegible]	[illegible]	[illegible]	[illegible]	[illegible]	[illegible]	[illegible]	[illegible]	[illegible]	[illegible]	[illegible]	[illegible]	[illegible]	[illegible]	[illegible]	[illegible]	[illegible]	[illegible]	[illegible]	[illegible]	[illegible]	[illegible]
Cocaine use disorders	[illegible]	[illegible]	[illegible]	[illegible]	[illegible]	[illegible]	[illegible]	[illegible]	[illegible]	[illegible]	[illegible]	[illegible]	[illegible]	[illegible]	[illegible]	[illegible]	[illegible]	[illegible]	[illegible]	[illegible]	[illegible]	[illegible]	[illegible]	[illegible]
Amphetamine use disorders	[illegible]	[illegible]	[illegible]	[illegible]	[illegible]	[illegible]	[illegible]	[illegible]	[illegible]	[illegible]	[illegible]	[illegible]	[illegible]	[illegible]	[illegible]	[illegible]	[illegible]	[illegible]	[illegible]	[illegible]	[illegible]	[illegible]	[illegible]	[illegible]
Cannabis use disorders	[illegible]	[illegible]	[illegible]	[illegible]	[illegible]	[illegible]	[illegible]	[illegible]	[illegible]	[illegible]	[illegible]	[illegible]	[illegible]	[illegible]	[illegible]	[illegible]	[illegible]	[illegible]	[illegible]	[illegible]	[illegible]	[illegible]	[illegible]	[illegible]
Other drug use disorders	[illegible]	[illegible]	[illegible]	[illegible]	[illegible]	[illegible]	[illegible]	[illegible]	[illegible]	[illegible]	[illegible]	[illegible]	[illegible]	[illegible]	[illegible]	[illegible]	[illegible]	[illegible]	[illegible]	[illegible]	[illegible]	[illegible]	[illegible]	[illegible]
Diabetes and kidney diseases	[illegible]	[illegible]	[illegible]	[illegible]	[illegible]	[illegible]	[illegible]	[illegible]	[illegible]	[illegible]	[illegible]	[illegible]	[illegible]	[illegible]	[illegible]	[illegible]	[illegible]	[illegible]	[illegible]	[illegible]	[illegible]	[illegible]	[illegible]	[illegible]
Diabetes mellitus	[illegible]	[illegible]	[illegible]	[illegible]	[illegible]	[illegible]	[illegible]	[illegible]	[illegible]	[illegible]	[illegible]	[illegible]	[illegible]	[illegible]	[illegible]	[illegible]	[illegible]	[illegible]	[illegible]	[illegible]	[illegible]	[illegible]	[illegible]	[illegible]
Diabetes mellitus type 1	[illegible]	[illegible]	[illegible]	[illegible]	[illegible]	[illegible]	[illegible]	[illegible]	[illegible]	[illegible]	[illegible]	[illegible]	[illegible]	[illegible]	[illegible]	[illegible]	[illegible]	[illegible]	[illegible]	[illegible]	[illegible]	[illegible]	[illegible]	[illegible]
Diabetes mellitus type 2	[illegible]	[illegible]	[illegible]	[illegible]	[illegible]	[illegible]	[illegible]	[illegible]	[illegible]	[illegible]	[illegible]	[illegible]	[illegible]	[illegible]	[illegible]	[illegible]	[illegible]	[illegible]	[illegible]	[illegible]	[illegible]	[illegible]	[illegible]	[illegible]
Chronic kidney disease	[illegible]	[illegible]	[illegible]	[illegible]	[illegible]	[illegible]	[illegible]	[illegible]	[illegible]	[illegible]	[illegible]	[illegible]	[illegible]	[illegible]	[illegible]	[illegible]	[illegible]	[illegible]	[illegible]	[illegible]	[illegible]	[illegible]	[illegible]	[illegible]
Chronic kidney disease due to diabetes mellitus type 1	[illegible]	[illegible]	[illegible]	[illegible]	[illegible]	[illegible]	[illegible]	[illegible]	[illegible]	[illegible]	[illegible]	[illegible]	[illegible]	[illegible]	[illegible]	[illegible]	[illegible]	[illegible]	[illegible]	[illegible]	[illegible]	[illegible]	[illegible]	[illegible]
Chronic kidney disease due to diabetes mellitus type 2	[illegible]	[illegible]	[illegible]	[illegible]	[illegible]	[illegible]	[illegible]	[illegible]	[illegible]	[illegible]	[illegible]	[illegible]	[illegible]	[illegible]	[illegible]	[illegible]	[illegible]	[illegible]	[illegible]	[illegible]	[illegible]	[illegible]	[illegible]	[illegible]
Chronic kidney disease due to hypertension	[illegible]	[illegible]	[illegible]	[illegible]	[illegible]	[illegible]	[illegible]	[illegible]	[illegible]	[illegible]	[illegible]	[illegible]	[illegible]	[illegible]	[illegible]	[illegible]	[illegible]	[illegible]	[illegible]	[illegible]	[illegible]	[illegible]	[illegible]	[illegible]
Chronic kidney disease due to glomerulonephritis	[illegible]	[illegible]	[illegible]	[illegible]	[illegible]	[illegible]	[illegible]	[illegible]	[illegible]	[illegible]	[illegible]	[illegible]	[illegible]	[illegible]	[illegible]	[illegible]	[illegible]	[illegible]	[illegible]	[illegible]	[illegible]	[illegible]	[illegible]	[illegible]
Chronic kidney disease due to other and unspecified causes	[illegible]	[illegible]	[illegible]	[illegible]	[illegible]	[illegible]	[illegible]	[illegible]	[illegible]	[illegible]	[illegible]	[illegible]	[illegible]	[illegible]	[illegible]	[illegible]	[illegible]	[illegible]	[illegible]	[illegible]	[illegible]	[illegible]	[illegible]	[illegible]
Acute glomerulonephritis	[illegible]	[illegible]	[illegible]	[illegible]	[illegible]	[illegible]	[illegible]	[illegible]	[illegible]	[illegible]	[illegible]	[illegible]	[illegible]	[illegible]	[illegible]	[illegible]	[illegible]	[illegible]	[illegible]	[illegible]	[illegible]	[illegible]	[illegible]	[illegible]
Skin and subcutaneous diseases	[illegible]	[illegible]	[illegible]	[illegible]	[illegible]	[illegible]	[illegible]	[illegible]	[illegible]	[illegible]	[illegible]	[illegible]	[illegible]	[illegible]	[illegible]	[illegible]	[illegible]	[illegible]	[illegible]	[illegible]	[illegible]	[illegible]	[illegible]	[illegible]
Dermatitis	[illegible]	[illegible]	[illegible]	[illegible]	[illegible]	[illegible]	[illegible]	[illegible]	[illegible]	[illegible]	[illegible]	[illegible]	[illegible]	[illegible]	[illegible]	[illegible]	[illegible]	[illegible]	[illegible]	[illegible]	[illegible]	[illegible]	[illegible]	[illegible]
Atopic dermatitis	[illegible]	[illegible]	[illegible]	[illegible]	[illegible]	[illegible]	[illegible]	[illegible]	[illegible]	[illegible]	[illegible]	[illegible]	[illegible]	[illegible]	[illegible]	[illegible]	[illegible]	[illegible]	[illegible]	[illegible]	[illegible]	[illegible]	[illegible]	[illegible]
Contact dermatitis	[illegible]	[illegible]	[illegible]	[illegible]	[illegible]	[illegible]	[illegible]	[illegible]	[illegible]	[illegible]	[illegible]	[illegible]	[illegible]	[illegible]	[illegible]	[illegible]	[illegible]	[illegible]	[illegible]	[illegible]	[illegible]	[illegible]	[illegible]	[illegible]
Seborrhoeic dermatitis	[illegible]	[illegible]	[illegible]	[illegible]	[illegible]	[illegible]	[illegible]	[illegible]	[illegible]	[illegible]	[illegible]	[illegible]	[illegible]	[illegible]	[illegible]	[illegible]	[illegible]	[illegible]	[illegible]	[illegible]	[illegible]	[illegible]	[illegible]	[illegible]
Psoriasis	[illegible]	[illegible]	[illegible]	[illegible]	[illegible]	[illegible]	[illegible]	[illegible]	[illegible]	[illegible]	[illegible]	[illegible]	[illegible]	[illegible]	[illegible]	[illegible]	[illegible]	[illegible]	[illegible]	[illegible]	[illegible]	[illegible]	[illegible]	[illegible]
Bacterial skin diseases	[illegible]	[illegible]	[illegible]	[illegible]	[illegible]	[illegible]	[illegible]	[illegible]	[illegible]	[illegible]	[illegible]	[illegible]	[illegible]	[illegible]	[illegible]	[illegible]	[illegible]	[illegible]	[illegible]	[illegible]	[illegible]	[illegible]	[illegible]	[illegible]
Cellulitis	[illegible]	[illegible]	[illegible]	[illegible]	[illegible]	[illegible]	[illegible]	[illegible]	[illegible]	[illegible]	[illegible]	[illegible]	[illegible]	[illegible]	[illegible]	[illegible]	[illegible]	[illegible]	[illegible]	[illegible]	[illegible]	[illegible]	[illegible]	[illegible]
Pyoderma	[illegible]	[illegible]	[illegible]	[illegible]	[illegible]	[illegible]	[illegible]	[illegible]	[illegible]	[illegible]	[illegible]	[illegible]	[illegible]	[illegible]	[illegible]	[illegible]	[illegible]	[illegible]	[illegible]	[illegible]	[illegible]	[illegible]	[illegible]	[illegible]
Scabies	[illegible]	[illegible]	[illegible]	[illegible]	[illegible]	[illegible]	[illegible]	[illegible]	[illegible]	[illegible]	[illegible]	[illegible]	[illegible]	[illegible]	[illegible]	[illegible]	[illegible]	[illegible]	[illegible]	[illegible]	[illegible]	[illegible]	[illegible]	[illegible]
Fungal skin diseases	[illegible]	[illegible]	[illegible]	[illegible]	[illegible]	[illegible]	[illegible]	[illegible]	[illegible]	[illegible]	[illegible]	[illegible]	[illegible]	[illegible]	[illegible]	[illegible]	[illegible]	[illegible]	[illegible]	[illegible]	[illegible]	[illegible]	[illegible]	[illegible]
Viral skin diseases	[illegible]	[illegible]	[illegible]	[illegible]	[illegible]	[illegible]	[illegible]	[illegible]	[illegible]	[illegible]	[illegible]	[illegible]	[illegible]	[illegible]	[illegible]	[illegible]	[illegible]	[illegible]	[illegible]	[illegible]	[illegible]	[illegible]	[illegible]	[illegible]
Acne vulgaris	[illegible]	[illegible]	[illegible]	[illegible]	[illegible]	[illegible]	[illegible]	[illegible]	[illegible]	[illegible]	[illegible]	[illegible]	[illegible]	[illegible]	[illegible]	[illegible]	[illegible]	[illegible]	[illegible]	[illegible]	[illegible]	[illegible]	[illegible]	[illegible]
Alopecia areata	[illegible]	[illegible]	[illegible]	[illegible]	[illegible]	[illegible]	[illegible]	[illegible]	[illegible]	[illegible]	[illegible]	[illegible]	[illegible]	[illegible]	[illegible]	[illegible]	[illegible]	[illegible]	[illegible]	[illegible]	[illegible]	[illegible]	[illegible]	[illegible]
Pruritus	[illegible]	[illegible]	[illegible]	[illegible]	[illegible]	[illegible]	[illegible]	[illegible]	[illegible]	[illegible]	[illegible]	[illegible]	[illegible]	[illegible]	[illegible]	[illegible]	[illegible]	[illegible]	[illegible]	[illegible]	[illegible]	[illegible]	[illegible]	[illegible]
Urticaria	[illegible]	[illegible]	[illegible]	[illegible]	[illegible]	[illegible]	[illegible]	[illegible]	[illegible]	[illegible]	[illegible]	[illegible]	[illegible]	[illegible]	[illegible]	[illegible]	[illegible]	[illegible]	[illegible]	[illegible]	[illegible]	[illegible]	[illegible]	[illegible]
Decubitus ulcer	[illegible]	[illegible]	[illegible]	[illegible]	[illegible]	[illegible]	[illegible]	[illegible]	[illegible]	[illegible]	[illegible]	[illegible]	[illegible]	[illegible]	[illegible]	[illegible]	[illegible]	[illegible]	[illegible]	[illegible]	[illegible]	[illegible]	[illegible]	[illegible]
Other skin and subcutaneous diseases	[illegible]	[illegible]	[illegible]	[illegible]	[illegible]	[illegible]	[illegible]	[illegible]	[illegible]	[illegible]	[illegible]	[illegible]	[illegible]	[illegible]	[illegible]	[illegible]	[illegible]	[illegible]	[illegible]	[illegible]	[illegible]	[illegible]	[illegible]	[illegible]
Sense organ diseases	[illegible]	[illegible]	[illegible]	[illegible]	[illegible]	[illegible]	[illegible]	[illegible]	[illegible]	[illegible]	[illegible]	[illegible]	[illegible]	[illegible]	[illegible]	[illegible]	[illegible]	[illegible]	[illegible]	[illegible]	[illegible]	[illegible]	[illegible]	[illegible]
Blindness and vision loss	[illegible]	[illegible]	[illegible]	[illegible]	[illegible]	[illegible]	[illegible]	[illegible]	[illegible]	[illegible]	[illegible]	[illegible]	[illegible]	[illegible]	[illegible]	[illegible]	[illegible]	[illegible]	[illegible]	[illegible]	[illegible]	[illegible]	[illegible]	[illegible]
Glaucoma	[illegible]	[illegible]	[illegible]	[illegible]	[illegible]	[illegible]	[illegible]	[illegible]	[illegible]	[illegible]	[illegible]	[illegible]	[illegible]	[illegible]	[illegible]	[illegible]	[illegible]	[illegible]	[illegible]	[illegible]	[illegible]	[illegible]	[illegible]	[illegible]
Cataract	[illegible]	[illegible]	[illegible]	[illegible]	[illegible]	[illegible]	[illegible]	[illegible]	[illegible]	[illegible]	[illegible]	[illegible]	[illegible]	[illegible]	[illegible]	[illegible]	[illegible]	[illegible]	[illegible]	[illegible]	[illegible]	[illegible]	[illegible]	[illegible]
Age-related macular degeneration	[illegible]	[illegible]	[illegible]	[illegible]	[illegible]	[illegible]	[illegible]	[illegible]	[illegible]	[illegible]	[illegible]	[illegible]	[illegible]	[illegible]	[illegible]	[illegible]	[illegible]	[illegible]	[illegible]	[illegible]	[illegible]	[illegible]	[illegible]	[illegible]
Refraction disorders	[illegible]	[illegible]	[illegible]	[illegible]	[illegible]	[illegible]	[illegible]	[illegible]	[illegible]	[illegible]	[illegible]	[illegible]	[illegible]	[illegible]	[illegible]	[illegible]	[illegible]	[illegible]	[illegible]	[illegible]	[illegible]	[illegible]	[illegible]	[illegible]
Near vision loss	[illegible]	[illegible]	[illegible]	[illegible]	[illegible]	[illegible]	[illegible]	[illegible]	[illegible]	[illegible]	[illegible]	[illegible]	[illegible]	[illegible]	[illegible]	[illegible]	[illegible]	[illegible]	[illegible]	[illegible]	[illegible]	[illegible]	[illegible]	[illegible]
Other vision loss	[illegible]	[illegible]	[illegible]	[illegible]	[illegible]	[illegible]	[illegible]	[illegible]	[illegible]	[illegible]	[illegible]	[illegible]	[illegible]	[illegible]	[illegible]	[illegible]	[illegible]	[illegible]	[illegible]	[illegible]	[illegible]	[illegible]	[illegible]	[illegible]
Age-related and other hearing loss	[illegible]	[illegible]	[illegible]	[illegible]	[illegible]	[illegible]	[illegible]	[illegible]	[illegible]	[illegible]	[illegible]	[illegible]	[illegible]	[illegible]	[illegible]	[illegible]	[illegible]	[illegible]	[illegible]	[illegible]	[illegible]	[illegible]	[illegible]	[illegible]
Other sense organ diseases	[illegible]	[illegible]	[illegible]	[illegible]	[illegible]	[illegible]	[illegible]	[illegible]	[illegible]	[illegible]	[illegible]	[illegible]	[illegible]	[illegible]	[illegible]	[illegible]	[illegible]	[illegible]	[illegible]	[illegible]	[illegible]	[illegible]	[illegible]	[illegible]
Musculoskeletal disorders	[illegible]	[illegible]	[illegible]	[illegible]	[illegible]	[illegible]	[illegible]	[illegible]	[illegible]	[illegible]	[illegible]	[illegible]	[illegible]	[illegible]	[illegible]	[illegible]	[illegible]	[illegible]	[illegible]	[illegible]	[illegible]	[illegible]	[illegible]	[illegible]
Rheumatoid arthritis	[illegible]	[illegible]	[illegible]	[illegible]	[illegible]	[illegible]	[illegible]	[illegible]	[illegible]	[illegible]	[illegible]	[illegible]	[illegible]	[illegible]	[illegible]	[illegible]	[illegible]	[illegible]	[illegible]	[illegible]	[illegible]	[illegible]	[illegible]	[illegible]
Osteoarthritis	[illegible]	[illegible]	[illegible]	[illegible]	[illegible]	[illegible]	[illegible]	[illegible]	[illegible]	[illegible]	[illegible]	[illegible]	[illegible]	[illegible]	[illegible]	[illegible]	[illegible]	[illegible]	[illegible]	[illegible]	[illegible]	[illegible]	[illegible]	[illegible]
Osteoarthritis hip	[illegible]	[illegible]	[illegible]	[illegible]	[illegible]	[illegible]	[illegible]	[illegible]	[illegible]	[illegible]	[illegible]	[illegible]	[illegible]	[illegible]	[illegible]	[illegible]	[illegible]	[illegible]	[illegible]	[illegible]	[illegible]	[illegible]	[illegible]	[illegible]
Osteoarthritis knee	[illegible]	[illegible]	[illegible]	[illegible]	[illegible]	[illegible]	[illegible]	[illegible]	[illegible]	[illegible]	[illegible]	[illegible]	[illegible]	[illegible]	[illegible]	[illegible]	[illegible]	[illegible]	[illegible]	[illegible]	[illegible]	[illegible]	[illegible]	[illegible]
Osteoarthritis hand	[illegible]	[illegible]	[illegible]	[illegible]	[illegible]	[illegible]	[illegible]	[illegible]	[illegible]	[illegible]	[illegible]	[illegible]	[illegible]	[illegible]	[illegible]	[illegible]	[illegible]	[illegible]	[illegible]	[illegible]	[illegible]	[illegible]	[illegible]	[illegible]

Osteoarthritis other	0 (0 to 0)	0 (0 to 0)	0 (0 to 0)	0 (0 to 0)	0 (0 to 0)	0 (0 to 0)	0 (0 to 0)	0 (0 to 0)	0 (0 to 0)	0·1 (0 to 0·2)	0·5 (0·2 to 1·3)	1·7 (0·7 to 3·9)	3·6 (1·5 to 8·2)	5·9 (2·6 to 12·7)	7·3 (3·4 to 15·7)	7·6 (3·5 to 16·2)	6·6 (3·2 to 14·2)	6·4 (3·1 to 13·2)	5 (2·5 to 10·1)	3·7 (1·9 to 7·4)	2·2 (1·1 to 4·3)	0·9 (0·5 to 1·8)	0·2 (0·1 to 0·4)	51·7
Low back pain	0 (0 to 0)	0 (0 to 0)	0 (0 to 0)	0 (0 to 0)	5·5 (2·6 to 10·1)	30·2 (17·7 to 48·7)	60·5 (36·2 to 97·3)	77·6 (46·1 to 123)	94 (54·7 to 146)	101 (58·2 to 155)	125 (74·3 to 190)	137 (81·3 to 214)	153 (90·8 to 240)	159 (97·9 to 250)	144 (89·4 to 218)	120 (76·6 to 180)	91·3 (58·1 to 141)	78·6 (48·9 to 117)	56·8 (35·1 to 85)	39·9 (25 to 59·5)	19·9 (12·4 to 29·3)	7 (4·3 to 10·4)	1·4 (0·9 to 2·2)	1,500
Neck pain	0 (0 to 0)	0 (0 to 0)	0 (0 to 0)	0 (0 to 0)	0·3 (0·1 to 0·7)	2 (0·9 to 3·8)	5 (2 to 9·3)	8·1 (3·6 to 16·5)	13 (6 to 25·2)	19·1 (9 to 36·4)	27·5 (13·9 to 47·3)	33·7 (17·2 to 60·6)	39 (19·8 to 69·9)	35·4 (18·1 to 67·1)	30·1 (14·7 to 54·4)	23·2 (11·6 to 40·6)	16·4 (8·8 to 27·7)	13·4 (7·1 to 22·8)	8·8 (4·4 to 16·2)	5·2 (2·6 to 9·4)	2·6 (1·4 to 4·4)	1 (0·5 to 1·7)	0·2 (0·1 to 0·4)	284
Gout	0 (0 to 0)	0 (0 to 0)	0 (0 to 0)	0 (0 to 0)	0 (0 to 0)	0 (0 to 0)	0 (0 to 0)	0·2 (0·1 to 0·3)	0·5 (0·1 to 0·9)	0·8 (0·2 to 1·5)	1·2 (0·5 to 2)	1·5 (0·7 to 2·7)	1·8 (0·9 to 3·2)	2 (1 to 3·6)	1·9 (0·9 to 3·3)	1·7 (0·8 to 3)	1·4 (0·7 to 2·4)	1·4 (0·7 to 2·3)	1·1 (0·5 to 1·8)	0·8 (0·4 to 1·4)	0·5 (0·2 to 0·8)	0·2 (0·1 to 0·3)	0 (0 to 0·1)	16·9
Other musculoskeletal disorders	0 (0 to 0)	0 (0 to 0)	0 (0 to 0)	0 (0 to 0)	0·5 (0·3 to 0·8)	1·8 (1 to 3·1)	14·1 (8·4 to 22·4)	36·2 (23·7 to 51·5)	67·2 (43·5 to 98·1)	83·7 (54·9 to 124)	96·9 (63·2 to 141)	105 (67·6 to 158)	114 (73·3 to 166)	113 (71·7 to 171)	100 (63·2 to 149)	79·8 (48·3 to 121)	57 (35·3 to 84·5)	45 (26·5 to 71·6)	26·6 (16 to 41·7)	15 (7·6 to 25)	11 (7·3 to 15·6)	4·3 (2·7 to 6·3)	0·9 (0·5 to 1·4)	972
Other non-communicable diseases	161 (105 to 243)	58·9 (39 to 84·5)	113 (75·8 to 164)	94·9 (68·9 to 131)	67·8 (54·4 to 85·7)	69·5 (54·4 to 87·9)	102 (78·8 to 134)	154 (115 to 209)	199 (147 to 271)	213 (157 to 287)	244 (182 to 334)	257 (187 to 353)	261 (190 to 352)	245 (181 to 322)	216 (158 to 291)	192 (142 to 255)	156 (116 to 205)	150 (113 to 193)	113 (87·6 to 146)	81·6 (63·6 to 103)	55·1 (44·4 to 67)	25·8 (20·9 to 31·4)	6 (4·8 to 7·5)	3,240
Congenital birth defects	157 (102 to 237)	50·5 (33 to 73·9)	71·4 (46·2 to 107)	52·6 (36·4 to 76·6)	27 (21·4 to 33·7)	24·3 (19·1 to 31)	24·6 (19·4 to 30·7)	22·5 (17·5 to 28·3)	20·9 (16·5 to 26·1)	16·8 (13·1 to 21)	16·2 (12·8 to 20·3)	14·8 (11·7 to 18·6)	14·4 (11·2 to 18·3)	12·7 (9·9 to 15·9)	9·8 (7·5 to 12·4)	6 (4·6 to 7·6)	3·7 (2·8 to 4·8)	1·5 (1 to 2·3)	0·9 (0·5 to 1·3)	0·5 (0·3 to 0·8)	0·2 (0·1 to 0·4)	0·1 (0·1 to 0·1)	0 (0 to 0)	548
Neural tube defects	12·7 (7 to 21·5)	4·1 (2 to 7·5)	3·7 (1·7 to 7·3)	3·8 (1·8 to 7·3)	1 (0·7 to 1·4)	1·4 (0·9 to 2·1)	1·2 (0·8 to 1·7)	0·7 (0·5 to 0·9)	0·8 (0·5 to 1·1)	0·7 (0·5 to 1)	0·5 (0·4 to 0·8)	0·5 (0·4 to 0·7)	0·3 (0·2 to 0·4)	0·2 (0·2 to 0·3)	0·2 (0·1 to 0·3)	0·1 (0·1 to 0·1)	0·1 (0·1 to 0·2)	0 (0 to 0)	0 (0 to 0)	0 (0 to 0)	0 (0 to 0)	0 (0 to 0)	0 (0 to 0)	32
Congenital heart anomalies	54·4 (31·1 to 87·5)	21·2 (12·6 to 32·8)	31·9 (19·1 to 50·5)	19·5 (11·9 to 31·3)	7·8 (5·5 to 10·6)	5·9 (4·1 to 8·1)	6·3 (4·5 to 8·4)	5·8 (4 to 8)	5·8 (4·2 to 7·9)	4·2 (3 to 5·7)	4·2 (3 to 5·8)	3·6 (2·6 to 5·1)	3·2 (2·2 to 4·3)	2·5 (1·7 to 3·4)	1·5 (1 to 2·1)	0·9 (0·6 to 1·3)	0·6 (0·4 to 0·8)	0·1 (0 to 0·2)	0·1 (0 to 0·1)	0 (0 to 0·1)	0 (0 to 0·1)	0 (0 to 0)	0 (0 to 0)	180
Orofacial clefts	0 (0 to 0·1)	0 (0 to 0)	0·1 (0·1 to 0·2)	0·3 (0·2 to 0·6)	0·3 (0·1 to 0·6)	0·4 (0·1 to 0·6)	0·4 (0·2 to 0·7)	0·4 (0·2 to 0·7)	0·4 (0·2 to 0·7)	0·3 (0·1 to 0·6)	0·4 (0·2 to 0·7)	0·3 (0·1 to 0·6)	0·3 (0·2 to 0·6)	0·3 (0·1 to 0·6)	0·3 (0·1 to 0·5)	0·2 (0·1 to 0·4)	0·1 (0·1 to 0·3)	0·1 (0 to 0·2)	0·1 (0 to 0·1)	0·1 (0 to 0·1)	0 (0 to 0)	0 (0 to 0)	0 (0 to 0)	5
Down syndrome	2·1 (1·1 to 3·7)	0·8 (0·4 to 1·4)	3·6 (1·9 to 6·3)	3·3 (1·8 to 5·8)	1·2 (0·9 to 1·7)	1·4 (0·9 to 2)	2·2 (1·4 to 3·2)	2·1 (1·4 to 3)	2·1 (1·4 to 3·2)	2·1 (1·3 to 3·1)	2·2 (1·5 to 3·2)	2·3 (1·6 to 3·4)	2·6 (1·8 to 3·8)	2·5 (1·7 to 3·6)	2·3 (1·6 to 3·4)	1·1 (0·7 to 1·6)	0·4 (0·3 to 0·6)	0 (0 to 0)	0 (0 to 0)	0 (0 to 0)	0 (0 to 0)	0 (0 to 0)	0 (0 to 0)	34·4
Turner syndrome	0 (0 to 0)	0 (0 to 0)	0 (0 to 0)	0 (0 to 0)	0 (0 to 0)	0 (0 to 0)	0 (0 to 0)	0 (0 to 0)	0 (0 to 0)	0 (0 to 0)	0 (0 to 0)	0 (0 to 0)	0 (0 to 0)	0 (0 to 0)	0 (0 to 0)	0 (0 to 0)	0 (0 to 0)	0 (0 to 0)	0 (0 to 0)	0 (0 to 0)	0 (0 to 0)	0 (0 to 0)	0 (0 to 0)	0·2
Klinefelter syndrome	0 (0 to 0)	0 (0 to 0)	0 (0 to 0)	0 (0 to 0)	0 (0 to 0)	0 (0 to 0)	0 (0 to 0)	0 (0 to 0)	0 (0 to 0)	0 (0 to 0)	0 (0 to 0)	0 (0 to 0)	0 (0 to 0)	0 (0 to 0)	0 (0 to 0)	0 (0 to 0)	0 (0 to 0)	0 (0 to 0)	0 (0 to 0)	0 (0 to 0)	0 (0 to 0)	0 (0 to 0)	0 (0 to 0)	0
Other chromosomal abnormalities	4·1 (2·1 to 7·1)	2·3 (1·2 to 3·8)	3·3 (2·1 to 5·1)	2 (1·4 to 2·9)	1·1 (0·8 to 1·6)	1 (0·7 to 1·5)	1·1 (0·8 to 1·6)	1 (0·7 to 1·3)	0·8 (0·6 to 1·1)	0·5 (0·4 to 0·7)	0·4 (0·3 to 0·5)	0·3 (0·2 to 0·4)	0·2 (0·1 to 0·3)	0·2 (0·1 to 0·2)	0·1 (0·1 to 0·1)	0 (0 to 0·1)	0 (0 to 0)	0 (0 to 0)	0 (0 to 0)	0 (0 to 0)	0 (0 to 0)	0 (0 to 0)	0 (0 to 0)	18·4
Congenital musculoskeletal and limb anomalies	4·7 (2·2 to 9·3)	1·2 (0·6 to 2·2)	4 (2·5 to 6·4)	5·9 (3·6 to 8·9)	6·4 (3·9 to 9·7)	7 (4·2 to 10·6)	6·7 (4 to 10·3)	5·1 (3·2 to 7·5)	4·8 (3 to 7·2)	4 (2·5 to 6)	4·3 (2·6 to 6·5)	4 (2·5 to 6·2)	4·1 (2·5 to 6·4)	3·9 (2·5 to 5·8)	3·2 (2 to 4·9)	2·4 (1·4 to 3·6)	1·4 (0·9 to 2·2)	0·9 (0·6 to 1·4)	0·5 (0·3 to 0·8)	0·3 (0·1 to 0·4)	0·1 (0·1 to 0·2)	0 (0 to 0·1)	0 (0 to 0)	75
Urogenital congenital anomalies	8·3 (4·5 to 14·6)	2·2 (1·2 to 3·9)	2 (1 to 3·5)	1·3 (0·8 to 1·9)	0·9 (0·6 to 1·2)	0·9 (0·6 to 1·3)	0·6 (0·4 to 0·8)	0·5 (0·3 to 0·8)	0·5 (0·3 to 0·7)	0·4 (0·2 to 0·6)	0·3 (0·2 to 0·4)	0·2 (0·1 to 0·3)	0·4 (0·2 to 0·5)	0·1 (0·1 to 0·2)	0·1 (0·1 to 0·2)	0·1 (0·1 to 0·2)	0·1 (0 to 0·1)	0 (0 to 0)	0 (0 to 0)	0 (0 to 0)	0 (0 to 0)	0 (0 to 0)	0 (0 to 0)	18·7
Digestive congenital anomalies	20·2 (11·7 to 32·5)	8·2 (4·5 to 13·4)	6·6 (3·7 to 11)	3·3 (1·9 to 5·9)	0·9 (0·6 to 1·4)	0·5 (0·3 to 0·7)	0·3 (0·2 to 0·4)	0·2 (0·1 to 0·3)	0·2 (0·1 to 0·2)	0·1 (0·1 to 0·2)	0·1 (0·1 to 0·1)	0·1 (0·1 to 0·2)	0·2 (0·1 to 0·2)	0·1 (0·1 to 0·1)	0·1 (0·1 to 0·2)	0·1 (0·1 to 0·1)	0 (0 to 0·1)	0 (0 to 0)	0 (0 to 0)	0 (0 to 0)	0 (0 to 0)	0 (0 to 0)	0 (0 to 0)	41·2
Other congenital birth defects	50·3 (31·2 to 77·6)	10·5 (6·3 to 16)	16·2 (9·9 to 25·3)	13·1 (7·8 to 20·7)	7·3 (5·2 to 10)	5·8 (4·1 to 8·1)	5·9 (4·2 to 8·2)	6·8 (4·7 to 9·5)	5·5 (3·8 to 7·8)	4·4 (3 to 6·2)	3·8 (2·6 to 5·5)	3·3 (2·3 to 4·9)	3·2 (2·1 to 4·8)	2·8 (2 to 4·2)	1·9 (1·2 to 3)	1·1 (0·7 to 1·9)	0·8 (0·5 to 1·4)	0·3 (0·1 to 0·8)	0·2 (0·1 to 0·5)	0·1 (0 to 0·4)	0·1 (0 to 0·2)	0 (0 to 0·1)	0 (0 to 0)	143
Urinary diseases and male infertility	0·3 (0·2 to 0·5)	0·4 (0·2 to 0·6)	1·6 (0·8 to 2·7)	1·4 (1 to 2·1)	1·1 (0·8 to 1·5)	1·5 (1·1 to 2·1)	2·4 (1·8 to 3·2)	5·5 (3·9 to 7·9)	7·1 (5 to 10·3)	9 (6·3 to 12·8)	12·2 (8·8 to 16·7)	14·3 (10·4 to 19)	16·7 (12·1 to 22·2)	21·4 (15·3 to 28·3)	26·9 (19·3 to 35·4)	32·9 (23·2 to 43·4)	31·6 (22·7 to 41·4)	39·1 (28·1 to 51·2)	31·7 (21·8 to 41·9)	25 (17·5 to 33·4)	18·3 (13·2 to 24)	9·1 (6·4 to 11·9)	2·1 (1·5 to 2·8)	311
Urolithiasis	0 (0 to 0)	0 (0 to 0)	0 (0 to 0)	0 (0 to 0)	0 (0 to 0·1)	0·1 (0 to 0·1)	0·1 (0·1 to 0·2)	0·3 (0·2 to 0·4)	0·5 (0·3 to 0·7)	0·9 (0·6 to 1·4)	1·4 (0·8 to 2·2)	1·5 (0·9 to 2·4)	2·4 (1·4 to 3·7)	2·2 (1·3 to 3·4)	2·3 (1·4 to 3·5)	1·8 (1·1 to 2·6)	1·4 (0·8 to 2·1)	0·9 (0·6 to 1·4)	0·6 (0·3 to 0·9)	0·3 (0·2 to 0·5)	0·3 (0·1 to 0·5)	0·1 (0·1 to 0·2)	0 (0 to 0·1)	17·1
Benign prostatic hyperplasia	0 (0 to 0)	0 (0 to 0)	0 (0 to 0)	0 (0 to 0)	0 (0 to 0)	0 (0 to 0)	0 (0 to 0)	0 (0 to 0)	0 (0 to 0)	0 (0 to 0)	0 (0 to 0)	0·1 (0 to 0·2)	0·5 (0·2 to 1)	2·1 (1 to 3·7)	6·1 (2·8 to 10·5)	9·8 (5 to 16·9)	11·3 (6·4 to 17·9)	12·5 (6·9 to 20·5)	7·9 (4·4 to 12·7)	4·2 (2·4 to 6·7)	1·5 (0·8 to 2·5)	0·4 (0·2 to 0·7)	0 (0 to 0·1)	56·3
Male infertility	0 (0 to 0)	0 (0 to 0)	0 (0 to 0)	0 (0 to 0)	0 (0 to 0)	0 (0 to 0)	0 (0 to 0)	1·3 (0·3 to 3·3)	1·6 (0·5 to 4)	1·6 (0·5 to 4·6)	1·7 (0·5 to 4·4)	1·6 (0·4 to 4)	0·2 (0 to 0·7)	0 (0 to 0)	0 (0 to 0)	0 (0 to 0)	0 (0 to 0)	0 (0 to 0)	0 (0 to 0)	0 (0 to 0)	0 (0 to 0)	0 (0 to 0)	0 (0 to 0)	8·1
Other urinary diseases	0·2 (0·1 to 0·3)	0·2 (0·1 to 0·4)	0·2 (0·1 to 0·4)	0·4 (0·2 to 0·6)	0·3 (0·2 to 0·5)	0·5 (0·3 to 0·8)	0·7 (0·5 to 1)	1·4 (0·8 to 2)	0·9 (0·5 to 1·3)	1·3 (0·8 to 2)	2·3 (1·3 to 3·7)	2·4 (1·4 to 3·7)	3·5 (1·8 to 5·6)	3·6 (2 to 5·9)	3·4 (1·7 to 5·4)	4·4 (2·4 to 7)	3·5 (1·8 to 5·7)	3·6 (1·7 to 6·2)	3·3 (1·4 to 5·7)	2·6 (1·1 to 4·8)	1·6 (0·7 to 2·9)	0·9 (0·4 to 1·6)	0·2 (0·1 to 0·3)	41·2
Gynecological diseases	0 (0 to 0)	0 (0 to 0)	0 (0 to 0)	0 (0 to 0)	0 (0 to 0)	8·5 (4·2 to 14·8)	24·4 (13·7 to 40·5)	57·1 (36·5 to 87·9)	83·6 (54·2 to 123)	93·4 (60·7 to 135)	106 (71·7 to 154)	104 (68·8 to 152)	90·5 (58·5 to 128)	60·1 (36 to 90·3)	33·1 (20·3 to 48·5)	19·9 (11·5 to 30·8)	13 (7·8 to 19·6)	9·5 (5·9 to 13·7)	6·3 (4 to 9·2)	4·1 (2·5 to 6·2)	2·4 (1·5 to 3·5)	1 (0·6 to 1·6)	0·3 (0·1 to 0·4)	717
Uterine fibroids	0 (0 to 0)	0 (0 to 0)	0 (0 to 0)	0 (0 to 0)	0 (0 to 0)	0·1 (0 to 0·3)	0·3 (0·1 to 0·7)	0·9 (0·4 to 2)	2·4 (1 to 4·9)	4·8 (2·1 to 9·8)	8·6 (3·8 to 16·6)	9·8 (4·8 to 18·2)	9·2 (4·6 to 17·4)	6·4 (3 to 12)	3·9 (1·8 to 7·4)	2·1 (1 to 4·3)	1·1 (0·5 to 2·2)	0·6 (0·3 to 1·3)	0·3 (0·2 to 0·7)	0·2 (0·1 to 0·3)	0·1 (0 to 0·1)	0 (0 to 0)	0 (0 to 0)	51
Polycystic ovarian syndrome	0 (0 to 0)	0 (0 to 0)	0 (0 to 0)	0 (0 to 0)	0 (0 to 0)	0·3 (0·1 to 0·6)	1·5 (0·6 to 3)	1·8 (0·7 to 3·8)	1·7 (0·7 to 3·6)	1·5 (0·6 to 3·2)	1·6 (0·7 to 3·2)	1·5 (0·6 to 3)	1·3 (0·5 to 2·7)	0·3 (0·1 to 0·7)	0 (0 to 0)	0 (0 to 0)	0 (0 to 0)	0 (0 to 0)	0 (0 to 0)	0 (0 to 0)	0 (0 to 0)	0 (0 to 0)	0 (0 to 0)	11·5
Female infertility	0 (0 to 0)	0 (0 to 0)	0 (0 to 0)	0 (0 to 0)	0 (0 to 0)	0 (0 to 0)	0 (0 to 0)	1·9 (0·4 to 5·4)	2·9 (0·7 to 7·7)	3·3 (0·7 to 9·7)	3·7 (0·4 to 10·7)	3·2 (0·4 to 9)	0·1 (0 to 0·4)	0 (0 to 0)	0 (0 to 0)	0 (0 to 0)	0 (0 to 0)	0 (0 to 0)	0 (0 to 0)	0 (0 to 0)	0 (0 to 0)	0 (0 to 0)	0 (0 to 0)	15·1
Endometriosis	0 (0 to 0)	0 (0 to 0)	0 (0 to 0)	0 (0 to 0)	0 (0 to 0)	0 (0 to 0)	1·9 (0·8 to 4)	8·4 (4 to 15·4)	9·7 (4·8 to 16·9)	7·4 (3·6 to 13·3)	6·8 (3·5 to 11·9)	6·5 (3·2 to 11·5)	6 (3·3 to 10·2)	2·8 (1·4 to 4·8)	0 (0 to 0)	0 (0 to 0)	0 (0 to 0)	0 (0 to 0)	0 (0 to 0)	0 (0 to 0)	0 (0 to 0)	0 (0 to 0)	0 (0 to 0)	49·6
Genital prolapse	0 (0 to 0)	0 (0 to 0)	0 (0 to 0)	0 (0 to 0)	0 (0 to 0)	0 (0 to 0)	0 (0 to 0)	0·1 (0 to 0·1)	0·2 (0·1 to 0·4)	0·3 (0·1 to 0·7)	0·6 (0·3 to 1·4)	1 (0·4 to 2·1)	1·4 (0·6 to 2·9)	1·6 (0·6 to 3·1)	1·5 (0·6 to 2·9)	1·2 (0·5 to 2·4)	0·9 (0·4 to 1·8)	0·7 (0·3 to 1·4)	0·5 (0·2 to 1)	0·4 (0·2 to 0·8)	0·2 (0·1 to 0·5)	0·1 (0 to 0·2)	0 (0 to 0·1)	10·7
Premenstrual syndrome	0 (0 to 0)	0 (0 to 0)	0 (0 to 0)	0 (0 to 0)	0 (0 to 0)	7·7 (3·7 to 13·8)	19·6 (9·8 to 34·5)	26·5 (13·5 to 47·5)	26·7 (15·3 to 43·6)	25·2 (13 to 41·2)	29·2 (16·4 to 46·3)	28·1 (16·1 to 45·4)	20·5 (11·2 to 34)	6·8 (3·3 to 12·2)	0 (0 to 0)	0 (0 to 0)	0 (0 to 0)	0 (0 to 0)	0 (0 to 0)	0 (0 to 0)	0 (0 to 0)	0 (0 to 0)	0 (0 to 0)	190
Other gynecological diseases	0 (0 to 0)	0 (0 to 0)	0 (0 to 0)	0 (0 to 0)	0 (0 to 0)	0·4 (0·1 to 0·8)	1 (0·4 to 2)	17·5 (9·8 to 27·5)	40 (22·1 to 63·3)	50·9 (30·1 to 78)	55·4 (32·2 to 87·3)	54·1 (29·9 to 85·7)	52·1 (29·3 to 81)	42·2 (20·6 to 68·1)	27·7 (16·4 to 41·7)	16·5 (8·8 to 26·2)	11 (6·2 to 17)	8·1 (4·9 to 12·1)	5·4 (3·2 to 8·4)	3·6 (2 to 5·6)	2·1 (1·2 to 3·2)	0·9 (0·5 to 1·4)	0·2 (0·1 to 0·4)	389
Hemoglobinopathies and hemolytic anemias	0·7 (0·4 to 1·1)	0·7 (0·5 to 1)	3·8 (2·5 to 5·7)	14·6 (9·5 to 21·7)	20·5 (15 to 27·8)	16·8 (12·1 to 22·4)	24·2 (17·6 to 32·3)	30·5 (20·3 to 42·8)	30·6 (20·7 to 44·1)	24·7 (17·1 to 35)	23·1 (16·9 to 30·7)	23·7 (17 to 31·4)	22·2 (16·1 to 29·3)	19·6 (14·5 to 25·9)	15·5 (11·3 to 20·6)	12·5 (8·9 to 17·2)	7·6 (5·5 to 10·2)	7·1 (5·2 to 9·4)	5·2 (4 to 6·9)	3·9 (2·9 to 5·1)	4·3 (3·3 to 5·6)	2·2 (1·7 to 2·8)	0·5 (0·3 to 0·6)	314
Thalassemias	0 (0 to 0)	0 (0 to 0)	0 (0 to 0)	0·1 (0 to 0·1)	0 (0 to 0·1)	0 (0 to 0)	0 (0 to 0)	0 (0 to 0)	0 (0 to 0)	0 (0 to 0)	0 (0 to 0)	0 (0 to 0)	0 (0 to 0)	0 (0 to 0)	0 (0 to 0)	0 (0 to 0)	0 (0 to 0)	0 (0 to 0)	0 (0 to 0)	0 (0 to 0)	0 (0 to 0)	0 (0 to 0)	0 (0 to 0)	0·2
Thalassemias trait	0 (0 to 0)	0 (0 to 0)	0·1 (0 to 0·1)	0·3 (0·1 to 0·6)	0·5 (0·2 to 0·9)	0·3 (0·1 to 0·6)	0·7 (0·3 to 1·2)	0·8 (0·4 to 1·4)	0·8 (0·4 to 1·4)	0·7 (0·3 to 1·3)	0·7 (0·3 to 1·3)	0·7 (0·3 to 1·2)	0·2 (0·1 to 0·5)	0·3 (0·1 to 0·5)	0·2 (0·1 to 0·5)	0·2 (0·1 to 0·3)	0·1 (0 to 0·2)	0·1 (0 to 0·2)	0·1 (0 to 0·1)	0·1 (0 to 0·1)	0 (0 to 0·1)	0 (0 to 0)	0 (0 to 0)	6·8
Sickle cell disorders	0·7 (0·4 to 1·1)	0·4 (0·2 to 0·7)	2·6 (1·5 to 4·3)	9·5 (5·7 to 15·3)	12·6 (9·1 to 16·4)	11·9 (8·5 to 16·1)	16·6 (11·5 to 22·5)	17·8 (12 to 25·1)	16·3 (11·3 to 22·6)	12·4 (8·4 to 17·7)	12 (7·9 to 17)	11·4 (7·4 to 16·3)	10·2 (6·7 to 15)	6·7 (4·2 to 10·3)	2·9 (1·7 to 4·6)	0·8 (0·4 to 1·4)	0·1 (0·1 to 0·3)	0 (0 to 0)	0 (0 to 0)	0 (0 to 0)	0 (0 to 0)	0 (0 to 0)	0 (0 to 0)	145
Sickle cell trait	0 (0 to 0)	0·2 (0·1 to 0·3)	0·6 (0·3 to 1·1)	2·9 (1·3 to 5)	4·5 (2·1 to 8·2)	2·6 (1·2 to 4·9)	1·4 (0·6 to 2·8)	6·4 (1·3 to 15·2)	7 (1·3 to 16·9)	5·1 (1·2 to 12·1)	1·3 (0·6 to 2·4)	2·1 (1 to 3·6)	2·6 (1·3 to 4·6)	2·7 (1·3 to 4·5)	2·4 (1·3 to 4·2)	1·6 (0·8 to 2·9)	0·9 (0·4 to 1·7)	0·8 (0·3 to 1·5)	0·7 (0·3 to 1·2)	0·5 (0·2 to 0·9)	0·4 (0·2 to 0·6)	0·1 (0·1 to 0·2)	0 (0 to 0)	47
G6PD deficiency	0 (0 to 0)	0 (0 to 0)	0 (0 to 0·1)	0·4 (0·2 to 0·7)	0·6 (0·3 to 1·2)	0·6 (0·3 to 1·3)	2·6 (1·5 to 4·1)	3·1 (1·2 to 6·2)	4 (1·8 to 6·9)	3·9 (1·8 to 6·7)	5·3 (3·1 to 8·5)	5·3 (2·7 to 8·3)	4·8 (2·4 to 7·6)	4·6 (2·6 to 6·9)	4·1 (2·5 to 6)	3 (1·8 to 4·6)	1·5 (0·9 to 2·3)	1·2 (0·6 to 1·8)	0·5 (0·3 to 0·8)	0·2 (0·1 to 0·3)	0·1 (0·1 to 0·2)	0 (0 to 0·1)	0 (0 to 0)	46·1
G6PD trait	0 (0 to 0)	0 (0 to 0)	0 (0 to 0)	0 (0 to 0)	0 (0 to 0)	0 (0 to 0)	0·1 (0·1 to 0·3)	0 (0 to 0)	0 (0 to 0)	0 (0 to 0)	0·2 (0·1 to 0·3)	0·2 (0·1 to 0·3)	0 (0 to 0)	0 (0 to 0)	0 (0 to 0)	0 (0 to 0)	0 (0 to 0)	0 (0 to 0)	0 (0 to 0)	0 (0 to 0)	0 (0 to 0)	0 (0 to 0)	0 (0 to 0)	0·5
Other hemoglobinopathies and hemolytic anemias	0 (0 to 0)	0 (0 to 0·1)	0·4 (0·2 to 0·7)	1·5 (0·7 to 3)	2·3 (0·9 to 4·4)	1·3 (0·5 to 2·5)	2·7 (1·2 to 5·1)	2·4 (1·2 to 4·4)	2·6 (1·4 to 4·4)	2·6 (1·4 to 4·7)	3·6 (2 to 6·1)	4·1 (2·2 to 6·7)	4·3 (2·5 to 6·7)	5·3 (3·4 to 7·9)	5·8 (3·9 to 8·4)	6·9 (4·6 to 10)	4·9 (3·5 to 6·8)	5 (3·7 to 6·8)	4 (3 to 5·2)	3·1 (2·3 to 4)	3·8 (2·9 to 4·9)	2 (1·5 to 2·6)	0·4 (0·3 to 0·5)	69
Endocrine, metabolic, blood, and immune disorders	3·4 (2 to 5·7)	3·1 (1·7 to 5·5)	19 (11 to 32·5)	21·7 (13·7 to 36·4)	13·1 (9·1 to 18·6)	11 (7·8 to 16·1)	14·6 (10·4 to 20·9)	17·9 (13 to 24·8)	26·6 (19·7 to 36·3)	33·3 (24·7 to 45·2)	42·2 (30·6 to 56·6)	50·4 (35·3 to 70·8)	59·4 (41·9 to 82·3)	65·4 (45·6 to 95·2)	63·1 (44·4 to 90·2)	55 (37·9 to 80·8)	42·7 (29·7 to 64·9)	39·4 (28·3 to 56·6)	29·9 (20·6 to 42·7)	21·3 (14·9 to 30·3)	15·6 (11·3 to 22·1)	7·8 (5·8 to 11·1)	2 (1·4 to 2·9)	658
Oral disorders	0 (0 to 0)	0 (0 to 0)	0 (0 to 0)	4·5 (2·6 to 7·5)	6·1 (3·3 to 10·4)	7·5 (3·9 to 13)	12·2 (6·6 to 21·4)	20·3 (10·8 to 37·4)	29·8 (15·1 to 55·9)	35·5 (16·9 to 66·8)	44·2 (20·7 to 82·5)	49·8 (24 to 90·8)	58·1 (29·8 to 104)	65·4 (35·7 to 114)	67·4 (38·8 to 108)	65·7 (39·4 to 104)	57·1 (34·8 to 88)	53·5 (33·5 to 81·5)	39·4 (24·6 to 58·4)	26·8 (17 to 39·4)	14·2 (8·9 to 20·8)	5·6 (3·5 to 8·3)	1·2 (0·8 to 1·8)	664
Caries of deciduous teeth	0 (0 to 0)	0 (0 to 0)	0 (0 to 0)	1·5 (0·6 to 3)	1·6 (0·6 to 3·5)	0·1 (0 to 0·3)	0 (0 to 0)	0 (0 to 0)	0 (0 to 0)	0 (0 to 0)	0 (0 to 0)	0 (0 to 0)	0 (0 to 0)	0 (0 to 0)	0 (0 to 0)	0 (0 to 0)	0 (0 to 0)	0 (0 to 0)	0 (0 to 0)	0 (0 to 0)	0 (0 to 0)	0 (0 to 0)	0 (0 to 0)	3·2
Caries of permanent teeth	0 (0 to 0)	0 (0 to 0)	0 (0 to 0)	0 (0 to 0)	1·4 (0·5 to 3·2)	3·6 (1·4 to 7·8)	4·3 (1·7 to 9·3)	5·1 (2·1 to 10·6)	5 (1·9 to 9·9)	4·3 (1·8 to 8·7)	4·5 (1·8 to 8·9)	4·1 (1·7 to 8·3)	4 (1·6 to 8·3)	3·7 (1·4 to 7·5)	2·9 (1·1 to 6·4)	2 (0·7 to 4·4)	1·2 (0·5 to 2·7)	1 (0·4 to 2)	0·6 (0·3 to 1·2)	0·4 (0·2 to 0·8)	0·2 (0·1 to 0·4)	0·1 (0 to 0·1)	0 (0 to 0)	48·2
Periodontal diseases	0 (0 to 0)	0 (0 to 0)	0 (0 to 0)	0 (0 to 0)	0 (0 to 0)	0 (0 to 0)	1·7 (0·5 to 4·3)	6·5 (1·9 to 16·2)	14·2 (4·5 to 34·5)	19·9 (6·9 to 45·7)	26·4 (9·3 to 59·3)	29·9 (11 to 64·5)	32·6 (12·3 to 68·7)	31·2 (12·2 to 65·8)	24·7 (9·7 to 52·5)	17·2 (6·8 to 35·5)	10·9 (4·2 to 22·7)	8·2 (3·2 to 16·6)	5·2 (2·1 to 10·3)	3·3 (1·3 to 6·5)	1·7 (0·6 to 3·5)	0·6 (0·2 to 1·4)	0·1 (0 to 0·3)	234
Other oral disorders	0 (0 to 0)	0 (0 to 0)	0 (0 to 0)	3 (1·8 to 4·7)	3·1 (1·8 to 4·8)	3·8 (2·2 to 6)	6·3 (3·8 to 9·7)	8 (4·8 to 12·2)	8·7 (5·2 to 13)	8·4 (5 to 12·8)	8·9 (5·3 to 13·6)	8·8 (5·4 to 13·5)	9·1 (5·5 to 13·8)	8·7 (5·4 to 13·2)	7·2 (4·3 to 10·7)	5·5 (3·4 to 8·2)	3·9 (2·3 to 5·9)	2·9 (1·8 to 4·4)	1·6 (1 to 2·4)	0·9 (0·5 to 1·3)	0·4 (0·2 to 0·5)	0·1 (0·1 to 0·2)	0 (0 to 0)	99·2
Sudden infant death syndrome	--	4·3 (2·5 to 6·7)	17·6 (9·4 to 29·3)	--	--	--	--	--	--	--	--	--	--	--	--	--	--	--	--	--	--	--	--	NA
Injuries	12·3 (7·9 to 18·1)	4·3 (2·8 to 6·5)	23·2 (15·1 to 35·1)	84 (59 to 117)	107 (91·5 to 125)	147 (125 to 172)	433 (367 to 509)	751 (648 to 860)	808 (712 to 906)	661 (582 to 755)	581 (499 to 675)	542 (459 to 632)	507 (426 to 604)	415 (348 to 490)	319 (267 to 377)	236 (197 to 278)	158 (133 to 187)	144 (121 to 170)	102 (85·9 to 119)	77·9 (66·8 to 90·6)	54·7 (46·6 to 62·6)	29·3 (24·9 to 33·9)	8 (6·8 to 9·1)	6,210
Transport injuries	0·7 (0·4 to 1)	0·3 (0·2 to 0·5)	2·1 (1·3 to 3·2)	16 (10·4 to 24·3)	29·6 (22·6 to 38·2)	39·9 (31·3 to 51·6)	113 (84·7 to 146)	194 (144 to 247)	196 (154 to 244)	167 (134 to 209)	155 (124 to 193)	151 (121 to 187)	145 (114 to 186)	119 (94·4 to 146)	93·4 (75·9 to 114)	67 (54·8 to 80·4)	43·6 (36·4 to 52·9)	36·5 (29·9 to 43·9)	23·3 (19 to 28·2)	14·3 (11·9 to 17·2)	7·7 (6·2 to 9·2)	3 (2·5 to 3·6)	0·7 (0·6 to 0·8)	1,620
Road injuries	0·5 (0·3 to 0·8)	0·2 (0·1 to 0·3)	1·6 (1 to 2·6)	14·1 (8·9 to 21·9)	27 (20·2 to 35·5)	35·5 (27·1 to 47·1)	103 (76·3 to 135)	178 (131 to 230)	179 (140 to 224)	151 (119 to 192)	141 (111 to 178)	136 (107 to 171)	133 (102 to 171)	109 (85·7 to 136)	84·2 (67·3 to 104)	61·7 (50·1 to 74·5)	40·7 (33·5 to 49·7)	33·2 (27·1 to 40·1)	21·5 (17·4 to 26·1)	13·4 (11 to 16·2)	7·1 (5·8 to 8·6)	2·8 (2·3 to 3·3)	0·6 (0·5 to 0·7)	1,470
Pedestrian road injuries	0·2 (0·1 to 0·3)	0·1 (0 to 0·1)	0·3 (0·2 to 0·5)	4·6 (2·8 to 7·2)	8·7 (6·4 to 11·7)	8·8 (6·6 to 11·9)	9·4 (7 to 12·2)	13 (9·8 to 16·6)	15·5 (12·1 to 19·3)	16 (12·8 to 20·4)	17·4 (13·7 to 22·1)	19·5 (15·2 to 24·7)	21·2 (16·1 to 27·3)	19·7 (15·2 to 24·8)	17·1 (13·5 to 21·6)	13·9 (11 to 17·2)	10·4 (8·4 to 12·7)	9·7 (7·8 to 11·9)	7·1 (5·7 to 8·7)	5 (4 to 6·1)	2·8 (2·3 to 3·5)	1 (0·8 to 1·2)	0·2 (0·2 to 0·3)	221
Cyclist road injuries	0 (0 to 0)	0 (0 to 0)	0 (0 to 0)	0·7 (0·4 to 1·3)	1·7 (1·1 to 2·6)	4·8 (2·9 to 7·6)	9·6 (5·8 to 15·4)	12·4 (8 to 18·9)	12·6 (8·7 to 17·4)	12 (8·5 to 17)	12·6 (8·9 to 17·4)	12·9 (9·4 to 17)	13·1 (9·5 to 17·8)	12·7 (9·4 to 16·5)	10·1 (7·5 to 13·4)	7·7 (5·7 to 10·1)	5·3 (4 to 7)	4·2 (3·1 to 5·6)	2·6 (1·9 to 3·5)	1·6 (1·1 to 2·1)	0·8 (0·6 to 1)	0·3 (0·2 to 0·4)	0·1 (0 to 0·1)	138
Motorcyclist road injuries	0 (0 to 0·1)	0 (0 to 0·1)	0·2 (0·1 to 0·2)	0·7 (0·5 to 1·1)	1·7 (1·2 to 2·4)	2·7 (2 to 3·6)	20·8 (14·5 to 28)	44·8 (32·3 to 58·8)	47·2 (36·1 to 61·1)	37·1 (28·8 to 48·2)	32·1 (25·1 to 41·4)	26·4 (20·7 to 33·8)	24·5 (18·7 to 31·8)	18·2 (14·1 to 23·3)	13·5 (10·5 to 17·1)	8·3 (6·6 to 10·4)	5·3 (4·1 to 6·8)	3·7 (2·7 to 4·9)	2·3 (1·7 to 3)	1·5 (1·1 to 2)	0·8 (0·5 to 1)	0·3 (0·2 to 0·4)	0·1 (0 to 0·1)	292
Motor vehicle road injuries	0·3 (0·2 to 0·4)	0·1 (0·1 to 0·2)	1·1 (0·7 to 1·8)	7·5 (4·7 to 11·9)	14·1 (10·5 to 18·8)	18·5 (13·9 to 24·6)	62·2 (45·2 to 82·2)	105 (77·1 to 137)	101 (78·4 to 128)	83·3 (65·5 to 107)	76 (59·2 to 97·5)	74·5 (57·4 to 94·7)	71·1 (53·1 to 93·9)	55·4 (42·7 to 71)	41·3 (32·5 to 51·2)	30·2 (24·1 to 36·9)	18·5 (15·2 to 22·5)	14·7 (11·9 to 17·9)	8·8 (7·2 to 10·6)	5 (4·1 to 6·1)	2·6 (2·1 to 3·1)	1·1 (0·9 to 1·3)	0·3 (0·2 to 0·3)	792
Other road injuries	0 (0 to 0)	0 (0 to 0)	0 (0 to 0·1)	0·5 (0·2 to 0·9)	0·7 (0·4 to 1·2)	0·7 (0·4 to 1)	1·3 (0·8 to 2·1)	2·4 (1·5 to 3·8)	2·8 (1·8 to 4·2)	2·6 (1·7 to 4)	2·7 (1·9 to 3·9)	2·7 (1·9 to 3·8)	3 (2·1 to 4·1)	2·7 (1·9 to 3·5)	2·2 (1·6 to 3)	1·7 (1·2 to 2·3)	1·3 (0·9 to 1·7)	1 (0·7 to 1·3)	0·7 (0·5 to 0·9)	0·4 (0·3 to 0·5)	0·2 (0·1 to 0·3)	0·1 (0·1 to 0·1)	0 (0 to 0)	29·7
Other transport injuries	0·2 (0·1 to 0·2)	0·1 (0·1 to 0·2)	0·5 (0·3 to 0·7)	1·9 (1·2 to 2·9)	2·7 (1·9 to 3·7)	4·4 (3 to 6·2)	9·3 (5·8 to 14)	16·1 (10·1 to 24·3)	16·8 (10·8 to 25·4)	15·8 (10·4 to 22·6)	14 (9·3 to 21·1)	15·3 (10·7 to 21·5)	12·5 (8·6 to 18·5)	10·4 (7·3 to 14·3)	9·2 (6·7 to 12·6)	5·3 (3·8 to 7·2)	2·9 (2·2 to 3·8)	3·3 (2·4 to 4·5)	1·8 (1·3 to 2·4)	1 (0·7 to 1·2)	0·5 (0·4 to 0·7)	0·3 (0·2 to 0·3)	0·1 (0·1 to 0·1)	144
Unintentional injuries	6·9 (4·4 to 10·4)	3·4 (2·2 to 5·3)	18·1 (11·2 to 28·2)	57·7 (39·1 to 82·3)	67·1 (53·5 to 82·7)	83·7 (67·2 to 103)	134 (105 to 168)	182 (142 to 230)	190 (151 to 233)	176 (143 to 214)	176 (142 to 215)	182 (147 to 223)	179 (142 to 218)	168 (135 to 209)	140 (112 to 172)	113 (90·6 to 139)	81·8 (65·3 to 101)	80·7 (65·9 to 98·2)	63·1 (52·3 to 75·4)	54·9 (46·5 to 64·9)	42·4 (35·5 to 49·1)	24·9 (21·1 to 28·9)	7 (6 to 8)	2,230
Falls	0·1 (0·1 to 0·2)	0 (0 to 0·1)	0·6 (0·4 to 0·9)	2·2 (1·6 to 3·2)	4 (2·9 to 5·3)	7·2 (5·2 to 9·6)	12·9 (9·8 to 16·9)	18 (13·4 to 23·2)	20·9 (16·1 to 27·2)	22 (17 to 28·3)	25·9 (19·9 to 33·6)	29·1 (22·7 to 37·3)	32·9 (25·4 to 41·9)	35·5 (27·8 to 44·6)	32·6 (25·3 to 41·3)	29·4 (23 to 36·9)	23·6 (18·7 to 29·4)	27·4 (22 to 33·1)	24·4 (19·7 to 29·4)	26·4 (21·8 to 31·6)	23·1 (19 to 27·1)	13·9 (11·7 to 16·3)	3·8 (3·2 to 4·4)	416
Drowning	0·6 (0·4 to 1)	0·2 (0·1 to 0·3)	2·1 (1·3 to 3·5)	21·9 (13·8 to 32·8)	28·5 (21·3 to 37·6)	33·8 (25 to 43·5)	54·3 (38·2 to 73·5)	62·3 (44·2 to 85·6)	56·4 (41·8 to 74)	46 (34·4 to 59·3)	39·7 (30·6 to 50·2)	37·1 (28·8 to 47·2)	30·5 (23·4 to 39·9)	24 (17·9 to 31·3)	16·1 (12·4 to 21)	10·6 (8·2 to 13·5)	5·6 (4·4 to 6·9)	4·1 (3·2 to 5·2)	2·4 (1·8 to 3)	1·1 (0·9 to 1·4)	0·8 (0·6 to 1)	0·5 (0·4 to 0·6)	0·1 (0·1 to 0·2)	479
Fire, heat, and hot substances	0·2 (0·1 to 0·3)	0·2 (0·1 to 0·3)	1·7 (1·1 to 2·7)	8·8 (5·7 to 13·1)	8·8 (6·9 to 11·1)	10 (7·7 to 13)	13·5 (10 to 18·2)	17·7 (13·3 to 23·1)	20·8 (15·9 to 26·9)	22·1 (16·8 to 29·1)	23·2 (17·7 to 30·5)	23·8 (17·9 to 31·3)	24·1 (18·1 to 31·8)	22·3 (16·5 to 29·6)	18·5 (13·9 to 24·7)	14·5 (10·9 to 19·6)	9·8 (7·3 to 13·3)	9 (6·7 to 11·9)	6·3 (4·8 to 8·3)	4·7 (3·6 to 6)	3·4 (2·8 to 4·2)	1·8 (1·5 to 2·3)	0·5 (0·4 to 0·6)	266
Poisonings	0 (0 to 0·1)	0·1 (0 to 0·1)	0·2 (0·1 to 0·3)	1·3 (0·8 to 2)	0·9 (0·6 to 1·2)	1 (0·7 to 1·5)	1·4 (1 to 2)	2 (1·5 to 2·6)	2·5 (1·9 to 3·3)	2·4 (1·8 to 3·1)	2·2 (1·6 to 2·9)	1·9 (1·5 to 2·5)	1·8 (1·3 to 2·4)	1·5 (1·1 to 2)	1·2 (0·9 to 1·6)	0·9 (0·6 to 1·2)	0·6 (0·4 to 0·8)	0·5 (0·4 to 0·7)	0·3 (0·3 to 0·5)	0·2 (0·2 to 0·3)	0·1 (0·1 to 0·2)	0·1 (0 to 0·1)	0 (0 to 0)	23·3
Poisoning by carbon monoxide	0 (0 to 0)	0 (0 to 0)	0·1 (0 to 0·1)	0·5 (0·2 to 0·8)	0·3 (0·2 to 0·5)	0·3 (0·2 to 0·5)	0·5 (0·4 to 0·8)	1 (0·7 to 1·4)	1·4 (0·9 to 1·9)	1·4 (0·9 to 1·9)	1·2 (0·8 to 1·7)	0·9 (0·7 to 1·3)	0·8 (0·6 to 1·1)	0·6 (0·4 to 0·8)	0·5 (0·3 to 0·6)	0·3 (0·2 to 0·5)	0·2 (0·1 to 0·3)	0·2 (0·1 to 0·2)	0·1 (0·1 to 0·1)	0·1 (0·1 to 0·1)	0 (0 to 0·1)	0 (0 to 0)	0 (0 to 0)	10·5
Poisoning by other means	0 (0 to 0)	0·1 (0 to 0·1)	0·1 (0·1 to 0·2)	0·8 (0·5 to 1·2)	0·6 (0·4 to 0·8)	0·7 (0·4 to 1·1)	0·9 (0·5 to 1·3)	1 (0·6 to 1·5)	1·1 (0·7 to 1·6)	1 (0·7 to 1·4)	1 (0·7 to 1·5)	1 (0·7 to 1·4)	1 (0·7 to 1·4)	0·9 (0·6 to 1·3)	0·7 (0·5 to 1)	0·5 (0·4 to 0·8)	0·4 (0·3 to 0·6)	0·3 (0·2 to 0·5)	0·2 (0·2 to 0·3)	0·2 (0·1 to 0·2)	0·1 (0·1 to 0·1)	0 (0 to 0·1)	0 (0 to 0)	12·7
Exposure to mechanical forces	0·2 (0·1 to 0·4)	0·2 (0·1 to 0·3)	0·8 (0·6 to 1·1)	6·6 (4·6 to 9·3)	9·8 (7·4 to 12·5)	12·6 (9·6 to 16·6)	26·7 (20·2 to 34·3)	44 (33·6 to 56·3)	45·9 (35·1 to 57·4)	42·9 (33·9 to 53·9)	42·7 (33·5 to 54·1)	44·5 (34·6 to 57)	42·6 (32·2 to 55·5)	39·8 (30·1 to 52·6)	32·5 (24·4 to 43·2)	24·3 (17·8 to 32·8)	16·6 (11·9 to 22·8)	14·3 (10·4 to 19·7)	9·6 (6·8 to 13)	6·7 (4·9 to 9)	4·1 (3·1 to 5·4)	2·3 (1·8 to 2·9)	0·7 (0·6 to 0·9)	470
Unintentional firearm injuries	0·1 (0 to 0·1)	0 (0 to 0·1)	0·1 (0·1 to 0·2)	0·8 (0·4 to 1·2)	1·3 (0·9 to 1·9)	2·8 (2 to 3·8)	11·3 (7·6 to 15·5)	22·1 (15·5 to 30·5)	20·3 (14·4 to 27·8)	16·9 (12·1 to 22·5)	14·6 (10·8 to 19)	14·1 (10·4 to 18·7)	11·3 (8·4 to 14·7)	8·5 (6·4 to 10·9)	6·2 (4·8 to 7·8)	4 (3·1 to 5)	2·4 (1·9 to 3·1)	2 (1·6 to 2·5)	1·3 (1 to 1·6)	1·1 (0·8 to 1·4)	0·9 (0·7 to 1·1)	0·7 (0·6 to 0·9)	0·3 (0·2 to 0·4)	143
Other exposure to mechanical forces	0·2 (0·1 to 0·3)	0·2 (0·1 to 0·3)	0·7 (0·5 to 1)	5·9 (4·1 to 8·3)	8·5 (6·3 to 10·9)	9·8 (7·2 to 13·3)	15·4 (11·1 to 21·4)	22 (15·8 to 29·7)	25·6 (18·6 to 33·9)	26 (19·1 to 35·4)	28·1 (20·4 to 38·6)	30·4 (22·3 to 41·2)	31·3 (22·6 to 43·1)	31·3 (22·8 to 43·1)	26·3 (19 to 36·2)	20·3 (14·4 to 28·3)	14·2 (9·9 to 19·7)	12·3 (8·6 to 17·2)	8·3 (5·8 to 11·5)	5·6 (3·9 to 7·7)	3·2 (2·3 to 4·3)	1·6 (1·2 to 2·1)	0·4 (0·3 to 0·6)	327
Adverse effects of medical treatment	1·8 (1·1 to 2·7)	1 (0·6 to 1·6)	2·8 (1·6 to 4·8)	3·2 (1·9 to 5·1)	2 (1·5 to 2·8)	2 (1·4 to 2·7)	2·8 (2 to 3·8)	6·2 (4·3 to 8·5)	7 (4·9 to 10)	6·7 (4·8 to 9·4)	7·2 (5·1 to 9·8)	8·7 (6·2 to 11·9)	10·3 (7·2 to 14·5)	11·2 (8 to 15·7)	10·7 (7·8 to 14·8)	10·2 (7·2 to 14·5)	8·5 (6·3 to 11·5)	9·1 (6·8 to 12·2)	8·1 (6·1 to 10·8)	6·5 (4·8 to 8·4)	5·1 (3·8 to 6·4)	2·7 (2 to 3·5)	0·7 (0·5 to 0·9)	134
Animal contact	0 (0 to 0)	0 (0 to 0)	0·1 (0·1 to 0·2)	0·6 (0·4 to 0·9)	0·8 (0·6 to 1·1)	1·2 (0·8 to 1·6)	1·5 (1·1 to 2·1)	1·8 (1·3 to 2·4)	2·4 (1·7 to 3)	1·9 (1·4 to 2·5)	2·4 (1·7 to 3·1)	2·5 (1·8 to 3·3)	2·9 (2·1 to 3·9)	2·8 (2·1 to 3·6)	2·5 (1·8 to 3·3)	2·2 (1·7 to 3)	1·7 (1·3 to 2·3)	1·7 (1·3 to 2·3)	1·3 (1 to 1·7)	0·9 (0·7 to 1·2)	0·7 (0·6 to 1)	0·7 (0·5 to 0·9)	0·2 (0·2 to 0·3)	32·9
Venomous animal contact	0 (0 to 0)	0 (0 to 0)	0·1 (0·1 to 0·1)	0·4 (0·3 to 0·6)	0·6 (0·4 to 0·8)	0·9 (0·6 to 1·3)	1·2 (0·8 to 1·7)	1·4 (1 to 1·8)	1·8 (1·3 to 2·5)	1·4 (1 to 1·9)	1·8 (1·3 to 2·4)	1·8 (1·3 to 2·5)	2·1 (1·6 to 2·9)	2 (1·5 to 2·7)	1·8 (1·3 to 2·4)	1·6 (1·2 to 2·2)	1·2 (0·9 to 1·6)	1·2 (0·8 to 1·6)	0·9 (0·7 to 1·2)	0·6 (0·5 to 0·8)	0·5 (0·4 to 0·7)	0·4 (0·3 to 0·6)	0·2 (0·1 to 0·2)	24
Non-venomous animal contact	0 (0 to 0)	0 (0 to 0)	0 (0 to 0·1)	0·2 (0·1 to 0·3)	0·2 (0·2 to 0·4)	0·3 (0·2 to 0·4)	0·4 (0·2 to 0·5)	0·4 (0·3 to 0·7)	0·5 (0·3 to 0·8)	0·5 (0·3 to 0·7)	0·5 (0·3 to 0·8)	0·6 (0·4 to 1)	0·8 (0·5 to 1·1)	0·8 (0·5 to 1·1)	0·7 (0·5 to 1)	0·6 (0·4 to 0·9)	0·5 (0·3 to 0·7)	0·6 (0·4 to 0·8)	0·4 (0·3 to 0·5)	0·3 (0·2 to 0·5)	0·3 (0·2 to 0·4)	0·2 (0·1 to 0·3)	0·1 (0 to 0·1)	8·9
Foreign body	3·6 (2·2 to 5·6)	1·6 (0·9 to 2·5)	9 (5·2 to 14·9)	9·1 (6 to 13·4)	5·3 (4·2 to 6·5)	6 (4·6 to 7·8)	4·7 (3·5 to 6)	6·9 (5·3 to 9)	8 (6·2 to 10·1)	7·2 (5·6 to 9)	7·4 (5·8 to 9·3)	8·3 (6·6 to 10·4)	8·7 (6·9 to 11)	8·8 (6·7 to 11·4)	7·3 (5·8 to 9·2)	6·7 (5·1 to 8·4)	5·2 (4·1 to 6·5)	5·7 (4·6 to 7)	4·5 (3·5 to 5·8)	3·9 (3 to 5)	2·3 (1·8 to 2·9)	1·5 (1·1 to 1·8)	0·5 (0·4 to 0·5)	132
Pulmonary aspiration and foreign body in airway	3·6 (2·2 to 5·6)	1·6 (0·9 to 2·5)	8·9 (5·1 to 14·8)	8·5 (5·5 to 12·7)	4·4 (3·4 to 5·6)	4·9 (3·6 to 6·5)	3·2 (2·4 to 4·2)	5·2 (3·9 to 6·9)	6·2 (4·7 to 7·9)	5·4 (4 to 7)	5·5 (4·2 to 7)	6·3 (4·9 to 8)	6·7 (5·1 to 8·7)	6·8 (5 to 9·1)	5·7 (4·4 to 7·5)	5·4 (3·9 to 7·1)	4·3 (3·2 to 5·4)	4·8 (3·7 to 6)	3·8 (2·9 to 5)	3·4 (2·5 to 4·4)	2 (1·5 to 2·5)	1·3 (1 to 1·6)	0·4 (0·3 to 0·5)	108
Foreign body in eyes	0 (0 to 0)	0 (0 to 0)	0 (0 to 0·1)	0·2 (0 to 0·4)	0·3 (0·1 to 0·7)	0·4 (0·1 to 1)	0·6 (0·2 to 1·4)	0·8 (0·3 to 1·8)	0·9 (0·3 to 1·9)	0·9 (0·3 to 1·8)	1 (0·4 to 2)	1 (0·4 to 2)	1 (0·5 to 1·9)	0·9 (0·5 to 1·6)	0·7 (0·4 to 1·1)	0·5 (0·3 to 0·8)	0·4 (0·2 to 0·6)	0·3 (0·2 to 0·5)	0·2 (0·1 to 0·3)	0·2 (0·1 to 0·2)	0·1 (0·1 to 0·1)	0 (0 to 0)	0 (0 to 0)	10·3
Foreign body in other body part	0 (0 to 0)	0 (0 to 0)	0·1 (0 to 0·1)	0·4 (0·3 to 0·6)	0·6 (0·4 to 0·8)	0·7 (0·5 to 1)	0·8 (0·6 to 1·1)	0·9 (0·7 to 1·3)	1 (0·7 to 1·3)	0·9 (0·6 to 1·2)	1 (0·7 to 1·3)	1 (0·7 to 1·4)	1·1 (0·8 to 1·5)	1·1 (0·8 to 1·4)	0·9 (0·7 to 1·2)	0·8 (0·6 to 1)	0·6 (0·4 to 0·8)	0·6 (0·4 to 0·7)	0·4 (0·3 to 0·6)	0·4 (0·3 to 0·5)	0·2 (0·2 to 0·3)	0·2 (0·1 to 0·2)	0·1 (0 to 0·1)	13·6
Environmental heat and cold exposure	0 (0 to 0)	0 (0 to 0)	0·1 (0·1 to 0·2)	0·7 (0·5 to 1)	1·4 (1 to 2·1)	2·1 (1·4 to 3·1)	3·3 (2·3 to 4·6)	4·5 (3·1 to 6·2)	5·1 (3·6 to 7·1)	5·3 (3·7 to 7·3)	5·8 (4 to 8·2)	6·2 (4·3 to 8·5)	6·6 (4·7 to 9)	6·7 (4·8 to 9·1)	5·9 (4·2 to 8)	4·9 (3·5 to 6·6)	3·7 (2·6 to 5)	3·5 (2·5 to 4·6)	2·6 (1·9 to 3·4)	2 (1·5 to 2·6)	1·4 (1·1 to 1·7)	0·9 (0·7 to 1·1)	0·3 (0·2 to 0·4)	73
Exposure to forces of nature	0 (0 to 0)	0 (0 to 0)	0 (0 to 0)	0 (0 to 0)	0·1 (0 to 0·1)	0·3 (0·2 to 0·4)	0·4 (0·3 to 0·7)	0·6 (0·4 to 1)	0·5 (0·3 to 0·8)	0·5 (0·3 to 0·8)	0·7 (0·5 to 1·1)	0·8 (0·5 to 1·2)	0·8 (0·5 to 1·2)	1 (0·7 to 1·4)	0·6 (0·4 to 0·9)	0·5 (0·3 to 0·7)	0·4 (0·3 to 0·5)	0·3 (0·2 to 0·4)	0·2 (0·1 to 0·2)	0·1 (0·1 to 0·2)	0·1 (0 to 0·1)	0 (0 to 0)	0 (0 to 0)	7·9
Other unintentional injuries	0·2 (0·1 to 0·4)	0·1 (0 to 0·1)	0·6 (0·4 to 0·9)	3·3 (2·3 to 4·5)	5·4 (4 to 7·3)	7·5 (5·7 to 10·1)	12·7 (9·5 to 17)	17·8 (13·5 to 23·5)	20 (15·5 to 25·8)	18·8 (14·5 to 24·4)	18·8 (14·4 to 23·9)	18·5 (14·3 to 23·6)	17·8 (13·4 to 23·4)	14·7 (10·8 to 20·1)	11·8 (8·8 to 16)	8·7 (6·3 to 12)	6·1 (4·3 to 8·5)	5·1 (3·7 to 7·2)	3·5 (2·5 to 4·9)	2·3 (1·7 to 3·3)	1·3 (0·9 to 1·8)	0·5 (0·4 to 0·7)	0·1 (0·1 to 0·2)	196
Self-harm and interpersonal violence	4·8 (2·7 to 7·6)	0·6 (0·3 to 1)	3 (1·7 to 5)	10·3 (5·7 to 17·9)	10·6 (7·8 to 14·8)	23·8 (18·2 to 30·7)	187 (142 to 237)	375 (301 to 456)	422 (352 to 503)	319 (257 to 390)	250 (197 to 318)	210 (162 to 265)	182 (142 to 237)	128 (98·6 to 161)	85·8 (66·5 to 110)	56·3 (44·3 to 70·6)	32·9 (26·2 to 40·4)	26·6 (21·6 to 32·9)	15·5 (12·7 to 18·8)	8·6 (7·1 to 10·4)	4·6 (3·8 to 5·5)	1·5 (1·2 to 1·7)	0·3 (0·2 to 0·3)	2,360
Self-harm	0 (0 to 0)	0 (0 to 0)	0 (0 to 0)	0 (0 to 0)	0 (0 to 0)	3·3 (2·2 to 4·8)	28 (19·3 to 38·9)	59·4 (43·1 to 81·5)	69·4 (50·5 to 91·2)	67·2 (49·5 to 89·2)	63·4 (46·5 to 82·6)	61·9 (45·8 to 82·5)	56·6 (40·7 to 77·1)	46·7 (34·2 to 61·7)	34·7 (25·6 to 45·5)	25·6 (18·8 to 33·2)	15·6 (11·6 to 20·1)	14·5 (10·9 to 19)	8·6 (6·5 to 11)	5·5 (4·2 to 7)	2·9 (2·3 to 3·7)	0·9 (0·7 to 1·1)	0·1 (0·1 to 0·2)	564
Self-harm by firearm	0 (0 to 0)	0 (0 to 0)	0 (0 to 0)	0 (0 to 0)	0 (0 to 0)	0·1 (0·1 to 0·1)	1·5 (0·8 to 2·7)	2·9 (1·6 to 5·3)	3·1 (1·6 to 5·1)	2·8 (1·6 to 4·6)	2·5 (1·4 to 4·1)	2·5 (1·4 to 4·1)	2·3 (1·2 to 3·8)	2·1 (1·1 to 3·4)	1·4 (0·8 to 2·2)	1·1 (0·6 to 1·7)	0·6 (0·3 to 0·9)	0·5 (0·3 to 0·8)	0·3 (0·2 to 0·5)	0·2 (0·1 to 0·3)	0·1 (0·1 to 0·1)	0 (0 to 0)	0 (0 to 0)	23·9
Self-harm by other specified means	0 (0 to 0)	0 (0 to 0)	0 (0 to 0)	0 (0 to 0)	0 (0 to 0)	3·2 (2·2 to 4·7)	26·5 (18·3 to 36·8)	56·5 (40·7 to 77·6)	66·4 (48·6 to 87·4)	64·5 (47·3 to 85·7)	60·9 (44·8 to 79·5)	59·4 (44·1 to 79·1)	54·3 (39 to 74·3)	44·7 (32·7 to 59·5)	33·4 (24·6 to 43·6)	24·6 (18 to 31·9)	15 (11·2 to 19·3)	13·9 (10·5 to 18·3)	8·2 (6·2 to 10·7)	5·3 (4 to 6·8)	2·8 (2·2 to 3·6)	0·9 (0·7 to 1·1)	0·1 (0·1 to 0·2)	541
Interpersonal violence	4·8 (2·7 to 7·6)	0·6 (0·3 to 1)	3 (1·7 to 4·9)	10·2 (5·6 to 17·8)	10·5 (7·7 to 14·7)	20·4 (15·1 to 26·5)	158 (118 to 204)	313 (245 to 388)	351 (285 to 425)	250 (195 to 314)	186 (138 to 243)	147 (109 to 194)	124 (90·7 to 167)	78·6 (57·1 to 105)	49·9 (36·9 to 66·6)	30·4 (22·6 to 40·5)	16·7 (12·6 to 22·3)	12 (9·3 to 15·4)	6·6 (5·1 to 8·4)	3·1 (2·5 to 3·8)	1·6 (1·3 to 2)	0·6 (0·4 to 0·7)	0·2 (0·1 to 0·2)	1,780
Physical violence by firearm	0·3 (0·2 to 0·4)	0 (0 to 0·1)	0·4 (0·2 to 0·7)	1·5 (0·8 to 2·7)	2·6 (1·8 to 3·8)	8 (5·7 to 10·7)	97·6 (71·6 to 129)	195 (150 to 242)	217 (174 to 264)	141 (108 to 178)	97·5 (71 to 128)	69·7 (50·1 to 93·8)	58·3 (41·3 to 79·6)	33·3 (23·2 to 45·6)	20·1 (14·2 to 27·6)	11·1 (7·6 to 15·5)	5·1 (3·6 to 7·1)	3 (2·1 to 4·1)	1·1 (0·8 to 1·5)	0·4 (0·3 to 0·5)	0·2 (0·2 to 0·3)	0·1 (0 to 0·1)	0 (0 to 0)	964
Physical violence by sharp object	0·4 (0·2 to 0·7)	0 (0 to 0·1)	0·2 (0·1 to 0·4)	1 (0·6 to 1·8)	1·4 (1·1 to 2)	3·5 (2·6 to 4·7)	43·5 (31·7 to 58·3)	90·6 (70·5 to 113)	102 (81·8 to 126)	81·3 (63 to 103)	62·7 (47 to 82·6)	51·7 (38·5 to 69·2)	41·4 (30·2 to 56)	25·8 (18·9 to 34·2)	14·8 (11·1 to 19·8)	8·8 (6·7 to 11·7)	4·5 (3·4 to 5·8)	3·6 (2·8 to 4·7)	1·9 (1·5 to 2·5)	1 (0·7 to 1·2)	0·5 (0·3 to 0·6)	0·2 (0·2 to 0·3)	0·1 (0 to 0·1)	541
Sexual violence	0 (0 to 0)	0 (0 to 0)	0 (0 to 0)	0·1 (0 to 0·1)	0·5 (0·3 to 0·8)	1·3 (0·8 to 2·1)	3·4 (1·9 to 5·4)	4·8 (2·8 to 7·3)	4·4 (2·5 to 7·1)	3·8 (2·1 to 6·3)	3·6 (2 to 5·8)	3 (1·7 to 4·8)	2·5 (1·5 to 4·2)	1·8 (1 to 3)	1·2 (0·7 to 1·9)	0·8 (0·5 to 1·3)	0·6 (0·4 to 0·9)	0·5 (0·3 to 0·7)	0·3 (0·2 to 0·5)	0·2 (0·1 to 0·3)	0·1 (0·1 to 0·2)	0 (0 to 0)	0 (0 to 0)	33
Physical violence by other means	4·1 (2·3 to 6·5)	0·5 (0·3 to 0·8)	2·3 (1·3 to 3·9)	7·5 (4·1 to 13·2)	5·9 (4·2 to 8·4)	7·5 (5·6 to 10·1)	13·4 (9·9 to 17·6)	22·8 (17·3 to 28·9)	26·7 (21·2 to 33·2)	23·4 (17·8 to 30)	22·1 (16·4 to 28·9)	22·6 (16·8 to 29·5)	21·9 (16·2 to 29·2)	17·7 (13 to 23·2)	13·8 (10·3 to 18·4)	9·7 (7·2 to 12·7)	6·6 (4·9 to 8·7)	4·9 (3·8 to 6·2)	3·2 (2·5 to 4·2)	1·5 (1·1 to 1·9)	0·8 (0·6 to 1·1)	0·3 (0·2 to 0·4)	0·1 (0·1 to 0·1)	239
Conflict and terrorism	0 (0 to 0)	0 (0 to 0)	0 (0 to 0)	0 (0 to 0)	0 (0 to 0)	0 (0 to 0)	0 (0 to 0)	0·1 (0·1 to 0·3)	0·1 (0·1 to 0·2)	0·1 (0·1 to 0·2)	0·2 (0·1 to 0·3)	0·2 (0·1 to 0·3)	1·1 (0·7 to 1·7)	2·3 (1·5 to 3·4)	1·2 (0·8 to 1·7)	0·3 (0·2 to 0·4)	0·7 (0·4 to 1)	0·1 (0·1 to 0·1)	0·4 (0·3 to 0·6)	0·1 (0 to 0·1)	0 (0 to 0·1)	0 (0 to 0)	0 (0 to 0)	6·8

SR Table 2c: Saint Vincent and the Grenadines DALYS by cause and age group for both sexes with 95% uncertainty intervals, 2019

Cause	Early Neonatal	Late Neonatal	Post Neonatal	1 to 4	5 to 9	10 to 14	15 to 19	20 to 24	25 to 29	30 to 34	35 to 39	40 to 44	45 to 49	50 to 54	55 to 59	60 to 64	65 to 69	70 to 74	75 to 79	80 to 84	85 to 89	90 to 94	95 plus	Total
All causes	944 (664 to 1,310)	364 (248 to 514)	522 (372 to 716)	506 (397 to 647)	496 (386 to 640)	745 (608 to 916)	1,170 (982 to 1,400)	1,410 (1,210 to 1,670)	1,610 (1,380 to 1,870)	1,700 (1,460 to 1,980)	2,000 (1,730 to 2,320)	2,330 (2,010 to 2,680)	2,900 (2,510 to 3,320)	3,240 (2,800 to 3,700)	3,420 (2,970 to 3,910)	3,360 (2,940 to 3,820)	2,910 (2,580 to 3,280)	2,730 (2,440 to 3,030)	2,410 (2,180 to 2,660)	2,090 (1,910 to 2,280)	1,230 (1,140 to 1,320)	435 (410 to 462)	101 (96·1 to 106)	38,600
Communicable, maternal, neonatal, and nutritional diseases	809 (568 to 1,130)	289 (198 to 407)	281 (200 to 384)	194 (146 to 256)	156 (107 to 223)	132 (101 to 174)	137 (109 to 174)	178 (150 to 211)	226 (200 to 257)	293 (266 to 327)	325 (295 to 369)	299 (265 to 337)	291 (257 to 336)	240 (210 to 281)	207 (175 to 248)	176 (149 to 210)	142 (119 to 168)	134 (107 to 165)	131 (110 to 157)	129 (105 to 160)	92·4 (75·7 to 111)	35·7 (29·5 to 42·2)	9 (7·1 to 10·7)	4,910
HIV/AIDS and sexually transmitted infections	1·5 (0·9 to 2·4)	0·4 (0·2 to 0·7)	9·5 (8·9 to 10·2)	6·3 (6 to 6·7)	7·4 (7 to 7·8)	8·4 (8 to 8·9)	13·3 (12·4 to 14·3)	50·1 (46·6 to 54·4)	97 (89·9 to 106)	165 (155 to 176)	186 (174 to 198)	151 (141 to 162)	134 (126 to 143)	78·6 (73·3 to 84·2)	49·4 (46·2 to 52·5)	30·2 (28·2 to 32·2)	16·4 (15·2 to 17·5)	5·5 (4·9 to 6)	2·9 (2·6 to 3·2)	1·6 (1·5 to 1·8)	0·7 (0·7 to 0·8)	0·1 (0·1 to 0·2)	0 (0 to 0)	1,010
HIV/AIDS	0 (0 to 0)	0 (0 to 0)	9·1 (8·6 to 9·6)	6·3 (6 to 6·7)	7·4 (7 to 7·8)	8·2 (7·8 to 8·6)	12·1 (11·4 to 12·8)	48 (44·6 to 52)	94·1 (87·6 to 102)	161 (151 to 173)	182 (171 to 194)	148 (138 to 158)	130 (123 to 139)	76·3 (71·2 to 81·6)	47·5 (44·5 to 50·5)	29 (27 to 30·9)	15·4 (14·3 to 16·3)	4·8 (4·4 to 5·2)	2·5 (2·3 to 2·7)	1·3 (1·2 to 1·4)	0·6 (0·5 to 0·6)	0·1 (0·1 to 0·1)	0 (0 to 0)	984
HIV/AIDS - Drug-susceptible Tuberculosis	0 (0 to 0)	0 (0 to 0)	0·5 (0·3 to 0·7)	0·3 (0·2 to 0·5)	0·3 (0·2 to 0·4)	0·4 (0·2 to 0·6)	0·6 (0·4 to 0·9)	2·5 (1·7 to 3·6)	4·8 (3·1 to 7)	7·6 (5 to 11·3)	8·6 (5·6 to 12·9)	7·2 (4·6 to 10·8)	6·8 (4·3 to 10·3)	4 (2·6 to 6·1)	2·5 (1·6 to 3·7)	1·4 (0·9 to 2·1)	0·8 (0·5 to 1·1)	0·2 (0·2 to 0·4)	0·1 (0·1 to 0·2)	0·1 (0 to 0·1)	0 (0 to 0)	0 (0 to 0)	0 (0 to 0)	48·7
HIV/AIDS - Multidrug-resistant Tuberculosis without extensive dru	0 (0 to 0)	0 (0 to 0)	0 (0 to 0)	0 (0 to 0)	0 (0 to 0)	0 (0 to 0)	0 (0 to 0)	0 (0 to 0·1)	0·1 (0 to 0·3)	0·1 (0 to 0·4)	0·1 (0 to 0·5)	0·1 (0 to 0·4)	0·1 (0 to 0·4)	0·1 (0 to 0·2)	0 (0 to 0·2)	0 (0 to 0·1)	0 (0 to 0)	0 (0 to 0)	0 (0 to 0)	0 (0 to 0)	0 (0 to 0)	0 (0 to 0)	0 (0 to 0)	0·7
HIV/AIDS - Extensively drug-resistant Tuberculosis	0 (0 to 0)	0 (0 to 0)	0 (0 to 0)	0 (0 to 0)	0 (0 to 0)	0 (0 to 0)	0 (0 to 0)	0 (0 to 0)	0 (0 to 0)	0 (0 to 0·1)	0 (0 to 0·1)	0 (0 to 0·1)	0 (0 to 0·1)	0 (0 to 0)	0 (0 to 0)	0 (0 to 0)	0 (0 to 0)	0 (0 to 0)	0 (0 to 0)	0 (0 to 0)	0 (0 to 0)	0 (0 to 0)	0 (0 to 0)	0·1
HIV/AIDS resulting in other diseases	0 (0 to 0)	0 (0 to 0)	8·6 (8·1 to 9·2)	6 (5·6 to 6·3)	7·1 (6·7 to 7·5)	7·8 (7·3 to 8·3)	11·4 (10·7 to 12·2)	45·4 (41·9 to 49·4)	89·3 (82·5 to 97·4)	154 (143 to 165)	173 (162 to 185)	140 (130 to 151)	124 (115 to 133)	72·2 (66·9 to 77·4)	45 (41·7 to 48·1)	27·6 (25·5 to 29·5)	14·6 (13·4 to 15·6)	4·6 (4·1 to 4·9)	2·4 (2·1 to 2·6)	1·2 (1·1 to 1·3)	0·6 (0·5 to 0·6)	0·1 (0·1 to 0·1)	0 (0 to 0)	935
Sexually transmitted infections excluding HIV	1·5 (0·9 to 2·4)	0·4 (0·2 to 0·7)	0·5 (0·2 to 1)	0 (0 to 0·1)	0 (0 to 0)	0·3 (0·2 to 0·4)	1·2 (0·7 to 1·9)	2·1 (1·3 to 3·3)	2·9 (1·8 to 4·7)	3·3 (2 to 5·5)	3·6 (2·2 to 6)	3·5 (2·1 to 6)	3·1 (1·9 to 5·2)	2·3 (1·3 to 4)	1·8 (1·1 to 3·2)	1·2 (0·7 to 2·2)	1 (0·6 to 1·7)	0·6 (0·4 to 1·1)	0·4 (0·2 to 0·7)	0·3 (0·2 to 0·5)	0·2 (0·1 to 0·3)	0 (0 to 0·1)	0 (0 to 0)	30·2
Syphilis	1·5 (0·9 to 2·4)	0·4 (0·2 to 0·7)	0·5 (0·2 to 1)	0 (0 to 0·1)	0 (0 to 0)	0·1 (0·1 to 0·1)	0·3 (0·1 to 0·5)	0·2 (0·1 to 0·4)	0·4 (0·2 to 0·6)	0·3 (0·2 to 0·6)	0·5 (0·3 to 0·9)	0·3 (0·2 to 0·5)	0·5 (0·2 to 0·8)	0·5 (0·3 to 0·9)	0·5 (0·3 to 0·9)	0·3 (0·2 to 0·6)	0·4 (0·2 to 0·7)	0·2 (0·1 to 0·4)	0·1 (0·1 to 0·2)	0·1 (0 to 0·2)	0 (0 to 0·1)	0 (0 to 0)	0 (0 to 0)	7·1
Chlamydial infection	0 (0 to 0)	0 (0 to 0)	0 (0 to 0)	0 (0 to 0)	0 (0 to 0)	0 (0 to 0)	0·1 (0·1 to 0·1)	0·3 (0·1 to 0·5)	0·4 (0·2 to 0·7)	0·4 (0·2 to 0·7)	0·4 (0·2 to 0·7)	0·5 (0·2 to 0·7)	0·3 (0·2 to 0·5)	0·1 (0 to 0·2)	0 (0 to 0·1)	0 (0 to 0)	0 (0 to 0)	0 (0 to 0)	0 (0 to 0)	0 (0 to 0)	0 (0 to 0)	0 (0 to 0)	0 (0 to 0)	2·6
Gonococcal infection	0 (0 to 0)	0 (0 to 0)	0 (0 to 0)	0 (0 to 0)	0 (0 to 0)	0 (0 to 0·1)	0·2 (0·1 to 0·5)	0·4 (0·2 to 0·7)	0·2 (0·1 to 0·4)	0·2 (0·1 to 0·4)	0·2 (0·2 to 0·3)	0·3 (0·2 to 0·4)	0·3 (0·2 to 0·4)	0·1 (0·1 to 0·2)	0·1 (0·1 to 0·1)	0·1 (0 to 0·1)	0·1 (0 to 0·1)	0 (0 to 0·1)	0 (0 to 0)	0 (0 to 0)	0 (0 to 0)	0 (0 to 0)	0 (0 to 0)	2·5
Trichomoniasis	0 (0 to 0)	0 (0 to 0)	0 (0 to 0)	0 (0 to 0)	0 (0 to 0)	0 (0 to 0)	0·1 (0 to 0·2)	0·4 (0·1 to 0·9)	0·7 (0·2 to 1·5)	0·8 (0·3 to 1·8)	0·8 (0·3 to 1·8)	0·8 (0·2 to 1·8)	0·7 (0·2 to 1·5)	0·5 (0·2 to 1·2)	0·4 (0·1 to 0·9)	0·2 (0·1 to 0·5)	0·1 (0·1 to 0·3)	0·1 (0 to 0·2)	0·1 (0 to 0·1)	0 (0 to 0·1)	0 (0 to 0)	0 (0 to 0)	0 (0 to 0)	5·6
Genital herpes	0 (0 to 0)	0 (0 to 0)	0 (0 to 0)	0 (0 to 0)	0 (0 to 0)	0 (0 to 0·1)	0·2 (0·1 to 0·4)	0·4 (0·1 to 0·9)	0·5 (0·2 to 1·3)	0·7 (0·2 to 1·6)	0·7 (0·2 to 1·8)	0·8 (0·3 to 1·9)	0·7 (0·2 to 1·8)	0·7 (0·2 to 1·7)	0·6 (0·2 to 1·5)	0·5 (0·2 to 1·2)	0·3 (0·1 to 0·8)	0·2 (0·1 to 0·6)	0·1 (0 to 0·4)	0·1 (0 to 0·2)	0 (0 to 0·1)	0 (0 to 0)	0 (0 to 0)	6·7
Other sexually transmitted infections	0 (0 to 0)	0 (0 to 0)	0 (0 to 0)	0 (0 to 0)	0 (0 to 0)	0·1 (0·1 to 0·2)	0·3 (0·2 to 0·6)	0·5 (0·3 to 0·7)	0·6 (0·4 to 1)	0·9 (0·5 to 1·3)	0·9 (0·5 to 1·4)	0·9 (0·6 to 1·4)	0·7 (0·4 to 1·1)	0·3 (0·2 to 0·5)	0·2 (0·1 to 0·3)	0·1 (0 to 0·1)	0·1 (0 to 0·1)	0 (0 to 0·1)	0·1 (0 to 0·1)	0·1 (0 to 0·2)	0·1 (0 to 0·1)	0 (0 to 0)	0 (0 to 0)	5·8
Respiratory infections and tuberculosis	21·3 (13·4 to 33·7)	21·3 (13 to 32·3)	74·5 (48·3 to 112)	40·1 (28·5 to 56·4)	21·3 (15·3 to 30·1)	21·8 (16·2 to 29·5)	23·1 (17 to 31·4)	27·9 (21 to 36·6)	31·4 (24·2 to 40·9)	33·4 (26 to 42)	37·7 (29·3 to 47·4)	43·8 (33 to 56·9)	54·3 (40·6 to 71·8)	59·6 (46·6 to 76·2)	62·2 (45·9 to 82·3)	66·3 (49·6 to 85·6)	65·1 (50·2 to 83)	73·7 (53·8 to 97·4)	81·8 (64·7 to 103)	87·6 (65·8 to 117)	66·7 (52 to 82·6)	26·2 (20·7 to 32·1)	6·5 (5 to 8·1)	1,050
Tuberculosis	0 (0 to 0)	0 (0 to 0)	0·7 (0·5 to 1·1)	1·1 (0·7 to 1·6)	1·1 (0·6 to 1·8)	1·1 (0·8 to 1·6)	2 (1·4 to 2·8)	5·5 (3·6 to 7·7)	7·1 (5·2 to 9·4)	8 (6·1 to 10·4)	8·3 (5·8 to 11·6)	9·2 (6·6 to 12·4)	9·6 (6·7 to 13·4)	9·6 (6·9 to 13·4)	8·2 (5·9 to 11·3)	7·6 (5·5 to 10·5)	5·6 (4 to 7·7)	4·8 (3·5 to 6·3)	3·7 (2·9 to 4·8)	2·8 (2 to 3·8)	1·6 (1·1 to 2·1)	0·4 (0·3 to 0·5)	0·1 (0 to 0·1)	98·2
Latent tuberculosis infection	0 (0 to 0)	0 (0 to 0)	0 (0 to 0)	0 (0 to 0)	0 (0 to 0)	0 (0 to 0)	0 (0 to 0)	0 (0 to 0)	0 (0 to 0)	0 (0 to 0)	0 (0 to 0)	0 (0 to 0)	0 (0 to 0)	0 (0 to 0)	0 (0 to 0)	0 (0 to 0)	0 (0 to 0)	0 (0 to 0)	0 (0 to 0)	0 (0 to 0)	0 (0 to 0)	0 (0 to 0)	0 (0 to 0)	0
Drug-susceptible tuberculosis	0 (0 to 0)	0 (0 to 0)	0·7 (0·5 to 1·1)	1·1 (0·7 to 1·6)	1·1 (0·6 to 1·8)	1·1 (0·8 to 1·6)	2 (1·4 to 2·8)	5·5 (3·6 to 7·6)	7 (5·1 to 9·4)	7·9 (6 to 10·3)	8·2 (5·8 to 11·4)	9·1 (6·5 to 12·3)	9·4 (6·7 to 13·2)	9·5 (6·8 to 13·2)	8·2 (5·8 to 11·2)	7·5 (5·4 to 10·4)	5·5 (4 to 7·6)	4·7 (3·5 to 6·2)	3·7 (2·8 to 4·7)	2·7 (2 to 3·7)	1·5 (1·1 to 2·1)	0·4 (0·3 to 0·5)	0·1 (0 to 0·1)	96·9
Multidrug-resistant tuberculosis without extensive drug resistanc	0 (0 to 0)	0 (0 to 0)	0 (0 to 0)	0 (0 to 0)	0 (0 to 0)	0 (0 to 0)	0 (0 to 0·1)	0·1 (0 to 0·2)	0·1 (0 to 0·3)	0·1 (0 to 0·3)	0·1 (0 to 0·4)	0·1 (0 to 0·4)	0·1 (0 to 0·4)	0·1 (0 to 0·4)	0·1 (0 to 0·4)	0·1 (0 to 0·3)	0·1 (0 to 0·3)	0·1 (0 to 0·2)	0 (0 to 0·2)	0 (0 to 0·1)	0 (0 to 0·1)	0 (0 to 0)	0 (0 to 0)	1·1
Extensively drug-resistant tuberculosis	0 (0 to 0)	0 (0 to 0)	0 (0 to 0)	0 (0 to 0)	0 (0 to 0)	0 (0 to 0)	0 (0 to 0)	0 (0 to 0)	0 (0 to 0)	0 (0 to 0·1)	0 (0 to 0·1)	0 (0 to 0·1)	0 (0 to 0·1)	0 (0 to 0·1)	0 (0 to 0·1)	0 (0 to 0·1)	0 (0 to 0)	0 (0 to 0)	0 (0 to 0)	0 (0 to 0)	0 (0 to 0)	0 (0 to 0)	0 (0 to 0)	0·2
Lower respiratory infections	21·3 (13·4 to 33·7)	21·1 (12·9 to 32·2)	70·5 (44·8 to 107)	26·9 (17·2 to 42·4)	6·9 (5·3 to 9)	9·2 (6·9 to 12·3)	10 (7 to 14)	12·4 (8·5 to 17)	15·7 (10·4 to 22·7)	17·7 (12·3 to 24·5)	21·5 (15·2 to 29·8)	26·9 (18·2 to 38·2)	37·6 (25·3 to 53·9)	43·5 (31·9 to 57·5)	48·1 (32·2 to 67·4)	54·2 (37·4 to 72·7)	56·5 (41·8 to 74·2)	66·9 (47·3 to 90·8)	76·7 (59·6 to 97·4)	83·9 (62·3 to 113)	64·7 (50·1 to 80·6)	25·7 (20·3 to 31·7)	6·5 (4·9 to 8·1)	824
Upper respiratory infections	0 (0 to 0)	0·1 (0·1 to 0·2)	2·9 (1·9 to 4·2)	8·9 (5·5 to 13·7)	9 (5 to 15·1)	8·3 (4·4 to 14·1)	8·2 (4·3 to 14·3)	7·6 (4·2 to 12·9)	6·8 (3·8 to 11·2)	6·1 (3·4 to 10·2)	6·3 (3·4 to 10·4)	6 (3·3 to 10·2)	5·4 (3 to 9·3)	4·8 (2·7 to 7·9)	4·3 (2·4 to 7)	3·5 (2 to 5·5)	2·5 (1·4 to 4·1)	1·7 (1 to 2·8)	1·1 (0·6 to 1·8)	0·7 (0·4 to 1·2)	0·3 (0·2 to 0·5)	0·1 (0·1 to 0·2)	0 (0 to 0)	94·5
Otitis media	0 (0 to 0)	0 (0 to 0)	0·3 (0·2 to 0·6)	3·2 (1·7 to 5·6)	4·3 (2·5 to 7·1)	3·1 (1·7 to 5·4)	2·9 (1·6 to 4·9)	2·5 (1·1 to 4·8)	1·9 (0·9 to 3·5)	1·5 (0·6 to 2·9)	1·6 (0·8 to 2·9)	1·7 (0·8 to 3·1)	1·8 (0·9 to 3·1)	1·7 (0·8 to 3·1)	1·5 (0·7 to 2·6)	1·1 (0·5 to 2·1)	0·6 (0·3 to 1·1)	0·4 (0·2 to 0·7)	0·3 (0·1 to 0·5)	0·2 (0·1 to 0·3)	0·1 (0 to 0·2)	0 (0 to 0·1)	0 (0 to 0)	30·6
Enteric infections	2·1 (1·4 to 3·3)	6·8 (3·7 to 10·9)	45·8 (28·9 to 69·3)	25·9 (18·5 to 35·4)	16·4 (10·3 to 24·9)	16·7 (10·2 to 25·5)	11·9 (7·8 to 17)	10 (6·9 to 14·1)	10·4 (7·2 to 14·5)	10·6 (7·5 to 14·3)	12·2 (8·8 to 16·2)	13·7 (9·9 to 18·2)	15·4 (11·2 to 20·6)	16·1 (12 to 21)	16·1 (12·1 to 21·2)	17·2 (12·8 to 22·3)	17·6 (12·9 to 23·3)	18·5 (13·3 to 24·1)	17·5 (13·4 to 22·4)	15·5 (11·5 to 20·9)	8·2 (6·3 to 10·3)	2·8 (2·1 to 3·9)	0·7 (0·5 to 0·9)	328
Diarrheal diseases	2·1 (1·4 to 3·3)	6·8 (3·7 to 10·9)	45·7 (28·8 to 69·2)	25·7 (18·3 to 35·2)	16·3 (10·2 to 24·8)	16·5 (10·1 to 25·4)	11·8 (7·7 to 16·9)	9·9 (6·8 to 14·1)	10·4 (7·2 to 14·4)	10·6 (7·4 to 14·3)	12·1 (8·8 to 16·2)	13·6 (9·9 to 18·1)	15·3 (11·1 to 20·5)	16 (12 to 21)	16 (12 to 21·2)	17·1 (12·7 to 22·3)	17·6 (12·9 to 23·3)	18·5 (13·3 to 24)	17·5 (13·3 to 22·3)	15·4 (11·5 to 20·9)	8·2 (6·3 to 10·3)	2·8 (2·1 to 3·9)	0·7 (0·5 to 0·9)	327
Typhoid and paratyphoid	0 (0 to 0)	0 (0 to 0)	0 (0 to 0)	0 (0 to 0)	0 (0 to 0)	0 (0 to 0)	0 (0 to 0·1)	0 (0 to 0)	0 (0 to 0)	0 (0 to 0)	0 (0 to 0)	0 (0 to 0)	0 (0 to 0)	0 (0 to 0)	0 (0 to 0)	0 (0 to 0)	0 (0 to 0)	0 (0 to 0)	0 (0 to 0)	0 (0 to 0)	0 (0 to 0)	0 (0 to 0)	0 (0 to 0)	0·1
Typhoid fever	0 (0 to 0)	0 (0 to 0)	0 (0 to 0)	0 (0 to 0)	0 (0 to 0)	0 (0 to 0)	0 (0 to 0)	0 (0 to 0)	0 (0 to 0)	0 (0 to 0)	0 (0 to 0)	0 (0 to 0)	0 (0 to 0)	0 (0 to 0)	0 (0 to 0)	0 (0 to 0)	0 (0 to 0)	0 (0 to 0)	0 (0 to 0)	0 (0 to 0)	0 (0 to 0)	0 (0 to 0)	0 (0 to 0)	0
Paratyphoid fever	0 (0 to 0)	0 (0 to 0)	0 (0 to 0)	0 (0 to 0)	0 (0 to 0)	0 (0 to 0)	0 (0 to 0·1)	0 (0 to 0)	0 (0 to 0)	0 (0 to 0)	0 (0 to 0)	0 (0 to 0)	0 (0 to 0)	0 (0 to 0)	0 (0 to 0)	0 (0 to 0)	0 (0 to 0)	0 (0 to 0)	0 (0 to 0)	0 (0 to 0)	0 (0 to 0)	0 (0 to 0)	0 (0 to 0)	0·1
Invasive Non-typhoidal Salmonella (iNTS)	0 (0 to 0)	0 (0 to 0)	0 (0 to 0)	0·1 (0 to 0·1)	0·1 (0 to 0·1)	0·1 (0 to 0·3)	0 (0 to 0)	0 (0 to 0)	0 (0 to 0·1)	0 (0 to 0)	0 (0 to 0)	0 (0 to 0·1)	0 (0 to 0)	0 (0 to 0)	0 (0 to 0)	0 (0 to 0·1)	0 (0 to 0)	0 (0 to 0)	0 (0 to 0)	0 (0 to 0)	0 (0 to 0)	0 (0 to 0)	0 (0 to 0)	0·5
Other intestinal infectious diseases	0 (0 to 0)	0 (0 to 0)	0·1 (0 to 0·2)	0·1 (0 to 0·2)	0 (0 to 0·1)	0·1 (0 to 0·2)	0 (0 to 0·1)	0 (0 to 0·1)	0 (0 to 0)	0 (0 to 0·1)	0 (0 to 0·1)	0 (0 to 0·1)	0 (0 to 0)	0 (0 to 0)	0 (0 to 0)	0 (0 to 0)	0 (0 to 0)	0 (0 to 0)	0 (0 to 0)	0 (0 to 0)	0 (0 to 0)	0 (0 to 0)	0 (0 to 0)	0·6
Neglected tropical diseases and malaria	0·1 (0 to 0·2)	0·3 (0·2 to 0·4)	2 (1·2 to 3·2)	6 (3·4 to 9·8)	7·8 (4·2 to 13·3)	5·4 (3 to 8·6)	5·1 (3 to 8)	5·7 (3·4 to 8·8)	5·9 (3·2 to 9·4)	6·4 (3·7 to 10·4)	7·3 (4 to 12·2)	8·1 (4·6 to 13·3)	8·5 (4·6 to 14·9)	9·4 (4·7 to 16·4)	10·1 (5·4 to 17·7)	9·7 (4·9 to 17·5)	8·3 (4·1 to 14·6)	7·2 (3·7 to 12·3)	5·7 (2·8 to 9·9)	4·3 (2 to 7·4)	2·1 (1·1 to 3·8)	0·7 (0·4 to 1·2)	0·2 (0·1 to 0·3)	126
Malaria	0 (0 to 0)	0 (0 to 0)	0 (0 to 0)	0 (0 to 0)	0 (0 to 0)	0 (0 to 0)	0 (0 to 0)	0 (0 to 0)	0 (0 to 0)	0 (0 to 0)	0 (0 to 0)	0 (0 to 0)	0 (0 to 0)	0 (0 to 0)	0 (0 to 0)	0 (0 to 0)	0 (0 to 0)	0 (0 to 0)	0 (0 to 0)	0 (0 to 0)	0 (0 to 0)	0 (0 to 0)	0 (0 to 0)	0
Chagas disease	0 (0 to 0)	0 (0 to 0)	0 (0 to 0)	0 (0 to 0)	0 (0 to 0)	0 (0 to 0)	0 (0 to 0)	0 (0 to 0)	0 (0 to 0)	0 (0 to 0)	0 (0 to 0)	0 (0 to 0)	0 (0 to 0)	0 (0 to 0)	0 (0 to 0)	0 (0 to 0)	0 (0 to 0)	0 (0 to 0)	0 (0 to 0)	0 (0 to 0)	0 (0 to 0)	0 (0 to 0)	0 (0 to 0)	0
Leishmaniasis	0 (0 to 0)	0 (0 to 0)	0 (0 to 0)	0 (0 to 0)	0 (0 to 0)	0 (0 to 0)	0 (0 to 0)	0 (0 to 0)	0 (0 to 0)	0 (0 to 0)	0 (0 to 0)	0 (0 to 0)	0 (0 to 0)	0 (0 to 0)	0 (0 to 0)	0 (0 to 0)	0 (0 to 0)	0 (0 to 0)	0 (0 to 0)	0 (0 to 0)	0 (0 to 0)	0 (0 to 0)	0 (0 to 0)	0
Visceral leishmaniasis	0 (0 to 0)	0 (0 to 0)	0 (0 to 0)	0 (0 to 0)	0 (0 to 0)	0 (0 to 0)	0 (0 to 0)	0 (0 to 0)	0 (0 to 0)	0 (0 to 0)	0 (0 to 0)	0 (0 to 0)	0 (0 to 0)	0 (0 to 0)	0 (0 to 0)	0 (0 to 0)	0 (0 to 0)	0 (0 to 0)	0 (0 to 0)	0 (0 to 0)	0 (0 to 0)	0 (0 to 0)	0 (0 to 0)	0
Cutaneous and mucocutaneous leishmaniasis	0 (0 to 0)	0 (0 to 0)	0 (0 to 0)	0 (0 to 0)	0 (0 to 0)	0 (0 to 0)	0 (0 to 0)	0 (0 to 0)	0 (0 to 0)	0 (0 to 0)	0 (0 to 0)	0 (0 to 0)	0 (0 to 0)	0 (0 to 0)	0 (0 to 0)	0 (0 to 0)	0 (0 to 0)	0 (0 to 0)	0 (0 to 0)	0 (0 to 0)	0 (0 to 0)	0 (0 to 0)	0 (0 to 0)	0
African trypanosomiasis	0 (0 to 0)	0 (0 to 0)	0 (0 to 0)	0 (0 to 0)	0 (0 to 0)	0 (0 to 0)	0 (0 to 0)	0 (0 to 0)	0 (0 to 0)	0 (0 to 0)	0 (0 to 0)	0 (0 to 0)	0 (0 to 0)	0 (0 to 0)	0 (0 to 0)	0 (0 to 0)	0 (0 to 0)	0 (0 to 0)	0 (0 to 0)	0 (0 to 0)	0 (0 to 0)	0 (0 to 0)	0 (0 to 0)	0
Schistosomiasis	0 (0 to 0)	0 (0 to 0)	0 (0 to 0)	0 (0 to 0)	0 (0 to 0)	0 (0 to 0)	0 (0 to 0)	0 (0 to 0)	0 (0 to 0)	0 (0 to 0)	0 (0 to 0)	0 (0 to 0)	0 (0 to 0)	0 (0 to 0)	0 (0 to 0)	0 (0 to 0)	0 (0 to 0)	0 (0 to 0)	0 (0 to 0)	0 (0 to 0)	0 (0 to 0)	0 (0 to 0)	0 (0 to 0)	0
Cysticercosis	0 (0 to 0)	0 (0 to 0)	0 (0 to 0)	0 (0 to 0)	0 (0 to 0)	0·1 (0 to 0·2)	0·5 (0·1 to 1·3)	1·6 (0·4 to 4)	2·7 (0·8 to 5·9)	3·2 (1·1 to 6·8)	3·9 (1·4 to 8·6)	4·8 (1·7 to 9·8)	5·6 (2·1 to 11·7)	6·7 (2·4 to 13·2)	7·7 (3·2 to 15)	8·2 (3·5 to 15·6)	7·3 (3·2 to 13·5)	6·3 (2·8 to 11·2)	5 (2·2 to 9)	3·8 (1·6 to 6·9)	1·8 (0·8 to 3·4)	0·5 (0·2 to 1·1)	0·1 (0 to 0·2)	69·7
Cystic echinococcosis	0 (0 to 0)	0 (0 to 0)	0 (0 to 0)	0 (0 to 0)	0 (0 to 0)	0 (0 to 0)	0 (0 to 0)	0 (0 to 0)	0 (0 to 0)	0 (0 to 0)	0 (0 to 0)	0 (0 to 0)	0 (0 to 0)	0 (0 to 0)	0 (0 to 0)	0 (0 to 0)	0 (0 to 0)	0 (0 to 0)	0 (0 to 0)	0 (0 to 0)	0 (0 to 0)	0 (0 to 0)	0 (0 to 0)	0
Lymphatic filariasis	0 (0 to 0)	0 (0 to 0)	0 (0 to 0)	0 (0 to 0)	0 (0 to 0)	0 (0 to 0)	0 (0 to 0)	0 (0 to 0)	0 (0 to 0)	0 (0 to 0)	0 (0 to 0)	0 (0 to 0)	0 (0 to 0)	0 (0 to 0)	0 (0 to 0)	0 (0 to 0)	0 (0 to 0)	0 (0 to 0)	0 (0 to 0)	0 (0 to 0)	0 (0 to 0)	0 (0 to 0)	0 (0 to 0)	0
Onchocerciasis	0 (0 to 0)	0 (0 to 0)	0 (0 to 0)	0 (0 to 0)	0 (0 to 0)	0 (0 to 0)	0 (0 to 0)	0 (0 to 0)	0 (0 to 0)	0 (0 to 0)	0 (0 to 0)	0 (0 to 0)	0 (0 to 0)	0 (0 to 0)	0 (0 to 0)	0 (0 to 0)	0 (0 to 0)	0 (0 to 0)	0 (0 to 0)	0 (0 to 0)	0 (0 to 0)	0 (0 to 0)	0 (0 to 0)	0
Trachoma	0 (0 to 0)	0 (0 to 0)	0 (0 to 0)	0 (0 to 0)	0 (0 to 0)	0 (0 to 0)	0 (0 to 0)	0 (0 to 0)	0 (0 to 0)	0 (0 to 0)	0 (0 to 0)	0 (0 to 0)	0 (0 to 0)	0 (0 to 0)	0 (0 to 0)	0 (0 to 0)	0 (0 to 0)	0 (0 to 0)	0 (0 to 0)	0 (0 to 0)	0 (0 to 0)	0 (0 to 0)	0 (0 to 0)	0
Dengue	0 (0 to 0)	0 (0 to 0)	0·3 (0·1 to 0·7)	0·5 (0·2 to 1)	1 (0·3 to 2·1)	1·1 (0·4 to 2·2)	1·1 (0·4 to 2·3)	1 (0·4 to 2·1)	0·9 (0·3 to 1·7)	0·8 (0·3 to 1·5)	0·7 (0·2 to 1·5)	0·7 (0·3 to 1·4)	0·6 (0·2 to 1·3)	0·6 (0·2 to 1·3)	0·6 (0·2 to 1·2)	0·5 (0·2 to 1)	0·3 (0·1 to 0·7)	0·3 (0·1 to 0·6)	0·2 (0 to 0·4)	0·1 (0 to 0·3)	0·1 (0 to 0·2)	0 (0 to 0·1)	0 (0 to 0)	11·6
Yellow fever	0 (0 to 0)	0 (0 to 0)	0 (0 to 0)	0 (0 to 0)	0 (0 to 0)	0 (0 to 0)	0 (0 to 0)	0 (0 to 0)	0 (0 to 0)	0 (0 to 0)	0 (0 to 0)	0 (0 to 0)	0 (0 to 0)	0 (0 to 0)	0 (0 to 0)	0 (0 to 0)	0 (0 to 0)	0 (0 to 0)	0 (0 to 0)	0 (0 to 0)	0 (0 to 0)	0 (0 to 0)	0 (0 to 0)	0
Rabies	0 (0 to 0)	0 (0 to 0)	0 (0 to 0)	0 (0 to 0·1)	0·1 (0 to 0·2)	0·1 (0 to 0·2)	0 (0 to 0·1)	0 (0 to 0)	0 (0 to 0)	0 (0 to 0)	0 (0 to 0)	0 (0 to 0)	0 (0 to 0·1)	0 (0 to 0)	0 (0 to 0)	0 (0 to 0)	0 (0 to 0)	0 (0 to 0)	0 (0 to 0)	0 (0 to 0)	0 (0 to 0)	0 (0 to 0)	0 (0 to 0)	0·4
Intestinal nematode infections	0 (0 to 0)	0 (0 to 0)	0 (0 to 0·1)	0·3 (0·1 to 0·6)	0·7 (0·4 to 1·2)	0·6 (0·3 to 1)	1·8 (0·8 to 3·4)	1·3 (0·6 to 2·5)	0·9 (0·4 to 1·6)	0·8 (0·3 to 1·4)	0·9 (0·4 to 1·6)	0·5 (0·3 to 0·8)	0·2 (0·1 to 0·4)	0·2 (0·1 to 0·4)	0·2 (0·1 to 0·3)	0·2 (0·1 to 0·3)	0·1 (0·1 to 0·2)	0·1 (0 to 0·1)	0·1 (0 to 0·1)	0 (0 to 0·1)	0 (0 to 0)	0 (0 to 0)	0 (0 to 0)	8·9
Ascariasis	0 (0 to 0)	0 (0 to 0)	0 (0 to 0·1)	0·1 (0 to 0·3)	0·2 (0 to 0·4)	0·1 (0 to 0·2)	0·1 (0 to 0·2)	0·1 (0 to 0·1)	0·1 (0 to 0·1)	0·1 (0 to 0·1)	0·1 (0 to 0·1)	0·1 (0 to 0·1)	0 (0 to 0·1)	0 (0 to 0·1)	0 (0 to 0·1)	0 (0 to 0·1)	0 (0 to 0)	0 (0 to 0)	0 (0 to 0)	0 (0 to 0)	0 (0 to 0)	0 (0 to 0)	0 (0 to 0)	1·1
Trichuriasis	0 (0 to 0)	0 (0 to 0)	0 (0 to 0)	0 (0 to 0)	0 (0 to 0)	0 (0 to 0)	0 (0 to 0)	0 (0 to 0)	0 (0 to 0)	0 (0 to 0)	0 (0 to 0)	0 (0 to 0)	0 (0 to 0)	0 (0 to 0)	0 (0 to 0)	0 (0 to 0)	0 (0 to 0)	0 (0 to 0)	0 (0 to 0)	0 (0 to 0)	0 (0 to 0)	0 (0 to 0)	0 (0 to 0)	0·1
Hookworm disease	0 (0 to 0)	0 (0 to 0)	0 (0 to 0)	0·2 (0·1 to 0·4)	0·5 (0·3 to 1)	0·5 (0·2 to 0·9)	1·7 (0·7 to 3·3)	1·2 (0·5 to 2·4)	0·8 (0·3 to 1·5)	0·7 (0·3 to 1·3)	0·8 (0·4 to 1·5)	0·5 (0·2 to 0·8)	0·1 (0·1 to 0·3)	0·2 (0·1 to 0·3)	0·2 (0·1 to 0·3)	0·1 (0·1 to 0·2)	0·1 (0 to 0·2)	0·1 (0 to 0·1)	0 (0 to 0·1)	0 (0 to 0·1)	0 (0 to 0)	0 (0 to 0)	0 (0 to 0)	7·8
Food-borne trematodiases	0 (0 to 0)	0 (0 to 0)	0 (0 to 0)	0 (0 to 0)	0 (0 to 0)	0 (0 to 0)	0 (0 to 0)	0 (0 to 0)	0 (0 to 0)	0 (0 to 0)	0 (0 to 0)	0 (0 to 0)	0 (0 to 0)	0 (0 to 0)	0 (0 to 0)	0 (0 to 0)	0 (0 to 0)	0 (0 to 0)	0 (0 to 0)	0 (0 to 0)	0 (0 to 0)	0 (0 to 0)	0 (0 to 0)	0
Leprosy	0 (0 to 0)	0 (0 to 0)	0 (0 to 0)	0 (0 to 0)	0 (0 to 0)	0 (0 to 0)	0 (0 to 0)	0 (0 to 0)	0 (0 to 0)	0 (0 to 0)	0 (0 to 0)	0 (0 to 0·1)	0 (0 to 0·1)	0·1 (0 to 0·1)	0·1 (0 to 0·1)	0·1 (0 to 0·1)	0 (0 to 0·1)	0 (0 to 0·1)	0 (0 to 0·1)	0 (0 to 0)	0 (0 to 0)	0 (0 to 0)	0 (0 to 0)	0·5
Ebola	0 (0 to 0)	0 (0 to 0)	0 (0 to 0)	0 (0 to 0)	0 (0 to 0)	0 (0 to 0)	0 (0 to 0)	0 (0 to 0)	0 (0 to 0)	0 (0 to 0)	0 (0 to 0)	0 (0 to 0)	0 (0 to 0)	0 (0 to 0)	0 (0 to 0)	0 (0 to 0)	0 (0 to 0)	0 (0 to 0)	0 (0 to 0)	0 (0 to 0)	0 (0 to 0)	0 (0 to 0)	0 (0 to 0)	0
Zika virus	0 (0 to 0)	0 (0 to 0)	0 (0 to 0·2)	0·2 (0 to 0·8)	0·2 (0 to 1·1)	0·3 (0 to 1·1)	0·3 (0 to 1·4)	0·3 (0 to 1·4)	0·3 (0 to 1·4)	0·3 (0 to 1·4)	0·3 (0 to 1·3)	0·2 (0 to 1·1)	0·2 (0 to 0·8)	0·2 (0 to 0·7)	0·1 (0 to 0·4)	0·1 (0 to 0·3)	0 (0 to 0·2)	0 (0 to 0·1)	0 (0 to 0)	0 (0 to 0)	0 (0 to 0)	0 (0 to 0)	0 (0 to 0)	3·1
Guinea worm disease	0 (0 to 0)	0 (0 to 0)	0 (0 to 0)	0 (0 to 0)	0 (0 to 0)	0 (0 to 0)	0 (0 to 0)	0 (0 to 0)	0 (0 to 0)	0 (0 to 0)	0 (0 to 0)	0 (0 to 0)	0 (0 to 0)	0 (0 to 0)	0 (0 to 0)	0 (0 to 0)	0 (0 to 0)	0 (0 to 0)	0 (0 to 0)	0 (0 to 0)	0 (0 to 0)	0 (0 to 0)	0 (0 to 0)	0
Other neglected tropical diseases	0·1 (0 to 0·2)	0·3 (0·2 to 0·4)	1·6 (0·9 to 2·6)	5 (2·6 to 8·4)	5·8 (2·7 to 10·6)	3·3 (1·5 to 5·9)	1·3 (0·6 to 2·3)	1·3 (0·7 to 2·2)	1 (0·5 to 1·9)	1·3 (0·7 to 2·3)	1·4 (0·7 to 2·5)	1·8 (0·9 to 2·9)	1·8 (1 to 3)	1·6 (0·9 to 2·7)	1·6 (0·8 to 2·6)	0·8 (0·4 to 1·2)	0·5 (0·3 to 0·8)	0·5 (0·3 to 0·9)	0·5 (0·3 to 0·8)	0·3 (0·2 to 0·4)	0·2 (0·1 to 0·3)	0·1 (0·1 to 0·1)	0 (0 to 0·1)	32
Other infectious diseases	2·7 (1·7 to 4)	5·5 (3·6 to 8·1)	19·9 (12·8 to 28·6)	16·1 (11·3 to 22·6)	10·4 (7·8 to 13·9)	11 (8·7 to 13·9)	12 (9·6 to 15)	10·1 (8 to 12·8)	11·2 (8·6 to 14·5)	11 (8·3 to 14·4)	12·8 (9·7 to 16·8)	14·7 (10·7 to 19·1)	19·3 (13·2 to 26·3)	17·7 (12·5 to 23·7)	15·5 (11·2 to 20·4)	12·1 (9 to 15·6)	8·5 (6·2 to 10·8)	6·8 (4·9 to 8·7)	4·6 (3·3 to 5·8)	3·5 (2·5 to 4·5)	2·6 (1·7 to 3·5)	0·6 (0·4 to 0·8)	0·1 (0·1 to 0·2)	229
Meningitis	1·6 (1 to 2·5)	4·1 (2·5 to 6·2)	10·7 (6 to 17·3)	6 (3·8 to 9·3)	2·7 (2·1 to 3·4)	3·5 (2·6 to 4·7)	3·5 (2·6 to 4·7)	2·5 (1·9 to 3·4)	2·5 (1·9 to 3·4)	2·6 (2 to 3·4)	2·8 (2·1 to 3·7)	3 (2·2 to 3·9)	3·8 (2·8 to 5)	3·7 (2·7 to 4·8)	3·1 (2·4 to 4)	2·6 (2 to 3·5)	1·9 (1·4 to 2·4)	1·5 (1·1 to 1·9)	1·2 (0·9 to 1·5)	0·9 (0·7 to 1·1)	0·5 (0·4 to 0·7)	0·1 (0·1 to 0·1)	0 (0 to 0)	64·7
Encephalitis	0·3 (0·2 to 0·4)	0·5 (0·3 to 0·8)	3·9 (2·2 to 6·2)	4·1 (2·3 to 6·8)	2 (1·4 to 3)	2·7 (1·9 to 3·7)	2·8 (2 to 3·9)	2·2 (1·6 to 3)	1·9 (1·4 to 2·5)	1·6 (1·2 to 2·2)	1·6 (1·2 to 2·2)	1·8 (1·4 to 2·5)	1·9 (1·4 to 2·6)	1·4 (1·1 to 2)	1·3 (1 to 1·8)	1 (0·8 to 1·4)	0·7 (0·5 to 1)	0·5 (0·4 to 0·8)	0·3 (0·2 to 0·4)	0·2 (0·2 to 0·3)	0·1 (0·1 to 0·1)	0 (0 to 0)	0 (0 to 0)	32·9
Diphtheria	0 (0 to 0)	0 (0 to 0)	0·1 (0 to 0·2)	0·2 (0 to 0·6)	0·3 (0·1 to 0·7)	0·1 (0 to 0·2)	0 (0 to 0)	0 (0 to 0)	0 (0 to 0)	0 (0 to 0·1)	0 (0 to 0)	0 (0 to 0)	0 (0 to 0)	0 (0 to 0)	0 (0 to 0)	0 (0 to 0)	0 (0 to 0)	0 (0 to 0)	0 (0 to 0)	0 (0 to 0)	0 (0 to 0)	0 (0 to 0)	0 (0 to 0)	0·7
Tetanus	0 (0 to 0)	0 (0 to 0)	0 (0 to 0)	0 (0 to 0)	0 (0 to 0)	0 (0 to 0·1)	0 (0 to 0·1)	0 (0 to 0·1)	0 (0 to 0·1)	0 (0 to 0·1)	0 (0 to 0·1)	0 (0 to 0·1)	0 (0 to 0·2)	0·1 (0 to 0·3)	0·1 (0 to 0·3)	0 (0 to 0·2)	0 (0 to 0·2)	0 (0 to 0·1)	0 (0 to 0·1)	0 (0 to 0·1)	0 (0 to 0)	0 (0 to 0)	0 (0 to 0)	0·5
Measles	0 (0 to 0)	0 (0 to 0)	0 (0 to 0)	0 (0 to 0)	0 (0 to 0)	0 (0 to 0)	0 (0 to 0)	0 (0 to 0)	0 (0 to 0)	0 (0 to 0)	0 (0 to 0)	0 (0 to 0)	0 (0 to 0)	0 (0 to 0)	0 (0 to 0)	0 (0 to 0)	0 (0 to 0)	0 (0 to 0)	0 (0 to 0)	0 (0 to 0)	0 (0 to 0)	0 (0 to 0)	0 (0 to 0)	0
Varicella and herpes zoster	0·1 (0 to 0·1)	0·1 (0·1 to 0·2)	0·4 (0·2 to 0·8)	0·2 (0·1 to 0·4)	0·3 (0·2 to 0·5)	0·3 (0·2 to 0·5)	0·2 (0·1 to 0·4)	0·3 (0·2 to 0·5)	0·3 (0·2 to 0·5)	0·3 (0·2 to 0·5)	0·4 (0·2 to 0·6)	0·4 (0·3 to 0·7)	0·4 (0·3 to 0·7)	0·4 (0·3 to 0·7)	0·4 (0·2 to 0·7)	0·4 (0·3 to 0·7)	0·3 (0·2 to 0·5)	0·3 (0·2 to 0·5)	0·2 (0·2 to 0·4)	0·2 (0·1 to 0·3)	0·1 (0·1 to 0·2)	0 (0 to 0·1)	0 (0 to 0)	6·4
Acute hepatitis	0 (0 to 0)	0 (0 to 0)	0·1 (0 to 0·1)	0·2 (0·1 to 0·3)	0·6 (0·4 to 1)	0·8 (0·5 to 1·2)	0·8 (0·5 to 1·2)	0·7 (0·5 to 1)	0·6 (0·4 to 0·9)	0·5 (0·3 to 0·7)	0·5 (0·3 to 0·7)	0·5 (0·3 to 0·6)	0·4 (0·3 to 0·6)	0·4 (0·3 to 0·5)	0·3 (0·2 to 0·4)	0·2 (0·1 to 0·2)	0·1 (0·1 to 0·1)	0·1 (0·1 to 0·1)	0·1 (0 to 0·1)	0 (0 to 0·1)	0 (0 to 0)	0 (0 to 0)	0 (0 to 0)	6·8
Acute hepatitis A	0 (0 to 0)	0 (0 to 0)	0 (0 to 0)	0·2 (0·1 to 0·3)	0·5 (0·3 to 0·9)	0·7 (0·4 to 1·1)	0·6 (0·4 to 1)	0·5 (0·3 to 0·8)	0·4 (0·2 to 0·6)	0·3 (0·2 to 0·5)	0·2 (0·1 to 0·4)	0·2 (0·1 to 0·3)	0·2 (0·1 to 0·3)	0·1 (0·1 to 0·2)	0·1 (0 to 0·1)	0 (0 to 0·1)	0 (0 to 0)	0 (0 to 0)	0 (0 to 0)	0 (0 to 0)	0 (0 to 0)	0 (0 to 0)	0 (0 to 0)	4·1
Acute hepatitis B	0 (0 to 0)	0 (0 to 0)	0 (0 to 0·1)	0 (0 to 0)	0 (0 to 0)	0 (0 to 0·1)	0·1 (0 to 0·1)	0·1 (0·1 to 0·2)	0·2 (0·1 to 0·3)	0·1 (0·1 to 0·2)	0·2 (0·1 to 0·2)	0·2 (0·1 to 0·3)	0·2 (0·1 to 0·2)	0·2 (0·1 to 0·3)	0·2 (0·1 to 0·2)	0·1 (0·1 to 0·1)	0·1 (0 to 0·1)	0·1 (0 to 0·1)	0 (0 to 0)	0 (0 to 0)	0 (0 to 0)	0 (0 to 0)	0 (0 to 0)	1·9
Acute hepatitis C	0 (0 to 0)	0 (0 to 0)	0 (0 to 0)	0 (0 to 0)	0 (0 to 0)	0 (0 to 0)	0 (0 to 0)	0 (0 to 0)	0 (0 to 0)	0 (0 to 0)	0 (0 to 0)	0 (0 to 0)	0 (0 to 0)	0 (0 to 0)	0 (0 to 0)	0 (0 to 0)	0 (0 to 0)	0 (0 to 0)	0 (0 to 0)	0 (0 to 0)	0 (0 to 0)	0 (0 to 0)	0 (0 to 0)	0·2
Acute hepatitis E	0 (0 to 0)	0 (0 to 0)	0 (0 to 0)	0 (0 to 0)	0 (0 to 0·1)	0·1 (0 to 0·1)	0·1 (0 to 0·2)	0·1 (0 to 0·1)	0·1 (0 to 0·1)	0 (0 to 0·1)	0 (0 to 0·1)	0 (0 to 0·1)	0·1 (0 to 0·1)	0 (0 to 0)	0 (0 to 0)	0 (0 to 0·1)	0 (0 to 0)	0 (0 to 0)	0 (0 to 0)	0 (0 to 0)	0 (0 to 0)	0 (0 to 0)	0 (0 to 0)	0·7
Other unspecified infectious diseases	0·7 (0·4 to 1·2)	0·9 (0·5 to 1·4)	4·1 (2·5 to 6·5)	4·9 (3 to 7·4)	4·4 (2·4 to 7·6)	3·6 (2·2 to 5·6)	4·7 (3 to 6·6)	4·3 (2·9 to 6·2)	5·8 (3·5 to 8·7)	5·8 (3·4 to 8·7)	7·5 (4·7 to 11·1)	8·9 (5·4 to 12·8)	12·7 (7·1 to 19·4)	11·7 (7 to 17·1)	10·4 (6·5 to 14·7)	7·7 (4·8 to 10·8)	5·5 (3·4 to 7·6)	4·3 (2·6 to 6·2)	2·8 (1·6 to 3·9)	2·2 (1·2 to 3·1)	1·8 (1 to 2·8)	0·4 (0·2 to 0·7)	0·1 (0·1 to 0·1)	115
Maternal and neonatal disorders	781 (549 to 1,090)	253 (171 to 357)	81·4 (53·3 to 120)	36·5 (28·1 to 47·3)	30·5 (22·6 to 39·5)	32·2 (24 to 42·2)	41·8 (33·4 to 52·5)	47·1 (38·6 to 57·2)	44·1 (36 to 53·7)	39·3 (31·8 to 48·1)	34 (27·6 to 41·5)	26·4 (21·1 to 33·2)	19·6 (15 to 25·1)	15·4 (11·5 to 20)	12 (8·9 to 16)	8·3 (6·1 to 11·1)	4·3 (3·1 to 5·8)	2 (1·4 to 2·7)	0·8 (0·5 to 1·1)	0·2 (0·1 to 0·3)	0 (0 to 0·1)	0 (0 to 0)	0 (0 to 0)	1,510
Maternal disorders	0 (0 to 0)	0 (0 to 0)	0 (0 to 0)	0 (0 to 0)	0 (0 to 0)	0·6 (0·5 to 0·7)	10·8 (8·6 to 13·4)	18·2 (14·1 to 22·8)	18·2 (14·1 to 22·8)	15·8 (12 to 20·3)	11·3 (8·7 to 14·4)	5·5 (4·2 to 6·9)	1·4 (1 to 1·8)	0·2 (0·1 to 0·2)	0 (0 to 0)	0 (0 to 0)	0 (0 to 0)	0 (0 to 0)	0 (0 to 0)	0 (0 to 0)	0 (0 to 0)	0 (0 to 0)	0 (0 to 0)	81·9
Maternal hemorrhage	0 (0 to 0)	0 (0 to 0)	0 (0 to 0)	0 (0 to 0)	0 (0 to 0)	0 (0 to 0)	1·2 (0·6 to 2·1)	1·5 (0·9 to 2·4)	1·5 (0·9 to 2·3)	1·4 (0·8 to 2·1)	1 (0·6 to 1·5)	0·4 (0·3 to 0·6)	0 (0 to 0·1)	0 (0 to 0)	0 (0 to 0)	0 (0 to 0)	0 (0 to 0)	0 (0 to 0)	0 (0 to 0)	0 (0 to 0)	0 (0 to 0)	0 (0 to 0)	0 (0 to 0)	7
Maternal sepsis and other maternal infections	0 (0 to 0)	0 (0 to 0)	0 (0 to 0)	0 (0 to 0)	0 (0 to 0)	0 (0 to 0)	0·3 (0·1 to 0·6)	0·4 (0·2 to 0·8)	0·3 (0·2 to 0·6)	0·2 (0·1 to 0·4)	0·2 (0·1 to 0·3)	0·1 (0 to 0·1)	0 (0 to 0)	0 (0 to 0)	0 (0 to 0)	0 (0 to 0)	0 (0 to 0)	0 (0 to 0)	0 (0 to 0)	0 (0 to 0)	0 (0 to 0)	0 (0 to 0)	0 (0 to 0)	1·6
Maternal hypertensive disorders	0 (0 to 0)	0 (0 to 0)	0 (0 to 0)	0 (0 to 0)	0 (0 to 0)	0·2 (0·2 to 0·3)	3·3 (2·5 to 4·4)	4·7 (3·4 to 6·3)	5 (3·7 to 6·8)	3·9 (2·8 to 5·2)	2·6 (1·8 to 3·4)	1·2 (0·9 to 1·7)	0·3 (0·2 to 0·4)	0 (0 to 0)	0 (0 to 0)	0 (0 to 0)	0 (0 to 0)	0 (0 to 0)	0 (0 to 0)	0 (0 to 0)	0 (0 to 0)	0 (0 to 0)	0 (0 to 0)	21·2
Maternal obstructed labor and uterine rupture	0 (0 to 0)	0 (0 to 0)	0 (0 to 0)	0 (0 to 0)	0 (0 to 0)	0 (0 to 0)	0·4 (0·2 to 0·6)	0·7 (0·3 to 1·1)	0·7 (0·4 to 1·3)	0·8 (0·4 to 1·3)	0·7 (0·4 to 1·2)	0·3 (0·2 to 0·5)	0·1 (0 to 0·1)	0 (0 to 0)	0 (0 to 0)	0 (0 to 0)	0 (0 to 0)	0 (0 to 0)	0 (0 to 0)	0 (0 to 0)	0 (0 to 0)	0 (0 to 0)	0 (0 to 0)	3·6
Maternal abortion and miscarriage	0 (0 to 0)	0 (0 to 0)	0 (0 to 0)	0 (0 to 0)	0 (0 to 0)	0 (0 to 0·1)	0·7 (0·4 to 1·1)	1·1 (0·6 to 1·8)	1 (0·5 to 1·7)	0·8 (0·4 to 1·4)	0·6 (0·3 to 1·1)	0·4 (0·2 to 0·7)	0·2 (0·1 to 0·3)	0 (0 to 0)	0 (0 to 0)	0 (0 to 0)	0 (0 to 0)	0 (0 to 0)	0 (0 to 0)	0 (0 to 0)	0 (0 to 0)	0 (0 to 0)	0 (0 to 0)	4·7
Ectopic pregnancy	0 (0 to 0)	0 (0 to 0)	0 (0 to 0)	0 (0 to 0)	0 (0 to 0)	0 (0 to 0)	0·6 (0·4 to 0·8)	1·7 (1·1 to 2·5)	1·9 (1·2 to 2·8)	1·8 (1·2 to 2·8)	1·2 (0·8 to 1·9)	0·5 (0·3 to 0·8)	0·2 (0·1 to 0·3)	0 (0 to 0)	0 (0 to 0)	0 (0 to 0)	0 (0 to 0)	0 (0 to 0)	0 (0 to 0)	0 (0 to 0)	0 (0 to 0)	0 (0 to 0)	0 (0 to 0)	8
Indirect maternal deaths	--	--	--	--	--	0·1 (0·1 to 0·2)	1·8 (1·1 to 2·6)	3·4 (2·2 to 5)	3 (1·8 to 4·7)	2·5 (1·5 to 3·8)	1·6 (1 to 2·3)	0·6 (0·4 to 1)	0·1 (0·1 to 0·2)	0 (0 to 0)	--	--	--	--	--	--	--	--	--	NA
Late maternal deaths	--	--	--	--	--	0 (0 to 0·1)	0·6 (0·4 to 0·9)	1·1 (0·7 to 1·7)	1·1 (0·6 to 1·7)	0·9 (0·5 to 1·4)	0·6 (0·4 to 1)	0·3 (0·2 to 0·5)	0·1 (0·1 to 0·1)	0 (0 to 0)	--	--	--	--	--	--	--	--	--	NA
Maternal deaths aggravated by HIV/AIDS	--	--	--	--	--	0 (0 to 0)	0 (0 to 0)	0 (0 to 0·1)	0 (0 to 0·1)	0 (0 to 0)	0 (0 to 0)	0 (0 to 0)	0 (0 to 0)	0 (0 to 0)	--	--	--	--	--	--	--	--	--	NA
Neonatal disorders	781 (549 to 1,090)	253 (171 to 357)	81·4 (53·3 to 120)	36·5 (28·1 to 47·3)	30·5 (22·6 to 39·5)	31·6 (23·4 to 41·5)	31·1 (23·1 to 41·1)	29 (21·3 to 37·3)	25·9 (19·2 to 33·9)	23·5 (17·4 to 30·9)	22·6 (16·8 to 29·4)	20·9 (15·6 to 27·4)	18·2 (13·7 to 23·7)	15·2 (11·3 to 19·9)	12 (8·9 to 16)	8·3 (6·1 to 11·1)	4·3 (3·1 to 5·8)	2 (1·4 to 2·7)	0·8 (0·5 to 1·1)	0·2 (0·1 to 0·3)	0 (0 to 0·1)	0 (0 to 0)	0 (0 to 0)	1,430
Neonatal preterm birth	401 (269 to 570)	90 (54·2 to 137)	35·1 (22·3 to 52·3)	12·1 (8·9 to 16)	14·3 (10·4 to 19)	14·8 (10·6 to 19·2)	14·3 (10·4 to 19·2)	13·1 (9·6 to 17·1)	11·6 (8·3 to 15·5)	10·3 (7·5 to 13·8)	9·8 (7·2 to 12·7)	8·8 (6·5 to 11·6)	7·5 (5·5 to 9·8)	5·9 (4·3 to 7·8)	4·4 (3·2 to 5·8)	2·8 (2·1 to 3·7)	1·4 (1 to 1·8)	0·6 (0·4 to 0·8)	0·2 (0·2 to 0·4)	0·1 (0 to 0·1)	0 (0 to 0)	0 (0 to 0)	0 (0 to 0)	658
Neonatal encephalopathy due to birth asphyxia and trauma	151 (98·9 to 223)	24·2 (14·1 to 39·3)	9·3 (5·7 to 14·6)	4·4 (2·9 to 6·2)	3·2 (1·9 to 4·9)	3·3 (1·9 to 5·1)	3·3 (1·8 to 5·3)	3·1 (1·7 to 4·9)	2·8 (1·6 to 4·3)	2·5 (1·4 to 4)	2·5 (1·3 to 3·9)	2·3 (1·3 to 3·6)	2 (1·1 to 3·2)	1·8 (1 to 2·8)	1·5 (0·9 to 2·4)	1·1 (0·7 to 1·7)	0·5 (0·3 to 0·7)	0·2 (0·1 to 0·2)	0 (0 to 0·1)	0 (0 to 0)	0 (0 to 0)	0 (0 to 0)	0 (0 to 0)	219
Neonatal sepsis and other neonatal infections	126 (77·1 to 196)	101 (62·6 to 149)	24·2 (14·7 to 37·5)	8·7 (5·7 to 12·7)	11·3 (7·1 to 16·8)	11·8 (7·3 to 18·1)	11·7 (7·2 to 17·9)	11·1 (7·1 to 16·5)	10 (6·2 to 15·1)	9·2 (5·7 to 14)	9 (5·6 to 13·3)	8·5 (5·3 to 12·6)	7·5 (4·7 to 11·3)	6·5 (4·1 to 9·6)	5·3 (3·2 to 8)	3·8 (2·2 to 5·7)	2·1 (1·2 to 3·2)	1·1 (0·6 to 1·7)	0·4 (0·2 to 0·7)	0·1 (0·1 to 0·2)	0 (0 to 0)	0 (0 to 0)	0 (0 to 0)	370
Hemolytic disease and other neonatal jaundice	4·7 (2·8 to 7·5)	2·1 (1·2 to 3·4)	0·8 (0·5 to 1·6)	1·4 (0·8 to 2)	1·7 (1 to 2·5)	1·8 (1 to 2·6)	1·8 (1·2 to 2·6)	1·7 (1·2 to 2·4)	1·5 (1·1 to 2)	1·4 (1·1 to 1·8)	1·4 (1 to 1·8)	1·3 (1 to 1·7)	1·2 (0·9 to 1·6)	1·1 (0·8 to 1·4)	0·9 (0·7 to 1·1)	0·6 (0·5 to 0·8)	0·4 (0·3 to 0·5)	0·2 (0·1 to 0·2)	0·1 (0·1 to 0·1)	0 (0 to 0)	0 (0 to 0)	0 (0 to 0)	0 (0 to 0)	25·9
Other neonatal disorders	98·8 (60·2 to 151)	35·2 (22·4 to 50·1)	11·9 (7·8 to 18)	9·8 (7·5 to 12·7)	0 (0 to 0)	0 (0 to 0)	0 (0 to 0)	0 (0 to 0)	0 (0 to 0)	0 (0 to 0)	0 (0 to 0)	0 (0 to 0)	0 (0 to 0)	0 (0 to 0)	0 (0 to 0)	0 (0 to 0)	0 (0 to 0)	0 (0 to 0)	0 (0 to 0)	0 (0 to 0)	0 (0 to 0)	0 (0 to 0)	0 (0 to 0)	156
Nutritional deficiencies	0·1 (0 to 0·1)	1·4 (0·8 to 2·4)	47·7 (31·1 to 69)	63·3 (36·9 to 99)	61·9 (29·4 to 112)	36·5 (18·8 to 63)	29·8 (15·6 to 52·3)	26·7 (14·6 to 47·3)	26·4 (14·9 to 45·5)	28 (15·6 to 46)	35·7 (20 to 59·3)	40·9 (23·4 to 64·3)	40·1 (23·4 to 64·9)	43·3 (26·4 to 66·7)	41·5 (25·1 to 64·9)	32·4 (21·1 to 48·3)	21·5 (14·9 to 30·2)	20·6 (14·9 to 28·6)	18·2 (13·6 to 24·6)	16·7 (12·6 to 22·1)	12 (8·7 to 16·1)	5·1 (3·7 to 6·8)	1·4 (1 to 1·9)	651
Protein-energy malnutrition	0 (0 to 0·1)	0·1 (0·1 to 0·2)	36·6 (22 to 56·3)	22·8 (13·3 to 36·3)	5·5 (3·7 to 7·9)	5·1 (3·5 to 7·2)	4·8 (3·2 to 6·9)	4·6 (3·2 to 6·3)	5·1 (3·4 to 7·4)	5·1 (3·9 to 6·6)	6·9 (5 to 9·5)	7·1 (5·2 to 9·4)	9·7 (6·7 to 13·8)	11 (7·9 to 14·6)	11·4 (8 to 16·1)	11·7 (8·2 to 16·6)	11 (7·8 to 15·1)	12·4 (8·9 to 16·3)	12·2 (9·2 to 16)	11·7 (8·6 to 15·7)	8·8 (6 to 11·9)	4 (2·8 to 5·4)	1·2 (0·8 to 1·6)	209
Iodine deficiency	0 (0 to 0)	0 (0 to 0)	0 (0 to 0)	0 (0 to 0)	0·2 (0·1 to 0·3)	0·4 (0·2 to 0·7)	0·5 (0·2 to 1)	0·6 (0·2 to 1·1)	0·6 (0·3 to 1·2)	0·6 (0·3 to 1·1)	0·6 (0·3 to 1·1)	0·6 (0·3 to 1·1)	0·6 (0·2 to 1)	0·5 (0·2 to 0·9)	0·4 (0·2 to 0·8)	0·3 (0·2 to 0·6)	0·2 (0·1 to 0·4)	0·2 (0·1 to 0·2)	0·1 (0·1 to 0·2)	0·1 (0 to 0·1)	0 (0 to 0·1)	0 (0 to 0)	0 (0 to 0)	6·6
Vitamin A deficiency	0 (0 to 0)	0·1 (0 to 0·2)	0·3 (0·1 to 0·5)	1·5 (0·8 to 2·6)	1·9 (0·9 to 3·5)	1·2 (0·6 to 2·2)	0·4 (0·1 to 0·7)	0·3 (0·1 to 0·5)	0·2 (0·1 to 0·4)	0·2 (0·1 to 0·3)	0·2 (0·1 to 0·3)	0·2 (0·1 to 0·3)	0·1 (0·1 to 0·3)	0·1 (0·1 to 0·2)	0·1 (0·1 to 0·2)	0·1 (0 to 0·1)	0·1 (0 to 0·1)	0 (0 to 0·1)	0 (0 to 0·1)	0 (0 to 0)	0 (0 to 0)	0 (0 to 0)	0 (0 to 0)	7·1
Dietary iron deficiency	0 (0 to 0)	1·2 (0·7 to 2·1)	9·9 (4·6 to 17·6)	38·3 (16·3 to 72·3)	54 (21·8 to 104)	29·3 (12·1 to 55·6)	22·9 (9·5 to 44·9)	19·8 (8·3 to 39·6)	19 (7·8 to 37·6)	20·9 (8·9 to 38·8)	26·7 (11·7 to 50·2)	31·1 (14·6 to 54·2)	27·7 (12·2 to 51·8)	29 (13·4 to 51·1)	27·2 (12·6 to 49·3)	17·9 (8·3 to 33·5)	8·1 (3·2 to 15·4)	6 (2·4 to 12)	4·3 (1·6 to 8·9)	3·3 (1·3 to 6·2)	2·2 (0·9 to 4·2)	0·7 (0·3 to 1·3)	0·1 (0 to 0·2)	400
Other nutritional deficiencies	0 (0 to 0)	0 (0 to 0)	0·9 (0·4 to 1·8)	0·6 (0·3 to 1·3)	0·4 (0·2 to 0·7)	0·5 (0·3 to 0·8)	1·1 (0·6 to 1·9)	1·4 (0·8 to 2·1)	1·4 (0·8 to 2·2)	1·2 (0·8 to 1·8)	1·4 (0·8 to 2·1)	2 (1·2 to 3·1)	2 (1·1 to 3·8)	2·7 (1·6 to 4·5)	2·4 (1·4 to 3·8)	2·3 (1·3 to 3·7)	2·1 (1·2 to 3·5)	2 (1·1 to 3·5)	1·5 (0·8 to 2·5)	1·7 (0·8 to 3)	1 (0·6 to 2)	0·4 (0·2 to 0·8)	0·1 (0 to 0·2)	29·2
Non-communicable diseases	126 (82·6 to 185)	70·7 (48·1 to 103)	207 (141 to 298)	246 (184 to 320)	279 (214 to 359)	484 (376 to 622)	703 (549 to 895)	813 (644 to 1,000)	935 (757 to 1,140)	1,050 (853 to 1,260)	1,320 (1,100 to 1,590)	1,710 (1,440 to 2,010)	2,280 (1,960 to 2,660)	2,730 (2,350 to 3,150)	2,990 (2,590 to 3,430)	3,020 (2,650 to 3,440)	2,650 (2,360 to 2,990)	2,500 (2,250 to 2,780)	2,200 (2,000 to 2,430)	1,900 (1,740 to 2,070)	1,100 (1,020 to 1,190)	385 (362 to 411)	88·9 (83·9 to 93·8)	29,800
Neoplasms	1·1 (0·7 to 1·7)	1·1 (0·8 to 1·6)	6·3 (4·1 to 9·6)	27·3 (18·2 to 40·8)	25·4 (20·9 to 30·6)	44·8 (37·5 to 54·1)	52·6 (42·8 to 63·2)	59·1 (49·1 to 70)	83·1 (68·8 to 99·3)	118 (97·7 to 145)	184 (151 to 220)	286 (240 to 344)	433 (360 to 512)	558 (478 to 666)	609 (516 to 713)	606 (524 to 704)	536 (469 to 623)	489 (421 to 561)	424 (369 to 485)	324 (279 to 371)	173 (144 to 199)	48·8 (39·4 to 57)	7·3 (5·7 to 8·5)	5,100
Lip and oral cavity cancer	0 (0 to 0)	0 (0 to 0)	0 (0 to 0)	0 (0 to 0)	0·1 (0·1 to 0·1)	0·2 (0·1 to 0·4)	0·4 (0·3 to 0·5)	0·6 (0·4 to 0·7)	0·9 (0·7 to 1·1)	1·4 (1 to 1·9)	2·8 (2 to 3·7)	6·2 (4·5 to 8·3)	13·7 (10·2 to 18·2)	19·8 (14·9 to 25·7)	22·4 (17·3 to 29·5)	18·9 (14·2 to 24·4)	15 (11·9 to 18·8)	11·8 (9·3 to 14·6)	9·4 (7·4 to 11·7)	6·4 (5·2 to 8)	2·8 (2·2 to 3·3)	0·8 (0·7 to 1)	0·1 (0·1 to 0·2)	134
Nasopharynx cancer	0 (0 to 0)	0 (0 to 0)	0 (0 to 0)	0 (0 to 0)	0·1 (0·1 to 0·2)	0·3 (0·2 to 0·3)	0·5 (0·4 to 0·6)	0·5 (0·4 to 0·7)	0·6 (0·5 to 0·8)	0·6 (0·4 to 0·8)	0·8 (0·5 to 1·1)	1·6 (1·2 to 2)	2·8 (2·1 to 3·7)	3·8 (2·7 to 5)	4 (3·1 to 5·1)	2·9 (2·1 to 3·7)	2·1 (1·6 to 2·8)	1·7 (1·2 to 2·2)	1 (0·7 to 1·3)	0·7 (0·5 to 0·9)	0·6 (0·4 to 0·7)	0·2 (0·1 to 0·2)	0 (0 to 0)	24·7
Other pharynx cancer	0 (0 to 0)	0 (0 to 0)	0 (0 to 0)	0 (0 to 0)	0 (0 to 0)	0 (0 to 0)	0 (0 to 0)	0·2 (0·2 to 0·3)	0·3 (0·2 to 0·4)	0·4 (0·3 to 0·5)	0·9 (0·6 to 1·2)	2·2 (1·5 to 3·2)	6·3 (4·4 to 8·7)	8·8 (6·1 to 11·8)	10·1 (7·4 to 13·5)	8·2 (6 to 11·3)	6 (4·5 to 8)	4·3 (3·2 to 5·7)	2·9 (2·2 to 4)	1·9 (1·4 to 2·6)	1 (0·7 to 1·2)	0·3 (0·2 to 0·4)	0 (0 to 0·1)	53·8
Esophageal cancer	0 (0 to 0)	0 (0 to 0)	0 (0 to 0)	0 (0 to 0)	0 (0 to 0)	0 (0 to 0)	0 (0 to 0)	0·1 (0·1 to 0·2)	0·2 (0·1 to 0·3)	0·4 (0·3 to 0·5)	1·2 (0·9 to 1·6)	3·4 (2·5 to 4·6)	8·8 (6·4 to 11·8)	13 (9·6 to 16·8)	15·1 (10·9 to 19·8)	12·6 (9·3 to 17·1)	10 (7·4 to 13)	7·8 (5·7 to 10·4)	5·7 (4·2 to 7·7)	3·6 (2·8 to 4·8)	1·8 (1·4 to 2·3)	0·4 (0·4 to 0·6)	0·1 (0 to 0·1)	84·2
Stomach cancer	0 (0 to 0)	0 (0 to 0)	0 (0 to 0)	0 (0 to 0)	0 (0 to 0)	0 (0 to 0)	0·7 (0·6 to 0·9)	1·3 (1·1 to 1·6)	3·1 (2·2 to 4·5)	5·8 (4·3 to 7·9)	9·6 (6·9 to 13·1)	17·2 (13·1 to 22·6)	28·4 (22·2 to 35·6)	40·4 (30·9 to 51·8)	45·1 (34·3 to 57·4)	45·8 (35 to 58·2)	41·6 (31·2 to 54·6)	36·4 (27·7 to 47)	30·9 (24·6 to 37·9)	22·3 (17·1 to 29)	11·6 (9·1 to 14·6)	3·2 (2·7 to 3·7)	0·5 (0·4 to 0·6)	344
Colon and rectum cancer	0 (0 to 0)	0 (0 to 0)	0 (0 to 0)	0 (0 to 0)	0·2 (0·1 to 0·2)	0·4 (0·3 to 0·5)	1·2 (1 to 1·6)	2·1 (1·6 to 2·6)	3·8 (2·7 to 5·1)	7 (5·3 to 9·1)	11·2 (8·4 to 14·3)	18·7 (15 to 23·5)	31·9 (24·9 to 40·3)	45 (35·3 to 56·6)	53·5 (43·1 to 67·8)	56·6 (45·2 to 70·8)	51·1 (41 to 63·3)	46·2 (37 to 57·5)	40·3 (32·5 to 49·9)	31·2 (25·1 to 38·5)	16 (13 to 19·3)	4·8 (4 to 5·7)	0·8 (0·6 to 1)	422
Liver cancer	0 (0 to 0)	0 (0 to 0)	0·1 (0·1 to 0·2)	0·3 (0·2 to 0·5)	0·3 (0·2 to 0·3)	0·6 (0·5 to 0·7)	0·4 (0·3 to 0·5)	0·7 (0·5 to 0·9)	1·2 (0·9 to 1·6)	1·4 (1 to 1·9)	2·8 (2 to 3·8)	5 (3·8 to 6·4)	8·8 (6·7 to 11·5)	12·7 (9·3 to 16·9)	15·9 (12·2 to 20·4)	14·4 (11·1 to 18·7)	12·5 (9·8 to 16)	10·8 (8·4 to 13·8)	8·5 (6·7 to 10·8)	5·6 (4·3 to 7)	1·8 (1·4 to 2·4)	0·4 (0·3 to 0·6)	0·1 (0 to 0·1)	105
Liver cancer due to hepatitis B	0 (0 to 0)	0 (0 to 0)	0 (0 to 0)	0 (0 to 0)	0 (0 to 0)	0·1 (0·1 to 0·2)	0·2 (0·1 to 0·3)	0·4 (0·2 to 0·5)	0·7 (0·4 to 1·1)	0·8 (0·5 to 1·2)	1·5 (0·9 to 2·3)	2·5 (1·5 to 3·6)	3·9 (2·3 to 6)	4·9 (2·7 to 8·1)	5·5 (3·2 to 8·6)	4·2 (2·3 to 6·9)	2·9 (1·5 to 4·8)	2·1 (1 to 3·6)	1·5 (0·8 to 2·4)	0·8 (0·4 to 1·4)	0·3 (0·1 to 0·4)	0·1 (0 to 0·1)	0 (0 to 0)	32·5
Liver cancer due to hepatitis C	0 (0 to 0)	0 (0 to 0)	0 (0 to 0)	0 (0 to 0)	0 (0 to 0)	0 (0 to 0)	0 (0 to 0)	0 (0 to 0)	0 (0 to 0·1)	0·1 (0 to 0·1)	0·2 (0·1 to 0·3)	0·4 (0·2 to 0·7)	0·9 (0·4 to 1·6)	1·7 (0·8 to 2·9)	2·5 (1·1 to 4·4)	2·6 (1·3 to 4·5)	2·4 (1·3 to 3·8)	2·3 (1·3 to 3·8)	2·2 (1·2 to 3·6)	1·8 (1·1 to 2·7)	0·7 (0·5 to 1)	0·2 (0·1 to 0·3)	0 (0 to 0)	17·9
Liver cancer due to alcohol use	0 (0 to 0)	0 (0 to 0)	0 (0 to 0)	0 (0 to 0)	0 (0 to 0)	0 (0 to 0)	0 (0 to 0)	0·1 (0 to 0·1)	0·2 (0·1 to 0·3)	0·3 (0·1 to 0·5)	0·7 (0·3 to 1·3)	1·4 (0·8 to 2·4)	2·9 (1·5 to 4·8)	4·6 (2·4 to 7·5)	6 (3·5 to 9·4)	5·9 (3·5 to 9)	5·7 (3·5 to 8·3)	4·9 (3 to 7·2)	3·5 (2·3 to 5)	1·9 (1·2 to 2·9)	0·6 (0·3 to 0·9)	0·1 (0·1 to 0·2)	0 (0 to 0)	38·8
Liver cancer due to NASH	0 (0 to 0)	0 (0 to 0)	0 (0 to 0)	0 (0 to 0)	0 (0 to 0)	0 (0 to 0)	0·1 (0 to 0·1)	0·1 (0·1 to 0·2)	0·1 (0·1 to 0·2)	0·1 (0·1 to 0·2)	0·2 (0·1 to 0·3)	0·3 (0·2 to 0·6)	0·6 (0·3 to 1)	0·9 (0·4 to 1·5)	1·1 (0·6 to 1·8)	1·1 (0·6 to 1·8)	1 (0·6 to 1·7)	1 (0·6 to 1·6)	1 (0·5 to 1·6)	0·7 (0·4 to 1·2)	0·2 (0·1 to 0·4)	0·1 (0 to 0·1)	0 (0 to 0)	8·4
Liver cancer due to other causes	0 (0 to 0)	0 (0 to 0)	0·1 (0·1 to 0·2)	0·3 (0·2 to 0·5)	0·3 (0·2 to 0·3)	0·4 (0·4 to 0·6)	0·1 (0·1 to 0·2)	0·2 (0·1 to 0·2)	0·2 (0·1 to 0·3)	0·2 (0·1 to 0·3)	0·2 (0·1 to 0·4)	0·4 (0·2 to 0·7)	0·6 (0·3 to 1)	0·7 (0·4 to 1·4)	0·8 (0·4 to 1·4)	0·7 (0·4 to 1·2)	0·5 (0·3 to 0·9)	0·5 (0·2 to 0·8)	0·4 (0·2 to 0·6)	0·3 (0·1 to 0·5)	0·1 (0 to 0·1)	0 (0 to 0)	0 (0 to 0)	7
Gallbladder and biliary tract cancer	0 (0 to 0)	0 (0 to 0)	0 (0 to 0)	0 (0 to 0)	0 (0 to 0)	0 (0 to 0)	0 (0 to 0)	0·1 (0·1 to 0·1)	0·2 (0·1 to 0·3)	0·3 (0·2 to 0·4)	0·7 (0·5 to 1)	1·4 (1·1 to 2·2)	2·6 (2 to 3·9)	4·1 (3·1 to 6·1)	5·1 (3·9 to 7·7)	5·3 (4 to 8·2)	4·7 (3·5 to 7·2)	4·2 (3·2 to 6·4)	3·3 (2·6 to 5)	2·4 (1·8 to 3·5)	1·1 (0·8 to 1·6)	0·3 (0·2 to 0·4)	0 (0 to 0·1)	35·8
Pancreatic cancer	0 (0 to 0)	0 (0 to 0)	0 (0 to 0)	0 (0 to 0)	0 (0 to 0)	0 (0 to 0)	0·3 (0·2 to 0·4)	0·4 (0·3 to 0·5)	0·8 (0·6 to 1·1)	1·9 (1·4 to 2·7)	3·9 (2·7 to 5·5)	8·7 (6·5 to 11·6)	16·8 (13·2 to 21·2)	23·4 (19·1 to 28·9)	28 (21·4 to 35·7)	28·9 (21·8 to 37·7)	25·4 (19·9 to 31·6)	22·8 (17 to 29·9)	17 (12·5 to 22)	12·3 (9·7 to 15·5)	5·8 (4·5 to 7·4)	1·5 (1·3 to 1·8)	0·2 (0·2 to 0·3)	198
Larynx cancer	0 (0 to 0)	0 (0 to 0)	0 (0 to 0)	0 (0 to 0)	0 (0 to 0)	0 (0 to 0)	0 (0 to 0)	0·2 (0·2 to 0·3)	0·2 (0·2 to 0·3)	0·4 (0·3 to 0·4)	1·1 (0·8 to 1·4)	3·1 (2·3 to 4·2)	9·1 (6·2 to 12·8)	15·2 (11·5 to 20·1)	18·1 (13·5 to 23·3)	15·9 (12·6 to 20·2)	13 (10·2 to 16·4)	9·7 (7·6 to 12·2)	7 (5·4 to 9·1)	4·1 (3·2 to 5·2)	1·8 (1·3 to 2·3)	0·4 (0·3 to 0·5)	0 (0 to 0)	99·4
Tracheal, bronchus, and lung cancer	0 (0 to 0)	0 (0 to 0)	0 (0 to 0)	0 (0 to 0)	0 (0 to 0)	0·1 (0·1 to 0·2)	0·7 (0·5 to 0·8)	0·8 (0·6 to 1)	1·2 (0·9 to 1·6)	2·7 (2 to 3·6)	6 (4·3 to 7·9)	13·2 (10·1 to 16·6)	29·2 (22·2 to 37·5)	44·3 (34·3 to 56)	53·3 (40·9 to 69·7)	54 (42·3 to 67·2)	44·9 (35·4 to 56)	37·3 (29·8 to 46·7)	26·7 (21·3 to 33·2)	16·7 (13 to 20·9)	7·7 (6 to 9·7)	1·8 (1·5 to 2·2)	0·3 (0·2 to 0·3)	341
Malignant skin melanoma	0 (0 to 0)	0 (0 to 0)	0·1 (0 to 0·1)	0·2 (0·1 to 0·5)	0·1 (0·1 to 0·1)	0·1 (0·1 to 0·2)	0·2 (0·2 to 0·3)	0·6 (0·4 to 0·9)	0·7 (0·5 to 1·1)	0·9 (0·6 to 1·3)	2 (1·3 to 2·9)	2 (1·4 to 2·8)	2·6 (1·8 to 3·6)	2·9 (2·1 to 4)	2·7 (2 to 3·8)	2·4 (1·7 to 3·4)	2 (1·5 to 2·7)	1·8 (1·3 to 2·5)	1·2 (0·9 to 1·7)	1·1 (0·8 to 1·6)	0·7 (0·5 to 1)	0·2 (0·2 to 0·3)	0·1 (0 to 0·1)	24·6
Non-melanoma skin cancer	0 (0 to 0)	0 (0 to 0)	0 (0 to 0)	0 (0 to 0)	0 (0 to 0)	0 (0 to 0)	0 (0 to 0)	0·2 (0·1 to 0·3)	0·5 (0·3 to 0·6)	0·6 (0·3 to 0·8)	0·8 (0·5 to 1·1)	1·5 (0·9 to 2)	1·9 (1·3 to 2·6)	2·9 (2 to 3·7)	3·1 (2·1 to 4·1)	2·9 (2·1 to 3·7)	2·8 (2 to 3·6)	2·6 (1·9 to 3·3)	2·7 (1·9 to 3·4)	2·7 (1·8 to 3·3)	1·9 (1·1 to 2·3)	0·9 (0·5 to 1·1)	0·2 (0·1 to 0·3)	28·1
Non-melanoma skin cancer (squamous-cell carcinoma)	0 (0 to 0)	0 (0 to 0)	0 (0 to 0)	0 (0 to 0)	0 (0 to 0)	0 (0 to 0)	0 (0 to 0)	0·2 (0·1 to 0·3)	0·5 (0·3 to 0·6)	0·6 (0·3 to 0·8)	0·8 (0·5 to 1·1)	1·5 (0·9 to 2)	1·9 (1·3 to 2·6)	2·9 (2 to 3·7)	3·1 (2·1 to 4·1)	2·9 (2·1 to 3·7)	2·8 (2 to 3·6)	2·6 (1·9 to 3·3)	2·7 (1·9 to 3·4)	2·7 (1·8 to 3·3)	1·9 (1·1 to 2·3)	0·9 (0·5 to 1·1)	0·2 (0·1 to 0·3)	28·1
Non-melanoma skin cancer (basal-cell carcinoma)	0 (0 to 0)	0 (0 to 0)	0 (0 to 0)	0 (0 to 0)	0 (0 to 0)	0 (0 to 0)	0 (0 to 0)	0 (0 to 0)	0 (0 to 0)	0 (0 to 0)	0 (0 to 0)	0 (0 to 0)	0 (0 to 0)	0 (0 to 0)	0 (0 to 0)	0 (0 to 0)	0 (0 to 0)	0 (0 to 0)	0 (0 to 0)	0 (0 to 0)	0 (0 to 0)	0 (0 to 0)	0 (0 to 0)	0
Breast cancer	0 (0 to 0)	0 (0 to 0)	0 (0 to 0)	0 (0 to 0)	0 (0 to 0)	0 (0 to 0)	0·7 (0·6 to 0·8)	1·6 (1·1 to 2·1)	6·5 (4·5 to 9·1)	20·5 (14·9 to 27·4)	36·3 (27·2 to 46·9)	61·2 (44·3 to 80·7)	77·7 (57·7 to 102)	93·9 (70·3 to 123)	77·2 (56·2 to 101)	67 (50·7 to 86·8)	48 (37 to 61·2)	36·8 (27·8 to 48)	26·9 (20·3 to 34·5)	22·3 (16·9 to 28·7)	11·9 (9·1 to 15)	3·7 (2·9 to 4·6)	0·9 (0·7 to 1·1)	593
Cervical cancer	0 (0 to 0)	0 (0 to 0)	0 (0 to 0)	0 (0 to 0)	0 (0 to 0)	0 (0 to 0)	0·9 (0·7 to 1·2)	4 (2·7 to 5·8)	11·5 (7·3 to 16·9)	22·7 (15·4 to 31·7)	38·9 (28·1 to 52·5)	51·9 (38·3 to 66·5)	60·4 (44·1 to 80·1)	56·2 (42·5 to 72)	49·4 (37·6 to 64·3)	39·8 (28·9 to 52·4)	28·1 (21·4 to 36·8)	22·7 (17·3 to 28·8)	15·6 (12·1 to 19·6)	11·6 (8·7 to 15·3)	4·7 (3·5 to 6·2)	1·4 (1·1 to 2)	0·3 (0·2 to 0·5)	420
Uterine cancer	0 (0 to 0)	0 (0 to 0)	0 (0 to 0)	0 (0 to 0)	0 (0 to 0)	0 (0 to 0)	0 (0 to 0)	0·7 (0·5 to 0·9)	1·6 (1·1 to 2·2)	3 (2·1 to 4·2)	4·9 (3·2 to 7·1)	8·2 (5·8 to 11·2)	12·9 (9·3 to 17·9)	15·6 (11 to 21·5)	16·4 (11·8 to 22)	20·9 (15·8 to 26·9)	16·8 (12·9 to 22·1)	14·1 (10·7 to 18·8)	9·2 (6·9 to 12·1)	7 (5 to 9·1)	2·7 (2·1 to 3·4)	0·9 (0·6 to 1·1)	0·2 (0·1 to 0·2)	135
Ovarian cancer	0 (0 to 0)	0 (0 to 0)	0 (0 to 0)	0 (0 to 0)	0·3 (0·1 to 0·4)	0·5 (0·3 to 0·9)	1·3 (0·8 to 1·9)	2·1 (1·3 to 3·1)	2·1 (1·3 to 3·1)	3·2 (1·9 to 4·7)	5·4 (3·5 to 7·7)	9·9 (6·8 to 13·4)	14 (9·2 to 19)	17 (11·6 to 23·6)	15·6 (11·1 to 20·4)	14 (9·5 to 18·7)	10·7 (7·3 to 14·7)	8·3 (5·9 to 11·1)	5·5 (3·7 to 7·3)	3·9 (2·7 to 5·2)	1·8 (1·2 to 2·3)	0·5 (0·3 to 0·7)	0·1 (0 to 0·1)	116
Prostate cancer	0 (0 to 0)	0 (0 to 0)	0 (0 to 0)	0 (0 to 0)	0 (0 to 0)	0 (0 to 0)	0 (0 to 0)	0·2 (0·2 to 0·3)	0·6 (0·4 to 0·8)	0·7 (0·4 to 1)	1 (0·6 to 1·4)	2·7 (1·7 to 3·8)	10·6 (6·7 to 15)	28 (19·1 to 37·8)	55·9 (38·5 to 74·3)	89·8 (66·4 to 124)	111 (81·3 to 152)	132 (96·6 to 179)	150 (111 to 195)	125 (92·1 to 163)	77·1 (52·9 to 96·1)	21·3 (13·8 to 27·5)	2·3 (1·4 to 3·2)	808
Testicular cancer	0 (0 to 0·4)	0 (0 to 0·1)	0 (0 to 0·2)	0·1 (0 to 0·2)	0·1 (0·1 to 0·2)	0·1 (0·1 to 0·2)	0·4 (0·3 to 0·7)	0·6 (0·4 to 0·8)	0·9 (0·5 to 1·3)	0·6 (0·4 to 0·9)	0·6 (0·4 to 0·8)	0·5 (0·3 to 0·7)	0·3 (0·2 to 0·5)	0·3 (0·2 to 0·4)	0·2 (0·1 to 0·3)	0·2 (0·1 to 0·2)	0·1 (0·1 to 0·2)	0·1 (0·1 to 0·2)	0·1 (0·1 to 0·2)	0·1 (0 to 0·1)	0 (0 to 0·1)	0 (0 to 0)	0 (0 to 0)	5·3
Kidney cancer	0 (0 to 0·1)	0 (0 to 0)	0·3 (0·2 to 0·5)	1·4 (0·8 to 2·1)	1 (0·7 to 1·3)	0·9 (0·6 to 1·2)	0·9 (0·7 to 1·1)	1·2 (0·9 to 1·5)	1·4 (1 to 1·9)	1·6 (1·1 to 2·2)	2·1 (1·5 to 3)	3·9 (2·7 to 5·2)	6·2 (4·7 to 7·9)	8·1 (6·2 to 10·6)	9 (7 to 11·2)	8·3 (6·2 to 10·7)	6·6 (5·2 to 8·3)	5·3 (4·2 to 6·5)	3·6 (2·8 to 4·6)	2·4 (1·9 to 3)	0·9 (0·7 to 1·1)	0·3 (0·2 to 0·4)	0 (0 to 0·1)	65·4
Bladder cancer	0 (0 to 0)	0 (0 to 0)	0 (0 to 0)	0 (0 to 0)	0 (0 to 0)	0 (0 to 0)	0·1 (0·1 to 0·1)	0·1 (0·1 to 0·1)	0·2 (0·1 to 0·2)	0·4 (0·3 to 0·6)	0·7 (0·5 to 0·9)	1·5 (1·1 to 2)	3 (2·1 to 4·2)	5·5 (4·2 to 7·1)	7·8 (5·9 to 9·9)	9·3 (7·2 to 11·7)	9·6 (7·6 to 12·2)	10 (7·7 to 12·6)	9·4 (7·2 to 11·7)	7·3 (5·5 to 9·6)	4·1 (3·1 to 5·4)	1·2 (1 to 1·5)	0·2 (0·1 to 0·2)	70·5
Brain and central nervous system cancer	0·2 (0·1 to 0·3)	0·2 (0·1 to 0·3)	1 (0·4 to 1·6)	3·5 (1·6 to 5·8)	3·9 (2·2 to 5·5)	5·4 (2·9 to 8)	3·3 (1·7 to 4·9)	3 (1·5 to 4·4)	3·6 (1·7 to 5·2)	3·8 (1·8 to 5·5)	5·2 (2·5 to 7·5)	6·6 (3·2 to 9·2)	8·3 (4 to 11·7)	9·5 (4·8 to 12·9)	9·6 (5 to 13·1)	7·1 (3·8 to 9·5)	5·4 (2·9 to 7·3)	3·7 (2 to 5)	2·4 (1·2 to 3·3)	1·3 (0·7 to 1·8)	0·5 (0·3 to 0·8)	0·1 (0·1 to 0·2)	0 (0 to 0)	87·5
Thyroid cancer	0 (0 to 0)	0 (0 to 0)	0 (0 to 0)	0 (0 to 0)	0·1 (0·1 to 0·1)	0·2 (0·1 to 0·2)	0·3 (0·2 to 0·4)	0·4 (0·3 to 0·5)	0·6 (0·5 to 0·8)	0·7 (0·5 to 0·9)	1·3 (0·9 to 1·7)	1·9 (1·4 to 2·5)	3·1 (2·3 to 4·1)	4·4 (3·2 to 5·9)	4·5 (3·4 to 5·9)	4·1 (3·2 to 5·1)	3·5 (2·7 to 4·5)	3·2 (2·5 to 4)	2·3 (1·8 to 2·9)	1·8 (1·4 to 2·3)	0·7 (0·6 to 0·9)	0·2 (0·2 to 0·3)	0 (0 to 0)	33·4
Mesothelioma	0 (0 to 0)	0 (0 to 0)	0 (0 to 0)	0 (0 to 0)	0 (0 to 0)	0 (0 to 0)	0 (0 to 0)	0·2 (0·1 to 0·3)	0·2 (0·1 to 0·3)	0·2 (0·1 to 0·3)	0·4 (0·3 to 0·5)	0·5 (0·4 to 0·7)	0·9 (0·7 to 1·2)	1·3 (1 to 1·7)	1·4 (1·1 to 1·8)	0·6 (0·4 to 0·7)	0·4 (0·3 to 0·5)	0·4 (0·4 to 0·6)	0·4 (0·3 to 0·5)	0·3 (0·2 to 0·4)	0·1 (0·1 to 0·2)	0 (0 to 0)	0 (0 to 0)	7·5
Hodgkin lymphoma	0 (0 to 0)	0 (0 to 0)	0 (0 to 0)	0·1 (0 to 0·1)	0·1 (0·1 to 0·2)	0·4 (0·3 to 0·5)	1·7 (1·2 to 2·4)	2·5 (1·8 to 3·4)	2·6 (1·8 to 3·6)	2 (1·4 to 2·9)	2·1 (1·5 to 2·8)	2·2 (1·6 to 3)	2·1 (1·5 to 2·9)	2·1 (1·6 to 3)	1·9 (1·4 to 2·6)	1·6 (1·2 to 2·1)	1·3 (1 to 1·8)	0·9 (0·7 to 1·2)	0·7 (0·6 to 1)	0·4 (0·3 to 0·6)	0·2 (0·1 to 0·3)	0 (0 to 0)	0 (0 to 0)	25
Non-Hodgkin lymphoma	0 (0 to 0)	0 (0 to 0)	0 (0 to 0)	3·9 (2·4 to 6·1)	4·1 (2·8 to 5·9)	8 (5·6 to 11·3)	10·4 (7·3 to 14·2)	12·1 (8·6 to 16·1)	14·6 (10·3 to 19·8)	14·8 (10·9 to 19·7)	16·8 (12 to 22·3)	19·3 (14·6 to 24·8)	27·4 (21·3 to 35·7)	30·4 (23·2 to 39·1)	31·3 (23·8 to 39·8)	27·4 (20·4 to 35·9)	22·3 (17·4 to 28·3)	18·5 (14·6 to 22·7)	13·2 (10 to 17·3)	8·3 (6·2 to 10·8)	3·1 (2·4 to 4)	0·8 (0·6 to 1)	0·1 (0·1 to 0·1)	287
Multiple myeloma	0 (0 to 0)	0 (0 to 0)	0 (0 to 0)	0 (0 to 0)	0 (0 to 0)	0 (0 to 0)	0 (0 to 0)	0·1 (0·1 to 0·2)	0·2 (0·1 to 0·2)	0·3 (0·2 to 0·4)	0·8 (0·5 to 1·1)	2 (1·5 to 2·6)	4·2 (3 to 5·5)	6·9 (5·2 to 9)	8·6 (6·6 to 11·2)	8·7 (6·8 to 11·6)	7·8 (6·1 to 10)	6·7 (5·3 to 8·3)	5 (3·9 to 6·3)	3·2 (2·5 to 4)	1·3 (1 to 1·6)	0·3 (0·2 to 0·3)	0 (0 to 0·1)	56·1
Leukemia	0·3 (0·2 to 0·5)	0·4 (0·2 to 0·5)	2·1 (1·3 to 3·2)	7·9 (4·9 to 12·2)	8·6 (6·5 to 11·4)	15·4 (11·3 to 20·3)	13·9 (10·5 to 18)	11·3 (8·6 to 14·4)	11·4 (8·7 to 14·5)	9·2 (7 to 11·9)	10·9 (8·3 to 14·3)	12·4 (9·8 to 15·9)	15·4 (11·8 to 19·4)	15·4 (12·3 to 19·3)	15·2 (12·3 to 19·3)	13·7 (11·1 to 17)	11·8 (9·5 to 14·4)	10·6 (8·5 to 12·8)	8·3 (6·7 to 10·1)	6·3 (5 to 7·7)	3·1 (2·5 to 3·8)	0·8 (0·7 to 1)	0·1 (0·1 to 0·2)	204
Acute lymphoid leukemia	0·1 (0 to 0·1)	0·1 (0 to 0·2)	0·6 (0·3 to 1)	3·5 (2·1 to 5·7)	4·3 (2·8 to 6)	6·2 (4 to 9·3)	4·9 (3·4 to 6·8)	2·3 (1·5 to 3·4)	1·5 (0·9 to 2·2)	1 (0·6 to 1·5)	0·9 (0·5 to 1·4)	1 (0·6 to 1·5)	0·9 (0·6 to 1·4)	1 (0·6 to 1·4)	0·8 (0·5 to 1·2)	0·6 (0·4 to 0·9)	0·4 (0·3 to 0·7)	0·3 (0·2 to 0·5)	0·3 (0·2 to 0·4)	0·2 (0·1 to 0·3)	0·1 (0 to 0·1)	0 (0 to 0)	0 (0 to 0)	30·8
Chronic lymphoid leukemia	0 (0 to 0)	0 (0 to 0)	0 (0 to 0)	0 (0 to 0)	0 (0 to 0)	0 (0 to 0)	0 (0 to 0)	0·3 (0·2 to 0·5)	0·3 (0·2 to 0·5)	0·2 (0·1 to 0·4)	0·4 (0·3 to 0·7)	0·7 (0·4 to 1·1)	0·7 (0·4 to 1)	1·8 (1·3 to 2·6)	2·7 (1·8 to 3·9)	2·9 (2 to 4·1)	2·9 (2 to 4)	2·4 (1·6 to 3·4)	2 (1·3 to 2·8)	1·5 (1 to 2)	0·7 (0·5 to 1)	0·2 (0·1 to 0·3)	0 (0 to 0)	19·9
Acute myeloid leukemia	0·1 (0 to 0·1)	0·1 (0·1 to 0·2)	0·5 (0·2 to 0·8)	2·1 (1·2 to 3·5)	1·8 (1·1 to 2·6)	3·8 (2·3 to 5·9)	4·1 (2·8 to 6)	3 (2·1 to 4·2)	3·5 (2·4 to 4·8)	2·4 (1·6 to 3·3)	3 (2·1 to 4·1)	2·6 (1·9 to 3·7)	3·7 (2·6 to 5·1)	3·3 (2·4 to 4·4)	2·9 (2·1 to 3·8)	2·3 (1·7 to 3·1)	1·7 (1·2 to 2·3)	1·3 (1 to 1·8)	0·9 (0·6 to 1·2)	0·6 (0·4 to 0·8)	0·2 (0·1 to 0·3)	0 (0 to 0·1)	0 (0 to 0)	43·9

Chronic myeloid leukemia	0 (0 to 0)	0 (0 to 0)	0·1 (0 to 0·1)	0·2 (0·1 to 0·4)	0·1 (0·1 to 0·2)	0·2 (0·2 to 0·4)	0·4 (0·2 to 0·6)	0·9 (0·5 to 1·4)	1·2 (0·7 to 1·9)	1·2 (0·7 to 1·8)	1·4 (0·9 to 2·1)	1·3 (0·8 to 2)	1·6 (1·1 to 2·4)	1·8 (1·2 to 2·6)	1·7 (1·2 to 2·5)	1·6 (1·1 to 2·4)	1·2 (0·8 to 1·7)	1 (0·7 to 1·5)	0·8 (0·5 to 1·2)	0·6 (0·4 to 0·9)	0·2 (0·2 to 0·4)	0·1 (0 to 0·1)	0 (0 to 0)	17·8
Other leukemia	0·2 (0·1 to 0·3)	0·1 (0·1 to 0·2)	1 (0·6 to 1·6)	2 (1·1 to 3·2)	2·4 (1·6 to 3·6)	5·2 (3·4 to 7·4)	4·5 (3·1 to 6·2)	4·8 (3·4 to 6·3)	4·9 (3·5 to 6·5)	4·4 (3·2 to 5·9)	5·2 (3·8 to 7)	6·8 (5·2 to 8·8)	8·5 (6·4 to 11)	7·4 (5·7 to 9·6)	7·2 (5·5 to 9·2)	6·3 (4·8 to 8·1)	5·5 (4·2 to 7)	5·5 (4·2 to 7)	4·4 (3·4 to 5·6)	3·4 (2·6 to 4·4)	1·9 (1·4 to 2·3)	0·5 (0·4 to 0·6)	0·1 (0·1 to 0·1)	92·2
Other malignant neoplasms	0·5 (0·3 to 0·7)	0·4 (0·3 to 0·7)	2·4 (1·6 to 3·7)	9·1 (5·9 to 13·8)	6 (4·7 to 7·6)	11·4 (9·2 to 14·1)	13·3 (10·5 to 16·4)	10·9 (8·8 to 13·5)	10·5 (8·7 to 13)	9·8 (7·8 to 12·3)	11·6 (9·3 to 14·4)	15·5 (12·4 to 19·6)	21·4 (16·9 to 26·5)	25 (19·8 to 31·4)	25·1 (20·2 to 31·6)	21·2 (17·3 to 26·1)	17·5 (14·4 to 21·4)	14·4 (11·8 to 17·3)	11·4 (9·6 to 13·7)	8·8 (7·2 to 10·4)	4·8 (4 to 5·8)	1·4 (1·1 to 1·7)	0·2 (0·2 to 0·3)	253
Other neoplasms	0·1 (0 to 0·1)	0·1 (0 to 0·2)	0·3 (0·2 to 0·6)	0·9 (0·5 to 1·5)	0·5 (0·3 to 0·6)	0·7 (0·5 to 1)	0·8 (0·5 to 1·1)	0·5 (0·4 to 0·7)	0·9 (0·7 to 1·3)	0·8 (0·5 to 1·1)	0·9 (0·6 to 1·3)	1·5 (1·1 to 2·1)	1·8 (1·2 to 2·7)	2·8 (1·9 to 4·1)	3·3 (2·3 to 5·3)	3·5 (2·6 to 5·8)	3·5 (2·5 to 5·3)	3·9 (2·8 to 6·3)	3·5 (2·6 to 5·4)	2·7 (2 to 3·8)	1·3 (1 to 2)	0·4 (0·2 to 0·5)	0 (0 to 0·1)	34·7
Myelodysplastic, myeloproliferative, and other hematopoietic neo	0·1 (0 to 0·1)	0·1 (0 to 0·2)	0·3 (0·2 to 0·6)	0·9 (0·5 to 1·5)	0·5 (0·3 to 0·6)	0·7 (0·5 to 1)	0·8 (0·5 to 1·1)	0·5 (0·4 to 0·7)	0·9 (0·7 to 1·3)	0·8 (0·5 to 1·1)	0·9 (0·6 to 1·3)	1·5 (1·1 to 2·1)	1·8 (1·2 to 2·7)	2·8 (1·9 to 4·1)	3·3 (2·3 to 5·3)	3·5 (2·6 to 5·8)	3·5 (2·5 to 5·3)	3·9 (2·8 to 6·3)	3·5 (2·6 to 5·4)	2·7 (2 to 3·8)	1·3 (1 to 2)	0·4 (0·2 to 0·5)	0 (0 to 0·1)	34·7
Benign and in situ intestinal neoplasms	0 (0 to 0)	0 (0 to 0)	0 (0 to 0)	0 (0 to 0)	0 (0 to 0)	0 (0 to 0)	0 (0 to 0)	0 (0 to 0)	0 (0 to 0)	0 (0 to 0)	0 (0 to 0)	0 (0 to 0)	0 (0 to 0)	0 (0 to 0)	0 (0 to 0)	0 (0 to 0)	0 (0 to 0)	0 (0 to 0)	0 (0 to 0)	0 (0 to 0)	0 (0 to 0)	0 (0 to 0)	0 (0 to 0)	0
Benign and in situ cervical and uterine neoplasms	0 (0 to 0)	0 (0 to 0)	0 (0 to 0)	0 (0 to 0)	0 (0 to 0)	0 (0 to 0)	0 (0 to 0)	0 (0 to 0)	0 (0 to 0)	0 (0 to 0)	0 (0 to 0)	0 (0 to 0)	0 (0 to 0)	0 (0 to 0)	0 (0 to 0)	0 (0 to 0)	0 (0 to 0)	0 (0 to 0)	0 (0 to 0)	0 (0 to 0)	0 (0 to 0)	0 (0 to 0)	0 (0 to 0)	0
Other benign and in situ neoplasms	0 (0 to 0)	0 (0 to 0)	0 (0 to 0)	0 (0 to 0)	0 (0 to 0)	0 (0 to 0)	0 (0 to 0)	0 (0 to 0)	0 (0 to 0)	0 (0 to 0)	0 (0 to 0)	0 (0 to 0)	0 (0 to 0)	0 (0 to 0)	0 (0 to 0)	0 (0 to 0)	0 (0 to 0)	0 (0 to 0)	0 (0 to 0)	0 (0 to 0)	0 (0 to 0)	0 (0 to 0)	0 (0 to 0)	0
Cardiovascular diseases	3·1 (1·9 to 4·7)	1·6 (1 to 2·5)	7·2 (4·3 to 10·9)	5·2 (3·7 to 7·1)	7·7 (5·9 to 9·7)	18·9 (14·8 to 23·5)	38·4 (30·8 to 46·9)	50·1 (41·2 to 59·5)	70·2 (55·8 to 88·5)	94·4 (74·2 to 119)	147 (116 to 181)	249 (206 to 301)	434 (352 to 525)	563 (459 to 676)	696 (591 to 818)	793 (675 to 929)	764 (670 to 868)	842 (748 to 948)	804 (711 to 897)	767 (682 to 852)	483 (424 to 536)	183 (158 to 203)	45·8 (37·7 to 50·6)	7,070
Rheumatic heart disease	0 (0 to 0)	0 (0 to 0)	0 (0 to 0)	0·5 (0·3 to 0·7)	2 (1·3 to 3·2)	6 (4·2 to 8·8)	9·4 (6·8 to 13·1)	9·8 (7 to 13·5)	11·4 (8·2 to 15·6)	11·1 (8·2 to 14·9)	12 (8·5 to 16·2)	13·1 (9·8 to 17·1)	13 (9·1 to 17·7)	11·1 (8·1 to 15)	9·5 (7·1 to 12·3)	6·4 (4·6 to 8·6)	4·3 (3·3 to 5·5)	3·1 (2·3 to 4)	2·3 (1·8 to 3·1)	1·5 (1·1 to 2)	0·8 (0·6 to 1)	0·3 (0·2 to 0·4)	0·1 (0·1 to 0·1)	128
Ischemic heart disease	0 (0 to 0)	[illegible]	[illegible]	[illegible]	[illegible]	[illegible]	[illegible]	[illegible]	[illegible]	[illegible]	[illegible]	[illegible]	[illegible]	[illegible]	[illegible]	[illegible]	[illegible]	[illegible]	[illegible]	[illegible]	[illegible]	[illegible]	22·9 (18·8 to 25·9)	2,940
Stroke	1·2 (0·7 to 2·1)	[illegible]	[illegible]	[illegible]	[illegible]	[illegible]	[illegible]	[illegible]	[illegible]	[illegible]	[illegible]	[illegible]	[illegible]	[illegible]	[illegible]	[illegible]	[illegible]	[illegible]	[illegible]	[illegible]	[illegible]	[illegible]	10·5 (8 to 12·8)	2,210
Ischemic stroke	0·2 (0·1 to 0·4)	[illegible]	[illegible]	[illegible]	[illegible]	[illegible]	[illegible]	[illegible]	[illegible]	[illegible]	[illegible]	[illegible]	[illegible]	[illegible]	[illegible]	[illegible]	[illegible]	[illegible]	[illegible]	[illegible]	[illegible]	[illegible]	8·6 (6·5 to 10·6)	971
Intracerebral hemorrhage	0·4 (0·2 to 0·7)	[illegible]	[illegible]	[illegible]	[illegible]	[illegible]	[illegible]	[illegible]	[illegible]	[illegible]	[illegible]	[illegible]	[illegible]	[illegible]	[illegible]	[illegible]	[illegible]	[illegible]	[illegible]	[illegible]	[illegible]	[illegible]	1·7 (1·2 to 2·2)	1,090
Subarachnoid hemorrhage	0·6 (0·3 to 1·1)	[illegible]	[illegible]	[illegible]	[illegible]	[illegible]	[illegible]	[illegible]	[illegible]	[illegible]	[illegible]	[illegible]	[illegible]	[illegible]	[illegible]	[illegible]	[illegible]	[illegible]	[illegible]	[illegible]	[illegible]	[illegible]	0·2 (0·1 to 0·2)	148
Hypertensive heart disease	0 (0 to 0)	[illegible]	[illegible]	[illegible]	[illegible]	[illegible]	[illegible]	[illegible]	[illegible]	[illegible]	[illegible]	[illegible]	[illegible]	[illegible]	[illegible]	[illegible]	[illegible]	[illegible]	[illegible]	[illegible]	[illegible]	[illegible]	6·8 (4·6 to 8·5)	879
Non-rheumatic valvular heart disease	0 (0 to 0)	[illegible]	[illegible]	[illegible]	[illegible]	[illegible]	[illegible]	[illegible]	[illegible]	[illegible]	[illegible]	[illegible]	[illegible]	[illegible]	[illegible]	[illegible]	[illegible]	[illegible]	[illegible]	[illegible]	[illegible]	[illegible]	0·1 (0·1 to 0·1)	58·3
Non-rheumatic calcific aortic valve disease	0 (0 to 0)	[illegible]	[illegible]	[illegible]	[illegible]	[illegible]	[illegible]	[illegible]	[illegible]	[illegible]	[illegible]	[illegible]	[illegible]	[illegible]	[illegible]	[illegible]	[illegible]	[illegible]	[illegible]	[illegible]	[illegible]	[illegible]	0·1 (0·1 to 0·1)	30·3
Non-rheumatic degenerative mitral valve disease	0 (0 to 0)	[illegible]	[illegible]	[illegible]	[illegible]	[illegible]	[illegible]	[illegible]	[illegible]	[illegible]	[illegible]	[illegible]	[illegible]	[illegible]	[illegible]	[illegible]	[illegible]	[illegible]	[illegible]	[illegible]	[illegible]	[illegible]	0 (0 to 0)	24·2
Other non-rheumatic valve diseases	0 (0 to 0)	[illegible]	[illegible]	[illegible]	[illegible]	[illegible]	[illegible]	[illegible]	[illegible]	[illegible]	[illegible]	[illegible]	[illegible]	[illegible]	[illegible]	[illegible]	[illegible]	[illegible]	[illegible]	[illegible]	[illegible]	[illegible]	0 (0 to 0)	3·8
Cardiomyopathy and myocarditis	0·5 (0·3 to 0·8)	[illegible]	[illegible]	[illegible]	[illegible]	[illegible]	[illegible]	[illegible]	[illegible]	[illegible]	[illegible]	[illegible]	[illegible]	[illegible]	[illegible]	[illegible]	[illegible]	[illegible]	[illegible]	[illegible]	[illegible]	[illegible]	0·4 (0·2 to 0·5)	164
Myocarditis	0·2 (0·1 to 0·3)	[illegible]	[illegible]	[illegible]	[illegible]	[illegible]	[illegible]	[illegible]	[illegible]	[illegible]	[illegible]	[illegible]	[illegible]	[illegible]	[illegible]	[illegible]	[illegible]	[illegible]	[illegible]	[illegible]	[illegible]	[illegible]	0·1 (0·1 to 0·1)	20
Alcoholic cardiomyopathy	0 (0 to 0)	[illegible]	[illegible]	[illegible]	[illegible]	[illegible]	[illegible]	[illegible]	[illegible]	[illegible]	[illegible]	[illegible]	[illegible]	[illegible]	[illegible]	[illegible]	[illegible]	[illegible]	[illegible]	[illegible]	[illegible]	[illegible]	0 (0 to 0)	53·1
Other cardiomyopathy	0·3 (0·2 to 0·5)	[illegible]	[illegible]	[illegible]	[illegible]	[illegible]	[illegible]	[illegible]	[illegible]	[illegible]	[illegible]	[illegible]	[illegible]	[illegible]	[illegible]	[illegible]	[illegible]	[illegible]	[illegible]	[illegible]	[illegible]	[illegible]	0·3 (0·2 to 0·4)	91
Atrial fibrillation and flutter	0 (0 to 0)	[illegible]	[illegible]	[illegible]	[illegible]	[illegible]	[illegible]	[illegible]	[illegible]	[illegible]	[illegible]	[illegible]	[illegible]	[illegible]	[illegible]	[illegible]	[illegible]	[illegible]	[illegible]	[illegible]	[illegible]	[illegible]	2·4 (1·9 to 3·2)	132
Aortic aneurysm	--	[illegible]	[illegible]	[illegible]	[illegible]	[illegible]	[illegible]	[illegible]	[illegible]	[illegible]	[illegible]	[illegible]	[illegible]	[illegible]	[illegible]	[illegible]	[illegible]	[illegible]	[illegible]	[illegible]	[illegible]	[illegible]	0·2 (0·2 to 0·3)	NA
Endocarditis	0·1 (0 to 0·1)	[illegible]	[illegible]	[illegible]	[illegible]	[illegible]	[illegible]	[illegible]	[illegible]	[illegible]	[illegible]	[illegible]	[illegible]	[illegible]	[illegible]	[illegible]	[illegible]	[illegible]	[illegible]	[illegible]	[illegible]	[illegible]	0·1 (0 to 0·1)	29·3
Other cardiovascular and circulatory diseases	1·3 (0·8 to 2)	[illegible]	[illegible]	[illegible]	[illegible]	[illegible]	[illegible]	[illegible]	[illegible]	[illegible]	[illegible]	[illegible]	[illegible]	[illegible]	[illegible]	[illegible]	[illegible]	[illegible]	[illegible]	[illegible]	[illegible]	[illegible]	1·9 (1·4 to 2·3)	405
Chronic respiratory diseases	0 (0 to 0)	[illegible]	[illegible]	[illegible]	[illegible]	[illegible]	[illegible]	[illegible]	[illegible]	[illegible]	[illegible]	[illegible]	[illegible]	[illegible]	[illegible]	[illegible]	[illegible]	[illegible]	[illegible]	[illegible]	[illegible]	[illegible]	1·7 (1·3 to 2)	727
Chronic obstructive pulmonary disease	0 (0 to 0)	[illegible]	[illegible]	[illegible]	[illegible]	[illegible]	[illegible]	[illegible]	[illegible]	[illegible]	[illegible]	[illegible]	[illegible]	[illegible]	[illegible]	[illegible]	[illegible]	[illegible]	[illegible]	[illegible]	[illegible]	[illegible]	1·4 (1·1 to 1·6)	316
Pneumoconiosis	0 (0 to 0)	[illegible]	[illegible]	[illegible]	[illegible]	[illegible]	[illegible]	[illegible]	[illegible]	[illegible]	[illegible]	[illegible]	[illegible]	[illegible]	[illegible]	[illegible]	[illegible]	[illegible]	[illegible]	[illegible]	[illegible]	[illegible]	0 (0 to 0)	4·1
Silicosis	0 (0 to 0)	[illegible]	[illegible]	[illegible]	[illegible]	[illegible]	[illegible]	[illegible]	[illegible]	[illegible]	[illegible]	[illegible]	[illegible]	[illegible]	[illegible]	[illegible]	[illegible]	[illegible]	[illegible]	[illegible]	[illegible]	[illegible]	0 (0 to 0)	0·6
Asbestosis	0 (0 to 0)	[illegible]	[illegible]	[illegible]	[illegible]	[illegible]	[illegible]	[illegible]	[illegible]	[illegible]	[illegible]	[illegible]	[illegible]	[illegible]	[illegible]	[illegible]	[illegible]	[illegible]	[illegible]	[illegible]	[illegible]	[illegible]	0 (0 to 0)	0·6
Coal workers pneumoconiosis	0 (0 to 0)	[illegible]	[illegible]	[illegible]	[illegible]	[illegible]	[illegible]	[illegible]	[illegible]	[illegible]	[illegible]	[illegible]	[illegible]	[illegible]	[illegible]	[illegible]	[illegible]	[illegible]	[illegible]	[illegible]	[illegible]	[illegible]	0 (0 to 0)	0·2
Other pneumoconiosis	0 (0 to 0)	[illegible]	[illegible]	[illegible]	[illegible]	[illegible]	[illegible]	[illegible]	[illegible]	[illegible]	[illegible]	[illegible]	[illegible]	[illegible]	[illegible]	[illegible]	[illegible]	[illegible]	[illegible]	[illegible]	[illegible]	[illegible]	0 (0 to 0)	2·7
Asthma	0 (0 to 0)	[illegible]	[illegible]	[illegible]	[illegible]	[illegible]	[illegible]	[illegible]	[illegible]	[illegible]	[illegible]	[illegible]	[illegible]	[illegible]	[illegible]	[illegible]	[illegible]	[illegible]	[illegible]	[illegible]	[illegible]	[illegible]	0·2 (0·2 to 0·3)	340
Interstitial lung disease and pulmonary sarcoidosis	0 (0 to 0)	[illegible]	[illegible]	[illegible]	[illegible]	[illegible]	[illegible]	[illegible]	[illegible]	[illegible]	[illegible]	[illegible]	[illegible]	[illegible]	[illegible]	[illegible]	[illegible]	[illegible]	[illegible]	[illegible]	[illegible]	[illegible]	0·1 (0 to 0·1)	33·1
Other chronic respiratory diseases	0 (0 to 0)	[illegible]	[illegible]	[illegible]	[illegible]	[illegible]	[illegible]	[illegible]	[illegible]	[illegible]	[illegible]	[illegible]	[illegible]	[illegible]	[illegible]	[illegible]	[illegible]	[illegible]	[illegible]	[illegible]	[illegible]	[illegible]	0 (0 to 0)	33·6
Digestive diseases	1·5 (1 to 2·1)	[illegible]	[illegible]	[illegible]	[illegible]	[illegible]	[illegible]	[illegible]	[illegible]	[illegible]	[illegible]	[illegible]	[illegible]	[illegible]	[illegible]	[illegible]	[illegible]	[illegible]	[illegible]	[illegible]	[illegible]	[illegible]	1·8 (1·4 to 2)	1,460
Cirrhosis and other chronic liver diseases	0 (0 to 0)	[illegible]	[illegible]	[illegible]	[illegible]	[illegible]	[illegible]	[illegible]	[illegible]	[illegible]	[illegible]	[illegible]	[illegible]	[illegible]	[illegible]	[illegible]	[illegible]	[illegible]	[illegible]	[illegible]	[illegible]	[illegible]	0·3 (0·2 to 0·3)	564
Cirrhosis and other chronic liver diseases due to hepatitis B	0 (0 to 0)	[illegible]	[illegible]	[illegible]	[illegible]	[illegible]	[illegible]	[illegible]	[illegible]	[illegible]	[illegible]	[illegible]	[illegible]	[illegible]	[illegible]	[illegible]	[illegible]	[illegible]	[illegible]	[illegible]	[illegible]	[illegible]	0 (0 to 0)	40·3
Cirrhosis and other chronic liver diseases due to hepatitis C	0 (0 to 0)	[illegible]	[illegible]	[illegible]	[illegible]	[illegible]	[illegible]	[illegible]	[illegible]	[illegible]	[illegible]	[illegible]	[illegible]	[illegible]	[illegible]	[illegible]	[illegible]	[illegible]	[illegible]	[illegible]	[illegible]	[illegible]	0 (0 to 0·1)	112
Cirrhosis and other chronic liver diseases due to alcohol use	0 (0 to 0)	[illegible]	[illegible]	[illegible]	[illegible]	[illegible]	[illegible]	[illegible]	[illegible]	[illegible]	[illegible]	[illegible]	[illegible]	[illegible]	[illegible]	[illegible]	[illegible]	[illegible]	[illegible]	[illegible]	[illegible]	[illegible]	0·1 (0 to 0·1)	256
Cirrhosis and other chronic liver diseases due to NAFLD	0 (0 to 0)	[illegible]	[illegible]	[illegible]	[illegible]	[illegible]	[illegible]	[illegible]	[illegible]	[illegible]	[illegible]	[illegible]	[illegible]	[illegible]	[illegible]	[illegible]	[illegible]	[illegible]	[illegible]	[illegible]	[illegible]	[illegible]	0·1 (0 to 0·1)	84·7
Cirrhosis and other chronic liver diseases due to other causes	0 (0 to 0)	[illegible]	[illegible]	[illegible]	[illegible]	[illegible]	[illegible]	[illegible]	[illegible]	[illegible]	[illegible]	[illegible]	[illegible]	[illegible]	[illegible]	[illegible]	[illegible]	[illegible]	[illegible]	[illegible]	[illegible]	[illegible]	0·1 (0 to 0·1)	71·3
Upper digestive system diseases	0 (0 to 0)	[illegible]	[illegible]	[illegible]	[illegible]	[illegible]	[illegible]	[illegible]	[illegible]	[illegible]	[illegible]	[illegible]	[illegible]	[illegible]	[illegible]	[illegible]	[illegible]	[illegible]	[illegible]	[illegible]	[illegible]	[illegible]	0·5 (0·4 to 0·6)	383
Peptic ulcer disease	0 (0 to 0)	[illegible]	[illegible]	[illegible]	[illegible]	[illegible]	[illegible]	[illegible]	[illegible]	[illegible]	[illegible]	[illegible]	[illegible]	[illegible]	[illegible]	[illegible]	[illegible]	[illegible]	[illegible]	[illegible]	[illegible]	[illegible]	0·3 (0·2 to 0·3)	125
Gastritis and duodenitis	0 (0 to 0)	[illegible]	[illegible]	[illegible]	[illegible]	[illegible]	[illegible]	[illegible]	[illegible]	[illegible]	[illegible]	[illegible]	[illegible]	[illegible]	[illegible]	[illegible]	[illegible]	[illegible]	[illegible]	[illegible]	[illegible]	[illegible]	0·1 (0·1 to 0·2)	103
Gastroesophageal reflux disease	0 (0 to 0)	[illegible]	[illegible]	[illegible]	[illegible]	[illegible]	[illegible]	[illegible]	[illegible]	[illegible]	[illegible]	[illegible]	[illegible]	[illegible]	[illegible]	[illegible]	[illegible]	[illegible]	[illegible]	[illegible]	[illegible]	[illegible]	0·1 (0 to 0·1)	155
Appendicitis	0 (0 to 0)	[illegible]	[illegible]	[illegible]	[illegible]	[illegible]	[illegible]	[illegible]	[illegible]	[illegible]	[illegible]	[illegible]	[illegible]	[illegible]	[illegible]	[illegible]	[illegible]	[illegible]	[illegible]	[illegible]	[illegible]	[illegible]	0 (0 to 0)	21·4
Paralytic ileus and intestinal obstruction	0·9 (0·6 to 1·3)	[illegible]	[illegible]	[illegible]	[illegible]	[illegible]	[illegible]	[illegible]	[illegible]	[illegible]	[illegible]	[illegible]	[illegible]	[illegible]	[illegible]	[illegible]	[illegible]	[illegible]	[illegible]	[illegible]	[illegible]	[illegible]	0·3 (0·2 to 0·4)	84·8
Inguinal, femoral, and abdominal hernia	0·6 (0·4 to 0·9)	[illegible]	[illegible]	[illegible]	[illegible]	[illegible]	[illegible]	[illegible]	[illegible]	[illegible]	[illegible]	[illegible]	[illegible]	[illegible]	[illegible]	[illegible]	[illegible]	[illegible]	[illegible]	[illegible]	[illegible]	[illegible]	0·1 (0·1 to 0·1)	98·7
Inflammatory bowel disease	0 (0 to 0)	[illegible]	[illegible]	[illegible]	[illegible]	[illegible]	[illegible]	[illegible]	[illegible]	[illegible]	[illegible]	[illegible]	[illegible]	[illegible]	[illegible]	[illegible]	[illegible]	[illegible]	[illegible]	[illegible]	[illegible]	[illegible]	0·1 (0 to 0·1)	49·1
Vascular intestinal disorders	0 (0 to 0)	[illegible]	[illegible]	[illegible]	[illegible]	[illegible]	[illegible]	[illegible]	[illegible]	[illegible]	[illegible]	[illegible]	[illegible]	[illegible]	[illegible]	[illegible]	[illegible]	[illegible]	[illegible]	[illegible]	[illegible]	[illegible]	0 (0 to 0)	10·6
Gallbladder and biliary diseases	0 (0 to 0)	[illegible]	[illegible]	[illegible]	[illegible]	[illegible]	[illegible]	[illegible]	[illegible]	[illegible]	[illegible]	[illegible]	[illegible]	[illegible]	[illegible]	[illegible]	[illegible]	[illegible]	[illegible]	[illegible]	[illegible]	[illegible]	0·1 (0·1 to 0·2)	82·3
Pancreatitis	0 (0 to 0)	[illegible]	[illegible]	[illegible]	[illegible]	[illegible]	[illegible]	[illegible]	[illegible]	[illegible]	[illegible]	[illegible]	[illegible]	[illegible]	[illegible]	[illegible]	[illegible]	[illegible]	[illegible]	[illegible]	[illegible]	[illegible]	0 (0 to 0·1)	60
Other digestive diseases	0 (0 to 0)	[illegible]	[illegible]	[illegible]	[illegible]	[illegible]	[illegible]	[illegible]	[illegible]	[illegible]	[illegible]	[illegible]	[illegible]	[illegible]	[illegible]	[illegible]	[illegible]	[illegible]	[illegible]	[illegible]	[illegible]	[illegible]	0·4 (0·2 to 0·5)	107
Neurological disorders	0 (0 to 0·1)	[illegible]	[illegible]	[illegible]	[illegible]	[illegible]	[illegible]	[illegible]	[illegible]	[illegible]	[illegible]	[illegible]	[illegible]	[illegible]	[illegible]	[illegible]	[illegible]	[illegible]	[illegible]	[illegible]	[illegible]	[illegible]	11·2 (5 to 24·7)	1,600
Alzheimer's disease and other dementias	0 (0 to 0)	[illegible]	[illegible]	[illegible]	[illegible]	[illegible]	[illegible]	[illegible]	[illegible]	[illegible]	[illegible]	[illegible]	[illegible]	[illegible]	[illegible]	[illegible]	[illegible]	[illegible]	[illegible]	[illegible]	[illegible]	[illegible]	10·4 (4·1 to 24)	351
Parkinson's disease	0 (0 to 0)	[illegible]	[illegible]	[illegible]	[illegible]	[illegible]	[illegible]	[illegible]	[illegible]	[illegible]	[illegible]	[illegible]	[illegible]	[illegible]	[illegible]	[illegible]	[illegible]	[illegible]	[illegible]	[illegible]	[illegible]	[illegible]	0·5 (0·4 to 0·6)	111
Idiopathic epilepsy	0 (0 to 0)	[illegible]	[illegible]	[illegible]	[illegible]	[illegible]	[illegible]	[illegible]	[illegible]	[illegible]	[illegible]	[illegible]	[illegible]	[illegible]	[illegible]	[illegible]	[illegible]	[illegible]	[illegible]	[illegible]	[illegible]	[illegible]	0·2 (0·1 to 0·3)	343
Multiple sclerosis	0 (0 to 0)	[illegible]	[illegible]	[illegible]	[illegible]	[illegible]	[illegible]	[illegible]	[illegible]	[illegible]	[illegible]	[illegible]	[illegible]	[illegible]	[illegible]	[illegible]	[illegible]	[illegible]	[illegible]	[illegible]	[illegible]	[illegible]	0 (0 to 0)	11·5
Motor neuron disease	0 (0 to 0)	[illegible]	[illegible]	[illegible]	[illegible]	[illegible]	[illegible]	[illegible]	[illegible]	[illegible]	[illegible]	[illegible]	[illegible]	[illegible]	[illegible]	[illegible]	[illegible]	[illegible]	[illegible]	[illegible]	[illegible]	[illegible]	0 (0 to 0)	21·8
Headache disorders	0 (0 to 0)	[illegible]	[illegible]	[illegible]	[illegible]	[illegible]	[illegible]	[illegible]	[illegible]	[illegible]	[illegible]	[illegible]	[illegible]	[illegible]	[illegible]	[illegible]	[illegible]	[illegible]	[illegible]	[illegible]	[illegible]	[illegible]	0·1 (0 to 0·2)	652
Migraine	0 (0 to 0)	[illegible]	[illegible]	[illegible]	[illegible]	[illegible]	[illegible]	[illegible]	[illegible]	[illegible]	[illegible]	[illegible]	[illegible]	[illegible]	[illegible]	[illegible]	[illegible]	[illegible]	[illegible]	[illegible]	[illegible]	[illegible]	0·1 (0 to 0·1)	588
Tension-type headache	0 (0 to 0)	[illegible]	[illegible]	[illegible]	[illegible]	[illegible]	[illegible]	[illegible]	[illegible]	[illegible]	[illegible]	[illegible]	[illegible]	[illegible]	[illegible]	[illegible]	[illegible]	[illegible]	[illegible]	[illegible]	[illegible]	[illegible]	0 (0 to 0·1)	63·6
Other neurological disorders	0 (0 to 0)	[illegible]	[illegible]	[illegible]	[illegible]	[illegible]	[illegible]	[illegible]	[illegible]	[illegible]	[illegible]	[illegible]	[illegible]	[illegible]	[illegible]	[illegible]	[illegible]	[illegible]	[illegible]	[illegible]	[illegible]	[illegible]	0 (0 to 0)	111
Mental disorders	0 (0 to 0)	[illegible]	[illegible]	[illegible]	[illegible]	[illegible]	[illegible]	[illegible]	[illegible]	[illegible]	[illegible]	[illegible]	[illegible]	[illegible]	[illegible]	[illegible]	[illegible]	[illegible]	[illegible]	[illegible]	[illegible]	[illegible]	0·7 (0·4 to 1)	2,060
Schizophrenia	0 (0 to 0)	[illegible]	[illegible]	[illegible]	[illegible]	[illegible]	[illegible]	[illegible]	[illegible]	[illegible]	[illegible]	[illegible]	[illegible]	[illegible]	[illegible]	[illegible]	[illegible]	[illegible]	[illegible]	[illegible]	[illegible]	[illegible]	0 (0 to 0)	219
Depressive disorders	0 (0 to 0)	[illegible]	[illegible]	[illegible]	[illegible]	[illegible]	[illegible]	[illegible]	[illegible]	[illegible]	[illegible]	[illegible]	[illegible]	[illegible]	[illegible]	[illegible]	[illegible]	[illegible]	[illegible]	[illegible]	[illegible]	[illegible]	0·4 (0·2 to 0·7)	737
Major depressive disorder	0 (0 to 0)	[illegible]	[illegible]	[illegible]	[illegible]	[illegible]	[illegible]	[illegible]	[illegible]	[illegible]	[illegible]	[illegible]	[illegible]	[illegible]	[illegible]	[illegible]	[illegible]	[illegible]	[illegible]	[illegible]	[illegible]	[illegible]	0·4 (0·2 to 0·7)	625
Dysthymia	0 (0 to 0)	[illegible]	[illegible]	[illegible]	[illegible]	[illegible]	[illegible]	[illegible]	[illegible]	[illegible]	[illegible]	[illegible]	[illegible]	[illegible]	[illegible]	[illegible]	[illegible]	[illegible]	[illegible]	[illegible]	[illegible]	[illegible]	0 (0 to 0·1)	112
Bipolar disorder	0 (0 to 0)	[illegible]	[illegible]	[illegible]	[illegible]	[illegible]	[illegible]	[illegible]	[illegible]	[illegible]	[illegible]	[illegible]	[illegible]	[illegible]	[illegible]	[illegible]	[illegible]	[illegible]	[illegible]	[illegible]	[illegible]	[illegible]	0 (0 to 0)	239
Anxiety disorders	0 (0 to 0)	[illegible]	[illegible]	[illegible]	[illegible]	[illegible]	[illegible]	[illegible]	[illegible]	[illegible]	[illegible]	[illegible]	[illegible]	[illegible]	[illegible]	[illegible]	[illegible]	[illegible]	[illegible]	[illegible]	[illegible]	[illegible]	0·1 (0·1 to 0·2)	491
Eating disorders	0 (0 to 0)	[illegible]	[illegible]	[illegible]	[illegible]	[illegible]	[illegible]	[illegible]	[illegible]	[illegible]	[illegible]	[illegible]	[illegible]	[illegible]	[illegible]	[illegible]	[illegible]	[illegible]	[illegible]	[illegible]	[illegible]	[illegible]	0 (0 to 0)	50·5
Anorexia nervosa	0 (0 to 0)	[illegible]	[illegible]	[illegible]	[illegible]	[illegible]	[illegible]	[illegible]	[illegible]	[illegible]	[illegible]	[illegible]	[illegible]	[illegible]	[illegible]	[illegible]	[illegible]	[illegible]	[illegible]	[illegible]	[illegible]	[illegible]	0 (0 to 0)	12·2
Bulimia nervosa	0 (0 to 0)	[illegible]	[illegible]	[illegible]	[illegible]	[illegible]	[illegible]	[illegible]	[illegible]	[illegible]	[illegible]	[illegible]	[illegible]	[illegible]	[illegible]	[illegible]	[illegible]	[illegible]	[illegible]	[illegible]	[illegible]	[illegible]	0 (0 to 0)	38·3
Autism spectrum disorders	0 (0 to 0)	[illegible]	[illegible]	[illegible]	[illegible]	[illegible]	[illegible]	[illegible]	[illegible]	[illegible]	[illegible]	[illegible]	[illegible]	[illegible]	[illegible]	[illegible]	[illegible]	[illegible]	[illegible]	[illegible]	[illegible]	[illegible]	0 (0 to 0)	58·1
Attention-deficit/hyperactivity disorder	0 (0 to 0)	[illegible]	[illegible]	[illegible]	[illegible]	[illegible]	[illegible]	[illegible]	[illegible]	[illegible]	[illegible]	[illegible]	[illegible]	[illegible]	[illegible]	[illegible]	[illegible]	[illegible]	[illegible]	[illegible]	[illegible]	[illegible]	0 (0 to 0)	39·8
Conduct disorder	0 (0 to 0)	[illegible]	[illegible]	[illegible]	[illegible]	[illegible]	[illegible]	[illegible]	[illegible]	[illegible]	[illegible]	[illegible]	[illegible]	[illegible]	[illegible]	[illegible]	[illegible]	[illegible]	[illegible]	[illegible]	[illegible]	[illegible]	0 (0 to 0)	68·4
Idiopathic developmental intellectual disability	0 (0 to 0)	[illegible]	[illegible]	[illegible]	[illegible]	[illegible]	[illegible]	[illegible]	[illegible]	[illegible]	[illegible]	[illegible]	[illegible]	[illegible]	[illegible]	[illegible]	[illegible]	[illegible]	[illegible]	[illegible]	[illegible]	[illegible]	0 (0 to 0)	17·1
Other mental disorders	0 (0 to 0)	[illegible]	[illegible]	[illegible]	[illegible]	[illegible]	[illegible]	[illegible]	[illegible]	[illegible]	[illegible]	[illegible]	[illegible]	[illegible]	[illegible]	[illegible]	[illegible]	[illegible]	[illegible]	[illegible]	[illegible]	[illegible]	0·1 (0·1 to 0·1)	137
Substance use disorders	0 (0 to 0)	[illegible]	[illegible]	[illegible]	[illegible]	[illegible]	[illegible]	[illegible]	[illegible]	[illegible]	[illegible]	[illegible]	[illegible]	[illegible]	[illegible]	[illegible]	[illegible]	[illegible]	[illegible]	[illegible]	[illegible]	[illegible]	0·2 (0·1 to 0·3)	659
Alcohol use disorders	0 (0 to 0)	[illegible]	[illegible]	[illegible]	[illegible]	[illegible]	[illegible]	[illegible]	[illegible]	[illegible]	[illegible]	[illegible]	[illegible]	[illegible]	[illegible]	[illegible]	[illegible]	[illegible]	[illegible]	[illegible]	[illegible]	[illegible]	0·2 (0·1 to 0·2)	528
Drug use disorders	0 (0 to 0)	[illegible]	[illegible]	[illegible]	[illegible]	[illegible]	[illegible]	[illegible]	[illegible]	[illegible]	[illegible]	[illegible]	[illegible]	[illegible]	[illegible]	[illegible]	[illegible]	[illegible]	[illegible]	[illegible]	[illegible]	[illegible]	0 (0 to 0)	132
Opioid use disorders	0 (0 to 0)	[illegible]	[illegible]	[illegible]	[illegible]	[illegible]	[illegible]	[illegible]	[illegible]	[illegible]	[illegible]	[illegible]	[illegible]	[illegible]	[illegible]	[illegible]	[illegible]	[illegible]	[illegible]	[illegible]	[illegible]	[illegible]	0 (0 to 0)	58·7
Cocaine use disorders	0 (0 to 0)	[illegible]	[illegible]	[illegible]	[illegible]	[illegible]	[illegible]	[illegible]	[illegible]	[illegible]	[illegible]	[illegible]	[illegible]	[illegible]	[illegible]	[illegible]	[illegible]	[illegible]	[illegible]	[illegible]	[illegible]	[illegible]	0 (0 to 0)	24·5
Amphetamine use disorders	0 (0 to 0)	[illegible]	[illegible]	[illegible]	[illegible]	[illegible]	[illegible]	[illegible]	[illegible]	[illegible]	[illegible]	[illegible]	[illegible]	[illegible]	[illegible]	[illegible]	[illegible]	[illegible]	[illegible]	[illegible]	[illegible]	[illegible]	0 (0 to 0)	7·7
Cannabis use disorders	0 (0 to 0)	[illegible]	[illegible]	[illegible]	[illegible]	[illegible]	[illegible]	[illegible]	[illegible]	[illegible]	[illegible]	[illegible]	[illegible]	[illegible]	[illegible]	[illegible]	[illegible]	[illegible]	[illegible]	[illegible]	[illegible]	[illegible]	0 (0 to 0)	20
Other drug use disorders	0 (0 to 0)	[illegible]	[illegible]	[illegible]	[illegible]	[illegible]	[illegible]	[illegible]	[illegible]	[illegible]	[illegible]	[illegible]	[illegible]	[illegible]	[illegible]	[illegible]	[illegible]	[illegible]	[illegible]	[illegible]	[illegible]	[illegible]	0 (0 to 0)	21
Diabetes and kidney diseases	0 (0 to 0)	[illegible]	[illegible]	[illegible]	[illegible]	[illegible]	[illegible]	[illegible]	[illegible]	[illegible]	[illegible]	[illegible]	[illegible]	[illegible]	[illegible]	[illegible]	[illegible]	[illegible]	[illegible]	[illegible]	[illegible]	[illegible]	10·4 (8·6 to 12)	5,070
Diabetes mellitus	0 (0 to 0)	[illegible]	[illegible]	[illegible]	[illegible]	[illegible]	[illegible]	[illegible]	[illegible]	[illegible]	[illegible]	[illegible]	[illegible]	[illegible]	[illegible]	[illegible]	[illegible]	[illegible]	[illegible]	[illegible]	[illegible]	[illegible]	7·1 (5·7 to 8·4)	3,690
Diabetes mellitus type 1	0 (0 to 0)	[illegible]	[illegible]	[illegible]	[illegible]	[illegible]	[illegible]	[illegible]	[illegible]	[illegible]	[illegible]	[illegible]	[illegible]	[illegible]	[illegible]	[illegible]	[illegible]	[illegible]	[illegible]	[illegible]	[illegible]	[illegible]	0·1 (0·1 to 0·2)	158
Diabetes mellitus type 2	0 (0 to 0)	[illegible]	[illegible]	[illegible]	[illegible]	[illegible]	[illegible]	[illegible]	[illegible]	[illegible]	[illegible]	[illegible]	[illegible]	[illegible]	[illegible]	[illegible]	[illegible]	[illegible]	[illegible]	[illegible]	[illegible]	[illegible]	7 (5·6 to 8·3)	3,530
Chronic kidney disease	0 (0 to 0)	[illegible]	[illegible]	[illegible]	[illegible]	[illegible]	[illegible]	[illegible]	[illegible]	[illegible]	[illegible]	[illegible]	[illegible]	[illegible]	[illegible]	[illegible]	[illegible]	[illegible]	[illegible]	[illegible]	[illegible]	[illegible]	3·3 (2·6 to 4·1)	1,380
Chronic kidney disease due to diabetes mellitus type 1	0 (0 to 0)	[illegible]	[illegible]	[illegible]	[illegible]	[illegible]	[illegible]	[illegible]	[illegible]	[illegible]	[illegible]	[illegible]	[illegible]	[illegible]	[illegible]	[illegible]	[illegible]	[illegible]	[illegible]	[illegible]	[illegible]	[illegible]	0 (0 to 0)	116
Chronic kidney disease due to diabetes mellitus type 2	0 (0 to 0)	[illegible]	[illegible]	[illegible]	[illegible]	[illegible]	[illegible]	[illegible]	[illegible]	[illegible]	[illegible]	[illegible]	[illegible]	[illegible]	[illegible]	[illegible]	[illegible]	[illegible]	[illegible]	[illegible]	[illegible]	[illegible]	0·9 (0·6 to 1·3)	332
Chronic kidney disease due to hypertension	0 (0 to 0)	[illegible]	[illegible]	[illegible]	[illegible]	[illegible]	[illegible]	[illegible]	[illegible]	[illegible]	[illegible]	[illegible]	[illegible]	[illegible]	[illegible]	[illegible]	[illegible]	[illegible]	[illegible]	[illegible]	[illegible]	[illegible]	1·3 (1 to 1·8)	268
Chronic kidney disease due to glomerulonephritis	0 (0 to 0)	[illegible]	[illegible]	[illegible]	[illegible]	[illegible]	[illegible]	[illegible]	[illegible]	[illegible]	[illegible]	[illegible]	[illegible]	[illegible]	[illegible]	[illegible]	[illegible]	[illegible]	[illegible]	[illegible]	[illegible]	[illegible]	0·2 (0·1 to 0·4)	303
Chronic kidney disease due to other and unspecified causes	0 (0 to 0)	[illegible]	[illegible]	[illegible]	[illegible]	[illegible]	[illegible]	[illegible]	[illegible]	[illegible]	[illegible]	[illegible]	[illegible]	[illegible]	[illegible]	[illegible]	[illegible]	[illegible]	[illegible]	[illegible]	[illegible]	[illegible]	0·8 (0·5 to 1·2)	363
Acute glomerulonephritis	0 (0 to 0)	[illegible]	[illegible]	[illegible]	[illegible]	[illegible]	[illegible]	[illegible]	[illegible]	[illegible]	[illegible]	[illegible]	[illegible]	[illegible]	[illegible]	[illegible]	[illegible]	[illegible]	[illegible]	[illegible]	[illegible]	[illegible]	0 (0 to 0)	2
Skin and subcutaneous diseases	0 (0 to 0)	[illegible]	[illegible]	[illegible]	[illegible]	[illegible]	[illegible]	[illegible]	[illegible]	[illegible]	[illegible]	[illegible]	[illegible]	[illegible]	[illegible]	[illegible]	[illegible]	[illegible]	[illegible]	[illegible]	[illegible]	[illegible]	1·8 (1·2 to 2·6)	721
Dermatitis	0 (0 to 0)	[illegible]	[illegible]	[illegible]	[illegible]	[illegible]	[illegible]	[illegible]	[illegible]	[illegible]	[illegible]	[illegible]	[illegible]	[illegible]	[illegible]	[illegible]	[illegible]	[illegible]	[illegible]	[illegible]	[illegible]	[illegible]	0 (0 to 0·1)	123
Atopic dermatitis	0 (0 to 0)	[illegible]	[illegible]	[illegible]	[illegible]	[illegible]	[illegible]	[illegible]	[illegible]	[illegible]	[illegible]	[illegible]	[illegible]	[illegible]	[illegible]	[illegible]	[illegible]	[illegible]	[illegible]	[illegible]	[illegible]	[illegible]	0 (0 to 0)	85·5
Contact dermatitis	0 (0 to 0)	[illegible]	[illegible]	[illegible]	[illegible]	[illegible]	[illegible]	[illegible]	[illegible]	[illegible]	[illegible]	[illegible]	[illegible]	[illegible]	[illegible]	[illegible]	[illegible]	[illegible]	[illegible]	[illegible]	[illegible]	[illegible]	0 (0 to 0)	33·6
Seborrhoeic dermatitis	0 (0 to 0)	[illegible]	[illegible]	[illegible]	[illegible]	[illegible]	[illegible]	[illegible]	[illegible]	[illegible]	[illegible]	[illegible]	[illegible]	[illegible]	[illegible]	[illegible]	[illegible]	[illegible]	[illegible]	[illegible]	[illegible]	[illegible]	0 (0 to 0)	4
Psoriasis	0 (0 to 0)	[illegible]	[illegible]	[illegible]	[illegible]	[illegible]	[illegible]	[illegible]	[illegible]	[illegible]	[illegible]	[illegible]	[illegible]	[illegible]	[illegible]	[illegible]	[illegible]	[illegible]	[illegible]	[illegible]	[illegible]	[illegible]	0 (0 to 0)	40·9
Bacterial skin diseases	0 (0 to 0)	[illegible]	[illegible]	[illegible]	[illegible]	[illegible]	[illegible]	[illegible]	[illegible]	[illegible]	[illegible]	[illegible]	[illegible]	[illegible]	[illegible]	[illegible]	[illegible]	[illegible]	[illegible]	[illegible]	[illegible]	[illegible]	0·3 (0·1 to 0·5)	78·1
Cellulitis	0 (0 to 0)	[illegible]	[illegible]	[illegible]	[illegible]	[illegible]	[illegible]	[illegible]	[illegible]	[illegible]	[illegible]	[illegible]	[illegible]	[illegible]	[illegible]	[illegible]	[illegible]	[illegible]	[illegible]	[illegible]	[illegible]	[illegible]	0 (0 to 0·1)	16·9
Pyoderma	0 (0 to 0)	[illegible]	[illegible]	[illegible]	[illegible]	[illegible]	[illegible]	[illegible]	[illegible]	[illegible]	[illegible]	[illegible]	[illegible]	[illegible]	[illegible]	[illegible]	[illegible]	[illegible]	[illegible]	[illegible]	[illegible]	[illegible]	0·3 (0·1 to 0·4)	61·2
Scabies	0 (0 to 0)	[illegible]	[illegible]	[illegible]	[illegible]	[illegible]	[illegible]	[illegible]	[illegible]	[illegible]	[illegible]	[illegible]	[illegible]	[illegible]	[illegible]	[illegible]	[illegible]	[illegible]	[illegible]	[illegible]	[illegible]	[illegible]	0 (0 to 0·1)	90·2
Fungal skin diseases	0 (0 to 0)	[illegible]	[illegible]	[illegible]	[illegible]	[illegible]	[illegible]	[illegible]	[illegible]	[illegible]	[illegible]	[illegible]	[illegible]	[illegible]	[illegible]	[illegible]	[illegible]	[illegible]	[illegible]	[illegible]	[illegible]	[illegible]	0·1 (0·1 to 0·3)	52·8
Viral skin diseases	0 (0 to 0)	[illegible]	[illegible]	[illegible]	[illegible]	[illegible]	[illegible]	[illegible]	[illegible]	[illegible]	[illegible]	[illegible]	[illegible]	[illegible]	[illegible]	[illegible]	[illegible]	[illegible]	[illegible]	[illegible]	[illegible]	[illegible]	0 (0 to 0)	47·2
Acne vulgaris	0 (0 to 0)	[illegible]	[illegible]	[illegible]	[illegible]	[illegible]	[illegible]	[illegible]	[illegible]	[illegible]	[illegible]	[illegible]	[illegible]	[illegible]	[illegible]	[illegible]	[illegible]	[illegible]	[illegible]	[illegible]	[illegible]	[illegible]	0 (0 to 0)	51·5
Alopecia areata	0 (0 to 0)	[illegible]	[illegible]	[illegible]	[illegible]	[illegible]	[illegible]	[illegible]	[illegible]	[illegible]	[illegible]	[illegible]	[illegible]	[illegible]	[illegible]	[illegible]	[illegible]	[illegible]	[illegible]	[illegible]	[illegible]	[illegible]	0 (0 to 0)	8·3
Pruritus	0 (0 to 0)	[illegible]	[illegible]	[illegible]	[illegible]	[illegible]	[illegible]	[illegible]	[illegible]	[illegible]	[illegible]	[illegible]	[illegible]	[illegible]	[illegible]	[illegible]	[illegible]	[illegible]	[illegible]	[illegible]	[illegible]	[illegible]	0 (0 to 0)	10·2
Urticaria	0 (0 to 0)	[illegible]	[illegible]	[illegible]	[illegible]	[illegible]	[illegible]	[illegible]	[illegible]	[illegible]	[illegible]	[illegible]	[illegible]	[illegible]	[illegible]	[illegible]	[illegible]	[illegible]	[illegible]	[illegible]	[illegible]	[illegible]	0 (0 to 0)	50·5
Decubitus ulcer	0 (0 to 0)	[illegible]	[illegible]	[illegible]	[illegible]	[illegible]	[illegible]	[illegible]	[illegible]	[illegible]	[illegible]	[illegible]	[illegible]	[illegible]	[illegible]	[illegible]	[illegible]	[illegible]	[illegible]	[illegible]	[illegible]	[illegible]	1·2 (0·8 to 1·9)	111
Other skin and subcutaneous diseases	0 (0 to 0)	[illegible]	[illegible]	[illegible]	[illegible]	[illegible]	[illegible]	[illegible]	[illegible]	[illegible]	[illegible]	[illegible]	[illegible]	[illegible]	[illegible]	[illegible]	[illegible]	[illegible]	[illegible]	[illegible]	[illegible]	[illegible]	0 (0 to 0·1)	57·9
Sense organ diseases	0 (0 to 0)	[illegible]	[illegible]	[illegible]	[illegible]	[illegible]	[illegible]	[illegible]	[illegible]	[illegible]	[illegible]	[illegible]	[illegible]	[illegible]	[illegible]	[illegible]	[illegible]	[illegible]	[illegible]	[illegible]	[illegible]	[illegible]	3·5 (2·5 to 4·7)	976
Blindness and vision loss	0 (0 to 0)	[illegible]	[illegible]	[illegible]	[illegible]	[illegible]	[illegible]	[illegible]	[illegible]	[illegible]	[illegible]	[illegible]	[illegible]	[illegible]	[illegible]	[illegible]	[illegible]	[illegible]	[illegible]	[illegible]	[illegible]	[illegible]	1·4 (1 to 2)	265
Glaucoma	0 (0 to 0)	[illegible]	[illegible]	[illegible]	[illegible]	[illegible]	[illegible]	[illegible]	[illegible]	[illegible]	[illegible]	[illegible]	[illegible]	[illegible]	[illegible]	[illegible]	[illegible]	[illegible]	[illegible]	[illegible]	[illegible]	[illegible]	0·2 (0·1 to 0·3)	15·3
Cataract	0 (0 to 0)	[illegible]	[illegible]	[illegible]	[illegible]	[illegible]	[illegible]	[illegible]	[illegible]	[illegible]	[illegible]	[illegible]	[illegible]	[illegible]	[illegible]	[illegible]	[illegible]	[illegible]	[illegible]	[illegible]	[illegible]	[illegible]	0·5 (0·3 to 0·7)	45
Age-related macular degeneration	0 (0 to 0)	[illegible]	[illegible]	[illegible]	[illegible]	[illegible]	[illegible]	[illegible]	[illegible]	[illegible]	[illegible]	[illegible]	[illegible]	[illegible]	[illegible]	[illegible]	[illegible]	[illegible]	[illegible]	[illegible]	[illegible]	[illegible]	0 (0 to 0)	1·9
Refraction disorders	0 (0 to 0)	[illegible]	[illegible]	[illegible]	[illegible]	[illegible]	[illegible]	[illegible]	[illegible]	[illegible]	[illegible]	[illegible]	[illegible]	[illegible]	[illegible]	[illegible]	[illegible]	[illegible]	[illegible]	[illegible]	[illegible]	[illegible]	0 (0 to 0·1)	66·1
Near vision loss	0 (0 to 0)	[illegible]	[illegible]	[illegible]	[illegible]	[illegible]	[illegible]	[illegible]	[illegible]	[illegible]	[illegible]	[illegible]	[illegible]	[illegible]	[illegible]	[illegible]	[illegible]	[illegible]	[illegible]	[illegible]	[illegible]	[illegible]	0·2 (0·1 to 0·4)	69·1
Other vision loss	0 (0 to 0)	[illegible]	[illegible]	[illegible]	[illegible]	[illegible]	[illegible]	[illegible]	[illegible]	[illegible]	[illegible]	[illegible]	[illegible]	[illegible]	[illegible]	[illegible]	[illegible]	[illegible]	[illegible]	[illegible]	[illegible]	[illegible]	0·5 (0·3 to 0·8)	67·6
Age-related and other hearing loss	0 (0 to 0)	[illegible]	[illegible]	[illegible]	[illegible]	[illegible]	[illegible]	[illegible]	[illegible]	[illegible]	[illegible]	[illegible]	[illegible]	[illegible]	[illegible]	[illegible]	[illegible]	[illegible]	[illegible]	[illegible]	[illegible]	[illegible]	2 (1·4 to 2·7)	665
Other sense organ diseases	0 (0 to 0)	[illegible]	[illegible]	[illegible]	[illegible]	[illegible]	[illegible]	[illegible]	[illegible]	[illegible]	[illegible]	[illegible]	[illegible]	[illegible]	[illegible]	[illegible]	[illegible]	[illegible]	[illegible]	[illegible]	[illegible]	[illegible]	0·1 (0 to 0·1)	45·7
Musculoskeletal disorders	0 (0 to 0)	[illegible]	[illegible]	[illegible]	[illegible]	[illegible]	[illegible]	[illegible]	[illegible]	[illegible]	[illegible]	[illegible]	[illegible]	[illegible]	[illegible]	[illegible]	[illegible]	[illegible]	[illegible]	[illegible]	[illegible]	[illegible]	1·7 (1·2 to 2·3)	1,970
Rheumatoid arthritis	0 (0 to 0)	[illegible]	[illegible]	[illegible]	[illegible]	[illegible]	[illegible]	[illegible]	[illegible]	[illegible]	[illegible]	[illegible]	[illegible]	[illegible]	[illegible]	[illegible]	[illegible]	[illegible]	[illegible]	[illegible]	[illegible]	[illegible]	0·1 (0 to 0·1)	33·5
Osteoarthritis	0 (0 to 0)	[illegible]	[illegible]	[illegible]	[illegible]	[illegible]	[illegible]	[illegible]	[illegible]	[illegible]	[illegible]	[illegible]	[illegible]	[illegible]	[illegible]	[illegible]	[illegible]	[illegible]	[illegible]	[illegible]	[illegible]	[illegible]	0·5 (0·2 to 0·9)	276
Osteoarthritis hip	0 (0 to 0)	[illegible]	[illegible]	[illegible]	[illegible]	[illegible]	[illegible]	[illegible]	[illegible]	[illegible]	[illegible]	[illegible]	[illegible]	[illegible]	[illegible]	[illegible]	[illegible]	[illegible]	[illegible]	[illegible]	[illegible]	[illegible]	0 (0 to 0·1)	14·3
Osteoarthritis knee	0 (0 to 0)	[illegible]	[illegible]	[illegible]	[illegible]	[illegible]	[illegible]	[illegible]	[illegible]	[illegible]	[illegible]	[illegible]	[illegible]	[illegible]	[illegible]	[illegible]	[illegible]	[illegible]	[illegible]	[illegible]	[illegible]	[illegible]	0·3 (0·1 to 0·5)	197
Osteoarthritis hand	0 (0 to 0)	[illegible]	[illegible]	[illegible]	[illegible]	[illegible]	[illegible]	[illegible]	[illegible]	[illegible]	[illegible]	[illegible]	[illegible]	[illegible]	[illegible]	[illegible]	[illegible]	[illegible]	[illegible]	[illegible]	[illegible]	[illegible]	0·1 (0 to 0·2)	32·2

Osteoarthritis other	0 (0 to 0)	0 (0 to 0)	0 (0 to 0)	0 (0 to 0)	0 (0 to 0)	0 (0 to 0)	0 (0 to 0)	0 (0 to 0)	0 (0 to 0)	0 (0 to 0·1)	0·3 (0·1 to 0·8)	1 (0·4 to 2·2)	2 (0·9 to 4·5)	3·4 (1·5 to 7·1)	4·6 (2·1 to 10·2)	5·2 (2·4 to 11)	4·5 (2·1 to 9·5)	3·9 (1·9 to 7·9)	3·1 (1·5 to 6·4)	2·4 (1·2 to 5)	1·3 (0·6 to 2·6)	0·4 (0·2 to 0·8)	0·1 (0 to 0·2)	32·3
Low back pain	0 (0 to 0)	0 (0 to 0)	0 (0 to 0)	0 (0 to 0)	4·3 (2·1 to 7·7)	21·7 (12·8 to 35·2)	39·9 (23·3 to 63·1)	48·2 (28·6 to 76·4)	54·1 (31·5 to 84·1)	59·9 (34·2 to 91)	71·9 (43·5 to 112)	79·7 (48 to 121)	83·7 (50 to 132)	88·4 (54·3 to 138)	88·9 (56 to 133)	80·8 (51·8 to 120)	60·6 (38·9 to 92·6)	46·7 (29·4 to 70·1)	33·9 (21·2 to 50·7)	24·9 (15·7 to 37·4)	11 (6·8 to 16·1)	3 (1·8 to 4·4)	0·6 (0·4 to 0·9)	902
Neck pain	0 (0 to 0)	0 (0 to 0)	0 (0 to 0)	0 (0 to 0)	0·2 (0·1 to 0·6)	1·5 (0·6 to 3)	3·4 (1·5 to 6·1)	5·2 (2·3 to 10·3)	7·7 (3·5 to 14·8)	11·6 (5·6 to 22·6)	16·3 (8·2 to 28·3)	20 (10·3 to 35·7)	22·1 (11·2 to 40)	20·3 (10·4 to 38·3)	19·1 (9·4 to 34·2)	16·1 (8·2 to 28·2)	11·2 (5·9 to 19)	8·2 (4·4 to 14·1)	5·4 (2·6 to 10)	3·4 (1·7 to 6)	1·5 (0·8 to 2·6)	0·4 (0·2 to 0·7)	0·1 (0·1 to 0·2)	174
Gout	0 (0 to 0)	0 (0 to 0)	0 (0 to 0)	0 (0 to 0)	0 (0 to 0)	0 (0 to 0)	0 (0 to 0)	0·1 (0 to 0·2)	0·3 (0·1 to 0·6)	0·4 (0·2 to 0·9)	0·7 (0·3 to 1·2)	0·9 (0·4 to 1·6)	1 (0·5 to 1·8)	1·2 (0·6 to 2·1)	1·2 (0·5 to 2·2)	1·2 (0·6 to 2·2)	1 (0·5 to 1·7)	0·9 (0·4 to 1·4)	0·7 (0·4 to 1·1)	0·5 (0·3 to 0·9)	0·3 (0·2 to 0·5)	0·1 (0·1 to 0·2)	0 (0 to 0)	10·4
Other musculoskeletal disorders	0 (0 to 0)	0 (0 to 0)	0 (0 to 0)	0 (0 to 0)	0·2 (0·2 to 0·4)	1·8 (1 to 2·9)	9·7 (5·7 to 15·2)	23·4 (15·3 to 33·5)	39·4 (26·2 to 57·3)	49·7 (32·6 to 72)	55·8 (36 to 82·4)	61·1 (38·5 to 90·6)	62·4 (40·9 to 91·8)	63·2 (40·1 to 96·6)	61·5 (38·7 to 91·5)	53 (31·6 to 84·1)	37·1 (22·2 to 56·3)	26 (14·9 to 41)	15·7 (9·1 to 24·2)	9·1 (4·4 to 15·1)	5·7 (3·5 to 8·2)	1·6 (1·1 to 2·4)	0·4 (0·2 to 0·6)	577
Other non-communicable diseases	120 (78·2 to 177)	66·3 (44·5 to 96·6)	164 (110 to 242)	80·9 (58·3 to 112)	50·5 (39·5 to 63·6)	67·3 (53·9 to 82·4)	88·4 (68·5 to 115)	109 (83·8 to 147)	132 (102 to 173)	141 (106 to 189)	158 (121 to 215)	168 (127 to 226)	166 (126 to 219)	161 (123 to 211)	154 (117 to 201)	144 (110 to 188)	117 (88·8 to 152)	101 (76·8 to 129)	81·4 (64·5 to 103)	64·6 (52 to 79·6)	38·2 (31·6 to 45·9)	12·7 (10·6 to 15·1)	2·8 (2·2 to 3·5)	2,390
Congenital birth defects	115 (74·7 to 171)	58·9 (39 to 86·5)	108 (70·2 to 160)	41·8 (29·3 to 59·7)	19·2 (15·1 to 24·2)	23·1 (18 to 29)	20·7 (16·2 to 26)	15·5 (12·3 to 19·2)	13·7 (10·8 to 17)	10·6 (8·2 to 13·2)	10·4 (8·2 to 13·1)	9·5 (7·6 to 11·9)	9·4 (7·4 to 11·9)	8·5 (6·8 to 10·6)	6·9 (5·4 to 8·6)	4·3 (3·3 to 5·4)	2·6 (2 to 3·3)	0·9 (0·6 to 1·3)	0·5 (0·3 to 0·8)	0·3 (0·2 to 0·5)	0·1 (0·1 to 0·2)	0 (0 to 0·1)	0 (0 to 0)	480
Neural tube defects	11·3 (6·4 to 18·6)	5·4 (2·8 to 10)	9·3 (4·6 to 18·2)	5·7 (2·8 to 10)	0·9 (0·6 to 1·2)	1·9 (1·2 to 2·8)	1·3 (0·9 to 1·8)	0·6 (0·4 to 0·8)	0·7 (0·5 to 1·1)	0·6 (0·4 to 0·8)	0·4 (0·3 to 0·6)	0·4 (0·3 to 0·5)	0·2 (0·1 to 0·3)	0·1 (0·1 to 0·2)	0·2 (0·1 to 0·2)	0·1 (0·1 to 0·1)	0·1 (0·1 to 0·1)	0 (0 to 0)	0 (0 to 0)	0 (0 to 0)	0 (0 to 0)	0 (0 to 0)	0 (0 to 0)	39·1
Congenital heart anomalies	42·3 (25·9 to 65·8)	27·4 (17·6 to 41·8)	51·1 (31·6 to 78·5)	15 (9 to 23·8)	5·4 (3·7 to 7·5)	6·7 (4·4 to 9·1)	6·1 (4·3 to 8·3)	4·6 (3·3 to 6·3)	4·3 (3 to 5·9)	2·9 (2 to 4·1)	3·2 (2·2 to 4·3)	2·7 (1·9 to 3·5)	2·5 (1·8 to 3·4)	2·1 (1·6 to 2·8)	1·2 (0·8 to 1·7)	0·8 (0·5 to 1·1)	0·5 (0·3 to 0·6)	0·1 (0 to 0·1)	0 (0 to 0·1)	0 (0 to 0·1)	0 (0 to 0)	0 (0 to 0)	0 (0 to 0)	179
Orofacial clefts	0·3 (0·1 to 0·7)	0·1 (0·1 to 0·2)	0·5 (0·2 to 1·2)	0·3 (0·2 to 0·6)	0·3 (0·1 to 0·5)	0·3 (0·1 to 0·5)	0·3 (0·1 to 0·5)	0·3 (0·1 to 0·5)	0·2 (0·1 to 0·4)	0·2 (0·1 to 0·4)	0·2 (0·1 to 0·4)	0·2 (0·1 to 0·4)	0·2 (0·1 to 0·4)	0·2 (0·1 to 0·4)	0·2 (0·1 to 0·3)	0·1 (0·1 to 0·3)	0·1 (0 to 0·2)	0·1 (0 to 0·1)	0·1 (0 to 0·1)	0 (0 to 0·1)	0 (0 to 0)	0 (0 to 0)	0 (0 to 0)	4·2
Down syndrome	1·8 (1 to 3·2)	0·9 (0·5 to 1·6)	4·4 (2·2 to 7·8)	2·3 (1·3 to 3·9)	0·7 (0·5 to 1·1)	1·2 (0·8 to 1·8)	1·9 (1·2 to 2·9)	1·2 (0·8 to 1·8)	1·3 (0·8 to 2)	1·2 (0·7 to 1·8)	1·4 (0·8 to 2·1)	1·4 (0·8 to 2·2)	1·7 (1·1 to 2·5)	1·6 (1 to 2·5)	1·7 (1·1 to 2·5)	0·7 (0·5 to 1·1)	0·3 (0·2 to 0·5)	0 (0 to 0)	0 (0 to 0)	0 (0 to 0)	0 (0 to 0)	0 (0 to 0)	0 (0 to 0)	25·8
Turner syndrome	0 (0 to 0)	0 (0 to 0)	0 (0 to 0)	0 (0 to 0)	0 (0 to 0)	0 (0 to 0)	0 (0 to 0)	0 (0 to 0)	0 (0 to 0)	0 (0 to 0)	0 (0 to 0)	0 (0 to 0)	0 (0 to 0)	0 (0 to 0)	0 (0 to 0)	0 (0 to 0)	0 (0 to 0)	0 (0 to 0)	0 (0 to 0)	0 (0 to 0)	0 (0 to 0)	0 (0 to 0)	0 (0 to 0)	0·1
Klinefelter syndrome	0 (0 to 0)	0 (0 to 0)	0 (0 to 0)	0 (0 to 0)	0 (0 to 0)	0 (0 to 0)	0 (0 to 0)	0 (0 to 0)	0 (0 to 0)	0 (0 to 0)	0 (0 to 0)	0 (0 to 0)	0 (0 to 0)	0 (0 to 0)	0 (0 to 0)	0 (0 to 0)	0 (0 to 0)	0 (0 to 0)	0 (0 to 0)	0 (0 to 0)	0 (0 to 0)	0 (0 to 0)	0 (0 to 0)	0
Other chromosomal abnormalities	3 (1·6 to 5·1)	1·4 (0·8 to 2·3)	2·9 (1·9 to 4·5)	1·4 (0·9 to 1·9)	0·8 (0·5 to 1·1)	0·8 (0·5 to 1·1)	0·8 (0·6 to 1·2)	0·6 (0·4 to 0·8)	0·5 (0·4 to 0·7)	0·3 (0·2 to 0·4)	0·3 (0·2 to 0·3)	0·2 (0·1 to 0·2)	0·1 (0·1 to 0·2)	0·1 (0·1 to 0·1)	0 (0 to 0·1)	0 (0 to 0)	0 (0 to 0)	0 (0 to 0)	0 (0 to 0)	0 (0 to 0)	0 (0 to 0)	0 (0 to 0)	0 (0 to 0)	13·1
Congenital musculoskeletal and limb anomalies	3·2 (1·8 to 5·4)	1·6 (0·9 to 2·8)	4·7 (3 to 7·1)	5·4 (3·3 to 8·2)	5·6 (3·4 to 8·5)	5·7 (3·4 to 8·9)	5 (2·9 to 7·6)	3·4 (2·1 to 4·9)	2·8 (1·8 to 4·2)	2·4 (1·5 to 3·7)	2·5 (1·6 to 3·6)	2·5 (1·5 to 3·6)	2·4 (1·5 to 3·6)	2·2 (1·4 to 3·3)	2 (1·3 to 3)	1·6 (0·9 to 2·3)	1 (0·6 to 1·4)	0·5 (0·3 to 0·8)	0·3 (0·2 to 0·4)	0·1 (0·1 to 0·2)	0·1 (0 to 0·1)	0 (0 to 0)	0 (0 to 0)	54·9
Urogenital congenital anomalies	5·2 (2·7 to 10)	1·8 (1 to 3·2)	2·1 (1·2 to 3·8)	1 (0·6 to 1·5)	0·6 (0·4 to 0·8)	0·6 (0·4 to 0·9)	0·3 (0·2 to 0·5)	0·3 (0·2 to 0·4)	0·3 (0·2 to 0·4)	0·2 (0·1 to 0·3)	0·1 (0·1 to 0·2)	0·1 (0·1 to 0·2)	0·2 (0·1 to 0·3)	0·1 (0·1 to 0·1)	0·1 (0·1 to 0·2)	0·1 (0 to 0·1)	0 (0 to 0·1)	0 (0 to 0)	0 (0 to 0)	0 (0 to 0)	0 (0 to 0)	0 (0 to 0)	0 (0 to 0)	13·2
Digestive congenital anomalies	16·8 (9·7 to 27·9)	9·6 (5·5 to 15·3)	11·6 (6·6 to 19·7)	2·4 (1·5 to 4)	0·7 (0·4 to 1)	0·5 (0·3 to 0·7)	0·3 (0·2 to 0·5)	0·1 (0·1 to 0·2)	0·1 (0·1 to 0·2)	0·1 (0·1 to 0·1)	0·1 (0 to 0·1)	0·1 (0·1 to 0·1)	0·1 (0·1 to 0·2)	0·1 (0·1 to 0·1)	0·1 (0 to 0·1)	0·1 (0·1 to 0·1)	0 (0 to 0·1)	0 (0 to 0)	0 (0 to 0)	0 (0 to 0)	0 (0 to 0)	0 (0 to 0)	0 (0 to 0)	42·7
Other congenital birth defects	31·4 (19 to 49·2)	10·7 (6·5 to 16·9)	20·9 (12·5 to 32·5)	8·3 (5·1 to 12·8)	4·4 (3·1 to 5·9)	5·5 (3·9 to 7·6)	4·6 (3·2 to 6·5)	4·4 (3·1 to 5·9)	3·3 (2·3 to 4·6)	2·6 (1·8 to 3·7)	2·3 (1·6 to 3·3)	2 (1·4 to 3)	2 (1·4 to 3)	2 (1·4 to 2·8)	1·4 (0·9 to 2·1)	0·8 (0·5 to 1·4)	0·6 (0·4 to 1)	0·2 (0·1 to 0·5)	0·1 (0 to 0·4)	0·1 (0 to 0·3)	0 (0 to 0·1)	0 (0 to 0)	0 (0 to 0)	108
Urinary diseases and male infertility	0·2 (0·1 to 0·3)	0·5 (0·3 to 0·8)	2·3 (1·4 to 3·6)	1·2 (0·8 to 1·8)	0·8 (0·6 to 1·1)	1·7 (1·3 to 2·2)	2·2 (1·7 to 2·8)	4·2 (3 to 5·9)	5·1 (3·7 to 7)	6·4 (4·7 to 9)	8·6 (6·2 to 11·4)	10·3 (7·7 to 13·3)	12·3 (9·2 to 16)	16 (12·2 to 20·4)	20·6 (15·5 to 25·9)	25 (18·8 to 32·6)	24·8 (18·9 to 31·8)	25·7 (19·6 to 32·7)	23·8 (17·8 to 31·4)	20·9 (15·9 to 27)	13 (9·8 to 16·7)	4·5 (3·2 to 5·7)	1 (0·7 to 1·3)	231
Urolithiasis	0 (0 to 0)	0 (0 to 0)	0 (0 to 0)	0 (0 to 0)	0 (0 to 0)	0 (0 to 0·1)	0·1 (0 to 0·1)	0·1 (0·1 to 0·2)	0·2 (0·1 to 0·3)	0·3 (0·2 to 0·6)	0·6 (0·3 to 1)	0·7 (0·4 to 1·2)	1·4 (0·8 to 2·2)	1·4 (0·8 to 2·1)	1·6 (0·9 to 2·5)	1·4 (0·8 to 2)	0·9 (0·5 to 1·4)	0·7 (0·4 to 1·1)	0·5 (0·3 to 0·9)	0·2 (0·1 to 0·4)	0·1 (0·1 to 0·2)	0 (0 to 0·1)	0 (0 to 0)	10·4
Benign prostatic hyperplasia	0 (0 to 0)	0 (0 to 0)	0 (0 to 0)	0 (0 to 0)	0 (0 to 0)	0 (0 to 0)	0 (0 to 0)	0 (0 to 0)	0 (0 to 0)	0 (0 to 0)	0 (0 to 0)	0 (0 to 0·1)	0·3 (0·1 to 0·7)	1·3 (0·6 to 2·3)	4·3 (2 to 7·5)	7·6 (3·9 to 13·2)	8·8 (5 to 14·1)	8·5 (4·7 to 13·8)	5·7 (3·2 to 9·2)	3·3 (1·8 to 5·3)	1·2 (0·6 to 1·9)	0·2 (0·1 to 0·4)	0 (0 to 0)	41·3
Male infertility	0 (0 to 0)	0 (0 to 0)	0 (0 to 0)	0 (0 to 0)	0 (0 to 0)	0 (0 to 0)	0 (0 to 0)	0·8 (0·2 to 2·3)	1 (0·3 to 2·4)	1 (0·3 to 2·7)	1 (0·3 to 2·6)	0·9 (0·3 to 2·4)	0·1 (0 to 0·4)	0 (0 to 0)	0 (0 to 0)	0 (0 to 0)	0 (0 to 0)	0 (0 to 0)	0 (0 to 0)	0 (0 to 0)	0 (0 to 0)	0 (0 to 0)	0 (0 to 0)	4·9
Other urinary diseases	0 (0 to 0·1)	0·1 (0·1 to 0·3)	0·1 (0 to 0·1)	0·3 (0·2 to 0·4)	0·2 (0·1 to 0·2)	0·3 (0·2 to 0·4)	0·3 (0·2 to 0·4)	0·3 (0·2 to 0·5)	0·2 (0·2 to 0·4)	0·4 (0·2 to 0·5)	0·6 (0·4 to 0·9)	0·6 (0·4 to 0·9)	0·9 (0·5 to 1·4)	0·8 (0·5 to 1·3)	0·8 (0·5 to 1·2)	1 (0·6 to 1·6)	0·8 (0·4 to 1·4)	0·6 (0·3 to 1)	0·6 (0·3 to 1·1)	0·5 (0·2 to 0·9)	0·3 (0·1 to 0·5)	0·1 (0·1 to 0·2)	0 (0 to 0)	9·9
Gynecological diseases	0 (0 to 0)	0 (0 to 0)	0 (0 to 0)	0 (0 to 0)	0 (0 to 0)	6·3 (3·1 to 11)	20·6 (11·9 to 33·3)	35·7 (22·6 to 54·7)	49·2 (32·5 to 72·1)	57 (37·3 to 82·1)	63·4 (42·4 to 91·1)	62·7 (41·3 to 90)	49·6 (32·4 to 70·3)	33·5 (20·4 to 49·6)	20·2 (12·6 to 29·2)	13·2 (7·6 to 20·2)	8·4 (5·2 to 12·6)	5·6 (3·5 to 8·2)	3·7 (2·3 to 5·5)	2·5 (1·5 to 3·8)	1·2 (0·8 to 1·8)	0·4 (0·3 to 0·6)	0·1 (0·1 to 0·2)	433
Uterine fibroids	0 (0 to 0)	0 (0 to 0)	0 (0 to 0)	0 (0 to 0)	0 (0 to 0)	0·1 (0 to 0·2)	0·2 (0·1 to 0·5)	0·6 (0·3 to 1·2)	1·4 (0·6 to 3)	3 (1·4 to 6)	5·2 (2·4 to 10·2)	6 (3 to 11·3)	5·2 (2·6 to 9·4)	3·6 (1·7 to 6·8)	2·4 (1·1 to 4·4)	1·4 (0·6 to 2·7)	0·7 (0·3 to 1·4)	0·4 (0·2 to 0·7)	0·2 (0·1 to 0·4)	0·1 (0 to 0·2)	0 (0 to 0·1)	0 (0 to 0)	0 (0 to 0)	30·5
Polycystic ovarian syndrome	0 (0 to 0)	0 (0 to 0)	0 (0 to 0)	0 (0 to 0)	0 (0 to 0)	0·2 (0·1 to 0·5)	1 (0·4 to 2·1)	1·2 (0·5 to 2·4)	1 (0·4 to 2·2)	1 (0·4 to 2)	0·9 (0·4 to 1·9)	0·9 (0·4 to 1·8)	0·7 (0·3 to 1·4)	0·2 (0·1 to 0·4)	0 (0 to 0)	0 (0 to 0)	0 (0 to 0)	0 (0 to 0)	0 (0 to 0)	0 (0 to 0)	0 (0 to 0)	0 (0 to 0)	0 (0 to 0)	7·1
Female infertility	0 (0 to 0)	0 (0 to 0)	0 (0 to 0)	0 (0 to 0)	0 (0 to 0)	0 (0 to 0)	0 (0 to 0)	1·2 (0·2 to 3·3)	1·6 (0·4 to 4·4)	2 (0·4 to 5·8)	2·2 (0·3 to 6·6)	1·9 (0·2 to 5·5)	0 (0 to 0·2)	0 (0 to 0)	0 (0 to 0)	0 (0 to 0)	0 (0 to 0)	0 (0 to 0)	0 (0 to 0)	0 (0 to 0)	0 (0 to 0)	0 (0 to 0)	0 (0 to 0)	8·9
Endometriosis	0 (0 to 0)	0 (0 to 0)	0 (0 to 0)	0 (0 to 0)	0 (0 to 0)	0 (0 to 0)	1·3 (0·5 to 2·7)	5·3 (2·5 to 9·9)	5·8 (2·8 to 10·5)	4·6 (2·3 to 8·4)	4·1 (2·2 to 7·4)	4 (2 to 7)	3·3 (1·8 to 5·4)	1·6 (0·8 to 2·7)	0 (0 to 0)	0 (0 to 0)	0 (0 to 0)	0 (0 to 0)	0 (0 to 0)	0 (0 to 0)	0 (0 to 0)	0 (0 to 0)	0 (0 to 0)	30·2
Genital prolapse	0 (0 to 0)	0 (0 to 0)	0 (0 to 0)	0 (0 to 0)	0 (0 to 0)	0 (0 to 0)	0 (0 to 0)	0 (0 to 0·1)	0·1 (0 to 0·2)	0·2 (0·1 to 0·5)	0·5 (0·2 to 0·9)	0·7 (0·3 to 1·6)	0·9 (0·4 to 1·9)	1·1 (0·4 to 2·1)	1·1 (0·5 to 2·1)	1 (0·4 to 1·9)	0·7 (0·3 to 1·4)	0·5 (0·3 to 1)	0·4 (0·2 to 0·7)	0·3 (0·1 to 0·5)	0·1 (0·1 to 0·3)	0·1 (0 to 0·1)	0 (0 to 0)	7·7
Premenstrual syndrome	0 (0 to 0)	0 (0 to 0)	0 (0 to 0)	0 (0 to 0)	0 (0 to 0)	5·7 (2·7 to 10·2)	13 (6·5 to 23·2)	16·2 (8·2 to 29)	15·4 (8·5 to 25·3)	15 (7·7 to 24·6)	17 (9·7 to 26·9)	16·6 (9·4 to 26·4)	11 (6·1 to 18·7)	3·7 (1·8 to 6·8)	0 (0 to 0)	0 (0 to 0)	0 (0 to 0)	0 (0 to 0)	0 (0 to 0)	0 (0 to 0)	0 (0 to 0)	0 (0 to 0)	0 (0 to 0)	114
Other gynecological diseases	0 (0 to 0)	0 (0 to 0)	0 (0 to 0)	0 (0 to 0)	0 (0 to 0)	0·3 (0·1 to 0·7)	5 (2 to 9·6)	11·2 (6·3 to 18)	23·8 (13·7 to 37·4)	31·2 (18·8 to 47·4)	33·5 (20·1 to 52·7)	32·6 (18·5 to 52)	28·5 (16·2 to 44·2)	23·4 (11·6 to 37·4)	16·7 (9·9 to 25·3)	10·8 (5·7 to 17·1)	7 (4 to 10·9)	4·7 (2·8 to 7)	3·1 (1·9 to 4·8)	2·1 (1·2 to 3·3)	1 (0·6 to 1·6)	0·4 (0·2 to 0·6)	0·1 (0 to 0·2)	235
Hemoglobinopathies and hemolytic anemias	0·5 (0·3 to 0·7)	0·7 (0·5 to 1)	4·6 (3 to 6·7)	11·2 (7·5 to 15·9)	13·9 (9·9 to 19·2)	16·9 (12·2 to 22·4)	21·6 (15·2 to 30·6)	23·7 (16·3 to 32·7)	22·5 (15·7 to 30·2)	16·9 (10·8 to 24·7)	14·7 (11 to 19·6)	15 (11·4 to 19·2)	14·4 (10·8 to 18·5)	13·2 (10·1 to 17)	10·9 (8·3 to 14·4)	9·1 (6·7 to 12·3)	5·7 (4·2 to 7·6)	4·7 (3·6 to 6·2)	3·8 (2·9 to 4·9)	3 (2·3 to 3·8)	2·7 (2·1 to 3·5)	0·9 (0·7 to 1·2)	0·2 (0·1 to 0·2)	231
Thalassemias	0 (0 to 0)	0 (0 to 0)	0 (0 to 0)	0·1 (0 to 0·1)	0 (0 to 0)	0 (0 to 0)	0 (0 to 0)	0 (0 to 0)	0 (0 to 0)	0 (0 to 0)	0 (0 to 0)	0 (0 to 0)	0 (0 to 0)	0 (0 to 0)	0 (0 to 0)	0 (0 to 0)	0 (0 to 0)	0 (0 to 0)	0 (0 to 0)	0 (0 to 0)	0 (0 to 0)	0 (0 to 0)	0 (0 to 0)	0·1
Thalassemias trait	0 (0 to 0)	0 (0 to 0)	0·1 (0 to 0·1)	0·3 (0·1 to 0·5)	0·4 (0·2 to 0·8)	0·2 (0·1 to 0·5)	0·6 (0·3 to 1)	0·5 (0·3 to 0·9)	0·5 (0·3 to 0·8)	0·5 (0·3 to 0·8)	0·5 (0·2 to 0·8)	0·5 (0·2 to 0·8)	0·1 (0·1 to 0·3)	0·2 (0·1 to 0·3)	0·2 (0·1 to 0·3)	0·1 (0·1 to 0·2)	0·1 (0 to 0·1)	0·1 (0 to 0·1)	0 (0 to 0·1)	0 (0 to 0·1)	0 (0 to 0)	0 (0 to 0)	0 (0 to 0)	4·9
Sickle cell disorders	0·4 (0·3 to 0·7)	0·4 (0·3 to 0·7)	3·5 (2·1 to 5·6)	6·6 (4·2 to 10·2)	7·2 (5·4 to 9·4)	12·8 (8·8 to 17·7)	13·8 (9·8 to 19)	12·4 (8·8 to 17·1)	11 (7·6 to 15·6)	7·8 (5·2 to 11)	7·8 (5·2 to 11·1)	7·1 (4·8 to 10)	6·4 (4·2 to 9·1)	4·4 (2·6 to 6·5)	1·8 (1·1 to 3)	0·5 (0·3 to 1)	0·1 (0 to 0·2)	0 (0 to 0)	0 (0 to 0)	0 (0 to 0)	0 (0 to 0)	0 (0 to 0)	0 (0 to 0)	104
Sickle cell trait	0 (0 to 0)	0·2 (0·1 to 0·3)	0·6 (0·3 to 1)	2·6 (1·3 to 4·4)	3·8 (1·8 to 6·5)	2·2 (1 to 4)	4·3 (1 to 10·8)	7·5 (2·2 to 14·1)	7·2 (2·1 to 13·1)	4·9 (1 to 10·9)	0·6 (0·2 to 1·2)	1·4 (0·7 to 2·3)	1·6 (0·8 to 2·7)	1·7 (0·9 to 2·8)	1·7 (0·9 to 2·8)	1·2 (0·6 to 2·1)	0·7 (0·3 to 1·3)	0·6 (0·3 to 1)	0·5 (0·2 to 0·8)	0·4 (0·2 to 0·6)	0·2 (0·1 to 0·4)	0·1 (0 to 0·1)	0 (0 to 0)	44
G6PD deficiency	0 (0 to 0)	0 (0 to 0)	0 (0 to 0·1)	0·3 (0·1 to 0·6)	0·3 (0·2 to 0·6)	0·6 (0·2 to 1·1)	1·2 (0·5 to 2·3)	1·6 (0·7 to 2·9)	2·1 (1 to 3·7)	1·9 (0·9 to 3·2)	3·3 (2 to 5)	3·1 (1·8 to 4·8)	3·3 (1·9 to 4·9)	3·2 (1·9 to 4·6)	2·9 (1·8 to 4·2)	2 (1·2 to 2·9)	1·1 (0·6 to 1·5)	0·6 (0·4 to 1)	0·3 (0·2 to 0·5)	0·1 (0·1 to 0·2)	0·1 (0 to 0·1)	0 (0 to 0)	0 (0 to 0)	28·2
G6PD trait	0 (0 to 0)	0 (0 to 0)	0 (0 to 0)	0 (0 to 0)	0 (0 to 0)	0 (0 to 0)	0 (0 to 0)	0 (0 to 0)	0 (0 to 0)	0 (0 to 0)	0·1 (0 to 0·2)	0·1 (0 to 0·2)	0 (0 to 0)	0 (0 to 0)	0 (0 to 0)	0 (0 to 0)	0 (0 to 0)	0 (0 to 0)	0 (0 to 0)	0 (0 to 0)	0 (0 to 0)	0 (0 to 0)	0 (0 to 0)	0·2
Other hemoglobinopathies and hemolytic anemias	0 (0 to 0)	0 (0 to 0·1)	0·4 (0·2 to 0·6)	1·4 (0·6 to 2·6)	2 (0·8 to 3·9)	1·1 (0·5 to 2·1)	1·7 (0·8 to 3·2)	1·6 (0·8 to 2·8)	1·7 (0·9 to 2·9)	1·8 (1 to 2·9)	2·5 (1·4 to 4)	2·9 (1·7 to 4·4)	3 (1·8 to 4·6)	3·8 (2·5 to 5·4)	4·3 (3 to 6·1)	5·3 (3·7 to 7·4)	3·7 (2·7 to 5·1)	3·4 (2·5 to 4·5)	2·9 (2·2 to 3·8)	2·5 (1·9 to 3·1)	2·4 (1·8 to 3)	0·9 (0·7 to 1·1)	0·2 (0·1 to 0·2)	49·2
Endocrine, metabolic, blood, and immune disorders	4·3 (2·6 to 6·9)	4·9 (2·9 to 7·9)	42·1 (25·2 to 66·9)	22·8 (14·2 to 36·9)	11·4 (8 to 15·9)	13·3 (9·7 to 18·9)	14·5 (10·6 to 19·8)	16·5 (12·3 to 22·3)	23·7 (17·7 to 31·9)	27·8 (20·8 to 37·5)	34·8 (26·2 to 45·7)	40·3 (29·7 to 54·8)	46·4 (34·4 to 62·5)	51·8 (38·8 to 69·5)	52 (37·9 to 71·9)	47·1 (33·8 to 67)	36·1 (26·2 to 53)	30·9 (22·6 to 43·7)	25·2 (18·5 to 34·5)	20·5 (15 to 28·5)	12·8 (9·6 to 17·9)	4·4 (3·3 to 6·2)	1 (0·7 to 1·6)	585
Oral disorders	0 (0 to 0)	0 (0 to 0)	0 (0 to 0)	3·9 (2·1 to 6·3)	5·3 (2·8 to 9·2)	6 (3·2 to 10·5)	8·8 (4·8 to 15·2)	13·4 (7 to 25·3)	18 (9 to 34·1)	22 (10·4 to 40·9)	26·6 (12·5 to 50·1)	30·2 (14·5 to 55·2)	33·3 (17·3 to 60·4)	37·9 (20·5 to 66·6)	43 (25 to 69·3)	45·6 (27·5 to 73·1)	39·2 (23·7 to 60·6)	32·7 (20·4 to 50·3)	24·4 (15·2 to 36·4)	17·4 (10·9 to 25·7)	8·2 (5·1 to 12·1)	2·5 (1·5 to 3·6)	0·5 (0·3 to 0·8)	419
Caries of deciduous teeth	0 (0 to 0)	0 (0 to 0)	0 (0 to 0)	1·3 (0·5 to 2·6)	1·4 (0·6 to 3)	0·1 (0 to 0·2)	0 (0 to 0)	0 (0 to 0)	0 (0 to 0)	0 (0 to 0)	0 (0 to 0)	0 (0 to 0)	0 (0 to 0)	0 (0 to 0)	0 (0 to 0)	0 (0 to 0)	0 (0 to 0)	0 (0 to 0)	0 (0 to 0)	0 (0 to 0)	0 (0 to 0)	0 (0 to 0)	0 (0 to 0)	2·8
Caries of permanent teeth	0 (0 to 0)	0 (0 to 0)	0 (0 to 0)	0 (0 to 0)	1·4 (0·5 to 3)	3·1 (1·3 to 6·8)	3·4 (1·4 to 7·3)	3·7 (1·5 to 7·6)	3·4 (1·4 to 7)	3·1 (1·3 to 6·4)	3·2 (1·3 to 6·4)	3 (1·3 to 6)	2·8 (1·2 to 5·8)	2·7 (1·1 to 5·7)	2·3 (0·9 to 5·1)	1·8 (0·7 to 3·9)	1·2 (0·4 to 2·4)	0·8 (0·3 to 1·7)	0·5 (0·2 to 1·1)	0·4 (0·1 to 0·7)	0·2 (0·1 to 0·3)	0 (0 to 0·1)	0 (0 to 0)	37
Periodontal diseases	0 (0 to 0)	0 (0 to 0)	0 (0 to 0)	0 (0 to 0)	0 (0 to 0)	0 (0 to 0)	1·1 (0·3 to 3)	4·1 (1·2 to 10·3)	8·4 (2·7 to 20·2)	12 (4·1 to 27·5)	15·5 (5·5 to 35·1)	17·8 (6·4 to 38·7)	18·5 (7 to 38·7)	17·9 (7 to 37·6)	15·8 (6·3 to 33·3)	12 (4·8 to 24·6)	7·5 (2·9 to 15·5)	5 (2 to 10·1)	3·3 (1·3 to 6·6)	2·2 (0·9 to 4·3)	1 (0·4 to 2·1)	0·3 (0·1 to 0·6)	0·1 (0 to 0·1)	142
Other oral disorders	0 (0 to 0)	0 (0 to 0)	0 (0 to 0)	2·5 (1·5 to 3·9)	2·5 (1·5 to 3·9)	2·8 (1·7 to 4·3)	4·3 (2·6 to 6·7)	5·1 (3·1 to 7·8)	5·2 (3 to 7·8)	5·1 (3·1 to 7·6)	5·2 (3·2 to 7·9)	5·3 (3·3 to 8)	5·2 (3·1 to 7·7)	5 (3·1 to 7·3)	4·5 (2·8 to 6·8)	3·8 (2·3 to 5·7)	2·6 (1·6 to 4)	1·8 (1·1 to 2·7)	1 (0·6 to 1·5)	0·6 (0·3 to 0·8)	0·2 (0·1 to 0·3)	0·1 (0 to 0·1)	0 (0 to 0)	62·6
Sudden infant death syndrome	--	1·3 (0·8 to 2)	7·7 (4·5 to 12·9)	--	--	--	--	--	--	--	--	--	--	--	--	--	--	--	--	--	--	--	--	NA
Injuries	8·8 (5·9 to 12·9)	4·8 (3·2 to 7·1)	33·4 (21 to 49·5)	66·1 (45·2 to 92·5)	61·5 (51·3 to 72·8)	128 (109 to 148)	326 (282 to 374)	422 (369 to 477)	447 (391 to 505)	359 (311 to 412)	350 (298 to 406)	322 (270 to 378)	326 (270 to 384)	270 (228 to 317)	219 (187 to 257)	163 (137 to 192)	116 (98·2 to 136)	91·3 (78·1 to 107)	73·5 (62·8 to 86·1)	61·5 (52·4 to 71)	38·7 (33·4 to 44·4)	14·6 (12·3 to 16·6)	3·2 (2·7 to 3·7)	3,910
Transport injuries	0·2 (0·2 to 0·4)	0·2 (0·1 to 0·3)	1·6 (1·1 to 2·4)	6·4 (4 to 9·8)	9·4 (7·2 to 12·2)	22·5 (17 to 29·6)	54·6 (40·2 to 72·3)	64·3 (48·5 to 82·9)	64·9 (51 to 80·1)	55·1 (43·6 to 68·8)	54·9 (43·6 to 68·7)	54·6 (43·2 to 67·4)	58·6 (45·1 to 72·2)	49·6 (40·1 to 60·5)	40·4 (33·3 to 48·6)	28·8 (23·6 to 34·2)	19·8 (16·3 to 23·8)	14 (11·6 to 16·7)	10 (8·2 to 12)	6·5 (5·2 to 7·8)	3·1 (2·5 to 3·7)	0·8 (0·7 to 1)	0·2 (0·1 to 0·2)	621
Road injuries	0·2 (0·1 to 0·3)	0·1 (0·1 to 0·2)	1·3 (0·8 to 2)	5·7 (3·5 to 9)	8·5 (6·2 to 11·2)	20·2 (15 to 26·8)	50·5 (36·8 to 67·8)	59·3 (44·3 to 77·3)	59·6 (46·4 to 74·6)	50·3 (39·6 to 63·3)	50·1 (39·2 to 62·9)	49·5 (38·9 to 60·9)	53·8 (41·4 to 66·8)	45·5 (36·6 to 55·9)	36·7 (29·9 to 44·1)	26·7 (21·8 to 31·8)	18·6 (15·4 to 22·3)	12·9 (10·6 to 15·4)	9·3 (7·6 to 11·1)	6·1 (4·9 to 7·3)	2·9 (2·3 to 3·5)	0·8 (0·6 to 0·9)	0·1 (0·1 to 0·2)	569
Pedestrian road injuries	0·1 (0·1 to 0·1)	0·1 (0 to 0·1)	0·4 (0·2 to 0·6)	2·5 (1·5 to 4)	3·6 (2·6 to 4·8)	7·1 (4·9 to 9·8)	6·8 (4·9 to 9·4)	7·1 (5·3 to 9·4)	8·1 (6·1 to 10·6)	8·2 (6·1 to 10·9)	9·3 (6·5 to 13·1)	10·1 (7·7 to 13·1)	12·1 (9·4 to 15·2)	11·4 (8·7 to 14·8)	10 (8·1 to 12·2)	7·7 (6·3 to 9·4)	6 (4·8 to 7·4)	4·5 (3·6 to 5·6)	3·7 (3 to 4·5)	2·6 (2·1 to 3·1)	1·3 (1 to 1·6)	0·3 (0·3 to 0·4)	0·1 (0 to 0·1)	123
Cyclist road injuries	0 (0 to 0)	0 (0 to 0)	0 (0 to 0)	0·4 (0·2 to 0·6)	0·7 (0·5 to 1·1)	3·1 (2 to 4·8)	6·6 (4·2 to 9·8)	6·2 (4·1 to 9·2)	6·2 (4·5 to 8·2)	5·9 (4·2 to 8)	6·4 (4·6 to 8·5)	6·6 (4·9 to 8·4)	6·9 (5·1 to 9·3)	6·8 (5·1 to 8·8)	5·8 (4·3 to 7·5)	4·5 (3·4 to 5·8)	3·2 (2·4 to 4)	2·1 (1·6 to 2·7)	1·4 (1 to 1·8)	0·9 (0·6 to 1·2)	0·4 (0·3 to 0·5)	0·1 (0·1 to 0·1)	0 (0 to 0)	74·1
Motorcyclist road injuries	0 (0 to 0)	0 (0 to 0)	0·1 (0·1 to 0·2)	0·3 (0·2 to 0·4)	0·5 (0·4 to 0·7)	1·2 (0·9 to 1·6)	9·1 (5·9 to 13·4)	13·1 (9·4 to 17·4)	14·1 (10 to 18·9)	11·1 (8 to 14·8)	10·1 (7·6 to 13·2)	8·5 (6·5 to 11)	8·8 (6·6 to 11·9)	6·7 (5·2 to 8·4)	5·2 (4·2 to 6·5)	3·4 (2·6 to 4·4)	2·3 (1·7 to 2·9)	1·5 (1·1 to 1·9)	1 (0·7 to 1·3)	0·7 (0·5 to 1)	0·3 (0·2 to 0·5)	0·1 (0·1 to 0·1)	0 (0 to 0)	98·3
Motor vehicle road injuries	0·1 (0·1 to 0·1)	0·1 (0 to 0·1)	0·8 (0·5 to 1·2)	2·4 (1·4 to 3·8)	3·6 (2·6 to 4·8)	8·5 (6 to 11·7)	27·5 (19·3 to 38)	32·2 (23·5 to 42·6)	30·4 (22·4 to 39·9)	24·3 (17·9 to 32·5)	23·5 (17·2 to 30·7)	23·3 (17·4 to 30·2)	24·9 (18·3 to 31·9)	19·6 (15·4 to 24·8)	14·7 (11·9 to 18)	10·2 (8·1 to 12·5)	6·5 (5·3 to 7·9)	4·3 (3·4 to 5·3)	2·9 (2·3 to 3·5)	1·7 (1·4 to 2)	0·8 (0·6 to 0·9)	0·2 (0·2 to 0·3)	0 (0 to 0·1)	263
Other road injuries	0 (0 to 0)	0 (0 to 0)	0 (0 to 0)	0·1 (0·1 to 0·2)	0·1 (0·1 to 0·2)	0·3 (0·2 to 0·4)	0·4 (0·3 to 0·6)	0·6 (0·5 to 0·9)	0·8 (0·6 to 1)	0·8 (0·6 to 1)	0·9 (0·6 to 1·1)	1 (0·7 to 1·3)	1·1 (0·8 to 1·4)	1 (0·8 to 1·4)	1 (0·7 to 1·3)	0·8 (0·6 to 1·1)	0·6 (0·4 to 0·8)	0·4 (0·3 to 0·6)	0·3 (0·2 to 0·4)	0·2 (0·1 to 0·3)	0·1 (0·1 to 0·1)	0 (0 to 0)	0 (0 to 0)	10·6
Other transport injuries	0·1 (0 to 0·1)	0·1 (0 to 0·1)	0·3 (0·2 to 0·5)	0·7 (0·5 to 1·1)	0·9 (0·7 to 1·2)	2·3 (1·8 to 3)	4·1 (2·8 to 6)	5 (3·5 to 7)	5·3 (3·8 to 7·3)	4·8 (3·2 to 6·9)	4·7 (3 to 6·9)	5·1 (3·7 to 7·1)	4·8 (3·1 to 6·8)	4 (2·9 to 5·7)	3·8 (2·7 to 5)	2·1 (1·6 to 2·8)	1·3 (1 to 1·6)	1·2 (0·9 to 1·5)	0·7 (0·6 to 1)	0·4 (0·3 to 0·5)	0·2 (0·2 to 0·2)	0·1 (0·1 to 0·1)	0 (0 to 0)	52
Unintentional injuries	4·6 (3 to 6·7)	3·8 (2·4 to 5·7)	26·3 (15·7 to 40)	50·1 (33·8 to 70·9)	44·4 (35·7 to 55·2)	80·5 (65 to 98·3)	120 (97·1 to 146)	119 (94·3 to 147)	125 (103 to 149)	114 (91·9 to 140)	122 (100 to 147)	127 (104 to 154)	135 (110 to 164)	128 (106 to 156)	114 (93·2 to 138)	93·9 (76·7 to 115)	70·2 (58 to 84·9)	60·4 (50·5 to 73·1)	52·1 (44·1 to 61·7)	48·7 (41 to 57·2)	32·6 (27·9 to 37·5)	13·1 (11·1 to 14·9)	3 (2·5 to 3·4)	1,690
Falls	0·1 (0·1 to 0·2)	0·1 (0·1 to 0·1)	1·2 (0·7 to 1·9)	2·9 (1·9 to 4·4)	3·5 (2·6 to 4·7)	7·4 (5·6 to 9·7)	13·9 (10·6 to 17·9)	15·6 (12·2 to 20)	17·7 (14 to 22·2)	18·3 (14·3 to 22·8)	22·9 (17·9 to 28·5)	26·2 (20·8 to 32·4)	32·1 (25·5 to 39·1)	35·4 (28·4 to 43·9)	34·5 (27·6 to 42·7)	31·4 (25·1 to 38·5)	25·9 (21·1 to 31·6)	25·8 (21 to 31·3)	24·6 (20·3 to 29·8)	27·3 (22·5 to 32·5)	20·2 (16·8 to 23·5)	8·2 (6·8 to 9·5)	1·7 (1·4 to 2)	397
Drowning	0·4 (0·3 to 0·6)	0·3 (0·2 to 0·4)	3·2 (1·8 to 5)	19·2 (12·1 to 28·4)	17·4 (12·8 to 23·2)	34·4 (26·2 to 43·6)	49·8 (37·5 to 64)	35·6 (25·1 to 48)	34·3 (25·7 to 44·8)	26·6 (19·6 to 34·9)	26·2 (20·3 to 32·8)	24·3 (18·4 to 31·9)	23·8 (17·9 to 31·2)	18·7 (13·6 to 24·9)	12·9 (9·3 to 17)	7·9 (6·2 to 10·1)	4·7 (3·6 to 6)	2·7 (2·1 to 3·4)	1·9 (1·5 to 2·4)	0·9 (0·7 to 1·1)	0·5 (0·4 to 0·7)	0·2 (0·2 to 0·2)	0·1 (0 to 0·1)	346
Fire, heat, and hot substances	0·2 (0·1 to 0·3)	0·3 (0·2 to 0·5)	3·3 (2 to 5·1)	9·1 (5·7 to 13·4)	7·2 (5·8 to 8·8)	10·7 (8·5 to 13·4)	12·5 (9·5 to 16)	14·8 (11·3 to 18·7)	16·5 (12·8 to 21·2)	17·1 (13·3 to 22·2)	18 (13·8 to 23·1)	18·4 (14·2 to 24·1)	18·7 (14·6 to 23·7)	17·2 (13·4 to 22·2)	15·3 (11·9 to 19·7)	12·7 (9·7 to 17)	8·6 (6·7 to 11·3)	7 (5·4 to 9·2)	5·5 (4·3 to 7)	4·3 (3·5 to 5·5)	2·8 (2·2 to 3·6)	1·1 (0·9 to 1·3)	0·3 (0·2 to 0·3)	222
Poisonings	0 (0 to 0)	0·1 (0 to 0·1)	0·2 (0·1 to 0·4)	0·8 (0·5 to 1·2)	0·5 (0·3 to 0·8)	0·7 (0·5 to 1·1)	0·9 (0·7 to 1·3)	1·1 (0·8 to 1·4)	1·2 (0·9 to 1·6)	1·2 (0·9 to 1·6)	1·1 (0·8 to 1·6)	1 (0·7 to 1·3)	1 (0·7 to 1·3)	0·8 (0·6 to 1·1)	0·7 (0·5 to 1)	0·6 (0·4 to 0·8)	0·4 (0·3 to 0·5)	0·3 (0·2 to 0·4)	0·2 (0·2 to 0·3)	0·2 (0·1 to 0·2)	0·1 (0·1 to 0·1)	0 (0 to 0)	0 (0 to 0)	13·2
Poisoning by carbon monoxide	0 (0 to 0)	0 (0 to 0)	0 (0 to 0·1)	0·1 (0·1 to 0·2)	0·1 (0·1 to 0·2)	0·2 (0·1 to 0·2)	0·2 (0·2 to 0·4)	0·3 (0·2 to 0·5)	0·4 (0·2 to 0·5)	0·3 (0·2 to 0·5)	0·3 (0·2 to 0·5)	0·3 (0·2 to 0·4)	0·2 (0·1 to 0·3)	0·2 (0·1 to 0·2)	0·2 (0·1 to 0·2)	0·2 (0·1 to 0·2)	0·1 (0·1 to 0·2)	0·1 (0·1 to 0·1)	0·1 (0·1 to 0·1)	0·1 (0 to 0·1)	0 (0 to 0·1)	0 (0 to 0)	0 (0 to 0)	3·3
Poisoning by other means	0 (0 to 0)	0 (0 to 0·1)	0·2 (0·1 to 0·3)	0·7 (0·4 to 1)	0·4 (0·3 to 0·6)	0·6 (0·4 to 0·8)	0·7 (0·5 to 1)	0·8 (0·5 to 1·1)	0·9 (0·7 to 1·2)	0·8 (0·6 to 1·1)	0·8 (0·6 to 1·1)	0·8 (0·5 to 1)	0·8 (0·6 to 1)	0·7 (0·5 to 0·9)	0·6 (0·4 to 0·8)	0·4 (0·3 to 0·6)	0·3 (0·2 to 0·4)	0·2 (0·2 to 0·3)	0·2 (0·1 to 0·2)	0·1 (0·1 to 0·2)	0·1 (0 to 0·1)	0 (0 to 0)	0 (0 to 0)	9·9
Exposure to mechanical forces	0·1 (0·1 to 0·2)	0·2 (0·1 to 0·3)	1 (0·7 to 1·4)	4·3 (3·1 to 5·9)	5·9 (4·6 to 7·7)	11 (8·5 to 13·9)	22·8 (18 to 28·5)	29·2 (22·8 to 36·1)	29·9 (23·5 to 37)	27·4 (21·5 to 34·8)	28·5 (22·3 to 36·4)	29·7 (23·3 to 37·9)	29·8 (23·2 to 38)	27·7 (21·3 to 36·2)	24·4 (18·5 to 32·2)	19·1 (14·2 to 25·8)	13 (9·5 to 17·7)	9·7 (7 to 13·3)	6·8 (5 to 9·3)	4·9 (3·6 to 6·6)	2·6 (1·9 to 3·5)	0·9 (0·7 to 1·2)	0·3 (0·2 to 0·3)	329
Unintentional firearm injuries	0 (0 to 0)	0 (0 to 0)	0·1 (0·1 to 0·2)	0·4 (0·2 to 0·6)	0·6 (0·4 to 0·9)	2·4 (1·6 to 3·4)	10·5 (7·5 to 13·9)	14·8 (10·5 to 19·6)	13·9 (10 to 18·6)	11 (7·8 to 15)	10·4 (7·3 to 13·9)	9·9 (7·3 to 13·2)	8·9 (6·4 to 11·9)	6·7 (4·8 to 8·7)	5·1 (3·9 to 6·5)	3·3 (2·5 to 4·1)	2 (1·5 to 2·6)	1·4 (1·1 to 1·7)	0·9 (0·7 to 1·1)	0·7 (0·5 to 0·9)	0·4 (0·4 to 0·6)	0·2 (0·2 to 0·3)	0·1 (0·1 to 0·1)	104
Other exposure to mechanical forces	0·1 (0·1 to 0·2)	0·2 (0·1 to 0·3)	0·9 (0·6 to 1·2)	3·9 (2·8 to 5·3)	5·3 (4 to 6·9)	8·6 (6·5 to 11·1)	12·3 (9·2 to 16·3)	14·4 (10·5 to 19·5)	16 (12 to 21·3)	16·4 (12·2 to 22·3)	18·1 (13·4 to 24·7)	19·8 (14·6 to 27)	20·9 (15·4 to 28·2)	21·1 (15·5 to 28·9)	19·3 (14 to 26·4)	15·8 (11·2 to 21·9)	11 (7·8 to 15·2)	8·3 (5·9 to 11·6)	5·9 (4·2 to 8·2)	4·2 (3 to 5·8)	2·1 (1·6 to 2·9)	0·7 (0·5 to 1)	0·2 (0·1 to 0·2)	226
Adverse effects of medical treatment	0·9 (0·6 to 1·4)	0·7 (0·5 to 1·1)	2·4 (1·4 to 3·8)	1·7 (1·1 to 2·6)	0·9 (0·7 to 1·2)	1·2 (0·9 to 1·7)	1·6 (1·1 to 2·1)	2·6 (1·8 to 3·5)	2·9 (2 to 3·9)	2·5 (1·8 to 3·5)	3 (2·2 to 4)	3·7 (2·7 to 4·9)	4·5 (3·2 to 6·2)	5·4 (3·8 to 7·4)	5·4 (4 to 7·2)	4·9 (3·5 to 6·7)	4·2 (3·1 to 5·5)	4 (3 to 5·3)	4 (3 to 5·2)	3·5 (2·7 to 4·4)	2·2 (1·7 to 2·7)	0·7 (0·6 to 0·9)	0·2 (0·1 to 0·2)	63
Animal contact	0 (0 to 0)	0 (0 to 0·1)	0·3 (0·1 to 0·4)	0·7 (0·5 to 1)	0·7 (0·5 to 1)	1·1 (0·8 to 1·5)	1·2 (0·9 to 1·7)	1·3 (0·9 to 1·7)	1·6 (1·2 to 2·1)	1·3 (0·9 to 1·7)	1·6 (1·2 to 2·1)	1·7 (1·3 to 2·2)	2·1 (1·6 to 2·7)	2 (1·5 to 2·5)	1·9 (1·5 to 2·5)	1·8 (1·4 to 2·3)	1·4 (1·1 to 1·9)	1·3 (1 to 1·6)	1·2 (0·9 to 1·5)	1·2 (0·8 to 1·6)	0·9 (0·6 to 1·3)	0·6 (0·4 to 0·8)	0·2 (0·2 to 0·3)	26·2
Venomous animal contact	0 (0 to 0)	0 (0 to 0)	0·1 (0 to 0·2)	0·3 (0·2 to 0·5)	0·4 (0·2 to 0·5)	0·7 (0·5 to 1)	0·7 (0·5 to 1·1)	0·7 (0·5 to 1·1)	0·9 (0·7 to 1·3)	0·8 (0·5 to 1)	1 (0·7 to 1·3)	0·9 (0·6 to 1·2)	1 (0·7 to 1·4)	1 (0·7 to 1·3)	1 (0·7 to 1·3)	0·9 (0·6 to 1·2)	0·7 (0·5 to 0·9)	0·5 (0·4 to 0·7)	0·5 (0·4 to 0·7)	0·5 (0·3 to 0·8)	0·4 (0·3 to 0·7)	0·3 (0·2 to 0·5)	0·1 (0·1 to 0·2)	13·5
Non-venomous animal contact	0 (0 to 0)	0 (0 to 0)	0·2 (0·1 to 0·3)	0·4 (0·2 to 0·6)	0·3 (0·2 to 0·5)	0·5 (0·3 to 0·7)	0·5 (0·4 to 0·7)	0·5 (0·4 to 0·7)	0·7 (0·5 to 0·9)	0·5 (0·4 to 0·7)	0·7 (0·5 to 0·9)	0·8 (0·6 to 1·1)	1 (0·7 to 1·4)	1 (0·7 to 1·3)	1 (0·7 to 1·3)	0·9 (0·6 to 1·2)	0·8 (0·5 to 1·1)	0·8 (0·5 to 1)	0·6 (0·4 to 0·9)	0·7 (0·4 to 1)	0·5 (0·3 to 0·7)	0·3 (0·2 to 0·4)	0·1 (0·1 to 0·2)	12·7
Foreign body	2·6 (1·7 to 3·8)	1·9 (1·2 to 3·1)	14 (7·7 to 22·4)	8·3 (5·5 to 12·1)	3·5 (2·7 to 4·5)	6·3 (4·8 to 8)	4·4 (3·4 to 5·5)	4·9 (3·8 to 6·2)	5·7 (4·6 to 7)	5 (3·9 to 6·2)	5·5 (4·1 to 7·2)	6·1 (4·9 to 7·6)	7·1 (5·5 to 9·2)	7·2 (5·6 to 9·1)	6·2 (4·8 to 7·9)	5·5 (4·2 to 7·2)	4·6 (3·7 to 5·6)	4 (3·2 to 5)	3·8 (2·9 to 4·8)	3·3 (2·5 to 4·2)	1·6 (1·3 to 1·9)	0·6 (0·5 to 0·7)	0·2 (0·1 to 0·2)	112
Pulmonary aspiration and foreign body in airway	2·6 (1·6 to 3·8)	1·9 (1·2 to 3·1)	13·8 (7·6 to 22·2)	7·8 (5 to 11·5)	2·8 (2·1 to 3·6)	5·3 (4 to 6·8)	3·4 (2·5 to 4·3)	3·7 (2·8 to 4·8)	4·4 (3·4 to 5·6)	3·8 (2·9 to 4·9)	4·2 (3·1 to 5·8)	4·7 (3·7 to 6)	5·6 (4·2 to 7·5)	5·8 (4·3 to 7·7)	4·9 (3·6 to 6·5)	4·4 (3·3 to 6)	3·7 (2·9 to 4·7)	3·3 (2·5 to 4·2)	3·2 (2·4 to 4·1)	2·8 (2 to 3·6)	1·3 (1·1 to 1·6)	0·5 (0·4 to 0·6)	0·1 (0·1 to 0·2)	94·3
Foreign body in eyes	0 (0 to 0)	0 (0 to 0)	0 (0 to 0·1)	0·1 (0 to 0·3)	0·2 (0·1 to 0·6)	0·3 (0·1 to 0·7)	0·4 (0·1 to 1)	0·5 (0·2 to 1·1)	0·5 (0·2 to 1·1)	0·5 (0·2 to 1·1)	0·6 (0·2 to 1·2)	0·6 (0·3 to 1·2)	0·6 (0·3 to 1·1)	0·5 (0·3 to 0·9)	0·4 (0·2 to 0·7)	0·4 (0·2 to 0·6)	0·3 (0·2 to 0·4)	0·2 (0·1 to 0·3)	0·1 (0·1 to 0·2)	0·1 (0·1 to 0·2)	0·1 (0 to 0·1)	0 (0 to 0)	0 (0 to 0)	6·5
Foreign body in other body part	0 (0 to 0)	0 (0 to 0)	0·1 (0·1 to 0·2)	0·4 (0·3 to 0·6)	0·5 (0·3 to 0·7)	0·7 (0·5 to 0·9)	0·6 (0·4 to 0·8)	0·7 (0·5 to 0·9)	0·8 (0·5 to 1·1)	0·6 (0·5 to 0·9)	0·7 (0·5 to 0·9)	0·8 (0·6 to 1·1)	0·9 (0·7 to 1·2)	0·8 (0·6 to 1·1)	0·8 (0·6 to 1·1)	0·7 (0·5 to 0·9)	0·6 (0·4 to 0·8)	0·5 (0·4 to 0·7)	0·4 (0·3 to 0·6)	0·4 (0·3 to 0·6)	0·2 (0·2 to 0·3)	0·1 (0·1 to 0·1)	0 (0 to 0)	11·3
Environmental heat and cold exposure	0 (0 to 0)	0 (0 to 0)	0·1 (0·1 to 0·2)	0·7 (0·5 to 1)	1·3 (0·8 to 1·9)	1·9 (1·3 to 2·7)	2·8 (2 to 3·8)	3·4 (2·4 to 4·6)	3·6 (2·6 to 5)	3·8 (2·7 to 5·2)	4·2 (3 to 5·7)	4·4 (3·1 to 6)	4·6 (3·4 to 6·2)	4·7 (3·4 to 6·2)	4·6 (3·3 to 6·1)	4 (2·9 to 5·4)	3 (2·2 to 4)	2·5 (1·9 to 3·3)	2 (1·5 to 2·6)	1·6 (1·3 to 2·1)	1 (0·8 to 1·2)	0·4 (0·3 to 0·5)	0·1 (0·1 to 0·2)	54·9
Exposure to forces of nature	0 (0 to 0)	0 (0 to 0)	0 (0 to 0)	0 (0 to 0)	0·1 (0·1 to 0·2)	0·2 (0·1 to 0·3)	0·5 (0·3 to 0·8)	0·5 (0·3 to 0·7)	0·6 (0·4 to 0·9)	0·5 (0·3 to 0·8)	0·5 (0·3 to 0·8)	0·5 (0·4 to 0·8)	0·5 (0·3 to 0·8)	0·5 (0·3 to 0·7)	0·4 (0·3 to 0·6)	0·4 (0·2 to 0·5)	0·3 (0·2 to 0·4)	0·2 (0·1 to 0·2)	0·1 (0·1 to 0·2)	0·1 (0 to 0·1)	0 (0 to 0·1)	0 (0 to 0)	0 (0 to 0)	5·8
Other unintentional injuries	0·1 (0·1 to 0·2)	0·1 (0 to 0·1)	0·6 (0·4 to 0·8)	2·4 (1·6 to 3·2)	3·4 (2·5 to 4·8)	5·6 (4·3 to 7·3)	9·1 (6·9 to 11·7)	9·8 (7·5 to 12·9)	10·8 (8·2 to 14·2)	10·1 (7·6 to 13·1)	10·6 (8·2 to 13·4)	10·5 (8·2 to 13·6)	10·7 (8·2 to 14·2)	8·7 (6·6 to 11·4)	7·5 (5·6 to 10·1)	5·8 (4·2 to 7·9)	4·1 (2·9 to 5·6)	3 (2·1 to 4·2)	2·2 (1·6 to 3)	1·5 (1·1 to 2)	0·7 (0·5 to 1)	0·2 (0·2 to 0·3)	0 (0 to 0·1)	118
Self-harm and interpersonal violence	4 (2·3 to 6·4)	0·8 (0·5 to 1·4)	5·4 (3·1 to 8·9)	9·6 (5·5 to 16·3)	7·6 (5·5 to 10·5)	25·3 (19·5 to 32·3)	152 (122 to 189)	239 (197 to 287)	257 (212 to 305)	190 (153 to 230)	173 (140 to 212)	141 (111 to 179)	133 (101 to 170)	92·3 (73 to 118)	64·9 (51·1 to 82)	40·7 (32·8 to 49·1)	25·5 (20·9 to 31·1)	16·8 (13·8 to 20·6)	11·3 (9·2 to 13·9)	6·3 (5·2 to 7·7)	3 (2·5 to 3·6)	0·7 (0·6 to 0·8)	0·1 (0·1 to 0·1)	1,600
Self-harm	0 (0 to 0)	0 (0 to 0)	0 (0 to 0)	0 (0 to 0)	0 (0 to 0)	2·8 (1·9 to 3·9)	21·1 (15·1 to 28·7)	31·2 (23·2 to 41·7)	38·5 (29·3 to 51)	35·2 (26·2 to 46·1)	35·6 (26·7 to 45·7)	34·3 (25·4 to 45·7)	36·9 (27·2 to 49·3)	31 (23·3 to 40·6)	23·6 (17·7 to 31·4)	16·2 (12·1 to 20·4)	11 (8·5 to 13·9)	8·1 (6·2 to 10·5)	5·8 (4·5 to 7·7)	3·7 (2·8 to 4·8)	1·8 (1·4 to 2·4)	0·4 (0·3 to 0·4)	0 (0 to 0)	337
Self-harm by firearm	0 (0 to 0)	0 (0 to 0)	0 (0 to 0)	0 (0 to 0)	0 (0 to 0)	0 (0 to 0·1)	0·6 (0·4 to 1·1)	0·9 (0·5 to 1·5)	0·9 (0·6 to 1·4)	0·9 (0·6 to 1·3)	0·8 (0·5 to 1·3)	0·8 (0·5 to 1·3)	0·8 (0·5 to 1·3)	0·8 (0·5 to 1·2)	0·5 (0·3 to 0·8)	0·4 (0·3 to 0·6)	0·3 (0·2 to 0·4)	0·2 (0·1 to 0·3)	0·1 (0·1 to 0·2)	0·1 (0·1 to 0·1)	0 (0 to 0·1)	0 (0 to 0)	0 (0 to 0)	8·2
Self-harm by other specified means	0 (0 to 0)	0 (0 to 0)	0 (0 to 0)	0 (0 to 0)	0 (0 to 0)	2·7 (1·9 to 3·9)	20·4 (14·6 to 27·7)	30·3 (22·6 to 40·6)	37·6 (28·6 to 49·8)	34·3 (25·4 to 45·1)	34·8 (26 to 44·7)	33·5 (24·8 to 44·5)	36·1 (26·5 to 48·3)	30·2 (22·7 to 39·8)	23·1 (17·3 to 30·6)	15·8 (11·8 to 19·9)	10·7 (8·3 to 13·6)	7·9 (6 to 10·3)	5·7 (4·4 to 7·5)	3·7 (2·8 to 4·8)	1·8 (1·4 to 2·4)	0·4 (0·3 to 0·4)	0 (0 to 0)	329
Interpersonal violence	4 (2·3 to 6·4)	0·8 (0·5 to 1·4)	5·4 (3·1 to 8·9)	9·5 (5·4 to 16·1)	7·5 (5·5 to 10·4)	22·4 (16·7 to 29·2)	131 (102 to 169)	208 (166 to 253)	217 (175 to 264)	155 (120 to 193)	137 (107 to 174)	106 (79 to 140)	95 (68·9 to 129)	60·9 (44·8 to 82·4)	40·9 (29·9 to 53·2)	23·8 (18·1 to 30·7)	14·2 (10·8 to 18·6)	8·5 (6·5 to 11·1)	5·4 (4 to 6·9)	2·5 (2 to 3·2)	1·2 (0·9 to 1·5)	0·3 (0·2 to 0·4)	0·1 (0·1 to 0·1)	1,260
Physical violence by firearm	0·1 (0·1 to 0·2)	0 (0 to 0·1)	0·4 (0·2 to 0·7)	1·1 (0·5 to 2)	1·3 (0·9 to 1·9)	6·6 (4·8 to 8·7)	70·6 (53·1 to 92·4)	116 (92·4 to 141)	119 (93·9 to 149)	78·4 (58·5 to 99·3)	64·4 (46·2 to 85·5)	44·1 (30·8 to 60·6)	38·5 (27·4 to 53·1)	21·7 (14·3 to 31·5)	13·5 (9·2 to 19·4)	6·7 (4·5 to 9·5)	3·3 (2·3 to 4·7)	1·6 (1·1 to 2·2)	0·6 (0·5 to 0·9)	0·2 (0·2 to 0·3)	0·1 (0·1 to 0·2)	0 (0 to 0)	0 (0 to 0)	588
Physical violence by sharp object	0·3 (0·2 to 0·6)	0 (0 to 0·1)	0·3 (0·2 to 0·6)	1 (0·6 to 1·6)	1·1 (0·8 to 1·5)	4·3 (3·2 to 5·5)	41·8 (29·9 to 57·5)	66·4 (52·2 to 81·5)	70·9 (53·1 to 91·8)	52·9 (38·5 to 69·8)	49·4 (36·9 to 65·5)	39·4 (28·9 to 52·6)	33·3 (23·9 to 45·2)	20·3 (14·9 to 27·8)	12·1 (8·8 to 16)	6·8 (5·1 to 8·8)	3·7 (2·8 to 4·8)	2·5 (1·9 to 3·2)	1·4 (1·1 to 1·8)	0·7 (0·6 to 1)	0·3 (0·2 to 0·4)	0·1 (0·1 to 0·1)	0 (0 to 0)	409
Sexual violence	0 (0 to 0)	0 (0 to 0)	0 (0 to 0)	0 (0 to 0·1)	0·4 (0·3 to 0·7)	1 (0·6 to 1·6)	2·3 (1·3 to 3·7)	3 (1·8 to 4·7)	2·6 (1·5 to 4·2)	2·3 (1·3 to 3·8)	2·1 (1·2 to 3·4)	1·8 (1 to 2·9)	1·4 (0·8 to 2·4)	1 (0·6 to 1·7)	0·7 (0·4 to 1·2)	0·6 (0·4 to 0·9)	0·4 (0·2 to 0·6)	0·3 (0·2 to 0·4)	0·2 (0·1 to 0·3)	0·1 (0·1 to 0·2)	0·1 (0 to 0·1)	0 (0 to 0)	0 (0 to 0)	20·5
Physical violence by other means	3·5 (2 to 5·8)	0·8 (0·4 to 1·2)	4·7 (2·6 to 7·7)	7·4 (4·2 to 12·7)	4·7 (3·3 to 6·7)	10·6 (7·8 to 14·1)	16·2 (12·2 to 21·2)	22·2 (15·7 to 30·5)	24·9 (19·3 to 31·5)	21 (16·2 to 26·4)	21·3 (16·3 to 27·5)	20·7 (15·4 to 27·9)	21·9 (15·9 to 29·5)	17·9 (13·2 to 24·1)	14·5 (10·5 to 19·2)	9·7 (7·2 to 12·7)	6·8 (5 to 9)	4·2 (3·2 to 5·6)	3·1 (2·3 to 4)	1·4 (1·1 to 1·8)	0·7 (0·5 to 0·8)	0·2 (0·1 to 0·2)	0 (0 to 0)	238
Conflict and terrorism	0 (0 to 0)	0 (0 to 0)	0 (0 to 0)	0 (0 to 0)	0 (0 to 0)	0 (0 to 0)	0 (0 to 0)	0 (0 to 0)	0 (0 to 0)	0·1 (0·1 to 0·2)	0·1 (0 to 0·1)	0·1 (0·1 to 0·2)	0·2 (0·1 to 0·3)	0·6 (0·4 to 0·9)	0·4 (0·2 to 0·5)	0·3 (0·2 to 0·5)	0·7 (0·4 to 0·9)	0·4 (0·2 to 0·5)	0·2 (0·1 to 0·3)	0·1 (0·1 to 0·1)	0 (0 to 0)	0 (0 to 0)	0 (0 to 0)	3·1

SR Table 2c: Suriname DALYS by cause and age group for both sexes with 95% uncertainty intervals, 2019

Cause	Early Neonatal	Late Neonatal	Post Neonatal	1 to 4	5 to 9	10 to 14	15 to 19	20 to 24	25 to 29	30 to 34	35 to 39	40 to 44
All causes	10,600 (7,430 to 14,700)	3,040 (2,160 to 4,210)	5,510 (4,030 to 7,420)	3,500 (2,820 to 4,300)	3,540 (2,840 to 4,410)	4,580 (3,750 to 5,570)	6,800 (5,750 to 8,130)	7,770 (6,610 to 9,190)	8,840 (7,580 to 10,300)	10,000 (8,550 to 11,700)	11,000 (9,450 to 12,700)	11,100 (9,500 to 12,900)
Communicable, maternal, neonatal, and nutritional diseases	9,190 (6,460 to 12,800)	2,420 (1,700 to 3,340)	3,140 (2,250 to 4,270)	1,440 (1,130 to 1,800)	1,150 (843 to 1,600)	885 (674 to 1,160)	924 (738 to 1,170)	1,070 (907 to 1,290)	1,400 (1,220 to 1,600)	1,810 (1,610 to 2,030)	1,730 (1,540 to 1,950)	1,540 (1,350 to 1,770)
HIV/AIDS and sexually transmitted infections	294 (81·3 to 682)	92·7 (26·2 to 214)	251 (149 to 432)	64·7 (59·4 to 72·2)	51·7 (48·4 to 55·8)	35·7 (33·7 to 38)	86·4 (79·7 to 93·9)	284 (262 to 310)	584 (545 to 635)	947 (888 to 1,010)	889 (833 to 953)	752 (707 to 803)
HIV/AIDS	0 (0 to 0)	0 (0 to 0)	109 (103 to 115)	59·6 (56·3 to 63·2)	49·4 (46·6 to 52·2)	34·4 (32·5 to 36·6)	80·8 (75·2 to 87·2)	274 (254 to 298)	570 (532 to 620)	930 (873 to 994)	872 (819 to 934)	736 (694 to 784)
HIV/AIDS - Drug-susceptible Tuberculosis	0 (0 to 0)	0 (0 to 0)	6·4 (4 to 9·9)	3·4 (2·1 to 5·2)	2·9 (1·8 to 4·4)	2·1 (1·3 to 3·2)	5 (3·3 to 7·5)	16·5 (11·1 to 24·4)	34·3 (22·4 to 51·6)	56 (35·9 to 84·6)	53·3 (34·4 to 79·9)	44 (28 to 66·5)
HIV/AIDS - Multidrug-resistant Tuberculosis without extensive	0 (0 to 0)	0 (0 to 0)	0·1 (0 to 0·3)	0 (0 to 0·1)	0 (0 to 0·1)	0 (0 to 0·1)	0·1 (0 to 0·2)	0·2 (0 to 0·6)	0·3 (0 to 1·3)	0·5 (0 to 2·1)	0·5 (0 to 2)	0·4 (0 to 1·7)
HIV/AIDS - Extensively drug-resistant Tuberculosis	0 (0 to 0)	0 (0 to 0)	0 (0 to 0)	0 (0 to 0)	0 (0 to 0)	0 (0 to 0)	0 (0 to 0)	0 (0 to 0·1)	0·1 (0 to 0·2)	0·1 (0 to 0·3)	0·1 (0 to 0·3)	0·1 (0 to 0·3)
HIV/AIDS resulting in other diseases	0 (0 to 0)	0 (0 to 0)	102 (94·9 to 109)	56·2 (52·4 to 60·2)	46·5 (43·3 to 49·6)	32·3 (30·1 to 34·7)	75·7 (70 to 82·3)	257 (237 to 281)	536 (496 to 587)	873 (811 to 939)	818 (760 to 880)	692 (643 to 745)
Sexually transmitted infections excluding HIV	294 (81·3 to 682)	92·7 (26·2 to 214)	142 (39·7 to 324)	5·1 (1·4 to 12)	2·3 (0·7 to 5·4)	1·3 (0·8 to 2·2)	5·6 (3·2 to 9·1)	9·9 (5·9 to 15·9)	13·5 (7·8 to 22·2)	17·1 (9·7 to 28·7)	17·3 (9·8 to 28·9)	16 (9 to 28·6)
Syphilis	294 (81·3 to 682)	92·7 (26·2 to 214)	142 (39·7 to 324)	5·1 (1·4 to 12)	2·3 (0·7 to 5·4)	0·3 (0·2 to 0·7)	0·6 (0·3 to 1·1)	0·4 (0·2 to 0·8)	0·7 (0·4 to 1·3)	0·9 (0·5 to 1·6)	1 (0·5 to 2·1)	0·5 (0·3 to 0·8)
Chlamydial infection	0 (0 to 0)	0 (0 to 0)	0 (0 to 0)	0 (0 to 0)	0 (0 to 0)	0·1 (0 to 0·1)	0·6 (0·3 to 0·9)	1·5 (0·8 to 2·7)	2 (1·1 to 3·5)	2·3 (1·1 to 4)	2·4 (1·3 to 4·1)	2·2 (1·3 to 3·7)
Gonococcal infection	0 (0 to 0)	0 (0 to 0)	0 (0 to 0)	0 (0 to 0)	0 (0 to 0)	0·2 (0·1 to 0·4)	1·3 (0·5 to 2·5)	1·8 (0·8 to 3·2)	1·3 (0·6 to 2·4)	1·4 (0·7 to 2·5)	1·5 (0·8 to 2·6)	1·5 (0·8 to 2·8)
Trichomoniasis	0 (0 to 0)	0 (0 to 0)	0 (0 to 0)	0 (0 to 0)	0 (0 to 0)	0 (0 to 0·1)	0·4 (0·1 to 1·1)	2 (0·6 to 4·8)	3·7 (1·2 to 8·4)	4·4 (1·4 to 10·3)	4 (1·3 to 9·4)	3·6 (1·2 to 9·1)
Genital herpes	0 (0 to 0)	0 (0 to 0)	0 (0 to 0)	0 (0 to 0)	0 (0 to 0)	0·2 (0·1 to 0·5)	0·9 (0·3 to 2·1)	1·8 (0·6 to 4·4)	2·7 (0·9 to 6·7)	3·6 (1·2 to 9)	3·8 (1·2 to 9·2)	3·6 (1·2 to 9·3)
Other sexually transmitted infections	0 (0 to 0)	0 (0 to 0)	0 (0 to 0)	0 (0 to 0)	0 (0 to 0)	0·5 (0·3 to 0·8)	1·9 (0·9 to 3·2)	2·4 (1·4 to 3·9)	3 (1·8 to 4·8)	4·5 (2·8 to 7)	4·7 (2·8 to 7·4)	4·4 (2·6 to 6·9)
Respiratory infections and tuberculosis	214 (128 to 350)	165 (94·5 to 269)	753 (467 to 1,170)	251 (172 to 344)	139 (101 to 189)	125 (92·5 to 173)	127 (94·7 to 169)	137 (104 to 178)	162 (127 to 206)	186 (146 to 240)	203 (156 to 262)	195 (149 to 248)
Tuberculosis	0 (0 to 0)	0 (0 to 0)	7·8 (4·6 to 12·1)	6·5 (4 to 9·6)	5·9 (3·3 to 9·2)	6·4 (4·4 to 9)	12 (8 to 17·7)	25·5 (16·6 to 36·2)	35·6 (24·9 to 48·9)	41·4 (28·6 to 56·9)	38·7 (26·7 to 54·1)	35·9 (24·2 to 51)
Latent tuberculosis infection	0 (0 to 0)	0 (0 to 0)	0 (0 to 0)	0 (0 to 0)	0 (0 to 0)	0 (0 to 0)	0 (0 to 0)	0 (0 to 0)	0 (0 to 0)	0 (0 to 0)	0 (0 to 0)	0 (0 to 0)
Drug-susceptible tuberculosis	0 (0 to 0)	0 (0 to 0)	7·7 (4·5 to 12)	6·5 (3·9 to 9·6)	5·8 (3·3 to 9·1)	6·3 (4·3 to 8·9)	11·9 (7·9 to 17·6)	25·3 (16·6 to 36)	35·3 (24·4 to 48·5)	41 (28·3 to 56·4)	38·4 (26·2 to 53·7)	35·6 (23·9 to 50·2)
Multidrug-resistant tuberculosis without extensive drug resista	0 (0 to 0)	0 (0 to 0)	0·1 (0 to 0·3)	0·1 (0 to 0·2)	0 (0 to 0·1)	0 (0 to 0·2)	0·1 (0 to 0·4)	0·2 (0 to 0·7)	0·3 (0 to 1)	0·3 (0 to 1·1)	0·3 (0 to 1·1)	0·3 (0 to 1·2)
Extensively drug-resistant tuberculosis	0 (0 to 0)	0 (0 to 0)	0 (0 to 0)	0 (0 to 0)	0 (0 to 0)	0 (0 to 0)	0 (0 to 0·1)	0 (0 to 0·1)	0 (0 to 0·1)	0 (0 to 0·2)	0 (0 to 0·2)	0 (0 to 0·2)
Lower respiratory infections	214 (128 to 350)	164 (93·8 to 269)	731 (447 to 1,150)	181 (110 to 272)	54·6 (39·5 to 72·8)	56·4 (41·8 to 74·3)	60·5 (43·2 to 83)	62·1 (43·7 to 85)	84·5 (60·7 to 117)	105 (72·4 to 146)	127 (89·3 to 176)	125 (86·1 to 172)
Upper respiratory infections	0·1 (0·1 to 0·2)	0·6 (0·3 to 1)	11·4 (6·6 to 18·2)	45·5 (25·8 to 74·7)	52·6 (28·5 to 88·9)	45·5 (24·9 to 79·9)	40·6 (20·7 to 71·1)	37·2 (19·9 to 62·5)	32·9 (18 to 54·3)	32·2 (17·3 to 55·1)	30·2 (15·2 to 50·9)	26·9 (14·4 to 46·8)
Otitis media	0 (0 to 0)	0·1 (0 to 0·2)	2·1 (1·1 to 3·8)	18·7 (10·2 to 33)	25·9 (14·6 to 42·7)	17·1 (9·1 to 29·7)	14·3 (7·7 to 24·4)	11·7 (5·1 to 23·1)	9·1 (4·2 to 17·3)	7·8 (3·4 to 15·2)	7·5 (3·6 to 14·2)	7·7 (3·6 to 14·3)
Enteric infections	43 (21·6 to 75·7)	96 (46·6 to 174)	599 (292 to 1,040)	228 (144 to 344)	117 (79·6 to 165)	106 (68·2 to 157)	68·2 (47·3 to 95·6)	57·8 (38·8 to 83·8)	63·9 (44·9 to 91·4)	71 (50·1 to 98·8)	81·2 (56 to 116)	85·4 (60 to 118)
Diarrheal diseases	43 (21·6 to 75·7)	96 (46·6 to 174)	598 (291 to 1,040)	226 (141 to 341)	115 (77·9 to 164)	104 (66·8 to 156)	67 (46 to 94·3)	56·9 (38·2 to 83)	63·3 (44·3 to 90·5)	70·4 (49·6 to 98·2)	80·7 (55·6 to 115)	84·9 (59·4 to 118)
Typhoid and paratyphoid	0 (0 to 0)	0 (0 to 0)	0·1 (0 to 0·2)	0·6 (0·2 to 1·6)	0·4 (0·1 to 1)	0·4 (0·1 to 1·1)	0·4 (0·1 to 1)	0·3 (0·1 to 0·7)	0·3 (0·1 to 0·7)	0·2 (0·1 to 0·5)	0·2 (0·1 to 0·4)	0·2 (0 to 0·4)
Typhoid fever	0 (0 to 0)	0 (0 to 0)	0·1 (0 to 0·2)	0·5 (0·1 to 1·3)	0·3 (0·1 to 0·7)	0·3 (0·1 to 0·8)	0·3 (0·1 to 0·7)	0·2 (0·1 to 0·6)	0·2 (0·1 to 0·5)	0·2 (0 to 0·4)	0·1 (0 to 0·4)	0·1 (0 to 0·3)
Paratyphoid fever	0 (0 to 0)	0 (0 to 0)	0 (0 to 0·1)	0·2 (0 to 0·4)	0·1 (0 to 0·3)	0·1 (0 to 0·4)	0·1 (0 to 0·3)	0·1 (0 to 0·2)	0·1 (0 to 0·2)	0 (0 to 0·1)	0 (0 to 0·1)	0 (0 to 0·1)
Invasive Non-typhoidal Salmonella (iNTS)	0 (0 to 0)	0 (0 to 0·1)	0·3 (0·1 to 0·9)	1 (0·2 to 2·3)	1 (0·3 to 2·2)	0·8 (0·2 to 2)	0·6 (0·1 to 1·5)	0·4 (0·1 to 1·1)	0·3 (0 to 0·8)	0·2 (0 to 0·6)	0·2 (0 to 0·5)	0·2 (0 to 0·5)
Other intestinal infectious diseases	0 (0 to 0)	0 (0 to 0)	0·8 (0 to 2·4)	0·6 (0·1 to 1·6)	0·4 (0·1 to 1)	0·4 (0·1 to 1)	0·2 (0 to 0·6)	0·2 (0 to 0·4)	0·1 (0 to 0·2)	0·2 (0·1 to 0·6)	0·1 (0 to 0·3)	0·1 (0 to 0·3)
Neglected tropical diseases and malaria	1 (0·4 to 2·1)	4·9 (1·9 to 11·3)	85·7 (24·4 to 199)	81·6 (45·3 to 134)	132 (74·3 to 221)	112 (58·6 to 191)	125 (65·8 to 230)	117 (62·4 to 208)	121 (66·4 to 198)	122 (68·8 to 197)	108 (65·4 to 171)	93·4 (55·9 to 147)
Malaria	0 (0 to 0)	0·3 (0 to 2)	1·2 (0 to 8·2)	2·4 (0·2 to 12·9)	2·8 (0·5 to 7·6)	2·2 (0·8 to 8·9)	5·4 (0·9 to 26·8)	4 (0·9 to 23·8)	3·6 (0·8 to 22·4)	3·3 (0·8 to 19·7)	3 (0·7 to 17·6)	2·3 (0·6 to 9·1)
Chagas disease	0 (0 to 0)	0 (0 to 0)	0 (0 to 0)	0 (0 to 0)	0 (0 to 0)	0 (0 to 0)	0 (0 to 0)	0 (0 to 0)	0 (0 to 0·1)	0 (0 to 0·1)	0·1 (0 to 0·1)	0·1 (0 to 0·3)
Leishmaniasis	0 (0 to 0)	0 (0 to 0)	0·6 (0·2 to 1·6)	5·1 (1·8 to 14·3)	4·7 (1·8 to 14·5)	2·5 (0·8 to 7·5)	1·9 (0·7 to 4·8)	1·6 (0·6 to 3·6)	1·6 (0·6 to 3·5)	1·6 (0·6 to 3·3)	1·6 (0·7 to 3·1)	1·6 (0·7 to 2·8)
Visceral leishmaniasis	0 (0 to 0)	0 (0 to 0)	0·3 (0 to 1·3)	2·1 (0 to 11·2)	1·6 (0 to 11·4)	0·8 (0 to 5·7)	0·6 (0 to 3·4)	0·4 (0 to 2·3)	0·5 (0 to 2·2)	0·4 (0 to 2)	0·4 (0 to 1·6)	0·3 (0 to 1·1)
Cutaneous and mucocutaneous leishmaniasis	0 (0 to 0)	0 (0 to 0)	0·3 (0·2 to 0·6)	3 (1·6 to 4·7)	3·1 (1·6 to 5·2)	1·7 (0·8 to 3)	1·3 (0·6 to 2·4)	1·2 (0·5 to 2·3)	1·1 (0·5 to 2)	1·2 (0·5 to 2)	1·2 (0·6 to 2·1)	1·3 (0·7 to 2·3)
African trypanosomiasis	0 (0 to 0)	0 (0 to 0)	0 (0 to 0)	0 (0 to 0)	0 (0 to 0)	0 (0 to 0)	0 (0 to 0)	0 (0 to 0)	0 (0 to 0)	0 (0 to 0)	0 (0 to 0)	0 (0 to 0)
Schistosomiasis	0 (0 to 0)	0 (0 to 0)	0 (0 to 0)	2·9 (0·7 to 7·7)	28 (8·4 to 69·8)	47·6 (14·5 to 117)	68 (25·1 to 160)	71·2 (28·5 to 150)	70·1 (27·2 to 140)	70·8 (29·9 to 138)	53·7 (23·9 to 104)	36·6 (12·4 to 81·7)
Cysticercosis	0 (0 to 0)	0 (0 to 0)	0 (0 to 0)	0 (0 to 0)	0 (0 to 0)	0·3 (0·1 to 0·8)	2·4 (0·3 to 5·8)	7·7 (2 to 17·6)	13·2 (4·4 to 28·7)	16·9 (5·5 to 37·2)	18·8 (6·8 to 39·2)	21·1 (7·2 to 46·4)
Cystic echinococcosis	0 (0 to 0)	0 (0 to 0)	0 (0 to 0)	0 (0 to 0)	0 (0 to 0)	0 (0 to 0·1)	0 (0 to 0·1)	0 (0 to 0)	0 (0 to 0·1)	0 (0 to 0·1)	0 (0 to 0·1)	0 (0 to 0·1)
Lymphatic filariasis	0 (0 to 0)	0 (0 to 0)	0 (0 to 0)	0 (0 to 0)	0 (0 to 0)	0 (0 to 0)	0 (0 to 0)	0 (0 to 0)	0 (0 to 0)	0 (0 to 0)	0 (0 to 0)	0 (0 to 0)
Onchocerciasis	0 (0 to 0)	0 (0 to 0)	0 (0 to 0)	0 (0 to 0)	0 (0 to 0)	0 (0 to 0)	0 (0 to 0)	0 (0 to 0)	0 (0 to 0)	0 (0 to 0)	0 (0 to 0)	0 (0 to 0)
Trachoma	0 (0 to 0)	0 (0 to 0)	0 (0 to 0)	0 (0 to 0)	0 (0 to 0)	0 (0 to 0)	0 (0 to 0)	0 (0 to 0)	0 (0 to 0)	0 (0 to 0)	0 (0 to 0)	0 (0 to 0)
Dengue	0 (0 to 0)	2·7 (0·4 to 8·7)	70·2 (10·2 to 180)	36·3 (7·4 to 87)	51·8 (12·2 to 114)	35 (9·4 to 73·7)	31·7 (9·4 to 70·8)	20·4 (7·2 to 44·6)	23·7 (6·9 to 54·9)	19·4 (6·5 to 43·7)	20·7 (5·6 to 46·9)	19·7 (5·7 to 41·8)
Yellow fever	0 (0 to 0)	0 (0 to 0)	0 (0 to 0·1)	0·3 (0 to 0·9)	0·6 (0·1 to 1·9)	0·5 (0·1 to 1·7)	0·5 (0·1 to 1·8)	0·4 (0·1 to 1·3)	0·3 (0·1 to 1·3)	0·3 (0 to 0·9)	0·2 (0 to 0·8)	0·2 (0 to 0·6)
Rabies	0 (0 to 0)	0 (0 to 0)	0·2 (0 to 0·8)	0·8 (0·1 to 2·5)	1·7 (0·3 to 4·8)	1·1 (0·2 to 3·1)	0·5 (0·1 to 1·5)	0·2 (0 to 0·7)	0·2 (0 to 0·6)	0 (0 to 0)	0·1 (0 to 0·4)	0·1 (0 to 0·4)
Intestinal nematode infections	0 (0 to 0)	0 (0 to 0)	0·3 (0 to 1·2)	1·6 (0·5 to 4)	3·2 (1·4 to 5·9)	2·2 (1·1 to 4)	7·2 (2·8 to 14·2)	4·5 (1·9 to 8·4)	2·8 (1·1 to 5·3)	2·5 (1·1 to 4·6)	3·1 (1·2 to 5·9)	2·1 (1·1 to 3·8)
Ascariasis	0 (0 to 0)	0 (0 to 0)	0·3 (0 to 1·1)	0·8 (0 to 3)	0·6 (0 to 2·1)	0·3 (0 to 1)	0·2 (0 to 0·6)	0·1 (0 to 0·3)	0·1 (0 to 0·3)	0·1 (0 to 0·3)	0·1 (0 to 0·3)	0·1 (0 to 0·2)
Trichuriasis	0 (0 to 0)	0 (0 to 0)	0 (0 to 0)	0 (0 to 0)	0 (0 to 0)	0 (0 to 0)	0 (0 to 0)	0 (0 to 0)	0 (0 to 0)	0 (0 to 0)	0 (0 to 0)	0 (0 to 0)
Hookworm disease	0 (0 to 0)	0 (0 to 0)	0 (0 to 0·1)	0·9 (0·4 to 1·7)	2·6 (1·2 to 4·8)	1·9 (0·9 to 3·5)	7 (2·5 to 14)	4·4 (1·9 to 8·3)	2·7 (1·1 to 5·2)	2·4 (1 to 4·5)	3 (1·2 to 5·9)	2·1 (1 to 3·7)
Food-borne trematodiases	0 (0 to 0)	0 (0 to 0)	0 (0 to 0)	0 (0 to 0)	0 (0 to 0)	0 (0 to 0)	0 (0 to 0)	0 (0 to 0)	0 (0 to 0)	0 (0 to 0)	0 (0 to 0)	0 (0 to 0)
Leprosy	0 (0 to 0)	0 (0 to 0)	0 (0 to 0)	0 (0 to 0)	0 (0 to 0)	0 (0 to 0)	0·1 (0 to 0·1)	0·1 (0·1 to 0·2)	0·1 (0·1 to 0·2)	0·2 (0·1 to 0·3)	0·2 (0·1 to 0·5)	0·3 (0·1 to 0·6)
Ebola	0 (0 to 0)	0 (0 to 0)	0 (0 to 0)	0 (0 to 0)	0 (0 to 0)	0 (0 to 0)	0 (0 to 0)	0 (0 to 0)	0 (0 to 0)	0 (0 to 0)	0 (0 to 0)	0 (0 to 0)
Zika virus	0 (0 to 0)	0 (0 to 0·1)	0·6 (0·1 to 2)	0·1 (0 to 0·7)	0·2 (0 to 0·9)	0·2 (0 to 1)	0·3 (0 to 1·2)	0·3 (0 to 1·2)	0·3 (0 to 1·3)	0·3 (0 to 1·3)	0·2 (0 to 1·2)	0·2 (0 to 1)
Guinea worm disease	0 (0 to 0)	0 (0 to 0)	0 (0 to 0)	0 (0 to 0)	0 (0 to 0)	0 (0 to 0)	0 (0 to 0)	0 (0 to 0)	0 (0 to 0)	0 (0 to 0)	0 (0 to 0)	0 (0 to 0)
Other neglected tropical diseases	0·9 (0·3 to 2·1)	1·8 (1 to 3·4)	12·4 (6·5 to 22·6)	32 (17 to 52·2)	38·8 (19·2 to 68·1)	19·9 (9·6 to 35·7)	6·7 (3·4 to 11·9)	6·3 (3·4 to 10·7)	5·1 (2·6 to 8·7)	6·9 (3·7 to 11·4)	6·5 (3 to 11·5)	9 (4·9 to 15·1)
Other infectious diseases	31·7 (18·9 to 50·2)	53·3 (30·6 to 88·3)	312 (177 to 517)	216 (128 to 384)	100 (72·5 to 135)	82·2 (63·4 to 106)	74·7 (56 to 98·2)	63·3 (47·5 to 85·4)	67·4 (49·3 to 98·1)	73·6 (52·2 to 111)	79·7 (55·9 to 117)	78·3 (55·4 to 114)
Meningitis	18·2 (9·3 to 32·9)	36·4 (17·9 to 63·3)	121 (65·5 to 197)	42·1 (24·5 to 67·5)	21·2 (14·7 to 29·9)	21·1 (14·9 to 28·9)	18·7 (12·9 to 27·1)	12·4 (8·9 to 17·2)	12·5 (8·8 to 17·4)	14·4 (9·9 to 20·5)	15·2 (10·5 to 22·4)	13 (8·9 to 18·5)
Encephalitis	4·8 (2·2 to 9·3)	7·1 (3·1 to 15)	68·5 (33·2 to 131)	52·7 (25·1 to 91·2)	30·4 (16·3 to 50·9)	27·6 (16·5 to 42·6)	23·6 (13·6 to 37)	19·1 (11·9 to 29·5)	16 (9·5 to 25·7)	14·8 (9·2 to 23)	13·2 (7·8 to 20·2)	13 (8 to 20)
Diphtheria	0 (0 to 0)	0 (0 to 0)	0·1 (0 to 0·4)	0·1 (0 to 0·4)	0·1 (0 to 0·3)	0·1 (0 to 0·2)	0 (0 to 0·1)	0 (0 to 0·1)	0 (0 to 0·1)	0 (0 to 0·1)	0 (0 to 0·1)	0 (0 to 0·1)
Tetanus	0·8 (0·3 to 1·6)	1·1 (0·3 to 2·8)	1·2 (0·5 to 2·5)	1·1 (0·4 to 6·4)	0·9 (0·4 to 2·2)	0·9 (0·4 to 2)	1 (0·5 to 2·5)	1 (0·5 to 1·9)	1 (0·4 to 2)	1·3 (0·6 to 2·7)	1·7 (0·9 to 3·6)	1·9 (0·9 to 4·1)
Measles	0 (0 to 0)	0 (0 to 0)	0 (0 to 0)	0 (0 to 0)	0 (0 to 0)	0 (0 to 0)	0 (0 to 0)	0 (0 to 0)	0 (0 to 0)	0 (0 to 0)	0 (0 to 0)	0 (0 to 0)
Varicella and herpes zoster	0·3 (0·1 to 0·8)	0·9 (0·2 to 2·1)	7·4 (1·7 to 17·9)	10·7 (2·5 to 25·8)	4·4 (1·2 to 10·1)	1·8 (0·7 to 3·8)	1·2 (0·5 to 2·2)	1·2 (0·6 to 2·2)	1·4 (0·6 to 2·5)	1·7 (0·8 to 2·9)	1·6 (0·7 to 2·7)	1·4 (0·6 to 2·4)
Acute hepatitis	0 (0 to 0)	0 (0 to 0)	1·7 (0·9 to 3·2)	1·8 (1 to 2·8)	4·5 (2·8 to 6·7)	5·6 (3·7 to 8·4)	5·4 (3·7 to 7·5)	5·2 (3·4 to 7·6)	5 (3·5 to 7·1)	4·5 (3·1 to 6·6)	4·4 (3 to 6·5)	4 (2·7 to 5·7)
Acute hepatitis A	0 (0 to 0)	0 (0 to 0)	0·1 (0 to 0·2)	1 (0·5 to 1·7)	3·4 (1·9 to 5·4)	3·9 (2·4 to 6·1)	3·4 (2·2 to 5·2)	2·6 (1·6 to 3·9)	1·9 (1·1 to 3)	1·7 (1 to 2·6)	1·2 (0·7 to 2)	1 (0·6 to 1·6)
Acute hepatitis B	0 (0 to 0)	0 (0 to 0)	1·5 (0·7 to 2·8)	0·6 (0·2 to 1·2)	0·7 (0·3 to 1·2)	1·2 (0·5 to 2·4)	1·3 (0·7 to 2·4)	2·1 (1·1 to 3·6)	2·7 (1·6 to 4·2)	2·5 (1·4 to 4·1)	2·8 (1·7 to 4·5)	2·6 (1·5 to 4·1)
Acute hepatitis C	0 (0 to 0)	0 (0 to 0)	0·1 (0 to 0·1)	0·1 (0·1 to 0·3)	0·1 (0 to 0·2)	0·1 (0 to 0·2)	0·1 (0·1 to 0·4)	0·1 (0·1 to 0·3)	0·1 (0·1 to 0·2)	0·1 (0·1 to 0·2)	0·1 (0·1 to 0·3)	0·2 (0·1 to 0·4)
Acute hepatitis E	0 (0 to 0)	0 (0 to 0)	0·1 (0 to 0·2)	0·1 (0 to 0·1)	0·3 (0·2 to 0·5)	0·4 (0·1 to 0·8)	0·5 (0·2 to 0·9)	0·4 (0·1 to 0·8)	0·3 (0·2 to 0·6)	0·3 (0·1 to 0·5)	0·2 (0·1 to 0·4)	0·2 (0·1 to 0·4)
Other unspecified infectious diseases	7·6 (3 to 16·8)	7·8 (3·5 to 15·9)	40·8 (17·4 to 90·8)	33·4 (18·9 to 58·3)	30·6 (17·2 to 50·9)	23·5 (14·2 to 37·7)	24·2 (14·2 to 39·3)	23·9 (13·7 to 42·8)	31·4 (17·6 to 58·2)	36·6 (19 to 71·3)	43·5 (23·4 to 76·6)	44·9 (25·9 to 77·7)
Maternal and neonatal disorders	8,610 (6,100 to 11,900)	2,000 (1,400 to 2,760)	897 (556 to 1,380)	261 (201 to 331)	227 (166 to 293)	226 (166 to 294)	312 (235 to 404)	311 (236 to 388)	308 (232 to 400)	297 (227 to 388)	236 (181 to 305)	157 (122 to 198)
Maternal disorders	0 (0 to 0)	0 (0 to 0)	0 (0 to 0)	0 (0 to 0)	0 (0 to 0)	5·5 (2·2 to 12·7)	117 (68·1 to 184)	132 (84·5 to 193)	149 (90·8 to 228)	143 (86·7 to 222)	98·9 (59·9 to 157)	38·1 (23·2 to 61·1)
Maternal hemorrhage	0 (0 to 0)	0 (0 to 0)	0 (0 to 0)	0 (0 to 0)	0 (0 to 0)	0·4 (0·2 to 0·9)	15·9 (8·8 to 26·1)	22·2 (13·1 to 35·4)	28·6 (16·5 to 44·7)	29·8 (16·7 to 50·2)	22·3 (12·3 to 38·6)	8·2 (4·3 to 14·8)
Maternal sepsis and other maternal infections	0 (0 to 0)	0 (0 to 0)	0 (0 to 0)	0 (0 to 0)	0 (0 to 0)	0·2 (0·1 to 0·4)	4·5 (2·4 to 7·8)	6·1 (3·4 to 9·7)	6·7 (3·6 to 11·5)	6 (3·2 to 10·9)	4 (2 to 7)	1·4 (0·7 to 2·5)
Maternal hypertensive disorders	0 (0 to 0)	0 (0 to 0)	0 (0 to 0)	0 (0 to 0)	0 (0 to 0)	1·7 (0·6 to 4)	31·1 (17 to 52·1)	26·2 (15·8 to 39)	29·4 (16·5 to 47·9)	25·1 (14·8 to 41)	15·8 (9·5 to 25·4)	5·8 (3·4 to 9·4)
Maternal obstructed labor and uterine rupture	0 (0 to 0)	0 (0 to 0)	0 (0 to 0)	0 (0 to 0)	0 (0 to 0)	0·1 (0·1 to 0·3)	2·7 (1·3 to 5·3)	3·2 (1·6 to 5·9)	3·9 (1·8 to 7·6)	4·2 (1·9 to 8·1)	3·5 (1·6 to 7)	1·3 (0·6 to 2·7)
Maternal abortion and miscarriage	0 (0 to 0)	0 (0 to 0)	0 (0 to 0)	0 (0 to 0)	0 (0 to 0)	0·2 (0·1 to 0·5)	3·9 (1·9 to 7·8)	4·5 (2·3 to 8·2)	4·5 (2·2 to 8·2)	4·1 (1·9 to 8)	3 (1·4 to 6·1)	1·5 (0·6 to 3·1)
Ectopic pregnancy	0 (0 to 0)	0 (0 to 0)	0 (0 to 0)	0 (0 to 0)	0 (0 to 0)	0·1 (0 to 0·3)	5·8 (2·9 to 10·6)	10·1 (5·3 to 17)	12·5 (6·5 to 21·2)	13 (6·7 to 22·9)	8·4 (4·3 to 15·3)	2·8 (1·4 to 5·2)
Indirect maternal deaths	--	--	--	--	--	1·3 (0·4 to 3·1)	24·5 (12·2 to 42·7)	27·2 (14·8 to 45·2)	27·1 (13·9 to 46·2)	23·6 (12·1 to 40·4)	14 (7·1 to 24·7)	4·6 (2·2 to 8·5)
Late maternal deaths	--	--	--	--	--	0·3 (0·1 to 0·6)	6·3 (2·9 to 11·8)	7 (3·4 to 13)	7·2 (3·3 to 13·3)	6·7 (3·3 to 12·6)	4·7 (2·2 to 8·9)	1·8 (0·9 to 3·2)
Maternal deaths aggravated by HIV/AIDS	--	--	--	--	--	0 (0 to 0)	0·2 (0·1 to 0·3)	0·5 (0·2 to 1)	0·8 (0·3 to 1·7)	1 (0·4 to 1·9)	0·7 (0·3 to 1·3)	0·2 (0·1 to 0·4)
Neonatal disorders	8,610 (6,100 to 11,900)	2,000 (1,400 to 2,760)	897 (556 to 1,380)	261 (201 to 331)	227 (166 to 293)	220 (160 to 289)	195 (141 to 254)	179 (128 to 236)	160 (117 to 212)	154 (113 to 200)	137 (101 to 181)	119 (88·9 to 157)
Neonatal preterm birth	4,620 (3,140 to 6,650)	706 (404 to 1,090)	393 (224 to 624)	82·8 (62 to 108)	100 (71·9 to 133)	96·6 (69·9 to 130)	84·2 (61·6 to 110)	75·4 (54·2 to 100)	67 (47·9 to 88·6)	63·2 (46·9 to 83·2)	55·3 (40·3 to 73·6)	46·8 (34·1 to 61·3)
Neonatal encephalopathy due to birth asphyxia and trauma	2,110 (1,320 to 3,130)	278 (157 to 452)	150 (74·2 to 263)	36·5 (23·8 to 54·9)	24·9 (13·6 to 39·2)	24·1 (12·8 to 38·1)	21·9 (11·9 to 34·9)	20·3 (11·3 to 32·3)	18 (10 to 28·9)	17·7 (9·9 to 28·5)	16 (8·6 to 25·6)	14·1 (7·8 to 22·9)
Neonatal sepsis and other neonatal infections	1,280 (647 to 2,170)	830 (506 to 1,210)	267 (137 to 457)	69·5 (43·9 to 99·4)	93·3 (57·4 to 135)	91·1 (58·1 to 137)	81·5 (49·7 to 122)	75·8 (45·7 to 113)	68·2 (41·9 to 102)	66·9 (42·2 to 101)	60 (37·2 to 91·3)	52·9 (32·7 to 79·9)
Hemolytic disease and other neonatal jaundice	49·4 (28·3 to 80·9)	14·4 (7·8 to 24·9)	7 (3·1 to 14·2)	6·8 (4·5 to 9·4)	8·6 (6·3 to 11·1)	8·5 (6·2 to 10·8)	7·8 (5·7 to 9·9)	7·2 (5·3 to 9·2)	6·5 (4·8 to 8·3)	6·5 (4·8 to 8·2)	5·9 (4·4 to 7·5)	5·2 (3·9 to 6·7)
Other neonatal disorders	553 (326 to 875)	170 (90·3 to 271)	79·9 (43·8 to 138)	65·9 (50 to 85)	0 (0 to 0)	0 (0 to 0)	0 (0 to 0)	0 (0 to 0)	0 (0 to 0)	0 (0 to 0)	0 (0 to 0)	0 (0 to 0)
Nutritional deficiencies	0·6 (0·3 to 0·9)	9 (5·3 to 14·3)	245 (148 to 384)	337 (195 to 543)	380 (187 to 695)	199 (92·3 to 362)	130 (62·6 to 242)	105 (53·9 to 194)	96·2 (47·2 to 164)	110 (58·7 to 188)	132 (65·1 to 233)	175 (90·3 to 297)
Protein-energy malnutrition	0·4 (0·3 to 0·6)	1·3 (0·8 to 1·8)	176 (94·1 to 301)	83·2 (51·5 to 134)	19·9 (12·9 to 29·2)	14·6 (9·4 to 22·2)	13·3 (9 to 19·4)	11·5 (8 to 15·8)	13 (8·8 to 18·5)	13·9 (9·7 to 19·8)	17·7 (12·2 to 24·2)	15·5 (11·3 to 21·1)
Iodine deficiency	0 (0 to 0)	0 (0 to 0)	0 (0 to 0)	0·1 (0 to 0·2)	0·8 (0·3 to 1·6)	1·9 (0·7 to 3·4)	2·5 (1 to 4·5)	2·7 (1·2 to 4·7)	2·7 (1·1 to 5·2)	2·8 (1·2 to 5·2)	2·7 (1·3 to 4·9)	2·5 (1·2 to 4·4)
Vitamin A deficiency	0 (0 to 0·1)	0·5 (0·2 to 1)	2 (1 to 3·6)	10·5 (5·4 to 18·5)	13·4 (6·8 to 23·2)	8 (3·8 to 13·6)	2·7 (1·2 to 4·7)	2·1 (0·9 to 4)	1·8 (0·8 to 3·4)	1·6 (0·8 to 2·7)	1·4 (0·8 to 2·3)	1·2 (0·6 to 2)
Dietary iron deficiency	0·1 (0 to 0·3)	7·2 (3·8 to 12·2)	62·3 (30·6 to 110)	241 (111 to 434)	344 (157 to 654)	173 (67·8 to 333)	108 (42·4 to 218)	85·2 (35·2 to 172)	75·4 (30·8 to 140)	88·7 (39 to 164)	107 (41·8 to 209)	152 (71·5 to 272)
Other nutritional deficiencies	0 (0 to 0)	0 (0 to 0)	4·3 (1·8 to 9)	2·2 (1·2 to 3·9)	1·4 (0·8 to 2·2)	1·4 (0·8 to 2·3)	3·4 (1·9 to 5·6)	2·9 (1·7 to 4·3)	3·3 (1·9 to 5·2)	2·9 (1·8 to 4·2)	3·1 (1·9 to 4·5)	3·3 (2·1 to 4·8)
Non-communicable diseases	1,260 (786 to 1,910)	561 (367 to 856)	1,900 (1,290 to 2,680)	1,560 (1,220 to 1,970)	1,860 (1,460 to 2,360)	2,770 (2,150 to 3,540)	3,810 (2,980 to 4,820)	4,230 (3,360 to 5,280)	4,860 (3,910 to 5,960)	5,780 (4,660 to 7,000)	6,940 (5,770 to 8,270)	7,740 (6,490 to 9,180)
Neoplasms	12·7 (6·3 to 23·6)	10·5 (5 to 19·4)	63·1 (36·4 to 99·1)	180 (121 to 260)	216 (166 to 275)	246 (191 to 315)	266 (206 to 333)	269 (210 to 333)	392 (312 to 486)	568 (455 to 702)	868 (684 to 1,070)	1,070 (859 to 1,330)
Lip and oral cavity cancer	0 (0 to 0)	0 (0 to 0)	0 (0 to 0)	0 (0 to 0)	0·4 (0·3 to 0·6)	0·7 (0·4 to 1·1)	1·2 (0·7 to 1·8)	1·3 (0·8 to 1·9)	2·4 (1·5 to 3·6)	3·5 (2·2 to 5)	6·1 (3·9 to 9·1)	11·1 (6·9 to 17)
Nasopharynx cancer	0 (0 to 0)	0 (0 to 0)	0 (0 to 0)	0 (0 to 0)	1·6 (0·9 to 2·8)	2·4 (1·4 to 4)	4·4 (2·6 to 6·9)	4 (2·3 to 6·5)	4·9 (3·1 to 7·4)	4·8 (3 to 7·3)	6·3 (4·1 to 9·4)	10·2 (6·7 to 14·5)
Other pharynx cancer	0 (0 to 0)	0 (0 to 0)	0 (0 to 0)	0 (0 to 0)	0 (0 to 0)	0 (0 to 0)	0 (0 to 0)	0·5 (0·3 to 0·9)	0·7 (0·4 to 1)	1 (0·7 to 1·5)	2 (1·3 to 3·1)	4·2 (2·7 to 6·5)
Esophageal cancer	0 (0 to 0)	0 (0 to 0)	0 (0 to 0)	0 (0 to 0)	0 (0 to 0)	0 (0 to 0)	0 (0 to 0)	0·4 (0·3 to 0·6)	0·8 (0·5 to 1·1)	1·6 (1 to 2·2)	4·3 (2·9 to 6·3)	9·5 (5·9 to 14·4)
Stomach cancer	0 (0 to 0)	0 (0 to 0)	0 (0 to 0)	0 (0 to 0)	0 (0 to 0)	0 (0 to 0)	3·1 (2·1 to 4·4)	4·8 (3·5 to 6·3)	12 (8·5 to 16·4)	23·8 (16·6 to 32·9)	39·4 (27·2 to 54)	54·4 (38·5 to 73·7)
Colon and rectum cancer	0 (0 to 0)	0 (0 to 0)	0 (0 to 0)	0 (0 to 0)	1·7 (1·1 to 2·5)	2·4 (1·5 to 3·6)	9·2 (5·8 to 13·9)	13·2 (8·9 to 18·3)	24·9 (17·3 to 34·6)	48·8 (33·8 to 70)	75·8 (50·5 to 106)	102 (71·9 to 141)
Liver cancer	0·3 (0·1 to 0·7)	0·2 (0·1 to 0·7)	1·1 (0·5 to 2·1)	1·7 (1 to 2·5)	1·9 (1·4 to 2·7)	3·3 (2·2 to 4·9)	1·9 (1 to 3·2)	3·5 (2 to 5·6)	5·7 (3·3 to 9·4)	7·6 (4·5 to 12·5)	13·7 (7·6 to 22·7)	20·7 (12·1 to 35·5)
Liver cancer due to hepatitis B	0 (0 to 0)	0 (0 to 0)	0 (0 to 0)	0 (0 to 0)	0 (0 to 0)	0·8 (0·4 to 1·7)	0·8 (0·4 to 1·5)	1·9 (1 to 3·2)	3·4 (1·7 to 6)	4·5 (2·3 to 7·7)	7·6 (3·6 to 14)	10·4 (5·2 to 18·8)
Liver cancer due to hepatitis C	0 (0 to 0)	0 (0 to 0)	0 (0 to 0)	0 (0 to 0)	0 (0 to 0)	0 (0 to 0)	0 (0 to 0·1)	0·1 (0 to 0·1)	0·2 (0·1 to 0·4)	0·3 (0·1 to 0·7)	0·8 (0·3 to 1·8)	1·7 (0·7 to 3·3)
Liver cancer due to alcohol use	0 (0 to 0)	0 (0 to 0)	0 (0 to 0)	0 (0 to 0)	0 (0 to 0)	0 (0 to 0)	0 (0 to 0·1)	0·3 (0·1 to 0·6)	0·7 (0·3 to 1·5)	1·3 (0·6 to 2·6)	3·1 (1·2 to 6·6)	5·5 (2·5 to 10·7)
Liver cancer due to NASH	0 (0 to 0)	0 (0 to 0)	0 (0 to 0)	0 (0 to 0)	0 (0 to 0)	0 (0 to 0)	0·4 (0·2 to 0·8)	0·5 (0·2 to 1)	0·6 (0·3 to 1·1)	0·6 (0·3 to 1·2)	0·9 (0·4 to 1·8)	1·4 (0·6 to 2·7)
Liver cancer due to other causes	0·3 (0·1 to 0·7)	0·2 (0·1 to 0·7)	1·1 (0·5 to 2·1)	1·7 (1 to 2·5)	1·9 (1·4 to 2·7)	2·5 (1·7 to 3·5)	0·6 (0·3 to 1·1)	0·8 (0·4 to 1·4)	0·9 (0·4 to 1·7)	0·9 (0·4 to 1·7)	1·3 (0·6 to 2·6)	1·7 (0·8 to 3·1)
Gallbladder and biliary tract cancer	0 (0 to 0)	0 (0 to 0)	0 (0 to 0)	0 (0 to 0)	0 (0 to 0)	0 (0 to 0)	0 (0 to 0)	0·5 (0·3 to 0·8)	1 (0·6 to 1·8)	2 (1·2 to 3·4)	4·4 (2·9 to 7·5)	7·5 (4·9 to 12·3)
Pancreatic cancer	0 (0 to 0)	0 (0 to 0)	0 (0 to 0)	0 (0 to 0)	0 (0 to 0)	0 (0 to 0)	2·3 (1·1 to 4·4)	2·4 (1·5 to 3·7)	5·5 (3·7 to 8·3)	13·6 (9·1 to 19·2)	26·7 (18·2 to 36·6)	48·6 (34 to 68)
Larynx cancer	0 (0 to 0)	0 (0 to 0)	0 (0 to 0)	0 (0 to 0)	0 (0 to 0)	0 (0 to 0)	0 (0 to 0)	0·5 (0·3 to 0·7)	0·5 (0·3 to 0·7)	0·8 (0·5 to 1·3)	2·2 (1·3 to 3·4)	4·9 (2·8 to 8·1)
Tracheal, bronchus, and lung cancer	0 (0 to 0)	0 (0 to 0)	0 (0 to 0)	0 (0 to 0)	0 (0 to 0)	1·2 (0·7 to 2)	6·4 (3·5 to 10·7)	6·3 (3·3 to 10·6)	10·2 (6·8 to 14·9)	24·2 (16·5 to 35·5)	50·9 (32·4 to 74·9)	92·4 (63·6 to 129)
Malignant skin melanoma	0 (0 to 0)	0 (0 to 0)	0·1 (0·1 to 0·2)	0·4 (0·3 to 0·7)	0·4 (0·3 to 0·7)	0·5 (0·3 to 0·8)	0·9 (0·5 to 1·4)	1·7 (1·1 to 2·6)	2·6 (1·7 to 3·9)	3·5 (2·1 to 5·2)	6·7 (4·2 to 10)	6·4 (4 to 9·7)
Non-melanoma skin cancer	0 (0 to 0)	0 (0 to 0)	0 (0 to 0)	0 (0 to 0)	0 (0 to 0)	0 (0 to 0)	0 (0 to 0)	0·6 (0·4 to 1)	1·4 (0·9 to 2·1)	1·8 (1·1 to 2·7)	2·3 (1·4 to 3·5)	3·4 (2·2 to 4·9)
Non-melanoma skin cancer (squamous-cell carcinoma)	0 (0 to 0)	0 (0 to 0)	0 (0 to 0)	0 (0 to 0)	0 (0 to 0)	0 (0 to 0)	0 (0 to 0)	0·6 (0·4 to 1)	1·4 (0·9 to 2·1)	1·8 (1·1 to 2·7)	2·3 (1·4 to 3·5)	3·4 (2·2 to 4·9)
Non-melanoma skin cancer (basal-cell carcinoma)	0 (0 to 0)	0 (0 to 0)	0 (0 to 0)	0 (0 to 0)	0 (0 to 0)	0 (0 to 0)	0 (0 to 0)	0 (0 to 0)	0 (0 to 0)	0 (0 to 0)	0 (0 to 0)	0 (0 to 0)
Breast cancer	0 (0 to 0)	0 (0 to 0)	0 (0 to 0)	0 (0 to 0)	0 (0 to 0)	0 (0 to 0)	3·6 (1·8 to 6·4)	5·2 (3·2 to 7·8)	25 (15·5 to 38·4)	77 (48·8 to 115)	135 (87·3 to 203)	164 (116 to 230)
Cervical cancer	0 (0 to 0)	0 (0 to 0)	0 (0 to 0)	0 (0 to 0)	0 (0 to 0)	0 (0 to 0)	6·2 (2·8 to 11·8)	16·7 (9·2 to 28·8)	55·1 (29·1 to 91)	108 (63·9 to 172)	194 (121 to 288)	198 (129 to 288)
Uterine cancer	0 (0 to 0)	0 (0 to 0)	0 (0 to 0)	0 (0 to 0)	0 (0 to 0)	0 (0 to 0)	0 (0 to 0)	1·2 (0·6 to 2·3)	3·2 (1·4 to 6·1)	6·1 (2·9 to 11·6)	9·9 (5·3 to 16·8)	12·3 (7 to 20·7)
Ovarian cancer	0 (0 to 0)	0 (0 to 0)	0 (0 to 0)	0 (0 to 0)	2·2 (0·8 to 4·2)	3·4 (1·2 to 6)	8·8 (2·9 to 16·7)	11·1 (4·6 to 20)	14·5 (6·2 to 24·8)	19·4 (8·6 to 32·2)	30·1 (14·4 to 49·9)	44·3 (22·7 to 70·3)
Prostate cancer	0 (0 to 0)	0 (0 to 0)	0 (0 to 0)	0 (0 to 0)	0 (0 to 0)	0 (0 to 0)	0 (0 to 0)	0·7 (0·5 to 1·1)	1·7 (1 to 2·4)	2·4 (1·3 to 3·4)	3·2 (1·9 to 5·1)	7·1 (4·2 to 11·8)
Testicular cancer	0·1 (0 to 0·2)	0·1 (0·1 to 0·2)	0·7 (0·4 to 1·2)	3·1 (1·7 to 5·5)	1·3 (0·6 to 2·2)	1·5 (0·7 to 2·9)	4·3 (2 to 8·2)	6·3 (3·3 to 10·6)	8·5 (4·7 to 14·2)	5·8 (3·1 to 9·4)	5·4 (2·8 to 9·3)	4 (2·1 to 6·8)
Kidney cancer	0·7 (0·2 to 1·5)	0·3 (0·1 to 0·7)	3·2 (1·7 to 5·5)	11·2 (5·9 to 19)	8·8 (5·8 to 13)	5·1 (3·1 to 7·9)	5·3 (3 to 8·7)	5·9 (3·3 to 9)	8·1 (5 to 12·3)	9·5 (5·8 to 14·8)	11·4 (7·2 to 17·5)	16·5 (10·5 to 23·8)
Bladder cancer	0 (0 to 0)	0 (0 to 0)	0 (0 to 0)	0 (0 to 0)	0 (0 to 0)	0 (0 to 0)	0·4 (0·3 to 0·6)	0·5 (0·3 to 0·7)	0·6 (0·4 to 0·9)	1·9 (1·2 to 2·7)	3·1 (2 to 4·5)	5·3 (3·5 to 7·8)
Brain and central nervous system cancer	3·9 (0·8 to 11·8)	3·2 (0·7 to 8·5)	18·2 (4·9 to 37·4)	50·1 (17·5 to 94·5)	66·5 (25·9 to 110)	59·1 (23·5 to 94·6)	32·7 (11·9 to 53·1)	29·4 (10·9 to 47)	37·2 (14·2 to 59·1)	38·9 (15·4 to 62·5)	50 (20·1 to 78·5)	54 (21·8 to 88·1)
Thyroid cancer	0 (0 to 0)	0 (0 to 0)	0 (0 to 0)	0 (0 to 0)	0·6 (0·4 to 1)	0·6 (0·4 to 0·9)	1·2 (0·6 to 2·2)	1·3 (0·6 to 2·4)	2·3 (1·2 to 4·6)	2·7 (1·4 to 4·5)	4·7 (2·9 to 7·3)	5·4 (3·3 to 8·4)
Mesothelioma	0 (0 to 0)	0 (0 to 0)	0 (0 to 0)	0 (0 to 0)	0 (0 to 0)	0 (0 to 0)	0 (0 to 0)	1·4 (0·5 to 3·6)	1·4 (0·5 to 3·2)	1·4 (0·6 to 3·1)	2·8 (1·1 to 6·4)	2·8 (1·4 to 5·4)
Hodgkin lymphoma	0 (0 to 0)	0 (0 to 0)	0 (0 to 0)	0·5 (0·2 to 1)	1·4 (0·7 to 2·7)	2·7 (1·5 to 4·9)	9·7 (5·7 to 15·8)	13·4 (8·6 to 20·8)	14·2 (9 to 21·6)	12·1 (7·7 to 18·3)	11·4 (7·2 to 17·1)	10·5 (6·8 to 15·4)
Non-Hodgkin lymphoma	0 (0 to 0)	0 (0 to 0)	0 (0 to 0)	16·3 (9·5 to 25·2)	21·7 (14·5 to 32·4)	27·9 (18·7 to 39·7)	33·8 (23·4 to 47·9)	36 (25·2 to 50)	44·3 (31·3 to 61·8)	46·9 (31·2 to 66·1)	52·4 (35·3 to 75·7)	47·8 (33·1 to 67·8)
Multiple myeloma	0 (0 to 0)	0 (0 to 0)	0 (0 to 0)	0 (0 to 0)	0 (0 to 0)	0 (0 to 0)	0 (0 to 0)	0·9 (0·5 to 1·6)	1·3 (0·8 to 2·1)	2·1 (1·3 to 3·3)	5·2 (3·2 to 8)	10·7 (7·3 to 15·8)
Leukemia	3·2 (1·3 to 6·9)	2·5 (1 to 5·2)	17 (9·5 to 28·5)	45·4 (27·2 to 74)	61·7 (41·6 to 89·8)	70·8 (47·7 to 98)	62·7 (42·1 to 90·3)	45·5 (31·4 to 62·9)	47·3 (32·7 to 66·4)	40·8 (28·6 to 56·4)	46·5 (31·7 to 65·4)	42·7 (30·5 to 58·6)
Acute lymphoid leukemia	0·4 (0·1 to 0·9)	0·3 (0·1 to 1)	3·4 (1·5 to 6·7)	18·7 (9·8 to 31·3)	28·2 (16·7 to 43·6)	25·3 (15·1 to 37·4)	21 (12·8 to 33)	8·7 (5·1 to 13·7)	5·5 (3·2 to 8·8)	4 (2·4 to 6·3)	3·6 (2·1 to 5·7)	3 (1·8 to 4·7)
Chronic lymphoid leukemia	0 (0 to 0)	0 (0 to 0)	0 (0 to 0)	0 (0 to 0)	0 (0 to 0)	0 (0 to 0)	0 (0 to 0)	0·6 (0·3 to 1)	0·6 (0·3 to 1·2)	0·4 (0·2 to 0·7)	0·9 (0·4 to 1·5)	1·3 (0·7 to 2·2)
Acute myeloid leukemia	0·6 (0·1 to 1·8)	1·1 (0·3 to 2·9)	4 (1·6 to 8·3)	13·2 (6·9 to 23·3)	14·2 (7·7 to 23·6)	18·9 (10·8 to 29·5)	18·4 (10·5 to 29·9)	12·9 (7·5 to 20·1)	15·4 (9·7 to 23·5)	11·2 (6·8 to 16·7)	12·7 (7·5 to 19·8)	9·5 (5·5 to 14·3)
Chronic myeloid leukemia	0 (0 to 0·1)	0 (0 to 0·1)	0·4 (0·1 to 0·8)	1 (0·4 to 2)	0·8 (0·4 to 1·3)	1·1 (0·6 to 1·9)	1·4 (0·7 to 2·5)	2·5 (1·3 to 4·1)	4 (2·2 to 6·4)	4·2 (2·4 to 6·5)	5 (2·7 to 8·1)	3·6 (2·1 to 5·9)
Other leukemia	2·2 (0·8 to 4·9)	1 (0·3 to 2·5)	9·2 (4·7 to 16·6)	12·5 (6 to 23·5)	18·5 (10 to 30·7)	25·5 (14·4 to 39·9)	21·8 (12·5 to 35·5)	20·8 (13 to 31·2)	21·8 (13·6 to 33·3)	21·1 (13·7 to 31·6)	24·4 (14·7 to 36·9)	25·4 (17·4 to 36·2)
Other malignant neoplasms	4·3 (2·2 to 8·2)	3·8 (1·6 to 8·3)	21·3 (13·1 to 33·5)	48·6 (32·7 to 67·8)	44·7 (34·8 to 55·5)	62·6 (48·4 to 83)	66·3 (49 to 89·6)	52·5 (40·6 to 68·6)	52·5 (41·1 to 66·8)	53·5 (40 to 68·4)	59·8 (43·7 to 80·4)	65·6 (47·7 to 84·5)

Cause	45 to 49	50 to 54	55 to 59	60 to 64	65 to 69	70 to 74	75 to 79	80 to 84	85 to 89	90 to 94	95 plus	Total
All causes	13,100 (11,100 to 15,300)	16,300 (13,900 to 19,000)	17,400 (14,800 to 20,100)	15,500 (13,400 to 17,900)	13,000 (11,300 to 14,800)	11,000 (9,640 to 12,500)	9,510 (8,420 to 10,700)	6,830 (6,080 to 7,590)	3,800 (3,420 to 4,180)	1,590 (1,440 to 1,730)	718 (666 to 771)	195,000
Communicable, maternal, neonatal, and nutritional diseases	1,380 (1,200 to 1,600)	1,290 (1,110 to 1,530)	1,100 (919 to 1,310)	839 (698 to 1,010)	664 (545 to 805)	595 (489 to 717)	568 (458 to 697)	448 (358 to 557)	292 (231 to 360)	130 (99·7 to 160)	62·4 (46·5 to 80)	34,100
HIV/AIDS and sexually transmitted infections	586 (549 to 624)	418 (392 to 446)	253 (237 to 270)	128 (119 to 137)	70·3 (65·5 to 75·5)	22·2 (20 to 24·7)	10·9 (9·7 to 12·4)	5·3 (4·7 to 6·1)	2·3 (2 to 2·7)	0·6 (0·5 to 0·7)	0·3 (0·2 to 0·3)	5,830
HIV/AIDS	572 (537 to 608)	409 (385 to 435)	246 (230 to 261)	124 (115 to 132)	67·3 (62·9 to 71·8)	20·2 (18·5 to 21·9)	9·6 (8·7 to 10·4)	4·5 (4·1 to 4·8)	1·9 (1·8 to 2·1)	0·4 (0·3 to 0·5)	0·2 (0·2 to 0·3)	5,170
HIV/AIDS - Drug-susceptible Tuberculosis	34·4 (22 to 52)	24·7 (15·6 to 37·1)	15 (9·8 to 22·4)	7·6 (5·1 to 11·2)	4·1 (2·8 to 6·1)	1·3 (0·9 to 1·8)	0·6 (0·4 to 0·9)	0·3 (0·2 to 0·4)	0·1 (0·1 to 0·2)	0 (0 to 0)	0 (0 to 0)	312
HIV/AIDS - Multidrug-resistant Tuberculosis without extensive	0·3 (0 to 1·3)	0·2 (0 to 1)	0·2 (0 to 0·6)	0·1 (0 to 0·3)	0 (0 to 0·2)	0 (0 to 0)	0 (0 to 0)	0 (0 to 0)	0 (0 to 0)	0 (0 to 0)	0 (0 to 0)	3·1
HIV/AIDS - Extensively drug-resistant Tuberculosis	0·1 (0 to 0·2)	0 (0 to 0·2)	0 (0 to 0·1)	0 (0 to 0)	0 (0 to 0)	0 (0 to 0)	0 (0 to 0)	0 (0 to 0)	0 (0 to 0)	0 (0 to 0)	0 (0 to 0)	0·5
HIV/AIDS resulting in other diseases	537 (500 to 574)	384 (357 to 410)	231 (213 to 246)	116 (107 to 125)	63·1 (58·3 to 67·7)	18·9 (17·2 to 20·6)	9 (8·1 to 9·8)	4·2 (3·8 to 4·6)	1·8 (1·6 to 2)	0·4 (0·3 to 0·4)	0·2 (0·2 to 0·2)	4,850
Sexually transmitted infections excluding HIV	13·8 (7·7 to 24·8)	9·8 (5·3 to 19)	7 (3·6 to 13·5)	4·3 (2·1 to 8·4)	3 (1·5 to 5·8)	2 (1 to 3·9)	1·3 (0·7 to 2·5)	0·8 (0·4 to 1·5)	0·4 (0·2 to 0·7)	0·2 (0·1 to 0·3)	0·1 (0 to 0·1)	659
Syphilis	0·7 (0·4 to 1·2)	0·7 (0·4 to 1·5)	0·6 (0·3 to 1·2)	0·4 (0·2 to 0·8)	0·4 (0·2 to 0·9)	0·3 (0·1 to 0·5)	0·1 (0·1 to 0·3)	0·1 (0·1 to 0·2)	0·1 (0 to 0·1)	0 (0 to 0)	0 (0 to 0)	544
Chlamydial infection	1·6 (0·8 to 2·7)	0·6 (0·3 to 1·1)	0·3 (0·2 to 0·5)	0·1 (0 to 0·2)	0 (0 to 0·1)	0 (0 to 0·1)	0 (0 to 0·1)	0 (0 to 0·1)	0 (0 to 0)	0 (0 to 0)	0 (0 to 0)	13·9
Gonococcal infection	1·2 (0·7 to 2·3)	0·7 (0·4 to 1·4)	0·4 (0·2 to 0·9)	0·3 (0·1 to 0·6)	0·3 (0·1 to 0·6)	0·2 (0·1 to 0·4)	0·1 (0 to 0·3)	0·1 (0 to 0·2)	0 (0 to 0·1)	0 (0 to 0)	0 (0 to 0)	12·4
Trichomoniasis	3·5 (1·1 to 8·4)	3 (0·9 to 6·7)	2 (0·6 to 4·6)	1·1 (0·4 to 2·5)	0·7 (0·2 to 1·6)	0·4 (0·2 to 1)	0·3 (0·1 to 0·6)	0·1 (0·1 to 0·3)	0·1 (0 to 0·1)	0 (0 to 0)	0 (0 to 0)	29·4
Genital herpes	3·7 (1·2 to 9·5)	3·6 (1·1 to 8·9)	3 (0·9 to 7·4)	2·1 (0·6 to 5·3)	1·4 (0·4 to 3·5)	1 (0·3 to 2·5)	0·7 (0·2 to 1·6)	0·4 (0·1 to 0·9)	0·2 (0·1 to 0·5)	0·1 (0 to 0·2)	0 (0 to 0·1)	32·9
Other sexually transmitted infections	3·1 (1·7 to 5·1)	1·2 (0·6 to 2)	0·7 (0·4 to 1·1)	0·3 (0·1 to 0·5)	0·1 (0·1 to 0·3)	0·1 (0 to 0·2)	0·1 (0 to 0·2)	0·1 (0 to 0·1)	0·1 (0 to 0·1)	0 (0 to 0)	0 (0 to 0)	27·2
Respiratory infections and tuberculosis	220 (167 to 288)	274 (201 to 361)	303 (226 to 399)	293 (216 to 390)	294 (216 to 394)	315 (235 to 411)	340 (251 to 450)	284 (210 to 374)	193 (142 to 252)	87·9 (63·5 to 115)	42·9 (29·8 to 58·3)	5,310
Tuberculosis	33·4 (22·6 to 47·9)	39·3 (26·5 to 56·9)	34·2 (23·3 to 47·1)	27·6 (18·7 to 39·7)	20·5 (14·1 to 29·1)	15·6 (10·8 to 22·1)	12·5 (8·9 to 17·2)	8·1 (5·6 to 11·8)	4·1 (2·9 to 5·7)	1·2 (0·8 to 1·6)	0·4 (0·3 to 0·6)	413
Latent tuberculosis infection	0 (0 to 0)	0 (0 to 0)	0 (0 to 0)	0 (0 to 0)	0 (0 to 0)	0 (0 to 0)	0 (0 to 0)	0 (0 to 0)	0 (0 to 0)	0 (0 to 0)	0 (0 to 0)	0
Drug-susceptible tuberculosis	33·1 (22·5 to 47·2)	39 (26·3 to 56·2)	33·8 (23·2 to 46·9)	27·3 (18·5 to 39·3)	20·3 (14·1 to 28·7)	15·5 (10·7 to 22)	12·4 (8·9 to 17·1)	8 (5·6 to 11·7)	4·1 (2·8 to 5·6)	1·2 (0·8 to 1·6)	0·4 (0·3 to 0·5)	409
Multidrug-resistant tuberculosis without extensive drug resista	0·3 (0 to 1·1)	0·3 (0 to 1·3)	0·3 (0 to 1·1)	0·2 (0 to 0·9)	0·2 (0 to 0·7)	0·1 (0 to 0·5)	0·1 (0 to 0·4)	0·1 (0 to 0·3)	0 (0 to 0·1)	0 (0 to 0)	0 (0 to 0)	3·2
Extensively drug-resistant tuberculosis	0 (0 to 0·2)	0·1 (0 to 0·2)	0 (0 to 0·2)	0 (0 to 0·1)	0 (0 to 0·1)	0 (0 to 0·1)	0 (0 to 0·1)	0 (0 to 0)	0 (0 to 0)	0 (0 to 0)	0 (0 to 0)	0·5
Lower respiratory infections	153 (108 to 210)	202 (139 to 280)	243 (171 to 335)	247 (174 to 342)	261 (186 to 362)	292 (212 to 388)	323 (234 to 427)	273 (199 to 362)	187 (136 to 246)	86 (62·1 to 112)	42·3 (29·2 to 57·6)	4,270
Upper respiratory infections	25·4 (13·5 to 44·8)	23·7 (13 to 40·3)	19·4 (10·5 to 32·9)	14 (7·4 to 23·2)	9·5 (5 to 16·1)	6·5 (3·5 to 11·1)	4·3 (2·3 to 7·2)	2·5 (1·3 to 4·2)	1·2 (0·6 to 1·9)	0·5 (0·3 to 0·8)	0·2 (0·1 to 0·4)	463
Otitis media	8·3 (4·1 to 14·9)	8·5 (4 to 15·5)	6·8 (3·4 to 12)	4·4 (2·1 to 8·6)	2·4 (1·2 to 4·6)	1·4 (0·6 to 2·8)	1 (0·5 to 1·9)	0·7 (0·3 to 1·3)	0·4 (0·2 to 0·7)	0·2 (0·1 to 0·3)	0·1 (0 to 0·1)	156
Enteric infections	99·5 (69·6 to 142)	114 (80·3 to 165)	117 (80·1 to 171)	115 (79 to 167)	109 (76·3 to 156)	112 (78·3 to 164)	106 (73·6 to 155)	83·5 (56·8 to 125)	47·1 (31·6 to 71·9)	20·3 (13 to 32·8)	9·5 (6 to 16)	2,550
Diarrheal diseases	99·1 (69·2 to 141)	113 (79·8 to 164)	116 (79·8 to 170)	115 (78·7 to 166)	109 (76·1 to 155)	112 (77·9 to 164)	106 (73·4 to 155)	83·4 (56·7 to 125)	47 (31·5 to 71·8)	20·3 (13 to 32·8)	9·5 (5·9 to 16)	2,540
Typhoid and paratyphoid	0·2 (0·1 to 0·4)	0·2 (0·1 to 0·4)	0·2 (0·1 to 0·4)	0·1 (0 to 0·3)	0·1 (0 to 0·2)	0·1 (0 to 0·1)	0·1 (0 to 0·1)	0 (0 to 0·1)	0 (0 to 0)	0 (0 to 0)	0 (0 to 0)	4
Typhoid fever	0·1 (0 to 0·3)	0·1 (0·1 to 0·3)	0·1 (0 to 0·3)	0·1 (0 to 0·2)	0·1 (0 to 0·1)	0 (0 to 0·1)	0 (0 to 0·1)	0 (0 to 0·1)	0 (0 to 0)	0 (0 to 0)	0 (0 to 0)	3
Paratyphoid fever	0 (0 to 0·1)	0 (0 to 0·1)	0 (0 to 0·1)	0 (0 to 0·1)	0 (0 to 0·1)	0 (0 to 0·1)	0 (0 to 0)	0 (0 to 0)	0 (0 to 0)	0 (0 to 0)	0 (0 to 0)	1·1
Invasive Non-typhoidal Salmonella (iNTS)	0·2 (0 to 0·5)	0·3 (0 to 0·6)	0·3 (0 to 0·7)	0·3 (0 to 0·6)	0·2 (0 to 0·5)	0·2 (0 to 0·4)	0·1 (0 to 0·3)	0·1 (0 to 0·2)	0 (0 to 0·1)	0 (0 to 0)	0 (0 to 0)	6·6
Other intestinal infectious diseases	0·1 (0 to 0·2)	0·1 (0 to 0·2)	0·1 (0 to 0·2)	0 (0 to 0·1)	0·1 (0 to 0·2)	0 (0 to 0·1)	0 (0 to 0·1)	0 (0 to 0·1)	0 (0 to 0)	0 (0 to 0)	0 (0 to 0)	3·6
Neglected tropical diseases and malaria	94·5 (57·2 to 145)	105 (63·1 to 159)	98·3 (58·7 to 150)	84·4 (48·1 to 129)	60·9 (35·8 to 92·4)	49·6 (29·7 to 76)	38·4 (22·6 to 60·9)	24·5 (13·9 to 39·2)	14 (8·7 to 21·4)	5·4 (3·4 to 8·3)	2·3 (1·3 to 3·5)	1,680
Malaria	2·1 (0·6 to 9·7)	2 (0·5 to 9·2)	1·7 (0·4 to 8·2)	1·1 (0·3 to 5·2)	0·7 (0·1 to 3·5)	0·5 (0·1 to 2·3)	0·2 (0 to 1·2)	0·1 (0 to 0·5)	0 (0 to 0·1)	0 (0 to 0)	0 (0 to 0)	38·9
Chagas disease	0·1 (0·1 to 0·3)	0·1 (0·1 to 0·3)	0·2 (0·1 to 0·6)	0·4 (0·2 to 0·8)	0·5 (0·2 to 0·8)	0·4 (0·2 to 0·8)	0·2 (0·1 to 0·4)	0·2 (0·1 to 0·4)	1·5 (0·7 to 4·1)	0·4 (0·2 to 1·3)	0·1 (0 to 0·4)	4·5
Leishmaniasis	1·8 (0·9 to 2·9)	2·1 (1·1 to 3·4)	2·2 (1·1 to 3·5)	1·9 (1·1 to 3)	1·5 (0·8 to 2·4)	1·2 (0·7 to 1·9)	1 (0·6 to 1·5)	0·6 (0·4 to 0·9)	0·3 (0·1 to 0·4)	0·1 (0 to 0·1)	0 (0 to 0)	35·6
Visceral leishmaniasis	0·2 (0 to 0·8)	0·2 (0 to 0·6)	0·2 (0 to 0·5)	0·1 (0 to 0·3)	0·1 (0 to 0·2)	0 (0 to 0·1)	0 (0 to 0·1)	0 (0 to 0)	0 (0 to 0)	0 (0 to 0)	0 (0 to 0)	8·3
Cutaneous and mucocutaneous leishmaniasis	1·6 (0·8 to 2·6)	1·9 (1 to 3·2)	2 (1·1 to 3·3)	1·8 (0·9 to 2·9)	1·4 (0·8 to 2·3)	1·2 (0·7 to 1·9)	1 (0·6 to 1·5)	0·6 (0·3 to 0·9)	0·3 (0·1 to 0·4)	0·1 (0 to 0·1)	0 (0 to 0)	27·3
African trypanosomiasis	0 (0 to 0)	0 (0 to 0)	0 (0 to 0)	0 (0 to 0)	0 (0 to 0)	0 (0 to 0)	0 (0 to 0)	0 (0 to 0)	0 (0 to 0)	0 (0 to 0)	0 (0 to 0)	0
Schistosomiasis	37·5 (14·6 to 78·6)	38·4 (14·7 to 78·3)	33·8 (12·3 to 73·1)	24·1 (8 to 52·2)	15·1 (4·9 to 33·8)	11·4 (3·4 to 26·8)	7·9 (2·2 to 19·8)	4·5 (1·2 to 10·6)	2·2 (0·7 to 5)	0·8 (0·3 to 1·7)	0·3 (0·1 to 0·6)	625
Cysticercosis	26 (9·5 to 56·2)	32·8 (11·7 to 66·4)	36·9 (14·7 to 73·2)	35·6 (13·9 to 67·8)	31·4 (12·8 to 58·5)	26·8 (11·5 to 50·1)	21·6 (9 to 40·3)	14·6 (5·8 to 27·5)	7·3 (3 to 13·2)	2·9 (1·2 to 5·6)	1·2 (0·4 to 2·3)	317
Cystic echinococcosis	0 (0 to 0·1)	0 (0 to 0·1)	0 (0 to 0·1)	0 (0 to 0·1)	0 (0 to 0·1)	0 (0 to 0)	0 (0 to 0)	0 (0 to 0)	0 (0 to 0)	0 (0 to 0)	0 (0 to 0)	0·4
Lymphatic filariasis	0 (0 to 0)	0 (0 to 0)	0 (0 to 0)	0 (0 to 0)	0 (0 to 0)	0 (0 to 0)	0 (0 to 0)	0 (0 to 0)	0 (0 to 0)	0 (0 to 0)	0 (0 to 0)	0
Onchocerciasis	0 (0 to 0)	0 (0 to 0)	0 (0 to 0)	0 (0 to 0)	0 (0 to 0)	0 (0 to 0)	0 (0 to 0)	0 (0 to 0)	0 (0 to 0)	0 (0 to 0)	0 (0 to 0)	0
Trachoma	0 (0 to 0)	0 (0 to 0)	0 (0 to 0)	0 (0 to 0)	0 (0 to 0)	0 (0 to 0)	0 (0 to 0)	0 (0 to 0)	0 (0 to 0)	0 (0 to 0)	0 (0 to 0)	0
Dengue	16·2 (5·3 to 34·7)	18·8 (5·5 to 38·2)	13·8 (4·4 to 27·9)	16·4 (3·8 to 37·9)	8·7 (2·3 to 19·9)	6·5 (1·8 to 13·1)	5 (1·2 to 10·8)	3·2 (0·9 to 6·5)	1·8 (0·5 to 3·7)	0·7 (0·2 to 1·3)	0·3 (0·1 to 0·7)	423
Yellow fever	0·2 (0 to 0·6)	0·1 (0 to 0·5)	0·1 (0 to 0·3)	0·1 (0 to 0·2)	0 (0 to 0·1)	0 (0 to 0·1)	0 (0 to 0)	0 (0 to 0)	0 (0 to 0)	0 (0 to 0)	0 (0 to 0)	3·9
Rabies	0·3 (0·1 to 0·9)	0·3 (0·1 to 0·6)	0 (0 to 0·1)	0·2 (0·1 to 0·6)	0·2 (0·1 to 0·5)	0·1 (0 to 0·2)	0 (0 to 0)	0 (0 to 0·1)	0 (0 to 0·1)	0 (0 to 0)	0 (0 to 0)	6·4
Intestinal nematode infections	0·6 (0·2 to 1)	0·7 (0·3 to 1·2)	0·6 (0·3 to 1·1)	0·5 (0·2 to 0·9)	0·3 (0·1 to 0·5)	0·2 (0·1 to 0·4)	0·2 (0·1 to 0·4)	0·1 (0 to 0·2)	0·1 (0 to 0·1)	0 (0 to 0·1)	0 (0 to 0)	32·9
Ascariasis	0·1 (0 to 0·2)	0·1 (0 to 0·3)	0·1 (0 to 0·3)	0·1 (0 to 0·4)	0 (0 to 0·1)	0·1 (0 to 0·2)	0 (0 to 0·2)	0 (0 to 0·1)	0 (0 to 0)	0 (0 to 0)	0 (0 to 0)	3·1
Trichuriasis	0 (0 to 0)	0 (0 to 0)	0 (0 to 0)	0 (0 to 0)	0 (0 to 0)	0 (0 to 0)	0 (0 to 0)	0 (0 to 0)	0 (0 to 0)	0 (0 to 0)	0 (0 to 0)	0
Hookworm disease	0·5 (0·2 to 0·9)	0·6 (0·3 to 1·1)	0·5 (0·2 to 1·1)	0·4 (0·2 to 0·8)	0·2 (0·1 to 0·5)	0·2 (0·1 to 0·4)	0·2 (0·1 to 0·3)	0·1 (0 to 0·2)	0 (0 to 0·1)	0 (0 to 0)	0 (0 to 0)	29·7
Food-borne trematodiases	0 (0 to 0)	0 (0 to 0)	0 (0 to 0)	0 (0 to 0)	0 (0 to 0)	0 (0 to 0)	0 (0 to 0)	0 (0 to 0)	0 (0 to 0)	0 (0 to 0)	0 (0 to 0)	0
Leprosy	0·4 (0·1 to 0·7)	0·4 (0·1 to 0·9)	0·5 (0·2 to 0·9)	0·4 (0·1 to 0·7)	0·3 (0·1 to 0·6)	0·3 (0·1 to 0·5)	0·2 (0·1 to 0·3)	0·1 (0·1 to 0·2)	0·1 (0 to 0·1)	0 (0 to 0·1)	0 (0 to 0)	3·8
Ebola	0 (0 to 0)	0 (0 to 0)	0 (0 to 0)	0 (0 to 0)	0 (0 to 0)	0 (0 to 0)	0 (0 to 0)	0 (0 to 0)	0 (0 to 0)	0 (0 to 0)	0 (0 to 0)	0
Zika virus	0·1 (0 to 0·7)	0·1 (0 to 0·6)	0·1 (0 to 0·4)	0·1 (0 to 0·3)	0 (0 to 0·2)	0 (0 to 0·1)	0 (0 to 0)	0 (0 to 0)	0 (0 to 0)	0 (0 to 0)	0 (0 to 0)	3·2
Guinea worm disease	0 (0 to 0)	0 (0 to 0)	0 (0 to 0)	0 (0 to 0)	0 (0 to 0)	0 (0 to 0)	0 (0 to 0)	0 (0 to 0)	0 (0 to 0)	0 (0 to 0)	0 (0 to 0)	0
Other neglected tropical diseases	9·1 (5 to 15·1)	8·9 (4·7 to 15)	8·3 (4·5 to 13·6)	3·7 (2·1 to 6·2)	2·1 (1·2 to 3·6)	2·3 (1·1 to 3·9)	2·1 (1·1 to 3·8)	1·1 (0·6 to 2)	0·8 (0·5 to 1·3)	0·4 (0·2 to 0·7)	0·3 (0·1 to 0·6)	186
Other infectious diseases	88 (62·1 to 128)	92·6 (62·2 to 139)	83·2 (58·1 to 121)	63·6 (45·2 to 89)	45·8 (31·6 to 67·7)	33·8 (23·7 to 49·5)	23·2 (16·4 to 33·6)	16·1 (11·2 to 23·8)	11·8 (7·8 to 17·9)	4·1 (2·5 to 6·4)	1·9 (1 to 3·4)	1,700
Meningitis	14·3 (9·9 to 21)	16·3 (10·9 to 23·6)	14·3 (10 to 21·1)	11·7 (7·9 to 17·1)	8·2 (5·4 to 12·4)	5·8 (3·8 to 8·8)	4·7 (3·1 to 6·7)	2·9 (1·9 to 4·2)	1·5 (1 to 2·1)	0·4 (0·2 to 0·5)	0·1 (0·1 to 0·2)	426
Encephalitis	12·4 (7·5 to 19·1)	10 (6·2 to 15·4)	9·7 (5·7 to 15·3)	7·8 (4·6 to 12)	4·9 (2·9 to 7·8)	3·7 (2·1 to 6)	2·1 (1·2 to 3·3)	1·3 (0·7 to 2·1)	0·5 (0·3 to 0·9)	0·2 (0·1 to 0·3)	0·1 (0 to 0·1)	343
Diphtheria	0 (0 to 0·1)	0 (0 to 0·1)	0 (0 to 0·1)	0 (0 to 0)	0 (0 to 0)	0 (0 to 0)	0 (0 to 0)	0 (0 to 0)	0 (0 to 0)	0 (0 to 0)	0 (0 to 0)	0·7
Tetanus	2·5 (1·1 to 5·7)	3·8 (1·7 to 7·6)	3·8 (1·8 to 8·5)	2·7 (1·2 to 6·5)	2 (1 to 4·6)	1·7 (0·8 to 3·8)	1·3 (0·6 to 2·9)	0·6 (0·3 to 1·3)	0·3 (0·2 to 0·7)	0·1 (0·1 to 0·2)	0 (0 to 0·1)	32·9
Measles	0 (0 to 0)	0 (0 to 0)	0 (0 to 0)	0 (0 to 0)	0 (0 to 0)	0 (0 to 0)	0 (0 to 0)	0 (0 to 0)	0 (0 to 0)	0 (0 to 0)	0 (0 to 0)	0
Varicella and herpes zoster	1·6 (0·7 to 2·6)	2 (1 to 3·2)	2·1 (1·1 to 3·5)	2 (1 to 3·4)	2·1 (1 to 3·8)	1·7 (0·8 to 3·1)	1·9 (0·8 to 3·8)	2·1 (0·7 to 4·5)	2 (0·6 to 4·6)	1·2 (0·3 to 2·9)	0·8 (0·2 to 2)	53·4
Acute hepatitis	3·6 (2·4 to 5·4)	3·8 (2·3 to 6·1)	2·9 (1·8 to 4·6)	1·9 (1·2 to 3)	1·3 (0·7 to 2·2)	0·9 (0·5 to 1·4)	0·5 (0·3 to 0·8)	0·3 (0·2 to 0·4)	0·2 (0·1 to 0·3)	0 (0 to 0·1)	0 (0 to 0)	57·7
Acute hepatitis A	0·8 (0·5 to 1·4)	0·6 (0·3 to 1)	0·4 (0·2 to 0·7)	0·2 (0·1 to 0·4)	0·1 (0 to 0·2)	0 (0 to 0·1)	0 (0 to 0·1)	0 (0 to 0·1)	0 (0 to 0)	0 (0 to 0)	0 (0 to 0)	22·6
Acute hepatitis B	2·2 (1·2 to 3·5)	2·8 (1·5 to 4·9)	2·1 (1·2 to 3·6)	1·3 (0·8 to 2·2)	1 (0·5 to 1·7)	0·6 (0·4 to 1·1)	0·3 (0·2 to 0·5)	0·2 (0·1 to 0·3)	0·1 (0·1 to 0·2)	0 (0 to 0·1)	0 (0 to 0)	28·6
Acute hepatitis C	0·3 (0·1 to 0·6)	0·3 (0·1 to 0·6)	0·3 (0·1 to 0·5)	0·2 (0·1 to 0·4)	0·1 (0·1 to 0·3)	0·1 (0 to 0·2)	0·1 (0 to 0·1)	0 (0 to 0·1)	0 (0 to 0)	0 (0 to 0)	0 (0 to 0)	2·6
Acute hepatitis E	0·3 (0·2 to 0·6)	0·1 (0·1 to 0·3)	0·1 (0·1 to 0·3)	0·2 (0·1 to 0·5)	0·1 (0 to 0·1)	0·1 (0 to 0·1)	0·1 (0 to 0·2)	0 (0 to 0·1)	0 (0 to 0·1)	0 (0 to 0)	0 (0 to 0)	4
Other unspecified infectious diseases	53·5 (30·8 to 91·2)	56·6 (31·3 to 99·4)	50·2 (28·1 to 84·6)	37·4 (21·1 to 61·3)	27·3 (14·8 to 47·3)	20 (11·1 to 35·5)	12·7 (7·1 to 22·4)	8·9 (4·8 to 15·8)	7·3 (3·9 to 12·8)	2·2 (1·1 to 4)	0·9 (0·4 to 1·7)	625
Maternal and neonatal disorders	121 (90·6 to 156)	98·7 (71·6 to 130)	71·8 (51·5 to 93·5)	43·2 (31·1 to 58·3)	22·5 (16·1 to 30·6)	10·7 (7·5 to 15)	4·4 (3 to 6·3)	1·2 (0·8 to 1·8)	0·3 (0·1 to 0·4)	0·1 (0 to 0·1)	0 (0 to 0)	14,200
Maternal disorders	10·8 (4·7 to 21·5)	1·4 (0·6 to 3)	0 (0 to 0)	0 (0 to 0)	0 (0 to 0)	0 (0 to 0)	0 (0 to 0)	0 (0 to 0)	0 (0 to 0)	0 (0 to 0)	0 (0 to 0)	695
Maternal hemorrhage	1·4 (0·6 to 2·9)	0·1 (0 to 0·2)	0 (0 to 0)	0 (0 to 0)	0 (0 to 0)	0 (0 to 0)	0 (0 to 0)	0 (0 to 0)	0 (0 to 0)	0 (0 to 0)	0 (0 to 0)	129
Maternal sepsis and other maternal infections	0·3 (0·1 to 0·7)	0 (0 to 0·1)	0 (0 to 0)	0 (0 to 0)	0 (0 to 0)	0 (0 to 0)	0 (0 to 0)	0 (0 to 0)	0 (0 to 0)	0 (0 to 0)	0 (0 to 0)	29·3
Maternal hypertensive disorders	1·6 (0·7 to 3)	0·2 (0·1 to 0·5)	0 (0 to 0)	0 (0 to 0)	0 (0 to 0)	0 (0 to 0)	0 (0 to 0)	0 (0 to 0)	0 (0 to 0)	0 (0 to 0)	0 (0 to 0)	137
Maternal obstructed labor and uterine rupture	0·5 (0·2 to 1)	0·1 (0 to 0·1)	0 (0 to 0)	0 (0 to 0)	0 (0 to 0)	0 (0 to 0)	0 (0 to 0)	0 (0 to 0)	0 (0 to 0)	0 (0 to 0)	0 (0 to 0)	19·5
Maternal abortion and miscarriage	0·7 (0·2 to 1·7)	0·1 (0 to 0·2)	0 (0 to 0)	0 (0 to 0)	0 (0 to 0)	0 (0 to 0)	0 (0 to 0)	0 (0 to 0)	0 (0 to 0)	0 (0 to 0)	0 (0 to 0)	22·5
Ectopic pregnancy	1·1 (0·4 to 2·2)	0·1 (0 to 0·1)	0 (0 to 0)	0 (0 to 0)	0 (0 to 0)	0 (0 to 0)	0 (0 to 0)	0 (0 to 0)	0 (0 to 0)	0 (0 to 0)	0 (0 to 0)	54·1
Indirect maternal deaths	1 (0·4 to 2·1)	0·1 (0 to 0·3)	--	--	--	--	--	--	--	--	--	NA
Late maternal deaths	0·5 (0·2 to 1)	0·1 (0 to 0·1)	--	--	--	--	--	--	--	--	--	NA
Maternal deaths aggravated by HIV/AIDS	0·1 (0 to 0·1)	0 (0 to 0)	--	--	--	--	--	--	--	--	--	NA
Neonatal disorders	110 (80·5 to 145)	97·3 (70 to 129)	71·8 (51·5 to 93·5)	43·2 (31·1 to 58·3)	22·5 (16·1 to 30·6)	10·7 (7·5 to 15)	4·4 (3 to 6·3)	1·2 (0·8 to 1·8)	0·3 (0·1 to 0·4)	0·1 (0 to 0·1)	0 (0 to 0)	13,500
Neonatal preterm birth	42 (30·9 to 56)	35·3 (25·5 to 47·9)	24·5 (17·8 to 32·6)	13·6 (9·8 to 18·3)	6·7 (5 to 8·7)	3 (2·2 to 4)	1·2 (0·8 to 1·8)	0·3 (0·2 to 0·5)	0·1 (0·1 to 0·3)	0·1 (0 to 0·1)	0 (0 to 0)	6,520
Neonatal encephalopathy due to birth asphyxia and trauma	13·3 (7·3 to 21·5)	11·8 (6·3 to 18·7)	9·2 (4·8 to 15·3)	5·8 (3 to 9·3)	2·8 (1·6 to 4·4)	0·9 (0·5 to 1·5)	0·2 (0·1 to 0·4)	0 (0 to 0·1)	0 (0 to 0)	0 (0 to 0)	0 (0 to 0)	2,780
Neonatal sepsis and other neonatal infections	50 (31·2 to 74·6)	45·6 (27·8 to 68·5)	34·6 (20·6 to 52·2)	21·5 (13 to 32·9)	11·8 (7 to 18·8)	6·1 (3·5 to 9·9)	2·7 (1·5 to 4·3)	0·8 (0·4 to 1·3)	0·1 (0·1 to 0·2)	0 (0 to 0)	0 (0 to 0)	3,210
Hemolytic disease and other neonatal jaundice	5 (3·7 to 6·4)	4·6 (3·4 to 5·9)	3·5 (2·6 to 4·5)	2·2 (1·6 to 2·8)	1·2 (0·9 to 1·6)	0·6 (0·5 to 0·8)	0·3 (0·2 to 0·4)	0·1 (0·1 to 0·1)	0 (0 to 0)	0 (0 to 0)	0 (0 to 0)	151
Other neonatal disorders	0 (0 to 0)	0 (0 to 0)	0 (0 to 0)	0 (0 to 0)	0 (0 to 0)	0 (0 to 0)	0 (0 to 0)	0 (0 to 0)	0 (0 to 0)	0 (0 to 0)	0 (0 to 0)	869
Nutritional deficiencies	170 (91·6 to 295)	190 (100 to 304)	171 (95 to 273)	111 (63·8 to 182)	61·6 (35·6 to 98·5)	51·8 (31·9 to 78·9)	44·6 (29·4 to 67·9)	33·5 (21·6 to 48·7)	23·2 (15·5 to 34·2)	11·3 (7·7 to 16·1)	5·5 (3·6 to 7·6)	2,790
Protein-energy malnutrition	18·2 (12·6 to 25·2)	23 (15·8 to 33·2)	24·3 (16·8 to 33·4)	23·6 (16·3 to 33·8)	21·7 (13·8 to 32·1)	21·6 (14·3 to 32)	21·7 (14·3 to 31·5)	17·3 (11·5 to 24·8)	12·2 (8·2 to 17·4)	6·5 (4·1 to 9·7)	4 (2·5 to 5·8)	574
Iodine deficiency	2·4 (1·1 to 4·3)	2·3 (1·1 to 4·1)	1·9 (0·9 to 3·3)	1·4 (0·7 to 2·2)	0·9 (0·5 to 1·4)	0·6 (0·3 to 1)	0·4 (0·2 to 0·6)	0·2 (0·1 to 0·3)	0·1 (0·1 to 0·2)	0 (0 to 0·1)	0 (0 to 0)	28·9
Vitamin A deficiency	1·1 (0·6 to 1·9)	1 (0·5 to 1·7)	0·8 (0·4 to 1·3)	0·5 (0·3 to 0·8)	0·3 (0·2 to 0·6)	0·2 (0·1 to 0·4)	0·2 (0·1 to 0·3)	0·1 (0·1 to 0·2)	0 (0 to 0·1)	0 (0 to 0)	0 (0 to 0)	49·5
Dietary iron deficiency	145 (67·2 to 263)	159 (71·1 to 268)	140 (65·6 to 237)	82·3 (37·1 to 149)	35·3 (13·8 to 69·2)	26·7 (10·4 to 53·5)	20·1 (7·5 to 40·7)	14·2 (5·4 to 27)	9·7 (4·2 to 18·8)	4·2 (1·8 to 7·5)	1·2 (0·5 to 2·2)	2,080
Other nutritional deficiencies	3·7 (2·3 to 5·5)	4·8 (3·2 to 6·9)	4·1 (2·7 to 6)	3·5 (2·3 to 5·1)	3·3 (2·1 to 5)	2·7 (1·7 to 4·2)	2·2 (1·4 to 3·8)	1·8 (1 to 3)	1·1 (0·6 to 1·9)	0·6 (0·3 to 1)	0·3 (0·1 to 0·4)	56·2
Non-communicable diseases	10,000 (8,400 to 11,700)	13,400 (11,200 to 15,700)	14,900 (12,700 to 17,300)	13,700 (11,800 to 15,800)	11,700 (10,100 to 13,400)	9,910 (8,660 to 11,300)	8,540 (7,520 to 9,610)	6,100 (5,430 to 6,790)	3,330 (2,990 to 3,680)	1,380 (1,250 to 1,520)	618 (570 to 670)	137,000
Neoplasms	1,490 (1,190 to 1,810)	2,150 (1,730 to 2,610)	2,540 (2,090 to 3,080)	2,350 (1,950 to 2,820)	1,970 (1,630 to 2,350)	1,670 (1,390 to 1,990)	1,340 (1,120 to 1,580)	835 (685 to 1,010)	402 (322 to 483)	131 (99·5 to 159)	42 (30·4 to 52·7)	19,100
Lip and oral cavity cancer	20 (12·9 to 29·5)	33·8 (22·6 to 50·6)	39·7 (26·8 to 56·4)	33·2 (23 to 45·6)	26·2 (18·5 to 36)	19·2 (13·6 to 26·3)	15·1 (10·7 to 20·8)	8·9 (6·3 to 12·2)	3·7 (2·5 to 5)	1·3 (0·9 to 1·8)	0·5 (0·3 to 0·7)	228
Nasopharynx cancer	15·9 (10·6 to 23·1)	23·8 (16·3 to 34·4)	27·7 (18·8 to 40)	19·3 (13·3 to 28·3)	13·5 (9 to 19·3)	9·8 (6·6 to 13·7)	5·6 (3·6 to 8)	3·3 (2·1 to 4·8)	2·5 (1·5 to 3·8)	0·8 (0·5 to 1·3)	0·2 (0·1 to 0·4)	161
Other pharynx cancer	9·7 (6·2 to 14·5)	15·4 (10·2 to 22·6)	18·6 (12·1 to 28·1)	15·4 (10·2 to 23·1)	10·7 (7·1 to 15·9)	7·2 (4·8 to 10·4)	4·7 (3·2 to 6·7)	2·6 (1·8 to 3·7)	1·2 (0·8 to 1·7)	0·4 (0·3 to 0·6)	0·2 (0·1 to 0·2)	94·6
Esophageal cancer	19·4 (12·8 to 28)	33·7 (22·5 to 48·9)	40·8 (28·2 to 56·7)	35·2 (24·3 to 49·7)	27·2 (18·9 to 37)	19·8 (14·2 to 26·1)	14·4 (10·3 to 19·8)	8·2 (5·8 to 11·1)	3·7 (2·7 to 4·9)	1·1 (0·8 to 1·5)	0·3 (0·2 to 0·5)	220
Stomach cancer	77 (55 to 107)	127 (89·1 to 179)	149 (107 to 202)	144 (106 to 193)	124 (94·8 to 161)	99·9 (75·9 to 130)	83·9 (64·4 to 107)	53·4 (41 to 69·7)	25·3 (18·5 to 33·1)	8 (5·6 to 10·6)	2·5 (1·7 to 3·4)	1,030
Colon and rectum cancer	153 (109 to 209)	249 (173 to 335)	318 (234 to 423)	306 (227 to 400)	278 (207 to 366)	241 (183 to 312)	201 (154 to 263)	125 (90·7 to 165)	60 (43·2 to 79·4)	20·9 (14·5 to 28·6)	6·5 (4·2 to 9·2)	2,240
Liver cancer	30·8 (17·9 to 50·1)	49·1 (29·8 to 79·8)	65·6 (41·9 to 99·7)	58·8 (37·8 to 86·6)	50·1 (34·3 to 72·1)	41·2 (28·2 to 58·5)	31·4 (21·7 to 43·7)	17·7 (12·3 to 24·6)	6·3 (4·3 to 8·7)	2·1 (1·4 to 3)	0·6 (0·3 to 0·8)	415
Liver cancer due to hepatitis B	13·6 (6·8 to 25·1)	19·1 (9·1 to 35·1)	22·9 (11·8 to 39·9)	17·6 (8·8 to 31·5)	12 (5·9 to 21·9)	8·1 (3·7 to 14·5)	5·4 (2·6 to 9·4)	2·6 (1·2 to 4·8)	0·9 (0·4 to 1·5)	0·3 (0·1 to 0·5)	0·1 (0 to 0·1)	132
Liver cancer due to hepatitis C	3·4 (1·4 to 6·7)	6·9 (3·1 to 13)	11·2 (4·9 to 21·4)	11·3 (4·9 to 21·2)	10·4 (5·5 to 17·4)	9·8 (5·2 to 16·5)	8·7 (4·5 to 14·5)	6·1 (3·4 to 9·9)	2·5 (1·4 to 3·9)	1 (0·6 to 1·6)	0·3 (0·1 to 0·5)	74·7
Liver cancer due to alcohol use	9·3 (3·7 to 18·4)	16·3 (7·4 to 31·7)	22·7 (11·3 to 40·4)	22·2 (11·7 to 38·1)	21·1 (12·3 to 33·2)	17·2 (10 to 27)	11·9 (7·2 to 19)	5·6 (3·2 to 9·1)	1·7 (1 to 2·7)	0·4 (0·2 to 0·8)	0·1 (0 to 0·2)	140
Liver cancer due to NASH	2·2 (1 to 4·4)	3·6 (1·7 to 7·5)	5 (2·4 to 9·1)	4·6 (2·4 to 8·3)	4·3 (2·2 to 8)	4·1 (2·1 to 7·5)	3·8 (2 to 6·6)	2·4 (1·3 to 4·1)	0·9 (0·4 to 1·5)	0·3 (0·1 to 0·5)	0·1 (0 to 0·2)	35·7
Liver cancer due to other causes	2·3 (1·1 to 4·1)	3·2 (1·5 to 6·1)	3·8 (1·8 to 7·1)	3·1 (1·5 to 5·9)	2·4 (1·1 to 4·3)	2 (0·9 to 3·5)	1·6 (0·7 to 2·7)	0·9 (0·5 to 1·6)	0·3 (0·2 to 0·6)	0·1 (0·1 to 0·2)	0 (0 to 0·1)	33·3
Gallbladder and biliary tract cancer	12·1 (7·8 to 20·4)	21·2 (14·1 to 35·4)	29 (19·3 to 48·7)	28·2 (18·9 to 46·6)	23·8 (15·8 to 40·9)	19·9 (13·1 to 32·7)	15·5 (10·8 to 26·2)	8·9 (6 to 15·3)	3·8 (2·5 to 6·6)	1·2 (0·7 to 2·1)	0·4 (0·2 to 0·7)	179
Pancreatic cancer	80·5 (57·9 to 112)	126 (90·2 to 172)	166 (118 to 228)	162 (119 to 217)	135 (99 to 179)	112 (83·8 to 145)	79·7 (60·1 to 106)	48·3 (35·4 to 64·1)	21·5 (15·2 to 29·1)	6·6 (4·5 to 9·2)	2 (1·3 to 2·8)	1,040
Larynx cancer	11·1 (6·7 to 17·3)	21·6 (13·6 to 31·9)	26·1 (16·8 to 37·8)	23·6 (16 to 33·4)	19·1 (12·8 to 27·8)	12·3 (8·5 to 17·3)	8·7 (6 to 12·1)	4·4 (3 to 6·1)	1·8 (1·2 to 2·6)	0·5 (0·3 to 0·7)	0·1 (0·1 to 0·2)	138
Tracheal, bronchus, and lung cancer	178 (123 to 244)	315 (226 to 429)	386 (287 to 516)	377 (281 to 499)	306 (228 to 399)	243 (188 to 306)	172 (130 to 225)	90·8 (64·6 to 120)	37·8 (24·7 to 55·3)	9·9 (6·3 to 14·4)	3 (2·1 to 4·1)	2,310
Malignant skin melanoma	7·7 (4·8 to 11·4)	10·2 (6·4 to 15·1)	10·6 (6·8 to 15·7)	8 (5·3 to 11·5)	6·2 (4·1 to 8·8)	5·2 (3·5 to 7·4)	3·8 (2·5 to 5·3)	2·7 (1·8 to 3·8)	1·5 (1 to 2·1)	0·5 (0·3 to 0·8)	0·2 (0·1 to 0·4)	79·9
Non-melanoma skin cancer	3·8 (2·5 to 5·5)	6·3 (4·1 to 9·2)	7 (4·8 to 10)	6·6 (4·3 to 9·2)	6·1 (3·9 to 8·8)	5·2 (3·5 to 7·5)	5·3 (3·5 to 7·5)	4·3 (2·9 to 6·1)	2·8 (1·8 to 4·1)	1·6 (1 to 2·6)	0·8 (0·5 to 1·2)	59·2
Non-melanoma skin cancer (squamous-cell carcinoma)	3·8 (2·5 to 5·5)	6·3 (4·1 to 9·2)	7 (4·8 to 10)	6·6 (4·3 to 9·2)	6·1 (3·9 to 8·8)	5·2 (3·5 to 7·5)	5·3 (3·5 to 7·5)	4·3 (2·9 to 6·1)	2·8 (1·8 to 4·1)	1·6 (1 to 2·6)	0·8 (0·5 to 1·2)	59·2
Non-melanoma skin cancer (basal-cell carcinoma)	0 (0 to 0)	0 (0 to 0)	0 (0 to 0)	0 (0 to 0)	0 (0 to 0)	0 (0 to 0)	0 (0 to 0)	0 (0 to 0)	0 (0 to 0)	0 (0 to 0)	0 (0 to 0)	0
Breast cancer	215 (149 to 297)	271 (192 to 368)	287 (205 to 389)	216 (156 to 288)	162 (116 to 216)	122 (90·6 to 160)	85·6 (62·4 to 117)	54·1 (37·7 to 75·4)	27·5 (18·7 to 38·5)	10 (6·4 to 14·2)	3·9 (2·2 to 6·2)	1,860
Cervical cancer	225 (152 to 314)	231 (151 to 336)	229 (151 to 332)	168 (111 to 249)	112 (74·2 to 162)	97·6 (67·5 to 141)	64·8 (42·8 to 93·7)	34·9 (22·2 to 53·8)	14 (9 to 20·4)	4·9 (2·9 to 7·4)	1·8 (1·1 to 2·7)	1,760
Uterine cancer	19·2 (11·5 to 30·5)	25·2 (15·7 to 38·9)	32·7 (19·9 to 52·1)	36·7 (22·9 to 57·7)	27·8 (17·1 to 43·1)	23·2 (14·7 to 35)	15·2 (9·6 to 22·7)	8·6 (5·3 to 13)	3·2 (2 to 4·8)	1·2 (0·7 to 1·8)	0·4 (0·2 to 0·6)	226
Ovarian cancer	61·7 (33·8 to 94·6)	77·1 (42·4 to 119)	86·9 (45·7 to 134)	70·5 (36·2 to 108)	57·2 (28·7 to 87·4)	42·6 (21·4 to 65·8)	27 (13·6 to 43·7)	15·1 (7·7 to 25·5)	6·6 (3·5 to 10·7)	2·3 (1·3 to 3·7)	0·5 (0·3 to 0·9)	581
Prostate cancer	22·1 (14·1 to 35·2)	65·2 (39·9 to 101)	124 (80·4 to 194)	212 (141 to 313)	241 (163 to 336)	274 (197 to 380)	305 (213 to 422)	229 (160 to 316)	127 (89·3 to 174)	41·5 (27·1 to 57·5)	13·1 (7·5 to 20·1)	1,670
Testicular cancer	2·4 (1·2 to 4·2)	2·4 (1·2 to 4·2)	1·7 (0·8 to 3·2)	1·4 (0·7 to 2·5)	1·2 (0·5 to 2·3)	0·9 (0·5 to 1·8)	0·9 (0·4 to 1·9)	0·5 (0·2 to 1)	0·3 (0·1 to 0·5)	0·1 (0 to 0·2)	0 (0 to 0·1)	52·8
Kidney cancer	25·3 (16·8 to 37)	40·2 (26·5 to 59·9)	47·3 (31·4 to 68·5)	40·5 (27·4 to 57·8)	31·7 (22·3 to 43·4)	22·9 (16·4 to 32)	15·2 (10·8 to 20·8)	7·9 (5·5 to 11)	2·8 (1·8 to 3·9)	0·9 (0·6 to 1·2)	0·3 (0·2 to 0·4)	321
Bladder cancer	9·1 (6·3 to 12·7)	19 (13·2 to 26·5)	28·6 (20·9 to 40·1)	32·9 (22·3 to 46·2)	31·9 (22·4 to 44·1)	30·2 (21·6 to 41·2)	27·4 (20·1 to 37·2)	17·7 (12·9 to 23·4)	9·2 (6·6 to 12·4)	3·2 (2·2 to 4·3)	1 (0·7 to 1·4)	222
Brain and central nervous system cancer	59·8 (24·3 to 92·2)	75·3 (31·1 to 118)	79·8 (33·6 to 128)	60·5 (24·2 to 95·2)	44·5 (18 to 69·5)	29 (11·6 to 45·8)	18·2 (7·4 to 28·8)	8·2 (3·3 to 13·6)	3·3 (1·4 to 5·8)	0·9 (0·4 to 1·5)	0·3 (0·1 to 0·5)	823
Thyroid cancer	8·1 (5·4 to 12·1)	13 (8·7 to 19·2)	14·9 (10·3 to 21·1)	12·5 (8·6 to 17·7)	10·6 (7·4 to 14·9)	9·2 (6·3 to 13·2)	6·7 (4·5 to 9·7)	4·1 (2·7 to 5·9)	1·6 (1 to 2·2)	0·5 (0·3 to 0·8)	0·2 (0·1 to 0·2)	100
Mesothelioma	3·8 (2 to 6·6)	5·8 (3·3 to 10·5)	6·6 (3·9 to 11)	2·4 (1·5 to 3·7)	1·8 (1·1 to 2·9)	1·8 (1 to 3·1)	1·4 (0·9 to 2·1)	0·9 (0·5 to 1·5)	0·4 (0·2 to 0·6)	0·1 (0·1 to 0·2)	0 (0 to 0·1)	34·4
Hodgkin lymphoma	8·5 (5·6 to 12·2)	10·4 (6·6 to 15·7)	9·7 (6·4 to 13·6)	7·7 (5·3 to 10·9)	6·2 (4·2 to 8·7)	4 (2·8 to 5·5)	3·1 (2·1 to 4·1)	1·6 (1·1 to 2·2)	0·7 (0·4 to 1)	0·1 (0·1 to 0·2)	0 (0 to 0·1)	128
Non-Hodgkin lymphoma	59·6 (41·9 to 84)	75·1 (51·7 to 104)	84·5 (60·3 to 115)	71·4 (52·5 to 95·3)	56 (41·1 to 74·8)	42·9 (31·2 to 56·7)	29·9 (21·8 to 40·3)	15·5 (11·1 to 21·7)	5·6 (3·8 to 7·7)	1·6 (1 to 2·2)	0·5 (0·3 to 0·7)	770
Multiple myeloma	21·2 (14·3 to 30·6)	40·3 (27·3 to 58·9)	51·6 (35·9 to 72)	50 (35·1 to 69·2)	43·5 (30·4 to 59·5)	33·5 (23·9 to 44·9)	24·1 (17·4 to 32·7)	12·9 (8·9 to 17·5)	4·8 (3·1 to 7·1)	1·1 (0·7 to 1·7)	0·3 (0·2 to 0·5)	304
Leukemia	47·6 (34 to 65·5)	54·7 (37·7 to 74·7)	59·4 (41·8 to 82·3)	50·8 (34·7 to 69·5)	41·3 (29·4 to 56·1)	34·6 (24·4 to 47·6)	26·3 (18·7 to 36·2)	16 (10·6 to 22·5)	7·4 (4·7 to 10·8)	2·3 (1·5 to 3·3)	0·7 (0·4 to 1·1)	827
Acute lymphoid leukemia	2·3 (1·3 to 3·8)	2·9 (1·7 to 4·6)	2·6 (1·6 to 4)	1·8 (1 to 2·9)	1·6 (0·9 to 2·4)	1·1 (0·7 to 1·7)	0·7 (0·4 to 1·2)	0·4 (0·2 to 0·7)	0·1 (0·1 to 0·2)	0 (0 to 0·1)	0 (0 to 0)	136
Chronic lymphoid leukemia	1 (0·5 to 1·6)	3·1 (1·8 to 5)	5·2 (3·1 to 8·3)	5·5 (3·1 to 8·8)	5·1 (3 to 8·3)	4·1 (2·4 to 6·5)	3·2 (1·8 to 5·3)	1·9 (1 to 3·1)	0·9 (0·5 to 1·5)	0·3 (0·2 to 0·5)	0·1 (0 to 0·1)	34·1
Acute myeloid leukemia	12 (7·1 to 18·1)	12·9 (7·8 to 19·8)	12·6 (7·4 to 19·6)	9·8 (5·5 to 15·5)	7·2 (4·1 to 11·6)	5·3 (3·1 to 8·3)	3·4 (1·8 to 5·5)	1·7 (0·9 to 2·8)	0·6 (0·3 to 1)	0·2 (0·1 to 0·3)	0·1 (0 to 0·1)	198
Chronic myeloid leukemia	3·9 (2·3 to 6·3)	5·8 (3·5 to 9)	5·9 (3·5 to 9·1)	5·4 (3·3 to 8·5)	3·8 (2·4 to 6)	2·8 (1·7 to 4·4)	2·4 (1·4 to 3·7)	1·4 (0·8 to 2·2)	0·5 (0·3 to 0·8)	0·2 (0·1 to 0·3)	0·1 (0 to 0·1)	56·2
Other leukemia	28·4 (19·2 to 40·7)	30 (19·2 to 43·9)	33·1 (22·1 to 48·6)	28·2 (17·8 to 41·6)	23·6 (15·6 to 32·9)	21·3 (14·4 to 30·8)	16·6 (11·3 to 23·8)	10·6 (6·8 to 15·5)	5·3 (3·4 to 8)	1·6 (1 to 2·4)	0·5 (0·3 to 0·8)	403
Other malignant neoplasms	78·3 (57·5 to 103)	103 (75·6 to 137)	110 (81·2 to 144)	88·7 (65·9 to 117)	71·4 (53 to 92·7)	55·4 (42·2 to 71·9)	41 (30·7 to 53·2)	25·7 (19·3 to 33·9)	13·5 (9·7 to 18)	4·6 (3·1 to 6·4)	1·4 (1 to 2·1)	1,130

Other neoplasms	0·3 (0·1 to 0·5)	0·4 (0·2 to 0·7)	1·6 (0·7 to 3)	3·1 (1·8 to 5·5)	1·6 (1 to 2·4)	[illegible]	[illegible]	[illegible]	[illegible]	[illegible]	[illegible]	[illegible]	[illegible]	[illegible]	[illegible]	[illegible]	[illegible]	[illegible]	[illegible]	[illegible]	[illegible]	0·6 (0·4 to 1)	0·2 (0·1 to 0·2)	71·9
Myelodysplastic, myeloproliferative, and other hematopoietic n	0·3 (0·1 to 0·5)	0·4 (0·2 to 0·7)	1·6 (0·7 to 3)	3·1 (1·8 to 5·5)	1·6 (1 to 2·4)	[illegible]	[illegible]	[illegible]	[illegible]	[illegible]	[illegible]	[illegible]	[illegible]	[illegible]	[illegible]	[illegible]	[illegible]	[illegible]	[illegible]	[illegible]	[illegible]	0·6 (0·4 to 1)	0·2 (0·1 to 0·2)	71·9
Benign and in situ intestinal neoplasms	0 (0 to 0)	0 (0 to 0)	0 (0 to 0)	0 (0 to 0)	0 (0 to 0)	[illegible]	[illegible]	[illegible]	[illegible]	[illegible]	[illegible]	[illegible]	[illegible]	[illegible]	[illegible]	[illegible]	[illegible]	[illegible]	[illegible]	[illegible]	[illegible]	0 (0 to 0)	0 (0 to 0)	0
Benign and in situ cervical and uterine neoplasms	0 (0 to 0)	0 (0 to 0)	0 (0 to 0)	0 (0 to 0)	0 (0 to 0)	[illegible]	[illegible]	[illegible]	[illegible]	[illegible]	[illegible]	[illegible]	[illegible]	[illegible]	[illegible]	[illegible]	[illegible]	[illegible]	[illegible]	[illegible]	[illegible]	0 (0 to 0)	0 (0 to 0)	0
Other benign and in situ neoplasms	0 (0 to 0)	0 (0 to 0)	0 (0 to 0)	0 (0 to 0)	0 (0 to 0)	[illegible]	[illegible]	[illegible]	[illegible]	[illegible]	[illegible]	[illegible]	[illegible]	[illegible]	[illegible]	[illegible]	[illegible]	[illegible]	[illegible]	[illegible]	[illegible]	0 (0 to 0)	0 (0 to 0)	0
Cardiovascular diseases	38·3 (20·8 to 66·6)	14·4 (8·1 to 24)	78·6 (43·2 to 135)	36·5 (23·6 to 53·5)	53·3 (40·6 to 67·3)	[illegible]	[illegible]	[illegible]	[illegible]	[illegible]	[illegible]	[illegible]	[illegible]	[illegible]	[illegible]	[illegible]	[illegible]	[illegible]	[illegible]	[illegible]	[illegible]	538 (422 to 638)	258 (193 to 303)	32,400
Rheumatic heart disease	0 (0 to 0)	0 (0 to 0)	0 (0 to 0)	2·5 (1·4 to 3·8)	12·1 (7·6 to 19·1)	[illegible]	[illegible]	[illegible]	[illegible]	[illegible]	[illegible]	[illegible]	[illegible]	[illegible]	[illegible]	[illegible]	[illegible]	[illegible]	[illegible]	[illegible]	[illegible]	0·8 (0·5 to 1·1)	0·4 (0·2 to 0·5)	468
Ischemic heart disease	0 (0 to 0)	0 (0 to 0)	0 (0 to 0)	0 (0 to 0)	0 (0 to 0)	[illegible]	[illegible]	[illegible]	[illegible]	[illegible]	[illegible]	[illegible]	[illegible]	[illegible]	[illegible]	[illegible]	[illegible]	[illegible]	[illegible]	[illegible]	[illegible]	233 (173 to 287)	117 (85 to 144)	13,500
Stroke	17·8 (7·7 to 34·8)	3·6 (1·6 to 7·2)	18·7 (8 to 37·4)	8·1 (5 to 12·9)	17·9 (12·5 to 24)	[illegible]	[illegible]	[illegible]	[illegible]	[illegible]	[illegible]	[illegible]	[illegible]	[illegible]	[illegible]	[illegible]	[illegible]	[illegible]	[illegible]	[illegible]	[illegible]	180 (134 to 230)	82·3 (59·4 to 104)	13,000
Ischemic stroke	2·3 (0·8 to 5)	0·2 (0·1 to 0·5)	0·9 (0·4 to 1·9)	2·5 (1·7 to 3·5)	5·8 (3·7 to 8·7)	[illegible]	[illegible]	[illegible]	[illegible]	[illegible]	[illegible]	[illegible]	[illegible]	[illegible]	[illegible]	[illegible]	[illegible]	[illegible]	[illegible]	[illegible]	[illegible]	132 (96·3 to 171)	62·4 (43·7 to 80·3)	4,480
Intracerebral hemorrhage	6·4 (1·9 to 14)	1·3 (0·5 to 2·7)	11·1 (4·3 to 23·4)	2·2 (1 to 4·4)	6·4 (4 to 9·8)	[illegible]	[illegible]	[illegible]	[illegible]	[illegible]	[illegible]	[illegible]	[illegible]	[illegible]	[illegible]	[illegible]	[illegible]	[illegible]	[illegible]	[illegible]	[illegible]	44·9 (31·7 to 61·4)	18·3 (12·4 to 24·6)	7,480
Subarachnoid hemorrhage	9·1 (3·1 to 19·5)	2·1 (0·8 to 4·3)	6·7 (2·5 to 14·1)	3·4 (1·7 to 6)	5·6 (3·5 to 8·4)	[illegible]	[illegible]	[illegible]	[illegible]	[illegible]	[illegible]	[illegible]	[illegible]	[illegible]	[illegible]	[illegible]	[illegible]	[illegible]	[illegible]	[illegible]	[illegible]	3·8 (2·5 to 5·7)	1·6 (1 to 2·5)	998
Hypertensive heart disease	0 (0 to 0)	0 (0 to 0)	0 (0 to 0)	0 (0 to 0)	0 (0 to 0)	[illegible]	[illegible]	[illegible]	[illegible]	[illegible]	[illegible]	[illegible]	[illegible]	[illegible]	[illegible]	[illegible]	[illegible]	[illegible]	[illegible]	[illegible]	[illegible]	60·9 (41·3 to 84·2)	30·9 (19·8 to 43·4)	2,590
Non-rheumatic valvular heart disease	0 (0 to 0)	0 (0 to 0)	0 (0 to 0)	0 (0 to 0)	0 (0 to 0)	[illegible]	[illegible]	[illegible]	[illegible]	[illegible]	[illegible]	[illegible]	[illegible]	[illegible]	[illegible]	[illegible]	[illegible]	[illegible]	[illegible]	[illegible]	[illegible]	2·7 (1·6 to 4·2)	0·9 (0·5 to 1·5)	255
Non-rheumatic calcific aortic valve disease	0 (0 to 0)	0 (0 to 0)	0 (0 to 0)	0 (0 to 0)	0 (0 to 0)	[illegible]	[illegible]	[illegible]	[illegible]	[illegible]	[illegible]	[illegible]	[illegible]	[illegible]	[illegible]	[illegible]	[illegible]	[illegible]	[illegible]	[illegible]	[illegible]	1·7 (1 to 2·7)	0·6 (0·3 to 1)	110
Non-rheumatic degenerative mitral valve disease	0 (0 to 0)	0 (0 to 0)	0 (0 to 0)	0 (0 to 0)	0 (0 to 0)	[illegible]	[illegible]	[illegible]	[illegible]	[illegible]	[illegible]	[illegible]	[illegible]	[illegible]	[illegible]	[illegible]	[illegible]	[illegible]	[illegible]	[illegible]	[illegible]	1 (0·5 to 1·6)	0·3 (0·2 to 0·6)	140
Other non-rheumatic valve diseases	0 (0 to 0)	[illegible]	[illegible]	[illegible]	[illegible]	[illegible]	[illegible]	[illegible]	[illegible]	[illegible]	[illegible]	[illegible]	[illegible]	[illegible]	[illegible]	[illegible]	[illegible]	[illegible]	[illegible]	[illegible]	[illegible]	0·1 (0 to 0·1)	0 (0 to 0)	5
Cardiomyopathy and myocarditis	6·6 (2·8 to 13·3)	[illegible]	[illegible]	[illegible]	[illegible]	[illegible]	[illegible]	[illegible]	[illegible]	[illegible]	[illegible]	[illegible]	[illegible]	[illegible]	[illegible]	[illegible]	[illegible]	[illegible]	[illegible]	[illegible]	[illegible]	8·8 (5·4 to 13·8)	3·7 (2·1 to 6)	941
Myocarditis	0·7 (0·2 to 2·1)	[illegible]	[illegible]	[illegible]	[illegible]	[illegible]	[illegible]	[illegible]	[illegible]	[illegible]	[illegible]	[illegible]	[illegible]	[illegible]	[illegible]	[illegible]	[illegible]	[illegible]	[illegible]	[illegible]	[illegible]	0·8 (0·3 to 1·7)	0·5 (0·2 to 1)	61·7
Alcoholic cardiomyopathy	0 (0 to 0)	[illegible]	[illegible]	[illegible]	[illegible]	[illegible]	[illegible]	[illegible]	[illegible]	[illegible]	[illegible]	[illegible]	[illegible]	[illegible]	[illegible]	[illegible]	[illegible]	[illegible]	[illegible]	[illegible]	[illegible]	0 (0 to 0·1)	0 (0 to 0)	47·8
Other cardiomyopathy	5·9 (2·5 to 12)	[illegible]	[illegible]	[illegible]	[illegible]	[illegible]	[illegible]	[illegible]	[illegible]	[illegible]	[illegible]	[illegible]	[illegible]	[illegible]	[illegible]	[illegible]	[illegible]	[illegible]	[illegible]	[illegible]	[illegible]	8 (5 to 12·3)	3·2 (1·8 to 5·2)	831
Atrial fibrillation and flutter	0 (0 to 0)	[illegible]	[illegible]	[illegible]	[illegible]	[illegible]	[illegible]	[illegible]	[illegible]	[illegible]	[illegible]	[illegible]	[illegible]	[illegible]	[illegible]	[illegible]	[illegible]	[illegible]	[illegible]	[illegible]	[illegible]	34·8 (26·3 to 44·3)	16·1 (11 to 21·4)	495
Aortic aneurysm	--	[illegible]	[illegible]	[illegible]	[illegible]	[illegible]	[illegible]	[illegible]	[illegible]	[illegible]	[illegible]	[illegible]	[illegible]	[illegible]	[illegible]	[illegible]	[illegible]	[illegible]	[illegible]	[illegible]	[illegible]	3·2 (2·2 to 4·3)	1·1 (0·7 to 1·6)	NA
[truncated: 2,148,529 more chars]
