# Supplementary material for: Global burden of 369 diseases and injuries in 204 countries and territories, 1990–2019: a systematic analysis for the Global Burden of Disease Study 2019
Source: Lancet. 2020 Oct 17;396(10258):1204–22. doi: 10.1016/S0140-6736(20)30925-9 (PMC7567026; doi:10.1016/S0140-6736(20)30925-9)
Supplement: Supplementary appendix 2 [file mmc2e.pdf]

Appendix 2E: supplementary results appendix to "Global burden of 369 diseases and injuries in 204 countries and territories, 1990–2019: a systematic analysis for the Global Burden of Disease Study 2019"

This supplement provides additional figures and more detailed results for "Global burden of 369 diseases and injuries in 204 countries and territories, 1990–2019: a systematic analysis for the Global Burden of Disease Study 2019."

List of supplementary results appendix figures and tables:

Appendix 2A

Figure S1: Leading GBD Level 3 causes of age-standardised YLDs rates by location for a) females and b) males, 2019. 1

Figure S2: Leading GBD Level 3 causes of age-standardised prevalence rates by location for a) females and b) males, 2019. 3

Figure S3: Trends of age-standardised YLD rates per 100,000 for the top eight Level 3 causes of non-fatal burden in 2019 by SDI quintile for both sexes, from 1990 to 2019. 5

Figure S4: Death counts due to fatal discontinuities by type, 1980–2019. 6

Figure S5: Leading level 3 causes of all-age DALY counts by location for females in 1990 (A) males in 1990 (B) females in 2019 (C) males in 2019 (D) 10

Figure S6: Leading 20 GBD cause hierarchy Level 3 causes of YLLs by SDI grouping and sex for 1990, 2010, and 2019, with percentage change in number of YLLs, and all-age and age-standardised rates. 14

Figure S7: Global distribution of total YLDs by 23 GBD age groups for Level 2 causes of non-fatal burden in 1990 and 2019 for a) females and b) males. 24

Figure S8: Global leading 30 level 3 causes of DALYs for 1990, 2010, and 2019, with percentage change in number of DALYs, and age-standardised rates, by sex and SDI quintile 26

Table S1: All-age DALYs counts and age standardised DALY rates per 100,000 individuals for all causes and level 1 cause groups by location for 1990, 2010, 2019 , both sexes combined. 36

Appendix 2B

Table S2a: Global DALYS by cause and age group for males with 95% uncertainty intervals, by location for 2019. 4734

Appendix 2C

Table S2b: Global DALYS by cause and age group for females with 95% uncertainty intervals, by location for 2019. 6708

Appendix 2D

Table S2c: Global DALYS by cause and age group for both sexes combined with 95% uncertainty intervals, by location for 2019. 8680

Appendix 2E

Table S3: Global deaths and YLL numbers, age-standardised rates per 100,000, and percentage change between 2010 and 2019 for males for all GBD causes and Levels 1 through 4 of the cause hierarchy 10630

Table S4: Global deaths by cause and age group for females (1990, 2010, 2019) 10635

Table S5: Global deaths by cause and age group for males (1990, 2010, 2019) 10642

Table S6: Global deaths by cause and age group for both sexes combined (1990, 2010, 2019) 10648

Table S7: Global YLLs by cause and age group for females. 10654

Table S8: Global YLLs by cause and age group for males. 10656

Table S9: Global YLLs by cause and age group for both sexes combined 10658

Table S10: Deaths due to fatal discontinuities by country, all ages, for both sexes combined 10660

Table S11: Diarrhoeal diseases and lower respiratory infection aetiologies deaths, mortality rates, and annual percentage change from 1990 to 2019. 10676

Table S12: Deaths by location, cause, and age group for males. 10677

Table S13: Deaths by location, cause, and age group for females. 13007

Table S14: Deaths by location, cause, and age group for both sexes combined. 15354

Table S15: Distribution of percentage change in age-standardised deaths for Level 1 causes by location from 1990-2010 and 2010-2019, both sexes. 17543

Table S16: Global deaths and YLL numbers, age-standardised rates per 100,000, and percentage change between 2010 and 2019 for females for all GBD causes and Levels 1 through 4 of the cause hierarchy. 17561

Table S17: Global prevalence, incidence and YLDs for 2019, percentage change of YLDs counts, and percentage change of YLD rates between 2010 and 2019 by cause and by age 17566

Table S18: Prevalence, incidence, and YLDs for 2019, percentage change of YLD counts, and percentage change of age-standardised YLD rates between 2010 and 2019 by cause and GBD location. 17707

SR Table 3: Global deaths and YLL numbers, age-standardised rates per 100,000, and percent change between 2010 and 2019 for males for all GBD causes and Levels 1 through 4 of the cause hierarchy. 95% UIs are in parentheses. UI=uncertainty interval. YLL=year of life lost.

Cause	All Age Deaths (thousands)		Age-standardised Death Rate (per 100,000)		All Age YLLs (thousands)		Age-standardised YLL Rate (per 100,000)	
	2019	Percent Change 2010-2019	2019	Percent Change 2010-2019	2019	Percent Change 2010-2019	2019	Percent Change 2010-2019
All causes	30700 (28800–32500)	6·27 (0·115–12·4)	874 (823–923)	-14·1 (-18·9–-9·34)	962000 (891000–1040000)	-8·13 (-14·1–-1·85)	25800 (23900–27900)	-19·6 (-24·8–-13·9)
Communicable, maternal, neonatal, and nutritional diseases	5340 (4800–5990)	-19 (-25·1–-12)	153 (137–171)	-26·9 (-32·4–-20·5)	299000 (262000–347000)	-26·7 (-34·1–-18·3)	8460 (7370–9860)	-29·8 (-37·1–-21·3)
HIV/AIDS and sexually transmitted infections	480 (445–532)	-28·1 (-30·5–-25·1)	12·1 (11·1–13·6)	-35·3 (-37·9–-32)	25400 (22500–29500)	-31·1 (-34·2–-27)	652 (569–780)	-36·6 (-40·2–-31·9)
HIV/AIDS	433 (417–453)	-30·1 (-32–-28·1)	10·7 (10·3–11·2)	-38·1 (-39·8–-36·3)	21300 (20500–22300)	-34·8 (-36·7–-32·7)	533 (512–558)	-41·4 (-43·2–-39·4)
HIV/AIDS - Drug-susceptible Tuberculosis	94·6 (68·4–121)	-40·5 (-44·1–-37·2)	2·34 (1·69–2·99)	-47·3 (-50·5–-44·4)	4730 (3450–6050)	-44·3 (-47·5–-41·2)	119 (86·7–151)	-49·8 (-52·8–-47·1)
HIV/AIDS - Multidrug-resistant Tuberculosis without extensive drug resistance	9·59 (4·3–18·1)	-33·2 (-51·8–-8·64)	0·238 (0·106–0·446)	-40·6 (-57·2–-18·9)	487 (219–921)	-36·4 (-54·5–-12·8)	12·3 (5·5–23·2)	-42·5 (-58·9–-20·9)
HIV/AIDS - Extensively drug-resistant Tuberculosis	0·487 (0·227–0·918)	-7·87 (-25·2–15·3)	0·012 (0·00556–0·0225)	-18·1 (-33·5–2·34)	24·1 (11·2–45·2)	-11·9 (-28·6–9·98)	0·598 (0·277–1·12)	-20·8 (-35·8–-1·26)
HIV/AIDS resulting in other diseases	329 (294–364)	-26·3 (-29–-23·3)	8·1 (7·24–8·95)	-34·8 (-37·1–-32·1)	16100 (14400–17800)	-31·3 (-34–-28·3)	402 (360–445)	-38·3 (-40·8–-35·6)
Sexually transmitted infections excluding HIV	46·7 (17–95·3)	-0·922 (-13·5–15·6)	1·39 (0·502–2·83)	-0·454 (-13·4–16)	4020 (1410–8260)	-0·977 (-14–16)	119 (41·8–246)	-0·0266 (-13·3–17·2)
Syphilis	46·2 (16·5–94·5)	-0·971 (-13·7–15·7)	1·37 (0·488–2·81)	-0·284 (-13·3–16·5)	4000 (1400–8250)	-0·955 (-14–16·1)	119 (41·4–246)	0·0514 (-13·3–17·3)
Chlamydial infection	0 (0–0)	--	0 (0–0)	--	0 (0–0)	--	0 (0–0)	--
Gonococcal infection	0·125 (0·0726–0·156)	-2·9 (-16–15)	0·00323 (0·00188–0·00403)	-17·5 (-28·9–-1·68)	4·99 (2·94–6·23)	-5·9 (-17·5–11·2)	0·126 (0·0744–0·156)	-16·2 (-26·8–-0·589)
Other sexually transmitted infections	0·368 (0·189–0·474)	6·38 (-12·1–30·7)	0·0115 (0·00562–0·0152)	-13·1 (-30·3–9·14)	11·4 (6·29–14·6)	-5·86 (-19·8–14·1)	0·301 (0·166–0·384)	-18·5 (-30·9–-1·79)
Respiratory infections and tuberculosis	2060 (1890–2240)	-7·31 (-13·6–-0·78)	59·6 (54·7–64·9)	-22·1 (-27·1–-17·1)	78700 (70100–89200)	-21·8 (-29·1–-13·8)	2150 (1910–2450)	-28·9 (-35·4–-21·3)
Tuberculosis	761 (688–836)	-13·2 (-21·5–-4·85)	19·7 (17·8–21·6)	-28·7 (-35·3–-21·8)	27500 (24800–30400)	-18 (-25·9–-10)	692 (623–765)	-29·2 (-36–-22·4)
Drug-susceptible tuberculosis	684 (590–771)	-14 (-23·5–-4·22)	17·7 (15·3–19·9)	-29·3 (-37·1–-21·5)	24700 (21200–28100)	-18·7 (-27·9–-10)	623 (535–708)	-29·9 (-37·7–-22·6)
Multidrug-resistant tuberculosis without extensive drug resistance	71·2 (28·9–137)	-6·32 (-41·9–54·8)	1·83 (0·746–3·51)	-22·9 (-51·9–27)	2570 (1060–4770)	-11·1 (-43·9–41·8)	64·3 (26·7–119)	-23·5 (-51·4–22)
Extensively drug-resistant tuberculosis	5·79 (2·79–10·2)	10·5 (-20·2–60·6)	0·147 (0·0706–0·261)	-8·48 (-33·7–33·8)	204 (101–356)	3·58 (-23·6–47·6)	5·05 (2·51–8·82)	-11·2 (-34·5–26·8)
Lower respiratory infections	1300 (1180–1420)	-3·4 (-9·86–3·69)	39·7 (36–43·6)	-18·3 (-23·3–-12·8)	50900 (44300–59300)	-23·7 (-31·9–-13·5)	1450 (1260–1700)	-28·7 (-36·5–-19)
Upper respiratory infections	5·31 (3·07–8·47)	-25 (-44·9–28·6)	0·154 (0·0909–0·244)	-33·4 (-49·3–4·35)	294 (117–584)	-33·4 (-50–9·5)	8·33 (3·27–16·7)	-36·6 (-51·9–-0·439)
Otitis media	0·428 (0·243–1·07)	-20·1 (-42–12·8)	0·0119 (0·00657–0·0309)	-27·3 (-46–0·274)	25·6 (10·9–79·1)	-26·3 (-48·8–9·72)	0·722 (0·288–2·28)	-29·3 (-52·5–5·87)
Enteric infections	825 (638–1220)	-23·7 (-33·6–-11·3)	23·9 (18·5–35·2)	-32·6 (-40·7–-22·6)	44200 (35400–56500)	-30·7 (-40·7–-19·1)	1250 (1000–1590)	-34·4 (-43·8–-23·2)
Diarrheal diseases	707 (521–1090)	-24·5 (-35·3–-10·6)	20·7 (15·3–31·5)	-34 (-42·8–-23·5)	35400 (27900–45700)	-33·2 (-43·8–-21·1)	1010 (797–1280)	-36·8 (-46·7–-25·3)
Typhoid and paratyphoid	73·9 (35·6–126)	-20·5 (-29·2–-11·2)	1·99 (0·965–3·39)	-24·6 (-32·8–-15·6)	5330 (2540–9140)	-21·5 (-30·3–-11·9)	145 (69·8–248)	-25 (-33·4–-15·6)
Typhoid fever	60·8 (29·2–107)	-22·6 (-31·1–-13·3)	1·64 (0·79–2·87)	-26·5 (-34·6–-17·8)	4410 (2090–7620)	-23·3 (-32·1–-13·9)	120 (56·9–207)	-26·5 (-35·1–-17·3)
Paratyphoid fever	13·1 (5·42–25·5)	-9·04 (-22·7–7·52)	0·35 (0·145–0·682)	-14·4 (-27·3–1·54)	918 (376–1800)	-12·1 (-26·1–5·04)	24·8 (10·2–48·4)	-16·5 (-29·7–-0·255)
Invasive Non-typhoidal Salmonella (iNTS)	44·2 (23·9–69)	-14·4 (-27·5–2·89)	1·24 (0·672–1·95)	-18·3 (-31·2–-1·75)	3400 (1840–5330)	-13·8 (-28·2–4·72)	96·6 (52·1–151)	-16·5 (-30·5–1·65)
Other intestinal infectious diseases	0·613 (0·379–0·916)	-27·8 (-58·7–27·3)	0·0174 (0·0106–0·0263)	-31·7 (-60·9–19·1)	44·3 (25·7–69·6)	-31·6 (-63·9–25·9)	1·26 (0·725–2)	-33·8 (-65·1–21·4)
Neglected tropical diseases and malaria	394 (217–653)	-26·9 (-47·8–0·976)	10·7 (5·92–17·8)	-32·5 (-52·1–-6·84)	25600 (13900–43200)	-31·3 (-51·6–-4·27)	713 (384–1210)	-34·4 (-53·9–-8·22)
Malaria	334 (156–600)	-28·9 (-53–3)	9·11 (4·3–16·3)	-34·1 (-56·5–-4·76)	22400 (10600–39800)	-32·6 (-55·5–-3·32)	628 (299–1120)	-35·5 (-57·4–-7·18)
Chagas disease	5·36 (2·46–10·2)	9·09 (-10·2–34·1)	0·148 (0·0684–0·287)	-17·1 (-30·9–0·71)	131 (65·1–246)	3·59 (-15·4–30·9)	3·32 (1·63–6·35)	-17·3 (-32·5–3·94)
Leishmaniasis	3·92 (1·17–13)	-55·6 (-68·3–-17·1)	0·106 (0·0323–0·353)	-58·2 (-70·2–-20·8)	277 (88·5–916)	-55·5 (-68·9–-17)	7·57 (2·45–25)	-57·5 (-70·5–-20·3)
Visceral leishmaniasis	3·92 (1·17–13)	-55·6 (-68·3–-17·1)	0·106 (0·0323–0·353)	-58·2 (-70·2–-20·8)	277 (88·5–916)	-55·5 (-68·9–-17)	7·57 (2·45–25)	-57·5 (-70·5–-20·3)
African trypanosomiasis	0·706 (0·295–1·39)	-75·4 (-87·8–-53·4)	0·0182 (0·00746–0·0358)	-77·3 (-88·8–-57·7)	42·5 (17·4–84·2)	-75·3 (-88·2–-52·2)	1·11 (0·449–2·2)	-76·8 (-88·9–-55·5)
Schistosomiasis	6·53 (5·63–7·59)	-13 (-23·3–-1·39)	0·166 (0·143–0·192)	-28·6 (-36·8–-19·5)	240 (205–283)	-14·1 (-25·3–-2·05)	5·98 (5·12–7·04)	-26·3 (-35·7–-16)
Cysticercosis	0·567 (0·311–0·827)	-8·71 (-50·7–63·9)	0·0142 (0·00791–0·0207)	-20·2 (-56·8–42·2)	27·5 (14·2–41)	-11·8 (-55·1–61·5)	0·688 (0·356–1·03)	-20·5 (-59·3–46)
Cystic echinococcosis	0·726 (0·455–1·03)	-8·03 (-44·6–53·5)	0·0191 (0·0121–0·0268)	-21·6 (-52·6–29)	32·2 (19–47)	-19·6 (-53·6–35·9)	0·841 (0·492–1·24)	-28 (-58·5–21·6)
Dengue	19 (3·67–23·9)	14 (-9·07–38·3)	0·515 (0·1–0·648)	1·31 (-18·7–22·3)	988 (189–1280)	1·83 (-19·4–28)	26·5 (5·09–34·6)	-5·41 (-25·2–19)
Yellow fever	3·09 (1·15–6·44)	-35 (-47·2–-20·9)	0·0816 (0·0305–0·171)	-39·1 (-50·5–-25·6)	209 (77·3–435)	-35·2 (-47·9–-19·8)	5·59 (2·06–11·6)	-38·5 (-50·6–-23·6)
Rabies	9·3 (3·17–13·3)	-23·3 (-39·2–-5·52)	0·244 (0·0826–0·355)	-30·5 (-44·4–-14·8)	550 (177–821)	-26·8 (-41·9–-7·44)	14·7 (4·71–22·2)	-31·7 (-45·6–-13)
Intestinal nematode infections	1·11 (0·87–1·41)	-42·8 (-53·6–-29·4)	0·0317 (0·0247–0·0403)	-45·2 (-55·6–-32·5)	87·6 (67·3–113)	-44·5 (-55·6–-30·4)	2·5 (1·91–3·23)	-46·3 (-57–-32·6)
Ascariasis	1·11 (0·87–1·41)	-42·8 (-53·6–-29·4)	0·0317 (0·0247–0·0403)	-45·2 (-55·6–-32·5)	87·6 (67·3–113)	-44·5 (-55·6–-30·4)	2·5 (1·91–3·23)	-46·3 (-57–-32·6)
Ebola	1·68 (1·38–1·99)	--	0·0422 (0·0346–0·0499)	--	89·5 (73·4–106)	--	2·28 (1·87–2·69)	--
Zika virus	0·00195 (0·00117–0·00297)	--	4·91e-05 (2·95e-05–7·51e-05)	--	0·105 (0·063–0·161)	--	0·00268 (0·00161–0·00411)	--
Other neglected tropical diseases	8·26 (5·17–23·8)	3·52 (-17·5–31·9)	0·23 (0·143–0·671)	-5·45 (-25·2–19·2)	522 (293–1720)	0·673 (-23·5–34·1)	14·7 (8·12–48·7)	-3·73 (-27·5–28·3)
Other infectious diseases	388 (322–477)	-28·5 (-38·1–-17·6)	10·8 (8·89–13·3)	-33·7 (-42·6–-23·5)	25200 (19900–32500)	-35·1 (-45·2–-22·6)	709 (552–919)	-37·7 (-47·4–-25·7)
Meningitis	129 (111–153)	-27·6 (-36·5–-16·9)	3·56 (3·05–4·27)	-32·6 (-40·9–-22·3)	8620 (7170–10600)	-32·6 (-42·1–-20·2)	242 (200–300)	-35·2 (-44·5–-22·9)
Encephalitis	47·4 (39·5–70)	3·07 (-8·87–19·7)	1·29 (1·08–1·9)	-9·74 (-20–4·74)	2350 (1920–3390)	-6·37 (-19·2–8·79)	63·7 (51·9–91·5)	-13·2 (-25·2–1·16)
Diphtheria	2·28 (1·47–3·44)	-42·7 (-64·7–-6·36)	0·0653 (0·0419–0·0989)	-44·2 (-65·7–-8·73)	190 (121–290)	-42·9 (-65·2–-5·39)	5·47 (3·47–8·37)	-44·3 (-66·2–-7·56)
Pertussis	53·4 (24·3–96·4)	-22 (-55·7–38·3)	1·55 (0·709–2·81)	-22·9 (-56·1–36·7)	4600 (2100–8300)	-22 (-55·6–38·2)	134 (61·3–242)	-22·8 (-56–36·8)
Tetanus	19·4 (13·5–28·7)	-44·2 (-57·3–-29·1)	0·536 (0·378–0·8)	-48·2 (-59·8–-33·3)	1270 (932–1970)	-47·8 (-61–-32·1)	36 (26·4–55·2)	-49·5 (-62·1–-33·7)
Measles	42·5 (15·7–92·2)	-59·9 (-67·2–-50·6)	1·23 (0·45–2·66)	-60·7 (-67·9–-51·7)	3570 (1270–7820)	-60·6 (-67·9–-51·6)	103 (36·7–226)	-61·4 (-68·5–-52·6)
Varicella and herpes zoster	6·87 (5·98–8·12)	-5·98 (-16·4–6·34)	0·212 (0·185–0·253)	-19 (-27·2–-9·18)	370 (308–449)	-13 (-27·1–3·64)	10·6 (8·83–12·9)	-17·4 (-30·6–-1·53)
Acute hepatitis	46·2 (36·1–56·1)	-22·1 (-38·7–-4·64)	1·2 (0·937–1·47)	-31·7 (-46·5–-16·3)	2270 (1720–2790)	-32 (-48·2–-15·3)	59·7 (45–73·6)	-37·8 (-52·9–-22·4)
Acute hepatitis A	21·1 (14·9–29)	-36·3 (-50·9–-18·7)	0·56 (0·396–0·77)	-42·9 (-56–-27·3)	1140 (798–1580)	-44·2 (-58·3–-28·6)	30·7 (21·3–42·3)	-48·2 (-61·6–-32·7)
Acute hepatitis B	21·5 (15·6–30·2)	-3·01 (-22·9–24·4)	0·551 (0·397–0·774)	-16·9 (-34·1–5·65)	954 (674–1330)	-10·8 (-30·5–17·6)	24·6 (17·3–34·4)	-19·8 (-37·6–6·04)
Acute hepatitis C	2·35 (1·32–4·46)	-13·6 (-44·8–22·8)	0·0606 (0·0342–0·115)	-25·7 (-52–4·81)	107 (56·9–201)	-23·1 (-52·5–17·3)	2·78 (1·46–5·21)	-30·8 (-57·1–3·59)
Acute hepatitis E	1·21 (0·608–1·94)	-4·97 (-36·7–23·6)	0·0322 (0·0161–0·0522)	-16·1 (-44·2–9·34)	61 (29·5–100)	-13·7 (-47·6–17·7)	1·64 (0·775–2·71)	-19·8 (-51·7–11·2)

Other unspecified infectious diseases	41 (27·1–50·4)	6·4 (-4·26–20·6)	1·13 (0·739–1·39)	-7·37 (-16·5–5·09)	2000 (1260–2570)	-3·41 (-16·1–15·1)	54·3 (34–70·2)	-10·4 (-22·5–7·21)
Maternal and neonatal disorders	1080 (906–1290)	-22·4 (-32·6–-10·9)	32·1 (27–38·4)	-21·4 (-31·7–-9·75)	94400 (79600–113000)	-22·4 (-32·6–-10·9)	2820 (2370–3370)	-21·4 (-31·7–-9·75)
Neonatal disorders	1080 (906–1290)	-22·4 (-32·6–-10·9)	32·1 (27–38·4)	-21·4 (-31·7–-9·75)	94400 (79600–113000)	-22·4 (-32·6–-10·9)	2820 (2370–3370)	-21·4 (-31·7–-9·75)
Neonatal preterm birth	384 (322–464)	-24·7 (-35·1–-12·5)	11·5 (9·6–13·8)	-23·7 (-34·3–-11·3)	33700 (28200–40700)	-24·7 (-35·1–-12·5)	1010 (843–1220)	-23·7 (-34·3–-11·3)
Neonatal encephalopathy due to birth asphyxia and trauma	331 (269–402)	-21·3 (-32·3–-9·21)	9·87 (8·02–12)	-20·3 (-31·5–-8·02)	29000 (23600–35300)	-21·3 (-32·3–-9·21)	866 (704–1050)	-20·3 (-31·5–-8·02)
Neonatal sepsis and other neonatal infections	126 (103–157)	-13·5 (-27·2–3·12)	3·77 (3·08–4·69)	-12·4 (-26·3–4·41)	11100 (9050–13800)	-13·5 (-27·2–3·12)	331 (270–411)	-12·4 (-26·3–4·41)
Hemolytic disease and other neonatal jaundice	30·4 (24·8–37·3)	-36·1 (-46·8–-24·4)	0·908 (0·739–1·11)	-35·3 (-46·2–-23·5)	2670 (2170–3280)	-36·1 (-46·8–-24·4)	79·7 (64·9–97·8)	-35·3 (-46·2–-23·5)
Other neonatal disorders	204 (167–250)	-22·2 (-34·8–-7·54)	6·09 (4·99–7·47)	-21·2 (-33·9–-6·35)	17900 (14700–22000)	-22·2 (-34·8–-7·54)	534 (438–655)	-21·2 (-33·9–-6·35)
Nutritional deficiencies	114 (99·2–129)	-26 (-33·6–-16·8)	3·45 (3·03–3·9)	-34·2 (-40·5–-26·6)	5690 (4650–6930)	-38·9 (-47·8–-27·8)	163 (133–199)	-41·7 (-50·4–-31)
Protein-energy malnutrition	98·5 (85·2–113)	-27·2 (-35·5–-17·2)	2·99 (2·6–3·43)	-34·5 (-41·5–-26·4)	5240 (4220–6410)	-39·5 (-49–-27·8)	151 (121–185)	-41·9 (-51·1–-30·7)
Other nutritional deficiencies	15 (13–18·3)	-16·7 (-28·5–-5·8)	0·456 (0·396–0·548)	-32·1 (-40·5–-24·1)	457 (379–545)	-32 (-47·8–-20·1)	12·6 (10·5–15·1)	-39·7 (-53·2–-29·5)
Non-communicable diseases	22400 (21000–23800)	17·3 (9·75–24·4)	644 (606–681)	-9·63 (-15·1–-4·57)	529000 (492000–565000)	9·64 (2·22–17·1)	13900 (13000–14900)	-11 (-16·7–-5·14)
Neoplasms	5720 (5280–6130)	20 (11·1–29·4)	157 (145–168)	-7·29 (-13·7–-0·444)	137000 (126000–148000)	14·4 (5·24–24)	3520 (3240–3790)	-8·35 (-15·4–-0·831)
Lip and oral cavity cancer	132 (118–145)	22·6 (9·29–35·9)	3·42 (3·06–3·77)	-3·35 (-13·5–6·89)	3670 (3250–4080)	18·1 (4·46–32)	90·5 (80·3–101)	-4·25 (-15·1–6·91)
Nasopharynx cancer	51·2 (46–57)	16·6 (2·96–31·3)	1·28 (1·15–1·43)	-5·98 (-16·7–5·55)	1610 (1450–1800)	12·6 (-0·875–27·2)	39·4 (35·3–43·8)	-6·65 (-17·7–5·39)
Other pharynx cancer	88 (78–98·7)	29·4 (14·1–44·2)	2·23 (1·98–2·5)	2·12 (-9·7–13·6)	2470 (2180–2780)	25·4 (9·66–40·5)	60·3 (53·3–68)	0·956 (-11·6–13·2)
Esophageal cancer	366 (315–415)	11·5 (-3·35–30·4)	9·68 (8·34–11)	-13·6 (-24·8–0·654)	8720 (7520–9980)	6·91 (-7·81–25·9)	219 (189–250)	-15·6 (-27·1–-0·813)
Stomach cancer	612 (544–678)	2·8 (-8·66–15·8)	16·6 (14·8–18·3)	-20·2 (-28·7–-10·6)	14300 (12700–16000)	-1·79 (-13·4–11·1)	363 (322–404)	-22 (-31–-11·9)
Colon and rectum cancer	594 (551–638)	27·8 (18·7–37·3)	16·6 (15·4–17·9)	-2·22 (-8·67–4·4)	13300 (12300–14400)	23·5 (13·7–34)	344 (317–371)	-2·56 (-10·1–5·46)
Liver cancer	334 (300–368)	26·7 (13·7–41·2)	8·73 (7·88–9·6)	-0·488 (-10·3–10·3)	8960 (7940–9990)	22·1 (7·89–38)	223 (198–248)	-1·18 (-12·4–11·1)
Liver cancer due to hepatitis B	157 (131–187)	24·1 (5·42–46·4)	3·95 (3·3–4·67)	-0·683 (-15·5–16·7)	4800 (4000–5710)	20 (1–42·5)	117 (97·7–139)	-1·17 (-16·7–17)
Liver cancer due to hepatitis C	74·4 (62·9–86·2)	23·9 (15·4–32·8)	2·09 (1·77–2·41)	-5·14 (-11–1·15)	1560 (1290–1850)	19·2 (9·71–28·9)	40·6 (33·8–47·6)	-7·11 (-14·1–0·0894)
Liver cancer due to alcohol use	71·7 (58·2–86·8)	32·6 (22·9–41·8)	1·88 (1·53–2·27)	2·35 (-4·76–9·51)	1730 (1400–2090)	29·3 (19·2–39·3)	43·2 (35·1–52·2)	1·75 (-6·07–9·35)
Liver cancer due to NASH	17·4 (14–22)	43 (30·2–55·9)	0·47 (0·38–0·593)	10·5 (1·15–20)	413 (332–515)	38·4 (24·6–52·5)	10·5 (8·4–13)	10 (-0·562–21·2)
Liver cancer due to other causes	13·2 (10·6–15·9)	26·1 (11·6–41·4)	0·345 (0·281–0·415)	2·93 (-8·15–14·9)	460 (383–538)	16·8 (3·08–31·5)	11·9 (9·92–13·8)	1·03 (-10·5–13·2)
Gallbladder and biliary tract cancer	73 (59·5–80·4)	21·8 (12–34)	2·06 (1·67–2·27)	-6·95 (-13·9–1·84)	1560 (1280–1730)	17·2 (6·74–30·7)	40·4 (33·2–44·6)	-8·22 (-16·2–2·03)
Pancreatic cancer	278 (258–299)	35·3 (25·9–45·4)	7·55 (7·01–8·1)	3·85 (-3·01–11·2)	6430 (5930–6950)	30·7 (20·6–41·6)	163 (150–176)	2·89 (-4·83–11·2)
Larynx cancer	106 (97·8–115)	16·7 (7·83–25·5)	2·75 (2·54–2·98)	-9·34 (-15·8–-2·77)	2700 (2500–2940)	13·2 (4·21–22·1)	66·9 (61·9–72·8)	-10·2 (-17·3–-3·28)
Tracheal, bronchus, and lung cancer	1390 (1260–1510)	19·1 (8·12–31·3)	37·4 (34·1–40·7)	-8·68 (-16·8–0·237)	31200 (28300–34300)	15 (3·73–27·8)	793 (719–869)	-9·98 (-18·7–-0·32)
Malignant skin melanoma	35·4 (22–42·7)	18·9 (10·1–27·3)	0·979 (0·603–1·18)	-7·15 (-13·9–-0·886)	905 (597–1140)	12·4 (4·14–21·8)	23 (15·1–28·8)	-8·5 (-15·3–-0·886)
Non-melanoma skin cancer	33·2 (30·3–35·6)	30·8 (22·7–39·1)	1·01 (0·908–1·08)	-1·83 (-7·39–3·7)	663 (613–711)	23·7 (15·1–32·3)	17·8 (16·4–19)	-3·12 (-9·53–3·19)
Non-melanoma skin cancer (squamous-cell carcinoma)	33·2 (30·3–35·6)	30·8 (22·7–39·1)	1·01 (0·908–1·08)	-1·83 (-7·39–3·7)	663 (613–711)	23·7 (15·1–32·3)	17·8 (16·4–19)	-3·12 (-9·53–3·19)
Breast cancer	12·1 (10·7–13·3)	21·8 (11–33·2)	0·327 (0·29–0·36)	-6·04 (-14·2–2·23)	297 (261–328)	17·2 (5·64–28·9)	7·48 (6·6–8·26)	-6·91 (-15·7–2·32)
Prostate cancer	487 (420–594)	28·8 (23·3–35)	15·3 (13–18·6)	-5·87 (-9·67–-1·66)	7740 (6710–9330)	25·6 (19·6–32·4)	220 (190–266)	-5·86 (-10·1–-1·09)
Testicular cancer	10·8 (9·96–11·9)	18·7 (10·7–27·4)	0·282 (0·259–0·309)	3·53 (-3·17–11)	508 (463–567)	13·7 (5·31–23·1)	12·9 (11·7–14·5)	3·2 (-4·27–11·6)
Kidney cancer	109 (102–116)	27·9 (20·6–35·7)	2·99 (2·78–3·18)	-1·24 (-6·67–4·52)	2640 (2450–2830)	22·1 (14·2–30·5)	67·6 (62·9–72·4)	-2·24 (-8·43–4·27)
Bladder cancer	169 (157–181)	26·4 (19–34)	5·09 (4·69–5·44)	-5·32 (-10·5–-0·157)	3110 (2890–3330)	21·4 (13·5–29·6)	84·5 (78·5–90·4)	-6·55 (-12·4–-0·587)
Brain and central nervous system cancer	139 (99·6–157)	18·3 (5·34–30·2)	3·58 (2·55–4·05)	-3·06 (-13–6·52)	4940 (3610–5670)	10·4 (-3·32–23·6)	126 (91·7–144)	-4·72 (-16·1–6·37)
Thyroid cancer	18·6 (16·8–20·2)	26·4 (15·5–36·6)	0·508 (0·456–0·551)	-1·3 (-9·5–6·55)	472 (427–514)	20·7 (9·5–31·4)	12 (10·9–13)	-2·15 (-11·1–6·21)
Mesothelioma	21·2 (20–22·5)	19·4 (14·8–24·1)	0·593 (0·556–0·628)	-8·17 (-11·6–-4·81)	462 (434–500)	14·3 (8·83–19·8)	12 (11·2–12·9)	-9·69 (-13·8–-5·66)
Hodgkin lymphoma	17·2 (13·9–21)	4·51 (-1·87–11·3)	0·442 (0·36–0·538)	-12 (-17·2–-6·53)	699 (558–862)	0·0946 (-6·82–7·18)	17·7 (14–21·8)	-11·5 (-17·7–-5·32)
Non-Hodgkin lymphoma	146 (136–155)	23·7 (16·1–31·8)	3·97 (3·71–4·23)	-2·57 (-8·38–3·5)	4050 (3770–4360)	16 (7·78–24·6)	104 (96·6–112)	-3·64 (-10·1–3·12)
Multiple myeloma	60·5 (50·7–67·1)	31·2 (22·8–38·6)	1·68 (1·4–1·84)	-0·349 (-6·19–4·9)	1320 (1120–1500)	27·4 (17·8–35·7)	34·1 (28·6–38·5)	-0·436 (-7·63–5·78)
Leukemia	188 (165–208)	12·3 (4·88–21)	5·2 (4·59–5·72)	-8·32 (-14–-1·9)	6470 (5520–7230)	1·53 (-7·82–12·4)	170 (145–190)	-11 (-19–-1·6)
Acute lymphoid leukemia	28·2 (22·2–31·9)	4·81 (-6·65–17·9)	0·75 (0·593–0·849)	-6·35 (-16·3–4·94)	1560 (1210–1790)	-1·87 (-14·2–12·6)	41·7 (32·1–48·1)	-8·43 (-20·1–5·13)
Chronic lymphoid leukemia	22·3 (20–26·3)	22·7 (15·5–30·7)	0·665 (0·591–0·785)	-7·8 (-13–-2·03)	441 (399–519)	18·1 (9·65–26·7)	11·8 (10·7–13·9)	-8·11 (-14·2–-1·68)
Acute myeloid leukemia	53·1 (43·7–60·3)	23·3 (16·6–31·2)	1·45 (1·18–1·63)	-0·753 (-6·03–5·43)	1710 (1430–2000)	14·2 (5·93–25·6)	44·6 (37–52·1)	-1·62 (-8·57–7·76)
Chronic myeloid leukemia	18·3 (15·6–21·3)	8·09 (-5·28–20·8)	0·491 (0·422–0·571)	-12 (-21·6–-2·88)	622 (514–738)	-0·733 (-18·7–18·1)	16 (13·2–19)	-14·3 (-29·1–0·783)
Other leukemia	66·7 (56·9–77·1)	6·22 (-4·15–17·9)	1·85 (1·59–2·12)	-13·4 (-21·1–-4·67)	2150 (1780–2550)	-6·46 (-19–6·34)	56·1 (47–66·8)	-18·5 (-29–-7·89)
Other malignant neoplasms	220 (180–249)	20·4 (11·8–29·7)	5·91 (4·84–6·68)	-2·66 (-9·28–4·73)	7320 (6190–8230)	12·6 (4·19–21·7)	189 (160–213)	-2·92 (-10·2–4·65)
Other neoplasms	32 (22·9–45)	35·2 (27–42·8)	1 (0·699–1·41)	-0·524 (-6·87–5·04)	569 (429–783)	28·7 (20·6–36·4)	15·9 (11·9–22)	-0·334 (-6·56–5·54)
Myelodysplastic, myeloproliferative, and other hematopoietic neoplasms	32 (22·9–45)	35·2 (27–42·8)	1 (0·699–1·41)	-0·524 (-6·87–5·04)	569 (429–783)	28·7 (20·6–36·4)	15·9 (11·9–22)	-0·334 (-6·56–5·54)
Cardiovascular diseases	9620 (8900–10300)	16·7 (9–24·1)	281 (259–300)	-11·1 (-16·5–-5·88)	207000 (191000–222000)	10·6 (2·77–18·1)	5440 (5040–5830)	-12·3 (-18·4–-6·47)
Rheumatic heart disease	132 (113–160)	-3·72 (-17·8–12·9)	3·62 (3·14–4·37)	-22·4 (-33·4–-9·3)	3950 (3400–4710)	-10·8 (-22·7–4·97)	101 (87·4–120)	-24·1 (-34·4–-11)
Ischemic heart disease	4970 (4590–5340)	18·6 (11–25·9)	145 (133–155)	-9·83 (-15·2–-4·83)	108000 (99600–116000)	13·2 (5·08–21)	2820 (2610–3040)	-10·5 (-16·7–-4·47)
Stroke	3330 (3040–3620)	12·3 (2·87–22·1)	96·3 (87·6–104)	-14·5 (-21·2–-7·64)	69600 (63200–75800)	7·38 (-2·28–17)	1840 (1670–2000)	-15·4 (-22·8–-8·08)
Ischemic stroke	1570 (1420–1710)	22·1 (12–32·3)	48·4 (43·7–52·5)	-9·4 (-16·3–-2·54)	26600 (23900–29300)	17·9 (6·78–28·8)	742 (668–813)	-10·1 (-18·1–-2·26)
Intracerebral hemorrhage	1570 (1410–1720)	3·9 (-5·88–13·6)	42·9 (38·6–46·9)	-20·1 (-27·4–-12·7)	37700 (33700–41300)	1·09 (-8·43–10·5)	961 (861–1050)	-19·6 (-27–-12·2)
Subarachnoid hemorrhage	186 (159–228)	13·1 (-1·23–34)	5·03 (4·31–6·05)	-10·4 (-21·2–5·44)	5240 (4430–6580)	6·76 (-6·22–26·1)	132 (113–165)	-11·5 (-22·1–4·07)
Hypertensive heart disease	496 (345–557)	32·5 (18·1–43·9)	14·9 (10·3–16·7)	-0·625 (-11–7·36)	9570 (6710–10800)	25·3 (12·2–36·8)	258 (180–291)	-2·17 (-12·3–6·37)
Non-rheumatic valvular heart disease	68·1 (61–73·3)	31·3 (25·4–37·3)	2·27 (1·98–2·45)	-3·81 (-7·74–1·13)	1200 (1110–1300)	22·7 (15·8–29·3)	34·2 (31·2–36·8)	-4·34 (-9·07–0·497)

Non-rheumatic calcific aortic valve disease	54·2 (47·8–58·7)	33·2 (27·4–40·6)	1·84 (1·58–2·01)	-3·35 (-7·49–2·48)	896 (819–984)	24·7 (17·9–32·3)	26 (23·4–28·3)	-3·96 (-8·52–1·54)
Non-rheumatic degenerative mitral valve disease	12·7 (9·91–15·2)	27·3 (19·2–35·9)	0·386 (0·293–0·463)	-3·68 (-9·02–1·93)	279 (223–326)	20·2 (11·2–30·6)	7·43 (5·87–8·77)	-3·51 (-10·3–4·32)
Other non-rheumatic valve diseases	1·21 (0·839–1·52)	-0·969 (-16·3–27·9)	0·0356 (0·0249–0·0453)	-23·7 (-35·5–-0·697)	28·7 (19·5–35·4)	-4·88 (-17·7–15·4)	0·762 (0·521–0·942)	-21·6 (-32·6–-3·51)
Cardiomyopathy and myocarditis	200 (159–224)	1·89 (-5·41–12·1)	5·62 (4·46–6·27)	-18 (-23·5–-10·5)	5790 (4670–6590)	-7·26 (-14·7–4·38)	149 (120–169)	-21·4 (-27·6–-12·4)
Myocarditis	16·5 (11·2–19·2)	10·8 (-1·6–26·8)	0·482 (0·32–0·565)	-11 (-21–4·23)	543 (405–669)	-2·46 (-14·6–13·1)	14·5 (10·7–17·6)	-14·2 (-24·5–0·512)
Alcoholic cardiomyopathy	58·4 (46·9–68·7)	-24·6 (-33·3–-9·51)	1·46 (1·16–1·71)	-37·6 (-44·7–-25·7)	1950 (1590–2280)	-29·2 (-38–-13·4)	47·5 (38·5–55·5)	-40·2 (-47·6–-27·2)
Other cardiomyopathy	125 (99·4–141)	20·4 (13–28·5)	3·68 (2·92–4·14)	-7·53 (-12·9–-1·55)	3290 (2640–3770)	12·5 (3·53–21·9)	86·8 (70–99·1)	-6·67 (-13·8–0·688)
Atrial fibrillation and flutter	121 (97·3–148)	43·7 (36·6–50)	4·33 (3·45–5·33)	1·73 (-2·76–5·79)	1600 (1300–1950)	36·2 (28·6–43)	49·9 (40·5–60·9)	0·527 (-4·64–5·17)
Aortic aneurysm	108 (100–115)	25·1 (17·8–32·7)	3·15 (2·89–3·35)	-5·5 (-10·6–-0·276)	2250 (2100–2400)	20·5 (12·5–29)	59·5 (55·3–63·3)	-5·54 (-11·5–0·641)
Lower extremity peripheral arterial disease	37·7 (17·6–79·5)	37·9 (26·1–48)	1·23 (0·561–2·62)	1·16 (-7·31–8·59)	602 (294–1240)	32·1 (20·4–43·2)	17·2 (8·27–35·8)	-0·306 (-8·9–8·02)
Endocarditis	32·6 (22·3–37·6)	21·8 (13·2–29·1)	0·94 (0·618–1·09)	-3·94 (-9·09–1·58)	958 (722–1100)	12·4 (3·71–21·6)	25 (18·6–28·8)	-4·53 (-11·1–2·82)
Other cardiovascular and circulatory diseases	131 (118–147)	15·6 (8·49–22·8)	3·76 (3·4–4·23)	-9·62 (-15·2–-4·43)	3480 (3140–3950)	5·92 (-1·21–13·7)	91·8 (83–104)	-11·5 (-17·2–-4·91)
Chronic respiratory diseases	2230 (2030–2450)	11·3 (2·24–22·1)	66·7 (60·5–73·1)	-16·2 (-22·6–-8·41)	41400 (37700–45700)	5·96 (-3–16·9)	1130 (1030–1240)	-17·7 (-24·4–-9·59)
Chronic obstructive pulmonary disease	1880 (1700–2070)	12 (2·43–23·3)	56·8 (51·1–62·5)	-16·2 (-23·1–-8·35)	32800 (29400–36300)	7·37 (-2·27–19)	902 (809–996)	-17·7 (-25–-9·25)
Pneumoconiosis	21 (18·5–24)	-0·78 (-12·5–15·4)	0·604 (0·534–0·689)	-23·9 (-32·2–-12·7)	440 (380–510)	-5·16 (-18·1–13·4)	11·5 (10·1–13·4)	-25 (-34·8–-11·3)
Silicosis	12·3 (10·2–15·4)	-6·45 (-20·7–13·3)	0·348 (0·293–0·437)	-28·1 (-38·2–-13·7)	269 (218–337)	-9·44 (-25·2–12·2)	6·98 (5·69–8·71)	-28·5 (-40·4–-12)
Asbestosis	3·12 (2·17–3·58)	18·9 (12·1–27·6)	0·0978 (0·0675–0·112)	-11·1 (-16–-4·9)	51·5 (36·1–60·8)	12 (5·2–21·9)	1·45 (1·02–1·7)	-13·6 (-18·7–-6·65)
Coal workers pneumoconiosis	2·78 (1·94–3·71)	0·147 (-12·7–17·7)	0·0816 (0·0592–0·107)	-23·8 (-33·1–-11·4)	54·1 (35·6–75·8)	-4·68 (-19·1–16·1)	1·45 (0·975–1·99)	-25·2 (-35·8–-9·77)
Other pneumoconiosis	2·74 (2·02–3·78)	7·26 (-7–29·9)	0·0764 (0·0575–0·103)	-16·5 (-27–0·263)	65 (46·9–92·7)	2·01 (-12·1–26)	1·66 (1·21–2·37)	-18·1 (-29–0·548)
Asthma	214 (174–260)	0·834 (-10·9–14·7)	5·93 (4·84–7·21)	-21·4 (-30·3–-11·3)	5470 (4540–6510)	-5·14 (-15·1–7·56)	142 (118–168)	-22·4 (-30·7–-12·2)
Interstitial lung disease and pulmonary sarcoidosis	93 (57·6–121)	28·3 (15·7–42·4)	2·72 (1·66–3·55)	-2·57 (-11·2–6·82)	1850 (1200–2400)	21·6 (8·01–36·8)	49·4 (31·5–63·9)	-4·66 (-14·6–6·49)
Other chronic respiratory diseases	24·7 (18·4–30·3)	15·3 (3·49–30·6)	0·657 (0·494–0·799)	-4·81 (-14·3–6·74)	898 (667–1120)	8·63 (-3·4–23·6)	22·7 (17–28·2)	-5·65 (-15·7–6·88)
Digestive diseases	1520 (1420–1640)	9·61 (2·98–17·1)	40·8 (38–43·8)	-12·2 (-17·3–-6·54)	46400 (42900–50200)	3·67 (-3·34–11·8)	1170 (1080–1260)	-13 (-18·8–-6·17)
Cirrhosis and other chronic liver diseases	969 (899–1050)	8·69 (1·39–16·6)	24·8 (23·1–26·7)	-12 (-17·8–-5·76)	31300 (29000–34000)	4·15 (-3·21–12·5)	773 (714–838)	-12·6 (-18·7–-5·69)
Cirrhosis and other chronic liver diseases due to hepatitis B	237 (197–281)	-6·54 (-15·1–3·02)	6 (5·02–7·08)	-23·7 (-30·8–-16·2)	7950 (6540–9440)	-9·2 (-18–0·438)	194 (161–230)	-23·5 (-31–-15·5)
Cirrhosis and other chronic liver diseases due to hepatitis C	268 (229–311)	15·1 (7·03–23·3)	6·87 (5·88–7·92)	-7·1 (-13·3–-0·788)	8530 (7190–9980)	10·3 (1·99–18·6)	209 (177–244)	-7·84 (-14·7–-1·03)
Cirrhosis and other chronic liver diseases due to alcohol use	275 (234–321)	13·9 (6·82–21·3)	6·93 (5·91–8·09)	-8·81 (-14·3–-2·99)	8420 (7140–9830)	10·6 (3·25–18·1)	205 (174–239)	-9·16 (-15–-2·86)
Cirrhosis and other chronic liver diseases due to NAFLD	72·4 (51·3–98)	25·3 (16·9–34·2)	1·91 (1·36–2·54)	-1·12 (-7·36–5·49)	2090 (1470–2870)	21·3 (12·8–30·3)	51·8 (36·6–71·1)	-0·54 (-7·15–6·5)
Cirrhosis and other chronic liver diseases due to other causes	117 (92·1–151)	10·2 (2–19)	3·1 (2·44–3·97)	-8·7 (-15–-1·53)	4360 (3520–5420)	2 (-7·39–12·5)	112 (91–138)	-10·4 (-18·1–-1·06)
Upper digestive system diseases	148 (135–166)	-2·83 (-11·3–5·93)	4·15 (3·79–4·63)	-23·5 (-29·7–-17)	3820 (3470–4250)	-9·02 (-17·4–-0·687)	98·3 (89·5–109)	-25·1 (-31·9–-18·4)
Peptic ulcer disease	128 (115–143)	-3·76 (-12·4–5·16)	3·57 (3·23–4)	-24·2 (-30·5–-17·5)	3270 (2960–3680)	-9·92 (-18·5–-1·07)	83·9 (76·1–94·6)	-26 (-32·7–-18·9)
Gastritis and duodenitis	21 (14·9–24·5)	3·21 (-11·9–20)	0·588 (0·422–0·682)	-18·8 (-30·3–-6·25)	556 (390–667)	-3·31 (-16·7–12)	14·4 (10·2–17·2)	-19·9 (-30·8–-7·51)
Appendicitis	16·3 (12·2–19·4)	-5·69 (-16–11·9)	0·442 (0·333–0·524)	-21·1 (-29–-7·39)	627 (455–743)	-13·9 (-24·2–5·19)	16·3 (11·8–19·2)	-23·5 (-32·6–-7·19)
Paralytic ileus and intestinal obstruction	125 (99·1–142)	18·1 (8·47–29·9)	3·64 (2·93–4·15)	-5·98 (-12·9–2·63)	3940 (3020–4730)	6·73 (-5·69–21·7)	106 (82·6–128)	-6·97 (-17·8–6·23)
Inguinal, femoral, and abdominal hernia	28·9 (25·3–33)	15·5 (5·35–29·3)	0·865 (0·767–0·986)	-9·68 (-17–0·093)	777 (668–892)	-0·294 (-13·2–17·4)	21·3 (18·3–24·4)	-14·5 (-24·4–-0·74)
Inflammatory bowel disease	18·6 (15·1–20·9)	19·1 (11·9–26·9)	0·555 (0·446–0·621)	-8·49 (-13·1–-2·78)	450 (369–507)	9·98 (0·523–19·9)	12 (9·82–13·4)	-9·61 (-16·4–-2·1)
Vascular intestinal disorders	45 (41·3–48·4)	22·3 (16·7–28·1)	1·35 (1·23–1·46)	-8·1 (-12·1–-4·05)	883 (813–955)	16·7 (10·4–23·8)	23·9 (21·9–25·9)	-8·72 (-13·3–-3·66)
Gallbladder and biliary diseases	52·7 (44·3–60·5)	26 (16·7–34·7)	1·64 (1·37–1·89)	-5·4 (-12·1–0·36)	1030 (855–1180)	18·3 (7·93–28·4)	28·2 (23·4–32·2)	-6·4 (-14·3–1·13)
Pancreatitis	72 (63·9–81·4)	11·6 (3·39–19)	1·88 (1·67–2·12)	-8·93 (-15·4–-3·1)	2390 (2130–2740)	7·01 (-1·29–14·6)	59·3 (52·9–67·8)	-9·12 (-16·1–-2·69)
Other digestive diseases	48·3 (38–56·1)	22·9 (12·4–33·3)	1·45 (1·13–1·71)	-6·41 (-13·8–0·586)	1170 (940–1360)	14·3 (1·36–28·2)	31·3 (25·2–36·3)	-6·31 (-15·7–4·22)
Neurological disorders	899 (475–1840)	35·5 (26·1–42·6)	31 (15·3–65·1)	-0·746 (-5·25–3·21)	14900 (9650–26200)	20·5 (10·1–29·4)	443 (273–819)	-4·02 (-9·7–1·26)
Alzheimer's disease and other dementias	562 (136–1510)	43·5 (37–50·7)	20·9 (5·09–55·6)	0·491 (-3–4·34)	6710 (1590–18500)	36 (28·8–43·7)	220 (52·8–601)	-1·14 (-5·55–3·53)
Parkinson's disease	208 (189–226)	32·8 (25·2–41)	6·66 (6·01–7·24)	-2·29 (-7·44–3·24)	2990 (2750–3240)	28·4 (20·2–37·1)	88 (80·3–95·2)	-3·29 (-9·18–2·93)
Idiopathic epilepsy	65·4 (58·7–75·8)	3·1 (-5·08–16·5)	1·71 (1·53–1·98)	-9·44 (-16·5–2·2)	3150 (2790–3710)	-3·97 (-12·6–11·2)	81·2 (72–96)	-12·2 (-20·2–1·9)
Multiple sclerosis	9·12 (7·67–12·4)	18·3 (5·41–26·8)	0·229 (0·191–0·308)	-4·26 (-14·2–2·38)	292 (251–401)	14·2 (1·61–23·4)	7·13 (6·11–9·73)	-4·86 (-15–2·79)
Motor neuron disease	21·6 (20·1–22·7)	29·4 (21·2–35·1)	0·571 (0·531–0·601)	0·54 (-5·83–4·94)	556 (521–585)	23·6 (16·3–29·3)	14·1 (13·2–14·8)	-0·174 (-6·03–4·5)
Other neurological disorders	32·5 (29·9–35·2)	21 (14–28)	0·884 (0·813–0·956)	0·787 (-4·54–6·17)	1220 (1100–1350)	7·65 (-1·14–16)	32·1 (28·8–35·5)	-3·85 (-11·6–3·79)
Mental disorders	0·0396 (0·0297–0·0595)	21·9 (-0·00169–48·8)	0·000977 (0·000732–0·00148)	13 (-6·51–37·9)	2·15 (1·6–3·32)	20·4 (-1·17–46·9)	0·0534 (0·0395–0·0831)	12·4 (-7·07–36·5)
Eating disorders	0·0396 (0·0297–0·0595)	21·9 (-0·00169–48·8)	0·000977 (0·000732–0·00148)	13 (-6·51–37·9)	2·15 (1·6–3·32)	20·4 (-1·17–46·9)	0·0534 (0·0395–0·0831)	12·4 (-7·07–36·5)
Anorexia nervosa	0·0258 (0·0205–0·0366)	16·2 (-5·72–43·4)	0·000637 (0·000505–0·000907)	7·71 (-12–32·6)	1·4 (1·11–2·04)	14·7 (-6·6–41·1)	0·0346 (0·0277–0·0511)	6·98 (-12·5–31·2)
Bulimia nervosa	0·0137 (0·00419–0·0341)	34·4 (3·51–75·8)	0·000339 (0·000104–0·000852)	24·6 (-3·35–61·3)	0·754 (0·232–1·97)	32·7 (2·7–73·1)	0·0188 (0·00584–0·0495)	23·9 (-3·04–60·3)
Substance use disorders	234 (212–248)	16·1 (9·51–23)	5·75 (5·22–6·1)	-1·25 (-6·82–4·64)	9730 (8830–10300)	15 (8·7–22·3)	237 (215–252)	0·429 (-5·09–6·86)
Alcohol use disorders	143 (121–154)	2·85 (-4·33–10·4)	3·5 (2·95–3·76)	-13·8 (-19·9–-7·61)	5410 (4530–5830)	-0·224 (-7·44–7·37)	131 (110–141)	-14·4 (-20·7–-7·94)
Drug use disorders	90·8 (87–95·8)	45·4 (39·7–53)	2·25 (2·15–2·37)	27·7 (22·6–34·4)	4320 (4120–4610)	42·2 (36·2–50·6)	106 (101–114)	27·7 (22·2–35·3)
Opioid use disorders	63·8 (61·1–67·6)	43·6 (37·6–51·6)	1·58 (1·51–1·67)	26·2 (20·9–33·3)	3060 (2920–3290)	41·5 (35–50·2)	75·5 (72–81·1)	27·1 (21·3–35·1)
Cocaine use disorders	9·26 (8·88–9·8)	74·2 (65·6–82·5)	0·225 (0·216–0·24)	51·9 (44·7–59·1)	429 (407–479)	71·3 (63·6–80·4)	10·5 (9·93–11·7)	52·2 (45·4–61·1)
Amphetamine use disorders	6·37 (5·94–6·83)	53·9 (43·5–65·7)	0·157 (0·146–0·168)	36·4 (27·3–46·8)	311 (286–340)	48·6 (37–62·7)	7·64 (7·04–8·38)	34 (23·4–46·9)
Other drug use disorders	11·4 (10·7–12·4)	32·8 (24·7–42·1)	0·289 (0·269–0·312)	16·6 (9·64–24·5)	517 (481–563)	25·5 (17·2–34·8)	12·8 (11·9–13·9)	12·8 (5·3–21)
Diabetes and kidney diseases	1500 (1400–1610)	30·6 (22·6–38)	42·7 (39·6–45·6)	-0·187 (-5·85–5·12)	35700 (33300–38400)	23·8 (16·2–31·5)	926 (864–995)	-0·485 (-6·44–5·54)
Diabetes mellitus	755 (704–809)	33·9 (26–41·6)	21 (19·5–22·5)	2·18 (-3·59–7·71)	17600 (16300–18900)	29·2 (21–37·4)	452 (420–485)	2·37 (-3·96–8·67)
Diabetes mellitus type 1	40·5 (35·5–51·3)	17·2 (5·68–28·4)	1·04 (0·915–1·32)	-0·93 (-10·7–8·22)	1650 (1460–2020)	11·6 (1·03–22)	42 (37·2–50·9)	-0·83 (-10·4–8·27)

Diabetes mellitus type 2	714 (666–764)	34·9 (26·8–42·9)	19·9 (18·5–21·3)	2·35 (-3·35–8)	15900 (14800–17100)	31·4 (23–40)	410 (381–440)	2·71 (-3·72–9·29)
Chronic kidney disease	741 (686–802)	27·7 (19·7–35·3)	21·6 (19·9–23·3)	-2·21 (-7·96–3·21)	18000 (16700–19600)	19·5 (11·6–27·7)	470 (435–511)	-2·76 (-8·96–3·72)
Chronic kidney disease due to diabetes mellitus type 1	46 (30·5–65·7)	17·9 (7·82–28)	1·13 (0·753–1·6)	-2·48 (-10·7–5·32)	1680 (1140–2350)	14·5 (4·83–24·6)	40·8 (27·8–56·5)	-2·32 (-10·3–5·57)
Chronic kidney disease due to diabetes mellitus type 2	205 (165–247)	29·7 (20·6–38·5)	5·9 (4·78–7·14)	-1·97 (-8·49–4·26)	4360 (3440–5290)	25·1 (15·1–34·6)	114 (90·6–138)	-2·42 (-9·76–4·82)
Chronic kidney disease due to hypertension	232 (197–270)	33·5 (24·6–42·2)	7·15 (6·05–8·24)	-0·991 (-7·04–4·85)	4540 (3810–5370)	26·8 (17·6–36·3)	123 (104–144)	-1·15 (-8·03–5·59)
Chronic kidney disease due to glomerulonephritis	103 (82·6–128)	25·6 (16·7–34·1)	2·84 (2·27–3·55)	0·633 (-5·8–6·76)	3200 (2620–3870)	16·6 (8·17–25·2)	82·2 (68·1–98·7)	-0·385 (-7·12–6·51)
Chronic kidney disease due to other and unspecified causes	155 (121–196)	21·5 (13·4–29·7)	4·55 (3·54–5·78)	-5·93 (-11·7–-0·163)	4170 (3340–5150)	11·1 (2·95–20·1)	110 (89·5–136)	-6·61 (-13–0·202)
Acute glomerulonephritis	5·67 (2·97–6·85)	-2·5 (-19·4–15·3)	0·156 (0·0837–0·187)	-20·4 (-34·3–-6·38)	168 (82·8–206)	-15·5 (-29·2–-0·844)	4·35 (2·15–5·32)	-27·2 (-39·2–-15)
Skin and subcutaneous diseases	43·4 (27·1–59·6)	28·2 (18·7–40)	1·31 (0·816–1·82)	-0·963 (-8·46–8·02)	1080 (676–1380)	15·9 (5·43–26·9)	29·1 (18·1–37·4)	-3·37 (-11·8–5·76)
Bacterial skin diseases	32·6 (19·2–45·2)	30·6 (19·7–44)	0·952 (0·563–1·34)	3·3 (-5·5–13·5)	891 (533–1130)	16·4 (4·59–28·3)	23·6 (14–30·3)	-1·43 (-11–8·78)
Cellulitis	8·31 (3·71–13·2)	25·9 (7·24–40·6)	0·241 (0·105–0·389)	-1·3 (-15·3–8·71)	219 (95·8–317)	14·1 (-3·72–30·5)	5·73 (2·49–8·32)	-4·5 (-19·4–8·23)
Pyoderma	24·3 (14–32·5)	32·2 (19·6–46·6)	0·711 (0·412–0·971)	4·97 (-4·09–16·1)	672 (395–844)	17·1 (2·62–31)	17·9 (10·5–22·6)	-0·407 (-12–11·2)
Decubitus ulcer	9·36 (5·64–14·8)	21·5 (12–33·3)	0·31 (0·185–0·494)	-11·5 (-18·1–-2·76)	159 (97–242)	14·9 (5·23–27·1)	4·54 (2·79–6·99)	-11·4 (-18·7–-2·22)
Other skin and subcutaneous diseases	1·4 (0·919–2·17)	22·8 (8–33·9)	0·0439 (0·028–0·0695)	-5·98 (-16·9–2·53)	32·5 (23–47·7)	8·9 (-5·31–20)	0·893 (0·63–1·32)	-9·02 (-19·6–-0·204)
Musculoskeletal disorders	36·3 (31·7–40·8)	28·3 (20–36·8)	1·06 (0·921–1·2)	-0·982 (-7·06–5·44)	815 (736–927)	17·3 (9·04–25·3)	21·6 (19·4–24·4)	-4·47 (-11·1–1·97)
Rheumatoid arthritis	13·8 (11·9–15·6)	26·9 (15·8–38·7)	0·404 (0·35–0·457)	-2·99 (-11·1–5·54)	265 (232–301)	17·8 (6·89–29·4)	7·11 (6·19–8·08)	-7·45 (-15·8–1·58)
Other musculoskeletal disorders	22·4 (19·3–25·5)	29·2 (21·3–37·6)	0·661 (0·556–0·751)	0·29 (-5·68–6·63)	550 (490–629)	17 (9·25–24·8)	14·5 (12·9–16·5)	-2·94 (-9·27–3·5)
Other non-communicable diseases	586 (512–674)	-3·22 (-13·9–11·4)	17·2 (15–19·8)	-11·4 (-20·4–0·943)	35300 (29400–43300)	-13·5 (-25·4–4·14)	1020 (841–1250)	-16 (-27·5–0·916)
Congenital birth defects	302 (240–400)	-18·1 (-30·6–1·15)	8·81 (6·98–11·7)	-18·5 (-31–0·774)	25500 (20000–34000)	-18·8 (-31·3–0·898)	747 (586–999)	-18·8 (-31·4–0·948)
Neural tube defects	41·1 (26–64·9)	-19·6 (-35·2–0·811)	1·21 (0·766–1·92)	-19·5 (-35·2–1·1)	3560 (2240–5640)	-19·7 (-35·3–0·594)	105 (66·1–166)	-19·6 (-35·3–1·03)
Congenital heart anomalies	122 (95·2–152)	-20·6 (-34·2–-2·62)	3·53 (2·75–4·41)	-21·3 (-34·8–-3·23)	10100 (7830–12700)	-21·4 (-35·1–-3·08)	296 (228–373)	-21·6 (-35·3–-3·1)
Orofacial clefts	1·4 (0·785–3·39)	-31·3 (-49·8–-5·79)	0·0417 (0·0233–0·101)	-30·5 (-49·3–-4·78)	123 (68·9–298)	-31·3 (-49·8–-5·79)	3·66 (2·05–8·85)	-30·5 (-49·3–-4·78)
Down syndrome	11·3 (9·14–18·8)	2·46 (-17·7–19·7)	0·313 (0·25–0·535)	-2·14 (-20·3–14·6)	827 (645–1490)	-1·19 (-20·7–17·8)	23·6 (18·3–42·9)	-3·36 (-22·7–15·3)
Other chromosomal abnormalities	10·6 (6·2–20)	-1·32 (-21·7–27·6)	0·309 (0·18–0·587)	-1·64 (-21·7–27·3)	892 (513–1710)	-2·18 (-22·3–27·5)	26·2 (15–50·2)	-2·04 (-22·2–27·9)
Congenital musculoskeletal and limb anomalies	6·29 (4·4–9·3)	-12·2 (-27·5–5·79)	0·183 (0·128–0·272)	-12·6 (-27·6–5·6)	528 (365–792)	-12·7 (-27·9–5·76)	15·5 (10·7–23·3)	-12·6 (-27·8–6·05)
Urogenital congenital anomalies	6·3 (3·78–9·06)	-15·9 (-29·2–5·6)	0·185 (0·111–0·267)	-15·9 (-29·2–5·78)	533 (318–775)	-16·7 (-30–5·1)	15·7 (9·36–22·9)	-16·3 (-29·7–5·72)
Digestive congenital anomalies	25·1 (14·9–38·2)	-17·8 (-32·7–7·16)	0·742 (0·439–1·13)	-17·3 (-32·5–7·75)	2170 (1280–3320)	-18 (-33·1–7·01)	64·5 (38–98·5)	-17·4 (-32·6–7·87)
Other congenital birth defects	78·3 (53·2–124)	-18 (-32·3–7·6)	2·3 (1·55–3·63)	-18·2 (-32·5–7·61)	6690 (4480–10700)	-18·5 (-32·9–7·42)	197 (132–314)	-18·4 (-32·8–7·69)
Urinary diseases and male infertility	146 (120–160)	28·6 (20·2–37·3)	4·47 (3·64–4·88)	-1·58 (-7·45–4·29)	3390 (2820–3740)	15·9 (7·51–25)	91·7 (77·1–101)	-4·28 (-11–2·79)
Urinary tract infections and interstitial nephritis	105 (82·4–117)	30·7 (21·5–39·2)	3·27 (2·54–3·65)	-0·667 (-7·24–5·27)	2350 (1900–2650)	16·8 (8·02–26·2)	64·2 (51·5–72)	-3·72 (-10·5–3·25)
Urolithiasis	6·93 (4·5–9·14)	16·3 (3·3–30·3)	0·199 (0·131–0·26)	-10·2 (-19·9–0·0169)	154 (95·7–203)	8·7 (-3·05–22)	4·01 (2·54–5·34)	-13·1 (-22·2–-2·63)
Other urinary diseases	34 (25·7–43·5)	25 (15·8–35)	0·993 (0·749–1·27)	-2·67 (-9·7–4·75)	882 (690–1120)	14·8 (5·19–24·9)	23·5 (18·4–29·6)	-4·16 (-11·9–4·09)
Hemoglobinopathies and hemolytic anemias	46·3 (36–62·3)	4·65 (-8·72–24·7)	1·28 (0·999–1·71)	-7·39 (-18·9–9·46)	2580 (1920–3550)	-0·73 (-17·5–26·5)	70·6 (52·4–97)	-6·86 (-22·8–19)
Thalassemias	5·23 (4·26–6·58)	-12·7 (-24·4–5·11)	0·143 (0·117–0·181)	-16·6 (-28–0·757)	388 (313–495)	-13·9 (-26·3–4·9)	10·8 (8·69–13·8)	-16·9 (-29·1–1·6)
Sickle cell disorders	20·8 (13·7–31·5)	2·21 (-19·9–41·6)	0·571 (0·375–0·865)	-1·86 (-23·4–36·7)	1570 (1030–2380)	0·456 (-22·5–42·6)	43·7 (28·5–65·9)	-2·97 (-25·4–39)
G6PD deficiency	8·62 (6·43–13)	15·4 (3·35–28·3)	0·217 (0·162–0·325)	-1·26 (-10·8–9·09)	383 (286–558)	8·87 (-3·16–22·7)	9·68 (7·26–14)	-2·79 (-13–9·15)
Other hemoglobinopathies and hemolytic anemias	11·6 (9·93–15)	11·8 (4·76–18·8)	0·347 (0·298–0·443)	-14·7 (-19·7–-9·85)	238 (200–311)	2·4 (-4·67–9·73)	6·44 (5·44–8·39)	-17·7 (-23·2–-12·2)
Endocrine, metabolic, blood, and immune disorders	76·9 (56·4–88·5)	28 (21·1–34·3)	2·2 (1·59–2·54)	3·54 (-1·69–8·81)	2540 (1950–3010)	12·5 (5·08–20·7)	68·1 (52·2–80·7)	-1·22 (-7·35–6·01)
Sudden infant death syndrome	15·3 (6·58–33·5)	-20·6 (-35·1–3·21)	0·454 (0·196–0·994)	-19·7 (-34·4–4·32)	1340 (578–2940)	-20·6 (-35·1–3·21)	39·9 (17·2–87·4)	-19·7 (-34·4–4·32)
Injuries	2940 (2670–3180)	-7·53 (-13·2–-1·92)	76·7 (69·7–83)	-19·3 (-24·1–-14·5)	133000 (121000–144000)	-14·4 (-19·6–-8·97)	3390 (3080–3670)	-22·4 (-27·1–-17·5)
Transport injuries	966 (820–1060)	-6·7 (-14·1–0·639)	24·4 (20·8–26·9)	-18 (-24·6–-11·7)	45900 (39300–50600)	-11·3 (-18·3–-4·69)	1160 (991–1270)	-19·4 (-25·6–-13·4)
Road injuries	901 (764–991)	-6·63 (-14·3–0·766)	22·8 (19·4–25·1)	-17·9 (-24·5–-11·6)	42800 (36600–47200)	-11·3 (-18·3–-4·54)	1080 (923–1190)	-19·4 (-25·7–-13·2)
Pedestrian road injuries	318 (254–367)	-10·8 (-21·3–2·02)	8·14 (6·52–9·4)	-22·9 (-31·9–-12·1)	14000 (11200–16200)	-16·6 (-26·4–-4·13)	355 (285–409)	-25 (-33·8–-14·1)
Cyclist road injuries	51·2 (41·3–59·2)	-3·24 (-18·1–9·41)	1·29 (1·04–1·49)	-17·4 (-30·1–-6·77)	2140 (1750–2460)	-10·3 (-23·4–0·355)	53·5 (44·1–61·6)	-20·4 (-32–-11)
Motorcyclist road injuries	190 (156–216)	-9·93 (-22·2–0·905)	4·74 (3·88–5·36)	-18·7 (-29·6–-8·83)	9820 (8160–11100)	-13·6 (-24·7–-3·62)	246 (204–278)	-20·3 (-30·5–-11·2)
Motor vehicle road injuries	332 (282–380)	-0·67 (-9·96–7·93)	8·38 (7·13–9·58)	-12·1 (-20·2–-4·46)	16400 (14100–18800)	-4·84 (-13·6–3·34)	415 (356–474)	-13·1 (-20·9–-5·65)
Other road injuries	9·29 (7·91–10·7)	-5·05 (-18–5·64)	0·238 (0·202–0·273)	-17·4 (-28·5–-8·26)	422 (360–487)	-10·8 (-23·5–-1·1)	10·7 (9·14–12·4)	-19·3 (-30·7–-10·5)
Other transport injuries	65·2 (52·2–73·1)	-7·62 (-14·3–0·595)	1·64 (1·32–1·84)	-18·7 (-24·7–-11·7)	3100 (2490–3480)	-11·8 (-18·3–-4·27)	78·1 (62·7–87·6)	-19·9 (-25·8–-13·1)
Unintentional injuries	1060 (932–1160)	-13·5 (-19·3–-7·4)	29·3 (25·7–32·1)	-24·9 (-29·7–-19·8)	43100 (37800–47900)	-26·3 (-31·6–-20·3)	1130 (987–1260)	-33·1 (-37·8–-27·6)
Falls	407 (340–454)	18·8 (9·76–28·4)	11·9 (10–13·3)	-4·72 (-11·2–2·6)	11600 (9760–13000)	3·99 (-5·02–15)	305 (257–343)	-10·5 (-18·1–-1·36)
Drowning	167 (148–188)	-18·1 (-24·3–-10·4)	4·39 (3·9–4·96)	-26·3 (-31·9–-19·6)	9210 (8140–10600)	-25·1 (-31·3–-17·9)	243 (215–280)	-30·5 (-36·2–-23·6)
Fire, heat, and hot substances	56·8 (44·1–67·3)	-8·31 (-14·6–3·02)	1·53 (1·19–1·81)	-21·4 (-26·7–-11·2)	2530 (1870–3130)	-14·9 (-22–1·03)	66·6 (49·2–82·8)	-22·9 (-29·5–-8·56)
Poisonings	49·1 (37·7–55·7)	-11·6 (-19·1–-3·07)	1·27 (0·974–1·45)	-23 (-29·5–-15·5)	2320 (1790–2720)	-16·9 (-24·2–-7·71)	60·3 (46·5–71·6)	-24·4 (-30·9–-15·6)
Poisoning by carbon monoxide	27·2 (20·1–31·3)	-9·2 (-17·6–-0·105)	0·694 (0·512–0·801)	-21·9 (-29·1–-14·2)	1170 (865–1380)	-13·2 (-21·4–-4·63)	29·7 (21·7–35)	-22·2 (-29·4–-14·5)
Poisoning by other means	21·9 (15·4–28)	-14·4 (-23·4–-3·5)	0·578 (0·408–0·733)	-24·3 (-32·3–-15)	1150 (856–1460)	-20·5 (-29·4–-8·26)	30·6 (23–39·1)	-26·4 (-34·7–-14·7)
Exposure to mechanical forces	91·7 (71·3–106)	-10·8 (-19·5–-1·56)	2·34 (1·81–2·71)	-21·2 (-28·7–-13·3)	4470 (3420–5290)	-14·1 (-22·6–-4·76)	114 (86·5–136)	-21·5 (-29·3–-12·6)
Unintentional firearm injuries	15·2 (10·6–19·6)	-6·72 (-17·9–9·46)	0·388 (0·269–0·502)	-16·4 (-26·4–-1·79)	811 (571–1070)	-9·21 (-20·7–7·25)	20·8 (14·5–27·5)	-16 (-26·8–-0·242)
Other exposure to mechanical forces	76·5 (57·8–88·6)	-11·5 (-21·1–-0·896)	1·95 (1·47–2·26)	-22·1 (-30·2–-13·2)	3660 (2690–4330)	-15·1 (-24·1–-4·83)	93·3 (68·1–111)	-22·6 (-30·8–-13·4)
Adverse effects of medical treatment	50·3 (40·4–57·9)	6·29 (-3·26–17·5)	1·4 (1·13–1·6)	-12·7 (-20·5–-3·59)	1780 (1400–2110)	-5·69 (-13·9–4·13)	47·6 (37–56·4)	-16·5 (-24–-8·14)
Animal contact	51·1 (35·9–63·9)	-8·28 (-20·6–8·48)	1·33 (0·932–1·67)	-20·3 (-30·8–-5·8)	2420 (1690–3100)	-16·6 (-27·6–1·89)	63·2 (44·3–81·2)	-24·3 (-34·1–-7·51)
Venomous animal contact	41·1 (27–51·3)	-9·46 (-23·2–8·2)	1·07 (0·697–1·34)	-21·3 (-32·9–-6·49)	1920 (1230–2500)	-18·2 (-30·5–-1·68)	49·8 (31·8–65)	-26 (-36·8–-10·7)

Non-venomous animal contact	9·95<br>(7·86–13·2)	-3·06<br>(-13·9–19·5)	0·263<br>(0·208–0·35)	-15·9<br>(-25·1–2·88)	503<br>(393–678)	-9·83<br>(-21·1–14·8)	13·4<br>(10·5–18)	-17·5<br>(-28·3–5·17)
Foreign body	68·3<br>(62·9–74·2)	2·35<br>(-4·77–9·19)	1·96<br>(1·8–2·12)	-11·9<br>(-17·8–-6·26)	3030<br>(2720–3390)	-10·9<br>(-19·2–-2·77)	82·8<br>(74–92·8)	-17·7<br>(-25·4–-10)
Pulmonary aspiration and foreign body in airway	64·6<br>(59·5–70)	2·28<br>(-4·7–9·02)	1·86<br>(1·71–2·01)	-12·1<br>(-17·7–-6·52)	2830<br>(2540–3150)	-11·2<br>(-19·3–-3)	77·3<br>(69·1–86·4)	-18·1<br>(-25·5–-10·3)
Foreign body in other body part	3·67<br>(2·88–4·58)	3·47<br>(-14·6–22·2)	0·1<br>(0·079–0·124)	-7·73<br>(-22·9–7·7)	203<br>(157–254)	-5·05<br>(-24·5–16·5)	5·52<br>(4·26–6·89)	-11·2<br>(-29·1–8·56)
Environmental heat and cold exposure	32·3<br>(22–36·6)	-22·3<br>(-30·6–-15·4)	0·843<br>(0·568–0·956)	-34·4<br>(-41·2–-28·8)	1170<br>(800–1340)	-27·3<br>(-34·2–-21·5)	29·5<br>(19·9–33·8)	-36·6<br>(-42·8–-31·4)
Exposure to forces of nature	3·72<br>(3·38–4·1)	-97·5<br>(-97·5–-97·5)	0·0962<br>(0·0873–0·106)	-97·8<br>(-97·8–-97·8)	209<br>(190–230)	-97·7<br>(-97·7–-97·7)	5·45<br>(4·95–6)	-97·8<br>(-97·8–-97·8)
Other unintentional injuries	85·8<br>(61·1–96·6)	-13·9<br>(-23·2–-4·52)	2·17<br>(1·55–2·44)	-23·3<br>(-31·4–-14·9)	4380<br>(3150–4940)	-17·3<br>(-25·9–-7·89)	111<br>(80–126)	-24·3<br>(-32·1–-15·6)
Self-harm and interpersonal violence	914<br>(835–989)	-0·521<br>(-6·04–5·84)	23·1<br>(21–25)	-12·4<br>(-17·3–-6·98)	44000<br>(40300–47500)	-2·64<br>(-8·01–3·49)	1100<br>(1010–1190)	-11·4<br>(-16·3–-5·89)
Self-harm	524<br>(447–583)	-1·45<br>(-8·14–6·88)	13·3<br>(11·3–14·7)	-15·3<br>(-21·1–-8·34)	22800<br>(19400–25500)	-3·86<br>(-10·3–5·15)	568<br>(484–634)	-14·1<br>(-19·9–-6·15)
Self-harm by firearm	45·1<br>(38·7–54·6)	0·881<br>(-3·89–6·4)	1·15<br>(0·992–1·39)	-14·6<br>(-18·5–-9·75)	1830<br>(1550–2260)	-3·41<br>(-8·57–2·52)	45·6<br>(38·6–56·2)	-14·4<br>(-19–-9·19)
Self-harm by other specified means	479<br>(403–534)	-1·66<br>(-8·67–7)	12·1<br>(10·2–13·5)	-15·4<br>(-21·5–-8·02)	21000<br>(17700–23500)	-3·9<br>(-10·7–5·36)	522<br>(441–585)	-14·1<br>(-20·1–-5·93)
Interpersonal violence	344<br>(320–372)	0·216<br>(-5·92–6·87)	8·6<br>(8–9·31)	-9·21<br>(-14·8–-3·21)	18600<br>(17300–20200)	-1·12<br>(-7·2–5·58)	468<br>(434–508)	-8·53<br>(-14·2–-2·23)
Physical violence by firearm	159<br>(147–173)	4·37<br>(-3·09–12)	3·97<br>(3·67–4·31)	-4·3<br>(-11·1–2·64)	8860<br>(8170–9620)	2·62<br>(-4·58–10·1)	223<br>(206–242)	-4·35<br>(-11·1–2·58)
Physical violence by sharp object	80·8<br>(65·4–92·3)	-5·72<br>(-12·3–2·32)	2·01<br>(1·63–2·3)	-14·9<br>(-20·8–-7·67)	4290<br>(3460–4900)	-6·94<br>(-13·6–0·839)	107<br>(86·6–123)	-14·3<br>(-20·3–-7)
Physical violence by other means	105<br>(93·7–117)	-0·949<br>(-8·14–7·35)	2·62<br>(2·35–2·93)	-11·5<br>(-18–-4·16)	5470<br>(4860–6120)	-2·12<br>(-9·86–6·77)	138<br>(123–155)	-10·2<br>(-17·3–-1·86)
Conflict and terrorism	38·3<br>(34·8–42·1)	0·973<br>(0·117–1·66)	1·02<br>(0·924–1·12)	-3·17<br>(-3·99–-2·51)	2170<br>(1970–2390)	-6·73<br>(-7·54–-6·08)	57·6<br>(52·3–63·4)	-9·5<br>(-10·3–-8·87)
Police conflict and executions	7·43<br>(6·45–8·52)	32·3<br>(16·8–49·4)	0·186<br>(0·162–0·213)	20·8<br>(6·7–36·3)	407<br>(352–468)	30·3<br>(14·5–46·9)	10·2<br>(8·85–11·7)	21<br>(6·51–36·2)

SR Table 4: Global deaths by cause and age group for females (with 95% uncertainty levels), 1990

Cause	Early Neonatal	Late Neonatal	Post Neonatal	1 to 4	5 to 9	10 to 14	15 to 19	20 to 24	25 to 29	30 to 34	35 to 39	40 to 44	45 to 49	50 to 54	55 to 59	60 to 64	65 to 69	70 to 74	75 to 79	80 to 84	85 to 89	90 to 94	95 plus
All causes	1300000 (1200000–1410000)	570000 (525000–616000)	1810000 (1710000–1920000)	1950000 (1840000–2060000)	381000 (369000–395000)	198000 (192000–205000)	284000 (275000–294000)	352000 (335000–369000)	356000 (338000–375000)	357000 (341000–376000)	405000 (385000–427000)	433000 (413000–455000)	495000 (473000–518000)	696000 (666000–728000)	903000 (864000–943000)	1230000 (1180000–1270000)	1510000 (1450000–1550000)	1750000 (1690000–1810000)	2130000 (2070000–2170000)	2090000 (2050000–2130000)	1470000 (1450000–1490000)	671000 (664000–677000)	249000 (247000–250000)
Communicable, maternal, neonatal, and nutritional diseases	1170000 (1080000–1270000)	504000 (459000–551000)	1510000 (1390000–1640000)	1580000 (1440000–1730000)	248000 (229000–268000)	104000 (94900–114000)	126000 (117000–136000)	170000 (159000–182000)	172000 (160000–184000)	156000 (146000–168000)	144000 (133000–155000)	119000 (109000–130000)	109000 (96900–124000)	123000 (107000–145000)	139000 (118000–168000)	174000 (143000–217000)	195000 (158000–247000)	233000 (187000–291000)	237000 (191000–297000)	227000 (184000–278000)	147000 (121000–175000)	64000 (51600–74800)	24100 (19000–28200)
HIV/AIDS and sexually transmitted infections	28200 (9660–58900)	7390 (2420–16200)	23800 (17700–34200)	20500 (18100–23000)	1680 (1290–2120)	293 (249–363)	6790 (5680–8140)	17300 (14600–20700)	25600 (21300–30700)	22700 (19000–26700)	16000 (13300–19300)	11800 (9950–13900)	10500 (8830–12400)	8460 (7240–9910)	5530 (4780–6440)	3400 (2970–3910)	2200 (1950–2500)	1450 (1250–1680)	818 (709–929)	257 (209–299)	150 (119–178)	64·1 (48·2–78)	21·9 (16·7–26·3)
HIV/AIDS	--	--	15500 (13800–17300)	20000 (17800–22500)	1470 (1140–1820)	215 (181–286)	6520 (5440–7870)	16900 (14100–20200)	24900 (20600–30000)	22100 (18600–26200)	15500 (12900–18800)	11300 (9470–13400)	10100 (8440–12000)	8010 (6800–9460)	5010 (4250–5900)	3030 (2600–3540)	1850 (1600–2140)	1160 (978–1380)	512 (429–615)	46·6 (36–59·3)	20·8 (16·2–26·3)	5·69 (4·48–7·13)	2·48 (2·24–2·77)
HIV/AIDS - Drug-susceptible Tuberculosis	--	--	6150 (5170–6980)	8180 (6850–9300)	535 (395–699)	62·8 (47·6–82·1)	2520 (2050–3020)	6560 (5330–7970)	9610 (7690–11800)	8450 (6810–10100)	5830 (4660–7160)	4310 (3450–5200)	4000 (3260–4830)	3230 (2680–3830)	2000 (1660–2380)	1180 (976–1390)	691 (572–817)	469 (387–554)	205 (169–245)	14·3 (10·3–19·1)	6·14 (4·48–8·21)	1·6 (1·18–2·13)	0·519 (0·411–0·647)
Tuberculosis without extensive drug resistance	--	--	30·1 (10·8–72·7)	38·6 (13·4–88·2)	2·51 (0·893–5·71)	0·563 (0·185–1·34)	12·6 (4·48–29·5)	29·7 (10·8–69·1)	46·2 (17·6–108)	42·5 (16·3–98·1)	30·8 (12·1–67·1)	22·8 (8·78–52·2)	20 (7·6–46·6)	16·1 (5·99–38·7)	10·7 (3·97–25·5)	7·12 (2·7–15·3)	4·46 (1·73–10)	2·38 (0·872–5·51)	1·32 (0·459–3·03)	0·172 (0·0586–0·405)	0·07 (0·0248–0·159)	0·017 (0·00644–0·0361)	0·00663 (0·00268–0·0136)
HIV/AIDS - Extensively drug-resistant Tuberculosis	--	--	0 (0–0)	0 (0–0)	0 (0–0)	0 (0–0)	0 (0–0)	0 (0–0)	0 (0–0)	0 (0–0)	0 (0–0)	0 (0–0)	0 (0–0)	0 (0–0)	0 (0–0)	0 (0–0)	0 (0–0)	0 (0–0)	0 (0–0)	0 (0–0)	0 (0–0)	0 (0–0)	0 (0–0)
HIV/AIDS resulting in other diseases	--	--	9310 (8090–10700)	11800 (10200–13700)	931 (702–1260)	152 (126–213)	3990 (3190–5240)	10300 (8320–13300)	15300 (12400–19500)	13600 (11200–17000)	9660 (7810–12400)	6940 (5670–8660)	6050 (4960–7610)	4760 (3970–5970)	3000 (2520–3720)	1850 (1570–2260)	1150 (985–1390)	692 (572–864)	306 (253–382)	32·1 (25–43·4)	14·6 (11·2–20)	4·07 (3·23–5·4)	1·95 (1·76–2·2)
Sexually transmitted infections excluding HIV	28200 (9660–58900)	7390 (2420–16200)	8330 (2720–18200)	478 (156–1070)	210 (65·6–479)	78 (50·1–97·2)	273 (202–334)	459 (300–562)	623 (361–763)	547 (357–665)	451 (323–542)	491 (340–585)	414 (290–492)	448 (325–557)	520 (362–655)	372 (266–459)	355 (273–428)	282 (230–337)	305 (240–364)	210 (162–251)	130 (98·6–157)	58·4 (42·5–71·9)	19·4 (14·2–23·7)
Syphilis	28200 (9660–58900)	7390 (2420–16200)	8330 (2720–18200)	478 (156–1070)	210 (65·6–479)	43·7 (28–54·5)	57·1 (41·5–70·4)	38·7 (27·7–45·9)	112 (63·1–138)	112 (70·1–138)	54·9 (37·3–67·2)	20·2 (13–24·5)	32·8 (21·6–39·7)	41·1 (28·3–52·4)	17 (11·2–21·8)	19·1 (12·7–24·3)	22·5 (15·8–28·3)	24·9 (18·5–31·3)	21 (14·8–26·7)	25·5 (16·3–33·5)	14 (8·37–18·9)	8·9 (4·54–12·6)	2·71 (1·34–3·9)
Chlamydial infection	--	--	--	--	--	7·82 (5·16–9·66)	28·4 (21·1–34·7)	100 (65·2–123)	95·1 (57·1–115)	39·8 (25–48·9)	63·3 (45·4–75·9)	79·7 (55·6–94·9)	67·5 (47·3–80·1)	65·9 (48·1–81·6)	84·5 (59·1–106)	61·6 (44·5–75·8)	37·6 (29–45·4)	41·6 (34·2–49·5)	48·3 (38·3–57·2)	42·5 (33·2–50·3)	24·4 (18·9–29)	10·7 (8·16–12·9)	3·9 (2·9–4·73)
Gonococcal infection	--	--	--	--	--	12·6 (8·17–15·6)	81·7 (60·7–99·8)	164 (107–201)	240 (138–295)	212 (141–255)	167 (121–200)	216 (151–257)	173 (123–205)	202 (147–250)	239 (167–301)	166 (122–202)	207 (161–249)	144 (119–171)	175 (140–207)	104 (82·9–122)	64·7 (50·1–76·9)	28·4 (21·9–34·1)	10·4 (7·99–12·1)
Other sexually transmitted infections	--	--	--	--	--	13·9 (8·78–17·5)	106 (78·4–129)	156 (101–192)	176 (103–215)	184 (120–223)	166 (119–199)	175 (120–209)	140 (98·3–167)	139 (101–173)	179 (125–226)	125 (86·9–156)	87·2 (66·7–106)	71·3 (58·3–85·2)	60·5 (46·8–72·7)	38·2 (29·7–45·4)	26·6 (20·1–32·6)	10·3 (7·63–12·6)	2·38 (1·66–2·98)
Respiratory infections and tuberculosis	102000 (88800–125000)	121000 (105000–141000)	556000 (479000–642000)	383000 (305000–472000)	47000 (37700–56200)	20200 (17100–23600)	27300 (23800–31200)	36900 (32100–42000)	41700 (36400–46900)	41300 (36500–46300)	44300 (38700–49800)	42600 (37200–47900)	44400 (38100–50300)	59000 (50700–67400)	67600 (57400–77300)	80300 (69000–92300)	89300 (76800–102000)	107000 (93000–123000)	114000 (101000–130000)	116000 (102000–130000)	86700 (74200–95300)	41900 (34500–46400)	17000 (13400–19000)
[illegible]	[illegible]	[illegible]	47200 [illegible]	59900 [illegible]	15500 [illegible]	9640 [illegible]	19000 [illegible]	28500 [illegible]	32700 [illegible]	33100 [illegible]	35100 [illegible]	33300 [illegible]	34200 [illegible]	44400 [illegible]	48100 [illegible]	50600 [illegible]	49900 [illegible]	52200 [illegible]	44000 [illegible]	32300 [illegible]	14600 [illegible]	4100 [illegible]	1290 [illegible]
[illegible]	[illegible]	[illegible]	[illegible]	[illegible]	[illegible]	[illegible]	[illegible]	[illegible]	[illegible]	[illegible]	[illegible]	[illegible]	[illegible]	[illegible]	[illegible]	[illegible]	[illegible]	[illegible]	[illegible]	[illegible]	[illegible]	[illegible]	[illegible]

Chronic respiratory diseases	0 (0–0)	0 (0–0)	0 (0–0)	13900 (6220–18800)	2830 (1860–3450)	2110 (1650–2450)	[illegible]
Chronic obstructive pulmonary disease	--	--	0 (0–0)	2160 (562–3880)	452 (197–663)	306 (160–420)	[illegible]
Pneumoconiosis	--	--	--	--	--	--	[illegible]
Silicosis	--	--	--	--	--	--	[illegible]
Asbestosis	--	--	--	--	--	--	[illegible]
Coal workers pneumoconiosis	--	--	--	--	--	--	[illegible]
Other pneumoconiosis	--	--	--	--	--	--	[illegible]
Asthma	--	--	--	10200 (4250–14500)	1970 (1280–2430)	1530 (1160–1830)	[illegible]
Interstitial lung disease and pulmonary sarcoidosis	--	--	--	146 (75·7–269)	67 (42·7–96·1)	46·4 (31·9–61·7)	[illegible]
Other chronic respiratory diseases	0 (0–0)	0 (0–0)	0 (0–0)	1380 (456–3040)	339 (194–629)	229 (145–384)	[illegible]
Digestive diseases	1460 (1320–1610)	1750 (1370–2100)	13000 (10100–16300)	19800 (12700–27300)	7060 (5200–9000)	5560 (4450–6650)	[illegible]
Cirrhosis and other chronic liver diseases	--	--	--	8380 (4570–13200)	3060 (2180–4310)	2780 (2180–3490)	[illegible]
Cirrhosis and other chronic liver diseases due to hepatitis B	--	--	--	39·8 (16·3–81·9)	35·9 (18·3–59·5)	64·1 (34·5–102)	[illegible]
Cirrhosis and other chronic liver diseases due to hepatitis C	--	--	--	42·2 (16·4–86·4)	43·9 (22·8–77)	75·6 (42·1–130)	[illegible]
Cirrhosis and other chronic liver diseases due to alcohol use	--	--	--	--	--	--	[illegible]
Cirrhosis and other chronic liver diseases due to NAFLD	--	--	--	--	--	--	[illegible]
Cirrhosis and other chronic liver diseases due to other causes	--	--	--	8300 (4540–13000)	2980 (2130–4210)	2640 (2060–3320)	[illegible]
Upper digestive system diseases	--	--	--	1700 (938–2560)	894 (608–1200)	573 (439–720)	[illegible]
Peptic ulcer disease	--	--	--	1520 (800–2340)	796 (533–1050)	512 (388–640)	[illegible]
Gastritis and duodenitis	--	--	--	[illegible]	[illegible]	[illegible]	[illegible]
Appendicitis	--	--	--	[illegible]	[illegible]	[illegible]	[illegible]
Paralytic ileus and intestinal obstruction	1070 (901–1250)	1470 (1150–1820)	12000 (9200–15000)	[illegible]	[illegible]	[illegible]	[illegible]
Inguinal, femoral, and abdominal hernia	388 (242–527)	273 (136–464)	1080 (356–2240)	[illegible]	[illegible]	[illegible]	[illegible]
Inflammatory bowel disease	--	--	--	[illegible]	[illegible]	[illegible]	[illegible]
Vascular intestinal disorders	--	--	--	[illegible]	[illegible]	[illegible]	[illegible]
Gallbladder and biliary diseases	--	--	--	[illegible]	[illegible]	[illegible]	[illegible]
Pancreatitis	--	--	--	[illegible]	[illegible]	[illegible]	[illegible]
Other digestive diseases	--	--	--	[illegible]	[illegible]	[illegible]	[illegible]
Neurological disorders	4·86 (3·52–7·69)	9·31 (6·82–14·8)	5030 (3420–6300)	[illegible]	[illegible]	[illegible]	[illegible]
Alzheimer's disease and other dementias	--	--	--	[illegible]	[illegible]	[illegible]	[illegible]
Parkinson's disease	--	--	--	[illegible]	[illegible]	[illegible]	[illegible]
Idiopathic epilepsy	--	--	3360 (1540–4570)	[illegible]	[illegible]	[illegible]	[illegible]
Multiple sclerosis	--	--	--	[illegible]	[illegible]	[illegible]	[illegible]
Motor neuron disease	4·86 (3·52–7·69)	9·31 (6·82–14·8)	656 (534–830)	[illegible]	[illegible]	[illegible]	[illegible]
Other neurological disorders	--	--	1010 (747–1370)	[illegible]	[illegible]	[illegible]	[illegible]
Mental disorders	--	--	--	[illegible]	[illegible]	[illegible]	[illegible]
Eating disorders	--	--	--	[illegible]	[illegible]	[illegible]	[illegible]
Anorexia nervosa	--	--	--	[illegible]	[illegible]	[illegible]	[illegible]
Bulimia nervosa	--	--	--	[illegible]	[illegible]	[illegible]	[illegible]
Substance use disorders	0 (0–0)	0 (0–0)	0 (0–0)	[illegible]	[illegible]	[illegible]	[illegible]
Alcohol use disorders	--	--	--	[illegible]	[illegible]	[illegible]	[illegible]
Drug use disorders	0 (0–0)	0 (0–0)	0 (0–0)	[illegible]	[illegible]	[illegible]	[illegible]
Opioid use disorders	0 (0–0)	0 (0–0)	0 (0–0)	[illegible]	[illegible]	[illegible]	[illegible]
Cocaine use disorders	--	--	--	[illegible]	[illegible]	[illegible]	[illegible]
Amphetamine use disorders	--	--	--	[illegible]	[illegible]	[illegible]	[illegible]
Other drug use disorders	--	--	--	[illegible]	[illegible]	[illegible]	[illegible]
Diabetes and kidney diseases	13·5 (7·48–20·3)	43 (25·6–65·8)	4610 (3580–5630)	[illegible]	[illegible]	[illegible]	[illegible]
Diabetes mellitus	13·5 (7·48–20·3)	43 (25·6–65·8)	918 (557–1200)	[illegible]	[illegible]	[illegible]	[illegible]
Diabetes mellitus type 1	13·5 (7·48–20·3)	43 (25·6–65·8)	918 (557–1200)	[illegible]	[illegible]	[illegible]	[illegible]
Diabetes mellitus type 2	--	--	--	[illegible]	[illegible]	[illegible]	[illegible]
Chronic kidney disease	--	--	3430 (2360–4340)	[illegible]	[illegible]	[illegible]	[illegible]
Chronic kidney disease due to diabetes mellitus type 1	--	--	24·7 (10·5–52·4)	[illegible]	[illegible]	[illegible]	[illegible]
Chronic kidney disease due to diabetes mellitus type 2	--	--	--	[illegible]	[illegible]	[illegible]	[illegible]
Chronic kidney disease due to hypertension	--	--	--	[illegible]	[illegible]	[illegible]	[illegible]
Chronic kidney disease due to glomerulonephritis	--	--	811 (429–1320)	[illegible]	[illegible]	[illegible]	[illegible]
Chronic kidney disease due to other and unspecified causes	--	--	2600 (1660–3450)	[illegible]	[illegible]	[illegible]	[illegible]
Acute glomerulonephritis	--	--	261 (160–375)	[illegible]	[illegible]	[illegible]	[illegible]
Skin and subcutaneous diseases	--	--	2290 (1010–3230)	[illegible]	[illegible]	[illegible]	[illegible]
Bacterial skin diseases	--	--	2260 (989–3190)	[illegible]	[illegible]	[illegible]	[illegible]
Cellulitis	--	--	220 (38·4–512)	[illegible]	[illegible]	[illegible]	[illegible]
Pyoderma	--	--	2040 (915–2990)	[illegible]	[illegible]	[illegible]	[illegible]
Decubitus ulcer	--	--	--	[illegible]	[illegible]	[illegible]	[illegible]
Other skin and subcutaneous diseases	--	--	37·6 (14·5–59·8)	[illegible]	[illegible]	[illegible]	[illegible]
Musculoskeletal disorders	--	--	--	[illegible]	[illegible]	[illegible]	[illegible]
Rheumatoid arthritis	--	--	--	[illegible]	[illegible]	[illegible]	[illegible]
Other musculoskeletal disorders	--	--	--	[illegible]	[illegible]	[illegible]	[illegible]
Other non-communicable diseases	109000 (90400–140000)	52300 (38300–70100)	182000 (110000–277000)	[illegible]	[illegible]	[illegible]	[illegible]
Congenital birth defects	108000 (89200–139000)	48700 (35600–66400)	151000 (92400–250000)	[illegible]	[illegible]	[illegible]	[illegible]
Neural tube defects	28900 (19500–42400)	9460 (5850–14600)	26100 (14800–45800)	[illegible]	[illegible]	[illegible]	[illegible]
Congenital heart anomalies	30900 (24100–39600)	19700 (13600–28900)	67900 (41800–109000)	[illegible]	[illegible]	[illegible]	[illegible]
Orofacial clefts	2230 (1440–4420)	936 (541–2060)	1890 (934–4800)	[illegible]	[illegible]	[illegible]	[illegible]
Down syndrome	2020 (1110–3740)	1030 (519–2200)	5060 (2270–11100)	[illegible]	[illegible]	[illegible]	[illegible]
Other chromosomal abnormalities	2590 (1570–6580)	1530 (840–4530)	3150 (1640–10000)	[illegible]	[illegible]	[illegible]	[illegible]
Congenital musculoskeletal and limb anomalies	2250 (1360–3680)	729 (364–1450)	1940 (836–4720)	[illegible]	[illegible]	[illegible]	[illegible]
Urogenital congenital anomalies	2590 (1610–4500)	830 (425–1810)	1450 (628–3640)	[illegible]	[illegible]	[illegible]	[illegible]
Digestive congenital anomalies	9970 (5570–18100)	5700 (2530–12400)	16700 (5630–41400)	[illegible]	[illegible]	[illegible]	[illegible]
Other congenital birth defects	26500 (16500–46000)	8790 (4340–19400)	26600 (11600–64400)	[illegible]	[illegible]	[illegible]	[illegible]
Urinary diseases and male infertility	199 (126–248)	189 (111–246)	1220 (768–1580)	[illegible]	[illegible]	[illegible]	[illegible]
Urolithiasis	--	--	--	[illegible]	[illegible]	[illegible]	[illegible]
Other urinary diseases	140 (87·8–180)	85·3 (49·5–131)	218 (109–383)	[illegible]	[illegible]	[illegible]	[illegible]
Gynecological diseases	--	--	--	[illegible]	[illegible]	[illegible]	[illegible]
Uterine fibroids	--	--	--	[illegible]	[illegible]	[illegible]	[illegible]
Endometriosis	--	--	--	[illegible]	[illegible]	[illegible]	[illegible]
Genital prolapse	--	--	--	[illegible]	[illegible]	[illegible]	[illegible]
Other gynecological diseases	--	--	--	[illegible]	[illegible]	[illegible]	[illegible]
Hemoglobinopathies and hemolytic anemias	365 (301–419)	280 (203–370)	2910 (1610–4650)	[illegible]	[illegible]	[illegible]	[illegible]
Thalassemias	207 (173–247)	161 (121–208)	1330 (706–2150)	[illegible]	[illegible]	[illegible]	[illegible]
Sickle cell disorders	126 (79·7–175)	110 (63·7–168)	1490 (732–2490)	[illegible]	[illegible]	[illegible]	[illegible]
G6PD deficiency	2·91 (1·7–4·34)	2·49 (1·39–3·96)	32·7 (16·4–58·3)	[illegible]	[illegible]	[illegible]	[illegible]
Other hemoglobinopathies and hemolytic anemias	28·3 (20·9–36·4)	6·63 (4·59–9·23)	54·8 (31·3–93)	[illegible]	[illegible]	[illegible]	[illegible]
Endocrine, metabolic, blood, and immune disorders	756 (484–885)	718 (422–905)	4190 (2370–6120)	[illegible]	[illegible]	[illegible]	[illegible]
Sudden infant death syndrome	--	2500 (1000–6090)	22600 (8100–56600)	[illegible]	[illegible]	[illegible]	[illegible]
Injuries	8980 (7590–10100)	8520 (6580–10500)	70200 (61200–79300)	[illegible]	[illegible]	[illegible]	[illegible]
Transport injuries	690 (591–826)	885 (723–1190)	10300 (8110–12400)	[illegible]	[illegible]	[illegible]	[illegible]
Road injuries	631 (539–754)	841 (685–1120)	9740 (7690–11900)	[illegible]	[illegible]	[illegible]	[illegible]
Pedestrian road injuries	333 (230–465)	436 (244–734)	5600 (3570–8140)	[illegible]	[illegible]	[illegible]	[illegible]
Cyclist road injuries	--	--	--	[illegible]	[illegible]	[illegible]	[illegible]
Motorcyclist road injuries	71·5 (43·5–125)	44·8 (20·8–85·1)	717 (319–1340)	[illegible]	[illegible]	[illegible]	[illegible]
Motor vehicle road injuries	215 (150–295)	352 (213–513)	3300 (1820–5040)	[illegible]	[illegible]	[illegible]	[illegible]
Other road injuries	11·7 (6·84–17)	8·01 (3·86–13·8)	123 (46·2–225)	[illegible]	[illegible]	[illegible]	[illegible]
Other transport injuries	58·9 (46·6–76·4)	43·6 (29·8–61·9)	553 (309–679)	[illegible]	[illegible]	[illegible]	[illegible]
Unintentional injuries	6150 (4840–7200)	6730 (4870–8640)	55200 (47200–62900)	[illegible]	[illegible]	[illegible]	[illegible]
Falls	912 (426–1250)	435 (211–610)	4870 (2180–7100)	[illegible]	[illegible]	[illegible]	[illegible]
Drowning	355 (306–410)	309 (262–361)	7940 (6570–9270)	[illegible]	[illegible]	[illegible]	[illegible]
Fire, heat, and hot substances	186 (105–252)	305 (125–489)	3560 (1330–5510)	[illegible]	[illegible]	[illegible]	[illegible]
Poisonings	167 (140–199)	214 (173–260)	2680 (2020–3320)	[illegible]	[illegible]	[illegible]	[illegible]
Poisoning by carbon monoxide	46·8 (28·3–69·7)	52·8 (31·6–74·3)	718 (344–1160)	[illegible]	[illegible]	[illegible]	[illegible]
Poisoning by other means	120 (90·8–147)	162 (114–204)	1960 (1240–2510)	[illegible]	[illegible]	[illegible]	[illegible]
Exposure to mechanical forces	583 (208–1090)	1360 (207–3000)	3050 (1450–4760)	[illegible]	[illegible]	[illegible]	[illegible]
Unintentional firearm injuries	114 (23·9–243)	284 (15·9–834)	695 (211–1210)	[illegible]	[illegible]	[illegible]	[illegible]
Other exposure to mechanical forces	469 (176–890)	1080 (184–2510)	2360 (1120–3820)	[illegible]	[illegible]	[illegible]	[illegible]
Adverse effects of medical treatment	772 (470–1050)	662 (367–849)	5190 (2180–7200)	[illegible]	[illegible]	[illegible]	[illegible]
Animal contact	199 (102–263)	226 (101–309)	2350 (1080–3330)	[illegible]	[illegible]	[illegible]	[illegible]
Venomous animal contact	172 (82·2–233)	175 (72·8–251)	1920 (810–2760)	[illegible]	[illegible]	[illegible]	[illegible]
Non-venomous animal contact	27 (18·7–34·8)	50·7 (27·3–63·5)	434 (234–581)	[illegible]	[illegible]	[illegible]	[illegible]
Foreign body	2460 (2090–2930)	2860 (2500–3310)	21900 (18800–25400)	[illegible]	[illegible]	[illegible]	[illegible]
Pulmonary aspiration and foreign body in airway	2360 (2000–2810)	2700 (2350–3120)	18600 (15700–21600)	[illegible]	[illegible]	[illegible]	[illegible]
Foreign body in other body part	104 (62·9–151)	165 (83·3–244)	3260 (1440–5310)	[illegible]	[illegible]	[illegible]	[illegible]
Environmental heat and cold exposure	132 (44–212)	106 (25·2–179)	1140 (235–1780)	[illegible]	[illegible]	[illegible]	[illegible]
Exposure to forces of nature	20·6 (18·7–22·7)	70·4 (64–77·5)	774 (703–852)	[illegible]	[illegible]	[illegible]	[illegible]
Other unintentional injuries	358 (285–439)	179 (142–218)	1780 (1410–2260)	[illegible]	[illegible]	[illegible]	[illegible]
Self-harm and interpersonal violence	2140 (2000–2290)	904 (825–998)	4720 (4160–5470)	[illegible]	[illegible]	[illegible]	[illegible]
Self-harm	--	--	--	[illegible]	[illegible]	[illegible]	[illegible]
Self-harm by firearm	--	--	--	[illegible]	[illegible]	[illegible]	[illegible]
Self-harm by other specified means	--	--	--	[illegible]	[illegible]	[illegible]	[illegible]
Interpersonal violence	1250 (1130–1400)	423 (359–506)	3160 (2620–3890)	[illegible]	[illegible]	[illegible]	[illegible]
Physical violence by firearm	90·3 (79·2–104)	41·3 (33·3–52·9)	266 (217–342)	[illegible]	[illegible]	[illegible]	[illegible]
Physical violence by sharp object	75·5 (68–85·5)	53·7 (40·8–68·2)	280 (180–366)	[illegible]	[illegible]	[illegible]	[illegible]
Physical violence by other means	1090 (977–1220)	328 (276–394)	2610 (2150–3220)	[illegible]	[illegible]	[illegible]	[illegible]
Conflict and terrorism	889 (830–963)	481 (449–521)	1550 (1450–1680)	[illegible]	[illegible]	[illegible]	[illegible]

SR Table 4: Global deaths by cause and age group for females (with 95% uncertainty levels), 2010

Cause	Early Neonatal	Late Neonatal	Post Neonatal	1 to 4	5 to 9	10 to 14	15 to 19	20 to 24	25 to 29	30 to 34	35 to 39	40 to 44	45 to 49	50 to 54	55 to 59	60 to 64	65 to 69	70 to 74	75 to 79	80 to 84	85 to 89	90 to 94	95 plus
All causes	1050000 (968000–1140000)	328000 (298000–360000)	983000 (894000–1070000)	1020000 (925000–1130000)	234000 (222000–246000)	167000 (161000–174000)	249000 (239000–258000)	333000 (318000–348000)	389000 (371000–407000)	435000 (418000–452000)	498000 (479000–518000)	579000 (557000–601000)	710000 (685000–736000)	847000 (819000–875000)	1080000 (1050000–1120000)	1340000 (1300000–1380000)	1620000 (1580000–1660000)	2220000 (2160000–2280000)	2600000 (2550000–2670000)	2880000 (2820000–2930000)	2360000 (2320000–2400000)	1270000 (1260000–1290000)	647000 (644000–650000)
Communicable, maternal, neonatal, and nutritional diseases	951000 (875000–1030000)	285000 (258000–314000)	798000 (719000–884000)	820000 (731000–919000)	139000 (128000–151000)	81400 (74400–89300)	106000 (97900–116000)	155000 (144000–166000)	202000 (190000–214000)	223000 (213000–236000)	219000 (209000–231000)	186000 (178000–198000)	163000 (153000–176000)	144000 (132000–162000)	139000 (124000–161000)	153000 (131000–187000)	182000 (152000–232000)	236000 (194000–302000)	242000 (197000–309000)	240000 (197000–302000)	190000 (155000–230000)	111000 (87100–130000)	60900 (46200–70000)
HIV/AIDS and sexually transmitted infections	23700 (8140–50200)	5790 (1940–12200)	30200 (24900–38700)	41400 (39200–43700)	12000 (11400–12600)	9280 (8850–9760)	19900 (17700–22200)	41700 (37700–46000)	89300 (82400–96500)	116000 (110000–123000)	115000 (109000–121000)	93200 (89700–96500)	73000 (69300–76800)	48000 (45900–50100)	28900 (27400–30400)	15900 (14900–16900)	9220 (8560–9860)	5550 (5140–5960)	3170 (2950–3400)	745 (686–801)	422 (381–458)	179 (153–199)	73·1 (59·5–82·7)
HIV/AIDS	--	--	22900 (21600–24200)	41000 (38800–43400)	11800 (11200–12400)	9210 (8780–9690)	19700 (17500–22000)	41300 (37300–45600)	88800 (81900–95900)	116000 (109000–123000)	114000 (108000–121000)	92700 (89200–96000)	72600 (68900–76300)	47600 (45500–49700)	28400 (26900–29900)	15600 (14500–16600)	8840 (8180–9490)	5220 (4810–5620)	2860 (2640–3090)	496 (452–543)	231 (211–253)	74·2 (67·6–81·6)	20·9 (19·5–22·5)
HIV/AIDS - Drug-susceptible Tuberculosis	--	--	6530 (4870–7990)	12400 (9200–15100)	3380 (2470–4120)	2650 (1960–3240)	5460 (3930–6970)	11700 (8360–14700)	24700 (17500–31200)	32200 (22900–40500)	31400 (22300–39400)	25700 (18200–32300)	20200 (14400–25700)	13400 (9710–17000)	8040 (5860–10200)	4410 (3210–5600)	2510 (1830–3200)	1560 (1130–1980)	847 (611–1070)	139 (101–176)	61·1 (43·2–79·4)	19·3 (13·4–25·5)	4·65 (3·32–6·07)
Tuberculosis without extensive drug resistance	--	--	612 (284–1120)	1060 (481–1950)	282 (130–522)	210 (95·4–389)	454 (205–865)	986 (441–1860)	2080 (949–3940)	2640 (1200–4900)	2510 (1150–4660)	2040 (933–3840)	1610 (733–3030)	1110 (504–2090)	679 (311–1270)	370 (167–697)	205 (92·7–385)	127 (57–240)	69·3 (30·5–132)	11·8 (5·11–22·2)	5·38 (2·3–10·2)	1·72 (0·718–3·29)	0·402 (0·17–0·755)
HIV/AIDS - Extensively drug-resistant Tuberculosis	--	--	11·8 (5·33–21·8)	12·5 (5·45–23·9)	4·77 (2·11–8·92)	3·46 (1·55–6·5)	6·72 (2·92–13)	17·7 (7·75–34·2)	46·6 (20·7–88·1)	62·2 (27·7–117)	59·3 (26·4–112)	48·7 (21·9–90·8)	35·3 (15·5–66·1)	23·3 (10·4–43·7)	14·5 (6·44–26·9)	7·73 (3·42–14·5)	4·32 (1·87–8·19)	2·16 (0·928–4·15)	1·25 (0·521–2·37)	0·223 (0·0919–0·421)	0·106 (0·0435–0·202)	0·035 (0·0144–0·0681)	0·0127 (0·00519–0·0248)
HIV/AIDS resulting in other diseases	--	--	15700 (14000–17900)	27600 (24500–31800)	8110 (7220–9190)	6340 (5650–7200)	13700 (11600–16300)	28600 (24400–33800)	62000 (53500–71800)	81000 (71100–92100)	80400 (70500–91500)	64900 (57300–73200)	50700 (44700–58000)	33000 (29200–37500)	19700 (17400–22300)	10800 (9450–12300)	6120 (5320–7050)	3530 (3050–4120)	1940 (1690–2260)	344 (297–405)	164 (141–193)	53·2 (45·5–62·9)	15·9 (14·1–18)
Sexually transmitted infections excluding HIV	23700 (8140–50200)	5790 (1940–12200)	7340 (2490–15800)	395 (134–878)	200 (63·9–441)	72·5 (53·7–89·8)	240 (191–289)	381 (293–446)	509 (375–592)	477 (363–557)	422 (325–486)	491 (386–572)	460 (363–514)	426 (355–499)	507 (388–607)	324 (256–381)	374 (296–448)	326 (270–372)	311 (271–348)	250 (203–281)	191 (152–220)	105 (78·3–123)	52·1 (38·2–61·5)
Syphilis	23700 (8140–50200)	5790 (1940–12200)	7340 (2490–15800)	395 (134–878)	200 (63·9–441)	40·6 (30·1–50·4)	50·4 (39·9–61·2)	32·5 (26·2–37·1)	91 (65·6–107)	97·1 (71·5–115)	51 (37·8–59·8)	19·9 (15–23·6)	34·9 (26·2–39·8)	38·3 (30·7–45·8)	16·5 (12·2–20)	17·1 (12·9–20·5)	26 (19·9–31·8)	30·9 (24·3–36·3)	23·5 (19·6–27·1)	31·5 (24·1–36·8)	20·1 (14·1–24·5)	15·6 (9·71–20)	6·16 (3·56–8·29)
Chlamydial infection	--	--	--	--	--	7·21 (5·41–8·88)	24·9 (20–29·9)	83·2 (63·7–97·6)	78·4 (58·6–90·4)	34·5 (25·5–40·7)	59·2 (45·8–68)	79·9 (63–92·8)	75 (59·4–83·9)	62·9 (52·7–73·5)	82·6 (63·4–98·6)	53·5 (42·6–62·7)	39·6 (31·4–47·4)	47·8 (39·8–54·4)	48·8 (42·6–54·4)	50·3 (41–56·4)	36·1 (28·9–41·1)	19·4 (14·6–22·6)	10·6 (7·82–12·4)
Gonococcal infection	--	--	--	--	--	11·6 (8·67–14·4)	71·7 (57·4–86·2)	136 (104–159)	196 (143–228)	185 (143–215)	157 (122–180)	217 (171–252)	194 (154–216)	193 (161–225)	233 (179–279)	143 (115–167)	216 (172–258)	165 (138–188)	176 (154–197)	123 (101–137)	95·7 (76·5–109)	51·4 (38·8–59·8)	29·1 (21·3–33·9)
Other sexually transmitted infections	--	--	--	--	--	13 (9·57–16·2)	92·8 (74·2–112)	129 (98·7–152)	144 (106–167)	160 (122–187)	155 (120–178)	175 (137–204)	156 (123–175)	132 (110–155)	175 (134–209)	110 (85·3–130)	92·6 (73·1–111)	82·4 (68·4–93·9)	62·6 (54–70·2)	45·3 (36·8–50·8)	39·2 (31·1–45·3)	18·5 (14–21·7)	6·28 (4·47–7·47)
Respiratory infections and tuberculosis	72400 (64600–81400)	62400 (55400–70000)	261000 (228000–299000)	163000 (132000–197000)	24600 (20300–28700)	14200 (12000–16200)	18800 (16700–21000)	26500 (24000–29200)	30900 (27800–34000)	32400 (29300–35500)	35500 (32400–38800)	36600 (33500–39800)	42000 (38700–45400)	51500 (47200–55900)	58300 (53200–63500)	68400 (62100–74400)	86300 (77900–93300)	113000 (103000–124000)	126000 (113000–137000)	135000 (118000–147000)	120000 (99100–131000)	76600 (59900–85400)	46200 (34600–52400)
Tuberculosis	--	--	23100 (19000–28400)	23500 (18000–30100)	7110 (5760–8570)	5830 (4830–6840)	11800 (10400–13500)	18800 (16800–20900)	22300 (19900–24700)	23900 (21500–26300)	25600 (23300–28200)	25300 (23000–27800)	28400 (25800–31100)	33000 (29800–36600)	34800 (31300–38800)	34900 (30900–39200)	38600 (34400–42900)	43600 (38700–48700)	38200 (33600–43300)	28500 (24800–32600)	14900 (12400–17200)	5070 (4010–5990)	1960 (1480–2390)
Drug-susceptible tuberculosis	--	--	21300 (17000–26500)	21800 (16600–27800)	6540 (5220–7980)	5340 (4370–6320)	10800 (9290–12500)	17100 (15100–19300)	20300 (17800–22900)	21700 (19100–24300)	23300 (20700–26100)	23000 (20300–25700)	25800 (22800–28500)	30100 (26700–33600)	31700 (27700–35900)	31800 (27700–36100)	35200 (30700–39700)	39900 (34900–45100)	35000 (30300–40100)	26200 (22300–30300)	13800 (11300–16100)	4710 (3720–5600)	1830 (1380–2270)
Multidrug-resistant tuberculosis without extensive drug resistance	--	--	1780 (831–3200)	1700 (743–3180)	553 (239–1040)	471 (205–885)	995 (429–1820)	1570 (721–2790)	1870 (872–3330)	2070 (957–3640)	2190 (1000–3850)	2180 (1000–3880)	2440 (1140–4320)	2770 (1280–4900)	2960 (1370–5290)	2900 (1310–5290)	3200 (1390–5810)	3570 (1580–6550)	3070 (1370–5570)	2210 (1000–4000)	1080 (505–1970)	339 (157–622)	119 (54·4–217)
Extensively drug-resistant tuberculosis	--	--	66·2 (31·9–118)	47·6 (21·7–89·5)	20·7 (9·3–39·2)	18·9 (8·5–34·9)	43 (20–79·6)	92·4 (46·6–161)	119 (61–202)	129 (65·4–220)	134 (67·5–228)	135 (67·3–233)	165 (82·2–283)	171 (85·6–298)	170 (82·9–298)	153 (71·7–274)	159 (71·5–289)	188 (85·7–343)	169 (77·2–308)	135 (62·4–244)	70·2 (32–128)	22 (10·1–41·1)	7·36 (3·28–13·9)
Lower respiratory infections	72200 (64500–81100)	62300 (55400–69900)	237000 (208000–272000)	137000 (112000–166000)	17300 (14000–20600)	8240 (6810–9420)	6930 (6020–7820)	7730 (6750–8930)	8620 (7490–9820)	8520 (7520–9510)	9810 (8730–11000)	11200 (10000–12500)	13600 (12300–15000)	18400 (16300–20300)	23400 (21000–25500)	33400 (28900–36300)	47600 (41200–51900)	69600 (61800–76500)	87100 (77300–96600)	106000 (91600–116000)	104000 (85900–114000)	71200 (55700–79500)	44100 (32900–50100)
Upper respiratory infections	19·3 (10–31·4)	59·1 (27·4–103)	1100 (267–2600)	1900 (194–5770)	207 (38·4–475)	72·3 (15·7–144)	40·1 (13·8–80·2)	40·4 (17·6–70·2)	52·2 (20·2–85·3)	50·6 (20·7–78·1)	56·1 (23·5–88·8)	55·2 (27·8–80·8)	64·1 (35·1–94·2)	67 (38·2–94·4)	81·6 (49–117)	110 (63·7–171)	114 (68·3–177)	207 (125–352)	266 (175–490)	418 (280–844)	422 (283–837)	259 (170–500)	123 (84·6–221)
Otitis media	101 (0·936–577)	8 (1·15–28·1)	28·8 (10·1–64·5)	13·4 (3·4–35·2)	11·5 (2·11–30·8)	8·21 (2·45–19·3)	3·99 (1·55–8·33)	2·48 (1·15–5·26)	3·07 (1·4–6·06)	3·1 (1·36–6·38)	4·52 (2·05–9·75)	5·83 (3·52–11·7)	4·99 (3·1–8·68)	6·75 (4–12·7)	12·3 (7·76–21·3)	9·95 (6·84–17·3)	13·9 (8·72–28·9)	13·3 (9·37–23·1)	11·9 (8·39–19·3)	11 (8·02–16·8)	8·68 (6·69–11·9)	4·09 (3·03–5·35)	2·61 (1·82–3·6)
Enteric infections	13400 (10700–18700)	26400 (21400–32800)	201000 (168000–236000)	202000 (160000–248000)	44900 (28900–65400)	27600 (18200–40600)	21200 (12400–33200)	17300 (9190–29500)	14500 (7800–25200)	13600 (6870–25200)	12700 (6100–24500)	12300 (5720–23600)	15100 (6820–29700)	19700 (8850–39100)	26000 (11300–50900)	40600 (16900–80600)	56100 (23800–111000)	82500 (33900–158000)	88700 (38800–165000)	85300 (40900–152000)	54300 (27600–94000)	24500 (12800–41100)	8880 (5230–14200)
Diarrheal diseases	13400 (10700–18700)	26000 (21000–32400)	194000 (162000–228000)	167000 (133000–207000)	23500 (9920–43500)	11200 (4490–21700)	10800 (4280–21800)	11300 (4230–23500)	10300 (3850–21300)	10700 (4260–22100)	10700 (4200–22600)	10700 (4010–21500)	13500 (5220–28200)	18200 (7340–37800)	24600 (9870–49200)	39400 (15800–79300)	55100 (22800–110000)	81700 (33100–157000)	88100 (38200–165000)	85000 (40600–152000)	54200 (27400–93900)	24400 (12700–41000)	8850 (5200–14200)
Typhoid and paratyphoid	--	--	64·1 (24·5–153)	13100 (4870–26300)	16800 (7210–31500)	14600 (6510–26600)	9540 (3580–18700)	5320 (2050–11100)	3530 (1490–7290)	2240 (905–4760)	1450 (599–2890)	1080 (455–2180)	990 (395–2020)	870 (355–1810)	773 (285–1710)	629 (231–1350)	489 (197–1030)	380 (157–798)	249 (95·1–493)	131 (49·6–269)	57 (26·6–102)	20·3 (9·23–44·9)	6·11 (2·92–13·3)
Typhoid fever	--	--	58·2 (22·1–139)	11500 (4310–23600)	14200 (6120–26800)	12300 (5260–22500)	8040 (2900–15800)	4460 (1680–9300)	2950 (1220–6120)	1870 (758–3960)	1200 (482–2420)	890 (373–1840)	806 (318–1700)	708 (286–1440)	628 (229–1400)	511 (190–1130)	398 (157–822)	309 (124–656)	204 (76·2–410)	109 (40·3–222)	47·4 (21·5–86·1)	17·1 (7·53–39·9)	4·93 (2·13–11·6)
Paratyphoid fever	--	--	5·87 (1·74–15·5)	1630 (499–3990)	2560 (927–5690)	2330 (884–4830)	1500 (491–3300)	858 (285–2080)	575 (199–1400)	372 (115–903)	250 (83·8–570)	193 (68·3–418)	184 (60·4–439)	162 (53·4–362)	144 (44·7–336)	119 (35·4–281)	91·2 (30·5–214)	70·8 (22·6–169)	45·2 (15·3–103)	22·5 (7·66–48·6)	9·58 (4·01–19·7)	3·21 (1·43–6·72)	1·18 (0·604–2·21)
Invasive Non-typhoidal Salmonella (iNTS)	--	413 (227–688)	6120 (3420–9600)	20800 (11300–34200)	4510 (2350–7550)	1740 (832–3140)	900 (412–1770)	708 (308–1430)	650 (263–1270)	574 (245–1100)	558 (250–1040)	561 (266–1000)	598 (295–1060)	652 (312–1190)	639 (319–1120)	560 (274–971)	498 (252–827)	407 (203–676)	310 (160–518)	205 (95·8–360)	94 (44·7–157)	33·5 (13·3–61·1)	13·3 (4·54–27·8)
Other intestinal infectious diseases	--	--	268 (127–454)	531 (287–879)	81·2 (26·5–168)	42·4 (7·51–106)	20·7 (1·13–58·9)	18·3 (0·927–55·5)	8·01 (0·0775–25·9)	17·5 (0·594–51·1)	9·41 (0·0805–29·3)	10·8 (0·0686–34·1)	7·46 (0·047–23·3)	7·64 (0·0426–25)	6·83 (0·0363–22·6)	6·77 (0·0429–22·1)	12·5 (0·125–37·8)	11·8 (0·0881–35·8)	10·1 (0·094–31·4)	13·4 (0·152–39·3)	19·1 (0·344–55·1)	8·91 (0·0657–28·4)	6·21 (0·0302–20·5)
Neglected tropical diseases and malaria	216 (125–643)	14600 (7420–24100)	95500 (50200–153000)	195000 (112000–296000)	20600 (13100–30000)	11800 (7500–17300)	10100 (6210–15100)	10300 (6160–15800)	8760 (5210–13600)	8530 (5130–13100)	9360 (5620–14500)	9290 (5480–14600)	10500 (6120–16500)	12400 (7150–19600)	13900 (7960–22100)	15300 (8630–24100)	14700 (8230–23000)	16500 (9720–24600)	5320 (3410–8150)	2110 (1450–2790)	1070 (765–1610)	422 (294–715)	175 (119–301)
Malaria	0 (0–0)	14300 (7080–23800)	92300 (47500–150000)	187000 (103000–290000)	16200 (8920–25200)	9500 (5310–14700)	8420 (4530–13300)	8670 (4540–14200)	7110 (3570–11900)	6800 (3390–11500)	7640 (3860–12700)	7520 (3720–13000)	8600 (4240–14600)	10500 (5180–17600)	11900 (5890–20000)	13200 (6590–22000)	12700 (6170–21200)	14200 (7390–22100)	3350 (1470–6080)	555 (234–1060)	33·4 (13·9–64·7)	0·595 (0·287–1·09)	0·0506 (0·00264–0·227)
Chagas disease	--	--	0·878 (0·393–2·34)	0·374 (0·197–1·08)	0·864 (0·374–2·21)	1·76 (0·658–3·42)	4·32 (1·84–9·78)	7·93 (3·7–19·1)	16·6 (8·42–38·6)	33·8 (14·4–69·8)	57·9 (22·8–118)	91·3 (37·8–192)	149 (67·7–316)	223 (84·1–470)	310 (110–561)	354 (128–697)	412 (142–755)	471 (144–853)	492 (163–788)	402 (125–690)	405 (221–881)	211 (106–482)	92·2 (50·8–206)
[illegible]	[illegible]	[illegible]	[illegible]	[illegible]	[illegible]	[illegible]	[illegible]	[illegible]	[illegible]	[illegible]	[illegible]	[illegible]	[illegible]	[illegible]	[illegible]	[illegible]	[illegible]	[illegible]	[illegible]	[illegible]	[illegible]	[illegible]	[illegible]

Myocarditis	55·4 (32·9–88)	55 (35·6–87·1)	713 (487–1050)	450 (288–732)	203 (146–265)	192 (145–255)	216 (159–281)	311 (221–391)	297 (219–372)	303 (217–386)	342 (254–420)	397 (282–503)	399 (287–481)	393 (273–500)	477 (324–576)	497 (304–603)	591 (373–711)	827 (474–996)	1170 (630–1440)	2050 (1030–2610)	2190 (1090–2900)	1540 (632–2190)	919 (344–1360)
Alcoholic cardiomyopathy	--	--	--	--	--	--	55·4 (41·5–67·3)	162 (92·3–182)	495 (301–541)	1020 (571–1110)	1490 (864–1590)	1910 (1260–2030)	2750 (1910–2910)	3470 (2470–3670)	3850 (2730–4060)	2540 (1950–2750)	1440 (1160–1710)	1570 (1170–1710)	1380 (797–1570)	1090 (496–1340)	588 (227–828)	258 (105–321)	128 (55–158)
Other cardiomyopathy	407 (275–518)	255 (180–331)	1800 (1330–2250)	872 (605–1230)	350 (265–442)	430 (334–531)	362 (278–422)	547 (397–630)	845 (636–983)	987 (763–1140)	1300 (1030–1470)	1640 (1330–1840)	2190 (1760–2470)	2750 (2260–3070)	3720 (3190–4290)	4220 (3620–4900)	4680 (4070–5570)	7010 (5940–8130)	10100 (8090–11100)	16300 (11600–18100)	17000 (12200–19300)	10500 (7160–12200)	6480 (4310–7890)
Atrial fibrillation and flutter	--	--	--	--	--	--	--	--	--	37·6 (30·5–45·9)	47·3 (41·8–53·9)	268 (239–307)	458 (411–526)	767 (696–874)	1660 (1510–1880)	2880 (2620–3210)	4650 (4200–5320)	10100 (9100–11400)	17000 (14900–19300)	30600 (25500–34900)	34000 (27100–41200)	24500 (18600–30800)	12400 (8960–16100)
Aortic aneurysm	--	--	--	--	--	--	62·7 (56–82·3)	117 (105–156)	172 (155–227)	263 (240–337)	397 (364–484)	612 (571–710)	947 (894–1080)	1380 (1310–1530)	2080 (1980–2290)	2830 (2690–3100)	3810 (3610–4230)	5840 (5490–6310)	7710 (7080–8260)	9040 (7740–9770)	7880 (6370–8700)	4190 (3160–4750)	1930 (1400–2220)
Endocarditis	38 (21·7–55·1)	44·3 (22·5–66·4)	440 (236–624)	346 (143–550)	135 (78–184)	168 (108–215)	267 (182–345)	433 (300–556)	606 (435–792)	549 (395–707)	630 (462–808)	753 (569–962)	762 (549–944)	911 (659–1110)	1130 (818–1430)	1260 (874–1500)	1610 (1130–1910)	2170 (1480–2530)	2880 (1790–3290)	3680 (2050–4400)	4190 (2030–5240)	2820 (1270–3670)	1820 (678–2460)
Other cardiovascular and circulatory diseases	478 (368–673)	408 (289–584)	2210 (1680–2890)	2200 (1460–2820)	1120 (896–1340)	1170 (1000–1370)	1020 (891–1160)	1520 (1320–1730)	1810 (1620–2010)	1990 (1800–2200)	2300 (2110–2530)	2910 (2670–3240)	3820 (3510–4230)	4820 (4440–5290)	5860 (5380–6490)	7660 (7010–8420)	9050 (8210–9990)	12500 (11400–14000)	15200 (13700–16900)	16400 (14300–18200)	14300 (11700–15900)	7590 (5850–8610)	4290 (3200–4910)
Chronic respiratory diseases	0 (0–0)	0 (0–0)	0 (0–0)	6840 (5070–8480)	1770 (1440–2100)	1670 (1380–1960)	2970 (2560–3520)	3940 (3370–4690)	4890 (4180–5540)	6350 (5480–7350)	9270 (8060–10700)	13400 (11800–15700)	22300 (18800–25300)	34400 (29000–37600)	56400 (48400–62500)	93700 (77900–103000)	138000 (116000–150000)	213000 (179000–235000)	262000 (214000–287000)	284000 (240000–313000)	208000 (169000–230000)	101000 (78100–112000)	39100 (28800–44500)
Chronic obstructive pulmonary disease	--	--	0 (0–0)	1050 (497–2000)	216 (129–309)	176 (120–241)	838 (584–1090)	1190 (853–1530)	1430 (1050–1810)	2070 (1560–2650)	3280 (2550–4170)	5560 (4540–6830)	10900 (8820–13500)	19800 (16400–23500)	36300 (30000–41900)	68400 (58100–77600)	106000 (91500–119000)	172000 (148000–193000)	220000 (183000–243000)	246000 (207000–273000)	184000 (151000–205000)	89900 (69800–101000)	34800 (26000–39500)
Pneumoconiosis	--	--	--	--	--	--	12·3 (10·3–15·3)	13·2 (11–16·5)	23 (18·7–29·1)	20·3 (17·2–24·8)	23·3 (20–29·5)	39·9 (33·9–52·4)	44·9 (38·2–57·7)	55·1 (46·9–68·7)	84·9 (72·2–112)	131 (106–170)	143 (117–185)	244 (202–307)	300 (252–367)	309 (248–376)	213 (166–258)	88·2 (65·4–107)	41·2 (28·2–49·4)
Silicosis	--	--	--	--	--	--	2·36 (1·49–3·56)	2·23 (1·39–3·4)	5·2 (2·72–8·63)	4·97 (3·13–7·33)	5·57 (3·93–8·43)	12·2 (8·24–17·5)	16 (12–23·2)	14·5 (9·91–19·8)	22 (16·2–31·3)	34·8 (24·6–50·4)	32·7 (23·4–49)	62·5 (45·7–88·1)	87·3 (68·4–123)	85·3 (64·4–123)	57·1 (42·85·1)	22·9 (16·4–33·8)	9·49 (6·36–14·5)
Asbestosis	--	--	--	--	--	--	1·78 (0·854–2·57)	2·65 (1·45–3·75)	3·29 (1·59–4·72)	3·2 (1·52–4·35)	4·06 (2·01–5·69)	5·38 (2·97–7·92)	7·36 (3·96–10·2)	11·9 (6·69–16·7)	19·1 (10·5–28)	27·1 (15–38·7)	30·9 (18–44·4)	54·2 (31·5–77·6)	64·5 (38·7–86·3)	71·8 (43·1–94·5)	59 (35·1–74·9)	22·7 (12·6–29·8)	11·1 (5·79–15·3)
Coal workers pneumoconiosis	--	--	--	--	--	--	1·57 (0·826–3·63)	1·32 (0·671–3·36)	1·96 (1·09–5·44)	2·25 (1·21–5·16)	2·06 (1·17–5·15)	4·87 (2·65–9·31)	5·32 (3·08–9·35)	6·93 (4·34–12·3)	9·83 (5·81–19·1)	13·4 (7·51–26·6)	18·7 (11–34·5)	25·8 (15·8–43·1)	32·9 (19·2–50·1)	30·5 (19·7–49·5)	22·2 (14·8–34·9)	10·7 (6·96–17·2)	6·08 (3·72–10)
Other pneumoconiosis	--	--	--	--	--	--	6·61 (4·95–9·49)	6·96 (5·13–9·71)	12·5 (8·08–17·3)	9·86 (7·34–13·6)	11·6 (8·48–16·1)	17·5 (13·5–25·5)	16·2 (12·2–23·2)	21·8 (16·5–30·9)	33·9 (25–48)	50·2 (37·2–80·4)	60·7 (44·7–84·2)	101 (76·9–132)	115 (84·2–151)	121 (85·9–158)	74·9 (53·6–98·2)	31·8 (22·2–42·1)	14·5 (9·01–19·2)
Asthma	--	--	--	4580 (3030–6210)	1140 (900–1390)	1140 (919–1370)	1780 (1430–2220)	2290 (1870–2770)	2800 (2250–3330)	3550 (2850–4190)	4770 (3800–5830)	6230 (4920–7800)	9170 (6940–11500)	11400 (8170–15500)	15500 (10800–22000)	20100 (13100–30700)	24500 (15600–36800)	31200 (20200–47500)	31400 (19700–48100)	27800 (18600–42400)	16600 (11700–23500)	7100 (5050–9930)	2610 (1850–3420)
Interstitial lung disease and pulmonary sarcoidosis	--	--	--	135 (88·2–232)	68·4 (45·9–96·8)	56·3 (38·5–76·4)	109 (74·4–142)	149 (102–198)	224 (159–287)	328 (229–433)	579 (427–793)	839 (631–1140)	1350 (979–1860)	2030 (1420–2740)	3040 (2130–4000)	4560 (3110–6130)	6180 (3910–8070)	8860 (5710–11300)	9980 (6080–12400)	9310 (5520–11600)	6830 (3660–8880)	3240 (1530–4420)	1460 (653–2060)
Other chronic respiratory diseases	0 (0–0)	0 (0–0)	0 (0–0)	1080 (454–2030)	345 (225–468)	308 (186–412)	227 (156–327)	299 (195–426)	409 (249–576)	379 (258–557)	610 (397–810)	755 (518–1050)	805 (566–1130)	1080 (768–1510)	1560 (1040–2180)	528 (327–683)	572 (362–750)	767 (462–1000)	888 (537–1120)	998 (594–1270)	781 (471–976)	437 (256–563)	212 (117–263)
Digestive diseases	1100 (967–1260)	920 (789–1070)	7140 (6130–8320)	10500 (8320–12800)	4950 (4110–5780)	5030 (4340–5740)	10600 (9490–11700)	13500 (12300–14700)	16000 (14700–17400)	18100 (16800–19400)	23500 (22000–25100)	30000 (28300–31700)	41100 (39000–43300)	51400 (48900–53900)	65400 (62200–68500)	72100 (68300–75900)	81200 (76300–85500)	96700 (90900–102000)	105000 (97100–111000)	106000 (92700–113000)	83300 (69700–90400)	43500 (34700–48400)	22200 (16600–25100)
Cirrhosis and other chronic liver diseases	--	--	--	4690 (3540–6210)	2160 (1750–2620)	2670 (2250–3120)	5660 (4960–6370)	7730 (6890–8610)	9560 (8640–10500)	12100 (11100–13000)	15800 (14700–16900)	21400 (20100–22900)	28400 (26900–30400)	35600 (33800–38400)	43700 (41500–46600)	45100 (42600–48000)	46200 (43200–49900)	50100 (46500–53500)	45100 (42400–50000)	37000 (32600–40600)	22700 (19000–25100)	9250 (7320–10600)	4210 (3150–5040)
Cirrhosis and other chronic liver diseases due to hepatitis B	--	--	--	24·4 (12·3–41·6)	24·2 (13·6–38·8)	57 (31·8–89·9)	193 (97·7–328)	936 (537–1460)	1970 (1260–2780)	3140 (2340–4010)	4400 (3450–5760)	6280 (4840–7800)	7580 (5790–9720)	8610 (6350–11500)	10000 (7280–13200)	9770 (6940–12900)	9550 (6910–12700)	10300 (7310–14000)	9730 (6880–12700)	7590 (5360–10000)	4250 (3150–5520)	1570 (1050–2150)	591 (331–922)
Cirrhosis and other chronic liver diseases due to hepatitis C	--	--	--	22·2 (12·2–38·5)	32 (18·3–50·9)	72·2 (42–118)	231 (122–391)	1160 (681–1710)	2370 (1620–3200)	3360 (2570–4250)	4660 (3520–5940)	6200 (4780–7750)	8060 (6120–10200)	9570 (7040–12400)	10900 (8060–14400)	10900 (7850–14800)	11300 (8520–14600)	11800 (9050–15200)	10800 (7840–14200)	8790 (6280–11600)	5650 (4070–7300)	2440 (1640–3320)	1220 (732–1770)
Cirrhosis and other chronic liver diseases due to alcohol use	--	--	--	--	--	--	11·5 (3·35–25·4)	192 (98·2–339)	523 (300–876)	1290 (910–1760)	2460 (1680–3410)	3950 (2840–5170)	6080 (4310–8040)	8570 (6050–11200)	11200 (8160–15000)	11300 (8090–15300)	10400 (7240–14000)	9990 (6710–13900)	7760 (5480–10400)	5460 (3680–7580)	3180 (2250–4340)	1260 (810–1900)	579 (304–1010)
Cirrhosis and other chronic liver diseases due to NAFLD	--	--	--	--	--	--	113 (45·7–223)	318 (152–556)	627 (301–1110)	932 (529–1500)	1190 (619–2050)	1810 (911–2900)	2690 (1300–4600)	3630 (1820–6000)	4370 (2230–7280)	4730 (2430–8270)	5290 (3340–7870)	6290 (3910–9220)	6320 (3530–9720)	5500 (3150–8270)	3550 (2160–5210)	1470 (818–2400)	669 (297–1170)
Cirrhosis and other chronic liver diseases due to other causes	--	--	--	4640 (3510–6150)	2100 (1700–2560)	2540 (2120–2970)	5110 (4380–5820)	5130 (3960–6340)	4080 (2780–5560)	3340 (2130–4950)	2880 (1640–4420)	3130 (1820–4990)	4040 (2320–6400)	5260 (3000–8240)	7180 (4100–11200)	8390 (4840–12800)	9640 (5490–15400)	11400 (6470–17400)	11500 (6760–17300)	9670 (5720–14500)	6080 (3890–8570)	2510 (1480–3670)	1150 (553–1890)
Upper digestive system diseases	--	--	--	994 (657–1720)	500 (391–644)	425 (352–505)	869 (733–1090)	1730 (1470–2110)	1640 (1420–2040)	1840 (1610–2190)	2440 (2160–2880)	2420 (2190–2760)	3890 (3500–4470)	4990 (4490–5510)	6300 (5720–6940)	8120 (7380–8920)	11500 (10300–12700)	14400 (13000–15700)	16200 (14600–18000)	16800 (14900–18400)	13300 (11300–14700)	6700 (5420–7560)	3020 (2330–3550)
Peptic ulcer disease	--	--	--	938 (599–1670)	436 (337–576)	379 (311–454)	819 (689–1040)	1540 (1300–1930)	1520 (1310–1880)	1660 (1440–1980)	2190 (1920–2610)	2150 (1940–2450)	3450 (3090–3950)	4490 (4010–5000)	5610 (5040–6230)	7190 (6480–7920)	10100 (9090–11400)	12700 (11500–13900)	14200 (12800–15600)	14400 (12700–15900)	11400 (9660–12800)	5620 (4510–6370)	2570 (1950–3030)
Gastritis and duodenitis	--	--	--	55·7 (32·5–86·1)	63·7 (38·8–90·9)	46·4 (32·63·1)	50·2 (33·9–71·9)	185 (111–277)	127 (80–184)	185 (128–261)	252 (184–348)	270 (223–335)	445 (357–564)	497 (397–628)	693 (577–826)	[illegible]	1310 (1090–1590)	1670 (1420–1940)	2020 (1740–2300)	2340 (1990–2660)	1850 (1520–2100)	1080 (860–1230)	455 (355–532)

Physical violence by other means	643 (577–728)	174 (154–198)	1240 (1100–1380)	2590 (2190–3090)	1590 (1370–1840)	1360 (1220–1500)	2740 (2500–2970)	3680 (3370–3990)	3320 (3030–3640)	3100 (2870–3330)	2730 (2540–2930)	2460 (2320–2600)	2360 (2240–2490)	1900 (1820–1980)	1750 (1680–1830)	1390 (1320–1460)	1230 (1160–1300)	1380 (1310–1450)	1230 (1150–1310)	415 (372–439)	207 (176–222)	63·1 (50·5–69·7)	21·6 (16·3–24·3)
Conflict and terrorism	299 (274–327)	163 (150–178)	540 (495–590)	3050 (2800–3330)	1750 (1600–1910)	1490 (1360–1620)	1820 (1670–1990)	1480 (1350–1620)	1000 (916–1100)	917 (839–1000)	775 (710–846)	576 (528–629)	500 (457–547)	463 (422–507)	410 (375–449)	359 (329–393)	316 (290–345)	244 (223–266)	184 (168–201)	141 (129–154)	78·8 (72–86·3)	15·8 (14·4–17·3)	30·9 (28·3–33·8)

SR Table 4: Global deaths by cause and age group for females (with 95% uncertainty levels), 2019

Cause	Early Neonatal	Late Neonatal	Post Neonatal	1 to 4	5 to 9	10 to 14	15 to 19	20 to 24	25 to 29	30 to 34	35 to 39	40 to 44	45 to 49
All causes	824000 (709000–961000)	228000 (196000–267000)	640000 (534000–768000)	619000 (530000–726000)	168000 (153000–184000)	129000 (119000–139000)	197000 (182000–212000)	255000 (232000–279000)	294000 (269000–321000)	357000 (330000–386000)	429000 (397000–464000)	533000 (492000–574000)	678000 (625000–730000)
Communicable, maternal, neonatal, and nutritional diseases	740000 (637000–864000)	194000 (166000–229000)	496000 (407000–607000)	478000 (403000–566000)	95700 (83700–108000)	61300 (53900–69400)	81800 (73000–91700)	109000 (98600–122000)	129000 (117000–142000)	147000 (135000–159000)	153000 (141000–166000)	143000 (132000–157000)	129000 (117000–144000)
HIV/AIDS and sexually transmitted infections	23000 (8000–47500)	5590 (1910–11600)	15500 (10300–23400)	14900 (13400–16700)	6060 (5600–6560)	7120 (6680–7560)	15000 (13500–16600)	23400 (21500–25500)	40600 (37100–44500)	57600 (54100–61500)	62800 (59800–66300)	58300 (56000–60600)	49200 (47100–51300)
HIV/AIDS	–	–	7930 (6970–9160)	14500 (13100–16200)	5850 (5410–6310)	7050 (6620–7490)	14800 (13300–16400)	23000 (21200–25200)	40100 (36700–44000)	57200 (53700–61100)	62400 (59400–65800)	57800 (55500–60200)	48800 (46700–50800)
HIV/AIDS - Drug-susceptible Tuberculosis	–	–	1990 (1460–2540)	3860 (2870–4750)	1590 (1220–1930)	1940 (1470–2380)	3700 (2660–4780)	5680 (4020–7250)	9380 (6560–12200)	13200 (9280–17100)	14500 (10300–18800)	13600 (9710–17700)	11300 (8020–14800)
resistant Tuberculosis without extensive drug resistance	–	–	242 (104–449)	454 (180–874)	178 (74·6–333)	199 (88·1–374)	404 (172–783)	603 (257–1160)	927 (402–1810)	1260 (554–2380)	1360 (600–2570)	1260 (561–2370)	1040 (460–1960)
HIV/AIDS - Extensively drug-resistant Tuberculosis	–	–	6·52 (2·9–12·1)	8·14 (3·33–15·8)	3·78 (1·69–7·31)	4·03 (1·76–7·67)	8·58 (3·63–16·2)	14·8 (6·39–27·7)	29·9 (13·5–56·3)	48·4 (22·9–88·8)	52·2 (24·9–95·7)	45·9 (21·8–83·6)	35·7 (16·9–65·8)
HIV/AIDS resulting in other diseases	–	–	5690 (4860–6890)	10200 (8780–12200)	4080 (3620–4600)	4900 (4370–5520)	10600 (9160–12600)	16700 (14500–19400)	29800 (25700–34400)	42600 (37500–48500)	46500 (41100–52100)	42800 (38000–47800)	36400 (32400–40500)
Sexually transmitted infections excluding HIV	23000 (8000–47500)	5590 (1910–11600)	7570 (2510–16000)	413 (138–898)	208 (65·3–457)	70·2 (52·8–87·3)	206 (160–260)	337 (262–411)	451 (332–561)	459 (345–560)	421 (321–511)	498 (375–626)	439 (354–511)
Syphilis	23000 (8000–47500)	5590 (1910–11600)	7570 (2510–16000)	413 (138–898)	208 (65·3–457)	39·3 (29·6–49)	43·5 (33·5–55·4)	27·6 (22·1–33)	81·4 (58·9–102)	94·6 (69·3–117)	51·7 (37·9–63·6)	20·7 (15·1–26·6)	34·7 (26·8–41)
Chlamydial infection	–	–	–	–	–	6·98 (5·31–8·64)	21·4 (16·7–26·9)	73·8 (57·2–90·2)	68·6 (51·7–84·4)	33·6 (24·7–41·4)	59 (45·2–71·4)	80·9 (61·1–101)	71·6 (57·8–83·4)
Gonococcal infection	–	–	–	–	–	11·3 (8·51–14)	61·7 (48·1–77·6)	120 (93·4–147)	174 (127–217)	177 (135–214)	156 (120–188)	219 (166–274)	184 (149–214)
Other sexually transmitted infections	–	–	–	–	–	12·6 (9·42–15·8)	79·8 (62·2–100)	115 (89–141)	127 (94·1–158)	154 (116–188)	155 (118–187)	178 (133–224)	149 (120–173)
Respiratory infections and tuberculosis	52300 (41700–63700)	39200 (32200–47100)	168000 (136000–207000)	98200 (76700–123000)	18100 (14700–21800)	11000 (9260–13000)	14100 (12300–16300)	19700 (17100–22700)	23600 (20400–27300)	26700 (22900–30400)	30800 (26800–35600)	34000 (29300–39100)	37300 (32400–42700)
Tuberculosis	–	–	12800 (9680–16600)	12200 (9400–15900)	4460 (3500–5540)	3930 (3230–4860)	8030 (6850–9590)	12900 (11000–15400)	15800 (13400–18700)	18200 (15500–21400)	20600 (17600–24600)	21900 (18500–26200)	23200 (19600–27900)
Drug-susceptible tuberculosis	–	–	11600 (8740–15200)	11200 (8500–14500)	4040 (3110–5130)	3550 (2850–4440)	7240 (5950–8800)	11600 (9670–14000)	14200 (11600–17000)	16300 (13400–19300)	18500 (15200–22100)	19600 (15900–23900)	20900 (17300–24900)
tuberculosis without extensive drug resistance	–	–	1110 (483–2040)	980 (432–1840)	403 (166–777)	362 (149–698)	746 (317–1390)	1210 (506–2330)	1470 (613–2850)	1760 (706–3470)	1990 (782–3910)	2180 (839–4330)	2210 (897–4260)
Extensively drug-resistant tuberculosis	–	–	52·7 (24·1–98·2)	37 (16·2–69·9)	20·8 (8·48–40·2)	19·3 (7·84–38·4)	41 (17–78·6)	80·9 (36·9–151)	110 (53·9–204)	136 (63·8–252)	150 (69·7–289)	161 (71·5–312)	175 (84–321)
Lower respiratory infections	52200 (41600–63600)	39200 (32200–47100)	154000 (125000–190000)	85000 (66400–106000)	13500 (10900–16400)	7050 (5800–8340)	6060 (5160–7130)	6760 (5720–8090)	7830 (6650–9190)	8430 (7200–9720)	10100 (8770–11700)	12000 (10200–13800)	14000 (12200–16000)
Upper respiratory infections	9·81 (5·15–15·4)	33·5 (17·1–56·2)	606 (195–1340)	1050 (169–2920)	134 (36·6–277)	50·7 (14·1–101)	27 (10·4–53·5)	25 (11·6–44·5)	35·8 (17·5–54·3)	39·2 (21·3–57)	44·2 (22–68·8)	42 (22·4–62·2)	49·6 (28·2–73·9)
Otitis media	60·8 (0·733–341)	3·8 (0·752–15·7)	18·7 (8·41–40·8)	8·42 (2·77–18·5)	8·4 (1·72–21·7)	6·46 (1·91–14·2)	3·21 (1·22–6·3)	2·09 (0·959–4·05)	2·56 (1·23–4·82)	2·96 (1·3–5·49)	4·36 (1·95–8·53)	5·45 (3·38–10·4)	4·8 (2·88–8·12)
Enteric infections	6400 (4710–8640)	13800 (10500–18000)	119000 (91900–155000)	127000 (95000–166000)	34500 (22100–51000)	21900 (14000–33200)	17200 (9980–26900)	14400 (7620–25000)	11800 (6190–21300)	11300 (5550–21100)	10700 (4930–21000)	10800 (4730–21900)	12700 (5560–25300)
Diarrheal diseases	6400 (4710–8640)	13500 (10300–17600)	114000 (87800–150000)	1e+05 (73800–133000)	17200 (7220–32500)	8430 (3200–16800)	8330 (3090–17200)	8970 (3230–19000)	8140 (2820–17000)	8730 (3230–18100)	8910 (3260–19100)	9340 (3480–20200)	11400 (4160–24000)
Typhoid and paratyphoid	–	–	59·8 (22·8–142)	9480 (3540–19200)	13100 (5490–24500)	11900 (5300–21400)	8110 (3050–15900)	4770 (1820–10000)	3120 (1310–6490)	2020 (799–4230)	1320 (555–2650)	995 (424–2080)	910 (352–1890)
Typhoid fever	–	–	54·1 (20·7–128)	8330 (3140–16900)	11100 (4680–20900)	9850 (4300–18200)	6630 (2450–13100)	3830 (1410–7950)	2500 (1020–5320)	1600 (637–3390)	1040 (410–2160)	778 (325–1620)	706 (269–1540)
Paratyphoid fever	–	–	5·76 (1·69–15·8)	1150 (348–2740)	2060 (745–4590)	2050 (766–4280)	1490 (498–3200)	933 (315–2210)	620 (209–1430)	411 (129–985)	277 (91·7–617)	217 (77–474)	204 (63·3–469)
Invasive Non-typhoidal Salmonella (iNTS)	–	288 (155–460)	4860 (2640–7960)	17400 (9570–28400)	4150 (2050–7260)	1540 (688–2770)	768 (335–1520)	609 (262–1220)	563 (226–1120)	505 (208–983)	475 (212–878)	447 (217–804)	441 (225–793)
Other intestinal infectious diseases	–	–	171 (67·6–336)	325 (149–566)	58·1 (14·5–136)	32·9 (3·95–87)	16·1 (0·519–48·9)	14·4 (0·339–43·7)	6·57 (0·0181–21·5)	15·4 (0·251–44·7)	8·39 (0·0206–29·1)	9·91 (0·0382–32·5)	6·78 (0·0193–20·8)
Neglected tropical diseases and malaria	208 (115–630)	9980 (4490–18800)	64900 (31200–114000)	115000 (59600–191000)	13900 (8380–21700)	8030 (4770–12600)	7350 (4090–11800)	7630 (4250–12600)	6790 (3800–11300)	7070 (3960–11800)	8030 (4490–13200)	8410 (4720–14400)	9660 (5420–16300)
Malaria	0 (0–0)	9700 (4110–18500)	62500 (29300–111000)	110000 (54200–185000)	10700 (5220–18600)	6260 (2950–10700)	6020 (2780–10500)	6190 (2790–11100)	5330 (2390–9900)	5510 (2440–10300)	6320 (2840–11700)	6570 (2880–12500)	7600 (3330–14200)
Chagas disease	–	–	0·597 (0·246–1·83)	0·257 (0·12–0·849)	0·618 (0·267–1·77)	1·24 (0·453–2·88)	3·4 (1·37–8·8)	6·49 (3·09–16·5)	12·8 (7·07–35·2)	25·6 (13·1–67·8)	53·3 (24·9–131)	86·2 (35·6–206)	135 (59·8–310)
Leishmaniasis	–	–	70·8 (19·1–205)	474 (161–1380)	376 (133–1290)	161 (52–573)	105 (29·3–390)	77 (18·4–292)	81 (16·1–302)	84·7 (15·2–307)	79·6 (12·8–275)	62·7 (9·48–207)	46·4 (6·58–139)
Visceral leishmaniasis	–	–	70·8 (19·1–205)	474 (161–1380)	376 (133–1290)	161 (52–573)	105 (29·3–390)	77 (18·4–292)	81 (16·1–302)	84·7 (15·2–307)	79·6 (12·8–275)	62·7 (9·48–207)	46·4 (6·58–139)
African trypanosomiasis	–	–	–	24·7 (2·72–98·9)	78·6 (11·3–287)	81·4 (12·7–286)	66·8 (11·2–221)	66·2 (10·5–235)	58·6 (8·87–241)	53·6 (8·4–205)	45·9 (7·22–178)	37·4 (6·31–144)	32·9 (4·95–122)
Schistosomiasis	–	–	0 (0–0)	78·9 (49·1–115)	56·7 (36·9–81·8)	73·4 (50–104)	81·6 (57·5–112)	113 (79·6–159)	150 (106–204)	184 (130–249)	250 (184–338)	298 (220–404)	341 (248–469)
Cysticercosis	–	–	–	8·78 (0·00223–30·3)	16·6 (0·853–48·2)	17·3 (1·09–48·2)	19·5 (1·27–59·7)	27·5 (2·54–76·9)	37·5 (5·31–92·1)	44·4 (6·73–110)	41·7 (5·17–101)	40·1 (4·79–107)	38·5 (4·09–103)
Cystic echinococcosis	–	–	–	56 (12·8–122)	36·8 (3·31–98·2)	20·7 (0·28–61·6)	11·2 (0·0528–36·8)	13 (0·079–41·6)	20·7 (0·813–59·5)	25·2 (1·03–66·4)	29 (1·28–84·3)	30·7 (1·78–85·8)	32·5 (2·55–83·3)
Dengue	0·000942 (0·000856–0·00104)	135 (6·61–233)	1260 (166–1940)	1740 (431–2620)	1370 (303–1970)	749 (161–1060)	527 (109–755)	675 (134–1030)	645 (126–966)	646 (131–982)	589 (114–885)	653 (131–970)	672 (120–1010)
Yellow fever	–	0·593 (0·198–1·34)	10·3 (3·43–22·5)	129 (44·7–295)	225 (76·1–507)	195 (68–430)	144 (49·5–325)	110 (36·1–261)	84·4 (28·6–200)	67·4 (23·5–150)	54·9 (19·7–126)	43·6 (14·6–99·2)	35·5 (12·2–80·2)
Rabies	–	–	64·2 (20·9–118)	585 (127–1050)	490 (137–824)	247 (66–393)	153 (44·3–252)	110 (41·7–180)	89·8 (27–144)	181 (59·4–276)	232 (67·7–363)	272 (87–432)	321 (99·5–490)
Intestinal nematode infections	–	–	58 (41·3–78·6)	611 (428–838)	141 (106–187)	29·4 (21·3–40)	8·44 (5·71–11·4)	6·13 (4·22–8·54)	6·14 (4·23–8·62)	7·45 (5·18–10·1)	7·36 (5·24–9·68)	6 (4·27–7·88)	6 (4·36–8·21)
Ascariasis	–	–	58 (41·3–78·6)	611 (428–838)	141 (106–187)	29·4 (21·3–40)	8·44 (5·71–11·4)	6·13 (4·22–8·54)	6·14 (4·23–8·62)	7·45 (5·18–10·1)	7·36 (5·24–9·68)	6 (4·27–7·88)	6 (4·36–8·21)
Ebola	0 (0–0)	0 (0–0)	20·8 (17·1–24·6)	167 (137–197)	55·5 (45·5–65·5)	48·6 (39·8–57·3)	97·2 (79·7–115)	139 (114–164)	174 (142–205)	139 (114–164)	208 (171–246)	187 (154–221)	287 (235–338)
Zika virus	2e-04 (8·3e-05–0·000358)	0·000433 (0·000172–0·000796)	0·0083 (0·00335–0·0151)	0·0313 (0·0124–0·0569)	0·0495 (0·0208–0·0865)	0·0549 (0·0239–0·0958)	0·0683 (0·0298–0·12)	0·0769 (0·0333–0·133)	0·0831 (0·0359–0·146)	0·095 (0·0412–0·168)	0·0934 (0·0406–0·163)	0·0865 (0·0377–0·152)	0·068 (0·0297–0·118)
Other neglected tropical diseases	208 (115–630)	138 (79·6–401)	850 (472–2340)	1480 (685–4730)	299 (172–856)	139 (82·6–413)	103 (67·8–246)	94·5 (62–224)	107 (61·8–221)	100 (54·2–189)	116 (60·3–211)	124 (67·8–225)	115 (65·6–217)
Other infectious diseases	7330 (5810–9450)	8130 (6160–10600)	68200 (47200–96700)	92600 (61200–138000)	19900 (14700–26800)	10700 (8520–13000)	8180 (6920–9460)	7890 (6690–9230)	6370 (5430–7410)	6490 (5570–7510)	6540 (5700–7580)	7190 (6260–8320)	7900 (6860–9040)
Meningitis	2610 (1980–3620)	3270 (2510–4300)	22200 (17000–29600)	21800 (15500–29000)	6100 (4770–7570)	4660 (3830–5490)	3880 (3270–4540)	3190 (2680–3740)	2930 (2450–3410)	2810 (2380–3230)	2740 (2330–3180)	2670 (2300–3070)	3130 (2680–3600)
Encephalitis	233 (164–300)	444 (306–617)	3310 (2450–4840)	3880 (2860–5730)	2830 (1940–4470)	1850 (1320–3030)	1270 (975–1960)	1440 (1080–2280)	1130 (866–1750)	1180 (913–1910)	1040 (807–1710)	1180 (900–1920)	1240 (968–1900)
Diphtheria	–	–	326 (187–556)	1460 (769–2440)	154 (94·7–255)	49·7 (31·6–76·6)	13·6 (8·88–20·1)	7·27 (4·81–10·9)	6·49 (4·35–9·52)	7·05 (4·85–10)	7·79 (5·38–11·2)	9·96 (6·92–14·7)	16·9 (11·8–23·6)
Tetanus	3080 (2100–4770)	3510 (2210–5490)	1150 (688–1990)	795 (382–2150)	377 (195–797)	283 (161–540)	348 (199–594)	303 (178–537)	285 (179–448)	344 (205–587)	340 (193–592)	444 (218–785)	463 (242–782)
Measles	92·8 (84·2–102)	20·5 (18·6–22·6)	8920 (3070–20000)	24800 (8620–54300)	3870 (1320–8520)	996 (340–2240)	357 (140–805)	209 (98·7–437)	149 (92·1–267)	136 (102–208)	134 (111–176)	131 (115–158)	127 (113–145)
Varicella and herpes zoster	50·4 (38·4–67·5)	130 (99·5–170)	864 (645–1150)	1490 (1110–1960)	550 (409–720)	176 (137–224)	85·9 (67·8–108)	76·5 (60·4–97·5)	85·2 (66·5–106)	112 (89·8–140)	95·7 (76·3–120)	64·4 (51·4–80)	71·2 (57·8–88·1)
Acute hepatitis	–	–	3000 (1980–4540)	2650 (1710–4300)	1690 (1080–2630)	1520 (1050–2300)	1380 (1050–1980)	1860 (1430–2590)	1040 (801–1400)	1160 (916–1570)	1430 (1160–1930)	1920 (1530–2620)	1840 (1450–2520)
Acute hepatitis A	–	–	1740 (820–3090)	1920 (1010–3440)	1250 (675–2230)	1080 (629–1780)	905 (522–1520)	1130 (638–1810)	590 (335–905)	615 (340–970)	752 (398–1120)	1020 (519–1620)	867 (463–1380)
Acute hepatitis B	–	–	986 (441–2020)	502 (248–903)	315 (135–640)	344 (156–672)	349 (175–628)	563 (296–983)	353 (215–581)	403 (226–690)	463 (259–815)	641 (297–1190)	675 (361–1170)
Acute hepatitis C	–	–	176 (49·2–392)	137 (37·7–291)	98·6 (28·5–204)	68·6 (18·5–154)	93·6 (36·3–179)	116 (54·9–209)	67·3 (32·5–114)	117 (60·7–202)	189 (111–316)	228 (123–409)	253 (118–456)
Acute hepatitis E	–	–	103 (34·9–218)	84·5 (25·7–182)	29·6 (9·69–63·6)	35·5 (12·6–69·9)	31·4 (10·9–58·6)	43 (16·3–77·8)	30·1 (12·6–49·9)	24 (9·1–41·6)	26·5 (10·2–44·6)	34·5 (13·3–64·8)	44 (17·3–74·6)
Other unspecified infectious diseases	1260 (747–1700)	749 (513–1000)	2860 (1880–3940)	2950 (1450–4490)	938 (592–1290)	527 (378–694)	644 (446–813)	635 (419–791)	647 (412–825)	668 (439–848)	696 (473–876)	713 (501–878)	965 (644–1190)
Maternal and neonatal disorders	651000 (558000–764000)	117000 (99700–138000)	36200 (28300–45200)	2810 (2100–3620)	–	978 (759–1240)	19000 (16000–22200)	35300 (30100–41300)	37800 (32300–43300)	35800 (31300–41000)	32600 (28500–37600)	22900 (19600–26700)	10700 (9020–12900)
Maternal disorders	–	–	–	–	–	978 (759–1240)	19000 (16000–22200)	35300 (30100–41300)	37800 (32300–43300)	35800 (31300–41000)	32600 (28500–37600)	22900 (19600–26700)	10700 (9020–12900)
Maternal hemorrhage	–	–	–	–	–	178 (130–242)	3900 (3120–4800)	8330 (6560–10300)	8900 (7190–10900)	8950 (7340–10600)	8580 (7140–10200)	5410 (4510–6460)	1960 (1630–2340)
Maternal sepsis and other maternal infections	–	–	–	–	–	75·5 (55·1–108)	1710 (1360–2100)	3030 (2420–3640)	3230 (2640–3880)	2930 (2420–3560)	2720 (2250–3310)	2060 (1660–2600)	985 (748–1300)
Maternal hypertensive disorders	–	–	–	–	–	170 (133–220)	3510 (2960–4140)	5480 (4660–6410)	6050 (5130–7000)	4870 (4150–5620)	3970 (3390–4660)	2570 (2180–3040)	1050 (879–1230)
Maternal obstructed labor and uterine rupture	–	–	–	–	–	33·7 (24–47·8)	826 (643–1050)	1810 (1360–2380)	1950 (1520–2490)	2000 (1570–2540)	1850 (1470–2350)	1320 (1050–1660)	713 (543–949)
Maternal abortion and miscarriage	–	–	–	–	–	210 (150–289)	1920 (1510–2350)	2690 (2160–3300)	2760 (2240–3390)	2890 (2300–3580)	3130 (2520–3900)	3240 (2600–3990)	2440 (1900–3120)
Ectopic pregnancy	–	–	–	–	–	44·8 (32·4–61·4)	665 (536–837)	1130 (916–1370)	1180 (977–1420)	1130 (930–1350)	945 (782–1140)	797 (645–987)	403 (323–490)
Indirect maternal deaths	–	–	–	–	–	91·2 (70·2–118)	2670 (2150–3240)	5300 (4290–6490)	5300 (4300–6390)	4740 (3900–5750)	3800 (3180–4610)	2240 (1880–2670)	929 (773–1120)
Late maternal deaths	–	–	–	–	–	62·4 (42·3–88·2)	1120 (768–1540)	2190 (1580–3040)	2700 (1930–3640)	2720 (1960–3630)	2460 (1800–3290)	1460 (1010–1990)	458 (352–598)
Maternal deaths aggravated by HIV/AIDS	–	–	–	–	–	2·27 (1·13–3·82)	61·4 (33·2–96·7)	143 (79·8–214)	248 (145–355)	322 (197–454)	338 (206–479)	289 (174–410)	169 (102–239)
Neonatal disorders	651000 (558000–764000)	117000 (99700–138000)	36200 (28300–45200)	2810 (2100–3620)	–	–	–	–	–	–	–	–	–
Neonatal preterm birth	236000 (201000–277000)	31700 (26500–37700)	10900 (8590–13600)	724 (522–980)	–	–	–	–	–	–	–	–	–
Neonatal encephalopathy due to birth asphyxia and trauma	212000 (179000–249000)	17800 (14400–21900)	5620 (4200–7530)	963 (738–1250)	–	–	–	–	–	–	–	–	–
Neonatal sepsis and other neonatal infections	59500 (48800–73100)	33300 (27400–40100)	7330 (5740–9290)	179 (126–249)	–	–	–	–	–	–	–	–	–
Hemolytic disease and other neonatal jaundice	13900 (11200–17500)	6790 (5370–8500)	1490 (953–2440)	107 (70·6–171)	–	–	–	–	–	–	–	–	–
Other neonatal disorders	130000 (107000–157000)	27400 (22800–33000)	10900 (8020–14700)	840 (581–1250)	–	–	–	–	–	–	–	–	–
Nutritional deficiencies	0 (0–0)	0 (0–0)	23800 (17700–32200)	27400 (20500–37100)	3250 (2440–4200)	1540 (1190–1960)	988 (780–1250)	1170 (927–1500)	1610 (1260–2090)	1590 (1280–2010)	1490 (1220–1880)	1930 (1590–2490)	1730 (1450–2190)
Protein-energy malnutrition	0 (0–0)	0 (0–0)	22700 (16900–30700)	26300 (19500–35800)	2510 (1840–3310)	1170 (887–1500)	601 (468–774)	633 (502–808)	798 (616–1070)	945 (747–1230)	940 (763–1190)	1220 (997–1580)	1180 (972–1490)
Other nutritional deficiencies	–	–	1070 (633–1650)	1060 (608–1610)	743 (454–1090)	375 (242–535)	388 (287–525)	541 (393–723)	813 (592–1060)	649 (457–861)	548 (396–719)	708 (513–969)	551 (410–728)
Non-communicable diseases	77500 (64500–94000)	30300 (25100–37000)	117000 (90700–152000)	82000 (59900–112000)	37900 (32400–43300)	38200 (33100–42800)	58300 (52400–64000)	78200 (70100–86700)	102000 (91400–112000)	149000 (135000–162000)	217000 (197000–236000)	330000 (3e+05–359000)	488000 (447000–531000)
Neoplasms	825 (645–1020)	732 (554–912)	4480 (3660–5510)	11800 (9660–14300)	11900 (10300–13500)	11900 (10400–13300)	14200 (12800–15500)	20100 (18000–22200)	30800 (27700–33900)	52900 (48100–57800)	85400 (77800–93300)	135000 (123000–148000)	2e+05 (183000–218000)
Lip and oral cavity cancer	–	–	–	–	72·7 (58·2–90·6)	117 (93·2–146)	200 (165–238)	406 (331–495)	589 (486–709)	1010 (854–1190)	1410 (1220–1630)	2460 (2120–2860)	3690 (3200–4250)
Nasopharynx cancer	–	–	–	–	59 (48·3–71·6)	105 (86·1–126)	140 (122–160)	205 (177–238)	297 (258–338)	499 (436–570)	761 (664–868)	1250 (1100–1430)	1710 (1510–1930)
Other pharynx cancer	–	–	–	–	–	–	–	200 (149–258)	286 (220–363)	380 (300–469)	671 (536–838)	1080 (876–1330)	1670 (1390–2000)
Esophageal cancer	–	–	–	–	–	–	–	172 (141–212)	320 (262–388)	547 (466–640)	1020 (881–1170)	2060 (1780–2340)	3900 (3400–4420)
Stomach cancer	–	–	–	–	–	–	333 (290–382)	932 (816–1060)	1850 (1630–2090)	3790 (3350–4230)	6030 (5400–6690)	9280 (8270–10400)	12500 (11300–14000)
Colon and rectum cancer	–	–	–	–	94·5 (78·6–111)	131 (113–149)	310 (280–343)	813 (726–912)	1560 (1400–1730)	3410 (3080–3770)	5370 (4860–5930)	8890 (7990–9780)	15200 (13900–16600)
Liver cancer	92·5 (59·5–136)	95·8 (57·6–158)	252 (168–352)	402 (296–520)	309 (244–373)	379 (309–456)	284 (236–339)	426 (355–500)	662 (563–770)	1170 (997–1360)	1840 (1590–2130)	3080 (2680–3500)	5110 (4430–5890)
Liver cancer due to hepatitis B	–	–	–	–	–	92·7 (65·6–126)	98·5 (73·2–129)	194 (149–241)	358 (272–453)	647 (504–808)	944 (717–1190)	1440 (1070–1830)	2090 (1570–2730)
Liver cancer due to hepatitis C	–	–	–	–	–	6·52 (3·39–11·7)	5·51 (2·89–9·57)	18·8 (11·6–28·3)	45·6 (25·4–73·9)	122 (79·7–178)	280 (175–421)	620 (421–849)	1300 (897–1770)
Liver cancer due to alcohol use	–	–	–	–	–	–	3·17 (1·32–5·78)	13·2 (8·34–20·4)	31·8 (18·1–53·2)	77·3 (49·7–115)	168 (99·6–265)	334 (208–495)	624 (363–977)
Liver cancer due to NASH	–	–	–	–	–	–	81·9 (59·4–112)	88·5 (64–118)	94·9 (62·5–136)	131 (90·3–181)	186 (126–254)	296 (203–409)	498 (339–721)
Liver cancer due to other causes	92·5 (59·5–136)	95·8 (57·6–158)	252 (168–352)	402 (296–520)	309 (244–373)	280 (226–341)	94·7 (70·9–123)	112 (84·3–146)	132 (90·2–190)	194 (135–267)	261 (179–359)	392 (271–545)	596 (419–817)
Gallbladder and biliary tract cancer	–	–	–	–	–	–	–	85·2 (62·3–102)	164 (122–196)	341 (259–400)	689 (536–800)	1320 (1040–1530)	2670 (2130–3090)
Pancreatic cancer	–	–	–	–	–	–	58·4 (52·7–63·9)	128 (114–142)	308 (275–344)	695 (625–767)	1400 (1260–1550)	2870 (2580–3170)	5700 (5170–6290)
Larynx cancer	–	–	–	–	–	–	–	68·4 (59·4–79·7)	108 (92·8–124)	160 (138–186)	294 (252–339)	525 (455–609)	879 (782–996)
Tracheal, bronchus, and lung cancer	–	–	–	–	–	103 (88·9–118)	274 (246–300)	562 (497–631)	1120 (989–1270)	2630 (2330–2960)	4880 (4370–5470)	10000 (8880–11300)	19200 (17200–21500)
Malignant skin melanoma	0·493 (0·318–0·951)	0·668 (0·499–1·09)	12·1 (5·96–23·5)	55 (25·6–119)	34·2 (29·3–39·7)	35·5 (30·9–40·7)	81·1 (63·8–115)	184 (142–250)	333 (267–424)	548 (421–693)	827 (653–1030)	1070 (815–1330)	1360 (988–1650)
Non-melanoma skin cancer	–	–	–	–	–	–	–	68·3 (60·1–77·1)	96·8 (84·6–109)	145 (124–165)	250 (221–281)	328 (288–370)	544 (474–619)
Non-melanoma skin cancer (squamous-cell carcinoma)	–	–	–	–	–	–	–	68·3 (60·1–77·1)	96·8 (84·6–109)	145 (124–165)	250 (221–281)	328 (288–370)	544 (474–619)
Breast cancer	–	–	–	–	–	–	678 (556–815)	1650 (1400–1900)	4490 (3970–5040)	12100 (10900–13200)	23900 (21700–26300)	38600 (35200–42300)	53900 (49400–58800)
Cervical cancer	–	–	–	–	–	–	236 (177–275)	1140 (921–1350)	3450 (2860–4000)	8200 (6810–9370)	14100 (12100–16300)	22000 (18500–25000)	27400 (22800–30900)
Uterine cancer	–	–	–	–	–	–	–	101 (68·4–115)	237 (175–272)	508 (379–581)	962 (752–1080)	1870 (1490–2100)	3440 (2900–3860)
Ovarian cancer	–	–	–	–	201 (157–257)	337 (273–413)	546 (447–637)	948 (771–1120)	1390 (1120–1610)	2270 (1910–2590)	3740 (3140–4320)	6800 (5810–7790)	12100 (10400–13700)
Kidney cancer	18·5 (14·9–22·8)	10·6 (8·54–13·1)	131 (109–158)	475 (386–581)	319 (277–365)	147 (131–164)	113 (102–125)	164 (147–182)	233 (209–259)	354 (320–393)	560 (504–624)	876 (802–974)	1560 (1430–1720)
Bladder cancer	–	–	–	–	–	–	37·5 (33·4–42)	48·8 (42·7–54·8)	89·5 (78·3–105)	157 (139–178)	298 (264–337)	575 (513–645)	935 (848–1030)
Brain and central nervous system cancer	106 (61·1–158)	76 (49·5–106)	717 (543–910)	2390 (1890–2970)	2780 (2080–3300)	2150 (1660–2530)	1530 (1160–1770)	1670 (1270–1950)	2240 (1610–2580)	2940 (2140–3430)	3580 (2610–4100)	4400 (3140–5010)	5830 (4360–6660)
Thyroid cancer	–	–	–	–	39·2 (32·1–47·5)	55·9 (47·2–67)	130 (108–158)	286 (219–366)	387 (300–480)	420 (337–504)	552 (457–632)	724 (576–824)	960 (799–1080)
Mesothelioma	–	–	–	–	–	–	–	53·7 (29·1–81·3)	95·7 (53·2–153)	106 (67·6–141)	211 (129–360)	305 (214–390)	312 (236–374)
Hodgkin lymphoma	–	–	–	59·8 (35–85·5)	130 (90·2–169)	267 (180–344)	467 (367–616)	706 (535–899)	821 (618–1040)	682 (505–845)	556 (410–669)	518 (397–625)	458 (357–574)
Non-Hodgkin lymphoma	–	–	–	679 (543–828)	697 (582–822)	707 (614–802)	1020 (913–1130)	1330 (1180–1490)	1560 (1390–1730)	1680 (1510–1870)	2010 (1820–2230)	2820 (2550–3130)	3750 (3390–4160)
Multiple myeloma	–	–	–	–	–	–	–	67·9 (43·2–84·1)	79·4 (51·9–97·9)	165 (116–196)	315 (235–366)	636 (496–725)	1230 (1000–1430)
Leukemia	241 (143–360)	205 (126–302)	1980 (1510–2600)	4400 (3530–5510)	3930 (3310–4560)	3530 (3100–3970)	3580 (3150–3990)	3500 (3050–3910)	3680 (3170–4150)	3840 (3350–4300)	4270 (3710–4790)	4850 (4240–5440)	5980 (5260–6690)
Acute lymphoid leukemia	53·9 (27·1–107)	40·8 (20·3–82·4)	564 (355–934)	1560 (1100–2340)	2150 (1720–2580)	1650 (1310–1980)	1270 (957–1530)	888 (638–1060)	851 (619–1050)	664 (449–786)	697 (497–849)	716 (514–868)	767 (544–930)
Chronic lymphoid leukemia	–	–	–	–	–	–	–	79·8 (63·7–98·5)	93·6 (72·5–117)	112 (84·8–143)	154 (118–191)	275 (221–339)	466 (386–580)
Acute myeloid leukemia	31·7 (14·5–53·7)	39·9 (20·5–69·1)	315 (210–494)	915 (680–1300)	726 (571–901)	876 (719–1050)	1060 (889–1260)	1000 (847–1160)	1090 (887–1250)	1150 (979–1340)	1340 (1140–1550)	1500 (1280–1720)	1900 (1630–2190)
Chronic myeloid leukemia	14·6 (5·97–31·1)	11·2 (4·46–28·6)	84·9 (42·1–158)	357 (177–610)	122 (84·5–156)	167 (125–211)	195 (148–249)	304 (241–377)	405 (316–498)	504 (413–615)	617 (501–745)	660 (538–798)	672 (545–814)
Other leukemia	141 (80·5–216)	113 (65·8–177)	1010 (605–1400)	1570 (992–2250)	924 (681–1260)	838 (629–1080)	1060 (775–1340)	1220 (950–1510)	1240 (943–1540)	1410 (1080–1710)	1460 (1150–1820)	1690 (1330–2090)	2180 (1740–2640)
Other malignant neoplasms	361 (272–493)	341 (237–481)	1350 (1060–1740)	3270 (2450–4530)	3200 (2660–3850)	3760 (3250–4330)	4110 (3620–4620)	4150 (3650–4720)	4310 (3810–4920)	4110 (3690–4600)	4760 (4270–5320)	5980 (5360–6640)	7790 (7010–8610)
Other neoplasms	4·98 (4·03–5·98)	3·17 (2·54–3·89)	35·4 (28·4–44·4)	70 (54·2–98·9)	50·1 (43·1–57·3)	55·4 (48·5–62·6)	53·1 (47·4–58·7)	63·6 (55·9–74·3)	82·8 (72·1–96·8)	93·9 (83·8–107)	132 (117–149)	200 (179–226)	289 (261–331)
myeloproliferative, and other hematopoietic neoplasms	4·98 (4·03–5·98)	3·17 (2·54–3·89)	35·4 (28·4–44·4)	70 (54·2–98·9)	50·1 (43·1–57·3)	55·4 (48·5–62·6)	53·1 (47·4–58·7)	63·6 (55·9–74·3)	82·8 (72·1–96·8)	93·9 (83·8–107)	132 (117–149)	200 (179–226)	289 (261–331)
Cardiovascular diseases	1200 (957–1550)	855 (650–1150)	5370 (4210–6900)	5260 (4120–6580)	3680 (3040–4410)	5050 (4310–5820)	12300 (10800–13800)	18600 (16200–21000)	24800 (21700–27900)	39500 (35100–43800)	60600 (54000–66700)	1e+05 (89400–110000)	156000 (140000–171000)
Rheumatic heart disease	–	–	–	803 (580–1080)	833 (642–1070)	1470 (1180–1760)	2500 (2140–2930)	3120 (2650–3740)	3620 (2960–4410)	4590 (3480–5830)	5770 (4150–7610)	7830 (5370–10600)	8040 (5770–10800)
Ischemic heart disease	–	–	–	–	–	–	3920 (3400–4480)	6620 (5710–7600)	8970 (7750–10300)	16200 (14200–18300)	25000 (21900–27900)	43100 (37900–48000)	69300 (61800–76900)
Stroke	427 (315–580)	295 (210–435)	1680 (1250–2280)	1500 (1090–2210)	1240 (1010–1510)	1700 (1430–1990)	3280 (2860–3720)	5150 (4480–5840)	6910 (5990–7870)	11300 (10000–12800)	19500 (17300–21800)	34600 (30900–38500)	57500 (51700–63600)
Ischemic stroke	124 (90·8–177)	99·8 (64–164)	354 (247–546)	182 (126–277)	140 (106–187)	149 (114–198)	488 (401–613)	806 (662–1020)	1030 (867–1250)	1710 (1480–1990)	2770 (2410–3210)	4960 (4330–5660)	8840 (7790–10100)
Intracerebral hemorrhage	215 (150–295)	138 (96–201)	1010 (720–1380)	985 (675–1640)	792 (626–999)	1120 (913–1350)	2090 (1790–2430)	3280 (2810–3820)	4400 (3790–5040)	7060 (6210–8050)	12300 (10800–13900)	22200 (19600–24900)	39400 (35000–43800)
Subarachnoid hemorrhage	88·1 (58·8–135)	57·4 (37·2–91·3)	318 (221–471)	338 (223–520)	308 (232–411)	435 (335–542)	699 (578–827)	1060 (844–1330)	1480 (1250–1740)	2570 (2200–3020)	4420 (3780–5190)	7430 (6330–8780)	9200 (7800–10900)
Hypertensive heart disease	–	–	–	–	–	–	389 (257–505)	533 (367–680)	983 (717–1230)	1840 (1380–2220)	3240 (2450–3900)	5460 (4110–6530)	9820 (7690–11600)
Non-rheumatic valvular heart disease	–	–	–	–	–	–	300 (210–401)	330 (229–448)	398 (291–522)	414 (323–515)	583 (460–716)	699 (574–838)	858 (718–1010)
Non-rheumatic calcific aortic valve disease	–	–	–	–	–	–	82·8 (62·2–125)	98·3 (71·8–162)	126 (96·6–192)	161 (129–221)	248 (198–342)	289 (239–386)	379 (320–491)
Non-rheumatic degenerative mitral valve disease	–	–	–	–	–	–	186 (125–263)	199 (132–289)	226 (159–312)	219 (165–291)	295 (224–391)	355 (283–464)	433 (349–555)
Other non-rheumatic valve diseases	–	–	–	–	–	–	31·2 (20·8–49·8)	32·8 (22·4–51)	46·1 (31·7–71·2)	33·3 (24·3–47·9)	40·5 (29–54·1)	54·4 (36·7–76·2)	46·4 (31·7–61·4)
Cardiomyopathy and myocarditis	382 (257–506)	242 (177–320)	1770 (1350–2340)	1020 (759–1370)	474 (380–599)	553 (448–706)	541 (428–656)	770 (593–907)	1300 (1030–1490)	1930 (1550–2230)	2700 (2190–3060)	3380 (2800–3840)	4030 (3400–4590)
Myocarditis	41·2 (26·7–70·7)	40·7 (27·9–66·2)	454 (338–677)	323 (238–478)	161 (128–219)	154 (120–224)	171 (133–238)	230 (175–311)	271 (207–358)	310 (243–430)	324 (258–429)	355 (282–500)	382 (282–505)
Alcoholic cardiomyopathy	–	–	–	–	–	–	39·7 (30·5–51·1)	63·5 (46–79·7)	182 (138–234)	486 (351–634)	821 (582–1070)	1090 (826–1410)	1260 (977–1670)
Other cardiomyopathy	341 (224–454)	201 (142–265)	1320 (958–1780)	694 (489–982)	314 (238–394)	399 (313–510)	330 (252–389)	477 (347–564)	843 (631–986)	1140 (811–1330)	1550 (1150–1810)	1930 (1500–2250)	2380 (1850–2760)
Atrial fibrillation and flutter	–	–	–	–	–	–	–	–	–	44·1 (35·3–50·6)	53·3 (46·2–61·7)	299 (258–345)	519 (451–604)
Aortic aneurysm	–	–	–	–	–	–	63·7 (56–77·4)	110 (96·1–137)	180 (159–218)	300 (267–359)	453 (407–528)	676 (615–762)	1030 (946–1150)
Endocarditis	28·6 (18–43·8)	33·7 (19·3–49·6)	358 (219–533)	278 (139–446)	125 (81·1–173)	162 (108–213)	261 (178–341)	424 (296–537)	610 (443–813)	599 (430–766)	712 (514–917)	852 (631–1080)	823 (588–1050)
Other cardiovascular and circulatory diseases	364 (272–554)	285 (202–419)	1560 (1160–2140)	1650 (1160–2150)	1010 (772–1250)	1160 (962–1400)	1030 (874–1240)	1500 (1260–1780)	1850 (1570–2150)	2220 (1920–2540)	2590 (2260–2910)	3240 (2880–3630)	4080 (3640–4540)
Chronic respiratory diseases	0 (0–0)	0 (0–0)	0 (0–0)	4840 (3590–6290)	1420 (1120–1870)	1460 (1210–1800)	2530 (2130–3090)	3360 (2820–4080)	4320 (3580–5070)	5970 (5000–7080)	8830 (7420–10500)	12900 (10700–15100)	21200 (17700–24800)
Chronic obstructive pulmonary disease	–	–	0 (0–0)	723 (373–1380)	165 (102–229)	143 (98·5–191)	723 (510–928)	971 (691–1250)	1230 (920–1560)	1940 (1490–2470)	2990 (2310–3890)	5010 (3860–6330)	10100 (7870–12700)
Pneumoconiosis	–	–	–	–	–	–	9·71 (7·93–12·7)	9·54 (7·8–12·4)	24 (18·5–32·4)	20·1 (16·8–25·8)	21·1 (17·6–27)	33 (26·3–41·6)	40·9 (33–53·1)
Silicosis	–	–	–	–	–	–	1·74 (1·07–2·79)	1·59 (0·951–2·38)	5·71 (2·69–9·64)	4·91 (3·11–7·39)	4·84 (3·4–7·21)	8·87 (5·32–12·4)	13·5 (9·03–19·5)
Asbestosis	–	–	–	–	–	–	1·51 (0·681–2·31)	1·78 (0·966–2·54)	3·17 (1·48–4·78)	3·22 (1·61–4·46)	3·71 (1·8–5·25)	4·83 (2·71–6·96)	6·87 (3·97–9·51)
Coal workers pneumoconiosis	–	–	–	–	–	–	1·11 (0·576–2·74)	1·02 (0·533–2·71)	1·72 (0·962–5·04)	2·05 (1·18–4·81)	1·91 (1·08–5·2)	3·65 (2·11–6·91)	5·09 (2·93–9·05)
Other pneumoconiosis	–	–	–	–	–	–	5·36 (3·97–7·8)	5·15 (3·83–7·43)	13·4 (8·54–20·7)	9·94 (7·32–14·1)	10·6 (7·87–15·2)	15·6 (11·7–22·9)	15·5 (11·8–23·1)

Cause	50 to 54	55 to 59	60 to 64	65 to 69	70 to 74	75 to 79	80 to 84	85 to 89	90 to 94	95 plus
All causes	967000 (888000–1050000)	1210000 (1120000–1310000)	1610000 (1500000–1720000)	2060000 (1920000–2200000)	2450000 (2300000–2600000)	2800000 (2650000–2950000)	3440000 (3290000–3600000)	2980000 (2860000–3100000)	1990000 (1940000–2050000)	987000 (973000–1e+06)
Communicable, maternal, neonatal, and nutritional diseases	133000 (117000–156000)	135000 (116000–161000)	159000 (133000–199000)	186000 (153000–239000)	228000 (184000–298000)	249000 (2e+05–328000)	3e+05 (238000–397000)	245000 (195000–310000)	167000 (129000–203000)	92200 (68700–108000)
HIV/AIDS and sexually transmitted infections	36800 (35200–38200)	23700 (22600–24700)	14600 (13900–15300)	8880 (8410–9410)	4540 (4260–4860)	2560 (2400–2700)	839 (758–909)	519 (453–572)	253 (207–291)	105 (82·8–122)
HIV/AIDS	36300 (34700–37600)	23100 (22100–24000)	14200 (13500–14900)	8430 (7990–8940)	4180 (3920–4490)	2200 (2070–2350)	518 (483–561)	256 (238–276)	86·4 (80·6–92·8)	26·5 (25–28·1)
HIV/AIDS - Drug-susceptible Tuberculosis	8550 (6080–11200)	5430 (3870–7140)	3330 (2370–4380)	1910 (1370–2500)	1010 (717–1310)	545 (384–711)	114 (80·5–147)	52·3 (36·2–69·4)	16·9 (11·8–22·6)	4·76 (3·35–6·32)
resistant Tuberculosis without extensive drug resistance	795 (348–1490)	505 (221–957)	308 (135–587)	176 (75·9–334)	92·9 (40–177)	49·9 (21·4–94·5)	10·8 (4·56–20·6)	4·92 (2·02–9·61)	1·58 (0·65–3·1)	0·423 (0·18–0·828)
HIV/AIDS - Extensively drug-resistant Tuberculosis	25·4 (11·8–47·2)	16·3 (7·5–30·3)	9·66 (4·36–18·7)	5·77 (2·49–11·7)	2·3 (0·942–4·71)	1·15 (0·465–2·4)	0·351 (0·131–0·764)	0·175 (0·0668–0·374)	0·0587 (0·0229–0·126)	0·0231 (0·00888–0·0484)
HIV/AIDS resulting in other diseases	26900 (23800–29800)	17100 (15100–19100)	10600 (9320–11800)	6340 (5620–7120)	3080 (2700–3500)	1610 (1400–1830)	393 (346–445)	198 (175–224)	67·9 (59·8–76·5)	21·2 (19·2–23·5)
Sexually transmitted infections excluding HIV	528 (382–679)	601 (424–795)	403 (301–502)	453 (351–561)	360 (288–428)	355 (294–413)	321 (252–376)	263 (197–311)	167 (121–205)	78·8 (56·8–94·8)
Syphilis	50·3 (35·2–65·9)	19·9 (13·6–26·8)	21·7 (15·4–27·4)	31·4 (23·4–39·8)	35·1 (26·9–43·1)	27·9 (22·1–33·3)	46 (33·6–56·5)	32·8 (21·9–40·8)	27·9 (17·1–38)	10·7 (6·23–14·6)
Chlamydial infection	77·4 (56·2–99·2)	97·7 (69·1–129)	66·5 (50–82·5)	47·9 (37·2–59·4)	52·7 (42·3–62·4)	55·4 (46·1–64·3)	64 (50·6–74·6)	48·3 (37–56·6)	30·1 (22·3–36·3)	15·9 (11·5–19)
Gonococcal infection	237 (172–303)	276 (195–365)	177 (135–218)	261 (204–322)	182 (146–215)	200 (166–232)	153 (123–178)	127 (98–150)	79·5 (59·2–95·6)	42·6 (30·8–50)
Other sexually transmitted infections	164 (119–211)	208 (146–275)	138 (101–173)	112 (86·7–139)	91 (72·9–108)	71·7 (59·1–83·7)	57·9 (45·7–67·7)	54·6 (40·7–64·7)	29·2 (21·5–35·6)	9·64 (6·9–11·8)
Respiratory infections and tuberculosis	53500 (45800–62600)	61700 (53800–70700)	78000 (68400–88400)	95800 (84200–108000)	119000 (106000–134000)	136000 (120000–151000)	168000 (144000–188000)	152000 (123000–170000)	114000 (86900–129000)	68400 (50100–78000)
Tuberculosis	31900 (26700–40200)	33400 (28000–40700)	34500 (29600–42300)	35400 (30600–43500)	37500 (32400–47400)	34400 (29300–43000)	31700 (26500–40500)	16900 (13800–21600)	6420 (4990–8370)	2480 (1830–3320)
Drug-susceptible tuberculosis	28600 (23100–36300)	29900 (24500–36800)	31000 (25600–38100)	31800 (26400–39300)	33900 (28400–42300)	31100 (26000–39100)	28600 (23400–36900)	15400 (12400–19700)	5890 (4560–7660)	2310 (1700–3060)
tuberculosis without extensive drug resistance	3120 (1170–6410)	3210 (1210–6590)	3270 (1220–6570)	3310 (1250–6770)	3440 (1280–7220)	3100 (1150–6250)	2890 (1060–6040)	1380 (544–2820)	488 (196–950)	165 (71·4–314)
Extensively drug-resistant tuberculosis	217 (94·4–428)	227 (102–440)	229 (99·4–443)	230 (95·2–448)	228 (90·3–455)	213 (86·2–417)	211 (86·8–415)	114 (49·3–223)	43·6 (19·5–84·1)	13·7 (6·07–26·5)
Lower respiratory infections	21500 (18200–24800)	28200 (24000–31900)	43300 (36700–48600)	60300 (51400–67700)	81600 (70300–91700)	102000 (88500–113000)	136000 (116000–151000)	135000 (109000–150000)	108000 (81700–122000)	65700 (48100–75100)
Upper respiratory infections	60·6 (36·2–85·4)	68·5 (42·4–98·6)	105 (62·3–153)	117 (71·8–191)	182 (113–311)	217 (138–416)	385 (235–894)	411 (223–1080)	297 (158–769)	165 (93·8–377)
Otitis media	7·55 (3·89–12·6)	12·9 (8·06–21·5)	11·4 (8·34–18·7)	15·6 (10·8–30·9)	14·7 (10·8–23·6)	12·7 (9·09–20·4)	13·1 (9·24–19·5)	10·1 (7·55–14·2)	5·84 (4·41–7·4)	3·27 (2·36–4·16)
Enteric infections	18300 (7520–39000)	23400 (9640–49200)	37400 (14000–78800)	50500 (19200–108000)	69900 (25400–142000)	81500 (32100–168000)	105000 (42700–207000)	71200 (31300–133000)	38400 (17300–70900)	15000 (8060–25300)
Diarrheal diseases	17000 (6050–37600)	22100 (8160–48200)	36300 (13100–77800)	49500 (18200–107000)	69200 (24600–142000)	81000 (31600–168000)	104000 (42300–206000)	71000 (31100–133000)	38300 (17200–70800)	15000 (8030–25200)
Typhoid and paratyphoid	862 (346–1800)	750 (273–1690)	637 (234–1380)	496 (200–1040)	362 (150–754)	251 (93·7–505)	167 (63–340)	79·5 (35·4–142)	31·4 (13·6–66·9)	9·62 (4·11–21·9)
Typhoid fever	667 (264–1370)	581 (212–1330)	492 (186–1100)	382 (155–811)	279 (112–589)	196 (73·4–406)	131 (45·6–269)	63·6 (28–118)	25·5 (10·8–57)	7·54 (2·89–17·8)
Paratyphoid fever	195 (61·9–445)	168 (50·5–393)	145 (44·2–344)	114 (38·4–268)	82·7 (27·4–197)	55·9 (18·3–123)	35·8 (11·8–79·8)	15·9 (6·34–32·8)	5·91 (2·39–13·4)	2·08 (0·948–4·58)
Invasive Non-typhoidal Salmonella (iNTS)	485 (237–878)	503 (248–854)	482 (241–853)	419 (210–726)	317 (155–532)	259 (130–447)	183 (84·6–333)	89·5 (40·3–156)	36·8 (15·5–69·9)	16·1 (6·03–33·7)
Other intestinal infectious diseases	8·17 (0·0244–27)	7·08 (0·017–21·5)	7·47 (0·02–25·5)	14·2 (0·0847–44·3)	12·1 (0·0531–35·9)	10·2 (0·0431–32)	15·3 (0·0826–44·4)	22·8 (0·242–72·2)	13·1 (0·0676–40·7)	8·84 (0·0269–28·1)
Neglected tropical diseases and malaria	12000 (6640–20800)	13600 (7400–23700)	15700 (8400–27500)	13900 (7280–24700)	15400 (8230–26400)	8140 (4730–13700)	4480 (2900–6730)	1920 (1240–2670)	706 (444–1140)	271 (168–448)
Malaria	9760 (4280–18500)	11300 (4960–21500)	13100 (5770–25100)	11500 (4980–22400)	12800 (5630–23900)	5860 (2470–11600)	2150 (924–4150)	371 (148–806)	38·6 (15·5–87·6)	1·53 (0·586–3·58)
Chagas disease	210 (82·2–544)	323 (119–637)	398 (144–885)	487 (171–985)	539 (163–1080)	553 (177–992)	453 (133–793)	479 (221–1080)	248 (104–656)	108 (47·2–268)
Leishmaniasis	38·3 (5·23–111)	33·9 (4·32–94·8)	30·7 (3·54–83·4)	26·4 (2·88–70·3)	19·6 (2·01–51·5)	13·3 (1·37–35·9)	8·57 (0·768–23·1)	4·22 (0·357–11·8)	1·86 (0·124–5·18)	0·73 (0·0338–2·05)
Visceral leishmaniasis	38·3 (5·23–111)	33·9 (4·32–94·8)	30·7 (3·54–83·4)	26·4 (2·88–70·3)	19·6 (2·01–51·5)	13·3 (1·37–35·9)	8·57 (0·768–23·1)	4·22 (0·357–11·8)	1·86 (0·124–5·18)	0·73 (0·0338–2·05)
African trypanosomiasis	28 (4·18–106)	24·5 (3·48–91·4)	19·5 (2·72–86·5)	14·3 (2·04–57·5)	9·59 (1·2–41·1)	6·13 (0·822–29·8)	3·72 (0·494–13·9)	1·98 (0·243–8·98)	0·584 (0·0789–2·77)	0·106 (0·0134–0·464)
Schistosomiasis	433 (324–584)	489 (369–666)	516 (389–687)	446 (344–597)	482 (370–624)	443 (352–554)	307 (238–391)	175 (132–221)	51·8 (36·9–70·1)	11·9 (7·48–17·5)
Cysticercosis	38 (3·41–98)	36 (3·3–98·6)	31·4 (2·36–81·2)	27·1 (1·44–72·8)	12·8 (0·0487–42·6)	16·9 (0·256–50·9)	13 (0·0418–39·3)	7·47 (0·00566–25·6)	3·07 (0·00151–11·2)	1·29 (0·000432–4·83)
Cystic echinococcosis	39·8 (3·95–103)	43·3 (4·81–107)	46·4 (6·36–123)	46·6 (6·84–120)	46 (6·1–114)	40·6 (5·13–106)	37·5 (4·54–97·2)	27·5 (2·88–83·1)	13·8 (0·981–41·7)	5·51 (0·295–17·2)
Dengue	740 (140–1110)	791 (115–1200)	904 (114–1440)	766 (126–1160)	1080 (215–1640)	798 (168–1160)	1260 (250–1890)	688 (157–1010)	271 (75–396)	90·2 (31–134)
Yellow fever	28·6 (9·65–65·7)	22·2 (7·87–51·5)	15·9 (5·71–35·7)	11·3 (3·97–25·5)	7·16 (2·43–15·9)	4·94 (1·76–10·9)	2·78 (0·983–6·35)	1·24 (0·413–2·86)	0·392 (0·127–0·933)	0·0875 (0·0289–0·222)
Rabies	405 (121–622)	278 (96·5–437)	257 (100–403)	295 (107–516)	177 (80·6–290)	162 (72·8–285)	53·1 (21·1–92)	42·1 (16–76·2)	15·3 (6·37–26·5)	8·78 (3·21–15·3)
Intestinal nematode infections	7·15 (5·21–9·66)	6·98 (5·1–9·15)	15·7 (12·1–20)	7·65 (5·51–10·1)	13·1 (9·79–16·7)	12·9 (9·93–16·4)	11·1 (8·07–14·3)	6·26 (4·41–8·24)	5·33 (3·78–7·22)	2·14 (1·4–3·02)
Ascariasis	7·15 (5·21–9·66)	6·98 (5·1–9·15)	15·7 (12·1–20)	7·65 (5·51–10·1)	13·1 (9·79–16·7)	12·9 (9·93–16·4)	11·1 (8·07–14·3)	6·26 (4·41–8·24)	5·33 (3·78–7·22)	2·14 (1·4–3·02)
Ebola	160 (131–188)	125 (102–147)	118 (96·8–139)	55·5 (45·5–65·5)	3·47 (2·85–4·1)	3·47 (2·85–4·1)	3·47 (2·85–4·1)	3·47 (2·85–4·1)	3·47 (2·85–4·1)	3·47 (2·85–4·1)
Zika virus	0·0666 (0·0292–0·116)	0·0515 (0·0224–0·0895)	0·038 (0·0167–0·0658)	0·0261 (0·0113–0·0452)	0·015 (0·00651–0·0261)	0·00957 (0·00414–0·0166)	0·00519 (0·00225–0·00911)	0·00244 (0·00104–0·00432)	0·000806 (0·000333–0·00144)	0·000222 (9·14e-05–0·000397)
Other neglected tropical diseases	149 (80·1–263)	147 (80–258)	171 (92·1–280)	217 (117–353)	214 (108–328)	222 (133–399)	178 (102–306)	111 (62·8–172)	52·5 (29–83·9)	36·9 (19·8–60·5)
Other infectious diseases	8800 (7660–10000)	9320 (8190–10800)	9810 (8600–11400)	11400 (9920–13100)	12200 (10500–14000)	11200 (9580–12800)	10600 (8730–12300)	7090 (5650–8180)	3280 (2510–3820)	1550 (1110–1850)
Meningitis	3280 (2820–3760)	3220 (2810–3680)	3190 (2790–3610)	3430 (3020–3870)	3830 (3380–4300)	2910 (2570–3230)	2740 (2370–3060)	1790 (1490–2030)	697 (548–798)	340 (258–399)
Encephalitis	1450 (1090–2350)	1750 (1310–2850)	2350 (1790–3750)	2820 (2110–4400)	3450 (2620–5200)	3390 (2610–4830)	3460 (2620–4610)	1920 (1400–2450)	686 (513–876)	225 (158–279)
Diphtheria	14·8 (10·7–20)	9·61 (7·07–13·1)	–	–	–	–	–	–	–	–
Tetanus	598 (318–909)	469 (267–714)	401 (250–576)	523 (273–810)	544 (292–807)	491 (247–717)	260 (112–391)	176 (76·5–243)	61 (28–84·6)	20 (10·1–28·8)
Measles	118 (106–133)	113 (102–126)	111 (101–122)	114 (104–126)	115 (104–126)	109 (99·4–120)	105 (95·5–116)	77·7 (70·5–85·5)	33·8 (30·7–37·2)	10·3 (9·36–11·3)
Varicella and herpes zoster	81·7 (66·4–99·7)	91·4 (74·9–114)	122 (101–152)	231 (190–290)	242 (198–307)	408 (333–527)	667 (535–859)	832 (654–1120)	680 (489–932)	485 (317–720)
Acute hepatitis	1980 (1600–2690)	2190 (1760–2890)	1880 (1510–2580)	2010 (1600–2750)	1780 (1430–2320)	1510 (1230–1950)	1190 (957–1590)	664 (521–869)	254 (190–338)	67·8 (49·6–89·2)
Acute hepatitis A	982 (509–1510)	1060 (511–1600)	742 (352–1210)	801 (382–1290)	738 (381–1160)	798 (453–1140)	660 (378–942)	359 (216–520)	139 (80·5–201)	38·2 (23–56)
Acute hepatitis B	664 (366–1150)	797 (451–1370)	876 (575–1360)	953 (634–1480)	817 (535–1240)	572 (380–910)	371 (217–628)	227 (143–372)	85 (49·9–150)	22·6 (13·8–36·7)
Acute hepatitis C	309 (181–525)	293 (166–517)	228 (128–411)	222 (107–411)	195 (100–348)	106 (54·6–207)	141 (85–229)	62·6 (37·8–100)	23·2 (13·8–38·6)	4·19 (2·56–6·74)
Acute hepatitis E	26·3 (10·6–47·1)	38·7 (17·5–70·9)	30·4 (12·6–51·6)	32·1 (13·4–54·4)	26·4 (11·7–44·5)	34·4 (14·3–54·3)	22·7 (9·18–37·5)	15·7 (6·75–26·6)	6·72 (3·41–11·1)	2·79 (1·26–5·14)
Other unspecified infectious diseases	1230 (822–1510)	1440 (983–1730)	1760 (1170–2090)	2290 (1530–2750)	2240 (1490–2680)	2370 (1520–2860)	2170 (1330–2690)	1620 (1010–2060)	866 (524–1130)	399 (259–506)
Maternal and neonatal disorders	1370 (1130–1660)	–	–	–	–	–	–	–	–	–
Maternal disorders	1370 (1130–1660)	–	–	–	–	–	–	–	–	–
Maternal hemorrhage	248 (199–307)	–	–	–	–	–	–	–	–	–
Maternal sepsis and other maternal infections	94·1 (71·6–124)	–	–	–	–	–	–	–	–	–
Maternal hypertensive disorders	166 (130–212)	–	–	–	–	–	–	–	–	–
Maternal obstructed labor and uterine rupture	37·1 (28·6–48·7)	–	–	–	–	–	–	–	–	–
Maternal abortion and miscarriage	293 (223–391)	–	–	–	–	–	–	–	–	–
Ectopic pregnancy	154 (113–211)	–	–	–	–	–	–	–	–	–
Indirect maternal deaths	129 (105–160)	–	–	–	–	–	–	–	–	–
Late maternal deaths	39 (29·9–50·5)	–	–	–	–	–	–	–	–	–
Maternal deaths aggravated by HIV/AIDS	16·2 (9·73–24·3)	–	–	–	–	–	–	–	–	–
Neonatal disorders	–	–	–	–	–	–	–	–	–	–
Neonatal preterm birth	–	–	–	–	–	–	–	–	–	–
Neonatal encephalopathy due to birth asphyxia and trauma	–	–	–	–	–	–	–	–	–	–
Neonatal sepsis and other neonatal infections	–	–	–	–	–	–	–	–	–	–
Hemolytic disease and other neonatal jaundice	–	–	–	–	–	–	–	–	–	–
Other neonatal disorders	–	–	–	–	–	–	–	–	–	–
Nutritional deficiencies	2290 (1890–2920)	3120 (2590–4040)	3930 (3300–4980)	5430 (4560–6860)	6400 (5570–7620)	9460 (8230–11200)	11300 (9680–13200)	12300 (10200–14200)	10300 (8210–11700)	6860 (5160–7890)
Protein-energy malnutrition	1350 (1110–1710)	2020 (1660–2600)	2760 (2330–3500)	3590 (3040–4470)	4610 (4030–5380)	6960 (6080–8070)	8770 (7450–9980)	10000 (8220–11500)	8630 (6740–9850)	5920 (4400–6870)
Other nutritional deficiencies	937 (704–1250)	1110 (820–1490)	1180 (852–1570)	1850 (1350–2510)	1790 (1370–2400)	2510 (1990–3250)	2580 (2060–3270)	2290 (1850–2880)	1660 (1280–2140)	943 (682–1390)
Non-communicable diseases	765000 (699000–832000)	1010000 (929000–1090000)	1370000 (1270000–1480000)	1790000 (1660000–1910000)	2140000 (2e+06–2280000)	2460000 (2310000–2610000)	3030000 (2870000–3190000)	2640000 (2510000–2760000)	1760000 (1700000–1830000)	865000 (841000–898000)
Neoplasms	302000 (276000–331000)	373000 (345000–405000)	453000 (422000–487000)	528000 (492000–568000)	545000 (505000–586000)	509000 (459000–547000)	473000 (405000–513000)	341000 (276000–377000)	183000 (141000–205000)	75800 (55000–86200)
Lip and oral cavity cancer	5310 (4480–6390)	6370 (5580–7350)	7850 (6930–8910)	7960 (7130–8960)	7440 (6650–8370)	7150 (6390–7950)	6630 (5650–7480)	5030 (4080–5650)	2900 (2260–3320)	1250 (908–1450)
Nasopharynx cancer	2290 (2000–2630)	2470 (2170–2800)	2390 (2110–2710)	2370 (2100–2680)	2140 (1890–2430)	1490 (1290–1690)	1140 (967–1300)	682 (548–787)	285 (216–329)	108 (80–126)
Other pharynx cancer	2730 (2210–3310)	3040 (2590–3590)	3680 (3100–4340)	3480 (2950–4110)	3100 (2620–3690)	2260 (1900–2640)	1920 (1560–2300)	1080 (870–1290)	456 (352–536)	149 (110–176)
Esophageal cancer	7220 (6330–8200)	10300 (9000–11700)	13600 (11800–15400)	18100 (15300–21000)	20800 (17000–24300)	19200 (15100–22300)	16900 (13000–19500)	11700 (8960–13500)	5150 (3880–5950)	1660 (1230–1920)
Stomach cancer	19500 (17300–22200)	24400 (21900–27500)	32400 (29200–36200)	41900 (37700–46800)	45500 (40800–50700)	45600 (40400–50700)	44300 (37700–49200)	32900 (26600–36900)	17200 (13300–19600)	7090 (5140–8150)
Colon and rectum cancer	24500 (21900–27200)	32500 (29700–35600)	43100 (39700–46700)	53500 (49600–57800)	60700 (55900–65500)	65500 (59200–70400)	69900 (60100–76200)	56400 (45500–62500)	34400 (26200–38800)	15400 (11100–17700)
Liver cancer	8830 (7620–10200)	11900 (10500–13500)	15800 (14000–18000)	20000 (17800–22500)	21200 (18900–23600)	20500 (18300–22600)	18500 (15500–20500)	12300 (9700–13900)	5640 (4190–6440)	2060 (1500–2400)
Liver cancer due to hepatitis B	3170 (2330–4200)	3820 (2760–5100)	4450 (3160–6080)	4900 (3430–6600)	4360 (2980–6020)	3460 (2410–4710)	2570 (1640–3610)	1410 (961–1940)	521 (324–759)	154 (81·5–259)
Liver cancer due to hepatitis C	2720 (2010–3520)	4260 (3160–5480)	6190 (4660–7960)	8360 (6720–10100)	9690 (7790–11500)	10400 (8240–12400)	10400 (8160–12300)	7710 (5850–9010)	3850 (2760–4590)	1510 (1030–1830)
Liver cancer due to alcohol use	1160 (720–1760)	1620 (1020–2360)	2310 (1520–3290)	3190 (2200–4500)	3280 (2240–4700)	2690 (1930–3590)	2000 (1400–2740)	1060 (733–1440)	394 (254–566)	110 (58·9–171)
Liver cancer due to NASH	876 (611–1240)	1200 (801–1690)	1660 (1150–2400)	2190 (1560–3090)	2530 (1860–3440)	2740 (1970–3720)	2480 (1700–3460)	1480 (1030–1980)	621 (397–894)	204 (115–321)
Liver cancer due to other causes	905 (622–1280)	1050 (724–1480)	1220 (824–1740)	1400 (990–1900)	1370 (970–1850)	1250 (895–1660)	1030 (715–1410)	608 (427–835)	250 (165–364)	83·5 (49·4–131)
Gallbladder and biliary tract cancer	5080 (4000–5890)	7240 (5970–8540)	10500 (8670–12300)	12700 (10700–15100)	13900 (11600–16800)	13700 (11300–16000)	13000 (10200–15100)	10100 (7450–11600)	5460 (3630–6480)	2550 (1580–3070)
Pancreatic cancer	10900 (9880–12100)	16900 (15400–18500)	26000 (23800–28200)	33800 (31300–36500)	38400 (35300–41500)	37100 (33200–39900)	34700 (29400–38100)	25400 (20200–28400)	13300 (9970–15200)	5180 (3690–6000)
Larynx cancer	1550 (1360–1790)	1880 (1690–2110)	2320 (2110–2590)	2540 (2300–2820)	2460 (2240–2730)	1950 (1740–2150)	1550 (1330–1730)	975 (796–1100)	404 (314–462)	135 (97·4–156)
Tracheal, bronchus, and lung cancer	36300 (32400–40900)	53300 (48500–58800)	74800 (68500–82200)	95300 (87200–105000)	102000 (93500–112000)	92000 (82400–1e+05)	79800 (67400–88000)	51300 (41200–57700)	23600 (18100–26900)	9040 (6630–10500)
Malignant skin melanoma	1770 (1330–2140)	2180 (1570–2670)	2480 (1680–2970)	2780 (1910–3270)	2890 (1920–3430)	2970 (1930–3470)	3060 (1910–3580)	2530 (1550–2970)	1510 (836–1790)	708 (401–862)
Non-melanoma skin cancer	817 (697–945)	1060 (910–1210)	1430 (1240–1640)	1880 (1630–2140)	2200 (1920–2480)	2430 (2140–2680)	3250 (2750–3600)	3530 (2810–3920)	2870 (2150–3230)	1920 (1360–2210)
Non-melanoma skin cancer (squamous-cell carcinoma)	817 (697–945)	1060 (910–1210)	1430 (1240–1640)	1880 (1630–2140)	2200 (1920–2480)	2430 (2140–2680)	3250 (2750–3600)	3530 (2810–3920)	2870 (2150–3230)	1920 (1360–2210)
Breast cancer	73800 (67100–80800)	79800 (73500–86900)	77500 (72000–83500)	75900 (70900–81400)	67200 (62300–71900)	57600 (52300–61600)	51600 (44300–56000)	37500 (30500–41300)	22300 (17300–25100)	10200 (7590–11700)
Cervical cancer	34600 (28600–39000)	34900 (29200–39200)	31700 (26900–35800)	29500 (25200–33400)	25000 (21900–28600)	18900 (16300–21400)	15000 (12400–16700)	9070 (7270–10300)	3790 (2940–4330)	1410 (1040–1640)
Uterine cancer	5910 (5090–6620)	8510 (7510–9560)	12600 (11300–13800)	14300 (13100–16000)	13200 (12100–14900)	10800 (9720–12200)	9200 (7860–10500)	5820 (4700–6680)	3030 (2330–3440)	1140 (851–1320)
Ovarian cancer	18900 (16200–21700)	22100 (19100–24600)	25300 (22100–28200)	26900 (23600–29600)	24600 (21700–26900)	19800 (17400–21700)	15900 (13300–17600)	10300 (8120–11500)	4750 (3540–5440)	1620 (1170–1900)
Kidney cancer	2650 (2450–2950)	3960 (3670–4330)	5740 (5370–6170)	7140 (6730–7640)	7610 (7070–8130)	7760 (7100–8310)	7610 (6400–8310)	5870 (4670–6560)	3160 (2430–3610)	1200 (866–1400)
Bladder cancer	1730 (1570–1920)	2590 (2370–2850)	3940 (3610–4270)	5540 (5120–6010)	7040 (6490–7610)	8870 (8020–9570)	10100 (8590–11100)	9110 (7360–10200)	5790 (4430–6590)	2640 (1940–3050)
Brain and central nervous system cancer	8230 (6130–9470)	10000 (7160–11400)	11600 (8100–13100)	12800 (8930–14600)	11600 (7500–13200)	9610 (5880–11200)	7170 (4120–8740)	4120 (2370–5080)	1550 (847–1950)	569 (315–723)
Thyroid cancer	1550 (1300–1740)	2020 (1720–2250)	2730 (2390–3000)	3310 (2920–3620)	3550 (3160–3890)	3390 (3010–3690)	3110 (2610–3430)	2160 (1720–2430)	1120 (835–1290)	446 (310–522)
Mesothelioma	480 (348–588)	692 (516–822)	796 (581–943)	984 (709–1140)	1100 (782–1270)	1050 (722–1200)	922 (626–1070)	572 (377–676)	259 (166–307)	90·2 (56·7–112)
Hodgkin lymphoma	520 (417–654)	617 (484–767)	726 (594–937)	887 (722–1190)	906 (715–1140)	850 (652–1070)	646 (465–794)	410 (278–500)	132 (77·1–164)	36·7 (19–48·5)
Non-Hodgkin lymphoma	5550 (4990–6180)	7120 (6530–7790)	9720 (9000–10500)	12200 (11400–13100)	13700 (12700–14600)	13500 (12300–14400)	13400 (11300–14500)	10100 (8100–11300)	5370 (4090–6100)	2000 (1440–2310)
Multiple myeloma	2380 (1960–2820)	3770 (3220–4410)	5650 (4880–6530)	7380 (6410–8460)	8210 (7170–9440)	8030 (6690–9000)	7070 (5450–7850)	4860 (3390–5570)	2450 (1630–2860)	732 (448–875)
Leukemia	7710 (6760–8730)	9360 (8410–10400)	11500 (10300–12500)	13600 (12200–14700)	14700 (13300–15800)	14300 (12700–15400)	13200 (11000–14500)	10100 (8140–11400)	5580 (4150–6370)	2250 (1640–2610)
Acute lymphoid leukemia	906 (628–1140)	1100 (797–1340)	1130 (806–1370)	1170 (817–1390)	1080 (765–1280)	839 (614–984)	698 (507–825)	431 (323–508)	194 (133–230)	57·4 (38·2–69·6)
Chronic lymphoid leukemia	908 (756–1100)	1360 (1170–1620)	2090 (1800–2420)	2460 (2180–2890)	2850 (2530–3330)	3220 (2860–3700)	3250 (2790–3680)	2630 (2100–3030)	1630 (1260–1920)	720 (522–857)
Acute myeloid leukemia	2130 (1800–2440)	2660 (2340–3020)	3350 (2950–3760)	4150 (3510–4590)	4570 (3780–5040)	4430 (3590–4910)	3640 (2590–4130)	2580 (1820–3020)	1150 (727–1370)	372 (202–480)
Chronic myeloid leukemia	911 (720–1120)	852 (719–1030)	891 (761–1030)	977 (851–1150)	989 (876–1160)	870 (763–1010)	855 (710–984)	673 (533–792)	383 (283–456)	158 (110–190)
Other leukemia	2860 (2260–3510)	3370 (2700–4020)	4000 (3190–4680)	4810 (3900–5560)	5200 (4360–6020)	4910 (4210–5560)	4730 (3870–5370)	3800 (3020–4390)	2220 (1630–2580)	946 (701–1120)
Other malignant neoplasms	10700 (9550–11900)	13400 (11900–14700)	16300 (14500–17900)	19500 (17300–21400)	20500 (18200–22500)	19400 (17100–21200)	18500 (15400–20400)	12900 (10200–14500)	6940 (5150–7950)	2690 (1900–3130)
Other neoplasms	473 (428–543)	711 (646–793)	1120 (1030–1250)	1730 (1580–1890)	2560 (2310–2760)	3520 (3130–3820)	4450 (3580–4890)	4530 (3440–5110)	3160 (2230–3650)	1460 (965–1730)
myeloproliferative, and other hematopoietic neoplasms	473 (428–543)	711 (646–793)	1120 (1030–1250)	1730 (1580–1890)	2560 (2310–2760)	3520 (3130–3820)	4450 (3580–4890)	4530 (3440–5110)	3160 (2230–3650)	1460 (965–1730)
Cardiovascular diseases	259000 (230000–287000)	361000 (325000–396000)	534000 (485000–581000)	752000 (688000–817000)	960000 (874000–1040000)	1200000 (1080000–1290000)	1580000 (1370000–1720000)	1420000 (1170000–1560000)	970000 (773000–1080000)	472000 (350000–533000)
Rheumatic heart disease	11500 (7400–15400)	12500 (9090–16400)	13400 (10400–16700)	16500 (13400–20000)	17000 (14000–20200)	18000 (14900–21100)	18800 (15100–22000)	14700 (11800–17000)	8930 (6870–10300)	4060 (3010–4760)
Ischemic heart disease	115000 (101000–128000)	165000 (148000–182000)	251000 (227000–273000)	350000 (320000–380000)	440000 (401000–478000)	549000 (495000–594000)	745000 (647000–807000)	672000 (553000–739000)	474000 (377000–527000)	233000 (175000–265000)
Stroke	1e+05 (89100–113000)	136000 (122000–152000)	204000 (184000–226000)	294000 (267000–325000)	379000 (341000–417000)	465000 (419000–509000)	577000 (5e+05–636000)	489000 (405000–542000)	299000 (237000–336000)	133000 (98400–152000)
Ischemic stroke	17600 (15400–20200)	27900 (24800–31600)	64400 (57800–72400)	106000 (94900–119000)	188000 (170000–208000)	252000 (228000–276000)	381000 (333000–420000)	339000 (281000–375000)	219000 (175000–246000)	102000 (76000–117000)
Intracerebral hemorrhage	69400 (61200–78200)	91500 (81300–101000)	123000 (109000–136000)	168000 (151000–185000)	171000 (151000–190000)	192000 (169000–212000)	175000 (150000–196000)	133000 (110000–150000)	70100 (56000–80100)	26200 (19900–30000)
Subarachnoid hemorrhage	13300 (11300–15700)	17100 (14700–20200)	16300 (14100–19400)	20700 (18100–23900)	20100 (17700–23000)	20900 (18400–23600)	20400 (17100–22700)	16200 (12900–18200)	9720 (7200–11000)	4270 (2920–4990)
Hypertensive heart disease	16800 (12800–20000)	24300 (18500–28400)	34800 (27300–40700)	50500 (40600–58100)	69000 (53000–79000)	87800 (70300–1e+05)	121000 (91900–137000)	112000 (80000–128000)	79900 (53500–92100)	41600 (25500–48600)
Non-rheumatic valvular heart disease	1330 (1100–1580)	1910 (1640–2220)	2680 (2370–3050)	3970 (3560–4440)	5980 (5430–6640)	8930 (7970–9870)	15400 (13000–17200)	20300 (15800–23400)	19400 (14000–22500)	12500 (8690–14800)
Non-rheumatic calcific aortic valve disease	631 (533–836)	918 (792–1140)	1490 (1310–1800)	2440 (2150–2890)	3870 (3410–4480)	6260 (5350–7170)	11900 (9790–13700)	16700 (12900–19400)	16400 (11900–19400)	10800 (7580–13000)
Non-rheumatic degenerative mitral valve disease	635 (502–822)	906 (735–1180)	1080 (903–1440)	1410 (1200–1890)	1920 (1620–2560)	2430 (1970–3330)	3210 (2390–4560)	3430 (2200–4990)	2830 (1600–4360)	1680 (814–3050)
Other non-rheumatic valve diseases	60·8 (42·7–79·2)	89·3 (61·5–114)	108 (72·2–141)	117 (74·5–149)	187 (131–231)	236 (167–289)	351 (245–432)	267 (178–334)	153 (99·6–198)	61·1 (37·7–81·7)
Cardiomyopathy and myocarditis	4800 (4070–5550)	6880 (6000–8150)	7790 (6780–9200)	8560 (7420–10200)	9690 (8340–11400)	12700 (10400–14400)	20100 (15100–22500)	22200 (16400–25300)	18300 (12500–21400)	10500 (7170–12400)
Myocarditis	443 (319–597)	503 (370–668)	547 (380–715)	757 (529–1010)	907 (589–1140)	1190 (707–1480)	2260 (1270–2880)	2640 (1460–3490)	2220 (1080–3030)	1240 (571–1730)
Alcoholic cardiomyopathy	1330 (1040–1780)	1910 (1500–2570)	1780 (1400–2460)	1420 (1120–1960)	869 (677–1120)	793 (545–1020)	586 (357–868)	333 (191–578)	224 (133–307)	92 (57·5–126)
Other cardiomyopathy	3020 (2460–3500)	4460 (3700–5260)	5470 (4620–6470)	6380 (5470–7590)	7910 (6820–9450)	10700 (8840–12200)	17300 (12900–19300)	19200 (14000–22200)	15900 (10800–18800)	9210 (6190–11100)
Atrial fibrillation and flutter	976 (844–1130)	2070 (1810–2400)	3850 (3410–4380)	6660 (5910–7670)	12500 (11100–14400)	20300 (17600–23200)	39400 (32900–45700)	45600 (36500–54700)	41200 (31500–52300)	20300 (14600–25800)
Aortic aneurysm	1630 (1490–1830)	2510 (2300–2720)	3650 (3370–3970)	5440 (5040–5840)	7340 (6770–7890)	9210 (8370–9880)	11300 (9430–12300)	10300 (8170–11500)	6740 (5010–7730)	3160 (2250–3700)
Endocarditis	1090 (768–1360)	1400 (993–1790)	1650 (1160–2030)	2200 (1560–2710)	2670 (1840–3130)	3260 (2090–3750)	4290 (2560–5110)	4960 (2530–6220)	4230 (1940–5550)	2670 (1010–3650)
Other cardiovascular and circulatory diseases	5790 (5100–6480)	7100 (6340–7890)	9970 (8950–11100)	12100 (10900–13300)	14800 (13400–16300)	17400 (15500–19200)	20400 (17400–22500)	17900 (14400–20000)	12100 (9360–13800)	6230 (4530–7210)
Chronic respiratory diseases	39700 (31400–47200)	61500 (50700–71200)	105000 (87400–120000)	158000 (134000–180000)	229000 (192000–260000)	270000 (221000–305000)	347000 (279000–396000)	258000 (205000–296000)	145000 (113000–167000)	60300 (43400–69600)
Chronic obstructive pulmonary disease	23300 (17900–28500)	39300 (31400–46800)	76200 (62300–89500)	123000 (102000–142000)	185000 (153000–214000)	225000 (185000–256000)	296000 (239000–341000)	226000 (181000–259000)	128000 (99800–148000)	53500 (39400–62500)
Pneumoconiosis	66·1 (52·9–82·3)	89·6 (73·3–114)	156 (126–196)	178 (145–221)	248 (205–296)	308 (250–369)	370 (283–441)	264 (203–310)	139 (100–169)	64·8 (43·1–78·5)
Silicosis	18·7 (11·3–25·7)	22·1 (14·7–30·1)	39·2 (25·3–54)	43·3 (27·5–59·2)	64·3 (44·6–88·2)	89·6 (62·9–120)	106 (74·7–140)	74·1 (52·1–103)	37 (25·6–51·7)	15·4 (9·99–22·3)
Asbestosis	13·1 (7·73–18·5)	20·5 (12–30·4)	32·2 (18·4–45·8)	39 (23·9–54)	58 (35·7–77)	64·8 (41·4–85·9)	80·8 (47·9–107)	68·7 (40·1–90·4)	36·3 (20–48·9)	17 (8·99–23·4)
Coal workers pneumoconiosis	8·01 (4·76–14·4)	10·2 (5·81–20)	15·6 (8·32–32·5)	22 (13·1–37·2)	26·7 (14·9–42·2)	34·2 (19–51·5)	37·7 (21·9–55·2)	29·4 (18·2–43·8)	17·2 (10·5–26·1)	9·91 (5·86–15·7)
Other pneumoconiosis	26·3 (18·9–37·7)	36·8 (28·1–51·7)	69 (47·2–94·2)	74 (55·1–101)	99·2 (74·1–134)	120 (86·8–158)	145 (106–191)	91·5 (67·3–120)	48·2 (33·8–63·5)	22·5 (14·1–30)

Asthma	–	–	–	3040 (2180–4240)	865 (655–1140)	944 (753–1190)	1490 (1200–1920)	1950 (1560–2470)	2400 (1940–2920)	3230 (2620–3910)	4500 (3570–5480)	6150 (4740–7470)	8720 (6700–10800)	12600 (8960–16200)	16700 (11900–21800)	22100 (14900–30200)	26000 (17700–35600)	31200 (21100–43400)	32300 (20800–44000)	36500 (24100–47700)	21900 (15400–28400)	10800 (7640–13900)	4100 (2740–5530)
Interstitial lung disease and pulmonary sarcoidosis	–	–	–	131 (83·3–214)	69·4 (46·5–101)	56·9 (39·3–79·5)	107 (74·2–142)	152 (105–208)	238 (169–315)	365 (258–500)	640 (476–890)	919 (681–1250)	1490 (1050–2080)	2430 (1630–3440)	3620 (2460–4930)	5640 (3870–7610)	8140 (5220–10600)	11100 (7060–14100)	12200 (7470–15200)	12600 (7360–15900)	9440 (5070–12400)	5190 (2470–7160)	2370 (1030–3450)
Other chronic respiratory diseases	0 (0–0)	0 (0–0)	0 (0–0)	940 (435–1600)	325 (218–450)	320 (201–437)	204 (148–293)	277 (187–386)	425 (268–574)	408 (281–590)	677 (445–899)	824 (559–1130)	829 (592–1200)	1270 (869–1830)	1800 (1210–2550)	651 (400–857)	744 (463–962)	924 (554–1220)	1030 (596–1320)	1310 (766–1690)	1060 (616–1360)	707 (414–912)	336 (186–432)
Digestive diseases	866 (681–1130)	704 (528–926)	5450 (3950–7480)	8050 (6150–10700)	4250 (3230–5500)	4590 (3750–5480)	9380 (7990–11100)	12200 (10300–14500)	14700 (12600–16900)	17700 (15700–20000)	23500 (21100–26100)	30200 (27100–33200)	40000 (36300–43900)	54700 (49200–60700)	71400 (65100–78200)	86200 (79500–93800)	1e+05 (92800–108000)	106000 (97900–114000)	114000 (103000–123000)	126000 (108000–137000)	103000 (84600–114000)	67000 (52500–75000)	33600 (24700–38300)
Cirrhosis and other chronic liver diseases	–	–	–	3300 (2330–4580)	1740 (1280–2270)	2320 (1840–2830)	4950 (4140–5820)	7030 (5850–8450)	8780 (7480–10100)	11900 (10500–13500)	15900 (14200–17600)	21600 (19500–24000)	27600 (25000–30400)	37300 (33400–42200)	47400 (43100–52700)	54600 (50100–60200)	57800 (53100–63900)	55000 (50300–61100)	50800 (45700–56900)	45100 (38800–50500)	29000 (23700–33100)	14200 (11100–16600)	6470 (4690–7950)
liver diseases due to hepatitis B	–	–	–	16·1 (8·18–28·5)	17·2 (8·89–28·4)	43 (23·7–68·3)	150 (76–261)	775 (404–1290)	1660 (944–2520)	2820 (1990–3810)	4160 (2880–5680)	5820 (4280–7680)	6820 (4900–9170)	8410 (5730–11800)	9380 (6460–13100)	9890 (6570–13900)	10000 (6740–13800)	9480 (6360–13300)	9080 (6180–12400)	8030 (5260–11100)	4800 (3360–6310)	2110 (1340–3030)	845 (437–1380)
liver diseases due to hepatitis C	–	–	–	14·8 (7·35–26·9)	26·2 (14·4–43·1)	63·8 (37–104)	205 (107–351)	1010 (564–1540)	2100 (1320–3060)	3280 (2370–4290)	4690 (3320–6250)	6320 (4710–8180)	7970 (5830–10500)	10400 (7360–14000)	12500 (9120–17000)	13800 (9700–19300)	14700 (10800–19300)	13800 (10200–17700)	12300 (8620–16500)	10800 (7650–14500)	7230 (5190–9460)	3700 (2440–5130)	1870 (1100–2770)
liver diseases due to alcohol use	–	–	–	–	–	–	9·71 (2·75–21·7)	169 (85·9–302)	471 (266–793)	1300 (898–1800)	2580 (1710–3600)	4110 (2890–5480)	5880 (4010–8080)	8710 (5900–11800)	12300 (8670–16800)	14100 (9770–19200)	13600 (9180–18600)	11100 (7500–15600)	8770 (6110–11800)	6950 (4610–9700)	4220 (2990–5830)	2050 (1300–3080)	905 (481–1580)
Cirrhosis and other chronic liver diseases due to NAFLD	–	–	–	–	–	–	102 (40·8–210)	312 (152–565)	636 (305–1140)	1030 (570–1670)	1360 (683–2360)	2040 (1030–3360)	2890 (1400–4940)	4110 (2080–6870)	5220 (2630–8720)	6290 (3220–10800)	7300 (4540–11200)	7600 (4660–11200)	7540 (4170–11700)	7120 (4100–10700)	4820 (2960–7070)	2380 (1330–3840)	1060 (469–1840)
liver diseases due to other causes	–	–	–	3260 (2310–4530)	1700 (1250–2210)	2210 (1760–2700)	4480 (3670–5310)	4770 (3490–6240)	3910 (2600–5360)	3480 (2170–5170)	3080 (1730–4730)	3360 (1920–5390)	4030 (2300–6450)	5610 (3060–8940)	8040 (4490–12700)	10400 (5970–15900)	12300 (6890–19500)	13000 (7380–20100)	13100 (7760–19800)	12200 (7230–18300)	7970 (5120–11300)	3950 (2340–5830)	1800 (852–2940)
Upper digestive system diseases	–	–	–	742 (467–1320)	416 (299–575)	392 (307–493)	622 (516–759)	1320 (1090–1610)	1320 (1100–1590)	1570 (1310–1890)	2140 (1790–2550)	2170 (1860–2550)	3270 (2820–3890)	5240 (4330–6250)	6250 (5390–7220)	8550 (7550–9770)	12000 (10500–13600)	13700 (12200–15400)	15800 (14000–17700)	19500 (16700–21800)	16000 (13100–18200)	9750 (7700–11300)	4370 (3230–5160)
Peptic ulcer disease	–	–	–	704 (437–1280)	361 (257–514)	353 (276–446)	582 (479–716)	1160 (951–1440)	1210 (1010–1480)	1400 (1160–1700)	1910 (1610–2300)	1950 (1660–2310)	2890 (2470–3460)	4680 (3830–5590)	5580 (4780–6520)	7610 (6680–8740)	10400 (9140–11900)	11900 (10500–13500)	13700 (12100–15300)	16700 (14300–18700)	13600 (11100–15500)	8190 (6430–9440)	3670 (2690–4350)
Gastritis and duodenitis	–	–	–	37·5 (24·5–60·1)	54·9 (34·4–80·1)	38·4 (27·1–54·3)	39·5 (28–54)	160 (107–229)	108 (77·2–143)	168 (128–218)	223 (167–293)	213 (171–264)	384 (304–480)	560 (433–701)	671 (537–810)	944 (752–1140)	1540 (1210–1830)	1790 (1410–2120)	2110 (1710–2480)	2770 (2270–3220)	2420 (1900–2790)	1570 (1220–1840)	701 (524–845)
Appendicitis	–	–	–	604 (301–976)	455 (309–610)	516 (378–665)	1190 (872–1520)	887 (654–1150)	1060 (747–1410)	573 (415–722)	687 (474–885)	549 (385–691)	771 (553–965)	697 (502–894)	1300 (899–1630)	1200 (880–1460)	1230 (912–1500)	1150 (837–1360)	1310 (1010–1530)	1220 (979–1420)	945 (747–1110)	522 (397–617)	206 (153–245)
Paralytic ileus and intestinal obstruction	656 (501–870)	621 (466–814)	5160 (3740–7020)	1390 (811–2050)	892 (587–1240)	827 (598–1040)	1770 (1170–2290)	1600 (1070–2110)	1700 (1140–2210)	1300 (838–1700)	1700 (1100–2170)	1970 (1180–2520)	2660 (1610–3370)	3700 (2130–4850)	5270 (3510–6690)	6410 (4570–7770)	8250 (6020–9840)	9210 (7220–10800)	11900 (9680–13700)	15100 (12000–17700)	14700 (11400–17100)	11300 (7950–13300)	6000 (3790–7390)
Inguinal, femoral, and abdominal hernia	210 (120–286)	82·7 (42·7–129)	295 (102–533)	98·2 (37·4–168)	62 (27·6–99·3)	48·5 (21·6–71·7)	60·6 (30·1–91·9)	67·5 (34·5–98·4)	68·3 (38–100)	83 (48·8–114)	116 (71·3–157)	177 (114–231)	247 (167–320)	549 (368–705)	819 (547–1020)	1170 (773–1510)	1490 (1060–1800)	1770 (1250–2220)	2310 (1710–2930)	3240 (2400–4060)	2960 (2270–3630)	2140 (1500–2660)	1060 (723–1360)
Inflammatory bowel disease	–	–	–	380 (173–695)	66·7 (40·9–108)	62·5 (41·4–86·8)	130 (86·9–176)	207 (138–284)	266 (184–347)	290 (218–355)	341 (268–399)	405 (321–471)	484 (406–557)	645 (544–738)	911 (768–1010)	1150 (958–1280)	1500 (1260–1640)	1910 (1630–2100)	2450 (2100–2700)	3380 (2700–3820)	3400 (2570–3940)	2780 (1960–3320)	1600 (1110–1990)
Vascular intestinal disorders	–	–	–	40·1 (25·7–64·4)	12·7 (9·46–17·6)	13·7 (10·3–19·5)	37·6 (29·2–51·3)	102 (76–136)	98·9 (75·5–140)	191 (150–240)	238 (197–299)	355 (304–434)	605 (527–720)	995 (870–1190)	1650 (1470–1910)	2770 (2480–3180)	4600 (4120–5220)	6800 (6090–7600)	9380 (8400–10400)	12400 (10700–14000)	10900 (8830–12400)	7210 (5600–8440)	3120 (2290–3830)
Gallbladder and biliary diseases	–	–	–	433 (200–697)	257 (154–366)	117 (78·7–150)	165 (115–199)	267 (192–325)	379 (291–450)	531 (401–626)	815 (613–969)	908 (714–1080)	1280 (1040–1530)	1980 (1650–2350)	2660 (2240–3150)	3940 (3320–4770)	5270 (4440–6300)	6370 (5470–7590)	8720 (7630–10500)	11900 (9700–14100)	11600 (8960–13700)	9120 (6550–10900)	5430 (3600–6720)
Pancreatitis	–	–	–	168 (38·6–321)	76·3 (48·5–108)	96·6 (66·8–126)	254 (188–318)	460 (344–582)	618 (499–782)	899 (731–1090)	1150 (922–1380)	1420 (1150–1770)	2220 (1730–2810)	2320 (1830–2850)	3190 (2610–4000)	3530 (2880–4220)	4420 (3570–5290)	4720 (3870–5600)	4580 (3670–5490)	5220 (4200–6280)	4120 (3290–4920)	2510 (1890–3040)	1100 (797–1370)
Other digestive diseases	–	–	–	894 (485–1540)	273 (191–358)	198 (142–256)	204 (141–269)	287 (205–372)	373 (269–479)	397 (301–490)	481 (365–583)	607 (471–752)	905 (703–1110)	1290 (1020–1540)	1920 (1470–2310)	2900 (2240–3420)	3580 (2790–4190)	4960 (3840–5940)	6540 (4970–7890)	8900 (6550–10700)	9640 (6940–11900)	7380 (5010–9920)	4200 (2780–5630)
Neurological disorders	3·07 (2·38–4·13)	6·43 (5·35–7·98)	2400 (1920–3000)	2960 (2350–3790)	2120 (1710–2610)	2640 (2150–3230)	3960 (3370–4620)	3910 (3220–4500)	3820 (3220–4360)	4340 (3750–4970)	4210 (3670–4830)	5260 (4610–6280)	7700 (5980–11500)	11900 (8470–20100)	17200 (10700–33000)	27800 (15400–57800)	48300 (24700–106000)	79800 (39800–173000)	129000 (59600–292000)	256000 (102000–592000)	307000 (108000–708000)	257000 (82100–605000)	150000 (43700–365000)
Alzheimer's disease and other dementias	–	–	–	–	–	–	–	–	–	–	–	287 (29·6–1040)	1700 (228–5370)	3820 (635–11700)	7800 (1490–22800)	15700 (3360–46100)	30600 (6830–89500)	52800 (12300–148000)	91200 (20800–258000)	208000 (51200–551000)	271000 (69000–677000)	239000 (62300–591000)	144000 (37200–361000)
Parkinson's disease	–	–	–	–	–	–	–	5·26 (4·16–6)	5·24 (4·5–5·99)	12·7 (10·8–14·5)	19·6 (17–21·8)	218 (191–243)	552 (490–615)	1210 (1070–1370)	1950 (1750–2170)	3770 (3390–4150)	8330 (7590–9090)	17500 (15800–19100)	29500 (26200–31800)	40600 (34300–44600)	30900 (25000–34200)	15200 (11900–17100)	4670 (3450–5310)
Idiopathic epilepsy	–	–	1470 (1060–2020)	2100 (1650–2640)	1550 (1240–1950)	1720 (1400–2070)	3050 (2500–3660)	3300 (2570–3890)	3100 (2520–3570)	3300 (2730–3860)	2700 (2190–3110)	2640 (2200–3060)	2230 (1850–2550)	2760 (2130–3230)	2280 (1690–2670)	2340 (1710–2760)	2570 (1940–3020)	2850 (2000–3400)	2240 (1540–2620)	2690 (1740–3180)	2050 (1200–2440)	1130 (613–1370)	476 (243–580)
Multiple sclerosis	–	–	–	–	59 (2·92–343)	7·21 (2·16–16·3)	29·9 (13·5–41·9)	121 (85·7–168)	206 (164–294)	386 (316–590)	653 (537–1030)	897 (752–1430)	1190 (992–1880)	1460 (1160–2200)	1680 (1260–2420)	1650 (1070–2260)	1520 (971–2050)	1270 (732–1690)	922 (543–1250)	659 (391–875)	411 (253–543)	157 (95·6–207)	45·9 (25·4–58·5)
Motor neuron disease	3·07 (2·38–4·13)	6·43 (5·35–7·98)	154 (133–181)	114 (94·2–139)	45·4 (38·7–53·4)	43·9 (38·8–49·3)	42·4 (38·1–47·4)	41·5 (37·2–46·2)	49·4 (45·2–54·3)	75·6 (69·3–82·8)	124 (115–134)	268 (248–288)	520 (485–556)	985 (913–1050)	1550 (1450–1640)	2160 (2020–2280)	2800 (2610–2990)	2880 (2630–3100)	2570 (2270–2820)	1780 (1440–1980)	896 (704–1020)	307 (227–354)	92·9 (67·5–108)
Other neurological disorders	–	–	771 (611–1010)	749 (486–1150)	463 (352–626)	866 (612–1260)	838 (620–1170)	437 (363–535)	456 (392–533)	558 (495–629)	708 (640–785)	955 (875–1040)	1510 (1350–1720)	1640 (1500–1790)	1890 (1760–2030)	2190 (2050–2350)	2480 (2320–2630)	2530 (2350–2690)	2520 (2280–2690)	2400 (2050–2610)	1750 (1400–1980)	1010 (768–1140)	446 (331–509)
Mental disorders	–	–	–	–	0·267 (0·201–0·409)	3·69 (2·59–7·33)	18·9 (15·3–27·6)	31·9 (27·1–41·9)	37·9 (32·2–48·3)	44·3 (37·6–56·4)	43·8 (38–51·9)	48·1 (42·6–54·5)	50 (44·6–56·2)	–	–	–	–	–	–	–	–	–	–
Eating disorders	–	–	–	–	0·267 (0·201–0·409)	3·69 (2·59–7·33)	18·9 (15·3–27·6)	31·9 (27·1–41·9)	37·9 (32·2–48·3)	44·3 (37·6–56·4)	43·8 (38–51·9)	48·1 (42·6–54·5)	50 (44·6–56·2)	–	–	–	–	–	–	–	–	–	–
Anorexia nervosa	–	–	–	–	0·143 (0·101–0·205)	3·32 (2·41–6·07)	17·6 (14·3–25·9)	26·6 (22·1–35·9)	32·9 (27·8–42·7)	38·9 (32·5–50·3)	38·5 (33·2–45·9)	40·7 (36–46·3)	44·1 (39·3–49·6)	–	–	–	–	–	–	–	–	–	–
Bulimia nervosa	–	–	–	–	0·123 (0·0875–0·243)	0·364 (0·148–2·14)	1·33 (0·879–2·89)	5·28 (3·94–7·44)	4·96 (3·84–6·44)	5·4 (4·15–7·11)	5·24 (4·16–6·74)	7·31 (5·91–9·1)	5·87 (4·84–7·2)	–	–	–	–	–	–	–	–	–	–
[illegible]	[illegible]	[illegible]	[illegible]	[illegible]	[illegible]	[illegible]	[illegible]	[illegible]	[illegible]	[illegible]	[illegible]	[illegible]	[illegible]	[illegible]	[illegible]	[illegible]	[illegible]	[illegible]	[illegible]	[illegible]	[illegible]	[illegible]	[illegible]

SR Table 5: Global deaths by cause and age group for males (with 95% uncertainty levels), 1990

Cause	Early Neonatal	Late Neonatal	Post Neonatal	1 to 4	5 to 9	10 to 14	15 to 19	20 to 24	25 to 29	30 to 34	35 to 39	40 to 44	45 to 49	50 to 54	55 to 59	60 to 64	65 to 69	70 to 74	75 to 79	80 to 84	85 to 89	90 to 94	95 plus
All causes	1730000 (1620000–1850000)	695000 (635000–755000)	2020000 (1900000–2150000)	1910000 (1770000–2040000)	441000 (424000–460000)	254000 (246000–264000)	389000 (375000–404000)	486000 (469000–502000)	487000 (474000–499000)	531000 (516000–544000)	640000 (616000–662000)	729000 (698000–759000)	849000 (810000–888000)	1190000 (1130000–1240000)	1510000 (1440000–1580000)	1950000 (1870000–2030000)	2070000 (1990000–2160000)	2090000 (2e+06–2170000)	2090000 (2030000–2150000)	1660000 (1630000–1700000)	926000 (914000–935000)	313000 (311000–315000)	88000 (87700–88300)
Communicable, maternal, neonatal, and nutritional diseases	1560000 (1450000–1680000)	610000 (552000–675000)	1660000 (1510000–1820000)	1490000 (1350000–1650000)	240000 (218000–263000)	109000 (98100–120000)	94700 (83800–108000)	97700 (89100–110000)	110000 (102000–121000)	127000 (120000–138000)	147000 (138000–160000)	150000 (141000–165000)	162000 (150000–180000)	186000 (172000–211000)	210000 (194000–242000)	249000 (228000–293000)	250000 (227000–299000)	261000 (236000–317000)	239000 (216000–292000)	203000 (180000–247000)	118000 (105000–138000)	41600 (36000–48200)	11900 (9780–13500)
HIV/AIDS and sexually transmitted infections	30700 (10100–64600)	10000 (3180–21800)	27900 (19600–42400)	20600 (17900–23300)	1770 (1410–2230)	455 (349–594)	1420 (1040–1890)	5410 (4740–6310)	17000 (15100–19500)	27100 (24300–30600)	29200 (25700–33500)	21800 (19400–24800)	14300 (12400–16700)	9290 (8070–10700)	6030 (5200–7040)	4100 (3580–4730)	2790 (2400–3250)	1610 (1310–1980)	1010 (823–1250)	235 (170–318)	145 (86·7–239)	47·4 (26·4–80·7)	18·2 (11·1–28·1)
HIV/AIDS	--	--	16100 (14400–18000)	20100 (17500–22700)	1560 (1240–1900)	331 (274–442)	1340 (958–1820)	5330 (4660–6240)	16900 (15000–19400)	26900 (24100–30400)	29000 (25400–33300)	21700 (19200–24700)	14200 (12200–16600)	9040 (7800–10500)	5710 (4890–6690)	3830 (3310–4450)	2620 (2230–3070)	1450 (1150–1800)	805 (644–1010)	114 (82·8–156)	49·6 (36–68·3)	12·6 (8·95–17·8)	4·42 (3·73–5·44)
HIV/AIDS - Drug-susceptible Tuberculosis	--	--	6340 (5330–7240)	8170 (6790–9390)	565 (409–729)	96·3 (72–126)	447 (307–603)	1710 (1370–2070)	4870 (3860–5970)	7740 (6210–9450)	8480 (6710–10300)	6360 (5060–7660)	4410 (3500–5550)	2950 (2390–3640)	1880 (1520–2330)	1280 (1050–1580)	898 (732–1110)	573 (454–724)	314 (250–394)	36·9 (26·3–51·2)	13·8 (9·86–19·3)	3·25 (2·3–4·48)	0·89 (0·663–1·19)
Tuberculosis without extensive drug resistance	--	--	31·2 (11–74·6)	39·4 (13·7–89·8)	2·73 (0·98–6·3)	0·765 (0·27–1·65)	2·88 (1·1–6·37)	12·1 (4·78–25·6)	39·2 (15·7–81·3)	60·8 (24·1–126)	63·8 (25·6–131)	49 (19·6–102)	32·1 (12·7–65·9)	21·7 (8·51–46·8)	14·8 (5·83–31·2)	10·9 (4·24–23·7)	7·47 (2·9–16·6)	3·54 (1·26–8·1)	2·76 (0·902–6·62)	0·423 (0·142–1·03)	0·15 (0·0513–0·354)	0·0304 (0·0112–0·0683)	0·0108 (0·00451–0·0221)
HIV/AIDS - Extensively drug-resistant Tuberculosis	--	--	0 (0–0)	0 (0–0)	0 (0–0)	0 (0–0)	0 (0–0)	0 (0–0)	0 (0–0)	0 (0–0)	0 (0–0)	0 (0–0)	0 (0–0)	0 (0–0)	0 (0–0)	0 (0–0)	0 (0–0)	0 (0–0)	0 (0–0)	0 (0–0)	0 (0–0)	0 (0–0)	0 (0–0)
HIV/AIDS resulting in other diseases	--	--	9730 (8510–11100)	11900 (10200–13900)	993 (773–1350)	233 (191–327)	888 (633–1290)	3610 (3050–4430)	12000 (10500–14400)	19100 (16900–22100)	20400 (17900–24400)	15300 (13600–18200)	9710 (8390–11700)	6080 (5240–7190)	3810 (3290–4540)	2530 (2200–2970)	1720 (1470–2030)	870 (684–1140)	488 (382–640)	76·4 (55·1–116)	35·7 (24·3–54·8)	9·3 (6·04–14·4)	3·52 (2·82–4·51)
Sexually transmitted infections excluding HIV	30700 (10100–64600)	10000 (3180–21800)	11800 (3770–25900)	472 (150–1050)	215 (69·1–489)	124 (50·9–205)	84·4 (45·1–124)	82·5 (42·2–114)	92 (52–121)	180 (75·2–266)	182 (79·3–263)	150 (71·6–204)	140 (64·8–203)	243 (102–383)	327 (126–558)	273 (144–401)	165 (115–244)	164 (112–232)	203 (123–302)	122 (64·7–195)	95·1 (39·1–193)	34·8 (14·1–68·3)	13·8 (6·71–23·7)
Syphilis	30700 (10100–64600)	10000 (3180–21800)	11800 (3770–25900)	472 (150–1050)	215 (69·1–489)	70·9 (29·3–117)	65·3 (34·2–96·3)	51·3 (26·3–70·9)	64 (35·9–84·8)	121 (50·9–179)	154 (64·5–225)	112 (53·2–153)	104 (51·1–148)	189 (85·1–290)	266 (109–446)	229 (124–332)	139 (99·6–200)	137 (94·7–190)	136 (90–194)	71·3 (43·5–105)	41·1 (22–72·2)	12·4 (6·69–20·5)	4·97 (3·21–7·1)
Chlamydial infection	--	--	--	--	--	0 (0–0)	0 (0–0)	0 (0–0)	0 (0–0)	0 (0–0)	0 (0–0)	0 (0–0)	0 (0–0)	0 (0–0)	0 (0–0)	0 (0–0)	0 (0–0)	0 (0–0)	0 (0–0)	0 (0–0)	0 (0–0)	0 (0–0)	0 (0–0)
Gonococcal infection	--	--	--	--	--	6·77 (3·38–10·4)	10·5 (5·95–14·8)	19·8 (10·1–27·4)	7·93 (5·08–9·93)	15·4 (8·34–20·9)	13·4 (7·21–17·8)	21·3 (11·3–28·1)	11·5 (4·44–17·4)	10·9 (3·75–18·5)	15·3 (4·58–27·9)	17·9 (7·41–28·2)	12·4 (7·64–20·7)	11·5 (6·85–17·9)	12·6 (6·49–20·6)	7·78 (3·17–14)	6·07 (1·92–13·5)	0·694 (0·229–1·45)	0·171 (0·112–0·242)
Other sexually transmitted infections	--	--	--	--	--	46·5 (18·2–77·7)	8·61 (4·7–12·4)	11·4 (5·83–15·7)	20 (10·9–26·9)	43·4 (15·6–66)	14·5 (6·83–20·4)	17·1 (8·45–23)	24·2 (8·56–37·6)	43·8 (14·2–74·8)	45·7 (13·6–83·1)	25·6 (10·5–40·5)	13·5 (8·33–22·7)	15·6 (9·32–24·4)	54 (27·7–88·1)	42·5 (17·3–76·9)	47·9 (15·1–107)	21·7 (6·84–46·3)	8·67 (3·17–16·5)
Respiratory infections and tuberculosis	149000 (125000–170000)	156000 (130000–193000)	619000 (543000–726000)	354000 (283000–439000)	41500 (34500–47900)	19600 (16900–22300)	26000 (23200–28800)	38600 (35200–42400)	50500 (46600–54800)	61200 (57100–65600)	73900 (68400–79200)	82700 (76500–88600)	95600 (87900–103000)	116000 (107000–125000)	131000 (120000–142000)	154000 (141000–167000)	151000 (138000–162000)	155000 (142000–167000)	143000 (132000–154000)	121000 (111000–130000)	73200 (65900–78500)	26300 (22700–28300)	7720 (6270–8510)
[illegible]	[illegible]	[illegible]	[illegible]	[illegible]	[illegible]	[illegible]	[illegible]	[illegible]	[illegible]	[illegible]	[illegible]	[illegible]	[illegible]	[illegible]	[illegible]	[illegible]	[illegible]	[illegible]	[illegible]	[illegible]	[illegible]	[illegible]	[illegible]

Cirrhosis and other chronic liver diseases	–	–	–	7540 (4300–12000)	4250 (2850–5870)	2730 (2170–3440)	5620 (4810–6480)	8870 (7660–10300)	15300 (13600–17000)	25800 (23400–27700)	40500 (36900–43300)	53900 (49300–57700)	62000 (57000–66500)	75700 (69900–80900)	81000 (74500–87000)	82100 (75500–88200)	69100 (63300–74200)	50400 (45700–54800)	38600 (35000–41900)	23500 (20700–26000)	10700 (9140–12000)	3170 (2580–3600)	805 (633–929)
Cirrhosis and other chronic liver diseases due to hepatitis B	–	–	–	85·2 (33·1–161)	127 (58·5–216)	152 (79·7–244)	478 (253–752)	2280 (1460–3150)	5460 (3880–7110)	9680 (7740–11600)	15200 (12000–18300)	19700 (16000–23900)	21400 (17100–26400)	24800 (19100–31600)	24800 (18400–32100)	23800 (17800–31100)	19300 (14200–25000)	14300 (10200–18800)	11000 (8110–14100)	6690 (4800–8650)	2900 (2150–3780)	815 (544–1120)	189 (109–285)
Cirrhosis and other chronic liver diseases due to hepatitis C	–	–	–	76·9 (28·2–151)	114 (53·8–202)	143 (76·7–239)	440 (237–730)	1710 (1080–2420)	3980 (2770–5380)	7080 (5490–8840)	11200 (8200–14700)	14800 (11300–18900)	16600 (12500–21100)	19600 (14400–25300)	20200 (15000–26900)	19600 (13700–26900)	17000 (12900–21800)	12800 (10000–16300)	9630 (7300–12400)	5960 (4400–7790)	2910 (2180–3740)	917 (646–1250)	243 (152–352)
Cirrhosis and other chronic liver diseases due to alcohol use	–	–	–	–	–	–	29 (8·68–65·2)	504 (300–808)	1530 (952–2330)	4230 (3200–5390)	8780 (6360–11400)	13200 (9960–16500)	16600 (12300–21400)	22000 (16000–28100)	25400 (19000–32500)	26600 (19700–34500)	21300 (15500–27800)	13700 (9850–18300)	9700 (7220–12500)	5290 (3720–7020)	2220 (1610–2880)	607 (399–874)	149 (84·5–244)
Cirrhosis and other chronic liver diseases due to NAFLD	–	–	–	–	–	–	149 (56·9–296)	392 (197–660)	835 (396–1470)	1370 (797–2150)	1950 (1000–3370)	2650 (1310–4440)	3430 (1630–6070)	4370 (2120–7700)	4660 (2300–8050)	5000 (2480–9020)	4770 (2890–7450)	3830 (2310–5720)	3280 (1780–5200)	2170 (1230–3300)	1070 (663–1640)	335 (186–580)	91·4 (38·8–177)
Cirrhosis and other chronic liver diseases due to other causes	–	–	–	7380 (4210–11800)	4010 (2700–5530)	2440 (1910–3100)	4520 (3680–5450)	3990 (2660–5570)	3550 (2170–5400)	3390 (1950–5620)	3340 (1790–5440)	3520 (1910–5980)	3880 (2180–6490)	4920 (2800–8020)	5970 (3330–10200)	7030 (4050–11500)	6780 (3640–11800)	5680 (3060–9230)	5000 (2770–8150)	3360 (1870–5470)	1620 (975–2450)	500 (272–857)	133 (57·7–252)
Upper digestive system diseases	–	–	–	1780 (1090–2890)	815 (635–1060)	506 (424–623)	1720 (1450–2030)	2330 (2030–2770)	3660 (3160–4230)	4570 (3950–5160)	7390 (6330–8300)	9080 (7830–10300)	11400 (9890–12700)	15000 (13100–16700)	18400 (16300–20400)	21200 (18800–23400)	21400 (19200–23600)	20700 (18400–22700)	19800 (17800–21600)	13800 (12200–15200)	7540 (6660–8240)	2300 (1950–2520)	598 (481–670)
Peptic ulcer disease	–	–	–	1150 (600–2220)	724 (551–975)	456 (374–573)	1540 (1300–1840)	2100 (1830–2520)	3270 (2850–3780)	3990 (3480–4540)	6590 (5630–7480)	8090 (6910–9260)	10200 (8710–11400)	13400 (11600–15000)	16500 (14500–18400)	19300 (16900–21500)	19400 (17100–21400)	18700 (16500–20700)	17900 (16000–19700)	12400 (11000–13600)	6730 (5920–7370)	2080 (1740–2290)	538 (429–603)
Gastritis and duodenitis	–	–	–	633 (305–1000)	90·8 (47·7–133)	50·7 (31·4–69·5)	182 (122–267)	229 (156–327)	396 (265–568)	581 (391–826)	801 (526–1180)	989 (665–1480)	1230 (804–1780)	1600 (1060–2300)	1910 (1260–2800)	1940 (1220–2890)	2040 (1330–2940)	2000 (1320–2980)	1920 (1340–2790)	1400 (1000–2020)	816 (592–1160)	222 (167–294)	59·2 (41·9–76·8)
Appendicitis	–	–	–	2190 (991–3530)	1510 (759–2150)	1000 (567–1310)	1320 (797–1690)	1070 (656–1360)	1350 (851–1670)	732 (468–876)	1000 (588–1210)	1310 (776–1600)	1120 (712–1360)	1160 (693–1400)	1280 (849–1540)	1500 (1030–1820)	1320 (967–1570)	1380 (955–1670)	1170 (895–1400)	889 (680–1070)	420 (307–507)	132 (95·7–164)	29·6 (20·5–39)
Paralytic ileus and intestinal obstruction	1290 (1020–1570)	1910 (1450–2420)	13800 (10500–17900)	2560 (1270–4830)	1570 (1020–2570)	773 (524–1060)	1600 (1050–1990)	1560 (976–2010)	1930 (1200–2420)	2000 (1180–2520)	2590 (1580–3230)	3120 (1940–3860)	3170 (2070–3880)	3810 (2650–4600)	4900 (3350–5940)	6110 (4340–7310)	6720 (5100–8100)	6450 (5100–7830)	6360 (5210–7960)	5770 (4580–7350)	3780 (3110–4840)	1390 (1120–1800)	383 (291–530)
Inguinal, femoral, and abdominal hernia	701 (440–976)	688 (329–1380)	3380 (1190–7530)	991 (309–2000)	271 (161–398)	139 (84·7–182)	267 (183–331)	287 (215–360)	323 (243–412)	292 (216–371)	458 (321–581)	564 (392–717)	673 (492–869)	1090 (844–1400)	1440 (1100–1840)	1890 (1500–2410)	2140 (1720–2700)	2330 (1880–3020)	2670 (2210–3480)	2570 (2100–3310)	1540 (1280–1950)	580 (465–743)	157 (120–208)
Inflammatory bowel disease	–	–	–	805 (251–1710)	136 (60·1–223)	76·1 (41·6–106)	107 (72·1–155)	174 (121–257)	283 (206–390)	328 (238–448)	373 (283–496)	463 (353–624)	517 (384–689)	661 (511–880)	804 (629–1060)	1010 (806–1310)	1070 (849–1390)	1130 (888–1470)	1260 (1000–1640)	1150 (889–1520)	678 (517–884)	241 (187–303)	68 (49·7–87·5)
Vascular intestinal disorders	–	–	–	354 (122–648)	71·8 (40·5–106)	28·5 (21·8–35·5)	45·3 (40·4–51)	88·3 (76·7–106)	132 (113–153)	142 (128–156)	233 (208–260)	370 (327–413)	557 (492–614)	1020 (907–1140)	1590 (1410–1750)	2410 (2220–2610)	3100 (2840–3320)	3350 (3070–3620)	3990 (3690–4280)	3360 (3030–3630)	1830 (1620–1990)	651 (551–726)	168 (134–196)
Gallbladder and biliary diseases	–	–	–	401 (132–572)	133 (80·3–184)	126 (72–164)	188 (106–230)	286 (163–361)	320 (198–380)	450 (289–544)	660 (415–805)	867 (546–1080)	1000 (646–1240)	1610 (1090–2000)	2160 (1480–2580)	3170 (2220–3760)	3790 (2690–4450)	4220 (3200–4940)	4990 (3930–5680)	4290 (3500–4870)	2510 (2050–2940)	939 (755–1100)	258 (195–305)
Pancreatitis	–	–	–	127 (51·7–252)	102 (63·2–172)	89·1 (67·1–123)	465 (372–646)	1020 (826–1370)	1940 (1600–2530)	2820 (2410–3600)	3620 (3100–4570)	3960 (3340–5060)	4230 (3460–5290)	3980 (3440–5020)	4420 (3670–5520)	3970 (3440–4950)	3590 (3110–4360)	2950 (2560–3640)	2740 (2340–3370)	1820 (1560–2240)	927 (779–1120)	279 (226–339)	64·9 (49·3–81·2)
Other digestive diseases	–	–	–	3370 (1720–5700)	579 (344–807)	292 (202–387)	328 (209–397)	329 (212–403)	460 (301–564)	602 (426–731)	808 (589–971)	1040 (760–1260)	1220 (938–1480)	1650 (1300–2100)	2000 (1600–2460)	2680 (2150–3280)	2820 (2300–3420)	3150 (2550–3760)	3280 (2700–4000)	2900 (2360–3550)	1730 (1350–2100)	648 (490–839)	208 (147–282)
Neurological disorders	4·66 (3·94–6·06)	13·3 (11·6–15·6)	6090 (4090–7330)	9580 (4780–12500)	3820 (2680–4540)	3830 (3060–4390)	6640 (5540–7330)	6120 (5270–6620)	4940 (4360–5280)	4670 (4200–4970)	5120 (4590–5490)	5250 (4730–5810)	5870 (4920–7820)	7490 (5870–11200)	10400 (7450–17100)	15400 (10000–28500)	22500 (13800–45000)	32900 (19600–66100)	50400 (29600–102000)	68200 (33000–156000)	54300 (22000–131000)	25100 (8440–62100)	9680 (2920–24400)
Alzheimer's disease and other dementias	–	–	–	–	–	–	–	–	–	–	–	167 (16·8–587)	829 (111–2690)	1760 (286–5510)	3520 (631–10200)	6640 (1350–19700)	11100 (2480–33600)	17100 (3750–49200)	27000 (5910–79500)	46000 (10500–135000)	42700 (9960–120000)	22100 (5190–59500)	9060 (2220–23900)
Parkinson's disease	–	–	–	–	–	–	–	9 (6·9–10·4)	11 (8·61–12·4)	21·7 (17·2–24·5)	29 (23·5–33)	281 (234–325)	541 (452–629)	1050 (907–1230)	1700 (1480–1970)	3380 (2990–3880)	6370 (5750–7150)	11900 (10900–13200)	19900 (18500–21700)	19900 (18300–21600)	10600 (9550–11400)	2740 (2360–2980)	553 (444–626)
Idiopathic epilepsy	–	–	3410 (1660–4280)	7160 (2940–9660)	3030 (1930–3660)	2930 (2230–3400)	4820 (3880–5400)	4790 (4020–5230)	4110 (3570–4420)	3840 (3410–4140)	3960 (3520–4270)	3350 (2970–3630)	2750 (2380–2990)	2400 (2090–2610)	2300 (1930–2510)	2140 (1790–2330)	1790 (1470–1960)	1350 (1170–1480)	1160 (1010–1260)	808 (675–893)	375 (326–420)	105 (89·3–119)	25·4 (20·5–28·9)
Multiple sclerosis	–	–	–	–	24·9 (1·32–79·7)	11·2 (1·38–21·7)	14·2 (4·37–22·3)	53·1 (34·3–67·3)	119 (84·7–162)	229 (168–330)	392 (295–567)	539 (400–805)	580 (444–842)	678 (534–1020)	707 (545–1090)	694 (546–1110)	554 (445–867)	366 (291–574)	276 (223–431)	147 (116–215)	55·9 (41–77)	12·4 (8·89–17·1)	2·24 (1·37–2·87)
Motor neuron disease	4·66 (3·94–6·06)	13·3 (11·6–15·6)	781 (620–1050)	151 (113–213)	39·4 (33·9–46·6)	52·2 (46–59·5)	153 (128–180)	111 (91·2–131)	66·3 (59·2–72·8)	99·1 (91–107)	185 (173–198)	298 (278–319)	440 (409–473)	667 (627–712)	1010 (951–1070)	1290 (1230–1350)	1440 (1380–1500)	1130 (1080–1160)	934 (897–968)	481 (450–504)	184 (166–195)	32·8 (28·3–35·6)	6·68 (5·42–7·37)
Other neurological disorders	–	–	1900 (1430–2500)	2270 (1510–3140)	730 (589–954)	841 (747–1000)	1650 (1490–1860)	1150 (1050–1270)	641 (596–707)	480 (449–528)	548 (511–609)	614 (571–686)	732 (681–818)	928 (861–1040)	1180 (1100–1300)	1240 (1170–1350)	1290 (1220–1380)	1130 (1080–1210)	1160 (1110–1220)	837 (779–884)	432 (389–462)	130 (111–141)	33·7 (27–38·4)
Mental disorders	–	–	–	–	0·246 (0·149–0·918)	0·337 (0·272–0·585)	2·15 (1·27–5·98)	3·7 (2·47–7·99)	2·38 (1·76–4·07)	2·86 (2·11–5·36)	2·62 (2·04–4·53)	3·2 (2·27–6·73)	2·82 (2·14–5·72)	–	–	–	–	–	–	–	–	–	–
Eating disorders	–	–	–	–	0·246 (0·149–0·918)	0·337 (0·272–0·585)	2·15 (1·27–5·98)	3·7 (2·47–7·99)	2·38 (1·76–4·07)	2·86 (2·11–5·36)	2·62 (2·04–4·53)	3·2 (2·27–6·73)	2·82 (2·14–5·72)	–	–	–	–	–	–	–	–	–	–
Anorexia nervosa	–	–	–	–	0·153 (0·1–0·582)	0·265 (0·218–0·378)	1·49 (1·1–3·53)	2·88 (2·19–5·57)	1·98 (1·59–2·92)	2·11 (1·71–3·27)	2·19 (1·85–3·44)	2·58 (2·04–5·06)	2·32 (1·93–4)	–	–	–	–	–	–	–	–	–	–
Bulimia nervosa	–	–	–	–	0·0934 (0·0418–0·39)	0·072 (0·0452–0·203)	0·658 (0·117–3·35)	0·821 (0·208–3·82)	0·402 (0·122–1·69)	0·745 (0·235–2·54)	0·428 (0·147–1·69)	0·614 (0·198–2·75)	0·497 (0·141–2·22)	–	–	–	–	–	–	–	–	–	–
Substance use disorders	0 (0–0)	0 (0–0)	1·13 (0·0464–4·21)	0 (0–0)	0 (0–0)	0 (0–0)	2780 (2450–3090)	6360 (5730–6900)	9350 (8610–9880)	13200 (12100–13900)	15900 (14700–16700)	16000 (14700–16900)	14600 (13400–15600)	15900 (14700–16700)	13400 (12400–14200)	10500 (9740–11100)	6290 (5850–6670)	3590 (3300–3840)	2620 (2380–2790)	1150 (1050–1220)	484 (434–518)	121 (102–131)	31·3 (24·4–35·3)
Alcohol use disorders	–	–	–	–	–	0 (0–0)	851 (737–935)	2110 (1850–2290)	4270 (3740–4550)	8020 (7180–8470)	11100 (10200–11700)	12200 (11100–12900)	12100 (11000–12900)	13500 (12300–14200)	11500 (10400–12100)	9050 (8220–9540)	5170 (4760–5450)	2780 (2490–2960)	2020 (1790–2150)	847 (770–898)	352 (311–378)	81·7 (68·5–89·1)	22·6 (17·1–25·7)
Drug use disorders	0 (0–0)	0 (0–0)	1·13 (0·0464–4·21)	0 (0–0)	0 (0–0)	0 (0–0)	1930 (1680–2190)	4250 (3800–4710)	5080 (4700–5460)	5160 (4780–5590)	4770 (4340–5250)	3790 (3380–4190)	2570 (2270–2890)	2400 (2110–2700)	1920 (1680–2170)	1480 (1320–1670)	1120 (998–1260)	802 (713–910)	604 (538–683)	305 (271–342)	132 (117–148)	39 (33·3–44·5)	8·71 (6·83–10·3)
Opioid use disorders	0 (0–0)	0 (0–0)	1·13 (0·0464–4·21)	0 (0–0)	0 (0–0)	0 (0–0)	1010 (877–1130)	2560 (2300–2830)	3320 (3070–3570)	3720 (3430–4040)	3380 (3050–3700)	2640 (2340–2920)	1760 (1540–1990)	1720 (1520–1930)	1360 (1190–1530)	1060 (948–1190)	794 (695–907)	642 (559–738)	492 (435–564)	231 (202–268)	99·7 (86·2–115)	27·6 (23–32·4)	5·87 (4·54–6·92)
Cocaine use disorders	–	–	–	–	–	0 (0–0)	77·6 (69–102)	167 (148–226)	213 (193–264)	282 (257–338)	257 (233–320)	194 (173–251)	135 (118–192)	135 (115–202)	103 (85·5–163)	72·9 (60·9–111)	54·6 (45·6–85·9)	27·9 (23–43·6)	18·2 (15·6–25·4)	3·33 (2·8–4·23)	1·64 (1·37–2·05)	0·573 (0·46–0·726)	0·198 (0·141–0·25)
Amphetamine use disorders	–	–	–	–	–	0 (0–0)	390 (306–509)	680 (524–948)	684 (567–909)	361 (307–491)	369 (304–518)	312 (250–448)	215 (168–313)	199 (154–288)	156 (117–230)	117 (87–172)	89·8 (68·9–134)	5·45 (4·27–8·26)	3·25 (2·67–4·57)	6·13 (5·3–7·18)	3·2 (2·78–3·65)	1·21 (1·03–1·38)	0·361 (0·29–0·421)
Other drug use disorders	–	–	–	–	–	0 (0–0)	452 (370–538)	842 (700–981)	866 (737–980)	792 (695–889)	773 (665–883)	647 (554–756)	457 (382–542)	349 (286–422)	308 (248–381)	230 (185–285)	177 (144–216)	127 (99·4–160)	90·6 (70·7–116)	64·1 (50–83·2)	27·4 (21–36·5)	9·61 (7·21–12·8)	2·28 (1·63–3·19)
Diabetes and kidney diseases	34·4 (25·1–46·2)	108 (80·3–144)	5710 (4150–6980)	5920 (3500–7750)	4230 (3220–5150)	2910 (2550–3280)	6350 (5730–6970)	8420 (7710–9160)	9000 (8330–9660)	12000 (11200–12800)	16300 (15100–17500)	21600 (20100–23100)	27200 (25300–28900)	40700 (38100–43400)	54600 (51000–58300)	71600 (67300–76100)	77000 (72400–81600)	76500 (71800–81500)	75700 (71000–80600)	57300 (52800–61600)	32400 (29000–35000)	11500 (9820–12600)	3290 (2640–3690)
Diabetes mellitus	34·4 (25·1–46·2)	108 (80·3–144)	1050 (659–1320)	802 (443–1170)	612 (397–773)	561 (354–742)	1260 (1150–1390)	2010 (1850–2190)	2450 (2290–2630)	3960 (3740–4210)	5550 (5230–5880)	8630 (8130–9180)	12800 (12100–13700)	20800 (19600–22200)	29300 (27500–31300)	39600 (37200–42000)	42700 (40400–45000)	41000 (38600–43400)	39000 (36800–41300)	27000 (24900–28700)	13600 (12200–14700)	4450 (3830–4850)	1140 (925–1250)
Diabetes mellitus type 1	34·4 (25·1–46·2)	108 (80·3–144)	1050 (659–1320)	802 (443–1170)	612 (397–773)	561 (354–742)	798 (677–945)	1360 (1180–1580)	1590 (1420–1820)	1640 (1400–1990)	2220 (1920–2670)	3120 (2670–3940)	892 (697–1330)	1330 (1060–1950)	1860 (1470–2690)	1860 (1440–2700)	2040 (1510–2940)	1440 (1080–2100)	1300 (936–1810)	485 (343–693)	265 (180–369)	95 (62·9–130)	25·1 (16·2–33·7)
Diabetes mellitus type 2	–	–	–	–	–	–	465 (350–577)	652 (495–807)	862 (679–1020)	2320 (1970–2610)	3330 (2800–3710)	5510 (4620–6130)	11900 (11100–12800)	19500 (18100–20800)	27400 (25600–29200)	37700 (35400–40100)	40700 (38300–42900)	39500 (37100–42000)	37700 (35400–40100)	26500 (24400–28300)	13400 (12000–14400)	4350 (3750–4750)	1110 (902–1230)
Chronic kidney disease	–	–	4360 (2830–5520)	4700 (2580–6220)	3150 (2220–3970)	2120 (1850–2410)	4800 (4280–5340)	6090 (5500–6720)	6250 (5720–6830)	7680 (7070–8370)	10300 (9440–11300)	12500 (11500–13700)	14000 (12800–15300)	19400 (17900–21300)	24700 (22700–27000)	31300 (29100–34100)	33600 (31000–36800)	34900 (32100–38600)	36200 (33400–39900)	30000 (27300–33400)	18600 (16600–20800)	6990 (5950–7890)	2140 (1690–2470)
Chronic kidney disease due to diabetes mellitus type 1	–	–	28·2 (11–60·3)	40·5 (14–85·7)	53·7 (18·2–125)	78·9 (29·1–163)	274 (104–551)	653 (287–1160)	1050 (523–1740)	1750 (996–2600)	2530 (1420–3810)	2910 (1560–4430)	2740 (1300–4370)	2760 (1170–4960)	2320 (831–4620)	2080 (800–4330)	1620 (666–3440)	1190 (499–2400)	771 (296–1670)	334 (128–738)	85·2 (29·7–204)	9·49 (3·03–23)	0·686 (0·194–1·87)
Chronic kidney disease due to diabetes mellitus type 2	–	–	–	–	–	–	146 (44–335)	295 (115–625)	390 (148–783)	634 (228–1220)	1070 (410–2020)	1740 (741–3070)	2750 (1390–4400)	5050 (2820–7360)	7480 (4430–10400)	9860 (5580–13300)	10600 (6650–14300)	10900 (7130–14600)	10500 (6730–14400)	7920 (5120–11000)	4430 (2860–6210)	1500 (919–2170)	423 (227–656)
Chronic kidney disease due to hypertension	–	–	–	–	–	–	369 (194–660)	689 (378–1120)	818 (477–1310)	1080 (673–1680)	1600 (1020–2400)	2190 (1440–3140)	2780 (1940–3900)	4500 (3220–6190)	6530 (4730–8920)	9300 (6820–12300)	10800 (8120–13900)	11800 (9110–14900)	13100 (10200–16700)	11700 (9010–14700)	8030 (6350–10100)	3260 (2520–4040)	1050 (765–1370)
Chronic kidney disease due to glomerulonephritis	–	–	1020 (508–1760)	1400 (611–2370)	982 (474–1610)	812 (439–1270)	1910 (1190–2720)	2170 (1290–3180)	2030 (1290–2880)	2100 (1200–3090)	2580 (1500–4030)	2840 (1410–4430)	2660 (1540–4210)	3330 (1860–5240)	3880 (2130–6370)	4450 (2400–7170)	4280 (2220–7200)	4160 (2270–6760)	4300 (2300–7090)	3390 (1690–5820)	1850 (940–3080)	622 (314–1070)	176 (75·9–354)
Chronic kidney disease due to other and unspecified causes	–	–	3320 (2000–4440)	3270 (1700–4620)	2110 (1350–2940)	1230 (775–1660)	2100 (1290–2960)	2280 (1330–3240)	1960 (1230–2770)	2120 (1250–3250)	2540 (1400–3960)	2860 (1520–4490)	3040 (1570–4700)	3770 (2200–5800)	4510 (2420–7400)	5660 (3190–9240)	6340 (3640–9860)	6870 (3970–10700)	7470 (4410–11500)	6630 (3810–10000)	4220 (2450–6160)	1600 (974–2330)	496 (266–759)
Acute glomerulonephritis	–	–	296 (153–436)	414 (219–630)	465 (263–656)	228 (166–309)	291 (222–393)	323 (244–420)	300 (237–383)	344 (276–428)	452 (359–579)	449 (354–573)	383 (298–501)	507 (404–679)	567 (445–744)	651 (504–854)	704 (543–923)	651 (501–952)	589 (468–785)	382 (298–562)	185 (145–277)	37·2 (29·2–50)	7·31 (5·61–9·32)
Skin and subcutaneous diseases	–	–	1480 (547–2370)	1110 (352–2210)	283 (110–425)	291 (130–388)	418 (209–523)	399 (195–492)	336 (191–415)	607 (344–741)	624 (370–770)	676 (464–891)	852 (573–1120)	1030 (689–1400)	1400 (928–1900)	1840 (1230–2520)	1900 (1340–2630)	2190 (1510–2990)	2690 (1850–3860)	2240 (1490–3330)	1590 (1050–2340)	656 (390–958)	206 (119–348)
Bacterial skin diseases	–	–	1450 (522–2320)	1060 (324–2110)	258 (93·6–391)	267 (112–359)	384 (184–483)	357 (162–446)	292 (155–360)	533 (280–654)	539 (290–664)	575 (357–724)	727 (452–936)	873 (535–1170)	1170 (680–1530)	1510 (887–1960)	1420 (825–1810)	1540 (888–1960)	1740 (1070–2230)	1220 (738–1610)	820 (451–1070)	320 (147–468)	87·1 (42·4–138)
Cellulitis	–	–	329 (94·8–665)	111 (16·7–306)	35·2 (10·8–69·4)	51·8 (20·6–89·9)	110 (36·1–179)	136 (56·8–204)	57 (27·9–94·4)	116 (56·1–198)	166 (83·1–266)	182 (99–317)	238 (114–408)	254 (140–471)	301 (159–536)	378 (204–712)	328 (164–628)	347 (177–654)	369 (176–734)	270 (133–538)	166 (80·6–351)	66·7 (33·9–143)	22·2 (9·46–48·5)
Pyoderma	–	–	1120 (357–2070)	949 (246–2060)	223 (72–360)	215 (82·2–311)	275 (134–375)	221 (86·9–307)	235 (108–296)	417 (180–527)	374 (157–489)	393 (194–505)	489 (238–642)	619 (296–784)	864 (397–1110)	1130 (535–1440)	1090 (536–1390)	1190 (548–1500)	1370 (742–1670)	954 (532–1180)	654 (329–862)	253 (105–368)	64·9 (29·4–99·9)
Decubitus ulcer	–	–	–	8·37 (1·69–21·7)	7·17 (2·34–12)	9·93 (4·02–14·8)	20 (8·58–29·9)	29·4 (13·7–46)	32·4 (15·2–55)	60·1 (37·3–98·6)	64·2 (38·8–119)	72·9 (41·9–146)	89·6 (51·5–179)	127 (73·5–262)	184 (110–387)	273 (150–581)	404 (223–774)	560 (291–1040)	842 (407–1530)	896 (427–1580)	686 (340–1170)	302 (142–534)	108 (49·3–203)
Other skin and subcutaneous diseases	–	–	35·1 (22·2–69·4)	42 (23·8–129)	17·4 (11·8–30·8)	13·4 (9·5–21)	13·8 (9·3–21·2)	12·2 (8·15–19·6)	11·6 (6·79–21·6)	14 (8·93–24·8)	20·7 (12·4–40·2)	27·9 (18·8–48·7)	35·5 (22·7–63)	32·3 (20·1–59·6)	50·8 (33·2–91·2)	61·3 (38·4–115)	74·5 (45·7–144)	84·8 (50·9–158)	114 (62·3–233)	116 (63·7–227)	85 (41·2–175)	33·7 (15·2–76·3)	9·88 (4·38–26)
Musculoskeletal disorders	–	–	–	–	181 (142–216)	258 (218–299)	341 (296–386)	354 (314–409)	358 (316–410)	381 (336–440)	468 (404–541)	559 (486–636)	587 (502–681)	765 (639–871)	1110 (906–1230)	1810 (1510–2020)	2070 (1710–2280)	2240 (1850–2490)	2320 (1880–2540)	1810 (1360–2030)	1270 (827–1440)	444 (276–510)	125 (82·5–147)
Rheumatoid arthritis	–	–	–	–	29 (20·5–38·8)	34·1 (23·5–45·7)	31·7 (25·1–37·7)	32·6 (26·4–39·5)	35·9 (29·3–41·7)	48·4 (40·2–55)	74·7 (61·3–86·5)	110 (91·1–127)	148 (120–171)	255 (209–291)	441 (358–503)	786 (633–897)	987 (785–1110)	1130 (885–1280)	1180 (926–1320)	790 (602–902)	489 (339–564)	131 (89–155)	32 (22·9–40)
Other musculoskeletal disorders	–	–	–	–	152 (118–181)	224 (189–258)	309 (268–352)	322 (286–372)	322 (285–370)	333 (293–388)	393 (342–460)	449 (391–527)	439 (379–518)	510 (427–590)	665 (543–761)	1030 (851–1200)	1080 (898–1260)	1110 (928–1300)	1140 (902–1340)	1030 (747–1180)	785 (487–902)	313 (183–365)	92·7 (58·2–109)
Other non-communicable diseases	151000 (117000–210000)	67700 (44100–99200)	221000 (129000–336000)	83200 (38800–146000)	18400 (13600–23700)	10700 (8990–12500)	9210 (8140–10300)	7630 (6830–8400)	6110 (5480–6760)	5350 (4780–5880)	5740 (5100–6340)	5930 (5190–6510)	5610 (4880–6170)	7080 (6050–7850)	7870 (6800–8690)	10400 (8760–11300)	11800 (10000–13000)	11800 (10000–13200)	12600 (10500–13800)	11100 (9390–12300)	6810 (5830–7650)	2750 (2280–3270)	901 (699–1120)
Congenital birth defects	149000 (115000–208000)	62900 (39800–95900)	182000 (106000–309000)	66200 (26100–127000)	13000 (8840–18000)	6920 (5560–8700)	5020 (4390–5750)	3690 (3230–4210)	2710 (2450–3080)	1820 (1630–2110)	1900 (1690–2160)	1680 (1490–1920)	905 (796–1050)	870 (760–1010)	840 (752–1030)	801 (715–990)	579 (495–682)	–	–	–	–	–	–
Neural tube defects	32200 (21400–49200)	10300 (5970–18300)	22900 (10900–40400)	13100 (3350–26700)	917 (505–1560)	575 (363–892)	317 (214–501)	206 (147–317)	147 (105–255)	111 (82·2–177)	104 (77·3–159)	101 (69–181)	58 (43·6–87·4)	47·4 (36·9–68·1)	45·4 (33·1–69·9)	37·4 (27·2–62·1)	27·9 (21·3–41·9)	–	–	–	–	–	–
Congenital heart anomalies	45300 (33700–60800)	26700 (16800–41500)	86100 (51200–148000)	27000 (12300–52900)	7540 (4880–10500)	4250 (3330–5150)	3230 (2750–3670)	2480 (2150–2820)	1740 (1530–1980)	1160 (1030–1350)	1210 (1060–1380)	1040 (910–1190)	522 (459–610)	491 (420–583)	461 (403–568)	450 (397–552)	321 (266–387)	–	–	–	–	–	–
Orofacial clefts	2910 (1240–5430)	887 (335–1630)	2590 (1000–4920)	172 (61·7–325)	–	–	–	–	–	–	–	–	–	–	–	–	–	–	–	–	–	–	–
Down syndrome	2240 (1680–4730)	1050 (692–2970)	5300 (3560–15500)	2040 (1140–8460)	337 (244–822)	180 (140–317)	167 (140–218)	143 (120–185)	141 (118–184)	121 (104–157)	145 (120–197)	143 (118–195)	109 (94·5–138)	125 (107–161)	130 (111–177)	99·5 (86–137)	47·4 (40·3–63·9)	–	–	–	–	–	–
Other chromosomal abnormalities	3280 (1500–8910)	1590 (611–5050)	2990 (1010–11100)	885 (186–3820)	151 (78·1–373)	85·5 (56–157)	84·9 (64·6–135)	68·7 (53·4–105)	77·1 (56·2–126)	36 (26·5–52·6)	52·6 (36·3–75·6)	31·1 (21–44·2)	19·3 (13·6–28·5)	16·9 (9·98–21·8)	17 (9·94–21·9)	12·9 (6·2–16·4)	10·6 (6·53–13·8)	–	–	–	–	–	–
Congenital musculoskeletal and limb anomalies	3280 (1970–5540)	883 (504–1600)	2180 (1180–4240)	721 (278–1920)	254 (159–423)	162 (111–215)	195 (141–250)	103 (71·2–133)	75·4 (54·4–98·6)	51·6 (39·3–64·6)	51·9 (37·6–67·5)	41·1 (29·1–53·8)	24·8 (17·6–30·6)	25·3 (16·7–31·3)	25·6 (17·1–35·4)	34·9 (23·6–47·2)	32·9 (16·8–40)	–	–	–	–	–	–
Urogenital congenital anomalies	4570 (2520–8340)	1260 (588–2410)	2150 (1040–3960)	519 (179–1090)	109 (65·8–174)	63·7 (45·1–97·1)	47·9 (33·4–58·5)	40·2 (28·8–47·7)	39 (27·7–46)	28·5 (22–33·4)	30·5 (23·9–35·8)	29·4 (23·8–35·3)	15·5 (12·3–18·2)	15·8 (13–20·6)	15·7 (12·7–21·5)	19·1 (15·8–28)	17·3 (14·1–26·9)	–	–	–	–	–	–
Digestive congenital anomalies	13000 (7200–21300)	6710 (3000–13200)	17600 (7140–37700)	3080 (786–8010)	311 (165–587)	133 (86–220)	53·6 (41·6–69·2)	34·4 (26·3–42·1)	38 (29–48·1)	23·7 (18–28·9)	29·3 (21·5–36·4)	27 (19·1–33·8)	21·8 (15·3–25·7)	25 (18·3–30·1)	32·1 (24·2–39·9)	36·2 (30·4–51·1)	36·2 (27·8–44·4)	–	–	–	–	–	–
Other congenital birth defects	42200 (23000–74600)	13500 (5530–26000)	40400 (16000–85200)	18700 (4670–46100)	3410 (1800–6100)	1470 (987–2380)	927 (753–1290)	620 (508–803)	451 (368–580)	283 (234–362)	285 (233–361)	264 (206–351)	135 (111–172)	124 (102–156)	113 (95·9–146)	111 (92·1–146)	85·6 (72·8–109)	–	–	–	–	–	–
Urinary diseases and male infertility	399 (286–471)	411 (264–536)	2100 (1450–2590)	2000 (961–2670)	1010 (684–1250)	551 (448–653)	968 (753–1120)	1230 (978–1390)	1130 (876–1280)	1390 (1090–1570)	1640 (1330–1860)	2080 (1610–2330)	2550 (1930–2880)	3480 (2710–3920)	4110 (3300–4590)	6000 (4730–6620)	7310 (5800–8160)	7860 (6530–8820)	8620 (7060–9530)	7960 (6680–9140)	4870 (4160–5840)	2040 (1690–2540)	688 (519–889)
Urolithiasis	–	–	–	77·2 (25·2–181)	56·4 (23·1–118)	33 (13–53·4)	36·6 (11·2–54·7)	50 (18·1–70·7)	68·4 (24·3–97·8)	81 (31·9–110)	147 (54·3–203)	198 (84·5–276)	243 (96·5–342)	397 (182–540)	469 (221–625)	713 (340–956)	836 (408–1130)	823 (404–1110)	870 (460–1140)	619 (327–831)	317 (178–421)	94·9 (51·6–127)	21·2 (13–27·7)
Other urinary diseases	286 (205–344)	193 (120–278)	405 (233–639)	696 (309–1050)	239 (156–352)	147 (107–200)	271 (203–328)	206 (137–290)	193 (130–243)	368 (275–458)	441 (324–572)	528 (378–674)	760 (556–970)	862 (561–1110)	1210 (889–1520)	1500 (1030–1910)	1910 (1360–2470)	2110 (1450–2690)	1830 (1280–2370)	1870 (1350–2340)	965 (743–1280)	390 (291–520)	106 (73·3–139)
Hemoglobinopathies and hemolytic anemias	622 (514–793)	535 (349–704)	4040 (1850–6900)	11800 (5800–19100)	3230 (2500–4360)	2380 (1990–3070)	2480 (2020–3380)	1950 (1640–2570)	1390 (1170–1840)	1060 (894–1430)	997 (831–1340)	887 (727–1220)	789 (644–1090)	1060 (868–1460)	1030 (854–1460)	1380 (1150–1870)	1510 (1270–2130)	1550 (1290–2230)	1530 (1300–2130)	1190 (1020–1590)	691 (590–905)	249 (205–324)	65·1 (51·2–83·5)
Thalassemias	338 (292–391)	297 (203–375)	1860 (974–2460)	3050 (1590–4150)	1130 (899–1340)	634 (533–762)	510 (395–667)	360 (268–483)	211 (155–278)	150 (105–206)	159 (111–219)	136 (95·1–192)	106 (72·8–150)	106 (68·8–150)	54·8 (30·5–85·4)	18·2 (9·12–31)	3·58 (1·72–6·57)	0·616 (0·271–1·2)	0·112 (0·0431–0·226)	0·0152 (0·00473–0·0376)	0·0015 (0·000383–0·00425)	8·79e-05 (1·9e-05–0·000241)	3·89e-06 (6·46e-07–1·21e-05)
Sickle cell disorders	229 (132–378)	216 (108–335)	2010 (681–4390)	8260 (3870–14600)	1770 (1260–2690)	1300 (989–1880)	1340 (999–1990)	964 (737–1390)	667 (507–1010)	448 (329–674)	401 (285–635)	314 (213–518)	196 (122–336)	143 (81·6–274)	32·7 (19·7–58·7)	13·7 (8·05–21·9)	3·83 (2·01–6·49)	0·937 (0·444–1·73)	0·218 (0·0877–0·439)	0·0447 (0·0161–0·0976)	0·00776 (0·00222–0·018)	0·000881 (0·000225–0·0022)	8·86e-05 (2·21e-05–0·000234)
G6PD deficiency	11·3 (6·69–17·1)	10·6 (5·79–16·4)	94·3 (43·8–158)	336 (143–566)	240 (135–351)	323 (198–455)	417 (252–594)	389 (242–546)	325 (209–443)	301 (194–426)	274 (167–381)	245 (151–357)	277 (171–436)	406 (234–664)	350 (201–611)	368 (209–616)	265 (130–474)	167 (84·8–311)	90·1 (41·3–171)	34·9 (16·9–65)	13 (6·37–22·8)	2·87 (1·42–5·08)	0·446 (0·181–0·827)
Other hemoglobinopathies and hemolytic anemias	43·4 (32·4–55·7)	11·8 (7·99–16·2)	78·1 (45·5–113)	119 (65·6–171)	88·9 (70–113)	127 (101–160)	212 (158–299)	237 (182–320)	186 (145–247)	163 (125–223)	163 (127–218)	192 (151–252)	210 (163–278)	406 (315–564)	594 (472–824)	975 (798–1310)	1240 (1030–1710)	1380 (1150–1960)	1440 (1230–1980)	1150 (993–1530)	678 (579–884)	246 (203–320)	64·6 (50·7–82·6)
Endocrine, metabolic, blood, and immune disorders	914 (555–1100)	933 (488–1270)	5110 (2620–8070)	3230 (1510–4980)	1150 (779–1590)	815 (611–1050)	737 (541–864)	751 (552–890)	891 (641–1030)	1070 (786–1240)	1200 (852–1380)	1290 (938–1490)	1360 (1000–1690)	1670 (1250–2060)	1880 (1390–2330)	2230 (1680–2720)	2450 (1860–3170)	2400 (1820–2940)	2420 (1750–3020)	1960 (1330–2360)	1250 (850–1500)	457 (318–577)	148 (107–210)
Sudden infant death syndrome	–	3000 (1200–6780)	27900 (11400–61600)	–	–	–	–	–	–	–	–	–	–	–	–	–	–	–	–	–	–	–	–
Injuries	10600 (8060–12300)	9470 (6720–13100)	77600 (66200–87700)	237000 (187000–277000)	133000 (118000–146000)	96300 (87900–104000)	213000 (201000–224000)	286000 (273000–298000)	251000 (241000–260000)	224000 (215000–232000)	212000 (2e+05–221000)	176000 (165000–186000)	148000 (138000–156000)	147000 (138000–156000)	131000 (123000–139000)	117000 (109000–124000)	95600 (88300–101000)	77300 (70300–82100)	67200 (61900–71200)	48900 (44300–52200)	26500 (23800–28300)	8670 (7460–9420)	2290 (1830–2570)
Transport injuries	1730 (608–2340)	1280 (539–1940)	12200 (8790–15400)	51800 (38200–65200)	41100 (35600–46900)	28100 (25500–31300)	78800 (73400–89600)	103000 (97000–117000)	87900 (83000–98900)	75500 (71000–83000)	72700 (67300–81700)	59300 (54400–67400)	48800 (44700–56300)	47300 (43200–54500)	41800 (38500–48400)	35400 (32600–40500)	28200 (25600–32900)	21100 (19000–24300)	16400 (15000–18900)	9700 (8830–10800)	4190 (3730–4640)	1040 (889–1150)	240 (192–273)
Road injuries	1560 (548–2120)	1160 (494–1780)	11500 (8200–14500)	48600 (36200–62200)	38900 (33700–44400)	26200 (23800–29300)	73600 (68700–84200)	96700 (90900–111000)	81700 (77200–92300)	70500 (66200–77900)	67500 (62500–76500)	54800 (50200–62300)	44900 (41200–51800)	43500 (39600–50300)	38500 (35400–44700)	32900 (30400–37800)	26400 (23900–30800)	19800 (17800–22900)	15400 (14100–17800)	9170 (8340–10200)	3970 (3550–4410)	980 (840–1090)	225 (180–255)
Pedestrian road injuries	715 (230–1040)	443 (191–612)	4840 (2370–6330)	26400 (15000–35600)	22600 (16700–27200)	13200 (10400–15800)	21800 (16500–27300)	28600 (21600–36500)	25500 (19600–32100)	24500 (19000–30700)	25800 (19700–32300)	21600 (16800–27200)	18300 (14300–22900)	19800 (15900–24400)	18000 (14600–22400)	16800 (14000–20500)	13700 (11500–16600)	10800 (8980–12900)	9150 (8010–10700)	5520 (4830–6280)	2470 (2150–2810)	575 (483–654)	133 (104–153)
Cyclist road injuries	–	–	–	2470 (1200–4890)	2330 (1700–3730)	2040 (1640–2920)	2940 (2310–4020)	2540 (2010–3480)	2170 (1690–2940)	2230 (1770–3000)	2340 (1790–3260)	2380 (1790–3360)	2290 (1720–3200)	2620 (1950–3670)	2340 (1740–3290)	2340 (1730–3180)	1930 (1450–2670)	1330 (998–1790)	964 (784–1220)	542 (457–637)	190 (159–223)	40 (32·8–47·2)	0·348 (0·269–0·407)
Motorcyclist road injuries	247 (45·1–438)	230 (42·5–508)	1730 (857–3320)	4740 (2080–9690)	2810 (2000–4320)	2910 (2280–3860)	19400 (16100–24100)	24700 (20400–30800)	18900 (15400–22800)	13900 (11200–17000)	12600 (10000–15800)	9830 (7580–12500)	7390 (5680–9440)	5830 (4380–7740)	4790 (3580–6330)	2940 (2180–3860)	2240 (1660–2930)	1260 (961–1630)	712 (563–891)	409 (319–508)	132 (100–169)	28·7 (21·7–37·8)	6·68 (4·94–8·86)
Motor vehicle road injuries	575 (244–873)	470 (198–780)	4730 (2630–7190)	14500 (8010–24900)	10800 (7600–15400)	7730 (6240–9640)	28800 (25700–33300)	40000 (35900–46300)	34300 (30400–39600)	29400 (25500–33500)	26200 (22200–30700)	20500 (17300–24400)	16400 (13800–19400)	14700 (12300–17600)	12900 (10700–15500)	10500 (8420–12600)	8200 (6730–9850)	6190 (5000–7440)	4420 (3720–5260)	2570 (2200–3050)	1130 (950–1330)	327 (271–386)	81·9 (63·7–97·6)
Other road injuries	24·5 (7·48–54·5)	19·7 (9·8–43·8)	169 (80·8–383)	503 (201–1320)	398 (269–655)	322 (253–451)	610 (483–781)	827 (681–1040)	724 (597–903)	528 (432–663)	498 (404–638)	442 (358–558)	451 (362–578)	504 (400–644)	434 (351–558)	340 (271–421)	312 (239–397)	253 (190–322)	188 (140–237)	120 (86·3–155)	50·5 (35·9–66·5)	9·96 (6·72–13·9)	3·24 (2·1–4·79)
Other transport injuries	171 (60·9–239)	116 (44·7–192)	773 (440–1050)	3180 (1640–5790)	2170 (1600–2710)	1920 (1530–2270)	5250 (4290–6250)	6450 (5540–7390)	6180 (5390–7100)	5040 (4420–5770)	5250 (4530–6070)	4560 (3850–5380)	3950 (3270–4660)	3840 (3260–4540)	3290 (2760–3850)	2420 (2110–2880)	1820 (1570–2210)	1270 (1080–1520)	969 (808–1160)	529 (449–627)	213 (182–257)	55·6 (46·8–67·9)	14·8 (11·5–19·1)
Unintentional injuries	7300 (5800–8510)	7620 (5400–10900)	61500 (52100–70500)	173000 (134000–204000)	83400 (70600–93500)	52300 (46400–58100)	60200 (53900–66700)	66500 (60400–73200)	57500 (53500–62600)	55000 (51500–59500)	57000 (53000–61500)	52100 (48500–56400)	47600 (43700–51600)	51700 (47900–55700)	49000 (45100–52900)	48400 (43900–52600)	41800 (37300–45800)	36600 (31900–40200)	34800 (30700–37900)	29100 (25700–31900)	17700 (15700–19300)	6600 (5670–7240)	1840 (1480–2070)
Falls	789 (508–991)	487 (247–684)	5630 (2740–7950)	12300 (5560–19300)	6620 (4170–8930)	4740 (3500–5760)	7020 (5840–8790)	10200 (8810–12300)	9980 (8710–11900)	10300 (8950–12500)	12200 (10600–14900)	12400 (10900–15400)	11500 (10000–13900)	13800 (12400–16200)	14700 (12900–17800)	16000 (13700–18900)	15600 (13200–18300)	16200 (13200–18600)	17700 (14900–20200)	17000 (14800–18800)	11300 (9930–12400)	4530 (3880–4970)	1270 (1020–1430)
Drowning	533 (384–647)	312 (190–385)	8230 (5530–9940)	91600 (68500–110000)	47700 (38600–54100)	26400 (23000–29700)	24300 (21000–28200)	19500 (16900–22500)	13400 (12100–15300)	11900 (10600–13600)	11000 (9820–12400)	9160 (8170–10400)	7290 (6410–8220)	7530 (6670–8590)	6120 (5450–6910)	5600 (4910–6430)	4170 (3630–4910)	3280 (2850–3950)	2420 (2160–2850)	1690 (1460–2060)	771 (651–951)	164 (137–200)	35·3 (28·5–41·5)
Fire, heat, and hot substances	302 (202–359)	276 (173–341)	4520 (2440–5630)	14100 (6660–18300)	3650 (2450–4310)	1770 (1250–2070)	2310 (1590–2660)	3320 (2390–3820)	3380 (2570–3770)	3470 (2760–3840)	3610 (2860–4000)	3120 (2580–3480)	2830 (2310–3200)	3220 (2730–3620)	2930 (2430–3280)	3020 (2500–3380)	2560 (2070–2870)	2410 (1900–2700)	2220 (1800–2480)	1880 (1520–2100)	1110 (919–1250)	370 (304–412)	103 (80·6–117)
Poisonings	318 (219–419)	394 (231–565)	3660 (2550–4530)	11000 (7250–13800)	2620 (2030–3040)	2090 (1620–2510)	2660 (2150–3260)	3290 (2720–3990)	3110 (2710–3680)	3100 (2720–3620)	3440 (3080–3930)	3320 (3000–3790)	2940 (2570–3400)	3560 (3160–4070)	2990 (2670–3460)	2790 (2410–3230)	1830 (1610–2180)	1420 (1230–1720)	1070 (956–1260)	672 (592–798)	295 (256–351)	71·2 (59·3–83·5)	15 (12·1–17·5)
Poisoning by carbon monoxide	56·5 (31·6–81)	61·7 (31·3–93·2)	737 (344–1080)	1960 (1040–2950)	885 (625–1140)	762 (544–1010)	1610 (1230–2010)	1910 (1490–2370)	1800 (1490–2190)	2000 (1730–2350)	2280 (1990–2620)	2190 (1920–2550)	1870 (1630–2210)	2390 (2150–2750)	1850 (1630–2200)	1700 (1460–2030)	1000 (833–1260)	767 (612–989)	635 (529–786)	397 (330–489)	180 (151–222)	41·7 (34·5–50·5)	7·28 (5·73–8·66)
Poisoning by other means	262 (171–359)	332 (175–488)	2920 (1890–3770)	8990 (5410–11800)	1730 (1170–2100)	1330 (954–1630)	1060 (739–1450)	1380 (977–1850)	1310 (1010–1720)	1100 (838–1460)	1160 (881–1490)	1130 (843–1450)	1070 (728–1390)	1180 (843–1560)	1140 (846–1520)	1090 (790–1420)	829 (602–1120)	654 (493–891)	440 (332–572)	275 (209–362)	115 (90–152)	29·5 (23·7–36·9)	7·72 (6·06–9·25)
Exposure to mechanical forces	628 (334–1080)	1750 (267–4740)	3680 (2010–5700)	6130 (3500–9360)	3880 (2980–4800)	3070 (2620–3570)	6950 (6040–8390)	9760 (8350–11900)	8640 (7570–10300)	7990 (7080–9680)	8400 (7360–10400)	6800 (5950–8560)	6030 (5240–7350)	5780 (5070–7000)	4820 (4180–5840)	3760 (3220–4470)	2690 (2210–3240)	1960 (1610–2410)	1520 (1260–1840)	837 (701–1050)	413 (349–537)	135 (111–171)	39·9 (30·7–53·9)
Unintentional firearm injuries	98·5 (24·9–211)	306 (16·1–994)	658 (167–1280)	1070 (518–1800)	677 (454–1000)	853 (669–1080)	2140 (1620–2630)	2990 (2100–3690)	2390 (1720–2970)	2010 (1400–2550)	1810 (1180–2290)	1420 (955–1820)	1240 (792–1610)	1030 (648–1340)	785 (495–1030)	623 (415–796)	465 (303–600)	361 (239–474)	282 (188–372)	169 (110–221)	81·2 (53·8–111)	27·6 (19·4–37·1)	7·79 (5·29–11·4)
Other exposure to mechanical forces	529 (266–925)	1440 (228–4000)	3020 (1640–4980)	5060 (2790–7840)	3200 (2410–4070)	2220 (1810–2680)	4810 (4060–6080)	6770 (5710–8570)	6250 (5310–7910)	5980 (5170–7480)	6590 (5660–8440)	5370 (4620–7150)	4790 (4130–6040)	4750 (4100–5970)	4030 (3460–5020)	3140 (2690–3790)	2230 (1820–2730)	1600 (1320–2030)	1230 (1010–1550)	668 (551–871)	332 (276–439)	107 (85·7–140)	32·1 (24·3–44·5)
Adverse effects of medical treatment	827 (560–1110)	621 (348–794)	4770 (2230–6520)	5380 (2030–8180)	1520 (998–1950)	919 (624–1240)	979 (576–1450)	1370 (827–1960)	1120 (712–1560)	1200 (772–1660)	1360 (896–1850)	1570 (1030–2150)	1580 (1070–2170)	1930 (1310–2600)	2580 (1800–3470)	3670 (2560–4930)	4160 (2890–5690)	3470 (2440–4780)	3270 (2380–4420)	2380 (1770–3230)	1310 (1000–1730)	446 (332–590)	119 (87·5–153)
Animal contact	453 (242–581)	517 (239–719)	3040 (1500–3990)	10100 (4360–14000)	6980 (3720–9040)	4190 (2520–5610)	2950 (1860–3810)	2810 (1670–3550)	2700 (1750–3520)	2500 (1600–3200)	2270 (1350–2870)	2700 (1710–3430)	3780 (2350–4750)	2930 (1890–3620)	3520 (2130–4410)	3340 (2030–4230)	3140 (1940–4000)	2220 (1390–2900)	1710 (1090–2250)	926 (590–1260)	306 (208–423)	69·4 (47·6–97·5)	17·3 (12·2–24·6)
Venomous animal contact	393 (196–517)	439 (184–639)	2190 (975–3010)	7340 (2960–10500)	5790 (2940–7690)	3620 (2130–4990)	2520 (1510–3310)	2380 (1370–3050)	2320 (1490–3070)	2130 (1340–2770)	1840 (1030–2350)	2260 (1350–2910)	3300 (1930–4180)	2310 (1380–2840)	2880 (1590–3600)	2680 (1460–3400)	2550 (1420–3240)	1760 (988–2260)	1350 (792–1730)	727 (429–957)	221 (138–295)	46·6 (30–61·6)	10·7 (7·2–14·3)
Non-venomous animal contact	59·1 (42·4–79·9)	77·6 (46·9–103)	852 (450–1190)	2730 (1150–4090)	1190 (688–1540)	564 (367–754)	430 (309–579)	433 (289–583)	383 (270–496)	367 (248–461)	434 (309–555)	439 (317–567)	480 (339–667)	611 (431–878)	644 (454–949)	662 (466–968)	591 (427–890)	460 (327–719)	359 (256–566)	199 (140–317)	85·3 (61·4–135)	22·7 (16·2–36·6)	6·53 (4·58–10·4)
Foreign body	2460 (2080–2860)	2770 (2300–3260)	22600 (18700–27100)	10000 (7420–12200)	2450 (2210–2720)	1770 (1650–1920)	1350 (1260–1440)	1790 (1680–1900)	1860 (1780–1950)	1950 (1880–2030)	2130 (2050–2220)	2120 (2040–2210)	2020 (1940–2110)	2430 (2330–2530)	2270 (2180–2370)	2300 (2200–2410)	1990 (1890–2080)	1790 (1700–1880)	1890 (1790–1960)	1710 (1590–1800)	1110 (993–1180)	447 (385–483)	135 (108–150)
Pulmonary aspiration and foreign body in airway	2390 (2020–2790)	2650 (2200–3110)	19500 (16000–23600)	7510 (5570–9450)	2160 (1940–2410)	1650 (1540–1790)	1210 (1130–1290)	1640 (1540–1750)	1740 (1660–1830)	1840 (1770–1920)	1970 (1900–2070)	1950 (1870–2030)	1880 (1800–1980)	2300 (2210–2410)	2160 (2070–2250)	2160 (2070–2260)	1860 (1770–1960)	1660 (1570–1740)	1760 (1670–1840)	1630 (1520–1720)	1060 (956–1140)	434 (374–469)	132 (106–146)
Foreign body in other body part	75·4 (41·8–110)	123 (52·1–207)	3110 (1210–5130)	2480 (1150–3950)	293 (164–416)	126 (80·4–165)	142 (100–176)	156 (109–200)	126 (92·4–157)	116 (85·4–143)	161 (122–200)	171 (127–209)	132 (94·5–169)	123 (89·9–153)	117 (86·5–147)	142 (103–180)	124 (90·1–160)	138 (100–178)	128 (92·5–160)	84·5 (60·5–108)	44 (31·2–56·5)	13·3 (9·44–16·8)	3·05 (2·23–3·72)
Environmental heat and cold exposure	244 (69·7–350)	194 (39·2–309)	1900 (394–2760)	1350 (425–1990)	537 (248–687)	393 (216–480)	1200 (793–1400)	1710 (1100–2010)	1980 (1460–2250)	2560 (2010–2900)	3100 (2400–3530)	3140 (2400–3550)	2850 (2180–3270)	4250 (3280–4850)	3520 (2610–4070)	3410 (2390–4020)	2280 (1340–2770)	1700 (930–2110)	1640 (890–1990)	1180 (646–1460)	633 (391–777)	188 (135–227)	46 (35·6–53·6)
Exposure to forces of nature	31·9 (29–35·1)	77·4 (70·3–85·1)	860 (781–947)	4200 (3810–4620)	2410 (2190–2650)	2790 (2540–3070)	2920 (2650–3220)	2750 (2490–3020)	2370 (2150–2600)	1860 (1690–2050)	1520 (1380–1670)	1230 (1120–1360)	1110 (1010–1220)	1590 (1440–1740)	1540 (1400–1700)	1350 (1220–1480)	1080 (984–1190)	768 (697–845)	288 (261–317)	129 (117–141)	98·4 (89·3–108)	36·4 (33·1–40·1)	6·53 (5·93–7·18)
Other unintentional injuries	708 (252–1110)	219 (147–300)	2590 (1560–3620)	7290 (4490–9620)	5080 (3110–6540)	4100 (2760–5390)	7550 (5690–9320)	9950 (7900–12400)	9000 (7490–11100)	8250 (6830–10100)	7920 (6630–9620)	6520 (5550–7990)	5670 (4400–7160)	4700 (3830–5840)	3950 (3190–4940)	3120 (2430–3950)	2260 (1740–2920)	1330 (1060–1710)	1120 (894–1450)	707 (552–945)	377 (298–518)	139 (98·7–218)	45·9 (30·6–75·2)
Self-harm and interpersonal violence	1620 (1500–1740)	571 (515–649)	3930 (3450–4740)	11900 (10600–14100)	8240 (7650–8850)	15900 (13500–17500)	73500 (64800–78300)	116000 (104000–122000)	106000 (96000–111000)	93000 (84200–97000)	81900 (72400–86200)	64600 (57300–68500)	51300 (45000–54800)	48400 (42300–51900)	40100 (34800–43700)	33700 (28700–36900)	25500 (21300–28200)	19600 (15900–21800)	16000 (13400–17600)	10100 (8430–11100)	4600 (3880–5000)	1040 (889–1130)	216 (176–239)
Self-harm	–	–	–	–	–	6660 (4560–7790)	31400 (24100–34800)	50200 (40000–54700)	48300 (40000–52300)	45400 (38200–48700)	44400 (36300–47700)	37900 (31700–41000)	32100 (26500–35300)	32900 (27300–36200)	28700 (23700–32200)	25100 (20600–28100)	19800 (16000–22400)	15800 (12400–17900)	13600 (11100–15200)	9210 (7610–10200)	4230 (3520–4620)	937 (794–1020)	153 (123–171)
Self-harm by firearm	–	–	–	–	–	323 (226–400)	2940 (2470–3310)	4820 (4050–5670)	4620 (3970–5500)	4430 (3820–5310)	3890 (3240–4500)	3450 (2790–4050)	3130 (2520–3670)	2850 (2360–3290)	2660 (2250–3080)	2530 (2230–2900)	2330 (2140–2750)	1880 (1780–2260)	1840 (1710–2260)	1260 (1130–1640)	588 (508–800)	151 (125–201)	29·3 (22·6–34·9)
Self-harm by other specified means	–	–	–	–	–	6340 (4310–7400)	28400 (20900–31800)	45400 (35300–49900)	43700 (35400–47600)	41000 (33900–44300)	40500 (32800–43700)	34400 (28300–37500)	29000 (23600–32100)	30000 (24600–33400)	26000 (21200–29600)	22600 (18100–25600)	17500 (13500–20100)	13900 (10300–16000)	11800 (9010–13300)	7950 (6110–8930)	3640 (2800–4020)	785 (646–865)	124 (100–139)
Interpersonal violence	1160 (1050–1280)	330 (280–405)	3170 (2700–3990)	5750 (4520–7970)	4170 (3690–4690)	5380 (4860–5890)	30600 (28300–32500)	47600 (44400–50000)	45400 (42500–47500)	39100 (36800–40900)	31600 (29700–33000)	23100 (21600–24200)	16700 (15500–17600)	13800 (12700–14500)	9980 (9170–10600)	7420 (6780–7910)	4860 (4390–5210)	3140 (2760–3410)	2020 (1810–2200)	631 (557–689)	265 (231–294)	64·1 (53·4–71·8)	45·5 (35·1–53·1)
Physical violence by firearm	143 (120–169)	44·2 (35·1–61·2)	341 (279–446)	806 (613–1160)	613 (533–742)	1490 (1380–1640)	13300 (12600–14000)	21500 (20500–22600)	19800 (18900–20800)	15900 (15100–16900)	12100 (11500–12900)	8480 (7970–8980)	5800 (5420–6190)	4320 (4000–4650)	3000 (2770–3250)	1920 (1770–2070)	1200 (1100–1300)	703 (628–770)	450 (399–502)	139 (119–159)	53·6 (45·2–60·5)	14·2 (11·5–16·4)	12 (8·97–14·3)
Physical violence by sharp object	115 (84·9–139)	33·9 (22·4–49·3)	374 (236–564)	1080 (731–1670)	785 (505–964)	1020 (682–1200)	8580 (6320–9670)	13400 (10300–14900)	13300 (10700–14600)	11600 (9270–12800)	9320 (7490–10200)	6500 (5140–7250)	4650 (3550–5190)	3780 (2900–4270)	2550 (1940–2910)	2060 (1550–2350)	1330 (988–1550)	770 (574–898)	485 (384–557)	172 (140–192)	68·1 (55·6–76·5)	17·9 (14·6–20·1)	13·8 (10·4–16·2)
Physical violence by other means	907 (806–1010)	252 (214–316)	2460 (2060–3200)	3860 (2990–5530)	2770 (2390–3270)	2870 (2490–3260)	8690 (7560–9870)	12700 (11100–14100)	12200 (11000–13500)	11600 (10400–12600)	10100 (9040–11000)	8100 (7270–8770)	6230 (5520–6760)	5670 (5130–6100)	4430 (3970–4810)	3430 (3050–3720)	2330 (2050–2550)	1670 (1420–1850)	1090 (948–1210)	319 (274–353)	143 (123–161)	32 (26·5–36·5)	19·7 (15·3–23·7)
Conflict and terrorism	455 (425–493)	241 (225–261)	730 (681–791)	6060 (5630–6590)	4020 (3730–4380)	3850 (3570–4200)	11200 (10300–12200)	17700 (16300–19400)	11200 (10400–12300)	7610 (7020–8340)	5220 (4810–5720)	3220 (2970–3530)	2210 (2040–2420)	1570 (1450–1720)	1310 (1210–1440)	1060 (980–1160)	797 (738–870)	594 (552–646)	384 (354–421)	224 (205–248)	103 (93·4–114)	37·5 (34·1–41·6)	16·2 (14·9–17·9)

SR Table 5: Global deaths by cause and age group for males (with 95% uncertainty levels), 2010

Cause	Early Neonatal	Late Neonatal	Post Neonatal	1 to 4
All causes	1430000 (1300000–1560000)	372000 (335000–414000)	1060000 (959000–1170000)	1060000 (947000–1180000)
Communicable, maternal, neonatal, and nutritional diseases	1290000 (1180000–1420000)	320000 (287000–358000)	840000 (750000–936000)	823000 (726000–931000)
HIV/AIDS and sexually transmitted infections	26000 (8810–54100)	7980 (2660–16900)	34500 (27000–46900)	43200 (40400–46000)
HIV/AIDS	--	--	23600 (22200–25000)	42800 (40100–45600)
HIV/AIDS - Drug-susceptible Tuberculosis	--	--	6660 (4950–8150)	12900 (9540–15800)
Tuberculosis without extensive drug resistance	--	--	626 (294–1150)	1100 (497–2040)
HIV/AIDS - Extensively drug-resistant Tuberculosis	--	--	13·3 (6·09–24·6)	13·2 (5·72–25)
HIV/AIDS resulting in other diseases	--	--	16300 (14500–18600)	28800 (25400–33500)
Sexually transmitted infections excluding HIV	26000 (8810–54100)	7980 (2660–16900)	10900 (3630–23500)	410 (139–916)
Syphilis	26000 (8810–54100)	7980 (2660–16900)	10900 (3630–23500)	410 (139–916)
Chlamydial infection	--	--	--	--
Gonococcal infection	--	--	--	--
Other sexually transmitted infections	--	--	--	--
Respiratory infections and tuberculosis	97400 (86000–111000)	66100 (57800–74900)	269000 (234000–313000)	156000 (126000–188000)
Tuberculosis	--	--	22000 (18400–26400)	22600 (17500–29000)
Drug-susceptible tuberculosis	--	--	20300 (16700–24400)	21000 (16100–27100)
Multidrug-resistant tuberculosis without extensive drug resistance	--	--	1650 (794–2920)	1550 (698–2860)
Extensively drug-resistant tuberculosis	--	--	66·6 (32·2–118)	45·3 (20·6–84·6)
Lower respiratory infections	97200 (85900–111000)	66000 (57700–74800)	245000 (214000–285000)	131000 (106000–160000)
Upper respiratory infections	34·9 (18–53)	78 (33·2–137)	1670 (396–3890)	2040 (248–5910)
Otitis media	154 (3·88–751)	50·7 (6·03–216)	45·8 (16·4–102)	27·4 (5·61–82·7)
Enteric infections	18400 (13700–23100)	30200 (23300–37500)	223000 (184000–261000)	221000 (171000–271000)
Diarrheal diseases	18400 (13700–23100)	29700 (22900–36900)	215000 (177000–251000)	181000 (138000–226000)
Typhoid and paratyphoid	--	--	76·6 (29·9–182)	15800 (5980–31400)
Typhoid fever	--	--	69 (26·8–161)	13700 (5410–27100)
Paratyphoid fever	--	--	7·57 (2·2–20)	2050 (641–4750)
Invasive Non-typhoidal Salmonella (iNTS)	--	521 (280–866)	7410 (4140–11900)	24000 (13200–38900)
Other intestinal infectious diseases	--	--	193 (74–371)	364 (174–619)
Neglected tropical diseases and malaria	292 (126–1150)	14100 (6890–24100)	85500 (43400–140000)	192000 (110000–298000)
Malaria	0 (0–0)	13700 (6410–23700)	82600 (40400–137000)	181000 (97100–289000)
Chagas disease	--	--	0·939 (0·279–1·57)	0·307 (0·138–0·815)
Leishmaniasis	--	--	270 (50·5–953)	2170 (498–8770)
Visceral leishmaniasis	--	--	270 (50·5–953)	2170 (498–8770)
African trypanosomiasis	--	--	--	93·1 (19·7–319)
Schistosomiasis	--	--	2·05 (0·261–5·5)	110 (72·4–157)
Cysticercosis	--	--	--	12 (0·552–35·9)
Cystic echinococcosis	--	--	--	118 (49–208)
Dengue	0·0251 (0·0228–0·0276)	206 (9·97–358)	1200 (169–1790)	2710 (533–3850)
Yellow fever	--	2·8 (0·956–6·25)	42·6 (14·5–95·1)	524 (170–1180)
Rabies	--	--	158 (43·5–276)	1620 (411–3730)
Intestinal nematode infections	--	--	120 (91·1–156)	1290 (956–1700)
Ascariasis	--	--	120 (91·1–156)	1290 (956–1700)
Ebola	0 (0–0)	0 (0–0)	0 (0–0)	0 (0–0)
Zika virus	0 (0–0)	0 (0–0)	0 (0–0)	0 (0–0)
Other neglected tropical diseases	292 (126–1150)	237 (115–837)	1190 (616–4140)	2200 (683–9890)
Other infectious diseases	17600 (14500–21900)	17200 (14400–20700)	121000 (90200–166000)	167000 (114000–244000)
Meningitis	6860 (5810–8170)	7170 (6070–8460)	45300 (38400–53400)	44400 (34900–54400)
Encephalitis	359 (288–455)	511 (404–678)	4870 (3880–6220)	5710 (4370–7770)
Diphtheria	--	--	591 (367–930)	2720 (1520–4500)
Tetanus	8560 (6060–12700)	8060 (6050–11000)	2410 (1750–3440)	1720 (829–5660)
Measles	0 (0–0)	0 (0–0)	26000 (8910–58400)	66100 (24000–135000)
Varicella and herpes zoster	69·5 (54·1–89)	183 (147–228)	1110 (860–1400)	1820 (1400–2340)
Acute hepatitis	--	--	6700 (5300–8320)	7240 (5040–9780)
Acute hepatitis A	--	--	4150 (2750–5970)	5630 (3620–8070)
Acute hepatitis B	--	--	2240 (1060–3630)	1010 (391–1840)
Acute hepatitis C	--	--	74·5 (23·4–150)	488 (100–1010)
Acute hepatitis E	--	--	238 (104–473)	107 (39·7–261)
Other unspecified infectious diseases	1780 (1020–2260)	1260 (752–1630)	3550 (2310–4640)	4170 (2160–6240)
Maternal and neonatal disorders	1130000 (1030000–1250000)	185000 (164000–207000)	61300 (53400–70900)	5050 (4110–6330)
Neonatal disorders	1130000 (1030000–1250000)	185000 (164000–207000)	61300 (53400–70900)	5050 (4110–6330)
Neonatal preterm birth	433000 (391000–479000)	53000 (46200–61000)	22700 (19200–26800)	1340 (1070–1820)
Neonatal encephalopathy due to birth asphyxia and trauma	376000 (322000–426000)	31000 (25100–37700)	10500 (8650–12600)	2100 (1690–2670)
Neonatal sepsis and other neonatal infections	88000 (72000–105000)	47600 (39300–55400)	10100 (8640–11800)	175 (131–233)
Hemolytic disease and other neonatal jaundice	30500 (25800–36200)	14100 (11500–17100)	2800 (1970–4310)	153 (103–243)
Other neonatal disorders	207000 (182000–233000)	39200 (33100–45400)	15200 (12800–18500)	1280 (965–1710)
Nutritional deficiencies	0 (0–0)	0 (0–0)	45400 (38100–53900)	39000 (30000–48700)
Protein-energy malnutrition	0 (0–0)	0 (0–0)	43900 (36700–52100)	37600 (29000–47000)
Other nutritional deficiencies	--	--	1460 (1060–2290)	1320 (816–2330)
Non-communicable diseases	131000 (105000–167000)	47000 (38200–59100)	178000 (131000–223000)	117000 (80000–160000)
Neoplasms	1090 (773–1390)	929 (670–1170)	6700 (5220–8370)	21700 (15900–28900)
Lip and oral cavity cancer	--	--	--	--
Nasopharynx cancer	--	--	--	--
Other pharynx cancer	--	--	--	--
Esophageal cancer	--	--	--	--
Stomach cancer	--	--	--	--
Colon and rectum cancer	--	--	--	--
Liver cancer	118 (78·6–171)	86 (55·3–134)	281 (220–357)	433 (338–573)
Liver cancer due to hepatitis B	--	--	--	--
Liver cancer due to hepatitis C	--	--	--	--
Liver cancer due to alcohol use	--	--	--	--
Liver cancer due to NASH	--	--	--	--
Liver cancer due to other causes	118 (78·6–171)	86 (55·3–134)	281 (220–357)	433 (338–573)
Gallbladder and biliary tract cancer	--	--	--	--
Pancreatic cancer	--	--	--	--
Larynx cancer	--	--	--	--
Tracheal, bronchus, and lung cancer	--	--	--	--
Malignant skin melanoma	1·23 (0·645–2·52)	1·44 (0·921–2·96)	12·4 (6·49–32·8)	64·1 (25·6–186)
Non-melanoma skin cancer	--	--	--	--
Non-melanoma skin cancer (squamous-cell carcinoma)	--	--	--	--
Breast cancer	--	--	--	--
Prostate cancer	--	--	--	--
Testicular cancer	19·8 (4·95–148)	12·1 (6·36–53·9)	55·1 (34·8–175)	242 (158–496)
Kidney cancer	31·1 (25·6–38·1)	17·8 (13·9–22·7)	193 (161–228)	945 (548–1310)
Bladder cancer	--	--	--	--
Brain and central nervous system cancer	219 (118–339)	233 (131–340)	1640 (1040–2350)	3750 (2500–5590)
Thyroid cancer	--	--	--	--
Mesothelioma	--	--	--	--
Hodgkin lymphoma	--	--	--	114 (45·4–193)
Non-Hodgkin lymphoma	--	--	--	1440 (933–1910)
Multiple myeloma	--	--	--	--
Leukemia	361 (215–537)	309 (186–451)	3080 (2180–3990)	7650 (4900–11000)
Acute lymphoid leukemia	136 (68·5–244)	98 (49·4–178)	1080 (636–1730)	2780 (1820–4510)
Chronic lymphoid leukemia	--	--	--	--
Acute myeloid leukemia	58·3 (21·9–103)	56·3 (21·5–102)	424 (241–590)	1840 (964–2820)
Chronic myeloid leukemia	15·9 (3·53–50·4)	23·3 (5·84–68)	433 (138–997)	794 (195–1940)
Other leukemia	152 (94·8–230)	132 (86·3–194)	1150 (852–1700)	2240 (1450–4370)
Other malignant neoplasms	325 (236–403)	265 (190–334)	1390 (1140–1670)	7010 (4850–9020)
Other neoplasms	11 (6·15–14·4)	4·59 (2·65–5·56)	46·2 (29·5–58·6)	87·9 (52·8–145)
[illegible] myeloproliferative, and other hematopoietic neoplasms	11 (6·15–14·4)	4·59 (2·65–5·56)	46·2 (29·5–58·6)	87·9 (52·8–145)
Cardiovascular diseases	2580 (1910–3300)	1720 (1270–2270)	9480 (7750–11300)	7630 (6360–9210)
Rheumatic heart disease	--	--	--	1280 (948–1740)
Ischemic heart disease	--	--	--	--
Stroke	1220 (840–1680)	754 (486–1110)	3840 (2840–4960)	2900 (2140–3860)
Ischemic stroke	204 (107–330)	117 (69·6–181)	361 (202–609)	543 (305–879)
Intracerebral hemorrhage	693 (471–986)	515 (315–798)	2800 (2030–3780)	1510 (928–2390)
Subarachnoid hemorrhage	327 (176–565)	122 (69·1–208)	672 (428–1030)	853 (509–1490)
Hypertensive heart disease	--	--	--	--
Non-rheumatic valvular heart disease	--	--	--	--
Non-rheumatic calcific aortic valve disease	--	--	--	--
Non-rheumatic degenerative mitral valve disease	--	--	--	--
Other non-rheumatic valve diseases	--	--	--	--
Cardiomyopathy and myocarditis	643 (451–844)	410 (295–554)	2780 (2100–3620)	1500 (1010–2110)
Myocarditis	84·7 (51·7–123)	105 (71·8–149)	894 (606–1230)	444 (295–676)
Alcoholic cardiomyopathy	--	--	--	--
Other cardiomyopathy	558 (394–733)	305 (217–419)	1880 (1430–2510)	1050 (681–1550)
Atrial fibrillation and flutter	--	--	--	--
Aortic aneurysm	--	--	--	--
Endocarditis	86·5 (50·6–119)	78·8 (42–112)	696 (406–984)	299 (135–473)
Other cardiovascular and circulatory diseases	625 (431–914)	481 (311–685)	2170 (1610–2840)	1650 (1200–2150)
Chronic respiratory diseases	0 (0–0)	0 (0–0)	0 (0–0)	6310 (5060–7760)
Chronic obstructive pulmonary disease	--	--	0 (0–0)	621 (406–1190)
Pneumoconiosis	--	--	--	--
Silicosis	--	--	--	--
Asbestosis	--	--	--	--
Coal workers pneumoconiosis	--	--	--	--
Other pneumoconiosis	--	--	--	--
Asthma	--	--	--	4600 (3430–5980)
Interstitial lung disease and pulmonary sarcoidosis	--	--	--	182 (91·5–347)
Other chronic respiratory diseases	0 (0–0)	0 (0–0)	0 (0–0)	904 (491–1480)
Digestive diseases	1570 (1360–1870)	1490 (1270–1770)	9790 (8460–11600)	11700 (9210–14300)

Cause	5 to 9	10 to 14	15 to 19	20 to 24	25 to 29	30 to 34	35 to 39	40 to 44	45 to 49	50 to 54	55 to 59	60 to 64	65 to 69	70 to 74	75 to 79	80 to 84	85 to 89	90 to 94	95 plus
All causes	290000 (273000–307000)	234000 (224000–244000)	369000 (356000–383000)	520000 (504000–536000)	566000 (551000–581000)	643000 (625000–660000)	808000 (784000–833000)	1010000 (972000–1040000)	1260000 (1220000–1310000)	1500000 (1450000–1550000)	1900000 (1830000–1960000)	2210000 (2140000–2290000)	2430000 (2350000–2510000)	2890000 (2810000–2990000)	3020000 (2930000–3110000)	2700000 (2640000–2760000)	1730000 (1700000–1750000)	648000 (643000–653000)	242000 (241000–243000)
Communicable, maternal, neonatal, and nutritional diseases	152000 (138000–168000)	93900 (83800–106000)	90000 (79300–104000)	97700 (88400–111000)	130000 (122000–142000)	171000 (162000–182000)	216000 (206000–230000)	227000 (216000–242000)	231000 (218000–247000)	213000 (2e+05–234000)	213000 (199000–236000)	217000 (201000–247000)	237000 (218000–277000)	264000 (242000–313000)	260000 (235000–308000)	239000 (213000–285000)	161000 (142000–187000)	71400 (60100–81700)	29500 (23600–33100)
HIV/AIDS and sexually transmitted infections	12100 (11600–12700)	9540 (9160–9940)	13300 (12000–14700)	13300 (12100–14600)	39500 (36300–42800)	72700 (67900–77700)	99900 (94100–106000)	1e+05 (96000–105000)	83200 (79100–87800)	50800 (48300–53300)	27800 (26200–29800)	15000 (14000–16300)	8810 (8140–9680)	5030 (4570–5580)	3180 (2930–3500)	702 (636–782)	309 (275–350)	103 (85·6–125)	41·4 (32·4–52·1)
HIV/AIDS	11900 (11400–12500)	9470 (9090–9860)	13300 (11900–14700)	13200 (12100–14600)	39400 (36300–42800)	72600 (67800–77500)	99800 (94000–106000)	1e+05 (95900–105000)	83100 (79000–87700)	50600 (48100–53200)	27600 (26000–29500)	14800 (13800–16200)	8720 (8060–9600)	4940 (4490–5490)	3080 (2830–3390)	635 (573–712)	256 (229–290)	73·1 (65·2–83)	23·6 (21·9–25·5)
HIV/AIDS - Drug-susceptible Tuberculosis	3440 (2520–4230)	2730 (2020–3320)	3620 (2640–4560)	3210 (2310–4080)	9120 (6410–11800)	17300 (12100–22200)	24600 (17500–31200)	25500 (18200–32400)	21200 (15200–27200)	13100 (9610–16600)	7140 (5240–9010)	3800 (2810–4770)	2250 (1660–2830)	1360 (993–1720)	796 (574–1020)	153 (109–199)	59·3 (40·9–78·7)	16·2 (10·9–21·9)	4·09 (2·79–5·58)
Tuberculosis without extensive drug resistance	282 (130–521)	217 (99·6–406)	293 (130–555)	307 (143–568)	937 (434–1730)	1700 (796–3180)	2270 (1050–4190)	2260 (1040–4180)	1840 (847–3370)	1150 (525–2120)	629 (285–1130)	334 (152–610)	195 (88·3–361)	119 (52·8–218)	71 (31·3–132)	14·1 (6·15–26·4)	5·55 (2·34–10·5)	1·51 (0·628–2·87)	0·398 (0·169–0·761)
HIV/AIDS - Extensively drug-resistant Tuberculosis	5·21 (2·32–9·71)	3·82 (1·68–7·18)	5·58 (2·42–10·7)	14·2 (6·13–27·1)	54·4 (24–100)	93·2 (41·9–173)	103 (46·7–191)	90·9 (41·5–169)	58·2 (26–111)	34·1 (15–64·4)	18 (8·03–34·7)	9·75 (4·25–19)	5·59 (2·44–10·9)	2·87 (1·24–5·59)	2·15 (0·888–4·35)	0·405 (0·167–0·82)	0·156 (0·0635–0·313)	0·0418 (0·0172–0·0817)	0·0245 (0·0102–0·0488)
HIV/AIDS resulting in other diseases	8200 (7320–9350)	6520 (5800–7320)	9360 (7860–11000)	9690 (8360–11300)	29300 (25600–33700)	53500 (46900–60700)	72900 (63900–82100)	72500 (63800–81200)	59900 (53100–67700)	36300 (32200–40700)	19800 (17600–22300)	10700 (9390–12300)	6270 (5500–7260)	3460 (2990–4070)	2210 (1920–2570)	467 (400–550)	191 (161–227)	55·3 (46·4–66·4)	19·1 (16·8–21·4)
Sexually transmitted infections excluding HIV	202 (65·2–441)	77·7 (45·4–122)	59·3 (37·5–83·4)	56·6 (36–77·5)	63·7 (42–83·6)	137 (61·7–219)	117 (53·8–174)	94 (51·8–127)	93·6 (45·8–134)	152 (63·5–239)	213 (82·8–337)	153 (82·9–226)	86·3 (62–149)	90·4 (68·1–152)	104 (78·5–182)	67·3 (47·5–97·8)	53·4 (28·9–78·7)	29·9 (13·8–52·2)	17·8 (8·41–28·8)
Syphilis	202 (65·2–441)	44·3 (25·9–69·5)	46·7 (29·6–65·8)	35·2 (22·4–48·1)	44·7 (29·5–58·7)	91·9 (41·6–147)	101 (45·8–151)	71 (38·8–96·7)	67 (33·6–94·6)	113 (48·9–176)	169 (67·3–266)	124 (68·4–183)	69·4 (50·4–118)	73·2 (55·8–126)	65·4 (50·6–111)	36·2 (27–50·7)	22·6 (14·5–31)	10·2 (6·17–15·4)	5·5 (3·58–7·64)
Chlamydial infection	--	0 (0–0)	0 (0–0)	0 (0–0)	0 (0–0)	0 (0–0)	0 (0–0)	0 (0–0)	0 (0–0)	0 (0–0)	0 (0–0)	0 (0–0)	0 (0–0)	0 (0–0)	0 (0–0)	0 (0–0)	0 (0–0)	0 (0–0)	0 (0–0)
Gonococcal infection	--	3·88 (2·35–6·01)	6·76 (4·34–9·41)	13·6 (8·66–18·7)	4·6 (3·17–5·87)	9·34 (4·81–14·3)	7·19 (3·76–10·3)	12·5 (7·09–16·6)	8·33 (3·81–12·1)	7·64 (2·92–12·3)	11 (3·91–17·8)	11·8 (5·95–17·8)	8·09 (5·48–14·6)	7·28 (5·22–12·8)	7·27 (5·28–13·1)	4·79 (3·21–7·17)	3·46 (1·59–5·44)	0·608 (0·253–1·11)	0·188 (0·123–0·258)
Other sexually transmitted infections	--	29·6 (17·2–46·8)	5·86 (3·73–8·21)	7·82 (4·98–10·7)	14·4 (9·44–18·9)	35·5 (15·9–57·6)	8·79 (4·21–12·9)	10·5 (5·85–14·2)	18·3 (8·09–26·8)	31·1 (11·6–50·4)	32·7 (11·6–53)	16·9 (8·53–25·6)	8·86 (6–16)	9·91 (7·1–17·5)	31·1 (22·5–56)	26·2 (17·5–39·3)	27·3 (12·6–43)	19·2 (7·7–35·3)	12·1 (4·83–20·9)
Respiratory infections and tuberculosis	23900 (20300–27300)	15500 [illegible]	20500 [illegible]	35100 [illegible]	50600 [illegible]	61100 [illegible]	74800 [illegible]	82400 [illegible]	98700 [illegible]	109000 [illegible]	123000 [illegible]	130000 [illegible]	144000 [illegible]	161000 [illegible]	166000 [illegible]	157000 [illegible]	111000 [illegible]	50800 [illegible]	22200 [illegible]
Tuberculosis	[illegible]	[illegible]	[illegible]	[illegible]	[illegible]	[illegible]	[illegible]	[illegible]	[illegible]	[illegible]	[illegible]	[illegible]	[illegible]	[illegible]	[illegible]	[illegible]	[illegible]	[illegible]	[illegible]
Drug-susceptible tuberculosis	[illegible]	[illegible]	[illegible]	[illegible]	[illegible]	[illegible]	[illegible]	[illegible]	[illegible]	[illegible]	[illegible]	[illegible]	[illegible]	[illegible]	[illegible]	[illegible]	[illegible]	[illegible]	[illegible]
Multidrug-resistant tuberculosis without extensive drug resistance	[illegible]	[illegible]	[illegible]	[illegible]	[illegible]	[illegible]	[illegible]	[illegible]	[illegible]	[illegible]	[illegible]	[illegible]	[illegible]	[illegible]	[illegible]	[illegible]	[illegible]	[illegible]	[illegible]
Extensively drug-resistant tuberculosis	[illegible]	[illegible]	[illegible]	[illegible]	[illegible]	[illegible]	[illegible]	[illegible]	[illegible]	[illegible]	[illegible]	[illegible]	[illegible]	[illegible]	[illegible]	[illegible]	[illegible]	[illegible]	[illegible]
Lower respiratory infections	[illegible]	[illegible]	[illegible]	[illegible]	[illegible]	[illegible]	[illegible]	[illegible]	[illegible]	[illegible]	[illegible]	[illegible]	[illegible]	[illegible]	[illegible]	[illegible]	[illegible]	[illegible]	[illegible]
Upper respiratory infections	[illegible]	[illegible]	[illegible]	[illegible]	[illegible]	[illegible]	[illegible]	[illegible]	[illegible]	[illegible]	[illegible]	[illegible]	[illegible]	[illegible]	[illegible]	[illegible]	[illegible]	[illegible]	[illegible]
Otitis media	[illegible]	[illegible]	[illegible]	[illegible]	[illegible]	[illegible]	[illegible]	[illegible]	[illegible]	[illegible]	[illegible]	[illegible]	[illegible]	[illegible]	[illegible]	[illegible]	[illegible]	[illegible]	[illegible]
Enteric infections	[illegible]	[illegible]	[illegible]	[illegible]	[illegible]	[illegible]	[illegible]	[illegible]	[illegible]	[illegible]	[illegible]	[illegible]	[illegible]	[illegible]	[illegible]	[illegible]	[illegible]	[illegible]	[illegible]
Diarrheal diseases	[illegible]	[illegible]	[illegible]	[illegible]	[illegible]	[illegible]	[illegible]	[illegible]	[illegible]	[illegible]	[illegible]	[illegible]	[illegible]	[illegible]	[illegible]	[illegible]	[illegible]	[illegible]	[illegible]
Typhoid and paratyphoid	[illegible]	[illegible]	[illegible]	[illegible]	[illegible]	[illegible]	[illegible]	[illegible]	[illegible]	[illegible]	[illegible]	[illegible]	[illegible]	[illegible]	[illegible]	[illegible]	[illegible]	[illegible]	[illegible]
Typhoid fever	[illegible]	[illegible]	[illegible]	[illegible]	[illegible]	[illegible]	[illegible]	[illegible]	[illegible]	[illegible]	[illegible]	[illegible]	[illegible]	[illegible]	[illegible]	[illegible]	[illegible]	[illegible]	[illegible]
Paratyphoid fever	[illegible]	[illegible]	[illegible]	[illegible]	[illegible]	[illegible]	[illegible]	[illegible]	[illegible]	[illegible]	[illegible]	[illegible]	[illegible]	[illegible]	[illegible]	[illegible]	[illegible]	[illegible]	[illegible]
Invasive Non-typhoidal Salmonella (iNTS)	[illegible]	[illegible]	[illegible]	[illegible]	[illegible]	[illegible]	[illegible]	[illegible]	[illegible]	[illegible]	[illegible]	[illegible]	[illegible]	[illegible]	[illegible]	[illegible]	[illegible]	[illegible]	[illegible]
Other intestinal infectious diseases	[illegible]	[illegible]	[illegible]	[illegible]	[illegible]	[illegible]	[illegible]	[illegible]	[illegible]	[illegible]	[illegible]	[illegible]	[illegible]	[illegible]	[illegible]	[illegible]	[illegible]	[illegible]	[illegible]
Neglected tropical diseases and malaria	[illegible]	[illegible]	[illegible]	[illegible]	[illegible]	[illegible]	[illegible]	[illegible]	[illegible]	[illegible]	[illegible]	[illegible]	[illegible]	[illegible]	[illegible]	[illegible]	[illegible]	[illegible]	[illegible]
Malaria	[illegible]	[illegible]	[illegible]	[illegible]	[illegible]	[illegible]	[illegible]	[illegible]	[illegible]	[illegible]	[illegible]	[illegible]	[illegible]	[illegible]	[illegible]	[illegible]	[illegible]	[illegible]	[illegible]
Chagas disease	[illegible]	[illegible]	[illegible]	[illegible]	[illegible]	[illegible]	[illegible]	[illegible]	[illegible]	[illegible]	[illegible]	[illegible]	[illegible]	[illegible]	[illegible]	[illegible]	[illegible]	[illegible]	[illegible]
Leishmaniasis	[illegible]	[illegible]	[illegible]	[illegible]	[illegible]	[illegible]	[illegible]	[illegible]	[illegible]	[illegible]	[illegible]	[illegible]	[illegible]	[illegible]	[illegible]	[illegible]	[illegible]	[illegible]	[illegible]
Visceral leishmaniasis	[illegible]	[illegible]	[illegible]	[illegible]	[illegible]	[illegible]	[illegible]	[illegible]	[illegible]	[illegible]	[illegible]	[illegible]	[illegible]	[illegible]	[illegible]	[illegible]	[illegible]	[illegible]	[illegible]
African trypanosomiasis	[illegible]	[illegible]	[illegible]	[illegible]	[illegible]	[illegible]	[illegible]	[illegible]	[illegible]	[illegible]	[illegible]	[illegible]	[illegible]	[illegible]	[illegible]	[illegible]	[illegible]	[illegible]	[illegible]
Schistosomiasis	[illegible]	[illegible]	[illegible]	[illegible]	[illegible]	[illegible]	[illegible]	[illegible]	[illegible]	[illegible]	[illegible]	[illegible]	[illegible]	[illegible]	[illegible]	[illegible]	[illegible]	[illegible]	[illegible]
Cysticercosis	[illegible]	[illegible]	[illegible]	[illegible]	[illegible]	[illegible]	[illegible]	[illegible]	[illegible]	[illegible]	[illegible]	[illegible]	[illegible]	[illegible]	[illegible]	[illegible]	[illegible]	[illegible]	[illegible]
Cystic echinococcosis	[illegible]	[illegible]	[illegible]	[illegible]	[illegible]	[illegible]	[illegible]	[illegible]	[illegible]	[illegible]	[illegible]	[illegible]	[illegible]	[illegible]	[illegible]	[illegible]	[illegible]	[illegible]	[illegible]
Dengue	[illegible]	[illegible]	[illegible]	[illegible]	[illegible]	[illegible]	[illegible]	[illegible]	[illegible]	[illegible]	[illegible]	[illegible]	[illegible]	[illegible]	[illegible]	[illegible]	[illegible]	[illegible]	[illegible]
Yellow fever	[illegible]	[illegible]	[illegible]	[illegible]	[illegible]	[illegible]	[illegible]	[illegible]	[illegible]	[illegible]	[illegible]	[illegible]	[illegible]	[illegible]	[illegible]	[illegible]	[illegible]	[illegible]	[illegible]
Rabies	[illegible]	[illegible]	[illegible]	[illegible]	[illegible]	[illegible]	[illegible]	[illegible]	[illegible]	[illegible]	[illegible]	[illegible]	[illegible]	[illegible]	[illegible]	[illegible]	[illegible]	[illegible]	[illegible]
Intestinal nematode infections	[illegible]	[illegible]	[illegible]	[illegible]	[illegible]	[illegible]	[illegible]	[illegible]	[illegible]	[illegible]	[illegible]	[illegible]	[illegible]	[illegible]	[illegible]	[illegible]	[illegible]	[illegible]	[illegible]
Ascariasis	[illegible]	[illegible]	[illegible]	[illegible]	[illegible]	[illegible]	[illegible]	[illegible]	[illegible]	[illegible]	[illegible]	[illegible]	[illegible]	[illegible]	[illegible]	[illegible]	[illegible]	[illegible]	[illegible]
Ebola	[illegible]	[illegible]	[illegible]	[illegible]	[illegible]	[illegible]	[illegible]	[illegible]	[illegible]	[illegible]	[illegible]	[illegible]	[illegible]	[illegible]	[illegible]	[illegible]	[illegible]	[illegible]	[illegible]
Zika virus	[illegible]	[illegible]	[illegible]	[illegible]	[illegible]	[illegible]	[illegible]	[illegible]	[illegible]	[illegible]	[illegible]	[illegible]	[illegible]	[illegible]	[illegible]	[illegible]	[illegible]	[illegible]	[illegible]
Other neglected tropical diseases	[illegible]	[illegible]	[illegible]	[illegible]	[illegible]	[illegible]	[illegible]	[illegible]	[illegible]	[illegible]	[illegible]	[illegible]	[illegible]	[illegible]	[illegible]	[illegible]	[illegible]	[illegible]	[illegible]
Other infectious diseases	[illegible]	[illegible]	[illegible]	[illegible]	[illegible]	[illegible]	[illegible]	[illegible]	[illegible]	[illegible]	[illegible]	[illegible]	[illegible]	[illegible]	[illegible]	[illegible]	[illegible]	[illegible]	[illegible]
Meningitis	[illegible]	[illegible]	[illegible]	[illegible]	[illegible]	[illegible]	[illegible]	[illegible]	[illegible]	[illegible]	[illegible]	[illegible]	[illegible]	[illegible]	[illegible]	[illegible]	[illegible]	[illegible]	[illegible]
Encephalitis	[illegible]	[illegible]	[illegible]	[illegible]	[illegible]	[illegible]	[illegible]	[illegible]	[illegible]	[illegible]	[illegible]	[illegible]	[illegible]	[illegible]	[illegible]	[illegible]	[illegible]	[illegible]	[illegible]
Diphtheria	[illegible]	[illegible]	[illegible]	[illegible]	[illegible]	[illegible]	[illegible]	[illegible]	[illegible]	[illegible]	[illegible]	[illegible]	[illegible]	[illegible]	[illegible]	[illegible]	[illegible]	[illegible]	[illegible]
Tetanus	[illegible]	[illegible]	[illegible]	[illegible]	[illegible]	[illegible]	[illegible]	[illegible]	[illegible]	[illegible]	[illegible]	[illegible]	[illegible]	[illegible]	[illegible]	[illegible]	[illegible]	[illegible]	[illegible]
Measles	[illegible]	[illegible]	[illegible]	[illegible]	[illegible]	[illegible]	[illegible]	[illegible]	[illegible]	[illegible]	[illegible]	[illegible]	[illegible]	[illegible]	[illegible]	[illegible]	[illegible]	[illegible]	[illegible]
Varicella and herpes zoster	[illegible]	[illegible]	[illegible]	[illegible]	[illegible]	[illegible]	[illegible]	[illegible]	[illegible]	[illegible]	[illegible]	[illegible]	[illegible]	[illegible]	[illegible]	[illegible]	[illegible]	[illegible]	[illegible]
Acute hepatitis	[illegible]	[illegible]	[illegible]	[illegible]	[illegible]	[illegible]	[illegible]	[illegible]	[illegible]	[illegible]	[illegible]	[illegible]	[illegible]	[illegible]	[illegible]	[illegible]	[illegible]	[illegible]	[illegible]
Acute hepatitis A	[illegible]	[illegible]	[illegible]	[illegible]	[illegible]	[illegible]	[illegible]	[illegible]	[illegible]	[illegible]	[illegible]	[illegible]	[illegible]	[illegible]	[illegible]	[illegible]	[illegible]	[illegible]	[illegible]
Acute hepatitis B	[illegible]	[illegible]	[illegible]	[illegible]	[illegible]	[illegible]	[illegible]	[illegible]	[illegible]	[illegible]	[illegible]	[illegible]	[illegible]	[illegible]	[illegible]	[illegible]	[illegible]	[illegible]	[illegible]
Acute hepatitis C	[illegible]	[illegible]	[illegible]	[illegible]	[illegible]	[illegible]	[illegible]	[illegible]	[illegible]	[illegible]	[illegible]	[illegible]	[illegible]	[illegible]	[illegible]	[illegible]	[illegible]	[illegible]	[illegible]
Acute hepatitis E	[illegible]	[illegible]	[illegible]	[illegible]	[illegible]	[illegible]	[illegible]	[illegible]	[illegible]	[illegible]	[illegible]	[illegible]	[illegible]	[illegible]	[illegible]	[illegible]	[illegible]	[illegible]	[illegible]
Other unspecified infectious diseases	[illegible]	[illegible]	[illegible]	[illegible]	[illegible]	[illegible]	[illegible]	[illegible]	[illegible]	[illegible]	[illegible]	[illegible]	[illegible]	[illegible]	[illegible]	[illegible]	[illegible]	[illegible]	[illegible]
Maternal and neonatal disorders	[illegible]	[illegible]	[illegible]	[illegible]	[illegible]	[illegible]	[illegible]	[illegible]	[illegible]	[illegible]	[illegible]	[illegible]	[illegible]	[illegible]	[illegible]	[illegible]	[illegible]	[illegible]	[illegible]
Neonatal disorders	[illegible]	[illegible]	[illegible]	[illegible]	[illegible]	[illegible]	[illegible]	[illegible]	[illegible]	[illegible]	[illegible]	[illegible]	[illegible]	[illegible]	[illegible]	[illegible]	[illegible]	[illegible]	[illegible]
Neonatal preterm birth	[illegible]	[illegible]	[illegible]	[illegible]	[illegible]	[illegible]	[illegible]	[illegible]	[illegible]	[illegible]	[illegible]	[illegible]	[illegible]	[illegible]	[illegible]	[illegible]	[illegible]	[illegible]	[illegible]
Neonatal encephalopathy due to birth asphyxia and trauma	[illegible]	[illegible]	[illegible]	[illegible]	[illegible]	[illegible]	[illegible]	[illegible]	[illegible]	[illegible]	[illegible]	[illegible]	[illegible]	[illegible]	[illegible]	[illegible]	[illegible]	[illegible]	[illegible]
Neonatal sepsis and other neonatal infections	[illegible]	[illegible]	[illegible]	[illegible]	[illegible]	[illegible]	[illegible]	[illegible]	[illegible]	[illegible]	[illegible]	[illegible]	[illegible]	[illegible]	[illegible]	[illegible]	[illegible]	[illegible]	[illegible]
Hemolytic disease and other neonatal jaundice	[illegible]	[illegible]	[illegible]	[illegible]	[illegible]	[illegible]	[illegible]	[illegible]	[illegible]	[illegible]	[illegible]	[illegible]	[illegible]	[illegible]	[illegible]	[illegible]	[illegible]	[illegible]	[illegible]
Other neonatal disorders	[illegible]	[illegible]	[illegible]	[illegible]	[illegible]	[illegible]	[illegible]	[illegible]	[illegible]	[illegible]	[illegible]	[illegible]	[illegible]	[illegible]	[illegible]	[illegible]	[illegible]	[illegible]	[illegible]
Nutritional deficiencies	[illegible]	[illegible]	[illegible]	[illegible]	[illegible]	[illegible]	[illegible]	[illegible]	[illegible]	[illegible]	[illegible]	[illegible]	[illegible]	[illegible]	[illegible]	[illegible]	[illegible]	[illegible]	[illegible]
Protein-energy malnutrition	[illegible]	[illegible]	[illegible]	[illegible]	[illegible]	[illegible]	[illegible]	[illegible]	[illegible]	[illegible]	[illegible]	[illegible]	[illegible]	[illegible]	[illegible]	[illegible]	[illegible]	[illegible]	[illegible]
Other nutritional deficiencies	[illegible]	[illegible]	[illegible]	[illegible]	[illegible]	[illegible]	[illegible]	[illegible]	[illegible]	[illegible]	[illegible]	[illegible]	[illegible]	[illegible]	[illegible]	[illegible]	[illegible]	[illegible]	[illegible]
Non-communicable diseases	[illegible]	[illegible]	[illegible]	[illegible]	[illegible]	[illegible]	[illegible]	[illegible]	[illegible]	[illegible]	[illegible]	[illegible]	[illegible]	[illegible]	[illegible]	[illegible]	[illegible]	[illegible]	[illegible]
Neoplasms	[illegible]	[illegible]	[illegible]	[illegible]	[illegible]	[illegible]	[illegible]	[illegible]	[illegible]	[illegible]	[illegible]	[illegible]	[illegible]	[illegible]	[illegible]	[illegible]	[illegible]	[illegible]	[illegible]
Lip and oral cavity cancer	[illegible]	[illegible]	[illegible]	[illegible]	[illegible]	[illegible]	[illegible]	[illegible]	[illegible]	[illegible]	[illegible]	[illegible]	[illegible]	[illegible]	[illegible]	[illegible]	[illegible]	[illegible]	[illegible]
Nasopharynx cancer	[illegible]	[illegible]	[illegible]	[illegible]	[illegible]	[illegible]	[illegible]	[illegible]	[illegible]	[illegible]	[illegible]	[illegible]	[illegible]	[illegible]	[illegible]	[illegible]	[illegible]	[illegible]	[illegible]
Other pharynx cancer	[illegible]	[illegible]	[illegible]	[illegible]	[illegible]	[illegible]	[illegible]	[illegible]	[illegible]	[illegible]	[illegible]	[illegible]	[illegible]	[illegible]	[illegible]	[illegible]	[illegible]	[illegible]	[illegible]
Esophageal cancer	[illegible]	[illegible]	[illegible]	[illegible]	[illegible]	[illegible]	[illegible]	[illegible]	[illegible]	[illegible]	[illegible]	[illegible]	[illegible]	[illegible]	[illegible]	[illegible]	[illegible]	[illegible]	[illegible]
Stomach cancer	[illegible]	[illegible]	[illegible]	[illegible]	[illegible]	[illegible]	[illegible]	[illegible]	[illegible]	[illegible]	[illegible]	[illegible]	[illegible]	[illegible]	[illegible]	[illegible]	[illegible]	[illegible]	[illegible]
Colon and rectum cancer	[illegible]	[illegible]	[illegible]	[illegible]	[illegible]	[illegible]	[illegible]	[illegible]	[illegible]	[illegible]	[illegible]	[illegible]	[illegible]	[illegible]	[illegible]	[illegible]	[illegible]	[illegible]	[illegible]
Liver cancer	[illegible]	[illegible]	[illegible]	[illegible]	[illegible]	[illegible]	[illegible]	[illegible]	[illegible]	[illegible]	[illegible]	[illegible]	[illegible]	[illegible]	[illegible]	[illegible]	[illegible]	[illegible]	[illegible]
Liver cancer due to hepatitis B	[illegible]	[illegible]	[illegible]	[illegible]	[illegible]	[illegible]	[illegible]	[illegible]	[illegible]	[illegible]	[illegible]	[illegible]	[illegible]	[illegible]	[illegible]	[illegible]	[illegible]	[illegible]	[illegible]
Liver cancer due to hepatitis C	[illegible]	[illegible]	[illegible]	[illegible]	[illegible]	[illegible]	[illegible]	[illegible]	[illegible]	[illegible]	[illegible]	[illegible]	[illegible]	[illegible]	[illegible]	[illegible]	[illegible]	[illegible]	[illegible]
Liver cancer due to alcohol use	[illegible]	[illegible]	[illegible]	[illegible]	[illegible]	[illegible]	[illegible]	[illegible]	[illegible]	[illegible]	[illegible]	[illegible]	[illegible]	[illegible]	[illegible]	[illegible]	[illegible]	[illegible]	[illegible]
Liver cancer due to NASH	[illegible]	[illegible]	[illegible]	[illegible]	[illegible]	[illegible]	[illegible]	[illegible]	[illegible]	[illegible]	[illegible]	[illegible]	[illegible]	[illegible]	[illegible]	[illegible]	[illegible]	[illegible]	[illegible]
Liver cancer due to other causes	[illegible]	[illegible]	[illegible]	[illegible]	[illegible]	[illegible]	[illegible]	[illegible]	[illegible]	[illegible]	[illegible]	[illegible]	[illegible]	[illegible]	[illegible]	[illegible]	[illegible]	[illegible]	[illegible]
Gallbladder and biliary tract cancer	[illegible]	[illegible]	[illegible]	[illegible]	[illegible]	[illegible]	[illegible]	[illegible]	[illegible]	[illegible]	[illegible]	[illegible]	[illegible]	[illegible]	[illegible]	[illegible]	[illegible]	[illegible]	[illegible]
Pancreatic cancer	[illegible]	[illegible]	[illegible]	[illegible]	[illegible]	[illegible]	[illegible]	[illegible]	[illegible]	[illegible]	[illegible]	[illegible]	[illegible]	[illegible]	[illegible]	[illegible]	[illegible]	[illegible]	[illegible]
Larynx cancer	[illegible]	[illegible]	[illegible]	[illegible]	[illegible]	[illegible]	[illegible]	[illegible]	[illegible]	[illegible]	[illegible]	[illegible]	[illegible]	[illegible]	[illegible]	[illegible]	[illegible]	[illegible]	[illegible]
Tracheal, bronchus, and lung cancer	[illegible]	[illegible]	[illegible]	[illegible]	[illegible]	[illegible]	[illegible]	[illegible]	[illegible]	[illegible]	[illegible]	[illegible]	[illegible]	[illegible]	[illegible]	[illegible]	[illegible]	[illegible]	[illegible]
Malignant skin melanoma	[illegible]	[illegible]	[illegible]	[illegible]	[illegible]	[illegible]	[illegible]	[illegible]	[illegible]	[illegible]	[illegible]	[illegible]	[illegible]	[illegible]	[illegible]	[illegible]	[illegible]	[illegible]	[illegible]
Non-melanoma skin cancer	[illegible]	[illegible]	[illegible]	[illegible]	[illegible]	[illegible]	[illegible]	[illegible]	[illegible]	[illegible]	[illegible]	[illegible]	[illegible]	[illegible]	[illegible]	[illegible]	[illegible]	[illegible]	[illegible]
Non-melanoma skin cancer (squamous-cell carcinoma)	[illegible]	[illegible]	[illegible]	[illegible]	[illegible]	[illegible]	[illegible]	[illegible]	[illegible]	[illegible]	[illegible]	[illegible]	[illegible]	[illegible]	[illegible]	[illegible]	[illegible]	[illegible]	[illegible]
Breast cancer	[illegible]	[illegible]	[illegible]	[illegible]	[illegible]	[illegible]	[illegible]	[illegible]	[illegible]	[illegible]	[illegible]	[illegible]	[illegible]	[illegible]	[illegible]	[illegible]	[illegible]	[illegible]	[illegible]
Prostate cancer	[illegible]	[illegible]	[illegible]	[illegible]	[illegible]	[illegible]	[illegible]	[illegible]	[illegible]	[illegible]	[illegible]	[illegible]	[illegible]	[illegible]	[illegible]	[illegible]	[illegible]	[illegible]	[illegible]
Testicular cancer	[illegible]	[illegible]	[illegible]	[illegible]	[illegible]	[illegible]	[illegible]	[illegible]	[illegible]	[illegible]	[illegible]	[illegible]	[illegible]	[illegible]	[illegible]	[illegible]	[illegible]	[illegible]	[illegible]
Kidney cancer	[illegible]	[illegible]	[illegible]	[illegible]	[illegible]	[illegible]	[illegible]	[illegible]	[illegible]	[illegible]	[illegible]	[illegible]	[illegible]	[illegible]	[illegible]	[illegible]	[illegible]	[illegible]	[illegible]
Bladder cancer	[illegible]	[illegible]	[illegible]	[illegible]	[illegible]	[illegible]	[illegible]	[illegible]	[illegible]	[illegible]	[illegible]	[illegible]	[illegible]	[illegible]	[illegible]	[illegible]	[illegible]	[illegible]	[illegible]
Brain and central nervous system cancer	[illegible]	[illegible]	[illegible]	[illegible]	[illegible]	[illegible]	[illegible]	[illegible]	[illegible]	[illegible]	[illegible]	[illegible]	[illegible]	[illegible]	[illegible]	[illegible]	[illegible]	[illegible]	[illegible]
Thyroid cancer	[illegible]	[illegible]	[illegible]	[illegible]	[illegible]	[illegible]	[illegible]	[illegible]	[illegible]	[illegible]	[illegible]	[illegible]	[illegible]	[illegible]	[illegible]	[illegible]	[illegible]	[illegible]	[illegible]
Mesothelioma	[illegible]	[illegible]	[illegible]	[illegible]	[illegible]	[illegible]	[illegible]	[illegible]	[illegible]	[illegible]	[illegible]	[illegible]	[illegible]	[illegible]	[illegible]	[illegible]	[illegible]	[illegible]	[illegible]
Hodgkin lymphoma	[illegible]	[illegible]	[illegible]	[illegible]	[illegible]	[illegible]	[illegible]	[illegible]	[illegible]	[illegible]	[illegible]	[illegible]	[illegible]	[illegible]	[illegible]	[illegible]	[illegible]	[illegible]	[illegible]
Non-Hodgkin lymphoma	[illegible]	[illegible]	[illegible]	[illegible]	[illegible]	[illegible]	[illegible]	[illegible]	[illegible]	[illegible]	[illegible]	[illegible]	[illegible]	[illegible]	[illegible]	[illegible]	[illegible]	[illegible]	[illegible]
Multiple myeloma	[illegible]	[illegible]	[illegible]	[illegible]	[illegible]	[illegible]	[illegible]	[illegible]	[illegible]	[illegible]	[illegible]	[illegible]	[illegible]	[illegible]	[illegible]	[illegible]	[illegible]	[illegible]	[illegible]
Leukemia	[illegible]	[illegible]	[illegible]	[illegible]	[illegible]	[illegible]	[illegible]	[illegible]	[illegible]	[illegible]	[illegible]	[illegible]	[illegible]	[illegible]	[illegible]	[illegible]	[illegible]	[illegible]	[illegible]
Acute lymphoid leukemia	[illegible]	[illegible]	[illegible]	[illegible]	[illegible]	[illegible]	[illegible]	[illegible]	[illegible]	[illegible]	[illegible]	[illegible]	[illegible]	[illegible]	[illegible]	[illegible]	[illegible]	[illegible]	[illegible]
Chronic lymphoid leukemia	[illegible]	[illegible]	[illegible]	[illegible]	[illegible]	[illegible]	[illegible]	[illegible]	[illegible]	[illegible]	[illegible]	[illegible]	[illegible]	[illegible]	[illegible]	[illegible]	[illegible]	[illegible]	[illegible]
Acute myeloid leukemia	[illegible]	[illegible]	[illegible]	[illegible]	[illegible]	[illegible]	[illegible]	[illegible]	[illegible]	[illegible]	[illegible]	[illegible]	[illegible]	[illegible]	[illegible]	[illegible]	[illegible]	[illegible]	[illegible]
Chronic myeloid leukemia	[illegible]	[illegible]	[illegible]	[illegible]	[illegible]	[illegible]	[illegible]	[illegible]	[illegible]	[illegible]	[illegible]	[illegible]	[illegible]	[illegible]	[illegible]	[illegible]	[illegible]	[illegible]	[illegible]
Other leukemia	[illegible]	[illegible]	[illegible]	[illegible]	[illegible]	[illegible]	[illegible]	[illegible]	[illegible]	[illegible]	[illegible]	[illegible]	[illegible]	[illegible]	[illegible]	[illegible]	[illegible]	[illegible]	[illegible]
Other malignant neoplasms	[illegible]	[illegible]	[illegible]	[illegible]	[illegible]	[illegible]	[illegible]	[illegible]	[illegible]	[illegible]	[illegible]	[illegible]	[illegible]	[illegible]	[illegible]	[illegible]	[illegible]	[illegible]	[illegible]
Other neoplasms	[illegible]	[illegible]	[illegible]	[illegible]	[illegible]	[illegible]	[illegible]	[illegible]	[illegible]	[illegible]	[illegible]	[illegible]	[illegible]	[illegible]	[illegible]	[illegible]	[illegible]	[illegible]	[illegible]
[illegible] myeloproliferative, and other hematopoietic neoplasms	[illegible]	[illegible]	[illegible]	[illegible]	[illegible]	[illegible]	[illegible]	[illegible]	[illegible]	[illegible]	[illegible]	[illegible]	[illegible]	[illegible]	[illegible]	[illegible]	[illegible]	[illegible]	[illegible]
Cardiovascular diseases	[illegible]	[illegible]	[illegible]	[illegible]	[illegible]	[illegible]	[illegible]	[illegible]	[illegible]	[illegible]	[illegible]	[illegible]	[illegible]	[illegible]	[illegible]	[illegible]	[illegible]	[illegible]	[illegible]
Rheumatic heart disease	[illegible]	[illegible]	[illegible]	[illegible]	[illegible]	[illegible]	[illegible]	[illegible]	[illegible]	[illegible]	[illegible]	[illegible]	[illegible]	[illegible]	[illegible]	[illegible]	[illegible]	[illegible]	[illegible]
Ischemic heart disease	[illegible]	[illegible]	[illegible]	[illegible]	[illegible]	[illegible]	[illegible]	[illegible]	[illegible]	[illegible]	[illegible]	[illegible]	[illegible]	[illegible]	[illegible]	[illegible]	[illegible]	[illegible]	[illegible]
Stroke	[illegible]	[illegible]	[illegible]	[illegible]	[illegible]	[illegible]	[illegible]	[illegible]	[illegible]	[illegible]	[illegible]	[illegible]	[illegible]	[illegible]	[illegible]	[illegible]	[illegible]	[illegible]	[illegible]
Ischemic stroke	[illegible]	[illegible]	[illegible]	[illegible]	[illegible]	[illegible]	[illegible]	[illegible]	[illegible]	[illegible]	[illegible]	[illegible]	[illegible]	[illegible]	[illegible]	[illegible]	[illegible]	[illegible]	[illegible]
Intracerebral hemorrhage	[illegible]	[illegible]	[illegible]	[illegible]	[illegible]	[illegible]	[illegible]	[illegible]	[illegible]	[illegible]	[illegible]	[illegible]	[illegible]	[illegible]	[illegible]	[illegible]	[illegible]	[illegible]	[illegible]
Subarachnoid hemorrhage	[illegible]	[illegible]	[illegible]	[illegible]	[illegible]	[illegible]	[illegible]	[illegible]	[illegible]	[illegible]	[illegible]	[illegible]	[illegible]	[illegible]	[illegible]	[illegible]	[illegible]	[illegible]	[illegible]
Hypertensive heart disease	[illegible]	[illegible]	[illegible]	[illegible]	[illegible]	[illegible]	[illegible]	[illegible]	[illegible]	[illegible]	[illegible]	[illegible]	[illegible]	[illegible]	[illegible]	[illegible]	[illegible]	[illegible]	[illegible]
Non-rheumatic valvular heart disease	[illegible]	[illegible]	[illegible]	[illegible]	[illegible]	[illegible]	[illegible]	[illegible]	[illegible]	[illegible]	[illegible]	[illegible]	[illegible]	[illegible]	[illegible]	[illegible]	[illegible]	[illegible]	[illegible]
Non-rheumatic calcific aortic valve disease	[illegible]	[illegible]	[illegible]	[illegible]	[illegible]	[illegible]	[illegible]	[illegible]	[illegible]	[illegible]	[illegible]	[illegible]	[illegible]	[illegible]	[illegible]	[illegible]	[illegible]	[illegible]	[illegible]
Non-rheumatic degenerative mitral valve disease	[illegible]	[illegible]	[illegible]	[illegible]	[illegible]	[illegible]	[illegible]	[illegible]	[illegible]	[illegible]	[illegible]	[illegible]	[illegible]	[illegible]	[illegible]	[illegible]	[illegible]	[illegible]	[illegible]
Other non-rheumatic valve diseases	[illegible]	[illegible]	[illegible]	[illegible]	[illegible]	[illegible]	[illegible]	[illegible]	[illegible]	[illegible]	[illegible]	[illegible]	[illegible]	[illegible]	[illegible]	[illegible]	[illegible]	[illegible]	[illegible]
Cardiomyopathy and myocarditis	[illegible]	[illegible]	[illegible]	[illegible]	[illegible]	[illegible]	[illegible]	[illegible]	[illegible]	[illegible]	[illegible]	[illegible]	[illegible]	[illegible]	[illegible]	[illegible]	[illegible]	[illegible]	[illegible]
Myocarditis	[illegible]	[illegible]	[illegible]	[illegible]	[illegible]	[illegible]	[illegible]	[illegible]	[illegible]	[illegible]	[illegible]	[illegible]	[illegible]	[illegible]	[illegible]	[illegible]	[illegible]	[illegible]	[illegible]
Alcoholic cardiomyopathy	[illegible]	[illegible]	[illegible]	[illegible]	[illegible]	[illegible]	[illegible]	[illegible]	[illegible]	[illegible]	[illegible]	[illegible]	[illegible]	[illegible]	[illegible]	[illegible]	[illegible]	[illegible]	[illegible]
Other cardiomyopathy	[illegible]	[illegible]	[illegible]	[illegible]	[illegible]	[illegible]	[illegible]	[illegible]	[illegible]	[illegible]	[illegible]	[illegible]	[illegible]	[illegible]	[illegible]	[illegible]	[illegible]	[illegible]	[illegible]
Atrial fibrillation and flutter	[illegible]	[illegible]	[illegible]	[illegible]	[illegible]	[illegible]	[illegible]	[illegible]	[illegible]	[illegible]	[illegible]	[illegible]	[illegible]	[illegible]	[illegible]	[illegible]	[illegible]	[illegible]	[illegible]
Aortic aneurysm	[illegible]	[illegible]	[illegible]	[illegible]	[illegible]	[illegible]	[illegible]	[illegible]	[illegible]	[illegible]	[illegible]	[illegible]	[illegible]	[illegible]	[illegible]	[illegible]	[illegible]	[illegible]	[illegible]
Endocarditis	[illegible]	[illegible]	[illegible]	[illegible]	[illegible]	[illegible]	[illegible]	[illegible]	[illegible]	[illegible]	[illegible]	[illegible]	[illegible]	[illegible]	[illegible]	[illegible]	[illegible]	[illegible]	[illegible]
Other cardiovascular and circulatory diseases	[illegible]	[illegible]	[illegible]	[illegible]	[illegible]	[illegible]	[illegible]	[illegible]	[illegible]	[illegible]	[illegible]	[illegible]	[illegible]	[illegible]	[illegible]	[illegible]	[illegible]	[illegible]	[illegible]
Chronic respiratory diseases	[illegible]	[illegible]	[illegible]	[illegible]	[illegible]	[illegible]	[illegible]	[illegible]	[illegible]	[illegible]	[illegible]	[illegible]	[illegible]	[illegible]	[illegible]	[illegible]	[illegible]	[illegible]	[illegible]
Chronic obstructive pulmonary disease	[illegible]	[illegible]	[illegible]	[illegible]	[illegible]	[illegible]	[illegible]	[illegible]	[illegible]	[illegible]	[illegible]	[illegible]	[illegible]	[illegible]	[illegible]	[illegible]	[illegible]	[illegible]	[illegible]
Pneumoconiosis	[illegible]	[illegible]	[illegible]	[illegible]	[illegible]	[illegible]	[illegible]	[illegible]	[illegible]	[illegible]	[illegible]	[illegible]	[illegible]	[illegible]	[illegible]	[illegible]	[illegible]	[illegible]	[illegible]
Silicosis	[illegible]	[illegible]	[illegible]	[illegible]	[illegible]	[illegible]	[illegible]	[illegible]	[illegible]	[illegible]	[illegible]	[illegible]	[illegible]	[illegible]	[illegible]	[illegible]	[illegible]	[illegible]	[illegible]
Asbestosis	[illegible]	[illegible]	[illegible]	[illegible]	[illegible]	[illegible]	[illegible]	[illegible]	[illegible]	[illegible]	[illegible]	[illegible]	[illegible]	[illegible]	[illegible]	[illegible]	[illegible]	[illegible]	[illegible]
Coal workers pneumoconiosis	[illegible]	[illegible]	[illegible]	[illegible]	[illegible]	[illegible]	[illegible]	[illegible]	[illegible]	[illegible]	[illegible]	[illegible]	[illegible]	[illegible]	[illegible]	[illegible]	[illegible]	[illegible]	[illegible]
Other pneumoconiosis	[illegible]	[illegible]	[illegible]	[illegible]	[illegible]	[illegible]	[illegible]	[illegible]	[illegible]	[illegible]	[illegible]	[illegible]	[illegible]	[illegible]	[illegible]	[illegible]	[illegible]	[illegible]	[illegible]
Asthma	[illegible]	[illegible]	[illegible]	[illegible]	[illegible]	[illegible]	[illegible]	[illegible]	[illegible]	[illegible]	[illegible]	[illegible]	[illegible]	[illegible]	[illegible]	[illegible]	[illegible]	[illegible]	[illegible]
Interstitial lung disease and pulmonary sarcoidosis	[illegible]	[illegible]	[illegible]	[illegible]	[illegible]	[illegible]	[illegible]	[illegible]	[illegible]	[illegible]	[illegible]	[illegible]	[illegible]	[illegible]	[illegible]	[illegible]	[illegible]	[illegible]	[illegible]
Other chronic respiratory diseases	[illegible]	[illegible]	[illegible]	[illegible]	[illegible]	[illegible]	[illegible]	[illegible]	[illegible]	[illegible]	[illegible]	[illegible]	[illegible]	[illegible]	[illegible]	[illegible]	[illegible]	[illegible]	[illegible]
Digestive diseases	[illegible]	[illegible]	[illegible]	[illegible]	[illegible]	[illegible]	[illegible]	[illegible]	[illegible]	[illegible]	[illegible]	[illegible]	[illegible]	[illegible]	[illegible]	[illegible]	[illegible]	[illegible]	[illegible]

Cirrhosis and other chronic liver diseases	--	--	--	4930 (3750–6360)	3280 (2650–3930)	[illegible]
Cirrhosis and other chronic liver diseases due to hepatitis B	--	--	--	61 (29·2–105)	102 (55·1–161)	[illegible]
Cirrhosis and other chronic liver diseases due to hepatitis C	--	--	--	48·1 (23·2–84·3)	89·3 (47·4–147)	[illegible]
Cirrhosis and other chronic liver diseases due to alcohol use	--	--	--	--	--	[illegible]
Cirrhosis and other chronic liver diseases due to NAFLD	--	--	--	--	--	[illegible]
Cirrhosis and other chronic liver diseases due to other causes	--	--	--	4820 (3690–6230)	3090 (2510–3700)	[illegible]
Upper digestive system diseases	--	--	--	1040 (648–1730)	444 (354–565)	[illegible]
Peptic ulcer disease	--	--	--	544 (293–1230)	391 (309–509)	[illegible]
Gastritis and duodenitis	--	--	--	496 (270–883)	52·1 (28·8–72·6)	[illegible]
Appendicitis	--	--	--	840 (320–1270)	801 (497–1030)	[illegible]
Paralytic ileus and intestinal obstruction	1020 (761–1400)	1130 (851–1470)	8290 (6920–9980)	1990 (1160–2880)	1540 (1090–1950)	[illegible]
Inguinal, femoral, and abdominal hernia	545 (378–746)	358 (227–562)	1500 (962–2480)	569 (271–898)	167 (113–213)	[illegible]
Inflammatory bowel disease	--	--	--	425 (205–768)	87·9 (53·5–122)	[illegible]
Vascular intestinal disorders	--	--	--	299 (119–554)	70·8 (43·8–108)	[illegible]
Gallbladder and biliary diseases	--	--	--	175 (86–275)	80·3 (55–101)	[illegible]
Pancreatitis	--	--	--	69·4 (31·9–111)	67·9 (50–89)	[illegible]
Other digestive diseases	--	--	--	1340 (682–2290)	353 (257–462)	[illegible]
Neurological disorders	4·13 (3·63–4·73)	8·26 (7·44–9·07)	3370 (2820–3990)	5890 (4450–7310)	2890 (2380–3320)	[illegible]
Alzheimer's disease and other dementias	--	--	--	--	--	[illegible]
Parkinson's disease	--	--	--	--	--	[illegible]
Idiopathic epilepsy	--	--	1830 (1360–2190)	4320 (3110–5450)	2150 (1740–2460)	[illegible]
Multiple sclerosis	--	--	--	--	17·3 (1·08–91·3)	[illegible]
Motor neuron disease	4·13 (3·63–4·73)	8·26 (7·44–9·07)	271 (238–314)	86·9 (69·2–111)	25·5 (22·8–29·5)	[illegible]
Other neurological disorders	--	--	1260 (1030–1570)	1480 (1110–2010)	698 (560–856)	[illegible]
Mental disorders	--	--	--	--	0·355 (0·219–0·832)	[illegible]
Eating disorders	--	--	--	--	0·355 (0·219–0·832)	[illegible]
Anorexia nervosa	--	--	--	--	0·218 (0·152–0·564)	[illegible]
Bulimia nervosa	--	--	--	--	0·138 (0·0635–0·449)	[illegible]
Substance use disorders	0 (0–0)	0 (0–0)	0 (0–0)	3·78 (1·04–8·57)	11·2 (5·61–18·7)	[illegible]
Alcohol use disorders	--	--	--	--	--	[illegible]
Drug use disorders	0 (0–0)	0 (0–0)	0 (0–0)	3·78 (1·04–8·57)	11·2 (5·61–18·7)	[illegible]
Opioid use disorders	0 (0–0)	0 (0–0)	0 (0–0)	3·78 (1·04–8·57)	11·2 (5·61–18·7)	[illegible]
Cocaine use disorders	--	--	--	--	--	[illegible]
Amphetamine use disorders	--	--	--	--	--	[illegible]
Other drug use disorders	--	--	--	--	--	[illegible]
Diabetes and kidney diseases	32·1 (25·7–39·8)	82·9 (63·8–105)	3690 (3150–4310)	3830 (3030–4670)	3400 (2940–3900)	[illegible]
Diabetes mellitus	32·1 (25·7–39·8)	82·9 (63·8–105)	908 (645–1230)	654 (456–935)	552 (450–739)	[illegible]
Diabetes mellitus type 1	32·1 (25·7–39·8)	82·9 (63·8–105)	908 (645–1230)	654 (456–935)	552 (450–739)	[illegible]
Diabetes mellitus type 2	--	--	--	--	--	[illegible]
Chronic kidney disease	--	--	2690 (2270–3180)	3070 (2410–3710)	2670 (2290–3080)	[illegible]
Chronic kidney disease due to diabetes mellitus type 1	--	--	15·3 (6·64–31·3)	23·8 (10·9–47·5)	37·4 (13·8–83)	[illegible]
Chronic kidney disease due to diabetes mellitus type 2	--	--	--	--	--	[illegible]
Chronic kidney disease due to hypertension	--	--	--	--	--	[illegible]
Chronic kidney disease due to glomerulonephritis	--	--	692 (382–1120)	1010 (576–1600)	896 (476–1380)	[illegible]
Chronic kidney disease due to other and unspecified causes	--	--	1990 (1540–2470)	2030 (1460–2640)	1730 (1250–2240)	[illegible]
Acute glomerulonephritis	--	--	91·9 (27–141)	110 (32·1–166)	182 (62·7–259)	[illegible]
Skin and subcutaneous diseases	--	--	884 (433–1170)	717 (354–1040)	186 (90–241)	[illegible]
Bacterial skin diseases	--	--	861 (414–1140)	687 (331–992)	168 (78·2–220)	[illegible]
Cellulitis	--	--	106 (31·2–177)	63·3 (11·8–148)	18·8 (7·31–30·1)	[illegible]
Pyoderma	--	--	755 (357–1040)	624 (293–949)	149 (65·3–198)	[illegible]
Decubitus ulcer	--	--	--	4·65 (1·14–11·3)	5·03 (1·81–7·42)	[illegible]
Other skin and subcutaneous diseases	--	--	23·1 (17·3–38)	24·8 (17·4–46·6)	12·7 (9·58–18·1)	[illegible]
Musculoskeletal disorders	--	--	--	--	137 (114–164)	[illegible]
Rheumatoid arthritis	--	--	--	--	16·9 (12·9–23·3)	[illegible]
Other musculoskeletal disorders	--	--	--	--	120 (98·7–142)	[illegible]
Other non-communicable diseases	125000 (99900–162000)	42800 (34100–55100)	144000 (99000–189000)	59400 (32500–96500)	15000 (12800–17800)	[illegible]
Congenital birth defects	124000 (98400–161000)	39700 (31500–53000)	118000 (82100–166000)	46000 (22300–81500)	9880 (8250–12700)	[illegible]
Neural tube defects	22600 (14800–34500)	4860 (3110–7780)	12200 (6250–22100)	9650 (2820–20300)	539 (329–868)	[illegible]
Congenital heart anomalies	37700 (28200–48300)	17900 (14200–22300)	55900 (41200–77700)	17200 (10000–29800)	5610 (4590–6660)	[illegible]
Orofacial clefts	922 (477–2060)	274 (157–605)	787 (393–1870)	54·3 (32·9–103)	--	[illegible]
Down syndrome	1730 (1310–3370)	733 (558–1460)	3680 (2670–8620)	1480 (894–5800)	319 (253–562)	[illegible]
Other chromosomal abnormalities	3930 (2030–7790)	1440 (815–3000)	3320 (1560–7820)	927 (266–2880)	185 (99·9–310)	[illegible]
Congenital musculoskeletal and limb anomalies	3120 (1990–4980)	684 (461–984)	1760 (1120–2870)	600 (267–1490)	216 (155–306)	[illegible]
Urogenital congenital anomalies	4300 (2540–6960)	888 (534–1390)	1400 (734–2300)	329 (115–673)	82 (51·3–119)	[illegible]
Digestive congenital anomalies	11400 (6310–17700)	4390 (2610–6870)	11500 (5840–20200)	2350 (683–5570)	253 (147–402)	[illegible]
Other congenital birth defects	37900 (23400–61200)	8520 (5420–13500)	27500 (15000–48900)	13400 (4880–28900)	2680 (1870–4270)	[illegible]
Urinary diseases and male infertility	421 (319–511)	272 (198–330)	1650 (1290–1990)	1280 (887–1650)	790 (607–946)	[illegible]
Urolithiasis	--	--	--	12·3 (5·5–24·7)	21·9 (11·1–39·3)	[illegible]
Other urinary diseases	313 (228–396)	131 (87·3–178)	330 (195–476)	590 (358–810)	210 (152–288)	[illegible]
Hemoglobinopathies and hemolytic anemias	499 (397–729)	315 (254–408)	2930 (1590–4970)	9550 (6000–14600)	3180 (2480–4450)	[illegible]
Thalassemias	221 (179–286)	153 (115–212)	816 (651–1010)	1480 (1140–1890)	934 (711–1320)	[illegible]
Sickle cell disorders	254 (139–486)	153 (93·4–238)	2020 (766–4000)	7800 (4460–12700)	1980 (1430–3210)	[illegible]
G6PD deficiency	10·3 (6·27–15·3)	6·51 (4·03–9·73)	77·7 (46·8–121)	245 (135–386)	231 (135–329)	[illegible]
Other hemoglobinopathies and hemolytic anemias	13·9 (10·5–18·7)	2·68 (1·98–3·67)	18·4 (14–23·9)	24·8 (17·6–33·6)	29·2 (22·5–38·4)	[illegible]
Endocrine, metabolic, blood, and immune disorders	763 (496–948)	645 (426–831)	4020 (2550–5250)	2540 (1560–3470)	1130 (800–1500)	[illegible]
Sudden infant death syndrome	--	1810 (862–3520)	17400 (6620–38400)	--	--	[illegible]
Injuries	6760 (5250–7980)	4940 (3460–6810)	40700 (35000–46400)	117000 (99500–136000)	85700 (75900–93900)	[illegible]
Transport injuries	750 (464–923)	492 (295–614)	5870 (4260–7020)	26300 (20500–32300)	25900 (21400–29100)	[illegible]
Road injuries	674 (415–830)	448 (268–556)	5540 (4000–6650)	24700 (19300–31000)	24500 (20200–27700)	[illegible]
Pedestrian road injuries	259 (153–326)	171 (98·6–215)	2080 (1370–2580)	11900 (8360–14900)	12800 (10100–14600)	[illegible]
Cyclist road injuries	--	--	--	1160 (684–1810)	1380 (1120–1720)	[illegible]
Motorcyclist road injuries	83·3 (40·5–125)	66·4 (26·8–99)	710 (472–972)	2240 (1460–3360)	1860 (1430–2370)	[illegible]
Motor vehicle road injuries	325 (200–416)	203 (123–263)	2660 (1770–3420)	9130 (6180–13000)	8160 (6290–10200)	[illegible]
Other road injuries	7·7 (4·61–11·7)	7·74 (5·32–11·3)	86 (62·4–131)	307 (184–614)	293 (223–389)	[illegible]
Other transport injuries	75·4 (46·1–98·5)	44·3 (26·1–60·3)	329 (250–420)	1570 (993–2400)	1390 (1130–1640)	[illegible]
Unintentional injuries	5080 (3800–6060)	4150 (2820–5920)	32500 (27800–37200)	83700 (71200–97200)	55000 (47700–61200)	[illegible]
Falls	686 (408–893)	294 (184–392)	3010 (2090–3700)	6620 (4710–8310)	4930 (3660–5870)	[illegible]
Drowning	319 (244–379)	137 (89·7–168)	3170 (2480–3730)	32100 (25200–38300)	22300 (18700–25300)	[illegible]
Fire, heat, and hot substances	252 (156–314)	166 (111–206)	2300 (1540–2820)	6830 (4490–8600)	2220 (1500–2620)	[illegible]
Poisonings	248 (160–313)	227 (121–316)	2130 (1480–2710)	5280 (3890–6440)	1740 (1280–2130)	[illegible]
Poisoning by carbon monoxide	39 (19·6–57·2)	33·9 (16·7–52·6)	295 (159–445)	918 (501–1320)	563 (360–735)	[illegible]
Poisoning by other means	209 (135–266)	193 (101–276)	1830 (1260–2340)	4370 (3240–5390)	1180 (853–1460)	[illegible]
Exposure to mechanical forces	482 (212–771)	1010 (183–2690)	2060 (1010–3340)	3560 (2510–4460)	2600 (1960–3130)	[illegible]
Unintentional firearm injuries	57·3 (15·7–129)	151 (10·3–606)	315 (126–644)	649 (411–947)	364 (263–517)	[illegible]
Other exposure to mechanical forces	425 (190–681)	855 (164–2180)	1740 (837–2810)	2910 (1990–3700)	2240 (1610–2730)	[illegible]
Adverse effects of medical treatment	548 (397–730)	300 (217–405)	2710 (1780–3600)	3490 (1680–5230)	1070 (788–1350)	[illegible]
Animal contact	307 (159–382)	230 (140–304)	1550 (916–1950)	4890 (3180–6460)	4030 (2520–5150)	[illegible]
Venomous animal contact	264 (119–336)	190 (103–258)	1100 (597–1460)	3090 (1590–4490)	3190 (1800–4210)	[illegible]
Non-venomous animal contact	42·6 (33·1–55·3)	40·7 (30·5–53·5)	457 (290–600)	1800 (1050–2430)	845 (607–1110)	[illegible]
Foreign body	1550 (1310–1780)	1300 (1110–1470)	11200 (9980–12400)	5140 (4410–6080)	1680 (1520–1860)	[illegible]
Pulmonary aspiration and foreign body in airway	1500 (1260–1720)	1280 (1090–1440)	10800 (9710–12000)	4260 (3580–5210)	1500 (1350–1680)	[illegible]
Foreign body in other body part	55·3 (33·4–76·4)	24·3 (13·8–34·3)	329 (199–484)	876 (468–1440)	177 (118–234)	[illegible]
Environmental heat and cold exposure	121 (31–179)	75·7 (17·7–119)	554 (159–841)	739 (321–994)	313 (142–413)	[illegible]
Exposure to forces of nature	138 (126–152)	298 (270–327)	2440 (2210–2680)	10800 (9770–11800)	10900 (9890–12000)	[illegible]
Other unintentional injuries	428 (175–642)	119 (77·9–162)	1480 (771–2080)	4300 (2780–5690)	3230 (1970–4180)	[illegible]
Self-harm and interpersonal violence	931 (840–1040)	298 (270–331)	2300 (2060–2580)	6640 (5930–7550)	4900 (4420–5380)	[illegible]
Self-harm	--	--	--	--	--	[illegible]
Self-harm by firearm	--	--	--	--	--	[illegible]
Self-harm by other specified means	--	--	--	--	--	[illegible]
Interpersonal violence	779 (685–880)	216 (188–247)	2030 (1790–2310)	4240 (3550–5110)	3210 (2750–3660)	[illegible]
Physical violence by firearm	145 (123–171)	43·1 (35·8–50·7)	366 (312–431)	920 (737–1160)	658 (577–741)	[illegible]
Physical violence by sharp object	84·5 (57·2–104)	26 (15·6–33·2)	200 (139–253)	754 (497–993)	447 (312–538)	[illegible]
Physical violence by other means	549 (481–625)	147 (127–167)	1460 (1300–1670)	2570 (2170–3080)	2100 (1750–2470)	[illegible]
Conflict and terrorism	152 (140–166)	81·9 (75·1–89·4)	256 (235–280)	2330 (2140–2540)	1640 (1500–1790)	[illegible]

SR Table 5: Global deaths by cause and age group for males (with 95% uncertainty levels), 2019

Cause	Early Neonatal	Late Neonatal	Post Neonatal	1 to 4	5 to 9	10 to 14	15 to 19	20 to 24	25 to 29	30 to 34	35 to 39	40 to 44	45 to 49	50 to 54	55 to 59	60 to 64	65 to 69	70 to 74	75 to 79	80 to 84	85 to 89	90 to 94	95 plus
All causes	1110000 (935000–1320000)	260000 (219000–309000)	692000 (569000–849000)	668000 (557000–807000)	210000 (190000–233000)	170000 (157000–184000)	302000 (281000–324000)	437000 (410000–466000)	496000 (469000–524000)	618000 (585000–654000)	742000 (698000–790000)	908000 (847000–972000)	1190000 (1100000–1280000)	1580000 (1460000–1710000)	2020000 (1870000–2170000)	2520000 (2340000–2700000)	2990000 (2780000–3200000)	3280000 (3060000–3500000)	3280000 (3080000–3470000)	3300000 (3130000–3460000)	2440000 (2360000–2520000)	1090000 (1070000–1110000)	377000 (371000–381000)
Communicable, maternal, neonatal, and nutritional diseases	998000 (842000–1190000)	219000 (184000–262000)	523000 (425000–654000)	497000 (408000–609000)	108000 (92600–126000)	68600 (58200–80200)	74400 (64100–87000)	82500 (73200–94900)	99600 (90800–111000)	133000 (123000–145000)	165000 (153000–180000)	180000 (166000–196000)	192000 (176000–211000)	193000 (176000–216000)	202000 (183000–228000)	217000 (197000–248000)	235000 (212000–274000)	261000 (234000–310000)	259000 (233000–305000)	268000 (235000–314000)	208000 (181000–241000)	111000 (91400–128000)	44500 (34400–51400)
HIV/AIDS and sexually transmitted infections	25400 (8510–53000)	7640 (2600–15800)	19700 (12000–31900)	15500 (13800–17600)	6190 (5740–6650)	7570 (7210–7940)	12900 (11800–14000)	11900 (11100–13000)	24300 (22200–26800)	45800 (42600–49500)	64000 (60200–68400)	67100 (64100–70500)	61000 (58200–64000)	45100 (43300–47200)	28300 (27100–29700)	17000 (16200–18000)	10600 (10100–11400)	5350 (4970–5870)	2940 (2740–3180)	1020 (931–1120)	454 (402–519)	146 (110–204)	58·2 (39·9–83·1)
HIV/AIDS	--	--	8290 (7270–9610)	15100 (13400–17000)	5980 (5580–6400)	7500 (7150–7870)	12800 (11800–13900)	11900 (11100–12900)	24200 (22100–26700)	45700 (42500–49400)	63900 (60100–68300)	67000 (64000–70400)	61000 (58100–63900)	45000 (43100–47000)	28100 (26900–29500)	16800 (16000–17900)	10600 (9990–11400)	5260 (4880–5780)	2840 (2650–3080)	949 (859–1050)	376 (343–411)	93·6 (86·6–102)	28·9 (27·3–31)
HIV/AIDS - Drug-susceptible Tuberculosis	--	--	2040 (1490–2620)	3960 (2920–4890)	1630 (1260–1980)	2090 (1590–2580)	3240 (2340–4160)	2710 (1960–3450)	5030 (3620–6550)	9170 (6490–12000)	13100 (9210–17000)	14500 (10400–18800)	13400 (9580–17300)	10100 (7340–13000)	6240 (4530–8080)	3630 (2650–4680)	2050 (1500–2660)	1020 (742–1320)	542 (386–706)	140 (98·4–186)	56·5 (38·6–76·4)	14·5 (9·73–20)	4·14 (2·81–5·67)
Tuberculosis without extensive drug resistance	--	--	249 (107–460)	466 (186–896)	175 (76·3–325)	208 (92·8–400)	343 (147–653)	288 (126–530)	533 (232–1020)	974 (432–1860)	1340 (610–2550)	1440 (645–2730)	1290 (572–2430)	974 (433–1800)	597 (263–1120)	346 (153–649)	196 (86–369)	99·2 (43·6–191)	52·3 (22·6–101)	13·9 (5·62–28·4)	5·55 (2·18–11·4)	1·41 (0·573–2·74)	0·377 (0·157–0·723)
HIV/AIDS - Extensively drug-resistant Tuberculosis	--	--	7·24 (3·22–13·5)	8·48 (3·49–16·5)	4·12 (1·81–7·79)	4·44 (1·95–8·37)	7·92 (3·41–15·2)	12·5 (5·33–24·4)	34·6 (15·7–65·6)	73·5 (34·6–135)	96·4 (46·3–175)	83·7 (39·2–156)	59·1 (27–112)	40·2 (18·2–76·5)	23·9 (10·7–46·9)	14·2 (6·26–28·2)	9·36 (3·8–19·4)	3·88 (1·47–8·46)	2·05 (0·734–4·57)	0·859 (0·245–2·18)	0·352 (0·107–0·872)	0·0747 (0·0288–0·16)	0·0338 (0·0137–0·0676)
HIV/AIDS resulting in other diseases	--	--	6000 (5130–7250)	10600 (9090–12800)	4170 (3720–4690)	5200 (4640–5820)	9250 (8060–10700)	8860 (7840–10200)	18600 (16200–21500)	35500 (31100–40300)	49400 (43800–55400)	51000 (45300–56800)	46300 (41300–51400)	33900 (30300–37500)	21300 (19100–23500)	12800 (11500–14200)	8300 (7470–9200)	4130 (3670–4690)	2240 (2000–2520)	794 (695–896)	313 (275–353)	77·6 (68·6–87·7)	24·3 (21·9–26·9)
Sexually transmitted infections excluding HIV	25400 (8510–53000)	7640 (2600–15800)	11400 (3930–23600)	450 (151–982)	209 (67·6–459)	63·3 (41·3–89·7)	55·2 (35·1–73·6)	54·2 (33·9–72·3)	59·6 (38·3–75·7)	123 (59·8–188)	103 (51·3–149)	90·7 (50·4–125)	89·2 (43·3–130)	142 (57·7–214)	193 (75·4–297)	151 (81·4–225)	87 (62·1–140)	89·1 (66·6–140)	98·6 (73·3–159)	72·3 (45·2–99·8)	78·5 (35·6–130)	52·3 (18·1–111)	29·4 (10·9–54·7)
Syphilis	25400 (8510–53000)	7640 (2600–15800)	11400 (3930–23600)	450 (151–982)	209 (67·6–459)	36·1 (23·5–51·1)	43·4 (27·6–58·1)	33·7 (21–44·9)	41·8 (26·8–53·2)	82·7 (40·2–126)	88·6 (44·3–129)	68·6 (37·9–94·7)	63·4 (31·5–91·1)	105 (43·7–158)	154 (61·1–235)	123 (67·1–182)	70·2 (50·5–111)	72·1 (54·5–113)	62·1 (47·5–97·1)	38 (25·3–50·8)	31·1 (16·9–47)	16·6 (7·93–30·9)	8·67 (4·52–13·9)
Chlamydial infection	--	--	--	--	--	0 (0–0)	0 (0–0)	[illegible]	[illegible]	[illegible]	[illegible]	[illegible]	[illegible]	[illegible]	[illegible]	[illegible]	[illegible]	[illegible]	[illegible]	[illegible]	[illegible]	[illegible]	[illegible]
Gonococcal infection	--	--	--	--	--	3·21 (2·08–4·37)	6·28 (4·01–8·34)	[illegible]	[illegible]	[illegible]	[illegible]	[illegible]	[illegible]	[illegible]	[illegible]	[illegible]	[illegible]	[illegible]	[illegible]	[illegible]	[illegible]	[illegible]	[illegible]
Other sexually transmitted infections	--	--	--	--	--	24 (15·5–34·3)	5·45 (3·47–7·24)	[illegible]	[illegible]	[illegible]	[illegible]	[illegible]	[illegible]	[illegible]	[illegible]	[illegible]	[illegible]	[illegible]	[illegible]	[illegible]	[illegible]	[illegible]	[illegible]
Respiratory infections and tuberculosis	65200 (51800–81800)	39900 (32600–49400)	167000 (133000–210000)	97300 (75400–123000)	17800 (14700–21300)	11400 (9690–13300)	17100 (15000–19700)	[illegible]	[illegible]	[illegible]	[illegible]	[illegible]	[illegible]	[illegible]	[illegible]	[illegible]	[illegible]	[illegible]	[illegible]	[illegible]	[illegible]	[illegible]	[illegible]
Tuberculosis	--	--	12600 (9780–16200)	12600 (9540–16400)	3550 (2910–4280)	3500 (2940–4140)	9560 (8140–11200)	[illegible]	[illegible]	[illegible]	[illegible]	[illegible]	[illegible]	[illegible]	[illegible]	[illegible]	[illegible]	[illegible]	[illegible]	[illegible]	[illegible]	[illegible]	[illegible]
Drug-susceptible tuberculosis	--	--	11500 (8800–14900)	11600 (8740–15100)	3240 (2610–3940)	3180 (2600–3860)	8670 (7250–10300)	[illegible]	[illegible]	[illegible]	[illegible]	[illegible]	[illegible]	[illegible]	[illegible]	[illegible]	[illegible]	[illegible]	[illegible]	[illegible]	[illegible]	[illegible]	[illegible]
Multidrug-resistant tuberculosis without extensive drug resistance	--	--	1050 (452–1890)	971 (425–1840)	288 (131–529)	305 (129–570)	850 (364–1570)	[illegible]	[illegible]	[illegible]	[illegible]	[illegible]	[illegible]	[illegible]	[illegible]	[illegible]	[illegible]	[illegible]	[illegible]	[illegible]	[illegible]	[illegible]	[illegible]
Extensively drug-resistant tuberculosis	--	--	50·4 (23·8–90·1)	37·4 (17·2–68·6)	15·2 (6·87–28·9)	16·1 (6·92–30·9)	42·2 (18·5–79·6)	[illegible]	[illegible]	[illegible]	[illegible]	[illegible]	[illegible]	[illegible]	[illegible]	[illegible]	[illegible]	[illegible]	[illegible]	[illegible]	[illegible]	[illegible]	[illegible]
Lower respiratory infections	65000 (51600–81500)	39800 (32500–49300)	153000 (122000–193000)	83400 (64900–105000)	14100 (11600–16900)	7790 (6640–9140)	7470 (6610–8710)	[illegible]	[illegible]	[illegible]	[illegible]	[illegible]	[illegible]	[illegible]	[illegible]	[illegible]	[illegible]	[illegible]	[illegible]	[illegible]	[illegible]	[illegible]	[illegible]
Upper respiratory infections	19·7 (10·2–30)	47·6 (21·8–81·8)	1020 (307–2140)	1230 (231–3190)	147 (49–282)	113 (35·7–200)	61·6 (23·8–113)	[illegible]	[illegible]	[illegible]	[illegible]	[illegible]	[illegible]	[illegible]	[illegible]	[illegible]	[illegible]	[illegible]	[illegible]	[illegible]	[illegible]	[illegible]	[illegible]
Otitis media	114 (2·47–547)	28·9 (3·95–130)	30·1 (12·7–68·1)	16 (4·5–45·9)	13·9 (5·05–32·6)	11·2 (4·62–25·6)	8·65 (4·53–20·8)	[illegible]	[illegible]	[illegible]	[illegible]	[illegible]	[illegible]	[illegible]	[illegible]	[illegible]	[illegible]	[illegible]	[illegible]	[illegible]	[illegible]	[illegible]	[illegible]
Enteric infections	8110 (6250–10100)	15600 (12100–19600)	136000 (105000–177000)	146000 (109000–194000)	40500 (29000–57600)	25400 (16500–37900)	20200 (12400–32000)	[illegible]	[illegible]	[illegible]	[illegible]	[illegible]	[illegible]	[illegible]	[illegible]	[illegible]	[illegible]	[illegible]	[illegible]	[illegible]	[illegible]	[illegible]	[illegible]
Diarrheal diseases	8110 (6250–10100)	15200 (11800–19200)	130000 (1e+05–167000)	113000 (83200–152000)	18500 (11000–31700)	8440 (4910–15900)	8460 (4710–16200)	[illegible]	[illegible]	[illegible]	[illegible]	[illegible]	[illegible]	[illegible]	[illegible]	[illegible]	[illegible]	[illegible]	[illegible]	[illegible]	[illegible]	[illegible]	[illegible]
Typhoid and paratyphoid	--	--	71·9 (27·2–170)	12100 (4680–24300)	16300 (7040–29800)	14800 (6550–26100)	10600 (3990–20200)	[illegible]	[illegible]	[illegible]	[illegible]	[illegible]	[illegible]	[illegible]	[illegible]	[illegible]	[illegible]	[illegible]	[illegible]	[illegible]	[illegible]	[illegible]	[illegible]
Typhoid fever	--	--	64·2 (24·3–152)	10500 (4100–21200)	13600 (5810–25200)	12200 (5190–22700)	8610 (3200–16800)	[illegible]	[illegible]	[illegible]	[illegible]	[illegible]	[illegible]	[illegible]	[illegible]	[illegible]	[illegible]	[illegible]	[illegible]	[illegible]	[illegible]	[illegible]	[illegible]
Paratyphoid fever	--	--	7·73 (2·28–20·4)	1560 (490–3760)	2640 (960–5690)	2600 (942–5480)	1990 (641–4310)	[illegible]	[illegible]	[illegible]	[illegible]	[illegible]	[illegible]	[illegible]	[illegible]	[illegible]	[illegible]	[illegible]	[illegible]	[illegible]	[illegible]	[illegible]	[illegible]
Invasive Non-typhoidal Salmonella (iNTS)	--	367 (197–617)	6010 (3090–9840)	20900 (11200–34100)	5710 (2970–9740)	2100 (963–3820)	1140 (496–2270)	[illegible]	[illegible]	[illegible]	[illegible]	[illegible]	[illegible]	[illegible]	[illegible]	[illegible]	[illegible]	[illegible]	[illegible]	[illegible]	[illegible]	[illegible]	[illegible]
Other intestinal infectious diseases	--	--	124 (37·8–257)	234 (91–432)	47·3 (8·38–119)	29 (2·17–79)	17·7 (0·333–51·5)	[illegible]	[illegible]	[illegible]	[illegible]	[illegible]	[illegible]	[illegible]	[illegible]	[illegible]	[illegible]	[illegible]	[illegible]	[illegible]	[illegible]	[illegible]	[illegible]
Neglected tropical diseases and malaria	278 (116–1080)	9360 (4130–18100)	58700 (27500–107000)	117000 (59600–196000)	19100 (11500–29600)	11600 (7120–18100)	12200 (6950–19800)	[illegible]	[illegible]	[illegible]	[illegible]	[illegible]	[illegible]	[illegible]	[illegible]	[illegible]	[illegible]	[illegible]	[illegible]	[illegible]	[illegible]	[illegible]	[illegible]
Malaria	0 (0–0)	8970 (3770–17800)	56200 (25000–104000)	109000 (52500–189000)	14300 (6880–24800)	8290 (3900–14600)	9520 (4240–17000)	[illegible]	[illegible]	[illegible]	[illegible]	[illegible]	[illegible]	[illegible]	[illegible]	[illegible]	[illegible]	[illegible]	[illegible]	[illegible]	[illegible]	[illegible]	[illegible]
Chagas disease	--	--	0·624 (0·17–1·21)	0·222 (0·091–0·69)	1·03 (0·514–2·91)	2·06 (0·881–4·64)	7·32 (3·62–20·1)	[illegible]	[illegible]	[illegible]	[illegible]	[illegible]	[illegible]	[illegible]	[illegible]	[illegible]	[illegible]	[illegible]	[illegible]	[illegible]	[illegible]	[illegible]	[illegible]
Leishmaniasis	--	--	103 (29·1–302)	908 (320–2680)	803 (298–2720)	443 (152–1570)	324 (98·3–1170)	[illegible]	[illegible]	[illegible]	[illegible]	[illegible]	[illegible]	[illegible]	[illegible]	[illegible]	[illegible]	[illegible]	[illegible]	[illegible]	[illegible]	[illegible]	[illegible]
Visceral leishmaniasis	--	--	103 (29·1–302)	908 (320–2680)	803 (298–2720)	443 (152–1570)	324 (98·3–1170)	[illegible]	[illegible]	[illegible]	[illegible]	[illegible]	[illegible]	[illegible]	[illegible]	[illegible]	[illegible]	[illegible]	[illegible]	[illegible]	[illegible]	[illegible]	[illegible]
African trypanosomiasis	--	--	--	30 (2·87–159)	75·5 (11–311)	81 (11–289)	77·9 (14·3–260)	[illegible]	[illegible]	[illegible]	[illegible]	[illegible]	[illegible]	[illegible]	[illegible]	[illegible]	[illegible]	[illegible]	[illegible]	[illegible]	[illegible]	[illegible]	[illegible]
Schistosomiasis	--	--	0 (0–0)	75·7 (45·4–119)	55·6 (37·9–80·7)	80 (56·3–111)	122 (87–165)	[illegible]	[illegible]	[illegible]	[illegible]	[illegible]	[illegible]	[illegible]	[illegible]	[illegible]	[illegible]	[illegible]	[illegible]	[illegible]	[illegible]	[illegible]	[illegible]
Cysticercosis	--	--	--	7·03 (0·000586–22·6)	13·8 (0·503–42·2)	15·9 (0·887–48·5)	26·2 (1·53–73·6)	[illegible]	[illegible]	[illegible]	[illegible]	[illegible]	[illegible]	[illegible]	[illegible]	[illegible]	[illegible]	[illegible]	[illegible]	[illegible]	[illegible]	[illegible]	[illegible]
Cystic echinococcosis	--	--	--	64·5 (16·1–143)	43·1 (5·33–108)	27 (0·957–75·2)	17·7 (0·208–52·3)	[illegible]	[illegible]	[illegible]	[illegible]	[illegible]	[illegible]	[illegible]	[illegible]	[illegible]	[illegible]	[illegible]	[illegible]	[illegible]	[illegible]	[illegible]	[illegible]
Dengue	0·00139 (0·00126–0·00152)	164 (7·67–307)	989 (119–1650)	2370 (533–3570)	[illegible]	[illegible]	[illegible]	[illegible]	[illegible]	[illegible]	[illegible]	[illegible]	[illegible]	[illegible]	[illegible]	[illegible]	[illegible]	[illegible]	[illegible]	[illegible]	[illegible]	[illegible]	[illegible]
Yellow fever	--	1·52 (0·534–3·53)	25·7 (8·43–57·7)	324 (111–768)	[illegible]	[illegible]	[illegible]	[illegible]	[illegible]	[illegible]	[illegible]	[illegible]	[illegible]	[illegible]	[illegible]	[illegible]	[illegible]	[illegible]	[illegible]	[illegible]	[illegible]	[illegible]	[illegible]
Rabies	--	--	86·3 (25·3–163)	1050 (275–2120)	[illegible]	[illegible]	[illegible]	[illegible]	[illegible]	[illegible]	[illegible]	[illegible]	[illegible]	[illegible]	[illegible]	[illegible]	[illegible]	[illegible]	[illegible]	[illegible]	[illegible]	[illegible]	[illegible]
Intestinal nematode infections	--	--	67·2 (48·2–94·4)	688 (491–935)	[illegible]	[illegible]	[illegible]	[illegible]	[illegible]	[illegible]	[illegible]	[illegible]	[illegible]	[illegible]	[illegible]	[illegible]	[illegible]	[illegible]	[illegible]	[illegible]	[illegible]	[illegible]	[illegible]
Ascariasis	--	--	67·2 (48·2–94·4)	688 (491–935)	[illegible]	[illegible]	[illegible]	[illegible]	[illegible]	[illegible]	[illegible]	[illegible]	[illegible]	[illegible]	[illegible]	[illegible]	[illegible]	[illegible]	[illegible]	[illegible]	[illegible]	[illegible]	[illegible]
Ebola	0 (0–0)	0 (0–0)	34·7 (28·5–41)	101 (83–119)	[illegible]	[illegible]	[illegible]	[illegible]	[illegible]	[illegible]	[illegible]	[illegible]	[illegible]	[illegible]	[illegible]	[illegible]	[illegible]	[illegible]	[illegible]	[illegible]	[illegible]	[illegible]	[illegible]
Zika virus	0·000391 (0·000221–0·00061)	0·000976 (0·000528–0·00159)	0·0175 (0·00985–0·0279)	0·0666 (0·0369–0·106)	[illegible]	[illegible]	[illegible]	[illegible]	[illegible]	[illegible]	[illegible]	[illegible]	[illegible]	[illegible]	[illegible]	[illegible]	[illegible]	[illegible]	[illegible]	[illegible]	[illegible]	[illegible]	[illegible]
Other neglected tropical diseases	278 (116–1080)	218 (107–792)	1220 (554–4150)	2120 (802–8480)	[illegible]	[illegible]	[illegible]	[illegible]	[illegible]	[illegible]	[illegible]	[illegible]	[illegible]	[illegible]	[illegible]	[illegible]	[illegible]	[illegible]	[illegible]	[illegible]	[illegible]	[illegible]	[illegible]
Other infectious diseases	9620 (7400–12700)	9730 (7410–13000)	73000 (51000–102000)	94600 (64100–138000)	[illegible]	[illegible]	[illegible]	[illegible]	[illegible]	[illegible]	[illegible]	[illegible]	[illegible]	[illegible]	[illegible]	[illegible]	[illegible]	[illegible]	[illegible]	[illegible]	[illegible]	[illegible]	[illegible]
Meningitis	4100 (3150–5630)	4030 (3160–5280)	27300 (20800–36800)	26900 (20200–35500)	[illegible]	[illegible]	[illegible]	[illegible]	[illegible]	[illegible]	[illegible]	[illegible]	[illegible]	[illegible]	[illegible]	[illegible]	[illegible]	[illegible]	[illegible]	[illegible]	[illegible]	[illegible]	[illegible]
Encephalitis	298 (215–409)	440 (316–606)	3620 (2650–5210)	4630 (3380–6450)	[illegible]	[illegible]	[illegible]	[illegible]	[illegible]	[illegible]	[illegible]	[illegible]	[illegible]	[illegible]	[illegible]	[illegible]	[illegible]	[illegible]	[illegible]	[illegible]	[illegible]	[illegible]	[illegible]
Diphtheria	--	--	361 (203–599)	1500 (784–2520)	[illegible]	[illegible]	[illegible]	[illegible]	[illegible]	[illegible]	[illegible]	[illegible]	[illegible]	[illegible]	[illegible]	[illegible]	[illegible]	[illegible]	[illegible]	[illegible]	[illegible]	[illegible]	[illegible]
Tetanus	3560 (2330–6080)	4080 (2500–7020)	1100 (705–2020)	1020 (372–4080)	[illegible]	[illegible]	[illegible]	[illegible]	[illegible]	[illegible]	[illegible]	[illegible]	[illegible]	[illegible]	[illegible]	[illegible]	[illegible]	[illegible]	[illegible]	[illegible]	[illegible]	[illegible]	[illegible]
Measles	120 (109–133)	22·9 (20·8–25·2)	10100 (3420–22500)	25800 (8860–56200)	[illegible]	[illegible]	[illegible]	[illegible]	[illegible]	[illegible]	[illegible]	[illegible]	[illegible]	[illegible]	[illegible]	[illegible]	[illegible]	[illegible]	[illegible]	[illegible]	[illegible]	[illegible]	[illegible]
Varicella and herpes zoster	54 (41–72·2)	143 (109–190)	919 (683–1230)	1570 (1170–2040)	[illegible]	[illegible]	[illegible]	[illegible]	[illegible]	[illegible]	[illegible]	[illegible]	[illegible]	[illegible]	[illegible]	[illegible]	[illegible]	[illegible]	[illegible]	[illegible]	[illegible]	[illegible]	[illegible]
Acute hepatitis	--	--	3480 (2230–5140)	3300 (2160–4850)	[illegible]	[illegible]	[illegible]	[illegible]	[illegible]	[illegible]	[illegible]	[illegible]	[illegible]	[illegible]	[illegible]	[illegible]	[illegible]	[illegible]	[illegible]	[illegible]	[illegible]	[illegible]	[illegible]
Acute hepatitis A	--	--	1780 (980–3070)	2300 (1360–3520)	[illegible]	[illegible]	[illegible]	[illegible]	[illegible]	[illegible]	[illegible]	[illegible]	[illegible]	[illegible]	[illegible]	[illegible]	[illegible]	[illegible]	[illegible]	[illegible]	[illegible]	[illegible]	[illegible]
Acute hepatitis B	--	--	1490 (807–2340)	650 (322–1080)	[illegible]	[illegible]	[illegible]	[illegible]	[illegible]	[illegible]	[illegible]	[illegible]	[illegible]	[illegible]	[illegible]	[illegible]	[illegible]	[illegible]	[illegible]	[illegible]	[illegible]	[illegible]	[illegible]
Acute hepatitis C	--	--	42·5 (16·3–94·2)	272 (92·4–504)	[illegible]	[illegible]	[illegible]	[illegible]	[illegible]	[illegible]	[illegible]	[illegible]	[illegible]	[illegible]	[illegible]	[illegible]	[illegible]	[illegible]	[illegible]	[illegible]	[illegible]	[illegible]	[illegible]
Acute hepatitis E	--	--	168 (63·5–334)	72·7 (26·3–146)	[illegible]	[illegible]	[illegible]	[illegible]	[illegible]	[illegible]	[illegible]	[illegible]	[illegible]	[illegible]	[illegible]	[illegible]	[illegible]	[illegible]	[illegible]	[illegible]	[illegible]	[illegible]	[illegible]
Other unspecified infectious diseases	1490 (868–2050)	1010 (612–1440)	3040 (1900–4370)	3530 (1890–5590)	[illegible]	[illegible]	[illegible]	[illegible]	[illegible]	[illegible]	[illegible]	[illegible]	[illegible]	[illegible]	[illegible]	[illegible]	[illegible]	[illegible]	[illegible]	[illegible]	[illegible]	[illegible]	[illegible]
Maternal and neonatal disorders	889000 (748000–1070000)	137000 (114000–163000)	45400 (35400–58000)	4160 (3090–5460)	[illegible]	[illegible]	[illegible]	[illegible]	[illegible]	[illegible]	[illegible]	[illegible]	[illegible]	[illegible]	[illegible]	[illegible]	[illegible]	[illegible]	[illegible]	[illegible]	[illegible]	[illegible]	[illegible]
Neonatal disorders	889000 (748000–1070000)	137000 (114000–163000)	45400 (35400–58000)	4160 (3090–5460)	[illegible]	[illegible]	[illegible]	[illegible]	[illegible]	[illegible]	[illegible]	[illegible]	[illegible]	[illegible]	[illegible]	[illegible]	[illegible]	[illegible]	[illegible]	[illegible]	[illegible]	[illegible]	[illegible]
Neonatal preterm birth	331000 (276000–402000)	36600 (30100–44100)	15400 (12000–20100)	1060 (769–1510)	[illegible]	[illegible]	[illegible]	[illegible]	[illegible]	[illegible]	[illegible]	[illegible]	[illegible]	[illegible]	[illegible]	[illegible]	[illegible]	[illegible]	[illegible]	[illegible]	[illegible]	[illegible]	[illegible]
Neonatal encephalopathy due to birth asphyxia and trauma	299000 (243000–363000)	22500 (17200–28500)	7580 (5630–10300)	1680 (1230–2250)	[illegible]	[illegible]	[illegible]	[illegible]	[illegible]	[illegible]	[illegible]	[illegible]	[illegible]	[illegible]	[illegible]	[illegible]	[illegible]	[illegible]	[illegible]	[illegible]	[illegible]	[illegible]	[illegible]
Neonatal sepsis and other neonatal infections	77200 (61600–97300)	40300 (32700–50200)	8560 (6670–10900)	198 (136–287)	[illegible]	[illegible]	[illegible]	[illegible]	[illegible]	[illegible]	[illegible]	[illegible]	[illegible]	[illegible]	[illegible]	[illegible]	[illegible]	[illegible]	[illegible]	[illegible]	[illegible]	[illegible]	[illegible]
Hemolytic disease and other neonatal jaundice	20100 (16200–24900)	8300 (6530–10500)	1890 (1170–3160)	142 (92·5–228)	[illegible]	[illegible]	[illegible]	[illegible]	[illegible]	[illegible]	[illegible]	[illegible]	[illegible]	[illegible]	[illegible]	[illegible]	[illegible]	[illegible]	[illegible]	[illegible]	[illegible]	[illegible]	[illegible]
Other neonatal disorders	162000 (132000–199000)	28800 (23400–35600)	12000 (8750–16400)	1080 (747–1590)	[illegible]	[illegible]	[illegible]	[illegible]	[illegible]	[illegible]	[illegible]	[illegible]	[illegible]	[illegible]	[illegible]	[illegible]	[illegible]	[illegible]	[illegible]	[illegible]	[illegible]	[illegible]	[illegible]
Nutritional deficiencies	0 (0–0)	0 (0–0)	23300 (17800–30500)	22800 (17100–29900)	[illegible]	[illegible]	[illegible]	[illegible]	[illegible]	[illegible]	[illegible]	[illegible]	[illegible]	[illegible]	[illegible]	[illegible]	[illegible]	[illegible]	[illegible]	[illegible]	[illegible]	[illegible]	[illegible]
Protein-energy malnutrition	0 (0–0)	0 (0–0)	22600 (17100–29600)	22100 (16600–29000)	[illegible]	[illegible]	[illegible]	[illegible]	[illegible]	[illegible]	[illegible]	[illegible]	[illegible]	[illegible]	[illegible]	[illegible]	[illegible]	[illegible]	[illegible]	[illegible]	[illegible]	[illegible]	[illegible]
Other nutritional deficiencies	--	--	742 (514–1050)	694 (465–983)	[illegible]	[illegible]	[illegible]	[illegible]	[illegible]	[illegible]	[illegible]	[illegible]	[illegible]	[illegible]	[illegible]	[illegible]	[illegible]	[illegible]	[illegible]	[illegible]	[illegible]	[illegible]	[illegible]
Non-communicable diseases	106000 (85300–132000)	37000 (30500–45700)	140000 (109000–179000)	96800 (70900–129000)	[illegible]	[illegible]	[illegible]	[illegible]	[illegible]	[illegible]	[illegible]	[illegible]	[illegible]	[illegible]	[illegible]	[illegible]	[illegible]	[illegible]	[illegible]	[illegible]	[illegible]	[illegible]	[illegible]
Neoplasms	949 (670–1210)	803 (557–1070)	5890 (4430–7410)	19000 (14500–23900)	[illegible]	[illegible]	[illegible]	[illegible]	[illegible]	[illegible]	[illegible]	[illegible]	[illegible]	[illegible]	[illegible]	[illegible]	[illegible]	[illegible]	[illegible]	[illegible]	[illegible]	[illegible]	[illegible]
Lip and oral cavity cancer	--	--	--	--	[illegible]	[illegible]	[illegible]	[illegible]	[illegible]	[illegible]	[illegible]	[illegible]	[illegible]	[illegible]	[illegible]	[illegible]	[illegible]	[illegible]	[illegible]	[illegible]	[illegible]	[illegible]	[illegible]
Nasopharynx cancer	--	--	--	--	[illegible]	[illegible]	[illegible]	[illegible]	[illegible]	[illegible]	[illegible]	[illegible]	[illegible]	[illegible]	[illegible]	[illegible]	[illegible]	[illegible]	[illegible]	[illegible]	[illegible]	[illegible]	[illegible]
Other pharynx cancer	--	--	--	--	[illegible]	[illegible]	[illegible]	[illegible]	[illegible]	[illegible]	[illegible]	[illegible]	[illegible]	[illegible]	[illegible]	[illegible]	[illegible]	[illegible]	[illegible]	[illegible]	[illegible]	[illegible]	[illegible]
Esophageal cancer	--	--	--	--	[illegible]	[illegible]	[illegible]	[illegible]	[illegible]	[illegible]	[illegible]	[illegible]	[illegible]	[illegible]	[illegible]	[illegible]	[illegible]	[illegible]	[illegible]	[illegible]	[illegible]	[illegible]	[illegible]
Stomach cancer	--	--	--	--	[illegible]	[illegible]	[illegible]	[illegible]	[illegible]	[illegible]	[illegible]	[illegible]	[illegible]	[illegible]	[illegible]	[illegible]	[illegible]	[illegible]	[illegible]	[illegible]	[illegible]	[illegible]	[illegible]
Colon and rectum cancer	--	--	--	--	[illegible]	[illegible]	[illegible]	[illegible]	[illegible]	[illegible]	[illegible]	[illegible]	[illegible]	[illegible]	[illegible]	[illegible]	[illegible]	[illegible]	[illegible]	[illegible]	[illegible]	[illegible]	[illegible]
Liver cancer	99·1 (57·6–162)	75 (43·4–135)	261 (178–359)	454 (340–568)	[illegible]	[illegible]	[illegible]	[illegible]	[illegible]	[illegible]	[illegible]	[illegible]	[illegible]	[illegible]	[illegible]	[illegible]	[illegible]	[illegible]	[illegible]	[illegible]	[illegible]	[illegible]	[illegible]
Liver cancer due to hepatitis B	--	--	--	--	[illegible]	[illegible]	[illegible]	[illegible]	[illegible]	[illegible]	[illegible]	[illegible]	[illegible]	[illegible]	[illegible]	[illegible]	[illegible]	[illegible]	[illegible]	[illegible]	[illegible]	[illegible]	[illegible]
Liver cancer due to hepatitis C	--	--	--	--	[illegible]	[illegible]	[illegible]	[illegible]	[illegible]	[illegible]	[illegible]	[illegible]	[illegible]	[illegible]	[illegible]	[illegible]	[illegible]	[illegible]	[illegible]	[illegible]	[illegible]	[illegible]	[illegible]
Liver cancer due to alcohol use	--	--	--	--	[illegible]	[illegible]	[illegible]	[illegible]	[illegible]	[illegible]	[illegible]	[illegible]	[illegible]	[illegible]	[illegible]	[illegible]	[illegible]	[illegible]	[illegible]	[illegible]	[illegible]	[illegible]	[illegible]
Liver cancer due to NASH	--	--	--	--	[illegible]	[illegible]	[illegible]	[illegible]	[illegible]	[illegible]	[illegible]	[illegible]	[illegible]	[illegible]	[illegible]	[illegible]	[illegible]	[illegible]	[illegible]	[illegible]	[illegible]	[illegible]	[illegible]
Liver cancer due to other causes	99·1 (57·6–162)	75 (43·4–135)	261 (178–359)	454 (340–568)	[illegible]	[illegible]	[illegible]	[illegible]	[illegible]	[illegible]	[illegible]	[illegible]	[illegible]	[illegible]	[illegible]	[illegible]	[illegible]	[illegible]	[illegible]	[illegible]	[illegible]	[illegible]	[illegible]
Gallbladder and biliary tract cancer	--	--	--	--	[illegible]	[illegible]	[illegible]	[illegible]	[illegible]	[illegible]	[illegible]	[illegible]	[illegible]	[illegible]	[illegible]	[illegible]	[illegible]	[illegible]	[illegible]	[illegible]	[illegible]	[illegible]	[illegible]
Pancreatic cancer	--	--	--	--	[illegible]	[illegible]	[illegible]	[illegible]	[illegible]	[illegible]	[illegible]	[illegible]	[illegible]	[illegible]	[illegible]	[illegible]	[illegible]	[illegible]	[illegible]	[illegible]	[illegible]	[illegible]	[illegible]
Larynx cancer	--	--	--	--	[illegible]	[illegible]	[illegible]	[illegible]	[illegible]	[illegible]	[illegible]	[illegible]	[illegible]	[illegible]	[illegible]	[illegible]	[illegible]	[illegible]	[illegible]	[illegible]	[illegible]	[illegible]	[illegible]
Tracheal, bronchus, and lung cancer	--	--	--	--	[illegible]	[illegible]	[illegible]	[illegible]	[illegible]	[illegible]	[illegible]	[illegible]	[illegible]	[illegible]	[illegible]	[illegible]	[illegible]	[illegible]	[illegible]	[illegible]	[illegible]	[illegible]	[illegible]
Malignant skin melanoma	1·01 (0·579–1·98)	1·21 (0·799–2·36)	11·7 (6·34–32·5)	59·3 (26·5–167)	[illegible]	[illegible]	[illegible]	[illegible]	[illegible]	[illegible]	[illegible]	[illegible]	[illegible]	[illegible]	[illegible]	[illegible]	[illegible]	[illegible]	[illegible]	[illegible]	[illegible]	[illegible]	[illegible]
Non-melanoma skin cancer	--	--	--	--	[illegible]	[illegible]	[illegible]	[illegible]	[illegible]	[illegible]	[illegible]	[illegible]	[illegible]	[illegible]	[illegible]	[illegible]	[illegible]	[illegible]	[illegible]	[illegible]	[illegible]	[illegible]	[illegible]
Non-melanoma skin cancer (squamous-cell carcinoma)	--	--	--	--	[illegible]	[illegible]	[illegible]	[illegible]	[illegible]	[illegible]	[illegible]	[illegible]	[illegible]	[illegible]	[illegible]	[illegible]	[illegible]	[illegible]	[illegible]	[illegible]	[illegible]	[illegible]	[illegible]
Breast cancer	--	--	--	--	[illegible]	[illegible]	[illegible]	[illegible]	[illegible]	[illegible]	[illegible]	[illegible]	[illegible]	[illegible]	[illegible]	[illegible]	[illegible]	[illegible]	[illegible]	[illegible]	[illegible]	[illegible]	[illegible]
Prostate cancer	--	--	--	--	[illegible]	[illegible]	[illegible]	[illegible]	[illegible]	[illegible]	[illegible]	[illegible]	[illegible]	[illegible]	[illegible]	[illegible]	[illegible]	[illegible]	[illegible]	[illegible]	[illegible]	[illegible]	[illegible]
Testicular cancer	16 (4·79–112)	11·1 (5·98–45·2)	52·1 (33·2–140)	244 (165–433)	[illegible]	[illegible]	[illegible]	[illegible]	[illegible]	[illegible]	[illegible]	[illegible]	[illegible]	[illegible]	[illegible]	[illegible]	[illegible]	[illegible]	[illegible]	[illegible]	[illegible]	[illegible]	[illegible]
Kidney cancer	29·4 (22·3–40)	16·2 (12·4–21·7)	169 (136–212)	905 (599–1230)	[illegible]	[illegible]	[illegible]	[illegible]	[illegible]	[illegible]	[illegible]	[illegible]	[illegible]	[illegible]	[illegible]	[illegible]	[illegible]	[illegible]	[illegible]	[illegible]	[illegible]	[illegible]	[illegible]
Bladder cancer	--	--	--	--	[illegible]	[illegible]	[illegible]	[illegible]	[illegible]	[illegible]	[illegible]	[illegible]	[illegible]	[illegible]	[illegible]	[illegible]	[illegible]	[illegible]	[illegible]	[illegible]	[illegible]	[illegible]	[illegible]
Brain and central nervous system cancer	192 (106–275)	206 (100–342)	1490 (932–1990)	3350 (2300–4360)	[illegible]	[illegible]	[illegible]	[illegible]	[illegible]	[illegible]	[illegible]	[illegible]	[illegible]	[illegible]	[illegible]	[illegible]	[illegible]	[illegible]	[illegible]	[illegible]	[illegible]	[illegible]	[illegible]
Thyroid cancer	--	--	--	--	[illegible]	[illegible]	[illegible]	[illegible]	[illegible]	[illegible]	[illegible]	[illegible]	[illegible]	[illegible]	[illegible]	[illegible]	[illegible]	[illegible]	[illegible]	[illegible]	[illegible]	[illegible]	[illegible]
Mesothelioma	--	--	--	--	[illegible]	[illegible]	[illegible]	[illegible]	[illegible]	[illegible]	[illegible]	[illegible]	[illegible]	[illegible]	[illegible]	[illegible]	[illegible]	[illegible]	[illegible]	[illegible]	[illegible]	[illegible]	[illegible]
Hodgkin lymphoma	--	--	--	90·2 (42–150)	[illegible]	[illegible]	[illegible]	[illegible]	[illegible]	[illegible]	[illegible]	[illegible]	[illegible]	[illegible]	[illegible]	[illegible]	[illegible]	[illegible]	[illegible]	[illegible]	[illegible]	[illegible]	[illegible]
Non-Hodgkin lymphoma	--	--	--	1240 (948–1570)	[illegible]	[illegible]	[illegible]	[illegible]	[illegible]	[illegible]	[illegible]	[illegible]	[illegible]	[illegible]	[illegible]	[illegible]	[illegible]	[illegible]	[illegible]	[illegible]	[illegible]	[illegible]	[illegible]
Multiple myeloma	--	--	--	--	[illegible]	[illegible]	[illegible]	[illegible]	[illegible]	[illegible]	[illegible]	[illegible]	[illegible]	[illegible]	[illegible]	[illegible]	[illegible]	[illegible]	[illegible]	[illegible]	[illegible]	[illegible]	[illegible]
Leukemia	310 (191–439)	255 (157–352)	2610 (1720–3490)	6350 (4740–8180)	[illegible]	[illegible]	[illegible]	[illegible]	[illegible]	[illegible]	[illegible]	[illegible]	[illegible]	[illegible]	[illegible]	[illegible]	[illegible]	[illegible]	[illegible]	[illegible]	[illegible]	[illegible]	[illegible]
Acute lymphoid leukemia	122 (64·8–198)	83·6 (46–133)	1030 (595–1440)	2460 (1610–3260)	[illegible]	[illegible]	[illegible]	[illegible]	[illegible]	[illegible]	[illegible]	[illegible]	[illegible]	[illegible]	[illegible]	[illegible]	[illegible]	[illegible]	[illegible]	[illegible]	[illegible]	[illegible]	[illegible]
Chronic lymphoid leukemia	--	--	--	--	[illegible]	[illegible]	[illegible]	[illegible]	[illegible]	[illegible]	[illegible]	[illegible]	[illegible]	[illegible]	[illegible]	[illegible]	[illegible]	[illegible]	[illegible]	[illegible]	[illegible]	[illegible]	[illegible]
Acute myeloid leukemia	55·8 (22·8–94·2)	51·6 (20·9–90·2)	416 (249–606)	1770 (1170–2540)	[illegible]	[illegible]	[illegible]	[illegible]	[illegible]	[illegible]	[illegible]	[illegible]	[illegible]	[illegible]	[illegible]	[illegible]	[illegible]	[illegible]	[illegible]	[illegible]	[illegible]	[illegible]	[illegible]
Chronic myeloid leukemia	12·6 (3·65–35·9)	19·8 (7·16–50·7)	308 (142–607)	509 (210–1010)	[illegible]	[illegible]	[illegible]	[illegible]	[illegible]	[illegible]	[illegible]	[illegible]	[illegible]	[illegible]	[illegible]	[illegible]	[illegible]	[illegible]	[illegible]	[illegible]	[illegible]	[illegible]	[illegible]
Other leukemia	119 (80·2–167)	99·5 (69·1–136)	859 (584–1230)	1610 (1210–2520)	[illegible]	[illegible]	[illegible]	[illegible]	[illegible]	[illegible]	[illegible]	[illegible]	[illegible]	[illegible]	[illegible]	[illegible]	[illegible]	[illegible]	[illegible]	[illegible]	[illegible]	[illegible]	[illegible]
Other malignant neoplasms	291 (205–397)	235 (168–313)	1250 (983–1590)	6190 (4310–8240)	[illegible]	[illegible]	[illegible]	[illegible]	[illegible]	[illegible]	[illegible]	[illegible]	[illegible]	[illegible]	[illegible]	[illegible]	[illegible]	[illegible]	[illegible]	[illegible]	[illegible]	[illegible]	[illegible]
Other neoplasms	10·1 (6·02–13)	4·07 (2·41–5·21)	41·5 (29–55·6)	83·4 (56·1–126)	[illegible]	[illegible]	[illegible]	[illegible]	[illegible]	[illegible]	[illegible]	[illegible]	[illegible]	[illegible]	[illegible]	[illegible]	[illegible]	[illegible]	[illegible]	[illegible]	[illegible]	[illegible]	[illegible]
[illegible] myeloproliferative, and other hematopoietic neoplasms	10·1 (6·02–13)	4·07 (2·41–5·21)	41·5 (29–55·6)	83·4 (56·1–126)	[illegible]	[illegible]	[illegible]	[illegible]	[illegible]	[illegible]	[illegible]	[illegible]	[illegible]	[illegible]	[illegible]	[illegible]	[illegible]	[illegible]	[illegible]	[illegible]	[illegible]	[illegible]	[illegible]
Cardiovascular diseases	1730 (1330–2350)	1220 (895–1690)	6460 (4940–8600)	5600 (4350–7520)	[illegible]	[illegible]	[illegible]	[illegible]	[illegible]	[illegible]	[illegible]	[illegible]	[illegible]	[illegible]	[illegible]	[illegible]	[illegible]	[illegible]	[illegible]	[illegible]	[illegible]	[illegible]	[illegible]
Rheumatic heart disease	--	--	--	852 (617–1180)	[illegible]	[illegible]	[illegible]	[illegible]	[illegible]	[illegible]	[illegible]	[illegible]	[illegible]	[illegible]	[illegible]	[illegible]	[illegible]	[illegible]	[illegible]	[illegible]	[illegible]	[illegible]	[illegible]
Ischemic heart disease	--	--	--	--	[illegible]	[illegible]	[illegible]	[illegible]	[illegible]	[illegible]	[illegible]	[illegible]	[illegible]	[illegible]	[illegible]	[illegible]	[illegible]	[illegible]	[illegible]	[illegible]	[illegible]	[illegible]	[illegible]
Stroke	718 (522–975)	496 (322–764)	2430 (1780–3380)	2050 (1500–2830)	[illegible]	[illegible]	[illegible]	[illegible]	[illegible]	[illegible]	[illegible]	[illegible]	[illegible]	[illegible]	[illegible]	[illegible]	[illegible]	[illegible]	[illegible]	[illegible]	[illegible]	[illegible]	[illegible]
Ischemic stroke	105 (66–163)	76·7 (47·7–128)	219 (124–394)	394 (227–606)	[illegible]	[illegible]	[illegible]	[illegible]	[illegible]	[illegible]	[illegible]	[illegible]	[illegible]	[illegible]	[illegible]	[illegible]	[illegible]	[illegible]	[illegible]	[illegible]	[illegible]	[illegible]	[illegible]
Intracerebral hemorrhage	435 (312–595)	334 (208–544)	1750 (1230–2530)	938 (531–1570)	[illegible]	[illegible]	[illegible]	[illegible]	[illegible]	[illegible]	[illegible]	[illegible]	[illegible]	[illegible]	[illegible]	[illegible]	[illegible]	[illegible]	[illegible]	[illegible]	[illegible]	[illegible]	[illegible]
Subarachnoid hemorrhage	178 (98·7–291)	84·9 (51·2–143)	456 (295–700)	716 (418–1250)	[illegible]	[illegible]	[illegible]	[illegible]	[illegible]	[illegible]	[illegible]	[illegible]	[illegible]	[illegible]	[illegible]	[illegible]	[illegible]	[illegible]	[illegible]	[illegible]	[illegible]	[illegible]	[illegible]
Hypertensive heart disease	--	--	--	--	[illegible]	[illegible]	[illegible]	[illegible]	[illegible]	[illegible]	[illegible]	[illegible]	[illegible]	[illegible]	[illegible]	[illegible]	[illegible]	[illegible]	[illegible]	[illegible]	[illegible]	[illegible]	[illegible]
Non-rheumatic valvular heart disease	--	--	--	--	[illegible]	[illegible]	[illegible]	[illegible]	[illegible]	[illegible]	[illegible]	[illegible]	[illegible]	[illegible]	[illegible]	[illegible]	[illegible]	[illegible]	[illegible]	[illegible]	[illegible]	[illegible]	[illegible]
Non-rheumatic calcific aortic valve disease	--	--	--	--	[illegible]	[illegible]	[illegible]	[illegible]	[illegible]	[illegible]	[illegible]	[illegible]	[illegible]	[illegible]	[illegible]	[illegible]	[illegible]	[illegible]	[illegible]	[illegible]	[illegible]	[illegible]	[illegible]
Non-rheumatic degenerative mitral valve disease	--	--	--	--	[illegible]	[illegible]	[illegible]	[illegible]	[illegible]	[illegible]	[illegible]	[illegible]	[illegible]	[illegible]	[illegible]	[illegible]	[illegible]	[illegible]	[illegible]	[illegible]	[illegible]	[illegible]	[illegible]
Other non-rheumatic valve diseases	--	--	--	--	[illegible]	[illegible]	[illegible]	[illegible]	[illegible]	[illegible]	[illegible]	[illegible]	[illegible]	[illegible]	[illegible]	[illegible]	[illegible]	[illegible]	[illegible]	[illegible]	[illegible]	[illegible]	[illegible]
Cardiomyopathy and myocarditis	531 (365–733)	317 (225–434)	1940 (1460–2670)	1240 (894–1790)	[illegible]	[illegible]	[illegible]	[illegible]	[illegible]	[illegible]	[illegible]	[illegible]	[illegible]	[illegible]	[illegible]	[illegible]	[illegible]	[illegible]	[illegible]	[illegible]	[illegible]	[illegible]	[illegible]
Myocarditis	59·1 (37·7–101)	79·2 (54·5–117)	584 (447–786)	337 (254–477)	[illegible]	[illegible]	[illegible]	[illegible]	[illegible]	[illegible]	[illegible]	[illegible]	[illegible]	[illegible]	[illegible]	[illegible]	[illegible]	[illegible]	[illegible]	[illegible]	[illegible]	[illegible]	[illegible]
Alcoholic cardiomyopathy	--	--	--	--	[illegible]	[illegible]	[illegible]	[illegible]	[illegible]	[illegible]	[illegible]	[illegible]	[illegible]	[illegible]	[illegible]	[illegible]	[illegible]	[illegible]	[illegible]	[illegible]	[illegible]	[illegible]	[illegible]
Other cardiomyopathy	471 (321–660)	238 (163–340)	1350 (988–1950)	906 (610–1400)	[illegible]	[illegible]	[illegible]	[illegible]	[illegible]	[illegible]	[illegible]	[illegible]	[illegible]	[illegible]	[illegible]	[illegible]	[illegible]	[illegible]	[illegible]	[illegible]	[illegible]	[illegible]	[illegible]
Atrial fibrillation and flutter	--	--	--	--	[illegible]	[illegible]	[illegible]	[illegible]	[illegible]	[illegible]	[illegible]	[illegible]	[illegible]	[illegible]	[illegible]	[illegible]	[illegible]	[illegible]	[illegible]	[illegible]	[illegible]	[illegible]	[illegible]
Aortic aneurysm	--	--	--	--	[illegible]	[illegible]	[illegible]	[illegible]	[illegible]	[illegible]	[illegible]	[illegible]	[illegible]	[illegible]	[illegible]	[illegible]	[illegible]	[illegible]	[illegible]	[illegible]	[illegible]	[illegible]	[illegible]
Endocarditis	75·1 (46·5–113)	69 (41–102)	598 (387–861)	251 (130–389)	[illegible]	[illegible]	[illegible]	[illegible]	[illegible]	[illegible]	[illegible]	[illegible]	[illegible]	[illegible]	[illegible]	[illegible]	[illegible]	[illegible]	[illegible]	[illegible]	[illegible]	[illegible]	[illegible]
Other cardiovascular and circulatory diseases	409 (293–645)	340 (229–511)	1500 (1080–2070)	1200 (873–1600)	[illegible]	[illegible]	[illegible]	[illegible]	[illegible]	[illegible]	[illegible]	[illegible]	[illegible]	[illegible]	[illegible]	[illegible]	[illegible]	[illegible]	[illegible]	[illegible]	[illegible]	[illegible]	[illegible]
Chronic respiratory diseases	0 (0–0)	0 (0–0)	0 (0–0)	4350 (3430–5570)	[illegible]	[illegible]	[illegible]	[illegible]	[illegible]	[illegible]	[illegible]	[illegible]	[illegible]	[illegible]	[illegible]	[illegible]	[illegible]	[illegible]	[illegible]	[illegible]	[illegible]	[illegible]	[illegible]
Chronic obstructive pulmonary disease	--	--	0 (0–0)	400 (270–621)	[illegible]	[illegible]	[illegible]	[illegible]	[illegible]	[illegible]	[illegible]	[illegible]	[illegible]	[illegible]	[illegible]	[illegible]	[illegible]	[illegible]	[illegible]	[illegible]	[illegible]	[illegible]	[illegible]
Pneumoconiosis	--	--	--	--	[illegible]	[illegible]	[illegible]	[illegible]	[illegible]	[illegible]	[illegible]	[illegible]	[illegible]	[illegible]	[illegible]	[illegible]	[illegible]	[illegible]	[illegible]	[illegible]	[illegible]	[illegible]	[illegible]
Silicosis	--	--	--	--	[illegible]	[illegible]	[illegible]	[illegible]	[illegible]	[illegible]	[illegible]	[illegible]	[illegible]	[illegible]	[illegible]	[illegible]	[illegible]	[illegible]	[illegible]	[illegible]	[illegible]	[illegible]	[illegible]
Asbestosis	--	--	--	--	[illegible]	[illegible]	[illegible]	[illegible]	[illegible]	[illegible]	[illegible]	[illegible]	[illegible]	[illegible]	[illegible]	[illegible]	[illegible]	[illegible]	[illegible]	[illegible]	[illegible]	[illegible]	[illegible]
Coal workers pneumoconiosis	--	--	--	--	[illegible]	[illegible]	[illegible]	[illegible]	[illegible]	[illegible]	[illegible]	[illegible]	[illegible]	[illegible]	[illegible]	[illegible]	[illegible]	[illegible]	[illegible]	[illegible]	[illegible]	[illegible]	[illegible]
Other pneumoconiosis	--	--	--	--	[illegible]	[illegible]	[illegible]	[illegible]	[illegible]	[illegible]	[illegible]	[illegible]	[illegible]	[illegible]	[illegible]	[illegible]	[illegible]	[illegible]	[illegible]	[illegible]	[illegible]	[illegible]	[illegible]
Asthma	--	--	--	3060 (2320–4070)	[illegible]	[illegible]	[illegible]	[illegible]	[illegible]	[illegible]	[illegible]	[illegible]	[illegible]	[illegible]	[illegible]	[illegible]	[illegible]	[illegible]	[illegible]	[illegible]	[illegible]	[illegible]	[illegible]
Interstitial lung disease and pulmonary sarcoidosis	--	--	--	148 (83·4–253)	[illegible]	[illegible]	[illegible]	[illegible]	[illegible]	[illegible]	[illegible]	[illegible]	[illegible]	[illegible]	[illegible]	[illegible]	[illegible]	[illegible]	[illegible]	[illegible]	[illegible]	[illegible]	[illegible]
Other chronic respiratory diseases	0 (0–0)	0 (0–0)	0 (0–0)	747 (433–1150)	[illegible]	[illegible]	[illegible]	[illegible]	[illegible]	[illegible]	[illegible]	[illegible]	[illegible]	[illegible]	[illegible]	[illegible]	[illegible]	[illegible]	[illegible]	[illegible]	[illegible]	[illegible]	[illegible]
Digestive diseases	1280 (980–1740)	1160 (858–1600)	7600 (5460–10600)	9690 (7230–13200)	[illegible]	[illegible]	[illegible]	[illegible]	[illegible]	[illegible]	[illegible]	[illegible]	[illegible]	[illegible]	[illegible]	[illegible]	[illegible]	[illegible]	[illegible]	[illegible]	[illegible]	[illegible]	[illegible]

Cirrhosis and other chronic liver diseases	–	–	–	4060 (2850–5880)	2780 (2070–3590)	2480 (1980–3080)	5950 (5000–7040)	10100 (8620–11700)	18800 (16800–21100)	32600 (29800–35800)	50600 (46200–55600)	69200 (63000–76000)	88700 (80900–97200)	107000 (98000–116000)	116000 (107000–126000)	117000 (108000–126000)	105000 (97400–114000)	83500 (77400–91200)	65200 (60000–71000)	47900 (42900–53100)	28000 (24300–31000)	10800 (8870–12200)	3580 (2770–4140)
Cirrhosis and other chronic liver diseases due to hepatitis B	–	–	–	44·8 (21·6–80·1)	72·7 (37·7–118)	118 (63·9–187)	416 (217–672)	2020 (1200–3060)	5300 (3480–7390)	10200 (7910–12900)	16500 (12000–21500)	21900 (16700–27900)	25900 (19000–33800)	28100 (19900–37800)	27000 (18800–36900)	25400 (17700–35500)	22200 (15700–30200)	17600 (12400–24300)	14500 (10200–18900)	10800 (7420–14300)	6010 (4450–7770)	2150 (1440–3000)	651 (348–1040)
Cirrhosis and other chronic liver diseases due to hepatitis C	–	–	–	38·9 (17–74·4)	76·2 (38·5–133)	127 (68–215)	474 (254–792)	2050 (1300–2970)	5260 (3490–7170)	9570 (7230–12100)	14700 (10500–19500)	20200 (14900–26100)	25700 (18900–33800)	30700 (22200–40800)	32000 (23600–42600)	31300 (22200–42300)	28700 (21600–37000)	23200 (17900–29100)	18200 (13700–23600)	13400 (9970–17500)	8170 (6120–10400)	3240 (2250–4300)	1120 (677–1630)
Cirrhosis and other chronic liver diseases due to alcohol use	–	–	–	–	–	–	29·7 (8·74–65·6)	561 (308–946)	1950 (1180–3030)	5760 (4340–7450)	11900 (8270–15800)	18100 (13400–23300)	25300 (18600–33100)	33100 (24100–42600)	39600 (29200–51000)	41100 (30300–53200)	34900 (25900–44900)	25100 (18400–32500)	17100 (12700–21600)	11300 (8160–14800)	6080 (4570–7860)	2270 (1560–3220)	716 (407–1180)
Cirrhosis and other chronic liver diseases due to NAFLD	–	–	–	–	–	–	158 (63·3–308)	464 (239–799)	1100 (521–1970)	1920 (1080–3070)	2750 (1360–4790)	3950 (2000–6530)	5710 (2730–9900)	7320 (3510–12700)	7890 (3910–13400)	8390 (4200–15100)	8470 (5170–13200)	7510 (4670–11200)	6490 (3530–10100)	5210 (2940–7980)	3250 (2020–4970)	1320 (717–2210)	468 (197–861)
Cirrhosis and other chronic liver diseases due to other causes	–	–	–	3980 (2790–5740)	2630 (1950–3410)	2240 (1770–2790)	4870 (3900–5930)	4960 (3400–6850)	5140 (3150–7790)	5140 (3000–8420)	4790 (2600–7820)	5040 (2830–8390)	6060 (3430–10300)	7540 (4230–12500)	9290 (5080–15700)	10800 (6090–17800)	11100 (6000–18800)	10100 (5510–16600)	8980 (4920–14600)	7260 (4080–11900)	4470 (2630–6710)	1810 (990–3030)	622 (268–1160)
Upper digestive system diseases	–	–	–	773 (445–1400)	328 (241–440)	254 (204–317)	926 (773–1110)	1480 (1250–1730)	2390 (2100–2830)	2960 (2630–3430)	4160 (3700–4880)	5280 (4610–6060)	7530 (6540–8760)	9760 (8620–11100)	11900 (10600–13500)	13500 (12000–15300)	16000 (14300–18200)	17300 (15600–19300)	18400 (16600–20500)	16900 (15100–19000)	12200 (10700–13400)	4890 (4090–5450)	1580 (1230–1820)
Peptic ulcer disease	–	–	–	358 (176–882)	289 (210–390)	226 (179–279)	790 (651–960)	1290 (1100–1530)	2040 (1780–2410)	2450 (2180–2870)	3600 (3160–4340)	4540 (3930–5330)	6440 (5560–7630)	8370 (7360–9560)	10400 (9170–11900)	11900 (10600–13500)	13900 (12400–15900)	14900 (13400–16900)	15800 (14200–17900)	14400 (12900–16300)	10200 (8950–11500)	4290 (3580–4820)	1400 (1090–1620)
Gastritis and duodenitis	–	–	–	414 (228–755)	39·1 (20·5–55·3)	27·9 (14·2–41·9)	136 (87·5–200)	191 (119–279)	352 (248–489)	507 (390–654)	555 (437–729)	740 (559–922)	1090 (738–1370)	1390 (900–1740)	1540 (1010–1880)	1600 (1120–1910)	2150 (1520–2580)	2400 (1550–2880)	2600 (1710–3080)	2520 (1720–2990)	1950 (1410–2260)	603 (486–700)	183 (140–219)
Appendicitis	–	–	–	557 (293–891)	575 (400–746)	539 (370–700)	723 (527–939)	647 (464–809)	819 (635–1020)	522 (380–648)	743 (549–923)	901 (654–1100)	866 (614–1060)	900 (664–1110)	1050 (779–1290)	1210 (878–1490)	1250 (924–1570)	1390 (973–1710)	1270 (959–1570)	1180 (933–1460)	751 (584–939)	344 (260–435)	105 (74·6–140)
Paralytic ileus and intestinal obstruction	848 (578–1260)	903 (618–1300)	6610 (4630–9460)	1940 (1080–3060)	1570 (1000–2210)	1060 (713–1450)	1920 (1300–2490)	1900 (1270–2450)	2360 (1660–2960)	2810 (1940–3460)	3770 (2550–4650)	4580 (3150–5680)	4670 (3330–5780)	5340 (3820–6430)	7060 (5200–8450)	8770 (6450–10600)	11200 (8420–13300)	11500 (9070–13700)	12100 (9950–14500)	13500 (10900–16000)	11700 (9450–13700)	6110 (4590–7080)	2280 (1500–2700)
Inguinal, femoral, and abdominal hernia	429 (312–571)	257 (181–359)	991 (690–1390)	458 (258–674)	143 (88·2–199)	101 (69·2–131)	188 (141–255)	261 (201–335)	313 (255–382)	279 (237–335)	432 (359–518)	560 (455–668)	759 (595–916)	1230 (964–1490)	1670 (1330–1960)	2090 (1670–2470)	2680 (2140–3120)	3130 (2670–3680)	3680 (3190–4340)	4030 (3480–4720)	3000 (2540–3500)	1640 (1350–1930)	604 (467–732)
Inflammatory bowel disease	–	–	–	337 (190–541)	74·6 (50·1–93·6)	57·8 (42·9–72)	100 (75·2–129)	155 (125–195)	269 (219–335)	359 (286–442)	421 (342–499)	560 (465–685)	689 (550–827)	879 (696–1040)	1130 (915–1320)	1430 (1100–1650)	1720 (1320–1990)	1970 (1560–2260)	2280 (1770–2600)	2540 (1980–2900)	2080 (1560–2360)	1150 (811–1330)	414 (272–505)
Vascular intestinal disorders	–	–	–	258 (134–476)	70·5 (49·2–110)	24·9 (20·4–31·4)	45·7 (39·6–54)	114 (94·9–137)	184 (156–215)	178 (160–199)	312 (277–354)	520 (464–580)	889 (789–989)	1500 (1340–1650)	2540 (2320–2780)	3580 (3280–3870)	5300 (4860–5740)	6400 (5920–6900)	6910 (6360–7400)	7330 (6580–8020)	5250 (4450–5830)	2740 (2260–3180)	893 (706–1060)
Gallbladder and biliary diseases	–	–	–	135 (66·3–227)	67·2 (42–87·7)	170 (87·8–244)	140 (93·3–179)	253 (174–313)	352 (242–436)	458 (334–555)	648 (493–772)	906 (678–1100)	1180 (893–1430)	1890 (1430–2290)	2480 (1950–2950)	3500 (2830–4150)	4710 (3890–5630)	6140 (5130–7390)	7720 (6410–9110)	8730 (7200–10100)	7560 (6030–8740)	4110 (3170–4790)	1580 (1130–1920)
Pancreatitis	–	–	–	53·2 (26·5–89·1)	54·6 (39·9–71)	67·8 (50·6–86·7)	469 (358–598)	1110 (904–1400)	2510 (2120–3100)	3830 (3360–4630)	5020 (4360–6100)	5940 (5080–7290)	7160 (5940–8650)	6020 (5100–7240)	7440 (6250–8720)	6370 (5420–7520)	6360 (5390–7460)	5680 (4870–6560)	5200 (4400–6160)	4270 (3690–4930)	2870 (2420–3320)	1190 (977–1390)	359 (278–421)
Other digestive diseases	–	–	–	1120 (696–1700)	336 (240–427)	245 (185–322)	265 (200–352)	322 (245–407)	510 (389–637)	703 (547–836)	917 (713–1090)	1330 (1040–1590)	1740 (1340–2080)	2370 (1800–2830)	3000 (2280–3550)	3950 (3040–4670)	4440 (3330–5240)	5180 (3900–6190)	5620 (4240–6790)	6390 (4730–7660)	5430 (3970–6680)	3070 (2190–3990)	1300 (867–1690)
Neurological disorders	3·6 (2·87–4·45)	7·7 (6·56–8·96)	2580 (2050–3480)	4650 (3690–6080)	2490 (2030–3110)	3180 (2710–3860)	5960 (5190–7040)	6270 (5590–7280)	6110 (5510–7010)	6350 (5730–7210)	6790 (6150–7660)	7680 (6880–8940)	10200 (8450–14100)	13400 (10100–21100)	19200 (13300–31700)	28900 (18300–55300)	47600 (28400–96900)	77900 (46600–154000)	117000 (68300–236000)	187000 (91900–410000)	188000 (80000–430000)	110000 (39400–258000)	48300 (15300–118000)
Alzheimer's disease and other dementias	–	–	–	–	–	–	–	–	–	–	–	283 (28·2–1020)	1640 (211–5220)	3480 (564–11000)	6770 (1240–19500)	13100 (2670–39500)	24500 (5350–73500)	40600 (9160–117000)	63200 (14200–184000)	125000 (29100–352000)	144000 (34300–390000)	94300 (22900–245000)	44600 (11200–115000)
Parkinson's disease	–	–	–	–	–	–	–	6·78 (5·89–7·52)	12·5 (10·8–13·7)	27·6 (24·5–30·6)	37 (33·2–41·2)	429 (382–475)	971 (852–1090)	1860 (1660–2070)	2970 (2650–3310)	5900 (5350–6580)	12700 (11600–14100)	27500 (25200–30100)	45000 (41400–48800)	54700 (49100–59500)	39100 (33900–42600)	13900 (11600–15300)	3230 (2480–3810)
Idiopathic epilepsy	–	–	1330 (1000–1980)	3340 (2610–4410)	1770 (1390–2320)	2290 (1900–2910)	4210 (3570–5200)	4730 (4170–5610)	4960 (4400–5800)	5060 (4470–5890)	5060 (4520–5840)	4660 (4100–5430)	4270 (3750–4930)	3660 (3240–4100)	3610 (3140–4070)	3290 (2900–3730)	3200 (2750–3680)	2690 (2410–3040)	2540 (2220–2850)	2350 (1980–2640)	1560 (1330–1730)	664 (548–745)	196 (154–224)
Multiple sclerosis	–	–	–	–	15·2 (0·906–75·9)	5·21 (1·2–14·2)	13·1 (4·75–21·7)	55·6 (40·4–69·5)	126 (105–191)	283 (239–451)	466 (391–738)	706 (592–1110)	907 (755–1310)	1070 (844–1520)	1220 (944–1760)	1190 (858–1670)	1030 (746–1350)	816 (552–1010)	573 (390–758)	376 (262–490)	190 (129–240)	59 (39·3–73)	13·9 (7·24–17·8)
Motor neuron disease	3·6 (2·87–4·45)	7·7 (6·56–8·96)	242 (203–294)	79·3 (61·8–106)	24·5 (21·2–30·4)	38·4 (33·9–43·8)	91·5 (80·9–104)	94·2 (82·3–110)	82·2 (74·1–91·3)	149 (135–163)	282 (259–305)	500 (463–535)	903 (836–967)	1410 (1310–1510)	2160 (2010–2290)	2820 (2650–2970)	3400 (3170–3580)	3430 (3170–3640)	2850 (2610–3040)	1820 (1600–1980)	879 (744–967)	250 (205–278)	61·2 (47·5–70·2)
Other neurological disorders	–	–	1010 (794–1280)	1220 (884–1600)	680 (528–812)	841 (709–996)	1640 (1440–1930)	1390 (1240–1600)	929 (847–1060)	827 (754–938)	940 (853–1060)	1110 (1010–1240)	1540 (1400–1720)	1900 (1730–2110)	2450 (2250–2700)	2530 (2350–2740)	2770 (2580–2990)	2870 (2690–3070)	2830 (2620–3050)	2460 (2190–2640)	1600 (1380–1750)	681 (560–747)	219 (170–262)
Mental disorders	–	–	–	–	0·388 (0·232–0·753)	0·284 (0·204–0·422)	2·97 (1·42–7·3)	5·29 (2·92–10·7)	3·9 (2·86–5·83)	5·75 (4·1–8·53)	4·68 (3·58–6·57)	8·51 (6·32–11·9)	7·8 (5·79–10·3)	–	–	–	–	–	–	–	–	–	–
Eating disorders	–	–	–	–	0·388 (0·232–0·753)	0·284 (0·204–0·422)	2·97 (1·42–7·3)	5·29 (2·92–10·7)	3·9 (2·86–5·83)	5·75 (4·1–8·53)	4·68 (3·58–6·57)	8·51 (6·32–11·9)	7·8 (5·79–10·3)	–	–	–	–	–	–	–	–	–	–
Anorexia nervosa	–	–	–	–	0·225 (0·152–0·493)	0·165 (0·12–0·247)	1·7 (1·1–3·78)	3·4 (2·29–6·86)	2·91 (2·13–4)	3·43 (2·39–5·44)	2·83 (2·24–3·99)	5·88 (4·29–8·33)	5·29 (3·7–7·86)	–	–	–	–	–	–	–	–	–	–
Bulimia nervosa	–	–	–	–	0·163 (0·0749–0·474)	0·119 (0·0736–0·25)	1·26 (0·248–5·06)	1·89 (0·495–6·8)	0·984 (0·27–3·04)	2·32 (0·417–5·32)	1·84 (1·06–3·72)	2·63 (0·62–6·09)	2·51 (0·422–5·14)	–	–	–	–	–	–	–	–	–	–
Substance use disorders	0 (0–0)	0 (0–0)	0 (0–0)	0 (0–0)	0 (0–0)	0 (0–0)	2430 (2250–2620)	9180 (8470–10200)	16200 (14900–17800)	23500 (21400–25600)	25600 (23100–27700)	25800 (23500–27600)	27900 (24900–30000)	25600 (22900–27400)	25100 (22600–26800)	19400 (17400–20800)	13500 (12000–14400)	8360 (7410–8990)	5670 (4940–6110)	3160 (2780–3410)	1700 (1470–1840)	639 (528–703)	205 (162–231)
Alcohol use disorders	–	–	–	–	–	0 (0–0)	592 (433–673)	1960 (1480–2210)	4490 (3650–4910)	9430 (7670–10300)	12800 (10600–13900)	15700 (13300–17100)	19100 (16000–20900)	18400 (15600–20100)	19000 (16500–20600)	15800 (13800–17100)	10900 (9430–11800)	6640 (5620–7200)	4380 (3640–4800)	2200 (1860–2380)	1070 (867–1160)	324 (258–359)	103 (75·6–118)
Drug use disorders	0 (0–0)	0 (0–0)	0 (0–0)	0 (0–0)	0 (0–0)	0 (0–0)	1840 (1680–2000)	7220 (6710–8050)	11700 (10900–13000)	14100 (13200–15700)	12700 (11900–14200)	10100 (9570–10700)	8760 (8210–9370)	7200 (6610–7690)	6060 (5510–6550)	3620 (3320–3890)	2580 (2380–2780)	1720 (1580–1860)	1290 (1190–1390)	964 (866–1060)	635 (549–701)	315 (259–359)	102 (80·3–116)
Opioid use disorders	0 (0–0)	0 (0–0)	0 (0–0)	0 (0–0)	0 (0–0)	0 (0–0)	1270 (1150–1390)	5330 (4920–6030)	8640 (7980–9900)	10000 (9190–11400)	9100 (8490–10300)	7090 (6710–7580)	6050 (5610–6650)	4760 (4350–5200)	4010 (3620–4390)	2420 (2210–2630)	1720 (1580–1860)	1150 (1050–1250)	892 (814–971)	627 (560–692)	414 (354–463)	194 (160–226)	61·5 (49·5–70·8)
Cocaine use disorders	–	–	–	–	–	0 (0–0)	182 (161–201)	552 (500–631)	851 (736–1350)	1400 (1220–2230)	1250 (1140–1440)	1000 (820–1090)	969 (727–1060)	1090 (786–1200)	916 (670–1030)	500 (370–565)	289 (235–320)	139 (116–156)	87·2 (72·8–97·1)	14·4 (12·4–16·5)	8·62 (7·12–9·81)	4·27 (3·38–4·82)	2·02 (1·53–2·33)
Amphetamine use disorders	–	–	–	–	–	0 (0–0)	135 (113–156)	512 (457–575)	947 (792–1210)	1060 (885–1390)	865 (783–972)	672 (564–769)	558 (436–657)	566 (413–676)	450 (325–536)	270 (198–318)	179 (144–208)	34·2 (23·8–40·8)	20·2 (14·5–23·9)	33·3 (24·8–46·4)	34·1 (24–50·4)	24 (16·5–34·2)	8·61 (5·89–11·9)
Other drug use disorders	–	–	–	–	–	0 (0–0)	253 (229–286)	827 (747–926)	1240 (1130–1470)	1580 (1440–1840)	1490 (1360–1660)	1320 (1220–1430)	1180 (1090–1310)	778 (710–852)	680 (615–749)	430 (390–473)	389 (352–433)	393 (358–433)	292 (262–326)	289 (260–321)	178 (156–196)	92·4 (75·7–104)	29·5 (22·6–36·1)
Diabetes and kidney diseases	28·9 (21·5–38·3)	72·8 (54·6–97·5)	2850 (2230–3680)	3270 (2520–4190)	3050 (2530–3640)	2860 (2450–3310)	7340 (6500–8340)	10600 (9500–11800)	12400 (11400–13500)	18300 (16900–19900)	25100 (23200–27400)	38500 (35200–42300)	56800 (51800–62100)	88300 (80500–96100)	124000 (113000–134000)	158000 (146000–171000)	183000 (170000–197000)	192000 (179000–206000)	189000 (177000–202000)	173000 (158000–186000)	128000 (112000–138000)	62300 (52100–67800)	22600 (17700–25200)
Diabetes mellitus	28·9 (21·5–38·3)	72·8 (54·6–97·5)	813 (579–1140)	640 (438–950)	554 (438–758)	606 (485–768)	1590 (1390–1820)	2730 (2440–3080)	3420 (3120–3770)	6340 (5790–6980)	9130 (8360–9980)	16800 (15300–18300)	28400 (25800–31200)	47000 (43100–51000)	68500 (62900–74300)	89300 (82500–96800)	102000 (95100–110000)	105000 (97500–113000)	1e+05 (93500–107000)	85000 (77100–91600)	55700 (49100–60200)	23800 (19900–26000)	7140 (5650–7940)
Diabetes mellitus type 1	28·9 (21·5–38·3)	72·8 (54·6–97·5)	813 (579–1140)	640 (438–950)	554 (438–758)	606 (485–768)	929 (808–1070)	1840 (1620–2110)	2140 (1940–2430)	2410 (2090–2850)	3250 (2860–3870)	5110 (4430–6460)	1640 (1340–2360)	2430 (1990–3510)	3490 (2870–5150)	3260 (2700–4630)	3690 (3040–5430)	2810 (2330–4030)	2440 (2000–3460)	1100 (899–1510)	761 (602–1050)	375 (279–517)	129 (87·5–183)
Diabetes mellitus type 2	–	–	–	–	–	–	658 (525–832)	890 (724–1120)	1280 (1100–1530)	3930 (3480–4470)	5880 (5250–6610)	11700 (10400–13000)	26800 (24300–29300)	44600 (40800–48400)	65000 (59600–70600)	86000 (79500–93200)	98600 (91500–106000)	102000 (94900–109000)	97800 (91200–104000)	83900 (76100–90400)	55000 (48300–59300)	23400 (19600–25500)	7010 (5520–7810)
Chronic kidney disease	–	–	1980 (1530–2590)	2560 (1960–3390)	2370 (1940–2900)	2190 (1850–2580)	5700 (4990–6540)	7810 (6940–8740)	8820 (8020–9760)	11800 (10800–13000)	15800 (14500–17500)	21500 (19600–23900)	28200 (25400–31300)	41000 (37100–45300)	54700 (49600–60600)	68100 (62400–75300)	80300 (74200–87500)	86500 (80000–93800)	88500 (82400–95300)	87700 (79200–94600)	71800 (62800–77700)	38400 (32100–41900)	15500 (12000–17400)
Chronic kidney disease due to diabetes mellitus type 1	–	–	11·3 (4·83–23·7)	19·5 (8·57–38·7)	32·6 (11·7–72·1)	61·4 (22·6–126)	260 (99·8–516)	675 (298–1220)	1240 (628–2090)	2350 (1260–3540)	3600 (1960–5520)	4990 (2700–7720)	5760 (2660–9410)	6060 (2640–11000)	5390 (1940–10600)	4780 (1870–9870)	4180 (1790–8770)	3180 (1330–6320)	2010 (804–4240)	1020 (394–2260)	345 (122–816)	55 (17·3–133)	5·4 (1·53–14)
Chronic kidney disease due to diabetes mellitus type 2	–	–	–	–	–	–	140 (40·2–320)	307 (116–661)	468 (177–923)	869 (307–1710)	1550 (579–2910)	3030 (1270–5360)	5870 (3000–9340)	11300 (6420–16500)	17600 (10800–24400)	22800 (13200–31000)	27200 (17500–36000)	28900 (19300–37700)	28200 (18400–37600)	25800 (16800–35100)	18900 (12300–26600)	8980 (5450–13100)	3500 (1860–5430)
Chronic kidney disease due to hypertension	–	–	–	–	–	–	397 (204–699)	804 (454–1310)	1100 (654–1740)	1600 (990–2430)	2410 (1520–3580)	3710 (2510–5300)	5550 (3930–7770)	9390 (6600–12800)	14000 (9950–19000)	19700 (14600–26100)	25000 (19000–32300)	28300 (22200–35900)	31200 (24600–40200)	33500 (26000–42200)	30400 (23900–37700)	17600 (13300–21800)	7360 (5280–9730)
Chronic kidney disease due to glomerulonephritis	–	–	536 (273–897)	881 (462–1420)	843 (441–1340)	917 (510–1400)	2460 (1580–3490)	3030 (1830–4330)	3090 (1970–4420)	3390 (1920–5150)	4120 (2360–6480)	4810 (2310–7620)	5030 (2990–8020)	6560 (3670–10400)	8210 (4640–13300)	9320 (5030–14800)	9820 (5140–16400)	9890 (5380–16000)	9910 (5380–16100)	9300 (4610–15800)	6670 (3380–11300)	3200 (1580–5470)	1110 (488–2220)
Chronic kidney disease due to other and unspecified causes	–	–	1440 (1030–1920)	1660 (1130–2310)	1500 (1020–2040)	1210 (747–1670)	2440 (1510–3400)	2990 (1830–4310)	2920 (1830–4060)	3550 (2060–5320)	4170 (2280–6450)	5000 (2680–7790)	5960 (3100–9390)	7610 (4220–12200)	9510 (5120–15700)	11400 (6170–19400)	14100 (7890–22300)	16200 (9380–25200)	17100 (9650–26700)	18100 (10300–27700)	15500 (8860–23000)	8600 (5330–12800)	3480 (1800–5420)
Acute glomerulonephritis	–	–	58·4 (17·4–91)	68·1 (20·6–101)	123 (38·5–177)	62·7 (21·3–88·2)	58 (29–78·5)	75·3 (39–99·7)	110 (51·8–136)	159 (78·7–200)	174 (85·5–221)	223 (104–287)	261 (133–339)	357 (172–466)	370 (194–487)	456 (236–579)	632 (312–806)	610 (338–777)	669 (329–847)	619 (316–766)	440 (244–530)	115 (71·7–137)	27·9 (20·7–33·9)
Skin and subcutaneous diseases	–	–	731 (363–1030)	612 (334–820)	158 (79·7–208)	229 (124–308)	436 (256–538)	408 (225–498)	379 (225–476)	867 (524–1050)	901 (523–1140)	1080 (702–1340)	1590 (977–2020)	1870 (1140–2370)	2660 (1620–3500)	3380 (2050–4320)	3860 (2380–5100)	4610 (2840–6430)	5520 (3600–8040)	5240 (3360–8220)	4720 (2940–7430)	2850 (1630–4480)	1260 (662–2060)
Bacterial skin diseases	–	–	713 (347–1000)	586 (312–792)	142 (69·4–192)	211 (113–285)	403 (243–499)	365 (199–446)	327 (195–400)	766 (450–932)	784 (438–989)	928 (582–1180)	1400 (841–1770)	1610 (957–2040)	2260 (1320–2950)	2850 (1660–3680)	3070 (1770–4110)	3470 (1990–4910)	3990 (2430–5890)	3320 (1950–5250)	2870 (1540–4760)	1770 (819–2960)	756 (327–1300)
Cellulitis	–	–	79·7 (21·8–135)	50·9 (10·6–116)	15·6 (5·62–23·6)	33·7 (14·8–47·9)	101 (27·6–157)	138 (53–190)	68·8 (28·5–97·7)	174 (71·2–247)	248 (106–359)	293 (138–430)	441 (190–629)	462 (215–700)	628 (269–952)	785 (355–1190)	800 (348–1240)	876 (383–1400)	905 (379–1660)	844 (330–1520)	709 (256–1430)	452 (179–845)	207 (63·2–380)
Pyoderma	–	–	633 (310–885)	535 (291–735)	127 (59·9–173)	177 (90·9–244)	302 (202–392)	227 (129–303)	258 (154–316)	592 (327–725)	537 (282–689)	635 (369–810)	957 (547–1230)	1150 (663–1460)	1630 (922–2110)	2070 (1160–2620)	2260 (1260–2970)	2590 (1420–3520)	3080 (1810–4320)	2480 (1420–3810)	2160 (1140–3360)	1320 (592–2130)	549 (240–913)
Decubitus ulcer	–	–	–	3·93 (1·1–8·87)	4·09 (1·44–6·18)	7·45 (2·8–10·3)	21·8 (6·59–31·1)	31·7 (11·5–46·2)	40 (16·3–58·5)	84·6 (52·4–120)	94·5 (52·6–136)	118 (74·8–172)	145 (84·9–224)	213 (127–299)	327 (188–492)	439 (239–644)	684 (397–996)	1000 (585–1510)	1360 (775–2340)	1710 (1040–2780)	1650 (974–2730)	965 (505–1650)	460 (216–826)
Other skin and subcutaneous diseases	–	–	18·5 (12·2–30·3)	21·8 (15·4–34·7)	11·6 (7·75–15·6)	10·9 (6·89–15)	11·4 (7·11–15·5)	10·9 (7·73–17·1)	11·4 (7·61–17·4)	16 (10·9–23)	22·1 (14·4–33·4)	35·1 (24·8–46·7)	49·5 (32·1–66·9)	46·6 (29·4–64·6)	70·4 (45·8–97·1)	85·4 (53·7–118)	107 (66·1–154)	137 (86·5–206)	165 (103–272)	205 (123–342)	201 (118–349)	115 (58–212)	46·6 (18·8–90·8)
Musculoskeletal disorders	–	–	–	–	120 (102–147)	214 (185–262)	303 (268–356)	376 (336–441)	457 (413–527)	555 (503–644)	642 (575–747)	829 (741–979)	1010 (896–1210)	1310 (1150–1550)	1890 (1670–2200)	3030 (2680–3530)	3930 (3500–4520)	4930 (4350–5660)	5180 (4480–5850)	5130 (4270–5810)	3930 (3160–4420)	1800 (1350–2070)	612 (419–728)
Rheumatoid arthritis	–	–	–	–	13 (10·6–18)	21·3 (16·8–27·7)	18·8 (15·9–23·1)	22·4 (18·8–27·5)	32·3 (27·8–38·8)	52·7 (45·9–63·6)	77 (66·5–89·9)	137 (118–163)	223 (190–268)	391 (337–465)	661 (570–766)	1170 (1010–1360)	1720 (1500–1960)	2310 (1990–2650)	2500 (2140–2850)	2240 (1860–2550)	1600 (1330–1820)	514 (406–604)	142 (103–177)
Other musculoskeletal disorders	–	–	–	–	107 (90·2–129)	193 (166–235)	284 (252–332)	353 (316–414)	424 (384–490)	502 (456–580)	565 (508–661)	692 (617–816)	785 (695–946)	914 (805–1090)	1230 (1070–1460)	1860 (1620–2190)	2220 (1940–2610)	2620 (2290–3070)	2680 (2250–3090)	2890 (2290–3290)	2340 (1830–2670)	1290 (919–1500)	469 (316–566)
Other non-communicable diseases	102000 (81700–128000)	33800 (27600–42300)	114000 (84100–150000)	49700 (30300–75200)	13600 (11300–16400)	9880 (8070–12000)	9000 (7480–11300)	9010 (7650–10800)	8160 (7110–9570)	8090 (7140–9200)	8790 (7650–9980)	9710 (8330–11000)	10600 (8920–11800)	13300 (11000–14800)	15800 (12700–17400)	19600 (16100–21500)	23800 (19500–26100)	26000 (21500–28500)	27200 (21900–29700)	29800 (23600–32500)	24400 (19100–26800)	14300 (10600–16000)	6250 (4210–7180)
Congenital birth defects	1e+05 (80500–126000)	31300 (25700–39700)	92800 (68300–131000)	37700 (20800–62400)	8690 (7300–10800)	5790 (4860–7040)	4060 (3380–4980)	3690 (3150–4400)	3310 (2880–3920)	2510 (2190–2910)	2640 (2270–3070)	2460 (2100–2920)	1540 (1280–1750)	1520 (1200–1740)	1580 (1190–1800)	1460 (1100–1650)	1040 (810–1170)	–	–	–	–	–	–
Neural tube defects	17100 (11200–25300)	3510 (2400–5150)	9870 (5700–17100)	9090 (4140–17100)	448 (281–684)	310 (204–470)	142 (92·6–254)	107 (76·9–165)	100 (76·8–145)	89·2 (69·8–123)	80·5 (66·5–105)	80·1 (63·6–111)	51·9 (42·3–68·3)	40·4 (32·9–50·7)	35·5 (28·4–45·9)	36·4 (28·9–49·4)	29·8 (24·1–36·9)	–	–	–	–	–	–
Congenital heart anomalies	30100 (21900–39800)	14100 (11000–17500)	42000 (31700–55300)	13000 (8120–21600)	4760 (3790–6000)	3450 (2830–4260)	2430 (2010–2960)	2360 (2010–2860)	2000 (1700–2360)	1510 (1310–1740)	1530 (1320–1820)	1410 (1210–1690)	783 (647–907)	713 (575–829)	664 (533–767)	616 (499–714)	459 (367–525)	–	–	–	–	–	–
Orofacial clefts	591 (308–1470)	181 (101–427)	582 (296–1460)	46·3 (31·8–83·7)	–	–	–	–	–	–	–	–	–	–	–	–	–	–	–	–	–	–	–
Down syndrome	1580 (1160–2670)	691 (518–1090)	3530 (2550–6560)	1350 (822–4260)	346 (271–508)	231 (187–319)	225 (179–286)	237 (198–292)	250 (206–313)	248 (208–320)	290 (238–370)	312 (254–389)	306 (249–358)	422 (311–480)	537 (357–615)	469 (300–535)	237 (156–268)	–	–	–	–	–	–
Other chromosomal abnormalities	3830 (2090–6980)	1370 (822–2390)	3210 (1760–6560)	835 (301–2490)	205 (129–317)	144 (92·3–193)	148 (105–184)	138 (99–173)	167 (123–213)	101 (59·6–119)	135 (89–164)	80·8 (47·8–100)	65·5 (35·2–78·5)	48·2 (21·4–58·6)	50·5 (20·8–62·3)	32·8 (11·1–42·5)	33·5 (14·2–41·1)	–	–	–	–	–	–
Congenital musculoskeletal and limb anomalies	2710 (1740–4100)	578 (401–789)	1530 (1000–2400)	511 (240–1230)	197 (143–263)	133 (99·1–174)	138 (99·6–188)	87·4 (63·5–113)	82·9 (60·7–110)	58·7 (46·4–71·3)	60·9 (46·4–77·5)	49·3 (35·8–63·6)	32 (21·2–38·5)	31·2 (21·3–37·5)	26·7 (17·1–32·6)	35 (25·1–43·7)	26·2 (19·2–36)	–	–	–	–	–	–
Urogenital congenital anomalies	3600 (2070–5560)	707 (440–1060)	1120 (617–1700)	261 (95·8–513)	73·2 (48–100)	55·6 (40·7–77·2)	51·9 (33·3–65)	59·5 (33·3–74·3)	66·2 (40·8–80·6)	51·3 (35·6–61·4)	53·5 (38·6–64·5)	54·6 (41·1–66·1)	30·3 (22·1–36·1)	28·7 (21·2–33·6)	27·7 (19·6–32·7)	33 (24·1–39)	31·9 (24·2–38·6)	–	–	–	–	–	–
Digestive congenital anomalies	9450 (5390–14400)	3530 (2310–4970)	9320 (5320–14800)	1900 (662–4290)	234 (150–329)	126 (89·3–168)	50·3 (36·2–68·8)	40·1 (27·8–52)	52·1 (37·2–69·1)	34·5 (25·7–42·5)	44·3 (30·9–54·2)	43 (28·7–54·2)	35·1 (23·2–41·8)	39·6 (24·9–46·7)	53 (34·3–63·1)	56·4 (38·8–66·6)	66·1 (42·8–78·2)	–	–	–	–	–	–
Other congenital birth defects	31200 (21300–47800)	6670 (4940–9750)	21600 (13700–35600)	10700 (4720–21900)	2420 (1900–3690)	1350 (1080–1930)	874 (669–1200)	661 (526–848)	596 (463–784)	417 (343–536)	439 (350–554)	430 (331–545)	234 (180–283)	196 (148–235)	188 (138–220)	179 (130–210)	155 (120–181)	–	–	–	–	–	–
Urinary diseases and male infertility	367 (262–506)	204 (146–281)	1320 (976–1830)	1010 (771–1280)	676 (508–857)	530 (428–638)	1190 (956–1410)	1750 (1490–2020)	1610 (1360–1840)	2190 (1860–2490)	2600 (2200–2980)	3390 (2780–3890)	4450 (3540–5070)	5870 (4610–6620)	7470 (5910–8360)	10200 (7970–11400)	13600 (10900–15300)	16300 (13000–18100)	17800 (14100–19700)	20900 (16100–23100)	17200 (13000–19000)	10600 (7850–11900)	4510 (3040–5210)
Urolithiasis	–	–	–	7·01 (3·51–12)	15·1 (8·22–25·6)	11·8 (5·98–18·3)	15·1 (5·75–25·3)	19·8 (10–28·3)	37·3 (19–55·4)	51·7 (28·9–72·1)	97·7 (48·4–128)	167 (92·3–227)	239 (118–325)	381 (202–499)	456 (257–607)	692 (383–899)	890 (541–1180)	930 (587–1280)	980 (614–1400)	909 (584–1240)	633 (420–864)	303 (201–440)	94·2 (63·1–143)
Other urinary diseases	276 (184–391)	98·7 (62·8–147)	265 (147–417)	510 (363–702)	190 (133–269)	155 (109–206)	388 (302–500)	293 (187–420)	333 (244–430)	663 (524–842)	782 (599–1040)	925 (698–1240)	1300 (991–1770)	1480 (981–2000)	2150 (1600–2830)	2570 (1780–3470)	3440 (2500–4650)	4040 (2850–5680)	3820 (2660–5220)	4840 (3500–6490)	3140 (2310–4130)	1730 (1250–2390)	592 (385–771)
Hemoglobinopathies and hemolytic anemias	416 (300–625)	249 (175–358)	2560 (1530–4440)	8690 (5630–13900)	3130 (2250–4710)	2610 (1910–3950)	2920 (2110–4670)	2560 (1860–3960)	1940 (1430–2990)	1580 (1200–2420)	1430 (1050–2210)	1340 (963–2130)	1240 (929–1930)	1540 (1170–2330)	1420 (1130–2220)	1840 (1490–2670)	2120 (1760–2990)	2250 (1900–3040)	2150 (1850–2880)	1960 (1690–2560)	1420 (1220–1780)	659 (542–797)	220 (170–259)
Thalassemias	169 (123–250)	117 (82·4–183)	606 (451–808)	1250 (959–1660)	862 (602–1350)	511 (366–770)	358 (250–550)	238 (167–339)	169 (122–237)	159 (110–226)	136 (95·9–187)	146 (105–200)	157 (111–211)	171 (114–237)	108 (65·5–162)	53·5 (29·5–85·5)	18·1 (9·04–30·8)	4·46 (2·18–8·01)	0·78 (0·353–1·46)	0·112 (0·0386–0·264)	0·0115 (0·00313–0·0313)	0·000642 (0·000147–0·00183)	2·97e-05 (4·78e-06–9·18e-05)
Sickle cell disorders	230 (132–428)	125 (76·2–208)	1880 (797–3670)	7230 (4360–12000)	2040 (1310–3280)	1710 (1180–2760)	1950 (1280–3410)	1640 (1050–2800)	1170 (740–2050)	768 (465–1420)	734 (418–1380)	617 (328–1180)	362 (183–740)	246 (117–489)	67·7 (39·1–126)	33·5 (20·7–53·9)	14·9 (9·3–22·1)	5·5 (3·35–8·66)	1·16 (0·588–2·12)	0·224 (0·0935–0·434)	0·0412 (0·0136–0·0882)	0·00635 (0·00173–0·0155)	0·000754 (0·000193–0·00193)
G6PD deficiency	8·48 (4·81–13·4)	5·09 (2·9–8·29)	57·8 (33·7–92·9)	199 (116–330)	208 (115–324)	337 (202–534)	524 (308–836)	544 (344–828)	508 (335–751)	542 (364–792)	456 (296–660)	450 (292–702)	550 (349–941)	801 (480–1360)	760 (470–1400)	872 (534–1460)	737 (426–1240)	534 (306–893)	316 (156–551)	144 (71·8–258)	51·4 (25·3–89·8)	11·7 (5·9–20·4)	1·86 (0·754–3·32)
Other hemoglobinopathies and hemolytic anemias	8·96 (6·22–12·9)	1·62 (1·1–2·47)	11·8 (8·7–16·1)	15·6 (11·4–21)	23·7 (17·4–33·8)	45·2 (31·8–65·2)	89·9 (58·8–149)	137 (92·1–218)	95 (65·5–146)	115 (78·2–176)	108 (76·7–161)	127 (92·1–183)	167 (120–241)	326 (232–486)	485 (359–704)	880 (671–1240)	1350 (1080–1830)	1710 (1410–2210)	1840 (1560–2400)	1810 (1560–2310)	1370 (1170–1690)	647 (535–778)	219 (169–256)
Endocrine, metabolic, blood, and immune disorders	688 (457–861)	583 (381–746)	3550 (2410–4800)	2300 (1560–3120)	1120 (831–1490)	951 (727–1260)	841 (643–1060)	1010 (794–1310)	1300 (1010–1680)	1810 (1410–2220)	2120 (1560–2500)	2520 (1810–2900)	3380 (2440–3980)	4370 (3060–5050)	5280 (3540–6090)	6100 (4230–6940)	7030 (4940–8470)	7480 (5450–8880)	7260 (5100–8470)	6900 (4600–7910)	5750 (3880–6670)	3030 (1850–3540)	1510 (769–1860)
Sudden infant death syndrome	–	1430 (758–2660)	13800 (5800–30800)	–	–	–	–	–	–	–	–	–	–	–	–	–	–	–	–	–	–	–	–
Injuries	6660 (5300–8200)	4170 (2740–6170)	28900 (23200–36200)	74300 (59600–93500)	55000 (47300–63800)	57900 (50600–65100)	153000 (139000–167000)	244000 (226000–261000)	244000 (228000–260000)	245000 (228000–262000)	229000 (208000–248000)	210000 (189000–228000)	205000 (182000–225000)	196000 (172000–216000)	181000 (160000–198000)	162000 (142000–176000)	151000 (130000–166000)	134000 (117000–146000)	120000 (105000–131000)	109000 (94800–119000)	80400 (68000–88400)	37300 (30600–41200)	12900 (10100–14700)
Transport injuries	515 (333–680)	300 (201–391)	3960 (2810–5210)	18100 (13700–24200)	19200 (15800–23000)	18700 (15400–21800)	59300 (51800–66300)	93100 (82500–102000)	90000 (78700–98400)	89400 (75700–97700)	83900 (70000–93000)	76300 (62500–85200)	75200 (60400–84700)	71900 (57200–81300)	63400 (51900–71200)	54000 (42900–60400)	47800 (37300–53900)	36800 (29600–40800)	28200 (23200–31200)	20200 (17000–22200)	11100 (9390–12300)	3440 (2800–3840)	924 (719–1090)
Road injuries	466 (298–616)	273 (182–356)	3740 (2640–4930)	17000 (12800–23100)	18100 (14900–21800)	17200 (14300–20200)	55100 (48100–61700)	87200 (77200–96100)	83600 (73300–91400)	83800 (71000–91600)	78000 (65000–86500)	71000 (58200–79500)	70000 (56400–78800)	66700 (53000–75600)	58700 (48100–65900)	50600 (40000–56600)	44800 (34900–50500)	34600 (27600–38400)	26400 (21700–29100)	18900 (16000–20800)	10400 (8850–11600)	3210 (2610–3590)	851 (663–989)
Pedestrian road injuries	171 (107–233)	103 (63·8–135)	1360 (881–1810)	7860 (5740–10800)	8850 (7070–10800)	7510 (5820–8970)	13600 (10500–15800)	21000 (16300–24400)	21800 (17200–25300)	24400 (19200–28500)	23500 (18300–27500)	22400 (17200–26500)	24100 (18200–28600)	25400 (18900–30100)	22600 (17100–26800)	22200 (16700–26100)	20800 (15600–24500)	17200 (13000–20000)	14700 (11800–16900)	10700 (8640–12200)	6000 (4900–6840)	1670 (1340–1920)	441 (346–519)
Cyclist road injuries	–	–	–	764 (520–1150)	969 (775–1220)	1310 (1070–1590)	2420 (1960–2820)	2670 (2200–3100)	2720 (2240–3150)	3240 (2600–3830)	3190 (2550–3730)	3620 (2790–4280)	4280 (3390–5100)	4910 (3850–5870)	4400 (3430–5260)	4710 (3590–5620)	4460 (3440–5330)	3330 (2590–3950)	2100 (1600–2530)	1320 (1040–1570)	673 (532–802)	187 (147–219)	1·42 (1·07–1·73)
Motorcyclist road injuries	46·6 (30·1–67·2)	29·9 (18·2–41·3)	440 (313–608)	1430 (1020–2040)	1350 (1040–1740)	2080 (1650–2530)	16400 (13700–18700)	27100 (23100–31000)	23300 (19900–27000)	21300 (17200–24200)	19300 (15700–22400)	16800 (13500–19600)	15600 (12000–18100)	13100 (10100–15400)	11100 (8050–13100)	7140 (5060–8540)	5800 (3910–6910)	3300 (2200–3920)	1720 (1200–2040)	1410 (970–1750)	624 (436–762)	183 (126–224)	48·8 (35–59·9)
Motor vehicle road injuries	243 (155–327)	135 (90–178)	1880 (1300–2560)	6740 (4690–9560)	6730 (5370–8410)	6050 (4980–7280)	22200 (19200–26200)	35600 (31300–40900)	34400 (29500–39900)	34300 (28600–39000)	31500 (26000–36200)	27600 (22000–32100)	25400 (20400–29200)	22500 (18000–26600)	19900 (15900–23100)	16000 (12600–18800)	13200 (10500–15400)	10200 (8430–12100)	7440 (6300–8820)	5200 (4420–6210)	2990 (2450–3580)	1130 (920–1340)	343 (266–418)
Other road injuries	5·28 (3·41–8·26)	4·99 (3·4–6·99)	58 (40·1–80·6)	198 (127–359)	231 (164–315)	275 (199–371)	513 (420–622)	801 (667–939)	757 (631–883)	618 (525–729)	607 (507–708)	623 (519–723)	707 (579–826)	757 (626–902)	667 (551–794)	528 (443–613)	560 (442–672)	486 (394–577)	373 (302–447)	304 (244–371)	160 (125–196)	38·8 (29·7–48·7)	16·4 (11·9–20·3)
Other transport injuries	49·4 (32·3–68)	27 (18·2–36·3)	224 (159–293)	1110 (650–1540)	1050 (818–1290)	1470 (1140–1760)	4220 (3310–5000)	5920 (4840–6890)	6400 (5280–7300)	5610 (4560–6340)	5840 (4670–6700)	5280 (4170–6070)	5260 (4070–6010)	5130 (3970–5880)	4760 (3650–5520)	3490 (2760–4010)	3030 (2400–3480)	2260 (1790–2570)	1830 (1440–2120)	1250 (1010–1430)	639 (530–731)	232 (188–268)	73·5 (56·7–90·5)
Unintentional injuries	3720 (2610–5000)	3060 (1750–5010)	21500 (17000–27400)	49800 (39900–63900)	31500 (26200–37300)	28000 (24000–32100)	34900 (30700–38800)	47700 (42600–52200)	48900 (44000–52700)	54300 (49000–58900)	56100 (49900–61300)	57800 (51000–63800)	61900 (52800–69000)	63500 (54200–71000)	63700 (54700–70500)	64400 (55600–71400)	66300 (55400–73900)	67200 (55800–75000)	67600 (56300–75300)	70800 (59200–78600)	58300 (47800–64900)	30500 (24900–33800)	11100 (8680–12700)
Falls	532 (307–780)	218 (131–332)	2290 (1430–3380)	5200 (3560–7290)	4100 (3030–5450)	4060 (3050–5120)	5190 (4330–6280)	8820 (7450–10500)	10200 (8830–12000)	12700 (10500–14800)	14800 (12400–17600)	17200 (14000–20200)	19200 (15400–22300)	22700 (18300–26100)	24600 (20200–28100)	27300 (22100–31200)	31000 (24100–35800)	36600 (28300–41900)	40700 (32200–46300)	46500 (37000–52900)	41500 (32500–47000)	23000 (18500–25800)	8380 (6620–9900)
Drowning	229 (167–305)	92·7 (61·5–126)	1960 (1420–2630)	19200 (15400–24900)	14200 (11800–17100)	11800 (10200–13700)	12100 (10500–13700)	12000 (10600–13700)	9910 (8860–11100)	10300 (9120–11400)	9640 (8440–10900)	9040 (7940–10100)	8670 (7560–9780)	8350 (7250–9480)	7350 (6310–8240)	6840 (5820–7620)	6420 (5400–7200)	5910 (4910–6640)	4670 (3860–5240)	4300 (3510–4830)	2710 (2210–3040)	788 (637–884)	194 (146–224)
Fire, heat, and hot substances	194 (118–293)	122 (79·2–179)	1660 (1080–2490)	5040 (3310–7260)	1770 (1170–2390)	1080 (727–1410)	1530 (989–1900)	2500 (1700–3020)	2800 (2050–3360)	3190 (2440–3840)	3450 (2650–4090)	3230 (2530–3740)	3410 (2660–3930)	3500 (2790–4070)	3540 (2920–4060)	3670 (2960–4210)	3400 (2720–3890)	3270 (2620–3800)	2910 (2330–3450)	3080 (2380–3630)	2240 (1820–2720)	946 (763–1130)	314 (241–379)
Poisonings	191 (121–269)	167 (89·4–257)	1580 (1070–2370)	3780 (2730–5480)	1370 (1010–1840)	1340 (983–1650)	1660 (1290–1920)	2530 (1920–2910)	2840 (2240–3210)	3080 (2370–3420)	3090 (2450–3460)	3270 (2620–3680)	3460 (2620–3930)	3680 (2770–4210)	3470 (2590–3980)	3430 (2520–3940)	2930 (2040–3390)	2510 (1640–2950)	1950 (1280–2280)	1610 (1090–1870)	934 (657–1070)	218 (170–245)	45 (35·8–51·2)
Poisoning by carbon monoxide	32·1 (14·9–52·3)	29·2 (13–49·1)	239 (122–436)	765 (415–1300)	478 (297–673)	511 (308–676)	1020 (735–1250)	1530 (1070–1800)	1730 (1270–1970)	2040 (1560–2310)	2030 (1580–2310)	2100 (1650–2400)	2200 (1730–2570)	2220 (1660–2590)	2110 (1620–2440)	2110 (1590–2480)	1740 (1230–2060)	1450 (915–1760)	1140 (720–1370)	971 (625–1170)	575 (380–686)	142 (105–164)	25·6 (19·9–29·5)
Poisoning by other means	159 (104–222)	138 (73·3–215)	1340 (902–1970)	3010 (2200–4360)	889 (666–1190)	834 (645–1060)	644 (460–877)	1010 (714–1420)	1110 (812–1540)	1040 (724–1420)	1060 (744–1430)	1170 (777–1530)	1260 (771–1680)	1460 (874–1930)	1360 (849–1780)	1320 (781–1760)	1190 (683–1680)	1060 (603–1490)	804 (451–1150)	637 (369–905)	359 (231–514)	75·5 (55·5–99)	19·4 (15–23·3)
Exposure to mechanical forces	422 (147–798)	1020 (126–2790)	1700 (645–3250)	2830 (1940–4050)	2040 (1450–2730)	1820 (1350–2290)	4440 (3430–5410)	7260 (5620–8620)	7610 (6020–8810)	8000 (6340–9130)	8090 (6310–9310)	7510 (5580–8730)	7960 (5780–9490)	7470 (5440–8840)	6060 (4460–7110)	4750 (3530–5480)	3830 (2750–4480)	2980 (2260–3430)	2460 (1830–2810)	1740 (1330–2000)	1110 (880–1240)	435 (353–492)	150 (115–186)
Unintentional firearm injuries	46·9 (10·2–107)	130 (7·19–496)	272 (90·6–596)	509 (285–822)	279 (181–456)	373 (283–540)	1270 (965–1760)	1900 (1450–2550)	1780 (1270–2290)	1520 (1090–1970)	1360 (902–1780)	1210 (815–1610)	1090 (680–1450)	814 (515–1060)	646 (426–859)	551 (350–727)	418 (267–558)	337 (213–445)	287 (194–379)	211 (133–270)	124 (79·8–161)	59·8 (39·3–80·3)	20·9 (14–30·2)
Other exposure to mechanical forces	375 (132–724)	887 (117–2520)	1430 (529–2790)	2320 (1570–3300)	1760 (1220–2330)	1440 (1010–1810)	3170 (2240–3820)	5360 (3880–6320)	5830 (4410–6740)	6470 (4950–7440)	6740 (5140–7780)	6310 (4630–7370)	6870 (4860–8240)	6650 (4720–7910)	5410 (3970–6380)	4200 (3040–4900)	3410 (2400–4020)	2640 (1970–3070)	2170 (1590–2500)	1530 (1160–1780)	983 (779–1110)	375 (305–422)	129 (98·5–156)
Adverse effects of medical treatment	402 (282–563)	214 (150–299)	2110 (1420–2860)	2720 (1520–4060)	889 (659–1140)	677 (495–890)	723 (501–1020)	1170 (871–1510)	1040 (767–1330)	1260 (955–1560)	1410 (1070–1740)	1710 (1300–2090)	1900 (1470–2300)	2240 (1750–2630)	3080 (2450–3560)	4260 (3350–5020)	5450 (4290–6380)	5050 (4040–5880)	4900 (3970–5650)	4350 (3550–5020)	2920 (2420–3350)	1400 (1100–1600)	480 (364–551)
Animal contact	202 (112–294)	136 (81·5–205)	995 (624–1630)	3160 (2140–4920)	2860 (1920–4120)	3050 (2030–4090)	2130 (1500–2720)	2370 (1630–2990)	2640 (1860–3320)	2720 (1950–3370)	2360 (1550–2900)	2850 (1950–3560)	3890 (2740–4900)	2990 (2240–3610)	3580 (2530–4420)	3270 (2260–4000)	3590 (2420–4440)	2890 (1940–3530)	2460 (1620–3020)	1800 (1070–2180)	796 (546–955)	268 (185–320)	81·3 (57·6–102)
Venomous animal contact	167 (79·6–254)	106 (55·6–166)	669 (365–1190)	1830 (999–3300)	2130 (1300–3250)	2570 (1610–3500)	1750 (1210–2300)	2010 (1300–2620)	2280 (1560–2930)	2360 (1630–2970)	1990 (1260–2520)	2410 (1540–3090)	3350 (2240–4310)	2340 (1630–2900)	2890 (1930–3670)	2570 (1670–3190)	2940 (1810–3670)	2360 (1480–2950)	2010 (1240–2530)	1490 (816–1860)	624 (389–769)	208 (132–255)	59 (39·3–74·9)
Non-venomous animal contact	34·2 (25·5–48·7)	29·9 (21·8–43·2)	326 (228–480)	1320 (923–1890)	725 (521–1040)	484 (345–699)	374 (267–522)	358 (265–489)	359 (274–466)	355 (273–462)	371 (282–505)	431 (319–597)	542 (399–759)	649 (501–910)	683 (521–939)	698 (548–972)	658 (522–882)	524 (417–701)	457 (358–611)	312 (242–413)	172 (138–217)	59·9 (46·9–73·7)	22·3 (16·8–27·3)
Foreign body	1160 (903–1440)	954 (761–1150)	7840 (6490–9260)	4110 (3370–5060)	1450 (1250–1690)	1260 (1100–1450)	1270 (1130–1430)	2100 (1870–2340)	2200 (2030–2400)	2480 (2290–2680)	2640 (2430–2860)	2850 (2610–3120)	3050 (2780–3350)	3280 (3000–3560)	3640 (3350–3960)	3800 (3520–4100)	3880 (3620–4140)	3860 (3610–4110)	3950 (3650–4190)	4530 (4030–4870)	4230 (3640–4580)	2620 (2140–2890)	1170 (895–1320)
Pulmonary aspiration and foreign body in airway	1110 (870–1390)	937 (747–1130)	7600 (6300–8980)	3390 (2730–4300)	1290 (1110–1510)	1160 (1030–1330)	1110 (981–1260)	1910 (1700–2130)	2030 (1870–2220)	2310 (2150–2500)	2460 (2270–2680)	2640 (2430–2890)	2880 (2630–3160)	3140 (2880–3410)	3510 (3230–3800)	3650 (3380–3930)	3750 (3480–4000)	3720 (3470–3960)	3790 (3480–4020)	4390 (3900–4690)	4130 (3560–4460)	2580 (2100–2840)	1160 (882–1300)
Foreign body in other body part	48·4 (29–70·3)	17·2 (11·1–24·7)	235 (160–332)	713 (420–1150)	161 (111–216)	98·3 (68·5–133)	160 (105–222)	188 (118–271)	172 (115–238)	167 (117–224)	181 (124–246)	206 (143–278)	169 (114–233)	136 (92·9–185)	132 (90·5–183)	152 (104–205)	134 (91·4–179)	145 (101–194)	159 (115–213)	143 (104–199)	98·4 (72·3–137)	43·8 (31·7–62)	14·1 (9·94–19·6)
Environmental heat and cold exposure	78·9 (19–133)	48 (10·6–88·7)	342 (107–595)	540 (260–874)	253 (122–375)	218 (105–297)	537 (309–707)	867 (524–1110)	1040 (702–1240)	1550 (1120–1750)	1920 (1470–2170)	2310 (1770–2650)	2620 (2050–2990)	3120 (2360–3600)	3310 (2530–3870)	3180 (2370–3700)	2700 (1710–3280)	2150 (1220–2630)	2040 (1060–2590)	1700 (927–2100)	1160 (690–1370)	494 (343–573)	142 (106–163)
Exposure to forces of nature	11·2 (10·1–12·3)	8·27 (7·51–9·1)	51·4 (46·7–56·5)	214 (194–236)	237 (215–261)	322 (292–354)	365 (331–401)	345 (313–380)	323 (294–356)	288 (261–317)	257 (233–283)	230 (209–253)	213 (193–234)	190 (172–209)	153 (139–169)	145 (132–160)	116 (105–127)	95·7 (86·9–105)	73·6 (66·8–80·9)	52·7 (47·8–58)	24·9 (22·6–27·4)	7·39 (6·71–8·13)	2 (1·81–2·2)
Other unintentional injuries	295 (129–453)	84·2 (53·8–122)	980 (538–1420)	3030 (2050–4180)	2330 (1520–3030)	2400 (1570–2920)	5010 (3590–5860)	7740 (5510–9080)	8270 (6150–9500)	8790 (6330–10000)	8390 (5950–9720)	7630 (5570–8760)	7500 (5120–8740)	5990 (4050–6930)	4910 (3390–5670)	3810 (2560–4360)	3030 (2020–3460)	1880 (1260–2130)	1440 (1030–1630)	1100 (776–1250)	706 (509–800)	309 (229–360)	115 (85·5–138)
Self-harm and interpersonal violence	2430 (2210–2670)	804 (736–881)	3390 (2980–3900)	6380 (5200–7700)	4270 (3610–4940)	11200 (9560–12800)	59200 (53700–65000)	103000 (94900–112000)	105000 (97500–113000)	102000 (93400–110000)	89300 (81800–96800)	76000 (69000–82800)	68200 (61200–74900)	60700 (54700–66800)	53700 (47600–58900)	43200 (38200–47300)	36800 (32100–40800)	30300 (26100–33800)	24300 (20600–26800)	18300 (15500–20500)	11000 (9240–12400)	3430 (2860–3810)	895 (709–1010)
Self-harm	–	–	–	–	–	4990 (3920–6090)	23700 (20000–27100)	44900 (38600–51700)	50800 (43800–57400)	52700 (45400–59300)	48600 (41400–54300)	43900 (37700–49200)	42700 (36200–48100)	40200 (34600–45300)	38000 (31900–42400)	31700 (26900–35400)	28100 (23500–31600)	24100 (20000–27300)	20000 (16300–22500)	16200 (13400–18400)	9750 (8050–11100)	2970 (2420–3350)	668 (524–778)
Self-harm by firearm	–	–	–	–	–	246 (200–306)	2010 (1740–2580)	3450 (2950–4540)	3490 (3000–4520)	3470 (2830–4440)	3340 (2650–4070)	3350 (2750–4110)	3680 (3060–4570)	3790 (3290–4600)	3960 (3520–4750)	3450 (3040–3990)	3010 (2580–3470)	2520 (2170–2770)	2070 (1750–2290)	1650 (1380–1860)	1080 (895–1250)	425 (348–508)	108 (84·3–140)
Self-harm by other specified means	–	–	–	–	–	4740 (3710–5830)	21700 (18100–24900)	41400 (34800–47700)	47300 (40600–53400)	49200 (41900–55400)	45300 (38500–50800)	40500 (34500–45500)	39000 (32400–44100)	36400 (30800–41100)	34000 (27900–38600)	28200 (23200–31700)	25100 (20500–28500)	21600 (17400–24700)	17900 (14300–20300)	14600 (11900–16700)	8670 (7050–10100)	2540 (2050–2890)	560 (431–652)
Interpersonal violence	639 (505–813)	185 (141–233)	1730 (1330–2180)	4060 (2910–5380)	2910 (2260–3570)	4870 (4080–5690)	31600 (28400–35300)	51900 (48200–56500)	49500 (46200–53300)	45000 (41800–48400)	37900 (35100–41000)	30000 (27600–32500)	23400 (21600–25600)	18600 (17200–20200)	14300 (13200–15500)	10300 (9590–11200)	7460 (6920–8080)	4980 (4610–5440)	3090 (2830–3390)	1010 (894–1120)	485 (415–539)	180 (146–201)	149 (113–175)
Physical violence by firearm	131 (97·8–169)	38·2 (28–49·3)	316 (237–405)	908 (619–1220)	602 (492–732)	1650 (1450–1880)	17600 (16000–19500)	28800 (26600–31200)	25600 (23800–27600)	21600 (20100–23400)	17800 (16300–19500)	13500 (12300–14800)	9660 (8690–10800)	7490 (6740–8320)	5260 (4720–5790)	3230 (2940–3590)	2030 (1840–2250)	1240 (1140–1370)	794 (709–878)	275 (237–315)	121 (99·7–135)	54·4 (42·7–62·2)	55·2 (40·5–67·3)
Physical violence by sharp object	69·5 (43·2–96·4)	22·4 (12·1–31·8)	175 (103–244)	749 (400–1070)	400 (244–511)	701 (440–863)	6530 (4880–7680)	11500 (8940–13300)	12000 (9980–13800)	11300 (9280–13100)	9260 (7740–10700)	7210 (5790–8230)	5840 (4620–6670)	4600 (3630–5220)	3300 (2700–3810)	2690 (2200–3110)	1950 (1590–2250)	1150 (941–1320)	729 (617–840)	288 (247–332)	134 (110–156)	52·1 (41·5–60)	42·5 (32·2–50·1)
Physical violence by other means	439 (346–553)	124 (94·2–158)	1240 (959–1580)	2410 (1760–3110)	1910 (1430–2410)	2520 (1980–3060)	7400 (6070–8840)	11600 (10000–13700)	11800 (10400–13500)	12100 (10800–13800)	10800 (9520–12100)	9230 (8190–10300)	7930 (7000–8830)	6520 (5850–7220)	5730 (5160–6370)	4400 (3970–4870)	3470 (3150–3850)	2590 (2330–2910)	1560 (1410–1760)	450 (395–514)	230 (198–260)	73·7 (60·1–83·5)	51·4 (39·3–59·4)
Conflict and terrorism	1790 (1630–1970)	619 (562–681)	1650 (1500–1810)	2230 (2020–2450)	1290 (1170–1420)	1250 (1130–1380)	3230 (2930–3550)	5180 (4700–5700)	3770 (3420–4150)	2810 (2550–3090)	2070 (1880–2280)	1550 (1410–1710)	1550 (1410–1700)	1470 (1330–1610)	1170 (1070–1290)	1070 (973–1180)	1180 (1070–1290)	1190 (1080–1310)	1150 (1040–1270)	1020 (922–1120)	715 (649–787)	267 (243–294)	71·7 (65·1–78·9)

SR Table 6: Global deaths by cause and age group for both sexes combined (with 95% uncertainty levels), 1990

Cause	Early Neonatal	Late Neonatal	Post Neonatal	1 to 4	5 to 9	10 to 14	15 to 19	20 to 24	25 to 29	30 to 34	35 to 39	40 to 44	45 to 49	50 to 54	55 to 59	60 to 64	65 to 69	70 to 74	75 to 79	80 to 84	85 to 89	90 to 94	95 plus
All causes	3030000 (2840000–3230000)	1260000 (1170000–1360000)	3830000 (3640000–4050000)	3860000 (3610000–4110000)	822000 (794000–854000)	453000 (439000–468000)	673000 (653000–694000)	838000 (811000–866000)	843000 (817000–870000)	888000 (863000–916000)	1050000 (1010000–1080000)	1160000 (1120000–1200000)	1340000 (1300000–1390000)	1890000 (1820000–1950000)	2420000 (2330000–2500000)	3180000 (3080000–3270000)	3580000 (3480000–3680000)	3840000 (3740000–3940000)	4220000 (4140000–4300000)	3760000 (3700000–3810000)	2400000 (2370000–2420000)	985000 (977000–991000)	337000 (335000–338000)
Communicable, maternal, neonatal, and nutritional diseases	2730000 (2560000–2920000)	1110000 (1020000–1210000)	3170000 (2920000–3450000)	3060000 (2800000–3370000)	488000 (449000–528000)	213000 (195000–232000)	221000 (203000–243000)	268000 (251000–287000)	282000 (266000–299000)	283000 (269000–299000)	290000 (275000–308000)	269000 (253000–287000)	271000 (252000–294000)	309000 (286000–338000)	350000 (321000–388000)	423000 (381000–477000)	445000 (397000–508000)	494000 (437000–566000)	475000 (420000–542000)	430000 (374000–486000)	265000 (228000–296000)	106000 (88900–119000)	36000 (28900–41100)
HIV/AIDS and sexually transmitted infections	58900 (19700–123000)	17400 (5560–38300)	51700 (37300–76900)	41100 (36000–46400)	3450 (2700–4330)	748 (613–950)	8210 (6960–9790)	22700 (19300–26700)	42600 (36600–49900)	49800 (43500–57000)	45100 (39200–52400)	33600 (29500–38400)	24800 (21400–28500)	17700 (15500–20200)	11600 (10200–13100)	7500 (6650–8480)	4990 (4440–5640)	3060 (2630–3570)	1830 (1580–2120)	492 (404–582)	295 (217–395)	111 (78·4–147)	40·1 (29·8–50·6)
HIV/AIDS	--	--	31600 (28200–35300)	40100 (35400–45200)	3030 (2390–3690)	546 (457–723)	7860 (6610–9440)	22200 (18900–26200)	41900 (35900–49200)	49000 (42900–56200)	44500 (38500–51800)	33000 (28900–37700)	24200 (20800–28000)	17100 (14900–19500)	10700 (9360–12300)	6860 (6040–7820)	4470 (3930–5110)	2610 (2200–3090)	1320 (1100–1570)	160 (122–206)	70·4 (53·3–91·2)	18·3 (13·7–23·8)	6·89 (6·03–7·96)
HIV/AIDS - Drug-susceptible Tuberculosis	--	--	12500 (10500–14200)	16300 (13600–18600)	1100 (806–1410)	159 (120–208)	2970 (2430–3560)	8270 (6770–10000)	14500 (11500–17600)	16200 (13100–19400)	14300 (11400–17400)	10700 (8620–12700)	8410 (6800–10200)	6180 (5160–7260)	3880 (3260–4580)	2460 (2080–2890)	1590 (1330–1880)	1040 (863–1250)	519 (429–625)	51·2 (37·1–69)	20 (14·5–26·8)	4·85 (3·52–6·48)	1·41 (1·1–1·79)
Tuberculosis without extensive drug resistance	--	--	61·3 (21·8–146)	78 (27–178)	5·24 (1·88–11·8)	1·33 (0·466–2·91)	15·4 (5·67–36)	41·8 (15·5–93·5)	85·5 (33·8–183)	103 (41–219)	94·6 (38·3–194)	71·8 (29–150)	52·1 (20·6–109)	37·8 (14·8–82)	25·6 (9·9–55·1)	18 (6·94–38·6)	11·9 (4·67–25·7)	5·92 (2·19–13·5)	4·08 (1·39–9·6)	0·595 (0·199–1·44)	0·22 (0·0769–0·522)	0·0474 (0·0176–0·105)	0·0174 (0·00722–0·0355)
HIV/AIDS - Extensively drug-resistant Tuberculosis	--	--	0 (0–0)	0 (0–0)	0 (0–0)	0 (0–0)	0 (0–0)	0 (0–0)	0 (0–0)	0 (0–0)	0 (0–0)	0 (0–0)	0 (0–0)	0 (0–0)	0 (0–0)	0 (0–0)	0 (0–0)	0 (0–0)	0 (0–0)	0 (0–0)	0 (0–0)	0 (0–0)	0 (0–0)
HIV/AIDS resulting in other diseases	--	--	19000 (16600–21900)	23700 (20400–27600)	1920 (1470–2590)	385 (317–537)	4880 (3960–6270)	13900 (11500–17600)	27300 (23100–33600)	32700 (28300–38800)	30100 (25800–36300)	22200 (19300–26200)	15800 (13500–18900)	10800 (9370–12700)	6810 (5910–7980)	4380 (3820–5110)	2870 (2490–3350)	1560 (1290–1920)	794 (653–984)	108 (82–151)	50·2 (36·1–71·2)	13·4 (9·42–18·9)	5·47 (4·63–6·55)
Sexually transmitted infections excluding HIV	58900 (19700–123000)	17400 (5560–38300)	20200 (6530–44600)	950 (305–2140)	425 (138–962)	202 (129–288)	357 (271–427)	542 (364–650)	715 (448–862)	727 (488–875)	633 (446–753)	641 (475–753)	554 (382–650)	691 (473–856)	847 (572–1110)	644 (469–790)	520 (427–630)	446 (377–539)	508 (406–626)	332 (243–410)	225 (148–322)	93·2 (61·2–129)	33·2 (22·8–43·8)
Syphilis	58900 (19700–123000)	17400 (5560–38300)	20200 (6530–44600)	950 (305–2140)	425 (138–962)	115 (73·2–163)	122 (87·7–156)	90·1 (61·7–111)	176 (120–212)	233 (152–298)	209 (117–280)	132 (73·6–174)	137 (81·4–181)	230 (124–331)	283 (128–461)	249 (147–348)	162 (122–222)	162 (123–214)	157 (110–216)	96·8 (63·5–129)	55·1 (31·5–85·4)	21·3 (11·9–29·8)	7·68 (4·82–9·92)
Chlamydial infection	--	--	--	--	--	7·82 (5·16–9·66)	28·4 (21·1–34·7)	100 (65·2–123)	95·1 (57·1–115)	39·8 (25–48·9)	63·3 (45·4–75·9)	79·7 (55·6–94·9)	67·5 (47·3–80·1)	65·9 (48·1–81·6)	84·5 (59·1–106)	61·6 (44·5–75·8)	37·6 (29–45·4)	41·6 (34·2–49·5)	48·3 (38·3–57·2)	42·5 (33·2–50·3)	24·4 (18·9–29)	10·7 (8·16–12·9)	3·9 (2·9–4·73)
Gonococcal infection	--	--	--	--	--	19·3 (14·5–24·7)	92·2 (68·4–110)	184 (122–221)	248 (145–302)	227 (153–269)	181 (130–215)	237 (172–277)	185 (130–215)	213 (154–260)	254 (177–315)	184 (138–221)	220 (175–263)	156 (130–183)	188 (153–222)	112 (88·7–130)	70·7 (53·9–85)	29·1 (22·2–34·7)	10·6 (8·14–12·3)
Other sexually transmitted infections	--	--	--	--	--	60·4 (32·4–92·5)	114 (84·9–137)	168 (109–203)	196 (121–237)	227 (153–272)	180 (129–214)	192 (138–225)	165 (116–194)	183 (127–225)	225 (156–281)	150 (109–182)	101 (81·5–122)	86·9 (72·7–103)	114 (84·5–150)	80·7 (53·7–115)	74·6 (39·5–134)	32 (16·5–56·4)	11·1 (5·51–18·9)
Respiratory infections and tuberculosis	251000 (220000–284000)	277000 (240000–332000)	1180000 (1040000–1360000)	737000 (6e+05–909000)	88500 (73700–104000)	39900 (34500–45400)	53300 (48300–58900)	75500 (69000–82100)	92300 (85100–99600)	102000 (95100–110000)	118000 (110000–126000)	125000 (116000–134000)	140000 (130000–150000)	175000 (162000–188000)	199000 (183000–214000)	235000 (217000–254000)	240000 (222000–258000)	261000 (242000–283000)	258000 (240000–278000)	237000 (217000–256000)	160000 (141000–172000)	68200 (57000–74300)	24700 (19800–27400)
[illegible]	[illegible]	[illegible]	[illegible]	[illegible]	[illegible]	[illegible]	[illegible]	[illegible]	[illegible]	[illegible]	[illegible]	[illegible]	[illegible]	[illegible]	[illegible]	[illegible]	[illegible]	[illegible]	[illegible]	[illegible]	[illegible]	[illegible]	[illegible]

Endocarditis	194 (87·8–278)	212 (73·7–322)	2090 (738–3150)	1080 (287–1860)	308 (143–421)	331 (190–413)	554 (377–652)	839 (651–985)	1060 (855–1350)	1040 (871–1350)	1180 (999–1540)	1260 (1070–1660)	1180 (998–1540)	1470 (1240–1890)	1730 (1460–2220)	1950 (1680–2560)	2250 (1940–2930)	2240 (1920–2940)	2510 (2130–3460)	2360 (1910–3460)	1730 (1370–2630)	869 (665–1300)	325 (231–517)
Other cardiovascular and circulatory diseases	1410 (1090–1910)	1460 (960–1960)	7850 (4660–10300)	6610 (3160–8930)	2730 (2140–3260)	2310 (2010–2630)	1900 (1670–2160)	2540 (2270–2880)	2980 (2720–3350)	3410 (3130–3830)	4140 (3800–4770)	5090 (4630–6080)	6160 (5580–7480)	8630 (7860–10400)	11300 (10300–13800)	15900 (14600–19100)	18400 (17000–21800)	19700 (18000–23800)	24400 (22400–29000)	22400 (20100–26400)	15900 (13900–18300)	6860 (5680–7920)	2240 (1760–2650)
Chronic respiratory diseases	0 (0–0)	0 (0–0)	0 (0–0)	27500 (17800–34200)	5950 (4850–6920)	4620 (4020–5160)	8320 (7150–9190)	10700 (9140–11700)	12400 (10300–13500)	16100 (13600–17500)	24000 (20100–26200)	34800 (28700–38000)	53900 (43900–59200)	105000 (85300–115000)	174000 (138000–191000)	3e+05 (242000–327000)	393000 (321000–427000)	517000 (427000–562000)	552000 (467000–595000)	482000 (406000–524000)	264000 (222000–287000)	83000 (68200–91900)	21600 (16900–24100)
Chronic obstructive pulmonary disease	–	–	0 (0–0)	3920 (2240–7090)	979 (699–1260)	661 (509–792)	2870 (2300–3320)	3910 (3130–4550)	4510 (3570–5210)	6660 (5370–7600)	11300 (9060–12800)	18600 (15100–21000)	31600 (26000–35400)	72100 (58600–80100)	127000 (101000–141000)	238000 (193000–260000)	324000 (269000–352000)	443000 (367000–480000)	478000 (406000–514000)	424000 (358000–458000)	235000 (198000–255000)	72900 (59700–80100)	18900 (14900–20900)
Pneumoconiosis	–	–	–	–	–	–	75·9 (54·4–99·5)	93·4 (65·7–125)	135 (96·7–173)	268 (193–357)	611 (408–821)	840 (549–1140)	913 (591–1270)	1410 (965–1820)	2040 (1430–2580)	2770 (2040–3380)	3500 (2700–4140)	3650 (2880–4220)	3640 (3020–4160)	2400 (1960–2720)	1060 (862–1190)	255 (212–285)	55·8 (44·4–63·2)
Silicosis	–	–	–	–	–	–	30·3 (17·2–43·7)	42·5 (24·4–63·3)	72·3 (44·6–100)	177 (115–249)	439 (279–616)	596 (364–848)	627 (379–910)	959 (606–1310)	1350 (871–1790)	1760 (1220–2250)	2280 (1650–2800)	2320 (1690–2840)	2280 (1730–2730)	1410 (1030–1680)	604 (445–730)	126 (99·8–145)	23 (18·2–27)
Asbestosis	–	–	–	–	–	–	6·73 (4–9·85)	8·43 (5·07–12·1)	7·8 (4·75–11·1)	11·3 (6·9–17·3)	18·1 (10·6–28·3)	22·3 (12·9–33·2)	28·2 (14·1–41·9)	63·1 (34·1–88·4)	110 (61·1–149)	175 (111–231)	201 (152–254)	240 (192–292)	245 (200–301)	183 (150–235)	97·2 (79·2–136)	32·4 (24·9–44·3)	8·21 (6·15–11)
Coal workers pneumoconiosis	–	–	–	–	–	–	8·58 (3·87–15·4)	9·3 (4·51–15·6)	20 (8·34–31·9)	28·1 (11·9–46·3)	66·3 (22·8–113)	117 (41·1–193)	132 (48·4–221)	197 (100–294)	297 (170–415)	470 (306–616)	678 (436–863)	762 (477–984)	807 (583–982)	575 (431–682)	258 (192–304)	66·9 (52·3–77)	16·6 (12–19·5)
Other pneumoconiosis	–	–	–	–	–	–	30·3 (19·5–47·4)	33·2 (20·8–52·9)	35·1 (22·7–56·5)	52·1 (33·4–91·4)	87·8 (50·1–152)	104 (60·2–169)	126 (64·2–214)	193 (101–305)	280 (155–441)	365 (214–572)	341 (209–521)	328 (210–486)	304 (208–420)	229 (160–310)	99 (72·3–127)	29·6 (22·6–37·7)	7·97 (6·12–9·79)
Asthma	–	–	–	20400 (11700–26600)	4100 (3190–4850)	3410 (2900–3820)	4560 (3760–5210)	5660 (4570–6440)	6620 (5290–7460)	7650 (6150–8650)	10100 (8210–11700)	12700 (10000–15100)	17800 (13800–22000)	26500 (19500–33900)	36800 (26300–48700)	50900 (35400–68300)	55300 (39400–76100)	59100 (41400–83100)	58100 (41900–81300)	46400 (32700–67100)	23500 (17300–33300)	7860 (5660–10900)	2040 (1430–3000)
Interstitial lung disease and pulmonary sarcoidosis	–	–	–	415 (212–760)	146 (105–216)	105 (82·6–148)	182 (148–244)	265 (213–349)	375 (304–481)	535 (431–672)	843 (678–1080)	1220 (933–1610)	1790 (1350–2390)	3020 (2250–4170)	4670 (3490–6290)	6850 (5230–9370)	8620 (6730–11500)	9850 (7760–12800)	10500 (8550–13600)	8090 (6480–10600)	4230 (3290–5910)	1470 (1090–2220)	446 (312–676)
Other chronic respiratory diseases	0 (0–0)	0 (0–0)	0 (0–0)	2730 (1230–5210)	724 (488–1190)	450 (333–686)	636 (481–883)	805 (589–1110)	782 (598–1050)	957 (711–1240)	1150 (864–1510)	1420 (1030–1840)	1840 (1290–2370)	2380 (1700–3080)	3520 (2540–4540)	1200 (881–1520)	1280 (957–1580)	1360 (949–1660)	1560 (1010–1850)	1630 (963–1910)	1080 (611–1280)	468 (262–564)	147 (80·7–182)
Digestive diseases	3450 (3120–3800)	4340 (3410–5300)	30200 (23200–37900)	39900 (26100–54700)	16500 (12400–20900)	11300 (9300–13200)	22500 (19600–25200)	29100 (25600–32100)	40800 (37300–43600)	53800 (50500–56700)	79400 (74600–83300)	99900 (93800–105000)	117000 (110000–123000)	149000 (141000–157000)	173000 (163000–181000)	192000 (182000–201000)	189000 (178000–199000)	172000 (162000–183000)	169000 (159000–179000)	136000 (125000–147000)	83000 (73100–90900)	33000 (27600–36600)	10400 (8260–11800)
Cirrhosis and other chronic liver diseases	–	–	–	15900 (9080–24200)	7310 (5100–9760)	5510 (4430–6730)	10700 (9270–12500)	15400 (13600–17600)	23100 (20800–25300)	35400 (32800–37800)	53800 (50200–57000)	70800 (66000–75100)	82300 (76700–87400)	104000 (97500–110000)	115000 (108000–123000)	121000 (113000–129000)	109000 (102000–116000)	87000 (80400–94200)	72800 (67100–78600)	48700 (43400–53800)	24500 (21200–27600)	8140 (6720–9330)	2360 (1860–2760)
Cirrhosis and other chronic liver diseases due to hepatitis B	–	–	–	125 (50·3–240)	163 (79·3–275)	216 (118–341)	674 (349–1070)	3220 (2000–4470)	7360 (5140–9730)	12500 (10000–15200)	19500 (15500–23500)	25200 (20600–30200)	27600 (22200–33800)	33000 (25500–41600)	34000 (25700–43700)	33500 (24700–43600)	28800 (21200–37100)	23400 (16900–30300)	19400 (14100–24800)	12700 (9130–16600)	5920 (4410–7750)	1800 (1220–2540)	455 (262–716)
Cirrhosis and other chronic liver diseases due to hepatitis C	–	–	–	119 (45–235)	158 (76·6–277)	219 (121–366)	652 (349–1090)	2610 (1600–3760)	5770 (3970–7830)	9650 (7500–12000)	14900 (11000–19400)	19500 (15000–24600)	22100 (16700–28000)	26900 (19900–34700)	28400 (21000–37700)	28700 (20100–39200)	26200 (20000–33600)	21300 (16600–26900)	17200 (12900–22500)	11600 (8510–15300)	6160 (4590–7970)	2160 (1500–2980)	643 (395–946)
Cirrhosis and other chronic liver diseases due to alcohol use	–	–	–	–	–	–	39·6 (11·8–88·7)	668 (379–1100)	1930 (1180–3030)	5200 (3950–6660)	10700 (7690–14100)	16100 (12100–20300)	20600 (15100–26500)	28000 (20200–35900)	33300 (24700–43000)	35800 (26200–46900)	30000 (21600–39400)	20400 (14300–27700)	15400 (11200–19900)	8970 (6240–12100)	4130 (2970–5470)	1280 (827–1880)	357 (192–612)
Cirrhosis and other chronic liver diseases due to NAFLD	–	–	–	–	–	–	261 (105–518)	714 (344–1220)	1420 (688–2510)	2190 (1280–3470)	3040 (1560–5300)	4120 (2070–6790)	5340 (2600–9340)	7110 (3490–12300)	7920 (3950–13400)	8890 (4410–15800)	9180 (5700–14200)	8130 (5010–12200)	7580 (4110–11900)	5570 (3140–8460)	3060 (1890–4610)	1070 (596–1810)	337 (148–628)
Cirrhosis and other chronic liver diseases due to other causes	–	–	–	15700 (8920–23900)	6990 (4870–9370)	5080 (4080–6200)	9090 (7680–10800)	8190 (5760–10900)	6640 (4170–9620)	5860 (3550–9310)	5630 (3170–8960)	5910 (3310–9540)	6650 (3810–10800)	8890 (5130–14200)	11400 (6560–18500)	14000 (8210–21900)	14900 (8340–24400)	13800 (7740–21800)	13300 (7700–20700)	9800 (5720–15100)	5220 (3300–7560)	1830 (1060–2800)	564 (267–929)
Upper digestive system diseases	–	–	–	3480 (2110–5180)	1710 (1290–2210)	1080 (902–1300)	3160 (2680–3730)	4650 (3940–5530)	6040 (5420–6890)	6980 (6360–7770)	10700 (9720–12100)	12200 (11000–13500)	15700 (14100–17400)	21400 (19500–23600)	26400 (24000–29100)	31800 (29200–34600)	34600 (31800–37500)	35400 (32600–38400)	36600 (33900–39300)	30000 (27200–32500)	19000 (16700–20800)	7240 (6070–8070)	2230 (1760–2530)
Peptic ulcer disease	–	–	–	2670 (1460–4240)	1520 (1120–1960)	968 (796–1170)	2900 (2450–3420)	4200 (3510–4940)	5460 (4880–6220)	6150 (5580–6880)	9630 (8650–10700)	10900 (9740–12200)	14100 (12600–15600)	19200 (17500–21200)	23700 (21600–26200)	28900 (26400–31700)	31200 (28500–34000)	32000 (29200–34600)	33000 (30400–35600)	26900 (24500–29300)	17000 (15100–18700)	6460 (5410–7210)	1980 (1550–2260)
Gastritis and duodenitis	–	–	–	815 (432–1290)	189 (110–282)	112 (76·9–153)	263 (186–366)	455 (307–665)	586 (400–783)	824 (586–1060)	1120 (790–1490)	1280 (909–1740)	1650 (1160–2210)	2180 (1580–2900)	2640 (1900–3530)	2910 (2140–3920)	3360 (2580–4420)	3440 (2640–4590)	3570 (2800–4690)	3070 (2460–4120)	2000 (1580–2560)	778 (602–951)	252 (193–300)
Appendicitis	–	–	–	4240 (2020–6500)	2640 (1450–3780)	1860 (1190–2480)	3290 (2250–4450)	2530 (1640–3450)	3020 (2230–3900)	1540 (1110–1940)	1960 (1390–2460)	1960 (1360–2360)	1980 (1420–2420)	1900 (1400–2320)	2540 (1940–3100)	2710 (2110–3230)	2440 (1930–2880)	2450 (1910–2930)	2390 (1980–2880)	1830 (1520–2190)	1030 (856–1250)	372 (299–460)	97 (75–129)
Paralytic ileus and intestinal obstruction	2360 (2050–2740)	3380 (2700–4100)	25800 (20000–32000)	4340 (2480–6780)	2450 (1650–3410)	1440 (1070–1770)	2960 (2040–3640)	2850 (2020–3480)	3260 (2150–3920)	2960 (1950–3520)	3830 (2580–4540)	4420 (2990–5220)	4830 (3330–5680)	6030 (4330–6940)	8010 (6050–9180)	10000 (7920–11400)	11300 (9180–12800)	11700 (10200–13600)	13500 (12000–16000)	13600 (11800–16400)	10100 (8500–12100)	4730 (3790–5700)	1580 (1190–1970)
Inguinal, femoral, and abdominal hernia	1090 (761–1440)	961 (516–1740)	4460 (1710–8960)	1260 (468–2460)	383 (252–528)	197 (139–246)	341 (251–411)	358 (270–435)	388 (302–476)	365 (289–441)	560 (409–681)	708 (515–855)	854 (635–1050)	1520 (1190–1840)	2120 (1630–2580)	2890 (2300–3520)	3360 (2700–4060)	3680 (2960–4530)	4670 (3820–5670)	4840 (3850–5920)	3280 (2650–3930)	1400 (1070–1690)	439 (315–526)
Inflammatory bowel disease	–	–	–	2090 (740–3640)	258 (139–380)	154 (97·6–201)	250 (162–333)	374 (246–501)	538 (396–691)	573 (448–718)	667 (539–820)	766 (629–958)	849 (699–1070)	1080 (913–1340)	1360 (1140–1690)	1750 (1490–2130)	2030 (1730–2450)	2300 (1950–2790)	2900 (2490–3480)	2970 (2490–3620)	2080 (1700–2490)	955 (764–1140)	337 (255–400)
Vascular intestinal disorders	–	–	–	443 (168–820)	93·5 (56·8–140)	45 (35·2–58)	79·5 (67·3–98·7)	163 (134–209)	214 (182–258)	272 (238–322)	398 (356–457)	603 (546–664)	912 (833–1000)	1630 (1500–1770)	2530 (2340–2710)	4130 (3870–4370)	5870 (5560–6210)	7210 (6830–7580)	10100 (9530–10700)	9890 (9010–10500)	6480 (5620–7040)	2720 (2230–3020)	800 (624–906)
Gallbladder and biliary diseases	–	–	–	1360 (439–2060)	397 (240–541)	269 (180–351)	409 (280–491)	611 (427–743)	784 (567–929)	1100 (794–1290)	1500 (1070–1760)	1810 (1280–2140)	2200 (1600–2600)	3560 (2610–4160)	4810 (3500–5490)	7020 (5310–7970)	8710 (6650–9730)	10000 (7890–11100)	12700 (10500–13900)	12100 (10400–13300)	8080 (6840–9130)	3740 (2960–4290)	1270 (963–1470)
Pancreatitis	–	–	–	350 (122–628)	220 (136–354)	200 (149–283)	762 (626–1030)	1500 (1270–1970)	2560 (2190–3310)	3610 (3170–4560)	4600 (4030–5640)	5080 (4430–6330)	5610 (4810–6820)	5590 (4980–6750)	6510 (5600–7960)	6440 (5750–7900)	6450 (5750–7860)	5920 (5190–7020)	5910 (5220–6960)	4600 (3970–5430)	2640 (2230–3080)	977 (792–1150)	283 (217–338)
Other digestive diseases	–	–	–	6440 (3240–11600)	1030 (588–1470)	567 (344–776)	545 (328–686)	652 (405–852)	862 (541–1120)	1010 (665–1270)	1280 (893–1580)	1560 (1150–1890)	1880 (1350–2300)	2590 (2020–3200)	3310 (2650–3990)	4590 (3710–5480)	5140 (4220–6100)	6390 (5150–7340)	7460 (6280–8910)	7680 (6320–8900)	5770 (4620–6790)	2710 (2050–3330)	1000 (718–1240)
Neurological disorders	9·52 (7·72–12·9)	22·6 (19·2–28·4)	11100 (8040–12900)	16400 (9060–21100)	7270 (5430–8680)	6760 (5620–7660)	10800 (9210–11800)	10200 (8640–11100)	8570 (7490–9250)	8330 (7290–8960)	8700 (7860–9270)	9060 (8230–10100)	10300 (8600–14100)	13900 (10700–21600)	19500 (13300–33600)	29900 (18400–58800)	46300 (26300–96800)	70400 (38600–147000)	116000 (59800–252000)	179000 (75700–433000)	166000 (59800–406000)	94500 (29200–232000)	40100 (11400–102000)
Alzheimer's disease and other dementias	–	–	–	–	–	–	–	–	–	–	–	336 (34·1–1170)	1680 (227–5390)	3660 (606–11300)	7540 (1390–21800)	14500 (3040–43400)	25500 (5630–76400)	41400 (9270–117000)	72700 (16100–211000)	137000 (32000–394000)	142000 (34100–384000)	87200 (21400–226000)	38400 (9540–101000)
Parkinson's disease	–	–	–	–	–	–	–	15·1 (12·6–17·1)	17·2 (14·6–19·2)	35·8 (29·3–40·2)	49 (40·9–55)	456 (391–521)	899 (793–1020)	1800 (1610–2050)	2920 (2650–3290)	5700 (5220–6360)	11100 (10200–12300)	21000 (19600–22900)	36000 (34000–38700)	37600 (34700–40300)	21700 (19200–23200)	6490 (5470–7040)	1440 (1140–1600)
Idiopathic epilepsy	–	–	6770 (4260–8530)	12600 (5970–17300)	5920 (4160–7270)	5290 (4160–6170)	8470 (6940–9420)	8480 (6930–9320)	7220 (6120–7880)	6780 (5730–7370)	6470 (5610–6980)	5440 (4760–5870)	4250 (3730–4590)	4070 (3570–4420)	3660 (3160–3960)	3540 (3010–3840)	3160 (2690–3470)	2740 (2340–3050)	2180 (1950–2390)	1710 (1500–1920)	886 (769–1000)	286 (235–331)	83·6 (65–98·4)
Multiple sclerosis	–	–	–	–	76·9 (4·72–200)	28·5 (4·36–51·9)	45·6 (17·8–69·6)	152 (112–217)	296 (238–383)	557 (448–727)	944 (755–1260)	1250 (995–1670)	1370 (1140–1840)	1600 (1370–2320)	1640 (1390–2360)	1600 (1380–2350)	1380 (1150–2020)	949 (811–1440)	769 (654–1230)	436 (362–684)	200 (155–300)	51·4 (39·3–75·6)	10·6 (7·22–14·8)
Motor neuron disease	9·52 (7·72–12·9)	22·6 (19·2–28·4)	1440 (1200–1790)	370 (289–482)	84·2 (74·5–97·5)	90·6 (82·3–100)	192 (166–220)	144 (123–165)	102 (94–110)	150 (140–160)	269 (254–287)	455 (428–482)	698 (655–745)	1120 (1070–1190)	1710 (1620–1800)	2270 (2180–2360)	2730 (2630–2820)	2210 (2130–2270)	1950 (1870–2020)	1040 (946–1090)	489 (426–525)	97 (79·9–106)	18·2 (14·3–20·2)
Other neurological disorders	–	–	2920 (2240–3420)	3410 (2280–4120)	1190 (967–1410)	1350 (1180–1530)	2110 (1930–2320)	1460 (1350–1580)	940 (883–1000)	808 (764–860)	966 (916–1040)	1120 (1050–1210)	1370 (1300–1480)	1660 (1570–1800)	2040 (1920–2210)	2260 (2140–2390)	2410 (2310–2540)	2190 (2090–2300)	2350 (2230–2470)	1870 (1690–1980)	1130 (993–1230)	416 (341–458)	129 (99·2–145)
Mental disorders	–	–	–	–	0·426 (0·273–1·12)	3·4 (2·61–4·96)	17·9 (14·5–24·4)	26·5 (22·5–33·5)	27·5 (23·8–32·5)	27·8 (23·8–33·8)	26 (22·5–30·3)	27 (23·6–31·8)	21·7 (19–25·6)	–	–	–	–	–	–	–	–	–	–
Eating disorders	–	–	–	–	0·426 (0·273–1·12)	3·4 (2·61–4·96)	17·9 (14·5–24·4)	26·5 (22·5–33·5)	27·5 (23·8–32·5)	27·8 (23·8–33·8)	26 (22·5–30·3)	27 (23·6–31·8)	21·7 (19–25·6)	–	–	–	–	–	–	–	–	–	–
Anorexia nervosa	–	–	–	–	0·252 (0·17–0·679)	3·12 (2·44–4·24)	16·8 (14·1–21·5)	24·1 (20·9–29·6)	25·8 (22·6–30·1)	25·5 (22·1–30·5)	24 (21–27·7)	24·7 (22–28·3)	20 (17·8–22·9)	–	–	–	–	–	–	–	–	–	–
Bulimia nervosa	–	–	–	–	0·174 (0·0956–0·49)	0·279 (0·147–0·816)	1·06 (0·371–3·85)	2·43 (1·17–5·5)	1·66 (0·881–3·05)	2·32 (1·36–4·21)	1·96 (1·25–3·33)	2·29 (1·4–4·53)	1·67 (1·01–3·35)	–	–	–	–	–	–	–	–	–	–
Substance use disorders	0 (0–0)	0 (0–0)	1·13 (0·0464–4·21)	0 (0–0)	0 (0–0)	0 (0–0)	4750 (4330–5200)	9080 (8280–9850)	12500 (11600–13300)	16600 (15300–17400)	20100 (18800–21100)	19700 (18400–20700)	17900 (16700–18900)	19900 (18600–20800)	16900 (15800–17800)	13700 (12900–14300)	8770 (8310–9190)	5170 (4850–5490)	4120 (3850–4360)	2010 (1860–2130)	968 (858–1030)	273 (230–296)	81·2 (63·5–90·5)
Alcohol use disorders	–	–	–	–	–	0 (0–0)	1190 (1080–1290)	2560 (2290–2750)	5010 (4450–5290)	9290 (8420–9750)	13000 (12000–13700)	14400 (13200–15100)	14200 (13100–15100)	16400 (15200–17100)	14000 (12800–14600)	11200 (10400–11700)	6720 (6300–7010)	3570 (3270–3750)	2780 (2550–2930)	1270 (1170–1340)	603 (533–645)	154 (130–168)	46·3 (36–52·2)
Drug use disorders	0 (0–0)	0 (0–0)	1·13 (0·0464–4·21)	0 (0–0)	0 (0–0)	0 (0–0)	3550 (3210–3940)	6520 (5890–7220)	7520 (6950–8130)	7270 (6770–7820)	7070 (6490–7660)	5350 (4900–5850)	3690 (3350–4050)	3480 (3170–3830)	2940 (2670–3230)	2480 (2280–2700)	2050 (1880–2230)	1600 (1460–1760)	1340 (1240–1460)	741 (672–804)	365 (319–396)	118 (99–130)	34·9 (27·4–39·2)
Opioid use disorders	0 (0–0)	0 (0–0)	1·13 (0·0464–4·21)	0 (0–0)	0 (0–0)	0 (0–0)	1770 (1580–1960)	3650 (3260–3990)	4500 (4130–4830)	5100 (4740–5500)	4840 (4430–5270)	3630 (3300–3970)	2480 (2230–2710)	2460 (2230–2700)	2050 (1850–2240)	1740 (1580–1890)	1430 (1280–1580)	1210 (1090–1340)	1040 (935–1140)	543 (481–599)	271 (234–298)	88 (72·9–97·8)	25·8 (20·3–29)
Cocaine use disorders	–	–	–	–	–	0 (0–0)	141 (127–170)	233 (209–304)	284 (261–344)	369 (341–432)	348 (320–416)	257 (233–316)	182 (163–240)	165 (143–233)	129 (109–188)	98 (84·9–135)	75·8 (66–107)	37·7 (32·4–52·6)	26·5 (23·4–33·1)	6·05 (5·24–6·93)	3·02 (2·51–3·52)	1·09 (0·888–1·25)	0·328 (0·237–0·398)
Amphetamine use disorders	–	–	–	–	–	0 (0–0)	769 (632–1080)	1240 (997–1860)	1280 (1050–1840)	614 (520–925)	656 (543–993)	499 (414–729)	342 (282–504)	315 (256–468)	272 (217–410)	224 (180–335)	189 (152–279)	66·7 (46·8–97·4)	53·7 (35·6–74·4)	59·6 (42·4–76·2)	26·1 (18·5–34·2)	5·62 (3·99–7·07)	1·3 (0·929–1·65)
Other drug use disorders	–	–	–	–	–	0 (0–0)	874 (679–1030)	1400 (1130–1640)	1450 (1120–1660)	1190 (1020–1320)	1230 (1070–1390)	967 (854–1100)	693 (599–797)	541 (466–626)	491 (420–572)	421 (364–481)	352 (305–399)	290 (251–332)	228 (198–258)	132 (115–150)	64·9 (55–74·1)	23·6 (19·3–27)	7·53 (5·93–8·95)
Diabetes and kidney diseases	47·8 (34·6–62·8)	151 (112–198)	10300 (7980–12100)	13000 (8010–16300)	7570 (6270–8870)	6250 (5470–7070)	11900 (11000–12900)	15900 (14800–16900)	17800 (16700–19000)	22400 (21100–23700)	30000 (28100–31900)	38700 (36400–41000)	49400 (46700–52000)	75800 (71500–79900)	103000 (97600–109000)	139000 (132000–145000)	155000 (148000–163000)	157000 (150000–166000)	167000 (158000–175000)	133000 (122000–142000)	80500 (70800–86100)	33200 (27900–36100)	11300 (8760–12500)
Diabetes mellitus	47·8 (34·6–62·8)	151 (112–198)	1970 (1320–2390)	1690 (1020–2360)	1480 (1010–2010)	1380 (927–1740)	3010 (2740–3280)	4100 (3810–4410)	5230 (4850–5630)	7350 (6850–7890)	10400 (9680–11200)	15800 (14900–16900)	23600 (22200–25100)	39500 (37100–42200)	57000 (53900–60600)	81500 (77200–85800)	92400 (88000–96700)	91600 (86900–96200)	94200 (89000–99200)	70800 (64900–75500)	38400 (33600–41200)	15000 (12600–16400)	4870 (3810–5420)
Diabetes mellitus type 1	47·8 (34·6–62·8)	151 (112–198)	1970 (1320–2390)	1690 (1020–2360)	1480 (1010–2010)	1380 (927–1740)	1780 (1330–2060)	2540 (2070–2870)	3140 (2570–3520)	3180 (2550–3650)	4220 (3410–4830)	5790 (4570–6770)	1950 (1390–2490)	2970 (2140–3770)	4170 (3070–5320)	4090 (3050–5160)	4750 (3560–5970)	3590 (2670–4540)	3670 (2670–4520)	1430 (1030–1770)	890 (609–1070)	395 (257–477)	142 (89–176)
Diabetes mellitus type 2	–	–	–	–	–	–	1230 (1010–1690)	1560 (1270–2000)	2090 (1690–2690)	4170 (3590–4860)	6180 (5370–7110)	10000 (8710–11400)	21600 (20200–23100)	36500 (34200–39000)	52800 (49700–56300)	77400 (73100–81600)	87700 (83300–92300)	88000 (83400–92800)	90600 (85400–95300)	69400 (63500–74100)	37500 (32800–40300)	14700 (12300–16000)	4730 (3670–5280)
Chronic kidney disease	–	–	7790 (5380–9310)	10300 (5650–13100)	5240 (4060–6260)	4320 (3820–4800)	8430 (7720–9110)	11200 (10200–12000)	12000 (11100–12900)	14400 (13500–15400)	18800 (17500–20100)	22100 (20600–23600)	25100 (23400–26700)	35500 (33100–37600)	45200 (42200–48100)	56000 (52600–59500)	61500 (57900–65700)	64400 (60400–69500)	71200 (66800–76700)	61600 (56000–67100)	41700 (36800–45400)	18000 (15100–19900)	6390 (4950–7230)
Chronic kidney disease due to diabetes mellitus type 1	–	–	52·9 (22·4–112)	90·7 (34·8–184)	95·2 (33·3–211)	169 (63·5–356)	539 (209–1070)	1260 (570–2220)	2090 (1080–3420)	3370 (1920–5010)	4700 (2680–7080)	5160 (2770–7800)	4910 (2310–7810)	5010 (2190–8880)	4220 (1500–8440)	3690 (1420–7630)	2970 (1220–6320)	2250 (935–4420)	1610 (622–3480)	756 (291–1670)	212 (74·5–503)	26·2 (8·33–63·3)	2·19 (0·623–5·79)
Chronic kidney disease due to diabetes mellitus type 2	–	–	–	–	–	–	284 (85·2–655)	563 (218–1190)	775 (293–1530)	1220 (433–2340)	2000 (765–3680)	3120 (1300–5430)	5010 (2560–7860)	9340 (5330–13500)	13900 (8530–19200)	17900 (10200–24300)	19800 (12500–26400)	20600 (13700–27000)	21500 (13800–28800)	17100 (11200–23400)	10500 (6840–14600)	4100 (2530–5880)	1360 (722–2080)
Chronic kidney disease due to hypertension	–	–	–	–	–	–	625 (325–1090)	1190 (660–1950)	1490 (872–2390)	1950 (1210–3010)	2840 (1780–4250)	3800 (2530–5400)	4960 (3550–6960)	8130 (5840–11100)	11800 (8560–16000)	16300 (12100–21800)	19400 (14700–24900)	21400 (16600–27000)	25200 (19600–32100)	23600 (18100–29800)	17700 (13900–21900)	8320 (6330–10400)	3120 (2230–4070)
Chronic kidney disease due to glomerulonephritis	–	–	1830 (997–3080)	3090 (1450–5020)	1640 (827–2600)	1630 (870–2520)	3200 (1980–4560)	3890 (2290–5640)	3800 (2360–5360)	3870 (2240–5710)	4570 (2650–7240)	4890 (2430–7600)	4640 (2760–7410)	5850 (3250–9250)	6750 (3700–11100)	7560 (4050–12200)	7340 (3770–12300)	7130 (3920–11700)	7850 (4160–13000)	6370 (3160–11000)	3790 (1940–6390)	1460 (734–2500)	479 (212–951)
Chronic kidney disease due to other and unspecified causes	–	–	5910 (3910–7520)	7070 (3660–9620)	3500 (2400–4690)	2520 (1620–3390)	3780 (2370–5190)	4260 (2560–5970)	3870 (2440–5370)	4020 (2380–6040)	4690 (2600–7170)	5160 (2850–7850)	5570 (2950–8520)	7130 (4130–10800)	8500 (4740–13600)	10500 (6020–16700)	12000 (6920–18200)	13000 (7670–19800)	15100 (8960–22900)	13800 (8120–20500)	9560 (5590–13800)	4140 (2580–5960)	1430 (787–2140)
Acute glomerulonephritis	–	–	557 (371–760)	1060 (565–1620)	851 (579–1180)	555 (398–751)	510 (413–627)	623 (504–770)	588 (476–717)	625 (522–738)	814 (675–978)	780 (646–939)	705 (580–861)	869 (737–1080)	1000 (840–1250)	1190 (1010–1480)	1280 (1070–1590)	1240 (1020–1620)	1140 (951–1410)	795 (655–1030)	410 (336–556)	110 (86·6–143)	28·2 (21·6–37·5)
Skin and subcutaneous diseases	–	–	3770 (2360–5350)	2580 (1630–4840)	652 (372–931)	548 (328–696)	737 (452–882)	702 (420–821)	600 (382–702)	918 (604–1080)	1040 (698–1230)	1140 (849–1400)	1430 (1090–1760)	1920 (1510–2430)	2530 (1940–3180)	3200 (2560–4090)	3620 (2890–4610)	4280 (3450–5350)	5760 (4590–7320)	5790 (4450–7590)	4650 (3370–6060)	2400 (1640–3110)	986 (637–1330)
Bacterial skin diseases	–	–	3700 (2300–5250)	2490 (1550–4620)	609 (328–871)	500 (284–645)	669 (400–802)	608 (349–719)	511 (311–601)	786 (489–927)	894 (537–1050)	959 (666–1150)	1200 (821–1460)	1610 (1170–1990)	2100 (1530–2570)	2580 (1900–3150)	2670 (1980–3230)	2910 (2140–3470)	3450 (2660–4040)	2860 (2220–3510)	2020 (1440–2450)	965 (643–1210)	372 (246–477)
Cellulitis	–	–	549 (152–971)	481 (61·4–1320)	60 (20·9–109)	79·4 (36·6–120)	215 (89·4–302)	235 (118–317)	156 (93·9–203)	241 (153–339)	264 (158–372)	296 (196–445)	379 (234–555)	475 (328–735)	568 (387–840)	696 (482–1090)	654 (450–1020)	702 (487–1040)	780 (526–1250)	668 (463–1070)	465 (304–763)	241 (145–358)	99·3 (57·4–145)
Pyoderma	–	–	3150 (1940–4720)	2010 (1030–4330)	549 (289–809)	421 (235–572)	454 (290–596)	373 (213–492)	355 (207–435)	545 (287–673)	630 (332–768)	663 (398–795)	825 (491–1020)	1140 (709–1370)	1540 (977–1850)	1890 (1200–2280)	2010 (1360–2390)	2210 (1470–2570)	2670 (1970–3040)	2190 (1690–2580)	1550 (1090–1880)	724 (484–904)	272 (180–346)
Decubitus ulcer	–	–	–	17 (5·25–32)	13·4 (6·85–18·8)	17·2 (9·47–22·4)	34·6 (18·6–45·8)	43·4 (25·7–60·8)	54·6 (33·7–79·8)	88 (64·2–127)	97·9 (70–157)	115 (79·5–190)	151 (109–259)	235 (168–383)	328 (237–562)	489 (343–850)	791 (565–1220)	1190 (827–1750)	2050 (1440–3070)	2620 (1840–3810)	2380 (1550–3320)	1310 (797–1790)	571 (326–797)
Other skin and subcutaneous diseases	–	–	72·7 (47–117)	68·7 (45·7–167)	30·3 (22·6–45·7)	30·4 (22·5–40·3)	34·3 (24·4–44·2)	50·6 (36·2–68·3)	34·1 (23·2–47)	44·1 (32–58·2)	50·8 (35·7–74·3)	66·7 (51·7–93·9)	72·3 (54·8–107)	75·9 (57·7–114)	99·8 (76·2–153)	128 (96·7–212)	160 (121–268)	184 (136–307)	259 (188–511)	311 (223–599)	250 (171–485)	123 (78·6–247)	43·7 (26·6–81·7)
Musculoskeletal disorders	–	–	–	–	389 (318–486)	674 (584–859)	1320 (1120–1730)	1490 (1250–2060)	1550 (1290–2100)	1600 (1330–2110)	1770 (1470–2380)	1960 (1630–2500)	1960 (1610–2490)	2320 (1880–2890)	3110 (2470–3760)	4660 (3720–5470)	5770 (4610–6810)	6250 (4960–7620)	6940 (5480–8610)	5850 (4320–7470)	4360 (2900–5050)	1810 (1130–2210)	612 (365–814)
Rheumatoid arthritis	–	–	–	–	59·7 (45·4–80·6)	66·4 (52·4–84·9)	78·3 (59·3–101)	88·8 (64·3–121)	110 (78–146)	155 (114–200)	222 (170–306)	326 (260–434)	482 (377–631)	790 (626–994)	1330 (1040–1620)	2290 (1780–2780)	3090 (2430–3720)	3520 (2780–4320)	3950 (3080–4950)	2970 (2270–3880)	1870 (1330–2330)	617 (420–817)	160 (107–225)
Other musculoskeletal disorders	–	–	–	–	330 (266–408)	608 (522–772)	1240 (1060–1630)	1400 (1170–1930)	1440 (1210–1960)	1440 (1200–1920)	1550 (1290–2110)	1640 (1360–2110)	1470 (1210–1860)	1530 (1230–1980)	1780 (1390–2210)	2370 (1900–2820)	2680 (2130–3230)	2720 (2120–3370)	3000 (2260–3760)	2880 (2030–3540)	2490 (1560–2900)	1200 (697–1460)	452 (256–589)
Other non-communicable diseases	260000 (212000–341000)	120000 (84200–168000)	403000 (242000–590000)	166000 (79100–271000)	34100 (25900–43200)	20300 (17300–23600)	17600 (16100–19400)	15400 (14200–16800)	12800 (11800–14000)	11300 (10400–12300)	11600 (10800–12500)	11600 (10600–12300)	11400 (10200–12200)	13900 (12500–14900)	16300 (14400–17600)	20500 (18000–22000)	22900 (20700–24500)	22600 (20400–24500)	26000 (23500–27800)	23700 (21200–25600)	16100 (13900–17700)	7650 (6280–8570)	3020 (2350–3470)
Congenital birth defects	257000 (209000–338000)	112000 (78000–163000)	333000 (205000–538000)	133000 (59100–230000)	24300 (17900–31700)	13400 (11200–16200)	9150 (8200–10400)	6990 (6210–8040)	5290 (4750–6070)	3560 (3240–3990)	3570 (3270–4010)	3200 (2950–3580)	1830 (1680–2050)	1730 (1580–1980)	1690 (1570–1970)	1510 (1410–1790)	1260 (1140–1410)	–	–	–	–	–	–
Neural tube defects	61100 (43400–83900)	19800 (13700–31100)	49100 (32900–82100)	29000 (13500–63500)	1980 (1290–3080)	1110 (765–1550)	610 (443–829)	401 (293–524)	270 (199–372)	202 (155–268)	175 (137–233)	169 (124–251)	95·1 (76·6–124)	81·8 (66·6–103)	79·2 (62·3–104)	65·2 (51·9–89·7)	57 (44·5–75·5)	–	–	–	–	–	–
Congenital heart anomalies	76200 (62200–97400)	46300 (32300–68400)	154000 (97900–248000)	53800 (27400–99700)	14200 (10900–18700)	8390 (7020–9920)	5910 (5250–6700)	4750 (4170–5400)	3540 (3160–4070)	2300 (2070–2590)	2350 (2140–2650)	2010 (1840–2260)	1050 (960–1190)	954 (873–1100)	950 (865–1120)	841 (774–1020)	717 (636–812)	–	–	–	–	–	–
Orofacial clefts	5140 (3190–7780)	1820 (1080–2890)	4470 (2370–7170)	338 (176–646)	–	–	–	–	–	–	–	–	–	–	–	–	–	–	–	–	–	–	–
Down syndrome	4260 (3170–7340)	2080 (1340–4590)	10400 (6610–22700)	4820 (2140–13200)	619 (428–1190)	381 (291–583)	326 (265–435)	302 (244–411)	289 (239–392)	257 (216–337)	266 (228–342)	310 (262–407)	242 (214–294)	289 (258–351)	275 (246–353)	211 (189–277)	107 (96·2–135)	–	–	–	–	–	–
Other chromosomal abnormalities	5870 (3380–13900)	3120 (1590–9090)	6140 (2960–19000)	1610 (495–6320)	311 (189–735)	184 (134–331)	155 (124–236)	122 (98·8–185)	123 (96·7–187)	67·2 (54·8–95)	77·3 (61·3–103)	58 (43–79·8)	34·5 (25·5–44·4)	31·3 (20–37·6)	31·3 (20·4–37·9)	26·6 (14·4–31·9)	22·3 (14·7–27·4)	–	–	–	–	–	–
Congenital musculoskeletal and limb anomalies	5540 (3550–8760)	1610 (919–2910)	4120 (2130–7790)	1490 (536–3540)	460 (290–724)	318 (222–420)	305 (219–383)	171 (121–224)	132 (92·9–176)	93·9 (69·6–122)	91·9 (68·4–118)	77·6 (58·1–95·1)	53·1 (40·6–62·5)	52·7 (40–61·3)	56·6 (45·9–69)	63·8 (51–77·9)	64·9 (45·7–73·9)	–	–	–	–	–	–
Urogenital congenital anomalies	7170 (4430–11100)	2090 (1070–3520)	3610 (1820–6720)	980 (393–2280)	211 (137–361)	127 (96–176)	90·7 (65·6–108)	75·3 (53·9–88·1)	67·8 (50·7–78·9)	53·5 (41–62)	54·6 (42·4–64·8)	60·1 (45·9–70·9)	35 (27·5–40·5)	34·7 (30·2–39·4)	36·5 (29·8–42·7)	34·9 (29·9–44·1)	35·8 (30·2–46·4)	–	–	–	–	–	–
Digestive congenital anomalies	23000 (13700–36800)	12400 (6040–23900)	34300 (13300–73200)	7540 (1710–24200)	601 (316–1250)	255 (169–429)	110 (83–159)	78 (57·4–119)	63 (48·3–86·7)	47·7 (35·5–67·3)	46·7 (34·9–61·1)	45·4 (34–59·2)	38·3 (29–46·8)	48·4 (36·7–59·5)	55·6 (45·9–67·6)	68·6 (58·2–92·2)	75·6 (60–94·7)	–	–	–	–	–	–
Other congenital birth defects	68700 (41700–118000)	22300 (10300–41800)	67000 (28700–135000)	33300 (8580–83900)	5950 (3280–11700)	2650 (1820–4260)	1640 (1280–2300)	1090 (842–1520)	802 (626–1080)	544 (433–713)	505 (413–643)	475 (377–609)	281 (231–341)	235 (199–280)	209 (180–254)	204 (176–251)	185 (159–219)	–	–	–	–	–	–
Urinary diseases and male infertility	598 (438–690)	600 (410–747)	3320 (2360–3990)	4560 (2280–6290)	1890 (1380–2330)	1180 (960–1380)	1940 (1580–2170)	2500 (2000–2760)	2200 (1760–2420)	2630 (2130–2860)	3090 (2510–3370)	3610 (2920–3970)	4320 (3440–4760)	6390 (5190–7010)	7810 (6480–8540)	10700 (8890–11700)	13000 (10900–14200)	13800 (12200–15100)	16300 (14600–17700)	15300 (13600–16900)	10500 (9150–11800)	5250 (4280–6030)	2180 (1690–2540)
Urolithiasis	–	–	–	133 (41·6–263)	99 (44·2–171)	64·1 (30·5–90·8)	70·7 (30·4–99·3)	103 (42–134)	132 (55·1–173)	180 (82·1–227)	283 (139–357)	349 (173–452)	516 (292–649)	688 (394–866)	892 (544–1110)	1250 (788–1530)	1480 (933–1830)	1420 (907–1800)	1650 (1090–2020)	1130 (732–1410)	606 (392–757)	221 (146–275)	69·9 (46·5–86·6)
Other urinary diseases	425 (316–501)	279 (184–387)	622 (382–904)	1420 (653–2420)	432 (293–635)	300 (226–382)	391 (296–464)	318 (225–393)	324 (231–383)	508 (386–605)	569 (427–702)	679 (499–829)	991 (714–1210)	1110 (755–1380)	1570 (1140–1890)	2060 (1480–2510)	2380 (1740–2920)	2600 (1830–3170)	2400 (1730–2910)	2340 (1700–2790)	1290 (1010–1560)	585 (432–725)	193 (126–256)
Gynecological diseases	–	–	–	–	–	102 (47·9–167)	257 (128–387)	307 (163–466)	426 (227–664)	460 (230–755)	533 (275–849)	419 (226–577)	939 (427–1460)	368 (225–582)	854 (368–1580)	930 (426–1600)	238 (143–353)	414 (242–702)	343 (202–527)	395 (233–621)	205 (122–318)	72 (45·7–108)	27·4 (17·2–40·3)
Uterine fibroids	–	–	–	–	–	12·9 (5·15–25·4)	53 (17–93·7)	87·6 (32·3–153)	222 (94·2–376)	249 (100–476)	304 (142–518)	230 (128–319)	671 (301–1080)	254 (139–404)	667 (270–1280)	659 (257–1190)	93·9 (52·8–164)	232 (105–451)	172 (88·6–312)	183 (84·7–348)	65·4 (30·2–134)	22·8 (12·3–43·9)	7·98 (3·63–14·5)
Endometriosis	–	–	–	–	–	4·11 (1·29–10·2)	5·47 (3·35–9·14)	9·33 (4·49–15·6)	11·5 (5·56–17·6)	15 (8·91–22·7)	55·6 (23–90·1)	23·1 (12·4–41·4)	21·6 (12·3–36·4)	9·37 (5·92–15·2)	–	–	–	–	–	–	–	–	–
Genital prolapse	–	–	–	–	–	67·1 (30·1–122)	61·5 (27·1–130)	56·8 (24·5–127)	52·8 (20·8–113)	87 (32·2–162)	66 (25·3–124)	23 (10–45·5)	98·2 (34·3–224)	26·5 (13·6–55)	114 (34·1–335)	185 (64·9–415)	54·1 (30·4–84·3)	121 (52–238)	95·4 (49·9–160)	107 (52·3–176)	62·3 (34–109)	19·1 (10·7–28·7)	7·58 (4·29–11·5)
Other gynecological diseases	–	–	–	–	–	17·8 (7·8–35·2)	137 (64·4–229)	153 (77·3–255)	139 (69·2–263)	109 (56·2–177)	107 (60·5–176)	142 (76·9–222)	148 (72·2–243)	78·3 (46·1–134)	72·8 (42·3–130)	86·9 (49·1–175)	90·3 (48–134)	60·6 (40·4–102)	75·1 (43·7–108)	106 (58·6–159)	77·1 (44·6–112)	30·1 (18·6–41·2)	11·8 (7·56–17·4)
Hemoglobinopathies and hemolytic anemias	987 (845–1160)	816 (580–1010)	6950 (3660–10300)	22100 (10800–34700)	5770 (4300–7520)	4040 (3320–4950)	4710 (3980–5590)	3920 (3290–4570)	2960 (2440–3460)	2470 (2070–2870)	1990 (1720–2330)	1870 (1640–2220)	1610 (1410–1960)	2010 (1770–2450)	2080 (1830–2600)	2560 (2270–3160)	2890 (2570–3610)	3040 (2700–3850)	3270 (2930–4040)	2750 (2430–3260)	1700 (1460–2000)	708 (576–829)	219 (169–258)
Thalassemias	545 (477–621)	458 (346–565)	3200 (1820–4490)	5350 (2940–7780)	2040 (1610–2550)	1170 (975–1410)	1060 (871–1270)	730 (572–897)	464 (356–600)	338 (249–448)	342 (253–447)	302 (225–399)	238 (173–309)	235 (164–313)	141 (86·9–210)	48·6 (25·5–79·2)	10·5 (5·19–18·4)	1·85 (0·827–3·51)	0·378 (0·152–0·76)	0·0505 (0·0175–0·116)	0·00534 (0·00147–0·0151)	0·000375 (8·85e-05–0·00108)	2·1e-05 (3·75e-06–6·39e-05)
Sickle cell disorders	356 (223–508)	326 (189–469)	3500 (1550–5980)	16000 (7220–26400)	3200 (2150–4530)	2170 (1600–2900)	2610 (2020–3340)	2130 (1630–2690)	1570 (1170–1990)	1210 (916–1570)	802 (614–1050)	664 (507–889)	411 (297–592)	266 (178–408)	97·9 (67·2–139)	36·6 (23·9–51·9)	14·3 (8·21–22)	3·99 (2·12–6·71)	1·43 (0·674–2·6)	0·366 (0·158–0·708)	0·0733 (0·0256–0·146)	0·00924 (0·0028–0·0206)	0·00095 (0·000303–0·0023)
G6PD deficiency	14·2 (8·65–21·2)	13·1 (7·4–19·9)	127 (62–206)	466 (198–764)	350 (201–514)	457 (286–626)	626 (389–849)	600 (366–810)	533 (348–708)	500 (316–671)	431 (266–583)	409 (253–565)	426 (261–624)	570 (330–874)	509 (292–831)	483 (274–779)	358 (183–585)	223 (116–388)	132 (65·2–222)	55·1 (27·9–91·3)	19·6 (9·83–32·1)	4·61 (2·28–7·67)	0·766 (0·316–1·35)
Other hemoglobinopathies and hemolytic anemias	71·7 (54·3–91·7)	18·4 (13·1–24·8)	133 (80·3–200)	224 (128–340)	179 (139–229)	246 (197–306)	408 (318–522)	465 (373–591)	393 (317–493)	423 (337–538)	416 (332–521)	491 (405–595)	531 (447–635)	938 (798–1150)	1330 (1140–1620)	1990 (1740–2400)	2500 (2210–3040)	2810 (2490–3510)	3140 (2810–3820)	2690 (2390–3170)	1680 (1440–1970)	704 (574–822)	218 (168–257)
Endocrine, metabolic, blood, and immune disorders	1670 (1190–1940)	1650 (1140–2060)	9300 (6720–13100)	6330 (3900–9360)	2180 (1690–2720)	1540 (1260–1860)	1540 (1210–1710)	1700 (1320–1900)	1920 (1460–2140)	2150 (1680–2380)	2420 (1930–2710)	2490 (1980–2720)	2700 (2170–3080)	3400 (2770–3830)	3870 (3200–4440)	4770 (4050–5560)	5490 (4660–6370)	5410 (4630–6300)	6040 (4920–6910)	5240 (4270–6070)	3660 (3010–4110)	1620 (1240–1900)	595 (437–744)
Sudden infant death syndrome	–	5500 (2380–12500)	50500 (19900–114000)	–	–	–	–	–	–	–	–	–	–	–	–	–	–	–	–	–	–	–	–
Injuries	19600 (16000–22000)	18000 (13700–22400)	148000 (130000–164000)	437000 (346000–504000)	210000 (187000–230000)	146000 (134000–157000)	303000 (288000–318000)	384000 (367000–401000)	334000 (321000–346000)	294000 (283000–304000)	280000 (266000–291000)	233000 (222000–243000)	196000 (186000–205000)	201000 (192000–211000)	184000 (175000–193000)	173000 (164000–180000)	151000 (143000–159000)	133000 (125000–140000)	128000 (120000–134000)	104000 (95600–110000)	65700 (58000–70300)	26200 (21900–28600)	8490 (6690–9480)
Transport injuries	2420 (1290–3100)	2160 (1340–3070)	22500 (17400–27400)	95900 (71000–118000)	64900 (57400–72300)	41600 (38100–45700)	103000 (96400–114000)	127000 (120000–141000)	108000 (103000–119000)	93900 (88700–101000)	91200 (85400–1e+05)	75700 (70500–84100)	63000 (58800–70600)	62900 (58800–70400)	56800 (53300–63400)	50200 (47200–55900)	41800 (39200–46700)	32800 (30700–36100)	27100 (25700–29800)	16600 (15300–18000)	7480 (6630–8140)	1960 (1660–2150)	498 (396–554)
Road injuries	2190 (1160–2830)	2000 (1250–2860)	21200 (16400–26100)	90200 (67500–111000)	61700 (54500–69100)	38900 (35700–42800)	96100 (90200–107000)	120000 (113000–134000)	101000 (96000–112000)	87900 (83000–95100)	85000 (79800–93900)	70300 (65700–78200)	58200 (54400–65200)	58200 (54400–65600)	52800 (49500–59300)	46900 (44100–52200)	39200 (36800–43900)	31000 (29100–34300)	25700 (24300–28200)	15800 (14500–17000)	7110 (6310–7730)	1860 (1570–2040)	467 (369–520)
Pedestrian road injuries	1050 (483–1410)	880 (471–1290)	10400 (7380–13700)	55600 (41300–72200)	37900 (31700–43900)	20600 (17700–23700)	30900 (26100–36600)	37900 (31500–45800)	33200 (27900–39700)	31900 (27000–38100)	34200 (28400–40400)	28900 (24200–34800)	24700 (20900–29500)	27400 (23600–31900)	25800 (22600–30300)	25200 (22500–29400)	21500 (19200–24800)	17900 (16200–20300)	16000 (14700–17700)	10100 (9110–11100)	4590 (4030–5020)	1110 (942–1230)	269 (212–302)
Cyclist road injuries	–	–	–	3050 (1770–5300)	2870 (2270–4180)	2590 (2160–3430)	3670 (3010–4690)	3100 (2510–4000)	2630 (2140–3370)	2740 (2240–3460)	2910 (2310–3780)	2960 (2330–3890)	2850 (2290–3720)	3220 (2540–4240)	2970 (2350–3880)	2900 (2290–3720)	2490 (1990–3210)	1740 (1390–2210)	1250 (1060–1500)	666 (570–771)	242 (206–278)	56·7 (46·7–65·2)	0·539 (0·419–0·62)
Motorcyclist road injuries	318 (113–507)	274 (68·5–555)	2450 (1250–4010)	6050 (2810–10900)	3670 (2660–5290)	3700 (2970–4780)	22000 (18800–27200)	27400 (23300–33800)	21100 (18000–25300)	15800 (13100–19200)	14300 (11700–17900)	11400 (9150–14200)	8600 (7010–10700)	7130 (5710–9190)	5900 (4700–7500)	3680 (2930–4690)	2830 (2270–3590)	1630 (1320–2050)	937 (785–1140)	529 (430–637)	192 (154–236)	49·2 (39·5–60·1)	14·1 (10·8–17·5)
Motor vehicle road injuries	790 (471–1080)	823 (492–1110)	8020 (5120–10400)	24600 (14000–35100)	16700 (12400–21200)	11600 (9540–13700)	38700 (35000–43900)	50000 (45200–56300)	43200 (39100–49100)	36700 (32800–41200)	33000 (28600–37400)	26500 (23100–30200)	21500 (18700–24500)	19800 (17200–22600)	17600 (15400–20100)	14700 (12800–16900)	12000 (10600–13700)	9390 (8220–10600)	7190 (6470–8050)	4310 (3820–4770)	2010 (1730–2250)	620 (515–691)	177 (140–200)
Other road injuries	36·2 (17·5–66·9)	27·7 (17–52·4)	292 (198–501)	834 (542–1680)	603 (460–896)	437 (361–574)	858 (704–1040)	1070 (903–1300)	913 (771–1110)	707 (592–849)	675 (563–820)	576 (477–695)	606 (498–746)	653 (528–797)	587 (482–710)	394 (316–473)	361 (283–446)	288 (220–355)	268 (210–320)	167 (128–202)	75·8 (59·2–93·3)	19·1 (14·3–24·1)	6·35 (4·57–8·24)
Other transport injuries	229 (122–299)	160 (88·6–239)	1330 (882–1670)	5640 (3240–7930)	3150 (2550–3670)	2710 (2310–3130)	6810 (5880–7890)	7740 (6860–8650)	7350 (6610–8280)	6000 (5400–6720)	6200 (5520–7040)	5450 (4740–6270)	4780 (4110–5520)	4740 (4150–5460)	4000 (3440–4590)	3250 (2940–3760)	2520 (2260–2950)	1790 (1590–2070)	1470 (1310–1700)	862 (767–999)	365 (323–431)	105 (88·5–126)	31·6 (24·8–39·4)
Unintentional injuries	13400 (10900–15300)	14300 (10700–18400)	117000 (102000–130000)	315000 (248000–368000)	129000 (112000–143000)	76600 (68900–83700)	82000 (74600–88400)	87600 (80400–94000)	75800 (70900–80100)	71900 (67500–75900)	73900 (69300–78100)	67600 (63700–71700)	62200 (57700–66100)	69300 (65000–73100)	68200 (63600–72500)	72300 (66500–76800)	68500 (62100–73100)	66500 (60000–71600)	71900 (65700–76900)	69400 (62700–74400)	49600 (43500–53500)	22300 (18500–24400)	7580 (5940–8510)
Falls	1700 (1120–2100)	923 (679–1190)	10500 (8120–14100)	21400 (13900–32300)	10100 (7940–13000)	6560 (5450–7880)	9310 (8260–11100)	12400 (11100–14700)	11900 (10700–13900)	12400 (11200–14600)	14700 (13300–17600)	14800 (13400–17700)	14200 (12800–16700)	17700 (16200–20300)	20300 (18300–23300)	24800 (22000–27800)	27100 (23700–30300)	31800 (27400–35400)	39600 (35000–43200)	44400 (39700–48100)	34600 (30100–37500)	16500 (13700–18200)	5710 (4460–6420)
Drowning	888 (728–1020)	621 (502–718)	16200 (12800–18500)	166000 (130000–196000)	69300 (58900–77700)	36700 (32600–40700)	30800 (27200–34700)	24000 (21100–26900)	16800 (15200–18700)	14800 (13400–16500)	13800 (12500–15300)	11500 (10400–12800)	9350 (8460–10300)	9960 (9030–11000)	8510 (7790–9330)	8090 (7390–8970)	6790 (6170–7630)	5680 (5150–6390)	4890 (4450–5460)	3540 (3150–4000)	1650 (1440–1860)	384 (326–432)	79·6 (64·7–90·4)
Fire, heat, and hot substances	488 (368–594)	582 (373–791)	8080 (5110–10700)	26400 (15000–38400)	6710 (4850–8590)	3600 (2570–4590)	5560 (3630–7430)	7320 (5000–9710)	6600 (4800–8360)	6370 (4840–7840)	5850 (4730–6890)	4830 (4090–5540)	4450 (3730–5100)	4810 (4190–5360)	4530 (3940–5060)	4780 (4180–5280)	4550 (3930–5080)	4500 (3910–5020)	4720 (4150–5180)	4340 (3790–4780)	2760 (2390–3020)	1000 (838–1110)	327 (257–368)
Poisonings	485 (370–597)	608 (419–777)	6340 (4820–7520)	20200 (13600–24900)	4380 (3610–4960)	3570 (3040–4070)	4160 (3670–4810)	4820 (4250–5550)	4410 (3930–5030)	4190 (3820–4690)	4630 (4250–5150)	4400 (4040–4890)	3840 (3450–4310)	4680 (4280–5190)	4040 (3700–4550)	3830 (3480–4300)	2840 (2580–3260)	2330 (2110–2690)	1990 (1840–2250)	1390 (1250–1570)	680 (601–770)	180 (152–203)	45 (36·2–52·5)
Poisoning by carbon monoxide	103 (70·5–135)	115 (74·2–151)	1450 (812–2010)	4260 (2420–5820)	1610 (1250–1980)	1370 (1070–1720)	2460 (2070–2910)	2760 (2290–3320)	2490 (2110–2930)	2590 (2270–2950)	2910 (2590–3280)	2800 (2530–3180)	2370 (2130–2720)	3070 (2820–3450)	2480 (2240–2850)	2380 (2140–2710)	1640 (1450–1910)	1320 (1160–1590)	1250 (1130–1450)	860 (769–988)	436 (382–491)	111 (93·9–125)	24 (19·1–28·9)
Poisoning by other means	382 (276–477)	493 (310–653)	4880 (3350–5970)	15900 (9740–20400)	2770 (2060–3300)	2190 (1760–2550)	1710 (1400–2100)	2070 (1710–2500)	1920 (1600–2290)	1600 (1340–1930)	1730 (1420–2040)	1600 (1290–1930)	1460 (1110–1780)	1610 (1290–1990)	1560 (1270–1920)	1450 (1140–1790)	1200 (1000–1500)	1000 (831–1230)	742 (622–896)	526 (447–621)	245 (207–291)	69·5 (57·7–80·8)	21 (16·8–24·5)
Exposure to mechanical forces	1210 (602–1920)	3110 (685–6890)	6740 (4930–9300)	10500 (7550–15100)	6120 (5190–7310)	4080 (3560–4620)	8310 (7270–9670)	11100 (9630–13200)	9960 (8780–11700)	9170 (8120–11000)	9670 (8560–11700)	7930 (7020–9750)	6970 (6130–8370)	6650 (5880–7920)	5910 (5220–6980)	4780 (4150–5500)	3660 (3200–4210)	2800 (2410–3260)	2290 (2000–2650)	1540 (1350–1780)	846 (729–995)	335 (274–392)	115 (89·3–143)
Unintentional firearm injuries	212 (61·6–390)	590 (46·2–1560)	1350 (555–2130)	1940 (1310–2810)	1020 (802–1350)	1100 (922–1350)	2450 (1910–2950)	3330 (2420–4040)	2710 (2020–3300)	2320 (1730–2870)	2060 (1430–2560)	1640 (1180–2040)	1430 (967–1820)	1210 (837–1530)	946 (678–1200)	792 (581–987)	632 (474–798)	518 (384–653)	457 (356–566)	314 (242–398)	174 (136–222)	76·6 (59·3–100)	27·7 (20·4–38·7)
Other exposure to mechanical forces	998 (518–1610)	2520 (619–5700)	5380 (3770–7800)	8540 (5980–12600)	5100 (4250–6180)	2980 (2480–3440)	5860 (4980–7070)	7770 (6600–9570)	7250 (6220–8860)	6850 (5940–8280)	7610 (6610–9460)	6290 (5500–8080)	5540 (4850–6780)	5440 (4780–6660)	4960 (4360–5940)	3990 (3500–4640)	3030 (2620–3530)	2280 (1940–2700)	1830 (1590–2150)	1220 (1070–1430)	672 (575–802)	259 (212–301)	87·8 (67·2–107)
Adverse effects of medical treatment	1600 (1130–2070)	1280 (841–1560)	9970 (5690–12900)	11400 (5910–16500)	2940 (2030–3590)	1780 (1260–2250)	2030 (1360–2670)	3180 (2190–3970)	3020 (2090–3660)	2740 (1930–3390)	3030 (2140–3710)	3330 (2390–4130)	3470 (2550–4370)	3780 (2810–4720)	4890 (3690–6170)	6960 (5380–8720)	7480 (5800–9470)	6780 (5230–8660)	6730 (5370–8450)	5410 (4350–6670)	3540 (2720–4290)	1450 (1080–1750)	482 (344–591)
Animal contact	651 (361–817)	743 (400–983)	5390 (3040–6960)	17600 (8640–24700)	12100 (7180–15800)	6800 (4270–8620)	4810 (3130–5880)	4530 (2920–5400)	4180 (2640–5000)	3950 (2450–4730)	3890 (2340–4610)	4530 (2870–5350)	5360 (3250–6410)	4960 (3180–5820)	5050 (3190–5930)	4990 (3070–5840)	4880 (2930–5800)	3670 (2310–4360)	2920 (1850–3490)	1490 (997–1800)	517 (379–630)	124 (91·1–154)	31·7 (23·9–41·7)
Venomous animal contact	565 (291–720)	615 (307–845)	4110 (2210–5530)	13600 (6950–19700)	10600 (6190–14100)	6080 (3750–7810)	4290 (2770–5290)	4010 (2510–4870)	3720 (2280–4510)	3500 (2140–4250)	3370 (1960–4050)	4010 (2420–4770)	4770 (2810–5750)	4190 (2480–5010)	4230 (2540–5070)	4200 (2410–5000)	4150 (2330–4990)	3090 (1810–3710)	2470 (1470–3010)	1210 (767–1470)	386 (263–468)	84·6 (59·7–106)	20·1 (14·5–26·9)
Non-venomous animal contact	86·2 (65·3–106)	128 (87·8–158)	1290 (745–1680)	3960 (1680–5640)	1530 (954–1930)	716 (494–901)	522 (384–659)	523 (371–655)	464 (335–571)	450 (313–537)	526 (383–637)	528 (389–645)	587 (433–761)	773 (573–1010)	820 (610–1110)	791 (592–1080)	726 (549–1030)	584 (443–837)	456 (352–648)	281 (218–398)	130 (102–182)	39·2 (30·9–54·3)	11·6 (8·8–16·3)
Foreign body	4930 (4300–5630)	5630 (4970–6420)	44400 (39300–50700)	18000 (15400–21400)	3990 (3630–4410)	2640 (2470–2850)	1910 (1800–2030)	2380 (2240–2520)	2470 (2360–2590)	2570 (2470–2670)	2800 (2700–2900)	2830 (2720–2930)	2660 (2560–2780)	3290 (3180–3420)	3190 (3070–3300)	3370 (3240–3510)	3120 (2980–3250)	3040 (2910–3170)	3570 (3390–3730)	3480 (3190–3680)	2570 (2250–2760)	1280 (1060–1400)	487 (377–547)
Pulmonary aspiration and foreign body in airway	4750 (4130–5430)	5350 (4730–6070)	38100 (33600–43600)	13900 (11700–16600)	3550 (3220–3930)	2440 (2290–2650)	1710 (1600–1820)	2150 (2030–2290)	2260 (2160–2380)	2380 (2290–2490)	2550 (2460–2660)	2600 (2500–2700)	2480 (2380–2600)	3110 (2990–3240)	3000 (2890–3130)	3160 (3040–3300)	2910 (2790–3040)	2810 (2690–2930)	3340 (3160–3500)	3300 (3030–3500)	2480 (2170–2670)	1240 (1030–1360)	475 (366–535)
Foreign body in other body part	179 (115–236)	288 (148–403)	6380 (3010–9470)	4060 (2040–6340)	441 (269–588)	198 (138–244)	206 (158–245)	230 (178–274)	203 (158–238)	186 (147–219)	248 (196–292)	230 (181–272)	181 (141–218)	186 (149–221)	185 (149–219)	204 (161–245)	206 (166–246)	230 (185–279)	232 (186–273)	175 (137–207)	95·2 (73·4–115)	35·5 (26·8–43·5)	12·1 (8·95–15·2)
Environmental heat and cold exposure	376 (129–520)	300 (87·2–450)	3040 (889–4340)	2290 (964–3430)	872 (433–1170)	531 (313–640)	1470 (1010–1690)	2010 (1350–2340)	2320 (1770–2620)	3020 (2400–3410)	3710 (2940–4200)	3820 (2990–4340)	3540 (2760–4030)	5340 (4270–6110)	4540 (3490–5220)	4620 (3360–5380)	3300 (2160–3910)	2680 (1650–3270)	2810 (1800–3350)	2300 (1520–2800)	1430 (1030–1690)	558 (429–650)	179 (138–209)
Exposure to forces of nature	52·5 (47·7–57·8)	148 (134–163)	1630 (1480–1800)	8190 (7440–9010)	4490 (4080–4940)	4660 (4230–5130)	4270 (3880–4700)	3920 (3560–4310)	3390 (3080–3730)	2730 (2480–3010)	2230 (2030–2460)	1870 (1700–2060)	1680 (1520–1840)	2410 (2190–2650)	2370 (2150–2610)	2100 (1900–2310)	1710 (1550–1880)	1240 (1130–1370)	551 (500–606)	285 (259–314)	219 (199–241)	86·9 (78·9–95·6)	17·4 (15·8–19·2)
Other unintentional injuries	1070 (570–1470)	397 (303–488)	4380 (3020–5520)	13100 (9040–16700)	7660 (5340–9280)	5720 (4250–7050)	9420 (7360–11100)	11900 (9650–14300)	10800 (9060–12900)	10000 (8490–11800)	9510 (8000–11200)	7760 (6690–9180)	6700 (5360–8150)	5750 (4810–6860)	4940 (4120–5900)	3970 (3270–4760)	3070 (2570–3690)	1900 (1640–2260)	1790 (1570–2080)	1280 (1120–1500)	776 (667–915)	319 (258–394)	114 (87·5–146)
Self-harm and interpersonal violence	3760 (3540–4000)	1470 (1350–1620)	8650 (7680–10100)	25900 (23000–30300)	16000 (15000–17400)	27900 (25300–30000)	118000 (109000–125000)	169000 (155000–179000)	150000 (139000–157000)	128000 (119000–134000)	115000 (105000–121000)	89500 (82000–94000)	71100 (64700–75000)	69100 (63400–73100)	58500 (53400–62400)	50600 (46500–53900)	41100 (37600–44000)	33500 (30600–35900)	28600 (26500–30500)	18400 (16700–19700)	8610 (7660–9240)	2000 (1710–2170)	412 (339–451)
Self-harm	–	–	–	–	–	12500 (10200–14100)	65300 (57600–70900)	91300 (80100–99900)	82100 (72300–89100)	71000 (63200–76200)	68900 (60200–74000)	56700 (50300–60800)	47300 (41600–50700)	49300 (44300–53000)	43700 (39000–47300)	39000 (35400–42200)	33000 (29800–35800)	27700 (25100–30100)	24400 (22500–26300)	16800 (15100–18000)	7870 (6960–8480)	1810 (1540–1970)	310 (253–342)
Self-harm by firearm	–	–	–	–	–	676 (516–986)	3730 (3260–4170)	5910 (5140–6780)	5630 (5010–6580)	5250 (4650–6100)	4620 (4000–5260)	4050 (3440–4650)	3610 (3010–4150)	3190 (2710–3640)	2970 (2570–3400)	2790 (2480–3170)	2580 (2380–2990)	2070 (1970–2440)	1980 (1850–2420)	1350 (1210–1730)	629 (543–848)	166 (137–216)	34·6 (27·2–41·4)
Self-harm by other specified means	–	–	–	–	–	11800 (9590–13300)	61600 (53800–67100)	85400 (74200–93800)	76400 (66600–83100)	65800 (58200–70800)	64300 (55600–69300)	52600 (46300–56600)	43700 (38100–47100)	46100 (41200–49700)	40700 (36100–44300)	36200 (32400–39400)	30400 (27100–33200)	25700 (22900–28000)	22400 (20400–24300)	15400 (13800–16600)	7240 (6370–7820)	1650 (1400–1800)	275 (225–305)
Interpersonal violence	2420 (2220–2630)	752 (648–885)	6330 (5400–7750)	11700 (9230–15800)	7680 (6810–8770)	8040 (7380–8700)	36900 (34700–38900)	55800 (52800–58200)	53400 (50500–55600)	46700 (44500–48600)	38200 (36300–39700)	28200 (26700–29400)	20400 (19300–21300)	17200 (16200–18000)	12700 (11900–13400)	9830 (9210–10300)	6760 (6290–7160)	4740 (4370–5030)	3540 (3310–3740)	1210 (1110–1290)	551 (486–594)	133 (112–146)	64·2 (49·5–73·5)
Physical violence by firearm	233 (206–264)	85·5 (70–108)	606 (509–742)	1550 (1190–2090)	1080 (945–1270)	2020 (1890–2180)	15200 (14400–15900)	23800 (22800–25000)	22100 (21200–23200)	17900 (17100–18900)	13800 (13200–14600)	9670 (9130–10200)	6650 (6270–7030)	5020 (4690–5360)	3530 (3290–3790)	2350 (2190–2500)	1480 (1370–1590)	910 (828–987)	633 (575–691)	208 (182–229)	85·6 (74–95·1)	21·8 (17·9–24·7)	14·6 (10·9–17·3)
Physical violence by sharp object	191 (157–218)	87·6 (72·7–109)	654 (518–847)	1920 (1430–2690)	1280 (1020–1490)	1560 (1230–1770)	10000 (7750–11100)	15700 (12700–17100)	15600 (13100–16900)	13900 (11700–15000)	11300 (9440–12300)	8110 (6740–8860)	5780 (4660–6340)	4800 (3930–5320)	3320 (2710–3710)	2740 (2210–3060)	1800 (1460–2050)	1210 (1010–1370)	838 (731–929)	331 (291–361)	137 (118–150)	33·7 (27·9–37·3)	18·2 (14–20·9)
Physical violence by other means	1990 (1810–2180)	579 (498–686)	5070 (4310–6270)	8250 (6510–11300)	5320 (4660–6290)	4450 (3990–4940)	11700 (10500–13000)	16300 (14800–17800)	15600 (14400–17000)	14900 (13800–16000)	13100 (12000–14000)	10400 (9580–11100)	8020 (7330–8570)	7340 (6850–7780)	5820 (5410–6190)	4740 (4390–5040)	3480 (3210–3710)	2610 (2380–2800)	2070 (1920–2200)	675 (614–718)	329 (292–354)	77·8 (64·8–85·4)	31·4 (24·4–36·2)
Conflict and terrorism	1340 (1250–1460)	722 (674–783)	2280 (2130–2470)	14100 (13100–15300)	8290 (7700–9030)	7270 (6740–7930)	15100 (13900–16500)	20900 (19400–22900)	13400 (12400–14600)	9430 (8710–10300)	6660 (6150–7300)	4210 (3880–4610)	3080 (2850–3380)	2370 (2190–2610)	1990 (1840–2190)	1660 (1530–1820)	1340 (1240–1460)	988 (916–1080)	638 (587–702)	381 (348–423)	179 (163–199)	50 (45·5–55·4)	36·3 (33·2–40)

SR Table 6: Global deaths by cause and age group for both sexes combined (with 95% uncertainty levels), 2010

Cause	Early Neonatal	Late Neonatal	Post Neonatal	1 to 4	5 to 9	10 to 14	15 to 19	20 to 24	25 to 29	30 to 34	35 to 39	40 to 44	45 to 49	50 to 54	55 to 59	60 to 64	65 to 69	70 to 74	75 to 79	80 to 84	85 to 89	90 to 94	95 plus
All causes	2480000 (2280000–2700000)	701000 (635000–773000)	2040000 (1850000–2240000)	2080000 (1870000–2300000)	524000 (495000–553000)	401000 (385000–417000)	618000 (596000–640000)	853000 (825000–879000)	955000 (925000–985000)	1080000 (1050000–1110000)	1310000 (1270000–1350000)	1580000 (1540000–1630000)	1970000 (1910000–2030000)	2340000 (2280000–2410000)	2980000 (2890000–3060000)	3550000 (3450000–3640000)	4050000 (3950000–4140000)	5110000 (5e+06–5220000)	5620000 (5510000–5730000)	5580000 (5490000–5660000)	4080000 (4040000–4130000)	1920000 (1910000–1940000)	889000 (885000–893000)
Communicable, maternal, neonatal, and nutritional diseases	2250000 (2060000–2440000)	605000 (548000–669000)	1640000 (1470000–1820000)	1640000 (1460000–1850000)	291000 (267000–318000)	175000 (159000–194000)	196000 (179000–217000)	252000 (235000–272000)	332000 (314000–352000)	394000 (376000–415000)	435000 (416000–458000)	414000 (396000–436000)	394000 (374000–420000)	357000 (336000–386000)	352000 (326000–387000)	370000 (338000–416000)	420000 (380000–483000)	501000 (449000–580000)	502000 (448000–582000)	480000 (422000–555000)	352000 (303000–399000)	182000 (149000–207000)	90400 (69900–103000)
HIV/AIDS and sexually transmitted infections	49800 (17000–104000)	13800 (4610–28900)	64700 (52000–85300)	84600 (79700–89900)	24100 (23000–25300)	18800 (18100–19700)	33200 (29800–36800)	54900 (50100–60100)	129000 (119000–139000)	189000 (179000–2e+05)	215000 (203000–226000)	194000 (187000–2e+05)	156000 (150000–163000)	98800 (95000–102000)	56700 (54300–59200)	30900 (29300–32600)	18000 (17000–19100)	10600 (9950–11300)	6350 (5990–6750)	1450 (1360–1550)	731 (672–789)	282 (247–314)	114 (95·3–130)
HIV/AIDS	--	--	46500 (43800–49200)	83800 (79200–89100)	23700 (22700–24800)	18700 (17900–19500)	32900 (29400–36500)	54500 (49700–59700)	128000 (119000–138000)	188000 (178000–199000)	214000 (203000–225000)	193000 (186000–2e+05)	156000 (149000–162000)	98200 (94500–102000)	56000 (53600–58500)	30400 (28900–32100)	17600 (16600–18700)	10200 (9540–10900)	5940 (5590–6330)	1130 (1050–1220)	487 (451–527)	147 (136–159)	44·5 (42·2–47·1)
HIV/AIDS - Drug-susceptible Tuberculosis	--	--	13200 (9830–16200)	25300 (18700–30800)	6820 (4990–8330)	5380 (4000–6550)	9080 (6560–11500)	14900 (10700–18700)	33800 (24000–42900)	49500 (34900–62500)	55900 (39800–70100)	51200 (36500–64600)	41500 (29500–52700)	26600 (19300–33700)	15200 (11100–19100)	8210 (6040–10300)	4760 (3480–5980)	2920 (2120–3650)	1640 (1190–2070)	292 (211–372)	120 (84–157)	35·5 (24·3–47)	8·74 (6·13–11·6)
Tuberculosis without extensive drug resistance	--	--	1240 (580–2260)	2160 (980–4000)	564 (259–1040)	427 (195–791)	747 (334–1440)	1290 (583–2430)	3020 (1390–5640)	4340 (2000–8030)	4780 (2190–8840)	4310 (1980–7940)	3450 (1590–6340)	2260 (1030–4200)	1310 (594–2440)	704 (320–1310)	401 (183–745)	246 (110–458)	140 (62·2–260)	25·9 (11·4–48·3)	10·9 (4·71–20·8)	3·24 (1·37–6·24)	0·8 (0·342–1·53)
HIV/AIDS - Extensively drug-resistant Tuberculosis	--	--	25·1 (11·4–46·4)	25·7 (11·2–48·9)	9·98 (4·41–18·7)	7·28 (3·24–13·7)	12·3 (5·35–23·6)	31·9 (14–61·7)	101 (45·2–190)	155 (69·6–290)	163 (73·7–301)	140 (63·2–263)	93·5 (41·3–178)	57·3 (25·6–108)	32·5 (14·4–60·8)	17·5 (7·71–33)	9·92 (4·32–18·8)	5·03 (2·21–9·54)	3·4 (1·44–6·62)	0·628 (0·263–1·23)	0·262 (0·108–0·515)	0·0767 (0·0318–0·15)	0·0372 (0·0156–0·0734)
HIV/AIDS resulting in other diseases	--	--	32000 (28600–36700)	56300 (49900–65000)	16300 (14600–18400)	12900 (11500–14500)	23100 (19500–27200)	38300 (32900–45000)	91300 (79300–105000)	134000 (118000–153000)	153000 (135000–173000)	137000 (122000–154000)	111000 (98100–125000)	69300 (61700–78100)	39500 (35200–44400)	21500 (19000–24200)	12400 (10900–14000)	6990 (6130–8030)	4150 (3650–4740)	812 (711–927)	355 (308–407)	108 (94–125)	34·9 (31·4–38·8)
Sexually transmitted infections excluding HIV	49800 (17000–104000)	13800 (4610–28900)	18200 (6140–39300)	805 (271–1790)	402 (129–879)	150 (116–193)	299 (243–353)	438 (349–505)	573 (435–662)	613 (452–721)	539 (416–624)	585 (473–673)	553 (438–625)	578 (449–686)	720 (527–875)	476 (376–568)	460 (387–550)	417 (363–493)	415 (368–503)	317 (264–359)	245 (196–282)	135 (99·8–163)	69·9 (51·2–85·3)
Syphilis	49800 (17000–104000)	13800 (4610–28900)	18200 (6140–39300)	805 (271–1790)	402 (129–879)	84·9 (65·7–109)	97·1 (75·7–118)	67·7 (52·8–81·2)	136 (105–158)	189 (130–245)	152 (94·9–201)	90·9 (58·5–118)	102 (67·2–131)	152 (85·2–215)	186 (85·4–283)	141 (85·6–200)	95·4 (75·3–143)	104 (85·7–152)	88·9 (72·7–133)	67·7 (56–81·7)	42·8 (33–52)	25·8 (17·4–32·4)	11·7 (7·77–14·7)
Chlamydial infection	--	--	--	--	--	7·21 (5·41–8·88)	24·9 (20–29·9)	83·2 (63·7–97·6)	78·4 (58·6–90·4)	34·5 (25·5–40·7)	59·2 (45·8–68)	79·9 (63–92·8)	75 (59·4–83·9)	62·9 (52·7–73·5)	82·6 (63·4–98·6)	53·5 (42·6–62·7)	39·6 (31·4–47·4)	47·8 (39·8–54·4)	48·8 (42·6–54·4)	50·3 (41–56·4)	36·1 (28·9–41·1)	19·4 (14·6–22·6)	10·6 (7·82–12·4)
Gonococcal infection	--	--	--	--	--	15·5 (12·6–18·8)	78·5 (63·7–93·1)	150 (118–174)	201 (147–233)	194 (150–225)	164 (128–187)	229 (183–265)	202 (160–226)	200 (166–232)	244 (186–289)	155 (127–180)	224 (180–265)	172 (147–196)	184 (161–206)	127 (105–142)	99·2 (79·7–112)	52 (39·6–60·4)	29·3 (21·5–34·1)
Other sexually transmitted infections	--	--	--	--	--	42·6 (30·8–59·9)	98·6 (79·8–118)	137 (107–160)	158 (120–183)	196 (145–228)	164 (127–188)	185 (147–215)	174 (137–196)	163 (128–191)	208 (153–247)	127 (101–149)	101 (82·2–120)	92·3 (79–107)	93·6 (81·8–120)	71·5 (58·8–86)	66·5 (49·4–83)	37·7 (24·8–54)	18·4 (10·8–27·5)
Respiratory infections and tuberculosis	170000 (152000–190000)	128000 (114000–143000)	531000 (466000–610000)	318000 (261000–384000)	48500 (41500–55100)	29600 (25900–33100)	39300 (35600–43000)	61700 (56900–66500)	81500 (75800–87500)	93500 (87800–99900)	110000 (104000–117000)	119000 (112000–127000)	141000 (133000–150000)	161000 (151000–170000)	181000 (172000–191000)	198000 (188000–209000)	230000 (218000–242000)	275000 (260000–289000)	291000 (273000–308000)	292000 (264000–311000)	231000 (198000–248000)	127000 (103000–140000)	68400 (52100–76900)
Tuberculosis	--	--	45200 (38000–53600)	46200 (36300–57400)	12800 (10800–15000)	11400 (9640–13000)	24000 (21500–26700)	44300 (40600–47900)	61500 (56900–65900)	71500 (66800–76600)	83700 (78400–89500)	87300 (81600–93300)	99600 (93200–107000)	108000 (101000–115000)	114000 (107000–122000)	108000 (102000–116000)	115000 (108000–122000)	116000 (109000–124000)	96200 (89900–103000)	70100 (63500–76400)	35400 (30900–38800)	11800 (9890–13200)	3620 (2870–4190)
Drug-susceptible tuberculosis	--	--	41600 (34500–49700)	42800 (33200–53500)	11800 (9840–13900)	10400 (8710–12100)	21900 (19100–24700)	40400 (36000–44400)	55900 (50000–61500)	64900 (58100–71000)	75900 (68200–82800)	79200 (71100–86600)	89600 (80300–98000)	97000 (87400–106000)	103000 (92600–113000)	98000 (87700–107000)	104000 (93700–113000)	106000 (95800–115000)	87800 (79500–96100)	64300 (57100–70900)	32600 (28300–36300)	11000 (9110–12400)	3380 (2650–3950)
Multidrug-resistant tuberculosis without extensive drug resistance	--	--	3420 (1630–6080)	3250 (1420–5910)	975 (442–1770)	909 (403–1670)	1970 (892–3550)	3680 (1750–6430)	5190 (2480–9080)	6190 (2960–10700)	7310 (3450–12800)	7650 (3640–13400)	9220 (4490–15900)	9720 (4760–16700)	10300 (4980–17900)	9580 (4420–17000)	9910 (4370–18000)	9670 (4390–17500)	7920 (3630–13900)	5490 (2520–9730)	2640 (1230–4740)	805 (381–1430)	223 (104–397)
Extensively drug-resistant tuberculosis	--	--	133 (64·6–234)	92·9 (42·5–173)	37·6 (17·5–70·3)	36·6 (16·5–67·6)	83·9 (40·1–155)	225 (116–387)	351 (180–590)	446 (230–742)	478 (244–809)	515 (263–886)	779 (402–1280)	809 (416–1350)	762 (388–1300)	608 (296–1070)	533 (244–972)	553 (257–1000)	454 (210–819)	331 (153–594)	161 (73·9–289)	48·7 (22·1–90·1)	14·2 (6·33–27)
Lower respiratory infections	169000 (151000–190000)	128000 (114000–143000)	482000 (425000–555000)	268000 (217000–324000)	35200 (30000–40200)	18000 (15700–19900)	15200 (13800–16800)	17200 (15800–19000)	19900 (18300–21700)	21800 (20400–23900)	26400 (24800–28700)	31500 (29600–33800)	40900 (38400–44100)	52700 (49600–55900)	66800 (62800–70800)	89600 (83700–94600)	115000 (107000–122000)	158000 (148000–168000)	195000 (180000–206000)	221000 (197000–235000)	195000 (166000–209000)	115000 (93300–127000)	64600 (49000–72700)
Upper respiratory infections	54·2 (28–82·1)	137 (62·3–233)	2770 (658–6290)	3940 (443–11200)	419 (90–876)	215 (50·8–398)	116 (42·3–212)	104 (49·7–170)	125 (57·7–186)	133 (64·8–190)	199 (92·2–283)	194 (104–276)	235 (124–327)	228 (129–306)	266 (161–365)	302 (182–449)	319 (202–487)	458 (287–771)	591 (404–1030)	818 (569–1590)	722 (507–1390)	359 (247–670)	149 (105–258)
Otitis media	256 (6·54–993)	58·7 (9·14–227)	74·6 (29·7–137)	40·8 (10·8–104)	30·3 (12·1–65·8)	22·6 (11·4–47·4)	14·5 (8·79–29·7)	9·75 (6·07–20·4)	9·38 (6·56–16·4)	7·32 (5·12–12·9)	10·1 (7·15–18·1)	13·9 (9·86–23·5)	33·2 (20·2–58·3)	47·9 (27·6–92·5)	29·6 (21·4–49·3)	32·4 (23·4–54·5)	36·1 (25·5–59·1)	30·1 (22·6–47·2)	25·5 (20–38·3)	22·5 (17·6–34·7)	15·5 (12·5–21·4)	5·69 (4·42–7·45)	3·26 (2·43–4·28)
Enteric infections	31800 (24900–40000)	56600 (45800–68700)	424000 (358000–495000)	423000 (334000–517000)	95500 (68900–130000)	60600 (40900–85700)	46300 (29800–68300)	36500 (23800–55100)	30200 (20100–45100)	27100 (17900–41900)	27900 (18800–45100)	28200 (18900–45700)	33700 (22000–55800)	41700 (26600–68900)	53300 (34200–87500)	77500 (47500–125000)	105000 (64300–176000)	144000 (86900–229000)	154000 (94300–246000)	148000 (94200–226000)	92100 (60400–138000)	39700 (26000–58400)	13600 (9320–19400)
Diarrheal diseases	31800 (24900–40000)	55700 (44900–67900)	410000 (345000–479000)	348000 (276000–428000)	46400 (28900–73300)	22000 (13000–36200)	21100 (12700–35800)	22100 (13300–36700)	20700 (12500–34700)	20800 (12600–35700)	23300 (14500–40500)	24400 (15200–41900)	30100 (18500–52800)	38300 (23200–65200)	50100 (31100–84100)	74800 (44700–123000)	102000 (62300–173000)	142000 (85200–228000)	153000 (93000–244000)	148000 (93700–226000)	91800 (60100–138000)	39600 (25900–58200)	13600 (9250–19400)
Typhoid and paratyphoid	--	--	141 (55–335)	28900 (10900–57500)	38400 (17100–71200)	34300 (15300–61400)	22800 (8700–44700)	12500 (4820–26400)	7910 (3320–15900)	4880 (2000–10300)	3170 (1340–6370)	2340 (986–4700)	2160 (868–4410)	1900 (768–3860)	1680 (619–3670)	1370 (511–2920)	1060 (427–2190)	771 (314–1600)	506 (189–977)	272 (105–551)	114 (53·1–198)	37·7 (17·1–75·1)	10·7 (5·04–22·4)
Typhoid fever	--	--	127 (49·3–300)	25200 (9690–50900)	32400 (14300–60100)	28900 (12400–53100)	19200 (7180–37300)	10500 (3930–22100)	6610 (2690–13700)	4070 (1670–8520)	2630 (1080–5290)	1940 (809–3920)	1770 (705–3720)	1560 (630–3150)	1380 (518–3100)	1120 (417–2450)	874 (348–1860)	640 (254–1340)	422 (159–819)	229 (86·6–458)	97 (44·2–174)	31·9 (13·9–66·5)	8·8 (3·86–19·9)
Paratyphoid fever	--	--	13·4 (4·07–35·4)	3680 (1140–8980)	6000 (2190–13300)	5460 (2110–11600)	3570 (1210–7750)	2010 (679–4730)	1300 (446–3100)	812 (251–1910)	540 (182–1240)	409 (143–884)	388 (128–911)	339 (110–768)	298 (94–711)	241 (75–581)	182 (61–430)	132 (41·9–317)	83·3 (29·5–181)	42·9 (15·2–90·1)	17·5 (7·44–35·6)	5·81 (2·6–12·2)	1·85 (0·965–3·68)
[illegible]	[illegible]	[illegible]	[illegible]	[illegible]	[illegible]	[illegible]	[illegible]	[illegible]	[illegible]	[illegible]	[illegible]	[illegible]	[illegible]	[illegible]	[illegible]	[illegible]	[illegible]	[illegible]	[illegible]	[illegible]	[illegible]	[illegible]	[illegible]

Endocarditis	124 (79·2–167)	123 (71·3–167)	1140 (701–1540)	645 (297–976)	239 (156–301)	357 (250–434)	591 (455–693)	1030 (841–1220)	1450 (1190–1780)	1520 (1230–1820)	1790 (1530–2200)	2110 (1810–2720)	2260 (1810–2760)	2600 (2100–3110)	3060 (2400–3530)	3280 (2470–3660)	3800 (2820–4260)	4540 (3260–5090)	5430 (3520–6200)	6250 (3710–7390)	6150 (3110–7640)	3730 (1720–4810)	2240 (878–3020)
Other cardiovascular and circulatory diseases	1100 (850–1490)	889 (650–1170)	4370 (3530–5430)	3850 (2800–4770)	2310 (1910–2700)	2420 (2110–2740)	2060 (1830–2320)	2940 (2680–3270)	3640 (3310–4070)	4160 (3800–4660)	5110 (4730–5680)	6770 (6240–7650)	8820 (8180–9850)	11100 (10300–12400)	14000 (13000–15700)	17300 (16000–19400)	19700 (18000–22000)	25300 (23200–28700)	29600 (26900–33200)	29000 (25700–32500)	22600 (19100–25100)	10900 (8560–12400)	5630 (4280–6400)
Chronic respiratory diseases	0 (0–0)	0 (0–0)	0 (0–0)	13100 (10700–16000)	3630 (3140–4140)	3690 (3250–4150)	6470 (5790–7310)	9160 (8340–10200)	11100 (10200–12200)	14500 (13500–15800)	21700 (20100–23800)	33800 (31500–37200)	57000 (53000–60800)	92000 (85000–97100)	155000 (143000–165000)	255000 (232000–269000)	370000 (337000–390000)	538000 (492000–566000)	637000 (578000–670000)	635000 (564000–674000)	426000 (367000–456000)	169000 (139000–184000)	59300 (46300–66300)
Chronic obstructive pulmonary disease	--	--	0 (0–0)	1670 (1070–2940)	462 (371–570)	396 (339–476)	1960 (1680–2410)	2810 (2410–3360)	3570 (3180–4140)	5390 (4830–6240)	8860 (7890–10400)	16500 (14900–19100)	32100 (29100–36300)	59700 (54200–65700)	109000 (99800–119000)	199000 (184000–213000)	3e+05 (277000–320000)	453000 (419000–482000)	548000 (503000–582000)	559000 (501000–597000)	380000 (328000–410000)	151000 (124000–165000)	52900 (41400–59200)
Pneumoconiosis	--	--	--	--	--	--	33·4 (28·7–39·9)	50 (43·4–58·9)	81·8 (71·6–95·8)	153 (136–177)	395 (340–459)	725 (629–844)	944 (795–1100)	1070 (883–1240)	1620 (1290–1880)	1910 (1540–2240)	2370 (1930–2750)	3240 (2780–3600)	3950 (3530–4360)	3540 (3180–3870)	2090 (1840–2290)	575 (475–643)	167 (131–188)
Silicosis	--	--	--	--	--	--	9·94 (7·32–13·4)	18·2 (13·3–23·9)	35·5 (27·6–48·1)	87·3 (68–110)	255 (204–321)	496 (400–628)	645 (505–806)	709 (555–878)	1070 (813–1300)	1180 (902–1430)	1440 (1090–1760)	1890 (1530–2250)	2360 (2010–2890)	2030 (1770–2420)	1140 (989–1380)	251 (208–302)	59·8 (46·9–74·6)
Asbestosis	--	--	--	--	--	--	4·85 (3·4–6·13)	7·08 (5·29–9·07)	7·89 (5·67–9·88)	11·8 (8·64–15·8)	20·2 (14·9–29·2)	30·1 (21·8–41·4)	41·8 (26·3–57·5)	77 (51·9–105)	122 (86·2–160)	194 (143–256)	263 (187–314)	434 (307–503)	565 (348–651)	609 (384–692)	435 (298–495)	151 (106–173)	47 (30·6–54·8)
Coal workers pneumoconiosis	--	--	--	--	--	--	3·13 (2·22–5·25)	4·4 (3·04–6·84)	8·15 (5·89–12·2)	12·5 (8·45–17·5)	35·3 (19·9–54·6)	81 (44·3–123)	105 (58·4–163)	116 (70·6–162)	168 (107–237)	215 (131–288)	293 (183–381)	468 (301–591)	556 (390–705)	493 (390–631)	297 (244–376)	87·8 (67·9–108)	29·8 (23–39·4)
Other pneumoconiosis	--	--	--	--	--	--	15·5 (12·7–21)	20·3 (16·6–27·6)	30·2 (24·1–41·7)	41·3 (33·3–61·5)	84·8 (63·1–132)	118 (88·3–178)	152 (106–226)	172 (122–246)	264 (184–394)	329 (226–480)	371 (253–541)	455 (347–606)	469 (372–632)	408 (330–519)	222 (170–275)	85·2 (64·1–104)	30·5 (22–37·3)
Asthma	--	--	--	9180 (6880–11700)	2320 (1940–2700)	2610 (2240–2990)	3500 (3060–4030)	4810 (4140–5440)	5840 (5100–6530)	6850 (5970–7670)	9490 (8220–10800)	12400 (10500–14500)	17900 (14800–21400)	23200 (18300–29100)	32000 (24600–41800)	41600 (30400–56600)	51300 (37500–69800)	60000 (44200–83700)	60400 (44400–84300)	50500 (37500–70800)	28600 (21800–37600)	11300 (8600–14700)	3730 (2800–4640)
Interstitial lung disease and pulmonary sarcoidosis	--	--	--	317 (199–497)	135 (100–180)	117 (92·2–156)	231 (182–288)	374 (294–480)	549 (446–693)	771 (609–956)	1290 (999–1620)	2010 (1600–2530)	3150 (2440–3920)	4900 (3730–6090)	7700 (5810–9670)	11100 (8190–13700)	15300 (10900–19500)	20200 (14100–24700)	22800 (15300–27700)	19900 (12800–24700)	13200 (7980–17000)	5540 (3050–7370)	2200 (1100–3080)
Other chronic respiratory diseases	0 (0–0)	0 (0–0)	0 (0–0)	1980 (1020–3320)	710 (516–916)	562 (396–713)	745 (584–931)	1120 (875–1400)	1050 (831–1290)	1340 (1040–1660)	1670 (1280–2050)	2160 (1680–2620)	2930 (2230–3610)	3210 (2440–3890)	4850 (3590–5910)	1540 (1120–1860)	1670 (1210–2040)	1990 (1360–2380)	2060 (1420–2420)	2210 (1480–2570)	1560 (1080–1830)	768 (523–899)	330 (205–392)
Digestive diseases	2660 (2350–3110)	2410 (2090–2810)	16900 (14800–19600)	22200 (17600–26800)	11800 (10100–13500)	10700 (9450–12000)	22300 (20200–24400)	31000 (28900–33300)	46800 (44300–49500)	61900 (58900–64900)	90800 (87100–95600)	122000 (117000–128000)	159000 (152000–166000)	182000 (176000–189000)	210000 (203000–218000)	213000 (205000–221000)	214000 (206000–222000)	224000 (215000–233000)	222000 (210000–231000)	198000 (178000–209000)	140000 (120000–150000)	65000 (52700–71300)	30300 (23100–33900)
Cirrhosis and other chronic liver diseases	--	--	--	9620 (7670–12200)	5440 (4580–6400)	5700 (4950–6480)	12300 (11000–13600)	18600 (17200–20100)	30000 (28200–32100)	44300 (42000–46700)	66600 (63700–70500)	92800 (88600–98300)	120000 (115000–126000)	137000 (132000–143000)	152000 (146000–159000)	146000 (140000–152000)	133000 (127000–139000)	124000 (118000–130000)	104000 (98100–110000)	75800 (68300–80600)	42000 (36300–45500)	15800 (12900–17500)	6530 (4970–7550)
Cirrhosis and other chronic liver diseases due to hepatitis B	--	--	--	85·4 (43·5–144)	126 (69–198)	223 (124–344)	723 (375–1140)	3440 (2140–4920)	8560 (6010–11500)	14500 (11600–17700)	23400 (18300–28400)	31800 (25600–38500)	37400 (29700–46500)	38100 (29300–49100)	39200 (29400–51000)	35900 (26600–47400)	31700 (23900–41600)	29000 (21000–38300)	25100 (18600–31700)	17800 (12900–22800)	8940 (6800–11400)	3020 (2090–4120)	1040 (581–1600)
Cirrhosis and other chronic liver diseases due to hepatitis C	--	--	--	70·2 (36·2–122)	121 (67·1–197)	226 (130–369)	744 (405–1230)	3300 (2080–4680)	7790 (5440–10300)	12400 (9800–15400)	18800 (14100–24200)	26300 (20400–33000)	33600 (25600–42500)	37300 (27700–47900)	39000 (29600–50900)	36100 (26300–48500)	33300 (25400–42500)	30800 (24300–38500)	26000 (19600–33200)	19200 (14200–24900)	11100 (8230–13900)	4410 (3040–5840)	1950 (1200–2800)
Cirrhosis and other chronic liver diseases due to alcohol use	--	--	--	--	--	--	45·9 (13·6–101)	820 (456–1340)	2670 (1690–4070)	6710 (5100–8670)	13500 (9760–17900)	21400 (16300–27100)	31100 (23200–39800)	39800 (29400–50300)	47000 (35900–60400)	45400 (33800–58900)	37900 (27800–49200)	31700 (23000–41900)	22500 (16600–28700)	14200 (10000–18900)	7240 (5360–9530)	2530 (1710–3690)	1020 (569–1730)
Cirrhosis and other chronic liver diseases due to NAFLD	--	--	--	--	--	--	285 (112–555)	770 (381–1300)	1650 (788–2970)	2560 (1450–4120)	3510 (1800–6060)	5280 (2630–8730)	7800 (3800–13600)	9760 (4830–16800)	10800 (5450–18600)	11100 (5610–19600)	11500 (7220–17400)	12300 (7630–18100)	11600 (6440–18300)	9390 (5460–14000)	5680 (3550–8400)	2240 (1240–3690)	966 (425–1710)
Cirrhosis and other chronic liver diseases due to other causes	--	--	--	9460 (7540–12100)	5200 (4320–6140)	5250 (4510–6000)	10500 (9020–12000)	10300 (7650–13000)	9340 (6070–13300)	8080 (4940–12700)	7380 (4190–11700)	8040 (4620–13000)	10000 (5840–16300)	12200 (7120–19400)	15500 (8940–25200)	17400 (10100–26900)	18500 (10400–30100)	20000 (11400–31300)	19200 (11200–29400)	15300 (8890–23300)	9070 (5790–13000)	3570 (2060–5430)	1550 (734–2640)
Upper digestive system diseases	--	--	--	2030 (1360–3380)	944 (763–1190)	758 (650–885)	2020 (1790–2430)	3490 (3120–4110)	4540 (4100–5520)	5120 (4710–6000)	7520 (7000–8780)	8920 (8300–9990)	13000 (12100–14400)	15600 (14600–16700)	19700 (18500–21300)	22600 (21200–24500)	27600 (25800–30000)	32700 (30700–34900)	35500 (33200–37700)	31900 (28800–34200)	22800 (19800–24900)	9940 (8250–11000)	4170 (3250–4840)
Peptic ulcer disease	--	--	--	1480 (927–2860)	828 (663–1080)	675 (574–794)	1830 (1610–2220)	3100 (2740–3700)	4040 (3650–4970)	4420 (4050–5130)	6650 (6140–7850)	7790 (7210–8770)	11400 (10600–12700)	13700 (12900–14700)	17300 (16200–18900)	19800 (18700–21600)	24200 (22600–26700)	28700 (26900–30600)	30900 (28800–33000)	27400 (24800–29700)	19600 (16900–21300)	8430 (6940–9420)	3580 (2780–4160)
Gastritis and duodenitis	--	--	--	552 (318–930)	116 (82·1–153)	83·1 (62·1–105)	194 (146–262)	388 (289–515)	499 (390–669)	707 (561–905)	876 (699–1130)	1130 (919–1350)	1640 (1330–1950)	1830 (1400–2160)	2400 (1870–2770)	2720 (2250–3070)	3320 (2700–3740)	3990 (3320–4510)	4590 (3710–5140)	4440 (3810–4880)	3270 (2790–3620)	1510 (1240–1710)	591 (467–683)
Appendicitis	--	--	--	1700 (793–2470)	1400 (993–1770)	1330 (994–1650)	2370 (1940–2850)	1860 (1560–2230)	2330 (1980–2750)	1200 (983–1400)	1600 (1320–1870)	1600 (1260–1840)	1770 (1380–2030)	1570 (1260–1840)	2250 (1790–2610)	2250 (1770–2590)	2330 (1840–2710)	2480 (1960–2830)	2500 (2070–2850)	2040 (1710–2330)	1330 (1130–1520)	572 (461–658)	208 (161–248)
Paralytic ileus and intestinal obstruction	1840 (1500–2360)	1930 (1580–2390)	15000 (12900–17900)	3440 (2190–4800)	2430 (1730–2940)	1730 (1330–2040)	3520 (2540–4110)	3360 (2410–3870)	3870 (2760–4390)	3630 (2590–4190)	4860 (3360–5510)	5810 (4030–6580)	6500 (4750–7330)	7720 (5540–8760)	10600 (7900–11900)	12600 (9600–14300)	15600 (12000–17300)	17700 (14700–19500)	21100 (18000–23300)	23400 (19600–26500)	20000 (16300–22400)	10900 (8060–12400)	5330 (3630–6320)
Inguinal, femoral, and abdominal hernia	826 (629–1050)	478 (327–701)	1950 (1300–2900)	692 (360–1030)	233 (171–290)	161 (124–196)	264 (219–322)	325 (271–396)	390 (335–480)	345 (300–412)	540 (473–647)	698 (607–805)	955 (815–1110)	1590 (1300–1840)	2140 (1780–2460)	2690 (2240–3120)	3330 (2840–3750)	4230 (3580–4850)	5250 (4530–6030)	5860 (4860–6780)	4490 (3760–5120)	2310 (1820–2660)	1080 (817–1280)
Inflammatory bowel disease	--	--	--	947 (476–1440)	166 (115–216)	126 (92·4–157)	227 (171–288)	363 (278–451)	524 (423–632)	590 (499–698)	710 (610–819)	927 (799–1070)	1170 (992–1320)	1390 (1210–1560)	1800 (1530–2020)	2160 (1780–2410)	2490 (2100–2770)	3360 (2810–3700)	4270 (3600–4720)	5080 (4130–5570)	4680 (3610–5170)	2520 (1850–2850)	1380 (984–1600)
Vascular intestinal disorders	--	--	--	349 (153–613)	85·5 (56·4–122)	41·4 (33·3–52)	82·1 (69·9–102)	198 (164–246)	263 (224–323)	319 (276–380)	483 (426–560)	812 (743–898)	1450 (1330–1590)	2340 (2200–2530)	3700 (3510–3940)	5160 (4880–5510)	7300 (6830–7950)	11700 (11000–12400)	14700 (13800–15700)	16500 (14800–17800)	12800 (10800–14200)	6010 (4820–6840)	2660 (2020–3170)
Gallbladder and biliary diseases	--	--	--	717 (308–1060)	345 (221–441)	272 (178–337)	306 (236–348)	516 (412–583)	716 (560–814)	949 (750–1070)	1360 (1130–1550)	1740 (1480–2010)	2370 (2060–2670)	3420 (2920–3890)	4520 (4000–5210)	6080 (5560–7100)	7770 (7170–8970)	10900 (9980–12700)	15000 (13400–17100)	17100 (14900–19300)	14400 (11900–16400)	8060 (6160–9320)	4370 (3140–5210)
Pancreatitis	--	--	--	260 (76·1–446)	156 (111–201)	185 (141–223)	772 (639–937)	1750 (1510–2080)	3320 (2900–4070)	4480 (4040–5350)	5890 (5180–6910)	7090 (6220–8370)	9180 (8060–10500)	8180 (7380–9230)	9590 (8350–10800)	8300 (7310–9350)	8360 (7420–9370)	9320 (8190–10200)	8910 (7790–9830)	7740 (6750–8640)	5210 (4420–5860)	2210 (1760–2530)	942 (712–1110)
Other digestive diseases	--	--	--	2400 (1260–4310)	630 (463–835)	424 (344–513)	417 (346–484)	559 (461–650)	807 (656–923)	977 (820–1100)	1240 (1060–1390)	1770 (1500–1990)	2480 (2080–2760)	3250 (2660–3600)	4180 (3390–4640)	5400 (4490–6130)	5990 (5000–6700)	8340 (6830–9370)	10600 (8510–11800)	12700 (10100–14200)	11800 (9130–13700)	6680 (4880–8330)	3650 (2550–4490)
Neurological disorders	7·8 (6·79–9·42)	15·7 (14·2–17·4)	6420 (5550–7320)	9820 (7710–11900)	5260 (4570–5960)	6500 (5750–7250)	10500 (9630–11500)	10900 (10100–11600)	9800 (9050–10400)	9610 (8940–10300)	10400 (9790–11100)	12500 (11600–14200)	16900 (14000–23700)	20900 (15900–33200)	30600 (20400–55000)	44900 (26900–90500)	69100 (38800–146000)	125000 (68600–259000)	207000 (108000–444000)	344000 (150000–796000)	354000 (133000–834000)	220000 (72700–525000)	121000 (35900–3e+05)

SR Table 6: Global deaths by cause and age group for both sexes combined (with 95% uncertainty levels), 2019

Cause	Early Neonatal	Late Neonatal	Post Neonatal	1 to 4	5 to 9	10 to 14	15 to 19	20 to 24	25 to 29	30 to 34	35 to 39	40 to 44	45 to 49	50 to 54	55 to 59	60 to 64	65 to 69	70 to 74	75 to 79	80 to 84	85 to 89	90 to 94	95 plus
All causes	1930000 (1650000–2280000)	488000 (415000–577000)	1330000 (1100000–1620000)	1290000 (1090000–1530000)	377000 (342000–418000)	290000 (277000–322000)	499000 (466000–531000)	692000 (647000–737000)	790000 (742000–837000)	975000 (922000–1030000)	1170000 (1100000–1240000)	1440000 (1350000–1530000)	1860000 (1740000–1980000)	2550000 (2390000–2710000)	3230000 (3030000–3430000)	4130000 (3890000–4360000)	5050000 (4770000–5300000)	5730000 (5440000–5990000)	6080000 (5810000–6330000)	6740000 (6490000–6960000)	5420000 (5260000–5560000)	3080000 (3020000–3150000)	1360000 (1350000–1380000)
Communicable, maternal, neonatal, and nutritional diseases	1740000 (1480000–2050000)	412000 (351000–491000)	1020000 (835000–1260000)	976000 (810000–1170000)	203000 (178000–233000)	130000 (113000–149000)	156000 (138000–177000)	192000 (173000–212000)	228000 (209000–248000)	279000 (260000–299000)	318000 (296000–342000)	323000 (3e+05–347000)	321000 (297000–349000)	326000 (298000–361000)	337000 (304000–374000)	377000 (335000–429000)	421000 (373000–484000)	488000 (430000–568000)	508000 (445000–593000)	568000 (486000–678000)	454000 (382000–526000)	278000 (222000–324000)	137000 (103000–157000)
HIV/AIDS and sexually transmitted infections	48400 (16400–101000)	13200 (4520–27400)	35200 (22000–55500)	30400 (27200–34300)	12300 (11400–13200)	14700 (14000–15400)	27900 (25500–30700)	35300 (32500–38200)	64900 (59800–70500)	103000 (97300–110000)	127000 (121000–134000)	125000 (121000–130000)	110000 (106000–115000)	81900 (79200–84700)	52000 (50200–53900)	31900 (30400–33000)	19500 (18700–20500)	9890 (9370–10600)	5490 (5220–5810)	1860 (1730–2000)	973 (875–1070)	399 (329–469)	163 (129–198)
HIV/AIDS	--	--	16200 (14200–18800)	29500 (26500–33000)	11800 (11100–12600)	14600 (13800–15300)	27600 (25200–30400)	34900 (32500–37800)	64400 (59300–70000)	103000 (96700–110000)	126000 (120000–133000)	125000 (121000–130000)	110000 (106000–114000)	81300 (78600–84100)	51200 (49400–53100)	31100 (29800–32500)	19000 (18200–20000)	9440 (8940–10100)	5040 (4790–5330)	1470 (1370–1580)	632 (592–675)	180 (170–192)	55·3 (53–58·1)
HIV/AIDS - Drug-susceptible Tuberculosis	--	--	4030 (2950–5160)	7820 (5770–9650)	3230 (2470–3910)	4030 (3070–4940)	6940 (5020–8970)	8380 (5930–10700)	14400 (10100–18700)	22400 (15800–29000)	27600 (19500–35700)	28100 (20100–36500)	24700 (17500–32000)	18600 (13400–24200)	11700 (8400–15200)	6960 (5020–9050)	3960 (2850–5150)	2030 (1460–2610)	1090 (771–1400)	254 (180–330)	109 (74·8–145)	31·4 (21·4–42·3)	8·89 (6·21–11·9)
Tuberculosis without extensive drug resistance	--	--	491 (211–909)	920 (366–1780)	352 (153–658)	407 (179–775)	747 (321–1430)	892 (389–1710)	1460 (634–2840)	2230 (988–4260)	2700 (1210–5110)	2700 (1220–5100)	2330 (1040–4360)	1770 (781–3290)	1100 (483–2050)	654 (285–1230)	372 (159–704)	192 (82·6–364)	102 (44·3–192)	24·7 (10·2–48)	10·5 (4·27–20·7)	2·99 (1·22–5·82)	0·799 (0·336–1·53)
HIV/AIDS - Extensively drug-resistant Tuberculosis	--	--	13·8 (6·13–25·6)	16·6 (6·8–32·3)	7·9 (3·49–15·1)	8·48 (3·71–16·1)	16·5 (7·06–31·1)	27·3 (12–52·5)	64·4 (29·1–121)	122 (58·2–225)	149 (72·1–270)	130 (61·8–239)	94·8 (44·2–178)	65·6 (30·4–125)	40·2 (18·3–77·1)	23·9 (10·6–46·5)	15·1 (6·28–31·1)	6·17 (2·4–12·9)	3·2 (1·22–6·82)	1·21 (0·389–2·89)	0·527 (0·178–1·24)	0·133 (0·0516–0·283)	0·0569 (0·0227–0·115)
HIV/AIDS resulting in other diseases	--	--	11700 (10000–14200)	20800 (17900–25000)	8250 (7360–9250)	10100 (9020–11300)	19900 (17300–23300)	25600 (22300–29400)	48400 (42100–55400)	78100 (69000–88200)	95900 (85300–107000)	93800 (83900–104000)	82600 (73800–91700)	60800 (54300–67200)	38400 (34300–42400)	23400 (21000–25900)	14600 (13200–16200)	7210 (6420–8090)	3850 (3420–4280)	1190 (1050–1320)	512 (454–568)	146 (130–162)	45·6 (41·5–49·8)
Sexually transmitted infections excluding HIV	48400 (16400–101000)	13200 (4520–27400)	18900 (6510–39600)	864 (289–1880)	417 (134–902)	134 ([illegible])	262 ([illegible])	391 ([illegible])	511 ([illegible])	582 ([illegible])	524 ([illegible])	589 ([illegible])	528 ([illegible])	670 ([illegible])	795 ([illegible])	554 ([illegible])	540 ([illegible])	450 ([illegible])	453 ([illegible])	393 ([illegible])	342 ([illegible])	219 ([illegible])	108 ([illegible])
Syphilis	48400 (16400–101000)	13200 (4520–27400)	18900 (6510–39600)	864 (289–1880)	417 (134–902)	[illegible]	[illegible]	[illegible]	[illegible]	[illegible]	[illegible]	[illegible]	[illegible]	[illegible]	[illegible]	[illegible]	[illegible]	[illegible]	[illegible]	[illegible]	[illegible]	[illegible]	[illegible]
Chlamydial infection	--	--	--	--	--	[illegible]	[illegible]	[illegible]	[illegible]	[illegible]	[illegible]	[illegible]	[illegible]	[illegible]	[illegible]	[illegible]	[illegible]	[illegible]	[illegible]	[illegible]	[illegible]	[illegible]	[illegible]
Gonococcal infection	--	--	--	--	--	[illegible]	[illegible]	[illegible]	[illegible]	[illegible]	[illegible]	[illegible]	[illegible]	[illegible]	[illegible]	[illegible]	[illegible]	[illegible]	[illegible]	[illegible]	[illegible]	[illegible]	[illegible]
Other sexually transmitted infections	--	--	--	--	--	[illegible]	[illegible]	[illegible]	[illegible]	[illegible]	[illegible]	[illegible]	[illegible]	[illegible]	[illegible]	[illegible]	[illegible]	[illegible]	[illegible]	[illegible]	[illegible]	[illegible]	[illegible]
Respiratory infections and tuberculosis	117000 (95000–144000)	79100 (66000–96200)	334000 (270000–415000)	196000 (155000–244000)	[illegible]	[illegible]	[illegible]	[illegible]	[illegible]	[illegible]	[illegible]	[illegible]	[illegible]	[illegible]	[illegible]	[illegible]	[illegible]	[illegible]	[illegible]	[illegible]	[illegible]	[illegible]	[illegible]
Tuberculosis	--	--	25300 (19600–32500)	[illegible]	[illegible]	[illegible]	[illegible]	[illegible]	[illegible]	[illegible]	[illegible]	[illegible]	[illegible]	[illegible]	[illegible]	[illegible]	[illegible]	[illegible]	[illegible]	[illegible]	[illegible]	[illegible]	[illegible]
Drug-susceptible tuberculosis	--	--	23100 (17700–30100)	[illegible]	[illegible]	[illegible]	[illegible]	[illegible]	[illegible]	[illegible]	[illegible]	[illegible]	[illegible]	[illegible]	[illegible]	[illegible]	[illegible]	[illegible]	[illegible]	[illegible]	[illegible]	[illegible]	[illegible]
Multidrug-resistant tuberculosis without extensive drug resistance	--	--	2160 (949–3850)	[illegible]	[illegible]	[illegible]	[illegible]	[illegible]	[illegible]	[illegible]	[illegible]	[illegible]	[illegible]	[illegible]	[illegible]	[illegible]	[illegible]	[illegible]	[illegible]	[illegible]	[illegible]	[illegible]	[illegible]
Extensively drug-resistant tuberculosis	--	--	103 (48·4–186)	[illegible]	[illegible]	[illegible]	[illegible]	[illegible]	[illegible]	[illegible]	[illegible]	[illegible]	[illegible]	[illegible]	[illegible]	[illegible]	[illegible]	[illegible]	[illegible]	[illegible]	[illegible]	[illegible]	[illegible]
Lower respiratory infections	117000 (95000–144000)	79000 (65900–96000)	307000 (249000–380000)	[illegible]	[illegible]	[illegible]	[illegible]	[illegible]	[illegible]	[illegible]	[illegible]	[illegible]	[illegible]	[illegible]	[illegible]	[illegible]	[illegible]	[illegible]	[illegible]	[illegible]	[illegible]	[illegible]	[illegible]
Upper respiratory infections	29·5 (15·5–45·4)	81·2 (39·1–135)	1630 (510–3500)	[illegible]	[illegible]	[illegible]	[illegible]	[illegible]	[illegible]	[illegible]	[illegible]	[illegible]	[illegible]	[illegible]	[illegible]	[illegible]	[illegible]	[illegible]	[illegible]	[illegible]	[illegible]	[illegible]	[illegible]
Otitis media	175 (4·24–726)	32·7 (5·74–132)	48·8 (23·6–94·6)	[illegible]	[illegible]	[illegible]	[illegible]	[illegible]	[illegible]	[illegible]	[illegible]	[illegible]	[illegible]	[illegible]	[illegible]	[illegible]	[illegible]	[illegible]	[illegible]	[illegible]	[illegible]	[illegible]	[illegible]
Enteric infections	14500 (11400–18000)	29400 (23100–36800)	256000 (201000–329000)	[illegible]	[illegible]	[illegible]	[illegible]	[illegible]	[illegible]	[illegible]	[illegible]	[illegible]	[illegible]	[illegible]	[illegible]	[illegible]	[illegible]	[illegible]	[illegible]	[illegible]	[illegible]	[illegible]	[illegible]
Diarrheal diseases	14500 (11400–18000)	28800 (22600–36000)	244000 (191000–315000)	[illegible]	[illegible]	[illegible]	[illegible]	[illegible]	[illegible]	[illegible]	[illegible]	[illegible]	[illegible]	[illegible]	[illegible]	[illegible]	[illegible]	[illegible]	[illegible]	[illegible]	[illegible]	[illegible]	[illegible]
Typhoid and paratyphoid	--	--	132 (49·7–307)	[illegible]	[illegible]	[illegible]	[illegible]	[illegible]	[illegible]	[illegible]	[illegible]	[illegible]	[illegible]	[illegible]	[illegible]	[illegible]	[illegible]	[illegible]	[illegible]	[illegible]	[illegible]	[illegible]	[illegible]
Typhoid fever	--	--	118 (45·1–280)	[illegible]	[illegible]	[illegible]	[illegible]	[illegible]	[illegible]	[illegible]	[illegible]	[illegible]	[illegible]	[illegible]	[illegible]	[illegible]	[illegible]	[illegible]	[illegible]	[illegible]	[illegible]	[illegible]	[illegible]
Paratyphoid fever	--	--	13·5 (3·95–37·2)	[illegible]	[illegible]	[illegible]	[illegible]	[illegible]	[illegible]	[illegible]	[illegible]	[illegible]	[illegible]	[illegible]	[illegible]	[illegible]	[illegible]	[illegible]	[illegible]	[illegible]	[illegible]	[illegible]	[illegible]
Invasive Non-typhoidal Salmonella (iNTS)	--	655 (355–1080)	10900 (5760–17700)	[illegible]	[illegible]	[illegible]	[illegible]	[illegible]	[illegible]	[illegible]	[illegible]	[illegible]	[illegible]	[illegible]	[illegible]	[illegible]	[illegible]	[illegible]	[illegible]	[illegible]	[illegible]	[illegible]	[illegible]
Other intestinal infectious diseases	--	--	295 (135–520)	[illegible]	[illegible]	[illegible]	[illegible]	[illegible]	[illegible]	[illegible]	[illegible]	[illegible]	[illegible]	[illegible]	[illegible]	[illegible]	[illegible]	[illegible]	[illegible]	[illegible]	[illegible]	[illegible]	[illegible]
Neglected tropical diseases and malaria	487 (246–1530)	19300 (8470–37100)	124000 (59000–221000)	[illegible]	[illegible]	[illegible]	[illegible]	[illegible]	[illegible]	[illegible]	[illegible]	[illegible]	[illegible]	[illegible]	[illegible]	[illegible]	[illegible]	[illegible]	[illegible]	[illegible]	[illegible]	[illegible]	[illegible]
Malaria	0 (0–0)	18700 (7870–36500)	119000 (54000–216000)	[illegible]	[illegible]	[illegible]	[illegible]	[illegible]	[illegible]	[illegible]	[illegible]	[illegible]	[illegible]	[illegible]	[illegible]	[illegible]	[illegible]	[illegible]	[illegible]	[illegible]	[illegible]	[illegible]	[illegible]
Chagas disease	--	--	1·22 (0·585–2·68)	[illegible]	[illegible]	[illegible]	[illegible]	[illegible]	[illegible]	[illegible]	[illegible]	[illegible]	[illegible]	[illegible]	[illegible]	[illegible]	[illegible]	[illegible]	[illegible]	[illegible]	[illegible]	[illegible]	[illegible]
Leishmaniasis	--	--	174 (48·2–500)	[illegible]	[illegible]	[illegible]	[illegible]	[illegible]	[illegible]	[illegible]	[illegible]	[illegible]	[illegible]	[illegible]	[illegible]	[illegible]	[illegible]	[illegible]	[illegible]	[illegible]	[illegible]	[illegible]	[illegible]
Visceral leishmaniasis	--	--	174 (48·2–500)	[illegible]	[illegible]	[illegible]	[illegible]	[illegible]	[illegible]	[illegible]	[illegible]	[illegible]	[illegible]	[illegible]	[illegible]	[illegible]	[illegible]	[illegible]	[illegible]	[illegible]	[illegible]	[illegible]	[illegible]
African trypanosomiasis	--	--	--	[illegible]	[illegible]	[illegible]	[illegible]	[illegible]	[illegible]	[illegible]	[illegible]	[illegible]	[illegible]	[illegible]	[illegible]	[illegible]	[illegible]	[illegible]	[illegible]	[illegible]	[illegible]	[illegible]	[illegible]
Schistosomiasis	--	--	0 (0–0)	[illegible]	[illegible]	[illegible]	[illegible]	[illegible]	[illegible]	[illegible]	[illegible]	[illegible]	[illegible]	[illegible]	[illegible]	[illegible]	[illegible]	[illegible]	[illegible]	[illegible]	[illegible]	[illegible]	[illegible]
Cysticercosis	--	--	--	[illegible]	[illegible]	[illegible]	[illegible]	[illegible]	[illegible]	[illegible]	[illegible]	[illegible]	[illegible]	[illegible]	[illegible]	[illegible]	[illegible]	[illegible]	[illegible]	[illegible]	[illegible]	[illegible]	[illegible]
Cystic echinococcosis	--	--	--	[illegible]	[illegible]	[illegible]	[illegible]	[illegible]	[illegible]	[illegible]	[illegible]	[illegible]	[illegible]	[illegible]	[illegible]	[illegible]	[illegible]	[illegible]	[illegible]	[illegible]	[illegible]	[illegible]	[illegible]
Dengue	0·00233 (0·00211–0·00256)	298 (21·8–493)	2250 (365–3410)	[illegible]	[illegible]	[illegible]	[illegible]	[illegible]	[illegible]	[illegible]	[illegible]	[illegible]	[illegible]	[illegible]	[illegible]	[illegible]	[illegible]	[illegible]	[illegible]	[illegible]	[illegible]	[illegible]	[illegible]
Yellow fever	--	2·11 (0·754–4·67)	36 (12·3–77·9)	[illegible]	[illegible]	[illegible]	[illegible]	[illegible]	[illegible]	[illegible]	[illegible]	[illegible]	[illegible]	[illegible]	[illegible]	[illegible]	[illegible]	[illegible]	[illegible]	[illegible]	[illegible]	[illegible]	[illegible]
Rabies	--	--	151 (58·5–247)	[illegible]	[illegible]	[illegible]	[illegible]	[illegible]	[illegible]	[illegible]	[illegible]	[illegible]	[illegible]	[illegible]	[illegible]	[illegible]	[illegible]	[illegible]	[illegible]	[illegible]	[illegible]	[illegible]	[illegible]
Intestinal nematode infections	--	--	125 (93·1–168)	[illegible]	[illegible]	[illegible]	[illegible]	[illegible]	[illegible]	[illegible]	[illegible]	[illegible]	[illegible]	[illegible]	[illegible]	[illegible]	[illegible]	[illegible]	[illegible]	[illegible]	[illegible]	[illegible]	[illegible]
Ascariasis	--	--	125 (93·1–168)	[illegible]	[illegible]	[illegible]	[illegible]	[illegible]	[illegible]	[illegible]	[illegible]	[illegible]	[illegible]	[illegible]	[illegible]	[illegible]	[illegible]	[illegible]	[illegible]	[illegible]	[illegible]	[illegible]	[illegible]
Ebola	0 (0–0)	0 (0–0)	55·5 (45·5–65·5)	[illegible]	[illegible]	[illegible]	[illegible]	[illegible]	[illegible]	[illegible]	[illegible]	[illegible]	[illegible]	[illegible]	[illegible]	[illegible]	[illegible]	[illegible]	[illegible]	[illegible]	[illegible]	[illegible]	[illegible]
Zika virus	0·000591 (0·000337–0·00093)	0·00141 (0·000762–0·00224)	0·0258 (0·0148–0·0409)	[illegible]	[illegible]	[illegible]	[illegible]	[illegible]	[illegible]	[illegible]	[illegible]	[illegible]	[illegible]	[illegible]	[illegible]	[illegible]	[illegible]	[illegible]	[illegible]	[illegible]	[illegible]	[illegible]	[illegible]
Other neglected tropical diseases	487 (246–1530)	356 (195–1080)	2060 (1120–5970)	[illegible]	[illegible]	[illegible]	[illegible]	[illegible]	[illegible]	[illegible]	[illegible]	[illegible]	[illegible]	[illegible]	[illegible]	[illegible]	[illegible]	[illegible]	[illegible]	[illegible]	[illegible]	[illegible]	[illegible]
Other infectious diseases	17000 (13400–21600)	17900 (13700–23000)	141000 (98100–197000)	[illegible]	[illegible]	[illegible]	[illegible]	[illegible]	[illegible]	[illegible]	[illegible]	[illegible]	[illegible]	[illegible]	[illegible]	[illegible]	[illegible]	[illegible]	[illegible]	[illegible]	[illegible]	[illegible]	[illegible]
Meningitis	6720 (5310–8870)	7300 (5840–9240)	49400 (38200–64000)	[illegible]	[illegible]	[illegible]	[illegible]	[illegible]	[illegible]	[illegible]	[illegible]	[illegible]	[illegible]	[illegible]	[illegible]	[illegible]	[illegible]	[illegible]	[illegible]	[illegible]	[illegible]	[illegible]	[illegible]
Encephalitis	531 (407–681)	884 (666–1160)	6940 (5290–9460)	[illegible]	[illegible]	[illegible]	[illegible]	[illegible]	[illegible]	[illegible]	[illegible]	[illegible]	[illegible]	[illegible]	[illegible]	[illegible]	[illegible]	[illegible]	[illegible]	[illegible]	[illegible]	[illegible]	[illegible]
Diphtheria	--	--	687 (432–1030)	[illegible]	[illegible]	[illegible]	[illegible]	[illegible]	[illegible]	[illegible]	[illegible]	[illegible]	[illegible]	[illegible]	[illegible]	[illegible]	[illegible]	[illegible]	[illegible]	[illegible]	[illegible]	[illegible]	[illegible]
Tetanus	6640 (4550–10100)	7590 (4890–11900)	2250 (1460–3780)	[illegible]	[illegible]	[illegible]	[illegible]	[illegible]	[illegible]	[illegible]	[illegible]	[illegible]	[illegible]	[illegible]	[illegible]	[illegible]	[illegible]	[illegible]	[illegible]	[illegible]	[illegible]	[illegible]	[illegible]
Measles	213 (194–235)	43·5 (39·5–47·8)	19000 (6520–42600)	[illegible]	[illegible]	[illegible]	[illegible]	[illegible]	[illegible]	[illegible]	[illegible]	[illegible]	[illegible]	[illegible]	[illegible]	[illegible]	[illegible]	[illegible]	[illegible]	[illegible]	[illegible]	[illegible]	[illegible]
Varicella and herpes zoster	104 (82·2–134)	273 (217–343)	1780 (1380–2290)	[illegible]	[illegible]	[illegible]	[illegible]	[illegible]	[illegible]	[illegible]	[illegible]	[illegible]	[illegible]	[illegible]	[illegible]	[illegible]	[illegible]	[illegible]	[illegible]	[illegible]	[illegible]	[illegible]	[illegible]
Acute hepatitis	--	--	6480 (4410–9430)	[illegible]	[illegible]	[illegible]	[illegible]	[illegible]	[illegible]	[illegible]	[illegible]	[illegible]	[illegible]	[illegible]	[illegible]	[illegible]	[illegible]	[illegible]	[illegible]	[illegible]	[illegible]	[illegible]	[illegible]
Acute hepatitis A	--	--	3520 (1950–5830)	[illegible]	[illegible]	[illegible]	[illegible]	[illegible]	[illegible]	[illegible]	[illegible]	[illegible]	[illegible]	[illegible]	[illegible]	[illegible]	[illegible]	[illegible]	[illegible]	[illegible]	[illegible]	[illegible]	[illegible]
Acute hepatitis B	--	--	2480 (1410–3990)	[illegible]	[illegible]	[illegible]	[illegible]	[illegible]	[illegible]	[illegible]	[illegible]	[illegible]	[illegible]	[illegible]	[illegible]	[illegible]	[illegible]	[illegible]	[illegible]	[illegible]	[illegible]	[illegible]	[illegible]
Acute hepatitis C	--	--	218 (73·7–434)	[illegible]	[illegible]	[illegible]	[illegible]	[illegible]	[illegible]	[illegible]	[illegible]	[illegible]	[illegible]	[illegible]	[illegible]	[illegible]	[illegible]	[illegible]	[illegible]	[illegible]	[illegible]	[illegible]	[illegible]
Acute hepatitis E	--	--	271 (129–500)	[illegible]	[illegible]	[illegible]	[illegible]	[illegible]	[illegible]	[illegible]	[illegible]	[illegible]	[illegible]	[illegible]	[illegible]	[illegible]	[illegible]	[illegible]	[illegible]	[illegible]	[illegible]	[illegible]	[illegible]
Other unspecified infectious diseases	2750 (1760–3650)	1760 (1220–2360)	5900 (4140–8070)	[illegible]	[illegible]	[illegible]	[illegible]	[illegible]	[illegible]	[illegible]	[illegible]	[illegible]	[illegible]	[illegible]	[illegible]	[illegible]	[illegible]	[illegible]	[illegible]	[illegible]	[illegible]	[illegible]	[illegible]
Maternal and neonatal disorders	1540000 (1310000–1830000)	254000 (214000–302000)	81700 (63600–102000)	[illegible]	[illegible]	[illegible]	[illegible]	[illegible]	[illegible]	[illegible]	[illegible]	[illegible]	[illegible]	[illegible]	[illegible]	[illegible]	[illegible]	[illegible]	[illegible]	[illegible]	[illegible]	[illegible]	[illegible]
Maternal disorders	--	--	--	[illegible]	[illegible]	[illegible]	[illegible]	[illegible]	[illegible]	[illegible]	[illegible]	[illegible]	[illegible]	[illegible]	[illegible]	[illegible]	[illegible]	[illegible]	[illegible]	[illegible]	[illegible]	[illegible]	[illegible]
Maternal hemorrhage	--	--	--	[illegible]	[illegible]	[illegible]	[illegible]	[illegible]	[illegible]	[illegible]	[illegible]	[illegible]	[illegible]	[illegible]	[illegible]	[illegible]	[illegible]	[illegible]	[illegible]	[illegible]	[illegible]	[illegible]	[illegible]
Maternal sepsis and other maternal infections	--	--	--	[illegible]	[illegible]	[illegible]	[illegible]	[illegible]	[illegible]	[illegible]	[illegible]	[illegible]	[illegible]	[illegible]	[illegible]	[illegible]	[illegible]	[illegible]	[illegible]	[illegible]	[illegible]	[illegible]	[illegible]
Maternal hypertensive disorders	--	--	--	[illegible]	[illegible]	[illegible]	[illegible]	[illegible]	[illegible]	[illegible]	[illegible]	[illegible]	[illegible]	[illegible]	[illegible]	[illegible]	[illegible]	[illegible]	[illegible]	[illegible]	[illegible]	[illegible]	[illegible]
Maternal obstructed labor and uterine rupture	--	--	--	[illegible]	[illegible]	[illegible]	[illegible]	[illegible]	[illegible]	[illegible]	[illegible]	[illegible]	[illegible]	[illegible]	[illegible]	[illegible]	[illegible]	[illegible]	[illegible]	[illegible]	[illegible]	[illegible]	[illegible]
Maternal abortion and miscarriage	--	--	--	[illegible]	[illegible]	[illegible]	[illegible]	[illegible]	[illegible]	[illegible]	[illegible]	[illegible]	[illegible]	[illegible]	[illegible]	[illegible]	[illegible]	[illegible]	[illegible]	[illegible]	[illegible]	[illegible]	[illegible]
Ectopic pregnancy	--	--	--	[illegible]	[illegible]	[illegible]	[illegible]	[illegible]	[illegible]	[illegible]	[illegible]	[illegible]	[illegible]	[illegible]	[illegible]	[illegible]	[illegible]	[illegible]	[illegible]	[illegible]	[illegible]	[illegible]	[illegible]
Indirect maternal deaths	--	--	--	[illegible]	[illegible]	[illegible]	[illegible]	[illegible]	[illegible]	[illegible]	[illegible]	[illegible]	[illegible]	[illegible]	[illegible]	[illegible]	[illegible]	[illegible]	[illegible]	[illegible]	[illegible]	[illegible]	[illegible]
Late maternal deaths	--	--	--	[illegible]	[illegible]	[illegible]	[illegible]	[illegible]	[illegible]	[illegible]	[illegible]	[illegible]	[illegible]	[illegible]	[illegible]	[illegible]	[illegible]	[illegible]	[illegible]	[illegible]	[illegible]	[illegible]	[illegible]
Maternal deaths aggravated by HIV/AIDS	--	--	--	[illegible]	[illegible]	[illegible]	[illegible]	[illegible]	[illegible]	[illegible]	[illegible]	[illegible]	[illegible]	[illegible]	[illegible]	[illegible]	[illegible]	[illegible]	[illegible]	[illegible]	[illegible]	[illegible]	[illegible]
Neonatal disorders	1540000 (1310000–1830000)	254000 (214000–302000)	81700 (63600–102000)	[illegible]	[illegible]	[illegible]	[illegible]	[illegible]	[illegible]	[illegible]	[illegible]	[illegible]	[illegible]	[illegible]	[illegible]	[illegible]	[illegible]	[illegible]	[illegible]	[illegible]	[illegible]	[illegible]	[illegible]
Neonatal preterm birth	567000 (476000–674000)	68300 (57100–81100)	26300 (20900–33200)	[illegible]	[illegible]	[illegible]	[illegible]	[illegible]	[illegible]	[illegible]	[illegible]	[illegible]	[illegible]	[illegible]	[illegible]	[illegible]	[illegible]	[illegible]	[illegible]	[illegible]	[illegible]	[illegible]	[illegible]
Neonatal encephalopathy due to birth asphyxia and trauma	511000 (429000–606000)	40300 (32600–49600)	13200 (10000–17100)	[illegible]	[illegible]	[illegible]	[illegible]	[illegible]	[illegible]	[illegible]	[illegible]	[illegible]	[illegible]	[illegible]	[illegible]	[illegible]	[illegible]	[illegible]	[illegible]	[illegible]	[illegible]	[illegible]	[illegible]
Neonatal sepsis and other neonatal infections	137000 (113000–168000)	73500 (60500–90300)	15900 (12700–19900)	[illegible]	[illegible]	[illegible]	[illegible]	[illegible]	[illegible]	[illegible]	[illegible]	[illegible]	[illegible]	[illegible]	[illegible]	[illegible]	[illegible]	[illegible]	[illegible]	[illegible]	[illegible]	[illegible]	[illegible]
Hemolytic disease and other neonatal jaundice	34000 (28000–41000)	15100 (12200–18500)	3390 (2180–5580)	[illegible]	[illegible]	[illegible]	[illegible]	[illegible]	[illegible]	[illegible]	[illegible]	[illegible]	[illegible]	[illegible]	[illegible]	[illegible]	[illegible]	[illegible]	[illegible]	[illegible]	[illegible]	[illegible]	[illegible]
Other neonatal disorders	292000 (241000–354000)	56300 (46400–68500)	22900 (17200–30700)	[illegible]	[illegible]	[illegible]	[illegible]	[illegible]	[illegible]	[illegible]	[illegible]	[illegible]	[illegible]	[illegible]	[illegible]	[illegible]	[illegible]	[illegible]	[illegible]	[illegible]	[illegible]	[illegible]	[illegible]
Nutritional deficiencies	0 (0–0)	0 (0–0)	47200 (36500–61300)	[illegible]	[illegible]	[illegible]	[illegible]	[illegible]	[illegible]	[illegible]	[illegible]	[illegible]	[illegible]	[illegible]	[illegible]	[illegible]	[illegible]	[illegible]	[illegible]	[illegible]	[illegible]	[illegible]	[illegible]
Protein-energy malnutrition	0 (0–0)	0 (0–0)	45300 (35200–59200)	[illegible]	[illegible]	[illegible]	[illegible]	[illegible]	[illegible]	[illegible]	[illegible]	[illegible]	[illegible]	[illegible]	[illegible]	[illegible]	[illegible]	[illegible]	[illegible]	[illegible]	[illegible]	[illegible]	[illegible]
Other nutritional deficiencies	--	--	1810 (1250–2600)	[illegible]	[illegible]	[illegible]	[illegible]	[illegible]	[illegible]	[illegible]	[illegible]	[illegible]	[illegible]	[illegible]	[illegible]	[illegible]	[illegible]	[illegible]	[illegible]	[illegible]	[illegible]	[illegible]	[illegible]
Non-communicable diseases	183000 (153000–227000)	67400 (56200–82200)	257000 (201000–325000)	[illegible]	[illegible]	[illegible]	[illegible]	[illegible]	[illegible]	[illegible]	[illegible]	[illegible]	[illegible]	[illegible]	[illegible]	[illegible]	[illegible]	[illegible]	[illegible]	[illegible]	[illegible]	[illegible]	[illegible]
Neoplasms	1770 (1380–2200)	1540 (1160–1920)	10400 (8410–12800)	[illegible]	[illegible]	[illegible]	[illegible]	[illegible]	[illegible]	[illegible]	[illegible]	[illegible]	[illegible]	[illegible]	[illegible]	[illegible]	[illegible]	[illegible]	[illegible]	[illegible]	[illegible]	[illegible]	[illegible]
Lip and oral cavity cancer	--	--	--	[illegible]	[illegible]	[illegible]	[illegible]	[illegible]	[illegible]	[illegible]	[illegible]	[illegible]	[illegible]	[illegible]	[illegible]	[illegible]	[illegible]	[illegible]	[illegible]	[illegible]	[illegible]	[illegible]	[illegible]
Nasopharynx cancer	--	--	--	[illegible]	[illegible]	[illegible]	[illegible]	[illegible]	[illegible]	[illegible]	[illegible]	[illegible]	[illegible]	[illegible]	[illegible]	[illegible]	[illegible]	[illegible]	[illegible]	[illegible]	[illegible]	[illegible]	[illegible]
Other pharynx cancer	--	--	--	[illegible]	[illegible]	[illegible]	[illegible]	[illegible]	[illegible]	[illegible]	[illegible]	[illegible]	[illegible]	[illegible]	[illegible]	[illegible]	[illegible]	[illegible]	[illegible]	[illegible]	[illegible]	[illegible]	[illegible]
Esophageal cancer	--	--	--	[illegible]	[illegible]	[illegible]	[illegible]	[illegible]	[illegible]	[illegible]	[illegible]	[illegible]	[illegible]	[illegible]	[illegible]	[illegible]	[illegible]	[illegible]	[illegible]	[illegible]	[illegible]	[illegible]	[illegible]
Stomach cancer	--	--	--	[illegible]	[illegible]	[illegible]	[illegible]	[illegible]	[illegible]	[illegible]	[illegible]	[illegible]	[illegible]	[illegible]	[illegible]	[illegible]	[illegible]	[illegible]	[illegible]	[illegible]	[illegible]	[illegible]	[illegible]
Colon and rectum cancer	--	--	--	[illegible]	[illegible]	[illegible]	[illegible]	[illegible]	[illegible]	[illegible]	[illegible]	[illegible]	[illegible]	[illegible]	[illegible]	[illegible]	[illegible]	[illegible]	[illegible]	[illegible]	[illegible]	[illegible]	[illegible]
Liver cancer	192 (123–286)	171 (109–277)	513 (351–699)	[illegible]	[illegible]	[illegible]	[illegible]	[illegible]	[illegible]	[illegible]	[illegible]	[illegible]	[illegible]	[illegible]	[illegible]	[illegible]	[illegible]	[illegible]	[illegible]	[illegible]	[illegible]	[illegible]	[illegible]
Liver cancer due to hepatitis B	--	--	--	[illegible]	[illegible]	[illegible]	[illegible]	[illegible]	[illegible]	[illegible]	[illegible]	[illegible]	[illegible]	[illegible]	[illegible]	[illegible]	[illegible]	[illegible]	[illegible]	[illegible]	[illegible]	[illegible]	[illegible]
Liver cancer due to hepatitis C	--	--	--	[illegible]	[illegible]	[illegible]	[illegible]	[illegible]	[illegible]	[illegible]	[illegible]	[illegible]	[illegible]	[illegible]	[illegible]	[illegible]	[illegible]	[illegible]	[illegible]	[illegible]	[illegible]	[illegible]	[illegible]
Liver cancer due to alcohol use	--	--	--	[illegible]	[illegible]	[illegible]	[illegible]	[illegible]	[illegible]	[illegible]	[illegible]	[illegible]	[illegible]	[illegible]	[illegible]	[illegible]	[illegible]	[illegible]	[illegible]	[illegible]	[illegible]	[illegible]	[illegible]
Liver cancer due to NASH	--	--	--	[illegible]	[illegible]	[illegible]	[illegible]	[illegible]	[illegible]	[illegible]	[illegible]	[illegible]	[illegible]	[illegible]	[illegible]	[illegible]	[illegible]	[illegible]	[illegible]	[illegible]	[illegible]	[illegible]	[illegible]
Liver cancer due to other causes	192 (123–286)	171 (109–277)	513 (351–699)	[illegible]	[illegible]	[illegible]	[illegible]	[illegible]	[illegible]	[illegible]	[illegible]	[illegible]	[illegible]	[illegible]	[illegible]	[illegible]	[illegible]	[illegible]	[illegible]	[illegible]	[illegible]	[illegible]	[illegible]
Gallbladder and biliary tract cancer	--	--	--	[illegible]	[illegible]	[illegible]	[illegible]	[illegible]	[illegible]	[illegible]	[illegible]	[illegible]	[illegible]	[illegible]	[illegible]	[illegible]	[illegible]	[illegible]	[illegible]	[illegible]	[illegible]	[illegible]	[illegible]
Pancreatic cancer	--	--	--	[illegible]	[illegible]	[illegible]	[illegible]	[illegible]	[illegible]	[illegible]	[illegible]	[illegible]	[illegible]	[illegible]	[illegible]	[illegible]	[illegible]	[illegible]	[illegible]	[illegible]	[illegible]	[illegible]	[illegible]
Larynx cancer	--	--	--	[illegible]	[illegible]	[illegible]	[illegible]	[illegible]	[illegible]	[illegible]	[illegible]	[illegible]	[illegible]	[illegible]	[illegible]	[illegible]	[illegible]	[illegible]	[illegible]	[illegible]	[illegible]	[illegible]	[illegible]
Tracheal, bronchus, and lung cancer	--	--	--	[illegible]	[illegible]	[illegible]	[illegible]	[illegible]	[illegible]	[illegible]	[illegible]	[illegible]	[illegible]	[illegible]	[illegible]	[illegible]	[illegible]	[illegible]	[illegible]	[illegible]	[illegible]	[illegible]	[illegible]
Malignant skin melanoma	1·5 (0·921–2·91)	1·87 (1·31–3·32)	23·8 (13·4–48·1)	[illegible]	[illegible]	[illegible]	[illegible]	[illegible]	[illegible]	[illegible]	[illegible]	[illegible]	[illegible]	[illegible]	[illegible]	[illegible]	[illegible]	[illegible]	[illegible]	[illegible]	[illegible]	[illegible]	[illegible]
Non-melanoma skin cancer	--	--	--	[illegible]	[illegible]	[illegible]	[illegible]	[illegible]	[illegible]	[illegible]	[illegible]	[illegible]	[illegible]	[illegible]	[illegible]	[illegible]	[illegible]	[illegible]	[illegible]	[illegible]	[illegible]	[illegible]	[illegible]
Non-melanoma skin cancer (squamous-cell carcinoma)	--	--	--	[illegible]	[illegible]	[illegible]	[illegible]	[illegible]	[illegible]	[illegible]	[illegible]	[illegible]	[illegible]	[illegible]	[illegible]	[illegible]	[illegible]	[illegible]	[illegible]	[illegible]	[illegible]	[illegible]	[illegible]
Breast cancer	--	--	--	[illegible]	[illegible]	[illegible]	[illegible]	[illegible]	[illegible]	[illegible]	[illegible]	[illegible]	[illegible]	[illegible]	[illegible]	[illegible]	[illegible]	[illegible]	[illegible]	[illegible]	[illegible]	[illegible]	[illegible]
Cervical cancer	--	--	--	[illegible]	[illegible]	[illegible]	[illegible]	[illegible]	[illegible]	[illegible]	[illegible]	[illegible]	[illegible]	[illegible]	[illegible]	[illegible]	[illegible]	[illegible]	[illegible]	[illegible]	[illegible]	[illegible]	[illegible]
Uterine cancer	--	--	--	[illegible]	[illegible]	[illegible]	[illegible]	[illegible]	[illegible]	[illegible]	[illegible]	[illegible]	[illegible]	[illegible]	[illegible]	[illegible]	[illegible]	[illegible]	[illegible]	[illegible]	[illegible]	[illegible]	[illegible]
Ovarian cancer	--	--	--	[illegible]	[illegible]	[illegible]	[illegible]	[illegible]	[illegible]	[illegible]	[illegible]	[illegible]	[illegible]	[illegible]	[illegible]	[illegible]	[illegible]	[illegible]	[illegible]	[illegible]	[illegible]	[illegible]	[illegible]
Prostate cancer	--	--	--	[illegible]	[illegible]	[illegible]	[illegible]	[illegible]	[illegible]	[illegible]	[illegible]	[illegible]	[illegible]	[illegible]	[illegible]	[illegible]	[illegible]	[illegible]	[illegible]	[illegible]	[illegible]	[illegible]	[illegible]
Testicular cancer	16 (4·79–112)	11·1 (5·98–45·2)	52·1 (33·2–140)	[illegible]	[illegible]	[illegible]	[illegible]	[illegible]	[illegible]	[illegible]	[illegible]	[illegible]	[illegible]	[illegible]	[illegible]	[illegible]	[illegible]	[illegible]	[illegible]	[illegible]	[illegible]	[illegible]	[illegible]
Kidney cancer	48 (38·1–61·2)	26·8 (21·4–33·5)	300 (252–365)	[illegible]	[illegible]	[illegible]	[illegible]	[illegible]	[illegible]	[illegible]	[illegible]	[illegible]	[illegible]	[illegible]	[illegible]	[illegible]	[illegible]	[illegible]	[illegible]	[illegible]	[illegible]	[illegible]	[illegible]
Bladder cancer	--	--	--	[illegible]	[illegible]	[illegible]	[illegible]	[illegible]	[illegible]	[illegible]	[illegible]	[illegible]	[illegible]	[illegible]	[illegible]	[illegible]	[illegible]	[illegible]	[illegible]	[illegible]	[illegible]	[illegible]	[illegible]
Brain and central nervous system cancer	299 (181–404)	282 (156–430)	2210 (1560–2840)	[illegible]	[illegible]	[illegible]	[illegible]	[illegible]	[illegible]	[illegible]	[illegible]	[illegible]	[illegible]	[illegible]	[illegible]	[illegible]	[illegible]	[illegible]	[illegible]	[illegible]	[illegible]	[illegible]	[illegible]
Thyroid cancer	--	--	--	[illegible]	[illegible]	[illegible]	[illegible]	[illegible]	[illegible]	[illegible]	[illegible]	[illegible]	[illegible]	[illegible]	[illegible]	[illegible]	[illegible]	[illegible]	[illegible]	[illegible]	[illegible]	[illegible]	[illegible]
Mesothelioma	--	--	--	[illegible]	[illegible]	[illegible]	[illegible]	[illegible]	[illegible]	[illegible]	[illegible]	[illegible]	[illegible]	[illegible]	[illegible]	[illegible]	[illegible]	[illegible]	[illegible]	[illegible]	[illegible]	[illegible]	[illegible]
Hodgkin lymphoma	--	--	--	[illegible]	[illegible]	[illegible]	[illegible]	[illegible]	[illegible]	[illegible]	[illegible]	[illegible]	[illegible]	[illegible]	[illegible]	[illegible]	[illegible]	[illegible]	[illegible]	[illegible]	[illegible]	[illegible]	[illegible]
Non-Hodgkin lymphoma	--	--	--	[illegible]	[illegible]	[illegible]	[illegible]	[illegible]	[illegible]	[illegible]	[illegible]	[illegible]	[illegible]	[illegible]	[illegible]	[illegible]	[illegible]	[illegible]	[illegible]	[illegible]	[illegible]	[illegible]	[illegible]
Multiple myeloma	--	--	--	[illegible]	[illegible]	[illegible]	[illegible]	[illegible]	[illegible]	[illegible]	[illegible]	[illegible]	[illegible]	[illegible]	[illegible]	[illegible]	[illegible]	[illegible]	[illegible]	[illegible]	[illegible]	[illegible]	[illegible]
Leukemia	551 (378–765)	460 (305–618)	4590 (3430–5890)	[illegible]	[illegible]	[illegible]	[illegible]	[illegible]	[illegible]	[illegible]	[illegible]	[illegible]	[illegible]	[illegible]	[illegible]	[illegible]	[illegible]	[illegible]	[illegible]	[illegible]	[illegible]	[illegible]	[illegible]
Acute lymphoid leukemia	176 (104–264)	124 (71·9–187)	1590 (1060–2190)	[illegible]	[illegible]	[illegible]	[illegible]	[illegible]	[illegible]	[illegible]	[illegible]	[illegible]	[illegible]	[illegible]	[illegible]	[illegible]	[illegible]	[illegible]	[illegible]	[illegible]	[illegible]	[illegible]	[illegible]
Chronic lymphoid leukemia	--	--	--	[illegible]	[illegible]	[illegible]	[illegible]	[illegible]	[illegible]	[illegible]	[illegible]	[illegible]	[illegible]	[illegible]	[illegible]	[illegible]	[illegible]	[illegible]	[illegible]	[illegible]	[illegible]	[illegible]	[illegible]
Acute myeloid leukemia	87·5 (49·2–130)	91·5 (51·6–141)	731 (524–1030)	[illegible]	[illegible]	[illegible]	[illegible]	[illegible]	[illegible]	[illegible]	[illegible]	[illegible]	[illegible]	[illegible]	[illegible]	[illegible]	[illegible]	[illegible]	[illegible]	[illegible]	[illegible]	[illegible]	[illegible]
Chronic myeloid leukemia	27·3 (12·4–58·3)	31 (14·6–68·2)	393 (203–729)	[illegible]	[illegible]	[illegible]	[illegible]	[illegible]	[illegible]	[illegible]	[illegible]	[illegible]	[illegible]	[illegible]	[illegible]	[illegible]	[illegible]	[illegible]	[illegible]	[illegible]	[illegible]	[illegible]	[illegible]
Other leukemia	260 (174–365)	213 (143–296)	1870 (1330–2480)	[illegible]	[illegible]	[illegible]	[illegible]	[illegible]	[illegible]	[illegible]	[illegible]	[illegible]	[illegible]	[illegible]	[illegible]	[illegible]	[illegible]	[illegible]	[illegible]	[illegible]	[illegible]	[illegible]	[illegible]
Other malignant neoplasms	652 (508–805)	576 (436–723)	2610 (2100–3270)	[illegible]	[illegible]	[illegible]	[illegible]	[illegible]	[illegible]	[illegible]	[illegible]	[illegible]	[illegible]	[illegible]	[illegible]	[illegible]	[illegible]	[illegible]	[illegible]	[illegible]	[illegible]	[illegible]	[illegible]
Other neoplasms	15·1 (10·7–18·6)	7·24 (5·34–8·77)	76·9 (61·8–95·1)	[illegible]	[illegible]	[illegible]	[illegible]	[illegible]	[illegible]	[illegible]	[illegible]	[illegible]	[illegible]	[illegible]	[illegible]	[illegible]	[illegible]	[illegible]	[illegible]	[illegible]	[illegible]	[illegible]	[illegible]
[illegible] myeloproliferative, and other hematopoietic neoplasms	15·1 (10·7–18·6)	7·24 (5·34–8·77)	76·9 (61·8–95·1)	[illegible]	[illegible]	[illegible]	[illegible]	[illegible]	[illegible]	[illegible]	[illegible]	[illegible]	[illegible]	[illegible]	[illegible]	[illegible]	[illegible]	[illegible]	[illegible]	[illegible]	[illegible]	[illegible]	[illegible]
Cardiovascular diseases	2930 (2350–3820)	2080 (1590–2750)	11800 (9390–15200)	[illegible]	[illegible]	[illegible]	[illegible]	[illegible]	[illegible]	[illegible]	[illegible]	[illegible]	[illegible]	[illegible]	[illegible]	[illegible]	[illegible]	[illegible]	[illegible]	[illegible]	[illegible]	[illegible]	[illegible]
Rheumatic heart disease	--	--	--	[illegible]	[illegible]	[illegible]	[illegible]	[illegible]	[illegible]	[illegible]	[illegible]	[illegible]	[illegible]	[illegible]	[illegible]	[illegible]	[illegible]	[illegible]	[illegible]	[illegible]	[illegible]	[illegible]	[illegible]
Ischemic heart disease	--	--	--	[illegible]	[illegible]	[illegible]	[illegible]	[illegible]	[illegible]	[illegible]	[illegible]	[illegible]	[illegible]	[illegible]	[illegible]	[illegible]	[illegible]	[illegible]	[illegible]	[illegible]	[illegible]	[illegible]	[illegible]
Stroke	1140 (889–1500)	791 (566–1160)	4110 (3130–5540)	[illegible]	[illegible]	[illegible]	[illegible]	[illegible]	[illegible]	[illegible]	[illegible]	[illegible]	[illegible]	[illegible]	[illegible]	[illegible]	[illegible]	[illegible]	[illegible]	[illegible]	[illegible]	[illegible]	[illegible]
Ischemic stroke	228 (170–328)	176 (122–273)	573 (409–885)	[illegible]	[illegible]	[illegible]	[illegible]	[illegible]	[illegible]	[illegible]	[illegible]	[illegible]	[illegible]	[illegible]	[illegible]	[illegible]	[illegible]	[illegible]	[illegible]	[illegible]	[illegible]	[illegible]	[illegible]
Intracerebral hemorrhage	650 (498–843)	472 (326–711)	2760 (2040–3800)	[illegible]	[illegible]	[illegible]	[illegible]	[illegible]	[illegible]	[illegible]	[illegible]	[illegible]	[illegible]	[illegible]	[illegible]	[illegible]	[illegible]	[illegible]	[illegible]	[illegible]	[illegible]	[illegible]	[illegible]
Subarachnoid hemorrhage	266 (167–401)	142 (95·4–213)	775 (544–1150)	[illegible]	[illegible]	[illegible]	[illegible]	[illegible]	[illegible]	[illegible]	[illegible]	[illegible]	[illegible]	[illegible]	[illegible]	[illegible]	[illegible]	[illegible]	[illegible]	[illegible]	[illegible]	[illegible]	[illegible]
Hypertensive heart disease	--	--	--	[illegible]	[illegible]	[illegible]	[illegible]	[illegible]	[illegible]	[illegible]	[illegible]	[illegible]	[illegible]	[illegible]	[illegible]	[illegible]	[illegible]	[illegible]	[illegible]	[illegible]	[illegible]	[illegible]	[illegible]
Non-rheumatic valvular heart disease	--	--	--	[illegible]	[illegible]	[illegible]	[illegible]	[illegible]	[illegible]	[illegible]	[illegible]	[illegible]	[illegible]	[illegible]	[illegible]	[illegible]	[illegible]	[illegible]	[illegible]	[illegible]	[illegible]	[illegible]	[illegible]
Non-rheumatic calcific aortic valve disease	--	--	--	[illegible]	[illegible]	[illegible]	[illegible]	[illegible]	[illegible]	[illegible]	[illegible]	[illegible]	[illegible]	[illegible]	[illegible]	[illegible]	[illegible]	[illegible]	[illegible]	[illegible]	[illegible]	[illegible]	[illegible]
Non-rheumatic degenerative mitral valve disease	--	--	--	[illegible]	[illegible]	[illegible]	[illegible]	[illegible]	[illegible]	[illegible]	[illegible]	[illegible]	[illegible]	[illegible]	[illegible]	[illegible]	[illegible]	[illegible]	[illegible]	[illegible]	[illegible]	[illegible]	[illegible]
Other non-rheumatic valve diseases	--	--	--	[illegible]	[illegible]	[illegible]	[illegible]	[illegible]	[illegible]	[illegible]	[illegible]	[illegible]	[illegible]	[illegible]	[illegible]	[illegible]	[illegible]	[illegible]	[illegible]	[illegible]	[illegible]	[illegible]	[illegible]
Cardiomyopathy and myocarditis	913 (658–1190)	558 (416–736)	3710 (2890–4890)	[illegible]	[illegible]	[illegible]	[illegible]	[illegible]	[illegible]	[illegible]	[illegible]	[illegible]	[illegible]	[illegible]	[illegible]	[illegible]	[illegible]	[illegible]	[illegible]	[illegible]	[illegible]	[illegible]	[illegible]
Myocarditis	100 (69·5–150)	120 (87·4–171)	1040 (813–1380)	[illegible]	[illegible]	[illegible]	[illegible]	[illegible]	[illegible]	[illegible]	[illegible]	[illegible]	[illegible]	[illegible]	[illegible]	[illegible]	[illegible]	[illegible]	[illegible]	[illegible]	[illegible]	[illegible]	[illegible]
Alcoholic cardiomyopathy	--	--	--	[illegible]	[illegible]	[illegible]	[illegible]	[illegible]	[illegible]	[illegible]	[illegible]	[illegible]	[illegible]	[illegible]	[illegible]	[illegible]	[illegible]	[illegible]	[illegible]	[illegible]	[illegible]	[illegible]	[illegible]
Other cardiomyopathy	813 (578–1060)	438 (314–578)	2670 (2010–3610)	[illegible]	[illegible]	[illegible]	[illegible]	[illegible]	[illegible]	[illegible]	[illegible]	[illegible]	[illegible]	[illegible]	[illegible]	[illegible]	[illegible]	[illegible]	[illegible]	[illegible]	[illegible]	[illegible]	[illegible]
Atrial fibrillation and flutter	--	--	--	[illegible]	[illegible]	[illegible]	[illegible]	[illegible]	[illegible]	[illegible]	[illegible]	[illegible]	[illegible]	[illegible]	[illegible]	[illegible]	[illegible]	[illegible]	[illegible]	[illegible]	[illegible]	[illegible]	[illegible]
Aortic aneurysm	--	--	--	[illegible]	[illegible]	[illegible]	[illegible]	[illegible]	[illegible]	[illegible]	[illegible]	[illegible]	[illegible]	[illegible]	[illegible]	[illegible]	[illegible]	[illegible]	[illegible]	[illegible]	[illegible]	[illegible]	[illegible]

Endocarditis	104 (69·7–150)	103 (66·1–143)	956 (653–1320)	529 (289–795)	216 (154–271)	340 (255–414)	576 (438–694)	1020 (820–1220)	1480 (1230–1840)	1710 (1370–2030)	2040 (1660–2500)	2340 (1920–2960)	2450 (1920–3020)	3020 (2390–3610)	3700 (2830–4340)	4200 (3180–4800)	5130 (3750–5810)	5680 (4010–6410)	6290 (4210–7210)	7530 (4660–8930)	7670 (4090–9560)	5840 (2750–7630)	3390 (1330–4650)
Other cardiovascular and circulatory diseases	773 (586–1120)	625 (452–870)	3060 (2330–4010)	2860 (2120–3660)	2040 (1620–2460)	2260 (1880–2700)	2090 (1790–2410)	2900 (2530–3330)	3720 (3290–4270)	4610 (4130–5250)	5620 (5100–6260)	7310 (6550–8260)	9290 (8370–10500)	12600 (11400–14000)	16200 (14700–17900)	21400 (19600–23500)	25400 (23200–27700)	29600 (26900–32500)	33300 (30100–36600)	35800 (31300–39200)	29400 (24500–32500)	17800 (14200–20100)	8280 (6150–9490)
Chronic respiratory diseases	0 (0–0)	0 (0–0)	0 (0–0)	9190 (7160–11600)	2820 (2370–3360)	3080 (2640–3620)	5670 (5020–6520)	8090 (7190–9190)	9860 (8890–11000)	14000 (12600–15600)	20400 (18300–22600)	31400 (28200–34800)	53800 (48400–59400)	1e+05 (88100–111000)	163000 (144000–180000)	272000 (244000–298000)	409000 (369000–445000)	573000 (517000–622000)	653000 (587000–708000)	757000 (667000–821000)	546000 (467000–597000)	251000 (204000–279000)	90500 (68900–103000)
Chronic obstructive pulmonary disease	–	–	0 (0–0)	1120 (734–1890)	343 (274–430)	309 (261–377)	1730 (1480–2140)	2350 (1990–2820)	3130 (2700–3620)	5190 (4510–6060)	8070 (6930–9350)	14600 (12700–16700)	29600 (25700–33600)	65400 (56400–73300)	114000 (99100–127000)	211000 (187000–234000)	333000 (299000–365000)	482000 (430000–528000)	558000 (498000–609000)	661000 (583000–724000)	486000 (415000–535000)	223000 (179000–250000)	80200 (61200–91800)
Pneumoconiosis	–	–	–	–	–	–	24·4 (20·5–29·4)	34·8 (29–41·8)	70·8 (60·3–86·5)	125 (106–152)	254 (216–309)	468 (384–568)	840 (661–1050)	1310 (1050–1640)	1550 (1270–1860)	1950 (1630–2330)	2480 (2090–2920)	3020 (2660–3450)	3420 (3050–3880)	3700 (3300–4190)	2600 (2270–2960)	929 (765–1040)	247 (191–279)
Silicosis	–	–	–	–	–	–	6·14 (4·59–8·32)	11·4 (8·38–16)	27·8 (21·4–38)	61·5 (47·8–84·9)	142 (112–191)	294 (222–378)	550 (400–731)	886 (649–1170)	1000 (762–1280)	1160 (902–1490)	1480 (1170–1870)	1650 (1340–2060)	1840 (1550–2400)	1940 (1650–2510)	1350 (1140–1790)	395 (324–494)	83·3 (63·4–102)
Asbestosis	–	–	–	–	–	–	4·14 (2·83–5·56)	5·68 (3·95–7·71)	7·56 (5·48–9·66)	12·3 (9·05–16·2)	18·5 (13·7–24·3)	27·2 (19·8–36·5)	41·1 (27·9–58·6)	81·5 (55·9–113)	125 (91·3–163)	206 (152–266)	293 (227–354)	497 (364–576)	604 (396–699)	715 (455–822)	587 (400–673)	272 (179–314)	74·9 (48·3–89·2)
Coal workers pneumoconiosis	–	–	–	–	–	–	2·28 (1·58–3·98)	3·01 (2·13–4·78)	6·6 (4·76–10·4)	10·6 (7·23–15·2)	23·4 (14·1–33)	51·4 (28·8–76)	97·5 (49·7–168)	140 (74·8–229)	149 (94·9–233)	216 (132–324)	294 (185–412)	419 (283–557)	507 (347–638)	555 (409–690)	368 (293–457)	124 (96·3–152)	40·2 (29·4–50)
Other pneumoconiosis	–	–	–	–	–	–	11·8 (9·37–16·1)	14·7 (11·7–19·9)	28·9 (22·5–39·9)	40·6 (32·1–55·6)	70·1 (52·8–100)	95 (71·6–133)	151 (101–237)	200 (141–322)	271 (191–401)	364 (261–526)	410 (298–580)	457 (346–620)	465 (376–622)	488 (402–625)	295 (233–371)	138 (105–165)	49 (35·3–59·4)
Asthma	–	–	–	6100 (4620–7980)	1700 (1400–2040)	2110 (1790–2510)	2970 (2540–3430)	4180 (3540–4790)	5010 (4330–5670)	6300 (5480–7120)	8830 (7580–10100)	11800 (9840–13500)	16700 (13900–19500)	24100 (18900–28600)	32900 (25700–39500)	43900 (33700–54200)	52500 (40100–66100)	59400 (45200–75700)	60600 (45700–77300)	62600 (47000–76900)	36700 (28300–44800)	16800 (12800–20700)	5690 (4060–7460)
Interstitial lung disease and pulmonary sarcoidosis	–	–	–	279 (184–420)	128 (97·3–166)	112 (90·2–147)	222 (175–273)	373 (298–465)	567 (467–699)	861 (699–1070)	1410 (1120–1760)	2160 (1720–2740)	3430 (2670–4350)	5700 (4330–7270)	8990 (6670–11500)	13400 (9880–16300)	19400 (13800–24100)	25600 (17500–31200)	28400 (18400–34800)	26800 (16800–33500)	18900 (11100–24500)	9400 (4960–12800)	3730 (1770–5410)
Other chronic respiratory diseases	0 (0–0)	0 (0–0)	0 (0–0)	1690 (918–2650)	651 (496–814)	545 (398–688)	723 (574–913)	1160 (915–1470)	1080 (872–1320)	1520 (1200–1900)	1810 (1420–2230)	2290 (1800–2840)	3180 (2400–4010)	3720 (2800–4770)	5640 (4230–7080)	1880 (1380–2290)	2150 (1560–2620)	2430 (1660–2880)	2410 (1690–2840)	2870 (1980–3410)	2180 (1530–2570)	1270 (873–1540)	566 (333–701)
Digestive diseases	2140 (1670–2850)	1860 (1400–2490)	13100 (9400–17800)	17700 (13500–23600)	10300 (7950–13000)	9590 (7930–11400)	20100 (17400–23400)	28500 (25100–32800)	43100 (39100–47700)	62500 (57800–67900)	90500 (83700–97900)	120000 (111000–130000)	154000 (143000–166000)	191000 (178000–204000)	225000 (210000–241000)	248000 (232000–264000)	259000 (244000–275000)	248000 (234000–263000)	242000 (226000–257000)	239000 (211000–256000)	182000 (154000–197000)	103000 (82700–114000)	46300 (34400–52500)
Cirrhosis and other chronic liver diseases	–	–	–	7350 (5340–10200)	4520 (3470–5790)	4800 (3920–5800)	10900 (9290–12700)	17100 (14900–19700)	27500 (24800–30800)	44500 (41100–48500)	66500 (61100–72400)	90800 (83500–99000)	116000 (107000–126000)	144000 (134000–156000)	163000 (152000–175000)	172000 (161000–184000)	163000 (153000–175000)	139000 (129000–149000)	116000 (107000–125000)	93000 (81900–102000)	57000 (48600–63600)	25000 (20000–28200)	10100 (7440–11800)
Cirrhosis and other chronic liver diseases due to hepatitis B	–	–	–	60·9 (31·1–107)	89·8 (47·1–146)	161 (89·1–253)	567 (293–908)	2800 (1630–4260)	6960 (4440–9930)	13000 (9860–16500)	20700 (14900–26900)	27700 (21100–35500)	32700 (24200–42500)	36500 (26300–48800)	36400 (25200–49800)	35300 (24100–49200)	32200 (22200–43700)	27100 (18600–37600)	23600 (16400–31000)	18800 (13000–25500)	10800 (7910–14100)	4250 (2800–5970)	1500 (796–2390)
Cirrhosis and other chronic liver diseases due to hepatitis C	–	–	–	53·7 (24·6–99·7)	102 (53·7–176)	191 (106–317)	679 (366–1120)	3060 (1900–4440)	7360 (4860–10100)	12800 (9660–16300)	19400 (14000–25600)	26500 (19900–34200)	33700 (24800–43500)	41100 (29800–54300)	44500 (32700–59300)	45100 (31900–61200)	43400 (32700–55900)	37000 (28300–46400)	30400 (22700–39700)	24200 (17600–31800)	15400 (11400–19700)	6940 (4650–9340)	2990 (1810–4360)
Cirrhosis and other chronic liver diseases due to alcohol use	–	–	–	–	–	–	39·4 (11·5–87·5)	730 (401–1240)	2420 (1450–3840)	7060 (5240–9260)	14400 (10000–19300)	22200 (16300–28700)	31200 (22800–41000)	41800 (30400–54100)	51900 (37900–67200)	55200 (40200–72300)	48400 (35000–63600)	36200 (26000–47900)	25800 (18800–33400)	18200 (12800–24400)	10300 (7550–13700)	4320 (2890–6290)	1620 (883–2740)
Cirrhosis and other chronic liver diseases due to NAFLD	–	–	–	–	–	–	261 (105–523)	776 (392–1330)	1740 (837–3090)	2950 (1680–4680)	4110 (2050–7160)	5990 (3060–9830)	8600 (4170–14700)	11400 (5670–19400)	13100 (6550–22200)	14700 (7490–25700)	15800 (9720–24000)	15100 (9360–22200)	14000 (7740–21700)	12300 (7070–18800)	8070 (5020–12000)	3700 (2040–6060)	1530 (665–2680)
Cirrhosis and other chronic liver diseases due to other causes	–	–	–	7240 (5250–10000)	4330 (3330–5540)	4450 (3620–5400)	9350 (7710–11100)	9720 (7010–12700)	9050 (5900–13000)	8630 (5260–13400)	7870 (4510–12500)	8400 (4810–13600)	10100 (5770–16800)	13100 (7530–21200)	17300 (9740–28300)	21200 (12200–33400)	23400 (12900–38100)	23100 (12900–36200)	22100 (12800–34200)	19500 (11400–29800)	12400 (7910–17700)	5770 (3350–8780)	2420 (1140–4110)
Upper digestive system diseases	–	–	–	1510 (945–2610)	744 (555–994)	645 (519–788)	1550 (1330–1800)	2800 (2430–3220)	3710 (3310–4280)	4530 (4120–5170)	6290 (5640–7260)	7440 (6670–8390)	10800 (9640–12200)	15000 (13500–16700)	18100 (16500–20200)	22000 (20100–24300)	28000 (25700–30800)	31000 (28600–33700)	34100 (31400–37400)	36400 (32300–40000)	28200 (24100–31400)	14600 (11800–16500)	5950 (4470–6940)
Peptic ulcer disease	–	–	–	1060 (637–2110)	650 (483–879)	579 (466–708)	1370 (1160–1610)	2450 (2120–2850)	3250 (2880–3790)	3860 (3470–4390)	5510 (4910–6400)	6490 (5780–7370)	9330 (8280–10700)	13000 (11800–14600)	15900 (14400–17900)	19500 (17800–21700)	24300 (22200–27100)	26800 (24700–29400)	29400 (27100–32500)	31100 (27500–34600)	23800 (20300–26900)	12500 (10100–14200)	5070 (3780–5950)
Gastritis and duodenitis	–	–	–	452 (255–790)	94 (61·4–126)	66·3 (44–86·8)	175 (125–243)	351 (256–466)	459 (351–607)	675 (539–840)	778 (625–964)	953 (758–1150)	1470 (1100–1780)	1950 (1390–2310)	2210 (1640–2560)	2540 (1930–2930)	3680 (2820–4210)	4190 (3220–4780)	4710 (3580–5360)	5290 (4290–6020)	4370 (3580–4920)	2170 (1740–2510)	884 (674–1060)
Appendicitis	–	–	–	1160 (705–1680)	1030 (777–1290)	1050 (783–1300)	1920 (1500–2340)	1530 (1230–1880)	1880 (1490–2260)	1100 (869–1280)	1430 (1140–1690)	1450 (1110–1690)	1640 (1280–1930)	1600 (1220–1880)	2350 (1860–2810)	2410 (1860–2830)	2470 (1910–2920)	2540 (1940–2960)	2570 (2110–2970)	2410 (2000–2770)	1700 (1410–1960)	866 (687–1010)	311 (233–368)
Paralytic ileus and intestinal obstruction	1500 (1120–2100)	1520 (1100–2080)	11800 (8470–16500)	3330 (2000–5100)	2460 (1680–3360)	1890 (1400–2400)	3680 (2700–4600)	3500 (2510–4330)	4060 (2990–4880)	4110 (3020–4880)	5470 (3890–6430)	6550 (4560–7710)	7330 (5260–8570)	9040 (6300–10600)	12300 (9250–14300)	15200 (11700–17300)	19500 (15100–21900)	20700 (17300–23200)	24000 (20500–26900)	28500 (23900–32800)	26400 (21400–30000)	17500 (12800–20000)	8280 (5510–9990)
Inguinal, femoral, and abdominal hernia	639 (482–818)	340 (248–447)	1290 (891–1760)	556 (345–795)	205 (134–284)	149 (108–192)	248 (191–328)	328 (259–419)	382 (315–464)	362 (303–426)	548 (463–653)	737 (622–863)	1010 (804–1180)	1780 (1420–2060)	2490 (1980–2840)	3260 (2650–3760)	4160 (3450–4770)	4900 (4100–5610)	5980 (5080–6830)	7280 (6120–8400)	5960 (4900–6870)	3780 (2990–4420)	1670 (1210–2030)
Inflammatory bowel disease	–	–	–	717 (403–1060)	141 (101–189)	120 (91·6–153)	230 (181–291)	361 (284–456)	535 (434–653)	649 (551–771)	762 (658–880)	965 (835–1120)	1170 (988–1340)	1520 (1300–1710)	2040 (1720–2270)	2590 (2170–2870)	3220 (2730–3550)	3880 (3310–4250)	4730 (4060–5210)	5920 (4930–6580)	5480 (4360–6180)	3930 (2920–4490)	2020 (1450–2390)
Vascular intestinal disorders	–	–	–	298 (165–517)	83·3 (60·5–123)	38·5 (32·1–47)	83·3 (70·9–99·5)	215 (177–257)	283 (245–331)	369 (319–426)	550 (483–637)	875 (782–983)	1490 (1350–1650)	2500 (2270–2780)	4190 (3870–4590)	6350 (5860–6910)	9900 (9140–10700)	13200 (12300–14200)	16300 (14900–17600)	19800 (17300–21600)	16100 (13400–18100)	9950 (7860–11400)	4010 (3000–4830)
Gallbladder and biliary diseases	–	–	–	568 (277–887)	325 (206–450)	287 (177–378)	306 (222–368)	521 (389–619)	732 (559–855)	990 (785–1130)	1460 (1170–1680)	1810 (1480–2090)	2460 (2010–2790)	3870 (3240–4450)	5140 (4340–5840)	7440 (6460–8460)	9970 (8700–11400)	12500 (11100–14400)	16400 (14300–18700)	20600 (17500–23200)	19200 (15400–22000)	13200 (9830–15300)	7010 (4790–8570)
Pancreatitis	–	–	–	221 (65·9–397)	131 (95·4–169)	164 (124–202)	722 (585–877)	1570 (1320–1900)	3130 (2700–3810)	4730 (4230–5530)	6170 (5460–7180)	7370 (6450–8760)	9380 (8090–11000)	8340 (7350–9560)	10600 (9310–12100)	9890 (8760–11100)	10800 (9560–12300)	10400 (9110–11700)	9780 (8550–11000)	9490 (8130–10800)	6990 (5790–7990)	3700 (2940–4280)	1460 (1100–1750)
Other digestive diseases	–	–	–	2020 (1300–3160)	609 (445–762)	443 (337–558)	469 (353–590)	609 (468–754)	883 (683–1070)	1100 (874–1280)	1400 (1130–1620)	1940 (1550–2240)	2650 (2140–3090)	3660 (2950–4210)	4920 (4000–5610)	6850 (5570–7840)	8020 (6410–9120)	10100 (8030–11600)	12200 (9570–14300)	15300 (11900–17500)	15100 (11600–17800)	10500 (7520–13400)	5500 (3790–6930)
Neurological disorders	6·66 (5·43–8·18)	14·1 (12·1–16·6)	4970 (4020–6310)	7610 (6090–9550)	4610 (3860–5520)	5820 (4970–6780)	9920 (8730–11400)	10200 (9170–11600)	9920 (8980–11100)	10700 (9680–11900)	11000 (10100–12200)	12900 (11700–15100)	17900 (14600–25500)	25300 (18800–40900)	36300 (24300–63600)	56600 (33800–113000)	95900 (53500–203000)	158000 (86500–327000)	246000 (129000–524000)	443000 (194000–995000)	495000 (188000–1140000)	367000 (122000–867000)	198000 (59200–485000)
Alzheimer's disease and other dementias	–	–	–	–	–	–	–	–	–	–	–	569 (59·1–2060)	3340 (444–10600)	7300 (1200–22600)	14600 (2690–42000)	28800 (5980–85400)	55100 (12200–163000)	93400 (21500–264000)	154000 (35100–437000)	334000 (80800–897000)	415000 (103000–1070000)	334000 (85300–840000)	189000 (48700–477000)
Parkinson's disease	–	–	–	–	–	–	–	12 (10·7–13·2)	17·7 (15·9–19·2)	40·3 (36·4–44·1)	56·6 (51·1–61·6)	646 (583–706)	1520 (1380–1670)	3070 (2800–3370)	4920 (4510–5310)	9670 (8960–10400)	21100 (19600–22600)	45000 (41900–47800)	74500 (69200–79700)	95300 (85000–102000)	70000 (59600–76100)	29100 (23400–32100)	7890 (5940–8980)
Idiopathic epilepsy	–	–	2800 (2110–3870)	5440 (4310–7020)	3320 (2710–4120)	4010 (3390–4830)	7260 (6300–8670)	8030 (7100–9370)	8060 (7160–9200)	8360 (7430–9490)	7760 (6960–8770)	7300 (6540–8290)	6500 (5760–7320)	6420 (5600–7160)	5880 (5030–6570)	5630 (4780–6320)	5770 (4810–6590)	5540 (4590–6320)	4770 (3840–5390)	5050 (3840–5700)	3620 (2620–4100)	1790 (1220–2100)	672 (428–795)
Multiple sclerosis	–	–	–	–	74·3 (4·01–369)	12·4 (3·65–25)	43 (19·4–59·4)	177 (137–224)	332 (282–456)	669 (571–970)	1120 (949–1660)	1600 (1380–2370)	2100 (1830–2970)	2530 (2220–3530)	2900 (2400–3840)	2840 (2230–3670)	2550 (1920–3140)	2090 (1430–2510)	1500 (1060–1880)	1040 (733–1250)	601 (421–729)	216 (150–266)	59·8 (35·7–74·2)
Motor neuron disease	6·66 (5·43–8·18)	14·1 (12·1–16·6)	397 (342–466)	193 (161–234)	69·9 (60·7–80·8)	82·4 (74·1–91·6)	134 (121–148)	136 (121–154)	132 (121–143)	224 (207–243)	405 (378–434)	768 (724–811)	1420 (1340–1500)	2400 (2260–2530)	3710 (3520–3890)	4980 (4740–5210)	6190 (5840–6510)	6310 (5890–6700)	5420 (4910–5810)	3610 (3080–3940)	1770 (1460–1970)	557 (430–629)	154 (115–177)
Other neurological disorders	–	–	1780 (1440–2200)	1970 (1420–2600)	1140 (892–1390)	1710 (1340–2210)	2480 (2090–2980)	1820 (1620–2090)	1380 (1250–1570)	1390 (1270–1550)	1650 (1510–1830)	2060 (1900–2250)	3050 (2790–3390)	3540 (3280–3840)	4350 (4070–4670)	4720 (4440–5050)	5250 (4940–5570)	5400 (5090–5720)	5350 (4950–5710)	4860 (4240–5230)	3350 (2780–3700)	1690 (1340–1880)	665 (500–758)
Mental disorders	–	–	–	–	0·655 (0·465–1·12)	3·97 (2·83–7·67)	21·9 (17·3–33·5)	37·2 (30·7–51·3)	41·8 (35·8–52·9)	50·1 (42·7–63·8)	48·4 (42·4–57·6)	56·6 (50·6–64·6)	57·8 (51·8–64·4)	–	–	–	–	–	–	–	–	–	–
Eating disorders	–	–	–	–	0·655 (0·465–1·12)	3·97 (2·83–7·67)	21·9 (17·3–33·5)	37·2 (30·7–51·3)	41·8 (35·8–52·9)	50·1 (42·7–63·8)	48·4 (42·4–57·6)	56·6 (50·6–64·6)	57·8 (51·8–64·4)	–	–	–	–	–	–	–	–	–	–
Anorexia nervosa	–	–	–	–	0·369 (0·278–0·675)	3·49 (2·56–6·32)	19·3 (15·8–29)	30 (25–42·7)	35·8 (30·5–45·9)	42·3 (36·3–53·8)	41·3 (35·9–49·2)	46·6 (41·8–53·3)	49·4 (44–55·5)	–	–	–	–	–	–	–	–	–	–
Bulimia nervosa	–	–	–	–	0·286 (0·171–0·61)	0·483 (0·233–2·28)	2·6 (1·21–6·81)	7·17 (4·71–12·4)	5·95 (4·31–8·6)	7·72 (4·9–11·2)	7·09 (5·51–9·54)	9·94 (7·01–13·7)	8·38 (5·59–11·3)	–	–	–	–	–	–	–	–	–	–
Substance use disorders	0 (0–0)	0 (0–0)	0 (0–0)	0 (0–0)	0 (0–0)	0 (0–0)	3540 (3300–3780)	11400 (10700–12500)	20000 (18800–21800)	28700 (26500–30900)	31400 (28800–33700)	31900 (29300–33800)	34700 (31600–36900)	32500 (29700–34500)	31700 (29000–33500)	24500 (22300–26000)	17400 (15900–18400)	10800 (9800–11500)	7840 (7040–8340)	4790 (4260–5140)	2970 (2540–3240)	1350 (1090–1510)	535 (405–606)
Alcohol use disorders	–	–	–	–	–	0 (0–0)	721 (561–807)	2200 (1720–2450)	5060 (4220–5490)	10600 (8830–11500)	14800 (12500–15900)	18100 (15500–19600)	22000 (18700–23700)	21500 (18600–23300)	22400 (19800–23900)	18700 (16500–20100)	13200 (11600–14100)	7910 (6830–8510)	5520 (4700–5950)	2910 (2470–3140)	1570 (1310–1720)	562 (441–626)	207 (157–236)
Drug use disorders	0 (0–0)	0 (0–0)	0 (0–0)	0 (0–0)	0 (0–0)	0 (0–0)	2820 (2610–3010)	9240 (8660–10100)	15000 (14100–16400)	18000 (17000–19800)	16600 (15700–18200)	13800 (13100–14500)	12700 (12000–13500)	11000 (10300–11600)	9280 (8630–9940)	5800 (5400–6160)	4240 (3980–4510)	2930 (2740–3130)	2320 (2150–2470)	1880 (1670–2040)	1400 (1160–1570)	791 (627–909)	328 (247–378)
Opioid use disorders	0 (0–0)	0 (0–0)	0 (0–0)	0 (0–0)	0 (0–0)	0 (0–0)	1870 (1710–2020)	6630 (6190–7330)	10800 (10100–12100)	12600 (11700–14000)	11600 (11000–12800)	9500 (9030–10100)	8610 (8050–9230)	7410 (6850–7950)	6260 (5730–6790)	3890 (3590–4180)	2800 (2610–2990)	1910 (1780–2050)	1560 (1450–1680)	1220 (1090–1350)	952 (770–1080)	546 (425–638)	229 (171–266)
Cocaine use disorders	–	–	–	–	–	0 (0–0)	272 (248–298)	744 (678–823)	1170 (1020–1640)	1930 (1690–2750)	1720 (1560–1950)	1450 (1320–1560)	1440 (1260–1570)	1460 (1190–1600)	1220 (998–1350)	672 (560–744)	395 (348–430)	162 (139–180)	107 (92·1–118)	20·1 (17·6–22·6)	12·6 (10·4–14·1)	6·5 (5·21–7·33)	3·04 (2·28–3·49)
Amphetamine use disorders	–	–	–	–	–	0 (0–0)	212 (191–237)	694 (636–764)	1270 (1110–1560)	1390 (1200–1760)	1160 (1050–1290)	938 (848–1060)	828 (722–952)	817 (685–956)	661 (550–774)	413 (344–475)	286 (248–321)	68·9 (54·4–81·8)	50·6 (41·4–59·1)	75·4 (61·9–92)	59·3 (46·5–77)	31·8 (23·7–42·5)	11·2 (8·18–14·6)
Other drug use disorders	–	–	–	–	–	0 (0–0)	464 (426–503)	1180 (1080–1290)	1780 (1620–2010)	2170 (1990–2440)	2100 (1930–2290)	1890 (1750–2030)	1800 (1660–1960)	1270 (1160–1390)	1140 (1040–1250)	818 (755–885)	758 (699–828)	786 (728–850)	600 (548–655)	558 (495–607)	377 (318–416)	206 (164–233)	85·1 (63·4–100)
Diabetes and kidney diseases	37·4 (28·3–48)	96·9 (74–125)	5160 (4170–6420)	6510 (5160–8020)	5350 (4520–6220)	5840 (5100–6660)	12600 (11300–14100)	18200 (16500–20000)	22200 (20300–24100)	31100 (28500–33700)	43100 (39500–46700)	66500 (61400–71600)	99800 (91900–108000)	161000 (150000–173000)	221000 (205000–236000)	294000 (276000–315000)	348000 (328000–369000)	369000 (348000–392000)	382000 (356000–406000)	372000 (332000–399000)	287000 (245000–312000)	164000 (132000–182000)	73100 (54600–82700)
Diabetes mellitus	37·4 (28·3–48)	96·9 (74–125)	1360 (1020–1780)	1210 (888–1650)	1270 (1020–1570)	1410 (1140–1710)	3310 (2940–3750)	4890 (4430–5430)	6600 (6040–7250)	10500 (9700–11400)	15900 (14700–17300)	29500 (27300–32000)	49900 (45900–54100)	86300 (80400–92700)	123000 (115000–131000)	171000 (160000–184000)	201000 (189000–214000)	209000 (197000–223000)	211000 (196000–225000)	196000 (175000–210000)	134000 (114000–145000)	67600 (55000–74700)	25400 (19200–28700)
Diabetes mellitus type 1	37·4 (28·3–48)	96·9 (74–125)	1360 (1020–1780)	1210 (888–1650)	1270 (1020–1570)	1410 (1140–1710)	1770 (1480–2020)	2940 (2580–3300)	3720 (3260–4180)	4010 (3470–4560)	5450 (4750–6270)	8710 (7470–10400)	3200 (2660–4130)	4910 (4030–6370)	6750 (5660–8910)	6290 (5210–8260)	7330 (6120–9590)	5800 (4880–7510)	5500 (4510–7060)	2740 (2270–3580)	2060 (1620–2620)	1170 (883–1500)	500 (344–661)
Diabetes mellitus type 2	–	–	–	–	–	–	1540 (1280–1970)	1950 (1680–2440)	2880 (2500–3490)	6510 (5840–7350)	10500 (9520–11600)	20800 (18900–22800)	46700 (42900–50700)	81300 (75700–87400)	116000 (108000–124000)	165000 (154000–177000)	194000 (183000–205000)	203000 (191000–216000)	206000 (191000–219000)	193000 (173000–208000)	132000 (112000–143000)	66400 (54100–73400)	24900 (18800–28100)
Chronic kidney disease	–	–	3690 (2960–4630)	5130 (4060–6350)	3860 (3240–4500)	4260 (3710–4850)	9170 (8230–10200)	13100 (11800–14500)	15400 (13900–16800)	20300 (18500–22100)	26900 (24500–29200)	36600 (33400–39600)	49400 (45100–53400)	74300 (67600–80400)	97000 (89200–105000)	122000 (113000–132000)	146000 (136000–155000)	159000 (149000–169000)	170000 (157000–181000)	175000 (155000–188000)	153000 (129000–166000)	95900 (76900–107000)	47500 (35400–54100)
Chronic kidney disease due to diabetes mellitus type 1	–	–	21·5 (9·16–44·6)	39·8 (17·8–77·3)	56·5 (20·8–123)	128 (48·8–263)	473 (184–925)	1200 (535–2140)	2250 (1170–3760)	4150 (2240–6180)	6110 (3410–9310)	8350 (4540–13000)	9840 (4620–15900)	10800 (4660–19500)	9390 (3300–18700)	8470 (3290–17500)	7580 (3150–15700)	5930 (2500–11700)	4030 (1610–8710)	2220 (845–4910)	813 (287–1940)	148 (48·2–359)	17·6 (5·02–45·4)
Chronic kidney disease due to diabetes mellitus type 2	–	–	–	–	–	–	251 (75–572)	542 (203–1170)	844 (322–1680)	1520 (547–2950)	2630 (1010–5020)	5130 (2180–9070)	10200 (5170–16000)	20500 (11800–29700)	31200 (19200–42500)	41300 (24100–55500)	49800 (32400–65600)	54000 (36100–69900)	55700 (36700–72900)	53800 (35600–72800)	42600 (27700–59600)	24300 (15000–34900)	11600 (6260–17600)
Chronic kidney disease due to hypertension	–	–	–	–	–	–	617 (322–1080)	1280 (717–2130)	1820 (1080–2920)	2670 (1660–4080)	3970 (2480–5960)	6130 (4150–8740)	9520 (6680–13400)	16500 (11800–22600)	24400 (17500–32800)	34600 (25700–45800)	44700 (34100–57500)	51400 (40000–65200)	59000 (46000–75300)	65700 (50600–82700)	63000 (48400–78400)	42600 (31300–53400)	21900 (15000–29300)
Chronic kidney disease due to glomerulonephritis	–	–	1050 (568–1710)	1770 (985–2830)	1370 (717–2100)	1750 (981–2630)	3790 (2420–5330)	4920 (2980–7160)	5180 (3220–7360)	5730 (3240–8710)	6850 (3900–10800)	8090 (3910–12800)	8790 (5180–13900)	11700 (6460–19000)	14200 (7880–23600)	16200 (8780–26000)	17000 (8870–28700)	17100 (9410–27900)	17900 (9400–29500)	17200 (8550–29500)	12900 (6380–21900)	7100 (3510–12300)	3000 (1320–6070)
Chronic kidney disease due to other and unspecified causes	–	–	2620 (1910–3420)	3320 (2320–4420)	2430 (1670–3210)	2390 (1520–3270)	4040 (2560–5600)	5160 (3160–7270)	5260 (3310–7280)	6220 (3680–9110)	7290 (4140–11100)	8920 (4880–13700)	11000 (5820–17000)	14800 (8380–23100)	17700 (9760–28400)	21700 (12200–35500)	26700 (15100–41100)	30200 (17500–47200)	33100 (19100–51100)	36300 (21200–55100)	33400 (19400–48700)	21800 (13500–32200)	11000 (5780–16600)
Acute glomerulonephritis	–	–	100 (44·8–145)	163 (63·4–240)	223 (101–304)	168 (69·1–238)	112 (69·4–143)	150 (99·5–184)	195 (122–236)	265 (174–314)	281 (185–337)	361 (228–433)	455 (301–553)	585 (389–709)	620 (399–743)	812 (532–974)	1130 (741–1330)	1140 (786–1370)	1220 (808–1460)	1210 (839–1410)	882 (634–1030)	337 (257–395)	111 (81·7–131)
Skin and subcutaneous diseases	–	–	2310 (1390–3080)	1410 (980–1830)	371 (223–499)	458 (295–575)	746 (508–885)	719 (476–837)	688 (484–807)	1280 (872–1500)	1510 (1010–1770)	1880 (1380–2220)	2650 (1930–3180)	3590 (2650–4370)	5090 (3730–6080)	6330 (4570–7750)	7860 (5850–9560)	9470 (6980–11800)	11900 (9260–15200)	13100 (10200–17400)	12600 (9270–17100)	9290 (6280–12400)	5170 (3150–6960)
Bacterial skin diseases	–	–	2270 (1340–3020)	1360 (939–1780)	342 (200–468)	420 (269–532)	682 (463–812)	626 (412–734)	594 (416–701)	1120 (749–1310)	1310 (854–1540)	1620 (1140–1910)	2300 (1620–2770)	3060 (2220–3720)	4350 (3140–5200)	5310 (3770–6450)	6230 (4480–7620)	7050 (5110–8770)	8400 (6420–10800)	8060 (5970–10900)	7360 (4960–10100)	5240 (3190–7340)	2800 (1630–4120)
Cellulitis	–	–	168 (59·4–260)	186 (58·3–363)	29·6 (15·5–40·2)	54·4 (29·7–71·4)	198 (83·1–272)	252 (124–329)	196 (108–255)	348 (210–440)	387 (214–494)	487 (282–630)	700 (401–910)	866 (540–1120)	1220 (740–1530)	1500 (925–1910)	1620 (958–2130)	1830 (1170–2440)	1970 (1190–2890)	2070 (1230–3020)	1840 (1070–2820)	1370 (739–2180)	773 (371–1220)
Pyoderma	–	–	2100 (1270–2810)	1170 (818–1570)	312 (182–434)	366 (234–467)	483 (368–586)	374 (273–468)	398 (294–483)	771 (507–917)	926 (618–1130)	1130 (802–1340)	1600 (1140–1920)	2200 (1600–2700)	3130 (2280–3820)	3810 (2700–4600)	4610 (3390–5600)	5220 (3760–6500)	6430 (5020–8130)	6000 (4580–8150)	5520 (3860–7570)	3870 (2440–5280)	2030 (1210–2850)
Decubitus ulcer	–	–	–	7·87 (3·15–12·7)	7·63 (4·08–10·5)	13·2 (6·6–16·8)	35·3 (15·9–45·3)	44·4 (22·3–58·8)	63 (34·1–80·5)	117 (78·2–151)	139 (91·4–183)	175 (117–226)	242 (163–330)	414 (269–518)	595 (378–744)	832 (534–1090)	1390 (898–1740)	2110 (1420–2660)	3090 (2130–4130)	4480 (3210–5870)	4750 (3280–6280)	3680 (2360–4810)	2190 (1300–2930)
Other skin and subcutaneous diseases	–	–	45·3 (32·2–63)	39 (29·3–54·4)	21·7 (17–27·6)	24·5 (18·2–30·3)	29·3 (22–36·5)	49 (39·3–61)	30·2 (23·7–41·2)	46·6 (37·7–60·5)	57·1 (41·9–75·1)	92·9 (73·8–114)	112 (85·8–139)	117 (86·5–144)	147 (109–185)	189 (143–241)	244 (187–321)	307 (236–407)	389 (298–577)	561 (409–834)	543 (382–849)	379 (239–595)	181 (94·4–290)
Musculoskeletal disorders	–	–	–	–	300 (251–365)	655 (541–798)	1470 (1160–1760)	1940 (1570–2370)	2300 (1800–2800)	2610 (2100–3170)	2780 (2300–3410)	3520 (2870–4240)	3970 (3300–4860)	4630 (3850–5580)	5940 (4910–7230)	8670 (7250–10400)	11700 (9750–13700)	13800 (11200–16300)	14600 (11700–16800)	15500 (11600–17800)	12500 (9170–14400)	7290 (4880–8670)	3300 (1970–4070)
Rheumatoid arthritis	–	–	–	–	32·8 (24·5–41·6)	43·8 (34·6–55·5)	50·1 (37·4–62·3)	59·6 (45·8–72·9)	91·3 (69·6–113)	154 (118–196)	216 (170–276)	398 (307–489)	745 (564–935)	1230 (946–1540)	2050 (1590–2470)	3610 (2810–4350)	5530 (4190–6610)	6920 (5380–8270)	7660 (5870–8850)	7530 (5280–8770)	5080 (3770–5990)	2250 (1540–2730)	758 (486–969)
Other musculoskeletal disorders	–	–	–	–	267 (222–329)	611 (500–743)	1420 (1120–1710)	1880 (1520–2300)	2210 (1730–2670)	2460 (1970–2990)	2570 (2100–3140)	3120 (2550–3760)	3230 (2680–3980)	3400 (2840–4250)	3890 (3230–4860)	5060 (4210–6160)	6220 (5100–7390)	6900 (5560–8300)	6970 (5540–8210)	7980 (5960–9300)	7390 (5210–8580)	5040 (3230–6030)	2540 (1480–3150)
Other non-communicable diseases	176000 (146000–219000)	61800 (51400–76000)	209000 (157000–274000)	94700 (59500–142000)	25500 (21700–30300)	18800 (16000–22000)	17100 (14800–20200)	17400 (15400–19800)	15800 (14200–17800)	15900 (14500–17500)	16600 (15000–18200)	18500 (16400–20300)	20200 (17800–22000)	25700 (22400–27900)	31100 (26300–33700)	38300 (32300–41200)	45800 (39600–49200)	49800 (43300–53200)	55600 (46700–59600)	63500 (52800–68900)	57200 (46200–63000)	40400 (30200–45600)	22100 (15300–25400)
Congenital birth defects	174000 (144000–217000)	57400 (47800–71400)	171000 (130000–229000)	71000 (41200–114000)	16300 (13900–19500)	11400 (9670–13400)	7450 (6410–8820)	6650 (5850–7620)	5950 (5280–6810)	4640 (4130–5170)	4650 (4150–5230)	4660 (4130–5250)	3020 (2590–3340)	2960 (2450–3280)	3090 (2480–3460)	2690 (2110–2990)	2200 (1810–2450)	–	–	–	–	–	–
Neural tube defects	33500 (24600–44800)	6900 (5010–9560)	22700 (15600–34200)	18700 (9660–34000)	1020 (696–1440)	641 (460–878)	310 (222–455)	234 (178–312)	199 (157–261)	170 (137–211)	146 (123–177)	153 (128–189)	99·4 (83·6–119)	83·1 (69·8–99·2)	73·5 (62·1–88·5)	68·2 (58·1–84·1)	75·2 (60–93·3)	–	–	–	–	–	–
Congenital heart anomalies	50200 (38400–65000)	25200 (20200–31700)	74800 (59300–96800)	24200 (16300–37700)	8980 (7330–11100)	6810 (5610–8320)	4410 (3730–5230)	4190 (3640–4850)	3660 (3220–4200)	2770 (2440–3110)	2770 (2460–3130)	2640 (2340–3010)	1500 (1300–1680)	1330 (1130–1500)	1310 (1120–1480)	1100 (930–1230)	986 (839–1110)	–	–	–	–	–	–
Orofacial clefts	1170 (696–2360)	413 (250–784)	1090 (582–2210)	96·9 (64·3–180)	–	–	–	–	–	–	–	–	–	–	–	–	–	–	–	–	–	–	–
Down syndrome	3060 (2310–4430)	1340 (1020–1910)	6820 (4730–11200)	3020 (1580–7270)	623 (493–875)	475 (398–600)	432 (364–517)	466 (401–555)	480 (411–582)	486 (425–573)	515 (447–611)	652 (559–749)	625 (538–699)	873 (688–962)	1060 (767–1170)	890 (619–992)	465 (334–518)	–	–	–	–	–	–
Other chromosomal abnormalities	6980 (4540–11400)	2940 (2060–4750)	6900 (4490–12500)	1500 (702–4000)	430 (317–658)	310 (226–410)	272 (205–329)	238 (182–283)	261 (199–314)	173 (114–198)	191 (129–219)	143 (91·2–170)	105 (60·2–121)	82·8 (40·9–97·4)	83·3 (38·7–100)	62·4 (24·1–78·1)	61·7 (29·7–72·8)	–	–	–	–	–	–
Congenital musculoskeletal and limb anomalies	4630 (3210–6680)	1040 (760–1410)	2770 (1860–4290)	933 (436–2010)	356 (269–456)	264 (202–324)	230 (172–290)	152 (111–184)	142 (105–174)	110 (83–129)	111 (84·4–132)	99·4 (75·7–118)	69·5 (50·9–79·8)	66 (47–75·4)	63·7 (48·1–72·7)	67·5 (51·2–77·1)	64·7 (50·5–74·7)	–	–	–	–	–	–
Urogenital congenital anomalies	5610 (3990–7670)	1190 (903–1560)	1830 (1240–2620)	403 (200–732)	138 (109–173)	113 (93·5–139)	98·7 (68·3–121)	106 (65·8–130)	107 (73·5–128)	92·5 (67·2–109)	89·5 (66·6–107)	113 (81·8–137)	67 (49·8–78·9)	62·1 (49·6–71·4)	60·8 (45–70·7)	59·8 (44·9–68·8)	66·6 (52·5–76·6)	–	–	–	–	–	–
Digestive congenital anomalies	17100 (11100–25100)	6620 (4630–9350)	17800 (11200–29400)	4150 (1560–10200)	442 (310–649)	243 (182–325)	106 (74·9–133)	87·9 (61·3–110)	81·9 (59·2–101)	66 (46·9–79·1)	67·8 (47·6–81·3)	72·7 (49·7–86·8)	62·2 (42·8–72·5)	77·8 (51·8–90)	90·9 (62·4–106)	109 (78·3–127)	135 (92·4–157)	–	–	–	–	–	–
Other congenital birth defects	51600 (37400–78900)	11800 (8860–16600)	36600 (24200–59600)	18000 (8240–36700)	4350 (3430–6240)	2510 (2100–3300)	1590 (1300–2020)	1180 (974–1430)	1030 (853–1270)	778 (655–902)	759 (639–898)	786 (644–917)	491 (392–572)	385 (292–444)	350 (268–405)	336 (262–385)	348 (275–404)	–	–	–	–	–	–
Urinary diseases and male infertility	529 (390–707)	312 (230–412)	2100 (1560–2860)	2170 (1680–2700)	1310 (1020–1580)	1140 (940–1330)	2270 (1910–2610)	3350 (2880–3800)	3010 (2580–3360)	3880 (3390–4300)	4640 (4040–5160)	5840 (5010–6480)	7510 (6290–8330)	11000 (9270–12100)	14200 (11700–15600)	19000 (15600–20900)	25200 (21200–27600)	29500 (25000–32100)	35100 (29200–38200)	42000 (34600–45700)	38600 (30300–42600)	28400 (21000–32200)	15600 (11100–17900)
Urolithiasis	–	–	–	14·4 (8·04–22·2)	24·9 (17·2–37)	25·5 (17·3–34·8)	34·2 (22·2–50·3)	44 (27·8–56·4)	68·8 (44·1–87·3)	117 (78·8–143)	191 (124–232)	294 (188–364)	527 (377–656)	634 (423–772)	856 (600–1040)	1180 (799–1400)	1550 (1140–1850)	1600 (1250–1990)	1860 (1450–2380)	1760 (1400–2250)	1300 (1030–1720)	795 (579–1160)	397 (268–656)
Other urinary diseases	383 (265–522)	142 (93·5–196)	376 (227–572)	869 (612–1170)	351 (263–460)	298 (232–366)	534 (414–652)	421 (286–528)	486 (363–592)	823 (652–1010)	942 (729–1190)	1140 (857–1450)	1680 (1250–2090)	1900 (1260–2390)	2790 (1980–3430)	3690 (2560–4520)	4450 (3120–5530)	5240 (3580–6510)	5270 (3500–6420)	6420 (4360–7670)	4490 (3220–5390)	2790 (1860–3450)	1160 (678–1530)
Gynecological diseases	–	–	–	–	–	86·2 (36·6–137)	223 (88·3–338)	247 (137–396)	301 (189–571)	344 (220–697)	437 (270–802)	440 (247–629)	810 (516–1450)	453 (267–616)	955 (444–1430)	1110 (555–1730)	345 (182–437)	722 (340–1020)	583 (279–818)	860 (373–1230)	436 (247–604)	199 (132–274)	90·2 (59·8–124)
Uterine fibroids	–	–	–	–	–	8 (3·37–14)	28·4 (9·95–44·4)	44·3 (21·1–78·9)	122 (73–253)	119 (62·8–301)	170 (103–372)	182 (121–281)	434 (295–874)	250 (153–378)	624 (294–981)	683 (318–1070)	127 (62·1–181)	416 (182–627)	311 (133–483)	437 (167–694)	152 (75·4–242)	63·5 (39·1–106)	23·4 (13·9–37·1)
Endometriosis	–	–	–	–	–	1·39 (0·644–2·65)	1·94 (1·13–3·13)	3·13 (1·86–5·02)	3·94 (2·57–5·95)	6·8 (4·47–10·8)	39·7 (14·1–74·3)	13·5 (7·32–25·4)	13·3 (8·52–20·2)	5·89 (3·78–8·73)	–	–	–	–	–	–	–	–	–
Genital prolapse	–	–	–	–	–	69·8 (28·1–112)	99·5 (38·9–170)	93·2 (46·1–172)	80·7 (39·2–177)	117 (63·7–296)	95·4 (55·1–233)	40·8 (20·9–78·8)	143 (81·3–320)	50·7 (27·9–93·3)	214 (75·2–427)	279 (120–519)	57·8 (34·9–90·7)	188 (74·4–317)	138 (54·2–223)	203 (79·6–334)	114 (60·2–182)	41 (24·5–66·1)	20 (11·6–30)
Other gynecological diseases	–	–	–	–	–	6·93 (3·1–10·7)	92·9 (36·6–131)	106 (51–161)	94·6 (55·4–162)	101 (51·7–134)	132 (65·3–181)	203 (93·7–279)	221 (93·9–292)	146 (69·9–197)	117 (66·7–170)	148 (84·3–205)	160 (77·2–198)	118 (61·7–148)	134 (64·5–166)	220 (106–279)	170 (96·6–218)	94·4 (60–121)	46·8 (30·2–60·1)
Hemoglobinopathies and hemolytic anemias	660 (510–898)	417 (314–564)	4660 (3040–7020)	17400 (11500–25000)	5870 (4540–7840)	4510 (3530–6080)	5580 (4460–7670)	5090 (4040–6790)	3940 (3100–5280)	3620 (2910–4690)	2740 (2180–3600)	2790 (2230–3740)	2480 (2030–3330)	2970 (2470–3910)	2890 (2450–3870)	3460 (2980–4420)	4120 (3600–5160)	4420 (3880–5370)	4610 (4050–5570)	4470 (3790–5360)	3370 (2750–3940)	1900 (1470–2260)	806 (581–982)
Thalassemias	282 (207–404)	204 (144–302)	1140 (860–1500)	2160 (1710–2690)	1530 (1150–2140)	935 (730–1220)	868 (657–1140)	583 (442–757)	386 (288–507)	383 (275–517)	290 (209–384)	324 (242–421)	342 (257–442)	375 (271–496)	243 (161–345)	126 (74·1–188)	48·1 (26–77·8)	11·1 (5·76–19·7)	2·23 (1·03–4·19)	0·317 (0·119–0·733)	0·0358 (0·011–0·0947)	0·00294 (0·000767–0·00772)	0·000201 (3·99e-05–0·000606)
Sickle cell disorders	352 (223–565)	203 (137–299)	3430 (1840–5560)	15000 (9090–22300)	3990 (2850–5760)	3000 (2120–4380)	3700 (2700–5480)	3390 (2450–4900)	2510 (1770–3630)	2010 (1400–2900)	1410 (949–2110)	1270 (820–1930)	788 (502–1260)	521 (319–842)	224 (146–330)	90·9 (62·8–125)	54·5 (37·3–75·9)	22 (14·4–32·6)	5·77 (3·14–9·61)	1·38 (0·632–2·44)	0·286 (0·113–0·545)	0·0489 (0·0159–0·102)	0·00653 (0·00206–0·0154)
G6PD deficiency	10·8 (6·2–16·8)	6·56 (3·77–10·3)	76·9 (46·5–120)	272 (159–428)	299 (170–447)	474 (287–691)	779 (474–1150)	829 (517–1180)	778 (507–1090)	887 (584–1220)	723 (466–998)	781 (506–1120)	876 (556–1340)	1220 (758–1880)	1170 (732–1950)	1220 (747–1930)	1080 (630–1690)	746 (430–1200)	481 (249–768)	229 (118–379)	80·4 (40·6–135)	20·1 (9·77–34·2)	3·5 (1·42–6·26)
Other hemoglobinopathies and hemolytic anemias	15·7 (11–21·9)	2·87 (1·94–4·27)	20·3 (15·1–27·3)	34·9 (26·1–46·6)	49·6 (38–65·3)	101 (76–134)	233 (168–327)	289 (215–403)	270 (205–358)	342 (260–455)	320 (244–422)	422 (327–545)	472 (368–607)	848 (664–1090)	1250 (999–1610)	2020 (1660–2530)	2940 (2490–3600)	3640 (3160–4380)	4120 (3590–4970)	4230 (3600–5030)	3290 (2690–3850)	1880 (1450–2240)	803 (578–977)
Endocrine, metabolic, blood, and immune disorders	1220 (902–1480)	1010 (746–1230)	6130 (4650–7720)	4120 (3220–5250)	1960 (1630–2420)	1660 (1400–2070)	1610 (1370–1920)	2020 (1740–2420)	2590 (2210–3070)	3430 (2880–4000)	4090 (3360–4720)	4720 (3790–5270)	6370 (5040–7230)	8310 (6640–9280)	9900 (7760–10900)	12000 (9410–13200)	13900 (11200–15300)	15100 (12200–16800)	15300 (11800–17000)	16200 (12200–18000)	14800 (11000–16700)	9830 (6780–11300)	5690 (3520–6710)
Sudden infant death syndrome	–	2620 (1480–4980)	25100 (10500–56100)	–	–	–	–	–	–	–	–	–	–	–	–	–	–	–	–	–	–	–	–
Injuries	12400 (9920–15000)	8220 (5570–11300)	56000 (45200–68800)	132000 (108000–162000)	88900 (76800–102000)	87500 (77400–97700)	210000 (193000–227000)	311000 (289000–333000)	308000 (287000–329000)	307000 (285000–327000)	289000 (265000–309000)	269000 (245000–290000)	266000 (240000–287000)	266000 (237000–287000)	248000 (225000–268000)	236000 (211000–255000)	234000 (206000–254000)	222000 (197000–240000)	214000 (190000–232000)	217000 (188000–237000)	174000 (144000–192000)	99100 (78600–111000)	43600 (32600–49700)
Transport injuries	767 (559–987)	558 (439–698)	7500 (6010–9590)	32000 (25600–40900)	31200 (26600–36900)	27600 (24000–31500)	75400 (66700–83600)	111000 (1e+05–122000)	108000 (96300–117000)	108000 (94600–117000)	102000 (88600–112000)	95500 (81600–105000)	95200 (80000–105000)	95300 (80200–105000)	85300 (73200–94200)	76400 (64300–83600)	69100 (58400–75900)	55100 (47600–60100)	43200 (38200–46700)	32500 (28700–35200)	18400 (15600–20200)	6490 (5270–7200)	2030 (1550–2320)
Road injuries	701 (510–903)	517 (408–648)	7100 (5690–9090)	30100 (24000–39000)	29700 (25300–35100)	25600 (22300–29200)	70000 (62200–77800)	105000 (94000–115000)	1e+05 (90000–110000)	101000 (88900–111000)	95700 (83000–105000)	89300 (76400–98400)	88900 (74900–98700)	89000 (74800–98300)	79700 (68400–87800)	71800 (60500–78500)	65100 (55100–71400)	52000 (45000–56700)	40700 (35900–44000)	30600 (27000–33100)	17400 (14700–19100)	6090 (4940–6750)	1880 (1440–2160)
Pedestrian road injuries	270 (195–358)	188 (141–239)	2630 (2030–3380)	14600 (11500–19000)	14800 (12600–17600)	11200 (9510–13100)	18000 (14800–20500)	26200 (21400–29800)	26900 (22200–30500)	30200 (25100–34500)	30000 (24600–34200)	29100 (23800–33400)	31400 (25500–36200)	34800 (28400–39900)	32100 (26800–36600)	33100 (27800–37500)	31700 (26200–35700)	27400 (23200–30600)	23200 (20300–25600)	18200 (15800–20100)	10300 (8710–11500)	3280 (2650–3670)	991 (760–1130)
Cyclist road injuries	–	–	–	1100 (809–1530)	1340 (1120–1620)	1740 (1480–2040)	2950 (2450–3360)	3200 (2710–3660)	3260 (2760–3710)	4000 (3280–4580)	3820 (3140–4360)	4440 (3610–5140)	5380 (4450–6200)	6250 (5190–7220)	5670 (4670–6580)	5920 (4760–6870)	5780 (4690–6700)	4300 (3500–4940)	2740 (2250–3180)	1660 (1370–1920)	883 (722–1020)	281 (226–323)	2·11 (1·61–2·5)
Motorcyclist road injuries	67·5 (50–89·9)	49·4 (35·2–64·4)	829 (624–1100)	2210 (1680–2950)	2000 (1620–2490)	2810 (2320–3340)	18600 (15800–21100)	29900 (25700–33900)	26500 (22700–29600)	24100 (19900–27100)	21700 (17900–24700)	19500 (15800–22400)	18100 (14200–20700)	16200 (12600–18700)	13500 (10300–15500)	9120 (6660–10600)	7420 (5260–8560)	4300 (3030–4960)	2360 (1750–2690)	1810 (1290–2160)	859 (629–1000)	284 (212–332)	88·3 (65·3–104)
Motor vehicle road injuries	354 (247–456)	271 (211–344)	3540 (2750–4610)	11900 (9040–15700)	11200 (9300–13500)	9430 (8120–11000)	29700 (26000–34200)	44200 (39500–50200)	42900 (38100–48700)	42300 (36600–47300)	39400 (33800–44700)	35500 (29700–40600)	33000 (28000–37700)	30800 (26000–35100)	27600 (23400–31000)	23100 (19600–25900)	19500 (16600–22000)	15500 (13600–17500)	11900 (10600–13400)	8530 (7510–9670)	5140 (4280–5860)	2180 (1760–2490)	773 (585–904)
Other road injuries	8·74 (6·5–12·3)	7·61 (5·65–9·97)	102 (76·5–136)	329 (240–506)	356 (278–456)	363 (279–466)	719 (610–846)	1020 (879–1180)	936 (806–1070)	799 (700–915)	807 (702–928)	796 (674–912)	932 (798–1060)	973 (826–1120)	883 (759–1020)	601 (509–689)	628 (508–740)	546 (453–633)	528 (449–611)	408 (340–481)	227 (187–272)	70 (56·5–85·6)	30·1 (22·6–36·9)
Other transport injuries	65·6 (47·1–85·7)	41·5 (31·5–54·2)	396 (303–503)	1870 (1120–2540)	1550 (1270–1860)	2040 (1690–2400)	5340 (4400–6210)	6840 (5770–7890)	7330 (6160–8300)	6440 (5370–7230)	6640 (5380–7510)	6200 (5080–7040)	6270 (5060–7060)	6330 (5120–7170)	5580 (4440–6400)	4590 (3840–5180)	4040 (3340–4540)	3030 (2540–3400)	2540 (2130–2860)	1920 (1670–2160)	1000 (849–1120)	403 (331–455)	144 (110–169)
Unintentional injuries	7240 (5170–9380)	6120 (3640–9090)	41700 (32700–51900)	87800 (70800–110000)	49800 (41700–58600)	42100 (36200–48100)	49000 (43000–54800)	63600 (56300–70400)	64600 (57100–70600)	71000 (63400–77300)	72800 (64700–79300)	75700 (66600–82900)	80900 (69300–89000)	87300 (74700–96200)	90200 (77800–98600)	99200 (85100–109000)	111000 (93400–123000)	121000 (102000–133000)	133000 (112000–146000)	156000 (130000–172000)	138000 (112000–154000)	86700 (68200–97300)	40000 (29900–45800)
Falls	1220 (655–1730)	479 (266–684)	5040 (3110–6940)	9090 (6270–12100)	6320 (4800–8050)	5630 (4390–6900)	7150 (6110–8310)	11000 (9470–12600)	12300 (10700–14000)	15300 (13000–17300)	17800 (15300–20600)	20600 (17000–23400)	23500 (19200–26700)	29300 (23500–33100)	33900 (28200–37800)	42400 (34100–47800)	53400 (42200–60300)	68200 (54300–76500)	82800 (67600–92200)	107000 (86200–119000)	102000 (80600–115000)	67400 (52800–76000)	31000 (23300–35700)
Drowning	388 (309–495)	182 (145–230)	3870 (3050–4910)	32100 (26400–39600)	20500 (17600–23900)	16200 (14100–18500)	14900 (13300–16700)	14300 (12800–16000)	12100 (10900–13500)	12500 (11200–13800)	11700 (10400–13100)	11100 (9930–12300)	10900 (9760–12100)	11300 (9910–12600)	10100 (8960–11200)	9870 (8780–10800)	10200 (9060–11200)	9640 (8560–10600)	8940 (7920–9840)	8550 (7310–9460)	5500 (4530–6170)	1900 (1480–2160)	521 (383–601)
Fire, heat, and hot substances	303 (197–436)	259 (164–359)	3370 (2230–4910)	10100 (7090–13900)	3440 (2430–4560)	2400 (1680–3110)	4110 (2670–5570)	6020 (4010–8270)	5950 (4220–7890)	6450 (4730–8090)	5990 (4580–7170)	5320 (4140–6190)	5620 (4390–6500)	5620 (4520–6500)	5750 (4760–6590)	6070 (4990–6960)	6280 (5150–7220)	6260 (5190–7330)	6100 (5060–7100)	7010 (5740–8200)	5190 (4220–6050)	2580 (2030–2980)	1080 (809–1280)
Poisonings	295 (213–399)	258 (167–364)	2910 (2140–4190)	7110 (5490–9580)	2280 (1830–2900)	2380 (1950–2800)	2680 (2270–3010)	3700 (3050–4150)	4040 (3390–4480)	4280 (3590–4690)	4300 (3590–4750)	4590 (3870–5070)	4840 (3910–5380)	5240 (4190–5870)	5030 (4080–5610)	4960 (3980–5530)	4720 (3730–5280)	4250 (3240–4800)	3530 (2730–3960)	3140 (2480–3500)	1920 (1530–2150)	581 (465–650)	147 (114–167)
Poisoning by carbon monoxide	60·1 (38·6–88)	49 (29·7–72·7)	485 (292–896)	1540 (951–2720)	858 (629–1150)	995 (691–1250)	1610 (1270–1880)	2200 (1690–2530)	2400 (1880–2710)	2710 (2180–3020)	2690 (2230–3000)	2830 (2350–3150)	2920 (2410–3290)	3000 (2420–3390)	2930 (2410–3310)	2980 (2410–3340)	2740 (2160–3120)	2430 (1860–2800)	2100 (1610–2400)	1920 (1500–2180)	1210 (940–1380)	386 (307–436)	87·1 (65·9–101)
Poisoning by other means	235 (164–314)	209 (133–300)	2430 (1780–3420)	5580 (4310–7300)	1420 (1130–1790)	1390 (1130–1710)	1070 (859–1330)	1500 (1210–1930)	1640 (1320–2120)	1570 (1210–2030)	1600 (1210–2060)	1750 (1290–2190)	1920 (1320–2400)	2240 (1560–2760)	2090 (1510–2610)	1980 (1320–2490)	1970 (1310–2540)	1810 (1160–2370)	1430 (929–1880)	1220 (838–1590)	719 (522–928)	195 (150–247)	60 (45·1–72·6)
Exposure to mechanical forces	933 (298–1640)	2020 (265–4600)	3580 (1370–5910)	4980 (3530–6770)	3400 (2500–4250)	2620 (2010–3180)	5610 (4340–6690)	8490 (6740–9950)	9010 (7210–10300)	9420 (7520–10700)	9560 (7550–10900)	9040 (6970–10400)	9430 (7090–11000)	8900 (6640–10300)	7740 (5910–8880)	6360 (4750–7220)	5590 (4200–6400)	4530 (3470–5090)	3910 (3060–4360)	3360 (2640–3770)	2310 (1850–2590)	1140 (909–1290)	474 (361–546)
Unintentional firearm injuries	126 (24·9–273)	258 (15–734)	600 (192–1160)	939 (604–1380)	518 (372–711)	542 (435–719)	1540 (1200–2030)	2190 (1690–2860)	2070 (1500–2610)	1810 (1330–2270)	1590 (1110–2030)	1440 (1020–1850)	1320 (881–1700)	1010 (694–1270)	833 (587–1070)	727 (499–920)	607 (440–761)	510 (369–624)	500 (376–596)	413 (303–501)	277 (205–330)	167 (125–202)	78·8 (58·2–98·1)
Other exposure to mechanical forces	807 (268–1440)	1760 (248–4010)	2980 (1160–4930)	4040 (2860–5520)	2880 (2070–3630)	2080 (1530–2530)	4070 (2980–4840)	6310 (4720–7380)	6950 (5360–7980)	7610 (5940–8660)	7960 (6180–9090)	7600 (5760–8740)	8110 (5960–9540)	7900 (5770–9230)	6910 (5200–7960)	5630 (4240–6420)	4980 (3690–5710)	4020 (3050–4550)	3410 (2640–3840)	2950 (2330–3340)	2030 (1630–2290)	975 (766–1100)	395 (297–456)
Adverse effects of medical treatment	800 (588–1060)	469 (347–607)	4260 (3080–5600)	5000 (3210–7190)	1620 (1290–2030)	1240 (963–1560)	1430 (1060–1870)	2670 (2070–3230)	2690 (2080–3150)	2720 (2150–3160)	3030 (2410–3440)	3690 (2920–4230)	4240 (3420–4810)	4590 (3790–5200)	6040 (5020–6780)	8750 (7240–9820)	10300 (8570–11500)	10100 (8450–11200)	10000 (8320–11100)	9610 (8020–10700)	7080 (5830–7950)	3880 (3040–4400)	1570 (1170–1810)
Animal contact	321 (187–458)	232 (135–345)	2340 (1350–3670)	6750 (4520–10300)	5770 (3810–8290)	5450 (3480–7330)	3890 (2640–4990)	4220 (2800–5340)	4390 (2880–5400)	4570 (2860–5620)	4410 (2740–5430)	5380 (3400–6600)	6040 (3980–7410)	6130 (4040–7470)	5780 (3970–6900)	5680 (3790–6820)	6620 (4180–8060)	5360 (3470–6470)	4730 (3110–5730)	3210 (2130–3850)	1480 (1040–1780)	543 (395–648)	175 (128–215)
Venomous animal contact	266 (135–382)	183 (93·9–278)	1750 (880–2890)	4520 (2630–8040)	4740 (2890–6990)	4790 (2930–6550)	3410 (2200–4460)	3760 (2440–4830)	3940 (2510–4900)	4120 (2490–5120)	3950 (2270–4920)	4850 (2960–6080)	5370 (3380–6690)	5270 (3280–6590)	4880 (3180–5980)	4850 (3100–5910)	5790 (3490–7170)	4670 (2880–5710)	4140 (2600–5090)	2750 (1720–3340)	1200 (820–1470)	428 (298–520)	129 (92·7–162)
Non-venomous animal contact	55·8 (42·2–75·9)	49·1 (36·8–67·7)	587 (415–821)	2230 (1560–3250)	1020 (767–1390)	653 (495–909)	480 (373–650)	462 (359–610)	448 (356–571)	453 (366–573)	465 (370–599)	534 (414–720)	677 (527–903)	856 (687–1130)	895 (720–1190)	836 (668–1120)	827 (670–1100)	690 (561–904)	590 (483–782)	456 (372–583)	279 (227–343)	115 (90·7–140)	45·4 (34–55·2)
Foreign body	2400 (1980–2890)	1980 (1680–2320)	14100 (12000–16600)	6810 (5670–8210)	2400 (2090–2760)	2100 (1860–2380)	1870 (1690–2090)	2760 (2490–3060)	2900 (2670–3170)	3240 (3000–3510)	3460 (3190–3740)	3820 (3540–4150)	4060 (3730–4420)	4600 (4270–4950)	5140 (4790–5500)	5560 (5220–5920)	5910 (5560–6250)	6290 (5940–6650)	7030 (6500–7430)	8440 (7400–9070)	8620 (7120–9430)	6680 (5200–7520)	4180 (3050–4790)
Pulmonary aspiration and foreign body in airway	2340 (1930–2820)	1960 (1660–2290)	13800 (11700–16200)	5810 (4750–6990)	2180 (1900–2480)	1940 (1710–2200)	1650 (1480–1860)	2510 (2260–2790)	2670 (2460–2920)	3020 (2810–3260)	3210 (2960–3480)	3560 (3300–3860)	3840 (3540–4180)	4390 (4090–4720)	4940 (4600–5290)	5340 (5010–5690)	5690 (5360–6030)	6050 (5710–6390)	6750 (6240–7130)	8170 (7160–8780)	8430 (6960–9210)	6570 (5110–7390)	4130 (3010–4730)
Foreign body in other body part	60·8 (40–84·4)	25·5 (18·5–33·9)	330 (247–443)	1000 (606–1540)	221 (167–283)	165 (127–212)	222 (166–292)	252 (181–339)	235 (174–306)	225 (171–286)	249 (189–320)	265 (201–342)	223 (165–292)	206 (159–260)	198 (151–252)	217 (163–273)	218 (165–271)	238 (182–292)	278 (214–339)	266 (203–340)	192 (144–253)	115 (83·9–164)	51·7 (36·8–75·3)
Environmental heat and cold exposure	135 (39·5–222)	83·4 (22·8–143)	660 (222–1150)	924 (469–1430)	453 (215–683)	330 (178–445)	724 (416–920)	1070 (654–1310)	1260 (855–1470)	1900 (1380–2170)	2380 (1820–2670)	2900 (2220–3300)	3330 (2570–3800)	4010 (3020–4630)	4370 (3270–5080)	4440 (3170–5250)	3860 (2460–4610)	3450 (1940–4320)	3470 (1840–4410)	3410 (1860–4310)	2480 (1530–3010)	1330 (904–1600)	493 (352–586)
Exposure to forces of nature	18·4 (16·7–20·3)	15·4 (14–17)	98·7 (89·6–109)	426 (387–469)	442 (401–486)	535 (486–589)	534 (485–588)	496 (450–545)	470 (427–518)	427 (387–469)	382 (347–420)	351 (319–386)	325 (295–358)	299 (271–329)	260 (236–286)	254 (231–280)	214 (194–235)	184 (167–202)	149 (135–164)	111 (101–123)	57·6 (52·3–63·4)	20·3 (18·4–22·3)	5·75 (5·22–6·33)
Other unintentional injuries	422 (250–593)	134 (101–178)	1480 (1030–1990)	4560 (3460–5910)	3250 (2470–3990)	3210 (2440–3810)	6090 (4730–7010)	8940 (6830–10300)	9560 (7550–10800)	10300 (7830–11600)	9750 (7460–11100)	8890 (6870–10000)	8660 (6320–9880)	7350 (5480–8290)	6110 (4580–6920)	4820 (3580–5400)	4170 (3240–4640)	2610 (2020–2860)	2240 (1830–2440)	1930 (1590–2120)	1350 (1110–1500)	702 (557–786)	293 (219–335)
Self-harm and interpersonal violence	4420 (4040–4850)	1540 (1410–1690)	6710 (5950–7600)	12600 (10500–15000)	7760 (6770–8840)	17800 (15700–19700)	85900 (78900–93100)	137000 (127000–147000)	135000 (126000–145000)	128000 (119000–138000)	114000 (105000–122000)	97900 (90100–106000)	89400 (82200–97200)	82900 (76200–90000)	72900 (66800–78900)	60700 (55600–65700)	54000 (49500–58700)	46000 (41700–50100)	37800 (34000–41100)	29100 (25700–32200)	17600 (15100–19500)	5870 (4870–6550)	1550 (1220–1750)
Self-harm	–	–	–	–	–	8330 (7070–9680)	43500 (39100–48700)	69700 (62800–77900)	73000 (66100–81200)	71200 (63800–79000)	65700 (58700–72000)	59400 (53400–65800)	58000 (51700–63900)	57100 (51400–63400)	52600 (47000–57900)	45300 (40500–49500)	41500 (37200–45600)	36300 (32000–40200)	30400 (26700–33500)	25300 (22100–28200)	15400 (13100–17300)	5070 (4120–5700)	1230 (948–1410)
Self-harm by firearm	–	–	–	–	–	653 (519–770)	2520 (2230–3160)	4250 (3760–5350)	4300 (3790–5350)	4160 (3530–5130)	4020 (3350–4740)	4010 (3410–4750)	4300 (3680–5200)	4330 (3810–5110)	4480 (4030–5270)	3860 (3440–4400)	3320 (2890–3770)	2750 (2410–3020)	2240 (1930–2470)	1770 (1510–1990)	1150 (953–1320)	461 (378–545)	126 (99·2–159)
Self-harm by other specified means	–	–	–	–	–	7670 (6450–8920)	41000 (36700–46000)	65400 (58800–73200)	68700 (61800–76500)	67100 (59800–74000)	61600 (54800–67800)	55400 (49400–61500)	53700 (47500–59500)	52800 (47100–58700)	48100 (42400–53200)	41400 (36700–45600)	38200 (33900–42200)	33500 (29400–37300)	28200 (24500–31200)	23500 (20500–26300)	14300 (12100–16000)	4610 (3730–5200)	1100 (853–1260)
Interpersonal violence	1280 (1020–1570)	375 (290–458)	3100 (2420–3860)	7590 (5520–9910)	4970 (3970–6000)	6920 (5910–7960)	36900 (33400–40800)	59100 (54700–64000)	56400 (52600–61000)	52000 (48400–55700)	44000 (41000–47400)	35400 (32800–38100)	28200 (26200–30600)	22700 (21100–24400)	17700 (16600–19100)	13300 (12400–14200)	10000 (9390–10700)	7220 (6780–7720)	5000 (4620–5360)	1750 (1560–1890)	877 (744–957)	333 (269–369)	203 (155–235)
Physical violence by firearm	202 (155–256)	61·1 (46·3–76·3)	515 (391–650)	1570 (1110–2080)	1020 (855–1210)	2170 (1920–2440)	19500 (17700–21500)	31200 (28800–33800)	27800 (25900–30100)	23700 (21900–25600)	19600 (18100–21500)	15000 (13700–16400)	10800 (9830–12100)	8520 (7750–9390)	6060 (5500–6670)	3880 (3570–4270)	2480 (2270–2710)	1570 (1460–1710)	1060 (970–1160)	372 (323–416)	174 (146–193)	73·6 (57·3–83·8)	65·5 (47·5–79·3)
Physical violence by sharp object	112 (79·8–147)	49·8 (33·7–66·6)	316 (210–431)	1410 (861–1990)	692 (487–862)	1100 (798–1320)	7650 (5870–8870)	13200 (10500–15000)	13800 (11700–15600)	13200 (11100–15100)	11000 (9420–12400)	8790 (7360–9890)	7210 (6000–8070)	5790 (4820–6440)	4250 (3670–4750)	3460 (2940–3880)	2550 (2180–2850)	1750 (1550–1930)	1180 (1060–1300)	516 (448–569)	248 (210–275)	92·4 (74·4–103)	56·8 (42·9–66)
Physical violence by other means	962 (768–1170)	264 (206–325)	2270 (1780–2820)	4610 (3420–5860)	3260 (2580–3990)	3650 (3010–4320)	9760 (8340–11300)	14700 (12900–16800)	14800 (13100–16500)	15100 (13600–16900)	13400 (12000–14800)	11600 (10400–12700)	10100 (9160–11100)	8340 (7650–9180)	7420 (6800–8090)	5910 (5440–6410)	4990 (4620–5400)	3890 (3590–4250)	2760 (2530–3000)	859 (757–946)	454 (386–501)	168 (134–186)	80·6 (61·6–91·9)
Conflict and terrorism	3140 (2850–3460)	1170 (1060–1280)	3580 (3250–3940)	4820 (4380–5310)	2660 (2410–2920)	2380 (2160–2620)	4670 (4240–5140)	6470 (5870–7120)	4770 (4330–5250)	3730 (3390–4100)	2940 (2670–3230)	2400 (2180–2640)	2720 (2470–2990)	2640 (2400–2910)	2140 (1950–2360)	1980 (1800–2180)	2310 (2100–2540)	2380 (2160–2620)	2310 (2100–2540)	1940 (1760–2130)	1280 (1170–1410)	446 (405–491)	112 (102–123)

SR Table 7: Global YLLs by cause and age group for females (with 95% confidence intervals), 2019

Cause	Early Neonatal	Late Neonatal	Post Neonatal	1 to 4	5 to 9	10 to 14	15 to 19	20 to 24	25 to 29	30 to 34	35 to 39	40 to 44	45 to 49	50 to 54	55 to 59	60 to 64	65 to 69	70 to 74	75 to 79	80 to 84	85 to 89	90 to 94	95 plus
All causes	72,300,000 (62 200 000–84 400 000)	20,000,000 (17 200 000–23 500 000)	56,200,000 (47 000 000–67 500 000)	53,200,000 (45 600 000–62 500 000)	13,700,000 (12 500 000–15 100 000)	9,860,000 (9 130 000–10 700 000)	14,100,000 (13 000 000–15 200 000)	17,000,000 (15 500 000–18 500 000)	18,100,000 (16 600 000–19 800 000)	20,200,000 (18 700 000–21 800 000)	22,200,000 (20 500 000–24 000 000)	24,900,000 (23 000 000–26 800 000)	28,400,000 (26 100 000–30 600 000)	35,900,000 (33 000 000–38 900 000)	39,300,000 (36 300 000–42 400 000)	44,800,000 (41 700 000–47 900 000)	48,100,000 (44 900 000–51 300 000)	46,800,000 (43 900 000–49 700 000)	42,200,000 (39 900 000–44 500 000)	39,800,000 (38 100 000–41 700 000)	26,300,000 (25 200 000–27 300 000)	13,600,000 (13 200 000–14 000 000)	5,060,000 (4 990 000–5 140 000)
Communicable, maternal, neonatal, and nutritional diseases	65,000,000 (55 900 000–75 800 000)	17,000,000 (14 600 000–20 100 000)	43,600,000 (35 800 000–53 400 000)	41,200,000 (34 700 000–48 700 000)	7,830,000 (6 850 000–8 840 000)	4,690,000 (4 120 000–5 300 000)	5,840,000 (5 220 000–6 560 000)	7,280,000 (6 560 000–8 110 000)	7,920,000 (7 180 000–8 730 000)	8,300,000 (7 660 000–9 020 000)	7,910,000 (7 300 000–8 570 000)	6,710,000 (6 160 000–7 330 000)	5,410,000 (4 910 000–6 050 000)	4,940,000 (4 370 000–5 780 000)	4,380,000 (3 770 000–5 240 000)	4,440,000 (3 700 000–5 530 000)	4,340,000 (3 570 000–5 580 000)	4,350,000 (3 510 000–5 690 000)	3,760,000 (3 020 000–4 960 000)	3,480,000 (2 760 000–4 610 000)	2,170,000 (1 720 000–2 740 000)	1,140,000 (882 000–1 390 000)	473,000 (353 000–554 000)
HIV/AIDS and sexually transmitted infections	2,020,000 (702 000–4 170 000)	491,000 (167 000–1 020 000)	1,360,000 (902 000–2 050 000)	1,280,000 (1 150 000–1 440 000)	496,000 (458 000–537 000)	544,000 (511 000–578 000)	1,070,000 (967 000–1 190 000)	1,550,000 (1 430 000–1 700 000)	2,500,000 (2 290 000–2 740 000)	3,260,000 (3 070 000–3 490 000)	3,250,000 (3 090 000–3 430 000)	2,730,000 (2 620 000–2 840 000)	2,060,000 (1 970 000–2 150 000)	1,370,000 (1 310 000–1 420 000)	769,000 (735 000–801 000)	408,000 (388 000–427 000)	207,000 (197 000–220 000)	86,800 (81 400–92 900)	38,600 (36 200–40 800)	9,730 (8 790–10 500)	4,590 (4 010–5 060)	1,730 (1 410–1 990)	540 (426–625)
HIV/AIDS	–	–	697,000 (612 000–806 000)	1,250,000 (1 120 000–1 390 000)	479,000 (443 000–516 000)	539,000 (506 000–572 000)	1,050,000 (953 000–1 180 000)	1,530,000 (1 410 000–1 680 000)	2,470,000 (2 260 000–2 710 000)	3,240,000 (3 040 000–3 460 000)	3,230,000 (3 070 000–3 400 000)	2,700,000 (2 600 000–2 820 000)	2,050,000 (1 960 000–2 130 000)	1,350,000 (1 290 000–1 400 000)	750,000 (717 000–780 000)	396,000 (377 000–416 000)	197,000 (187 000–209 000)	79,900 (75 000–85 900)	33,300 (31 200–35 400)	6,010 (5 600–6 510)	2,270 (2 110–2 450)	593 (553–637)	137 (130–146)
HIV/AIDS - Drug-susceptible Tuberculosis	–	–	175,000 (128 000–223 000)	332,000 (247 000–408 000)	130,000 (99 600–158 000)	149,000 (113 000–182 000)	265,000 (190 000–342 000)	378,000 (267 000–483 000)	578,000 (404 000–753 000)	749,000 (526 000–969 000)	749,000 (532 000–973 000)	638,000 (455 000–827 000)	475,000 (336 000–620 000)	318,000 (226 000–415 000)	176,000 (126 000–232 000)	92,700 (66 100–122 000)	44,500 (32 000–58 500)	19,300 (13 700–25 100)	8,240 (5 800–10 700)	1,320 (936–1 710)	464 (327–616)	116 (80·7–155)	24·8 (17·5–32·9)
HIV/AIDS - Multidrug-resistant Tuberculosis without extensive drug resistance	–	–	21,300 (9 120–39 500)	39,000 (15 500–75 200)	14,500 (6 100–27 300)	15,200 (6 730–28 600)	28,900 (12 300–55 900)	40,200 (17 100–77 200)	57,100 (24 700–111 000)	71,100 (31 400–135 000)	70,100 (31 000–133 000)	59,200 (26 300–111 000)	43,700 (19 300–82 300)	29,600 (13 000–55 400)	16,400 (7 160–31 100)	8,590 (3 760–16 400)	4,120 (1 780–7 810)	1,780 (764–3 380)	754 (324–1 430)	126 (53–239)	43·7 (18–85·3)	10·9 (4·47–21·3)	2·21 (0·94–4·33)
HIV/AIDS - Extensively drug-resistant Tuberculosis	–	–	573 (255–1 060)	700 (286–1 360)	309 (139–598)	308 (135–586)	613 (260–1 150)	987 (425–1 840)	1,840 (832–3 470)	2,740 (1 290–5 030)	2,700 (1 280–4 950)	2,150 (1 020–3 910)	1,500 (710–2 760)	944 (440–1 750)	529 (243–983)	269 (121–520)	135 (58·2–273)	43·9 (18–90)	17·4 (7·03–36·2)	4·07 (1·52–8·85)	1·55 (0·591–3·31)	0·402 (0·157–0·864)	0·119 (0·0456–0·249)
HIV/AIDS resulting in other diseases	–	–	500,000 (427 000–606 000)	875,000 (755 000–1 050 000)	334,000 (296 000–377 000)	375,000 (334 000–422 000)	760,000 (654 000–898 000)	1,110,000 (964 000–1 290 000)	1,840,000 (1 580 000–2 120 000)	2,420,000 (2 120 000–2 750 000)	2,400,000 (2 130 000–2 700 000)	2,000,000 (1 780 000–2 240 000)	1,520,000 (1 360 000–1 700 000)	1,000,000 (886 000–1 110 000)	556,000 (491 000–621 000)	295,000 (259 000–330 000)	148,000 (131 000–166 000)	58,800 (51 600–66 800)	24,200 (21 200–27 600)	4,560 (4 010–5 170)	1,760 (1 550–1 980)	465 (410–524)	110 (99·3–122)
Sexually transmitted infections excluding HIV	2,020,000 (702 000–4 170 000)	491,000 (167 000–1 020 000)	666,000 (221 000–1 410 000)	35,600 (11 900–77 200)	17,000 (5 340–37 400)	5,360 (4 040–6 680)	14,800 (11 500–18 600)	22,400 (17 400–27 400)	27,800 (20 500–34 600)	26,000 (19 600–31 700)	21,800 (16 600–26 400)	23,300 (17 600–29 300)	18,400 (14 800–21 400)	19,600 (14 200–25 200)	19,500 (13 800–25 800)	11,200 (8 390–14 000)	10,600 (8 200–13 100)	6,880 (5 500–8 170)	5,350 (4 430–6 230)	3,720 (2 920–4 360)	2,320 (1 730–2 750)	1,140 (827–1 400)	403 (290–486)
Syphilis	2,020,000 (702 000–4 170 000)	491,000 (167 000–1 020 000)	666,000 (221 000–1 410 000)	35,600 (11 900–77 200)	17,000 (5 340–37 400)	3,010 (2 260–3 740)	3,110 (2 390–3 960)	1,840 (1 470–2 190)	5,020 (3 630–6 290)	5,360 (3 920–6 620)	2,670 (1 960–3 280)	970 (706–1 240)	1,450 (1 120–1 720)	1,870 (1 310–2 450)	647 (442–869)	603 (430–762)	733 (546–930)	671 (514–824)	421 (334–502)	534 (389–656)	290 (194–361)	191 (117–260)	55·4 (32·2–75·6)
Chlamydial infection	–	–	–	–	–	534 (406–660)	1,530 (1 190–1 920)	4,910 (3 810–6 000)	4,230 (3 180–5 200)	1,900 (1 400–2 350)	3,050 (2 330–3 690)	3,780 (2 860–4 740)	3,000 (2 420–3 490)	2,870 (2 090–3 680)	3,170 (2 240–4 190)	1,850 (1 390–2 300)	1,120 (869–1 390)	1,010 (808–1 190)	835 (695–969)	741 (585–865)	426 (326–500)	205 (152–247)	81·2 (58·6–97·3)
Gonococcal infection	–	–	–	–	–	860 (651–1 070)	4,410 (3 430–5 540)	8,010 (6 220–9 800)	10,700 (7 850–13 300)	10,000 (7 670–12 100)	8,050 (6 200–9 720)	10,200 (7 750–12 800)	7,690 (6 250–8 960)	8,790 (6 390–11 300)	8,960 (6 340–11 800)	4,930 (3 760–6 070)	6,100 (4 760–7 530)	3,470 (2 790–4 100)	3,010 (2 510–3 490)	1,770 (1 420–2 060)	1,120 (865–1 320)	542 (404–652)	217 (157–255)
Trichomoniasis	–	–	–	–	–	–	–	–	–	–	–	–	–	–	–	–	–	–	–	–	–	–	–
Genital herpes	–	–	–	–	–	–	–	–	–	–	–	–	–	–	–	–	–	–	–	–	–	–	–
Other sexually transmitted infections	–	–	–	–	–	964 (720–1 200)	5,700 (4 440–7 180)	7,660 (5 920–9 390)	7,840 (5 800–9 720)	8,720 (6 580–10 600)	8,000 (6 110–9 690)	8,320 (6 240–10 500)	6,230 (5 030–7 260)	6,090 (4 400–7 820)	6,740 (4 740–8 920)	3,840 (2 810–4 820)	2,620 (2 020–3 250)	1,740 (1 390–2 060)	1,080 (891–1 260)	671 (529–784)	482 (359–572)	199 (146–243)	49·4 (35·4–60·4)
Respiratory infections and tuberculosis	4,590,000 (3 660 000–5 590 000)	3,440,000 (2 830 000–4 140 000)	14,700,000 (11 900 000–18 200 000)	8,450,000 (6 590 000–10 600 000)	1,480,000 [illegible]	844,000 [illegible]	1,010,000 [illegible]	1,310,000 [illegible]	1,460,000 [illegible]	1,510,000 [illegible]	1,590,000 [illegible]	1,590,000 [illegible]	1,560,000 [illegible]	1,990,000 [illegible]	2,000,000 [illegible]	2,170,000 [illegible]	2,240,000 [illegible]	2,280,000 [illegible]	2,060,000 [illegible]	1,950,000 [illegible]	1,350,000 [illegible]	780,000 [illegible]	349,000 [illegible]

Anorexia nervosa	–	–	–	–
Bulimia nervosa	–	–	–	–
Autism spectrum disorders	–	–	–	–
Attention-deficit/hyperactivity disorder	–	–	–	–
Conduct disorder	–	–	–	–
Idiopathic developmental intellectual disability	–	–	–	–
Other mental disorders	–	–	–	–
Substance use disorders	0 (0–0)	0 (0–0)	0 (0–0)	0 (0–0)
Alcohol use disorders	–	–	–	–
Drug use disorders	0 (0–0)	0 (0–0)	0 (0–0)	0 (0–0)
Opioid use disorders	0 (0–0)	0 (0–0)	0 (0–0)	0 (0–0)
Cocaine use disorders	–	–	–	–
Amphetamine use disorders	–	–	–	–
Cannabis use disorders	–	–	–	–
Other drug use disorders	–	–	–	–
Diabetes and kidney diseases	751 (538–955)	2,110 (1 500–2 830)	202,000 (163 000–250 000)	279,000 (219 000–342 000)
Diabetes mellitus	751 (538–955)	2,110 (1 500–2 830)	48,200 (34 100–64 300)	49,100 (35 200–66 600)
Diabetes mellitus type 1	751 (538–955)	2,110 (1 500–2 830)	48,200 (34 100–64 300)	49,100 (35 200–66 600)
Diabetes mellitus type 2	–	–	–	–
Chronic kidney disease	–	–	150,000 (120 000–187 000)	221,000 (174 000–271 000)
Chronic kidney disease due to diabetes mellitus type 1	–	–	898 (367–1 910)	1,750 (784–3 460)
Chronic kidney disease due to diabetes mellitus type 2	–	–	–	–
Chronic kidney disease due to hypertension	–	–	–	–
Chronic kidney disease due to glomerulonephritis	–	–	45,300 (24 900–73 200)	76,700 (43 200–122 000)
Chronic kidney disease due to other and unspecified causes	–	–	104,000 (74 700–136 000)	143,000 (96 900–189 000)
Acute glomerulonephritis	–	–	3,660 (1 290–6 100)	8,170 (3 120–13 400)
Skin and subcutaneous diseases	–	–	139,000 (84 500–191 000)	68,400 (45 900–93 500)
Dermatitis	–	–	–	–
Atopic dermatitis	–	–	–	–
Contact dermatitis	–	–	–	–
Seborrhoeic dermatitis	–	–	–	–
Psoriasis	–	–	–	–
Bacterial skin diseases	–	–	137,000 (83 000–187 000)	66,600 (44 600–91 500)
Cellulitis	–	–	7,750 (2 390–13 500)	11,700 (3 500–24 900)
Pyoderma	–	–	129,000 (78 100–180 000)	54,900 (37 400–77 800)
Scabies	–	–	–	–
Fungal skin diseases	–	–	–	–
Viral skin diseases	–	–	–	–
Acne vulgaris	–	–	–	–
Alopecia areata	–	–	–	–
Pruritus	–	–	–	–
Urticaria	–	–	–	–
Decubitus ulcer	–	–	–	339 (127–544)
Other skin and subcutaneous diseases	–	–	2,360 (1 450–3 690)	1,480 (1 070–2 040)
Sense organ diseases	–	–	–	–
Blindness and vision loss	–	–	–	–
Glaucoma	–	–	–	–
Cataract	–	–	–	–
Age-related macular degeneration	–	–	–	–
Refraction disorders	–	–	–	–
Near vision loss	–	–	–	–
Other vision loss	–	–	–	–
Age-related and other hearing loss	–	–	–	–
Other sense organ diseases	–	–	–	–
Musculoskeletal disorders	–	–	–	–
Rheumatoid arthritis	–	–	–	–
Osteoarthritis	–	–	–	–
Osteoarthritis hip	–	–	–	–
Osteoarthritis knee	–	–	–	–
Osteoarthritis hand	–	–	–	–
Osteoarthritis other	–	–	–	–
Low back pain	–	–	–	–
Neck pain	–	–	–	–
Gout	–	–	–	–
Other musculoskeletal disorders	–	–	–	–
Other non-communicable diseases	6,550,000 (5 440 000–7 980 000)	2,460,000 (2 030 000–3 030 000)	8,370,000 (6 190 000–11 300 000)	3,870,000 (2 430 000–6 060 000)
Congenital birth defects	6,470,000 (5 370 000–7 910 000)	2,290,000 (1 910 000–2 840 000)	6,910,000 (5 180 000–9 430 000)	2,870,000 (1 690 000–4 680 000)
Neural tube defects	1,440,000 (994 000–2 020 000)	297,000 (190 000–462 000)	1,120,000 (696 000–1 880 000)	829,000 (403 000–1 620 000)
Congenital heart anomalies	1,770,000 (1 340 000–2 280 000)	979,000 (759 000–1 260 000)	2,880,000 (2 260 000–3 760 000)	959,000 (641 000–1 490 000)
Orofacial clefts	50,800 (27 400–109 000)	20,400 (10 500–46 200)	44,500 (20 200–120 000)	4,350 (2 620–11 200)
Down syndrome	130,000 (88 900–206 000)	57,100 (40 300–88 300)	290,000 (178 000–517 000)	144,000 (59 000–386 000)
Turner syndrome	–	–	–	–
Klinefelter syndrome	–	–	–	–
Other chromosomal abnormalities	277,000 (176 000–488 000)	137,000 (92 700–235 000)	325,000 (207 000–697 000)	56,800 (29 600–191 000)
Congenital musculoskeletal and limb anomalies	168,000 (104 000–254 000)	40,600 (26 600–57 600)	109,000 (61 700–194 000)	36,300 (14 500–91 900)
Urogenital congenital anomalies	177,000 (100 000–271 000)	42,800 (28 400–59 100)	63,200 (35 800–102 000)	12,300 (5 950–26 000)
Digestive congenital anomalies	672,000 (418 000–1 040 000)	271,000 (170 000–397 000)	746,000 (415 000–1 390 000)	194,000 (62 800–587 000)
Other congenital birth defects	1,780,000 (1 220 000–2 720 000)	448,000 (316 000–659 000)	1,320,000 (834 000–2 370 000)	631,000 (279 000–1 600 000)
Urinary diseases and male infertility	14,200 (9 880–18 600)	9,520 (6 600–12 600)	68,000 (48 100–97 300)	99,100 (73 700–127 000)
Urolithiasis	–	–	–	633 (302–1 140)
Benign prostatic hyperplasia	–	–	–	–
Male infertility	–	–	–	–
Other urinary diseases	9,390 (6 020–12 800)	3,760 (2 360–5 260)	9,780 (5 560–15 900)	30,900 (17 300–47 600)
Gynecological diseases	–	–	–	–
Uterine fibroids	–	–	–	–
Polycystic ovarian syndrome	–	–	–	–
Female infertility	–	–	–	–
Endometriosis	–	–	–	–
Genital prolapse	–	–	–	–
Premenstrual syndrome	–	–	–	–
Other gynecological diseases	–	–	–	–
Hemoglobinopathies and hemolytic anemias	21,500 (17 600–26 200)	14,800 (11 400–20 000)	185,000 (125 000–254 000)	752,000 (435 000–1 170 000)
Thalassemias	9,950 (6 790–14 300)	7,680 (4 880–12 000)	46,500 (34 200–63 600)	78,400 (59 800–99 100)
Thalassemias trait	–	–	–	–
Sickle cell disorders	10,700 (6 860–14 800)	6,840 (4 640–9 250)	136,000 (81 200–199 000)	666,000 (363 000–1 060 000)
Sickle cell trait	–	–	–	–
G6PD deficiency	204 (118–303)	129 (74·8–199)	1,680 (1 030–2 570)	6,280 (3 450–10 300)
G6PD trait	–	–	–	–
Other hemoglobinopathies and hemolytic anemias	594 (415–827)	110 (71·2–167)	747 (542–1 000)	1,660 (1 180–2 260)
Endocrine, metabolic, blood, and immune disorders	46,800 (33 100–58 700)	37,900 (27 800–47 800)	227,000 (172 000–289 000)	157,000 (121 000–200 000)
Oral disorders	–	–	–	–
Caries of deciduous teeth	–	–	–	–
Caries of permanent teeth	–	–	–	–
Periodontal diseases	–	–	–	–
Other oral disorders	–	–	–	–
Sudden infant death syndrome	–	105,000 (54 000–228 000)	987,000 (385 000–2 430 000)	–
Injuries	505,000 (401 000–615 000)	356,000 (245 000–500 000)	2,380,000 (1 930 000–2 910 000)	5,000,000 (4 090 000–6 060 000)
Transport injuries	22,100 (17 900–28 500)	22,700 (18 200–28 400)	311,000 (250 000–389 000)	1,200,000 (963 000–1 500 000)
Road injuries	20,600 (16 700–26 800)	21,400 (17 200–26 800)	296,000 (238 000–372 000)	1,130,000 (911 000–1 420 000)
Pedestrian road injuries	8,710 (6 810–11 800)	7,470 (5 970–9 570)	111,000 (85 300–144 000)	581,000 (452 000–762 000)
Cyclist road injuries	–	–	–	28,700 (22 500–36 800)
Motorcyclist road injuries	1,840 (1 460–2 530)	1,710 (1 260–2 290)	34,200 (25 200–47 100)	67,100 (50 200–95 700)
Motor vehicle road injuries	9,780 (7 470–12 400)	12,000 (9 570–15 100)	146,000 (113 000–187 000)	444,000 (335 000–584 000)
Other road injuries	303 (230–406)	229 (168–316)	3,870 (2 710–5 360)	11,300 (8 020–15 500)
Other transport injuries	1,420 (1 120–1 860)	1,280 (966–1 670)	15,100 (11 100–19 900)	65,600 (38 700–92 600)
Unintentional injuries	309,000 (219 000–403 000)	268,000 (162 000–408 000)	1,780,000 (1 390 000–2 220 000)	3,270,000 (2 620 000–4 010 000)
Falls	60,800 (25 900–89 400)	22,900 (10 700–34 200)	241,000 (129 000–351 000)	335,000 (207 000–455 000)
Drowning	14,000 (11 700–17 300)	7,870 (6 450–9 760)	168,000 (134 000–215 000)	1,110,000 (920 000–1 350 000)
Fire, heat, and hot substances	9,580 (6 060–13 800)	11,900 (6 640–17 500)	150,000 (89 100–228 000)	436,000 (279 000–639 000)
Poisonings	9,140 (7 110–12 000)	7,990 (6 000–10 100)	117,000 (85 200–162 000)	287,000 (219 000–375 000)
Poisoning by carbon monoxide	2,460 (1 590–3 710)	1,740 (1 170–2 400)	21,600 (12 400–43 400)	66,500 (37 400–127 000)
Poisoning by other means	6,690 (4 990–8 670)	6,250 (4 640–8 040)	95,800 (68 400–128 000)	221,000 (164 000–285 000)
Exposure to mechanical forces	44,900 (11 600–91 200)	88,000 (10 900–216 000)	165,000 (58 700–285 000)	185,000 (126 000–254 000)
Unintentional firearm injuries	6,930 (1 150–18 500)	11,200 (541–38 000)	28,900 (8 070–63 000)	37,000 (22 600–59 600)
Other exposure to mechanical forces	37,900 (10 200–80 000)	76,800 (10 300–189 000)	136,000 (49 500–234 000)	148,000 (100 000–205 000)
Adverse effects of medical treatment	34,900 (23 200–46 300)	22,400 (15 600–29 800)	190,000 (123 000–259 000)	196,000 (108 000–293 000)
Animal contact	10,500 (5 540–15 500)	8,440 (3 980–13 700)	118,000 (57 600–197 000)	309,000 (175 000–495 000)
Venomous animal contact	8,610 (4 100–13 000)	6,750 (2 830–11 200)	95,100 (41 000–164 000)	232,000 (122 000–408 000)
Non-venomous animal contact	1,890 (1 320–2 750)	1,690 (1 120–2 520)	22,900 (14 500–37 500)	77,900 (46 300–137 000)
Foreign body	108,000 (89 500–133 000)	90,500 (76 200–107 000)	554,000 (469 000–651 000)	233,000 (195 000–277 000)
Pulmonary aspiration and foreign body in airway	107,000 (88 600–132 000)	89,800 (75 600–106 000)	546,000 (462 000–643 000)	208,000 (172 000–248 000)
Foreign body in eyes	–	–	–	–
Foreign body in other body part	1,090 (733–1 490)	734 (514–1 040)	8,310 (5 790–12 000)	25,100 (12 700–42 400)
Environmental heat and cold exposure	4,930 (1 210–9 410)	3,110 (716–5 870)	28,000 (7 220–53 800)	33,000 (12 900–53 600)
Exposure to forces of nature	638 (579–702)	627 (569–690)	4,160 (3 780–4 580)	18,200 (16 500–20 000)
Other unintentional injuries	11,200 (9 050–14 000)	4,410 (3 560–5 490)	44,300 (35 000–55 700)	132,000 (103 000–167 000)
Self-harm and interpersonal violence	174,000 (159 000–193 000)	64,700 (58 900–71 400)	292,000 (257 000–330 000)	535,000 (442 000–639 000)
Self-harm	–	–	–	–
Self-harm by firearm	–	–	–	–
Self-harm by other specified means	–	–	–	–
Interpersonal violence	55,900 (43 200–69 100)	16,700 (12 500–21 100)	121,000 (92 000–153 000)	303,000 (215 000–405 000)
Physical violence by firearm	6,230 (4 720–7 970)	2,010 (1 500–2 610)	17,500 (13 000–22 900)	56,900 (40 100–78 200)
Physical violence by sharp object	3,770 (2 960–4 860)	2,410 (1 690–3 420)	12,400 (8 810–17 500)	56,800 (35 900–83 800)
Sexual violence	–	–	–	–
Physical violence by other means	45,900 (35 600–57 100)	12,300 (9 210–15 400)	91,000 (70 000–113 000)	190,000 (137 000–248 000)
Conflict and terrorism	118,000 (108 000–130 000)	48,000 (43 600–52 800)	170,000 (154 000–187 000)	223,000 (203 000–246 000)

SR Table 8: Global YLLs by cause and age group for males (with 95% confidence intervals), 2019

Cause	Early Neonatal	Late Neonatal	Post Neonatal	1 to 4	5 to 9	10 to 14	15 to 19	20 to 24	25 to 29	30 to 34	35 to 39	40 to 44	45 to 49	50 to 54	55 to 59	60 to 64	65 to 69	70 to 74	75 to 79	80 to 84	85 to 89	90 to 94	95 plus
All causes	97,400,000 (82 100 000–116 000 000)	22,800,000 (19 200 000–27 100 000)	60,800,000 (50 000 000–74 600 000)	57,500,000 (47 900 000–69 400 000)	17,100,000 (15 500 000–19 100 000)	13,000,000 (12 000 000–14 000 000)	21,600,000 (20 100 000–23 200 000)	29,100,000 (27 300 000–31 000 000)	30,500,000 (28 900 000–32 300 000)	35,000,000 (33 100 000–37 000 000)	38,400,000 (36 100 000–40 800 000)	42,500,000 (39 600 000–45 500 000)	49,700,000 (45 900 000–53 400 000)	58,800,000 (54 100 000–63 400 000)	65,600,000 (60 600 000–70 500 000)	70,300,000 (65 300 000–75 200 000)	70,000,000 (65 100 000–74 800 000)	62,600,000 (58 400 000–66 800 000)	49,500,000 (46 500 000–52 400 000)	38,300,000 (36 300 000–40 200 000)	21,700,000 (20 900 000–22 300 000)	7,500,000 (7 330 000–7 650 000)	1,940,000 (1 920 000–1 970 000)
Communicable, maternal, neonatal, and nutritional diseases	87,600,000 (73 900 000–104 000 000)	19,200,000 (16 200 000–23 000 000)	46,000,000 (37 400 000–57 500 000)	42,800,000 (35 100 000–52 400 000)	8,800,000 (7 570 000–10 300 000)	5,250,000 (4 450 000–6 130 000)	5,310,000 (4 580 000–6 210 000)	5,490,000 (4 870 000–6 310 000)	6,130,000 (5 590 000–6 850 000)	7,510,000 (6 980 000–8 230 000)	8,550,000 (7 930 000–9 280 000)	8,400,000 (7 760 000–9 180 000)	8,040,000 (7 380 000–8 840 000)	7,180,000 (6 550 000–8 020 000)	6,550,000 (5 930 000–7 400 000)	6,060,000 (5 490 000–6 920 000)	5,510,000 (4 950 000–6 420 000)	4,990,000 (4 480 000–5 940 000)	3,930,000 (3 530 000–4 610 000)	3,110,000 (2 730 000–3 650 000)	1,850,000 (1 600 000–2 140 000)	761,000 (628 000–880 000)	230,000 (178 000–266 000)
HIV/AIDS and sexually transmitted infections	2,230,000 (747 000–4 650 000)	671,000 (228 000–1 390 000)	1,730,000 (1 050 000–2 800 000)	1,330,000 (1 190 000–1 510 000)	506,000 (469 000–544 000)	578,000 (551 000–607 000)	921,000 (846 000–999 000)	793,000 (741 000–864 000)	1,500,000 (1 370 000–1 650 000)	2,600,000 (2 410 000–2 800 000)	3,310,000 (3 110 000–3 530 000)	3,140,000 (3 000 000–3 300 000)	2,560,000 (2 440 000–2 680 000)	1,680,000 (1 610 000–1 750 000)	920,000 (880 000–965 000)	473,000 (451 000–503 000)	249,000 (236 000–268 000)	102,000 (95 100–112 000)	44,500 (41 500–48 200)	11,900 (10 800–13 000)	4,040 (3 570–4 620)	1,010 (764–1 410)	303 (207–433)
HIV/AIDS	–	–	729,000 (639 000–845 000)	1,290,000 (1 160 000–1 460 000)	489,000 (456 000–523 000)	573,000 (546 000–602 000)	917,000 (842 000–995 000)	790,000 (737 000–860 000)	1,490,000 (1 360 000–1 650 000)	2,590,000 (2 410 000–2 800 000)	3,300,000 (3 110 000–3 530 000)	3,140,000 (3 000 000–3 290 000)	2,560,000 (2 440 000–2 680 000)	1,670,000 (1 600 000–1 750 000)	913,000 (874 000–959 000)	469,000 (446 000–498 000)	247,000 (234 000–266 000)	101,000 (93 400–111 000)	43,000 (40 200–46 600)	11,000 (10 000–12 200)	3,350 (3 050–3 660)	648 (599–710)	150 (142–161)
HIV/AIDS - Drug-susceptible Tuberculosis	–	–	180,000 (131 000–230 000)	341,000 (251 000–421 000)	134,000 (103 000–162 000)	160,000 (121 000–197 000)	231,000 (167 000–297 000)	180,000 (130 000–230 000)	310,000 (223 000–403 000)	519,000 (367 000–679 000)	675,000 (476 000–879 000)	680,000 (486 000–880 000)	560,000 (402 000–727 000)	375,000 (273 000–485 000)	203,000 (147 000–263 000)	101,000 (74 000–130 000)	48,100 (35 100–62 300)	19,600 (14 200–25 400)	8,220 (5 860–10 700)	1,630 (1 150–2 160)	503 (344–681)	99·9 (67·2–138)	21·6 (14·7–29·6)
HIV/AIDS - Multidrug-resistant Tuberculosis without extensive drug resistance	–	–	21,900 (9 420–40 400)	40,100 (16 000–77 100)	14,300 (6 240–26 600)	15,900 (7 090–30 600)	24,500 (10 500–46 600)	19,200 (8 390–35 200)	32,800 (14 300–63 000)	55,200 (24 500–105 000)	69,300 (31 500–132 000)	67,300 (30 200–128 000)	54,000 (24 000–102 000)	36,200 (16 100–67 100)	19,400 (8 540–36 400)	9,650 (4 260–18 100)	4,590 (2 020–8 650)	1,900 (836–3 670)	793 (343–1 530)	162 (65·7–331)	49·5 (19·5–102)	9·72 (3·96–19)	1·97 (0·819–3·79)
HIV/AIDS - Extensively drug-resistant Tuberculosis	–	–	637 (283–1 190)	729 (300–1 420)	337 (148–637)	340 (149–640)	565 (244–1 090)	831 (354–1 620)	2,130 (969–4 040)	4,160 (1 980–7 620)	4,990 (2 390–9 040)	3,910 (1 830–7 280)	2,480 (1 130–4 710)	1,490 (678–2 840)	775 (348–1 520)	397 (175–787)	219 (88·8–454)	74·1 (28·1–162)	31·1 (11·1–69·2)	9·97 (2·85–25·3)	3·13 (0·95–7·76)	0·517 (0·199–1·1)	0·173 (0·0708–0·348)
HIV/AIDS resulting in other diseases	–	–	527,000 (451 000–637 000)	913,000 (782 000–1 100 000)	340,000 (304 000–383 000)	397,000 (355 000–445 000)	660,000 (576 000–764 000)	590,000 (522 000–679 000)	1,150,000 (998 000–1 330 000)	2,010,000 (1 760 000–2 280 000)	2,550,000 (2 270 000–2 870 000)	2,390,000 (2 120 000–2 660 000)	1,940,000 (1 730 000–2 150 000)	1,260,000 (1 130 000–1 390 000)	690,000 (619 000–763 000)	358,000 (321 000–396 000)	194,000 (175 000–215 000)	79,000 (70 200–89 900)	34,000 (30 200–38 100)	9,240 (8 080–10 400)	2,790 (2 450–3 140)	538 (475–607)	126 (114–140)
Sexually transmitted infections excluding HIV	2,230,000 (747 000–4 650 000)	671,000 (228 000–1 390 000)	1,000,000 (346 000–2 070 000)	38,700 (13 000–84 400)	17,100 (5 520–37 500)	4,840 (3 150–6 860)	3,940 (2 510–5 250)	3,610 (2 250–4 810)	3,670 (2 360–4 660)	6,970 (3 390–10 600)	5,310 (2 650–7 680)	4,240 (2 360–5 840)	3,740 (1 810–5 430)	5,280 (2 140–7 940)	6,280 (2 450–9 650)	4,210 (2 270–6 280)	2,040 (1 450–3 270)	1,700 (1 270–2 680)	1,490 (1 110–2 410)	839 (524–1 160)	696 (315–1 150)	359 (124–764)	153 (56·3–285)
Syphilis	2,230,000 (747 000–4 650 000)	671,000 (228 000–1 390 000)	1,000,000 (346 000–2 070 000)	38,700 (13 000–84 400)	17,100 (5 520–37 500)	2,760 (1 800–3 910)	3,100 (1 970–4 150)	2,240 (1 400–2 990)	2,580 (1 650–3 280)	4,680 (2 280–7 140)	4,580 (2 290–6 640)	3,210 (1 770–4 430)	2,660 (1 320–3 820)	3,910 (1 620–5 870)	4,990 (1 980–7 630)	3,420 (1 870–5 080)	1,640 (1 180–2 590)	1,380 (1 040–2 170)	940 (718–1 470)	441 (293–589)	276 (150–417)	114 (54·4–212)	45 (23·4–72·3)
Chlamydial infection	–	–	–	–	–	0 (0–0)	0 (0–0)	0 (0–0)	0 (0–0)	0 (0–0)	0 (0–0)	0 (0–0)	0 (0–0)	0 (0–0)	0 (0–0)	0 (0–0)	0 (0–0)	0 (0–0)	0 (0–0)	0 (0–0)	0 (0–0)	0 (0–0)	0 (0–0)
Gonococcal infection	–	–	–	–	–	245 [illegible]	448 [illegible]	869 [illegible]	262 [illegible]	476 [illegible]	329 [illegible]	558 [illegible]	337 [illegible]	269 [illegible]	324 [illegible]	324 [illegible]	188 [illegible]	137 [illegible]	105 [illegible]	61·4 [illegible]	47·2 [illegible]	7·5 [illegible]	1·53 [illegible]
Trichomoniasis	–	–	–	–	–	–	–	–	–	–	–	–	–	–	–	–	–	–	–	–	–	–	–
Genital herpes	–	–	–	–	[illegible]	[illegible]	[illegible]	[illegible]	[illegible]	[illegible]	[illegible]	[illegible]	[illegible]	[illegible]	[illegible]	[illegible]	[illegible]	[illegible]	[illegible]	[illegible]	[illegible]	[illegible]	[illegible]
Other sexually transmitted infections	–	–	–	–	[illegible]	[illegible]	[illegible]	[illegible]	[illegible]	[illegible]	[illegible]	[illegible]	[illegible]	[illegible]	[illegible]	[illegible]	[illegible]	[illegible]	[illegible]	[illegible]	[illegible]	[illegible]	[illegible]
Respiratory infections and tuberculosis	5,720,000 (4 550 000–7 180 000)	3,500,000 (2 860 000–4 340 000)	14,700,000 (11 700 000–18 500 000)	8,370,000 (6 490 000–10 600 000)	[illegible]	[illegible]	[illegible]	[illegible]	[illegible]	[illegible]	[illegible]	[illegible]	[illegible]	[illegible]	[illegible]	[illegible]	[illegible]	[illegible]	[illegible]	[illegible]	[illegible]	[illegible]	[illegible]
Tuberculosis	–	–	1,100,000 (859 000–1 420 000)	1,090,000 (820 000–1 410 000)	[illegible]	[illegible]	[illegible]	[illegible]	[illegible]	[illegible]	[illegible]	[illegible]	[illegible]	[illegible]	[illegible]	[illegible]	[illegible]	[illegible]	[illegible]	[illegible]	[illegible]	[illegible]	[illegible]
Latent tuberculosis infection	–	–	–	–	[illegible]	[illegible]	[illegible]	[illegible]	[illegible]	[illegible]	[illegible]	[illegible]	[illegible]	[illegible]	[illegible]	[illegible]	[illegible]	[illegible]	[illegible]	[illegible]	[illegible]	[illegible]	[illegible]
Drug-susceptible tuberculosis	–	–	1,010,000 (774 000–1 310 000)	1,000,000 (752 000–1 300 000)	[illegible]	[illegible]	[illegible]	[illegible]	[illegible]	[illegible]	[illegible]	[illegible]	[illegible]	[illegible]	[illegible]	[illegible]	[illegible]	[illegible]	[illegible]	[illegible]	[illegible]	[illegible]	[illegible]
Multidrug-resistant tuberculosis without extensive drug resistance	–	–	91,900 (39 800–166 000)	83,500 (36 500–158 000)	[illegible]	[illegible]	[illegible]	[illegible]	[illegible]	[illegible]	[illegible]	[illegible]	[illegible]	[illegible]	[illegible]	[illegible]	[illegible]	[illegible]	[illegible]	[illegible]	[illegible]	[illegible]	[illegible]
Extensively drug-resistant tuberculosis	–	–	4,430 (2 090–7 920)	3,220 (1 480–5 900)	[illegible]	[illegible]	[illegible]	[illegible]	[illegible]	[illegible]	[illegible]	[illegible]	[illegible]	[illegible]	[illegible]	[illegible]	[illegible]	[illegible]	[illegible]	[illegible]	[illegible]	[illegible]	[illegible]
Lower respiratory infections	5,710,000 (4 530 000–7 160 000)	3,490,000 (2 850 000–4 330 000)	13,500,000 (10 700 000–16 900 000)	7,180,000 (5 580 000–9 040 000)	[illegible]	[illegible]	[illegible]	[illegible]	[illegible]	[illegible]	[illegible]	[illegible]	[illegible]	[illegible]	[illegible]	[illegible]	[illegible]	[illegible]	[illegible]	[illegible]	[illegible]	[illegible]	[illegible]
[illegible]	1,730 [illegible]	4,180 [illegible]	89,900 [illegible]	105,000 [illegible]	[illegible]	[illegible]	[illegible]	[illegible]	[illegible]	[illegible]	[illegible]	[illegible]	[illegible]	[illegible]	[illegible]	[illegible]	[illegible]	[illegible]	[illegible]	[illegible]	[illegible]	[illegible]	[illegible]

[illegible]

Anorexia nervosa	–	–	–	–	18·4 (12·5–40·3)	12·6 (9·15–18·9)	121 (78·6–270)	226 (152–456)	180 (131–247)	194 (135–308)	146 (115–206)	275 (201–390)	221 (155–329)	–	–	–	–	–	–	–	–	–	–
Bulimia nervosa	–	–	–	–	13·3 (6·12–38·7)	9·11 (5·62–19·1)	90·2 (17·7–362)	126 (32·9–452)	60·6 (16·7–187)	131 (23·6–301)	95·2 (55–192)	123 (29–285)	105 (17·7–216)	–	–	–	–	–	–	–	–	–	–
Autism spectrum disorders	–	–	–	–	–	–	–	–	–	–	–	–	–	–	–	–	–	–	–	–	–	–	–
Attention-deficit/hyperactivity disorder	–	–	–	–	–	–	–	–	–	–	–	–	–	–	–	–	–	–	–	–	–	–	–
Conduct disorder	–	–	–	–	–	–	–	–	–	–	–	–	–	–	–	–	–	–	–	–	–	–	–
Idiopathic developmental intellectual disability	–	–	–	–	–	–	–	–	–	–	–	–	–	–	–	–	–	–	–	–	–	–	–
Other mental disorders	–	–	–	–	–	–	–	–	–	–	–	–	–	–	–	–	–	–	–	–	–	–	–
Substance use disorders	0 (0–0)	0 (0–0)	0 (0–0)	0 (0–0)	0 (0–0)	0 (0–0)	173,000 (161,000–187,000)	611,000 (563,000–676,000)	996,000 (921,000–1,100,000)	1,330,000 (1,210,000–1,450,000)	1,320,000 (1,190,000–1,430,000)	1,210,000 (1,100,000–1,290,000)	1,170,000 (1,050,000–1,260,000)	950,000 (850,000–1,020,000)	814,000 (733,000–871,000)	541,000 (486,000–580,000)	316,000 (281,000–337,000)	160,000 (142,000–172,000)	85,700 (74,600–92,400)	36,700 (32,200–39,500)	15,100 (13,000–16,300)	4,380 (3,620–4,830)	1,060 (838–1,190)
Alcohol use disorders	–	–	–	–	–	0 (0–0)	42,200 (30,900–48,000)	130,000 (98,200–147,000)	276,000 (225,000–302,000)	534,000 (434,000–580,000)	664,000 (550,000–721,000)	736,000 (622,000–802,000)	800,000 (670,000–875,000)	683,000 (580,000–745,000)	617,000 (535,000–667,000)	440,000 (384,000–477,000)	255,000 (220,000–275,000)	127,000 (108,000–138,000)	66,200 (54,900–72,500)	25,500 (21,500–27,700)	9,440 (7,680–10,300)	2,220 (1,770–2,470)	533 (390–609)
Drug use disorders	0 (0–0)	0 (0–0)	0 (0–0)	0 (0–0)	0 (0–0)	0 (0–0)	131,000 (120,000–142,000)	480,000 (447,000–536,000)	719,000 (672,000–803,000)	796,000 (746,000–890,000)	657,000 (614,000–734,000)	471,000 (447,000–498,000)	367,000 (344,000–392,000)	267,000 (246,000–286,000)	196,000 (179,000–213,000)	101,000 (92,400–108,000)	60,300 (55,600–65,100)	32,900 (30,300–35,600)	19,500 (17,900–21,100)	11,200 (10,100–12,200)	5,620 (4,860–6,210)	2,160 (1,780–2,460)	526 (416–602)
Opioid use disorders	0 (0–0)	0 (0–0)	0 (0–0)	0 (0–0)	0 (0–0)	0 (0–0)	90,400 (81,700–98,900)	355,000 (327,000–401,000)	532,000 (492,000–610,000)	568,000 (521,000–647,000)	471,000 (439,000–530,000)	332,000 (314,000–354,000)	253,000 (235,000–279,000)	177,000 (162,000–193,000)	130,000 (117,000–143,000)	67,600 (61,500–73,300)	40,200 (37,000–43,500)	22,000 (20,100–23,900)	13,500 (12,300–14,700)	7,270 (6,490–8,030)	3,660 (3,140–4,100)	1,330 (1,090–1,550)	318 (257–367)
Cocaine use disorders	–	–	–	–	–	0 (0–0)	13,000 (11,500–14,400)	36,700 (33,300–41,900)	52,400 (45,400–63,400)	79,500 (69,200–126,000)	64,700 (59,100–74,500)	46,800 (38,300–51,100)	40,600 (30,400–44,400)	40,500 (29,200–44,600)	29,700 (21,700–33,400)	13,900 (10,300–15,700)	6,770 (5,510–7,480)	2,660 (2,210–2,980)	1,320 (1,100–1,470)	167 (144–191)	76·3 (63–86·8)	29·2 (23·1–33)	10·2 (7·77–11·8)
Amphetamine use disorders	–	–	–	–	–	0 (0–0)	9,620 (8,060–11,200)	34,100 (30,400–38,200)	58,300 (48,800–74,700)	59,800 (50,100–78,500)	44,700 (40,500–50,200)	31,400 (26,400–35,900)	23,400 (18,300–27,500)	21,000 (15,300–25,100)	14,600 (10,500–17,400)	7,530 (5,520–8,870)	4,180 (3,360–4,860)	654 (455–779)	306 (218–362)	385 (287–535)	300 (211–444)	164 (113–233)	44·8 (30·7–62·1)
Cannabis use disorders	–	–	–	–	–	–	–	–	–	–	–	–	–	–	–	–	–	–	–	–	–	–	–
Other drug use disorders	–	–	–	–	–	0 (0–0)	18,000 (16,300–20,400)	55,000 (49,700–61,600)	76,400 (69,500–90,400)	89,300 (81,800–104,000)	77,200 (70,500–85,600)	61,700 (57,200–66,800)	49,600 (45,600–54,800)	28,900 (26,400–31,600)	22,100 (20,000–24,300)	12,000 (10,900–13,200)	9,090 (8,240–10,100)	7,500 (6,830–8,280)	4,410 (3,950–4,930)	3,360 (3,020–3,730)	1,580 (1,390–1,740)	635 (520–713)	153 (117–186)
Diabetes and kidney diseases	2,530 (1,890–3,360)	6,400 (4,790–8,560)	251,000 (196,000–323,000)	281,000 (217,000–361,000)	249,000 (207,000–297,000)	218,000 (187,000–253,000)	524,000 (464,000–595,000)	706,000 (632,000–785,000)	761,000 (699,000–834,000)	1,030,000 (955,000–1,130,000)	1,300,000 (1,200,000–1,420,000)	1,800,000 (1,650,000–1,980,000)	2,380,000 (2,170,000–2,600,000)	3,280,000 (2,990,000–3,570,000)	4,010,000 (3,670,000–4,360,000)	4,400,000 (4,070,000–4,770,000)	4,280,000 (3,990,000–4,610,000)	3,670,000 (3,430,000–3,940,000)	2,860,000 (2,680,000–3,060,000)	2,010,000 (1,830,000–2,160,000)	1,130,000 (995,000–1,220,000)	427,000 (358,000–465,000)	117,000 (91,300–130,000)
Diabetes mellitus	2,530 (1,890–3,360)	6,400 (4,790–8,560)	71,400 (50,900–100,000)	55,000 (37,700–81,700)	45,300 (35,800–61,900)	46,300 (37,000–58,700)	113,000 (99,300–130,000)	181,000 (162,000–205,000)	211,000 (193,000–232,000)	359,000 (328,000–395,000)	472,000 (432,000–516,000)	785,000 (714,000–857,000)	1,190,000 (1,080,000–1,310,000)	1,750,000 (1,600,000–1,890,000)	2,220,000 (2,040,000–2,410,000)	2,490,000 (2,300,000–2,700,000)	2,390,000 (2,220,000–2,570,000)	2,010,000 (1,860,000–2,160,000)	1,520,000 (1,410,000–1,620,000)	987,000 (895,000–1,060,000)	494,000 (436,000–534,000)	163,000 (137,000–178,000)	36,900 (29,200–41,000)
Diabetes mellitus type 1	2,530 (1,890–3,360)	6,400 (4,790–8,560)	71,400 (50,900–100,000)	55,000 (37,700–81,700)	45,300 (35,800–61,900)	46,300 (37,000–58,700)	66,300 (57,700–76,600)	122,000 (108,000–140,000)	132,000 (119,000–150,000)	136,000 (118,000–161,000)	168,000 (148,000–200,000)	239,000 (207,000–302,000)	68,500 (56,300–98,700)	90,100 (73,900–130,000)	113,000 (93,200–167,000)	90,800 (75,300–129,000)	86,400 (71,100–127,000)	53,800 (44,600–77,100)	36,900 (30,300–52,400)	12,800 (10,400–17,500)	6,750 (5,340–9,270)	2,570 (1,920–3,550)	666 (453–946)
Diabetes mellitus type 2	–	–	–	–	–	–	47,000 (37,400–59,400)	59,200 (48,200–74,700)	78,900 (67,800–94,000)	222,000 (197,000–253,000)	304,000 (271,000–342,000)	546,000 (485,000–607,000)	1,120,000 (1,020,000–1,230,000)	1,660,000 (1,510,000–1,800,000)	2,110,000 (1,930,000–2,290,000)	2,400,000 (2,210,000–2,600,000)	2,310,000 (2,140,000–2,490,000)	1,950,000 (1,810,000–2,090,000)	1,480,000 (1,380,000–1,580,000)	975,000 (883,000–1,050,000)	487,000 (428,000–526,000)	160,000 (135,000–175,000)	36,200 (28,600–40,300)
Chronic kidney disease	–	–	174,000 (134,000–227,000)	220,000 (168,000–292,000)	194,000 (158,000–237,000)	167,000 (141,000–197,000)	407,000 (357,000–467,000)	519,000 (462,000–581,000)	544,000 (494,000–601,000)	666,000 (610,000–735,000)	819,000 (748,000–906,000)	1,010,000 (917,000–1,120,000)	1,180,000 (1,060,000–1,310,000)	1,520,000 (1,380,000–1,680,000)	1,780,000 (1,610,000–1,970,000)	1,900,000 (1,740,000–2,100,000)	1,880,000 (1,740,000–2,050,000)	1,650,000 (1,530,000–1,790,000)	1,340,000 (1,250,000–1,440,000)	1,020,000 (919,000–1,100,000)	636,000 (556,000–688,000)	264,000 (220,000–287,000)	79,500 (61,900–89,400)
Chronic kidney disease due to diabetes mellitus type 1	–	–	996 (424–2,090)	1,670 (737–3,330)	2,670 (995–5,890)	4,690 (1,720–9,660)	18,600 (7,130–36,900)	44,900 (19,900–81,000)	76,400 (38,200–129,000)	133,000 (71,300–200,000)	186,000 (101,000–285,000)	233,000 (125,000–361,000)	241,000 (111,000–394,000)	225,000 (97,900–407,000)	175,000 (67,900–345,000)	133,000 (52,000–275,000)	97,800 (41,800–205,000)	60,800 (25,400–121,000)	30,400 (12,200–64,100)	11,900 (4,570–26,200)	3,050 (1,080–7,720)	377 (118–917)	27·9 (7·9–77·6)
Chronic kidney disease due to diabetes mellitus type 2	–	–	–	–	–	–	9,960 (2,870–22,800)	20,500 (7,730–44,000)	28,800 (10,900–56,900)	49,200 (17,400–96,700)	79,800 (29,900–150,000)	142,000 (59,500–250,000)	246,000 (126,000–391,000)	421,000 (238,000–612,000)	570,000 (350,000–793,000)	636,000 (368,000–864,000)	635,000 (410,000–841,000)	553,000 (369,000–722,000)	427,000 (279,000–568,000)	299,000 (195,000–408,000)	167,000 (109,000–235,000)	61,600 (37,400–90,200)	18,000 (9,550–27,900)
Chronic kidney disease due to hypertension	–	–	–	–	–	–	28,300 (14,500–49,900)	53,500 (30,200–87,200)	67,800 (40,300–107,000)	90,700 (56,000–137,000)	125,000 (78,800–185,000)	173,000 (117,000–248,000)	233,000 (165,000–326,000)	349,000 (245,000–476,000)	455,000 (323,000–616,000)	550,000 (408,000–727,000)	584,000 (443,000–754,000)	541,000 (424,000–686,000)	472,000 (372,000–609,000)	389,000 (301,000–490,000)	270,000 (211,000–334,000)	121,000 (91,500–150,000)	37,900 (27,200–50,100)
Chronic kidney disease due to glomerulonephritis	–	–	47,100 (24,000–78,900)	75,800 (39,700–122,000)	68,900 (36,000–109,000)	70,100 (38,900–107,000)	176,000 (113,000–250,000)	201,000 (122,000–288,000)	191,000 (121,000–273,000)	192,000 (109,000–292,000)	213,000 (122,000–335,000)	225,000 (108,000–356,000)	211,000 (125,000–336,000)	244,000 (136,000–386,000)	267,000 (151,000–432,000)	260,000 (140,000–413,000)	230,000 (120,000–384,000)	189,000 (103,000–306,000)	150,000 (81,400–244,000)	108,000 (53,500–183,000)	59,000 (29,900–99,600)	22,000 (10,900–37,500)	5,710 (2,520–11,400)
Chronic kidney disease due to other and unspecified causes	–	–	126,000 (90,600–169,000)	143,000 (97,600–199,000)	122,000 (83,000–166,000)	92,500 (57,100–128,000)	174,000 (108,000–241,000)	199,000 (122,000–287,000)	180,000 (113,000–250,000)	201,000 (117,000–301,000)	215,000 (118,000–332,000)	234,000 (175,000–364,000)	250,000 (130,000–394,000)	282,000 (157,000–451,000)	309,000 (166,000–511,000)	318,000 (172,000–542,000)	331,000 (184,000–520,000)	310,000 (179,000–481,000)	258,000 (146,000–403,000)	210,000 (119,000–321,000)	137,000 (78,400–204,000)	59,000 (36,600–87,500)	17,900 (9,260–27,900)
Acute glomerulonephritis	–	–	5,130 (1,530–8,000)	5,860 (1,780–8,680)	10,000 (3,150–14,500)	4,790 (1,630–6,740)	4,140 (2,070–5,600)	5,010 (2,590–6,630)	6,780 (3,190–8,350)	9,000 (4,450–11,300)	8,980 (4,410–11,400)	10,400 (4,850–13,400)	10,900 (5,590–14,200)	13,300 (6,400–17,300)	12,000 (6,290–15,800)	12,700 (6,570–16,100)	14,800 (7,280–18,800)	11,600 (6,450–14,800)	10,100 (4,970–12,800)	7,190 (3,660–8,890)	3,910 (2,170–4,720)	794 (495–948)	145 (108–175)
Skin and subcutaneous diseases	–	–	64,300 (31,900–90,200)	52,600 (28,700–70,500)	12,900 (6,510–17,000)	17,500 (9,490–23,500)	31,100 (18,300–38,400)	27,100 (15,000–33,200)	23,300 (13,800–29,300)	49,100 (29,700–59,400)	46,600 (27,000–59,000)	50,500 (32,800–62,700)	66,700 (41,000–84,500)	69,500 (42,300–88,200)	86,400 (52,700–114,000)	94,100 (57,200–120,000)	90,200 (55,700–119,000)	88,200 (54,400–123,000)	83,400 (54,400–122,000)	60,700 (39,000–95,300)	41,800 (26,000–65,700)	19,500 (11,200–30,700)	6,490 (3,410–10,600)
Dermatitis	–	–	–	–	–	–	–	–	–	–	–	–	–	–	–	–	–	–	–	–	–	–	–
Atopic dermatitis	–	–	–	–	–	–	–	–	–	–	–	–	–	–	–	–	–	–	–	–	–	–	–
Contact dermatitis	–	–	–	–	–	–	–	–	–	–	–	–	–	–	–	–	–	–	–	–	–	–	–
Seborrhoeic dermatitis	–	–	–	–	–	–	–	–	–	–	–	–	–	–	–	–	–	–	–	–	–	–	–
Psoriasis	–	–	–	–	–	–	–	–	–	–	–	–	–	–	–	–	–	–	–	–	–	–	–
Bacterial skin diseases	–	–	62,600 (30,500–88,100)	50,400 (26,800–68,100)	11,600 (5,670–15,700)	16,100 (8,620–21,700)	28,800 (17,300–35,600)	24,300 (13,200–29,700)	20,200 (12,000–24,700)	43,400 (25,500–52,800)	40,500 (22,600–51,100)	43,400 (27,200–55,000)	58,600 (35,300–74,100)	59,900 (35,500–75,700)	73,400 (43,000–95,700)	79,500 (46,400–103,000)	71,700 (41,400–96,000)	66,300 (38,100–93,900)	60,300 (36,700–89,100)	38,500 (22,600–60,900)	25,400 (13,600–42,100)	12,100 (5,620–20,300)	3,890 (1,680–6,670)
Cellulitis	–	–	7,010 (1,920–11,900)	4,380 (908–9,970)	1,270 (459–1,930)	2,580 (1,130–3,660)	7,200 (1,970–11,200)	9,190 (3,530–12,700)	4,240 (1,760–6,020)	9,850 (4,030–14,000)	12,800 (5,500–18,600)	13,700 (6,470–20,100)	18,500 (7,970–26,400)	17,200 (7,970–26,000)	20,400 (8,730–30,900)	21,900 (9,900–33,200)	18,700 (8,130–29,100)	16,700 (7,310–26,700)	13,700 (5,720–25,000)	9,790 (3,840–17,600)	6,270 (2,270–12,700)	3,090 (1,230–5,780)	1,060 (324–1,940)
Pyoderma	–	–	55,600 (27,300–77,800)	46,000 (25,000–63,200)	10,400 (4,900–14,200)	13,500 (6,950–18,600)	21,600 (14,400–28,000)	15,100 (8,600–20,200)	15,900 (9,490–19,500)	33,500 (18,500–41,000)	27,700 (14,600–35,600)	29,700 (17,300–37,900)	40,100 (22,900–51,700)	42,700 (24,600–54,300)	53,100 (29,900–68,400)	57,600 (32,200–73,000)	53,000 (29,400–69,400)	49,600 (27,100–67,300)	46,600 (27,400–65,300)	28,700 (16,400–44,200)	19,100 (10,100–29,800)	9,030 (4,060–14,600)	2,830 (1,240–4,690)
Scabies	–	–	–	–	–	–	–	–	–	–	–	–	–	–	–	–	–	–	–	–	–	–	–
Fungal skin diseases	–	–	–	–	–	–	–	–	–	–	–	–	–	–	–	–	–	–	–	–	–	–	–
Viral skin diseases	–	–	–	–	–	–	–	–	–	–	–	–	–	–	–	–	–	–	–	–	–	–	–
Acne vulgaris	–	–	–	–	–	–	–	–	–	–	–	–	–	–	–	–	–	–	–	–	–	–	–
Alopecia areata	–	–	–	–	–	–	–	–	–	–	–	–	–	–	–	–	–	–	–	–	–	–	–
Pruritus	–	–	–	–	–	–	–	–	–	–	–	–	–	–	–	–	–	–	–	–	–	–	–
Urticaria	–	–	–	–	–	–	–	–	–	–	–	–	–	–	–	–	–	–	–	–	–	–	–
Decubitus ulcer	–	–	–	338 (94·7–763)	335 (117–505)	569 (214–785)	1,550 (470–2,220)	2,110 (764–3,070)	2,470 (1,010–3,600)	4,790 (2,970–6,780)	4,880 (2,720–7,030)	5,510 (3,500–8,020)	6,060 (3,560–9,380)	7,910 (4,720–11,100)	10,600 (6,100–16,000)	12,200 (6,660–17,900)	16,000 (9,290–23,300)	19,200 (11,200–28,900)	20,600 (11,700–35,400)	19,800 (12,100–32,200)	14,600 (8,630–26,200)	6,620 (3,470–11,300)	2,360 (1,110–4,240)
Other skin and subcutaneous diseases	–	–	1,630 (1,070–2,660)	1,870 (1,320–2,990)	947 (633–1,280)	829 (526–1,150)	812 (507–1,100)	724 (514–1,130)	703 (469–1,070)	908 (617–1,300)	1,140 (743–1,730)	1,640 (1,160–2,180)	2,070 (1,350–2,800)	1,730 (1,090–2,400)	2,280 (1,490–3,150)	2,380 (1,500–3,300)	2,490 (1,540–3,600)	2,620 (1,650–3,930)	2,500 (1,560–4,100)	2,380 (1,430–3,960)	1,780 (1,040–3,080)	788 (398–1,450)	239 (97·1–465)
Sense organ diseases	–	–	–	–	–	–	–	–	–	–	–	–	–	–	–	–	–	–	–	–	–	–	–
Blindness and vision loss	–	–	–	–	–	–	–	–	–	–	–	–	–	–	–	–	–	–	–	–	–	–	–
Glaucoma	–	–	–	–	–	–	–	–	–	–	–	–	–	–	–	–	–	–	–	–	–	–	–
Cataract	–	–	–	–	–	–	–	–	–	–	–	–	–	–	–	–	–	–	–	–	–	–	–
Age-related macular degeneration	–	–	–	–	–	–	–	–	–	–	–	–	–	–	–	–	–	–	–	–	–	–	–
Refraction disorders	–	–	–	–	–	–	–	–	–	–	–	–	–	–	–	–	–	–	–	–	–	–	–
Near vision loss	–	–	–	–	–	–	–	–	–	–	–	–	–	–	–	–	–	–	–	–	–	–	–
Other vision loss	–	–	–	–	–	–	–	–	–	–	–	–	–	–	–	–	–	–	–	–	–	–	–
Age-related and other hearing loss	–	–	–	–	–	–	–	–	–	–	–	–	–	–	–	–	–	–	–	–	–	–	–
Other sense organ diseases	–	–	–	–	–	–	–	–	–	–	–	–	–	–	–	–	–	–	–	–	–	–	–
Musculoskeletal disorders	–	–	–	–	9,780 (8,290–12,000)	16,300 (14,100–20,000)	21,600 (19,100–25,400)	25,000 (22,300–29,300)	28,100 (25,400–32,500)	31,400 (28,500–36,500)	33,200 (29,700–38,600)	38,700 (34,700–45,800)	42,200 (37,500–50,600)	48,500 (42,900–57,500)	61,400 (54,300–71,400)	84,500 (74,700–98,400)	91,900 (81,700–106,000)	94,200 (83,100–108,000)	78,300 (67,700–88,300)	59,500 (49,500–67,400)	34,800 (28,000–39,200)	12,400 (9,240–14,200)	3,170 (2,170–3,770)
Rheumatoid arthritis	–	–	–	–	1,060 (869–1,470)	1,630 (1,280–2,110)	1,340 (1,130–1,650)	1,490 (1,250–1,830)	1,990 (1,710–2,390)	2,980 (2,600–3,600)	3,980 (3,440–4,640)	6,400 (5,510–7,610)	9,350 (7,970–11,200)	14,500 (12,500–17,300)	21,400 (18,500–24,900)	32,600 (28,000–37,900)	40,100 (34,900–45,700)	44,000 (38,000–50,600)	37,800 (32,300–43,100)	26,000 (21,600–29,600)	14,200 (11,800–16,200)	3,540 (2,800–4,160)	737 (532–917)
Osteoarthritis	–	–	–	–	–	–	–	–	–	–	–	–	–	–	–	–	–	–	–	–	–	–	–
Osteoarthritis hip	–	–	–	–	–	–	–	–	–	–	–	–	–	–	–	–	–	–	–	–	–	–	–
Osteoarthritis knee	–	–	–	–	–	–	–	–	–	–	–	–	–	–	–	–	–	–	–	–	–	–	–
Osteoarthritis hand	–	–	–	–	–	–	–	–	–	–	–	–	–	–	–	–	–	–	–	–	–	–	–
Osteoarthritis other	–	–	–	–	–	–	–	–	–	–	–	–	–	–	–	–	–	–	–	–	–	–	–
Low back pain	–	–	–	–	–	–	–	–	–	–	–	–	–	–	–	–	–	–	–	–	–	–	–
Neck pain	–	–	–	–	–	–	–	–	–	–	–	–	–	–	–	–	–	–	–	–	–	–	–
Gout	–	–	–	–	–	–	–	–	–	–	–	–	–	–	–	–	–	–	–	–	–	–	–
Other musculoskeletal disorders	–	–	–	–	8,720 (7,370–10,600)	14,700 (12,600–17,900)	20,200 (18,000–23,700)	23,500 (21,000–27,500)	26,100 (23,700–30,200)	28,400 (25,800–32,900)	29,200 (26,200–34,200)	32,300 (28,900–38,200)	32,900 (29,100–39,600)	33,900 (29,900–40,600)	40,000 (34,900–47,400)	51,900 (45,100–61,000)	51,800 (45,300–61,100)	50,200 (43,800–58,700)	40,500 (34,000–46,800)	33,500 (26,600–38,200)	20,700 (16,200–23,700)	8,810 (6,300–10,300)	2,430 (1,640–2,920)
Other non-communicable diseases	8,920,000 (7,170,000–11,200,000)	2,960,000 (2,430,000–3,720,000)	10,000,000 (7,390,000–13,200,000)	4,280,000 (2,600,000–6,470,000)	1,110,000 (921,000–1,340,000)	755,000 (616,000–915,000)	643,000 (534,000–806,000)	599,000 (509,000–718,000)	503,000 (438,000–589,000)	458,000 (404,000–521,000)	454,000 (395,000–516,000)	454,000 (389,000–515,000)	444,000 (374,000–496,000)	494,000 (409,000–548,000)	511,000 (414,000–565,000)	547,000 (447,000–599,000)	557,000 (456,000–609,000)	497,000 (411,000–546,000)	412,000 (331,000–449,000)	345,000 (274,000–377,000)	216,000 (169,000–237,000)	98,200 (72,600–110,000)	32,100 (21,700–36,900)
Congenital birth defects	8,790,000 (7,060,000–11,100,000)	2,750,000 (2,250,000–3,490,000)	8,150,000 (6,000,000–11,500,000)	3,240,000 (1,790,000–5,370,000)	710,000 (596,000–880,000)	443,000 (371,000–538,000)	289,000 (242,000–356,000)	245,000 (210,000–293,000)	204,000 (178,000–241,000)	140,000 (124,000–165,000)	136,000 (117,000–159,000)	115,000 (98,300–137,000)	64,400 (53,600–73,400)	56,400 (44,500–64,400)	51,400 (38,500–58,500)	40,600 (30,700–46,000)	24,300 (18,900–27,400)	–	–	–	–	–	–
Neural tube defects	1,500,000 (986,000–2,220,000)	309,000 (211,000–452,000)	868,000 (501,000–1,510,000)	782,000 (356,000–1,470,000)	36,600 (22,900–55,900)	23,700 (15,600–35,900)	10,200 (6,610–18,100)	7,130 (5,120–11,000)	6,170 (4,730–8,920)	5,050 (3,950–6,950)	4,160 (3,440–5,410)	3,740 (2,970–5,190)	2,170 (1,770–2,880)	1,500 (1,220–1,880)	1,150 (923–1,490)	1,010 (806–1,380)	696 (563–863)	–	–	–	–	–	–
Congenital heart anomalies	2,640,000 (1,920,000–3,490,000)	1,230,000 (969,000–1,540,000)	3,690,000 (2,780,000–4,860,000)	1,120,000 (698,000–1,880,000)	389,000 (310,000–490,000)	263,000 (216,000–326,000)	173,000 (143,000–212,000)	157,000 (134,000–190,000)	123,000 (105,000–145,000)	85,600 (73,900–98,800)	79,200 (68,000–93,800)	66,100 (56,400–79,200)	32,800 (27,100–38,000)	26,500 (21,400–30,800)	21,500 (17,300–24,900)	17,200 (13,900–19,900)	10,700 (8,580–12,300)	–	–	–	–	–	–
Orofacial clefts	51,900 (27,100–129,000)	15,900 (8,850–37,500)	51,200 (26,000–128,000)	3,980 (2,730–7,200)	–	–	–	–	–	–	–	–	–	–	–	–	–	–	–	–	–	–	–
Down syndrome	139,000 (102,000–234,000)	60,700 (45,500–95,700)	310,000 (224,000–576,000)	116,000 (70,700–366,000)	28,300 (22,200–41,500)	17,700 (14,300–24,400)	16,000 (12,800–20,400)	15,800 (13,200–19,400)	15,400 (12,700–19,300)	14,000 (11,800–18,200)	15,000 (12,300–19,100)	14,600 (11,900–18,200)	12,800 (10,400–15,000)	15,700 (11,600–17,800)	17,400 (11,600–20,000)	13,100 (8,340–14,900)	5,530 (3,640–6,250)	–	–	–	–	–	–
Turner syndrome	–	–	–	–	–	–	–	–	–	–	–	–	–	–	–	–	–	–	–	–	–	–	–
Klinefelter syndrome	–	–	–	–	–	–	–	–	–	–	–	–	–	–	–	–	–	–	–	–	–	–	–
Other chromosomal abnormalities	336,000 (183,000–613,000)	121,000 (72,200–210,000)	282,000 (154,000–577,000)	71,800 (25,900–214,000)	16,700 (10,500–25,900)	11,000 (7,040–14,700)	10,600 (7,460–13,200)	9,190 (6,590–11,500)	10,300 (7,550–13,100)	5,710 (3,370–6,760)	6,990 (4,600–8,480)	3,780 (2,230–4,680)	2,740 (1,470–3,290)	1,790 (796–2,180)	1,640 (674–2,020)	914 (308–1,180)	783 (331–962)	–	–	–	–	–	–
Congenital musculoskeletal and limb anomalies	238,000 (153,000–359,000)	50,700 (35,200–69,300)	135,000 (87,900–211,000)	43,900 (20,700–106,000)	16,100 (11,700–21,500)	10,100 (7,570–13,500)	9,880 (7,110–12,400)	5,820 (4,220–7,540)	5,110 (3,740–6,790)	3,320 (2,650–4,040)	3,150 (2,450–4,000)	2,300 (1,620–2,970)	1,340 (889–1,610)	1,160 (789–1,390)	865 (556–1,060)	974 (668–1,220)	613 (450–840)	–	–	–	–	–	–
Urogenital congenital anomalies	316,000 (182,000–488,000)	62,100 (38,700–93,400)	98,000 (54,200–149,000)	22,400 (8,240–44,100)	5,980 (3,920–8,190)	4,250 (3,110–5,900)	3,710 (2,380–4,640)	3,960 (2,210–4,950)	4,080 (2,520–4,970)	2,910 (2,020–3,470)	2,760 (2,000–3,330)	2,550 (1,920–3,090)	1,270 (926–1,510)	1,070 (786–1,250)	899 (636–1,060)	920 (671–1,090)	746 (565–903)	–	–	–	–	–	–
Digestive congenital anomalies	830,000 (473,000–1,260,000)	310,000 (203,000–437,000)	819,000 (468,000–1,300,000)	163,000 (57,000–369,000)	19,100 (12,300–26,900)	9,620 (6,820–12,800)	3,590 (2,590–4,910)	2,670 (1,850–3,460)	3,210 (2,290–4,260)	1,950 (1,460–2,410)	2,290 (1,600–2,800)	2,060 (1,340–2,540)	1,470 (974–1,750)	1,470 (925–1,740)	1,720 (1,110–2,050)	1,570 (1,080–1,860)	1,540 (1,000–1,830)	–	–	–	–	–	–
Other congenital birth defects	2,740,000 (1,870,000–4,190,000)	585,000 (434,000–856,000)	1,900,000 (1,210,000–3,130,000)	918,000 (406,000–1,880,000)	198,000 (155,000–332,000)	103,000 (82,800–148,000)	62,400 (47,700–85,900)	44,000 (35,000–56,500)	36,700 (28,500–48,300)	23,600 (19,400–30,300)	22,700 (18,100–28,600)	20,100 (15,500–25,500)	9,800 (7,540–11,900)	7,260 (5,510–8,720)	6,100 (4,460–7,150)	4,980 (3,610–5,850)	3,620 (2,810–4,220)	–	–	–	–	–	–
Urinary diseases and male infertility	32,200 (23,000–44,400)	17,900 (12,800–24,700)	116,000 (85,800–161,000)	87,300 (66,300–110,000)	55,200 (41,500–70,000)	40,500 (32,700–48,700)	84,600 (68,200–101,000)	116,000 (99,000–134,000)	99,100 (83,900–113,000)	124,000 (105,000–141,000)	134,000 (113,000–154,000)	158,000 (130,000–182,000)	187,000 (148,000–212,000)	218,000 (171,000–246,000)	242,000 (192,000–271,000)	285,000 (222,000–318,000)	318,000 (254,000–357,000)	311,000 (249,000–347,000)	269,000 (214,000–297,000)	243,000 (187,000–268,000)	152,000 (115,000–168,000)	72,900 (53,800–81,800)	23,200 (15,700–26,700)
Urolithiasis	–	–	–	603 (302–1,020)	1,230 (672–2,090)	904 (457–1,400)	1,080 (410–1,800)	1,320 (668–1,880)	2,300 (1,170–3,410)	2,930 (1,630–4,090)	5,050 (2,500–6,600)	7,820 (4,320–10,600)	10,000 (4,940–13,600)	14,100 (7,490–18,500)	14,800 (8,350–19,700)	19,300 (10,700–25,000)	20,800 (12,700–27,500)	17,800 (11,200–24,600)	14,800 (9,290–21,200)	10,600 (6,780–14,400)	5,620 (3,730–7,660)	2,080 (1,380–3,020)	485 (325–731)
Benign prostatic hyperplasia	–	–	–	–	–	–	–	–	–	–	–	–	–	–	–	–	–	–	–	–	–	–	–
Male infertility	–	–	–	–	–	–	–	–	–	–	–	–	–	–	–	–	–	–	–	–	–	–	–
Other urinary diseases	24,200 (16,200–34,300)	8,670 (5,510–12,900)	23,300 (12,900–36,600)	43,900 (31,200–60,400)	15,500 (10,900–22,000)	11,800 (8,360–15,700)	27,700 (21,600–35,700)	19,500 (12,400–27,900)	20,500 (15,100–26,500)	37,500 (29,700–47,700)	40,400 (31,000–53,600)	43,300 (32,700–58,100)	54,600 (41,500–74,300)	55,200 (36,400–74,200)	69,700 (51,900–92,000)	71,600 (49,700–95,700)	80,400 (58,400–109,000)	77,400 (54,500–109,000)	57,800 (40,300–79,000)	56,300 (40,800–75,500)	27,900 (20,500–36,700)	11,900 (8,570–16,400)	3,050 (1,990–3,970)
Gynecological diseases	–	–	–	–	–	–	–	–	–	–	–	–	–	–	–	–	–	–	–	–	–	–	–
Uterine fibroids	–	–	–	–	–	–	–	–	–	–	–	–	–	–	–	–	–	–	–	–	–	–	–
Polycystic ovarian syndrome	–	–	–	–	–	–	–	–	–	–	–	–	–	–	–	–	–	–	–	–	–	–	–
Female infertility	–	–	–	–	–	–	–	–	–	–	–	–	–	–	–	–	–	–	–	–	–	–	–
Endometriosis	–	–	–	–	–	–	–	–	–	–	–	–	–	–	–	–	–	–	–	–	–	–	–
Genital prolapse	–	–	–	–	–	–	–	–	–	–	–	–	–	–	–	–	–	–	–	–	–	–	–
Premenstrual syndrome	–	–	–	–	–	–	–	–	–	–	–	–	–	–	–	–	–	–	–	–	–	–	–
Other gynecological diseases	–	–	–	–	–	–	–	–	–	–	–	–	–	–	–	–	–	–	–	–	–	–	–
Hemoglobinopathies and hemolytic anemias	36,500 (26,300–54,900)	21,800 (15,400–31,400)	225,000 (134,000–391,000)	748,000 (485,000–1,190,000)	256,000 (184,000–385,000)	199,000 (146,000–302,000)	209,000 (150,000–320,000)	170,000 (124,000–264,000)	120,000 (88,100–184,000)	89,700 (67,800–137,000)	74,100 (54,100–114,000)	62,700 (45,100–99,500)	51,800 (38,900–81,000)	57,400 (43,600–86,600)	46,100 (36,600–72,000)	51,200 (41,500–74,300)	49,600 (41,200–69,900)	43,000 (36,200–58,200)	32,600 (28,000–43,600)	22,700 (19,600–29,800)	12,600 (10,800–15,800)	4,520 (3,720–5,470)	1,140 (879–1,340)
Thalassemias	14,800 (10,800–22,000)	10,300 (7,230–16,100)	53,300 (39,600–71,000)	107,000 (82,500–142,000)	70,400 (49,200–111,000)	39,100 (28,000–58,900)	25,600 (17,800–39,300)	15,800 (11,100–22,600)	10,400 (7,500–14,600)	9,020 (6,220–12,800)	7,020 (4,950–9,660)	6,820 (4,920–9,350)	6,580 (4,650–8,840)	6,350 (4,250–8,780)	3,500 (2,120–5,270)	1,490 (821–2,380)	421 (211–718)	85·1 (41·6–153)	11·8 (5·33–22·1)	1·29 (0·447–3·06)	0·102 (0·0277–0·278)	0·00441 (0·00102–0·0126)	0·000154 (0·0000248–0·000476)
Thalassemias trait	–	–	–	–	–	–	–	–	–	–	–	–	–	–	–	–	–	–	–	–	–	–	–
Sickle cell disorders	20,200 (11,600–37,600)	11,000 (6,690–18,300)	165,000 (70,000–323,000)	622,000 (375,000–1,030,000)	166,000 (107,000–268,000)	131,000 (90,400–211,000)	139,000 (91,300–243,000)	109,000 (70,000–186,000)	72,000 (45,600–126,000)	43,500 (26,300–80,200)	37,900 (21,600–71,500)	28,900 (15,400–55,100)	15,200 (7,650–31,000)	9,150 (4,340–18,200)	2,200 (1,270–4,070)	933 (577–1,500)	348 (217–517)	105 (64–166)	17·5 (8·87–32·1)	2·6 (1·08–5·02)	0·363 (0·12–0·778)	0·0434 (0·0118–0·106)	0·0039 (0·000996–0·00999)
Sickle cell trait	–	–	–	–	–	–	–	–	–	–	–	–	–	–	–	–	–	–	–	–	–	–	–
G6PD deficiency	745 (423–1,170)	447 (254–728)	5,080 (2,960–8,160)	17,100 (10,000–28,400)	17,000 (9,410–26,500)	25,700 (15,400–40,800)	37,400 (22,000–59,700)	36,200 (22,900–55,100)	31,300 (20,600–46,300)	30,700 (20,600–44,800)	23,500 (15,300–34,100)	21,000 (13,600–32,800)	23,000 (14,600–39,400)	29,800 (17,800–50,500)	24,700 (15,300–45,500)	24,300 (14,900–40,700)	17,200 (9,960–29,100)	10,200 (5,850–17,100)	4,790 (2,360–8,360)	1,680 (834–3,010)	456 (225–799)	80·2 (40·5–140)	9·66 (3·91–17·4)
G6PD trait	–	–	–	–	–	–	–	–	–	–	–	–	–	–	–	–	–	–	–	–	–	–	–
Other hemoglobinopathies and hemolytic anemias	787 (546–1,140)	142 (96·4–217)	1,040 (764–1,420)	1,340 (983–1,810)	1,940 (1,420–2,760)	3,450 (2,430–4,990)	6,420 (4,200–10,600)	9,090 (6,130–14,500)	5,850 (4,040–8,970)	6,500 (4,430–9,970)	5,600 (3,960–8,310)	5,950 (4,310–8,560)	6,980 (5,010–10,100)	12,100 (8,620–18,100)	15,700 (11,700–22,800)	24,500 (18,700–34,500)	31,600 (25,200–42,900)	32,600 (26,900–42,300)	27,800 (23,600–36,300)	21,000 (18,100–26,900)	12,200 (10,400–15,000)	4,440 (3,670–5,340)	1,130 (873–1,330)
Endocrine, metabolic, blood, and immune disorders	60,400 (40,100–75,600)	51,200 (33,500–65,500)	312,000 (212,000–422,000)	198,000 (135,000–269,000)	91,700 (67,900–121,000)	72,600 (55,500–96,100)	60,000 (45,900–75,900)	67,400 (52,800–86,800)	80,100 (62,200–104,000)	102,000 (80,000–126,000)	110,000 (80,400–129,000)	118,000 (84,900–135,000)	142,000 (102,000–167,000)	162,000 (114,000–187,000)	171,000 (115,000–198,000)	170,000 (118,000–193,000)	164,000 (116,000–198,000)	143,000 (104,000–170,000)	110,000 (77,100–128,000)	79,900 (53,300–91,600)	50,800 (34,300–59,000)	20,700 (12,700–24,300)	7,770 (3,950–9,550)
Oral disorders	–	–	–	–	–	–	–	–	–	–	–	–	–	–	–	–	–	–	–	–	–	–	–
Caries of deciduous teeth	–	–	–	–	–	–	–	–	–	–	–	–	–	–	–	–	–	–	–	–	–	–	–
Caries of permanent teeth	–	–	–	–	–	–	–	–	–	–	–	–	–	–	–	–	–	–	–	–	–	–	–
Periodontal diseases	–	–	–	–	–	–	–	–	–	–	–	–	–	–	–	–	–	–	–	–	–	–	–
Other oral disorders	–	–	–	–	–	–	–	–	–	–	–	–	–	–	–	–	–	–	–	–	–	–	–
Sudden infant death syndrome	–	126,000 (66,500–233,000)	1,220,000 (510,000–2,710,000)	–	–	–	–	–	–	–	–	–	–	–	–	–	–	–	–	–	–	–	–
Injuries	585,000 (465,000–720,000)	366,000 (241,000–541,000)	2,540,000 (2,040,000–3,180,000)	6,390,000 (5,130,000–8,040,000)	4,490,000 (3,870,000–5,220,000)	4,430,000 (3,860,000–4,970,000)	11,000,000 (9,950,000–11,900,000)	16,200,000 (15,000,000–17,400,000)	15,000,000 (14,000,000–16,000,000)	13,900,000 (12,900,000–14,800,000)	11,900,000 (10,800,000–12,800,000)	9,830,000 (8,820,000–10,700,000)	8,600,000 (7,620,000–9,430,000)	7,280,000 (6,400,000–8,010,000)	5,870,000 (5,190,000–6,420,000)	4,500,000 (3,950,000–4,900,000)	3,530,000 (3,050,000–3,870,000)	2,570,000 (2,240,000–2,800,000)	1,810,000 (1,590,000–1,980,000)	1,270,000 (1,100,000–1,380,000)	713,000 (603,000–783,000)	256,000 (210,000–283,000)	66,600 (52,400–76,100)
Transport injuries	45,200 (29,200–59,700)	26,300 (17,600–34,300)	348,000 (247,000–458,000)	1,560,000 (1,180,000–2,080,000)	1,570,000 (1,290,000–1,880,000)	1,430,000 (1,180,000–1,670,000)	4,240,000 (3,700,000–4,730,000)	6,190,000 (5,490,000–6,820,000)	5,540,000 (4,850,000–6,060,000)	5,060,000 (4,290,000–5,530,000)	4,330,000 (3,620,000–4,800,000)	3,570,000 (2,920,000–3,990,000)	3,150,000 (2,530,000–3,550,000)	2,670,000 (2,120,000–3,020,000)	2,060,000 (1,680,000–2,310,000)	1,510,000 (1,190,000–1,680,000)	1,120,000 (872,000–1,260,000)	704,000 (566,000–781,000)	426,000 (350,000–471,000)	234,000 (198,000–257,000)	98,300 (83,300–109,000)	23,700 (19,200–26,400)	4,780 (3,720–5,600)
Road injuries	40,900 (26,100–54,000)	24,000 (16,000–31,200)	329,000 (232,000–434,000)	1,460,000 (1,100,000–1,980,000)	1,480,000 (1,220,000–1,780,000)	1,320,000 (1,090,000–1,540,000)	3,930,000 (3,430,000–4,410,000)	5,800,000 (5,130,000–6,390,000)	5,150,000 (4,520,000–5,630,000)	4,740,000 (4,020,000–5,190,000)	4,030,000 (3,360,000–4,470,000)	3,320,000 (2,720,000–3,720,000)	2,930,000 (2,360,000–3,300,000)	2,480,000 (1,970,000–2,810,000)	1,900,000 (1,560,000–2,140,000)	1,410,000 (1,110,000–1,580,000)	1,050,000 (816,000–1,180,000)	661,000 (528,000–734,000)	399,000 (328,000–440,000)	220,000 (186,000–242,000)	92,600 (78,500–103,000)	22,100 (17,900–24,700)	4,400 (3,430–5,150)
Pedestrian road injuries	15,000 (9,370–20,500)	9,040 (5,600–11,900)	119,000 (77,500–159,000)	676,000 (494,000–932,000)	723,000 (577,000–888,000)	574,000 (444,000–685,000)	970,000 (748,000–1,130,000)	1,400,000 (1,090,000–1,630,000)	1,340,000 (1,060,000–1,560,000)	1,380,000 (1,090,000–1,610,000)	1,210,000 (948,000–1,420,000)	1,050,000 (805,000–1,240,000)	1,010,000 (762,000–1,200,000)	942,000 (703,000–1,120,000)	732,000 (555,000–868,000)	617,000 (466,000–726,000)	486,000 (365,000–573,000)	329,000 (248,000–382,000)	223,000 (179,000–256,000)	124,000 (100,000–142,000)	53,200 (43,400–60,700)	11,500 (9,200–13,200)	2,280 (1,790–2,680)
Cyclist road injuries	–	–	–	65,700 (44,700–99,000)	79,100 (63,300–100,000)	99,900 (81,900–121,000)	172,000 (140,000–201,000)	178,000 (147,000–206,000)	167,000 (138,000–194,000)	184,000 (147,000–217,000)	165,000 (132,000–193,000)	169,000 (130,000–200,000)	179,000 (142,000–214,000)	182,000 (143,000–218,000)	143,000 (111,000–171,000)	131,000 (100,000–156,000)	104,000 (80,400–125,000)	63,600 (49,500–75,400)	31,700 (24,200–38,200)	15,300 (12,100–18,200)	5,960 (4,710–7,110)	1,290 (1,010–1,510)	7·32 (5·55–8·94)
Motorcyclist road injuries	4,090 (2,640–5,900)	2,630 (1,600–3,620)	38,700 (27,500–53,400)	123,000 (87,600–175,000)	110,000 (84,800–142,000)	159,000 (126,000–194,000)	1,170,000 (981,000–1,330,000)	1,800,000 (1,540,000–2,060,000)	1,470,000 (1,230,000–1,670,000)	1,200,000 (973,000–1,370,000)	996,000 (809,000–1,160,000)	787,000 (632,000–918,000)	652,000 (501,000–757,000)	488,000 (375,000–573,000)	361,000 (261,000–426,000)	199,000 (141,000–238,000)	135,000 (91,300–161,000)	63,100 (42,100–74,800)	26,000 (18,200–30,900)	16,400 (11,300–20,300)	5,540 (3,870–6,770)	1,260 (864–1,530)	252 (181–308)
Motor vehicle road injuries	21,300 (13,600–28,700)	11,900 (7,900–15,600)	165,000 (115,000–225,000)	579,000 (403,000–823,000)	550,000 (439,000–688,000)	462,000 (381,000–556,000)	1,580,000 (1,370,000–1,870,000)	2,370,000 (2,080,000–2,720,000)	2,120,000 (1,820,000–2,460,000)	1,940,000 (1,620,000–2,210,000)	1,630,000 (1,340,000–1,870,000)	1,290,000 (1,030,000–1,500,000)	1,060,000 (854,000–1,220,000)	837,000 (667,000–989,000)	647,000 (514,000–750,000)	446,000 (351,000–524,000)	309,000 (246,000–359,000)	196,000 (161,000–231,000)	113,000 (95,300–134,000)	60,400 (51,300–72,100)	26,500 (21,700–31,700)	7,750 (6,320–9,220)	1,780 (1,380–2,160)
Other road injuries	464 (300–725)	438 (298–614)	5,100 (3,520–7,080)	17,000 (10,900–30,800)	18,900 (13,400–25,700)	21,000 (15,200–28,400)	36,600 (30,000–44,400)	53,300 (44,400–62,800)	46,600 (38,900–54,400)	35,000 (29,700–41,300)	31,400 (26,200–36,600)	29,100 (24,300–33,500)	29,600 (24,200–34,600)	28,100 (23,300–33,500)	21,700 (17,900–25,700)	14,700 (12,300–17,100)	13,100 (10,300–15,700)	9,300 (7,540–11,000)	5,650 (4,560–6,780)	3,530 (2,830–4,310)	1,420 (1,110–1,740)	267 (204–375)	84·5 (61·6–105)
Other transport injuries	4,340 (2,830–5,970)	2,370 (1,600–3,190)	19,700 (13,900–25,700)	95,300 (55,900–132,000)	85,500 (66,900–106,000)	112,000 (87,100–134,000)	301,000 (236,000–357,000)	394,000 (322,000–458,000)	394,000 (325,000–449,000)	318,000 (258,000–359,000)	302,000 (241,000–346,000)	247,000 (195,000–284,000)	220,000 (171,000–252,000)	191,000 (148,000–218,000)	155,000 (118,000–179,000)	97,100 (76,700–112,000)	70,700 (56,000–81,300)	43,200 (34,300–49,100)	27,700 (21,800–32,100)	14,500 (11,800–16,700)	5,670 (4,700–6,490)	1,590 (1,290–1,840)	381 (294–467)
Unintentional injuries	326,000 (229,000–439,000)	269,000 (153,000–439,000)	1,890,000 (1,490,000–2,410,000)	4,280,000 (3,430,000–5,500,000)	2,580,000 (2,140,000–3,050,000)	2,140,000 (1,830,000–2,450,000)	2,490,000 (2,190,000–2,770,000)	3,170,000 (2,830,000–3,470,000)	3,010,000 (2,710,000–3,250,000)	3,080,000 (2,780,000–3,340,000)	2,900,000 (2,580,000–3,170,000)	2,700,000 (2,380,000–2,980,000)	2,590,000 (2,210,000–2,890,000)	2,360,000 (2,010,000–2,640,000)	2,070,000 (1,780,000–2,290,000)	1,790,000 (1,550,000–1,990,000)	1,550,000 (1,300,000–1,730,000)	1,290,000 (1,070,000–1,430,000)	1,020,000 (852,000–1,140,000)	822,000 (687,000–912,000)	517,000 (423,000–575,000)	209,000 (171,000–232,000)	57,200 (44,900–65,800)
Falls	46,700 (27,000–68,400)	19,200 (11,500–29,200)	202,000 (126,000–297,000)	447,000 (306,000–627,000)	335,000 (247,000–445,000)	310,000 (233,000–391,000)	370,000 (309,000–448,000)	587,000 (496,000–701,000)	630,000 (544,000–741,000)	720,000 (592,000–839,000)	766,000 (641,000–909,000)	804,000 (655,000–943,000)	805,000 (644,000–933,000)	843,000 (678,000–970,000)	799,000 (654,000–913,000)	759,000 (614,000–870,000)	724,000 (564,000–836,000)	700,000 (540,000–802,000)	615,000 (486,000–700,000)	540,000 (429,000–614,000)	368,000 (288,000–417,000)	158,000 (127,000–177,000)	43,300 (34,300–51,100)
Drowning	20,100 (14,700–26,800)	8,140 (5,400–11,100)	172,000 (125,000–231,000)	1,650,000 (1,320,000–2,140,000)	1,160,000 (968,000–1,400,000)	902,000 (781,000–1,050,000)	862,000 (752,000–976,000)	798,000 (703,000–912,000)	610,000 (546,000–682,000)	581,000 (516,000–647,000)	498,000 (436,000–563,000)	423,000 (371,000–473,000)	363,000 (317,000–410,000)	310,000 (269,000–352,000)	239,000 (205,000–267,000)	190,000 (162,000–212,000)	150,000 (126,000–168,000)	113,000 (93,900–127,000)	70,600 (58,300–79,200)	49,900 (40,700–56,000)	24,100 (19,600–27,000)	5,420 (4,380–6,080)	993 (750–1,150)
Fire, heat, and hot substances	17,000 (10,300–25,700)	10,800 (6,950–15,700)	146,000 (94,700–219,000)	433,000 (285,000–624,000)	145,000 (98,500–195,000)	82,300 (55,500–108,000)	109,000 (70,600–136,000)	166,000 (113,000–201,000)	172,000 (126,000–207,000)	178,000 (138,000–217,000)	178,000 (137,000–211,000)	151,000 (118,000–175,000)	143,000 (112,000–165,000)	130,000 (104,000–151,000)	115,000 (94,700–132,000)	102,000 (82,500–117,000)	79,500 (63,800–90,900)	62,500 (50,100–72,800)	44,000 (35,200–52,100)	35,800 (27,700–42,100)	19,900 (16,100–24,100)	6,510 (5,250–7,780)	1,620 (1,250–1,980)
Poisonings	16,800 (10,700–23,600)	14,700 (7,850–22,600)	139,000 (93,700–208,000)	325,000 (234,000–472,000)	112,000 (82,300–150,000)	103,000 (75,100–126,000)	119,000 (91,900–137,000)	168,000 (128,000–194,000)	175,000 (138,000–198,000)	174,000 (134,000–194,000)	160,000 (127,000–179,000)	153,000 (122,000–172,000)	145,000 (110,000–165,000)	136,000 (103,000–156,000)	113,000 (83,900–129,000)	95,500 (70,300–110,000)	68,500 (47,700–79,300)	48,000 (31,400–56,300)	29,500 (19,400–34,400)	18,700 (12,600–21,700)	8,300 (5,840–9,480)	1,500 (1,170–1,700)	235 (187–267)
Poisoning by carbon monoxide	2,820 (1,310–4,590)	2,560 (1,140–4,310)	21,000 (10,700–38,400)	65,800 (35,700–119,000)	39,100 (24,300–55,900)	39,000 (23,500–51,700)	72,700 (52,400–89,400)	101,000 (71,400–120,000)	106,000 (77,900–122,000)	115,000 (88,500–131,000)	105,000 (81,800–119,000)	98,100 (77,300–112,000)	92,000 (72,400–108,000)	82,400 (61,500–96,400)	68,400 (52,400–79,300)	58,800 (44,400–69,100)	40,700 (28,800–48,100)	27,700 (17,500–33,100)	17,300 (10,900–20,800)	11,300 (7,260–13,600)	5,110 (3,380–6,100)	983 (773–1,130)	134 (105–154)
Poisoning by other means	13,900 (9,090–19,500)	12,100 (6,440–18,900)	117,000 (79,300–173,000)	259,000 (189,000–375,000)	72,700 (54,400–97,500)	63,700 (49,300–81,300)	46,600 (32,800–62,600)	67,000 (47,500–94,800)	68,400 (50,000–94,700)	58,900 (41,000–80,300)	54,600 (38,500–73,600)	54,800 (36,300–71,700)	52,900 (32,300–70,500)	54,100 (32,500–71,700)	44,200 (27,600–57,600)	36,700 (21,800–48,900)	27,800 (16,000–39,200)	20,300 (11,500–28,600)	12,200 (6,820–17,500)	7,400 (4,280–10,500)	3,190 (2,050–4,570)	522 (382–685)	101 (77·9–121)
Exposure to mechanical forces	37,000 (12,900–70,100)	89,300 (11,100–245,000)	150,000 (56,700–285,000)	243,000 (167,000–348,000)	167,000 (118,000–223,000)	139,000 (103,000–175,000)	317,000 (245,000–386,000)	483,000 (374,000–574,000)	469,000 (371,000–543,000)	453,000 (359,000–517,000)	418,000 (326,000–481,000)	351,000 (261,000–408,000)	333,000 (242,000–397,000)	277,000 (202,000–328,000)	197,000 (145,000–231,000)	132,000 (98,300–153,000)	89,500 (64,300–105,000)	56,900 (43,200–65,600)	37,200 (27,700–42,500)	20,200 (15,500–23,200)	9,820 (7,800–11,000)	2,990 (2,420–3,390)	774 (593–962)
Unintentional firearm injuries	4,120 (895–9,350)	11,400 (631–43,600)	23,900 (7,960–52,400)	43,800 (24,500–70,700)	22,800 (14,800–37,300)	28,500 (21,600–41,200)	90,600 (68,900–125,000)	126,000 (96,600–170,000)	110,000 (78,400–141,000)	86,300 (61,900–111,000)	70,000 (46,600–92,000)	56,500 (38,100–75,200)	45,500 (28,500–60,900)	30,200 (19,100–39,300)	21,000 (13,800–27,900)	15,400 (9,740–20,300)	9,770 (6,260–13,100)	6,450 (4,080–8,530)	4,350 (2,940–5,750)	2,460 (1,540–3,140)	1,100 (707–1,430)	411 (270–552)	108 (72·6–157)
Other exposure to mechanical forces	32,900 (11,600–63,600)	77,900 (10,200–221,000)	126,000 (46,500–245,000)	199,000 (135,000–283,000)	144,000 (99,900–190,000)	110,000 (77,700–138,000)	226,000 (160,000–273,000)	357,000 (258,000–421,000)	359,000 (272,000–415,000)	367,000 (280,000–421,000)	348,000 (268,000–402,000)	295,000 (216,000–345,000)	288,000 (204,000–345,000)	247,000 (175,000–294,000)	176,000 (129,000–207,000)	117,000 (84,700–136,000)	79,700 (56,200–93,800)	50,500 (37,700–58,600)	32,800 (24,000–37,800)	17,800 (13,400–20,600)	8,720 (6,900–9,820)	2,580 (2,100–2,900)	666 (510–809)
Adverse effects of medical treatment	35,300 (24,700–49,400)	18,700 (13,100–26,200)	185,000 (125,000–251,000)	234,000 (131,000–349,000)	72,600 (53,800–93,500)	51,700 (37,800–68,000)	51,600 (35,700–73,000)	78,000 (58,000–101,000)	63,800 (47,300–82,200)	71,200 (54,100–88,300)	73,100 (55,300–89,700)	79,800 (61,000–97,800)	79,800 (61,500–96,500)	83,000 (64,900–97,600)	99,900 (79,600–115,000)	119,000 (93,300–140,000)	127,000 (100,000–149,000)	96,600 (77,200–112,000)	74,200 (60,100–85,400)	50,500 (41,200–58,300)	25,900 (21,400–29,700)	9,620 (7,530–11,000)	2,480 (1,890–2,850)
Animal contact	17,700 (9,850–25,800)	11,900 (7,150–18,000)	87,500 (54,900–143,000)	272,000 (184,000–423,000)	234,000 (157,000–337,000)	233,000 (155,000–313,000)	152,000 (107,000–194,000)	158,000 (108,000–199,000)	163,000 (115,000–205,000)	154,000 (110,000–191,000)	122,000 (80,300–150,000)	133,000 (91,100–167,000)	163,000 (115,000–205,000)	111,000 (83,000–134,000)	116,000 (82,000–143,000)	91,200 (62,900–111,000)	84,100 (56,600–104,000)	55,300 (37,200–67,700)	37,300 (24,600–45,700)	21,000 (12,400–25,400)	7,080 (4,880–8,500)	1,840 (1,270–2,200)	424 (298–530)
Venomous animal contact	14,700 (6,990–22,300)	9,310 (4,880–14,500)	58,800 (32,100–105,000)	158,000 (85,900–284,000)	174,000 (106,000–265,000)	196,000 (123,000–267,000)	125,000 (86,300–164,000)	134,000 (86,200–174,000)	140,000 (96,000–181,000)	134,000 (92,400–168,000)	103,000 (64,900–130,000)	113,000 (72,100–145,000)	140,000 (93,900–181,000)	86,800 (60,400–108,000)	93,900 (62,800–119,000)	71,800 (46,600–88,900)	68,700 (42,300–85,900)	45,300 (28,400–56,500)	30,400 (18,800–38,300)	17,400 (9,510–21,700)	5,550 (3,460–6,840)	1,430 (906–1,750)	308 (205–390)
Non-venomous animal contact	3,010 (2,240–4,270)	2,630 (1,910–3,800)	28,700 (20,100–42,200)	114,000 (79,400–163,000)	59,300 (42,500–84,900)	37,000 (26,400–53,400)	26,700 (19,000–37,300)	23,800 (17,600–32,600)	22,100 (16,900–28,700)	20,100 (15,500–26,200)	19,200 (14,600–26,100)	20,200 (14,900–27,900)	22,700 (16,700–31,800)	24,100 (18,600–33,800)	22,200 (16,900–30,500)	19,400 (15,300–27,100)	15,400 (12,200–20,700)	10,000 (7,970–13,400)	6,920 (5,420–9,270)	3,630 (2,820–4,810)	1,530 (1,230–1,930)	412 (322–507)	116 (87·5–142)
Foreign body	102,000 (79,300–127,000)	83,800 (66,800–101,000)	689,000 (571,000–814,000)	353,000 (290,000–435,000)	119,000 (102,000–138,000)	96,200 (84,200–110,000)	90,600 (80,500–102,000)	139,000 (125,000–155,000)	136,000 (125,000–148,000)	140,000 (130,000–152,000)	137,000 (125,000–148,000)	133,000 (122,000–146,000)	128,000 (116,000–140,000)	122,000 (111,000–132,000)	118,000 (109,000–128,000)	106,000 (98,100–114,000)	90,800 (84,600–96,800)	73,800 (68,900–78,400)	59,600 (55,100–63,300)	52,500 (46,600–56,400)	37,300 (32,100–40,400)	18,000 (14,700–19,800)	6,000 (4,590–6,770)
Pulmonary aspiration and foreign body in airway	97,700 (76,400–122,000)	82,300 (65,500–99,000)	668,000 (553,000–790,000)	292,000 (235,000–370,000)	106,000 (90,600–124,000)	88,700 (78,300–102,000)	79,200 (70,000–90,300)	127,000 (113,000–142,000)	125,000 (115,000–137,000)	131,000 (122,000–142,000)	127,000 (117,000–139,000)	124,000 (114,000–135,000)	121,000 (110,000–133,000)	117,000 (107,000–127,000)	114,000 (105,000–123,000)	102,000 (94,300–110,000)	87,700 (81,500–93,600)	71,100 (66,300–75,600)	57,200 (52,500–60,700)	50,800 (45,200–54,300)	36,500 (31,400–39,400)	17,700 (14,400–19,500)	5,920 (4,530–6,680)
Foreign body in eyes	–	–	–	–	–	–	–	–	–	–	–	–	–	–	–	–	–	–	–	–	–	–	–
Foreign body in other body part	4,250 (2,550–6,170)	1,510 (973–2,170)	20,700 (14,100–29,200)	61,300 (36,200–99,200)	13,100 (9,100–17,700)	7,510 (5,230–10,100)	11,400 (7,500–15,900)	12,500 (7,830–18,000)	10,600 (7,060–14,600)	9,450 (6,640–12,700)	9,360 (6,420–12,700)	9,640 (6,670–13,000)	7,080 (4,760–9,770)	5,050 (3,450–6,870)	4,300 (2,940–5,940)	4,230 (2,900–5,720)	3,120 (2,140–4,190)	2,770 (1,940–3,710)	2,410 (1,740–3,220)	1,670 (1,210–2,320)	874 (643–1,210)	302 (218–427)	73 (51·5–100)
Environmental heat and cold exposure	6,920 (1,670–11,700)	4,210 (934–7,790)	30,100 (9,400–52,300)	46,500 (22,400–75,200)	20,700 (9,950–30,600)	16,600 (8,050–22,700)	38,300 (22,100–50,500)	57,600 (34,900–73,600)	64,000 (43,200–76,400)	87,800 (63,200–99,300)	99,500 (75,800–112,000)	108,000 (82,900–124,000)	110,000 (85,900–125,000)	116,000 (87,500–134,000)	107,000 (82,100–126,000)	88,800 (66,200–103,000)	63,100 (40,000–76,800)	41,100 (23,200–50,300)	30,900 (16,000–39,200)	19,700 (10,700–24,400)	10,300 (6,100–12,100)	3,390 (2,350–3,930)	735 (549–844)
Exposure to forces of nature	980 (890–1,080)	726 (659–799)	4,520 (4,100–4,970)	18,400 (16,700–20,300)	19,400 (17,600–21,300)	24,600 (22,300–27,000)	26,000 (23,600–28,600)	23,000 (20,800–25,300)	19,900 (18,100–21,900)	16,300 (14,800–17,900)	13,300 (12,100–14,600)	10,800 (9,770–11,800)	8,910 (8,090–9,800)	7,040 (6,400–7,750)	4,980 (4,520–5,480)	4,050 (3,680–4,460)	2,710 (2,460–2,980)	1,830 (1,660–2,020)	1,110 (1,010–1,230)	613 (557–675)	221 (201–244)	50·8 (46·1–55·9)	10·4 (9·49–11·5)
Other unintentional injuries	25,900 (11,300–39,800)	7,400 (4,720–10,700)	86,100 (47,300–125,000)	261,000 (176,000–360,000)	191,000 (124,000–247,000)	183,000 (120,000–223,000)	358,000 (256,000–418,000)	515,000 (367,000–604,000)	509,000 (379,000–586,000)	498,000 (358,000–567,000)	433,000 (307,000–502,000)	357,000 (260,000–410,000)	314,000 (215,000–366,000)	223,000 (151,000–257,000)	159,000 (110,000–184,000)	106,000 (71,300–122,000)	70,900 (47,400–80,900)	35,900 (24,200–40,700)	21,800 (15,600–24,600)	12,800 (9,020–14,600)	6,270 (4,510–7,100)	2,130 (1,570–2,470)	598 (441–718)
Self-harm and interpersonal violence	213,000 (194,000–235,000)	70,600 (64,600–77,400)	298,000 (262,000–343,000)	549,000 (447,000–662,000)	349,000 (295,000–404,000)	856,000 (731,000–975,000)	4,220,000 (3,830,000–4,630,000)	6,870,000 (6,310,000–7,440,000)	6,480,000 (6,010,000–6,980,000)	5,750,000 (5,290,000–6,220,000)	4,620,000 (4,230,000–5,000,000)	3,550,000 (3,230,000–3,870,000)	2,860,000 (2,570,000–3,140,000)	2,250,000 (2,030,000–2,480,000)	1,740,000 (1,550,000–1,910,000)	1,200,000 (1,060,000–1,320,000)	861,000 (750,000–955,000)	580,000 (499,000–647,000)	367,000 (312,000–406,000)	212,000 (180,000–238,000)	97,300 (81,900–110,000)	23,600 (19,700–26,200)	4,640 (3,680–5,250)
Self-harm	–	–	–	–	–	381,000 (300,000–465,000)	1,690,000 (1,430,000–1,930,000)	2,990,000 (2,570,000–3,440,000)	3,130,000 (2,700,000–3,540,000)	2,960,000 (2,570,000–3,360,000)	2,510,000 (2,140,000–2,810,000)	2,050,000 (1,760,000–2,300,000)	1,750,000 (1,520,000–2,010,000)	1,490,000 (1,290,000–1,680,000)	1,230,000 (1,040,000–1,380,000)	883,000 (748,000–985,000)	657,000 (548,000–738,000)	460,000 (382,000–522,000)	302,000 (246,000–339,000)	188,000 (156,000–213,000)	86,400 (71,300–98,400)	20,400 (16,700–23,000)	3,450 (2,720–4,020)
Self-harm by firearm	–	–	–	–	–	18,800 (15,300–23,400)	143,000 (124,000–184,000)	230,000 (196,000–302,000)	215,000 (185,000–279,000)	197,000 (160,000–252,000)	172,000 (137,000–210,000)	157,000 (128,000–197,000)	154,000 (128,000–191,000)	141,000 (122,000–171,000)	129,000 (114,000–154,000)	96,100 (86,600–111,000)	70,500 (60,300–81,000)	48,100 (41,500–57,000)	31,300 (26,500–34,600)	19,100 (16,000–21,600)	9,510 (7,910–11,000)	2,910 (2,380–3,480)	554 (434–721)
Self-harm by other specified means	–	–	–	–	–	362,000 (283,000–446,000)	1,550,000 (1,290,000–1,780,000)	2,760,000 (2,310,000–3,170,000)	2,910,000 (2,500,000–3,290,000)	2,790,000 (2,370,000–3,130,000)	2,340,000 (1,990,000–2,630,000)	1,900,000 (1,610,000–2,130,000)	1,640,000 (1,360,000–1,850,000)	1,350,000 (1,140,000–1,530,000)	1,100,000 (907,000–1,250,000)	787,000 (647,000–882,000)	586,000 (479,000–666,000)	412,000 (333,000–471,000)	271,000 (217,000–307,000)	169,000 (138,000–193,000)	76,900 (62,400–89,200)	17,500 (14,100–19,900)	2,900 (2,240–3,370)
Interpersonal violence	56,100 (44,300–71,300)	16,200 (12,400–20,500)	152,000 (117,000–192,000)	349,000 (250,000–463,000)	238,000 (184,000–292,000)	372,000 (312,000–435,000)	2,250,000 (2,030,000–2,520,000)	3,450,000 (3,200,000–3,760,000)	3,050,000 (2,850,000–3,280,000)	2,550,000 (2,370,000–2,740,000)	1,960,000 (1,820,000–2,120,000)	1,400,000 (1,290,000–1,520,000)	982,000 (907,000–1,070,000)	691,000 (638,000–752,000)	464,000 (429,000–504,000)	288,000 (267,000–313,000)	175,000 (162,000–189,000)	95,200 (88,100–104,000)	46,700 (42,800–51,300)	11,800 (10,400–13,100)	4,300 (3,680–4,790)	1,240 (1,000–1,380)	763 (579–893)
Physical violence by firearm	11,500 (8,580–14,900)	3,360 (2,450–4,330)	27,800 (20,800–35,600)	78,100 (53,300–105,000)	49,200 (40,200–59,800)	126,000 (111,000–144,000)	1,260,000 (1,140,000–1,390,000)	1,910,000 (1,770,000–2,070,000)	1,580,000 (1,470,000–1,700,000)	1,220,000 (1,140,000–1,330,000)	921,000 (844,000–1,010,000)	632,000 (574,000–693,000)	405,000 (364,000–453,000)	278,000 (250,000–309,000)	171,000 (153,000–188,000)	90,100 (81,900–100,000)	47,500 (43,200–52,600)	23,800 (21,700–26,200)	12,000 (10,700–13,300)	3,200 (2,760–3,670)	1,070 (884–1,200)	373 (292–426)	279 (205–339)
Physical violence by sharp object	6,100 (3,790–8,460)	1,970 (1,060–2,790)	15,400 (9,020–21,500)	64,400 (34,400–92,000)	32,700 (19,900–41,800)	53,600 (33,600–65,900)	466,000 (348,000–548,000)	767,000 (595,000–884,000)	742,000 (615,000–850,000)	639,000 (526,000–741,000)	479,000 (400,000–554,000)	337,000 (271,000–385,000)	245,000 (194,000–280,000)	171,000 (135,000–194,000)	107,000 (87,600–124,000)	75,100 (61,200–86,600)	45,700 (37,200–52,700)	21,900 (18,000–25,200)	11,000 (9,350–12,700)	3,350 (2,870–3,860)	1,190 (977–1,380)	357 (285–412)	218 (165–257)
Sexual violence	–	–	–	–	–	–	–	–	–	–	–	–	–	–	–	–	–	–	–	–	–	–	–
Physical violence by other means	38,500 (30,400–48,500)	10,900 (8,270–13,900)	109,000 (84,300–139,000)	207,000 (152,000–267,000)	156,000 (117,000–197,000)	192,000 (152,000–234,000)	528,000 (433,000–631,000)	773,000 (668,000–909,000)	730,000 (638,000–834,000)	685,000 (609,000–784,000)	558,000 (492,000–626,000)	432,000 (383,000–480,000)	332,000 (293,000–370,000)	242,000 (217,000–268,000)	186,000 (167,000–207,000)	123,000 (111,000–136,000)	81,200 (73,800–90,100)	49,500 (44,600–55,700)	23,600 (21,400–26,700)	5,230 (4,600–5,980)	2,040 (1,750–2,310)	505 (412–573)	266 (203–307)
Conflict and terrorism	157,000 (143,000–173,000)	54,400 (49,400–59,800)	145,000 (132,000–159,000)	192,000 (174,000–211,000)	105,000 (95,700–116,000)	95,500 (86,800–105,000)	231,000 (210,000–254,000)	345,000 (313,000–379,000)	232,000 (211,000–255,000)	159,000 (144,000–175,000)	107,000 (97,100–118,000)	72,600 (65,900–79,900)	64,900 (58,900–71,400)	54,500 (49,500–59,900)	38,100 (34,600–41,900)	29,900 (27,100–32,900)	27,600 (25,000–30,300)	22,900 (20,800–25,200)	17,400 (15,800–19,200)	11,800 (10,800–13,000)	6,370 (5,780–7,010)	1,850 (1,680–2,030)	384 (349–423)

SR Table 9: Global YLLs by cause and age group for both sexes combined (with 95% confidence intervals), 2019

Cause	Early Neonatal	Late Neonatal	Post Neonatal	1 to 4	5 to 9	10 to 14	15 to 19	20 to 24	25 to 29	30 to 34	35 to 39	40 to 44	45 to 49	50 to 54	55 to 59	60 to 64	65 to 69	70 to 74	75 to 79	80 to 84	85 to 89	90 to 94	95 plus
All causes	170,000,000 (144.000.000–200.000.000)	42,900,000 (36.500.000–50.700.000)	117,000,000 (96.900.000–142.000.000)	111,000,000 (93.600.000–132.000.000)	30,800,000 (28.000.000–34.200.000)	22,800,000 (21.200.000–24.600.000)	35,600,000 (33.300.000–37.900.000)	46,100,000 (43.100.000–49.000.000)	48,700,000 (45.700.000–51.600.000)	55,200,000 (52.200.000–58.300.000)	60,500,000 (57.100.000–64.100.000)	67,400,000 (63.200.000–71.500.000)	78,100,000 (73.000.000–83.000.000)	94,700,000 (88.600.000–101.000.000)	105,000,000 (98.400.000–111.000.000)	115,000,000 (108.000.000–121.000.000)	118,000,000 (112.000.000–124.000.000)	109,000,000 (104.000.000–114.000.000)	91,700,000 (87.700.000–95.500.000)	78,100,000 (75.200.000–80.700.000)	47,900,000 (46.500.000–49.200.000)	21,100,000 (20.600.000–21.500.000)	7,010,000 (6.920.000–7.100.000)
Communicable, maternal, neonatal, and nutritional diseases	153,000,000 (130.000.000–180.000.000)	36,200,000 (30.800.000–43.100.000)	89,600,000 (73.400.000–111.000.000)	83,900,000 (69 700 000–101 000 000)	16,600,000 (14.500.000–19.000.000)	9,930,000 (8.660.000–11.400.000)	11,200,000 (9.840.000–12.600.000)	12,800,000 (11.500.000–14.100.000)	14,100,000 (12.900.000–15.300.000)	15,800,000 (14.700.000–17.000.000)	16,500,000 (15.300.000–17.700.000)	15,100,000 (14.000.000–16.200.000)	13,500,000 (12.500.000–14.600.000)	12,100,000 (11.100.000–13.400.000)	10,900,000 (9.860.000–12.100.000)	10,500,000 (9.340.000–11.900.000)	9,850,000 (8.730.000–11.300.000)	9,340,000 (8.230.000–10.900.000)	7,690,000 (6.730.000–8.960.000)	6,590,000 (5.640.000–7.880.000)	4,020,000 (3.390.000–4.660.000)	1,900,000 (1.520.000–2.210.000)	702,000 (531.000–808.000)
HIV/AIDS and sexually transmitted infections	4,250,000 (1.440.000–8.900.000)	1,160,000 (396 000–2 410 000)	3,090,000 (1 940 000–4 880 000)	2,620,000 (2 340 000–2 950 000)	1,000,000 (933 000–1 080 000)	1,120,000 (1 070 000–1 180 000)	1,990,000 (1 820 000–2 190 000)	2,350,000 (2 190 000–2 540 000)	4,000,000 (3 680 000–4 340 000)	5,860,000 (5 510 000–6 250 000)	6,560,000 (6 240 000–6 930 000)	5,870,000 (5 660 000–6 100 000)	4,620,000 (4 450 000–4 810 000)	3,050,000 (2 940 000–3 150 000)	1,690,000 (1 630 000–1 750 000)	881,000 (848 000–919 000)	457,000 (438 000–480 000)	189,000 (179 000–202 000)	83,100 (79 000–87 900)	21,600 (20 100–23 200)	8,630 (7 760–9 480)	2,740 (2 260–3 220)	843 (666–1 020)
HIV/AIDS	–	–	1,430,000 (1 250 000–1 650 000)	2,540,000 (2 280 000–2 840 000)	967,000 (906 000–1 030 000)	1,110,000 (1 060 000–1 170 000)	1,970,000 (1 800 000–2 170 000)	2,320,000 (2 160 000–2 520 000)	3,960,000 (3 650 000–4 310 000)	5,830,000 (5 480 000–6 220 000)	6,530,000 (6 210 000–6 900 000)	5,840,000 (5 630 000–6 070 000)	4,600,000 (4 430 000–4 790 000)	3,020,000 (2 920 000–3 130 000)	1,660,000 (1 600 000–1 720 000)	866,000 (831 000–904 000)	444,000 (425 000–468 000)	181,000 (171 000–193 000)	76,300 (72 400–80 700)	17,100 (15 900–18 400)	5,610 (5 260–6 000)	1,240 (1 170–1 330)	287 (275–302)
HIV/AIDS - Drug-susceptible Tuberculosis	–	–	355,000 (260 000–453 000)	673,000 (496 000–830 000)	264,000 (202 000–320 000)	308,000 (235 000–378 000)	496,000 (359 000–641 000)	558,000 (395 000–712 000)	887,000 (625 000–1 150 000)	1,270,000 (895 000–1 650 000)	1,420,000 (1 010 000–1 850 000)	1,320,000 (939 000–1 710 000)	1,040,000 (735 000–1 340 000)	693,000 (498 000–900 000)	379,000 (273 000–493 000)	194,000 (140 000–252 000)	92,600 (66 700–121 000)	38,900 (27 900–49 900)	16,500 (11 700–21 200)	2,950 (2 090–3 840)	967 (665–1 290)	216 (147–291)	46·4 (32·4–62·2)
HIV/AIDS - Multidrug-resistant Tuberculosis without extensive drug resistance	–	–	43,200 (18 600–79 900)	79,100 (31 400–153 000)	28,800 (12.500–53.800)	31,100 (13.700–59.200)	53,300 (23.000–102.000)	59,300 (25.900–113.000)	90,000 (39.100–175.000)	126,000 (56.000–241.000)	139,000 (62.600–264.000)	126,000 (57.100–239.000)	97,800 (43.700–183.000)	65,800 (29.000–122.000)	35,800 (15.700–66.700)	18,200 (7.950–34.200)	8,710 (3.730–16.500)	3,680 (1.580–6.970)	1,550 (672–2.900)	287 (119–558)	93·2 (38–184)	20·6 (8·39–40·1)	4·18 (1·75–7·96)
HIV/AIDS - Extensively drug-resistant Tuberculosis	–	–	1,210 (539–2.250)	1,430 (585–2.780)	646 (285–1.240)	648 (284–1.230)	1,180 (505–2.220)	1,820 (802–3.490)	3,970 (1.790–7.480)	6,900 (3.290–12.700)	7,680 (3.730–14.000)	6,060 (2.890–11.200)	3,970 (1.850–7.440)	2,440 (1.130–4.630)	1,300 (593–2.500)	666 (295–1.300)	354 (147–726)	118 (46–246)	48·4 (18·4–103)	14 (4·52–33·6)	4·68 (1·58–11)	0·919 (0·356–1·95)	0·293 (0·117–0·594)
HIV/AIDS resulting in other diseases	–	–	1,030,000 (879.000–1.240.000)	1,790,000 (1.540.000–2.150.000)	674,000 (602.000–756.000)	772,000 (689.000–865.000)	1,420,000 (1.230.000–1.660.000)	1,700,000 (1.490.000–1.960.000)	2,980,000 (2.590.000–3.410.000)	4,430,000 (3.910.000–5.000.000)	4,960,000 (4.410.000–5.530.000)	4,390,000 (3.930.000–4.870.000)	3,470,000 (3.090.000–3.850.000)	2,260,000 (2.020.000–2.500.000)	1,250,000 (1.120.000–1.380.000)	653,000 (586.000–721.000)	342,000 (309.000–378.000)	138,000 (123.000–155.000)	58,200 (51.800–64.700)	13,800 (12.200–15.300)	4,550 (4.030–5.050)	1,000 (896–1.110)	236 (215–258)
Sexually transmitted infections excluding HIV	4,250,000 (1.440.000–8.900.000)	1,160,000 (396 000–2 410 000)	1,670,000 (573 000–3 480 000)	74,300 (24 900–162 000)	34,100 (10 900–73 700)	10,200 (8 240–12 600)	18,700 (15 000–22 800)	26,000 (20 500–31 300)	31,500 (23 500–38 400)	33,000 (24 000–40 100)	27,100 (20 200–32 400)	27,600 (21 200–33 700)	22,100 (17 300–25 600)	24,900 (17 400–31 000)	25,800 (17 400–32 900)	15,400 (11 700–18 600)	12,600 (10 100–15 400)	8,590 (7 190–10 100)	6,840 (5 850–8 070)	4,560 (3 640–5 320)	3,020 (2 250–3 690)	1,500 (1 040–1 980)	556 (377–730)
Syphilis	4,250,000 (1.440.000–8.900.000)	1,160,000 (396 000–2 410 000)	1,670,000 (573 000–3 480 000)	74,300 (24 900–162 000)	34,100 (10 900–73 700)	5,760 (4 660–7 130)	6,210 (4 730–7 590)	4,080 (3 030–4 930)	7,590 (5 720–9 040)	10,000 (8 850–12 700)	7,250 (4 680–9 450)	4,180 (2 650–5 470)	4,110 (2 710–5 340)	5,780 (3 330–7 680)	5,630 (2 630–8 220)	4,020 (2 420–5 650)	2,370 (1 890–3 300)	2,050 (1 690–2 730)	1,360 (1 110–1 890)	975 (771–1 170)	566 (395–740)	305 (185–425)	100 (61·5–138)
Chlamydial infection	–	–	–	–	–	534 (406–660)	1,530 (1 190–1 920)	4,910 (3 810–6 000)	4,230 (3 180–5 200)	1,900 (1 400–2 350)	3,050 (2 330–3 690)	3,780 (2 860–4 740)	3,000 (2 420–3 490)	2,870 (2 090–3 680)	3,170 (2 240–4 190)	1,850 (1 390–2 300)	1,120 (869–1 390)	1,010 (808–1 190)	835 (695–969)	741 (585–865)	426 (326–500)	205 (152–247)	81·2 (58·6–97·3)
Gonococcal infection	–	–	–	–	–	1,110 (882–1 340)	4,860 (3 850–6 020)	8,880 (6 930–10 800)	11,000 (8 100–13 600)	10,500 (7 990–12 600)	8,380 (6 480–10 100)	10,800 (8 330–13 400)	8,030 (6 440–9 300)	9,060 (6 520–11 500)	9,280 (6 540–12 200)	5,260 (4 030–6 370)	6,290 (4 930–7 740)	3,610 (2 930–4 230)	3,120 (2 600–3 600)	1,840 (1 480–2 130)	1,170 (893–1 370)	549 (407–658)	219 (158–257)
Trichomoniasis	–	–	–	–	–	–	–	–	–	–	–	–	–	–	–	–	–	–	–	–	–	–	–

Cause	[illegible]
Anorexia nervosa	–
Bulimia nervosa	–
Autism spectrum disorders	–
Attention-deficit/hyperactivity disorder	–
Conduct disorder	–
Idiopathic developmental intellectual disability	–
Other mental disorders	–
Substance use disorders	0 (0–0)
Alcohol use disorders	–
Drug use disorders	0 (0–0)
Opioid use disorders	0 (0–0)
Cocaine use disorders	–
Amphetamine use disorders	–
Cannabis use disorders	–
Other drug use disorders	–
Diabetes and kidney diseases	3,280 (2,490–4,220)
Diabetes mellitus	3,280 (2,490–4,220)
Diabetes mellitus type 1	3,280 (2,490–4,220)
Diabetes mellitus type 2	–
Chronic kidney disease	–
Chronic kidney disease due to diabetes mellitus type 1	–
Chronic kidney disease due to diabetes mellitus type 2	–
Chronic kidney disease due to hypertension	–
Chronic kidney disease due to glomerulonephritis	–
Chronic kidney disease due to other and unspecified causes	–
Acute glomerulonephritis	–
Skin and subcutaneous diseases	–
Dermatitis	–
Atopic dermatitis	–
Contact dermatitis	–
Seborrhoeic dermatitis	–
Psoriasis	–
Bacterial skin diseases	–
Cellulitis	–
Pyoderma	–
Scabies	–
Fungal skin diseases	–
Viral skin diseases	–
Acne vulgaris	–
Alopecia areata	–
Pruritus	–
Urticaria	–
Decubitus ulcer	–
Other skin and subcutaneous diseases	–
Sense organ diseases	–
Blindness and vision loss	–
Glaucoma	–
Cataract	–
Age-related macular degeneration	–
Refraction disorders	–
Near vision loss	–
Other vision loss	–
Age-related and other hearing loss	–
Other sense organ diseases	–
Musculoskeletal disorders	–
Rheumatoid arthritis	–
Osteoarthritis	–
Osteoarthritis hip	–
Osteoarthritis knee	–
Osteoarthritis hand	–
Osteoarthritis other	–
Low back pain	–
Neck pain	–
Gout	–
Other musculoskeletal disorders	–
Other non-communicable diseases	15,500,000 [illegible]
Congenital birth defects	15,300,000 [illegible]
Neural tube defects	2,940,000 [illegible]
Congenital heart anomalies	4,410,000 [illegible]
Orofacial clefts	103,000 [illegible]
Down syndrome	269,000 [illegible]
Turner syndrome	–
Klinefelter syndrome	–
Other chromosomal abnormalities	612,000 [illegible]
Congenital musculoskeletal and limb anomalies	406,000 [illegible]
Urogenital congenital anomalies	493,000 [illegible]
Digestive congenital anomalies	1,500,000 [illegible]
Other congenital birth defects	4,530,000 [illegible]
Urinary diseases and male infertility	46,400 [illegible]
Urolithiasis	–
Benign prostatic hyperplasia	–
Male infertility	–
Other urinary diseases	33,600 [illegible]
Gynecological diseases	–
Uterine fibroids	–
Polycystic ovarian syndrome	–
Female infertility	–
Endometriosis	–
Genital prolapse	–
Premenstrual syndrome	–
Other gynecological diseases	–
Hemoglobinopathies and hemolytic anemias	58,000 [illegible]
Thalassemias	24,800 [illegible]
Thalassemias trait	–
Sickle cell disorders	30,900 [illegible]
Sickle cell trait	–
G6PD deficiency	948 (544–1,470)
G6PD trait	–
Other hemoglobinopathies and hemolytic anemias	1,380 (965–1,930)
Endocrine, metabolic, blood, and immune disorders	107,000 [illegible]
Oral disorders	–
Caries of deciduous teeth	–
Caries of permanent teeth	–
Periodontal diseases	–
Other oral disorders	–
Sudden infant death syndrome	–
Injuries	1,090,000 [illegible]
Transport injuries	67,300 [illegible]
Road injuries	61,500 [illegible]
Pedestrian road injuries	23,700 [illegible]
Cyclist road injuries	–
Motorcyclist road injuries	5,930 [illegible]
Motor vehicle road injuries	31,100 [illegible]
Other road injuries	767 [illegible]
Other transport injuries	5,760 [illegible]
Unintentional injuries	635,000 [illegible]
Falls	107,000 [illegible]
Drowning	34,100 [illegible]
Fire, heat, and hot substances	26,600 [illegible]
Poisonings	25,900 [illegible]
Poisoning by carbon monoxide	5,270 [illegible]
Poisoning by other means	20,600 [illegible]
Exposure to mechanical forces	81,900 [illegible]
Unintentional firearm injuries	11,000 [illegible]
Other exposure to mechanical forces	70,800 [illegible]
Adverse effects of medical treatment	70,200 [illegible]
Animal contact	28,200 [illegible]
Venomous animal contact	23,300 [illegible]
Non-venomous animal contact	4,900 [illegible]
Foreign body	210,000 [illegible]
Pulmonary aspiration and foreign body in airway	205,000 [illegible]
Foreign body in eyes	–
Foreign body in other body part	5,340 [illegible]
Environmental heat and cold exposure	11,900 [illegible]
Exposure to forces of nature	1,620 [illegible]
Other unintentional injuries	37,100 [illegible]
Self-harm and interpersonal violence	388,000 [illegible]
Self-harm	–
Self-harm by firearm	–
Self-harm by other specified means	–
Interpersonal violence	112,000 [illegible]
Physical violence by firearm	17,700 [illegible]
Physical violence by sharp object	9,870 [illegible]
Sexual violence	–
Physical violence by other means	84,400 [illegible]
Conflict and terrorism	276,000 [illegible]

SR Table 10: Deaths due to fatal discontinuities by country, all ages, for both sexes combined, 1980-2019

Country	1980	1981	1982	1983	1984	1985	1986	1987	1988	1989	1990	1991	1992	1993	1994	1995	1996	1997	1998	1999	2000	2001	2002	2003	2004	2005	2006	2007	2008	2009	2010	2011	2012	2013	2014	2015	2016	2017	2018	2019
Afghanistan	35000	30000	35500	55012	80001	65000	60000	45070	30128	5482	1772	5118	5048	5336	9356	6645	3756	7079	19535	5461	6038	6210	5412	1267	1195	2885	6121	8572	8259	7768	8902	9342	11803	12083	18600	24691	28625	20443	28034	24513
Albania	0	0	1	0	0	125	0	2	0	22	22	60	12	2	44	6	16	2027	5	13	1	5	8	0	28	15	18	18	46	0	16	0	18	0	1	1	2	7	1	51
Algeria	2653	43	167	9	54	56	0	33	116	49	48	76	551	863	2702	3526	1632	5571	4559	1568	2604	2328	1777	3496	720	367	443	813	633	740	347	382	378	281	245	189	153	135	361	23
American Samoa	0	0	0	0	0	0	0	0	0	0	0	0	0	0	0	0	0	0	0	0	0	0	0	7	1	2	2	2	1	34	0	0	0	2	0	0	0	0	0	0
Andorra	0	0	0	0	0	0	0	0	0	0	0	0	0	0	0	0	0	0	0	0	0	0	0	0	0	0	0	0	0	0	0	0	0	0	0	0	0	0	0	0
Angola	5873	5858	5851	6257	6029	5862	6003	5915	7635	4075	3818	1556	3000	12893	4988	882	135	2922	14199	7321	3714	3450	834	129	269	278	2099	1014	387	279	78	264	122	120	29	111	505	49	82	48
Antigua and Barbuda	0	0	0	0	0	0	0	0	0	2	0	1	0	0	0	2	0	32	2	2	0	0	0	0	0	0	0	0	0	0	0	0	0	0	0	0	0	1	0	0
Argentina	241	450	50	50	190	248	225	160	208	220	185	114	189	158	132	54	53	22	23	115	101	122	29	32	15	48	5	49	14	22	64	84	108	128	8	17	11	77	2	0
Armenia	0	0	0	0	0	0	0	0	25000	0	25	0	6	12	45	10	2	4	0	8	0	1	0	0	1	1	3	16	8	7	5	6	11	7	22	51	120	0	1	0
Australia	13	12	7	88	43	22	16	2	28	52	64	45	12	16	59	13	71	40	7	16	18	8	34	22	3	13	4	16	5	193	15	64	9	7	24	12	10	12	2	4
Austria	2	8	16	1	46	19	1	7	1	6	3	10	0	0	2	13	4	3	2	53	14	1	18	33	5	27	22	19	47	25	24	1	20	24	6	30	9	11	0	11
Azerbaijan	0	1	5	0	0	2	6	4	3	69	63	100	2496	1344	1587	453	7	107	35	19	65	8	48	16	9	47	5	37	35	8	23	21	21	11	46	107	138	36	41	0
Bahamas	0	1	0	0	0	1	0	0	0	0	0	0	4	5	6	0	1	1	2	2	0	0	1	0	12	1	0	0	0	0	3	0	1	0	0	33	0	0	0	78
Bahrain	0	0	0	0	0	0	74	0	0	0	10	2	0	0	0	1	8	5	0	0	143	0	0	0	0	0	69	0	2	0	1	26	14	16	9	9	4	18	0	0
Bangladesh	743	1162	2812	1517	2473	16627	2687	3939	4498	2160	1544	145688	1400	797	833	2666	1271	927	1717	874	811	822	1017	1997	1678	1070	549	5811	148	526	667	733	1081	1559	659	628	562	671	207	579
Barbados	0	0	0	1	3	1	0	1	0	0	0	0	6	4	0	0	0	0	0	0	0	0	0	1	1	0	0	0	0	0	0	0	0	0	0	0	0	1	0	0
Belarus	0	31	24	0	0	16	0	0	0	0	0	0	0	0	0	0	0	6	0	2	0	0	14	50	0	0	0	15	18	16	11	35	0	10	9	0	1	45	4	0
Belgium	20	7	13	3	1	13	4	192	17	17	38	54	1	13	22	16	0	7	1	4	1	0	3	41	11	5	65	2	0	0	16	5	7	5	4	15	72	0	5	0
Belize	0	0	0	0	0	0	0	0	0	12	6	18	1	7	14	7	1	0	9	0	14	30	0	0	0	3	0	0	8	0	0	0	0	0	0	0	0	0	0	0
Benin	29	55	27	45	26	113	56	162	56	496	58	348	17	2	175	32	279	133	210	113	414	408	171	231	107	81	122	129	53	126	71	70	136	110	100	55	31	15	25	26
Bermuda	0	0	0	0	0	0	0	0	0	0	0	0	0	0	0	0	0	0	0	0	7	0	0	4	0	0	0	0	0	0	0	0	0	0	0	0	0	0	0	0
Bhutan	0	0	0	0	0	61	21	0	0	0	0	19	6	0	39	0	0	4	0	0	200	0	0	0	0	0	4	0	5	23	0	1	0	0	0	0	0	0	0	0
Bolivia (Plurinational State of)	11	1	20	250	1	29	30	26	2	92	6	361	469	275	52	54	143	78	189	101	159	50	169	171	94	0	91	66	153	37	94	148	43	137	126	40	5	0	37	75
Bosnia and Herzegovina	0	0	0	0	0	686	697	671	607	607	657	512	7686	5812	3140	9757	2	17	2	1	51	0	0	0	0	4	0	1	13	1	6	0	4	0	28	4	0	0	1	1
Botswana	7	4	8	1	4	0	3	4	196	4	11	3	0	0	0	20	0	0	0	0	3	0	0	0	0	0	470	1	2	3	1	0	2	13	0	0	1	0	3	0
Brazil	160	484	146	701	564	381	230	434	1073	740	541	758	841	2247	3343	5373	4635	4224	3831	3512	3735	3590	3960	3742	3737	3302	2974	2141	1658	1104	1652	2814	420	1659	1834	1437	1728	784	585	691
Brunei Darussalam	0	0	0	0	0	0	0	0	0	0	0	0	0	0	0	0	0	0	0	0	0	0	0	0	0	0	0	0	0	0	0	0	0	0	0	3	0	0	0	0
Bulgaria	0	0	2	0	2	0	3	0	4	20	1	2	4	2	16	3	10	3	38	5	27	2	2	2	30	1	31	18	11	0	15	0	98	0	5	8	0	10	16	0
Burkina Faso	306	1593	752	927	1119	749	458	99	533	0	338	357	426	0	22	434	8535	4721	902	671	793	1777	1307	1141	679	779	873	1542	414	697	861	636	800	363	389	69	185	135	342	1006
Burundi	35	36	22	57	29	18	25	9	18	131	11	395	893	5	12806	13144	13909	14970	13146	13398	14575	13751	13435	13640	12825	13097	286	105	305	527	283	231	35	42	166	833	489	331	351	124
Cabo Verde	6	0	4	1	29	0	0	7	10	21	3	40	39	28	37	208	3	0	1	18	0	0	0	2	0	0	0	57	0	9	0	0	0	0	0	24	0	0	0	0
Cambodia	6971	6971	6971	6971	6972	6972	6972	1055	200	827	377	535	307	588	1592	993	1140	750	800	64	363	61	33	2	1	33	9	209	1	46	402	276	27	213	55	8	6	3	17	4
Cameroon	23	221	167	306	873	338	1981	160	342	356	415	714	1145	1102	256	390	411	355	774	352	126	64	128	280	48	148	232	270	221	197	742	895	101	84	1321	1885	1067	939	1764	757
Canada	185	132	40	13	17	359	22	32	13	84	26	17	9	38	40	41	61	22	50	38	25	39	14	31	6	11	46	48	60	46	27	19	7	28	24	8	2	2	86	1
Central African Republic	31	27	34	13	15	17	51	120	218	221	170	150	204	115	2	0	85	218	79	252	474	613	504	211	158	61	305	449	244	374	422	350	251	4820	5971	1158	1007	2474	900	348
Chad	1505	1632	2248	2440	2268	783	1558	6448	919	51	688	1436	1544	545	565	55	208	871	1319	774	1838	962	489	196	499	721	2812	639	1227	252	300	579	125	92	88	622	51	179	430	363
Chile	6	19	2	20	164	210	64	173	19	45	45	327	23	134	13	70	2	63	4	1	17	40	21	0	5	58	42	55	26	23	612	15	25	34	44	227	13	42	0	17
China	6689	2809	742	1835	6409	2187	1409	3672	5152	6579	1850	5940	1958	3458	5285	3295	6598	3077	5711	3299	2766	2812	3010	2997	2531	3122	3104	2315	89454	1748	8027	1530	1219	2111	2484	2188	1738	789	534	668
Colombia	427	988	628	1027	972	23569	1468	1802	2542	1351	1408	1855	2208	1259	1601	1801	2045	3670	6015	8016	10152	11570	13954	3632	3027	1818	924	762	500	642	1053	883	568	379	510	484	104	792	195	238
Comoros	0	0	0	33	0	2	0	24	9	32	0	0	1	0	0	0	199	56	121	14	91	4	62	0	0	26	41	104	0	184	0	120	40	35	0	0	0	0	0	8
Congo	32	27	15	0	6	6	0	0	7	0	23	110	23	502	174	2	1	10489	3826	1005	552	48	237	60	52	29	16	57	27	100	273	66	383	58	11	4	69	23	66	18
Cook Islands	0	0	0	0	0	0	0	5	0	0	8	0	0	0	0	0	0	19	0	0	0	0	0	0	0	0	0	0	0	0	0	0	0	0	0	0	0	0	0	0
Costa Rica	1	4	4	12	2	0	0	0	36	2	4	48	0	12	3	0	61	6	13	17	48	3	11	3	10	8	0	19	12	22	51	4	7	0	1	0	9	25	1	19
Croatia	0	0	0	0	0	4	12	3	6	6	4	3490	2181	756	226	966	156	41	28	16	17	4	8	14	11	21	1	18	4	5	5	2	15	3	2	2	3	0	0	1
Cuba	151	214	245	250	244	237	225	182	220	159	151	99	74	120	73	40	46	114	38	40	6	8	9	3	8	36	9	13	10	15	32	0	20	2	0	3	1	10	121	6
Cyprus	3	0	0	0	6	3	1	1	6	1	0	0	0	0	2	2	3	0	52	0	5	0	0	0	1	31	2	4	0	1	0	13	0	0	0	0	0	0	50	71
Czechia	0	0	0	0	0	0	0	2	0	0	11	11	0	0	13	1	3	19	5	3	0	2	7	7	4	2	32	1	4	10	55	1	20	2	6	0	0	1	16	0
Côte d'Ivoire	204	107	130	71	25	105	63	195	322	531	20	16	8	66	79	227	60	253	371	671	482	270	1421	1210	811	458	96	73	69	77	219	2076	180	81	134	117	114	60	36	27
Democratic People's Republic of Korea	0	0	0	0	0	0	0	435	0	0	0	110	0	229	3	97668	73316	73200	73250	73242	73246	73324	73203	0	185	207	280	722	15	0	90	104	153	51	0	33	552	200	268	0
Democratic Republic of the Congo	81	806	700	249	16	48	126	1036	60	120	275	829	182	3463	4553	680	35428	15293	11601	11737	5721	3552	12242	8415	4044	2139	1719	2018	5453	7412	1926	2010	4111	4350	3210	1921	3635	8234	3613	9493
Denmark	0	9	0	2	0	1	0	0	0	0	1	0	1	0	0	4	0	0	0	10	4	4	1	4	0	3	0	2	13	3	0	3	0	2	0	4	1	0	0	0
Djibouti	0	25	0	0	0	7	0	10	10	21	60	82	357	122	200	17	0	51	65	39	4	0	3	0	51	0	143	6	9	17	22	4	1	6	9	27	0	0	2	104
Dominica	1	6	0	0	6	2	0	0	0	0	0	1	0	0	0	2	0	0	11	0	0	0	0	2	2	0	0	5	0	0	3	0	0	0	0	36	0	64	0	0
Dominican Republic	8	20	9	4	33	67	12	113	2	53	63	11	56	30	72	13	263	8	386	24	3	5	32	61	800	170	28	191	53	42	39	139	251	54	22	23	49	5	0	0
Ecuador	13	10	70	458	61	4	21	5244	24	77	35	379	231	538	43	87	116	181	254	17	150	110	142	86	27	25	95	80	105	33	143	34	95	50	73	45	736	62	48	31
Egypt	189	167	270	351	285	230	233	310	454	11	44	757	893	220	956	544	405	364	147	228	206	152	638	121	367	370	1313	85	381	123	136	1394	424	2326	777	1767	882	1763	1576	717
El Salvador	2782	14579	7069	10667	7315	5013	5430	3428	1416	5284	1175	1420	130	39	60	84	40	12	484	55	94	1168	34	307	70	172	6	14	49	401	49	49	3248	2999	2959	3298	0	10	4	5
Equatorial Guinea	0	0	0	0	31	1	0	15	0	0	0	0	1	0	0	0	0	0	6	0	1	1	0	3	17	124	0	3	26	3	4	0	1	0	1	0	0	1	4	0
Eritrea	10410	10409	10409	10409	10410	10410	10410	10410	10410	10410	10410	10410	0	49	0	33	92	32	878	19079	1779	1	0	78	56	217	2	14	245	10	2	20	1	38	10	2	27	1	1	0
Estonia	0	12	12	0	0	15	4	2	3	2	0	0	0	0	290	2	1	0	0	0	1	3	1	0	0	0	1	2	1	5	1	5	18	0	0	0	0	0	7	0
Eswatini	0	17	13	500	53	0	0	4	0	2	0	0	30	0	0	0	80	0	2	0	33	138	2	14	38	0	1	2	2	0	1	3	2	0	11	15	0	0	0	0
Ethiopia	2391	3997	2130	122667	181749	3040	1060	2217	8988	26976	49982	12766	1663	163	513	1616	254	488	1290	37346	49278	975	1578	2566	996	898	2451	1143	414	1839	652	222	660	580	185	640	2317	1043	1285	254
Fiji	18	0	0	9	0	32	20	1	0	10	0	0	2	21	0	0	0	25	0	29	11	2	7	17	16	0	5	7	7	15	2	0	17	0	0	0	47	0	7	0
Finland	2	3	3	1	4	2	0	176	3	4	7	2	1	2	4	5	1	4	26	2	6	5	11	1	2	3	1	14	3	2	2	4	6	3	2	0	1	0	0	0
France	108	54	66	148	77	240	126	128	110	147	72	269	183	44	51	44	53	37	76	115	198	116	139	1853	79	144	244	37	28	143	167	47	164	37	23	354	187	20	37	1513
Gabon	0	0	6	10	4	4	0	26	0	34	27	72	0	30	0	0	0	8	0	4	0	0	15	19	28	0	8	12	32	0	0	13	1	30	2	0	25	0	0	2
Gambia	19	650	0	0	0	0	0	0	0	18	0	0	100	0	13	0	0	241	0	53	25	20	14	3	3	24	0	47	10	5	10	0	26	36	41	0	4	6	3	1
Georgia	49	0	8	0	0	9	2	141	3	100	0	61	820	1925	12	58	1	42	189	46	25	82	156	20	28	49	39	11	621	6	6	25	62	13	13	18	1	11	0	0
Germany	48	46	25	31	17	22	105	29	208	173	163	50	49	35	37	30	143	16	177	27	65	28	93	27	15	20	107	23	11	40	82	18	18	17	19	10	46	27	6	1
Ghana	80	161	1263	876	483	178	154	108	113	90	187	222	440	321	2247	414	278	1762	295	271	391	681	466	245	198	200	379	429	223	176	150	217	106	104	165	366	170	109	59	105
Greece	6	29	2	19	5	80	26	1074	271	36	7	76	2	2	18	12	3	65	5	116	92	12	29	13	14	106	4	130	2	1	5	0	8	2	46	279	212	42	123	0
Greenland	0	0	0	0	0	0	0	0	0	0	0	0	0	0	0	0	0	0	0	0	0	0	0	1	0	0	0	0	0	0	0	1	0	0	0	0	0	0	0	0
Grenada	9	0	0	176	2	0	0	0	1	0	0	0	3	0	0	0	0	0	0	0	0	0	0	0	18	1	0	0	0	0	0	0	0	0	0	0	0	0	0	0
Guam	0	0	0	0	0	0	0	0	0	0	0	1	1	0	0	0	0	228	0	0	0	0	1	0	2	1	0	3	4	1	2	0	0	2	0	0	0	0	0	0
Guatemala	3528	7381	11202	2530	1771	1737	1662	1295	640	548	749	583	503	378	517	260	39	53	489	88	63	224	98	57	33	808	36	28	252	1076	177	102	9	15	18	10	10	61	427	19
Guinea	1	0	0	275	0	189	58	38	50	106	39	0	425	183	464	699	61	4	134	151	1309	711	118	37	138	91	201	325	124	346	52	87	248	337	150	74	19	47	41	36
Guinea-Bissau	0	3	0	0	0	0	50	69	0	0	0	0	2	0	195	14	961	781	836	1006	8	13	6	5	13	419	68	39	226	79	23	1	60	28	45	12	0	0	32	0
Guyana	1	1	0	0	94	0	0	0	0	1	0	0	2	0	0	0	0	0	1	0	0	0	4	0	0	34	6	0	52	0	0	0	0	0	0	0	0	0	0	0
Haiti	220	2	0	5	0	0	349	37	84	48	15	407	121	1850	1283	119	259	489	246	41	48	71	47	188	5660	129	98	179	849	89	224040	5577	207	621	383	216	1026	126	39	14
Honduras	9	8	154	75	5	28	60	21	51	161	43	18	22	516	261	122	33	15	14617	46	31	21	20	143	173	61	4	7	108	17	269	67	4940	4330	4338	4920	19	21	6	155
Hungary	2	0	0	0	4	0	1	1	1	0	1	1	5	0	63	0	2	3	0	48	1	68	14	7	0	9	28	0	0	2	2	18	2	5	0	5	0	0	0	25
Iceland	0	0	0	4	0	0	0	0	0	2	0	1	0	0	1	69	0	0	0	2	0	0	0	0	1	0	1	0	0	0	0	0	0	0	0	0	0	1	0	0
India	5719	2497	5515	3076	19544	5288	3756	5456	12937	7800	14008	8759	7451	14393	5378	5661	6418	6292	12652	15427	9100	29099	9381	6232	21326	9066	5279	5349	5716	5325	4157	3326	2530	8740	2846	6813	3664	6598	3910	3263
Indonesia	5760	7262	6455	5968	5806	5501	6249	6159	5833	578	734	1231	2996	400	691	373	918	2336	1750	2917	2311	2187	1541	1808	168163	1976	8176	1564	238	2092	1390	617	311	313	583	576	414	760	6080	733
Iran (Islamic Republic of)	11246	17659	34529	27316	14409	17675	45960	23299	23790	1505	41449	1589	934	1713	924	516	503	3282	606	750	573	1330	999	27786	1864	1560	760	597	825	835	618	696	741	499	436	955	747	1439	1608	482
Iraq	19516	25368	63714	50341	26417	32491	84077	41231	40705	2759	2031	1427	1403	968	1749	1436	619	876	412	426	589	330	385	14923	16345	24400	39926	37990	15194	9132	7106	6911	8340	18990	37450	29926	31944	28198	2453	2437
Ireland	12	2	2	15	1	4	11	6	4	3	2	3	0	3	7	1	3	8	4	3	10	0	0	0	0	2	1	0	0	0	0	3	0	0	1	2	3	0	2	0
Israel	30	27	6	95	38	52	20	32	41	125	115	81	110	95	181	96	206	77	25	10	122	505	1230	625	573	256	682	320	753	707	84	94	87	14	1734	16	28	31	95	7
Italy	2228	51	41	22	52	642	28	133	35	19	37	33	26	14	108	4	28	35	295	19	68	10	27	51	25	14	20	10	21	382	55	34	45	42	38	44	321	84	78	0
Jamaica	19	6	8	15	11	18	57	43	49	28	24	36	27	9	6	1	1	10	0	0	1	1	13	0	16	6	1	5	13	0	15	0	1	1	0	0	0	0	0	0
Japan	250	223	978	447	153	817	400	32	153	189	151	409	166	517	186	5721	113	73	88	264	134	412	27	78	547	339	166	87	177	228	196	18438	305	357	328	309	312	127	464	286
Jordan	1	4	0	0	1	0	0	9	0	0	0	11	17	0	32	1	0	2	3	0	23	53	8	22	13	121	11	0	21	0	4	0	1	22	12	11	93	30	879	6
Kazakhstan	0	0	0	0	0	0	0	0	0	0	0	0	9	10	0	115	0	2	2	4	6	20	0	3	172	43	34	35	37	25	86	39	57	5	15	17	16	15	52	13
Kenya	154	215	531	89	0	102	45	38	77	197	61	337	740	738	1414	90	181	1548	2082	2430	1438	948	567	274	519	756	1058	1235	2621	1024	630	492	707	1187	602	905	507	907	609	93
Kiribati	0	0	0	0	0	0	0	0	0	0	0	2	1	0	3	7	1	0	0	0	0	0	0	0	0	0	0	0	0	0	0	0	0	0	0	0	0	0	80	0
Kuwait	4	3	0	1	7	15	12	1	2	0	1169	2472	2	18	6	8	15	11	8	9	7	5	6	6	9	12	18	12	1	52	0	1	0	1	1	54	1	7	16	0
Kyrgyzstan	0	0	0	0	0	0	0	0	0	0	0	0	58	0	162	0	0	0	1	15	183	17	15	44	50	23	20	3	99	26	446	44	18	9	10	19	12	84	0	0
Lao People's Democratic Republic	0	0	0	0	14	0	8	70	20	32	255	0	212	324	555	137	139	2	40	2	109	0	6	15	4	0	73	5	6	26	4	48	11	149	0	48	10	2	136	17
Latvia	0	9	10	0	0	0	0	0	0	0	0	1	0	0	1	0	0	0	1	6	1	32	0	0	1	0	94	2	1	2	0	0	21	52	0	0	0	0	0	0
Lebanon	90	264	34433	6576	6002	6644	6247	178	176	1210	2061	166	235	185	200	160	269	134	102	97	77	4	2	42	1	25	756	42	180	61	92	13	61	179	335	316	293	366	853	17
Lesotho	0	10	0	30	1	0	0	18	4	0	22	0	0	0	0	0	0	7	109	69	37	1	2	1	0	0	2	45	0	4	1	26	8	0	1	4	0	4	0	4
Liberia	563	112	104	62	15	22	0	43	1	45	3731	618	490	2679	4302	2419	3163	13	65	171	152	482	2346	3320	23	45	21	11	28	4	24	14	0	13	26	5	18	7	2	33
Libya	6	6	0	1	20	2	1	3551	30	78	0	0	182	0	0	2	2521	16	2	0	75	1	82	0	27	1	18	9	194	225	122	7049	507	245	5318	4702	5212	2063	1914	1280
Lithuania	0	16	6	0	0	13	2	5	0	0	0	6	0	12	9	1	0	2	7	32	0	72	1	0	0	1	2	0	2	0	80	0	42	0	0	0	0	0	5	0
Luxembourg	0	1	0	0	0	0	0	0	1	0	0	0	0	1	0	0	0	0	0	0	2	0	3	0	0	0	0	0	0	0	0	0	0	0	0	0	0	0	0	0
Madagascar	0	115	92	42	121	0	99	0	32	88	42	118	58	0	212	38	10	174	0	783	352	462	811	151	410	109	4	96	125	183	122	46	227	330	510	313	218	462	805	725
Malawi	0	125	0	49	0	0	0	159	29	439	68	717	40	524	11	1	29	10	95	648	138	908	1063	46	33	71	29	34	60	113	72	28	51	8	11	308	36	40	23	59
Malaysia	10	114	17	48	201	2	33	21	98	62	37	444	13	192	72	79	346	141	122	63	95	38	30	24	100	14	19	108	10	9	38	39	10	180	318	92	61	260	7	4
Maldives	0	0	0	0	0	0	0	0	0	0	0	0	0	0	0	0	0	0	1	10	0	0	0	0	133	0	0	0	0	0	0	4	0	0	0	0	0	0	0	0
Mali	76	910	358	170	1085	1159	306	333	76	72	677	342	118	0	860	490	1097	7261	10288	2190	2239	2140	2126	1661	166	91	357	124	208	173	113	159	507	1302	453	673	274	889	2243	1537
Malta	0	0	0	0	0	0	3	0	0	0	0	1	1	0	0	2	0	1	4	3	2	0	1	0	2	0	0	0	0	0	0	0	0	1	0	0	0	0	0	6
Marshall Islands	0	0	0	0	0	0	0	0	0	0	0	0	0	0	0	0	0	0	0	0	6	0	0	0	0	0	0	0	0	0	0	0	0	0	0	0	0	0	0	0
Mauritania	0	0	5	9	15	31	114	149	50	164	53	4	0	0	80	0	46	12	49	53	55	17	31	46	0	147	26	64	24	3	15	42	25	23	1	15	0	20	14	1
Mauritius	0	0	0	1	2	0	0	1	0	1	0	0	0	0	2	0	3	0	0	0	0	0	3	0	0	0	0	2	0	0	0	0	0	21	0	0	0	0	0	0
Mexico	123	28	450	480	355	3680	269	328	478	323	201	267	63	139	328	293	269	373	910	752	289	80	129	74	106	151	147	127	115	147	369	472	212	413	92	129	294	810	85	273
Micronesia (Federated States of)	0	0	11	0	0	0	0	5	0	0	1	0	0	0	0	0	0	0	0	0	19	0	47	0	1	0	0	0	0	0	0	0	0	0	0	5	0	0	0	0
Monaco	0	0	0	0	0	0	0	0	0	0	0	0	0	0	0	0	0	0	0	0	0	0	0	0	0	0	0	0	0	0	0	0	0	0	0	0	0	0	0	0
Mongolia	0	0	0	0	0	0	0	0	0	0	87	6	0	47	161	41	74	0	28	0	23	26	2	17	0	0	0	14	53	27	15	0	0	0	0	0	12	17	0	12
Montenegro	0	0	0	0	0	0	0	0	0	0	0	0	0	0	0	1	1	0	4	2	1	1	1	0	1	0	3	0	0	0	0	0	1	13	0	0	0	0	1	0

Morocco	1300	606	607	607	607	607	609	942	591	207	0	66	34	15	81	808	64	120	152	138	97	26	209	142	718	72	155	69	148	30	131	139	83	46	89	82	6	41	19	1
Mozambique	587	25664	25751	25725	46190	45973	26531	26546	3402	2404	2660	2660	1263	609	267	133	51	431	759	1262	1267	287	394	274	78	53	359	304	168	366	171	90	54	187	63	271	246	172	414	807
Myanmar	2755	3315	3603	3310	3250	3250	3276	3697	6879	1203	1050	1588	1908	332	949	1584	676	907	639	388	735	459	568	274	515	485	511	795	138520	341	383	1136	1318	732	379	1248	600	9486	258	795
Namibia	519	517	519	521	533	581	522	519	642	1	0	23	0	0	224	4	2	0	30	23	133	162	29	16	0	0	20	14	46	97	8	109	1	53	18	4	0	2	1	0
Nauru	0	0	0	0	0	0	0	0	0	0	0	0	0	0	0	0	0	0	0	0	0	0	0	0	0	0	0	0	0	0	0	0	0	0	0	0	0	0	0	0
Nepal	100	750	138	207	346	52	23	214	984	110	219	2327	1036	1367	4	511	1010	167	927	727	986	921	5585	2129	3400	1938	977	350	324	555	326	323	159	265	689	9114	251	257	140	142
Netherlands	2	1	1	1	1	0	2	0	4	11	10	10	56	2	0	15	67	2	0	0	0	11	4	2	1	14	1	14	6	11	8	0	1	3	186	1	3	4	27	0
New Zealand	5	7	1	1	1	9	1	1	9	5	3	4	3	0	2	0	3	6	10	2	2	2	2	5	13	0	4	1	11	7	1	188	2	4	3	5	2	0	0	2
Nicaragua	37	372	525	6978	9117	7475	5295	6749	3238	574	66	57	291	303	174	226	172	43	4131	68	35	27	18	14	36	9	45	200	30	9	93	18	9	35	57	3	15	23	38	15
Niger	293	187	111	74	390	71	790	175	160	758	417	3289	764	1080	1240	5610	2399	924	589	563	1199	628	374	1124	509	207	88	226	273	185	128	91	255	159	128	862	408	543	687	528
Nigeria	76	56	86	559	343	63	2583	1817	754	1503	359	15530	2366	1355	626	242	18703	1489	3643	5257	7939	6259	5539	3069	4929	2245	2339	1913	2877	7103	7095	5554	4970	8260	17878	16771	6725	7711	7739	3570
Niue	0	0	0	0	0	0	0	0	0	0	0	0	0	0	0	0	0	0	0	1	0	0	0	0	1	0	0	0	0	0	0	0	0	0	0	0	0	0	0	0
North Macedonia	0	0	0	12	0	0	0	5	0	0	0	29	1	203	0	4	1	0	0	6	6	132	23	17	15	1	6	13	3	16	0	1	6	1	0	21	22	3	1	13
Northern Mariana Islands	0	0	0	0	0	0	0	0	0	0	0	0	0	0	0	0	0	0	0	0	0	0	0	0	4	2	0	2	0	0	0	0	0	0	0	0	0	0	2	0
Norway	0	1	2	0	0	0	21	9	9	10	5	2	5	2	7	4	5	7	4	4	2	10	6	0	6	8	2	5	8	3	16	91	3	6	7	7	2	1	0	0
Oman	0	26	0	0	0	0	0	0	0	0	0	0	0	5	1	2	0	0	1	0	7	0	53	30	21	0	0	88	0	4	16	14	0	0	0	2	20	3	6	0
Pakistan	110	408	71	168	122	5	177	272	622	475	1125	1549	2102	1187	929	2014	867	1240	1612	1220	503	456	499	1007	751	74930	1248	3813	5771	8697	14825	7079	8641	6722	7271	7575	3309	1833	1900	955
Palau	0	0	0	0	0	0	0	0	0	0	0	0	0	0	0	0	1	0	0	0	0	0	0	0	0	0	0	0	0	0	0	0	0	0	0	0	0	0	0	0
Palestine	287	262	21088	265	261	256	260	678	750	492	482	463	513	421	240	164	138	130	163	246	2330	3268	4390	3301	3759	2476	4928	2724	6432	6130	673	747	1130	21	76	164	153	154	347	86
Panama	26	1	1	0	1	5	9	15	46	891	2	44	15	0	21	4	9	10	4	6	11	0	6	5	13	1	15	7	36	2	8	5	8	0	23	1	5	25	0	0
Papua New Guinea	0	0	0	66	0	0	0	1	76	27	33	529	365	372	367	303	165	52	2341	83	15	65	190	13	0	2	16	172	10	224	95	30	368	0	0	24	12	0	181	10
Paraguay	3	1	1	77	1	0	0	3	1	150	1	0	0	0	7	8	29	34	55	5	48	3	4	2	391	3	14	12	13	9	15	46	12	17	8	25	29	0	0	17
Peru	89	179	1260	4328	5913	2067	1459	2169	2544	3887	2929	12791	2792	1470	833	552	512	683	669	321	312	532	309	518	263	172	196	793	203	755	749	240	501	251	673	180	126	209	113	93
Philippines	5909	6542	5336	4957	7764	5834	5039	10103	5963	2114	5302	8843	1291	1566	1431	2435	677	519	1282	728	3148	2071	1415	1769	2928	662	4281	873	2874	3595	1717	3250	3381	15374	1058	1495	2541	4371	1845	1673
Poland	0	0	0	0	7	2	5	1	5	2	1	0	6	0	1	0	0	57	9	4	2	35	15	14	26	5	6	21	11	13	20	16	1	6	0	6	2	6	9	0
Portugal	1168	1249	1130	1146	4	57	41	2	3	181	0	0	42	6	4	1	12	59	10	44	4	50	5	90	45	0	0	43	3	0	7	1	0	6	2	1	13	107	13	0
Puerto Rico	0	26	0	1	2	600	8	0	0	0	0	0	19	0	0	1	13	12	0	3	3	2	0	3	13	6	4	9	1	1	0	6	2	1	1	0	0	2364	0	0
Qatar	0	0	1	0	0	0	0	0	0	3	0	0	0	0	0	0	0	0	0	0	0	1	0	0	0	2	0	0	0	30	0	0	19	0	15	0	11	0	0	0
Republic of Korea	169	178	69	1	251	76	76	820	79	165	243	336	41	312	165	749	206	87	648	192	107	113	448	591	50	66	110	41	61	31	128	169	31	11	403	42	17	44	37	1
Republic of Moldova	0	30	48	0	0	43	43	34	29	30	21	22	845	199	61	5	3	9	2	0	22	0	1	0	1	0	30	0	3	0	1	0	42	0	0	0	0	0	0	0
Romania	147	155	145	145	142	149	150	149	156	1482	110	142	45	53	169	115	114	99	122	27	16	15	69	6	52	56	11	13	6	33	61	0	142	5	34	39	2	139	2	0
Russian Federation	301	95	93	100	522	151	100	94	43	132	298	393	154	298	797	2416	3070	239	212	7236	6734	4620	3032	1273	2271	1173	488	350	644	944	5507	610	1725	1421	395	641	401	392	389	65
Rwanda	35	37	10	17	21	8	38	40	93	284	2668	1169	1187	993	513532	4412	448	6629	2767	603	231	2065	202	517	30	5	84	74	57	1912	260	161	111	11	5	14	287	40	129	15
Saint Kitts and Nevis	0	0	0	0	0	0	0	0	0	1	0	0	0	0	0	0	0	0	5	0	0	0	0	0	0	0	0	0	0	0	0	0	0	0	0	0	0	0	0	0
Saint Lucia	9	0	0	2	0	0	0	3	1	1	1	1	1	2	5	0	0	0	1	0	2	0	0	0	0	0	0	2	0	0	14	0	0	6	0	0	0	0	0	0
Saint Vincent and the Grenadines	0	0	0	0	0	0	0	6	0	0	0	0	3	0	0	3	1	0	0	0	0	0	8	0	0	2	0	0	0	0	0	0	0	12	0	0	0	0	0	0
Samoa	0	0	0	0	0	0	0	0	0	0	8	13	0	0	0	0	0	0	0	0	0	0	0	0	1	9	0	0	0	148	0	0	12	0	0	0	0	0	0	0
San Marino	0	0	0	0	0	0	0	0	0	0	0	0	0	0	0	0	0	0	0	0	0	0	0	0	0	0	0	0	0	0	0	0	0	0	0	0	0	0	0	0
Sao Tome and Principe	1	0	0	5	6	3	151	2	0	32	9	1	0	0	0	0	0	0	0	3	0	0	0	0	0	25	9	13	24	0	0	0	0	0	0	0	0	0	0	0
Saudi Arabia	301	45	0	0	0	33	132	0	0	1	1426	388	0	0	182	205	80	465	118	164	305	151	121	227	410	181	457	221	132	235	52	109	150	119	217	2945	465	174	160	12
Senegal	66	97	149	122	206	344	92	306	10	121	104	21	330	285	5	464	76	475	1034	855	95	301	1364	86	68	352	277	89	68	35	26	68	114	72	30	76	23	106	23	2
Serbia	0	70	0	2	33	0	0	22	71	92	0	3298	344	2	0	11	21	18	1557	9464	540	134	84	37	62	21	49	9	6	0	12	3	32	2	60	2	0	6	9	1
Seychelles	0	0	11	0	0	3	5	4	0	5	4	5	5	9	5	0	0	5	0	0	0	0	0	0	3	0	0	1	0	0	0	0	0	0	0	0	0	0	0	0
Sierra Leone	32	3	0	50	63	352	669	92	0	0	0	1353	652	642	2948	3834	1447	1444	5287	4930	1444	103	3	15	93	20	111	280	176	262	16	43	279	48	13	12	8	1354	20	15
Singapore	0	0	0	0	6	0	0	0	0	0	0	4	0	0	1	0	0	0	1	0	0	0	0	0	0	0	0	0	1	0	0	0	0	0	0	2	2	18	13	0
Slovakia	0	0	0	0	0	10	0	0	0	0	0	0	0	0	1	16	2	2	56	4	8	6	1	12	5	3	1	12	1	42	67	61	5	2	3	0	0	0	0	0
Slovenia	0	0	0	0	0	1	0	0	0	0	1	26	1	2	2	2	3	1	1	2	0	1	0	6	2	1	2	10	3	4	7	0	1	3	8	1	0	0	0	0
Solomon Islands	0	0	0	0	0	0	101	0	1	0	0	33	0	4	0	0	0	0	0	2	0	0	1	1	0	0	0	52	0	21	2	0	0	18	51	9	0	0	0	0
Somalia	1	5	512	619	512	1262	1819	1155	11666	1155	2706	8591	4787	483	2132	684	1040	3789	831	798	911	845	895	954	2457	847	1479	3638	2585	2672	13900	13972	4318	2464	1931	1943	3476	5386	3984	1752
South Africa	569	1201	1287	1211	623	1488	3070	1753	1527	524	1668	1176	1654	1100	1308	520	567	234	365	415	416	349	357	232	130	158	155	212	231	175	172	227	357	244	192	307	154	146	129	144
South Sudan	0	0	0	2045	2045	2045	2045	2045	2044	0	0	0	0	0	0	0	0	7000	5000	3000	2000	1000	1000	1000	200	90	1761	950	325	2500	800	2259	1411	3822	4517	2632	2210	2618	1918	887
Spain	198	43	121	421	114	213	58	76	46	65	72	96	54	13	93	118	184	97	74	184	227	194	224	417	663	253	249	182	286	52	73	72	37	100	3	7	8	20	24	326
Sri Lanka	38	90	813	52	2202	2278	2461	3351	3896	3487	6403	7729	5446	2253	898	6114	4944	3573	4593	2173	3070	1370	46	291	35607	294	3214	3029	8862	10969	8551	266	58	133	320	36	278	690	60	285
Sudan	2	2	0	44066	56371	56305	2134	4288	4330	9121	6214	4610	4127	6066	1007	1530	3813	5741	8606	4072	4464	4241	7280	9081	13964	3066	4213	3448	2461	4278	2413	5448	2299	3582	2909	3384	2862	1685	1424	311
Suriname	0	0	0	0	0	0	68	73	73	183	0	1	1	0	0	0	0	0	0	0	0	10	0	0	0	0	0	0	20	0	0	0	0	0	0	0	0	0	0	0
Sweden	0	0	0	2	0	11	1	24	8	2	2	3	2	1	510	1	2	4	58	8	1	4	1	7	2	8	4	4	0	0	3	0	5	11	0	4	3	6	0	0
Switzerland	0	2	0	0	0	4	0	2	0	0	6	0	6	2	0	10	15	22	5	30	49	70	19	10	10	25	12	9	11	12	16	13	23	16	13	19	10	8	20	4
Syrian Arab Republic	601	704	13598	3	0	31	8	0	0	54	54	0	0	0	46	0	15	102	57	69	20	74	213	80	37	97	1036	552	196	62	27	12943	60179	49731	75357	61012	54443	32055	11615	7172
Taiwan (Province of China)	0	10	10	20	43	2	29	9	0	3	10	43	23	1	7	3	37	54	20	2352	38	140	4	3	46	25	9	7	15	600	46	1	20	4	0	9	115	38	110	0
Tajikistan	38	17	92	0	0	21	15	143	183	125	128	0	5573	15994	414	446	2847	348	712	36	57	129	87	17	20	46	51	52	1	41	223	28	56	3	28	36	5	13	13	0
Thailand	1082	737	550	129	42	36	157	396	1172	835	347	571	210	406	384	361	157	420	151	81	349	340	274	56	8792	733	1038	1012	572	639	929	1442	693	788	507	208	335	281	348	93
Timor-Leste	1008	650	569	602	423	358	341	244	260	325	211	358	293	374	293	374	0	455	472	2618	82	99	114	57	0	22	50	37	3	0	0	0	0	0	2	0	0	0	0	0
Togo	10	0	1	30	9	5	38	36	58	147	0	157	66	50	52	7	239	751	327	103	175	235	244	94	79	579	62	137	73	71	99	82	36	37	101	17	18	18	16	1
Tokelau	0	0	0	0	0	0	0	0	0	0	0	0	0	0	0	0	0	0	0	0	0	0	0	0	0	0	0	0	0	0	0	0	0	0	0	0	0	0	0	0
Tonga	0	0	7	0	0	0	0	0	0	0	1	0	0	0	0	0	0	0	0	0	0	0	0	0	0	0	0	0	0	84	0	0	0	0	1	0	0	0	0	0
Trinidad and Tobago	0	3	1	5	4	2	11	12	21	19	39	9	12	15	20	22	23	19	5	0	0	0	0	2	4	2	0	0	2	0	5	1	2	0	0	0	0	0	0	0
Tunisia	559	0	117	0	0	0	23	0	8	0	57	4	0	0	22	7	0	0	0	4	18	3	49	250	93	0	19	44	24	110	3	389	15	77	114	178	76	59	120	16
Turkey	323	47	9	1488	206	233	262	401	380	534	1012	870	4304	2795	5303	3611	3478	2483	2313	19307	239	259	202	691	571	457	476	678	562	225	364	1153	1175	1356	1535	1674	2807	624	1630	322
Turkmenistan	0	0	0	0	0	0	0	0	0	0	0	458	360	105	0	0	0	0	0	5	5	21	7	7	11	35	5	4	6	6	80	22	50	6	11	13	0	0	0	0
Tuvalu	0	0	0	0	0	0	0	0	0	0	0	0	0	0	0	0	0	0	0	0	18	0	0	0	0	0	0	0	0	0	0	0	0	0	0	0	0	0	0	0
Uganda	14956	14838	14913	15026	15095	14889	14886	2222	1194	1285	1113	1312	503	504	312	748	2376	1992	1701	1844	1632	428	2897	3762	2692	1428	745	466	388	316	906	220	200	145	1268	304	292	321	669	89
Ukraine	0	292	358	0	0	493	102	0	0	11	0	135	58	9	138	224	197	29	109	50	90	103	137	17	66	93	1060	219	193	98	115	101	254	12	5845	2155	436	516	552	247
United Arab Emirates	1	2	0	223	0	0	0	0	0	1	4	0	3	0	0	0	0	85	3	12	0	14	29	0	43	0	0	0	12	0	12	1	1	22	16	11	0	1	0	0
United Kingdom	227	214	845	171	225	288	240	275	696	166	212	295	173	61	280	75	30	105	208	113	30	82	49	73	39	99	68	122	70	215	107	54	56	52	17	22	16	93	285	2
United Republic of Tanzania	687	418	438	310	371	260	215	90	580	262	445	687	2256	396	225	58	1152	2608	2635	857	286	296	968	164	310	38	268	326	199	167	155	366	222	145	60	318	292	492	352	122
United States Virgin Islands	6	0	0	0	0	0	0	0	0	0	0	0	0	0	0	7	0	0	0	3	0	0	0	0	0	0	1	0	0	0	1	0	0	0	0	0	0	7	0	0
United States of America	1167	175	417	1084	655	500	466	677	303	697	695	629	307	975	706	912	912	374	561	932	905	5330	870	1315	1333	5223	1699	2025	1097	939	929	1604	604	977	798	973	1049	434	388	194
Uruguay	0	0	0	1	1	2	3	0	0	3	0	0	0	0	0	0	1	81	1	1	0	0	2	0	0	1	0	2	0	1	14	0	0	1	0	8	13	0	0	0
Uzbekistan	0	0	20	0	0	14	112	100	67	45	13	50	10	135	26	0	0	0	0	93	298	20	0	0	100	50	0	2	1	19	82	31	54	5	10	14	4	0	0	0
Vanuatu	0	0	0	0	0	9	0	48	1	12	0	0	2	4	0	0	0	100	0	44	0	2	0	0	2	0	0	0	0	0	0	0	0	0	12	11	0	0	0	0
Venezuela (Bolivarian Republic of)	12	66	78	13	2	38	39	200	168	169	64	77	303	196	452	36	55	136	46	30391	54	54	43	70	95	88	57	15	50	34	85	36	26	12	1	1	14	191	19	0
Viet Nam	839	285	126	892	217	968	693	276	253	1181	399	929	513	429	486	415	1120	3780	864	893	649	469	207	297	333	431	616	478	450	427	291	266	112	266	58	76	207	325	235	6
Yemen	570	513	2501	0	0	0	11500	0	0	63	0	11	171	31	1503	135	386	11	258	21	65	100	58	175	69	99	54	332	241	332	686	2200	3444	1295	3641	8290	16556	18822	32077	14179
Zambia	83	76	270	19	0	51	44	950	436	489	1269	1881	1948	488	0	0	158	35	1	484	165	3267	12	140	122	123	23	68	108	182	59	120	59	66	33	42	31	82	65	29
Zimbabwe	43	17	111	54	4	139	106	143	38	177	29	170	266	332	0	43	1311	10	109	80	255	61	319	143	32	63	113	132	2224	2609	55	128	29	155	29	60	68	324	119	750

SR Table 10a: Deaths due to conflict & terror by country, all ages, for both sexes combined, 1980-2019

Country	1980	1981	1982	1983	1984	1985	1986	1987	1988	1989	1990	1991	1992	1993	1994	1995	1996	1997	1998	1999	2000	2001	2002	2003	2004	2005	2006	2007	2008	2009	2010	2011	2012	2013	2014	2015	2016	2017	2018	2019
Afghanistan	35000	30000	35000	55000	80000	65000	60000	45000	30128	5184	1490	3370	4344	4096	8959	5525	3255	6723	12073	5104	5429	5689	977	864	1049	2114	5677	8220	6895	7617	8273	9142	11350	11829	18008	23667	28051	19356	27079	24295
Albania	0	0	0	0	0	0	0	0	0	0	0	0	1	2	18	2	8	2026	5	12	1	5	1	0	9	10	18	18	8	0	0	0	0	0	1	0	0	0	0	0
Algeria	0	0	0	0	0	0	0	0	0	0	4	76	510	826	2428	3425	1620	4905	4410	1519	2534	1330	1597	932	577	324	406	700	507	637	318	342	290	216	114	112	92	107	76	13
American Samoa	0	0	0	0	0	0	0	0	0	0	0	0	0	0	0	0	0	0	0	0	0	0	0	1	1	2	2	2	1	0	0	0	0	1	0	0	0	0	0	0
Andorra	0	0	0	0	0	0	0	0	0	0	0	0	0	0	0	0	0	0	0	0	0	0	0	0	0	0	0	0	0	0	0	0	0	0	0	0	0	0	0	0
Angola	5848	5852	5851	6131	6029	5839	5970	5839	5979	2961	2756	595	2425	12398	4117	267	93	2922	14070	7100	3420	3068	600	81	50	11	12	34	5	28	23	7	6	4	0	1	36	27	31	16
Antigua and Barbuda	0	0	0	0	0	0	0	0	0	0	0	1	0	0	0	0	0	0	0	0	0	0	0	0	0	0	0	0	0	0	0	0	0	0	0	0	0	0	0	0
Argentina	0	0	0	0	160	205	221	159	183	220	162	114	143	125	132	40	53	3	0	0	0	0	0	0	0	0	0	0	0	0	0	0	0	0	0	0	1	0	0	0
Armenia	0	0	0	0	0	0	0	0	0	0	25	0	6	12	45	10	2	0	0	8	0	1	0	0	0	1	2	16	6	5	4	5	9	6	22	51	120	0	1	0
Australia	6	1	0	5	3	3	1	0	1	0	2	4	2	0	6	0	2	0	0	0	1	4	0	0	0	0	0	0	0	2	10	11	1	0	4	3	1	0	2	0
Austria	0	3	0	1	2	4	0	0	0	3	0	0	0	0	0	6	0	0	0	0	0	0	0	0	0	0	5	9	7	1	0	0	0	2	0	0	0	0	0	0
Azerbaijan	0	0	0	0	0	0	0	0	0	6	31	64	2430	1197	1519	54	7	30	34	18	13	7	5	15	8	24	5	12	35	8	20	21	16	11	46	63	138	36	16	0
Bahamas	0	1	0	0	0	1	0	0	0	0	0	0	0	5	6	0	1	1	2	0	0	0	0	0	0	0	0	0	0	0	0	0	0	0	0	0	0	0	0	0
Bahrain	0	0	0	0	0	0	74	0	0	0	0	2	0	0	0	1	8	5	0	0	0	0	0	0	0	0	0	0	1	0	0	0	4	3	9	9	3	13	0	0
Bangladesh	6	6	0	0	1	0	54	26	87	211	158	164	325	12	56	41	127	19	5	31	25	61	26	10	82	123	97	19	22	20	127	402	317	141	299	163	231	139	59	155
Barbados	0	0	0	1	3	1	0	1	0	0	0	0	6	4	0	0	0	0	0	0	0	0	0	0	0	0	0	0	0	0	0	0	0	0	0	0	0	0	0	0
Belarus	0	0	0	0	0	0	0	0	0	0	0	0	0	0	0	0	0	1	0	0	0	0	11	9	0	0	0	12	12	11	8	26	0	7	6	0	1	0	0	0
Belgium	20	7	1	2	1	13	4	3	17	15	22	54	1	0	0	0	0	4	0	2	0	0	0	0	1	2	0	0	0	0	0	0	1	0	4	0	70	0	4	0
Belize	0	0	0	0	0	0	0	0	0	12	6	18	1	7	14	7	1	0	0	0	0	0	0	0	0	0	0	0	0	0	0	0	0	0	0	0	0	0	0	0
Benin	0	0	0	0	0	0	0	0	0	0	0	0	0	0	0	0	0	0	0	0	0	0	0	0	0	0	0	0	0	1	0	0	0	0	0	3	13	13	0	1
Bermuda	0	0	0	0	0	0	0	0	0	0	0	0	0	0	0	0	0	0	0	0	0	0	0	0	0	0	0	0	0	0	0	0	0	0	0	0	0	0	0	0
Bhutan	0	0	0	0	0	0	0	0	0	0	0	0	0	0	0	0	0	4	0	0	0	0	0	0	0	0	4	0	5	0	0	0	0	0	0	0	0	0	0	0
Bolivia (Plurinational State of)	11	1	0	0	1	0	1	1	2	2	6	7	5	0	1	0	0	4	0	0	0	0	0	0	2	0	0	0	0	0	0	0	0	0	0	0	0	0	0	0
Bosnia and Herzegovina	0	0	0	0	0	686	697	671	577	607	488	512	7686	5812	3140	9757	2	5	2	1	1	0	0	0	0	0	0	1	2	0	1	0	0	0	0	4	0	0	0	0
Botswana	0	0	0	0	0	0	0	3	1	0	7	0	0	0	0	0	0	0	0	0	0	0	0	0	0	0	0	0	0	1	0	0	0	0	0	0	0	0	0	0
Brazil	19	2	27	1	0	0	14	0	0	1	43	31	51	48	33	36	53	36	1	5	0	4	0	0	0	34	1	7	62	14	0	2	4	18	64	36	15	0	0	0
Brunei Darussalam	0	0	0	0	0	0	0	0	0	0	0	0	0	0	0	0	0	0	0	0	0	0	0	0	0	0	0	0	0	0	0	0	0	0	0	0	0	0	0	0
Bulgaria	0	0	2	0	0	0	0	0	0	0	0	0	0	0	1	3	9	0	0	0	4	0	1	0	23	0	0	0	0	0	0	0	8	0	1	0	0	0	0	0
Burkina Faso	0	0	0	0	1	93	0	0	0	0	0	1	0	0	0	0	0	0	0	0	1	0	0	65	76	6	16	0	14	0	3	2	27	3	7	25	88	96	246	707
Burundi	0	0	0	0	0	0	0	0	0	0	7	395	122	5	12765	13083	13871	13822	13105	13314	14144	13673	13247	13415	12764	12921	237	33	292	481	273	203	29	25	58	666	369	284	252	80
Cabo Verde	0	0	0	0	0	0	0	0	0	0	0	0	0	0	0	0	0	0	0	0	0	0	0	0	0	0	0	0	0	0	0	0	0	0	0	0	0	0	0	0
Cambodia	6971	6971	6971	6971	6972	6972	6972	1000	200	825	377	338	257	484	797	747	890	544	310	1	16	5	4	2	0	1	1	2	1	0	25	23	4	6	3	2	5	3	8	2
Cameroon	0	0	0	0	500	0	0	0	0	0	6	36	6	0	99	5	3	0	0	1	21	0	0	0	2	48	31	66	40	3	6	17	5	14	1117	1714	970	726	1571	626
Canada	9	9	10	13	17	347	0	1	0	16	0	0	1	0	1	2	0	0	2	7	0	7	1	0	3	4	36	30	32	32	16	0	1	0	7	0	2	0	0	1
Central African Republic	0	3	0	0	0	0	0	0	0	0	0	3	4	4	2	0	56	140	6	7	9	265	296	34	75	8	222	271	123	322	308	171	185	4169	4526	701	761	1230	570	340
Chad	1505	1505	1511	1520	1497	0	1498	6448	0	51	688	135	1360	313	522	53	5	242	229	437	1012	263	369	37	176	417	2368	378	1057	138	37	15	17	42	53	543	41	90	350	276
Chile	6	19	2	16	37	30	22	9	19	24	8	16	12	0	0	1	2	0	0	1	1	0	0	0	0	0	0	0	0	0	0	0	0	2	1	0	0	0	0	0
China	196	344	2	122	475	3	485	2319	96	22	26	4	16	6	21	6	23	42	100	305	201	525	102	2	16	25	0	88	114	392	19	67	55	213	425	211	20	14	5	0
Colombia	359	705	498	687	865	1435	1071	855	2194	1135	1365	1614	2131	257	1429	1464	2012	2057	2049	2208	2154	2420	3202	1266	1603	1657	662	426	337	526	498	270	395	277	299	247	76	34	149	0
Comoros	0	0	0	0	0	0	0	0	0	29	0	0	1	0	0	0	0	56	0	0	0	0	0	0	0	0	0	0	0	0	0	0	0	0	0	0	0	0	0	0
Congo	0	0	15	0	0	0	0	0	0	0	0	0	2	135	32	2	1	10447	3784	981	500	3	235	44	9	15	0	2	1	100	0	1	2	42	11	0	63	20	13	0
Cook Islands	0	0	0	0	0	0	0	0	0	0	0	0	0	0	0	0	0	0	0	0	0	0	0	0	0	0	0	0	0	0	0	0	0	0	0	0	0	0	0	0
Costa Rica	0	4	4	0	2	0	0	0	1	2	1	0	0	0	1	0	1	2	0	0	0	1	0	0	0	0	0	0	0	0	0	0	0	0	0	0	0	0	0	0
Croatia	0	0	0	0	0	0	0	0	0	0	0	3486	2178	755	225	964	156	41	28	15	9	4	2	4	11	5	1	3	3	4	4	2	4	3	0	1	3	0	0	0
Cuba	35	34	34	34	32	26	32	29	31	32	34	34	38	42	28	23	29	2	0	0	0	0	0	0	0	0	0	1	0	0	0	0	0	0	0	0	0	0	0	0
Cyprus	3	0	0	0	6	3	1	1	6	1	0	0	0	0	2	0	3	0	0	0	0	0	0	0	0	0	0	0	0	1	0	0	0	0	0	0	0	0	1	0
Czechia	0	0	0	0	0	0	0	2	0	0	0	2	0	0	0	1	2	1	0	2	0	1	0	0	1	0	0	0	0	0	0	0	0	1	6	0	0	0	0	0
Côte d'Ivoire	0	0	0	0	0	0	0	0	0	0	9	0	1	1	8	45	10	16	9	51	108	32	1209	986	466	148	44	3	14	2	82	1821	43	22	29	15	18	24	12	10
Democratic People's Republic of Korea	0	0	0	0	0	0	0	0	0	0	0	0	0	0	3	0	0	0	0	0	0	0	0	0	0	0	0	0	0	0	0	0	0	0	0	0	0	0	0	0
Democratic Republic of the Congo	0	0	0	0	2	0	0	0	28	28	147	35	77	106	184	101	34541	14347	9946	8637	2608	2136	6233	3745	897	556	448	934	3641	6654	1352	410	2109	2830	1151	1367	1776	6422	1293	1630
Denmark	0	0	0	0	0	1	0	0	0	0	0	0	1	0	0	0	0	0	0	1	0	0	0	1	0	1	0	2	13	3	0	3	0	0	0	4	1	0	0	0
Djibouti	0	0	0	0	0	0	0	10	0	6	60	67	357	33	45	0	0	22	19	38	0	0	2	0	0	0	0	0	9	4	3	0	1	0	6	27	0	0	0	0
Dominica	1	6	0	0	4	2	0	0	0	0	0	1	0	0	0	0	0	0	0	0	0	0	0	0	0	0	0	0	0	0	0	0	0	0	0	0	0	0	0	0
Dominican Republic	1	0	9	4	33	55	0	10	2	3	3	1	2	0	2	0	3	3	0	0	0	0	0	0	0	0	1	0	0	0	0	0	0	1	0	0	0	0	0	0
Ecuador	5	10	2	0	1	4	21	0	4	12	7	0	2	1	0	54	8	1	0	0	0	0	0	0	0	0	0	0	25	0	0	0	0	1	0	0	1	0	0	0
Egypt	22	67	0	0	0	2	12	1	1	1	25	8	62	108	335	365	246	236	31	6	2	4	23	0	62	130	24	0	16	10	39	116	117	168	293	1125	484	1305	1520	682
El Salvador	2782	14579	6549	10667	7315	5013	4293	3421	1361	5274	1142	1261	83	3	22	4	26	1	0	0	0	0	0	0	0	0	0	0	0	0	0	0	0	0	0	0	0	0	0	0
Equatorial Guinea	0	0	0	0	0	0	0	0	0	0	0	0	1	0	0	0	0	0	6	0	0	0	0	1	12	0	0	0	0	3	0	0	1	0	0	0	0	0	0	0
Eritrea	10410	10409	10409	10409	10410	10410	10410	10410	10410	10410	10410	10410	0	21	0	0	20	31	878	19078	1779	1	0	78	56	161	2	1	245	9	1	17	0	0	10	2	25	0	1	0
Estonia	0	0	0	0	0	0	0	0	0	0	0	0	0	0	0	0	1	0	0	0	0	1	0	0	0	0	0	2	1	4	1	2	0	0	0	0	0	0	0	0
Eswatini	0	0	0	0	0	0	0	4	0	2	0	0	0	0	0	0	0	0	2	0	0	0	0	0	0	0	0	0	2	0	0	0	0	0	0	0	0	0	0	0
Ethiopia	2023	2023	1511	2667	1712	1512	1040	1769	1512	25370	49873	12425	811	62	487	446	187	144	503	37194	48344	105	624	699	625	244	228	721	52	1287	573	203	391	411	130	368	601	304	357	165
Fiji	0	0	0	0	0	0	0	0	0	0	0	0	1	0	0	0	0	0	0	0	7	0	0	0	0	0	0	0	0	0	0	0	0	0	0	0	0	0	0	0
Finland	2	3	3	1	4	2	0	176	3	4	7	2	1	2	4	5	1	3	1	2	3	3	11	1	2	3	1	12	3	1	1	2	5	2	1	0	1	0	0	0
France	41	44	39	44	53	47	50	36	35	15	8	12	17	7	13	27	23	4	6	14	18	14	7	7	8	6	8	10	11	11	17	26	12	9	4	295	182	2	14	0
Gabon	0	0	0	0	0	0	0	0	0	0	0	0	0	0	0	0	0	8	0	2	0	0	0	0	0	0	0	0	0	0	0	0	0	0	0	0	0	0	0	2
Gambia	0	649	0	0	0	0	0	0	0	0	0	0	0	0	13	0	0	1	0	0	2	2	0	0	1	11	0	0	0	0	0	0	8	0	0	0	0	3	0	0
Georgia	0	0	0	0	0	0	0	0	0	0	0	53	819	1826	12	57	1	24	172	46	25	82	150	20	27	49	31	10	621	4	4	18	60	11	7	12	1	0	0	0
Germany	48	46	25	31	14	22	36	29	17	63	67	32	41	24	22	24	15	16	9	8	12	6	10	4	6	6	6	3	6	3	6	9	2	1	4	3	39	3	1	0
Ghana	0	0	0	0	0	0	0	0	0	0	0	0	0	0	13	2	3	2	4	0	33	18	0	7	2	2	3	6	29	18	4	5	15	16	12	25	28	56	11	20
Greece	5	3	2	4	5	65	6	2	16	4	3	15	2	0	3	0	2	2	0	1	2	0	0	0	0	0	0	0	0	1	4	0	1	2	0	1	66	0	0	0
Greenland	0	0	0	0	0	0	0	0	0	0	0	0	0	0	0	0	0	0	0	0	0	0	0	0	0	0	0	0	0	0	0	0	0	0	0	0	0	0	0	0
Grenada	9	0	0	176	2	0	0	0	1	0	0	0	3	0	0	0	0	0	0	0	0	0	0	0	0	0	0	0	0	0	0	0	0	0	0	0	0	0	0	0
Guam	0	0	0	0	0	0	0	0	0	0	0	1	0	0	0	0	0	0	0	0	0	0	0	0	1	1	0	3	4	1	2	0	0	2	0	0	0	0	0	0
Guatemala	3528	7381	10562	2530	1771	1737	1654	1211	635	534	749	499	421	218	309	162	36	53	31	25	12	18	23	30	27	2	5	5	0	0	0	27	2	4	0	0	0	0	0	0
Guinea	1	0	0	0	0	0	0	0	0	0	0	0	0	0	2	0	29	0	31	83	842	471	14	0	4	4	17	45	5	160	2	5	11	96	0	8	1	28	8	13
Guinea-Bissau	0	0	0	0	0	0	0	0	0	0	0	0	2	0	0	0	0	0	605	200	8	13	0	3	6	5	68	0	5	5	0	1	8	0	22	0	0	0	0	0
Guyana	1	1	0	0	94	0	0	0	0	1	0	0	0	0	0	0	0	0	1	0	0	0	4	0	0	0	6	0	52	0	0	0	0	0	0	0	0	0	0	0
Haiti	0	2	0	5	0	0	0	4	3	48	15	367	18	37	131	17	14	9	0	14	5	0	0	4	238	41	82	1	6	1	0	0	0	0	0	0	7	0	22	0
Honduras	7	8	24	75	5	7	10	21	32	10	5	18	5	0	10	8	11	14	0	0	0	0	0	23	56	0	0	0	0	2	0	2	0	5	0	0	2	0	0	0
Hungary	1	0	0	0	0	0	1	1	0	0	1	1	0	0	0	0	0	3	0	0	0	1	1	0	0	0	0	0	0	2	1	0	0	0	0	0	0	0	0	0
Iceland	0	0	0	0	0	0	0	0	0	0	0	0	0	0	0	0	0	0	0	0	0	0	0	0	0	0	0	0	0	0	0	0	0	0	0	0	0	0	0	0
India	1529	149	3088	860	2431	761	1613	2565	4035	4237	7966	4721	4840	1896	1773	1359	1712	2018	1790	2592	4534	6567	3431	2694	2097	2442	2787	2269	2580	2319	1640	1368	1074	624	1219	724	1533	1112	2061	688
Indonesia	5357	5361	5357	5357	5359	5364	5357	5358	5358	92	212	450	86	27	13	104	64	182	106	1196	570	461	982	1105	1084	308	19	37	7	53	16	74	16	43	32	34	41	16	94	57
Iran (Islamic Republic of)	10551	13915	34473	27167	14286	17563	45414	22252	21949	1098	1106	757	517	455	474	289	261	194	162	146	182	290	246	387	475	351	367	96	143	255	211	224	29	29	29	77	210	65	1143	38
Iraq	19457	25368	63714	50341	26417	32491	84077	41231	40679	2009	2031	1400	1058	843	817	692	489	454	309	296	339	310	300	14835	16158	23172	39762	37739	15122	8999	7044	6837	8202	18796	37209	29814	31752	28043	2379	1779
Ireland	12	2	2	5	1	2	0	6	1	3	0	3	0	0	4	1	1	0	1	1	0	0	0	0	0	0	0	0	0	0	0	1	0	0	0	0	3	0	0	0
Israel	30	27	6	90	33	52	20	30	41	120	100	76	109	89	177	96	206	57	25	8	119	495	1213	625	573	256	682	320	751	707	68	94	87	10	1734	16	28	27	79	6
Italy	130	30	16	8	26	35	6	5	11	2	1	0	17	1	4	1	0	0	3	1	0	0	1	17	1	2	0	0	1	0	12	1	0	3	2	1	1	0	0	0
Jamaica	13	6	8	15	11	11	3	34	0	28	24	21	27	0	2	1	1	10	0	0	0	0	0	0	0	0	0	0	0	0	0	0	0	0	0	0	0	0	0	0
Japan	4	5	8	8	3	7	8	5	4	7	6	4	2	2	11	15	0	0	0	0	0	0	0	0	0	0	0	0	0	0	0	0	0	0	0	0	38	0	0	0
Jordan	1	0	0	0	1	0	0	0	0	0	0	1	2	0	0	1	0	0	0	0	0	1	3	0	1	121	1	0	0	0	2	0	1	5	1	8	73	15	844	4
Kazakhstan	0	0	0	0	0	0	0	0	0	0	0	0	9	0	0	0	0	2	0	4	6	20	0	0	34	35	27	28	29	20	80	33	52	2	7	12	16	0	0	0
Kenya	15	0	0	0	0	0	0	0	0	7	7	0	158	1	46	6	5	344	329	351	464	472	367	174	139	307	280	566	1876	326	169	130	327	707	354	516	201	416	213	68
Kiribati	0	0	0	0	0	0	0	0	0	0	0	2	1	0	3	7	1	0	0	0	0	0	0	0	0	0	0	0	0	0	0	0	0	0	0	0	0	0	0	0
Kuwait	4	3	0	1	7	15	12	1	2	0	1169	2472	2	18	6	8	15	9	8	9	7	5	6	6	9	12	18	12	1	3	0	1	0	1	1	54	1	0	0	0
Kyrgyzstan	0	0	0	0	0	0	0	0	0	0	0	0	0	0	0	0	0	0	0	15	175	1	3	1	0	0	7	0	0	0	424	40	1	1	1	2	1	0	0	0
Lao People's Democratic Republic	0	0	0	0	0	0	8	7	20	32	255	0	3	3	2	0	5	2	0	2	8	0	4	15	4	0	73	0	0	0	0	0	11	0	0	0	5	1	0	0
Latvia	0	0	0	0	0	0	0	0	0	0	0	1	0	0	1	0	0	0	0	0	1	0	0	0	1	0	2	0	1	2	0	0	0	0	0	0	0	0	0	0
Lebanon	90	264	34433	6556	6002	6644	6247	178	176	893	878	143	180	136	152	124	250	118	71	66	38	4	2	4	1	25	756	41	142	17	2	13	9	52	260	190	258	143	838	14
Lesotho	0	10	0	30	1	0	0	0	4	0	0	0	0	0	0	0	0	6	107	16	0	0	2	0	0	0	2	0	0	4	1	0	5	0	1	3	0	4	0	0
Liberia	0	0	0	0	0	0	0	0	0	45	3686	272	435	2565	3788	2267	1101	13	15	132	107	482	2286	3282	18	3	0	1	25	0	1	14	0	1	4	1	14	5	0	0
Libya	6	6	0	1	16	0	1	3551	0	0	0	0	0	0	0	0	2451	8	2	0	52	1	1	0	0	1	1	0	40	0	0	5782	35	100	3632	1599	3220	884	848	1039
Lithuania	0	0	0	0	0	0	0	0	0	0	0	6	0	6	3	1	0	2	0	0	0	0	0	0	0	0	0	0	1	0	0	0	0	0	0	0	0	0	0	0
Luxembourg	0	0	0	0	0	0	0	0	1	0	0	0	0	1	0	0	0	0	0	0	0	0	0	0	0	0	0	0	0	0	0	0	0	0	0	0	0	0	0	0
Madagascar	0	0	0	0	0	0	0	0	0	0	0	4	16	0	0	4	1	0	0	0	1	0	6	0	1	2	0	1	0	71	2	0	23	108	440	169	174	55	126	139
Malawi	0	0	0	0	0	0	0	0	0	0	0	0	32	0	0	0	0	0	0	0	1	2	1	0	0	0	0	0	3	0	0	1	0	2	3	13	22	12	0	0
Malaysia	0	100	0	0	0	0	20	0	9	0	0	0	0	0	0	0	0	12	0	0	5	3	0	9	0	2	2	2	0	1	1	0	3	72	1	3	6	1	0	0
Maldives	0	0	0	0	0	0	0	0	0	0	0	0	0	0	0	0	0	0	0	0	0	0	0	0	0	0	0	0	0	0	0	0	0	0	0	0	0	0	0	0
Mali	0	0	0	0	0	87	0	0	0	0	596	290	91	0	830	6	0	5000	10000	2009	2003	2002	2005	1513	1	12	200	59	117	137	34	35	333	1127	233	264	162	647	1899	1170
Malta	0	0	0	0	0	0	3	0	0	0	0	1	1	0	0	1	0	0	0	0	0	0	0	0	0	0	0	0	0	0	0	0	0	0	0	0	0	0	0	0
Marshall Islands	0	0	0	0	0	0	0	0	0	0	0	0	0	0	0	0	0	0	0	0	0	0	0	0	0	0	0	0	0	0	0	0	0	0	0	0	0	0	0	0
Mauritania	0	0	0	0	0	0	1	0	0	26	30	0	0	0	0	0	0	3	0	0	0	0	0	18	0	49	0	4	24	2	2	14	5	0	0	1	0	2	2	0
Mauritius	0	0	0	0	1	0	0	1	0	0	0	0	0	0	0	0	0	0	0	0	0	0	0	0	0	0	0	0	0	0	0	0	0	0	0	0	0	0	0	0
Mexico	3	0	1	0	13	6	1	5	7	14	14	20	10	5	236	82	139	68	107	0	21	13	4	2	1	2	7	30	27	5	88	241	30	102	38	23	12	0	6	0
Micronesia (Federated States of)	0	0	0	0	0	0	0	0	0	0	0	0	0	0	0	0	0	0	0	0	0	0	0	0	0	0	0	0	0	0	0	0	0	0	0	0	0	0	0	0
Monaco	0	0	0	0	0	0	0	0	0	0	0	0	0	0	0	0	0	0	0	0	0	0	0	0	0	0	0	0	0	0	0	0	0	0	0	0	0	0	0	0
Mongolia	0	0	0	0	0	0	0	0	0	0	0	0	0	0	156	0	0	0	0	0	0	0	0	0	0	0	0	0	0	0	0	0	0	0	0	0	0	0	0	0
Montenegro	0	0	0	0	0	0	0	0	0	0	0	0	0	0	0	0	0	0	0	0	1	1	0	0	0	0	0	0	0	0	0	0	0	0	0	0	0	0	1	0

Morocco	1300	606	606	607	607	607	609	850	390	207	0	22	2	0	0	0	0	6	10	0	0	0	1	77	0	0	2	2	0	0	14	18	1	2	3	4	3	2	2	0
Mozambique	512	5506	5518	5505	26056	25904	26365	26362	3133	2262	1944	2122	676	0	0	15	0	0	1	0	1	0	7	0	3	12	3	6	1	0	7	0	8	51	33	4	57	120	344	159
Myanmar	2705	3299	3302	3271	3250	3250	3250	3250	6744	1105	616	1096	1888	143	793	480	611	804	495	359	671	339	547	265	178	358	450	664	114	267	220	865	994	396	315	724	269	2569	145	617
Namibia	519	517	519	521	533	581	522	519	542	1	0	3	0	0	0	0	2	0	30	23	119	11	10	11	0	0	0	0	0	0	0	0	1	0	2	3	0	1	0	0
Nauru	0	0	0	0	0	0	0	0	0	0	0	0	0	0	0	0	0	0	0	0	0	0	0	0	0	0	0	0	0	0	0	0	0	0	0	0	0	0	0	0
Nepal	0	0	0	0	0	6	0	0	0	0	14	2	9	3	4	0	83	63	357	318	456	741	4918	1754	3086	1610	699	45	133	29	27	13	14	4	5	8	7	13	11	21
Netherlands	2	1	1	0	1	0	2	0	4	0	4	0	0	0	0	4	0	0	0	0	0	1	2	0	1	0	1	7	6	11	3	0	0	1	186	0	3	0	0	0
New Zealand	0	0	0	0	1	0	0	0	0	0	0	0	0	0	0	0	0	0	0	0	0	0	0	0	0	0	0	0	0	0	0	0	0	0	0	0	0	0	0	0
Nicaragua	37	372	454	6877	9117	7475	5295	6749	3108	574	62	55	61	40	35	6	21	7	0	0	2	0	0	1	0	0	0	0	0	0	0	0	0	0	5	0	0	0	34	0
Niger	0	0	0	0	0	2	0	0	0	235	199	45	52	6	86	82	15	78	251	12	1	3	3	0	4	1	2	110	59	13	20	26	6	57	43	614	319	300	551	395
Nigeria	0	0	0	348	0	1	0	0	4	106	81	13	147	3	90	9	83	452	943	2692	3553	1155	2101	1027	1445	360	589	628	913	2668	1497	1650	3091	6260	14156	14635	5064	5077	5440	3086
Niue	0	0	0	0	0	0	0	0	0	0	0	0	0	0	0	0	0	0	0	0	0	0	0	0	0	0	0	0	0	0	0	0	0	0	0	0	0	0	0	0
North Macedonia	0	0	0	0	0	0	0	0	0	0	0	27	1	1	0	3	0	0	0	6	6	107	23	15	0	0	6	12	3	0	0	0	5	0	0	0	0	0	0	0
Northern Mariana Islands	0	0	0	0	0	0	0	0	0	0	0	0	0	0	0	0	0	0	0	0	0	0	0	0	1	2	0	2	0	0	0	0	0	0	0	0	0	0	0	0
Norway	0	1	2	0	0	0	0	1	1	1	0	1	0	0	0	0	0	0	0	1	0	1	0	0	0	0	0	1	1	1	5	77	0	0	0	0	0	0	0	0
Oman	0	0	0	0	0	0	0	0	0	0	0	0	0	0	0	0	0	0	0	0	0	0	0	0	0	0	0	0	0	0	0	0	0	0	0	0	0	0	0	0
Pakistan	17	4	3	10	19	0	60	194	136	56	261	264	155	5	394	766	477	498	182	541	119	115	150	274	477	220	524	2150	4676	8105	11537	5833	6963	5527	6325	5041	2450	1224	1482	500
Palau	0	0	0	0	0	0	0	0	0	0	0	0	0	0	0	0	0	0	0	0	0	0	0	0	0	0	0	0	0	0	0	0	0	0	0	0	0	0	0	0
Palestine	31	6	20832	9	5	0	4	423	430	492	482	463	513	421	240	164	138	130	163	232	2330	3268	4390	3301	3759	2472	4928	2719	6432	6130	668	744	1124	12	75	160	139	126	174	60
Panama	26	1	0	0	0	5	7	15	16	891	2	2	3	0	21	3	9	10	0	0	0	0	0	0	0	0	0	0	0	1	0	0	0	0	0	0	0	0	0	0
Papua New Guinea	0	0	0	0	0	0	0	0	0	27	33	13	47	21	26	27	104	5	0	0	0	4	0	0	0	0	0	0	0	0	0	0	0	0	0	0	0	0	0	0
Paraguay	3	1	1	1	1	0	0	3	1	150	1	0	0	0	5	8	0	1	0	2	0	0	0	0	0	0	0	0	0	0	2	0	1	12	6	13	18	0	0	0
Peru	12	21	725	3705	5885	1897	1285	1725	2088	3436	2368	2715	2007	779	450	338	179	163	40	117	9	37	22	20	9	22	18	50	40	73	29	15	40	9	18	15	22	7	0	0
Philippines	4382	4660	4427	4389	4550	4740	4611	4693	4180	1377	1809	1980	840	275	569	518	361	420	206	258	2051	1333	887	1407	676	536	459	598	786	1155	527	510	516	969	485	1057	753	2089	519	419
Poland	0	0	0	0	0	0	0	0	0	0	1	0	4	0	0	0	0	2	0	0	0	0	0	0	14	0	1	1	6	0	0	13	0	0	0	0	0	0	0	0
Portugal	1099	1217	1127	1125	3	1	3	0	2	2	0	0	0	0	4	1	0	1	0	1	0	0	1	0	1	0	0	0	1	0	0	1	0	0	0	0	0	0	0	0
Puerto Rico	0	0	0	1	2	0	1	0	0	0	0	0	1	0	0	0	0	12	0	1	0	0	0	1	4	6	4	8	1	1	0	5	2	1	0	0	0	9	0	0
Qatar	0	0	1	0	0	0	0	0	0	3	0	0	0	0	0	0	0	0	0	0	0	1	0	0	0	2	0	0	0	0	0	0	0	0	3	0	0	0	0	0
Republic of Korea	0	0	1	1	0	6	18	11	14	25	29	28	29	15	45	26	21	42	1	0	0	0	5	2	1	0	0	1	0	1	50	0	0	1	2	0	0	0	0	0
Republic of Moldova	0	0	0	0	0	0	0	0	0	0	0	16	840	196	8	4	1	0	0	0	1	0	0	0	0	0	2	0	0	0	0	0	1	0	0	0	0	0	0	0
Romania	147	155	145	145	142	149	150	149	156	1059	100	26	45	49	166	93	65	42	35	0	0	4	1	1	0	1	2	0	0	0	0	0	1	0	1	1	0	0	0	0
Russian Federation	3	0	0	0	0	0	0	0	0	21	192	271	36	147	277	2055	2901	75	57	7145	6617	4513	2644	1185	2175	1043	398	299	574	883	1139	564	1347	1339	266	415	225	187	136	20
Rwanda	0	0	0	0	0	0	0	0	0	0	2644	1102	1084	959	503532	4342	438	6357	2610	365	168	2047	5	503	16	5	13	18	9	1844	226	120	103	11	5	11	166	37	38	1
Saint Kitts and Nevis	0	0	0	0	0	0	0	0	0	0	0	0	0	0	0	0	0	0	0	0	0	0	0	0	0	0	0	0	0	0	0	0	0	0	0	0	0	0	0	0
Saint Lucia	0	0	0	2	0	0	0	3	1	1	1	1	1	2	1	0	0	0	0	0	2	0	0	0	0	0	0	0	0	0	0	0	0	0	0	0	0	0	0	0
Saint Vincent and the Grenadines	0	0	0	0	0	0	0	6	0	0	0	0	0	0	0	3	1	0	0	0	0	0	0	0	0	0	0	0	0	0	0	0	0	0	0	0	0	0	0	0
Samoa	0	0	0	0	0	0	0	0	0	0	0	0	0	0	0	0	0	0	0	0	0	0	0	0	0	0	0	0	0	0	0	0	0	0	0	0	0	0	0	0
San Marino	0	0	0	0	0	0	0	0	0	0	0	0	0	0	0	0	0	0	0	0	0	0	0	0	0	0	0	0	0	0	0	0	0	0	0	0	0	0	0	0
Sao Tome and Principe	0	0	0	0	0	0	0	0	0	0	0	0	0	0	0	0	0	0	0	0	0	0	0	0	0	0	0	0	0	0	0	0	0	0	0	0	0	0	0	0
Saudi Arabia	0	0	0	0	0	1	62	0	0	1	0	29	0	0	0	14	19	0	0	0	1	2	1	81	154	32	15	5	2	5	1	3	4	1	20	323	275	3	11	0
Senegal	0	6	0	0	0	0	0	0	2	2	104	21	199	285	5	347	5	440	373	38	81	78	83	40	3	46	5	5	14	28	23	52	24	12	16	1	17	0	5	2
Serbia	0	0	0	2	2	0	0	0	0	0	0	3026	146	2	0	2	10	18	1503	9429	532	128	71	29	51	16	2	9	5	0	2	2	6	2	3	0	0	0	8	0
Seychelles	0	0	11	0	0	0	0	0	0	0	0	0	0	0	0	0	0	0	0	0	0	0	0	0	0	0	0	0	0	0	0	0	0	0	0	0	0	0	0	0
Sierra Leone	0	0	0	0	0	0	0	0	0	0	0	1344	551	640	2300	3194	999	1249	5162	4553	1162	91	3	5	1	0	0	4	6	0	0	0	0	3	0	0	3	0	0	0
Singapore	0	0	0	0	0	0	0	0	0	0	0	4	0	0	1	0	0	0	0	0	0	0	0	0	0	0	0	0	0	0	0	0	0	0	0	0	0	0	0	0
Slovakia	0	0	0	0	0	0	0	0	0	0	0	0	0	0	0	3	1	2	1	0	0	0	0	0	0	0	0	0	0	0	0	0	0	0	0	0	0	0	0	0
Slovenia	0	0	0	0	0	1	0	0	0	0	0	26	1	0	1	0	0	0	0	0	0	0	0	0	0	0	0	0	0	0	0	0	0	0	0	0	0	0	0	0
Solomon Islands	0	0	0	0	0	0	0	0	0	0	0	0	0	0	0	0	0	0	0	2	0	0	1	1	0	0	0	0	0	0	0	0	0	0	0	0	0	0	0	0
Somalia	1	0	512	619	512	0	512	555	11666	1091	2656	8461	1252	25	315	134	466	516	206	79	29	306	168	472	1322	301	615	2301	2311	1919	3202	2609	3660	2040	1779	1713	2586	4647	3616	1686
South Africa	553	535	536	555	539	636	680	684	716	116	366	472	828	239	540	105	82	47	96	45	46	16	19	23	31	10	6	8	25	26	24	21	167	63	101	137	85	64	58	38
South Sudan	0	0	0	2045	2045	2045	2045	2045	2044	0	0	0	0	0	0	0	0	7000	5000	3000	2000	1000	1000	1000	200	90	750	450	175	2500	800	1276	831	3515	3657	1833	1622	1869	1425	470
Spain	122	39	42	42	51	67	57	62	25	46	51	90	52	12	28	31	9	17	17	5	50	53	15	20	384	18	2	2	6	6	1	1	0	0	0	0	0	16	0	1
Sri Lanka	34	26	738	47	2151	2278	2357	3274	3885	2809	6289	7680	5428	2120	869	5991	4924	3388	4510	2156	3018	1357	11	53	54	223	3088	2963	8799	10614	8485	5	1	11	3	4	2	1	18	282
Sudan	0	2	0	2066	2371	2066	2074	2066	2125	5528	5298	3596	3086	2032	827	1400	2390	5026	6071	1756	3354	3771	6409	8258	12666	1897	2548	1631	1999	2059	1504	3656	1886	1465	1743	2305	2344	564	1145	101
Suriname	0	0	0	0	0	0	2	6	6	14	0	1	0	0	0	0	0	0	0	0	0	0	0	0	0	0	0	0	0	0	0	0	0	0	0	0	0	0	0	0
Sweden	0	0	0	2	0	0	1	1	1	1	1	2	2	0	0	1	0	1	0	3	0	0	0	0	0	2	0	1	0	0	2	0	1	0	0	4	2	5	0	0
Switzerland	0	2	0	0	0	0	0	1	0	0	1	0	6	0	0	1	1	0	0	0	0	14	0	1	0	0	0	0	0	0	0	0	0	0	0	0	0	0	0	0
Syrian Arab Republic	601	704	13598	3	0	31	8	0	0	54	54	0	0	0	0	0	15	102	20	7	10	20	100	80	9	35	1003	499	193	32	10	12918	60179	49731	75356	60942	54433	31830	10815	6900
Taiwan (Province of China)	0	0	0	0	0	0	0	0	0	0	0	7	17	0	1	0	9	0	0	0	0	2	0	0	0	0	0	0	0	0	0	0	0	0	0	0	0	0	0	0
Tajikistan	0	0	0	0	0	0	0	0	0	0	0	0	3391	15873	414	446	2847	140	629	6	57	128	50	10	20	1	8	3	1	17	102	28	53	3	8	24	1	0	7	0
Thailand	897	678	524	1	0	2	105	93	290	237	42	11	39	1	18	25	24	27	15	3	15	36	39	32	342	624	735	782	496	530	515	579	527	547	281	174	174	115	236	60
Timor-Leste	1008	650	569	602	423	358	341	244	260	325	211	358	293	374	293	374	0	455	472	2618	82	98	114	54	0	0	50	36	3	0	0	0	0	0	0	0	0	0	0	0
Togo	0	0	0	0	0	1	0	0	0	0	0	45	17	48	47	0	3	4	10	4	0	0	0	0	0	436	0	0	1	2	0	3	0	6	0	0	0	12	0	0
Tokelau	0	0	0	0	0	0	0	0	0	0	0	0	0	0	0	0	0	0	0	0	0	0	0	0	0	0	0	0	0	0	0	0	0	0	0	0	0	0	0	0
Tonga	0	0	0	0	0	0	0	0	0	0	0	0	0	0	0	0	0	0	0	0	0	0	0	0	0	0	0	0	0	0	0	0	0	0	0	0	0	0	0	0
Trinidad and Tobago	0	3	1	5	4	2	11	12	19	19	39	9	11	10	19	22	23	19	5	0	0	0	0	0	0	0	0	0	0	0	0	0	0	0	0	0	0	0	0	0
Tunisia	41	0	0	0	0	0	0	0	0	0	0	4	0	0	1	7	0	0	0	0	5	3	22	0	0	0	2	28	0	0	0	20	9	73	92	106	74	11	17	15
Turkey	111	11	9	2	200	174	199	360	275	505	824	703	3012	2529	5059	3318	3432	2366	1983	1286	133	104	28	173	231	400	269	485	474	144	218	451	990	1309	1120	1549	2555	451	1509	309
Turkmenistan	0	0	0	0	0	0	0	0	0	0	0	458	360	105	0	0	0	0	0	4	4	20	6	6	10	35	4	4	5	5	79	18	50	4	10	12	0	0	0	0
Tuvalu	0	0	0	0	0	0	0	0	0	0	0	0	0	0	0	0	0	0	0	0	0	0	0	0	0	0	0	0	0	0	0	0	0	0	0	0	0	0	0	0
Uganda	14769	14826	14857	14978	15092	14889	14859	2001	1160	1129	893	1020	74	0	182	682	2266	1649	1517	1241	924	237	2401	3110	2475	1252	460	192	123	211	311	133	31	71	920	228	177	254	361	61
Ukraine	0	0	0	0	0	0	0	0	0	0	0	0	1	0	4	0	0	2	0	1	0	0	1	0	0	59	56	54	51	40	39	34	34	1	5774	2079	362	478	281	227
United Arab Emirates	1	2	0	111	0	0	0	0	0	1	0	0	0	0	0	0	0	0	0	0	0	0	0	0	0	0	0	0	0	0	1	0	0	0	1	0	0	0	0	0
United Kingdom	209	200	840	164	140	122	129	204	478	111	126	218	136	52	89	21	22	30	130	57	14	14	5	55	24	81	68	95	56	112	104	51	47	14	9	3	11	0	0	2
United Republic of Tanzania	88	88	0	0	0	0	0	0	0	0	0	0	1	0	1	0	0	20	17	13	37	161	164	3	11	9	2	6	7	2	21	19	30	36	27	30	42	5	17	19
United States Virgin Islands	6	0	0	0	0	0	0	0	0	0	0	0	0	0	0	0	0	0	0	0	0	0	0	0	0	0	0	0	0	0	0	0	0	0	0	0	0	0	0	0
United States of America	37	31	17	306	60	26	14	52	42	51	21	159	78	72	44	190	43	32	197	46	41	4279	66	538	927	969	948	1031	487	502	579	489	326	170	91	74	57	17	2	31
Uruguay	0	0	0	1	1	2	3	0	0	3	0	0	0	0	0	0	1	0	0	0	0	0	0	0	0	0	0	0	0	0	0	0	0	0	0	0	2	0	0	0
Uzbekistan	0	0	0	0	0	0	0	0	0	0	2	0	0	128	26	0	0	0	0	69	298	20	0	0	73	45	0	2	1	19	81	18	51	2	8	12	0	0	0	0
Vanuatu	0	0	0	0	0	0	0	0	0	0	0	0	0	0	0	0	0	0	0	0	0	0	0	0	0	0	0	0	0	0	0	0	0	0	0	0	0	0	0	0
Venezuela (Bolivarian Republic of)	12	6	41	13	2	0	36	57	91	169	64	77	230	79	234	36	46	6	10	26	25	27	20	25	31	30	22	3	1	10	0	1	0	0	1	1	14	28	0	0
Viet Nam	10	19	0	7	26	0	27	130	5	0	0	0	0	0	0	0	0	1	0	0	0	7	0	29	0	6	5	5	0	2	2	2	4	10	4	6	4	8	3	0
Yemen	550	513	512	0	0	0	11500	0	0	0	0	1	24	0	1503	4	3	11	8	11	19	2	5	16	0	12	14	24	95	141	520	1743	3386	1206	3008	7747	15117	16809	30779	13402
Zambia	0	0	0	0	0	0	2	0	9	45	52	0	0	1	0	0	0	1	1	4	1	11	0	4	0	0	5	18	0	9	0	10	11	8	6	7	3	1	5	1
Zimbabwe	37	13	22	11	4	109	106	141	14	39	25	0	0	0	0	8	0	0	4	2	27	31	45	5	1	4	1	6	372	15	7	5	10	8	5	13	6	5	9	11

SR Table 10b: Deaths due to epidemics by country, all ages, for both sexes combined, 1980-2019

Country	1980	1981	1982	1983	1984	1985	1986	1987	1988	1989	1990	1991	1992	1993	1994	1995	1996	1997	1998	1999	2000	2001	2002	2003	2004	2005	2006	2007	2008	2009	2010	2011	2012	2013	2014	2015	2016	2017	2018	2019
Afghanistan	0	0	0	0	0	0	0	0	0	0	0	0	0	931	118	624	0	125	185	287	557	154	2794	10	0	0	0	0	17	11	10	14	0	14	4	8	0	0	0	0
Albania	0	0	0	0	0	0	0	0	0	22	22	0	0	0	25	0	7	0	0	0	0	0	0	0	0	0	0	0	0	0	0	0	0	0	0	0	0	0	0	0
Algeria	18	0	0	9	0	0	0	0	116	0	20	0	0	0	4	0	0	1	6	0	0	0	0	0	0	0	0	0	0	0	0	0	0	0	0	0	0	0	0	0
American Samoa	0	0	0	0	0	0	0	0	0	0	0	0	0	0	0	0	0	0	0	0	0	0	0	0	0	0	0	0	0	0	0	0	0	0	0	0	0	0	0	0
Andorra	0	0	0	0	0	0	0	0	0	0	0	0	0	0	0	0	0	0	0	0	0	0	0	0	0	0	0	0	0	0	0	0	0	0	0	0	0	0	0	0
Angola	25	6	0	0	0	23	33	76	1630	1114	992	903	548	495	571	248	42	0	115	193	18	39	176	8	82	247	2069	868	324	155	30	110	98	86	3	35	349	0	2	0
Antigua and Barbuda	0	0	0	0	0	0	0	0	0	0	0	0	0	0	0	0	0	0	0	0	0	0	0	0	0	0	0	0	0	0	0	0	0	0	0	0	0	0	0	0
Argentina	0	0	0	0	0	0	0	0	0	0	0	0	0	18	0	0	0	0	0	0	0	0	0	0	0	0	0	1	0	0	0	0	3	0	0	0	0	0	0	0
Armenia	0	0	0	0	0	0	0	0	0	0	0	0	0	0	0	0	0	0	0	0	0	0	0	0	0	0	0	0	0	0	0	0	0	0	0	0	0	0	0	0
Australia	0	0	0	0	0	0	0	0	0	0	0	0	0	0	0	0	0	0	0	0	0	0	0	0	0	0	0	0	0	0	0	0	0	0	0	0	0	0	0	0
Austria	0	0	0	0	0	0	0	0	0	0	0	0	0	0	0	0	4	0	2	0	0	1	0	0	0	0	0	0	0	0	0	0	0	0	0	0	0	0	0	0
Azerbaijan	0	1	5	0	0	2	6	4	3	6	2	0	7	6	18	8	0	0	0	0	0	0	0	0	0	0	0	0	0	0	0	0	0	0	0	0	0	0	0	0
Bahamas	0	0	0	0	0	0	0	0	0	0	0	0	0	0	0	0	0	0	0	0	0	0	0	0	0	0	0	0	0	0	0	0	0	0	0	0	0	0	0	0
Bahrain	0	0	0	0	0	0	0	0	0	0	0	0	0	0	0	0	0	0	0	0	0	0	0	0	0	0	0	0	0	0	0	0	0	0	0	0	0	0	0	0
Bangladesh	0	0	2696	500	950	900	165	773	43	22	4	5624	673	38	41	461	20	159	177	63	47	0	96	0	32	0	0	86	0	0	0	0	0	0	0	0	0	15	0	104
Barbados	0	0	0	0	0	0	0	0	0	0	0	0	0	0	0	0	0	0	0	0	0	0	0	0	0	0	0	0	0	0	0	0	0	0	0	0	0	0	0	0
Belarus	0	31	24	0	0	16	0	0	0	0	0	0	0	0	0	0	0	0	0	0	0	0	0	0	0	0	0	0	0	0	0	0	0	0	0	0	0	0	0	0
Belgium	0	0	0	0	0	0	0	0	0	0	0	0	0	0	0	0	0	0	0	0	0	0	0	0	0	0	0	0	0	0	0	0	0	0	0	0	0	0	0	0
Belize	0	0	0	0	0	0	0	0	0	0	0	0	0	0	0	0	0	0	0	0	0	0	0	0	0	0	0	0	0	0	0	0	0	0	0	0	0	0	0	0
Benin	29	55	27	45	26	52	56	162	56	496	58	345	17	2	175	12	268	133	209	110	362	378	156	92	105	81	68	107	33	65	25	54	115	88	100	0	9	0	0	0
Bermuda	0	0	0	0	0	0	0	0	0	0	0	0	0	0	0	0	0	0	0	0	0	0	0	0	0	0	0	0	0	0	0	0	0	0	0	0	0	0	0	0
Bhutan	0	0	0	0	0	61	21	0	0	0	0	19	6	0	0	0	0	0	0	0	0	0	0	0	0	0	0	0	0	0	0	0	0	0	0	0	0	0	0	0
Bolivia (Plurinational State of)	0	0	0	0	0	0	0	0	0	67	0	329	383	254	46	54	68	18	5	33	0	0	0	0	0	0	0	1	9	18	4	25	0	0	0	0	0	0	14	9
Bosnia and Herzegovina	0	0	0	0	0	0	0	0	0	0	0	0	0	0	0	0	0	0	0	0	0	0	0	0	0	0	0	0	0	0	0	0	0	0	0	0	0	0	0	0
Botswana	7	4	8	1	4	0	3	1	187	4	4	3	0	0	0	0	0	0	0	0	0	0	0	0	0	0	470	0	1	1	0	0	0	0	0	0	0	0	0	0
Brazil	0	0	0	106	230	122	146	179	242	287	360	404	480	685	623	544	513	490	435	370	303	307	281	215	243	225	0	98	99	147	120	90	101	0	0	0	20	324	38	0
Brunei Darussalam	0	0	0	0	0	0	0	0	0	0	0	0	0	0	0	0	0	0	0	0	0	0	0	0	0	0	0	0	0	0	0	0	0	0	0	0	0	0	0	0
Bulgaria	0	0	0	0	0	0	0	0	0	0	0	0	0	0	12	0	0	0	0	0	0	0	0	0	0	0	0	0	0	0	0	0	0	0	0	0	0	0	0	0
Burkina Faso	306	1593	752	927	1118	656	458	0	517	0	338	356	426	0	0	434	8535	4721	898	662	791	1777	1201	1058	528	773	784	1490	292	647	841	590	716	339	353	0	0	18	0	0
Burundi	35	36	22	57	29	18	25	9	18	19	4	0	771	0	41	61	38	21	41	83	329	39	146	21	4	18	17	2	4	0	1	12	5	17	9	0	1	0	0	0
Cabo Verde	6	0	1	1	0	0	0	7	10	21	3	40	39	28	37	208	3	0	1	0	0	0	0	2	0	0	0	0	0	6	0	0	0	0	0	0	0	0	0	0
Cambodia	0	0	0	0	0	0	0	55	0	2	0	97	50	104	258	246	167	117	490	56	0	0	0	0	1	3	3	183	0	0	1	0	0	0	0	0	0	0	0	0
Cameroon	23	221	167	306	336	338	235	160	280	356	409	626	1114	1055	117	11	408	355	602	298	51	31	124	137	46	42	19	8	0	158	659	804	69	68	171	6	1	0	57	0
Canada	0	0	0	0	0	0	0	0	0	0	0	0	0	14	4	0	0	9	0	10	11	12	6	8	0	0	0	0	0	0	0	0	0	0	0	0	0	0	0	0
Central African Republic	31	24	34	13	15	17	20	120	218	221	170	147	200	111	0	0	22	78	60	245	452	343	102	56	55	28	40	152	89	48	106	62	31	31	41	0	21	0	1	0
Chad	0	7	17	200	51	63	60	0	865	0	0	1262	184	232	43	0	203	628	1090	337	754	568	120	158	291	259	178	105	170	108	239	557	88	40	22	0	0	58	72	18
Chile	0	0	0	0	0	0	0	0	0	0	5	10	12	15	10	0	0	0	0	0	0	0	0	0	0	0	0	0	0	0	0	0	12	3	12	0	0	0	0	0
China	0	0	0	0	5790	0	0	0	87	58	5	1075	1	398	618	388	281	251	200	136	1	0	107	263	1	47	8	3	0	0	0	0	0	1	0	0	0	0	0	0
Colombia	0	0	0	0	0	0	0	0	0	0	0	52	54	4	6	3	10	47	11	0	0	0	0	0	0	0	0	0	0	0	0	0	0	0	0	0	0	0	0	169
Comoros	0	0	0	0	0	0	0	0	9	3	0	0	0	0	0	0	0	0	40	14	91	4	46	0	0	0	0	29	0	0	0	0	0	0	0	0	0	0	0	0
Congo	32	27	0	0	6	6	0	0	7	0	0	0	0	0	0	0	0	42	41	15	26	0	2	6	0	0	10	40	26	0	219	65	42	16	0	0	3	0	0	0
Cook Islands	0	0	0	0	0	0	0	0	0	0	7	0	0	0	0	0	0	0	0	0	0	0	0	0	0	0	0	0	0	0	0	0	0	0	0	0	0	0	0	0
Costa Rica	0	0	0	0	0	0	0	0	0	0	0	0	0	0	0	0	0	0	0	0	0	1	0	0	0	2	0	0	0	0	0	0	0	0	0	0	0	0	0	0
Croatia	0	0	0	0	0	4	8	1	5	5	4	3	3	1	0	0	0	0	0	0	0	0	0	0	0	0	0	0	0	0	0	0	0	0	0	0	0	0	0	0
Cuba	114	180	188	201	211	206	194	152	165	126	113	65	36	28	22	16	13	6	2	17	4	2	0	2	4	6	0	0	0	0	2	0	3	0	0	0	0	0	0	0
Cyprus	0	0	0	0	0	0	0	0	0	0	0	0	0	0	0	0	0	0	0	0	0	0	0	0	0	0	0	0	0	0	0	0	0	0	0	0	0	0	0	0
Czechia	0	0	0	0	0	0	0	0	0	0	0	0	0	0	13	0	0	0	0	0	0	0	0	0	0	0	0	0	0	0	0	0	0	0	0	0	0	0	0	0
Côte d'Ivoire	204	107	130	71	25	105	63	144	322	483	11	16	7	42	55	150	22	214	324	565	63	217	162	54	83	146	42	31	45	48	28	49	78	36	37	6	1	2	0	0
Democratic People's Republic of Korea	0	0	0	0	0	0	0	0	0	0	0	0	0	0	0	0	0	0	0	0	0	0	0	0	0	0	2	2	0	0	0	0	0	0	0	0	0	0	0	0
Democratic Republic of the Congo	81	806	700	249	14	48	126	513	32	6	61	794	59	175	4184	51	213	218	1029	1351	1894	519	2643	3334	2694	1258	1033	606	1233	209	56	1097	1631	1350	1366	276	827	22	21	7498
Denmark	0	0	0	0	0	0	0	0	0	0	0	0	0	0	0	4	0	0	0	2	4	4	0	3	0	0	0	0	0	0	0	0	0	0	0	0	0	0	0	0
Djibouti	0	0	0	0	0	7	0	0	10	5	0	0	0	89	10	0	0	29	46	0	4	0	0	0	0	0	11	6	0	0	19	1	0	0	0	0	0	0	0	0
Dominica	0	0	0	0	0	0	0	0	0	0	0	0	0	0	0	0	0	0	0	0	0	0	0	0	0	0	0	0	0	0	0	0	0	0	0	0	0	0	0	0
Dominican Republic	0	0	0	0	0	0	0	0	0	0	0	0	20	0	0	2	0	0	0	16	0	0	0	0	18	21	26	15	21	32	30	127	186	52	21	21	34	0	0	0
Ecuador	0	0	0	0	0	0	0	0	0	0	0	347	207	77	19	30	12	3	13	0	9	0	0	0	0	0	0	0	0	0	4	0	11	0	0	0	0	0	0	0
Egypt	167	100	170	155	187	212	171	172	353	0	0	244	233	0	0	0	0	0	62	0	0	0	0	0	0	0	8	8	0	0	0	0	0	0	0	0	0	0	0	0
El Salvador	0	0	0	0	0	0	0	7	0	0	0	159	45	14	38	7	2	0	1	0	26	0	6	304	0	0	0	0	0	7	0	0	0	0	4	0	0	0	0	5
Equatorial Guinea	0	0	0	0	31	1	0	0	0	0	0	0	0	0	0	0	0	0	0	0	0	0	0	2	5	44	0	0	0	0	0	0	0	0	0	0	0	0	0	0
Eritrea	0	0	0	0	0	0	0	0	0	0	0	0	0	0	0	0	0	1	0	0	0	0	0	0	0	0	0	12	0	0	0	0	0	0	0	0	0	0	0	0
Estonia	0	12	12	0	0	15	4	2	3	2	0	0	0	0	0	2	0	0	0	0	0	0	0	0	0	0	0	0	0	0	0	0	0	0	0	0	0	0	0	0
Eswatini	0	17	13	0	0	0	0	0	0	0	0	0	30	0	0	0	80	0	0	0	32	107	2	13	16	0	0	0	0	0	0	0	0	0	0	0	0	0	0	0
Ethiopia	368	1974	619	0	37	1127	20	27	7400	1512	109	0	0	0	0	10	0	8	0	35	427	390	51	1189	146	131	919	312	61	489	37	0	0	110	0	0	0	0	0	0
Fiji	0	0	0	0	0	0	0	0	0	0	0	0	0	0	0	0	0	0	0	0	0	0	0	0	0	0	0	0	0	0	0	0	0	0	0	0	0	0	0	0
Finland	0	0	0	0	0	0	0	0	0	0	0	0	0	0	0	0	0	0	0	0	0	0	0	0	0	0	0	0	0	0	0	0	0	0	0	0	0	0	0	0
France	0	0	0	0	0	0	0	0	0	0	0	0	0	0	0	0	0	0	0	0	8	0	7	0	0	0	0	0	0	0	0	0	0	0	0	0	0	0	0	0
Gabon	0	0	6	10	4	4	0	26	0	34	27	0	0	0	0	0	0	0	0	0	0	0	1	2	1	0	7	0	0	0	0	0	0	0	0	0	0	0	0	0
Gambia	19	1	0	0	0	0	0	0	0	18	0	0	0	0	0	0	0	240	0	0	8	13	5	0	0	13	0	13	0	0	1	0	9	34	38	0	0	0	0	0
Georgia	0	0	8	0	0	9	2	5	3	2	0	0	0	0	0	0	0	0	0	0	0	0	0	0	0	0	0	0	0	0	0	0	0	0	0	0	0	0	0	0
Germany	0	0	0	0	0	0	0	0	0	0	0	0	0	0	2	0	0	0	0	0	0	14	23	14	7	0	1	0	0	0	0	0	2	4	2	0	0	0	0	0
Ghana	80	111	1263	850	416	174	154	108	113	83	127	103	377	289	230	112	275	1757	291	159	314	312	302	238	171	133	214	314	114	72	30	158	76	59	151	143	57	0	0	0
Greece	0	0	0	0	0	0	0	0	0	0	0	0	0	0	0	0	0	0	0	0	0	0	0	0	0	0	0	0	0	0	0	0	0	0	0	0	0	0	0	0
Greenland	0	0	0	0	0	0	0	0	0	0	0	0	0	0	0	0	0	0	0	0	0	0	0	0	0	0	0	0	0	0	0	0	0	0	0	0	0	0	0	0
Grenada	0	0	0	0	0	0	0	0	0	0	0	0	0	0	0	0	0	0	0	0	0	0	0	0	0	0	0	0	0	0	0	0	0	0	0	0	0	0	0	0
Guam	0	0	0	0	0	0	0	0	0	0	0	0	0	0	0	0	0	0	0	0	0	0	0	0	0	0	0	0	0	0	0	0	0	0	0	0	0	0	0	0
Guatemala	0	0	0	0	0	0	0	0	0	0	0	34	82	160	198	67	3	0	24	0	4	0	0	0	0	0	0	0	0	0	0	0	0	0	0	0	0	0	0	0
Guinea	0	0	0	0	0	189	58	38	50	89	39	0	69	183	462	599	32	4	63	46	226	27	69	35	100	72	129	98	90	8	11	0	118	83	55	0	0	0	0	0
Guinea-Bissau	0	3	0	0	0	0	50	68	0	0	0	0	0	0	195	0	961	781	28	806	0	0	6	2	3	399	0	8	221	0	0	0	22	28	2	0	0	0	0	0
Guyana	0	0	0	0	0	0	0	0	0	0	0	0	2	0	0	0	0	0	0	0	0	0	0	0	0	0	0	0	0	0	0	0	0	0	0	0	0	0	0	0
Haiti	0	0	0	0	0	0	0	0	0	0	0	0	0	0	0	55	0	0	0	0	0	0	0	40	0	0	0	0	0	0	1382	5526	50	593	296	170	420	0	0	0
Honduras	0	0	0	0	0	0	0	0	0	0	0	0	17	103	100	82	14	1	17	3	3	0	8	0	0	0	0	0	0	7	67	0	0	27	0	0	0	0	0	128
Hungary	0	0	0	0	0	0	0	0	0	0	0	0	0	0	0	0	0	0	0	0	0	0	0	0	0	0	0	0	0	0	0	4	0	5	0	5	0	0	0	0
Iceland	0	0	0	0	0	0	0	0	0	0	0	0	0	0	0	0	0	0	0	0	0	0	0	0	0	0	0	0	0	0	0	0	0	0	0	0	0	0	0	0
India	1284	200	335	432	4567	2008	336	314	3911	272	235	331	55	53	85	5	389	96	822	287	1251	1058	1041	625	612	1104	315	310	543	790	350	255	414	251	156	2339	0	2398	0	121
Indonesia	150	463	483	466	164	85	441	18	0	0	50	294	0	1	0	0	117	197	1449	56	25	6	17	0	658	51	33	425	27	0	0	0	0	0	0	0	0	493	0	0
Iran (Islamic Republic of)	32	108	0	0	5	9	1	4	15	85	3	32	3	5	0	59	0	9	1	21	79	0	0	0	1	11	0	0	4	0	0	12	0	7	0	1	0	0	0	0
Iraq	0	0	0	0	0	0	0	0	0	0	0	7	0	0	0	3	0	0	1	30	4	0	0	0	0	0	2	24	11	0	0	0	4	0	0	2	0	0	0	0
Ireland	0	0	0	0	0	0	0	0	0	0	0	0	0	0	0	0	0	8	2	0	9	0	0	0	0	0	0	0	0	0	0	0	0	0	0	0	0	0	0	0
Israel	0	0	0	4	4	0	0	2	0	4	3	0	0	7	4	0	0	4	0	0	0	0	0	0	0	0	0	0	0	0	0	0	0	0	0	0	0	0	0	0
Italy	0	0	0	0	0	0	0	0	0	0	0	0	0	0	0	0	0	0	0	0	0	0	0	0	0	0	0	0	0	0	0	0	0	0	0	0	0	0	0	0
Jamaica	0	0	0	0	0	0	0	0	0	0	0	0	0	0	0	0	0	0	0	0	0	0	0	0	0	0	0	0	0	0	0	0	0	0	0	0	0	0	0	0
Japan	0	0	0	0	0	0	0	0	0	0	0	0	0	0	0	0	0	0	0	0	0	0	0	0	0	0	0	0	0	0	0	0	0	0	0	0	0	0	0	0
Jordan	0	4	0	0	0	0	0	0	0	0	0	0	0	0	0	0	0	0	3	0	0	0	0	0	0	0	0	0	0	0	0	0	0	0	0	0	0	0	0	0
Kazakhstan	0	0	0	0	0	0	0	0	0	0	0	0	0	0	0	0	0	0	0	0	0	0	0	0	0	0	0	0	0	0	0	0	0	0	0	0	0	0	0	0
Kenya	139	215	138	89	0	102	45	12	0	32	0	26	80	0	1028	28	14	804	1284	1831	50	40	12	0	23	38	137	203	43	251	109	11	0	0	12	108	20	76	0	0
Kiribati	0	0	0	0	0	0	0	0	0	0	0	0	0	0	0	0	0	0	0	0	0	0	0	0	0	0	0	0	0	0	0	0	0	0	0	0	0	0	0	0
Kuwait	0	0	0	0	0	0	0	0	0	0	0	0	0	0	0	0	0	0	0	0	0	0	0	0	0	0	0	0	0	0	0	0	0	0	0	0	0	0	0	0
Kyrgyzstan	0	0	0	0	0	0	0	0	0	0	0	0	0	0	0	0	0	0	0	0	0	0	0	0	0	0	0	0	0	0	0	0	0	0	0	0	0	0	0	0
Lao People's Democratic Republic	0	0	0	0	0	0	0	63	0	0	0	0	177	313	553	111	104	0	0	0	58	0	0	0	0	0	0	5	0	0	4	0	0	77	0	0	0	0	0	0
Latvia	0	9	10	0	0	0	0	0	0	0	0	0	0	0	0	0	0	0	0	0	0	0	0	0	0	0	0	0	0	0	0	0	0	0	0	0	0	0	0	0
Lebanon	0	0	0	0	0	0	0	0	0	0	0	0	0	23	0	0	0	0	0	0	0	0	0	0	0	0	0	0	0	0	0	0	0	0	0	0	0	0	0	0
Lesotho	0	0	0	0	0	0	0	0	0	0	0	0	0	0	0	0	0	0	0	19	37	0	0	0	0	0	0	5	0	0	0	0	0	0	0	0	0	0	0	0
Liberia	536	112	58	62	15	22	0	43	1	0	0	40	0	6	25	129	62	0	36	39	13	0	0	38	4	29	21	9	3	2	0	0	0	0	0	0	2	0	0	0
Libya	0	0	0	0	0	0	0	0	0	0	0	0	0	0	0	2	0	0	0	0	0	0	0	0	0	0	0	0	0	0	0	0	0	0	0	0	0	0	0	0
Lithuania	0	16	6	0	0	13	2	5	0	0	0	0	0	0	6	0	0	0	0	0	0	0	0	0	0	0	0	0	0	0	0	0	0	0	0	0	0	0	0	0
Luxembourg	0	0	0	0	0	0	0	0	0	0	0	0	0	0	0	0	0	0	0	0	0	0	0	0	0	0	0	0	0	0	0	0	0	0	0	0	0	0	0	0
Madagascar	0	8	0	0	0	0	0	0	0	0	0	0	0	0	0	0	0	0	0	783	198	413	671	0	0	0	0	0	20	0	0	0	0	85	28	0	0	207	571	570
Malawi	0	125	0	49	0	0	0	159	29	405	68	245	8	524	11	0	29	10	74	648	83	826	520	34	4	11	20	5	33	87	72	4	2	0	0	11	0	3	3	0
Malaysia	7	14	17	38	1	2	2	18	32	14	37	268	13	13	0	27	15	88	84	62	5	11	9	2	0	0	2	58	2	2	6	11	1	1	1	2	0	171	0	0
Maldives	0	0	0	0	0	0	0	0	0	0	0	0	0	0	0	0	0	0	1	0	0	0	0	0	0	0	0	0	0	0	0	4	0	0	0	0	0	0	0	0
Mali	76	910	358	170	1085	1021	306	333	59	71	81	0	0	0	0	484	1097	2236	285	120	144	136	96	148	88	66	84	47	90	10	34	66	31	9	4	0	0	0	0	0
Malta	0	0	0	0	0	0	0	0	0	0	0	0	0	0	0	1	0	1	3	3	2	0	1	0	2	0	0	0	0	0	0	0	0	0	0	0	0	0	0	0
Marshall Islands	0	0	0	0	0	0	0	0	0	0	0	0	0	0	0	0	0	0	0	0	6	0	0	0	0	0	0	0	0	0	0	0	0	0	0	0	0	0	0	0
Mauritania	0	0	5	9	15	31	113	149	50	36	0	0	0	0	0	0	46	9	9	52	41	6	5	11	0	55	0	0	0	0	0	3	6	5	0	0	0	0	0	0
Mauritius	0	0	0	0	1	0	0	0	0	0	0	0	0	0	0	0	0	0	0	0	0	0	0	0	0	0	0	0	0	0	0	0	0	0	0	0	0	0	0	0
Mexico	0	0	0	0	0	0	0	0	0	0	0	43	9	18	6	15	0	0	0	0	0	0	0	0	0	0	0	0	0	0	0	0	0	0	0	0	0	0	0	0
Micronesia (Federated States of)	0	0	11	0	0	0	0	0	0	0	1	0	0	0	0	0	0	0	0	0	19	0	0	0	0	0	0	0	0	0	0	0	0	0	0	0	0	0	0	0
Monaco	0	0	0	0	0	0	0	0	0	0	0	0	0	0	0	0	0	0	0	0	0	0	0	0	0	0	0	0	0	0	0	0	0	0	0	0	0	0	0	0
Mongolia	0	0	0	0	0	0	0	0	0	0	0	0	0	0	0	0	8	0	0	0	0	0	0	0	0	0	0	0	0	0	0	0	0	0	0	0	0	0	0	0
Montenegro	0	0	0	0	0	0	0	0	0	0	0	0	0	0	0	0	0	0	0	0	0	0	0	0	0	0	0	0	0	0	0	0	0	0	0	0	0	0	0	0

Morocco	0	0	0	0	0	0	0	92	170	0	0	0	2	0	0	0	0	0	41	0	0	0	0	0	0	0	0	0	0	0	0	0	0	0	0	0	0	0	0	0
Mozambique	75	159	233	221	25	3	89	134	169	43	716	366	587	507	27	118	0	396	671	1239	402	198	160	216	64	24	325	98	83	318	56	13	7	2	11	32	0	1	0	0
Myanmar	50	3	0	39	0	0	0	0	0	11	0	39	15	52	4	6	0	0	0	0	0	0	0	0	0	0	0	30	1	0	0	0	0	0	0	12	34	0	0	0
Namibia	0	0	0	0	0	0	0	0	100	0	0	0	0	0	224	4	0	0	0	0	14	151	0	0	0	0	15	7	4	5	0	0	0	17	13	0	0	0	0	0
Nauru	0	0	0	0	0	0	0	0	0	0	0	0	0	0	0	0	0	0	0	0	0	0	0	0	0	0	0	0	0	0	0	0	0	0	0	0	0	0	0	0
Nepal	0	0	46	0	46	0	0	0	0	2	150	2207	655	0	0	126	119	84	217	150	319	26	32	0	0	0	0	0	0	314	73	0	0	0	2	0	0	0	0	0
Netherlands	0	0	0	0	0	0	0	0	0	0	0	2	0	1	0	9	0	0	0	0	0	0	0	0	0	0	0	0	0	0	0	0	0	0	0	0	0	0	0	0
New Zealand	0	0	0	0	0	0	0	0	0	0	0	0	0	0	0	0	0	0	0	0	0	0	0	0	0	0	0	0	0	0	0	0	0	0	0	0	0	0	0	0
Nicaragua	0	0	0	0	0	0	0	0	0	0	0	2	50	226	139	182	107	36	67	13	24	11	15	12	7	2	1	2	0	8	16	0	0	3	0	0	0	0	0	15
Niger	293	187	110	74	390	69	790	175	140	353	218	3244	712	1074	1094	5528	2384	821	301	537	1194	573	316	1117	491	206	62	108	173	169	103	48	153	57	33	130	28	118	68	0
Nigeria	76	56	86	63	243	62	2406	1747	505	1087	60	14999	776	0	313	87	18164	134	876	681	607	1042	638	618	172	767	564	490	670	3990	3685	1375	102	276	836	259	298	1040	90	15
Niue	0	0	0	0	0	0	0	0	0	0	0	0	0	0	0	0	0	0	0	1	0	0	0	0	0	0	0	0	0	0	0	0	0	0	0	0	0	0	0	0
North Macedonia	0	0	0	0	0	0	0	0	0	0	0	2	0	3	0	1	0	0	0	0	0	0	0	0	0	0	0	0	0	0	0	0	0	0	0	0	0	0	0	0
Northern Mariana Islands	0	0	0	0	0	0	0	0	0	0	0	0	0	0	0	0	0	0	0	0	0	0	0	0	0	0	0	0	0	0	0	0	0	0	0	0	0	0	0	0
Norway	0	0	0	0	0	0	0	0	0	0	0	0	0	0	0	0	0	0	0	0	0	0	0	0	0	0	0	0	0	0	0	0	0	0	0	0	0	0	0	0
Oman	0	0	0	0	0	0	0	0	0	0	0	0	0	5	1	2	0	0	1	0	7	0	0	0	0	0	0	0	0	0	0	0	0	0	0	0	0	0	0	0
Pakistan	0	0	0	0	0	0	0	0	0	0	0	0	0	206	0	0	0	0	83	0	109	0	10	0	2	42	0	0	0	0	210	219	8	23	6	0	0	83	0	0
Palau	0	0	0	0	0	0	0	0	0	0	0	0	0	0	0	0	0	0	0	0	0	0	0	0	0	0	0	0	0	0	0	0	0	0	0	0	0	0	0	0
Palestine	0	0	0	0	0	0	0	0	0	0	0	0	0	0	0	0	0	0	0	0	0	0	0	0	0	0	0	0	0	0	0	0	0	0	0	0	0	0	0	0
Panama	0	0	0	0	0	0	0	0	0	0	0	0	0	0	0	0	0	0	0	0	0	0	0	0	0	0	0	0	0	0	0	0	0	0	0	0	0	0	0	0
Papua New Guinea	0	0	0	0	0	0	0	0	0	0	0	316	318	283	297	261	0	0	114	0	0	0	142	0	0	0	0	0	0	192	95	2	0	0	0	0	0	0	0	0
Paraguay	0	0	0	0	0	0	0	0	0	0	0	0	0	0	2	0	0	0	0	0	0	0	0	0	0	0	13	4	8	8	0	44	0	0	0	0	0	0	0	0
Peru	0	0	0	0	0	0	0	0	230	0	0	9726	690	575	199	171	21	1	41	6	6	0	0	0	0	0	0	0	0	0	2	11	11	0	0	0	24	2	0	0
Philippines	1086	298	256	163	296	353	0	0	0	0	21	0	271	205	27	4	30	0	219	10	7	0	0	2	27	10	1	0	0	0	739	59	44	0	118	0	0	657	202	825
Poland	0	0	0	0	0	0	0	0	0	0	0	0	0	0	0	0	0	0	0	0	0	0	0	0	0	0	0	0	0	0	0	0	0	0	0	0	0	0	0	0
Portugal	0	0	0	0	0	0	0	0	0	0	0	0	0	0	0	0	0	0	0	0	0	0	0	0	0	0	0	0	0	0	0	0	0	0	0	0	0	0	0	0
Puerto Rico	0	0	0	0	0	0	0	0	0	0	0	0	0	0	0	0	0	0	0	0	0	0	0	0	0	0	0	0	0	0	0	0	0	0	0	0	0	0	0	0
Qatar	0	0	0	0	0	0	0	0	0	0	0	0	0	0	0	0	0	0	0	0	0	0	0	0	0	0	0	0	0	0	0	0	0	0	0	0	0	0	0	0
Republic of Korea	4	0	0	0	0	0	0	0	0	0	0	0	7	4	5	5	0	0	13	0	9	4	0	0	0	0	0	0	0	0	0	0	0	0	0	36	0	0	0	0
Republic of Moldova	0	30	48	0	0	43	43	34	29	30	21	6	5	3	3	1	0	0	0	0	0	0	0	0	0	0	0	0	0	0	0	0	0	0	0	0	0	0	0	0
Romania	0	0	0	0	0	0	0	0	0	0	1	8	0	1	1	0	0	0	0	0	0	0	0	0	0	0	0	0	0	0	0	0	0	0	0	0	0	127	0	0
Russian Federation	82	95	93	100	122	122	97	94	0	0	0	0	0	0	61	61	0	0	0	0	0	0	0	0	0	0	0	0	0	0	0	0	0	0	0	0	0	0	0	0
Rwanda	35	37	10	17	21	8	38	40	45	47	24	67	103	34	10000	0	10	20	82	169	18	6	83	1	4	0	44	25	0	67	0	0	0	0	0	0	0	0	0	0
Saint Kitts and Nevis	0	0	0	0	0	0	0	0	0	0	0	0	0	0	0	0	0	0	0	0	0	0	0	0	0	0	0	0	0	0	0	0	0	0	0	0	0	0	0	0
Saint Lucia	0	0	0	0	0	0	0	0	0	0	0	0	0	0	0	0	0	0	0	0	0	0	0	0	0	0	0	0	0	0	0	0	0	0	0	0	0	0	0	0
Saint Vincent and the Grenadines	0	0	0	0	0	0	0	0	0	0	0	0	0	0	0	0	0	0	0	0	0	0	0	0	0	0	0	0	0	0	0	0	0	0	0	0	0	0	0	0
Samoa	0	0	0	0	0	0	0	0	0	0	0	0	0	0	0	0	0	0	0	0	0	0	0	0	0	0	0	0	0	0	0	0	0	0	0	0	0	0	0	0
San Marino	0	0	0	0	0	0	0	0	0	0	0	0	0	0	0	0	0	0	0	0	0	0	0	0	0	0	0	0	0	0	0	0	0	0	0	0	0	0	0	0
Sao Tome and Principe	1	0	0	5	6	3	151	2	0	32	9	1	0	0	0	0	0	0	0	3	0	0	0	0	0	25	9	0	4	0	0	0	0	0	0	0	0	0	0	0
Saudi Arabia	0	0	0	0	0	0	3	0	0	0	0	0	0	0	0	0	0	0	0	0	133	35	0	0	0	0	0	0	0	0	0	0	0	0	0	0	0	0	0	0
Senegal	66	91	149	122	206	344	92	306	8	0	0	0	0	0	0	117	71	15	595	631	0	204	28	8	3	306	20	22	20	0	0	0	28	10	12	0	0	0	0	0
Serbia	0	0	0	0	0	0	0	0	0	0	0	0	0	0	0	0	0	0	0	0	0	0	0	0	0	0	0	0	0	0	0	0	0	0	0	0	0	0	0	0
Seychelles	0	0	0	0	0	3	5	4	0	5	4	5	5	9	5	0	0	0	0	0	0	0	0	0	0	0	0	1	0	0	0	0	0	0	0	0	0	0	0	0
Sierra Leone	32	3	0	0	3	352	669	30	0	0	0	0	0	0	623	550	152	125	55	45	126	12	0	10	56	0	100	84	170	0	0	0	276	24	0	0	0	0	0	0
Singapore	0	0	0	0	6	0	0	0	0	0	0	0	0	0	0	0	0	0	1	0	0	0	0	0	0	0	0	0	0	0	0	0	0	0	0	0	0	0	0	0
Slovakia	0	0	0	0	0	0	0	0	0	0	0	0	0	0	1	1	0	0	0	0	0	0	0	0	0	0	0	0	0	0	0	0	0	0	0	0	0	0	0	0
Slovenia	0	0	0	0	0	0	0	0	0	0	0	0	0	0	0	0	0	0	0	0	0	0	0	0	0	0	0	0	0	0	0	0	0	0	0	0	0	0	0	0
Solomon Islands	0	0	0	0	0	0	0	0	0	0	0	0	0	0	0	0	0	0	0	0	0	0	0	0	0	0	0	0	0	0	0	0	0	8	4	0	0	0	0	0
Somalia	0	0	0	0	0	1262	1307	0	0	0	0	0	0	0	1298	191	62	685	295	201	688	149	101	56	26	13	110	1167	13	3	103	1130	200	140	0	84	497	302	0	0
South Africa	16	52	224	63	20	4	1	0	0	0	0	0	85	72	29	35	22	45	29	17	121	124	143	63	34	35	0	0	19	46	27	20	0	5	0	8	0	0	0	0
South Sudan	0	0	0	0	0	0	0	0	0	0	0	0	0	0	0	0	0	0	0	0	0	0	0	0	0	0	1011	500	133	0	0	0	0	14	151	47	78	5	0	0
Spain	0	0	0	0	0	0	0	0	0	0	0	0	0	0	0	0	0	0	0	0	0	0	0	0	0	0	0	0	0	0	0	0	0	0	0	0	0	0	0	0
Sri Lanka	4	57	21	2	0	0	0	53	11	0	0	2	3	0	0	0	0	10	26	6	2	0	1	0	88	0	0	0	0	346	0	167	0	0	0	0	0	320	0	0
Sudan	2	0	0	0	0	239	0	2127	2088	1493	691	12	0	0	0	0	700	0	1791	1685	654	336	504	185	273	333	1137	907	27	52	98	0	162	83	5	0	19	657	0	0
Suriname	0	0	0	0	0	0	0	0	0	0	0	0	1	0	0	0	0	0	0	0	0	0	0	0	0	0	0	0	0	0	0	0	0	0	0	0	0	0	0	0
Sweden	0	0	0	0	0	0	0	0	0	0	0	0	0	0	0	0	0	0	0	0	0	0	0	0	0	0	0	0	0	0	0	0	0	0	0	0	0	0	0	0
Switzerland	0	0	0	0	0	0	0	0	0	0	0	0	0	0	0	0	0	0	0	2	2	0	0	0	0	0	0	0	0	0	0	0	0	0	0	0	0	0	0	0
Syrian Arab Republic	0	0	0	0	0	0	0	0	0	0	0	0	0	0	0	0	0	0	0	0	0	0	0	0	0	0	0	0	0	0	0	0	0	0	0	0	0	0	0	0
Taiwan (Province of China)	0	0	0	0	0	0	0	0	0	0	0	0	0	0	0	0	0	0	0	0	0	0	0	0	0	0	0	0	0	0	0	0	0	0	0	0	0	0	0	0
Tajikistan	0	17	92	0	0	21	15	59	183	125	85	0	99	5	0	0	0	168	0	3	0	0	0	0	0	0	0	0	0	3	21	0	0	0	0	0	0	0	0	0
Thailand	90	4	19	48	25	16	10	47	42	6	5	3	11	4	13	27	20	20	34	19	119	22	20	14	22	14	5	17	5	6	22	33	3	2	4	3	3	0	0	0
Timor-Leste	0	0	0	0	0	0	0	0	0	0	0	0	0	0	0	0	0	0	0	0	0	0	0	0	0	22	0	0	0	0	0	0	0	0	2	0	0	0	0	0
Togo	10	0	1	30	9	4	8	36	58	147	0	81	49	2	5	4	236	747	316	99	174	235	244	92	77	61	62	66	66	50	63	43	36	29	25	9	18	0	0	0
Tokelau	0	0	0	0	0	0	0	0	0	0	0	0	0	0	0	0	0	0	0	0	0	0	0	0	0	0	0	0	0	0	0	0	0	0	0	0	0	0	0	0
Tonga	0	0	0	0	0	0	0	0	0	0	0	0	0	0	0	0	0	0	0	0	0	0	0	0	0	0	0	0	0	0	0	0	0	0	0	0	0	0	0	0
Trinidad and Tobago	0	0	0	0	0	0	0	0	0	0	0	0	0	0	0	0	0	0	0	0	0	0	0	0	0	0	0	0	0	0	0	0	0	0	0	0	0	0	0	0
Tunisia	0	0	0	0	0	0	0	0	0	0	0	0	0	0	0	0	0	0	0	0	0	0	0	0	0	0	0	0	0	0	0	0	0	0	0	0	0	0	0	0
Turkey	0	0	0	0	0	0	0	11	0	0	0	0	0	0	0	0	0	0	0	0	0	0	0	0	0	0	24	0	0	0	0	0	0	0	0	0	0	0	0	0
Turkmenistan	0	0	0	0	0	0	0	0	0	0	0	0	0	0	0	0	0	0	0	0	0	0	0	0	0	0	0	0	0	0	0	0	0	0	0	0	0	0	0	0
Tuvalu	0	0	0	0	0	0	0	0	0	0	0	0	0	0	0	0	0	0	0	0	0	0	0	0	0	0	0	0	0	0	0	0	0	0	0	0	0	0	0	0
Uganda	187	12	56	48	3	0	27	50	34	156	197	292	351	474	39	66	60	188	30	137	47	36	183	107	3	14	122	101	127	24	126	0	118	28	9	33	0	3	31	6
Ukraine	0	292	358	0	0	413	0	0	0	0	0	0	0	0	71	214	0	0	0	0	0	0	0	0	0	0	0	0	0	0	0	0	0	0	0	0	0	0	16	0
United Arab Emirates	0	0	0	0	0	0	0	0	0	0	4	0	3	0	0	0	0	0	3	0	0	0	0	0	0	0	0	0	0	0	0	0	0	0	0	0	0	0	0	0
United Kingdom	0	0	0	0	0	0	0	0	0	0	0	0	0	2	0	4	2	0	5	0	0	63	0	0	0	0	0	0	0	0	0	2	0	4	0	2	0	0	0	0
United Republic of Tanzania	599	330	438	290	338	260	215	90	580	232	256	574	2231	342	141	55	240	2355	2461	699	125	39	306	72	272	1	75	219	88	12	59	11	4	17	0	132	217	217	16	0
United States Virgin Islands	0	0	0	0	0	0	0	0	0	0	0	0	0	0	0	0	0	0	0	0	0	0	0	0	0	0	0	0	0	0	0	0	0	0	0	0	0	0	0	0
United States of America	0	0	0	0	0	0	0	0	0	0	0	0	0	0	0	0	0	0	0	0	0	0	0	0	0	0	0	0	0	0	0	0	0	0	0	0	0	0	0	0
Uruguay	0	0	0	0	0	0	0	0	0	0	0	0	0	0	0	0	0	0	0	0	0	0	0	0	0	0	0	0	0	0	0	0	0	0	0	0	0	0	0	0
Uzbekistan	0	0	20	0	0	14	112	100	67	45	11	0	0	8	0	0	0	0	0	0	0	0	0	0	0	0	0	0	0	0	0	0	0	0	0	0	0	0	0	0
Vanuatu	0	0	0	0	0	0	0	0	0	12	0	0	0	0	0	0	0	0	0	0	0	0	0	0	0	0	0	0	0	0	0	0	0	0	0	0	0	0	0	0
Venezuela (Bolivarian Republic of)	0	0	0	0	0	0	0	0	0	0	0	0	73	9	0	0	0	34	0	6	3	0	0	0	0	0	0	0	0	0	0	0	0	0	0	0	0	0	0	0
Viet Nam	427	89	56	151	3	77	66	23	41	0	215	109	168	50	125	102	79	0	376	0	0	0	0	12	30	23	13	27	0	1	0	0	0	0	0	0	27	32	0	0
Yemen	20	0	0	0	0	0	0	0	0	0	0	0	0	0	0	0	0	0	0	0	32	0	0	0	0	0	0	0	0	3	4	134	0	0	0	3	385	1573	847	763
Zambia	83	76	270	19	0	51	42	550	427	444	1188	1881	1948	487	0	0	42	34	0	462	163	3137	12	59	120	12	14	16	69	141	50	7	2	0	0	0	0	80	29	0
Zimbabwe	6	4	29	12	0	30	0	2	24	27	4	83	266	332	0	0	1311	8	22	57	113	14	196	40	9	26	61	58	1809	2515	28	45	1	0	0	0	0	0	12	0

SR Table 10c: Deaths due to famine by country, all ages, for both sexes combined, 1980-2019																																								
Country	1980	1981	1982	1983	1984	1985	1986	1987	1988	1989	1990	1991	1992	1993	1994	1995	1996	1997	1998	1999	2000	2001	2002	2003	2004	2005	2006	2007	2008	2009	2010	2011	2012	2013	2014	2015	2016	2017	2018	2019
Afghanistan	0	0	0	0	0	0	0	0	0	0	0	0	0	0	0	0	0	0	0	0	9	14	14	0	0	0	0	0	0	0	0	0	0	0	0	0	0	0	0	0
Albania	0	0	0	0	0	0	0	0	0	0	0	0	0	0	0	0	0	0	0	0	0	0	0	0	0	0	0	0	0	0	0	0	0	0	0	0	0	0	0	0
Algeria	0	0	0	0	0	0	0	0	0	0	0	0	0	0	0	0	0	0	0	0	0	0	0	0	0	0	0	0	0	0	0	0	0	0	0	0	0	0	0	0
American Samoa	0	0	0	0	0	0	0	0	0	0	0	0	0	0	0	0	0	0	0	0	0	0	0	0	0	0	0	0	0	0	0	0	0	0	0	0	0	0	0	0
Andorra	0	0	0	0	0	0	0	0	0	0	0	0	0	0	0	0	0	0	0	0	0	0	0	0	0	0	0	0	0	0	0	0	0	0	0	0	0	0	0	0
Angola	0	0	0	0	0	0	0	0	0	0	0	0	0	0	0	0	0	0	0	0	0	58	0	0	0	0	0	0	0	0	0	0	0	0	0	0	0	0	0	0
Antigua and Barbuda	0	0	0	0	0	0	0	0	0	0	0	0	0	0	0	0	0	0	0	0	0	0	0	0	0	0	0	0	0	0	0	0	0	0	0	0	0	0	0	0
Argentina	0	0	0	0	0	0	0	0	0	0	0	0	0	0	0	0	0	0	0	0	0	0	0	0	0	0	0	0	0	0	0	0	0	0	0	0	0	0	0	0
Armenia	0	0	0	0	0	0	0	0	0	0	0	0	0	0	0	0	0	0	0	0	0	0	0	0	0	0	0	0	0	0	0	0	0	0	0	0	0	0	0	0
Australia	0	0	0	0	0	0	0	0	0	0	0	0	0	0	0	0	0	0	0	0	0	0	0	0	0	0	0	0	0	0	0	0	0	0	0	0	0	0	0	0
Austria	0	0	0	0	0	0	0	0	0	0	0	0	0	0	0	0	0	0	0	0	0	0	0	0	0	0	0	0	0	0	0	0	0	0	0	0	0	0	0	0
Azerbaijan	0	0	0	0	0	0	0	0	0	0	0	0	0	0	0	0	0	0	0	0	0	0	0	0	0	0	0	0	0	0	0	0	0	0	0	0	0	0	0	0
Bahamas	0	0	0	0	0	0	0	0	0	0	0	0	0	0	0	0	0	0	0	0	0	0	0	0	0	0	0	0	0	0	0	0	0	0	0	0	0	0	0	0
Bahrain	0	0	0	0	0	0	0	0	0	0	0	0	0	0	0	0	0	0	0	0	0	0	0	0	0	0	0	0	0	0	0	0	0	0	0	0	0	0	0	0
Bangladesh	0	0	0	0	0	0	0	0	0	0	0	0	0	0	0	0	0	0	0	0	0	0	0	0	0	0	0	0	0	0	0	0	0	0	0	0	0	0	0	0
Barbados	0	0	0	0	0	0	0	0	0	0	0	0	0	0	0	0	0	0	0	0	0	0	0	0	0	0	0	0	0	0	0	0	0	0	0	0	0	0	0	0
Belarus	0	0	0	0	0	0	0	0	0	0	0	0	0	0	0	0	0	0	0	0	0	0	0	0	0	0	0	0	0	0	0	0	0	0	0	0	0	0	0	0
Belgium	0	0	0	0	0	0	0	0	0	0	0	0	0	0	0	0	0	0	0	0	0	0	0	0	0	0	0	0	0	0	0	0	0	0	0	0	0	0	0	0
Belize	0	0	0	0	0	0	0	0	0	0	0	0	0	0	0	0	0	0	0	0	0	0	0	0	0	0	0	0	0	0	0	0	0	0	0	0	0	0	0	0
Benin	0	0	0	0	0	0	0	0	0	0	0	0	0	0	0	0	0	0	0	0	0	0	0	0	0	0	0	0	0	0	0	0	0	0	0	0	0	0	0	0
Bermuda	0	0	0	0	0	0	0	0	0	0	0	0	0	0	0	0	0	0	0	0	0	0	0	0	0	0	0	0	0	0	0	0	0	0	0	0	0	0	0	0
Bhutan	0	0	0	0	0	0	0	0	0	0	0	0	0	0	0	0	0	0	0	0	0	0	0	0	0	0	0	0	0	0	0	0	0	0	0	0	0	0	0	0
Bolivia (Plurinational State of)	0	0	0	0	0	0	0	0	0	0	0	0	0	0	0	0	0	0	0	0	0	0	0	0	0	0	0	0	0	0	0	0	0	0	0	0	0	0	0	0
Bosnia and Herzegovina	0	0	0	0	0	0	0	0	0	0	0	0	0	0	0	0	0	0	0	0	0	0	0	0	0	0	0	0	0	0	0	0	0	0	0	0	0	0	0	0
Botswana	0	0	0	0	0	0	0	0	0	0	0	0	0	0	0	0	0	0	0	0	0	0	0	0	0	0	0	0	0	0	0	0	0	0	0	0	0	0	0	0
Brazil	0	0	0	364	0	0	0	0	0	0	0	0	0	0	0	0	0	0	0	0	0	0	0	0	0	0	0	0	0	0	0	0	0	0	0	0	0	0	0	0
Brunei Darussalam	0	0	0	0	0	0	0	0	0	0	0	0	0	0	0	0	0	0	0	0	0	0	0	0	0	0	0	0	0	0	0	0	0	0	0	0	0	0	0	0
Bulgaria	0	0	0	0	0	0	0	0	0	0	0	0	0	0	0	0	0	0	0	0	0	0	0	0	0	0	0	0	0	0	0	0	0	0	0	0	0	0	0	0
Burkina Faso	0	0	0	0	0	0	0	0	0	0	0	0	0	0	0	0	0	0	0	0	0	0	0	0	0	0	0	0	0	0	0	0	0	0	0	0	0	0	0	0
Burundi	0	0	0	0	0	0	0	0	0	0	0	0	0	0	0	0	0	0	0	1	3	3	0	0	0	110	10	0	0	0	0	0	0	0	0	0	0	0	0	0
Cabo Verde	0	0	0	0	0	0	0	0	0	0	0	0	0	0	0	0	0	0	0	0	0	0	0	0	0	0	0	0	0	0	0	0	0	0	0	0	0	0	0	0
Cambodia	0	0	0	0	0	0	0	0	0	0	0	0	0	0	0	0	0	0	0	0	0	0	0	0	0	0	0	0	0	0	0	0	0	0	0	0	0	0	0	0
Cameroon	0	0	0	0	0	0	0	0	0	0	0	0	0	0	0	0	0	0	0	0	0	0	0	0	0	0	0	0	0	0	0	0	0	0	0	0	0	0	0	0
Canada	0	0	0	0	0	0	0	0	0	0	0	0	0	0	0	0	0	0	0	0	0	0	0	0	0	0	0	0	0	0	0	0	0	0	0	0	0	0	0	0
Central African Republic	0	0	0	0	0	0	0	0	0	0	0	0	0	0	0	0	0	0	0	0	0	0	0	0	0	0	0	0	0	0	0	0	0	0	0	0	0	0	0	0
Chad	0	120	720	720	720	720	0	0	0	0	0	0	0	0	0	0	0	0	0	0	0	0	0	0	0	0	0	0	0	0	0	0	0	0	0	0	0	0	0	0
Chile	0	0	0	0	0	0	0	0	0	0	0	0	0	0	0	0	0	0	0	0	0	0	0	0	0	0	0	0	0	0	0	0	0	0	0	0	0	0	0	0
China	0	0	0	0	0	0	0	0	1400	0	0	2000	0	0	0	0	0	0	0	0	0	0	0	0	0	0	131	0	0	0	0	0	0	0	0	0	0	0	0	0
Colombia	0	0	0	0	0	0	0	0	0	0	0	0	0	0	0	0	0	0	0	0	0	0	0	0	0	0	0	0	0	0	0	0	0	0	0	0	0	0	0	0
Comoros	0	0	0	0	0	0	0	0	0	0	0	0	0	0	0	0	0	0	0	0	0	0	0	0	0	0	0	0	0	0	0	0	0	0	0	0	0	0	0	0
Congo	0	0	0	0	0	0	0	0	0	0	0	0	0	0	0	0	0	0	0	0	0	0	0	0	0	0	0	0	0	0	0	0	0	0	0	0	0	0	0	0
Cook Islands	0	0	0	0	0	0	0	0	0	0	0	0	0	0	0	0	0	0	0	0	0	0	0	0	0	0	0	0	0	0	0	0	0	0	0	0	0	0	0	0
Costa Rica	0	0	0	0	0	0	0	0	0	0	0	0	0	0	0	0	0	0	0	0	0	0	0	0	0	0	0	0	0	0	0	0	0	0	0	0	0	0	0	0
Croatia	0	0	0	0	0	0	0	0	0	0	0	0	0	0	0	0	0	0	0	0	0	0	0	0	0	0	0	0	0	0	0	0	0	0	0	0	0	0	0	0
Cuba	0	0	0	0	0	0	0	0	0	0	0	0	0	0	0	0	0	0	0	0	0	0	0	0	0	0	0	0	0	0	0	0	0	0	0	0	0	0	0	0
Cyprus	0	0	0	0	0	0	0	0	0	0	0	0	0	0	0	0	0	0	0	0	0	0	0	0	0	0	0	0	0	0	0	0	0	0	0	0	0	0	0	0
Czechia	0	0	0	0	0	0	0	0	0	0	0	0	0	0	0	0	0	0	0	0	0	0	0	0	0	0	0	0	0	0	0	0	0	0	0	0	0	0	0	0
Côte d'Ivoire	0	0	0	0	0	0	0	0	0	0	0	0	0	0	0	0	0	0	0	0	0	0	0	0	0	0	0	0	0	0	0	0	0	0	0	0	0	0	0	0
Democratic People's Republic of Korea	0	0	0	0	0	0	0	0	0	0	0	0	0	0	0	97600	73200	73200	73200	73200	73200	73200	73200	0	0	0	0	0	0	0	0	0	0	0	0	0	0	0	0	0
Democratic Republic of the Congo	0	0	0	0	0	0	0	0	0	0	0	0	0	0	0	0	0	0	0	0	0	0	0	0	0	0	0	0	0	0	0	0	0	0	0	0	0	0	0	0
Denmark	0	0	0	0	0	0	0	0	0	0	0	0	0	0	0	0	0	0	0	0	0	0	0	0	0	0	0	0	0	0	0	0	0	0	0	0	0	0	0	0
Djibouti	0	0	0	0	0	0	0	0	0	0	0	0	0	0	0	0	0	0	0	0	0	0	0	0	0	0	0	0	0	0	0	0	0	0	0	0	0	0	0	0
Dominica	0	0	0	0	0	0	0	0	0	0	0	0	0	0	0	0	0	0	0	0	0	0	0	0	0	0	0	0	0	0	0	0	0	0	0	0	0	0	0	0
Dominican Republic	0	0	0	0	0	0	0	0	0	0	0	0	0	0	0	0	0	0	0	0	0	0	0	0	0	0	0	0	0	0	0	0	0	0	0	0	0	0	0	0
Ecuador	0	0	0	0	0	0	0	0	0	0	0	0	0	0	0	0	0	0	0	0	0	0	0	0	0	0	0	0	0	0	0	0	0	0	0	0	0	0	0	0
Egypt	0	0	0	0	0	0	0	0	0	0	0	0	0	0	0	0	0	0	0	0	0	0	0	0	0	0	0	0	0	0	0	0	0	0	0	0	0	0	0	0
El Salvador	0	0	0	0	0	0	0	0	0	0	0	0	0	0	0	0	0	0	0	0	0	0	0	0	0	0	0	0	0	0	0	0	0	0	0	0	0	0	0	0
Equatorial Guinea	0	0	0	0	0	0	0	0	0	0	0	0	0	0	0	0	0	0	0	0	0	0	0	0	0	0	0	0	0	0	0	0	0	0	0	0	0	0	0	0
Eritrea	0	0	0	0	0	0	0	0	0	0	0	0	0	0	0	0	0	0	0	0	0	0	0	0	0	0	0	0	0	0	0	0	0	0	0	0	0	0	0	0
Estonia	0	0	0	0	0	0	0	0	0	0	0	0	0	0	0	0	0	0	0	0	0	0	0	0	0	0	0	0	0	0	0	0	0	0	0	0	0	0	0	0
Eswatini	0	0	0	500	0	0	0	0	0	0	0	0	0	0	0	0	0	0	0	0	0	0	0	0	0	0	0	0	0	0	0	0	0	0	0	0	0	0	0	0
Ethiopia	0	0	0	120000	180000	0	0	367	0	0	0	0	0	0	0	0	0	0	0	0	0	0	0	0	0	0	0	0	0	0	0	0	0	0	0	0	0	0	0	0
Fiji	0	0	0	0	0	0	0	0	0	0	0	0	0	0	0	0	0	0	0	0	0	0	0	0	0	0	0	0	0	0	0	0	0	0	0	0	0	0	0	0
Finland	0	0	0	0	0	0	0	0	0	0	0	0	0	0	0	0	0	0	0	0	0	0	0	0	0	0	0	0	0	0	0	0	0	0	0	0	0	0	0	0
France	0	0	0	0	0	0	0	0	0	0	0	0	0	0	0	0	0	0	0	0	0	0	0	0	0	0	0	0	0	0	0	0	0	0	0	0	0	0	0	0
Gabon	0	0	0	0	0	0	0	0	0	0	0	0	0	0	0	0	0	0	0	0	0	0	0	0	0	0	0	0	0	0	0	0	0	0	0	0	0	0	0	0
Gambia	0	0	0	0	0	0	0	0	0	0	0	0	0	0	0	0	0	0	0	0	0	0	0	0	0	0	0	0	0	0	0	0	0	0	0	0	0	0	0	0
Georgia	0	0	0	0	0	0	0	0	0	0	0	0	0	0	0	0	0	0	0	0	0	0	0	0	0	0	0	0	0	0	0	0	0	0	0	0	0	0	0	0
Germany	0	0	0	0	0	0	0	0	0	0	0	0	0	0	0	0	0	0	0	0	0	0	0	0	0	0	0	0	0	0	0	0	0	0	0	0	0	0	0	0
Ghana	0	0	0	0	0	0	0	0	0	0	0	0	0	0	0	0	0	0	0	0	0	0	0	0	0	0	0	0	0	0	0	0	0	0	0	0	0	0	0	0
Greece	0	0	0	0	0	0	0	0	0	0	0	0	0	0	0	0	0	0	0	0	0	0	0	0	0	0	0	0	0	0	0	0	0	0	0	0	0	0	0	0
Greenland	0	0	0	0	0	0	0	0	0	0	0	0	0	0	0	0	0	0	0	0	0	0	0	0	0	0	0	0	0	0	0	0	0	0	0	0	0	0	0	0
Grenada	0	0	0	0	0	0	0	0	0	0	0	0	0	0	0	0	0	0	0	0	0	0	0	0	0	0	0	0	0	0	0	0	0	0	0	0	0	0	0	0
Guam	0	0	0	0	0	0	0	0	0	0	0	0	0	0	0	0	0	0	0	0	0	0	0	0	0	0	0	0	0	0	0	0	0	0	0	0	0	0	0	0
Guatemala	0	0	0	0	0	0	0	0	0	0	0	0	0	0	0	0	0	0	0	0	0	196	0	0	0	0	0	0	0	0	0	0	0	0	0	0	0	0	0	0
Guinea	0	0	0	0	0	0	0	0	0	0	0	0	0	0	0	0	0	0	12	0	0	0	0	0	0	0	0	0	0	0	0	0	0	0	0	0	0	0	0	0
Guinea-Bissau	0	0	0	0	0	0	0	0	0	0	0	0	0	0	0	0	0	0	0	0	0	0	0	0	0	0	0	0	0	0	0	0	0	0	0	0	0	0	0	0
Guyana	0	0	0	0	0	0	0	0	0	0	0	0	0	0	0	0	0	0	0	0	0	0	0	0	0	0	0	0	0	0	0	0	0	0	0	0	0	0	0	0
Haiti	0	0	0	0	0	0	0	0	0	0	0	0	0	0	0	0	0	0	0	0	0	0	0	0	0	0	0	0	0	0	0	0	0	0	0	0	0	0	0	0
Honduras	0	0	0	0	0	0	0	0	0	0	0	0	0	0	0	0	0	0	0	0	0	0	0	0	0	0	0	0	0	0	0	0	0	0	0	0	0	0	0	0
Hungary	0	0	0	0	0	0	0	0	0	0	0	0	0	0	0	0	0	0	0	0	0	0	0	0	0	0	0	0	0	0	0	0	0	0	0	0	0	0	0	0
Iceland	0	0	0	0	0	0	0	0	0	0	0	0	0	0	0	0	0	0	0	0	0	0	0	0	0	0	0	0	0	0	0	0	0	0	0	0	0	0	0	0
India	0	0	0	0	0	0	0	300	0	0	0	0	0	0	0	0	0	0	0	0	9	11	0	0	0	0	0	0	0	0	0	0	0	0	0	0	0	0	0	0
Indonesia	0	0	280	0	230	0	84	0	0	0	0	0	0	0	0	0	0	672	0	0	0	0	0	0	0	0	0	0	0	0	0	0	0	0	0	11	0	0	0	0
Iran (Islamic Republic of)	0	0	0	0	0	0	0	0	0	0	0	0	0	0	0	0	0	0	0	0	0	0	0	0	0	0	0	0	0	0	0	0	0	0	0	0	0	0	0	0
Iraq	0	0	0	0	0	0	0	0	0	0	0	0	0	0	0	0	0	0	0	0	0	0	0	0	0	0	0	0	0	0	0	0	0	0	0	0	0	0	0	0
Ireland	0	0	0	0	0	0	0	0	0	0	0	0	0	0	0	0	0	0	0	0	0	0	0	0	0	0	0	0	0	0	0	0	0	0	0	0	0	0	0	0
Israel	0	0	0	0	0	0	0	0	0	0	0	0	0	0	0	0	0	0	0	0	0	0	0	0	0	0	0	0	0	0	0	0	0	0	0	0	0	0	0	0
Italy	0	0	0	0	0	0	0	0	0	0	0	0	0	0	0	0	0	0	0	0	0	0	0	0	0	0	0	0	0	0	0	0	0	0	0	0	0	0	0	0
Jamaica	0	0	0	0	0	0	0	0	0	0	0	0	0	0	0	0	0	0	0	0	0	0	0	0	0	0	0	0	0	0	0	0	0	0	0	0	0	0	0	0
Japan	0	0	0	0	0	0	0	0	0	0	0	0	0	0	0	0	0	0	0	0	0	0	0	0	0	0	0	0	0	0	0	0	0	0	0	0	0	0	0	0
Jordan	0	0	0	0	0	0	0	0	0	0	0	0	0	0	0	0	0	0	0	0	0	0	0	0	0	0	0	0	0	0	0	0	0	0	0	0	0	0	0	0
Kazakhstan	0	0	0	0	0	0	0	0	0	0	0	0	0	0	0	0	0	0	0	0	0	0	0	0	0	0	0	0	0	0	0	0	0	0	0	0	0	0	0	0
Kenya	0	0	0	0	0	0	0	0	0	0	0	0	0	0	0	0	0	0	0	3	27	27	27	0	80	2	25	0	1	3	0	0	0	0	0	0	0	0	0	0
Kiribati	0	0	0	0	0	0	0	0	0	0	0	0	0	0	0	0	0	0	0	0	0	0	0	0	0	0	0	0	0	0	0	0	0	0	0	0	0	0	0	0
Kuwait	0	0	0	0	0	0	0	0	0	0	0	0	0	0	0	0	0	0	0	0	0	0	0	0	0	0	0	0	0	0	0	0	0	0	0	0	0	0	0	0
Kyrgyzstan	0	0	0	0	0	0	0	0	0	0	0	0	0	0	0	0	0	0	0	0	0	0	0	0	0	0	0	0	0	0	0	0	0	0	0	0	0	0	0	0
Lao People's Democratic Republic	0	0	0	0	0	0	0	0	0	0	0	0	0	0	0	0	0	0	0	0	0	0	0	0	0	0	0	0	0	0	0	0	0	0	0	0	0	0	0	0
Latvia	0	0	0	0	0	0	0	0	0	0	0	0	0	0	0	0	0	0	0	0	0	0	0	0	0	0	0	0	0	0	0	0	0	0	0	0	0	0	0	0
Lebanon	0	0	0	0	0	0	0	0	0	0	0	0	0	0	0	0	0	0	0	0	0	0	0	0	0	0	0	0	0	0	0	0	0	0	0	0	0	0	0	0
Lesotho	0	0	0	0	0	0	0	0	0	0	0	0	0	0	0	0	0	0	0	0	0	0	0	0	0	0	0	0	0	0	0	0	0	0	0	0	0	0	0	0
Liberia	0	0	0	0	0	0	0	0	0	0	0	0	0	0	0	0	0	0	0	0	0	0	0	0	0	0	0	0	0	0	0	0	0	0	0	0	0	0	0	0
Libya	0	0	0	0	0	0	0	0	0	0	0	0	0	0	0	0	0	0	0	0	0	0	0	0	0	0	0	0	0	0	0	0	0	0	0	0	0	0	0	0
Lithuania	0	0	0	0	0	0	0	0	0	0	0	0	0	0	0	0	0	0	0	0	0	0	0	0	0	0	0	0	0	0	0	0	0	0	0	0	0	0	0	0
Luxembourg	0	0	0	0	0	0	0	0	0	0	0	0	0	0	0	0	0	0	0	0	0	0	0	0	0	0	0	0	0	0	0	0	0	0	0	0	0	0	0	0
Madagascar	0	0	0	0	0	0	0	0	32	42	42	42	42	0	0	0	0	0	0	0	0	0	0	0	0	0	0	0	0	0	0	0	0	0	0	0	0	0	0	0
Malawi	0	0	0	0	0	0	0	0	0	0	0	0	0	0	0	0	0	0	0	0	0	0	500	0	0	0	0	0	0	0	0	0	0	0	0	0	0	0	0	0
Malaysia	0	0	0	0	0	0	0	0	0	0	0	0	0	0	0	0	0	0	0	0	0	0	0	0	0	0	0	0	0	0	0	0	0	0	0	0	0	0	0	0
Maldives	0	0	0	0	0	0	0	0	0	0	0	0	0	0	0	0	0	0	0	0	0	0	0	0	0	0	0	0	0	0	0	0	0	0	0	0	0	0	0	0
Mali	0	0	0	0	0	0	0	0	0	0	0	0	0	0	0	0	0	0	0	0	0	0	0	0	0	0	0	0	0	0	0	0	0	0	0	0	0	0	0	0
Malta	0	0	0	0	0	0	0	0	0	0	0	0	0	0	0	0	0	0	0	0	0	0	0	0	0	0	0	0	0	0	0	0	0	0	0	0	0	0	0	0
Marshall Islands	0	0	0	0	0	0	0	0	0	0	0	0	0	0	0	0	0	0	0	0	0	0	0	0	0	0	0	0	0	0	0	0	0	0	0	0	0	0	0	0
Mauritania	0	0	0	0	0	0	0	0	0	0	0	0	0	0	0	0	0	0	0	0	0	0	0	0	0	0	0	0	0	0	0	0	0	0	0	0	0	0	0	0
Mauritius	0	0	0	0	0	0	0	0	0	0	0	0	0	0	0	0	0	0	0	0	0	0	0	0	0	0	0	0	0	0	0	0	0	0	0	0	0	0	0	0
Mexico	0	0	0	0	0	0	0	0	0	0	0	0	0	0	0	0	0	0	0	0	0	0	0	0	0	0	0	0	0	0	0	0	0	0	0	0	0	0	0	0
Micronesia (Federated States of)	0	0	0	0	0	0	0	0	0	0	0	0	0	0	0	0	0	0	0	0	0	0	0	0	0	0	0	0	0	0	0	0	0	0	0	0	0	0	0	0
Monaco	0	0	0	0	0	0	0	0	0	0	0	0	0	0	0	0	0	0	0	0	0	0	0	0	0	0	0	0	0	0	0	0	0	0	0	0	0	0	0	0
Mongolia	0	0	0	0	0	0	0	0	0	0	0	0	0	0	0	0	0	0	0	0	0	0	0	0	0	0	0	0	0	0	0	0	0	0	0	0	0	0	0	0
Montenegro	0	0	0	0	0	0	0	0	0	0	0	0	0	0	0	0	0	0	0	0	0	0	0	0	0	0	0	0	0	0	0	0	0	0	0	0	0	0	0	0

Morocco	0	0	0	0	0	0	0	0	0	0	0	0	0	0	0	0	0	0	0	0	0	0	0	0	0	0	0	0	0	0	0	0	0	0	0	0	0	0	0	0
Mozambique	0	20000	20000	20000	20000	20000	0	50	0	0	0	0	0	0	0	0	0	0	0	0	0	0	9	9	0	0	0	0	0	0	0	0	0	0	0	0	0	0	0	0
Myanmar	0	0	0	0	0	0	0	0	0	0	0	0	0	0	0	0	0	0	0	0	0	0	0	0	0	0	0	0	0	0	0	0	0	0	0	0	0	0	0	0
Namibia	0	0	0	0	0	0	0	0	0	0	0	0	0	0	0	0	0	0	0	0	0	0	0	0	0	0	0	0	0	0	0	0	0	0	0	0	0	0	0	0
Nauru	0	0	0	0	0	0	0	0	0	0	0	0	0	0	0	0	0	0	0	0	0	0	0	0	0	0	0	0	0	0	0	0	0	0	0	0	0	0	0	0
Nepal	0	0	0	0	0	0	0	0	0	0	0	0	0	0	0	0	0	0	0	0	0	0	0	0	0	0	0	0	0	0	0	0	0	0	0	0	0	0	0	0
Netherlands	0	0	0	0	0	0	0	0	0	0	0	0	0	0	0	0	0	0	0	0	0	0	0	0	0	0	0	0	0	0	0	0	0	0	0	0	0	0	0	0
New Zealand	0	0	0	0	0	0	0	0	0	0	0	0	0	0	0	0	0	0	0	0	0	0	0	0	0	0	0	0	0	0	0	0	0	0	0	0	0	0	0	0
Nicaragua	0	0	0	0	0	0	0	0	0	0	0	0	0	0	0	0	0	0	0	0	0	0	0	0	0	0	0	0	0	0	0	0	0	0	0	0	0	0	0	0
Niger	0	0	0	0	0	0	0	0	0	0	0	0	0	0	0	0	0	0	0	0	0	0	0	0	0	0	0	0	0	0	0	0	0	0	0	0	0	0	0	0
Nigeria	0	0	0	0	0	0	0	0	0	0	0	0	0	0	0	0	0	0	0	0	0	0	0	0	0	0	0	0	0	0	0	0	0	0	0	0	0	0	0	0
Niue	0	0	0	0	0	0	0	0	0	0	0	0	0	0	0	0	0	0	0	0	0	0	0	0	0	0	0	0	0	0	0	0	0	0	0	0	0	0	0	0
North Macedonia	0	0	0	0	0	0	0	0	0	0	0	0	0	0	0	0	0	0	0	0	0	0	0	0	0	0	0	0	0	0	0	0	0	0	0	0	0	0	0	0
Northern Mariana Islands	0	0	0	0	0	0	0	0	0	0	0	0	0	0	0	0	0	0	0	0	0	0	0	0	0	0	0	0	0	0	0	0	0	0	0	0	0	0	0	0
Norway	0	0	0	0	0	0	0	0	0	0	0	0	0	0	0	0	0	0	0	0	0	0	0	0	0	0	0	0	0	0	0	0	0	0	0	0	0	0	0	0
Oman	0	0	0	0	0	0	0	0	0	0	0	0	0	0	0	0	0	0	0	0	0	0	0	0	0	0	0	0	0	0	0	0	0	0	0	0	0	0	0	0
Pakistan	0	0	0	0	0	0	0	0	0	0	0	0	0	0	0	0	0	0	0	6	34	34	34	34	0	0	0	0	0	0	0	0	0	0	0	0	0	0	0	0
Palau	0	0	0	0	0	0	0	0	0	0	0	0	0	0	0	0	0	0	0	0	0	0	0	0	0	0	0	0	0	0	0	0	0	0	0	0	0	0	0	0
Palestine	0	0	0	0	0	0	0	0	0	0	0	0	0	0	0	0	0	0	0	0	0	0	0	0	0	0	0	0	0	0	0	0	0	0	0	0	0	0	0	0
Panama	0	0	0	0	0	0	0	0	0	0	0	0	0	0	0	0	0	0	0	0	0	0	0	0	0	0	0	0	0	0	0	0	0	0	0	0	0	0	0	0
Papua New Guinea	0	0	0	0	0	0	0	0	0	0	0	0	0	0	0	0	0	15	45	0	0	0	0	0	0	0	0	0	0	0	0	0	0	0	0	24	0	0	0	0
Paraguay	0	0	0	0	0	0	0	0	0	0	0	0	0	0	0	0	0	0	0	2	3	3	3	0	0	0	0	0	4	0	0	0	0	0	0	0	0	0	0	0
Peru	0	0	0	0	0	0	0	0	0	0	0	0	0	0	0	0	0	0	0	0	0	0	0	0	0	0	0	0	0	0	0	0	0	0	0	0	0	0	0	0
Philippines	0	0	0	0	0	0	0	0	0	0	0	0	0	0	0	0	0	0	8	0	0	0	0	0	0	0	0	0	0	0	0	0	0	0	0	0	0	0	0	0
Poland	0	0	0	0	0	0	0	0	0	0	0	0	0	0	0	0	0	0	0	0	0	0	0	0	0	0	0	0	0	0	0	0	0	0	0	0	0	0	0	0
Portugal	0	0	0	0	0	0	0	0	0	0	0	0	0	0	0	0	0	0	0	0	0	0	0	0	0	0	0	0	0	0	0	0	0	0	0	0	0	0	0	0
Puerto Rico	0	0	0	0	0	0	0	0	0	0	0	0	0	0	0	0	0	0	0	0	0	0	0	0	0	0	0	0	0	0	0	0	0	0	0	0	0	0	0	0
Qatar	0	0	0	0	0	0	0	0	0	0	0	0	0	0	0	0	0	0	0	0	0	0	0	0	0	0	0	0	0	0	0	0	0	0	0	0	0	0	0	0
Republic of Korea	0	0	0	0	0	0	0	0	0	0	0	0	0	0	0	0	0	0	0	0	0	0	0	0	0	0	0	0	0	0	0	0	0	0	0	0	0	0	0	0
Republic of Moldova	0	0	0	0	0	0	0	0	0	0	0	0	0	0	0	0	0	0	0	0	0	0	0	0	0	0	0	0	0	0	0	0	0	0	0	0	0	0	0	0
Romania	0	0	0	0	0	0	0	0	0	0	0	0	0	0	0	0	0	0	0	0	0	0	0	0	0	0	0	0	0	0	0	0	0	0	0	0	0	0	0	0
Russian Federation	0	0	0	0	0	0	0	0	0	0	0	0	0	0	0	0	0	0	0	0	0	0	0	0	0	0	0	0	0	0	0	0	0	0	0	0	0	0	0	0
Rwanda	0	0	0	0	0	0	0	0	0	237	0	0	0	0	0	0	0	0	0	0	0	0	0	0	0	0	0	0	0	0	0	0	0	0	0	0	0	0	0	0
Saint Kitts and Nevis	0	0	0	0	0	0	0	0	0	0	0	0	0	0	0	0	0	0	0	0	0	0	0	0	0	0	0	0	0	0	0	0	0	0	0	0	0	0	0	0
Saint Lucia	0	0	0	0	0	0	0	0	0	0	0	0	0	0	0	0	0	0	0	0	0	0	0	0	0	0	0	0	0	0	0	0	0	0	0	0	0	0	0	0
Saint Vincent and the Grenadines	0	0	0	0	0	0	0	0	0	0	0	0	0	0	0	0	0	0	0	0	0	0	0	0	0	0	0	0	0	0	0	0	0	0	0	0	0	0	0	0
Samoa	0	0	0	0	0	0	0	0	0	0	0	0	0	0	0	0	0	0	0	0	0	0	0	0	0	0	0	0	0	0	0	0	0	0	0	0	0	0	0	0
San Marino	0	0	0	0	0	0	0	0	0	0	0	0	0	0	0	0	0	0	0	0	0	0	0	0	0	0	0	0	0	0	0	0	0	0	0	0	0	0	0	0
Sao Tome and Principe	0	0	0	0	0	0	0	0	0	0	0	0	0	0	0	0	0	0	0	0	0	0	0	0	0	0	0	0	0	0	0	0	0	0	0	0	0	0	0	0
Saudi Arabia	0	0	0	0	0	0	0	0	0	0	0	0	0	0	0	0	0	0	0	0	0	0	0	0	0	0	0	0	0	0	0	0	0	0	0	0	0	0	0	0
Senegal	0	0	0	0	0	0	0	0	0	0	0	0	0	0	0	0	0	0	0	0	0	0	0	0	0	0	0	0	0	0	0	0	0	0	0	0	0	0	0	0
Serbia	0	0	0	0	0	0	0	0	0	0	0	0	0	0	0	0	0	0	0	0	0	0	0	0	0	0	0	0	0	0	0	0	0	0	0	0	0	0	0	0
Seychelles	0	0	0	0	0	0	0	0	0	0	0	0	0	0	0	0	0	0	0	0	0	0	0	0	0	0	0	0	0	0	0	0	0	0	0	0	0	0	0	0
Sierra Leone	0	0	0	0	0	0	0	0	0	0	0	0	0	0	0	0	0	0	0	0	0	0	0	0	0	0	0	0	0	0	0	0	0	0	0	0	0	0	0	0
Singapore	0	0	0	0	0	0	0	0	0	0	0	0	0	0	0	0	0	0	0	0	0	0	0	0	0	0	0	0	0	0	0	0	0	0	0	0	0	0	0	0
Slovakia	0	0	0	0	0	0	0	0	0	0	0	0	0	0	0	0	0	0	0	0	0	0	0	0	0	0	0	0	0	0	0	0	0	0	0	0	0	0	0	0
Slovenia	0	0	0	0	0	0	0	0	0	0	0	0	0	0	0	0	0	0	0	0	0	0	0	0	0	0	0	0	0	0	0	0	0	0	0	0	0	0	0	0
Solomon Islands	0	0	0	0	0	0	0	0	0	0	0	0	0	0	0	0	0	0	0	0	0	0	0	0	0	0	0	0	0	0	0	0	0	0	0	0	0	0	0	0
Somalia	0	0	0	0	0	0	0	600	0	0	0	0	0	0	0	0	0	0	0	0	11	10	0	0	0	2	0	0	0	0	10000	10000	0	0	0	0	0	0	0	0
South Africa	0	0	0	0	0	0	0	0	0	0	0	0	0	0	0	0	0	0	0	0	0	0	0	0	0	0	0	0	0	0	0	0	0	0	0	0	0	0	0	0
South Sudan	0	0	0	0	0	0	0	0	0	0	0	0	0	0	0	0	0	0	0	0	0	0	0	0	0	0	0	0	0	0	0	0	0	0	0	0	0	0	0	0
Spain	0	0	0	0	0	0	0	0	0	0	0	0	0	0	0	0	0	0	0	0	0	0	0	0	0	0	0	0	0	0	0	0	0	0	0	0	0	0	0	0
Sri Lanka	0	0	0	0	0	0	0	0	0	0	0	0	0	0	0	0	0	0	0	0	0	0	0	0	0	0	0	0	0	0	0	0	0	0	0	0	0	0	0	0
Sudan	0	0	0	42000	54000	54000	0	0	0	0	0	0	0	0	0	0	0	0	0	0	0	0	0	0	0	0	0	0	0	0	0	0	0	0	0	0	0	0	0	0
Suriname	0	0	0	0	0	0	0	0	0	0	0	0	0	0	0	0	0	0	0	0	0	0	0	0	0	0	0	0	0	0	0	0	0	0	0	0	0	0	0	0
Sweden	0	0	0	0	0	0	0	0	0	0	0	0	0	0	0	0	0	0	0	0	0	0	0	0	0	0	0	0	0	0	0	0	0	0	0	0	0	0	0	0
Switzerland	0	0	0	0	0	0	0	0	0	0	0	0	0	0	0	0	0	0	0	0	0	0	0	0	0	0	0	0	0	0	0	0	0	0	0	0	0	0	0	0
Syrian Arab Republic	0	0	0	0	0	0	0	0	0	0	0	0	0	0	0	0	0	0	0	0	0	0	0	0	0	0	0	0	0	0	0	0	0	0	0	0	0	0	0	0
Taiwan (Province of China)	0	0	0	0	0	0	0	0	0	0	0	0	0	0	0	0	0	0	0	0	0	0	0	0	0	0	0	0	0	0	0	0	0	0	0	0	0	0	0	0
Tajikistan	0	0	0	0	0	0	0	0	0	0	0	0	0	0	0	0	0	0	0	0	0	0	0	0	0	0	0	0	0	0	0	0	0	0	0	0	0	0	0	0
Thailand	0	0	0	0	0	0	0	0	0	0	0	0	0	0	0	0	0	0	0	0	0	0	0	0	0	0	0	0	0	0	0	0	0	0	0	0	0	0	0	0
Timor-Leste	0	0	0	0	0	0	0	0	0	0	0	0	0	0	0	0	0	0	0	0	0	0	0	0	0	0	0	0	0	0	0	0	0	0	0	0	0	0	0	0
Togo	0	0	0	0	0	0	0	0	0	0	0	0	0	0	0	0	0	0	0	0	0	0	0	0	0	0	0	0	0	0	0	0	0	0	0	0	0	0	0	0
Tokelau	0	0	0	0	0	0	0	0	0	0	0	0	0	0	0	0	0	0	0	0	0	0	0	0	0	0	0	0	0	0	0	0	0	0	0	0	0	0	0	0
Tonga	0	0	0	0	0	0	0	0	0	0	0	0	0	0	0	0	0	0	0	0	0	0	0	0	0	0	0	0	0	0	0	0	0	0	0	0	0	0	0	0
Trinidad and Tobago	0	0	0	0	0	0	0	0	0	0	0	0	0	0	0	0	0	0	0	0	0	0	0	0	0	0	0	0	0	0	0	0	0	0	0	0	0	0	0	0
Tunisia	0	0	0	0	0	0	0	0	0	0	0	0	0	0	0	0	0	0	0	0	0	0	0	0	0	0	0	0	0	0	0	0	0	0	0	0	0	0	0	0
Turkey	0	0	0	0	0	0	0	0	0	0	0	0	0	0	0	0	0	0	0	0	0	0	0	0	0	0	0	0	0	0	0	0	0	0	0	0	0	0	0	0
Turkmenistan	0	0	0	0	0	0	0	0	0	0	0	0	0	0	0	0	0	0	0	0	0	0	0	0	0	0	0	0	0	0	0	0	0	0	0	0	0	0	0	0
Tuvalu	0	0	0	0	0	0	0	0	0	0	0	0	0	0	0	0	0	0	0	0	0	0	0	0	0	0	0	0	0	0	0	0	0	0	0	0	0	0	0	0
Uganda	0	0	0	0	0	0	0	0	0	0	0	0	0	0	0	0	0	0	0	21	47	47	79	0	0	0	0	0	0	0	0	0	0	0	0	0	0	0	0	0
Ukraine	0	0	0	0	0	0	0	0	0	0	0	0	0	0	0	0	0	0	0	0	0	0	0	0	0	0	0	0	0	0	0	0	0	0	0	0	0	0	0	0
United Arab Emirates	0	0	0	0	0	0	0	0	0	0	0	0	0	0	0	0	0	0	0	0	0	0	0	0	0	0	0	0	0	0	0	0	0	0	0	0	0	0	0	0
United Kingdom	0	0	0	0	0	0	0	0	0	0	0	0	0	0	0	0	0	0	0	0	0	0	0	0	0	0	0	0	0	0	0	0	0	0	0	0	0	0	0	0
United Republic of Tanzania	0	0	0	0	0	0	0	0	0	0	0	0	0	0	0	0	0	0	0	0	0	0	0	0	0	0	0	0	0	0	0	0	0	0	0	0	0	0	0	0
United States Virgin Islands	0	0	0	0	0	0	0	0	0	0	0	0	0	0	0	0	0	0	0	0	0	0	0	0	0	0	0	0	0	0	0	0	0	0	0	0	0	0	0	0
United States of America	0	0	0	0	0	0	0	0	0	0	0	0	0	0	0	0	0	0	0	0	0	0	0	0	0	0	0	0	0	0	0	0	0	0	0	0	0	0	0	0
Uruguay	0	0	0	0	0	0	0	0	0	0	0	0	0	0	0	0	0	0	0	0	0	0	0	0	0	0	0	0	0	0	0	0	0	0	0	0	0	0	0	0
Uzbekistan	0	0	0	0	0	0	0	0	0	0	0	0	0	0	0	0	0	0	0	0	0	0	0	0	0	0	0	0	0	0	0	0	0	0	0	0	0	0	0	0
Vanuatu	0	0	0	0	0	0	0	0	0	0	0	0	0	0	0	0	0	0	0	0	0	0	0	0	0	0	0	0	0	0	0	0	0	0	0	0	0	0	0	0
Venezuela (Bolivarian Republic of)	0	0	0	0	0	0	0	0	0	0	0	0	0	0	0	0	0	0	0	0	0	0	0	0	0	0	0	0	0	0	0	0	0	0	0	0	0	0	0	0
Viet Nam	0	0	0	0	0	0	0	0	0	0	0	0	0	0	0	0	0	0	0	0	0	0	0	0	0	0	0	0	0	0	0	0	0	0	0	0	0	0	0	0
Yemen	0	0	0	0	0	0	0	0	0	0	0	0	0	0	0	0	0	0	0	0	0	0	0	0	0	0	0	0	0	0	0	0	0	0	0	0	0	0	0	0
Zambia	0	0	0	0	0	0	0	0	0	0	0	0	0	0	0	0	0	0	0	0	0	0	0	0	0	0	0	0	0	0	0	0	0	0	0	0	0	0	0	0
Zimbabwe	0	0	0	0	0	0	0	0	0	0	0	0	0	0	0	0	0	0	0	0	0	0	0	0	0	0	0	0	0	0	0	0	0	0	0	0	0	0	0	0

SR Table 10d: Deaths due to natural disaster by country, all ages, for both sexes combined, 1980-2019

Country	1980	1981	1982	1983	1984	1985	1986	1987	1988	1989	1990	1991	1992	1993	1994	1995	1996	1997	1998	1999	2000	2001	2002	2003	2004	2005	2006	2007	2008	2009	2010	2011	2012	2013	2014	2015	2016	2017	2018	2019
Afghanistan	0	0	500	12	1	0	0	70	0	0	0	1347	614	225	160	381	70	175	7123	70	0	4	1286	137	18	561	382	296	1317	101	350	83	333	155	575	446	84	226	123	128
Albania	0	0	1	0	0	57	0	2	0	0	0	0	11	0	1	4	1	1	0	1	0	0	7	0	1	5	0	0	0	0	2	0	0	0	0	1	2	1	0	51
Algeria	2635	43	37	0	54	56	0	1	0	22	0	0	21	37	211	0	4	4	0	34	48	921	48	2329	22	22	5	82	93	48	0	10	69	0	6	12	0	0	6	0
American Samoa	0	0	0	0	0	0	0	0	0	0	0	0	0	0	0	0	0	0	0	0	0	0	0	6	0	0	0	0	0	34	0	0	0	1	0	0	0	0	0	0
Andorra	0	0	0	0	0	0	0	0	0	0	0	0	0	0	0	0	0	0	0	0	0	0	0	0	0	0	0	0	0	0	0	0	0	0	0	0	0	0	0	0
Angola	0	0	0	0	0	0	0	0	0	0	0	0	0	0	0	0	0	0	0	0	59	57	8	15	28	0	0	105	7	62	25	130	0	9	0	75	113	11	8	30
Antigua and Barbuda	0	0	0	0	0	0	0	0	0	2	0	0	0	0	0	2	0	32	2	2	0	0	0	0	0	0	0	0	0	0	0	0	0	0	0	0	0	1	0	0
Argentina	41	0	0	0	30	43	4	1	25	0	21	0	45	15	0	6	0	19	23	11	19	33	21	32	7	14	3	14	11	22	7	2	4	58	8	17	10	1	2	0
Armenia	0	0	0	0	0	0	0	0	25000	0	0	0	0	0	0	0	0	4	0	0	0	0	0	0	1	0	1	0	1	1	1	1	2	1	0	0	0	0	0	0
Australia	7	11	7	10	40	1	15	2	27	52	16	14	10	3	1	5	23	40	7	12	3	4	1	3	3	4	4	5	5	6	5	34	8	4	5	9	9	12	0	4
Austria	2	5	16	0	44	15	1	7	1	3	3	10	0	0	2	7	0	3	0	53	14	0	18	21	5	27	17	9	17	15	24	1	13	22	6	18	9	11	0	11
Azerbaijan	0	0	0	0	0	0	0	0	0	0	0	0	0	141	0	5	0	11	1	1	42	1	1	1	1	0	0	0	0	0	3	0	0	0	0	0	0	0	0	0
Bahamas	0	0	0	0	0	0	0	0	0	0	0	0	4	0	0	0	0	0	0	2	0	0	1	0	12	1	0	0	0	0	3	0	1	0	0	33	0	0	0	50
Bahrain	0	0	0	0	0	0	0	0	0	0	0	0	0	0	0	0	0	0	0	0	0	0	0	0	0	0	0	0	0	0	0	0	0	0	0	0	0	0	0	0
Bangladesh	666	1085	0	930	1200	15155	214	2292	3506	996	905	139252	40	646	321	1494	681	527	1371	330	142	247	132	314	1002	232	154	5505	68	213	107	40	272	50	79	159	198	323	68	131
Barbados	0	0	0	0	0	0	0	0	0	0	0	0	0	0	0	0	0	0	0	0	0	0	0	1	1	0	0	0	0	0	0	0	0	0	0	0	0	1	0	0
Belarus	0	0	0	0	0	0	0	0	0	0	0	0	0	0	0	0	0	5	0	2	0	0	4	3	0	0	0	3	5	5	4	6	0	3	3	0	0	0	0	0
Belgium	0	0	0	1	0	0	0	0	0	0	16	0	0	0	0	6	0	0	1	2	1	0	3	0	0	1	0	2	0	0	4	5	0	0	0	0	2	0	1	0
Belize	0	0	0	0	0	0	0	0	0	0	0	0	0	0	0	0	0	0	9	0	14	30	0	0	0	3	0	0	8	0	0	0	0	0	0	0	0	0	0	0
Benin	0	0	0	0	0	61	0	0	0	0	0	3	0	0	0	20	11	0	0	3	0	0	0	0	0	0	0	10	15	10	46	0	4	0	0	0	0	0	0	0
Bermuda	0	0	0	0	0	0	0	0	0	0	0	0	0	0	0	0	0	0	0	0	2	0	0	4	0	0	0	0	0	0	0	0	0	0	0	0	0	0	0	0
Bhutan	0	0	0	0	0	0	0	0	0	0	0	0	0	0	39	0	0	0	0	0	200	0	0	0	0	0	0	0	0	23	0	1	0	0	0	0	0	0	0	0
Bolivia (Plurinational State of)	0	0	20	250	0	0	29	25	0	0	0	0	53	0	5	0	40	56	155	0	31	41	96	108	34	0	25	65	50	0	26	52	13	88	126	40	4	0	6	58
Bosnia and Herzegovina	0	0	0	0	0	0	0	0	0	0	0	0	0	0	0	0	0	0	0	0	6	0	0	0	0	4	0	0	0	0	3	0	0	0	28	0	0	0	0	0
Botswana	0	0	0	0	0	0	0	0	8	0	0	0	0	0	0	20	0	0	0	0	3	0	0	0	0	0	0	0	0	0	0	0	0	12	0	0	0	0	0	0
Brazil	141	118	34	167	289	153	44	122	695	327	35	56	205	4	21	268	239	114	42	58	150	121	157	230	197	88	90	140	487	165	728	2562	77	185	119	77	94	63	22	22
Brunei Darussalam	0	0	0	0	0	0	0	0	0	0	0	0	0	0	0	0	0	0	0	0	0	0	0	0	0	0	0	0	0	0	0	0	0	0	0	3	0	0	0	0
Bulgaria	0	0	0	0	0	0	3	0	0	0	1	2	4	2	3	0	1	3	3	5	10	2	1	2	7	1	1	13	3	0	1	0	6	0	4	8	0	0	0	0
Burkina Faso	0	0	0	0	0	0	0	0	16	0	0	0	0	0	22	0	0	0	0	6	0	0	0	0	0	0	0	52	6	9	16	0	18	2	0	8	15	2	0	0
Burundi	0	0	0	0	0	0	0	0	0	12	0	0	0	0	0	0	0	0	0	0	0	0	0	0	3	0	22	5	9	0	9	12	0	0	96	37	26	6	0	10
Cabo Verde	0	0	3	0	29	0	0	0	0	0	0	0	0	0	0	0	0	0	0	0	0	0	0	0	0	0	0	0	0	3	0	0	0	0	0	9	0	0	0	0
Cambodia	0	0	0	0	0	0	0	0	0	0	0	100	0	0	506	0	59	25	0	7	347	56	29	0	0	16	5	2	0	19	8	247	14	200	45	0	0	0	0	0
Cameroon	0	0	0	0	37	0	1746	0	2	0	0	2	0	0	6	0	0	0	0	24	3	30	0	20	0	2	0	8	9	0	13	0	25	0	0	4	0	3	0	43
Canada	0	0	0	0	0	12	15	31	13	6	26	3	8	6	8	14	19	13	48	21	14	20	7	23	3	7	10	18	21	14	11	19	6	6	11	8	0	2	0	0
Central African Republic	0	0	0	0	0	0	0	0	0	0	0	0	0	0	0	0	7	0	0	0	0	3	0	0	3	1	0	1	0	4	3	1	0	0	0	1	0	0	0	0
Chad	0	0	0	0	0	0	0	0	54	0	0	39	0	0	0	2	0	0	0	0	0	100	0	0	0	0	52	29	0	6	24	0	20	0	0	0	0	0	0	0
Chile	0	0	0	4	127	180	39	164	0	21	0	223	0	120	3	30	0	33	4	0	16	9	21	0	5	58	18	15	10	3	508	1	1	4	3	227	13	31	0	6
China	6493	2465	582	944	144	1664	686	690	2569	3173	1324	2339	1456	1573	3100	2408	5615	1674	4503	1706	679	922	1419	1094	773	1029	1999	1180	88421	611	7175	844	871	1402	1684	551	1340	620	354	295
Colombia	68	283	130	340	107	22023	368	947	229	118	43	154	23	261	166	131	23	65	55	913	212	97	134	160	61	135	262	312	163	116	458	588	173	102	127	180	28	701	26	14
Comoros	0	0	0	33	0	2	0	24	0	0	0	0	0	0	0	0	0	0	0	0	0	0	0	0	0	1	0	0	0	2	0	0	4	0	0	0	0	0	0	8
Congo	0	0	0	0	0	0	0	0	0	0	0	0	0	0	0	0	0	0	0	2	0	0	0	0	0	1	6	0	0	0	0	0	19	0	0	0	0	0	0	0
Cook Islands	0	0	0	0	0	0	0	5	0	0	1	0	0	0	0	0	0	19	0	0	0	0	0	0	0	0	0	0	0	0	0	0	0	0	0	0	0	0	0	0
Costa Rica	1	0	0	12	0	0	0	0	35	0	1	48	0	12	2	0	60	4	13	17	2	1	11	3	10	6	0	19	12	22	51	4	7	0	1	0	9	13	1	0
Croatia	0	0	0	0	0	0	4	2	1	1	0	1	0	0	1	2	0	0	0	1	0	0	0	0	0	2	0	0	1	1	1	0	0	0	2	1	0	0	0	0
Cuba	3	0	24	15	0	4	0	0	23	0	4	0	0	51	14	2	3	0	4	12	2	6	1	1	4	16	9	4	10	3	4	0	17	2	0	3	1	10	9	6
Cyprus	0	0	0	0	0	0	0	0	0	0	0	0	0	0	0	2	0	0	0	0	0	0	0	0	1	0	2	0	0	0	0	0	0	0	0	0	0	0	0	0
Czechia	0	0	0	0	0	0	0	0	0	0	0	1	0	0	0	0	1	18	5	1	0	1	7	0	3	2	7	1	4	10	17	1	0	1	0	0	0	1	0	0
Côte d'Ivoire	0	0	0	0	0	0	0	0	0	0	0	0	0	0	0	0	28	0	0	0	0	0	0	0	0	0	0	0	6	27	18	0	0	0	46	16	20	15	20	0
Democratic People's Republic of Korea	0	0	0	0	0	0	0	315	0	0	0	0	0	6	0	68	116	0	50	42	46	124	3	0	24	193	278	610	0	0	20	74	147	51	0	33	552	0	232	0
Democratic Republic of the Congo	0	0	0	0	0	0	0	0	0	0	23	0	9	0	39	0	0	0	0	2	0	62	240	29	0	14	4	32	36	0	46	0	1	0	165	52	50	504	95	0
Denmark	0	9	0	2	0	0	0	0	0	0	1	0	0	0	0	0	0	0	0	7	0	1	0	0	0	2	0	0	0	0	0	0	0	2	0	0	0	0	0	0
Djibouti	0	25	0	0	0	0	0	0	0	10	0	0	0	0	145	0	0	0	0	0	0	0	0	0	51	0	0	0	0	0	0	0	0	0	0	0	0	0	2	0
Dominica	0	0	0	0	2	0	0	0	0	0	0	0	0	0	0	2	0	0	0	0	0	0	0	2	2	0	0	5	0	0	3	0	0	0	0	36	0	64	0	0
Dominican Republic	7	20	0	0	0	12	12	3	0	0	0	10	0	30	0	11	31	5	361	8	3	5	4	21	704	13	1	176	14	10	9	12	9	1	1	2	15	5	0	0
Ecuador	8	0	68	339	0	0	0	5102	0	35	4	0	22	460	0	3	36	174	139	17	81	108	95	22	27	25	49	18	66	33	45	34	52	37	20	45	713	29	0	14
Egypt	0	0	0	0	0	0	0	30	0	0	0	1	552	34	600	17	12	22	0	0	2	1	25	2	16	6	2	4	98	2	62	2	5	7	5	41	54	0	0	0
El Salvador	0	0	520	0	0	0	1100	0	55	10	0	0	2	22	0	8	12	11	483	9	8	1168	6	3	2	172	5	14	36	394	22	49	1	5	9	13	0	10	4	0
Equatorial Guinea	0	0	0	0	0	0	0	0	0	0	0	0	0	0	0	0	0	0	0	0	0	0	0	0	0	0	0	0	0	0	0	0	0	0	0	0	0	0	0	0
Eritrea	0	0	0	0	0	0	0	0	0	0	0	0	0	3	0	0	0	0	0	0	0	0	0	0	0	0	0	0	0	0	0	0	0	0	0	0	0	0	0	0
Estonia	0	0	0	0	0	0	0	0	0	0	0	0	0	0	0	0	0	0	0	0	1	0	1	0	0	0	1	0	0	1	0	0	1	0	0	0	0	0	0	0
Eswatini	0	0	0	0	53	0	0	0	0	0	0	0	0	0	0	0	0	0	0	0	0	0	0	0	0	0	1	0	0	0	0	0	0	0	11	0	0	0	0	0
Ethiopia	0	0	0	0	0	9	0	0	45	0	0	13	0	2	26	27	40	326	0	48	73	5	22	119	0	211	951	22	45	0	19	0	0	0	0	0	228	115	44	0
Fiji	18	0	0	9	0	32	20	1	0	10	0	0	1	21	0	0	0	25	0	12	4	2	7	17	16	0	5	7	7	15	2	0	17	0	0	0	47	0	7	0
Finland	0	0	0	0	0	0	0	0	0	0	0	0	0	0	0	0	0	1	2	0	3	2	0	0	0	0	0	2	0	1	1	2	1	1	1	0	0	0	0	0
France	67	10	27	55	24	27	24	65	24	8	33	32	60	18	38	17	30	23	43	101	23	29	49	26	25	30	47	27	17	26	71	21	12	28	19	25	5	18	20	0
Gabon	0	0	0	0	0	0	0	0	0	0	0	0	0	0	0	0	0	0	0	0	0	0	0	0	0	0	0	0	0	0	0	0	1	0	0	0	0	0	0	0
Gambia	0	0	0	0	0	0	0	0	0	0	0	0	0	0	0	0	0	0	0	53	0	1	0	3	2	0	0	0	0	5	9	0	0	2	0	0	0	0	0	0
Georgia	49	0	0	0	0	0	0	136	0	98	0	8	1	98	0	1	0	7	0	0	0	0	6	0	1	0	8	1	0	2	2	7	2	2	6	6	0	0	0	0
Germany	0	0	0	0	3	0	0	0	6	0	64	5	1	11	13	6	1	0	3	19	1	9	33	2	2	6	8	20	5	8	7	9	1	11	10	7	7	5	5	1
Ghana	0	0	0	0	0	0	0	0	0	7	0	5	0	0	0	145	0	0	0	52	0	12	4	0	0	0	0	56	0	40	63	20	0	5	0	25	10	20	34	0
Greece	1	24	0	0	0	0	20	52	0	2	4	2	0	2	15	12	1	9	5	115	1	12	2	1	0	1	4	2	2	0	1	0	1	0	7	7	6	26	0	0
Greenland	0	0	0	0	0	0	0	0	0	0	0	0	0	0	0	0	0	0	0	0	0	0	0	1	0	0	0	0	0	0	0	1	0	0	0	0	0	0	0	0
Grenada	0	0	0	0	0	0	0	0	0	0	0	0	0	0	0	0	0	0	0	0	0	0	0	0	18	1	0	0	0	0	0	0	0	0	0	0	0	0	0	0
Guam	0	0	0	0	0	0	0	0	0	0	0	0	1	0	0	0	0	0	0	0	0	1	0	1	0	0	0	0	0	0	0	0	0	0	0	0	0	0	0	0
Guatemala	0	0	640	0	0	0	0	84	5	10	0	37	0	0	10	31	0	0	435	14	41	7	71	27	6	324	20	23	62	2	177	75	7	11	18	7	10	21	427	0
Guinea	0	0	0	275	0	0	0	0	0	0	0	0	0	0	0	0	0	0	0	0	4	9	0	0	0	0	0	0	0	2	8	0	0	0	0	16	0	0	0	0
Guinea-Bissau	0	0	0	0	0	0	0	1	0	0	0	0	0	0	0	0	0	0	0	0	0	0	0	0	3	0	0	0	0	0	2	0	0	0	0	0	0	0	3	0
Guyana	0	0	0	0	0	0	0	0	0	0	0	0	0	0	0	0	0	0	0	0	0	0	0	0	0	34	0	0	0	0	0	0	0	0	0	0	0	0	0	0
Haiti	220	0	0	0	0	0	148	33	81	0	0	0	0	13	1122	0	84	0	190	14	16	26	37	90	5422	88	16	163	698	21	222641	39	130	6	12	11	599	35	17	0
Honduras	2	0	130	0	0	0	0	0	19	10	5	0	0	413	151	32	8	0	14600	34	0	21	11	10	0	61	4	7	67	8	144	33	1	0	4	0	7	21	6	0
Hungary	1	0	0	0	4	0	0	0	1	0	0	0	0	0	0	0	2	0	0	48	1	1	0	7	0	4	5	0	0	0	1	0	0	0	0	0	0	0	0	0
Iceland	0	0	0	4	0	0	0	0	0	2	0	1	0	0	1	69	0	0	0	2	0	0	0	0	1	0	1	0	0	0	0	0	0	0	0	0	0	1	0	0
India	2727	1570	1676	1634	1284	1834	800	1480	3063	1788	2097	2641	977	11251	2234	2376	2876	2476	5976	11032	2273	20783	881	641	17737	3782	1384	2051	1738	1773	994	826	335	6562	892	1152	822	2082	1335	2065
Indonesia	253	1007	323	41	28	52	175	459	229	144	230	173	2632	134	609	230	294	21	38	106	539	580	280	460	166020	1198	7342	694	132	1497	1294	129	107	173	243	54	270	99	5493	621
Iran (Islamic Republic of)	306	3458	0	33	0	1	266	867	91	100	40042	385	68	805	53	27	58	2781	211	102	14	598	291	26817	705	737	80	24	58	0	20	2	324	69	54	63	39	846	11	126
Iraq	0	0	0	0	0	0	0	0	0	0	0	20	0	0	0	0	0	0	0	0	0	0	0	0	0	0	20	0	5	2	0	6	5	12	0	58	0	10	21	10
Ireland	0	0	0	10	0	0	11	0	3	0	2	0	0	3	0	0	2	0	1	2	1	0	0	0	0	2	0	0	0	0	0	2	0	0	1	2	0	0	2	0
Israel	0	0	0	0	0	0	0	0	0	0	0	5	0	0	0	0	0	17	0	2	3	2	2	0	0	0	0	0	0	0	2	0	0	4	0	0	0	0	12	0
Italy	2098	21	25	14	26	607	22	128	24	17	36	33	9	13	104	3	28	35	292	18	68	10	26	34	24	12	20	10	20	382	43	33	45	39	36	42	320	44	22	0
Jamaica	6	0	0	0	0	7	54	9	49	0	0	15	0	9	4	0	0	0	0	0	1	1	13	0	16	6	1	5	13	0	15	0	1	1	0	0	0	0	0	0
Japan	85	218	861	439	150	131	115	27	66	78	145	161	39	515	24	5521	75	73	88	166	50	43	27	78	358	113	147	50	47	89	30	18397	223	103	321	41	107	110	319	80
Jordan	0	0	0	0	0	0	0	9	0	0	0	10	0	0	0	0	0	2	0	0	9	0	5	0	0	0	0	0	0	0	2	0	0	0	0	1	0	0	34	0
Kazakhstan	0	0	0	0	0	0	0	0	0	0	0	0	0	10	0	112	0	0	0	0	0	0	0	3	8	7	7	6	6	5	5	5	5	3	8	5	0	15	0	0
Kenya	0	0	75	0	0	0	0	0	50	0	44	0	0	0	0	0	0	86	40	0	0	4	101	40	55	26	212	39	65	36	224	33	73	128	0	82	98	26	134	0
Kiribati	0	0	0	0	0	0	0	0	0	0	0	0	0	0	0	0	0	0	0	0	0	0	0	0	0	0	0	0	0	0	0	0	0	0	0	0	0	0	0	0
Kuwait	0	0	0	0	0	0	0	0	0	0	0	0	0	0	0	0	0	2	0	0	0	0	0	0	0	0	0	0	0	0	0	0	0	0	0	0	0	0	1	0
Kyrgyzstan	0	0	0	0	0	0	0	0	0	0	0	0	58	0	162	0	0	0	1	0	8	7	12	43	47	23	13	3	80	26	11	4	12	8	9	17	11	45	0	0
Lao People's Democratic Republic	0	0	0	0	14	0	0	0	0	0	0	0	32	8	0	26	30	0	0	0	15	0	2	0	0	0	0	0	6	26	0	48	0	23	0	4	5	0	136	16
Latvia	0	0	0	0	0	0	0	0	0	0	0	0	0	0	0	0	0	0	1	6	0	0	0	0	0	0	0	2	0	0	0	0	0	1	0	0	0	0	0	0
Lebanon	0	0	0	20	0	0	0	0	0	0	0	0	25	0	0	0	0	0	0	0	0	0	0	0	0	0	0	0	0	0	0	0	0	0	0	5	0	0	15	3
Lesotho	0	0	0	0	0	0	0	18	0	0	22	0	0	0	0	0	0	0	0	0	0	1	0	0	0	0	0	0	0	0	0	26	0	0	0	0	0	0	0	0
Liberia	0	0	46	0	0	0	0	0	0	0	0	0	0	0	0	0	0	0	10	0	0	0	0	0	0	0	0	1	0	2	1	0	0	0	0	0	0	0	0	0
Libya	0	0	0	0	0	0	0	0	0	0	0	0	0	0	0	0	0	0	0	0	0	0	0	0	0	0	0	0	0	0	0	0	0	16	0	0	0	0	0	0
Lithuania	0	0	0	0	0	0	0	0	0	0	0	0	0	6	0	0	0	0	1	3	0	1	1	0	0	1	2	0	1	0	4	0	0	0	0	0	0	0	0	0
Luxembourg	0	1	0	0	0	0	0	0	0	0	0	0	0	0	0	0	0	0	0	0	2	0	0	0	0	0	0	0	0	0	0	0	0	0	0	0	0	0	0	0
Madagascar	0	107	92	42	68	0	99	0	0	46	0	36	0	0	212	0	9	174	0	0	153	0	23	105	395	107	4	91	105	27	120	35	112	51	17	121	0	81	94	0
Malawi	0	0	0	0	0	0	0	0	0	22	0	472	0	0	0	1	0	0	4	0	0	59	9	12	0	11	9	2	0	4	0	4	1	6	0	283	12	0	5	59
Malaysia	0	0	0	10	0	0	11	3	27	0	0	0	0	102	0	20	320	2	0	1	17	10	21	13	100	12	15	48	7	6	4	28	6	5	19	25	0	9	2	1
Maldives	0	0	0	0	0	0	0	0	0	0	0	0	0	0	0	0	0	0	0	0	0	0	0	0	102	0	0	0	0	0	0	0	0	0	0	0	0	0	0	0
Mali	0	0	0	0	0	0	0	0	17	1	0	0	0	0	0	0	0	0	3	2	15	2	2	0	0	0	1	18	0	25	0	1	0	37	0	5	13	0	0	0
Malta	0	0	0	0	0	0	0	0	0	0	0	0	0	0	0	0	0	0	1	0	0	0	0	0	0	0	0	0	0	0	0	0	0	0	0	0	0	0	0	0
Marshall Islands	0	0	0	0	0	0	0	0	0	0	0	0	0	0	0	0	0	0	0	0	0	0	0	0	0	0	0	0	0	0	0	0	0	0	0	0	0	0	0	0
Mauritania	0	0	0	0	0	0	0	0	0	0	0	4	0	0	0	0	0	0	0	1	0	0	25	9	0	0	7	3	0	1	0	0	0	8	0	0	0	18	0	0
Mauritius	0	0	0	1	0	0	0	0	0	1	0	0	0	0	2	0	3	0	0	0	0	0	3	0	0	0	0	2	0	0	0	0	0	21	0	0	0	0	0	0
Mexico	120	26	374	332	14	3667	70	25	291	58	121	55	9	116	5	163	25	302	748	567	184	41	72	55	91	100	55	71	50	52	206	149	32	220	54	53	225	732	40	0
Micronesia (Federated States of)	0	0	0	0	0	0	0	5	0	0	0	0	0	0	0	0	0	0	0	0	0	0	47	0	1	0	0	0	0	0	0	0	0	0	0	5	0	0	0	0
Monaco	0	0	0	0	0	0	0	0	0	0	0	0	0	0	0	0	0	0	0	0	0	0	0	0	0	0	0	0	0	0	0	0	0	0	0	0	0	0	0	0
Mongolia	0	0	0	0	0	0	0	0	0	0	36	6	0	7	5	0	41	0	0	0	23	16	2	17	0	0	0	0	52	26	11	0	0	0	0	0	12	17	0	12
Montenegro	0	0	0	0	0	0	0	0	0	0	0	0	0	0	0	0	0	0	0	1	0	0	0	0	0	0	0	0	0	0	0	0	0	0	0	0	0	0	0	0

Morocco	0	0	1	0	0	0	0	0	31	0	0	0	0	0	0	791	39	60	0	0	6	15	80	35	628	3	17	2	39	30	42	0	0	0	60	0	0	0	0	0
Mozambique	0	0	0	0	109	8	0	0	100	2	0	0	0	0	240	0	11	35	87	23	832	79	3	49	0	5	15	54	31	12	5	23	33	125	16	160	63	19	28	643
Myanmar	0	0	11	0	0	0	0	0	0	0	0	23	5	0	17	51	0	68	0	22	0	51	21	0	307	17	59	5	138366	24	113	225	40	23	0	213	97	3	67	115
Namibia	0	0	0	0	0	0	0	0	0	0	0	0	0	0	0	0	0	0	0	0	0	0	0	2	0	0	5	7	42	92	8	108	0	0	0	0	0	0	0	0
Nauru	0	0	0	0	0	0	0	0	0	0	0	0	0	0	0	0	0	0	0	0	0	0	0	0	0	0	0	0	0	0	0	0	0	0	0	0	0	0	0	0
Nepal	100	750	92	207	200	46	23	188	912	49	30	57	0	1364	0	320	808	20	310	170	144	144	472	239	185	69	157	214	115	127	150	140	83	195	573	9034	163	154	15	119
Netherlands	0	0	0	1	0	0	0	0	0	11	6	0	1	1	0	3	3	2	0	0	0	3	2	0	0	0	0	7	0	0	5	0	0	2	0	1	0	4	3	0
New Zealand	5	7	1	1	0	9	1	1	9	5	3	4	3	0	2	0	3	6	10	2	2	2	2	5	13	0	4	1	11	7	1	188	2	4	3	5	2	0	0	2
Nicaragua	0	0	71	0	0	0	0	0	130	0	4	0	180	37	0	38	44	0	4064	11	9	16	3	1	29	7	0	198	30	1	77	18	9	32	15	3	2	23	4	0
Niger	0	0	0	0	0	0	0	0	20	0	0	0	0	0	60	0	0	0	0	14	2	4	4	7	0	0	4	7	7	3	5	16	91	32	36	27	61	72	36	0
Nigeria	0	0	0	0	0	0	0	0	130	0	0	0	0	0	30	0	0	0	15	189	36	200	0	16	94	60	40	91	0	31	40	185	378	19	15	94	46	20	300	0
Niue	0	0	0	0	0	0	0	0	0	0	0	0	0	0	0	0	0	0	0	0	0	0	0	0	1	0	0	0	0	0	0	0	0	0	0	0	0	0	0	0
North Macedonia	0	0	0	12	0	0	0	0	0	0	0	0	0	1	0	0	1	0	0	0	0	0	0	2	0	1	0	0	0	1	0	1	0	1	0	7	22	0	0	0
Northern Mariana Islands	0	0	0	0	0	0	0	0	0	0	0	0	0	0	0	0	0	0	0	0	0	0	0	0	3	0	0	0	0	0	0	0	0	0	0	0	0	0	2	0
Norway	0	0	0	0	0	0	21	8	8	9	5	1	5	2	7	4	5	7	4	3	2	9	6	0	6	8	2	4	7	2	11	14	3	6	7	7	2	1	0	0
Oman	0	26	0	0	0	0	0	0	0	0	0	0	0	0	0	0	0	0	0	0	0	0	7	30	0	0	0	76	0	0	16	14	0	0	0	0	2	3	6	0
Pakistan	93	394	68	158	43	5	11	0	282	20	22	349	1541	682	352	1063	155	262	1026	292	0	257	90	329	29	74056	429	911	249	102	2186	511	671	762	271	663	409	215	60	225
Palau	0	0	0	0	0	0	0	0	0	0	0	0	0	0	0	0	0	0	0	0	0	0	0	0	0	0	0	0	0	0	0	0	0	0	0	0	0	0	0	0
Palestine	0	0	0	0	0	0	0	0	0	0	0	0	0	0	0	0	0	0	0	0	0	0	0	0	0	0	0	0	0	0	0	0	0	6	0	4	0	0	0	0
Panama	0	0	0	0	1	0	2	0	30	0	0	42	12	0	0	1	0	0	4	6	2	0	6	5	13	1	15	3	33	1	8	5	8	0	23	1	5	7	0	0
Papua New Guinea	0	0	0	66	0	0	0	1	76	0	0	200	0	68	5	0	42	32	2182	2	2	0	41	13	0	2	16	172	10	19	0	0	65	0	0	0	0	0	181	10
Paraguay	0	0	0	76	0	0	0	0	0	0	0	0	0	0	0	0	6	33	55	1	0	0	1	2	1	3	1	0	1	1	1	2	11	5	2	12	11	0	0	17
Peru	77	158	535	606	7	150	118	268	118	38	294	64	0	86	85	20	21	430	433	116	38	186	100	41	23	7	1	626	60	187	207	53	92	77	61	30	7	200	2	76
Philippines	264	1552	596	158	2679	567	179	1094	842	521	3330	6793	180	671	418	1725	83	67	594	370	747	630	320	222	2045	39	3689	209	1174	2297	359	2597	2773	14246	384	288	87	233	313	8
Poland	0	0	0	0	7	2	5	1	5	2	0	0	2	0	1	0	0	55	9	4	2	35	15	14	12	5	5	20	5	13	20	3	1	6	0	6	2	6	0	0
Portugal	69	32	3	21	1	2	0	2	1	2	0	0	2	6	0	0	12	58	10	1	4	13	4	2	6	0	0	0	2	0	7	0	0	1	2	1	0	0	2	0
Puerto Rico	0	26	0	0	0	600	0	0	0	0	0	0	18	0	0	1	13	0	0	2	3	2	0	2	1	0	0	1	0	0	0	1	0	0	1	0	0	2355	0	0
Qatar	0	0	0	0	0	0	0	0	0	0	0	0	0	0	0	0	0	0	0	0	0	0	0	0	0	0	0	0	0	0	0	0	0	0	0	0	0	0	0	0
Republic of Korea	165	113	68	0	213	57	33	669	25	109	154	126	5	43	28	124	144	31	607	115	67	109	283	156	49	42	95	26	21	13	22	147	26	10	52	1	10	0	0	0
Republic of Moldova	0	0	0	0	0	0	0	0	0	0	0	0	0	0	50	0	2	9	2	0	0	0	1	0	1	0	0	0	3	0	1	0	0	0	0	0	0	0	0	0
Romania	0	0	0	0	0	0	0	0	0	0	9	108	0	3	2	1	1	32	31	27	16	11	14	5	14	8	9	13	6	11	3	0	0	5	5	1	2	12	0	0
Russian Federation	216	0	0	0	400	29	3	0	0	100	86	116	114	151	162	203	154	128	122	91	103	89	340	77	86	83	79	51	68	51	66	35	197	43	48	90	110	133	10	0
Rwanda	0	0	0	0	0	0	0	0	48	0	0	0	0	0	0	0	0	0	0	0	0	12	114	0	0	0	24	30	36	0	21	14	5	0	0	2	121	3	76	0
Saint Kitts and Nevis	0	0	0	0	0	0	0	0	0	1	0	0	0	0	0	0	0	0	5	0	0	0	0	0	0	0	0	0	0	0	0	0	0	0	0	0	0	0	0	0
Saint Lucia	9	0	0	0	0	0	0	0	0	0	0	0	0	0	4	0	0	0	1	0	0	0	0	0	0	0	0	2	0	0	14	0	0	6	0	0	0	0	0	0
Saint Vincent and the Grenadines	0	0	0	0	0	0	0	0	0	0	0	0	3	0	0	0	0	0	0	0	0	0	8	0	0	2	0	0	0	0	0	0	0	12	0	0	0	0	0	0
Samoa	0	0	0	0	0	0	0	0	0	0	8	13	0	0	0	0	0	0	0	0	0	0	0	0	1	9	0	0	0	148	0	0	12	0	0	0	0	0	0	0
San Marino	0	0	0	0	0	0	0	0	0	0	0	0	0	0	0	0	0	0	0	0	0	0	0	0	0	0	0	0	0	0	0	0	0	0	0	0	0	0	0	0
Sao Tome and Principe	0	0	0	0	0	0	0	0	0	0	0	0	0	0	0	0	0	0	0	0	0	0	0	0	0	0	0	0	0	0	0	0	0	0	0	0	0	0	0	0
Saudi Arabia	0	0	0	0	0	32	0	0	0	0	0	0	0	0	0	0	0	0	0	0	0	0	19	12	5	63	0	0	0	161	24	11	19	39	0	23	24	4	0	12
Senegal	0	0	0	0	0	0	0	0	0	0	0	0	0	0	0	0	0	0	0	187	0	0	28	8	2	0	0	8	1	6	2	0	19	8	0	0	5	0	0	0
Serbia	0	70	0	0	0	0	0	0	0	0	0	0	1	0	0	0	0	0	1	12	5	6	4	8	0	5	1	0	1	0	5	1	1	0	57	2	0	0	0	0
Seychelles	0	0	0	0	0	0	0	0	0	0	0	0	0	0	0	0	0	5	0	0	0	0	0	0	3	0	0	0	0	0	0	0	0	0	0	0	0	0	0	0
Sierra Leone	0	0	0	0	60	0	0	0	0	0	0	0	0	0	0	0	12	0	0	13	0	0	0	0	10	20	0	0	0	103	16	21	0	0	0	10	0	1352	0	7
Singapore	0	0	0	0	0	0	0	0	0	0	0	0	0	0	0	0	0	0	0	0	0	0	0	0	0	0	0	0	0	0	0	0	0	0	0	0	0	0	0	0
Slovakia	0	0	0	0	0	10	0	0	0	0	0	0	0	0	0	1	1	0	55	4	2	6	1	1	5	3	1	0	1	10	6	0	0	2	3	0	0	0	0	0
Slovenia	0	0	0	0	0	0	0	0	0	0	1	0	0	2	1	2	3	1	1	2	0	1	0	1	2	1	2	10	1	4	7	0	1	3	3	1	0	0	0	0
Solomon Islands	0	0	0	0	0	0	101	0	1	0	0	0	0	4	0	0	0	0	0	0	0	0	0	0	0	0	0	52	0	21	2	0	0	10	47	9	0	0	0	0
Somalia	0	5	0	0	0	0	0	0	0	34	0	0	0	0	130	20	3	2311	0	0	0	0	0	1	298	12	102	25	0	0	11	0	31	169	0	0	9	0	58	0
South Africa	0	104	17	10	64	0	0	515	33	0	12	0	0	19	2	103	172	20	19	53	98	19	41	4	3	2	12	0	17	35	20	66	16	5	34	0	13	23	0	68
South Sudan	0	0	0	0	0	0	0	0	0	0	0	0	0	0	0	0	0	0	0	0	0	0	0	0	0	0	0	0	17	0	0	0	47	99	0	0	0	0	0	110
Spain	0	1	79	53	9	4	1	6	21	19	10	1	0	1	16	14	175	47	0	7	10	6	33	7	9	26	7	13	15	7	11	16	13	11	3	6	8	0	15	0
Sri Lanka	0	7	54	3	51	0	43	0	0	325	33	27	15	73	0	3	0	10	2	7	10	0	2	235	35405	6	29	33	57	4	37	87	53	117	316	31	274	329	34	0
Sudan	0	0	0	0	0	0	0	0	96	0	0	0	0	24	8	0	152	8	103	38	0	3	33	20	0	5	27	150	0	20	36	20	35	76	39	20	171	25	46	0
Suriname	0	0	0	0	0	0	0	0	0	0	0	0	0	0	0	0	0	0	0	0	0	0	0	0	0	0	0	0	0	0	0	0	0	0	0	0	0	0	0	0
Sweden	0	0	0	0	0	11	0	1	7	1	1	1	0	1	0	0	2	3	1	5	1	4	1	7	2	6	4	3	0	0	1	0	4	11	0	0	1	1	0	0
Switzerland	0	0	0	0	0	0	0	1	0	0	5	0	0	2	0	9	14	22	5	27	29	20	19	9	10	25	12	9	11	12	16	13	11	16	13	19	10	8	0	4
Syrian Arab Republic	0	0	0	0	0	0	0	0	0	0	0	0	0	0	0	0	0	0	0	0	0	27	80	0	5	0	6	0	0	0	0	0	0	0	0	13	0	0	0	0
Taiwan (Province of China)	0	10	10	20	43	2	29	9	0	3	10	36	6	1	6	3	28	54	20	2352	38	138	4	3	46	25	9	7	15	600	46	1	20	4	0	9	115	6	91	0
Tajikistan	38	0	0	0	0	0	0	52	0	0	43	0	1601	5	0	0	0	40	62	27	0	1	35	7	0	24	25	49	0	21	75	0	2	0	20	12	4	13	6	0
Thailand	57	55	0	50	17	18	42	24	664	458	49	55	6	45	130	258	100	212	1	24	109	222	190	9	8375	68	298	53	39	18	297	796	141	85	33	4	33	150	0	7
Timor-Leste	0	0	0	0	0	0	0	0	0	0	0	0	0	0	0	0	0	0	0	0	0	1	0	3	0	0	0	1	0	0	0	0	0	0	0	0	0	0	0	0
Togo	0	0	0	0	0	0	0	0	0	0	0	0	0	0	0	3	0	0	0	0	0	0	0	0	0	0	0	41	6	0	22	0	0	0	0	0	0	0	0	0
Tokelau	0	0	0	0	0	0	0	0	0	0	0	0	0	0	0	0	0	0	0	0	0	0	0	0	0	0	0	0	0	0	0	0	0	0	0	0	0	0	0	0
Tonga	0	0	7	0	0	0	0	0	0	0	1	0	0	0	0	0	0	0	0	0	0	0	0	0	0	0	0	0	0	9	0	0	0	0	1	0	0	0	0	0
Trinidad and Tobago	0	0	0	0	0	0	0	0	0	0	0	0	0	5	0	0	0	0	0	0	0	0	0	2	4	2	0	0	2	0	5	1	2	0	0	0	0	0	0	0
Tunisia	0	0	117	0	0	0	23	0	0	0	37	0	0	0	0	0	0	0	0	0	0	0	0	12	0	0	0	16	0	17	0	0	0	0	0	0	0	0	13	0
Turkey	115	10	0	1346	6	0	15	0	77	0	69	43	914	135	30	231	0	0	239	17982	5	21	86	186	75	26	59	16	0	62	64	656	15	11	4	18	1	0	24	0
Turkmenistan	0	0	0	0	0	0	0	0	0	0	0	0	0	0	0	0	0	0	0	1	1	1	1	1	1	1	1	1	1	1	1	1	1	1	1	1	0	0	0	0
Tuvalu	0	0	0	0	0	0	0	0	0	0	0	0	0	0	0	0	0	0	0	0	0	0	0	0	0	0	0	0	0	0	0	0	0	0	0	0	0	0	0	0
Uganda	0	0	0	0	0	0	0	0	0	0	0	0	0	0	7	0	0	120	0	0	0	12	17	25	0	0	3	33	49	0	388	50	18	13	0	0	19	17	51	0
Ukraine	0	0	0	0	0	0	0	0	0	0	0	41	0	9	0	10	0	11	18	0	10	9	0	0	0	21	21	18	50	14	15	13	12	10	14	10	9	1	0	0
United Arab Emirates	0	0	0	0	0	0	0	0	0	0	0	0	0	0	0	0	0	0	0	0	0	0	0	0	0	0	0	0	0	0	0	0	0	0	0	0	0	0	0	0
United Kingdom	0	14	5	7	5	5	6	24	2	25	86	52	2	7	15	17	7	1	40	15	16	4	15	0	2	18	0	27	14	8	3	1	8	20	8	4	5	13	0	0
United Republic of Tanzania	0	0	0	0	0	0	0	0	0	10	189	0	0	54	35	3	0	83	61	0	37	18	11	0	10	3	0	0	73	29	9	37	10	0	31	59	33	7	15	0
United States Virgin Islands	0	0	0	0	0	0	0	0	0	0	0	0	0	0	0	7	0	0	0	3	0	0	0	0	0	0	1	0	0	0	1	0	0	0	0	0	0	7	0	0
United States of America	246	144	400	142	595	302	83	167	75	200	230	125	123	254	303	133	266	261	283	259	140	154	249	251	180	3923	185	240	231	125	177	828	235	216	235	287	252	243	220	105
Uruguay	0	0	0	0	0	0	0	0	0	0	0	0	0	0	0	0	0	1	1	1	0	0	2	0	0	1	0	2	0	1	0	0	0	1	0	2	11	0	0	0
Uzbekistan	0	0	0	0	0	0	0	0	0	0	0	50	10	0	0	0	0	0	0	24	0	0	0	0	0	5	0	0	0	0	1	13	3	3	2	2	4	0	0	0
Vanuatu	0	0	0	0	0	9	0	48	1	0	0	0	2	4	0	0	0	100	0	44	0	2	0	0	2	0	0	0	0	0	0	0	0	0	12	11	0	0	0	0
Venezuela (Bolivarian Republic of)	0	35	0	0	0	38	3	136	77	0	0	0	0	100	44	0	9	96	36	30342	26	14	23	36	50	44	35	12	17	9	67	29	22	12	0	0	0	0	2	0
Viet Nam	402	0	70	734	188	891	600	123	157	959	184	492	287	270	361	269	1011	3692	413	799	592	392	147	128	189	308	579	326	406	360	221	138	72	215	38	52	162	284	134	5
Yemen	0	0	1989	0	0	0	0	0	0	63	0	10	0	31	0	0	345	0	70	0	0	63	53	15	0	87	30	99	90	11	22	0	0	40	0	26	40	50	49	0
Zambia	0	0	0	0	0	0	0	0	0	0	0	0	0	0	0	0	0	0	0	0	0	5	0	4	2	0	0	7	11	31	9	0	0	0	0	0	0	0	0	4
Zimbabwe	0	0	0	0	0	0	0	0	0	0	0	0	0	0	0	0	0	0	36	0	70	13	0	10	0	0	0	15	12	0	0	0	0	125	8	16	31	251	0	660

SR Table 10e: Deaths due to other inuries by country, all ages, for both sexes combined, 1980-2019

Country	1980	1981	1982	1983	1984	1985	1986	1987	1988	1989	1990	1991	1992	1993	1994	1995	1996	1997	1998	1999	2000	2001	2002	2003	2004	2005	2006	2007	2008	2009	2010	2011	2012	2013	2014	2015	2016	2017	2018	2019
Afghanistan	0	0	0	0	0	0	0	0	0	61	246	150	0	76	0	0	60	54	45	0	40	349	95	13	79	210	26	40	14	39	170	76	96	73	0	35	108	15	66	0
Albania	0	0	0	0	0	68	0	0	0	0	0	60	0	0	0	0	0	0	0	0	0	0	0	0	18	0	0	0	38	0	14	0	18	0	0	0	0	6	0	0
Algeria	0	0	130	0	0	0	0	32	0	27	24	0	20	0	44	50	8	0	0	12	22	0	125	194	82	21	32	8	31	55	29	0	14	0	107	64	58	17	271	8
American Samoa	0	0	0	0	0	0	0	0	0	0	0	0	0	0	0	0	0	0	0	0	0	0	0	0	0	0	0	0	0	0	0	0	0	0	0	0	0	0	0	0
Andorra	0	0	0	0	0	0	0	0	0	0	0	0	0	0	0	0	0	0	0	0	0	0	0	0	0	0	0	0	0	0	0	0	0	0	0	0	0	0	0	0
Angola	0	0	0	126	0	0	0	0	26	0	70	58	27	0	300	367	0	0	14	28	217	206	50	25	90	20	18	5	51	32	0	17	16	16	26	0	0	0	17	0
Antigua and Barbuda	0	0	0	0	0	0	0	0	0	0	0	0	0	0	0	0	0	0	0	0	0	0	0	0	0	0	0	0	0	0	0	0	0	0	0	0	0	0	0	0
Argentina	0	350	0	0	0	0	0	0	0	0	2	0	1	0	0	8	0	0	0	103	82	89	8	0	8	35	2	34	3	0	57	82	102	71	0	0	0	76	0	0
Armenia	0	0	0	0	0	0	0	0	0	0	0	0	0	0	0	0	0	0	0	0	0	0	0	0	0	0	0	0	0	0	0	0	0	0	0	0	0	0	0	0
Australia	0	0	0	72	0	18	0	0	0	0	45	27	0	13	52	8	46	0	0	4	14	0	33	19	0	9	0	11	0	185	0	18	0	3	15	0	0	0	0	0
Austria	0	0	0	0	0	0	0	0	0	0	0	0	0	0	0	0	0	0	0	0	0	0	0	11	0	0	0	1	23	9	0	0	7	0	0	12	0	0	0	0
Azerbaijan	0	0	0	0	0	0	0	0	0	57	30	36	59	0	50	386	0	66	0	0	10	0	42	0	0	23	0	25	0	0	0	0	5	0	0	44	0	0	25	0
Bahamas	0	0	0	0	0	0	0	0	0	0	0	0	0	0	0	0	0	0	0	0	0	0	0	0	0	0	0	0	0	0	0	0	0	0	0	0	0	0	0	28
Bahrain	0	0	0	0	0	0	0	0	0	0	10	0	0	0	0	0	0	0	0	0	143	0	0	0	0	0	69	0	0	0	0	0	10	13	0	0	0	0	0	0
Bangladesh	0	0	45	15	250	500	2182	776	790	930	475	636	361	70	409	662	435	211	148	437	507	456	762	1673	560	715	280	195	53	282	330	125	444	1189	241	241	73	156	34	121
Barbados	0	0	0	0	0	0	0	0	0	0	0	0	0	0	0	0	0	0	0	0	0	0	0	0	0	0	0	0	0	0	0	0	0	0	0	0	0	0	0	0
Belarus	0	0	0	0	0	0	0	0	0	0	0	0	0	0	0	0	0	0	0	0	0	0	0	38	0	0	0	0	0	0	0	0	0	0	0	0	0	43	0	0
Belgium	0	0	12	0	0	0	0	189	0	2	0	0	0	13	22	10	0	3	0	0	0	0	0	41	10	2	65	0	0	0	12	0	6	5	0	15	0	0	0	0
Belize	0	0	0	0	0	0	0	0	0	0	0	0	0	0	0	0	0	0	0	0	0	0	0	0	0	0	0	0	0	0	0	0	0	0	0	0	0	0	0	0
Benin	0	0	0	0	0	0	0	0	0	0	0	0	0	0	0	0	0	0	0	0	51	26	15	139	0	0	54	10	5	50	0	15	17	22	0	52	0	0	0	0
Bermuda	0	0	0	0	0	0	0	0	0	0	0	0	0	0	0	0	0	0	0	0	5	0	0	0	0	0	0	0	0	0	0	0	0	0	0	0	0	0	0	0
Bhutan	0	0	0	0	0	0	0	0	0	0	0	0	0	0	0	0	0	0	0	0	0	0	0	0	0	0	0	0	0	0	0	0	0	0	0	0	0	0	0	0
Bolivia (Plurinational State of)	0	0	0	0	0	29	0	0	0	23	0	25	28	21	0	0	35	0	27	68	78	9	73	62	58	0	66	0	94	19	64	71	30	49	0	0	0	0	17	0
Bosnia and Herzegovina	0	0	0	0	0	0	0	0	30	0	169	0	0	0	0	0	0	12	0	0	44	0	0	0	0	0	0	0	11	1	2	0	4	0	0	0	0	0	0	0
Botswana	0	0	0	0	0	0	0	0	0	0	0	0	0	0	0	0	0	0	0	0	0	0	0	0	0	0	0	0	0	0	0	0	0	0	0	0	0	0	0	0
Brazil	0	364	85	63	44	106	26	133	136	124	94	267	0	35	140	198	296	0	68	106	185	160	139	205	331	158	279	124	79	179	71	120	141	402	61	75	14	88	84	669
Brunei Darussalam	0	0	0	0	0	0	0	0	0	0	0	0	0	0	0	0	0	0	0	0	0	0	0	0	0	0	0	0	0	0	0	0	0	0	0	0	0	0	0	0
Bulgaria	0	0	0	0	2	0	0	0	4	20	0	0	0	0	0	0	0	0	35	0	13	0	0	0	0	0	30	4	8	0	14	0	84	0	0	0	0	10	16	0
Burkina Faso	0	0	0	0	0	0	0	0	0	0	0	0	0	0	0	0	0	0	0	0	0	0	0	17	72	0	71	0	102	41	0	36	35	14	0	22	39	0	0	0
Burundi	0	0	0	0	0	0	0	0	0	100	0	0	0	0	0	0	0	0	0	0	0	12	41	160	0	48	0	12	0	44	0	0	0	0	0	0	40	0	0	0
Cabo Verde	0	0	0	0	0	0	0	0	0	0	0	0	0	0	0	0	0	0	0	18	0	0	0	0	0	0	0	57	0	0	0	0	0	0	0	15	0	0	0	0
Cambodia	0	0	0	0	0	0	0	0	0	0	0	0	0	0	31	0	24	64	0	0	0	0	0	0	0	13	0	22	0	27	358	0	0	0	0	0	0	0	0	0
Cameroon	0	0	0	0	0	0	0	0	60	0	0	0	0	0	0	374	0	0	120	29	51	0	0	119	0	54	182	183	67	36	64	74	0	0	0	106	75	54	0	0
Canada	176	123	30	0	0	0	7	0	0	62	0	13	0	18	0	0	0	0	0	0	0	0	0	0	0	0	0	0	6	0	0	0	0	22	7	0	0	0	86	0
Central African Republic	0	0	0	0	0	0	31	0	0	0	0	0	0	0	0	0	0	0	0	0	12	0	104	64	25	23	11	12	31	0	0	38	28	62	149	132	0	87	0	0
Chad	0	0	0	0	0	0	0	0	0	0	0	0	0	0	0	0	0	0	0	0	0	29	0	0	30	14	0	0	0	0	0	0	0	0	0	52	0	0	0	0
Chile	0	0	0	0	0	0	3	0	0	0	33	79	0	0	0	39	0	30	0	0	0	32	0	0	0	0	24	40	16	20	105	14	12	26	28	0	0	11	0	0
China	0	0	158	769	0	520	238	664	1000	731	496	522	485	1481	1546	493	680	1109	908	1152	1885	1365	1383	1638	1742	2021	966	1043	920	549	832	619	291	494	376	1426	374	148	175	363
Colombia	0	0	0	0	0	111	30	0	119	98	0	35	0	0	0	202	0	0	171	0	41	2	0	0	0	26	0	23	0	0	96	25	0	0	84	57	0	57	20	52
Comoros	0	0	0	0	0	0	0	0	0	0	0	0	0	0	0	0	199	0	41	0	0	0	16	0	0	25	41	75	0	182	0	120	36	35	0	0	0	0	0	0
Congo	0	0	0	0	0	0	0	0	0	0	23	110	21	367	142	0	0	0	0	0	11	45	0	0	42	12	0	15	0	0	54	0	318	0	0	0	0	0	26	18
Cook Islands	0	0	0	0	0	0	0	0	0	0	0	0	0	0	0	0	0	0	0	0	0	0	0	0	0	0	0	0	0	0	0	0	0	0	0	0	0	0	0	0
Costa Rica	0	0	0	0	0	0	0	0	0	0	2	0	0	0	0	0	0	0	0	0	46	0	0	0	0	0	0	0	0	0	0	0	0	0	0	0	0	12	0	19
Croatia	0	0	0	0	0	0	0	0	0	0	0	0	0	0	0	0	0	0	0	0	8	0	6	10	0	14	0	15	0	0	0	0	10	0	0	0	0	0	0	0
Cuba	0	0	0	0	0	0	0	0	1	1	0	0	1	0	8	0	0	106	32	12	0	0	8	0	0	14	0	8	0	12	27	0	0	0	0	0	0	0	112	0
Cyprus	0	0	0	0	0	0	0	0	0	0	0	0	0	0	0	0	0	0	52	0	5	0	0	0	0	31	0	4	0	0	0	13	0	0	0	0	0	0	49	71
Czechia	0	0	0	0	0	0	0	0	0	0	11	8	0	0	0	0	0	0	0	0	0	0	0	7	0	0	25	0	0	0	38	0	20	0	0	0	0	0	16	0
Côte d'Ivoire	0	0	0	0	0	0	0	51	0	48	0	0	0	23	16	0	0	21	37	53	209	19	0	0	49	0	8	39	0	0	0	94	36	0	18	71	0	0	0	0
Democratic People's Republic of Korea	0	0	0	0	0	0	0	120	0	0	0	110	0	223	0	0	0	0	0	0	0	0	0	0	161	14	0	110	0	0	10	0	0	0	0	0	0	200	36	0
Democratic Republic of the Congo	0	0	0	0	0	0	0	523	0	86	44	0	37	0	0	63	547	46	200	39	170	244	133	382	351	278	226	380	455	463	450	413	109	16	334	144	57	169	229	297
Denmark	0	0	0	0	0	0	0	0	0	0	0	0	0	0	0	0	0	0	0	0	0	0	0	0	0	0	0	0	0	0	0	0	0	0	0	0	0	0	0	0
Djibouti	0	0	0	0	0	0	0	0	0	0	0	0	0	0	0	0	0	0	0	0	0	0	1	0	0	0	132	0	0	13	0	0	0	0	0	0	0	0	0	101
Dominica	0	0	0	0	0	0	0	0	0	0	0	0	0	0	0	0	0	0	11	0	0	0	0	0	0	0	0	0	0	0	0	0	0	0	0	0	0	0	0	0
Dominican Republic	0	0	0	0	0	0	0	100	0	50	60	0	34	0	70	0	229	0	25	0	0	0	28	40	78	136	0	0	18	0	0	0	56	0	0	0	0	0	0	0
Ecuador	0	0	0	119	60	0	0	142	20	30	24	32	0	0	24	0	60	3	102	0	60	0	47	38	0	0	46	62	14	0	94	0	32	12	53	0	22	33	48	17
Egypt	0	0	100	196	98	16	50	107	100	10	19	504	46	78	21	156	147	85	45	222	163	147	589	119	288	136	1278	73	252	103	11	106	92	134	214	388	273	81	12	20
El Salvador	0	0	0	0	0	0	37	0	0	0	33	0	0	0	0	65	0	0	0	46	60	0	22	0	68	0	1	0	13	0	27	0	0	0	0	0	0	0	0	0
Equatorial Guinea	0	0	0	0	0	0	0	15	0	0	0	0	0	0	0	0	0	0	0	0	1	1	0	0	0	80	0	0	23	0	0	0	0	0	0	0	0	0	0	0
Eritrea	0	0	0	0	0	0	0	0	0	0	0	0	0	25	0	33	72	0	0	0	0	0	0	0	0	56	0	0	0	0	0	0	0	0	0	0	0	0	0	0
Estonia	0	0	0	0	0	0	0	0	0	0	0	0	0	0	290	0	0	0	0	0	0	2	0	0	0	0	0	0	0	0	0	2	16	0	0	0	0	0	7	0
Eswatini	0	0	0	0	0	0	0	0	0	0	0	0	0	0	0	0	0	0	0	0	0	30	0	0	22	0	0	2	0	0	0	0	0	0	0	12	0	0	0	0
Ethiopia	0	0	0	0	0	392	0	54	31	78	0	150	0	30	0	133	9	0	21	11	24	37	71	25	45	50	17	0	0	0	0	0	0	0	0	53	75	0	18	0
Fiji	0	0	0	0	0	0	0	0	0	0	0	0	0	0	0	0	0	0	0	17	0	0	0	0	0	0	0	0	0	0	0	0	0	0	0	0	0	0	0	0
Finland	0	0	0	0	0	0	0	0	0	0	0	0	0	0	0	0	0	0	23	0	0	0	0	0	0	0	0	0	0	0	0	0	0	0	0	0	0	0	0	0
France	0	0	0	48	0	166	52	28	52	125	31	226	106	19	0	0	0	9	27	0	157	65	83	1813	46	108	190	0	0	106	79	0	140	0	0	34	0	0	3	1513
Gabon	0	0	0	0	0	0	0	0	0	0	0	72	0	30	0	0	0	0	0	0	0	0	14	17	19	0	0	12	32	0	0	13	0	30	0	0	18	0	0	0
Gambia	0	0	0	0	0	0	0	0	0	0	0	0	100	0	0	0	0	0	0	0	0	0	9	0	0	0	0	32	10	0	0	0	0	0	0	0	0	0	0	0
Georgia	0	0	0	0	0	0	0	0	0	0	0	0	0	0	0	0	0	0	0	0	0	0	0	0	0	0	0	0	0	0	0	0	0	0	0	0	0	11	0	0
Germany	0	0	0	0	0	0	69	0	185	110	32	13	7	0	0	0	126	0	165	0	53	0	28	8	0	8	92	0	0	29	69	0	14	0	4	0	0	18	0	0
Ghana	0	0	0	0	67	4	0	0	0	0	60	84	0	30	0	0	0	0	0	60	13	163	124	0	24	58	162	48	25	38	52	34	10	17	0	167	61	22	0	76
Greece	0	2	0	15	0	15	0	1020	255	30	0	59	0	0	0	0	0	54	0	0	89	0	27	12	14	105	0	128	0	0	0	0	6	0	39	271	140	16	116	0
Greenland	0	0	0	0	0	0	0	0	0	0	0	0	0	0	0	0	0	0	0	0	0	0	0	0	0	0	0	0	0	0	0	0	0	0	0	0	0	0	0	0
Grenada	0	0	0	0	0	0	0	0	0	0	0	0	0	0	0	0	0	0	0	0	0	0	0	0	0	0	0	0	0	0	0	0	0	0	0	0	0	0	0	0
Guam	0	0	0	0	0	0	0	0	0	0	0	0	0	0	0	0	0	228	0	0	0	0	0	0	0	0	0	0	0	0	0	0	0	0	0	0	0	0	0	0
Guatemala	0	0	0	0	0	0	8	0	0	4	0	13	0	0	0	0	0	0	0	49	6	3	4	0	0	0	11	0	0	0	0	0	0	0	0	0	0	40	0	19
Guinea	0	0	0	0	0	0	0	0	0	17	0	0	356	0	0	100	0	0	0	20	70	116	35	0	33	10	43	116	27	13	19	56	110	25	83	22	15	0	18	17
Guinea-Bissau	0	0	0	0	0	0	0	0	0	0	0	0	0	0	0	14	0	0	203	0	0	0	0	0	0	12	0	29	0	73	21	0	29	0	21	11	0	0	29	0
Guyana	0	0	0	0	0	0	0	0	0	0	0	0	0	0	0	0	0	0	0	0	0	0	0	0	0	0	0	0	0	0	0	0	0	0	0	0	0	0	0	0
Haiti	0	0	0	0	0	0	201	0	0	0	0	0	103	1800	30	47	161	480	56	13	27	45	10	54	0	0	0	15	145	67	17	12	27	22	75	35	0	91	0	10
Honduras	0	0	0	0	0	21	50	0	0	141	33	0	0	0	0	0	0	0	0	0	22	0	0	86	115	0	0	0	23	0	0	14	379	0	14	0	0	0	0	27
Hungary	0	0	0	0	0	0	0	0	0	0	0	0	5	0	63	0	0	0	0	0	0	66	13	0	0	5	23	0	0	0	0	14	2	0	0	0	0	0	0	25
Iceland	0	0	0	0	0	0	0	0	0	0	0	0	0	0	0	0	0	0	0	0	0	0	0	0	0	0	0	0	0	0	0	0	0	0	0	0	0	0	0	0
India	179	578	416	150	7261	685	1007	797	1928	942	1832	961	1109	765	1227	1825	1256	1496	3903	1486	922	613	2821	2183	873	1702	703	643	767	367	1167	836	566	1221	576	2597	1024	845	371	278
Indonesia	0	431	12	104	25	0	192	324	246	342	242	314	278	238	69	39	443	903	157	883	684	634	236	243	352	418	781	408	62	542	80	407	188	92	308	452	96	147	458	13
Iran (Islamic Republic of)	218	50	0	0	0	0	103	0	290	0	21	26	66	158	198	3	0	123	58	189	109	254	379	502	607	356	181	67	174	234	71	140	72	83	39	519	91	80	182	15
Iraq	59	0	0	0	0	0	0	0	26	750	0	0	0	0	25	73	0	0	0	0	15	0	0	22	61	1225	86	46	16	11	61	0	0	0	0	0	88	20	0	100
Ireland	0	0	0	0	0	2	0	0	0	0	0	0	0	0	3	0	0	0	0	0	0	0	0	0	0	0	0	0	0	0	0	0	0	0	0	0	0	0	0	0
Israel	0	0	0	0	0	0	0	0	0	0	12	0	1	0	0	0	0	0	0	0	0	8	0	0	0	0	0	0	2	0	14	0	0	0	0	0	0	0	0	0
Italy	0	0	0	0	0	0	0	0	0	0	0	0	0	0	0	0	0	0	0	0	0	0	0	0	0	0	0	0	0	0	0	0	0	0	0	0	0	40	56	0
Jamaica	0	0	0	0	0	0	0	0	0	0	0	0	0	0	0	0	0	0	0	0	0	0	0	0	0	0	0	0	0	0	0	0	0	0	0	0	0	0	0	0
Japan	161	0	109	0	0	679	277	0	82	104	0	245	125	0	151	185	38	0	0	99	84	369	0	0	189	226	19	31	120	136	165	41	80	250	8	268	167	13	130	173
Jordan	0	0	0	0	0	0	0	0	0	0	0	0	15	0	32	0	0	0	0	0	14	52	0	22	12	0	10	0	21	0	0	0	0	17	0	0	14	0	0	0
Kazakhstan	0	0	0	0	0	0	0	0	0	0	0	0	0	0	0	3	0	0	2	0	0	0	0	0	130	0	0	0	2	0	0	0	0	0	0	0	0	0	52	13
Kenya	0	0	2	0	0	0	0	26	27	98	10	294	146	449	312	0	0	36	105	67	354	140	0	34	166	142	127	67	15	254	0	173	28	95	0	12	124	83	93	0
Kiribati	0	0	0	0	0	0	0	0	0	0	0	0	0	0	0	0	0	0	0	0	0	0	0	0	0	0	0	0	0	0	0	0	0	0	0	0	0	0	80	0
Kuwait	0	0	0	0	0	0	0	0	0	0	0	0	0	0	0	0	0	0	0	0	0	0	0	0	0	0	0	0	0	49	0	0	0	0	0	0	0	0	15	0
Kyrgyzstan	0	0	0	0	0	0	0	0	0	0	0	0	0	0	0	0	0	0	0	0	0	9	0	0	3	0	0	0	19	0	0	0	5	0	0	0	0	39	0	0
Lao People's Democratic Republic	0	0	0	0	0	0	0	0	0	0	0	0	0	0	0	0	0	0	40	0	28	0	0	0	0	0	0	0	0	0	0	0	0	49	0	23	0	0	0	0
Latvia	0	0	0	0	0	0	0	0	0	0	0	0	0	0	0	0	0	0	0	0	0	32	0	0	0	0	93	0	0	0	0	0	21	51	0	0	0	0	0	0
Lebanon	0	0	0	0	0	0	0	0	0	0	45	0	0	0	0	0	0	0	0	0	10	0	0	38	0	0	0	1	0	44	90	0	0	0	0	0	0	0	0	0
Lesotho	0	0	0	0	0	0	0	0	0	0	0	0	0	0	0	0	0	0	0	0	0	0	0	0	0	0	0	40	0	0	0	0	0	0	0	0	0	0	0	0
Liberia	0	0	0	0	0	0	0	0	0	0	0	0	0	0	0	0	0	0	0	0	0	0	60	0	0	0	0	0	0	0	11	0	0	10	0	0	0	0	0	28
Libya	0	0	0	0	0	0	0	0	30	78	0	0	182	0	0	0	70	0	0	0	23	0	81	0	27	0	0	0	140	221	104	100	55	25	389	1788	1363	450	425	207
Lithuania	0	0	0	0	0	0	0	0	0	0	0	0	0	0	0	0	0	0	6	29	0	71	0	0	0	0	0	0	0	0	76	0	42	0	0	0	0	0	5	0
Luxembourg	0	0	0	0	0	0	0	0	0	0	0	0	0	0	0	0	0	0	0	0	0	0	3	0	0	0	0	0	0	0	0	0	0	0	0	0	0	0	0	0
Madagascar	0	0	0	0	53	0	0	0	0	0	0	36	0	0	0	34	0	0	0	0	0	47	0	45	14	0	0	0	0	0	0	11	0	14	0	0	41	81	0	0
Malawi	0	0	0	0	0	0	0	0	0	12	0	0	0	0	0	0	0	0	17	0	51	15	33	0	27	49	0	26	24	22	0	0	47	0	0	0	0	18	0	0
Malaysia	3	0	0	0	200	0	0	0	30	48	0	176	0	77	72	32	11	39	38	0	68	14	0	0	0	0	0	0	0	0	26	0	0	100	297	61	46	75	0	0
Maldives	0	0	0	0	0	0	0	0	0	0	0	0	0	0	0	0	0	0	0	10	0	0	0	0	31	0	0	0	0	0	0	0	0	0	0	0	0	0	0	0
Mali	0	0	0	0	0	51	0	0	0	0	0	52	27	0	0	0	0	0	0	0	72	0	23	0	57	13	72	0	0	0	43	54	0	85	116	322	0	0	46	13
Malta	0	0	0	0	0	0	0	0	0	0	0	0	0	0	0	0	0	0	0	0	0	0	0	0	0	0	0	0	0	0	0	0	0	1	0	0	0	0	0	5
Marshall Islands	0	0	0	0	0	0	0	0	0	0	0	0	0	0	0	0	0	0	0	0	0	0	0	0	0	0	0	0	0	0	0	0	0	0	0	0	0	0	0	0
Mauritania	0	0	0	0	0	0	0	0	0	0	23	0	0	0	80	0	0	0	39	0	13	11	0	0	0	43	19	56	0	0	13	22	10	10	0	14	0	0	11	0
Mauritius	0	0	0	0	0	0	0	0	0	0	0	0	0	0	0	0	0	0	0	0	0	0	0	0	0	0	0	0	0	0	0	0	0	0	0	0	0	0	0	0
Mexico	0	2	75	148	328	7	198	298	180	251	66	149	35	0	72	33	102	2	55	185	84	26	52	17	14	49	85	26	38	90	75	82	149	92	0	53	57	78	39	273
Micronesia (Federated States of)	0	0	0	0	0	0	0	0	0	0	0	0	0	0	0	0	0	0	0	0	0	0	0	0	0	0	0	0	0	0	0	0	0	0	0	0	0	0	0	0
Monaco	0	0	0	0	0	0	0	0	0	0	0	0	0	0	0	0	0	0	0	0	0	0	0	0	0	0	0	0	0	0	0	0	0	0	0	0	0	0	0	0
Mongolia	0	0	0	0	0	0	0	0	0	0	51	0	0	40	0	41	25	0	28	0	0	10	0	0	0	0	0	14	0	1	4	0	0	0	0	0	0	0	0	0
Montenegro	0	0	0	0	0	0	0	0	0	0	0	0	0	0	0	1	1	0	4	2	0	0	1	0	1	0	3	0	0	0	0	0	1	13	0	0	0	0	0	0

Morocco	0	0	0	0	0	0	0	0	0	0	0	44	30	15	81	17	25	51	101	136	91	11	128	30	88	66	136	65	109	0	46	106	80	39	23	76	0	37	11	0
Mozambique	0	0	0	0	0	58	77	0	0	97	0	109	0	102	0	0	40	0	0	0	32	10	215	0	11	0	16	146	49	31	84	51	0	0	0	69	93	21	23	0
Myanmar	0	13	290	0	0	0	26	447	135	87	200	75	0	137	127	1	0	35	58	0	0	16	0	0	30	0	0	51	38	50	36	15	0	198	0	191	106	217	0	0
Namibia	0	0	0	0	0	0	0	0	0	0	0	20	0	0	0	0	0	0	0	0	0	0	19	0	0	0	0	0	0	0	0	0	0	33	0	0	0	0	0	0
Nauru	0	0	0	0	0	0	0	0	0	0	0	0	0	0	0	0	0	0	0	0	0	0	0	0	0	0	0	0	0	0	0	0	0	0	0	0	0	0	0	0
Nepal	0	0	0	0	100	0	0	26	72	59	25	61	372	0	0	65	0	0	43	89	67	10	163	136	129	259	121	61	76	85	68	169	55	64	108	30	80	87	113	0
Netherlands	0	0	0	0	0	0	0	0	0	0	0	9	55	0	0	0	64	0	0	0	0	8	0	2	0	14	0	0	0	0	0	0	1	0	0	0	0	0	24	0
New Zealand	0	0	0	0	0	0	0	0	0	0	0	0	0	0	0	0	0	0	0	0	0	0	0	0	0	0	0	0	0	0	0	0	0	0	0	0	0	0	0	0
Nicaragua	0	0	0	101	0	0	0	0	0	0	0	0	0	0	0	0	0	0	0	44	0	0	0	0	0	0	44	0	0	0	0	0	0	0	37	0	13	0	0	0
Niger	0	0	1	0	0	0	0	0	0	170	0	0	0	0	0	0	0	13	30	0	0	47	0	0	14	0	20	0	34	0	0	0	0	12	12	78	0	26	0	118
Nigeria	0	0	0	148	100	0	177	70	115	310	209	138	501	170	60	46	374	280	1392	359	1555	778	1935	884	360	777	900	523	226	242	333	155	386	402	216	359	323	111	112	39
Niue	0	0	0	0	0	0	0	0	0	0	0	0	0	0	0	0	0	0	0	0	0	0	0	0	0	0	0	0	0	0	0	0	0	0	0	0	0	0	0	0
North Macedonia	0	0	0	0	0	0	0	5	0	0	0	0	0	198	0	0	0	0	0	0	0	25	0	0	15	0	0	1	0	15	0	0	1	0	0	14	0	3	0	13
Northern Mariana Islands	0	0	0	0	0	0	0	0	0	0	0	0	0	0	0	0	0	0	0	0	0	0	0	0	0	0	0	0	0	0	0	0	0	0	0	0	0	0	0	0
Norway	0	0	0	0	0	0	0	0	0	0	0	0	0	0	0	0	0	0	0	0	0	0	0	0	0	0	0	0	0	0	0	0	0	0	0	0	0	0	0	0
Oman	0	0	0	0	0	0	0	0	0	0	0	0	0	0	0	0	0	0	0	0	0	0	46	0	21	0	0	12	0	0	0	0	0	0	0	0	18	0	0	0
Pakistan	0	10	0	0	60	0	106	78	204	285	529	901	334	249	88	143	230	407	283	381	240	49	215	370	243	612	213	77	53	142	280	135	454	67	432	1419	300	248	260	96
Palau	0	0	0	0	0	0	0	0	0	0	0	0	0	0	0	0	1	0	0	0	0	0	0	0	0	0	0	0	0	0	0	0	0	0	0	0	0	0	0	0
Palestine	0	0	0	0	0	0	0	0	0	0	0	0	0	0	0	0	0	0	0	14	0	0	0	0	0	0	0	5	0	0	0	0	0	0	0	0	0	0	0	0
Panama	0	0	1	0	0	0	0	0	0	0	0	0	0	0	0	0	0	0	0	0	8	0	0	0	0	0	0	4	4	0	0	0	0	0	0	0	0	18	0	0
Papua New Guinea	0	0	0	0	0	0	0	0	0	0	0	0	0	0	39	15	19	0	0	21	0	0	0	0	0	0	0	0	0	13	0	28	246	0	0	0	12	0	0	0
Paraguay	0	0	0	0	0	0	0	0	0	0	0	0	0	0	0	0	23	0	0	0	45	0	0	0	390	0	0	8	0	0	12	0	0	0	0	0	0	0	0	0
Peru	0	0	0	17	21	20	56	176	108	402	261	236	95	30	99	23	291	90	154	82	259	309	187	457	231	143	177	117	103	496	511	161	358	165	594	135	73	0	111	17
Philippines	177	32	57	247	239	174	249	4316	941	216	142	70	0	372	410	188	203	32	225	84	343	108	207	138	180	77	97	66	914	99	92	53	42	159	72	133	2	98	10	51
Poland	0	0	0	0	0	0	0	0	0	0	0	0	0	0	0	0	0	0	0	0	0	0	0	0	0	0	0	0	0	0	0	0	0	0	0	0	0	0	9	0
Portugal	0	0	0	0	0	54	38	0	0	177	0	0	40	0	0	0	0	0	0	42	0	36	0	88	38	0	0	43	0	0	0	0	0	6	0	0	13	107	11	0
Puerto Rico	0	0	0	0	0	0	7	0	0	0	0	0	0	0	0	0	0	0	0	0	0	0	0	0	8	0	0	0	0	0	0	0	0	0	0	0	0	0	0	0
Qatar	0	0	0	0	0	0	0	0	0	0	0	0	0	0	0	0	0	0	0	0	0	0	0	0	0	0	0	0	0	30	0	0	19	0	12	0	11	0	0	0
Republic of Korea	0	65	0	0	38	13	25	140	40	31	60	182	0	250	87	594	41	14	27	77	31	0	160	433	0	24	15	14	40	17	56	22	5	0	349	5	6	44	37	1
Republic of Moldova	0	0	0	0	0	0	0	0	0	0	0	0	0	0	0	0	0	0	0	0	0	0	0	0	0	0	28	0	0	0	0	0	41	0	0	0	0	0	0	0
Romania	0	0	0	0	0	0	0	0	0	423	0	0	0	0	0	21	48	25	56	0	0	0	54	0	38	47	0	0	0	22	58	0	142	0	28	37	0	0	2	0
Russian Federation	0	0	0	0	0	0	0	0	43	0	4	0	4	0	54	97	4	36	33	0	13	18	48	11	10	48	11	0	2	10	4302	11	181	39	81	126	66	72	225	39
Rwanda	0	0	0	0	0	0	0	0	0	0	0	0	0	0	0	70	0	0	22	68	39	0	0	13	0	0	0	0	12	0	11	27	0	0	0	0	0	0	0	14
Saint Kitts and Nevis	0	0	0	0	0	0	0	0	0	0	0	0	0	0	0	0	0	0	0	0	0	0	0	0	0	0	0	0	0	0	0	0	0	0	0	0	0	0	0	0
Saint Lucia	0	0	0	0	0	0	0	0	0	0	0	0	0	0	0	0	0	0	0	0	0	0	0	0	0	0	0	0	0	0	0	0	0	0	0	0	0	0	0	0
Saint Vincent and the Grenadines	0	0	0	0	0	0	0	0	0	0	0	0	0	0	0	0	0	0	0	0	0	0	0	0	0	0	0	0	0	0	0	0	0	0	0	0	0	0	0	0
Samoa	0	0	0	0	0	0	0	0	0	0	0	0	0	0	0	0	0	0	0	0	0	0	0	0	0	0	0	0	0	0	0	0	0	0	0	0	0	0	0	0
San Marino	0	0	0	0	0	0	0	0	0	0	0	0	0	0	0	0	0	0	0	0	0	0	0	0	0	0	0	0	0	0	0	0	0	0	0	0	0	0	0	0
Sao Tome and Principe	0	0	0	0	0	0	0	0	0	0	0	0	0	0	0	0	0	0	0	0	0	0	0	0	0	0	0	13	20	0	0	0	0	0	0	0	0	0	0	0
Saudi Arabia	301	45	0	0	0	0	67	0	0	0	1426	359	0	0	182	0	61	343	118	61	48	35	98	134	251	0	442	73	28	0	0	13	48	0	15	2442	12	21	0	0
Senegal	0	0	0	0	0	0	0	0	0	0	0	0	131	0	0	0	0	20	18	0	13	18	1225	30	60	0	126	40	33	0	0	15	33	41	0	75	0	105	0	0
Serbia	0	0	0	0	31	0	0	22	71	92	0	0	0	0	0	9	11	0	53	22	3	0	9	0	11	0	46	0	0	0	5	0	25	0	0	0	0	6	0	0
Seychelles	0	0	0	0	0	0	0	0	0	0	0	0	0	0	0	0	0	0	0	0	0	0	0	0	0	0	0	0	0	0	0	0	0	0	0	0	0	0	0	0
Sierra Leone	0	0	0	50	0	0	0	62	0	0	0	0	100	0	0	50	280	70	70	220	150	0	0	0	24	0	11	190	0	154	0	20	0	18	11	0	0	0	11	0
Singapore	0	0	0	0	0	0	0	0	0	0	0	0	0	0	0	0	0	0	0	0	0	0	0	0	0	0	0	0	0	0	0	0	0	0	0	0	0	10	0	0
Slovakia	0	0	0	0	0	0	0	0	0	0	0	0	0	0	0	11	0	0	0	0	6	0	0	11	0	0	0	12	0	32	61	61	5	0	0	0	0	0	0	0
Slovenia	0	0	0	0	0	0	0	0	0	0	0	0	0	0	0	0	0	0	0	0	0	0	0	5	0	0	0	0	2	0	0	0	0	0	5	0	0	0	0	0
Solomon Islands	0	0	0	0	0	0	0	0	0	0	0	33	0	0	0	0	0	0	0	0	0	0	0	0	0	0	0	0	0	0	0	0	0	0	0	0	0	0	0	0
Somalia	0	0	0	0	0	0	0	0	0	30	0	130	50	0	0	0	0	0	0	12	0	130	101	0	118	200	12	22	0	15	0	0	55	0	0	0	106	0	0	0
South Africa	0	0	0	73	0	0	245	243	80	181	47	45	75	101	292	134	135	66	163	253	143	187	138	142	58	110	128	198	163	61	99	110	87	138	10	113	11	28	46	18
South Sudan	0	0	0	0	0	0	0	0	0	0	0	0	0	0	0	0	0	0	0	0	0	0	0	0	0	0	0	0	0	0	0	0	0	0	56	244	0	0	20	50
Spain	76	0	0	326	53	142	0	8	0	0	11	5	0	0	49	73	0	32	57	171	167	134	176	390	271	209	240	166	265	39	62	55	24	89	0	1	0	4	9	325
Sri Lanka	0	0	0	0	0	0	61	24	0	52	25	20	0	60	0	120	20	165	55	0	40	13	23	0	0	33	0	22	0	0	25	0	0	0	0	0	0	40	0	0
Sudan	0	0	0	0	0	0	60	95	21	100	190	0	0	0	22	30	186	62	159	49	50	50	141	241	72	88	20	0	126	11	85	239	101	234	61	46	16	0	23	0
Suriname	0	0	0	0	0	0	0	0	0	169	0	0	0	0	0	0	0	0	0	0	0	10	0	0	0	0	0	0	20	0	0	0	0	0	0	0	0	0	0	0
Sweden	0	0	0	0	0	0	0	23	0	0	0	0	0	0	510	0	0	0	57	0	0	0	0	0	0	0	0	0	0	0	0	0	0	0	0	0	0	0	0	0
Switzerland	0	0	0	0	0	4	0	0	0	0	0	0	0	0	0	0	0	0	0	0	18	36	0	0	0	0	0	0	0	0	0	0	12	0	0	0	0	0	20	0
Syrian Arab Republic	0	0	0	0	0	0	0	0	0	0	0	0	0	0	46	0	0	0	37	62	10	27	33	0	23	62	22	46	0	22	0	25	0	0	0	57	0	0	0	11
Taiwan (Province of China)	0	0	0	0	0	0	0	0	0	0	0	0	0	0	0	0	0	0	0	0	0	0	0	0	0	0	0	0	0	0	0	0	0	0	0	0	0	32	18	0
Tajikistan	0	0	0	0	0	0	0	32	0	0	0	0	0	112	0	0	0	0	21	0	0	0	0	0	0	21	18	0	0	0	25	0	1	0	0	0	0	0	0	0
Thailand	38	0	7	30	0	0	0	232	176	134	251	500	154	356	223	48	12	161	101	35	106	60	25	0	53	27	0	160	32	83	0	12	12	120	148	13	107	0	93	0
Timor-Leste	0	0	0	0	0	0	0	0	0	0	0	0	0	0	0	0	0	0	0	0	0	0	0	0	0	0	0	0	0	0	0	0	0	0	0	0	0	0	0	0
Togo	0	0	0	0	0	0	0	0	0	0	0	0	0	0	0	0	0	0	0	0	0	0	0	0	0	0	0	30	0	19	14	36	0	0	76	0	0	0	11	0
Tokelau	0	0	0	0	0	0	0	0	0	0	0	0	0	0	0	0	0	0	0	0	0	0	0	0	0	0	0	0	0	0	0	0	0	0	0	0	0	0	0	0
Tonga	0	0	0	0	0	0	0	0	0	0	0	0	0	0	0	0	0	0	0	0	0	0	0	0	0	0	0	0	0	75	0	0	0	0	0	0	0	0	0	0
Trinidad and Tobago	0	0	0	0	0	0	0	0	2	0	0	0	0	0	0	0	0	0	0	0	0	0	0	0	0	0	0	0	0	0	0	0	0	0	0	0	0	0	0	0
Tunisia	0	0	0	0	0	0	0	0	8	0	20	0	0	0	21	0	0	0	0	0	13	0	27	238	93	0	17	0	20	93	0	150	0	0	11	33	0	46	87	0
Turkey	97	26	0	140	0	59	48	30	28	29	119	124	378	131	214	62	46	111	91	39	101	134	88	332	265	31	124	177	88	19	82	45	170	28	410	106	177	75	62	10
Turkmenistan	0	0	0	0	0	0	0	0	0	0	0	0	0	0	0	0	0	0	0	0	0	0	0	0	0	0	0	0	0	0	0	3	0	0	0	0	0	0	0	0
Tuvalu	0	0	0	0	0	0	0	0	0	0	0	0	0	0	0	0	0	0	0	0	18	0	0	0	0	0	0	0	0	0	0	0	0	0	0	0	0	0	0	0
Uganda	0	0	0	0	0	0	0	171	0	0	23	0	74	30	84	0	50	0	0	50	180	43	185	116	151	44	80	67	49	11	65	19	29	31	308	31	50	14	168	0
Ukraine	0	0	0	0	0	80	102	0	0	11	0	94	57	0	63	0	30	16	91	49	80	94	136	17	66	13	983	147	92	45	61	54	209	0	12	66	64	37	10	0
United Arab Emirates	0	0	0	112	0	0	0	0	0	0	0	0	0	0	0	0	0	85	0	12	0	14	29	0	43	0	0	0	11	0	11	0	0	22	15	10	0	0	0	0
United Kingdom	18	0	0	0	80	160	105	48	216	30	0	25	35	0	176	33	0	74	32	41	0	0	29	18	13	0	0	0	0	95	0	0	0	13	0	12	0	80	285	0
United Republic of Tanzania	0	0	0	20	33	0	0	0	0	20	0	113	24	0	48	0	902	134	95	145	61	68	486	89	11	16	185	101	31	119	66	282	144	84	0	95	0	45	289	100
United States Virgin Islands	0	0	0	0	0	0	0	0	0	0	0	0	0	0	0	0	0	0	0	0	0	0	0	0	0	0	0	0	0	0	0	0	0	0	0	0	0	0	0	0
United States of America	817	0	0	635	0	172	369	458	186	447	445	345	106	649	359	590	602	81	81	627	724	897	555	526	227	332	566	754	369	297	159	257	7	564	389	599	721	62	141	46
Uruguay	0	0	0	0	0	0	0	0	0	0	0	0	0	0	0	0	0	80	0	0	0	0	0	0	0	0	0	0	0	0	14	0	0	0	0	6	0	0	0	0
Uzbekistan	0	0	0	0	0	0	0	0	0	0	0	0	0	0	0	0	0	0	0	0	0	0	0	0	27	0	0	0	0	0	0	0	0	0	0	0	0	0	0	0
Vanuatu	0	0	0	0	0	0	0	0	0	0	0	0	0	0	0	0	0	0	0	0	0	0	0	0	0	0	0	0	0	0	0	0	0	0	0	0	0	0	0	0
Venezuela (Bolivarian Republic of)	0	25	37	0	0	0	0	7	0	0	0	0	0	8	0	0	0	0	0	17	0	12	0	9	13	14	0	0	32	15	18	6	4	0	0	0	0	0	17	0
Viet Nam	0	177	0	0	0	0	0	0	50	222	0	328	58	109	0	44	30	87	75	94	57	70	60	63	50	94	19	95	25	54	64	63	35	34	16	18	13	0	13	0
Yemen	0	0	0	0	0	0	0	0	0	0	0	0	147	0	0	131	38	0	180	10	14	35	0	138	41	0	10	194	43	147	61	279	15	36	173	35	60	21	62	0
Zambia	0	0	0	0	0	0	0	400	0	0	29	0	0	0	0	0	116	0	0	18	0	113	0	69	0	111	0	15	17	0	0	89	45	53	26	34	24	0	20	20
Zimbabwe	0	0	60	31	0	0	0	0	0	111	0	87	0	0	0	35	0	0	40	15	28	0	74	88	21	32	51	46	14	62	18	74	15	21	11	24	30	64	89	65

SR Table 10f: Deaths due to executions and police conflict by country, all ages, for both sexes combined, 1980-2019

Country	1980	1981	1982	1983	1984	1985	1986	1987	1988	1989	1990	1991	1992	1993	1994	1995	1996	1997	1998	1999	2000	2001	2002	2003	2004	2005	2006	2007	2008	2009	2010	2011	2012	2013	2014	2015	2016	2017	2018	2019
Afghanistan	0	0	0	0	0	0	0	0	0	0	0	0	0	0	0	0	0	0	0	0	0	0	0	0	0	0	0	15	17	0	0	2	14	2	0	1	6	426	134	88
Albania	0	0	0	0	0	0	0	0	0	0	0	0	0	0	0	0	0	0	0	0	0	0	0	0	0	0	0	0	0	0	0	0	0	0	0	0	0	0	1	0
Algeria	0	0	0	0	0	0	0	0	0	0	0	0	0	0	0	0	0	411	0	0	0	77	7	41	39	0	0	23	2	0	0	30	5	38	18	1	3	11	8	2
American Samoa	0	0	0	0	0	0	0	0	0	0	0	0	0	0	0	0	0	0	0	0	0	0	0	0	0	0	0	0	0	0	0	0	0	0	0	0	0	0	0	0
Andorra	0	0	0	0	0	0	0	0	0	0	0	0	0	0	0	0	0	0	0	0	0	0	0	0	0	0	0	0	0	0	0	0	0	0	0	0	0	0	0	0
Angola	0	0	0	0	0	0	0	0	0	0	0	0	0	0	0	0	0	0	0	0	0	22	0	0	19	0	0	2	0	2	0	0	2	5	0	0	7	11	24	2
Antigua and Barbuda	0	0	0	0	0	0	0	0	0	0	0	0	0	0	0	0	0	0	0	0	0	0	0	0	0	0	0	0	0	0	0	0	0	0	0	0	0	0	0	0
Argentina	200	100	50	50	0	0	0	0	0	0	0	0	0	0	0	0	0	0	0	0	0	0	0	0	0	0	0	0	0	0	0	0	0	0	0	0	0	0	0	0
Armenia	0	0	0	0	0	0	0	0	0	0	0	0	0	0	0	0	0	0	0	0	0	0	0	0	0	0	0	0	0	0	0	0	0	0	0	0	0	0	0	0
Australia	0	0	0	0	0	0	0	0	0	0	0	0	0	0	0	0	0	0	0	0	0	0	0	0	0	0	0	0	0	0	0	0	0	0	0	0	0	0	0	0
Austria	0	0	0	0	0	0	0	0	0	0	0	0	0	0	0	0	0	0	0	0	0	0	0	0	0	0	0	0	0	0	0	0	0	0	0	0	0	0	0	0
Azerbaijan	0	0	0	0	0	0	0	0	0	0	0	0	0	0	0	0	0	0	0	0	0	0	0	0	0	0	0	0	0	0	0	0	0	0	0	0	0	0	0	0
Bahamas	0	0	0	0	0	0	0	0	0	0	0	0	0	0	0	0	0	0	0	0	0	0	0	0	0	0	0	0	0	0	0	0	0	0	0	0	0	0	0	0
Bahrain	0	0	0	0	0	0	0	0	0	0	0	0	0	0	0	0	0	0	0	0	0	0	0	0	0	0	0	0	1	0	1	26	0	0	0	0	1	5	0	0
Bangladesh	71	71	71	72	72	72	72	72	72	0	0	0	0	0	0	0	0	0	0	0	0	0	0	0	0	0	0	6	5	3	99	59	46	135	38	63	51	21	23	19
Barbados	0	0	0	0	0	0	0	0	0	0	0	0	0	0	0	0	0	0	0	0	0	0	0	0	0	0	0	0	0	0	0	0	0	0	0	0	0	0	0	0
Belarus	0	0	0	0	0	0	0	0	0	0	0	0	0	0	0	0	0	0	0	0	0	0	0	0	0	0	0	0	1	0	0	3	0	0	0	0	0	2	4	0
Belgium	0	0	0	0	0	0	0	0	0	0	0	0	0	0	0	0	0	0	0	0	0	0	0	0	0	0	0	0	0	0	0	0	0	0	0	0	0	0	0	0
Belize	0	0	0	0	0	0	0	0	0	0	0	0	0	0	0	0	0	0	0	0	0	0	0	0	0	0	0	0	0	0	0	0	0	0	0	0	0	0	0	0
Benin	0	0	0	0	0	0	0	0	0	0	0	0	0	0	0	0	0	0	1	0	1	0	0	0	2	0	0	2	0	0	0	1	0	0	0	0	9	2	0	0
Bermuda	0	0	0	0	0	0	0	0	0	0	0	0	0	0	0	0	0	0	0	0	0	0	0	0	0	0	0	0	0	0	0	0	0	0	0	0	0	0	0	0
Bhutan	0	0	0	0	0	0	0	0	0	0	0	0	0	0	0	0	0	0	0	0	0	0	0	0	0	0	0	0	0	0	0	0	0	0	0	0	0	0	0	0
Bolivia (Plurinational State of)	0	0	0	0	0	0	0	0	0	0	0	0	0	0	0	0	0	0	0	0	0	0	0	0	0	0	0	0	0	0	0	0	0	0	0	0	1	0	0	8
Bosnia and Herzegovina	0	0	0	0	0	0	0	0	0	0	0	0	0	0	0	0	0	0	0	0	0	0	0	0	0	0	0	0	0	0	0	0	0	0	0	0	0	0	0	0
Botswana	0	0	0	0	0	0	0	0	0	0	0	0	0	0	0	0	0	0	0	0	0	0	0	0	0	0	0	1	1	1	1	0	2	1	0	0	1	0	2	0
Brazil	0	0	0	0	0	0	0	0	0	0	9	0	104	0	2	2	7	0	0	0	0	0	0	0	0	0	0	0	15	0	0	0	0	818	1172	1244	1582	0	0	0
Brunei Darussalam	0	0	0	0	0	0	0	0	0	0	0	0	0	0	0	0	0	0	0	0	0	0	0	0	0	0	0	0	0	0	0	0	0	0	0	0	0	0	0	0
Bulgaria	0	0	0	0	0	0	0	0	0	0	0	0	0	0	0	0	0	0	0	0	0	0	0	0	0	0	0	0	0	0	0	0	0	0	0	0	0	0	0	0
Burkina Faso	0	0	0	0	0	0	0	99	0	0	0	0	0	0	0	0	0	0	4	3	1	0	105	1	3	0	2	0	0	0	1	8	4	5	29	14	43	19	86	233
Burundi	0	0	0	0	0	0	0	0	0	0	0	0	0	0	0	0	0	913	0	0	0	25	1	1	0	0	0	1	0	2	0	4	1	0	3	131	53	41	37	16
Cabo Verde	0	0	0	0	0	0	0	0	0	0	0	0	0	0	0	0	0	0	0	0	0	0	0	0	0	0	0	0	0	0	0	0	0	0	0	0	0	0	0	0
Cambodia	0	0	0	0	0	0	0	0	0	0	0	0	0	0	0	0	0	0	0	0	0	0	0	0	0	0	0	0	0	0	9	4	5	5	5	6	1	0	8	0
Cameroon	0	0	0	0	0	0	0	0	0	0	0	0	0	0	0	0	0	0	1	0	0	3	4	4	0	2	0	5	105	0	0	0	2	2	33	55	17	94	136	88
Canada	0	0	0	0	0	0	0	0	0	0	0	0	0	0	0	0	0	0	0	0	0	0	0	0	0	0	0	0	0	0	0	0	0	0	0	0	0	0	0	0
Central African Republic	0	0	0	0	0	0	0	0	0	0	0	0	0	0	0	0	0	0	13	0	1	2	2	57	0	1	32	9	1	0	5	14	7	182	168	47	11	76	18	4
Chad	0	0	0	0	0	0	0	0	0	0	0	0	0	0	0	0	0	1	0	0	0	1	0	0	2	31	0	1	0	0	0	7	0	8	2	24	6	3	0	3
Chile	0	0	0	0	0	0	0	0	0	0	0	0	0	0	0	0	0	0	0	0	0	0	0	0	0	0	0	0	0	0	0	0	0	0	0	0	0	0	0	11
China	0	0	0	0	0	0	0	0	0	2595	0	0	0	0	0	0	0	0	0	0	0	0	0	0	0	0	0	0	0	0	0	0	1	1	0	0	1	6	0	2
Colombia	0	0	0	0	0	0	0	0	0	0	0	0	0	0	0	0	0	0	0	0	0	0	0	0	0	0	0	0	0	0	0	0	0	0	0	0	0	0	0	3
Comoros	0	0	0	0	0	0	0	0	0	0	0	0	0	0	0	0	0	0	0	0	0	0	0	0	0	0	0	0	0	0	0	0	0	0	0	0	0	0	0	0
Congo	0	0	0	0	0	0	0	0	0	0	0	0	0	0	0	0	0	0	0	7	15	0	0	10	1	1	0	0	0	0	0	0	2	0	0	4	3	3	26	0
Cook Islands	0	0	0	0	0	0	0	0	0	0	0	0	0	0	0	0	0	0	0	0	0	0	0	0	0	0	0	0	0	0	0	0	0	0	0	0	0	0	0	0
Costa Rica	0	0	0	0	0	0	0	0	0	0	0	0	0	0	0	0	0	0	0	0	0	0	0	0	0	0	0	0	0	0	0	0	0	0	0	0	0	0	0	0
Croatia	0	0	0	0	0	0	0	0	0	0	0	0	0	0	0	0	0	0	0	0	0	0	0	0	0	0	0	0	0	0	0	0	0	0	0	0	0	0	0	0
Cuba	0	0	0	0	0	0	0	0	0	0	0	0	0	0	0	0	0	0	0	0	0	0	0	0	0	0	0	0	0	0	0	0	0	0	0	0	0	0	0	0
Cyprus	0	0	0	0	0	0	0	0	0	0	0	0	0	0	0	0	0	0	0	0	0	0	0	0	0	0	0	0	0	0	0	0	0	0	0	0	0	0	0	0
Czechia	0	0	0	0	0	0	0	0	0	0	0	0	0	0	0	0	0	0	0	0	0	0	0	0	0	0	0	0	0	0	0	0	0	0	0	0	0	0	0	0
Côte d'Ivoire	0	0	0	0	0	0	0	0	0	0	0	0	0	0	0	0	0	2	1	2	71	0	0	5	16	20	0	0	4	0	66	54	21	23	4	9	42	19	3	17
Democratic People's Republic of Korea	0	0	0	0	0	0	0	0	0	0	0	0	0	0	0	0	0	0	0	0	0	0	0	0	0	0	0	0	15	0	60	30	6	0	0	0	0	0	0	0
Democratic Republic of the Congo	0	0	0	0	0	0	0	0	0	0	0	0	0	0	0	0	0	530	100	17	0	33	25	2	51	34	0	32	1	76	22	43	17	39	44	77	219	380	281	62
Denmark	0	0	0	0	0	0	0	0	0	0	0	0	0	0	0	0	0	0	0	0	0	0	0	0	0	0	0	0	0	0	0	0	0	0	0	0	0	0	0	0
Djibouti	0	0	0	0	0	0	0	0	0	0	0	0	0	0	0	0	0	0	0	1	0	0	0	0	0	0	0	0	0	0	0	3	0	6	3	0	0	0	0	3
Dominica	0	0	0	0	0	0	0	0	0	0	0	0	0	0	0	0	0	0	0	0	0	0	0	0	0	0	0	0	0	0	0	0	0	0	0	0	0	0	0	0
Dominican Republic	0	0	0	0	0	0	0	0	0	0	0	0	0	0	0	0	0	0	0	0	0	0	0	0	0	0	0	0	0	0	0	0	0	0	0	0	0	0	0	0
Ecuador	0	0	0	0	0	0	0	0	0	0	0	0	0	0	0	0	0	0	0	0	0	0	0	0	0	0	0	0	0	0	0	0	0	0	0	0	0	0	0	0
Egypt	0	0	0	0	0	0	0	0	0	0	0	0	0	0	0	0	0	21	9	0	36	0	1	0	0	98	0	0	13	7	24	1138	136	1887	224	213	70	377	43	15
El Salvador	0	0	0	0	0	0	0	0	0	0	0	0	0	0	0	0	0	0	0	0	0	0	0	0	0	0	0	0	0	0	0	0	0	0	0	0	0	0	0	0
Equatorial Guinea	0	0	0	0	0	0	0	0	0	0	0	0	0	0	0	0	0	0	0	0	0	0	0	0	0	0	0	3	3	0	4	0	0	0	1	0	0	1	4	0
Eritrea	0	0	0	0	0	0	0	0	0	0	0	0	0	0	0	0	0	0	0	0	0	0	0	0	0	0	0	1	0	1	0	3	1	38	0	0	2	1	0	0
Estonia	0	0	0	0	0	0	0	0	0	0	0	0	0	0	0	0	0	0	0	0	0	0	0	0	0	0	0	0	0	0	0	0	0	0	0	0	0	0	0	0
Eswatini	0	0	0	0	0	0	0	0	0	0	0	0	0	0	0	0	0	0	0	0	1	0	0	1	0	0	0	0	0	0	1	3	2	0	0	3	0	0	0	0
Ethiopia	0	0	0	0	0	0	0	0	0	0	0	0	0	0	0	0	0	4	0	2	1	90	87	2	5	94	8	3	0	2	22	4	22	58	55	220	1235	323	568	87
Fiji	0	0	0	0	0	0	0	0	0	0	0	0	0	0	0	0	0	0	0	0	0	0	0	0	0	0	0	0	0	0	0	0	0	0	0	0	0	0	0	0
Finland	0	0	0	0	0	0	0	0	0	0	0	0	0	0	0	0	0	0	0	0	0	0	0	0	0	0	0	0	0	0	0	0	0	0	0	0	0	0	0	0
France	0	0	0	0	0	0	0	0	0	0	0	0	0	0	0	0	0	0	0	0	0	0	0	0	0	0	0	0	0	0	0	0	0	0	0	0	0	0	0	0
Gabon	0	0	0	0	0	0	0	0	0	0	0	0	0	0	0	0	0	0	0	2	0	0	0	0	8	0	1	0	0	0	0	0	0	0	2	0	7	0	0	0
Gambia	0	0	0	0	0	0	0	0	0	0	0	0	0	0	0	0	0	0	0	0	15	4	0	0	0	0	0	2	0	0	0	0	9	0	3	0	4	3	3	0
Georgia	0	0	0	0	0	0	0	0	0	0	0	0	0	0	0	0	0	0	0	0	0	0	0	0	0	0	0	0	0	0	0	0	0	0	0	0	0	0	0	0
Germany	0	0	0	0	0	0	0	0	0	0	0	0	0	0	0	0	0	0	0	0	0	0	0	0	0	0	0	0	0	0	0	0	0	0	0	0	0	1	0	0
Ghana	0	50	0	26	0	0	0	0	0	0	0	0	0	0	0	0	0	3	0	0	1	126	0	0	1	7	0	5	0	0	0	0	5	7	2	7	14	11	8	6
Greece	0	0	0	0	0	0	0	0	0	0	0	0	0	0	0	0	0	0	0	0	0	0	0	0	0	0	0	0	0	0	0	0	0	0	0	0	0	0	0	0
Greenland	0	0	0	0	0	0	0	0	0	0	0	0	0	0	0	0	0	0	0	0	0	0	0	0	0	0	0	0	0	0	0	0	0	0	0	0	0	0	0	0
Grenada	0	0	0	0	0	0	0	0	0	0	0	0	0	0	0	0	0	0	0	0	0	0	0	0	0	0	0	0	0	0	0	0	0	0	0	0	0	0	0	0
Guam	0	0	0	0	0	0	0	0	0	0	0	0	0	0	0	0	0	0	0	0	0	0	0	0	0	0	0	0	0	0	0	0	0	0	0	0	0	0	0	0
Guatemala	0	0	0	0	0	0	0	0	0	0	0	0	0	0	0	0	0	0	0	0	0	0	0	0	0	0	0	0	0	0	0	0	0	0	0	0	0	0	0	0
Guinea	0	0	0	0	0	0	0	0	0	0	0	0	0	0	0	0	0	0	29	2	136	88	0	2	1	5	12	66	2	163	12	1	9	35	12	28	3	19	15	4
Guinea-Bissau	0	0	0	0	0	0	0	0	0	0	0	0	0	0	0	0	0	0	0	0	0	0	0	0	1	3	0	2	0	1	0	0	1	0	0	1	0	0	0	0
Guyana	0	0	0	0	0	0	0	0	0	0	0	0	0	0	0	0	0	0	0	0	0	0	0	0	0	0	0	0	0	0	0	0	0	0	0	0	0	0	0	0
Haiti	0	0	0	0	0	0	0	0	0	0	0	0	0	0	0	0	0	0	0	0	0	0	0	0	0	0	0	0	0	0	0	0	0	0	0	0	0	0	0	4
Honduras	0	0	0	0	0	0	0	0	0	0	0	0	0	0	0	0	0	0	0	0	0	0	0	0	0	0	0	0	0	0	0	0	0	0	0	0	0	0	0	0
Hungary	0	0	0	0	0	0	0	0	0	0	0	0	0	0	0	0	0	0	0	0	0	0	0	0	0	0	0	0	0	0	0	0	0	0	0	0	0	0	0	0
Iceland	0	0	0	0	0	0	0	0	0	0	0	0	0	0	0	0	0	0	0	0	0	0	0	0	0	0	0	0	0	0	0	0	0	0	0	0	0	0	0	0
India	0	0	0	0	0	0	0	0	0	0	0	0	0	0	0	0	0	0	0	0	0	0	0	0	0	0	0	0	0	0	4	3	1	3	0	1	250	150	140	63
Indonesia	0	0	0	0	0	0	0	0	0	0	0	0	0	0	0	0	0	0	0	0	0	0	0	0	0	0	0	1	10	0	0	0	0	5	0	25	7	4	35	41
Iran (Islamic Republic of)	139	128	57	116	118	102	177	176	1446	221	277	389	280	289	199	138	184	175	174	291	189	189	83	81	76	105	132	410	446	346	316	318	316	312	314	295	406	448	272	304
Iraq	0	0	0	0	0	0	0	0	0	0	0	0	0	0	199	0	0	100	100	100	100	0	0	0	0	2	54	33	40	120	1	68	129	169	177	26	88	125	53	517
Ireland	0	0	0	0	0	0	0	0	0	0	0	0	0	0	0	0	0	0	0	0	0	0	0	0	0	0	0	0	0	0	0	0	0	0	0	0	0	0	0	0
Israel	0	0	0	0	0	0	0	0	0	0	0	0	0	0	0	0	0	0	0	0	0	0	0	0	0	0	0	0	0	0	0	0	0	0	0	0	0	4	3	1
Italy	0	0	0	0	0	0	0	0	0	0	0	0	0	0	0	0	0	0	0	0	0	0	0	0	0	0	0	0	0	0	0	0	0	0	0	0	0	0	0	0
Jamaica	0	0	0	0	0	0	0	0	0	0	0	0	0	0	0	0	0	0	0	0	0	0	0	0	0	0	0	0	0	0	0	0	0	0	0	0	0	0	0	0
Japan	0	0	0	0	0	0	0	0	0	0	0	0	0	0	0	0	0	0	0	0	0	0	0	0	0	0	0	6	10	2	2	0	2	4	0	0	1	4	15	0
Jordan	0	0	0	0	0	0	0	0	0	0	0	0	0	0	0	0	0	0	0	0	0	0	0	0	0	0	0	0	0	0	0	0	0	0	11	2	5	15	1	2
Kazakhstan	0	0	0	0	0	0	0	0	0	0	0	0	0	0	0	0	0	0	0	0	0	0	0	0	0	0	0	0	0	0	0	0	0	0	0	0	0	0	0	0
Kenya	0	0	316	0	0	0	0	0	0	0	0	0	0	0	0	0	0	76	2	1	18	83	28	19	6	13	51	306	134	8	44	12	33	82	80	20	19	237	103	12
Kiribati	0	0	0	0	0	0	0	0	0	0	0	0	0	0	0	0	0	0	0	0	0	0	0	0	0	0	0	0	0	0	0	0	0	0	0	0	0	0	0	0
Kuwait	0	0	0	0	0	0	0	0	0	0	0	0	0	0	0	0	0	0	0	0	0	0	0	0	0	0	0	0	0	0	0	0	0	0	0	0	0	7	0	0
Kyrgyzstan	0	0	0	0	0	0	0	0	0	0	0	0	0	0	0	0	0	0	0	0	0	0	0	0	0	0	0	0	0	0	0	0	0	0	0	0	0	0	0	0
Lao People's Democratic Republic	0	0	0	0	0	0	0	0	0	0	0	0	0	0	0	0	0	0	0	0	0	0	0	0	0	0	0	0	0	0	0	0	0	0	0	20	0	1	0	1
Latvia	0	0	0	0	0	0	0	0	0	0	0	0	0	0	0	0	0	0	0	0	0	0	0	0	0	0	0	0	0	0	0	0	0	0	0	0	0	0	0	0
Lebanon	0	0	0	0	0	0	0	0	0	0	0	0	0	0	0	0	0	0	0	0	0	0	0	0	0	0	0	0	0	0	0	0	0	1	0	0	1	0	0	0
Lesotho	0	0	0	0	0	0	0	0	0	0	0	0	0	0	0	0	0	1	2	34	0	0	0	1	0	0	0	0	0	0	0	0	3	0	0	1	0	0	0	0
Liberia	27	0	0	0	0	0	0	0	0	0	0	0	0	0	0	0	0	0	4	0	32	0	0	0	1	13	0	0	0	0	10	0	0	2	22	4	2	2	0	0
Libya	0	0	0	0	4	2	0	0	0	0	0	0	0	0	0	0	0	8	0	0	0	0	0	0	0	0	17	9	8	4	18	1167	41	99	127	115	38	39	22	25
Lithuania	0	0	0	0	0	0	0	0	0	0	0	0	0	0	0	0	0	0	0	0	0	0	0	0	0	0	0	0	0	0	0	0	0	0	0	0	0	0	0	0
Luxembourg	0	0	0	0	0	0	0	0	0	0	0	0	0	0	0	0	0	0	0	0	0	0	0	0	0	0	0	0	0	0	0	0	0	0	0	0	0	0	0	0
Madagascar	0	0	0	0	0	0	0	0	0	0	0	0	0	0	0	0	0	0	0	0	0	2	32	1	0	0	0	4	0	3	0	0	92	73	25	23	3	38	5	10
Malawi	0	0	0	0	0	0	0	0	0	0	0	0	0	0	0	0	0	0	0	0	3	6	0	0	2	0	0	1	0	0	0	19	1	0	8	1	2	7	0	0
Malaysia	0	0	0	0	0	0	0	0	0	0	0	0	0	0	0	0	0	0	0	0	0	0	0	0	0	0	0	0	1	0	1	0	0	2	0	1	9	4	3	0
Maldives	0	0	0	0	0	0	0	0	0	0	0	0	0	0	0	0	0	0	0	0	0	0	0	0	0	0	0	0	0	0	0	0	0	0	0	0	0	0	0	0
Mali	0	0	0	0	0	0	0	0	0	0	0	0	0	0	0	0	0	3	0	0	5	0	0	0	0	0	0	0	1	1	2	3	25	16	5	13	46	73	93	33
Malta	0	0	0	0	0	0	0	0	0	0	0	0	0	0	0	0	0	0	0	0	0	0	0	0	0	0	0	0	0	0	0	0	0	0	0	0	0	0	0	1
Marshall Islands	0	0	0	0	0	0	0	0	0	0	0	0	0	0	0	0	0	0	0	0	0	0	0	0	0	0	0	0	0	0	0	0	0	0	0	0	0	0	0	0
Mauritania	0	0	0	0	0	0	0	0	0	0	0	0	0	0	0	0	0	0	1	0	1	0	1	8	0	0	0	1	0	0	0	3	5	0	1	0	0	0	0	1
Mauritius	0	0	0	0	0	0	0	0	0	0	0	0	0	0	0	0	0	0	0	0	0	0	0	0	0	0	0	0	0	0	0	0	0	0	0	0	0	0	0	0
Mexico	0	0	0	0	0	0	0	0	0	0	0	0	0	0	10	0	3	0	0	0	0	0	0	0	0	0	0	0	0	0	0	0	0	0	0	0	0	0	0	0
Micronesia (Federated States of)	0	0	0	0	0	0	0	0	0	0	0	0	0	0	0	0	0	0	0	0	0	0	0	0	0	0	0	0	0	0	0	0	0	0	0	0	0	0	0	0
Monaco	0	0	0	0	0	0	0	0	0	0	0	0	0	0	0	0	0	0	0	0	0	0	0	0	0	0	0	0	0	0	0	0	0	0	0	0	0	0	0	0
Mongolia	0	0	0	0	0	0	0	0	0	0	0	0	0	0	0	0	0	0	0	0	0	0	0	0	0	0	0	0	1	0	0	0	0	0	0	0	0	0	0	0
Montenegro	0	0	0	0	0	0	0	0	0	0	0	0	0	0	0	0	0	0	0	0	0	0	0	0	0	0	0	0	0	0	0	0	0	0	0	0	0	0	0	0

Morocco	0	0	0	0	0	0	0	0	0	0	0	0	0	0	0	0	0	3	0	2	0	0	0	0	2	3	0	0	0	0	29	15	2	5	3	2	3	2	5	1
Mozambique	0	0	0	0	0	0	0	0	0	0	0	0	0	0	0	0	0	0	0	0	0	0	0	0	0	12	0	0	4	4	19	3	6	9	4	6	33	11	18	2
Myanmar	0	0	0	0	0	0	0	0	0	0	0	0	0	0	0	0	0	0	0	0	0	0	0	0	0	0	0	0	0	0	14	30	150	65	62	108	90	6691	39	57
Namibia	0	0	0	0	0	0	0	0	0	0	0	0	0	0	0	0	0	0	0	0	0	0	0	3	0	0	0	0	0	0	0	1	0	3	3	1	0	1	0	0
Nauru	0	0	0	0	0	0	0	0	0	0	0	0	0	0	0	0	0	0	0	0	0	0	0	0	0	0	0	0	0	0	0	0	0	0	0	0	0	0	0	0
Nepal	0	0	0	0	0	0	0	0	0	0	0	0	0	0	0	0	0	0	0	0	0	0	0	0	0	0	0	0	0	0	7	1	7	2	1	42	1	3	1	2
Netherlands	0	0	0	0	0	0	0	0	0	0	0	0	0	0	0	0	0	0	0	0	0	0	0	0	0	0	0	0	0	0	0	0	0	0	0	0	0	0	0	0
New Zealand	0	0	0	0	0	0	0	0	0	0	0	0	0	0	0	0	0	0	0	0	0	0	0	0	0	0	0	0	0	0	0	0	0	0	0	0	0	0	0	0
Nicaragua	0	0	0	0	0	0	0	0	0	0	0	0	0	0	0	0	0	0	0	0	0	0	0	0	0	0	0	0	0	0	0	0	0	0	0	0	0	0	0	0
Niger	0	0	0	0	0	0	0	0	0	0	0	0	0	0	0	0	0	0	7	0	1	1	51	0	0	0	0	1	0	0	0	1	5	1	0	7	0	20	2	4
Nigeria	0	0	0	0	0	0	0	0	0	0	0	0	0	0	100	100	0	32	122	322	126	1053	365	176	929	77	40	33	788	62	671	821	478	399	1289	584	228	526	311	244
Niue	0	0	0	0	0	0	0	0	0	0	0	0	0	0	0	0	0	0	0	0	0	0	0	0	0	0	0	0	0	0	0	0	0	0	0	0	0	0	0	0
North Macedonia	0	0	0	0	0	0	0	0	0	0	0	0	0	0	0	0	0	0	0	0	0	0	0	0	0	0	0	0	0	0	0	0	0	0	0	0	0	0	0	0
Northern Mariana Islands	0	0	0	0	0	0	0	0	0	0	0	0	0	0	0	0	0	0	0	0	0	0	0	0	0	0	0	0	0	0	0	0	0	0	0	0	0	0	0	0
Norway	0	0	0	0	0	0	0	0	0	0	0	0	0	0	0	0	0	0	0	0	0	0	0	0	0	0	0	0	0	0	0	0	0	0	0	0	0	0	0	0
Oman	0	0	0	0	0	0	0	0	0	0	0	0	0	0	0	0	0	0	0	0	0	0	0	0	0	0	0	0	0	4	0	0	0	0	0	2	0	0	0	0
Pakistan	0	0	0	0	0	0	0	0	0	0	0	0	0	0	0	0	0	0	0	0	0	0	0	0	0	0	82	135	36	0	189	60	228	99	63	337	97	60	47	70
Palau	0	0	0	0	0	0	0	0	0	0	0	0	0	0	0	0	0	0	0	0	0	0	0	0	0	0	0	0	0	0	0	0	0	0	0	0	0	0	0	0
Palestine	256	256	256	256	256	256	256	256	321	0	0	0	0	0	0	0	0	0	0	0	0	0	0	0	0	4	0	0	0	0	5	3	6	3	0	0	14	28	170	26
Panama	0	0	0	0	0	0	0	0	0	0	0	0	0	0	0	0	0	0	0	0	0	0	0	0	0	0	0	0	0	0	0	0	0	0	0	0	0	0	0	0
Papua New Guinea	0	0	0	0	0	0	0	0	0	0	0	0	0	0	0	0	0	0	0	0	0	0	0	0	0	0	0	0	0	0	0	0	0	0	0	0	0	0	0	0
Paraguay	0	0	0	0	0	0	0	0	0	0	0	0	0	0	0	0	0	0	0	0	0	0	0	0	0	0	0	0	0	0	0	0	0	0	0	0	0	0	0	0
Peru	0	0	0	0	0	0	0	0	0	0	0	0	0	0	0	0	0	0	0	0	0	0	0	0	0	0	0	0	0	0	0	0	0	0	0	0	0	0	0	0
Philippines	0	0	0	0	0	0	0	0	0	0	0	0	0	0	0	0	0	0	0	0	0	0	0	0	0	0	0	0	0	0	0	0	0	0	0	0	1647	1045	771	354
Poland	0	0	0	0	0	0	0	0	0	0	0	0	0	0	0	0	0	0	0	0	0	0	0	0	0	0	0	0	0	0	0	0	0	0	0	0	0	0	0	0
Portugal	0	0	0	0	0	0	0	0	0	0	0	0	0	0	0	0	0	0	0	0	0	0	0	0	0	0	0	0	0	0	0	0	0	0	0	0	0	0	0	0
Puerto Rico	0	0	0	0	0	0	0	0	0	0	0	0	0	0	0	0	0	0	0	0	0	0	0	0	0	0	0	0	0	0	0	0	0	0	0	0	0	0	0	0
Qatar	0	0	0	0	0	0	0	0	0	0	0	0	0	0	0	0	0	0	0	0	0	0	0	0	0	0	0	0	0	0	0	0	0	0	0	0	0	0	0	0
Republic of Korea	0	0	0	0	0	0	0	0	0	0	0	0	0	0	0	0	0	0	0	0	0	0	0	0	0	0	0	0	0	0	0	0	0	0	0	0	1	0	0	0
Republic of Moldova	0	0	0	0	0	0	0	0	0	0	0	0	0	0	0	0	0	0	0	0	0	0	0	0	0	0	0	0	0	0	0	0	0	0	0	0	0	0	0	0
Romania	0	0	0	0	0	0	0	0	0	0	0	0	0	0	0	0	0	0	0	0	0	0	0	0	0	0	0	0	0	0	0	0	0	0	0	0	0	0	0	0
Russian Federation	0	0	0	0	0	0	0	0	0	0	0	0	0	0	0	0	11	0	0	0	0	0	0	0	0	0	0	0	0	0	0	0	0	0	0	11	0	0	13	5
Rwanda	0	0	0	0	0	0	0	0	0	0	0	0	0	0	0	0	0	253	54	1	6	0	0	0	10	0	3	1	0	1	2	0	3	0	0	1	0	0	15	0
Saint Kitts and Nevis	0	0	0	0	0	0	0	0	0	0	0	0	0	0	0	0	0	0	0	0	0	0	0	0	0	0	0	0	0	0	0	0	0	0	0	0	0	0	0	0
Saint Lucia	0	0	0	0	0	0	0	0	0	0	0	0	0	0	0	0	0	0	0	0	0	0	0	0	0	0	0	0	0	0	0	0	0	0	0	0	0	0	0	0
Saint Vincent and the Grenadines	0	0	0	0	0	0	0	0	0	0	0	0	0	0	0	0	0	0	0	0	0	0	0	0	0	0	0	0	0	0	0	0	0	0	0	0	0	0	0	0
Samoa	0	0	0	0	0	0	0	0	0	0	0	0	0	0	0	0	0	0	0	0	0	0	0	0	0	0	0	0	0	0	0	0	0	0	0	0	0	0	0	0
San Marino	0	0	0	0	0	0	0	0	0	0	0	0	0	0	0	0	0	0	0	0	0	0	0	0	0	0	0	0	0	0	0	0	0	0	0	0	0	0	0	0
Sao Tome and Principe	0	0	0	0	0	0	0	0	0	0	0	0	0	0	0	0	0	0	0	0	0	0	0	0	0	0	0	0	0	0	0	0	0	0	0	0	0	0	0	0
Saudi Arabia	0	0	0	0	0	0	0	0	0	0	0	0	0	0	0	191	0	122	0	103	123	79	3	0	0	86	0	143	102	69	27	82	79	79	182	158	154	146	149	0
Senegal	0	0	0	0	0	0	0	0	0	0	0	0	0	0	0	0	0	0	41	0	1	1	0	0	0	0	2	14	0	1	1	1	10	1	2	0	1	1	5	0
Serbia	0	0	0	0	0	0	0	0	0	0	0	0	0	0	0	0	0	0	0	0	0	0	0	0	0	0	0	0	0	0	0	0	0	0	0	0	0	0	0	0
Seychelles	0	0	0	0	0	0	0	0	0	0	0	0	0	0	0	0	0	0	0	0	0	0	0	0	0	0	0	0	0	0	0	0	0	0	0	0	0	0	0	0
Sierra Leone	0	0	0	0	0	0	0	0	0	0	0	0	0	0	0	0	0	0	0	100	6	0	0	0	2	0	0	2	0	5	0	2	3	3	2	2	5	2	7	8
Singapore	0	0	0	0	0	0	0	0	0	0	0	0	0	0	0	0	0	0	0	0	0	0	0	0	0	0	0	0	1	0	0	0	0	0	0	2	2	8	13	0
Slovakia	0	0	0	0	0	0	0	0	0	0	0	0	0	0	0	0	0	0	0	0	0	0	0	0	0	0	0	0	0	0	0	0	0	0	0	0	0	0	0	0
Slovenia	0	0	0	0	0	0	0	0	0	0	0	0	0	0	0	0	0	0	0	0	0	0	0	0	0	0	0	0	0	0	0	0	0	0	0	0	0	0	0	0
Solomon Islands	0	0	0	0	0	0	0	0	0	0	0	0	0	0	0	0	0	0	0	0	0	0	0	0	0	0	0	0	0	0	0	0	0	0	0	0	0	0	0	0
Somalia	0	0	0	0	0	0	0	0	0	0	0	0	0	0	0	0	0	0	0	0	0	0	0	3	23	10	14	103	116	55	30	124	91	114	143	145	237	388	196	56
South Africa	0	511	511	511	0	848	2144	311	698	0	0	0	0	0	0	0	0	26	14	28	8	4	15	0	4	1	9	6	7	7	2	10	87	33	47	49	42	31	21	18
South Sudan	0	0	0	0	0	0	0	0	0	0	0	0	0	0	0	0	0	0	0	0	0	0	0	0	0	0	0	0	0	0	0	9	5	4	622	218	199	249	28	7
Spain	0	3	0	0	0	0	0	0	0	0	0	0	2	0	0	0	0	0	0	0	0	0	0	0	0	0	0	0	0	0	0	0	0	0	0	0	0	0	0	0
Sri Lanka	0	0	0	0	0	0	0	0	0	0	0	0	0	0	0	0	0	0	0	0	0	0	0	0	0	0	0	0	0	0	3	3	4	3	1	1	2	0	6	3
Sudan	0	0	0	0	0	0	0	0	0	0	0	0	0	0	0	0	0	0	75	0	5	11	20	191	742	243	65	7	21	9	38	551	52	389	92	96	94	123	70	204
Suriname	0	0	0	0	0	0	66	67	67	0	0	0	0	0	0	0	0	0	0	0	0	0	0	0	0	0	0	0	0	0	0	0	0	0	0	0	0	0	0	0
Sweden	0	0	0	0	0	0	0	0	0	0	0	0	0	0	0	0	0	0	0	0	0	0	0	0	0	0	0	0	0	0	0	0	0	0	0	0	0	0	0	0
Switzerland	0	0	0	0	0	0	0	0	0	0	0	0	0	0	0	0	0	0	0	0	0	0	0	0	0	0	0	0	0	0	0	0	0	0	0	0	0	0	0	0
Syrian Arab Republic	0	0	0	0	0	0	0	0	0	0	0	0	0	0	0	0	0	0	0	0	0	0	0	0	0	0	5	7	3	8	17	0	0	0	1	0	10	194	800	260
Taiwan (Province of China)	0	0	0	0	0	0	0	0	0	0	0	0	0	0	0	0	0	0	0	0	0	0	0	0	0	0	0	0	0	0	0	0	0	0	0	0	0	0	1	0
Tajikistan	0	0	0	0	0	0	0	0	0	0	0	0	0	0	0	0	0	0	0	0	0	0	2	0	0	0	0	0	0	0	0	0	0	0	0	0	0	0	0	0
Thailand	0	0	0	0	0	0	0	0	0	0	0	0	0	0	0	0	0	0	0	0	0	0	0	0	0	0	0	0	0	2	95	15	8	28	41	13	17	10	11	20
Timor-Leste	0	0	0	0	0	0	0	0	0	0	0	0	0	0	0	0	0	0	0	0	0	0	0	0	0	0	0	0	0	0	0	0	0	0	0	0	0	0	0	0
Togo	0	0	0	0	0	0	30	0	0	0	0	0	0	0	0	0	0	0	1	0	1	0	0	2	2	82	0	0	0	0	0	0	0	2	0	8	0	6	5	1
Tokelau	0	0	0	0	0	0	0	0	0	0	0	0	0	0	0	0	0	0	0	0	0	0	0	0	0	0	0	0	0	0	0	0	0	0	0	0	0	0	0	0
Tonga	0	0	0	0	0	0	0	0	0	0	0	0	0	0	0	0	0	0	0	0	0	0	0	0	0	0	0	0	0	0	0	0	0	0	0	0	0	0	0	0
Trinidad and Tobago	0	0	0	0	0	0	0	0	0	0	0	0	0	0	0	0	0	0	0	0	0	0	0	0	0	0	0	0	0	0	0	0	0	0	0	0	0	0	0	0
Tunisia	518	0	0	0	0	0	0	0	0	0	0	0	0	0	0	0	0	0	0	4	0	0	0	0	0	0	0	0	4	0	3	219	6	4	10	39	2	2	3	1
Turkey	0	0	0	0	0	0	0	0	0	0	0	0	0	0	0	0	0	0	0	0	0	0	0	0	0	0	0	0	0	0	0	1	0	8	1	0	74	98	35	3
Turkmenistan	0	0	0	0	0	0	0	0	0	0	0	0	0	0	0	0	0	0	0	0	0	0	0	0	0	0	0	0	0	0	0	0	0	0	0	0	0	0	0	0
Tuvalu	0	0	0	0	0	0	0	0	0	0	0	0	0	0	0	0	0	0	0	0	0	0	0	0	0	0	0	0	0	0	0	0	0	0	0	0	0	0	0	0
Uganda	0	0	0	0	0	0	0	0	0	0	0	0	0	0	0	0	0	35	16	7	4	10	15	43	45	28	15	30	9	32	16	18	4	2	31	13	16	33	38	16
Ukraine	0	0	0	0	0	0	0	0	0	0	0	0	0	0	0	0	167	0	0	0	0	0	0	0	0	0	0	0	0	0	0	0	0	0	0	0	0	0	245	20
United Arab Emirates	0	0	0	0	0	0	0	0	0	0	0	0	0	0	0	0	0	0	0	0	0	0	0	0	0	0	0	0	1	0	0	1	1	0	0	1	0	1	0	0
United Kingdom	0	0	0	0	0	0	0	0	0	0	0	0	0	0	0	0	0	0	0	0	0	0	0	0	0	0	0	0	0	0	0	0	0	0	0	0	0	0	0	0
United Republic of Tanzania	0	0	0	0	0	0	0	0	0	0	0	0	0	0	0	0	0	6	1	0	26	10	1	0	6	9	6	0	0	5	0	17	24	8	2	2	0	218	13	2
United States Virgin Islands	0	0	0	0	0	0	0	0	0	0	0	0	0	0	0	0	0	0	0	0	0	0	0	0	0	0	0	0	0	0	0	0	0	0	0	0	0	0	0	0
United States of America	67	0	0	0	0	0	0	0	0	0	0	0	0	0	0	0	0	0	0	0	0	0	0	0	0	0	0	0	9	15	14	29	35	27	83	13	19	23	25	0
Uruguay	0	0	0	0	0	0	0	0	0	0	0	0	0	0	0	0	0	0	0	0	0	0	0	0	0	0	0	0	0	0	0	0	0	0	0	0	0	0	0	0
Uzbekistan	0	0	0	0	0	0	0	0	0	0	0	0	0	0	0	0	0	0	0	0	0	0	0	0	0	0	0	0	0	0	0	0	0	0	0	0	0	0	0	0
Vanuatu	0	0	0	0	0	0	0	0	0	0	0	0	0	0	0	0	0	0	0	0	0	0	0	0	0	0	0	0	0	0	0	0	0	0	0	0	0	0	0	0
Venezuela (Bolivarian Republic of)	0	0	0	0	0	0	0	0	0	0	0	0	0	0	0	0	0	0	0	0	0	0	0	0	0	0	0	0	0	0	0	0	0	0	0	0	0	163	0	0
Viet Nam	0	0	0	0	0	0	0	0	0	0	0	0	0	0	0	0	0	0	0	0	0	0	0	64	64	0	0	25	19	9	3	63	1	7	0	0	0	1	85	0
Yemen	0	0	0	0	0	0	0	0	0	0	0	0	0	0	0	0	0	0	0	0	0	0	0	0	0	0	0	15	13	30	53	41	28	13	0	102	3	3	17	9
Zambia	0	0	0	0	0	0	0	0	0	0	0	0	0	0	0	0	0	0	0	0	1	1	0	4	0	0	4	12	11	1	0	14	1	5	1	1	4	1	5	3
Zimbabwe	0	0	0	0	0	0	0	0	0	0	0	0	0	0	0	0	0	2	7	6	17	3	4	0	1	1	1	8	17	17	2	4	3	1	4	7	1	4	9	14

SR Table 10g: Deaths due to homicide by country, all ages, for both sexes combined, 1980-2019

Country	1980	1981	1982	1983	1984	1985	1986	1987	1988	1989	1990	1991	1992	1993	1994	1995	1996	1997	1998	1999	2000	2001	2002	2003	2004	2005	2006	2007	2008	2009	2010	2011	2012	2013	2014	2015	2016	2017	2018	2019
Afghanistan	0	0	0	0	0	0	0	0	0	237	36	251	90	8	119	115	371	2	109	0	3	0	246	243	49	0	36	1	0	0	99	25	10	10	13	534	376	419	631	2
Albania	0	0	0	0	0	0	0	0	0	0	0	0	0	0	0	0	0	0	0	0	0	0	0	0	0	0	0	0	0	0	0	0	0	0	0	0	0	0	0	0
Algeria	0	0	0	0	0	0	0	0	0	0	0	0	0	0	15	51	0	250	143	3	0	0	0	0	0	0	0	0	0	0	0	0	0	27	0	0	0	0	0	0
American Samoa	0	0	0	0	0	0	0	0	0	0	0	0	0	0	0	0	0	0	0	0	0	0	0	0	0	0	0	0	0	0	0	0	0	0	0	0	0	0	0	0
Andorra	0	0	0	0	0	0	0	0	0	0	0	0	0	0	0	0	0	0	0	0	0	0	0	0	0	0	0	0	0	0	0	0	0	0	0	0	0	0	0	0
Angola	0	0	0	0	0	0	0	0	0	0	0	0	0	0	0	0	0	0	0	0	0	0	0	0	0	0	0	0	0	0	0	0	0	0	0	0	0	0	0	0
Antigua and Barbuda	0	0	0	0	0	0	0	0	0	0	0	0	0	0	0	0	0	0	0	0	0	0	0	0	0	0	0	0	0	0	0	0	0	0	0	0	0	0	0	0
Argentina	0	0	0	0	0	0	0	0	0	0	0	0	0	0	0	0	0	0	0	0	0	0	0	0	0	0	0	0	0	0	0	0	0	0	0	0	0	0	0	0
Armenia	0	0	0	0	0	0	0	0	0	0	0	0	0	0	0	0	0	0	0	0	0	0	0	0	0	0	0	0	0	0	0	0	0	0	0	0	0	0	0	0
Australia	0	0	0	0	0	0	0	0	0	0	0	0	0	0	0	0	0	0	0	0	0	0	0	0	0	0	0	0	0	0	0	0	0	0	0	0	0	0	0	0
Austria	0	0	0	0	0	0	0	0	0	0	0	0	0	0	0	0	0	0	0	0	0	0	0	0	0	0	0	0	0	0	0	0	0	0	0	0	0	0	0	0
Azerbaijan	0	0	0	0	0	0	0	0	0	0	0	0	0	0	0	0	0	0	0	0	0	0	0	0	0	0	0	0	0	0	0	0	0	0	0	0	0	0	0	0
Bahamas	0	0	0	0	0	0	0	0	0	0	0	0	0	0	0	0	0	0	0	0	0	0	0	0	0	0	0	0	0	0	0	0	0	0	0	0	0	0	0	0
Bahrain	0	0	0	0	0	0	0	0	0	0	0	0	0	0	0	0	0	0	0	0	0	0	0	0	0	0	0	0	0	0	0	0	0	0	0	0	0	0	0	0
Bangladesh	0	0	0	0	0	0	0	0	0	1	2	12	1	31	6	8	8	11	16	13	90	58	1	1	2	0	18	0	0	8	3	107	2	44	2	2	10	17	23	49
Barbados	0	0	0	0	0	0	0	0	0	0	0	0	0	0	0	0	0	0	0	0	0	0	0	0	0	0	0	0	0	0	0	0	0	0	0	0	0	0	0	0
Belarus	0	0	0	0	0	0	0	0	0	0	0	0	0	0	0	0	0	0	0	0	0	0	0	0	0	0	0	0	0	0	0	0	0	0	0	0	0	0	0	0
Belgium	0	0	0	0	0	0	0	0	0	0	0	0	0	0	0	0	0	0	0	0	0	0	0	0	0	0	0	0	0	0	0	0	0	0	0	0	0	0	0	0
Belize	0	0	0	0	0	0	0	0	0	0	0	0	0	0	0	0	0	0	0	0	0	0	0	0	0	0	0	0	0	0	0	0	0	0	0	0	0	0	0	0
Benin	0	0	0	0	0	0	0	0	0	0	0	0	0	0	0	0	0	0	0	0	0	4	0	0	0	0	0	0	0	0	0	0	0	0	0	0	0	0	25	25
Bermuda	0	0	0	0	0	0	0	0	0	0	0	0	0	0	0	0	0	0	0	0	0	0	0	0	0	0	0	0	0	0	0	0	0	0	0	0	0	0	0	0
Bhutan	0	0	0	0	0	0	0	0	0	0	0	0	0	0	0	0	0	0	0	0	0	0	0	0	0	0	0	0	0	0	0	0	0	0	0	0	0	0	0	0
Bolivia (Plurinational State of)	0	0	0	0	0	0	0	0	0	0	0	0	0	0	0	0	0	0	2	0	50	0	0	0	0	0	0	0	0	0	0	0	0	0	0	0	0	0	0	0
Bosnia and Herzegovina	0	0	0	0	0	0	0	0	0	0	0	0	0	0	0	0	0	0	0	0	0	0	0	0	0	0	0	0	0	0	0	0	0	0	0	0	0	0	1	1
Botswana	0	0	0	0	0	0	0	0	0	0	0	0	0	0	0	0	0	0	0	0	0	0	0	0	0	0	0	0	0	0	0	0	0	0	0	0	0	0	1	0
Brazil	0	0	0	0	0	0	0	0	0	0	0	0	0	1475	2524	4325	3528	3584	3285	2973	3098	2998	3382	3092	2966	2797	2604	1772	917	599	732	41	97	236	417	6	5	309	440	0
Brunei Darussalam	0	0	0	0	0	0	0	0	0	0	0	0	0	0	0	0	0	0	0	0	0	0	0	0	0	0	0	0	0	0	0	0	0	0	0	0	0	0	0	0
Bulgaria	0	0	0	0	0	0	0	0	0	0	0	0	0	0	0	0	0	0	0	0	0	0	0	0	0	0	0	0	0	0	0	0	0	0	0	0	0	0	0	0
Burkina Faso	0	0	0	0	0	0	0	0	0	0	0	0	0	0	0	0	0	0	0	0	0	0	0	0	0	0	0	0	0	0	0	0	0	0	0	0	0	0	11	66
Burundi	0	0	0	0	0	0	0	0	0	0	0	0	0	0	0	0	0	214	0	0	100	0	0	43	54	0	0	52	0	0	0	0	0	0	0	0	0	0	61	18
Cabo Verde	0	0	0	0	0	0	0	0	0	0	0	0	0	0	0	0	0	0	0	0	0	0	0	0	0	0	0	0	0	0	0	0	0	0	0	0	0	0	0	0
Cambodia	0	0	0	0	0	0	0	0	0	0	0	0	0	0	0	0	0	0	0	0	0	0	0	0	0	0	0	0	0	0	1	3	4	2	1	0	0	0	1	2
Cameroon	0	0	0	0	0	0	0	0	0	0	0	50	25	47	34	0	0	0	51	0	0	0	0	0	0	0	0	0	0	0	0	0	0	0	0	0	4	62	1	0
Canada	0	0	0	0	0	0	0	0	0	0	0	0	0	0	26	24	42	0	0	0	0	0	0	0	0	0	0	0	0	0	0	0	0	0	0	0	0	0	0	0
Central African Republic	0	0	0	0	0	0	0	0	0	0	0	0	0	0	0	0	0	0	0	0	0	0	0	0	0	0	0	4	0	0	0	64	0	376	1087	277	214	1082	311	4
Chad	0	0	0	0	0	0	0	0	0	0	0	0	0	0	0	0	0	0	0	0	72	0	0	0	0	0	214	126	0	0	0	0	0	2	10	3	4	28	8	66
Chile	0	0	0	0	0	0	0	0	0	0	0	0	0	0	0	0	0	0	0	0	0	0	0	0	0	0	0	0	0	0	0	0	0	0	0	0	0	0	0	0
China	0	0	0	0	0	0	0	0	0	0	0	0	0	0	0	0	0	0	0	0	0	0	0	0	0	0	0	0	0	195	0	0	1	0	0	0	3	1	0	8
Colombia	0	0	0	0	0	0	0	0	0	0	0	0	0	737	0	0	0	1500	3729	4894	7744	9050	10618	2206	1363	0	0	0	0	0	0	0	0	0	0	0	0	0	0	0
Comoros	0	0	0	0	0	0	0	0	0	0	0	0	0	0	0	0	0	0	40	0	0	0	0	0	0	0	0	0	0	0	0	0	0	0	0	0	0	0	0	0
Congo	0	0	0	0	0	0	0	0	0	0	0	0	0	0	0	0	0	0	0	0	0	0	0	0	0	0	0	0	0	0	0	0	0	0	0	0	0	0	1	0
Cook Islands	0	0	0	0	0	0	0	0	0	0	0	0	0	0	0	0	0	0	0	0	0	0	0	0	0	0	0	0	0	0	0	0	0	0	0	0	0	0	0	0
Costa Rica	0	0	0	0	0	0	0	0	0	0	0	0	0	0	0	0	0	0	0	0	0	0	0	0	0	0	0	0	0	0	0	0	0	0	0	0	0	0	0	0
Croatia	0	0	0	0	0	0	0	0	0	0	0	0	0	0	0	0	0	0	0	0	0	0	0	0	0	0	0	0	0	0	0	0	0	0	0	0	0	0	0	1
Cuba	0	0	0	0	0	0	0	0	0	0	0	0	0	0	0	0	0	0	0	0	0	0	0	0	0	0	0	0	0	0	0	0	0	0	0	0	0	0	0	0
Cyprus	0	0	0	0	0	0	0	0	0	0	0	0	0	0	0	0	0	0	0	0	0	0	0	0	0	0	0	0	0	0	0	0	0	0	0	0	0	0	0	0
Czechia	0	0	0	0	0	0	0	0	0	0	0	0	0	0	0	0	0	0	0	0	0	0	0	0	0	0	0	0	0	0	0	0	0	0	0	0	0	0	0	0
Côte d'Ivoire	0	0	0	0	0	0	0	0	0	0	0	0	0	0	0	32	0	0	0	0	31	2	50	165	197	144	2	0	0	0	25	59	2	0	0	0	33	0	1	0
Democratic People's Republic of Korea	0	0	0	0	0	0	0	0	0	0	0	0	0	0	0	0	0	0	0	0	0	0	0	0	0	0	0	0	0	0	0	0	0	0	0	0	0	0	0	0
Democratic Republic of the Congo	0	0	0	0	0	0	0	0	0	0	0	0	0	3182	146	465	127	153	326	1691	1049	558	2969	923	51	0	8	34	87	10	0	48	244	115	150	5	706	738	1693	7
Denmark	0	0	0	0	0	0	0	0	0	0	0	0	0	0	0	0	0	0	0	0	0	0	0	0	0	0	0	0	0	0	0	0	0	0	0	0	0	0	0	0
Djibouti	0	0	0	0	0	0	0	0	0	0	0	15	0	0	0	17	0	0	0	0	0	0	0	0	0	0	0	0	0	0	0	0	0	0	0	0	0	0	0	0
Dominica	0	0	0	0	0	0	0	0	0	0	0	0	0	0	0	0	0	0	0	0	0	0	0	0	0	0	0	0	0	0	0	0	0	0	0	0	0	0	0	0
Dominican Republic	0	0	0	0	0	0	0	0	0	0	0	0	0	0	0	0	0	0	0	0	0	0	0	0	0	0	0	0	0	0	0	0	0	0	0	0	0	0	0	0
Ecuador	0	0	0	0	0	0	0	0	0	0	0	0	0	0	0	0	0	0	0	0	0	2	0	26	0	0	0	0	0	0	0	0	0	0	0	0	0	0	0	0
Egypt	0	0	0	0	0	0	0	0	0	0	0	0	0	0	0	6	0	0	0	0	3	0	0	0	1	0	2	0	2	1	0	31	75	130	41	0	1	0	1	0
El Salvador	0	0	0	0	0	0	0	0	0	0	0	0	0	0	0	0	0	0	0	0	0	0	0	0	0	0	0	0	0	0	0	0	3247	2994	2946	3285	0	0	0	0
Equatorial Guinea	0	0	0	0	0	0	0	0	0	0	0	0	0	0	0	0	0	0	0	0	0	0	0	0	0	0	0	0	0	0	0	0	0	0	0	0	0	0	0	0
Eritrea	0	0	0	0	0	0	0	0	0	0	0	0	0	0	0	0	0	0	0	0	0	0	0	0	0	0	0	0	0	0	0	0	0	0	0	0	0	0	0	0
Estonia	0	0	0	0	0	0	0	0	0	0	0	0	0	0	0	0	0	0	0	0	0	0	0	0	0	0	0	0	0	0	0	0	0	0	0	0	0	0	0	0
Eswatini	0	0	0	0	0	0	0	0	0	0	0	0	0	0	0	0	0	0	0	0	0	1	0	0	0	0	0	0	0	0	0	0	0	0	0	0	0	0	0	0
Ethiopia	0	0	0	0	0	0	0	0	0	16	0	178	852	69	0	1000	18	6	766	57	409	348	724	532	175	169	329	85	255	61	1	15	247	0	0	0	178	302	297	2
Fiji	0	0	0	0	0	0	0	0	0	0	0	0	0	0	0	0	0	0	0	0	0	0	0	0	0	0	0	0	0	0	0	0	0	0	0	0	0	0	0	0
Finland	0	0	0	0	0	0	0	0	0	0	0	0	0	0	0	0	0	0	0	0	0	0	0	0	0	0	0	0	0	0	0	0	0	0	0	0	0	0	0	0
France	0	0	0	0	0	0	0	0	0	0	0	0	0	0	0	0	0	0	0	0	0	0	0	0	0	0	0	0	0	0	0	0	0	0	0	0	0	0	0	0
Gabon	0	0	0	0	0	0	0	0	0	0	0	0	0	0	0	0	0	0	0	0	0	0	0	0	0	0	0	0	0	0	0	0	0	0	0	0	0	0	0	0
Gambia	0	0	0	0	0	0	0	0	0	0	0	0	0	0	0	0	0	0	0	0	0	0	0	0	0	0	0	0	0	0	0	0	0	0	0	0	0	0	0	1
Georgia	0	0	0	0	0	0	0	0	0	0	0	0	0	0	0	0	0	11	17	0	0	0	0	0	0	0	0	0	0	0	0	0	0	0	0	0	0	0	0	0
Germany	0	0	0	0	0	0	0	0	0	0	0	0	0	0	0	0	0	0	0	0	0	0	0	0	0	0	0	0	0	0	0	0	0	0	0	0	0	0	0	0
Ghana	0	0	0	0	0	0	0	0	0	0	0	30	63	2	2004	155	0	0	0	0	30	50	36	0	0	0	0	0	55	8	1	0	0	0	0	0	0	0	6	3
Greece	0	0	0	0	0	0	0	0	0	0	0	0	0	0	0	0	0	0	0	0	0	0	0	0	0	0	0	0	0	0	0	0	0	0	0	0	0	0	7	0
Greenland	0	0	0	0	0	0	0	0	0	0	0	0	0	0	0	0	0	0	0	0	0	0	0	0	0	0	0	0	0	0	0	0	0	0	0	0	0	0	0	0
Grenada	0	0	0	0	0	0	0	0	0	0	0	0	0	0	0	0	0	0	0	0	0	0	0	0	0	0	0	0	0	0	0	0	0	0	0	0	0	0	0	0
Guam	0	0	0	0	0	0	0	0	0	0	0	0	0	0	0	0	0	0	0	0	0	0	0	0	0	0	0	0	0	0	0	0	0	0	0	0	0	0	0	0
Guatemala	0	0	0	0	0	0	0	0	0	0	0	0	0	0	0	0	0	0	0	0	0	0	0	0	0	481	0	0	190	1074	0	0	0	0	0	3	0	0	0	0
Guinea	0	0	0	0	0	0	0	0	0	0	0	0	0	0	0	0	0	0	0	0	31	0	0	0	0	0	0	0	0	0	0	25	0	98	0	0	0	0	0	2
Guinea-Bissau	0	0	0	0	0	0	0	0	0	0	0	0	0	0	0	0	0	0	0	0	0	0	0	0	0	0	0	0	0	0	0	0	0	0	0	0	0	0	0	0
Guyana	0	0	0	0	0	0	0	0	0	0	0	0	0	0	0	0	0	0	0	0	0	0	0	0	0	0	0	0	0	0	0	0	0	0	0	0	0	0	0	0
Haiti	0	0	0	0	0	0	0	0	0	0	0	40	0	0	0	0	0	0	0	0	0	0	0	0	0	0	0	0	0	0	0	0	0	0	0	0	0	0	0	0
Honduras	0	0	0	0	0	0	0	0	0	0	0	0	0	0	0	0	0	0	0	9	6	0	1	24	2	0	0	0	18	0	58	18	4560	4298	4320	4920	10	0	0	0
Hungary	0	0	0	0	0	0	0	0	0	0	0	0	0	0	0	0	0	0	0	0	0	0	0	0	0	0	0	0	0	0	0	0	0	0	0	0	0	0	0	0
Iceland	0	0	0	0	0	0	0	0	0	0	0	0	0	0	0	0	0	0	0	0	0	0	0	0	0	0	0	0	0	0	0	0	0	0	0	0	0	0	0	0
India	0	0	0	0	4000	0	0	0	0	561	1878	105	470	428	59	96	185	206	161	30	112	67	1207	89	7	36	90	77	87	76	3	38	140	79	3	0	35	11	3	48
Indonesia	0	0	0	0	0	0	0	0	0	0	0	0	0	0	0	0	0	361	0	676	493	507	26	0	49	0	0	0	0	0	0	7	0	0	0	0	0	1	0	1
Iran (Islamic Republic of)	0	0	0	0	0	0	0	0	0	0	0	0	0	0	0	0	0	0	0	0	0	0	0	0	0	0	0	0	0	0	0	0	0	0	0	0	0	0	0	0
Iraq	0	0	0	0	0	0	0	0	0	0	0	0	345	125	708	668	130	322	3	0	132	20	85	66	126	1	2	148	0	0	0	0	0	13	64	26	16	0	0	31
Ireland	0	0	0	0	0	0	0	0	0	0	0	0	0	0	0	0	0	0	0	0	0	0	0	0	0	0	0	0	0	0	0	0	0	0	0	0	0	0	0	0
Israel	0	0	0	0	0	0	0	0	0	0	0	0	0	0	0	0	0	0	0	0	0	0	15	0	0	0	0	0	0	0	0	0	0	0	0	0	0	0	1	0
Italy	0	0	0	0	0	0	0	0	0	0	0	0	0	0	0	0	0	0	0	0	0	0	0	0	0	0	0	0	0	0	0	0	0	0	0	0	0	0	0	0
Jamaica	0	0	0	0	0	0	0	0	0	0	0	0	0	0	0	0	0	0	0	0	0	0	0	0	0	0	0	0	0	0	0	0	0	0	0	0	0	0	0	0
Japan	0	0	0	0	0	0	0	0	0	0	0	0	0	0	0	0	0	0	0	0	0	0	0	0	0	0	0	0	0	0	0	0	0	0	0	0	0	0	0	33
Jordan	0	0	0	0	0	0	0	0	0	0	0	0	0	0	0	0	0	0	0	0	0	0	0	0	0	0	0	0	0	0	0	0	0	0	0	0	1	0	0	0
Kazakhstan	0	0	0	0	0	0	0	0	0	0	0	0	0	0	0	0	0	0	0	0	0	0	0	0	0	0	0	0	0	0	0	0	0	0	0	0	0	0	0	0
Kenya	0	0	0	0	0	0	0	0	0	60	0	17	356	288	28	56	162	202	322	176	524	181	32	7	50	227	226	54	488	145	85	133	247	175	156	167	45	69	66	13
Kiribati	0	0	0	0	0	0	0	0	0	0	0	0	0	0	0	0	0	0	0	0	0	0	0	0	0	0	0	0	0	0	0	0	0	0	0	0	0	0	0	0
Kuwait	0	0	0	0	0	0	0	0	0	0	0	0	0	0	0	0	0	0	0	0	0	0	0	0	0	0	0	0	0	0	0	0	0	0	0	0	0	0	0	0
Kyrgyzstan	0	0	0	0	0	0	0	0	0	0	0	0	0	0	0	0	0	0	0	0	0	0	0	0	0	0	0	0	0	0	11	0	0	0	0	0	0	0	0	0
Lao People's Democratic Republic	0	0	0	0	0	0	0	0	0	0	0	0	0	0	0	0	0	0	0	0	0	0	0	0	0	0	0	0	0	0	0	0	0	0	0	1	0	0	0	0
Latvia	0	0	0	0	0	0	0	0	0	0	0	0	0	0	0	0	0	0	0	0	0	0	0	0	0	0	0	0	0	0	0	0	0	0	0	0	0	0	0	0
Lebanon	0	0	0	0	0	0	0	0	0	317	1138	23	30	26	48	36	19	16	31	31	29	0	0	0	0	0	0	0	38	0	0	0	52	126	75	121	34	223	0	0
Lesotho	0	0	0	0	0	0	0	0	0	0	0	0	0	0	0	0	0	0	0	0	0	0	0	0	0	0	0	0	0	0	0	0	0	0	0	0	0	0	0	4
Liberia	0	0	0	0	0	0	0	0	0	0	45	306	55	108	489	23	2000	0	0	0	0	0	0	0	0	0	0	0	0	0	1	0	0	0	0	0	0	0	2	5
Libya	0	0	0	0	0	0	0	0	0	0	0	0	0	0	0	0	0	0	0	0	0	0	0	0	0	0	0	0	6	0	0	0	377	5	1169	1201	591	691	620	9
Lithuania	0	0	0	0	0	0	0	0	0	0	0	0	0	0	0	0	0	0	0	0	0	0	0	0	0	0	0	0	0	0	0	0	0	0	0	0	0	0	0	0
Luxembourg	0	0	0	0	0	0	0	0	0	0	0	0	0	0	0	0	0	0	0	0	0	0	0	0	0	0	0	0	0	0	0	0	0	0	0	0	0	0	0	0
Madagascar	0	0	0	0	0	0	0	0	0	0	0	0	0	0	0	0	0	0	0	0	0	0	79	0	0	0	0	0	0	82	0	0	0	0	0	0	0	0	9	5
Malawi	0	0	0	0	0	0	0	0	0	0	0	0	0	0	0	0	0	0	0	0	0	0	0	0	0	0	0	0	0	0	0	0	0	0	0	0	0	0	15	0
Malaysia	0	0	0	0	0	0	0	0	0	0	0	0	0	0	0	0	0	0	0	0	0	0	0	0	0	0	0	0	0	0	0	0	0	0	0	0	0	0	3	2
Maldives	0	0	0	0	0	0	0	0	0	0	0	0	0	0	0	0	0	0	0	0	0	0	0	0	0	0	0	0	0	0	0	0	0	0	0	0	0	0	0	0
Mali	0	0	0	0	0	0	0	0	0	0	0	0	0	0	30	0	0	22	0	59	0	0	0	0	20	0	0	0	0	0	0	0	118	28	95	69	53	169	205	321
Malta	0	0	0	0	0	0	0	0	0	0	0	0	0	0	0	0	0	0	0	0	0	0	0	0	0	0	0	0	0	0	0	0	0	0	0	0	0	0	0	0
Marshall Islands	0	0	0	0	0	0	0	0	0	0	0	0	0	0	0	0	0	0	0	0	0	0	0	0	0	0	0	0	0	0	0	0	0	0	0	0	0	0	0	0
Mauritania	0	0	0	0	0	0	0	0	0	102	0	0	0	0	0	0	0	0	0	0	0	0	0	0	0	0	0	0	0	0	0	0	0	0	0	0	0	0	1	0
Mauritius	0	0	0	0	0	0	0	0	0	0	0	0	0	0	0	0	0	0	0	0	0	0	0	0	0	0	0	0	0	0	0	0	0	0	0	0	0	0	0	0
Mexico	0	0	0	0	0	0	0	0	0	0	0	0	0	0	0	0	0	0	0	0	0	0	0	0	0	0	0	0	0	0	0	0	0	0	0	0	0	0	0	0
Micronesia (Federated States of)	0	0	0	0	0	0	0	0	0	0	0	0	0	0	0	0	0	0	0	0	0	0	0	0	0	0	0	0	0	0	0	0	0	0	0	0	0	0	0	0
Monaco	0	0	0	0	0	0	0	0	0	0	0	0	0	0	0	0	0	0	0	0	0	0	0	0	0	0	0	0	0	0	0	0	0	0	0	0	0	0	0	0
Mongolia	0	0	0	0	0	0	0	0	0	0	0	0	0	0	0	0	0	0	0	0	0	0	0	0	0	0	0	0	0	0	0	0	0	0	0	0	0	0	0	0
Montenegro	0	0	0	0	0	0	0	0	0	0	0	0	0	0	0	0	0	0	0	0	0	0	0	0	0	0	0	0	0	0	0	0	0	0	0	0	0	0	0	0

Morocco	0	0	0	0	0	0	0	0	0	0	0	0	0	0	0	0	0	0	0	0	0	0	0	0	0	0	0	0	0	0	0	0	0	0	0	0	0	0	1	0
Mozambique	0	0	0	0	0	0	0	0	0	0	0	63	0	0	0	0	0	0	0	0	0	0	0	0	0	0	0	0	0	0	0	0	0	0	0	0	0	0	1	3
Myanmar	0	0	0	0	0	0	0	0	0	0	234	355	0	0	8	1046	65	0	86	7	64	53	0	9	0	110	2	45	1	0	0	1	134	50	2	0	4	6	6	5
Namibia	0	0	0	0	0	0	0	0	0	0	0	0	0	0	0	0	0	0	0	0	0	0	0	0	0	0	0	0	0	0	0	0	0	0	0	0	0	0	1	0
Nauru	0	0	0	0	0	0	0	0	0	0	0	0	0	0	0	0	0	0	0	0	0	0	0	0	0	0	0	0	0	0	0	0	0	0	0	0	0	0	0	0
Nepal	0	0	0	0	0	0	0	0	0	0	0	0	0	0	0	0	0	0	0	0	0	0	0	0	0	0	0	30	0	0	1	0	0	0	0	0	0	0	0	0
Netherlands	0	0	0	0	0	0	0	0	0	0	0	0	0	0	0	0	0	0	0	0	0	0	0	0	0	0	0	0	0	0	0	0	0	0	0	0	0	0	0	0
New Zealand	0	0	0	0	0	0	0	0	0	0	0	0	0	0	0	0	0	0	0	0	0	0	0	0	0	0	0	0	0	0	0	0	0	0	0	0	0	0	0	0
Nicaragua	0	0	0	0	0	0	0	0	0	0	0	0	0	0	0	0	0	0	0	0	0	0	0	0	0	0	0	0	0	0	0	0	0	0	0	0	0	0	0	0
Niger	0	0	0	0	0	0	0	0	0	0	0	0	0	0	0	0	0	12	0	0	0	0	0	0	0	0	0	0	0	0	0	0	0	0	5	6	0	7	29	12
Nigeria	0	0	0	0	0	0	0	0	0	0	9	380	942	1182	34	0	82	591	295	1014	2062	2031	500	347	1929	204	206	148	280	111	869	1368	535	903	1366	841	766	937	1487	186
Niue	0	0	0	0	0	0	0	0	0	0	0	0	0	0	0	0	0	0	0	0	0	0	0	0	0	0	0	0	0	0	0	0	0	0	0	0	0	0	0	0
North Macedonia	0	0	0	0	0	0	0	0	0	0	0	0	0	0	0	0	0	0	0	0	0	0	0	0	0	0	0	0	0	0	0	0	0	0	0	0	0	0	1	0
Northern Mariana Islands	0	0	0	0	0	0	0	0	0	0	0	0	0	0	0	0	0	0	0	0	0	0	0	0	0	0	0	0	0	0	0	0	0	0	0	0	0	0	0	0
Norway	0	0	0	0	0	0	0	0	0	0	0	0	0	0	0	0	0	0	0	0	0	0	0	0	0	0	0	0	0	0	0	0	0	0	0	0	0	0	0	0
Oman	0	0	0	0	0	0	0	0	0	0	0	0	0	0	0	0	0	0	0	0	0	0	0	0	0	0	0	0	0	0	0	0	0	0	0	0	0	0	0	0
Pakistan	0	0	0	0	0	0	0	0	0	114	313	35	72	45	95	42	5	73	38	0	0	0	0	0	0	0	0	540	757	348	422	321	317	244	174	115	53	3	51	64
Palau	0	0	0	0	0	0	0	0	0	0	0	0	0	0	0	0	0	0	0	0	0	0	0	0	0	0	0	0	0	0	0	0	0	0	0	0	0	0	0	0
Palestine	0	0	0	0	0	0	0	0	0	0	0	0	0	0	0	0	0	0	0	0	0	0	0	0	0	0	0	0	0	0	0	0	0	0	0	0	0	0	3	0
Panama	0	0	0	0	0	0	0	0	0	0	0	0	0	0	0	0	0	0	0	0	0	0	0	0	0	0	0	0	0	0	0	0	0	0	0	0	0	0	0	0
Papua New Guinea	0	0	0	0	0	0	0	0	0	0	0	0	0	0	0	0	0	0	0	60	13	61	7	0	0	0	0	0	0	0	0	0	57	0	0	0	0	0	0	0
Paraguay	0	0	0	0	0	0	0	0	0	0	0	0	0	0	0	0	0	0	0	0	0	0	0	0	0	0	0	0	0	0	0	0	0	0	0	0	0	0	0	0
Peru	0	0	0	0	0	0	0	0	0	11	6	50	0	0	0	0	0	0	0	0	0	0	0	0	0	0	0	0	0	0	0	0	0	0	0	0	0	0	0	0
Philippines	0	0	0	0	0	0	0	0	0	0	0	0	0	43	7	0	0	0	30	6	0	0	1	0	0	0	35	0	0	44	0	31	6	0	0	17	52	249	30	16
Poland	0	0	0	0	0	0	0	0	0	0	0	0	0	0	0	0	0	0	0	0	0	0	0	0	0	0	0	0	0	0	0	0	0	0	0	0	0	0	0	0
Portugal	0	0	0	0	0	0	0	0	0	0	0	0	0	0	0	0	0	0	0	0	0	0	0	0	0	0	0	0	0	0	0	0	0	0	0	0	0	0	0	0
Puerto Rico	0	0	0	0	0	0	0	0	0	0	0	0	0	0	0	0	0	0	0	0	0	0	0	0	0	0	0	0	0	0	0	0	0	0	0	0	0	0	0	0
Qatar	0	0	0	0	0	0	0	0	0	0	0	0	0	0	0	0	0	0	0	0	0	0	0	0	0	0	0	0	0	0	0	0	0	0	0	0	0	0	0	0
Republic of Korea	0	0	0	0	0	0	0	0	0	0	0	0	0	0	0	0	0	0	0	0	0	0	0	0	0	0	0	0	0	0	0	0	0	0	0	0	0	0	0	0
Republic of Moldova	0	0	0	0	0	0	0	0	0	0	0	0	0	0	0	0	0	0	0	0	21	0	0	0	0	0	0	0	0	0	0	0	0	0	0	0	0	0	0	0
Romania	0	0	0	0	0	0	0	0	0	0	0	0	0	0	0	0	0	0	0	0	0	0	0	0	0	0	0	0	0	0	0	0	0	0	0	0	0	0	0	0
Russian Federation	0	0	0	0	0	0	0	0	0	10	15	7	0	0	244	0	0	0	0	0	0	0	0	0	0	0	0	0	0	0	0	0	0	0	0	0	0	0	5	1
Rwanda	0	0	0	0	0	0	0	0	0	0	0	0	0	0	0	0	0	0	0	0	0	0	0	0	0	0	0	0	0	0	0	0	0	0	0	0	0	0	0	0
Saint Kitts and Nevis	0	0	0	0	0	0	0	0	0	0	0	0	0	0	0	0	0	0	0	0	0	0	0	0	0	0	0	0	0	0	0	0	0	0	0	0	0	0	0	0
Saint Lucia	0	0	0	0	0	0	0	0	0	0	0	0	0	0	0	0	0	0	0	0	0	0	0	0	0	0	0	0	0	0	0	0	0	0	0	0	0	0	0	0
Saint Vincent and the Grenadines	0	0	0	0	0	0	0	0	0	0	0	0	0	0	0	0	0	0	0	0	0	0	0	0	0	0	0	0	0	0	0	0	0	0	0	0	0	0	0	0
Samoa	0	0	0	0	0	0	0	0	0	0	0	0	0	0	0	0	0	0	0	0	0	0	0	0	0	0	0	0	0	0	0	0	0	0	0	0	0	0	0	0
San Marino	0	0	0	0	0	0	0	0	0	0	0	0	0	0	0	0	0	0	0	0	0	0	0	0	0	0	0	0	0	0	0	0	0	0	0	0	0	0	0	0
Sao Tome and Principe	0	0	0	0	0	0	0	0	0	0	0	0	0	0	0	0	0	0	0	0	0	0	0	0	0	0	0	0	0	0	0	0	0	0	0	0	0	0	0	0
Saudi Arabia	0	0	0	0	0	0	0	0	0	0	0	0	0	0	0	0	0	0	0	0	0	0	0	0	0	0	0	0	0	0	0	0	0	0	0	0	0	0	0	0
Senegal	0	0	0	0	0	0	0	0	0	119	0	0	0	0	0	0	0	0	7	0	0	0	0	0	0	0	124	0	0	0	0	0	0	0	0	0	0	0	14	0
Serbia	0	0	0	0	0	0	0	0	0	0	0	272	197	0	0	0	0	0	0	0	0	0	0	0	0	0	0	0	0	0	0	0	0	0	0	0	0	0	1	1
Seychelles	0	0	0	0	0	0	0	0	0	0	0	0	0	0	0	0	0	0	0	0	0	0	0	0	0	0	0	0	0	0	0	0	0	0	0	0	0	0	0	0
Sierra Leone	0	0	0	0	0	0	0	0	0	0	0	9	1	2	25	40	4	0	0	0	0	0	0	0	0	0	0	0	0	0	0	0	0	0	0	0	0	0	2	0
Singapore	0	0	0	0	0	0	0	0	0	0	0	0	0	0	0	0	0	0	0	0	0	0	0	0	0	0	0	0	0	0	0	0	0	0	0	0	0	0	0	0
Slovakia	0	0	0	0	0	0	0	0	0	0	0	0	0	0	0	0	0	0	0	0	0	0	0	0	0	0	0	0	0	0	0	0	0	0	0	0	0	0	0	0
Slovenia	0	0	0	0	0	0	0	0	0	0	0	0	0	0	0	0	0	0	0	0	0	0	0	0	0	0	0	0	0	0	0	0	0	0	0	0	0	0	0	0
Solomon Islands	0	0	0	0	0	0	0	0	0	0	0	0	0	0	0	0	0	0	0	0	0	0	0	0	0	0	0	0	0	0	0	0	0	0	0	0	0	0	0	0
Somalia	0	0	0	0	0	0	0	0	0	0	50	0	3485	458	389	339	509	277	330	505	183	250	524	422	670	309	626	20	145	681	554	109	281	1	9	2	41	49	114	10
South Africa	0	0	0	0	0	0	0	0	0	227	1243	659	666	669	445	143	156	30	44	19	0	0	0	0	0	0	0	0	0	0	0	0	0	0	0	0	3	0	4	2
South Sudan	0	0	0	0	0	0	0	0	0	0	0	0	0	0	0	0	0	0	0	0	0	0	0	0	0	0	0	0	0	0	0	974	528	190	30	290	311	495	446	250
Spain	0	0	0	0	0	0	0	0	0	0	0	0	0	0	0	0	0	0	0	0	0	0	0	0	0	0	0	0	0	0	0	0	0	0	0	0	0	0	0	0
Sri Lanka	0	0	0	0	0	0	0	0	0	301	56	0	0	0	29	0	0	0	0	4	0	0	9	3	60	32	97	11	6	5	1	4	0	2	0	0	0	0	2	0
Sudan	0	0	0	0	0	0	0	0	0	2000	35	1002	1041	4010	150	100	385	645	407	544	401	70	173	186	212	500	417	754	289	2127	652	983	64	1335	969	917	218	316	139	6
Suriname	0	0	0	0	0	0	0	0	0	0	0	0	0	0	0	0	0	0	0	0	0	0	0	0	0	0	0	0	0	0	0	0	0	0	0	0	0	0	0	0
Sweden	0	0	0	0	0	0	0	0	0	0	0	0	0	0	0	0	0	0	0	0	0	0	0	0	0	0	0	0	0	0	0	0	0	0	0	0	0	0	0	0
Switzerland	0	0	0	0	0	0	0	0	0	0	0	0	0	0	0	0	0	0	0	0	0	0	0	0	0	0	0	0	0	0	0	0	0	0	0	0	0	0	0	0
Syrian Arab Republic	0	0	0	0	0	0	0	0	0	0	0	0	0	0	0	0	0	0	0	0	0	0	0	0	0	0	0	0	0	0	0	0	0	0	0	0	0	31	0	1
Taiwan (Province of China)	0	0	0	0	0	0	0	0	0	0	0	0	0	0	0	0	0	0	0	0	0	0	0	0	0	0	0	0	0	0	0	0	0	0	0	0	0	0	0	0
Tajikistan	0	0	0	0	0	0	0	0	0	0	0	0	482	0	0	0	0	0	0	0	0	0	0	0	0	0	0	0	0	0	0	0	0	0	0	0	0	0	0	0
Thailand	0	0	0	0	0	0	0	0	0	0	0	2	0	0	0	3	1	0	0	0	0	0	0	0	0	0	0	0	0	0	0	7	3	6	1	0	1	6	8	7
Timor-Leste	0	0	0	0	0	0	0	0	0	0	0	0	0	0	0	0	0	0	0	0	0	0	0	0	0	0	0	0	0	0	0	0	0	0	0	0	0	0	0	0
Togo	0	0	0	0	0	0	0	0	0	0	0	31	0	0	0	0	0	0	0	0	0	0	0	0	0	0	0	0	0	0	0	0	0	0	0	0	0	0	0	0
Tokelau	0	0	0	0	0	0	0	0	0	0	0	0	0	0	0	0	0	0	0	0	0	0	0	0	0	0	0	0	0	0	0	0	0	0	0	0	0	0	0	0
Tonga	0	0	0	0	0	0	0	0	0	0	0	0	0	0	0	0	0	0	0	0	0	0	0	0	0	0	0	0	0	0	0	0	0	0	0	0	0	0	0	0
Trinidad and Tobago	0	0	0	0	0	0	0	0	0	0	0	0	0	0	0	0	0	0	0	0	0	0	0	0	0	0	0	0	0	0	0	0	0	0	0	0	0	0	0	0
Tunisia	0	0	0	0	0	0	0	0	0	0	0	0	0	0	0	0	0	0	0	0	0	0	0	0	0	0	0	0	0	0	0	0	0	0	1	0	0	0	0	0
Turkey	0	0	0	0	0	0	0	0	0	0	0	0	0	0	0	0	0	6	0	0	0	0	0	0	0	0	0	0	0	0	0	0	0	0	0	0	0	0	0	0
Turkmenistan	0	0	0	0	0	0	0	0	0	0	0	0	0	0	0	0	0	0	0	0	0	0	0	0	0	0	0	0	0	0	0	0	0	0	0	0	0	0	0	0
Tuvalu	0	0	0	0	0	0	0	0	0	0	0	0	0	0	0	0	0	0	0	0	0	0	0	0	0	0	0	0	0	0	0	0	0	0	0	0	0	0	0	0
Uganda	0	0	0	0	0	0	0	0	0	0	0	0	4	0	0	0	0	0	138	388	430	43	17	361	18	91	65	43	31	38	0	0	0	0	0	0	30	0	21	6
Ukraine	0	0	0	0	0	0	0	0	0	0	0	0	0	0	0	0	0	0	0	0	0	0	0	0	0	0	0	0	0	0	0	0	0	0	45	0	0	0	0	0
United Arab Emirates	0	0	0	0	0	0	0	0	0	0	0	0	0	0	0	0	0	0	0	0	0	0	0	0	0	0	0	0	0	0	0	0	0	0	0	0	0	0	0	0
United Kingdom	0	0	0	0	0	0	0	0	0	0	0	0	0	0	0	0	0	0	0	0	0	0	0	0	0	0	0	0	0	0	0	0	0	0	0	0	0	0	0	0
United Republic of Tanzania	0	0	0	0	0	0	0	0	0	0	0	0	0	0	0	0	10	10	0	0	0	0	0	0	0	0	0	0	0	0	0	0	10	0	0	0	0	0	2	1
United States Virgin Islands	0	0	0	0	0	0	0	0	0	0	0	0	0	0	0	0	0	0	0	0	0	0	0	0	0	0	0	0	0	0	0	0	0	0	0	0	0	0	0	0
United States of America	0	0	0	0	0	0	0	0	0	0	0	0	0	0	0	0	0	0	0	0	0	0	0	0	0	0	0	0	0	0	0	0	0	0	0	0	0	90	0	12
Uruguay	0	0	0	0	0	0	0	0	0	0	0	0	0	0	0	0	0	0	0	0	0	0	0	0	0	0	0	0	0	0	0	0	0	0	0	0	0	0	0	0
Uzbekistan	0	0	0	0	0	0	0	0	0	0	0	0	0	0	0	0	0	0	0	0	0	0	0	0	0	0	0	0	0	0	0	0	0	0	0	0	0	0	0	0
Vanuatu	0	0	0	0	0	0	0	0	0	0	0	0	0	0	0	0	0	0	0	0	0	0	0	0	0	0	0	0	0	0	0	0	0	0	0	0	0	0	0	0
Venezuela (Bolivarian Republic of)	0	0	0	0	0	0	0	0	0	0	0	0	0	0	175	0	0	0	0	0	0	0	0	0	0	0	0	0	0	0	0	0	0	0	0	0	0	0	0	0
Viet Nam	0	0	0	0	0	0	0	0	0	0	0	0	0	0	0	0	0	0	0	0	0	0	0	0	0	0	0	0	0	0	1	0	0	0	0	1	1	0	0	1
Yemen	0	0	0	0	0	0	0	0	0	0	0	0	0	0	0	0	0	0	0	0	0	0	0	6	28	0	0	0	0	0	26	3	15	0	460	378	951	365	322	5
Zambia	0	0	0	0	0	0	0	0	0	0	0	0	0	0	0	0	0	0	0	0	0	0	0	0	0	0	0	0	0	0	0	0	0	0	0	0	0	0	6	1
Zimbabwe	0	0	0	0	0	0	0	0	0	0	0	0	0	0	0	0	0	0	0	0	0	0	0	0	0	0	0	0	0	0	0	0	0	0	0	0	0	0	0	0

SR Table 11: Diarrhoeal Diseases and Lower Respiratory Infection Aetiologies deaths, mortality rate and annual percent change from 1990 to 2019

Aetiologies	Deaths among all ages (95% UI)	Deaths per 100,000 all ages (95% UI)	Percent change 1990-2019 all ages (95% UI)	Deaths among children under-5 (95% UI)	Deaths per 100,000 among children under-5 (95% UI)	Percent change 1990-2019 among children under-5 (95% UI)	Deaths among adults 70+ (95% UI)	Deaths per 100,000 among adults 70+ (95% UI)	Percent change 1990-2019 among adults 70+ (95% UI)
Adenovirus	107000 (63500–173000)	1·4 (0·8–2·2)	-69·1 (-77·6–-55·6)	83500 (43900–144000)	12·6 (6·6–21·7)	-62·4 (-73·8–-43·7)	12700 (5900–23600)	2·7 (1·3–5·1)	-53 (-67·7–-28·9)
Aeromonas	28000 (12900–50300)	0·4 (0·2–0·7)	-67·3 (-75·5–-54·6)	19700 (7870–39000)	3 (1·2–5·9)	-61·9 (-72·6–-46·2)	4770 (1910–10400)	1 (0·4–2·2)	-61 (-73–-40·6)
Campylobacter	139000 (47000–305000)	1·8 (0·6–3·9)	-61·9 (-74·1–-44)	58900 (24000–116000)	8·9 (3·6–17·5)	-65 (-74·8–-51·2)	55000 (9630–148000)	11·9 (2·1–32)	-57·4 (-70·7–-37·1)
Cholera	117000 (71100–178000)	1·5 (0·9–2·3)	-2·4 (-36·4–52·5)	55700 (28000–93900)	8·4 (4·2–14·2)	16·5 (-32·8–84·6)	22500 (14600–34300)	4·9 (3·2–7·4)	-29·8 (-51·9–9·4)
Clostridium difficile	32100 (28100–36600)	0·4 (0·4–0·5)	167 (121–219)	2100 (1310–3220)	0·3 (0·2–0·5)	32·2 (14–52·7)	20400 (16700–24100)	4·4 (3·6–5·2)	270 (242–301)
Cryptosporidium	133000 (26400–360000)	1·7 (0·3–4·7)	-64·6 (-74·2–-51·1)	77500 (16000–190000)	11·7 (2·4–28·7)	-63·4 (-72·8–-47·9)	41900 (5610–148000)	9 (1·2–31·8)	-51·4 (-66·5–-27·5)
Entamoeba	33400 (10500–82400)	0·4 (0·1–1·1)	-73·2 (-79·3–-63·4)	19000 (4950–50300)	2·9 (0·7–7·6)	-72·5 (-79·2–-63·4)	2970 (780–8350)	0·6 (0·2–1·8)	-54·4 (-68·8–-30·4)
Enteropathogenic E coli	20600 (10100–37200)	0·3 (0·1–0·5)	-70·8 (-78·2–-59·5)	15800 (7450–30000)	2·4 (1·1–4·5)	-64·4 (-74·1–-49·2)	1410 (479–2930)	0·3 (0·1–0·6)	-55·2 (-69·2–-33·8)
Enterotoxigenic E coli	39800 (18000–77000)	0·5 (0·2–1)	-61·2 (-73·5–-42·9)	12400 (4980–26400)	1·9 (0·8–4)	-72·7 (-79·6–-63·2)	22100 (8720–47400)	4·8 (1·9–10·2)	-50·3 (-66·1–-25·3)
H influenzae type B	75400 (30100–131000)	1 (0·4–1·7)	-84·9 (-88·7–-80·2)	65800 (26100–114000)	9·9 (3·9–17·3)	-79·6 (-84·9–-72·3)	1130 (226–3130)	0·2 (0–0·7)	--
Influenza	244000 (96000–460000)	3·1 (1·2–5·9)	-34·5 (-53–-11·7)	37100 (12100–84400)	5·6 (1·8–12·7)	-73·6 (-80–-66·2)	138000 (50900–276000)	29·9 (11–59·5)	-13·7 (-21·8–-5·2)
Meningococcal meningitis	53700 (17300–107000)	0·7 (0·2–1·4)	-54·4 (-63·8–-44·6)	23900 (7100–49200)	3·6 (1·1–7·4)	-54·7 (-66·4–-41·1)	5130 (1460–11000)	1·1 (0·3–2·4)	-46·5 (-57·6–-36·4)
Non-typhoidal Salmonella	61600 (4380–191000)	0·8 (0·1–2·5)	-68·8 (-83·1–-52·6)	39500 (4380–108000)	6 (0·7–16·3)	-66·1 (-76·8–-48·9)	10700 (0–44400)	2·3 (0–9·6)	-55 (-65·8–NA)
Norovirus	136000 (25100–304000)	1·8 (0·3–3·9)	-61·4 (-75·1–-46·2)	43500 (11800–99200)	6·6 (1·8–15)	-69·7 (-77·6–-58·2)	54700 (5910–137000)	11·8 (1·3–29·5)	-54·9 (-67·4–-35·3)
Pneumococcal pneumonia	1470000 (971000–2e+06)	18·9 (12·6–25·9)	-49·3 (-58·5–-39·5)	376000 (246000–526000)	56·7 (37·1–79·4)	-65·5 (-74–-55)	702000 (406000–1060000)	152 (87·6–228)	-32·2 (-41·3–-23·2)
Respiratory syncytial virus	338000 (127000–667000)	4·4 (1·6–8·6)	-56·7 (-68·3–-45·9)	124000 (54400–212000)	18·7 (8·2–32)	-71·5 (-78·1–-62·3)	160000 (45600–350000)	34·5 (9·8–75·4)	-8 (-14·2–-1·2)
Rotavirus	235000 (110000–415000)	3 (1·4–5·4)	-75·3 (-81–-67)	152000 (70600–266000)	22·9 (10·6–40·2)	-73·3 (-79·8–-63·4)	48100 (18300–101000)	10·4 (4–21·7)	-56·6 (-69·8–-33·7)
Shigella	148000 (62000–284000)	1·9 (0·8–3·7)	-65·9 (-74·6–-52·8)	93800 (35900–186000)	14·2 (5·4–28·1)	-62·3 (-72·6–-45·5)	28800 (9650–67600)	6·2 (2·1–14·6)	-56·1 (-68·9–-36·7)

SR Table 12: Global deaths by cause and age group for males

Cause	Early Neonatal	Late Neonatal	Post Neonatal	1 to 4	5 to 9	10 to 14	15 to 19	20 to 24	25 to 29	30 to 34	35 to 39	40 to 44	45 to 49	50 to 54	55 to 59	60 to 64	65 to 69	70 to 74	75 to 79	80 to 84	85 to 89	90 to 94	95 plus
All causes	1,109,838 (935,413–1,318,929)	259,895 (219,190–309,173)	692,118 (569,083–848,686)	668,445 (556,866–806,846)	209,627 (189,775–233,192)	169,718 (156,953–183,671)	302,316 (281,348–324,456)	437,258 (409,760–466,367)	495,786 (469,135–523,540)	618,435 (585,326–653,793)	742,497 (697,586–789,989)	908,469 (846,974–972,364)	1,185,466 (1,095,063–1,275,700)	1,584,393 (1,456,886–1,708,039)	2,021,077 (1,866,233–2,173,553)	2,521,938 (2,344,804–2,698,053)	2,994,059 (2,783,601–3,198,844)	3,275,740 (3,055,501–3,495,038)	3,276,003 (3,077,753–3,466,763)	3,295,861 (3,126,252–3,460,099)	2,442,983 (2,362,339–2,518,601)	1,091,911 (1,067,817–1,113,929)	--
Sexually transmitted infections excluding HIV	25,415 (8,506–52,998)	7,639 (2,595–15,803)	11,371 (3,934–23,606)	450 (151–982)	209 (68–459)	63 (41–90)	55 (35–74)	54 (34–72)	60 (38–76)	123 (60–188)	103 (51–149)	91 (50–125)	89 (43–130)	142 (58–214)	193 (75–297)	151 (81–225)	87 (62–140)	89 (67–140)	99 (73–159)	72 (45–100)	79 (36–130)	52 (18–111)	--
Syphilis	25,415 (8,506–52,998)	7,639 (2,595–15,803)	11,371 (3,934–23,606)	450 (151–982)	209 (68–459)	36 (24–51)	43 (28–58)	34 (21–45)	42 (27–53)	83 (40–126)	89 (44–129)	69 (38–95)	63 (31–91)	105 (44–158)	154 (61–235)	123 (67–182)	70 (51–111)	72 (55–113)	62 (47–97)	38 (25–51)	31 (17–47)	17 (8–31)	--
Chlamydial infection	--	--	--	--	--	0 (0–0)	0 (0–0)	0 (0–0)	0 (0–0)	0 (0–0)	0 (0–0)	0 (0–0)	0 (0–0)	0 (0–0)	0 (0–0)	0 (0–0)	0 (0–0)	0 (0–0)	0 (0–0)	0 (0–0)	0 (0–0)	0 (0–0)	--
Gonococcal infection	--	--	--	--	--	3 (2–4)	6 (4–8)	13 (8–17)	4 (3–5)	8 (5–12)	6 (4–9)	12 (7–16)	8 (4–12)	7 (3–11)	10 (4–16)	12 (6–18)	8 (5–14)	7 (5–12)	7 (5–12)	5 (3–8)	5 (2–9)	1 (0–2)	--
Other sexually transmitted infections	--	--	--	--	--	24 (16–34)	5 (3–7)	7 (5–10)	13 (9–17)	32 (15–49)	8 (4–11)	10 (6–14)	18 (8–26)	30 (11–45)	30 (11–47)	17 (8–25)	9 (6–15)	10 (7–16)	30 (21–50)	29 (16–42)	42 (16–73)	35 (10–78)	--
Tuberculosis	--	--	12,569 (9,775–16,160)	12,634 (9,536–16,351)	3,547 (2,913–4,282)	3,503 (2,936–4,142)	9,563 (8,141–11,163)	20,385 (17,796–23,305)	30,722 (27,281–34,449)	39,947 (35,659–44,478)	47,816 (42,577–53,989)	52,358 (46,253–59,091)	60,697 (53,747–68,718)	65,957 (58,147–74,383)	71,802 (63,555–80,531)	68,443 (60,720–76,918)	68,459 (61,106–76,537)	65,007 (58,038–72,570)	51,680 (46,404–57,463)	42,439 (37,637–46,919)	22,877 (20,050–25,144)	8,344 (6,889–9,263)	--
Drug-susceptible tuberculosis	--	--	11,473 (8,803–14,928)	11,626 (8,742–15,074)	3,245 (2,614–3,939)	3,182 (2,603–3,858)	8,671 (7,246–10,320)	18,459 (15,546–21,404)	27,753 (23,732–31,842)	35,888 (30,748–41,065)	42,911 (36,299–49,514)	46,951 (39,476–54,394)	54,081 (45,371–62,193)	58,958 (50,092–68,237)	64,074 (54,045–73,802)	61,199 (52,210–70,805)	61,454 (52,195–70,418)	58,534 (50,380–66,528)	46,584 (39,950–52,919)	38,380 (32,933–43,483)	20,828 (18,005–23,372)	7,646 (6,322–8,630)	--
Multidrug-resistant tuberculosis without extensive drug resistance	--	--	1,045 (452–1,893)	971 (425–1,837)	288 (131–529)	305 (129–570)	850 (364–1,567)	1,811 (792–3,348)	2,760 (1,197–5,179)	3,747 (1,591–6,992)	4,558 (1,845–8,728)	5,017 (2,048–9,681)	6,034 (2,503–11,498)	6,389 (2,610–12,366)	7,056 (2,863–13,785)	6,636 (2,564–13,112)	6,485 (2,410–13,023)	6,039 (2,203–11,642)	4,753 (1,790–9,356)	3,782 (1,449–7,445)	1,904 (769–3,531)	647 (259–1,218)	--
Extensively drug-resistant tuberculosis	--	--	50 (24–90)	37 (17–69)	15 (7–29)	16 (7–31)	42 (18–80)	115 (56–207)	208 (105–361)	312 (159–532)	347 (169–622)	389 (190–696)	582 (301–1,006)	611 (314–1,049)	672 (345–1,167)	608 (298–1,081)	520 (232–968)	434 (182–850)	343 (142–678)	277 (114–542)	145 (62–276)	51 (22–96)	--
Lower respiratory infections	65,032 (51,636–81,512)	39,801 (32,459–49,349)	153,062 (122,142–192,595)	83,420 (64,907–105,074)	14,050 (11,560–16,877)	7,794 (6,644–9,136)	7,473 (6,608–8,705)	8,934 (7,838–10,084)	10,648 (9,568–11,991)	13,493 (12,217–15,100)	16,982 (15,465–18,851)	20,612 (18,734–22,933)	26,644 (24,138–29,656)	34,577 (31,558–37,995)	46,472 (42,280–50,878)	65,812 (60,196–71,777)	81,324 (74,617–88,337)	103,180 (94,996–111,805)	120,913 (111,570–130,952)	142,955 (127,507–154,941)	127,366 (110,578–138,384)	73,365 (60,311–80,452)	--
Upper respiratory infections	20 (10–30)	48 (22–82)	1,023 (307–2,136)	1,226 (231–3,193)	147 (49–282)	113 (36–200)	62 (24–113)	48 (23–78)	58 (33–84)	73 (45–101)	127 (66–178)	114 (67–152)	148 (83–198)	151 (92–208)	163 (104–231)	184 (114–275)	215 (138–397)	239 (163–416)	279 (185–567)	383 (243–916)	342 (205–844)	118 (78–255)	--
Otitis media	114 (2–547)	29 (4–130)	30 (13–68)	16 (4–46)	14 (5–33)	11 (5–26)	9 (5–21)	6 (4–16)	6 (4–10)	4 (3–6)	5 (4–8)	8 (5–13)	23 (14–39)	36 (20–67)	16 (11–25)	23 (15–42)	22 (14–36)	17 (12–26)	14 (10–21)	13 (9–20)	9 (7–13)	3 (2–3)	--
Diarrhoeal diseases	8,110 (6,252–10,083)	15,218 (11,770–19,172)	130,034 (100,474–167,351)	112,926 (83,152–152,256)	18,481 (11,028–31,719)	8,440 (4,914–15,931)	8,457 (4,706–16,154)	9,250 (5,165–18,347)	8,693 (4,778–16,950)	8,879 (4,749–17,049)	10,674 (6,025–20,536)	11,926 (6,638–23,477)	14,213 (8,428–28,040)	17,346 (10,122–36,323)	21,608 (12,748–43,250)	30,071 (17,374–61,171)	39,468 (23,487–79,643)	50,805 (30,311–102,034)	55,240 (31,817–101,420)	57,872 (34,334–109,990)	40,777 (25,551–73,188)	20,844 (13,114–36,878)	--
Typhoid fever	--	--	64 (24–152)	10,487 (4,099–21,213)	13,635	12,194	8,610	4,950	3,000	1,930	1,216	880	818	740	649	541	417	288	189	115	56	20	[illegible]
Paratyphoid fever	--	--	8 (2–20)	1,564 (490–3,755)	[illegible]	[illegible]	[illegible]	[illegible]	[illegible]	[illegible]	[illegible]	[illegible]	[illegible]	[illegible]	[illegible]	[illegible]	[illegible]	[illegible]	[illegible]	[illegible]	[illegible]	[illegible]	[illegible]

Upper digestive system diseases	--	--	--	773 (445–1,402)	328 (241–440)	254 (204–317)	926 (773–1,113)	1,483 (1,246–1,731)	2,389 (2,103–2,831)	2,958 (2,635–3,430)	4,155 (3,698–4,875)	5,277 (4,610–6,062)	7,527 (6,537–8,755)	9,763 (8,621–11,082)	11,898 (10,554–13,463)	13,488 (11,967–15,251)	16,030 (14,258–18,155)	17,278 (15,577–19,332)	18,368 (16,618–20,529)	16,914 (15,117–18,952)	12,203 (10,727–13,432)	4,889 (4,095–5,454)	--
Peptic ulcer disease	--	--	--	358 (176–882)	289 (210–390)	226 (179–279)	790 (651–960)	1,292 (1,098–1,528)	2,037 (1,782–2,414)	2,452 (2,176–2,869)	3,600 (3,158–4,335)	4,537 (3,934–5,330)	6,439 (5,557–7,631)	8,371 (7,361–9,558)	10,363 (9,167–11,854)	11,891 (10,606–13,506)	13,885 (12,382–15,893)	14,883 (13,389–16,860)	15,770 (14,225–17,869)	14,395 (12,854–16,336)	10,248 (8,947–11,517)	4,286 (3,582–4,820)	--
Gastritis and duodenitis	--	--	--	414 (228–755)	39 (21–55)	28 (14–42)	136 (88–200)	191 (119–279)	352 (248–489)	507 (390–654)	555 (437–729)	740 (559–922)	1,088 (738–1,372)	1,392 (900–1,738)	1,535 (1,006–1,880)	1,597 (1,116–1,908)	2,145 (1,523–2,583)	2,396 (1,555–2,878)	2,598 (1,711–3,075)	2,519 (1,719–2,994)	1,955 (1,411–2,256)	603 (486–700)	--
Appendicitis	--	--	--	557 (293–891)	575 (400–746)	539 (370–700)	723 (527–939)	647 (464–809)	819 (635–1,023)	522 (380–648)	743 (549–923)	901 (654–1,095)	866 (614–1,055)	900 (664–1,110)	1,049 (779–1,287)	1,210 (878–1,486)	1,247 (924–1,575)	1,387 (973–1,708)	1,265 (959–1,569)	1,183 (933–1,457)	751 (584–939)	344 (260–435)	--
Paralytic ileus and intestinal obstruction	848 (578–1,256)	903 (618–1,298)	6,608 (4,632–9,456)	1,936 (1,076–3,063)	1,569 (1,003–2,209)	1,061 (713–1,449)	1,918 (1,300–2,491)	1,902 (1,265–2,455)	2,361 (1,658–2,961)	2,811 (1,943–3,462)	3,765 (2,551–4,652)	4,579 (3,147–5,677)	4,668 (3,333–5,778)	5,338 (3,819–6,433)	7,064 (5,199–8,452)	8,774 (6,448–10,550)	11,226 (8,419–13,273)	11,529 (9,070–13,705)	12,126 (9,953–14,462)	13,476 (10,934–16,040)	11,731 (9,447–13,690)	6,114 (4,592–7,085)	--
Inguinal, femoral, and abdominal hernia	429 (312–571)	257 (181–359)	991 (690–1,387)	458 (258–674)	143 (88–199)	101 (69–131)	188 (141–255)	261 (201–335)	313 (255–382)	279 (237–335)	432 (359–518)	560 (455–668)	759 (595–916)	1,234 (964–1,489)	1,670 (1,330–1,956)	2,094 (1,675–2,469)	2,677 (2,144–3,116)	3,132 (2,667–3,680)	3,676 (3,190–4,339)	4,032 (3,482–4,715)	2,995 (2,539–3,501)	1,644 (1,346–1,929)	--
Inflammatory bowel disease	--	--	--	337 (190–541)	75 (50–94)	58 (43–72)	100 (75–129)	155 (125–195)	269 (219–335)	359 (286–442)	421 (342–499)	560 (465–685)	689 (550–827)	879 (696–1,044)	1,133 (915–1,320)	1,432 (1,100–1,647)	1,724 (1,320–1,987)	1,968 (1,561–2,257)	2,281 (1,769–2,599)	2,544 (1,982–2,897)	2,084 (1,564–2,362)	1,149 (811–1,331)	--
Vascular intestinal disorders	--	--	--	258 (134–476)	71 (49–110)	25 (20–31)	46 (40–54)	114 (95–137)	184 (156–215)	178 (160–199)	312 (277–354)	520 (464–580)	889 (789–989)	1,500 (1,344–1,651)	2,544 (2,319–2,782)	3,579 (3,284–3,871)	5,300 (4,855–5,737)	6,401 (5,920–6,897)	6,906 (6,359–7,398)	7,330 (6,577–8,020)	5,245 (4,453–5,835)	2,739 (2,259–3,180)	--
Gallbladder and biliary diseases	--	--	--	135 (66–227)	67 (42–88)	170 (88–244)	140 (93–179)	253 (174–313)	352 (242–436)	458 (334–555)	648 (493–772)	906 (678–1,096)	1,179 (893–1,429)	1,890 (1,425–2,295)	2,483 (1,947–2,947)	3,496 (2,830–4,155)	4,706 (3,886–5,630)	6,139 (5,129–7,391)	7,724 (6,407–9,114)	8,730 (7,201–10,079)	7,558 (6,035–8,738)	4,108 (3,170–4,792)	--
Pancreatitis	--	--	--	53 (27–89)	55 (40–71)	68 (51–87)	469 (358–598)	1,113 (904–1,396)	2,508 (2,119–3,101)	3,833 (3,356–4,631)	5,021 (4,359–6,097)	5,945 (5,076–7,286)	7,164 (5,940–8,651)	6,020 (5,101–7,238)	7,438 (6,250–8,718)	6,365 (5,418–7,525)	6,359 (5,392–7,457)	5,682 (4,870–6,558)	5,201 (4,396–6,158)	4,268 (3,693–4,927)	2,872 (2,423–3,319)	1,188 (977–1,387)	--
Other digestive diseases	--	--	--	1,123 (696–1,705)	336 (240–427)	245 (185–322)	265 (200–352)	322 (245–407)	510 (389–637)	703 (547–836)	917 (713–1,087)	1,330 (1,035–1,593)	1,744 (1,342–2,077)	2,370 (1,801–2,833)	2,998 (2,276–3,545)	3,952 (3,036–4,666)	4,445 (3,330–5,236)	5,182 (3,896–6,187)	5,620 (4,238–6,791)	6,395 (4,727–7,657)	5,434 (3,967–6,684)	3,069 (2,187–3,991)	--
Alzheimer's disease and other dementias	--	--	--	--	--	--	--	--	--	--	--	283 (28–1,022)	1,640 (211–5,216)	3,483 (564–10,967)	6,770 (1,244–19,511)	13,123 (2,669–39,478)	24,482 (5,345–73,527)	40,597 (9,163–117,298)	63,161 (14,154–183,665)	125,391 (29,145–352,373)	144,321 (34,303–390,077)	94,297 (22,906–245,220)	--
Parkinson's disease	--	--	--	--	--	--	--	7 (6–8)	12 (11–14)	28 (25–31)	37 (33–41)	429 (382–475)	971 (852–1,092)	1,861 (1,656–2,075)	2,967 (2,652–3,308)	5,903 (5,348–6,584)	12,727 (11,642–14,058)	27,506 (25,220–30,069)	45,044 (41,410–48,794)	54,669 (49,087–59,465)	39,141 (33,906–42,628)	13,877 (11,561–15,265)	--
Idiopathic epilepsy	--	--	1,329 (1,002–1,983)	3,344 (2,613–4,412)	1,773 (1,391–2,321)	2,292 (1,897–2,913)	4,211 (3,569–5,203)	4,730 (4,165–5,612)	4,956 (4,402–5,796)	5,061 (4,472–5,888)	5,061 (4,523–5,839)	4,659 (4,100–5,427)	4,265 (3,750–4,929)	3,661 (3,244–4,103)	3,608 (3,143–4,070)	3,292 (2,900–3,729)	3,201 (2,754–3,681)	2,690 (2,411–3,044)	2,538 (2,221–2,848)	2,352 (1,977–2,638)	1,564 (1,329–1,731)	664 (548–745)	--
Multiple sclerosis	--	--	--	--	15 (1–76)	5 (1–14)	13 (5–22)	56 (40–69)	126 (105–191)	283 (239–451)	466 (391–738)	706 (592–1,109)	907 (755–1,306)	1,070 (844–1,515)	1,217 (944–1,757)	1,192 (858–1,671)	1,033 (746–1,354)	816 (552–1,012)	573 (390–758)	376 (262–490)	190 (129–240)	59 (39–73)	--
Motor neuron disease	4 (3–4)	8 (7–9)	242 (203–294)	79 (62–106)	25 (21–30)	38 (34–44)	92 (81–104)	94 (82–110)	82 (74–91)	149 (135–163)	282 (259–305)	500 (463–535)	903 (836–967)	1,412 (1,308–1,510)	2,160 (2,011–2,288)	2,821 (2,655–2,971)	3,397 (3,169–3,583)	3,435 (3,175–3,644)	2,849 (2,611–3,043)	1,824 (1,595–1,981)	879 (744–967)	250 (205–278)	--
Other neurological disorders	--	--	1,006 (794–1,280)	1,224 (884–1,596)	680 (528–812)	841 (709–996)	1,644 (1,438–1,930)	1,385 (1,237–1,599)	929 (847–1,057)	827 (754–938)	940 (853–1,063)	1,106 (1,006–1,239)	1,544 (1,404–1,716)	1,904 (1,731–2,106)	2,454 (2,249–2,703)	2,531 (2,349–2,744)	2,773 (2,578–2,988)	2,873 (2,687–3,074)	2,831 (2,621–3,047)	2,457 (2,191–2,643)	1,602 (1,384–1,746)	681 (560–747)	--
Eating disorders	--	--	--	--	0 (0–1)	0 (0–0)	3 (1–7)	5 (3–11)	4 (3–6)	6 (4–9)	5 (4–7)	9 (6–12)	8 (6–10)	--	--	--	--	--	--	--	--	--	--
Anorexia nervosa	--	--	--	--	0 (0–0)	0 (0–0)	2 (1–4)	3 (2–7)	3 (2–4)	3 (2–5)	3 (2–4)	6 (4–8)	5 (4–8)	--	--	--	--	--	--	--	--	--	--
Bulimia nervosa	--	--	--	--	0 (0–0)	0 (0–0)	1 (0–5)	2 (0–7)	1 (0–3)	2 (0–5)	2 (1–4)	3 (1–6)	3 (0–5)	--	--	--	--	--	--	--	--	--	--
Alcohol use disorders	--	--	--	--	--	0 (0–0)	592 (433–673)	1,961 (1,477–2,209)	4,487 (3,653–4,908)	9,428 (7,665–10,255)	12,842 (10,643–13,949)	15,740 (13,306–17,142)	19,094 (15,983–20,879)	18,387 (15,605–20,055)	19,025 (16,478–20,558)	15,805 (13,763–17,106)	10,922 (9,425–11,757)	6,636 (5,625–7,195)	4,381 (3,635–4,796)	2,200 (1,855–2,385)	1,065 (867–1,163)	324 (258–359)	--
Drug use disorders	0 (0–0)	0 (0–0)	0 (0–0)	0 (0–0)	0 (0–0)	0 (0–0)	1,837 (1,683–1,996)	7,223 (6,714–8,052)	11,674 (10,912–13,030)	14,056 (13,169–15,708)	12,717 (11,872–14,208)	10,083 (9,567–10,656)	8,760 (8,208–9,367)	7,197 (6,615–7,694)	6,055 (5,515–6,549)	3,625 (3,316–3,894)	2,578 (2,379–2,782)	1,720 (1,584–1,861)	1,291 (1,186–1,394)	964 (866–1,055)	635 (549–701)	315 (259–359)	--
Opioid use disorders	0 (0–0)	0 (0–0)	0 (0–0)	0 (0–0)	0 (0–0)	0 (0–0)	1,268 (1,147–1,387)	5,333 (4,924–6,025)	8,636 (7,978–9,904)	10,020 (9,192–11,421)	9,105 (8,494–10,261)	7,090 (6,709–7,578)	6,048 (5,606–6,650)	4,763 (4,351–5,203)	4,009 (3,618–4,393)	2,425 (2,207–2,630)	1,721 (1,584–1,861)	1,153 (1,054–1,249)	892 (814–971)	627 (560–692)	414 (354–463)	194 (160–226)	--
Cocaine use disorders	--	--	--	--	--	0 (0–0)	182 (161–201)	552 (500–631)	851 (736–1,354)	1,403 (1,221–2,226)	1,252 (1,143–1,440)	1,000 (820–1,093)	969 (727–1,060)	1,090 (786–1,200)	916 (670–1,031)	500 (370–565)	289 (235–320)	139 (116–156)	87 (73–97)	14 (12–16)	9 (7–10)	4 (3–5)	--
Amphetamine use disorders	--	--	--	--	--	0 (0–0)	135 (113–156)	512 (457–575)	947 (792–1,213)	1,056 (885–1,385)	865 (783–972)	672 (564–769)	558 (436–657)	566 (413–676)	450 (325–536)	270 (198–318)	179 (144–208)	34 (24–41)	20 (14–24)	33 (25–46)	34 (24–50)	24 (17–34)	--
Other drug use disorders	--	--	--	--	--	0 (0–0)	253 (229–286)	827 (747–926)	1,240 (1,128–1,467)	1,577 (1,444–1,840)	1,495 (1,364–1,656)	1,320 (1,224–1,430)	1,184 (1,088–1,308)	778 (710–852)	680 (615–749)	430 (390–473)	389 (352–433)	393 (358–433)	292 (262–326)	289 (260–321)	178 (156–196)	92 (76–104)	--
Diabetes mellitus	29 (22–38)	73 (55–97)	813 (579–1,138)	640 (438–950)	554 (438–758)	606 (485–768)	1,587 (1,390–1,820)	2,727 (2,441–3,081)	3,421 (3,124–3,767)	6,336 (5,791–6,976)	9,128 (8,356–9,978)	16,784 (15,270–18,329)	28,398 (25,824–31,221)	47,036 (43,070–50,961)	68,510 (62,852–74,287)	89,280 (82,534–96,824)	102,277 (95,087–110,041)	104,853 (97,521–112,843)	100,205 (93,489–106,912)	85,024 (77,107–91,560)	55,747 (49,136–60,191)	23,762 (19,933–25,960)	--
Diabetes mellitus type 1	29 (22–38)	73 (55–97)	813 (579–1,138)	640 (438–950)	554 (438–758)	606 (485–768)	929 (808–1,073)	1,838 (1,619–2,111)	2,140 (1,936–2,430)	2,410 (2,089–2,846)	3,251 (2,860–3,870)	5,108 (4,431–6,460)	1,635 (1,344–2,357)	2,427 (1,991–3,507)	3,492 (2,872–5,155)	3,260 (2,701–4,629)	3,693 (3,040–5,426)	2,813 (2,330–4,029)	2,441 (2,000–3,461)	1,099 (899–1,509)	761 (602–1,046)	375 (279–517)	--
Diabetes mellitus type 2	--	--	--	--	--	--	658 (525–832)	890 (724–1,123)	1,280 (1,100–1,525)	3,926 (3,478–4,466)	5,877 (5,245–6,611)	11,676 (10,385–12,990)	26,763 (24,294–29,272)	44,609 (40,753–48,414)	65,017 (59,571–70,590)	86,020 (79,457–93,237)	98,585 (91,543–106,358)	102,040 (94,914–109,499)	97,764 (91,197–104,499)	83,925 (76,073–90,408)	54,986 (48,341–59,300)	23,387 (19,600–25,542)	--
Chronic kidney disease	--	--	1,982 (1,528–2,585)	2,561 (1,957–3,392)	2,374 (1,939–2,899)	2,188 (1,850–2,576)	5,696 (4,995–6,542)	7,807 (6,940–8,740)	8,824 (8,019–9,756)	11,760 (10,769–12,988)	15,843 (14,474–17,535)	21,537 (19,605–23,881)	28,177 (25,392–31,294)	40,950 (37,108–45,268)	54,722 (49,624–60,640)	68,115 (62,437–75,251)	80,271 (74,215–87,538)	86,466 (79,951–93,772)	88,469 (82,429–95,344)	87,705 (79,189–94,585)	71,849 (62,755–77,723)	38,441 (32,099–41,918)	--
Chronic kidney disease due to diabetes mellitus type 1	--	--	11 (5–24)	19 (9–39)	33 (12–72)	61 (23–126)	260 (100–516)	675 (298–1,217)	1,239 (628–2,087)	2,353 (1,260–3,539)	3,601 (1,958–5,524)	4,992 (2,702–7,725)	5,756 (2,661–9,413)	6,059 (2,637–10,968)	5,393 (1,939–10,643)	4,778 (1,867–9,871)	4,183 (1,788–8,774)	3,178 (1,328–6,317)	2,008 (804–4,238)	1,025 (394–2,255)	345 (122–816)	55 (17–133)	--
Chronic kidney disease due to diabetes mellitus type 2	--	--	--	--	--	--	140 (40–320)	307 (116–661)	468 (177–923)	869 (307–1,708)	1,545 (579–2,911)	3,029 (1,273–5,358)	5,871 (3,000–9,343)	11,334 (6,417–16,487)	17,580 (10,784–24,430)	22,847 (13,213–31,010)	27,158 (17,542–35,971)	28,927 (19,281–37,745)	28,233 (18,424–37,582)	25,767 (16,773–35,128)	18,858 (12,294–26,567)	8,976 (5,452–13,148)	--
Chronic kidney disease due to hypertension	--	--	--	--	--	--	397 (204–699)	804 (454–1,310)	1,100 (654–1,742)	1,601 (990–2,426)	2,413 (1,525–3,579)	3,710 (2,510–5,297)	5,554 (3,931–7,771)	9,386 (6,604–12,814)	14,028 (9,946–18,983)	19,744 (14,634–26,096)	24,966 (18,955–32,260)	28,275 (22,179–35,864)	31,226 (24,632–40,245)	33,494 (25,954–42,215)	30,450 (23,853–37,730)	17,604 (13,344–21,810)	--
Chronic kidney disease due to glomerulonephritis	--	--	536 (273–897)	881 (462–1,424)	843 (441–1,338)	917 (510–1,399)	2,463 (1,580–3,495)	3,026 (1,828–4,327)	3,093 (1,966–4,425)	3,388 (1,923–5,150)	4,116 (2,365–6,484)	4,810 (2,308–7,615)	5,034 (2,990–8,015)	6,562 (3,671–10,387)	8,214 (4,639–13,314)	9,316 (5,032–14,824)	9,817 (5,140–16,405)	9,893 (5,378–15,994)	9,908 (5,381–16,121)	9,303 (4,607–15,791)	6,669 (3,380–11,254)	3,203 (1,584–5,469)	--
Chronic kidney disease due to other and unspecified causes	--	--	1,435 (1,031–1,922)	1,661 (1,134–2,311)	1,498 (1,016–2,037)	1,210 (747–1,674)	2,438 (1,511–3,399)	2,995 (1,829–4,309)	2,923 (1,833–4,058)	3,548 (2,063–5,315)	4,167 (2,277–6,447)	4,997 (2,678–7,787)	5,963 (3,102–9,393)	7,608 (4,222–12,158)	9,508 (5,116–15,732)	11,431 (6,172–19,441)	14,146 (7,889–22,258)	16,192 (9,379–25,242)	17,094 (9,651–26,676)	18,116 (10,300–27,691)	15,528 (8,858–22,997)	8,603 (5,334–12,760)	--
Acute glomerulonephritis	--	--	58 (17–91)	68 (21–101)	123 (39–177)	63 (21–88)	58 (29–79)	75 (39–100)	110 (52–136)	159 (79–200)	174 (85–221)	223 (104–287)	261 (133–339)	357 (172–466)	370 (194–487)	456 (236–579)	632 (312–806)	610 (338–777)	669 (329–847)	619 (316–766)	440 (244–530)	115 (72–137)	--
Skin and subcutaneous diseases	--	--	731 (363–1,026)	612 (334–820)	158 (80–208)	229 (124–308)	436 (256–538)	408 (225–498)	379 (225–476)	867 (524–1,048)	901 (523–1,142)	1,081 (702–1,340)	1,593 (977–2,016)	1,872 (1,140–2,374)	2,661 (1,625–3,503)	3,377 (2,054–4,324)	3,856 (2,381–5,098)	4,611 (2,843–6,426)	5,517 (3,595–8,044)	5,235 (3,356–8,219)	4,719 (2,938–7,431)	2,850 (1,628–4,481)	--
Bacterial skin diseases	--	--	713 (347–1,002)	586 (312–792)	142 (69–192)	211 (113–285)	403 (243–499)	365 (199–446)	327 (195–400)	766 (450–932)	784 (438–989)	928 (582–1,175)	1,398 (841–1,769)	1,612 (957–2,039)	2,263 (1,325–2,949)	2,853 (1,664–3,682)	3,065 (1,772–4,106)	3,469 (1,992–4,910)	3,990 (2,429–5,893)	3,320 (1,950–5,252)	2,871 (1,542–4,757)	1,769 (819–2,962)	--
Cellulitis	--	--	80 (22–135)	51 (11–116)	16 (6–24)	34 (15–48)	101 (28–157)	138 (53–190)	69 (29–98)	174 (71–247)	248 (106–359)	293 (138–430)	441 (190–629)	462 (215–700)	628 (269–952)	785 (355–1,190)	800 (348–1,243)	876 (383–1,398)	905 (379–1,657)	844 (330–1,517)	709 (256–1,433)	452 (179–845)	--
Pyoderma	--	--	633 (310–885)	535 (291–735)	127 (60–173)	177 (91–244)	302 (202–392)	227 (129–303)	258 (154–316)	592 (327–725)	537 (282–689)	635 (369–810)	957 (547–1,233)	1,150 (663–1,462)	1,635 (922–2,106)	2,068 (1,156–2,621)	2,265 (1,257–2,967)	2,594 (1,417–3,521)	3,084 (1,812–4,316)	2,476 (1,417–3,814)	2,161 (1,144–3,364)	1,318 (592–2,130)	--
Decubitus ulcer	--	--	--	4 (1–9)	4 (1–6)	7 (3–10)	22 (7–31)	32 (11–46)	40 (16–58)	85 (52–120)	95 (53–136)	118 (75–172)	145 (85–224)	213 (127–299)	327 (188–492)	439 (239–644)	684 (397–996)	1,005 (585–1,512)	1,362 (775–2,341)	1,710 (1,044–2,780)	1,647 (974–2,729)	965 (505–1,645)	--
Other skin and subcutaneous diseases	--	--	18 (12–30)	22 (15–35)	12 (8–16)	11 (7–15)	11 (7–15)	11 (8–17)	11 (8–17)	16 (11–23)	22 (14–33)	35 (25–47)	49 (32–67)	47 (29–65)	70 (46–97)	85 (54–118)	107 (66–154)	137 (87–206)	165 (103–272)	205 (123–342)	201 (118–349)	115 (58–212)	--
Musculoskeletal disorders	--	--	--	--	120 (102–147)	214 (185–262)	303 (268–356)	376 (336–441)	457 (413–527)	555 (503–644)	642 (575–747)	829 (741–979)	1,008 (896–1,209)	1,305 (1,155–1,548)	1,893 (1,674–2,201)	3,033 (2,683–3,532)	3,932 (3,496–4,523)	4,930 (4,350–5,660)	5,184 (4,480–5,846)	5,126 (4,267–5,805)	3,933 (3,165–4,418)	1,800 (1,346–2,067)	--
Rheumatoid arthritis	--	--	--	--	13 (11–18)	21 (17–28)	19 (16–23)	22 (19–28)	32 (28–39)	53 (46–64)	77 (67–90)	137 (118–163)	223 (190–268)	391 (337–465)	661 (570–766)	1,172 (1,007–1,360)	1,716 (1,495–1,958)	2,305 (1,986–2,647)	2,502 (2,135–2,854)	2,237 (1,862–2,548)	1,597 (1,333–1,821)	514 (406–604)	--
Other musculoskeletal disorders	--	--	--	--	107 (90–129)	193 (166–235)	284 (252–332)	353 (316–414)	424 (384–490)	502 (456–580)	565 (508–661)	692 (617–816)	785 (695–946)	914 (805–1,093)	1,233 (1,075–1,462)	1,862 (1,619–2,191)	2,216 (1,939–2,612)	2,625 (2,293–3,069)	2,682 (2,251–3,095)	2,888 (2,295–3,295)	2,336 (1,826–2,674)	1,286 (919–1,496)	--
Congenital anomalies	100,159 (80,464–126,197)	31,304 (25,676–39,720)	92,762 (68,257–130,980)	37,699 (20,820–62,404)	8,691 (7,298–10,773)	5,795 (4,861–7,041)	4,056 (3,385–4,984)	3,688 (3,152–4,404)	3,310 (2,883–3,916)	2,512 (2,193–2,913)	2,636 (2,267–3,074)	2,465 (2,103–2,924)	1,538 (1,279–1,752)	1,519 (1,199–1,735)	1,583 (1,187–1,803)	1,458 (1,102–1,650)	1,037 (810–1,170)	--	--	--	--	--	--
Neural tube defects	17,074 (11,235–25,300)	3,515 (2,401–5,145)	9,871 (5,697–17,128)	9,086 (4,142–17,060)	448 (281–684)	310 (204–470)	142 (93–254)	107 (77–165)	100 (77–145)	89 (70–123)	81 (67–105)	80 (64–111)	52 (42–68)	40 (33–51)	35 (28–46)	36 (29–49)	30 (24–37)	--	--	--	--	--	--
Congenital heart anomalies	30,083 (21,899–39,767)	14,061 (11,032–17,539)	42,007 (31,672–55,337)	13,039 (8,116–21,632)	4,764 (3,795–5,998)	3,447 (2,826–4,265)	2,426 (2,005–2,965)	2,358 (2,009–2,856)	1,995 (1,705–2,361)	1,512 (1,305–1,744)	1,532 (1,316–1,816)	1,415 (1,207–1,693)	783 (647–907)	713 (575–829)	664 (533–767)	616 (499–714)	459 (367–525)	--	--	--	--	--	--
Orofacial clefts	591 (308–1,468)	181 (101–427)	582 (296–1,457)	46 (32–84)	--	--	--	--	--	--	--	--	--	--	--	--	--	--	--	--	--	--	--
Down's syndrome	1,578 (1,164–2,666)	691 (518–1,091)	3,525 (2,550–6,556)	1,352 (822–4,256)	346 (271–508)	231 (187–319)	225 (179–286)	237 (198–292)	250 (206–313)	248 (208–320)	290 (238–370)	312 (254–389)	306 (249–358)	422 (311–480)	537 (357–615)	469 (300–535)	237 (156–268)	--	--	--	--	--	--
Other chromosomal abnormalities	3,826 (2,087–6,981)	1,373 (822–2,390)	3,205 (1,756–6,564)	835 (301–2,487)	205 (129–317)	144 (92–193)	148 (105–184)	138 (99–173)	167 (123–213)	101 (60–119)	135 (89–164)	81 (48–100)	65 (35–78)	48 (21–59)	51 (21–62)	33 (11–42)	34 (14–41)	--	--	--	--	--	--
Congenital musculoskeletal and limb anomalies	2,711 (1,741–4,095)	578 (401–789)	1,533 (1,000–2,400)	511 (240–1,232)	197 (143–263)	133 (99–174)	138 (100–188)	87 (64–113)	83 (61–110)	59 (46–71)	61 (46–77)	49 (36–64)	32 (21–38)	31 (21–38)	27 (17–33)	35 (25–44)	26 (19–36)	--	--	--	--	--	--
Urogenital congenital anomalies	3,595 (2,069–5,560)	707 (440–1,064)	1,115 (617–1,699)	261 (96–513)	73 (48–100)	56 (41–77)	52 (33–65)	60 (33–74)	66 (41–81)	51 (36–61)	53 (39–64)	55 (41–66)	30 (22–36)	29 (21–34)	28 (20–33)	33 (24–39)	32 (24–39)	--	--	--	--	--	--
Digestive congenital anomalies	9,454 (5,388–14,394)	3,533 (2,311–4,974)	9,318 (5,321–14,796)	1,899 (662–4,290)	234 (150–329)	126 (89–168)	50 (36–69)	40 (28–52)	52 (37–69)	34 (26–43)	44 (31–54)	44 (29–54)	35 (23–42)	40 (25–47)	53 (34–63)	56 (39–67)	66 (43–78)	--	--	--	--	--	--
Other congenital anomalies	31,248 (21,316–47,780)	6,666 (4,944–9,747)	21,605 (13,720–35,626)	10,669 (4,721–21,889)	2,423 (1,899–3,690)	1,348 (1,084–1,934)	874 (669–1,204)	661 (526–848)	596 (463–784)	417 (343–536)	439 (350–554)	430 (331–545)	234 (180–283)	196 (148–235)	188 (138–220)	179 (130–210)	155 (120–181)	--	--	--	--	--	--
Urinary diseases and male infertility	367 (262–506)	204 (146–281)	1,321 (976–1,829)	1,015 (771–1,284)	676 (508–857)	530 (428–638)	1,185 (956–1,408)	1,748 (1,488–2,016)	1,609 (1,362–1,838)	2,188 (1,861–2,487)	2,599 (2,196–2,979)	3,388 (2,780–3,886)	4,453 (3,535–5,067)	5,874 (4,606–6,619)	7,470 (5,907–8,361)	10,227 (7,965–11,418)	13,614 (10,855–15,281)	16,269 (13,029–18,113)	17,814 (14,122–19,659)	20,905 (16,086–23,080)	17,181 (12,987–19,001)	10,642 (7,853–11,939)	--
Urinary tract infections and interstitial nephritis	91 (54–137)	105 (58–151)	1,057 (748–1,470)	497 (336–654)	471 (337–615)	364 (274–445)	783 (619–952)	1,436 (1,206–1,696)	1,238 (1,029–1,453)	1,474 (1,192–1,727)	1,720 (1,377–2,019)	2,295 (1,807–2,721)	2,912 (2,236–3,462)	4,008 (3,180–4,718)	4,866 (3,696–5,633)	6,967 (5,334–8,123)	9,288 (7,105–10,776)	11,296 (8,600–12,909)	13,018 (9,704–14,770)	15,159 (10,969–17,113)	13,405 (9,676–15,079)	8,612 (5,920–9,763)	--
Urolithiasis	--	--	--	7 (4–12)	15 (8–26)	12 (6–18)	15 (6–25)	20 (10–28)	37 (19–55)	52 (29–72)	98 (48–128)	167 (92–227)	239 (118–325)	381 (202–499)	456 (257–607)	692 (383–899)	890 (541–1,175)	930 (587–1,284)	980 (614–1,398)	909 (584–1,243)	633 (420–864)	303 (201–440)	--
Other urinary diseases	276 (184–391)	99 (63–147)	265 (147–417)	510 (363–702)	190 (133–269)	155 (109–206)	388 (302–500)	293 (187–420)	333 (244–430)	663 (524–842)	782 (599–1,036)	925 (698–1,242)	1,302 (991–1,774)	1,485 (981–1,996)	2,148 (1,599–2,833)	2,569 (1,783–3,471)	3,436 (2,496–4,648)	4,044 (2,851–5,680)	3,816 (2,665–5,217)	4,837 (3,504–6,491)	3,143 (2,306–4,131)	1,727 (1,248–2,386)	--
Gynecological diseases	--	--	--	--	--	--	--	--	--	--	--	--	--	--	--	--	--	--	--	--	--	--	--
Uterine fibroids	--	--	--	--	--	--	--	--	--	--	--	--	--	--	--	--	--	--	--	--	--	--	--
Endometriosis	--	--	--	--	--	--	--	--	--	--	--	--	--	--	--	--	--	--	--	--	--	--	--
Genital prolapse	--	--	--	--	--	--	--	--	--	--	--	--	--	--	--	--	--	--	--	--	--	--	--
Other gynecological diseases	--	--	--	--	--	--	--	--	--	--	--	--	--	--	--	--	--	--	--	--	--	--	--
Hemoglobinopathies and hemolytic anaemias	416 (300–625)	249 (175–358)	2,558 (1,529–4,445)	8,694 (5,634–13,853)	3,130 (2,252–4,708)	2,608 (1,910–3,949)	2,920 (2,106–4,667)	2,556 (1,857–3,963)	1,940 (1,430–2,992)	1,584 (1,197–2,417)	1,434 (1,047–2,211)	1,340 (963–2,128)	1,236 (929–1,934)	1,545 (1,174–2,330)	1,420 (1,128–2,218)	1,839 (1,491–2,667)	2,120 (1,762–2,989)	2,250 (1,896–3,042)	2,155 (1,851–2,878)	1,956 (1,692–2,564)	1,424 (1,217–1,779)	659 (542–797)	--
Thalassemias	169 (123–250)	117 (82–183)	606 (451–808)	1,247 (959–1,656)	862 (602–1,354)	511 (366–770)	358 (250–550)	238 (167–339)	169 (122–237)	159 (110–226)	136 (96–187)	146 (105–200)	157 (111–211)	171 (114–237)	108 (65–162)	53 (29–85)	18 (9–31)	4 (2–8)	1 (0–1)	0 (0–0)	0 (0–0)	0 (0–0)	--
Sickle cell disorders	230 (132–428)	125 (76–208)	1,883 (797–3,670)	7,232 (4,364–11,957)	2,037 (1,314–3,281)	1,715 (1,183–2,764)	1,948 (1,278–3,406)	1,637 (1,052–2,797)	1,168 (740–2,051)	768 (465–1,415)	734 (418–1,384)	617 (328–1,178)	362 (183–740)	246 (117–489)	68 (39–126)	33 (21–54)	15 (9–22)	5 (3–9)	1 (1–2)	0 (0–0)	0 (0–0)	0 (0–0)	--
G6PD deficiency	8 (5–13)	5 (3–8)	58 (34–93)	199 (116–330)	208 (115–324)	337 (202–534)	524 (308–836)	544 (344–828)	508 (335–751)	542 (364–792)	456 (296–660)	450 (292–702)	550 (349–941)	801 (480–1,360)	760 (470–1,403)	872 (534–1,460)	737 (426–1,243)	534 (306–893)	316 (156–551)	144 (72–258)	51 (25–90)	12 (6–20)	--
Other hemoglobinopathies and hemolytic anaemias	9 (6–13)	2 (1–2)	12 (9–16)	16 (11–21)	24 (17–34)	45 (32–65)	90 (59–149)	137 (92–218)	95 (66–146)	115 (78–176)	108 (77–161)	127 (92–183)	167 (120–241)	326 (232–486)	485 (359–704)	880 (671–1,239)	1,350 (1,078–1,834)	1,705 (1,407–2,210)	1,836 (1,561–2,400)	1,811 (1,564–2,311)	1,373 (1,174–1,689)	647 (535–778)	--
Endocrine, metabolic, blood, and immune disorders	688 (457–861)	583 (381–746)	3,548 (2,411–4,800)	2,297 (1,564–3,124)	1,122 (831–1,487)	951 (727–1,259)	841 (643–1,065)	1,013 (794–1,305)	1,300 (1,009–1,683)	1,807 (1,412–2,218)	2,125 (1,556–2,504)	2,518 (1,815–2,895)	3,379 (2,445–3,979)	4,372 (3,057–5,048)	5,284 (3,545–6,090)	6,103 (4,226–6,938)	7,028 (4,944–8,466)	7,485 (5,445–8,884)	7,256 (5,103–8,467)	6,896 (4,597–7,910)	5,747 (3,878–6,667)	3,025 (1,853–3,543)	--
Sudden infant death syndrome	--	1,431 (758–2,658)	13,838 (5,800–30,798)	--	--	--	--	--	--	--	--	--	--	--	--	--	--	--	--	--	--	--	--
Transport injuries	515 (333–680)	300 (201–391)	3,963 (2,810–5,210)	18,099 (13,715–24,178)	19,176 (15,806–23,008)	18,691 (15,424–21,847)	59,348 (51,785–66,272)	93,105 (82,545–102,447)	89,972 (78,673–98,379)	89,409 (75,702–97,696)	83,886 (69,964–92,976)	76,309 (62,515–85,247)	75,219 (60,386–84,655)	71,864 (57,172–81,302)	63,424 (51,869–71,226)	54,045 (42,856–60,440)	47,835 (37,288–53,906)	36,825 (29,628–40,837)	28,203 (23,166–31,173)	20,165 (17,043–22,153)	11,080 (9,389–12,250)	3,443 (2,796–3,841)	--
Road injuries	466 (298–616)	273 (182–356)	3,738 (2,638–4,933)	16,991 (12,759–23,074)	18,129 (14,940–21,840)	17,224 (14,289–20,189)	55,127 (48,087–61,733)	87,189 (77,152–96,070)	83,569 (73,336–91,371)	83,796 (71,006–91,648)	78,041 (65,012–86,516)	71,026 (58,168–79,495)	69,964 (56,422–78,848)	66,732 (53,033–75,646)	58,662 (48,080–65,859)	50,558 (39,998–56,580)	44,809 (34,903–50,508)	34,563 (27,594–38,411)	26,369 (21,678–29,095)	18,918 (16,011–20,809)	10,440 (8,848–11,558)	3,210 (2,606–3,593)	--
Pedestrian road injuries	171 (107–233)	103 (64–135)	1,358 (881–1,808)	7,859 (5,740–10,840)	8,850 (7,065–10,808)	7,510 (5,816–8,967)	13,593 (10,487–15,796)	21,039 (16,315–24,429)	21,827 (17,206–25,276)	24,368 (19,168–28,494)	23,509 (18,350–27,450)	22,373 (17,217–26,525)	24,063 (18,177–28,560)	25,377 (18,922–30,125)	22,550 (17,107–26,752)	22,165 (16,734–26,052)	20,805 (15,612–24,509)	17,214 (12,953–19,979)	14,738 (11,842–16,919)	10,689 (8,643–12,203)	5,997 (4,897–6,839)	1,672 (1,339–1,923)	--
Cyclist road injuries	--	--	--	764 (520–1,151)	969 (775–1,224)	1,308 (1,073–1,588)	2,416 (1,964–2,818)	2,670 (2,202–3,099)	2,717 (2,244–3,152)	3,244 (2,602–3,829)	3,186 (2,549–3,731)	3,617 (2,786–4,285)	4,278 (3,387–5,098)	4,908 (3,854–5,873)	4,399 (3,427–5,259)	4,710 (3,594–5,617)	4,456 (3,443–5,335)	3,332 (2,589–3,946)	2,097 (1,602–2,531)	1,317 (1,044–1,574)	673 (532–802)	187 (147–219)	--
Motorcyclist road injuries	47 (30–67)	30 (18–41)	440 (313–608)	1,435 (1,018–2,036)	1,351 (1,037–1,742)	2,080 (1,654–2,533)	16,434 (13,743–18,699)	27,071 (23,074–30,996)	23,889 (19,899–27,026)	21,262 (17,178–24,211)	19,276 (15,655–22,353)	16,841 (13,525–19,640)	15,561 (11,956–18,074)	13,148 (10,090–15,421)	11,111 (8,054–13,139)	7,138 (5,058–8,543)	5,796 (3,908–6,908)	3,303 (2,203–3,916)	1,723 (1,204–2,044)	1,411 (970–1,751)	624 (436–762)	183 (126–224)	--
Motor vehicle road injuries	243 (155–327)	135 (90–178)	1,882 (1,304–2,560)	6,735 (4,690–9,563)	6,728 (5,368–8,413)	6,052 (4,981–7,281)	22,170 (19,202–26,238)	35,609 (31,261–40,897)	34,379 (29,511–39,940)	34,305 (28,569–39,008)	31,464 (25,982–36,160)	27,572 (21,994–32,055)	25,355 (20,397–29,207)	22,543 (17,967–26,633)	19,934 (15,850–23,124)	16,017 (12,612–18,815)	13,192 (10,506–15,356)	10,228 (8,429–12,055)	7,438 (6,298–8,823)	5,197 (4,422–6,207)	2,987 (2,453–3,577)	1,129 (920–1,343)	--
Other road injuries	5 (3–8)	5 (3–7)	58 (40–81)	198 (127–359)	231 (164–315)	275 (199–371)	513 (420–622)	801 (667–939)	757 (631–883)	618 (525–729)	607 (507–708)	623 (519–723)	707 (579–826)	757 (626–902)	667 (551–794)	528 (443–613)	560 (442–672)	486 (394–577)	373 (302–447)	304 (244–371)	160 (125–196)	39 (30–49)	--
Other transport injuries	49 (32–68)	27 (18–36)	224 (159–293)	1,108 (650–1,538)	1,047 (818–1,291)	1,467 (1,141–1,756)	4,221 (3,309–5,002)	5,916 (4,840–6,887)	6,403 (5,278–7,296)	5,612 (4,559–6,339)	5,844 (4,668–6,703)	5,283 (4,168–6,075)	5,255 (4,074–6,013)	5,132 (3,973–5,883)	4,762 (3,648–5,519)	3,487 (2,755–4,010)	3,025 (2,395–3,479)	2,262 (1,795–2,571)	1,834 (1,440–2,121)	1,247 (1,014–1,435)	639 (530–731)	232 (188–268)	--
Falls	532 (307–780)	218 (131–332)	2,293 (1,431–3,380)	5,200 (3,562–7,288)	4,102 (3,026–5,445)	4,061 (3,053–5,118)	5,185 (4,325–6,282)	8,822 (7,449–10,542)	10,226 (8,829–12,032)	12,716 (10,451–14,820)	14,819 (12,410–17,594)	17,188 (14,017–20,168)	19,214 (15,372–22,263)	22,693 (18,269–26,135)	24,625 (20,163–28,124)	27,258 (22,053–31,216)	30,973 (24,138–35,758)	36,641 (28,256–41,940)	40,688 (32,152–46,305)	46,548 (36,995–52,880)	41,512 (32,493–47,009)	22,962 (18,492–25,839)	--
Drowning	229 (167–305)	93 (62–126)	1,956 (1,424–2,628)	19,206 (15,394–24,850)	14,225 (11,843–17,133)	11,815 (10,230–13,730)	12,081 (10,536–13,681)	11,995 (10,565–13,714)	9,908 (8,857–11,068)	10,259 (9,116–11,425)	9,640 (8,436–10,894)	9,040 (7,937–10,125)	8,668 (7,557–9,779)	8,351 (7,254–9,476)	7,354 (6,307–8,241)	6,837 (5,818–7,624)	6,418 (5,399–7,203)	5,906 (4,911–6,645)	4,674 (3,858–5,242)	4,304 (3,507–4,830)	2,713 (2,211–3,043)	788 (637–884)	--
Fire, heat, and hot substances	194 (118–293)	122 (79–179)	1,663 (1,077–2,488)	5,038 (3,310–7,260)	1,772 (1,169–2,389)	1,077 (727–1,410)	1,532 (989–1,903)	2,499 (1,704–3,017)	2,797 (2,045–3,364)	3,190 (2,444–3,841)	3,445 (2,654–4,090)	3,234 (2,527–3,736)	3,411 (2,662–3,927)	3,497 (2,788–4,069)	3,539 (2,919–4,057)	3,671 (2,961–4,209)	3,398 (2,719–3,887)	3,268 (2,619–3,804)	2,911 (2,329–3,447)	3,081 (2,383–3,630)	2,244 (1,817–2,721)	946 (763–1,131)	--
Poisonings	191 (121–269)	167 (89–257)	1,576 (1,066–2,368)	3,776 (2,725–5,484)	1,367 (1,007–1,839)	1,345 (983–1,650)	1,663 (1,288–1,922)	2,532 (1,923–2,910)	2,835 (2,236–3,206)	3,077 (2,373–3,422)	3,090 (2,453–3,455)	3,268 (2,618–3,676)	3,460 (2,618–3,933)	3,675 (2,772–4,207)	3,471 (2,586–3,975)	3,428 (2,522–3,943)	2,929 (2,040–3,391)	2,514 (1,644–2,948)	1,949 (1,284–2,277)	1,608 (1,089–1,872)	934 (657–1,067)	218 (170–245)	--
Poisoning by carbon monoxide	32 (15–52)	29 (13–49)	239 (122–436)	765 (415–1,304)	478 (297–673)	511 (308–676)	1,019 (735–1,252)	1,526 (1,074–1,802)	1,726 (1,266–1,975)	2,037 (1,564–2,313)	2,032 (1,582–2,308)	2,097 (1,653–2,399)	2,196 (1,727–2,574)	2,219 (1,655–2,595)	2,109 (1,615–2,444)	2,111 (1,594–2,480)	1,739 (1,230–2,058)	1,450 (915–1,762)	1,144 (720–1,375)	971 (625–1,170)	575 (380–686)	142 (105–164)	--
Poisoning by other means	159 (104–222)	138 (73–215)	1,337 (902–1,968)	3,011 (2,199–4,359)	889 (666–1,193)	834 (645–1,064)	644 (460–877)	1,007 (714–1,425)	1,109 (812–1,537)	1,040 (724–1,419)	1,058 (744–1,425)	1,171 (777–1,534)	1,263 (771–1,683)	1,456 (874–1,932)	1,362 (849–1,775)	1,316 (781–1,757)	1,190 (683–1,677)	1,064 (603–1,495)	804 (451–1,155)	637 (369–905)	359 (231–514)	76 (55–99)	--
Exposure to mechanical forces	422 (147–798)	1,017 (126–2,789)	1,702 (645–3,246)	2,828 (1,937–4,049)	2,042 (1,446–2,726)	1,817 (1,348–2,289)	4,438 (3,429–5,405)	7,259 (5,621–8,620)	7,611 (6,017–8,813)	7,998 (6,337–9,130)	8,091 (6,307–9,311)	7,515 (5,579–8,729)	7,957 (5,779–9,488)	7,468 (5,436–8,845)	6,057 (4,459–7,111)	4,748 (3,530–5,478)	3,830 (2,750–4,482)	2,978 (2,258–3,434)	2,457 (1,833–2,812)	1,741 (1,331–2,000)	1,107 (880–1,244)	435 (353–492)	--
Unintentional firearm injuries	47 (10–107)	130 (7–496)	272 (91–596)	509 (285–822)	279 (181–456)	373 (283–540)	1,270 (965–1,757)	1,896 (1,452–2,555)	1,784 (1,272–2,293)	1,524 (1,093–1,967)	1,355 (902–1,780)	1,209 (815–1,609)	1,087 (680–1,454)	814 (515–1,059)	646 (426–859)	551 (350–727)	418 (267–558)	337 (213–445)	287 (194–379)	211 (133–270)	124 (80–161)	60 (39–80)	--
Other exposure to mechanical forces	375 (132–724)	887 (117–2,521)	1,430 (529–2,788)	2,319 (1,571–3,295)	1,763 (1,223–2,326)	1,444 (1,011–1,813)	3,168 (2,239–3,819)	5,363 (3,883–6,322)	5,827 (4,413–6,738)	6,474 (4,951–7,444)	6,735 (5,141–7,781)	6,306 (4,630–7,375)	6,870 (4,858–8,243)	6,654 (4,724–7,906)	5,411 (3,967–6,380)	4,197 (3,042–4,896)	3,412 (2,404–4,016)	2,641 (1,973–3,068)	2,170 (1,589–2,497)	1,530 (1,155–1,777)	983 (779–1,107)	375 (305–422)	--
Adverse effects of medical treatment	402 (282–563)	214 (150–299)	2,105 (1,424–2,861)	2,716 (1,518–4,056)	889 (659–1,144)	677 (495–890)	723 (501–1,023)	1,173 (871–1,511)	1,035 (767–1,335)	1,258 (955–1,558)	1,414 (1,070–1,736)	1,707 (1,305–2,092)	1,904 (1,469–2,303)	2,235 (1,748–2,629)	3,078 (2,451–3,558)	4,259 (3,349–5,022)	5,447 (4,291–6,376)	5,049 (4,037–5,877)	4,905 (3,974–5,645)	4,347 (3,550–5,019)	2,921 (2,421–3,348)	1,402 (1,098–1,604)	--
Animal contact	202 (112–294)	136 (81–205)	995 (624–1,632)	3,156 (2,135–4,920)	2,858 (1,925–4,120)	3,050 (2,034–4,092)	2,129 (1,496–2,715)	2,371 (1,628–2,995)	2,640 (1,861–3,323)	2,717 (1,949–3,373)	2,361 (1,553–2,903)	2,846 (1,948–3,564)	3,887 (2,742–4,903)	2,986 (2,236–3,610)	3,575 (2,527–4,417)	3,272 (2,257–3,996)	3,593 (2,420–4,441)	2,888 (1,941–3,534)	2,464 (1,624–3,016)	1,801 (1,068–2,183)	796 (546–955)	268 (185–320)	--
Venomous animal contact	167 (80–254)	106 (56–166)	669 (365–1,191)	1,831 (999–3,305)	2,132 (1,297–3,245)	2,566 (1,614–3,498)	1,755 (1,209–2,295)	2,013 (1,297–2,617)	2,281 (1,558–2,934)	2,362 (1,632–2,973)	1,990 (1,256–2,520)	2,415 (1,541–3,094)	3,346 (2,240–4,311)	2,337 (1,627–2,895)	2,892 (1,935–3,672)	2,574 (1,671–3,189)	2,935 (1,807–3,670)	2,364 (1,483–2,951)	2,007 (1,240–2,532)	1,489 (816–1,863)	624 (389–769)	208 (132–255)	--
Non-venomous animal contact	34 (26–49)	30 (22–43)	326 (228–480)	1,325 (923–1,891)	725 (521–1,038)	484 (345–699)	374 (267–522)	358 (265–489)	359 (274–466)	355 (273–462)	371 (282–505)	431 (319–597)	542 (399–759)	649 (501–910)	683 (521–939)	698 (548–972)	658 (522–882)	524 (417–701)	457 (358–611)	312 (242–413)	172 (138–217)	60 (47–74)	--
Foreign body	1,162 (903–1,444)	954 (761–1,149)	7,838 (6,495–9,259)	4,107 (3,368–5,060)	1,453 (1,246–1,695)	1,260 (1,102–1,445)	1,270 (1,128–1,427)	2,095 (1,874–2,337)	2,200 (2,028–2,399)	2,479 (2,293–2,677)	2,644 (2,426–2,862)	2,848 (2,612–3,122)	3,050 (2,775–3,347)	3,276 (2,997–3,555)	3,640 (3,348–3,959)	3,798 (3,523–4,102)	3,883 (3,618–4,141)	3,864 (3,605–4,105)	3,947 (3,646–4,191)	4,531 (4,027–4,868)	4,226 (3,638–4,578)	2,624 (2,141–2,894)	--
Pulmonary aspiration and foreign body in airway	1,114 (870–1,386)	937 (747–1,128)	7,603 (6,296–8,985)	3,394 (2,729–4,305)	1,292 (1,109–1,512)	1,161 (1,025–1,331)	1,110 (981–1,265)	1,907 (1,697–2,127)	2,029 (1,870–2,222)	2,312 (2,149–2,503)	2,463 (2,267–2,682)	2,642 (2,428–2,895)	2,881 (2,634–3,163)	3,140 (2,882–3,412)	3,508 (3,226–3,795)	3,646 (3,384–3,931)	3,749 (3,484–4,003)	3,719 (3,468–3,959)	3,788 (3,476–4,019)	4,387 (3,903–4,693)	4,127 (3,557–4,461)	2,580 (2,101–2,842)	--
Foreign body in other body part	48 (29–70)	17 (11–25)	235 (160–332)	713 (420–1,153)	161 (111–216)	98 (69–133)	160 (105–222)	188 (118–271)	172 (115–238)	167 (117–224)	181 (124–246)	206 (143–278)	169 (114–233)	136 (93–185)	132 (90–183)	152 (104–205)	134 (91–179)	145 (101–194)	159 (115–213)	143 (104–199)	98 (72–137)	44 (32–62)	--
Environmental heat and cold exposure	79 (19–133)	48 (11–89)	342 (107–595)	540 (260–874)	253 (122–375)	218 (105–297)	537 (309–707)	867 (524–1,106)	1,039 (702–1,241)	1,551 (1,116–1,754)	1,924 (1,467–2,175)	2,313 (1,772–2,647)	2,619 (2,049–2,988)	3,118 (2,356–3,604)	3,310 (2,528–3,871)	3,185 (2,374–3,703)	2,698 (1,710–3,281)	2,147 (1,216–2,632)	2,044 (1,061–2,590)	1,699 (927–2,100)	1,164 (690–1,369)	494 (343–573)	--
Other unintentional injuries	295 (129–453)	84 (54–122)	980 (538–1,425)	3,029 (2,051–4,185)	2,335 (1,515–3,026)	2,399 (1,567–2,918)	5,011 (3,594–5,862)	7,743 (5,513–9,081)	8,267 (6,146–9,505)	8,791 (6,330–10,022)	8,386 (5,949–9,720)	7,634 (5,569–8,760)	7,505 (5,120–8,743)	5,995 (4,054–6,935)	4,910 (3,390–5,672)	3,812 (2,561–4,362)	3,031 (2,025–3,458)	1,879 (1,264–2,128)	1,443 (1,034–1,628)	1,101 (776–1,252)	706 (509–800)	309 (229–360)	--
Self-harm	--	--	--	--	--	4,988 (3,924–6,088)	23,689 (20,008–27,056)	44,889 (38,608–51,662)	50,771 (43,775–57,400)	52,701 (45,450–59,261)	48,592 (41,388–54,328)	43,884 (37,722–49,150)	42,711 (36,175–48,079)	40,224 (34,623–45,336)	37,966 (31,898–42,410)	31,685 (26,852–35,350)	28,099 (23,459–31,580)	24,089 (19,988–27,295)	19,980 (16,284–22,451)	16,243 (13,443–18,357)	9,747 (8,045–11,097)	2,970 (2,424–3,347)	--
Self-harm by firearm	--	--	--	--	--	246 (200–306)	2,006 (1,735–2,576)	3,454 (2,951–4,541)	3,493 (2,999–4,520)	3,474 (2,832–4,444)	3,335 (2,650–4,068)	3,350 (2,747–4,111)	3,683 (3,062–4,569)	3,793 (3,290–4,598)	3,964 (3,518–4,747)	3,450 (3,038–3,994)	3,013 (2,579–3,466)	2,517 (2,174–2,773)	2,072 (1,753–2,294)	1,647 (1,385–1,862)	1,077 (895–1,249)	425 (348–508)	--
Self-harm by other specified means	--	--	--	--	--	4,742 (3,707–5,833)	21,682 (18,069–24,924)	41,435 (34,753–47,696)	47,278 (40,628–53,448)	49,227 (41,864–55,360)	45,257 (38,482–50,812)	40,534 (34,538–45,549)	39,028 (32,444–44,100)	36,431 (30,760–41,134)	34,002 (27,948–38,641)	28,235 (23,240–31,679)	25,086 (20,487–28,511)	21,572 (17,414–24,670)	17,908 (14,332–20,297)	14,596 (11,908–16,664)	8,669 (7,045–10,054)	2,545 (2,048–2,886)	--
Interpersonal violence	639 (505–813)	185 (141–233)	1,728 (1,328–2,180)	4,062 (2,910–5,383)	2,910 (2,257–3,574)	4,870 (4,084–5,691)	31,582 (28,448–35,293)	51,923 (48,157–56,454)	49,475 (46,167–53,261)	44,989 (41,828–48,445)	37,868 (35,143–41,044)	29,958 (27,622–32,507)	23,432 (21,634–25,558)	18,607 (17,182–20,247)	14,285 (13,218–15,519)	10,332 (9,591–11,217)	7,458 (6,920–8,082)	4,977 (4,608–5,444)	3,085 (2,826–3,391)	1,012 (894–1,124)	485 (415–539)	180 (146–201)	--
Physical violence by firearm	131 (98–169)	38 (28–49)	316 (237–405)	908 (619–1,225)	602 (492–732)	1,654 (1,451–1,885)	17,646 (16,006–19,456)	28,760 (26,606–31,175)	25,587 (23,797–27,643)	21,613 (20,089–23,396)	17,806 (16,324–19,489)	13,516 (12,276–14,811)	9,657 (8,690–10,821)	7,486 (6,740–8,325)	5,259 (4,720–5,794)	3,233 (2,939–3,589)	2,032 (1,845–2,249)	1,244 (1,136–1,370)	794 (709–878)	275 (237–315)	121 (100–135)	54 (43–62)	--
Physical violence by sharp object	69 (43–96)	22 (12–32)	175 (103–244)	749 (400–1,069)	400 (244–511)	701 (440–863)	6,533 (4,883–7,680)	11,538 (8,944–13,289)	12,045 (9,983–13,798)	11,281 (9,284–13,073)	9,260 (7,742–10,725)	7,207 (5,792–8,225)	5,843 (4,619–6,673)	4,597 (3,629–5,223)	3,302 (2,700–3,814)	2,694 (2,196–3,108)	1,955 (1,589–2,252)	1,147 (941–1,316)	729 (617–840)	288 (247–332)	134 (110–156)	52 (42–60)	--
Physical violence by other means	439 (346–553)	124 (94–158)	1,237 (959–1,576)	2,406 (1,762–3,109)	1,907 (1,429–2,406)	2,515 (1,983–3,064)	7,403 (6,074–8,844)	11,625 (10,044–13,664)	11,843 (10,355–13,534)	12,094 (10,758–13,837)	10,802 (9,516–12,119)	9,234 (8,191–10,257)	7,932 (6,998–8,830)	6,523 (5,852–7,224)	5,725 (5,159–6,372)	4,405 (3,968–4,866)	3,471 (3,153–3,849)	2,587 (2,330–2,912)	1,563 (1,411–1,762)	450 (395–514)	230 (198–260)	74 (60–83)	--
Executions and police conflict	0 (0–0)	0 (0–0)	18 (9–45)	90 (81–103)	69 (62–78)	93 (85–105)	682 (563–855)	1,218 (964–1,509)	1,148 (923–1,430)	1,056 (847–1,329)	801 (658–1,003)	622 (488–824)	467 (362–687)	410 (295–667)	308 (223–503)	156 (122–210)	111 (86–161)	68 (52–92)	45 (36–60)	34 (28–44)	23 (18–30)	9 (7–13)	--

SR Table 12: Cambodia deaths by cause and age group for males

Cause	Early Neonatal	Late Neonatal	Post Neonatal	1 to 4	5 to 9	10 to 14	15 to 19	20 to 24	25 to 29	30 to 34	35 to 39	40 to 44	45 to 49	50 to 54	55 to 59	60 to 64	65 to 69	70 to 74	75 to 79	80 to 84	85 to 89	90 to 94	95 plus
All causes	2,825 (2,129–3,780)	690 (520–923)	2,135 (1,610–2,846)	909 (691–1,204)	503 (440–588)	448 (392–511)	956 (744–1,044)	1,345 (1,022–1,526)	1,361 (1,107–1,668)	1,487 (1,226–1,879)	1,705 (1,379–2,186)	1,969 (1,569–2,587)	2,568 (2,047–3,315)	3,913 (3,097–4,890)	4,953 (3,888–5,975)	5,517 (4,350–6,392)	5,535 (4,403–6,193)	5,705 (4,721–6,222)	5,037 (4,320–5,348)	4,255 (3,791–4,475)	2,745 (2,421–2,969)	1,025 (878–1,110)	--
Sexually transmitted infections excluding HIV	5 (1–11)	1 (0–3)	2 (1–4)	0 (0–0)	0 (0–0)	0 (0–0)	0 (0–0)	0 (0–0)	0 (0–0)	0 (0–0)	0 (0–1)	0 (0–0)	0 (0–0)	0 (0–1)	0 (0–0)	0 (0–1)	0 (0–1)	0 (0–1)	0 (0–1)	0 (0–0)	0 (0–0)	0 (0–0)	--
Syphilis	5 (1–11)	1 (0–3)	2 (1–4)	0 (0–0)	0 (0–0)	0 (0–0)	0 (0–0)	0 (0–0)	0 (0–0)	0 (0–0)	0 (0–0)	0 (0–0)	0 (0–0)	0 (0–0)	0 (0–0)	0 (0–1)	0 (0–0)	0 (0–1)	0 (0–0)	0 (0–0)	0 (0–0)	0 (0–0)	--
Chlamydial infection	--	--	--	--	--	0 (0–0)	0 (0–0)	0 (0–0)	0 (0–0)	0 (0–0)	0 (0–0)	0 (0–0)	0 (0–0)	0 (0–0)	0 (0–0)	0 (0–0)	0 (0–0)	0 (0–0)	0 (0–0)	0 (0–0)	0 (0–0)	0 (0–0)	--
Gonococcal infection	--	--	--	--	--	0 (0–0)	0 (0–0)	0 (0–0)	0 (0–0)	0 (0–0)	0 (0–0)	0 (0–0)	0 (0–0)	0 (0–0)	0 (0–0)	0 (0–0)	0 (0–0)	0 (0–0)	0 (0–0)	0 (0–0)	0 (0–0)	0 (0–0)	--
Other sexually transmitted infections	--	--	--	--	--	0 (0–0)	0 (0–0)	0 (0–0)	0 (0–0)	0 (0–0)	0 (0–0)	0 (0–0)	0 (0–0)	0 (0–0)	0 (0–0)	0 (0–0)	0 (0–0)	0 (0–0)	0 (0–0)	0 (0–0)	0 (0–0)	0 (0–0)	--
Tuberculosis	--	--	42 (22–73)	20 (11–36)	20 (11–33)	16 (10–26)	27 (13–49)	82 (41–142)	150 (82–252)	155 (91–244)	176 (98–282)	187 (102–301)	228 (136–374)	338 (193–540)	382 (223–579)	401 (244–613)	407 (240–634)	378 (222–585)	285 (163–452)	217 (122–354)	102 (59–164)	36 (21–56)	--
Drug-susceptible tuberculosis	--	--	41 (22–72)	20 (11–35)	20 (11–33)	16 (9–25)	27 (13–47)	81 (40–140)	148 (80–248)	152 (90–240)	172 (96–276)	184 (101–298)	224 (133–370)	332 (189–531)	375 (217–567)	394 (239–603)	399 (233–622)	371 (214–573)	279 (159–445)	213 (120–348)	100 (57–161)	35 (21–55)	--
Multidrug-resistant tuberculosis without extensive drug resistance	--	--	1 (0–3)	0 (0–1)	0 (0–1)	0 (0–1)	0 (0–2)	1 (0–5)	2 (0–9)	2 (0–9)	3 (0–11)	3 (0–12)	4 (0–14)	5 (1–21)	6 (1–25)	6 (1–25)	7 (1–26)	6 (1–25)	5 (0–17)	4 (0–14)	2 (0–7)	1 (0–2)	--
Extensively drug-resistant tuberculosis	--	--	0 (0–0)	0 (0–0)	0 (0–0)	0 (0–0)	0 (0–0)	0 (0–1)	0 (0–2)	0 (0–2)	1 (0–2)	1 (0–2)	1 (0–3)	1 (0–4)	1 (0–5)	1 (0–5)	1 (0–5)	1 (0–5)	1 (0–3)	1 (0–3)	0 (0–1)	0 (0–0)	--
Lower respiratory infections	365 (197–589)	225 (136–334)	1,056 (750–1,493)	271 (170–404)	59 (39–85)	38 (25–54)	59 (36–89)	77 (43–122)	76 (46–115)	89 (54–136)	99 (64–150)	103 (62–162)	119 (71–186)	190 (114–293)	260 (160–397)	413 (257–608)	449 (277–660)	502 (327–717)	485 (317–712)	493 (329–681)	366 (256–505)	149 (102–200)	--
Upper respiratory infections	0 (0–0)	0 (0–0)	1 (0–3)	1 (0–3)	0 (0–1)	0 (0–0)	0 (0–1)	0 (0–0)	0 (0–1)	0 (0–0)	0 (0–1)	0 (0–1)	0 (0–2)	1 (0–2)	0 (0–1)	1 (0–2)	1 (0–1)	0 (0–1)	0 (0–1)	0 (0–0)	0 (0–1)	0 (0–0)	--
Otitis media	0 (0–0)	0 (0–0)	0 (0–0)	0 (0–0)	0 (0–0)	0 (0–0)	0 (0–0)	0 (0–0)	0 (0–0)	0 (0–0)	0 (0–0)	0 (0–0)	0 (0–0)	0 (0–0)	0 (0–0)	0 (0–0)	0 (0–0)	0 (0–0)	0 (0–0)	0 (0–0)	0 (0–0)	0 (0–0)	--
Diarrhoeal diseases	5 (2–10)	15 (5–36)	137 (46–301)	50 (19–110)	15 (5–32)	9 (4–17)	7 (3–15)	7 (2–15)	11 (4–22)	12 (5–25)	14 (6–29)	14 (6–32)	21 (9–45)	32 (15–75)	40 (18–86)	46 (21–103)	54 (24–115)	64 (31–135)	75 (33–153)	80 (36–165)	63 (30–117)	28 (12–53)	--
Typhoid fever	--	--	0 (0–0)	22 (7–49)	26 (10–54)	29 (11–59)	28 (9–59)	16 (6–35)	9 (3–20)	5 (2–12)	3 (1–7)	2 (1–5)	2 (1–4)	2 (1–5)	2 (1–5)	2 (1–4)	1 (0–2)	1 (0–2)	0 (0–1)	0 (0–1)	0 (0–0)	0 (0–0)	--
Paratyphoid fever	--	--	0 (0–0)	4 (1–11)	5 (2–12)	5 (2–12)	5 (1–11)	3 (1–7)	1 (0–4)	1 (0–2)	1 (0–1)	0 (0–1)	0 (0–1)	0 (0–1)	0 (0–1)	0 (0–1)	0 (0–0)	0 (0–0)	0 (0–0)	0 (0–0)	0 (0–0)	0 (0–0)	--
iNTS	--	0 (0–0)	1 (1–2)	4 (2–7)	3 (2–6)	2 (1–4)	2 (1–4)	1 (0–3)	1 (0–2)	1 (0–1)	0 (0–1)	0 (0–1)	0 (0–1)	1 (0–1)	1 (0–2)	1 (0–2)	1 (0–2)	1 (0–2)	1 (0–1)	0 (0–1)	0 (0–0)	0 (0–0)	--
Other intestinal infectious diseases	--	--	0 (0–1)	0 (0–1)	0 (0–0)	0 (0–0)	0 (0–0)	0 (0–0)	0 (0–0)	0 (0–0)	0 (0–0)	0 (0–0)	0 (0–0)	0 (0–0)	0 (0–0)	0 (0–0)	0 (0–0)	0 (0–0)	0 (0–0)	0 (0–0)	0 (0–0)	0 (0–0)	--
Malaria	0 (0–0)	0 (0–0)	1 (0–4)	2 (0–6)	1 (0–4)	1 (0–2)	5 (1–14)	4 (1–10)	2 (0–5)	1 (0–3)	1 (0–2)	1 (0–3)	1 (0–4)	3 (0–8)	3 (0–8)	2 (0–5)	2 (0–4)	1 (0–3)	0 (0–1)	0 (0–0)	0 (0–0)	0 (0–0)	--
Chagas disease	--	--	0 (0–0)	0 (0–0)	0 (0–0)	0 (0–0)	0 (0–0)	0 (0–0)	0 (0–0)	0 (0–0)	0 (0–0)	0 (0–0)	0 (0–0)	0 (0–0)	0 (0–0)	0 (0–0)	0 (0–0)	0 (0–0)	0 (0–0)	0 (0–0)	0 (0–0)	0 (0–0)	--
Visceral leishmaniasis	--	--	0 (0–0)	0 (0–0)	0 (0–0)	0 (0–0)	0 (0–0)	0 (0–0)	0 (0–0)	0 (0–0)	0 (0–0)	0 (0–0)	0 (0–0)	0 (0–0)	0 (0–0)	0 (0–0)	0 (0–0)	0 (0–0)	0 (0–0)	0 (0–0)	0 (0–0)	0 (0–0)	--
African trypanosomiasis	--	--	--	0 (0–0)	0 (0–0)	0 (0–0)	0 (0–0)	0 (0–0)	0 (0–0)	0 (0–0)	0 (0–0)	0 (0–0)	0 (0–0)	0 (0–0)	0 (0–0)	0 (0–0)	0 (0–0)	0 (0–0)	0 (0–0)	0 (0–0)	0 (0–0)	0 (0–0)	--
Schistosomiasis	--	--	0 (0–0)	0 (0–0)	0 (0–0)	0 (0–0)	0 (0–1)	0 (0–2)	0 (0–2)	1 (0–2)	1 (0–3)	1 (0–3)	1 (0–4)	1 (0–5)	2 (0–7)	2 (0–6)	1 (0–5)	1 (0–5)	1 (0–4)	1 (0–3)	0 (0–1)	0 (0–0)	--
Cysticercosis	--	--	--	0 (0–0)	0 (0–0)	0 (0–0)	0 (0–0)	0 (0–0)	0 (0–0)	0 (0–0)	0 (0–0)	0 (0–0)	0 (0–0)	0 (0–0)	0 (0–0)	0 (0–0)	0 (0–0)	0 (0–0)	0 (0–0)	0 (0–0)	0 (0–0)	0 (0–0)	--
Cystic echinococcosis	--	--	--	0 (0–0)	0 (0–0)	0 (0–0)	0 (0–0)	0 (0–0)	0 (0–0)	0 (0–0)	0 (0–0)	0 (0–0)	0 (0–0)	0 (0–0)	0 (0–0)	0 (0–0)	0 (0–0)	0 (0–0)	0 (0–0)	0 (0–0)	0 (0–0)	0 (0–0)	--
Dengue	0 (0–0)	0 (0–0)	1 (0–5)	7 (1–24)	4 (1–14)	2 (0–8)	2 (0–7)	2 (0–9)	2 (0–9)	1 (0–4)	1 (0–3)	1 (0–3)	1 (0–3)	1 (0–2)	1 (0–3)	1 (0–2)	0 (0–2)	0 (0–1)	0 (0–1)	0 (0–1)	0 (0–1)	0 (0–0)	--
Yellow fever	--	0 (0–0)	0 (0–0)	0 (0–0)	0 (0–0)	0 (0–0)	0 (0–0)	0 (0–0)	0 (0–0)	0 (0–0)	0 (0–0)	0 (0–0)	0 (0–0)	0 (0–0)	0 (0–0)	0 (0–0)	0 (0–0)	0 (0–0)	0 (0–0)	0 (0–0)	0 (0–0)	0 (0–0)	--
Rabies	--	--	0 (0–1)	1 (0–3)	4 (0–19)	4 (0–17)	1 (0–5)	2 (0–6)	1 (0–4)	2 (0–5)	2 (0–5)	2 (0–5)	2 (0–6)	2 (0–6)	2 (0–7)	1 (0–4)	1 (0–3)	1 (0–2)	0 (0–1)	0 (0–1)	0 (0–1)	0 (0–0)	--
Ascariasis	--	--	0 (0–1)	1 (0–2)	1 (0–2)	0 (0–1)	0 (0–0)	0 (0–0)	0 (0–0)	0 (0–0)	0 (0–0)	0 (0–0)	0 (0–0)	0 (0–0)	0 (0–0)	0 (0–0)	0 (0–0)	0 (0–0)	0 (0–0)	0 (0–0)	0 (0–0)	0 (0–0)	--
Zika virus disease	0 (0–0)	0 (0–0)	0 (0–0)	0 (0–0)	0 (0–0)	0 (0–0)	0 (0–0)	0 (0–0)	0 (0–0)	0 (0–0)	0 (0–0)	0 (0–0)	0 (0–0)	0 (0–0)	0 (0–0)	0 (0–0)	0 (0–0)	0 (0–0)	0 (0–0)	0 (0–0)	0 (0–0)	0 (0–0)	--
Other neglected tropical diseases	0 (0–0)	0 (0–0)	0 (0–1)	1 (0–3)	0 (0–1)	0 (0–1)	0 (0–0)	0 (0–0)	0 (0–0)	0 (0–0)	0 (0–0)	0 (0–0)	0 (0–0)	0 (0–0)	1 (0–1)	0 (0–1)	0 (0–0)	1 (0–1)	0 (0–1)	0 (0–1)	0 (0–0)	0 (0–0)	--
Meningitis	1 (0–3)	4 (1–8)	43 (20–83)	16 (8–30)	2 (1–3)	1 (1–2)	1 (1–2)	2 (1–3)	1 (1–2)	1 (1–2)	1 (0–2)	1 (0–1)	1 (0–1)	1 (0–1)	1 (0–1)	1 (0–1)	0 (0–1)	0 (0–1)	0 (0–1)	0 (0–0)	0 (0–0)	0 (0–0)	--
Encephalitis	0 (0–0)	1 (0–2)	12 (4–31)	17 (6–42)	8 (3–19)	5 (2–11)	5 (2–12)	4 (2–8)	4 (2–8)	5 (2–9)	4 (1–7)	3 (1–7)	4 (2–8)	3 (1–7)	3 (1–8)	4 (1–9)	3 (1–6)	3 (1–7)	2 (1–7)	1 (0–2)	1 (0–2)	0 (0–0)	--
Diphtheria	--	--	0 (0–0)	0 (0–0)	0 (0–0)	0 (0–0)	0 (0–0)	0 (0–0)	0 (0–0)	0 (0–0)	0 (0–0)	0 (0–0)	0 (0–0)	0 (0–0)	0 (0–0)	--	--	--	--	--	--	--	--
Whooping cough	--	--	38 (4–129)	36 (4–116)	5 (0–16)	1 (0–3)	0 (0–2)	0 (0–1)	0 (0–1)	0 (0–1)	0 (0–0)	0 (0–0)	0 (0–0)	0 (0–0)	0 (0–1)	--	--	--	--	--	--	--	--
Tetanus	4 (0–15)	5 (1–20)	1 (0–4)	2 (0–10)	2 (0–6)	2 (0–5)	2 (0–5)	2 (0–6)	2 (0–5)	4 (1–11)	4 (1–11)	5 (1–13)	7 (1–15)	7 (1–19)	9 (1–22)	6 (1–14)	5 (1–13)	4 (1–9)	3 (1–8)	1 (0–3)	1 (0–2)	0 (0–1)	--
Measles	0 (0–0)	0 (0–0)	16 (5–38)	37 (12–82)	7 (2–17)	2 (1–4)	1 (0–2)	0 (0–1)	0 (0–0)	0 (0–0)	0 (0–0)	0 (0–0)	0 (0–0)	0 (0–0)	0 (0–0)	0 (0–0)	0 (0–0)	0 (0–0)	0 (0–0)	0 (0–0)	0 (0–0)	0 (0–0)	--
Varicella and herpes zoster	0 (0–0)	0 (0–1)	2 (0–7)	3 (0–9)	1 (0–4)	0 (0–1)	0 (0–1)	0 (0–1)	0 (0–1)	0 (0–1)	0 (0–1)	0 (0–1)	0 (0–1)	0 (0–1)	0 (0–1)	0 (0–1)	1 (0–2)	1 (0–2)	1 (0–3)	1 (0–4)	1 (0–4)	1 (0–2)	--
Acute hepatitis	--	--	2 (1–4)	1 (0–2)	2 (1–4)	1 (0–3)	4 (1–8)	7 (3–17)	9 (3–19)	10 (4–21)	12 (5–25)	16 (6–34)	15 (6–32)	20 (8–44)	25 (10–54)	21 (8–45)	13 (5–28)	9 (4–20)	8 (3–18)	4 (1–9)	4 (2–8)	1 (0–1)	--
Acute hepatitis A	--	--	1 (0–2)	0 (0–1)	1 (0–2)	1 (0–2)	2 (0–4)	3 (1–8)	5 (1–13)	3 (0–7)	3 (1–9)	5 (1–16)	3 (0–9)	6 (1–20)	9 (2–26)	7 (1–20)	3 (0–11)	3 (0–9)	3 (0–10)	2 (0–5)	1 (0–3)	0 (0–1)	--
Acute hepatitis B	--	--	1 (0–2)	0 (0–0)	0 (0–1)	0 (0–1)	2 (0–4)	3 (1–9)	3 (1–8)	5 (1–13)	7 (2–16)	7 (2–17)	9 (3–20)	11 (3–27)	12 (2–31)	11 (2–28)	7 (2–17)	5 (1–13)	4 (1–11)	2 (0–4)	2 (1–5)	0 (0–1)	--
Acute hepatitis C	--	--	0 (0–0)	0 (0–0)	0 (0–1)	0 (0–0)	0 (0–1)	1 (0–2)	1 (0–2)	2 (0–5)	2 (0–6)	2 (0–8)	3 (1–8)	3 (1–10)	4 (1–10)	3 (1–10)	2 (1–7)	1 (0–2)	1 (0–2)	0 (0–1)	0 (0–1)	0 (0–0)	--
Acute hepatitis E	--	--	0 (0–0)	0 (0–0)	0 (0–0)	0 (0–0)	0 (0–1)	1 (0–2)	0 (0–1)	0 (0–1)	0 (0–1)	1 (0–3)	0 (0–1)	0 (0–1)	0 (0–1)	0 (0–1)	0 (0–1)	0 (0–1)	0 (0–0)	0 (0–0)	0 (0–0)	0 (0–0)	--
Other unspecified infectious diseases	10 (1–28)	9 (2–30)	15 (3–50)	3 (1–11)	2 (1–4)	1 (0–2)	6 (3–14)	11 (4–26)	12 (5–26)	13 (5–27)	12 (5–27)	10 (4–20)	11 (5–23)	14 (6–30)	14 (6–29)	12 (5–22)	11 (5–23)	10 (4–22)	6 (3–13)	5 (2–10)	3 (1–8)	1 (0–2)	--
Maternal disorders	--	--	--	--	--	--	--	--	--	--	--	--	--	--	--	--	--	--	--	--	--	--	--
Maternal haemorrhage	--	--	--	--	--	--	--	--	--	--	--	--	--	--	--	--	--	--	--	--	--	--	--
Maternal sepsis and other pregnancy related infections	--	--	--	--	--	--	--	--	--	--	--	--	--	--	--	--	--	--	--	--	--	--	--
Maternal hypertensive disorders	--	--	--	--	--	--	--	--	--	--	--	--	--	--	--	--	--	--	--	--	--	--	--
Maternal obstructed labour and uterine rupture	--	--	--	--	--	--	--	--	--	--	--	--	--	--	--	--	--	--	--	--	--	--	--
Maternal abortive outcome	--	--	--	--	--	--	--	--	--	--	--	--	--	--	--	--	--	--	--	--	--	--	--
Ectopic pregnancy	--	--	--	--	--	--	--	--	--	--	--	--	--	--	--	--	--	--	--	--	--	--	--
Indirect maternal deaths	--	--	--	--	--	--	--	--	--	--	--	--	--	--	--	--	--	--	--	--	--	--	--
Late maternal deaths	--	--	--	--	--	--	--	--	--	--	--	--	--	--	--	--	--	--	--	--	--	--	--
Maternal deaths aggravated by HIV/AIDS	--	--	--	--	--	--	--	--	--	--	--	--	--	--	--	--	--	--	--	--	--	--	--
Other maternal disorders	--	--	--	--	--	--	--	--	--	--	--	--	--	--	--	--	--	--	--	--	--	--	--
Neonatal disorders	2,045 (1,465–2,807)	301 (193–439)	118 (57–218)	3 (1–8)	--	--	--	--	--	--	--	--	--	--	--	--	--	--	--	--	--	--	--
Neonatal preterm birth	1,103 (688–1,617)	96 (42–177)	47 (20–99)	1 (0–1)	--	--	--	--	--	--	--	--	--	--	--	--	--	--	--	--	--	--	--
Neonatal encephalopathy due to birth asphyxia and trauma	568 (291–985)	56 (23–121)	28 (9–66)	2 (1–6)	--	--	--	--	--	--	--	--	--	--	--	--	--	--	--	--	--	--	--
Neonatal sepsis and other neonatal infections	214 (76–480)	112 (48–200)	25 (9–62)	0 (0–0)	--	--	--	--	--	--	--	--	--	--	--	--	--	--	--	--	--	--	--
Hemolytic disease and other neonatal jaundice	17 (8–34)	4 (2–9)	1 (0–2)	0 (0–0)	--	--	--	--	--	--	--	--	--	--	--	--	--	--	--	--	--	--	--
Other neonatal disorders	143 (62–269)	34 (15–63)	16 (5–36)	1 (0–2)	--	--	--	--	--	--	--	--	--	--	--	--	--	--	--	--	--	--	--
Nutritional deficiencies	0 (0–0)	0 (0–0)	33 (13–72)	15 (6–30)	5 (2–8)	3 (2–5)	3 (1–5)	3 (1–5)	3 (1–5)	3 (1–5)	3 (1–6)	3 (2–6)	5 (2–9)	7 (4–14)	8 (4–14)	11 (5–20)	14 (6–26)	18 (9–33)	28 (14–47)	32 (17–55)	28 (14–48)	14 (7–23)	--
Protein-energy malnutrition	0 (0–0)	0 (0–0)	31 (12–67)	14 (5–29)	5 (2–8)	3 (1–5)	3 (1–5)	3 (1–5)	3 (1–5)	2 (1–5)	3 (1–5)	3 (2–6)	4 (2–8)	5 (2–10)	7 (4–12)	9 (4–16)	12 (5–23)	14 (7–26)	23 (12–40)	26 (14–45)	23 (11–40)	11 (5–18)	--
Other nutritional deficiencies	--	--	2 (1–7)	0 (0–1)	0 (0–1)	1 (0–2)	0 (0–1)	0 (0–1)	0 (0–1)	0 (0–1)	0 (0–1)	0 (0–1)	1 (0–1)	2 (1–5)	1 (0–3)	2 (1–5)	2 (0–3)	4 (1–9)	4 (1–10)	6 (3–14)	5 (2–12)	3 (1–6)	--
Lip and oral cavity cancer	--	--	--	--	0 (0–0)	0 (0–0)	0 (0–1)	1 (1–2)	2 (1–3)	3 (1–5)	4 (2–7)	7 (3–12)	13 (7–22)	22 (12–36)	29 (17–47)	31 (19–48)	28 (17–43)	24 (14–37)	18 (11–27)	12 (6–19)	6 (3–9)	2 (1–3)	--
Nasopharynx cancer	--	--	--	--	0 (0–0)	0 (0–1)	2 (1–3)	3 (1–5)	2 (1–4)	3 (2–6)	6 (3–10)	8 (5–14)	14 (8–25)	22 (12–37)	28 (15–46)	24 (13–39)	20 (12–32)	18 (10–29)	9 (5–15)	5 (3–9)	2 (1–4)	1 (0–1)	--
Other pharynx cancer	--	--	--	--	--	--	--	0 (0–0)	0 (0–0)	0 (0–0)	1 (0–1)	1 (1–2)	3 (2–5)	6 (3–10)	9 (5–14)	9 (5–15)	7 (4–12)	7 (4–12)	4 (2–6)	2 (1–4)	1 (1–1)	0 (0–1)	--
Oesophageal cancer	--	--	--	--	--	--	--	1 (0–1)	1 (0–1)	1 (1–2)	3 (2–5)	5 (3–9)	11 (6–18)	21 (12–34)	32 (19–49)	34 (20–55)	33 (21–52)	27 (17–42)	18 (11–28)	11 (7–19)	6 (4–9)	1 (1–2)	--
Stomach cancer	--	--	--	--	--	--	1 (1–2)	3 (2–4)	4 (2–7)	8 (5–14)	12 (7–20)	19 (11–29)	28 (17–43)	52 (32–79)	73 (47–107)	81 (52–118)	81 (55–113)	78 (54–107)	60 (43–82)	42 (29–58)	26 (18–35)	7 (4–10)	--
Colon and rectum cancer	--	--	--	--	0 (0–1)	0 (0–1)	3 (1–5)	5 (3–8)	8 (5–14)	14 (8–23)	20 (11–35)	26 (14–43)	42 (25–67)	74 (46–108)	101 (64–148)	111 (71–161)	112 (73–161)	102 (67–140)	76 (53–104)	56 (36–82)	29 (18–42)	7 (4–11)	--
Liver cancer due to hepatitis B	--	--	--	--	--	5 (2–12)	7 (3–13)	7 (3–12)	8 (4–15)	9 (4–18)	10 (4–22)	12 (5–24)	15 (6–30)	24 (11–46)	31 (14–58)	28 (13–52)	22 (11–39)	18 (8–33)	12 (6–21)	7 (3–14)	4 (2–7)	1 (0–2)	--
Liver cancer due to hepatitis C	--	--	--	--	--	0 (0–0)	0 (0–0)	0 (0–1)	0 (0–1)	1 (0–2)	1 (0–3)	2 (1–5)	4 (1–10)	10 (4–22)	18 (7–35)	22 (9–44)	23 (12–39)	24 (12–43)	20 (11–34)	17 (10–27)	11 (6–18)	4 (2–6)	--
Liver cancer due to alcohol use	--	--	--	--	--	--	0 (0–0)	0 (0–1)	1 (0–2)	1 (0–3)	2 (1–5)	3 (1–8)	6 (2–13)	11 (4–23)	17 (7–32)	21 (10–38)	23 (11–40)	21 (10–37)	14 (7–23)	8 (4–14)	4 (2–7)	1 (0–2)	--
Liver cancer due to NASH	--	--	--	--	--	--	1 (0–2)	1 (0–1)	0 (0–1)	0 (0–1)	0 (0–1)	1 (0–1)	1 (0–2)	2 (1–4)	3 (1–6)	3 (1–6)	3 (1–7)	3 (2–7)	3 (1–6)	2 (1–4)	1 (1–3)	0 (0–1)	--
	0 (0–0)	0 (0–0)	0 (0–0)	1 (0–1)	0 (0–1)	0 (0–1)	--	--	--	--	--	--	--	--	--	--	--	--	--	--	--	--	--
Liver cancer due to other causes	7 (1–25)	5 (0–20)	16 (4–41)	7 (2–20)	3 (1–6)	1 (0–2)	1 (0–2)	1 (0–1)	0 (0–1)	0 (0–1)	0 (0–1)	0 (0–1)	1 (0–1)	1 (0–2)	1 (0–3)	1 (1–2)	1 (0–2)	1 (0–2)	1 (0–1)	0 (0–1)	0 (0–0)	0 (0–0)	--
Gallbladder and biliary tract cancer	--	--	--	--	--	--	--	0 (0–0)	0 (0–0)	0 (0–1)	1 (0–1)	1 (1–3)	3 (1–4)	5 (3–10)	7 (4–13)	9 (5–15)	9 (5–16)	9 (5–16)	8 (4–13)	5 (3–9)	3 (1–5)	1 (0–1)	--
Pancreatic cancer	--	--	--	--	--	--	0 (0–1)	1 (0–1)	1 (1–2)	3 (1–4)	4 (3–7)	6 (4–10)	10 (6–17)	19 (12–31)	29 (18–44)	30 (19–45)	30 (20–45)	27 (19–39)	21 (14–30)	14 (9–21)	6 (4–10)	2 (1–3)	--
Larynx cancer	--	--	--	--	--	--	--	0 (0–0)	0 (0–1)	1 (0–1)	1 (1–2)	2 (1–4)	6 (3–10)	14 (8–22)	22 (13–34)	26 (15–39)	25 (16–39)	23 (14–37)	16 (10–26)	10 (6–16)	4 (2–7)	1 (1–1)	--
Tracheal, bronchus, and lung cancer	--	--	--	--	--	0 (0–1)	3 (1–5)	4 (2–8)	6 (3–10)	11 (6–18)	20 (11–34)	34 (19–55)	74 (47–115)	170 (106–245)	280 (184–397)	359 (243–499)	377 (262–527)	356 (240–531)	247 (163–373)	136 (87–208)	70 (45–108)	15 (9–22)	--
Malignant skin melanoma	0 (0–0)	0 (0–0)	0 (0–0)	0 (0–0)	0 (0–0)	0 (0–0)	0 (0–0)	0 (0–1)	0 (0–1)	1 (0–1)	1 (0–1)	1 (1–2)	1 (1–2)	2 (1–3)	3 (1–4)	2 (1–3)	2 (1–4)	2 (1–3)	2 (1–3)	1 (1–2)	1 (0–1)	0 (0–0)	--
Non-melanoma skin cancer (squamous-cell carcinoma)	--	--	--	--	--	--	--	0 (0–1)	0 (0–1)	1 (0–1)	1 (0–1)	1 (1–2)	2 (1–3)	4 (2–6)	6 (3–9)	8 (4–12)	8 (4–13)	8 (4–14)	7 (4–12)	6 (3–10)	4 (2–6)	2 (1–3)	--
	0 (0–0)	0 (0–0)	0 (0–1)	1 (0–2)	1 (0–2)	1 (0–2)	1 (1–3)	1 (0–2)	1 (1–2)	1 (0–2)	1 (0–2)	1 (1–2)	2 (1–3)	2 (1–5)	2 (1–4)	3 (1–6)	2 (1–5)	2 (1–4)	1 (1–3)	1 (0–2)	1 (0–1)	0 (0–0)	--
	--	--	--	1 (1–2)	2 (1–4)	4 (2–8)	8 (3–19)	7 (3–15)	5 (2–10)	4 (1–8)	4 (1–8)	4 (1–9)	5 (1–12)	9 (2–20)	10 (2–25)	12 (3–27)	11 (2–25)	8 (2–19)	6 (1–15)	3 (1–8)	1 (0–3)	0 (0–1)	--
Breast cancer	--	--	--	--	--	--	0 (0–0)	0 (0–0)	0 (0–0)	0 (0–1)	0 (0–1)	1 (0–2)	1 (0–3)	2 (1–4)	3 (1–6)	3 (1–6)	3 (1–6)	3 (1–6)	2 (1–4)	1 (1–3)	1 (0–2)	0 (0–1)	--
Cervical cancer	--	--	--	--	--	--	--	--	--	--	--	--	--	--	--	--	--	--	--	--	--	--	--
Uterine cancer	--	--	--	--	--	--	--	--	--	--	--	--	--	--	--	--	--	--	--	--	--	--	--
Ovarian cancer	--	--	--	--	--	--	--	--	--	--	--	--	--	--	--	--	--	--	--	--	--	--	--
Prostate cancer	--	--	--	--	--	--	--	0 (0–0)	0 (0–1)	0 (0–1)	1 (0–1)	1 (1–2)	4 (2–6)	13 (8–21)	30 (18–44)	61 (39–91)	83 (54–122)	116 (77–169)	135 (91–194)	118 (80–164)	71 (48–103)	22 (14–31)	--
Testicular cancer	0 (0–0)	0 (0–0)	0 (0–0)	0 (0–1)	0 (0–0)	0 (0–0)	1 (0–1)	1 (1–3)	2 (1–4)	2 (1–3)	1 (1–3)	1 (1–3)	1 (0–2)	1 (0–1)	1 (0–2)	1 (0–2)	1 (0–2)	1 (0–1)	1 (0–1)	1 (0–1)	0 (0–0)	0 (0–0)	--
Kidney cancer	0 (0–0)	0 (0–0)	1 (0–1)	1 (1–3)	1 (0–1)	0 (0–1)	0 (0–1)	0 (0–1)	1 (0–1)	1 (0–2)	2 (1–3)	2 (1–4)	4 (2–8)	8 (4–14)	11 (6–18)	12 (7–18)	11 (7–18)	9 (6–15)	7 (4–11)	4 (3–7)	2 (1–4)	1 (0–1)	--
Bladder cancer	--	--	--	--	--	--	0 (0–0)	0 (0–0)	0 (0–0)	1 (0–1)	1 (1–2)	2 (1–3)	3 (2–5)	7 (5–12)	12 (7–19)	17 (10–27)	18 (11–29)	21 (13–32)	21 (13–32)	16 (10–25)	10 (6–15)	3 (2–4)	--
Brain and nervous system cancer	0 (0–0)	0 (0–0)	2 (0–4)	6 (2–12)	10 (6–18)	7 (4–13)	6 (3–11)	7 (3–12)	8 (4–14)	9 (5–15)	10 (6–18)	11 (6–19)	12 (6–22)	18 (9–31)	21 (10–38)	19 (9–35)	16 (7–28)	12 (6–21)	8 (4–14)	4 (2–8)	2 (1–4)	1 (0–1)	--
	0 (0–0)	0 (0–0)	0 (0–0)	2 (1–3)	1 (0–1)	0 (0–1)	0 (0–1)	0 (0–1)	0 (0–1)	0 (0–0)	0 (0–0)	0 (0–0)	0 (0–0)	0 (0–1)	0 (0–1)	0 (0–1)	0 (0–1)	0 (0–1)	0 (0–1)	0 (0–0)	0 (0–0)	0 (0–0)	--
	0 (0–0)	0 (0–0)	0 (0–0)	1 (0–2)	0 (0–1)	0 (0–0)	0 (0–0)	--	--	--	--	--	--	--	--	--	--	--	--	--	--	--	--
	--	--	--	0 (0–1)	0 (0–0)	0 (0–0)	0 (0–0)	0 (0–1)	0 (0–1)	0 (0–0)	0 (0–0)	0 (0–0)	0 (0–0)	0 (0–1)	0 (0–1)	0 (0–1)	0 (0–1)	0 (0–1)	0 (0–1)	0 (0–0)	0 (0–0)	0 (0–0)	--
	0 (0–0)	0 (0–0)	0 (0–1)	1 (0–3)	1 (0–2)	0 (0–1)	0 (0–1)	0 (0–1)	0 (0–1)	0 (0–1)	0 (0–0)	0 (0–0)	0 (0–1)	0 (0–1)	0 (0–1)	0 (0–1)	0 (0–1)	0 (0–0)	0 (0–0)	0 (0–0)	0 (0–0)	0 (0–0)	--
Thyroid cancer	--	--	--	--	0 (0–0)	0 (0–0)	0 (0–0)	0 (0–1)	0 (0–1)	1 (0–1)	1 (0–1)	1 (0–2)	2 (1–3)	4 (2–6)	5 (3–9)	6 (4–10)	6 (3–9)	5 (3–8)	4 (2–6)	2 (1–4)	1 (1–2)	0 (0–0)	--
Mesothelioma	--	--	--	--	--	--	--	0 (0–1)	0 (0–1)	0 (0–1)	1 (0–1)	1 (0–2)	2 (1–4)	3 (2–5)	5 (3–7)	1 (1–2)	1 (1–2)	1 (0–1)	2 (1–2)	1 (1–2)	1 (0–1)	0 (0–0)	--
Hodgkin lymphoma	--	--	--	0 (0–0)	0 (0–0)	1 (0–1)	1 (0–2)	1 (1–3)	2 (1–4)	1 (1–2)	1 (1–3)	1 (1–3)	1 (1–3)	2 (1–4)	2 (1–4)	2 (1–3)	2 (1–4)	2 (1–4)	1 (0–1)	1 (0–1)	0 (0–1)	0 (0–0)	--
Non-Hodgkin's lymphoma	--	--	--	2 (1–4)	4 (2–6)	3 (2–5)	6 (4–10)	7 (4–12)	7 (4–11)	7 (4–11)	6 (3–11)	9 (5–14)	10 (6–17)	18 (11–28)	23 (15–36)	24 (15–36)	22 (14–33)	20 (13–29)	12 (8–18)	8 (5–12)	3 (2–5)	1 (1–2)	--
	--	--	--	0 (0–1)	0 (0–1)	0 (0–1)	0 (0–1)	0 (0–1)	0 (0–1)	0 (0–0)	0 (0–0)	0 (0–0)	0 (0–0)	0 (0–0)	0 (0–0)	0 (0–0)	0 (0–0)	0 (0–0)	0 (0–0)	0 (0–0)	0 (0–0)	0 (0–0)	--
	--	--	--	2 (1–4)	3 (2–6)	3 (1–5)	6 (3–9)	7 (4–12)	7 (4–11)	6 (4–11)	6 (3–10)	8 (5–14)	10 (6–16)	18 (11–28)	23 (15–36)	24 (15–36)	22 (14–32)	20 (13–29)	12 (8–18)	8 (5–12)	3 (2–5)	1 (1–2)	--
Multiple myeloma	--	--	--	--	--	--	--	0 (0–0)	0 (0–0)	0 (0–1)	1 (0–1)	1 (1–2)	2 (1–4)	5 (3–8)	7 (4–12)	9 (5–15)	9 (5–15)	8 (5–14)	5 (3–8)	3 (2–6)	1 (1–3)	0 (0–1)	--
Leukaemia	0 (0–1)	0 (0–1)	4 (1–9)	22 (11–39)	25 (14–41)	23 (13–35)	25 (14–41)	20 (11–33)	17 (9–26)	14 (8–22)	14 (8–23)	14 (8–22)	17 (10–26)	24 (14–36)	32 (19–49)	34 (21–53)	33 (20–49)	31 (20–46)	25 (16–36)	16 (10–25)	10 (6–15)	3 (2–4)	--

Acute lymphoid leukaemia	0 (0–0)	0 (0–0)	2 (0–4)	7 (2–14)	9 (3–17)	8 (3–15)	7 (3–15)	4 (2–9)	2 (1–5)	2 (1–4)	2 (1–3)	1 (0–3)	2 (1–4)	3 (1–5)	2 (1–4)	2 (1–4)	2 (1–4)	2 (1–4)	1 (0–2)	1 (0–2)	1 (0–2)	0 (0–0)	--
Chronic lymphoid leukaemia	--	--	--	--	--	--	--	0 (0–0)	0 (0–0)	0 (0–0)	0 (0–0)	0 (0–0)	0 (0–1)	1 (0–2)	1 (1–3)	2 (1–4)	2 (1–5)	1 (1–3)	2 (1–3)	2 (1–4)	1 (0–1)	0 (0–0)	--
Acute myeloid leukaemia	0 (0–0)	0 (0–0)	1 (0–1)	3 (1–7)	3 (1–8)	2 (1–5)	5 (2–9)	3 (1–6)	2 (1–5)	3 (1–5)	3 (1–6)	3 (1–6)	2 (1–5)	4 (2–9)	6 (2–12)	4 (1–10)	4 (1–8)	4 (1–8)	2 (1–5)	2 (1–4)	1 (0–2)	0 (0–0)	--
Chronic myeloid leukaemia	0 (0–0)	0 (0–0)	0 (0–1)	0 (0–1)	0 (0–1)	1 (0–2)	1 (0–1)	1 (0–3)	1 (0–2)	1 (0–2)	1 (0–4)	1 (0–3)	2 (1–4)	2 (1–5)	3 (1–7)	3 (1–7)	4 (1–8)	3 (1–6)	3 (1–7)	1 (1–3)	1 (0–2)	0 (0–1)	--
Other leukaemia	0 (0–1)	0 (0–0)	2 (1–5)	12 (5–23)	12 (5–24)	12 (5–22)	13 (6–24)	11 (6–21)	11 (6–19)	8 (4–15)	8 (4–15)	8 (4–15)	10 (6–17)	13 (7–22)	19 (10–31)	22 (13–37)	20 (12–33)	21 (13–34)	17 (10–26)	10 (6–16)	7 (4–11)	2 (1–3)	--
Other malignant cancers	0 (0–1)	0 (0–1)	1 (1–3)	4 (2–8)	4 (2–6)	5 (2–8)	7 (3–10)	8 (4–12)	7 (3–11)	7 (2–12)	8 (3–14)	9 (3–16)	12 (5–22)	19 (8–34)	25 (12–44)	24 (12–42)	24 (12–40)	23 (11–39)	19 (9–32)	13 (6–23)	6 (3–11)	2 (1–3)	--
Myelodysplastic, myeloproliferative, and other hematopoietic neoplasms	0 (0–0)	0 (0–0)	0 (0–0)	0 (0–0)	0 (0–0)	0 (0–0)	0 (0–0)	0 (0–0)	0 (0–0)	0 (0–0)	0 (0–0)	0 (0–1)	1 (0–1)	1 (1–2)	2 (1–3)	2 (1–4)	3 (2–5)	3 (2–6)	3 (1–5)	1 (1–3)	1 (0–2)	0 (0–0)	--
Cardiovascular diseases	15 (4–33)	7 (2–15)	54 (20–101)	19 (9–34)	6 (4–9)	6 (3–11)	32 (19–51)	60 (33–95)	90 (52–136)	145 (90–222)	225 (142–348)	334 (215–503)	565 (373–825)	1,041 (730–1,421)	1,477 (1,043–1,942)	1,731 (1,270–2,187)	1,762 (1,300–2,212)	1,947 (1,467–2,388)	1,799 (1,409–2,187)	1,538 (1,190–1,872)	968 (749–1,175)	362 (273–445)	--
Rheumatic heart disease	--	--	--	4 (1–9)	2 (1–4)	3 (1–6)	8 (4–16)	9 (4–19)	10 (4–20)	9 (4–18)	9 (4–19)	9 (4–19)	8 (4–18)	10 (5–21)	13 (6–25)	11 (5–19)	9 (4–15)	8 (5–14)	6 (4–11)	5 (3–8)	3 (2–5)	1 (1–2)	--
Ischaemic heart disease	--	--	--	--	--	--	9 (5–16)	22 (11–39)	42 (22–68)	73 (42–117)	109 (65–175)	162 (97–254)	272 (167–412)	486 (310–712)	681 (448–943)	762 (523–1,042)	753 (526–1,028)	804 (557–1,085)	739 (533–976)	648 (456–866)	424 (295–560)	169 (118–221)	--
Stroke	11 (2–24)	3 (1–6)	23 (4–50)	8 (3–16)	2 (1–3)	2 (1–3)	9 (5–16)	19 (10–32)	25 (13–42)	45 (26–72)	79 (43–127)	124 (72–197)	224 (139–344)	429 (279–623)	615 (400–888)	756 (504–1,026)	800 (542–1,085)	906 (610–1,193)	842 (607–1,118)	703 (506–940)	428 (304–572)	146 (102–196)	--
Ischaemic stroke	2 (0–6)	1 (0–2)	1 (0–3)	1 (0–2)	0 (0–0)	0 (0–0)	1 (0–2)	1 (0–2)	2 (1–3)	3 (1–6)	5 (2–10)	8 (4–14)	19 (9–34)	47 (24–78)	74 (39–129)	183 (100–298)	213 (115–339)	369 (211–558)	375 (215–570)	445 (287–622)	271 (171–380)	94 (60–134)	--
Intracerebral hemorrhage	6 (1–15)	1 (0–3)	18 (3–39)	5 (2–9)	1 (1–3)	1 (1–3)	7 (4–13)	14 (7–25)	19 (9–33)	36 (19–57)	64 (35–103)	102 (59–164)	187 (117–289)	352 (224–521)	499 (324–723)	546 (366–765)	560 (373–788)	515 (321–741)	449 (285–645)	242 (146–373)	149 (88–228)	48 (29–74)	--
Subarachnoid hemorrhage	2 (0–6)	1 (0–2)	4 (1–12)	3 (1–6)	0 (0–1)	0 (0–1)	2 (0–4)	3 (1–8)	5 (2–9)	6 (3–12)	10 (4–21)	14 (6–29)	18 (8–38)	31 (14–65)	42 (20–83)	27 (12–58)	27 (12–59)	21 (9–44)	19 (8–39)	16 (7–34)	9 (4–19)	4 (2–7)	--
Hypertensive heart disease	--	--	--	--	--	--	1 (0–1)	2 (1–3)	3 (1–5)	7 (3–13)	12 (5–22)	22 (9–41)	39 (16–73)	80 (36–147)	122 (54–225)	153 (65–287)	148 (70–268)	169 (76–293)	152 (73–263)	120 (61–198)	68 (34–114)	26 (14–43)	--
Non-rheumatic valvular heart disease	--	--	--	--	--	--	0 (0–0)	0 (0–1)	0 (0–1)	0 (0–1)	1 (0–1)	1 (0–1)	1 (0–2)	1 (1–3)	2 (1–3)	2 (1–4)	3 (1–5)	3 (1–5)	2 (1–4)	2 (1–4)	1 (1–3)	0 (0–1)	--
Non-rheumatic calcific aortic valve disease	--	--	--	--	--	--	0 (0–0)	0 (0–0)	0 (0–0)	0 (0–0)	0 (0–1)	0 (0–1)	1 (0–1)	1 (0–2)	1 (1–2)	2 (1–3)	2 (1–3)	2 (1–4)	1 (1–3)	1 (1–3)	1 (0–2)	0 (0–1)	--
Non-rheumatic degenerative mitral valve disease	--	--	--	--	--	--	0 (0–0)	0 (0–0)	0 (0–0)	0 (0–0)	0 (0–0)	0 (0–0)	0 (0–1)	0 (0–1)	1 (0–1)	0 (0–1)	1 (0–1)	1 (0–1)	1 (0–1)	1 (0–1)	0 (0–1)	0 (0–0)	--
Other non-rheumatic valve diseases	--	--	--	--	--	--	0 (0–0)	0 (0–0)	0 (0–0)	0 (0–0)	0 (0–0)	0 (0–0)	0 (0–0)	0 (0–0)	0 (0–0)	0 (0–1)	0 (0–1)	0 (0–1)	0 (0–1)	0 (0–0)	0 (0–0)	0 (0–0)	--
Cardiomyopathy and myocarditis	3 (1–9)	2 (1–6)	16 (5–40)	3 (1–6)	0 (0–1)	0 (0–1)	1 (1–3)	2 (1–5)	3 (1–6)	4 (2–8)	7 (3–13)	7 (3–15)	10 (4–21)	15 (6–31)	19 (9–40)	20 (9–41)	19 (9–36)	22 (11–37)	22 (12–34)	22 (11–39)	15 (6–30)	7 (3–13)	--
Myocarditis	1 (0–3)	1 (0–3)	6 (1–17)	1 (0–3)	0 (0–1)	0 (0–0)	0 (0–1)	0 (0–1)	1 (0–1)	1 (0–1)	1 (0–2)	1 (0–2)	1 (0–2)	1 (0–3)	1 (0–5)	2 (0–5)	1 (0–3)	1 (0–3)	2 (1–3)	2 (1–5)	2 (0–4)	1 (0–2)	--
Alcoholic cardiomyopathy	--	--	--	--	--	--	0 (0–0)	0 (0–0)	0 (0–1)	0 (0–1)	1 (0–2)	1 (0–3)	1 (0–3)	1 (0–3)	2 (1–4)	1 (0–3)	1 (0–2)	1 (0–2)	0 (0–1)	0 (0–1)	0 (0–1)	0 (0–1)	--
Other cardiomyopathy	2 (1–7)	1 (0–4)	10 (3–26)	1 (0–4)	0 (0–1)	0 (0–1)	1 (0–2)	2 (1–4)	2 (1–5)	3 (1–6)	5 (2–10)	5 (2–12)	7 (3–17)	12 (5–26)	16 (7–34)	17 (8–35)	17 (8–33)	20 (10–34)	19 (11–31)	19 (10–35)	13 (6–26)	6 (2–11)	--
Pulmonary Arterial Hypertension	0 (0–4)	0 (0–1)	2 (0–6)	1 (0–2)	0 (0–0)	0 (0–0)	0 (0–1)	0 (0–1)	0 (0–1)	0 (0–1)	0 (0–1)	0 (0–1)	0 (0–1)	0 (0–1)	0 (0–1)	0 (0–1)	0 (0–1)	0 (0–1)	0 (0–1)	0 (0–1)	0 (0–1)	0 (0–0)	--
Atrial fibrillation and flutter	--	--	--	--	--	--	--	--	--	0 (0–0)	0 (0–1)	0 (0–1)	1 (1–1)	2 (1–3)	3 (2–5)	4 (3–7)	6 (4–9)	8 (5–12)	11 (7–15)	17 (11–24)	15 (10–22)	9 (5–13)	--
Aortic aneurysm	--	--	--	--	--	--	0 (0–1)	0 (0–1)	1 (0–1)	1 (0–1)	1 (1–2)	1 (1–2)	2 (1–4)	3 (2–6)	6 (4–11)	8 (5–13)	10 (6–15)	11 (7–17)	11 (7–17)	9 (6–14)	5 (3–8)	2 (1–3)	--
Peripheral vascular disease	--	--	--	--	--	--	--	--	--	--	--	0 (0–0)	0 (0–0)	0 (0–1)	1 (0–2)	1 (0–2)	1 (0–2)	1 (1–3)	1 (0–2)	1 (1–2)	1 (0–1)	0 (0–1)	--
Endocarditis	0 (0–0)	0 (0–0)	4 (1–11)	0 (0–1)	0 (0–0)	0 (0–0)	1 (0–2)	2 (1–3)	3 (1–6)	3 (1–6)	3 (1–7)	4 (1–9)	3 (1–7)	4 (2–9)	4 (2–9)	3 (2–6)	3 (2–7)	3 (1–6)	3 (1–5)	2 (1–4)	1 (1–2)	0 (0–1)	--
Other cardiovascular and circulatory diseases	1 (0–2)	1 (0–3)	9 (3–21)	3 (1–6)	1 (0–1)	0 (0–1)	1 (0–2)	3 (1–5)	2 (1–4)	3 (1–5)	3 (2–6)	4 (2–8)	5 (3–11)	8 (4–16)	9 (5–20)	10 (6–21)	9 (5–19)	10 (6–19)	9 (6–17)	9 (6–16)	6 (3–10)	2 (1–4)	--
Chronic respiratory diseases	0 (0–0)	0 (0–0)	0 (0–0)	11 (5–21)	5 (3–8)	5 (3–8)	13 (7–21)	20 (12–30)	20 (12–31)	20 (13–31)	23 (14–36)	30 (19–47)	54 (35–82)	117 (74–171)	205 (131–298)	303 (201–434)	372 (246–520)	466 (311–645)	463 (318–644)	425 (295–584)	284 (197–385)	101 (70–139)	--
Chronic obstructive pulmonary disease	--	--	0 (0–0)	0 (0–1)	0 (0–1)	0 (0–1)	3 (1–6)	8 (4–14)	7 (4–12)	8 (5–15)	10 (6–17)	14 (8–24)	31 (19–49)	75 (45–114)	134 (80–203)	220 (139–329)	279 (177–399)	351 (219–498)	348 (224–493)	340 (228–480)	223 (150–311)	76 (49–109)	--
Pneumoconiosis	--	--	--	--	--	--	0 (0–0)	0 (0–0)	0 (0–0)	0 (0–0)	0 (0–0)	0 (0–0)	0 (0–0)	0 (0–0)	0 (0–1)	0 (0–0)	0 (0–1)	0 (0–1)	0 (0–1)	0 (0–0)	0 (0–0)	0 (0–0)	--
Silicosis	--	--	--	--	--	--	0 (0–0)	0 (0–0)	0 (0–0)	0 (0–0)	0 (0–0)	0 (0–0)	0 (0–0)	0 (0–0)	0 (0–0)	0 (0–0)	0 (0–0)	0 (0–0)	0 (0–0)	0 (0–0)	0 (0–0)	0 (0–0)	--
Asbestosis	--	--	--	--	--	--	0 (0–0)	0 (0–0)	0 (0–0)	0 (0–0)	0 (0–0)	0 (0–0)	0 (0–0)	0 (0–0)	0 (0–0)	0 (0–0)	0 (0–0)	0 (0–0)	0 (0–0)	0 (0–0)	0 (0–0)	0 (0–0)	--
Coal workers pneumoconiosis	--	--	--	--	--	--	0 (0–0)	0 (0–0)	0 (0–0)	0 (0–0)	0 (0–0)	0 (0–0)	0 (0–0)	0 (0–0)	0 (0–0)	0 (0–0)	0 (0–0)	0 (0–0)	0 (0–0)	0 (0–0)	0 (0–0)	0 (0–0)	--
Other pneumoconiosis	--	--	--	--	--	--	0 (0–0)	0 (0–0)	0 (0–0)	0 (0–0)	0 (0–0)	0 (0–0)	0 (0–0)	0 (0–0)	0 (0–0)	0 (0–0)	0 (0–0)	0 (0–0)	0 (0–0)	0 (0–0)	0 (0–0)	0 (0–0)	--
Asthma	--	--	--	10 (5–18)	4 (2–6)	4 (3–7)	8 (5–14)	11 (6–17)	11 (6–18)	10 (6–16)	11 (6–19)	14 (7–23)	20 (11–34)	37 (19–60)	61 (34–101)	79 (42–134)	89 (48–148)	110 (56–180)	110 (58–183)	81 (43–137)	58 (32–95)	25 (13–39)	--
Interstitial lung disease and pulmonary sarcoidosis	--	--	--	0 (0–0)	0 (0–0)	0 (0–0)	0 (0–0)	0 (0–1)	0 (0–1)	0 (0–1)	0 (0–0)	1 (0–2)	1 (0–3)	2 (1–6)	4 (1–10)	3 (1–9)	4 (1–10)	5 (1–13)	4 (1–12)	3 (1–8)	2 (1–7)	1 (0–1)	--
Other chronic respiratory diseases	0 (0–0)	0 (0–0)	0 (0–0)	1 (0–3)	0 (0–1)	0 (0–1)	1 (0–3)	2 (1–4)	1 (0–3)	2 (1–3)	2 (0–3)	2 (1–4)	2 (1–4)	3 (1–6)	5 (2–12)	1 (0–1)	1 (0–1)	0 (0–1)	0 (0–1)	1 (0–1)	0 (0–0)	0 (0–0)	--
Digestive diseases	6 (3–14)	11 (5–24)	67 (23–157)	50 (18–122)	30 (18–47)	25 (16–39)	71 (42–111)	121 (75–180)	184 (118–279)	284 (195–408)	382 (263–558)	455 (311–675)	544 (369–770)	703 (496–989)	767 (504–1,050)	709 (478–972)	652 (442–886)	571 (391–785)	457 (312–631)	333 (224–457)	202 (143–273)	73 (50–102)	--
Cirrhosis and other chronic liver diseases	--	--	--	19 (5–52)	9 (4–17)	12 (7–20)	47 (26–77)	98 (56–149)	151 (94–234)	247 (167–361)	334 (227–491)	399 (269–593)	471 (318–666)	583 (409–824)	605 (400–849)	518 (334–720)	435 (291–615)	325 (208–467)	222 (139–330)	149 (90–228)	76 (48–113)	22 (13–36)	--
Cirrhosis and other chronic liver diseases due to hepatitis B	--	--	--	0 (0–0)	0 (0–0)	0 (0–1)	2 (1–6)	16 (7–31)	36 (14–69)	71 (36–119)	106 (54–184)	126 (67–224)	139 (73–238)	160 (80–281)	151 (68–267)	120 (54–220)	97 (47–167)	73 (35–126)	53 (25–91)	36 (17–64)	19 (10–31)	6 (2–11)	--
Cirrhosis and other chronic liver diseases due to hepatitis C	--	--	--	0 (0–1)	0 (0–1)	1 (0–1)	4 (1–9)	23 (10–44)	51 (24–94)	89 (49–145)	117 (62–201)	137 (73–238)	159 (83–262)	191 (103–323)	190 (100–318)	158 (76–263)	135 (73–220)	102 (55–164)	69 (35–117)	46 (24–80)	24 (13–40)	7 (3–13)	--
Cirrhosis and other chronic liver diseases due to alcohol use	--	--	--	--	--	--	0 (0–1)	5 (2–12)	13 (5–28)	34 (18–60)	62 (28–118)	85 (42–154)	112 (57–203)	153 (75–264)	175 (87–302)	153 (75–267)	121 (56–207)	81 (37–143)	47 (23–81)	27 (12–48)	13 (6–23)	3 (1–7)	--
Cirrhosis and other chronic liver diseases due to NAFLD	--	--	--	--	--	--	2 (1–4)	6 (2–13)	12 (5–25)	18 (9–33)	20 (8–42)	24 (10–47)	30 (12–61)	40 (16–79)	42 (18–82)	39 (16–77)	37 (19–66)	31 (15–55)	24 (10–45)	17 (8–33)	9 (4–17)	3 (1–6)	--
Cirrhosis and other chronic liver diseases due to other causes	--	--	--	19 (5–51)	9 (4–17)	11 (7–18)	39 (21–64)	47 (24–80)	38 (17–71)	34 (16–62)	28 (12–55)	26 (11–54)	30 (13–60)	39 (16–75)	47 (20–88)	47 (21–95)	45 (21–84)	39 (16–75)	30 (13–54)	22 (9–42)	12 (5–21)	3 (1–7)	--
Upper digestive system diseases	--	--	--	7 (2–21)	3 (1–6)	4 (2–7)	9 (4–18)	11 (5–22)	13 (6–25)	18 (8–36)	26 (13–46)	32 (16–59)	46 (24–82)	79 (43–135)	107 (53–172)	132 (75–214)	152 (88–241)	175 (100–284)	171 (99–272)	128 (73–200)	89 (54–135)	37 (23–53)	--
Peptic ulcer disease	--	--	--	5 (1–13)	3 (1–6)	3 (1–7)	8 (3–17)	10 (4–21)	12 (5–25)	18 (8–35)	24 (12–45)	31 (15–57)	44 (22–78)	76 (41–132)	104 (52–167)	129 (72–210)	149 (87–239)	170 (98–277)	166 (97–266)	124 (71–195)	87 (53–133)	36 (22–51)	--
Gastritis and duodenitis	--	--	--	3 (0–9)	0 (0–1)	0 (0–0)	1 (0–2)	1 (0–2)	1 (0–2)	0 (0–1)	1 (0–3)	1 (0–3)	2 (1–4)	2 (1–6)	3 (1–8)	3 (1–7)	3 (1–7)	5 (1–12)	4 (1–10)	4 (1–9)	2 (0–4)	1 (0–3)	--
Appendicitis	--	--	--	3 (1–7)	5 (2–9)	3 (1–6)	4 (1–8)	3 (1–5)	3 (1–5)	1 (0–3)	1 (0–3)	1 (0–3)	2 (1–4)	2 (1–4)	3 (1–6)	3 (1–5)	2 (1–4)	2 (1–4)	2 (1–3)	1 (1–3)	1 (0–2)	0 (0–1)	--
Paralytic ileus and intestinal obstruction	4 (2–9)	10 (4–20)	59 (20–139)	15 (4–43)	10 (4–21)	4 (1–9)	8 (3–19)	4 (1–8)	6 (2–12)	3 (1–7)	6 (2–12)	6 (3–13)	8 (4–16)	14 (6–27)	16 (7–32)	20 (10–38)	23 (12–41)	27 (13–48)	26 (12–46)	23 (12–43)	16 (8–28)	6 (3–11)	--
Inguinal, femoral, and abdominal hernia	2 (1–5)	2 (0–5)	7 (1–23)	2 (0–6)	0 (0–1)	0 (0–1)	0 (0–1)	1 (0–1)	1 (0–2)	1 (0–1)	1 (0–2)	1 (0–2)	1 (1–3)	3 (1–6)	5 (2–11)	5 (2–9)	7 (3–13)	8 (4–14)	7 (4–13)	7 (3–13)	5 (3–9)	2 (1–4)	--
Inflammatory bowel disease	--	--	--	1 (0–2)	0 (0–1)	0 (0–0)	0 (0–1)	0 (0–1)	1 (0–2)	0 (0–1)	0 (0–1)	1 (0–1)	1 (0–1)	1 (0–2)	1 (1–3)	2 (1–3)	2 (1–4)	2 (1–3)	2 (1–3)	2 (1–3)	1 (0–1)	0 (0–1)	--
Vascular intestinal disorders	--	--	--	0 (0–0)	0 (0–0)	0 (0–0)	0 (0–0)	0 (0–0)	0 (0–0)	0 (0–0)	0 (0–0)	0 (0–1)	1 (0–1)	1 (1–2)	2 (1–2)	2 (1–3)	3 (2–5)	4 (2–6)	3 (2–5)	3 (2–5)	2 (1–3)	1 (0–1)	--
Gallbladder and biliary diseases	--	--	--	1 (0–2)	0 (0–1)	0 (0–1)	0 (0–1)	1 (0–2)	2 (0–4)	2 (1–5)	2 (1–5)	3 (1–6)	3 (1–6)	5 (2–11)	8 (3–17)	10 (4–21)	9 (4–19)	12 (6–21)	10 (5–18)	8 (4–14)	5 (3–9)	2 (1–4)	--
Pancreatitis	--	--	--	0 (0–0)	0 (0–1)	0 (0–0)	2 (1–4)	4 (1–9)	7 (2–16)	9 (3–19)	10 (4–22)	10 (4–21)	10 (4–21)	12 (6–24)	13 (6–26)	11 (5–22)	11 (6–20)	10 (5–18)	7 (4–13)	4 (2–7)	2 (1–4)	1 (0–1)	--
Other digestive diseases	--	--	--	3 (1–8)	1 (0–3)	1 (0–2)	1 (0–2)	1 (0–2)	1 (0–3)	1 (1–3)	2 (1–4)	2 (1–4)	3 (1–6)	4 (2–8)	6 (3–12)	5 (3–11)	7 (3–13)	7 (3–14)	7 (3–14)	7 (3–14)	5 (2–9)	2 (1–4)	--
Alzheimer's disease and other dementias	--	--	--	--	--	--	--	--	--	--	--	1 (0–2)	3 (0–11)	8 (1–24)	17 (3–52)	32 (6–94)	50 (11–150)	71 (16–203)	90 (20–262)	156 (37–450)	162 (36–438)	83 (19–221)	--
Parkinson's disease	--	--	--	--	--	--	--	0 (0–0)	0 (0–0)	0 (0–0)	0 (0–0)	1 (1–2)	2 (1–3)	4 (3–6)	6 (4–8)	10 (7–14)	17 (12–22)	32 (23–42)	45 (33–59)	54 (41–69)	28 (21–37)	5 (3–9)	--
Idiopathic epilepsy	--	--	2 (1–5)	3 (1–6)	3 (1–6)	3 (2–6)	4 (3–8)	6 (4–11)	6 (3–10)	5 (3–8)	5 (3–9)	5 (3–9)	5 (3–9)	4 (2–8)	4 (3–8)	3 (2–6)	2 (2–4)	2 (2–4)	2 (1–3)	1 (1–2)	1 (0–1)	0 (0–0)	--
Multiple sclerosis	--	--	--	--	0 (0–0)	0 (0–0)	0 (0–0)	0 (0–0)	0 (0–0)	0 (0–1)	0 (0–1)	1 (0–1)	1 (1–2)	1 (1–2)	1 (1–2)	1 (1–2)	1 (0–1)	0 (0–1)	0 (0–0)	0 (0–0)	0 (0–0)	0 (0–0)	--
Motor neuron disease	0 (0–0)	0 (0–0)	0 (0–0)	0 (0–0)	0 (0–0)	0 (0–0)	0 (0–0)	0 (0–0)	0 (0–0)	0 (0–0)	0 (0–0)	0 (0–0)	0 (0–1)	0 (0–1)	1 (0–2)	1 (0–2)	1 (0–1)	0 (0–1)	0 (0–0)	0 (0–0)	0 (0–0)	0 (0–0)	--
Other neurological disorders	--	--	2 (1–3)	1 (0–1)	1 (0–1)	2 (1–2)	3 (2–5)	3 (2–4)	1 (1–2)	1 (0–1)	1 (1–1)	1 (1–2)	1 (1–2)	2 (1–3)	2 (1–3)	1 (1–2)	1 (1–2)	1 (1–2)	1 (1–2)	1 (0–1)	0 (0–1)	0 (0–0)	--
Eating disorders	--	--	--	--	0 (0–0)	0 (0–0)	0 (0–0)	0 (0–0)	0 (0–0)	0 (0–0)	0 (0–0)	0 (0–0)	0 (0–0)	--	--	--	--	--	--	--	--	--	--
Anorexia nervosa	--	--	--	--	0 (0–0)	0 (0–0)	0 (0–0)	0 (0–0)	0 (0–0)	0 (0–0)	0 (0–0)	0 (0–0)	0 (0–0)	--	--	--	--	--	--	--	--	--	--
Bulimia nervosa	--	--	--	--	0 (0–0)	0 (0–0)	0 (0–0)	0 (0–0)	0 (0–0)	0 (0–0)	0 (0–0)	0 (0–0)	0 (0–0)	--	--	--	--	--	--	--	--	--	--
Alcohol use disorders	--	--	--	--	--	0 (0–0)	1 (0–2)	2 (1–4)	5 (2–8)	8 (4–15)	13 (7–22)	14 (8–23)	17 (10–29)	17 (10–27)	16 (9–25)	12 (7–20)	8 (5–13)	5 (3–8)	3 (2–4)	1 (1–2)	1 (0–1)	0 (0–0)	--
Drug use disorders	0 (0–0)	0 (0–0)	0 (0–0)	0 (0–0)	0 (0–0)	0 (0–0)	1 (1–2)	3 (1–6)	3 (1–6)	3 (2–6)	3 (2–5)	2 (1–4)	2 (1–3)	2 (1–3)	1 (1–3)	1 (0–2)	1 (0–1)	1 (0–1)	0 (0–1)	0 (0–1)	0 (0–0)	0 (0–0)	--
Opioid use disorders	0 (0–0)	0 (0–0)	0 (0–0)	0 (0–0)	0 (0–0)	0 (0–0)	1 (0–2)	2 (1–4)	2 (1–5)	2 (1–4)	2 (1–4)	2 (1–3)	1 (1–2)	1 (1–2)	1 (0–2)	1 (0–1)	1 (0–1)	0 (0–1)	0 (0–0)	0 (0–0)	0 (0–0)	0 (0–0)	--
Cocaine use disorders	--	--	--	--	--	0 (0–0)	0 (0–0)	0 (0–0)	0 (0–0)	0 (0–1)	0 (0–0)	0 (0–0)	0 (0–0)	0 (0–0)	0 (0–0)	0 (0–0)	0 (0–0)	0 (0–0)	0 (0–0)	0 (0–0)	0 (0–0)	0 (0–0)	--
Amphetamine use disorders	--	--	--	--	--	0 (0–0)	0 (0–0)	0 (0–0)	0 (0–1)	0 (0–1)	0 (0–0)	0 (0–0)	0 (0–0)	0 (0–0)	0 (0–0)	0 (0–0)	0 (0–0)	0 (0–0)	0 (0–0)	0 (0–0)	0 (0–0)	0 (0–0)	--
Other drug use disorders	--	--	--	--	--	0 (0–0)	0 (0–0)	0 (0–1)	1 (0–1)	0 (0–1)	0 (0–1)	0 (0–0)	0 (0–0)	0 (0–0)	0 (0–0)	0 (0–0)	0 (0–0)	0 (0–0)	0 (0–0)	0 (0–0)	0 (0–0)	0 (0–0)	--
Diabetes mellitus	0 (0–0)	0 (0–0)	2 (1–4)	2 (1–5)	2 (1–4)	3 (1–5)	7 (4–11)	12 (6–20)	15 (8–25)	21 (12–36)	29 (16–47)	43 (26–68)	70 (42–110)	137 (86–204)	195 (120–304)	214 (135–320)	204 (134–293)	192 (123–275)	161 (105–235)	114 (72–166)	65 (42–96)	23 (15–34)	--
Diabetes mellitus type 1	0 (0–0)	0 (0–0)	2 (1–4)	2 (1–5)	2 (1–4)	3 (1–5)	4 (2–6)	6 (3–10)	7 (4–13)	6 (2–11)	7 (3–14)	9 (4–20)	5 (2–11)	8 (3–19)	11 (4–26)	9 (3–22)	9 (4–21)	7 (3–18)	5 (2–13)	3 (1–7)	2 (1–4)	1 (0–2)	--
Diabetes mellitus type 2	--	--	--	--	--	--	3 (2–6)	6 (3–10)	8 (4–14)	16 (8–27)	22 (11–36)	34 (18–56)	66 (40–106)	129 (80–195)	184 (109–291)	205 (128–313)	195 (128–282)	184 (119–267)	156 (100–228)	111 (70–161)	64 (41–93)	22 (14–33)	--
Chronic kidney disease	--	--	5 (3–9)	5 (3–10)	5 (4–9)	6 (4–8)	15 (9–24)	30 (18–47)	29 (18–45)	35 (22–53)	41 (25–67)	47 (28–75)	60 (37–94)	87 (53–129)	109 (65–168)	109 (70–159)	105 (67–157)	97 (62–141)	86 (55–121)	70 (46–100)	47 (32–65)	20 (13–27)	--
Chronic kidney disease due to diabetes mellitus type 1	--	--	0 (0–0)	0 (0–0)	0 (0–1)	1 (0–1)	2 (1–5)	7 (3–14)	11 (5–19)	16 (8–26)	19 (10–33)	19 (9–33)	19 (8–33)	18 (7–36)	14 (4–30)	10 (3–23)	7 (3–16)	5 (2–10)	3 (1–6)	1 (0–2)	0 (0–1)	0 (0–0)	--
Chronic kidney disease due to diabetes mellitus type 2	--	--	--	--	--	--	1 (0–3)	3 (1–7)	4 (1–8)	5 (2–11)	7 (2–15)	10 (4–21)	18 (8–33)	32 (17–56)	46 (24–76)	47 (25–75)	44 (25–69)	40 (24–61)	34 (20–52)	27 (16–40)	17 (10–25)	7 (4–10)	--
Chronic kidney disease due to hypertension	--	--	--	--	--	--	3 (1–5)	7 (3–12)	6 (3–12)	7 (3–13)	9 (4–17)	11 (6–21)	17 (9–29)	28 (15–46)	39 (21–66)	43 (25–68)	44 (26–70)	42 (26–64)	39 (23–58)	33 (20–50)	23 (14–33)	10 (6–14)	--
Chronic kidney disease due to glomerulonephritis	--	--	1 (0–2)	1 (0–2)	1 (0–3)	2 (1–3)	4 (2–8)	7 (3–13)	5 (2–9)	4 (1–7)	3 (1–8)	3 (1–7)	3 (1–7)	4 (2–9)	4 (2–9)	4 (2–8)	3 (1–7)	3 (1–6)	3 (1–6)	3 (1–5)	2 (1–4)	1 (0–1)	--
Chronic kidney disease due to other and unspecified causes	--	--	4 (2–8)	4 (2–8)	4 (2–6)	3 (2–5)	5 (2–9)	7 (3–14)	4 (2–9)	3 (1–7)	3 (1–7)	3 (1–6)	3 (1–7)	5 (2–9)	5 (2–11)	6 (2–11)	6 (3–12)	7 (3–13)	7 (3–13)	7 (3–13)	5 (2–10)	2 (1–4)	--
Acute glomerulonephritis	--	--	1 (0–3)	1 (0–3)	2 (0–7)	1 (0–3)	0 (0–1)	0 (0–1)	1 (0–2)	0 (0–1)	0 (0–1)	1 (0–2)	0 (0–1)	1 (0–4)	1 (0–2)	1 (0–3)	1 (0–4)	1 (0–3)	1 (0–3)	1 (0–3)	1 (0–2)	0 (0–1)	--
Skin and subcutaneous diseases	--	--	1 (0–3)	1 (0–2)	0 (0–1)	1 (0–2)	2 (1–3)	2 (1–4)	2 (1–4)	2 (1–4)	3 (1–5)	3 (1–7)	4 (2–8)	6 (3–11)	9 (4–16)	10 (5–17)	9 (4–16)	12 (6–20)	13 (7–23)	14 (7–24)	9 (4–16)	4 (2–7)	--
Bacterial skin diseases	--	--	1 (0–3)	1 (0–2)	0 (0–1)	0 (0–1)	1 (0–3)	2 (1–3)	2 (1–3)	1 (0–3)	2 (1–4)	2 (1–5)	3 (1–5)	4 (2–8)	5 (2–10)	5 (2–11)	4 (2–8)	5 (2–11)	6 (2–13)	5 (2–11)	3 (1–7)	1 (0–3)	--
Cellulitis	--	--	0 (0–0)	0 (0–0)	0 (0–0)	0 (0–1)	0 (0–0)	0 (0–1)	0 (0–0)	0 (0–0)	0 (0–1)	1 (0–2)	1 (0–2)	1 (0–3)	1 (0–3)	2 (0–4)	2 (0–4)	2 (0–4)	1 (0–3)	1 (0–3)	1 (0–2)	0 (0–1)	--
Pyoderma	--	--	1 (0–3)	1 (0–2)	0 (0–1)	0 (0–1)	1 (0–2)	1 (0–2)	1 (1–3)	1 (0–2)	1 (0–3)	2 (1–3)	2 (1–4)	3 (1–6)	4 (1–7)	4 (1–8)	2 (1–5)	3 (1–7)	4 (1–9)	4 (2–9)	2 (1–5)	1 (0–2)	--
Decubitus ulcer	--	--	--	0 (0–0)	0 (0–0)	0 (0–0)	0 (0–1)	0 (0–1)	0 (0–1)	1 (0–3)	1 (0–2)	1 (0–2)	1 (0–3)	2 (0–4)	3 (1–7)	4 (1–8)	5 (2–10)	6 (2–12)	7 (2–14)	8 (3–15)	6 (2–11)	2 (1–5)	--
Other skin and subcutaneous diseases	--	--	0 (0–0)	0 (0–0)	0 (0–0)	0 (0–0)	0 (0–0)	0 (0–0)	0 (0–0)	0 (0–0)	0 (0–0)	0 (0–1)	0 (0–1)	0 (0–1)	0 (0–1)	0 (0–1)	0 (0–1)	0 (0–1)	0 (0–1)	0 (0–1)	0 (0–0)	0 (0–0)	--
Musculoskeletal disorders	--	--	--	--	0 (0–1)	1 (0–1)	1 (1–2)	1 (1–2)	1 (1–2)	1 (1–2)	1 (1–2)	1 (1–2)	2 (1–3)	2 (1–3)	2 (1–4)	3 (2–5)	3 (2–4)	3 (1–4)	2 (1–3)	1 (1–2)	1 (1–2)	0 (0–1)	--
Rheumatoid arthritis	--	--	--	--	0 (0–0)	0 (0–0)	0 (0–0)	0 (0–0)	0 (0–0)	0 (0–0)	0 (0–0)	0 (0–1)	0 (0–0)	0 (0–1)	1 (0–1)	1 (0–2)	1 (0–2)	1 (0–2)	1 (0–1)	0 (0–1)	1 (0–1)	0 (0–0)	--
Other musculoskeletal disorders	--	--	--	--	0 (0–1)	1 (0–1)	1 (1–2)	1 (1–2)	1 (1–2)	1 (1–2)	1 (1–2)	1 (1–2)	1 (1–2)	2 (1–3)	2 (1–3)	2 (1–4)	2 (1–3)	2 (1–3)	1 (1–2)	1 (0–2)	0 (0–1)	0 (0–0)	--
Congenital anomalies	351 (163–610)	89 (45–149)	292 (106–558)	69 (35–117)	30 (15–50)	22 (12–35)	20 (10–33)	14 (7–27)	10 (5–19)	6 (3–10)	4 (2–7)	4 (2–6)	2 (1–4)	2 (1–3)	2 (1–3)	2 (1–3)	1 (1–2)	--	--	--	--	--	--
Neural tube defects	58 (15–143)	6 (1–16)	14 (3–43)	5 (0–19)	1 (0–2)	0 (0–1)	0 (0–0)	0 (0–0)	0 (0–0)	0 (0–0)	0 (0–0)	0 (0–0)	0 (0–0)	0 (0–0)	0 (0–0)	0 (0–0)	0 (0–0)	--	--	--	--	--	--
Congenital heart anomalies	129 (48–256)	49 (23–86)	171 (56–344)	39 (17–70)	19 (9–32)	16 (9–27)	15 (8–27)	11 (5–20)	9 (4–16)	5 (2–8)	3 (2–5)	3 (2–5)	1 (1–3)	1 (1–2)	1 (1–2)	1 (1–2)	1 (0–1)	--	--	--	--	--	--
Orofacial clefts	2 (0–6)	1 (0–2)	2 (0–7)	0 (0–0)	--	--	--	--	--	--	--	--	--	--	--	--	--	--	--	--	--	--	--
Down's syndrome	2 (1–4)	1 (0–2)	4 (1–13)	2 (0–4)	1 (0–2)	0 (0–1)	0 (0–1)	1 (0–1)	0 (0–1)	0 (0–1)	0 (0–1)	0 (0–0)	0 (0–1)	0 (0–1)	0 (0–0)	0 (0–0)	0 (0–0)	--	--	--	--	--	--
Other chromosomal abnormalities	5 (1–15)	1 (0–4)	3 (1–9)	0 (0–1)	0 (0–0)	0 (0–0)	0 (0–0)	0 (0–0)	0 (0–0)	0 (0–0)	0 (0–0)	0 (0–0)	0 (0–0)	0 (0–0)	0 (0–0)	0 (0–0)	0 (0–0)	--	--	--	--	--	--
Congenital musculoskeletal and limb anomalies	5 (2–11)	1 (0–2)	2 (0–5)	0 (0–1)	0 (0–1)	0 (0–0)	0 (0–1)	0 (0–0)	0 (0–0)	0 (0–0)	0 (0–0)	0 (0–0)	0 (0–0)	0 (0–0)	0 (0–0)	0 (0–0)	0 (0–0)	--	--	--	--	--	--
Urogenital congenital anomalies	6 (2–16)	1 (0–2)	1 (0–4)	0 (0–1)	0 (0–0)	0 (0–0)	0 (0–0)	0 (0–0)	0 (0–0)	0 (0–0)	0 (0–0)	0 (0–0)	0 (0–0)	0 (0–0)	0 (0–0)	0 (0–0)	0 (0–0)	--	--	--	--	--	--
Digestive congenital anomalies	37 (9–87)	13 (4–27)	43 (10–102)	7 (2–16)	1 (0–3)	1 (0–2)	0 (0–1)	0 (0–0)	0 (0–0)	0 (0–0)	0 (0–0)	0 (0–0)	0 (0–0)	0 (0–0)	0 (0–0)	0 (0–0)	0 (0–0)	--	--	--	--	--	--
Other congenital anomalies	108 (33–252)	16 (6–38)	52 (12–155)	14 (5–36)	8 (3–20)	3 (1–9)	3 (1–8)	2 (1–6)	0 (0–1)	0 (0–1)	0 (0–1)	0 (0–0)	0 (0–1)	0 (0–0)	0 (0–1)	0 (0–1)	0 (0–1)	--	--	--	--	--	--
Urinary diseases and male infertility	0 (0–0)	0 (0–0)	3 (1–5)	1 (1–3)	2 (1–3)	2 (1–3)	3 (1–4)	4 (2–7)	4 (2–7)	5 (3–9)	6 (3–12)	8 (4–14)	12 (6–21)	19 (10–35)	25 (13–44)	29 (16–49)	31 (17–55)	34 (19–55)	30 (16–53)	31 (17–51)	18 (10–32)	7 (4–12)	--
Urinary tract infections and interstitial nephritis	0 (0–0)	0 (0–0)	2 (1–4)	1 (1–2)	1 (1–2)	1 (1–2)	2 (1–4)	3 (1–5)	3 (2–5)	4 (2–7)	4 (2–8)	6 (3–11)	8 (4–14)	13 (6–23)	16 (7–29)	18 (9–31)	19 (9–34)	22 (11–37)	21 (11–39)	18 (9–32)	12 (6–21)	4 (2–7)	--
Urolithiasis	--	--	--	0 (0–0)	0 (0–0)	0 (0–0)	0 (0–0)	0 (0–0)	0 (0–1)	0 (0–1)	1 (0–2)	1 (0–2)	1 (0–4)	2 (0–6)	2 (0–6)	4 (0–10)	4 (0–10)	3 (0–8)	4 (0–10)	3 (0–7)	1 (0–3)	0 (0–1)	--
Other urinary diseases	0 (0–0)	0 (0–0)	1 (0–1)	0 (0–1)	0 (0–1)	0 (0–1)	0 (0–1)	2 (0–3)	0 (0–1)	1 (0–2)	1 (0–3)	1 (0–3)	3 (1–7)	4 (1–9)	7 (3–15)	7 (3–14)	8 (3–17)	9 (4–18)	5 (2–12)	10 (4–20)	5 (2–10)	3 (1–5)	--
Gynecological diseases	--	--	--	--	--	--	--	--	--	--	--	--	--	--	--	--	--	--	--	--	--	--	--
Uterine fibroids	--	--	--	--	--	--	--	--	--	--	--	--	--	--	--	--	--	--	--	--	--	--	--

Endometriosis	--	--	--	--	--	--	--	--	--	--	--	--	--	--	--	--	--	--	--	--	--	--	--
Genital prolapse	--	--	--	--	--	--	--	--	--	--	--	--	--	--	--	--	--	--	--	--	--	--	--
Other gynecological diseases	--	--	--	--	--	--	--	--	--	--	--	--	--	--	--	--	--	--	--	--	--	--	--
Hemoglobinopathies and hemolytic anaemias	0 (0–1)	0 (0–0)	3 (1–6)	3 (2–6)	3 (2–6)	4 (2–7)	5 (3–9)	5 (3–9)	4 (2–7)	3 (2–5)	3 (1–4)	3 (1–4)	3 (2–4)	5 (3–8)	6 (4–9)	6 (4–9)	6 (4–10)	6 (4–10)	5 (3–7)	3 (2–5)	2 (1–4)	1 (0–1)	--
Thalassemias	0 (0–1)	0 (0–0)	3 (1–6)	3 (2–6)	3 (2–5)	4 (2–6)	4 (2–6)	2 (1–3)	1 (0–2)	0 (0–1)	0 (0–1)	0 (0–1)	0 (0–0)	0 (0–0)	0 (0–0)	0 (0–0)	0 (0–0)	0 (0–0)	0 (0–0)	0 (0–0)	0 (0–0)	0 (0–0)	--
Sickle cell disorders	0 (0–0)	0 (0–0)	0 (0–0)	0 (0–0)	0 (0–0)	0 (0–0)	0 (0–0)	0 (0–0)	0 (0–0)	0 (0–0)	0 (0–0)	0 (0–0)	0 (0–0)	0 (0–0)	0 (0–0)	0 (0–0)	0 (0–0)	0 (0–0)	0 (0–0)	0 (0–0)	0 (0–0)	0 (0–0)	--
G6PD deficiency	0 (0–0)	0 (0–0)	0 (0–0)	0 (0–0)	0 (0–0)	1 (0–1)	2 (1–3)	3 (1–5)	3 (2–5)	2 (1–4)	2 (1–4)	2 (1–3)	2 (1–4)	4 (2–6)	5 (3–8)	4 (2–7)	4 (2–6)	2 (1–4)	1 (1–2)	1 (0–1)	0 (0–0)	0 (0–0)	--
Other hemoglobinopathies and hemolytic anaemias	0 (0–0)	0 (0–0)	0 (0–0)	0 (0–0)	0 (0–0)	0 (0–0)	0 (0–0)	0 (0–0)	0 (0–0)	0 (0–0)	0 (0–0)	0 (0–0)	0 (0–1)	1 (0–1)	1 (1–2)	2 (1–3)	3 (2–5)	4 (2–6)	4 (2–6)	3 (2–4)	2 (1–4)	1 (0–1)	--
Endocrine, metabolic, blood, and immune disorders	1 (1–2)	1 (0–2)	9 (4–19)	3 (2–6)	2 (1–3)	2 (1–4)	2 (1–3)	2 (1–4)	3 (1–5)	3 (2–6)	3 (2–6)	3 (2–6)	5 (2–8)	7 (3–12)	7 (3–13)	7 (3–12)	7 (4–13)	9 (5–15)	8 (4–13)	7 (4–13)	4 (2–8)	2 (1–4)	--
Sudden infant death syndrome	--	8 (3–17)	46 (13–124)	--	--	--	--	--	--	--	--	--	--	--	--	--	--	--	--	--	--	--	--
Transport injuries	1 (0–3)	1 (0–1)	11 (5–22)	29 (15–50)	53 (31–78)	60 (38–87)	260 (174–355)	372 (250–504)	267 (174–384)	197 (130–286)	156 (99–239)	134 (86–210)	135 (84–211)	155 (98–233)	147 (92–220)	107 (66–160)	83 (49–125)	66 (41–103)	46 (30–69)	29 (19–44)	14 (9–22)	4 (2–5)	--
Road injuries	1 (0–3)	1 (0–1)	10 (5–20)	27 (14–46)	49 (30–74)	56 (36–82)	244 (160–335)	348 (234–471)	248 (158–360)	182 (121–264)	145 (91–222)	125 (80–196)	125 (79–192)	144 (91–216)	137 (85–203)	98 (62–149)	76 (44–116)	60 (38–94)	42 (27–64)	27 (17–41)	13 (8–20)	3 (2–5)	--
Pedestrian road injuries	0 (0–1)	0 (0–0)	3 (1–7)	9 (3–17)	17 (8–29)	17 (8–29)	25 (12–45)	36 (17–63)	25 (12–45)	26 (14–49)	22 (11–41)	20 (10–38)	26 (13–47)	34 (17–59)	34 (18–57)	33 (18–56)	26 (13–44)	23 (12–39)	24 (15–37)	15 (9–23)	7 (4–11)	1 (1–2)	--
Cyclist road injuries	--	--	--	3 (1–7)	6 (3–13)	7 (3–13)	11 (5–21)	14 (6–27)	10 (4–21)	13 (5–24)	10 (4–19)	8 (3–16)	15 (7–28)	19 (8–34)	19 (8–37)	16 (7–30)	12 (5–23)	9 (3–17)	1 (0–2)	1 (0–2)	1 (0–1)	0 (0–1)	--
Motorcyclist road injuries	0 (0–1)	0 (0–0)	3 (1–7)	7 (3–14)	11 (5–20)	16 (7–28)	127 (76–191)	184 (109–268)	126 (73–195)	77 (43–123)	59 (33–100)	49 (26–82)	40 (21–69)	41 (21–75)	37 (17–66)	14 (6–28)	10 (4–19)	6 (2–12)	2 (1–4)	1 (0–3)	1 (0–1)	0 (0–0)	--
Motor vehicle road injuries	0 (0–1)	0 (0–0)	4 (1–8)	7 (3–14)	14 (7–24)	15 (7–26)	77 (39–127)	108 (56–172)	83 (45–138)	63 (34–106)	51 (28–87)	44 (24–76)	41 (23–71)	46 (25–79)	45 (23–78)	32 (17–57)	26 (13–45)	21 (11–36)	15 (8–25)	9 (5–16)	4 (2–7)	1 (1–2)	--
Other road injuries	0 (0–0)	0 (0–0)	0 (0–0)	0 (0–1)	1 (0–2)	1 (0–2)	3 (1–7)	5 (2–10)	4 (2–8)	3 (1–6)	2 (1–5)	2 (1–5)	2 (1–4)	3 (1–7)	2 (1–5)	2 (1–4)	2 (0–4)	1 (0–3)	1 (0–3)	1 (0–2)	0 (0–1)	0 (0–0)	--
Other transport injuries	0 (0–0)	0 (0–0)	1 (0–2)	2 (1–6)	3 (1–7)	4 (2–8)	16 (7–32)	24 (11–45)	19 (9–38)	15 (6–29)	11 (5–23)	10 (4–20)	10 (4–21)	12 (5–23)	10 (5–21)	9 (4–17)	7 (3–14)	5 (2–11)	4 (2–8)	2 (1–5)	2 (1–3)	0 (0–1)	--
Falls	2 (1–4)	2 (1–4)	14 (5–32)	27 (13–54)	21 (13–33)	20 (12–30)	48 (27–75)	69 (40–110)	68 (41–102)	65 (40–100)	69 (43–107)	70 (41–119)	67 (36–114)	96 (52–175)	106 (60–184)	119 (73–179)	102 (64–150)	102 (66–151)	91 (60–128)	77 (48–113)	57 (35–89)	26 (15–40)	--
Drowning	1 (0–2)	0 (0–1)	9 (3–20)	74 (35–134)	69 (40–110)	58 (34–93)	89 (47–151)	81 (46–131)	60 (32–97)	49 (27–81)	43 (24–73)	38 (21–66)	36 (19–62)	38 (22–62)	35 (19–62)	27 (16–47)	22 (12–35)	17 (10–27)	12 (7–20)	7 (4–11)	4 (2–8)	1 (1–2)	--
Fire, heat, and hot substances	0 (0–0)	0 (0–0)	5 (2–10)	10 (5–17)	4 (2–7)	3 (2–6)	6 (3–10)	9 (4–17)	9 (4–16)	7 (3–12)	6 (3–11)	6 (3–11)	5 (3–10)	7 (3–12)	7 (4–13)	7 (4–12)	8 (4–13)	6 (3–9)	5 (3–8)	4 (3–7)	4 (2–5)	1 (1–2)	--
Poisonings	0 (0–0)	0 (0–1)	1 (0–3)	4 (2–10)	2 (1–4)	2 (1–3)	4 (1–7)	5 (2–10)	5 (2–9)	4 (2–7)	3 (2–6)	3 (1–6)	3 (2–6)	3 (2–6)	3 (1–5)	2 (1–4)	1 (1–3)	1 (0–2)	1 (0–1)	1 (0–1)	1 (0–1)	0 (0–0)	--
Poisoning by carbon monoxide	0 (0–0)	0 (0–0)	0 (0–0)	0 (0–0)	0 (0–0)	0 (0–0)	0 (0–1)	1 (0–1)	0 (0–1)	0 (0–1)	0 (0–1)	0 (0–1)	0 (0–1)	0 (0–1)	0 (0–1)	0 (0–0)	0 (0–0)	0 (0–0)	0 (0–0)	0 (0–0)	0 (0–0)	0 (0–0)	--
Poisoning by other means	0 (0–0)	0 (0–1)	1 (0–3)	4 (1–9)	2 (1–4)	1 (1–3)	3 (1–6)	5 (2–9)	5 (2–8)	3 (2–6)	3 (1–6)	3 (1–5)	3 (1–5)	3 (1–6)	3 (1–5)	2 (1–4)	1 (1–3)	1 (0–2)	1 (0–1)	1 (0–1)	0 (0–1)	0 (0–0)	--
Exposure to mechanical forces	0 (0–1)	1 (0–8)	3 (0–15)	5 (2–12)	6 (3–10)	5 (2–9)	12 (6–22)	20 (10–33)	17 (8–29)	15 (8–28)	13 (7–24)	11 (6–21)	12 (6–23)	12 (6–21)	12 (6–22)	8 (4–12)	6 (3–10)	5 (3–8)	3 (2–5)	2 (1–3)	1 (1–2)	1 (0–1)	--
Unintentional firearm injuries	0 (0–0)	0 (0–1)	0 (0–2)	0 (0–1)	0 (0–1)	0 (0–1)	1 (0–4)	3 (1–8)	2 (0–5)	2 (0–5)	2 (0–5)	1 (0–4)	1 (0–4)	1 (0–3)	1 (0–2)	1 (0–1)	0 (0–1)	0 (0–1)	0 (0–0)	0 (0–0)	0 (0–0)	0 (0–0)	--
Other exposure to mechanical forces	0 (0–1)	1 (0–6)	3 (0–14)	4 (1–12)	5 (3–10)	5 (2–8)	10 (5–20)	17 (8–29)	15 (7–26)	13 (7–24)	11 (6–21)	10 (5–19)	10 (5–21)	11 (5–20)	11 (6–21)	7 (4–12)	5 (3–9)	5 (3–8)	3 (2–5)	2 (1–3)	1 (1–2)	1 (0–1)	--
Adverse effects of medical treatment	1 (0–2)	0 (0–1)	5 (2–10)	2 (1–4)	2 (1–4)	2 (1–3)	2 (1–4)	4 (2–6)	3 (2–5)	3 (1–5)	3 (1–6)	3 (2–6)	5 (2–9)	5 (3–9)	7 (3–14)	8 (3–15)	7 (3–14)	6 (3–11)	5 (2–9)	4 (2–7)	2 (1–4)	1 (0–2)	--
Animal contact	0 (0–0)	0 (0–0)	2 (1–4)	5 (2–9)	6 (3–12)	5 (2–10)	5 (2–10)	5 (2–11)	5 (2–9)	4 (2–7)	5 (2–9)	4 (2–8)	5 (2–10)	7 (3–13)	7 (3–14)	6 (3–11)	5 (2–9)	4 (2–8)	3 (2–6)	3 (1–5)	1 (1–3)	0 (0–1)	--
Venomous animal contact	0 (0–0)	0 (0–0)	0 (0–0)	0 (0–1)	1 (0–1)	1 (0–1)	1 (0–2)	1 (0–2)	1 (0–1)	0 (0–1)	0 (0–1)	0 (0–1)	0 (0–1)	0 (0–1)	0 (0–1)	0 (0–0)	0 (0–0)	0 (0–0)	0 (0–0)	0 (0–0)	0 (0–0)	0 (0–0)	--
Non-venomous animal contact	0 (0–0)	0 (0–0)	2 (1–4)	4 (2–9)	6 (2–11)	4 (2–8)	4 (2–8)	5 (2–10)	4 (2–8)	3 (1–7)	4 (2–8)	4 (2–8)	5 (2–9)	7 (3–13)	7 (3–14)	6 (3–11)	5 (2–9)	4 (2–8)	3 (2–6)	3 (1–5)	1 (1–2)	0 (0–1)	--
Foreign body	1 (0–3)	1 (1–3)	24 (9–53)	8 (4–14)	4 (2–7)	4 (2–7)	4 (2–7)	7 (4–12)	6 (4–10)	5 (3–8)	4 (2–7)	4 (2–6)	4 (2–6)	5 (3–7)	5 (3–8)	5 (2–8)	4 (2–6)	5 (3–7)	4 (3–7)	4 (2–6)	3 (1–4)	1 (1–2)	--
Pulmonary aspiration and foreign body in airway	1 (0–3)	1 (1–3)	23 (9–52)	7 (4–13)	4 (2–7)	4 (2–7)	4 (2–6)	7 (4–12)	6 (4–10)	5 (3–8)	4 (2–6)	4 (2–6)	4 (2–6)	5 (3–7)	5 (3–8)	4 (2–8)	4 (2–6)	4 (3–7)	4 (3–7)	4 (2–6)	2 (1–4)	1 (1–2)	--
Foreign body in other body part	0 (0–0)	0 (0–0)	0 (0–1)	0 (0–1)	0 (0–0)	0 (0–0)	0 (0–0)	0 (0–0)	0 (0–0)	0 (0–0)	0 (0–0)	0 (0–0)	0 (0–0)	0 (0–0)	0 (0–0)	0 (0–0)	0 (0–0)	0 (0–0)	0 (0–0)	0 (0–0)	0 (0–0)	0 (0–0)	--
Environmental heat and cold exposure	0 (0–0)	0 (0–0)	0 (0–0)	0 (0–0)	0 (0–0)	0 (0–0)	0 (0–0)	0 (0–0)	0 (0–0)	0 (0–0)	0 (0–0)	0 (0–0)	0 (0–0)	0 (0–1)	0 (0–1)	0 (0–1)	0 (0–0)	0 (0–0)	0 (0–0)	0 (0–0)	0 (0–0)	0 (0–0)	--
Other unintentional injuries	0 (0–0)	0 (0–0)	0 (0–0)	0 (0–1)	0 (0–1)	1 (0–1)	2 (1–4)	2 (1–5)	2 (1–4)	2 (1–3)	1 (0–3)	1 (0–2)	1 (0–2)	1 (0–2)	1 (0–2)	1 (0–1)	0 (0–1)	0 (0–0)	0 (0–0)	0 (0–0)	0 (0–0)	0 (0–0)	--
Self-harm	--	--	--	--	--	7 (3–13)	45 (27–70)	73 (43–108)	66 (41–98)	65 (40–99)	53 (34–83)	43 (27–66)	37 (22–59)	38 (24–57)	37 (22–55)	34 (19–51)	26 (14–39)	21 (11–30)	12 (7–18)	5 (3–8)	2 (2–4)	1 (0–1)	--
Self-harm by firearm	--	--	--	--	--	0 (0–0)	1 (0–2)	2 (1–5)	2 (1–4)	2 (1–3)	1 (1–3)	1 (0–3)	1 (1–3)	1 (0–2)	1 (0–2)	1 (0–1)	0 (0–1)	0 (0–1)	0 (0–0)	0 (0–0)	0 (0–0)	0 (0–0)	--
Self-harm by other specified means	--	--	--	--	--	7 (3–13)	44 (26–68)	71 (42–106)	64 (40–94)	63 (39–96)	52 (33–82)	42 (26–65)	36 (22–57)	37 (23–56)	36 (21–54)	33 (18–50)	26 (14–39)	20 (11–30)	12 (7–17)	5 (3–8)	2 (2–4)	1 (0–1)	--
Interpersonal violence	1 (0–3)	0 (0–1)	2 (1–5)	4 (1–10)	3 (2–6)	6 (3–11)	46 (22–83)	79 (40–139)	65 (35–110)	51 (28–87)	39 (19–66)	29 (15–51)	22 (12–37)	19 (10–32)	14 (7–24)	8 (4–14)	4 (2–8)	3 (1–5)	2 (1–3)	0 (0–1)	0 (0–0)	0 (0–0)	--
Physical violence by firearm	0 (0–0)	0 (0–0)	0 (0–1)	1 (0–2)	1 (0–1)	1 (0–3)	14 (5–30)	28 (10–58)	21 (9–43)	20 (8–39)	16 (6–32)	12 (6–25)	8 (3–16)	7 (3–14)	5 (2–10)	3 (1–6)	1 (0–3)	1 (0–1)	0 (0–1)	0 (0–0)	0 (0–0)	0 (0–0)	--
Physical violence by sharp object	0 (0–1)	0 (0–0)	0 (0–1)	1 (0–3)	1 (0–2)	2 (0–4)	25 (8–49)	42 (16–78)	36 (14–67)	25 (10–49)	18 (6–34)	13 (4–26)	11 (4–21)	8 (2–17)	6 (2–12)	3 (1–6)	2 (1–4)	1 (0–2)	1 (0–1)	0 (0–0)	0 (0–0)	0 (0–0)	--
Physical violence by other means	1 (0–2)	0 (0–0)	2 (1–4)	2 (1–5)	2 (1–3)	3 (1–6)	7 (3–16)	10 (4–23)	8 (3–18)	6 (2–14)	5 (2–11)	4 (1–9)	3 (1–7)	3 (1–6)	3 (1–6)	2 (1–4)	1 (0–3)	1 (0–2)	1 (0–1)	0 (0–0)	0 (0–0)	0 (0–0)	--
Executions and police conflict	0 (0–0)	0 (0–0)	0 (0–0)	0 (0–0)	0 (0–0)	0 (0–0)	0 (0–1)	1 (0–3)	1 (0–4)	1 (0–3)	1 (0–2)	0 (0–1)	0 (0–1)	0 (0–1)	0 (0–0)	0 (0–0)	0 (0–0)	0 (0–0)	0 (0–0)	0 (0–0)	0 (0–0)	0 (0–0)	--

SR Table 12: High-income North America deaths by cause and age group for males

Cause	Early Neonatal	Late Neonatal	Post Neonatal	1 to 4	5 to 9	10 to 14	15 to 19
All causes	6,764 (6,149–7,438)	1,611 (1,466–1,774)	4,240 (3,911–4,599)	2,224 (2,058–2,405)	1,351 (1,318–1,386)	1,939 (1,907–1,973)	8,094 (7,989–8,198)
Sexually transmitted infections excluding HIV	1 (1–1)	0 (0–0)	0 (0–0)	0 (0–0)	0 (0–0)	0 (0–0)	0 (0–0)
Syphilis	1 (1–1)	0 (0–0)	0 (0–0)	0 (0–0)	0 (0–0)	0 (0–0)	0 (0–0)
Chlamydial infection	--	--	--	--	--	0 (0–0)	0 (0–0)
Gonococcal infection	--	--	--	--	--	0 (0–0)	0 (0–0)
Other sexually transmitted infections	--	--	--	--	--	0 (0–0)	0 (0–0)
Tuberculosis	--	--	1 (0–1)	1 (1–1)	0 (0–0)	0 (0–0)	1 (1–1)
Drug-susceptible tuberculosis	--	--	0 (0–1)	1 (1–1)	0 (0–0)	0 (0–0)	1 (1–1)
Multidrug-resistant tuberculosis without extensive drug resistance	--	--	0 (0–0)	0 (0–0)	0 (0–0)	0 (0–0)	0 (0–0)
Extensively drug-resistant tuberculosis	--	--	0 (0–0)	0 (0–0)	0 (0–0)	0 (0–0)	0 (0–0)
Lower respiratory infections	16 (13–18)	25 (22–28)	205 (179–233)	81 (71–91)	30 (28–32)	29 (27–31)	42 (39–46)
Upper respiratory infections	0 (0–0)	0 (0–0)	5 (4–6)	5 (4–6)	1 (1–1)	1 (1–1)	1 (1–1)
Otitis media	0 (0–0)	0 (0–0)	1 (1–2)	1 (0–1)	0 (0–0)	0 (0–0)	0 (0–0)
Diarrhoeal diseases	3 (3–4)	4 (3–4)	23 (19–27)	9 (7–10)	3 (3–4)	3 (3–3)	3 (3–4)
Typhoid fever	--	--	0 (0–0)	0 (0–0)	0 (0–0)	0 (0–0)	0 (0–0)
Paratyphoid fever	--	--	0 (0–0)	0 (0–0)	0 (0–0)	0 (0–0)	0 (0–0)
iNTS	--	0 (0–1)	1 (1–2)	1 (0–2)	0 (0–1)	0 (0–1)	0 (0–0)
Other intestinal infectious diseases	--	--	0 (0–2)	0 (0–2)	0 (0–1)	0 (0–1)	0 (0–1)
Malaria	0 (0–0)	0 (0–0)	0 (0–0)	0 (0–0)	0 (0–0)	0 (0–0)	0 (0–0)
Chagas disease	--	--	0 (0–0)	0 (0–0)	0 (0–0)	0 (0–0)	0 (0–0)
Visceral leishmaniasis	--	--	0 (0–0)	0 (0–0)	0 (0–0)	0 (0–0)	0 (0–0)
African trypanosomiasis	--	--	--	0 (0–0)	0 (0–0)	0 (0–0)	0 (0–0)
Schistosomiasis	--	--	0 (0–0)	0 (0–0)	0 (0–0)	0 (0–0)	0 (0–0)
Cysticercosis	--	--	--	0 (0–0)	0 (0–0)	0 (0–0)	0 (0–0)
Cystic echinococcosis	--	--	--	0 (0–0)	0 (0–0)	0 (0–0)	0 (0–0)
Dengue	0 (0–0)	0 (0–0)	0 (0–0)	0 (0–0)	0 (0–0)	0 (0–0)	0 (0–0)
Yellow fever	--	0 (0–0)	0 (0–0)	0 (0–0)	0 (0–0)	0 (0–0)	0 (0–0)
Rabies	--	--	0 (0–0)	0 (0–0)	0 (0–0)	0 (0–0)	0 (0–0)
Ascariasis	--	--	0 (0–0)	0 (0–0)	0 (0–0)	0 (0–0)	0 (0–0)
Zika virus disease	0 (0–0)	0 (0–0)	0 (0–0)	0 (0–0)	0 (0–0)	0 (0–0)	0 (0–0)
Other neglected tropical diseases	0 (0–0)	1 (0–1)	2 (0–3)	2 (1–3)	2 (0–2)	2 (0–2)	1 (0–1)
Meningitis	3 (2–3)	9 (8–10)	39 (33–45)	23 (20–26)	10 (9–11)	9 (9–10)	14 (12–15)
Encephalitis	1 (1–1)	2 (2–3)	11 (9–13)	14 (12–17)	8 (7–9)	8 (7–9)	7 (6–7)
Diphtheria	--	--	0 (0–0)	0 (0–0)	0 (0–0)	0 (0–0)	0 (0–0)
Whooping cough	--	--	5 (1–7)	0 (0–0)	0 (0–0)	0 (0–0)	0 (0–0)
Tetanus	0 (0–0)	0 (0–0)	0 (0–0)	0 (0–0)	0 (0–0)	0 (0–0)	0 (0–0)
Measles	0 (0–0)	0 (0–0)	0 (0–0)	0 (0–0)	0 (0–0)	0 (0–0)	0 (0–0)
Varicella and herpes zoster	0 (0–1)	1 (0–3)	2 (1–5)	3 (1–7)	3 (2–8)	1 (1–4)	1 (0–2)
Acute hepatitis	--	--	1 (1–1)	0 (0–1)	0 (0–0)	0 (0–0)	0 (0–1)
Acute hepatitis A	--	--	0 (0–0)	0 (0–0)	0 (0–0)	0 (0–0)	0 (0–0)
Acute hepatitis B	--	--	1 (0–1)	0 (0–0)	0 (0–0)	0 (0–0)	0 (0–0)
Acute hepatitis C	--	--	0 (0–0)	0 (0–0)	0 (0–0)	0 (0–0)	0 (0–0)
Acute hepatitis E	--	--	0 (0–0)	0 (0–0)	0 (0–0)	0 (0–0)	0 (0–0)
Other unspecified infectious diseases	10 (6–16)	32 (17–45)	68 (41–116)	20 (13–37)	12 (8–22)	11 (7–20)	15 (10–28)
Maternal disorders	--	--	--	--	--	--	--
Maternal haemorrhage	--	--	--	--	--	--	--
Maternal sepsis and other pregnancy related infections	--	--	--	--	--	--	--
Maternal hypertensive disorders	--	--	--	--	--	--	--
Maternal obstructed labour and uterine rupture	--	--	--	--	--	--	--
Maternal abortive outcome	--	--	--	--	--	--	--
Ectopic pregnancy	--	--	--	--	--	--	--
Indirect maternal deaths	--	--	--	--	--	--	--
Late maternal deaths	--	--	--	--	--	--	--
Maternal deaths aggravated by HIV/AIDS	--	--	--	--	--	--	--
Other maternal disorders	--	--	--	--	--	--	--
Neonatal disorders	5,183 (4,609–5,763)	895 (789–996)	519 (452–594)	54 (47–62)	--	--	--
Neonatal preterm birth	3,137 (2,797–3,501)	371 (323–418)	324 (279–374)	21 (18–25)	--	--	--
Neonatal encephalopathy due to birth asphyxia and trauma	795 (667–916)	110 (93–129)	58 (48–68)	20 (17–23)	--	--	--
Neonatal sepsis and other neonatal infections	171 (148–194)	187 (161–212)	50 (43–58)	0 (0–0)	--	--	--
Hemolytic disease and other neonatal jaundice	4 (4–5)	1 (1–2)	1 (1–1)	2 (1–2)	--	--	--
Other neonatal disorders	1,076 (938–1,229)	226 (198–260)	87 (73–103)	10 (9–12)	--	--	--
Nutritional deficiencies	0 (0–0)	0 (0–0)	6 (5–7)	2 (2–3)	1 (1–1)	1 (1–1)	1 (1–2)
Protein-energy malnutrition	0 (0–0)	0 (0–0)	5 (5–6)	1 (1–2)	1 (1–1)	1 (1–1)	1 (1–1)
Other nutritional deficiencies	--	--	1 (0–1)	1 (1–1)	1 (0–1)	1 (1–1)	1 (0–1)
Lip and oral cavity cancer	--	--	--	--	1 (1–1)	1 (1–1)	2 (1–2)
Nasopharynx cancer	--	--	--	--	1 (1–1)	1 (1–1)	3 (2–3)
Other pharynx cancer	--	--	--	--	--	--	--
Oesophageal cancer	--	--	--	--	--	--	--
Stomach cancer	--	--	--	--	--	--	2 (2–2)
Colon and rectum cancer	--	--	--	--	2 (2–3)	3 (3–3)	8 (8–9)
Liver cancer due to hepatitis B	--	--	--	--	--	2 (1–2)	3 (2–4)
Liver cancer due to hepatitis C	--	--	--	--	--	0 (0–0)	0 (0–0)
Liver cancer due to alcohol use	--	--	--	--	--	--	0 (0–1)
Liver cancer due to NASH	--	--	--	--	--	--	2 (1–3)
	0 (0–0)	0 (0–0)	2 (1–2)	8 (5–10)	5 (4–6)	5 (4–6)	--
Liver cancer due to other causes	1 (0–1)	0 (0–0)	2 (1–2)	5 (3–6)	3 (3–4)	3 (2–4)	4 (3–5)
Gallbladder and biliary tract cancer	--	--	--	--	--	--	--
Pancreatic cancer	--	--	--	--	--	--	2 (2–2)
Larynx cancer	--	--	--	--	--	--	--
Tracheal, bronchus, and lung cancer	--	--	--	--	--	2 (2–2)	6 (5–6)
Malignant skin melanoma	0 (0–0)	0 (0–0)	1 (0–7)	4 (1–28)	1 (1–1)	2 (1–3)	6 (4–10)
Non-melanoma skin cancer (squamous-cell carcinoma)	--	--	--	--	--	--	--
	0 (0–0)	0 (0–0)	3 (2–4)	13 (8–19)	15 (9–22)	19 (11–28)	39 (23–55)
	--	--	--	3 (2–4)	8 (6–10)	28 (19–34)	71 (47–85)
Breast cancer	--	--	--	--	--	--	1 (1–1)
Cervical cancer	--	--	--	--	--	--	--
Uterine cancer	--	--	--	--	--	--	--
Ovarian cancer	--	--	--	--	--	--	--
Prostate cancer	--	--	--	--	--	--	--
Testicular cancer	1 (0–7)	0 (0–2)	1 (0–5)	2 (1–2)	2 (2–2)	2 (2–3)	10 (9–12)
Kidney cancer	0 (0–0)	0 (0–0)	2 (2–2)	9 (8–10)	10 (9–12)	5 (4–5)	5 (5–6)
Bladder cancer	--	--	--	--	--	--	1 (1–1)
Brain and nervous system cancer	2 (1–2)	1 (1–1)	14 (10–20)	76 (57–103)	121 (87–153)	108 (80–143)	88 (68–117)
	0 (0–0)	0 (0–0)	1 (1–1)	4 (3–5)	4 (3–6)	4 (3–6)	4 (3–6)
	0 (0–0)	0 (0–0)	1 (1–1)	2 (1–3)	2 (1–3)	2 (1–3)	2 (1–3)
	--	--	--	2 (1–2)	2 (1–3)	2 (2–3)	2 (2–3)
	0 (0–0)	1 (1–1)	4 (3–4)	37 (30–45)	22 (12–29)	7 (4–9)	5 (3–6)
Thyroid cancer	--	--	--	--	1 (1–1)	1 (1–1)	1 (1–2)
Mesothelioma	--	--	--	--	--	--	--
Hodgkin lymphoma	--	--	--	1 (1–1)	1 (1–1)	2 (2–3)	9 (7–14)
Non-Hodgkin's lymphoma	--	--	--	8 (7–10)	15 (13–17)	21 (19–23)	36 (33–40)
	--	--	--	3 (2–3)	4 (2–6)	5 (3–7)	6 (3–8)
	--	--	--	6 (5–7)	11 (9–13)	16 (14–18)	30 (27–33)
Multiple myeloma	--	--	--	--	--	--	--
Leukaemia	2 (1–2)	2 (1–2)	14 (12–17)	72 (63–82)	79 (71–89)	93 (84–104)	125 (114–139)
Acute lymphoid leukaemia	0 (0–1)	0 (0–0)	4 (3–5)	26 (21–32)	44 (38–56)	50 (43–60)	58 (49–70)
Chronic lymphoid leukaemia	--	--	--	--	--	--	--
Acute myeloid leukaemia	0 (0–0)	0 (0–0)	6 (5–8)	34 (25–40)	21 (14–26)	28 (20–34)	43 (34–53)
Chronic myeloid leukaemia	0 (0–0)	0 (0–0)	1 (0–1)	1 (1–2)	1 (1–1)	1 (1–2)	3 (2–3)
Other leukaemia	1 (1–1)	1 (1–1)	4 (3–5)	11 (8–13)	13 (8–16)	14 (9–17)	22 (15–26)
Other malignant cancers	4 (3–5)	2 (1–2)	8 (7–11)	28 (24–38)	28 (25–37)	31 (27–37)	62 (57–69)
Myelodysplastic, myeloproliferative, and other hematopoietic neoplasms	1 (1–2)	1 (0–1)	3 (2–5)	3 (2–5)	2 (1–3)	3 (2–5)	3 (2–6)
Cardiovascular diseases	25 (21–28)	24 (21–27)	151 (134–170)	79 (70–88)	45 (41–48)	78 (73–84)	238 (222–258)
Rheumatic heart disease	--	--	--	2 (1–2)	1 (1–1)	2 (1–2)	3 (3–4)
Ischaemic heart disease	--	--	--	--	--	--	50 (45–57)
Stroke	4 (2–5)	4 (3–4)	36 (30–47)	15 (12–18)	12 (10–15)	22 (18–25)	44 (39–53)
Ischaemic stroke	3 (1–3)	2 (2–3)	4 (3–6)	2 (2–3)	1 (1–2)	1 (1–2)	4 (3–5)
Intracerebral hemorrhage	0 (0–0)	0 (0–0)	19 (15–26)	4 (3–5)	4 (4–5)	7 (5–8)	20 (17–24)
Subarachnoid hemorrhage	1 (1–2)	1 (1–1)	14 (11–17)	9 (7–10)	7 (6–8)	13 (11–16)	21 (18–25)
Hypertensive heart disease	--	--	--	--	--	--	6 (3–7)
Non-rheumatic valvular heart disease	--	--	--	--	--	--	7 (7–9)
Non-rheumatic calcific aortic valve disease	--	--	--	--	--	--	4 (3–5)
Non-rheumatic degenerative mitral valve disease	--	--	--	--	--	--	2 (2–3)
Other non-rheumatic valve diseases	--	--	--	--	--	--	0 (0–1)
Cardiomyopathy and myocarditis	12 (6–15)	13 (8–15)	66 (54–78)	41 (34–48)	18 (14–21)	37 (31–42)	96 (78–107)
Myocarditis	1 (0–2)	4 (2–5)	22 (13–28)	20 (12–25)	8 (5–10)	10 (6–13)	25 (16–31)
Alcoholic cardiomyopathy	--	--	--	--	--	--	3 (3–4)
Other cardiomyopathy	11 (6–14)	9 (6–11)	44 (36–54)	21 (17–28)	10 (8–13)	27 (23–32)	68 (56–79)
Pulmonary Arterial Hypertension	4 (2–12)	4 (3–9)	32 (17–40)	12 (6–16)	6 (3–10)	6 (3–12)	8 (5–13)
Atrial fibrillation and flutter	--	--	--	--	--	--	--

Cause	20 to 24	25 to 29	30 to 34	35 to 39	40 to 44	45 to 49	50 to 54	55 to 59
All causes	15,849 (15,691–16,019)	18,954 (18,802–19,118)	20,334 (20,165–20,514)	23,256 (23,021–23,499)	27,692 (27,368–28,027)	41,943 (41,396–42,522)	66,440 (65,564–67,369)	106,998 (105,563–108,514)
Sexually transmitted infections excluding HIV	0 (0–0)	0 (0–1)	1 (1–1)	1 (1–1)	1 (1–1)	1 (1–2)	2 (1–2)	3 (2–3)
Syphilis	0 (0–0)	0 (0–0)	1 (0–1)	1 (0–1)	1 (0–1)	1 (1–2)	2 (1–2)	3 (2–3)
Chlamydial infection	0 (0–0)	0 (0–0)	0 (0–0)	0 (0–0)	0 (0–0)	0 (0–0)	0 (0–0)	0 (0–0)
Gonococcal infection	0 (0–0)	0 (0–0)	0 (0–0)	0 (0–0)	0 (0–0)	0 (0–0)	0 (0–0)	0 (0–0)
Other sexually transmitted infections	0 (0–0)	0 (0–0)	0 (0–0)	0 (0–0)	0 (0–0)	0 (0–0)	0 (0–0)	0 (0–0)
Tuberculosis	2 (2–2)	5 (4–6)	7 (6–8)	11 (10–12)	14 (13–15)	24 (21–26)	38 (34–42)	56 (50–61)
Drug-susceptible tuberculosis	2 (2–2)	5 (4–5)	7 (6–8)	11 (10–12)	13 (12–15)	23 (20–25)	37 (33–41)	54 (48–59)
Multidrug-resistant tuberculosis without extensive drug resistance	0 (0–0)	0 (0–0)	0 (0–0)	0 (0–1)	0 (0–1)	1 (0–2)	1 (0–3)	2 (0–4)
Extensively drug-resistant tuberculosis	0 (0–0)	0 (0–0)	0 (0–0)	0 (0–0)	0 (0–0)	0 (0–0)	0 (0–1)	0 (0–1)
Lower respiratory infections	79 (72–86)	115 (102–127)	163 (144–178)	229 (212–247)	333 (309–359)	527 (499–557)	901 (842–962)	1,521 (1,429–1,619)
Upper respiratory infections	1 (1–1)	1 (1–1)	2 (2–2)	2 (2–3)	3 (2–3)	3 (3–3)	3 (3–4)	4 (3–4)
Otitis media	0 (0–0)	0 (0–0)	0 (0–0)	1 (0–1)	0 (0–1)	1 (1–1)	1 (0–1)	1 (1–1)
Diarrhoeal diseases	5 (4–5)	6 (6–7)	10 (9–12)	14 (12–16)	22 (20–25)	39 (35–44)	84 (75–95)	171 (154–191)
Typhoid fever	0 (0–0)	0 (0–0)	0 (0–0)	0 (0–0)	0 (0–0)	0 (0–0)	0 (0–0)	0 (0–0)
Paratyphoid fever	0 (0–0)	0 (0–0)	0 (0–0)	0 (0–0)	0 (0–0)	0 (0–0)	0 (0–0)	0 (0–0)
iNTS	0 (0–0)	0 (0–1)	1 (0–1)	0 (0–1)	1 (0–1)	1 (0–2)	1 (1–3)	1 (1–3)
Other intestinal infectious diseases	0 (0–1)	0 (0–1)	0 (0–2)	0 (0–1)	0 (0–1)	0 (0–1)	0 (0–1)	0 (0–1)
Malaria	0 (0–0)	0 (0–0)	0 (0–0)	0 (0–0)	0 (0–0)	0 (0–0)	0 (0–0)	0 (0–0)
Chagas disease	0 (0–0)	0 (0–0)	0 (0–0)	0 (0–0)	0 (0–0)	0 (0–0)	0 (0–0)	0 (0–0)
Visceral leishmaniasis	0 (0–0)	0 (0–0)	0 (0–0)	0 (0–0)	0 (0–0)	0 (0–0)	0 (0–0)	0 (0–0)
African trypanosomiasis	0 (0–0)	0 (0–0)	0 (0–0)	0 (0–0)	0 (0–0)	0 (0–0)	0 (0–0)	0 (0–0)
Schistosomiasis	0 (0–0)	0 (0–0)	0 (0–0)	0 (0–0)	0 (0–0)	0 (0–0)	0 (0–0)	0 (0–0)
Cysticercosis	0 (0–0)	0 (0–0)	0 (0–0)	0 (0–0)	0 (0–0)	0 (0–0)	0 (0–0)	0 (0–0)
Cystic echinococcosis	0 (0–0)	0 (0–0)	0 (0–0)	0 (0–0)	0 (0–0)	0 (0–0)	0 (0–1)	0 (0–1)
Dengue	0 (0–0)	0 (0–0)	0 (0–0)	0 (0–0)	0 (0–0)	0 (0–0)	0 (0–0)	0 (0–0)
Yellow fever	0 (0–0)	0 (0–0)	0 (0–0)	0 (0–0)	0 (0–0)	0 (0–0)	0 (0–0)	0 (0–0)
Rabies	0 (0–0)	0 (0–0)	0 (0–0)	0 (0–0)	0 (0–0)	0 (0–0)	0 (0–0)	0 (0–0)
Ascariasis	0 (0–0)	0 (0–0)	0 (0–0)	0 (0–0)	0 (0–0)	0 (0–0)	0 (0–0)	0 (0–0)
Zika virus disease	0 (0–0)	0 (0–0)	0 (0–0)	0 (0–0)	0 (0–0)	0 (0–0)	0 (0–0)	0 (0–0)
Other neglected tropical diseases	1 (0–1)	1 (0–1)	1 (0–2)	2 (0–2)	2 (0–3)	3 (1–4)	3 (1–5)	6 (1–8)
Meningitis	18 (15–20)	21 (16–24)	24 (19–27)	29 (24–33)	33 (30–36)	44 (40–48)	42 (38–45)	48 (45–52)
Encephalitis	11 (9–12)	11 (9–13)	11 (9–13)	12 (10–13)	11 (10–12)	15 (13–17)	19 (17–20)	26 (23–28)
Diphtheria	0 (0–0)	0 (0–0)	0 (0–0)	0 (0–0)	0 (0–0)	0 (0–0)	0 (0–0)	0 (0–0)
Whooping cough	0 (0–0)	0 (0–0)	0 (0–0)	0 (0–0)	0 (0–0)	0 (0–0)	0 (0–0)	0 (0–0)
Tetanus	0 (0–0)	0 (0–0)	0 (0–0)	0 (0–0)	0 (0–0)	0 (0–1)	0 (0–1)	0 (0–1)
Measles	0 (0–0)	0 (0–0)	0 (0–0)	0 (0–0)	0 (0–0)	0 (0–0)	0 (0–0)	0 (0–0)
Varicella and herpes zoster	1 (1–3)	2 (1–4)	2 (1–5)	2 (1–4)	2 (1–4)	2 (1–4)	2 (1–6)	3 (2–9)
Acute hepatitis	1 (1–1)	1 (1–2)	2 (1–2)	3 (2–3)	4 (4–5)	8 (7–9)	9 (8–10)	11 (10–12)
Acute hepatitis A	0 (0–1)	0 (0–1)	1 (0–1)	1 (1–2)	1 (1–2)	2 (1–4)	2 (1–4)	3 (2–6)
Acute hepatitis B	0 (0–1)	1 (1–1)	1 (0–1)	1 (1–2)	2 (1–3)	4 (2–5)	4 (2–5)	5 (2–6)
Acute hepatitis C	0 (0–0)	0 (0–0)	0 (0–0)	0 (0–1)	1 (0–2)	2 (1–4)	2 (1–5)	3 (1–6)
Acute hepatitis E	0 (0–0)	0 (0–0)	0 (0–0)	0 (0–0)	0 (0–1)	1 (0–1)	0 (0–0)	0 (0–1)
Other unspecified infectious diseases	18 (12–34)	23 (14–50)	31 (17–71)	49 (29–108)	54 (36–112)	83 (58–168)	123 (90–232)	204 (139–379)
Maternal disorders	--	--	--	--	--	--	--	--
Maternal haemorrhage	--	--	--	--	--	--	--	--
Maternal sepsis and other pregnancy related infections	--	--	--	--	--	--	--	--
Maternal hypertensive disorders	--	--	--	--	--	--	--	--
Maternal obstructed labour and uterine rupture	--	--	--	--	--	--	--	--
Maternal abortive outcome	--	--	--	--	--	--	--	--
Ectopic pregnancy	--	--	--	--	--	--	--	--
Indirect maternal deaths	--	--	--	--	--	--	--	--
Late maternal deaths	--	--	--	--	--	--	--	--
Maternal deaths aggravated by HIV/AIDS	--	--	--	--	--	--	--	--
Other maternal disorders	--	--	--	--	--	--	--	--
Neonatal disorders	--	--	--	--	--	--	--	--
Neonatal preterm birth	--	--	--	--	--	--	--	--
Neonatal encephalopathy due to birth asphyxia and trauma	--	--	--	--	--	--	--	--
Neonatal sepsis and other neonatal infections	--	--	--	--	--	--	--	--
Hemolytic disease and other neonatal jaundice	--	--	--	--	--	--	--	--
Other neonatal disorders	--	--	--	--	--	--	--	--
Nutritional deficiencies	3 (2–3)	4 (4–5)	6 (5–6)	8 (7–8)	11 (10–11)	22 (20–23)	37 (34–40)	75 (70–81)
Protein-energy malnutrition	2 (1–2)	3 (3–4)	5 (4–6)	6 (6–7)	10 (8–10)	20 (17–22)	34 (29–38)	71 (63–77)
Other nutritional deficiencies	1 (1–1)	1 (1–2)	1 (1–2)	1 (1–2)	1 (1–2)	2 (1–3)	2 (1–6)	5 (3–12)
Lip and oral cavity cancer	4 (4–5)	9 (8–11)	18 (16–20)	35 (31–39)	70 (63–77)	166 (151–181)	345 (321–372)	644 (602–691)
Nasopharynx cancer	5 (4–6)	6 (5–7)	7 (6–8)	15 (13–17)	27 (25–30)	50 (46–55)	90 (83–98)	149 (137–161)
Other pharynx cancer	1 (1–1)	1 (1–1)	3 (3–4)	10 (9–12)	29 (26–32)	95 (86–104)	219 (202–236)	407 (376–441)
Oesophageal cancer	4 (4–5)	11 (9–12)	27 (23–30)	70 (60–78)	184 (170–199)	442 (415–470)	994 (936–1,050)	1,990 (1,880–2,107)
Stomach cancer	8 (7–8)	23 (20–25)	53 (48–60)	105 (95–115)	178 (165–193)	323 (303–347)	606 (569–647)	1,039 (977–1,102)
Colon and rectum cancer	28 (26–30)	74 (66–81)	172 (156–191)	341 (316–366)	628 (584–673)	1,270 (1,200–1,344)	2,396 (2,280–2,536)	4,142 (3,954–4,346)
Liver cancer due to hepatitis B	5 (4–7)	12 (8–17)	20 (14–28)	33 (23–44)	54 (37–74)	96 (67–131)	192 (134–267)	356 (254–491)
Liver cancer due to hepatitis C	0 (0–1)	1 (1–2)	3 (2–5)	8 (5–12)	23 (15–33)	82 (55–115)	282 (202–369)	741 (557–958)
Liver cancer due to alcohol use	2 (1–3)	6 (4–9)	15 (10–20)	32 (23–44)	71 (50–92)	175 (125–227)	465 (344–601)	999 (764–1,272)
Liver cancer due to NASH	2 (1–3)	3 (2–4)	3 (2–5)	5 (3–7)	9 (6–13)	24 (16–34)	68 (47–95)	159 (109–219)
	--	--	--	--	--	--	--	--
Liver cancer due to other causes	4 (3–5)	5 (3–7)	7 (4–10)	9 (6–13)	16 (11–22)	35 (24–48)	84 (59–117)	166 (117–225)
Gallbladder and biliary tract cancer	1 (1–1)	1 (1–2)	4 (3–5)	9 (8–12)	18 (16–23)	40 (36–50)	86 (78–102)	166 (149–202)
Pancreatic cancer	5 (4–5)	13 (11–14)	41 (36–44)	112 (102–121)	255 (240–271)	633 (596–672)	1,448 (1,367–1,531)	2,844 (2,689–3,010)
Larynx cancer	1 (1–1)	1 (1–1)	3 (2–3)	9 (8–11)	28 (25–31)	87 (80–94)	225 (210–241)	464 (437–493)
Tracheal, bronchus, and lung cancer	13 (12–15)	30 (26–34)	83 (73–92)	210 (190–229)	540 (508–577)	1,642 (1,561–1,730)	4,453 (4,238–4,684)	9,878 (9,446–10,340)
Malignant skin melanoma	20 (15–38)	49 (37–91)	88 (67–167)	137 (106–262)	209 (168–371)	300 (229–529)	477 (359–801)	769 (551–1,239)
Non-melanoma skin cancer (squamous-cell carcinoma)	2 (2–2)	4 (3–5)	7 (6–9)	13 (11–16)	23 (21–28)	51 (46–55)	116 (107–127)	217 (199–236)
	47 (32–68)	55 (36–78)	60 (39–90)	73 (47–106)	92 (64–131)	121 (80–178)	178 (114–245)	254 (161–351)
	67 (52–76)	44 (36–52)	30 (24–35)	29 (24–35)	31 (26–36)	38 (32–46)	55 (46–65)	78 (65–93)
Breast cancer	1 (1–1)	1 (1–1)	1 (1–2)	4 (3–4)	7 (6–8)	17 (15–20)	31 (27–36)	55 (49–61)
Cervical cancer	--	--	--	--	--	--	--	--
Uterine cancer	--	--	--	--	--	--	--	--
Ovarian cancer	--	--	--	--	--	--	--	--
Prostate cancer	3 (3–4)	4 (4–6)	6 (5–8)	8 (7–11)	22 (18–30)	113 (92–149)	442 (362–579)	1,290 (1,087–1,761)
Testicular cancer	38 (33–44)	55 (47–65)	50 (43–59)	47 (41–56)	44 (39–49)	39 (35–44)	36 (32–41)	36 (32–41)
Kidney cancer	12 (10–13)	17 (14–20)	31 (25–36)	68 (57–76)	144 (131–157)	351 (315–388)	699 (633–765)	1,332 (1,210–1,446)
Bladder cancer	1 (1–1)	2 (2–3)	8 (7–9)	18 (16–20)	46 (42–50)	128 (118–140)	334 (311–359)	747 (695–801)
Brain and nervous system cancer	92 (78–125)	128 (106–176)	187 (156–257)	271 (230–358)	391 (334–511)	581 (484–739)	915 (737–1,175)	1,457 (1,139–1,752)
	4 (3–5)	4 (3–5)	4 (3–5)	4 (3–5)	5 (3–6)	7 (5–8)	14 (10–18)	22 (16–28)
	--	--	--	--	--	--	--	--
	4 (3–5)	4 (3–5)	4 (3–5)	4 (3–5)	5 (3–6)	7 (5–8)	14 (10–18)	22 (16–28)
	6 (5–7)	6 (5–8)	6 (5–8)	6 (5–9)	6 (5–8)	8 (6–10)	11 (9–13)	15 (13–19)
Thyroid cancer	2 (2–3)	4 (3–5)	5 (4–6)	8 (7–10)	16 (15–18)	33 (30–37)	62 (56–68)	104 (94–116)
Mesothelioma	0 (0–1)	1 (1–2)	3 (2–3)	6 (6–7)	11 (10–12)	22 (20–24)	50 (46–53)	114 (107–122)
Hodgkin lymphoma	23 (20–37)	33 (28–56)	35 (29–59)	34 (29–57)	37 (33–55)	41 (37–59)	54 (49–71)	75 (68–96)
Non-Hodgkin's lymphoma	58 (52–63)	74 (62–82)	100 (84–111)	141 (124–153)	214 (202–228)	350 (329–373)	649 (612–687)	1,205 (1,137–1,276)
	6 (3–8)	5 (3–7)	6 (3–9)	7 (4–10)	8 (4–11)	11 (5–16)	15 (7–20)	26 (12–34)
	52 (46–58)	69 (57–77)	94 (78–105)	133 (116–146)	206 (194–220)	338 (318–363)	634 (596–673)	1,179 (1,110–1,251)
Multiple myeloma	2 (2–2)	3 (3–4)	9 (8–13)	26 (22–36)	67 (59–95)	157 (141–215)	350 (315–477)	678 (618–912)
Leukaemia	146 (127–163)	150 (124–169)	151 (125–171)	178 (153–200)	224 (206–244)	326 (302–352)	552 (511–595)	1,004 (929–1,089)
Acute lymphoid leukaemia	59 (48–71)	51 (36–61)	38 (27–47)	39 (27–48)	42 (28–50)	44 (29–51)	57 (40–68)	80 (55–95)
Chronic lymphoid leukaemia	1 (1–1)	1 (1–1)	1 (1–2)	3 (3–4)	9 (7–11)	27 (23–37)	68 (58–93)	165 (141–228)
Acute myeloid leukaemia	53 (43–71)	59 (45–87)	67 (51–94)	83 (66–111)	110 (95–142)	163 (143–200)	277 (237–338)	500 (426–584)
Chronic myeloid leukaemia	5 (4–7)	10 (8–12)	15 (12–18)	18 (14–21)	18 (15–22)	25 (20–29)	35 (29–42)	54 (45–65)
Other leukaemia	27 (21–33)	28 (22–35)	29 (23–36)	36 (29–43)	45 (36–52)	67 (54–78)	115 (89–135)	204 (170–246)
Other malignant cancers	64 (58–72)	57 (49–67)	64 (55–75)	84 (75–96)	114 (103–126)	180 (165–198)	289 (265–318)	476 (438–530)
Myelodysplastic, myeloproliferative, and other hematopoietic neoplasms	3 (2–5)	6 (3–9)	7 (4–10)	10 (6–18)	17 (10–29)	28 (18–50)	66 (39–114)	156 (96–283)
Cardiovascular diseases	480 (444–514)	874 (784–936)	1,620 (1,446–1,733)	3,039 (2,813–3,198)	5,484 (5,140–5,776)	10,162 (9,703–10,546)	18,071 (17,412–18,674)	30,556 (29,523–31,490)
Rheumatic heart disease	5 (5–6)	11 (9–13)	15 (12–18)	19 (17–21)	25 (22–28)	42 (38–47)	65 (59–72)	109 (100–120)
Ischaemic heart disease	134 (123–155)	293 (267–331)	675 (611–751)	1,474 (1,377–1,626)	3,010 (2,808–3,248)	6,134 (5,818–6,548)	11,661 (11,109–12,323)	20,227 (19,331–21,147)
Stroke	82 (73–96)	139 (121–162)	237 (209–275)	428 (388–485)	739 (677–823)	1,274 (1,163–1,411)	2,225 (2,074–2,435)	3,779 (3,522–4,089)
Ischaemic stroke	8 (6–9)	12 (10–15)	22 (17–25)	34 (29–39)	58 (50–65)	133 (119–151)	251 (228–282)	479 (434–540)
Intracerebral hemorrhage	36 (32–43)	65 (56–77)	121 (105–140)	228 (204–258)	414 (373–469)	741 (666–846)	1,325 (1,220–1,485)	2,237 (2,056–2,495)
Subarachnoid hemorrhage	38 (33–48)	61 (52–75)	95 (83–116)	166 (148–200)	267 (240–311)	400 (360–454)	649 (582–732)	1,064 (922–1,187)
Hypertensive heart disease	34 (15–40)	88 (39–107)	199 (92–235)	378 (173–436)	669 (312–762)	1,121 (535–1,257)	1,740 (837–1,933)	2,537 (1,276–2,807)
Non-rheumatic valvular heart disease	11 (10–13)	19 (15–23)	28 (22–32)	41 (35–46)	60 (51–67)	95 (86–105)	165 (151–181)	284 (261–309)
Non-rheumatic calcific aortic valve disease	7 (5–8)	12 (10–15)	19 (15–23)	28 (23–34)	44 (37–51)	71 (61–82)	130 (114–147)	224 (195–251)
Non-rheumatic degenerative mitral valve disease	4 (3–5)	6 (4–9)	8 (6–12)	12 (9–18)	15 (11–22)	22 (17–33)	34 (25–51)	59 (46–92)
Other non-rheumatic valve diseases	1 (0–1)	1 (1–2)	1 (1–1)	1 (1–2)	1 (1–2)	1 (1–2)	1 (1–1)	1 (1–3)
Cardiomyopathy and myocarditis	148 (117–168)	202 (153–242)	265 (190–323)	388 (295–462)	506 (393–625)	757 (614–892)	1,069 (933–1,405)	1,614 (1,419–2,216)
Myocarditis	32 (20–40)	32 (20–43)	30 (17–42)	33 (20–43)	31 (17–40)	40 (22–52)	38 (20–49)	38 (19–50)
Alcoholic cardiomyopathy	6 (5–9)	19 (14–29)	37 (27–58)	72 (55–104)	134 (102–182)	279 (206–345)	468 (349–598)	782 (577–1,014)
Other cardiomyopathy	110 (86–131)	151 (111–190)	198 (140–247)	283 (209–342)	342 (262–418)	437 (354–546)	564 (476–795)	794 (668–1,215)
Pulmonary Arterial Hypertension	8 (5–13)	12 (8–19)	15 (10–25)	24 (15–39)	31 (18–48)	50 (26–74)	73 (36–99)	116 (52–147)
Atrial fibrillation and flutter	--	--	2 (1–3)	4 (2–5)	29 (19–41)	62 (42–89)	138 (99–197)	327 (233–461)

Cause	60 to 64	65 to 69	70 to 74	75 to 79	80 to 84	85 to 89	90 to 94	95 plus
All causes	143,511 (141,735–145,402)	169,678 (167,697–171,789)	200,425 (198,212–202,787)	207,424 (205,322–209,666)	207,789 (205,950–209,773)	193,815 (192,473–195,258)	133,185 (132,374–134,056)	--
Sexually transmitted infections excluding HIV	3 (2–3)	4 (3–5)	4 (3–6)	5 (4–6)	3 (3–5)	4 (3–5)	2 (2–3)	--
Syphilis	3 (2–3)	4 (3–5)	4 (3–6)	5 (4–6)	3 (3–5)	4 (3–5)	2 (2–3)	--
Chlamydial infection	0 (0–0)	0 (0–0)	0 (0–0)	0 (0–0)	0 (0–0)	0 (0–0)	0 (0–0)	--
Gonococcal infection	0 (0–0)	0 (0–0)	0 (0–0)	0 (0–0)	0 (0–0)	0 (0–0)	0 (0–0)	--
Other sexually transmitted infections	0 (0–0)	0 (0–0)	0 (0–0)	0 (0–0)	0 (0–0)	0 (0–0)	0 (0–0)	--
Tuberculosis	70 (64–76)	75 (69–82)	84 (78–90)	86 (79–94)	82 (74–90)	74 (64–83)	40 (33–45)	--
Drug-susceptible tuberculosis	67 (61–73)	73 (65–80)	81 (75–88)	83 (75–91)	79 (70–87)	72 (61–81)	38 (31–44)	--
Multidrug-resistant tuberculosis without extensive drug resistance	2 (1–4)	2 (1–5)	2 (1–6)	2 (1–6)	2 (1–5)	2 (1–5)	1 (0–3)	--
Extensively drug-resistant tuberculosis	1 (0–1)	1 (0–1)	1 (0–1)	1 (0–2)	1 (0–1)	1 (0–1)	0 (0–1)	--
Lower respiratory infections	2,219 (2,087–2,364)	2,904 (2,731–3,087)	4,046 (3,767–4,312)	5,212 (4,832–5,605)	6,501 (5,866–7,041)	7,582 (6,588–8,201)	6,358 (5,310–6,996)	--
Upper respiratory infections	3 (3–4)	3 (3–4)	3 (3–4)	3 (3–4)	4 (3–4)	3 (3–4)	2 (2–3)	--
Otitis media	1 (1–1)	1 (1–2)	2 (1–2)	1 (1–2)	2 (1–2)	2 (1–2)	1 (1–1)	--
Diarrhoeal diseases	278 (252–309)	414 (372–461)	625 (561–698)	875 (782–978)	965 (830–1,096)	982 (823–1,129)	702 (571–809)	--
Typhoid fever	0 (0–0)	0 (0–0)	0 (0–0)	0 (0–0)	0 (0–0)	0 (0–0)	0 (0–0)	--
Paratyphoid fever	0 (0–0)	0 (0–0)	0 (0–0)	0 (0–0)	0 (0–0)	0 (0–0)	0 (0–0)	--
iNTS	2 (1–4)	2 (2–6)	3 (2–6)	3 (2–7)	3 (2–6)	3 (2–6)	2 (1–4)	--
Other intestinal infectious diseases	0 (0–1)	1 (0–3)	1 (0–2)	0 (0–2)	1 (0–2)	1 (0–3)	1 (0–2)	--
Malaria	0 (0–0)	0 (0–0)	0 (0–0)	0 (0–0)	0 (0–0)	0 (0–0)	0 (0–0)	--
Chagas disease	0 (0–0)	0 (0–0)	0 (0–0)	0 (0–0)	0 (0–0)	0 (0–0)	0 (0–0)	--
Visceral leishmaniasis	0 (0–0)	0 (0–0)	0 (0–0)	0 (0–0)	0 (0–0)	0 (0–0)	0 (0–0)	--
African trypanosomiasis	0 (0–0)	0 (0–0)	0 (0–0)	0 (0–0)	0 (0–0)	0 (0–0)	0 (0–0)	--
Schistosomiasis	0 (0–0)	0 (0–0)	0 (0–0)	0 (0–0)	0 (0–0)	0 (0–0)	0 (0–0)	--
Cysticercosis	0 (0–0)	0 (0–0)	0 (0–0)	0 (0–0)	0 (0–0)	0 (0–0)	0 (0–0)	--
Cystic echinococcosis	0 (0–1)	0 (0–1)	0 (0–1)	0 (0–1)	0 (0–1)	0 (0–0)	0 (0–0)	--
Dengue	0 (0–0)	0 (0–0)	0 (0–0)	0 (0–0)	0 (0–0)	0 (0–0)	0 (0–0)	--
Yellow fever	0 (0–0)	0 (0–0)	0 (0–0)	0 (0–0)	0 (0–0)	0 (0–0)	0 (0–0)	--
Rabies	0 (0–0)	0 (0–0)	0 (0–0)	0 (0–0)	0 (0–0)	0 (0–0)	0 (0–0)	--
Ascariasis	0 (0–0)	0 (0–0)	0 (0–0)	0 (0–0)	0 (0–0)	0 (0–0)	0 (0–0)	--
Zika virus disease	0 (0–0)	0 (0–0)	0 (0–0)	0 (0–0)	0 (0–0)	0 (0–0)	0 (0–0)	--
Other neglected tropical diseases	8 (2–11)	13 (3–18)	18 (4–24)	18 (3–25)	16 (3–22)	10 (2–13)	6 (1–8)	--
Meningitis	49 (45–54)	48 (44–52)	47 (44–51)	45 (41–48)	41 (36–45)	34 (29–37)	19 (15–21)	--
Encephalitis	32 (28–35)	39 (35–43)	43 (36–47)	44 (36–49)	41 (32–47)	29 (18–34)	14 (10–17)	--
Diphtheria	--	--	--	--	--	--	--	--
Whooping cough	--	--	--	--	--	--	--	--
Tetanus	0 (0–1)	0 (0–1)	0 (0–1)	0 (0–1)	1 (0–1)	0 (0–1)	0 (0–1)	--
Measles	0 (0–0)	0 (0–0)	0 (0–0)	0 (0–0)	0 (0–0)	0 (0–0)	0 (0–0)	--
Varicella and herpes zoster	6 (3–14)	8 (4–20)	12 (6–25)	14 (6–30)	20 (9–41)	28 (11–53)	22 (7–38)	--
Acute hepatitis	11 (10–11)	10 (9–10)	9 (8–9)	7 (6–7)	4 (3–4)	3 (2–3)	2 (1–2)	--
Acute hepatitis A	3 (2–6)	3 (2–5)	3 (2–5)	2 (2–4)	2 (1–3)	2 (1–2)	1 (1–1)	--
Acute hepatitis B	5 (3–6)	5 (2–6)	4 (2–5)	3 (2–4)	2 (1–2)	1 (0–1)	0 (0–0)	--
Acute hepatitis C	2 (1–4)	2 (1–4)	1 (0–3)	1 (0–2)	0 (0–1)	0 (0–1)	0 (0–0)	--
Acute hepatitis E	0 (0–1)	0 (0–1)	0 (0–1)	0 (0–0)	0 (0–0)	0 (0–0)	0 (0–0)	--
Other unspecified infectious diseases	282 (184–495)	359 (230–639)	358 (231–632)	296 (172–517)	202 (123–334)	135 (84–226)	67 (40–110)	--
Maternal disorders	--	--	--	--	--	--	--	--
Maternal haemorrhage	--	--	--	--	--	--	--	--
Maternal sepsis and other pregnancy related infections	--	--	--	--	--	--	--	--
Maternal hypertensive disorders	--	--	--	--	--	--	--	--
Maternal obstructed labour and uterine rupture	--	--	--	--	--	--	--	--
Maternal abortive outcome	--	--	--	--	--	--	--	--
Ectopic pregnancy	--	--	--	--	--	--	--	--
Indirect maternal deaths	--	--	--	--	--	--	--	--
Late maternal deaths	--	--	--	--	--	--	--	--
Maternal deaths aggravated by HIV/AIDS	--	--	--	--	--	--	--	--
Other maternal disorders	--	--	--	--	--	--	--	--
Neonatal disorders	--	--	--	--	--	--	--	--
Neonatal preterm birth	--	--	--	--	--	--	--	--
Neonatal encephalopathy due to birth asphyxia and trauma	--	--	--	--	--	--	--	--
Neonatal sepsis and other neonatal infections	--	--	--	--	--	--	--	--
Hemolytic disease and other neonatal jaundice	--	--	--	--	--	--	--	--
Other neonatal disorders	--	--	--	--	--	--	--	--
Nutritional deficiencies	118 (109–128)	165 (155–176)	227 (208–246)	284 (264–304)	352 (315–384)	437 (380–477)	399 (333–440)	--
Protein-energy malnutrition	109 (96–119)	154 (137–166)	213 (192–233)	266 (238–286)	329 (282–361)	406 (334–446)	372 (293–413)	--
Other nutritional deficiencies	9 (5–22)	11 (6–28)	14 (7–28)	18 (9–35)	23 (10–42)	30 (15–70)	27 (13–61)	--
Lip and oral cavity cancer	895 (836–957)	916 (859–978)	853 (793–910)	718 (661–772)	555 (498–605)	395 (340–438)	221 (183–247)	--
Nasopharynx cancer	138 (128–150)	134 (124–145)	119 (109–129)	63 (56–69)	42 (37–48)	26 (21–29)	11 (9–12)	--
Other pharynx cancer	490 (450–533)	474 (434–518)	402 (366–440)	264 (239–292)	163 (143–183)	95 (82–106)	42 (35–49)	--
Oesophageal cancer	2,851 (2,704–2,999)	3,274 (3,079–3,443)	3,218 (3,033–3,408)	2,565 (2,389–2,734)	1,843 (1,652–1,996)	1,195 (1,037–1,307)	539 (448–598)	--
Stomach cancer	1,428 (1,356–1,512)	1,655 (1,570–1,740)	1,938 (1,831–2,044)	1,924 (1,808–2,032)	1,649 (1,485–1,770)	1,325 (1,163–1,437)	713 (587–790)	--
Colon and rectum cancer	5,750 (5,506–6,028)	6,603 (6,318–6,911)	7,227 (6,871–7,604)	6,814 (6,404–7,174)	5,878 (5,260–6,345)	4,779 (4,131–5,196)	2,726 (2,258–3,028)	--
Liver cancer due to hepatitis B	418 (284–593)	316 (215–441)	249 (164–350)	190 (134–256)	125 (82–175)	68 (48–92)	29 (19–41)	--
Liver cancer due to hepatitis C	1,082 (801–1,414)	909 (693–1,145)	784 (585–980)	647 (483–823)	497 (375–631)	329 (248–415)	162 (118–209)	--
Liver cancer due to alcohol use	1,414 (1,071–1,809)	1,351 (1,042–1,653)	1,208 (930–1,480)	900 (698–1,096)	558 (432–696)	290 (220–359)	110 (78–144)	--
Liver cancer due to NASH	240 (163–334)	236 (161–332)	242 (167–332)	241 (171–319)	187 (130–251)	110 (78–146)	49 (33–68)	--
	--	--	--	--	--	--	--	--
Liver cancer due to other causes	207 (144–288)	167 (117–222)	141 (99–191)	118 (83–154)	82 (58–111)	46 (33–62)	20 (13–28)	--
Gallbladder and biliary tract cancer	267 (237–314)	334 (300–415)	422 (375–512)	416 (368–508)	336 (287–412)	264 (222–316)	130 (104–160)	--
Pancreatic cancer	4,253 (4,024–4,486)	5,164 (4,872–5,430)	5,502 (5,196–5,797)	4,689 (4,375–4,975)	3,599 (3,243–3,879)	2,414 (2,114–2,625)	1,098 (907–1,223)	--
Larynx cancer	648 (611–692)	735 (691–782)	721 (674–773)	573 (531–613)	429 (385–470)	270 (234–301)	134 (112–150)	--
Tracheal, bronchus, and lung cancer	15,707 (15,120–16,368)	20,515 (19,814–21,207)	24,227 (23,262–25,180)	20,184 (19,118–21,110)	14,815 (13,416–15,829)	9,254 (8,089–10,090)	3,859 (3,219–4,237)	--
Malignant skin melanoma	994 (652–1,385)	1,128 (675–1,450)	1,206 (677–1,513)	1,103 (583–1,297)	961 (469–1,134)	696 (335–834)	367 (179–453)	--
Non-melanoma skin cancer (squamous-cell carcinoma)	336 (312–361)	405 (363–432)	453 (394–485)	495 (415–534)	515 (426–560)	472 (368–522)	323 (230–364)	--
	330 (205–440)	368 (220–504)	399 (231–506)	354 (202–460)	294 (158–379)	200 (108–260)	96 (51–129)	--
	96 (77–114)	108 (80–124)	115 (85–133)	103 (75–120)	87 (62–102)	63 (43–77)	36 (22–45)	--
Breast cancer	88 (78–98)	103 (92–116)	109 (96–123)	81 (70–92)	74 (64–87)	54 (44–64)	29 (23–35)	--
Cervical cancer	--	--	--	--	--	--	--	--
Uterine cancer	--	--	--	--	--	--	--	--
Ovarian cancer	--	--	--	--	--	--	--	--
Prostate cancer	3,107 (2,750–4,375)	4,974 (4,396–7,567)	7,196 (6,333–11,201)	9,519 (8,380–14,940)	10,109 (8,501–15,507)	9,480 (7,703–13,673)	6,115 (4,657–8,277)	--
Testicular cancer	31 (27–35)	31 (26–36)	25 (22–29)	25 (21–29)	24 (20–28)	18 (15–21)	10 (8–12)	--
Kidney cancer	1,859 (1,711–2,008)	2,194 (2,031–2,362)	2,286 (2,126–2,453)	1,967 (1,814–2,114)	1,565 (1,385–1,716)	1,146 (979–1,260)	572 (474–641)	--
Bladder cancer	1,339 (1,261–1,427)	1,909 (1,790–2,028)	2,576 (2,415–2,744)	3,023 (2,809–3,229)	3,085 (2,750–3,345)	2,830 (2,448–3,113)	1,741 (1,448–1,940)	--
Brain and nervous system cancer	1,759 (1,330–2,130)	1,898 (1,366–2,240)	1,824 (1,247–2,069)	1,389 (938–1,577)	908 (564–1,071)	516 (288–616)	212 (111–259)	--
	31 (23–40)	37 (27–47)	40 (29–50)	34 (24–43)	30 (22–40)	20 (13–23)	12 (8–15)	--
	--	--	--	--	--	--	--	--
	31 (23–40)	37 (27–47)	40 (29–50)	34 (24–43)	30 (22–40)	20 (13–23)	12 (8–15)	--
	15 (13–19)	16 (13–20)	16 (13–19)	13 (10–15)	9 (7–11)	7 (5–8)	3 (2–4)	--
Thyroid cancer	137 (121–149)	173 (145–189)	187 (160–203)	198 (166–217)	155 (124–173)	110 (86–124)	59 (44–67)	--
Mesothelioma	227 (213–243)	381 (359–406)	572 (530–614)	627 (572–676)	530 (471–577)	344 (295–383)	129 (106–146)	--
Hodgkin lymphoma	86 (77–106)	103 (93–116)	117 (102–129)	101 (80–111)	80 (61–90)	58 (42–66)	21 (13–26)	--
Non-Hodgkin's lymphoma	1,748 (1,659–1,844)	2,275 (2,165–2,397)	2,845 (2,695–2,997)	2,838 (2,652–3,021)	2,554 (2,278–2,772)	2,006 (1,741–2,195)	952 (794–1,057)	--
	30 (14–40)	39 (18–52)	43 (20–58)	39 (20–53)	32 (17–47)	26 (14–39)	14 (9–22)	--
	1,718 (1,629–1,813)	2,236 (2,125–2,359)	2,803 (2,654–2,957)	2,798 (2,618–2,974)	2,522 (2,247–2,742)	1,980 (1,718–2,171)	938 (784–1,043)	--
Multiple myeloma	1,107 (1,011–1,481)	1,533 (1,373–1,923)	1,884 (1,624–2,216)	1,794 (1,480–1,996)	1,552 (1,165–1,728)	1,085 (795–1,219)	478 (336–552)	--
Leukaemia	1,621 (1,504–1,740)	2,359 (2,200–2,523)	3,118 (2,909–3,329)	3,272 (3,017–3,538)	2,942 (2,599–3,234)	2,280 (1,949–2,537)	1,198 (975–1,352)	--
Acute lymphoid leukaemia	92 (65–114)	108 (81–132)	113 (85–150)	104 (81–132)	77 (61–103)	58 (43–74)	26 (19–33)	--
Chronic lymphoid leukaemia	301 (256–414)	442 (378–606)	612 (527–788)	693 (593–895)	716 (595–911)	625 (518–792)	393 (309–490)	--
Acute myeloid leukaemia	805 (649–927)	1,163 (936–1,311)	1,488 (1,123–1,666)	1,431 (1,048–1,627)	1,132 (785–1,330)	735 (508–873)	322 (214–394)	--
Chronic myeloid leukaemia	71 (58–86)	98 (83–121)	127 (106–160)	145 (123–184)	143 (119–180)	117 (95–148)	64 (50–79)	--
Other leukaemia	352 (297–425)	547 (459–663)	777 (668–948)	899 (771–1,104)	875 (705–1,075)	745 (590–916)	394 (301–475)	--
Other malignant cancers	648 (596–712)	798 (737–859)	951 (851–1,031)	901 (787–981)	726 (626–801)	534 (436–598)	289 (222–327)	--
Myelodysplastic, myeloproliferative, and other hematopoietic neoplasms	319 (185–558)	568 (351–934)	933 (542–1,501)	1,208 (694–1,956)	1,354 (775–2,211)	1,238 (676–1,984)	716 (373–1,094)	--
Cardiovascular diseases	42,351 (40,756–43,708)	50,286 (48,623–51,760)	60,275 (57,797–62,288)	65,860 (62,286–68,275)	71,980 (65,439–75,879)	73,992 (64,674–78,925)	56,372 (47,293–60,995)	--
Rheumatic heart disease	163 (148–177)	208 (191–229)	278 (255–305)	330 (301–361)	408 (360–451)	436 (373–486)	321 (264–364)	--
Ischaemic heart disease	28,461 (27,130–29,581)	33,633 (32,309–34,818)	39,306 (37,529–40,754)	41,443 (39,036–43,235)	44,183 (39,863–46,822)	45,001 (39,127–48,295)	34,137 (28,524–37,220)	--
Stroke	5,483 (5,114–5,897)	6,968 (6,521–7,484)	9,659 (8,986–10,375)	11,838 (10,923–12,695)	13,616 (12,139–14,761)	14,069 (12,020–15,435)	10,322 (8,534–11,497)	--
Ischaemic stroke	1,437 (1,308–1,586)	1,985 (1,812–2,198)	4,010 (3,683–4,392)	5,325 (4,884–5,808)	8,833 (7,852–9,751)	9,760 (8,327–10,867)	7,408 (6,103–8,426)	--
Intracerebral hemorrhage	3,100 (2,870–3,410)	3,883 (3,581–4,238)	4,606 (4,259–5,000)	5,419 (4,948–5,887)	3,687 (3,221–4,102)	3,227 (2,714–3,639)	2,121 (1,710–2,457)	--
Subarachnoid hemorrhage	946 (767–1,050)	1,100 (906–1,209)	1,043 (807–1,151)	1,094 (772–1,231)	1,096 (723–1,257)	1,082 (617–1,285)	792 (413–957)	--
Hypertensive heart disease	2,893 (1,501–3,205)	2,723 (1,515–3,006)	2,498 (1,582–2,748)	2,400 (1,487–2,653)	2,384 (1,553–2,653)	2,510 (1,553–2,831)	2,089 (1,149–2,389)	--
Non-rheumatic valvular heart disease	439 (406–473)	633 (585–682)	966 (899–1,037)	1,379 (1,270–1,479)	2,071 (1,814–2,245)	2,773 (2,343–3,038)	2,507 (1,992–2,803)	--
Non-rheumatic calcific aortic valve disease	358 (313–396)	526 (454–576)	823 (723–898)	1,198 (1,063–1,295)	1,865 (1,625–2,031)	2,538 (2,138–2,797)	2,325 (1,843–2,605)	--
Non-rheumatic degenerative mitral valve disease	80 (64–129)	106 (82–174)	141 (107–232)	180 (138–287)	205 (152–331)	233 (163–347)	181 (121–263)	--
Other non-rheumatic valve diseases	1 (1–2)	1 (1–2)	2 (1–3)	2 (1–3)	2 (1–3)	1 (1–2)	2 (1–3)	--
Cardiomyopathy and myocarditis	1,954 (1,728–2,789)	2,202 (1,942–3,059)	2,443 (2,082–3,203)	2,474 (1,913–3,074)	2,419 (1,769–2,743)	2,242 (1,482–2,603)	1,597 (992–1,908)	--
Myocarditis	36 (20–47)	34 (18–43)	28 (13–37)	27 (13–35)	24 (11–32)	22 (11–30)	20 (10–27)	--
Alcoholic cardiomyopathy	955 (715–1,245)	1,018 (702–1,324)	875 (628–1,131)	727 (505–958)	131 (92–179)	54 (39–72)	17 (12–24)	--
Other cardiomyopathy	962 (790–1,570)	1,150 (974–1,807)	1,539 (1,320–2,114)	1,720 (1,313–2,150)	2,264 (1,655–2,567)	2,166 (1,428–2,517)	1,560 (966–1,863)	--
Pulmonary Arterial Hypertension	165 (68–208)	215 (82–271)	263 (94–331)	277 (96–357)	277 (88–368)	255 (62–350)	165 (41–228)	--
Atrial fibrillation and flutter	620 (406–870)	959 (610–1,281)	1,544 (1,003–2,097)	2,117 (1,353–2,902)	2,983 (1,947–3,934)	3,233 (2,153–4,482)	2,871 (1,900–3,880)	--

Aortic aneurysm	--	--	--	--	--	--	6 (5–7)	[illegible]
Peripheral vascular disease	--	--	--	--	--	--	--	[illegible]
Endocarditis	0 (0–1)	1 (0–1)	2 (1–3)	2 (1–2)	1 (1–1)	2 (1–2)	4 (2–5)	[illegible]
Other cardiovascular and circulatory diseases	3 (2–4)	4 (3–4)	14 (12–18)	7 (6–9)	6 (5–7)	10 (8–11)	13 (12–15)	[illegible]
Chronic respiratory diseases	0 (0–0)	0 (0–0)	0 (0–0)	56 (50–62)	44 (40–47)	59 (55–65)	64 (60–69)	[illegible]
Chronic obstructive pulmonary disease	--	--	0 (0–0)	5 (4–6)	2 (2–3)	2 (2–2)	8 (7–9)	[illegible]
Pneumoconiosis	--	--	--	--	--	--	0 (0–0)	[illegible]
Silicosis	--	--	--	--	--	--	0 (0–0)	[illegible]
Asbestosis	--	--	--	--	--	--	0 (0–0)	[illegible]
Coal workers pneumoconiosis	--	--	--	--	--	--	0 (0–0)	[illegible]
Other pneumoconiosis	--	--	--	--	--	--	0 (0–0)	[illegible]
Asthma	--	--	--	16 (13–19)	28 (25–31)	43 (39–48)	38 (35–42)	[illegible]
Interstitial lung disease and pulmonary sarcoidosis	--	--	--	6 (4–10)	3 (2–5)	4 (2–6)	6 (4–8)	[illegible]
Other chronic respiratory diseases	0 (0–0)	0 (0–0)	0 (0–0)	28 (23–33)	11 (9–13)	10 (8–12)	11 (9–14)	[illegible]
Digestive diseases	10 (9–12)	14 (12–16)	149 (130–169)	55 (49–61)	25 (23–26)	30 (28–32)	52 (49–56)	[illegible]
Cirrhosis and other chronic liver diseases	--	--	--	9 (7–11)	4 (3–4)	4 (4–5)	15 (13–17)	[illegible]
Cirrhosis and other chronic liver diseases due to hepatitis B	--	--	--	0 (0–0)	0 (0–0)	0 (0–0)	0 (0–0)	[illegible]
Cirrhosis and other chronic liver diseases due to hepatitis C	--	--	--	0 (0–0)	0 (0–0)	0 (0–1)	2 (1–3)	[illegible]
Cirrhosis and other chronic liver diseases due to alcohol use	--	--	--	--	--	--	0 (0–0)	[illegible]
Cirrhosis and other chronic liver diseases due to NAFLD	--	--	--	--	--	--	0 (0–1)	[illegible]
Cirrhosis and other chronic liver diseases due to other causes	--	[illegible]	[illegible]	[illegible]	[illegible]	[illegible]	[illegible]	[illegible]
Upper digestive system diseases	--	[illegible]	[illegible]	[illegible]	[illegible]	[illegible]	[illegible]	[illegible]
Peptic ulcer disease	--	[illegible]	[illegible]	[illegible]	[illegible]	[illegible]	[illegible]	[illegible]
Gastritis and duodenitis	--	[illegible]	[illegible]	[illegible]	[illegible]	[illegible]	[illegible]	[illegible]
Appendicitis	--	[illegible]	[illegible]	[illegible]	[illegible]	[illegible]	[illegible]	[illegible]
Paralytic ileus and intestinal obstruction	4 (3–5)	[illegible]	[illegible]	[illegible]	[illegible]	[illegible]	[illegible]	[illegible]
Inguinal, femoral, and abdominal hernia	7 (5–8)	[illegible]	[illegible]	[illegible]	[illegible]	[illegible]	[illegible]	[illegible]
Inflammatory bowel disease	--	[illegible]	[illegible]	[illegible]	[illegible]	[illegible]	[illegible]	[illegible]
Vascular intestinal disorders	--	[illegible]	[illegible]	[illegible]	[illegible]	[illegible]	[illegible]	[illegible]
Gallbladder and biliary diseases	--	[illegible]	[illegible]	[illegible]	[illegible]	[illegible]	[illegible]	[illegible]
Pancreatitis	--	[illegible]	[illegible]	[illegible]	[illegible]	[illegible]	[illegible]	[illegible]
Other digestive diseases	--	[illegible]	[illegible]	[illegible]	[illegible]	[illegible]	[illegible]	[illegible]
Alzheimer's disease and other dementias	--	[illegible]	[illegible]	[illegible]	[illegible]	[illegible]	[illegible]	[illegible]
Parkinson's disease	--	[illegible]	[illegible]	[illegible]	[illegible]	[illegible]	[illegible]	[illegible]
Idiopathic epilepsy	--	[illegible]	[illegible]	[illegible]	[illegible]	[illegible]	[illegible]	[illegible]
Multiple sclerosis	--	[illegible]	[illegible]	[illegible]	[illegible]	[illegible]	[illegible]	[illegible]
Motor neuron disease	1 (1–1)	[illegible]	[illegible]	[illegible]	[illegible]	[illegible]	[illegible]	[illegible]
Other neurological disorders	--	[illegible]	[illegible]	[illegible]	[illegible]	[illegible]	[illegible]	[illegible]
Eating disorders	--	[illegible]	[illegible]	[illegible]	[illegible]	[illegible]	[illegible]	[illegible]
Anorexia nervosa	--	[illegible]	[illegible]	[illegible]	[illegible]	[illegible]	[illegible]	[illegible]
Bulimia nervosa	--	[illegible]	[illegible]	[illegible]	[illegible]	[illegible]	[illegible]	[illegible]
Alcohol use disorders	--	[illegible]	[illegible]	[illegible]	[illegible]	[illegible]	[illegible]	[illegible]
Drug use disorders	0 (0–0)	[illegible]	[illegible]	[illegible]	[illegible]	[illegible]	[illegible]	[illegible]
Opioid use disorders	0 (0–0)	[illegible]	[illegible]	[illegible]	[illegible]	[illegible]	[illegible]	[illegible]
Cocaine use disorders	--	[illegible]	[illegible]	[illegible]	[illegible]	[illegible]	[illegible]	[illegible]
Amphetamine use disorders	--	[illegible]	[illegible]	[illegible]	[illegible]	[illegible]	[illegible]	[illegible]
Other drug use disorders	--	[illegible]	[illegible]	[illegible]	[illegible]	[illegible]	[illegible]	[illegible]
Diabetes mellitus	0 (0–1)	[illegible]	[illegible]	[illegible]	[illegible]	[illegible]	[illegible]	[illegible]
Diabetes mellitus type 1	0 (0–1)	[illegible]	[illegible]	[illegible]	[illegible]	[illegible]	[illegible]	[illegible]
Diabetes mellitus type 2	--	[illegible]	[illegible]	[illegible]	[illegible]	[illegible]	[illegible]	[illegible]
Chronic kidney disease	--	[illegible]	[illegible]	[illegible]	[illegible]	[illegible]	[illegible]	[illegible]
Chronic kidney disease due to diabetes mellitus type 1	--	[illegible]	[illegible]	[illegible]	[illegible]	[illegible]	[illegible]	[illegible]
Chronic kidney disease due to diabetes mellitus type 2	--	[illegible]	[illegible]	[illegible]	[illegible]	[illegible]	[illegible]	[illegible]
Chronic kidney disease due to hypertension	--	[illegible]	[illegible]	[illegible]	[illegible]	[illegible]	[illegible]	[illegible]
Chronic kidney disease due to glomerulonephritis	--	[illegible]	[illegible]	[illegible]	[illegible]	[illegible]	[illegible]	[illegible]
Chronic kidney disease due to other and unspecified causes	--	[illegible]	[illegible]	[illegible]	[illegible]	[illegible]	[illegible]	[illegible]
Acute glomerulonephritis	--	[illegible]	[illegible]	[illegible]	[illegible]	[illegible]	[illegible]	[illegible]
Skin and subcutaneous diseases	--	[illegible]	[illegible]	[illegible]	[illegible]	[illegible]	[illegible]	[illegible]
Bacterial skin diseases	--	[illegible]	[illegible]	[illegible]	[illegible]	[illegible]	[illegible]	[illegible]
Cellulitis	--	[illegible]	[illegible]	[illegible]	[illegible]	[illegible]	[illegible]	[illegible]
Pyoderma	--	[illegible]	[illegible]	[illegible]	[illegible]	[illegible]	[illegible]	[illegible]
Decubitus ulcer	--	[illegible]	[illegible]	[illegible]	[illegible]	[illegible]	[illegible]	[illegible]
Other skin and subcutaneous diseases	--	[illegible]	[illegible]	[illegible]	[illegible]	[illegible]	[illegible]	[illegible]
Musculoskeletal disorders	--	[illegible]	[illegible]	[illegible]	[illegible]	[illegible]	[illegible]	[illegible]
Rheumatoid arthritis	--	[illegible]	[illegible]	[illegible]	[illegible]	[illegible]	[illegible]	[illegible]
Other musculoskeletal disorders	--	[illegible]	[illegible]	[illegible]	[illegible]	[illegible]	[illegible]	[illegible]
Congenital anomalies	1,421 (1,207–1,880)	[illegible]	[illegible]	[illegible]	[illegible]	[illegible]	[illegible]	[illegible]
Neural tube defects	144 (102–187)	[illegible]	[illegible]	[illegible]	[illegible]	[illegible]	[illegible]	[illegible]
Congenital heart anomalies	225 (177–342)	[illegible]	[illegible]	[illegible]	[illegible]	[illegible]	[illegible]	[illegible]
Orofacial clefts	1 (0–1)	[illegible]	[illegible]	[illegible]	[illegible]	[illegible]	[illegible]	[illegible]
Down's syndrome	22 (16–32)	[illegible]	[illegible]	[illegible]	[illegible]	[illegible]	[illegible]	[illegible]
Other chromosomal abnormalities	289 (206–433)	[illegible]	[illegible]	[illegible]	[illegible]	[illegible]	[illegible]	[illegible]
Congenital musculoskeletal and limb anomalies	65 (28–90)	[illegible]	[illegible]	[illegible]	[illegible]	[illegible]	[illegible]	[illegible]
Urogenital congenital anomalies	162 (99–242)	[illegible]	[illegible]	[illegible]	[illegible]	[illegible]	[illegible]	[illegible]
Digestive congenital anomalies	113 (82–161)	[illegible]	[illegible]	[illegible]	[illegible]	[illegible]	[illegible]	[illegible]
Other congenital anomalies	400 (328–588)	[illegible]	[illegible]	[illegible]	[illegible]	[illegible]	[illegible]	[illegible]
Urinary diseases and male infertility	4 (3–7)	[illegible]	[illegible]	[illegible]	[illegible]	[illegible]	[illegible]	[illegible]
Urinary tract infections and interstitial nephritis	1 (1–2)	[illegible]	[illegible]	[illegible]	[illegible]	[illegible]	[illegible]	[illegible]
Urolithiasis	--	[illegible]	[illegible]	[illegible]	[illegible]	[illegible]	[illegible]	[illegible]
Other urinary diseases	3 (2–5)	[illegible]	[illegible]	[illegible]	[illegible]	[illegible]	[illegible]	[illegible]
Gynecological diseases	--	[illegible]	[illegible]	[illegible]	[illegible]	[illegible]	[illegible]	[illegible]
Uterine fibroids	--	[illegible]	[illegible]	[illegible]	[illegible]	[illegible]	[illegible]	[illegible]
Endometriosis	--	[illegible]	[illegible]	[illegible]	[illegible]	[illegible]	[illegible]	[illegible]
Genital prolapse	--	[illegible]	[illegible]	[illegible]	[illegible]	[illegible]	[illegible]	[illegible]
Other gynecological diseases	--	[illegible]	[illegible]	[illegible]	[illegible]	[illegible]	[illegible]	[illegible]
Hemoglobinopathies and hemolytic anaemias	2 (2–2)	[illegible]	[illegible]	[illegible]	[illegible]	[illegible]	[illegible]	[illegible]
Thalassemias	1 (0–1)	[illegible]	[illegible]	[illegible]	[illegible]	[illegible]	[illegible]	[illegible]
Sickle cell disorders	1 (1–2)	[illegible]	[illegible]	[illegible]	[illegible]	[illegible]	[illegible]	[illegible]
G6PD deficiency	0 (0–0)	[illegible]	[illegible]	[illegible]	[illegible]	[illegible]	[illegible]	[illegible]
Other hemoglobinopathies and hemolytic anaemias	0 (0–0)	[illegible]	[illegible]	[illegible]	[illegible]	[illegible]	[illegible]	[illegible]
Endocrine, metabolic, blood, and immune disorders	40 (31–69)	[illegible]	[illegible]	[illegible]	[illegible]	[illegible]	[illegible]	[illegible]
Sudden infant death syndrome	--	[illegible]	[illegible]	[illegible]	[illegible]	[illegible]	[illegible]	[illegible]
Transport injuries	4 (3–4)	[illegible]	[illegible]	[illegible]	[illegible]	[illegible]	[illegible]	[illegible]
Road injuries	3 (3–4)	[illegible]	[illegible]	[illegible]	[illegible]	[illegible]	[illegible]	[illegible]
Pedestrian road injuries	1 (1–1)	[illegible]	[illegible]	[illegible]	[illegible]	[illegible]	[illegible]	[illegible]
Cyclist road injuries	--	[illegible]	[illegible]	[illegible]	[illegible]	[illegible]	[illegible]	[illegible]
Motorcyclist road injuries	0 (0–0)	[illegible]	[illegible]	[illegible]	[illegible]	[illegible]	[illegible]	[illegible]
Motor vehicle road injuries	2 (2–2)	[illegible]	[illegible]	[illegible]	[illegible]	[illegible]	[illegible]	[illegible]
Other road injuries	0 (0–0)	[illegible]	[illegible]	[illegible]	[illegible]	[illegible]	[illegible]	[illegible]
Other transport injuries	1 (1–1)	[illegible]	[illegible]	[illegible]	[illegible]	[illegible]	[illegible]	[illegible]
Falls	1 (1–1)	[illegible]	[illegible]	[illegible]	[illegible]	[illegible]	[illegible]	[illegible]
Drowning	1 (1–2)	[illegible]	[illegible]	[illegible]	[illegible]	[illegible]	[illegible]	[illegible]
Fire, heat, and hot substances	0 (0–0)	[illegible]	[illegible]	[illegible]	[illegible]	[illegible]	[illegible]	[illegible]
Poisonings	1 (1–1)	[illegible]	[illegible]	[illegible]	[illegible]	[illegible]	[illegible]	[illegible]
Poisoning by carbon monoxide	0 (0–0)	[illegible]	[illegible]	[illegible]	[illegible]	[illegible]	[illegible]	[illegible]
Poisoning by other means	1 (1–1)	[illegible]	[illegible]	[illegible]	[illegible]	[illegible]	[illegible]	[illegible]
Exposure to mechanical forces	1 (1–1)	[illegible]	[illegible]	[illegible]	[illegible]	[illegible]	[illegible]	[illegible]
Unintentional firearm injuries	0 (0–0)	[illegible]	[illegible]	[illegible]	[illegible]	[illegible]	[illegible]	[illegible]
Other exposure to mechanical forces	0 (0–0)	[illegible]	[illegible]	[illegible]	[illegible]	[illegible]	[illegible]	[illegible]
Adverse effects of medical treatment	4 (3–5)	[illegible]	[illegible]	[illegible]	[illegible]	[illegible]	[illegible]	[illegible]
Animal contact	0 (0–0)	[illegible]	[illegible]	[illegible]	[illegible]	[illegible]	[illegible]	[illegible]
Venomous animal contact	0 (0–0)	[illegible]	[illegible]	[illegible]	[illegible]	[illegible]	[illegible]	[illegible]
Non-venomous animal contact	0 (0–0)	[illegible]	[illegible]	[illegible]	[illegible]	[illegible]	[illegible]	[illegible]
Foreign body	9 (8–10)	[illegible]	[illegible]	[illegible]	[illegible]	[illegible]	[illegible]	[illegible]
Pulmonary aspiration and foreign body in airway	9 (8–10)	[illegible]	[illegible]	[illegible]	[illegible]	[illegible]	[illegible]	[illegible]
Foreign body in other body part	0 (0–0)	[illegible]	[illegible]	[illegible]	[illegible]	[illegible]	[illegible]	[illegible]
Environmental heat and cold exposure	0 (0–0)	[illegible]	[illegible]	[illegible]	[illegible]	[illegible]	[illegible]	[illegible]
Other unintentional injuries	0 (0–0)	[illegible]	[illegible]	[illegible]	[illegible]	[illegible]	[illegible]	[illegible]
Self-harm	--	[illegible]	[illegible]	[illegible]	[illegible]	[illegible]	[illegible]	[illegible]
Self-harm by firearm	--	[illegible]	[illegible]	[illegible]	[illegible]	[illegible]	[illegible]	[illegible]
Self-harm by other specified means	--	[illegible]	[illegible]	[illegible]	[illegible]	[illegible]	[illegible]	[illegible]
Interpersonal violence	11 (9–13)	[illegible]	[illegible]	[illegible]	[illegible]	[illegible]	[illegible]	[illegible]
Physical violence by firearm	1 (1–1)	[illegible]	[illegible]	[illegible]	[illegible]	[illegible]	[illegible]	[illegible]
Physical violence by sharp object	0 (0–1)	[illegible]	[illegible]	[illegible]	[illegible]	[illegible]	[illegible]	[illegible]
Physical violence by other means	10 (9–12)	[illegible]	[illegible]	[illegible]	[illegible]	[illegible]	[illegible]	[illegible]
Executions and police conflict	0 (0–0)	[illegible]	[illegible]	[illegible]	[illegible]	[illegible]	[illegible]	[illegible]

SR Table 12: Canada deaths by cause and age group for males

Cause	Early Neonatal	Late Neonatal	Post Neonatal	1 to 4	5 to 9	10 to 14	15 to 19	20 to 24	25 to 29	30 to 34	35 to 39	40 to 44	45 to 49	50 to 54	55 to 59	60 to 64	65 to 69	70 to 74	75 to 79	80 to 84	85 to 89	90 to 94	95 plus
All causes	489 (430–557)	110 (97–125)	244 (218–273)	146 (135–158)	90 (86–94)	133 (129–138)	462 (451–473)	863 (853–873)	1,083 (1,073–1,094)	1,167 (1,155–1,180)	1,347 (1,331–1,365)	1,746 (1,721–1,772)	2,635 (2,595–2,677)	4,354 (4,289–4,422)	7,686 (7,566–7,812)	11,100 (10,936–11,270)	14,227 (14,025–14,437)	18,112 (17,870–18,370)	19,800 (19,550–20,063)	21,678 (21,436–21,933)	21,321 (21,133–21,518)	13,431 (13,332–13,535)	--
Sexually transmitted infections excluding HIV	0 (0–0)	0 (0–0)	0 (0–0)	0 (0–0)	0 (0–0)	0 (0–0)	0 (0–0)	0 (0–0)	0 (0–0)	0 (0–0)	0 (0–0)	0 (0–0)	0 (0–0)	0 (0–0)	0 (0–0)	0 (0–0)	0 (0–0)	0 (0–0)	0 (0–0)	0 (0–0)	0 (0–0)	0 (0–0)	--
Syphilis	0 (0–0)	0 (0–0)	0 (0–0)	0 (0–0)	0 (0–0)	0 (0–0)	0 (0–0)	0 (0–0)	0 (0–0)	0 (0–0)	0 (0–0)	0 (0–0)	0 (0–0)	0 (0–0)	0 (0–0)	0 (0–0)	0 (0–0)	0 (0–0)	0 (0–0)	0 (0–0)	0 (0–0)	0 (0–0)	--
Chlamydial infection	--	--	--	--	--	0 (0–0)	0 (0–0)	0 (0–0)	0 (0–0)	0 (0–0)	0 (0–0)	0 (0–0)	0 (0–0)	0 (0–0)	0 (0–0)	0 (0–0)	0 (0–0)	0 (0–0)	0 (0–0)	0 (0–0)	0 (0–0)	0 (0–0)	--
Gonococcal infection	--	--	--	--	--	0 (0–0)	0 (0–0)	0 (0–0)	0 (0–0)	0 (0–0)	0 (0–0)	0 (0–0)	0 (0–0)	0 (0–0)	0 (0–0)	0 (0–0)	0 (0–0)	0 (0–0)	0 (0–0)	0 (0–0)	0 (0–0)	0 (0–0)	--
Other sexually transmitted infections	--	--	--	--	--	0 (0–0)	0 (0–0)	0 (0–0)	0 (0–0)	0 (0–0)	0 (0–0)	0 (0–0)	0 (0–0)	0 (0–0)	0 (0–0)	0 (0–0)	0 (0–0)	0 (0–0)	0 (0–0)	0 (0–0)	0 (0–0)	0 (0–0)	--
Tuberculosis	--	--	0 (0–0)	0 (0–0)	0 (0–0)	0 (0–0)	0 (0–0)	0 (0–0)	0 (0–1)	1 (0–1)	1 (1–1)	1 (1–1)	1 (1–2)	2 (2–3)	4 (3–6)	6 (4–7)	8 (6–11)	10 (8–13)	13 (9–17)	13 (10–18)	15 (10–20)	8 (6–11)	--
Drug-susceptible tuberculosis	--	--	0 (0–0)	0 (0–0)	0 (0–0)	0 (0–0)	0 (0–0)	0 (0–0)	0 (0–1)	1 (0–1)	1 (1–1)	1 (1–1)	1 (1–2)	2 (1–3)	4 (3–6)	5 (4–7)	8 (5–10)	10 (7–13)	12 (9–16)	13 (10–17)	14 (10–19)	8 (5–10)	--
Multidrug-resistant tuberculosis without extensive drug resistance	--	--	0 (0–0)	0 (0–0)	0 (0–0)	0 (0–0)	0 (0–0)	0 (0–0)	0 (0–0)	0 (0–0)	0 (0–0)	0 (0–0)	0 (0–0)	0 (0–0)	0 (0–0)	0 (0–0)	0 (0–1)	0 (0–1)	0 (0–1)	0 (0–1)	0 (0–1)	0 (0–1)	--
Extensively drug-resistant tuberculosis	--	--	0 (0–0)	0 (0–0)	0 (0–0)	0 (0–0)	0 (0–0)	0 (0–0)	0 (0–0)	0 (0–0)	0 (0–0)	0 (0–0)	0 (0–0)	0 (0–0)	0 (0–0)	0 (0–0)	0 (0–0)	0 (0–0)	0 (0–0)	0 (0–0)	0 (0–0)	0 (0–0)	--
Lower respiratory infections	1 (1–2)	1 (1–2)	9 (6–13)	5 (4–7)	1 (1–2)	2 (1–2)	2 (2–3)	4 (3–6)	6 (4–9)	9 (6–13)	12 (9–16)	18 (12–24)	30 (22–40)	49 (36–66)	90 (65–121)	129 (98–166)	190 (141–251)	305 (225–395)	441 (323–580)	674 (505–860)	982 (763–1,226)	756 (580–953)	--
Upper respiratory infections	0 (0–0)	0 (0–0)	0 (0–0)	0 (0–1)	0 (0–0)	0 (0–0)	0 (0–0)	0 (0–0)	0 (0–0)	0 (0–0)	0 (0–0)	0 (0–0)	0 (0–0)	0 (0–0)	0 (0–1)	0 (0–1)	0 (0–1)	1 (0–1)	0 (0–1)	1 (0–1)	1 (0–1)	0 (0–1)	--
Otitis media	0 (0–0)	0 (0–0)	0 (0–1)	0 (0–0)	0 (0–0)	0 (0–0)	0 (0–0)	0 (0–0)	0 (0–0)	0 (0–0)	0 (0–0)	0 (0–0)	0 (0–0)	0 (0–0)	0 (0–0)	0 (0–0)	0 (0–0)	0 (0–0)	0 (0–0)	0 (0–1)	0 (0–1)	0 (0–0)	--
Diarrhoeal diseases	0 (0–0)	0 (0–0)	3 (2–5)	1 (1–2)	0 (0–0)	0 (0–0)	0 (0–1)	0 (0–1)	1 (0–1)	1 (1–1)	1 (1–2)	2 (1–3)	3 (2–5)	5 (3–8)	13 (8–19)	22 (15–31)	31 (20–46)	60 (36–91)	103 (64–153)	131 (80–198)	148 (88–233)	111 (71–167)	--
Typhoid fever	--	--	0 (0–0)	0 (0–0)	0 (0–0)	0 (0–0)	0 (0–0)	0 (0–0)	0 (0–0)	0 (0–0)	0 (0–0)	0 (0–0)	0 (0–0)	0 (0–0)	0 (0–0)	0 (0–0)	0 (0–0)	0 (0–0)	0 (0–0)	0 (0–0)	0 (0–0)	0 (0–0)	--
Paratyphoid fever	--	--	0 (0–0)	0 (0–0)	0 (0–0)	0 (0–0)	0 (0–0)	0 (0–0)	0 (0–0)	0 (0–0)	0 (0–0)	0 (0–0)	0 (0–0)	0 (0–0)	0 (0–0)	0 (0–0)	0 (0–0)	0 (0–0)	0 (0–0)	0 (0–0)	0 (0–0)	0 (0–0)	--
iNTS	--	0 (0–0)	0 (0–0)	0 (0–0)	0 (0–0)	0 (0–0)	0 (0–0)	0 (0–0)	0 (0–0)	0 (0–0)	0 (0–0)	0 (0–0)	0 (0–0)	0 (0–0)	0 (0–0)	0 (0–0)	0 (0–0)	0 (0–0)	0 (0–0)	0 (0–0)	0 (0–0)	0 (0–0)	--
Other intestinal infectious diseases	--	--	0 (0–0)	0 (0–0)	0 (0–0)	0 (0–0)	0 (0–0)	0 (0–0)	0 (0–0)	0 (0–0)	0 (0–0)	0 (0–0)	0 (0–0)	0 (0–0)	0 (0–0)	0 (0–0)	0 (0–0)	0 (0–0)	0 (0–0)	0 (0–0)	0 (0–0)	0 (0–0)	--
Malaria	0 (0–0)	0 (0–0)	0 (0–0)	0 (0–0)	0 (0–0)	0 (0–0)	0 (0–0)	0 (0–0)	0 (0–0)	0 (0–0)	0 (0–0)	0 (0–0)	0 (0–0)	0 (0–0)	0 (0–0)	0 (0–0)	0 (0–0)	0 (0–0)	0 (0–0)	0 (0–0)	0 (0–0)	0 (0–0)	--
Chagas disease	--	--	0 (0–0)	0 (0–0)	0 (0–0)	0 (0–0)	0 (0–0)	0 (0–0)	0 (0–0)	0 (0–0)	0 (0–0)	0 (0–0)	0 (0–0)	0 (0–0)	0 (0–0)	0 (0–0)	0 (0–0)	0 (0–0)	0 (0–0)	0 (0–0)	0 (0–0)	0 (0–0)	--
Visceral leishmaniasis	--	--	0 (0–0)	0 (0–0)	0 (0–0)	0 (0–0)	0 (0–0)	0 (0–0)	0 (0–0)	0 (0–0)	0 (0–0)	0 (0–0)	0 (0–0)	0 (0–0)	0 (0–0)	0 (0–0)	0 (0–0)	0 (0–0)	0 (0–0)	0 (0–0)	0 (0–0)	0 (0–0)	--
African trypanosomiasis	--	--	--	0 (0–0)	0 (0–0)	0 (0–0)	0 (0–0)	0 (0–0)	0 (0–0)	0 (0–0)	0 (0–0)	0 (0–0)	0 (0–0)	0 (0–0)	0 (0–0)	0 (0–0)	0 (0–0)	0 (0–0)	0 (0–0)	0 (0–0)	0 (0–0)	0 (0–0)	--
Schistosomiasis	--	--	0 (0–0)	0 (0–0)	0 (0–0)	0 (0–0)	0 (0–0)	0 (0–0)	0 (0–0)	0 (0–0)	0 (0–0)	0 (0–0)	0 (0–0)	0 (0–0)	0 (0–0)	0 (0–0)	0 (0–0)	0 (0–0)	0 (0–0)	0 (0–0)	0 (0–0)	0 (0–0)	--
Cysticercosis	--	--	--	0 (0–0)	0 (0–0)	0 (0–0)	0 (0–0)	0 (0–0)	0 (0–0)	0 (0–0)	0 (0–0)	0 (0–0)	0 (0–0)	0 (0–0)	0 (0–0)	0 (0–0)	0 (0–0)	0 (0–0)	0 (0–0)	0 (0–0)	0 (0–0)	0 (0–0)	--
Cystic echinococcosis	--	--	--	0 (0–0)	0 (0–0)	0 (0–0)	0 (0–0)	0 (0–0)	0 (0–0)	0 (0–0)	0 (0–0)	0 (0–0)	0 (0–0)	0 (0–0)	0 (0–0)	0 (0–0)	0 (0–0)	0 (0–0)	0 (0–0)	0 (0–0)	0 (0–0)	0 (0–0)	--
Dengue	0 (0–0)	0 (0–0)	0 (0–0)	0 (0–0)	0 (0–0)	0 (0–0)	0 (0–0)	0 (0–0)	0 (0–0)	0 (0–0)	0 (0–0)	0 (0–0)	0 (0–0)	0 (0–0)	0 (0–0)	0 (0–0)	0 (0–0)	0 (0–0)	0 (0–0)	0 (0–0)	0 (0–0)	0 (0–0)	--
Yellow fever	--	0 (0–0)	0 (0–0)	0 (0–0)	0 (0–0)	0 (0–0)	0 (0–0)	0 (0–0)	0 (0–0)	0 (0–0)	0 (0–0)	0 (0–0)	0 (0–0)	0 (0–0)	0 (0–0)	0 (0–0)	0 (0–0)	0 (0–0)	0 (0–0)	0 (0–0)	0 (0–0)	0 (0–0)	--
Rabies	--	--	0 (0–0)	0 (0–0)	0 (0–0)	0 (0–0)	0 (0–0)	0 (0–0)	0 (0–0)	0 (0–0)	0 (0–0)	0 (0–0)	0 (0–0)	0 (0–0)	0 (0–0)	0 (0–0)	0 (0–0)	0 (0–0)	0 (0–0)	0 (0–0)	0 (0–0)	0 (0–0)	--
Ascariasis	--	--	0 (0–0)	0 (0–0)	0 (0–0)	0 (0–0)	0 (0–0)	0 (0–0)	0 (0–0)	0 (0–0)	0 (0–0)	0 (0–0)	0 (0–0)	0 (0–0)	0 (0–0)	0 (0–0)	0 (0–0)	0 (0–0)	0 (0–0)	0 (0–0)	0 (0–0)	0 (0–0)	--
Zika virus disease	0 (0–0)	0 (0–0)	0 (0–0)	0 (0–0)	0 (0–0)	0 (0–0)	0 (0–0)	0 (0–0)	0 (0–0)	0 (0–0)	0 (0–0)	0 (0–0)	0 (0–0)	0 (0–0)	0 (0–0)	0 (0–0)	0 (0–0)	0 (0–0)	0 (0–0)	0 (0–0)	0 (0–0)	0 (0–0)	--
Other neglected tropical diseases	0 (0–0)	0 (0–0)	0 (0–0)	0 (0–0)	0 (0–0)	0 (0–0)	0 (0–0)	0 (0–0)	0 (0–0)	0 (0–0)	0 (0–0)	0 (0–0)	0 (0–0)	0 (0–0)	0 (0–0)	0 (0–1)	0 (0–1)	0 (0–1)	0 (0–1)	0 (0–1)	0 (0–0)	0 (0–0)	--
Meningitis	0 (0–0)	1 (0–1)	4 (3–6)	3 (2–4)	1 (1–1)	1 (1–1)	1 (1–2)	1 (1–2)	1 (1–2)	1 (1–2)	1 (1–2)	2 (1–2)	2 (2–3)	3 (2–4)	3 (2–4)	4 (3–6)	4 (3–5)	4 (3–6)	4 (3–6)	4 (3–5)	3 (2–4)	2 (1–2)	--
Encephalitis	0 (0–0)	0 (0–0)	1 (1–2)	1 (1–1)	0 (0–1)	0 (0–1)	0 (0–1)	1 (0–1)	1 (1–1)	1 (0–1)	1 (1–1)	1 (1–1)	1 (1–2)	1 (1–2)	2 (1–3)	2 (2–4)	3 (2–5)	4 (3–6)	4 (3–7)	4 (2–7)	3 (1–5)	1 (1–2)	--
Diphtheria	--	--	0 (0–0)	0 (0–0)	0 (0–0)	0 (0–0)	0 (0–0)	0 (0–0)	0 (0–0)	0 (0–0)	0 (0–0)	0 (0–0)	0 (0–0)	0 (0–0)	0 (0–0)	--	--	--	--	--	--	--	--
Whooping cough	--	--	0 (0–1)	0 (0–0)	0 (0–0)	0 (0–0)	0 (0–0)	0 (0–0)	0 (0–0)	0 (0–0)	0 (0–0)	0 (0–0)	0 (0–0)	0 (0–0)	0 (0–0)	--	--	--	--	--	--	--	--
Tetanus	0 (0–0)	0 (0–0)	0 (0–0)	0 (0–0)	0 (0–0)	0 (0–0)	0 (0–0)	0 (0–0)	0 (0–0)	0 (0–0)	0 (0–0)	0 (0–0)	0 (0–0)	0 (0–0)	0 (0–0)	0 (0–0)	0 (0–0)	0 (0–0)	0 (0–0)	0 (0–0)	0 (0–0)	0 (0–0)	--
Measles	0 (0–0)	0 (0–0)	0 (0–0)	0 (0–0)	0 (0–0)	0 (0–0)	0 (0–0)	0 (0–0)	0 (0–0)	0 (0–0)	0 (0–0)	0 (0–0)	0 (0–0)	0 (0–0)	0 (0–0)	0 (0–0)	0 (0–0)	0 (0–0)	0 (0–0)	0 (0–0)	0 (0–0)	0 (0–0)	--
Varicella and herpes zoster	0 (0–0)	0 (0–0)	0 (0–1)	0 (0–0)	0 (0–1)	0 (0–0)	0 (0–0)	0 (0–0)	0 (0–0)	0 (0–0)	0 (0–0)	0 (0–0)	0 (0–0)	0 (0–0)	0 (0–1)	1 (0–1)	1 (0–2)	1 (0–2)	2 (0–3)	3 (1–6)	6 (1–10)	5 (1–9)	--
Acute hepatitis	--	--	0 (0–0)	0 (0–0)	0 (0–0)	0 (0–0)	0 (0–0)	0 (0–0)	0 (0–0)	0 (0–0)	0 (0–0)	0 (0–1)	1 (0–1)	1 (1–1)	1 (1–1)	1 (1–1)	1 (1–1)	1 (1–1)	1 (0–1)	0 (0–1)	0 (0–0)	0 (0–0)	--
Acute hepatitis A	--	--	0 (0–0)	0 (0–0)	0 (0–0)	0 (0–0)	0 (0–0)	0 (0–0)	0 (0–0)	0 (0–0)	0 (0–0)	0 (0–0)	0 (0–0)	0 (0–0)	0 (0–0)	0 (0–0)	0 (0–0)	0 (0–0)	0 (0–0)	0 (0–0)	0 (0–0)	0 (0–0)	--
Acute hepatitis B	--	--	0 (0–0)	0 (0–0)	0 (0–0)	0 (0–0)	0 (0–0)	0 (0–0)	0 (0–0)	0 (0–0)	0 (0–0)	0 (0–0)	0 (0–1)	0 (0–1)	1 (0–1)	1 (0–1)	1 (0–1)	1 (0–1)	0 (0–1)	0 (0–0)	0 (0–0)	0 (0–0)	--
Acute hepatitis C	--	--	0 (0–0)	0 (0–0)	0 (0–0)	0 (0–0)	0 (0–0)	0 (0–0)	0 (0–0)	0 (0–0)	0 (0–0)	0 (0–0)	0 (0–0)	0 (0–0)	0 (0–1)	0 (0–0)	0 (0–0)	0 (0–0)	0 (0–0)	0 (0–0)	0 (0–0)	0 (0–0)	--
Acute hepatitis E	--	--	0 (0–0)	0 (0–0)	0 (0–0)	0 (0–0)	0 (0–0)	0 (0–0)	0 (0–0)	0 (0–0)	0 (0–0)	0 (0–0)	0 (0–0)	0 (0–0)	0 (0–0)	0 (0–0)	0 (0–0)	0 (0–0)	0 (0–0)	0 (0–0)	0 (0–0)	0 (0–0)	--
Other unspecified infectious diseases	1 (0–1)	1 (1–2)	4 (2–7)	1 (1–2)	1 (0–1)	1 (0–1)	1 (1–2)	1 (1–2)	2 (1–3)	2 (1–5)	3 (1–7)	3 (2–7)	5 (2–10)	7 (4–14)	13 (7–23)	18 (11–31)	22 (14–39)	24 (15–40)	22 (11–33)	16 (8–23)	11 (6–16)	5 (2–8)	--
Maternal disorders	--	--	--	--	--	--	--	--	--	--	--	--	--	--	--	--	--	--	--	--	--	--	--
Maternal haemorrhage	--	--	--	--	--	--	--	--	--	--	--	--	--	--	--	--	--	--	--	--	--	--	--
Maternal sepsis and other pregnancy related infections	--	--	--	--	--	--	--	--	--	--	--	--	--	--	--	--	--	--	--	--	--	--	--
Maternal hypertensive disorders	--	--	--	--	--	--	--	--	--	--	--	--	--	--	--	--	--	--	--	--	--	--	--
Maternal obstructed labour and uterine rupture	--	--	--	--	--	--	--	--	--	--	--	--	--	--	--	--	--	--	--	--	--	--	--
Maternal abortive outcome	--	--	--	--	--	--	--	--	--	--	--	--	--	--	--	--	--	--	--	--	--	--	--
Ectopic pregnancy	--	--	--	--	--	--	--	--	--	--	--	--	--	--	--	--	--	--	--	--	--	--	--
Indirect maternal deaths	--	--	--	--	--	--	--	--	--	--	--	--	--	--	--	--	--	--	--	--	--	--	--
Late maternal deaths	--	--	--	--	--	--	--	--	--	--	--	--	--	--	--	--	--	--	--	--	--	--	--
Maternal deaths aggravated by HIV/AIDS	--	--	--	--	--	--	--	--	--	--	--	--	--	--	--	--	--	--	--	--	--	--	--
Other maternal disorders	--	--	--	--	--	--	--	--	--	--	--	--	--	--	--	--	--	--	--	--	--	--	--
Neonatal disorders	380 (322–445)	66 (55–77)	42 (30–56)	4 (3–6)	--	--	--	--	--	--	--	--	--	--	--	--	--	--	--	--	--	--	--
Neonatal preterm birth	189 (153–228)	24 (18–31)	21 (15–28)	1 (1–2)	--	--	--	--	--	--	--	--	--	--	--	--	--	--	--	--	--	--	--
Neonatal encephalopathy due to birth asphyxia and trauma	88 (66–115)	12 (8–17)	7 (4–10)	1 (1–2)	--	--	--	--	--	--	--	--	--	--	--	--	--	--	--	--	--	--	--
Neonatal sepsis and other neonatal infections	10 (7–15)	9 (6–12)	4 (3–5)	0 (0–0)	--	--	--	--	--	--	--	--	--	--	--	--	--	--	--	--	--	--	--
Hemolytic disease and other neonatal jaundice	0 (0–1)	0 (0–0)	0 (0–0)	0 (0–0)	--	--	--	--	--	--	--	--	--	--	--	--	--	--	--	--	--	--	--
Other neonatal disorders	92 (69–119)	21 (16–26)	11 (8–16)	1 (1–2)	--	--	--	--	--	--	--	--	--	--	--	--	--	--	--	--	--	--	--
Nutritional deficiencies	0 (0–0)	0 (0–0)	0 (0–0)	0 (0–0)	0 (0–0)	0 (0–0)	0 (0–0)	0 (0–0)	0 (0–0)	0 (0–0)	0 (0–0)	0 (0–1)	1 (1–1)	1 (1–2)	3 (2–4)	4 (3–6)	8 (6–11)	11 (7–16)	16 (12–22)	19 (13–27)	28 (20–37)	25 (18–33)	--
Protein-energy malnutrition	0 (0–0)	0 (0–0)	0 (0–0)	0 (0–0)	0 (0–0)	0 (0–0)	0 (0–0)	0 (0–0)	0 (0–0)	0 (0–0)	0 (0–0)	0 (0–0)	1 (0–1)	1 (1–2)	2 (1–3)	4 (2–5)	7 (5–9)	10 (6–14)	14 (9–20)	16 (10–23)	23 (16–32)	21 (14–28)	--
Other nutritional deficiencies	--	--	0 (0–0)	0 (0–0)	0 (0–0)	0 (0–0)	0 (0–0)	0 (0–0)	0 (0–0)	0 (0–0)	0 (0–0)	0 (0–0)	0 (0–0)	0 (0–0)	0 (0–1)	1 (0–1)	1 (0–2)	1 (1–3)	2 (1–5)	3 (1–6)	4 (2–9)	4 (2–10)	--
Lip and oral cavity cancer	--	--	--	--	0 (0–0)	0 (0–0)	0 (0–0)	1 (0–1)	1 (1–2)	2 (1–4)	5 (3–7)	9 (6–13)	17 (11–24)	33 (24–43)	70 (52–92)	97 (72–128)	104 (79–138)	98 (73–127)	88 (67–114)	72 (53–93)	54 (39–74)	31 (22–42)	--
Nasopharynx cancer	--	--	--	--	0 (0–0)	0 (0–0)	--	0 (0–1)	1 (0–1)	1 (1–1)	2 (1–3)	3 (2–5)	6 (4–8)	9 (6–13)	14 (9–19)	14 (10–19)	15 (11–21)	14 (9–19)	7 (4–10)	5 (3–8)	3 (2–5)	1 (1–2)	--
Other pharynx cancer	--	--	--	--	--	--	--	0 (0–0)	0 (0–0)	0 (0–0)	1 (1–2)	2 (2–3)	8 (6–12)	21 (15–28)	40 (28–55)	56 (39–78)	54 (37–78)	48 (32–69)	33 (23–47)	22 (15–32)	12 (9–17)	5 (3–8)	--
Oesophageal cancer	--	--	--	--	--	--	--	0 (0–0)	1 (0–1)	2 (2–3)	6 (4–9)	16 (10–23)	37 (28–50)	87 (66–108)	190 (148–240)	267 (209–338)	303 (239–385)	320 (248–400)	261 (203–330)	207 (152–270)	144 (112–181)	65 (49–81)	--
Stomach cancer	--	--	--	--	--	--	0 (0–0)	1 (0–1)	2 (1–2)	4 (3–6)	9 (6–12)	15 (11–20)	35 (27–44)	64 (49–80)	116 (91–146)	185 (150–229)	223 (182–271)	286 (235–343)	292 (239–348)	266 (214–326)	223 (175–276)	120 (89–154)	--
Colon and rectum cancer	--	--	--	--	0 (0–0)	0 (0–0)	1 (0–1)	2 (1–3)	6 (4–9)	15 (11–20)	26 (18–36)	49 (35–65)	95 (72–124)	194 (153–249)	391 (308–485)	609 (491–751)	778 (630–950)	939 (753–1,160)	942 (755–1,141)	867 (654–1,117)	701 (541–883)	371 (267–494)	--
Liver cancer due to hepatitis B	--	--	--	--	--	0 (0–0)	0 (0–0)	0 (0–1)	1 (0–2)	1 (1–3)	2 (1–4)	3 (1–6)	4 (2–8)	8 (4–17)	16 (8–32)	20 (9–39)	17 (8–33)	17 (7–33)	14 (7–25)	9 (4–18)	6 (3–10)	2 (1–4)	--
Liver cancer due to hepatitis C	--	--	--	--	--	0 (0–0)	0 (0–0)	0 (0–0)	0 (0–0)	0 (0–0)	0 (0–1)	1 (0–2)	2 (1–5)	7 (3–14)	20 (9–39)	30 (13–58)	29 (15–51)	32 (16–56)	31 (15–54)	26 (13–46)	20 (11–35)	10 (5–18)	--
Liver cancer due to alcohol use	--	--	--	--	--	--	0 (0–0)	0 (0–1)	2 (1–3)	3 (2–6)	6 (3–10)	12 (7–18)	24 (14–39)	55 (34–81)	120 (79–170)	177 (124–248)	197 (141–262)	214 (156–283)	181 (132–234)	119 (84–157)	67 (46–92)	25 (16–36)	--
Liver cancer due to NASH	--	--	--	--	--	--	0 (0–0)	0 (0–0)	0 (0–1)	0 (0–1)	1 (0–1)	1 (0–2)	2 (1–4)	5 (2–9)	11 (5–21)	18 (9–32)	19 (10–36)	24 (13–42)	26 (14–45)	22 (11–37)	14 (8–23)	6 (3–11)	--
	0 (0–0)	0 (0–0)	0 (0–0)	1 (0–1)	0 (0–1)	0 (0–1)	--	--	--	--	--	--	--	--	--	--	--	--	--	--	--	--	--
Liver cancer due to other causes	0 (0–0)	0 (0–0)	0 (0–0)	0 (0–1)	0 (0–0)	0 (0–1)	0 (0–0)	0 (0–1)	0 (0–1)	1 (0–1)	1 (0–1)	1 (0–2)	2 (1–3)	4 (2–7)	7 (3–13)	9 (4–17)	8 (4–14)	8 (4–15)	8 (4–14)	6 (3–11)	4 (2–7)	2 (1–3)	--
Gallbladder and biliary tract cancer	--	--	--	--	--	--	--	0 (0–0)	0 (0–0)	0 (0–1)	1 (1–2)	2 (2–3)	5 (3–7)	11 (8–15)	22 (16–29)	33 (24–45)	47 (35–63)	60 (44–82)	58 (43–81)	50 (35–71)	44 (32–60)	22 (15–31)	--
Pancreatic cancer	--	--	--	--	--	--	0 (0–0)	0 (0–1)	1 (1–2)	4 (3–5)	11 (8–14)	23 (17–31)	49 (36–63)	121 (92–151)	240 (186–305)	355 (277–452)	469 (371–587)	519 (414–638)	444 (348–549)	369 (288–469)	254 (195–323)	113 (80–152)	--
Larynx cancer	--	--	--	--	--	--	--	0 (0–0)	0 (0–0)	0 (0–0)	1 (0–1)	2 (1–4)	7 (4–10)	15 (11–21)	34 (25–45)	53 (40–69)	67 (51–88)	73 (55–95)	62 (47–80)	52 (38–69)	35 (25–47)	15 (11–20)	--
Tracheal, bronchus, and lung cancer	--	--	--	--	--	0 (0–0)	0 (0–1)	1 (1–2)	3 (2–4)	7 (4–11)	18 (12–27)	42 (31–56)	119 (93–147)	350 (280–427)	868 (719–1,023)	1,514 (1,276–1,765)	2,051 (1,751–2,364)	2,518 (2,179–2,870)	2,185 (1,847–2,537)	1,718 (1,367–2,133)	1,097 (822–1,390)	426 (320–534)	--
Malignant skin melanoma	0 (0–0)	0 (0–0)	0 (0–1)	0 (0–2)	0 (0–0)	0 (0–0)	0 (0–1)	2 (1–3)	6 (3–8)	10 (5–14)	13 (8–21)	19 (11–28)	27 (15–40)	43 (25–63)	72 (41–106)	90 (51–132)	102 (55–146)	111 (61–158)	104 (55–145)	95 (48–136)	67 (35–96)	35 (18–51)	--
Non-melanoma skin cancer (squamous-cell carcinoma)	--	--	--	--	--	--	--	0 (0–0)	0 (0–1)	1 (1–1)	1 (1–1)	2 (1–3)	3 (2–4)	6 (5–8)	13 (10–18)	21 (17–26)	31 (23–38)	38 (26–49)	47 (28–60)	60 (32–75)	59 (29–75)	43 (17–58)	--
	0 (0–0)	0 (0–0)	0 (0–0)	1 (1–2)	1 (1–2)	2 (1–3)	3 (1–5)	4 (2–7)	5 (3–9)	6 (3–10)	6 (3–11)	7 (4–12)	10 (5–16)	15 (7–25)	20 (11–35)	27 (15–45)	33 (17–56)	34 (19–55)	31 (17–54)	26 (14–43)	20 (10–36)	9 (4–19)	--
	--	--	--	0 (0–0)	1 (0–1)	2 (1–4)	6 (3–10)	6 (3–8)	4 (3–6)	3 (2–5)	3 (2–5)	3 (2–4)	3 (2–5)	5 (3–8)	8 (5–13)	10 (6–15)	11 (7–18)	12 (8–18)	10 (6–16)	9 (5–14)	7 (4–11)	3 (2–6)	--
Breast cancer	--	--	--	--	--	--	0 (0–0)	0 (0–0)	0 (0–0)	0 (0–0)	0 (0–0)	0 (0–1)	1 (0–2)	2 (1–3)	3 (2–6)	5 (3–9)	7 (4–12)	8 (4–13)	7 (4–12)	6 (3–12)	5 (2–10)	3 (1–5)	--
Cervical cancer	--	--	--	--	--	--	--	--	--	--	--	--	--	--	--	--	--	--	--	--	--	--	--
Uterine cancer	--	--	--	--	--	--	--	--	--	--	--	--	--	--	--	--	--	--	--	--	--	--	--
Ovarian cancer	--	--	--	--	--	--	--	--	--	--	--	--	--	--	--	--	--	--	--	--	--	--	--
Prostate cancer	--	--	--	--	--	--	--	0 (0–0)	0 (0–1)	1 (0–1)	1 (1–1)	2 (1–3)	10 (6–15)	39 (27–63)	116 (79–191)	294 (209–488)	514 (368–818)	823 (616–1,288)	1,110 (849–1,725)	1,320 (1,009–1,851)	1,306 (959–1,753)	755 (526–1,020)	--
Testicular cancer	0 (0–1)	0 (0–0)	0 (0–1)	0 (0–0)	0 (0–0)	0 (0–0)	1 (1–2)	3 (2–5)	5 (3–8)	4 (3–7)	4 (3–6)	3 (2–5)	3 (2–5)	2 (1–4)	2 (1–4)	2 (1–4)	2 (1–4)	2 (1–4)	2 (1–4)	3 (1–4)	2 (1–3)	1 (1–2)	--
Kidney cancer	0 (0–0)	0 (0–0)	0 (0–0)	1 (1–1)	1 (1–1)	0 (0–1)	0 (0–1)	1 (0–1)	1 (1–2)	2 (1–4)	6 (4–9)	12 (8–17)	30 (21–40)	58 (41–78)	118 (87–157)	163 (122–219)	212 (161–277)	237 (180–307)	204 (153–259)	179 (133–234)	142 (104–188)	67 (48–91)	--
Bladder cancer	--	--	--	--	--	--	0 (0–0)	0 (0–0)	0 (0–1)	1 (1–1)	2 (1–3)	5 (3–7)	13 (9–17)	32 (24–40)	72 (54–93)	141 (108–182)	213 (165–271)	309 (242–384)	363 (284–453)	381 (294–478)	372 (282–476)	207 (153–264)	--
Brain and nervous system cancer	0 (0–0)	0 (0–0)	2 (1–2)	8 (5–11)	11 (7–15)	10 (6–14)	8 (4–11)	10 (6–14)	15 (10–20)	23 (15–32)	31 (21–42)	46 (30–62)	61 (41–82)	100 (65–133)	157 (106–207)	198 (130–263)	198 (138–267)	199 (135–272)	146 (102–199)	94 (63–137)	54 (35–79)	19 (12–28)	--
	0 (0–0)	0 (0–0)	0 (0–0)	0 (0–1)	0 (0–1)	0 (0–1)	0 (0–0)	0 (0–1)	0 (0–1)	0 (0–1)	0 (0–1)	0 (0–1)	1 (0–1)	2 (1–3)	3 (2–4)	3 (2–5)	4 (2–6)	5 (3–8)	4 (3–7)	4 (2–5)	3 (1–4)	2 (1–3)	--
	0 (0–0)	0 (0–0)	0 (0–0)	0 (0–0)	0 (0–0)	0 (0–0)	0 (0–0)	--	--	--	--	--	--	--	--	--	--	--	--	--	--	--	--
	--	--	--	0 (0–0)	0 (0–0)	0 (0–0)	0 (0–0)	0 (0–1)	0 (0–1)	0 (0–1)	0 (0–1)	0 (0–1)	1 (0–1)	2 (1–3)	3 (2–4)	3 (2–5)	4 (2–6)	5 (3–8)	4 (3–7)	4 (2–5)	3 (1–4)	2 (1–3)	--
	0 (0–0)	0 (0–0)	0 (0–1)	4 (2–6)	2 (1–5)	1 (0–1)	0 (0–1)	1 (0–1)	1 (0–1)	1 (0–1)	1 (0–1)	1 (0–1)	1 (0–1)	1 (1–2)	1 (1–2)	2 (1–2)	2 (1–3)	2 (1–3)	2 (1–3)	1 (1–2)	1 (0–1)	0 (0–1)	--
Thyroid cancer	--	--	--	--	0 (0–0)	0 (0–0)	0 (0–0)	0 (0–1)	1 (0–1)	1 (0–1)	1 (0–1)	2 (1–3)	3 (2–4)	5 (4–8)	10 (6–13)	12 (8–17)	17 (11–24)	20 (14–27)	20 (14–28)	15 (10–22)	12 (8–17)	6 (4–9)	--
Mesothelioma	--	--	--	--	--	--	--	0 (0–0)	0 (0–0)	0 (0–0)	1 (1–1)	2 (1–2)	4 (3–5)	10 (8–12)	22 (18–27)	40 (31–49)	69 (54–86)	92 (73–115)	110 (87–137)	87 (69–107)	56 (42–73)	22 (16–30)	--
Hodgkin lymphoma	--	--	--	0 (0–0)	0 (0–0)	0 (0–0)	1 (0–1)	2 (1–3)	4 (2–5)	3 (2–5)	3 (2–5)	3 (2–5)	4 (2–5)	5 (3–7)	6 (4–9)	8 (5–11)	9 (6–12)	10 (7–14)	9 (6–12)	6 (4–9)	4 (3–7)	1 (1–3)	--
Non-Hodgkin's lymphoma	--	--	--	1 (1–1)	2 (1–3)	2 (2–3)	4 (3–5)	7 (5–9)	8 (5–11)	11 (8–16)	16 (11–21)	23 (17–31)	35 (25–46)	63 (47–82)	123 (93–160)	179 (136–229)	239 (184–303)	297 (230–370)	306 (239–393)	305 (226–408)	222 (166–291)	97 (70–130)	--
	--	--	--	0 (0–0)	0 (0–1)	1 (0–1)	1 (0–1)	1 (0–1)	1 (0–1)	1 (0–1)	1 (0–2)	1 (0–1)	1 (0–2)	1 (0–3)	2 (1–4)	3 (1–5)	4 (2–8)	5 (2–10)	4 (2–7)	4 (2–6)	3 (1–5)	1 (1–3)	--
	--	--	--	1 (0–1)	1 (1–2)	2 (1–3)	3 (2–5)	6 (4–8)	7 (5–10)	11 (7–15)	15 (10–20)	22 (17–30)	34 (25–45)	62 (46–80)	121 (91–157)	176 (133–226)	235 (181–298)	292 (225–366)	302 (235–388)	302 (223–404)	219 (163–287)	96 (69–129)	--
Multiple myeloma	--	--	--	--	--	--	--	0 (0–0)	0 (0–0)	1 (1–2)	3 (2–4)	6 (4–9)	15 (11–21)	31 (23–43)	65 (48–88)	103 (77–139)	153 (112–202)	194 (145–254)	185 (133–240)	163 (112–223)	118 (77–162)	48 (31–71)	--
Leukaemia	0 (0–0)	0 (0–0)	1 (1–2)	6 (4–8)	7 (4–10)	8 (5–11)	10 (7–14)	11 (8–16)	14 (10–19)	14 (10–20)	16 (11–21)	18 (13–25)	27 (21–36)	47 (34–63)	91 (66–121)	147 (107–198)	213 (155–276)	305 (229–395)	323 (235–427)	308 (217–422)	229 (159–313)	121 (82–167)	--

Acute lymphoid leukaemia	0 (0–0)	0 (0–0)	0 (0–1)	2 (1–3)	4 (2–6)	4 (2–6)	4 (2–7)	4 (2–7)	4 (2–6)	3 (2–5)	3 (1–5)	3 (2–5)	3 (2–5)	4 (2–6)	6 (4–10)	7 (4–12)	9 (5–14)	10 (6–16)	10 (5–16)	8 (4–13)	6 (3–10)	2 (1–5)	--
Chronic lymphoid leukaemia	--	--	--	--	--	--	--	0 (0–0)	0 (0–0)	0 (0–0)	0 (0–1)	1 (1–2)	4 (2–6)	9 (5–13)	18 (11–30)	35 (21–53)	51 (30–78)	72 (46–111)	82 (51–123)	87 (54–138)	70 (42–109)	46 (27–70)	--
Acute myeloid leukaemia	0 (0–0)	0 (0–0)	1 (0–1)	3 (2–4)	2 (1–3)	2 (1–4)	3 (2–6)	4 (2–7)	6 (4–9)	7 (4–10)	8 (5–11)	8 (5–12)	13 (8–18)	23 (15–34)	44 (28–63)	67 (43–98)	95 (58–139)	128 (81–189)	123 (74–178)	103 (60–158)	62 (35–97)	28 (14–49)	--
Chronic myeloid leukaemia	0 (0–0)	0 (0–0)	0 (0–0)	0 (0–0)	0 (0–0)	0 (0–0)	0 (0–1)	0 (0–1)	1 (1–2)	1 (1–3)	2 (1–3)	2 (1–3)	2 (1–3)	2 (1–4)	4 (2–8)	6 (3–11)	8 (4–14)	12 (7–21)	13 (7–23)	14 (7–24)	12 (7–21)	6 (3–10)	--
Other leukaemia	0 (0–0)	0 (0–0)	0 (0–1)	1 (1–2)	1 (0–2)	1 (1–2)	2 (1–3)	2 (1–4)	3 (1–4)	3 (2–5)	3 (2–6)	4 (2–7)	6 (4–10)	9 (5–15)	18 (10–28)	31 (19–51)	51 (31–78)	82 (48–124)	95 (53–148)	95 (55–148)	79 (47–122)	39 (24–61)	--
Other malignant cancers	0 (0–0)	0 (0–0)	1 (1–1)	3 (2–4)	3 (2–3)	3 (2–4)	5 (4–6)	6 (5–8)	6 (5–7)	7 (5–8)	9 (7–11)	11 (8–15)	17 (12–22)	28 (20–38)	47 (35–62)	66 (48–87)	81 (59–105)	99 (75–128)	97 (72–126)	84 (64–110)	65 (47–85)	35 (24–46)	--
Myelodysplastic, myeloproliferative, and other hematopoietic neoplasms	0 (0–0)	0 (0–0)	0 (0–0)	0 (0–0)	0 (0–0)	0 (0–0)	0 (0–1)	0 (0–1)	1 (0–1)	1 (0–2)	1 (1–2)	2 (1–3)	2 (1–4)	6 (3–10)	13 (8–26)	28 (17–53)	49 (32–89)	85 (56–158)	119 (72–214)	143 (86–243)	132 (77–231)	67 (37–121)	--
Cardiovascular diseases	1 (1–1)	1 (1–1)	5 (3–6)	3 (3–4)	2 (1–2)	4 (3–5)	9 (7–12)	22 (17–27)	45 (36–56)	80 (63–98)	150 (122–179)	289 (243–335)	542 (467–617)	1,035 (922–1,150)	1,900 (1,713–2,084)	2,817 (2,535–3,093)	3,654 (3,312–4,020)	4,740 (4,282–5,196)	5,547 (5,068–6,023)	6,612 (5,780–7,270)	7,182 (6,144–7,968)	4,871 (4,077–5,435)	--
Rheumatic heart disease	--	--	--	0 (0–0)	0 (0–0)	0 (0–0)	0 (0–0)	0 (0–1)	1 (1–1)	1 (1–2)	2 (1–3)	2 (1–3)	4 (2–5)	6 (4–8)	10 (7–13)	16 (12–22)	24 (18–32)	34 (25–44)	40 (31–50)	51 (38–66)	53 (39–68)	32 (24–42)	--
Ischaemic heart disease	--	--	--	--	--	--	2 (1–2)	5 (4–7)	17 (14–21)	39 (31–46)	86 (71–101)	184 (159–210)	376 (332–418)	755 (683–828)	1,395 (1,268–1,523)	2,055 (1,876–2,234)	2,590 (2,376–2,814)	3,155 (2,857–3,416)	3,496 (3,201–3,780)	4,030 (3,522–4,415)	4,326 (3,687–4,737)	2,901 (2,434–3,250)	--
Stroke	0 (0–0)	0 (0–0)	1 (0–1)	1 (0–1)	0 (0–1)	1 (1–1)	2 (1–3)	4 (3–7)	8 (5–13)	13 (8–19)	21 (13–31)	38 (26–54)	64 (45–90)	114 (83–151)	210 (155–275)	325 (242–422)	485 (366–637)	776 (592–1,013)	1,062 (840–1,314)	1,390 (1,060–1,760)	1,548 (1,161–1,957)	1,069 (816–1,325)	--
Ischaemic stroke	0 (0–0)	0 (0–0)	0 (0–0)	0 (0–0)	0 (0–0)	0 (0–0)	0 (0–0)	0 (0–1)	1 (0–1)	1 (1–2)	1 (1–2)	3 (2–4)	7 (4–10)	14 (9–19)	29 (19–41)	93 (65–131)	155 (110–218)	365 (267–489)	535 (406–687)	960 (712–1,220)	1,138 (848–1,483)	814 (613–1,013)	--
Intracerebral hemorrhage	0 (0–0)	0 (0–0)	0 (0–1)	0 (0–0)	0 (0–0)	0 (0–0)	1 (0–1)	1 (1–2)	3 (1–4)	4 (2–6)	8 (5–12)	16 (10–24)	29 (19–41)	56 (39–76)	106 (76–144)	165 (118–221)	248 (183–331)	330 (240–448)	435 (319–576)	332 (243–447)	310 (221–420)	193 (133–263)	--
Subarachnoid hemorrhage	0 (0–0)	0 (0–0)	0 (0–1)	0 (0–1)	0 (0–0)	1 (0–1)	1 (1–2)	3 (2–4)	5 (3–8)	8 (4–12)	12 (7–18)	20 (13–29)	28 (18–41)	45 (29–63)	74 (51–106)	67 (45–97)	83 (56–118)	81 (55–114)	92 (62–127)	98 (61–142)	101 (54–159)	62 (34–95)	--
Hypertensive heart disease	--	--	--	--	--	--	0 (0–0)	0 (0–1)	1 (1–2)	2 (1–3)	4 (3–7)	9 (5–13)	16 (11–24)	26 (17–37)	42 (27–58)	52 (35–72)	60 (38–83)	64 (42–85)	72 (41–99)	86 (47–113)	105 (51–142)	75 (35–102)	--
Non-rheumatic valvular heart disease	--	--	--	--	--	--	0 (0–1)	1 (1–1)	2 (1–2)	2 (1–3)	3 (2–5)	4 (3–6)	8 (5–11)	13 (9–18)	22 (16–29)	35 (26–46)	55 (41–73)	91 (69–117)	150 (113–190)	202 (153–259)	282 (211–362)	198 (141–256)	--
Non-rheumatic calcific aortic valve disease	--	--	--	--	--	--	0 (0–0)	1 (0–1)	1 (1–2)	2 (1–3)	3 (2–4)	3 (2–5)	6 (4–9)	11 (8–15)	19 (13–25)	30 (22–40)	48 (36–63)	81 (61–105)	134 (101–172)	186 (140–239)	261 (195–336)	184 (131–238)	--
Non-rheumatic degenerative mitral valve disease	--	--	--	--	--	--	0 (0–0)	0 (0–0)	0 (0–1)	0 (0–1)	1 (0–1)	1 (0–2)	1 (1–2)	2 (1–4)	3 (2–6)	5 (3–9)	7 (4–12)	10 (6–18)	15 (9–25)	17 (10–27)	20 (11–34)	14 (8–23)	--
Other non-rheumatic valve diseases	--	--	--	--	--	--	0 (0–0)	0 (0–0)	0 (0–0)	0 (0–0)	0 (0–0)	0 (0–0)	0 (0–0)	0 (0–0)	0 (0–0)	0 (0–0)	0 (0–0)	0 (0–0)	0 (0–0)	0 (0–0)	0 (0–0)	0 (0–0)	--
Cardiomyopathy and myocarditis	1 (0–1)	0 (0–1)	3 (2–4)	2 (1–2)	1 (0–1)	2 (1–2)	4 (3–5)	7 (5–9)	9 (6–13)	13 (8–18)	19 (12–26)	25 (17–35)	33 (23–47)	49 (35–69)	82 (58–121)	101 (70–164)	111 (78–181)	129 (91–207)	123 (87–197)	111 (72–174)	97 (64–155)	66 (44–104)	--
Myocarditis	0 (0–0)	0 (0–0)	1 (0–1)	1 (0–1)	0 (0–1)	1 (0–1)	1 (1–2)	2 (1–3)	2 (1–3)	3 (1–5)	3 (1–5)	3 (1–6)	3 (1–5)	3 (1–5)	4 (1–7)	3 (1–6)	3 (1–5)	3 (1–6)	2 (1–5)	2 (1–4)	2 (1–5)	2 (1–4)	--
Alcoholic cardiomyopathy	--	--	--	--	--	--	0 (0–1)	1 (0–2)	2 (1–3)	2 (1–4)	5 (3–8)	8 (5–13)	15 (9–22)	23 (14–33)	45 (29–66)	60 (36–88)	54 (32–80)	65 (37–95)	52 (27–80)	14 (6–26)	5 (2–10)	3 (1–6)	--
Other cardiomyopathy	0 (0–1)	0 (0–0)	2 (1–2)	1 (1–1)	0 (0–1)	1 (1–2)	2 (1–3)	4 (3–6)	5 (3–8)	8 (5–11)	11 (7–17)	13 (8–20)	16 (10–25)	24 (15–38)	33 (20–60)	38 (19–82)	54 (32–106)	62 (32–123)	68 (38–133)	95 (59–157)	90 (58–146)	61 (40–96)	--
Pulmonary Arterial Hypertension	0 (0–1)	0 (0–0)	1 (0–2)	1 (0–1)	0 (0–1)	0 (0–1)	0 (0–1)	1 (0–1)	1 (0–2)	1 (1–2)	2 (1–3)	2 (1–3)	2 (1–4)	3 (2–6)	6 (3–10)	9 (4–15)	12 (5–20)	18 (6–30)	21 (7–39)	21 (6–38)	17 (5–31)	10 (3–18)	--
Atrial fibrillation and flutter	--	--	--	--	--	--	--	--	--	0 (0–0)	0 (0–0)	2 (1–3)	3 (2–6)	8 (6–13)	21 (15–33)	41 (28–64)	67 (46–102)	119 (82–176)	175 (117–255)	274 (185–378)	313 (215–450)	266 (169–362)	--
Aortic aneurysm	--	--	--	--	--	--	0 (0–1)	1 (1–1)	2 (1–3)	3 (2–5)	5 (3–6)	8 (6–11)	14 (10–18)	22 (16–29)	47 (35–61)	81 (60–104)	117 (89–154)	173 (132–230)	203 (154–260)	213 (161–276)	211 (158–264)	104 (77–133)	--
Peripheral vascular disease	--	--	--	--	--	--	--	--	--	--	--	1 (0–3)	3 (1–8)	9 (3–21)	19 (6–47)	39 (13–100)	57 (18–142)	86 (28–219)	102 (34–241)	117 (37–290)	120 (38–302)	84 (28–199)	--
Endocarditis	0 (0–0)	0 (0–0)	0 (0–0)	0 (0–0)	0 (0–0)	0 (0–0)	0 (0–1)	1 (0–2)	2 (1–3)	3 (1–5)	4 (2–7)	6 (2–10)	7 (3–11)	10 (4–16)	15 (6–23)	20 (7–32)	25 (9–41)	34 (11–52)	36 (12–56)	44 (14–70)	46 (14–77)	29 (9–47)	--
Other cardiovascular and circulatory diseases	0 (0–0)	0 (0–0)	0 (0–1)	0 (0–0)	0 (0–0)	0 (0–0)	0 (0–0)	1 (1–2)	2 (1–3)	3 (2–5)	5 (3–7)	8 (5–11)	12 (9–16)	20 (15–26)	33 (25–42)	42 (32–53)	51 (39–64)	62 (48–78)	67 (52–85)	73 (55–95)	65 (49–83)	37 (28–46)	--
Chronic respiratory diseases	0 (0–0)	0 (0–0)	0 (0–0)	2 (1–2)	1 (1–2)	2 (2–3)	2 (2–3)	4 (3–5)	6 (4–8)	6 (5–9)	9 (7–12)	15 (12–18)	35 (27–44)	91 (72–111)	230 (179–285)	481 (383–580)	817 (657–982)	1,323 (1,075–1,563)	1,630 (1,357–1,930)	1,866 (1,530–2,215)	1,836 (1,461–2,220)	1,081 (842–1,305)	--
Chronic obstructive pulmonary disease	--	--	0 (0–0)	0 (0–0)	0 (0–0)	0 (0–0)	0 (0–1)	1 (0–1)	1 (0–1)	1 (1–2)	2 (1–4)	6 (4–8)	18 (13–24)	62 (47–78)	173 (132–218)	383 (296–477)	655 (509–812)	1,081 (864–1,322)	1,344 (1,090–1,626)	1,576 (1,260–1,913)	1,591 (1,249–1,964)	956 (734–1,182)	--
Pneumoconiosis	--	--	--	--	--	--	0 (0–0)	0 (0–0)	0 (0–0)	0 (0–0)	0 (0–0)	0 (0–1)	0 (0–1)	1 (1–2)	2 (1–3)	4 (3–7)	10 (6–14)	17 (11–25)	24 (17–34)	28 (19–41)	25 (17–37)	12 (7–17)	--
Silicosis	--	--	--	--	--	--	0 (0–0)	0 (0–0)	0 (0–0)	0 (0–0)	0 (0–0)	0 (0–0)	0 (0–1)	1 (0–1)	1 (0–2)	1 (0–4)	2 (1–6)	4 (1–9)	5 (2–14)	6 (2–16)	6 (2–16)	3 (1–7)	--
Asbestosis	--	--	--	--	--	--	0 (0–0)	0 (0–0)	0 (0–0)	0 (0–0)	0 (0–0)	0 (0–0)	0 (0–0)	0 (0–1)	1 (0–2)	3 (1–5)	7 (3–11)	13 (6–19)	18 (8–28)	21 (8–32)	17 (5–28)	8 (2–13)	--
Coal workers pneumoconiosis	--	--	--	--	--	--	0 (0–0)	0 (0–0)	0 (0–0)	0 (0–0)	0 (0–0)	0 (0–0)	0 (0–0)	0 (0–0)	0 (0–0)	0 (0–0)	0 (0–1)	1 (0–2)	1 (0–2)	1 (1–4)	1 (1–3)	1 (0–2)	--
Other pneumoconiosis	--	--	--	--	--	--	0 (0–0)	0 (0–0)	0 (0–0)	0 (0–0)	0 (0–0)	0 (0–0)	0 (0–0)	0 (0–0)	0 (0–0)	0 (0–0)	0 (0–0)	0 (0–0)	0 (0–0)	0 (0–0)	0 (0–0)	0 (0–0)	--
Asthma	--	--	--	0 (0–0)	0 (0–1)	1 (1–2)	1 (1–2)	2 (1–2)	2 (1–3)	2 (1–2)	2 (1–3)	2 (1–2)	3 (2–4)	3 (2–5)	5 (3–8)	6 (4–9)	6 (4–10)	8 (5–12)	10 (7–15)	12 (8–19)	16 (10–23)	9 (6–14)	--
Interstitial lung disease and pulmonary sarcoidosis	--	--	--	1 (0–1)	0 (0–0)	0 (0–1)	0 (0–1)	1 (0–1)	2 (1–3)	3 (2–4)	3 (2–5)	5 (3–7)	10 (6–15)	19 (11–28)	42 (22–64)	80 (39–119)	137 (64–215)	207 (91–328)	240 (107–399)	237 (104–418)	193 (79–350)	97 (36–179)	--
Other chronic respiratory diseases	0 (0–0)	0 (0–0)	0 (0–0)	1 (1–1)	0 (0–1)	1 (0–1)	0 (0–1)	1 (0–1)	1 (1–2)	1 (1–2)	2 (1–3)	2 (1–4)	3 (2–5)	6 (4–9)	9 (5–14)	8 (4–14)	9 (5–15)	10 (6–16)	11 (6–16)	12 (6–21)	11 (5–21)	7 (3–13)	--
Digestive diseases	1 (0–1)	1 (0–1)	3 (2–5)	4 (3–5)	1 (1–2)	2 (1–2)	3 (2–3)	6 (5–8)	15 (11–20)	31 (23–39)	53 (42–65)	98 (80–117)	181 (151–214)	336 (283–391)	569 (484–660)	715 (607–836)	737 (623–871)	773 (652–904)	746 (631–865)	770 (623–917)	771 (631–915)	497 (407–578)	--
Cirrhosis and other chronic liver diseases	--	--	--	1 (1–1)	0 (0–0)	0 (0–0)	1 (0–1)	2 (2–3)	8 (6–11)	19 (14–25)	38 (29–47)	75 (61–91)	145 (120–172)	270 (225–318)	450 (381–529)	539 (447–636)	487 (401–583)	418 (338–504)	306 (243–371)	239 (186–307)	180 (131–242)	103 (74–139)	--
Cirrhosis and other chronic liver diseases due to hepatitis B	--	--	--	0 (0–0)	0 (0–0)	0 (0–0)	0 (0–0)	0 (0–0)	0 (0–1)	1 (1–2)	3 (1–5)	6 (3–10)	11 (5–19)	18 (8–33)	26 (12–49)	27 (12–52)	23 (11–43)	20 (9–36)	16 (8–28)	13 (6–23)	9 (5–16)	5 (2–10)	--
Cirrhosis and other chronic liver diseases due to hepatitis C	--	--	--	0 (0–0)	0 (0–0)	0 (0–0)	0 (0–0)	1 (0–1)	4 (2–6)	10 (7–14)	19 (13–27)	37 (25–52)	70 (45–96)	124 (77–178)	193 (117–285)	220 (124–327)	198 (122–289)	170 (105–248)	125 (76–184)	99 (58–146)	76 (46–113)	44 (25–67)	--
Cirrhosis and other chronic liver diseases due to alcohol use	--	--	--	--	--	--	0 (0–0)	0 (0–1)	2 (1–3)	5 (3–8)	11 (6–18)	25 (14–37)	50 (27–77)	99 (56–146)	178 (103–264)	221 (122–334)	194 (111–288)	156 (91–230)	104 (62–154)	74 (41–115)	51 (30–80)	27 (14–46)	--
Cirrhosis and other chronic liver diseases due to NAFLD	--	--	--	--	--	--	0 (0–0)	0 (0–0)	0 (0–1)	1 (0–2)	2 (1–3)	3 (1–6)	7 (3–13)	13 (6–25)	22 (10–39)	27 (11–51)	26 (14–45)	24 (13–41)	19 (9–33)	16 (8–28)	13 (7–23)	8 (4–15)	--
Cirrhosis and other chronic liver diseases due to other causes	--	--	--	1 (1–1)	0 (0–0)	0 (0–0)	1 (0–1)	1 (1–2)	2 (1–3)	2 (1–4)	3 (1–5)	4 (2–8)	8 (4–15)	16 (7–28)	31 (14–56)	44 (21–77)	47 (22–85)	48 (23–82)	42 (21–75)	38 (18–65)	31 (16–51)	19 (9–34)	--
Upper digestive system diseases	--	--	--	0 (0–0)	0 (0–0)	0 (0–0)	0 (0–0)	0 (0–0)	1 (0–1)	1 (1–2)	2 (1–3)	3 (2–4)	6 (4–8)	11 (8–16)	20 (14–29)	28 (20–38)	36 (26–48)	44 (31–59)	49 (35–66)	61 (44–82)	66 (47–92)	45 (33–61)	--
Peptic ulcer disease	--	--	--	0 (0–0)	0 (0–0)	0 (0–0)	0 (0–0)	0 (0–0)	1 (0–1)	1 (1–2)	1 (1–2)	2 (2–4)	5 (3–7)	10 (6–13)	17 (12–24)	25 (18–34)	31 (23–42)	40 (28–54)	45 (32–61)	57 (40–76)	61 (43–83)	40 (29–56)	--
Gastritis and duodenitis	--	--	--	0 (0–0)	0 (0–0)	0 (0–0)	0 (0–0)	0 (0–0)	0 (0–0)	0 (0–0)	1 (0–1)	1 (0–1)	1 (1–2)	2 (1–3)	3 (2–5)	3 (2–6)	4 (2–7)	4 (2–7)	4 (2–6)	5 (3–8)	6 (3–10)	5 (2–7)	--
Appendicitis	--	--	--	0 (0–0)	0 (0–0)	0 (0–0)	0 (0–0)	0 (0–0)	0 (0–0)	0 (0–0)	0 (0–1)	0 (0–1)	1 (0–1)	1 (1–2)	2 (1–3)	3 (2–4)	3 (2–5)	5 (3–7)	5 (3–8)	6 (3–9)	6 (3–9)	4 (2–5)	--
Paralytic ileus and intestinal obstruction	0 (0–0)	0 (0–1)	3 (2–4)	1 (0–1)	0 (0–1)	0 (0–1)	1 (0–1)	1 (1–2)	1 (1–2)	2 (1–3)	2 (1–3)	3 (2–4)	4 (3–6)	8 (5–11)	14 (9–21)	23 (15–34)	34 (22–49)	58 (37–79)	83 (48–112)	111 (63–155)	136 (68–193)	93 (46–132)	--
Inguinal, femoral, and abdominal hernia	0 (0–0)	0 (0–0)	0 (0–1)	0 (0–0)	0 (0–0)	0 (0–0)	0 (0–0)	0 (0–0)	0 (0–0)	0 (0–0)	0 (0–1)	1 (0–1)	1 (1–2)	3 (2–4)	5 (3–7)	7 (5–10)	11 (8–15)	16 (11–21)	23 (17–32)	31 (22–41)	41 (29–58)	30 (20–43)	--
Inflammatory bowel disease	--	--	--	1 (0–1)	0 (0–0)	0 (0–0)	0 (0–0)	0 (0–1)	1 (1–2)	1 (1–2)	2 (1–3)	2 (1–3)	3 (2–5)	5 (3–7)	8 (5–12)	12 (7–18)	14 (9–22)	20 (13–32)	23 (15–40)	24 (15–43)	26 (16–45)	18 (12–28)	--
Vascular intestinal disorders	--	--	--	0 (0–0)	0 (0–0)	0 (0–0)	0 (0–0)	0 (0–0)	1 (0–1)	1 (1–1)	1 (1–2)	2 (1–3)	4 (3–6)	9 (7–13)	18 (12–26)	31 (23–41)	53 (38–69)	72 (54–96)	89 (67–118)	88 (64–117)	85 (62–116)	50 (36–70)	--
Gallbladder and biliary diseases	--	--	--	0 (0–0)	0 (0–0)	0 (0–0)	0 (0–0)	0 (0–0)	0 (0–1)	1 (0–1)	1 (1–2)	1 (1–2)	2 (1–4)	5 (3–8)	10 (7–15)	16 (11–23)	25 (17–35)	41 (27–57)	60 (37–81)	79 (48–109)	85 (54–118)	58 (37–82)	--
Pancreatitis	--	--	--	0 (0–0)	0 (0–0)	0 (0–0)	0 (0–0)	1 (0–1)	2 (1–3)	3 (2–5)	5 (3–7)	6 (4–10)	9 (6–13)	13 (9–19)	22 (15–31)	26 (18–35)	33 (24–44)	39 (29–52)	35 (25–46)	37 (25–51)	32 (21–46)	18 (12–26)	--
Other digestive diseases	--	--	--	1 (0–1)	0 (0–0)	0 (0–0)	0 (0–1)	1 (0–1)	1 (1–2)	2 (1–3)	2 (1–3)	4 (2–6)	6 (3–9)	11 (6–16)	20 (10–31)	29 (16–44)	41 (23–60)	60 (33–89)	73 (43–104)	92 (54–129)	112 (64–158)	78 (46–111)	--
Alzheimer's disease and other dementias	--	--	--	--	--	--	--	--	--	--	--	2 (0–7)	10 (1–32)	18 (3–57)	43 (8–127)	87 (18–251)	157 (34–468)	310 (72–869)	537 (127–1,524)	1,115 (270–3,025)	1,464 (354–4,098)	1,153 (285–3,128)	--
Parkinson's disease	--	--	--	--	--	--	--	0 (0–0)	0 (0–0)	0 (0–0)	0 (0–0)	1 (1–1)	4 (3–5)	10 (9–12)	22 (19–26)	51 (45–59)	121 (106–138)	296 (262–331)	514 (451–577)	669 (567–774)	578 (481–660)	275 (223–324)	--
Idiopathic epilepsy	--	--	1 (0–1)	2 (1–2)	1 (1–2)	2 (1–2)	4 (3–5)	8 (6–11)	11 (8–14)	11 (8–14)	11 (8–14)	12 (9–15)	12 (9–16)	13 (10–17)	14 (11–18)	13 (10–18)	12 (9–15)	13 (10–16)	11 (8–15)	10 (7–15)	8 (6–12)	5 (3–7)	--
Multiple sclerosis	--	--	--	--	0 (0–0)	0 (0–0)	0 (0–0)	0 (0–0)	1 (0–1)	2 (1–3)	4 (2–6)	7 (3–9)	12 (4–17)	18 (7–25)	26 (10–36)	32 (11–45)	27 (11–38)	21 (9–30)	17 (6–26)	10 (3–15)	5 (2–8)	2 (1–3)	--
Motor neuron disease	0 (0–0)	0 (0–0)	3 (1–4)	1 (1–2)	0 (0–0)	0 (0–0)	0 (0–1)	1 (0–1)	1 (1–2)	2 (2–3)	4 (3–6)	9 (7–13)	15 (11–20)	31 (23–40)	55 (44–69)	86 (69–106)	108 (83–138)	119 (90–150)	99 (72–134)	70 (50–97)	34 (24–48)	11 (8–15)	--
Other neurological disorders	--	--	8 (5–12)	3 (2–5)	2 (1–3)	3 (2–4)	6 (4–9)	9 (7–13)	9 (7–12)	7 (5–9)	8 (6–10)	10 (8–14)	16 (12–21)	26 (20–33)	39 (29–51)	53 (40–70)	61 (46–81)	73 (55–96)	78 (57–104)	68 (49–94)	47 (34–65)	23 (17–30)	--
Eating disorders	--	--	--	--	0 (0–0)	0 (0–0)	0 (0–0)	0 (0–0)	0 (0–0)	0 (0–0)	0 (0–0)	0 (0–0)	0 (0–0)	--	--	--	--	--	--	--	--	--	--
Anorexia nervosa	--	--	--	--	0 (0–0)	0 (0–0)	0 (0–0)	0 (0–0)	0 (0–0)	0 (0–0)	0 (0–0)	0 (0–0)	0 (0–0)	--	--	--	--	--	--	--	--	--	--
Bulimia nervosa	--	--	--	--	0 (0–0)	0 (0–0)	0 (0–0)	0 (0–0)	0 (0–0)	0 (0–0)	0 (0–0)	0 (0–0)	0 (0–0)	--	--	--	--	--	--	--	--	--	--
Alcohol use disorders	--	--	--	--	--	0 (0–0)	3 (2–5)	8 (6–12)	16 (11–23)	25 (18–34)	36 (25–50)	57 (41–81)	85 (59–121)	109 (79–148)	151 (112–203)	150 (110–195)	132 (97–173)	104 (76–138)	68 (50–90)	43 (31–59)	24 (17–33)	9 (7–12)	--
Drug use disorders	0 (0–0)	0 (0–0)	0 (0–0)	0 (0–0)	0 (0–0)	0 (0–0)	27 (16–43)	118 (79–167)	212 (154–286)	237 (178–310)	231 (175–300)	219 (156–299)	212 (140–306)	176 (120–243)	141 (87–213)	75 (45–112)	38 (25–55)	22 (14–32)	16 (11–22)	12 (8–17)	9 (6–14)	6 (4–9)	--
Opioid use disorders	0 (0–0)	0 (0–0)	0 (0–0)	0 (0–0)	0 (0–0)	0 (0–0)	22 (13–35)	98 (66–140)	172 (125–232)	173 (129–227)	168 (123–225)	161 (115–221)	156 (104–227)	121 (83–168)	96 (59–145)	51 (31–76)	27 (17–38)	14 (9–20)	10 (7–14)	10 (6–14)	8 (5–11)	5 (3–8)	--
Cocaine use disorders	--	--	--	--	--	0 (0–0)	2 (1–3)	9 (6–12)	16 (11–22)	33 (24–45)	34 (20–51)	30 (22–42)	29 (19–43)	33 (23–46)	27 (16–41)	14 (9–22)	7 (4–10)	4 (2–6)	3 (1–4)	0 (0–0)	0 (0–0)	0 (0–0)	--
Amphetamine use disorders	--	--	--	--	--	0 (0–0)	1 (1–2)	5 (3–7)	11 (8–16)	16 (12–22)	14 (9–21)	13 (9–18)	12 (8–17)	13 (9–18)	10 (6–16)	6 (3–9)	3 (2–5)	1 (1–2)	1 (1–1)	0 (0–0)	0 (0–0)	0 (0–0)	--
Other drug use disorders	--	--	--	--	--	0 (0–0)	2 (1–3)	7 (4–9)	13 (9–18)	16 (12–22)	15 (9–22)	15 (10–20)	15 (9–22)	9 (6–12)	7 (5–11)	3 (2–5)	2 (1–3)	3 (2–4)	2 (1–3)	2 (1–3)	2 (1–3)	1 (1–2)	--
Diabetes mellitus	0 (0–0)	0 (0–0)	0 (0–1)	0 (0–0)	0 (0–0)	0 (0–1)	1 (1–2)	4 (3–5)	9 (8–11)	15 (12–18)	24 (20–28)	37 (30–44)	60 (49–73)	112 (90–137)	213 (167–265)	329 (253–416)	453 (353–569)	554 (423–711)	574 (447–717)	586 (458–748)	515 (399–631)	342 (268–427)	--
Diabetes mellitus type 1	0 (0–0)	0 (0–0)	0 (0–1)	0 (0–0)	0 (0–0)	0 (0–1)	1 (1–1)	4 (3–4)	9 (7–11)	12 (9–15)	19 (15–23)	28 (21–35)	13 (7–18)	23 (11–33)	41 (22–61)	37 (18–55)	50 (27–75)	43 (22–69)	40 (20–65)	17 (7–27)	15 (6–23)	10 (4–15)	--
Diabetes mellitus type 2	--	--	--	--	--	--	0 (0–0)	0 (0–0)	0 (0–1)	3 (2–5)	5 (3–9)	9 (5–14)	48 (38–59)	90 (70–112)	172 (131–221)	292 (222–372)	404 (310–514)	511 (388–661)	534 (416–670)	569 (446–723)	500 (386–615)	332 (260–415)	--
Chronic kidney disease	--	--	6 (4–9)	1 (1–1)	0 (0–1)	0 (0–1)	1 (1–1)	2 (1–2)	4 (3–6)	6 (4–8)	9 (7–12)	13 (10–17)	22 (17–28)	43 (33–55)	85 (66–107)	154 (118–197)	234 (184–293)	364 (295–444)	501 (388–627)	657 (524–811)	753 (595–912)	536 (415–661)	--
Chronic kidney disease due to diabetes mellitus type 1	--	--	0 (0–0)	0 (0–0)	0 (0–0)	0 (0–0)	0 (0–0)	0 (0–0)	0 (0–0)	0 (0–1)	1 (0–2)	1 (0–2)	2 (1–3)	2 (1–5)	3 (1–6)	4 (1–9)	4 (1–10)	5 (2–11)	5 (2–13)	5 (2–13)	3 (1–7)	1 (0–2)	--
Chronic kidney disease due to diabetes mellitus type 2	--	--	--	--	--	--	0 (0–0)	0 (0–0)	0 (0–0)	0 (0–1)	1 (0–1)	1 (1–3)	4 (2–6)	9 (4–15)	19 (9–32)	36 (17–61)	54 (28–87)	79 (41–128)	94 (48–162)	102 (50–186)	95 (51–166)	58 (30–102)	--
Chronic kidney disease due to hypertension	--	--	--	--	--	--	0 (0–0)	0 (0–0)	0 (0–1)	1 (0–2)	2 (1–2)	3 (1–4)	5 (3–8)	11 (7–18)	25 (16–37)	51 (32–73)	84 (56–122)	141 (96–192)	216 (146–295)	317 (221–421)	407 (295–533)	308 (225–406)	--
Chronic kidney disease due to glomerulonephritis	--	--	2 (1–3)	0 (0–0)	0 (0–0)	0 (0–0)	0 (0–1)	1 (0–1)	2 (1–3)	2 (1–4)	3 (2–5)	4 (2–7)	6 (3–11)	11 (5–18)	19 (9–32)	30 (14–52)	40 (19–71)	55 (28–94)	69 (32–122)	79 (35–150)	73 (34–133)	41 (18–77)	--
Chronic kidney disease due to other and unspecified causes	--	--	4 (3–7)	1 (0–1)	0 (0–0)	0 (0–0)	0 (0–1)	1 (0–1)	2 (1–2)	2 (1–3)	3 (1–5)	3 (2–6)	6 (3–10)	10 (5–17)	19 (9–32)	33 (17–58)	51 (26–84)	84 (44–139)	117 (59–197)	154 (80–255)	175 (92–279)	128 (71–205)	--
Acute glomerulonephritis	--	--	0 (0–0)	0 (0–0)	0 (0–0)	0 (0–0)	0 (0–0)	0 (0–0)	0 (0–0)	0 (0–0)	0 (0–0)	0 (0–0)	0 (0–0)	0 (0–0)	0 (0–0)	0 (0–0)	0 (0–0)	0 (0–0)	0 (0–1)	0 (0–1)	1 (0–1)	0 (0–1)	--
Skin and subcutaneous diseases	--	--	0 (0–1)	0 (0–0)	0 (0–0)	0 (0–0)	0 (0–0)	0 (0–1)	1 (0–1)	1 (0–2)	2 (1–3)	2 (1–4)	3 (1–7)	7 (3–12)	11 (4–19)	16 (6–28)	21 (7–37)	29 (11–54)	37 (13–72)	45 (16–78)	45 (17–79)	31 (10–48)	--
Bacterial skin diseases	--	--	0 (0–1)	0 (0–0)	0 (0–0)	0 (0–0)	0 (0–0)	0 (0–1)	1 (0–1)	1 (0–2)	2 (1–3)	2 (1–4)	3 (1–7)	7 (2–11)	10 (3–17)	15 (5–26)	19 (6–35)	26 (9–49)	33 (11–64)	38 (12–69)	37 (13–68)	25 (8–41)	--
Cellulitis	--	--	0 (0–0)	0 (0–0)	0 (0–0)	0 (0–0)	0 (0–0)	0 (0–0)	0 (0–1)	1 (0–1)	1 (0–2)	2 (1–3)	3 (1–5)	5 (1–8)	7 (2–12)	10 (3–17)	13 (3–22)	15 (4–29)	18 (5–33)	21 (5–36)	21 (6–39)	14 (3–25)	--
Pyoderma	--	--	0 (0–0)	0 (0–0)	0 (0–0)	0 (0–0)	0 (0–0)	0 (0–0)	0 (0–0)	0 (0–1)	0 (0–1)	1 (0–1)	1 (0–2)	2 (0–4)	3 (1–8)	5 (1–11)	7 (1–17)	10 (2–23)	15 (3–35)	17 (3–39)	17 (4–35)	11 (2–25)	--
Decubitus ulcer	--	--	--	0 (0–0)	0 (0–0)	0 (0–0)	0 (0–0)	0 (0–0)	0 (0–0)	0 (0–0)	0 (0–0)	0 (0–0)	0 (0–0)	0 (0–0)	0 (0–1)	1 (0–2)	1 (0–2)	2 (0–4)	3 (0–7)	3 (1–8)	4 (0–9)	3 (0–6)	--
Other skin and subcutaneous diseases	--	--	0 (0–0)	0 (0–0)	0 (0–0)	0 (0–0)	0 (0–0)	0 (0–0)	0 (0–0)	0 (0–0)	0 (0–0)	0 (0–0)	0 (0–1)	0 (0–1)	1 (0–1)	1 (0–1)	1 (0–2)	2 (0–3)	2 (1–5)	3 (1–7)	4 (1–8)	2 (1–5)	--
Musculoskeletal disorders	--	--	--	--	0 (0–0)	0 (0–1)	1 (0–1)	1 (1–2)	2 (1–3)	2 (1–4)	3 (2–5)	4 (3–7)	6 (4–10)	11 (7–16)	19 (12–26)	26 (17–37)	37 (22–50)	50 (27–66)	49 (26–67)	48 (22–67)	40 (17–57)	25 (9–39)	--
Rheumatoid arthritis	--	--	--	--	0 (0–0)	0 (0–0)	0 (0–0)	0 (0–0)	0 (0–0)	0 (0–0)	0 (0–0)	0 (0–1)	1 (0–2)	2 (1–3)	5 (3–7)	8 (4–12)	12 (7–19)	18 (10–27)	19 (10–29)	16 (7–25)	10 (4–18)	5 (2–10)	--
Other musculoskeletal disorders	--	--	--	--	0 (0–0)	0 (0–1)	1 (0–1)	1 (1–2)	1 (1–3)	2 (1–4)	3 (2–5)	4 (2–6)	5 (3–8)	9 (6–13)	14 (9–20)	19 (12–26)	25 (14–34)	32 (17–44)	30 (15–42)	32 (14–46)	29 (13–43)	20 (7–31)	--
Congenital anomalies	98 (73–150)	32 (25–41)	84 (62–106)	26 (19–35)	8 (6–13)	9 (6–13)	9 (7–14)	13 (9–18)	15 (9–21)	14 (9–19)	13 (9–18)	13 (9–17)	15 (10–20)	20 (12–27)	29 (16–40)	27 (16–37)	20 (14–27)	--	--	--	--	--	--
Neural tube defects	6 (3–10)	2 (1–3)	4 (2–9)	5 (3–8)	0 (0–1)	0 (0–0)	0 (0–1)	1 (0–1)	1 (0–1)	1 (0–1)	1 (0–1)	0 (0–1)	0 (0–1)	0 (0–1)	0 (0–1)	0 (0–0)	0 (0–1)	--	--	--	--	--	--
Congenital heart anomalies	22 (12–34)	11 (8–15)	27 (17–37)	8 (5–11)	3 (2–5)	3 (2–5)	4 (3–7)	5 (3–8)	6 (4–9)	6 (4–9)	6 (4–9)	6 (4–8)	5 (3–7)	4 (2–7)	5 (2–8)	4 (2–6)	5 (3–8)	--	--	--	--	--	--
Orofacial clefts	0 (0–0)	0 (0–0)	0 (0–0)	0 (0–0)	--	--	--	--	--	--	--	--	--	--	--	--	--	--	--	--	--	--	--
Down's syndrome	5 (3–9)	2 (1–3)	7 (4–10)	1 (1–2)	0 (0–1)	1 (0–1)	1 (0–1)	2 (1–3)	2 (1–4)	2 (1–4)	2 (1–4)	3 (2–5)	6 (3–9)	12 (7–17)	20 (11–29)	20 (12–28)	10 (6–14)	--	--	--	--	--	--
Other chromosomal abnormalities	20 (11–46)	7 (4–14)	16 (10–26)	3 (2–6)	1 (1–2)	2 (1–3)	1 (1–2)	2 (1–3)	2 (1–3)	2 (1–3)	2 (1–3)	1 (1–2)	1 (0–2)	1 (0–2)	1 (0–3)	1 (0–2)	1 (0–2)	--	--	--	--	--	--
Congenital musculoskeletal and limb anomalies	4 (2–7)	1 (0–2)	3 (1–5)	1 (0–1)	0 (0–1)	0 (0–0)	0 (0–1)	0 (0–1)	1 (0–1)	0 (0–1)	0 (0–1)	0 (0–1)	0 (0–1)	0 (0–1)	1 (0–1)	1 (0–1)	1 (0–2)	--	--	--	--	--	--
Urogenital congenital anomalies	8 (4–21)	1 (1–2)	3 (1–6)	0 (0–0)	0 (0–0)	0 (0–0)	0 (0–0)	0 (0–0)	0 (0–0)	0 (0–0)	0 (0–0)	0 (0–0)	0 (0–0)	0 (0–0)	0 (0–0)	0 (0–0)	0 (0–0)	--	--	--	--	--	--
Digestive congenital anomalies	9 (6–14)	3 (2–5)	7 (3–11)	2 (1–2)	0 (0–0)	0 (0–0)	0 (0–0)	0 (0–0)	0 (0–0)	0 (0–0)	0 (0–0)	0 (0–0)	0 (0–0)	0 (0–0)	0 (0–1)	0 (0–1)	1 (0–1)	--	--	--	--	--	--
Other congenital anomalies	23 (14–44)	5 (4–8)	17 (10–24)	7 (4–10)	3 (2–4)	2 (1–4)	2 (1–4)	3 (2–5)	3 (2–5)	2 (1–4)	2 (1–3)	2 (1–3)	2 (1–2)	1 (1–2)	1 (1–2)	1 (1–2)	1 (1–2)	--	--	--	--	--	--
Urinary diseases and male infertility	0 (0–1)	0 (0–0)	0 (0–1)	0 (0–0)	0 (0–0)	0 (0–1)	0 (0–0)	1 (0–1)	1 (1–1)	1 (1–2)	2 (1–2)	2 (1–3)	4 (2–5)	8 (5–11)	15 (10–19)	25 (18–33)	37 (27–51)	65 (46–88)	112 (76–154)	161 (108–225)	217 (136–300)	168 (96–237)	--
Urinary tract infections and interstitial nephritis	0 (0–0)	0 (0–0)	0 (0–1)	0 (0–0)	0 (0–0)	0 (0–0)	0 (0–0)	0 (0–0)	1 (0–1)	1 (0–1)	1 (1–2)	2 (1–2)	3 (2–4)	6 (4–8)	11 (7–15)	19 (13–25)	28 (20–39)	55 (38–74)	99 (64–136)	143 (93–200)	195 (118–274)	152 (83–216)	--
Urolithiasis	--	--	--	0 (0–0)	0 (0–0)	0 (0–0)	0 (0–0)	0 (0–0)	0 (0–0)	0 (0–0)	0 (0–0)	0 (0–0)	0 (0–1)	1 (0–1)	1 (1–2)	2 (1–3)	3 (1–5)	3 (2–6)	5 (2–9)	7 (4–13)	7 (3–13)	5 (2–11)	--
Other urinary diseases	0 (0–1)	0 (0–0)	0 (0–0)	0 (0–0)	0 (0–0)	0 (0–0)	0 (0–0)	0 (0–0)	0 (0–0)	0 (0–0)	0 (0–1)	0 (0–1)	1 (0–1)	2 (1–3)	3 (1–4)	4 (2–6)	5 (3–9)	7 (4–13)	9 (4–17)	12 (6–21)	15 (7–31)	11 (5–23)	--
Gynecological diseases	--	--	--	--	--	--	--	--	--	--	--	--	--	--	--	--	--	--	--	--	--	--	--
Uterine fibroids	--	--	--	--	--	--	--	--	--	--	--	--	--	--	--	--	--	--	--	--	--	--	--

Endometriosis	--	--	--	--	--	--	--	--	--	--	--	--	--	--	--	--	--	--	--	--	--	--	--
Genital prolapse	--	--	--	--	--	--	--	--	--	--	--	--	--	--	--	--	--	--	--	--	--	--	--
Other gynecological diseases	--	--	--	--	--	--	--	--	--	--	--	--	--	--	--	--	--	--	--	--	--	--	--
Hemoglobinopathies and hemolytic anaemias	0 (0–0)	0 (0–0)	0 (0–0)	1 (1–1)	0 (0–1)	0 (0–1)	1 (0–1)	1 (1–1)	1 (1–2)	1 (1–1)	1 (1–1)	1 (1–1)	1 (1–2)	2 (1–2)	3 (2–4)	4 (3–5)	7 (5–10)	9 (7–12)	11 (8–13)	16 (12–19)	15 (11–19)	10 (7–12)	--
Thalassemias	0 (0–0)	0 (0–0)	0 (0–0)	0 (0–0)	0 (0–0)	0 (0–0)	0 (0–0)	0 (0–0)	0 (0–0)	0 (0–0)	0 (0–0)	0 (0–0)	0 (0–0)	0 (0–0)	0 (0–0)	0 (0–0)	0 (0–0)	0 (0–0)	0 (0–0)	0 (0–0)	0 (0–0)	0 (0–0)	--
Sickle cell disorders	0 (0–0)	0 (0–0)	0 (0–0)	0 (0–0)	0 (0–0)	0 (0–0)	0 (0–0)	0 (0–0)	0 (0–0)	0 (0–0)	0 (0–0)	0 (0–0)	0 (0–0)	0 (0–0)	0 (0–0)	0 (0–0)	0 (0–0)	0 (0–0)	0 (0–0)	0 (0–0)	0 (0–0)	0 (0–0)	--
G6PD deficiency	0 (0–0)	0 (0–0)	0 (0–0)	0 (0–1)	0 (0–0)	0 (0–1)	0 (0–1)	1 (0–1)	1 (1–1)	1 (1–1)	1 (1–1)	1 (1–1)	1 (1–1)	1 (1–1)	2 (1–2)	2 (1–3)	2 (1–3)	2 (1–3)	1 (1–2)	1 (0–2)	0 (0–1)	0 (0–0)	--
Other hemoglobinopathies and hemolytic anaemias	0 (0–0)	0 (0–0)	0 (0–0)	0 (0–0)	0 (0–0)	0 (0–0)	0 (0–0)	0 (0–0)	0 (0–0)	0 (0–0)	0 (0–0)	0 (0–0)	0 (0–0)	1 (0–1)	1 (1–2)	2 (1–3)	5 (3–7)	7 (5–10)	9 (7–12)	14 (11–18)	14 (11–18)	9 (7–12)	--
Endocrine, metabolic, blood, and immune disorders	3 (2–5)	2 (1–3)	13 (8–20)	11 (7–16)	6 (4–9)	7 (4–11)	7 (4–13)	10 (6–20)	15 (9–28)	17 (11–35)	22 (14–44)	29 (19–57)	40 (25–78)	63 (43–118)	100 (72–167)	119 (84–197)	153 (104–229)	163 (112–243)	158 (102–235)	148 (99–214)	131 (82–186)	80 (44–113)	--
Sudden infant death syndrome	--	2 (1–3)	26 (16–38)	--	--	--	--	--	--	--	--	--	--	--	--	--	--	--	--	--	--	--	--
Transport injuries	0 (0–0)	0 (0–0)	3 (2–4)	15 (10–20)	17 (12–22)	24 (18–30)	135 (110–164)	215 (177–256)	188 (150–230)	147 (116–183)	121 (95–152)	117 (93–146)	126 (99–159)	134 (107–168)	157 (123–195)	138 (110–170)	116 (90–147)	111 (87–141)	100 (76–128)	83 (65–106)	58 (43–75)	27 (21–35)	--
Road injuries	0 (0–0)	0 (0–0)	3 (2–4)	13 (9–18)	14 (10–18)	18 (13–24)	117 (91–143)	183 (148–219)	157 (123–194)	118 (92–149)	95 (71–121)	89 (68–113)	99 (76–127)	106 (83–136)	125 (96–157)	112 (86–141)	94 (72–122)	93 (72–119)	86 (65–111)	76 (60–98)	54 (40–71)	26 (19–33)	--
Pedestrian road injuries	0 (0–0)	0 (0–0)	1 (0–1)	7 (5–10)	5 (4–8)	5 (3–6)	18 (13–22)	26 (20–32)	22 (17–29)	19 (14–24)	16 (12–22)	16 (12–20)	19 (15–25)	22 (17–28)	26 (20–34)	28 (21–35)	25 (19–32)	25 (19–34)	31 (23–40)	30 (23–38)	22 (16–29)	11 (8–14)	--
Cyclist road injuries	--	--	--	1 (0–1)	3 (1–4)	4 (2–6)	5 (3–9)	4 (2–7)	4 (3–7)	4 (2–6)	4 (2–6)	4 (2–7)	5 (3–9)	6 (4–10)	8 (5–13)	8 (5–13)	7 (4–11)	7 (3–11)	4 (2–8)	3 (1–5)	1 (1–2)	1 (0–1)	--
Motorcyclist road injuries	0 (0–0)	0 (0–0)	0 (0–0)	0 (0–0)	0 (0–1)	2 (1–3)	9 (5–14)	23 (15–35)	22 (14–33)	19 (12–28)	14 (9–21)	13 (8–19)	14 (9–20)	15 (9–23)	17 (10–25)	11 (6–18)	8 (4–12)	4 (2–6)	2 (1–3)	1 (1–2)	1 (0–1)	0 (0–1)	--
Motor vehicle road injuries	0 (0–0)	0 (0–0)	2 (1–3)	5 (3–8)	5 (3–8)	7 (5–10)	81 (63–100)	122 (96–149)	101 (79–127)	73 (56–92)	57 (42–75)	52 (40–67)	56 (43–72)	58 (45–75)	69 (53–89)	61 (47–77)	52 (39–68)	56 (43–72)	47 (35–62)	41 (32–52)	29 (21–38)	14 (10–18)	--
Other road injuries	0 (0–0)	0 (0–0)	0 (0–0)	0 (0–0)	0 (0–1)	1 (0–1)	4 (2–8)	8 (3–15)	7 (3–13)	4 (2–8)	4 (2–7)	4 (2–7)	4 (2–7)	5 (2–8)	4 (2–7)	3 (2–6)	3 (1–5)	2 (1–4)	2 (1–4)	2 (1–4)	1 (0–1)	0 (0–0)	--
Other transport injuries	0 (0–0)	0 (0–0)	0 (0–0)	1 (1–2)	3 (2–4)	6 (4–9)	19 (12–27)	32 (22–44)	31 (20–43)	29 (20–41)	26 (18–37)	28 (20–38)	27 (19–38)	28 (20–39)	32 (22–45)	26 (18–37)	22 (15–30)	18 (12–25)	13 (9–20)	7 (4–10)	3 (2–5)	1 (1–2)	--
Falls	0 (0–0)	0 (0–0)	1 (1–2)	3 (2–4)	1 (1–2)	2 (2–3)	8 (6–11)	16 (12–21)	17 (12–21)	17 (13–21)	20 (15–25)	25 (20–31)	35 (28–43)	56 (44–69)	93 (72–117)	119 (94–146)	156 (124–192)	238 (185–304)	341 (263–429)	531 (412–670)	684 (512–864)	518 (390–650)	--
Drowning	0 (0–0)	0 (0–0)	1 (1–2)	12 (9–15)	6 (4–7)	6 (4–8)	13 (10–18)	20 (15–28)	19 (14–24)	15 (12–20)	12 (9–15)	13 (10–16)	13 (10–16)	15 (12–18)	16 (13–20)	15 (12–18)	14 (11–17)	12 (10–15)	9 (7–10)	6 (5–7)	4 (3–5)	2 (2–2)	--
Fire, heat, and hot substances	0 (0–0)	0 (0–0)	1 (1–1)	5 (4–7)	3 (2–3)	2 (2–3)	3 (2–4)	5 (4–7)	7 (5–9)	7 (5–10)	7 (5–9)	7 (5–9)	8 (7–11)	10 (8–12)	12 (10–15)	12 (9–16)	12 (10–15)	12 (10–16)	12 (10–15)	13 (11–16)	13 (10–16)	8 (7–10)	--
Poisonings	0 (0–0)	0 (0–0)	0 (0–0)	1 (0–1)	0 (0–0)	1 (1–2)	2 (1–3)	5 (4–7)	8 (5–10)	7 (5–10)	8 (5–11)	8 (6–10)	8 (6–10)	8 (6–11)	9 (7–12)	8 (6–10)	5 (4–7)	4 (3–5)	3 (2–4)	2 (2–3)	2 (1–2)	1 (1–1)	--
Poisoning by carbon monoxide	0 (0–0)	0 (0–0)	0 (0–0)	0 (0–1)	0 (0–0)	1 (0–1)	2 (1–3)	4 (3–6)	6 (4–9)	6 (4–9)	6 (4–9)	6 (4–9)	6 (4–9)	6 (4–9)	7 (5–10)	6 (4–9)	4 (3–6)	3 (2–4)	2 (1–3)	2 (1–2)	1 (1–2)	1 (0–1)	--
Poisoning by other means	0 (0–0)	0 (0–0)	0 (0–0)	0 (0–0)	0 (0–0)	0 (0–1)	0 (0–0)	1 (0–1)	1 (1–2)	1 (1–1)	1 (1–2)	1 (1–2)	1 (1–2)	2 (1–3)	2 (1–3)	2 (1–2)	1 (1–1)	1 (1–1)	1 (0–1)	1 (0–1)	1 (0–1)	0 (0–0)	--
Exposure to mechanical forces	0 (0–0)	0 (0–0)	0 (0–0)	2 (2–3)	2 (1–2)	4 (2–5)	6 (4–8)	10 (7–13)	12 (8–16)	13 (9–17)	13 (10–17)	12 (9–17)	15 (12–20)	18 (14–23)	21 (17–27)	21 (17–25)	18 (14–22)	14 (11–17)	11 (9–14)	9 (7–11)	7 (5–9)	4 (3–5)	--
Unintentional firearm injuries	0 (0–0)	0 (0–0)	0 (0–0)	0 (0–0)	0 (0–0)	1 (1–2)	1 (1–3)	2 (1–3)	1 (1–2)	1 (1–2)	1 (1–2)	1 (0–1)	1 (0–2)	1 (1–2)	1 (1–2)	1 (1–2)	1 (1–1)	1 (0–1)	0 (0–1)	0 (0–1)	0 (0–0)	0 (0–0)	--
Other exposure to mechanical forces	0 (0–0)	0 (0–0)	0 (0–0)	2 (2–3)	1 (1–2)	2 (2–4)	4 (3–6)	9 (6–11)	10 (7–14)	12 (8–15)	12 (9–16)	12 (8–16)	14 (11–19)	16 (13–22)	20 (16–25)	20 (16–24)	17 (13–21)	13 (10–17)	11 (9–13)	9 (7–11)	7 (5–8)	4 (3–5)	--
Adverse effects of medical treatment	0 (0–1)	0 (0–0)	2 (1–3)	1 (1–2)	0 (0–1)	0 (0–1)	0 (0–1)	1 (1–1)	1 (1–2)	1 (1–2)	2 (1–3)	2 (1–3)	3 (2–5)	5 (4–8)	8 (6–13)	13 (9–19)	18 (13–25)	24 (17–34)	27 (19–36)	29 (21–39)	25 (19–33)	14 (10–19)	--
Animal contact	0 (0–0)	0 (0–0)	0 (0–0)	0 (0–0)	0 (0–0)	0 (0–0)	0 (0–0)	0 (0–0)	0 (0–1)	0 (0–1)	0 (0–0)	1 (0–1)	1 (0–1)	1 (1–2)	1 (1–2)	2 (1–2)	1 (1–2)	1 (1–2)	1 (1–1)	1 (0–1)	0 (0–1)	0 (0–0)	--
Venomous animal contact	0 (0–0)	0 (0–0)	0 (0–0)	0 (0–0)	0 (0–0)	0 (0–0)	0 (0–0)	0 (0–0)	0 (0–0)	0 (0–0)	0 (0–0)	0 (0–1)	0 (0–1)	1 (0–1)	1 (0–1)	1 (0–1)	1 (0–1)	0 (0–1)	0 (0–0)	0 (0–0)	0 (0–0)	0 (0–0)	--
Non-venomous animal contact	0 (0–0)	0 (0–0)	0 (0–0)	0 (0–0)	0 (0–0)	0 (0–0)	0 (0–0)	0 (0–0)	0 (0–0)	0 (0–0)	0 (0–0)	0 (0–0)	0 (0–1)	1 (0–1)	1 (0–1)	1 (1–1)	1 (1–1)	1 (1–1)	1 (0–1)	1 (0–1)	0 (0–1)	0 (0–0)	--
Foreign body	1 (0–1)	1 (1–1)	14 (9–20)	5 (4–7)	2 (1–2)	4 (3–5)	3 (2–4)	5 (4–6)	7 (5–8)	6 (5–8)	7 (5–9)	8 (6–9)	9 (7–11)	12 (10–16)	17 (14–21)	19 (15–25)	22 (17–28)	26 (20–32)	28 (23–34)	33 (26–42)	41 (32–52)	31 (23–38)	--
Pulmonary aspiration and foreign body in airway	1 (0–1)	1 (1–1)	14 (9–20)	5 (4–7)	2 (1–2)	4 (3–5)	3 (2–4)	5 (4–6)	7 (5–8)	6 (5–8)	7 (5–9)	8 (6–9)	9 (7–11)	12 (10–15)	17 (14–20)	19 (15–24)	21 (17–27)	25 (20–32)	28 (23–34)	33 (26–41)	41 (31–52)	30 (23–38)	--
Foreign body in other body part	0 (0–0)	0 (0–0)	0 (0–0)	0 (0–0)	0 (0–0)	0 (0–0)	0 (0–0)	0 (0–0)	0 (0–0)	0 (0–0)	0 (0–0)	0 (0–0)	0 (0–0)	0 (0–0)	0 (0–0)	0 (0–0)	0 (0–0)	0 (0–1)	0 (0–1)	1 (0–1)	0 (0–1)	0 (0–0)	--
Environmental heat and cold exposure	0 (0–0)	0 (0–0)	0 (0–0)	0 (0–0)	0 (0–0)	0 (0–0)	2 (1–3)	3 (2–5)	4 (3–5)	3 (2–5)	3 (2–5)	4 (3–6)	5 (4–7)	6 (4–9)	8 (6–11)	7 (5–10)	7 (5–10)	7 (5–10)	7 (5–10)	7 (5–10)	7 (5–9)	4 (3–6)	--
Other unintentional injuries	0 (0–0)	0 (0–0)	0 (0–0)	0 (0–0)	0 (0–0)	1 (1–1)	1 (1–2)	3 (2–4)	4 (3–6)	4 (3–6)	4 (3–5)	3 (3–4)	3 (3–5)	3 (2–4)	4 (3–5)	3 (2–4)	2 (2–3)	2 (1–3)	2 (1–3)	2 (1–3)	2 (1–3)	1 (1–2)	--
Self-harm	--	--	--	--	--	19 (13–26)	138 (115–163)	240 (197–282)	285 (240–335)	280 (229–333)	282 (228–331)	292 (236–347)	342 (280–413)	346 (282–421)	370 (291–460)	283 (226–350)	193 (156–238)	149 (120–181)	111 (87–138)	83 (65–104)	51 (40–65)	23 (17–28)	--
Self-harm by firearm	--	--	--	--	--	3 (2–5)	23 (15–35)	34 (21–52)	41 (27–59)	37 (24–53)	39 (24–56)	43 (29–63)	48 (32–77)	61 (42–95)	75 (50–111)	74 (50–111)	52 (36–73)	51 (37–70)	35 (24–50)	25 (17–36)	12 (7–17)	5 (3–7)	--
Self-harm by other specified means	--	--	--	--	--	16 (11–22)	115 (93–140)	206 (166–246)	244 (201–290)	243 (196–292)	243 (197–286)	249 (199–299)	293 (238–359)	285 (227–346)	294 (228–374)	209 (161–264)	141 (110–179)	98 (75–124)	75 (58–98)	57 (43–74)	40 (30–51)	18 (13–22)	--
Interpersonal violence	1 (0–1)	0 (0–0)	3 (2–5)	5 (3–7)	2 (1–3)	4 (3–6)	26 (19–35)	56 (42–71)	59 (43–77)	44 (33–57)	38 (29–49)	30 (23–39)	28 (21–36)	26 (19–34)	28 (20–37)	20 (15–26)	14 (11–19)	11 (8–14)	7 (5–10)	3 (2–4)	2 (2–3)	1 (1–2)	--
Physical violence by firearm	0 (0–0)	0 (0–0)	0 (0–0)	0 (0–1)	0 (0–1)	1 (1–2)	11 (8–15)	28 (21–36)	28 (20–37)	19 (14–24)	14 (10–18)	10 (7–13)	8 (6–10)	6 (4–7)	6 (4–8)	4 (3–5)	2 (2–3)	2 (1–3)	1 (1–2)	0 (0–1)	0 (0–0)	0 (0–0)	--
Physical violence by sharp object	0 (0–0)	0 (0–0)	0 (0–0)	0 (0–1)	0 (0–1)	1 (0–1)	11 (8–15)	19 (14–24)	21 (15–27)	15 (12–20)	14 (10–18)	10 (8–14)	9 (7–12)	8 (6–11)	8 (6–11)	6 (4–8)	5 (3–6)	3 (2–5)	2 (1–2)	1 (1–1)	1 (0–1)	0 (0–0)	--
Physical violence by other means	1 (0–1)	0 (0–0)	3 (2–4)	4 (3–6)	1 (1–2)	2 (2–3)	4 (3–6)	9 (6–11)	10 (7–14)	10 (8–13)	11 (8–14)	10 (8–13)	11 (9–15)	12 (9–15)	13 (10–18)	10 (8–14)	7 (6–10)	5 (4–7)	4 (3–6)	2 (1–3)	1 (1–2)	1 (0–1)	--
Executions and police conflict	0 (0–0)	0 (0–0)	0 (0–0)	0 (0–0)	0 (0–0)	0 (0–0)	1 (0–2)	2 (1–6)	3 (1–7)	3 (1–7)	2 (1–7)	2 (0–5)	2 (0–4)	1 (0–2)	0 (0–1)	0 (0–1)	0 (0–0)	0 (0–0)	0 (0–0)	0 (0–0)	0 (0–0)	0 (0–0)	--

SR Table 12: USA deaths by cause and age group for males

Cause	Early Neonatal	Late Neonatal	Post Neonatal	1 to 4	5 to 9	10 to 14	15 to 19	20 to 24	25 to 29	30 to 34	35 to 39	40 to 44	45 to 49	50 to 54	55 to 59	60 to 64	65 to 69	70 to 74	75 to 79	80 to 84	85 to 89	90 to 94	95 plus
All causes	6,273 (5,718–6,879)	1,501 (1,370–1,649)	3,994 (3,692–4,324)	2,077 (1,922–2,245)	1,261 (1,232–1,292)	1,806 (1,777–1,835)	7,630 (7,532–7,731)	14,982 (14,821–15,151)	17,865 (17,713–18,027)	19,162 (18,992–19,340)	21,903 (21,665–22,143)	25,938 (25,612–26,276)	39,295 (38,739–39,877)	62,058 (61,178–62,980)	99,274 (97,827–100,799)	132,371 (130,580–134,258)	155,413 (153,414–157,537)	182,274 (180,032–184,653)	187,590 (185,450–189,847)	186,085 (184,200–188,058)	172,476 (171,115–173,894)	119,746 (118,917–120,610)	--
Sexually transmitted infections excluding HIV	1 (1–1)	0 (0–0)	0 (0–0)	0 (0–0)	0 (0–0)	0 (0–0)	0 (0–0)	0 (0–0)	0 (0–1)	1 (1–1)	1 (1–1)	1 (1–1)	1 (1–2)	2 (1–2)	3 (2–3)	3 (2–3)	4 (3–5)	4 (3–5)	5 (4–6)	3 (3–5)	4 (3–5)	2 (1–3)	--
Syphilis	1 (1–1)	0 (0–0)	0 (0–0)	0 (0–0)	0 (0–0)	0 (0–0)	0 (0–0)	0 (0–0)	0 (0–0)	1 (0–1)	1 (0–1)	1 (0–1)	1 (1–2)	2 (1–2)	3 (2–3)	3 (2–3)	4 (3–5)	4 (3–5)	5 (4–6)	3 (3–5)	4 (3–5)	2 (1–3)	--
Chlamydial infection	--	--	--	--	--	0 (0–0)	0 (0–0)	0 (0–0)	0 (0–0)	0 (0–0)	0 (0–0)	0 (0–0)	0 (0–0)	0 (0–0)	0 (0–0)	0 (0–0)	0 (0–0)	0 (0–0)	0 (0–0)	0 (0–0)	0 (0–0)	0 (0–0)	--
Gonococcal infection	--	--	--	--	--	0 (0–0)	0 (0–0)	0 (0–0)	0 (0–0)	0 (0–0)	0 (0–0)	0 (0–0)	0 (0–0)	0 (0–0)	0 (0–0)	0 (0–0)	0 (0–0)	0 (0–0)	0 (0–0)	0 (0–0)	0 (0–0)	0 (0–0)	--
Other sexually transmitted infections	--	--	--	--	--	0 (0–0)	0 (0–0)	0 (0–0)	0 (0–0)	0 (0–0)	0 (0–0)	0 (0–0)	0 (0–0)	0 (0–0)	0 (0–0)	0 (0–0)	0 (0–0)	0 (0–0)	0 (0–0)	0 (0–0)	0 (0–0)	0 (0–0)	--
Tuberculosis	--	--	0 (0–1)	1 (1–1)	0 (0–0)	0 (0–0)	1 (1–1)	2 (2–2)	5 (4–5)	6 (6–7)	10 (9–12)	13 (12–14)	22 (20–24)	36 (32–39)	51 (46–56)	64 (58–70)	67 (61–73)	74 (68–79)	73 (67–80)	69 (61–75)	59 (51–65)	32 (26–35)	--
Drug-susceptible tuberculosis	--	--	0 (0–1)	1 (1–1)	0 (0–0)	0 (0–0)	1 (0–1)	2 (2–2)	4 (4–5)	6 (5–7)	10 (9–11)	13 (11–14)	21 (19–24)	35 (31–38)	49 (44–54)	62 (55–68)	65 (58–71)	71 (65–77)	71 (64–77)	66 (58–72)	57 (48–64)	31 (25–34)	--
Multidrug-resistant tuberculosis without extensive drug resistance	--	--	0 (0–0)	0 (0–0)	0 (0–0)	0 (0–0)	0 (0–0)	0 (0–0)	0 (0–0)	0 (0–0)	0 (0–1)	0 (0–1)	1 (0–1)	1 (0–2)	1 (0–3)	2 (1–4)	2 (1–5)	2 (1–5)	2 (1–5)	2 (1–5)	2 (0–4)	1 (0–2)	--
Extensively drug-resistant tuberculosis	--	--	0 (0–0)	0 (0–0)	0 (0–0)	0 (0–0)	0 (0–0)	0 (0–0)	0 (0–0)	0 (0–0)	0 (0–0)	0 (0–0)	0 (0–0)	0 (0–1)	0 (0–1)	0 (0–1)	0 (0–1)	1 (0–1)	1 (0–1)	1 (0–1)	0 (0–1)	0 (0–1)	--
Lower respiratory infections	14 (12–16)	24 (21–27)	196 (171–223)	76 (67–86)	29 (27–31)	27 (25–30)	40 (37–43)	75 (68–82)	109 (97–121)	154 (136–169)	216 (200–233)	315 (290–340)	498 (470–526)	852 (797–912)	1,431 (1,343–1,532)	2,089 (1,966–2,224)	2,713 (2,557–2,886)	3,740 (3,478–4,004)	4,770 (4,437–5,145)	5,826 (5,263–6,297)	6,598 (5,742–7,147)	5,601 (4,703–6,171)	--
Upper respiratory infections	0 (0–0)	0 (0–0)	5 (4–6)	5 (4–6)	1 (1–1)	1 (1–1)	1 (1–1)	1 (1–1)	1 (1–1)	2 (2–2)	2 (2–3)	2 (2–3)	3 (2–3)	3 (3–3)	3 (3–4)	3 (3–3)	3 (3–3)	3 (2–3)	3 (3–4)	3 (3–3)	3 (2–3)	2 (2–2)	--
Otitis media	0 (0–0)	0 (0–0)	1 (1–2)	1 (0–1)	0 (0–0)	0 (0–0)	0 (0–0)	0 (0–0)	0 (0–0)	0 (0–0)	1 (0–1)	0 (0–1)	1 (1–1)	0 (0–1)	1 (1–1)	1 (1–1)	1 (1–1)	2 (1–2)	1 (1–1)	1 (1–2)	1 (1–1)	1 (0–1)	--
Diarrhoeal diseases	3 (3–4)	3 (3–4)	20 (17–23)	7 (6–9)	3 (3–3)	3 (2–3)	3 (3–3)	4 (4–5)	6 (5–7)	9 (8–11)	13 (11–15)	21 (18–23)	36 (32–40)	79 (70–89)	159 (143–176)	256 (232–283)	383 (345–426)	565 (507–634)	772 (693–862)	834 (719–932)	834 (699–948)	591 (478–674)	--
Typhoid fever	--	--	0 (0–0)	0 (0–0)	0 (0–0)	0 (0–0)	0 (0–0)	0 (0–0)	0 (0–0)	0 (0–0)	0 (0–0)	0 (0–0)	0 (0–0)	0 (0–0)	0 (0–0)	0 (0–0)	0 (0–0)	0 (0–0)	0 (0–0)	0 (0–0)	0 (0–0)	0 (0–0)	--
Paratyphoid fever	--	--	0 (0–0)	0 (0–0)	0 (0–0)	0 (0–0)	0 (0–0)	0 (0–0)	0 (0–0)	0 (0–0)	0 (0–0)	0 (0–0)	0 (0–0)	0 (0–0)	0 (0–0)	0 (0–0)	0 (0–0)	0 (0–0)	0 (0–0)	0 (0–0)	0 (0–0)	0 (0–0)	--
iNTS	--	0 (0–1)	1 (0–2)	1 (0–2)	0 (0–1)	0 (0–1)	0 (0–0)	0 (0–0)	0 (0–1)	1 (0–1)	0 (0–1)	1 (0–1)	1 (0–2)	1 (1–3)	1 (1–3)	2 (1–4)	2 (1–6)	3 (2–6)	3 (2–7)	3 (2–6)	3 (2–6)	2 (1–4)	--
Other intestinal infectious diseases	--	--	0 (0–2)	0 (0–2)	0 (0–1)	0 (0–1)	0 (0–1)	0 (0–1)	0 (0–1)	0 (0–2)	0 (0–1)	0 (0–1)	0 (0–1)	0 (0–1)	0 (0–1)	0 (0–1)	1 (0–2)	1 (0–2)	0 (0–2)	1 (0–2)	1 (0–3)	1 (0–2)	--
Malaria	0 (0–0)	0 (0–0)	0 (0–0)	0 (0–0)	0 (0–0)	0 (0–0)	0 (0–0)	0 (0–0)	0 (0–0)	0 (0–0)	0 (0–0)	0 (0–0)	0 (0–0)	0 (0–0)	0 (0–0)	0 (0–0)	0 (0–0)	0 (0–0)	0 (0–0)	0 (0–0)	0 (0–0)	0 (0–0)	--
Chagas disease	--	--	0 (0–0)	0 (0–0)	0 (0–0)	0 (0–0)	0 (0–0)	0 (0–0)	0 (0–0)	0 (0–0)	0 (0–0)	0 (0–0)	0 (0–0)	0 (0–0)	0 (0–0)	0 (0–0)	0 (0–0)	0 (0–0)	0 (0–0)	0 (0–0)	0 (0–0)	0 (0–0)	--
Visceral leishmaniasis	--	--	0 (0–0)	0 (0–0)	0 (0–0)	0 (0–0)	0 (0–0)	0 (0–0)	0 (0–0)	0 (0–0)	0 (0–0)	0 (0–0)	0 (0–0)	0 (0–0)	0 (0–0)	0 (0–0)	0 (0–0)	0 (0–0)	0 (0–0)	0 (0–0)	0 (0–0)	0 (0–0)	--
African trypanosomiasis	--	--	--	0 (0–0)	0 (0–0)	0 (0–0)	0 (0–0)	0 (0–0)	0 (0–0)	0 (0–0)	0 (0–0)	0 (0–0)	0 (0–0)	0 (0–0)	0 (0–0)	0 (0–0)	0 (0–0)	0 (0–0)	0 (0–0)	0 (0–0)	0 (0–0)	0 (0–0)	--
Schistosomiasis	--	--	0 (0–0)	0 (0–0)	0 (0–0)	0 (0–0)	0 (0–0)	0 (0–0)	0 (0–0)	0 (0–0)	0 (0–0)	0 (0–0)	0 (0–0)	0 (0–0)	0 (0–0)	0 (0–0)	0 (0–0)	0 (0–0)	0 (0–0)	0 (0–0)	0 (0–0)	0 (0–0)	--
Cysticercosis	--	--	--	0 (0–0)	0 (0–0)	0 (0–0)	0 (0–0)	0 (0–0)	0 (0–0)	0 (0–0)	0 (0–0)	0 (0–0)	0 (0–0)	0 (0–0)	0 (0–0)	0 (0–0)	0 (0–0)	0 (0–0)	0 (0–0)	0 (0–0)	0 (0–0)	0 (0–0)	--
Cystic echinococcosis	--	--	--	0 (0–0)	0 (0–0)	0 (0–0)	0 (0–0)	0 (0–0)	0 (0–0)	0 (0–0)	0 (0–0)	0 (0–0)	0 (0–0)	0 (0–1)	0 (0–1)	0 (0–1)	0 (0–1)	0 (0–1)	0 (0–1)	0 (0–0)	0 (0–0)	0 (0–0)	--
Dengue	0 (0–0)	0 (0–0)	0 (0–0)	0 (0–0)	0 (0–0)	0 (0–0)	0 (0–0)	0 (0–0)	0 (0–0)	0 (0–0)	0 (0–0)	0 (0–0)	0 (0–0)	0 (0–0)	0 (0–0)	0 (0–0)	0 (0–0)	0 (0–0)	0 (0–0)	0 (0–0)	0 (0–0)	0 (0–0)	--
Yellow fever	--	0 (0–0)	0 (0–0)	0 (0–0)	0 (0–0)	0 (0–0)	0 (0–0)	0 (0–0)	0 (0–0)	0 (0–0)	0 (0–0)	0 (0–0)	0 (0–0)	0 (0–0)	0 (0–0)	0 (0–0)	0 (0–0)	0 (0–0)	0 (0–0)	0 (0–0)	0 (0–0)	0 (0–0)	--
Rabies	--	--	0 (0–0)	0 (0–0)	0 (0–0)	0 (0–0)	0 (0–0)	0 (0–0)	0 (0–0)	0 (0–0)	0 (0–0)	0 (0–0)	0 (0–0)	0 (0–0)	0 (0–0)	0 (0–0)	0 (0–0)	0 (0–0)	0 (0–0)	0 (0–0)	0 (0–0)	0 (0–0)	--
Ascariasis	--	--	0 (0–0)	0 (0–0)	0 (0–0)	0 (0–0)	0 (0–0)	0 (0–0)	0 (0–0)	0 (0–0)	0 (0–0)	0 (0–0)	0 (0–0)	0 (0–0)	0 (0–0)	0 (0–0)	0 (0–0)	0 (0–0)	0 (0–0)	0 (0–0)	0 (0–0)	0 (0–0)	--
Zika virus disease	0 (0–0)	0 (0–0)	0 (0–0)	0 (0–0)	0 (0–0)	0 (0–0)	0 (0–0)	0 (0–0)	0 (0–0)	0 (0–0)	0 (0–0)	0 (0–0)	0 (0–0)	0 (0–0)	0 (0–0)	0 (0–0)	0 (0–0)	0 (0–0)	0 (0–0)	0 (0–0)	0 (0–0)	0 (0–0)	--
Other neglected tropical diseases	0 (0–0)	1 (0–1)	2 (0–3)	2 (1–3)	2 (0–2)	2 (0–2)	1 (0–1)	1 (0–1)	1 (0–1)	1 (0–2)	2 (0–2)	2 (0–3)	3 (1–4)	3 (1–5)	6 (1–8)	8 (2–11)	13 (3–17)	18 (4–24)	18 (3–24)	16 (3–21)	9 (2–13)	6 (1–8)	--
Meningitis	3 (2–3)	8 (7–9)	35 (30–40)	20 (18–23)	9 (9–10)	9 (8–9)	13 (11–14)	17 (14–19)	20 (16–23)	23 (18–26)	28 (23–31)	31 (28–34)	42 (38–45)	39 (36–42)	45 (41–48)	45 (41–49)	44 (40–47)	43 (39–46)	41 (37–44)	37 (33–41)	31 (26–34)	17 (14–19)	--
Encephalitis	1 (1–1)	2 (2–2)	10 (8–12)	13 (11–16)	8 (7–9)	7 (6–8)	6 (6–7)	10 (8–11)	10 (8–12)	10 (9–12)	11 (9–12)	10 (9–11)	14 (12–15)	17 (15–19)	24 (21–26)	29 (26–32)	36 (31–39)	39 (33–43)	40 (33–44)	37 (29–43)	26 (17–31)	13 (9–15)	--
Diphtheria	--	--	0 (0–0)	0 (0–0)	0 (0–0)	0 (0–0)	0 (0–0)	0 (0–0)	0 (0–0)	0 (0–0)	0 (0–0)	0 (0–0)	0 (0–0)	0 (0–0)	0 (0–0)	--	--	--	--	--	--	--	--
Whooping cough	--	--	4 (1–6)	0 (0–0)	0 (0–0)	0 (0–0)	0 (0–0)	0 (0–0)	0 (0–0)	0 (0–0)	0 (0–0)	0 (0–0)	0 (0–0)	0 (0–0)	0 (0–0)	--	--	--	--	--	--	--	--
Tetanus	0 (0–0)	0 (0–0)	0 (0–0)	0 (0–0)	0 (0–0)	0 (0–0)	0 (0–0)	0 (0–0)	0 (0–0)	0 (0–0)	0 (0–0)	0 (0–0)	0 (0–1)	0 (0–1)	0 (0–1)	0 (0–1)	0 (0–1)	0 (0–1)	0 (0–1)	0 (0–1)	0 (0–1)	0 (0–1)	--
Measles	0 (0–0)	0 (0–0)	0 (0–0)	0 (0–0)	0 (0–0)	0 (0–0)	0 (0–0)	0 (0–0)	0 (0–0)	0 (0–0)	0 (0–0)	0 (0–0)	0 (0–0)	0 (0–0)	0 (0–0)	0 (0–0)	0 (0–0)	0 (0–0)	0 (0–0)	0 (0–0)	0 (0–0)	0 (0–0)	--
Varicella and herpes zoster	0 (0–1)	1 (0–3)	2 (1–4)	2 (1–6)	2 (2–7)	1 (1–4)	1 (0–2)	1 (1–3)	1 (1–4)	1 (1–4)	2 (1–4)	1 (1–4)	2 (1–4)	2 (1–5)	3 (2–8)	5 (3–13)	7 (4–18)	10 (5–23)	12 (5–28)	17 (8–37)	22 (10–47)	17 (7–34)	--
Acute hepatitis	--	--	1 (1–1)	0 (0–0)	0 (0–0)	0 (0–0)	0 (0–0)	1 (1–1)	1 (1–1)	2 (1–2)	3 (2–3)	4 (4–4)	8 (7–8)	8 (8–9)	10 (9–11)	10 (9–11)	9 (8–10)	8 (7–8)	6 (5–6)	3 (3–4)	3 (2–3)	1 (1–2)	--
Acute hepatitis A	--	--	0 (0–0)	0 (0–0)	0 (0–0)	0 (0–0)	0 (0–0)	0 (0–1)	0 (0–1)	1 (0–1)	1 (1–2)	1 (1–2)	2 (1–4)	2 (1–4)	3 (2–5)	3 (2–5)	3 (2–5)	3 (2–5)	2 (1–4)	2 (1–2)	1 (1–2)	1 (1–1)	--
Acute hepatitis B	--	--	1 (0–1)	0 (0–0)	0 (0–0)	0 (0–0)	0 (0–0)	0 (0–1)	1 (0–1)	1 (0–1)	1 (1–2)	2 (1–2)	3 (2–4)	4 (2–5)	4 (2–6)	4 (2–6)	4 (2–5)	3 (2–5)	3 (1–4)	1 (1–2)	1 (0–1)	0 (0–0)	--
Acute hepatitis C	--	--	0 (0–0)	0 (0–0)	0 (0–0)	0 (0–0)	0 (0–0)	0 (0–0)	0 (0–0)	0 (0–0)	0 (0–1)	1 (0–2)	2 (1–4)	2 (1–4)	2 (1–5)	2 (1–4)	1 (1–3)	1 (0–3)	1 (0–2)	0 (0–1)	0 (0–1)	0 (0–0)	--
Acute hepatitis E	--	--	0 (0–0)	0 (0–0)	0 (0–0)	0 (0–0)	0 (0–0)	0 (0–0)	0 (0–0)	0 (0–0)	0 (0–0)	0 (0–1)	0 (0–1)	0 (0–0)	0 (0–1)	0 (0–1)	0 (0–1)	0 (0–1)	0 (0–0)	0 (0–0)	0 (0–0)	0 (0–0)	--
Other unspecified infectious diseases	9 (5–15)	31 (16–43)	65 (39–109)	19 (13–35)	11 (7–21)	10 (7–19)	14 (9–26)	17 (11–32)	22 (13–46)	28 (16–66)	46 (27–102)	51 (34–105)	78 (55–158)	116 (85–218)	191 (130–356)	264 (172–465)	337 (216–600)	334 (216–591)	274 (161–483)	186 (115–311)	124 (79–211)	62 (37–103)	--
Maternal disorders	--	--	--	--	--	--	--	--	--	--	--	--	--	--	--	--	--	--	--	--	--	--	--
Maternal haemorrhage	--	--	--	--	--	--	--	--	--	--	--	--	--	--	--	--	--	--	--	--	--	--	--
Maternal sepsis and other pregnancy related infections	--	--	--	--	--	--	--	--	--	--	--	--	--	--	--	--	--	--	--	--	--	--	--
Maternal hypertensive disorders	--	--	--	--	--	--	--	--	--	--	--	--	--	--	--	--	--	--	--	--	--	--	--
Maternal obstructed labour and uterine rupture	--	--	--	--	--	--	--	--	--	--	--	--	--	--	--	--	--	--	--	--	--	--	--
Maternal abortive outcome	--	--	--	--	--	--	--	--	--	--	--	--	--	--	--	--	--	--	--	--	--	--	--
Ectopic pregnancy	--	--	--	--	--	--	--	--	--	--	--	--	--	--	--	--	--	--	--	--	--	--	--
Indirect maternal deaths	--	--	--	--	--	--	--	--	--	--	--	--	--	--	--	--	--	--	--	--	--	--	--
Late maternal deaths	--	--	--	--	--	--	--	--	--	--	--	--	--	--	--	--	--	--	--	--	--	--	--
Maternal deaths aggravated by HIV/AIDS	--	--	--	--	--	--	--	--	--	--	--	--	--	--	--	--	--	--	--	--	--	--	--
Other maternal disorders	--	--	--	--	--	--	--	--	--	--	--	--	--	--	--	--	--	--	--	--	--	--	--
Neonatal disorders	4,802 (4,278–5,330)	829 (732–919)	477 (416–545)	50 (43–57)	--	--	--	--	--	--	--	--	--	--	--	--	--	--	--	--	--	--	--
Neonatal preterm birth	2,946 (2,632–3,278)	347 (303–389)	303 (261–349)	20 (17–24)	--	--	--	--	--	--	--	--	--	--	--	--	--	--	--	--	--	--	--
Neonatal encephalopathy due to birth asphyxia and trauma	707 (593–809)	98 (83–115)	51 (43–61)	19 (16–22)	--	--	--	--	--	--	--	--	--	--	--	--	--	--	--	--	--	--	--
Neonatal sepsis and other neonatal infections	160 (140–182)	178 (154–202)	47 (40–54)	0 (0–0)	--	--	--	--	--	--	--	--	--	--	--	--	--	--	--	--	--	--	--
Hemolytic disease and other neonatal jaundice	4 (3–5)	1 (1–2)	1 (1–1)	2 (1–2)	--	--	--	--	--	--	--	--	--	--	--	--	--	--	--	--	--	--	--
Other neonatal disorders	984 (861–1,122)	205 (179–236)	75 (63–90)	9 (8–11)	--	--	--	--	--	--	--	--	--	--	--	--	--	--	--	--	--	--	--
Nutritional deficiencies	0 (0–0)	0 (0–0)	6 (5–6)	2 (2–3)	1 (1–1)	1 (1–1)	1 (1–2)	2 (2–3)	4 (4–5)	6 (5–6)	7 (7–8)	10 (10–11)	21 (19–22)	36 (33–39)	73 (68–79)	114 (105–123)	157 (148–167)	216 (199–233)	268 (248–287)	333 (296–363)	409 (356–449)	374 (313–412)	--
Protein-energy malnutrition	0 (0–0)	0 (0–0)	5 (5–6)	1 (1–2)	1 (1–1)	1 (1–1)	1 (1–1)	2 (1–2)	3 (3–4)	5 (4–5)	6 (5–7)	9 (8–10)	19 (17–21)	33 (28–37)	69 (61–75)	106 (92–115)	147 (132–158)	203 (182–222)	251 (225–271)	313 (269–343)	383 (315–423)	351 (277–390)	--
Other nutritional deficiencies	--	--	1 (0–1)	1 (1–1)	1 (0–1)	1 (0–1)	1 (0–1)	1 (0–1)	1 (1–2)	1 (1–2)	1 (0–2)	1 (1–2)	1 (1–3)	2 (1–6)	4 (2–11)	8 (5–21)	10 (6–25)	12 (6–26)	16 (8–31)	20 (9–38)	26 (13–60)	23 (11–51)	--
Lip and oral cavity cancer	--	--	--	--	1 (1–1)	1 (1–1)	1 (1–2)	4 (3–4)	8 (7–9)	16 (14–18)	30 (27–33)	61 (56–68)	149 (135–163)	311 (290–336)	574 (535–615)	797 (747–850)	812 (760–867)	755 (703–806)	630 (580–674)	483 (434–525)	341 (296–375)	190 (157–213)	--
Nasopharynx cancer	--	--	--	--	1 (1–1)	1 (1–1)	3 (2–3)	5 (4–6)	5 (4–6)	6 (5–7)	13 (11–15)	24 (22–26)	45 (41–49)	81 (74–88)	135 (124–146)	124 (115–134)	118 (109–128)	105 (96–114)	56 (50–61)	37 (33–42)	23 (19–26)	9 (8–11)	--
Other pharynx cancer	--	--	--	--	--	--	--	1 (1–1)	1 (1–1)	3 (2–3)	9 (8–11)	27 (24–29)	86 (79–94)	197 (181–212)	367 (339–398)	433 (400–468)	420 (389–456)	353 (325–384)	231 (211–252)	141 (125–156)	82 (71–92)	37 (30–43)	--
Oesophageal cancer	--	--	--	--	--	--	--	4 (3–4)	10 (8–11)	25 (20–27)	64 (55–71)	168 (156–182)	404 (378–431)	906 (853–958)	1,799 (1,703–1,905)	2,583 (2,455–2,707)	2,970 (2,799–3,132)	2,897 (2,738–3,072)	2,303 (2,147–2,452)	1,636 (1,472–1,768)	1,050 (910–1,147)	475 (396–530)	--
Stomach cancer	--	--	--	--	--	--	2 (1–2)	7 (7–8)	21 (19–23)	49 (43–55)	96 (87–106)	162 (151–176)	288 (269–309)	542 (508–580)	923 (867–985)	1,243 (1,180–1,313)	1,432 (1,361–1,510)	1,651 (1,566–1,739)	1,632 (1,537–1,723)	1,382 (1,251–1,481)	1,102 (966–1,191)	593 (493–655)	--
Colon and rectum cancer	--	--	--	--	2 (2–2)	3 (2–3)	8 (7–9)	26 (24–28)	68 (60–75)	158 (142–175)	314 (290–340)	579 (541–625)	1,174 (1,107–1,247)	2,201 (2,093–2,318)	3,750 (3,571–3,948)	5,140 (4,933–5,382)	5,823 (5,579–6,072)	6,286 (5,974–6,588)	5,871 (5,537–6,159)	5,010 (4,503–5,378)	4,078 (3,571–4,396)	2,354 (1,953–2,598)	--
Liver cancer due to hepatitis B	--	--	--	--	--	2 (1–2)	3 (2–4)	5 (4–7)	11 (8–16)	19 (13–26)	31 (22–42)	52 (35–69)	92 (65–124)	183 (129–254)	340 (243–464)	398 (273–561)	299 (204–414)	232 (156–319)	177 (125–234)	116 (77–159)	63 (44–83)	26 (17–37)	--
Liver cancer due to hepatitis C	--	--	--	--	--	0 (0–0)	0 (0–0)	0 (0–1)	1 (1–2)	3 (2–4)	8 (5–12)	22 (15–31)	80 (54–111)	274 (197–358)	721 (544–929)	1,052 (783–1,360)	879 (673–1,100)	752 (562–936)	617 (463–785)	471 (355–596)	309 (234–388)	153 (111–194)	--
Liver cancer due to alcohol use	--	--	--	--	--	--	0 (0–0)	1 (1–2)	5 (3–7)	11 (8–16)	26 (18–37)	58 (41–77)	151 (107–199)	410 (298–534)	878 (664–1,130)	1,237 (922–1,587)	1,154 (881–1,424)	994 (755–1,250)	719 (552–890)	438 (333–554)	222 (168–278)	86 (60–113)	--
Liver cancer due to NASH	--	--	--	--	--	--	2 (1–2)	2 (1–2)	2 (1–3)	3 (2–4)	5 (3–6)	8 (6–12)	22 (15–31)	63 (44–87)	147 (103–202)	223 (151–307)	217 (148–301)	218 (151–295)	214 (153–282)	165 (117–222)	96 (68–127)	43 (29–59)	--
	0 (0–0)	0 (0–0)	1 (1–2)	7 (4–9)	5 (4–6)	4 (4–6)	--	--	--	--	--	--	--	--	--	--	--	--	--	--	--	--	--
Liver cancer due to other causes	0 (0–1)	0 (0–0)	2 (1–2)	4 (3–6)	3 (2–4)	3 (2–4)	4 (3–5)	4 (3–5)	5 (3–7)	6 (4–9)	9 (6–12)	15 (10–20)	33 (23–45)	81 (57–111)	159 (113–214)	198 (140–273)	159 (112–212)	133 (93–180)	110 (78–143)	76 (54–103)	43 (31–57)	18 (12–26)	--
Gallbladder and biliary tract cancer	--	--	--	--	--	--	--	1 (1–1)	1 (1–2)	3 (3–4)	8 (7–10)	16 (14–20)	35 (31–44)	74 (67–91)	144 (131–175)	234 (208–271)	286 (257–359)	362 (320–434)	358 (314–432)	286 (246–345)	221 (185–264)	108 (86–135)	--
Pancreatic cancer	--	--	--	--	--	--	2 (1–2)	4 (4–5)	11 (10–13)	37 (33–40)	102 (92–110)	232 (217–247)	584 (547–619)	1,327 (1,253–1,403)	2,603 (2,464–2,761)	3,896 (3,699–4,104)	4,694 (4,424–4,947)	4,983 (4,717–5,251)	4,244 (3,953–4,502)	3,229 (2,911–3,490)	2,159 (1,885–2,347)	985 (813–1,103)	--
Larynx cancer	--	--	--	--	--	--	--	1 (1–1)	1 (1–1)	2 (2–3)	8 (7–10)	26 (23–29)	80 (73–87)	210 (195–225)	429 (405–457)	595 (561–633)	668 (627–713)	648 (606–693)	511 (474–550)	377 (337–412)	235 (203–262)	119 (100–133)	--
Tracheal, bronchus, and lung cancer	--	--	--	--	--	2 (2–2)	5 (5–6)	12 (11–14)	27 (23–31)	75 (66–84)	191 (174–209)	497 (468–533)	1,522 (1,450–1,606)	4,100 (3,902–4,314)	9,005 (8,601–9,452)	14,187 (13,686–14,770)	18,458 (17,877–19,062)	21,703 (20,868–22,541)	17,995 (17,061–18,810)	13,094 (11,910–13,933)	8,156 (7,097–8,843)	3,433 (2,846–3,780)	--
Malignant skin melanoma	0 (0–0)	0 (0–0)	1 (0–6)	3 (1–26)	1 (1–1)	2 (1–3)	5 (4–9)	18 (13–36)	43 (32–85)	78 (59–155)	123 (94–242)	190 (154–341)	273 (214–482)	434 (336–742)	697 (504–1,140)	904 (602–1,270)	1,026 (614–1,331)	1,096 (616–1,373)	999 (526–1,174)	866 (419–1,019)	629 (298–753)	333 (161–410)	--
Non-melanoma skin cancer (squamous-cell carcinoma)	--	--	--	--	--	--	--	2 (2–2)	3 (3–5)	6 (5–9)	12 (10–15)	22 (20–26)	48 (44–52)	110 (101–120)	204 (187–222)	315 (292–339)	374 (341–401)	414 (369–444)	448 (386–483)	455 (384–495)	413 (338–456)	280 (208–315)	--
	0 (0–0)	0 (0–0)	3 (2–4)	12 (7–18)	14 (8–20)	17 (10–26)	36 (22–51)	43 (30–63)	49 (33–72)	55 (36–83)	66 (43–100)	85 (59–122)	112 (74–167)	163 (106–228)	234 (151–325)	303 (188–406)	336 (202–461)	365 (212–464)	323 (185–420)	268 (144–347)	180 (98–234)	86 (47–117)	--
	--	--	--	3 (2–3)	8 (6–9)	26 (18–31)	65 (44–78)	62 (48–71)	40 (33–48)	27 (22–33)	26 (22–32)	28 (24–33)	34 (30–42)	50 (42–58)	71 (59–84)	86 (70–103)	97 (72–112)	103 (76–119)	92 (67–107)	78 (55–93)	56 (37–68)	32 (20–41)	--
Breast cancer	--	--	--	--	--	--	1 (1–1)	1 (1–1)	1 (1–1)	1 (1–1)	4 (3–4)	6 (5–7)	16 (14–19)	29 (26–33)	51 (46–57)	82 (73–92)	96 (85–108)	101 (89–114)	74 (65–84)	68 (58–79)	48 (40–58)	26 (21–32)	--
Cervical cancer	--	--	--	--	--	--	--	--	--	--	--	--	--	--	--	--	--	--	--	--	--	--	--
Uterine cancer	--	--	--	--	--	--	--	--	--	--	--	--	--	--	--	--	--	--	--	--	--	--	--
Ovarian cancer	--	--	--	--	--	--	--	--	--	--	--	--	--	--	--	--	--	--	--	--	--	--	--
Prostate cancer	--	--	--	--	--	--	--	3 (2–4)	4 (3–6)	5 (4–7)	7 (6–10)	20 (16–27)	103 (82–137)	403 (320–515)	1,174 (969–1,562)	2,812 (2,443–3,935)	4,459 (3,941–6,744)	6,373 (5,608–9,867)	8,409 (7,390–13,233)	8,788 (7,441–13,632)	8,173 (6,620–11,886)	5,360 (4,097–7,409)	--
Testicular cancer	1 (0–7)	0 (0–2)	1 (0–4)	2 (1–2)	2 (2–2)	2 (2–2)	9 (8–11)	34 (30–40)	50 (42–59)	45 (39–54)	43 (37–51)	40 (36–46)	36 (32–41)	34 (30–39)	34 (29–39)	29 (25–33)	28 (25–33)	23 (20–26)	23 (19–26)	21 (18–25)	16 (13–18)	9 (7–11)	--
Kidney cancer	0 (0–0)	0 (0–0)	2 (2–2)	8 (7–9)	10 (9–11)	4 (4–5)	5 (4–6)	11 (9–12)	16 (13–18)	29 (23–33)	62 (52–70)	132 (120–145)	322 (290–355)	641 (579–702)	1,214 (1,100–1,320)	1,695 (1,562–1,826)	1,981 (1,835–2,134)	2,049 (1,907–2,197)	1,764 (1,631–1,899)	1,386 (1,233–1,511)	1,004 (864–1,105)	505 (414–565)	--
Bladder cancer	--	--	--	--	--	--	1 (1–1)	1 (1–1)	2 (2–2)	7 (6–8)	16 (14–18)	41 (37–45)	116 (106–126)	302 (281–326)	675 (630–727)	1,198 (1,129–1,276)	1,696 (1,590–1,798)	2,267 (2,132–2,414)	2,660 (2,491–2,848)	2,704 (2,436–2,936)	2,457 (2,130–2,703)	1,534 (1,275–1,717)	--
Brain and nervous system cancer	1 (1–2)	1 (1–1)	13 (9–18)	69 (52–93)	110 (80–138)	99 (73–130)	80 (62–106)	81 (69–112)	113 (93–158)	164 (136–229)	240 (203–323)	345 (301–465)	519 (437–665)	814 (660–1,049)	1,300 (1,035–1,568)	1,560 (1,198–1,907)	1,700 (1,219–1,991)	1,625 (1,108–1,832)	1,243 (829–1,401)	814 (496–953)	461 (254–548)	193 (99–236)	--
	0 (0–0)	0 (0–0)	1 (1–1)	3 (3–5)	4 (3–5)	4 (3–5)	4 (3–5)	4 (3–5)	3 (3–5)	3 (3–5)	4 (3–5)	4 (3–5)	6 (4–7)	12 (9–16)	20 (14–25)	28 (21–35)	33 (24–43)	35 (26–45)	30 (22–38)	27 (19–35)	17 (11–20)	11 (7–13)	--
	0 (0–0)	0 (0–0)	1 (1–1)	2 (1–3)	2 (1–3)	2 (1–3)	2 (1–3)	--	--	--	--	--	--	--	--	--	--	--	--	--	--	--	--
	--	--	--	2 (1–2)	2 (1–3)	2 (1–3)	2 (1–3)	4 (3–5)	3 (3–5)	3 (3–5)	4 (3–5)	4 (3–5)	6 (4–7)	12 (9–16)	20 (14–25)	28 (21–35)	33 (24–43)	35 (26–45)	30 (22–38)	27 (19–35)	17 (11–20)	11 (7–13)	--
	0 (0–0)	1 (0–1)	3 (3–4)	33 (27–41)	20 (11–26)	7 (4–9)	4 (3–5)	5 (4–7)	5 (4–7)	5 (4–7)	6 (5–8)	5 (5–7)	7 (6–9)	10 (8–12)	14 (11–17)	14 (11–17)	14 (12–17)	14 (12–17)	11 (9–13)	8 (6–10)	6 (5–7)	3 (2–3)	--
Thyroid cancer	--	--	--	--	1 (1–1)	1 (1–1)	1 (1–1)	2 (2–2)	4 (3–4)	5 (4–6)	8 (6–9)	14 (13–16)	30 (28–34)	56 (51–62)	95 (86–104)	124 (110–135)	157 (131–171)	167 (143–183)	178 (148–194)	140 (108–156)	98 (76–110)	53 (39–61)	--
Mesothelioma	--	--	--	--	--	--	--	0 (0–0)	1 (1–1)	2 (2–3)	5 (5–6)	9 (9–10)	18 (17–20)	40 (38–43)	92 (86–97)	188 (177–199)	313 (296–332)	480 (446–512)	517 (474–556)	442 (396–480)	288 (249–318)	106 (88–120)	--
Hodgkin lymphoma	--	--	--	1 (1–1)	1 (1–1)	2 (2–3)	8 (7–13)	21 (18–35)	30 (25–52)	32 (26–55)	30 (26–53)	34 (30–52)	38 (33–55)	50 (44–66)	69 (63–89)	78 (70–98)	94 (85–107)	107 (93–118)	92 (73–102)	74 (54–83)	53 (37–61)	20 (11–24)	--
Non-Hodgkin's lymphoma	--	--	--	7 (7–8)	13 (12–15)	18 (17–20)	32 (29–36)	51 (46–56)	66 (55–73)	89 (74–99)	125 (109–136)	191 (180–204)	314 (296–336)	586 (550–619)	1,081 (1,022–1,142)	1,569 (1,488–1,651)	2,036 (1,943–2,137)	2,548 (2,413–2,686)	2,531 (2,364–2,681)	2,249 (2,029–2,427)	1,784 (1,554–1,947)	854 (716–947)	--
	--	--	--	2 (1–3)	4 (2–5)	4 (2–6)	5 (2–7)	5 (3–7)	4 (2–6)	6 (3–8)	7 (3–9)	7 (3–10)	11 (5–14)	14 (6–19)	24 (11–31)	28 (13–37)	35 (17–47)	38 (18–50)	35 (18–48)	29 (15–41)	23 (13–35)	12 (8–20)	--
	--	--	--	5 (4–6)	9 (8–11)	14 (12–16)	27 (24–30)	46 (41–51)	62 (51–69)	83 (69–93)	118 (102–130)	184 (172–196)	304 (285–324)	572 (538–606)	1,058 (997–1,123)	1,542 (1,459–1,626)	2,001 (1,903–2,103)	2,510 (2,377–2,647)	2,496 (2,332–2,650)	2,220 (1,999–2,397)	1,761 (1,533–1,923)	842 (706–935)	--
Multiple myeloma	--	--	--	--	--	--	--	2 (1–2)	3 (2–4)	8 (7–12)	23 (19–32)	61 (54–88)	142 (127–199)	319 (287–434)	613 (560–832)	1,003 (916–1,347)	1,380 (1,250–1,743)	1,690 (1,471–1,994)	1,609 (1,337–1,799)	1,388 (1,036–1,546)	967 (706–1,082)	430 (301–493)	--
Leukaemia	1 (1–2)	1 (1–2)	13 (11–15)	66 (58–75)	73 (66–81)	85 (77–95)	116 (105–128)	134 (117–151)	136 (112–154)	137 (112–155)	163 (139–183)	206 (190–224)	299 (277–322)	505 (469–543)	913 (845–992)	1,473 (1,368–1,576)	2,145 (1,991–2,294)	2,813 (2,631–3,002)	2,949 (2,735–3,182)	2,634 (2,351–2,883)	2,051 (1,745–2,275)	1,077 (886–1,218)	--
Acute lymphoid leukaemia	0 (0–1)	0 (0–0)	3 (3–4)	23 (20–29)	41 (35–51)	46 (40–55)	53 (46–65)	55 (43–65)	47 (33–57)	35 (24–44)	36 (25–45)	39 (25–46)	41 (27–47)	53 (36–63)	74 (49–88)	85 (58–104)	100 (73–120)	103 (76–134)	94 (72–120)	69 (54–92)	52 (39–68)	23 (17–29)	--
Chronic lymphoid leukaemia	--	--	--	--	--	--	--	1 (1–1)	1 (1–1)	1 (1–2)	3 (2–4)	8 (6–10)	24 (20–32)	59 (50–82)	147 (127–201)	266 (225–368)	391 (337–540)	539 (464–702)	611 (524–791)	628 (525–798)	554 (455–702)	348 (274–430)	--
Acute myeloid leukaemia	0 (0–0)	0 (0–0)	6 (4–7)	32 (23–37)	19 (13–23)	26 (19–31)	40 (32–49)	49 (39–66)	53 (39–80)	60 (46–87)	75 (60–102)	102 (88–132)	150 (132–186)	254 (219–310)	456 (391–534)	738 (598–845)	1,068 (865–1,202)	1,360 (1,037–1,531)	1,308 (947–1,475)	1,028 (707–1,205)	674 (460–797)	294 (195–362)	--
Chronic myeloid leukaemia	0 (0–0)	0 (0–0)	1 (0–1)	1 (1–2)	1 (1–1)	1 (1–2)	2 (2–3)	5 (4–6)	9 (7–11)	14 (10–17)	16 (13–19)	17 (13–20)	23 (18–27)	33 (26–39)	50 (41–60)	64 (53–77)	90 (76–109)	115 (97–145)	132 (112–168)	129 (107–164)	105 (86–135)	58 (45–71)	--
Other leukaemia	1 (1–1)	1 (1–1)	3 (3–4)	10 (7–12)	12 (7–14)	12 (8–15)	20 (14–24)	25 (20–30)	26 (20–32)	26 (21–33)	32 (26–39)	41 (32–47)	61 (49–70)	106 (81–124)	187 (155–227)	321 (274–387)	496 (412–597)	695 (598–852)	805 (696–989)	780 (629–958)	665 (529–824)	354 (273–427)	--
Other malignant cancers	3 (3–4)	1 (1–2)	7 (6–10)	25 (21–34)	25 (23–33)	28 (25–34)	56 (52–63)	57 (52–65)	51 (44–60)	57 (49–67)	75 (67–87)	103 (93–114)	164 (149–179)	261 (240–288)	429 (396–474)	582 (535–638)	717 (661–769)	851 (758–920)	804 (702–873)	642 (549–706)	469 (383–525)	254 (197–288)	--
Myelodysplastic, myeloproliferative, and other hematopoietic neoplasms	1 (1–2)	1 (0–1)	3 (1–4)	3 (2–4)	2 (1–3)	2 (2–4)	3 (2–5)	3 (2–5)	5 (3–9)	6 (3–10)	9 (5–16)	16 (9–27)	25 (16–46)	60 (35–104)	143 (88–256)	291 (167–504)	519 (316–847)	848 (488–1,357)	1,088 (621–1,744)	1,211 (695–1,941)	1,105 (603–1,740)	648 (338–983)	--
Cardiovascular diseases	24 (21–27)	24 (20–27)	147 (130–164)	76 (67–84)	43 (39–46)	74 (69–80)	229 (214–249)	459 (422–491)	828 (743–891)	1,539 (1,361–1,652)	2,889 (2,669–3,044)	5,194 (4,859–5,477)	9,617 (9,191–9,974)	17,029 (16,402–17,592)	28,646 (27,642–29,564)	39,523 (37,959–40,745)	46,621 (45,124–48,057)	55,524 (53,158–57,347)	60,302 (56,958–62,604)	65,358 (59,484–68,891)	66,802 (58,720–71,368)	51,497 (43,101–55,651)	--
Rheumatic heart disease	--	--	--	2 (1–2)	1 (1–1)	2 (1–2)	3 (2–3)	5 (4–6)	10 (8–11)	14 (11–16)	17 (15–19)	23 (20–26)	38 (35–43)	59 (54–65)	100 (91–110)	147 (133–160)	184 (168–202)	244 (225–268)	291 (265–319)	357 (316–395)	383 (327–428)	288 (236–329)	--
Ischaemic heart disease	--	--	--	--	--	--	48 (44–55)	128 (117–148)	276 (251–312)	636 (576–709)	1,388 (1,292–1,533)	2,825 (2,634–3,048)	5,757 (5,460–6,171)	10,902 (10,390–11,553)	18,827 (18,018–19,724)	26,399 (25,159–27,546)	31,037 (29,778–32,154)	36,145 (34,499–37,508)	37,941 (35,655–39,580)	40,149 (36,303–42,628)	40,672 (35,419–43,621)	31,234 (26,239–34,029)	--
Stroke	4 (2–5)	3 (3–4)	36 (30–46)	14 (12–17)	12 (10–14)	21 (17–24)	43 (37–50)	78 (69–91)	130 (113–153)	225 (198–261)	407 (367–459)	700 (637–782)	1,209 (1,108–1,342)	2,109 (1,963–2,302)	3,567 (3,316–3,872)	5,155 (4,798–5,568)	6,480 (6,061–6,962)	8,880 (8,294–9,515)	10,772 (9,942–11,555)	12,222 (10,908–13,261)	12,518 (10,731–13,664)	9,252 (7,633–10,257)	--
Ischaemic stroke	2 (1–3)	2 (2–3)	4 (3–5)	2 (2–3)	1 (1–1)	1 (1–2)	4 (3–4)	7 (6–9)	12 (9–14)	21 (16–24)	33 (28–38)	55 (47–63)	126 (112–143)	237 (215–269)	450 (404–511)	1,343 (1,215–1,489)	1,830 (1,676–2,025)	3,644 (3,345–3,989)	4,788 (4,370–5,224)	7,871 (7,019–8,700)	8,620 (7,414–9,639)	6,593 (5,406–7,518)	--
Intracerebral hemorrhage	0 (0–0)	0 (0–0)	18 (15–26)	4 (3–5)	4 (3–5)	6 (5–8)	19 (17–23)	35 (30–41)	63 (54–74)	116 (101–137)	220 (197–250)	398 (356–453)	712 (642–812)	1,268 (1,163–1,426)	2,129 (1,957–2,383)	2,934 (2,708–3,228)	3,633 (3,342–3,977)	4,274 (3,960–4,639)	4,982 (4,535–5,414)	3,354 (2,956–3,729)	2,916 (2,450–3,288)	1,927 (1,564–2,219)	--
Subarachnoid hemorrhage	1 (1–2)	1 (1–1)	14 (11–17)	8 (7–10)	7 (5–8)	13 (11–15)	20 (17–24)	35 (30–45)	56 (48–69)	88 (76–108)	154 (137–184)	247 (222–286)	372 (335–421)	604 (536–676)	989 (848–1,102)	878 (699–974)	1,017 (818–1,119)	961 (740–1,063)	1,002 (695–1,123)	997 (653–1,153)	981 (559–1,166)	731 (379–884)	--
Hypertensive heart disease	--	--	--	--	--	--	6 (3–7)	33 (15–40)	87 (38–107)	197 (90–233)	374 (168–433)	660 (304–752)	1,104 (524–1,236)	1,713 (820–1,906)	2,495 (1,245–2,763)	2,841 (1,466–3,153)	2,663 (1,475–2,943)	2,434 (1,534–2,674)	2,327 (1,447–2,574)	2,298 (1,501–2,562)	2,405 (1,499–2,711)	2,014 (1,116–2,308)	--
Non-rheumatic valvular heart disease	--	--	--	--	--	--	7 (6–8)	10 (9–12)	17 (14–21)	26 (20–30)	38 (32–43)	56 (47–62)	87 (78–96)	152 (139–167)	263 (242–286)	404 (373–436)	578 (536–621)	875 (813–936)	1,229 (1,132–1,318)	1,868 (1,652–2,032)	2,491 (2,118–2,730)	2,309 (1,840–2,586)	--
Non-rheumatic calcific aortic valve disease	--	--	--	--	--	--	4 (3–5)	6 (5–7)	11 (9–14)	17 (13–21)	26 (21–31)	41 (34–48)	65 (55–75)	119 (104–135)	205 (178–231)	327 (286–364)	478 (414–523)	742 (653–808)	1,063 (947–1,152)	1,679 (1,468–1,834)	2,277 (1,916–2,513)	2,141 (1,696–2,399)	--
Non-rheumatic degenerative mitral valve disease	--	--	--	--	--	--	2 (2–3)	3 (3–5)	6 (4–8)	8 (5–11)	11 (9–16)	14 (11–21)	21 (16–31)	32 (24–49)	56 (44–86)	75 (60–121)	99 (77–164)	131 (101–214)	165 (127–269)	188 (140–307)	213 (150–323)	167 (112–244)	--
Other non-rheumatic valve diseases	--	--	--	--	--	--	0 (0–1)	1 (0–1)	1 (1–2)	1 (0–1)	1 (1–2)	1 (1–2)	1 (1–2)	1 (0–1)	1 (1–3)	1 (1–2)	1 (1–2)	1 (1–3)	2 (1–3)	2 (1–3)	1 (1–2)	1 (1–3)	--

Cardiomyopathy and myocarditis	12 (6–15)	12 (8–15)	64 (52–75)	40 (33–46)	17 (14–20)	36 (30–40)	93 (75–103)	141 (112–160)	193 (143–232)	252 (180–310)	369 (280–441)	481 (373–589)	723 (582–857)	1,019 (888–1,335)	1,532 (1,348–2,107)	1,852 (1,636–2,634)	2,091 (1,839–2,883)	2,312 (1,947–3,002)	2,351 (1,790–2,871)	2,307 (1,661–2,610)	2,145 (1,381–2,486)	1,530 (934–1,836)	--
Myocarditis	1 (0–2)	4 (2–5)	21 (12–27)	19 (11–24)	8 (4–9)	10 (6–12)	24 (15–30)	30 (18–38)	30 (18–41)	27 (16–39)	30 (18–40)	28 (16–37)	37 (20–48)	35 (19–45)	34 (17–45)	33 (18–43)	31 (16–39)	25 (12–32)	24 (12–32)	21 (10–29)	20 (10–26)	18 (9–25)	--
Alcoholic cardiomyopathy	--	--	--	--	--	--	3 (2–4)	5 (4–7)	18 (13–27)	34 (25–55)	67 (51–97)	125 (94–172)	265 (195–330)	445 (332–568)	737 (548–956)	895 (668–1,160)	964 (668–1,254)	810 (579–1,048)	675 (466–890)	117 (82–158)	49 (36–67)	13 (10–19)	--
Other cardiomyopathy	11 (6–13)	9 (6–10)	42 (35–52)	20 (17–27)	10 (8–13)	26 (22–31)	66 (54–77)	106 (83–127)	146 (105–184)	191 (133–239)	271 (201–329)	328 (251–403)	421 (342–521)	539 (457–764)	760 (638–1,154)	923 (762–1,500)	1,096 (927–1,707)	1,477 (1,265–2,000)	1,652 (1,234–2,014)	2,169 (1,555–2,452)	2,076 (1,332–2,410)	1,499 (909–1,797)	--
Pulmonary Arterial Hypertension	4 (2–12)	4 (3–8)	31 (16–39)	11 (6–15)	6 (3–10)	6 (3–11)	7 (5–13)	7 (4–12)	11 (7–17)	13 (9–23)	23 (14–37)	29 (17–45)	48 (24–71)	70 (34–93)	110 (49–140)	157 (64–197)	204 (75–255)	245 (86–310)	256 (90–330)	256 (83–339)	239 (57–328)	155 (38–216)	--
Atrial fibrillation and flutter	--	--	--	--	--	--	--	--	--	2 (1–3)	4 (2–4)	28 (18–39)	58 (39–84)	130 (92–181)	307 (213–431)	579 (375–813)	891 (561–1,184)	1,425 (919–1,920)	1,942 (1,241–2,643)	2,709 (1,770–3,557)	2,920 (1,926–4,042)	2,606 (1,731–3,550)	--
Aortic aneurysm	--	--	--	--	--	--	6 (5–6)	12 (10–14)	25 (20–29)	47 (36–54)	84 (71–95)	127 (108–140)	199 (179–217)	286 (260–309)	497 (455–533)	729 (664–784)	923 (853–989)	1,119 (1,028–1,198)	1,140 (1,042–1,222)	1,070 (949–1,166)	913 (785–1,011)	560 (461–628)	--
Peripheral vascular disease	--	--	--	--	--	--	--	--	--	--	--	11 (4–27)	34 (11–86)	92 (29–217)	207 (66–493)	386 (125–925)	590 (184–1,462)	802 (262–1,904)	972 (324–2,258)	1,036 (340–2,507)	1,083 (366–2,708)	838 (275–2,002)	--
Endocarditis	0 (0–1)	1 (0–1)	2 (1–3)	2 (1–2)	1 (1–1)	2 (1–2)	4 (2–5)	13 (6–17)	27 (13–36)	44 (21–58)	60 (32–74)	80 (42–97)	112 (57–137)	159 (79–190)	240 (113–289)	284 (135–342)	327 (152–397)	386 (175–463)	429 (180–526)	493 (196–615)	521 (197–671)	379 (137–503)	--
Other cardiovascular and circulatory diseases	3 (2–4)	3 (2–4)	14 (12–18)	7 (6–9)	6 (5–7)	9 (8–11)	13 (11–15)	30 (27–35)	51 (42–60)	84 (65–98)	126 (110–143)	175 (151–196)	247 (227–271)	338 (315–368)	503 (469–540)	591 (551–633)	653 (613–695)	657 (613–700)	653 (602–701)	592 (529–642)	514 (446–564)	331 (272–368)	--
Chronic respiratory diseases	0 (0–0)	0 (0–0)	0 (0–0)	54 (48–60)	42 (39–46)	57 (53–62)	62 (58–66)	87 (80–95)	106 (93–116)	144 (124–157)	210 (190–227)	344 (319–366)	749 (709–792)	1,873 (1,774–1,968)	4,319 (4,107–4,526)	8,008 (7,626–8,375)	12,288 (11,763–12,809)	17,866 (17,069–18,600)	19,386 (18,271–20,265)	18,312 (16,505–19,477)	15,268 (13,241–16,466)	9,062 (7,418–9,907)	--
Chronic obstructive pulmonary disease	--	--	0 (0–0)	5 (4–6)	2 (2–2)	2 (2–2)	8 (7–9)	11 (10–13)	16 (14–19)	32 (27–38)	67 (59–78)	165 (153–183)	479 (444–522)	1,444 (1,354–1,553)	3,585 (3,396–3,831)	6,917 (6,551–7,408)	10,664 (10,090–11,449)	15,574 (14,635–16,739)	16,880 (15,742–18,184)	15,924 (14,215–17,373)	13,348 (11,448–14,746)	7,986 (6,569–8,968)	--
Pneumoconiosis	--	--	--	--	--	--	0 (0–0)	0 (0–0)	0 (0–0)	1 (0–1)	1 (1–1)	1 (1–2)	3 (2–3)	8 (7–9)	21 (18–23)	45 (41–51)	80 (71–89)	153 (138–169)	183 (164–204)	219 (190–243)	192 (163–215)	111 (91–126)	--
Silicosis	--	--	--	--	--	--	0 (0–0)	0 (0–0)	0 (0–0)	0 (0–0)	0 (0–1)	1 (0–1)	1 (1–1)	2 (2–3)	4 (3–5)	7 (5–9)	9 (6–11)	14 (11–20)	15 (11–22)	16 (12–27)	12 (9–23)	5 (3–10)	--
Asbestosis	--	--	--	--	--	--	0 (0–0)	0 (0–0)	0 (0–0)	0 (0–0)	0 (0–0)	0 (0–0)	1 (1–1)	3 (2–3)	8 (7–11)	20 (17–26)	43 (37–50)	92 (79–104)	120 (100–135)	146 (116–166)	129 (96–147)	71 (49–83)	--
Coal workers pneumoconiosis	--	--	--	--	--	--	0 (0–0)	0 (0–0)	0 (0–0)	0 (0–0)	0 (0–0)	0 (0–0)	1 (0–1)	3 (2–3)	6 (4–8)	16 (12–20)	26 (20–32)	43 (33–55)	46 (35–61)	55 (42–72)	50 (37–72)	33 (24–47)	--
Other pneumoconiosis	--	--	--	--	--	--	0 (0–0)	0 (0–0)	0 (0–0)	0 (0–0)	0 (0–0)	0 (0–0)	0 (0–1)	1 (1–1)	2 (1–2)	3 (2–3)	3 (2–4)	3 (2–4)	4 (3–5)	2 (2–4)	1 (1–3)	2 (2–4)	--
Asthma	--	--	--	16 (13–19)	27 (25–31)	42 (38–47)	37 (34–41)	54 (48–59)	60 (50–67)	63 (51–72)	70 (60–78)	74 (64–83)	87 (78–95)	108 (98–118)	126 (115–138)	124 (113–135)	109 (101–117)	103 (93–113)	94 (84–103)	97 (85–108)	92 (78–105)	73 (59–84)	--
Interstitial lung disease and pulmonary sarcoidosis	--	--	--	6 (4–9)	3 (2–4)	3 (2–5)	6 (4–7)	9 (7–13)	15 (11–22)	28 (21–41)	45 (35–62)	65 (48–83)	120 (81–150)	227 (146–275)	449 (285–545)	797 (467–961)	1,294 (687–1,569)	1,882 (945–2,355)	2,086 (1,041–2,629)	1,939 (931–2,559)	1,513 (724–2,037)	806 (386–1,112)	--
Other chronic respiratory diseases	0 (0–0)	0 (0–0)	0 (0–0)	28 (22–32)	10 (8–12)	9 (8–11)	11 (9–13)	13 (10–15)	15 (11–17)	20 (13–23)	27 (19–31)	39 (26–45)	60 (42–69)	86 (57–97)	138 (100–159)	124 (76–143)	143 (89–163)	154 (100–174)	143 (92–163)	133 (87–156)	123 (76–147)	86 (48–102)	--
Digestive diseases	10 (9–11)	13 (11–15)	146 (128–165)	52 (46–57)	23 (22–25)	28 (27–30)	50 (47–53)	101 (93–107)	233 (199–255)	506 (434–547)	964 (885–1,022)	1,709 (1,601–1,801)	3,254 (3,110–3,396)	5,766 (5,540–5,995)	8,527 (8,222–8,854)	9,298 (8,955–9,669)	8,092 (7,819–8,397)	7,320 (7,014–7,604)	6,381 (6,053–6,669)	5,609 (5,068–5,962)	4,904 (4,251–5,268)	3,281 (2,753–3,574)	--
Cirrhosis and other chronic liver diseases	--	--	--	8 (6–10)	4 (3–4)	4 (3–5)	14 (13–16)	41 (34–45)	139 (112–157)	365 (304–404)	731 (647–787)	1,388 (1,293–1,467)	2,718 (2,583–2,843)	4,879 (4,677–5,088)	7,084 (6,798–7,389)	7,289 (6,992–7,629)	5,555 (5,260–5,814)	4,158 (3,875–4,385)	2,848 (2,575–3,044)	1,858 (1,615–2,036)	1,222 (1,036–1,353)	647 (524–740)	--
Cirrhosis and other chronic liver diseases due to hepatitis B	--	--	--	0 (0–0)	0 (0–0)	0 (0–0)	0 (0–0)	1 (0–1)	4 (2–5)	12 (8–16)	28 (19–38)	53 (38–69)	95 (68–132)	153 (106–214)	191 (135–265)	176 (122–251)	129 (90–179)	103 (72–141)	82 (56–110)	56 (38–77)	36 (26–48)	19 (12–27)	--
Cirrhosis and other chronic liver diseases due to hepatitis C	--	--	--	0 (0–0)	0 (0–0)	0 (0–1)	2 (1–3)	15 (9–20)	73 (54–92)	210 (169–250)	426 (353–496)	801 (682–911)	1,528 (1,290–1,770)	2,616 (2,209–3,075)	3,538 (2,851–4,211)	3,437 (2,704–4,177)	2,544 (2,082–3,023)	1,854 (1,521–2,197)	1,237 (990–1,495)	799 (623–970)	528 (409–634)	280 (202–361)	--
Cirrhosis and other chronic liver diseases due to alcohol use	--	--	--	--	--	--	0 (0–0)	3 (2–5)	19 (11–28)	66 (49–86)	162 (113–216)	337 (248–431)	692 (496–906)	1,354 (963–1,755)	2,201 (1,632–2,893)	2,383 (1,749–3,131)	1,788 (1,330–2,257)	1,281 (957–1,638)	818 (611–1,032)	493 (357–632)	300 (226–383)	149 (102–205)	--
Cirrhosis and other chronic liver diseases due to NAFLD	--	--	--	--	--	--	0 (0–1)	2 (1–3)	7 (3–13)	19 (10–32)	39 (19–71)	80 (39–133)	182 (85–323)	343 (164–594)	474 (239–831)	489 (232–883)	393 (237–623)	312 (184–470)	227 (119–364)	158 (88–250)	111 (66–178)	62 (33–113)	--
Cirrhosis and other chronic liver diseases due to other causes	--	--	--	8 (6–10)	3 (3–4)	4 (3–4)	12 (10–14)	20 (14–27)	37 (21–54)	58 (32–96)	77 (42–126)	117 (62–196)	220 (120–359)	413 (230–668)	680 (361–1,179)	804 (469–1,318)	701 (381–1,183)	609 (333–975)	485 (269–777)	352 (202–555)	247 (151–367)	137 (76–226)	--
Upper digestive system diseases	--	--	--	1 (1–1)	1 (0–1)	1 (1–1)	2 (1–2)	3 (3–4)	6 (6–8)	12 (10–15)	24 (21–29)	37 (34–41)	72 (65–79)	118 (108–129)	198 (179–217)	253 (232–275)	270 (247–295)	306 (280–330)	334 (297–364)	349 (301–392)	337 (283–382)	240 (198–273)	--
Peptic ulcer disease	--	--	--	1 (1–1)	0 (0–1)	1 (1–1)	1 (1–1)	2 (2–3)	5 (4–6)	9 (8–12)	20 (17–24)	31 (27–35)	62 (55–68)	106 (95–116)	179 (160–197)	233 (212–254)	249 (227–273)	288 (261–312)	314 (277–343)	327 (280–367)	315 (262–356)	222 (179–254)	--
Gastritis and duodenitis	--	--	--	0 (0–0)	0 (0–0)	0 (0–0)	0 (0–1)	1 (1–1)	1 (1–2)	2 (2–3)	4 (3–6)	6 (5–9)	10 (8–15)	12 (10–18)	19 (16–29)	20 (17–29)	20 (18–30)	18 (15–28)	19 (16–31)	22 (17–34)	23 (17–36)	18 (13–27)	--
Appendicitis	--	--	--	2 (1–3)	3 (1–4)	4 (2–5)	3 (2–5)	3 (2–5)	3 (2–5)	4 (2–6)	7 (4–10)	8 (5–11)	12 (7–17)	18 (11–27)	26 (16–40)	34 (21–52)	41 (25–64)	48 (30–72)	44 (25–67)	44 (26–67)	36 (22–57)	23 (13–35)	--
Paralytic ileus and intestinal obstruction	3 (3–4)	9 (7–10)	128 (113–144)	13 (10–17)	7 (5–9)	7 (6–10)	9 (7–11)	11 (8–13)	11 (7–14)	13 (9–17)	23 (17–30)	31 (22–39)	54 (39–71)	95 (67–127)	163 (114–206)	256 (180–328)	353 (261–462)	508 (389–649)	663 (486–836)	799 (578–1,003)	841 (561–1,015)	641 (415–754)	--
Inguinal, femoral, and abdominal hernia	6 (5–7)	5 (3–6)	18 (11–24)	1 (1–2)	1 (1–1)	1 (1–1)	1 (1–1)	1 (1–1)	2 (2–2)	3 (3–4)	6 (5–6)	8 (7–9)	14 (13–16)	31 (29–33)	47 (43–51)	65 (59–70)	73 (67–79)	93 (84–101)	102 (90–111)	122 (104–136)	138 (116–156)	106 (88–122)	--
Inflammatory bowel disease	--	--	--	12 (9–16)	4 (2–4)	4 (3–5)	5 (4–6)	9 (6–10)	12 (8–14)	15 (10–18)	23 (16–27)	31 (22–36)	48 (32–55)	79 (50–90)	125 (79–144)	182 (117–209)	230 (144–261)	290 (193–328)	320 (211–367)	318 (212–365)	293 (195–339)	198 (127–234)	--
Vascular intestinal disorders	--	--	--	3 (2–4)	1 (1–1)	2 (2–2)	3 (3–4)	6 (5–6)	7 (6–8)	10 (8–12)	18 (15–20)	29 (26–32)	57 (53–62)	123 (113–132)	223 (206–242)	354 (327–380)	468 (438–500)	596 (553–636)	624 (563–669)	597 (520–658)	518 (437–577)	323 (262–366)	--
Gallbladder and biliary diseases	--	--	--	1 (1–1)	0 (0–0)	1 (0–1)	1 (1–1)	4 (3–4)	7 (5–8)	11 (8–13)	17 (12–20)	22 (18–29)	37 (29–46)	74 (58–92)	132 (100–160)	198 (155–236)	291 (213–351)	387 (281–476)	471 (360–557)	520 (391–638)	519 (383–624)	366 (274–462)	--
Pancreatitis	--	--	--	1 (1–1)	1 (1–1)	1 (1–2)	5 (4–5)	15 (13–17)	33 (27–39)	54 (44–65)	87 (76–104)	105 (94–117)	156 (141–172)	199 (181–218)	267 (240–291)	304 (279–334)	340 (311–371)	336 (308–364)	267 (234–296)	212 (183–237)	167 (138–189)	101 (80–117)	--
Other digestive diseases	--	--	--	9 (6–13)	3 (2–5)	4 (2–6)	7 (5–10)	9 (7–14)	13 (9–20)	18 (13–31)	30 (22–51)	49 (38–81)	86 (66–147)	151 (111–250)	262 (187–417)	362 (263–595)	470 (362–776)	596 (467–1,002)	708 (564–1,143)	789 (610–1,270)	832 (632–1,301)	637 (468–1,000)	--
Alzheimer's disease and other dementias	--	--	--	--	--	--	--	--	--	--	--	15 (2–50)	79 (10–256)	125 (20–377)	275 (51–813)	562 (114–1,615)	1,162 (255–3,415)	2,384 (557–6,634)	4,246 (957–12,265)	8,624 (2,038–24,262)	11,800 (2,930–31,301)	10,604 (2,662–27,592)	--
Parkinson's disease	--	--	--	--	--	--	--	0 (0–0)	0 (0–0)	0 (0–0)	1 (1–1)	10 (9–11)	39 (37–41)	96 (89–100)	198 (185–206)	434 (408–454)	985 (940–1,026)	2,242 (2,137–2,328)	3,862 (3,628–4,016)	4,779 (4,258–5,074)	4,234 (3,641–4,553)	2,269 (1,880–2,482)	--
Idiopathic epilepsy	--	--	8 (7–10)	18 (16–21)	13 (11–14)	17 (16–19)	34 (31–36)	59 (54–64)	65 (53–74)	64 (52–73)	73 (64–81)	74 (69–80)	90 (85–96)	94 (89–100)	113 (108–120)	104 (97–111)	86 (81–92)	78 (73–83)	70 (64–75)	55 (48–60)	39 (34–43)	24 (20–27)	--
Multiple sclerosis	--	--	--	--	0 (0–0)	0 (0–0)	1 (1–2)	2 (2–4)	5 (3–8)	14 (10–23)	29 (21–49)	47 (34–79)	85 (58–130)	138 (87–198)	210 (117–305)	247 (116–311)	231 (119–271)	196 (86–232)	128 (57–153)	67 (29–80)	32 (14–39)	10 (5–12)	--
Motor neuron disease	1 (1–1)	2 (2–3)	45 (40–51)	10 (8–11)	4 (3–4)	3 (2–3)	3 (3–4)	4 (4–5)	8 (7–9)	17 (15–19)	40 (37–43)	84 (77–92)	155 (145–167)	271 (252–290)	459 (433–490)	677 (640–720)	854 (810–898)	928 (871–983)	795 (729–862)	507 (448–561)	244 (204–275)	78 (64–89)	--
Other neurological disorders	--	--	21 (18–24)	18 (16–20)	14 (12–15)	27 (25–29)	78 (72–84)	94 (86–101)	76 (63–84)	66 (53–73)	74 (66–80)	93 (86–99)	152 (143–162)	199 (188–210)	288 (272–305)	347 (327–367)	404 (380–430)	471 (441–503)	506 (466–542)	455 (409–494)	338 (293–373)	175 (145–195)	--
Eating disorders	--	--	--	--	0 (0–0)	0 (0–0)	0 (0–0)	1 (0–1)	1 (0–1)	1 (0–1)	0 (0–1)	0 (0–0)	1 (0–1)	--	--	--	--	--	--	--	--	--	--
Anorexia nervosa	--	--	--	--	0 (0–0)	0 (0–0)	0 (0–0)	0 (0–1)	0 (0–1)	0 (0–0)	0 (0–0)	0 (0–0)	0 (0–1)	--	--	--	--	--	--	--	--	--	--
Bulimia nervosa	--	--	--	--	0 (0–0)	0 (0–0)	0 (0–0)	0 (0–0)	0 (0–0)	0 (0–0)	0 (0–0)	0 (0–0)	0 (0–0)	--	--	--	--	--	--	--	--	--	--
Alcohol use disorders	--	--	--	--	--	0 (0–0)	44 (40–48)	128 (117–140)	224 (193–255)	355 (313–415)	550 (501–605)	746 (682–810)	1,202 (1,107–1,303)	1,451 (1,359–1,553)	1,765 (1,666–1,883)	1,547 (1,460–1,639)	1,091 (1,024–1,158)	643 (600–684)	361 (334–386)	182 (161–199)	95 (81–105)	36 (30–41)	--
Drug use disorders	0 (0–0)	0 (0–0)	0 (0–0)	0 (0–0)	0 (0–0)	0 (0–0)	687 (565–785)	3,829 (3,466–4,536)	6,553 (6,003–7,716)	7,009 (6,484–8,121)	6,265 (5,798–7,131)	4,698 (4,279–5,163)	4,672 (4,120–5,222)	4,140 (3,531–4,550)	3,465 (2,897–3,888)	1,792 (1,467–1,994)	858 (723–955)	403 (334–450)	206 (173–230)	127 (104–145)	96 (76–111)	54 (42–62)	--
Opioid use disorders	0 (0–0)	0 (0–0)	0 (0–0)	0 (0–0)	0 (0–0)	0 (0–0)	563 (465–659)	3,192 (2,865–3,801)	5,380 (4,805–6,501)	5,192 (4,568–6,277)	4,687 (4,236–5,381)	3,447 (3,125–3,955)	3,378 (2,931–3,996)	2,754 (2,307–3,220)	2,280 (1,885–2,668)	1,172 (944–1,370)	559 (466–644)	234 (192–264)	134 (111–151)	102 (84–117)	77 (61–89)	42 (33–49)	--
Cocaine use disorders	--	--	--	--	--	0 (0–0)	46 (25–54)	239 (200–307)	446 (344–936)	861 (694–1,672)	769 (678–937)	596 (416–669)	628 (374–708)	804 (493–913)	674 (413–788)	351 (212–414)	162 (99–187)	67 (40–80)	37 (22–43)	0 (0–0)	0 (0–0)	0 (0–0)	--
Amphetamine use disorders	--	--	--	--	--	0 (0–0)	30 (16–38)	185 (161–231)	385 (268–637)	544 (409–857)	409 (366–482)	287 (193–373)	263 (151–355)	327 (187–435)	276 (157–357)	146 (80–190)	64 (37–83)	22 (13–28)	13 (7–16)	0 (0–0)	0 (0–0)	0 (0–0)	--
Other drug use disorders	--	--	--	--	--	0 (0–0)	49 (30–57)	212 (185–259)	342 (292–535)	411 (358–610)	401 (356–466)	368 (259–413)	402 (259–460)	255 (159–291)	236 (158–279)	123 (80–145)	73 (48–86)	80 (55–92)	23 (16–27)	24 (17–28)	18 (12–22)	12 (9–14)	--
Diabetes mellitus	0 (0–1)	1 (1–2)	7 (4–8)	4 (3–6)	4 (3–5)	11 (7–13)	30 (28–32)	79 (74–84)	140 (127–150)	239 (219–258)	391 (368–411)	637 (605–675)	1,115 (1,066–1,165)	1,936 (1,849–2,029)	3,314 (3,150–3,496)	4,633 (4,392–4,870)	5,388 (5,100–5,679)	5,926 (5,584–6,270)	5,474 (5,099–5,818)	4,752 (4,296–5,076)	3,899 (3,417–4,223)	2,333 (1,956–2,556)	--
Diabetes mellitus type 1	0 (0–1)	1 (1–2)	7 (4–8)	4 (3–6)	4 (3–5)	11 (7–13)	26 (23–29)	70 (62–75)	123 (111–133)	139 (98–161)	224 (158–264)	352 (238–402)	104 (50–139)	169 (79–223)	286 (143–389)	228 (114–308)	267 (138–386)	213 (105–302)	152 (80–241)	49 (24–75)	40 (19–59)	23 (12–36)	--
Diabetes mellitus type 2	--	--	--	--	--	--	3 (2–6)	9 (6–16)	17 (11–29)	100 (80–144)	168 (134–236)	285 (227–390)	1,010 (956–1,072)	1,767 (1,672–1,880)	3,028 (2,843–3,243)	4,405 (4,158–4,667)	5,121 (4,827–5,438)	5,712 (5,359–6,048)	5,321 (4,960–5,659)	4,703 (4,268–5,025)	3,859 (3,371–4,179)	2,310 (1,930–2,529)	--
Chronic kidney disease	--	--	26 (23–29)	8 (7–9)	5 (4–5)	5 (5–6)	14 (13–15)	37 (34–39)	81 (72–88)	159 (141–173)	295 (272–316)	473 (445–509)	839 (793–885)	1,441 (1,360–1,533)	2,526 (2,400–2,662)	3,882 (3,687–4,104)	4,936 (4,709–5,189)	6,395 (6,089–6,708)	7,326 (6,886–7,734)	8,035 (7,230–8,585)	7,988 (6,981–8,572)	5,944 (5,002–6,496)	--
Chronic kidney disease due to diabetes mellitus type 1	--	--	0 (0–0)	0 (0–0)	0 (0–0)	0 (0–0)	0 (0–0)	0 (0–1)	3 (1–7)	13 (5–24)	33 (14–63)	57 (23–108)	89 (35–171)	114 (45–228)	144 (49–308)	172 (64–367)	177 (72–374)	185 (79–368)	156 (63–338)	102 (40–221)	40 (14–95)	7 (2–17)	--
Chronic kidney disease due to diabetes mellitus type 2	--	--	--	--	--	--	0 (0–0)	0 (0–1)	2 (1–4)	8 (3–18)	27 (11–55)	71 (32–128)	187 (94–292)	409 (237–585)	805 (464–1,129)	1,304 (722–1,798)	1,712 (1,044–2,321)	2,237 (1,405–3,029)	2,436 (1,513–3,381)	2,387 (1,470–3,515)	1,952 (1,166–3,006)	1,176 (672–1,886)	--
Chronic kidney disease due to hypertension	--	--	--	--	--	--	1 (0–1)	3 (1–5)	9 (5–15)	23 (14–38)	50 (31–78)	92 (59–138)	185 (121–267)	355 (242–521)	689 (475–975)	1,179 (817–1,593)	1,614 (1,164–2,183)	2,231 (1,622–2,997)	2,837 (2,073–3,776)	3,536 (2,605–4,523)	4,054 (3,058–5,001)	3,267 (2,483–4,030)	--
Chronic kidney disease due to glomerulonephritis	--	--	7 (4–11)	2 (1–3)	1 (1–2)	2 (1–3)	5 (3–7)	14 (7–21)	31 (19–46)	58 (34–85)	101 (59–155)	141 (68–221)	199 (111–330)	285 (140–474)	437 (223–753)	565 (273–980)	599 (301–1,070)	667 (331–1,166)	683 (326–1,184)	660 (295–1,146)	545 (261–962)	350 (160–658)	--
Chronic kidney disease due to other and unspecified causes	--	--	19 (15–23)	6 (5–7)	3 (3–4)	4 (3–5)	9 (6–11)	19 (12–26)	35 (22–48)	56 (32–85)	83 (42–136)	111 (55–186)	179 (87–292)	278 (144–452)	452 (220–751)	662 (342–1,135)	833 (433–1,379)	1,074 (562–1,767)	1,214 (644–2,048)	1,349 (660–2,280)	1,398 (725–2,227)	1,145 (644–1,801)	--
Acute glomerulonephritis	--	--	0 (0–0)	0 (0–0)	0 (0–0)	0 (0–0)	0 (0–0)	0 (0–0)	0 (0–0)	1 (1–1)	1 (1–1)	1 (1–1)	1 (1–1)	2 (2–3)	5 (4–6)	7 (6–8)	10 (9–12)	16 (13–19)	17 (14–20)	23 (19–27)	22 (18–26)	16 (12–19)	--
Skin and subcutaneous diseases	--	--	4 (1–7)	2 (1–3)	1 (1–2)	2 (1–3)	2 (1–4)	4 (2–7)	10 (4–16)	16 (6–27)	28 (11–50)	42 (15–67)	71 (24–126)	119 (43–185)	188 (65–290)	271 (94–426)	318 (124–605)	380 (153–800)	461 (195–1,067)	458 (206–1,083)	461 (233–1,107)	314 (150–719)	--
Bacterial skin diseases	--	--	4 (1–6)	1 (1–2)	1 (0–1)	1 (1–3)	2 (1–4)	3 (1–6)	8 (3–14)	14 (5–24)	24 (8–42)	38 (13–61)	63 (20–113)	105 (34–163)	161 (50–258)	224 (77–383)	257 (85–495)	292 (89–610)	335 (123–795)	320 (114–751)	324 (122–773)	220 (84–534)	--
Cellulitis	--	--	1 (0–1)	0 (0–0)	0 (0–0)	1 (0–1)	1 (0–2)	2 (1–3)	5 (2–9)	10 (4–17)	16 (5–27)	26 (9–42)	43 (13–72)	67 (18–103)	102 (28–155)	139 (39–231)	151 (45–269)	165 (42–342)	184 (52–416)	169 (47–404)	180 (60–453)	123 (43–291)	--
Pyoderma	--	--	3 (1–5)	1 (1–2)	1 (0–1)	1 (0–2)	1 (0–2)	1 (0–3)	3 (1–6)	4 (1–9)	8 (2–16)	12 (3–23)	20 (5–42)	38 (10–72)	59 (15–110)	85 (22–160)	106 (31–239)	127 (37–304)	151 (52–407)	150 (45–380)	143 (54–358)	97 (33–268)	--
Decubitus ulcer	--	--	--	0 (0–0)	0 (0–0)	0 (0–0)	0 (0–0)	0 (0–1)	1 (0–2)	1 (0–3)	3 (0–6)	3 (0–7)	6 (1–12)	12 (2–22)	22 (3–44)	38 (6–73)	52 (10–122)	78 (17–192)	113 (26–318)	124 (35–358)	120 (33–350)	81 (24–247)	--
Other skin and subcutaneous diseases	--	--	0 (0–1)	1 (1–1)	0 (0–1)	0 (0–1)	0 (0–1)	1 (0–1)	1 (0–1)	1 (0–1)	1 (0–2)	1 (0–3)	2 (1–4)	3 (1–5)	5 (2–8)	8 (3–15)	9 (3–17)	10 (3–21)	14 (5–33)	15 (5–37)	18 (7–44)	12 (5–30)	--
Musculoskeletal disorders	--	--	--	--	2 (2–4)	6 (5–11)	12 (9–17)	21 (15–30)	24 (18–34)	29 (22–42)	41 (32–65)	51 (43–79)	81 (68–133)	116 (97–190)	186 (158–304)	249 (213–435)	327 (278–558)	375 (322–618)	365 (305–608)	325 (245–511)	255 (187–373)	163 (107–216)	--
Rheumatoid arthritis	--	--	--	--	0 (0–0)	0 (0–1)	0 (0–0)	0 (0–1)	1 (1–1)	1 (1–2)	2 (2–4)	4 (3–6)	9 (7–15)	20 (17–33)	46 (38–77)	78 (65–134)	117 (97–196)	153 (129–254)	152 (124–249)	133 (99–203)	85 (61–123)	37 (24–49)	--
Other musculoskeletal disorders	--	--	--	--	2 (2–4)	6 (5–10)	11 (9–17)	20 (15–29)	23 (17–33)	28 (21–41)	38 (30–61)	47 (40–74)	72 (61–119)	96 (81–157)	140 (119–229)	170 (144–298)	210 (179–361)	222 (190–370)	213 (178–357)	192 (147–303)	170 (124–253)	126 (82–168)	--
Congenital anomalies	1,323 (1,125–1,734)	415 (358–503)	878 (705–1,070)	270 (212–359)	102 (73–133)	104 (75–133)	125 (92–159)	130 (106–177)	126 (94–180)	118 (88–165)	121 (95–161)	121 (91–154)	146 (106–177)	191 (125–219)	252 (158–290)	243 (156–281)	173 (126–209)	--	--	--	--	--	--
Neural tube defects	138 (99–182)	19 (14–24)	47 (33–60)	74 (50–96)	3 (2–5)	4 (3–5)	4 (3–5)	6 (4–8)	6 (4–9)	6 (4–9)	6 (4–8)	5 (3–6)	5 (3–6)	4 (2–5)	2 (1–2)	2 (1–3)	2 (1–3)	--	--	--	--	--	--
Congenital heart anomalies	203 (164–316)	174 (146–227)	377 (274–472)	79 (60–115)	37 (26–51)	44 (31–60)	64 (47–86)	62 (50–93)	64 (46–96)	64 (46–96)	64 (49–91)	63 (46–82)	61 (44–76)	59 (38–72)	66 (40–78)	59 (35–71)	61 (42–81)	--	--	--	--	--	--
Orofacial clefts	0 (0–1)	0 (0–0)	1 (0–1)	0 (0–0)	--	--	--	--	--	--	--	--	--	--	--	--	--	--	--	--	--	--	--
Down's syndrome	17 (13–24)	7 (5–9)	32 (23–41)	8 (6–11)	3 (2–4)	5 (3–7)	7 (5–10)	10 (8–16)	11 (8–19)	11 (8–20)	15 (11–22)	22 (16–31)	45 (33–58)	94 (63–109)	147 (92–170)	146 (93–171)	72 (48–86)	--	--	--	--	--	--
Other chromosomal abnormalities	269 (183–390)	85 (64–120)	138 (85–187)	25 (13–35)	12 (6–17)	12 (5–16)	11 (4–15)	13 (5–17)	11 (4–17)	11 (4–16)	11 (4–15)	9 (3–12)	11 (4–14)	9 (3–11)	9 (4–12)	7 (3–10)	8 (3–10)	--	--	--	--	--	--
Congenital musculoskeletal and limb anomalies	61 (25–83)	10 (5–13)	24 (13–37)	5 (3–9)	2 (1–4)	2 (1–4)	3 (2–5)	3 (2–6)	3 (2–5)	3 (1–4)	3 (1–4)	3 (1–4)	3 (2–5)	4 (2–5)	4 (2–7)	5 (3–9)	4 (3–12)	--	--	--	--	--	--
Urogenital congenital anomalies	153 (91–224)	11 (7–19)	16 (10–26)	2 (1–3)	1 (1–2)	1 (1–2)	1 (1–2)	1 (1–2)	1 (1–2)	1 (1–2)	1 (1–1)	1 (1–1)	1 (1–1)	1 (1–1)	1 (1–1)	1 (1–1)	1 (1–2)	--	--	--	--	--	--
Digestive congenital anomalies	104 (76–147)	43 (26–56)	75 (43–96)	12 (8–18)	4 (2–5)	3 (2–4)	2 (1–3)	3 (2–4)	2 (2–3)	1 (1–2)	2 (1–2)	1 (1–2)	2 (1–3)	3 (2–4)	3 (2–4)	4 (2–5)	7 (4–8)	--	--	--	--	--	--
Other congenital anomalies	377 (310–554)	65 (52–89)	168 (133–220)	65 (51–93)	39 (27–52)	33 (22–43)	33 (22–43)	32 (24–45)	27 (19–40)	22 (15–33)	20 (15–29)	17 (12–24)	18 (11–23)	18 (10–22)	19 (10–24)	18 (9–22)	18 (11–22)	--	--	--	--	--	--
Urinary diseases and male infertility	4 (3–6)	2 (2–4)	6 (5–9)	3 (3–4)	4 (3–5)	4 (3–5)	4 (3–5)	6 (5–9)	11 (9–15)	15 (12–20)	23 (17–30)	37 (28–45)	65 (46–77)	138 (96–161)	237 (162–272)	386 (271–442)	548 (393–655)	801 (619–1,014)	1,155 (951–1,452)	1,400 (1,184–1,921)	1,582 (1,282–2,242)	1,287 (1,018–1,811)	--
Urinary tract infections and interstitial nephritis	1 (1–2)	1 (1–1)	4 (3–6)	2 (2–3)	2 (1–2)	2 (2–3)	2 (2–3)	4 (3–6)	8 (6–11)	12 (9–15)	18 (13–22)	29 (21–35)	51 (36–61)	113 (80–132)	201 (135–228)	332 (228–372)	478 (329–557)	714 (537–865)	1,061 (846–1,305)	1,293 (1,084–1,770)	1,472 (1,188–2,017)	1,202 (947–1,665)	--
Urolithiasis	--	--	--	0 (0–0)	0 (0–0)	0 (0–0)	0 (0–0)	1 (0–1)	1 (0–1)	1 (1–2)	2 (1–2)	3 (2–4)	3 (2–5)	6 (4–9)	9 (6–14)	15 (10–23)	20 (13–29)	26 (17–39)	28 (17–40)	34 (21–49)	33 (20–52)	23 (15–36)	--
Other urinary diseases	3 (2–5)	1 (1–3)	2 (1–4)	1 (1–2)	2 (1–2)	2 (1–2)	1 (1–2)	1 (1–2)	2 (1–4)	2 (1–5)	4 (2–7)	6 (3–9)	10 (5–15)	19 (10–29)	28 (16–46)	40 (23–70)	50 (31–91)	61 (38–115)	66 (38–139)	73 (43–151)	76 (44–167)	62 (38–141)	--
Gynecological diseases	--	--	--	--	--	--	--	--	--	--	--	--	--	--	--	--	--	--	--	--	--	--	--
Uterine fibroids	--	--	--	--	--	--	--	--	--	--	--	--	--	--	--	--	--	--	--	--	--	--	--
Endometriosis	--	--	--	--	--	--	--	--	--	--	--	--	--	--	--	--	--	--	--	--	--	--	--
Genital prolapse	--	--	--	--	--	--	--	--	--	--	--	--	--	--	--	--	--	--	--	--	--	--	--
Other gynecological diseases	--	--	--	--	--	--	--	--	--	--	--	--	--	--	--	--	--	--	--	--	--	--	--
Hemoglobinopathies and hemolytic anaemias	2 (2–2)	1 (1–1)	5 (4–6)	11 (10–12)	8 (7–9)	8 (7–9)	15 (13–16)	29 (26–32)	33 (28–38)	34 (28–38)	34 (30–37)	35 (32–37)	37 (35–40)	40 (38–42)	52 (49–55)	65 (61–69)	77 (73–82)	95 (89–101)	105 (98–112)	109 (98–117)	98 (85–106)	70 (58–78)	--
Thalassemias	0 (0–1)	0 (0–0)	2 (1–2)	5 (4–6)	4 (3–5)	2 (2–3)	3 (2–4)	4 (3–6)	3 (2–4)	1 (1–2)	1 (0–1)	0 (0–1)	0 (0–1)	0 (0–1)	0 (0–1)	0 (0–0)	0 (0–0)	0 (0–0)	0 (0–0)	0 (0–0)	0 (0–0)	0 (0–0)	--
Sickle cell disorders	1 (1–2)	0 (0–1)	3 (2–4)	5 (4–6)	2 (2–3)	2 (2–3)	5 (4–7)	12 (9–15)	15 (11–20)	15 (11–20)	14 (11–19)	13 (10–18)	13 (10–18)	13 (9–17)	15 (11–19)	12 (9–16)	9 (6–12)	4 (2–6)	1 (0–1)	0 (0–0)	0 (0–0)	0 (0–0)	--
G6PD deficiency	0 (0–0)	0 (0–0)	0 (0–0)	1 (1–1)	2 (1–3)	3 (2–4)	6 (4–7)	11 (7–14)	14 (9–18)	15 (10–20)	16 (10–21)	16 (11–21)	18 (12–23)	19 (12–23)	22 (15–28)	25 (16–32)	22 (13–29)	19 (11–26)	14 (7–19)	8 (4–11)	4 (2–5)	1 (1–2)	--
Other hemoglobinopathies and hemolytic anaemias	0 (0–0)	0 (0–0)	0 (0–0)	0 (0–0)	0 (0–0)	0 (0–0)	1 (1–1)	2 (2–3)	2 (2–3)	3 (2–4)	3 (3–5)	4 (3–6)	5 (4–7)	8 (7–11)	15 (12–18)	28 (23–35)	46 (40–55)	71 (64–81)	91 (83–100)	101 (92–109)	94 (81–103)	69 (57–76)	--
Endocrine, metabolic, blood, and immune disorders	37 (28–64)	24 (18–38)	101 (78–180)	101 (81–168)	62 (51–100)	73 (61–132)	87 (73–147)	127 (101–210)	165 (121–246)	208 (149–302)	317 (215–418)	405 (261–532)	641 (391–796)	932 (575–1,144)	1,375 (788–1,646)	1,681 (947–1,903)	1,909 (1,114–2,264)	1,938 (1,132–2,213)	1,824 (1,023–2,133)	1,647 (881–1,887)	1,460 (711–1,708)	1,011 (421–1,222)	--
Sudden infant death syndrome	--	59 (50–69)	851 (742–959)	--	--	--	--	--	--	--	--	--	--	--	--	--	--	--	--	--	--	--	--
Transport injuries	4 (3–4)	3 (3–4)	55 (49–63)	251 (227–280)	260 (243–277)	369 (347–389)	2,049 (1,881–2,160)	3,148 (2,860–3,341)	2,581 (2,143–2,787)	2,102 (1,704–2,290)	2,027 (1,774–2,171)	1,950 (1,782–2,077)	2,165 (2,044–2,286)	2,339 (2,217–2,457)	2,544 (2,416–2,667)	2,277 (2,136–2,387)	1,883 (1,790–1,973)	1,565 (1,483–1,640)	1,246 (1,170–1,316)	946 (852–1,014)	666 (576–728)	303 (255–332)	--
Road injuries	3 (3–3)	3 (2–3)	54 (47–61)	240 (216–267)	232 (217–248)	306 (286–324)	1,918 (1,762–2,027)	2,976 (2,708–3,164)	2,416 (2,010–2,606)	1,948 (1,581–2,122)	1,865 (1,634–2,001)	1,788 (1,636–1,906)	1,977 (1,865–2,094)	2,134 (2,019–2,246)	2,322 (2,201–2,440)	2,068 (1,941–2,177)	1,703 (1,616–1,789)	1,425 (1,347–1,498)	1,147 (1,074–1,210)	891 (802–956)	635 (548–694)	291 (245–319)	--
Pedestrian road injuries	1 (1–1)	0 (0–0)	7 (6–8)	115 (103–128)	79 (73–85)	78 (72–83)	199 (185–211)	315 (286–336)	298 (249–321)	285 (232–311)	300 (266–321)	315 (290–337)	376 (352–403)	453 (423–484)	502 (471–537)	441 (412–469)	352 (331–373)	308 (289–328)	268 (249–286)	204 (183–221)	147 (126–162)	68 (56–76)	--
Cyclist road injuries	--	--	--	5 (4–5)	21 (19–23)	42 (37–47)	51 (45–57)	43 (36–49)	42 (30–49)	44 (30–52)	47 (37–54)	59 (49–67)	83 (74–94)	105 (95–117)	121 (108–135)	106 (93–120)	87 (79–96)	58 (51–66)	39 (34–44)	21 (17–24)	13 (11–15)	4 (3–5)	--
Motorcyclist road injuries	0 (0–0)	0 (0–0)	1 (1–1)	2 (1–2)	4 (4–4)	15 (14–16)	142 (134–151)	455 (413–486)	451 (377–485)	355 (286–384)	349 (308–375)	346 (316–374)	385 (352–421)	400 (369–437)	423 (389–460)	324 (295–355)	225 (204–247)	100 (90–110)	45 (39–51)	20 (18–21)	8 (7–9)	5 (4–5)	--
Motor vehicle road injuries	2 (2–2)	2 (2–2)	45 (40–50)	116 (104–131)	126 (117–135)	167 (156–177)	1,521 (1,381–1,614)	2,158 (1,941–2,300)	1,619 (1,322–1,756)	1,260 (997–1,380)	1,166 (1,011–1,255)	1,062 (946–1,138)	1,124 (1,059–1,189)	1,165 (1,099–1,230)	1,265 (1,199–1,334)	1,186 (1,103–1,251)	1,029 (978–1,082)	951 (898–1,000)	788 (737–832)	642 (578–690)	464 (399–506)	213 (180–234)	--
Other road injuries	0 (0–0)	0 (0–0)	1 (1–1)	2 (2–2)	2 (2–3)	4 (3–4)	5 (4–5)	5 (5–6)	6 (5–8)	5 (4–5)	4 (3–5)	5 (4–6)	8 (7–9)	10 (9–12)	12 (10–14)	11 (9–13)	10 (9–12)	9 (8–10)	7 (6–8)	5 (4–6)	2 (2–3)	1 (1–1)	--
Other transport injuries	1 (1–1)	0 (0–0)	2 (1–2)	11 (10–13)	27 (25–30)	63 (58–69)	131 (115–145)	172 (150–190)	165 (124–187)	154 (110–175)	162 (134–181)	162 (137–178)	188 (173–203)	206 (191–221)	221 (205–238)	209 (190–227)	180 (168–192)	139 (129–151)	99 (90–108)	55 (48–61)	32 (27–35)	12 (10–14)	--
Falls	1 (1–1)	1 (1–1)	10 (8–11)	23 (20–26)	10 (9–11)	16 (15–18)	66 (62–71)	127 (118–135)	135 (115–146)	153 (129–166)	198 (181–211)	268 (252–283)	411 (389–434)	650 (616–689)	978 (924–1,036)	1,255 (1,193–1,317)	1,507 (1,429–1,604)	2,051 (1,941–2,174)	2,644 (2,479–2,817)	3,398 (3,058–3,639)	3,761 (3,206–4,108)	2,958 (2,459–3,313)	--
Drowning	1 (1–1)	0 (0–0)	26 (23–29)	240 (215–265)	92 (85–98)	86 (81–92)	228 (213–245)	232 (215–247)	189 (170–206)	162 (147–177)	159 (149–169)	152 (144–163)	164 (155–173)	178 (168–188)	188 (179–197)	181 (172–190)	142 (135–149)	121 (115–127)	90 (85–95)	65 (59–70)	41 (35–44)	19 (16–21)	--
Fire, heat, and hot substances	0 (0–0)	1 (1–1)	14 (13–16)	75 (67–84)	46 (43–49)	29 (27–31)	27 (25–29)	44 (41–47)	52 (47–57)	53 (48–58)	63 (59–67)	71 (66–76)	97 (92–103)	135 (128–142)	182 (173–192)	209 (198–222)	192 (183–204)	181 (171–193)	167 (157–178)	149 (135–159)	122 (106–132)	81 (68–89)	--
Poisonings	1 (1–1)	1 (0–1)	7 (7–8)	21 (19–24)	7 (6–8)	17 (16–18)	32 (30–34)	68 (57–74)	91 (73–100)	74 (59–82)	78 (64–86)	78 (70–84)	69 (62–75)	88 (80–94)	88 (78–94)	68 (60–73)	46 (42–49)	38 (34–41)	28 (26–31)	28 (25–31)	25 (21–27)	13 (10–14)	--
Poisoning by carbon monoxide	0 (0–0)	0 (0–0)	2 (2–3)	8 (7–9)	4 (3–4)	7 (6–8)	23 (21–25)	46 (39–51)	61 (49–68)	62 (49–69)	66 (54–73)	66 (59–72)	58 (52–63)	60 (53–65)	62 (54–68)	50 (44–55)	36 (32–39)	26 (23–29)	21 (19–23)	19 (17–21)	18 (15–20)	8 (7–9)	--
Poisoning by other means	1 (1–1)	0 (0–1)	5 (5–6)	13 (12–15)	3 (3–3)	10 (9–11)	9 (8–10)	22 (18–24)	30 (24–33)	12 (9–14)	12 (10–14)	12 (10–13)	11 (10–12)	28 (25–30)	26 (23–28)	18 (16–19)	10 (9–10)	12 (11–13)	7 (7–8)	9 (8–10)	7 (6–8)	4 (4–5)	--
Exposure to mechanical forces	1 (0–1)	0 (0–0)	5 (5–6)	40 (36–44)	27 (25–28)	42 (39–44)	96 (89–105)	139 (129–150)	133 (119–148)	130 (118–146)	138 (129–151)	146 (136–162)	172 (162–185)	203 (192–219)	238 (226–255)	251 (239–271)	219 (210–230)	197 (187–206)	149 (141–156)	106 (96–112)	68 (60–73)	36 (30–40)	--
Unintentional firearm injuries	0 (0–0)	0 (0–0)	0 (0–1)	10 (6–12)	10 (8–12)	26 (24–32)	61 (54–71)	68 (59–86)	49 (41–67)	38 (31–53)	34 (28–50)	30 (25–45)	34 (26–46)	34 (25–45)	38 (27–52)	32 (21–38)	29 (18–36)	23 (14–27)	16 (11–20)	12 (8–17)	7 (5–10)	3 (2–4)	--
Other exposure to mechanical forces	0 (0–0)	0 (0–0)	5 (4–5)	30 (26–34)	17 (14–19)	15 (10–17)	35 (23–41)	71 (50–81)	83 (65–99)	92 (75–108)	104 (88–115)	116 (100–130)	138 (124–149)	169 (154–183)	199 (185–215)	220 (207–237)	190 (179–202)	174 (165–185)	134 (124–141)	93 (83–100)	61 (53–66)	33 (28–36)	--
Adverse effects of medical treatment	4 (3–5)	3 (3–4)	22 (18–28)	15 (12–18)	8 (7–10)	9 (8–11)	10 (9–11)	16 (14–18)	19 (17–23)	24 (20–28)	33 (30–38)	43 (39–48)	77 (70–85)	112 (103–123)	182 (168–197)	230 (211–256)	293 (268–328)	322 (295–351)	306 (276–359)	267 (236–326)	215 (183–265)	128 (105–160)	--
Animal contact	0 (0–0)	0 (0–1)	1 (1–2)	6 (5–7)	4 (3–4)	2 (2–2)	2 (2–2)	3 (2–3)	4 (3–4)	4 (4–5)	7 (6–7)	9 (8–10)	12 (11–13)	16 (14–17)	20 (18–21)	22 (20–24)	20 (19–22)	19 (18–21)	13 (11–14)	10 (9–12)	6 (5–7)	3 (2–3)	--
Venomous animal contact	0 (0–0)	0 (0–0)	0 (0–0)	1 (1–1)	1 (1–1)	1 (1–1)	1 (1–1)	1 (1–1)	2 (1–2)	2 (2–3)	4 (3–4)	6 (5–6)	7 (6–8)	9 (8–10)	10 (9–11)	11 (10–13)	9 (8–11)	7 (6–8)	3 (3–4)	3 (2–3)	1 (1–1)	1 (1–1)	--
Non-venomous animal contact	0 (0–0)	0 (0–0)	1 (1–1)	5 (5–6)	3 (3–3)	1 (1–1)	1 (1–1)	2 (1–2)	2 (2–2)	2 (2–2)	3 (3–3)	4 (3–4)	5 (4–5)	6 (6–7)	9 (8–10)	11 (10–12)	11 (10–12)	12 (11–13)	9 (8–10)	8 (7–8)	5 (4–5)	2 (2–2)	--
Foreign body	8 (7–9)	38 (33–42)	538 (482–594)	94 (85–103)	24 (23–26)	36 (34–38)	33 (31–35)	48 (44–51)	58 (53–63)	67 (61–73)	79 (74–84)	90 (86–95)	120 (115–126)	157 (149–166)	224 (214–235)	283 (269–299)	308 (294–324)	386 (366–406)	451 (423–473)	509 (460–543)	549 (476–591)	438 (369–479)	--
Pulmonary aspiration and foreign body in airway	8 (7–9)	38 (33–42)	537 (482–594)	92 (83–102)	23 (22–25)	35 (33–37)	32 (30–34)	47 (43–50)	57 (52–62)	66 (60–71)	78 (73–82)	89 (85–94)	119 (113–125)	155 (147–164)	222 (212–233)	281 (267–297)	306 (292–322)	384 (364–404)	448 (420–470)	507 (458–541)	548 (474–589)	436 (368–478)	--
Foreign body in other body part	0 (0–0)	0 (0–0)	0 (0–1)	2 (1–2)	1 (1–1)	1 (1–1)	1 (1–1)	1 (1–1)	1 (1–1)	1 (1–1)	1 (1–2)	1 (1–1)	1 (1–2)	2 (2–2)	2 (2–3)	2 (2–3)	2 (2–3)	2 (2–3)	3 (2–3)	2 (2–3)	2 (1–2)	1 (1–2)	--
Environmental heat and cold exposure	0 (0–0)	0 (0–0)	4 (4–5)	6 (5–7)	2 (1–2)	2 (2–3)	11 (10–12)	18 (16–20)	24 (22–26)	30 (27–33)	37 (35–40)	45 (41–49)	60 (55–64)	87 (80–94)	102 (95–109)	100 (92–109)	82 (77–88)	77 (71–83)	76 (70–81)	63 (56–68)	55 (48–61)	33 (27–37)	--
Other unintentional injuries	0 (0–0)	0 (0–0)	2 (2–2)	4 (4–5)	4 (4–5)	8 (8–9)	20 (18–22)	38 (35–42)	43 (38–48)	48 (43–55)	49 (45–54)	45 (42–50)	49 (45–54)	44 (41–49)	43 (40–48)	33 (31–36)	26 (24–28)	18 (17–19)	15 (14–16)	12 (10–13)	11 (9–12)	6 (5–7)	--
Self-harm	--	--	--	--	--	235 (218–255)	1,558 (1,478–1,657)	2,566 (2,393–2,717)	2,503 (2,269–2,653)	2,338 (2,135–2,490)	2,548 (2,394–2,695)	2,625 (2,491–2,778)	3,002 (2,857–3,162)	3,300 (3,137–3,451)	3,505 (3,345–3,683)	2,857 (2,729–3,003)	2,208 (2,105–2,325)	1,883 (1,792–1,992)	1,446 (1,349–1,564)	1,071 (968–1,168)	723 (622–793)	322 (269–360)	--
Self-harm by firearm	--	--	--	--	--	95 (84–118)	809 (738–939)	1,326 (1,190–1,607)	1,252 (1,114–1,483)	1,136 (992–1,316)	1,208 (1,071–1,426)	1,286 (1,155–1,515)	1,476 (1,358–1,803)	1,781 (1,644–2,202)	2,040 (1,893–2,471)	1,844 (1,727–2,147)	1,560 (1,444–1,730)	1,441 (1,306–1,553)	1,135 (994–1,236)	847 (727–930)	553 (466–615)	229 (186–260)	--

Self-harm by other specified means	--	--	--	--	--	140<br>(113–155)	749<br>(598–823)	1,240<br>(1,009–1,375)	1,251<br>(1,065–1,394)	1,202<br>(1,020–1,352)	1,340<br>(1,128–1,483)	1,339<br>(1,066–1,463)	1,525<br>(1,170–1,666)	1,519<br>(1,132–1,656)	1,465<br>(1,057–1,603)	1,013<br>(720–1,116)	648<br>(490–733)	442<br>(364–545)	311<br>(265–411)	224<br>(190–311)	170<br>(139–233)	93<br>(72–122)	--
Interpersonal violence	10<br>(9–12)	6<br>(5–7)	183<br>(162–205)	214<br>(192–236)	80<br>(75–86)	148<br>(139–157)	1,424<br>(1,352–1,515)	2,395<br>(2,172–2,540)	1,994<br>(1,697–2,147)	1,557<br>(1,314–1,672)	1,256<br>(1,094–1,342)	954<br>(898–1,007)	827<br>(783–871)	725<br>(684–764)	631<br>(590–666)	463<br>(434–488)	322<br>(305–339)	218<br>(206–230)	140<br>(132–148)	45<br>(41–49)	21<br>(19–23)	9<br>(7–10)	--
Physical violence by firearm	1<br>(1–1)	1<br>(1–1)	11<br>(10–13)	45<br>(40–51)	38<br>(35–41)	108<br>(101–115)	1,287<br>(1,220–1,367)	2,145<br>(1,944–2,274)	1,744<br>(1,483–1,882	1,322<br>(1,114–1,422)	1,019<br>(887–1,092)	742<br>(698–785)	609<br>(573–642)	491<br>(462–519)	403<br>(375–428)	286<br>(267–303)	187<br>(175–197)	119<br>(111–125)	73<br>(68–77)	20<br>(19–22)	8<br>(7–9)	3<br>(2–3)	--
Physical violence by sharp object	0<br>(0–0)	0<br>(0–0)	2<br>(2–2)	5<br>(5–6)	5<br>(4–5)	8<br>(8–9)	94<br>(87–102)	176<br>(160–191)	165<br>(141–183)	143<br>(122–160)	134<br>(119–145)	113<br>(106–122)	108<br>(115–101)	113<br>(106–120)	102<br>(95–109)	75<br>(71–80)	56<br>(53–59)	39<br>(37–41)	24<br>(22–26)	9<br>(8–9)	4<br>(4–5)	2<br>(2–3)	--
Physical violence by other means	9<br>(8–11)	5<br>(4–6)	169<br>(150–190)	163<br>(146–181)	38<br>(35–41)	31<br>(29–34)	43<br>(41–47)	74<br>(67–80)	85<br>(72–93)	92<br>(77–101)	102<br>(89–110)	98<br>(93–105)	111<br>(105–117)	121<br>(114–128)	126<br>(117–134)	101<br>(95–107)	80<br>(75–84)	61<br>(57–64)	44<br>(41–47)	16<br>(15–17)	9<br>(8–10)	4<br>(3–4)	--
Executions and police conflict	0<br>(0–0)	0<br>(0–0)	1<br>(0–1)	3<br>(3–4)	2<br>(2–2)	3<br>(3–3)	48<br>(35–68)	132<br>(95–181)	172<br>(122–234)	166<br>(125–221)	154<br>(120–206)	113<br>(88–150)	98<br>(78–124)	72<br>(59–91)	54<br>(44–67)	37<br>(31–44)	16<br>(14–19)	10<br>(8–11)	5<br>(4–6)	3<br>(2–3)	2<br>(1–2)	1<br>(0–1)	--

SR Table 12: Latin America and Caribbean deaths by cause and age group for males

Cause	Early Neonatal	Late Neonatal	Post Neonatal	1 to 4	5 to 9	10 to 14	15 to 19	20 to 24	25 to 29	30 to 34	35 to 39	40 to 44	45 to 49	50 to 54	55 to 59	60 to 64	65 to 69	70 to 74	75 to 79	80 to 84	85 to 89	90 to 94	95 plus
All causes	41,007 (32,243–51,546)	12,805 (10,154–15,957)	35,145 (27,217–45,125)	16,060 (12,894–19,988)	8,654 (7,584–9,890)	9,658 (8,515–10,940)	33,276 (29,684–37,353)	53,701 (49,172–58,767)	54,879 (50,948–59,374)	56,392 (52,454–60,864)	62,040 (57,138–67,664)	69,953 (63,858–77,029)	83,474 (75,580–92,752)	107,921 (97,597–119,914)	134,638 (121,903–149,400)	159,489 (145,022–176,019)	177,792 (162,491–195,232)	194,634 (178,722–212,495)	198,422 (183,280–215,182)	190,560 (177,177–205,103)	148,076 (139,382–157,270)	86,948 (82,227–91,979)	--
Sexually transmitted infections excluding HIV	598 (249–1,203)	207 (85–402)	291 (122–569)	10 (4–20)	5 (2–10)	3 (2–5)	3 (2–4)	4 (2–7)	4 (2–8)	5 (3–8)	8 (5–14)	6 (4–9)	7 (5–11)	9 (6–15)	9 (6–14)	11 (7–17)	12 (8–18)	12 (8–16)	9 (7–13)	9 (6–13)	6 (4–10)	4 (2–6)	--
Syphilis	598 (249–1,203)	207 (85–402)	291 (122–569)	10 (4–20)	5 (2–10)	2 (1–3)	2 (1–3)	2 (2–4)	3 (2–6)	3 (2–5)	7 (4–12)	5 (3–7)	5 (3–7)	7 (4–11)	7 (5–11)	9 (6–13)	9 (6–14)	9 (7–12)	5 (4–7)	4 (3–6)	2 (1–3)	1 (0–1)	--
Chlamydial infection	--	--	--	--	--	0 (0–0)	0 (0–0)	0 (0–0)	0 (0–0)	0 (0–0)	0 (0–0)	0 (0–0)	0 (0–0)	0 (0–0)	0 (0–0)	0 (0–0)	0 (0–0)	0 (0–0)	0 (0–0)	0 (0–0)	0 (0–0)	0 (0–0)	--
Gonococcal infection	--	--	--	--	--	0 (0–0)	0 (0–0)	1 (1–2)	0 (0–1)	0 (0–0)	0 (0–1)	1 (1–1)	1 (0–1)	1 (0–1)	1 (0–1)	1 (1–1)	1 (1–2)	1 (1–2)	1 (1–1)	1 (1–1)	0 (0–1)	0 (0–0)	--
Other sexually transmitted infections	--	--	--	--	--	1 (1–2)	0 (0–0)	1 (0–1)	1 (1–2)	1 (1–2)	1 (0–1)	1 (0–1)	1 (1–2)	2 (1–3)	2 (1–2)	1 (1–2)	1 (1–2)	2 (1–2)	3 (2–4)	4 (3–6)	4 (2–6)	3 (2–4)	--
Tuberculosis	--	--	131 (94–175)	110 (80–147)	49 (39–61)	47 (37–58)	134 (107–167)	343 (281–426)	468 (402–548)	584 (503–679)	702 (607–815)	818 (709–947)	930 (813–1,075)	1,034 (900–1,198)	1,098 (963–1,249)	1,083 (947–1,223)	1,013 (879–1,171)	988 (858–1,141)	875 (753–1,014)	667 (559–772)	425 (349–495)	184 (143–217)	--
Drug-susceptible tuberculosis	--	--	123 (87–164)	104 (75–140)	45 (36–57)	43 (33–53)	122 (97–153)	314 (254–389)	431 (364–510)	541 (454–636)	650 (553–758)	758 (651–885)	861 (734–1,004)	957 (815–1,107)	1,015 (872–1,173)	1,000 (857–1,143)	934 (799–1,088)	909 (770–1,065)	802 (683–944)	610 (506–713)	389 (311–460)	168 (129–201)	--
Multidrug-resistant tuberculosis without extensive drug resistance	--	--	8 (3–17)	5 (2–11)	3 (1–7)	3 (1–7)	10 (4–22)	25 (10–53)	32 (14–63)	37 (15–76)	45 (18–96)	51 (20–109)	59 (23–127)	66 (26–144)	72 (27–156)	71 (27–149)	68 (27–143)	68 (27–142)	63 (25–126)	48 (19–98)	31 (13–64)	14 (5–28)	--
Extensively drug-resistant tuberculosis	--	--	1 (0–3)	1 (0–2)	1 (0–1)	1 (0–1)	2 (1–3)	4 (2–9)	5 (2–11)	6 (2–13)	7 (3–15)	8 (3–18)	10 (4–21)	11 (4–24)	12 (4–25)	12 (4–25)	11 (4–24)	11 (4–23)	10 (4–22)	8 (3–16)	5 (2–11)	2 (1–5)	--
Lower respiratory infections	1,109 (800–1,492)	1,015 (762–1,312)	6,589 (4,892–8,675)	2,304 (1,756–2,959)	589 (483–697)	430 (359–505)	575 (491–669)	806 (707–916)	1,026 (915–1,144)	1,288 (1,160–1,431)	1,719 (1,548–1,910)	2,165 (1,941–2,434)	2,653 (2,348–2,978)	3,461 (3,080–3,892)	4,398 (3,896–4,926)	5,457 (4,877–6,138)	6,674 (5,957–7,444)	8,682 (7,766–9,623)	10,616 (9,456–11,784)	12,418 (10,784–13,874)	11,597 (9,553–12,970)	7,672 (6,108–8,780)	--
Upper respiratory infections	2 (1–3)	4 (3–6)	50 (33–75)	35 (20–68)	8 (5–12)	3 (3–4)	4 (3–5)	4 (3–5)	4 (4–5)	4 (3–4)	5 (4–6)	4 (3–5)	6 (5–7)	6 (5–7)	7 (6–9)	8 (7–10)	9 (7–11)	9 (8–11)	9 (8–11)	11 (9–13)	8 (6–10)	6 (5–8)	--
Otitis media	1 (0–3)	1 (0–6)	4 (2–7)	1 (1–2)	1 (0–1)	1 (1–2)	2 (1–2)	1 (1–2)	1 (1–2)	1 (1–1)	1 (1–1)	1 (1–1)	1 (1–1)	1 (1–2)	1 (1–2)	1 (1–2)	1 (1–2)	2 (1–2)	1 (1–2)	1 (1–1)	1 (1–1)	0 (0–0)	--
Diarrhoeal diseases	95 (50–182)	260 (132–502)	3,321 (2,231–4,849)	1,450 (920–2,298)	231 (171–336)	114 (86–163)	96 (73–136)	122 (96–165)	149 (122–196)	164 (133–217)	196 (161–258)	248 (200–334)	282 (226–382)	359 (295–474)	437 (361–570)	542 (454–706)	671 (563–861)	846 (716–1,106)	976 (823–1,234)	1,093 (896–1,369)	901 (733–1,120)	536 (412–655)	--
Typhoid fever	--	--	2 (1–4)	3 (1–6)	2 (1–3)	2 (1–4)	3 (1–10)	4 (1–8)	3 (1–7)	3 (1–8)	3 (1–8)	3 (1–9)	3 (1–11)	3 (1–11)	3 (1–9)	3 (2–9)	3 (2–11)	3 (2–9)	3 (2–9)	5 (2–17)	3 (1–9)	1 (1–6)	--
Paratyphoid fever	--	--	0 (0–0)	0 (0–1)	0 (0–1)	0 (0–1)	0 (0–0)	0 (0–0)	0 (0–0)	0 (0–0)	0 (0–0)	0 (0–0)	0 (0–0)	0 (0–0)	0 (0–0)	0 (0–0)	0 (0–0)	0 (0–0)	0 (0–0)	0 (0–0)	0 (0–0)	0 (0–0)	--
iNTS	--	0 (0–1)	6 (3–12)	10 (4–18)	9 (4–21)	6 (2–17)	3 (1–7)	3 (1–6)	2 (1–5)	2 (1–4)	2 (1–4)	2 (1–4)	2 (1–4)	2 (1–5)	3 (1–6)	3 (1–6)	3 (2–6)	4 (2–7)	3 (1–5)	2 (1–4)	2 (1–3)	1 (0–2)	--
Other intestinal infectious diseases	--	--	4 (0–15)	4 (0–14)	2 (0–6)	1 (0–5)	1 (0–5)	2 (0–5)	1 (0–3)	1 (0–3)	1 (0–3)	1 (0–3)	0 (0–2)	1 (0–2)	0 (0–2)	0 (0–2)	1 (0–3)	1 (0–2)	0 (0–2)	1 (0–2)	1 (0–3)	0 (0–1)	--
Malaria	0 (0–0)	3 (1–8)	18 (5–44)	33 (9–86)	21 (7–48)	20 (6–45)	65 (20–154)	74 (23–179)	54 (17–127)	52 (17–121)	38 (12–89)	49 (15–119)	43 (13–104)	43 (13–102)	40 (12–96)	36 (11–86)	31 (9–73)	29 (9–68)	10 (3–23)	5 (1–11)	1 (0–1)	0 (0–0)	--
Chagas disease	--	--	0 (0–1)	0 (0–1)	1 (0–3)	2 (1–5)	7 (3–20)	13 (7–35)	20 (11–63)	53 (28–146)	102 (53–236)	158 (67–383)	223 (81–475)	337 (145–753)	471 (189–1,013)	618 (235–1,295)	699 (254–1,339)	651 (215–1,249)	563 (173–908)	384 (113–686)	322 (102–603)	149 (51–336)	--
Visceral leishmaniasis	--	--	23 (0–80)	102 (0–427)	92 (0–502)	60 (0–321)	51 (0–244)	45 (0–197)	53 (0–206)	56 (0–202)	52 (0–173)	39 (0–123)	29 (0–80)	24 (0–64)	22 (0–57)	20 (0–49)	17 (0–41)	13 (0–30)	8 (0–21)	5 (0–14)	3 (0–7)	1 (0–4)	--
African trypanosomiasis	--	--	--	0 (0–0)	0 (0–0)	0 (0–0)	0 (0–0)	0 (0–0)	0 (0–0)	0 (0–0)	0 (0–0)	0 (0–0)	0 (0–0)	0 (0–0)	0 (0–0)	0 (0–0)	0 (0–0)	0 (0–0)	0 (0–0)	0 (0–0)	0 (0–0)	0 (0–0)	--
Schistosomiasis	--	--	0 (0–0)	0 (0–0)	0 (0–1)	1 (0–2)	4 (2–8)	7 (4–12)	9 (4–14)	10 (5–17)	15 (8–24)	19 (10–29)	22 (12–35)	30 (18–46)	37 (22–56)	39 (24–61)	34 (20–52)	39 (24–59)	32 (18–50)	22 (13–35)	12 (6–19)	4 (1–8)	--
Cysticercosis	--	--	--	0 (0–1)	2 (0–6)	3 (0–11)	8 (0–24)	14 (1–38)	19 (2–50)	20 (2–53)	18 (1–48)	17 (0–45)	15 (0–43)	14 (0–42)	14 (0–40)	11 (0–33)	10 (0–29)	4 (0–15)	6 (0–18)	4 (0–14)	2 (0–8)	1 (0–4)	--
Cystic echinococcosis	--	--	--	0 (0–0)	0 (0–1)	0 (0–1)	0 (0–1)	0 (0–1)	0 (0–1)	0 (0–2)	0 (0–2)	0 (0–2)	0 (0–2)	1 (0–2)	1 (0–2)	1 (0–2)	1 (0–2)	1 (0–2)	0 (0–2)	0 (0–1)	0 (0–1)	0 (0–1)	--
Dengue	0 (0–0)	1 (0–1)	37 (16–55)	61 (42–81)	57 (34–77)	42 (21–57)	29 (11–41)	25 (9–37)	25 (8–36)	24 (6–32)	25 (7–33)	26 (6–35)	28 (7–36)	32 (7–42)	33 (6–45)	36 (7–54)	42 (7–59)	41 (8–57)	39 (7–53)	35 (7–46)	25 (5–34)	13 (3–18)	--
Yellow fever	--	0 (0–0)	0 (0–1)	3 (1–9)	6 (2–17)	6 (2–18)	6 (2–18)	6 (1–18)	5 (1–15)	5 (1–13)	4 (1–11)	4 (1–10)	3 (1–9)	2 (1–7)	2 (1–6)	2 (0–4)	1 (0–3)	1 (0–3)	1 (0–2)	0 (0–1)	0 (0–1)	0 (0–0)	--
Rabies	--	--	0 (0–0)	1 (0–1)	1 (0–2)	0 (0–1)	0 (0–1)	0 (0–0)	0 (0–1)	1 (0–2)	0 (0–1)	1 (0–2)	1 (1–2)	0 (0–1)	1 (0–2)	1 (0–2)	0 (0–1)	0 (0–1)	0 (0–1)	0 (0–1)	0 (0–0)	0 (0–0)	--
Ascariasis	--	--	2 (1–4)	8 (4–13)	5 (3–8)	2 (1–3)	1 (1–3)	1 (1–2)	1 (1–2)	1 (1–2)	1 (1–2)	1 (0–1)	1 (0–1)	1 (0–1)	1 (0–1)	2 (1–3)	1 (0–1)	1 (1–2)	1 (1–2)	1 (1–2)	1 (0–1)	1 (0–1)	--
Zika virus disease	0 (0–0)	0 (0–0)	0 (0–0)	0 (0–0)	0 (0–0)	0 (0–0)	0 (0–0)	0 (0–0)	0 (0–0)	0 (0–0)	0 (0–0)	0 (0–0)	0 (0–0)	0 (0–0)	0 (0–0)	0 (0–0)	0 (0–0)	0 (0–0)	0 (0–0)	0 (0–0)	0 (0–0)	0 (0–0)	--
Other neglected tropical diseases	8 (3–13)	7 (3–12)	43 (16–79)	38 (19–91)	17 (9–32)	10 (4–15)	9 (3–13)	8 (3–12)	8 (3–12)	10 (3–14)	10 (4–14)	11 (4–16)	12 (5–17)	13 (4–17)	12 (5–17)	13 (5–18)	14 (5–18)	14 (6–19)	12 (5–17)	12 (4–16)	9 (3–13)	6 (2–8)	--
Meningitis	59 (35–99)	107 (65–171)	650 (450–924)	359 (253–519)	147 (120–179)	109 (92–129)	112 (90–133)	115 (97–134)	114 (99–130)	121 (104–139)	133 (115–153)	145 (124–169)	149 (127–174)	143 (122–168)	151 (127–175)	146 (122–169)	130 (111–153)	114 (97–133)	95 (81–110)	72 (59–84)	50 (39–59)	20 (15–24)	--
Encephalitis	15 (10–21)	22 (15–33)	222 (149–311)	181 (127–258)	106 (83–140)	78 (62–99)	66 (54–82)	73 (60–90)	72 (59–89)	75 (61–91)	80 (65–98)	74 (59–90)	76 (60–93)	76 (61–93)	78 (62–96)	76 (59–92)	72 (58–87)	66 (52–77)	52 (42–62)	39 (31–48)	25 (19–30)	10 (7–13)	--
Diphtheria	--	--	1 (0–3)	2 (1–5)	1 (0–2)	0 (0–1)	0 (0–0)	0 (0–0)	0 (0–0)	0 (0–0)	0 (0–0)	0 (0–0)	0 (0–1)	0 (0–1)	0 (0–0)	--	--	--	--	--	--	--	--
Whooping cough	--	--	480 (208–1,010)	403 (156–819)	42 (18–86)	8 (4–17)	4 (2–7)	2 (1–5)	2 (1–3)	2 (1–3)	1 (1–2)	1 (1–2)	1 (1–2)	1 (1–2)	2 (1–3)	--	--	--	--	--	--	--	--
Tetanus	14 (5–33)	20 (6–66)	4 (2–9)	6 (2–42)	6 (3–20)	6 (4–22)	5 (3–15)	6 (4–17)	6 (4–19)	8 (5–23)	11 (7–29)	14 (9–36)	17 (10–47)	22 (13–59)	23 (14–60)	23 (14–61)	22 (13–61)	21 (12–60)	16 (10–51)	11 (6–34)	6 (4–19)	3 (2–8)	--
Measles	0 (0–0)	0 (0–0)	0 (0–0)	0 (0–0)	0 (0–0)	0 (0–0)	0 (0–0)	0 (0–0)	0 (0–0)	0 (0–0)	0 (0–0)	0 (0–0)	0 (0–0)	0 (0–0)	0 (0–0)	0 (0–0)	0 (0–0)	0 (0–0)	0 (0–0)	0 (0–0)	0 (0–0)	0 (0–0)	--
Varicella and herpes zoster	3 (1–6)	8 (4–15)	55 (30–98)	66 (38–116)	27 (15–40)	11 (5–18)	6 (3–9)	6 (2–8)	7 (3–11)	9 (5–18)	14 (8–29)	8 (4–15)	11 (5–19)	11 (4–17)	13 (5–19)	11 (5–16)	20 (11–34)	21 (11–36)	24 (14–45)	28 (18–54)	24 (15–42)	18 (11–31)	--
Acute hepatitis	--	--	27 (19–38)	26 (20–36)	11 (9–14)	10 (8–13)	9 (7–11)	12 (9–16)	14 (12–17)	18 (15–21)	22 (19–26)	28 (24–33)	33 (28–39)	35 (29–42)	37 (31–43)	34 (29–40)	31 (26–37)	28 (24–33)	22 (19–26)	16 (14–19)	11 (9–14)	5 (4–6)	--
Acute hepatitis A	--	--	9 (5–15)	18 (12–25)	7 (5–9)	5 (4–7)	5 (3–7)	6 (4–9)	5 (4–8)	6 (5–10)	6 (5–10)	7 (5–12)	8 (5–13)	9 (6–14)	9 (6–15)	8 (6–13)	10 (7–14)	9 (7–15)	8 (6–12)	7 (5–10)	6 (4–8)	2 (2–3)	--
Acute hepatitis B	--	--	15 (9–21)	5 (2–8)	3 (2–4)	3 (2–5)	3 (2–4)	4 (2–6)	7 (5–9)	8 (6–11)	10 (7–13)	13 (9–17)	14 (9–19)	16 (11–21)	18 (12–23)	15 (9–20)	14 (8–17)	12 (8–16)	9 (5–12)	6 (3–8)	3 (2–5)	2 (1–2)	--
Acute hepatitis C	--	--	1 (0–1)	3 (2–7)	1 (1–2)	1 (1–2)	1 (1–2)	2 (1–3)	2 (1–3)	2 (1–4)	5 (3–8)	6 (3–10)	9 (5–15)	9 (5–15)	8 (4–14)	9 (5–15)	7 (4–12)	6 (3–9)	4 (2–6)	3 (2–6)	2 (1–3)	1 (0–1)	--
Acute hepatitis E	--	--	3 (1–6)	1 (0–1)	0 (0–1)	0 (0–1)	0 (0–1)	0 (0–0)	0 (0–1)	0 (0–1)	1 (0–2)	1 (1–2)	1 (1–3)	1 (0–2)	1 (1–3)	1 (1–3)	1 (1–2)	1 (0–2)	1 (1–2)	0 (0–1)	0 (0–1)	0 (0–1)	--
Other unspecified infectious diseases	40 (27–67)	40 (26–70)	164 (98–292)	72 (41–130)	38 (26–64)	42 (29–68)	56 (38–87)	69 (45–106)	81 (54–129)	99 (66–162)	119 (81–197)	145 (99–243)	163 (112–277)	179 (128–299)	187 (129–310)	169 (118–276)	151 (107–249)	131 (87–204)	104 (68–165)	69 (44–100)	58 (38–88)	32 (20–49)	--
Maternal disorders	--	--	--	--	--	--	--	--	--	--	--	--	--	--	--	--	--	--	--	--	--	--	--
Maternal haemorrhage	--	--	--	--	--	--	--	--	--	--	--	--	--	--	--	--	--	--	--	--	--	--	--
Maternal sepsis and other pregnancy related infections	--	--	--	--	--	--	--	--	--	--	--	--	--	--	--	--	--	--	--	--	--	--	--
Maternal hypertensive disorders	--	--	--	--	--	--	--	--	--	--	--	--	--	--	--	--	--	--	--	--	--	--	--
Maternal obstructed labour and uterine rupture	--	--	--	--	--	--	--	--	--	--	--	--	--	--	--	--	--	--	--	--	--	--	--
Maternal abortive outcome	--	--	--	--	--	--	--	--	--	--	--	--	--	--	--	--	--	--	--	--	--	--	--
Ectopic pregnancy	--	--	--	--	--	--	--	--	--	--	--	--	--	--	--	--	--	--	--	--	--	--	--
Indirect maternal deaths	--	--	--	--	--	--	--	--	--	--	--	--	--	--	--	--	--	--	--	--	--	--	--
Late maternal deaths	--	--	--	--	--	--	--	--	--	--	--	--	--	--	--	--	--	--	--	--	--	--	--
Maternal deaths aggravated by HIV/AIDS	--	--	--	--	--	--	--	--	--	--	--	--	--	--	--	--	--	--	--	--	--	--	--
Other maternal disorders	--	--	--	--	--	--	--	--	--	--	--	--	--	--	--	--	--	--	--	--	--	--	--
Neonatal disorders	30,985 (23,959–39,073)	7,900 (6,148–9,896)	5,788 (4,377–7,517)	193 (146–248)	--	--	--	--	--	--	--	--	--	--	--	--	--	--	--	--	--	--	--
Neonatal preterm birth	13,516 (10,319–17,183)	2,337 (1,798–2,981)	1,971 (1,477–2,595)	32 (23–43)	--	--	--	--	--	--	--	--	--	--	--	--	--	--	--	--	--	--	--
Neonatal encephalopathy due to birth asphyxia and trauma	7,508 (5,718–9,480)	1,163 (869–1,504)	996 (734–1,314)	103 (76–132)	--	--	--	--	--	--	--	--	--	--	--	--	--	--	--	--	--	--	--
Neonatal sepsis and other neonatal infections	4,963 (3,750–6,419)	2,958 (2,278–3,753)	1,688 (1,258–2,220)	7 (5–10)	--	--	--	--	--	--	--	--	--	--	--	--	--	--	--	--	--	--	--
Hemolytic disease and other neonatal jaundice	210 (158–271)	68 (52–87)	18 (12–27)	4 (3–5)	--	--	--	--	--	--	--	--	--	--	--	--	--	--	--	--	--	--	--
Other neonatal disorders	4,787 (3,622–6,204)	1,375 (1,040–1,785)	1,115 (813–1,476)	47 (34–64)	--	--	--	--	--	--	--	--	--	--	--	--	--	--	--	--	--	--	--
Nutritional deficiencies	0 (0–0)	0 (0–0)	1,366 (976–1,839)	687 (494–935)	138 (113–166)	90 (75–106)	86 (73–100)	110 (94–129)	130 (114–148)	158 (140–176)	209 (185–237)	255 (224–288)	304 (267–347)	388 (339–443)	485 (428–550)	603 (531–684)	754 (671–845)	1,007 (893–1,135)	1,336 (1,193–1,487)	1,573 (1,365–1,773)	1,524 (1,272–1,706)	1,065 (850–1,202)	--
Protein-energy malnutrition	0 (0–0)	0 (0–0)	1,333 (952–1,796)	667 (480–914)	131 (107–159)	84 (70–99)	76 (63–90)	97 (82–114)	115 (99–133)	142 (123–159)	189 (163–215)	227 (196–258)	272 (234–313)	349 (302–401)	433 (378–493)	540 (465–617)	684 (598–772)	918 (809–1,035)	1,214 (1,061–1,362)	1,443 (1,208–1,630)	1,407 (1,126–1,580)	974 (727–1,106)	--
Other nutritional deficiencies	--	--	33 (23–46)	20 (14–28)	7 (4–10)	6 (4–9)	10 (6–18)	13 (7–18)	15 (10–25)	16 (9–26)	21 (11–33)	28 (16–45)	32 (15–53)	39 (20–57)	52 (26–78)	63 (33–93)	70 (37–110)	89 (44–129)	122 (57–186)	130 (67–214)	117 (66–230)	91 (53–184)	--
Lip and oral cavity cancer	--	--	--	--	3 (2–3)	4 (3–4)	7 (6–9)	13 (12–15)	21 (19–24)	43 (39–48)	96 (87–106)	214 (193–238)	426 (383–473)	708 (644–774)	950 (866–1,037)	997 (908–1,090)	903 (826–987)	785 (708–861)	636 (573–699)	473 (411–523)	291 (239–330)	137 (107–156)	--
Nasopharynx cancer	--	--	--	--	4 (3–4)	7 (6–9)	14 (12–16)	14 (12–17)	12 (11–14)	15 (14–17)	26 (23–29)	50 (45–56)	74 (65–82)	119 (105–133)	151 (134–170)	109 (95–124)	104 (91–118)	92 (81–105)	63 (54–71)	46 (38–53)	33 (26–38)	16 (12–18)	--
Other pharynx cancer	--	--	--	--	--	--	--	7 (6–8)	9 (7–10)	17 (15–19)	51 (45–58)	145 (130–162)	324 (294–356)	559 (507–618)	743 (672–825)	752 (679–829)	648 (585–717)	481 (431–533)	332 (298–366)	209 (180–234)	124 (102–139)	49 (38–58)	--
Oesophageal cancer	--	--	--	--	--	--	--	14 (12–15)	23 (21–26)	62 (57–68)	151 (137–166)	378 (343–417)	799 (733–868)	1,456 (1,343–1,586)	2,048 (1,894–2,217)	2,200 (2,030–2,375)	2,135 (1,970–2,326)	1,908 (1,744–2,079)	1,540 (1,393–1,675)	1,109 (963–1,221)	659 (544–740)	278 (219–315)	--
Stomach cancer	--	--	--	--	--	--	42 (35–50)	95 (82–109)	191 (167–217)	384 (341–437)	655 (578–749)	1,048 (920–1,205)	1,606 (1,404–1,850)	2,561 (2,242–2,920)	3,577 (3,152–4,073)	4,640 (4,135–5,233)	5,309 (4,730–5,991)	5,618 (4,999–6,316)	5,062 (4,488–5,637)	3,907 (3,349–4,407)	2,568 (2,109–2,927)	1,045 (827–1,201)	--
Colon and rectum cancer	--	--	--	--	7 (6–9)	9 (8–11)	49 (42–58)	106 (94–121)	190 (171–212)	353 (318–389)	562 (504–627)	863 (771–970)	1,314 (1,171–1,492)	2,010 (1,784–2,265)	2,857 (2,561–3,201)	3,713 (3,349–4,152)	4,142 (3,730–4,600)	4,356 (3,952–4,814)	4,034 (3,628–4,468)	3,301 (2,856–3,679)	2,180 (1,819–2,441)	960 (766–1,096)	--
Liver cancer due to hepatitis B	--	--	--	--	--	8 (6–10)	10 (8–14)	18 (14–23)	31 (23–38)	42 (33–53)	60 (43–77)	85 (60–113)	123 (86–169)	200 (140–276)	291 (205–405)	329 (219–463)	310 (202–438)	277 (172–413)	223 (149–315)	141 (86–206)	70 (46–98)	26 (16–39)	--
Liver cancer due to hepatitis C	--	--	--	--	--	0 (0–0)	0 (0–1)	1 (1–1)	2 (1–4)	4 (3–6)	10 (6–16)	22 (14–31)	51 (32–75)	123 (85–171)	237 (160–338)	341 (233–491)	411 (305–544)	471 (348–616)	484 (361–621)	395 (299–493)	227 (178–280)	96 (69–122)	--
Liver cancer due to alcohol use	--	--	--	--	--	--	1 (0–1)	3 (2–5)	8 (4–12)	15 (10–22)	32 (21–47)	61 (42–86)	118 (79–165)	240 (162–325)	397 (281–530)	557 (403–727)	706 (546–901)	739 (563–936)	596 (461–744)	364 (273–467)	163 (121–209)	55 (37–75)	--
Liver cancer due to NASH	--	--	--	--	--	--	4 (3–6)	4 (3–6)	4 (2–6)	4 (3–6)	6 (4–9)	9 (6–13)	17 (11–25)	32 (22–47)	53 (35–78)	73 (49–105)	88 (60–132)	104 (72–155)	113 (78–156)	87 (59–127)	45 (31–62)	17 (11–27)	--
	0 (0–0)	0 (0–0)	7 (4–11)	29 (18–42)	14 (11–17)	11 (9–15)	--	--	--	--	--	--	--	--	--	--	--	--	--	--	--	--	--
Liver cancer due to other causes	1 (1–2)	1 (1–1)	7 (4–9)	7 (5–10)	6 (5–8)	6 (4–8)	6 (4–8)	6 (4–8)	6 (4–9)	6 (4–9)	8 (5–12)	11 (7–16)	17 (11–25)	29 (19–42)	40 (27–60)	47 (31–70)	48 (32–69)	49 (32–69)	45 (31–64)	32 (21–46)	16 (11–22)	6 (4–9)	--
Gallbladder and biliary tract cancer	--	--	--	--	--	--	--	6 (5–8)	13 (11–16)	26 (22–31)	51 (44–60)	95 (83–113)	162 (139–193)	295 (252–352)	429 (364–499)	560 (481–650)	638 (546–748)	684 (597–803)	644 (559–758)	499 (416–581)	324 (261–383)	141 (108–175)	--
Pancreatic cancer	--	--	--	--	--	--	8 (7–9)	19 (17–22)	36 (32–40)	92 (82–102)	188 (170–211)	361 (320–407)	650 (573–736)	1,166 (1,034–1,318)	1,755 (1,565–1,981)	2,213 (1,986–2,487)	2,401 (2,155–2,668)	2,398 (2,154–2,660)	2,024 (1,799–2,253)	1,500 (1,294–1,685)	859 (716–969)	344 (268–396)	--
Larynx cancer	--	--	--	--	--	--	--	8 (7–9)	11 (10–12)	22 (19–24)	59 (52–66)	178 (157–201)	406 (364–455)	801 (726–881)	1,166 (1,061–1,278)	1,344 (1,226–1,471)	1,316 (1,201–1,440)	1,219 (1,106–1,340)	940 (847–1,035)	661 (568–733)	359 (296–405)	145 (114–167)	--
Tracheal, bronchus, and lung cancer	--	--	--	--	--	7 (6–8)	45 (38–52)	75 (66–87)	99 (88–112)	200 (178–225)	343 (303–386)	649 (573–730)	1,422 (1,260–1,616)	2,882 (2,574–3,253)	4,850 (4,335–5,400)	6,742 (6,074–7,462)	7,807 (7,079–8,648)	8,207 (7,471–9,056)	6,952 (6,289–7,618)	4,912 (4,266–5,416)	2,757 (2,277–3,081)	1,077 (852–1,220)	--
Malignant skin melanoma	0 (0–0)	0 (0–0)	1 (1–6)	5 (2–19)	3 (3–4)	6 (4–9)	9 (6–14)	20 (15–34)	33 (25–60)	64 (49–109)	98 (75–165)	138 (107–234)	162 (120–267)	230 (167–363)	285 (210–447)	299 (209–445)	312 (208–450)	310 (207–451)	276 (188–396)	237 (155–333)	167 (104–224)	83 (53–120)	--
Non-melanoma skin cancer (squamous-cell carcinoma)	--	--	--	--	--	--	--	14 (12–16)	21 (18–23)	30 (27–34)	50 (44–57)	76 (67–86)	116 (102–133)	174 (153–196)	243 (212–276)	342 (303–381)	394 (348–440)	454 (403–505)	503 (438–557)	532 (451–596)	475 (382–532)	320 (240–369)	--
	1 (0–2)	1 (0–1)	12 (7–18)	35 (21–52)	39 (27–57)	36 (25–51)	61 (42–80)	71 (50–95)	70 (49–97)	80 (55–109)	85 (56–112)	106 (74–143)	114 (77–157)	139 (96–183)	172 (116–223)	180 (123–242)	185 (120–234)	177 (117–227)	148 (98–191)	119 (77–154)	72 (46–92)	31 (19–40)	--
	--	--	--	13 (9–17)	40 (33–52)	101 (82–121)	210 (174–254)	175 (149–225)	121 (102–163)	87 (74–112)	92 (78–120)	110 (95–136)	137 (112–163)	188 (150–218)	235 (180–278)	275 (198–324)	294 (214–346)	284 (189–344)	239 (158–292)	189 (120–235)	106 (65–136)	41 (25–54)	--
Breast cancer	--	--	--	--	--	--	2 (2–3)	2 (2–3)	3 (3–3)	5 (4–6)	8 (7–10)	13 (11–16)	35 (30–42)	56 (48–65)	70 (61–82)	95 (84–109)	104 (90–119)	95 (82–110)	56 (47–66)	46 (38–55)	40 (31–49)	22 (17–27)	--
Cervical cancer	--	--	--	--	--	--	--	--	--	--	--	--	--	--	--	--	--	--	--	--	--	--	--
Uterine cancer	--	--	--	--	--	--	--	--	--	--	--	--	--	--	--	--	--	--	--	--	--	--	--
Ovarian cancer	--	--	--	--	--	--	--	--	--	--	--	--	--	--	--	--	--	--	--	--	--	--	--
Prostate cancer	--	--	--	--	--	--	--	14 (11–17)	19 (15–22)	26 (19–30)	40 (31–47)	92 (73–117)	274 (229–359)	768 (635–1,003)	1,785 (1,465–2,314)	4,027 (3,356–5,282)	6,350 (5,371–8,345)	9,232 (7,943–12,371)	11,572 (9,651–15,172)	11,618 (9,293–15,420)	9,222 (7,242–12,409)	4,659 (3,486–6,182)	--
Testicular cancer	7 (0–71)	3 (0–27)	11 (3–70)	22 (14–41)	14 (10–18)	17 (13–20)	111 (92–132)	255 (214–292)	285 (246–324)	221 (195–250)	157 (137–179)	112 (97–129)	69 (59–81)	55 (47–65)	49 (42–57)	51 (43–59)	53 (45–63)	57 (47–66)	53 (44–63)	49 (40–59)	39 (31–46)	20 (15–25)	--
Kidney cancer	4 (2–7)	2 (1–3)	20 (15–27)	64 (50–82)	47 (38–56)	20 (17–23)	20 (17–24)	30 (26–35)	41 (36–47)	62 (54–70)	108 (96–123)	198 (171–227)	380 (327–440)	644 (562–744)	925 (807–1,063)	1,080 (949–1,232)	1,116 (992–1,270)	998 (883–1,121)	845 (747–951)	602 (515–682)	344 (283–396)	136 (105–157)	--
Bladder cancer	--	--	--	--	--	--	3 (3–4)	4 (4–5)	7 (6–8)	16 (14–18)	28 (25–32)	58 (51–66)	112 (99–127)	226 (202–254)	418 (375–464)	683 (617–761)	936 (845–1,040)	1,150 (1,040–1,267)	1,290 (1,159–1,421)	1,173 (1,015–1,302)	860 (722–961)	413 (321–469)	--
Brain and nervous system cancer	7 (3–13)	5 (2–8)	64 (36–92)	252 (142–360)	363 (226–464)	270 (181–328)	192 (140–227)	219 (158–255)	280 (202–325)	369 (268–426)	466 (342–538)	584 (412–683)	694 (474–807)	901 (591–1,051)	1,107 (708–1,299)	1,148 (681–1,345)	1,108 (647–1,300)	977 (537–1,167)	704 (377–849)	444 (229–544)	217 (111–271)	76 (40–95)	--
	0 (0–0)	0 (0–0)	3 (2–4)	57 (40–77)	16 (12–20)	10 (8–12)	8 (6–10)	9 (7–11)	9 (7–12)	9 (7–12)	9 (8–13)	10 (8–13)	12 (9–14)	14 (10–17)	20 (14–24)	23 (16–27)	25 (18–30)	29 (20–34)	33 (23–39)	30 (20–36)	25 (16–30)	14 (9–17)	--
	0 (0–0)	0 (0–0)	3 (2–4)	47 (33–64)	10 (7–12)	5 (4–6)	4 (3–5)	--	--	--	--	--	--	--	--	--	--	--	--	--	--	--	--
	--	--	--	9 (6–14)	6 (4–8)	5 (3–6)	4 (3–5)	9 (7–11)	9 (7–12)	9 (7–12)	9 (8–13)	10 (8–13)	12 (9–14)	14 (10–17)	20 (14–24)	23 (16–27)	25 (18–30)	29 (20–34)	33 (23–39)	30 (20–36)	25 (16–30)	14 (9–17)	--
	0 (0–1)	1 (0–1)	15 (9–21)	72 (48–108)	39 (23–52)	14 (10–17)	12 (10–15)	12 (10–18)	13 (11–19)	13 (11–18)	11 (9–17)	12 (10–16)	11 (9–14)	15 (12–18)	16 (13–20)	15 (11–18)	13 (10–15)	12 (9–15)	9 (6–11)	6 (4–7)	3 (2–4)	2 (1–2)	--
Thyroid cancer	--	--	--	--	4 (3–5)	5 (4–6)	6 (5–7)	11 (10–14)	16 (14–20)	17 (14–22)	25 (21–30)	38 (33–46)	68 (59–80)	113 (98–132)	155 (135–180)	160 (137–184)	171 (149–197)	178 (156–203)	187 (163–210)	144 (120–164)	87 (71–101)	39 (29–45)	--
Mesothelioma	--	--	--	--	--	--	--	7 (6–8)	11 (9–13)	13 (12–14)	24 (21–28)	40 (35–47)	80 (72–88)	130 (118–141)	185 (169–203)	82 (73–92)	91 (81–103)	86 (77–97)	111 (101–123)	79 (69–88)	46 (39–52)	18 (14–21)	--
Hodgkin lymphoma	--	--	--	5 (3–6)	19 (15–24)	23 (19–30)	46 (39–60)	68 (58–83)	78 (67–99)	77 (67–102)	73 (63–91)	74 (64–94)	76 (64–92)	91 (78–110)	107 (92–130)	115 (96–137)	124 (104–145)	116 (96–134)	98 (79–112)	67 (48–78)	42 (30–49)	12 (6–15)	--
Non-Hodgkin's lymphoma	--	--	--	89 (67–114)	120 (100–147)	112 (96–132)	158 (136–182)	199 (176–226)	229 (207–254)	270 (243–300)	322 (287–363)	407 (360–463)	501 (440–573)	714 (630–814)	921 (813–1,048)	1,050 (937–1,190)	1,143 (1,018–1,284)	1,172 (1,049–1,325)	951 (843–1,062)	707 (611–795)	395 (327–449)	156 (121–182)	--
	--	--	--	17 (7–29)	23 (10–36)	16 (8–23)	15 (9–19)	12 (7–16)	12 (7–16)	11 (7–15)	11 (6–15)	10 (5–14)	12 (6–16)	13 (6–18)	13 (7–18)	14 (8–19)	13 (7–18)	15 (8–21)	11 (6–15)	8 (4–10)	6 (3–8)	2 (1–3)	--
	--	--	--	72 (52–95)	98 (78–119)	97 (82–114)	144 (123–167)	187 (164–214)	217 (196–242)	259 (232–288)	310 (275–352)	396 (350–451)	489 (430–563)	701 (616–800)	907 (800–1,033)	1,035 (923–1,175)	1,130 (1,005–1,273)	1,157 (1,034–1,306)	940 (832–1,054)	700 (604–787)	389 (322–442)	154 (119–180)	--
Multiple myeloma	--	--	--	--	--	--	--	6 (5–7)	10 (8–12)	21 (18–24)	45 (39–55)	97 (82–120)	191 (159–227)	362 (298–429)	543 (429–639)	698 (549–814)	754 (620–875)	741 (597–850)	574 (470–668)	401 (315–466)	206 (159–244)	68 (50–84)	--
Leukaemia	13 (9–20)	10 (7–13)	117 (85–162)	493 (371–644)	707 (582–859)	640 (545–753)	685 (589–800)	603 (532–688)	523 (466–587)	478 (427–538)	505 (442–574)	531 (463–607)	554 (487–639)	649 (564–750)	777 (684–890)	928 (822–1,043)	1,076 (961–1,210)	1,160 (1,034–1,297)	1,097 (972–1,231)	913 (774–1,028)	588 (485–674)	243 (189–281)	--
Acute lymphoid leukaemia	5 (3–8)	3 (2–4)	50 (33–74)	295 (210–391)	488 (381–597)	405 (322–487)	406 (318–481)	276 (214–328)	192 (144–224)	142 (104–167)	131 (95–154)	130 (91–155)	113 (80–137)	113 (82–137)	112 (84–135)	102 (75–124)	110 (81–129)	103 (77–122)	91 (66–109)	62 (44–76)	44 (30–54)	16 (11–20)	--
Chronic lymphoid leukaemia	--	--	--	--	--	--	--	5 (4–6)	5 (4–6)	7 (5–9)	7 (5–9)	12 (10–16)	18 (15–23)	39 (32–49)	66 (55–81)	105 (89–133)	138 (115–172)	157 (133–192)	164 (136–199)	152 (119–187)	101 (77–123)	45 (34–56)	--
Acute myeloid leukaemia	2 (1–5)	3 (2–5)	31 (20–45)	111 (73–155)	117 (79–156)	144 (107–182)	157 (126–202)	151 (125–189)	155 (130–192)	160 (130–190)	180 (150–217)	187 (157–229)	198 (165–238)	237 (188–282)	277 (220–330)	329 (251–388)	369 (290–435)	370 (286–439)	314 (234–375)	237 (171–284)	133 (91–161)	48 (33–61)	--
Chronic myeloid leukaemia	0 (0–0)	0 (0–1)	3 (2–8)	12 (5–35)	6 (5–10)	6 (5–8)	11 (9–14)	23 (19–30)	31 (26–38)	41 (34–51)	49 (41–59)	54 (45–65)	60 (50–71)	69 (57–83)	80 (68–97)	93 (77–112)	101 (85–120)	110 (93–130)	114 (95–136)	90 (75–108)	60 (48–72)	25 (19–30)	--
Other leukaemia	5 (4–8)	4 (2–6)	33 (23–45)	74 (49–115)	95 (71–129)	85 (67–112)	111 (87–148)	147 (119–185)	140 (119–176)	128 (108–155)	138 (116–166)	147 (122–178)	164 (136–199)	192 (160–235)	242 (204–290)	299 (254–356)	358 (305–423)	420 (358–487)	414 (352–487)	371 (304–437)	250 (201–293)	109 (82–128)	--
Other malignant cancers	13 (8–19)	11 (7–17)	62 (44–86)	122 (87–168)	103 (83–127)	120 (100–143)	148 (126–179)	170 (148–202)	174 (155–197)	192 (172–218)	251 (224–287)	352 (308–399)	448 (391–512)	624 (538–712)	830 (718–953)	926 (791–1,053)	992 (860–1,122)	977 (858–1,102)	901 (783–1,024)	712 (594–811)	471 (384–543)	225 (171–262)	--
Myelodysplastic, myeloproliferative, and other hematopoietic neoplasms	2 (1–3)	1 (0–1)	11 (5–18)	18 (9–26)	13 (7–17)	14 (8–19)	16 (9–22)	20 (11–28)	23 (12–34)	20 (10–26)	29 (15–44)	43 (25–66)	49 (26–70)	75 (38–103)	116 (61–168)	167 (92–252)	212 (123–317)	262 (149–380)	280 (156–417)	238 (126–330)	168 (90–246)	76 (40–114)	--
Cardiovascular diseases	90 (66–125)	53 (39–72)	564 (416–741)	317 (240–408)	241 (201–283)	357 (308–413)	1,047 (913–1,188)	1,716 (1,534–1,918)	2,471 (2,242–2,728)	3,995 (3,632–4,367)	6,426 (5,844–7,066)	10,434 (9,403–11,496)	16,395 (14,718–18,208)	26,008 (23,413–28,946)	36,459 (32,996–40,296)	47,079 (42,762–52,013)	55,479 (50,641–60,823)	62,874 (57,534–68,750)	65,375 (59,167–71,279)	64,468 (56,318–70,886)	50,164 (42,367–55,478)	31,637 (25,060–35,482)	--
Rheumatic heart disease	--	--	--	18 (10–33)	22 (16–31)	41 (32–53)	55 (45–69)	57 (47–70)	73 (63–90)	84 (71–103)	102 (87–123)	127 (108–150)	138 (119–162)	172 (151–196)	209 (184–236)	213 (190–237)	229 (206–257)	215 (193–241)	200 (177–224)	137 (117–157)	98 (81–112)	53 (41–62)	--
Ischaemic heart disease	--	--	--	--	--	--	334 (287–384)	711 (629–807)	1,106 (996–1,226)	2,023 (1,829–2,218)	3,386 (3,056–3,724)	5,736 (5,156–6,399)	9,508 (8,444–10,685)	15,313 (13,670–17,149)	21,594 (19,304–24,102)	27,211 (24,457–30,202)	31,283 (28,332–34,675)	34,089 (30,939–37,498)	34,099 (30,547–37,674)	33,282 (29,113–36,963)	26,277 (21,988–29,368)	17,353 (13,731–19,595)	--
Stroke	24 (14–41)	14 (9–21)	134 (84–212)	79 (54–114)	86 (70–106)	134 (112–162)	348 (295–407)	506 (438–584)	677 (596–773)	998 (886–1,123)	1,615 (1,427–1,813)	2,652 (2,348–2,979)	4,069 (3,591–4,556)	6,498 (5,784–7,275)	9,074 (8,193–10,052)	12,385 (11,193–13,739)	15,267 (13,844–16,839)	18,409 (16,751–20,185)	20,108 (18,236–21,934)	19,420 (16,998–21,298)	14,298 (11,966–15,846)	8,099 (6,325–9,134)	--
Ischaemic stroke	3 (2–4)	3 (2–5)	7 (4–13)	11 (6–22)	6 (5–8)	7 (6–9)	28 (23–35)	44 (37–53)	61 (52–72)	89 (76–102)	145 (126–166)	243 (210–277)	592 (517–668)	1,089 (959–1,231)	1,693 (1,511–1,899)	4,557 (4,094–5,053)	6,143 (5,532–6,783)	10,300 (9,361–11,305)	11,849 (10,678–12,961)	14,658 (12,772–16,079)	10,976 (9,100–12,094)	6,226 (4,872–7,031)	--
Intracerebral hemorrhage	5 (2–13)	4 (3–7)	77 (46–129)	26 (16–45)	31 (24–41)	40 (32–51)	163 (135–197)	236 (202–278)	320 (277–372)	542 (476–615)	917 (805–1,040)	1,586 (1,392–1,805)	2,619 (2,300–2,977)	4,167 (3,701–4,679)	5,766 (5,159–6,470)	6,727 (5,987–7,584)	7,971 (7,098–8,880)	7,167 (6,382–7,993)	7,379 (6,570–8,208)	3,977 (3,409–4,493)	2,773 (2,309–3,178)	1,578 (1,232–1,834)	--
Subarachnoid hemorrhage	16 (9–29)	7 (4–12)	50 (31–78)	41 (27–60)	49 (39–61)	87 (72–106)	157 (131–185)	226 (193–267)	295 (254–349)	367 (319–429)	553 (482–640)	823 (714–961)	858 (731–995)	1,242 (1,067–1,454)	1,615 (1,385–1,877)	1,100 (838–1,283)	1,153 (924–1,335)	943 (727–1,099)	880 (650–1,023)	785 (588–917)	549 (389–658)	295 (208–357)	--
Hypertensive heart disease	--	--	--	--	--	--	20 (16–25)	44 (36–56)	66 (56–89)	130 (111–172)	229 (197–315)	409 (347–542)	682 (578–907)	1,133 (967–1,498)	1,686 (1,459–2,224)	2,432 (2,084–3,368)	3,012 (2,614–3,828)	3,766 (3,275–4,789)	4,211 (3,639–5,592)	4,673 (3,919–5,924)	3,808 (3,015–4,529)	2,477 (1,845–2,971)	--
Non-rheumatic valvular heart disease	--	--	--	--	--	--	31 (27–37)	35 (31–41)	50 (44–58)	65 (57–74)	88 (78–100)	124 (108–141)	155 (134–178)	239 (207–273)	340 (300–386)	457 (402–515)	555 (491–623)	595 (530–667)	586 (514–656)	492 (415–551)	340 (273–387)	178 (135–205)	--
Non-rheumatic calcific aortic valve disease	--	--	--	--	--	--	18 (15–22)	22 (19–26)	33 (28–39)	41 (35–50)	58 (50–69)	83 (71–99)	109 (93–132)	171 (147–204)	248 (214–295)	345 (300–408)	426 (371–498)	463 (402–541)	460 (394–533)	383 (321–444)	273 (218–317)	141 (107–167)	--
Non-rheumatic degenerative mitral valve disease	--	--	--	--	--	--	12 (9–15)	12 (9–16)	16 (13–21)	22 (17–28)	29 (22–36)	38 (29–48)	43 (30–54)	65 (44–80)	89 (59–110)	108 (69–135)	125 (76–155)	128 (78–156)	122 (73–151)	103 (66–129)	63 (40–79)	34 (22–44)	--
Other non-rheumatic valve diseases	--	--	--	--	--	--	1 (1–2)	1 (1–2)	2 (1–2)	2 (1–2)	2 (1–2)	3 (2–4)	2 (1–3)	2 (1–3)	3 (2–4)	4 (2–6)	5 (3–7)	4 (3–6)	4 (3–6)	6 (3–9)	5 (3–7)	3 (1–4)	--
Cardiomyopathy and myocarditis	32 (22–46)	18 (13–26)	262 (192–347)	113 (80–152)	61 (42–76)	85 (59–102)	125 (91–146)	167 (128–192)	242 (190–277)	352 (261–408)	519 (374–599)	692 (523–797)	904 (661–1,057)	1,242 (967–1,459)	1,545 (1,210–1,840)	1,683 (1,392–2,036)	1,783 (1,511–2,254)	1,820 (1,536–2,334)	1,767 (1,472–2,323)	1,579 (1,280–2,004)	1,161 (919–1,455)	666 (484–856)	--
Myocarditis	3 (1–5)	3 (2–5)	52 (34–83)	24 (15–41)	12 (8–18)	11 (7–15)	15 (11–23)	18 (13–29)	21 (16–33)	22 (16–36)	26 (19–38)	27 (20–41)	27 (20–42)	33 (24–52)	35 (26–58)	36 (26–59)	36 (26–59)	39 (27–61)	47 (33–75)	45 (29–65)	43 (26–59)	34 (21–48)	--
Alcoholic cardiomyopathy	--	--	--	--	--	--	6 (5–9)	10 (7–13)	27 (21–35)	69 (52–90)	111 (83–142)	159 (123–205)	229 (168–288)	285 (218–355)	326 (239–399)	283 (217–356)	268 (204–333)	163 (120–202)	121 (85–154)	49 (34–66)	22 (15–32)	13 (8–19)	--
Other cardiomyopathy	30 (20–43)	15 (11–22)	210 (147–280)	89 (61–119)	49 (34–62)	74 (49–89)	104 (72–122)	140 (101–161)	194 (143–225)	261 (187–302)	381 (260–446)	505 (373–587)	648 (462–754)	925 (702–1,081)	1,185 (911–1,410)	1,364 (1,122–1,639)	1,479 (1,261–1,888)	1,619 (1,357–2,082)	1,599 (1,331–2,123)	1,485 (1,213–1,903)	1,096 (866–1,380)	619 (452–792)	--
Pulmonary Arterial Hypertension	21 (14–31)	10 (7–15)	67 (32–99)	27 (17–40)	16 (11–27)	16 (11–29)	16 (11–27)	16 (10–26)	19 (13–32)	19 (12–32)	23 (15–38)	25 (15–39)	29 (16–45)	36 (19–51)	42 (22–61)	50 (22–71)	54 (23–74)	59 (24–81)	59 (23–83)	64 (25–90)	47 (17–70)	28 (12–41)	--
Atrial fibrillation and flutter	--	--	--	--	--	--	--	--	--	6 (4–7)	10 (7–12)	46 (32–60)	76 (54–103)	139 (100–187)	239 (172–323)	376 (269–511)	521 (375–699)	838 (599–1,119)	1,219 (897–1,635)	2,025 (1,420–2,661)	2,116 (1,511–2,870)	1,683 (1,156–2,271)	--

Aortic aneurysm	--	--	--	--	--	--	20 (17–23)	33 (29–37)	52 (47–59)	85 (76–94)	144 (130–160)	215 (194–237)	326 (292–363)	513 (463–568)	784 (706–867)	1,109 (1,000–1,232)	1,427 (1,281–1,596)	1,553 (1,395–1,729)	1,528 (1,354–1,698)	1,238 (1,070–1,379)	779 (644–875)	369 (290–418)	--
Peripheral vascular disease	--	--	--	--	--	--	--	--	--	--	--	17 (6–39)	30 (10–72)	65 (22–150)	102 (36–239)	163 (56–389)	224 (78–535)	310 (110–719)	368 (132–865)	398 (144–940)	341 (124–799)	220 (78–512)	--
Endocarditis	2 (1–3)	2 (1–4)	45 (30–65)	30 (19–45)	19 (13–26)	29 (21–40)	41 (32–60)	61 (47–80)	69 (54–100)	82 (61–113)	104 (74–142)	131 (92–170)	145 (100–186)	187 (125–235)	231 (151–291)	243 (150–299)	257 (150–311)	246 (138–300)	215 (124–260)	160 (92–196)	100 (60–124)	53 (33–66)	--
Other cardiovascular and circulatory diseases	10 (6–16)	8 (5–14)	56 (35–90)	50 (32–75)	37 (30–46)	53 (44–63)	57 (47–67)	87 (74–100)	117 (102–133)	150 (133–169)	205 (181–232)	261 (230–291)	332 (294–376)	472 (420–529)	614 (544–684)	758 (682–838)	866 (784–955)	973 (867–1,075)	1,014 (900–1,125)	1,000 (864–1,115)	798 (658–904)	459 (356–526)	--
Chronic respiratory diseases	0 (0–0)	0 (0–0)	0 (0–0)	524 (346–733)	134 (109–166)	118 (99–138)	198 (170–229)	278 (245–315)	353 (315–393)	452 (406–504)	627 (560–699)	882 (792–989)	1,366 (1,223–1,530)	2,405 (2,164–2,670)	3,997 (3,602–4,457)	6,452 (5,835–7,121)	9,180 (8,371–10,065)	12,646 (11,543–13,869)	15,082 (13,762–16,472)	16,075 (14,078–17,751)	13,248 (11,127–14,715)	7,775 (6,143–8,818)	--
Chronic obstructive pulmonary disease	--	--	0 (0–0)	61 (38–106)	18 (14–24)	16 (13–20)	77 (65–93)	116 (99–137)	146 (127–172)	209 (184–243)	325 (285–377)	513 (455–591)	905 (805–1,030)	1,794 (1,601–2,020)	3,161 (2,834–3,558)	5,467 (4,910–6,103)	7,931 (7,189–8,774)	11,121 (10,091–12,183)	13,392 (12,138–14,676)	14,454 (12,676–16,056)	11,963 (10,049–13,374)	7,061 (5,595–8,068)	--
Pneumoconiosis	--	--	--	--	--	--	2 (1–3)	4 (3–5)	7 (6–9)	10 (9–12)	19 (17–22)	27 (23–31)	36 (31–42)	45 (39–51)	64 (55–74)	76 (66–88)	86 (75–100)	87 (76–99)	92 (80–105)	79 (66–94)	50 (40–58)	22 (18–26)	--
Silicosis	--	--	--	--	--	--	0 (0–1)	2 (1–2)	3 (3–4)	5 (4–6)	12 (10–14)	18 (15–21)	24 (21–28)	30 (26–36)	42 (36–50)	49 (42–57)	54 (47–63)	55 (47–63)	56 (49–64)	37 (31–44)	19 (15–24)	8 (6–9)	--
Asbestosis	--	--	--	--	--	--	0 (0–1)	0 (0–0)	1 (1–1)	1 (0–1)	1 (1–1)	1 (1–2)	2 (1–3)	3 (1–3)	4 (2–6)	5 (3–6)	7 (4–9)	8 (5–10)	7 (4–10)	9 (6–12)	6 (4–8)	3 (2–4)	--
Coal workers pneumoconiosis	--	--	--	--	--	--	0 (0–1)	0 (0–0)	1 (0–1)	1 (1–2)	2 (2–3)	3 (2–3)	3 (2–4)	4 (3–5)	5 (4–7)	8 (6–11)	8 (6–11)	8 (6–10)	10 (8–13)	11 (8–14)	7 (5–9)	3 (2–3)	--
Other pneumoconiosis	--	--	--	--	--	--	1 (1–1)	2 (1–2)	3 (2–3)	3 (3–4)	4 (4–6)	5 (4–6)	7 (6–9)	8 (6–10)	12 (9–16)	14 (11–19)	16 (12–22)	16 (12–23)	19 (13–27)	22 (17–28)	17 (13–23)	8 (6–11)	--
Asthma	--	--	--	250 (139–418)	60 (44–83)	48 (37–59)	44 (34–55)	54 (45–65)	68 (56–81)	80 (66–96)	93 (76–112)	117 (96–140)	148 (124–178)	190 (159–225)	230 (190–276)	293 (240–349)	329 (275–394)	401 (341–478)	397 (338–459)	406 (342–468)	306 (249–353)	183 (142–215)	--
Interstitial lung disease and pulmonary sarcoidosis	--	--	--	26 (15–45)	11 (8–14)	9 (6–11)	21 (13–27)	36 (24–47)	50 (33–63)	67 (43–85)	95 (60–121)	122 (80–155)	164 (104–211)	244 (155–312)	379 (239–493)	527 (326–688)	729 (448–960)	924 (562–1,226)	1,085 (662–1,422)	1,013 (596–1,377)	835 (488–1,123)	443 (259–622)	--
Other chronic respiratory diseases	0 (0–0)	0 (0–0)	0 (0–0)	187 (123–291)	45 (36–58)	45 (37–55)	55 (45–67)	68 (55–80)	82 (68–97)	85 (71–100)	95 (76–111)	103 (85–125)	112 (90–135)	134 (104–162)	164 (130–197)	89 (55–109)	106 (64–128)	113 (67–136)	115 (70–145)	122 (72–161)	95 (49–127)	65 (32–90)	--
Digestive diseases	62 (42–89)	56 (40–75)	777 (552–1,056)	662 (474–876)	350 (290–418)	302 (257–356)	503 (429–583)	938 (821–1,056)	1,773 (1,590–1,975)	3,284 (2,970–3,617)	5,529 (4,986–6,091)	8,238 (7,409–9,138)	10,823 (9,653–12,218)	13,939 (12,406–15,711)	15,558 (13,801–17,477)	15,708 (14,016–17,620)	14,940 (13,397–16,661)	14,005 (12,554–15,546)	12,524 (11,172–13,856)	10,588 (9,220–11,737)	7,348 (6,197–8,161)	3,628 (2,863–4,073)	--
Cirrhosis and other chronic liver diseases	--	--	--	158 (114–223)	92 (74–114)	93 (74–112)	200 (159–241)	437 (347–511)	1,075 (950–1,203)	2,317 (2,091–2,579)	4,161 (3,719–4,605)	6,472 (5,809–7,224)	8,636 (7,690–9,787)	11,079 (9,852–12,517)	11,964 (10,576–13,520)	11,386 (10,067–12,908)	9,902 (8,764–11,209)	8,193 (7,228–9,223)	6,284 (5,539–7,115)	4,525 (3,889–5,126)	2,736 (2,284–3,098)	1,185 (938–1,356)	--
Cirrhosis and other chronic liver diseases due to hepatitis B	--	--	--	0 (0–1)	0 (0–1)	1 (0–2)	4 (2–6)	22 (13–34)	77 (47–118)	199 (146–263)	402 (283–552)	628 (462–841)	793 (562–1,096)	931 (647–1,308)	862 (598–1,208)	715 (474–1,039)	571 (387–820)	462 (297–667)	362 (240–515)	261 (167–377)	153 (106–216)	65 (40–97)	--
Cirrhosis and other chronic liver diseases due to hepatitis C	--	--	--	1 (1–3)	2 (1–4)	4 (2–7)	16 (8–28)	93 (56–138)	329 (226–453)	734 (560–943)	1,247 (911–1,661)	1,831 (1,364–2,402)	2,255 (1,629–2,996)	2,646 (1,861–3,601)	2,603 (1,840–3,557)	2,323 (1,588–3,281)	1,974 (1,454–2,681)	1,609 (1,203–2,121)	1,231 (866–1,701)	902 (618–1,245)	558 (395–742)	249 (158–351)	--
Cirrhosis and other chronic liver diseases due to alcohol use	--	--	--	--	--	--	2 (0–3)	57 (33–91)	233 (156–333)	736 (582–897)	1,660 (1,264–2,054)	2,811 (2,241–3,420)	3,929 (3,130–4,915)	5,320 (4,261–6,521)	6,086 (4,896–7,432)	5,831 (4,694–7,113)	4,849 (3,896–5,918)	3,742 (2,936–4,583)	2,583 (1,967–3,218)	1,691 (1,233–2,160)	959 (718–1,241)	392 (273–535)	--
Cirrhosis and other chronic liver diseases due to NAFLD	--	--	--	--	--	--	12 (5–22)	50 (25–86)	159 (77–279)	320 (186–492)	496 (257–850)	770 (407–1,251)	1,107 (543–1,887)	1,448 (718–2,465)	1,527 (790–2,562)	1,541 (796–2,603)	1,526 (943–2,290)	1,426 (911–2,091)	1,241 (686–1,875)	972 (580–1,433)	615 (388–880)	275 (156–421)	--
Cirrhosis and other chronic liver diseases due to other causes	--	--	--	156 (112–220)	90 (72–111)	87 (70–107)	167 (133–206)	215 (139–293)	276 (163–417)	328 (187–525)	354 (187–589)	432 (230–734)	552 (301–934)	733 (391–1,242)	887 (468–1,512)	976 (547–1,602)	982 (517–1,662)	955 (510–1,568)	868 (471–1,438)	699 (382–1,147)	450 (258–690)	203 (107–344)	--
Upper digestive system diseases	--	--	--	24 (14–41)	14 (11–18)	16 (13–20)	42 (35–52)	78 (67–92)	104 (90–121)	152 (133–174)	230 (202–262)	320 (281–364)	430 (377–491)	611 (535–699)	775 (680–885)	945 (836–1,080)	1,100 (975–1,248)	1,269 (1,126–1,442)	1,375 (1,208–1,572)	1,388 (1,180–1,589)	1,095 (893–1,256)	607 (475–707)	--
Peptic ulcer disease	--	--	--	18 (10–30)	11 (9–15)	13 (11–17)	35 (28–43)	66 (56–78)	85 (73–99)	123 (106–142)	188 (164–214)	262 (230–301)	353 (308–405)	522 (457–599)	657 (574–758)	802 (705–916)	928 (825–1,060)	1,064 (940–1,215)	1,152 (1,008–1,330)	1,145 (974–1,320)	897 (728–1,038)	487 (380–572)	--
Gastritis and duodenitis	--	--	--	6 (3–13)	3 (2–4)	3 (2–4)	8 (6–10)	13 (10–15)	20 (16–24)	29 (24–35)	43 (35–52)	58 (47–72)	77 (64–97)	89 (72–110)	118 (97–145)	144 (116–175)	172 (140–213)	205 (168–245)	223 (180–267)	243 (196–293)	197 (150–238)	120 (87–141)	--
Appendicitis	--	--	--	84 (50–140)	69 (52–92)	64 (49–83)	60 (46–83)	72 (54–99)	75 (52–100)	83 (59–117)	101 (67–138)	118 (80–158)	127 (77–168)	146 (93–194)	166 (103–218)	187 (117–246)	195 (118–248)	202 (118–249)	192 (111–238)	162 (99–208)	114 (68–146)	53 (32–68)	--
Paralytic ileus and intestinal obstruction	43 (28–67)	43 (30–61)	675 (475–923)	192 (129–267)	85 (65–106)	57 (44–71)	57 (43–72)	84 (64–104)	99 (73–121)	112 (82–136)	152 (116–192)	207 (155–258)	263 (195–331)	371 (276–465)	509 (369–623)	659 (481–792)	822 (598–990)	1,007 (748–1,197)	1,112 (797–1,321)	1,147 (804–1,368)	865 (612–1,034)	454 (312–548)	--
Inguinal, femoral, and abdominal hernia	19 (12–30)	13 (8–21)	102 (59–188)	30 (16–55)	14 (10–19)	8 (6–10)	12 (10–16)	18 (15–22)	22 (18–26)	28 (24–32)	38 (33–44)	58 (50–66)	78 (67–90)	137 (118–158)	186 (162–213)	235 (203–269)	284 (248–319)	348 (305–393)	418 (366–470)	432 (368–492)	348 (286–399)	189 (147–219)	--
Inflammatory bowel disease	--	--	--	22 (14–34)	7 (5–8)	5 (4–7)	9 (6–11)	19 (14–23)	27 (21–32)	33 (25–39)	44 (35–51)	52 (42–63)	60 (47–73)	73 (55–88)	89 (67–109)	100 (76–120)	105 (82–125)	106 (86–128)	107 (86–132)	95 (74–117)	66 (51–80)	33 (24–42)	--
Vascular intestinal disorders	--	--	--	8 (6–12)	5 (4–6)	5 (4–6)	12 (10–14)	16 (13–18)	20 (18–23)	32 (28–37)	48 (42–54)	75 (66–85)	126 (110–143)	206 (183–234)	313 (277–355)	416 (369–471)	541 (478–613)	680 (598–771)	747 (653–850)	729 (622–842)	530 (437–615)	277 (214–322)	--
Gallbladder and biliary diseases	--	--	--	6 (4–10)	5 (3–7)	6 (4–8)	17 (13–21)	29 (21–35)	44 (34–53)	64 (50–75)	94 (69–109)	139 (101–164)	195 (145–228)	291 (211–342)	412 (301–486)	551 (411–642)	703 (519–822)	850 (619–989)	959 (720–1,103)	945 (716–1,091)	722 (507–844)	380 (277–450)	--
Pancreatitis	--	--	--	9 (6–12)	9 (6–11)	13 (10–16)	51 (42–63)	127 (107–149)	236 (204–270)	365 (316–413)	521 (463–581)	610 (528–686)	657 (576–743)	664 (583–758)	683 (598–785)	651 (565–746)	636 (559–740)	612 (537–700)	559 (476–648)	434 (364–507)	270 (217–318)	116 (90–140)	--
Other digestive diseases	--	--	--	129 (80–203)	51 (33–76)	35 (23–48)	42 (30–55)	58 (41–74)	72 (51–93)	99 (69–133)	140 (97–183)	188 (128–254)	250 (170–345)	361 (239–487)	460 (301–605)	578 (374–739)	652 (430–816)	739 (476–948)	771 (498–964)	731 (461–915)	601 (385–739)	334 (214–423)	--
Alzheimer's disease and other dementias	--	--	--	--	--	--	--	--	--	--	--	21 (2–76)	119 (16–389)	251 (41–793)	517 (97–1,505)	1,076 (224–3,203)	2,128 (468–6,340)	3,814 (858–10,773)	5,904 (1,338–16,583)	10,790 (2,563–29,119)	12,252 (3,041–31,638)	9,692 (2,400–24,136)	--
Parkinson's disease	--	--	--	--	--	--	--	1 (1–1)	2 (1–2)	2 (2–2)	4 (4–5)	26 (23–29)	62 (55–70)	119 (105–135)	193 (172–219)	380 (339–429)	792 (715–886)	1,738 (1,575–1,941)	3,010 (2,721–3,314)	3,527 (3,078–3,908)	2,500 (2,085–2,798)	1,006 (805–1,151)	--
Idiopathic epilepsy	--	--	115 (83–156)	197 (145–270)	132 (108–162)	159 (136–187)	263 (225–310)	339 (297–390)	399 (356–444)	423 (382–477)	488 (436–546)	492 (440–553)	479 (425–540)	423 (372–481)	375 (332–425)	302 (268–337)	254 (226–285)	207 (186–232)	189 (167–210)	145 (125–164)	97 (81–110)	52 (41–60)	--
Multiple sclerosis	--	--	--	--	1 (0–3)	1 (0–2)	2 (1–3)	5 (3–6)	9 (6–11)	17 (12–25)	29 (20–43)	42 (31–66)	51 (37–75)	56 (39–84)	62 (41–99)	55 (37–89)	45 (32–66)	38 (26–56)	29 (20–43)	22 (15–35)	14 (10–22)	6 (4–9)	--
Motor neuron disease	0 (0–0)	0 (0–1)	22 (15–30)	8 (6–11)	4 (3–5)	5 (4–6)	11 (9–12)	10 (8–11)	9 (8–11)	20 (17–23)	40 (34–46)	76 (65–87)	108 (92–123)	181 (155–208)	218 (186–249)	233 (196–265)	245 (211–277)	186 (161–211)	127 (108–142)	61 (51–69)	26 (21–30)	7 (5–8)	--
Other neurological disorders	--	--	72 (53–94)	67 (50–86)	46 (38–55)	95 (82–108)	207 (181–233)	165 (148–183)	111 (101–121)	104 (94–114)	115 (104–127)	137 (122–154)	166 (147–187)	197 (174–220)	226 (199–254)	229 (205–255)	216 (194–241)	200 (177–223)	171 (153–190)	127 (110–141)	77 (64–87)	36 (28–42)	--
Eating disorders	--	--	--	--	0 (0–0)	0 (0–0)	0 (0–1)	0 (0–1)	0 (0–1)	0 (0–1)	0 (0–1)	1 (0–1)	0 (0–1)	--	--	--	--	--	--	--	--	--	--
Anorexia nervosa	--	--	--	--	0 (0–0)	0 (0–0)	0 (0–1)	0 (0–1)	0 (0–1)	0 (0–0)	0 (0–1)	0 (0–1)	0 (0–0)	--	--	--	--	--	--	--	--	--	--
Bulimia nervosa	--	--	--	--	0 (0–0)	0 (0–0)	0 (0–0)	0 (0–0)	0 (0–0)	0 (0–0)	0 (0–0)	0 (0–0)	0 (0–0)	--	--	--	--	--	--	--	--	--	--
Alcohol use disorders	--	--	--	--	--	0 (0–0)	84 (70–101)	253 (219–293)	577 (514–654)	1,123 (1,015–1,243)	1,749 (1,579–1,933)	2,328 (2,097–2,596)	2,707 (2,420–3,028)	2,493 (2,221–2,786)	2,349 (2,118–2,588)	1,840 (1,651–2,043)	1,428 (1,286–1,573)	977 (871–1,078)	593 (527–656)	327 (283–366)	161 (133–180)	57 (45–65)	--
Drug use disorders	0 (0–0)	0 (0–0)	0 (0–0)	0 (0–0)	0 (0–0)	0 (0–0)	135 (117–155)	235 (205–269)	258 (228–288)	241 (213–268)	231 (205–261)	204 (177–238)	172 (146–200)	134 (115–158)	109 (92–129)	84 (71–101)	71 (60–85)	59 (50–69)	54 (47–63)	44 (38–51)	23 (18–26)	12 (10–14)	--
Opioid use disorders	0 (0–0)	0 (0–0)	0 (0–0)	0 (0–0)	0 (0–0)	0 (0–0)	14 (12–17)	27 (22–32)	29 (25–34)	47 (40–55)	51 (43–59)	51 (43–61)	35 (28–43)	34 (28–41)	29 (23–35)	25 (20–30)	22 (18–27)	15 (13–19)	15 (12–18)	13 (11–15)	5 (4–6)	3 (2–4)	--
Cocaine use disorders	--	--	--	--	--	0 (0–0)	92 (79–108)	156 (136–179)	170 (152–190)	147 (131–164)	136 (121–152)	112 (99–129)	103 (88–118)	63 (55–73)	50 (42–59)	36 (31–42)	28 (24–34)	27 (23–32)	24 (21–29)	10 (9–12)	7 (5–8)	3 (2–3)	--
Amphetamine use disorders	--	--	--	--	--	0 (0–0)	5 (4–5)	10 (8–11)	12 (10–13)	15 (13–17)	15 (13–18)	14 (11–17)	11 (9–14)	14 (11–17)	12 (9–15)	10 (7–12)	8 (6–10)	0 (0–0)	0 (0–0)	0 (0–0)	0 (0–0)	0 (0–0)	--
Other drug use disorders	--	--	--	--	--	0 (0–0)	24 (20–27)	42 (36–49)	47 (40–53)	32 (27–36)	30 (26–35)	27 (23–32)	23 (19–27)	24 (21–28)	19 (16–22)	14 (12–17)	13 (11–16)	17 (14–19)	15 (13–17)	21 (18–25)	11 (9–13)	6 (5–7)	--
Diabetes mellitus	1 (1–2)	3 (2–5)	52 (34–73)	39 (27–55)	28 (20–35)	36 (25–46)	111 (97–127)	242 (216–270)	422 (384–464)	726 (656–798)	1,265 (1,138–1,409)	2,228 (1,976–2,522)	3,765 (3,283–4,332)	6,213 (5,443–7,077)	8,940 (7,839–10,174)	11,528 (10,268–12,982)	13,116 (11,760–14,594)	13,763 (12,412–15,297)	12,870 (11,551–14,240)	10,290 (8,985–11,404)	6,497 (5,402–7,187)	2,976 (2,344–3,358)	--
Diabetes mellitus type 1	1 (1–2)	3 (2–5)	52 (34–73)	39 (27–55)	28 (20–35)	36 (25–46)	68 (50–80)	146 (105–170)	238 (168–276)	201 (117–243)	296 (188–387)	432 (280–569)	135 (87–219)	200 (129–318)	275 (188–438)	280 (190–460)	327 (239–564)	266 (180–462)	237 (159–418)	100 (59–160)	75 (42–113)	41 (22–64)	--
Diabetes mellitus type 2	--	--	--	--	--	--	44 (35–64)	96 (78–136)	184 (154–256)	525 (460–616)	970 (845–1,117)	1,796 (1,556–2,051)	3,631 (3,158–4,173)	6,013 (5,245–6,876)	8,665 (7,586–9,878)	11,248 (9,996–12,683)	12,788 (11,444–14,245)	13,497 (12,180–14,977)	12,632 (11,314–13,993)	10,190 (8,898–11,286)	6,422 (5,338–7,096)	2,935 (2,312–3,314)	--
Chronic kidney disease	--	--	183 (130–242)	155 (112–202)	130 (108–154)	150 (128–174)	377 (324–434)	721 (633–815)	904 (803–1,002)	1,118 (1,005–1,238)	1,563 (1,391–1,753)	2,464 (2,154–2,803)	3,874 (3,332–4,473)	6,297 (5,421–7,307)	8,785 (7,626–10,112)	11,023 (9,689–12,589)	11,920 (10,522–13,457)	12,194 (10,934–13,692)	11,809 (10,498–13,079)	10,684 (9,317–11,920)	7,878 (6,513–8,849)	4,359 (3,429–4,939)	--
Chronic kidney disease due to diabetes mellitus type 1	--	--	0 (0–2)	1 (0–2)	1 (1–3)	3 (1–7)	14 (5–30)	50 (21–98)	114 (53–211)	208 (105–333)	330 (173–516)	507 (273–802)	671 (308–1,101)	786 (336–1,442)	715 (251–1,524)	679 (252–1,456)	560 (228–1,257)	421 (175–858)	256 (102–544)	110 (41–242)	28 (9–68)	3 (1–9)	--
Chronic kidney disease due to diabetes mellitus type 2	--	--	--	--	--	--	8 (2–18)	22 (8–52)	41 (14–88)	73 (24–147)	135 (49–262)	295 (121–539)	657 (329–1,092)	1,406 (757–2,168)	2,289 (1,307–3,387)	3,048 (1,624–4,329)	3,384 (2,079–4,722)	3,538 (2,299–4,758)	3,457 (2,230–4,623)	3,127 (2,031–4,214)	2,237 (1,461–3,125)	1,187 (725–1,698)	--
Chronic kidney disease due to hypertension	--	--	--	--	--	--	13 (6–22)	34 (19–58)	56 (32–93)	83 (48–134)	135 (79–213)	258 (162–387)	502 (327–750)	1,006 (662–1,452)	1,685 (1,102–2,407)	2,489 (1,665–3,394)	3,012 (2,115–4,012)	3,358 (2,482–4,383)	3,609 (2,722–4,796)	3,648 (2,712–4,731)	3,049 (2,304–3,865)	1,847 (1,327–2,339)	--
Chronic kidney disease due to glomerulonephritis	--	--	56 (29–93)	52 (29–88)	53 (29–81)	72 (42–105)	186 (121–255)	345 (225–473)	396 (269–538)	430 (275–593)	555 (355–767)	815 (467–1,172)	1,179 (752–1,700)	1,807 (1,034–2,654)	2,392 (1,425–3,600)	2,726 (1,506–4,066)	2,578 (1,347–4,098)	2,328 (1,295–3,655)	2,024 (1,061–3,211)	1,571 (780–2,625)	926 (439–1,549)	428 (200–736)	--
Chronic kidney disease due to other and unspecified causes	--	--	126 (83–176)	102 (67–144)	76 (50–106)	75 (44–110)	157 (94–226)	269 (157–393)	296 (181–430)	324 (186–500)	407 (214–643)	590 (306–928)	864 (435–1,368)	1,292 (703–2,077)	1,682 (884–2,784)	2,080 (1,135–3,385)	2,386 (1,302–3,703)	2,550 (1,490–3,857)	2,463 (1,376–3,774)	2,227 (1,247–3,390)	1,638 (903–2,507)	893 (514–1,403)	--
Acute glomerulonephritis	--	--	2 (1–3)	4 (3–7)	4 (3–6)	2 (2–3)	2 (2–3)	3 (2–3)	3 (2–3)	3 (3–4)	4 (3–4)	7 (6–9)	6 (5–8)	9 (7–11)	12 (9–16)	16 (12–20)	19 (14–24)	19 (14–24)	22 (18–28)	20 (16–25)	15 (12–18)	8 (6–10)	--
Skin and subcutaneous diseases	--	--	87 (42–179)	41 (20–95)	21 (10–37)	28 (14–53)	33 (17–60)	50 (25–95)	66 (30–114)	92 (39–157)	128 (51–221)	176 (69–269)	233 (92–387)	327 (133–506)	435 (166–649)	518 (203–769)	587 (242–913)	679 (283–1,093)	750 (310–1,277)	765 (333–1,322)	602 (270–1,079)	354 (156–650)	--
Bacterial skin diseases	--	--	82 (39–167)	36 (18–84)	18 (9–31)	25 (12–45)	26 (13–46)	37 (18–74)	47 (19–83)	58 (28–118)	100 (36–169)	142 (51–219)	188 (65–310)	267 (95–411)	354 (121–526)	414 (144–635)	449 (153–693)	501 (177–825)	513 (192–997)	515 (189–886)	390 (152–739)	229 (86–444)	--
Cellulitis	--	--	16 (6–35)	8 (3–18)	5 (2–9)	7 (3–13)	9 (3–16)	10 (4–22)	14 (5–26)	19 (6–34)	30 (9–52)	38 (12–62)	54 (16–90)	69 (22–117)	82 (24–134)	93 (26–154)	99 (28–179)	100 (28–182)	87 (27–179)	90 (28–175)	67 (20–150)	39 (13–86)	--
Pyoderma	--	--	66 (30–139)	28 (13–67)	13 (6–23)	17 (8–33)	17 (8–32)	27 (12–55)	33 (12–63)	40 (19–90)	70 (23–123)	104 (37–167)	134 (42–233)	198 (66–315)	271 (85–389)	321 (100–492)	350 (113–558)	401 (137–687)	442 (155–831)	423 (152–771)	323 (124–597)	190 (70–375)	--
Decubitus ulcer	--	--	--	2 (0–6)	1 (0–2)	2 (1–4)	2 (1–5)	10 (3–21)	15 (4–30)	19 (5–42)	22 (5–46)	28 (7–56)	34 (9–68)	44 (14–88)	66 (19–123)	87 (25–150)	118 (38–225)	158 (48–274)	204 (64–392)	235 (74–427)	197 (63–373)	117 (36–230)	--
Other skin and subcutaneous diseases	--	--	5 (2–16)	3 (1–8)	2 (1–5)	2 (1–5)	2 (1–4)	2 (1–5)	4 (2–8)	4 (2–7)	6 (2–12)	6 (2–10)	11 (4–19)	12 (5–21)	16 (6–26)	18 (7–30)	21 (8–35)	20 (8–36)	17 (7–35)	17 (7–30)	15 (6–31)	7 (3–16)	--
Musculoskeletal disorders	--	--	--	--	26 (20–38)	47 (35–66)	45 (33–59)	54 (39–69)	65 (45–83)	66 (47–82)	82 (56–106)	100 (70–127)	112 (76–141)	140 (97–177)	180 (117–222)	209 (135–252)	220 (137–261)	233 (149–273)	245 (166–295)	230 (153–286)	247 (184–336)	145 (104–201)	--
Rheumatoid arthritis	--	--	--	--	2 (2–4)	3 (2–4)	3 (2–5)	4 (3–5)	5 (4–7)	5 (4–7)	10 (7–13)	16 (11–21)	23 (16–30)	39 (28–50)	55 (37–70)	76 (50–92)	88 (56–108)	109 (70–131)	122 (83–149)	118 (77–143)	121 (90–164)	68 (48–94)	--
Other musculoskeletal disorders	--	--	--	--	24 (18–34)	44 (33–62)	42 (31–55)	50 (35–63)	60 (41–76)	61 (43–76)	72 (49–92)	84 (60–107)	89 (60–112)	102 (69–128)	125 (80–153)	133 (85–161)	132 (82–156)	123 (79–144)	123 (80–149)	112 (75–142)	126 (94–172)	77 (55–108)	--
Congenital anomalies	7,466 (5,438–9,950)	2,679 (2,022–3,489)	8,963 (6,506–11,895)	1,864 (1,245–2,516)	589 (402–787)	448 (316–566)	367 (252–461)	326 (213–402)	280 (180–340)	218 (142–267)	205 (136–254)	179 (118–223)	158 (102–197)	152 (95–192)	168 (106–207)	137 (89–166)	108 (71–129)	--	--	--	--	--	--
Neural tube defects	800 (554–1,160)	140 (96–220)	359 (235–563)	353 (217–560)	18 (11–36)	17 (11–32)	14 (9–23)	11 (7–16)	8 (5–13)	6 (4–9)	4 (3–6)	4 (3–6)	5 (4–7)	4 (3–6)	5 (4–7)	4 (3–5)	4 (3–6)	--	--	--	--	--	--
Congenital heart anomalies	2,637 (1,841–3,605)	1,329 (952–1,748)	4,525 (3,076–6,138)	778 (499–1,099)	296 (203–408)	238 (170–302)	191 (134–238)	169 (113–205)	149 (98–179)	107 (70–132)	104 (67–128)	90 (59–112)	62 (39–78)	57 (33–73)	60 (34–75)	55 (32–69)	50 (31–61)	--	--	--	--	--	--
Orofacial clefts	19 (13–28)	7 (5–10)	29 (19–44)	2 (1–4)	--	--	--	--	--	--	--	--	--	--	--	--	--	--	--	--	--	--	--
Down's syndrome	128 (92–178)	57 (43–79)	555 (394–816)	127 (84–182)	39 (28–53)	34 (25–45)	37 (27–52)	37 (27–50)	37 (27–48)	40 (29–54)	38 (28–50)	37 (26–50)	47 (32–62)	48 (32–64)	59 (37–79)	37 (24–49)	15 (10–21)	--	--	--	--	--	--
Other chromosomal abnormalities	273 (136–378)	86 (45–125)	343 (134–479)	38 (15–64)	18 (7–26)	13 (5–18)	15 (6–21)	14 (4–19)	14 (5–19)	11 (3–14)	10 (3–14)	7 (2–10)	6 (1–8)	4 (1–5)	4 (1–6)	3 (1–4)	3 (0–4)	--	--	--	--	--	--
Congenital musculoskeletal and limb anomalies	136 (87–202)	29 (19–48)	90 (55–141)	22 (13–39)	12 (7–20)	12 (7–19)	9 (6–16)	6 (3–9)	6 (3–9)	5 (3–6)	5 (3–8)	4 (2–5)	3 (1–4)	4 (2–6)	4 (2–6)	5 (2–7)	4 (2–5)	--	--	--	--	--	--
Urogenital congenital anomalies	362 (227–647)	122 (81–207)	110 (69–213)	16 (9–31)	10 (6–16)	9 (6–15)	16 (6–22)	26 (6–38)	19 (3–27)	11 (2–16)	7 (2–10)	6 (2–9)	5 (2–7)	4 (2–5)	3 (2–4)	4 (2–5)	3 (2–4)	--	--	--	--	--	--
Digestive congenital anomalies	1,003 (697–1,393)	469 (336–634)	1,129 (731–1,560)	105 (60–153)	27 (16–39)	15 (10–20)	8 (5–11)	6 (4–8)	5 (3–7)	5 (3–6)	6 (3–7)	7 (4–9)	6 (3–8)	8 (4–11)	10 (5–13)	11 (6–14)	11 (6–14)	--	--	--	--	--	--
Other congenital anomalies	2,109 (1,451–2,865)	439 (311–593)	1,823 (1,255–2,474)	425 (241–618)	169 (97–235)	111 (64–149)	77 (44–101)	57 (32–75)	42 (24–55)	33 (20–44)	30 (17–39)	24 (14–32)	23 (13–31)	22 (12–30)	21 (11–27)	17 (9–21)	17 (10–22)	--	--	--	--	--	--
Urinary diseases and male infertility	5 (3–8)	6 (4–9)	156 (102–210)	57 (41–80)	27 (21–34)	30 (24–36)	48 (38–59)	77 (59–90)	99 (70–113)	132 (87–150)	196 (122–227)	275 (178–320)	368 (208–430)	564 (333–659)	798 (435–928)	1,091 (632–1,266)	1,428 (843–1,656)	1,915 (1,136–2,222)	2,415 (1,434–2,800)	2,662 (1,567–3,102)	2,310 (1,392–2,714)	1,392 (855–1,689)	--
Urinary tract infections and interstitial nephritis	4 (2–5)	4 (3–6)	149 (97–199)	51 (37–71)	22 (17–28)	25 (20–31)	41 (32–51)	64 (49–76)	82 (58–95)	107 (69–122)	154 (92–177)	211 (133–248)	284 (150–333)	442 (245–519)	640 (337–755)	899 (488–1,051)	1,212 (667–1,419)	1,665 (904–1,949)	2,146 (1,195–2,515)	2,400 (1,332–2,835)	2,115 (1,210–2,493)	1,266 (732–1,545)	--
Urolithiasis	--	--	--	1 (0–3)	1 (0–1)	1 (1–1)	1 (1–2)	3 (2–5)	5 (4–8)	9 (6–13)	12 (8–19)	23 (16–34)	27 (17–41)	40 (26–62)	53 (35–90)	59 (40–102)	65 (43–110)	68 (46–113)	67 (45–113)	52 (34–89)	33 (22–52)	22 (14–37)	--
Other urinary diseases	2 (1–3)	2 (1–4)	7 (4–11)	5 (3–8)	4 (3–6)	4 (3–6)	6 (4–9)	9 (5–14)	12 (6–17)	17 (9–25)	29 (15–43)	41 (20–64)	57 (24–87)	82 (39–129)	105 (47–176)	133 (66–230)	150 (89–255)	183 (117–312)	201 (123–335)	209 (127–347)	162 (102–302)	105 (65–198)	--
Gynecological diseases	--	--	--	--	--	--	--	--	--	--	--	--	--	--	--	--	--	--	--	--	--	--	--
Uterine fibroids	--	--	--	--	--	--	--	--	--	--	--	--	--	--	--	--	--	--	--	--	--	--	--
Endometriosis	--	--	--	--	--	--	--	--	--	--	--	--	--	--	--	--	--	--	--	--	--	--	--
Genital prolapse	--	--	--	--	--	--	--	--	--	--	--	--	--	--	--	--	--	--	--	--	--	--	--
Other gynecological diseases	--	--	--	--	--	--	--	--	--	--	--	--	--	--	--	--	--	--	--	--	--	--	--
Hemoglobinopathies and hemolytic anaemias	6 (5–8)	5 (4–7)	84 (58–118)	131 (80–193)	110 (87–139)	105 (89–125)	100 (86–115)	115 (102–131)	102 (92–116)	102 (90–115)	98 (88–110)	99 (87–112)	100 (89–113)	110 (97–125)	123 (110–139)	135 (120–151)	141 (127–157)	149 (134–166)	140 (124–156)	119 (102–132)	93 (75–105)	48 (38–55)	--
Thalassemias	0 (0–1)	0 (0–1)	8 (4–13)	11 (6–17)	5 (3–8)	2 (1–4)	1 (0–1)	0 (0–1)	0 (0–1)	0 (0–0)	0 (0–0)	0 (0–0)	0 (0–0)	0 (0–0)	0 (0–0)	0 (0–0)	0 (0–0)	0 (0–0)	0 (0–0)	0 (0–0)	0 (0–0)	0 (0–0)	--
Sickle cell disorders	4 (3–6)	4 (3–6)	60 (37–93)	92 (46–147)	66 (46–91)	54 (41–69)	45 (34–58)	44 (32–59)	35 (25–47)	34 (24–47)	29 (20–41)	27 (18–37)	23 (15–33)	16 (10–24)	8 (5–13)	2 (1–4)	0 (0–1)	0 (0–0)	0 (0–0)	0 (0–0)	0 (0–0)	0 (0–0)	--
G6PD deficiency	0 (0–1)	0 (0–1)	11 (7–16)	24 (15–35)	32 (20–43)	38 (25–50)	41 (28–55)	51 (35–67)	52 (37–66)	51 (35–64)	49 (33–64)	49 (33–62)	50 (33–64)	52 (35–67)	55 (37–69)	47 (31–61)	33 (20–45)	23 (13–31)	14 (7–19)	6 (3–9)	3 (1–4)	1 (0–1)	--
Other hemoglobinopathies and hemolytic anaemias	1 (1–2)	0 (0–1)	5 (3–7)	4 (3–6)	7 (5–10)	11 (8–14)	13 (9–18)	19 (14–27)	15 (11–21)	17 (13–23)	20 (14–27)	24 (18–32)	27 (21–36)	41 (32–53)	61 (48–77)	86 (71–105)	107 (93–126)	126 (111–144)	127 (112–142)	112 (97–126)	90 (73–103)	47 (37–54)	--
Endocrine, metabolic, blood, and immune disorders	69 (49–101)	53 (38–79)	517 (361–761)	298 (207–456)	120 (93–170)	109 (81–152)	106 (76–134)	137 (90–168)	200 (126–238)	296 (177–347)	381 (211–452)	466 (236–555)	563 (280–679)	650 (322–776)	743 (352–892)	849 (416–1,011)	856 (423–1,015)	911 (491–1,071)	850 (463–997)	748 (425–877)	594 (371–708)	323 (205–391)	--
Sudden infant death syndrome	--	89 (68–117)	582 (397–840)	--	--	--	--	--	--	--	--	--	--	--	--	--	--	--	--	--	--	--	--
Transport injuries	28 (19–40)	19 (14–26)	281 (209–370)	923 (693–1,193)	1,224 (1,012–1,464)	1,434 (1,235–1,669)	5,509 (4,755–6,300)	10,169 (9,088–11,235)	9,699 (8,769–10,671)	8,828 (7,953–9,690)	8,317 (7,432–9,208)	7,453 (6,670–8,384)	6,724 (5,979–7,635)	6,125 (5,376–6,958)	5,461 (4,835–6,187)	4,508 (3,990–5,072)	3,654 (3,250–4,158)	2,967 (2,655–3,336)	2,344 (2,069–2,620)	1,697 (1,461–1,923)	1,005 (831–1,148)	373 (291–428)	--
Road injuries	25 (17–36)	17 (12–23)	264 (196–346)	881 (661–1,137)	1,169 (966–1,403)	1,361 (1,171–1,587)	5,240 (4,529–6,005)	9,667 (8,642–10,672)	9,187 (8,314–10,092)	8,348 (7,523–9,150)	7,856 (7,019–8,699)	7,032 (6,313–7,869)	6,340 (5,641–7,172)	5,765 (5,062–6,538)	5,148 (4,564–5,831)	4,274 (3,783–4,802)	3,472 (3,093–3,945)	2,829 (2,535–3,167)	2,236 (1,978–2,498)	1,631 (1,407–1,848)	967 (801–1,106)	357 (277–410)	--
Pedestrian road injuries	12 (8–17)	7 (5–9)	91 (65–122)	499 (365–649)	631 (519–756)	596 (502–702)	1,148 (966–1,351)	2,006 (1,752–2,307)	2,044 (1,797–2,316)	2,219 (1,955–2,507)	2,251 (1,968–2,551)	2,167 (1,901–2,494)	2,109 (1,817–2,459)	2,170 (1,855–2,533)	2,070 (1,780–2,424)	2,024 (1,753–2,330)	1,771 (1,548–2,039)	1,657 (1,460–1,896)	1,485 (1,299–1,682)	1,163 (995–1,322)	706 (578–808)	262 (205–302)	--
Cyclist road injuries	--	--	--	39 (25–62)	76 (60–97)	145 (122–175)	210 (176–251)	251 (216–289)	241 (212–275)	245 (217–276)	262 (232–298)	259 (230–294)	275 (243–312)	317 (279–362)	327 (287–374)	344 (303–393)	298 (263–341)	192 (168–221)	90 (77–103)	51 (43–59)	19 (16–23)	6 (5–7)	--
Motorcyclist road injuries	3 (2–6)	2 (2–3)	31 (22–42)	57 (40–87)	70 (55–91)	157 (134–186)	2,051 (1,789–2,336)	3,936 (3,529–4,364)	3,441 (3,121–3,749)	2,710 (2,476–2,950)	2,384 (2,172–2,591)	1,948 (1,774–2,149)	1,629 (1,481–1,782)	1,202 (1,098–1,321)	971 (886–1,065)	523 (473–573)	355 (318–396)	183 (163–208)	74 (65–84)	41 (35–47)	19 (15–23)	10 (8–12)	--
Motor vehicle road injuries	8 (6–12)	7 (5–10)	133 (99–175)	265 (192–361)	353 (289–437)	412 (356–487)	1,711 (1,466–1,973)	3,255 (2,823–3,638)	3,244 (2,847–3,593)	2,981 (2,636–3,295)	2,777 (2,447–3,100)	2,482 (2,183–2,814)	2,162 (1,908–2,473)	1,907 (1,667–2,167)	1,630 (1,425–1,863)	1,248 (1,093–1,424)	937 (822–1,085)	699 (615–792)	506 (444–573)	318 (269–365)	189 (150–224)	74 (57–88)	--
Other road injuries	1 (1–2)	1 (1–1)	9 (6–12)	21 (15–29)	38 (31–49)	50 (42–62)	120 (100–144)	219 (187–253)	218 (190–254)	193 (165–227)	181 (157–214)	176 (151–207)	165 (141–193)	168 (141–199)	151 (128–180)	134 (115–156)	110 (95–130)	97 (85–113)	81 (70–96)	56 (48–68)	34 (27–40)	4 (3–5)	--
Other transport injuries	3 (2–5)	2 (2–4)	18 (13–24)	42 (31–59)	54 (44–68)	73 (61–89)	269 (220–323)	503 (425–581)	512 (441–587)	480 (411–552)	462 (392–536)	421 (355–501)	385 (325–461)	359 (305–429)	313 (266–368)	234 (196–274)	182 (155–216)	139 (117–165)	108 (90–129)	66 (54–79)	38 (30–46)	16 (13–19)	--
Falls	11 (8–15)	6 (4–8)	112 (84–151)	178 (131–232)	166 (139–197)	175 (150–206)	411 (352–480)	721 (639–822)	804 (720–896)	888 (809–984)	1,074 (974–1,194)	1,225 (1,108–1,359)	1,349 (1,209–1,515)	1,798 (1,607–2,021)	1,869 (1,676–2,090)	2,049 (1,831–2,284)	1,982 (1,798–2,194)	2,166 (1,952–2,398)	2,225 (2,008–2,447)	2,575 (2,233–2,842)	2,255 (1,897–2,508)	1,576 (1,241–1,788)	--
Drowning	12 (8–17)	6 (4–8)	106 (67–163)	1,017 (705–1,394)	707 (555–870)	783 (657–920)	1,342 (1,171–1,545)	1,287 (1,150–1,464)	1,095 (995–1,227)	997 (908–1,105)	973 (884–1,078)	907 (820–1,007)	790 (708–887)	717 (645–804)	586 (526–656)	477 (428–535)	379 (339–423)	274 (246–307)	196 (174–217)	119 (101–133)	78 (63–90)	33 (26–38)	--
Fire, heat, and hot substances	4 (3–6)	5 (3–6)	91 (60–130)	267 (167–377)	113 (88–146)	67 (55–83)	82 (69–98)	138 (121–161)	156 (138–178)	160 (143–185)	166 (147–189)	176 (155–200)	175 (155–199)	179 (157–205)	177 (156–200)	175 (153–200)	163 (144–185)	178 (158–203)	173 (154–195)	174 (155–197)	140 (117–160)	85 (67–98)	--
Poisonings	7 (4–13)	7 (4–16)	35 (22–55)	116 (77–178)	43 (33–65)	38 (31–50)	57 (45–72)	105 (85–126)	103 (85–125)	91 (74–114)	83 (68–104)	78 (63–96)	70 (57–87)	67 (54–83)	63 (51–76)	51 (42–62)	43 (35–52)	38 (32–46)	35 (29–41)	26 (21–32)	17 (14–21)	7 (5–8)	--
Poisoning by carbon monoxide	1 (0–1)	1 (0–2)	5 (3–7)	18 (11–31)	11 (8–15)	10 (8–14)	20 (16–25)	36 (30–43)	38 (31–47)	34 (28–47)	32 (26–42)	28 (23–36)	24 (20–31)	21 (17–26)	22 (18–27)	16 (13–19)	15 (12–18)	14 (11–17)	13 (11–16)	11 (9–14)	7 (5–9)	3 (2–4)	--
Poisoning by other means	7 (4–12)	6 (3–14)	30 (19–49)	98 (64–158)	32 (24–50)	28 (22–36)	37 (28–48)	69 (54–88)	65 (52–83)	56 (44–72)	51 (41–65)	50 (39–62)	46 (36–60)	46 (36–58)	41 (32–52)	36 (28–45)	29 (23–36)	24 (20–30)	22 (18–27)	15 (12–19)	11 (8–13)	4 (3–4)	--
Exposure to mechanical forces	11 (4–41)	22 (3–200)	55 (35–124)	125 (87–178)	149 (114–209)	194 (155–252)	606 (493–753)	961 (810–1,162)	939 (809–1,118)	827 (717–986)	771 (664–914)	684 (578–829)	582 (496–697)	523 (447–627)	442 (380–526)	331 (292–386)	243 (213–282)	182 (160–207)	136 (122–154)	95 (83–108)	65 (54–75)	34 (27–39)	--
Unintentional firearm injuries	5 (1–25)	13 (1–130)	18 (6–70)	40 (18–77)	53 (31–98)	94 (67–141)	389 (291–521)	599 (476–790)	557 (439–725)	459 (356–601)	388 (288–515)	324 (229–445)	245 (174–338)	190 (137–271)	144 (102–211)	99 (70–142)	69 (49–98)	43 (29–60)	31 (22–44)	21 (14–28)	15 (11–20)	7 (5–9)	--
Other exposure to mechanical forces	6 (2–20)	10 (2–55)	37 (27–61)	84 (65–119)	96 (77–128)	100 (83–125)	217 (185–275)	361 (309–460)	382 (332–490)	368 (321–462)	382 (333–463)	360 (314–432)	337 (294–397)	332 (290–392)	298 (260–347)	233 (205–268)	173 (153–200)	139 (123–158)	105 (93–119)	74 (64–85)	50 (41–57)	27 (21–31)	--
Adverse effects of medical treatment	28 (20–40)	21 (14–30)	156 (109–219)	88 (60–128)	48 (37–62)	44 (36–54)	37 (31–46)	57 (48–69)	65 (56–81)	75 (64–88)	98 (86–120)	112 (96–133)	139 (119–162)	176 (151–208)	223 (193–258)	290 (252–337)	317 (280–364)	341 (301–388)	336 (299–382)	293 (251–338)	209 (173–244)	111 (88–130)	--
Animal contact	2 (1–3)	2 (1–3)	17 (11–24)	49 (36–65)	44 (36–55)	36 (30–43)	29 (24–35)	34 (28–40)	38 (33–44)	40 (35–46)	47 (41–54)	54 (47–63)	64 (56–74)	87 (74–100)	98 (85–112)	112 (97–129)	104 (90–119)	111 (96–127)	98 (85–112)	78 (66–90)	50 (41–58)	24 (18–28)	--
Venomous animal contact	1 (1–2)	2 (1–2)	13 (9–18)	38 (28–51)	33 (26–40)	25 (21–30)	19 (15–24)	22 (18–27)	27 (23–31)	26 (22–31)	32 (27–37)	38 (32–44)	44 (38–52)	61 (52–73)	70 (60–81)	78 (66–91)	69 (60–81)	75 (64–89)	65 (55–74)	56 (47–65)	37 (29–43)	18 (13–22)	--
Non-venomous animal contact	1 (0–1)	0 (0–1)	4 (3–6)	10 (7–15)	12 (9–15)	11 (8–13)	10 (8–12)	11 (10–14)	11 (10–13)	14 (12–16)	15 (13–17)	17 (14–20)	20 (17–23)	25 (21–29)	28 (24–32)	34 (29–40)	35 (29–40)	35 (30–42)	33 (28–38)	22 (18–26)	14 (11–16)	6 (4–7)	--
Foreign body	103 (75–143)	107 (82–139)	1,917 (1,409–2,536)	623 (461–824)	270 (220–326)	281 (234–338)	229 (193–274)	370 (313–431)	397 (348–455)	421 (368–481)	468 (407–541)	477 (408–559)	469 (397–554)	491 (418–580)	498 (426–589)	478 (405–562)	436 (379–504)	416 (366–481)	385 (339–434)	354 (306–398)	241 (200–272)	151 (119–172)	--
Pulmonary aspiration and foreign body in airway	102 (75–142)	106 (81–138)	1,911 (1,405–2,529)	612 (452–808)	264 (215–318)	275 (229–332)	226 (189–270)	366 (310–426)	392 (343–450)	417 (364–477)	463 (403–536)	473 (404–554)	463 (392–548)	485 (413–574)	492 (421–582)	471 (399–556)	430 (373–498)	410 (361–475)	378 (333–426)	346 (299–389)	237 (195–267)	148 (116–169)	--
Foreign body in other body part	1 (0–1)	1 (0–1)	6 (4–12)	11 (6–23)	6 (4–9)	5 (4–8)	4 (3–5)	4 (4–5)	5 (4–6)	4 (3–5)	5 (4–6)	5 (4–6)	6 (5–7)	5 (4–7)	6 (5–8)	7 (5–9)	7 (5–9)	6 (5–8)	7 (6–9)	7 (6–10)	4 (3–6)	3 (2–4)	--
Environmental heat and cold exposure	2 (1–5)	1 (1–2)	7 (4–13)	11 (6–22)	8 (5–16)	6 (4–11)	11 (7–23)	18 (12–34)	19 (13–34)	21 (15–37)	23 (17–38)	25 (18–42)	27 (19–44)	38 (28–56)	37 (27–57)	32 (23–52)	28 (20–45)	32 (23–53)	31 (23–53)	28 (20–49)	23 (16–37)	15 (11–24)	--
Other unintentional injuries	14 (9–21)	5 (3–8)	52 (38–69)	118 (83–171)	139 (109–184)	195 (161–241)	476 (400–575)	780 (673–904)	816 (720–937)	797 (704–909)	787 (696–899)	730 (632–847)	660 (566–771)	525 (457–609)	457 (396–527)	307 (268–359)	241 (209–275)	165 (144–188)	137 (120–156)	101 (86–116)	71 (58–82)	41 (32–48)	--
Self-harm	--	--	--	--	--	--	317 (265–375)	2,099 (1,807–2,406)	3,481 (3,086–3,898)	3,439 (3,107–3,777)	3,150 (2,857–3,460)	2,938 (2,659–3,226)	2,600 (2,320–2,907)	2,338 (2,085–2,637)	2,118 (1,891–2,368)	1,883 (1,674–2,108)	1,524 (1,354–1,711)	1,247 (1,119–1,400)	1,003 (900–1,116)	752 (675–832)	537 (467–598)	328 (271–372)	127 (101–145)
Self-harm by firearm	--	--	--	--	--	--	17 (13–28)	280 (217–396)	456 (365–640)	426 (345–587)	386 (315–537)	345 (284–481)	295 (241–416)	273 (221–370)	257 (212–343)	213 (174–287)	191 (158–253)	166 (139–219)	135 (113–178)	105 (87–131)	78 (64–100)	46 (36–57)	18 (14–23)
Self-harm by other specified means	--	--	--	--	--	--	301 (245–356)	1,819 (1,545–2,086)	3,025 (2,660–3,377)	3,014 (2,702–3,308)	2,765 (2,497–3,039)	2,593 (2,342–2,851)	2,305 (2,052–2,574)	2,065 (1,837–2,327)	1,861 (1,664–2,077)	1,670 (1,479–1,876)	1,333 (1,179–1,501)	1,081 (960–1,216)	868 (768–971)	647 (575–717)	459 (394–514)	282 (221–321)	109 (87–125)
Interpersonal violence	108 (82–140)	29 (22–38)	273 (203–359)	445 (337–579)	449 (369–543)	1,388 (1,196–1,614)	14,925 (13,306–16,678)	24,241 (22,213–26,546)	21,652 (19,912–23,563)	18,123 (16,623–19,738)	15,103 (13,666–16,732)	11,297 (10,126–12,606)	8,383 (7,442–9,502)	6,205 (5,495–6,997)	4,454 (3,959–5,034)	3,004 (2,680–3,354)	1,978 (1,770–2,211)	1,285 (1,158–1,439)	835 (744–930)	304 (262–341)	156 (129–178)	70 (54–82)	--
Physical violence by firearm	26 (19–34)	8 (6–11)	96 (72–128)	148 (112–196)	199 (165–240)	932 (798–1,084)	12,083 (10,798–13,511)	19,358 (17,720–21,249)	16,855 (15,509–18,393)	13,651 (12,508–14,918)	11,034 (9,952–12,290)	7,901 (7,050–8,870)	5,585 (4,922–6,376)	3,916 (3,437–4,461)	2,633 (2,307–3,027)	1,666 (1,471–1,880)	1,003 (890–1,138)	580 (516–663)	342 (303–388)	113 (96–130)	56 (45–64)	32 (24–38)	--
Physical violence by sharp object	15 (11–20)	3 (2–4)	21 (15–28)	30 (22–44)	40 (32–56)	141 (119–171)	1,755 (1,533–2,051)	3,046 (2,721–3,453)	2,954 (2,673–3,334)	2,683 (2,435–3,019)	2,332 (2,092–2,631)	1,836 (1,640–2,078)	1,433 (1,272–1,639)	1,145 (1,011–1,304)	842 (745–958)	602 (533–686)	414 (368–471)	284 (252–320)	186 (163–212)	84 (72–96)	41 (34–48)	15 (12–18)	--
Physical violence by other means	68 (52–89)	17 (13–23)	156 (115–207)	267 (196–361)	211 (166–267)	316 (254–390)	1,086 (947–1,252)	1,837 (1,648–2,049)	1,844 (1,645–2,057)	1,789 (1,604–1,980)	1,736 (1,537–1,959)	1,560 (1,365–1,775)	1,365 (1,187–1,562)	1,143 (1,002–1,301)	978 (860–1,113)	736 (647–830)	560 (492–627)	421 (377–470)	307 (270–341)	106 (92–120)	59 (49–67)	23 (18–27)	--
Executions and police conflict	0 (0–0)	0 (0–0)	2 (1–3)	5 (3–6)	3 (3–4)	14 (10–20)	279 (173–443)	475 (272–712)	333 (193–501)	219 (129–322)	114 (78–158)	62 (42–92)	35 (24–51)	17 (13–24)	12 (9–17)	7 (5–10)	5 (4–7)	2 (2–3)	2 (1–2)	1 (1–2)	1 (1–1)	1 (0–1)	--

SR Table 12: Caribbean deaths by cause and age group for males

Cause	Early Neonatal	Late Neonatal	Post Neonatal	1 to 4	5 to 9	10 to 14	15 to 19	20 to 24	25 to 29	30 to 34	35 to 39
All causes	6,663 (5,120–8,494)	2,061 (1,593–2,618)	5,177 (3,836–6,852)	4,186 (3,449–5,053)	1,459 (1,232–1,667)	1,090 (949–1,238)	2,012 (1,710–2,343)	3,223 (2,790–3,709)	3,734 (3,315–4,190)	4,341 (3,838–4,871)	4,834 (4,212–5,543)
Sexually transmitted infections excluding HIV	295 (99–615)	101 (35–211)	129 (42–278)	4 (2–9)	2 (1–4)	1 (0–2)	1 (0–3)	1 (0–4)	2 (1–5)	2 (1–5)	3 (1–10)
Syphilis	295 (99–615)	101 (35–211)	129 (42–278)	4 (2–9)	2 (1–4)	0 (0–1)	1 (0–2)	1 (0–2)	1 (0–4)	2 (1–4)	3 (1–9)
Chlamydial infection	--	--	--	--	--	0 (0–0)	0 (0–0)	0 (0–0)	0 (0–0)	0 (0–0)	0 (0–0)
Gonococcal infection	--	--	--	--	--	0 (0–0)	0 (0–0)	0 (0–1)	0 (0–0)	0 (0–0)	0 (0–1)
Other sexually transmitted infections	--	--	--	--	--	0 (0–1)	0 (0–0)	0 (0–1)	0 (0–1)	1 (0–1)	0 (0–1)
Tuberculosis	--	--	39 (20–67)	47 (28–76)	12 (8–18)	8 (5–12)	18 (11–28)	52 (33–77)	74 (50–106)	104 (71–146)	101 (68–140)
Drug-susceptible tuberculosis	--	--	39 (20–66)	46 (28–76)	12 (7–18)	8 (5–12)	18 (11–27)	51 (32–76)	74 (49–104)	103 (70–144)	99 (67–139)
Multidrug-resistant tuberculosis without extensive drug resistance	--	--	0 (0–2)	0 (0–2)	0 (0–0)	0 (0–0)	0 (0–1)	1 (0–2)	1 (0–3)	1 (0–4)	1 (0–4)
Extensively drug-resistant tuberculosis	--	--	0 (0–0)	0 (0–0)	0 (0–0)	0 (0–0)	0 (0–0)	0 (0–0)	0 (0–0)	0 (0–1)	0 (0–1)
Lower respiratory infections	342 (183–605)	242 (128–418)	994 (629–1,497)	540 (323–822)	84 (55–121)	40 (27–57)	34 (24–51)	47 (33–67)	62 (45–86)	89 (67–119)	101 (74–135)
Upper respiratory infections	0 (0–0)	0 (0–0)	5 (1–19)	10 (1–44)	2 (0–6)	0 (0–1)	0 (0–1)	0 (0–1)	0 (0–0)	0 (0–0)	0 (0–1)
Otitis media	1 (0–3)	1 (0–5)	0 (0–1)	0 (0–0)	0 (0–0)	0 (0–0)	0 (0–0)	0 (0–0)	0 (0–0)	0 (0–0)	0 (0–0)
Diarrhoeal diseases	52 (16–135)	166 (48–385)	1,130 (420–2,402)	510 (157–1,233)	42 (18–85)	14 (6–30)	11 (5–26)	15 (7–34)	18 (9–41)	21 (10–45)	25 (13–55)
Typhoid fever	--	--	0 (0–0)	0 (0–1)	0 (0–1)	0 (0–1)	0 (0–0)	0 (0–0)	0 (0–0)	0 (0–0)	0 (0–0)
Paratyphoid fever	--	--	0 (0–0)	0 (0–1)	0 (0–1)	0 (0–1)	0 (0–0)	0 (0–0)	0 (0–0)	0 (0–0)	0 (0–0)
iNTS	--	0 (0–0)	0 (0–1)	1 (0–2)	1 (0–2)	0 (0–1)	0 (0–1)	0 (0–1)	0 (0–0)	0 (0–0)	0 (0–0)
Other intestinal infectious diseases	--	--	1 (0–3)	1 (0–4)	0 (0–1)	0 (0–1)	0 (0–0)	0 (0–0)	0 (0–0)	0 (0–0)	0 (0–0)
Malaria	0 (0–0)	2 (0–6)	6 (1–23)	18 (2–60)	9 (1–29)	6 (1–20)	28 (4–93)	31 (4–109)	20 (3–68)	16 (2–52)	13 (2–43)
Chagas disease	--	--	0 (0–0)	0 (0–0)	0 (0–0)	0 (0–0)	0 (0–0)	0 (0–0)	0 (0–0)	0 (0–0)	0 (0–0)
Visceral leishmaniasis	--	--	0 (0–0)	0 (0–0)	0 (0–0)	0 (0–0)	0 (0–0)	0 (0–0)	0 (0–0)	0 (0–0)	0 (0–0)
African trypanosomiasis	--	--	--	0 (0–0)	0 (0–0)	0 (0–0)	0 (0–0)	0 (0–0)	0 (0–0)	0 (0–0)	0 (0–0)
Schistosomiasis	--	--	0 (0–0)	0 (0–0)	0 (0–0)	0 (0–0)	0 (0–1)	0 (0–2)	1 (0–3)	1 (0–3)	1 (0–4)
Cysticercosis	--	--	--	0 (0–0)	0 (0–1)	0 (0–1)	0 (0–2)	1 (0–3)	1 (0–4)	2 (0–4)	2 (0–3)
Cystic echinococcosis	--	--	--	0 (0–0)	0 (0–0)	0 (0–0)	0 (0–0)	0 (0–0)	0 (0–0)	0 (0–0)	0 (0–0)
Dengue	0 (0–0)	0 (0–0)	3 (1–11)	2 (0–8)	2 (1–5)	1 (0–2)	1 (0–3)	1 (0–2)	1 (0–2)	1 (0–2)	1 (0–2)
Yellow fever	--	0 (0–0)	0 (0–0)	0 (0–0)	0 (0–0)	0 (0–0)	0 (0–0)	0 (0–0)	0 (0–0)	0 (0–0)	0 (0–0)
Rabies	--	--	0 (0–0)	0 (0–1)	1 (0–1)	0 (0–1)	0 (0–0)	0 (0–0)	0 (0–0)	0 (0–0)	0 (0–0)
Ascariasis	--	--	0 (0–2)	2 (1–5)	1 (0–2)	0 (0–1)	0 (0–0)	0 (0–0)	0 (0–0)	0 (0–0)	0 (0–0)
Zika virus disease	0 (0–0)	0 (0–0)	0 (0–0)	0 (0–0)	0 (0–0)	0 (0–0)	0 (0–0)	0 (0–0)	0 (0–0)	0 (0–0)	0 (0–0)
Other neglected tropical diseases	0 (0–0)	0 (0–0)	1 (0–3)	3 (1–9)	1 (0–3)	0 (0–1)	0 (0–0)	0 (0–1)	0 (0–0)	0 (0–1)	0 (0–0)
Meningitis	39 (18–76)	70 (33–129)	249 (113–451)	192 (103–340)	35 (21–54)	15 (10–23)	11 (7–17)	9 (6–13)	10 (7–13)	12 (9–17)	13 (9–18)
Encephalitis	2 (1–3)	3 (1–7)	24 (10–50)	26 (12–56)	14 (7–27)	9 (5–15)	7 (4–12)	7 (4–11)	6 (4–9)	6 (4–10)	6 (4–9)
Diphtheria	--	--	1 (0–2)	1 (0–4)	0 (0–2)	0 (0–0)	0 (0–0)	0 (0–0)	0 (0–0)	0 (0–0)	0 (0–0)
Whooping cough	--	--	215 (42–644)	204 (42–543)	19 (4–52)	3 (1–10)	1 (0–3)	1 (0–2)	1 (0–2)	1 (0–1)	0 (0–1)
Tetanus	10 (2–29)	16 (2–61)	2 (1–6)	3 (0–24)	2 (1–8)	1 (0–4)	1 (0–4)	1 (0–3)	1 (1–3)	2 (1–4)	2 (1–5)
Measles	0 (0–0)	0 (0–0)	0 (0–0)	0 (0–0)	0 (0–0)	0 (0–0)	0 (0–0)	0 (0–0)	0 (0–0)	0 (0–0)	0 (0–0)
Varicella and herpes zoster	0 (0–1)	1 (0–4)	7 (1–21)	11 (2–32)	4 (1–11)	1 (0–3)	1 (0–1)	1 (0–1)	1 (0–1)	1 (0–2)	1 (0–2)
Acute hepatitis	--	--	4 (1–10)	4 (1–10)	2 (1–5)	2 (1–5)	2 (1–4)	3 (2–6)	4 (2–6)	4 (2–7)	4 (2–7)
Acute hepatitis A	--	--	2 (0–6)	3 (1–8)	2 (1–4)	1 (0–3)	1 (1–2)	2 (1–4)	1 (0–2)	1 (1–3)	1 (0–3)
Acute hepatitis B	--	--	2 (0–4)	0 (0–1)	0 (0–1)	1 (0–2)	1 (0–1)	1 (0–2)	2 (1–3)	2 (1–4)	2 (1–4)
Acute hepatitis C	--	--	0 (0–0)	0 (0–1)	0 (0–0)	0 (0–1)	0 (0–1)	1 (0–2)	1 (0–2)	1 (0–2)	1 (0–2)
Acute hepatitis E	--	--	0 (0–1)	0 (0–0)	0 (0–0)	0 (0–0)	0 (0–0)	0 (0–0)	0 (0–0)	0 (0–0)	0 (0–0)
Other unspecified infectious diseases	3 (1–7)	3 (1–9)	21 (5–75)	16 (3–55)	5 (2–11)	4 (2–7)	6 (3–10)	7 (4–12)	11 (7–17)	14 (8–23)	16 (10–25)
Maternal disorders	--	--	--	--	--	--	--	--	--	--	--
Maternal haemorrhage	--	--	--	--	--	--	--	--	--	--	--
Maternal sepsis and other pregnancy related infections	--	--	--	--	--	--	--	--	--	--	--
Maternal hypertensive disorders	--	--	--	--	--	--	--	--	--	--	--
Maternal obstructed labour and uterine rupture	--	--	--	--	--	--	--	--	--	--	--
Maternal abortive outcome	--	--	--	--	--	--	--	--	--	--	--
Ectopic pregnancy	--	--	--	--	--	--	--	--	--	--	--
Indirect maternal deaths	--	--	--	--	--	--	--	--	--	--	--
Late maternal deaths	--	--	--	--	--	--	--	--	--	--	--
Maternal deaths aggravated by HIV/AIDS	--	--	--	--	--	--	--	--	--	--	--
Other maternal disorders	--	--	--	--	--	--	--	--	--	--	--
Neonatal disorders	4,891 (3,657–6,373)	974 (670–1,308)	303 (183–499)	20 (12–34)	--	--	--	--	--	--	--
Neonatal preterm birth	1,874 (1,279–2,657)	234 (139–355)	67 (38–122)	2 (1–5)	--	--	--	--	--	--	--
Neonatal encephalopathy due to birth asphyxia and trauma	1,330 (851–1,934)	170 (93–271)	76 (34–152)	11 (6–19)	--	--	--	--	--	--	--
Neonatal sepsis and other neonatal infections	691 (384–1,135)	319 (197–477)	66 (35–115)	1 (1–3)	--	--	--	--	--	--	--
Hemolytic disease and other neonatal jaundice	42 (24–72)	11 (6–20)	3 (1–10)	0 (0–0)	--	--	--	--	--	--	--
Other neonatal disorders	954 (449–1,572)	240 (111–415)	91 (36–186)	6 (3–12)	--	--	--	--	--	--	--
Nutritional deficiencies	0 (0–0)	0 (0–0)	294 (130–584)	203 (100–384)	27 (17–42)	9 (6–12)	6 (4–9)	7 (5–11)	8 (5–12)	11 (7–17)	14 (10–20)
Protein-energy malnutrition	0 (0–0)	0 (0–0)	291 (129–579)	200 (99–380)	26 (16–40)	8 (6–12)	5 (3–8)	5 (3–8)	7 (5–10)	10 (6–14)	12 (8–17)
Other nutritional deficiencies	--	--	3 (1–7)	2 (1–5)	1 (1–3)	1 (0–1)	1 (0–1)	2 (1–3)	1 (1–2)	1 (1–3)	2 (1–4)
Lip and oral cavity cancer	--	--	--	--	0 (0–1)	1 (0–1)	1 (0–1)	2 (1–2)	2 (1–3)	4 (3–6)	9 (6–12)
Nasopharynx cancer	--	--	--	--	1 (0–1)	1 (1–2)	1 (1–2)	2 (1–2)	2 (1–2)	2 (1–3)	3 (2–4)
Other pharynx cancer	--	--	--	--	--	--	--	1 (0–1)	1 (1–1)	2 (1–2)	4 (3–5)
Oesophageal cancer	--	--	--	--	--	--	--	1 (1–2)	2 (1–2)	4 (3–6)	10 (8–14)
Stomach cancer	--	--	--	--	--	--	2 (1–3)	4 (3–5)	9 (7–12)	23 (17–31)	38 (28–50)
Colon and rectum cancer	--	--	--	--	1 (1–2)	1 (1–1)	3 (2–5)	7 (5–9)	13 (10–17)	30 (23–38)	45 (34–60)
Liver cancer due to hepatitis B	--	--	--	--	--	1 (0–2)	1 (0–2)	2 (1–4)	4 (2–7)	6 (3–11)	9 (5–17)
Liver cancer due to hepatitis C	--	--	--	--	--	0 (0–0)	0 (0–0)	0 (0–0)	0 (0–0)	0 (0–1)	1 (0–1)
Liver cancer due to alcohol use	--	--	--	--	--	--	0 (0–0)	0 (0–1)	1 (0–2)	2 (1–3)	4 (2–8)
Liver cancer due to NASH	--	--	--	--	--	--	0 (0–0)	0 (0–1)	0 (0–1)	0 (0–1)	1 (0–1)
	0 (0–0)	0 (0–0)	0 (0–1)	1 (1–3)	1 (1–2)	1 (1–1)	--	--	--	--	--
Liver cancer due to other causes	0 (0–1)	0 (0–0)	1 (0–2)	1 (0–2)	1 (0–1)	1 (0–1)	0 (0–1)	0 (0–1)	1 (0–1)	1 (0–1)	1 (0–2)
Gallbladder and biliary tract cancer	--	--	--	--	--	--	--	1 (0–1)	1 (1–2)	3 (2–4)	5 (3–7)
Pancreatic cancer	--	--	--	--	--	--	1 (0–1)	1 (1–1)	1 (1–2)	6 (5–8)	11 (9–14)
Larynx cancer	--	--	--	--	--	--	--	1 (1–2)	1 (1–2)	2 (2–3)	6 (5–9)
Tracheal, bronchus, and lung cancer	--	--	--	--	--	1 (0–1)	4 (3–5)	6 (4–8)	8 (6–10)	20 (15–27)	38 (28–50)
Malignant skin melanoma	0 (0–0)	0 (0–0)	0 (0–0)	1 (0–2)	0 (0–0)	0 (0–1)	0 (0–1)	1 (1–1)	2 (1–3)	3 (2–4)	4 (3–6)
Non-melanoma skin cancer (squamous-cell carcinoma)	--	--	--	--	--	--	--	1 (0–1)	2 (1–3)	4 (3–5)	4 (3–6)
	0 (0–0)	0 (0–0)	3 (1–6)	8 (3–14)	5 (3–8)	4 (3–5)	4 (3–5)	4 (3–5)	4 (3–5)	5 (4–7)	5 (3–7)
	--	--	--	2 (1–4)	3 (2–5)	6 (4–8)	7 (5–11)	8 (5–11)	6 (4–8)	5 (3–7)	5 (3–7)
Breast cancer	--	--	--	--	--	--	0 (0–0)	0 (0–0)	0 (0–1)	1 (0–1)	1 (1–2)
Cervical cancer	--	--	--	--	--	--	--	--	--	--	--
Uterine cancer	--	--	--	--	--	--	--	--	--	--	--
Ovarian cancer	--	--	--	--	--	--	--	--	--	--	--
Prostate cancer	--	--	--	--	--	--	--	1 (1–1)	2 (2–3)	3 (2–4)	4 (3–6)
Testicular cancer	0 (0–1)	0 (0–0)	0 (0–1)	1 (1–3)	1 (0–2)	1 (0–1)	2 (1–3)	4 (3–5)	6 (4–7)	4 (3–5)	3 (2–5)
Kidney cancer	1 (0–1)	0 (0–0)	2 (1–3)	11 (5–20)	7 (5–9)	2 (1–3)	2 (2–3)	3 (2–4)	5 (4–7)	7 (5–10)	7 (5–10)
Bladder cancer	--	--	--	--	--	--	0 (0–0)	0 (0–0)	1 (0–1)	2 (1–2)	3 (2–4)
Brain and nervous system cancer	1 (0–2)	0 (0–1)	7 (2–20)	31 (10–94)	28 (14–62)	17 (10–29)	9 (6–14)	9 (6–13)	12 (7–17)	18 (11–24)	23 (14–32)
	0 (0–0)	0 (0–0)	1 (0–1)	10 (5–18)	2 (1–3)	1 (1–2)	1 (1–1)	1 (1–1)	1 (1–1)	1 (1–1)	1 (1–2)
	0 (0–0)	0 (0–0)	1 (0–1)	7 (3–13)	1 (1–2)	1 (0–1)	0 (0–1)	--	--	--	--
	--	--	--	3 (1–6)	1 (1–1)	1 (0–1)	0 (0–1)	1 (1–1)	1 (1–1)	1 (1–1)	1 (1–2)
	0 (0–0)	0 (0–0)	3 (1–6)	18 (6–43)	7 (4–14)	2 (1–3)	1 (1–1)	1 (1–1)	1 (1–1)	2 (1–2)	1 (1–1)
Thyroid cancer	--	--	--	--	1 (0–1)	0 (0–0)	0 (0–0)	1 (0–1)	1 (1–2)	1 (1–2)	2 (2–3)
Mesothelioma	--	--	--	--	--	--	--	0 (0–1)	1 (1–1)	1 (1–1)	1 (1–2)
Hodgkin lymphoma	--	--	--	1 (0–1)	1 (1–3)	1 (1–2)	4 (2–6)	6 (4–9)	8 (5–11)	8 (6–11)	8 (5–10)
Non-Hodgkin's lymphoma	--	--	--	19 (7–38)	19 (11–30)	13 (9–18)	14 (11–18)	19 (15–25)	25 (19–33)	33 (25–42)	34 (26–44)
	--	--	--	2 (0–14)	2 (1–5)	2 (1–3)	1 (1–2)	1 (1–2)	1 (1–2)	1 (1–2)	1 (1–2)
	--	--	--	17 (6–34)	17 (10–28)	11 (8–15)	13 (10–17)	18 (14–23)	24 (18–31)	31 (24–40)	33 (25–43)
Multiple myeloma	--	--	--	--	--	--	--	1 (0–1)	1 (1–1)	2 (1–2)	3 (3–5)
Leukaemia	2 (1–4)	2 (1–4)	14 (5–33)	71 (27–158)	70 (42–114)	46 (31–67)	37 (26–50)	35 (26–47)	34 (25–46)	35 (26–46)	37 (27–48)
Acute lymphoid leukaemia	0 (0–1)	0 (0–0)	4 (1–9)	33 (12–78)	40 (22–72)	21 (13–34)	17 (12–24)	11 (7–15)	6 (5–9)	6 (4–8)	5 (4–7)
Chronic lymphoid leukaemia	--	--	--	--	--	--	--	0 (0–1)	0 (0–1)	0 (0–1)	0 (0–1)
Acute myeloid leukaemia	0 (0–1)	1 (0–2)	3 (1–7)	12 (4–28)	9 (5–16)	10 (6–16)	10 (7–14)	10 (7–14)	12 (9–17)	12 (8–16)	12 (8–16)
Chronic myeloid leukaemia	0 (0–0)	0 (0–0)	2 (0–7)	7 (1–31)	2 (1–5)	1 (1–2)	1 (1–1)	2 (1–3)	4 (2–5)	4 (3–6)	5 (3–7)
Other leukaemia	1 (1–3)	1 (0–2)	6 (2–17)	19 (4–60)	18 (6–42)	14 (7–25)	9 (5–15)	12 (8–19)	11 (8–17)	13 (8–19)	14 (10–21)
Other malignant cancers	3 (1–5)	2 (1–5)	7 (3–17)	23 (10–61)	14 (9–26)	13 (9–19)	15 (11–22)	16 (12–22)	18 (14–24)	20 (16–26)	24 (18–31)
Myelodysplastic, myeloproliferative, and other hematopoietic neoplasms	0 (0–0)	0 (0–0)	0 (0–1)	1 (1–3)	1 (1–2)	1 (1–1)	1 (0–1)	1 (0–1)	1 (1–2)	1 (1–1)	2 (1–2)
Cardiovascular diseases	30 (16–56)	16 (8–30)	98 (38–196)	90 (41–151)	49 (33–70)	47 (30–69)	90 (65–125)	152 (108–213)	244 (181–332)	438 (334–558)	708 (545–918)
Rheumatic heart disease	--	--	--	11 (3–25)	10 (5–18)	15 (8–25)	14 (7–26)	15 (8–28)	19 (10–34)	23 (12–41)	26 (14–46)
Ischaemic heart disease	--	--	--	--	--	--	20 (13–30)	49 (32–73)	96 (68–134)	197 (143–260)	344 (259–441)
Stroke	15 (7–28)	5 (2–11)	38 (11–88)	25 (8–56)	16 (9–27)	12 (8–20)	25 (16–38)	45 (30–69)	64 (44–97)	117 (83–163)	186 (132–259)
Ischaemic stroke	1 (0–2)	1 (0–2)	3 (1–8)	6 (1–17)	1 (0–2)	1 (1–2)	3 (2–5)	5 (3–7)	8 (5–13)	15 (10–22)	22 (15–32)
Intracerebral hemorrhage	4 (1–11)	1 (0–3)	26 (7–65)	11 (3–28)	9 (5–17)	5 (3–9)	12 (7–20)	23 (15–38)	36 (24–55)	75 (51–106)	119 (82–173)
Subarachnoid hemorrhage	10 (4–21)	3 (1–8)	9 (2–30)	8 (2–24)	6 (3–12)	6 (3–11)	10 (6–17)	17 (10–30)	20 (12–35)	28 (17–47)	44 (26–77)

Cause	40 to 44	45 to 49	50 to 54	55 to 59	60 to 64	65 to 69	70 to 74	75 to 79	80 to 84	85 to 89	90 to 94	95 plus
All causes	5,980 (5,148–6,948)	7,882 (6,732–9,239)	10,858 (9,240–12,701)	13,643 (11,605–15,952)	15,986 (13,689–18,486)	18,169 (15,779–20,837)	21,113 (18,389–23,975)	20,908 (18,485–23,491)	19,880 (17,821–22,059)	15,801 (14,440–17,168)	9,596 (8,845–10,319)	--
Sexually transmitted infections excluding HIV	2 (1–4)	3 (1–6)	3 (1–8)	4 (2–8)	3 (1–7)	4 (2–10)	3 (1–6)	2 (1–4)	2 (1–5)	2 (1–4)	1 (0–2)	--
Syphilis	1 (1–3)	2 (1–4)	2 (1–6)	3 (1–6)	3 (1–6)	3 (1–8)	2 (1–5)	1 (0–2)	1 (0–2)	1 (0–1)	0 (0–0)	--
Chlamydial infection	0 (0–0)	0 (0–0)	0 (0–0)	0 (0–0)	0 (0–0)	0 (0–0)	0 (0–0)	0 (0–0)	0 (0–0)	0 (0–0)	0 (0–0)	--
Gonococcal infection	0 (0–1)	0 (0–1)	0 (0–0)	0 (0–0)	0 (0–1)	0 (0–1)	0 (0–1)	0 (0–0)	0 (0–0)	0 (0–0)	0 (0–0)	--
Other sexually transmitted infections	0 (0–0)	1 (0–1)	1 (0–2)	1 (0–1)	0 (0–1)	1 (0–1)	0 (0–1)	1 (0–1)	1 (0–2)	1 (0–3)	1 (0–1)	--
Tuberculosis	106 (72–146)	96 (67–138)	98 (69–139)	92 (64–129)	95 (69–129)	87 (64–120)	82 (58–111)	70 (51–96)	58 (42–76)	42 (30–57)	18 (13–23)	--
Drug-susceptible tuberculosis	104 (71–144)	95 (66–136)	96 (67–137)	91 (63–128)	93 (67–128)	86 (63–118)	81 (57–109)	69 (50–94)	57 (41–75)	42 (30–56)	17 (12–23)	--
Multidrug-resistant tuberculosis without extensive drug resistance	1 (0–4)	1 (0–3)	1 (0–4)	1 (0–3)	1 (0–4)	1 (0–3)	1 (0–3)	1 (0–3)	1 (0–2)	1 (0–2)	0 (0–1)	--
Extensively drug-resistant tuberculosis	0 (0–1)	0 (0–1)	0 (0–1)	0 (0–1)	0 (0–1)	0 (0–1)	0 (0–1)	0 (0–0)	0 (0–0)	0 (0–0)	0 (0–0)	--
Lower respiratory infections	135 (101–178)	177 (134–226)	248 (191–316)	335 (261–426)	434 (339–536)	564 (448–706)	817 (652–1,012)	1,009 (812–1,253)	1,178 (958–1,426)	1,132 (926–1,326)	810 (643–965)	--
Upper respiratory infections	0 (0–1)	0 (0–1)	0 (0–1)	1 (0–1)	0 (0–1)	1 (0–1)	0 (0–1)	1 (0–1)	0 (0–0)	0 (0–0)	0 (0–0)	--
Otitis media	0 (0–0)	0 (0–0)	0 (0–0)	0 (0–0)	0 (0–0)	0 (0–0)	0 (0–0)	0 (0–0)	0 (0–0)	0 (0–0)	0 (0–0)	--
Diarrhoeal diseases	36 (18–79)	40 (21–83)	50 (27–99)	59 (34–116)	72 (39–141)	79 (44–153)	102 (58–194)	104 (60–200)	110 (65–200)	85 (53–143)	46 (29–73)	--
Typhoid fever	0 (0–0)	0 (0–0)	0 (0–0)	0 (0–0)	0 (0–0)	0 (0–0)	0 (0–0)	0 (0–0)	0 (0–0)	0 (0–0)	0 (0–0)	--
Paratyphoid fever	0 (0–0)	0 (0–0)	0 (0–0)	0 (0–0)	0 (0–0)	0 (0–0)	0 (0–0)	0 (0–0)	0 (0–0)	0 (0–0)	0 (0–0)	--
iNTS	0 (0–0)	0 (0–0)	0 (0–0)	0 (0–0)	0 (0–1)	0 (0–1)	0 (0–0)	0 (0–0)	0 (0–0)	0 (0–0)	0 (0–0)	--
Other intestinal infectious diseases	0 (0–0)	0 (0–0)	0 (0–0)	0 (0–0)	0 (0–0)	0 (0–0)	0 (0–0)	0 (0–0)	0 (0–0)	0 (0–0)	0 (0–0)	--
Malaria	15 (2–52)	14 (2–46)	15 (2–51)	10 (1–33)	12 (2–40)	11 (1–37)	11 (2–38)	4 (1–13)	1 (0–4)	0 (0–0)	0 (0–0)	--
Chagas disease	0 (0–0)	0 (0–0)	0 (0–0)	0 (0–0)	0 (0–0)	0 (0–0)	0 (0–0)	0 (0–0)	0 (0–0)	0 (0–1)	0 (0–0)	--
Visceral leishmaniasis	0 (0–0)	0 (0–0)	0 (0–0)	0 (0–0)	0 (0–0)	0 (0–0)	0 (0–0)	0 (0–0)	0 (0–0)	0 (0–0)	0 (0–0)	--
African trypanosomiasis	0 (0–0)	0 (0–0)	0 (0–0)	0 (0–0)	0 (0–0)	0 (0–0)	0 (0–0)	0 (0–0)	0 (0–0)	0 (0–0)	0 (0–0)	--
Schistosomiasis	1 (0–4)	1 (0–5)	2 (0–5)	2 (0–5)	2 (0–6)	2 (0–5)	2 (0–6)	1 (0–5)	1 (0–3)	1 (0–2)	0 (0–1)	--
Cysticercosis	1 (0–3)	1 (0–3)	1 (0–3)	1 (0–3)	1 (0–2)	1 (0–2)	0 (0–1)	0 (0–1)	0 (0–1)	0 (0–0)	0 (0–0)	--
Cystic echinococcosis	0 (0–0)	0 (0–0)	0 (0–0)	0 (0–0)	0 (0–0)	0 (0–0)	0 (0–0)	0 (0–0)	0 (0–0)	0 (0–0)	0 (0–0)	--
Dengue	1 (0–2)	1 (0–2)	1 (0–2)	1 (0–1)	2 (1–4)	1 (1–3)	1 (1–2)	1 (0–2)	1 (0–1)	1 (0–1)	0 (0–1)	--
Yellow fever	0 (0–0)	0 (0–0)	0 (0–0)	0 (0–0)	0 (0–0)	0 (0–0)	0 (0–0)	0 (0–0)	0 (0–0)	0 (0–0)	0 (0–0)	--
Rabies	0 (0–0)	0 (0–1)	0 (0–1)	0 (0–0)	0 (0–1)	0 (0–1)	0 (0–1)	0 (0–0)	0 (0–0)	0 (0–0)	0 (0–0)	--
Ascariasis	0 (0–0)	0 (0–0)	0 (0–0)	0 (0–0)	0 (0–0)	0 (0–0)	0 (0–0)	0 (0–0)	0 (0–0)	0 (0–0)	0 (0–0)	--
Zika virus disease	0 (0–0)	0 (0–0)	0 (0–0)	0 (0–0)	0 (0–0)	0 (0–0)	0 (0–0)	0 (0–0)	0 (0–0)	0 (0–0)	0 (0–0)	--
Other neglected tropical diseases	0 (0–1)	1 (0–1)	0 (0–1)	0 (0–1)	1 (0–1)	0 (0–1)	1 (1–2)	1 (0–1)	0 (0–1)	1 (0–1)	0 (0–1)	--
Meningitis	13 (9–18)	15 (11–21)	17 (13–23)	17 (13–22)	20 (15–27)	17 (13–23)	18 (13–23)	14 (11–18)	11 (8–14)	9 (7–12)	3 (2–4)	--
Encephalitis	7 (5–11)	7 (5–11)	6 (4–9)	7 (5–10)	7 (5–10)	6 (4–8)	6 (4–8)	4 (3–6)	3 (2–5)	2 (1–2)	1 (1–1)	--
Diphtheria	0 (0–0)	0 (0–0)	0 (0–0)	0 (0–0)	--	--	--	--	--	--	--	--
Whooping cough	1 (0–1)	0 (0–1)	0 (0–1)	0 (0–1)	--	--	--	--	--	--	--	--
Tetanus	3 (1–8)	5 (2–11)	6 (2–16)	7 (3–19)	7 (3–19)	6 (2–15)	6 (2–15)	4 (1–11)	2 (1–6)	1 (1–3)	1 (0–1)	--
Measles	0 (0–0)	0 (0–0)	0 (0–0)	0 (0–0)	0 (0–0)	0 (0–0)	0 (0–0)	0 (0–0)	0 (0–0)	0 (0–0)	0 (0–0)	--
Varicella and herpes zoster	1 (0–1)	1 (0–2)	1 (0–2)	1 (0–2)	1 (1–2)	2 (1–4)	2 (1–5)	3 (1–6)	4 (1–8)	4 (1–9)	2 (1–5)	--
Acute hepatitis	5 (3–8)	4 (3–7)	6 (4–11)	5 (3–7)	4 (3–8)	4 (2–6)	3 (2–5)	2 (1–3)	2 (1–3)	2 (1–3)	1 (0–1)	--
Acute hepatitis A	1 (0–3)	2 (1–3)	2 (1–4)	1 (0–2)	1 (0–3)	1 (0–3)	1 (0–2)	0 (0–1)	0 (0–1)	0 (0–1)	0 (0–0)	--
Acute hepatitis B	2 (1–3)	1 (1–2)	2 (1–4)	2 (1–4)	1 (1–2)	1 (1–2)	1 (1–3)	0 (0–1)	0 (0–1)	1 (0–1)	0 (0–0)	--
Acute hepatitis C	1 (0–3)	2 (1–4)	2 (1–6)	1 (1–3)	2 (1–4)	1 (0–3)	1 (0–3)	1 (0–1)	1 (0–2)	1 (0–1)	0 (0–1)	--
Acute hepatitis E	0 (0–1)	0 (0–0)	0 (0–0)	0 (0–0)	1 (0–1)	0 (0–0)	0 (0–0)	0 (0–1)	0 (0–0)	0 (0–0)	0 (0–0)	--
Other unspecified infectious diseases	19 (12–29)	26 (17–39)	26 (17–39)	25 (16–37)	25 (16–37)	23 (15–33)	22 (14–32)	14 (9–20)	12 (8–17)	13 (8–19)	5 (3–7)	--
Maternal disorders	--	--	--	--	--	--	--	--	--	--	--	--
Maternal haemorrhage	--	--	--	--	--	--	--	--	--	--	--	--
Maternal sepsis and other pregnancy related infections	--	--	--	--	--	--	--	--	--	--	--	--
Maternal hypertensive disorders	--	--	--	--	--	--	--	--	--	--	--	--
Maternal obstructed labour and uterine rupture	--	--	--	--	--	--	--	--	--	--	--	--
Maternal abortive outcome	--	--	--	--	--	--	--	--	--	--	--	--
Ectopic pregnancy	--	--	--	--	--	--	--	--	--	--	--	--
Indirect maternal deaths	--	--	--	--	--	--	--	--	--	--	--	--
Late maternal deaths	--	--	--	--	--	--	--	--	--	--	--	--
Maternal deaths aggravated by HIV/AIDS	--	--	--	--	--	--	--	--	--	--	--	--
Other maternal disorders	--	--	--	--	--	--	--	--	--	--	--	--
Neonatal disorders	--	--	--	--	--	--	--	--	--	--	--	--
Neonatal preterm birth	--	--	--	--	--	--	--	--	--	--	--	--
Neonatal encephalopathy due to birth asphyxia and trauma	--	--	--	--	--	--	--	--	--	--	--	--
Neonatal sepsis and other neonatal infections	--	--	--	--	--	--	--	--	--	--	--	--
Hemolytic disease and other neonatal jaundice	--	--	--	--	--	--	--	--	--	--	--	--
Other neonatal disorders	--	--	--	--	--	--	--	--	--	--	--	--
Nutritional deficiencies	14 (10–20)	16 (11–23)	24 (16–33)	26 (19–36)	37 (26–51)	42 (30–59)	53 (39–70)	54 (42–68)	56 (43–72)	53 (40–71)	37 (27–49)	--
Protein-energy malnutrition	10 (8–14)	14 (10–19)	19 (13–26)	22 (16–30)	31 (22–43)	36 (26–50)	46 (34–61)	49 (38–63)	50 (38–65)	49 (36–65)	33 (24–45)	--
Other nutritional deficiencies	4 (2–6)	3 (1–5)	5 (2–8)	4 (2–7)	6 (3–10)	6 (3–10)	7 (4–11)	5 (3–8)	6 (4–8)	4 (3–7)	3 (2–6)	--
Lip and oral cavity cancer	19 (14–26)	43 (32–57)	84 (65–109)	117 (90–150)	120 (95–149)	119 (93–148)	119 (95–146)	99 (81–119)	79 (63–96)	46 (36–58)	23 (18–29)	--
Nasopharynx cancer	6 (5–8)	11 (8–15)	21 (16–28)	30 (23–38)	24 (19–31)	24 (18–30)	22 (17–28)	14 (11–18)	10 (8–13)	9 (7–13)	5 (3–6)	--
Other pharynx cancer	9 (6–12)	26 (20–33)	47 (36–61)	68 (51–86)	70 (52–90)	67 (51–85)	61 (47–78)	44 (35–55)	32 (25–42)	22 (17–28)	10 (7–13)	--
Oesophageal cancer	31 (23–39)	78 (61–99)	147 (116–183)	222 (175–279)	213 (170–259)	208 (166–254)	205 (167–249)	151 (125–180)	116 (94–143)	70 (57–84)	33 (26–41)	--
Stomach cancer	69 (53–89)	98 (76–125)	180 (141–225)	246 (194–299)	301 (243–364)	332 (273–401)	358 (298–419)	305 (256–357)	234 (195–272)	154 (128–179)	70 (54–83)	--
Colon and rectum cancer	74 (57–95)	124 (96–157)	214 (167–266)	312 (245–392)	415 (332–506)	487 (402–583)	574 (471–686)	561 (466–672)	456 (375–548)	310 (253–368)	155 (120–189)	--
Liver cancer due to hepatitis B	14 (8–25)	20 (11–33)	30 (16–50)	43 (25–68)	46 (25–75)	42 (21–68)	38 (19–64)	29 (17–49)	19 (10–33)	9 (5–16)	3 (2–6)	--
Liver cancer due to hepatitis C	1 (1–3)	3 (1–6)	7 (3–12)	14 (6–26)	19 (8–36)	23 (12–39)	28 (15–46)	29 (14–48)	26 (15–41)	16 (10–25)	7 (4–11)	--
Liver cancer due to alcohol use	8 (4–15)	14 (6–25)	26 (13–42)	42 (23–67)	58 (33–90)	75 (49–106)	84 (54–120)	66 (44–91)	43 (28–61)	20 (12–28)	6 (4–10)	--
Liver cancer due to NASH	1 (0–2)	2 (1–4)	4 (2–6)	6 (3–10)	8 (4–14)	9 (5–17)	12 (7–21)	13 (7–21)	11 (6–17)	6 (3–9)	2 (1–4)	--
	--	--	--	--	--	--	--	--	--	--	--	--
Liver cancer due to other causes	1 (1–3)	2 (1–4)	3 (1–5)	4 (2–8)	5 (2–9)	5 (2–8)	5 (2–9)	5 (2–8)	4 (2–6)	2 (1–3)	1 (0–1)	--
Gallbladder and biliary tract cancer	9 (6–13)	13 (9–18)	24 (17–32)	34 (25–46)	44 (32–59)	49 (37–64)	52 (40–68)	44 (35–58)	36 (28–46)	25 (19–33)	11 (8–15)	--
Pancreatic cancer	27 (21–34)	55 (43–71)	99 (77–125)	151 (119–191)	177 (140–219)	193 (155–236)	216 (176–264)	181 (148–217)	138 (112–167)	80 (65–96)	37 (28–46)	--
Larynx cancer	18 (13–25)	53 (40–69)	120 (93–155)	171 (133–216)	193 (152–240)	205 (162–256)	198 (159–249)	162 (130–199)	114 (92–142)	62 (48–78)	29 (22–37)	--
Tracheal, bronchus, and lung cancer	86 (65–112)	209 (163–265)	468 (374–581)	777 (617–964)	958 (780–1,162)	1,113 (923–1,330)	1,185 (983–1,399)	999 (829–1,213)	708 (576–861)	445 (360–535)	196 (157–238)	--
Malignant skin melanoma	7 (5–10)	9 (6–13)	14 (10–20)	18 (12–26)	14 (10–21)	15 (11–22)	17 (12–25)	16 (12–24)	15 (11–23)	10 (7–15)	5 (3–7)	--
Non-melanoma skin cancer (squamous-cell carcinoma)	8 (6–11)	13 (9–17)	22 (17–28)	30 (23–38)	36 (28–46)	45 (36–57)	52 (42–64)	60 (47–74)	66 (52–82)	61 (46–76)	48 (34–59)	--
	7 (4–9)	8 (5–11)	12 (8–17)	14 (9–21)	12 (8–16)	13 (8–19)	16 (10–22)	10 (6–14)	10 (6–14)	6 (4–8)	3 (2–4)	--
	6 (4–9)	8 (5–13)	13 (7–21)	16 (8–26)	20 (9–30)	21 (10–32)	19 (8–31)	19 (8–31)	15 (7–24)	8 (3–13)	4 (2–7)	--
Breast cancer	2 (1–3)	5 (3–7)	7 (5–10)	9 (6–13)	10 (7–14)	13 (9–17)	12 (9–17)	11 (8–15)	9 (6–12)	10 (7–16)	6 (4–8)	--
Cervical cancer	--	--	--	--	--	--	--	--	--	--	--	--
Uterine cancer	--	--	--	--	--	--	--	--	--	--	--	--
Ovarian cancer	--	--	--	--	--	--	--	--	--	--	--	--
Prostate cancer	11 (8–15)	38 (28–49)	121 (90–157)	276 (208–358)	606 (473–767)	959 (744–1,212)	1,484 (1,160–1,881)	1,828 (1,437–2,292)	1,840 (1,444–2,265)	1,490 (1,169–1,845)	823 (603–1,030)	--
Testicular cancer	3 (2–4)	2 (2–3)	3 (2–4)	2 (1–3)	2 (2–3)	3 (2–4)	3 (2–5)	4 (3–6)	3 (2–4)	3 (2–4)	1 (1–2)	--
Kidney cancer	13 (9–17)	24 (17–31)	47 (36–61)	67 (51–84)	72 (56–91)	77 (61–97)	79 (63–96)	63 (50–77)	47 (38–58)	26 (20–32)	12 (9–15)	--
Bladder cancer	6 (5–8)	12 (9–15)	29 (23–37)	56 (45–69)	87 (69–107)	123 (99–152)	166 (136–199)	180 (143–223)	156 (127–187)	120 (97–146)	66 (51–83)	--
Brain and nervous system cancer	30 (17–41)	42 (21–59)	60 (29–83)	76 (34–106)	68 (31–94)	70 (29–98)	65 (26–90)	45 (17–64)	28 (11–41)	13 (6–19)	6 (2–8)	--
	1 (1–2)	2 (1–2)	2 (1–2)	2 (1–2)	2 (1–3)	2 (1–2)	2 (1–3)	2 (1–4)	3 (1–4)	2 (1–2)	1 (1–2)	--
	--	--	--	--	--	--	--	--	--	--	--	--
	1 (1–2)	2 (1–2)	2 (1–2)	2 (1–2)	2 (1–3)	2 (1–2)	2 (1–3)	2 (1–4)	3 (1–4)	2 (1–2)	1 (1–2)	--
	1 (1–1)	1 (1–2)	1 (1–2)	1 (1–2)	1 (1–2)	1 (1–1)	1 (1–1)	1 (0–1)	0 (0–0)	0 (0–0)	0 (0–0)	--
Thyroid cancer	3 (2–5)	7 (5–10)	12 (9–16)	15 (11–20)	15 (12–20)	15 (12–20)	16 (13–20)	14 (11–18)	12 (9–16)	7 (5–9)	4 (3–5)	--
Mesothelioma	2 (1–3)	4 (3–5)	7 (5–9)	10 (8–12)	5 (4–6)	5 (4–6)	6 (5–8)	8 (6–9)	6 (5–7)	4 (3–4)	1 (1–1)	--
Hodgkin lymphoma	9 (6–12)	9 (5–12)	12 (7–17)	13 (8–17)	13 (8–17)	14 (8–18)	12 (7–16)	11 (6–15)	7 (3–10)	4 (2–6)	2 (1–3)	--
Non-Hodgkin's lymphoma	42 (33–54)	59 (46–75)	81 (64–100)	109 (88–135)	109 (89–131)	119 (98–143)	127 (103–152)	101 (84–119)	71 (59–86)	36 (29–43)	14 (11–18)	--
	1 (1–1)	1 (0–1)	1 (0–1)	1 (1–1)	1 (1–1)	1 (0–1)	1 (0–1)	1 (0–1)	0 (0–1)	0 (0–0)	0 (0–0)	--
	41 (32–53)	59 (45–74)	81 (63–99)	108 (87–134)	108 (88–130)	118 (98–142)	126 (103–151)	100 (84–118)	71 (58–85)	36 (29–43)	14 (11–17)	--
Multiple myeloma	9 (7–13)	23 (17–30)	47 (36–60)	70 (52–89)	81 (62–102)	100 (77–125)	99 (78–123)	86 (68–107)	61 (48–78)	34 (26–43)	12 (9–16)	--
Leukaemia	42 (32–56)	55 (43–69)	68 (53–87)	81 (62–103)	93 (74–116)	107 (85–134)	119 (95–145)	117 (93–144)	96 (76–120)	66 (51–83)	28 (21–35)	--
Acute lymphoid leukaemia	5 (4–7)	5 (3–6)	5 (4–7)	6 (4–8)	5 (3–7)	8 (6–11)	8 (5–11)	5 (3–7)	5 (3–7)	3 (2–5)	1 (1–2)	--
Chronic lymphoid leukaemia	1 (1–2)	2 (1–2)	5 (3–7)	9 (6–12)	12 (9–16)	16 (11–22)	18 (13–24)	18 (13–24)	18 (13–24)	13 (9–18)	6 (4–9)	--
Acute myeloid leukaemia	11 (8–16)	17 (12–22)	21 (15–28)	22 (16–29)	23 (16–31)	25 (18–34)	26 (20–36)	26 (18–36)	17 (12–25)	10 (7–14)	4 (3–6)	--
Chronic myeloid leukaemia	5 (3–7)	7 (5–10)	9 (7–13)	12 (8–16)	15 (10–20)	16 (11–22)	15 (11–21)	17 (12–24)	13 (9–18)	7 (4–9)	3 (2–5)	--
Other leukaemia	20 (14–29)	24 (18–33)	28 (20–38)	33 (24–44)	38 (28–50)	42 (31–54)	51 (40–67)	50 (39–64)	43 (33–55)	33 (25–43)	13 (10–17)	--
Other malignant cancers	31 (23–41)	47 (35–62)	65 (50–83)	83 (64–106)	82 (63–105)	95 (76–119)	94 (75–115)	88 (71–108)	68 (55–83)	55 (43–68)	27 (21–33)	--
Myelodysplastic, myeloproliferative, and other hematopoietic neoplasms	2 (1–2)	3 (2–4)	6 (4–8)	9 (5–13)	12 (7–17)	14 (9–20)	21 (13–30)	21 (13–31)	17 (11–24)	13 (7–18)	6 (3–9)	--
Cardiovascular diseases	1,235 (977–1,523)	2,104 (1,695–2,559)	3,409 (2,807–4,053)	4,684 (3,894–5,560)	5,939 (4,991–6,930)	7,007 (5,966–8,143)	8,308 (7,169–9,571)	8,212 (7,158–9,340)	7,995 (6,892–9,026)	6,318 (5,308–7,101)	3,990 (3,250–4,485)	--
Rheumatic heart disease	31 (18–54)	31 (19–51)	32 (21–51)	36 (24–55)	30 (20–45)	27 (18–40)	23 (16–33)	21 (15–29)	11 (8–16)	8 (6–11)	5 (4–6)	--
Ischaemic heart disease	627 (485–802)	1,138 (892–1,395)	1,901 (1,536–2,317)	2,631 (2,161–3,172)	3,298 (2,725–3,906)	3,832 (3,203–4,505)	4,451 (3,804–5,169)	4,280 (3,670–4,925)	4,198 (3,626–4,825)	3,394 (2,851–3,852)	2,220 (1,792–2,516)	--
Stroke	339 (250–451)	569 (421–744)	930 (730–1,165)	1,277 (1,015–1,585)	1,714 (1,371–2,118)	2,090 (1,705–2,515)	2,572 (2,102–3,065)	2,609 (2,192–3,079)	2,424 (1,985–2,825)	1,782 (1,460–2,092)	1,009 (809–1,182)	--
Ischaemic stroke	41 (29–61)	78 (56–110)	161 (121–210)	258 (199–337)	527 (411–680)	727 (572–911)	1,282 (1,039–1,571)	1,408 (1,163–1,697)	1,729 (1,421–2,051)	1,313 (1,071–1,542)	769 (613–908)	--
Intracerebral hemorrhage	232 (168–309)	411 (303–551)	667 (517–852)	884 (696–1,126)	1,082 (842–1,356)	1,250 (997–1,538)	1,190 (944–1,474)	1,116 (910–1,345)	622 (496–763)	421 (332–510)	215 (168–262)	--
Subarachnoid hemorrhage	66 (38–108)	80 (50–133)	102 (66–157)	134 (87–202)	105 (66–171)	114 (75–181)	99 (68–150)	85 (58–127)	74 (50–107)	48 (34–67)	25 (18–34)	--

Hypertensive heart disease	--	--	--	--	--	--	2 (1–4)	5 (3–7)	9 (6–13)	21 (14–31)	41 (28–61)	79 (56–110)	145 (107–201)	227 (172–299)	321 (247–424)	422 (326–557)	494 (380–646)	583 (454–738)	599 (458–743)	623 (475–773)	496 (352–619)	313 (215–389)	--
Non-rheumatic valvular heart disease	--	--	--	--	--	--	3 (2–5)	4 (3–5)	6 (4–9)	8 (6–11)	9 (6–13)	14 (10–19)	19 (14–25)	25 (19–33)	36 (28–45)	38 (30–49)	50 (39–62)	52 (42–64)	49 (39–60)	51 (40–62)	37 (29–46)	22 (16–27)	--
Non-rheumatic calcific aortic valve disease	--	--	--	--	--	--	2 (1–3)	2 (2–3)	4 (2–5)	4 (3–6)	5 (3–7)	8 (6–11)	12 (8–16)	16 (12–21)	23 (17–30)	25 (19–33)	37 (28–47)	39 (31–49)	38 (30–47)	42 (33–53)	30 (23–38)	18 (13–22)	--
Non-rheumatic degenerative mitral valve disease	--	--	--	--	--	--	1 (1–1)	1 (1–2)	3 (2–4)	4 (3–5)	4 (3–6)	6 (4–9)	7 (5–10)	9 (6–13)	13 (9–17)	13 (9–17)	13 (9–18)	12 (9–16)	11 (7–14)	8 (6–12)	7 (4–9)	4 (2–6)	--
Other non-rheumatic valve diseases	--	--	--	--	--	--	0 (0–0)	0 (0–1)	0 (0–0)	0 (0–1)	0 (0–1)	0 (0–0)	0 (0–0)	0 (0–0)	0 (0–0)	0 (0–1)	0 (0–0)	0 (0–1)	0 (0–0)	0 (0–1)	0 (0–1)	0 (0–0)	--
Cardiomyopathy and myocarditis	7 (3–17)	5 (2–12)	29 (9–80)	22 (8–56)	10 (5–19)	8 (4–13)	12 (8–18)	15 (10–22)	24 (17–35)	35 (25–50)	52 (37–74)	74 (51–104)	109 (81–146)	146 (112–192)	179 (138–235)	167 (127–224)	169 (133–221)	181 (142–233)	149 (117–189)	120 (90–150)	93 (69–118)	48 (34–61)	--
Myocarditis	1 (0–3)	1 (0–3)	8 (2–31)	6 (1–21)	2 (1–5)	1 (1–2)	2 (1–4)	2 (1–3)	3 (2–5)	4 (2–6)	4 (3–7)	5 (3–8)	4 (2–6)	5 (3–8)	5 (3–7)	4 (3–6)	5 (3–8)	4 (2–6)	4 (2–6)	3 (2–5)	3 (2–4)	1 (1–2)	--
Alcoholic cardiomyopathy	--	--	--	--	--	--	1 (1–1)	1 (1–2)	4 (2–6)	10 (7–15)	16 (11–23)	28 (20–39)	58 (43–79)	86 (64–113)	106 (78–141)	93 (69–126)	95 (72–124)	95 (65–125)	73 (48–99)	22 (12–36)	11 (6–20)	6 (3–10)	--
Other cardiomyopathy	6 (2–14)	4 (1–9)	21 (6–58)	16 (6–39)	8 (4–15)	6 (4–11)	9 (6–14)	11 (8–17)	17 (12–26)	21 (14–32)	32 (21–49)	40 (26–63)	47 (31–72)	55 (37–81)	68 (47–101)	70 (47–104)	68 (47–103)	83 (60–115)	72 (53–98)	95 (71–120)	79 (58–101)	41 (29–53)	--
Pulmonary Arterial Hypertension	3 (1–10)	1 (0–3)	6 (2–18)	4 (2–8)	1 (1–3)	1 (1–2)	1 (1–2)	1 (1–2)	1 (1–2)	1 (1–2)	2 (1–3)	2 (1–3)	3 (2–4)	3 (1–4)	3 (2–5)	3 (2–5)	4 (2–6)	5 (2–7)	5 (2–8)	4 (2–7)	3 (1–6)	1 (0–4)	--
Atrial fibrillation and flutter	--	--	--	--	--	--	--	--	--	1 (1–1)	1 (1–1)	4 (3–5)	7 (5–10)	13 (10–17)	23 (17–30)	34 (25–44)	49 (36–63)	81 (60–104)	115 (86–151)	186 (134–236)	212 (152–279)	202 (143–267)	--
Aortic aneurysm	--	--	--	--	--	--	1 (1–2)	1 (1–2)	3 (2–4)	4 (3–6)	7 (5–10)	13 (9–17)	18 (14–24)	34 (26–43)	63 (50–77)	94 (73–117)	131 (103–160)	169 (134–205)	177 (144–221)	153 (121–190)	106 (84–131)	55 (41–67)	--
Peripheral vascular disease	--	--	--	--	--	--	--	--	--	--	--	2 (1–4)	5 (2–12)	13 (5–31)	19 (7–44)	29 (12–66)	44 (17–99)	65 (26–143)	76 (30–169)	84 (33–183)	71 (28–163)	49 (18–112)	--
Endocarditis	1 (0–2)	1 (0–2)	6 (2–16)	7 (2–17)	2 (1–4)	3 (1–4)	4 (2–6)	5 (3–8)	7 (5–10)	10 (7–14)	12 (8–16)	16 (11–24)	17 (11–22)	20 (14–27)	20 (13–26)	19 (13–25)	18 (12–24)	18 (13–23)	14 (9–18)	12 (9–16)	10 (6–12)	5 (3–6)	--
Other cardiovascular and circulatory diseases	5 (2–10)	4 (2–9)	19 (6–48)	21 (7–46)	9 (5–16)	8 (5–13)	8 (5–11)	12 (8–18)	16 (11–23)	21 (14–30)	28 (20–40)	34 (25–47)	44 (33–60)	63 (48–85)	78 (59–106)	91 (71–119)	100 (79–132)	109 (87–140)	118 (96–151)	127 (102–160)	106 (83–134)	60 (47–75)	--
Chronic respiratory diseases	0 (0–0)	0 (0–0)	0 (0–0)	243 (67–432)	43 (24–68)	27 (16–38)	26 (17–39)	33 (23–47)	44 (31–62)	55 (39–75)	68 (47–92)	98 (71–129)	145 (107–188)	248 (190–315)	396 (308–504)	600 (474–746)	803 (641–990)	1,095 (883–1,321)	1,162 (966–1,374)	1,100 (902–1,300)	848 (689–995)	493 (396–573)	--
Chronic obstructive pulmonary disease	--	--	0 (0–0)	25 (5–67)	3 (1–6)	2 (1–3)	5 (3–8)	8 (5–14)	11 (7–17)	14 (9–22)	22 (14–34)	40 (28–58)	74 (55–101)	153 (116–197)	292 (227–374)	481 (379–597)	678 (541–833)	948 (770–1,148)	1,030 (853–1,220)	981 (803–1,161)	773 (630–904)	454 (363–527)	--
Pneumoconiosis	--	--	--	--	--	--	1 (0–1)	0 (0–0)	0 (0–1)	0 (0–0)	0 (0–1)	0 (0–0)	1 (0–2)	1 (0–2)	1 (0–2)	1 (1–3)	3 (1–6)	2 (1–4)	2 (1–3)	2 (1–4)	2 (1–3)	1 (1–2)	--
Silicosis	--	--	--	--	--	--	0 (0–0)	0 (0–0)	0 (0–1)	0 (0–0)	0 (0–0)	0 (0–0)	0 (0–1)	0 (0–1)	0 (0–1)	1 (0–1)	1 (0–2)	1 (0–2)	1 (0–2)	1 (0–2)	1 (0–2)	0 (0–1)	--
Asbestosis	--	--	--	--	--	--	0 (0–1)	0 (0–0)	0 (0–0)	0 (0–0)	0 (0–0)	0 (0–0)	0 (0–0)	0 (0–0)	0 (0–0)	0 (0–1)	0 (0–1)	1 (0–1)	0 (0–1)	0 (0–1)	1 (0–1)	0 (0–1)	--
Coal workers pneumoconiosis	--	--	--	--	--	--	0 (0–0)	0 (0–0)	0 (0–0)	0 (0–0)	0 (0–0)	0 (0–0)	0 (0–0)	0 (0–1)	0 (0–0)	0 (0–1)	0 (0–1)	0 (0–1)	0 (0–0)	0 (0–0)	0 (0–0)	0 (0–0)	--
Other pneumoconiosis	--	--	--	--	--	--	0 (0–1)	0 (0–0)	0 (0–0)	0 (0–0)	0 (0–0)	0 (0–0)	0 (0–1)	0 (0–0)	0 (0–1)	0 (0–1)	1 (1–3)	1 (0–2)	1 (0–1)	1 (0–1)	0 (0–1)	0 (0–1)	--
Asthma	--	--	--	158 (38–318)	34 (18–56)	21 (12–30)	15 (9–23)	18 (12–26)	23 (16–34)	29 (20–41)	32 (21–46)	40 (27–56)	48 (33–69)	64 (46–91)	67 (46–98)	87 (59–127)	85 (57–129)	97 (66–145)	81 (57–116)	77 (54–110)	45 (31–64)	25 (17–34)	--
Interstitial lung disease and pulmonary sarcoidosis	--	--	--	8 (3–27)	1 (0–1)	1 (0–1)	1 (1–2)	2 (1–3)	2 (1–2)	4 (3–6)	5 (3–8)	9 (5–13)	9 (6–14)	15 (9–21)	20 (12–29)	23 (14–33)	30 (18–45)	39 (24–55)	41 (25–58)	32 (19–47)	21 (12–31)	11 (6–16)	--
Other chronic respiratory diseases	0 (0–0)	0 (0–0)	0 (0–0)	52 (13–143)	5 (2–13)	4 (2–8)	4 (2–7)	4 (3–7)	7 (5–12)	8 (5–12)	9 (5–15)	10 (6–16)	12 (7–20)	15 (10–23)	15 (10–25)	7 (4–13)	7 (4–12)	9 (5–14)	8 (5–14)	8 (5–12)	6 (4–10)	3 (2–5)	--
Digestive diseases	15 (7–29)	12 (5–25)	120 (39–288)	151 (49–334)	54 (33–81)	35 (23–49)	42 (27–60)	65 (44–92)	103 (72–145)	179 (128–241)	292 (208–386)	467 (352–613)	717 (554–899)	1,008 (801–1,238)	1,155 (928–1,420)	1,175 (934–1,446)	1,099 (886–1,340)	1,083 (890–1,294)	918 (769–1,085)	755 (630–881)	541 (443–634)	283 (228–337)	--
Cirrhosis and other chronic liver diseases	--	--	--	37 (10–84)	17 (9–28)	13 (8–20)	19 (11–30)	30 (18–47)	54 (33–79)	106 (71–153)	188 (128–259)	331 (240–448)	541 (409–689)	765 (600–957)	858 (682–1,051)	837 (650–1,048)	717 (563–891)	639 (508–790)	465 (377–570)	337 (272–411)	224 (177–280)	100 (75–128)	--
Cirrhosis and other chronic liver diseases due to hepatitis B	--	--	--	0 (0–0)	0 (0–0)	0 (0–0)	0 (0–1)	2 (1–4)	5 (2–10)	12 (6–22)	25 (10–49)	44 (22–81)	67 (34–120)	85 (40–153)	80 (38–144)	69 (32–131)	54 (26–98)	46 (21–84)	34 (17–59)	25 (12–43)	16 (9–27)	7 (3–12)	--
Cirrhosis and other chronic liver diseases due to hepatitis C	--	--	--	0 (0–1)	0 (0–1)	0 (0–1)	1 (0–3)	5 (2–10)	13 (6–25)	28 (14–48)	47 (23–85)	79 (43–131)	120 (67–199)	158 (85–267)	165 (90–283)	152 (75–267)	128 (72–207)	112 (66–179)	80 (43–129)	59 (32–96)	40 (23–66)	19 (10–32)	--
Cirrhosis and other chronic liver diseases due to alcohol use	--	--	--	--	--	--	0 (0–0)	3 (1–8)	11 (4–22)	33 (18–55)	75 (40–120)	145 (88–215)	251 (151–363)	374 (236–532)	443 (288–602)	436 (288–614)	361 (237–488)	305 (199–416)	205 (135–293)	137 (84–196)	86 (55–124)	36 (21–55)	--
Cirrhosis and other chronic liver diseases due to NAFLD	--	--	--	--	--	--	1 (0–2)	3 (1–7)	8 (3–16)	15 (7–27)	22 (10–45)	38 (17–72)	66 (31–126)	94 (44–172)	103 (48–188)	105 (49–194)	101 (57–163)	101 (58–163)	82 (42–134)	65 (35–102)	45 (27–71)	21 (11–37)	--
Cirrhosis and other chronic liver diseases due to other causes	--	--	--	37 (10–84)	17 (8–27)	12 (7–19)	17 (10–26)	16 (9–28)	17 (8–30)	18 (9–33)	19 (8–36)	25 (11–48)	37 (18–69)	54 (26–95)	67 (33–120)	75 (39–130)	73 (35–129)	76 (38–129)	64 (33–109)	52 (27–87)	36 (20–59)	17 (8–30)	--
Upper digestive system diseases	--	--	--	11 (3–26)	4 (2–7)	3 (1–4)	5 (3–7)	10 (6–15)	14 (9–21)	23 (15–32)	33 (22–46)	43 (29–63)	59 (41–83)	88 (64–118)	105 (75–144)	123 (90–165)	138 (101–180)	150 (117–193)	150 (118–190)	130 (103–164)	100 (77–125)	59 (45–74)	--
Peptic ulcer disease	--	--	--	7 (2–19)	3 (1–6)	2 (1–4)	4 (2–6)	8 (5–13)	11 (7–17)	18 (12–27)	27 (18–40)	37 (24–52)	47 (32–68)	76 (55–103)	89 (63–122)	105 (77–140)	118 (86–156)	128 (97–165)	129 (100–165)	110 (85–140)	85 (65–109)	48 (37–61)	--
Gastritis and duodenitis	--	--	--	3 (0–10)	1 (0–2)	0 (0–0)	1 (1–2)	1 (1–3)	2 (1–4)	4 (2–7)	6 (3–10)	7 (4–12)	12 (7–19)	12 (7–20)	16 (10–26)	18 (11–29)	20 (13–33)	22 (14–33)	22 (14–32)	20 (13–29)	15 (10–22)	11 (8–15)	--
Appendicitis	--	--	--	34 (10–83)	14 (7–24)	8 (5–13)	6 (3–10)	7 (4–11)	7 (4–12)	8 (5–13)	11 (6–17)	11 (7–19)	12 (8–18)	15 (9–22)	14 (9–22)	14 (9–21)	18 (12–26)	18 (13–27)	13 (9–19)	11 (7–16)	9 (6–12)	5 (3–6)	--
Paralytic ileus and intestinal obstruction	8 (4–16)	9 (4–19)	85 (31–208)	23 (7–71)	8 (4–15)	5 (3–8)	4 (2–6)	5 (3–8)	5 (4–8)	7 (5–11)	12 (8–18)	15 (10–23)	21 (15–30)	33 (23–45)	44 (32–60)	49 (35–68)	61 (44–81)	78 (60–102)	85 (64–110)	85 (61–107)	62 (47–77)	33 (23–41)	--
Inguinal, femoral, and abdominal hernia	7 (2–16)	4 (1–9)	35 (5–116)	12 (2–38)	3 (1–7)	1 (0–2)	1 (1–3)	2 (1–3)	3 (2–5)	3 (2–5)	4 (2–6)	6 (4–11)	8 (5–13)	12 (8–19)	16 (10–24)	19 (12–29)	21 (13–32)	26 (18–36)	30 (21–42)	28 (20–38)	24 (18–31)	15 (11–19)	--
Inflammatory bowel disease	--	--	--	5 (1–16)	1 (0–2)	0 (0–1)	1 (1–2)	1 (1–2)	3 (2–5)	5 (3–8)	5 (3–7)	5 (3–8)	6 (4–9)	7 (5–10)	10 (7–14)	8 (6–13)	9 (6–14)	12 (8–17)	10 (7–14)	9 (6–13)	6 (4–8)	3 (2–4)	--
Vascular intestinal disorders	--	--	--	1 (0–2)	0 (0–0)	0 (0–0)	0 (0–1)	0 (0–0)	1 (1–1)	2 (1–3)	3 (2–4)	4 (3–5)	8 (6–11)	14 (11–19)	20 (15–26)	24 (19–31)	32 (25–42)	41 (32–52)	46 (35–59)	45 (35–57)	33 (25–41)	21 (16–27)	--
Gallbladder and biliary diseases	--	--	--	1 (0–2)	0 (0–0)	0 (0–1)	1 (0–1)	1 (1–2)	2 (1–3)	4 (2–6)	4 (3–6)	7 (5–10)	8 (6–12)	15 (10–21)	21 (15–30)	24 (18–34)	32 (23–43)	41 (31–54)	42 (32–53)	41 (31–51)	29 (22–37)	17 (13–21)	--
Pancreatitis	--	--	--	1 (0–4)	0 (0–0)	1 (0–1)	2 (1–3)	4 (3–6)	9 (7–13)	16 (11–23)	25 (18–36)	33 (24–46)	37 (27–53)	38 (28–52)	41 (30–55)	41 (31–54)	35 (27–46)	34 (27–45)	31 (24–41)	25 (19–32)	15 (11–18)	7 (5–9)	--
Other digestive diseases	--	--	--	27 (7–76)	6 (3–13)	3 (2–5)	3 (2–4)	4 (3–7)	5 (3–8)	7 (4–10)	8 (5–13)	11 (7–16)	17 (12–23)	21 (14–29)	27 (18–38)	35 (23–49)	36 (23–49)	44 (28–60)	46 (28–63)	43 (26–57)	40 (24–52)	24 (14–32)	--
Alzheimer's disease and other dementias	--	--	--	--	--	--	--	--	--	--	--	2 (0–7)	11 (1–36)	22 (4–71)	42 (8–126)	80 (17–236)	160 (35–487)	301 (69–852)	471 (106–1,345)	898 (207–2,527)	1,086 (264–2,860)	848 (206–2,203)	--
Parkinson's disease	--	--	--	--	--	--	--	0 (0–0)	0 (0–0)	0 (0–0)	0 (0–1)	2 (2–3)	6 (5–7)	13 (10–15)	22 (18–27)	43 (35–51)	89 (73–104)	182 (153–213)	299 (256–345)	346 (296–396)	258 (216–295)	117 (92–136)	--
Idiopathic epilepsy	--	--	13 (4–25)	45 (14–78)	21 (11–32)	19 (13–26)	25 (18–34)	34 (25–45)	39 (29–51)	41 (30–54)	45 (33–58)	46 (35–61)	50 (39–66)	45 (34–59)	42 (34–53)	34 (26–43)	28 (22–34)	22 (17–26)	19 (15–23)	16 (13–19)	10 (8–13)	5 (4–7)	--
Multiple sclerosis	--	--	--	--	0 (0–1)	0 (0–0)	0 (0–0)	1 (0–1)	1 (1–1)	2 (1–2)	3 (2–4)	4 (3–6)	5 (3–7)	5 (3–7)	7 (4–9)	6 (3–8)	5 (3–6)	4 (2–5)	3 (2–4)	1 (1–2)	1 (0–1)	0 (0–0)	--
Motor neuron disease	0 (0–0)	0 (0–0)	4 (1–10)	1 (1–4)	0 (0–0)	0 (0–0)	1 (1–1)	1 (1–1)	1 (1–1)	2 (1–2)	4 (3–5)	7 (5–10)	10 (7–13)	18 (13–24)	22 (17–28)	21 (16–27)	24 (19–30)	20 (15–25)	12 (9–15)	5 (4–7)	3 (2–3)	1 (0–1)	--
Other neurological disorders	--	--	15 (7–28)	15 (7–27)	7 (5–11)	11 (8–14)	15 (11–20)	13 (10–16)	10 (8–13)	10 (8–12)	10 (8–12)	14 (11–17)	16 (12–21)	24 (19–31)	28 (22–35)	25 (20–32)	23 (19–29)	22 (17–28)	19 (15–24)	16 (13–21)	8 (7–11)	5 (3–6)	--
Eating disorders	--	--	--	--	0 (0–0)	0 (0–0)	0 (0–0)	0 (0–0)	0 (0–0)	0 (0–0)	0 (0–0)	0 (0–0)	0 (0–0)	--	--	--	--	--	--	--	--	--	--
Anorexia nervosa	--	--	--	--	0 (0–0)	0 (0–0)	0 (0–0)	0 (0–0)	0 (0–0)	0 (0–0)	0 (0–0)	0 (0–0)	0 (0–0)	--	--	--	--	--	--	--	--	--	--
Bulimia nervosa	--	--	--	--	0 (0–0)	0 (0–0)	0 (0–0)	0 (0–0)	0 (0–0)	0 (0–0)	0 (0–0)	0 (0–0)	0 (0–0)	--	--	--	--	--	--	--	--	--	--
Alcohol use disorders	--	--	--	--	--	0 (0–0)	4 (2–6)	10 (7–14)	20 (14–28)	43 (29–60)	74 (51–102)	113 (83–151)	173 (130–227)	202 (153–260)	215 (168–272)	165 (130–210)	134 (105–168)	83 (64–102)	46 (37–56)	19 (15–24)	9 (7–11)	3 (2–4)	--
Drug use disorders	0 (0–0)	0 (0–0)	0 (0–0)	0 (0–0)	0 (0–0)	0 (0–0)	3 (2–4)	9 (6–12)	13 (9–17)	17 (12–22)	21 (15–28)	21 (15–29)	19 (13–26)	13 (9–19)	11 (8–16)	8 (5–11)	6 (4–8)	5 (4–7)	5 (4–7)	5 (3–6)	2 (2–3)	1 (1–2)	--
Opioid use disorders	0 (0–0)	0 (0–0)	0 (0–0)	0 (0–0)	0 (0–0)	0 (0–0)	1 (1–2)	3 (2–4)	3 (2–5)	6 (4–8)	7 (5–10)	7 (5–10)	5 (3–8)	4 (3–6)	3 (2–5)	2 (2–4)	2 (1–3)	1 (1–2)	1 (1–2)	1 (1–2)	1 (0–1)	0 (0–0)	--
Cocaine use disorders	--	--	--	--	--	0 (0–0)	1 (1–1)	3 (2–5)	6 (4–8)	6 (5–8)	8 (6–11)	8 (6–12)	9 (6–12)	5 (4–7)	5 (3–6)	3 (2–4)	2 (1–3)	2 (1–3)	2 (1–2)	1 (0–1)	0 (0–0)	0 (0–0)	--
Amphetamine use disorders	--	--	--	--	--	0 (0–0)	0 (0–0)	0 (0–1)	1 (1–1)	1 (1–2)	2 (1–2)	2 (1–3)	2 (1–2)	2 (1–3)	1 (1–2)	1 (1–2)	1 (0–1)	0 (0–0)	0 (0–0)	0 (0–0)	0 (0–0)	0 (0–0)	--
Other drug use disorders	--	--	--	--	--	0 (0–0)	1 (0–1)	2 (1–3)	3 (2–4)	3 (2–5)	4 (2–5)	4 (3–5)	3 (2–5)	2 (2–4)	2 (1–3)	1 (1–2)	1 (1–2)	2 (1–2)	2 (1–3)	3 (2–4)	1 (1–2)	1 (1–1)	--
Diabetes mellitus	0 (0–0)	1 (0–1)	9 (4–20)	11 (4–22)	5 (3–9)	7 (4–11)	12 (8–16)	25 (17–36)	43 (32–57)	67 (49–89)	102 (74–133)	176 (133–232)	290 (222–376)	512 (394–655)	734 (570–961)	953 (748–1,198)	1,115 (891–1,378)	1,218 (989–1,497)	1,113 (917–1,347)	882 (725–1,058)	574 (469–682)	292 (231–348)	--
Diabetes mellitus type 1	0 (0–0)	1 (0–1)	9 (4–20)	11 (4–22)	5 (3–9)	7 (4–11)	7 (5–10)	14 (10–21)	26 (18–35)	22 (15–30)	33 (23–47)	57 (40–79)	18 (12–27)	29 (20–43)	42 (28–62)	43 (29–62)	50 (34–77)	41 (28–61)	38 (26–59)	22 (15–34)	15 (10–24)	9 (5–14)	--
Diabetes mellitus type 2	--	--	--	--	--	--	5 (3–7)	11 (7–17)	17 (11–27)	45 (32–64)	68 (47–94)	119 (85–164)	272 (208–354)	482 (370–616)	692 (536–901)	910 (715–1,145)	1,065 (851–1,322)	1,177 (952–1,449)	1,075 (885–1,303)	860 (707–1,035)	558 (456–663)	284 (224–339)	--
Chronic kidney disease	--	--	30 (12–56)	33 (13–55)	24 (16–33)	15 (11–19)	27 (19–35)	38 (29–51)	61 (48–76)	91 (71–114)	131 (101–165)	196 (153–253)	302 (238–383)	441 (351–553)	585 (464–742)	661 (532–810)	747 (605–915)	827 (681–998)	764 (641–906)	699 (581–830)	510 (413–612)	306 (243–363)	--
Chronic kidney disease due to diabetes mellitus type 1	--	--	0 (0–0)	0 (0–1)	0 (0–1)	0 (0–1)	1 (0–2)	2 (1–5)	7 (3–13)	16 (8–27)	28 (14–47)	41 (21–69)	53 (25–92)	56 (23–109)	49 (16–103)	40 (14–87)	35 (13–78)	30 (11–63)	18 (7–40)	9 (3–20)	2 (1–6)	0 (0–1)	--
Chronic kidney disease due to diabetes mellitus type 2	--	--	--	--	--	--	0 (0–1)	1 (0–2)	2 (1–5)	5 (2–12)	11 (3–22)	23 (9–43)	51 (24–87)	100 (51–157)	158 (84–236)	192 (107–274)	223 (136–327)	251 (158–353)	234 (147–326)	213 (140–302)	151 (96–214)	87 (53–126)	--
Chronic kidney disease due to hypertension	--	--	--	--	--	--	1 (0–2)	2 (1–3)	4 (2–6)	6 (3–11)	11 (5–19)	20 (11–32)	38 (23–60)	70 (43–108)	112 (67–171)	151 (92–222)	193 (122–283)	233 (161–319)	240 (167–331)	247 (173–331)	205 (149–270)	135 (97–176)	--
Chronic kidney disease due to glomerulonephritis	--	--	9 (3–19)	11 (4–21)	9 (5–16)	7 (4–11)	13 (8–19)	19 (11–27)	27 (17–39)	36 (21–53)	47 (27–70)	66 (34–100)	92 (53–139)	125 (69–193)	153 (84–247)	154 (79–254)	150 (74–257)	144 (75–239)	118 (58–204)	91 (42–168)	52 (23–90)	26 (11–47)	--
Chronic kidney disease due to other and unspecified causes	--	--	21 (8–41)	22 (9–39)	14 (8–22)	7 (4–11)	11 (6–18)	15 (8–23)	21 (12–32)	27 (13–44)	34 (17–57)	47 (24–81)	68 (33–117)	91 (47–155)	112 (55–195)	124 (63–216)	146 (78–244)	169 (93–278)	154 (83–253)	139 (72–221)	99 (51–160)	58 (32–96)	--
Acute glomerulonephritis	--	--	0 (0–0)	1 (0–3)	1 (0–3)	0 (0–1)	0 (0–1)	0 (0–0)	0 (0–0)	0 (0–0)	0 (0–0)	0 (0–1)	1 (0–1)	0 (0–1)	1 (0–1)	0 (0–1)	0 (0–1)	1 (0–2)	1 (0–1)	1 (0–1)	0 (0–1)	0 (0–0)	--
Skin and subcutaneous diseases	--	--	21 (5–69)	8 (2–25)	5 (2–12)	5 (3–9)	4 (2–6)	5 (3–8)	8 (4–12)	8 (5–13)	10 (6–17)	16 (10–25)	21 (12–35)	31 (19–49)	36 (21–59)	43 (25–69)	62 (36–99)	73 (40–119)	92 (50–167)	95 (52–167)	76 (40–138)	50 (25–95)	--
Bacterial skin diseases	--	--	19 (5–63)	6 (2–20)	5 (2–10)	4 (2–8)	3 (2–5)	3 (1–5)	5 (3–8)	6 (3–10)	6 (4–11)	11 (6–18)	14 (8–25)	20 (12–34)	25 (14–43)	25 (14–44)	33 (18–62)	41 (21–78)	50 (25–99)	51 (25–103)	40 (20–87)	28 (13–61)	--
Cellulitis	--	--	5 (1–19)	2 (0–8)	1 (0–4)	1 (0–3)	1 (0–2)	1 (0–2)	2 (1–3)	2 (1–4)	2 (1–4)	3 (1–6)	5 (2–10)	7 (3–14)	7 (3–15)	8 (4–17)	9 (4–20)	11 (5–25)	11 (5–31)	11 (5–30)	10 (4–29)	7 (3–20)	--
Pyoderma	--	--	15 (3–55)	5 (1–18)	3 (1–8)	3 (1–6)	2 (1–3)	2 (1–3)	3 (1–5)	4 (2–6)	4 (2–7)	7 (4–12)	10 (5–17)	14 (7–22)	18 (9–29)	18 (9–29)	25 (12–44)	30 (14–54)	39 (18–74)	40 (19–77)	31 (15–61)	21 (9–41)	--
Decubitus ulcer	--	--	--	1 (0–5)	0 (0–1)	1 (0–2)	1 (0–1)	2 (1–3)	3 (1–5)	2 (1–5)	3 (1–6)	4 (2–8)	6 (2–10)	10 (5–17)	11 (5–18)	16 (8–27)	25 (13–42)	30 (15–48)	39 (18–65)	42 (21–66)	34 (16–57)	21 (9–38)	--
Other skin and subcutaneous diseases	--	--	2 (0–10)	0 (0–2)	0 (0–0)	0 (0–0)	0 (0–0)	0 (0–0)	0 (0–1)	0 (0–1)	1 (0–1)	0 (0–1)	1 (0–1)	1 (1–3)	1 (0–1)	1 (1–3)	3 (2–7)	2 (1–5)	3 (1–8)	2 (1–4)	2 (1–4)	1 (1–3)	--
Musculoskeletal disorders	--	--	--	--	1 (1–1)	2 (1–2)	2 (1–2)	3 (2–5)	4 (3–5)	6 (4–7)	7 (5–10)	8 (6–10)	7 (5–10)	8 (6–10)	9 (7–12)	9 (7–11)	10 (8–13)	10 (7–13)	10 (7–13)	9 (7–13)	47 (33–68)	27 (19–40)	--
Rheumatoid arthritis	--	--	--	--	0 (0–0)	0 (0–0)	0 (0–0)	0 (0–0)	0 (0–0)	0 (0–0)	1 (0–1)	0 (0–1)	1 (1–1)	1 (1–2)	2 (1–3)	2 (1–3)	3 (2–5)	4 (3–6)	4 (3–6)	4 (3–6)	18 (11–28)	10 (7–17)	--
Other musculoskeletal disorders	--	--	--	--	1 (1–1)	1 (1–2)	2 (1–2)	3 (2–5)	3 (3–5)	6 (4–7)	7 (5–9)	7 (5–10)	6 (5–8)	7 (5–9)	7 (5–10)	7 (5–9)	7 (5–9)	6 (5–9)	5 (4–8)	5 (4–7)	29 (20–43)	17 (11–25)	--
Congenital anomalies	883 (509–1,471)	360 (216–566)	724 (395–1,290)	357 (197–678)	107 (47–254)	61 (28–137)	42 (21–94)	36 (19–76)	34 (19–68)	32 (18–63)	29 (16–58)	25 (14–51)	26 (15–52)	29 (17–54)	28 (15–51)	18 (10–32)	13 (8–24)	--	--	--	--	--	--
Neural tube defects	107 (28–311)	31 (7–96)	65 (13–206)	61 (7–224)	5 (1–22)	4 (1–19)	3 (1–11)	1 (0–6)	2 (1–7)	1 (0–5)	0 (0–1)	1 (0–3)	0 (0–2)	0 (0–0)	0 (0–1)	0 (0–1)	0 (0–0)	--	--	--	--	--	--
Congenital heart anomalies	350 (167–620)	174 (87–291)	343 (138–655)	151 (45–329)	55 (20–138)	30 (13–71)	20 (9–45)	17 (8–38)	16 (9–32)	15 (8–31)	13 (7–27)	11 (6–24)	10 (6–21)	9 (5–18)	6 (3–12)	6 (3–11)	5 (3–9)	--	--	--	--	--	--
Orofacial clefts	2 (0–8)	1 (0–3)	1 (0–6)	0 (0–1)	--	--	--	--	--	--	--	--	--	--	--	--	--	--	--	--	--	--	--
Down's syndrome	11 (4–25)	6 (2–15)	31 (10–90)	20 (5–62)	5 (1–15)	5 (2–14)	6 (3–16)	5 (2–11)	5 (2–11)	6 (3–14)	7 (4–16)	8 (4–17)	9 (5–19)	11 (6–22)	15 (8–29)	8 (4–16)	5 (3–9)	--	--	--	--	--	--
Other chromosomal abnormalities	38 (13–95)	17 (5–40)	28 (5–79)	10 (1–30)	3 (1–9)	2 (1–5)	2 (1–5)	1 (0–3)	2 (1–4)	1 (0–2)	1 (0–3)	0 (0–1)	0 (0–1)	0 (0–1)	0 (0–1)	0 (0–0)	0 (0–0)	--	--	--	--	--	--
Congenital musculoskeletal and limb anomalies	17 (6–37)	6 (2–14)	11 (3–30)	6 (1–18)	3 (1–10)	2 (0–5)	1 (0–2)	1 (0–2)	1 (0–3)	0 (0–1)	1 (0–2)	0 (0–1)	0 (0–1)	1 (0–2)	1 (0–2)	0 (0–1)	0 (0–1)	--	--	--	--	--	--
Urogenital congenital anomalies	22 (9–46)	8 (3–18)	11 (3–33)	4 (1–14)	1 (0–4)	1 (0–2)	0 (0–1)	0 (0–1)	0 (0–1)	0 (0–1)	0 (0–0)	0 (0–1)	0 (0–1)	1 (0–0)	0 (0–1)	0 (0–1)	0 (0–1)	--	--	--	--	--	--
Digestive congenital anomalies	133 (57–236)	60 (24–106)	90 (24–195)	23 (4–57)	6 (2–14)	2 (1–5)	1 (0–2)	0 (0–0)	0 (0–1)	0 (0–1)	0 (0–0)	0 (0–1)	0 (0–1)	1 (0–1)	0 (0–0)	0 (0–1)	1 (0–1)	--	--	--	--	--	--
Other congenital anomalies	204 (78–376)	57 (21–112)	145 (35–376)	82 (15–198)	30 (9–76)	16 (7–40)	10 (5–24)	11 (6–25)	8 (4–17)	7 (4–15)	5 (3–12)	5 (3–10)	5 (3–10)	7 (4–14)	5 (2–9)	2 (1–4)	2 (1–4)	--	--	--	--	--	--
Urinary diseases and male infertility	1 (1–2)	2 (1–4)	16 (5–35)	8 (3–15)	3 (2–4)	2 (1–3)	2 (1–3)	4 (3–6)	5 (3–6)	8 (6–10)	12 (9–17)	15 (10–20)	21 (14–28)	34 (21–46)	50 (31–68)	73 (47–99)	90 (58–119)	122 (80–157)	145 (92–190)	151 (91–191)	134 (84–171)	75 (48–94)	--
Urinary tract infections and interstitial nephritis	1 (0–1)	1 (0–2)	14 (4–32)	7 (2–12)	2 (1–3)	1 (1–2)	1 (1–2)	3 (2–4)	4 (3–5)	6 (4–8)	8 (6–12)	10 (7–14)	13 (9–18)	24 (14–32)	36 (22–49)	54 (34–74)	68 (43–93)	100 (64–129)	118 (72–155)	129 (78–166)	119 (72–154)	64 (40–81)	--
Urolithiasis	--	--	--	0 (0–2)	0 (0–0)	0 (0–0)	0 (0–0)	0 (0–0)	0 (0–0)	0 (0–0)	1 (0–1)	1 (1–2)	3 (2–5)	4 (2–7)	7 (4–12)	7 (4–12)	7 (4–12)	8 (5–13)	8 (5–13)	4 (2–6)	3 (2–4)	2 (1–3)	--
Other urinary diseases	0 (0–1)	1 (0–3)	2 (0–5)	1 (0–2)	1 (1–2)	1 (1–1)	1 (1–1)	2 (1–3)	1 (0–1)	1 (1–2)	3 (2–5)	3 (2–5)	5 (3–7)	6 (3–9)	7 (4–11)	12 (7–18)	15 (9–22)	14 (8–20)	19 (10–28)	18 (10–26)	12 (7–18)	9 (5–14)	--
Gynecological diseases	--	--	--	--	--	--	--	--	--	--	--	--	--	--	--	--	--	--	--	--	--	--	--
Uterine fibroids	--	--	--	--	--	--	--	--	--	--	--	--	--	--	--	--	--	--	--	--	--	--	--
Endometriosis	--	--	--	--	--	--	--	--	--	--	--	--	--	--	--	--	--	--	--	--	--	--	--
Genital prolapse	--	--	--	--	--	--	--	--	--	--	--	--	--	--	--	--	--	--	--	--	--	--	--
Other gynecological diseases	--	--	--	--	--	--	--	--	--	--	--	--	--	--	--	--	--	--	--	--	--	--	--
Hemoglobinopathies and hemolytic anaemias	2 (1–4)	2 (1–4)	28 (8–59)	65 (18–119)	44 (25–66)	27 (18–39)	21 (14–30)	25 (18–35)	23 (17–32)	24 (17–34)	22 (16–30)	24 (18–33)	22 (16–28)	21 (16–28)	20 (16–27)	19 (14–25)	16 (12–22)	17 (13–22)	15 (12–19)	12 (10–16)	14 (11–18)	8 (6–9)	--
Thalassemias	0 (0–0)	0 (0–0)	0 (0–0)	0 (0–1)	0 (0–0)	0 (0–0)	0 (0–0)	0 (0–0)	0 (0–0)	0 (0–0)	0 (0–0)	0 (0–0)	0 (0–0)	0 (0–0)	0 (0–0)	0 (0–0)	0 (0–0)	0 (0–0)	0 (0–0)	0 (0–0)	0 (0–0)	0 (0–0)	--
Sickle cell disorders	2 (1–4)	2 (1–4)	28 (8–58)	61 (17–111)	40 (22–60)	24 (16–34)	17 (11–25)	17 (12–25)	14 (10–21)	14 (8–20)	11 (7–17)	11 (6–17)	8 (5–13)	5 (3–9)	3 (1–5)	1 (0–1)	0 (0–0)	0 (0–0)	0 (0–0)	0 (0–0)	0 (0–0)	0 (0–0)	--
G6PD deficiency	0 (0–0)	0 (0–0)	0 (0–1)	3 (1–7)	4 (1–7)	3 (1–6)	4 (2–8)	7 (3–12)	8 (4–14)	10 (5–16)	10 (5–16)	12 (7–18)	12 (7–18)	13 (8–18)	14 (9–19)	12 (8–17)	8 (5–12)	6 (4–10)	4 (2–6)	2 (1–3)	1 (1–2)	0 (0–1)	--
Other hemoglobinopathies and hemolytic anaemias	0 (0–0)	0 (0–0)	0 (0–0)	0 (0–0)	0 (0–0)	0 (0–0)	0 (0–0)	1 (0–1)	1 (0–1)	1 (0–1)	1 (1–1)	1 (1–2)	2 (1–2)	3 (2–4)	4 (3–6)	6 (4–9)	8 (5–11)	10 (8–14)	11 (8–14)	11 (8–14)	13 (10–17)	7 (6–9)	--
Endocrine, metabolic, blood, and immune disorders	17 (9–28)	13 (8–21)	124 (58–218)	73 (34–129)	20 (13–29)	13 (9–18)	11 (7–15)	16 (10–23)	26 (18–37)	37 (26–51)	45 (32–63)	49 (35–67)	51 (37–72)	68 (49–98)	71 (51–99)	82 (58–113)	80 (55–108)	91 (66–126)	88 (64–120)	88 (64–129)	88 (61–128)	53 (34–77)	--
Sudden infant death syndrome	--	9 (4–21)	86 (21–242)	--	--	--	--	--	--	--	--	--	--	--	--	--	--	--	--	--	--	--	--
Transport injuries	7 (3–15)	4 (2–8)	27 (12–47)	119 (65–225)	130 (84–245)	124 (86–213)	304 (220–452)	559 (421–773)	583 (442–763)	565 (438–743)	481 (365–637)	449 (341–607)	419 (314–573)	407 (305–539)	355 (272–471)	294 (223–389)	230 (180–303)	188 (150–242)	148 (117–187)	100 (80–124)	72 (55–93)	27 (20–34)	--
Road injuries	6 (2–13)	3 (1–7)	23 (11–43)	112 (60–213)	125 (79–232)	117 (81–196)	289 (208–431)	534 (399–730)	556 (421–726)	536 (413–702)	455 (343–599)	422 (319–568)	395 (294–541)	384 (286–507)	330 (252–436)	280 (212–368)	220 (172–289)	177 (140–227)	141 (112–178)	96 (77–119)	70 (53–91)	26 (19–32)	--
Pedestrian road injuries	2 (1–5)	1 (0–2)	5 (2–11)	36 (12–80)	44 (23–85)	30 (18–51)	25 (17–39)	35 (25–52)	43 (32–61)	50 (36–71)	51 (37–70)	60 (44–83)	69 (52–94)	79 (59–105)	85 (65–111)	76 (58–98)	71 (55–91)	70 (54–90)	68 (53–85)	52 (40–64)	37 (28–47)	14 (10–17)	--
Cyclist road injuries	--	--	--	11 (3–31)	10 (4–26)	17 (9–36)	23 (14–43)	26 (17–45)	24 (17–39)	26 (18–42)	25 (17–41)	25 (18–38)	26 (18–37)	31 (22–43)	30 (21–43)	26 (18–36)	23 (16–33)	16 (11–23)	11 (7–16)	5 (3–7)	1 (1–2)	0 (0–1)	--
Motorcyclist road injuries	1 (0–3)	1 (0–2)	6 (2–14)	18 (6–44)	14 (5–37)	11 (5–28)	60 (36–123)	128 (86–217)	135 (93–230)	120 (84–193)	96 (66–165)	77 (54–148)	66 (46–123)	54 (38–98)	41 (28–75)	24 (16–44)	16 (10–29)	6 (4–11)	3 (2–5)	1 (1–2)	1 (1–2)	0 (0–0)	--
Motor vehicle road injuries	2 (1–5)	2 (1–4)	12 (5–25)	46 (20–100)	56 (34–102)	57 (38–95)	179 (126–265)	341 (246–458)	348 (256–461)	335 (250–444)	278 (202–373)	255 (184–351)	226 (163–316)	212 (151–301)	166 (120–231)	150 (106–209)	105 (76–151)	81 (59–109)	56 (41–76)	36 (27–49)	30 (20–44)	11 (7–15)	--
Other road injuries	0 (0–0)	0 (0–0)	0 (0–1)	1 (0–2)	1 (0–2)	1 (1–2)	2 (1–3)	5 (3–7)	5 (3–8)	5 (3–8)	5 (3–8)	6 (4–9)	9 (5–14)	8 (5–13)	8 (4–13)	4 (3–7)	5 (3–8)	5 (3–8)	4 (2–6)	2 (1–4)	1 (1–2)	0 (0–0)	--
Other transport injuries	1 (0–3)	1 (0–2)	3 (1–7)	7 (3–16)	6 (3–13)	8 (5–15)	14 (10–25)	25 (18–39)	27 (20–43)	29 (21–45)	25 (19–40)	27 (20–42)	24 (18–35)	23 (18–32)	24 (19–36)	14 (11–21)	10 (7–14)	11 (9–17)	7 (5–9)	4 (3–5)	2 (2–3)	1 (1–1)	--
Falls	1 (1–2)	1 (0–1)	13 (4–31)	31 (8–74)	17 (9–27)	17 (11–24)	23 (17–32)	33 (23–44)	37 (27–48)	42 (31–54)	49 (36–65)	59 (46–76)	78 (61–98)	110 (87–138)	123 (99–152)	132 (106–161)	148 (119–181)	216 (175–265)	271 (215–329)	369 (291–454)	389 (308–477)	324 (251–403)	--
Drowning	4 (2–7)	2 (1–4)	39 (10–87)	323 (106–582)	206 (123–307)	145 (93–202)	145 (95–220)	138 (94–204)	128 (93–186)	122 (91–160)	106 (76–143)	102 (76–140)	82 (62–110)	79 (62–105)	62 (48–82)	50 (39–66)	37 (29–48)	25 (20–31)	19 (15–23)	10 (7–12)	9 (5–12)	4 (3–6)	--
Fire, heat, and hot substances	1 (1–2)	2 (1–4)	36 (9–70)	130 (32–230)	40 (17–64)	18 (9–28)	9 (6–14)	14 (9–21)	17 (11–24)	21 (14–30)	18 (12–26)	19 (13–27)	20 (14–28)	18 (13–25)	18 (14–24)	20 (14–26)	18 (13–24)	21 (16–27)	20 (16–25)	20 (16–26)	19 (15–23)	11 (9–14)	--
Poisonings	2 (1–7)	3 (1–12)	12 (4–29)	55 (23–111)	12 (5–32)	6 (3–16)	5 (2–13)	8 (4–21)	12 (7–32)	13 (7–36)	12 (6–32)	9 (5–25)	8 (5–24)	7 (4–17)	5 (3–15)	5 (3–13)	3 (2–9)	3 (2–6)	2 (1–5)	2 (1–5)	1 (1–3)	1 (0–1)	--
Poisoning by carbon monoxide	0 (0–1)	0 (0–2)	0 (0–1)	5 (1–17)	2 (1–6)	2 (1–6)	2 (1–7)	4 (2–12)	5 (3–17)	6 (3–21)	6 (3–18)	4 (2–14)	3 (1–11)	2 (1–7)	2 (1–7)	1 (1–6)	1 (0–4)	1 (0–3)	1 (0–2)	1 (0–2)	0 (0–1)	0 (0–0)	--
Poisoning by other means	2 (1–6)	3 (0–11)	12 (4–29)	51 (21–104)	10 (4–25)	4 (2–10)	2 (1–6)	4 (2–10)	7 (3–17)	7 (4–18)	6 (3–15)	5 (3–13)	5 (3–14)	5 (3–11)	3 (2–8)	3 (2–8)	2 (1–5)	2 (1–4)	2 (1–3)	1 (1–3)	1 (1–2)	1 (0–1)	--
Exposure to mechanical forces	7 (1–35)	18 (0–171)	17 (1–82)	39 (13–79)	38 (13–85)	34 (12–75)	79 (31–178)	118 (57–240)	116 (58–241)	114 (60–217)	97 (49–189)	96 (47–198)	77 (38–159)	70 (36–144)	60 (31–123)	42 (22–84)	27 (13–54)	19 (10–34)	12 (7–23)	8 (5–15)	7 (4–11)	3 (2–5)	--
Unintentional firearm injuries	4 (0–24)	12 (0–130)	12 (1–61)	26 (5–60)	24 (6–65)	25 (7–65)	67 (24–164)	102 (47–228)	99 (46–225)	95 (47–200)	80 (37–173)	77 (34–171)	58 (26–134)	48 (20–113)	39 (15–100)	28 (12–66)	17 (7–43)	10 (4–25)	6 (2–16)	4 (2–10)	3 (2–7)	1 (1–3)	--
Other exposure to mechanical forces	2 (0–17)	6 (0–44)	5 (0–25)	13 (3–42)	15 (4–47)	9 (3–29)	12 (6–37)	16 (8–47)	17 (9–48)	19 (10–52)	17 (9–50)	19 (10–56)	19 (11–49)	22 (13–50)	21 (13–48)	15 (9–33)	10 (6–23)	8 (5–16)	6 (4–13)	4 (3–8)	4 (2–6)	2 (2–3)	--
Adverse effects of medical treatment	10 (6–16)	9 (4–17)	36 (12–76)	26 (9–51)	12 (7–18)	7 (5–10)	6 (4–8)	11 (8–16)	13 (9–18)	13 (9–19)	16 (11–24)	18 (13–27)	20 (14–27)	30 (22–42)	34 (24–46)	42 (31–56)	46 (34–61)	50 (38–64)	52 (41–67)	50 (39–65)	38 (30–49)	21 (16–26)	--

Animal contact	0 (0–0)	0 (0–1)	1 (0–3)	6 (2–14)	2 (1–4)	2 (1–3)	1 (1–2)	1 (1–2)	3 (2–4)	2 (1–3)	3 (2–4)	3 (2–5)	5 (3–7)	6 (4–8)	6 (4–9)	7 (5–10)	7 (5–11)	7 (5–10)	7 (5–10)	4 (3–6)	3 (2–4)	2 (1–3)	--
Venomous animal contact	0 (0–0)	0 (0–0)	1 (0–2)	5 (1–11)	1 (1–3)	1 (1–2)	1 (1–2)	1 (1–1)	2 (1–3)	1 (1–2)	2 (1–4)	2 (1–3)	3 (2–4)	3 (2–5)	3 (2–5)	3 (2–6)	3 (2–6)	3 (2–5)	3 (2–5)	2 (1–3)	2 (1–2)	2 (1–2)	--
Non-venomous animal contact	0 (0–0)	0 (0–0)	0 (0–1)	1 (0–4)	1 (0–1)	1 (0–1)	0 (0–1)	0 (0–1)	1 (1–2)	1 (0–1)	1 (0–1)	1 (1–2)	2 (1–3)	3 (2–4)	3 (2–4)	3 (2–5)	4 (3–6)	5 (3–7)	4 (3–6)	2 (1–4)	1 (1–2)	0 (0–1)	--
Foreign body	19 (10–33)	15 (9–25)	159 (65–294)	122 (53–194)	32 (20–48)	28 (19–37)	13 (9–18)	21 (14–29)	26 (18–35)	26 (18–34)	25 (18–35)	29 (20–40)	30 (21–40)	36 (26–49)	35 (26–46)	39 (28–52)	40 (30–53)	42 (32–54)	39 (31–48)	40 (32–49)	25 (20–30)	17 (13–20)	--
Pulmonary aspiration and foreign body in airway	18 (10–33)	15 (8–25)	158 (64–290)	118 (52–190)	31 (20–47)	27 (19–36)	12 (8–17)	21 (14–28)	25 (17–35)	25 (17–34)	25 (17–34)	28 (20–38)	29 (21–39)	35 (26–48)	34 (25–45)	38 (28–51)	39 (29–52)	40 (30–51)	36 (29–45)	37 (30–45)	23 (18–28)	15 (12–18)	--
Foreign body in other body part	0 (0–0)	0 (0–0)	1 (0–6)	3 (0–13)	1 (0–3)	1 (0–2)	0 (0–1)	1 (0–1)	1 (1–1)	1 (0–1)	1 (0–1)	1 (1–1)	1 (1–2)	1 (1–1)	1 (1–2)	1 (1–2)	1 (1–2)	2 (1–3)	2 (2–3)	3 (2–4)	2 (1–3)	2 (1–3)	--
Environmental heat and cold exposure	1 (0–5)	1 (0–2)	3 (1–8)	6 (2–17)	4 (1–11)	2 (1–7)	4 (1–16)	6 (2–22)	6 (2–21)	7 (2–23)	7 (2–21)	7 (2–24)	7 (2–24)	8 (2–24)	9 (2–29)	8 (2–28)	7 (2–23)	10 (3–32)	9 (3–30)	10 (3–31)	7 (3–22)	5 (2–13)	--
Other unintentional injuries	7 (3–14)	2 (1–5)	11 (5–20)	34 (15–71)	30 (14–63)	31 (17–60)	45 (27–77)	64 (39–100)	76 (51–114)	78 (52–116)	70 (45–104)	68 (45–98)	61 (42–90)	46 (33–65)	38 (26–56)	25 (17–38)	19 (14–28)	14 (9–20)	10 (7–14)	6 (4–9)	4 (3–6)	2 (2–3)	--
Self-harm	--	--	--	--	--	18 (11–27)	109 (79–149)	220 (167–291)	264 (210–341)	299 (239–378)	282 (223–356)	288 (229–358)	309 (246–387)	318 (255–391)	294 (234–360)	241 (195–296)	204 (165–250)	198 (161–242)	171 (138–209)	140 (111–171)	94 (75–116)	35 (28–42)	--
Self-harm by firearm	--	--	--	--	--	1 (0–1)	11 (7–19)	23 (14–40)	26 (17–44)	25 (16–43)	21 (13–34)	22 (14–34)	19 (12–30)	19 (12–29)	15 (10–22)	14 (9–20)	10 (7–14)	9 (6–13)	6 (4–9)	5 (3–7)	3 (2–4)	1 (0–1)	--
Self-harm by other specified means	--	--	--	--	--	17 (11–26)	97 (70–133)	197 (150–260)	238 (191–304)	273 (221–344)	260 (207–329)	267 (212–331)	290 (230–364)	299 (240–368)	279 (222–342)	228 (185–280)	194 (157–239)	189 (154–233)	165 (133–203)	135 (107–166)	91 (72–113)	34 (27–41)	--
Interpersonal violence	13 (7–23)	4 (2–7)	23 (11–42)	76 (36–143)	62 (25–110)	102 (43–174)	593 (328–837)	1,123 (736–1,475)	1,149 (763–1,510)	1,009 (671–1,350)	808 (499–1,110)	640 (374–935)	501 (309–721)	374 (227–549)	277 (170–409)	189 (116–269)	123 (74–179)	80 (51–116)	48 (32–70)	19 (13–27)	10 (7–14)	3 (2–4)	--
Physical violence by firearm	2 (1–3)	1 (0–1)	2 (1–5)	7 (3–18)	10 (5–21)	29 (15–55)	348 (190–519)	732 (483–995)	720 (493–973)	589 (410–810)	438 (296–624)	308 (197–447)	222 (146–330)	152 (102–223)	103 (69–154)	68 (47–97)	40 (28–59)	23 (16–33)	10 (7–14)	4 (3–6)	2 (2–3)	1 (0–1)	--
Physical violence by sharp object	1 (0–3)	0 (0–1)	1 (0–3)	5 (2–16)	6 (2–19)	13 (6–33)	149 (78–304)	254 (151–482)	266 (158–495)	261 (157–474)	209 (116–381)	177 (100–327)	141 (88–249)	101 (62–184)	64 (40–111)	40 (24–73)	22 (14–41)	19 (12–31)	11 (8–19)	5 (4–8)	3 (2–4)	2 (1–2)	--
Physical violence by other means	10 (6–18)	3 (1–6)	19 (9–38)	65 (28–125)	46 (16–88)	60 (18–119)	96 (26–196)	137 (44–271)	162 (55–305)	160 (51–304)	161 (50–314)	155 (48–314)	138 (50–262)	121 (49–235)	110 (47–202)	81 (34–140)	60 (28–105)	39 (19–65)	27 (15–44)	10 (5–16)	5 (3–8)	1 (1–1)	--
Executions and police conflict	0 (0–0)	0 (0–0)	0 (0–0)	0 (0–0)	0 (0–0)	0 (0–0)	1 (1–2)	5 (2–10)	6 (2–13)	5 (2–12)	3 (1–8)	1 (1–3)	1 (1–3)	1 (0–1)	0 (0–0)	0 (0–0)	0 (0–0)	0 (0–0)	0 (0–0)	0 (0–0)	0 (0–0)	0 (0–0)	--

SR Table 12: Antigua and Barbuda deaths by cause and age group for males

Cause	Early Neonatal	Late Neonatal	Post Neonatal	1 to 4	5 to 9	10 to 14	15 to 19	20 to 24	25 to 29	30 to 34	35 to 39	40 to 44	45 to 49	50 to 54	55 to 59	60 to 64	65 to 69	70 to 74	75 to 79	80 to 84	85 to 89	90 to 94	95 plus
All causes	2 (1–3)	1 (1–2)	1 (1–2)	1 (1–2)	1 (1–1)	1 (1–1)	2 (2–2)	5 (4–6)	6 (5–6)	5 (4–5)	6 (6–7)	7 (6–8)	10 (8–12)	16 (14–20)	24 (20–28)	31 (27–37)	30 (26–35)	36 (31–41)	38 (33–43)	42 (37–46)	27 (25–30)	15 (14–16)	--
Sexually transmitted infections excluding HIV	0 (0–0)	0 (0–0)	0 (0–0)	0 (0–0)	0 (0–0)	0 (0–0)	0 (0–0)	0 (0–0)	0 (0–0)	0 (0–0)	0 (0–0)	0 (0–0)	0 (0–0)	0 (0–0)	0 (0–0)	0 (0–0)	0 (0–0)	0 (0–0)	0 (0–0)	0 (0–0)	0 (0–0)	0 (0–0)	--
Syphilis	0 (0–0)	0 (0–0)	0 (0–0)	0 (0–0)	0 (0–0)	0 (0–0)	0 (0–0)	0 (0–0)	0 (0–0)	0 (0–0)	0 (0–0)	0 (0–0)	0 (0–0)	0 (0–0)	0 (0–0)	0 (0–0)	0 (0–0)	0 (0–0)	0 (0–0)	0 (0–0)	0 (0–0)	0 (0–0)	--
Chlamydial infection	--	--	--	--	--	0 (0–0)	0 (0–0)	0 (0–0)	0 (0–0)	0 (0–0)	0 (0–0)	0 (0–0)	0 (0–0)	0 (0–0)	0 (0–0)	0 (0–0)	0 (0–0)	0 (0–0)	0 (0–0)	0 (0–0)	0 (0–0)	0 (0–0)	--
Gonococcal infection	--	--	--	--	--	0 (0–0)	0 (0–0)	0 (0–0)	0 (0–0)	0 (0–0)	0 (0–0)	0 (0–0)	0 (0–0)	0 (0–0)	0 (0–0)	0 (0–0)	0 (0–0)	0 (0–0)	0 (0–0)	0 (0–0)	0 (0–0)	0 (0–0)	--
Other sexually transmitted infections	--	--	--	--	--	0 (0–0)	0 (0–0)	0 (0–0)	0 (0–0)	0 (0–0)	0 (0–0)	0 (0–0)	0 (0–0)	0 (0–0)	0 (0–0)	0 (0–0)	0 (0–0)	0 (0–0)	0 (0–0)	0 (0–0)	0 (0–0)	0 (0–0)	--
Tuberculosis	--	--	0 (0–0)	0 (0–0)	0 (0–0)	0 (0–0)	0 (0–0)	0 (0–0)	0 (0–0)	0 (0–0)	0 (0–0)	0 (0–0)	0 (0–0)	0 (0–0)	0 (0–0)	0 (0–0)	0 (0–0)	0 (0–0)	0 (0–0)	0 (0–0)	0 (0–0)	0 (0–0)	--
Drug-susceptible tuberculosis	--	--	0 (0–0)	0 (0–0)	0 (0–0)	0 (0–0)	0 (0–0)	0 (0–0)	0 (0–0)	0 (0–0)	0 (0–0)	0 (0–0)	0 (0–0)	0 (0–0)	0 (0–0)	0 (0–0)	0 (0–0)	0 (0–0)	0 (0–0)	0 (0–0)	0 (0–0)	0 (0–0)	--
Multidrug-resistant tuberculosis without extensive drug resistance	--	--	0 (0–0)	0 (0–0)	0 (0–0)	0 (0–0)	0 (0–0)	0 (0–0)	0 (0–0)	0 (0–0)	0 (0–0)	0 (0–0)	0 (0–0)	0 (0–0)	0 (0–0)	0 (0–0)	0 (0–0)	0 (0–0)	0 (0–0)	0 (0–0)	0 (0–0)	0 (0–0)	--
Extensively drug-resistant tuberculosis	--	--	0 (0–0)	0 (0–0)	0 (0–0)	0 (0–0)	0 (0–0)	0 (0–0)	0 (0–0)	0 (0–0)	0 (0–0)	0 (0–0)	0 (0–0)	0 (0–0)	0 (0–0)	0 (0–0)	0 (0–0)	0 (0–0)	0 (0–0)	0 (0–0)	0 (0–0)	0 (0–0)	--
Lower respiratory infections	0 (0–0)	0 (0–0)	0 (0–0)	0 (0–0)	0 (0–0)	0 (0–0)	0 (0–0)	0 (0–0)	0 (0–0)	0 (0–0)	0 (0–0)	0 (0–0)	0 (0–0)	0 (0–1)	1 (0–1)	1 (1–1)	1 (1–1)	1 (1–2)	2 (1–3)	3 (2–3)	2 (2–3)	1 (1–2)	--
Upper respiratory infections	0 (0–0)	0 (0–0)	0 (0–0)	0 (0–0)	0 (0–0)	0 (0–0)	0 (0–0)	0 (0–0)	0 (0–0)	0 (0–0)	0 (0–0)	0 (0–0)	0 (0–0)	0 (0–0)	0 (0–0)	0 (0–0)	0 (0–0)	0 (0–0)	0 (0–0)	0 (0–0)	0 (0–0)	0 (0–0)	--
Otitis media	0 (0–0)	0 (0–0)	0 (0–0)	0 (0–0)	0 (0–0)	0 (0–0)	0 (0–0)	0 (0–0)	0 (0–0)	0 (0–0)	0 (0–0)	0 (0–0)	0 (0–0)	0 (0–0)	0 (0–0)	0 (0–0)	0 (0–0)	0 (0–0)	0 (0–0)	0 (0–0)	0 (0–0)	0 (0–0)	--
Diarrhoeal diseases	0 (0–0)	0 (0–0)	0 (0–0)	0 (0–0)	0 (0–0)	0 (0–0)	0 (0–0)	0 (0–0)	0 (0–0)	0 (0–0)	0 (0–0)	0 (0–0)	0 (0–0)	0 (0–0)	0 (0–0)	0 (0–0)	0 (0–0)	0 (0–0)	0 (0–0)	0 (0–0)	0 (0–0)	0 (0–0)	--
Typhoid fever	--	--	0 (0–0)	0 (0–0)	0 (0–0)	0 (0–0)	0 (0–0)	0 (0–0)	0 (0–0)	0 (0–0)	0 (0–0)	0 (0–0)	0 (0–0)	0 (0–0)	0 (0–0)	0 (0–0)	0 (0–0)	0 (0–0)	0 (0–0)	0 (0–0)	0 (0–0)	0 (0–0)	--
Paratyphoid fever	--	--	0 (0–0)	0 (0–0)	0 (0–0)	0 (0–0)	0 (0–0)	0 (0–0)	0 (0–0)	0 (0–0)	0 (0–0)	0 (0–0)	0 (0–0)	0 (0–0)	0 (0–0)	0 (0–0)	0 (0–0)	0 (0–0)	0 (0–0)	0 (0–0)	0 (0–0)	0 (0–0)	--
iNTS	--	0 (0–0)	0 (0–0)	0 (0–0)	0 (0–0)	0 (0–0)	0 (0–0)	0 (0–0)	0 (0–0)	0 (0–0)	0 (0–0)	0 (0–0)	0 (0–0)	0 (0–0)	0 (0–0)	0 (0–0)	0 (0–0)	0 (0–0)	0 (0–0)	0 (0–0)	0 (0–0)	0 (0–0)	--
Other intestinal infectious diseases	--	--	0 (0–0)	0 (0–0)	0 (0–0)	0 (0–0)	0 (0–0)	0 (0–0)	0 (0–0)	0 (0–0)	0 (0–0)	0 (0–0)	0 (0–0)	0 (0–0)	0 (0–0)	0 (0–0)	0 (0–0)	0 (0–0)	0 (0–0)	0 (0–0)	0 (0–0)	0 (0–0)	--
Malaria	0 (0–0)	0 (0–0)	0 (0–0)	0 (0–0)	0 (0–0)	0 (0–0)	0 (0–0)	0 (0–0)	0 (0–0)	0 (0–0)	0 (0–0)	0 (0–0)	0 (0–0)	0 (0–0)	0 (0–0)	0 (0–0)	0 (0–0)	0 (0–0)	0 (0–0)	0 (0–0)	0 (0–0)	0 (0–0)	--
Chagas disease	--	--	0 (0–0)	0 (0–0)	0 (0–0)	0 (0–0)	0 (0–0)	0 (0–0)	0 (0–0)	0 (0–0)	0 (0–0)	0 (0–0)	0 (0–0)	0 (0–0)	0 (0–0)	0 (0–0)	0 (0–0)	0 (0–0)	0 (0–0)	0 (0–0)	0 (0–0)	0 (0–0)	--
Visceral leishmaniasis	--	--	0 (0–0)	0 (0–0)	0 (0–0)	0 (0–0)	0 (0–0)	0 (0–0)	0 (0–0)	0 (0–0)	0 (0–0)	0 (0–0)	0 (0–0)	0 (0–0)	0 (0–0)	0 (0–0)	0 (0–0)	0 (0–0)	0 (0–0)	0 (0–0)	0 (0–0)	0 (0–0)	--
African trypanosomiasis	--	--	--	0 (0–0)	0 (0–0)	0 (0–0)	0 (0–0)	0 (0–0)	0 (0–0)	0 (0–0)	0 (0–0)	0 (0–0)	0 (0–0)	0 (0–0)	0 (0–0)	0 (0–0)	0 (0–0)	0 (0–0)	0 (0–0)	0 (0–0)	0 (0–0)	0 (0–0)	--
Schistosomiasis	--	--	0 (0–0)	0 (0–0)	0 (0–0)	0 (0–0)	0 (0–0)	0 (0–0)	0 (0–0)	0 (0–0)	0 (0–0)	0 (0–0)	0 (0–0)	0 (0–0)	0 (0–0)	0 (0–0)	0 (0–0)	0 (0–0)	0 (0–0)	0 (0–0)	0 (0–0)	0 (0–0)	--
Cysticercosis	--	--	--	0 (0–0)	0 (0–0)	0 (0–0)	0 (0–0)	0 (0–0)	0 (0–0)	0 (0–0)	0 (0–0)	0 (0–0)	0 (0–0)	0 (0–0)	0 (0–0)	0 (0–0)	0 (0–0)	0 (0–0)	0 (0–0)	0 (0–0)	0 (0–0)	0 (0–0)	--
Cystic echinococcosis	--	--	--	0 (0–0)	0 (0–0)	0 (0–0)	0 (0–0)	0 (0–0)	0 (0–0)	0 (0–0)	0 (0–0)	0 (0–0)	0 (0–0)	0 (0–0)	0 (0–0)	0 (0–0)	0 (0–0)	0 (0–0)	0 (0–0)	0 (0–0)	0 (0–0)	0 (0–0)	--
Dengue	0 (0–0)	0 (0–0)	0 (0–0)	0 (0–0)	0 (0–0)	0 (0–0)	0 (0–0)	0 (0–0)	0 (0–0)	0 (0–0)	0 (0–0)	0 (0–0)	0 (0–0)	0 (0–0)	0 (0–0)	0 (0–0)	0 (0–0)	0 (0–0)	0 (0–0)	0 (0–0)	0 (0–0)	0 (0–0)	--
Yellow fever	--	0 (0–0)	0 (0–0)	0 (0–0)	0 (0–0)	0 (0–0)	0 (0–0)	0 (0–0)	0 (0–0)	0 (0–0)	0 (0–0)	0 (0–0)	0 (0–0)	0 (0–0)	0 (0–0)	0 (0–0)	0 (0–0)	0 (0–0)	0 (0–0)	0 (0–0)	0 (0–0)	0 (0–0)	--
Rabies	--	--	0 (0–0)	0 (0–0)	0 (0–0)	0 (0–0)	0 (0–0)	0 (0–0)	0 (0–0)	0 (0–0)	0 (0–0)	0 (0–0)	0 (0–0)	0 (0–0)	0 (0–0)	0 (0–0)	0 (0–0)	0 (0–0)	0 (0–0)	0 (0–0)	0 (0–0)	0 (0–0)	--
Ascariasis	--	--	0 (0–0)	0 (0–0)	0 (0–0)	0 (0–0)	0 (0–0)	0 (0–0)	0 (0–0)	0 (0–0)	0 (0–0)	0 (0–0)	0 (0–0)	0 (0–0)	0 (0–0)	0 (0–0)	0 (0–0)	0 (0–0)	0 (0–0)	0 (0–0)	0 (0–0)	0 (0–0)	--
Zika virus disease	0 (0–0)	0 (0–0)	0 (0–0)	0 (0–0)	0 (0–0)	0 (0–0)	0 (0–0)	0 (0–0)	0 (0–0)	0 (0–0)	0 (0–0)	0 (0–0)	0 (0–0)	0 (0–0)	0 (0–0)	0 (0–0)	0 (0–0)	0 (0–0)	0 (0–0)	0 (0–0)	0 (0–0)	0 (0–0)	--
Other neglected tropical diseases	0 (0–0)	0 (0–0)	0 (0–0)	0 (0–0)	0 (0–0)	0 (0–0)	0 (0–0)	0 (0–0)	0 (0–0)	0 (0–0)	0 (0–0)	0 (0–0)	0 (0–0)	0 (0–0)	0 (0–0)	0 (0–0)	0 (0–0)	0 (0–0)	0 (0–0)	0 (0–0)	0 (0–0)	0 (0–0)	--
Meningitis	0 (0–0)	0 (0–0)	0 (0–0)	0 (0–0)	0 (0–0)	0 (0–0)	0 (0–0)	0 (0–0)	0 (0–0)	0 (0–0)	0 (0–0)	0 (0–0)	0 (0–0)	0 (0–0)	0 (0–0)	0 (0–0)	0 (0–0)	0 (0–0)	0 (0–0)	0 (0–0)	0 (0–0)	0 (0–0)	--
Encephalitis	0 (0–0)	0 (0–0)	0 (0–0)	0 (0–0)	0 (0–0)	0 (0–0)	0 (0–0)	0 (0–0)	0 (0–0)	0 (0–0)	0 (0–0)	0 (0–0)	0 (0–0)	0 (0–0)	0 (0–0)	0 (0–0)	0 (0–0)	0 (0–0)	0 (0–0)	0 (0–0)	0 (0–0)	0 (0–0)	--
Diphtheria	--	--	0 (0–0)	0 (0–0)	0 (0–0)	0 (0–0)	0 (0–0)	0 (0–0)	0 (0–0)	0 (0–0)	0 (0–0)	0 (0–0)	0 (0–0)	0 (0–0)	0 (0–0)	--	--	--	--	--	--	--	--
Whooping cough	--	--	0 (0–0)	0 (0–0)	0 (0–0)	0 (0–0)	0 (0–0)	0 (0–0)	0 (0–0)	0 (0–0)	0 (0–0)	0 (0–0)	0 (0–0)	0 (0–0)	0 (0–0)	--	--	--	--	--	--	--	--
Tetanus	0 (0–0)	0 (0–0)	0 (0–0)	0 (0–0)	0 (0–0)	0 (0–0)	0 (0–0)	0 (0–0)	0 (0–0)	0 (0–0)	0 (0–0)	0 (0–0)	0 (0–0)	0 (0–0)	0 (0–0)	0 (0–0)	0 (0–0)	0 (0–0)	0 (0–0)	0 (0–0)	0 (0–0)	0 (0–0)	--
Measles	0 (0–0)	0 (0–0)	0 (0–0)	0 (0–0)	0 (0–0)	0 (0–0)	0 (0–0)	0 (0–0)	0 (0–0)	0 (0–0)	0 (0–0)	0 (0–0)	0 (0–0)	0 (0–0)	0 (0–0)	0 (0–0)	0 (0–0)	0 (0–0)	0 (0–0)	0 (0–0)	0 (0–0)	0 (0–0)	--
Varicella and herpes zoster	0 (0–0)	0 (0–0)	0 (0–0)	0 (0–0)	0 (0–0)	0 (0–0)	0 (0–0)	0 (0–0)	0 (0–0)	0 (0–0)	0 (0–0)	0 (0–0)	0 (0–0)	0 (0–0)	0 (0–0)	0 (0–0)	0 (0–0)	0 (0–0)	0 (0–0)	0 (0–0)	0 (0–0)	0 (0–0)	--
Acute hepatitis	--	--	0 (0–0)	0 (0–0)	0 (0–0)	0 (0–0)	0 (0–0)	0 (0–0)	0 (0–0)	0 (0–0)	0 (0–0)	0 (0–0)	0 (0–0)	0 (0–0)	0 (0–0)	0 (0–0)	0 (0–0)	0 (0–0)	0 (0–0)	0 (0–0)	0 (0–0)	0 (0–0)	--
Acute hepatitis A	--	--	0 (0–0)	0 (0–0)	0 (0–0)	0 (0–0)	0 (0–0)	0 (0–0)	0 (0–0)	0 (0–0)	0 (0–0)	0 (0–0)	0 (0–0)	0 (0–0)	0 (0–0)	0 (0–0)	0 (0–0)	0 (0–0)	0 (0–0)	0 (0–0)	0 (0–0)	0 (0–0)	--
Acute hepatitis B	--	--	0 (0–0)	0 (0–0)	0 (0–0)	0 (0–0)	0 (0–0)	0 (0–0)	0 (0–0)	0 (0–0)	0 (0–0)	0 (0–0)	0 (0–0)	0 (0–0)	0 (0–0)	0 (0–0)	0 (0–0)	0 (0–0)	0 (0–0)	0 (0–0)	0 (0–0)	0 (0–0)	--
Acute hepatitis C	--	--	0 (0–0)	0 (0–0)	0 (0–0)	0 (0–0)	0 (0–0)	0 (0–0)	0 (0–0)	0 (0–0)	0 (0–0)	0 (0–0)	0 (0–0)	0 (0–0)	0 (0–0)	0 (0–0)	0 (0–0)	0 (0–0)	0 (0–0)	0 (0–0)	0 (0–0)	0 (0–0)	--
Acute hepatitis E	--	--	0 (0–0)	0 (0–0)	0 (0–0)	0 (0–0)	0 (0–0)	0 (0–0)	0 (0–0)	0 (0–0)	0 (0–0)	0 (0–0)	0 (0–0)	0 (0–0)	0 (0–0)	0 (0–0)	0 (0–0)	0 (0–0)	0 (0–0)	0 (0–0)	0 (0–0)	0 (0–0)	--
Other unspecified infectious diseases	0 (0–0)	0 (0–0)	0 (0–0)	0 (0–0)	0 (0–0)	0 (0–0)	0 (0–0)	0 (0–0)	0 (0–0)	0 (0–0)	0 (0–0)	0 (0–0)	0 (0–0)	0 (0–0)	0 (0–0)	0 (0–0)	0 (0–0)	0 (0–0)	0 (0–0)	0 (0–0)	0 (0–0)	0 (0–0)	--
Maternal disorders	--	--	--	--	--	--	--	--	--	--	--	--	--	--	--	--	--	--	--	--	--	--	--
Maternal haemorrhage	--	--	--	--	--	--	--	--	--	--	--	--	--	--	--	--	--	--	--	--	--	--	--
Maternal sepsis and other pregnancy related infections	--	--	--	--	--	--	--	--	--	--	--	--	--	--	--	--	--	--	--	--	--	--	--
Maternal hypertensive disorders	--	--	--	--	--	--	--	--	--	--	--	--	--	--	--	--	--	--	--	--	--	--	--
Maternal obstructed labour and uterine rupture	--	--	--	--	--	--	--	--	--	--	--	--	--	--	--	--	--	--	--	--	--	--	--
Maternal abortive outcome	--	--	--	--	--	--	--	--	--	--	--	--	--	--	--	--	--	--	--	--	--	--	--
Ectopic pregnancy	--	--	--	--	--	--	--	--	--	--	--	--	--	--	--	--	--	--	--	--	--	--	--
Indirect maternal deaths	--	--	--	--	--	--	--	--	--	--	--	--	--	--	--	--	--	--	--	--	--	--	--
Late maternal deaths	--	--	--	--	--	--	--	--	--	--	--	--	--	--	--	--	--	--	--	--	--	--	--
Maternal deaths aggravated by HIV/AIDS	--	--	--	--	--	--	--	--	--	--	--	--	--	--	--	--	--	--	--	--	--	--	--
Other maternal disorders	--	--	--	--	--	--	--	--	--	--	--	--	--	--	--	--	--	--	--	--	--	--	--
Neonatal disorders	2 (1–2)	1 (1–1)	0 (0–0)	0 (0–0)	--	--	--	--	--	--	--	--	--	--	--	--	--	--	--	--	--	--	--
Neonatal preterm birth	1 (1–1)	0 (0–1)	0 (0–0)	0 (0–0)	--	--	--	--	--	--	--	--	--	--	--	--	--	--	--	--	--	--	--
Neonatal encephalopathy due to birth asphyxia and trauma	0 (0–0)	0 (0–0)	0 (0–0)	0 (0–0)	--	--	--	--	--	--	--	--	--	--	--	--	--	--	--	--	--	--	--
Neonatal sepsis and other neonatal infections	0 (0–0)	0 (0–0)	0 (0–0)	0 (0–0)	--	--	--	--	--	--	--	--	--	--	--	--	--	--	--	--	--	--	--
Hemolytic disease and other neonatal jaundice	0 (0–0)	0 (0–0)	0 (0–0)	0 (0–0)	--	--	--	--	--	--	--	--	--	--	--	--	--	--	--	--	--	--	--
Other neonatal disorders	0 (0–0)	0 (0–0)	0 (0–0)	0 (0–0)	--	--	--	--	--	--	--	--	--	--	--	--	--	--	--	--	--	--	--
Nutritional deficiencies	0 (0–0)	0 (0–0)	0 (0–0)	0 (0–0)	0 (0–0)	0 (0–0)	0 (0–0)	0 (0–0)	0 (0–0)	0 (0–0)	0 (0–0)	0 (0–0)	0 (0–0)	0 (0–0)	0 (0–0)	0 (0–0)	0 (0–0)	0 (0–0)	0 (0–0)	0 (0–0)	0 (0–0)	0 (0–0)	--
Protein-energy malnutrition	0 (0–0)	0 (0–0)	0 (0–0)	0 (0–0)	0 (0–0)	0 (0–0)	0 (0–0)	0 (0–0)	0 (0–0)	0 (0–0)	0 (0–0)	0 (0–0)	0 (0–0)	0 (0–0)	0 (0–0)	0 (0–0)	0 (0–0)	0 (0–0)	0 (0–0)	0 (0–0)	0 (0–0)	0 (0–0)	--
Other nutritional deficiencies	--	--	0 (0–0)	0 (0–0)	0 (0–0)	0 (0–0)	0 (0–0)	0 (0–0)	0 (0–0)	0 (0–0)	0 (0–0)	0 (0–0)	0 (0–0)	0 (0–0)	0 (0–0)	0 (0–0)	0 (0–0)	0 (0–0)	0 (0–0)	0 (0–0)	0 (0–0)	0 (0–0)	--
Lip and oral cavity cancer	--	--	--	--	0 (0–0)	0 (0–0)	0 (0–0)	0 (0–0)	0 (0–0)	0 (0–0)	0 (0–0)	0 (0–0)	0 (0–0)	0 (0–0)	0 (0–0)	0 (0–0)	0 (0–0)	0 (0–0)	0 (0–0)	0 (0–0)	0 (0–0)	0 (0–0)	--
Nasopharynx cancer	--	--	--	--	0 (0–0)	0 (0–0)	0 (0–0)	0 (0–0)	0 (0–0)	0 (0–0)	0 (0–0)	0 (0–0)	0 (0–0)	0 (0–0)	0 (0–0)	0 (0–0)	0 (0–0)	0 (0–0)	0 (0–0)	0 (0–0)	0 (0–0)	0 (0–0)	--
Other pharynx cancer	--	--	--	--	--	--	--	0 (0–0)	0 (0–0)	0 (0–0)	0 (0–0)	0 (0–0)	0 (0–0)	0 (0–0)	0 (0–0)	0 (0–0)	0 (0–0)	0 (0–0)	0 (0–0)	0 (0–0)	0 (0–0)	0 (0–0)	--
Oesophageal cancer	--	--	--	--	--	--	--	0 (0–0)	0 (0–0)	0 (0–0)	0 (0–0)	0 (0–0)	0 (0–0)	0 (0–0)	0 (0–0)	0 (0–0)	0 (0–0)	0 (0–0)	0 (0–0)	0 (0–0)	0 (0–0)	0 (0–0)	--
Stomach cancer	--	--	--	--	--	--	0 (0–0)	0 (0–0)	0 (0–0)	0 (0–0)	0 (0–0)	0 (0–0)	0 (0–0)	0 (0–1)	1 (0–1)	1 (1–1)	1 (1–1)	1 (1–1)	1 (1–1)	1 (1–1)	0 (0–1)	0 (0–0)	--
Colon and rectum cancer	--	--	--	--	0 (0–0)	0 (0–0)	0 (0–0)	0 (0–0)	0 (0–0)	0 (0–0)	0 (0–0)	0 (0–0)	0 (0–0)	0 (0–0)	1 (0–1)	1 (1–1)	1 (1–1)	1 (1–1)	1 (1–1)	1 (1–1)	0 (0–1)	0 (0–0)	--

Liver cancer due to hepatitis B	--	--	--	--	--	0 (0–0)	0 (0–0)	0 (0–0)	0 (0–0)	0 (0–0)	0 (0–0)	0 (0–0)	0 (0–0)	0 (0–0)	0 (0–0)	0 (0–0)	0 (0–0)	0 (0–0)	0 (0–0)	0 (0–0)	0 (0–0)	0 (0–0)	--
Liver cancer due to hepatitis C	--	--	--	--	--	0 (0–0)	0 (0–0)	0 (0–0)	0 (0–0)	0 (0–0)	0 (0–0)	0 (0–0)	0 (0–0)	0 (0–0)	0 (0–0)	0 (0–0)	0 (0–0)	0 (0–0)	0 (0–0)	0 (0–0)	0 (0–0)	0 (0–0)	--
Liver cancer due to alcohol use	--	--	--	--	--	--	0 (0–0)	0 (0–0)	0 (0–0)	0 (0–0)	0 (0–0)	0 (0–0)	0 (0–0)	0 (0–0)	0 (0–0)	0 (0–0)	0 (0–0)	0 (0–0)	0 (0–0)	0 (0–0)	0 (0–0)	0 (0–0)	--
Liver cancer due to NASH	--	--	--	--	--	--	0 (0–0)	0 (0–0)	0 (0–0)	0 (0–0)	0 (0–0)	0 (0–0)	0 (0–0)	0 (0–0)	0 (0–0)	0 (0–0)	0 (0–0)	0 (0–0)	0 (0–0)	0 (0–0)	0 (0–0)	0 (0–0)	--
	0 (0–0)	0 (0–0)	0 (0–0)	0 (0–0)	0 (0–0)	0 (0–0)	--	--	--	--	--	--	--	--	--	--	--	--	--	--	--	--	--
Liver cancer due to other causes	0 (0–0)	0 (0–0)	0 (0–0)	0 (0–0)	0 (0–0)	0 (0–0)	0 (0–0)	0 (0–0)	0 (0–0)	0 (0–0)	0 (0–0)	0 (0–0)	0 (0–0)	0 (0–0)	0 (0–0)	0 (0–0)	0 (0–0)	0 (0–0)	0 (0–0)	0 (0–0)	0 (0–0)	0 (0–0)	--
Gallbladder and biliary tract cancer	--	--	--	--	--	--	--	0 (0–0)	0 (0–0)	0 (0–0)	0 (0–0)	0 (0–0)	0 (0–0)	0 (0–0)	0 (0–0)	0 (0–0)	0 (0–0)	0 (0–0)	0 (0–0)	0 (0–0)	0 (0–0)	0 (0–0)	--
Pancreatic cancer	--	--	--	--	--	--	0 (0–0)	0 (0–0)	0 (0–0)	0 (0–0)	0 (0–0)	0 (0–0)	0 (0–0)	0 (0–0)	0 (0–1)	0 (0–1)	0 (0–1)	0 (0–1)	0 (0–1)	0 (0–1)	0 (0–0)	0 (0–0)	--
Larynx cancer	--	--	--	--	--	--	--	0 (0–0)	0 (0–0)	0 (0–0)	0 (0–0)	0 (0–0)	0 (0–0)	0 (0–0)	0 (0–0)	0 (0–0)	0 (0–0)	0 (0–0)	0 (0–0)	0 (0–0)	0 (0–0)	0 (0–0)	--
Tracheal, bronchus, and lung cancer	--	--	--	--	--	0 (0–0)	0 (0–0)	0 (0–0)	0 (0–0)	0 (0–0)	0 (0–0)	0 (0–0)	0 (0–0)	0 (0–1)	1 (0–1)	1 (1–1)	1 (1–1)	1 (1–1)	1 (1–1)	1 (0–1)	0 (0–0)	0 (0–0)	--
Malignant skin melanoma	0 (0–0)	0 (0–0)	0 (0–0)	0 (0–0)	0 (0–0)	0 (0–0)	0 (0–0)	0 (0–0)	0 (0–0)	0 (0–0)	0 (0–0)	0 (0–0)	0 (0–0)	0 (0–0)	0 (0–0)	0 (0–0)	0 (0–0)	0 (0–0)	0 (0–0)	0 (0–0)	0 (0–0)	0 (0–0)	--
Non-melanoma skin cancer (squamous-cell carcinoma)	--	--	--	--	--	--	--	0 (0–0)	0 (0–0)	0 (0–0)	0 (0–0)	0 (0–0)	0 (0–0)	0 (0–0)	0 (0–0)	0 (0–0)	0 (0–0)	0 (0–0)	0 (0–0)	0 (0–0)	0 (0–0)	0 (0–0)	--
	0 (0–0)	0 (0–0)	0 (0–0)	0 (0–0)	0 (0–0)	0 (0–0)	0 (0–0)	0 (0–0)	0 (0–0)	0 (0–0)	0 (0–0)	0 (0–0)	0 (0–0)	0 (0–0)	0 (0–0)	0 (0–0)	0 (0–0)	0 (0–0)	0 (0–0)	0 (0–0)	0 (0–0)	0 (0–0)	--
	--	--	--	0 (0–0)	0 (0–0)	0 (0–0)	0 (0–0)	0 (0–0)	0 (0–0)	0 (0–0)	0 (0–0)	0 (0–0)	0 (0–0)	0 (0–0)	0 (0–0)	0 (0–0)	0 (0–0)	0 (0–0)	0 (0–0)	0 (0–0)	0 (0–0)	0 (0–0)	--
Breast cancer	--	--	--	--	--	--	0 (0–0)	0 (0–0)	0 (0–0)	0 (0–0)	0 (0–0)	0 (0–0)	0 (0–0)	0 (0–0)	0 (0–0)	0 (0–0)	0 (0–0)	0 (0–0)	0 (0–0)	0 (0–0)	0 (0–0)	0 (0–0)	--
Cervical cancer	--	--	--	--	--	--	--	--	--	--	--	--	--	--	--	--	--	--	--	--	--	--	--
Uterine cancer	--	--	--	--	--	--	--	--	--	--	--	--	--	--	--	--	--	--	--	--	--	--	--
Ovarian cancer	--	--	--	--	--	--	--	--	--	--	--	--	--	--	--	--	--	--	--	--	--	--	--
Prostate cancer	--	--	--	--	--	--	--	0 (0–0)	0 (0–0)	0 (0–0)	0 (0–0)	0 (0–0)	0 (0–0)	0 (0–1)	1 (1–1)	2 (2–3)	3 (2–4)	5 (3–6)	6 (4–8)	6 (4–9)	4 (3–6)	2 (1–3)	--
Testicular cancer	0 (0–0)	0 (0–0)	0 (0–0)	0 (0–0)	0 (0–0)	0 (0–0)	0 (0–0)	0 (0–0)	0 (0–0)	0 (0–0)	0 (0–0)	0 (0–0)	0 (0–0)	0 (0–0)	0 (0–0)	0 (0–0)	0 (0–0)	0 (0–0)	0 (0–0)	0 (0–0)	0 (0–0)	0 (0–0)	--
Kidney cancer	0 (0–0)	0 (0–0)	0 (0–0)	0 (0–0)	0 (0–0)	0 (0–0)	0 (0–0)	0 (0–0)	0 (0–0)	0 (0–0)	0 (0–0)	0 (0–0)	0 (0–0)	0 (0–0)	0 (0–0)	0 (0–0)	0 (0–0)	0 (0–0)	0 (0–0)	0 (0–0)	0 (0–0)	0 (0–0)	--
Bladder cancer	--	--	--	--	--	--	0 (0–0)	0 (0–0)	0 (0–0)	0 (0–0)	0 (0–0)	0 (0–0)	0 (0–0)	0 (0–0)	0 (0–0)	0 (0–0)	0 (0–0)	0 (0–0)	0 (0–0)	0 (0–0)	0 (0–0)	0 (0–0)	--
Brain and nervous system cancer	0 (0–0)	0 (0–0)	0 (0–0)	0 (0–0)	0 (0–0)	0 (0–0)	0 (0–0)	0 (0–0)	0 (0–0)	0 (0–0)	0 (0–0)	0 (0–0)	0 (0–0)	0 (0–0)	0 (0–0)	0 (0–0)	0 (0–0)	0 (0–0)	0 (0–0)	0 (0–0)	0 (0–0)	0 (0–0)	--
	0 (0–0)	0 (0–0)	0 (0–0)	0 (0–0)	0 (0–0)	0 (0–0)	0 (0–0)	0 (0–0)	0 (0–0)	0 (0–0)	0 (0–0)	0 (0–0)	0 (0–0)	0 (0–0)	0 (0–0)	0 (0–0)	0 (0–0)	0 (0–0)	0 (0–0)	0 (0–0)	0 (0–0)	0 (0–0)	--
	0 (0–0)	0 (0–0)	0 (0–0)	0 (0–0)	0 (0–0)	0 (0–0)	0 (0–0)	--	--	--	--	--	--	--	--	--	--	--	--	--	--	--	--
	--	--	--	0 (0–0)	0 (0–0)	0 (0–0)	0 (0–0)	0 (0–0)	0 (0–0)	0 (0–0)	0 (0–0)	0 (0–0)	0 (0–0)	0 (0–0)	0 (0–0)	0 (0–0)	0 (0–0)	0 (0–0)	0 (0–0)	0 (0–0)	0 (0–0)	0 (0–0)	--
	0 (0–0)	0 (0–0)	0 (0–0)	0 (0–0)	0 (0–0)	0 (0–0)	0 (0–0)	0 (0–0)	0 (0–0)	0 (0–0)	0 (0–0)	0 (0–0)	0 (0–0)	0 (0–0)	0 (0–0)	0 (0–0)	0 (0–0)	0 (0–0)	0 (0–0)	0 (0–0)	0 (0–0)	0 (0–0)	--
Thyroid cancer	--	--	--	--	0 (0–0)	0 (0–0)	0 (0–0)	0 (0–0)	0 (0–0)	0 (0–0)	0 (0–0)	0 (0–0)	0 (0–0)	0 (0–0)	0 (0–0)	0 (0–0)	0 (0–0)	0 (0–0)	0 (0–0)	0 (0–0)	0 (0–0)	0 (0–0)	--
Mesothelioma	--	--	--	--	--	--	--	0 (0–0)	0 (0–0)	0 (0–0)	0 (0–0)	0 (0–0)	0 (0–0)	0 (0–0)	0 (0–0)	0 (0–0)	0 (0–0)	0 (0–0)	0 (0–0)	0 (0–0)	0 (0–0)	0 (0–0)	--
Hodgkin lymphoma	--	--	--	0 (0–0)	0 (0–0)	0 (0–0)	0 (0–0)	0 (0–0)	0 (0–0)	0 (0–0)	0 (0–0)	0 (0–0)	0 (0–0)	0 (0–0)	0 (0–0)	0 (0–0)	0 (0–0)	0 (0–0)	0 (0–0)	0 (0–0)	0 (0–0)	0 (0–0)	--
Non-Hodgkin's lymphoma	--	--	--	0 (0–0)	0 (0–0)	0 (0–0)	0 (0–0)	0 (0–0)	0 (0–0)	0 (0–0)	0 (0–0)	0 (0–0)	0 (0–0)	0 (0–0)	0 (0–0)	0 (0–0)	0 (0–0)	0 (0–0)	0 (0–0)	0 (0–0)	0 (0–0)	0 (0–0)	--
	--	--	--	0 (0–0)	0 (0–0)	0 (0–0)	0 (0–0)	0 (0–0)	0 (0–0)	0 (0–0)	0 (0–0)	0 (0–0)	0 (0–0)	0 (0–0)	0 (0–0)	0 (0–0)	0 (0–0)	0 (0–0)	0 (0–0)	0 (0–0)	0 (0–0)	0 (0–0)	--
	--	--	--	0 (0–0)	0 (0–0)	0 (0–0)	0 (0–0)	0 (0–0)	0 (0–0)	0 (0–0)	0 (0–0)	0 (0–0)	0 (0–0)	0 (0–0)	0 (0–0)	0 (0–0)	0 (0–0)	0 (0–0)	0 (0–0)	0 (0–0)	0 (0–0)	0 (0–0)	--
Multiple myeloma	--	--	--	--	--	--	--	0 (0–0)	0 (0–0)	0 (0–0)	0 (0–0)	0 (0–0)	0 (0–0)	0 (0–0)	0 (0–0)	0 (0–0)	0 (0–0)	0 (0–0)	0 (0–0)	0 (0–0)	0 (0–0)	0 (0–0)	--
Leukaemia	0 (0–0)	0 (0–0)	0 (0–0)	0 (0–0)	0 (0–0)	0 (0–0)	0 (0–0)	0 (0–0)	0 (0–0)	0 (0–0)	0 (0–0)	0 (0–0)	0 (0–0)	0 (0–0)	0 (0–0)	0 (0–0)	0 (0–0)	0 (0–0)	0 (0–0)	0 (0–0)	0 (0–0)	0 (0–0)	--
Acute lymphoid leukaemia	0 (0–0)	0 (0–0)	0 (0–0)	0 (0–0)	0 (0–0)	0 (0–0)	0 (0–0)	0 (0–0)	0 (0–0)	0 (0–0)	0 (0–0)	0 (0–0)	0 (0–0)	0 (0–0)	0 (0–0)	0 (0–0)	0 (0–0)	0 (0–0)	0 (0–0)	0 (0–0)	0 (0–0)	0 (0–0)	--
Chronic lymphoid leukaemia	--	--	--	--	--	--	--	0 (0–0)	0 (0–0)	0 (0–0)	0 (0–0)	0 (0–0)	0 (0–0)	0 (0–0)	0 (0–0)	0 (0–0)	0 (0–0)	0 (0–0)	0 (0–0)	0 (0–0)	0 (0–0)	0 (0–0)	--
Acute myeloid leukaemia	0 (0–0)	0 (0–0)	0 (0–0)	0 (0–0)	0 (0–0)	0 (0–0)	0 (0–0)	0 (0–0)	0 (0–0)	0 (0–0)	0 (0–0)	0 (0–0)	0 (0–0)	0 (0–0)	0 (0–0)	0 (0–0)	0 (0–0)	0 (0–0)	0 (0–0)	0 (0–0)	0 (0–0)	0 (0–0)	--
Chronic myeloid leukaemia	0 (0–0)	0 (0–0)	0 (0–0)	0 (0–0)	0 (0–0)	0 (0–0)	0 (0–0)	0 (0–0)	0 (0–0)	0 (0–0)	0 (0–0)	0 (0–0)	0 (0–0)	0 (0–0)	0 (0–0)	0 (0–0)	0 (0–0)	0 (0–0)	0 (0–0)	0 (0–0)	0 (0–0)	0 (0–0)	--
Other leukaemia	0 (0–0)	0 (0–0)	0 (0–0)	0 (0–0)	0 (0–0)	0 (0–0)	0 (0–0)	0 (0–0)	0 (0–0)	0 (0–0)	0 (0–0)	0 (0–0)	0 (0–0)	0 (0–0)	0 (0–0)	0 (0–0)	0 (0–0)	0 (0–0)	0 (0–0)	0 (0–0)	0 (0–0)	0 (0–0)	--
Other malignant cancers	0 (0–0)	0 (0–0)	0 (0–0)	0 (0–0)	0 (0–0)	0 (0–0)	0 (0–0)	0 (0–0)	0 (0–0)	0 (0–0)	0 (0–0)	0 (0–0)	0 (0–0)	0 (0–0)	0 (0–0)	0 (0–0)	0 (0–0)	0 (0–0)	0 (0–0)	0 (0–0)	0 (0–0)	0 (0–0)	--
Myelodysplastic, myeloproliferative, and other hematopoietic neoplasms	0 (0–0)	0 (0–0)	0 (0–0)	0 (0–0)	0 (0–0)	0 (0–0)	0 (0–0)	0 (0–0)	0 (0–0)	0 (0–0)	0 (0–0)	0 (0–0)	0 (0–0)	0 (0–0)	0 (0–0)	0 (0–0)	0 (0–0)	0 (0–0)	0 (0–0)	0 (0–0)	0 (0–0)	0 (0–0)	--
Cardiovascular diseases	0 (0–0)	0 (0–0)	0 (0–0)	0 (0–0)	0 (0–0)	0 (0–0)	0 (0–0)	0 (0–0)	0 (0–0)	0 (0–1)	1 (1–1)	1 (1–1)	2 (2–3)	5 (4–6)	8 (6–9)	10 (8–12)	10 (8–12)	12 (10–14)	13 (10–15)	14 (12–17)	10 (8–11)	5 (4–6)	--
Rheumatic heart disease	--	--	--	0 (0–0)	0 (0–0)	0 (0–0)	0 (0–0)	0 (0–0)	0 (0–0)	0 (0–0)	0 (0–0)	0 (0–0)	0 (0–0)	0 (0–0)	0 (0–0)	0 (0–0)	0 (0–0)	0 (0–0)	0 (0–0)	0 (0–0)	0 (0–0)	0 (0–0)	--
Ischaemic heart disease	--	--	--	--	--	--	0 (0–0)	0 (0–0)	0 (0–0)	0 (0–0)	0 (0–0)	0 (0–1)	1 (1–1)	2 (2–2)	3 (2–4)	4 (3–5)	4 (3–5)	5 (4–6)	5 (4–6)	6 (5–7)	4 (3–5)	2 (2–3)	--
Stroke	0 (0–0)	0 (0–0)	0 (0–0)	0 (0–0)	0 (0–0)	0 (0–0)	0 (0–0)	0 (0–0)	0 (0–0)	0 (0–0)	0 (0–0)	0 (0–0)	1 (0–1)	1 (1–2)	2 (2–3)	3 (2–4)	3 (2–4)	4 (3–5)	4 (3–5)	4 (3–6)	3 (2–4)	2 (1–2)	--
Ischaemic stroke	0 (0–0)	0 (0–0)	0 (0–0)	0 (0–0)	0 (0–0)	0 (0–0)	0 (0–0)	0 (0–0)	0 (0–0)	0 (0–0)	0 (0–0)	0 (0–0)	0 (0–0)	0 (0–0)	0 (0–0)	1 (0–1)	1 (1–1)	1 (1–2)	2 (1–2)	3 (2–4)	2 (1–3)	1 (1–1)	--
Intracerebral hemorrhage	0 (0–0)	0 (0–0)	0 (0–0)	0 (0–0)	0 (0–0)	0 (0–0)	0 (0–0)	0 (0–0)	0 (0–0)	0 (0–0)	0 (0–0)	0 (0–0)	0 (0–1)	1 (1–1)	2 (1–2)	2 (1–3)	2 (2–3)	2 (2–3)	2 (2–3)	2 (1–2)	1 (1–1)	0 (0–1)	--
Subarachnoid hemorrhage	0 (0–0)	0 (0–0)	0 (0–0)	0 (0–0)	0 (0–0)	0 (0–0)	0 (0–0)	0 (0–0)	0 (0–0)	0 (0–0)	0 (0–0)	0 (0–0)	0 (0–0)	0 (0–0)	0 (0–0)	0 (0–0)	0 (0–0)	0 (0–0)	0 (0–0)	0 (0–0)	0 (0–0)	0 (0–0)	--
Hypertensive heart disease	--	--	--	--	--	--	0 (0–0)	0 (0–0)	0 (0–0)	0 (0–0)	0 (0–0)	0 (0–0)	0 (0–0)	1 (0–1)	1 (1–1)	1 (1–2)	1 (1–2)	1 (1–2)	2 (1–2)	2 (1–2)	1 (1–2)	1 (0–1)	--
Non-rheumatic valvular heart disease	--	--	--	--	--	--	0 (0–0)	0 (0–0)	0 (0–0)	0 (0–0)	0 (0–0)	0 (0–0)	0 (0–0)	0 (0–0)	0 (0–0)	0 (0–0)	0 (0–0)	0 (0–0)	0 (0–0)	0 (0–0)	0 (0–0)	0 (0–0)	--
Non-rheumatic calcific aortic valve disease	--	--	--	--	--	--	0 (0–0)	0 (0–0)	0 (0–0)	0 (0–0)	0 (0–0)	0 (0–0)	0 (0–0)	0 (0–0)	0 (0–0)	0 (0–0)	0 (0–0)	0 (0–0)	0 (0–0)	0 (0–0)	0 (0–0)	0 (0–0)	--
Non-rheumatic degenerative mitral valve disease	--	--	--	--	--	--	0 (0–0)	0 (0–0)	0 (0–0)	0 (0–0)	0 (0–0)	0 (0–0)	0 (0–0)	0 (0–0)	0 (0–0)	0 (0–0)	0 (0–0)	0 (0–0)	0 (0–0)	0 (0–0)	0 (0–0)	0 (0–0)	--
Other non-rheumatic valve diseases	--	--	--	--	--	--	0 (0–0)	0 (0–0)	0 (0–0)	0 (0–0)	0 (0–0)	0 (0–0)	0 (0–0)	0 (0–0)	0 (0–0)	0 (0–0)	0 (0–0)	0 (0–0)	0 (0–0)	0 (0–0)	0 (0–0)	0 (0–0)	--
Cardiomyopathy and myocarditis	0 (0–0)	0 (0–0)	0 (0–0)	0 (0–0)	0 (0–0)	0 (0–0)	0 (0–0)	0 (0–0)	0 (0–0)	0 (0–0)	0 (0–0)	0 (0–0)	0 (0–0)	0 (0–0)	0 (0–1)	0 (0–1)	0 (0–1)	0 (0–1)	0 (0–0)	0 (0–0)	0 (0–0)	0 (0–0)	--
Myocarditis	0 (0–0)	0 (0–0)	0 (0–0)	0 (0–0)	0 (0–0)	0 (0–0)	0 (0–0)	0 (0–0)	0 (0–0)	0 (0–0)	0 (0–0)	0 (0–0)	0 (0–0)	0 (0–0)	0 (0–0)	0 (0–0)	0 (0–0)	0 (0–0)	0 (0–0)	0 (0–0)	0 (0–0)	0 (0–0)	--
Alcoholic cardiomyopathy	--	--	--	--	--	--	0 (0–0)	0 (0–0)	0 (0–0)	0 (0–0)	0 (0–0)	0 (0–0)	0 (0–0)	0 (0–0)	0 (0–0)	0 (0–0)	0 (0–0)	0 (0–0)	0 (0–0)	0 (0–0)	0 (0–0)	0 (0–0)	--
Other cardiomyopathy	0 (0–0)	0 (0–0)	0 (0–0)	0 (0–0)	0 (0–0)	0 (0–0)	0 (0–0)	0 (0–0)	0 (0–0)	0 (0–0)	0 (0–0)	0 (0–0)	0 (0–0)	0 (0–0)	0 (0–0)	0 (0–0)	0 (0–0)	0 (0–0)	0 (0–0)	0 (0–0)	0 (0–0)	0 (0–0)	--
Pulmonary Arterial Hypertension	0 (0–0)	0 (0–0)	0 (0–0)	0 (0–0)	0 (0–0)	0 (0–0)	0 (0–0)	0 (0–0)	0 (0–0)	0 (0–0)	0 (0–0)	0 (0–0)	0 (0–0)	0 (0–0)	0 (0–0)	0 (0–0)	0 (0–0)	0 (0–0)	0 (0–0)	0 (0–0)	0 (0–0)	0 (0–0)	--
Atrial fibrillation and flutter	--	--	--	--	--	--	--	--	--	0 (0–0)	0 (0–0)	0 (0–0)	0 (0–0)	0 (0–0)	0 (0–0)	0 (0–0)	0 (0–0)	0 (0–0)	0 (0–0)	0 (0–1)	0 (0–1)	0 (0–1)	--
Aortic aneurysm	--	--	--	--	--	--	0 (0–0)	0 (0–0)	0 (0–0)	0 (0–0)	0 (0–0)	0 (0–0)	0 (0–0)	0 (0–0)	0 (0–0)	0 (0–0)	0 (0–0)	0 (0–0)	0 (0–0)	0 (0–0)	0 (0–0)	0 (0–0)	--
Peripheral vascular disease	--	--	--	--	--	--	--	--	--	--	--	0 (0–0)	0 (0–0)	0 (0–0)	0 (0–0)	0 (0–0)	0 (0–0)	0 (0–0)	0 (0–0)	0 (0–0)	0 (0–0)	0 (0–0)	--
Endocarditis	0 (0–0)	0 (0–0)	0 (0–0)	0 (0–0)	0 (0–0)	0 (0–0)	0 (0–0)	0 (0–0)	0 (0–0)	0 (0–0)	0 (0–0)	0 (0–0)	0 (0–0)	0 (0–0)	0 (0–0)	0 (0–0)	0 (0–0)	0 (0–0)	0 (0–0)	0 (0–0)	0 (0–0)	0 (0–0)	--
Other cardiovascular and circulatory diseases	0 (0–0)	0 (0–0)	0 (0–0)	0 (0–0)	0 (0–0)	0 (0–0)	0 (0–0)	0 (0–0)	0 (0–0)	0 (0–0)	0 (0–0)	0 (0–0)	0 (0–0)	0 (0–0)	0 (0–0)	0 (0–1)	0 (0–1)	0 (0–1)	1 (0–1)	1 (0–1)	0 (0–1)	0 (0–0)	--
Chronic respiratory diseases	0 (0–0)	0 (0–0)	0 (0–0)	0 (0–0)	0 (0–0)	0 (0–0)	0 (0–0)	0 (0–0)	0 (0–0)	0 (0–0)	0 (0–0)	0 (0–0)	0 (0–0)	0 (0–0)	0 (0–0)	1 (0–1)	1 (0–1)	1 (1–1)	1 (1–1)	1 (1–1)	1 (1–1)	0 (0–1)	--
Chronic obstructive pulmonary disease	--	--	--	0 (0–0)	0 (0–0)	0 (0–0)	0 (0–0)	0 (0–0)	0 (0–0)	0 (0–0)	0 (0–0)	0 (0–0)	0 (0–0)	0 (0–0)	0 (0–0)	0 (0–1)	0 (0–1)	1 (0–1)	1 (1–1)	1 (1–1)	1 (0–1)	0 (0–0)	--
Pneumoconiosis	--	--	--	--	--	--	0 (0–0)	0 (0–0)	0 (0–0)	0 (0–0)	0 (0–0)	0 (0–0)	0 (0–0)	0 (0–0)	0 (0–0)	0 (0–0)	0 (0–0)	0 (0–0)	0 (0–0)	0 (0–0)	0 (0–0)	0 (0–0)	--
Silicosis	--	--	--	--	--	--	0 (0–0)	0 (0–0)	0 (0–0)	0 (0–0)	0 (0–0)	0 (0–0)	0 (0–0)	0 (0–0)	0 (0–0)	0 (0–0)	0 (0–0)	0 (0–0)	0 (0–0)	0 (0–0)	0 (0–0)	0 (0–0)	--
Asbestosis	--	--	--	--	--	--	0 (0–0)	0 (0–0)	0 (0–0)	0 (0–0)	0 (0–0)	0 (0–0)	0 (0–0)	0 (0–0)	0 (0–0)	0 (0–0)	0 (0–0)	0 (0–0)	0 (0–0)	0 (0–0)	0 (0–0)	0 (0–0)	--
Coal workers pneumoconiosis	--	--	--	--	--	--	0 (0–0)	0 (0–0)	0 (0–0)	0 (0–0)	0 (0–0)	0 (0–0)	0 (0–0)	0 (0–0)	0 (0–0)	0 (0–0)	0 (0–0)	0 (0–0)	0 (0–0)	0 (0–0)	0 (0–0)	0 (0–0)	--
Other pneumoconiosis	--	--	--	--	--	--	0 (0–0)	0 (0–0)	0 (0–0)	0 (0–0)	0 (0–0)	0 (0–0)	0 (0–0)	0 (0–0)	0 (0–0)	0 (0–0)	0 (0–0)	0 (0–0)	0 (0–0)	0 (0–0)	0 (0–0)	0 (0–0)	--
Asthma	--	--	--	0 (0–0)	0 (0–0)	0 (0–0)	0 (0–0)	0 (0–0)	0 (0–0)	0 (0–0)	0 (0–0)	0 (0–0)	0 (0–0)	0 (0–0)	0 (0–0)	0 (0–0)	0 (0–0)	0 (0–0)	0 (0–0)	0 (0–0)	0 (0–0)	0 (0–0)	--

Interstitial lung disease and pulmonary sarcoidosis	--	--	--	0 (0–0)	0 (0–0)	0 (0–0)	0 (0–0)	0 (0–0)	0 (0–0)	0 (0–0)	0 (0–0)	0 (0–0)	0 (0–0)	0 (0–0)	0 (0–0)	0 (0–0)	0 (0–0)	0 (0–0)	0 (0–0)	0 (0–0)	0 (0–0)	0 (0–0)	--
Other chronic respiratory diseases	0 (0–0)	0 (0–0)	0 (0–0)	0 (0–0)	0 (0–0)	0 (0–0)	0 (0–0)	0 (0–0)	0 (0–0)	0 (0–0)	0 (0–0)	0 (0–0)	0 (0–0)	0 (0–0)	0 (0–0)	0 (0–0)	0 (0–0)	0 (0–0)	0 (0–0)	0 (0–0)	0 (0–0)	0 (0–0)	--
Digestive diseases	0 (0–0)	0 (0–0)	0 (0–0)	0 (0–0)	0 (0–0)	0 (0–0)	0 (0–0)	0 (0–0)	0 (0–0)	0 (0–0)	0 (0–0)	0 (0–1)	1 (1–1)	1 (1–2)	2 (1–2)	2 (1–2)	1 (1–2)	1 (1–2)	1 (1–2)	1 (1–2)	1 (1–1)	0 (0–0)	--
Cirrhosis and other chronic liver diseases	--	--	--	0 (0–0)	0 (0–0)	0 (0–0)	0 (0–0)	0 (0–0)	0 (0–0)	0 (0–0)	0 (0–0)	0 (0–0)	1 (0–1)	1 (1–1)	1 (1–1)	1 (1–2)	1 (1–1)	1 (1–1)	1 (0–1)	1 (0–1)	0 (0–0)	0 (0–0)	--
Cirrhosis and other chronic liver diseases due to hepatitis B	--	--	--	0 (0–0)	0 (0–0)	0 (0–0)	0 (0–0)	0 (0–0)	0 (0–0)	0 (0–0)	0 (0–0)	0 (0–0)	0 (0–0)	0 (0–0)	0 (0–0)	0 (0–0)	0 (0–0)	0 (0–0)	0 (0–0)	0 (0–0)	0 (0–0)	0 (0–0)	--
Cirrhosis and other chronic liver diseases due to hepatitis C	--	--	--	0 (0–0)	0 (0–0)	0 (0–0)	0 (0–0)	0 (0–0)	0 (0–0)	0 (0–0)	0 (0–0)	0 (0–0)	0 (0–0)	0 (0–0)	0 (0–0)	0 (0–0)	0 (0–0)	0 (0–0)	0 (0–0)	0 (0–0)	0 (0–0)	0 (0–0)	--
Cirrhosis and other chronic liver diseases due to alcohol use	--	--	--	--	--	--	0 (0–0)	0 (0–0)	0 (0–0)	0 (0–0)	0 (0–0)	0 (0–0)	0 (0–0)	0 (0–1)	1 (0–1)	1 (0–1)	0 (0–1)	0 (0–1)	0 (0–0)	0 (0–0)	0 (0–0)	0 (0–0)	--
Cirrhosis and other chronic liver diseases due to NAFLD	--	--	--	--	--	--	0 (0–0)	0 (0–0)	0 (0–0)	0 (0–0)	0 (0–0)	0 (0–0)	0 (0–0)	0 (0–0)	0 (0–0)	0 (0–0)	0 (0–0)	0 (0–0)	0 (0–0)	0 (0–0)	0 (0–0)	0 (0–0)	--
Cirrhosis and other chronic liver diseases due to other causes	--	--	--	0 (0–0)	0 (0–0)	0 (0–0)	0 (0–0)	0 (0–0)	0 (0–0)	0 (0–0)	0 (0–0)	0 (0–0)	0 (0–0)	0 (0–0)	0 (0–0)	0 (0–0)	0 (0–0)	0 (0–0)	0 (0–0)	0 (0–0)	0 (0–0)	0 (0–0)	--
Upper digestive system diseases	--	--	--	0 (0–0)	0 (0–0)	0 (0–0)	0 (0–0)	0 (0–0)	0 (0–0)	0 (0–0)	0 (0–0)	0 (0–0)	0 (0–0)	0 (0–0)	0 (0–0)	0 (0–0)	0 (0–0)	0 (0–0)	0 (0–0)	0 (0–0)	0 (0–0)	0 (0–0)	--
Peptic ulcer disease	--	--	--	0 (0–0)	0 (0–0)	0 (0–0)	0 (0–0)	0 (0–0)	0 (0–0)	0 (0–0)	0 (0–0)	0 (0–0)	0 (0–0)	0 (0–0)	0 (0–0)	0 (0–0)	0 (0–0)	0 (0–0)	0 (0–0)	0 (0–0)	0 (0–0)	0 (0–0)	--
Gastritis and duodenitis	--	--	--	0 (0–0)	0 (0–0)	0 (0–0)	0 (0–0)	0 (0–0)	0 (0–0)	0 (0–0)	0 (0–0)	0 (0–0)	0 (0–0)	0 (0–0)	0 (0–0)	0 (0–0)	0 (0–0)	0 (0–0)	0 (0–0)	0 (0–0)	0 (0–0)	0 (0–0)	--
Appendicitis	--	--	--	0 (0–0)	0 (0–0)	0 (0–0)	0 (0–0)	0 (0–0)	0 (0–0)	0 (0–0)	0 (0–0)	0 (0–0)	0 (0–0)	0 (0–0)	0 (0–0)	0 (0–0)	0 (0–0)	0 (0–0)	0 (0–0)	0 (0–0)	0 (0–0)	0 (0–0)	--
Paralytic ileus and intestinal obstruction	0 (0–0)	0 (0–0)	0 (0–0)	0 (0–0)	0 (0–0)	0 (0–0)	0 (0–0)	0 (0–0)	0 (0–0)	0 (0–0)	0 (0–0)	0 (0–0)	0 (0–0)	0 (0–0)	0 (0–0)	0 (0–0)	0 (0–0)	0 (0–0)	0 (0–0)	0 (0–0)	0 (0–0)	0 (0–0)	--
Inguinal, femoral, and abdominal hernia	0 (0–0)	0 (0–0)	0 (0–0)	0 (0–0)	0 (0–0)	0 (0–0)	0 (0–0)	0 (0–0)	0 (0–0)	0 (0–0)	0 (0–0)	0 (0–0)	0 (0–0)	0 (0–0)	0 (0–0)	0 (0–0)	0 (0–0)	0 (0–0)	0 (0–0)	0 (0–0)	0 (0–0)	0 (0–0)	--
Inflammatory bowel disease	--	--	--	0 (0–0)	0 (0–0)	0 (0–0)	0 (0–0)	0 (0–0)	0 (0–0)	0 (0–0)	0 (0–0)	0 (0–0)	0 (0–0)	0 (0–0)	0 (0–0)	0 (0–0)	0 (0–0)	0 (0–0)	0 (0–0)	0 (0–0)	0 (0–0)	0 (0–0)	--
Vascular intestinal disorders	--	--	--	0 (0–0)	0 (0–0)	0 (0–0)	0 (0–0)	0 (0–0)	0 (0–0)	0 (0–0)	0 (0–0)	0 (0–0)	0 (0–0)	0 (0–0)	0 (0–0)	0 (0–0)	0 (0–0)	0 (0–0)	0 (0–0)	0 (0–0)	0 (0–0)	0 (0–0)	--
Gallbladder and biliary diseases	--	--	--	0 (0–0)	0 (0–0)	0 (0–0)	0 (0–0)	0 (0–0)	0 (0–0)	0 (0–0)	0 (0–0)	0 (0–0)	0 (0–0)	0 (0–0)	0 (0–0)	0 (0–0)	0 (0–0)	0 (0–0)	0 (0–0)	0 (0–0)	0 (0–0)	0 (0–0)	--
Pancreatitis	--	--	--	0 (0–0)	0 (0–0)	0 (0–0)	0 (0–0)	0 (0–0)	0 (0–0)	0 (0–0)	0 (0–0)	0 (0–0)	0 (0–0)	0 (0–0)	0 (0–0)	0 (0–0)	0 (0–0)	0 (0–0)	0 (0–0)	0 (0–0)	0 (0–0)	0 (0–0)	--
Other digestive diseases	--	--	--	0 (0–0)	0 (0–0)	0 (0–0)	0 (0–0)	0 (0–0)	0 (0–0)	0 (0–0)	0 (0–0)	0 (0–0)	0 (0–0)	0 (0–0)	0 (0–0)	0 (0–0)	0 (0–0)	0 (0–0)	0 (0–0)	0 (0–0)	0 (0–0)	0 (0–0)	--
Alzheimer's disease and other dementias	--	--	--	--	--	--	--	--	--	--	--	0 (0–0)	0 (0–0)	0 (0–0)	0 (0–0)	0 (0–1)	0 (0–1)	1 (0–2)	1 (0–2)	1 (0–4)	1 (0–4)	1 (0–3)	--
Parkinson's disease	--	--	--	--	--	--	--	0 (0–0)	0 (0–0)	0 (0–0)	0 (0–0)	0 (0–0)	0 (0–0)	0 (0–0)	0 (0–0)	0 (0–0)	0 (0–0)	0 (0–0)	1 (1–1)	1 (1–1)	1 (0–1)	0 (0–0)	--
Idiopathic epilepsy	--	--	0 (0–0)	0 (0–0)	0 (0–0)	0 (0–0)	0 (0–0)	0 (0–0)	0 (0–0)	0 (0–0)	0 (0–0)	0 (0–0)	0 (0–0)	0 (0–0)	0 (0–0)	0 (0–0)	0 (0–0)	0 (0–0)	0 (0–0)	0 (0–0)	0 (0–0)	0 (0–0)	--
Multiple sclerosis	--	--	--	--	0 (0–0)	0 (0–0)	0 (0–0)	0 (0–0)	0 (0–0)	0 (0–0)	0 (0–0)	0 (0–0)	0 (0–0)	0 (0–0)	0 (0–0)	0 (0–0)	0 (0–0)	0 (0–0)	0 (0–0)	0 (0–0)	0 (0–0)	0 (0–0)	--
Motor neuron disease	0 (0–0)	0 (0–0)	0 (0–0)	0 (0–0)	0 (0–0)	0 (0–0)	0 (0–0)	0 (0–0)	0 (0–0)	0 (0–0)	0 (0–0)	0 (0–0)	0 (0–0)	0 (0–0)	0 (0–0)	0 (0–0)	0 (0–0)	0 (0–0)	0 (0–0)	0 (0–0)	0 (0–0)	0 (0–0)	--
Other neurological disorders	--	--	0 (0–0)	0 (0–0)	0 (0–0)	0 (0–0)	0 (0–0)	0 (0–0)	0 (0–0)	0 (0–0)	0 (0–0)	0 (0–0)	0 (0–0)	0 (0–0)	0 (0–0)	0 (0–0)	0 (0–0)	0 (0–0)	0 (0–0)	0 (0–0)	0 (0–0)	0 (0–0)	--
Eating disorders	--	--	--	--	0 (0–0)	0 (0–0)	0 (0–0)	0 (0–0)	0 (0–0)	0 (0–0)	0 (0–0)	0 (0–0)	0 (0–0)	--	--	--	--	--	--	--	--	--	--
Anorexia nervosa	--	--	--	--	0 (0–0)	0 (0–0)	0 (0–0)	0 (0–0)	0 (0–0)	0 (0–0)	0 (0–0)	0 (0–0)	0 (0–0)	--	--	--	--	--	--	--	--	--	--
Bulimia nervosa	--	--	--	--	0 (0–0)	0 (0–0)	0 (0–0)	0 (0–0)	0 (0–0)	0 (0–0)	0 (0–0)	0 (0–0)	0 (0–0)	--	--	--	--	--	--	--	--	--	--
Alcohol use disorders	--	--	--	--	--	0 (0–0)	0 (0–0)	0 (0–0)	0 (0–0)	0 (0–0)	0 (0–0)	0 (0–1)	1 (0–1)	1 (1–1)	1 (1–1)	1 (1–1)	1 (0–1)	0 (0–1)	0 (0–0)	0 (0–0)	0 (0–0)	0 (0–0)	--
Drug use disorders	0 (0–0)	0 (0–0)	0 (0–0)	0 (0–0)	0 (0–0)	0 (0–0)	0 (0–0)	0 (0–0)	0 (0–0)	0 (0–0)	0 (0–0)	0 (0–0)	0 (0–0)	0 (0–0)	0 (0–0)	0 (0–0)	0 (0–0)	0 (0–0)	0 (0–0)	0 (0–0)	0 (0–0)	0 (0–0)	--
Opioid use disorders	0 (0–0)	0 (0–0)	0 (0–0)	0 (0–0)	0 (0–0)	0 (0–0)	0 (0–0)	0 (0–0)	0 (0–0)	0 (0–0)	0 (0–0)	0 (0–0)	0 (0–0)	0 (0–0)	0 (0–0)	0 (0–0)	0 (0–0)	0 (0–0)	0 (0–0)	0 (0–0)	0 (0–0)	0 (0–0)	--
Cocaine use disorders	--	--	--	--	--	0 (0–0)	0 (0–0)	0 (0–0)	0 (0–0)	0 (0–0)	0 (0–0)	0 (0–0)	0 (0–0)	0 (0–0)	0 (0–0)	0 (0–0)	0 (0–0)	0 (0–0)	0 (0–0)	0 (0–0)	0 (0–0)	0 (0–0)	--
Amphetamine use disorders	--	--	--	--	--	0 (0–0)	0 (0–0)	0 (0–0)	0 (0–0)	0 (0–0)	0 (0–0)	0 (0–0)	0 (0–0)	0 (0–0)	0 (0–0)	0 (0–0)	0 (0–0)	0 (0–0)	0 (0–0)	0 (0–0)	0 (0–0)	0 (0–0)	--
Other drug use disorders	--	--	--	--	--	0 (0–0)	0 (0–0)	0 (0–0)	0 (0–0)	0 (0–0)	0 (0–0)	0 (0–0)	0 (0–0)	0 (0–0)	0 (0–0)	0 (0–0)	0 (0–0)	0 (0–0)	0 (0–0)	0 (0–0)	0 (0–0)	0 (0–0)	--
Diabetes mellitus	0 (0–0)	0 (0–0)	0 (0–0)	0 (0–0)	0 (0–0)	0 (0–0)	0 (0–0)	0 (0–0)	0 (0–0)	0 (0–0)	0 (0–0)	0 (0–0)	1 (0–1)	1 (1–2)	2 (1–3)	3 (2–4)	3 (2–4)	3 (2–4)	4 (3–5)	3 (3–4)	2 (1–2)	1 (1–1)	--
Diabetes mellitus type 1	0 (0–0)	0 (0–0)	0 (0–0)	0 (0–0)	0 (0–0)	0 (0–0)	0 (0–0)	0 (0–0)	0 (0–0)	0 (0–0)	0 (0–0)	0 (0–0)	0 (0–0)	0 (0–0)	0 (0–0)	0 (0–0)	0 (0–0)	0 (0–0)	0 (0–0)	0 (0–0)	0 (0–0)	0 (0–0)	--
Diabetes mellitus type 2	--	--	--	--	--	--	0 (0–0)	0 (0–0)	0 (0–0)	0 (0–0)	0 (0–0)	0 (0–0)	1 (0–1)	1 (1–2)	2 (1–3)	3 (2–4)	3 (2–4)	3 (2–4)	3 (3–4)	3 (3–4)	2 (1–2)	1 (1–1)	--
Chronic kidney disease	--	--	0 (0–0)	0 (0–0)	0 (0–0)	0 (0–0)	0 (0–0)	0 (0–0)	0 (0–0)	0 (0–0)	0 (0–0)	0 (0–1)	1 (0–1)	1 (1–2)	2 (1–2)	2 (2–3)	2 (1–3)	2 (2–3)	2 (2–3)	3 (2–4)	2 (1–2)	1 (1–1)	--
Chronic kidney disease due to diabetes mellitus type 1	--	--	0 (0–0)	0 (0–0)	0 (0–0)	0 (0–0)	0 (0–0)	0 (0–0)	0 (0–0)	0 (0–0)	0 (0–0)	0 (0–0)	0 (0–0)	0 (0–0)	0 (0–0)	0 (0–0)	0 (0–0)	0 (0–0)	0 (0–0)	0 (0–0)	0 (0–0)	0 (0–0)	--
Chronic kidney disease due to diabetes mellitus type 2	--	--	--	--	--	--	0 (0–0)	0 (0–0)	0 (0–0)	0 (0–0)	0 (0–0)	0 (0–0)	0 (0–0)	0 (0–0)	0 (0–1)	1 (0–1)	1 (0–1)	1 (0–1)	1 (0–1)	1 (1–1)	0 (0–1)	0 (0–0)	--
Chronic kidney disease due to hypertension	--	--	--	--	--	--	0 (0–0)	0 (0–0)	0 (0–0)	0 (0–0)	0 (0–0)	0 (0–0)	0 (0–0)	0 (0–0)	0 (0–1)	0 (0–1)	0 (0–1)	1 (0–1)	1 (0–1)	1 (1–1)	1 (0–1)	0 (0–1)	--
Chronic kidney disease due to glomerulonephritis	--	--	0 (0–0)	0 (0–0)	0 (0–0)	0 (0–0)	0 (0–0)	0 (0–0)	0 (0–0)	0 (0–0)	0 (0–0)	0 (0–0)	0 (0–0)	0 (0–1)	0 (0–1)	1 (0–1)	0 (0–1)	0 (0–1)	0 (0–1)	0 (0–1)	0 (0–0)	0 (0–0)	--
Chronic kidney disease due to other and unspecified causes	--	--	0 (0–0)	0 (0–0)	0 (0–0)	0 (0–0)	0 (0–0)	0 (0–0)	0 (0–0)	0 (0–0)	0 (0–0)	0 (0–0)	0 (0–0)	0 (0–0)	0 (0–1)	0 (0–1)	0 (0–1)	0 (0–1)	0 (0–1)	1 (0–1)	0 (0–0)	0 (0–0)	--
Acute glomerulonephritis	--	--	0 (0–0)	0 (0–0)	0 (0–0)	0 (0–0)	0 (0–0)	0 (0–0)	0 (0–0)	0 (0–0)	0 (0–0)	0 (0–0)	0 (0–0)	0 (0–0)	0 (0–0)	0 (0–0)	0 (0–0)	0 (0–0)	0 (0–0)	0 (0–0)	0 (0–0)	0 (0–0)	--
Skin and subcutaneous diseases	--	--	0 (0–0)	0 (0–0)	0 (0–0)	0 (0–0)	0 (0–0)	0 (0–0)	0 (0–0)	0 (0–0)	0 (0–0)	0 (0–0)	0 (0–0)	0 (0–0)	0 (0–0)	0 (0–1)	0 (0–1)	0 (0–1)	0 (0–1)	1 (0–1)	0 (0–1)	0 (0–0)	--
Bacterial skin diseases	--	--	0 (0–0)	0 (0–0)	0 (0–0)	0 (0–0)	0 (0–0)	0 (0–0)	0 (0–0)	0 (0–0)	0 (0–0)	0 (0–0)	0 (0–0)	0 (0–0)	0 (0–0)	0 (0–0)	0 (0–0)	0 (0–0)	0 (0–0)	0 (0–0)	0 (0–0)	0 (0–0)	--
Cellulitis	--	--	0 (0–0)	0 (0–0)	0 (0–0)	0 (0–0)	0 (0–0)	0 (0–0)	0 (0–0)	0 (0–0)	0 (0–0)	0 (0–0)	0 (0–0)	0 (0–0)	0 (0–0)	0 (0–0)	0 (0–0)	0 (0–0)	0 (0–0)	0 (0–0)	0 (0–0)	0 (0–0)	--
Pyoderma	--	--	0 (0–0)	0 (0–0)	0 (0–0)	0 (0–0)	0 (0–0)	0 (0–0)	0 (0–0)	0 (0–0)	0 (0–0)	0 (0–0)	0 (0–0)	0 (0–0)	0 (0–0)	0 (0–0)	0 (0–0)	0 (0–0)	0 (0–0)	0 (0–0)	0 (0–0)	0 (0–0)	--
Decubitus ulcer	--	--	--	0 (0–0)	0 (0–0)	0 (0–0)	0 (0–0)	0 (0–0)	0 (0–0)	0 (0–0)	0 (0–0)	0 (0–0)	0 (0–0)	0 (0–0)	0 (0–0)	0 (0–0)	0 (0–0)	0 (0–1)	0 (0–1)	0 (0–1)	0 (0–1)	0 (0–0)	--
Other skin and subcutaneous diseases	--	--	0 (0–0)	0 (0–0)	0 (0–0)	0 (0–0)	0 (0–0)	0 (0–0)	0 (0–0)	0 (0–0)	0 (0–0)	0 (0–0)	0 (0–0)	0 (0–0)	0 (0–0)	0 (0–0)	0 (0–0)	0 (0–0)	0 (0–0)	0 (0–0)	0 (0–0)	0 (0–0)	--
Musculoskeletal disorders	--	--	--	--	0 (0–0)	0 (0–0)	0 (0–0)	0 (0–0)	0 (0–0)	0 (0–0)	0 (0–0)	0 (0–0)	0 (0–0)	0 (0–0)	0 (0–0)	0 (0–0)	0 (0–0)	0 (0–0)	0 (0–0)	0 (0–0)	0 (0–0)	0 (0–0)	--
Rheumatoid arthritis	--	--	--	--	0 (0–0)	0 (0–0)	0 (0–0)	0 (0–0)	0 (0–0)	0 (0–0)	0 (0–0)	0 (0–0)	0 (0–0)	0 (0–0)	0 (0–0)	0 (0–0)	0 (0–0)	0 (0–0)	0 (0–0)	0 (0–0)	0 (0–0)	0 (0–0)	--
Other musculoskeletal disorders	--	--	--	--	0 (0–0)	0 (0–0)	0 (0–0)	0 (0–0)	0 (0–0)	0 (0–0)	0 (0–0)	0 (0–0)	0 (0–0)	0 (0–0)	0 (0–0)	0 (0–0)	0 (0–0)	0 (0–0)	0 (0–0)	0 (0–0)	0 (0–0)	0 (0–0)	--
Congenital anomalies	0 (0–0)	0 (0–0)	0 (0–0)	0 (0–0)	0 (0–0)	0 (0–0)	0 (0–0)	0 (0–0)	0 (0–0)	0 (0–0)	0 (0–0)	0 (0–0)	0 (0–0)	0 (0–0)	0 (0–0)	0 (0–0)	0 (0–0)	--	--	--	--	--	--
Neural tube defects	0 (0–0)	0 (0–0)	0 (0–0)	0 (0–0)	0 (0–0)	0 (0–0)	0 (0–0)	0 (0–0)	0 (0–0)	0 (0–0)	0 (0–0)	0 (0–0)	0 (0–0)	0 (0–0)	0 (0–0)	0 (0–0)	0 (0–0)	--	--	--	--	--	--
Congenital heart anomalies	0 (0–0)	0 (0–0)	0 (0–0)	0 (0–0)	0 (0–0)	0 (0–0)	0 (0–0)	0 (0–0)	0 (0–0)	0 (0–0)	0 (0–0)	0 (0–0)	0 (0–0)	0 (0–0)	0 (0–0)	0 (0–0)	0 (0–0)	--	--	--	--	--	--
Orofacial clefts	0 (0–0)	0 (0–0)	0 (0–0)	0 (0–0)	--	--	--	--	--	--	--	--	--	--	--	--	--	--	--	--	--	--	--
Down's syndrome	0 (0–0)	0 (0–0)	0 (0–0)	0 (0–0)	0 (0–0)	0 (0–0)	0 (0–0)	0 (0–0)	0 (0–0)	0 (0–0)	0 (0–0)	0 (0–0)	0 (0–0)	0 (0–0)	0 (0–0)	0 (0–0)	0 (0–0)	--	--	--	--	--	--
Other chromosomal abnormalities	0 (0–0)	0 (0–0)	0 (0–0)	0 (0–0)	0 (0–0)	0 (0–0)	0 (0–0)	0 (0–0)	0 (0–0)	0 (0–0)	0 (0–0)	0 (0–0)	0 (0–0)	0 (0–0)	0 (0–0)	0 (0–0)	0 (0–0)	--	--	--	--	--	--
Congenital musculoskeletal and limb anomalies	0 (0–0)	0 (0–0)	0 (0–0)	0 (0–0)	0 (0–0)	0 (0–0)	0 (0–0)	0 (0–0)	0 (0–0)	0 (0–0)	0 (0–0)	0 (0–0)	0 (0–0)	0 (0–0)	0 (0–0)	0 (0–0)	0 (0–0)	--	--	--	--	--	--
Urogenital congenital anomalies	0 (0–0)	0 (0–0)	0 (0–0)	0 (0–0)	0 (0–0)	0 (0–0)	0 (0–0)	0 (0–0)	0 (0–0)	0 (0–0)	0 (0–0)	0 (0–0)	0 (0–0)	0 (0–0)	0 (0–0)	0 (0–0)	0 (0–0)	--	--	--	--	--	--
Digestive congenital anomalies	0 (0–0)	0 (0–0)	0 (0–0)	0 (0–0)	0 (0–0)	0 (0–0)	0 (0–0)	0 (0–0)	0 (0–0)	0 (0–0)	0 (0–0)	0 (0–0)	0 (0–0)	0 (0–0)	0 (0–0)	0 (0–0)	0 (0–0)	--	--	--	--	--	--
Other congenital anomalies	0 (0–0)	0 (0–0)	0 (0–0)	0 (0–0)	0 (0–0)	0 (0–0)	0 (0–0)	0 (0–0)	0 (0–0)	0 (0–0)	0 (0–0)	0 (0–0)	0 (0–0)	0 (0–0)	0 (0–0)	0 (0–0)	0 (0–0)	--	--	--	--	--	--
Urinary diseases and male infertility	0 (0–0)	0 (0–0)	0 (0–0)	0 (0–0)	0 (0–0)	0 (0–0)	0 (0–0)	0 (0–0)	0 (0–0)	0 (0–0)	0 (0–0)	0 (0–0)	0 (0–0)	0 (0–0)	0 (0–0)	0 (0–0)	0 (0–0)	0 (0–0)	0 (0–1)	0 (0–1)	0 (0–0)	0 (0–0)	--
Urinary tract infections and interstitial nephritis	0 (0–0)	0 (0–0)	0 (0–0)	0 (0–0)	0 (0–0)	0 (0–0)	0 (0–0)	0 (0–0)	0 (0–0)	0 (0–0)	0 (0–0)	0 (0–0)	0 (0–0)	0 (0–0)	0 (0–0)	0 (0–0)	0 (0–0)	0 (0–0)	0 (0–0)	0 (0–1)	0 (0–0)	0 (0–0)	--
Urolithiasis	--	--	--	0 (0–0)	0 (0–0)	0 (0–0)	0 (0–0)	0 (0–0)	0 (0–0)	0 (0–0)	0 (0–0)	0 (0–0)	0 (0–0)	0 (0–0)	0 (0–0)	0 (0–0)	0 (0–0)	0 (0–0)	0 (0–0)	0 (0–0)	0 (0–0)	0 (0–0)	--
Other urinary diseases	0 (0–0)	0 (0–0)	0 (0–0)	0 (0–0)	0 (0–0)	0 (0–0)	0 (0–0)	0 (0–0)	0 (0–0)	0 (0–0)	0 (0–0)	0 (0–0)	0 (0–0)	0 (0–0)	0 (0–0)	0 (0–0)	0 (0–0)	0 (0–0)	0 (0–0)	0 (0–0)	0 (0–0)	0 (0–0)	--
Gynecological diseases	--	--	--	--	--	--	--	--	--	--	--	--	--	--	--	--	--	--	--	--	--	--	--
Uterine fibroids	--	--	--	--	--	--	--	--	--	--	--	--	--	--	--	--	--	--	--	--	--	--	--
Endometriosis	--	--	--	--	--	--	--	--	--	--	--	--	--	--	--	--	--	--	--	--	--	--	--
Genital prolapse	--	--	--	--	--	--	--	--	--	--	--	--	--	--	--	--	--	--	--	--	--	--	--

Other gynecological diseases	--	--	--	--	--	--	--	--	--	--	--	--	--	--	--	--	--	--	--	--	--	--	--
Hemoglobinopathies and hemolytic anaemias	0 (0–0)	0 (0–0)	0 (0–0)	0 (0–0)	0 (0–0)	0 (0–0)	0 (0–0)	0 (0–0)	0 (0–0)	0 (0–0)	0 (0–0)	0 (0–0)	0 (0–0)	0 (0–0)	0 (0–0)	0 (0–0)	0 (0–0)	0 (0–0)	0 (0–0)	0 (0–0)	0 (0–0)	0 (0–0)	--
Thalassemias	0 (0–0)	0 (0–0)	0 (0–0)	0 (0–0)	0 (0–0)	0 (0–0)	0 (0–0)	0 (0–0)	0 (0–0)	0 (0–0)	0 (0–0)	0 (0–0)	0 (0–0)	0 (0–0)	0 (0–0)	0 (0–0)	0 (0–0)	0 (0–0)	0 (0–0)	0 (0–0)	0 (0–0)	0 (0–0)	--
Sickle cell disorders	0 (0–0)	0 (0–0)	0 (0–0)	0 (0–0)	0 (0–0)	0 (0–0)	0 (0–0)	0 (0–0)	0 (0–0)	0 (0–0)	0 (0–0)	0 (0–0)	0 (0–0)	0 (0–0)	0 (0–0)	0 (0–0)	0 (0–0)	0 (0–0)	0 (0–0)	0 (0–0)	0 (0–0)	0 (0–0)	--
G6PD deficiency	0 (0–0)	0 (0–0)	0 (0–0)	0 (0–0)	0 (0–0)	0 (0–0)	0 (0–0)	0 (0–0)	0 (0–0)	0 (0–0)	0 (0–0)	0 (0–0)	0 (0–0)	0 (0–0)	0 (0–0)	0 (0–0)	0 (0–0)	0 (0–0)	0 (0–0)	0 (0–0)	0 (0–0)	0 (0–0)	--
Other hemoglobinopathies and hemolytic anaemias	0 (0–0)	0 (0–0)	0 (0–0)	0 (0–0)	0 (0–0)	0 (0–0)	0 (0–0)	0 (0–0)	0 (0–0)	0 (0–0)	0 (0–0)	0 (0–0)	0 (0–0)	0 (0–0)	0 (0–0)	0 (0–0)	0 (0–0)	0 (0–0)	0 (0–0)	0 (0–0)	0 (0–0)	0 (0–0)	--
Endocrine, metabolic, blood, and immune disorders	0 (0–0)	0 (0–0)	0 (0–0)	0 (0–0)	0 (0–0)	0 (0–0)	0 (0–0)	0 (0–0)	0 (0–0)	0 (0–0)	0 (0–0)	0 (0–0)	0 (0–0)	0 (0–0)	0 (0–0)	0 (0–1)	0 (0–0)	0 (0–1)	0 (0–0)	0 (0–0)	0 (0–0)	0 (0–0)	--
Sudden infant death syndrome	--	0 (0–0)	0 (0–0)	--	--	--	--	--	--	--	--	--	--	--	--	--	--	--	--	--	--	--	--
Transport injuries	0 (0–0)	0 (0–0)	0 (0–0)	0 (0–0)	0 (0–0)	0 (0–0)	0 (0–0)	1 (1–1)	1 (0–1)	0 (0–1)	0 (0–1)	0 (0–0)	0 (0–1)	0 (0–1)	0 (0–1)	0 (0–1)	0 (0–0)	0 (0–0)	0 (0–0)	0 (0–0)	0 (0–0)	0 (0–0)	--
Road injuries	0 (0–0)	0 (0–0)	0 (0–0)	0 (0–0)	0 (0–0)	0 (0–0)	0 (0–0)	1 (0–1)	1 (0–1)	0 (0–1)	0 (0–1)	0 (0–0)	0 (0–0)	0 (0–1)	0 (0–1)	0 (0–0)	0 (0–0)	0 (0–0)	0 (0–0)	0 (0–0)	0 (0–0)	0 (0–0)	--
Pedestrian road injuries	0 (0–0)	0 (0–0)	0 (0–0)	0 (0–0)	0 (0–0)	0 (0–0)	0 (0–0)	0 (0–0)	0 (0–0)	0 (0–0)	0 (0–0)	0 (0–0)	0 (0–0)	0 (0–0)	0 (0–0)	0 (0–0)	0 (0–0)	0 (0–0)	0 (0–0)	0 (0–0)	0 (0–0)	0 (0–0)	--
Cyclist road injuries	--	--	--	0 (0–0)	0 (0–0)	0 (0–0)	0 (0–0)	0 (0–0)	0 (0–0)	0 (0–0)	0 (0–0)	0 (0–0)	0 (0–0)	0 (0–0)	0 (0–0)	0 (0–0)	0 (0–0)	0 (0–0)	0 (0–0)	0 (0–0)	0 (0–0)	0 (0–0)	--
Motorcyclist road injuries	0 (0–0)	0 (0–0)	0 (0–0)	0 (0–0)	0 (0–0)	0 (0–0)	0 (0–0)	0 (0–0)	0 (0–0)	0 (0–0)	0 (0–0)	0 (0–0)	0 (0–0)	0 (0–0)	0 (0–0)	0 (0–0)	0 (0–0)	0 (0–0)	0 (0–0)	0 (0–0)	0 (0–0)	0 (0–0)	--
Motor vehicle road injuries	0 (0–0)	0 (0–0)	0 (0–0)	0 (0–0)	0 (0–0)	0 (0–0)	0 (0–0)	0 (0–1)	0 (0–0)	0 (0–0)	0 (0–0)	0 (0–0)	0 (0–0)	0 (0–0)	0 (0–0)	0 (0–0)	0 (0–0)	0 (0–0)	0 (0–0)	0 (0–0)	0 (0–0)	0 (0–0)	--
Other road injuries	0 (0–0)	0 (0–0)	0 (0–0)	0 (0–0)	0 (0–0)	0 (0–0)	0 (0–0)	0 (0–0)	0 (0–0)	0 (0–0)	0 (0–0)	0 (0–0)	0 (0–0)	0 (0–0)	0 (0–0)	0 (0–0)	0 (0–0)	0 (0–0)	0 (0–0)	0 (0–0)	0 (0–0)	0 (0–0)	--
Other transport injuries	0 (0–0)	0 (0–0)	0 (0–0)	0 (0–0)	0 (0–0)	0 (0–0)	0 (0–0)	0 (0–0)	0 (0–0)	0 (0–0)	0 (0–0)	0 (0–0)	0 (0–0)	0 (0–0)	0 (0–0)	0 (0–0)	0 (0–0)	0 (0–0)	0 (0–0)	0 (0–0)	0 (0–0)	0 (0–0)	--
Falls	0 (0–0)	0 (0–0)	0 (0–0)	0 (0–0)	0 (0–0)	0 (0–0)	0 (0–0)	0 (0–0)	0 (0–0)	0 (0–0)	0 (0–0)	0 (0–0)	0 (0–0)	0 (0–0)	0 (0–0)	0 (0–0)	0 (0–0)	0 (0–0)	0 (0–0)	0 (0–0)	0 (0–0)	0 (0–0)	--
Drowning	0 (0–0)	0 (0–0)	0 (0–0)	0 (0–0)	0 (0–0)	0 (0–0)	0 (0–0)	0 (0–1)	0 (0–0)	0 (0–0)	0 (0–0)	0 (0–0)	0 (0–0)	0 (0–0)	0 (0–0)	0 (0–0)	0 (0–0)	0 (0–0)	0 (0–0)	0 (0–0)	0 (0–0)	0 (0–0)	--
Fire, heat, and hot substances	0 (0–0)	0 (0–0)	0 (0–0)	0 (0–0)	0 (0–0)	0 (0–0)	0 (0–0)	0 (0–0)	0 (0–0)	0 (0–0)	0 (0–0)	0 (0–0)	0 (0–0)	0 (0–0)	0 (0–0)	0 (0–0)	0 (0–0)	0 (0–0)	0 (0–0)	0 (0–0)	0 (0–0)	0 (0–0)	--
Poisonings	0 (0–0)	0 (0–0)	0 (0–0)	0 (0–0)	0 (0–0)	0 (0–0)	0 (0–0)	0 (0–0)	0 (0–0)	0 (0–0)	0 (0–0)	0 (0–0)	0 (0–0)	0 (0–0)	0 (0–0)	0 (0–0)	0 (0–0)	0 (0–0)	0 (0–0)	0 (0–0)	0 (0–0)	0 (0–0)	--
Poisoning by carbon monoxide	0 (0–0)	0 (0–0)	0 (0–0)	0 (0–0)	0 (0–0)	0 (0–0)	0 (0–0)	0 (0–0)	0 (0–0)	0 (0–0)	0 (0–0)	0 (0–0)	0 (0–0)	0 (0–0)	0 (0–0)	0 (0–0)	0 (0–0)	0 (0–0)	0 (0–0)	0 (0–0)	0 (0–0)	0 (0–0)	--
Poisoning by other means	0 (0–0)	0 (0–0)	0 (0–0)	0 (0–0)	0 (0–0)	0 (0–0)	0 (0–0)	0 (0–0)	0 (0–0)	0 (0–0)	0 (0–0)	0 (0–0)	0 (0–0)	0 (0–0)	0 (0–0)	0 (0–0)	0 (0–0)	0 (0–0)	0 (0–0)	0 (0–0)	0 (0–0)	0 (0–0)	--
Exposure to mechanical forces	0 (0–0)	0 (0–0)	0 (0–0)	0 (0–0)	0 (0–0)	0 (0–0)	0 (0–0)	1 (0–1)	1 (0–1)	0 (0–1)	0 (0–1)	0 (0–0)	0 (0–0)	0 (0–1)	0 (0–0)	0 (0–0)	0 (0–0)	0 (0–0)	0 (0–0)	0 (0–0)	0 (0–0)	0 (0–0)	--
Unintentional firearm injuries	0 (0–0)	0 (0–0)	0 (0–0)	0 (0–0)	0 (0–0)	0 (0–0)	0 (0–0)	1 (0–1)	1 (0–1)	0 (0–0)	0 (0–0)	0 (0–0)	0 (0–0)	0 (0–0)	0 (0–0)	0 (0–0)	0 (0–0)	0 (0–0)	0 (0–0)	0 (0–0)	0 (0–0)	0 (0–0)	--
Other exposure to mechanical forces	0 (0–0)	0 (0–0)	0 (0–0)	0 (0–0)	0 (0–0)	0 (0–0)	0 (0–0)	0 (0–0)	0 (0–0)	0 (0–0)	0 (0–0)	0 (0–0)	0 (0–0)	0 (0–0)	0 (0–0)	0 (0–0)	0 (0–0)	0 (0–0)	0 (0–0)	0 (0–0)	0 (0–0)	0 (0–0)	--
Adverse effects of medical treatment	0 (0–0)	0 (0–0)	0 (0–0)	0 (0–0)	0 (0–0)	0 (0–0)	0 (0–0)	0 (0–0)	0 (0–0)	0 (0–0)	0 (0–0)	0 (0–0)	0 (0–0)	0 (0–0)	0 (0–0)	0 (0–0)	0 (0–0)	0 (0–0)	0 (0–0)	0 (0–0)	0 (0–0)	0 (0–0)	--
Animal contact	0 (0–0)	0 (0–0)	0 (0–0)	0 (0–0)	0 (0–0)	0 (0–0)	0 (0–0)	0 (0–0)	0 (0–0)	0 (0–0)	0 (0–0)	0 (0–0)	0 (0–0)	0 (0–0)	0 (0–0)	0 (0–0)	0 (0–0)	0 (0–0)	0 (0–0)	0 (0–0)	0 (0–0)	0 (0–0)	--
Venomous animal contact	0 (0–0)	0 (0–0)	0 (0–0)	0 (0–0)	0 (0–0)	0 (0–0)	0 (0–0)	0 (0–0)	0 (0–0)	0 (0–0)	0 (0–0)	0 (0–0)	0 (0–0)	0 (0–0)	0 (0–0)	0 (0–0)	0 (0–0)	0 (0–0)	0 (0–0)	0 (0–0)	0 (0–0)	0 (0–0)	--
Non-venomous animal contact	0 (0–0)	0 (0–0)	0 (0–0)	0 (0–0)	0 (0–0)	0 (0–0)	0 (0–0)	0 (0–0)	0 (0–0)	0 (0–0)	0 (0–0)	0 (0–0)	0 (0–0)	0 (0–0)	0 (0–0)	0 (0–0)	0 (0–0)	0 (0–0)	0 (0–0)	0 (0–0)	0 (0–0)	0 (0–0)	--
Foreign body	0 (0–0)	0 (0–0)	0 (0–0)	0 (0–0)	0 (0–0)	0 (0–0)	0 (0–0)	0 (0–0)	0 (0–0)	0 (0–0)	0 (0–0)	0 (0–0)	0 (0–0)	0 (0–0)	0 (0–0)	0 (0–0)	0 (0–0)	0 (0–0)	0 (0–0)	0 (0–0)	0 (0–0)	0 (0–0)	--
Pulmonary aspiration and foreign body in airway	0 (0–0)	0 (0–0)	0 (0–0)	0 (0–0)	0 (0–0)	0 (0–0)	0 (0–0)	0 (0–0)	0 (0–0)	0 (0–0)	0 (0–0)	0 (0–0)	0 (0–0)	0 (0–0)	0 (0–0)	0 (0–0)	0 (0–0)	0 (0–0)	0 (0–0)	0 (0–0)	0 (0–0)	0 (0–0)	--
Foreign body in other body part	0 (0–0)	0 (0–0)	0 (0–0)	0 (0–0)	0 (0–0)	0 (0–0)	0 (0–0)	0 (0–0)	0 (0–0)	0 (0–0)	0 (0–0)	0 (0–0)	0 (0–0)	0 (0–0)	0 (0–0)	0 (0–0)	0 (0–0)	0 (0–0)	0 (0–0)	0 (0–0)	0 (0–0)	0 (0–0)	--
Environmental heat and cold exposure	0 (0–0)	0 (0–0)	0 (0–0)	0 (0–0)	0 (0–0)	0 (0–0)	0 (0–0)	0 (0–0)	0 (0–0)	0 (0–0)	0 (0–0)	0 (0–0)	0 (0–0)	0 (0–0)	0 (0–0)	0 (0–0)	0 (0–0)	0 (0–0)	0 (0–0)	0 (0–0)	0 (0–0)	0 (0–0)	--
Other unintentional injuries	0 (0–0)	0 (0–0)	0 (0–0)	0 (0–0)	0 (0–0)	0 (0–0)	0 (0–0)	0 (0–0)	0 (0–0)	0 (0–0)	0 (0–0)	0 (0–0)	0 (0–0)	0 (0–0)	0 (0–0)	0 (0–0)	0 (0–0)	0 (0–0)	0 (0–0)	0 (0–0)	0 (0–0)	0 (0–0)	--
Self-harm	--	--	--	--	--	0 (0–0)	0 (0–0)	0 (0–0)	0 (0–1)	0 (0–0)	0 (0–0)	0 (0–0)	0 (0–0)	0 (0–0)	0 (0–0)	0 (0–0)	0 (0–0)	0 (0–0)	0 (0–0)	0 (0–0)	0 (0–0)	0 (0–0)	--
Self-harm by firearm	--	--	--	--	--	0 (0–0)	0 (0–0)	0 (0–0)	0 (0–0)	0 (0–0)	0 (0–0)	0 (0–0)	0 (0–0)	0 (0–0)	0 (0–0)	0 (0–0)	0 (0–0)	0 (0–0)	0 (0–0)	0 (0–0)	0 (0–0)	0 (0–0)	--
Self-harm by other specified means	--	--	--	--	--	0 (0–0)	0 (0–0)	0 (0–0)	0 (0–1)	0 (0–0)	0 (0–0)	0 (0–0)	0 (0–0)	0 (0–0)	0 (0–0)	0 (0–0)	0 (0–0)	0 (0–0)	0 (0–0)	0 (0–0)	0 (0–0)	0 (0–0)	--
Interpersonal violence	0 (0–0)	0 (0–0)	0 (0–0)	0 (0–0)	0 (0–0)	0 (0–0)	0 (0–0)	1 (1–1)	1 (1–1)	0 (0–1)	0 (0–0)	0 (0–0)	0 (0–0)	0 (0–0)	0 (0–0)	0 (0–0)	0 (0–0)	0 (0–0)	0 (0–0)	0 (0–0)	0 (0–0)	0 (0–0)	--
Physical violence by firearm	0 (0–0)	0 (0–0)	0 (0–0)	0 (0–0)	0 (0–0)	0 (0–0)	0 (0–0)	0 (0–1)	0 (0–0)	0 (0–0)	0 (0–0)	0 (0–0)	0 (0–0)	0 (0–0)	0 (0–0)	0 (0–0)	0 (0–0)	0 (0–0)	0 (0–0)	0 (0–0)	0 (0–0)	0 (0–0)	--
Physical violence by sharp object	0 (0–0)	0 (0–0)	0 (0–0)	0 (0–0)	0 (0–0)	0 (0–0)	0 (0–0)	0 (0–0)	0 (0–0)	0 (0–0)	0 (0–0)	0 (0–0)	0 (0–0)	0 (0–0)	0 (0–0)	0 (0–0)	0 (0–0)	0 (0–0)	0 (0–0)	0 (0–0)	0 (0–0)	0 (0–0)	--
Physical violence by other means	0 (0–0)	0 (0–0)	0 (0–0)	0 (0–0)	0 (0–0)	0 (0–0)	0 (0–0)	0 (0–0)	0 (0–0)	0 (0–0)	0 (0–0)	0 (0–0)	0 (0–0)	0 (0–0)	0 (0–0)	0 (0–0)	0 (0–0)	0 (0–0)	0 (0–0)	0 (0–0)	0 (0–0)	0 (0–0)	--
Executions and police conflict	0 (0–0)	0 (0–0)	0 (0–0)	0 (0–0)	0 (0–0)	0 (0–0)	0 (0–0)	0 (0–0)	0 (0–0)	0 (0–0)	0 (0–0)	0 (0–0)	0 (0–0)	0 (0–0)	0 (0–0)	0 (0–0)	0 (0–0)	0 (0–0)	0 (0–0)	0 (0–0)	0 (0–0)	0 (0–0)	--

SR Table 12: The Bahamas deaths by cause and age group for males

Cause	Early Neonatal	Late Neonatal	Post Neonatal	1 to 4	5 to 9	10 to 14	15 to 19	20 to 24	25 to 29	30 to 34	35 to 39	40 to 44	45 to 49	50 to 54	55 to 59	60 to 64	65 to 69	70 to 74	75 to 79	80 to 84	85 to 89	90 to 94	95 plus
All causes	9 (7–11)	4 (2–7)	8 (6–9)	6 (4–8)	5 (5–6)	8 (7–9)	19 (16–23)	40 (35–47)	51 (46–58)	56 (49–64)	68 (59–80)	83 (69–100)	99 (81–123)	121 (98–151)	136 (110–171)	146 (119–180)	141 (117–172)	132 (110–158)	131 (112–155)	117 (101–136)	85 (76–95)	50 (46–55)	--
Sexually transmitted infections excluding HIV	0 (0–0)	0 (0–0)	0 (0–0)	0 (0–0)	0 (0–0)	0 (0–0)	0 (0–0)	0 (0–0)	0 (0–0)	0 (0–0)	0 (0–0)	0 (0–0)	0 (0–0)	0 (0–0)	0 (0–0)	0 (0–0)	0 (0–0)	0 (0–0)	0 (0–0)	0 (0–0)	0 (0–0)	0 (0–0)	--
Syphilis	0 (0–0)	0 (0–0)	0 (0–0)	0 (0–0)	0 (0–0)	0 (0–0)	0 (0–0)	0 (0–0)	0 (0–0)	0 (0–0)	0 (0–0)	0 (0–0)	0 (0–0)	0 (0–0)	0 (0–0)	0 (0–0)	0 (0–0)	0 (0–0)	0 (0–0)	0 (0–0)	0 (0–0)	0 (0–0)	--
Chlamydial infection	--	--	--	--	--	0 (0–0)	0 (0–0)	0 (0–0)	0 (0–0)	0 (0–0)	0 (0–0)	0 (0–0)	0 (0–0)	0 (0–0)	0 (0–0)	0 (0–0)	0 (0–0)	0 (0–0)	0 (0–0)	0 (0–0)	0 (0–0)	0 (0–0)	--
Gonococcal infection	--	--	--	--	--	0 (0–0)	0 (0–0)	0 (0–0)	0 (0–0)	0 (0–0)	0 (0–0)	0 (0–0)	0 (0–0)	0 (0–0)	0 (0–0)	0 (0–0)	0 (0–0)	0 (0–0)	0 (0–0)	0 (0–0)	0 (0–0)	0 (0–0)	--
Other sexually transmitted infections	--	--	--	--	--	0 (0–0)	0 (0–0)	0 (0–0)	0 (0–0)	0 (0–0)	0 (0–0)	0 (0–0)	0 (0–0)	0 (0–0)	0 (0–0)	0 (0–0)	0 (0–0)	0 (0–0)	0 (0–0)	0 (0–0)	0 (0–0)	0 (0–0)	--
Tuberculosis	--	--	0 (0–0)	0 (0–0)	0 (0–0)	0 (0–0)	0 (0–0)	0 (0–0)	0 (0–0)	0 (0–0)	0 (0–1)	0 (0–1)	0 (0–1)	1 (0–1)	1 (0–1)	0 (0–1)	0 (0–1)	0 (0–1)	0 (0–0)	0 (0–0)	0 (0–0)	0 (0–0)	--
Drug-susceptible tuberculosis	--	--	0 (0–0)	0 (0–0)	0 (0–0)	0 (0–0)	0 (0–0)	0 (0–0)	0 (0–0)	0 (0–0)	0 (0–1)	0 (0–1)	0 (0–1)	1 (0–1)	0 (0–1)	0 (0–1)	0 (0–1)	0 (0–0)	0 (0–0)	0 (0–0)	0 (0–0)	0 (0–0)	--
Multidrug-resistant tuberculosis without extensive drug resistance	--	--	0 (0–0)	0 (0–0)	0 (0–0)	0 (0–0)	0 (0–0)	0 (0–0)	0 (0–0)	0 (0–0)	0 (0–0)	0 (0–0)	0 (0–0)	0 (0–0)	0 (0–0)	0 (0–0)	0 (0–0)	0 (0–0)	0 (0–0)	0 (0–0)	0 (0–0)	0 (0–0)	--
Extensively drug-resistant tuberculosis	--	--	0 (0–0)	0 (0–0)	0 (0–0)	0 (0–0)	0 (0–0)	0 (0–0)	0 (0–0)	0 (0–0)	0 (0–0)	0 (0–0)	0 (0–0)	0 (0–0)	0 (0–0)	0 (0–0)	0 (0–0)	0 (0–0)	0 (0–0)	0 (0–0)	0 (0–0)	0 (0–0)	--
Lower respiratory infections	0 (0–0)	0 (0–1)	1 (1–2)	1 (0–1)	0 (0–0)	0 (0–0)	0 (0–0)	1 (0–1)	1 (1–1)	1 (1–2)	1 (1–2)	2 (1–3)	2 (1–3)	3 (2–4)	3 (2–4)	3 (2–5)	4 (3–6)	5 (3–7)	5 (4–7)	6 (4–8)	5 (4–7)	4 (3–5)	--
Upper respiratory infections	0 (0–0)	0 (0–0)	0 (0–0)	0 (0–0)	0 (0–0)	0 (0–0)	0 (0–0)	0 (0–0)	0 (0–0)	0 (0–0)	0 (0–0)	0 (0–0)	0 (0–0)	0 (0–0)	0 (0–0)	0 (0–0)	0 (0–0)	0 (0–0)	0 (0–0)	0 (0–0)	0 (0–0)	0 (0–0)	--
Otitis media	0 (0–0)	0 (0–0)	0 (0–0)	0 (0–0)	0 (0–0)	0 (0–0)	0 (0–0)	0 (0–0)	0 (0–0)	0 (0–0)	0 (0–0)	0 (0–0)	0 (0–0)	0 (0–0)	0 (0–0)	0 (0–0)	0 (0–0)	0 (0–0)	0 (0–0)	0 (0–0)	0 (0–0)	0 (0–0)	--
Diarrhoeal diseases	0 (0–0)	0 (0–0)	0 (0–0)	0 (0–0)	0 (0–0)	0 (0–0)	0 (0–0)	0 (0–0)	0 (0–0)	0 (0–0)	0 (0–0)	0 (0–0)	0 (0–0)	0 (0–0)	0 (0–0)	0 (0–0)	0 (0–0)	0 (0–0)	0 (0–0)	0 (0–0)	0 (0–0)	0 (0–0)	--
Typhoid fever	--	--	0 (0–0)	0 (0–0)	0 (0–0)	0 (0–0)	0 (0–0)	0 (0–0)	0 (0–0)	0 (0–0)	0 (0–0)	0 (0–0)	0 (0–0)	0 (0–0)	0 (0–0)	0 (0–0)	0 (0–0)	0 (0–0)	0 (0–0)	0 (0–0)	0 (0–0)	0 (0–0)	--
Paratyphoid fever	--	--	0 (0–0)	0 (0–0)	0 (0–0)	0 (0–0)	0 (0–0)	0 (0–0)	0 (0–0)	0 (0–0)	0 (0–0)	0 (0–0)	0 (0–0)	0 (0–0)	0 (0–0)	0 (0–0)	0 (0–0)	0 (0–0)	0 (0–0)	0 (0–0)	0 (0–0)	0 (0–0)	--
iNTS	--	0 (0–0)	0 (0–0)	0 (0–0)	0 (0–0)	0 (0–0)	0 (0–0)	0 (0–0)	0 (0–0)	0 (0–0)	0 (0–0)	0 (0–0)	0 (0–0)	0 (0–0)	0 (0–0)	0 (0–0)	0 (0–0)	0 (0–0)	0 (0–0)	0 (0–0)	0 (0–0)	0 (0–0)	--
Other intestinal infectious diseases	--	--	0 (0–0)	0 (0–0)	0 (0–0)	0 (0–0)	0 (0–0)	0 (0–0)	0 (0–0)	0 (0–0)	0 (0–0)	0 (0–0)	0 (0–0)	0 (0–0)	0 (0–0)	0 (0–0)	0 (0–0)	0 (0–0)	0 (0–0)	0 (0–0)	0 (0–0)	0 (0–0)	--
Malaria	0 (0–0)	0 (0–0)	0 (0–0)	0 (0–0)	0 (0–0)	0 (0–0)	0 (0–0)	0 (0–0)	0 (0–0)	0 (0–0)	0 (0–0)	0 (0–0)	0 (0–0)	0 (0–0)	0 (0–0)	0 (0–0)	0 (0–0)	0 (0–0)	0 (0–0)	0 (0–0)	0 (0–0)	0 (0–0)	--
Chagas disease	--	--	0 (0–0)	0 (0–0)	0 (0–0)	0 (0–0)	0 (0–0)	0 (0–0)	0 (0–0)	0 (0–0)	0 (0–0)	0 (0–0)	0 (0–0)	0 (0–0)	0 (0–0)	0 (0–0)	0 (0–0)	0 (0–0)	0 (0–0)	0 (0–0)	0 (0–0)	0 (0–0)	--
Visceral leishmaniasis	--	--	0 (0–0)	0 (0–0)	0 (0–0)	0 (0–0)	0 (0–0)	0 (0–0)	0 (0–0)	0 (0–0)	0 (0–0)	0 (0–0)	0 (0–0)	0 (0–0)	0 (0–0)	0 (0–0)	0 (0–0)	0 (0–0)	0 (0–0)	0 (0–0)	0 (0–0)	0 (0–0)	--
African trypanosomiasis	--	--	--	0 (0–0)	0 (0–0)	0 (0–0)	0 (0–0)	0 (0–0)	0 (0–0)	0 (0–0)	0 (0–0)	0 (0–0)	0 (0–0)	0 (0–0)	0 (0–0)	0 (0–0)	0 (0–0)	0 (0–0)	0 (0–0)	0 (0–0)	0 (0–0)	0 (0–0)	--
Schistosomiasis	--	--	0 (0–0)	0 (0–0)	0 (0–0)	0 (0–0)	0 (0–0)	0 (0–0)	0 (0–0)	0 (0–0)	0 (0–0)	0 (0–0)	0 (0–0)	0 (0–0)	0 (0–0)	0 (0–0)	0 (0–0)	0 (0–0)	0 (0–0)	0 (0–0)	0 (0–0)	0 (0–0)	--
Cysticercosis	--	--	--	0 (0–0)	0 (0–0)	0 (0–0)	0 (0–0)	0 (0–0)	0 (0–0)	0 (0–0)	0 (0–0)	0 (0–0)	0 (0–0)	0 (0–0)	0 (0–0)	0 (0–0)	0 (0–0)	0 (0–0)	0 (0–0)	0 (0–0)	0 (0–0)	0 (0–0)	--
Cystic echinococcosis	--	--	--	0 (0–0)	0 (0–0)	0 (0–0)	0 (0–0)	0 (0–0)	0 (0–0)	0 (0–0)	0 (0–0)	0 (0–0)	0 (0–0)	0 (0–0)	0 (0–0)	0 (0–0)	0 (0–0)	0 (0–0)	0 (0–0)	0 (0–0)	0 (0–0)	0 (0–0)	--
Dengue	0 (0–0)	0 (0–0)	0 (0–0)	0 (0–0)	0 (0–0)	0 (0–0)	0 (0–0)	0 (0–0)	0 (0–0)	0 (0–0)	0 (0–0)	0 (0–0)	0 (0–0)	0 (0–0)	0 (0–0)	0 (0–0)	0 (0–0)	0 (0–0)	0 (0–0)	0 (0–0)	0 (0–0)	0 (0–0)	--
Yellow fever	--	0 (0–0)	0 (0–0)	0 (0–0)	0 (0–0)	0 (0–0)	0 (0–0)	0 (0–0)	0 (0–0)	0 (0–0)	0 (0–0)	0 (0–0)	0 (0–0)	0 (0–0)	0 (0–0)	0 (0–0)	0 (0–0)	0 (0–0)	0 (0–0)	0 (0–0)	0 (0–0)	0 (0–0)	--
Rabies	--	--	0 (0–0)	0 (0–0)	0 (0–0)	0 (0–0)	0 (0–0)	0 (0–0)	0 (0–0)	0 (0–0)	0 (0–0)	0 (0–0)	0 (0–0)	0 (0–0)	0 (0–0)	0 (0–0)	0 (0–0)	0 (0–0)	0 (0–0)	0 (0–0)	0 (0–0)	0 (0–0)	--
Ascariasis	--	--	0 (0–0)	0 (0–0)	0 (0–0)	0 (0–0)	0 (0–0)	0 (0–0)	0 (0–0)	0 (0–0)	0 (0–0)	0 (0–0)	0 (0–0)	0 (0–0)	0 (0–0)	0 (0–0)	0 (0–0)	0 (0–0)	0 (0–0)	0 (0–0)	0 (0–0)	0 (0–0)	--
Zika virus disease	0 (0–0)	0 (0–0)	0 (0–0)	0 (0–0)	0 (0–0)	0 (0–0)	0 (0–0)	0 (0–0)	0 (0–0)	0 (0–0)	0 (0–0)	0 (0–0)	0 (0–0)	0 (0–0)	0 (0–0)	0 (0–0)	0 (0–0)	0 (0–0)	0 (0–0)	0 (0–0)	0 (0–0)	0 (0–0)	--
Other neglected tropical diseases	0 (0–0)	0 (0–0)	0 (0–0)	0 (0–0)	0 (0–0)	0 (0–0)	0 (0–0)	0 (0–0)	0 (0–0)	0 (0–0)	0 (0–0)	0 (0–0)	0 (0–0)	0 (0–0)	0 (0–0)	0 (0–0)	0 (0–0)	0 (0–0)	0 (0–0)	0 (0–0)	0 (0–0)	0 (0–0)	--
Meningitis	0 (0–0)	0 (0–0)	0 (0–0)	0 (0–0)	0 (0–0)	0 (0–0)	0 (0–0)	0 (0–0)	0 (0–0)	0 (0–0)	0 (0–0)	0 (0–0)	0 (0–0)	0 (0–0)	0 (0–0)	0 (0–0)	0 (0–0)	0 (0–0)	0 (0–0)	0 (0–0)	0 (0–0)	0 (0–0)	--
Encephalitis	0 (0–0)	0 (0–0)	0 (0–0)	0 (0–0)	0 (0–0)	0 (0–0)	0 (0–0)	0 (0–0)	0 (0–0)	0 (0–0)	0 (0–0)	0 (0–0)	0 (0–0)	0 (0–0)	0 (0–0)	0 (0–0)	0 (0–0)	0 (0–0)	0 (0–0)	0 (0–0)	0 (0–0)	0 (0–0)	--
Diphtheria	--	--	0 (0–0)	0 (0–0)	0 (0–0)	0 (0–0)	0 (0–0)	0 (0–0)	0 (0–0)	0 (0–0)	0 (0–0)	0 (0–0)	0 (0–0)	0 (0–0)	0 (0–0)	--	--	--	--	--	--	--	--
Whooping cough	--	--	0 (0–0)	0 (0–0)	0 (0–0)	0 (0–0)	0 (0–0)	0 (0–0)	0 (0–0)	0 (0–0)	0 (0–0)	0 (0–0)	0 (0–0)	0 (0–0)	0 (0–0)	--	--	--	--	--	--	--	--
Tetanus	0 (0–0)	0 (0–0)	0 (0–0)	0 (0–0)	0 (0–0)	0 (0–0)	0 (0–0)	0 (0–0)	0 (0–0)	0 (0–0)	0 (0–0)	0 (0–0)	0 (0–0)	0 (0–0)	0 (0–0)	0 (0–0)	0 (0–0)	0 (0–0)	0 (0–0)	0 (0–0)	0 (0–0)	0 (0–0)	--
Measles	0 (0–0)	0 (0–0)	0 (0–0)	0 (0–0)	0 (0–0)	0 (0–0)	0 (0–0)	0 (0–0)	0 (0–0)	0 (0–0)	0 (0–0)	0 (0–0)	0 (0–0)	0 (0–0)	0 (0–0)	0 (0–0)	0 (0–0)	0 (0–0)	0 (0–0)	0 (0–0)	0 (0–0)	0 (0–0)	--
Varicella and herpes zoster	0 (0–0)	0 (0–0)	0 (0–0)	0 (0–0)	0 (0–0)	0 (0–0)	0 (0–0)	0 (0–0)	0 (0–0)	0 (0–0)	0 (0–0)	0 (0–0)	0 (0–0)	0 (0–0)	0 (0–0)	0 (0–0)	0 (0–0)	0 (0–0)	0 (0–0)	0 (0–0)	0 (0–0)	0 (0–0)	--
Acute hepatitis	--	--	0 (0–0)	0 (0–0)	0 (0–0)	0 (0–0)	0 (0–0)	0 (0–0)	0 (0–0)	0 (0–0)	0 (0–0)	0 (0–0)	0 (0–0)	0 (0–0)	0 (0–0)	0 (0–0)	0 (0–0)	0 (0–0)	0 (0–0)	0 (0–0)	0 (0–0)	0 (0–0)	--
Acute hepatitis A	--	--	0 (0–0)	0 (0–0)	0 (0–0)	0 (0–0)	0 (0–0)	0 (0–0)	0 (0–0)	0 (0–0)	0 (0–0)	0 (0–0)	0 (0–0)	0 (0–0)	0 (0–0)	0 (0–0)	0 (0–0)	0 (0–0)	0 (0–0)	0 (0–0)	0 (0–0)	0 (0–0)	--
Acute hepatitis B	--	--	0 (0–0)	0 (0–0)	0 (0–0)	0 (0–0)	0 (0–0)	0 (0–0)	0 (0–0)	0 (0–0)	0 (0–0)	0 (0–0)	0 (0–0)	0 (0–0)	0 (0–0)	0 (0–0)	0 (0–0)	0 (0–0)	0 (0–0)	0 (0–0)	0 (0–0)	0 (0–0)	--
Acute hepatitis C	--	--	0 (0–0)	0 (0–0)	0 (0–0)	0 (0–0)	0 (0–0)	0 (0–0)	0 (0–0)	0 (0–0)	0 (0–0)	0 (0–0)	0 (0–0)	0 (0–0)	0 (0–0)	0 (0–0)	0 (0–0)	0 (0–0)	0 (0–0)	0 (0–0)	0 (0–0)	0 (0–0)	--
Acute hepatitis E	--	--	0 (0–0)	0 (0–0)	0 (0–0)	0 (0–0)	0 (0–0)	0 (0–0)	0 (0–0)	0 (0–0)	0 (0–0)	0 (0–0)	0 (0–0)	0 (0–0)	0 (0–0)	0 (0–0)	0 (0–0)	0 (0–0)	0 (0–0)	0 (0–0)	0 (0–0)	0 (0–0)	--
Other unspecified infectious diseases	0 (0–0)	0 (0–0)	0 (0–0)	0 (0–0)	0 (0–0)	0 (0–0)	0 (0–0)	0 (0–0)	0 (0–0)	0 (0–0)	0 (0–0)	0 (0–0)	0 (0–0)	0 (0–0)	0 (0–0)	0 (0–0)	0 (0–0)	0 (0–0)	0 (0–0)	0 (0–0)	0 (0–0)	0 (0–0)	--
Maternal disorders	--	--	--	--	--	--	--	--	--	--	--	--	--	--	--	--	--	--	--	--	--	--	--
Maternal haemorrhage	--	--	--	--	--	--	--	--	--	--	--	--	--	--	--	--	--	--	--	--	--	--	--
Maternal sepsis and other pregnancy related infections	--	--	--	--	--	--	--	--	--	--	--	--	--	--	--	--	--	--	--	--	--	--	--
Maternal hypertensive disorders	--	--	--	--	--	--	--	--	--	--	--	--	--	--	--	--	--	--	--	--	--	--	--
Maternal obstructed labour and uterine rupture	--	--	--	--	--	--	--	--	--	--	--	--	--	--	--	--	--	--	--	--	--	--	--
Maternal abortive outcome	--	--	--	--	--	--	--	--	--	--	--	--	--	--	--	--	--	--	--	--	--	--	--
Ectopic pregnancy	--	--	--	--	--	--	--	--	--	--	--	--	--	--	--	--	--	--	--	--	--	--	--
Indirect maternal deaths	--	--	--	--	--	--	--	--	--	--	--	--	--	--	--	--	--	--	--	--	--	--	--
Late maternal deaths	--	--	--	--	--	--	--	--	--	--	--	--	--	--	--	--	--	--	--	--	--	--	--
Maternal deaths aggravated by HIV/AIDS	--	--	--	--	--	--	--	--	--	--	--	--	--	--	--	--	--	--	--	--	--	--	--
Other maternal disorders	--	--	--	--	--	--	--	--	--	--	--	--	--	--	--	--	--	--	--	--	--	--	--
Neonatal disorders	7 (6–9)	3 (2–4)	1 (1–2)	0 (0–0)	--	--	--	--	--	--	--	--	--	--	--	--	--	--	--	--	--	--	--
Neonatal preterm birth	3 (2–4)	1 (0–1)	0 (0–1)	0 (0–0)	--	--	--	--	--	--	--	--	--	--	--	--	--	--	--	--	--	--	--
Neonatal encephalopathy due to birth asphyxia and trauma	2 (1–3)	0 (0–1)	0 (0–0)	0 (0–0)	--	--	--	--	--	--	--	--	--	--	--	--	--	--	--	--	--	--	--
Neonatal sepsis and other neonatal infections	1 (1–2)	1 (1–2)	0 (0–1)	0 (0–0)	--	--	--	--	--	--	--	--	--	--	--	--	--	--	--	--	--	--	--
Hemolytic disease and other neonatal jaundice	0 (0–0)	0 (0–0)	0 (0–0)	0 (0–0)	--	--	--	--	--	--	--	--	--	--	--	--	--	--	--	--	--	--	--
Other neonatal disorders	1 (1–2)	1 (0–1)	0 (0–0)	0 (0–0)	--	--	--	--	--	--	--	--	--	--	--	--	--	--	--	--	--	--	--
Nutritional deficiencies	0 (0–0)	0 (0–0)	0 (0–0)	0 (0–0)	0 (0–0)	0 (0–0)	0 (0–0)	0 (0–0)	0 (0–0)	0 (0–0)	0 (0–0)	0 (0–0)	0 (0–0)	0 (0–0)	0 (0–0)	0 (0–0)	0 (0–0)	0 (0–0)	0 (0–0)	0 (0–0)	0 (0–0)	0 (0–0)	--
Protein-energy malnutrition	0 (0–0)	0 (0–0)	0 (0–0)	0 (0–0)	0 (0–0)	0 (0–0)	0 (0–0)	0 (0–0)	0 (0–0)	0 (0–0)	0 (0–0)	0 (0–0)	0 (0–0)	0 (0–0)	0 (0–0)	0 (0–0)	0 (0–0)	0 (0–0)	0 (0–0)	0 (0–0)	0 (0–0)	0 (0–0)	--
Other nutritional deficiencies	--	--	0 (0–0)	0 (0–0)	0 (0–0)	0 (0–0)	0 (0–0)	0 (0–0)	0 (0–0)	0 (0–0)	0 (0–0)	0 (0–0)	0 (0–0)	0 (0–0)	0 (0–0)	0 (0–0)	0 (0–0)	0 (0–0)	0 (0–0)	0 (0–0)	0 (0–0)	0 (0–0)	--
Lip and oral cavity cancer	--	--	--	--	0 (0–0)	0 (0–0)	0 (0–0)	0 (0–0)	0 (0–0)	0 (0–0)	0 (0–0)	0 (0–1)	1 (0–1)	1 (1–2)	1 (1–2)	1 (1–2)	1 (1–1)	1 (1–1)	1 (1–1)	1 (0–1)	0 (0–0)	0 (0–0)	--
Nasopharynx cancer	--	--	--	--	0 (0–0)	0 (0–0)	0 (0–0)	0 (0–0)	0 (0–0)	0 (0–0)	0 (0–0)	0 (0–0)	0 (0–0)	0 (0–0)	0 (0–0)	0 (0–0)	0 (0–0)	0 (0–0)	0 (0–0)	0 (0–0)	0 (0–0)	0 (0–0)	--
Other pharynx cancer	--	--	--	--	--	--	--	0 (0–0)	0 (0–0)	0 (0–0)	0 (0–0)	0 (0–0)	0 (0–1)	1 (0–1)	1 (1–1)	1 (1–1)	1 (0–1)	0 (0–1)	0 (0–0)	0 (0–0)	0 (0–0)	0 (0–0)	--
Oesophageal cancer	--	--	--	--	--	--	--	0 (0–0)	0 (0–0)	0 (0–0)	0 (0–0)	1 (0–1)	1 (1–2)	2 (1–3)	3 (2–4)	3 (2–4)	2 (2–3)	2 (1–3)	1 (1–2)	1 (1–1)	1 (0–1)	0 (0–0)	--
Stomach cancer	--	--	--	--	--	--	0 (0–0)	0 (0–0)	0 (0–0)	0 (0–0)	0 (0–1)	1 (1–1)	1 (1–2)	2 (1–2)	2 (2–3)	3 (2–4)	3 (2–3)	2 (2–3)	2 (2–3)	2 (1–2)	1 (1–1)	0 (0–1)	--
Colon and rectum cancer	--	--	--	--	0 (0–0)	0 (0–0)	0 (0–0)	0 (0–0)	0 (0–0)	1 (0–1)	1 (1–1)	1 (1–2)	2 (1–3)	3 (2–4)	4 (3–6)	5 (4–6)	5 (4–7)	4 (3–6)	4 (3–6)	3 (2–4)	2 (1–2)	1 (1–1)	--
Liver cancer due to hepatitis B	--	--	--	--	--	0 (0–0)	0 (0–0)	0 (0–0)	0 (0–0)	0 (0–0)	0 (0–0)	0 (0–0)	0 (0–0)	0 (0–1)	0 (0–1)	0 (0–1)	0 (0–0)	0 (0–0)	0 (0–0)	0 (0–0)	0 (0–0)	0 (0–0)	--
Liver cancer due to hepatitis C	--	--	--	--	--	0 (0–0)	0 (0–0)	0 (0–0)	0 (0–0)	0 (0–0)	0 (0–0)	0 (0–0)	0 (0–0)	0 (0–0)	0 (0–0)	0 (0–0)	0 (0–0)	0 (0–0)	0 (0–0)	0 (0–0)	0 (0–0)	0 (0–0)	--
Liver cancer due to alcohol use	--	--	--	--	--	--	0 (0–0)	0 (0–0)	0 (0–0)	0 (0–0)	0 (0–0)	0 (0–0)	0 (0–0)	0 (0–1)	0 (0–1)	0 (0–1)	1 (0–1)	0 (0–1)	0 (0–1)	0 (0–0)	0 (0–0)	0 (0–0)	--
Liver cancer due to NASH	--	--	--	--	--	--	0 (0–0)	0 (0–0)	0 (0–0)	0 (0–0)	0 (0–0)	0 (0–0)	0 (0–0)	0 (0–0)	0 (0–0)	0 (0–0)	0 (0–0)	0 (0–0)	0 (0–0)	0 (0–0)	0 (0–0)	0 (0–0)	--
	0 (0–0)	0 (0–0)	0 (0–0)	0 (0–0)	0 (0–0)	0 (0–0)	--	--	--	--	--	--	--	--	--	--	--	--	--	--	--	--	--

Liver cancer due to other causes	0 (0–0)	0 (0–0)	0 (0–0)	0 (0–0)	0 (0–0)	0 (0–0)	0 (0–0)	0 (0–0)	0 (0–0)	0 (0–0)	0 (0–0)	0 (0–0)	0 (0–0)	0 (0–0)	0 (0–0)	0 (0–0)	0 (0–0)	0 (0–0)	0 (0–0)	0 (0–0)	0 (0–0)	0 (0–0)	--
Gallbladder and biliary tract cancer	--	--	--	--	--	--	--	0 (0–0)	0 (0–0)	0 (0–0)	0 (0–0)	0 (0–0)	0 (0–0)	0 (0–0)	0 (0–1)	0 (0–1)	0 (0–1)	0 (0–1)	0 (0–1)	0 (0–1)	0 (0–0)	0 (0–0)	--
Pancreatic cancer	--	--	--	--	--	--	0 (0–0)	0 (0–0)	0 (0–0)	0 (0–0)	0 (0–0)	0 (0–1)	1 (0–1)	1 (1–1)	1 (1–2)	1 (1–2)	1 (1–2)	1 (1–2)	1 (1–1)	1 (0–1)	0 (0–0)	0 (0–0)	--
Larynx cancer	--	--	--	--	--	--	--	0 (0–0)	0 (0–0)	0 (0–0)	0 (0–0)	0 (0–0)	1 (0–1)	1 (1–1)	1 (1–2)	1 (1–2)	1 (1–2)	1 (1–1)	1 (1–1)	1 (0–1)	0 (0–0)	0 (0–0)	--
Tracheal, bronchus, and lung cancer	--	--	--	--	--	0 (0–0)	0 (0–0)	0 (0–0)	0 (0–0)	0 (0–0)	1 (0–1)	1 (1–2)	2 (2–3)	4 (3–6)	6 (4–8)	6 (4–8)	6 (4–8)	5 (3–6)	4 (3–5)	3 (2–4)	1 (1–2)	1 (0–1)	--
Malignant skin melanoma	0 (0–0)	0 (0–0)	0 (0–0)	0 (0–0)	0 (0–0)	0 (0–0)	0 (0–0)	0 (0–0)	0 (0–0)	0 (0–0)	0 (0–0)	0 (0–0)	0 (0–0)	0 (0–0)	0 (0–1)	0 (0–0)	0 (0–0)	0 (0–0)	0 (0–0)	0 (0–0)	0 (0–0)	0 (0–0)	--
Non-melanoma skin cancer (squamous-cell carcinoma)	--	--	--	--	--	--	--	0 (0–0)	0 (0–0)	0 (0–0)	0 (0–0)	0 (0–0)	0 (0–0)	0 (0–0)	0 (0–0)	0 (0–0)	0 (0–0)	0 (0–0)	0 (0–0)	0 (0–0)	0 (0–0)	0 (0–0)	--
	0 (0–0)	0 (0–0)	0 (0–0)	0 (0–0)	0 (0–0)	0 (0–0)	0 (0–0)	0 (0–0)	0 (0–0)	0 (0–0)	0 (0–0)	0 (0–0)	0 (0–0)	0 (0–0)	0 (0–0)	0 (0–0)	0 (0–0)	0 (0–0)	0 (0–0)	0 (0–0)	0 (0–0)	0 (0–0)	--
	--	--	--	0 (0–0)	0 (0–0)	0 (0–0)	0 (0–0)	0 (0–0)	0 (0–0)	0 (0–0)	0 (0–0)	0 (0–0)	0 (0–0)	0 (0–0)	0 (0–0)	0 (0–0)	0 (0–0)	0 (0–0)	0 (0–0)	0 (0–0)	0 (0–0)	0 (0–0)	--
Breast cancer	--	--	--	--	--	--	0 (0–0)	0 (0–0)	0 (0–0)	0 (0–0)	0 (0–0)	0 (0–0)	0 (0–0)	0 (0–0)	0 (0–0)	0 (0–0)	0 (0–0)	0 (0–0)	0 (0–0)	0 (0–0)	0 (0–0)	0 (0–0)	--
Cervical cancer	--	--	--	--	--	--	--	--	--	--	--	--	--	--	--	--	--	--	--	--	--	--	--
Uterine cancer	--	--	--	--	--	--	--	--	--	--	--	--	--	--	--	--	--	--	--	--	--	--	--
Ovarian cancer	--	--	--	--	--	--	--	--	--	--	--	--	--	--	--	--	--	--	--	--	--	--	--
Prostate cancer	--	--	--	--	--	--	--	0 (0–0)	0 (0–0)	0 (0–0)	0 (0–0)	0 (0–0)	1 (0–1)	2 (1–3)	4 (3–6)	8 (6–12)	11 (8–16)	15 (11–21)	18 (13–26)	18 (13–25)	12 (9–18)	7 (5–10)	--
Testicular cancer	0 (0–0)	0 (0–0)	0 (0–0)	0 (0–0)	0 (0–0)	0 (0–0)	0 (0–0)	0 (0–0)	0 (0–0)	0 (0–0)	0 (0–0)	0 (0–0)	0 (0–0)	0 (0–0)	0 (0–0)	0 (0–0)	0 (0–0)	0 (0–0)	0 (0–0)	0 (0–0)	0 (0–0)	0 (0–0)	--
Kidney cancer	0 (0–0)	0 (0–0)	0 (0–0)	0 (0–0)	0 (0–0)	0 (0–0)	0 (0–0)	0 (0–0)	0 (0–0)	0 (0–0)	0 (0–0)	0 (0–0)	0 (0–0)	1 (0–1)	1 (0–1)	1 (1–1)	1 (0–1)	1 (0–1)	0 (0–1)	0 (0–0)	0 (0–0)	0 (0–0)	--
Bladder cancer	--	--	--	--	--	--	0 (0–0)	0 (0–0)	0 (0–0)	0 (0–0)	0 (0–0)	0 (0–0)	0 (0–0)	0 (0–0)	0 (0–0)	1 (0–1)	1 (0–1)	1 (0–1)	1 (0–1)	0 (0–1)	0 (0–0)	0 (0–0)	--
Brain and nervous system cancer	0 (0–0)	0 (0–0)	0 (0–0)	0 (0–0)	0 (0–0)	0 (0–0)	0 (0–0)	0 (0–0)	0 (0–0)	0 (0–0)	0 (0–0)	0 (0–1)	0 (0–1)	0 (0–1)	1 (0–1)	0 (0–1)	0 (0–1)	0 (0–0)	0 (0–0)	0 (0–0)	0 (0–0)	0 (0–0)	--
	0 (0–0)	0 (0–0)	0 (0–0)	0 (0–0)	0 (0–0)	0 (0–0)	0 (0–0)	0 (0–0)	0 (0–0)	0 (0–0)	0 (0–0)	0 (0–0)	0 (0–0)	0 (0–0)	0 (0–0)	0 (0–0)	0 (0–0)	0 (0–0)	0 (0–0)	0 (0–0)	0 (0–0)	0 (0–0)	--
	0 (0–0)	0 (0–0)	0 (0–0)	0 (0–0)	0 (0–0)	0 (0–0)	0 (0–0)	--	--	--	--	--	--	--	--	--	--	--	--	--	--	--	--
	--	--	--	0 (0–0)	0 (0–0)	0 (0–0)	0 (0–0)	0 (0–0)	0 (0–0)	0 (0–0)	0 (0–0)	0 (0–0)	0 (0–0)	0 (0–0)	0 (0–0)	0 (0–0)	0 (0–0)	0 (0–0)	0 (0–0)	0 (0–0)	0 (0–0)	0 (0–0)	--
	0 (0–0)	0 (0–0)	0 (0–0)	0 (0–0)	0 (0–0)	0 (0–0)	0 (0–0)	0 (0–0)	0 (0–0)	0 (0–0)	0 (0–0)	0 (0–0)	0 (0–0)	0 (0–0)	0 (0–0)	0 (0–0)	0 (0–0)	0 (0–0)	0 (0–0)	0 (0–0)	0 (0–0)	0 (0–0)	--
Thyroid cancer	--	--	--	--	0 (0–0)	0 (0–0)	0 (0–0)	0 (0–0)	0 (0–0)	0 (0–0)	0 (0–0)	0 (0–0)	0 (0–0)	0 (0–0)	0 (0–0)	0 (0–0)	0 (0–0)	0 (0–0)	0 (0–0)	0 (0–0)	0 (0–0)	0 (0–0)	--
Mesothelioma	--	--	--	--	--	--	--	0 (0–0)	0 (0–0)	0 (0–0)	0 (0–0)	0 (0–0)	0 (0–0)	0 (0–0)	0 (0–0)	0 (0–0)	0 (0–0)	0 (0–0)	0 (0–0)	0 (0–0)	0 (0–0)	0 (0–0)	--
Hodgkin lymphoma	--	--	--	0 (0–0)	0 (0–0)	0 (0–0)	0 (0–0)	0 (0–0)	0 (0–0)	0 (0–0)	0 (0–0)	0 (0–0)	0 (0–0)	0 (0–0)	0 (0–0)	0 (0–0)	0 (0–0)	0 (0–0)	0 (0–0)	0 (0–0)	0 (0–0)	0 (0–0)	--
Non-Hodgkin's lymphoma	--	--	--	0 (0–0)	0 (0–0)	0 (0–0)	0 (0–0)	0 (0–0)	0 (0–1)	0 (0–1)	1 (0–1)	1 (0–1)	1 (0–1)	1 (1–1)	1 (1–1)	1 (1–1)	1 (1–1)	1 (0–1)	1 (0–1)	0 (0–0)	0 (0–0)	0 (0–0)	--
	--	--	--	0 (0–0)	0 (0–0)	0 (0–0)	0 (0–0)	0 (0–0)	0 (0–0)	0 (0–0)	0 (0–0)	0 (0–0)	0 (0–0)	0 (0–0)	0 (0–0)	0 (0–0)	0 (0–0)	0 (0–0)	0 (0–0)	0 (0–0)	0 (0–0)	0 (0–0)	--
	--	--	--	0 (0–0)	0 (0–0)	0 (0–0)	0 (0–0)	0 (0–0)	0 (0–1)	0 (0–1)	1 (0–1)	1 (0–1)	1 (0–1)	1 (1–1)	1 (1–1)	1 (1–1)	1 (1–1)	1 (0–1)	1 (0–1)	0 (0–0)	0 (0–0)	0 (0–0)	--
Multiple myeloma	--	--	--	--	--	--	--	0 (0–0)	0 (0–0)	0 (0–0)	0 (0–0)	0 (0–0)	1 (0–1)	1 (1–1)	1 (1–2)	1 (1–2)	1 (1–2)	1 (1–2)	1 (1–1)	1 (1–1)	0 (0–0)	0 (0–0)	--
Leukaemia	0 (0–0)	0 (0–0)	0 (0–0)	0 (0–0)	0 (0–0)	0 (0–0)	0 (0–0)	0 (0–0)	0 (0–1)	0 (0–1)	0 (0–1)	0 (0–1)	0 (0–1)	1 (0–1)	1 (0–1)	1 (0–1)	1 (0–1)	1 (0–1)	0 (0–1)	0 (0–0)	0 (0–0)	0 (0–0)	--
Acute lymphoid leukaemia	0 (0–0)	0 (0–0)	0 (0–0)	0 (0–0)	0 (0–0)	0 (0–0)	0 (0–0)	0 (0–0)	0 (0–0)	0 (0–0)	0 (0–0)	0 (0–0)	0 (0–0)	0 (0–0)	0 (0–0)	0 (0–0)	0 (0–0)	0 (0–0)	0 (0–0)	0 (0–0)	0 (0–0)	0 (0–0)	--
Chronic lymphoid leukaemia	--	--	--	--	--	--	--	0 (0–0)	0 (0–0)	0 (0–0)	0 (0–0)	0 (0–0)	0 (0–0)	0 (0–0)	0 (0–0)	0 (0–0)	0 (0–0)	0 (0–0)	0 (0–0)	0 (0–0)	0 (0–0)	0 (0–0)	--
Acute myeloid leukaemia	0 (0–0)	0 (0–0)	0 (0–0)	0 (0–0)	0 (0–0)	0 (0–0)	0 (0–0)	0 (0–0)	0 (0–0)	0 (0–0)	0 (0–0)	0 (0–0)	0 (0–0)	0 (0–0)	0 (0–0)	0 (0–0)	0 (0–0)	0 (0–0)	0 (0–0)	0 (0–0)	0 (0–0)	0 (0–0)	--
Chronic myeloid leukaemia	0 (0–0)	0 (0–0)	0 (0–0)	0 (0–0)	0 (0–0)	0 (0–0)	0 (0–0)	0 (0–0)	0 (0–0)	0 (0–0)	0 (0–0)	0 (0–0)	0 (0–0)	0 (0–0)	0 (0–0)	0 (0–0)	0 (0–0)	0 (0–0)	0 (0–0)	0 (0–0)	0 (0–0)	0 (0–0)	--
Other leukaemia	0 (0–0)	0 (0–0)	0 (0–0)	0 (0–0)	0 (0–0)	0 (0–0)	0 (0–0)	0 (0–0)	0 (0–0)	0 (0–0)	0 (0–0)	0 (0–0)	0 (0–0)	0 (0–0)	0 (0–0)	0 (0–0)	0 (0–0)	0 (0–0)	0 (0–0)	0 (0–0)	0 (0–0)	0 (0–0)	--
Other malignant cancers	0 (0–0)	0 (0–0)	0 (0–0)	0 (0–0)	0 (0–0)	0 (0–0)	0 (0–0)	0 (0–0)	0 (0–0)	0 (0–0)	0 (0–0)	0 (0–1)	1 (0–1)	1 (0–1)	1 (1–1)	1 (1–1)	1 (1–1)	1 (0–1)	1 (0–1)	0 (0–1)	0 (0–0)	0 (0–0)	--
Myelodysplastic, myeloproliferative, and other hematopoietic neoplasms	0 (0–0)	0 (0–0)	0 (0–0)	0 (0–0)	0 (0–0)	0 (0–0)	0 (0–0)	0 (0–0)	0 (0–0)	0 (0–0)	0 (0–0)	0 (0–0)	0 (0–0)	0 (0–0)	0 (0–0)	0 (0–0)	0 (0–0)	0 (0–0)	0 (0–0)	0 (0–0)	0 (0–0)	0 (0–0)	--
Cardiovascular diseases	0 (0–0)	0 (0–0)	0 (0–0)	0 (0–0)	0 (0–0)	0 (0–0)	1 (0–1)	1 (1–2)	3 (2–4)	5 (4–6)	10 (7–12)	17 (12–22)	26 (20–35)	38 (30–49)	49 (38–62)	55 (44–68)	53 (43–65)	49 (40–59)	49 (41–59)	44 (37–52)	33 (27–38)	19 (16–23)	--
Rheumatic heart disease	--	--	--	0 (0–0)	0 (0–0)	0 (0–0)	0 (0–0)	0 (0–0)	0 (0–0)	0 (0–0)	0 (0–0)	0 (0–0)	0 (0–0)	0 (0–0)	0 (0–0)	0 (0–0)	0 (0–0)	0 (0–0)	0 (0–0)	0 (0–0)	0 (0–0)	0 (0–0)	--
Ischaemic heart disease	--	--	--	--	--	--	0 (0–0)	0 (0–0)	1 (0–1)	1 (1–2)	3 (2–4)	6 (4–8)	11 (8–15)	17 (13–22)	22 (17–28)	25 (19–31)	24 (19–29)	20 (17–25)	19 (15–23)	17 (14–21)	13 (10–16)	7 (6–9)	--
Stroke	0 (0–0)	0 (0–0)	0 (0–0)	0 (0–0)	0 (0–0)	0 (0–0)	0 (0–0)	0 (0–1)	1 (0–1)	1 (1–2)	2 (1–3)	3 (2–5)	5 (3–8)	8 (5–12)	10 (7–13)	12 (9–16)	12 (9–16)	12 (9–16)	14 (10–18)	11 (8–14)	8 (6–10)	4 (3–5)	--
Ischaemic stroke	0 (0–0)	0 (0–0)	0 (0–0)	0 (0–0)	0 (0–0)	0 (0–0)	0 (0–0)	0 (0–0)	0 (0–0)	0 (0–0)	0 (0–0)	0 (0–1)	1 (0–1)	1 (1–2)	2 (1–2)	3 (2–4)	3 (2–5)	5 (4–7)	6 (4–9)	7 (5–10)	5 (4–7)	3 (2–4)	--
Intracerebral hemorrhage	0 (0–0)	0 (0–0)	0 (0–0)	0 (0–0)	0 (0–0)	0 (0–0)	0 (0–0)	0 (0–0)	0 (0–1)	1 (0–1)	1 (1–2)	2 (2–4)	4 (3–6)	6 (4–9)	7 (5–10)	8 (6–11)	8 (6–12)	6 (5–9)	7 (5–10)	4 (2–5)	2 (2–3)	1 (1–2)	--
Subarachnoid hemorrhage	0 (0–0)	0 (0–0)	0 (0–0)	0 (0–0)	0 (0–0)	0 (0–0)	0 (0–0)	0 (0–0)	0 (0–0)	0 (0–0)	0 (0–1)	1 (0–1)	1 (0–1)	1 (1–1)	1 (1–2)	1 (0–1)	1 (0–1)	0 (0–1)	0 (0–1)	0 (0–1)	0 (0–0)	0 (0–0)	--
Hypertensive heart disease	--	--	--	--	--	--	0 (0–0)	0 (0–0)	0 (0–0)	1 (0–1)	2 (1–2)	3 (2–4)	6 (4–8)	8 (5–11)	10 (7–14)	11 (8–15)	11 (7–15)	10 (7–14)	10 (7–14)	10 (7–14)	7 (5–10)	5 (3–6)	--
Non-rheumatic valvular heart disease	--	--	--	--	--	--	0 (0–0)	0 (0–0)	0 (0–0)	0 (0–0)	0 (0–0)	0 (0–0)	0 (0–0)	0 (0–0)	0 (0–0)	0 (0–0)	0 (0–0)	0 (0–0)	0 (0–0)	0 (0–0)	0 (0–0)	0 (0–0)	--
Non-rheumatic calcific aortic valve disease	--	--	--	--	--	--	0 (0–0)	0 (0–0)	0 (0–0)	0 (0–0)	0 (0–0)	0 (0–0)	0 (0–0)	0 (0–0)	0 (0–0)	0 (0–0)	0 (0–0)	0 (0–0)	0 (0–0)	0 (0–0)	0 (0–0)	0 (0–0)	--
Non-rheumatic degenerative mitral valve disease	--	--	--	--	--	--	0 (0–0)	0 (0–0)	0 (0–0)	0 (0–0)	0 (0–0)	0 (0–0)	0 (0–0)	0 (0–0)	0 (0–0)	0 (0–0)	0 (0–0)	0 (0–0)	0 (0–0)	0 (0–0)	0 (0–0)	0 (0–0)	--
Other non-rheumatic valve diseases	--	--	--	--	--	--	0 (0–0)	0 (0–0)	0 (0–0)	0 (0–0)	0 (0–0)	0 (0–0)	0 (0–0)	0 (0–0)	0 (0–0)	0 (0–0)	0 (0–0)	0 (0–0)	0 (0–0)	0 (0–0)	0 (0–0)	0 (0–0)	--
Cardiomyopathy and myocarditis	0 (0–0)	0 (0–0)	0 (0–0)	0 (0–0)	0 (0–0)	0 (0–0)	0 (0–0)	0 (0–0)	1 (0–1)	1 (1–1)	2 (1–2)	2 (1–3)	3 (2–4)	3 (2–4)	3 (2–5)	3 (2–4)	2 (2–3)	2 (1–3)	2 (1–2)	1 (1–2)	1 (1–2)	1 (0–1)	--
Myocarditis	0 (0–0)	0 (0–0)	0 (0–0)	0 (0–0)	0 (0–0)	0 (0–0)	0 (0–0)	0 (0–0)	0 (0–0)	0 (0–0)	0 (0–0)	0 (0–0)	0 (0–0)	0 (0–0)	0 (0–0)	0 (0–0)	0 (0–0)	0 (0–0)	0 (0–0)	0 (0–0)	0 (0–0)	0 (0–0)	--
Alcoholic cardiomyopathy	--	--	--	--	--	--	0 (0–0)	0 (0–0)	0 (0–0)	0 (0–0)	0 (0–1)	1 (0–1)	1 (1–2)	1 (1–2)	1 (1–2)	1 (1–2)	1 (1–1)	1 (0–1)	0 (0–1)	0 (0–0)	0 (0–0)	0 (0–0)	--
Other cardiomyopathy	0 (0–0)	0 (0–0)	0 (0–0)	0 (0–0)	0 (0–0)	0 (0–0)	0 (0–0)	0 (0–0)	0 (0–1)	1 (0–1)	1 (1–2)	1 (1–2)	1 (1–2)	2 (1–2)	2 (1–3)	2 (1–2)	1 (1–2)	1 (1–2)	1 (1–2)	1 (1–2)	1 (1–1)	0 (0–1)	--
Pulmonary Arterial Hypertension	0 (0–0)	0 (0–0)	0 (0–0)	0 (0–0)	0 (0–0)	0 (0–0)	0 (0–0)	0 (0–0)	0 (0–0)	0 (0–0)	0 (0–0)	0 (0–0)	0 (0–0)	0 (0–0)	0 (0–0)	0 (0–0)	0 (0–0)	0 (0–0)	0 (0–0)	0 (0–0)	0 (0–0)	0 (0–0)	--
Atrial fibrillation and flutter	--	--	--	--	--	--	--	--	--	0 (0–0)	0 (0–0)	0 (0–0)	0 (0–0)	0 (0–0)	0 (0–0)	0 (0–1)	1 (0–1)	1 (0–1)	1 (1–1)	1 (1–2)	1 (1–2)	1 (1–2)	--
Aortic aneurysm	--	--	--	--	--	--	0 (0–0)	0 (0–0)	0 (0–0)	0 (0–0)	0 (0–0)	0 (0–0)	0 (0–0)	0 (0–1)	1 (0–1)	1 (1–1)	1 (1–1)	1 (1–1)	1 (1–1)	1 (1–1)	1 (0–1)	0 (0–0)	--
Peripheral vascular disease	--	--	--	--	--	--	--	--	--	--	--	0 (0–0)	0 (0–0)	0 (0–0)	0 (0–1)	0 (0–1)	0 (0–1)	0 (0–1)	1 (0–1)	1 (0–2)	0 (0–1)	0 (0–1)	--
Endocarditis	0 (0–0)	0 (0–0)	0 (0–0)	0 (0–0)	0 (0–0)	0 (0–0)	0 (0–0)	0 (0–0)	0 (0–0)	0 (0–0)	0 (0–0)	0 (0–1)	0 (0–1)	0 (0–1)	0 (0–1)	0 (0–0)	0 (0–0)	0 (0–0)	0 (0–0)	0 (0–0)	0 (0–0)	0 (0–0)	--
Other cardiovascular and circulatory diseases	0 (0–0)	0 (0–0)	0 (0–0)	0 (0–0)	0 (0–0)	0 (0–0)	0 (0–0)	0 (0–0)	0 (0–0)	0 (0–1)	1 (0–1)	1 (0–1)	1 (1–1)	1 (1–2)	1 (1–2)	1 (1–2)	1 (1–2)	1 (1–2)	1 (1–2)	1 (1–2)	1 (1–1)	1 (0–1)	--
Chronic respiratory diseases	0 (0–0)	0 (0–0)	0 (0–0)	0 (0–0)	0 (0–0)	0 (0–0)	0 (0–0)	0 (0–0)	0 (0–1)	0 (0–1)	1 (0–1)	1 (1–1)	1 (1–2)	2 (1–2)	2 (2–3)	3 (2–4)	3 (2–5)	4 (3–5)	4 (3–6)	4 (3–5)	3 (2–3)	2 (1–2)	--
Chronic obstructive pulmonary disease	--	--	0 (0–0)	0 (0–0)	0 (0–0)	0 (0–0)	0 (0–0)	0 (0–0)	0 (0–0)	0 (0–0)	0 (0–0)	0 (0–0)	0 (0–1)	1 (1–1)	1 (1–2)	2 (1–3)	3 (2–4)	3 (2–4)	4 (3–5)	3 (2–4)	2 (2–3)	1 (1–2)	--
Pneumoconiosis	--	--	--	--	--	--	0 (0–0)	0 (0–0)	0 (0–0)	0 (0–0)	0 (0–0)	0 (0–0)	0 (0–0)	0 (0–0)	0 (0–0)	0 (0–0)	0 (0–0)	0 (0–0)	0 (0–0)	0 (0–0)	0 (0–0)	0 (0–0)	--
Silicosis	--	--	--	--	--	--	0 (0–0)	0 (0–0)	0 (0–0)	0 (0–0)	0 (0–0)	0 (0–0)	0 (0–0)	0 (0–0)	0 (0–0)	0 (0–0)	0 (0–0)	0 (0–0)	0 (0–0)	0 (0–0)	0 (0–0)	0 (0–0)	--
Asbestosis	--	--	--	--	--	--	0 (0–0)	0 (0–0)	0 (0–0)	0 (0–0)	0 (0–0)	0 (0–0)	0 (0–0)	0 (0–0)	0 (0–0)	0 (0–0)	0 (0–0)	0 (0–0)	0 (0–0)	0 (0–0)	0 (0–0)	0 (0–0)	--
Coal workers pneumoconiosis	--	--	--	--	--	--	0 (0–0)	0 (0–0)	0 (0–0)	0 (0–0)	0 (0–0)	0 (0–0)	0 (0–0)	0 (0–0)	0 (0–0)	0 (0–0)	0 (0–0)	0 (0–0)	0 (0–0)	0 (0–0)	0 (0–0)	0 (0–0)	--
Other pneumoconiosis	--	--	--	--	--	--	0 (0–0)	0 (0–0)	0 (0–0)	0 (0–0)	0 (0–0)	0 (0–0)	0 (0–0)	0 (0–0)	0 (0–0)	0 (0–0)	0 (0–0)	0 (0–0)	0 (0–0)	0 (0–0)	0 (0–0)	0 (0–0)	--
Asthma	--	--	--	0 (0–0)	0 (0–0)	0 (0–0)	0 (0–0)	0 (0–0)	0 (0–0)	0 (0–0)	0 (0–0)	0 (0–0)	0 (0–0)	0 (0–1)	0 (0–1)	0 (0–1)	0 (0–1)	0 (0–1)	0 (0–1)	0 (0–0)	0 (0–0)	0 (0–0)	--
Interstitial lung disease and pulmonary sarcoidosis	--	--	--	0 (0–0)	0 (0–0)	0 (0–0)	0 (0–0)	0 (0–0)	0 (0–0)	0 (0–0)	0 (0–0)	0 (0–0)	0 (0–0)	0 (0–0)	0 (0–0)	0 (0–0)	0 (0–0)	0 (0–1)	0 (0–1)	0 (0–0)	0 (0–0)	0 (0–0)	--
Other chronic respiratory diseases	0 (0–0)	0 (0–0)	0 (0–0)	0 (0–0)	0 (0–0)	0 (0–0)	0 (0–0)	0 (0–0)	0 (0–0)	0 (0–0)	0 (0–0)	0 (0–0)	0 (0–0)	0 (0–0)	0 (0–0)	0 (0–0)	0 (0–0)	0 (0–0)	0 (0–0)	0 (0–0)	0 (0–0)	0 (0–0)	--
Digestive diseases	0 (0–0)	0 (0–0)	0 (0–0)	0 (0–0)	0 (0–0)	0 (0–0)	0 (0–0)	1 (0–1)	1 (1–2)	2 (1–3)	4 (3–5)	5 (4–7)	7 (5–10)	10 (7–13)	9 (7–12)	9 (7–12)	8 (6–10)	6 (5–8)	5 (4–7)	4 (3–5)	2 (2–3)	1 (1–2)	--
Cirrhosis and other chronic liver diseases	--	--	--	0 (0–0)	0 (0–0)	0 (0–0)	0 (0–0)	0 (0–0)	0 (0–1)	1 (1–1)	2 (1–3)	3 (2–5)	5 (3–6)	7 (5–9)	6 (4–8)	6 (4–7)	4 (3–6)	3 (2–4)	2 (2–3)	1 (1–2)	1 (0–1)	0 (0–0)	--
Cirrhosis and other chronic liver diseases due to hepatitis B	--	--	--	0 (0–0)	0 (0–0)	0 (0–0)	0 (0–0)	0 (0–0)	0 (0–0)	0 (0–0)	0 (0–0)	0 (0–1)	0 (0–1)	1 (0–1)	0 (0–1)	0 (0–1)	0 (0–0)	0 (0–0)	0 (0–0)	0 (0–0)	0 (0–0)	0 (0–0)	--
Cirrhosis and other chronic liver diseases due to hepatitis C	--	--	--	0 (0–0)	0 (0–0)	0 (0–0)	0 (0–0)	0 (0–0)	0 (0–0)	0 (0–0)	1 (0–1)	1 (0–1)	1 (1–2)	1 (1–2)	1 (1–2)	1 (0–2)	1 (0–1)	1 (0–1)	0 (0–1)	0 (0–0)	0 (0–0)	0 (0–0)	--
Cirrhosis and other chronic liver diseases due to alcohol use	--	--	--	--	--	--	0 (0–0)	0 (0–0)	0 (0–0)	0 (0–1)	1 (0–1)	2 (1–2)	2 (1–3)	3 (2–5)	3 (2–4)	3 (2–4)	2 (1–3)	1 (1–2)	1 (1–1)	1 (0–1)	0 (0–0)	0 (0–0)	--
Cirrhosis and other chronic liver diseases due to NAFLD	--	--	--	--	--	--	0 (0–0)	0 (0–0)	0 (0–0)	0 (0–0)	0 (0–0)	0 (0–1)	1 (0–1)	1 (0–2)	1 (0–1)	1 (0–1)	1 (0–1)	0 (0–1)	0 (0–1)	0 (0–0)	0 (0–0)	0 (0–0)	--
Cirrhosis and other chronic liver diseases due to other causes	--	--	--	0 (0–0)	0 (0–0)	0 (0–0)	0 (0–0)	0 (0–0)	0 (0–0)	0 (0–0)	0 (0–0)	0 (0–1)	0 (0–1)	0 (0–1)	0 (0–1)	1 (0–1)	0 (0–1)	0 (0–1)	0 (0–1)	0 (0–0)	0 (0–0)	0 (0–0)	--
Upper digestive system diseases	--	--	--	0 (0–0)	0 (0–0)	0 (0–0)	0 (0–0)	0 (0–0)	0 (0–0)	0 (0–0)	0 (0–0)	0 (0–1)	1 (0–1)	1 (1–1)	1 (1–1)	1 (1–1)	1 (1–1)	1 (1–1)	1 (1–1)	1 (0–1)	0 (0–1)	0 (0–0)	--

Peptic ulcer disease	--	--	--	0 (0–0)	0 (0–0)	0 (0–0)	0 (0–0)	0 (0–0)	0 (0–0)	0 (0–0)	0 (0–0)	0 (0–1)	0 (0–1)	1 (0–1)	1 (0–1)	1 (1–1)	1 (1–1)	1 (0–1)	1 (0–1)	1 (0–1)	0 (0–1)	0 (0–0)	--
Gastritis and duodenitis	--	--	--	0 (0–0)	0 (0–0)	0 (0–0)	0 (0–0)	0 (0–0)	0 (0–0)	0 (0–0)	0 (0–0)	0 (0–0)	0 (0–0)	0 (0–0)	0 (0–0)	0 (0–0)	0 (0–0)	0 (0–0)	0 (0–0)	0 (0–0)	0 (0–0)	0 (0–0)	--
Appendicitis	--	--	--	0 (0–0)	0 (0–0)	0 (0–0)	0 (0–0)	0 (0–0)	0 (0–0)	0 (0–0)	0 (0–0)	0 (0–0)	0 (0–0)	0 (0–0)	0 (0–0)	0 (0–0)	0 (0–0)	0 (0–0)	0 (0–0)	0 (0–0)	0 (0–0)	0 (0–0)	--
Paralytic ileus and intestinal obstruction	0 (0–0)	0 (0–0)	0 (0–0)	0 (0–0)	0 (0–0)	0 (0–0)	0 (0–0)	0 (0–0)	0 (0–0)	0 (0–0)	0 (0–0)	0 (0–0)	0 (0–1)	0 (0–1)	1 (0–1)	1 (0–1)	1 (0–1)	1 (0–1)	1 (0–1)	1 (0–1)	0 (0–1)	0 (0–0)	--
Inguinal, femoral, and abdominal hernia	0 (0–0)	0 (0–0)	0 (0–0)	0 (0–0)	0 (0–0)	0 (0–0)	0 (0–0)	0 (0–0)	0 (0–0)	0 (0–0)	0 (0–0)	0 (0–0)	0 (0–0)	0 (0–0)	0 (0–0)	0 (0–0)	0 (0–0)	0 (0–0)	0 (0–0)	0 (0–0)	0 (0–0)	0 (0–0)	--
Inflammatory bowel disease	--	--	--	0 (0–0)	0 (0–0)	0 (0–0)	0 (0–0)	0 (0–0)	0 (0–0)	0 (0–0)	0 (0–0)	0 (0–0)	0 (0–0)	0 (0–0)	0 (0–0)	0 (0–0)	0 (0–0)	0 (0–0)	0 (0–0)	0 (0–0)	0 (0–0)	0 (0–0)	--
Vascular intestinal disorders	--	--	--	0 (0–0)	0 (0–0)	0 (0–0)	0 (0–0)	0 (0–0)	0 (0–0)	0 (0–0)	0 (0–0)	0 (0–0)	0 (0–0)	0 (0–0)	0 (0–0)	0 (0–0)	0 (0–0)	0 (0–0)	0 (0–0)	0 (0–0)	0 (0–0)	0 (0–0)	--
Gallbladder and biliary diseases	--	--	--	0 (0–0)	0 (0–0)	0 (0–0)	0 (0–0)	0 (0–0)	0 (0–0)	0 (0–0)	0 (0–0)	0 (0–0)	0 (0–0)	0 (0–0)	0 (0–0)	0 (0–0)	0 (0–1)	0 (0–1)	0 (0–1)	0 (0–0)	0 (0–0)	0 (0–0)	--
Pancreatitis	--	--	--	0 (0–0)	0 (0–0)	0 (0–0)	0 (0–0)	0 (0–0)	0 (0–0)	0 (0–0)	1 (0–1)	1 (0–1)	1 (0–1)	1 (0–1)	1 (0–1)	1 (0–1)	0 (0–1)	0 (0–0)	0 (0–0)	0 (0–0)	0 (0–0)	0 (0–0)	--
Other digestive diseases	--	--	--	0 (0–0)	0 (0–0)	0 (0–0)	0 (0–0)	0 (0–0)	0 (0–0)	0 (0–0)	0 (0–0)	0 (0–0)	0 (0–1)	0 (0–1)	0 (0–1)	1 (0–1)	0 (0–1)	0 (0–1)	0 (0–1)	0 (0–1)	0 (0–1)	0 (0–0)	--
Alzheimer's disease and other dementias	--	--	--	--	--	--	--	--	--	--	--	0 (0–0)	0 (0–0)	0 (0–1)	0 (0–1)	1 (0–2)	1 (0–3)	2 (0–5)	3 (1–8)	5 (1–14)	5 (1–15)	4 (1–12)	--
Parkinson's disease	--	--	--	--	--	--	--	0 (0–0)	0 (0–0)	0 (0–0)	0 (0–0)	0 (0–0)	0 (0–0)	0 (0–0)	0 (0–0)	0 (0–1)	1 (1–1)	1 (1–2)	2 (2–2)	2 (2–3)	2 (1–2)	1 (1–1)	--
Idiopathic epilepsy	--	--	0 (0–0)	0 (0–0)	0 (0–0)	0 (0–0)	0 (0–0)	0 (0–0)	0 (0–1)	0 (0–1)	0 (0–1)	0 (0–1)	0 (0–1)	0 (0–1)	0 (0–0)	0 (0–0)	0 (0–0)	0 (0–0)	0 (0–0)	0 (0–0)	0 (0–0)	0 (0–0)	--
Multiple sclerosis	--	--	--	--	0 (0–0)	0 (0–0)	0 (0–0)	0 (0–0)	0 (0–0)	0 (0–0)	0 (0–0)	0 (0–0)	0 (0–0)	0 (0–0)	0 (0–0)	0 (0–0)	0 (0–0)	0 (0–0)	0 (0–0)	0 (0–0)	0 (0–0)	0 (0–0)	--
Motor neuron disease	0 (0–0)	0 (0–0)	0 (0–0)	0 (0–0)	0 (0–0)	0 (0–0)	0 (0–0)	0 (0–0)	0 (0–0)	0 (0–0)	0 (0–0)	0 (0–0)	0 (0–0)	0 (0–0)	0 (0–0)	0 (0–0)	0 (0–0)	0 (0–0)	0 (0–0)	0 (0–0)	0 (0–0)	0 (0–0)	--
Other neurological disorders	--	--	0 (0–0)	0 (0–0)	0 (0–0)	0 (0–0)	0 (0–0)	0 (0–0)	0 (0–0)	0 (0–0)	0 (0–0)	0 (0–0)	0 (0–0)	0 (0–0)	0 (0–0)	0 (0–0)	0 (0–0)	0 (0–0)	0 (0–0)	0 (0–0)	0 (0–0)	0 (0–0)	--
Eating disorders	--	--	--	--	0 (0–0)	0 (0–0)	0 (0–0)	0 (0–0)	0 (0–0)	0 (0–0)	0 (0–0)	0 (0–0)	0 (0–0)	--	--	--	--	--	--	--	--	--	--
Anorexia nervosa	--	--	--	--	0 (0–0)	0 (0–0)	0 (0–0)	0 (0–0)	0 (0–0)	0 (0–0)	0 (0–0)	0 (0–0)	0 (0–0)	--	--	--	--	--	--	--	--	--	--
Bulimia nervosa	--	--	--	--	0 (0–0)	0 (0–0)	0 (0–0)	0 (0–0)	0 (0–0)	0 (0–0)	0 (0–0)	0 (0–0)	0 (0–0)	--	--	--	--	--	--	--	--	--	--
Alcohol use disorders	--	--	--	--	--	0 (0–0)	0 (0–0)	0 (0–0)	0 (0–1)	1 (0–1)	1 (1–2)	2 (1–3)	2 (1–3)	2 (1–3)	2 (1–3)	2 (1–2)	1 (1–2)	1 (0–1)	0 (0–0)	0 (0–0)	0 (0–0)	0 (0–0)	--
Drug use disorders	0 (0–0)	0 (0–0)	0 (0–0)	0 (0–0)	0 (0–0)	0 (0–0)	0 (0–0)	0 (0–0)	0 (0–0)	0 (0–0)	0 (0–0)	0 (0–0)	0 (0–0)	0 (0–0)	0 (0–0)	0 (0–0)	0 (0–0)	0 (0–0)	0 (0–0)	0 (0–0)	0 (0–0)	0 (0–0)	--
Opioid use disorders	0 (0–0)	0 (0–0)	0 (0–0)	0 (0–0)	0 (0–0)	0 (0–0)	0 (0–0)	0 (0–0)	0 (0–0)	0 (0–0)	0 (0–0)	0 (0–0)	0 (0–0)	0 (0–0)	0 (0–0)	0 (0–0)	0 (0–0)	0 (0–0)	0 (0–0)	0 (0–0)	0 (0–0)	0 (0–0)	--
Cocaine use disorders	--	--	--	--	--	0 (0–0)	0 (0–0)	0 (0–0)	0 (0–0)	0 (0–0)	0 (0–0)	0 (0–0)	0 (0–0)	0 (0–0)	0 (0–0)	0 (0–0)	0 (0–0)	0 (0–0)	0 (0–0)	0 (0–0)	0 (0–0)	0 (0–0)	--
Amphetamine use disorders	--	--	--	--	--	0 (0–0)	0 (0–0)	0 (0–0)	0 (0–0)	0 (0–0)	0 (0–0)	0 (0–0)	0 (0–0)	0 (0–0)	0 (0–0)	0 (0–0)	0 (0–0)	0 (0–0)	0 (0–0)	0 (0–0)	0 (0–0)	0 (0–0)	--
Other drug use disorders	--	--	--	--	--	0 (0–0)	0 (0–0)	0 (0–0)	0 (0–0)	0 (0–0)	0 (0–0)	0 (0–0)	0 (0–0)	0 (0–0)	0 (0–0)	0 (0–0)	0 (0–0)	0 (0–0)	0 (0–0)	0 (0–0)	0 (0–0)	0 (0–0)	--
Diabetes mellitus	0 (0–0)	0 (0–0)	0 (0–0)	0 (0–0)	0 (0–0)	0 (0–0)	0 (0–0)	0 (0–0)	1 (0–1)	1 (1–1)	1 (1–2)	2 (2–3)	3 (2–5)	6 (4–8)	6 (4–9)	9 (6–12)	9 (7–13)	9 (6–12)	7 (5–10)	6 (4–8)	3 (3–4)	2 (1–2)	--
Diabetes mellitus type 1	0 (0–0)	0 (0–0)	0 (0–0)	0 (0–0)	0 (0–0)	0 (0–0)	0 (0–0)	0 (0–0)	0 (0–1)	0 (0–0)	0 (0–1)	1 (0–1)	0 (0–0)	0 (0–1)	0 (0–1)	0 (0–1)	0 (0–1)	0 (0–1)	0 (0–0)	0 (0–0)	0 (0–0)	0 (0–0)	--
Diabetes mellitus type 2	--	--	--	--	--	--	0 (0–0)	0 (0–0)	0 (0–0)	1 (0–1)	1 (1–1)	1 (1–2)	3 (2–5)	5 (4–8)	6 (4–8)	8 (6–12)	9 (6–12)	8 (6–12)	7 (5–9)	6 (4–8)	3 (2–4)	2 (1–2)	--
Chronic kidney disease	--	--	0 (0–0)	0 (0–0)	0 (0–0)	0 (0–0)	0 (0–0)	1 (0–1)	1 (1–1)	1 (1–2)	2 (1–3)	3 (2–4)	4 (3–5)	5 (3–7)	6 (4–9)	6 (4–9)	7 (5–9)	6 (4–8)	6 (4–7)	5 (4–6)	3 (3–4)	2 (1–3)	--
Chronic kidney disease due to diabetes mellitus type 1	--	--	0 (0–0)	0 (0–0)	0 (0–0)	0 (0–0)	0 (0–0)	0 (0–0)	0 (0–0)	0 (0–0)	0 (0–1)	1 (0–1)	1 (0–1)	1 (0–1)	1 (0–1)	0 (0–1)	0 (0–1)	0 (0–0)	0 (0–0)	0 (0–0)	0 (0–0)	0 (0–0)	--
Chronic kidney disease due to diabetes mellitus type 2	--	--	--	--	--	--	0 (0–0)	0 (0–0)	0 (0–0)	0 (0–0)	0 (0–0)	0 (0–1)	1 (0–1)	1 (1–2)	2 (1–3)	2 (1–3)	2 (1–3)	2 (1–3)	2 (1–3)	1 (1–2)	1 (1–2)	1 (0–1)	--
Chronic kidney disease due to hypertension	--	--	--	--	--	--	0 (0–0)	0 (0–0)	0 (0–0)	0 (0–0)	0 (0–0)	0 (0–0)	0 (0–1)	1 (0–1)	1 (1–2)	1 (1–2)	2 (1–3)	2 (1–2)	2 (1–3)	2 (1–2)	1 (1–2)	1 (1–1)	--
Chronic kidney disease due to glomerulonephritis	--	--	0 (0–0)	0 (0–0)	0 (0–0)	0 (0–0)	0 (0–0)	0 (0–0)	0 (0–1)	0 (0–1)	1 (0–1)	1 (0–2)	1 (1–2)	1 (1–2)	2 (1–3)	1 (1–2)	1 (1–2)	1 (1–2)	1 (0–2)	1 (0–1)	0 (0–1)	0 (0–0)	--
Chronic kidney disease due to other and unspecified causes	--	--	0 (0–0)	0 (0–0)	0 (0–0)	0 (0–0)	0 (0–0)	0 (0–0)	0 (0–1)	0 (0–1)	0 (0–1)	1 (0–1)	1 (0–2)	1 (0–2)	1 (1–2)	1 (1–2)	1 (1–2)	1 (1–2)	1 (1–2)	1 (0–2)	1 (0–1)	0 (0–1)	--
Acute glomerulonephritis	--	--	0 (0–0)	0 (0–0)	0 (0–0)	0 (0–0)	0 (0–0)	0 (0–0)	0 (0–0)	0 (0–0)	0 (0–0)	0 (0–0)	0 (0–0)	0 (0–0)	0 (0–0)	0 (0–0)	0 (0–0)	0 (0–0)	0 (0–0)	0 (0–0)	0 (0–0)	0 (0–0)	--
Skin and subcutaneous diseases	--	--	0 (0–0)	0 (0–0)	0 (0–0)	0 (0–0)	0 (0–0)	0 (0–0)	0 (0–0)	0 (0–0)	0 (0–0)	0 (0–1)	0 (0–1)	1 (0–1)	1 (0–1)	1 (0–1)	1 (0–2)	1 (0–2)	1 (0–2)	1 (0–2)	1 (0–2)	0 (0–1)	--
Bacterial skin diseases	--	--	0 (0–0)	0 (0–0)	0 (0–0)	0 (0–0)	0 (0–0)	0 (0–0)	0 (0–0)	0 (0–0)	0 (0–0)	0 (0–0)	0 (0–0)	0 (0–0)	0 (0–0)	0 (0–0)	0 (0–0)	0 (0–0)	0 (0–0)	0 (0–0)	0 (0–0)	0 (0–0)	--
Cellulitis	--	--	0 (0–0)	0 (0–0)	0 (0–0)	0 (0–0)	0 (0–0)	0 (0–0)	0 (0–0)	0 (0–0)	0 (0–0)	0 (0–0)	0 (0–0)	0 (0–0)	0 (0–0)	0 (0–0)	0 (0–0)	0 (0–0)	0 (0–0)	0 (0–0)	0 (0–0)	0 (0–0)	--
Pyoderma	--	--	0 (0–0)	0 (0–0)	0 (0–0)	0 (0–0)	0 (0–0)	0 (0–0)	0 (0–0)	0 (0–0)	0 (0–0)	0 (0–0)	0 (0–0)	0 (0–0)	0 (0–0)	0 (0–0)	0 (0–0)	0 (0–0)	0 (0–0)	0 (0–0)	0 (0–0)	0 (0–0)	--
Decubitus ulcer	--	--	--	0 (0–0)	0 (0–0)	0 (0–0)	0 (0–0)	0 (0–0)	0 (0–0)	0 (0–0)	0 (0–0)	0 (0–0)	0 (0–1)	0 (0–1)	0 (0–1)	1 (0–1)	1 (0–1)	1 (0–1)	1 (0–2)	1 (0–2)	1 (0–2)	0 (0–1)	--
Other skin and subcutaneous diseases	--	--	0 (0–0)	0 (0–0)	0 (0–0)	0 (0–0)	0 (0–0)	0 (0–0)	0 (0–0)	0 (0–0)	0 (0–0)	0 (0–0)	0 (0–0)	0 (0–0)	0 (0–0)	0 (0–0)	0 (0–0)	0 (0–0)	0 (0–0)	0 (0–0)	0 (0–0)	0 (0–0)	--
Musculoskeletal disorders	--	--	--	--	0 (0–0)	0 (0–0)	0 (0–0)	0 (0–0)	0 (0–0)	0 (0–0)	0 (0–0)	0 (0–0)	0 (0–0)	0 (0–0)	0 (0–0)	0 (0–0)	0 (0–0)	0 (0–0)	0 (0–0)	0 (0–0)	0 (0–1)	0 (0–0)	--
Rheumatoid arthritis	--	--	--	--	0 (0–0)	0 (0–0)	0 (0–0)	0 (0–0)	0 (0–0)	0 (0–0)	0 (0–0)	0 (0–0)	0 (0–0)	0 (0–0)	0 (0–0)	0 (0–0)	0 (0–0)	0 (0–0)	0 (0–0)	0 (0–0)	0 (0–0)	0 (0–0)	--
Other musculoskeletal disorders	--	--	--	--	0 (0–0)	0 (0–0)	0 (0–0)	0 (0–0)	0 (0–0)	0 (0–0)	0 (0–0)	0 (0–0)	0 (0–0)	0 (0–0)	0 (0–0)	0 (0–0)	0 (0–0)	0 (0–0)	0 (0–0)	0 (0–0)	0 (0–0)	0 (0–0)	--
Congenital anomalies	1 (1–2)	1 (0–1)	1 (1–2)	0 (0–1)	0 (0–0)	0 (0–0)	0 (0–0)	0 (0–0)	0 (0–0)	0 (0–0)	0 (0–0)	0 (0–0)	0 (0–0)	0 (0–0)	0 (0–0)	0 (0–0)	0 (0–0)	--	--	--	--	--	--
Neural tube defects	0 (0–0)	0 (0–0)	0 (0–0)	0 (0–0)	0 (0–0)	0 (0–0)	0 (0–0)	0 (0–0)	0 (0–0)	0 (0–0)	0 (0–0)	0 (0–0)	0 (0–0)	0 (0–0)	0 (0–0)	0 (0–0)	0 (0–0)	--	--	--	--	--	--
Congenital heart anomalies	0 (0–1)	0 (0–1)	1 (0–1)	0 (0–0)	0 (0–0)	0 (0–0)	0 (0–0)	0 (0–0)	0 (0–0)	0 (0–0)	0 (0–0)	0 (0–0)	0 (0–0)	0 (0–0)	0 (0–0)	0 (0–0)	0 (0–0)	--	--	--	--	--	--
Orofacial clefts	0 (0–0)	0 (0–0)	0 (0–0)	0 (0–0)	--	--	--	--	--	--	--	--	--	--	--	--	--	--	--	--	--	--	--
Down's syndrome	0 (0–0)	0 (0–0)	0 (0–0)	0 (0–0)	0 (0–0)	0 (0–0)	0 (0–0)	0 (0–0)	0 (0–0)	0 (0–0)	0 (0–0)	0 (0–0)	0 (0–0)	0 (0–0)	0 (0–0)	0 (0–0)	0 (0–0)	--	--	--	--	--	--
Other chromosomal abnormalities	0 (0–0)	0 (0–0)	0 (0–0)	0 (0–0)	0 (0–0)	0 (0–0)	0 (0–0)	0 (0–0)	0 (0–0)	0 (0–0)	0 (0–0)	0 (0–0)	0 (0–0)	0 (0–0)	0 (0–0)	0 (0–0)	0 (0–0)	--	--	--	--	--	--
Congenital musculoskeletal and limb anomalies	0 (0–0)	0 (0–0)	0 (0–0)	0 (0–0)	0 (0–0)	0 (0–0)	0 (0–0)	0 (0–0)	0 (0–0)	0 (0–0)	0 (0–0)	0 (0–0)	0 (0–0)	0 (0–0)	0 (0–0)	0 (0–0)	0 (0–0)	--	--	--	--	--	--
Urogenital congenital anomalies	0 (0–0)	0 (0–0)	0 (0–0)	0 (0–0)	0 (0–0)	0 (0–0)	0 (0–0)	0 (0–0)	0 (0–0)	0 (0–0)	0 (0–0)	0 (0–0)	0 (0–0)	0 (0–0)	0 (0–0)	0 (0–0)	0 (0–0)	--	--	--	--	--	--
Digestive congenital anomalies	0 (0–0)	0 (0–0)	0 (0–0)	0 (0–0)	0 (0–0)	0 (0–0)	0 (0–0)	0 (0–0)	0 (0–0)	0 (0–0)	0 (0–0)	0 (0–0)	0 (0–0)	0 (0–0)	0 (0–0)	0 (0–0)	0 (0–0)	--	--	--	--	--	--
Other congenital anomalies	0 (0–1)	0 (0–0)	0 (0–0)	0 (0–0)	0 (0–0)	0 (0–0)	0 (0–0)	0 (0–0)	0 (0–0)	0 (0–0)	0 (0–0)	0 (0–0)	0 (0–0)	0 (0–0)	0 (0–0)	0 (0–0)	0 (0–0)	--	--	--	--	--	--
Urinary diseases and male infertility	0 (0–0)	0 (0–0)	0 (0–0)	0 (0–0)	0 (0–0)	0 (0–0)	0 (0–0)	0 (0–0)	0 (0–0)	0 (0–0)	0 (0–0)	0 (0–0)	0 (0–1)	1 (0–1)	1 (0–1)	1 (1–1)	1 (1–2)	1 (1–2)	2 (1–2)	1 (1–2)	1 (1–2)	1 (0–1)	--
Urinary tract infections and interstitial nephritis	0 (0–0)	0 (0–0)	0 (0–0)	0 (0–0)	0 (0–0)	0 (0–0)	0 (0–0)	0 (0–0)	0 (0–0)	0 (0–0)	0 (0–0)	0 (0–0)	0 (0–1)	0 (0–1)	1 (0–1)	1 (1–1)	1 (1–1)	1 (1–2)	1 (1–2)	1 (1–2)	1 (1–2)	1 (0–1)	--
Urolithiasis	--	--	--	0 (0–0)	0 (0–0)	0 (0–0)	0 (0–0)	0 (0–0)	0 (0–0)	0 (0–0)	0 (0–0)	0 (0–0)	0 (0–0)	0 (0–0)	0 (0–0)	0 (0–0)	0 (0–0)	0 (0–0)	0 (0–0)	0 (0–0)	0 (0–0)	0 (0–0)	--
Other urinary diseases	0 (0–0)	0 (0–0)	0 (0–0)	0 (0–0)	0 (0–0)	0 (0–0)	0 (0–0)	0 (0–0)	0 (0–0)	0 (0–0)	0 (0–0)	0 (0–0)	0 (0–0)	0 (0–0)	0 (0–0)	0 (0–0)	0 (0–0)	0 (0–0)	0 (0–0)	0 (0–0)	0 (0–0)	0 (0–0)	--
Gynecological diseases	--	--	--	--	--	--	--	--	--	--	--	--	--	--	--	--	--	--	--	--	--	--	--
Uterine fibroids	--	--	--	--	--	--	--	--	--	--	--	--	--	--	--	--	--	--	--	--	--	--	--
Endometriosis	--	--	--	--	--	--	--	--	--	--	--	--	--	--	--	--	--	--	--	--	--	--	--
Genital prolapse	--	--	--	--	--	--	--	--	--	--	--	--	--	--	--	--	--	--	--	--	--	--	--
Other gynecological diseases	--	--	--	--	--	--	--	--	--	--	--	--	--	--	--	--	--	--	--	--	--	--	--
Hemoglobinopathies and hemolytic anaemias	0 (0–0)	0 (0–0)	0 (0–0)	0 (0–0)	0 (0–0)	0 (0–0)	0 (0–0)	0 (0–1)	0 (0–1)	0 (0–1)	0 (0–1)	0 (0–1)	0 (0–0)	0 (0–0)	0 (0–0)	0 (0–0)	0 (0–0)	0 (0–0)	0 (0–0)	0 (0–0)	0 (0–0)	0 (0–0)	--
Thalassemias	0 (0–0)	0 (0–0)	0 (0–0)	0 (0–0)	0 (0–0)	0 (0–0)	0 (0–0)	0 (0–0)	0 (0–0)	0 (0–0)	0 (0–0)	0 (0–0)	0 (0–0)	0 (0–0)	0 (0–0)	0 (0–0)	0 (0–0)	0 (0–0)	0 (0–0)	0 (0–0)	0 (0–0)	0 (0–0)	--
Sickle cell disorders	0 (0–0)	0 (0–0)	0 (0–0)	0 (0–0)	0 (0–0)	0 (0–0)	0 (0–0)	0 (0–0)	0 (0–0)	0 (0–0)	0 (0–0)	0 (0–0)	0 (0–0)	0 (0–0)	0 (0–0)	0 (0–0)	0 (0–0)	0 (0–0)	0 (0–0)	0 (0–0)	0 (0–0)	0 (0–0)	--
G6PD deficiency	0 (0–0)	0 (0–0)	0 (0–0)	0 (0–0)	0 (0–0)	0 (0–0)	0 (0–0)	0 (0–0)	0 (0–0)	0 (0–0)	0 (0–0)	0 (0–0)	0 (0–0)	0 (0–0)	0 (0–0)	0 (0–0)	0 (0–0)	0 (0–0)	0 (0–0)	0 (0–0)	0 (0–0)	0 (0–0)	--
Other hemoglobinopathies and hemolytic anaemias	0 (0–0)	0 (0–0)	0 (0–0)	0 (0–0)	0 (0–0)	0 (0–0)	0 (0–0)	0 (0–0)	0 (0–0)	0 (0–0)	0 (0–0)	0 (0–0)	0 (0–0)	0 (0–0)	0 (0–0)	0 (0–0)	0 (0–0)	0 (0–0)	0 (0–0)	0 (0–0)	0 (0–0)	0 (0–0)	--
Endocrine, metabolic, blood, and immune disorders	0 (0–0)	0 (0–0)	1 (1–2)	0 (0–1)	0 (0–0)	0 (0–0)	0 (0–0)	0 (0–1)	1 (0–1)	1 (1–2)	2 (1–2)	2 (1–3)	2 (1–3)	2 (1–3)	2 (1–3)	2 (1–3)	2 (1–3)	1 (1–3)	1 (1–3)	1 (1–3)	1 (1–2)	1 (0–1)	--
Sudden infant death syndrome	--	0 (0–0)	0 (0–0)	--	--	--	--	--	--	--	--	--	--	--	--	--	--	--	--	--	--	--	--
Transport injuries	0 (0–0)	0 (0–0)	0 (0–0)	0 (0–1)	1 (1–1)	1 (1–1)	4 (3–5)	8 (6–10)	9 (7–11)	8 (7–10)	8 (6–10)	7 (6–9)	7 (6–9)	6 (5–7)	5 (4–6)	3 (3–4)	2 (2–3)	2 (1–2)	1 (1–2)	1 (1–1)	0 (0–1)	0 (0–0)	--
Road injuries	0 (0–0)	0 (0–0)	0 (0–0)	0 (0–0)	0 (0–0)	1 (0–1)	2 (1–3)	5 (4–7)	6 (4–7)	5 (4–7)	5 (3–7)	4 (3–5)	4 (3–5)	3 (2–5)	3 (2–4)	2 (1–3)	1 (1–2)	1 (1–1)	1 (1–1)	0 (0–1)	0 (0–0)	0 (0–0)	--
Pedestrian road injuries	0 (0–0)	0 (0–0)	0 (0–0)	0 (0–0)	0 (0–0)	0 (0–0)	0 (0–0)	0 (0–1)	1 (0–1)	1 (1–1)	1 (1–1)	1 (1–1)	1 (1–1)	1 (1–1)	1 (1–1)	1 (1–1)	1 (0–1)	0 (0–1)	0 (0–1)	0 (0–0)	0 (0–0)	0 (0–0)	--
Cyclist road injuries	--	--	--	0 (0–0)	0 (0–0)	0 (0–0)	0 (0–0)	0 (0–0)	0 (0–0)	0 (0–0)	0 (0–0)	0 (0–0)	0 (0–0)	0 (0–0)	0 (0–0)	0 (0–0)	0 (0–0)	0 (0–0)	0 (0–0)	0 (0–0)	0 (0–0)	0 (0–0)	--
Motorcyclist road injuries	0 (0–0)	0 (0–0)	0 (0–0)	0 (0–0)	0 (0–0)	0 (0–0)	1 (0–1)	1 (1–2)	2 (1–2)	1 (1–2)	1 (1–2)	1 (1–1)	1 (0–1)	0 (0–1)	0 (0–0)	0 (0–0)	0 (0–0)	0 (0–0)	0 (0–0)	0 (0–0)	0 (0–0)	0 (0–0)	--
Motor vehicle road injuries	0 (0–0)	0 (0–0)	0 (0–0)	0 (0–0)	0 (0–0)	0 (0–0)	1 (1–2)	3 (2–4)	3 (2–4)	3 (2–4)	3 (2–4)	2 (1–3)	2 (1–3)	2 (1–2)	1 (1–2)	1 (1–1)	1 (0–1)	0 (0–1)	0 (0–0)	0 (0–0)	0 (0–0)	0 (0–0)	--
Other road injuries	0 (0–0)	0 (0–0)	0 (0–0)	0 (0–0)	0 (0–0)	0 (0–0)	0 (0–0)	0 (0–0)	0 (0–0)	0 (0–0)	0 (0–0)	0 (0–0)	0 (0–0)	0 (0–0)	0 (0–0)	0 (0–0)	0 (0–0)	0 (0–0)	0 (0–0)	0 (0–0)	0 (0–0)	0 (0–0)	--

Other transport injuries	0 (0–0)	0 (0–0)	0 (0–0)	0 (0–0)	0 (0–0)	1 (1–1)	2 (2–2)	3 (3–4)	3 (3–4)	3 (3–4)	3 (3–4)	3 (3–4)	3 (3–3)	3 (2–3)	2 (2–3)	1 (1–2)	1 (1–1)	1 (1–1)	0 (0–1)	0 (0–0)	0 (0–0)	0 (0–0)	--
Falls	0 (0–0)	0 (0–0)	0 (0–0)	0 (0–0)	0 (0–0)	0 (0–0)	0 (0–0)	0 (0–0)	0 (0–1)	0 (0–1)	1 (0–1)	1 (0–1)	1 (0–1)	1 (0–1)	1 (0–1)	1 (0–1)	1 (0–1)	1 (1–1)	1 (1–1)	1 (1–1)	1 (1–1)	1 (1–1)	--
Drowning	0 (0–0)	0 (0–0)	0 (0–0)	0 (0–1)	1 (0–1)	1 (1–1)	1 (1–2)	2 (1–3)	2 (2–3)	2 (2–3)	2 (2–3)	2 (2–3)	2 (1–2)	1 (1–2)	1 (1–1)	1 (1–1)	0 (0–1)	0 (0–0)	0 (0–0)	0 (0–0)	0 (0–0)	0 (0–0)	--
Fire, heat, and hot substances	0 (0–0)	0 (0–0)	0 (0–0)	0 (0–0)	0 (0–0)	0 (0–0)	0 (0–0)	0 (0–0)	0 (0–1)	0 (0–1)	0 (0–1)	0 (0–1)	0 (0–1)	0 (0–1)	0 (0–1)	0 (0–1)	0 (0–0)	0 (0–0)	0 (0–0)	0 (0–0)	0 (0–0)	0 (0–0)	--
Poisonings	0 (0–0)	0 (0–0)	0 (0–0)	0 (0–0)	0 (0–0)	0 (0–0)	0 (0–0)	0 (0–0)	0 (0–0)	0 (0–0)	0 (0–0)	0 (0–0)	0 (0–0)	0 (0–0)	0 (0–0)	0 (0–0)	0 (0–0)	0 (0–0)	0 (0–0)	0 (0–0)	0 (0–0)	0 (0–0)	--
Poisoning by carbon monoxide	0 (0–0)	0 (0–0)	0 (0–0)	0 (0–0)	0 (0–0)	0 (0–0)	0 (0–0)	0 (0–0)	0 (0–0)	0 (0–0)	0 (0–0)	0 (0–0)	0 (0–0)	0 (0–0)	0 (0–0)	0 (0–0)	0 (0–0)	0 (0–0)	0 (0–0)	0 (0–0)	0 (0–0)	0 (0–0)	--
Poisoning by other means	0 (0–0)	0 (0–0)	0 (0–0)	0 (0–0)	0 (0–0)	0 (0–0)	0 (0–0)	0 (0–0)	0 (0–0)	0 (0–0)	0 (0–0)	0 (0–0)	0 (0–0)	0 (0–0)	0 (0–0)	0 (0–0)	0 (0–0)	0 (0–0)	0 (0–0)	0 (0–0)	0 (0–0)	0 (0–0)	--
Exposure to mechanical forces	0 (0–0)	0 (0–0)	0 (0–0)	0 (0–0)	0 (0–0)	0 (0–0)	0 (0–0)	0 (0–0)	0 (0–0)	0 (0–0)	0 (0–0)	0 (0–0)	0 (0–0)	0 (0–0)	0 (0–0)	0 (0–0)	0 (0–0)	0 (0–0)	0 (0–0)	0 (0–0)	0 (0–0)	0 (0–0)	--
Unintentional firearm injuries	0 (0–0)	0 (0–0)	0 (0–0)	0 (0–0)	0 (0–0)	0 (0–0)	0 (0–0)	0 (0–0)	0 (0–0)	0 (0–0)	0 (0–0)	0 (0–0)	0 (0–0)	0 (0–0)	0 (0–0)	0 (0–0)	0 (0–0)	0 (0–0)	0 (0–0)	0 (0–0)	0 (0–0)	0 (0–0)	--
Other exposure to mechanical forces	0 (0–0)	0 (0–0)	0 (0–0)	0 (0–0)	0 (0–0)	0 (0–0)	0 (0–0)	0 (0–0)	0 (0–0)	0 (0–0)	0 (0–0)	0 (0–0)	0 (0–0)	0 (0–0)	0 (0–0)	0 (0–0)	0 (0–0)	0 (0–0)	0 (0–0)	0 (0–0)	0 (0–0)	0 (0–0)	--
Adverse effects of medical treatment	0 (0–0)	0 (0–0)	0 (0–0)	0 (0–0)	0 (0–0)	0 (0–0)	0 (0–0)	0 (0–0)	0 (0–0)	0 (0–0)	0 (0–0)	0 (0–0)	0 (0–0)	0 (0–1)	0 (0–1)	0 (0–1)	0 (0–1)	0 (0–1)	0 (0–1)	0 (0–1)	0 (0–0)	0 (0–0)	--
Animal contact	0 (0–0)	0 (0–0)	0 (0–0)	0 (0–0)	0 (0–0)	0 (0–0)	0 (0–0)	0 (0–0)	0 (0–0)	0 (0–0)	0 (0–0)	0 (0–0)	0 (0–0)	0 (0–0)	0 (0–0)	0 (0–0)	0 (0–0)	0 (0–0)	0 (0–0)	0 (0–0)	0 (0–0)	0 (0–0)	--
Venomous animal contact	0 (0–0)	0 (0–0)	0 (0–0)	0 (0–0)	0 (0–0)	0 (0–0)	0 (0–0)	0 (0–0)	0 (0–0)	0 (0–0)	0 (0–0)	0 (0–0)	0 (0–0)	0 (0–0)	0 (0–0)	0 (0–0)	0 (0–0)	0 (0–0)	0 (0–0)	0 (0–0)	0 (0–0)	0 (0–0)	--
Non-venomous animal contact	0 (0–0)	0 (0–0)	0 (0–0)	0 (0–0)	0 (0–0)	0 (0–0)	0 (0–0)	0 (0–0)	0 (0–0)	0 (0–0)	0 (0–0)	0 (0–0)	0 (0–0)	0 (0–0)	0 (0–0)	0 (0–0)	0 (0–0)	0 (0–0)	0 (0–0)	0 (0–0)	0 (0–0)	0 (0–0)	--
Foreign body	0 (0–0)	0 (0–0)	0 (0–0)	0 (0–0)	0 (0–0)	0 (0–0)	0 (0–0)	0 (0–0)	0 (0–0)	0 (0–0)	0 (0–0)	0 (0–0)	0 (0–0)	0 (0–1)	0 (0–1)	0 (0–1)	0 (0–0)	0 (0–0)	0 (0–0)	0 (0–0)	0 (0–0)	0 (0–0)	--
Pulmonary aspiration and foreign body in airway	0 (0–0)	0 (0–0)	0 (0–0)	0 (0–0)	0 (0–0)	0 (0–0)	0 (0–0)	0 (0–0)	0 (0–0)	0 (0–0)	0 (0–0)	0 (0–0)	0 (0–0)	0 (0–1)	0 (0–0)	0 (0–0)	0 (0–0)	0 (0–0)	0 (0–0)	0 (0–0)	0 (0–0)	0 (0–0)	--
Foreign body in other body part	0 (0–0)	0 (0–0)	0 (0–0)	0 (0–0)	0 (0–0)	0 (0–0)	0 (0–0)	0 (0–0)	0 (0–0)	0 (0–0)	0 (0–0)	0 (0–0)	0 (0–0)	0 (0–0)	0 (0–0)	0 (0–0)	0 (0–0)	0 (0–0)	0 (0–0)	0 (0–0)	0 (0–0)	0 (0–0)	--
Environmental heat and cold exposure	0 (0–0)	0 (0–0)	0 (0–0)	0 (0–0)	0 (0–0)	0 (0–0)	0 (0–0)	0 (0–0)	0 (0–0)	0 (0–0)	0 (0–0)	0 (0–0)	0 (0–0)	0 (0–0)	0 (0–0)	0 (0–0)	0 (0–0)	0 (0–0)	0 (0–0)	0 (0–0)	0 (0–0)	0 (0–0)	--
Other unintentional injuries	0 (0–0)	0 (0–0)	0 (0–0)	0 (0–0)	0 (0–0)	0 (0–0)	0 (0–0)	0 (0–0)	0 (0–0)	0 (0–0)	0 (0–0)	0 (0–0)	0 (0–0)	0 (0–0)	0 (0–0)	0 (0–0)	0 (0–0)	0 (0–0)	0 (0–0)	0 (0–0)	0 (0–0)	0 (0–0)	--
Self-harm	--	--	--	--	--	0 (0–0)	0 (0–0)	1 (1–1)	1 (1–1)	1 (1–2)	1 (1–2)	1 (1–2)	1 (1–1)	1 (1–1)	1 (0–1)	1 (0–1)	0 (0–1)	0 (0–0)	0 (0–0)	0 (0–0)	0 (0–0)	0 (0–0)	--
Self-harm by firearm	--	--	--	--	--	0 (0–0)	0 (0–0)	0 (0–0)	0 (0–0)	0 (0–0)	0 (0–0)	0 (0–0)	0 (0–0)	0 (0–0)	0 (0–0)	0 (0–0)	0 (0–0)	0 (0–0)	0 (0–0)	0 (0–0)	0 (0–0)	0 (0–0)	--
Self-harm by other specified means	--	--	--	--	--	0 (0–0)	0 (0–0)	1 (0–1)	1 (1–1)	1 (1–1)	1 (1–1)	1 (1–1)	1 (1–1)	1 (1–1)	1 (0–1)	1 (0–1)	0 (0–1)	0 (0–0)	0 (0–0)	0 (0–0)	0 (0–0)	0 (0–0)	--
Interpersonal violence	0 (0–0)	0 (0–0)	0 (0–0)	0 (0–0)	0 (0–0)	1 (0–1)	6 (4–8)	16 (13–20)	19 (16–23)	15 (12–19)	13 (10–17)	10 (7–13)	6 (4–9)	4 (3–6)	3 (2–4)	2 (1–3)	1 (1–2)	1 (0–1)	0 (0–1)	0 (0–0)	0 (0–0)	0 (0–0)	--
Physical violence by firearm	0 (0–0)	0 (0–0)	0 (0–0)	0 (0–0)	0 (0–0)	0 (0–0)	5 (3–6)	14 (11–17)	16 (13–19)	12 (9–15)	10 (8–13)	7 (5–9)	4 (3–6)	3 (2–4)	2 (1–3)	1 (1–2)	1 (0–1)	0 (0–1)	0 (0–0)	0 (0–0)	0 (0–0)	0 (0–0)	--
Physical violence by sharp object	0 (0–0)	0 (0–0)	0 (0–0)	0 (0–0)	0 (0–0)	0 (0–0)	1 (1–1)	2 (1–3)	2 (2–3)	2 (2–3)	2 (1–3)	2 (1–3)	1 (1–2)	1 (1–1)	1 (0–1)	0 (0–1)	0 (0–0)	0 (0–0)	0 (0–0)	0 (0–0)	0 (0–0)	0 (0–0)	--
Physical violence by other means	0 (0–0)	0 (0–0)	0 (0–0)	0 (0–0)	0 (0–0)	0 (0–0)	0 (0–0)	0 (0–1)	1 (0–1)	1 (0–1)	1 (0–1)	1 (0–1)	1 (0–1)	1 (0–1)	1 (0–1)	0 (0–1)	0 (0–0)	0 (0–0)	0 (0–0)	0 (0–0)	0 (0–0)	0 (0–0)	--
Executions and police conflict	0 (0–0)	0 (0–0)	0 (0–0)	0 (0–0)	0 (0–0)	0 (0–0)	0 (0–0)	0 (0–0)	0 (0–0)	0 (0–0)	0 (0–0)	0 (0–0)	0 (0–0)	0 (0–0)	0 (0–0)	0 (0–0)	0 (0–0)	0 (0–0)	0 (0–0)	0 (0–0)	0 (0–0)	0 (0–0)	--

SR Table 12: Barbados deaths by cause and age group for males

Cause	Early Neonatal	Late Neonatal	Post Neonatal	1 to 4	5 to 9	10 to 14	15 to 19	20 to 24	25 to 29	30 to 34	35 to 39	40 to 44	45 to 49	50 to 54	55 to 59	60 to 64	65 to 69	70 to 74	75 to 79	80 to 84	85 to 89	90 to 94	95 plus
All causes	11 (7–16)	2 (2–4)	4 (2–5)	2 (1–2)	2 (1–2)	2 (2–3)	8 (6–10)	10 (9–12)	15 (13–18)	19 (16–22)	20 (17–24)	25 (21–31)	41 (33–51)	54 (43–67)	93 (74–114)	131 (107–157)	156 (129–183)	200 (167–231)	203 (173–230)	208 (182–230)	184 (167–198)	110 (102–116)	--
Sexually transmitted infections excluding HIV	0 (0–0)	0 (0–0)	0 (0–0)	0 (0–0)	0 (0–0)	0 (0–0)	0 (0–0)	0 (0–0)	0 (0–0)	0 (0–0)	0 (0–0)	0 (0–0)	0 (0–0)	0 (0–0)	0 (0–0)	0 (0–0)	0 (0–0)	0 (0–0)	0 (0–0)	0 (0–0)	0 (0–0)	0 (0–0)	--
Syphilis	0 (0–0)	0 (0–0)	0 (0–0)	0 (0–0)	0 (0–0)	0 (0–0)	0 (0–0)	0 (0–0)	0 (0–0)	0 (0–0)	0 (0–0)	0 (0–0)	0 (0–0)	0 (0–0)	0 (0–0)	0 (0–0)	0 (0–0)	0 (0–0)	0 (0–0)	0 (0–0)	0 (0–0)	0 (0–0)	--
Chlamydial infection	--	--	--	--	--	0 (0–0)	0 (0–0)	0 (0–0)	0 (0–0)	0 (0–0)	0 (0–0)	0 (0–0)	0 (0–0)	0 (0–0)	0 (0–0)	0 (0–0)	0 (0–0)	0 (0–0)	0 (0–0)	0 (0–0)	0 (0–0)	0 (0–0)	--
Gonococcal infection	--	--	--	--	--	0 (0–0)	0 (0–0)	0 (0–0)	0 (0–0)	0 (0–0)	0 (0–0)	0 (0–0)	0 (0–0)	0 (0–0)	0 (0–0)	0 (0–0)	0 (0–0)	0 (0–0)	0 (0–0)	0 (0–0)	0 (0–0)	0 (0–0)	--
Other sexually transmitted infections	--	--	--	--	--	0 (0–0)	0 (0–0)	0 (0–0)	0 (0–0)	0 (0–0)	0 (0–0)	0 (0–0)	0 (0–0)	0 (0–0)	0 (0–0)	0 (0–0)	0 (0–0)	0 (0–0)	0 (0–0)	0 (0–0)	0 (0–0)	0 (0–0)	--
Tuberculosis	--	--	0 (0–0)	0 (0–0)	0 (0–0)	0 (0–0)	0 (0–0)	0 (0–0)	0 (0–0)	0 (0–0)	0 (0–0)	0 (0–0)	0 (0–0)	0 (0–0)	0 (0–0)	0 (0–0)	0 (0–0)	0 (0–0)	0 (0–0)	0 (0–0)	0 (0–0)	0 (0–0)	--
Drug-susceptible tuberculosis	--	--	0 (0–0)	0 (0–0)	0 (0–0)	0 (0–0)	0 (0–0)	0 (0–0)	0 (0–0)	0 (0–0)	0 (0–0)	0 (0–0)	0 (0–0)	0 (0–0)	0 (0–0)	0 (0–0)	0 (0–0)	0 (0–0)	0 (0–0)	0 (0–0)	0 (0–0)	0 (0–0)	--
Multidrug-resistant tuberculosis without extensive drug resistance	--	--	0 (0–0)	0 (0–0)	0 (0–0)	0 (0–0)	0 (0–0)	0 (0–0)	0 (0–0)	0 (0–0)	0 (0–0)	0 (0–0)	0 (0–0)	0 (0–0)	0 (0–0)	0 (0–0)	0 (0–0)	0 (0–0)	0 (0–0)	0 (0–0)	0 (0–0)	0 (0–0)	--
Extensively drug-resistant tuberculosis	--	--	0 (0–0)	0 (0–0)	0 (0–0)	0 (0–0)	0 (0–0)	0 (0–0)	0 (0–0)	0 (0–0)	0 (0–0)	0 (0–0)	0 (0–0)	0 (0–0)	0 (0–0)	0 (0–0)	0 (0–0)	0 (0–0)	0 (0–0)	0 (0–0)	0 (0–0)	0 (0–0)	--
Lower respiratory infections	0 (0–0)	0 (0–0)	1 (0–1)	0 (0–0)	0 (0–0)	0 (0–0)	0 (0–0)	0 (0–0)	0 (0–0)	0 (0–1)	0 (0–1)	1 (0–1)	1 (1–1)	1 (1–2)	2 (2–3)	4 (2–5)	5 (3–7)	8 (5–11)	10 (7–14)	14 (10–19)	14 (11–19)	11 (8–14)	--
Upper respiratory infections	0 (0–0)	0 (0–0)	0 (0–0)	0 (0–0)	0 (0–0)	0 (0–0)	0 (0–0)	0 (0–0)	0 (0–0)	0 (0–0)	0 (0–0)	0 (0–0)	0 (0–0)	0 (0–0)	0 (0–0)	0 (0–0)	0 (0–0)	0 (0–0)	0 (0–0)	0 (0–0)	0 (0–0)	0 (0–0)	--
Otitis media	0 (0–0)	0 (0–0)	0 (0–0)	0 (0–0)	0 (0–0)	0 (0–0)	0 (0–0)	0 (0–0)	0 (0–0)	0 (0–0)	0 (0–0)	0 (0–0)	0 (0–0)	0 (0–0)	0 (0–0)	0 (0–0)	0 (0–0)	0 (0–0)	0 (0–0)	0 (0–0)	0 (0–0)	0 (0–0)	--
Diarrhoeal diseases	0 (0–0)	0 (0–0)	0 (0–0)	0 (0–0)	0 (0–0)	0 (0–0)	0 (0–0)	0 (0–0)	0 (0–0)	0 (0–0)	0 (0–0)	0 (0–0)	0 (0–0)	0 (0–0)	0 (0–0)	0 (0–0)	0 (0–0)	0 (0–0)	0 (0–0)	0 (0–1)	0 (0–1)	0 (0–0)	--
Typhoid fever	--	--	0 (0–0)	0 (0–0)	0 (0–0)	0 (0–0)	0 (0–0)	0 (0–0)	0 (0–0)	0 (0–0)	0 (0–0)	0 (0–0)	0 (0–0)	0 (0–0)	0 (0–0)	0 (0–0)	0 (0–0)	0 (0–0)	0 (0–0)	0 (0–0)	0 (0–0)	0 (0–0)	--
Paratyphoid fever	--	--	0 (0–0)	0 (0–0)	0 (0–0)	0 (0–0)	0 (0–0)	0 (0–0)	0 (0–0)	0 (0–0)	0 (0–0)	0 (0–0)	0 (0–0)	0 (0–0)	0 (0–0)	0 (0–0)	0 (0–0)	0 (0–0)	0 (0–0)	0 (0–0)	0 (0–0)	0 (0–0)	--
iNTS	--	0 (0–0)	0 (0–0)	0 (0–0)	0 (0–0)	0 (0–0)	0 (0–0)	0 (0–0)	0 (0–0)	0 (0–0)	0 (0–0)	0 (0–0)	0 (0–0)	0 (0–0)	0 (0–0)	0 (0–0)	0 (0–0)	0 (0–0)	0 (0–0)	0 (0–0)	0 (0–0)	0 (0–0)	--
Other intestinal infectious diseases	--	--	0 (0–0)	0 (0–0)	0 (0–0)	0 (0–0)	0 (0–0)	0 (0–0)	0 (0–0)	0 (0–0)	0 (0–0)	0 (0–0)	0 (0–0)	0 (0–0)	0 (0–0)	0 (0–0)	0 (0–0)	0 (0–0)	0 (0–0)	0 (0–0)	0 (0–0)	0 (0–0)	--
Malaria	0 (0–0)	0 (0–0)	0 (0–0)	0 (0–0)	0 (0–0)	0 (0–0)	0 (0–0)	0 (0–0)	0 (0–0)	0 (0–0)	0 (0–0)	0 (0–0)	0 (0–0)	0 (0–0)	0 (0–0)	0 (0–0)	0 (0–0)	0 (0–0)	0 (0–0)	0 (0–0)	0 (0–0)	0 (0–0)	--
Chagas disease	--	--	0 (0–0)	0 (0–0)	0 (0–0)	0 (0–0)	0 (0–0)	0 (0–0)	0 (0–0)	0 (0–0)	0 (0–0)	0 (0–0)	0 (0–0)	0 (0–0)	0 (0–0)	0 (0–0)	0 (0–0)	0 (0–0)	0 (0–0)	0 (0–0)	0 (0–0)	0 (0–0)	--
Visceral leishmaniasis	--	--	0 (0–0)	0 (0–0)	0 (0–0)	0 (0–0)	0 (0–0)	0 (0–0)	0 (0–0)	0 (0–0)	0 (0–0)	0 (0–0)	0 (0–0)	0 (0–0)	0 (0–0)	0 (0–0)	0 (0–0)	0 (0–0)	0 (0–0)	0 (0–0)	0 (0–0)	0 (0–0)	--
African trypanosomiasis	--	--	--	0 (0–0)	0 (0–0)	0 (0–0)	0 (0–0)	0 (0–0)	0 (0–0)	0 (0–0)	0 (0–0)	0 (0–0)	0 (0–0)	0 (0–0)	0 (0–0)	0 (0–0)	0 (0–0)	0 (0–0)	0 (0–0)	0 (0–0)	0 (0–0)	0 (0–0)	--
Schistosomiasis	--	--	0 (0–0)	0 (0–0)	0 (0–0)	0 (0–0)	0 (0–0)	0 (0–0)	0 (0–0)	0 (0–0)	0 (0–0)	0 (0–0)	0 (0–0)	0 (0–0)	0 (0–0)	0 (0–0)	0 (0–0)	0 (0–0)	0 (0–0)	0 (0–0)	0 (0–0)	0 (0–0)	--
Cysticercosis	--	--	--	0 (0–0)	0 (0–0)	0 (0–0)	0 (0–0)	0 (0–0)	0 (0–0)	0 (0–0)	0 (0–0)	0 (0–0)	0 (0–0)	0 (0–0)	0 (0–0)	0 (0–0)	0 (0–0)	0 (0–0)	0 (0–0)	0 (0–0)	0 (0–0)	0 (0–0)	--
Cystic echinococcosis	--	--	--	0 (0–0)	0 (0–0)	0 (0–0)	0 (0–0)	0 (0–0)	0 (0–0)	0 (0–0)	0 (0–0)	0 (0–0)	0 (0–0)	0 (0–0)	0 (0–0)	0 (0–0)	0 (0–0)	0 (0–0)	0 (0–0)	0 (0–0)	0 (0–0)	0 (0–0)	--
Dengue	0 (0–0)	0 (0–0)	0 (0–0)	0 (0–0)	0 (0–0)	0 (0–0)	0 (0–0)	0 (0–0)	0 (0–0)	0 (0–0)	0 (0–0)	0 (0–0)	0 (0–0)	0 (0–0)	0 (0–0)	0 (0–0)	0 (0–0)	0 (0–0)	0 (0–0)	0 (0–0)	0 (0–0)	0 (0–0)	--
Yellow fever	--	0 (0–0)	0 (0–0)	0 (0–0)	0 (0–0)	0 (0–0)	0 (0–0)	0 (0–0)	0 (0–0)	0 (0–0)	0 (0–0)	0 (0–0)	0 (0–0)	0 (0–0)	0 (0–0)	0 (0–0)	0 (0–0)	0 (0–0)	0 (0–0)	0 (0–0)	0 (0–0)	0 (0–0)	--
Rabies	--	--	0 (0–0)	0 (0–0)	0 (0–0)	0 (0–0)	0 (0–0)	0 (0–0)	0 (0–0)	0 (0–0)	0 (0–0)	0 (0–0)	0 (0–0)	0 (0–0)	0 (0–0)	0 (0–0)	0 (0–0)	0 (0–0)	0 (0–0)	0 (0–0)	0 (0–0)	0 (0–0)	--
Ascariasis	--	--	0 (0–0)	0 (0–0)	0 (0–0)	0 (0–0)	0 (0–0)	0 (0–0)	0 (0–0)	0 (0–0)	0 (0–0)	0 (0–0)	0 (0–0)	0 (0–0)	0 (0–0)	0 (0–0)	0 (0–0)	0 (0–0)	0 (0–0)	0 (0–0)	0 (0–0)	0 (0–0)	--
Zika virus disease	0 (0–0)	0 (0–0)	0 (0–0)	0 (0–0)	0 (0–0)	0 (0–0)	0 (0–0)	0 (0–0)	0 (0–0)	0 (0–0)	0 (0–0)	0 (0–0)	0 (0–0)	0 (0–0)	0 (0–0)	0 (0–0)	0 (0–0)	0 (0–0)	0 (0–0)	0 (0–0)	0 (0–0)	0 (0–0)	--
Other neglected tropical diseases	0 (0–0)	0 (0–0)	0 (0–0)	0 (0–0)	0 (0–0)	0 (0–0)	0 (0–0)	0 (0–0)	0 (0–0)	0 (0–0)	0 (0–0)	0 (0–0)	0 (0–0)	0 (0–0)	0 (0–0)	0 (0–0)	0 (0–0)	0 (0–0)	0 (0–0)	0 (0–0)	0 (0–0)	0 (0–0)	--
Meningitis	0 (0–0)	0 (0–0)	0 (0–0)	0 (0–0)	0 (0–0)	0 (0–0)	0 (0–0)	0 (0–0)	0 (0–0)	0 (0–0)	0 (0–0)	0 (0–0)	0 (0–0)	0 (0–0)	0 (0–0)	0 (0–0)	0 (0–0)	0 (0–0)	0 (0–0)	0 (0–0)	0 (0–0)	0 (0–0)	--
Encephalitis	0 (0–0)	0 (0–0)	0 (0–0)	0 (0–0)	0 (0–0)	0 (0–0)	0 (0–0)	0 (0–0)	0 (0–0)	0 (0–0)	0 (0–0)	0 (0–0)	0 (0–0)	0 (0–0)	0 (0–0)	0 (0–0)	0 (0–0)	0 (0–0)	0 (0–0)	0 (0–0)	0 (0–0)	0 (0–0)	--
Diphtheria	--	--	0 (0–0)	0 (0–0)	0 (0–0)	0 (0–0)	0 (0–0)	0 (0–0)	0 (0–0)	0 (0–0)	0 (0–0)	0 (0–0)	0 (0–0)	0 (0–0)	0 (0–0)	--	--	--	--	--	--	--	--
Whooping cough	--	--	0 (0–0)	0 (0–0)	0 (0–0)	0 (0–0)	0 (0–0)	0 (0–0)	0 (0–0)	0 (0–0)	0 (0–0)	0 (0–0)	0 (0–0)	0 (0–0)	0 (0–0)	--	--	--	--	--	--	--	--
Tetanus	0 (0–0)	0 (0–0)	0 (0–0)	0 (0–0)	0 (0–0)	0 (0–0)	0 (0–0)	0 (0–0)	0 (0–0)	0 (0–0)	0 (0–0)	0 (0–0)	0 (0–0)	0 (0–0)	0 (0–0)	0 (0–0)	0 (0–0)	0 (0–0)	0 (0–0)	0 (0–0)	0 (0–0)	0 (0–0)	--
Measles	0 (0–0)	0 (0–0)	0 (0–0)	0 (0–0)	0 (0–0)	0 (0–0)	0 (0–0)	0 (0–0)	0 (0–0)	0 (0–0)	0 (0–0)	0 (0–0)	0 (0–0)	0 (0–0)	0 (0–0)	0 (0–0)	0 (0–0)	0 (0–0)	0 (0–0)	0 (0–0)	0 (0–0)	0 (0–0)	--
Varicella and herpes zoster	0 (0–0)	0 (0–0)	0 (0–0)	0 (0–0)	0 (0–0)	0 (0–0)	0 (0–0)	0 (0–0)	0 (0–0)	0 (0–0)	0 (0–0)	0 (0–0)	0 (0–0)	0 (0–0)	0 (0–0)	0 (0–0)	0 (0–0)	0 (0–0)	0 (0–0)	0 (0–0)	0 (0–0)	0 (0–0)	--
Acute hepatitis	--	--	0 (0–0)	0 (0–0)	0 (0–0)	0 (0–0)	0 (0–0)	0 (0–0)	0 (0–0)	0 (0–0)	0 (0–0)	0 (0–0)	0 (0–0)	0 (0–0)	0 (0–0)	0 (0–0)	0 (0–0)	0 (0–0)	0 (0–0)	0 (0–0)	0 (0–0)	0 (0–0)	--
Acute hepatitis A	--	--	0 (0–0)	0 (0–0)	0 (0–0)	0 (0–0)	0 (0–0)	0 (0–0)	0 (0–0)	0 (0–0)	0 (0–0)	0 (0–0)	0 (0–0)	0 (0–0)	0 (0–0)	0 (0–0)	0 (0–0)	0 (0–0)	0 (0–0)	0 (0–0)	0 (0–0)	0 (0–0)	--
Acute hepatitis B	--	--	0 (0–0)	0 (0–0)	0 (0–0)	0 (0–0)	0 (0–0)	0 (0–0)	0 (0–0)	0 (0–0)	0 (0–0)	0 (0–0)	0 (0–0)	0 (0–0)	0 (0–0)	0 (0–0)	0 (0–0)	0 (0–0)	0 (0–0)	0 (0–0)	0 (0–0)	0 (0–0)	--
Acute hepatitis C	--	--	0 (0–0)	0 (0–0)	0 (0–0)	0 (0–0)	0 (0–0)	0 (0–0)	0 (0–0)	0 (0–0)	0 (0–0)	0 (0–0)	0 (0–0)	0 (0–0)	0 (0–0)	0 (0–0)	0 (0–0)	0 (0–0)	0 (0–0)	0 (0–0)	0 (0–0)	0 (0–0)	--
Acute hepatitis E	--	--	0 (0–0)	0 (0–0)	0 (0–0)	0 (0–0)	0 (0–0)	0 (0–0)	0 (0–0)	0 (0–0)	0 (0–0)	0 (0–0)	0 (0–0)	0 (0–0)	0 (0–0)	0 (0–0)	0 (0–0)	0 (0–0)	0 (0–0)	0 (0–0)	0 (0–0)	0 (0–0)	--
Other unspecified infectious diseases	0 (0–0)	0 (0–0)	0 (0–0)	0 (0–0)	0 (0–0)	0 (0–0)	0 (0–0)	0 (0–0)	0 (0–0)	0 (0–0)	0 (0–0)	0 (0–0)	0 (0–1)	0 (0–1)	0 (0–1)	1 (0–1)	1 (0–1)	1 (0–1)	0 (0–1)	0 (0–1)	0 (0–1)	0 (0–0)	--
Maternal disorders	--	--	--	--	--	--	--	--	--	--	--	--	--	--	--	--	--	--	--	--	--	--	--
Maternal haemorrhage	--	--	--	--	--	--	--	--	--	--	--	--	--	--	--	--	--	--	--	--	--	--	--
Maternal sepsis and other pregnancy related infections	--	--	--	--	--	--	--	--	--	--	--	--	--	--	--	--	--	--	--	--	--	--	--
Maternal hypertensive disorders	--	--	--	--	--	--	--	--	--	--	--	--	--	--	--	--	--	--	--	--	--	--	--
Maternal obstructed labour and uterine rupture	--	--	--	--	--	--	--	--	--	--	--	--	--	--	--	--	--	--	--	--	--	--	--
Maternal abortive outcome	--	--	--	--	--	--	--	--	--	--	--	--	--	--	--	--	--	--	--	--	--	--	--
Ectopic pregnancy	--	--	--	--	--	--	--	--	--	--	--	--	--	--	--	--	--	--	--	--	--	--	--
Indirect maternal deaths	--	--	--	--	--	--	--	--	--	--	--	--	--	--	--	--	--	--	--	--	--	--	--
Late maternal deaths	--	--	--	--	--	--	--	--	--	--	--	--	--	--	--	--	--	--	--	--	--	--	--
Maternal deaths aggravated by HIV/AIDS	--	--	--	--	--	--	--	--	--	--	--	--	--	--	--	--	--	--	--	--	--	--	--
Other maternal disorders	--	--	--	--	--	--	--	--	--	--	--	--	--	--	--	--	--	--	--	--	--	--	--
Neonatal disorders	8 (6–12)	2 (1–2)	1 (0–1)	0 (0–0)	--	--	--	--	--	--	--	--	--	--	--	--	--	--	--	--	--	--	--
Neonatal preterm birth	3 (2–5)	0 (0–1)	0 (0–0)	0 (0–0)	--	--	--	--	--	--	--	--	--	--	--	--	--	--	--	--	--	--	--
Neonatal encephalopathy due to birth asphyxia and trauma	3 (2–4)	0 (0–1)	0 (0–0)	0 (0–0)	--	--	--	--	--	--	--	--	--	--	--	--	--	--	--	--	--	--	--
Neonatal sepsis and other neonatal infections	1 (1–2)	1 (0–1)	0 (0–0)	0 (0–0)	--	--	--	--	--	--	--	--	--	--	--	--	--	--	--	--	--	--	--
Hemolytic disease and other neonatal jaundice	0 (0–0)	0 (0–0)	0 (0–0)	0 (0–0)	--	--	--	--	--	--	--	--	--	--	--	--	--	--	--	--	--	--	--
Other neonatal disorders	1 (1–2)	0 (0–0)	0 (0–0)	0 (0–0)	--	--	--	--	--	--	--	--	--	--	--	--	--	--	--	--	--	--	--
Nutritional deficiencies	0 (0–0)	0 (0–0)	0 (0–0)	0 (0–0)	0 (0–0)	0 (0–0)	0 (0–0)	0 (0–0)	0 (0–0)	0 (0–0)	0 (0–0)	0 (0–0)	0 (0–0)	0 (0–0)	0 (0–0)	0 (0–0)	0 (0–0)	0 (0–1)	0 (0–1)	1 (0–1)	1 (0–1)	0 (0–1)	--
Protein-energy malnutrition	0 (0–0)	0 (0–0)	0 (0–0)	0 (0–0)	0 (0–0)	0 (0–0)	0 (0–0)	0 (0–0)	0 (0–0)	0 (0–0)	0 (0–0)	0 (0–0)	0 (0–0)	0 (0–0)	0 (0–0)	0 (0–0)	0 (0–0)	0 (0–1)	0 (0–1)	0 (0–1)	0 (0–1)	0 (0–0)	--
Other nutritional deficiencies	--	--	0 (0–0)	0 (0–0)	0 (0–0)	0 (0–0)	0 (0–0)	0 (0–0)	0 (0–0)	0 (0–0)	0 (0–0)	0 (0–0)	0 (0–0)	0 (0–0)	0 (0–0)	0 (0–0)	0 (0–0)	0 (0–0)	0 (0–0)	0 (0–0)	0 (0–0)	0 (0–0)	--
Lip and oral cavity cancer	--	--	--	--	0 (0–0)	0 (0–0)	0 (0–0)	0 (0–0)	0 (0–0)	0 (0–0)	0 (0–0)	0 (0–0)	0 (0–0)	0 (0–1)	1 (1–1)	1 (1–1)	1 (1–1)	1 (1–1)	1 (1–1)	1 (1–1)	0 (0–1)	0 (0–0)	--
Nasopharynx cancer	--	--	--	--	0 (0–0)	0 (0–0)	0 (0–0)	0 (0–0)	0 (0–0)	0 (0–0)	0 (0–0)	0 (0–0)	0 (0–0)	0 (0–0)	0 (0–0)	0 (0–0)	0 (0–0)	0 (0–0)	0 (0–0)	0 (0–0)	0 (0–0)	0 (0–0)	--
Other pharynx cancer	--	--	--	--	--	--	--	0 (0–0)	0 (0–0)	0 (0–0)	0 (0–0)	0 (0–0)	0 (0–0)	0 (0–1)	1 (1–1)	1 (1–1)	1 (1–1)	1 (1–1)	1 (0–1)	0 (0–1)	0 (0–0)	0 (0–0)	--
Oesophageal cancer	--	--	--	--	--	--	--	0 (0–0)	0 (0–0)	0 (0–0)	0 (0–0)	0 (0–0)	1 (1–1)	1 (1–2)	2 (1–3)	3 (2–4)	3 (2–4)	3 (2–4)	2 (2–3)	2 (1–2)	1 (1–2)	1 (0–1)	--
Stomach cancer	--	--	--	--	--	--	0 (0–0)	0 (0–0)	0 (0–0)	0 (0–0)	0 (0–0)	0 (0–1)	1 (1–1)	1 (1–2)	2 (2–3)	3 (2–4)	4 (3–5)	5 (3–6)	4 (3–5)	3 (3–5)	3 (2–3)	1 (1–2)	--
Colon and rectum cancer	--	--	--	--	0 (0–0)	0 (0–0)	0 (0–0)	0 (0–0)	0 (0–0)	0 (0–0)	0 (0–1)	1 (0–1)	1 (1–2)	2 (1–3)	4 (3–6)	7 (5–9)	7 (5–10)	9 (7–11)	8 (6–11)	7 (6–9)	5 (4–6)	2 (2–3)	--
Liver cancer due to hepatitis B	--	--	--	--	--	0 (0–0)	0 (0–0)	0 (0–0)	0 (0–0)	0 (0–0)	0 (0–0)	0 (0–0)	0 (0–0)	0 (0–0)	0 (0–0)	0 (0–0)	0 (0–0)	0 (0–0)	0 (0–0)	0 (0–0)	0 (0–0)	0 (0–0)	--
Liver cancer due to hepatitis C	--	--	--	--	--	0 (0–0)	0 (0–0)	0 (0–0)	0 (0–0)	0 (0–0)	0 (0–0)	0 (0–0)	0 (0–0)	0 (0–0)	0 (0–0)	0 (0–0)	0 (0–0)	0 (0–0)	0 (0–0)	0 (0–0)	0 (0–0)	0 (0–0)	--
Liver cancer due to alcohol use	--	--	--	--	--	--	0 (0–0)	0 (0–0)	0 (0–0)	0 (0–0)	0 (0–0)	0 (0–0)	0 (0–0)	0 (0–0)	0 (0–0)	0 (0–1)	1 (0–1)	1 (0–1)	1 (0–1)	0 (0–1)	0 (0–0)	0 (0–0)	--
Liver cancer due to NASH	--	--	--	--	--	--	0 (0–0)	0 (0–0)	0 (0–0)	0 (0–0)	0 (0–0)	0 (0–0)	0 (0–0)	0 (0–0)	0 (0–0)	0 (0–0)	0 (0–0)	0 (0–0)	0 (0–0)	0 (0–0)	0 (0–0)	0 (0–0)	--
	0 (0–0)	0 (0–0)	0 (0–0)	0 (0–0)	0 (0–0)	0 (0–0)	--	--	--	--	--	--	--	--	--	--	--	--	--	--	--	--	--

Liver cancer due to other causes	0 (0–0)	0 (0–0)	0 (0–0)	0 (0–0)	0 (0–0)	0 (0–0)	0 (0–0)	0 (0–0)	0 (0–0)	0 (0–0)	0 (0–0)	0 (0–0)	0 (0–0)	0 (0–0)	0 (0–0)	0 (0–0)	0 (0–0)	0 (0–0)	0 (0–0)	0 (0–0)	0 (0–0)	0 (0–0)	--
Gallbladder and biliary tract cancer	--	--	--	--	--	--	--	0 (0–0)	0 (0–0)	0 (0–0)	0 (0–0)	0 (0–0)	0 (0–0)	0 (0–0)	0 (0–0)	0 (0–1)	0 (0–1)	0 (0–1)	0 (0–1)	0 (0–1)	0 (0–0)	0 (0–0)	--
Pancreatic cancer	--	--	--	--	--	--	0 (0–0)	0 (0–0)	0 (0–0)	0 (0–0)	0 (0–0)	0 (0–0)	1 (0–1)	1 (1–1)	2 (1–3)	3 (2–4)	3 (2–4)	3 (2–4)	3 (2–3)	2 (2–3)	2 (1–2)	1 (1–1)	--
Larynx cancer	--	--	--	--	--	--	--	0 (0–0)	0 (0–0)	0 (0–0)	0 (0–0)	0 (0–0)	0 (0–0)	0 (0–0)	1 (0–1)	1 (1–1)	1 (1–1)	1 (1–1)	1 (1–1)	1 (0–1)	0 (0–0)	0 (0–0)	--
Tracheal, bronchus, and lung cancer	--	--	--	--	--	0 (0–0)	0 (0–0)	0 (0–0)	0 (0–0)	0 (0–0)	0 (0–0)	0 (0–0)	1 (1–1)	1 (1–2)	3 (2–4)	4 (3–6)	5 (3–7)	5 (4–7)	4 (3–5)	3 (2–4)	2 (2–3)	1 (1–1)	--
Malignant skin melanoma	0 (0–0)	0 (0–0)	0 (0–0)	0 (0–0)	0 (0–0)	0 (0–0)	0 (0–0)	0 (0–0)	0 (0–0)	0 (0–0)	0 (0–0)	0 (0–0)	0 (0–0)	0 (0–0)	0 (0–0)	0 (0–0)	0 (0–0)	0 (0–0)	0 (0–0)	0 (0–0)	0 (0–0)	0 (0–0)	--
Non-melanoma skin cancer (squamous-cell carcinoma)	--	--	--	--	--	--	--	0 (0–0)	0 (0–0)	0 (0–0)	0 (0–0)	0 (0–0)	0 (0–0)	0 (0–0)	0 (0–0)	0 (0–0)	0 (0–0)	0 (0–0)	0 (0–0)	0 (0–0)	0 (0–0)	0 (0–0)	--
	0 (0–0)	0 (0–0)	0 (0–0)	0 (0–0)	0 (0–0)	0 (0–0)	0 (0–0)	0 (0–0)	0 (0–0)	0 (0–0)	0 (0–0)	0 (0–0)	0 (0–0)	0 (0–0)	0 (0–0)	0 (0–0)	0 (0–0)	0 (0–0)	0 (0–0)	0 (0–0)	0 (0–0)	0 (0–0)	--
	--	--	--	0 (0–0)	0 (0–0)	0 (0–0)	0 (0–0)	0 (0–0)	0 (0–0)	0 (0–0)	0 (0–0)	0 (0–0)	0 (0–0)	0 (0–0)	0 (0–0)	0 (0–0)	0 (0–0)	0 (0–0)	0 (0–0)	0 (0–0)	0 (0–0)	0 (0–0)	--
Breast cancer	--	--	--	--	--	--	0 (0–0)	0 (0–0)	0 (0–0)	0 (0–0)	0 (0–0)	0 (0–0)	0 (0–0)	0 (0–0)	0 (0–0)	0 (0–0)	0 (0–0)	0 (0–0)	0 (0–0)	0 (0–0)	0 (0–0)	0 (0–0)	--
Cervical cancer	--	--	--	--	--	--	--	--	--	--	--	--	--	--	--	--	--	--	--	--	--	--	--
Uterine cancer	--	--	--	--	--	--	--	--	--	--	--	--	--	--	--	--	--	--	--	--	--	--	--
Ovarian cancer	--	--	--	--	--	--	--	--	--	--	--	--	--	--	--	--	--	--	--	--	--	--	--
Prostate cancer	--	--	--	--	--	--	--	0 (0–0)	0 (0–0)	0 (0–0)	0 (0–0)	0 (0–0)	0 (0–1)	1 (1–2)	4 (3–6)	11 (7–15)	17 (11–22)	27 (19–36)	33 (24–43)	33 (24–43)	29 (21–37)	16 (11–21)	--
Testicular cancer	0 (0–0)	0 (0–0)	0 (0–0)	0 (0–0)	0 (0–0)	0 (0–0)	0 (0–0)	0 (0–0)	0 (0–0)	0 (0–0)	0 (0–0)	0 (0–0)	0 (0–0)	0 (0–0)	0 (0–0)	0 (0–0)	0 (0–0)	0 (0–0)	0 (0–0)	0 (0–0)	0 (0–0)	0 (0–0)	--
Kidney cancer	0 (0–0)	0 (0–0)	0 (0–0)	0 (0–0)	0 (0–0)	0 (0–0)	0 (0–0)	0 (0–0)	0 (0–0)	0 (0–0)	0 (0–0)	0 (0–0)	0 (0–0)	0 (0–1)	1 (1–1)	1 (1–1)	1 (1–2)	1 (1–2)	1 (1–1)	1 (1–1)	0 (0–1)	0 (0–0)	--
Bladder cancer	--	--	--	--	--	--	0 (0–0)	0 (0–0)	0 (0–0)	0 (0–0)	0 (0–0)	0 (0–0)	0 (0–0)	0 (0–0)	0 (0–1)	1 (1–1)	1 (1–1)	1 (1–2)	1 (1–2)	1 (1–2)	1 (1–2)	1 (0–1)	--
Brain and nervous system cancer	0 (0–0)	0 (0–0)	0 (0–0)	0 (0–0)	0 (0–0)	0 (0–0)	0 (0–0)	0 (0–0)	0 (0–0)	0 (0–0)	0 (0–0)	0 (0–0)	0 (0–0)	0 (0–1)	1 (0–1)	1 (0–1)	1 (0–1)	1 (0–1)	0 (0–1)	0 (0–0)	0 (0–0)	0 (0–0)	--
	0 (0–0)	0 (0–0)	0 (0–0)	0 (0–0)	0 (0–0)	0 (0–0)	0 (0–0)	0 (0–0)	0 (0–0)	0 (0–0)	0 (0–0)	0 (0–0)	0 (0–0)	0 (0–0)	0 (0–0)	0 (0–0)	0 (0–0)	0 (0–0)	0 (0–0)	0 (0–0)	0 (0–0)	0 (0–0)	--
	0 (0–0)	0 (0–0)	0 (0–0)	0 (0–0)	0 (0–0)	0 (0–0)	0 (0–0)	--	--	--	--	--	--	--	--	--	--	--	--	--	--	--	--
	--	--	--	0 (0–0)	0 (0–0)	0 (0–0)	0 (0–0)	0 (0–0)	0 (0–0)	0 (0–0)	0 (0–0)	0 (0–0)	0 (0–0)	0 (0–0)	0 (0–0)	0 (0–0)	0 (0–0)	0 (0–0)	0 (0–0)	0 (0–0)	0 (0–0)	0 (0–0)	--
	0 (0–0)	0 (0–0)	0 (0–0)	0 (0–0)	0 (0–0)	0 (0–0)	0 (0–0)	0 (0–0)	0 (0–0)	0 (0–0)	0 (0–0)	0 (0–0)	0 (0–0)	0 (0–0)	0 (0–0)	0 (0–0)	0 (0–0)	0 (0–0)	0 (0–0)	0 (0–0)	0 (0–0)	0 (0–0)	--
Thyroid cancer	--	--	--	--	0 (0–0)	0 (0–0)	0 (0–0)	0 (0–0)	0 (0–0)	0 (0–0)	0 (0–0)	0 (0–0)	0 (0–0)	0 (0–0)	0 (0–0)	0 (0–0)	0 (0–0)	0 (0–0)	0 (0–0)	0 (0–0)	0 (0–0)	0 (0–0)	--
Mesothelioma	--	--	--	--	--	--	--	0 (0–0)	0 (0–0)	0 (0–0)	0 (0–0)	0 (0–0)	0 (0–0)	0 (0–0)	0 (0–0)	0 (0–0)	0 (0–0)	0 (0–0)	0 (0–0)	0 (0–0)	0 (0–0)	0 (0–0)	--
Hodgkin lymphoma	--	--	--	0 (0–0)	0 (0–0)	0 (0–0)	0 (0–0)	0 (0–0)	0 (0–0)	0 (0–0)	0 (0–0)	0 (0–0)	0 (0–0)	0 (0–0)	0 (0–0)	0 (0–0)	0 (0–0)	0 (0–0)	0 (0–0)	0 (0–0)	0 (0–0)	0 (0–0)	--
Non-Hodgkin's lymphoma	--	--	--	0 (0–0)	0 (0–0)	0 (0–0)	0 (0–0)	0 (0–0)	0 (0–0)	0 (0–0)	0 (0–0)	0 (0–1)	1 (0–1)	1 (1–1)	1 (1–2)	1 (1–2)	1 (1–2)	2 (1–2)	1 (1–2)	1 (1–1)	1 (0–1)	0 (0–0)	--
	--	--	--	0 (0–0)	0 (0–0)	0 (0–0)	0 (0–0)	0 (0–0)	0 (0–0)	0 (0–0)	0 (0–0)	0 (0–0)	0 (0–0)	0 (0–0)	0 (0–0)	0 (0–0)	0 (0–0)	0 (0–0)	0 (0–0)	0 (0–0)	0 (0–0)	0 (0–0)	--
	--	--	--	0 (0–0)	0 (0–0)	0 (0–0)	0 (0–0)	0 (0–0)	0 (0–0)	0 (0–0)	0 (0–0)	0 (0–1)	1 (0–1)	1 (1–1)	1 (1–2)	1 (1–2)	1 (1–2)	2 (1–2)	1 (1–2)	1 (1–1)	1 (0–1)	0 (0–0)	--
Multiple myeloma	--	--	--	--	--	--	--	0 (0–0)	0 (0–0)	0 (0–0)	0 (0–0)	0 (0–0)	0 (0–1)	1 (0–1)	1 (1–2)	2 (1–3)	2 (1–3)	2 (2–3)	2 (1–3)	1 (1–2)	1 (1–1)	0 (0–0)	--
Leukaemia	0 (0–0)	0 (0–0)	0 (0–0)	0 (0–0)	0 (0–0)	0 (0–0)	0 (0–0)	0 (0–0)	0 (0–0)	0 (0–0)	0 (0–0)	0 (0–0)	0 (0–1)	0 (0–1)	1 (1–1)	1 (1–1)	1 (1–2)	1 (1–2)	1 (1–2)	1 (1–2)	1 (1–1)	0 (0–0)	--
Acute lymphoid leukaemia	0 (0–0)	0 (0–0)	0 (0–0)	0 (0–0)	0 (0–0)	0 (0–0)	0 (0–0)	0 (0–0)	0 (0–0)	0 (0–0)	0 (0–0)	0 (0–0)	0 (0–0)	0 (0–0)	0 (0–0)	0 (0–0)	0 (0–0)	0 (0–0)	0 (0–0)	0 (0–0)	0 (0–0)	0 (0–0)	--
Chronic lymphoid leukaemia	--	--	--	--	--	--	--	0 (0–0)	0 (0–0)	0 (0–0)	0 (0–0)	0 (0–0)	0 (0–0)	0 (0–0)	0 (0–0)	0 (0–0)	0 (0–0)	0 (0–0)	0 (0–0)	0 (0–0)	0 (0–0)	0 (0–0)	--
Acute myeloid leukaemia	0 (0–0)	0 (0–0)	0 (0–0)	0 (0–0)	0 (0–0)	0 (0–0)	0 (0–0)	0 (0–0)	0 (0–0)	0 (0–0)	0 (0–0)	0 (0–0)	0 (0–0)	0 (0–0)	0 (0–0)	0 (0–0)	0 (0–0)	0 (0–0)	0 (0–0)	0 (0–0)	0 (0–0)	0 (0–0)	--
Chronic myeloid leukaemia	0 (0–0)	0 (0–0)	0 (0–0)	0 (0–0)	0 (0–0)	0 (0–0)	0 (0–0)	0 (0–0)	0 (0–0)	0 (0–0)	0 (0–0)	0 (0–0)	0 (0–0)	0 (0–0)	0 (0–0)	0 (0–0)	0 (0–0)	0 (0–0)	0 (0–0)	0 (0–0)	0 (0–0)	0 (0–0)	--
Other leukaemia	0 (0–0)	0 (0–0)	0 (0–0)	0 (0–0)	0 (0–0)	0 (0–0)	0 (0–0)	0 (0–0)	0 (0–0)	0 (0–0)	0 (0–0)	0 (0–0)	0 (0–0)	0 (0–0)	0 (0–0)	0 (0–1)	1 (0–1)	1 (0–1)	1 (0–1)	1 (0–1)	0 (0–1)	0 (0–0)	--
Other malignant cancers	0 (0–0)	0 (0–0)	0 (0–0)	0 (0–0)	0 (0–0)	0 (0–0)	0 (0–0)	0 (0–0)	0 (0–0)	0 (0–0)	0 (0–0)	0 (0–0)	0 (0–0)	0 (0–1)	1 (0–1)	1 (1–1)	1 (1–1)	1 (1–1)	1 (1–1)	1 (1–1)	1 (0–1)	0 (0–0)	--
Myelodysplastic, myeloproliferative, and other hematopoietic neoplasms	0 (0–0)	0 (0–0)	0 (0–0)	0 (0–0)	0 (0–0)	0 (0–0)	0 (0–0)	0 (0–0)	0 (0–0)	0 (0–0)	0 (0–0)	0 (0–0)	0 (0–0)	0 (0–0)	0 (0–0)	0 (0–0)	0 (0–0)	0 (0–0)	0 (0–0)	0 (0–0)	0 (0–0)	0 (0–0)	--
Cardiovascular diseases	0 (0–0)	0 (0–0)	0 (0–0)	0 (0–0)	0 (0–0)	0 (0–0)	0 (0–0)	0 (0–1)	1 (1–1)	2 (1–2)	2 (2–3)	4 (3–6)	9 (7–12)	14 (11–18)	26 (20–32)	36 (28–44)	45 (36–54)	57 (46–69)	57 (46–66)	58 (47–68)	54 (45–62)	34 (28–39)	--
Rheumatic heart disease	--	--	--	0 (0–0)	0 (0–0)	0 (0–0)	0 (0–0)	0 (0–0)	0 (0–0)	0 (0–0)	0 (0–0)	0 (0–0)	0 (0–0)	0 (0–0)	0 (0–0)	0 (0–0)	0 (0–0)	0 (0–0)	0 (0–0)	0 (0–0)	0 (0–0)	0 (0–0)	--
Ischaemic heart disease	--	--	--	--	--	--	0 (0–0)	0 (0–0)	0 (0–0)	0 (0–1)	1 (1–1)	1 (1–2)	4 (3–5)	6 (5–8)	11 (9–14)	15 (12–19)	19 (16–23)	24 (19–28)	22 (18–26)	22 (18–26)	22 (18–25)	14 (11–16)	--
Stroke	0 (0–0)	0 (0–0)	0 (0–0)	0 (0–0)	0 (0–0)	0 (0–0)	0 (0–0)	0 (0–0)	0 (0–0)	0 (0–1)	1 (0–1)	1 (1–2)	3 (2–4)	4 (3–6)	8 (5–11)	12 (8–16)	15 (11–20)	20 (15–26)	21 (16–27)	21 (15–27)	19 (14–24)	11 (8–14)	--
Ischaemic stroke	0 (0–0)	0 (0–0)	0 (0–0)	0 (0–0)	0 (0–0)	0 (0–0)	0 (0–0)	0 (0–0)	0 (0–0)	0 (0–0)	0 (0–0)	0 (0–0)	0 (0–1)	1 (0–1)	1 (1–2)	4 (2–5)	5 (4–7)	11 (7–14)	11 (8–15)	15 (11–20)	14 (11–19)	9 (6–11)	--
Intracerebral hemorrhage	0 (0–0)	0 (0–0)	0 (0–0)	0 (0–0)	0 (0–0)	0 (0–0)	0 (0–0)	0 (0–0)	0 (0–0)	0 (0–0)	0 (0–1)	1 (0–1)	2 (1–3)	3 (2–4)	5 (3–7)	7 (5–10)	9 (6–11)	9 (6–12)	9 (6–12)	5 (3–7)	4 (2–5)	2 (1–3)	--
Subarachnoid hemorrhage	0 (0–0)	0 (0–0)	0 (0–0)	0 (0–0)	0 (0–0)	0 (0–0)	0 (0–0)	0 (0–0)	0 (0–0)	0 (0–0)	0 (0–0)	0 (0–1)	1 (0–1)	1 (0–1)	1 (1–2)	1 (1–2)	1 (1–2)	1 (1–2)	1 (1–1)	1 (1–1)	1 (0–1)	0 (0–1)	--
Hypertensive heart disease	--	--	--	--	--	--	0 (0–0)	0 (0–0)	0 (0–0)	0 (0–0)	0 (0–0)	0 (0–0)	1 (0–1)	1 (1–2)	2 (1–3)	3 (2–4)	3 (2–4)	4 (2–6)	4 (3–6)	4 (3–6)	4 (3–5)	2 (2–3)	--
Non-rheumatic valvular heart disease	--	--	--	--	--	--	0 (0–0)	0 (0–0)	0 (0–0)	0 (0–0)	0 (0–0)	0 (0–0)	0 (0–0)	0 (0–0)	0 (0–0)	0 (0–0)	0 (0–1)	0 (0–1)	0 (0–1)	1 (0–1)	0 (0–1)	0 (0–0)	--
Non-rheumatic calcific aortic valve disease	--	--	--	--	--	--	0 (0–0)	0 (0–0)	0 (0–0)	0 (0–0)	0 (0–0)	0 (0–0)	0 (0–0)	0 (0–0)	0 (0–0)	0 (0–0)	0 (0–1)	0 (0–1)	0 (0–1)	0 (0–1)	0 (0–0)	0 (0–0)	--
Non-rheumatic degenerative mitral valve disease	--	--	--	--	--	--	0 (0–0)	0 (0–0)	0 (0–0)	0 (0–0)	0 (0–0)	0 (0–0)	0 (0–0)	0 (0–0)	0 (0–0)	0 (0–0)	0 (0–0)	0 (0–0)	0 (0–0)	0 (0–0)	0 (0–0)	0 (0–0)	--
Other non-rheumatic valve diseases	--	--	--	--	--	--	0 (0–0)	0 (0–0)	0 (0–0)	0 (0–0)	0 (0–0)	0 (0–0)	0 (0–0)	0 (0–0)	0 (0–0)	0 (0–0)	0 (0–0)	0 (0–0)	0 (0–0)	0 (0–0)	0 (0–0)	0 (0–0)	--
Cardiomyopathy and myocarditis	0 (0–0)	0 (0–0)	0 (0–0)	0 (0–0)	0 (0–0)	0 (0–0)	0 (0–0)	0 (0–0)	0 (0–0)	0 (0–0)	0 (0–1)	1 (0–1)	1 (1–2)	1 (1–2)	2 (1–3)	3 (2–4)	3 (2–4)	3 (2–4)	2 (1–3)	2 (1–3)	2 (1–3)	1 (1–2)	--
Myocarditis	0 (0–0)	0 (0–0)	0 (0–0)	0 (0–0)	0 (0–0)	0 (0–0)	0 (0–0)	0 (0–0)	0 (0–0)	0 (0–0)	0 (0–0)	0 (0–0)	0 (0–0)	0 (0–0)	0 (0–0)	0 (0–0)	0 (0–0)	0 (0–0)	0 (0–0)	0 (0–0)	0 (0–0)	0 (0–0)	--
Alcoholic cardiomyopathy	--	--	--	--	--	--	0 (0–0)	0 (0–0)	0 (0–0)	0 (0–0)	0 (0–0)	0 (0–0)	1 (0–1)	1 (0–1)	1 (1–2)	1 (1–2)	1 (1–2)	1 (1–2)	1 (0–1)	0 (0–0)	0 (0–0)	0 (0–0)	--
Other cardiomyopathy	0 (0–0)	0 (0–0)	0 (0–0)	0 (0–0)	0 (0–0)	0 (0–0)	0 (0–0)	0 (0–0)	0 (0–0)	0 (0–0)	0 (0–0)	0 (0–0)	1 (0–1)	1 (0–1)	1 (1–2)	1 (1–2)	1 (1–2)	2 (1–3)	1 (1–2)	2 (1–3)	2 (1–2)	1 (1–1)	--
Pulmonary Arterial Hypertension	0 (0–0)	0 (0–0)	0 (0–0)	0 (0–0)	0 (0–0)	0 (0–0)	0 (0–0)	0 (0–0)	0 (0–0)	0 (0–0)	0 (0–0)	0 (0–0)	0 (0–0)	0 (0–0)	0 (0–0)	0 (0–0)	0 (0–0)	0 (0–0)	0 (0–0)	0 (0–0)	0 (0–0)	0 (0–0)	--
Atrial fibrillation and flutter	--	--	--	--	--	--	--	--	--	0 (0–0)	0 (0–0)	0 (0–0)	0 (0–0)	0 (0–0)	0 (0–0)	0 (0–1)	0 (0–1)	1 (1–1)	1 (1–2)	2 (1–3)	3 (2–4)	2 (2–4)	--
Aortic aneurysm	--	--	--	--	--	--	0 (0–0)	0 (0–0)	0 (0–0)	0 (0–0)	0 (0–0)	0 (0–0)	0 (0–0)	0 (0–0)	0 (0–1)	1 (1–1)	1 (1–2)	1 (1–2)	1 (1–2)	1 (1–2)	1 (1–1)	1 (0–1)	--
Peripheral vascular disease	--	--	--	--	--	--	--	--	--	--	--	0 (0–0)	0 (0–0)	0 (0–1)	0 (0–1)	1 (0–2)	1 (0–2)	2 (1–4)	2 (1–4)	2 (1–5)	2 (1–4)	1 (0–3)	--
Endocarditis	0 (0–0)	0 (0–0)	0 (0–0)	0 (0–0)	0 (0–0)	0 (0–0)	0 (0–0)	0 (0–0)	0 (0–0)	0 (0–0)	0 (0–0)	0 (0–0)	0 (0–0)	0 (0–1)	0 (0–1)	0 (0–1)	0 (0–1)	0 (0–1)	0 (0–1)	0 (0–1)	0 (0–0)	0 (0–0)	--
Other cardiovascular and circulatory diseases	0 (0–0)	0 (0–0)	0 (0–0)	0 (0–0)	0 (0–0)	0 (0–0)	0 (0–0)	0 (0–0)	0 (0–0)	0 (0–0)	0 (0–0)	0 (0–0)	0 (0–1)	0 (0–1)	1 (1–1)	1 (1–1)	1 (1–2)	1 (1–2)	2 (1–2)	2 (1–3)	2 (1–2)	1 (1–1)	--
Chronic respiratory diseases	0 (0–0)	0 (0–0)	0 (0–0)	0 (0–0)	0 (0–0)	0 (0–0)	0 (0–0)	0 (0–0)	0 (0–0)	0 (0–0)	0 (0–0)	0 (0–0)	1 (0–1)	1 (1–1)	2 (1–2)	3 (2–4)	4 (3–5)	5 (4–7)	6 (4–8)	6 (5–8)	5 (4–7)	3 (2–4)	--
Chronic obstructive pulmonary disease	--	--	0 (0–0)	0 (0–0)	0 (0–0)	0 (0–0)	0 (0–0)	0 (0–0)	0 (0–0)	0 (0–0)	0 (0–0)	0 (0–0)	0 (0–0)	0 (0–0)	1 (0–1)	2 (1–2)	2 (2–3)	4 (3–5)	4 (3–6)	5 (3–6)	4 (3–6)	2 (2–3)	--
Pneumoconiosis	--	--	--	--	--	--	0 (0–0)	0 (0–0)	0 (0–0)	0 (0–0)	0 (0–0)	0 (0–0)	0 (0–0)	0 (0–0)	0 (0–0)	0 (0–0)	0 (0–0)	0 (0–0)	0 (0–0)	0 (0–0)	0 (0–0)	0 (0–0)	--
Silicosis	--	--	--	--	--	--	0 (0–0)	0 (0–0)	0 (0–0)	0 (0–0)	0 (0–0)	0 (0–0)	0 (0–0)	0 (0–0)	0 (0–0)	0 (0–0)	0 (0–0)	0 (0–0)	0 (0–0)	0 (0–0)	0 (0–0)	0 (0–0)	--
Asbestosis	--	--	--	--	--	--	0 (0–0)	0 (0–0)	0 (0–0)	0 (0–0)	0 (0–0)	0 (0–0)	0 (0–0)	0 (0–0)	0 (0–0)	0 (0–0)	0 (0–0)	0 (0–0)	0 (0–0)	0 (0–0)	0 (0–0)	0 (0–0)	--
Coal workers pneumoconiosis	--	--	--	--	--	--	0 (0–0)	0 (0–0)	0 (0–0)	0 (0–0)	0 (0–0)	0 (0–0)	0 (0–0)	0 (0–0)	0 (0–0)	0 (0–0)	0 (0–0)	0 (0–0)	0 (0–0)	0 (0–0)	0 (0–0)	0 (0–0)	--
Other pneumoconiosis	--	--	--	--	--	--	0 (0–0)	0 (0–0)	0 (0–0)	0 (0–0)	0 (0–0)	0 (0–0)	0 (0–0)	0 (0–0)	0 (0–0)	0 (0–0)	0 (0–0)	0 (0–0)	0 (0–0)	0 (0–0)	0 (0–0)	0 (0–0)	--
Asthma	--	--	--	0 (0–0)	0 (0–0)	0 (0–0)	0 (0–0)	0 (0–0)	0 (0–0)	0 (0–0)	0 (0–0)	0 (0–0)	0 (0–0)	0 (0–0)	1 (0–1)	1 (0–1)	1 (1–1)	1 (1–2)	1 (1–1)	1 (1–2)	1 (0–1)	0 (0–1)	--
Interstitial lung disease and pulmonary sarcoidosis	--	--	--	0 (0–0)	0 (0–0)	0 (0–0)	0 (0–0)	0 (0–0)	0 (0–0)	0 (0–0)	0 (0–0)	0 (0–0)	0 (0–0)	0 (0–0)	0 (0–0)	0 (0–0)	0 (0–0)	0 (0–1)	0 (0–1)	0 (0–1)	0 (0–1)	0 (0–0)	--
Other chronic respiratory diseases	0 (0–0)	0 (0–0)	0 (0–0)	0 (0–0)	0 (0–0)	0 (0–0)	0 (0–0)	0 (0–0)	0 (0–0)	0 (0–0)	0 (0–0)	0 (0–0)	0 (0–0)	0 (0–0)	0 (0–0)	0 (0–0)	0 (0–0)	0 (0–0)	0 (0–0)	0 (0–0)	0 (0–0)	0 (0–0)	--
Digestive diseases	0 (0–0)	0 (0–0)	0 (0–0)	0 (0–0)	0 (0–0)	0 (0–0)	0 (0–0)	0 (0–0)	0 (0–1)	1 (1–1)	1 (1–1)	2 (1–2)	3 (2–4)	4 (3–6)	6 (5–9)	8 (6–11)	8 (6–10)	9 (6–11)	7 (5–9)	7 (5–9)	5 (4–7)	3 (2–3)	--
Cirrhosis and other chronic liver diseases	--	--	--	0 (0–0)	0 (0–0)	0 (0–0)	0 (0–0)	0 (0–0)	0 (0–0)	0 (0–1)	1 (0–1)	1 (1–2)	2 (2–3)	3 (2–4)	4 (3–6)	5 (4–7)	5 (3–6)	4 (3–6)	3 (2–4)	2 (2–3)	2 (1–2)	1 (1–1)	--
Cirrhosis and other chronic liver diseases due to hepatitis B	--	--	--	0 (0–0)	0 (0–0)	0 (0–0)	0 (0–0)	0 (0–0)	0 (0–0)	0 (0–0)	0 (0–0)	0 (0–0)	0 (0–0)	0 (0–1)	0 (0–1)	0 (0–1)	0 (0–1)	0 (0–0)	0 (0–0)	0 (0–0)	0 (0–0)	0 (0–0)	--
Cirrhosis and other chronic liver diseases due to hepatitis C	--	--	--	0 (0–0)	0 (0–0)	0 (0–0)	0 (0–0)	0 (0–0)	0 (0–0)	0 (0–0)	0 (0–0)	0 (0–0)	0 (0–1)	1 (0–1)	1 (0–1)	1 (0–2)	1 (0–1)	1 (0–1)	0 (0–1)	0 (0–1)	0 (0–0)	0 (0–0)	--
Cirrhosis and other chronic liver diseases due to alcohol use	--	--	--	--	--	--	0 (0–0)	0 (0–0)	0 (0–0)	0 (0–0)	0 (0–0)	1 (0–1)	1 (1–2)	1 (1–2)	2 (1–3)	3 (2–4)	2 (2–3)	2 (1–3)	1 (1–2)	1 (1–1)	1 (0–1)	0 (0–0)	--
Cirrhosis and other chronic liver diseases due to NAFLD	--	--	--	--	--	--	0 (0–0)	0 (0–0)	0 (0–0)	0 (0–0)	0 (0–0)	0 (0–0)	0 (0–1)	0 (0–1)	1 (0–1)	1 (0–1)	1 (0–1)	1 (0–1)	1 (0–1)	0 (0–1)	0 (0–1)	0 (0–0)	--
Cirrhosis and other chronic liver diseases due to other causes	--	--	--	0 (0–0)	0 (0–0)	0 (0–0)	0 (0–0)	0 (0–0)	0 (0–0)	0 (0–0)	0 (0–0)	0 (0–0)	0 (0–0)	0 (0–0)	0 (0–1)	0 (0–1)	0 (0–1)	0 (0–1)	0 (0–1)	0 (0–1)	0 (0–0)	0 (0–0)	--
Upper digestive system diseases	--	--	--	0 (0–0)	0 (0–0)	0 (0–0)	0 (0–0)	0 (0–0)	0 (0–0)	0 (0–0)	0 (0–0)	0 (0–0)	0 (0–0)	0 (0–1)	1 (0–1)	1 (0–1)	1 (1–1)	1 (1–1)	1 (1–1)	1 (1–1)	1 (1–1)	1 (0–1)	--

Peptic ulcer disease	--	--	--	0 (0–0)	0 (0–0)	0 (0–0)	0 (0–0)	0 (0–0)	0 (0–0)	0 (0–0)	0 (0–0)	0 (0–0)	0 (0–0)	0 (0–0)	0 (0–1)	1 (0–1)	1 (1–1)	1 (1–1)	1 (1–1)	1 (1–1)	1 (1–1)	0 (0–1)	--
Gastritis and duodenitis	--	--	--	0 (0–0)	0 (0–0)	0 (0–0)	0 (0–0)	0 (0–0)	0 (0–0)	0 (0–0)	0 (0–0)	0 (0–0)	0 (0–0)	0 (0–0)	0 (0–0)	0 (0–0)	0 (0–0)	0 (0–0)	0 (0–0)	0 (0–0)	0 (0–0)	0 (0–0)	--
Appendicitis	--	--	--	0 (0–0)	0 (0–0)	0 (0–0)	0 (0–0)	0 (0–0)	0 (0–0)	0 (0–0)	0 (0–0)	0 (0–0)	0 (0–0)	0 (0–0)	0 (0–0)	0 (0–0)	0 (0–0)	0 (0–0)	0 (0–0)	0 (0–0)	0 (0–0)	0 (0–0)	--
Paralytic ileus and intestinal obstruction	0 (0–0)	0 (0–0)	0 (0–0)	0 (0–0)	0 (0–0)	0 (0–0)	0 (0–0)	0 (0–0)	0 (0–0)	0 (0–0)	0 (0–0)	0 (0–0)	0 (0–0)	0 (0–1)	1 (0–1)	1 (0–1)	1 (1–1)	1 (1–2)	1 (1–2)	2 (1–2)	1 (1–2)	1 (0–1)	--
Inguinal, femoral, and abdominal hernia	0 (0–0)	0 (0–0)	0 (0–0)	0 (0–0)	0 (0–0)	0 (0–0)	0 (0–0)	0 (0–0)	0 (0–0)	0 (0–0)	0 (0–0)	0 (0–0)	0 (0–0)	0 (0–0)	0 (0–0)	0 (0–0)	0 (0–0)	0 (0–0)	0 (0–0)	0 (0–0)	0 (0–0)	0 (0–0)	--
Inflammatory bowel disease	--	--	--	0 (0–0)	0 (0–0)	0 (0–0)	0 (0–0)	0 (0–0)	0 (0–0)	0 (0–0)	0 (0–0)	0 (0–0)	0 (0–0)	0 (0–0)	0 (0–0)	0 (0–0)	0 (0–0)	0 (0–0)	0 (0–0)	0 (0–0)	0 (0–0)	0 (0–0)	--
Vascular intestinal disorders	--	--	--	0 (0–0)	0 (0–0)	0 (0–0)	0 (0–0)	0 (0–0)	0 (0–0)	0 (0–0)	0 (0–0)	0 (0–0)	0 (0–0)	0 (0–0)	0 (0–0)	0 (0–0)	0 (0–0)	0 (0–0)	0 (0–0)	0 (0–0)	0 (0–0)	0 (0–0)	--
Gallbladder and biliary diseases	--	--	--	0 (0–0)	0 (0–0)	0 (0–0)	0 (0–0)	0 (0–0)	0 (0–0)	0 (0–0)	0 (0–0)	0 (0–0)	0 (0–0)	0 (0–0)	0 (0–0)	0 (0–0)	0 (0–0)	0 (0–0)	0 (0–0)	0 (0–0)	0 (0–0)	0 (0–0)	--
Pancreatitis	--	--	--	0 (0–0)	0 (0–0)	0 (0–0)	0 (0–0)	0 (0–0)	0 (0–0)	0 (0–0)	0 (0–0)	0 (0–0)	0 (0–0)	0 (0–0)	0 (0–1)	0 (0–1)	0 (0–1)	0 (0–1)	0 (0–0)	0 (0–0)	0 (0–0)	0 (0–0)	--
Other digestive diseases	--	--	--	0 (0–0)	0 (0–0)	0 (0–0)	0 (0–0)	0 (0–0)	0 (0–0)	0 (0–0)	0 (0–0)	0 (0–0)	0 (0–0)	0 (0–0)	0 (0–1)	1 (0–1)	1 (0–1)	1 (0–1)	1 (0–1)	1 (0–1)	1 (0–1)	0 (0–1)	--
Alzheimer's disease and other dementias	--	--	--	--	--	--	--	--	--	--	--	0 (0–0)	0 (0–0)	0 (0–0)	0 (0–1)	1 (0–2)	2 (0–5)	3 (1–9)	5 (1–14)	9 (2–25)	11 (3–31)	8 (2–22)	--
Parkinson's disease	--	--	--	--	--	--	--	0 (0–0)	0 (0–0)	0 (0–0)	0 (0–0)	0 (0–0)	0 (0–0)	0 (0–0)	0 (0–0)	0 (0–0)	1 (1–1)	2 (1–2)	3 (2–4)	4 (3–4)	3 (2–4)	1 (1–2)	--
Idiopathic epilepsy	--	--	0 (0–0)	0 (0–0)	0 (0–0)	0 (0–0)	0 (0–0)	0 (0–0)	0 (0–0)	0 (0–0)	0 (0–0)	0 (0–0)	0 (0–1)	0 (0–1)	0 (0–1)	0 (0–1)	0 (0–0)	0 (0–0)	0 (0–0)	0 (0–0)	0 (0–0)	0 (0–0)	--
Multiple sclerosis	--	--	--	--	0 (0–0)	0 (0–0)	0 (0–0)	0 (0–0)	0 (0–0)	0 (0–0)	0 (0–0)	0 (0–0)	0 (0–0)	0 (0–0)	0 (0–0)	0 (0–0)	0 (0–0)	0 (0–0)	0 (0–0)	0 (0–0)	0 (0–0)	0 (0–0)	--
Motor neuron disease	0 (0–0)	0 (0–0)	0 (0–0)	0 (0–0)	0 (0–0)	0 (0–0)	0 (0–0)	0 (0–0)	0 (0–0)	0 (0–0)	0 (0–0)	0 (0–0)	0 (0–0)	0 (0–0)	0 (0–0)	0 (0–1)	0 (0–1)	0 (0–1)	0 (0–0)	0 (0–0)	0 (0–0)	0 (0–0)	--
Other neurological disorders	--	--	0 (0–0)	0 (0–0)	0 (0–0)	0 (0–0)	0 (0–0)	0 (0–0)	0 (0–0)	0 (0–0)	0 (0–0)	0 (0–0)	0 (0–0)	0 (0–0)	0 (0–1)	0 (0–1)	0 (0–1)	0 (0–1)	0 (0–0)	0 (0–0)	0 (0–0)	0 (0–0)	--
Eating disorders	--	--	--	--	0 (0–0)	0 (0–0)	0 (0–0)	0 (0–0)	0 (0–0)	0 (0–0)	0 (0–0)	0 (0–0)	0 (0–0)	--	--	--	--	--	--	--	--	--	--
Anorexia nervosa	--	--	--	--	0 (0–0)	0 (0–0)	0 (0–0)	0 (0–0)	0 (0–0)	0 (0–0)	0 (0–0)	0 (0–0)	0 (0–0)	--	--	--	--	--	--	--	--	--	--
Bulimia nervosa	--	--	--	--	0 (0–0)	0 (0–0)	0 (0–0)	0 (0–0)	0 (0–0)	0 (0–0)	0 (0–0)	0 (0–0)	0 (0–0)	--	--	--	--	--	--	--	--	--	--
Alcohol use disorders	--	--	--	--	--	0 (0–0)	0 (0–0)	0 (0–0)	0 (0–0)	0 (0–0)	0 (0–1)	1 (0–1)	1 (0–1)	1 (1–1)	1 (1–2)	1 (1–2)	1 (1–1)	1 (0–1)	0 (0–0)	0 (0–0)	0 (0–0)	0 (0–0)	--
Drug use disorders	0 (0–0)	0 (0–0)	0 (0–0)	0 (0–0)	0 (0–0)	0 (0–0)	0 (0–0)	0 (0–0)	0 (0–0)	0 (0–0)	0 (0–0)	0 (0–0)	0 (0–0)	0 (0–0)	0 (0–0)	0 (0–0)	0 (0–0)	0 (0–0)	0 (0–0)	0 (0–0)	0 (0–0)	0 (0–0)	--
Opioid use disorders	0 (0–0)	0 (0–0)	0 (0–0)	0 (0–0)	0 (0–0)	0 (0–0)	0 (0–0)	0 (0–0)	0 (0–0)	0 (0–0)	0 (0–0)	0 (0–0)	0 (0–0)	0 (0–0)	0 (0–0)	0 (0–0)	0 (0–0)	0 (0–0)	0 (0–0)	0 (0–0)	0 (0–0)	0 (0–0)	--
Cocaine use disorders	--	--	--	--	--	0 (0–0)	0 (0–0)	0 (0–0)	0 (0–0)	0 (0–0)	0 (0–0)	0 (0–0)	0 (0–0)	0 (0–0)	0 (0–0)	0 (0–0)	0 (0–0)	0 (0–0)	0 (0–0)	0 (0–0)	0 (0–0)	0 (0–0)	--
Amphetamine use disorders	--	--	--	--	--	0 (0–0)	0 (0–0)	0 (0–0)	0 (0–0)	0 (0–0)	0 (0–0)	0 (0–0)	0 (0–0)	0 (0–0)	0 (0–0)	0 (0–0)	0 (0–0)	0 (0–0)	0 (0–0)	0 (0–0)	0 (0–0)	0 (0–0)	--
Other drug use disorders	--	--	--	--	--	0 (0–0)	0 (0–0)	0 (0–0)	0 (0–0)	0 (0–0)	0 (0–0)	0 (0–0)	0 (0–0)	0 (0–0)	0 (0–0)	0 (0–0)	0 (0–0)	0 (0–0)	0 (0–0)	0 (0–0)	0 (0–0)	0 (0–0)	--
Diabetes mellitus	0 (0–0)	0 (0–0)	0 (0–0)	0 (0–0)	0 (0–0)	0 (0–0)	0 (0–0)	0 (0–0)	0 (0–1)	1 (0–1)	1 (1–1)	1 (1–2)	3 (2–4)	4 (3–6)	8 (6–11)	12 (9–16)	16 (12–21)	19 (14–25)	20 (15–27)	18 (14–24)	14 (11–18)	7 (6–9)	--
Diabetes mellitus type 1	0 (0–0)	0 (0–0)	0 (0–0)	0 (0–0)	0 (0–0)	0 (0–0)	0 (0–0)	0 (0–0)	0 (0–0)	0 (0–0)	0 (0–0)	0 (0–1)	0 (0–0)	0 (0–0)	1 (0–1)	1 (0–1)	1 (0–1)	1 (0–1)	1 (0–1)	0 (0–1)	0 (0–1)	0 (0–0)	--
Diabetes mellitus type 2	--	--	--	--	--	--	0 (0–0)	0 (0–0)	0 (0–0)	0 (0–1)	1 (0–1)	1 (1–1)	2 (2–3)	4 (3–5)	8 (5–11)	12 (8–16)	15 (11–20)	19 (14–24)	20 (15–26)	18 (14–24)	13 (10–17)	7 (5–9)	--
Chronic kidney disease	--	--	0 (0–0)	0 (0–0)	0 (0–0)	0 (0–0)	0 (0–0)	0 (0–0)	0 (0–1)	1 (0–1)	1 (1–1)	1 (1–2)	2 (1–3)	3 (2–4)	4 (3–6)	6 (4–8)	7 (5–9)	8 (6–11)	7 (5–10)	8 (6–11)	6 (5–8)	4 (3–5)	--
Chronic kidney disease due to diabetes mellitus type 1	--	--	0 (0–0)	0 (0–0)	0 (0–0)	0 (0–0)	0 (0–0)	0 (0–0)	0 (0–0)	0 (0–0)	0 (0–0)	0 (0–0)	0 (0–1)	0 (0–1)	0 (0–1)	0 (0–1)	0 (0–1)	0 (0–1)	0 (0–0)	0 (0–0)	0 (0–0)	0 (0–0)	--
Chronic kidney disease due to diabetes mellitus type 2	--	--	--	--	--	--	0 (0–0)	0 (0–0)	0 (0–0)	0 (0–0)	0 (0–0)	0 (0–0)	0 (0–1)	1 (0–1)	1 (1–2)	2 (1–3)	2 (1–3)	3 (2–4)	2 (1–3)	3 (2–4)	2 (1–3)	1 (1–2)	--
Chronic kidney disease due to hypertension	--	--	--	--	--	--	0 (0–0)	0 (0–0)	0 (0–0)	0 (0–0)	0 (0–0)	0 (0–0)	0 (0–0)	0 (0–1)	1 (0–1)	1 (1–2)	2 (1–3)	2 (1–3)	2 (1–3)	3 (2–4)	2 (2–3)	2 (1–2)	--
Chronic kidney disease due to glomerulonephritis	--	--	0 (0–0)	0 (0–0)	0 (0–0)	0 (0–0)	0 (0–0)	0 (0–0)	0 (0–0)	0 (0–0)	0 (0–0)	0 (0–1)	1 (0–1)	1 (0–1)	1 (1–2)	1 (1–2)	1 (1–2)	1 (1–2)	1 (1–2)	1 (0–2)	1 (0–1)	0 (0–1)	--
Chronic kidney disease due to other and unspecified causes	--	--	0 (0–0)	0 (0–0)	0 (0–0)	0 (0–0)	0 (0–0)	0 (0–0)	0 (0–0)	0 (0–0)	0 (0–0)	0 (0–0)	0 (0–1)	1 (0–1)	1 (0–2)	1 (1–2)	1 (1–2)	2 (1–3)	1 (1–2)	2 (1–3)	1 (1–2)	1 (0–1)	--
Acute glomerulonephritis	--	--	0 (0–0)	0 (0–0)	0 (0–0)	0 (0–0)	0 (0–0)	0 (0–0)	0 (0–0)	0 (0–0)	0 (0–0)	0 (0–0)	0 (0–0)	0 (0–0)	0 (0–0)	0 (0–0)	0 (0–0)	0 (0–0)	0 (0–0)	0 (0–0)	0 (0–0)	0 (0–0)	--
Skin and subcutaneous diseases	--	--	0 (0–0)	0 (0–0)	0 (0–0)	0 (0–0)	0 (0–0)	0 (0–0)	0 (0–0)	0 (0–0)	0 (0–1)	0 (0–1)	1 (0–1)	1 (0–2)	1 (1–3)	2 (1–4)	3 (1–6)	4 (2–9)	5 (2–11)	5 (3–12)	5 (2–11)	3 (1–7)	--
Bacterial skin diseases	--	--	0 (0–0)	0 (0–0)	0 (0–0)	0 (0–0)	0 (0–0)	0 (0–0)	0 (0–0)	0 (0–0)	0 (0–0)	0 (0–1)	0 (0–1)	0 (0–1)	1 (0–2)	1 (0–2)	1 (0–3)	1 (0–3)	1 (0–4)	1 (0–4)	1 (0–4)	1 (0–2)	--
Cellulitis	--	--	0 (0–0)	0 (0–0)	0 (0–0)	0 (0–0)	0 (0–0)	0 (0–0)	0 (0–0)	0 (0–0)	0 (0–0)	0 (0–0)	0 (0–0)	0 (0–0)	0 (0–1)	0 (0–1)	0 (0–1)	0 (0–1)	0 (0–1)	0 (0–1)	0 (0–1)	0 (0–1)	--
Pyoderma	--	--	0 (0–0)	0 (0–0)	0 (0–0)	0 (0–0)	0 (0–0)	0 (0–0)	0 (0–0)	0 (0–0)	0 (0–0)	0 (0–0)	0 (0–1)	0 (0–1)	0 (0–1)	1 (0–1)	1 (0–2)	1 (0–2)	1 (0–3)	1 (0–3)	1 (0–2)	1 (0–2)	--
Decubitus ulcer	--	--	--	0 (0–0)	0 (0–0)	0 (0–0)	0 (0–0)	0 (0–0)	0 (0–0)	0 (0–0)	0 (0–0)	0 (0–0)	0 (0–1)	0 (0–1)	1 (0–2)	1 (0–3)	2 (1–5)	3 (1–6)	3 (1–9)	4 (2–9)	4 (2–8)	2 (1–5)	--
Other skin and subcutaneous diseases	--	--	0 (0–0)	0 (0–0)	0 (0–0)	0 (0–0)	0 (0–0)	0 (0–0)	0 (0–0)	0 (0–0)	0 (0–0)	0 (0–0)	0 (0–0)	0 (0–0)	0 (0–0)	0 (0–0)	0 (0–0)	0 (0–0)	0 (0–0)	0 (0–0)	0 (0–0)	0 (0–0)	--
Musculoskeletal disorders	--	--	--	--	0 (0–0)	0 (0–0)	0 (0–0)	0 (0–0)	0 (0–0)	0 (0–0)	0 (0–0)	0 (0–0)	0 (0–0)	0 (0–0)	0 (0–0)	0 (0–0)	0 (0–0)	0 (0–0)	0 (0–0)	0 (0–0)	1 (0–1)	0 (0–1)	--
Rheumatoid arthritis	--	--	--	--	0 (0–0)	0 (0–0)	0 (0–0)	0 (0–0)	0 (0–0)	0 (0–0)	0 (0–0)	0 (0–0)	0 (0–0)	0 (0–0)	0 (0–0)	0 (0–0)	0 (0–0)	0 (0–0)	0 (0–0)	0 (0–0)	0 (0–0)	0 (0–0)	--
Other musculoskeletal disorders	--	--	--	--	0 (0–0)	0 (0–0)	0 (0–0)	0 (0–0)	0 (0–0)	0 (0–0)	0 (0–0)	0 (0–0)	0 (0–0)	0 (0–0)	0 (0–0)	0 (0–0)	0 (0–0)	0 (0–0)	0 (0–0)	0 (0–0)	0 (0–1)	0 (0–0)	--
Congenital anomalies	2 (1–3)	0 (0–1)	1 (1–1)	0 (0–0)	0 (0–0)	0 (0–0)	0 (0–0)	0 (0–0)	0 (0–0)	0 (0–0)	0 (0–0)	0 (0–0)	0 (0–0)	0 (0–0)	0 (0–0)	0 (0–0)	0 (0–0)	--	--	--	--	--	--
Neural tube defects	0 (0–0)	0 (0–0)	0 (0–0)	0 (0–0)	0 (0–0)	0 (0–0)	0 (0–0)	0 (0–0)	0 (0–0)	0 (0–0)	0 (0–0)	0 (0–0)	0 (0–0)	0 (0–0)	0 (0–0)	0 (0–0)	0 (0–0)	--	--	--	--	--	--
Congenital heart anomalies	1 (0–1)	0 (0–0)	0 (0–1)	0 (0–0)	0 (0–0)	0 (0–0)	0 (0–0)	0 (0–0)	0 (0–0)	0 (0–0)	0 (0–0)	0 (0–0)	0 (0–0)	0 (0–0)	0 (0–0)	0 (0–0)	0 (0–0)	--	--	--	--	--	--
Orofacial clefts	0 (0–0)	0 (0–0)	0 (0–0)	0 (0–0)	--	--	--	--	--	--	--	--	--	--	--	--	--	--	--	--	--	--	--
Down's syndrome	0 (0–0)	0 (0–0)	0 (0–0)	0 (0–0)	0 (0–0)	0 (0–0)	0 (0–0)	0 (0–0)	0 (0–0)	0 (0–0)	0 (0–0)	0 (0–0)	0 (0–0)	0 (0–0)	0 (0–0)	0 (0–0)	0 (0–0)	--	--	--	--	--	--
Other chromosomal abnormalities	0 (0–0)	0 (0–0)	0 (0–0)	0 (0–0)	0 (0–0)	0 (0–0)	0 (0–0)	0 (0–0)	0 (0–0)	0 (0–0)	0 (0–0)	0 (0–0)	0 (0–0)	0 (0–0)	0 (0–0)	0 (0–0)	0 (0–0)	--	--	--	--	--	--
Congenital musculoskeletal and limb anomalies	0 (0–0)	0 (0–0)	0 (0–0)	0 (0–0)	0 (0–0)	0 (0–0)	0 (0–0)	0 (0–0)	0 (0–0)	0 (0–0)	0 (0–0)	0 (0–0)	0 (0–0)	0 (0–0)	0 (0–0)	0 (0–0)	0 (0–0)	--	--	--	--	--	--
Urogenital congenital anomalies	0 (0–0)	0 (0–0)	0 (0–0)	0 (0–0)	0 (0–0)	0 (0–0)	0 (0–0)	0 (0–0)	0 (0–0)	0 (0–0)	0 (0–0)	0 (0–0)	0 (0–0)	0 (0–0)	0 (0–0)	0 (0–0)	0 (0–0)	--	--	--	--	--	--
Digestive congenital anomalies	0 (0–0)	0 (0–0)	0 (0–0)	0 (0–0)	0 (0–0)	0 (0–0)	0 (0–0)	0 (0–0)	0 (0–0)	0 (0–0)	0 (0–0)	0 (0–0)	0 (0–0)	0 (0–0)	0 (0–0)	0 (0–0)	0 (0–0)	--	--	--	--	--	--
Other congenital anomalies	0 (0–1)	0 (0–0)	0 (0–0)	0 (0–0)	0 (0–0)	0 (0–0)	0 (0–0)	0 (0–0)	0 (0–0)	0 (0–0)	0 (0–0)	0 (0–0)	0 (0–0)	0 (0–0)	0 (0–0)	0 (0–0)	0 (0–0)	--	--	--	--	--	--
Urinary diseases and male infertility	0 (0–0)	0 (0–0)	0 (0–0)	0 (0–0)	0 (0–0)	0 (0–0)	0 (0–0)	0 (0–0)	0 (0–0)	0 (0–0)	0 (0–0)	0 (0–0)	0 (0–1)	1 (0–1)	1 (1–2)	2 (1–3)	3 (2–4)	5 (2–7)	5 (3–8)	6 (3–10)	6 (3–9)	4 (2–6)	--
Urinary tract infections and interstitial nephritis	0 (0–0)	0 (0–0)	0 (0–0)	0 (0–0)	0 (0–0)	0 (0–0)	0 (0–0)	0 (0–0)	0 (0–0)	0 (0–0)	0 (0–0)	0 (0–0)	0 (0–1)	1 (0–1)	1 (1–2)	2 (1–3)	3 (1–4)	4 (2–6)	5 (3–8)	6 (3–9)	6 (3–9)	4 (2–5)	--
Urolithiasis	--	--	--	0 (0–0)	0 (0–0)	0 (0–0)	0 (0–0)	0 (0–0)	0 (0–0)	0 (0–0)	0 (0–0)	0 (0–0)	0 (0–0)	0 (0–0)	0 (0–0)	0 (0–0)	0 (0–0)	0 (0–0)	0 (0–0)	0 (0–0)	0 (0–0)	0 (0–0)	--
Other urinary diseases	0 (0–0)	0 (0–0)	0 (0–0)	0 (0–0)	0 (0–0)	0 (0–0)	0 (0–0)	0 (0–0)	0 (0–0)	0 (0–0)	0 (0–0)	0 (0–0)	0 (0–0)	0 (0–0)	0 (0–0)	0 (0–0)	0 (0–0)	0 (0–0)	0 (0–1)	0 (0–1)	0 (0–1)	0 (0–0)	--
Gynecological diseases	--	--	--	--	--	--	--	--	--	--	--	--	--	--	--	--	--	--	--	--	--	--	--
Uterine fibroids	--	--	--	--	--	--	--	--	--	--	--	--	--	--	--	--	--	--	--	--	--	--	--
Endometriosis	--	--	--	--	--	--	--	--	--	--	--	--	--	--	--	--	--	--	--	--	--	--	--
Genital prolapse	--	--	--	--	--	--	--	--	--	--	--	--	--	--	--	--	--	--	--	--	--	--	--
Other gynecological diseases	--	--	--	--	--	--	--	--	--	--	--	--	--	--	--	--	--	--	--	--	--	--	--
Hemoglobinopathies and hemolytic anaemias	0 (0–0)	0 (0–0)	0 (0–0)	0 (0–0)	0 (0–0)	0 (0–0)	0 (0–0)	0 (0–0)	0 (0–0)	0 (0–0)	0 (0–0)	0 (0–0)	0 (0–0)	0 (0–0)	0 (0–0)	0 (0–0)	0 (0–0)	0 (0–0)	0 (0–0)	0 (0–0)	0 (0–0)	0 (0–0)	--
Thalassemias	0 (0–0)	0 (0–0)	0 (0–0)	0 (0–0)	0 (0–0)	0 (0–0)	0 (0–0)	0 (0–0)	0 (0–0)	0 (0–0)	0 (0–0)	0 (0–0)	0 (0–0)	0 (0–0)	0 (0–0)	0 (0–0)	0 (0–0)	0 (0–0)	0 (0–0)	0 (0–0)	0 (0–0)	0 (0–0)	--
Sickle cell disorders	0 (0–0)	0 (0–0)	0 (0–0)	0 (0–0)	0 (0–0)	0 (0–0)	0 (0–0)	0 (0–0)	0 (0–0)	0 (0–0)	0 (0–0)	0 (0–0)	0 (0–0)	0 (0–0)	0 (0–0)	0 (0–0)	0 (0–0)	0 (0–0)	0 (0–0)	0 (0–0)	0 (0–0)	0 (0–0)	--
G6PD deficiency	0 (0–0)	0 (0–0)	0 (0–0)	0 (0–0)	0 (0–0)	0 (0–0)	0 (0–0)	0 (0–0)	0 (0–0)	0 (0–0)	0 (0–0)	0 (0–0)	0 (0–0)	0 (0–0)	0 (0–0)	0 (0–0)	0 (0–0)	0 (0–0)	0 (0–0)	0 (0–0)	0 (0–0)	0 (0–0)	--
Other hemoglobinopathies and hemolytic anaemias	0 (0–0)	0 (0–0)	0 (0–0)	0 (0–0)	0 (0–0)	0 (0–0)	0 (0–0)	0 (0–0)	0 (0–0)	0 (0–0)	0 (0–0)	0 (0–0)	0 (0–0)	0 (0–0)	0 (0–0)	0 (0–0)	0 (0–0)	0 (0–0)	0 (0–0)	0 (0–0)	0 (0–0)	0 (0–0)	--
Endocrine, metabolic, blood, and immune disorders	0 (0–0)	0 (0–0)	0 (0–1)	0 (0–0)	0 (0–0)	0 (0–0)	0 (0–0)	0 (0–0)	0 (0–1)	0 (0–1)	1 (0–1)	1 (0–1)	1 (0–1)	1 (1–2)	1 (1–2)	2 (1–3)	1 (1–3)	2 (1–3)	2 (1–3)	2 (1–3)	2 (1–4)	1 (1–2)	--
Sudden infant death syndrome	--	0 (0–0)	0 (0–0)	--	--	--	--	--	--	--	--	--	--	--	--	--	--	--	--	--	--	--	--
Transport injuries	0 (0–0)	0 (0–0)	0 (0–0)	0 (0–0)	0 (0–0)	0 (0–0)	1 (1–2)	2 (1–2)	2 (1–3)	2 (1–3)	2 (1–2)	2 (1–2)	2 (1–3)	2 (1–2)	2 (1–3)	2 (1–3)	2 (1–2)	1 (1–2)	1 (1–2)	1 (1–1)	1 (0–1)	0 (0–0)	--
Road injuries	0 (0–0)	0 (0–0)	0 (0–0)	0 (0–0)	0 (0–0)	0 (0–0)	1 (1–2)	2 (1–2)	2 (1–3)	2 (1–3)	2 (1–2)	2 (1–2)	2 (1–3)	2 (1–2)	2 (1–3)	2 (1–3)	2 (1–2)	1 (1–2)	1 (1–2)	1 (1–1)	1 (0–1)	0 (0–0)	--
Pedestrian road injuries	0 (0–0)	0 (0–0)	0 (0–0)	0 (0–0)	0 (0–0)	0 (0–0)	0 (0–0)	0 (0–0)	0 (0–0)	0 (0–0)	0 (0–0)	0 (0–0)	0 (0–0)	0 (0–0)	0 (0–1)	0 (0–1)	0 (0–1)	0 (0–1)	0 (0–1)	0 (0–1)	0 (0–0)	0 (0–0)	--
Cyclist road injuries	--	--	--	0 (0–0)	0 (0–0)	0 (0–0)	0 (0–0)	0 (0–0)	0 (0–0)	0 (0–0)	0 (0–0)	0 (0–0)	0 (0–0)	0 (0–0)	0 (0–0)	0 (0–0)	0 (0–0)	0 (0–0)	0 (0–0)	0 (0–0)	0 (0–0)	0 (0–0)	--
Motorcyclist road injuries	0 (0–0)	0 (0–0)	0 (0–0)	0 (0–0)	0 (0–0)	0 (0–0)	0 (0–0)	0 (0–1)	1 (0–1)	0 (0–1)	0 (0–1)	0 (0–0)	0 (0–0)	0 (0–0)	0 (0–0)	0 (0–0)	0 (0–0)	0 (0–0)	0 (0–0)	0 (0–0)	0 (0–0)	0 (0–0)	--
Motor vehicle road injuries	0 (0–0)	0 (0–0)	0 (0–0)	0 (0–0)	0 (0–0)	0 (0–0)	1 (0–1)	1 (1–2)	1 (1–2)	1 (1–2)	1 (1–1)	1 (1–1)	1 (1–2)	1 (1–2)	1 (1–2)	1 (1–2)	1 (1–1)	1 (1–1)	1 (0–1)	0 (0–1)	0 (0–0)	0 (0–0)	--
Other road injuries	0 (0–0)	0 (0–0)	0 (0–0)	0 (0–0)	0 (0–0)	0 (0–0)	0 (0–0)	0 (0–0)	0 (0–0)	0 (0–0)	0 (0–0)	0 (0–0)	0 (0–0)	0 (0–0)	0 (0–0)	0 (0–0)	0 (0–0)	0 (0–0)	0 (0–0)	0 (0–0)	0 (0–0)	0 (0–0)	--

Other transport injuries	0 (0–0)	0 (0–0)	0 (0–0)	0 (0–0)	0 (0–0)	0 (0–0)	0 (0–0)	0 (0–0)	0 (0–0)	0 (0–0)	0 (0–0)	0 (0–0)	0 (0–0)	0 (0–0)	0 (0–0)	0 (0–0)	0 (0–0)	0 (0–0)	0 (0–0)	0 (0–0)	0 (0–0)	0 (0–0)	--
Falls	0 (0–0)	0 (0–0)	0 (0–0)	0 (0–0)	0 (0–0)	0 (0–0)	0 (0–0)	0 (0–0)	0 (0–0)	0 (0–0)	0 (0–0)	0 (0–0)	0 (0–0)	0 (0–0)	0 (0–0)	0 (0–1)	0 (0–1)	1 (1–1)	1 (1–1)	1 (1–2)	1 (1–2)	1 (1–1)	--
Drowning	0 (0–0)	0 (0–0)	0 (0–0)	0 (0–0)	0 (0–0)	0 (0–0)	1 (1–1)	1 (0–1)	1 (1–1)	1 (1–1)	1 (0–1)	1 (0–1)	1 (0–1)	1 (0–1)	1 (0–1)	1 (0–1)	0 (0–1)	0 (0–0)	0 (0–0)	0 (0–0)	0 (0–0)	0 (0–0)	--
Fire, heat, and hot substances	0 (0–0)	0 (0–0)	0 (0–0)	0 (0–0)	0 (0–0)	0 (0–0)	0 (0–0)	0 (0–0)	0 (0–0)	0 (0–0)	0 (0–0)	0 (0–0)	0 (0–0)	0 (0–0)	0 (0–0)	0 (0–0)	0 (0–0)	0 (0–0)	0 (0–0)	0 (0–0)	0 (0–0)	0 (0–0)	--
Poisonings	0 (0–0)	0 (0–0)	0 (0–0)	0 (0–0)	0 (0–0)	0 (0–0)	0 (0–0)	0 (0–0)	0 (0–0)	0 (0–0)	0 (0–0)	0 (0–0)	0 (0–0)	0 (0–0)	0 (0–0)	0 (0–0)	0 (0–0)	0 (0–0)	0 (0–0)	0 (0–0)	0 (0–0)	0 (0–0)	--
Poisoning by carbon monoxide	0 (0–0)	0 (0–0)	0 (0–0)	0 (0–0)	0 (0–0)	0 (0–0)	0 (0–0)	0 (0–0)	0 (0–0)	0 (0–0)	0 (0–0)	0 (0–0)	0 (0–0)	0 (0–0)	0 (0–0)	0 (0–0)	0 (0–0)	0 (0–0)	0 (0–0)	0 (0–0)	0 (0–0)	0 (0–0)	--
Poisoning by other means	0 (0–0)	0 (0–0)	0 (0–0)	0 (0–0)	0 (0–0)	0 (0–0)	0 (0–0)	0 (0–0)	0 (0–0)	0 (0–0)	0 (0–0)	0 (0–0)	0 (0–0)	0 (0–0)	0 (0–0)	0 (0–0)	0 (0–0)	0 (0–0)	0 (0–0)	0 (0–0)	0 (0–0)	0 (0–0)	--
Exposure to mechanical forces	0 (0–0)	0 (0–0)	0 (0–0)	0 (0–0)	0 (0–0)	0 (0–0)	0 (0–0)	0 (0–0)	0 (0–0)	0 (0–0)	0 (0–0)	0 (0–0)	0 (0–0)	0 (0–0)	0 (0–0)	0 (0–0)	0 (0–0)	0 (0–0)	0 (0–0)	0 (0–0)	0 (0–0)	0 (0–0)	--
Unintentional firearm injuries	0 (0–0)	0 (0–0)	0 (0–0)	0 (0–0)	0 (0–0)	0 (0–0)	0 (0–0)	0 (0–0)	0 (0–0)	0 (0–0)	0 (0–0)	0 (0–0)	0 (0–0)	0 (0–0)	0 (0–0)	0 (0–0)	0 (0–0)	0 (0–0)	0 (0–0)	0 (0–0)	0 (0–0)	0 (0–0)	--
Other exposure to mechanical forces	0 (0–0)	0 (0–0)	0 (0–0)	0 (0–0)	0 (0–0)	0 (0–0)	0 (0–0)	0 (0–0)	0 (0–0)	0 (0–0)	0 (0–0)	0 (0–0)	0 (0–0)	0 (0–0)	0 (0–0)	0 (0–0)	0 (0–0)	0 (0–0)	0 (0–0)	0 (0–0)	0 (0–0)	0 (0–0)	--
Adverse effects of medical treatment	0 (0–0)	0 (0–0)	0 (0–0)	0 (0–0)	0 (0–0)	0 (0–0)	0 (0–0)	0 (0–0)	0 (0–0)	0 (0–0)	0 (0–0)	0 (0–0)	0 (0–0)	0 (0–0)	0 (0–0)	0 (0–1)	1 (0–1)	1 (0–1)	1 (1–1)	1 (1–1)	1 (1–1)	0 (0–1)	--
Animal contact	0 (0–0)	0 (0–0)	0 (0–0)	0 (0–0)	0 (0–0)	0 (0–0)	0 (0–0)	0 (0–0)	0 (0–0)	0 (0–0)	0 (0–0)	0 (0–0)	0 (0–0)	0 (0–0)	0 (0–0)	0 (0–0)	0 (0–0)	0 (0–0)	0 (0–0)	0 (0–0)	0 (0–0)	0 (0–0)	--
Venomous animal contact	0 (0–0)	0 (0–0)	0 (0–0)	0 (0–0)	0 (0–0)	0 (0–0)	0 (0–0)	0 (0–0)	0 (0–0)	0 (0–0)	0 (0–0)	0 (0–0)	0 (0–0)	0 (0–0)	0 (0–0)	0 (0–0)	0 (0–0)	0 (0–0)	0 (0–0)	0 (0–0)	0 (0–0)	0 (0–0)	--
Non-venomous animal contact	0 (0–0)	0 (0–0)	0 (0–0)	0 (0–0)	0 (0–0)	0 (0–0)	0 (0–0)	0 (0–0)	0 (0–0)	0 (0–0)	0 (0–0)	0 (0–0)	0 (0–0)	0 (0–0)	0 (0–0)	0 (0–0)	0 (0–0)	0 (0–0)	0 (0–0)	0 (0–0)	0 (0–0)	0 (0–0)	--
Foreign body	0 (0–0)	0 (0–0)	0 (0–0)	0 (0–0)	0 (0–0)	0 (0–0)	0 (0–0)	0 (0–0)	0 (0–0)	0 (0–0)	0 (0–0)	0 (0–0)	0 (0–0)	0 (0–0)	0 (0–0)	0 (0–0)	0 (0–0)	0 (0–0)	0 (0–0)	0 (0–0)	0 (0–0)	0 (0–0)	--
Pulmonary aspiration and foreign body in airway	0 (0–0)	0 (0–0)	0 (0–0)	0 (0–0)	0 (0–0)	0 (0–0)	0 (0–0)	0 (0–0)	0 (0–0)	0 (0–0)	0 (0–0)	0 (0–0)	0 (0–0)	0 (0–0)	0 (0–0)	0 (0–0)	0 (0–0)	0 (0–0)	0 (0–0)	0 (0–0)	0 (0–0)	0 (0–0)	--
Foreign body in other body part	0 (0–0)	0 (0–0)	0 (0–0)	0 (0–0)	0 (0–0)	0 (0–0)	0 (0–0)	0 (0–0)	0 (0–0)	0 (0–0)	0 (0–0)	0 (0–0)	0 (0–0)	0 (0–0)	0 (0–0)	0 (0–0)	0 (0–0)	0 (0–0)	0 (0–0)	0 (0–0)	0 (0–0)	0 (0–0)	--
Environmental heat and cold exposure	0 (0–0)	0 (0–0)	0 (0–0)	0 (0–0)	0 (0–0)	0 (0–0)	0 (0–0)	0 (0–0)	0 (0–0)	0 (0–0)	0 (0–0)	0 (0–0)	0 (0–0)	0 (0–0)	0 (0–0)	0 (0–0)	0 (0–0)	0 (0–0)	0 (0–0)	0 (0–0)	0 (0–0)	0 (0–0)	--
Other unintentional injuries	0 (0–0)	0 (0–0)	0 (0–0)	0 (0–0)	0 (0–0)	0 (0–0)	0 (0–0)	0 (0–0)	0 (0–0)	0 (0–0)	0 (0–0)	0 (0–0)	0 (0–0)	0 (0–0)	0 (0–0)	0 (0–0)	0 (0–0)	0 (0–0)	0 (0–0)	0 (0–0)	0 (0–0)	0 (0–0)	--
Self-harm	--	--	--	--	--	0 (0–0)	0 (0–0)	1 (0–1)	1 (1–1)	1 (1–1)	1 (1–1)	1 (1–1)	1 (1–1)	1 (1–1)	1 (1–1)	1 (1–2)	1 (1–1)	1 (1–1)	1 (1–1)	1 (1–1)	1 (0–1)	0 (0–0)	--
Self-harm by firearm	--	--	--	--	--	0 (0–0)	0 (0–0)	0 (0–0)	0 (0–0)	0 (0–0)	0 (0–0)	0 (0–0)	0 (0–0)	0 (0–0)	0 (0–0)	0 (0–0)	0 (0–0)	0 (0–0)	0 (0–0)	0 (0–0)	0 (0–0)	0 (0–0)	--
Self-harm by other specified means	--	--	--	--	--	0 (0–0)	0 (0–0)	0 (0–1)	1 (1–1)	1 (1–1)	1 (1–1)	1 (1–1)	1 (1–1)	1 (1–1)	1 (1–1)	1 (1–1)	1 (1–1)	1 (1–1)	1 (1–1)	1 (0–1)	0 (0–1)	0 (0–0)	--
Interpersonal violence	0 (0–0)	0 (0–0)	0 (0–0)	0 (0–0)	0 (0–0)	0 (0–0)	2 (1–3)	3 (2–4)	4 (3–5)	4 (3–5)	3 (2–3)	2 (1–3)	2 (1–3)	1 (1–2)	1 (1–2)	1 (1–2)	1 (1–1)	1 (0–1)	0 (0–1)	0 (0–0)	0 (0–0)	0 (0–0)	--
Physical violence by firearm	0 (0–0)	0 (0–0)	0 (0–0)	0 (0–0)	0 (0–0)	0 (0–0)	1 (1–1)	2 (1–2)	2 (2–3)	2 (2–3)	1 (1–2)	1 (1–1)	1 (0–1)	0 (0–1)	0 (0–1)	0 (0–1)	0 (0–0)	0 (0–0)	0 (0–0)	0 (0–0)	0 (0–0)	0 (0–0)	--
Physical violence by sharp object	0 (0–0)	0 (0–0)	0 (0–0)	0 (0–0)	0 (0–0)	0 (0–0)	1 (0–1)	1 (1–1)	1 (1–2)	1 (1–2)	1 (1–1)	1 (1–1)	1 (0–1)	0 (0–1)	0 (0–1)	0 (0–0)	0 (0–0)	0 (0–0)	0 (0–0)	0 (0–0)	0 (0–0)	0 (0–0)	--
Physical violence by other means	0 (0–0)	0 (0–0)	0 (0–0)	0 (0–0)	0 (0–0)	0 (0–0)	0 (0–0)	0 (0–0)	1 (0–1)	1 (0–1)	1 (0–1)	0 (0–1)	1 (0–1)	0 (0–1)	1 (0–1)	1 (0–1)	0 (0–1)	0 (0–1)	0 (0–0)	0 (0–0)	0 (0–0)	0 (0–0)	--
Executions and police conflict	0 (0–0)	0 (0–0)	0 (0–0)	0 (0–0)	0 (0–0)	0 (0–0)	0 (0–0)	0 (0–0)	0 (0–0)	0 (0–0)	0 (0–0)	0 (0–0)	0 (0–0)	0 (0–0)	0 (0–0)	0 (0–0)	0 (0–0)	0 (0–0)	0 (0–0)	0 (0–0)	0 (0–0)	0 (0–0)	--

SR Table 12: Belize deaths by cause and age group for males

Cause	Early Neonatal	Late Neonatal	Post Neonatal	1 to 4	5 to 9	10 to 14	15 to 19	20 to 24	25 to 29	30 to 34	35 to 39	40 to 44	45 to 49	50 to 54	55 to 59	60 to 64	65 to 69	70 to 74	75 to 79	80 to 84	85 to 89	90 to 94	95 plus
All causes	24 (19–30)	12 (9–15)	19 (15–23)	10 (8–12)	6 (5–7)	9 (8–9)	31 (28–35)	50 (45–56)	61 (56–67)	62 (57–68)	57 (51–63)	58 (51–66)	66 (57–76)	72 (63–84)	94 (81–109)	95 (83–109)	92 (81–105)	92 (81–104)	86 (76–96)	95 (86–105)	71 (66–77)	43 (40–46)	--
Sexually transmitted infections excluding HIV	0 (0–0)	0 (0–0)	0 (0–0)	0 (0–0)	0 (0–0)	0 (0–0)	0 (0–0)	0 (0–0)	0 (0–0)	0 (0–0)	0 (0–0)	0 (0–0)	0 (0–0)	0 (0–0)	0 (0–0)	0 (0–0)	0 (0–0)	0 (0–0)	0 (0–0)	0 (0–0)	0 (0–0)	0 (0–0)	--
Syphilis	0 (0–0)	0 (0–0)	0 (0–0)	0 (0–0)	0 (0–0)	0 (0–0)	0 (0–0)	0 (0–0)	0 (0–0)	0 (0–0)	0 (0–0)	0 (0–0)	0 (0–0)	0 (0–0)	0 (0–0)	0 (0–0)	0 (0–0)	0 (0–0)	0 (0–0)	0 (0–0)	0 (0–0)	0 (0–0)	--
Chlamydial infection	--	--	--	--	--	0 (0–0)	0 (0–0)	0 (0–0)	0 (0–0)	0 (0–0)	0 (0–0)	0 (0–0)	0 (0–0)	0 (0–0)	0 (0–0)	0 (0–0)	0 (0–0)	0 (0–0)	0 (0–0)	0 (0–0)	0 (0–0)	0 (0–0)	--
Gonococcal infection	--	--	--	--	--	0 (0–0)	0 (0–0)	0 (0–0)	0 (0–0)	0 (0–0)	0 (0–0)	0 (0–0)	0 (0–0)	0 (0–0)	0 (0–0)	0 (0–0)	0 (0–0)	0 (0–0)	0 (0–0)	0 (0–0)	0 (0–0)	0 (0–0)	--
Other sexually transmitted infections	--	--	--	--	--	0 (0–0)	0 (0–0)	0 (0–0)	0 (0–0)	0 (0–0)	0 (0–0)	0 (0–0)	0 (0–0)	0 (0–0)	0 (0–0)	0 (0–0)	0 (0–0)	0 (0–0)	0 (0–0)	0 (0–0)	0 (0–0)	0 (0–0)	--
Tuberculosis	--	--	0 (0–0)	0 (0–0)	0 (0–0)	0 (0–0)	0 (0–0)	1 (0–1)	1 (1–1)	1 (1–1)	1 (1–1)	1 (1–1)	1 (1–1)	1 (1–1)	1 (1–1)	1 (1–1)	1 (0–1)	1 (0–1)	1 (0–1)	1 (0–1)	0 (0–0)	0 (0–0)	--
Drug-susceptible tuberculosis	--	--	0 (0–0)	0 (0–0)	0 (0–0)	0 (0–0)	0 (0–0)	1 (0–1)	1 (1–1)	1 (1–1)	1 (1–1)	1 (1–1)	1 (1–1)	1 (1–1)	1 (1–1)	1 (1–1)	1 (0–1)	1 (0–1)	1 (0–1)	1 (0–1)	0 (0–0)	0 (0–0)	--
Multidrug-resistant tuberculosis without extensive drug resistance	--	--	0 (0–0)	0 (0–0)	0 (0–0)	0 (0–0)	0 (0–0)	0 (0–0)	0 (0–0)	0 (0–0)	0 (0–0)	0 (0–0)	0 (0–0)	0 (0–0)	0 (0–0)	0 (0–0)	0 (0–0)	0 (0–0)	0 (0–0)	0 (0–0)	0 (0–0)	0 (0–0)	--
Extensively drug-resistant tuberculosis	--	--	0 (0–0)	0 (0–0)	0 (0–0)	0 (0–0)	0 (0–0)	0 (0–0)	0 (0–0)	0 (0–0)	0 (0–0)	0 (0–0)	0 (0–0)	0 (0–0)	0 (0–0)	0 (0–0)	0 (0–0)	0 (0–0)	0 (0–0)	0 (0–0)	0 (0–0)	0 (0–0)	--
Lower respiratory infections	1 (1–1)	1 (1–2)	4 (3–5)	1 (1–2)	0 (0–0)	0 (0–0)	1 (0–1)	1 (1–1)	1 (1–2)	2 (1–2)	2 (1–2)	2 (1–3)	2 (1–3)	2 (2–3)	3 (2–4)	4 (3–5)	4 (2–5)	4 (3–6)	5 (4–7)	8 (6–11)	7 (6–9)	5 (4–7)	--
Upper respiratory infections	0 (0–0)	0 (0–0)	0 (0–0)	0 (0–0)	0 (0–0)	0 (0–0)	0 (0–0)	0 (0–0)	0 (0–0)	0 (0–0)	0 (0–0)	0 (0–0)	0 (0–0)	0 (0–0)	0 (0–0)	0 (0–0)	0 (0–0)	0 (0–0)	0 (0–0)	0 (0–0)	0 (0–0)	0 (0–0)	--
Otitis media	0 (0–0)	0 (0–0)	0 (0–0)	0 (0–0)	0 (0–0)	0 (0–0)	0 (0–0)	0 (0–0)	0 (0–0)	0 (0–0)	0 (0–0)	0 (0–0)	0 (0–0)	0 (0–0)	0 (0–0)	0 (0–0)	0 (0–0)	0 (0–0)	0 (0–0)	0 (0–0)	0 (0–0)	0 (0–0)	--
Diarrhoeal diseases	0 (0–0)	0 (0–0)	2 (1–3)	0 (0–1)	0 (0–0)	0 (0–0)	0 (0–0)	0 (0–0)	0 (0–0)	0 (0–0)	0 (0–0)	0 (0–0)	0 (0–0)	0 (0–0)	0 (0–0)	0 (0–0)	0 (0–0)	0 (0–1)	0 (0–1)	0 (0–1)	0 (0–1)	0 (0–0)	--
Typhoid fever	--	--	0 (0–0)	0 (0–0)	0 (0–0)	0 (0–0)	0 (0–0)	0 (0–0)	0 (0–0)	0 (0–0)	0 (0–0)	0 (0–0)	0 (0–0)	0 (0–0)	0 (0–0)	0 (0–0)	0 (0–0)	0 (0–0)	0 (0–0)	0 (0–0)	0 (0–0)	0 (0–0)	--
Paratyphoid fever	--	--	0 (0–0)	0 (0–0)	0 (0–0)	0 (0–0)	0 (0–0)	0 (0–0)	0 (0–0)	0 (0–0)	0 (0–0)	0 (0–0)	0 (0–0)	0 (0–0)	0 (0–0)	0 (0–0)	0 (0–0)	0 (0–0)	0 (0–0)	0 (0–0)	0 (0–0)	0 (0–0)	--
iNTS	--	0 (0–0)	0 (0–0)	0 (0–0)	0 (0–0)	0 (0–0)	0 (0–0)	0 (0–0)	0 (0–0)	0 (0–0)	0 (0–0)	0 (0–0)	0 (0–0)	0 (0–0)	0 (0–0)	0 (0–0)	0 (0–0)	0 (0–0)	0 (0–0)	0 (0–0)	0 (0–0)	0 (0–0)	--
Other intestinal infectious diseases	--	--	0 (0–0)	0 (0–0)	0 (0–0)	0 (0–0)	0 (0–0)	0 (0–0)	0 (0–0)	0 (0–0)	0 (0–0)	0 (0–0)	0 (0–0)	0 (0–0)	0 (0–0)	0 (0–0)	0 (0–0)	0 (0–0)	0 (0–0)	0 (0–0)	0 (0–0)	0 (0–0)	--
Malaria	0 (0–0)	0 (0–0)	0 (0–0)	0 (0–0)	0 (0–0)	0 (0–0)	0 (0–0)	0 (0–0)	0 (0–0)	0 (0–0)	0 (0–0)	0 (0–0)	0 (0–0)	0 (0–0)	0 (0–0)	0 (0–0)	0 (0–0)	0 (0–0)	0 (0–0)	0 (0–0)	0 (0–0)	0 (0–0)	--
Chagas disease	--	--	0 (0–0)	0 (0–0)	0 (0–0)	0 (0–0)	0 (0–0)	0 (0–0)	0 (0–0)	0 (0–0)	0 (0–0)	0 (0–0)	0 (0–0)	0 (0–0)	0 (0–0)	0 (0–0)	0 (0–0)	0 (0–0)	0 (0–0)	0 (0–0)	0 (0–0)	0 (0–0)	--
Visceral leishmaniasis	--	--	0 (0–0)	0 (0–0)	0 (0–0)	0 (0–0)	0 (0–0)	0 (0–0)	0 (0–0)	0 (0–0)	0 (0–0)	0 (0–0)	0 (0–0)	0 (0–0)	0 (0–0)	0 (0–0)	0 (0–0)	0 (0–0)	0 (0–0)	0 (0–0)	0 (0–0)	0 (0–0)	--
African trypanosomiasis	--	--	--	0 (0–0)	0 (0–0)	0 (0–0)	0 (0–0)	0 (0–0)	0 (0–0)	0 (0–0)	0 (0–0)	0 (0–0)	0 (0–0)	0 (0–0)	0 (0–0)	0 (0–0)	0 (0–0)	0 (0–0)	0 (0–0)	0 (0–0)	0 (0–0)	0 (0–0)	--
Schistosomiasis	--	--	0 (0–0)	0 (0–0)	0 (0–0)	0 (0–0)	0 (0–0)	0 (0–0)	0 (0–0)	0 (0–0)	0 (0–0)	0 (0–0)	0 (0–0)	0 (0–0)	0 (0–0)	0 (0–0)	0 (0–0)	0 (0–0)	0 (0–0)	0 (0–0)	0 (0–0)	0 (0–0)	--
Cysticercosis	--	--	--	0 (0–0)	0 (0–0)	0 (0–0)	0 (0–0)	0 (0–0)	0 (0–0)	0 (0–0)	0 (0–0)	0 (0–0)	0 (0–0)	0 (0–0)	0 (0–0)	0 (0–0)	0 (0–0)	0 (0–0)	0 (0–0)	0 (0–0)	0 (0–0)	0 (0–0)	--
Cystic echinococcosis	--	--	--	0 (0–0)	0 (0–0)	0 (0–0)	0 (0–0)	0 (0–0)	0 (0–0)	0 (0–0)	0 (0–0)	0 (0–0)	0 (0–0)	0 (0–0)	0 (0–0)	0 (0–0)	0 (0–0)	0 (0–0)	0 (0–0)	0 (0–0)	0 (0–0)	0 (0–0)	--
Dengue	0 (0–0)	0 (0–0)	0 (0–0)	0 (0–0)	0 (0–0)	0 (0–0)	0 (0–0)	0 (0–0)	0 (0–0)	0 (0–0)	0 (0–0)	0 (0–0)	0 (0–0)	0 (0–0)	0 (0–0)	0 (0–0)	0 (0–0)	0 (0–0)	0 (0–0)	0 (0–0)	0 (0–0)	0 (0–0)	--
Yellow fever	--	0 (0–0)	0 (0–0)	0 (0–0)	0 (0–0)	0 (0–0)	0 (0–0)	0 (0–0)	0 (0–0)	0 (0–0)	0 (0–0)	0 (0–0)	0 (0–0)	0 (0–0)	0 (0–0)	0 (0–0)	0 (0–0)	0 (0–0)	0 (0–0)	0 (0–0)	0 (0–0)	0 (0–0)	--
Rabies	--	--	0 (0–0)	0 (0–0)	0 (0–0)	0 (0–0)	0 (0–0)	0 (0–0)	0 (0–0)	0 (0–0)	0 (0–0)	0 (0–0)	0 (0–0)	0 (0–0)	0 (0–0)	0 (0–0)	0 (0–0)	0 (0–0)	0 (0–0)	0 (0–0)	0 (0–0)	0 (0–0)	--
Ascariasis	--	--	0 (0–0)	0 (0–0)	0 (0–0)	0 (0–0)	0 (0–0)	0 (0–0)	0 (0–0)	0 (0–0)	0 (0–0)	0 (0–0)	0 (0–0)	0 (0–0)	0 (0–0)	0 (0–0)	0 (0–0)	0 (0–0)	0 (0–0)	0 (0–0)	0 (0–0)	0 (0–0)	--
Zika virus disease	0 (0–0)	0 (0–0)	0 (0–0)	0 (0–0)	0 (0–0)	0 (0–0)	0 (0–0)	0 (0–0)	0 (0–0)	0 (0–0)	0 (0–0)	0 (0–0)	0 (0–0)	0 (0–0)	0 (0–0)	0 (0–0)	0 (0–0)	0 (0–0)	0 (0–0)	0 (0–0)	0 (0–0)	0 (0–0)	--
Other neglected tropical diseases	0 (0–0)	0 (0–0)	0 (0–0)	0 (0–0)	0 (0–0)	0 (0–0)	0 (0–0)	0 (0–0)	0 (0–0)	0 (0–0)	0 (0–0)	0 (0–0)	0 (0–0)	0 (0–0)	0 (0–0)	0 (0–0)	0 (0–0)	0 (0–0)	0 (0–0)	0 (0–0)	0 (0–0)	0 (0–0)	--
Meningitis	0 (0–0)	0 (0–0)	0 (0–0)	0 (0–0)	0 (0–0)	0 (0–0)	0 (0–0)	0 (0–0)	0 (0–0)	0 (0–0)	0 (0–0)	0 (0–0)	0 (0–0)	0 (0–0)	0 (0–0)	0 (0–0)	0 (0–0)	0 (0–0)	0 (0–0)	0 (0–0)	0 (0–0)	0 (0–0)	--
Encephalitis	0 (0–0)	0 (0–0)	0 (0–0)	0 (0–0)	0 (0–0)	0 (0–0)	0 (0–0)	0 (0–0)	0 (0–0)	0 (0–0)	0 (0–0)	0 (0–0)	0 (0–0)	0 (0–0)	0 (0–0)	0 (0–0)	0 (0–0)	0 (0–0)	0 (0–0)	0 (0–0)	0 (0–0)	0 (0–0)	--
Diphtheria	--	--	0 (0–0)	0 (0–0)	0 (0–0)	0 (0–0)	0 (0–0)	0 (0–0)	0 (0–0)	0 (0–0)	0 (0–0)	0 (0–0)	0 (0–0)	0 (0–0)	0 (0–0)	--	--	--	--	--	--	--	--
Whooping cough	--	--	0 (0–0)	0 (0–0)	0 (0–0)	0 (0–0)	0 (0–0)	0 (0–0)	0 (0–0)	0 (0–0)	0 (0–0)	0 (0–0)	0 (0–0)	0 (0–0)	0 (0–0)	--	--	--	--	--	--	--	--
Tetanus	0 (0–0)	0 (0–0)	0 (0–0)	0 (0–0)	0 (0–0)	0 (0–0)	0 (0–0)	0 (0–0)	0 (0–0)	0 (0–0)	0 (0–0)	0 (0–0)	0 (0–0)	0 (0–0)	0 (0–0)	0 (0–0)	0 (0–0)	0 (0–0)	0 (0–0)	0 (0–0)	0 (0–0)	0 (0–0)	--
Measles	0 (0–0)	0 (0–0)	0 (0–0)	0 (0–0)	0 (0–0)	0 (0–0)	0 (0–0)	0 (0–0)	0 (0–0)	0 (0–0)	0 (0–0)	0 (0–0)	0 (0–0)	0 (0–0)	0 (0–0)	0 (0–0)	0 (0–0)	0 (0–0)	0 (0–0)	0 (0–0)	0 (0–0)	0 (0–0)	--
Varicella and herpes zoster	0 (0–0)	0 (0–0)	0 (0–0)	0 (0–0)	0 (0–0)	0 (0–0)	0 (0–0)	0 (0–0)	0 (0–0)	0 (0–0)	0 (0–0)	0 (0–0)	0 (0–0)	0 (0–0)	0 (0–0)	0 (0–0)	0 (0–0)	0 (0–0)	0 (0–0)	0 (0–0)	0 (0–0)	0 (0–0)	--
Acute hepatitis	--	--	0 (0–0)	0 (0–0)	0 (0–0)	0 (0–0)	0 (0–0)	0 (0–0)	0 (0–0)	0 (0–0)	0 (0–0)	0 (0–0)	0 (0–0)	0 (0–0)	0 (0–0)	0 (0–0)	0 (0–0)	0 (0–0)	0 (0–0)	0 (0–0)	0 (0–0)	0 (0–0)	--
Acute hepatitis A	--	--	0 (0–0)	0 (0–0)	0 (0–0)	0 (0–0)	0 (0–0)	0 (0–0)	0 (0–0)	0 (0–0)	0 (0–0)	0 (0–0)	0 (0–0)	0 (0–0)	0 (0–0)	0 (0–0)	0 (0–0)	0 (0–0)	0 (0–0)	0 (0–0)	0 (0–0)	0 (0–0)	--
Acute hepatitis B	--	--	0 (0–0)	0 (0–0)	0 (0–0)	0 (0–0)	0 (0–0)	0 (0–0)	0 (0–0)	0 (0–0)	0 (0–0)	0 (0–0)	0 (0–0)	0 (0–0)	0 (0–0)	0 (0–0)	0 (0–0)	0 (0–0)	0 (0–0)	0 (0–0)	0 (0–0)	0 (0–0)	--
Acute hepatitis C	--	--	0 (0–0)	0 (0–0)	0 (0–0)	0 (0–0)	0 (0–0)	0 (0–0)	0 (0–0)	0 (0–0)	0 (0–0)	0 (0–0)	0 (0–0)	0 (0–0)	0 (0–0)	0 (0–0)	0 (0–0)	0 (0–0)	0 (0–0)	0 (0–0)	0 (0–0)	0 (0–0)	--
Acute hepatitis E	--	--	0 (0–0)	0 (0–0)	0 (0–0)	0 (0–0)	0 (0–0)	0 (0–0)	0 (0–0)	0 (0–0)	0 (0–0)	0 (0–0)	0 (0–0)	0 (0–0)	0 (0–0)	0 (0–0)	0 (0–0)	0 (0–0)	0 (0–0)	0 (0–0)	0 (0–0)	0 (0–0)	--
Other unspecified infectious diseases	0 (0–0)	0 (0–0)	0 (0–0)	0 (0–0)	0 (0–0)	0 (0–0)	0 (0–0)	0 (0–0)	0 (0–0)	0 (0–0)	0 (0–0)	0 (0–0)	0 (0–0)	0 (0–0)	0 (0–0)	0 (0–0)	0 (0–0)	0 (0–0)	0 (0–0)	0 (0–0)	0 (0–0)	0 (0–0)	--
Maternal disorders	--	--	--	--	--	--	--	--	--	--	--	--	--	--	--	--	--	--	--	--	--	--	--
Maternal haemorrhage	--	--	--	--	--	--	--	--	--	--	--	--	--	--	--	--	--	--	--	--	--	--	--
Maternal sepsis and other pregnancy related infections	--	--	--	--	--	--	--	--	--	--	--	--	--	--	--	--	--	--	--	--	--	--	--
Maternal hypertensive disorders	--	--	--	--	--	--	--	--	--	--	--	--	--	--	--	--	--	--	--	--	--	--	--
Maternal obstructed labour and uterine rupture	--	--	--	--	--	--	--	--	--	--	--	--	--	--	--	--	--	--	--	--	--	--	--
Maternal abortive outcome	--	--	--	--	--	--	--	--	--	--	--	--	--	--	--	--	--	--	--	--	--	--	--
Ectopic pregnancy	--	--	--	--	--	--	--	--	--	--	--	--	--	--	--	--	--	--	--	--	--	--	--
Indirect maternal deaths	--	--	--	--	--	--	--	--	--	--	--	--	--	--	--	--	--	--	--	--	--	--	--
Late maternal deaths	--	--	--	--	--	--	--	--	--	--	--	--	--	--	--	--	--	--	--	--	--	--	--
Maternal deaths aggravated by HIV/AIDS	--	--	--	--	--	--	--	--	--	--	--	--	--	--	--	--	--	--	--	--	--	--	--
Other maternal disorders	--	--	--	--	--	--	--	--	--	--	--	--	--	--	--	--	--	--	--	--	--	--	--
Neonatal disorders	19 (15–24)	8 (6–10)	3 (2–4)	0 (0–0)	--	--	--	--	--	--	--	--	--	--	--	--	--	--	--	--	--	--	--
Neonatal preterm birth	9 (7–12)	3 (2–4)	1 (1–1)	0 (0–0)	--	--	--	--	--	--	--	--	--	--	--	--	--	--	--	--	--	--	--
Neonatal encephalopathy due to birth asphyxia and trauma	5 (4–7)	1 (1–2)	0 (0–1)	0 (0–0)	--	--	--	--	--	--	--	--	--	--	--	--	--	--	--	--	--	--	--
Neonatal sepsis and other neonatal infections	2 (2–4)	2 (2–4)	1 (0–1)	0 (0–0)	--	--	--	--	--	--	--	--	--	--	--	--	--	--	--	--	--	--	--
Hemolytic disease and other neonatal jaundice	1 (0–1)	0 (0–1)	0 (0–0)	0 (0–0)	--	--	--	--	--	--	--	--	--	--	--	--	--	--	--	--	--	--	--
Other neonatal disorders	2 (1–3)	1 (1–2)	0 (0–1)	0 (0–0)	--	--	--	--	--	--	--	--	--	--	--	--	--	--	--	--	--	--	--
Nutritional deficiencies	0 (0–0)	0 (0–0)	1 (1–2)	1 (0–1)	0 (0–0)	0 (0–0)	0 (0–0)	0 (0–0)	0 (0–0)	0 (0–0)	0 (0–0)	0 (0–0)	0 (0–0)	0 (0–1)	0 (0–1)	0 (0–1)	1 (0–1)	1 (0–1)	1 (0–1)	1 (0–1)	1 (0–1)	0 (0–1)	--
Protein-energy malnutrition	0 (0–0)	0 (0–0)	1 (1–2)	1 (0–1)	0 (0–0)	0 (0–0)	0 (0–0)	0 (0–0)	0 (0–0)	0 (0–0)	0 (0–0)	0 (0–0)	0 (0–0)	0 (0–0)	0 (0–1)	0 (0–1)	0 (0–1)	1 (0–1)	1 (0–1)	1 (0–1)	1 (0–1)	0 (0–1)	--
Other nutritional deficiencies	--	--	0 (0–0)	0 (0–0)	0 (0–0)	0 (0–0)	0 (0–0)	0 (0–0)	0 (0–0)	0 (0–0)	0 (0–0)	0 (0–0)	0 (0–0)	0 (0–0)	0 (0–0)	0 (0–0)	0 (0–0)	0 (0–0)	0 (0–0)	0 (0–0)	0 (0–0)	0 (0–0)	--
Lip and oral cavity cancer	--	--	--	--	0 (0–0)	0 (0–0)	0 (0–0)	0 (0–0)	0 (0–0)	0 (0–0)	0 (0–0)	0 (0–0)	0 (0–0)	0 (0–0)	0 (0–1)	0 (0–0)	0 (0–0)	0 (0–0)	0 (0–0)	0 (0–0)	0 (0–0)	0 (0–0)	--
Nasopharynx cancer	--	--	--	--	0 (0–0)	0 (0–0)	0 (0–0)	0 (0–0)	0 (0–0)	0 (0–0)	0 (0–0)	0 (0–0)	0 (0–0)	0 (0–0)	0 (0–0)	0 (0–0)	0 (0–0)	0 (0–0)	0 (0–0)	0 (0–0)	0 (0–0)	0 (0–0)	--
Other pharynx cancer	--	--	--	--	--	--	--	0 (0–0)	0 (0–0)	0 (0–0)	0 (0–0)	0 (0–0)	0 (0–0)	0 (0–0)	0 (0–0)	0 (0–0)	0 (0–0)	0 (0–0)	0 (0–0)	0 (0–0)	0 (0–0)	0 (0–0)	--
Oesophageal cancer	--	--	--	--	--	--	--	0 (0–0)	0 (0–0)	0 (0–0)	0 (0–0)	0 (0–0)	0 (0–1)	1 (0–1)	1 (1–1)	1 (1–1)	1 (1–1)	1 (0–1)	0 (0–1)	0 (0–0)	0 (0–0)	0 (0–0)	--
Stomach cancer	--	--	--	--	--	--	0 (0–0)	0 (0–0)	0 (0–0)	0 (0–0)	0 (0–1)	1 (1–1)	1 (1–1)	1 (1–2)	2 (2–3)	2 (2–3)	2 (2–3)	2 (1–3)	2 (1–2)	2 (1–2)	1 (1–1)	0 (0–1)	--
Colon and rectum cancer	--	--	--	--	0 (0–0)	0 (0–0)	0 (0–0)	0 (0–0)	0 (0–0)	0 (0–0)	0 (0–1)	1 (0–1)	1 (1–1)	1 (1–2)	2 (1–2)	2 (2–3)	2 (1–2)	2 (1–2)	2 (1–2)	1 (1–2)	1 (1–1)	0 (0–1)	--
Liver cancer due to hepatitis B	--	--	--	--	--	0 (0–0)	0 (0–0)	0 (0–0)	0 (0–0)	0 (0–0)	0 (0–0)	0 (0–0)	0 (0–0)	0 (0–0)	0 (0–0)	0 (0–0)	0 (0–0)	0 (0–0)	0 (0–0)	0 (0–0)	0 (0–0)	0 (0–0)	--

Liver cancer due to hepatitis C	--	--	--	--	--	0 (0–0)	0 (0–0)	0 (0–0)	0 (0–0)	0 (0–0)	0 (0–0)	0 (0–0)	0 (0–0)	0 (0–0)	0 (0–0)	0 (0–0)	0 (0–0)	0 (0–0)	0 (0–0)	0 (0–0)	0 (0–0)	0 (0–0)	--
Liver cancer due to alcohol use	--	--	--	--	--	--	0 (0–0)	0 (0–0)	0 (0–0)	0 (0–0)	0 (0–0)	0 (0–0)	0 (0–0)	0 (0–0)	0 (0–0)	0 (0–0)	0 (0–1)	0 (0–1)	0 (0–0)	0 (0–0)	0 (0–0)	0 (0–0)	--
Liver cancer due to NASH	--	--	--	--	--	--	0 (0–0)	0 (0–0)	0 (0–0)	0 (0–0)	0 (0–0)	0 (0–0)	0 (0–0)	0 (0–0)	0 (0–0)	0 (0–0)	0 (0–0)	0 (0–0)	0 (0–0)	0 (0–0)	0 (0–0)	0 (0–0)	--
	0 (0–0)	0 (0–0)	0 (0–0)	0 (0–0)	0 (0–0)	0 (0–0)	--	--	--	--	--	--	--	--	--	--	--	--	--	--	--	--	--
Liver cancer due to other causes	0 (0–0)	0 (0–0)	0 (0–0)	0 (0–0)	0 (0–0)	0 (0–0)	0 (0–0)	0 (0–0)	0 (0–0)	0 (0–0)	0 (0–0)	0 (0–0)	0 (0–0)	0 (0–0)	0 (0–0)	0 (0–0)	0 (0–0)	0 (0–0)	0 (0–0)	0 (0–0)	0 (0–0)	0 (0–0)	--
Gallbladder and biliary tract cancer	--	--	--	--	--	--	--	0 (0–0)	0 (0–0)	0 (0–0)	0 (0–0)	0 (0–0)	0 (0–0)	0 (0–0)	0 (0–0)	0 (0–0)	0 (0–0)	0 (0–0)	0 (0–0)	0 (0–0)	0 (0–0)	0 (0–0)	--
Pancreatic cancer	--	--	--	--	--	--	0 (0–0)	0 (0–0)	0 (0–0)	0 (0–0)	0 (0–0)	0 (0–0)	1 (0–1)	1 (1–1)	1 (1–1)	1 (1–1)	1 (1–1)	1 (1–1)	1 (1–1)	1 (0–1)	0 (0–0)	0 (0–0)	--
Larynx cancer	--	--	--	--	--	--	--	0 (0–0)	0 (0–0)	0 (0–0)	0 (0–0)	0 (0–0)	0 (0–0)	0 (0–1)	1 (1–1)	1 (1–1)	1 (0–1)	0 (0–1)	0 (0–0)	0 (0–0)	0 (0–0)	0 (0–0)	--
Tracheal, bronchus, and lung cancer	--	--	--	--	--	0 (0–0)	0 (0–0)	0 (0–0)	0 (0–0)	0 (0–0)	0 (0–1)	1 (1–1)	2 (1–2)	2 (2–3)	4 (3–5)	4 (3–6)	4 (3–5)	3 (3–4)	2 (2–3)	2 (1–3)	1 (1–1)	0 (0–1)	--
Malignant skin melanoma	0 (0–0)	0 (0–0)	0 (0–0)	0 (0–0)	0 (0–0)	0 (0–0)	0 (0–0)	0 (0–0)	0 (0–0)	0 (0–0)	0 (0–0)	0 (0–0)	0 (0–0)	0 (0–0)	0 (0–0)	0 (0–0)	0 (0–0)	0 (0–0)	0 (0–0)	0 (0–0)	0 (0–0)	0 (0–0)	--
Non-melanoma skin cancer (squamous-cell carcinoma)	--	--	--	--	--	--	--	0 (0–0)	0 (0–0)	0 (0–0)	0 (0–0)	0 (0–0)	0 (0–0)	0 (0–0)	0 (0–0)	0 (0–0)	0 (0–0)	0 (0–0)	0 (0–0)	0 (0–0)	0 (0–0)	0 (0–0)	--
	0 (0–0)	0 (0–0)	0 (0–0)	0 (0–0)	0 (0–0)	0 (0–0)	0 (0–0)	0 (0–0)	0 (0–0)	0 (0–0)	0 (0–0)	0 (0–0)	0 (0–0)	0 (0–0)	0 (0–0)	0 (0–0)	0 (0–0)	0 (0–0)	0 (0–0)	0 (0–0)	0 (0–0)	0 (0–0)	--
	--	--	--	0 (0–0)	0 (0–0)	0 (0–0)	0 (0–0)	0 (0–0)	0 (0–0)	0 (0–0)	0 (0–0)	0 (0–0)	0 (0–0)	0 (0–0)	0 (0–0)	0 (0–0)	0 (0–0)	0 (0–0)	0 (0–0)	0 (0–0)	0 (0–0)	0 (0–0)	--
Breast cancer	--	--	--	--	--	--	0 (0–0)	0 (0–0)	0 (0–0)	0 (0–0)	0 (0–0)	0 (0–0)	0 (0–0)	0 (0–0)	0 (0–0)	0 (0–0)	0 (0–0)	0 (0–0)	0 (0–0)	0 (0–0)	0 (0–0)	0 (0–0)	--
Cervical cancer	--	--	--	--	--	--	--	--	--	--	--	--	--	--	--	--	--	--	--	--	--	--	--
Uterine cancer	--	--	--	--	--	--	--	--	--	--	--	--	--	--	--	--	--	--	--	--	--	--	--
Ovarian cancer	--	--	--	--	--	--	--	--	--	--	--	--	--	--	--	--	--	--	--	--	--	--	--
Prostate cancer	--	--	--	--	--	--	--	0 (0–0)	0 (0–0)	0 (0–0)	0 (0–0)	0 (0–0)	0 (0–0)	1 (1–1)	2 (1–3)	4 (3–5)	5 (3–7)	6 (5–9)	8 (6–10)	9 (6–12)	6 (5–9)	3 (2–5)	--
Testicular cancer	0 (0–0)	0 (0–0)	0 (0–0)	0 (0–0)	0 (0–0)	0 (0–0)	0 (0–0)	0 (0–0)	0 (0–0)	0 (0–0)	0 (0–0)	0 (0–0)	0 (0–0)	0 (0–0)	0 (0–0)	0 (0–0)	0 (0–0)	0 (0–0)	0 (0–0)	0 (0–0)	0 (0–0)	0 (0–0)	--
Kidney cancer	0 (0–0)	0 (0–0)	0 (0–0)	0 (0–0)	0 (0–0)	0 (0–0)	0 (0–0)	0 (0–0)	0 (0–0)	0 (0–0)	0 (0–0)	0 (0–0)	0 (0–0)	0 (0–0)	0 (0–1)	0 (0–1)	0 (0–0)	0 (0–0)	0 (0–0)	0 (0–0)	0 (0–0)	0 (0–0)	--
Bladder cancer	--	--	--	--	--	--	0 (0–0)	0 (0–0)	0 (0–0)	0 (0–0)	0 (0–0)	0 (0–0)	0 (0–0)	0 (0–0)	0 (0–0)	0 (0–0)	0 (0–1)	0 (0–1)	0 (0–1)	0 (0–1)	0 (0–0)	0 (0–0)	--
Brain and nervous system cancer	0 (0–0)	0 (0–0)	0 (0–0)	0 (0–0)	0 (0–0)	0 (0–0)	0 (0–0)	0 (0–0)	0 (0–0)	0 (0–0)	0 (0–0)	0 (0–0)	0 (0–0)	0 (0–0)	0 (0–1)	0 (0–0)	0 (0–0)	0 (0–0)	0 (0–0)	0 (0–0)	0 (0–0)	0 (0–0)	--
	0 (0–0)	0 (0–0)	0 (0–0)	0 (0–0)	0 (0–0)	0 (0–0)	0 (0–0)	0 (0–0)	0 (0–0)	0 (0–0)	0 (0–0)	0 (0–0)	0 (0–0)	0 (0–0)	0 (0–0)	0 (0–0)	0 (0–0)	0 (0–0)	0 (0–0)	0 (0–0)	0 (0–0)	0 (0–0)	--
	0 (0–0)	0 (0–0)	0 (0–0)	0 (0–0)	0 (0–0)	0 (0–0)	0 (0–0)	--	--	--	--	--	--	--	--	--	--	--	--	--	--	--	--
	--	--	--	0 (0–0)	0 (0–0)	0 (0–0)	0 (0–0)	0 (0–0)	0 (0–0)	0 (0–0)	0 (0–0)	0 (0–0)	0 (0–0)	0 (0–0)	0 (0–0)	0 (0–0)	0 (0–0)	0 (0–0)	0 (0–0)	0 (0–0)	0 (0–0)	0 (0–0)	--
	0 (0–0)	0 (0–0)	0 (0–0)	0 (0–0)	0 (0–0)	0 (0–0)	0 (0–0)	0 (0–0)	0 (0–0)	0 (0–0)	0 (0–0)	0 (0–0)	0 (0–0)	0 (0–0)	0 (0–0)	0 (0–0)	0 (0–0)	0 (0–0)	0 (0–0)	0 (0–0)	0 (0–0)	0 (0–0)	--
Thyroid cancer	--	--	--	--	0 (0–0)	0 (0–0)	0 (0–0)	0 (0–0)	0 (0–0)	0 (0–0)	0 (0–0)	0 (0–0)	0 (0–0)	0 (0–0)	0 (0–0)	0 (0–0)	0 (0–0)	0 (0–0)	0 (0–0)	0 (0–0)	0 (0–0)	0 (0–0)	--
Mesothelioma	--	--	--	--	--	--	--	0 (0–0)	0 (0–0)	0 (0–0)	0 (0–0)	0 (0–0)	0 (0–0)	0 (0–0)	0 (0–0)	0 (0–0)	0 (0–0)	0 (0–0)	0 (0–0)	0 (0–0)	0 (0–0)	0 (0–0)	--
Hodgkin lymphoma	--	--	--	0 (0–0)	0 (0–0)	0 (0–0)	0 (0–0)	0 (0–0)	0 (0–0)	0 (0–0)	0 (0–0)	0 (0–0)	0 (0–0)	0 (0–0)	0 (0–0)	0 (0–0)	0 (0–0)	0 (0–0)	0 (0–0)	0 (0–0)	0 (0–0)	0 (0–0)	--
Non-Hodgkin's lymphoma	--	--	--	0 (0–0)	0 (0–0)	0 (0–0)	0 (0–0)	0 (0–0)	0 (0–0)	0 (0–0)	0 (0–0)	0 (0–0)	0 (0–0)	0 (0–0)	0 (0–1)	0 (0–0)	0 (0–0)	0 (0–0)	0 (0–0)	0 (0–0)	0 (0–0)	0 (0–0)	--
	--	--	--	0 (0–0)	0 (0–0)	0 (0–0)	0 (0–0)	0 (0–0)	0 (0–0)	0 (0–0)	0 (0–0)	0 (0–0)	0 (0–0)	0 (0–0)	0 (0–0)	0 (0–0)	0 (0–0)	0 (0–0)	0 (0–0)	0 (0–0)	0 (0–0)	0 (0–0)	--
	--	--	--	0 (0–0)	0 (0–0)	0 (0–0)	0 (0–0)	0 (0–0)	0 (0–0)	0 (0–0)	0 (0–0)	0 (0–0)	0 (0–0)	0 (0–0)	0 (0–1)	0 (0–0)	0 (0–0)	0 (0–0)	0 (0–0)	0 (0–0)	0 (0–0)	0 (0–0)	--
Multiple myeloma	--	--	--	--	--	--	--	0 (0–0)	0 (0–0)	0 (0–0)	0 (0–0)	0 (0–0)	0 (0–0)	0 (0–0)	0 (0–0)	0 (0–0)	0 (0–0)	0 (0–0)	0 (0–0)	0 (0–0)	0 (0–0)	0 (0–0)	--
Leukaemia	0 (0–0)	0 (0–0)	0 (0–0)	0 (0–0)	0 (0–0)	0 (0–0)	0 (0–1)	0 (0–1)	0 (0–1)	0 (0–0)	0 (0–0)	0 (0–0)	0 (0–0)	0 (0–0)	0 (0–1)	0 (0–1)	0 (0–1)	0 (0–1)	0 (0–0)	0 (0–0)	0 (0–0)	0 (0–0)	--
Acute lymphoid leukaemia	0 (0–0)	0 (0–0)	0 (0–0)	0 (0–0)	0 (0–0)	0 (0–0)	0 (0–0)	0 (0–0)	0 (0–0)	0 (0–0)	0 (0–0)	0 (0–0)	0 (0–0)	0 (0–0)	0 (0–0)	0 (0–0)	0 (0–0)	0 (0–0)	0 (0–0)	0 (0–0)	0 (0–0)	0 (0–0)	--
Chronic lymphoid leukaemia	--	--	--	--	--	--	--	0 (0–0)	0 (0–0)	0 (0–0)	0 (0–0)	0 (0–0)	0 (0–0)	0 (0–0)	0 (0–0)	0 (0–0)	0 (0–0)	0 (0–0)	0 (0–0)	0 (0–0)	0 (0–0)	0 (0–0)	--
Acute myeloid leukaemia	0 (0–0)	0 (0–0)	0 (0–0)	0 (0–0)	0 (0–0)	0 (0–0)	0 (0–0)	0 (0–0)	0 (0–0)	0 (0–0)	0 (0–0)	0 (0–0)	0 (0–0)	0 (0–0)	0 (0–0)	0 (0–0)	0 (0–0)	0 (0–0)	0 (0–0)	0 (0–0)	0 (0–0)	0 (0–0)	--
Chronic myeloid leukaemia	0 (0–0)	0 (0–0)	0 (0–0)	0 (0–0)	0 (0–0)	0 (0–0)	0 (0–0)	0 (0–0)	0 (0–0)	0 (0–0)	0 (0–0)	0 (0–0)	0 (0–0)	0 (0–0)	0 (0–0)	0 (0–0)	0 (0–0)	0 (0–0)	0 (0–0)	0 (0–0)	0 (0–0)	0 (0–0)	--
Other leukaemia	0 (0–0)	0 (0–0)	0 (0–0)	0 (0–0)	0 (0–0)	0 (0–0)	0 (0–0)	0 (0–0)	0 (0–0)	0 (0–0)	0 (0–0)	0 (0–0)	0 (0–0)	0 (0–0)	0 (0–0)	0 (0–0)	0 (0–0)	0 (0–0)	0 (0–0)	0 (0–0)	0 (0–0)	0 (0–0)	--
Other malignant cancers	0 (0–0)	0 (0–0)	0 (0–0)	0 (0–0)	0 (0–0)	0 (0–0)	0 (0–0)	0 (0–0)	0 (0–0)	0 (0–0)	0 (0–0)	0 (0–0)	0 (0–0)	0 (0–0)	0 (0–1)	0 (0–1)	0 (0–1)	0 (0–1)	0 (0–0)	0 (0–0)	0 (0–0)	0 (0–0)	--
Myelodysplastic, myeloproliferative, and other hematopoietic neoplasms	0 (0–0)	0 (0–0)	0 (0–0)	0 (0–0)	0 (0–0)	0 (0–0)	0 (0–0)	0 (0–0)	0 (0–0)	0 (0–0)	0 (0–0)	0 (0–0)	0 (0–0)	0 (0–0)	0 (0–0)	0 (0–0)	0 (0–0)	0 (0–0)	0 (0–0)	0 (0–0)	0 (0–0)	0 (0–0)	--
Cardiovascular diseases	0 (0–0)	0 (0–0)	0 (0–0)	0 (0–0)	0 (0–0)	0 (0–0)	1 (1–1)	2 (1–2)	2 (2–3)	4 (3–4)	5 (4–6)	7 (6–9)	12 (9–14)	16 (13–19)	25 (20–30)	28 (23–33)	29 (24–34)	29 (25–34)	29 (25–33)	31 (26–35)	25 (20–29)	15 (12–17)	--
Rheumatic heart disease	--	--	--	0 (0–0)	0 (0–0)	0 (0–0)	0 (0–0)	0 (0–0)	0 (0–0)	0 (0–0)	0 (0–0)	0 (0–0)	0 (0–0)	0 (0–0)	0 (0–0)	0 (0–0)	0 (0–0)	0 (0–0)	0 (0–0)	0 (0–0)	0 (0–0)	0 (0–0)	--
Ischaemic heart disease	--	--	--	--	--	--	0 (0–0)	0 (0–0)	1 (0–1)	1 (1–2)	2 (1–2)	3 (2–4)	5 (4–7)	8 (6–10)	13 (10–16)	14 (11–16)	15 (13–17)	14 (12–17)	14 (12–16)	15 (12–17)	12 (10–14)	7 (6–8)	--
Stroke	0 (0–0)	0 (0–0)	0 (0–0)	0 (0–0)	0 (0–0)	0 (0–0)	0 (0–0)	0 (0–1)	1 (0–1)	1 (1–1)	1 (1–2)	2 (1–3)	3 (2–4)	4 (3–6)	7 (5–9)	8 (6–11)	8 (6–11)	9 (7–12)	9 (7–12)	9 (7–12)	7 (5–9)	4 (3–5)	--
Ischaemic stroke	0 (0–0)	0 (0–0)	0 (0–0)	0 (0–0)	0 (0–0)	0 (0–0)	0 (0–0)	0 (0–0)	0 (0–0)	0 (0–0)	0 (0–0)	0 (0–0)	0 (0–1)	1 (0–1)	1 (1–2)	2 (1–3)	2 (2–3)	4 (3–5)	4 (3–5)	6 (5–8)	5 (3–6)	3 (2–4)	--
Intracerebral hemorrhage	0 (0–0)	0 (0–0)	0 (0–0)	0 (0–0)	0 (0–0)	0 (0–0)	0 (0–0)	0 (0–0)	0 (0–1)	1 (0–1)	1 (1–1)	1 (1–2)	2 (2–3)	3 (2–4)	5 (4–7)	6 (4–8)	6 (4–7)	5 (4–7)	5 (4–7)	3 (2–4)	2 (1–3)	1 (1–2)	--
Subarachnoid hemorrhage	0 (0–0)	0 (0–0)	0 (0–0)	0 (0–0)	0 (0–0)	0 (0–0)	0 (0–0)	0 (0–0)	0 (0–0)	0 (0–0)	0 (0–0)	0 (0–0)	0 (0–1)	0 (0–1)	1 (0–1)	0 (0–1)	0 (0–1)	0 (0–1)	0 (0–0)	0 (0–0)	0 (0–0)	0 (0–0)	--
Hypertensive heart disease	--	--	--	--	--	--	0 (0–0)	0 (0–0)	0 (0–0)	0 (0–0)	0 (0–1)	1 (0–1)	1 (1–2)	2 (1–2)	2 (1–3)	3 (2–4)	3 (2–4)	3 (2–4)	3 (2–4)	3 (2–4)	3 (2–3)	2 (1–2)	--
Non-rheumatic valvular heart disease	--	--	--	--	--	--	0 (0–0)	0 (0–0)	0 (0–0)	0 (0–0)	0 (0–0)	0 (0–0)	0 (0–0)	0 (0–0)	0 (0–0)	0 (0–0)	0 (0–0)	0 (0–0)	0 (0–0)	0 (0–0)	0 (0–0)	0 (0–0)	--
Non-rheumatic calcific aortic valve disease	--	--	--	--	--	--	0 (0–0)	0 (0–0)	0 (0–0)	0 (0–0)	0 (0–0)	0 (0–0)	0 (0–0)	0 (0–0)	0 (0–0)	0 (0–0)	0 (0–0)	0 (0–0)	0 (0–0)	0 (0–0)	0 (0–0)	0 (0–0)	--
Non-rheumatic degenerative mitral valve disease	--	--	--	--	--	--	0 (0–0)	0 (0–0)	0 (0–0)	0 (0–0)	0 (0–0)	0 (0–0)	0 (0–0)	0 (0–0)	0 (0–0)	0 (0–0)	0 (0–0)	0 (0–0)	0 (0–0)	0 (0–0)	0 (0–0)	0 (0–0)	--
Other non-rheumatic valve diseases	--	--	--	--	--	--	0 (0–0)	0 (0–0)	0 (0–0)	0 (0–0)	0 (0–0)	0 (0–0)	0 (0–0)	0 (0–0)	0 (0–0)	0 (0–0)	0 (0–0)	0 (0–0)	0 (0–0)	0 (0–0)	0 (0–0)	0 (0–0)	--
Cardiomyopathy and myocarditis	0 (0–0)	0 (0–0)	0 (0–0)	0 (0–0)	0 (0–0)	0 (0–0)	0 (0–0)	0 (0–0)	0 (0–0)	0 (0–1)	1 (0–1)	1 (0–1)	1 (1–1)	1 (1–1)	1 (1–2)	1 (1–2)	1 (1–1)	1 (1–1)	1 (0–1)	1 (0–1)	1 (0–1)	0 (0–0)	--
Myocarditis	0 (0–0)	0 (0–0)	0 (0–0)	0 (0–0)	0 (0–0)	0 (0–0)	0 (0–0)	0 (0–0)	0 (0–0)	0 (0–0)	0 (0–0)	0 (0–0)	0 (0–0)	0 (0–0)	0 (0–0)	0 (0–0)	0 (0–0)	0 (0–0)	0 (0–0)	0 (0–0)	0 (0–0)	0 (0–0)	--
Alcoholic cardiomyopathy	--	--	--	--	--	--	0 (0–0)	0 (0–0)	0 (0–0)	0 (0–0)	0 (0–0)	0 (0–0)	0 (0–0)	0 (0–0)	0 (0–0)	0 (0–0)	0 (0–0)	0 (0–0)	0 (0–0)	0 (0–0)	0 (0–0)	0 (0–0)	--
Other cardiomyopathy	0 (0–0)	0 (0–0)	0 (0–0)	0 (0–0)	0 (0–0)	0 (0–0)	0 (0–0)	0 (0–0)	0 (0–0)	0 (0–1)	0 (0–1)	1 (0–1)	1 (0–1)	1 (0–1)	1 (1–1)	1 (1–1)	1 (1–1)	1 (0–1)	1 (0–1)	1 (0–1)	1 (0–1)	0 (0–0)	--
Pulmonary Arterial Hypertension	0 (0–0)	0 (0–0)	0 (0–0)	0 (0–0)	0 (0–0)	0 (0–0)	0 (0–0)	0 (0–0)	0 (0–0)	0 (0–0)	0 (0–0)	0 (0–0)	0 (0–0)	0 (0–0)	0 (0–0)	0 (0–0)	0 (0–0)	0 (0–0)	0 (0–0)	0 (0–0)	0 (0–0)	0 (0–0)	--
Atrial fibrillation and flutter	--	--	--	--	--	--	--	--	--	0 (0–0)	0 (0–0)	0 (0–0)	0 (0–0)	0 (0–0)	0 (0–0)	0 (0–0)	0 (0–0)	0 (0–1)	1 (0–1)	1 (1–1)	1 (1–2)	1 (1–1)	--
Aortic aneurysm	--	--	--	--	--	--	0 (0–0)	0 (0–0)	0 (0–0)	0 (0–0)	0 (0–0)	0 (0–0)	0 (0–0)	0 (0–0)	0 (0–0)	0 (0–0)	0 (0–0)	0 (0–0)	0 (0–0)	0 (0–0)	0 (0–0)	0 (0–0)	--
Peripheral vascular disease	--	--	--	--	--	--	--	--	--	--	--	0 (0–0)	0 (0–0)	0 (0–0)	0 (0–0)	0 (0–0)	0 (0–0)	0 (0–0)	0 (0–0)	0 (0–0)	0 (0–0)	0 (0–0)	--
Endocarditis	0 (0–0)	0 (0–0)	0 (0–0)	0 (0–0)	0 (0–0)	0 (0–0)	0 (0–0)	0 (0–0)	0 (0–0)	0 (0–0)	0 (0–0)	0 (0–0)	0 (0–0)	0 (0–0)	0 (0–0)	0 (0–0)	0 (0–0)	0 (0–0)	0 (0–0)	0 (0–0)	0 (0–0)	0 (0–0)	--
Other cardiovascular and circulatory diseases	0 (0–0)	0 (0–0)	0 (0–0)	0 (0–0)	0 (0–0)	0 (0–0)	0 (0–0)	0 (0–0)	0 (0–0)	0 (0–0)	0 (0–0)	0 (0–1)	1 (0–1)	1 (0–1)	1 (1–1)	1 (1–1)	1 (1–1)	1 (1–1)	1 (1–1)	1 (1–1)	1 (1–1)	0 (0–1)	--
Chronic respiratory diseases	0 (0–0)	0 (0–0)	0 (0–0)	1 (0–1)	0 (0–0)	0 (0–0)	0 (0–1)	1 (0–1)	1 (1–1)	1 (1–1)	1 (1–1)	1 (1–2)	2 (1–2)	2 (2–3)	4 (3–5)	5 (4–7)	6 (4–7)	7 (5–9)	6 (5–8)	7 (6–9)	5 (3–6)	3 (2–3)	--
Chronic obstructive pulmonary disease	--	--	0 (0–0)	0 (0–0)	0 (0–0)	0 (0–0)	0 (0–0)	0 (0–0)	0 (0–0)	0 (0–0)	0 (0–0)	0 (0–1)	1 (0–1)	1 (1–2)	2 (2–3)	4 (3–5)	4 (3–5)	5 (4–7)	5 (4–6)	6 (4–7)	4 (3–5)	2 (2–3)	--
Pneumoconiosis	--	--	--	--	--	--	0 (0–0)	0 (0–0)	0 (0–0)	0 (0–0)	0 (0–0)	0 (0–0)	0 (0–0)	0 (0–0)	0 (0–0)	0 (0–0)	0 (0–0)	0 (0–0)	0 (0–0)	0 (0–0)	0 (0–0)	0 (0–0)	--
Silicosis	--	--	--	--	--	--	0 (0–0)	0 (0–0)	0 (0–0)	0 (0–0)	0 (0–0)	0 (0–0)	0 (0–0)	0 (0–0)	0 (0–0)	0 (0–0)	0 (0–0)	0 (0–0)	0 (0–0)	0 (0–0)	0 (0–0)	0 (0–0)	--
Asbestosis	--	--	--	--	--	--	0 (0–0)	0 (0–0)	0 (0–0)	0 (0–0)	0 (0–0)	0 (0–0)	0 (0–0)	0 (0–0)	0 (0–0)	0 (0–0)	0 (0–0)	0 (0–0)	0 (0–0)	0 (0–0)	0 (0–0)	0 (0–0)	--
Coal workers pneumoconiosis	--	--	--	--	--	--	0 (0–0)	0 (0–0)	0 (0–0)	0 (0–0)	0 (0–0)	0 (0–0)	0 (0–0)	0 (0–0)	0 (0–0)	0 (0–0)	0 (0–0)	0 (0–0)	0 (0–0)	0 (0–0)	0 (0–0)	0 (0–0)	--
Other pneumoconiosis	--	--	--	--	--	--	0 (0–0)	0 (0–0)	0 (0–0)	0 (0–0)	0 (0–0)	0 (0–0)	0 (0–0)	0 (0–0)	0 (0–0)	0 (0–0)	0 (0–0)	0 (0–0)	0 (0–0)	0 (0–0)	0 (0–0)	0 (0–0)	--
Asthma	--	--	--	0 (0–0)	0 (0–0)	0 (0–0)	0 (0–0)	0 (0–0)	0 (0–0)	0 (0–0)	0 (0–0)	0 (0–0)	0 (0–0)	0 (0–1)	0 (0–1)	0 (0–1)	0 (0–1)	0 (0–1)	0 (0–0)	0 (0–1)	0 (0–0)	0 (0–0)	--
Interstitial lung disease and pulmonary sarcoidosis	--	--	--	0 (0–0)	0 (0–0)	0 (0–0)	0 (0–0)	0 (0–0)	0 (0–0)	0 (0–0)	0 (0–1)	0 (0–1)	0 (0–1)	1 (0–1)	1 (0–1)	1 (1–1)	1 (1–1)	1 (1–2)	1 (1–2)	1 (1–2)	1 (0–1)	0 (0–0)	--
Other chronic respiratory diseases	0 (0–0)	0 (0–0)	0 (0–0)	0 (0–0)	0 (0–0)	0 (0–0)	0 (0–0)	0 (0–0)	0 (0–0)	0 (0–0)	0 (0–0)	0 (0–0)	0 (0–0)	0 (0–0)	0 (0–0)	0 (0–0)	0 (0–0)	0 (0–0)	0 (0–0)	0 (0–0)	0 (0–0)	0 (0–0)	--

Digestive diseases	0 (0–0)	0 (0–0)	0 (0–1)	0 (0–1)	0 (0–0)	0 (0–0)	1 (0–1)	1 (1–1)	1 (1–2)	2 (2–3)	3 (2–4)	4 (3–6)	6 (5–8)	8 (6–10)	9 (7–11)	8 (6–9)	6 (5–7)	5 (4–6)	4 (3–5)	4 (3–5)	2 (2–3)	1 (1–2)	--
Cirrhosis and other chronic liver diseases	--	--	--	0 (0–0)	0 (0–0)	0 (0–0)	0 (0–0)	0 (0–1)	1 (1–1)	1 (1–2)	2 (2–3)	3 (3–4)	5 (4–7)	6 (5–8)	7 (6–9)	6 (4–7)	4 (3–5)	3 (3–4)	2 (2–3)	2 (1–2)	1 (1–1)	0 (0–1)	--
Cirrhosis and other chronic liver diseases due to hepatitis B	--	--	--	0 (0–0)	0 (0–0)	0 (0–0)	0 (0–0)	0 (0–0)	0 (0–0)	0 (0–0)	0 (0–0)	0 (0–1)	1 (0–1)	1 (0–1)	1 (0–1)	0 (0–1)	0 (0–1)	0 (0–0)	0 (0–0)	0 (0–0)	0 (0–0)	0 (0–0)	--
Cirrhosis and other chronic liver diseases due to hepatitis C	--	--	--	0 (0–0)	0 (0–0)	0 (0–0)	0 (0–0)	0 (0–0)	0 (0–0)	0 (0–1)	1 (0–1)	1 (0–1)	1 (1–2)	1 (1–2)	1 (1–2)	1 (1–2)	1 (0–1)	1 (0–1)	0 (0–1)	0 (0–1)	0 (0–0)	0 (0–0)	--
Cirrhosis and other chronic liver diseases due to alcohol use	--	--	--	--	--	--	0 (0–0)	0 (0–0)	0 (0–0)	0 (0–1)	1 (0–1)	1 (1–2)	2 (1–3)	3 (2–4)	4 (2–5)	3 (2–4)	2 (1–3)	2 (1–2)	1 (1–1)	1 (0–1)	0 (0–1)	0 (0–0)	--
Cirrhosis and other chronic liver diseases due to NAFLD	--	--	--	--	--	--	0 (0–0)	0 (0–0)	0 (0–0)	0 (0–0)	0 (0–1)	0 (0–1)	1 (0–1)	1 (0–1)	1 (0–2)	1 (0–1)	1 (0–1)	1 (0–1)	0 (0–1)	0 (0–1)	0 (0–0)	0 (0–0)	--
Cirrhosis and other chronic liver diseases due to other causes	--	--	--	0 (0–0)	0 (0–0)	0 (0–0)	0 (0–0)	0 (0–0)	0 (0–0)	0 (0–0)	0 (0–0)	0 (0–0)	0 (0–1)	0 (0–1)	1 (0–1)	1 (0–1)	0 (0–1)	0 (0–1)	0 (0–1)	0 (0–1)	0 (0–0)	0 (0–0)	--
Upper digestive system diseases	--	--	--	0 (0–0)	0 (0–0)	0 (0–0)	0 (0–0)	0 (0–0)	0 (0–0)	0 (0–0)	0 (0–0)	0 (0–0)	0 (0–0)	0 (0–1)	1 (0–1)	1 (0–1)	0 (0–1)	0 (0–1)	0 (0–1)	0 (0–1)	0 (0–0)	0 (0–0)	--
Peptic ulcer disease	--	--	--	0 (0–0)	0 (0–0)	0 (0–0)	0 (0–0)	0 (0–0)	0 (0–0)	0 (0–0)	0 (0–0)	0 (0–0)	0 (0–0)	0 (0–0)	0 (0–1)	0 (0–1)	0 (0–1)	0 (0–1)	0 (0–0)	0 (0–1)	0 (0–0)	0 (0–0)	--
Gastritis and duodenitis	--	--	--	0 (0–0)	0 (0–0)	0 (0–0)	0 (0–0)	0 (0–0)	0 (0–0)	0 (0–0)	0 (0–0)	0 (0–0)	0 (0–0)	0 (0–0)	0 (0–0)	0 (0–0)	0 (0–0)	0 (0–0)	0 (0–0)	0 (0–0)	0 (0–0)	0 (0–0)	--
Appendicitis	--	--	--	0 (0–0)	0 (0–0)	0 (0–0)	0 (0–0)	0 (0–0)	0 (0–0)	0 (0–0)	0 (0–0)	0 (0–0)	0 (0–0)	0 (0–0)	0 (0–0)	0 (0–0)	0 (0–0)	0 (0–0)	0 (0–0)	0 (0–0)	0 (0–0)	0 (0–0)	--
Paralytic ileus and intestinal obstruction	0 (0–0)	0 (0–0)	0 (0–1)	0 (0–0)	0 (0–0)	0 (0–0)	0 (0–0)	0 (0–0)	0 (0–0)	0 (0–0)	0 (0–0)	0 (0–0)	0 (0–0)	0 (0–0)	0 (0–1)	0 (0–1)	0 (0–1)	0 (0–1)	0 (0–1)	1 (0–1)	0 (0–1)	0 (0–0)	--
Inguinal, femoral, and abdominal hernia	0 (0–0)	0 (0–0)	0 (0–0)	0 (0–0)	0 (0–0)	0 (0–0)	0 (0–0)	0 (0–0)	0 (0–0)	0 (0–0)	0 (0–0)	0 (0–0)	0 (0–0)	0 (0–0)	0 (0–0)	0 (0–0)	0 (0–0)	0 (0–0)	0 (0–0)	0 (0–0)	0 (0–0)	0 (0–0)	--
Inflammatory bowel disease	--	--	--	0 (0–0)	0 (0–0)	0 (0–0)	0 (0–0)	0 (0–0)	0 (0–0)	0 (0–0)	0 (0–0)	0 (0–0)	0 (0–0)	0 (0–0)	0 (0–0)	0 (0–0)	0 (0–0)	0 (0–0)	0 (0–0)	0 (0–0)	0 (0–0)	0 (0–0)	--
Vascular intestinal disorders	--	--	--	0 (0–0)	0 (0–0)	0 (0–0)	0 (0–0)	0 (0–0)	0 (0–0)	0 (0–0)	0 (0–0)	0 (0–0)	0 (0–0)	0 (0–0)	0 (0–0)	0 (0–0)	0 (0–0)	0 (0–0)	0 (0–0)	0 (0–0)	0 (0–0)	0 (0–0)	--
Gallbladder and biliary diseases	--	--	--	0 (0–0)	0 (0–0)	0 (0–0)	0 (0–0)	0 (0–0)	0 (0–0)	0 (0–0)	0 (0–0)	0 (0–0)	0 (0–0)	0 (0–0)	0 (0–0)	0 (0–0)	0 (0–0)	0 (0–0)	0 (0–0)	0 (0–0)	0 (0–0)	0 (0–0)	--
Pancreatitis	--	--	--	0 (0–0)	0 (0–0)	0 (0–0)	0 (0–0)	0 (0–0)	0 (0–0)	0 (0–0)	0 (0–0)	0 (0–0)	0 (0–0)	0 (0–0)	0 (0–0)	0 (0–0)	0 (0–0)	0 (0–0)	0 (0–0)	0 (0–0)	0 (0–0)	0 (0–0)	--
Other digestive diseases	--	--	--	0 (0–0)	0 (0–0)	0 (0–0)	0 (0–0)	0 (0–0)	0 (0–0)	0 (0–0)	0 (0–0)	0 (0–0)	0 (0–0)	0 (0–0)	0 (0–0)	0 (0–0)	0 (0–0)	0 (0–0)	0 (0–0)	0 (0–0)	0 (0–0)	0 (0–0)	--
Alzheimer's disease and other dementias	--	--	--	--	--	--	--	--	--	--	--	0 (0–0)	0 (0–0)	0 (0–0)	0 (0–1)	1 (0–1)	1 (0–3)	2 (0–4)	2 (1–6)	5 (1–13)	5 (1–14)	4 (1–12)	--
Parkinson's disease	--	--	--	--	--	--	--	0 (0–0)	0 (0–0)	0 (0–0)	0 (0–0)	0 (0–0)	0 (0–0)	0 (0–0)	0 (0–0)	0 (0–0)	1 (0–1)	1 (1–1)	1 (1–2)	2 (1–2)	1 (1–1)	1 (0–1)	--
Idiopathic epilepsy	--	--	0 (0–0)	0 (0–0)	0 (0–0)	0 (0–0)	0 (0–0)	0 (0–1)	1 (0–1)	1 (0–1)	0 (0–1)	0 (0–1)	0 (0–1)	0 (0–0)	0 (0–0)	0 (0–0)	0 (0–0)	0 (0–0)	0 (0–0)	0 (0–0)	0 (0–0)	0 (0–0)	--
Multiple sclerosis	--	--	--	--	0 (0–0)	0 (0–0)	0 (0–0)	0 (0–0)	0 (0–0)	0 (0–0)	0 (0–0)	0 (0–0)	0 (0–0)	0 (0–0)	0 (0–0)	0 (0–0)	0 (0–0)	0 (0–0)	0 (0–0)	0 (0–0)	0 (0–0)	0 (0–0)	--
Motor neuron disease	0 (0–0)	0 (0–0)	0 (0–0)	0 (0–0)	0 (0–0)	0 (0–0)	0 (0–0)	0 (0–0)	0 (0–0)	0 (0–0)	0 (0–0)	0 (0–0)	0 (0–0)	0 (0–0)	0 (0–0)	0 (0–0)	0 (0–0)	0 (0–0)	0 (0–0)	0 (0–0)	0 (0–0)	0 (0–0)	--
Other neurological disorders	--	--	0 (0–0)	0 (0–0)	0 (0–0)	0 (0–0)	0 (0–0)	0 (0–0)	0 (0–0)	0 (0–0)	0 (0–0)	0 (0–0)	0 (0–0)	0 (0–0)	0 (0–0)	0 (0–0)	0 (0–0)	0 (0–0)	0 (0–0)	0 (0–0)	0 (0–0)	0 (0–0)	--
Eating disorders	--	--	--	--	0 (0–0)	0 (0–0)	0 (0–0)	0 (0–0)	0 (0–0)	0 (0–0)	0 (0–0)	0 (0–0)	0 (0–0)	--	--	--	--	--	--	--	--	--	--
Anorexia nervosa	--	--	--	--	0 (0–0)	0 (0–0)	0 (0–0)	0 (0–0)	0 (0–0)	0 (0–0)	0 (0–0)	0 (0–0)	0 (0–0)	--	--	--	--	--	--	--	--	--	--
Bulimia nervosa	--	--	--	--	0 (0–0)	0 (0–0)	0 (0–0)	0 (0–0)	0 (0–0)	0 (0–0)	0 (0–0)	0 (0–0)	0 (0–0)	--	--	--	--	--	--	--	--	--	--
Alcohol use disorders	--	--	--	--	--	0 (0–0)	0 (0–0)	0 (0–0)	0 (0–0)	1 (0–1)	1 (1–1)	1 (1–2)	2 (1–2)	1 (1–2)	2 (1–2)	1 (1–1)	1 (0–1)	0 (0–1)	0 (0–0)	0 (0–0)	0 (0–0)	0 (0–0)	--
Drug use disorders	0 (0–0)	0 (0–0)	0 (0–0)	0 (0–0)	0 (0–0)	0 (0–0)	0 (0–0)	0 (0–0)	0 (0–0)	0 (0–0)	0 (0–0)	0 (0–0)	0 (0–0)	0 (0–0)	0 (0–0)	0 (0–0)	0 (0–0)	0 (0–0)	0 (0–0)	0 (0–0)	0 (0–0)	0 (0–0)	--
Opioid use disorders	0 (0–0)	0 (0–0)	0 (0–0)	0 (0–0)	0 (0–0)	0 (0–0)	0 (0–0)	0 (0–0)	0 (0–0)	0 (0–0)	0 (0–0)	0 (0–0)	0 (0–0)	0 (0–0)	0 (0–0)	0 (0–0)	0 (0–0)	0 (0–0)	0 (0–0)	0 (0–0)	0 (0–0)	0 (0–0)	--
Cocaine use disorders	--	--	--	--	--	0 (0–0)	0 (0–0)	0 (0–0)	0 (0–0)	0 (0–0)	0 (0–0)	0 (0–0)	0 (0–0)	0 (0–0)	0 (0–0)	0 (0–0)	0 (0–0)	0 (0–0)	0 (0–0)	0 (0–0)	0 (0–0)	0 (0–0)	--
Amphetamine use disorders	--	--	--	--	--	0 (0–0)	0 (0–0)	0 (0–0)	0 (0–0)	0 (0–0)	0 (0–0)	0 (0–0)	0 (0–0)	0 (0–0)	0 (0–0)	0 (0–0)	0 (0–0)	0 (0–0)	0 (0–0)	0 (0–0)	0 (0–0)	0 (0–0)	--
Other drug use disorders	--	--	--	--	--	0 (0–0)	0 (0–0)	0 (0–0)	0 (0–0)	0 (0–0)	0 (0–0)	0 (0–0)	0 (0–0)	0 (0–0)	0 (0–0)	0 (0–0)	0 (0–0)	0 (0–0)	0 (0–0)	0 (0–0)	0 (0–0)	0 (0–0)	--
Diabetes mellitus	0 (0–0)	0 (0–0)	0 (0–0)	0 (0–0)	0 (0–0)	0 (0–0)	0 (0–0)	0 (0–1)	1 (1–1)	1 (1–2)	1 (1–2)	2 (2–3)	4 (3–5)	6 (4–7)	9 (7–12)	10 (7–13)	10 (7–12)	9 (7–12)	7 (5–9)	7 (5–8)	4 (3–5)	2 (1–2)	--
Diabetes mellitus type 1	0 (0–0)	0 (0–0)	0 (0–0)	0 (0–0)	0 (0–0)	0 (0–0)	0 (0–0)	0 (0–1)	0 (0–1)	0 (0–1)	0 (0–1)	1 (0–1)	0 (0–0)	0 (0–0)	0 (0–1)	0 (0–1)	0 (0–1)	0 (0–0)	0 (0–0)	0 (0–0)	0 (0–0)	0 (0–0)	--
Diabetes mellitus type 2	--	--	--	--	--	--	0 (0–0)	0 (0–0)	0 (0–0)	1 (1–1)	1 (1–1)	2 (1–2)	4 (3–5)	5 (4–7)	9 (6–11)	9 (7–12)	9 (7–12)	9 (7–12)	7 (5–9)	7 (5–8)	4 (3–5)	2 (1–2)	--
Chronic kidney disease	--	--	0 (0–0)	0 (0–0)	0 (0–0)	0 (0–0)	1 (0–1)	1 (1–1)	1 (1–2)	2 (1–2)	2 (1–3)	3 (2–3)	4 (3–5)	5 (4–6)	7 (5–9)	6 (5–8)	6 (5–8)	6 (4–7)	5 (4–7)	6 (4–7)	4 (3–4)	2 (2–3)	--
Chronic kidney disease due to diabetes mellitus type 1	--	--	0 (0–0)	0 (0–0)	0 (0–0)	0 (0–0)	0 (0–0)	0 (0–0)	0 (0–0)	0 (0–1)	0 (0–1)	1 (0–1)	1 (0–1)	1 (0–1)	1 (0–1)	0 (0–1)	0 (0–1)	0 (0–0)	0 (0–0)	0 (0–0)	0 (0–0)	0 (0–0)	--
Chronic kidney disease due to diabetes mellitus type 2	--	--	--	--	--	--	0 (0–0)	0 (0–0)	0 (0–0)	0 (0–0)	0 (0–1)	0 (0–1)	1 (0–1)	1 (1–2)	2 (1–3)	2 (1–3)	2 (1–3)	2 (1–3)	2 (1–2)	2 (1–3)	1 (1–2)	1 (0–1)	--
Chronic kidney disease due to hypertension	--	--	--	--	--	--	0 (0–0)	0 (0–0)	0 (0–0)	0 (0–0)	0 (0–0)	0 (0–0)	0 (0–1)	1 (0–1)	1 (1–2)	1 (1–2)	2 (1–2)	2 (1–2)	2 (1–2)	2 (1–3)	1 (1–2)	1 (1–1)	--
Chronic kidney disease due to glomerulonephritis	--	--	0 (0–0)	0 (0–0)	0 (0–0)	0 (0–0)	0 (0–1)	0 (0–1)	1 (0–1)	1 (0–1)	1 (0–1)	1 (0–1)	1 (1–2)	1 (1–2)	2 (1–3)	1 (1–2)	1 (1–2)	1 (1–2)	1 (0–1)	1 (0–1)	0 (0–1)	0 (0–0)	--
Chronic kidney disease due to other and unspecified causes	--	--	0 (0–0)	0 (0–0)	0 (0–0)	0 (0–0)	0 (0–0)	0 (0–1)	0 (0–1)	0 (0–1)	1 (0–1)	1 (0–1)	1 (0–1)	1 (0–2)	1 (1–2)	1 (1–2)	1 (1–2)	1 (1–2)	1 (1–2)	1 (1–2)	1 (0–1)	0 (0–1)	--
Acute glomerulonephritis	--	--	0 (0–0)	0 (0–0)	0 (0–0)	0 (0–0)	0 (0–0)	0 (0–0)	0 (0–0)	0 (0–0)	0 (0–0)	0 (0–0)	0 (0–0)	0 (0–0)	0 (0–0)	0 (0–0)	0 (0–0)	0 (0–0)	0 (0–0)	0 (0–0)	0 (0–0)	0 (0–0)	--
Skin and subcutaneous diseases	--	--	0 (0–0)	0 (0–0)	0 (0–0)	0 (0–0)	0 (0–0)	0 (0–0)	0 (0–0)	0 (0–0)	0 (0–0)	0 (0–0)	0 (0–0)	0 (0–0)	0 (0–0)	0 (0–0)	0 (0–0)	0 (0–0)	0 (0–1)	0 (0–1)	0 (0–1)	0 (0–0)	--
Bacterial skin diseases	--	--	0 (0–0)	0 (0–0)	0 (0–0)	0 (0–0)	0 (0–0)	0 (0–0)	0 (0–0)	0 (0–0)	0 (0–0)	0 (0–0)	0 (0–0)	0 (0–0)	0 (0–0)	0 (0–0)	0 (0–0)	0 (0–0)	0 (0–0)	0 (0–0)	0 (0–0)	0 (0–0)	--
Cellulitis	--	--	0 (0–0)	0 (0–0)	0 (0–0)	0 (0–0)	0 (0–0)	0 (0–0)	0 (0–0)	0 (0–0)	0 (0–0)	0 (0–0)	0 (0–0)	0 (0–0)	0 (0–0)	0 (0–0)	0 (0–0)	0 (0–0)	0 (0–0)	0 (0–0)	0 (0–0)	0 (0–0)	--
Pyoderma	--	--	0 (0–0)	0 (0–0)	0 (0–0)	0 (0–0)	0 (0–0)	0 (0–0)	0 (0–0)	0 (0–0)	0 (0–0)	0 (0–0)	0 (0–0)	0 (0–0)	0 (0–0)	0 (0–0)	0 (0–0)	0 (0–0)	0 (0–0)	0 (0–0)	0 (0–0)	0 (0–0)	--
Decubitus ulcer	--	--	--	0 (0–0)	0 (0–0)	0 (0–0)	0 (0–0)	0 (0–0)	0 (0–0)	0 (0–0)	0 (0–0)	0 (0–0)	0 (0–0)	0 (0–0)	0 (0–0)	0 (0–0)	0 (0–0)	0 (0–0)	0 (0–0)	0 (0–0)	0 (0–0)	0 (0–0)	--
Other skin and subcutaneous diseases	--	--	0 (0–0)	0 (0–0)	0 (0–0)	0 (0–0)	0 (0–0)	0 (0–0)	0 (0–0)	0 (0–0)	0 (0–0)	0 (0–0)	0 (0–0)	0 (0–0)	0 (0–0)	0 (0–0)	0 (0–0)	0 (0–0)	0 (0–0)	0 (0–0)	0 (0–0)	0 (0–0)	--
Musculoskeletal disorders	--	--	--	--	0 (0–0)	0 (0–0)	0 (0–0)	0 (0–0)	0 (0–0)	0 (0–0)	0 (0–0)	0 (0–0)	0 (0–0)	0 (0–0)	0 (0–0)	0 (0–0)	0 (0–0)	0 (0–0)	0 (0–0)	0 (0–0)	0 (0–0)	0 (0–0)	--
Rheumatoid arthritis	--	--	--	--	0 (0–0)	0 (0–0)	0 (0–0)	0 (0–0)	0 (0–0)	0 (0–0)	0 (0–0)	0 (0–0)	0 (0–0)	0 (0–0)	0 (0–0)	0 (0–0)	0 (0–0)	0 (0–0)	0 (0–0)	0 (0–0)	0 (0–0)	0 (0–0)	--
Other musculoskeletal disorders	--	--	--	--	0 (0–0)	0 (0–0)	0 (0–0)	0 (0–0)	0 (0–0)	0 (0–0)	0 (0–0)	0 (0–0)	0 (0–0)	0 (0–0)	0 (0–0)	0 (0–0)	0 (0–0)	0 (0–0)	0 (0–0)	0 (0–0)	0 (0–0)	0 (0–0)	--
Congenital anomalies	3 (2–5)	2 (1–2)	3 (2–5)	1 (1–1)	0 (0–0)	0 (0–0)	0 (0–1)	0 (0–1)	0 (0–1)	0 (0–0)	0 (0–0)	0 (0–0)	0 (0–0)	0 (0–0)	0 (0–0)	0 (0–0)	0 (0–0)	--	--	--	--	--	--
Neural tube defects	0 (0–0)	0 (0–0)	0 (0–0)	0 (0–0)	0 (0–0)	0 (0–0)	0 (0–0)	0 (0–0)	0 (0–0)	0 (0–0)	0 (0–0)	0 (0–0)	0 (0–0)	0 (0–0)	0 (0–0)	0 (0–0)	0 (0–0)	--	--	--	--	--	--
Congenital heart anomalies	1 (1–2)	1 (1–1)	2 (1–2)	0 (0–1)	0 (0–0)	0 (0–0)	0 (0–0)	0 (0–0)	0 (0–0)	0 (0–0)	0 (0–0)	0 (0–0)	0 (0–0)	0 (0–0)	0 (0–0)	0 (0–0)	0 (0–0)	--	--	--	--	--	--
Orofacial clefts	0 (0–0)	0 (0–0)	0 (0–0)	0 (0–0)	--	--	--	--	--	--	--	--	--	--	--	--	--	--	--	--	--	--	--
Down's syndrome	0 (0–0)	0 (0–0)	0 (0–0)	0 (0–0)	0 (0–0)	0 (0–0)	0 (0–0)	0 (0–0)	0 (0–0)	0 (0–0)	0 (0–0)	0 (0–0)	0 (0–0)	0 (0–0)	0 (0–0)	0 (0–0)	0 (0–0)	--	--	--	--	--	--
Other chromosomal abnormalities	0 (0–0)	0 (0–0)	0 (0–0)	0 (0–0)	0 (0–0)	0 (0–0)	0 (0–0)	0 (0–0)	0 (0–0)	0 (0–0)	0 (0–0)	0 (0–0)	0 (0–0)	0 (0–0)	0 (0–0)	0 (0–0)	0 (0–0)	--	--	--	--	--	--
Congenital musculoskeletal and limb anomalies	0 (0–0)	0 (0–0)	0 (0–0)	0 (0–0)	0 (0–0)	0 (0–0)	0 (0–0)	0 (0–0)	0 (0–0)	0 (0–0)	0 (0–0)	0 (0–0)	0 (0–0)	0 (0–0)	0 (0–0)	0 (0–0)	0 (0–0)	--	--	--	--	--	--
Urogenital congenital anomalies	0 (0–0)	0 (0–0)	0 (0–0)	0 (0–0)	0 (0–0)	0 (0–0)	0 (0–0)	0 (0–0)	0 (0–0)	0 (0–0)	0 (0–0)	0 (0–0)	0 (0–0)	0 (0–0)	0 (0–0)	0 (0–0)	0 (0–0)	--	--	--	--	--	--
Digestive congenital anomalies	1 (0–1)	0 (0–1)	0 (0–1)	0 (0–0)	0 (0–0)	0 (0–0)	0 (0–0)	0 (0–0)	0 (0–0)	0 (0–0)	0 (0–0)	0 (0–0)	0 (0–0)	0 (0–0)	0 (0–0)	0 (0–0)	0 (0–0)	--	--	--	--	--	--
Other congenital anomalies	1 (1–1)	0 (0–0)	1 (0–1)	0 (0–0)	0 (0–0)	0 (0–0)	0 (0–0)	0 (0–0)	0 (0–0)	0 (0–0)	0 (0–0)	0 (0–0)	0 (0–0)	0 (0–0)	0 (0–0)	0 (0–0)	0 (0–0)	--	--	--	--	--	--
Urinary diseases and male infertility	0 (0–0)	0 (0–0)	0 (0–0)	0 (0–0)	0 (0–0)	0 (0–0)	0 (0–0)	0 (0–0)	0 (0–0)	0 (0–0)	0 (0–0)	0 (0–0)	0 (0–0)	0 (0–1)	1 (0–1)	1 (0–1)	1 (1–1)	1 (1–1)	1 (1–2)	1 (1–2)	1 (1–2)	1 (0–1)	--
Urinary tract infections and interstitial nephritis	0 (0–0)	0 (0–0)	0 (0–0)	0 (0–0)	0 (0–0)	0 (0–0)	0 (0–0)	0 (0–0)	0 (0–0)	0 (0–0)	0 (0–0)	0 (0–0)	0 (0–0)	0 (0–0)	0 (0–1)	1 (0–1)	1 (0–1)	1 (1–1)	1 (1–1)	1 (1–2)	1 (1–1)	1 (0–1)	--
Urolithiasis	--	--	--	0 (0–0)	0 (0–0)	0 (0–0)	0 (0–0)	0 (0–0)	0 (0–0)	0 (0–0)	0 (0–0)	0 (0–0)	0 (0–0)	0 (0–0)	0 (0–0)	0 (0–0)	0 (0–0)	0 (0–0)	0 (0–0)	0 (0–0)	0 (0–0)	0 (0–0)	--
Other urinary diseases	0 (0–0)	0 (0–0)	0 (0–0)	0 (0–0)	0 (0–0)	0 (0–0)	0 (0–0)	0 (0–0)	0 (0–0)	0 (0–0)	0 (0–0)	0 (0–0)	0 (0–0)	0 (0–0)	0 (0–0)	0 (0–0)	0 (0–0)	0 (0–0)	0 (0–0)	0 (0–0)	0 (0–0)	0 (0–0)	--
Gynecological diseases	--	--	--	--	--	--	--	--	--	--	--	--	--	--	--	--	--	--	--	--	--	--	--
Uterine fibroids	--	--	--	--	--	--	--	--	--	--	--	--	--	--	--	--	--	--	--	--	--	--	--
Endometriosis	--	--	--	--	--	--	--	--	--	--	--	--	--	--	--	--	--	--	--	--	--	--	--
Genital prolapse	--	--	--	--	--	--	--	--	--	--	--	--	--	--	--	--	--	--	--	--	--	--	--
Other gynecological diseases	--	--	--	--	--	--	--	--	--	--	--	--	--	--	--	--	--	--	--	--	--	--	--
Hemoglobinopathies and hemolytic anaemias	0 (0–0)	0 (0–0)	0 (0–0)	0 (0–0)	0 (0–0)	0 (0–0)	0 (0–0)	0 (0–0)	0 (0–0)	0 (0–0)	0 (0–0)	0 (0–0)	0 (0–0)	0 (0–0)	0 (0–0)	0 (0–0)	0 (0–0)	0 (0–0)	0 (0–0)	0 (0–0)	0 (0–0)	0 (0–0)	--
Thalassemias	0 (0–0)	0 (0–0)	0 (0–0)	0 (0–0)	0 (0–0)	0 (0–0)	0 (0–0)	0 (0–0)	0 (0–0)	0 (0–0)	0 (0–0)	0 (0–0)	0 (0–0)	0 (0–0)	0 (0–0)	0 (0–0)	0 (0–0)	0 (0–0)	0 (0–0)	0 (0–0)	0 (0–0)	0 (0–0)	--

Sickle cell disorders	0 (0–0)	0 (0–0)	0 (0–0)	0 (0–0)	0 (0–0)	0 (0–0)	0 (0–0)	0 (0–0)	0 (0–0)	0 (0–0)	0 (0–0)	0 (0–0)	0 (0–0)	0 (0–0)	0 (0–0)	0 (0–0)	0 (0–0)	0 (0–0)	0 (0–0)	0 (0–0)	0 (0–0)	0 (0–0)	--
G6PD deficiency	0 (0–0)	0 (0–0)	0 (0–0)	0 (0–0)	0 (0–0)	0 (0–0)	0 (0–0)	0 (0–0)	0 (0–0)	0 (0–0)	0 (0–0)	0 (0–0)	0 (0–0)	0 (0–0)	0 (0–0)	0 (0–0)	0 (0–0)	0 (0–0)	0 (0–0)	0 (0–0)	0 (0–0)	0 (0–0)	--
Other hemoglobinopathies and hemolytic anaemias	0 (0–0)	0 (0–0)	0 (0–0)	0 (0–0)	0 (0–0)	0 (0–0)	0 (0–0)	0 (0–0)	0 (0–0)	0 (0–0)	0 (0–0)	0 (0–0)	0 (0–0)	0 (0–0)	0 (0–0)	0 (0–0)	0 (0–0)	0 (0–0)	0 (0–0)	0 (0–0)	0 (0–0)	0 (0–0)	--
Endocrine, metabolic, blood, and immune disorders	0 (0–0)	0 (0–0)	1 (1–2)	0 (0–1)	0 (0–0)	0 (0–0)	0 (0–0)	0 (0–0)	0 (0–1)	1 (0–1)	1 (0–1)	1 (0–1)	1 (0–1)	1 (0–1)	1 (0–1)	1 (0–1)	1 (0–1)	1 (0–1)	0 (0–1)	1 (0–1)	1 (0–1)	0 (0–1)	--
Sudden infant death syndrome	--	0 (0–0)	1 (0–1)	--	--	--	--	--	--	--	--	--	--	--	--	--	--	--	--	--	--	--	--
Transport injuries	0 (0–0)	0 (0–0)	0 (0–0)	1 (0–1)	1 (1–1)	2 (1–2)	6 (5–8)	10 (7–13)	11 (9–14)	9 (7–12)	7 (5–9)	6 (4–8)	5 (4–7)	4 (3–5)	4 (3–5)	3 (2–3)	2 (1–2)	1 (1–2)	1 (1–1)	1 (0–1)	0 (0–1)	0 (0–0)	--
Road injuries	0 (0–0)	0 (0–0)	0 (0–0)	1 (0–1)	1 (1–1)	1 (1–2)	6 (4–8)	9 (7–12)	10 (8–13)	8 (6–11)	6 (5–8)	5 (4–7)	5 (4–6)	4 (3–5)	3 (2–4)	2 (2–3)	2 (1–2)	1 (1–1)	1 (1–1)	1 (0–1)	0 (0–1)	0 (0–0)	--
Pedestrian road injuries	0 (0–0)	0 (0–0)	0 (0–0)	0 (0–0)	0 (0–0)	0 (0–1)	1 (0–1)	1 (0–1)	1 (1–1)	1 (1–1)	1 (1–1)	1 (1–1)	1 (1–1)	1 (1–1)	1 (1–1)	1 (0–1)	0 (0–1)	0 (0–1)	0 (0–0)	0 (0–0)	0 (0–0)	0 (0–0)	--
Cyclist road injuries	--	--	--	0 (0–0)	0 (0–0)	0 (0–0)	1 (0–1)	1 (0–1)	1 (0–1)	1 (0–1)	1 (0–1)	0 (0–1)	0 (0–1)	0 (0–1)	0 (0–1)	0 (0–0)	0 (0–0)	0 (0–0)	0 (0–0)	0 (0–0)	0 (0–0)	0 (0–0)	--
Motorcyclist road injuries	0 (0–0)	0 (0–0)	0 (0–0)	0 (0–0)	0 (0–0)	0 (0–0)	1 (1–2)	3 (2–4)	3 (2–4)	2 (2–3)	2 (1–2)	1 (1–1)	1 (1–1)	1 (0–1)	0 (0–1)	0 (0–0)	0 (0–0)	0 (0–0)	0 (0–0)	0 (0–0)	0 (0–0)	0 (0–0)	--
Motor vehicle road injuries	0 (0–0)	0 (0–0)	0 (0–0)	0 (0–0)	0 (0–1)	1 (0–1)	3 (2–4)	5 (4–6)	5 (4–7)	4 (3–6)	3 (2–4)	3 (2–4)	3 (2–4)	2 (1–3)	2 (1–2)	1 (1–2)	1 (1–1)	1 (0–1)	0 (0–0)	0 (0–0)	0 (0–0)	0 (0–0)	--
Other road injuries	0 (0–0)	0 (0–0)	0 (0–0)	0 (0–0)	0 (0–0)	0 (0–0)	0 (0–0)	0 (0–0)	0 (0–0)	0 (0–0)	0 (0–0)	0 (0–0)	0 (0–0)	0 (0–0)	0 (0–0)	0 (0–0)	0 (0–0)	0 (0–0)	0 (0–0)	0 (0–0)	0 (0–0)	0 (0–0)	--
Other transport injuries	0 (0–0)	0 (0–0)	0 (0–0)	0 (0–0)	0 (0–0)	0 (0–0)	1 (0–1)	1 (1–1)	1 (1–1)	1 (1–1)	1 (0–1)	1 (0–1)	0 (0–1)	0 (0–1)	0 (0–1)	0 (0–0)	0 (0–0)	0 (0–0)	0 (0–0)	0 (0–0)	0 (0–0)	0 (0–0)	--
Falls	0 (0–0)	0 (0–0)	0 (0–0)	0 (0–0)	0 (0–0)	0 (0–0)	0 (0–0)	0 (0–1)	0 (0–1)	0 (0–1)	0 (0–1)	0 (0–1)	0 (0–1)	1 (0–1)	1 (0–1)	1 (0–1)	1 (0–1)	1 (0–1)	1 (0–1)	1 (1–1)	1 (1–1)	1 (1–1)	--
Drowning	0 (0–0)	0 (0–0)	0 (0–0)	1 (1–1)	1 (1–1)	1 (1–2)	3 (2–4)	3 (2–4)	3 (2–4)	2 (2–3)	2 (1–2)	1 (1–2)	1 (1–1)	1 (1–1)	1 (1–1)	0 (0–1)	0 (0–0)	0 (0–0)	0 (0–0)	0 (0–0)	0 (0–0)	0 (0–0)	--
Fire, heat, and hot substances	0 (0–0)	0 (0–0)	0 (0–0)	0 (0–0)	0 (0–0)	0 (0–0)	0 (0–0)	0 (0–0)	0 (0–0)	0 (0–0)	0 (0–0)	0 (0–0)	0 (0–0)	0 (0–0)	0 (0–0)	0 (0–0)	0 (0–0)	0 (0–0)	0 (0–0)	0 (0–0)	0 (0–0)	0 (0–0)	--
Poisonings	0 (0–0)	0 (0–0)	0 (0–0)	0 (0–0)	0 (0–0)	0 (0–0)	0 (0–0)	0 (0–0)	0 (0–0)	0 (0–0)	0 (0–0)	0 (0–0)	0 (0–0)	0 (0–0)	0 (0–0)	0 (0–0)	0 (0–0)	0 (0–0)	0 (0–0)	0 (0–0)	0 (0–0)	0 (0–0)	--
Poisoning by carbon monoxide	0 (0–0)	0 (0–0)	0 (0–0)	0 (0–0)	0 (0–0)	0 (0–0)	0 (0–0)	0 (0–0)	0 (0–0)	0 (0–0)	0 (0–0)	0 (0–0)	0 (0–0)	0 (0–0)	0 (0–0)	0 (0–0)	0 (0–0)	0 (0–0)	0 (0–0)	0 (0–0)	0 (0–0)	0 (0–0)	--
Poisoning by other means	0 (0–0)	0 (0–0)	0 (0–0)	0 (0–0)	0 (0–0)	0 (0–0)	0 (0–0)	0 (0–0)	0 (0–0)	0 (0–0)	0 (0–0)	0 (0–0)	0 (0–0)	0 (0–0)	0 (0–0)	0 (0–0)	0 (0–0)	0 (0–0)	0 (0–0)	0 (0–0)	0 (0–0)	0 (0–0)	--
Exposure to mechanical forces	0 (0–0)	0 (0–0)	0 (0–0)	0 (0–0)	0 (0–0)	0 (0–0)	1 (1–2)	2 (1–3)	2 (1–3)	2 (1–2)	1 (1–2)	1 (1–2)	1 (1–1)	1 (1–1)	1 (1–1)	0 (0–1)	0 (0–0)	0 (0–0)	0 (0–0)	0 (0–0)	0 (0–0)	0 (0–0)	--
Unintentional firearm injuries	0 (0–0)	0 (0–0)	0 (0–0)	0 (0–0)	0 (0–0)	0 (0–0)	1 (1–2)	2 (1–2)	2 (1–2)	1 (1–2)	1 (1–2)	1 (1–1)	1 (1–1)	1 (0–1)	0 (0–1)	0 (0–0)	0 (0–0)	0 (0–0)	0 (0–0)	0 (0–0)	0 (0–0)	0 (0–0)	--
Other exposure to mechanical forces	0 (0–0)	0 (0–0)	0 (0–0)	0 (0–0)	0 (0–0)	0 (0–0)	0 (0–0)	0 (0–1)	0 (0–1)	0 (0–1)	0 (0–1)	0 (0–1)	0 (0–0)	0 (0–0)	0 (0–0)	0 (0–0)	0 (0–0)	0 (0–0)	0 (0–0)	0 (0–0)	0 (0–0)	0 (0–0)	--
Adverse effects of medical treatment	0 (0–0)	0 (0–0)	0 (0–1)	0 (0–0)	0 (0–0)	0 (0–0)	0 (0–0)	0 (0–1)	0 (0–1)	0 (0–1)	0 (0–1)	0 (0–1)	0 (0–1)	1 (0–1)	1 (0–1)	1 (0–1)	1 (0–1)	1 (0–1)	1 (0–1)	1 (1–1)	1 (0–1)	0 (0–0)	--
Animal contact	0 (0–0)	0 (0–0)	0 (0–0)	0 (0–0)	0 (0–0)	0 (0–0)	0 (0–0)	0 (0–0)	0 (0–0)	0 (0–0)	0 (0–0)	0 (0–0)	0 (0–0)	0 (0–0)	0 (0–0)	0 (0–0)	0 (0–0)	0 (0–0)	0 (0–0)	0 (0–0)	0 (0–0)	0 (0–0)	--
Venomous animal contact	0 (0–0)	0 (0–0)	0 (0–0)	0 (0–0)	0 (0–0)	0 (0–0)	0 (0–0)	0 (0–0)	0 (0–0)	0 (0–0)	0 (0–0)	0 (0–0)	0 (0–0)	0 (0–0)	0 (0–0)	0 (0–0)	0 (0–0)	0 (0–0)	0 (0–0)	0 (0–0)	0 (0–0)	0 (0–0)	--
Non-venomous animal contact	0 (0–0)	0 (0–0)	0 (0–0)	0 (0–0)	0 (0–0)	0 (0–0)	0 (0–0)	0 (0–0)	0 (0–0)	0 (0–0)	0 (0–0)	0 (0–0)	0 (0–0)	0 (0–0)	0 (0–0)	0 (0–0)	0 (0–0)	0 (0–0)	0 (0–0)	0 (0–0)	0 (0–0)	0 (0–0)	--
Foreign body	0 (0–0)	0 (0–0)	1 (0–1)	0 (0–1)	0 (0–0)	0 (0–0)	0 (0–0)	0 (0–0)	0 (0–0)	0 (0–0)	0 (0–0)	0 (0–0)	0 (0–0)	0 (0–0)	0 (0–0)	0 (0–0)	0 (0–0)	0 (0–0)	0 (0–0)	0 (0–0)	0 (0–0)	0 (0–0)	--
Pulmonary aspiration and foreign body in airway	0 (0–0)	0 (0–0)	1 (0–1)	0 (0–1)	0 (0–0)	0 (0–0)	0 (0–0)	0 (0–0)	0 (0–0)	0 (0–0)	0 (0–0)	0 (0–0)	0 (0–0)	0 (0–0)	0 (0–0)	0 (0–0)	0 (0–0)	0 (0–0)	0 (0–0)	0 (0–0)	0 (0–0)	0 (0–0)	--
Foreign body in other body part	0 (0–0)	0 (0–0)	0 (0–0)	0 (0–0)	0 (0–0)	0 (0–0)	0 (0–0)	0 (0–0)	0 (0–0)	0 (0–0)	0 (0–0)	0 (0–0)	0 (0–0)	0 (0–0)	0 (0–0)	0 (0–0)	0 (0–0)	0 (0–0)	0 (0–0)	0 (0–0)	0 (0–0)	0 (0–0)	--
Environmental heat and cold exposure	0 (0–0)	0 (0–0)	0 (0–0)	0 (0–0)	0 (0–0)	0 (0–0)	0 (0–0)	0 (0–0)	0 (0–0)	0 (0–0)	0 (0–0)	0 (0–0)	0 (0–0)	0 (0–0)	0 (0–0)	0 (0–0)	0 (0–0)	0 (0–0)	0 (0–0)	0 (0–0)	0 (0–0)	0 (0–0)	--
Other unintentional injuries	0 (0–0)	0 (0–0)	0 (0–0)	0 (0–0)	0 (0–0)	0 (0–0)	0 (0–0)	0 (0–1)	1 (0–1)	0 (0–1)	0 (0–0)	0 (0–0)	0 (0–0)	0 (0–0)	0 (0–0)	0 (0–0)	0 (0–0)	0 (0–0)	0 (0–0)	0 (0–0)	0 (0–0)	0 (0–0)	--
Self-harm	--	--	--	--	--	0 (0–0)	1 (1–2)	3 (2–4)	3 (2–4)	3 (2–5)	3 (2–3)	2 (1–3)	2 (1–2)	2 (1–2)	1 (1–2)	1 (1–2)	1 (1–1)	1 (0–1)	0 (0–1)	0 (0–1)	0 (0–0)	0 (0–0)	--
Self-harm by firearm	--	--	--	--	--	0 (0–0)	0 (0–0)	0 (0–1)	0 (0–1)	0 (0–1)	0 (0–0)	0 (0–0)	0 (0–0)	0 (0–0)	0 (0–0)	0 (0–0)	0 (0–0)	0 (0–0)	0 (0–0)	0 (0–0)	0 (0–0)	0 (0–0)	--
Self-harm by other specified means	--	--	--	--	--	0 (0–0)	1 (1–2)	2 (2–3)	3 (2–4)	3 (2–4)	2 (2–3)	2 (1–2)	2 (1–2)	1 (1–2)	1 (1–2)	1 (1–1)	1 (1–1)	1 (0–1)	0 (0–1)	0 (0–1)	0 (0–0)	0 (0–0)	--
Interpersonal violence	0 (0–0)	0 (0–0)	0 (0–0)	0 (0–1)	0 (0–0)	1 (1–1)	10 (8–13)	19 (16–22)	20 (17–23)	16 (13–20)	11 (9–14)	8 (6–10)	6 (4–8)	4 (3–5)	3 (2–4)	2 (1–2)	1 (1–1)	1 (0–1)	0 (0–0)	0 (0–0)	0 (0–0)	0 (0–0)	--
Physical violence by firearm	0 (0–0)	0 (0–0)	0 (0–0)	0 (0–0)	0 (0–0)	0 (0–1)	7 (5–8)	13 (11–16)	14 (11–16)	10 (9–13)	7 (5–9)	4 (3–6)	3 (2–4)	2 (1–2)	1 (1–2)	1 (0–1)	0 (0–0)	0 (0–0)	0 (0–0)	0 (0–0)	0 (0–0)	0 (0–0)	--
Physical violence by sharp object	0 (0–0)	0 (0–0)	0 (0–0)	0 (0–0)	0 (0–0)	0 (0–0)	3 (2–3)	4 (3–5)	5 (4–6)	4 (3–5)	3 (2–4)	2 (2–3)	2 (1–2)	1 (1–1)	1 (0–1)	0 (0–1)	0 (0–0)	0 (0–0)	0 (0–0)	0 (0–0)	0 (0–0)	0 (0–0)	--
Physical violence by other means	0 (0–0)	0 (0–0)	0 (0–0)	0 (0–1)	0 (0–0)	0 (0–0)	1 (1–1)	1 (1–2)	2 (1–2)	2 (1–2)	1 (1–2)	1 (1–2)	1 (1–2)	1 (1–1)	1 (1–1)	1 (0–1)	0 (0–1)	0 (0–0)	0 (0–0)	0 (0–0)	0 (0–0)	0 (0–0)	--
Executions and police conflict	0 (0–0)	0 (0–0)	0 (0–0)	0 (0–0)	0 (0–0)	0 (0–0)	0 (0–0)	0 (0–0)	0 (0–0)	0 (0–0)	0 (0–0)	0 (0–0)	0 (0–0)	0 (0–0)	0 (0–0)	0 (0–0)	0 (0–0)	0 (0–0)	0 (0–0)	0 (0–0)	0 (0–0)	0 (0–0)	--

SR Table 12: Cuba deaths by cause and age group for males

Cause	Early Neonatal	Late Neonatal	Post Neonatal	1 to 4	5 to 9	10 to 14	15 to 19	20 to 24	25 to 29	30 to 34	35 to 39	40 to 44	45 to 49	50 to 54	55 to 59	60 to 64	65 to 69	70 to 74	75 to 79	80 to 84	85 to 89	90 to 94	95 plus
All causes	89 (70–111)	48 (38–59)	91 (73–111)	57 (48–68)	57 (51–63)	69 (59–82)	168 (137–205)	260 (219–308)	365 (318–417)	485 (420–557)	514 (434–605)	827 (678–1,000)	1,702 (1,357–2,108)	2,947 (2,339–3,670)	4,146 (3,283–5,162)	4,339 (3,495–5,317)	5,555 (4,527–6,735)	7,108 (5,853–8,531)	7,969 (6,663–9,424)	7,685 (6,557–8,917)	6,269 (5,530–7,052)	4,288 (3,863–4,727)	--
Sexually transmitted infections excluding HIV	0 (0–0)	0 (0–0)	0 (0–0)	0 (0–0)	0 (0–0)	0 (0–0)	0 (0–0)	0 (0–0)	0 (0–0)	0 (0–0)	0 (0–0)	0 (0–0)	0 (0–0)	0 (0–0)	0 (0–0)	0 (0–0)	0 (0–0)	0 (0–0)	0 (0–0)	0 (0–0)	0 (0–0)	0 (0–0)	--
Syphilis	0 (0–0)	0 (0–0)	0 (0–0)	0 (0–0)	0 (0–0)	0 (0–0)	0 (0–0)	0 (0–0)	0 (0–0)	0 (0–0)	0 (0–0)	0 (0–0)	0 (0–0)	0 (0–0)	0 (0–0)	0 (0–0)	0 (0–0)	0 (0–0)	0 (0–0)	0 (0–0)	0 (0–0)	0 (0–0)	--
Chlamydial infection	--	--	--	--	--	0 (0–0)	0 (0–0)	0 (0–0)	0 (0–0)	0 (0–0)	0 (0–0)	0 (0–0)	0 (0–0)	0 (0–0)	0 (0–0)	0 (0–0)	0 (0–0)	0 (0–0)	0 (0–0)	0 (0–0)	0 (0–0)	0 (0–0)	--
Gonococcal infection	--	--	--	--	--	0 (0–0)	0 (0–0)	0 (0–0)	0 (0–0)	0 (0–0)	0 (0–0)	0 (0–0)	0 (0–0)	0 (0–0)	0 (0–0)	0 (0–0)	0 (0–0)	0 (0–0)	0 (0–0)	0 (0–0)	0 (0–0)	0 (0–0)	--
Other sexually transmitted infections	--	--	--	--	--	0 (0–0)	0 (0–0)	0 (0–0)	0 (0–0)	0 (0–0)	0 (0–0)	0 (0–0)	0 (0–0)	0 (0–0)	0 (0–0)	0 (0–0)	0 (0–0)	0 (0–0)	0 (0–0)	0 (0–0)	0 (0–0)	0 (0–0)	--
Tuberculosis	--	--	0 (0–0)	0 (0–0)	0 (0–0)	0 (0–0)	0 (0–0)	0 (0–0)	0 (0–0)	0 (0–1)	1 (0–1)	1 (1–1)	2 (1–2)	3 (2–4)	3 (2–5)	3 (2–5)	3 (2–5)	4 (3–5)	4 (3–6)	3 (2–4)	2 (2–3)	1 (1–2)	--
Drug-susceptible tuberculosis	--	--	0 (0–0)	0 (0–0)	0 (0–0)	0 (0–0)	0 (0–0)	0 (0–0)	0 (0–0)	0 (0–1)	0 (0–1)	1 (1–1)	1 (1–2)	3 (2–4)	3 (2–5)	3 (2–5)	3 (2–5)	4 (2–5)	4 (3–5)	3 (2–4)	2 (2–3)	1 (1–2)	--
Multidrug-resistant tuberculosis without extensive drug resistance	--	--	0 (0–0)	0 (0–0)	0 (0–0)	0 (0–0)	0 (0–0)	0 (0–0)	0 (0–0)	0 (0–0)	0 (0–0)	0 (0–0)	0 (0–0)	0 (0–0)	0 (0–1)	0 (0–1)	0 (0–1)	0 (0–1)	0 (0–1)	0 (0–0)	0 (0–0)	0 (0–0)	--
Extensively drug-resistant tuberculosis	--	--	0 (0–0)	0 (0–0)	0 (0–0)	0 (0–0)	0 (0–0)	0 (0–0)	0 (0–0)	0 (0–0)	0 (0–0)	0 (0–0)	0 (0–0)	0 (0–0)	0 (0–0)	0 (0–0)	0 (0–0)	0 (0–0)	0 (0–0)	0 (0–0)	0 (0–0)	0 (0–0)	--
Lower respiratory infections	4 (3–6)	4 (3–6)	13 (9–18)	5 (4–7)	2 (1–2)	1 (1–2)	3 (2–4)	4 (3–6)	6 (4–8)	10 (7–14)	11 (8–16)	21 (14–29)	43 (29–62)	77 (54–108)	123 (85–171)	154 (109–205)	219 (152–297)	372 (266–507)	530 (388–717)	635 (482–825)	628 (489–783)	509 (393–636)	--
Upper respiratory infections	0 (0–0)	0 (0–0)	0 (0–0)	0 (0–0)	0 (0–0)	0 (0–0)	0 (0–0)	0 (0–0)	0 (0–0)	0 (0–0)	0 (0–0)	0 (0–0)	0 (0–0)	0 (0–0)	0 (0–0)	0 (0–0)	0 (0–0)	0 (0–0)	0 (0–1)	0 (0–0)	0 (0–0)	0 (0–0)	--
Otitis media	0 (0–0)	0 (0–0)	0 (0–0)	0 (0–0)	0 (0–0)	0 (0–0)	0 (0–0)	0 (0–0)	0 (0–0)	0 (0–0)	0 (0–0)	0 (0–0)	0 (0–0)	0 (0–0)	0 (0–0)	0 (0–0)	0 (0–0)	0 (0–0)	0 (0–0)	0 (0–0)	0 (0–0)	0 (0–0)	--
Diarrhoeal diseases	0 (0–0)	0 (0–1)	3 (2–5)	1 (0–1)	0 (0–0)	0 (0–0)	0 (0–0)	0 (0–1)	0 (0–1)	1 (0–1)	1 (1–1)	2 (1–3)	4 (2–6)	7 (4–11)	8 (5–13)	9 (6–13)	10 (6–16)	16 (9–25)	18 (11–28)	18 (11–28)	13 (8–21)	9 (5–13)	--
Typhoid fever	--	--	0 (0–0)	0 (0–0)	0 (0–0)	0 (0–0)	0 (0–0)	0 (0–0)	0 (0–0)	0 (0–0)	0 (0–0)	0 (0–0)	0 (0–0)	0 (0–0)	0 (0–0)	0 (0–0)	0 (0–0)	0 (0–0)	0 (0–0)	0 (0–0)	0 (0–0)	0 (0–0)	--
Paratyphoid fever	--	--	0 (0–0)	0 (0–0)	0 (0–0)	0 (0–0)	0 (0–0)	0 (0–0)	0 (0–0)	0 (0–0)	0 (0–0)	0 (0–0)	0 (0–0)	0 (0–0)	0 (0–0)	0 (0–0)	0 (0–0)	0 (0–0)	0 (0–0)	0 (0–0)	0 (0–0)	0 (0–0)	--
iNTS	--	0 (0–0)	0 (0–0)	0 (0–0)	0 (0–0)	0 (0–0)	0 (0–0)	0 (0–0)	0 (0–0)	0 (0–0)	0 (0–0)	0 (0–0)	0 (0–0)	0 (0–0)	0 (0–0)	0 (0–0)	0 (0–0)	0 (0–0)	0 (0–0)	0 (0–0)	0 (0–0)	0 (0–0)	--
Other intestinal infectious diseases	--	--	0 (0–0)	0 (0–0)	0 (0–0)	0 (0–0)	0 (0–0)	0 (0–0)	0 (0–0)	0 (0–0)	0 (0–0)	0 (0–0)	0 (0–0)	0 (0–0)	0 (0–0)	0 (0–0)	0 (0–0)	0 (0–0)	0 (0–0)	0 (0–0)	0 (0–0)	0 (0–0)	--
Malaria	0 (0–0)	0 (0–0)	0 (0–0)	0 (0–0)	0 (0–0)	0 (0–0)	0 (0–0)	0 (0–0)	0 (0–0)	0 (0–0)	0 (0–0)	0 (0–0)	0 (0–0)	0 (0–0)	0 (0–0)	0 (0–0)	0 (0–0)	0 (0–0)	0 (0–0)	0 (0–0)	0 (0–0)	0 (0–0)	--
Chagas disease	--	--	0 (0–0)	0 (0–0)	0 (0–0)	0 (0–0)	0 (0–0)	0 (0–0)	0 (0–0)	0 (0–0)	0 (0–0)	0 (0–0)	0 (0–0)	0 (0–0)	0 (0–0)	0 (0–0)	0 (0–0)	0 (0–0)	0 (0–0)	0 (0–0)	0 (0–0)	0 (0–0)	--
Visceral leishmaniasis	--	--	0 (0–0)	0 (0–0)	0 (0–0)	0 (0–0)	0 (0–0)	0 (0–0)	0 (0–0)	0 (0–0)	0 (0–0)	0 (0–0)	0 (0–0)	0 (0–0)	0 (0–0)	0 (0–0)	0 (0–0)	0 (0–0)	0 (0–0)	0 (0–0)	0 (0–0)	0 (0–0)	--
African trypanosomiasis	--	--	--	0 (0–0)	0 (0–0)	0 (0–0)	0 (0–0)	0 (0–0)	0 (0–0)	0 (0–0)	0 (0–0)	0 (0–0)	0 (0–0)	0 (0–0)	0 (0–0)	0 (0–0)	0 (0–0)	0 (0–0)	0 (0–0)	0 (0–0)	0 (0–0)	0 (0–0)	--
Schistosomiasis	--	--	0 (0–0)	0 (0–0)	0 (0–0)	0 (0–0)	0 (0–0)	0 (0–0)	0 (0–0)	0 (0–0)	0 (0–0)	0 (0–0)	0 (0–0)	0 (0–0)	0 (0–0)	0 (0–0)	0 (0–0)	0 (0–0)	0 (0–0)	0 (0–0)	0 (0–0)	0 (0–0)	--
Cysticercosis	--	--	--	0 (0–0)	0 (0–0)	0 (0–0)	0 (0–0)	0 (0–0)	0 (0–0)	0 (0–0)	0 (0–0)	0 (0–0)	0 (0–0)	0 (0–0)	0 (0–0)	0 (0–0)	0 (0–0)	0 (0–0)	0 (0–0)	0 (0–0)	0 (0–0)	0 (0–0)	--
Cystic echinococcosis	--	--	--	0 (0–0)	0 (0–0)	0 (0–0)	0 (0–0)	0 (0–0)	0 (0–0)	0 (0–0)	0 (0–0)	0 (0–0)	0 (0–0)	0 (0–0)	0 (0–0)	0 (0–0)	0 (0–0)	0 (0–0)	0 (0–0)	0 (0–0)	0 (0–0)	0 (0–0)	--
Dengue	0 (0–0)	0 (0–0)	0 (0–0)	0 (0–0)	0 (0–0)	0 (0–0)	0 (0–0)	0 (0–0)	0 (0–0)	0 (0–0)	0 (0–0)	0 (0–0)	0 (0–0)	0 (0–0)	0 (0–0)	0 (0–0)	0 (0–0)	0 (0–0)	0 (0–0)	0 (0–0)	0 (0–0)	0 (0–0)	--
Yellow fever	--	0 (0–0)	0 (0–0)	0 (0–0)	0 (0–0)	0 (0–0)	0 (0–0)	0 (0–0)	0 (0–0)	0 (0–0)	0 (0–0)	0 (0–0)	0 (0–0)	0 (0–0)	0 (0–0)	0 (0–0)	0 (0–0)	0 (0–0)	0 (0–0)	0 (0–0)	0 (0–0)	0 (0–0)	--
Rabies	--	--	0 (0–0)	0 (0–0)	0 (0–0)	0 (0–0)	0 (0–0)	0 (0–0)	0 (0–0)	0 (0–0)	0 (0–0)	0 (0–0)	0 (0–0)	0 (0–0)	0 (0–0)	0 (0–0)	0 (0–0)	0 (0–0)	0 (0–0)	0 (0–0)	0 (0–0)	0 (0–0)	--
Ascariasis	--	--	0 (0–0)	0 (0–0)	0 (0–0)	0 (0–0)	0 (0–0)	0 (0–0)	0 (0–0)	0 (0–0)	0 (0–0)	0 (0–0)	0 (0–0)	0 (0–0)	0 (0–0)	0 (0–0)	0 (0–0)	0 (0–0)	0 (0–0)	0 (0–0)	0 (0–0)	0 (0–0)	--
Zika virus disease	0 (0–0)	0 (0–0)	0 (0–0)	0 (0–0)	0 (0–0)	0 (0–0)	0 (0–0)	0 (0–0)	0 (0–0)	0 (0–0)	0 (0–0)	0 (0–0)	0 (0–0)	0 (0–0)	0 (0–0)	0 (0–0)	0 (0–0)	0 (0–0)	0 (0–0)	0 (0–0)	0 (0–0)	0 (0–0)	--
Other neglected tropical diseases	0 (0–0)	0 (0–0)	0 (0–0)	0 (0–0)	0 (0–0)	0 (0–0)	0 (0–0)	0 (0–0)	0 (0–0)	0 (0–0)	0 (0–0)	0 (0–0)	0 (0–0)	0 (0–0)	0 (0–0)	0 (0–0)	0 (0–0)	0 (0–0)	0 (0–0)	0 (0–0)	0 (0–0)	0 (0–0)	--
Meningitis	0 (0–0)	1 (1–1)	3 (2–5)	2 (1–3)	1 (1–2)	1 (1–1)	1 (1–2)	1 (0–1)	1 (1–1)	1 (1–2)	1 (1–2)	2 (1–2)	3 (2–4)	4 (3–7)	5 (4–8)	4 (3–7)	4 (3–6)	5 (3–7)	4 (3–6)	3 (2–5)	2 (1–3)	1 (1–1)	--
Encephalitis	0 (0–0)	0 (0–0)	1 (0–2)	1 (1–2)	1 (0–1)	1 (0–1)	1 (0–1)	1 (0–1)	1 (0–1)	1 (1–1)	1 (0–1)	1 (1–1)	1 (1–2)	1 (1–2)	1 (1–2)	1 (1–2)	1 (1–2)	2 (1–2)	1 (1–2)	1 (0–1)	0 (0–1)	0 (0–0)	--
Diphtheria	--	--	0 (0–0)	0 (0–0)	0 (0–0)	0 (0–0)	0 (0–0)	0 (0–0)	0 (0–0)	0 (0–0)	0 (0–0)	0 (0–0)	0 (0–0)	0 (0–0)	0 (0–0)	--	--	--	--	--	--	--	--
Whooping cough	--	--	0 (0–0)	0 (0–0)	0 (0–0)	0 (0–0)	0 (0–0)	0 (0–0)	0 (0–0)	0 (0–0)	0 (0–0)	0 (0–0)	0 (0–0)	0 (0–0)	0 (0–0)	--	--	--	--	--	--	--	--
Tetanus	0 (0–0)	0 (0–0)	0 (0–0)	0 (0–0)	0 (0–0)	0 (0–0)	0 (0–0)	0 (0–0)	0 (0–0)	0 (0–0)	0 (0–0)	0 (0–0)	0 (0–0)	0 (0–0)	0 (0–0)	0 (0–0)	0 (0–0)	0 (0–0)	0 (0–0)	0 (0–0)	0 (0–0)	0 (0–0)	--
Measles	0 (0–0)	0 (0–0)	0 (0–0)	0 (0–0)	0 (0–0)	0 (0–0)	0 (0–0)	0 (0–0)	0 (0–0)	0 (0–0)	0 (0–0)	0 (0–0)	0 (0–0)	0 (0–0)	0 (0–0)	0 (0–0)	0 (0–0)	0 (0–0)	0 (0–0)	0 (0–0)	0 (0–0)	0 (0–0)	--
Varicella and herpes zoster	0 (0–0)	0 (0–0)	0 (0–0)	0 (0–0)	0 (0–0)	0 (0–0)	0 (0–0)	0 (0–0)	0 (0–0)	0 (0–0)	0 (0–0)	0 (0–0)	0 (0–1)	0 (0–1)	0 (0–1)	0 (0–1)	0 (0–1)	1 (0–2)	1 (0–2)	1 (0–2)	0 (0–1)	0 (0–1)	--
Acute hepatitis	--	--	0 (0–0)	0 (0–0)	0 (0–0)	0 (0–0)	0 (0–0)	0 (0–0)	0 (0–0)	0 (0–0)	0 (0–0)	0 (0–0)	0 (0–0)	0 (0–0)	0 (0–0)	0 (0–0)	0 (0–0)	0 (0–0)	0 (0–0)	0 (0–0)	0 (0–0)	0 (0–0)	--
Acute hepatitis A	--	--	0 (0–0)	0 (0–0)	0 (0–0)	0 (0–0)	0 (0–0)	0 (0–0)	0 (0–0)	0 (0–0)	0 (0–0)	0 (0–0)	0 (0–0)	0 (0–0)	0 (0–0)	0 (0–0)	0 (0–0)	0 (0–0)	0 (0–0)	0 (0–0)	0 (0–0)	0 (0–0)	--
Acute hepatitis B	--	--	0 (0–0)	0 (0–0)	0 (0–0)	0 (0–0)	0 (0–0)	0 (0–0)	0 (0–0)	0 (0–0)	0 (0–0)	0 (0–0)	0 (0–0)	0 (0–0)	0 (0–0)	0 (0–0)	0 (0–0)	0 (0–0)	0 (0–0)	0 (0–0)	0 (0–0)	0 (0–0)	--
Acute hepatitis C	--	--	0 (0–0)	0 (0–0)	0 (0–0)	0 (0–0)	0 (0–0)	0 (0–0)	0 (0–0)	0 (0–0)	0 (0–0)	0 (0–0)	0 (0–0)	0 (0–0)	0 (0–0)	0 (0–0)	0 (0–0)	0 (0–0)	0 (0–0)	0 (0–0)	0 (0–0)	0 (0–0)	--
Acute hepatitis E	--	--	0 (0–0)	0 (0–0)	0 (0–0)	0 (0–0)	0 (0–0)	0 (0–0)	0 (0–0)	0 (0–0)	0 (0–0)	0 (0–0)	0 (0–0)	0 (0–0)	0 (0–0)	0 (0–0)	0 (0–0)	0 (0–0)	0 (0–0)	0 (0–0)	0 (0–0)	0 (0–0)	--
Other unspecified infectious diseases	0 (0–0)	0 (0–0)	1 (0–2)	0 (0–1)	0 (0–0)	0 (0–0)	1 (0–1)	1 (0–2)	2 (1–3)	2 (1–3)	2 (1–3)	3 (1–4)	5 (3–9)	6 (3–10)	6 (3–10)	5 (3–8)	5 (3–8)	4 (3–8)	3 (2–6)	2 (1–5)	3 (1–6)	1 (1–2)	--
Maternal disorders	--	--	--	--	--	--	--	--	--	--	--	--	--	--	--	--	--	--	--	--	--	--	--
Maternal haemorrhage	--	--	--	--	--	--	--	--	--	--	--	--	--	--	--	--	--	--	--	--	--	--	--
Maternal sepsis and other pregnancy related infections	--	--	--	--	--	--	--	--	--	--	--	--	--	--	--	--	--	--	--	--	--	--	--
Maternal hypertensive disorders	--	--	--	--	--	--	--	--	--	--	--	--	--	--	--	--	--	--	--	--	--	--	--
Maternal obstructed labour and uterine rupture	--	--	--	--	--	--	--	--	--	--	--	--	--	--	--	--	--	--	--	--	--	--	--
Maternal abortive outcome	--	--	--	--	--	--	--	--	--	--	--	--	--	--	--	--	--	--	--	--	--	--	--
Ectopic pregnancy	--	--	--	--	--	--	--	--	--	--	--	--	--	--	--	--	--	--	--	--	--	--	--
Indirect maternal deaths	--	--	--	--	--	--	--	--	--	--	--	--	--	--	--	--	--	--	--	--	--	--	--
Late maternal deaths	--	--	--	--	--	--	--	--	--	--	--	--	--	--	--	--	--	--	--	--	--	--	--
Maternal deaths aggravated by HIV/AIDS	--	--	--	--	--	--	--	--	--	--	--	--	--	--	--	--	--	--	--	--	--	--	--
Other maternal disorders	--	--	--	--	--	--	--	--	--	--	--	--	--	--	--	--	--	--	--	--	--	--	--
Neonatal disorders	64 (39–83)	27 (20–34)	13 (9–19)	1 (1–2)	--	--	--	--	--	--	--	--	--	--	--	--	--	--	--	--	--	--	--
Neonatal preterm birth	21 (13–30)	6 (4–9)	4 (3–6)	0 (0–0)	--	--	--	--	--	--	--	--	--	--	--	--	--	--	--	--	--	--	--
Neonatal encephalopathy due to birth asphyxia and trauma	15 (9–22)	4 (3–7)	4 (2–6)	1 (0–1)	--	--	--	--	--	--	--	--	--	--	--	--	--	--	--	--	--	--	--
Neonatal sepsis and other neonatal infections	9 (5–13)	7 (5–10)	1 (0–1)	0 (0–0)	--	--	--	--	--	--	--	--	--	--	--	--	--	--	--	--	--	--	--
Hemolytic disease and other neonatal jaundice	1 (0–1)	0 (0–0)	0 (0–0)	0 (0–0)	--	--	--	--	--	--	--	--	--	--	--	--	--	--	--	--	--	--	--
Other neonatal disorders	19 (10–27)	9 (6–12)	4 (3–7)	0 (0–0)	--	--	--	--	--	--	--	--	--	--	--	--	--	--	--	--	--	--	--
Nutritional deficiencies	0 (0–0)	0 (0–0)	1 (1–1)	0 (0–1)	0 (0–0)	0 (0–0)	0 (0–0)	0 (0–0)	0 (0–0)	0 (0–0)	0 (0–1)	1 (0–1)	1 (1–1)	2 (1–3)	2 (1–3)	2 (2–4)	4 (3–6)	5 (3–8)	6 (4–8)	5 (3–8)	5 (3–6)	3 (2–4)	--
Protein-energy malnutrition	0 (0–0)	0 (0–0)	1 (1–1)	0 (0–1)	0 (0–0)	0 (0–0)	0 (0–0)	0 (0–0)	0 (0–0)	0 (0–0)	0 (0–0)	0 (0–1)	1 (0–1)	1 (1–2)	2 (1–3)	2 (1–3)	3 (2–5)	4 (3–7)	5 (3–7)	4 (3–7)	4 (2–5)	2 (1–4)	--
Other nutritional deficiencies	--	--	0 (0–0)	0 (0–0)	0 (0–0)	0 (0–0)	0 (0–0)	0 (0–0)	0 (0–0)	0 (0–0)	0 (0–0)	0 (0–0)	0 (0–0)	0 (0–1)	0 (0–1)	0 (0–1)	1 (0–1)	1 (0–2)	1 (0–1)	1 (0–2)	1 (0–2)	1 (0–2)	--
Lip and oral cavity cancer	--	--	--	--	0 (0–0)	0 (0–0)	0 (0–0)	0 (0–0)	0 (0–1)	1 (1–1)	2 (1–3)	6 (3–8)	18 (11–27)	43 (30–62)	61 (42–85)	56 (40–77)	55 (39–75)	56 (40–75)	50 (37–64)	37 (27–51)	23 (16–32)	12 (8–16)	--
Nasopharynx cancer	--	--	--	--	0 (0–0)	0 (0–0)	0 (0–0)	0 (0–1)	0 (0–1)	0 (0–1)	1 (0–1)	2 (1–2)	4 (3–6)	10 (6–14)	13 (9–20)	10 (6–14)	10 (6–15)	9 (6–13)	7 (4–10)	4 (3–7)	4 (2–6)	2 (1–3)	--
Other pharynx cancer	--	--	--	--	--	--	--	0 (0–0)	0 (0–0)	0 (0–0)	1 (0–1)	2 (1–4)	10 (7–15)	22 (15–31)	32 (20–46)	27 (17–40)	27 (17–39)	25 (17–36)	20 (13–28)	12 (8–18)	8 (5–11)	4 (2–6)	--
Oesophageal cancer	--	--	--	--	--	--	--	0 (0–0)	0 (0–0)	1 (0–1)	3 (2–4)	13 (8–19)	43 (30–60)	88 (64–117)	136 (96–186)	112 (80–150)	105 (75–141)	105 (77–140)	76 (57–101)	60 (44–82)	35 (27–46)	18 (13–24)	--
Stomach cancer	--	--	--	--	--	--	0 (0–0)	0 (0–0)	1 (0–1)	2 (1–3)	3 (2–5)	8 (6–11)	19 (13–26)	47 (33–65)	67 (49–90)	70 (51–94)	88 (65–115)	104 (80–132)	100 (76–130)	72 (55–92)	47 (36–60)	25 (18–33)	--
Colon and rectum cancer	--	--	--	--	0 (0–0)	0 (0–0)	1 (0–1)	1 (1–2)	2 (1–3)	5 (3–7)	7 (5–10)	15 (10–22)	38 (26–53)	72 (50–99)	107 (76–144)	134 (97–177)	181 (134–235)	236 (178–308)	263 (198–342)	220 (163–289)	143 (109–185)	79 (56–105)	--
Liver cancer due to hepatitis B	--	--	--	--	--	0 (0–0)	0 (0–0)	0 (0–0)	0 (0–1)	0 (0–1)	1 (0–1)	1 (1–2)	3 (2–6)	7 (3–12)	11 (6–19)	11 (5–18)	12 (6–20)	12 (6–21)	11 (6–19)	7 (4–13)	4 (2–7)	1 (1–3)	--
Liver cancer due to hepatitis C	--	--	--	--	--	0 (0–0)	0 (0–0)	0 (0–0)	0 (0–0)	0 (0–0)	0 (0–0)	0 (0–0)	1 (0–1)	2 (1–3)	4 (1–7)	4 (2–9)	7 (3–12)	9 (4–16)	11 (5–19)	10 (5–16)	7 (4–11)	3 (2–5)	--
Liver cancer due to alcohol use	--	--	--	--	--	--	0 (0–0)	0 (0–0)	0 (0–0)	0 (0–0)	0 (0–1)	1 (0–1)	2 (1–5)	6 (3–10)	11 (6–19)	14 (8–23)	22 (14–34)	28 (17–42)	25 (16–37)	17 (10–24)	8 (5–13)	3 (2–5)	--
Liver cancer due to NASH	--	--	--	--	--	--	0 (0–0)	0 (0–0)	0 (0–0)	0 (0–0)	0 (0–0)	0 (0–0)	0 (0–1)	1 (0–2)	2 (1–3)	2 (1–4)	3 (1–6)	4 (2–8)	5 (3–9)	4 (2–7)	2 (1–4)	1 (0–2)	--
	0 (0–0)	0 (0–0)	0 (0–0)	0 (0–0)	0 (0–0)	0 (0–0)	--	--	--	--	--	--	--	--	--	--	--	--	--	--	--	--	--
Liver cancer due to other causes	0 (0–0)	0 (0–0)	0 (0–0)	0 (0–0)	0 (0–0)	0 (0–0)	0 (0–0)	0 (0–0)	0 (0–0)	0 (0–0)	0 (0–0)	0 (0–0)	0 (0–1)	1 (0–1)	1 (0–2)	1 (0–2)	1 (1–2)	2 (1–3)	2 (1–3)	1 (1–3)	1 (0–1)	0 (0–1)	--
Gallbladder and biliary tract cancer	--	--	--	--	--	--	--	0 (0–0)	0 (0–0)	0 (0–0)	0 (0–1)	1 (0–2)	2 (1–4)	5 (3–8)	8 (5–14)	8 (5–15)	12 (8–19)	14 (9–23)	13 (8–23)	11 (7–19)	7 (5–13)	4 (3–7)	--
Pancreatic cancer	--	--	--	--	--	--	0 (0–0)	0 (0–0)	1 (0–1)	2 (1–2)	3 (2–4)	8 (6–11)	23 (16–32)	44 (29–60)	66 (47–91)	68 (49–93)	79 (57–106)	92 (68–120)	83 (62–111)	66 (47–87)	37 (28–48)	18 (12–25)	--
Larynx cancer	--	--	--	--	--	--	--	0 (0–0)	0 (0–0)	1 (0–1)	1 (1–2)	7 (4–11)	29 (19–43)	77 (54–105)	110 (76–151)	116 (82–158)	127 (91–172)	126 (92–169)	107 (80–141)	75 (56–100)	40 (29–55)	21 (15–28)	--
Tracheal, bronchus, and lung cancer	--	--	--	--	--	0 (0–0)	0 (0–1)	1 (0–1)	1 (1–2)	4 (2–6)	9 (6–14)	25 (17–35)	85 (62–116)	242 (176–323)	438 (319–581)	512 (391–652)	646 (494–820)	687 (527–863)	624 (483–790)	432 (326–565)	280 (213–359)	133 (101–168)	--
Malignant skin melanoma	0 (0–0)	0 (0–0)	0 (0–0)	0 (0–0)	0 (0–0)	0 (0–0)	0 (0–0)	0 (0–0)	1 (0–1)	1 (0–1)	1 (1–2)	2 (1–3)	4 (2–6)	6 (4–10)	8 (5–12)	5 (3–9)	6 (4–10)	7 (4–12)	8 (5–12)	7 (4–12)	5 (3–8)	3 (2–4)	--
Non-melanoma skin cancer (squamous-cell carcinoma)	--	--	--	--	--	--	--	0 (0–0)	0 (0–1)	1 (0–1)	1 (1–1)	2 (1–3)	4 (3–6)	10 (7–14)	14 (10–20)	15 (11–21)	22 (16–30)	28 (20–37)	36 (25–48)	40 (28–53)	38 (26–49)	32 (20–42)	--
	0 (0–0)	0 (0–0)	0 (0–0)	0 (0–0)	0 (0–1)	0 (0–1)	1 (0–1)	1 (0–1)	1 (0–1)	1 (1–2)	1 (0–2)	2 (1–3)	3 (1–5)	4 (2–8)	6 (3–11)	4 (2–7)	5 (2–8)	6 (3–11)	4 (2–7)	4 (2–7)	2 (1–4)	1 (0–2)	--
	--	--	--	0 (0–0)	1 (0–1)	1 (1–2)	2 (1–5)	2 (1–4)	2 (1–3)	2 (1–3)	1 (1–2)	2 (1–4)	4 (2–7)	7 (2–14)	9 (3–17)	11 (3–20)	11 (4–21)	11 (3–20)	11 (3–21)	8 (3–16)	5 (1–9)	2 (1–5)	--
Breast cancer	--	--	--	--	--	--	0 (0–0)	0 (0–0)	0 (0–0)	0 (0–0)	0 (0–0)	0 (0–0)	1 (0–1)	1 (1–3)	2 (1–3)	2 (1–4)	3 (2–6)	3 (2–5)	3 (2–6)	3 (1–6)	4 (2–8)	2 (1–4)	--
Cervical cancer	--	--	--	--	--	--	--	--	--	--	--	--	--	--	--	--	--	--	--	--	--	--	--
Uterine cancer	--	--	--	--	--	--	--	--	--	--	--	--	--	--	--	--	--	--	--	--	--	--	--
Ovarian cancer	--	--	--	--	--	--	--	--	--	--	--	--	--	--	--	--	--	--	--	--	--	--	--
Prostate cancer	--	--	--	--	--	--	--	0 (0–0)	0 (0–0)	0 (0–1)	0 (0–1)	2 (1–2)	8 (5–12)	33 (20–48)	82 (48–115)	156 (92–214)	281 (164–396)	483 (283–654)	659 (388–879)	668 (383–883)	553 (335–728)	339 (190–452)	--
Testicular cancer	0 (0–0)	0 (0–0)	0 (0–0)	0 (0–0)	0 (0–0)	0 (0–0)	1 (0–1)	1 (1–1)	2 (1–2)	1 (1–2)	1 (1–2)	1 (1–2)	1 (1–2)	1 (1–2)	1 (1–2)	1 (0–2)	1 (1–2)	2 (1–3)	2 (1–4)	1 (1–3)	1 (1–2)	1 (0–1)	--
Kidney cancer	0 (0–0)	0 (0–0)	0 (0–0)	1 (1–1)	1 (0–1)	0 (0–0)	0 (0–1)	1 (0–1)	1 (1–2)	1 (1–2)	1 (1–2)	3 (2–5)	9 (6–13)	20 (13–29)	30 (20–43)	30 (20–42)	33 (23–46)	35 (25–48)	28 (20–38)	21 (15–30)	11 (7–16)	5 (3–7)	--
Bladder cancer	--	--	--	--	--	--	0 (0–0)	0 (0–0)	0 (0–0)	0 (0–1)	1 (0–1)	2 (1–3)	5 (3–7)	15 (10–21)	29 (20–41)	43 (29–58)	68 (49–94)	95 (72–124)	109 (80–148)	90 (67–118)	65 (48–87)	39 (28–52)	--
Brain and nervous system cancer	0 (0–0)	0 (0–0)	1 (0–1)	3 (1–5)	4 (2–7)	4 (1–6)	3 (1–5)	3 (1–5)	4 (1–7)	7 (2–10)	7 (3–12)	12 (4–18)	22 (7–35)	34 (11–53)	43 (13–68)	37 (10–58)	39 (11–61)	37 (10–57)	27 (7–43)	16 (5–27)	7 (3–12)	3 (1–5)	--
	0 (0–0)	0 (0–0)	0 (0–0)	0 (0–1)	0 (0–0)	0 (0–0)	0 (0–0)	0 (0–0)	0 (0–0)	0 (0–0)	0 (0–0)	0 (0–1)	1 (0–1)	1 (0–1)	1 (0–1)	1 (0–2)	1 (0–2)	1 (0–2)	2 (1–3)	2 (1–3)	1 (0–2)	1 (0–2)	--

	0 (0–0)	0 (0–0)	0 (0–0)	0 (0–0)	0 (0–0)	0 (0–0)	0 (0–0)	--	--	--	--	--	--	--	--	--	--	--	--	--	--	--	--
	--	--	--	0 (0–0)	0 (0–0)	0 (0–0)	0 (0–0)	0 (0–0)	0 (0–0)	0 (0–0)	0 (0–0)	0 (0–1)	1 (0–1)	1 (0–1)	1 (0–1)	1 (0–2)	1 (0–2)	1 (0–2)	2 (1–3)	2 (1–3)	1 (0–2)	1 (0–2)	--
	0 (0–0)	0 (0–0)	0 (0–1)	1 (1–2)	1 (0–1)	0 (0–0)	0 (0–0)	0 (0–0)	0 (0–0)	0 (0–1)	0 (0–0)	0 (0–0)	1 (0–1)	1 (0–1)	1 (0–1)	1 (0–1)	0 (0–1)	0 (0–1)	0 (0–0)	0 (0–0)	0 (0–0)	0 (0–0)	--
Thyroid cancer	--	--	--	--	0 (0–0)	0 (0–0)	0 (0–0)	0 (0–0)	0 (0–0)	0 (0–0)	0 (0–1)	1 (0–1)	2 (1–3)	4 (3–7)	6 (3–9)	5 (3–7)	5 (3–8)	6 (4–9)	6 (4–9)	5 (3–8)	3 (2–5)	2 (1–3)	--
Mesothelioma	--	--	--	--	--	--	--	0 (0–0)	0 (0–0)	0 (0–0)	0 (0–0)	0 (0–0)	1 (1–1)	1 (1–2)	2 (2–3)	1 (1–2)	1 (1–1)	2 (1–2)	2 (1–2)	1 (1–2)	1 (1–1)	0 (0–0)	--
Hodgkin lymphoma	--	--	--	0 (0–0)	0 (0–0)	0 (0–0)	1 (0–2)	2 (0–3)	3 (1–4)	3 (1–4)	2 (1–3)	3 (1–5)	4 (1–7)	6 (2–10)	7 (3–10)	6 (2–10)	7 (3–10)	7 (3–10)	6 (2–10)	4 (1–7)	3 (1–4)	1 (0–2)	--
Non-Hodgkin's lymphoma	--	--	--	1 (1–2)	2 (1–3)	2 (1–3)	3 (2–4)	4 (3–5)	5 (4–7)	6 (5–9)	6 (5–8)	9 (7–13)	17 (12–24)	27 (20–35)	43 (30–59)	38 (27–50)	46 (34–60)	53 (39–70)	45 (34–59)	30 (23–39)	14 (10–17)	5 (4–7)	--
	--	--	--	0 (0–0)	0 (0–0)	0 (0–0)	0 (0–0)	0 (0–0)	0 (0–0)	0 (0–0)	0 (0–0)	0 (0–0)	0 (0–0)	0 (0–0)	0 (0–0)	0 (0–0)	0 (0–0)	0 (0–0)	0 (0–0)	0 (0–0)	0 (0–0)	0 (0–0)	--
	--	--	--	1 (1–2)	2 (1–3)	2 (1–3)	3 (2–4)	4 (3–5)	5 (3–7)	6 (5–8)	6 (4–8)	9 (7–13)	17 (12–24)	26 (20–34)	42 (30–59)	37 (27–49)	46 (33–60)	53 (39–69)	45 (34–59)	30 (22–39)	13 (10–17)	5 (4–7)	--
Multiple myeloma	--	--	--	--	--	--	--	0 (0–0)	0 (0–0)	0 (0–0)	1 (0–1)	2 (1–3)	7 (5–10)	16 (11–23)	25 (16–38)	24 (16–35)	34 (23–48)	32 (22–45)	31 (22–45)	21 (14–33)	12 (7–18)	4 (3–7)	--
Leukaemia	0 (0–0)	0 (0–0)	1 (0–1)	4 (2–5)	6 (4–8)	5 (3–8)	7 (4–10)	6 (4–10)	7 (5–9)	7 (5–10)	6 (4–9)	8 (6–11)	17 (12–23)	23 (16–32)	28 (18–39)	31 (21–44)	39 (27–58)	42 (29–59)	51 (35–70)	39 (26–56)	25 (16–36)	11 (7–17)	--
Acute lymphoid leukaemia	0 (0–0)	0 (0–0)	0 (0–0)	2 (1–3)	4 (2–6)	3 (2–5)	4 (2–6)	3 (2–4)	2 (1–3)	1 (1–2)	1 (1–2)	1 (1–2)	2 (1–3)	2 (1–3)	2 (1–4)	2 (1–3)	3 (2–5)	3 (2–5)	3 (1–4)	3 (2–5)	2 (1–3)	1 (0–1)	--
Chronic lymphoid leukaemia	--	--	--	--	--	--	--	0 (0–0)	0 (0–0)	0 (0–0)	0 (0–0)	0 (0–1)	1 (0–1)	2 (1–4)	4 (2–7)	5 (3–8)	8 (5–13)	9 (5–14)	11 (7–16)	10 (6–16)	7 (4–11)	4 (2–6)	--
Acute myeloid leukaemia	0 (0–0)	0 (0–0)	0 (0–1)	1 (1–2)	1 (1–2)	2 (1–3)	2 (1–4)	2 (1–4)	3 (2–5)	3 (2–5)	3 (2–4)	4 (2–5)	8 (5–11)	10 (6–15)	10 (6–16)	11 (7–17)	12 (7–18)	12 (7–19)	13 (8–22)	8 (5–14)	5 (3–8)	2 (1–4)	--
Chronic myeloid leukaemia	0 (0–0)	0 (0–0)	0 (0–0)	0 (0–0)	0 (0–0)	0 (0–0)	0 (0–0)	1 (0–1)	1 (1–2)	1 (1–2)	1 (1–2)	1 (1–2)	4 (2–5)	5 (3–7)	6 (4–9)	8 (5–13)	9 (5–14)	9 (6–14)	11 (7–17)	8 (5–13)	4 (2–6)	2 (1–3)	--
Other leukaemia	0 (0–0)	0 (0–0)	0 (0–0)	0 (0–0)	0 (0–1)	0 (0–1)	0 (0–1)	1 (0–1)	1 (0–1)	1 (0–1)	1 (0–1)	1 (1–2)	3 (2–5)	4 (3–6)	5 (3–7)	5 (3–8)	7 (5–11)	9 (6–14)	13 (8–18)	10 (6–15)	8 (4–12)	3 (2–5)	--
Other malignant cancers	0 (0–0)	0 (0–0)	0 (0–1)	1 (1–2)	1 (1–2)	1 (1–2)	2 (1–3)	2 (2–3)	3 (2–4)	3 (2–5)	4 (3–5)	6 (4–9)	15 (10–21)	23 (16–33)	31 (21–43)	27 (19–38)	36 (25–51)	34 (24–48)	35 (24–49)	26 (18–37)	21 (15–30)	11 (8–16)	--
Myelodysplastic, myeloproliferative, and other hematopoietic neoplasms	0 (0–0)	0 (0–0)	0 (0–0)	0 (0–0)	0 (0–0)	0 (0–0)	0 (0–0)	0 (0–0)	0 (0–1)	0 (0–0)	0 (0–1)	0 (0–1)	1 (0–1)	1 (1–3)	3 (1–5)	3 (1–6)	5 (2–7)	6 (3–11)	7 (3–12)	4 (2–9)	3 (2–7)	1 (1–3)	--
Cardiovascular diseases	0 (0–0)	0 (0–0)	1 (1–2)	1 (1–1)	1 (1–1)	2 (2–3)	8 (6–10)	14 (10–18)	27 (21–33)	55 (45–69)	83 (66–103)	174 (138–219)	435 (335–556)	854 (664–1,077)	1,298 (1,018–1,653)	1,457 (1,165–1,809)	1,971 (1,591–2,426)	2,668 (2,160–3,255)	3,067 (2,533–3,710)	3,091 (2,560–3,679)	2,551 (2,101–2,974)	1,808 (1,482–2,092)	--
Rheumatic heart disease	--	--	--	0 (0–0)	0 (0–0)	0 (0–1)	1 (1–1)	1 (1–2)	2 (1–2)	2 (1–3)	3 (2–4)	4 (2–6)	6 (4–9)	8 (5–12)	10 (7–15)	7 (5–10)	7 (5–9)	5 (4–8)	5 (4–7)	3 (2–4)	2 (1–2)	1 (1–2)	--
Ischaemic heart disease	--	--	--	--	--	--	1 (1–1)	4 (3–5)	10 (8–13)	25 (20–31)	41 (32–52)	90 (70–112)	240 (185–306)	484 (377–613)	754 (588–958)	860 (687–1,066)	1,146 (932–1,417)	1,527 (1,240–1,860)	1,708 (1,403–2,078)	1,725 (1,417–2,070)	1,446 (1,199–1,698)	1,081 (884–1,260)	--
Stroke	0 (0–0)	0 (0–0)	0 (0–0)	0 (0–0)	0 (0–0)	1 (0–1)	2 (1–3)	4 (2–6)	6 (4–9)	13 (9–19)	20 (13–27)	43 (31–60)	103 (72–141)	203 (140–274)	307 (221–406)	352 (260–467)	499 (370–653)	705 (527–906)	869 (672–1,092)	869 (675–1,091)	693 (524–867)	425 (324–529)	--
Ischaemic stroke	0 (0–0)	0 (0–0)	0 (0–0)	0 (0–0)	0 (0–0)	0 (0–0)	0 (0–0)	0 (0–1)	1 (0–1)	2 (1–3)	3 (2–4)	7 (4–10)	20 (13–29)	53 (35–74)	98 (67–137)	153 (110–210)	243 (174–331)	425 (314–555)	556 (411–727)	656 (501–836)	537 (405–684)	342 (261–426)	--
Intracerebral hemorrhage	0 (0–0)	0 (0–0)	0 (0–0)	0 (0–0)	0 (0–0)	0 (0–0)	1 (1–1)	2 (1–3)	3 (2–5)	7 (5–11)	12 (8–17)	28 (19–39)	69 (48–95)	129 (89–176)	181 (129–245)	178 (127–242)	231 (161–309)	256 (181–346)	291 (213–382)	193 (136–265)	142 (101–189)	75 (52–100)	--
Subarachnoid hemorrhage	0 (0–0)	0 (0–0)	0 (0–0)	0 (0–0)	0 (0–0)	0 (0–1)	1 (1–2)	2 (1–3)	2 (1–4)	4 (3–6)	5 (3–8)	8 (5–12)	14 (9–21)	21 (13–32)	28 (18–44)	21 (14–30)	26 (17–38)	23 (15–33)	23 (16–32)	20 (13–28)	14 (9–21)	8 (5–12)	--
Hypertensive heart disease	--	--	--	--	--	--	0 (0–0)	0 (0–1)	1 (0–2)	2 (1–3)	4 (2–5)	9 (5–13)	23 (13–35)	45 (23–66)	68 (35–95)	80 (40–113)	105 (54–148)	140 (68–194)	166 (82–230)	171 (85–230)	144 (66–195)	100 (47–134)	--
Non-rheumatic valvular heart disease	--	--	--	--	--	--	0 (0–1)	1 (0–1)	1 (1–2)	2 (1–2)	2 (1–3)	3 (2–5)	6 (4–8)	10 (7–15)	15 (10–21)	15 (10–21)	21 (15–29)	22 (16–30)	21 (15–28)	19 (13–26)	13 (9–18)	8 (6–12)	--
Non-rheumatic calcific aortic valve disease	--	--	--	--	--	--	0 (0–0)	1 (0–1)	1 (0–1)	1 (0–1)	1 (1–1)	2 (1–3)	4 (2–5)	7 (4–10)	11 (7–15)	11 (7–16)	16 (10–22)	17 (12–24)	16 (11–23)	16 (11–22)	10 (7–14)	7 (4–9)	--
Non-rheumatic degenerative mitral valve disease	--	--	--	--	--	--	0 (0–0)	0 (0–0)	0 (0–1)	1 (0–1)	1 (0–1)	1 (1–2)	2 (1–3)	3 (2–6)	5 (3–8)	4 (2–7)	5 (3–8)	5 (3–8)	4 (3–7)	3 (2–6)	3 (1–5)	2 (1–3)	--
Other non-rheumatic valve diseases	--	--	--	--	--	--	0 (0–0)	0 (0–0)	0 (0–0)	0 (0–0)	0 (0–0)	0 (0–0)	0 (0–0)	0 (0–0)	0 (0–0)	0 (0–0)	0 (0–0)	0 (0–0)	0 (0–0)	0 (0–0)	0 (0–0)	0 (0–0)	--
Cardiomyopathy and myocarditis	0 (0–0)	0 (0–0)	1 (1–1)	1 (0–1)	0 (0–1)	1 (0–1)	2 (1–2)	2 (1–3)	4 (3–6)	7 (5–10)	10 (6–14)	17 (12–24)	37 (25–55)	61 (42–85)	78 (54–111)	60 (42–92)	65 (45–93)	82 (56–111)	68 (46–95)	51 (31–72)	38 (23–55)	21 (12–30)	--
Myocarditis	0 (0–0)	0 (0–0)	0 (0–1)	0 (0–0)	0 (0–0)	0 (0–0)	0 (0–1)	0 (0–0)	0 (0–1)	0 (0–1)	0 (0–1)	1 (0–1)	1 (0–1)	1 (0–2)	1 (0–1)	1 (0–1)	1 (0–2)	1 (0–1)	1 (0–2)	1 (0–2)	1 (0–2)	0 (0–1)	--
Alcoholic cardiomyopathy	--	--	--	--	--	--	0 (0–1)	1 (0–1)	2 (1–3)	5 (3–7)	7 (4–10)	13 (9–19)	33 (22–47)	55 (36–75)	70 (48–98)	54 (38–77)	57 (38–79)	72 (47–98)	57 (34–80)	18 (8–30)	9 (4–17)	5 (2–8)	--
Other cardiomyopathy	0 (0–0)	0 (0–0)	1 (0–1)	0 (0–1)	0 (0–1)	0 (0–1)	1 (1–2)	1 (1–2)	2 (1–3)	2 (1–3)	2 (1–4)	3 (2–6)	4 (2–9)	6 (3–13)	7 (3–17)	6 (2–16)	7 (3–17)	10 (4–24)	10 (4–23)	32 (18–49)	28 (16–41)	16 (9–23)	--
Pulmonary Arterial Hypertension	0 (0–0)	0 (0–0)	0 (0–0)	0 (0–0)	0 (0–0)	0 (0–0)	0 (0–0)	0 (0–0)	0 (0–0)	0 (0–0)	0 (0–0)	0 (0–0)	0 (0–1)	0 (0–1)	0 (0–2)	0 (0–2)	0 (0–2)	1 (0–3)	0 (0–3)	0 (0–3)	1 (0–3)	0 (0–2)	--
Atrial fibrillation and flutter	--	--	--	--	--	--	--	--	--	0 (0–0)	0 (0–0)	1 (0–1)	2 (1–3)	4 (2–6)	7 (4–10)	9 (6–13)	14 (9–22)	26 (17–39)	42 (26–61)	69 (43–99)	81 (50–117)	88 (50–126)	--
Aortic aneurysm	--	--	--	--	--	--	0 (0–0)	0 (0–0)	1 (0–1)	1 (0–1)	1 (1–2)	2 (1–3)	5 (4–7)	13 (9–18)	27 (19–36)	36 (25–50)	61 (44–84)	90 (64–121)	105 (76–143)	91 (66–123)	63 (45–84)	35 (25–45)	--
Peripheral vascular disease	--	--	--	--	--	--	--	--	--	--	--	1 (0–2)	3 (1–7)	8 (2–21)	11 (3–28)	16 (5–40)	25 (8–59)	37 (12–87)	45 (14–105)	49 (15–117)	40 (13–100)	28 (9–67)	--
Endocarditis	0 (0–0)	0 (0–0)	0 (0–0)	0 (0–0)	0 (0–0)	0 (0–0)	0 (0–1)	1 (0–1)	1 (0–1)	1 (1–2)	1 (1–2)	2 (1–3)	3 (2–5)	5 (3–8)	5 (3–8)	4 (2–6)	5 (3–7)	5 (3–7)	4 (2–6)	4 (2–5)	3 (2–4)	1 (1–2)	--
Other cardiovascular and circulatory diseases	0 (0–0)	0 (0–0)	0 (0–0)	0 (0–0)	0 (0–0)	0 (0–0)	0 (0–1)	1 (0–1)	1 (1–1)	1 (1–2)	2 (1–2)	3 (2–3)	6 (4–8)	12 (8–17)	16 (11–22)	17 (12–23)	23 (16–31)	28 (21–37)	33 (24–46)	40 (29–52)	27 (20–36)	18 (13–23)	--
Chronic respiratory diseases	0 (0–0)	0 (0–0)	0 (0–0)	1 (1–1)	0 (0–1)	1 (1–1)	2 (1–2)	2 (2–3)	4 (3–5)	5 (4–8)	6 (4–9)	11 (8–15)	25 (18–35)	61 (44–84)	121 (87–166)	167 (123–224)	270 (200–355)	392 (300–509)	454 (352–578)	414 (317–529)	317 (243–398)	203 (157–252)	--
Chronic obstructive pulmonary disease	--	--	0 (0–0)	0 (0–0)	0 (0–0)	0 (0–0)	0 (0–0)	0 (0–1)	1 (0–1)	1 (1–2)	2 (1–3)	6 (4–8)	16 (11–22)	44 (32–61)	103 (74–142)	151 (111–202)	251 (185–331)	372 (284–487)	435 (335–557)	399 (305–510)	307 (236–387)	197 (153–246)	--
Pneumoconiosis	--	--	--	--	--	--	0 (0–0)	0 (0–0)	0 (0–0)	0 (0–0)	0 (0–0)	0 (0–0)	0 (0–0)	0 (0–0)	0 (0–0)	0 (0–0)	0 (0–0)	0 (0–0)	0 (0–1)	0 (0–1)	0 (0–0)	0 (0–0)	--
Silicosis	--	--	--	--	--	--	0 (0–0)	0 (0–0)	0 (0–0)	0 (0–0)	0 (0–0)	0 (0–0)	0 (0–0)	0 (0–0)	0 (0–0)	0 (0–0)	0 (0–0)	0 (0–0)	0 (0–0)	0 (0–1)	0 (0–0)	0 (0–0)	--
Asbestosis	--	--	--	--	--	--	0 (0–0)	0 (0–0)	0 (0–0)	0 (0–0)	0 (0–0)	0 (0–0)	0 (0–0)	0 (0–0)	0 (0–0)	0 (0–0)	0 (0–0)	0 (0–0)	0 (0–0)	0 (0–0)	0 (0–0)	0 (0–0)	--
Coal workers pneumoconiosis	--	--	--	--	--	--	0 (0–0)	0 (0–0)	0 (0–0)	0 (0–0)	0 (0–0)	0 (0–0)	0 (0–0)	0 (0–0)	0 (0–0)	0 (0–0)	0 (0–0)	0 (0–0)	0 (0–0)	0 (0–0)	0 (0–0)	0 (0–0)	--
Other pneumoconiosis	--	--	--	--	--	--	0 (0–0)	0 (0–0)	0 (0–0)	0 (0–0)	0 (0–0)	0 (0–0)	0 (0–0)	0 (0–0)	0 (0–0)	0 (0–0)	0 (0–0)	0 (0–0)	0 (0–0)	0 (0–0)	0 (0–0)	0 (0–0)	--
Asthma	--	--	--	0 (0–0)	0 (0–0)	1 (0–1)	1 (1–1)	1 (1–2)	2 (2–3)	3 (2–5)	3 (2–5)	4 (2–5)	7 (4–10)	12 (7–18)	11 (7–18)	12 (7–19)	14 (8–21)	15 (9–23)	12 (8–19)	10 (6–15)	5 (3–8)	3 (2–5)	--
Interstitial lung disease and pulmonary sarcoidosis	--	--	--	0 (0–1)	0 (0–0)	0 (0–0)	0 (0–0)	0 (0–0)	0 (0–0)	0 (0–1)	0 (0–1)	1 (0–1)	1 (1–2)	2 (1–4)	3 (2–5)	3 (2–5)	4 (2–7)	5 (3–9)	6 (3–11)	4 (2–8)	3 (2–6)	2 (1–3)	--
Other chronic respiratory diseases	0 (0–0)	0 (0–0)	0 (0–0)	0 (0–1)	0 (0–0)	0 (0–0)	0 (0–0)	0 (0–0)	0 (0–1)	1 (0–1)	1 (0–1)	1 (0–2)	2 (1–3)	3 (1–5)	3 (1–5)	1 (0–1)	1 (0–1)	1 (0–1)	1 (0–2)	1 (0–2)	1 (0–1)	0 (0–1)	--
Digestive diseases	0 (0–0)	0 (0–0)	1 (1–2)	2 (1–2)	1 (1–1)	1 (1–1)	3 (2–4)	5 (4–7)	11 (8–14)	20 (15–26)	32 (25–42)	77 (60–99)	177 (133–229)	284 (214–365)	326 (248–419)	274 (211–348)	273 (214–348)	296 (231–376)	288 (226–355)	238 (186–291)	172 (136–212)	105 (83–126)	--
Cirrhosis and other chronic liver diseases	--	--	--	1 (0–1)	0 (0–1)	1 (0–1)	1 (1–2)	2 (1–3)	4 (3–6)	10 (8–14)	20 (15–26)	57 (44–73)	137 (103–177)	219 (163–281)	245 (183–321)	189 (143–245)	161 (122–208)	147 (110–190)	114 (86–148)	70 (52–91)	43 (31–55)	22 (16–28)	--
Cirrhosis and other chronic liver diseases due to hepatitis B	--	--	--	0 (0–0)	0 (0–0)	0 (0–0)	0 (0–0)	0 (0–0)	0 (0–1)	1 (0–2)	2 (1–4)	6 (3–10)	13 (7–25)	19 (9–37)	18 (8–35)	12 (5–24)	9 (4–18)	8 (4–16)	7 (3–12)	4 (2–8)	3 (1–4)	1 (1–2)	--
Cirrhosis and other chronic liver diseases due to hepatitis C	--	--	--	0 (0–0)	0 (0–0)	0 (0–0)	0 (0–0)	0 (0–1)	1 (0–2)	3 (2–4)	5 (3–9)	14 (8–22)	30 (16–51)	45 (23–75)	46 (23–82)	34 (16–60)	28 (15–48)	25 (14–42)	19 (10–32)	12 (6–20)	8 (4–12)	4 (2–7)	--
Cirrhosis and other chronic liver diseases due to alcohol use	--	--	--	--	--	--	0 (0–0)	0 (0–0)	1 (0–2)	3 (2–5)	8 (5–13)	26 (17–38)	67 (40–97)	112 (70–159)	131 (83–184)	102 (65–144)	83 (53–117)	72 (46–102)	52 (33–76)	29 (17–43)	17 (11–25)	8 (5–12)	--
Cirrhosis and other chronic liver diseases due to NAFLD	--	--	--	--	--	--	0 (0–0)	0 (0–0)	1 (0–1)	2 (1–3)	3 (1–5)	7 (3–12)	18 (8–34)	28 (13–54)	31 (14–56)	25 (11–47)	24 (13–40)	24 (13–40)	21 (10–35)	14 (7–23)	9 (5–14)	5 (2–8)	--
Cirrhosis and other chronic liver diseases due to other causes	--	--	--	1 (0–1)	0 (0–1)	0 (0–1)	1 (1–2)	1 (1–2)	1 (1–2)	2 (1–3)	2 (1–4)	4 (2–8)	9 (4–17)	15 (7–29)	19 (8–35)	17 (8–30)	16 (7–30)	17 (8–30)	15 (7–27)	10 (5–19)	7 (4–11)	4 (2–7)	--
Upper digestive system diseases	--	--	--	0 (0–0)	0 (0–0)	0 (0–0)	0 (0–0)	0 (0–1)	1 (0–1)	1 (1–2)	2 (1–3)	3 (2–5)	8 (5–12)	16 (11–24)	21 (14–31)	23 (16–34)	30 (21–43)	41 (29–57)	50 (36–67)	47 (34–62)	38 (28–50)	27 (20–35)	--
Peptic ulcer disease	--	--	--	0 (0–0)	0 (0–0)	0 (0–0)	0 (0–0)	0 (0–1)	1 (0–1)	1 (1–2)	1 (1–2)	2 (1–4)	6 (4–9)	13 (8–20)	17 (11–26)	20 (14–29)	25 (18–36)	34 (24–47)	41 (28–56)	38 (27–51)	31 (23–42)	21 (16–28)	--
Gastritis and duodenitis	--	--	--	0 (0–0)	0 (0–0)	0 (0–0)	0 (0–0)	0 (0–0)	0 (0–0)	0 (0–1)	0 (0–1)	1 (0–1)	2 (1–4)	3 (2–5)	4 (2–6)	4 (2–6)	5 (3–8)	8 (4–12)	9 (4–15)	9 (5–14)	7 (4–10)	6 (3–9)	--
Appendicitis	--	--	--	0 (0–0)	0 (0–0)	0 (0–0)	1 (0–1)	1 (0–1)	1 (0–1)	1 (1–2)	1 (1–2)	2 (1–3)	3 (1–5)	4 (2–7)	5 (2–8)	4 (2–7)	6 (3–10)	7 (4–11)	6 (3–10)	5 (3–9)	4 (2–7)	3 (1–4)	--
Paralytic ileus and intestinal obstruction	0 (0–0)	0 (0–0)	1 (1–2)	0 (0–1)	0 (0–0)	0 (0–0)	0 (0–1)	1 (0–1)	1 (1–2)	1 (1–2)	2 (1–3)	3 (2–4)	6 (3–9)	10 (5–15)	13 (7–20)	13 (7–20)	20 (11–30)	27 (16–39)	33 (18–47)	35 (19–50)	26 (15–35)	15 (8–21)	--
Inguinal, femoral, and abdominal hernia	0 (0–0)	0 (0–0)	0 (0–1)	0 (0–0)	0 (0–0)	0 (0–0)	0 (0–0)	0 (0–0)	0 (0–1)	0 (0–1)	0 (0–1)	1 (1–1)	2 (1–3)	3 (2–5)	5 (3–7)	5 (3–7)	7 (5–11)	11 (8–16)	15 (10–21)	14 (9–19)	12 (8–16)	8 (5–11)	--
Inflammatory bowel disease	--	--	--	0 (0–0)	0 (0–0)	0 (0–0)	0 (0–0)	0 (0–0)	0 (0–0)	0 (0–0)	0 (0–0)	1 (0–1)	1 (1–2)	2 (1–3)	2 (1–3)	1 (1–2)	2 (1–3)	3 (2–4)	2 (1–4)	2 (1–3)	2 (1–2)	1 (1–1)	--
Vascular intestinal disorders	--	--	--	0 (0–0)	0 (0–0)	0 (0–0)	0 (0–0)	0 (0–0)	0 (0–1)	1 (0–1)	1 (0–1)	1 (1–2)	4 (3–6)	8 (5–11)	11 (7–16)	13 (9–18)	19 (13–27)	25 (17–34)	30 (21–41)	30 (21–40)	21 (15–29)	15 (10–20)	--
Gallbladder and biliary diseases	--	--	--	0 (0–0)	0 (0–0)	0 (0–0)	0 (0–0)	0 (0–0)	0 (0–0)	0 (0–1)	0 (0–1)	1 (1–2)	2 (1–3)	4 (2–6)	6 (3–9)	6 (4–9)	9 (6–14)	12 (8–20)	15 (10–22)	17 (12–24)	12 (9–17)	8 (5–11)	--
Pancreatitis	--	--	--	0 (0–0)	0 (0–0)	0 (0–0)	0 (0–0)	1 (0–1)	2 (1–3)	3 (2–5)	4 (3–6)	8 (5–11)	12 (7–18)	14 (8–20)	14 (8–22)	13 (8–19)	12 (7–16)	12 (8–18)	11 (8–16)	9 (6–13)	5 (3–7)	2 (1–3)	--
Other digestive diseases	--	--	--	0 (0–0)	0 (0–0)	0 (0–0)	0 (0–0)	0 (0–0)	0 (0–1)	1 (0–1)	1 (0–1)	1 (0–2)	2 (1–4)	4 (1–6)	5 (2–8)	6 (2–9)	7 (3–11)	9 (4–15)	11 (5–17)	10 (4–15)	9 (4–14)	6 (3–9)	--
Alzheimer's disease and other dementias	--	--	--	--	--	--	--	--	--	--	--	0 (0–2)	4 (0–12)	8 (1–25)	15 (3–44)	24 (5–71)	52 (11–156)	104 (23–295)	178 (40–501)	335 (76–973)	401 (96–1,088)	339 (81–934)	--
Parkinson's disease	--	--	--	--	--	--	--	0 (0–0)	0 (0–0)	0 (0–0)	0 (0–0)	0 (0–1)	1 (1–2)	4 (3–5)	7 (5–9)	12 (9–17)	29 (22–37)	64 (51–80)	115 (90–142)	134 (108–167)	104 (83–126)	55 (42–68)	--
Idiopathic epilepsy	--	--	0 (0–0)	1 (0–1)	1 (0–1)	1 (1–1)	2 (1–3)	3 (2–4)	3 (2–5)	4 (3–5)	4 (3–5)	5 (4–7)	7 (5–10)	8 (5–11)	8 (6–11)	5 (3–7)	4 (3–5)	3 (2–5)	3 (2–4)	2 (1–3)	1 (1–2)	1 (1–1)	--
Multiple sclerosis	--	--	--	--	0 (0–0)	0 (0–0)	0 (0–0)	0 (0–0)	0 (0–0)	0 (0–1)	1 (0–1)	1 (0–2)	1 (1–2)	2 (1–3)	3 (1–4)	2 (1–3)	2 (1–3)	2 (1–2)	1 (1–2)	1 (0–1)	0 (0–1)	0 (0–0)	--
Motor neuron disease	0 (0–0)	0 (0–0)	0 (0–1)	0 (0–0)	0 (0–0)	0 (0–0)	0 (0–1)	0 (0–1)	0 (0–0)	1 (0–1)	1 (1–1)	2 (2–3)	4 (2–5)	7 (5–10)	9 (7–13)	9 (6–12)	11 (8–16)	9 (7–13)	5 (3–8)	3 (2–4)	1 (1–2)	0 (0–1)	--
Other neurological disorders	--	--	1 (1–2)	1 (1–2)	1 (1–2)	2 (2–3)	5 (3–7)	4 (3–6)	3 (2–5)	3 (2–5)	3 (2–4)	4 (3–6)	6 (4–9)	11 (8–16)	14 (9–20)	11 (8–16)	11 (7–15)	10 (7–14)	8 (5–11)	6 (4–9)	2 (2–3)	1 (1–1)	--
Eating disorders	--	--	--	--	0 (0–0)	0 (0–0)	0 (0–0)	0 (0–0)	0 (0–0)	0 (0–0)	0 (0–0)	0 (0–0)	0 (0–0)	--	--	--	--	--	--	--	--	--	--
Anorexia nervosa	--	--	--	--	0 (0–0)	0 (0–0)	0 (0–0)	0 (0–0)	0 (0–0)	0 (0–0)	0 (0–0)	0 (0–0)	0 (0–0)	--	--	--	--	--	--	--	--	--	--
Bulimia nervosa	--	--	--	--	0 (0–0)	0 (0–0)	0 (0–0)	0 (0–0)	0 (0–0)	0 (0–0)	0 (0–0)	0 (0–0)	0 (0–0)	--	--	--	--	--	--	--	--	--	--
Alcohol use disorders	--	--	--	--	--	0 (0–0)	1 (0–1)	1 (1–2)	3 (2–5)	7 (5–11)	14 (9–20)	29 (19–41)	71 (45–108)	97 (64–140)	109 (74–151)	77 (52–107)	64 (43–87)	38 (26–54)	22 (15–29)	7 (4–10)	3 (2–4)	1 (1–2)	--
Drug use disorders	0 (0–0)	0 (0–0)	0 (0–0)	0 (0–0)	0 (0–0)	0 (0–0)	0 (0–1)	1 (1–1)	1 (1–2)	1 (1–2)	1 (1–2)	2 (1–3)	2 (1–3)	2 (1–3)	2 (1–3)	1 (1–2)	1 (1–2)	1 (1–2)	1 (1–2)	1 (1–2)	1 (0–1)	1 (0–1)	--
Opioid use disorders	0 (0–0)	0 (0–0)	0 (0–0)	0 (0–0)	0 (0–0)	0 (0–0)	0 (0–0)	0 (0–1)	0 (0–1)	1 (0–1)	1 (0–1)	1 (0–1)	1 (0–1)	1 (0–1)	1 (0–1)	0 (0–1)	0 (0–1)	0 (0–1)	0 (0–1)	1 (0–1)	0 (0–0)	0 (0–0)	--
Cocaine use disorders	--	--	--	--	--	0 (0–0)	0 (0–0)	0 (0–1)	1 (0–1)	1 (0–1)	1 (0–1)	1 (0–1)	1 (1–2)	1 (0–1)	1 (0–1)	0 (0–1)	0 (0–0)	0 (0–1)	0 (0–1)	0 (0–0)	0 (0–0)	0 (0–0)	--
Amphetamine use disorders	--	--	--	--	--	0 (0–0)	0 (0–0)	0 (0–0)	0 (0–0)	0 (0–0)	0 (0–0)	0 (0–0)	0 (0–0)	0 (0–0)	0 (0–0)	0 (0–0)	0 (0–0)	0 (0–0)	0 (0–0)	0 (0–0)	0 (0–0)	0 (0–0)	--
Other drug use disorders	--	--	--	--	--	0 (0–0)	0 (0–0)	0 (0–0)	0 (0–0)	0 (0–0)	0 (0–0)	0 (0–0)	0 (0–1)	0 (0–1)	0 (0–1)	0 (0–0)	0 (0–0)	1 (0–1)	1 (0–1)	1 (0–1)	0 (0–1)	0 (0–0)	--
Diabetes mellitus	0 (0–0)	0 (0–0)	0 (0–0)	0 (0–0)	0 (0–0)	0 (0–0)	1 (0–1)	1 (1–2)	3 (2–4)	4 (3–6)	5 (4–7)	9 (6–12)	23 (16–32)	47 (34–64)	70 (50–97)	75 (54–102)	107 (76–146)	130 (95–175)	140 (103–185)	105 (77–134)	70 (53–89)	34 (26–44)	--
Diabetes mellitus type 1	0 (0–0)	0 (0–0)	0 (0–0)	0 (0–0)	0 (0–0)	0 (0–0)	0 (0–0)	1 (1–1)	2 (1–2)	1 (1–2)	2 (1–3)	3 (1–4)	1 (0–2)	2 (1–3)	2 (1–5)	2 (1–4)	3 (1–6)	2 (1–5)	2 (1–6)	1 (0–3)	1 (0–2)	0 (0–1)	--
Diabetes mellitus type 2	--	--	--	--	--	--	0 (0–0)	1 (0–1)	1 (1–2)	3 (2–4)	3 (2–5)	6 (4–9)	22 (16–31)	45 (32–63)	68 (48–94)	73 (52–100)	104 (74–143)	128 (94–171)	138 (101–180)	104 (76–133)	69 (53–88)	34 (26–44)	--
Chronic kidney disease	--	--	1 (0–1)	0 (0–1)	1 (1–1)	1 (1–1)	3 (2–4)	4 (3–6)	8 (6–12)	13 (10–18)	15 (11–20)	27 (20–36)	56 (40–75)	85 (61–114)	117 (83–157)	112 (82–152)	143 (106–190)	180 (136–237)	191 (143–253)	173 (132–224)	121 (94–152)	83 (62–105)	--
Chronic kidney disease due to diabetes mellitus type 1	--	--	0 (0–0)	0 (0–0)	0 (0–0)	0 (0–0)	0 (0–0)	0 (0–0)	1 (0–2)	2 (1–4)	3 (1–5)	5 (2–9)	9 (4–17)	10 (4–21)	9 (3–21)	6 (2–15)	6 (2–15)	6 (2–14)	4 (2–9)	2 (1–5)	0 (0–1)	0 (0–0)	--
Chronic kidney disease due to diabetes mellitus type 2	--	--	--	--	--	--	0 (0–0)	0 (0–0)	0 (0–1)	1 (0–2)	1 (0–2)	3 (1–6)	9 (4–16)	18 (9–31)	30 (15–49)	30 (14–49)	40 (21–62)	52 (30–79)	56 (32–85)	49 (29–78)	33 (19–50)	21 (12–32)	--
Chronic kidney disease due to hypertension	--	--	--	--	--	--	0 (0–0)	0 (0–0)	0 (0–1)	1 (0–2)	1 (1–2)	3 (2–5)	7 (4–12)	14 (8–22)	23 (13–37)	26 (15–41)	38 (23–60)	52 (33–76)	62 (40–92)	63 (41–91)	51 (35–72)	38 (26–52)	--
Chronic kidney disease due to glomerulonephritis	--	--	0 (0–0)	0 (0–0)	0 (0–1)	0 (0–1)	1 (1–2)	2 (1–3)	4 (2–6)	6 (3–8)	6 (3–9)	9 (5–15)	17 (10–27)	25 (13–40)	31 (16–51)	27 (13–47)	30 (14–52)	32 (16–56)	30 (14–54)	23 (10–42)	13 (6–22)	7 (3–13)	--
Chronic kidney disease due to other and unspecified causes	--	--	0 (0–1)	0 (0–0)	0 (0–1)	0 (0–1)	1 (1–2)	2 (1–3)	3 (2–5)	4 (2–7)	4 (2–7)	7 (3–12)	13 (6–22)	18 (9–32)	23 (11–41)	22 (10–38)	29 (14–50)	38 (20–63)	39 (21–64)	35 (18–59)	24 (12–40)	16 (9–28)	--
Acute glomerulonephritis	--	--	0 (0–0)	0 (0–0)	0 (0–0)	0 (0–0)	0 (0–0)	0 (0–0)	0 (0–0)	0 (0–0)	0 (0–0)	0 (0–0)	0 (0–0)	0 (0–0)	0 (0–0)	0 (0–0)	0 (0–0)	0 (0–0)	0 (0–0)	0 (0–0)	0 (0–0)	0 (0–0)	--
Skin and subcutaneous diseases	--	--	1 (0–2)	0 (0–1)	0 (0–1)	0 (0–1)	1 (0–1)	1 (0–2)	1 (0–3)	1 (1–3)	1 (1–4)	2 (1–6)	5 (2–12)	8 (4–18)	9 (4–22)	9 (4–23)	14 (7–37)	18 (9–46)	24 (12–69)	24 (12–69)	18 (8–58)	14 (7–46)	--
Bacterial skin diseases	--	--	1 (0–2)	0 (0–1)	0 (0–1)	0 (0–1)	1 (0–1)	1 (0–2)	1 (0–2)	1 (1–3)	1 (1–3)	2 (1–5)	4 (2–11)	7 (3–16)	8 (4–21)	8 (4–20)	11 (5–30)	14 (7–39)	18 (8–56)	18 (8–56)	14 (6–47)	10 (4–35)	--
Cellulitis	--	--	0 (0–1)	0 (0–0)	0 (0–0)	0 (0–1)	0 (0–1)	0 (0–1)	0 (0–1)	1 (0–1)	1 (0–2)	1 (0–3)	2 (1–5)	3 (1–8)	3 (1–9)	3 (1–9)	3 (1–12)	5 (2–16)	6 (2–23)	6 (2–22)	5 (2–22)	4 (1–16)	--

Pyoderma	--	--	1 (0–1)	0 (0–1)	0 (0–0)	0 (0–0)	0 (0–1)	0 (0–1)	1 (0–2)	1 (0–2)	1 (0–2)	1 (1–3)	3 (1–6)	4 (2–9)	6 (2–13)	4 (2–11)	7 (3–19)	9 (4–24)	12 (5–35)	13 (6–37)	8 (3–28)	7 (3–22)	--
Decubitus ulcer	--	--	--	0 (0–0)	0 (0–0)	0 (0–0)	0 (0–0)	0 (0–0)	0 (0–0)	0 (0–0)	0 (0–0)	0 (0–0)	0 (0–1)	0 (0–2)	1 (0–2)	1 (0–3)	2 (0–5)	3 (0–9)	5 (1–15)	5 (1–15)	4 (1–15)	3 (1–11)	--
Other skin and subcutaneous diseases	--	--	0 (0–0)	0 (0–0)	0 (0–0)	0 (0–0)	0 (0–0)	0 (0–0)	0 (0–0)	0 (0–0)	0 (0–0)	0 (0–0)	0 (0–0)	0 (0–1)	0 (0–1)	0 (0–1)	1 (0–4)	1 (0–4)	2 (1–6)	1 (0–3)	1 (0–2)	1 (0–3)	--
Musculoskeletal disorders	--	--	--	--	0 (0–0)	0 (0–0)	0 (0–0)	0 (0–1)	0 (0–1)	0 (0–1)	1 (0–1)	1 (1–1)	1 (1–2)	2 (1–3)	2 (1–3)	2 (1–3)	2 (1–4)	2 (1–4)	3 (1–4)	2 (1–3)	8 (5–13)	5 (3–8)	--
Rheumatoid arthritis	--	--	--	--	0 (0–0)	0 (0–0)	0 (0–0)	0 (0–0)	0 (0–0)	0 (0–0)	0 (0–0)	0 (0–0)	0 (0–1)	0 (0–1)	1 (0–1)	1 (0–1)	1 (0–2)	1 (1–2)	1 (1–2)	1 (0–2)	2 (1–4)	2 (1–3)	--
Other musculoskeletal disorders	--	--	--	--	0 (0–0)	0 (0–0)	0 (0–0)	0 (0–1)	0 (0–1)	0 (0–1)	1 (0–1)	1 (0–1)	1 (1–2)	1 (1–2)	2 (1–3)	1 (1–2)	1 (1–2)	1 (1–2)	1 (1–2)	1 (1–2)	5 (3–9)	4 (2–6)	--
Congenital anomalies	18 (11–45)	13 (9–21)	30 (22–44)	10 (6–18)	5 (3–9)	5 (3–8)	6 (4–9)	6 (4–9)	6 (4–9)	7 (4–10)	5 (3–8)	6 (4–8)	8 (6–12)	10 (7–15)	11 (7–16)	6 (4–9)	4 (3–7)	--	--	--	--	--	--
Neural tube defects	0 (0–1)	0 (0–1)	1 (1–2)	2 (1–5)	0 (0–0)	0 (0–0)	0 (0–1)	0 (0–0)	0 (0–0)	0 (0–0)	0 (0–0)	0 (0–0)	0 (0–0)	0 (0–0)	0 (0–0)	0 (0–0)	0 (0–0)	--	--	--	--	--	--
Congenital heart anomalies	7 (3–19)	7 (4–12)	14 (9–22)	3 (2–6)	2 (1–4)	2 (1–3)	2 (1–4)	2 (1–3)	2 (1–3)	3 (2–4)	2 (1–3)	2 (1–3)	3 (2–4)	3 (2–4)	2 (1–3)	1 (1–3)	1 (1–2)	--	--	--	--	--	--
Orofacial clefts	0 (0–0)	0 (0–0)	0 (0–0)	0 (0–0)	--	--	--	--	--	--	--	--	--	--	--	--	--	--	--	--	--	--	--
Down's syndrome	0 (0–1)	0 (0–0)	1 (1–2)	0 (0–1)	0 (0–0)	0 (0–0)	0 (0–1)	0 (0–1)	1 (0–1)	1 (1–2)	1 (1–2)	1 (1–2)	2 (1–4)	3 (2–6)	5 (3–8)	3 (2–4)	2 (1–3)	--	--	--	--	--	--
Other chromosomal abnormalities	1 (0–2)	0 (0–1)	1 (1–2)	0 (0–1)	0 (0–0)	0 (0–0)	0 (0–0)	0 (0–0)	0 (0–1)	0 (0–0)	0 (0–1)	0 (0–0)	0 (0–0)	0 (0–0)	0 (0–0)	0 (0–0)	0 (0–0)	--	--	--	--	--	--
Congenital musculoskeletal and limb anomalies	0 (0–2)	0 (0–1)	1 (0–1)	0 (0–1)	0 (0–0)	0 (0–0)	0 (0–0)	0 (0–0)	0 (0–0)	0 (0–0)	0 (0–0)	0 (0–0)	0 (0–0)	0 (0–0)	0 (0–1)	0 (0–0)	0 (0–0)	--	--	--	--	--	--
Urogenital congenital anomalies	1 (0–3)	0 (0–1)	0 (0–1)	0 (0–0)	0 (0–0)	0 (0–0)	0 (0–0)	0 (0–0)	0 (0–0)	0 (0–0)	0 (0–0)	0 (0–0)	0 (0–0)	0 (0–0)	0 (0–0)	0 (0–0)	0 (0–0)	--	--	--	--	--	--
Digestive congenital anomalies	4 (2–11)	3 (2–5)	6 (3–10)	1 (0–2)	0 (0–1)	0 (0–0)	0 (0–0)	0 (0–0)	0 (0–0)	0 (0–0)	0 (0–0)	0 (0–0)	0 (0–0)	0 (0–0)	0 (0–0)	0 (0–0)	0 (0–1)	--	--	--	--	--	--
Other congenital anomalies	4 (2–10)	2 (1–4)	6 (3–10)	2 (1–5)	2 (1–4)	2 (1–3)	3 (2–4)	3 (2–5)	3 (1–4)	3 (1–4)	2 (1–3)	2 (1–3)	3 (1–4)	4 (2–5)	2 (1–4)	1 (1–2)	1 (0–1)	--	--	--	--	--	--
Urinary diseases and male infertility	0 (0–0)	0 (0–0)	0 (0–1)	0 (0–0)	0 (0–0)	0 (0–0)	0 (0–0)	0 (0–1)	1 (0–1)	1 (0–1)	1 (1–2)	2 (1–3)	5 (2–7)	8 (4–13)	13 (6–18)	17 (8–24)	21 (11–31)	31 (17–44)	40 (23–59)	36 (22–53)	32 (21–44)	18 (12–27)	--
Urinary tract infections and interstitial nephritis	0 (0–0)	0 (0–0)	0 (0–1)	0 (0–0)	0 (0–0)	0 (0–0)	0 (0–0)	0 (0–0)	0 (0–1)	1 (0–1)	1 (0–1)	1 (0–2)	2 (1–4)	5 (2–7)	7 (3–11)	10 (4–16)	14 (6–21)	22 (11–33)	29 (15–44)	29 (17–43)	27 (17–37)	15 (9–21)	--
Urolithiasis	--	--	--	0 (0–0)	0 (0–0)	0 (0–0)	0 (0–0)	0 (0–0)	0 (0–0)	0 (0–0)	0 (0–0)	0 (0–1)	1 (1–2)	2 (1–3)	3 (1–5)	3 (1–5)	3 (1–5)	4 (2–6)	4 (2–8)	2 (1–3)	1 (1–2)	1 (1–2)	--
Other urinary diseases	0 (0–0)	0 (0–0)	0 (0–0)	0 (0–0)	0 (0–0)	0 (0–0)	0 (0–0)	0 (0–0)	0 (0–0)	0 (0–0)	0 (0–1)	0 (0–1)	1 (0–2)	2 (1–3)	3 (1–5)	4 (1–6)	4 (2–8)	5 (2–8)	7 (3–12)	5 (3–11)	4 (2–9)	3 (1–7)	--
Gynecological diseases	--	--	--	--	--	--	--	--	--	--	--	--	--	--	--	--	--	--	--	--	--	--	--
Uterine fibroids	--	--	--	--	--	--	--	--	--	--	--	--	--	--	--	--	--	--	--	--	--	--	--
Endometriosis	--	--	--	--	--	--	--	--	--	--	--	--	--	--	--	--	--	--	--	--	--	--	--
Genital prolapse	--	--	--	--	--	--	--	--	--	--	--	--	--	--	--	--	--	--	--	--	--	--	--
Other gynecological diseases	--	--	--	--	--	--	--	--	--	--	--	--	--	--	--	--	--	--	--	--	--	--	--
Hemoglobinopathies and hemolytic anaemias	0 (0–0)	0 (0–0)	0 (0–0)	0 (0–1)	1 (0–1)	1 (1–1)	1 (1–2)	2 (1–3)	2 (1–2)	2 (1–3)	2 (1–3)	3 (2–4)	4 (2–5)	3 (2–5)	4 (2–6)	3 (2–4)	2 (2–4)	3 (2–4)	3 (2–4)	2 (1–3)	2 (2–3)	1 (1–2)	--
Thalassemias	0 (0–0)	0 (0–0)	0 (0–0)	0 (0–0)	0 (0–0)	0 (0–0)	0 (0–0)	0 (0–0)	0 (0–0)	0 (0–0)	0 (0–0)	0 (0–0)	0 (0–0)	0 (0–0)	0 (0–0)	0 (0–0)	0 (0–0)	0 (0–0)	0 (0–0)	0 (0–0)	0 (0–0)	0 (0–0)	--
Sickle cell disorders	0 (0–0)	0 (0–0)	0 (0–0)	0 (0–1)	1 (0–1)	1 (0–1)	1 (1–2)	1 (1–2)	1 (0–1)	1 (0–1)	1 (0–1)	1 (0–2)	1 (1–2)	1 (0–1)	0 (0–1)	0 (0–0)	0 (0–0)	0 (0–0)	0 (0–0)	0 (0–0)	0 (0–0)	0 (0–0)	--
G6PD deficiency	0 (0–0)	0 (0–0)	0 (0–0)	0 (0–0)	0 (0–0)	0 (0–0)	0 (0–1)	1 (0–1)	1 (0–1)	1 (1–2)	1 (1–2)	1 (1–2)	2 (1–3)	2 (1–3)	2 (1–3)	1 (1–2)	1 (0–1)	1 (0–1)	0 (0–1)	0 (0–0)	0 (0–0)	0 (0–0)	--
Other hemoglobinopathies and hemolytic anaemias	0 (0–0)	0 (0–0)	0 (0–0)	0 (0–0)	0 (0–0)	0 (0–0)	0 (0–0)	0 (0–0)	0 (0–0)	0 (0–0)	0 (0–0)	0 (0–1)	1 (0–1)	1 (1–1)	1 (1–2)	1 (1–2)	2 (1–2)	2 (1–3)	3 (2–4)	2 (1–3)	2 (2–3)	1 (1–2)	--
Endocrine, metabolic, blood, and immune disorders	0 (0–1)	1 (0–1)	5 (3–8)	3 (2–4)	1 (1–2)	1 (1–2)	1 (1–2)	1 (1–3)	2 (1–5)	3 (2–7)	3 (2–8)	4 (2–9)	7 (4–15)	10 (6–21)	11 (6–22)	10 (6–19)	11 (7–22)	12 (7–26)	11 (6–29)	10 (5–30)	9 (4–29)	6 (3–17)	--
Sudden infant death syndrome	--	0 (0–0)	2 (1–3)	--	--	--	--	--	--	--	--	--	--	--	--	--	--	--	--	--	--	--	--
Transport injuries	0 (0–0)	0 (0–0)	1 (0–1)	3 (2–5)	7 (5–10)	11 (8–14)	27 (19–37)	46 (34–61)	65 (50–83)	68 (51–87)	57 (42–75)	67 (49–89)	87 (63–119)	95 (68–128)	93 (65–128)	62 (44–83)	59 (42–77)	53 (38–73)	47 (34–63)	33 (23–44)	19 (13–26)	9 (6–11)	--
Road injuries	0 (0–0)	0 (0–0)	1 (0–1)	3 (2–4)	7 (5–9)	10 (7–13)	26 (18–35)	43 (32–57)	62 (47–78)	64 (47–82)	53 (39–69)	62 (45–83)	81 (58–110)	88 (62–119)	85 (59–116)	58 (42–78)	56 (40–74)	49 (35–68)	45 (32–60)	31 (22–42)	19 (13–25)	8 (5–11)	--
Pedestrian road injuries	0 (0–0)	0 (0–0)	0 (0–0)	1 (1–2)	4 (3–5)	4 (3–6)	5 (4–8)	6 (5–9)	9 (7–12)	10 (7–13)	10 (7–13)	13 (10–18)	22 (15–31)	25 (17–35)	29 (20–39)	22 (15–29)	24 (17–33)	26 (18–36)	28 (20–38)	23 (16–31)	16 (11–21)	7 (5–9)	--
Cyclist road injuries	--	--	--	0 (0–1)	1 (0–1)	3 (2–4)	7 (4–11)	8 (5–12)	8 (5–12)	9 (5–13)	8 (5–12)	10 (6–14)	12 (7–19)	16 (10–23)	18 (10–27)	14 (8–20)	13 (8–19)	11 (6–17)	8 (4–13)	3 (2–6)	1 (1–2)	0 (0–0)	--
Motorcyclist road injuries	0 (0–0)	0 (0–0)	0 (0–0)	0 (0–0)	0 (0–0)	0 (0–0)	2 (2–4)	9 (6–12)	16 (12–20)	17 (13–22)	12 (9–15)	10 (7–13)	13 (9–18)	12 (9–17)	10 (7–14)	5 (3–7)	4 (2–6)	1 (1–2)	1 (1–1)	0 (0–0)	0 (0–0)	0 (0–0)	--
Motor vehicle road injuries	0 (0–0)	0 (0–0)	0 (0–0)	1 (1–1)	2 (1–3)	2 (1–3)	10 (6–14)	17 (12–24)	25 (18–33)	25 (16–33)	20 (14–27)	25 (17–34)	28 (18–39)	28 (18–40)	21 (13–30)	15 (9–20)	11 (7–15)	7 (4–10)	5 (3–7)	3 (1–4)	1 (1–2)	1 (0–1)	--
Other road injuries	0 (0–0)	0 (0–0)	0 (0–0)	0 (0–1)	0 (0–1)	1 (0–1)	1 (1–3)	3 (2–5)	4 (2–7)	4 (2–7)	4 (2–6)	5 (2–8)	7 (4–12)	7 (3–12)	7 (3–11)	4 (2–6)	5 (2–7)	4 (2–7)	4 (2–6)	2 (1–3)	1 (0–1)	0 (0–0)	--
Other transport injuries	0 (0–0)	0 (0–0)	0 (0–0)	0 (0–0)	0 (0–0)	1 (0–1)	1 (1–2)	3 (2–4)	4 (2–5)	4 (3–7)	4 (2–6)	5 (3–8)	6 (4–9)	7 (4–11)	8 (5–12)	3 (2–5)	3 (2–4)	4 (2–6)	2 (1–3)	1 (1–2)	1 (0–1)	0 (0–0)	--
Falls	0 (0–0)	0 (0–0)	0 (0–1)	1 (1–1)	1 (1–2)	2 (1–3)	5 (3–7)	6 (4–9)	7 (5–10)	9 (6–12)	10 (7–14)	15 (11–20)	29 (21–39)	46 (33–62)	55 (40–76)	52 (38–69)	70 (53–92)	116 (84–157)	171 (124–223)	246 (183–324)	271 (203–343)	244 (180–312)	--
Drowning	0 (0–0)	0 (0–0)	0 (0–1)	4 (3–5)	8 (6–10)	10 (8–13)	17 (12–23)	15 (11–20)	17 (13–22)	17 (13–22)	13 (10–17)	17 (13–22)	18 (13–24)	22 (16–29)	17 (12–22)	13 (10–16)	10 (8–13)	8 (6–10)	7 (5–8)	3 (2–4)	2 (1–2)	1 (1–1)	--
Fire, heat, and hot substances	0 (0–0)	0 (0–0)	0 (0–0)	1 (0–1)	1 (1–1)	0 (0–1)	1 (0–1)	1 (1–1)	1 (1–1)	2 (1–2)	1 (1–2)	1 (1–2)	3 (2–4)	3 (2–3)	3 (2–4)	3 (2–4)	3 (2–4)	4 (3–5)	4 (3–6)	5 (4–6)	4 (3–5)	3 (2–4)	--
Poisonings	0 (0–0)	0 (0–0)	0 (0–0)	0 (0–1)	0 (0–0)	0 (0–0)	0 (0–0)	0 (0–0)	0 (0–1)	0 (0–1)	0 (0–1)	0 (0–1)	1 (0–1)	1 (0–1)	1 (0–1)	1 (0–1)	1 (0–1)	0 (0–1)	0 (0–1)	0 (0–0)	0 (0–0)	0 (0–0)	--
Poisoning by carbon monoxide	0 (0–0)	0 (0–0)	0 (0–0)	0 (0–0)	0 (0–0)	0 (0–0)	0 (0–0)	0 (0–0)	0 (0–0)	0 (0–0)	0 (0–0)	0 (0–0)	0 (0–0)	0 (0–0)	0 (0–0)	0 (0–0)	0 (0–0)	0 (0–0)	0 (0–0)	0 (0–0)	0 (0–0)	0 (0–0)	--
Poisoning by other means	0 (0–0)	0 (0–0)	0 (0–0)	0 (0–1)	0 (0–0)	0 (0–0)	0 (0–0)	0 (0–0)	0 (0–0)	0 (0–0)	0 (0–0)	0 (0–0)	0 (0–1)	0 (0–1)	0 (0–1)	0 (0–1)	0 (0–1)	0 (0–0)	0 (0–0)	0 (0–0)	0 (0–0)	0 (0–0)	--
Exposure to mechanical forces	0 (0–0)	0 (0–0)	0 (0–0)	1 (0–1)	1 (1–1)	1 (1–2)	3 (2–5)	4 (3–6)	4 (3–6)	5 (3–6)	4 (3–5)	5 (3–7)	6 (4–9)	8 (6–12)	8 (6–11)	5 (3–6)	4 (3–5)	3 (2–4)	2 (2–3)	1 (1–2)	1 (1–1)	1 (1–1)	--
Unintentional firearm injuries	0 (0–0)	0 (0–0)	0 (0–0)	0 (0–0)	0 (0–0)	0 (0–0)	1 (0–1)	1 (0–1)	0 (0–1)	0 (0–1)	0 (0–1)	0 (0–1)	0 (0–1)	0 (0–1)	0 (0–1)	0 (0–1)	0 (0–1)	0 (0–0)	0 (0–0)	0 (0–0)	0 (0–0)	0 (0–0)	--
Other exposure to mechanical forces	0 (0–0)	0 (0–0)	0 (0–0)	1 (0–1)	1 (1–1)	1 (1–2)	3 (2–4)	4 (2–5)	4 (3–5)	4 (3–6)	4 (2–5)	5 (3–7)	6 (4–9)	8 (6–11)	8 (5–10)	4 (3–6)	3 (2–4)	3 (2–4)	2 (2–3)	1 (1–2)	1 (1–1)	1 (1–1)	--
Adverse effects of medical treatment	0 (0–0)	0 (0–0)	0 (0–0)	0 (0–0)	0 (0–0)	0 (0–0)	0 (0–0)	0 (0–0)	0 (0–1)	0 (0–1)	0 (0–1)	1 (0–1)	1 (1–2)	2 (1–3)	2 (1–4)	3 (2–4)	3 (2–5)	4 (3–6)	5 (3–8)	5 (4–8)	4 (3–6)	3 (2–4)	--
Animal contact	0 (0–0)	0 (0–0)	0 (0–0)	0 (0–0)	0 (0–0)	0 (0–0)	0 (0–0)	0 (0–0)	0 (0–1)	0 (0–1)	0 (0–1)	1 (0–1)	1 (1–2)	2 (1–3)	2 (1–4)	2 (1–4)	3 (2–5)	3 (2–5)	4 (2–6)	2 (1–4)	2 (1–2)	1 (1–2)	--
Venomous animal contact	0 (0–0)	0 (0–0)	0 (0–0)	0 (0–0)	0 (0–0)	0 (0–0)	0 (0–0)	0 (0–0)	0 (0–0)	0 (0–0)	0 (0–0)	0 (0–0)	0 (0–1)	0 (0–1)	0 (0–1)	0 (0–1)	0 (0–1)	0 (0–1)	1 (0–1)	1 (0–1)	1 (0–1)	1 (1–1)	--
Non-venomous animal contact	0 (0–0)	0 (0–0)	0 (0–0)	0 (0–0)	0 (0–0)	0 (0–0)	0 (0–0)	0 (0–0)	0 (0–1)	0 (0–0)	0 (0–1)	1 (0–1)	1 (1–2)	2 (1–3)	2 (1–3)	2 (1–3)	3 (2–4)	3 (2–5)	3 (2–5)	2 (1–3)	1 (0–1)	0 (0–0)	--
Foreign body	0 (0–0)	0 (0–1)	4 (3–6)	2 (1–3)	1 (1–1)	1 (1–2)	1 (1–1)	1 (1–1)	2 (1–2)	2 (1–2)	2 (1–2)	3 (2–4)	5 (3–6)	8 (5–11)	8 (6–11)	7 (5–10)	9 (6–12)	10 (7–13)	11 (8–14)	13 (9–16)	8 (6–10)	6 (5–8)	--
Pulmonary aspiration and foreign body in airway	0 (0–0)	0 (0–1)	4 (3–6)	2 (1–3)	1 (1–1)	1 (1–1)	1 (1–1)	1 (1–1)	1 (1–2)	2 (1–2)	2 (1–2)	2 (2–3)	4 (3–6)	7 (5–10)	8 (6–10)	7 (5–9)	8 (6–11)	9 (7–12)	10 (7–12)	11 (8–15)	7 (5–9)	5 (4–7)	--
Foreign body in other body part	0 (0–0)	0 (0–0)	0 (0–0)	0 (0–0)	0 (0–0)	0 (0–0)	0 (0–0)	0 (0–0)	0 (0–0)	0 (0–0)	0 (0–0)	0 (0–0)	0 (0–1)	0 (0–1)	0 (0–1)	0 (0–1)	1 (0–1)	1 (0–1)	1 (1–2)	1 (1–2)	1 (1–2)	1 (1–2)	--
Environmental heat and cold exposure	0 (0–0)	0 (0–0)	0 (0–0)	0 (0–0)	0 (0–0)	0 (0–0)	0 (0–0)	0 (0–0)	0 (0–0)	0 (0–0)	0 (0–0)	0 (0–0)	0 (0–0)	0 (0–0)	0 (0–0)	0 (0–0)	0 (0–0)	1 (0–1)	1 (0–1)	1 (1–1)	1 (1–1)	1 (1–1)	--
Other unintentional injuries	0 (0–0)	0 (0–0)	0 (0–0)	1 (1–1)	2 (1–2)	3 (2–4)	7 (5–10)	9 (7–13)	12 (9–15)	13 (10–18)	10 (7–13)	12 (9–16)	15 (11–21)	13 (9–19)	11 (8–16)	6 (4–9)	6 (4–8)	4 (3–5)	3 (2–5)	2 (1–3)	1 (1–2)	1 (1–1)	--
Self-harm	--	--	--	--	--	2 (1–3)	20 (14–27)	39 (28–51)	54 (42–69)	71 (55–89)	68 (51–86)	85 (63–113)	126 (91–170)	149 (106–198)	144 (105–192)	111 (81–151)	102 (76–135)	113 (83–148)	110 (82–146)	93 (69–121)	64 (49–83)	25 (19–32)	--
Self-harm by firearm	--	--	--	--	--	0 (0–0)	2 (1–3)	2 (1–3)	2 (1–3)	2 (1–3)	1 (1–2)	2 (1–2)	2 (1–3)	2 (1–4)	2 (1–3)	2 (1–3)	1 (1–2)	2 (1–3)	1 (1–2)	1 (0–1)	0 (0–0)	0 (0–0)	--
Self-harm by other specified means	--	--	--	--	--	2 (1–3)	18 (13–25)	37 (27–49)	52 (41–66)	69 (54–87)	66 (50–85)	83 (62–111)	124 (90–168)	147 (104–195)	142 (103–190)	110 (81–149)	101 (74–133)	111 (82–146)	109 (81–145)	92 (69–121)	63 (48–82)	25 (19–32)	--
Interpersonal violence	1 (0–1)	0 (0–0)	1 (0–1)	1 (1–2)	1 (1–1)	2 (2–3)	24 (16–33)	46 (35–60)	58 (45–75)	62 (46–80)	49 (37–63)	48 (34–64)	58 (41–81)	47 (33–64)	39 (27–53)	22 (15–30)	16 (11–22)	13 (9–19)	10 (7–15)	4 (2–5)	2 (1–2)	1 (0–1)	--
Physical violence by firearm	0 (0–0)	0 (0–0)	0 (0–0)	0 (0–0)	0 (0–0)	0 (0–1)	3 (2–4)	4 (3–6)	4 (2–6)	4 (2–6)	2 (1–3)	2 (1–3)	2 (1–3)	2 (1–2)	2 (1–3)	1 (1–2)	1 (0–1)	0 (0–1)	0 (0–1)	0 (0–0)	0 (0–0)	0 (0–0)	--
Physical violence by sharp object	0 (0–0)	0 (0–0)	0 (0–0)	0 (0–0)	0 (0–1)	1 (1–1)	19 (13–26)	37 (28–47)	48 (36–62)	50 (37–64)	39 (29–51)	37 (26–49)	42 (30–59)	30 (21–41)	19 (13–26)	10 (7–14)	6 (4–8)	5 (3–7)	3 (2–4)	1 (1–2)	1 (0–1)	0 (0–1)	--
Physical violence by other means	1 (0–1)	0 (0–0)	1 (0–1)	1 (0–1)	1 (0–1)	1 (1–1)	2 (1–3)	5 (3–7)	7 (5–10)	8 (5–11)	7 (5–10)	9 (6–12)	14 (10–19)	15 (11–21)	18 (13–25)	11 (7–15)	9 (7–13)	8 (5–11)	7 (5–10)	2 (2–3)	1 (1–2)	0 (0–0)	--
Executions and police conflict	0 (0–0)	0 (0–0)	0 (0–0)	0 (0–0)	0 (0–0)	0 (0–0)	0 (0–0)	0 (0–0)	0 (0–0)	0 (0–0)	0 (0–0)	0 (0–0)	0 (0–0)	0 (0–0)	0 (0–0)	0 (0–0)	0 (0–0)	0 (0–0)	0 (0–0)	0 (0–0)	0 (0–0)	0 (0–0)	--

SR Table 12: Indonesia deaths by cause and age group for males

Cause	Early Neonatal	Late Neonatal	Post Neonatal	1 to 4	5 to 9	10 to 14	15 to 19	20 to 24	25 to 29	30 to 34	35 to 39	40 to 44	45 to 49	50 to 54	55 to 59	60 to 64	65 to 69	70 to 74	75 to 79	80 to 84	85 to 89	90 to 94	95 plus
All causes	25,949 (20,199–32,528)	5,900 (4,580–7,412)	14,608 (10,018–20,896)	11,518 (9,353–14,085)	5,919 (5,050–6,716)	5,312 (4,508–6,260)	12,312 (9,633–16,017)	15,093 (11,917–20,073)	13,826 (11,318–18,087)	15,799 (12,841–20,495)	21,982 (17,813–29,005)	31,815 (24,715–42,372)	45,222 (34,412–59,825)	63,749 (47,830–82,945)	83,871 (63,544–107,014)	104,367 (82,207–128,357)	111,148 (89,035–132,350)	103,047 (83,964–120,178)	91,797 (77,166–104,620)	74,411 (64,321–83,160)	45,623 (40,338–50,784)	17,808 (15,431–19,379)	--
Sexually transmitted infections excluding HIV	1,203 (348–2,706)	488 (148–1,061)	406 (123–921)	20 (6–47)	8 (2–19)	5 (1–11)	7 (1–15)	8 (1–17)	10 (1–19)	5 (1–10)	17 (1–44)	4 (1–13)	8 (1–25)	9 (2–30)	6 (2–17)	7 (2–20)	5 (2–12)	5 (1–13)	3 (1–7)	1 (0–2)	2 (1–5)	2 (0–4)	--
Syphilis	1,203 (348–2,706)	488 (148–1,061)	406 (123–921)	20 (6–47)	8 (2–19)	3 (1–6)	6 (1–12)	5 (1–11)	7 (0–14)	3 (0–7)	15 (1–38)	3 (1–10)	5 (1–17)	7 (1–22)	5 (1–13)	5 (2–16)	4 (1–9)	4 (1–10)	2 (1–4)	0 (0–1)	1 (0–2)	0 (0–1)	--
Chlamydial infection	--	--	--	--	--	0 (0–0)	0 (0–0)	0 (0–0)	0 (0–0)	0 (0–0)	0 (0–0)	0 (0–0)	0 (0–0)	0 (0–0)	0 (0–0)	0 (0–0)	0 (0–0)	0 (0–0)	0 (0–0)	0 (0–0)	0 (0–0)	0 (0–0)	--
Gonococcal infection	--	--	--	--	--	0 (0–0)	1 (0–2)	2 (0–4)	1 (0–1)	0 (0–1)	1 (0–2)	1 (0–2)	1 (0–2)	0 (0–2)	0 (0–1)	1 (0–2)	1 (0–1)	0 (0–1)	0 (0–1)	0 (0–0)	0 (0–0)	0 (0–0)	--
Other sexually transmitted infections	--	--	--	--	--	2 (0–4)	1 (0–1)	1 (0–2)	2 (0–4)	1 (0–3)	1 (0–3)	0 (0–1)	2 (0–5)	2 (0–7)	1 (0–3)	1 (0–2)	1 (0–1)	1 (0–2)	1 (1–3)	0 (0–1)	1 (0–3)	1 (0–3)	--
Tuberculosis	--	--	388 (251–573)	302 (222–393)	175 (138–219)	197 (151–249)	550 (398–745)	1,683 (1,266–2,326)	2,215 (1,752–2,992)	2,280 (1,770–3,081)	2,602 (2,025–3,443)	2,775 (2,083–3,838)	3,199 (2,388–4,245)	3,794 (2,795–4,996)	4,260 (3,098–5,626)	4,927 (3,749–6,291)	5,388 (4,149–6,602)	4,769 (3,700–5,871)	3,541 (2,801–4,285)	3,119 (2,493–3,821)	1,680 (1,357–2,046)	717 (566–868)	--
Drug-susceptible tuberculosis	--	--	384 (247–564)	299 (219–390)	174 (135–217)	195 (149–247)	545 (395–737)	1,667 (1,256–2,310)	2,195 (1,718–2,956)	2,260 (1,757–3,044)	2,578 (2,000–3,434)	2,749 (2,071–3,820)	3,167 (2,367–4,171)	3,756 (2,759–4,972)	4,216 (3,048–5,560)	4,876 (3,690–6,248)	5,332 (4,097–6,556)	4,720 (3,666–5,800)	3,504 (2,755–4,235)	3,087 (2,457–3,765)	1,663 (1,345–2,031)	710 (555–861)	--
Multidrug-resistant tuberculosis without extensive drug resistance	--	--	3 (0–14)	3 (0–12)	2 (0–6)	2 (0–7)	5 (0–20)	13 (1–54)	17 (2–72)	17 (2–71)	20 (2–79)	22 (2–92)	26 (2–106)	32 (3–136)	37 (3–150)	43 (4–180)	47 (4–199)	41 (4–171)	31 (3–137)	28 (2–116)	15 (1–63)	6 (1–27)	--
Extensively drug-resistant tuberculosis	--	--	1 (0–3)	0 (0–2)	0 (0–1)	0 (0–1)	1 (0–4)	3 (0–11)	3 (0–14)	3 (0–14)	4 (0–16)	4 (0–18)	5 (0–22)	6 (1–27)	7 (1–30)	8 (1–35)	9 (1–39)	8 (1–34)	6 (1–28)	5 (0–23)	3 (0–12)	1 (0–5)	--
Lower respiratory infections	519 (375–710)	656 (487–845)	2,719 (1,805–3,965)	908 (643–1,194)	185 (144–231)	107 (81–139)	134 (98–184)	216 (156–299)	170 (130–234)	163 (121–241)	240 (180–355)	338 (243–506)	483 (353–686)	741 (534–1,008)	1,045 (756–1,391)	1,924 (1,433–2,483)	2,385 (1,816–3,013)	2,753 (2,083–3,444)	2,541 (1,944–3,071)	2,629 (2,106–3,195)	1,888 (1,503–2,311)	807 (629–991)	--
Upper respiratory infections	0 (0–0)	0 (0–0)	3 (1–6)	6 (2–11)	1 (0–2)	0 (0–1)	1 (0–3)	0 (0–1)	1 (0–4)	1 (0–3)	2 (1–5)	3 (1–8)	5 (2–12)	5 (2–14)	4 (1–10)	8 (2–19)	6 (2–14)	2 (1–7)	5 (1–12)	1 (0–3)	2 (0–5)	1 (0–2)	--
Otitis media	0 (0–0)	0 (0–0)	0 (0–0)	0 (0–0)	0 (0–0)	0 (0–0)	0 (0–0)	0 (0–0)	0 (0–0)	0 (0–0)	0 (0–0)	0 (0–0)	0 (0–0)	0 (0–1)	0 (0–0)	0 (0–1)	0 (0–0)	0 (0–0)	0 (0–0)	0 (0–0)	0 (0–0)	0 (0–0)	--
Diarrhoeal diseases	108 (77–153)	542 (393–729)	2,822 (1,851–4,254)	1,347 (931–1,822)	334 (189–638)	205 (109–388)	174 (83–309)	143 (60–231)	232 (99–362)	283 (123–486)	417 (234–836)	521 (289–1,158)	765 (440–1,718)	1,103 (627–2,381)	1,400 (756–2,892)	1,872 (983–3,515)	2,567 (1,191–4,267)	3,064 (1,315–4,703)	3,704 (1,497–5,545)	4,115 (1,442–6,175)	2,981 (1,086–4,373)	1,374 (490–2,020)	--
Typhoid fever	--	--	2 (1–4)	278 (91–620)	596 (228–1,164)	754 (322–1,351)	719 (272–1,532)	353 (124–822)	176 (71–375)	116 (44–252)	88 (34–185)	74 (30–155)	72 (28–157)	71 (26–153)	66 (23–153)	55 (19–122)	37 (14–76)	22 (8–49)	13 (5–26)	8 (2–16)	4 (1–7)	1 (0–2)	--
Paratyphoid fever	--	--	0 (0–0)	19 (5–49)	41 (14–93)	50 (18–106)	47 (14–111)	22 (7–57)	11 (4–26)	7 (2–17)	5 (2–12)	4 (1–10)	4 (1–10)	4 (1–9)	3 (1–8)	3 (1–6)	2 (1–4)	1 (0–2)	1 (0–1)	0 (0–1)	0 (0–0)	0 (0–0)	--
iNTS	--	1 (0–1)	7 (3–12)	37 (19–66)	32 (15–61)	21 (9–44)	18 (7–38)	11 (4–25)	6 (2–13)	5 (2–10)	5 (2–11)	6 (2–12)	7 (3–13)	8 (4–16)	11 (5–21)	14 (6–26)	14 (6–26)	11 (5–21)	8 (4–16)	5 (2–10)	3 (1–5)	1 (0–2)	--
Other intestinal infectious diseases	--	--	2 (0–6)	2 (0–8)	1 (0–4)	1 (0–3)	1 (0–2)	1 (0–2)	0 (0–1)	0 (0–2)	0 (0–1)	0 (0–1)	0 (0–1)	0 (0–1)	0 (0–1)	0 (0–1)	0 (0–2)	0 (0–1)	0 (0–1)	0 (0–1)	0 (0–1)	0 (0–0)	--
Malaria	0 (0–0)	2 (0–7)	8 (2–26)	8 (2–24)	4 (1–10)	2 (0–5)	32 (8–91)	29 (7–82)	22 (5–62)	19 (4–53)	20 (5–58)	35 (8–101)	31 (7–86)	25 (5–72)	20 (4–59)	31 (6–100)	22 (5–70)	16 (3–50)	5 (1–13)	3 (1–9)	0 (0–1)	0 (0–0)	--
Chagas disease	--	--	0 (0–0)	0 (0–0)	0 (0–0)	0 (0–0)	0 (0–0)	0 (0–0)	0 (0–0)	0 (0–0)	0 (0–0)	0 (0–0)	0 (0–0)	0 (0–0)	0 (0–0)	0 (0–0)	0 (0–0)	0 (0–0)	0 (0–0)	0 (0–0)	0 (0–0)	0 (0–0)	--
Visceral leishmaniasis	--	--	0 (0–0)	0 (0–0)	0 (0–0)	0 (0–0)	0 (0–0)	0 (0–0)	0 (0–0)	0 (0–0)	0 (0–0)	0 (0–0)	0 (0–0)	0 (0–0)	0 (0–0)	0 (0–0)	0 (0–0)	0 (0–0)	0 (0–0)	0 (0–0)	0 (0–0)	0 (0–0)	--
African trypanosomiasis	--	--	--	0 (0–0)	0 (0–0)	0 (0–0)	0 (0–0)	0 (0–0)	0 (0–0)	0 (0–0)	0 (0–0)	0 (0–0)	0 (0–0)	0 (0–0)	0 (0–0)	0 (0–0)	0 (0–0)	0 (0–0)	0 (0–0)	0 (0–0)	0 (0–0)	0 (0–0)	--
Schistosomiasis	--	--	0 (0–0)	0 (0–0)	0 (0–0)	0 (0–0)	0 (0–0)	0 (0–1)	0 (0–1)	0 (0–1)	0 (0–1)	0 (0–2)	0 (0–2)	1 (0–3)	1 (0–3)	1 (0–3)	0 (0–2)	0 (0–2)	0 (0–2)	0 (0–1)	0 (0–0)	0 (0–0)	--
Cysticercosis	--	--	--	0 (0–0)	0 (0–0)	0 (0–0)	0 (0–0)	0 (0–0)	0 (0–0)	0 (0–0)	0 (0–0)	0 (0–0)	0 (0–0)	0 (0–0)	0 (0–0)	0 (0–0)	0 (0–0)	0 (0–0)	0 (0–0)	0 (0–0)	0 (0–0)	0 (0–0)	--
Cystic echinococcosis	--	--	--	1 (0–3)	1 (0–3)	1 (0–2)	1 (0–2)	1 (0–2)	1 (0–3)	1 (0–4)	1 (0–5)	2 (0–5)	2 (0–6)	2 (0–7)	2 (0–7)	2 (0–6)	2 (0–6)	1 (0–5)	1 (0–4)	1 (0–3)	0 (0–2)	0 (0–1)	--
Dengue	0 (0–0)	122 (3–247)	553 (29–1,062)	1,251 (262–2,088)	400 (89–626)	178 (57–395)	249 (61–457)	360 (79–665)	330 (100–612)	159 (51–327)	197 (53–369)	222 (46–422)	257 (47–499)	222 (35–388)	283 (35–509)	283 (35–536)	193 (24–367)	112 (25–215)	117 (24–231)	89 (21–158)	116 (25–205)	48 (9–80)	--
Yellow fever	--	0 (0–0)	0 (0–0)	0 (0–0)	0 (0–0)	0 (0–0)	0 (0–0)	0 (0–0)	0 (0–0)	0 (0–0)	0 (0–0)	0 (0–0)	0 (0–0)	0 (0–0)	0 (0–0)	0 (0–0)	0 (0–0)	0 (0–0)	0 (0–0)	0 (0–0)	0 (0–0)	0 (0–0)	--
Rabies	--	--	1 (0–2)	2 (0–5)	9 (1–20)	12 (0–25)	2 (0–4)	2 (0–4)	1 (0–2)	1 (0–2)	3 (0–7)	3 (0–10)	6 (0–15)	6 (0–13)	9 (0–16)	3 (0–5)	2 (0–3)	3 (0–5)	1 (0–1)	0 (0–1)	0 (0–1)	0 (0–0)	--
Ascariasis	--	--	1 (0–2)	6 (3–11)	4 (2–6)	1 (0–2)	1 (0–1)	0 (0–1)	0 (0–1)	1 (0–1)	1 (0–1)	0 (0–1)	0 (0–1)	0 (0–1)	0 (0–1)	1 (0–2)	0 (0–1)	1 (0–1)	1 (0–1)	0 (0–1)	0 (0–0)	0 (0–0)	--
Zika virus disease	0 (0–0)	0 (0–0)	0 (0–0)	0 (0–0)	0 (0–0)	0 (0–0)	0 (0–0)	0 (0–0)	0 (0–0)	0 (0–0)	0 (0–0)	0 (0–0)	0 (0–0)	0 (0–0)	0 (0–0)	0 (0–0)	0 (0–0)	0 (0–0)	0 (0–0)	0 (0–0)	0 (0–0)	0 (0–0)	--
Other neglected tropical diseases	1 (0–1)	0 (0–1)	1 (0–3)	9 (3–18)	3 (1–5)	3 (1–5)	1 (0–2)	1 (0–1)	1 (0–1)	1 (0–1)	1 (0–1)	2 (1–3)	1 (0–2)	1 (0–2)	7 (3–13)	5 (2–8)	2 (1–4)	8 (4–16)	4 (2–9)	5 (3–11)	3 (1–5)	1 (0–2)	--
Meningitis	72 (49–113)	114 (80–170)	840 (541–1,299)	456 (323–656)	249 (187–313)	118 (87–153)	143 (101–195)	164 (118–223)	90 (67–125)	86 (63–119)	89 (64–123)	95 (69–133)	83 (58–114)	72 (51–98)	89 (62–120)	85 (62–112)	79 (57–106)	74 (55–95)	79 (58–102)	43 (32–55)	23 (18–30)	6 (5–8)	--
Encephalitis	6 (1–10)	30 (2–52)	246 (24–435)	245 (75–391)	54 (35–79)	64 (23–94)	53 (26–79)	38 (17–57)	18 (12–28)	47 (25–67)	50 (28–72)	42 (29–60)	35 (23–64)	27 (17–53)	36 (24–57)	28 (16–75)	36 (24–59)	50 (36–68)	56 (34–79)	11 (7–17)	9 (6–14)	3 (2–4)	--
Diphtheria	--	--	2 (1–4)	3 (2–6)	2 (1–4)	1 (1–2)	1 (0–1)	0 (0–1)	0 (0–0)	0 (0–1)	0 (0–1)	1 (0–1)	1 (1–2)	1 (1–2)	1 (0–1)	--	--	--	--	--	--	--	--
Whooping cough	--	--	678 (73–2,034)	962 (109–2,728)	101 (10–335)	23 (2–78)	11 (1–37)	8 (1–27)	5 (0–16)	4 (0–14)	3 (0–11)	5 (0–17)	4 (0–14)	3 (0–10)	4 (0–15)	--	--	--	--	--	--	--	--
Tetanus	34 (23–53)	59 (37–93)	26 (14–42)	66 (17–151)	30 (16–65)	10 (3–55)	25 (13–59)	33 (12–60)	35 (8–58)	60 (13–124)	93 (20–173)	159 (26–271)	265 (34–431)	218 (36–357)	287 (39–455)	200 (40–331)	140 (32–283)	134 (27–230)	118 (19–197)	41 (14–73)	25 (8–56)	9 (3–22)	--
Measles	0 (0–0)	0 (0–0)	75 (22–181)	267 (82–624)	40 (12–94)	11 (3–27)	5 (1–11)	2 (1–5)	1 (0–2)	1 (0–2)	0 (0–1)	0 (0–1)	0 (0–1)	0 (0–1)	0 (0–1)	0 (0–0)	0 (0–0)	0 (0–0)	0 (0–0)	0 (0–0)	0 (0–0)	0 (0–0)	--
Varicella and herpes zoster	1 (1–2)	5 (3–7)	19 (10–32)	43 (26–69)	15 (10–23)	5 (3–8)	3 (2–5)	3 (2–5)	3 (2–5)	5 (3–8)	4 (3–7)	3 (2–5)	4 (2–6)	4 (2–6)	5 (3–7)	6 (3–9)	10 (6–16)	9 (5–14)	12 (7–20)	18 (10–28)	18 (11–30)	10 (6–16)	--
Acute hepatitis	--	--	24 (15–37)	14 (10–20)	37 (27–50)	21 (15–29)	70 (48–100)	105 (67–161)	108 (78–156)	146 (105–202)	226 (166–317)	355 (251–496)	320 (229–446)	446 (304–632)	464 (321–633)	456 (322–630)	305 (209–418)	211 (153–288)	197 (140–272)	80 (58–111)	50 (36–66)	12 (9–15)	--
Acute hepatitis A	--	--	7 (3–16)	8 (4–12)	20 (11–32)	14 (9–21)	23 (9–44)	35 (14–69)	53 (25–89)	29 (7–74)	44 (10–113)	98 (22–222)	41 (5–133)	109 (19–272)	139 (26–318)	128 (23–298)	57 (7–159)	60 (10–141)	65 (14–141)	32 (11–60)	15 (5–33)	3 (1–7)	--
Acute hepatitis B	--	--	13 (6–22)	4 (1–7)	10 (3–18)	5 (1–9)	34 (13–54)	53 (20–89)	42 (13–71)	90 (44–130)	145 (73–219)	182 (75–287)	223 (132–334)	271 (117–417)	259 (97–405)	264 (101–417)	194 (96–301)	131 (52–206)	114 (43–194)	40 (13–68)	30 (12–45)	8 (4–11)	--
Acute hepatitis C	--	--	1 (0–2)	2 (1–4)	5 (2–10)	1 (0–2)	4 (2–8)	6 (2–12)	8 (3–17)	20 (8–43)	30 (11–72)	44 (17–120)	44 (17–102)	57 (22–132)	54 (21–133)	57 (22–126)	46 (19–91)	16 (6–31)	12 (5–26)	5 (2–9)	2 (1–5)	0 (0–1)	--
Acute hepatitis E	--	--	2 (1–5)	1 (0–2)	1 (1–2)	1 (0–2)	10 (4–17)	11 (4–22)	6 (2–10)	7 (2–12)	7 (2–14)	32 (11–57)	11 (2–20)	8 (1–16)	11 (2–20)	8 (2–15)	8 (1–14)	5 (1–9)	4 (1–8)	3 (1–6)	3 (1–6)	1 (0–2)	--
Other unspecified infectious diseases	96 (6–176)	86 (7–160)	134 (13–260)	46 (8–79)	22 (6–36)	7 (5–10)	24 (15–35)	20 (11–41)	16 (7–49)	22 (9–62)	30 (13–98)	27 (9–97)	40 (17–123)	49 (27–106)	54 (34–94)	38 (22–82)	37 (23–74)	42 (26–60)	30 (16–44)	32 (11–49)	28 (7–45)	10 (3–15)	--
Maternal disorders	--	--	--	--	--	--	--	--	--	--	--	--	--	--	--	--	--	--	--	--	--	--	--
Maternal haemorrhage	--	--	--	--	--	--	--	--	--	--	--	--	--	--	--	--	--	--	--	--	--	--	--
Maternal sepsis and other pregnancy related infections	--	--	--	--	--	--	--	--	--	--	--	--	--	--	--	--	--	--	--	--	--	--	--
Maternal hypertensive disorders	--	--	--	--	--	--	--	--	--	--	--	--	--	--	--	--	--	--	--	--	--	--	--
Maternal obstructed labour and uterine rupture	--	--	--	--	--	--	--	--	--	--	--	--	--	--	--	--	--	--	--	--	--	--	--
Maternal abortive outcome	--	--	--	--	--	--	--	--	--	--	--	--	--	--	--	--	--	--	--	--	--	--	--
Ectopic pregnancy	--	--	--	--	--	--	--	--	--	--	--	--	--	--	--	--	--	--	--	--	--	--	--
Indirect maternal deaths	--	--	--	--	--	--	--	--	--	--	--	--	--	--	--	--	--	--	--	--	--	--	--
Late maternal deaths	--	--	--	--	--	--	--	--	--	--	--	--	--	--	--	--	--	--	--	--	--	--	--
Maternal deaths aggravated by HIV/AIDS	--	--	--	--	--	--	--	--	--	--	--	--	--	--	--	--	--	--	--	--	--	--	--
Other maternal disorders	--	--	--	--	--	--	--	--	--	--	--	--	--	--	--	--	--	--	--	--	--	--	--
Neonatal disorders	20,449 (15,841–26,012)	2,386 (1,728–3,122)	602 (385–903)	20 (14–28)	--	--	--	--	--	--	--	--	--	--	--	--	--	--	--	--	--	--	--
Neonatal preterm birth	9,878 (7,338–13,397)	531 (352–741)	140 (86–214)	3 (2–5)	--	--	--	--	--	--	--	--	--	--	--	--	--	--	--	--	--	--	--
Neonatal encephalopathy due to birth asphyxia and trauma	6,841 (3,859–9,656)	379 (255–525)	110 (64–175)	8 (5–14)	--	--	--	--	--	--	--	--	--	--	--	--	--	--	--	--	--	--	--
Neonatal sepsis and other neonatal infections	2,130 (1,419–3,069)	1,102 (794–1,456)	221 (138–344)	1 (0–1)	--	--	--	--	--	--	--	--	--	--	--	--	--	--	--	--	--	--	--
Hemolytic disease and other neonatal jaundice	131 (90–181)	33 (21–48)	4 (2–7)	1 (0–1)	--	--	--	--	--	--	--	--	--	--	--	--	--	--	--	--	--	--	--
Other neonatal disorders	1,469 (800–2,192)	341 (204–489)	127 (75–207)	8 (5–12)	--	--	--	--	--	--	--	--	--	--	--	--	--	--	--	--	--	--	--
Nutritional deficiencies	0 (0–0)	0 (0–0)	263 (167–392)	211 (149–293)	51 (40–65)	43 (33–55)	53 (38–73)	39 (28–53)	46 (34–62)	51 (38–69)	57 (43–81)	75 (55–103)	118 (86–161)	141 (100–193)	193 (138–257)	379 (274–501)	770 (572–1,015)	1,176 (873–1,492)	1,460 (1,139–1,856)	1,492 (1,176–1,883)	1,156 (913–1,450)	596 (457–745)	--
Protein-energy malnutrition	0 (0–0)	0 (0–0)	248 (158–368)	206 (145–286)	50 (38–63)	38 (27–49)	48 (34–66)	34 (25–48)	30 (22–42)	41 (31–57)	49 (36–70)	68 (49–93)	109 (79–147)	105 (73–146)	169 (120–225)	321 (227–427)	723 (538–956)	1,057 (783–1,343)	1,353 (1,049–1,729)	1,370 (1,068–1,735)	1,057 (825–1,337)	546 (414–687)	--
Other nutritional deficiencies	--	--	15 (9–25)	5 (3–8)	2 (1–3)	5 (3–11)	5 (3–8)	4 (3–6)	16 (8–23)	9 (6–13)	8 (5–12)	7 (5–11)	10 (6–14)	36 (22–52)	24 (15–34)	58 (38–84)	47 (29–71)	119 (78–174)	107 (70–167)	122 (83–183)	99 (64–150)	50 (32–80)	--
Lip and oral cavity cancer	--	--	--	--	1 (1–2)	2 (1–3)	6 (4–9)	10 (6–14)	15 (10–25)	23 (14–40)	44 (27–75)	90 (55–148)	175 (112–269)	257 (169–376)	345 (233–489)	414 (290–592)	406 (279–563)	319 (221–443)	237 (163–332)	163 (109–234)	74 (45–108)	22 (13–32)	--
Nasopharynx cancer	--	--	--	--	3 (2–4)	5 (3–7)	18 (12–26)	22 (15–33)	17 (12–25)	28 (19–42)	63 (42–95)	114 (76–179)	195 (129–301)	274 (175–424)	328 (214–500)	326 (219–479)	293 (201–424)	230 (161–334)	116 (81–160)	68 (49–92)	25 (18–34)	7 (5–10)	--
Other pharynx cancer	--	--	--	--	--	--	--	2 (1–3)	2 (1–3)	3 (2–6)	11 (7–22)	25 (15–51)	54 (32–109)	101 (60–206)	141 (88–290)	162 (102–319)	140 (89–274)	119 (77–242)	68 (45–129)	40 (26–68)	14 (9–24)	6 (4–9)	--
Oesophageal cancer	--	--	--	--	--	--	--	7 (5–10)	8 (5–11)	13 (9–18)	39 (28–55)	90 (63–134)	196 (135–300)	331 (225–518)	482 (336–744)	577 (406–869)	586 (424–911)	441 (316–677)	295 (219–449)	180 (135–279)	90 (67–145)	24 (17–39)	--
Stomach cancer	--	--	--	--	--	--	14 (10–19)	22 (17–30)	33 (25–45)	70 (53–95)	128 (99–182)	242 (181–332)	381 (279–529)	613 (444–826)	860 (632–1,140)	1,095 (839–1,392)	1,162 (908–1,453)	1,019 (803–1,243)	788 (641–951)	558 (451–675)	336 (272–404)	92 (73–114)	--
Colon and rectum cancer	--	--	--	--	6 (4–7)	8 (5–10)	49 (30–75)	69 (46–97)	104 (70–146)	200 (133–292)	344 (229–479)	570 (384–811)	932 (627–1,335)	1,430 (934–2,044)	1,906 (1,232–2,700)	2,377 (1,509–3,277)	2,501 (1,590–3,395)	2,105 (1,336–2,887)	1,592 (1,001–2,125)	1,127 (690–1,509)	583 (332–790)	157 (83–214)	--
Liver cancer due to hepatitis B	--	--	--	--	--	7 (5–10)	10 (7–14)	9 (6–12)	12 (9–16)	13 (10–18)	22 (15–32)	33 (22–50)	46 (30–70)	72 (47–108)	102 (63–151)	111 (69–165)	97 (61–139)	71 (46–103)	47 (32–65)	24 (15–34)	10 (7–13)	2 (1–3)	--
Liver cancer due to hepatitis C	--	--	--	--	--	0 (0–0)	0 (0–1)	1 (0–1)	1 (1–2)	2 (1–3)	5 (3–8)	11 (7–17)	26 (16–40)	61 (40–91)	122 (77–179)	175 (116–245)	204 (148–267)	200 (148–260)	173 (132–222)	119 (92–151)	59 (46–75)	16 (12–21)	--
Liver cancer due to alcohol use	--	--	--	--	--	--	0 (0–1)	1 (1–1)	2 (1–3)	3 (2–4)	7 (4–11)	14 (9–22)	27 (16–41)	54 (33–83)	91 (58–135)	125 (84–177)	149 (107–207)	130 (92–177)	85 (62–113)	43 (31–55)	16 (11–21)	3 (2–5)	--
Liver cancer due to NASH	--	--	--	--	--	--	5 (3–7)	2 (1–4)	2 (1–3)	2 (1–3)	3 (2–4)	5 (3–7)	9 (5–13)	17 (11–25)	27 (17–41)	36 (23–53)	42 (28–60)	41 (29–57)	36 (25–51)	23 (16–32)	10 (7–13)	2 (2–3)	--
	0 (0–0)	0 (0–0)	2 (1–3)	9 (5–14)	7 (4–9)	6 (3–8)	--	--	--	--	--	--	--	--	--	--	--	--	--	--	--	--	--
Liver cancer due to other causes	4 (2–7)	4 (2–6)	10 (6–16)	13 (8–19)	6 (4–8)	3 (2–5)	4 (2–5)	2 (1–3)	1 (1–2)	1 (1–2)	2 (1–3)	3 (2–5)	4 (3–7)	7 (4–11)	10 (7–15)	12 (8–16)	11 (7–16)	9 (6–13)	7 (5–10)	4 (3–6)	2 (1–2)	0 (0–1)	--
Gallbladder and biliary tract cancer	--	--	--	--	--	--	--	1 (1–2)	2 (1–2)	4 (3–6)	10 (7–15)	22 (15–34)	39 (25–62)	72 (45–124)	96 (64–166)	131 (85–231)	135 (87–237)	124 (83–224)	107 (70–196)	70 (48–128)	36 (24–69)	10 (7–20)	--
Pancreatic cancer	--	--	--	--	--	--	5 (3–9)	10 (6–15)	17 (11–25)	44 (27–70)	93 (57–143)	163 (105–250)	280 (185–419)	452 (301–675)	653 (432–988)	768 (506–1,172)	800 (526–1,205)	641 (416–958)	481 (307–727)	327 (206–506)	143 (84–220)	45 (25–72)	--
Larynx cancer	--	--	--	--	--	--	--	2 (1–3)	3 (2–4)	5 (4–8)	11 (7–16)	30 (20–47)	73 (49–114)	148 (101–219)	233 (158–343)	307 (214–444)	307 (218–439)	256 (178–391)	180 (129–266)	111 (80–162)	46 (34–67)	10 (7–14)	--
Tracheal, bronchus, and lung cancer	--	--	--	--	--	4 (3–6)	41 (29–61)	60 (40–88)	75 (47–130)	139 (84–242)	312 (187–523)	645 (409–1,016)	1,409 (926–2,107)	2,710 (1,834–3,957)	4,330 (2,989–6,144)	5,972 (4,300–7,998)	6,281 (4,651–8,078)	5,220 (3,885–6,973)	3,503 (2,637–4,519)	1,867 (1,390–2,420)	910 (654–1,243)	202 (142–273)	--
Malignant skin melanoma	0 (0–0)	0 (0–0)	0 (0–0)	1 (1–2)	1 (1–1)	1 (1–1)	3 (2–4)	3 (2–4)	3 (2–5)	5 (4–9)	8 (5–13)	17 (11–28)	17 (10–28)	24 (15–39)	34 (21–57)	30 (19–47)	33 (22–54)	28 (19–46)	23 (15–36)	18 (13–30)	10 (7–16)	4 (3–6)	--
Non-melanoma skin cancer (squamous-cell carcinoma)	--	--	--	--	--	--	--	7 (5–10)	7 (5–10)	9 (6–13)	13 (9–19)	26 (18–37)	44 (29–63)	71 (47–103)	101 (67–141)	152 (102–214)	155 (105–213)	146 (96–208)	127 (87–170)	101 (73–129)	57 (41–73)	24 (17–32)	--
	0 (0–0)	0 (0–0)	4 (2–10)	12 (7–28)	11 (7–22)	12 (8–23)	20 (13–33)	11 (7–18)	13 (9–22)	11 (7–18)	16 (11–28)	21 (13–34)	28 (18–45)	39 (24–63)	30 (19–46)	49 (32–79)	45 (29–73)	29 (19–46)	23 (15–37)	20 (13–33)	11 (7–16)	2 (1–3)	--
	--	--	--	14 (9–20)	28 (17–41)	47 (22–70)	96 (37–152)	79 (32–120)	48 (20–73)	38 (14–61)	45 (14–74)	70 (19–118)	90 (21–150)	126 (27–219)	152 (29–269)	192 (39–333)	182 (37–311)	138 (29–242)	101 (22–169)	55 (12–91)	18 (4–30)	3 (1–6)	--
Breast cancer	--	--	--	--	--	--	1 (1–2)	2 (1–2)	2 (1–2)	2 (1–4)	5 (2–8)	7 (4–12)	18 (9–30)	22 (12–35)	30 (17–48)	36 (21–60)	38 (22–60)	32 (19–52)	26 (15–41)	19 (11–31)	8 (4–15)	4 (2–7)	--
Cervical cancer	--	--	--	--	--	--	--	--	--	--	--	--	--	--	--	--	--	--	--	--	--	--	--
Uterine cancer	--	--	--	--	--	--	--	--	--	--	--	--	--	--	--	--	--	--	--	--	--	--	--
Ovarian cancer	--	--	--	--	--	--	--	--	--	--	--	--	--	--	--	--	--	--	--	--	--	--	--
Prostate cancer	--	--	--	--	--	--	--	4 (2–6)	5 (4–8)	6 (4–8)	10 (7–13)	21 (13–30)	76 (46–106)	223 (139–311)	488 (307–656)	1,123 (729–1,479)	1,585 (1,040–2,082)	1,981 (1,350–2,538)	2,303 (1,586–2,924)	1,952 (1,358–2,511)	1,130 (777–1,490)	374 (258–501)	--
Testicular cancer	0 (0–0)	0 (0–0)	1 (1–2)	7 (5–10)	3 (2–4)	3 (2–5)	10 (7–17)	21 (15–31)	29 (21–42)	26 (18–38)	25 (18–36)	30 (21–43)	19 (13–28)	9 (6–14)	15 (9–22)	18 (11–26)	14 (9–22)	12 (8–18)	11 (7–17)	9 (6–16)	2 (1–3)	1 (1–2)	--
Kidney cancer	1 (0–1)	0 (0–1)	9 (4–17)	31 (18–50)	14 (9–20)	8 (5–12)	8 (5–13)	6 (4–10)	11 (7–17)	17 (10–27)	35 (21–55)	64 (40–102)	126 (79–198)	200 (122–310)	264 (164–394)	305 (196–460)	303 (195–446)	226 (147–328)	170 (113–245)	100 (66–143)	51 (34–74)	16 (10–23)	--
Bladder cancer	--	--	--	--	--	--	2 (1–3)	2 (2–4)	3 (2–5)	8 (5–13)	14 (9–23)	35 (22–58)	64 (39–106)	124 (76–208)	190 (117–322)	299 (188–499)	342 (221–573)	346 (222–583)	347 (233–582)	261 (184–419)	149 (107–232)	44 (31–69)	--
Brain and nervous system cancer	1 (1–2)	2 (1–4)	13 (6–24)	78 (48–126)	115 (70–173)	90 (52–137)	75 (42–116)	75 (36–119)	82 (38–138)	98 (44–173)	136 (63–235)	184 (90–311)	206 (103–335)	255 (132–407)	290 (159–444)	295 (160–444)	248 (131–357)	174 (98–248)	112 (66–161)	63 (39–91)	29 (18–42)	8 (5–11)	--
	0 (0–0)	0 (0–0)	2 (1–3)	23 (15–36)	8 (5–12)	4 (3–6)	4 (3–6)	5 (3–7)	3 (2–5)	3 (2–5)	3 (2–5)	4 (2–6)	4 (2–6)	6 (3–8)	5 (2–7)	6 (3–8)	6 (3–8)	4 (2–6)	5 (2–7)	2 (1–3)	1 (1–2)	0 (0–1)	--
	0 (0–0)	0 (0–0)	2 (1–3)	17 (10–27)	5 (3–7)	2 (1–4)	2 (1–4)	--	--	--	--	--	--	--	--	--	--	--	--	--	--	--	--
	--	--	--	6 (3–11)	3 (2–5)	2 (1–3)	2 (1–3)	5 (3–7)	3 (2–5)	3 (2–5)	3 (2–5)	4 (2–6)	4 (2–6)	6 (3–8)	5 (2–7)	6 (3–8)	6 (3–8)	4 (2–6)	5 (2–7)	2 (1–3)	1 (1–2)	0 (0–1)	--
	0 (0–0)	0 (0–0)	2 (1–4)	21 (12–43)	10 (6–15)	5 (3–7)	5 (3–8)	6 (4–9)	4 (3–6)	3 (2–5)	4 (3–6)	4 (3–7)	6 (4–10)	5 (3–8)	5 (3–7)	5 (4–8)	5 (4–8)	3 (2–4)	2 (2–4)	1 (1–2)	1 (0–1)	0 (0–0)	--
Thyroid cancer	--	--	--	--	2 (1–2)	2 (1–3)	3 (2–5)	4 (3–6)	5 (3–7)	6 (4–9)	10 (7–15)	19 (13–27)	34 (22–47)	64 (42–91)	88 (57–122)	114 (79–154)	109 (75–145)	87 (63–115)	64 (45–86)	41 (30–55)	19 (14–25)	5 (3–6)	--
Mesothelioma	--	--	--	--	--	--	--	4 (2–6)	4 (2–7)	4 (2–6)	7 (4–12)	12 (7–20)	23 (15–38)	31 (19–46)	50 (32–72)	15 (10–20)	15 (11–21)	8 (6–12)	20 (15–26)	14 (10–19)	7 (5–10)	1 (1–2)	--
Hodgkin lymphoma	--	--	--	2 (1–4)	2 (1–4)	6 (4–13)	14 (8–30)	15 (9–30)	15 (9–33)	11 (7–23)	15 (8–34)	19 (11–43)	20 (12–42)	27 (16–55)	28 (17–55)	28 (18–51)	34 (22–62)	29 (19–53)	13 (9–22)	8 (5–13)	5 (3–9)	1 (0–1)	--
Non-Hodgkin's lymphoma	--	--	--	38 (24–57)	48 (34–69)	42 (30–61)	90 (61–138)	92 (62–140)	74 (52–111)	78 (54–115)	91 (60–130)	152 (100–219)	191 (126–273)	293 (187–415)	367 (245–513)	435 (293–589)	418 (286–554)	335 (232–441)	212 (147–279)	131 (93–174)	56 (40–75)	19 (13–25)	--
	--	--	--	4 (2–7)	5 (2–7)	4 (2–6)	6 (3–9)	5 (3–8)	3 (2–5)	3 (1–4)	3 (2–5)	3 (1–5)	3 (1–6)	3 (1–5)	3 (1–5)	3 (1–4)	3 (1–6)	2 (1–3)	1 (0–2)	1 (0–1)	0 (0–0)	0 (0–0)	--
	--	--	--	33 (21–52)	44 (31–64)	39 (27–56)	85 (57–129)	88 (59–134)	71 (50–107)	75 (53–111)	87 (59–126)	149 (99–214)	187 (124–268)	290 (186–411)	364 (243–509)	433 (292–585)	414 (284–550)	333 (230–438)	210 (146–277)	130 (92–174)	56 (40–75)	18 (13–25)	--
Multiple myeloma	--	--	--	--	--	--	--	1 (1–2)	2 (1–2)	3 (2–5)	8 (5–12)	21 (14–34)	35 (24–55)	68 (44–107)	97 (65–155)	129 (86–208)	135 (90–217)	111 (75–180)	66 (45–108)	43 (29–75)	17 (11–31)	4 (3–8)	--
Leukaemia	4 (2–8)	3 (2–5)	35 (17–76)	300 (199–495)	297 (219–417)	268 (193–361)	302 (205–430)	200 (135–287)	151 (111–215)	135 (95–191)	176 (126–253)	215 (153–309)	274 (196–390)	334 (227–465)	443 (305–619)	536 (375–726)	542 (396–708)	469 (341–610)	373 (286–494)	238 (179–309)	144 (109–189)	40 (30–53)	--
Acute lymphoid leukaemia	1 (0–1)	1 (0–1)	14 (7–31)	97 (64–158)	126 (83–182)	105 (72–161)	90 (59–136)	45 (27–72)	23 (15–42)	19 (12–31)	23 (15–37)	23 (15–38)	32 (20–54)	42 (26–66)	37 (22–57)	41 (24–63)	47 (29–70)	33 (20–51)	15 (9–23)	19 (12–29)	14 (9–22)	2 (1–4)	--
Chronic lymphoid leukaemia	--	--	--	--	--	--	--	2 (1–3)	1 (1–2)	1 (1–2)	1 (1–2)	4 (2–6)	7 (4–12)	14 (8–21)	23 (14–36)	39 (23–61)	50 (32–76)	28 (17–42)	31 (19–47)	32 (21–47)	13 (8–19)	4 (3–6)	--
Acute myeloid leukaemia	1 (0–1)	1 (0–1)	6 (2–16)	56 (28–131)	49 (28–103)	41 (23–78)	76 (42–136)	42 (24–69)	32 (18–57)	38 (22–66)	50 (29–93)	60 (35–111)	60 (34–109)	86 (50–158)	114 (64–211)	100 (58–188)	91 (53–163)	81 (46–141)	52 (29–93)	38 (22–69)	17 (10–30)	4 (2–7)	--
Chronic myeloid leukaemia	0 (0–0)	0 (0–0)	1 (0–3)	3 (2–5)	3 (2–4)	6 (3–9)	4 (3–7)	10 (6–18)	6 (4–11)	6 (4–11)	12 (8–20)	13 (8–21)	18 (10–29)	23 (12–36)	27 (15–42)	31 (18–49)	38 (24–57)	27 (16–42)	32 (20–47)	15 (9–23)	8 (5–13)	3 (2–5)	--
Other leukaemia	2 (1–5)	1 (1–2)	14 (6–33)	144 (90–239)	120 (73–172)	115 (71–160)	130 (73–191)	101 (56–151)	88 (54–127)	71 (38–104)	90 (49–132)	116 (65–172)	157 (91–229)	170 (95–243)	242 (144–349)	324 (207–442)	315 (209–423)	300 (203–400)	244 (174–325)	134 (93–179)	91 (66–122)	26 (18–36)	--
Other malignant cancers	9 (4–15)	8 (3–15)	31 (10–55)	127 (65–182)	93 (48–125)	113 (47–158)	178 (71–268)	178 (63–280)	137 (39–221)	152 (43–248)	223 (66–368)	308 (106–509)	405 (143–654)	542 (213–842)	668 (287–992)	702 (315–1,005)	688 (319–963)	602 (300–834)	476 (239–649)	328 (167–437)	156 (79–218)	51 (25–74)	--
Myelodysplastic, myeloproliferative, and other hematopoietic neoplasms	0 (0–0)	0 (0–0)	1 (0–2)	2 (1–5)	1 (1–3)	1 (1–2)	1 (1–2)	1 (1–2)	2 (1–3)	2 (1–3)	3 (2–5)	7 (4–11)	11 (6–19)	19 (11–33)	33 (18–55)	43 (25–68)	61 (36–100)	59 (35–98)	49 (29–80)	24 (14–41)	18 (10–31)	4 (2–7)	--
Cardiovascular diseases	15 (10–34)	16 (11–23)	101 (62–171)	86 (60–162)	63 (47–127)	81 (60–150)	670 (503–895)	1,246 (862–1,687)	1,814 (1,261–2,491)	3,096 (2,172–4,166)	5,831 (4,205–7,974)	10,664 (7,765–14,481)	17,029 (12,203–22,750)	25,686 (18,902–33,834)	35,087 (26,306–45,249)	44,184 (34,576–54,246)	47,095 (37,170–56,753)	43,782 (35,119–51,859)	39,872 (33,131–46,250)	31,714 (26,578–36,465)	18,977 (15,694–21,870)	7,226 (5,832–8,373)	--
Rheumatic heart disease	--	--	--	3 (2–5)	4 (3–8)	9 (6–15)	16 (11–23)	23 (16–33)	25 (17–36)	30 (21–41)	31 (22–44)	32 (23–45)	35 (25–49)	31 (22–42)	45 (32–61)	60 (44–78)	48 (36–63)	49 (37–64)	44 (34–57)	46 (36–58)	21 (16–27)	9 (7–12)	--
Ischaemic heart disease	--	--	--	--	--	--	262 (192–354)	563 (394–771)	992 (698–1,352)	1,729 (1,259–2,347)	3,152 (2,353–4,257)	5,766 (4,263–7,691)	8,615 (6,210–11,527)	12,225 (9,026–16,215)	15,919 (11,970–20,496)	18,142 (14,009–22,614)	18,293 (14,202–22,379)	16,204 (12,820–19,644)	15,013 (12,310–17,689)	11,373 (9,391–13,327)	7,251 (5,927–8,562)	2,908 (2,344–3,447)	--
Stroke	11 (7–19)	7 (5–11)	46 (25–76)	51 (35–77)	36 (26–64)	49 (36–88)	285 (210–382)	483 (330–676)	571 (378–812)	1,011 (700–1,404)	2,056 (1,463–2,876)	3,861 (2,718–5,349)	6,795 (4,890–9,252)	10,972 (7,985–14,624)	15,743 (11,457–20,585)	21,529 (16,497–26,872)	24,035 (18,522–29,288)	22,684 (17,924–26,924)	20,320 (16,548–23,921)	16,466 (13,577–19,209)	9,401 (7,802–11,020)	3,305 (2,643–3,868)	--
Ischaemic stroke	3 (2–5)	3 (1–4)	3 (1–6)	7 (4–12)	2 (1–3)	2 (1–3)	37 (21–59)	50 (23–83)	63 (30–103)	111 (52–181)	204 (101–341)	370 (187–608)	887 (455–1,444)	1,791 (961–2,905)	2,806 (1,527–4,547)	7,017 (4,195–10,238)	8,286 (5,129–11,762)	11,096 (7,266–15,038)	10,534 (7,163–14,018)	11,285 (8,716–13,961)	6,429 (4,936–7,954)	2,303 (1,747–2,833)	--
Intracerebral hemorrhage	5 (3–9)	3 (2–4)	34 (18–55)	30 (20–45)	25 (18–43)	33 (23–60)	198 (142–271)	329 (218–475)	394 (254–571)	743 (495–1,047)	1,550 (1,094–2,205)	2,992 (2,063–4,155)	5,308 (3,768–7,209)	8,325 (5,880–11,116)	11,775 (8,141–15,555)	13,741 (9,755–18,058)	14,963 (10,771–19,405)	11,091 (7,336–15,243)	9,374 (6,462–12,857)	4,875 (3,210–6,895)	2,807 (1,907–3,962)	938 (603–1,327)	--
Subarachnoid hemorrhage	2 (1–6)	2 (1–3)	9 (4–17)	14 (7–26)	9 (5–16)	13 (8–23)	50 (29–77)	104 (55–174)	114 (62–192)	157 (84–259)	301 (163–505)	499 (262–862)	601 (300–1,094)	856 (441–1,607)	1,162 (567–2,217)	771 (368–1,566)	786 (367–1,648)	496 (217–1,015)	412 (180–826)	306 (131–659)	164 (68–348)	64 (27–146)	--
Hypertensive heart disease	--	--	--	--	--	--	11 (6–18)	30 (13–44)	48 (17–72)	118 (42–172)	246 (85–365)	539 (201–808)	976 (378–1,467)	1,682 (682–2,540)	2,379 (992–3,505)	3,255 (1,393–4,729)	3,415 (1,581–4,915)	3,501 (1,674–4,940)	3,158 (1,533–4,330)	2,476 (1,284–3,222)	1,354 (708–1,747)	536 (310–688)	--
Non-rheumatic valvular heart disease	--	--	--	--	--	--	3 (2–6)	5 (3–8)	5 (3–8)	7 (5–11)	14 (9–25)	17 (11–30)	24 (15–42)	33 (19–59)	44 (26–78)	56 (33–94)	68 (41–112)	61 (35–106)	53 (33–85)	45 (29–77)	27 (18–45)	10 (6–16)	--
Non-rheumatic calcific aortic valve disease	--	--	--	--	--	--	1 (1–3)	2 (1–4)	3 (2–5)	4 (2–6)	9 (5–17)	13 (8–23)	14 (8–26)	23 (13–41)	28 (14–49)	39 (22–67)	47 (26–80)	42 (23–76)	33 (19–57)	27 (16–49)	16 (10–29)	6 (4–10)	--
Non-rheumatic degenerative mitral valve disease	--	--	--	--	--	--	1 (1–3)	2 (1–4)	1 (1–2)	2 (1–5)	2 (1–6)	3 (1–6)	7 (4–16)	8 (4–15)	14 (7–27)	12 (6–23)	17 (9–33)	16 (8–29)	16 (9–28)	15 (8–27)	9 (5–15)	3 (2–5)	--
Other non-rheumatic valve diseases	--	--	--	--	--	--	1 (0–1)	1 (0–2)	0 (0–1)	1 (0–2)	3 (1–5)	2 (0–3)	2 (0–5)	2 (0–5)	2 (0–4)	5 (1–11)	4 (0–9)	3 (0–7)	4 (0–10)	3 (0–7)	2 (0–5)	1 (0–1)	--

Cardiomyopathy and myocarditis	3 (1–12)	5 (3–10)	29 (13–81)	12 (6–38)	6 (3–18)	9 (5–20)	33 (22–53)	50 (34–77)	62 (40–96)	82 (50–134)	150 (86–270)	200 (106–390)	269 (139–555)	341 (176–723)	429 (217–889)	479 (254–937)	465 (269–822)	482 (290–775)	463 (291–655)	440 (238–642)	284 (134–422)	133 (56–198)	--
Myocarditis	1 (0–2)	2 (1–4)	11 (4–29)	6 (3–16)	3 (2–9)	3 (2–8)	8 (4–15)	10 (5–17)	10 (5–17)	10 (5–19)	16 (8–36)	19 (9–48)	24 (11–64)	25 (10–73)	31 (13–90)	38 (16–106)	27 (13–61)	29 (14–53)	35 (16–56)	40 (13–72)	29 (6–58)	17 (3–34)	--
Alcoholic cardiomyopathy	--	--	--	--	--	--	1 (1–2)	2 (1–4)	4 (2–7)	9 (4–17)	20 (9–41)	27 (12–60)	33 (14–75)	33 (14–81)	40 (16–95)	25 (10–57)	17 (8–36)	16 (6–34)	10 (4–21)	5 (3–10)	4 (2–9)	4 (2–8)	--
Other cardiomyopathy	2 (1–9)	3 (2–7)	18 (8–52)	7 (3–22)	3 (2–9)	5 (3–14)	24 (16–39)	38 (25–60)	48 (30–76)	63 (38–104)	114 (63–207)	153 (79–297)	212 (106–427)	283 (145–566)	358 (180–727)	417 (221–796)	420 (238–747)	438 (262–693)	419 (266–596)	394 (216–576)	250 (123–371)	112 (50–170)	--
Pulmonary Arterial Hypertension	1 (0–5)	1 (0–3)	5 (2–10)	6 (3–12)	6 (2–11)	6 (2–9)	13 (4–21)	13 (4–21)	13 (4–21)	15 (5–24)	18 (6–29)	14 (6–22)	15 (7–23)	13 (7–19)	11 (7–17)	13 (8–19)	7 (4–16)	6 (3–16)	4 (2–16)	2 (1–15)	2 (0–10)	1 (0–4)	--
Atrial fibrillation and flutter	--	--	--	--	--	--	--	--	--	1 (0–2)	7 (3–10)	13 (7–19)	25 (16–36)	45 (28–66)	67 (43–96)	114 (73–163)	155 (103–214)	197 (130–268)	259 (179–344)	394 (277–521)	351 (247–463)	211 (147–281)	--
Aortic aneurysm	--	--	--	--	--	--	8 (4–13)	8 (4–13)	11 (6–17)	13 (7–21)	27 (14–43)	37 (20–59)	57 (31–93)	82 (46–132)	148 (85–234)	209 (125–318)	265 (161–391)	274 (167–404)	268 (167–392)	215 (137–313)	127 (79–188)	45 (28–68)	--
Peripheral vascular disease	--	--	--	--	--	--	--	--	--	--	--	4 (2–6)	4 (2–7)	13 (7–22)	25 (13–41)	32 (17–54)	34 (19–54)	42 (22–72)	33 (18–57)	30 (19–47)	18 (12–28)	9 (6–14)	--
Endocarditis	0 (0–0)	0 (0–1)	7 (4–13)	2 (1–5)	2 (1–3)	2 (1–4)	19 (11–30)	31 (19–46)	48 (29–80)	47 (27–89)	66 (35–136)	93 (47–201)	87 (44–188)	83 (42–176)	85 (46–165)	74 (42–134)	82 (47–150)	65 (37–119)	57 (35–103)	39 (24–72)	26 (16–46)	10 (6–19)	--
Other cardiovascular and circulatory diseases	1 (0–2)	2 (1–4)	15 (8–27)	11 (7–24)	10 (7–20)	7 (5–12)	19 (13–27)	41 (28–58)	39 (27–57)	44 (31–66)	65 (46–101)	88 (58–147)	125 (81–214)	165 (106–282)	192 (124–331)	221 (148–369)	228 (156–372)	217 (153–341)	200 (147–311)	188 (142–293)	115 (87–171)	50 (37–73)	--
Chronic respiratory diseases	0 (0–0)	0 (0–0)	0 (0–0)	171 (115–362)	70 (52–134)	109 (84–139)	292 (209–391)	442 (262–605)	393 (238–533)	452 (294–614)	617 (463–832)	913 (662–1,237)	1,597 (1,164–2,151)	2,792 (2,018–3,686)	4,374 (3,228–5,704)	7,350 (5,634–9,266)	9,497 (7,365–11,698)	10,577 (8,301–12,750)	9,529 (7,728–11,599)	8,137 (6,645–9,683)	5,119 (4,200–6,186)	1,877 (1,476–2,272)	--
Chronic obstructive pulmonary disease	--	--	0 (0–0)	8 (5–13)	7 (4–11)	10 (6–14)	72 (42–107)	155 (78–226)	131 (70–193)	182 (106–261)	275 (178–393)	446 (294–642)	940 (630–1,299)	1,838 (1,276–2,495)	2,919 (2,047–3,911)	5,498 (4,042–7,037)	7,367 (5,516–9,255)	8,245 (6,329–10,094)	7,389 (5,818–9,030)	6,601 (5,322–7,996)	4,127 (3,321–4,990)	1,505 (1,163–1,826)	--
Pneumoconiosis	--	--	--	--	--	--	0 (0–0)	0 (0–0)	0 (0–0)	0 (0–0)	0 (0–1)	1 (0–1)	1 (1–2)	3 (2–5)	4 (3–7)	4 (2–5)	6 (4–9)	5 (3–8)	4 (3–7)	3 (2–5)	3 (2–5)	1 (1–2)	--
Silicosis	--	--	--	--	--	--	0 (0–0)	0 (0–0)	0 (0–0)	0 (0–0)	0 (0–0)	0 (0–0)	0 (0–1)	1 (0–2)	1 (0–2)	1 (0–2)	1 (0–3)	1 (0–3)	1 (0–2)	0 (0–1)	1 (0–2)	0 (0–1)	--
Asbestosis	--	--	--	--	--	--	0 (0–0)	0 (0–0)	0 (0–0)	0 (0–0)	0 (0–0)	0 (0–0)	0 (0–0)	1 (0–2)	1 (0–2)	1 (0–2)	1 (0–2)	2 (1–3)	1 (0–1)	1 (0–1)	1 (0–1)	0 (0–1)	--
Coal workers pneumoconiosis	--	--	--	--	--	--	0 (0–0)	0 (0–0)	0 (0–0)	0 (0–0)	0 (0–0)	0 (0–0)	0 (0–0)	1 (0–1)	1 (0–2)	0 (0–1)	1 (0–1)	0 (0–1)	1 (0–1)	0 (0–1)	0 (0–1)	0 (0–0)	--
Other pneumoconiosis	--	--	--	--	--	--	0 (0–0)	0 (0–0)	0 (0–0)	0 (0–0)	0 (0–0)	0 (0–1)	1 (0–1)	1 (0–2)	2 (1–3)	2 (1–3)	2 (1–5)	2 (1–3)	2 (1–5)	2 (1–3)	2 (1–3)	1 (0–1)	--
Asthma	--	--	--	144 (98–281)	56 (41–101)	86 (65–111)	185 (126–254)	238 (135–344)	225 (133–311)	222 (143–316)	285 (198–398)	371 (258–535)	539 (369–770)	795 (536–1,131)	1,196 (809–1,698)	1,721 (1,181–2,440)	1,981 (1,397–2,763)	2,177 (1,525–3,181)	2,012 (1,428–2,874)	1,454 (1,040–2,137)	931 (670–1,352)	352 (244–506)	--
Interstitial lung disease and pulmonary sarcoidosis	--	--	--	1 (0–3)	0 (0–1)	1 (1–2)	4 (2–8)	6 (3–14)	6 (3–12)	7 (4–16)	6 (3–13)	29 (13–67)	39 (16–96)	70 (28–179)	104 (40–259)	110 (43–283)	122 (45–304)	138 (50–358)	116 (42–296)	66 (24–175)	55 (19–145)	13 (5–37)	--
Other chronic respiratory diseases	0 (0–0)	0 (0–0)	0 (0–0)	18 (8–63)	7 (4–19)	12 (7–25)	31 (16–64)	43 (23–86)	31 (17–59)	40 (22–81)	51 (28–106)	67 (38–137)	76 (45–145)	86 (51–161)	151 (90–268)	18 (11–30)	22 (13–37)	12 (8–20)	8 (5–12)	13 (8–24)	4 (3–8)	5 (3–10)	--
Digestive diseases	56 (38–83)	126 (86–183)	550 (345–848)	621 (434–846)	386 (306–483)	286 (223–358)	885 (664–1,176)	1,214 (906–1,643)	1,346 (1,059–1,823)	1,928 (1,516–2,540)	2,860 (2,193–3,912)	3,903 (2,957–5,279)	5,079 (3,748–6,815)	6,208 (4,578–8,197)	7,145 (5,348–9,152)	8,265 (6,331–10,323)	8,258 (6,444–10,160)	6,875 (5,471–8,401)	6,311 (5,136–7,548)	4,746 (3,873–5,665)	2,689 (2,193–3,169)	1,040 (825–1,226)	--
Cirrhosis and other chronic liver diseases	--	--	--	229 (151–332)	89 (63–158)	132 (98–174)	606 (427–823)	997 (719–1,366)	1,053 (798–1,435)	1,662 (1,291–2,210)	2,445 (1,876–3,362)	3,352 (2,539–4,584)	4,383 (3,226–5,979)	5,175 (3,814–6,886)	5,777 (4,313–7,478)	6,505 (4,949–8,184)	6,074 (4,663–7,614)	4,635 (3,610–5,801)	3,998 (3,132–4,924)	2,731 (2,087–3,377)	1,382 (1,040–1,688)	484 (346–605)	--
Cirrhosis and other chronic liver diseases due to hepatitis B	--	--	--	1 (1–2)	1 (1–3)	4 (2–6)	27 (13–46)	145 (77–232)	229 (140–352)	446 (310–629)	736 (491–1,084)	1,018 (693–1,463)	1,256 (842–1,836)	1,377 (894–2,019)	1,392 (917–2,015)	1,445 (948–2,119)	1,275 (859–1,851)	974 (647–1,424)	889 (598–1,208)	619 (418–849)	312 (218–415)	109 (72–154)	--
Cirrhosis and other chronic liver diseases due to hepatitis C	--	--	--	3 (1–5)	3 (2–6)	8 (4–13)	57 (28–98)	258 (147–406)	396 (255–572)	676 (481–935)	990 (687–1,411)	1,340 (956–1,905)	1,718 (1,178–2,399)	1,979 (1,334–2,781)	2,143 (1,455–2,976)	2,370 (1,647–3,259)	2,233 (1,616–3,010)	1,698 (1,239–2,253)	1,442 (1,038–1,879)	982 (705–1,279)	500 (362–649)	176 (118–244)	--
Cirrhosis and other chronic liver diseases due to alcohol use	--	--	--	--	--	--	2 (1–4)	32 (17–57)	58 (32–97)	158 (107–234)	324 (204–503)	516 (339–782)	765 (480–1,177)	1,014 (639–1,548)	1,273 (797–1,860)	1,474 (958–2,168)	1,277 (874–1,782)	864 (567–1,237)	625 (431–871)	366 (241–514)	169 (116–229)	54 (33–79)	--
Cirrhosis and other chronic liver diseases due to NAFLD	--	--	--	--	--	--	18 (7–37)	54 (25–100)	73 (33–141)	113 (63–197)	151 (73–285)	215 (102–385)	318 (140–586)	401 (178–724)	454 (212–802)	550 (260–1,006)	585 (335–931)	496 (290–760)	467 (251–760)	344 (185–550)	183 (110–289)	67 (36–121)	--
Cirrhosis and other chronic liver diseases due to other causes	--	--	--	225 (148–325)	84 (60–151)	120 (88–157)	502 (348–686)	508 (307–769)	296 (166–486)	270 (149–482)	243 (121–422)	261 (128–459)	326 (164–592)	403 (192–713)	516 (255–905)	667 (344–1,145)	705 (365–1,249)	575 (303–1,028)	575 (305–964)	419 (224–696)	218 (123–347)	78 (40–138)	--
Upper digestive system diseases	--	--	--	8 (5–12)	5 (4–7)	3 (2–4)	9 (6–17)	11 (7–21)	7 (5–11)	8 (6–12)	9 (7–13)	13 (9–18)	22 (13–30)	32 (19–45)	47 (29–65)	75 (48–99)	98 (66–131)	113 (79–149)	136 (101–172)	130 (93–167)	98 (70–126)	44 (29–58)	--
Peptic ulcer disease	--	--	--	5 (2–9)	4 (3–6)	3 (2–4)	8 (5–17)	10 (6–21)	7 (5–11)	8 (6–11)	9 (6–12)	12 (8–17)	21 (13–29)	31 (18–43)	45 (29–61)	72 (47–96)	95 (65–128)	108 (77–142)	131 (98–165)	124 (91–160)	95 (68–123)	42 (28–55)	--
Gastritis and duodenitis	--	--	--	3 (0–5)	1 (0–1)	0 (0–0)	1 (0–2)	1 (0–2)	1 (0–1)	0 (0–1)	1 (0–1)	1 (0–2)	1 (0–3)	1 (0–4)	2 (1–5)	3 (1–7)	3 (1–7)	5 (1–12)	5 (1–14)	6 (1–14)	4 (1–8)	2 (1–5)	--
Appendicitis	--	--	--	27 (14–48)	47 (25–70)	36 (21–53)	47 (24–87)	34 (17–67)	26 (14–42)	13 (6–20)	15 (6–24)	19 (7–30)	30 (12–48)	28 (11–45)	46 (21–72)	53 (26–83)	46 (24–69)	38 (21–58)	37 (21–57)	36 (21–53)	23 (13–33)	11 (6–16)	--
Paralytic ileus and intestinal obstruction	33 (11–56)	90 (38–143)	422 (241–674)	213 (115–329)	173 (99–236)	56 (37–78)	147 (77–223)	66 (29–106)	97 (43–147)	72 (28–108)	142 (56–223)	190 (80–293)	212 (102–309)	368 (175–525)	396 (218–575)	570 (368–892)	612 (411–1,117)	715 (483–1,262)	736 (498–1,372)	599 (365–1,277)	428 (268–895)	186 (119–379)	--
Inguinal, femoral, and abdominal hernia	23 (13–43)	36 (18–78)	127 (49–273)	94 (45–145)	42 (9–62)	37 (14–51)	26 (11–44)	21 (14–39)	42 (28–63)	23 (14–33)	41 (22–59)	54 (27–79)	96 (40–137)	195 (95–274)	313 (168–443)	371 (216–507)	590 (359–790)	516 (349–704)	570 (421–745)	510 (367–674)	292 (210–463)	112 (77–177)	--
Inflammatory bowel disease	--	--	--	8 (3–13)	4 (2–7)	3 (2–5)	5 (2–7)	7 (3–12)	12 (7–22)	6 (4–10)	7 (4–12)	16 (10–27)	18 (10–30)	21 (11–36)	33 (18–55)	42 (23–69)	65 (39–102)	51 (31–81)	57 (35–85)	58 (37–89)	23 (13–34)	11 (6–17)	--
Vascular intestinal disorders	--	--	--	1 (1–2)	0 (0–0)	0 (0–1)	1 (1–2)	2 (1–3)	2 (1–3)	3 (2–4)	4 (3–6)	7 (5–10)	15 (8–22)	21 (11–31)	34 (19–49)	49 (28–71)	88 (55–127)	99 (65–147)	103 (76–149)	111 (80–150)	61 (42–79)	29 (19–38)	--
Gallbladder and biliary diseases	--	--	--	6 (1–10)	4 (1–6)	3 (1–5)	5 (1–9)	10 (2–17)	17 (5–28)	22 (8–36)	29 (10–45)	48 (15–77)	50 (13–80)	83 (24–132)	140 (50–218)	226 (96–335)	228 (104–333)	276 (150–384)	278 (183–369)	228 (146–307)	161 (100–210)	69 (42–92)	--
Pancreatitis	--	--	--	1 (1–3)	5 (3–9)	3 (2–4)	26 (14–62)	53 (28–145)	73 (42–168)	101 (59–206)	135 (85–276)	160 (99–313)	187 (117–369)	202 (125–407)	229 (140–459)	233 (150–476)	254 (167–475)	230 (156–369)	183 (127–280)	108 (76–164)	60 (41–88)	22 (14–32)	--
Other digestive diseases	--	--	--	35 (20–55)	17 (9–25)	13 (9–19)	15 (10–23)	14 (9–24)	17 (12–26)	19 (13–28)	32 (21–51)	44 (26–67)	66 (33–98)	83 (41–127)	129 (66–195)	141 (75–215)	204 (114–303)	202 (114–296)	214 (138–311)	237 (149–342)	161 (102–225)	70 (44–97)	--
Alzheimer's disease and other dementias	--	--	--	--	--	--	--	--	--	--	--	9 (1–29)	56 (7–178)	134 (21–421)	286 (54–839)	554 (114–1,622)	888 (192–2,596)	1,170 (257–3,388)	1,471 (308–4,372)	2,450 (557–7,148)	2,436 (545–6,867)	1,335 (309–3,680)	--
Parkinson's disease	--	--	--	--	--	--	--	0 (0–0)	0 (0–0)	2 (1–3)	1 (0–1)	25 (15–35)	43 (28–60)	67 (44–92)	96 (66–133)	197 (137–268)	346 (246–459)	602 (433–789)	851 (629–1,107)	912 (685–1,157)	516 (391–653)	126 (88–165)	--
Idiopathic epilepsy	--	--	14 (8–22)	16 (11–30)	12 (9–23)	14 (11–25)	24 (18–33)	35 (26–47)	33 (23–45)	31 (20–42)	44 (29–60)	49 (34–67)	49 (34–65)	38 (27–52)	34 (24–45)	28 (21–36)	20 (15–25)	18 (14–23)	12 (10–15)	10 (8–13)	5 (4–6)	2 (1–2)	--
Multiple sclerosis	--	--	--	--	0 (0–0)	0 (0–0)	0 (0–0)	1 (1–2)	2 (1–3)	4 (2–5)	7 (4–12)	14 (8–23)	18 (10–29)	19 (10–31)	21 (12–35)	18 (11–30)	12 (7–19)	8 (5–11)	5 (3–8)	4 (2–5)	2 (1–3)	0 (0–1)	--
Motor neuron disease	0 (0–0)	0 (0–0)	2 (1–4)	0 (0–1)	0 (0–0)	0 (0–1)	1 (0–2)	0 (0–1)	1 (0–1)	1 (1–2)	4 (2–6)	5 (3–8)	12 (7–19)	14 (8–24)	25 (15–40)	23 (14–38)	22 (13–34)	11 (7–18)	4 (2–7)	1 (1–2)	0 (0–0)	0 (0–0)	--
Other neurological disorders	--	--	17 (10–29)	12 (7–19)	9 (6–14)	24 (16–38)	52 (35–83)	38 (26–59)	18 (12–27)	11 (7–17)	15 (10–23)	20 (14–32)	30 (20–47)	35 (23–54)	36 (24–56)	30 (21–47)	31 (20–46)	24 (16–37)	25 (17–39)	10 (7–16)	10 (7–15)	3 (2–4)	--
Eating disorders	--	--	--	--	0 (0–0)	0 (0–0)	0 (0–0)	0 (0–0)	0 (0–0)	0 (0–0)	0 (0–0)	0 (0–0)	0 (0–0)	--	--	--	--	--	--	--	--	--	--
Anorexia nervosa	--	--	--	--	0 (0–0)	0 (0–0)	0 (0–0)	0 (0–0)	0 (0–0)	0 (0–0)	0 (0–0)	0 (0–0)	0 (0–0)	--	--	--	--	--	--	--	--	--	--
Bulimia nervosa	--	--	--	--	0 (0–0)	0 (0–0)	0 (0–0)	0 (0–0)	0 (0–0)	0 (0–0)	0 (0–0)	0 (0–0)	0 (0–0)	--	--	--	--	--	--	--	--	--	--
Alcohol use disorders	--	--	--	--	--	0 (0–0)	26 (2–37)	30 (6–43)	33 (14–46)	37 (28–51)	56 (40–100)	71 (48–155)	84 (55–201)	69 (45–171)	61 (40–160)	54 (36–143)	37 (25–100)	23 (16–52)	14 (11–24)	10 (8–13)	9 (4–12)	3 (1–4)	--
Drug use disorders	0 (0–0)	0 (0–0)	0 (0–0)	0 (0–0)	0 (0–0)	0 (0–0)	23 (16–32)	51 (37–70)	36 (27–51)	42 (32–59)	48 (36–67)	45 (33–63)	31 (22–43)	27 (20–38)	24 (17–34)	18 (13–23)	24 (18–32)	14 (11–17)	15 (11–19)	9 (7–12)	3 (3–4)	1 (1–2)	--
Opioid use disorders	0 (0–0)	0 (0–0)	0 (0–0)	0 (0–0)	0 (0–0)	0 (0–0)	16 (11–23)	34 (24–50)	23 (17–34)	28 (21–41)	32 (24–44)	32 (23–44)	20 (14–28)	18 (13–24)	16 (11–22)	12 (9–16)	16 (11–21)	7 (5–9)	8 (6–10)	5 (4–7)	1 (1–2)	1 (0–1)	--
Cocaine use disorders	--	--	--	--	--	0 (0–0)	1 (1–2)	3 (2–5)	2 (1–4)	4 (2–6)	4 (3–6)	4 (2–6)	3 (2–4)	4 (2–6)	4 (2–6)	3 (2–4)	3 (2–5)	2 (1–3)	1 (1–2)	0 (0–0)	0 (0–0)	0 (0–0)	--
Amphetamine use disorders	--	--	--	--	--	0 (0–0)	1 (1–2)	4 (2–7)	3 (2–5)	4 (2–6)	4 (2–6)	3 (2–5)	2 (1–3)	2 (1–3)	1 (1–2)	1 (1–1)	1 (1–2)	1 (0–1)	0 (0–1)	0 (0–0)	1 (0–0)	0 (0–0)	--
Other drug use disorders	--	--	--	--	--	0 (0–0)	4 (2–6)	10 (6–15)	8 (5–11)	6 (4–9)	8 (6–11)	7 (5–9)	5 (4–8)	4 (3–5)	3 (2–4)	2 (2–3)	4 (3–5)	5 (3–6)	6 (4–8)	4 (3–5)	2 (1–2)	1 (1–1)	--
Diabetes mellitus	0 (0–0)	1 (0–1)	3 (2–5)	4 (3–6)	5 (3–7)	16 (4–24)	127 (94–173)	194 (142–267)	280 (220–381)	514 (398–705)	1,017 (785–1,389)	2,104 (1,577–2,895)	3,494 (2,608–4,790)	5,507 (4,090–7,320)	7,891 (5,948–10,254)	8,406 (6,466–10,574)	7,324 (5,757–9,010)	5,418 (4,280–6,624)	4,433 (3,507–5,309)	2,706 (2,201–3,273)	1,361 (1,112–1,648)	481 (375–583)	--
Diabetes mellitus type 1	0 (0–0)	1 (0–1)	3 (2–5)	4 (3–6)	5 (3–7)	16 (4–24)	62 (43–86)	95 (67–133)	133 (97–190)	131 (88–210)	247 (158–438)	457 (277–907)	324 (116–504)	307 (160–719)	438 (224–1,028)	350 (184–813)	324 (176–754)	208 (115–484)	140 (80–325)	65 (37–138)	36 (20–78)	14 (8–29)	--
Diabetes mellitus type 2	--	--	--	--	--	--	64 (47–92)	99 (69–138)	147 (104–203)	383 (287–518)	770 (555–1,068)	1,648 (1,130–2,285)	3,272 (2,403–4,463)	5,200 (3,828–6,913)	7,453 (5,545–9,685)	8,057 (6,214–10,171)	7,000 (5,446–8,608)	5,210 (4,130–6,351)	4,293 (3,409–5,148)	2,641 (2,155–3,212)	1,326 (1,083–1,609)	467 (366–564)	--
Chronic kidney disease	--	--	58 (39–88)	100 (73–133)	105 (82–131)	117 (91–147)	341 (258–451)	583 (439–792)	487 (386–656)	639 (496–864)	940 (724–1,295)	1,271 (966–1,767)	1,650 (1,228–2,214)	2,152 (1,576–2,879)	2,633 (1,948–3,387)	3,000 (2,320–3,794)	3,104 (2,419–3,831)	2,514 (2,010–3,052)	2,003 (1,596–2,398)	1,404 (1,126–1,683)	837 (668–1,014)	342 (268–410)	--
Chronic kidney disease due to diabetes mellitus type 1	--	--	1 (0–3)	3 (1–6)	6 (2–14)	16 (6–31)	62 (24–120)	156 (72–272)	177 (97–273)	268 (158–400)	383 (230–587)	454 (255–717)	461 (219–753)	413 (181–753)	330 (117–669)	270 (101–575)	207 (85–433)	116 (51–230)	54 (22–115)	16 (6–37)	3 (1–8)	0 (0–1)	--
Chronic kidney disease due to diabetes mellitus type 2	--	--	--	--	--	--	34 (11–73)	72 (27–151)	68 (27–134)	99 (37–190)	165 (62–324)	273 (110–506)	457 (223–775)	739 (409–1,135)	1,010 (590–1,475)	1,171 (680–1,692)	1,209 (782–1,675)	976 (655–1,317)	765 (519–1,022)	523 (355–697)	301 (203–403)	117 (75–159)	--
Chronic kidney disease due to hypertension	--	--	--	--	--	--	50 (24–93)	107 (53–194)	94 (48–161)	126 (68–219)	204 (115–337)	317 (196–506)	476 (298–715)	706 (440–1,043)	958 (610–1,419)	1,187 (825–1,706)	1,276 (901–1,722)	1,049 (758–1,410)	856 (629–1,117)	612 (453–804)	375 (283–488)	158 (119–203)	--
Chronic kidney disease due to glomerulonephritis	--	--	10 (4–21)	19 (8–35)	25 (9–46)	35 (15–64)	84 (41–148)	121 (57–230)	76 (36–136)	74 (32–145)	97 (42–200)	116 (45–229)	122 (59–237)	138 (62–256)	154 (70–298)	155 (72–280)	148 (66–269)	119 (53–213)	99 (48–186)	71 (32–132)	40 (18–73)	16 (8–29)	--
Chronic kidney disease due to other and unspecified causes	--	--	47 (29–73)	78 (53–110)	74 (50–102)	67 (39–97)	111 (52–188)	128 (58–233)	72 (35–125)	71 (32–138)	91 (38–188)	111 (46–222)	134 (55–257)	156 (70–295)	181 (77–352)	216 (96–389)	264 (116–480)	253 (120–434)	230 (119–393)	182 (94–318)	117 (60–192)	50 (27–82)	--
Acute glomerulonephritis	--	--	7 (0–15)	9 (0–16)	27 (0–49)	10 (0–20)	3 (0–5)	3 (0–5)	6 (0–11)	4 (0–7)	4 (0–8)	9 (0–17)	7 (0–13)	17 (0–33)	10 (0–20)	14 (0–26)	19 (1–37)	13 (0–25)	17 (1–34)	13 (1–24)	10 (1–17)	3 (0–6)	--
Skin and subcutaneous diseases	--	--	14 (1–24)	8 (2–13)	6 (1–9)	10 (3–14)	19 (7–28)	43 (8–65)	31 (8–45)	35 (12–51)	63 (18–93)	63 (34–92)	92 (44–133)	86 (52–121)	104 (69–142)	125 (82–172)	123 (82–161)	142 (95–186)	132 (92–185)	153 (106–197)	92 (59–120)	45 (27–59)	--
Bacterial skin diseases	--	--	14 (1–24)	7 (1–11)	5 (1–7)	7 (2–10)	16 (6–24)	41 (8–63)	30 (8–44)	33 (11–47)	61 (17–91)	58 (29–85)	87 (39–125)	81 (45–114)	95 (59–129)	105 (65–142)	98 (59–130)	116 (73–151)	112 (69–150)	118 (72–154)	63 (38–85)	32 (16–42)	--
Cellulitis	--	--	2 (0–4)	0 (0–1)	1 (0–2)	4 (1–7)	2 (0–3)	13 (2–24)	2 (0–4)	4 (1–7)	11 (2–19)	16 (5–28)	21 (5–38)	24 (7–44)	24 (8–40)	31 (12–50)	43 (19–64)	41 (14–63)	28 (9–47)	28 (8–46)	16 (4–25)	9 (2–14)	--
Pyoderma	--	--	12 (1–21)	6 (1–10)	4 (1–6)	3 (1–6)	15 (5–22)	28 (5–46)	28 (7–42)	29 (9–42)	50 (15–74)	42 (22–63)	66 (31–98)	56 (32–84)	71 (43–100)	74 (44–107)	55 (29–87)	76 (46–112)	83 (51–124)	90 (57–128)	47 (29–71)	23 (13–33)	--
Decubitus ulcer	--	--	--	1 (0–1)	0 (0–1)	1 (0–2)	1 (0–3)	1 (0–2)	0 (0–1)	2 (1–4)	1 (1–3)	1 (1–4)	2 (1–6)	3 (1–10)	5 (2–16)	16 (10–28)	21 (13–33)	21 (13–36)	15 (9–44)	30 (19–51)	27 (17–39)	13 (8–19)	--
Other skin and subcutaneous diseases	--	--	0 (0–0)	1 (0–2)	1 (0–1)	2 (0–3)	2 (1–3)	1 (0–1)	0 (0–0)	1 (0–1)	1 (0–1)	4 (2–7)	4 (1–7)	3 (1–5)	5 (2–8)	5 (2–8)	5 (3–9)	5 (2–9)	6 (3–9)	5 (3–8)	2 (1–3)	1 (0–1)	--
Musculoskeletal disorders	--	--	--	--	5 (3–6)	12 (8–16)	14 (10–20)	18 (12–25)	12 (9–17)	12 (9–17)	15 (11–21)	23 (16–32)	29 (20–41)	32 (22–45)	36 (25–49)	55 (39–75)	48 (34–65)	41 (30–55)	35 (26–46)	21 (15–27)	16 (12–21)	6 (4–8)	--
Rheumatoid arthritis	--	--	--	--	0 (0–1)	2 (1–3)	0 (0–1)	0 (0–0)	0 (0–1)	2 (1–3)	1 (1–1)	4 (3–7)	4 (2–7)	6 (4–10)	11 (6–17)	17 (11–26)	18 (12–27)	15 (10–23)	11 (8–17)	5 (4–8)	8 (5–11)	2 (1–2)	--
Other musculoskeletal disorders	--	--	--	--	4 (2–5)	10 (6–13)	14 (10–19)	17 (12–24)	12 (8–16)	11 (8–15)	14 (10–19)	18 (13–27)	25 (17–35)	26 (18–37)	25 (17–35)	38 (27–53)	30 (21–41)	25 (18–35)	23 (17–31)	16 (11–21)	8 (6–11)	4 (3–6)	--
Congenital anomalies	3,207 (2,192–4,629)	943 (676–1,366)	2,387 (1,463–3,939)	822 (543–1,471)	350 (258–535)	255 (185–364)	237 (166–322)	142 (100–204)	88 (64–126)	55 (40–79)	47 (33–68)	55 (37–82)	38 (25–55)	28 (18–43)	30 (19–43)	29 (19–41)	23 (15–32)	--	--	--	--	--	--
Neural tube defects	580 (291–880)	63 (33–99)	121 (49–220)	65 (25–129)	8 (4–13)	6 (3–9)	2 (1–3)	1 (1–2)	1 (0–1)	1 (1–1)	1 (1–1)	1 (1–2)	1 (1–2)	1 (1–1)	1 (1–1)	1 (1–1)	1 (0–1)	--	--	--	--	--	--
Congenital heart anomalies	1,123 (637–1,670)	522 (354–737)	1,382 (832–2,192)	447 (284–673)	214 (141–299)	188 (128–256)	174 (113–245)	103 (67–146)	74 (54–104)	42 (30–59)	34 (23–50)	43 (28–64)	21 (12–32)	17 (10–27)	18 (11–27)	19 (11–27)	12 (8–17)	--	--	--	--	--	--
Orofacial clefts	15 (5–35)	7 (2–17)	16 (4–45)	2 (1–4)	--	--	--	--	--	--	--	--	--	--	--	--	--	--	--	--	--	--	--
Down's syndrome	26 (16–44)	13 (8–21)	69 (37–127)	29 (17–56)	15 (10–23)	7 (4–11)	9 (5–14)	9 (6–15)	5 (3–10)	5 (3–7)	5 (3–8)	5 (3–9)	7 (5–11)	5 (3–9)	3 (2–4)	1 (1–2)	1 (0–1)	--	--	--	--	--	--
Other chromosomal abnormalities	61 (29–139)	21 (11–46)	29 (13–86)	7 (3–20)	2 (1–5)	1 (1–3)	3 (1–5)	2 (1–4)	1 (1–3)	0 (0–1)	1 (1–2)	0 (0–1)	1 (0–1)	0 (0–0)	0 (0–0)	0 (0–0)	0 (0–0)	--	--	--	--	--	--
Congenital musculoskeletal and limb anomalies	46 (26–77)	7 (4–13)	17 (8–35)	3 (2–7)	4 (2–8)	2 (1–5)	6 (3–11)	1 (0–1)	0 (0–1)	0 (0–1)	0 (0–1)	1 (0–1)	0 (0–1)	1 (0–1)	0 (0–1)	0 (0–0)	1 (0–1)	--	--	--	--	--	--
Urogenital congenital anomalies	54 (30–94)	7 (4–12)	10 (5–19)	3 (2–6)	1 (1–2)	1 (1–2)	1 (1–2)	1 (1–2)	1 (1–1)	1 (1–1)	1 (1–1)	1 (1–2)	1 (1–2)	1 (1–2)	1 (1–2)	1 (1–2)	1 (1–2)	--	--	--	--	--	--
Digestive congenital anomalies	278 (99–534)	116 (59–191)	292 (127–574)	73 (26–149)	13 (4–23)	9 (3–16)	2 (1–4)	1 (0–2)	1 (0–2)	0 (0–1)	1 (0–1)	1 (0–1)	1 (0–2)	1 (0–1)	2 (0–3)	1 (0–2)	3 (1–5)	--	--	--	--	--	--
Other congenital anomalies	1,024 (576–2,116)	187 (99–407)	452 (195–1,179)	192 (93–534)	94 (49–247)	42 (20–110)	40 (18–110)	25 (12–59)	4 (2–9)	5 (2–11)	3 (2–7)	3 (2–6)	5 (3–10)	3 (1–4)	5 (2–7)	5 (3–8)	5 (3–7)	--	--	--	--	--	--
Urinary diseases and male infertility	2 (0–4)	7 (1–12)	15 (9–29)	15 (10–27)	21 (16–29)	30 (22–38)	41 (26–49)	58 (38–81)	41 (29–56)	69 (51–94)	103 (74–142)	216 (123–301)	375 (179–519)	479 (249–660)	643 (321–875)	919 (431–1,215)	935 (504–1,219)	817 (506–1,038)	660 (454–846)	597 (452–746)	298 (232–399)	129 (97–186)	--
Urinary tract infections and interstitial nephritis	2 (0–2)	6 (1–10)	13 (7–22)	12 (8–20)	15 (10–21)	21 (12–29)	29 (19–40)	36 (17–55)	34 (25–48)	54 (37–76)	71 (47–101)	161 (94–229)	248 (117–357)	318 (175–446)	415 (204–576)	577 (285–784)	566 (307–775)	526 (321–692)	465 (323–626)	348 (239–461)	195 (140–270)	75 (50–105)	--
Urolithiasis	--	--	--	0 (0–0)	1 (0–2)	1 (0–3)	1 (0–3)	1 (0–3)	4 (0–8)	2 (0–4)	10 (0–19)	24 (1–45)	41 (2–77)	62 (3–113)	58 (3–107)	128 (10–224)	135 (12–237)	79 (8–137)	81 (11–140)	56 (8–94)	19 (3–32)	7 (1–13)	--
Other urinary diseases	1 (0–1)	1 (0–3)	3 (1–8)	3 (1–8)	5 (2–11)	7 (4–16)	5 (3–12)	21 (12–38)	3 (2–6)	12 (7–24)	21 (12–47)	31 (17–71)	86 (47–177)	84 (55–200)	170 (96–313)	214 (115–392)	234 (144–420)	212 (129–371)	113 (68–199)	192 (127–325)	84 (54–154)	47 (30–89)	--
Gynecological diseases	--	--	--	--	--	--	--	--	--	--	--	--	--	--	--	--	--	--	--	--	--	--	--
Uterine fibroids	--	--	--	--	--	--	--	--	--	--	--	--	--	--	--	--	--	--	--	--	--	--	--
Endometriosis	--	--	--	--	--	--	--	--	--	--	--	--	--	--	--	--	--	--	--	--	--	--	--
Genital prolapse	--	--	--	--	--	--	--	--	--	--	--	--	--	--	--	--	--	--	--	--	--	--	--
Other gynecological diseases	--	--	--	--	--	--	--	--	--	--	--	--	--	--	--	--	--	--	--	--	--	--	--
Hemoglobinopathies and hemolytic anaemias	38 (29–53)	15 (11–23)	81 (53–120)	94 (66–128)	53 (42–66)	37 (29–48)	57 (42–77)	55 (45–76)	31 (24–43)	28 (21–38)	31 (22–43)	34 (25–49)	39 (29–55)	55 (38–76)	77 (55–103)	100 (75–130)	108 (82–139)	95 (72–122)	84 (67–106)	60 (47–76)	49 (39–62)	18 (14–22)	--
Thalassemias	37 (27–50)	15 (10–23)	79 (51–116)	91 (64–123)	49 (39–61)	29 (22–39)	34 (23–49)	22 (14–35)	9 (6–15)	7 (4–12)	7 (4–13)	8 (4–13)	8 (4–13)	7 (3–12)	4 (2–7)	1 (0–2)	0 (0–1)	0 (0–0)	0 (0–0)	0 (0–0)	0 (0–0)	0 (0–0)	--
Sickle cell disorders	0 (0–1)	0 (0–0)	1 (0–1)	1 (0–1)	0 (0–1)	0 (0–0)	0 (0–0)	0 (0–0)	0 (0–0)	0 (0–0)	0 (0–0)	0 (0–0)	0 (0–0)	0 (0–0)	0 (0–0)	0 (0–0)	0 (0–0)	0 (0–0)	0 (0–0)	0 (0–0)	0 (0–0)	0 (0–0)	--
G6PD deficiency	1 (0–1)	0 (0–0)	1 (1–2)	2 (1–4)	4 (2–6)	7 (4–11)	20 (11–31)	27 (16–40)	18 (12–27)	17 (11–25)	19 (11–28)	21 (13–31)	24 (15–35)	32 (20–48)	43 (27–62)	45 (27–64)	33 (18–47)	19 (10–29)	10 (5–16)	4 (2–6)	2 (1–3)	0 (0–1)	--
Other hemoglobinopathies and hemolytic anaemias	1 (0–1)	0 (0–0)	0 (0–0)	0 (0–0)	0 (0–1)	1 (1–1)	3 (2–4)	6 (4–9)	3 (2–5)	3 (2–5)	4 (3–7)	6 (4–9)	8 (5–12)	16 (10–25)	31 (20–45)	54 (38–77)	75 (55–101)	76 (57–102)	73 (58–93)	56 (44–71)	47 (38–60)	17 (14–21)	--
Endocrine, metabolic, blood, and immune disorders	30 (4–56)	17 (3–33)	71 (22–120)	34 (12–51)	16 (9–22)	18 (10–25)	16 (9–23)	14 (8–20)	22 (10–33)	20 (13–30)	23 (15–39)	26 (18–46)	37 (24–67)	47 (30–90)	51 (34–83)	50 (33–83)	72 (45–98)	88 (48–119)	60 (40–81)	42 (29–71)	17 (10–38)	8 (4–19)	--
Sudden infant death syndrome	--	114 (65–204)	487 (153–1,022)	--	--	--	--	--	--	--	--	--	--	--	--	--	--	--	--	--	--	--	--
Transport injuries	31 (11–54)	26 (5–41)	210 (85–332)	577 (398–773)	809 (629–1,024)	1,031 (753–1,313)	4,169 (3,139–5,477)	4,262 (3,243–5,742)	2,406 (1,829–3,503)	1,915 (1,436–2,890)	1,911 (1,435–2,871)	1,979 (1,417–3,145)	2,101 (1,512–3,347)	2,191 (1,537–3,433)	1,966 (1,400–3,298)	1,689 (1,243–2,755)	1,461 (1,073–2,353)	1,129 (854–1,768)	868 (670–1,293)	547 (432–797)	249 (191–389)	61 (45–107)	--
Road injuries	27 (10–47)	23 (5–36)	191 (76–303)	524 (359–708)	753 (588–951)	956 (703–1,202)	3,874 (2,917–5,104)	3,946 (2,947–5,340)	2,212 (1,672–3,225)	1,755 (1,312–2,676)	1,753 (1,303–2,621)	1,817 (1,297–2,868)	1,926 (1,374–3,035)	2,005 (1,396–3,127)	1,809 (1,282–3,032)	1,536 (1,133–2,498)	1,329 (970–2,130)	1,028 (772–1,611)	787 (599–1,158)	495 (390–714)	219 (166–336)	52 (38–90)	--
Pedestrian road injuries	11 (4–21)	7 (1–12)	65 (25–110)	181 (96–265)	301 (188–412)	321 (201–465)	451 (241–766)	472 (245–820)	266 (137–469)	299 (165–505)	312 (173–536)	345 (191–592)	461 (269–796)	559 (324–921)	516 (306–869)	592 (370–925)	528 (341–852)	447 (298–687)	477 (346–691)	298 (211–444)	128 (90–207)	25 (16–43)	--
Cyclist road injuries	--	--	--	53 (22–90)	73 (36–115)	89 (46–147)	145 (68–246)	125 (57–212)	70 (31–117)	95 (44–156)	93 (42–156)	89 (40–154)	183 (86–312)	203 (95–349)	195 (91–338)	199 (96–334)	168 (79–286)	115 (54–200)	14 (6–26)	10 (5–18)	10 (5–17)	3 (1–6)	--
Motorcyclist road injuries	9 (2–20)	8 (1–14)	62 (17–111)	162 (82–241)	174 (100–247)	300 (178–421)	2,165 (1,475–2,945)	2,209 (1,547–3,176)	1,185 (829–1,721)	784 (526–1,175)	756 (487–1,127)	766 (461–1,213)	673 (388–1,068)	617 (345–973)	526 (287–870)	244 (119–461)	188 (94–342)	110 (52–185)	38 (17–65)	25 (12–40)	13 (7–20)	4 (2–7)	--
Motor vehicle road injuries	7 (3–13)	7 (2–12)	60 (23–101)	119 (67–189)	194 (132–284)	232 (156–329)	1,056 (713–1,534)	1,082 (709–1,666)	655 (425–1,178)	545 (367–965)	560 (384–973)	580 (388–1,056)	574 (371–1,085)	580 (373–1,112)	536 (343–1,093)	468 (302–953)	415 (272–821)	332 (220–614)	234 (153–417)	146 (96–254)	61 (40–107)	18 (12–33)	--
Other road injuries	1 (0–1)	0 (0–1)	4 (1–7)	9 (5–16)	11 (6–18)	15 (8–26)	57 (33–95)	57 (35–96)	36 (22–60)	33 (20–54)	33 (19–53)	36 (20–62)	36 (20–61)	47 (25–79)	36 (20–59)	33 (17–55)	29 (15–49)	23 (11–40)	24 (12–41)	16 (9–27)	7 (4–11)	1 (1–2)	--
Other transport injuries	3 (1–8)	3 (1–6)	20 (7–36)	53 (30–86)	55 (33–82)	75 (42–119)	296 (177–496)	316 (193–511)	195 (120–335)	160 (98–268)	158 (99–275)	162 (100–282)	175 (105–312)	185 (110–324)	157 (94–295)	153 (92–289)	132 (80–241)	101 (62–180)	82 (51–137)	52 (33–90)	30 (20–53)	9 (6–18)	--
Falls	3 (2–7)	11 (3–21)	97 (19–187)	236 (86–370)	157 (86–215)	125 (92–163)	258 (178–429)	287 (194–500)	298 (226–426)	308 (228–440)	387 (280–611)	533 (383–792)	535 (371–847)	733 (508–1,099)	733 (503–1,218)	859 (623–1,278)	822 (583–1,107)	813 (541–1,052)	766 (473–986)	660 (379–878)	469 (248–639)	216 (112–294)	--
Drowning	2 (1–2)	13 (1–21)	144 (15–236)	822 (289–1,160)	466 (238–599)	284 (201–365)	391 (284–527)	230 (169–326)	140 (107–216)	131 (99–194)	161 (121–234)	138 (100–235)	109 (75–227)	99 (68–207)	89 (61–175)	83 (61–153)	64 (45–134)	78 (60–99)	70 (52–87)	39 (29–48)	26 (20–33)	7 (5–8)	--
Fire, heat, and hot substances	4 (0–8)	2 (0–4)	63 (10–119)	83 (33–126)	16 (10–29)	12 (7–26)	34 (22–51)	40 (27–73)	47 (29–67)	35 (24–51)	36 (24–56)	51 (31–75)	45 (29–66)	57 (31–82)	59 (35–84)	57 (36–77)	66 (43–90)	36 (26–54)	29 (21–44)	38 (20–52)	19 (13–30)	6 (4–12)	--
Poisonings	1 (1–2)	1 (1–3)	4 (2–7)	52 (25–88)	33 (9–53)	33 (8–51)	74 (23–118)	66 (26–100)	60 (23–87)	46 (19–68)	70 (21–105)	88 (25–133)	81 (26–126)	48 (24–70)	39 (19–57)	43 (19–61)	11 (7–18)	12 (7–17)	10 (5–13)	4 (2–9)	2 (1–6)	1 (0–2)	--
Poisoning by carbon monoxide	0 (0–0)	0 (0–1)	0 (0–1)	8 (1–24)	6 (1–16)	5 (1–12)	19 (3–38)	16 (4–31)	12 (3–23)	14 (3–25)	24 (3–45)	30 (5–53)	20 (5–39)	11 (4–20)	9 (3–16)	11 (2–21)	2 (1–4)	2 (1–4)	1 (1–3)	1 (0–2)	0 (0–2)	0 (0–0)	--
Poisoning by other means	1 (0–1)	1 (0–2)	4 (2–6)	44 (22–75)	27 (8–46)	28 (7–43)	55 (17–90)	49 (21–76)	48 (20–71)	32 (15–49)	46 (17–74)	59 (17–92)	60 (20–94)	37 (18–53)	31 (15–44)	32 (16–47)	9 (6–15)	10 (5–14)	8 (4–12)	3 (2–7)	2 (1–5)	1 (0–1)	--
Exposure to mechanical forces	11 (0–32)	49 (0–181)	8 (3–23)	17 (10–37)	23 (15–45)	33 (23–45)	88 (58–124)	132 (78–196)	98 (63–142)	111 (56–162)	107 (59–157)	73 (51–110)	101 (66–141)	89 (58–124)	100 (61–138)	66 (46–88)	54 (36–72)	53 (31–70)	31 (20–40)	12 (8–20)	7 (4–14)	3 (2–6)	--
Unintentional firearm injuries	0 (0–2)	2 (0–8)	1 (0–3)	2 (1–3)	1 (1–2)	1 (1–2)	3 (1–5)	8 (2–14)	2 (1–4)	2 (1–5)	2 (1–6)	2 (1–6)	2 (1–6)	1 (1–3)	1 (1–2)	1 (1–2)	1 (0–1)	1 (0–2)	0 (0–1)	0 (0–1)	0 (0–1)	0 (0–0)	--
Other exposure to mechanical forces	11 (0–31)	47 (0–174)	7 (3–20)	15 (8–35)	22 (14–43)	32 (22–43)	85 (56–120)	124 (73–184)	96 (61–140)	110 (55–160)	105 (57–155)	71 (49–107)	99 (64–139)	88 (57–122)	98 (61–137)	66 (45–87)	53 (36–71)	52 (31–70)	30 (20–40)	12 (8–20)	7 (4–14)	3 (2–6)	--
Adverse effects of medical treatment	6 (3–11)	4 (2–9)	30 (10–67)	24 (12–47)	20 (10–36)	15 (9–26)	20 (11–33)	30 (16–50)	22 (12–36)	21 (11–36)	31 (18–53)	39 (23–68)	55 (32–97)	52 (31–93)	77 (43–139)	89 (50–164)	88 (50–157)	70 (42–123)	57 (34–100)	45 (26–80)	27 (15–45)	11 (6–19)	--
Animal contact	1 (1–2)	2 (1–2)	6 (4–12)	44 (30–63)	59 (40–78)	39 (25–53)	52 (28–73)	36 (24–49)	31 (21–44)	27 (19–38)	26 (18–48)	26 (17–53)	31 (21–66)	60 (42–89)	77 (51–107)	65 (46–88)	87 (52–117)	73 (40–98)	46 (30–60)	55 (25–71)	36 (14–48)	13 (5–17)	--
Venomous animal contact	1 (0–1)	0 (0–1)	1 (0–2)	13 (8–21)	27 (17–38)	18 (11–26)	22 (11–33)	15 (10–23)	8 (4–12)	11 (7–18)	10 (7–18)	12 (7–23)	12 (7–24)	27 (17–41)	40 (25–58)	29 (18–42)	45 (24–66)	34 (17–50)	22 (13–32)	30 (11–41)	21 (7–30)	8 (3–10)	--
Non-venomous animal contact	0 (0–1)	1 (0–2)	5 (3–10)	31 (21–44)	32 (22–43)	21 (13–28)	30 (18–42)	20 (13–29)	24 (16–34)	15 (10–23)	15 (10–30)	14 (9–30)	19 (12–43)	33 (22–50)	38 (24–54)	37 (26–50)	42 (27–56)	38 (22–52)	24 (17–31)	25 (13–33)	16 (7–21)	5 (3–7)	--
Foreign body	6 (4–12)	35 (17–50)	186 (117–295)	51 (36–81)	15 (11–33)	19 (14–29)	15 (11–24)	23 (16–39)	22 (16–33)	18 (13–29)	19 (14–31)	25 (18–39)	29 (21–43)	41 (29–56)	41 (29–56)	54 (40–72)	75 (45–98)	101 (44–132)	86 (40–108)	60 (38–76)	52 (23–68)	27 (11–34)	--
Pulmonary aspiration and foreign body in airway	6 (4–12)	35 (17–49)	184 (116–292)	49 (34–78)	15 (11–32)	18 (14–28)	15 (10–24)	22 (16–38)	21 (16–33)	18 (13–28)	19 (14–30)	25 (18–38)	29 (20–43)	40 (28–55)	41 (29–55)	52 (38–69)	73 (45–96)	98 (42–129)	84 (39–105)	58 (37–73)	51 (22–67)	26 (11–34)	--
Foreign body in other body part	0 (0–0)	0 (0–0)	2 (1–5)	2 (1–4)	0 (0–1)	0 (0–1)	0 (0–1)	0 (0–1)	0 (0–1)	0 (0–1)	0 (0–1)	1 (0–1)	1 (0–1)	1 (0–1)	1 (0–1)	2 (1–4)	1 (1–2)	3 (1–5)	2 (1–4)	2 (1–3)	2 (1–3)	1 (0–1)	--
Environmental heat and cold exposure	1 (0–2)	1 (0–2)	7 (0–16)	4 (1–9)	2 (1–5)	2 (1–3)	6 (1–12)	13 (1–26)	6 (1–12)	5 (1–10)	6 (1–11)	7 (2–15)	8 (2–17)	9 (2–19)	7 (2–14)	7 (3–14)	4 (2–8)	5 (2–8)	4 (2–6)	3 (2–5)	2 (1–3)	1 (0–1)	--
Other unintentional injuries	8 (2–13)	1 (0–2)	6 (3–15)	20 (11–47)	53 (29–71)	69 (37–95)	206 (113–297)	278 (143–415)	257 (107–380)	210 (100–304)	176 (92–255)	124 (87–177)	175 (89–254)	151 (73–218)	142 (68–206)	112 (51–160)	52 (37–69)	23 (16–34)	13 (9–25)	6 (4–15)	3 (2–9)	1 (1–3)	--
Self-harm	--	--	--	--	--	51 (31–68)	500 (342–678)	758 (516–1,058)	674 (456–949)	647 (447–904)	630 (419–863)	603 (405–815)	498 (340–689)	412 (287–553)	339 (241–461)	285 (206–385)	213 (157–301)	157 (118–229)	115 (86–150)	86 (57–106)	47 (30–58)	14 (10–18)	--
Self-harm by firearm	--	--	--	--	--	1 (1–1)	10 (6–18)	17 (9–34)	14 (7–25)	13 (7–23)	13 (7–26)	15 (8–32)	17 (10–43)	10 (5–24)	10 (6–28)	6 (3–15)	3 (2–9)	3 (2–9)	2 (1–4)	3 (2–7)	2 (1–3)	1 (0–2)	--

Self-harm by other specified means	--	--	--	--	--	50 (30–67)	490 (334–664)	740 (505–1,031)	659 (445–926)	635 (436–890)	617 (407–846)	588 (396–793)	481 (329–657)	402 (280–537)	329 (232–445)	280 (202–376)	210 (155–294)	154 (115–222)	113 (84–147)	83 (54–102)	45 (29–55)	13 (9–16)	--
Interpersonal violence	18 (12–25)	6 (4–9)	30 (18–46)	32 (21–45)	35 (25–45)	45 (34–59)	268 (186–375)	295 (224–397)	193 (149–263)	160 (124–228)	104 (78–147)	123 (90–178)	86 (61–121)	71 (50–100)	89 (64–121)	58 (42–77)	50 (37–65)	22 (16–30)	22 (16–28)	3 (2–3)	1 (1–2)	0 (0–1)	--
Physical violence by firearm	1 (0–2)	1 (0–1)	1 (0–2)	3 (2–5)	3 (2–6)	3 (2–7)	11 (6–26)	15 (10–25)	11 (8–17)	21 (14–33)	12 (8–19)	12 (8–18)	7 (5–11)	6 (4–9)	7 (4–10)	4 (3–6)	3 (2–4)	2 (1–3)	2 (1–3)	1 (0–1)	0 (0–0)	0 (0–0)	--
Physical violence by sharp object	4 (1–6)	1 (0–1)	1 (1–2)	5 (3–8)	14 (3–19)	21 (4–30)	185 (101–260)	189 (121–264)	125 (89–174)	95 (65–132)	58 (40–81)	71 (47–101)	54 (35–76)	40 (25–56)	36 (24–53)	16 (10–30)	18 (13–26)	5 (3–10)	3 (2–6)	1 (1–1)	0 (0–1)	0 (0–0)	--
Physical violence by other means	13 (9–19)	5 (3–7)	28 (17–43)	23 (15–33)	18 (13–26)	21 (15–37)	72 (45–149)	90 (63–139)	57 (41–89)	45 (32–70)	33 (23–50)	40 (27–63)	25 (15–39)	25 (16–38)	46 (31–64)	38 (25–51)	29 (20–39)	15 (11–21)	17 (11–22)	1 (1–2)	1 (0–1)	0 (0–0)	--
Executions and police conflict	0 (0–0)	0 (0–0)	0 (0–0)	1 (1–2)	1 (1–2)	2 (1–2)	6 (5–7)	11 (7–17)	11 (6–18)	11 (5–17)	10 (5–15)	9 (4–13)	7 (3–12)	5 (2–8)	4 (2–5)	2 (1–3)	1 (1–1)	1 (0–1)	0 (0–1)	0 (0–0)	0 (0–0)	0 (0–0)	--

SR Table 12: Dominica deaths by cause and age group for males

Cause	Early Neonatal	Late Neonatal	Post Neonatal	1 to 4	5 to 9	10 to 14	15 to 19	20 to 24	25 to 29	30 to 34	35 to 39	40 to 44	45 to 49	50 to 54	55 to 59	60 to 64	65 to 69	70 to 74	75 to 79	80 to 84	85 to 89	90 to 94	95 plus
All causes	6 (4–9)	2 (1–2)	3 (2–4)	2 (1–2)	1 (1–1)	1 (1–2)	3 (3–4)	4 (3–5)	5 (4–6)	6 (5–7)	7 (6–9)	7 (6–9)	10 (8–13)	20 (16–25)	29 (23–36)	34 (27–42)	40 (33–49)	45 (38–53)	51 (45–59)	60 (54–67)	39 (36–43)	20 (18–22)	--
Sexually transmitted infections excluding HIV	0 (0–0)	0 (0–0)	0 (0–0)	0 (0–0)	0 (0–0)	0 (0–0)	0 (0–0)	0 (0–0)	0 (0–0)	0 (0–0)	0 (0–0)	0 (0–0)	0 (0–0)	0 (0–0)	0 (0–0)	0 (0–0)	0 (0–0)	0 (0–0)	0 (0–0)	0 (0–0)	0 (0–0)	0 (0–0)	--
Syphilis	0 (0–0)	0 (0–0)	0 (0–0)	0 (0–0)	0 (0–0)	0 (0–0)	0 (0–0)	0 (0–0)	0 (0–0)	0 (0–0)	0 (0–0)	0 (0–0)	0 (0–0)	0 (0–0)	0 (0–0)	0 (0–0)	0 (0–0)	0 (0–0)	0 (0–0)	0 (0–0)	0 (0–0)	0 (0–0)	--
Chlamydial infection	--	--	--	--	--	0 (0–0)	0 (0–0)	0 (0–0)	0 (0–0)	0 (0–0)	0 (0–0)	0 (0–0)	0 (0–0)	0 (0–0)	0 (0–0)	0 (0–0)	0 (0–0)	0 (0–0)	0 (0–0)	0 (0–0)	0 (0–0)	0 (0–0)	--
Gonococcal infection	--	--	--	--	--	0 (0–0)	0 (0–0)	0 (0–0)	0 (0–0)	0 (0–0)	0 (0–0)	0 (0–0)	0 (0–0)	0 (0–0)	0 (0–0)	0 (0–0)	0 (0–0)	0 (0–0)	0 (0–0)	0 (0–0)	0 (0–0)	0 (0–0)	--
Other sexually transmitted infections	--	--	--	--	--	0 (0–0)	0 (0–0)	0 (0–0)	0 (0–0)	0 (0–0)	0 (0–0)	0 (0–0)	0 (0–0)	0 (0–0)	0 (0–0)	0 (0–0)	0 (0–0)	0 (0–0)	0 (0–0)	0 (0–0)	0 (0–0)	0 (0–0)	--
Tuberculosis	--	--	0 (0–0)	0 (0–0)	0 (0–0)	0 (0–0)	0 (0–0)	0 (0–0)	0 (0–0)	0 (0–0)	0 (0–0)	0 (0–0)	0 (0–0)	0 (0–0)	0 (0–0)	0 (0–0)	0 (0–0)	0 (0–0)	0 (0–0)	0 (0–0)	0 (0–0)	0 (0–0)	--
Drug-susceptible tuberculosis	--	--	0 (0–0)	0 (0–0)	0 (0–0)	0 (0–0)	0 (0–0)	0 (0–0)	0 (0–0)	0 (0–0)	0 (0–0)	0 (0–0)	0 (0–0)	0 (0–0)	0 (0–0)	0 (0–0)	0 (0–0)	0 (0–0)	0 (0–0)	0 (0–0)	0 (0–0)	0 (0–0)	--
Multidrug-resistant tuberculosis without extensive drug resistance	--	--	0 (0–0)	0 (0–0)	0 (0–0)	0 (0–0)	0 (0–0)	0 (0–0)	0 (0–0)	0 (0–0)	0 (0–0)	0 (0–0)	0 (0–0)	0 (0–0)	0 (0–0)	0 (0–0)	0 (0–0)	0 (0–0)	0 (0–0)	0 (0–0)	0 (0–0)	0 (0–0)	--
Extensively drug-resistant tuberculosis	--	--	0 (0–0)	0 (0–0)	0 (0–0)	0 (0–0)	0 (0–0)	0 (0–0)	0 (0–0)	0 (0–0)	0 (0–0)	0 (0–0)	0 (0–0)	0 (0–0)	0 (0–0)	0 (0–0)	0 (0–0)	0 (0–0)	0 (0–0)	0 (0–0)	0 (0–0)	0 (0–0)	--
Lower respiratory infections	0 (0–0)	0 (0–0)	0 (0–1)	0 (0–0)	0 (0–0)	0 (0–0)	0 (0–0)	0 (0–0)	0 (0–0)	0 (0–0)	0 (0–0)	0 (0–0)	0 (0–0)	0 (0–1)	1 (0–1)	1 (0–1)	1 (1–2)	2 (1–2)	2 (1–3)	3 (2–4)	2 (2–3)	2 (1–2)	--
Upper respiratory infections	0 (0–0)	0 (0–0)	0 (0–0)	0 (0–0)	0 (0–0)	0 (0–0)	0 (0–0)	0 (0–0)	0 (0–0)	0 (0–0)	0 (0–0)	0 (0–0)	0 (0–0)	0 (0–0)	0 (0–0)	0 (0–0)	0 (0–0)	0 (0–0)	0 (0–0)	0 (0–0)	0 (0–0)	0 (0–0)	--
Otitis media	0 (0–0)	0 (0–0)	0 (0–0)	0 (0–0)	0 (0–0)	0 (0–0)	0 (0–0)	0 (0–0)	0 (0–0)	0 (0–0)	0 (0–0)	0 (0–0)	0 (0–0)	0 (0–0)	0 (0–0)	0 (0–0)	0 (0–0)	0 (0–0)	0 (0–0)	0 (0–0)	0 (0–0)	0 (0–0)	--
Diarrhoeal diseases	0 (0–0)	0 (0–0)	0 (0–0)	0 (0–0)	0 (0–0)	0 (0–0)	0 (0–0)	0 (0–0)	0 (0–0)	0 (0–0)	0 (0–0)	0 (0–0)	0 (0–0)	0 (0–0)	0 (0–0)	0 (0–0)	0 (0–0)	0 (0–0)	0 (0–0)	0 (0–0)	0 (0–0)	0 (0–0)	--
Typhoid fever	--	--	0 (0–0)	0 (0–0)	0 (0–0)	0 (0–0)	0 (0–0)	0 (0–0)	0 (0–0)	0 (0–0)	0 (0–0)	0 (0–0)	0 (0–0)	0 (0–0)	0 (0–0)	0 (0–0)	0 (0–0)	0 (0–0)	0 (0–0)	0 (0–0)	0 (0–0)	0 (0–0)	--
Paratyphoid fever	--	--	0 (0–0)	0 (0–0)	0 (0–0)	0 (0–0)	0 (0–0)	0 (0–0)	0 (0–0)	0 (0–0)	0 (0–0)	0 (0–0)	0 (0–0)	0 (0–0)	0 (0–0)	0 (0–0)	0 (0–0)	0 (0–0)	0 (0–0)	0 (0–0)	0 (0–0)	0 (0–0)	--
iNTS	--	0 (0–0)	0 (0–0)	0 (0–0)	0 (0–0)	0 (0–0)	0 (0–0)	0 (0–0)	0 (0–0)	0 (0–0)	0 (0–0)	0 (0–0)	0 (0–0)	0 (0–0)	0 (0–0)	0 (0–0)	0 (0–0)	0 (0–0)	0 (0–0)	0 (0–0)	0 (0–0)	0 (0–0)	--
Other intestinal infectious diseases	--	--	0 (0–0)	0 (0–0)	0 (0–0)	0 (0–0)	0 (0–0)	0 (0–0)	0 (0–0)	0 (0–0)	0 (0–0)	0 (0–0)	0 (0–0)	0 (0–0)	0 (0–0)	0 (0–0)	0 (0–0)	0 (0–0)	0 (0–0)	0 (0–0)	0 (0–0)	0 (0–0)	--
Malaria	0 (0–0)	0 (0–0)	0 (0–0)	0 (0–0)	0 (0–0)	0 (0–0)	0 (0–0)	0 (0–0)	0 (0–0)	0 (0–0)	0 (0–0)	0 (0–0)	0 (0–0)	0 (0–0)	0 (0–0)	0 (0–0)	0 (0–0)	0 (0–0)	0 (0–0)	0 (0–0)	0 (0–0)	0 (0–0)	--
Chagas disease	--	--	0 (0–0)	0 (0–0)	0 (0–0)	0 (0–0)	0 (0–0)	0 (0–0)	0 (0–0)	0 (0–0)	0 (0–0)	0 (0–0)	0 (0–0)	0 (0–0)	0 (0–0)	0 (0–0)	0 (0–0)	0 (0–0)	0 (0–0)	0 (0–0)	0 (0–0)	0 (0–0)	--
Visceral leishmaniasis	--	--	0 (0–0)	0 (0–0)	0 (0–0)	0 (0–0)	0 (0–0)	0 (0–0)	0 (0–0)	0 (0–0)	0 (0–0)	0 (0–0)	0 (0–0)	0 (0–0)	0 (0–0)	0 (0–0)	0 (0–0)	0 (0–0)	0 (0–0)	0 (0–0)	0 (0–0)	0 (0–0)	--
African trypanosomiasis	--	--	--	0 (0–0)	0 (0–0)	0 (0–0)	0 (0–0)	0 (0–0)	0 (0–0)	0 (0–0)	0 (0–0)	0 (0–0)	0 (0–0)	0 (0–0)	0 (0–0)	0 (0–0)	0 (0–0)	0 (0–0)	0 (0–0)	0 (0–0)	0 (0–0)	0 (0–0)	--
Schistosomiasis	--	--	0 (0–0)	0 (0–0)	0 (0–0)	0 (0–0)	0 (0–0)	0 (0–0)	0 (0–0)	0 (0–0)	0 (0–0)	0 (0–0)	0 (0–0)	0 (0–0)	0 (0–0)	0 (0–0)	0 (0–0)	0 (0–0)	0 (0–0)	0 (0–0)	0 (0–0)	0 (0–0)	--
Cysticercosis	--	--	--	0 (0–0)	0 (0–0)	0 (0–0)	0 (0–0)	0 (0–0)	0 (0–0)	0 (0–0)	0 (0–0)	0 (0–0)	0 (0–0)	0 (0–0)	0 (0–0)	0 (0–0)	0 (0–0)	0 (0–0)	0 (0–0)	0 (0–0)	0 (0–0)	0 (0–0)	--
Cystic echinococcosis	--	--	--	0 (0–0)	0 (0–0)	0 (0–0)	0 (0–0)	0 (0–0)	0 (0–0)	0 (0–0)	0 (0–0)	0 (0–0)	0 (0–0)	0 (0–0)	0 (0–0)	0 (0–0)	0 (0–0)	0 (0–0)	0 (0–0)	0 (0–0)	0 (0–0)	0 (0–0)	--
Dengue	0 (0–0)	0 (0–0)	0 (0–0)	0 (0–0)	0 (0–0)	0 (0–0)	0 (0–0)	0 (0–0)	0 (0–0)	0 (0–0)	0 (0–0)	0 (0–0)	0 (0–0)	0 (0–0)	0 (0–0)	0 (0–0)	0 (0–0)	0 (0–0)	0 (0–0)	0 (0–0)	0 (0–0)	0 (0–0)	--
Yellow fever	--	0 (0–0)	0 (0–0)	0 (0–0)	0 (0–0)	0 (0–0)	0 (0–0)	0 (0–0)	0 (0–0)	0 (0–0)	0 (0–0)	0 (0–0)	0 (0–0)	0 (0–0)	0 (0–0)	0 (0–0)	0 (0–0)	0 (0–0)	0 (0–0)	0 (0–0)	0 (0–0)	0 (0–0)	--
Rabies	--	--	0 (0–0)	0 (0–0)	0 (0–0)	0 (0–0)	0 (0–0)	0 (0–0)	0 (0–0)	0 (0–0)	0 (0–0)	0 (0–0)	0 (0–0)	0 (0–0)	0 (0–0)	0 (0–0)	0 (0–0)	0 (0–0)	0 (0–0)	0 (0–0)	0 (0–0)	0 (0–0)	--
Ascariasis	--	--	0 (0–0)	0 (0–0)	0 (0–0)	0 (0–0)	0 (0–0)	0 (0–0)	0 (0–0)	0 (0–0)	0 (0–0)	0 (0–0)	0 (0–0)	0 (0–0)	0 (0–0)	0 (0–0)	0 (0–0)	0 (0–0)	0 (0–0)	0 (0–0)	0 (0–0)	0 (0–0)	--
Zika virus disease	0 (0–0)	0 (0–0)	0 (0–0)	0 (0–0)	0 (0–0)	0 (0–0)	0 (0–0)	0 (0–0)	0 (0–0)	0 (0–0)	0 (0–0)	0 (0–0)	0 (0–0)	0 (0–0)	0 (0–0)	0 (0–0)	0 (0–0)	0 (0–0)	0 (0–0)	0 (0–0)	0 (0–0)	0 (0–0)	--
Other neglected tropical diseases	0 (0–0)	0 (0–0)	0 (0–0)	0 (0–0)	0 (0–0)	0 (0–0)	0 (0–0)	0 (0–0)	0 (0–0)	0 (0–0)	0 (0–0)	0 (0–0)	0 (0–0)	0 (0–0)	0 (0–0)	0 (0–0)	0 (0–0)	0 (0–0)	0 (0–0)	0 (0–0)	0 (0–0)	0 (0–0)	--
Meningitis	0 (0–0)	0 (0–0)	0 (0–0)	0 (0–0)	0 (0–0)	0 (0–0)	0 (0–0)	0 (0–0)	0 (0–0)	0 (0–0)	0 (0–0)	0 (0–0)	0 (0–0)	0 (0–0)	0 (0–0)	0 (0–0)	0 (0–0)	0 (0–0)	0 (0–0)	0 (0–0)	0 (0–0)	0 (0–0)	--
Encephalitis	0 (0–0)	0 (0–0)	0 (0–0)	0 (0–0)	0 (0–0)	0 (0–0)	0 (0–0)	0 (0–0)	0 (0–0)	0 (0–0)	0 (0–0)	0 (0–0)	0 (0–0)	0 (0–0)	0 (0–0)	0 (0–0)	0 (0–0)	0 (0–0)	0 (0–0)	0 (0–0)	0 (0–0)	0 (0–0)	--
Diphtheria	--	--	0 (0–0)	0 (0–0)	0 (0–0)	0 (0–0)	0 (0–0)	0 (0–0)	0 (0–0)	0 (0–0)	0 (0–0)	0 (0–0)	0 (0–0)	0 (0–0)	0 (0–0)	--	--	--	--	--	--	--	--
Whooping cough	--	--	0 (0–0)	0 (0–0)	0 (0–0)	0 (0–0)	0 (0–0)	0 (0–0)	0 (0–0)	0 (0–0)	0 (0–0)	0 (0–0)	0 (0–0)	0 (0–0)	0 (0–0)	--	--	--	--	--	--	--	--
Tetanus	0 (0–0)	0 (0–0)	0 (0–0)	0 (0–0)	0 (0–0)	0 (0–0)	0 (0–0)	0 (0–0)	0 (0–0)	0 (0–0)	0 (0–0)	0 (0–0)	0 (0–0)	0 (0–0)	0 (0–0)	0 (0–0)	0 (0–0)	0 (0–0)	0 (0–0)	0 (0–0)	0 (0–0)	0 (0–0)	--
Measles	0 (0–0)	0 (0–0)	0 (0–0)	0 (0–0)	0 (0–0)	0 (0–0)	0 (0–0)	0 (0–0)	0 (0–0)	0 (0–0)	0 (0–0)	0 (0–0)	0 (0–0)	0 (0–0)	0 (0–0)	0 (0–0)	0 (0–0)	0 (0–0)	0 (0–0)	0 (0–0)	0 (0–0)	0 (0–0)	--
Varicella and herpes zoster	0 (0–0)	0 (0–0)	0 (0–0)	0 (0–0)	0 (0–0)	0 (0–0)	0 (0–0)	0 (0–0)	0 (0–0)	0 (0–0)	0 (0–0)	0 (0–0)	0 (0–0)	0 (0–0)	0 (0–0)	0 (0–0)	0 (0–0)	0 (0–0)	0 (0–0)	0 (0–0)	0 (0–0)	0 (0–0)	--
Acute hepatitis	--	--	0 (0–0)	0 (0–0)	0 (0–0)	0 (0–0)	0 (0–0)	0 (0–0)	0 (0–0)	0 (0–0)	0 (0–0)	0 (0–0)	0 (0–0)	0 (0–0)	0 (0–0)	0 (0–0)	0 (0–0)	0 (0–0)	0 (0–0)	0 (0–0)	0 (0–0)	0 (0–0)	--
Acute hepatitis A	--	--	0 (0–0)	0 (0–0)	0 (0–0)	0 (0–0)	0 (0–0)	0 (0–0)	0 (0–0)	0 (0–0)	0 (0–0)	0 (0–0)	0 (0–0)	0 (0–0)	0 (0–0)	0 (0–0)	0 (0–0)	0 (0–0)	0 (0–0)	0 (0–0)	0 (0–0)	0 (0–0)	--
Acute hepatitis B	--	--	0 (0–0)	0 (0–0)	0 (0–0)	0 (0–0)	0 (0–0)	0 (0–0)	0 (0–0)	0 (0–0)	0 (0–0)	0 (0–0)	0 (0–0)	0 (0–0)	0 (0–0)	0 (0–0)	0 (0–0)	0 (0–0)	0 (0–0)	0 (0–0)	0 (0–0)	0 (0–0)	--
Acute hepatitis C	--	--	0 (0–0)	0 (0–0)	0 (0–0)	0 (0–0)	0 (0–0)	0 (0–0)	0 (0–0)	0 (0–0)	0 (0–0)	0 (0–0)	0 (0–0)	0 (0–0)	0 (0–0)	0 (0–0)	0 (0–0)	0 (0–0)	0 (0–0)	0 (0–0)	0 (0–0)	0 (0–0)	--
Acute hepatitis E	--	--	0 (0–0)	0 (0–0)	0 (0–0)	0 (0–0)	0 (0–0)	0 (0–0)	0 (0–0)	0 (0–0)	0 (0–0)	0 (0–0)	0 (0–0)	0 (0–0)	0 (0–0)	0 (0–0)	0 (0–0)	0 (0–0)	0 (0–0)	0 (0–0)	0 (0–0)	0 (0–0)	--
Other unspecified infectious diseases	0 (0–0)	0 (0–0)	0 (0–0)	0 (0–0)	0 (0–0)	0 (0–0)	0 (0–0)	0 (0–0)	0 (0–0)	0 (0–0)	0 (0–0)	0 (0–0)	0 (0–0)	0 (0–0)	0 (0–0)	0 (0–0)	0 (0–0)	0 (0–0)	0 (0–0)	0 (0–0)	0 (0–0)	0 (0–0)	--
Maternal disorders	--	--	--	--	--	--	--	--	--	--	--	--	--	--	--	--	--	--	--	--	--	--	--
Maternal haemorrhage	--	--	--	--	--	--	--	--	--	--	--	--	--	--	--	--	--	--	--	--	--	--	--
Maternal sepsis and other pregnancy related infections	--	--	--	--	--	--	--	--	--	--	--	--	--	--	--	--	--	--	--	--	--	--	--
Maternal hypertensive disorders	--	--	--	--	--	--	--	--	--	--	--	--	--	--	--	--	--	--	--	--	--	--	--
Maternal obstructed labour and uterine rupture	--	--	--	--	--	--	--	--	--	--	--	--	--	--	--	--	--	--	--	--	--	--	--
Maternal abortive outcome	--	--	--	--	--	--	--	--	--	--	--	--	--	--	--	--	--	--	--	--	--	--	--
Ectopic pregnancy	--	--	--	--	--	--	--	--	--	--	--	--	--	--	--	--	--	--	--	--	--	--	--
Indirect maternal deaths	--	--	--	--	--	--	--	--	--	--	--	--	--	--	--	--	--	--	--	--	--	--	--
Late maternal deaths	--	--	--	--	--	--	--	--	--	--	--	--	--	--	--	--	--	--	--	--	--	--	--
Maternal deaths aggravated by HIV/AIDS	--	--	--	--	--	--	--	--	--	--	--	--	--	--	--	--	--	--	--	--	--	--	--
Other maternal disorders	--	--	--	--	--	--	--	--	--	--	--	--	--	--	--	--	--	--	--	--	--	--	--
Neonatal disorders	5 (3–7)	1 (1–1)	0 (0–1)	0 (0–0)	--	--	--	--	--	--	--	--	--	--	--	--	--	--	--	--	--	--	--
Neonatal preterm birth	2 (1–2)	0 (0–0)	0 (0–0)	0 (0–0)	--	--	--	--	--	--	--	--	--	--	--	--	--	--	--	--	--	--	--
Neonatal encephalopathy due to birth asphyxia and trauma	2 (1–3)	0 (0–0)	0 (0–0)	0 (0–0)	--	--	--	--	--	--	--	--	--	--	--	--	--	--	--	--	--	--	--
Neonatal sepsis and other neonatal infections	1 (0–1)	0 (0–1)	0 (0–0)	0 (0–0)	--	--	--	--	--	--	--	--	--	--	--	--	--	--	--	--	--	--	--
Hemolytic disease and other neonatal jaundice	0 (0–0)	0 (0–0)	0 (0–0)	0 (0–0)	--	--	--	--	--	--	--	--	--	--	--	--	--	--	--	--	--	--	--
Other neonatal disorders	1 (0–1)	0 (0–0)	0 (0–0)	0 (0–0)	--	--	--	--	--	--	--	--	--	--	--	--	--	--	--	--	--	--	--
Nutritional deficiencies	0 (0–0)	0 (0–0)	0 (0–0)	0 (0–0)	0 (0–0)	0 (0–0)	0 (0–0)	0 (0–0)	0 (0–0)	0 (0–0)	0 (0–0)	0 (0–0)	0 (0–0)	0 (0–0)	0 (0–0)	0 (0–0)	0 (0–0)	0 (0–0)	0 (0–0)	0 (0–0)	0 (0–0)	0 (0–0)	--
Protein-energy malnutrition	0 (0–0)	0 (0–0)	0 (0–0)	0 (0–0)	0 (0–0)	0 (0–0)	0 (0–0)	0 (0–0)	0 (0–0)	0 (0–0)	0 (0–0)	0 (0–0)	0 (0–0)	0 (0–0)	0 (0–0)	0 (0–0)	0 (0–0)	0 (0–0)	0 (0–0)	0 (0–0)	0 (0–0)	0 (0–0)	--
Other nutritional deficiencies	--	--	0 (0–0)	0 (0–0)	0 (0–0)	0 (0–0)	0 (0–0)	0 (0–0)	0 (0–0)	0 (0–0)	0 (0–0)	0 (0–0)	0 (0–0)	0 (0–0)	0 (0–0)	0 (0–0)	0 (0–0)	0 (0–0)	0 (0–0)	0 (0–0)	0 (0–0)	0 (0–0)	--
Lip and oral cavity cancer	--	--	--	--	0 (0–0)	0 (0–0)	0 (0–0)	0 (0–0)	0 (0–0)	0 (0–0)	0 (0–0)	0 (0–0)	0 (0–0)	0 (0–0)	0 (0–0)	0 (0–0)	0 (0–0)	0 (0–0)	0 (0–0)	0 (0–0)	0 (0–0)	0 (0–0)	--
Nasopharynx cancer	--	--	--	--	0 (0–0)	0 (0–0)	0 (0–0)	0 (0–0)	0 (0–0)	0 (0–0)	0 (0–0)	0 (0–0)	0 (0–0)	0 (0–0)	0 (0–0)	0 (0–0)	0 (0–0)	0 (0–0)	0 (0–0)	0 (0–0)	0 (0–0)	0 (0–0)	--
Other pharynx cancer	--	--	--	--	--	--	--	0 (0–0)	0 (0–0)	0 (0–0)	0 (0–0)	0 (0–0)	0 (0–0)	0 (0–0)	0 (0–0)	0 (0–0)	0 (0–0)	0 (0–0)	0 (0–0)	0 (0–0)	0 (0–0)	0 (0–0)	--
Oesophageal cancer	--	--	--	--	--	--	--	0 (0–0)	0 (0–0)	0 (0–0)	0 (0–0)	0 (0–0)	0 (0–0)	0 (0–0)	0 (0–1)	0 (0–1)	0 (0–1)	0 (0–1)	0 (0–1)	0 (0–1)	0 (0–0)	0 (0–0)	--

Stomach cancer	--	--	--	--	--	--	0 (0–0)	0 (0–0)	0 (0–0)	0 (0–0)	0 (0–0)	0 (0–0)	0 (0–0)	1 (0–1)	1 (1–1)	1 (1–2)	1 (1–2)	1 (1–2)	1 (1–2)	1 (1–2)	1 (1–2)	1 (1–1)	0 (0–0)	--
Colon and rectum cancer	--	--	--	--	0 (0–0)	0 (0–0)	0 (0–0)	0 (0–0)	0 (0–0)	0 (0–0)	0 (0–0)	0 (0–0)	0 (0–0)	0 (0–1)	1 (0–1)	1 (1–1)	1 (1–1)	1 (1–1)	1 (1–1)	1 (1–1)	1 (1–1)	1 (0–1)	0 (0–0)	--
Liver cancer due to hepatitis B	--	--	--	--	--	0 (0–0)	0 (0–0)	0 (0–0)	0 (0–0)	0 (0–0)	0 (0–0)	0 (0–0)	0 (0–0)	0 (0–0)	0 (0–0)	0 (0–0)	0 (0–0)	0 (0–0)	0 (0–0)	0 (0–0)	0 (0–0)	0 (0–0)	0 (0–0)	--
Liver cancer due to hepatitis C	--	--	--	--	--	0 (0–0)	0 (0–0)	0 (0–0)	0 (0–0)	0 (0–0)	0 (0–0)	0 (0–0)	0 (0–0)	0 (0–0)	0 (0–0)	0 (0–0)	0 (0–0)	0 (0–0)	0 (0–0)	0 (0–0)	0 (0–0)	0 (0–0)	0 (0–0)	--
Liver cancer due to alcohol use	--	--	--	--	--	--	0 (0–0)	0 (0–0)	0 (0–0)	0 (0–0)	0 (0–0)	0 (0–0)	0 (0–0)	0 (0–0)	0 (0–0)	0 (0–0)	0 (0–0)	0 (0–0)	0 (0–0)	0 (0–0)	0 (0–0)	0 (0–0)	0 (0–0)	--
Liver cancer due to NASH	--	--	--	--	--	--	0 (0–0)	0 (0–0)	0 (0–0)	0 (0–0)	0 (0–0)	0 (0–0)	0 (0–0)	0 (0–0)	0 (0–0)	0 (0–0)	0 (0–0)	0 (0–0)	0 (0–0)	0 (0–0)	0 (0–0)	0 (0–0)	0 (0–0)	--
	0 (0–0)	0 (0–0)	0 (0–0)	0 (0–0)	0 (0–0)	0 (0–0)	--	--	--	--	--	--	--	--	--	--	--	--	--	--	--	--	--	--
Liver cancer due to other causes	0 (0–0)	0 (0–0)	0 (0–0)	0 (0–0)	0 (0–0)	0 (0–0)	0 (0–0)	0 (0–0)	0 (0–0)	0 (0–0)	0 (0–0)	0 (0–0)	0 (0–0)	0 (0–0)	0 (0–0)	0 (0–0)	0 (0–0)	0 (0–0)	0 (0–0)	0 (0–0)	0 (0–0)	0 (0–0)	--
Gallbladder and biliary tract cancer	--	--	--	--	--	--	--	0 (0–0)	0 (0–0)	0 (0–0)	0 (0–0)	0 (0–0)	0 (0–0)	0 (0–0)	0 (0–0)	0 (0–0)	0 (0–0)	0 (0–0)	0 (0–0)	0 (0–0)	0 (0–0)	0 (0–0)	0 (0–0)	--
Pancreatic cancer	--	--	--	--	--	--	0 (0–0)	0 (0–0)	0 (0–0)	0 (0–0)	0 (0–0)	0 (0–0)	0 (0–0)	0 (0–0)	0 (0–1)	1 (0–1)	1 (0–1)	1 (0–1)	1 (0–1)	1 (0–1)	0 (0–0)	0 (0–0)	--
Larynx cancer	--	--	--	--	--	--	--	0 (0–0)	0 (0–0)	0 (0–0)	0 (0–0)	0 (0–0)	0 (0–0)	0 (0–0)	0 (0–0)	0 (0–0)	0 (0–0)	0 (0–0)	0 (0–0)	0 (0–0)	0 (0–0)	0 (0–0)	0 (0–0)	--
Tracheal, bronchus, and lung cancer	--	--	--	--	--	0 (0–0)	0 (0–0)	0 (0–0)	0 (0–0)	0 (0–0)	0 (0–0)	0 (0–0)	0 (0–0)	1 (1–1)	1 (1–2)	1 (1–2)	2 (1–2)	2 (1–2)	1 (1–2)	1 (1–2)	1 (0–1)	0 (0–0)	--
Malignant skin melanoma	0 (0–0)	0 (0–0)	0 (0–0)	0 (0–0)	0 (0–0)	0 (0–0)	0 (0–0)	0 (0–0)	0 (0–0)	0 (0–0)	0 (0–0)	0 (0–0)	0 (0–0)	0 (0–0)	0 (0–0)	0 (0–0)	0 (0–0)	0 (0–0)	0 (0–0)	0 (0–0)	0 (0–0)	0 (0–0)	--
Non-melanoma skin cancer (squamous-cell carcinoma)	--	--	--	--	--	--	--	0 (0–0)	0 (0–0)	0 (0–0)	0 (0–0)	0 (0–0)	0 (0–0)	0 (0–0)	0 (0–0)	0 (0–0)	0 (0–0)	0 (0–0)	0 (0–0)	0 (0–0)	0 (0–0)	0 (0–0)	0 (0–0)	--
	0 (0–0)	0 (0–0)	0 (0–0)	0 (0–0)	0 (0–0)	0 (0–0)	0 (0–0)	0 (0–0)	0 (0–0)	0 (0–0)	0 (0–0)	0 (0–0)	0 (0–0)	0 (0–0)	0 (0–0)	0 (0–0)	0 (0–0)	0 (0–0)	0 (0–0)	0 (0–0)	0 (0–0)	0 (0–0)	--
	--	--	--	0 (0–0)	0 (0–0)	0 (0–0)	0 (0–0)	0 (0–0)	0 (0–0)	0 (0–0)	0 (0–0)	0 (0–0)	0 (0–0)	0 (0–0)	0 (0–0)	0 (0–0)	0 (0–0)	0 (0–0)	0 (0–0)	0 (0–0)	0 (0–0)	0 (0–0)	--
Breast cancer	--	--	--	--	--	--	0 (0–0)	0 (0–0)	0 (0–0)	0 (0–0)	0 (0–0)	0 (0–0)	0 (0–0)	0 (0–0)	0 (0–0)	0 (0–0)	0 (0–0)	0 (0–0)	0 (0–0)	0 (0–0)	0 (0–0)	0 (0–0)	--
Cervical cancer	--	--	--	--	--	--	--	--	--	--	--	--	--	--	--	--	--	--	--	--	--	--	--
Uterine cancer	--	--	--	--	--	--	--	--	--	--	--	--	--	--	--	--	--	--	--	--	--	--	--
Ovarian cancer	--	--	--	--	--	--	--	--	--	--	--	--	--	--	--	--	--	--	--	--	--	--	--
Prostate cancer	--	--	--	--	--	--	--	0 (0–0)	0 (0–0)	0 (0–0)	0 (0–0)	0 (0–0)	0 (0–0)	1 (0–1)	1 (1–2)	3 (2–4)	5 (3–6)	7 (5–9)	9 (7–12)	12 (9–16)	8 (6–11)	4 (3–5)	--
Testicular cancer	0 (0–0)	0 (0–0)	0 (0–0)	0 (0–0)	0 (0–0)	0 (0–0)	0 (0–0)	0 (0–0)	0 (0–0)	0 (0–0)	0 (0–0)	0 (0–0)	0 (0–0)	0 (0–0)	0 (0–0)	0 (0–0)	0 (0–0)	0 (0–0)	0 (0–0)	0 (0–0)	0 (0–0)	0 (0–0)	--
Kidney cancer	0 (0–0)	0 (0–0)	0 (0–0)	0 (0–0)	0 (0–0)	0 (0–0)	0 (0–0)	0 (0–0)	0 (0–0)	0 (0–0)	0 (0–0)	0 (0–0)	0 (0–0)	0 (0–0)	0 (0–0)	0 (0–0)	0 (0–0)	0 (0–0)	0 (0–0)	0 (0–0)	0 (0–0)	0 (0–0)	--
Bladder cancer	--	--	--	--	--	--	0 (0–0)	0 (0–0)	0 (0–0)	0 (0–0)	0 (0–0)	0 (0–0)	0 (0–0)	0 (0–0)	0 (0–0)	0 (0–0)	0 (0–0)	0 (0–0)	0 (0–1)	0 (0–1)	0 (0–0)	0 (0–0)	--
Brain and nervous system cancer	0 (0–0)	0 (0–0)	0 (0–0)	0 (0–0)	0 (0–0)	0 (0–0)	0 (0–0)	0 (0–0)	0 (0–0)	0 (0–0)	0 (0–0)	0 (0–0)	0 (0–0)	0 (0–0)	0 (0–0)	0 (0–0)	0 (0–0)	0 (0–0)	0 (0–0)	0 (0–0)	0 (0–0)	0 (0–0)	--
	0 (0–0)	0 (0–0)	0 (0–0)	0 (0–0)	0 (0–0)	0 (0–0)	0 (0–0)	0 (0–0)	0 (0–0)	0 (0–0)	0 (0–0)	0 (0–0)	0 (0–0)	0 (0–0)	0 (0–0)	0 (0–0)	0 (0–0)	0 (0–0)	0 (0–0)	0 (0–0)	0 (0–0)	0 (0–0)	--
	0 (0–0)	0 (0–0)	0 (0–0)	0 (0–0)	0 (0–0)	0 (0–0)	0 (0–0)	--	--	--	--	--	--	--	--	--	--	--	--	--	--	--	--
	--	--	--	0 (0–0)	0 (0–0)	0 (0–0)	0 (0–0)	0 (0–0)	0 (0–0)	0 (0–0)	0 (0–0)	0 (0–0)	0 (0–0)	0 (0–0)	0 (0–0)	0 (0–0)	0 (0–0)	0 (0–0)	0 (0–0)	0 (0–0)	0 (0–0)	0 (0–0)	--
	0 (0–0)	0 (0–0)	0 (0–0)	0 (0–0)	0 (0–0)	0 (0–0)	0 (0–0)	0 (0–0)	0 (0–0)	0 (0–0)	0 (0–0)	0 (0–0)	0 (0–0)	0 (0–0)	0 (0–0)	0 (0–0)	0 (0–0)	0 (0–0)	0 (0–0)	0 (0–0)	0 (0–0)	0 (0–0)	--
Thyroid cancer	--	--	--	--	0 (0–0)	0 (0–0)	0 (0–0)	0 (0–0)	0 (0–0)	0 (0–0)	0 (0–0)	0 (0–0)	0 (0–0)	0 (0–0)	0 (0–0)	0 (0–0)	0 (0–0)	0 (0–0)	0 (0–0)	0 (0–0)	0 (0–0)	0 (0–0)	--
Mesothelioma	--	--	--	--	--	--	--	0 (0–0)	0 (0–0)	0 (0–0)	0 (0–0)	0 (0–0)	0 (0–0)	0 (0–0)	0 (0–0)	0 (0–0)	0 (0–0)	0 (0–0)	0 (0–0)	0 (0–0)	0 (0–0)	0 (0–0)	--
Hodgkin lymphoma	--	--	--	0 (0–0)	0 (0–0)	0 (0–0)	0 (0–0)	0 (0–0)	0 (0–0)	0 (0–0)	0 (0–0)	0 (0–0)	0 (0–0)	0 (0–0)	0 (0–0)	0 (0–0)	0 (0–0)	0 (0–0)	0 (0–0)	0 (0–0)	0 (0–0)	0 (0–0)	--
Non-Hodgkin's lymphoma	--	--	--	0 (0–0)	0 (0–0)	0 (0–0)	0 (0–0)	0 (0–0)	0 (0–0)	0 (0–0)	0 (0–0)	0 (0–0)	0 (0–0)	0 (0–0)	0 (0–1)	0 (0–1)	0 (0–1)	0 (0–1)	0 (0–0)	0 (0–0)	0 (0–0)	0 (0–0)	--
	--	--	--	0 (0–0)	0 (0–0)	0 (0–0)	0 (0–0)	0 (0–0)	0 (0–0)	0 (0–0)	0 (0–0)	0 (0–0)	0 (0–0)	0 (0–0)	0 (0–0)	0 (0–0)	0 (0–0)	0 (0–0)	0 (0–0)	0 (0–0)	0 (0–0)	0 (0–0)	--
	--	--	--	0 (0–0)	0 (0–0)	0 (0–0)	0 (0–0)	0 (0–0)	0 (0–0)	0 (0–0)	0 (0–0)	0 (0–0)	0 (0–0)	0 (0–0)	0 (0–1)	0 (0–1)	0 (0–1)	0 (0–1)	0 (0–0)	0 (0–0)	0 (0–0)	0 (0–0)	--
Multiple myeloma	--	--	--	--	--	--	--	0 (0–0)	0 (0–0)	0 (0–0)	0 (0–0)	0 (0–0)	0 (0–0)	0 (0–0)	0 (0–0)	0 (0–1)	0 (0–1)	0 (0–1)	0 (0–1)	0 (0–1)	0 (0–0)	0 (0–0)	--
Leukaemia	0 (0–0)	0 (0–0)	0 (0–0)	0 (0–0)	0 (0–0)	0 (0–0)	0 (0–0)	0 (0–0)	0 (0–0)	0 (0–0)	0 (0–0)	0 (0–0)	0 (0–0)	0 (0–0)	0 (0–0)	0 (0–0)	0 (0–0)	0 (0–0)	0 (0–0)	0 (0–0)	0 (0–0)	0 (0–0)	--
Acute lymphoid leukaemia	0 (0–0)	0 (0–0)	0 (0–0)	0 (0–0)	0 (0–0)	0 (0–0)	0 (0–0)	0 (0–0)	0 (0–0)	0 (0–0)	0 (0–0)	0 (0–0)	0 (0–0)	0 (0–0)	0 (0–0)	0 (0–0)	0 (0–0)	0 (0–0)	0 (0–0)	0 (0–0)	0 (0–0)	0 (0–0)	--
Chronic lymphoid leukaemia	--	--	--	--	--	--	--	0 (0–0)	0 (0–0)	0 (0–0)	0 (0–0)	0 (0–0)	0 (0–0)	0 (0–0)	0 (0–0)	0 (0–0)	0 (0–0)	0 (0–0)	0 (0–0)	0 (0–0)	0 (0–0)	0 (0–0)	--
Acute myeloid leukaemia	0 (0–0)	0 (0–0)	0 (0–0)	0 (0–0)	0 (0–0)	0 (0–0)	0 (0–0)	0 (0–0)	0 (0–0)	0 (0–0)	0 (0–0)	0 (0–0)	0 (0–0)	0 (0–0)	0 (0–0)	0 (0–0)	0 (0–0)	0 (0–0)	0 (0–0)	0 (0–0)	0 (0–0)	0 (0–0)	--
Chronic myeloid leukaemia	0 (0–0)	0 (0–0)	0 (0–0)	0 (0–0)	0 (0–0)	0 (0–0)	0 (0–0)	0 (0–0)	0 (0–0)	0 (0–0)	0 (0–0)	0 (0–0)	0 (0–0)	0 (0–0)	0 (0–0)	0 (0–0)	0 (0–0)	0 (0–0)	0 (0–0)	0 (0–0)	0 (0–0)	0 (0–0)	--
Other leukaemia	0 (0–0)	0 (0–0)	0 (0–0)	0 (0–0)	0 (0–0)	0 (0–0)	0 (0–0)	0 (0–0)	0 (0–0)	0 (0–0)	0 (0–0)	0 (0–0)	0 (0–0)	0 (0–0)	0 (0–0)	0 (0–0)	0 (0–0)	0 (0–0)	0 (0–0)	0 (0–0)	0 (0–0)	0 (0–0)	--
Other malignant cancers	0 (0–0)	0 (0–0)	0 (0–0)	0 (0–0)	0 (0–0)	0 (0–0)	0 (0–0)	0 (0–0)	0 (0–0)	0 (0–0)	0 (0–0)	0 (0–0)	0 (0–0)	0 (0–0)	0 (0–0)	0 (0–0)	0 (0–0)	0 (0–0)	0 (0–0)	0 (0–0)	0 (0–0)	0 (0–0)	--
Myelodysplastic, myeloproliferative, and other hematopoietic neoplasms	0 (0–0)	0 (0–0)	0 (0–0)	0 (0–0)	0 (0–0)	0 (0–0)	0 (0–0)	0 (0–0)	0 (0–0)	0 (0–0)	0 (0–0)	0 (0–0)	0 (0–0)	0 (0–0)	0 (0–0)	0 (0–0)	0 (0–0)	0 (0–0)	0 (0–0)	0 (0–0)	0 (0–0)	0 (0–0)	--
Cardiovascular diseases	0 (0–0)	0 (0–0)	0 (0–0)	0 (0–0)	0 (0–0)	0 (0–0)	0 (0–0)	0 (0–0)	0 (0–1)	1 (0–1)	1 (1–1)	1 (1–2)	2 (2–3)	5 (4–7)	9 (6–12)	11 (8–14)	13 (10–16)	15 (12–19)	16 (13–21)	19 (14–23)	12 (10–15)	6 (5–8)	--
Rheumatic heart disease	--	--	--	0 (0–0)	0 (0–0)	0 (0–0)	0 (0–0)	0 (0–0)	0 (0–0)	0 (0–0)	0 (0–0)	0 (0–0)	0 (0–0)	0 (0–0)	0 (0–0)	0 (0–0)	0 (0–0)	0 (0–0)	0 (0–0)	0 (0–0)	0 (0–0)	0 (0–0)	--
Ischaemic heart disease	--	--	--	--	--	--	0 (0–0)	0 (0–0)	0 (0–0)	0 (0–0)	0 (0–0)	0 (0–1)	1 (0–1)	2 (1–2)	3 (2–4)	4 (3–5)	5 (3–6)	5 (4–7)	5 (4–7)	6 (4–8)	4 (3–6)	2 (2–3)	--
Stroke	0 (0–0)	0 (0–0)	0 (0–0)	0 (0–0)	0 (0–0)	0 (0–0)	0 (0–0)	0 (0–0)	0 (0–0)	0 (0–0)	0 (0–0)	0 (0–0)	0 (0–1)	1 (1–2)	2 (1–3)	3 (2–4)	3 (2–5)	4 (3–6)	5 (3–7)	5 (4–7)	3 (2–5)	2 (1–2)	--
Ischaemic stroke	0 (0–0)	0 (0–0)	0 (0–0)	0 (0–0)	0 (0–0)	0 (0–0)	0 (0–0)	0 (0–0)	0 (0–0)	0 (0–0)	0 (0–0)	0 (0–0)	0 (0–0)	0 (0–0)	0 (0–1)	1 (0–1)	1 (1–2)	2 (1–3)	2 (2–4)	4 (3–5)	2 (2–3)	1 (1–2)	--
Intracerebral hemorrhage	0 (0–0)	0 (0–0)	0 (0–0)	0 (0–0)	0 (0–0)	0 (0–0)	0 (0–0)	0 (0–0)	0 (0–0)	0 (0–0)	0 (0–0)	0 (0–0)	0 (0–0)	1 (1–1)	1 (1–2)	2 (1–2)	2 (1–3)	2 (1–3)	2 (1–3)	1 (1–2)	1 (0–1)	0 (0–1)	--
Subarachnoid hemorrhage	0 (0–0)	0 (0–0)	0 (0–0)	0 (0–0)	0 (0–0)	0 (0–0)	0 (0–0)	0 (0–0)	0 (0–0)	0 (0–0)	0 (0–0)	0 (0–0)	0 (0–0)	0 (0–0)	0 (0–1)	0 (0–0)	0 (0–0)	0 (0–0)	0 (0–0)	0 (0–0)	0 (0–0)	0 (0–0)	--
Hypertensive heart disease	--	--	--	--	--	--	0 (0–0)	0 (0–0)	0 (0–0)	0 (0–0)	0 (0–0)	0 (0–0)	0 (0–1)	1 (1–1)	1 (1–2)	2 (1–3)	2 (1–3)	2 (1–4)	3 (2–4)	3 (2–5)	2 (1–3)	1 (1–2)	--
Non-rheumatic valvular heart disease	--	--	--	--	--	--	0 (0–0)	0 (0–0)	0 (0–0)	0 (0–0)	0 (0–0)	0 (0–0)	0 (0–0)	0 (0–0)	0 (0–0)	0 (0–0)	0 (0–0)	0 (0–0)	0 (0–0)	0 (0–0)	0 (0–0)	0 (0–0)	--
Non-rheumatic calcific aortic valve disease	--	--	--	--	--	--	0 (0–0)	0 (0–0)	0 (0–0)	0 (0–0)	0 (0–0)	0 (0–0)	0 (0–0)	0 (0–0)	0 (0–0)	0 (0–0)	0 (0–0)	0 (0–0)	0 (0–0)	0 (0–0)	0 (0–0)	0 (0–0)	--
Non-rheumatic degenerative mitral valve disease	--	--	--	--	--	--	0 (0–0)	0 (0–0)	0 (0–0)	0 (0–0)	0 (0–0)	0 (0–0)	0 (0–0)	0 (0–0)	0 (0–0)	0 (0–0)	0 (0–0)	0 (0–0)	0 (0–0)	0 (0–0)	0 (0–0)	0 (0–0)	--
Other non-rheumatic valve diseases	--	--	--	--	--	--	0 (0–0)	0 (0–0)	0 (0–0)	0 (0–0)	0 (0–0)	0 (0–0)	0 (0–0)	0 (0–0)	0 (0–0)	0 (0–0)	0 (0–0)	0 (0–0)	0 (0–0)	0 (0–0)	0 (0–0)	0 (0–0)	--
Cardiomyopathy and myocarditis	0 (0–0)	0 (0–0)	0 (0–0)	0 (0–0)	0 (0–0)	0 (0–0)	0 (0–0)	0 (0–0)	0 (0–0)	0 (0–0)	0 (0–0)	0 (0–1)	1 (0–1)	1 (1–2)	1 (1–2)	2 (1–2)	2 (1–2)	1 (1–2)	1 (1–2)	1 (1–2)	1 (1–2)	0 (0–1)	--
Myocarditis	0 (0–0)	0 (0–0)	0 (0–0)	0 (0–0)	0 (0–0)	0 (0–0)	0 (0–0)	0 (0–0)	0 (0–0)	0 (0–0)	0 (0–0)	0 (0–0)	0 (0–0)	0 (0–0)	0 (0–0)	0 (0–0)	0 (0–0)	0 (0–0)	0 (0–0)	0 (0–0)	0 (0–0)	0 (0–0)	--
Alcoholic cardiomyopathy	--	--	--	--	--	--	0 (0–0)	0 (0–0)	0 (0–0)	0 (0–0)	0 (0–0)	0 (0–0)	0 (0–0)	0 (0–0)	0 (0–0)	0 (0–0)	0 (0–0)	0 (0–0)	0 (0–0)	0 (0–0)	0 (0–0)	0 (0–0)	--
Other cardiomyopathy	0 (0–0)	0 (0–0)	0 (0–0)	0 (0–0)	0 (0–0)	0 (0–0)	0 (0–0)	0 (0–0)	0 (0–0)	0 (0–0)	0 (0–0)	0 (0–0)	0 (0–1)	1 (1–1)	1 (1–2)	1 (1–2)	1 (1–2)	1 (1–2)	1 (1–2)	1 (1–2)	1 (1–2)	0 (0–1)	--
Pulmonary Arterial Hypertension	0 (0–0)	0 (0–0)	0 (0–0)	0 (0–0)	0 (0–0)	0 (0–0)	0 (0–0)	0 (0–0)	0 (0–0)	0 (0–0)	0 (0–0)	0 (0–0)	0 (0–0)	0 (0–0)	0 (0–0)	0 (0–0)	0 (0–0)	0 (0–0)	0 (0–0)	0 (0–0)	0 (0–0)	0 (0–0)	--
Atrial fibrillation and flutter	--	--	--	--	--	--	--	--	--	0 (0–0)	0 (0–0)	0 (0–0)	0 (0–0)	0 (0–0)	0 (0–0)	0 (0–0)	0 (0–0)	0 (0–0)	0 (0–1)	1 (1–1)	1 (1–1)	1 (0–1)	--
Aortic aneurysm	--	--	--	--	--	--	0 (0–0)	0 (0–0)	0 (0–0)	0 (0–0)	0 (0–0)	0 (0–0)	0 (0–0)	0 (0–0)	0 (0–0)	0 (0–0)	0 (0–0)	0 (0–0)	0 (0–1)	0 (0–1)	0 (0–0)	0 (0–0)	--
Peripheral vascular disease	--	--	--	--	--	--	--	--	--	--	--	0 (0–0)	0 (0–0)	0 (0–0)	0 (0–0)	0 (0–0)	0 (0–0)	0 (0–0)	0 (0–0)	0 (0–0)	0 (0–0)	0 (0–0)	--
Endocarditis	0 (0–0)	0 (0–0)	0 (0–0)	0 (0–0)	0 (0–0)	0 (0–0)	0 (0–0)	0 (0–0)	0 (0–0)	0 (0–0)	0 (0–0)	0 (0–0)	0 (0–0)	0 (0–0)	0 (0–0)	0 (0–0)	0 (0–0)	0 (0–0)	0 (0–0)	0 (0–0)	0 (0–0)	0 (0–0)	--
Other cardiovascular and circulatory diseases	0 (0–0)	0 (0–0)	0 (0–0)	0 (0–0)	0 (0–0)	0 (0–0)	0 (0–0)	0 (0–0)	0 (0–0)	0 (0–0)	0 (0–0)	0 (0–0)	0 (0–0)	0 (0–0)	0 (0–0)	0 (0–1)	0 (0–1)	0 (0–1)	1 (0–1)	1 (0–1)	1 (0–1)	0 (0–0)	--
Chronic respiratory diseases	0 (0–0)	0 (0–0)	0 (0–0)	0 (0–0)	0 (0–0)	0 (0–0)	0 (0–0)	0 (0–0)	0 (0–0)	0 (0–0)	0 (0–0)	0 (0–0)	0 (0–0)	0 (0–1)	1 (1–1)	1 (1–2)	2 (1–2)	2 (1–3)	2 (2–3)	3 (2–4)	2 (1–3)	1 (1–1)	--
Chronic obstructive pulmonary disease	--	--	0 (0–0)	0 (0–0)	0 (0–0)	0 (0–0)	0 (0–0)	0 (0–0)	0 (0–0)	0 (0–0)	0 (0–0)	0 (0–0)	0 (0–0)	0 (0–0)	0 (0–1)	1 (0–1)	1 (1–2)	2 (1–2)	2 (1–3)	2 (2–3)	2 (1–2)	1 (1–1)	--
Pneumoconiosis	--	--	--	--	--	--	0 (0–0)	0 (0–0)	0 (0–0)	0 (0–0)	0 (0–0)	0 (0–0)	0 (0–0)	0 (0–0)	0 (0–0)	0 (0–0)	0 (0–0)	0 (0–0)	0 (0–0)	0 (0–0)	0 (0–0)	0 (0–0)	--
Silicosis	--	--	--	--	--	--	0 (0–0)	0 (0–0)	0 (0–0)	0 (0–0)	0 (0–0)	0 (0–0)	0 (0–0)	0 (0–0)	0 (0–0)	0 (0–0)	0 (0–0)	0 (0–0)	0 (0–0)	0 (0–0)	0 (0–0)	0 (0–0)	--

Asbestosis	--	--	--	--	--	--	0 (0–0)	0 (0–0)	0 (0–0)	0 (0–0)	0 (0–0)	0 (0–0)	0 (0–0)	0 (0–0)	0 (0–0)	0 (0–0)	0 (0–0)	0 (0–0)	0 (0–0)	0 (0–0)	0 (0–0)	0 (0–0)	--
Coal workers pneumoconiosis	--	--	--	--	--	--	0 (0–0)	0 (0–0)	0 (0–0)	0 (0–0)	0 (0–0)	0 (0–0)	0 (0–0)	0 (0–0)	0 (0–0)	0 (0–0)	0 (0–0)	0 (0–0)	0 (0–0)	0 (0–0)	0 (0–0)	0 (0–0)	--
Other pneumoconiosis	--	--	--	--	--	--	0 (0–0)	0 (0–0)	0 (0–0)	0 (0–0)	0 (0–0)	0 (0–0)	0 (0–0)	0 (0–0)	0 (0–0)	0 (0–0)	0 (0–0)	0 (0–0)	0 (0–0)	0 (0–0)	0 (0–0)	0 (0–0)	--
Asthma	--	--	--	0 (0–0)	0 (0–0)	0 (0–0)	0 (0–0)	0 (0–0)	0 (0–0)	0 (0–0)	0 (0–0)	0 (0–0)	0 (0–0)	0 (0–0)	0 (0–0)	0 (0–0)	0 (0–0)	0 (0–1)	0 (0–1)	0 (0–1)	0 (0–0)	0 (0–0)	--
Interstitial lung disease and pulmonary sarcoidosis	--	--	--	0 (0–0)	0 (0–0)	0 (0–0)	0 (0–0)	0 (0–0)	0 (0–0)	0 (0–0)	0 (0–0)	0 (0–0)	0 (0–0)	0 (0–0)	0 (0–0)	0 (0–0)	0 (0–0)	0 (0–0)	0 (0–0)	0 (0–0)	0 (0–0)	0 (0–0)	--
Other chronic respiratory diseases	0 (0–0)	0 (0–0)	0 (0–0)	0 (0–0)	0 (0–0)	0 (0–0)	0 (0–0)	0 (0–0)	0 (0–0)	0 (0–0)	0 (0–0)	0 (0–0)	0 (0–0)	0 (0–0)	0 (0–0)	0 (0–0)	0 (0–0)	0 (0–0)	0 (0–0)	0 (0–0)	0 (0–0)	0 (0–0)	--
Digestive diseases	0 (0–0)	0 (0–0)	0 (0–0)	0 (0–0)	0 (0–0)	0 (0–0)	0 (0–0)	0 (0–0)	0 (0–0)	0 (0–0)	0 (0–1)	0 (0–1)	1 (0–1)	1 (1–2)	2 (1–2)	2 (1–2)	2 (1–2)	2 (1–2)	1 (1–2)	1 (1–2)	1 (1–1)	0 (0–1)	--
Cirrhosis and other chronic liver diseases	--	--	--	0 (0–0)	0 (0–0)	0 (0–0)	0 (0–0)	0 (0–0)	0 (0–0)	0 (0–0)	0 (0–0)	0 (0–0)	0 (0–1)	1 (1–1)	1 (1–1)	1 (1–1)	1 (1–1)	1 (0–1)	1 (0–1)	0 (0–1)	0 (0–0)	0 (0–0)	--
Cirrhosis and other chronic liver diseases due to hepatitis B	--	--	--	0 (0–0)	0 (0–0)	0 (0–0)	0 (0–0)	0 (0–0)	0 (0–0)	0 (0–0)	0 (0–0)	0 (0–0)	0 (0–0)	0 (0–0)	0 (0–0)	0 (0–0)	0 (0–0)	0 (0–0)	0 (0–0)	0 (0–0)	0 (0–0)	0 (0–0)	--
Cirrhosis and other chronic liver diseases due to hepatitis C	--	--	--	0 (0–0)	0 (0–0)	0 (0–0)	0 (0–0)	0 (0–0)	0 (0–0)	0 (0–0)	0 (0–0)	0 (0–0)	0 (0–0)	0 (0–0)	0 (0–0)	0 (0–0)	0 (0–0)	0 (0–0)	0 (0–0)	0 (0–0)	0 (0–0)	0 (0–0)	--
Cirrhosis and other chronic liver diseases due to alcohol use	--	--	--	--	--	--	0 (0–0)	0 (0–0)	0 (0–0)	0 (0–0)	0 (0–0)	0 (0–0)	0 (0–0)	0 (0–1)	1 (0–1)	1 (0–1)	0 (0–1)	0 (0–1)	0 (0–0)	0 (0–0)	0 (0–0)	0 (0–0)	--
Cirrhosis and other chronic liver diseases due to NAFLD	--	--	--	--	--	--	0 (0–0)	0 (0–0)	0 (0–0)	0 (0–0)	0 (0–0)	0 (0–0)	0 (0–0)	0 (0–0)	0 (0–0)	0 (0–0)	0 (0–0)	0 (0–0)	0 (0–0)	0 (0–0)	0 (0–0)	0 (0–0)	--
Cirrhosis and other chronic liver diseases due to other causes	--	--	--	0 (0–0)	0 (0–0)	0 (0–0)	0 (0–0)	0 (0–0)	0 (0–0)	0 (0–0)	0 (0–0)	0 (0–0)	0 (0–0)	0 (0–0)	0 (0–0)	0 (0–0)	0 (0–0)	0 (0–0)	0 (0–0)	0 (0–0)	0 (0–0)	0 (0–0)	--
Upper digestive system diseases	--	--	--	0 (0–0)	0 (0–0)	0 (0–0)	0 (0–0)	0 (0–0)	0 (0–0)	0 (0–0)	0 (0–0)	0 (0–0)	0 (0–0)	0 (0–0)	0 (0–0)	0 (0–0)	0 (0–0)	0 (0–0)	0 (0–0)	0 (0–0)	0 (0–0)	0 (0–0)	--
Peptic ulcer disease	--	--	--	0 (0–0)	0 (0–0)	0 (0–0)	0 (0–0)	0 (0–0)	0 (0–0)	0 (0–0)	0 (0–0)	0 (0–0)	0 (0–0)	0 (0–0)	0 (0–0)	0 (0–0)	0 (0–0)	0 (0–0)	0 (0–0)	0 (0–0)	0 (0–0)	0 (0–0)	--
Gastritis and duodenitis	--	--	--	0 (0–0)	0 (0–0)	0 (0–0)	0 (0–0)	0 (0–0)	0 (0–0)	0 (0–0)	0 (0–0)	0 (0–0)	0 (0–0)	0 (0–0)	0 (0–0)	0 (0–0)	0 (0–0)	0 (0–0)	0 (0–0)	0 (0–0)	0 (0–0)	0 (0–0)	--
Appendicitis	--	--	--	0 (0–0)	0 (0–0)	0 (0–0)	0 (0–0)	0 (0–0)	0 (0–0)	0 (0–0)	0 (0–0)	0 (0–0)	0 (0–0)	0 (0–0)	0 (0–0)	0 (0–0)	0 (0–0)	0 (0–0)	0 (0–0)	0 (0–0)	0 (0–0)	0 (0–0)	--
Paralytic ileus and intestinal obstruction	0 (0–0)	0 (0–0)	0 (0–0)	0 (0–0)	0 (0–0)	0 (0–0)	0 (0–0)	0 (0–0)	0 (0–0)	0 (0–0)	0 (0–0)	0 (0–0)	0 (0–0)	0 (0–0)	0 (0–0)	0 (0–0)	0 (0–0)	0 (0–0)	0 (0–0)	0 (0–0)	0 (0–0)	0 (0–0)	--
Inguinal, femoral, and abdominal hernia	0 (0–0)	0 (0–0)	0 (0–0)	0 (0–0)	0 (0–0)	0 (0–0)	0 (0–0)	0 (0–0)	0 (0–0)	0 (0–0)	0 (0–0)	0 (0–0)	0 (0–0)	0 (0–0)	0 (0–0)	0 (0–0)	0 (0–0)	0 (0–0)	0 (0–0)	0 (0–0)	0 (0–0)	0 (0–0)	--
Inflammatory bowel disease	--	--	--	0 (0–0)	0 (0–0)	0 (0–0)	0 (0–0)	0 (0–0)	0 (0–0)	0 (0–0)	0 (0–0)	0 (0–0)	0 (0–0)	0 (0–0)	0 (0–0)	0 (0–0)	0 (0–0)	0 (0–0)	0 (0–0)	0 (0–0)	0 (0–0)	0 (0–0)	--
Vascular intestinal disorders	--	--	--	0 (0–0)	0 (0–0)	0 (0–0)	0 (0–0)	0 (0–0)	0 (0–0)	0 (0–0)	0 (0–0)	0 (0–0)	0 (0–0)	0 (0–0)	0 (0–0)	0 (0–0)	0 (0–0)	0 (0–0)	0 (0–0)	0 (0–0)	0 (0–0)	0 (0–0)	--
Gallbladder and biliary diseases	--	--	--	0 (0–0)	0 (0–0)	0 (0–0)	0 (0–0)	0 (0–0)	0 (0–0)	0 (0–0)	0 (0–0)	0 (0–0)	0 (0–0)	0 (0–0)	0 (0–0)	0 (0–0)	0 (0–0)	0 (0–0)	0 (0–0)	0 (0–0)	0 (0–0)	0 (0–0)	--
Pancreatitis	--	--	--	0 (0–0)	0 (0–0)	0 (0–0)	0 (0–0)	0 (0–0)	0 (0–0)	0 (0–0)	0 (0–0)	0 (0–0)	0 (0–0)	0 (0–0)	0 (0–0)	0 (0–0)	0 (0–0)	0 (0–0)	0 (0–0)	0 (0–0)	0 (0–0)	0 (0–0)	--
Other digestive diseases	--	--	--	0 (0–0)	0 (0–0)	0 (0–0)	0 (0–0)	0 (0–0)	0 (0–0)	0 (0–0)	0 (0–0)	0 (0–0)	0 (0–0)	0 (0–0)	0 (0–0)	0 (0–0)	0 (0–0)	0 (0–0)	0 (0–0)	0 (0–0)	0 (0–0)	0 (0–0)	--
Alzheimer's disease and other dementias	--	--	--	--	--	--	--	--	--	--	--	0 (0–0)	0 (0–0)	0 (0–0)	0 (0–0)	0 (0–1)	0 (0–1)	0 (0–1)	1 (0–3)	2 (0–6)	2 (0–6)	1 (0–4)	--
Parkinson's disease	--	--	--	--	--	--	--	0 (0–0)	0 (0–0)	0 (0–0)	0 (0–0)	0 (0–0)	0 (0–0)	0 (0–0)	0 (0–0)	0 (0–0)	0 (0–0)	0 (0–0)	1 (1–1)	1 (1–1)	1 (0–1)	0 (0–0)	--
Idiopathic epilepsy	--	--	0 (0–0)	0 (0–0)	0 (0–0)	0 (0–0)	0 (0–0)	0 (0–0)	0 (0–0)	0 (0–0)	0 (0–0)	0 (0–0)	0 (0–0)	0 (0–0)	0 (0–0)	0 (0–0)	0 (0–0)	0 (0–0)	0 (0–0)	0 (0–0)	0 (0–0)	0 (0–0)	--
Multiple sclerosis	--	--	--	--	0 (0–0)	0 (0–0)	0 (0–0)	0 (0–0)	0 (0–0)	0 (0–0)	0 (0–0)	0 (0–0)	0 (0–0)	0 (0–0)	0 (0–0)	0 (0–0)	0 (0–0)	0 (0–0)	0 (0–0)	0 (0–0)	0 (0–0)	0 (0–0)	--
Motor neuron disease	0 (0–0)	0 (0–0)	0 (0–0)	0 (0–0)	0 (0–0)	0 (0–0)	0 (0–0)	0 (0–0)	0 (0–0)	0 (0–0)	0 (0–0)	0 (0–0)	0 (0–0)	0 (0–0)	0 (0–0)	0 (0–0)	0 (0–0)	0 (0–0)	0 (0–0)	0 (0–0)	0 (0–0)	0 (0–0)	--
Other neurological disorders	--	--	0 (0–0)	0 (0–0)	0 (0–0)	0 (0–0)	0 (0–0)	0 (0–0)	0 (0–0)	0 (0–0)	0 (0–0)	0 (0–0)	0 (0–0)	0 (0–0)	0 (0–0)	0 (0–0)	0 (0–0)	0 (0–0)	0 (0–0)	0 (0–0)	0 (0–0)	0 (0–0)	--
Eating disorders	--	--	--	--	0 (0–0)	0 (0–0)	0 (0–0)	0 (0–0)	0 (0–0)	0 (0–0)	0 (0–0)	0 (0–0)	0 (0–0)	--	--	--	--	--	--	--	--	--	--
Anorexia nervosa	--	--	--	--	0 (0–0)	0 (0–0)	0 (0–0)	0 (0–0)	0 (0–0)	0 (0–0)	0 (0–0)	0 (0–0)	0 (0–0)	--	--	--	--	--	--	--	--	--	--
Bulimia nervosa	--	--	--	--	0 (0–0)	0 (0–0)	0 (0–0)	0 (0–0)	0 (0–0)	0 (0–0)	0 (0–0)	0 (0–0)	0 (0–0)	--	--	--	--	--	--	--	--	--	--
Alcohol use disorders	--	--	--	--	--	0 (0–0)	0 (0–0)	0 (0–0)	0 (0–0)	0 (0–0)	0 (0–0)	0 (0–0)	0 (0–1)	1 (0–1)	1 (0–1)	0 (0–1)	0 (0–1)	0 (0–0)	0 (0–0)	0 (0–0)	0 (0–0)	0 (0–0)	--
Drug use disorders	0 (0–0)	0 (0–0)	0 (0–0)	0 (0–0)	0 (0–0)	0 (0–0)	0 (0–0)	0 (0–0)	0 (0–0)	0 (0–0)	0 (0–0)	0 (0–0)	0 (0–0)	0 (0–0)	0 (0–0)	0 (0–0)	0 (0–0)	0 (0–0)	0 (0–0)	0 (0–0)	0 (0–0)	0 (0–0)	--
Opioid use disorders	0 (0–0)	0 (0–0)	0 (0–0)	0 (0–0)	0 (0–0)	0 (0–0)	0 (0–0)	0 (0–0)	0 (0–0)	0 (0–0)	0 (0–0)	0 (0–0)	0 (0–0)	0 (0–0)	0 (0–0)	0 (0–0)	0 (0–0)	0 (0–0)	0 (0–0)	0 (0–0)	0 (0–0)	0 (0–0)	--
Cocaine use disorders	--	--	--	--	--	0 (0–0)	0 (0–0)	0 (0–0)	0 (0–0)	0 (0–0)	0 (0–0)	0 (0–0)	0 (0–0)	0 (0–0)	0 (0–0)	0 (0–0)	0 (0–0)	0 (0–0)	0 (0–0)	0 (0–0)	0 (0–0)	0 (0–0)	--
Amphetamine use disorders	--	--	--	--	--	0 (0–0)	0 (0–0)	0 (0–0)	0 (0–0)	0 (0–0)	0 (0–0)	0 (0–0)	0 (0–0)	0 (0–0)	0 (0–0)	0 (0–0)	0 (0–0)	0 (0–0)	0 (0–0)	0 (0–0)	0 (0–0)	0 (0–0)	--
Other drug use disorders	--	--	--	--	--	0 (0–0)	0 (0–0)	0 (0–0)	0 (0–0)	0 (0–0)	0 (0–0)	0 (0–0)	0 (0–0)	0 (0–0)	0 (0–0)	0 (0–0)	0 (0–0)	0 (0–0)	0 (0–0)	0 (0–0)	0 (0–0)	0 (0–0)	--
Diabetes mellitus	0 (0–0)	0 (0–0)	0 (0–0)	0 (0–0)	0 (0–0)	0 (0–0)	0 (0–0)	0 (0–0)	0 (0–0)	0 (0–0)	0 (0–0)	0 (0–0)	1 (0–1)	1 (1–2)	2 (1–3)	2 (2–3)	3 (2–4)	3 (2–5)	4 (2–5)	4 (2–5)	2 (1–3)	1 (1–1)	--
Diabetes mellitus type 1	0 (0–0)	0 (0–0)	0 (0–0)	0 (0–0)	0 (0–0)	0 (0–0)	0 (0–0)	0 (0–0)	0 (0–0)	0 (0–0)	0 (0–0)	0 (0–0)	0 (0–0)	0 (0–0)	0 (0–0)	0 (0–0)	0 (0–0)	0 (0–0)	0 (0–0)	0 (0–0)	0 (0–0)	0 (0–0)	--
Diabetes mellitus type 2	--	--	--	--	--	--	0 (0–0)	0 (0–0)	0 (0–0)	0 (0–0)	0 (0–0)	0 (0–0)	1 (0–1)	1 (1–2)	2 (1–3)	2 (1–3)	3 (2–4)	3 (2–5)	3 (2–5)	4 (2–5)	2 (1–3)	1 (1–1)	--
Chronic kidney disease	--	--	0 (0–0)	0 (0–0)	0 (0–0)	0 (0–0)	0 (0–0)	0 (0–0)	0 (0–0)	0 (0–0)	0 (0–1)	0 (0–1)	1 (0–1)	1 (1–2)	2 (1–3)	2 (1–3)	3 (2–4)	3 (2–4)	3 (2–4)	3 (2–5)	2 (1–3)	1 (1–2)	--
Chronic kidney disease due to diabetes mellitus type 1	--	--	0 (0–0)	0 (0–0)	0 (0–0)	0 (0–0)	0 (0–0)	0 (0–0)	0 (0–0)	0 (0–0)	0 (0–0)	0 (0–0)	0 (0–0)	0 (0–0)	0 (0–0)	0 (0–0)	0 (0–0)	0 (0–0)	0 (0–0)	0 (0–0)	0 (0–0)	0 (0–0)	--
Chronic kidney disease due to diabetes mellitus type 2	--	--	--	--	--	--	0 (0–0)	0 (0–0)	0 (0–0)	0 (0–0)	0 (0–0)	0 (0–0)	0 (0–0)	0 (0–1)	1 (0–1)	1 (0–1)	1 (0–1)	1 (0–1)	1 (1–1)	1 (1–2)	1 (0–1)	0 (0–1)	--
Chronic kidney disease due to hypertension	--	--	--	--	--	--	0 (0–0)	0 (0–0)	0 (0–0)	0 (0–0)	0 (0–0)	0 (0–0)	0 (0–0)	0 (0–0)	0 (0–1)	0 (0–1)	1 (0–1)	1 (0–1)	1 (1–1)	1 (1–2)	1 (0–1)	0 (0–1)	--
Chronic kidney disease due to glomerulonephritis	--	--	0 (0–0)	0 (0–0)	0 (0–0)	0 (0–0)	0 (0–0)	0 (0–0)	0 (0–0)	0 (0–0)	0 (0–0)	0 (0–0)	0 (0–0)	0 (0–1)	1 (0–1)	1 (0–1)	0 (0–1)	0 (0–1)	0 (0–1)	0 (0–1)	0 (0–0)	0 (0–0)	--
Chronic kidney disease due to other and unspecified causes	--	--	0 (0–0)	0 (0–0)	0 (0–0)	0 (0–0)	0 (0–0)	0 (0–0)	0 (0–0)	0 (0–0)	0 (0–0)	0 (0–0)	0 (0–0)	0 (0–1)	0 (0–1)	0 (0–1)	0 (0–1)	1 (0–1)	1 (0–1)	1 (0–1)	0 (0–1)	0 (0–0)	--
Acute glomerulonephritis	--	--	0 (0–0)	0 (0–0)	0 (0–0)	0 (0–0)	0 (0–0)	0 (0–0)	0 (0–0)	0 (0–0)	0 (0–0)	0 (0–0)	0 (0–0)	0 (0–0)	0 (0–0)	0 (0–0)	0 (0–0)	0 (0–0)	0 (0–0)	0 (0–0)	0 (0–0)	0 (0–0)	--
Skin and subcutaneous diseases	--	--	0 (0–0)	0 (0–0)	0 (0–0)	0 (0–0)	0 (0–0)	0 (0–0)	0 (0–0)	0 (0–0)	0 (0–0)	0 (0–0)	0 (0–0)	0 (0–0)	0 (0–0)	0 (0–0)	0 (0–1)	0 (0–1)	0 (0–1)	1 (0–1)	0 (0–1)	0 (0–0)	--
Bacterial skin diseases	--	--	0 (0–0)	0 (0–0)	0 (0–0)	0 (0–0)	0 (0–0)	0 (0–0)	0 (0–0)	0 (0–0)	0 (0–0)	0 (0–0)	0 (0–0)	0 (0–0)	0 (0–0)	0 (0–0)	0 (0–0)	0 (0–0)	0 (0–0)	0 (0–0)	0 (0–0)	0 (0–0)	--
Cellulitis	--	--	0 (0–0)	0 (0–0)	0 (0–0)	0 (0–0)	0 (0–0)	0 (0–0)	0 (0–0)	0 (0–0)	0 (0–0)	0 (0–0)	0 (0–0)	0 (0–0)	0 (0–0)	0 (0–0)	0 (0–0)	0 (0–0)	0 (0–0)	0 (0–0)	0 (0–0)	0 (0–0)	--
Pyoderma	--	--	0 (0–0)	0 (0–0)	0 (0–0)	0 (0–0)	0 (0–0)	0 (0–0)	0 (0–0)	0 (0–0)	0 (0–0)	0 (0–0)	0 (0–0)	0 (0–0)	0 (0–0)	0 (0–0)	0 (0–0)	0 (0–0)	0 (0–0)	0 (0–0)	0 (0–0)	0 (0–0)	--
Decubitus ulcer	--	--	--	0 (0–0)	0 (0–0)	0 (0–0)	0 (0–0)	0 (0–0)	0 (0–0)	0 (0–0)	0 (0–0)	0 (0–0)	0 (0–0)	0 (0–0)	0 (0–0)	0 (0–0)	0 (0–0)	0 (0–0)	0 (0–1)	0 (0–1)	0 (0–1)	0 (0–0)	--
Other skin and subcutaneous diseases	--	--	0 (0–0)	0 (0–0)	0 (0–0)	0 (0–0)	0 (0–0)	0 (0–0)	0 (0–0)	0 (0–0)	0 (0–0)	0 (0–0)	0 (0–0)	0 (0–0)	0 (0–0)	0 (0–0)	0 (0–0)	0 (0–0)	0 (0–0)	0 (0–0)	0 (0–0)	0 (0–0)	--
Musculoskeletal disorders	--	--	--	--	0 (0–0)	0 (0–0)	0 (0–0)	0 (0–0)	0 (0–0)	0 (0–0)	0 (0–0)	0 (0–0)	0 (0–0)	0 (0–0)	0 (0–0)	0 (0–0)	0 (0–0)	0 (0–0)	0 (0–0)	0 (0–0)	0 (0–0)	0 (0–0)	--
Rheumatoid arthritis	--	--	--	--	0 (0–0)	0 (0–0)	0 (0–0)	0 (0–0)	0 (0–0)	0 (0–0)	0 (0–0)	0 (0–0)	0 (0–0)	0 (0–0)	0 (0–0)	0 (0–0)	0 (0–0)	0 (0–0)	0 (0–0)	0 (0–0)	0 (0–0)	0 (0–0)	--
Other musculoskeletal disorders	--	--	--	--	0 (0–0)	0 (0–0)	0 (0–0)	0 (0–0)	0 (0–0)	0 (0–0)	0 (0–0)	0 (0–0)	0 (0–0)	0 (0–0)	0 (0–0)	0 (0–0)	0 (0–0)	0 (0–0)	0 (0–0)	0 (0–0)	0 (0–0)	0 (0–0)	--
Congenital anomalies	1 (0–1)	0 (0–0)	1 (0–1)	0 (0–0)	0 (0–0)	0 (0–0)	0 (0–0)	0 (0–0)	0 (0–0)	0 (0–0)	0 (0–0)	0 (0–0)	0 (0–0)	0 (0–0)	0 (0–0)	0 (0–0)	0 (0–0)	--	--	--	--	--	--
Neural tube defects	0 (0–0)	0 (0–0)	0 (0–0)	0 (0–0)	0 (0–0)	0 (0–0)	0 (0–0)	0 (0–0)	0 (0–0)	0 (0–0)	0 (0–0)	0 (0–0)	0 (0–0)	0 (0–0)	0 (0–0)	0 (0–0)	0 (0–0)	--	--	--	--	--	--
Congenital heart anomalies	0 (0–0)	0 (0–0)	0 (0–0)	0 (0–0)	0 (0–0)	0 (0–0)	0 (0–0)	0 (0–0)	0 (0–0)	0 (0–0)	0 (0–0)	0 (0–0)	0 (0–0)	0 (0–0)	0 (0–0)	0 (0–0)	0 (0–0)	--	--	--	--	--	--
Orofacial clefts	0 (0–0)	0 (0–0)	0 (0–0)	0 (0–0)	--	--	--	--	--	--	--	--	--	--	--	--	--	--	--	--	--	--	--
Down's syndrome	0 (0–0)	0 (0–0)	0 (0–0)	0 (0–0)	0 (0–0)	0 (0–0)	0 (0–0)	0 (0–0)	0 (0–0)	0 (0–0)	0 (0–0)	0 (0–0)	0 (0–0)	0 (0–0)	0 (0–0)	0 (0–0)	0 (0–0)	--	--	--	--	--	--
Other chromosomal abnormalities	0 (0–0)	0 (0–0)	0 (0–0)	0 (0–0)	0 (0–0)	0 (0–0)	0 (0–0)	0 (0–0)	0 (0–0)	0 (0–0)	0 (0–0)	0 (0–0)	0 (0–0)	0 (0–0)	0 (0–0)	0 (0–0)	0 (0–0)	--	--	--	--	--	--
Congenital musculoskeletal and limb anomalies	0 (0–0)	0 (0–0)	0 (0–0)	0 (0–0)	0 (0–0)	0 (0–0)	0 (0–0)	0 (0–0)	0 (0–0)	0 (0–0)	0 (0–0)	0 (0–0)	0 (0–0)	0 (0–0)	0 (0–0)	0 (0–0)	0 (0–0)	--	--	--	--	--	--
Urogenital congenital anomalies	0 (0–0)	0 (0–0)	0 (0–0)	0 (0–0)	0 (0–0)	0 (0–0)	0 (0–0)	0 (0–0)	0 (0–0)	0 (0–0)	0 (0–0)	0 (0–0)	0 (0–0)	0 (0–0)	0 (0–0)	0 (0–0)	0 (0–0)	--	--	--	--	--	--
Digestive congenital anomalies	0 (0–0)	0 (0–0)	0 (0–0)	0 (0–0)	0 (0–0)	0 (0–0)	0 (0–0)	0 (0–0)	0 (0–0)	0 (0–0)	0 (0–0)	0 (0–0)	0 (0–0)	0 (0–0)	0 (0–0)	0 (0–0)	0 (0–0)	--	--	--	--	--	--
Other congenital anomalies	0 (0–0)	0 (0–0)	0 (0–0)	0 (0–0)	0 (0–0)	0 (0–0)	0 (0–0)	0 (0–0)	0 (0–0)	0 (0–0)	0 (0–0)	0 (0–0)	0 (0–0)	0 (0–0)	0 (0–0)	0 (0–0)	0 (0–0)	--	--	--	--	--	--
Urinary diseases and male infertility	0 (0–0)	0 (0–0)	0 (0–0)	0 (0–0)	0 (0–0)	0 (0–0)	0 (0–0)	0 (0–0)	0 (0–0)	0 (0–0)	0 (0–0)	0 (0–0)	0 (0–0)	0 (0–0)	0 (0–0)	0 (0–0)	0 (0–0)	0 (0–0)	0 (0–0)	0 (0–1)	0 (0–0)	0 (0–0)	--
Urinary tract infections and interstitial nephritis	0 (0–0)	0 (0–0)	0 (0–0)	0 (0–0)	0 (0–0)	0 (0–0)	0 (0–0)	0 (0–0)	0 (0–0)	0 (0–0)	0 (0–0)	0 (0–0)	0 (0–0)	0 (0–0)	0 (0–0)	0 (0–0)	0 (0–0)	0 (0–0)	0 (0–0)	0 (0–0)	0 (0–0)	0 (0–0)	--

Urolithiasis	--	--	--	0 (0–0)	0 (0–0)	0 (0–0)	0 (0–0)	0 (0–0)	0 (0–0)	0 (0–0)	0 (0–0)	0 (0–0)	0 (0–0)	0 (0–0)	0 (0–0)	0 (0–0)	0 (0–0)	0 (0–0)	0 (0–0)	0 (0–0)	0 (0–0)	0 (0–0)	--
Other urinary diseases	0 (0–0)	0 (0–0)	0 (0–0)	0 (0–0)	0 (0–0)	0 (0–0)	0 (0–0)	0 (0–0)	0 (0–0)	0 (0–0)	0 (0–0)	0 (0–0)	0 (0–0)	0 (0–0)	0 (0–0)	0 (0–0)	0 (0–0)	0 (0–0)	0 (0–0)	0 (0–0)	0 (0–0)	0 (0–0)	--
Gynecological diseases	--	--	--	--	--	--	--	--	--	--	--	--	--	--	--	--	--	--	--	--	--	--	--
Uterine fibroids	--	--	--	--	--	--	--	--	--	--	--	--	--	--	--	--	--	--	--	--	--	--	--
Endometriosis	--	--	--	--	--	--	--	--	--	--	--	--	--	--	--	--	--	--	--	--	--	--	--
Genital prolapse	--	--	--	--	--	--	--	--	--	--	--	--	--	--	--	--	--	--	--	--	--	--	--
Other gynecological diseases	--	--	--	--	--	--	--	--	--	--	--	--	--	--	--	--	--	--	--	--	--	--	--
Hemoglobinopathies and hemolytic anaemias	0 (0–0)	0 (0–0)	0 (0–0)	0 (0–0)	0 (0–0)	0 (0–0)	0 (0–0)	0 (0–0)	0 (0–0)	0 (0–0)	0 (0–0)	0 (0–0)	0 (0–0)	0 (0–0)	0 (0–0)	0 (0–0)	0 (0–0)	0 (0–0)	0 (0–0)	0 (0–0)	0 (0–0)	0 (0–0)	--
Thalassemias	0 (0–0)	0 (0–0)	0 (0–0)	0 (0–0)	0 (0–0)	0 (0–0)	0 (0–0)	0 (0–0)	0 (0–0)	0 (0–0)	0 (0–0)	0 (0–0)	0 (0–0)	0 (0–0)	0 (0–0)	0 (0–0)	0 (0–0)	0 (0–0)	0 (0–0)	0 (0–0)	0 (0–0)	0 (0–0)	--
Sickle cell disorders	0 (0–0)	0 (0–0)	0 (0–0)	0 (0–0)	0 (0–0)	0 (0–0)	0 (0–0)	0 (0–0)	0 (0–0)	0 (0–0)	0 (0–0)	0 (0–0)	0 (0–0)	0 (0–0)	0 (0–0)	0 (0–0)	0 (0–0)	0 (0–0)	0 (0–0)	0 (0–0)	0 (0–0)	0 (0–0)	--
G6PD deficiency	0 (0–0)	0 (0–0)	0 (0–0)	0 (0–0)	0 (0–0)	0 (0–0)	0 (0–0)	0 (0–0)	0 (0–0)	0 (0–0)	0 (0–0)	0 (0–0)	0 (0–0)	0 (0–0)	0 (0–0)	0 (0–0)	0 (0–0)	0 (0–0)	0 (0–0)	0 (0–0)	0 (0–0)	0 (0–0)	--
Other hemoglobinopathies and hemolytic anaemias	0 (0–0)	0 (0–0)	0 (0–0)	0 (0–0)	0 (0–0)	0 (0–0)	0 (0–0)	0 (0–0)	0 (0–0)	0 (0–0)	0 (0–0)	0 (0–0)	0 (0–0)	0 (0–0)	0 (0–0)	0 (0–0)	0 (0–0)	0 (0–0)	0 (0–0)	0 (0–0)	0 (0–0)	0 (0–0)	--
Endocrine, metabolic, blood, and immune disorders	0 (0–0)	0 (0–0)	0 (0–0)	0 (0–0)	0 (0–0)	0 (0–0)	0 (0–0)	0 (0–0)	0 (0–0)	0 (0–0)	0 (0–0)	0 (0–0)	0 (0–0)	0 (0–0)	0 (0–0)	0 (0–0)	0 (0–0)	0 (0–1)	0 (0–1)	0 (0–1)	0 (0–1)	0 (0–0)	--
Sudden infant death syndrome	--	0 (0–0)	0 (0–0)	--	--	--	--	--	--	--	--	--	--	--	--	--	--	--	--	--	--	--	--
Transport injuries	0 (0–0)	0 (0–0)	0 (0–0)	0 (0–0)	0 (0–0)	0 (0–0)	1 (0–1)	1 (1–1)	1 (1–1)	1 (1–1)	1 (1–1)	1 (0–1)	1 (0–1)	1 (1–1)	1 (0–1)	1 (0–1)	1 (0–1)	0 (0–1)	0 (0–1)	0 (0–0)	0 (0–0)	0 (0–0)	--
Road injuries	0 (0–0)	0 (0–0)	0 (0–0)	0 (0–0)	0 (0–0)	0 (0–0)	1 (0–1)	1 (1–1)	1 (1–1)	1 (1–1)	1 (1–1)	1 (0–1)	1 (0–1)	1 (0–1)	1 (0–1)	1 (0–1)	0 (0–1)	0 (0–1)	0 (0–1)	0 (0–0)	0 (0–0)	0 (0–0)	--
Pedestrian road injuries	0 (0–0)	0 (0–0)	0 (0–0)	0 (0–0)	0 (0–0)	0 (0–0)	0 (0–0)	0 (0–0)	0 (0–0)	0 (0–0)	0 (0–0)	0 (0–0)	0 (0–0)	0 (0–0)	0 (0–0)	0 (0–0)	0 (0–0)	0 (0–0)	0 (0–0)	0 (0–0)	0 (0–0)	0 (0–0)	--
Cyclist road injuries	--	--	--	0 (0–0)	0 (0–0)	0 (0–0)	0 (0–0)	0 (0–0)	0 (0–0)	0 (0–0)	0 (0–0)	0 (0–0)	0 (0–0)	0 (0–0)	0 (0–0)	0 (0–0)	0 (0–0)	0 (0–0)	0 (0–0)	0 (0–0)	0 (0–0)	0 (0–0)	--
Motorcyclist road injuries	0 (0–0)	0 (0–0)	0 (0–0)	0 (0–0)	0 (0–0)	0 (0–0)	0 (0–0)	0 (0–1)	0 (0–1)	0 (0–0)	0 (0–0)	0 (0–0)	0 (0–0)	0 (0–0)	0 (0–0)	0 (0–0)	0 (0–0)	0 (0–0)	0 (0–0)	0 (0–0)	0 (0–0)	0 (0–0)	--
Motor vehicle road injuries	0 (0–0)	0 (0–0)	0 (0–0)	0 (0–0)	0 (0–0)	0 (0–0)	0 (0–0)	0 (0–1)	0 (0–1)	0 (0–1)	0 (0–1)	0 (0–0)	0 (0–0)	0 (0–1)	0 (0–1)	0 (0–0)	0 (0–0)	0 (0–0)	0 (0–0)	0 (0–0)	0 (0–0)	0 (0–0)	--
Other road injuries	0 (0–0)	0 (0–0)	0 (0–0)	0 (0–0)	0 (0–0)	0 (0–0)	0 (0–0)	0 (0–0)	0 (0–0)	0 (0–0)	0 (0–0)	0 (0–0)	0 (0–0)	0 (0–0)	0 (0–0)	0 (0–0)	0 (0–0)	0 (0–0)	0 (0–0)	0 (0–0)	0 (0–0)	0 (0–0)	--
Other transport injuries	0 (0–0)	0 (0–0)	0 (0–0)	0 (0–0)	0 (0–0)	0 (0–0)	0 (0–0)	0 (0–0)	0 (0–0)	0 (0–0)	0 (0–0)	0 (0–0)	0 (0–0)	0 (0–0)	0 (0–0)	0 (0–0)	0 (0–0)	0 (0–0)	0 (0–0)	0 (0–0)	0 (0–0)	0 (0–0)	--
Falls	0 (0–0)	0 (0–0)	0 (0–0)	0 (0–0)	0 (0–0)	0 (0–0)	0 (0–0)	0 (0–0)	0 (0–0)	0 (0–0)	0 (0–0)	0 (0–0)	0 (0–0)	0 (0–0)	0 (0–0)	0 (0–0)	0 (0–0)	0 (0–0)	0 (0–0)	1 (0–1)	0 (0–1)	0 (0–0)	--
Drowning	0 (0–0)	0 (0–0)	0 (0–0)	0 (0–0)	0 (0–0)	0 (0–0)	0 (0–1)	0 (0–1)	0 (0–1)	0 (0–0)	0 (0–0)	0 (0–0)	0 (0–0)	0 (0–0)	0 (0–0)	0 (0–0)	0 (0–0)	0 (0–0)	0 (0–0)	0 (0–0)	0 (0–0)	0 (0–0)	--
Fire, heat, and hot substances	0 (0–0)	0 (0–0)	0 (0–0)	0 (0–0)	0 (0–0)	0 (0–0)	0 (0–0)	0 (0–0)	0 (0–0)	0 (0–0)	0 (0–0)	0 (0–0)	0 (0–0)	0 (0–0)	0 (0–0)	0 (0–0)	0 (0–0)	0 (0–0)	0 (0–0)	0 (0–0)	0 (0–0)	0 (0–0)	--
Poisonings	0 (0–0)	0 (0–0)	0 (0–0)	0 (0–0)	0 (0–0)	0 (0–0)	0 (0–0)	0 (0–0)	0 (0–0)	0 (0–0)	0 (0–0)	0 (0–0)	0 (0–0)	0 (0–0)	0 (0–0)	0 (0–0)	0 (0–0)	0 (0–0)	0 (0–0)	0 (0–0)	0 (0–0)	0 (0–0)	--
Poisoning by carbon monoxide	0 (0–0)	0 (0–0)	0 (0–0)	0 (0–0)	0 (0–0)	0 (0–0)	0 (0–0)	0 (0–0)	0 (0–0)	0 (0–0)	0 (0–0)	0 (0–0)	0 (0–0)	0 (0–0)	0 (0–0)	0 (0–0)	0 (0–0)	0 (0–0)	0 (0–0)	0 (0–0)	0 (0–0)	0 (0–0)	--
Poisoning by other means	0 (0–0)	0 (0–0)	0 (0–0)	0 (0–0)	0 (0–0)	0 (0–0)	0 (0–0)	0 (0–0)	0 (0–0)	0 (0–0)	0 (0–0)	0 (0–0)	0 (0–0)	0 (0–0)	0 (0–0)	0 (0–0)	0 (0–0)	0 (0–0)	0 (0–0)	0 (0–0)	0 (0–0)	0 (0–0)	--
Exposure to mechanical forces	0 (0–0)	0 (0–0)	0 (0–0)	0 (0–0)	0 (0–0)	0 (0–0)	0 (0–0)	0 (0–0)	0 (0–0)	0 (0–0)	0 (0–0)	0 (0–0)	0 (0–0)	0 (0–0)	0 (0–0)	0 (0–0)	0 (0–0)	0 (0–0)	0 (0–0)	0 (0–0)	0 (0–0)	0 (0–0)	--
Unintentional firearm injuries	0 (0–0)	0 (0–0)	0 (0–0)	0 (0–0)	0 (0–0)	0 (0–0)	0 (0–0)	0 (0–0)	0 (0–0)	0 (0–0)	0 (0–0)	0 (0–0)	0 (0–0)	0 (0–0)	0 (0–0)	0 (0–0)	0 (0–0)	0 (0–0)	0 (0–0)	0 (0–0)	0 (0–0)	0 (0–0)	--
Other exposure to mechanical forces	0 (0–0)	0 (0–0)	0 (0–0)	0 (0–0)	0 (0–0)	0 (0–0)	0 (0–0)	0 (0–0)	0 (0–0)	0 (0–0)	0 (0–0)	0 (0–0)	0 (0–0)	0 (0–0)	0 (0–0)	0 (0–0)	0 (0–0)	0 (0–0)	0 (0–0)	0 (0–0)	0 (0–0)	0 (0–0)	--
Adverse effects of medical treatment	0 (0–0)	0 (0–0)	0 (0–0)	0 (0–0)	0 (0–0)	0 (0–0)	0 (0–0)	0 (0–0)	0 (0–0)	0 (0–0)	0 (0–0)	0 (0–0)	0 (0–0)	0 (0–0)	0 (0–0)	0 (0–0)	0 (0–0)	0 (0–0)	0 (0–0)	0 (0–0)	0 (0–0)	0 (0–0)	--
Animal contact	0 (0–0)	0 (0–0)	0 (0–0)	0 (0–0)	0 (0–0)	0 (0–0)	0 (0–0)	0 (0–0)	0 (0–0)	0 (0–0)	0 (0–0)	0 (0–0)	0 (0–0)	0 (0–0)	0 (0–0)	0 (0–0)	0 (0–0)	0 (0–0)	0 (0–0)	0 (0–0)	0 (0–0)	0 (0–0)	--
Venomous animal contact	0 (0–0)	0 (0–0)	0 (0–0)	0 (0–0)	0 (0–0)	0 (0–0)	0 (0–0)	0 (0–0)	0 (0–0)	0 (0–0)	0 (0–0)	0 (0–0)	0 (0–0)	0 (0–0)	0 (0–0)	0 (0–0)	0 (0–0)	0 (0–0)	0 (0–0)	0 (0–0)	0 (0–0)	0 (0–0)	--
Non-venomous animal contact	0 (0–0)	0 (0–0)	0 (0–0)	0 (0–0)	0 (0–0)	0 (0–0)	0 (0–0)	0 (0–0)	0 (0–0)	0 (0–0)	0 (0–0)	0 (0–0)	0 (0–0)	0 (0–0)	0 (0–0)	0 (0–0)	0 (0–0)	0 (0–0)	0 (0–0)	0 (0–0)	0 (0–0)	0 (0–0)	--
Foreign body	0 (0–0)	0 (0–0)	0 (0–0)	0 (0–0)	0 (0–0)	0 (0–0)	0 (0–0)	0 (0–0)	0 (0–0)	0 (0–0)	0 (0–0)	0 (0–0)	0 (0–0)	0 (0–0)	0 (0–0)	0 (0–0)	0 (0–0)	0 (0–0)	0 (0–0)	0 (0–0)	0 (0–0)	0 (0–0)	--
Pulmonary aspiration and foreign body in airway	0 (0–0)	0 (0–0)	0 (0–0)	0 (0–0)	0 (0–0)	0 (0–0)	0 (0–0)	0 (0–0)	0 (0–0)	0 (0–0)	0 (0–0)	0 (0–0)	0 (0–0)	0 (0–0)	0 (0–0)	0 (0–0)	0 (0–0)	0 (0–0)	0 (0–0)	0 (0–0)	0 (0–0)	0 (0–0)	--
Foreign body in other body part	0 (0–0)	0 (0–0)	0 (0–0)	0 (0–0)	0 (0–0)	0 (0–0)	0 (0–0)	0 (0–0)	0 (0–0)	0 (0–0)	0 (0–0)	0 (0–0)	0 (0–0)	0 (0–0)	0 (0–0)	0 (0–0)	0 (0–0)	0 (0–0)	0 (0–0)	0 (0–0)	0 (0–0)	0 (0–0)	--
Environmental heat and cold exposure	0 (0–0)	0 (0–0)	0 (0–0)	0 (0–0)	0 (0–0)	0 (0–0)	0 (0–0)	0 (0–0)	0 (0–0)	0 (0–0)	0 (0–0)	0 (0–0)	0 (0–0)	0 (0–0)	0 (0–0)	0 (0–0)	0 (0–0)	0 (0–0)	0 (0–0)	0 (0–0)	0 (0–0)	0 (0–0)	--
Other unintentional injuries	0 (0–0)	0 (0–0)	0 (0–0)	0 (0–0)	0 (0–0)	0 (0–0)	0 (0–0)	0 (0–0)	0 (0–0)	0 (0–0)	0 (0–0)	0 (0–0)	0 (0–0)	0 (0–0)	0 (0–0)	0 (0–0)	0 (0–0)	0 (0–0)	0 (0–0)	0 (0–0)	0 (0–0)	0 (0–0)	--
Self-harm	--	--	--	--	--	0 (0–0)	0 (0–0)	0 (0–0)	0 (0–1)	0 (0–0)	0 (0–0)	0 (0–0)	0 (0–0)	0 (0–0)	0 (0–0)	0 (0–0)	0 (0–0)	0 (0–0)	0 (0–0)	0 (0–0)	0 (0–0)	0 (0–0)	--
Self-harm by firearm	--	--	--	--	--	0 (0–0)	0 (0–0)	0 (0–0)	0 (0–0)	0 (0–0)	0 (0–0)	0 (0–0)	0 (0–0)	0 (0–0)	0 (0–0)	0 (0–0)	0 (0–0)	0 (0–0)	0 (0–0)	0 (0–0)	0 (0–0)	0 (0–0)	--
Self-harm by other specified means	--	--	--	--	--	0 (0–0)	0 (0–0)	0 (0–0)	0 (0–0)	0 (0–0)	0 (0–0)	0 (0–0)	0 (0–0)	0 (0–0)	0 (0–0)	0 (0–0)	0 (0–0)	0 (0–0)	0 (0–0)	0 (0–0)	0 (0–0)	0 (0–0)	--
Interpersonal violence	0 (0–0)	0 (0–0)	0 (0–0)	0 (0–0)	0 (0–0)	0 (0–0)	1 (0–1)	1 (1–1)	1 (1–2)	1 (0–1)	1 (0–1)	0 (0–1)	0 (0–1)	0 (0–1)	0 (0–1)	0 (0–0)	0 (0–0)	0 (0–0)	0 (0–0)	0 (0–0)	0 (0–0)	0 (0–0)	--
Physical violence by firearm	0 (0–0)	0 (0–0)	0 (0–0)	0 (0–0)	0 (0–0)	0 (0–0)	0 (0–0)	0 (0–1)	0 (0–1)	0 (0–1)	0 (0–0)	0 (0–0)	0 (0–0)	0 (0–0)	0 (0–0)	0 (0–0)	0 (0–0)	0 (0–0)	0 (0–0)	0 (0–0)	0 (0–0)	0 (0–0)	--
Physical violence by sharp object	0 (0–0)	0 (0–0)	0 (0–0)	0 (0–0)	0 (0–0)	0 (0–0)	0 (0–0)	0 (0–1)	0 (0–1)	0 (0–1)	0 (0–1)	0 (0–0)	0 (0–0)	0 (0–0)	0 (0–0)	0 (0–0)	0 (0–0)	0 (0–0)	0 (0–0)	0 (0–0)	0 (0–0)	0 (0–0)	--
Physical violence by other means	0 (0–0)	0 (0–0)	0 (0–0)	0 (0–0)	0 (0–0)	0 (0–0)	0 (0–0)	0 (0–0)	0 (0–0)	0 (0–0)	0 (0–0)	0 (0–0)	0 (0–0)	0 (0–0)	0 (0–0)	0 (0–0)	0 (0–0)	0 (0–0)	0 (0–0)	0 (0–0)	0 (0–0)	0 (0–0)	--
Executions and police conflict	0 (0–0)	0 (0–0)	0 (0–0)	0 (0–0)	0 (0–0)	0 (0–0)	0 (0–0)	0 (0–0)	0 (0–0)	0 (0–0)	0 (0–0)	0 (0–0)	0 (0–0)	0 (0–0)	0 (0–0)	0 (0–0)	0 (0–0)	0 (0–0)	0 (0–0)	0 (0–0)	0 (0–0)	0 (0–0)	--

SR Table 12: Dominican Republic deaths by cause and age group for males

Cause	Early Neonatal	Late Neonatal	Post Neonatal	1 to 4	5 to 9	10 to 14	15 to 19	20 to 24	25 to 29	30 to 34	35 to 39	40 to 44	45 to 49	50 to 54	55 to 59	60 to 64	65 to 69	70 to 74	75 to 79	80 to 84	85 to 89	90 to 94	95 plus
All causes	1,887 (1,338–2,612)	426 (301–586)	653 (465–890)	465 (349–607)	241 (205–279)	220 (179–270)	505 (386–642)	987 (765–1,220)	1,104 (897–1,313)	1,260 (1,026–1,490)	1,350 (1,074–1,648)	1,598 (1,239–2,026)	1,930 (1,468–2,512)	2,514 (1,896–3,317)	2,738 (2,041–3,635)	3,545 (2,675–4,584)	3,503 (2,707–4,464)	4,109 (3,278–5,107)	3,495 (2,910–4,257)	3,456 (3,019–4,050)	2,994 (2,689–3,368)	1,576 (1,416–1,742)	--
Sexually transmitted infections excluding HIV	51 (16–113)	19 (6–43)	28 (8–64)	1 (0–2)	0 (0–1)	0 (0–1)	1 (0–2)	1 (0–3)	1 (0–4)	1 (0–4)	2 (0–8)	1 (0–3)	1 (0–4)	1 (0–5)	2 (0–4)	1 (0–4)	2 (1–6)	1 (0–3)	1 (0–2)	1 (0–3)	1 (0–3)	0 (0–2)	--
Syphilis	51 (16–113)	19 (6–43)	28 (8–64)	1 (0–2)	0 (0–1)	0 (0–1)	0 (0–2)	1 (0–2)	1 (0–3)	1 (0–3)	2 (0–7)	1 (0–2)	1 (0–3)	1 (0–3)	1 (0–3)	1 (0–3)	2 (0–4)	1 (0–3)	0 (0–1)	1 (0–1)	0 (0–1)	0 (0–0)	--
Chlamydial infection	--	--	--	--	--	0 (0–0)	0 (0–0)	0 (0–0)	0 (0–0)	0 (0–0)	0 (0–0)	0 (0–0)	0 (0–0)	0 (0–0)	0 (0–0)	0 (0–0)	0 (0–0)	0 (0–0)	0 (0–0)	0 (0–0)	0 (0–0)	0 (0–0)	--
Gonococcal infection	--	--	--	--	--	0 (0–0)	0 (0–0)	0 (0–1)	0 (0–0)	0 (0–0)	0 (0–0)	0 (0–0)	0 (0–0)	0 (0–0)	0 (0–0)	0 (0–0)	0 (0–1)	0 (0–0)	0 (0–0)	0 (0–0)	0 (0–0)	0 (0–0)	--
Other sexually transmitted infections	--	--	--	--	--	0 (0–0)	0 (0–0)	0 (0–0)	0 (0–1)	0 (0–1)	0 (0–1)	0 (0–0)	0 (0–1)	0 (0–1)	0 (0–1)	0 (0–0)	0 (0–1)	0 (0–0)	0 (0–1)	1 (0–2)	1 (0–2)	0 (0–1)	--
Tuberculosis	--	--	5 (2–9)	6 (3–11)	3 (2–5)	3 (2–5)	9 (4–15)	27 (15–47)	38 (21–62)	52 (30–81)	46 (27–75)	46 (27–76)	38 (21–64)	37 (21–60)	33 (19–52)	33 (19–56)	29 (17–46)	30 (18–49)	25 (15–40)	22 (13–35)	18 (11–28)	8 (5–13)	--
Drug-susceptible tuberculosis	--	--	5 (2–9)	6 (3–11)	3 (2–5)	3 (2–5)	9 (4–15)	27 (14–47)	37 (21–60)	51 (29–80)	46 (27–73)	45 (27–75)	38 (21–63)	36 (21–60)	32 (19–51)	32 (19–55)	28 (17–45)	30 (17–49)	25 (15–39)	22 (13–34)	18 (11–27)	8 (5–13)	--
Multidrug-resistant tuberculosis without extensive drug resistance	--	--	0 (0–0)	0 (0–0)	0 (0–0)	0 (0–0)	0 (0–1)	0 (0–1)	0 (0–2)	1 (0–3)	1 (0–3)	1 (0–2)	0 (0–2)	0 (0–2)	0 (0–2)	0 (0–2)	0 (0–2)	0 (0–2)	0 (0–1)	0 (0–1)	0 (0–1)	0 (0–0)	--
Extensively drug-resistant tuberculosis	--	--	0 (0–0)	0 (0–0)	0 (0–0)	0 (0–0)	0 (0–0)	0 (0–0)	0 (0–0)	0 (0–0)	0 (0–0)	0 (0–0)	0 (0–0)	0 (0–0)	0 (0–0)	0 (0–0)	0 (0–0)	0 (0–0)	0 (0–0)	0 (0–0)	0 (0–0)	0 (0–0)	--
Lower respiratory infections	27 (13–50)	15 (7–28)	77 (33–141)	38 (18–68)	17 (8–24)	10 (5–15)	10 (5–16)	13 (7–21)	15 (9–25)	20 (12–32)	20 (12–33)	26 (15–43)	34 (20–57)	41 (24–68)	46 (27–76)	66 (38–108)	75 (42–119)	104 (61–171)	95 (58–147)	108 (70–163)	115 (72–169)	64 (40–96)	--
Upper respiratory infections	0 (0–0)	0 (0–0)	0 (0–1)	0 (0–1)	0 (0–0)	0 (0–0)	0 (0–0)	0 (0–0)	0 (0–0)	0 (0–0)	0 (0–0)	0 (0–0)	0 (0–0)	0 (0–0)	0 (0–0)	0 (0–0)	0 (0–0)	0 (0–0)	0 (0–0)	0 (0–0)	0 (0–0)	0 (0–0)	--
Otitis media	0 (0–0)	0 (0–0)	0 (0–0)	0 (0–0)	0 (0–0)	0 (0–0)	0 (0–0)	0 (0–0)	0 (0–0)	0 (0–0)	0 (0–0)	0 (0–0)	0 (0–0)	0 (0–0)	0 (0–0)	0 (0–0)	0 (0–0)	0 (0–0)	0 (0–0)	0 (0–0)	0 (0–0)	0 (0–0)	--
Diarrhoeal diseases	4 (1–12)	9 (2–27)	77 (22–188)	32 (8–81)	7 (2–18)	2 (1–5)	2 (1–4)	3 (1–6)	3 (1–7)	3 (1–7)	4 (2–8)	5 (2–11)	6 (3–12)	7 (3–15)	8 (4–18)	11 (5–23)	10 (4–21)	16 (7–32)	15 (6–31)	19 (8–38)	21 (9–41)	12 (5–22)	--
Typhoid fever	--	--	0 (0–0)	0 (0–0)	0 (0–0)	0 (0–0)	0 (0–0)	0 (0–0)	0 (0–0)	0 (0–0)	0 (0–0)	0 (0–0)	0 (0–0)	0 (0–0)	0 (0–0)	0 (0–0)	0 (0–0)	0 (0–0)	0 (0–0)	0 (0–0)	0 (0–0)	0 (0–0)	--
Paratyphoid fever	--	--	0 (0–0)	0 (0–0)	0 (0–0)	0 (0–0)	0 (0–0)	0 (0–0)	0 (0–0)	0 (0–0)	0 (0–0)	0 (0–0)	0 (0–0)	0 (0–0)	0 (0–0)	0 (0–0)	0 (0–0)	0 (0–0)	0 (0–0)	0 (0–0)	0 (0–0)	0 (0–0)	--
iNTS	--	0 (0–0)	0 (0–0)	0 (0–1)	0 (0–0)	0 (0–0)	0 (0–0)	0 (0–0)	0 (0–0)	0 (0–0)	0 (0–0)	0 (0–0)	0 (0–0)	0 (0–0)	0 (0–0)	0 (0–0)	0 (0–0)	0 (0–0)	0 (0–0)	0 (0–0)	0 (0–0)	0 (0–0)	--
Other intestinal infectious diseases	--	--	0 (0–0)	0 (0–1)	0 (0–0)	0 (0–0)	0 (0–0)	0 (0–0)	0 (0–0)	0 (0–0)	0 (0–0)	0 (0–0)	0 (0–0)	0 (0–0)	0 (0–0)	0 (0–0)	0 (0–0)	0 (0–0)	0 (0–0)	0 (0–0)	0 (0–0)	0 (0–0)	--
Malaria	0 (0–0)	0 (0–0)	0 (0–0)	0 (0–0)	0 (0–0)	0 (0–0)	0 (0–1)	0 (0–2)	0 (0–1)	0 (0–1)	0 (0–0)	0 (0–1)	0 (0–1)	0 (0–1)	0 (0–0)	0 (0–1)	0 (0–1)	0 (0–1)	0 (0–0)	0 (0–0)	0 (0–0)	0 (0–0)	--
Chagas disease	--	--	0 (0–0)	0 (0–0)	0 (0–0)	0 (0–0)	0 (0–0)	0 (0–0)	0 (0–0)	0 (0–0)	0 (0–0)	0 (0–0)	0 (0–0)	0 (0–0)	0 (0–0)	0 (0–0)	0 (0–0)	0 (0–0)	0 (0–0)	0 (0–0)	0 (0–0)	0 (0–0)	--
Visceral leishmaniasis	--	--	0 (0–0)	0 (0–0)	0 (0–0)	0 (0–0)	0 (0–0)	0 (0–0)	0 (0–0)	0 (0–0)	0 (0–0)	0 (0–0)	0 (0–0)	0 (0–0)	0 (0–0)	0 (0–0)	0 (0–0)	0 (0–0)	0 (0–0)	0 (0–0)	0 (0–0)	0 (0–0)	--
African trypanosomiasis	--	--	--	0 (0–0)	0 (0–0)	0 (0–0)	0 (0–0)	0 (0–0)	0 (0–0)	0 (0–0)	0 (0–0)	0 (0–0)	0 (0–0)	0 (0–0)	0 (0–0)	0 (0–0)	0 (0–0)	0 (0–0)	0 (0–0)	0 (0–0)	0 (0–0)	0 (0–0)	--
Schistosomiasis	--	--	0 (0–0)	0 (0–0)	0 (0–0)	0 (0–0)	0 (0–1)	0 (0–2)	1 (0–3)	1 (0–3)	1 (0–4)	1 (0–4)	1 (0–4)	1 (0–5)	1 (0–5)	2 (0–6)	1 (0–5)	2 (0–6)	1 (0–4)	1 (0–3)	0 (0–2)	0 (0–1)	--
Cysticercosis	--	--	--	0 (0–0)	0 (0–0)	0 (0–0)	0 (0–0)	0 (0–1)	0 (0–1)	0 (0–1)	0 (0–1)	0 (0–1)	0 (0–1)	0 (0–1)	0 (0–1)	0 (0–1)	0 (0–1)	0 (0–0)	0 (0–0)	0 (0–0)	0 (0–0)	0 (0–0)	--
Cystic echinococcosis	--	--	--	0 (0–0)	0 (0–0)	0 (0–0)	0 (0–0)	0 (0–0)	0 (0–0)	0 (0–0)	0 (0–0)	0 (0–0)	0 (0–0)	0 (0–0)	0 (0–0)	0 (0–0)	0 (0–0)	0 (0–0)	0 (0–0)	0 (0–0)	0 (0–0)	0 (0–0)	--
Dengue	0 (0–0)	0 (0–0)	1 (0–3)	0 (0–2)	1 (0–2)	0 (0–1)	0 (0–1)	0 (0–1)	0 (0–1)	0 (0–1)	0 (0–1)	0 (0–1)	0 (0–1)	0 (0–1)	0 (0–0)	0 (0–1)	0 (0–1)	0 (0–1)	0 (0–1)	0 (0–0)	0 (0–0)	0 (0–0)	--
Yellow fever	--	0 (0–0)	0 (0–0)	0 (0–0)	0 (0–0)	0 (0–0)	0 (0–0)	0 (0–0)	0 (0–0)	0 (0–0)	0 (0–0)	0 (0–0)	0 (0–0)	0 (0–0)	0 (0–0)	0 (0–0)	0 (0–0)	0 (0–0)	0 (0–0)	0 (0–0)	0 (0–0)	0 (0–0)	--
Rabies	--	--	0 (0–0)	0 (0–1)	0 (0–1)	0 (0–0)	0 (0–0)	0 (0–0)	0 (0–0)	0 (0–0)	0 (0–0)	0 (0–0)	0 (0–1)	0 (0–1)	0 (0–0)	0 (0–1)	0 (0–1)	0 (0–1)	0 (0–0)	0 (0–0)	0 (0–0)	0 (0–0)	--
Ascariasis	--	--	0 (0–0)	0 (0–1)	0 (0–0)	0 (0–0)	0 (0–0)	0 (0–0)	0 (0–0)	0 (0–0)	0 (0–0)	0 (0–0)	0 (0–0)	0 (0–0)	0 (0–0)	0 (0–0)	0 (0–0)	0 (0–0)	0 (0–0)	0 (0–0)	0 (0–0)	0 (0–0)	--
Zika virus disease	0 (0–0)	0 (0–0)	0 (0–0)	0 (0–0)	0 (0–0)	0 (0–0)	0 (0–0)	0 (0–0)	0 (0–0)	0 (0–0)	0 (0–0)	0 (0–0)	0 (0–0)	0 (0–0)	0 (0–0)	0 (0–0)	0 (0–0)	0 (0–0)	0 (0–0)	0 (0–0)	0 (0–0)	0 (0–0)	--
Other neglected tropical diseases	0 (0–0)	0 (0–0)	0 (0–0)	0 (0–1)	0 (0–0)	0 (0–0)	0 (0–0)	0 (0–0)	0 (0–0)	0 (0–0)	0 (0–0)	0 (0–0)	0 (0–0)	0 (0–0)	0 (0–0)	0 (0–0)	0 (0–0)	0 (0–0)	0 (0–0)	0 (0–0)	0 (0–0)	0 (0–0)	--
Meningitis	6 (3–14)	10 (5–21)	24 (10–48)	14 (6–27)	7 (4–11)	4 (2–7)	4 (2–7)	3 (2–6)	3 (2–5)	4 (2–7)	4 (2–7)	4 (2–7)	4 (2–7)	4 (2–8)	4 (2–7)	7 (3–12)	5 (3–10)	6 (3–11)	4 (2–7)	3 (2–6)	4 (2–6)	1 (1–2)	--
Encephalitis	1 (0–1)	1 (0–2)	8 (2–19)	8 (3–22)	3 (1–8)	2 (1–4)	2 (1–4)	2 (1–4)	1 (1–3)	1 (1–3)	1 (1–3)	2 (1–4)	2 (1–4)	1 (1–3)	2 (1–4)	2 (1–4)	1 (1–3)	1 (1–3)	1 (0–2)	1 (0–2)	1 (0–1)	0 (0–1)	--
Diphtheria	--	--	0 (0–0)	0 (0–1)	0 (0–0)	0 (0–0)	0 (0–0)	0 (0–0)	0 (0–0)	0 (0–0)	0 (0–0)	0 (0–0)	0 (0–0)	0 (0–0)	0 (0–0)	--	--	--	--	--	--	--	--
Whooping cough	--	--	49 (5–136)	46 (5–126)	4 (0–13)	1 (0–2)	0 (0–1)	0 (0–1)	0 (0–1)	0 (0–0)	0 (0–0)	0 (0–0)	0 (0–0)	0 (0–0)	0 (0–0)	--	--	--	--	--	--	--	--
Tetanus	0 (0–1)	0 (0–1)	0 (0–1)	1 (0–4)	0 (0–1)	0 (0–1)	0 (0–1)	0 (0–1)	0 (0–1)	1 (0–2)	1 (0–2)	1 (0–3)	1 (0–4)	1 (0–5)	2 (0–5)	1 (0–5)	1 (0–4)	1 (0–5)	1 (0–3)	1 (0–2)	1 (0–2)	0 (0–1)	--
Measles	0 (0–0)	0 (0–0)	0 (0–0)	0 (0–0)	0 (0–0)	0 (0–0)	0 (0–0)	0 (0–0)	0 (0–0)	0 (0–0)	0 (0–0)	0 (0–0)	0 (0–0)	0 (0–0)	0 (0–0)	0 (0–0)	0 (0–0)	0 (0–0)	0 (0–0)	0 (0–0)	0 (0–0)	0 (0–0)	--
Varicella and herpes zoster	0 (0–0)	0 (0–0)	1 (0–3)	1 (0–5)	0 (0–2)	0 (0–0)	0 (0–0)	0 (0–0)	0 (0–0)	0 (0–0)	0 (0–0)	0 (0–0)	0 (0–0)	0 (0–0)	0 (0–0)	0 (0–0)	0 (0–1)	0 (0–1)	0 (0–1)	1 (0–2)	1 (0–3)	1 (0–2)	--
Acute hepatitis	--	--	1 (0–2)	1 (0–2)	1 (0–2)	1 (0–2)	1 (0–2)	2 (0–5)	2 (1–4)	2 (1–5)	2 (1–5)	2 (1–5)	2 (1–5)	3 (1–8)	2 (1–5)	2 (1–5)	2 (0–4)	2 (0–4)	1 (0–2)	1 (0–2)	1 (0–2)	0 (0–1)	--
Acute hepatitis A	--	--	0 (0–0)	0 (0–1)	0 (0–1)	0 (0–1)	0 (0–1)	1 (0–3)	0 (0–1)	0 (0–1)	0 (0–1)	0 (0–1)	0 (0–1)	0 (0–1)	0 (0–0)	0 (0–0)	0 (0–1)	0 (0–0)	0 (0–0)	0 (0–0)	0 (0–0)	0 (0–0)	--
Acute hepatitis B	--	--	1 (0–2)	0 (0–1)	0 (0–1)	0 (0–1)	0 (0–1)	0 (0–1)	1 (0–3)	1 (0–3)	1 (0–3)	1 (0–2)	0 (0–1)	1 (0–2)	1 (0–3)	0 (0–1)	1 (0–2)	1 (0–2)	0 (0–0)	0 (0–0)	0 (0–1)	0 (0–0)	--
Acute hepatitis C	--	--	0 (0–0)	0 (0–1)	0 (0–0)	0 (0–0)	0 (0–1)	1 (0–2)	1 (0–2)	0 (0–1)	1 (0–2)	1 (0–3)	1 (0–3)	2 (1–5)	1 (0–3)	1 (0–3)	1 (0–2)	1 (0–2)	0 (0–1)	1 (0–2)	1 (0–1)	0 (0–1)	--
Acute hepatitis E	--	--	0 (0–0)	0 (0–0)	0 (0–0)	0 (0–0)	0 (0–0)	0 (0–0)	0 (0–0)	0 (0–0)	0 (0–0)	0 (0–0)	0 (0–0)	0 (0–0)	0 (0–0)	0 (0–1)	0 (0–0)	0 (0–0)	0 (0–1)	0 (0–0)	0 (0–0)	0 (0–0)	--
Other unspecified infectious diseases	0 (0–1)	0 (0–1)	1 (0–4)	1 (0–3)	0 (0–1)	0 (0–1)	1 (0–1)	1 (0–2)	1 (0–3)	1 (0–3)	1 (0–4)	2 (1–4)	2 (1–5)	3 (1–6)	3 (1–5)	3 (1–6)	3 (1–5)	3 (1–7)	2 (1–4)	2 (1–4)	3 (1–7)	1 (0–3)	--
Maternal disorders	--	--	--	--	--	--	--	--	--	--	--	--	--	--	--	--	--	--	--	--	--	--	--
Maternal haemorrhage	--	--	--	--	--	--	--	--	--	--	--	--	--	--	--	--	--	--	--	--	--	--	--
Maternal sepsis and other pregnancy related infections	--	--	--	--	--	--	--	--	--	--	--	--	--	--	--	--	--	--	--	--	--	--	--
Maternal hypertensive disorders	--	--	--	--	--	--	--	--	--	--	--	--	--	--	--	--	--	--	--	--	--	--	--
Maternal obstructed labour and uterine rupture	--	--	--	--	--	--	--	--	--	--	--	--	--	--	--	--	--	--	--	--	--	--	--
Maternal abortive outcome	--	--	--	--	--	--	--	--	--	--	--	--	--	--	--	--	--	--	--	--	--	--	--
Ectopic pregnancy	--	--	--	--	--	--	--	--	--	--	--	--	--	--	--	--	--	--	--	--	--	--	--
Indirect maternal deaths	--	--	--	--	--	--	--	--	--	--	--	--	--	--	--	--	--	--	--	--	--	--	--
Late maternal deaths	--	--	--	--	--	--	--	--	--	--	--	--	--	--	--	--	--	--	--	--	--	--	--
Maternal deaths aggravated by HIV/AIDS	--	--	--	--	--	--	--	--	--	--	--	--	--	--	--	--	--	--	--	--	--	--	--
Other maternal disorders	--	--	--	--	--	--	--	--	--	--	--	--	--	--	--	--	--	--	--	--	--	--	--
Neonatal disorders	1,592 (1,111–2,181)	301 (201–424)	69 (34–129)	3 (1–5)	--	--	--	--	--	--	--	--	--	--	--	--	--	--	--	--	--	--	--
Neonatal preterm birth	804 (476–1,202)	87 (39–157)	22 (9–48)	1 (0–2)	--	--	--	--	--	--	--	--	--	--	--	--	--	--	--	--	--	--	--
Neonatal encephalopathy due to birth asphyxia and trauma	248 (118–448)	23 (10–47)	7 (2–15)	1 (0–2)	--	--	--	--	--	--	--	--	--	--	--	--	--	--	--	--	--	--	--
Neonatal sepsis and other neonatal infections	384 (175–716)	163 (88–259)	35 (14–70)	1 (0–2)	--	--	--	--	--	--	--	--	--	--	--	--	--	--	--	--	--	--	--
Hemolytic disease and other neonatal jaundice	12 (6–18)	2 (1–4)	0 (0–1)	0 (0–0)	--	--	--	--	--	--	--	--	--	--	--	--	--	--	--	--	--	--	--
Other neonatal disorders	145 (73–254)	26 (11–50)	5 (2–12)	0 (0–1)	--	--	--	--	--	--	--	--	--	--	--	--	--	--	--	--	--	--	--
Nutritional deficiencies	0 (0–0)	0 (0–0)	39 (14–86)	35 (14–71)	7 (4–12)	3 (2–5)	2 (1–5)	3 (2–6)	4 (2–7)	5 (3–10)	7 (3–12)	6 (3–10)	6 (3–11)	9 (4–17)	9 (5–16)	13 (6–25)	13 (6–25)	16 (8–30)	16 (8–27)	17 (9–27)	21 (11–36)	15 (8–25)	--
Protein-energy malnutrition	0 (0–0)	0 (0–0)	39 (14–84)	34 (14–70)	7 (4–11)	3 (2–4)	2 (1–4)	2 (1–4)	3 (1–6)	5 (2–9)	5 (3–9)	3 (2–6)	4 (2–8)	6 (3–12)	7 (4–13)	11 (5–19)	11 (5–21)	14 (7–25)	14 (7–24)	16 (9–26)	19 (11–33)	14 (7–23)	--
Other nutritional deficiencies	--	--	0 (0–1)	1 (0–1)	0 (0–1)	0 (0–0)	0 (0–1)	1 (0–3)	1 (0–2)	1 (0–2)	2 (1–3)	2 (1–5)	2 (1–3)	3 (1–6)	2 (1–4)	3 (1–6)	2 (1–6)	3 (1–6)	2 (1–4)	1 (0–3)	1 (0–3)	1 (0–2)	--
Lip and oral cavity cancer	--	--	--	--	0 (0–0)	0 (0–0)	0 (0–0)	1 (0–1)	1 (0–2)	2 (1–3)	3 (2–5)	5 (2–9)	9 (4–15)	13 (7–22)	16 (9–27)	22 (12–37)	23 (13–37)	25 (15–38)	18 (11–28)	18 (10–27)	10 (6–16)	5 (3–8)	--
Nasopharynx cancer	--	--	--	--	0 (0–0)	0 (0–0)	0 (0–0)	0 (0–1)	0 (0–1)	0 (0–1)	1 (0–1)	1 (0–1)	1 (1–2)	2 (1–4)	3 (2–5)	3 (2–5)	3 (2–5)	3 (2–5)	2 (1–3)	2 (1–3)	2 (1–4)	1 (0–2)	--
Other pharynx cancer	--	--	--	--	--	--	--	0 (0–1)	0 (0–1)	1 (0–1)	1 (1–3)	3 (1–5)	6 (3–11)	9 (5–15)	12 (6–20)	17 (8–29)	16 (8–27)	16 (9–26)	10 (6–16)	10 (6–16)	8 (4–13)	3 (2–5)	--
Oesophageal cancer	--	--	--	--	--	--	--	1 (0–1)	1 (0–1)	2 (1–3)	3 (1–6)	6 (3–11)	10 (5–17)	17 (8–28)	21 (12–34)	27 (15–43)	25 (14–39)	24 (14–37)	17 (10–26)	14 (9–21)	10 (6–15)	4 (2–5)	--
Stomach cancer	--	--	--	--	--	--	1 (0–1)	2 (1–3)	4 (2–6)	8 (5–14)	13 (7–21)	22 (13–34)	25 (15–40)	42 (25–68)	52 (33–82)	68 (43–105)	65 (43–101)	70 (45–103)	50 (34–74)	40 (28–58)	29 (20–41)	11 (8–17)	--
Colon and rectum cancer	--	--	--	--	0 (0–1)	0 (0–1)	1 (1–2)	2 (1–4)	5 (3–8)	10 (5–16)	16 (8–27)	21 (12–35)	30 (16–48)	44 (24–70)	51 (29–81)	73 (40–115)	67 (39–99)	85 (51–127)	67 (43–99)	56 (36–81)	42 (27–60)	17 (10–26)	--
Liver cancer due to hepatitis B	--	--	--	--	--	0 (0–1)	0 (0–1)	1 (0–3)	2 (1–5)	3 (1–7)	4 (1–11)	7 (2–17)	7 (3–18)	9 (3–22)	11 (5–24)	13 (5–28)	10 (4–22)	9 (3–19)	6 (3–13)	4 (2–8)	2 (1–4)	1 (0–1)	--
Liver cancer due to hepatitis C	--	--	--	--	--	0 (0–0)	0 (0–0)	0 (0–0)	0 (0–0)	0 (0–0)	0 (0–1)	1 (0–2)	1 (0–3)	2 (1–5)	4 (1–9)	5 (2–14)	6 (2–13)	7 (3–14)	6 (2–12)	6 (3–11)	4 (2–6)	1 (1–2)	--
Liver cancer due to alcohol use	--	--	--	--	--	--	0 (0–0)	0 (0–0)	0 (0–1)	1 (0–2)	2 (1–5)	4 (1–10)	5 (2–15)	8 (3–20)	12 (5–25)	17 (7–37)	19 (9–38)	21 (9–41)	14 (6–26)	10 (5–18)	5 (2–8)	1 (1–2)	--
Liver cancer due to NASH	--	--	--	--	--	--	0 (0–0)	0 (0–1)	0 (0–1)	0 (0–1)	0 (0–1)	1 (0–1)	1 (0–2)	1 (0–3)	1 (1–3)	2 (1–5)	2 (1–5)	3 (1–6)	2 (1–5)	2 (1–4)	1 (1–2)	0 (0–1)	--
	0 (0–0)	0 (0–0)	0 (0–0)	0 (0–1)	0 (0–1)	0 (0–0)	--	--	--	--	--	--	--	--	--	--	--	--	--	--	--	--	--
Liver cancer due to other causes	0 (0–1)	0 (0–0)	0 (0–1)	0 (0–1)	0 (0–0)	0 (0–1)	0 (0–0)	0 (0–1)	0 (0–1)	0 (0–1)	0 (0–1)	1 (0–2)	1 (0–2)	1 (0–2)	1 (0–3)	1 (0–3)	1 (0–3)	1 (0–3)	1 (0–2)	1 (0–2)	0 (0–1)	0 (0–0)	--
Gallbladder and biliary tract cancer	--	--	--	--	--	--	--	0 (0–1)	1 (0–2)	2 (0–3)	3 (1–5)	5 (2–9)	6 (3–10)	10 (5–17)	13 (6–22)	18 (9–28)	18 (9–29)	18 (10–28)	13 (7–21)	11 (6–18)	9 (4–15)	3 (2–6)	--
Pancreatic cancer	--	--	--	--	--	--	0 (0–0)	0 (0–0)	0 (0–1)	1 (1–2)	2 (1–4)	6 (3–10)	9 (5–16)	17 (9–27)	23 (12–38)	29 (15–47)	27 (14–44)	26 (15–41)	19 (11–29)	14 (9–21)	8 (5–12)	3 (2–5)	--
Larynx cancer	--	--	--	--	--	--	--	0 (0–1)	0 (0–1)	1 (0–2)	2 (1–4)	4 (2–7)	8 (4–13)	12 (7–21)	18 (10–28)	24 (14–37)	23 (14–33)	23 (14–35)	16 (10–24)	13 (8–19)	8 (5–12)	3 (2–4)	--
Tracheal, bronchus, and lung cancer	--	--	--	--	--	0 (0–0)	2 (1–3)	3 (1–5)	3 (2–5)	7 (3–12)	12 (6–19)	22 (12–36)	41 (22–65)	71 (41–113)	93 (54–144)	136 (79–216)	133 (87–198)	141 (92–202)	105 (71–153)	83 (53–124)	55 (34–79)	19 (12–28)	--
Malignant skin melanoma	0 (0–0)	0 (0–0)	0 (0–0)	0 (0–0)	0 (0–0)	0 (0–0)	0 (0–0)	0 (0–0)	0 (0–0)	0 (0–1)	0 (0–1)	1 (0–1)	1 (0–1)	1 (1–2)	1 (1–2)	1 (1–2)	1 (0–2)	1 (1–2)	1 (1–2)	1 (1–2)	1 (0–1)	0 (0–0)	--
Non-melanoma skin cancer (squamous-cell carcinoma)	--	--	--	--	--	--	--	0 (0–1)	1 (0–2)	2 (1–3)	2 (1–3)	3 (2–6)	5 (3–8)	6 (3–10)	8 (5–13)	11 (6–17)	11 (6–18)	11 (7–18)	10 (6–15)	9 (6–14)	9 (6–15)	5 (3–8)	--
	0 (0–0)	0 (0–0)	0 (0–1)	1 (0–2)	1 (0–1)	1 (0–1)	1 (0–2)	1 (0–1)	1 (0–1)	1 (0–2)	1 (0–2)	1 (1–2)	1 (0–2)	2 (1–4)	2 (1–3)	2 (1–3)	2 (1–4)	2 (1–4)	1 (0–2)	1 (0–2)	1 (0–2)	0 (0–1)	--
	--	--	--	1 (0–2)	1 (0–2)	1 (1–3)	2 (1–4)	2 (1–5)	2 (1–3)	1 (1–3)	2 (1–3)	1 (1–3)	2 (1–3)	2 (1–4)	2 (1–5)	3 (1–6)	3 (1–6)	3 (1–6)	2 (1–5)	2 (1–4)	1 (0–1)	0 (0–0)	--
Breast cancer	--	--	--	--	--	--	0 (0–0)	0 (0–0)	0 (0–0)	0 (0–1)	0 (0–1)	0 (0–1)	1 (0–3)	1 (0–3)	1 (0–3)	2 (1–4)	2 (1–4)	2 (1–4)	1 (1–3)	1 (0–3)	2 (1–4)	1 (0–2)	--
Cervical cancer	--	--	--	--	--	--	--	--	--	--	--	--	--	--	--	--	--	--	--	--	--	--	--
Uterine cancer	--	--	--	--	--	--	--	--	--	--	--	--	--	--	--	--	--	--	--	--	--	--	--
Ovarian cancer	--	--	--	--	--	--	--	--	--	--	--	--	--	--	--	--	--	--	--	--	--	--	--
Prostate cancer	--	--	--	--	--	--	--	0 (0–1)	1 (0–1)	1 (1–2)	1 (1–3)	3 (2–6)	9 (5–17)	27 (14–48)	50 (26–87)	119 (64–215)	165 (88–288)	265 (155–446)	286 (171–485)	304 (187–510)	285 (171–474)	129 (75–209)	--
Testicular cancer	0 (0–0)	0 (0–0)	0 (0–0)	0 (0–0)	0 (0–0)	0 (0–0)	0 (0–1)	0 (0–1)	1 (0–1)	0 (0–1)	0 (0–1)	0 (0–1)	0 (0–0)	0 (0–1)	0 (0–0)	0 (0–1)	0 (0–1)	0 (0–1)	0 (0–1)	0 (0–0)	0 (0–1)	0 (0–0)	--
Kidney cancer	0 (0–1)	0 (0–0)	1 (0–1)	3 (1–6)	2 (1–4)	1 (0–1)	1 (0–1)	1 (0–1)	2 (1–3)	2 (1–5)	2 (1–4)	4 (2–8)	6 (3–11)	10 (4–18)	12 (5–22)	13 (6–23)	13 (6–22)	12 (6–20)	9 (4–14)	7 (4–11)	5 (3–7)	2 (1–3)	--
Bladder cancer	--	--	--	--	--	--	0 (0–0)	0 (0–0)	0 (0–0)	0 (0–1)	1 (0–1)	1 (0–2)	2 (1–3)	3 (2–5)	5 (3–8)	8 (4–13)	9 (5–14)	12 (7–18)	11 (7–17)	12 (8–17)	11 (7–17)	5 (3–7)	--
Brain and nervous system cancer	0 (0–0)	0 (0–0)	1 (0–1)	2 (1–5)	2 (1–5)	2 (1–4)	1 (0–2)	1 (1–2)	1 (1–3)	2 (1–4)	3 (1–6)	3 (1–7)	4 (2–7)	4 (2–8)	4 (2–9)	4 (2–9)	4 (2–8)	3 (2–6)	2 (1–4)	2 (1–3)	1 (0–2)	0 (0–0)	--
	0 (0–0)	0 (0–0)	0 (0–0)	1 (0–2)	0 (0–0)	0 (0–0)	0 (0–0)	0 (0–0)	0 (0–0)	0 (0–0)	0 (0–0)	0 (0–0)	0 (0–1)	0 (0–1)	0 (0–0)	0 (0–1)	0 (0–0)	0 (0–0)	0 (0–0)	0 (0–0)	0 (0–0)	0 (0–0)	--
	0 (0–0)	0 (0–0)	0 (0–0)	0 (0–1)	0 (0–0)	0 (0–0)	0 (0–0)	--	--	--	--	--	--	--	--	--	--	--	--	--	--	--	--
	--	--	--	0 (0–1)	0 (0–0)	0 (0–0)	0 (0–0)	0 (0–0)	0 (0–0)	0 (0–0)	0 (0–0)	0 (0–0)	0 (0–1)	0 (0–1)	0 (0–0)	0 (0–1)	0 (0–0)	0 (0–0)	0 (0–0)	0 (0–0)	0 (0–0)	0 (0–0)	--
	0 (0–0)	0 (0–0)	0 (0–1)	3 (1–6)	1 (0–3)	0 (0–1)	0 (0–0)	0 (0–1)	0 (0–1)	0 (0–1)	0 (0–0)	0 (0–0)	0 (0–0)	0 (0–0)	0 (0–0)	0 (0–0)	0 (0–0)	0 (0–0)	0 (0–0)	0 (0–0)	0 (0–0)	0 (0–0)	--
Thyroid cancer	--	--	--	--	0 (0–0)	0 (0–0)	0 (0–0)	0 (0–0)	0 (0–1)	0 (0–1)	1 (0–2)	1 (1–2)	2 (1–4)	3 (2–6)	4 (2–7)	4 (2–7)	4 (2–6)	4 (2–6)	2 (1–4)	2 (1–3)	1 (1–2)	1 (0–1)	--
Mesothelioma	--	--	--	--	--	--	--	0 (0–0)	0 (0–1)	0 (0–0)	0 (0–1)	0 (0–1)	1 (0–1)	1 (0–1)	1 (1–2)	0 (0–1)	0 (0–1)	1 (0–1)	1 (1–2)	1 (0–1)	0 (0–1)	0 (0–0)	--
Hodgkin lymphoma	--	--	--	0 (0–0)	0 (0–0)	0 (0–0)	0 (0–0)	0 (0–1)	0 (0–1)	0 (0–1)	0 (0–1)	1 (0–1)	0 (0–1)	1 (0–1)	1 (0–1)	1 (0–1)	1 (0–1)	1 (0–1)	0 (0–1)	0 (0–0)	0 (0–0)	0 (0–0)	--
Non-Hodgkin's lymphoma	--	--	--	2 (1–5)	3 (2–5)	2 (1–4)	3 (2–5)	5 (2–8)	6 (2–10)	7 (3–12)	7 (3–13)	8 (4–13)	10 (5–18)	13 (7–22)	14 (8–23)	16 (9–27)	15 (8–23)	15 (8–22)	10 (6–16)	7 (4–11)	5 (3–8)	2 (1–3)	--
	--	--	--	0 (0–1)	1 (0–1)	0 (0–1)	0 (0–1)	1 (0–1)	1 (0–1)	1 (0–1)	0 (0–1)	0 (0–1)	0 (0–1)	0 (0–0)	0 (0–0)	0 (0–0)	0 (0–0)	0 (0–0)	0 (0–0)	0 (0–0)	0 (0–0)	0 (0–0)	--
	--	--	--	2 (1–4)	3 (1–4)	2 (1–3)	2 (1–4)	4 (2–7)	5 (2–9)	7 (3–11)	7 (3–12)	8 (4–13)	10 (5–18)	12 (7–22)	14 (8–23)	16 (9–26)	15 (8–23)	14 (8–22)	10 (6–15)	7 (4–11)	5 (3–7)	2 (1–3)	--
Multiple myeloma	--	--	--	--	--	--	--	0 (0–0)	0 (0–1)	0 (0–1)	1 (0–1)	2 (1–3)	4 (2–6)	8 (4–12)	9 (6–16)	12 (7–18)	11 (7–17)	12 (7–17)	8 (5–12)	6 (3–9)	3 (2–5)	1 (0–1)	--
Leukaemia	1 (0–2)	0 (0–1)	3 (1–5)	11 (5–20)	13 (7–21)	10 (6–16)	9 (5–16)	10 (5–18)	9 (5–17)	9 (5–17)	10 (5–18)	11 (6–20)	12 (7–22)	15 (8–26)	16 (9–27)	19 (10–33)	18 (10–31)	19 (11–30)	13 (7–21)	12 (7–19)	9 (5–15)	4 (2–7)	--

Acute lymphoid leukaemia	0 (0–1)	0 (0–0)	1 (0–3)	6 (2–11)	7 (3–13)	4 (2–7)	4 (2–7)	2 (1–5)	2 (1–3)	1 (1–3)	1 (0–3)	1 (1–2)	1 (0–2)	1 (0–2)	1 (1–3)	1 (0–2)	2 (1–4)	2 (1–3)	1 (0–2)	1 (0–1)	1 (0–1)	0 (0–0)	--
Chronic lymphoid leukaemia	--	--	--	--	--	--	--	0 (0–0)	0 (0–0)	0 (0–0)	0 (0–0)	0 (0–1)	0 (0–1)	1 (0–1)	1 (0–2)	2 (1–4)	2 (1–3)	2 (1–4)	1 (0–2)	1 (1–3)	1 (1–3)	1 (0–1)	--
Acute myeloid leukaemia	0 (0–0)	0 (0–0)	1 (0–1)	3 (1–6)	2 (1–5)	2 (1–5)	2 (1–5)	2 (1–5)	3 (1–7)	3 (1–7)	3 (1–7)	3 (1–5)	3 (1–6)	3 (1–7)	3 (1–6)	3 (1–8)	3 (1–6)	3 (1–6)	2 (1–5)	2 (1–3)	1 (0–1)	1 (0–1)	--
Chronic myeloid leukaemia	0 (0–0)	0 (0–0)	0 (0–0)	0 (0–1)	0 (0–1)	0 (0–0)	0 (0–0)	0 (0–1)	1 (0–2)	1 (0–1)	1 (0–2)	1 (0–2)	1 (0–2)	1 (0–3)	1 (0–3)	1 (0–3)	1 (0–2)	1 (0–2)	1 (0–2)	1 (0–1)	1 (0–1)	0 (0–0)	--
Other leukaemia	0 (0–1)	0 (0–0)	1 (0–2)	2 (1–5)	3 (1–7)	3 (1–6)	3 (1–5)	5 (2–9)	4 (2–7)	4 (2–8)	5 (2–9)	6 (3–12)	7 (4–14)	8 (4–15)	9 (5–16)	11 (6–20)	10 (5–18)	12 (6–19)	8 (4–14)	8 (4–13)	6 (3–10)	2 (1–4)	--
Other malignant cancers	1 (0–3)	1 (0–2)	2 (1–5)	5 (2–11)	3 (2–6)	3 (2–6)	4 (3–7)	5 (3–8)	5 (3–9)	6 (3–9)	7 (4–12)	9 (5–16)	10 (5–19)	14 (7–25)	15 (8–27)	16 (8–29)	16 (8–27)	16 (9–28)	15 (9–24)	13 (8–21)	13 (7–21)	6 (3–9)	--
Myelodysplastic, myeloproliferative, and other hematopoietic neoplasms	0 (0–0)	0 (0–0)	0 (0–0)	1 (0–1)	0 (0–1)	0 (0–1)	0 (0–1)	0 (0–0)	0 (0–1)	0 (0–0)	0 (0–1)	0 (0–1)	1 (0–1)	1 (1–2)	2 (1–3)	2 (1–4)	2 (1–4)	3 (1–6)	2 (1–5)	2 (1–4)	2 (1–3)	1 (0–1)	--
Cardiovascular diseases	5 (2–10)	2 (1–4)	9 (4–18)	9 (4–15)	9 (6–13)	11 (6–18)	29 (17–46)	59 (33–95)	88 (51–135)	153 (92–228)	237 (151–338)	400 (270–567)	638 (430–907)	972 (668–1,359)	1,158 (809–1,610)	1,604 (1,134–2,160)	1,649 (1,202–2,199)	1,988 (1,519–2,574)	1,692 (1,302–2,171)	1,689 (1,352–2,082)	1,388 (1,081–1,674)	795 (618–942)	--
Rheumatic heart disease	--	--	--	1 (0–2)	1 (1–3)	3 (1–5)	2 (1–5)	3 (1–5)	3 (1–6)	3 (1–6)	3 (1–6)	3 (1–7)	3 (1–7)	3 (1–7)	3 (1–6)	3 (1–6)	3 (1–5)	3 (2–6)	3 (2–6)	2 (1–3)	2 (1–3)	1 (1–2)	--
Ischaemic heart disease	--	--	--	--	--	--	11 (6–19)	30 (16–50)	48 (26–75)	89 (52–136)	148 (92–222)	248 (163–365)	403 (263–584)	600 (404–860)	711 (476–997)	967 (659–1,328)	970 (688–1,327)	1,137 (827–1,512)	950 (706–1,279)	973 (755–1,248)	807 (609–1,010)	470 (343–583)	--
Stroke	3 (1–6)	1 (0–2)	2 (1–6)	2 (1–4)	3 (1–5)	3 (1–5)	7 (4–13)	15 (7–27)	21 (10–37)	40 (19–68)	57 (30–94)	106 (57–177)	170 (92–273)	278 (161–424)	331 (203–503)	480 (299–718)	510 (322–762)	640 (424–910)	539 (366–752)	477 (336–653)	354 (243–486)	188 (131–255)	--
Ischaemic stroke	0 (0–0)	0 (0–0)	0 (0–1)	0 (0–1)	0 (0–1)	0 (0–1)	1 (0–2)	2 (1–4)	4 (2–7)	6 (2–11)	8 (3–14)	14 (7–26)	24 (12–44)	45 (23–77)	61 (32–103)	139 (77–229)	159 (93–251)	294 (187–444)	257 (169–368)	317 (220–441)	243 (163–335)	134 (91–186)	--
Intracerebral hemorrhage	1 (1–3)	0 (0–1)	1 (0–3)	0 (0–1)	1 (1–3)	1 (1–3)	4 (2–7)	9 (4–16)	12 (6–22)	27 (13–47)	39 (19–65)	78 (43–128)	131 (72–213)	212 (122–331)	244 (147–369)	323 (195–493)	332 (207–496)	330 (203–502)	271 (172–395)	148 (93–217)	104 (67–156)	51 (31–73)	--
Subarachnoid hemorrhage	1 (0–3)	0 (0–1)	1 (0–3)	1 (0–2)	1 (0–2)	1 (0–2)	2 (1–5)	5 (2–9)	5 (2–10)	7 (3–13)	10 (4–18)	14 (6–27)	15 (7–29)	21 (9–38)	25 (11–46)	19 (9–35)	18 (9–32)	16 (8–29)	12 (6–21)	12 (6–20)	8 (4–13)	3 (2–6)	--
Hypertensive heart disease	--	--	--	--	--	--	1 (0–2)	2 (1–3)	2 (1–5)	5 (2–11)	9 (3–18)	16 (7–31)	28 (13–51)	46 (22–83)	61 (31–106)	87 (44–156)	99 (51–172)	128 (68–222)	123 (66–214)	143 (76–231)	122 (68–196)	71 (40–114)	--
Non-rheumatic valvular heart disease	--	--	--	--	--	--	1 (0–2)	1 (0–2)	2 (1–3)	2 (1–3)	2 (1–3)	3 (2–4)	3 (2–6)	5 (3–7)	5 (3–9)	6 (3–10)	7 (4–11)	7 (5–12)	6 (4–10)	7 (4–12)	7 (4–12)	3 (2–6)	--
Non-rheumatic calcific aortic valve disease	--	--	--	--	--	--	1 (0–2)	1 (0–1)	1 (0–2)	1 (0–2)	1 (1–2)	2 (1–3)	2 (1–4)	3 (2–5)	3 (2–6)	4 (2–7)	5 (3–8)	6 (3–9)	5 (3–8)	6 (4–10)	5 (3–10)	3 (1–5)	--
Non-rheumatic degenerative mitral valve disease	--	--	--	--	--	--	0 (0–1)	0 (0–0)	1 (0–1)	1 (0–1)	1 (0–2)	1 (1–2)	1 (1–2)	2 (1–3)	2 (1–3)	2 (1–3)	2 (1–3)	2 (1–3)	1 (1–2)	1 (0–2)	1 (0–2)	1 (0–1)	--
Other non-rheumatic valve diseases	--	--	--	--	--	--	0 (0–0)	0 (0–0)	0 (0–0)	0 (0–0)	0 (0–0)	0 (0–0)	0 (0–0)	0 (0–0)	0 (0–0)	0 (0–0)	0 (0–0)	0 (0–0)	0 (0–0)	0 (0–0)	0 (0–0)	0 (0–0)	--
Cardiomyopathy and myocarditis	1 (0–3)	1 (0–1)	3 (1–6)	2 (1–4)	1 (1–3)	1 (1–3)	2 (1–4)	2 (1–4)	3 (2–7)	5 (2–8)	6 (3–12)	8 (4–14)	10 (5–19)	12 (6–21)	14 (7–23)	15 (7–25)	13 (7–22)	14 (7–23)	11 (5–18)	11 (5–18)	11 (5–19)	6 (2–11)	--
Myocarditis	0 (0–0)	0 (0–0)	0 (0–1)	0 (0–1)	0 (0–0)	0 (0–0)	0 (0–1)	0 (0–1)	0 (0–1)	0 (0–1)	0 (0–1)	0 (0–1)	0 (0–0)	0 (0–1)	0 (0–1)	0 (0–0)	0 (0–0)	0 (0–1)	0 (0–0)	0 (0–0)	0 (0–1)	0 (0–0)	--
Alcoholic cardiomyopathy	--	--	--	--	--	--	0 (0–0)	0 (0–0)	1 (0–1)	1 (1–3)	2 (1–4)	3 (1–6)	5 (2–9)	6 (3–11)	6 (3–11)	6 (3–11)	6 (2–10)	4 (2–9)	3 (1–6)	0 (0–1)	0 (0–0)	0 (0–0)	--
Other cardiomyopathy	1 (0–2)	0 (0–1)	2 (1–5)	2 (1–3)	1 (1–2)	1 (0–2)	2 (1–3)	2 (1–3)	3 (1–5)	3 (1–5)	4 (2–7)	5 (2–9)	6 (2–11)	6 (3–11)	8 (3–14)	9 (4–15)	7 (3–13)	9 (4–17)	8 (4–13)	10 (5–17)	11 (5–19)	6 (2–10)	--
Pulmonary Arterial Hypertension	0 (0–1)	0 (0–0)	1 (0–2)	0 (0–1)	0 (0–1)	0 (0–1)	0 (0–0)	0 (0–1)	0 (0–1)	0 (0–0)	0 (0–0)	0 (0–0)	0 (0–1)	0 (0–1)	0 (0–1)	0 (0–1)	0 (0–1)	0 (0–1)	0 (0–1)	0 (0–1)	0 (0–1)	0 (0–1)	--
Atrial fibrillation and flutter	--	--	--	--	--	--	--	--	--	0 (0–0)	0 (0–0)	1 (0–1)	1 (1–2)	3 (2–4)	4 (3–7)	7 (4–10)	9 (5–14)	15 (9–22)	20 (11–30)	34 (19–49)	41 (25–56)	31 (20–45)	--
Aortic aneurysm	--	--	--	--	--	--	0 (0–1)	0 (0–1)	1 (0–1)	1 (1–2)	2 (1–3)	4 (2–7)	5 (2–8)	7 (4–12)	10 (6–16)	15 (10–23)	16 (11–23)	17 (12–24)	15 (10–21)	14 (10–19)	12 (8–16)	5 (3–8)	--
Peripheral vascular disease	--	--	--	--	--	--	--	--	--	--	--	0 (0–0)	0 (0–0)	0 (0–1)	0 (0–1)	1 (0–2)	1 (1–2)	2 (1–3)	2 (1–3)	2 (1–4)	2 (1–4)	2 (1–3)	--
Endocarditis	0 (0–0)	0 (0–0)	1 (0–2)	1 (0–3)	1 (0–2)	1 (0–3)	1 (0–3)	2 (1–3)	2 (1–4)	3 (1–5)	3 (1–5)	3 (2–6)	3 (1–6)	4 (2–7)	3 (2–6)	3 (2–5)	3 (1–5)	3 (2–4)	2 (1–3)	2 (1–3)	2 (1–3)	1 (0–2)	--
Other cardiovascular and circulatory diseases	1 (0–2)	1 (0–1)	2 (1–5)	2 (1–5)	2 (1–4)	3 (1–5)	3 (1–5)	4 (1–9)	6 (2–10)	6 (2–11)	7 (3–12)	8 (3–13)	10 (4–17)	13 (6–22)	16 (7–26)	19 (9–30)	18 (8–29)	20 (10–32)	20 (11–31)	23 (13–33)	28 (16–43)	17 (9–25)	--
Chronic respiratory diseases	0 (0–0)	0 (0–0)	0 (0–0)	22 (10–41)	6 (3–9)	4 (2–7)	5 (3–9)	9 (5–15)	10 (6–17)	12 (7–20)	13 (7–21)	20 (12–33)	30 (18–48)	45 (27–71)	59 (35–92)	98 (60–154)	101 (63–159)	148 (93–229)	139 (88–209)	141 (92–211)	128 (83–187)	62 (41–94)	--
Chronic obstructive pulmonary disease	--	--	0 (0–0)	2 (1–3)	0 (0–1)	0 (0–1)	2 (1–3)	3 (1–6)	4 (2–6)	4 (2–7)	5 (2–9)	9 (4–15)	14 (8–24)	26 (15–43)	39 (22–62)	74 (43–118)	79 (49–126)	118 (72–183)	111 (69–166)	115 (74–170)	108 (69–158)	52 (34–78)	--
Pneumoconiosis	--	--	--	--	--	--	0 (0–0)	0 (0–0)	0 (0–0)	0 (0–0)	0 (0–0)	0 (0–0)	0 (0–0)	0 (0–0)	0 (0–0)	0 (0–0)	0 (0–0)	0 (0–0)	0 (0–0)	0 (0–0)	0 (0–0)	0 (0–0)	--
Silicosis	--	--	--	--	--	--	0 (0–0)	0 (0–0)	0 (0–0)	0 (0–0)	0 (0–0)	0 (0–0)	0 (0–0)	0 (0–0)	0 (0–0)	0 (0–0)	0 (0–0)	0 (0–0)	0 (0–0)	0 (0–0)	0 (0–0)	0 (0–0)	--
Asbestosis	--	--	--	--	--	--	0 (0–0)	0 (0–0)	0 (0–0)	0 (0–0)	0 (0–0)	0 (0–0)	0 (0–0)	0 (0–0)	0 (0–0)	0 (0–0)	0 (0–0)	0 (0–0)	0 (0–0)	0 (0–0)	0 (0–0)	0 (0–0)	--
Coal workers pneumoconiosis	--	--	--	--	--	--	0 (0–0)	0 (0–0)	0 (0–0)	0 (0–0)	0 (0–0)	0 (0–0)	0 (0–0)	0 (0–0)	0 (0–0)	0 (0–0)	0 (0–0)	0 (0–0)	0 (0–0)	0 (0–0)	0 (0–0)	0 (0–0)	--
Other pneumoconiosis	--	--	--	--	--	--	0 (0–0)	0 (0–0)	0 (0–0)	0 (0–0)	0 (0–0)	0 (0–0)	0 (0–0)	0 (0–0)	0 (0–0)	0 (0–0)	0 (0–0)	0 (0–0)	0 (0–0)	0 (0–0)	0 (0–0)	0 (0–0)	--
Asthma	--	--	--	14 (6–27)	5 (3–7)	3 (2–5)	3 (1–5)	4 (2–8)	5 (3–9)	6 (3–11)	7 (3–11)	9 (5–16)	12 (7–22)	15 (8–25)	16 (8–27)	21 (11–37)	18 (9–31)	25 (12–45)	23 (12–43)	22 (12–40)	17 (9–29)	9 (5–16)	--
Interstitial lung disease and pulmonary sarcoidosis	--	--	--	1 (0–1)	0 (0–0)	0 (0–0)	0 (0–0)	0 (0–1)	0 (0–0)	0 (0–1)	1 (0–1)	1 (1–3)	1 (1–3)	2 (1–4)	2 (1–5)	2 (1–4)	2 (1–5)	3 (1–5)	3 (1–6)	2 (1–5)	2 (1–3)	1 (0–1)	--
Other chronic respiratory diseases	0 (0–0)	0 (0–0)	0 (0–0)	5 (2–11)	1 (0–2)	1 (0–1)	1 (0–2)	1 (0–2)	1 (1–3)	1 (1–3)	1 (0–2)	1 (0–2)	2 (1–3)	2 (1–4)	2 (1–3)	1 (0–3)	1 (0–3)	1 (0–4)	1 (0–4)	1 (0–3)	1 (0–3)	0 (0–1)	--
Digestive diseases	3 (1–7)	3 (1–5)	28 (10–68)	36 (14–79)	17 (11–28)	12 (7–20)	16 (8–28)	26 (13–46)	37 (17–63)	57 (28–93)	86 (45–140)	114 (62–183)	161 (84–252)	218 (115–344)	232 (130–350)	274 (151–424)	242 (137–360)	258 (152–379)	193 (126–281)	171 (120–239)	145 (100–204)	66 (43–98)	--
Cirrhosis and other chronic liver diseases	--	--	--	14 (4–33)	8 (4–13)	6 (3–11)	10 (5–19)	16 (7–31)	25 (11–44)	42 (20–72)	66 (33–108)	92 (48–151)	135 (71–216)	183 (97–289)	195 (107–299)	227 (124–360)	198 (113–298)	204 (121–304)	148 (95–216)	127 (87–182)	103 (68–152)	44 (25–70)	--
Cirrhosis and other chronic liver diseases due to hepatitis B	--	--	--	0 (0–0)	0 (0–0)	0 (0–0)	0 (0–0)	1 (0–2)	2 (1–5)	4 (2–9)	8 (3–17)	11 (4–23)	16 (6–31)	19 (8–39)	18 (7–34)	18 (7–36)	14 (6–29)	14 (6–29)	11 (5–21)	10 (4–17)	8 (4–14)	3 (1–7)	--
Cirrhosis and other chronic liver diseases due to hepatitis C	--	--	--	0 (0–0)	0 (0–0)	0 (0–1)	1 (0–2)	3 (1–6)	6 (2–13)	11 (4–21)	17 (6–34)	22 (10–43)	30 (13–60)	38 (16–76)	38 (16–75)	42 (17–86)	36 (16–66)	36 (18–66)	26 (12–46)	22 (11–41)	19 (10–34)	8 (4–17)	--
Cirrhosis and other chronic liver diseases due to alcohol use	--	--	--	--	--	--	0 (0–0)	2 (1–5)	5 (2–12)	13 (5–26)	27 (12–49)	41 (19–71)	63 (29–108)	90 (42–151)	100 (51–163)	118 (61–199)	99 (52–161)	97 (51–151)	65 (37–103)	51 (28–82)	39 (21–64)	16 (7–28)	--
Cirrhosis and other chronic liver diseases due to NAFLD	--	--	--	--	--	--	1 (0–1)	2 (1–4)	4 (1–9)	6 (2–12)	8 (3–17)	11 (4–23)	16 (6–35)	22 (8–45)	23 (9–47)	28 (11–57)	27 (12–50)	32 (15–57)	25 (12–46)	24 (11–40)	20 (11–35)	9 (4–18)	--
Cirrhosis and other chronic liver diseases due to other causes	--	--	--	14 (4–33)	8 (4–13)	6 (3–10)	9 (4–16)	9 (4–18)	8 (3–16)	7 (3–15)	7 (2–15)	7 (3–16)	10 (4–20)	13 (5–27)	16 (7–31)	21 (9–40)	21 (8–39)	25 (10–48)	21 (9–40)	20 (9–37)	17 (8–29)	8 (3–15)	--
Upper digestive system diseases	--	--	--	1 (0–3)	1 (0–1)	1 (0–1)	2 (1–3)	4 (1–7)	4 (2–8)	6 (3–12)	9 (4–17)	9 (4–17)	12 (5–22)	17 (8–30)	18 (9–31)	23 (11–40)	23 (11–40)	26 (13–47)	21 (11–36)	18 (9–32)	19 (10–34)	10 (5–19)	--
Peptic ulcer disease	--	--	--	1 (0–2)	1 (0–1)	1 (0–1)	1 (1–3)	3 (1–6)	4 (1–7)	6 (2–10)	8 (3–15)	9 (4–15)	11 (5–20)	16 (7–29)	16 (8–29)	21 (10–36)	21 (10–37)	25 (12–44)	19 (10–33)	17 (9–29)	17 (9–32)	9 (5–18)	--
Gastritis and duodenitis	--	--	--	0 (0–1)	0 (0–0)	0 (0–0)	0 (0–1)	0 (0–1)	1 (0–1)	1 (0–2)	1 (0–2)	1 (0–2)	1 (0–3)	1 (0–3)	2 (1–4)	2 (1–5)	2 (1–4)	2 (1–4)	2 (1–3)	1 (1–3)	1 (1–3)	1 (0–2)	--
Appendicitis	--	--	--	7 (2–19)	5 (2–10)	3 (1–6)	2 (1–4)	2 (1–5)	2 (1–4)	2 (1–4)	2 (1–5)	2 (1–5)	2 (1–4)	2 (1–4)	2 (1–3)	2 (1–4)	2 (1–4)	2 (1–4)	1 (1–3)	1 (1–3)	1 (0–2)	0 (0–1)	--
Paralytic ileus and intestinal obstruction	2 (1–5)	2 (1–4)	23 (8–57)	5 (1–13)	1 (1–3)	1 (0–1)	1 (0–1)	1 (0–1)	1 (0–1)	1 (0–1)	1 (0–2)	1 (0–3)	1 (0–3)	2 (1–5)	3 (1–5)	3 (1–7)	3 (1–7)	5 (2–11)	4 (2–10)	5 (2–11)	5 (2–10)	2 (1–5)	--
Inguinal, femoral, and abdominal hernia	1 (0–2)	1 (0–1)	5 (1–14)	2 (0–5)	1 (0–1)	0 (0–0)	0 (0–1)	0 (0–1)	0 (0–1)	0 (0–1)	0 (0–1)	1 (0–2)	1 (0–2)	1 (0–2)	1 (1–2)	2 (1–3)	1 (1–2)	2 (1–3)	2 (1–3)	2 (1–3)	2 (1–4)	1 (0–2)	--
Inflammatory bowel disease	--	--	--	1 (0–2)	0 (0–0)	0 (0–0)	0 (0–0)	0 (0–0)	0 (0–1)	1 (0–1)	1 (0–1)	0 (0–1)	1 (0–1)	1 (0–1)	1 (0–2)	1 (0–2)	1 (0–1)	1 (1–2)	1 (0–1)	1 (0–2)	1 (0–2)	0 (0–1)	--
Vascular intestinal disorders	--	--	--	0 (0–0)	0 (0–0)	0 (0–0)	0 (0–0)	0 (0–0)	0 (0–1)	1 (0–1)	1 (0–2)	1 (0–2)	2 (1–3)	3 (1–5)	3 (1–4)	3 (2–5)	4 (2–6)	5 (3–7)	5 (3–8)	4 (3–7)	3 (2–6)	1 (1–3)	--
Gallbladder and biliary diseases	--	--	--	0 (0–0)	0 (0–0)	0 (0–0)	0 (0–0)	0 (0–1)	1 (0–1)	1 (0–2)	1 (0–2)	1 (0–3)	1 (0–3)	2 (1–5)	3 (1–6)	4 (2–8)	4 (2–8)	6 (3–11)	5 (2–9)	5 (3–9)	4 (2–6)	2 (1–3)	--
Pancreatitis	--	--	--	0 (0–0)	0 (0–0)	0 (0–0)	0 (0–1)	1 (0–2)	2 (1–3)	3 (1–5)	4 (2–7)	4 (2–8)	4 (2–8)	4 (2–7)	4 (2–7)	5 (2–8)	4 (2–7)	4 (2–6)	3 (2–5)	3 (1–4)	2 (1–3)	1 (0–1)	--
Other digestive diseases	--	--	--	7 (2–20)	2 (1–4)	1 (0–2)	1 (0–2)	1 (0–3)	1 (0–3)	1 (0–3)	2 (0–4)	2 (1–5)	3 (1–6)	3 (1–6)	3 (1–7)	4 (2–9)	3 (1–7)	4 (2–9)	4 (2–8)	4 (2–9)	5 (2–11)	3 (1–6)	--
Alzheimer's disease and other dementias	--	--	--	--	--	--	--	--	--	--	--	0 (0–2)	3 (0–8)	5 (1–17)	9 (2–28)	18 (4–54)	33 (7–96)	60 (13–175)	85 (19–242)	172 (38–482)	231 (53–612)	148 (36–406)	--
Parkinson's disease	--	--	--	--	--	--	--	0 (0–0)	0 (0–0)	0 (0–0)	0 (0–0)	1 (0–1)	2 (1–2)	3 (2–5)	5 (3–7)	10 (7–14)	18 (13–25)	38 (27–51)	55 (39–74)	67 (49–90)	49 (35–68)	15 (10–24)	--
Idiopathic epilepsy	--	--	1 (0–3)	4 (2–9)	3 (2–5)	4 (2–7)	7 (4–11)	10 (5–17)	10 (5–15)	9 (5–14)	10 (6–15)	9 (5–15)	10 (5–16)	10 (5–15)	8 (5–13)	7 (4–12)	6 (3–9)	5 (3–8)	4 (2–6)	4 (2–6)	3 (2–5)	2 (1–3)	--
Multiple sclerosis	--	--	--	--	0 (0–0)	0 (0–0)	0 (0–0)	0 (0–0)	0 (0–0)	0 (0–1)	1 (0–1)	1 (0–2)	1 (0–2)	1 (0–2)	1 (0–2)	1 (0–2)	1 (0–1)	0 (0–1)	0 (0–0)	0 (0–0)	0 (0–0)	0 (0–0)	--
Motor neuron disease	0 (0–0)	0 (0–0)	0 (0–1)	0 (0–1)	0 (0–0)	0 (0–0)	0 (0–0)	0 (0–0)	0 (0–0)	0 (0–1)	1 (0–2)	2 (1–4)	2 (1–5)	4 (1–9)	4 (2–8)	4 (2–8)	3 (1–5)	2 (1–4)	1 (0–2)	0 (0–1)	0 (0–0)	0 (0–0)	--
Other neurological disorders	--	--	1 (0–2)	1 (0–1)	1 (0–1)	1 (1–2)	2 (1–4)	2 (1–4)	2 (1–3)	2 (1–3)	2 (1–3)	2 (1–3)	2 (1–3)	2 (2–4)	2 (1–4)	2 (1–4)	3 (2–5)	2 (1–4)	2 (1–3)	1 (1–2)	1 (0–1)	0 (0–1)	--
Eating disorders	--	--	--	--	0 (0–0)	0 (0–0)	0 (0–0)	0 (0–0)	0 (0–0)	0 (0–0)	0 (0–0)	0 (0–0)	0 (0–0)	--	--	--	--	--	--	--	--	--	--
Anorexia nervosa	--	--	--	--	0 (0–0)	0 (0–0)	0 (0–0)	0 (0–0)	0 (0–0)	0 (0–0)	0 (0–0)	0 (0–0)	0 (0–0)	--	--	--	--	--	--	--	--	--	--
Bulimia nervosa	--	--	--	--	0 (0–0)	0 (0–0)	0 (0–0)	0 (0–0)	0 (0–0)	0 (0–0)	0 (0–0)	0 (0–0)	0 (0–0)	--	--	--	--	--	--	--	--	--	--
Alcohol use disorders	--	--	--	--	--	0 (0–0)	1 (0–2)	3 (1–5)	5 (2–9)	10 (5–18)	15 (8–26)	18 (10–30)	20 (11–34)	21 (12–35)	17 (10–30)	13 (8–22)	8 (4–14)	5 (3–9)	3 (2–5)	2 (1–3)	1 (0–2)	0 (0–1)	--
Drug use disorders	0 (0–0)	0 (0–0)	0 (0–0)	0 (0–0)	0 (0–0)	0 (0–0)	1 (0–1)	2 (1–4)	3 (1–6)	4 (2–7)	5 (3–10)	6 (3–11)	5 (2–10)	4 (2–7)	4 (2–7)	3 (1–6)	2 (1–4)	2 (1–3)	2 (1–3)	1 (1–2)	0 (0–1)	0 (0–0)	--
Opioid use disorders	0 (0–0)	0 (0–0)	0 (0–0)	0 (0–0)	0 (0–0)	0 (0–0)	0 (0–1)	1 (0–2)	1 (0–2)	2 (1–4)	2 (1–4)	2 (1–5)	2 (1–4)	1 (0–3)	1 (0–2)	1 (0–2)	1 (0–2)	0 (0–1)	0 (0–1)	0 (0–1)	0 (0–0)	0 (0–0)	--
Cocaine use disorders	--	--	--	--	--	0 (0–0)	0 (0–0)	1 (0–1)	1 (0–2)	1 (0–1)	1 (1–3)	2 (1–4)	2 (1–4)	1 (1–3)	1 (1–2)	1 (0–2)	1 (0–2)	1 (0–1)	1 (0–1)	0 (0–0)	0 (0–0)	0 (0–0)	--
Amphetamine use disorders	--	--	--	--	--	0 (0–0)	0 (0–0)	0 (0–0)	0 (0–0)	0 (0–0)	0 (0–1)	1 (0–1)	0 (0–1)	1 (0–1)	0 (0–1)	0 (0–1)	0 (0–1)	0 (0–0)	0 (0–0)	0 (0–0)	0 (0–0)	0 (0–0)	--
Other drug use disorders	--	--	--	--	--	0 (0–0)	0 (0–0)	1 (0–1)	1 (0–2)	1 (0–2)	1 (1–3)	1 (1–3)	1 (0–2)	1 (0–2)	1 (0–1)	1 (0–1)	1 (0–1)	1 (0–1)	1 (0–1)	1 (0–2)	0 (0–1)	0 (0–0)	--
Diabetes mellitus	0 (0–0)	0 (0–0)	2 (1–4)	2 (1–4)	1 (1–2)	1 (1–3)	6 (3–9)	12 (6–21)	19 (10–31)	27 (16–46)	39 (22–62)	62 (36–98)	84 (51–135)	135 (80–208)	155 (92–252)	201 (120–313)	198 (123–304)	203 (125–312)	155 (95–236)	114 (69–175)	71 (42–112)	31 (18–47)	--
Diabetes mellitus type 1	0 (0–0)	0 (0–0)	2 (1–4)	2 (1–4)	1 (1–2)	1 (1–3)	3 (1–5)	6 (2–10)	9 (4–16)	6 (3–11)	9 (4–17)	14 (7–27)	4 (2–8)	7 (3–13)	8 (4–16)	9 (4–17)	10 (5–18)	8 (4–15)	7 (3–12)	4 (2–7)	3 (1–6)	1 (1–3)	--
Diabetes mellitus type 2	--	--	--	--	--	--	3 (1–5)	6 (3–12)	10 (5–18)	21 (11–38)	30 (15–49)	47 (26–77)	80 (48–129)	128 (76–199)	147 (87–239)	192 (115–299)	188 (117–287)	195 (119–301)	149 (91–223)	111 (66–169)	68 (40–107)	30 (18–45)	--
Chronic kidney disease	--	--	5 (2–10)	6 (3–13)	6 (3–10)	4 (2–6)	7 (4–13)	11 (5–19)	16 (8–28)	23 (11–39)	34 (17–59)	49 (25–86)	72 (38–121)	101 (54–167)	115 (66–188)	134 (76–212)	134 (77–222)	143 (88–224)	114 (71–173)	105 (65–169)	81 (52–124)	41 (26–63)	--
Chronic kidney disease due to diabetes mellitus type 1	--	--	0 (0–0)	0 (0–0)	0 (0–0)	0 (0–0)	0 (0–0)	1 (0–1)	2 (1–4)	4 (1–8)	7 (3–14)	10 (4–19)	12 (4–24)	12 (4–27)	9 (2–23)	8 (2–19)	6 (2–15)	5 (2–12)	3 (1–6)	1 (0–3)	0 (0–1)	0 (0–0)	--
Chronic kidney disease due to diabetes mellitus type 2	--	--	--	--	--	--	0 (0–0)	0 (0–1)	1 (0–1)	1 (0–3)	3 (1–6)	5 (2–12)	12 (4–23)	22 (9–42)	30 (12–56)	37 (16–67)	38 (18–67)	41 (21–71)	33 (17–57)	30 (16–53)	22 (11–39)	11 (5–19)	--
Chronic kidney disease due to hypertension	--	--	--	--	--	--	0 (0–0)	0 (0–1)	1 (0–2)	2 (1–4)	3 (1–6)	5 (2–10)	9 (4–18)	16 (8–31)	23 (11–41)	31 (16–56)	36 (18–63)	41 (22–70)	37 (21–61)	38 (22–63)	34 (20–55)	19 (11–31)	--
Chronic kidney disease due to glomerulonephritis	--	--	1 (0–3)	2 (1–5)	2 (1–4)	2 (1–3)	4 (2–7)	5 (2–10)	7 (3–14)	9 (4–17)	13 (6–23)	17 (7–32)	22 (10–41)	29 (13–53)	31 (14–59)	32 (14–59)	28 (11–57)	26 (11–49)	18 (7–35)	14 (6–29)	9 (3–17)	4 (1–7)	--
Chronic kidney disease due to other and unspecified causes	--	--	3 (1–7)	4 (2–9)	3 (2–6)	2 (1–4)	3 (1–6)	4 (2–8)	6 (2–10)	7 (3–13)	9 (3–18)	12 (5–25)	17 (7–33)	21 (9–41)	23 (9–46)	26 (11–50)	27 (12–53)	30 (14–54)	23 (11–45)	21 (10–39)	16 (8–30)	8 (4–15)	--
Acute glomerulonephritis	--	--	0 (0–0)	0 (0–0)	0 (0–0)	0 (0–0)	0 (0–0)	0 (0–0)	0 (0–0)	0 (0–0)	0 (0–0)	0 (0–0)	0 (0–0)	0 (0–0)	0 (0–0)	0 (0–0)	0 (0–0)	0 (0–0)	0 (0–0)	0 (0–0)	0 (0–0)	0 (0–0)	--
Skin and subcutaneous diseases	--	--	1 (0–1)	0 (0–0)	0 (0–0)	0 (0–0)	0 (0–0)	0 (0–0)	0 (0–1)	0 (0–1)	0 (0–1)	1 (0–1)	1 (0–2)	2 (1–3)	1 (1–3)	1 (1–3)	3 (1–5)	2 (1–3)	2 (1–4)	3 (1–4)	3 (1–5)	1 (1–3)	--
Bacterial skin diseases	--	--	1 (0–1)	0 (0–0)	0 (0–0)	0 (0–0)	0 (0–0)	0 (0–0)	0 (0–1)	0 (0–1)	0 (0–1)	1 (0–1)	1 (0–2)	1 (1–2)	1 (1–2)	1 (1–2)	2 (1–3)	1 (1–3)	1 (1–3)	2 (1–3)	2 (1–3)	1 (0–2)	--
Cellulitis	--	--	0 (0–0)	0 (0–0)	0 (0–0)	0 (0–0)	0 (0–0)	0 (0–0)	0 (0–0)	0 (0–0)	0 (0–0)	0 (0–0)	0 (0–0)	0 (0–0)	0 (0–0)	0 (0–0)	0 (0–0)	0 (0–0)	0 (0–0)	0 (0–0)	0 (0–0)	0 (0–0)	--
Pyoderma	--	--	1 (0–1)	0 (0–0)	0 (0–0)	0 (0–0)	0 (0–0)	0 (0–0)	0 (0–1)	0 (0–1)	0 (0–1)	1 (0–1)	1 (0–2)	1 (1–2)	1 (1–2)	1 (1–2)	2 (1–3)	1 (1–2)	1 (1–3)	2 (1–3)	2 (1–3)	1 (0–2)	--
Decubitus ulcer	--	--	--	0 (0–0)	0 (0–0)	0 (0–0)	0 (0–0)	0 (0–0)	0 (0–0)	0 (0–0)	0 (0–0)	0 (0–0)	0 (0–0)	0 (0–1)	0 (0–0)	0 (0–1)	1 (0–2)	1 (0–1)	1 (0–1)	1 (0–2)	1 (0–2)	1 (0–1)	--
Other skin and subcutaneous diseases	--	--	0 (0–0)	0 (0–0)	0 (0–0)	0 (0–0)	0 (0–0)	0 (0–0)	0 (0–0)	0 (0–0)	0 (0–0)	0 (0–0)	0 (0–0)	0 (0–0)	0 (0–0)	0 (0–0)	0 (0–0)	0 (0–0)	0 (0–0)	0 (0–0)	0 (0–0)	0 (0–0)	--
Musculoskeletal disorders	--	--	--	--	0 (0–0)	0 (0–1)	0 (0–1)	1 (1–2)	1 (0–2)	2 (1–3)	2 (1–3)	2 (1–3)	2 (1–3)	2 (1–3)	2 (1–3)	2 (1–4)	3 (1–5)	3 (2–6)	3 (1–5)	3 (1–6)	22 (8–36)	12 (4–20)	--
Rheumatoid arthritis	--	--	--	--	0 (0–0)	0 (0–0)	0 (0–0)	0 (0–0)	0 (0–0)	0 (0–0)	0 (0–0)	0 (0–0)	0 (0–0)	0 (0–1)	0 (0–1)	0 (0–1)	1 (0–1)	1 (1–2)	1 (0–2)	1 (1–2)	8 (3–16)	5 (2–9)	--
Other musculoskeletal disorders	--	--	--	--	0 (0–0)	0 (0–1)	0 (0–1)	1 (1–2)	1 (0–1)	2 (1–3)	2 (1–3)	2 (1–3)	2 (1–3)	2 (1–3)	2 (1–3)	2 (1–4)	2 (1–4)	2 (1–4)	2 (1–3)	2 (1–4)	14 (4–24)	7 (2–13)	--
Congenital anomalies	171 (84–349)	49 (24–97)	108 (46–226)	35 (14–79)	11 (6–22)	7 (4–13)	5 (3–9)	5 (3–9)	4 (2–7)	3 (2–6)	3 (2–5)	3 (2–4)	2 (1–4)	3 (1–5)	2 (1–3)	1 (1–2)	1 (1–2)	--	--	--	--	--	--
Neural tube defects	9 (2–29)	2 (0–5)	3 (1–9)	1 (0–5)	0 (0–0)	0 (0–0)	0 (0–0)	0 (0–0)	0 (0–0)	0 (0–0)	0 (0–0)	0 (0–0)	0 (0–0)	0 (0–0)	0 (0–0)	0 (0–0)	0 (0–0)	--	--	--	--	--	--
Congenital heart anomalies	82 (35–174)	23 (10–44)	58 (22–120)	19 (7–44)	7 (3–14)	5 (2–9)	3 (1–5)	3 (1–5)	3 (1–5)	2 (1–3)	2 (1–3)	2 (1–3)	1 (1–2)	1 (1–2)	1 (0–1)	1 (0–2)	1 (0–1)	--	--	--	--	--	--
Orofacial clefts	0 (0–1)	0 (0–0)	0 (0–0)	0 (0–0)	--	--	--	--	--	--	--	--	--	--	--	--	--	--	--	--	--	--	--
Down's syndrome	4 (1–9)	2 (1–4)	9 (3–22)	5 (1–12)	1 (0–2)	1 (0–2)	1 (0–2)	1 (0–2)	1 (0–2)	1 (0–2)	1 (0–2)	1 (0–1)	1 (0–1)	1 (0–1)	1 (0–2)	0 (0–1)	0 (0–0)	--	--	--	--	--	--
Other chromosomal abnormalities	2 (1–5)	1 (0–2)	1 (0–3)	0 (0–1)	0 (0–0)	0 (0–0)	0 (0–0)	0 (0–0)	0 (0–0)	0 (0–0)	0 (0–0)	0 (0–0)	0 (0–0)	0 (0–0)	0 (0–0)	0 (0–0)	0 (0–0)	--	--	--	--	--	--
Congenital musculoskeletal and limb anomalies	1 (1–2)	1 (0–1)	1 (0–2)	0 (0–1)	0 (0–0)	0 (0–0)	0 (0–0)	0 (0–0)	0 (0–0)	0 (0–0)	0 (0–0)	0 (0–0)	0 (0–0)	0 (0–0)	0 (0–0)	0 (0–0)	0 (0–0)	--	--	--	--	--	--
Urogenital congenital anomalies	2 (1–6)	1 (0–2)	1 (0–3)	0 (0–1)	0 (0–0)	0 (0–0)	0 (0–0)	0 (0–0)	0 (0–0)	0 (0–0)	0 (0–0)	0 (0–0)	0 (0–0)	0 (0–0)	0 (0–0)	0 (0–0)	0 (0–0)	--	--	--	--	--	--
Digestive congenital anomalies	32 (11–69)	11 (4–25)	15 (4–37)	2 (1–6)	1 (0–2)	0 (0–1)	0 (0–0)	0 (0–0)	0 (0–0)	0 (0–0)	0 (0–0)	0 (0–0)	0 (0–0)	0 (0–0)	0 (0–0)	0 (0–0)	0 (0–0)	--	--	--	--	--	--
Other congenital anomalies	38 (10–87)	9 (2–24)	21 (5–57)	7 (2–19)	3 (1–6)	1 (1–3)	1 (0–2)	1 (0–2)	0 (0–1)	0 (0–1)	0 (0–1)	0 (0–0)	0 (0–0)	0 (0–1)	0 (0–1)	0 (0–0)	0 (0–0)	--	--	--	--	--	--
Urinary diseases and male infertility	0 (0–0)	0 (0–0)	0 (0–1)	0 (0–1)	0 (0–1)	0 (0–1)	0 (0–1)	1 (0–2)	1 (0–1)	1 (0–2)	1 (1–2)	1 (1–3)	2 (1–3)	3 (1–6)	3 (2–6)	5 (2–9)	7 (3–11)	10 (5–16)	11 (5–19)	11 (6–19)	10 (5–17)	5 (3–8)	--
Urinary tract infections and interstitial nephritis	0 (0–0)	0 (0–0)	0 (0–1)	0 (0–0)	0 (0–0)	0 (0–0)	0 (0–1)	0 (0–1)	1 (0–1)	1 (0–1)	1 (0–1)	1 (0–2)	1 (0–2)	2 (1–4)	3 (1–5)	4 (2–7)	5 (2–9)	8 (4–13)	8 (4–15)	9 (5–15)	8 (4–14)	4 (2–6)	--
Urolithiasis	--	--	--	0 (0–0)	0 (0–0)	0 (0–0)	0 (0–0)	0 (0–0)	0 (0–0)	0 (0–0)	0 (0–0)	0 (0–0)	0 (0–0)	0 (0–0)	0 (0–1)	0 (0–0)	0 (0–0)	0 (0–1)	0 (0–1)	0 (0–0)	0 (0–0)	0 (0–0)	--
Other urinary diseases	0 (0–0)	0 (0–0)	0 (0–0)	0 (0–0)	0 (0–0)	0 (0–0)	0 (0–0)	0 (0–1)	0 (0–1)	0 (0–1)	1 (0–1)	1 (0–1)	1 (0–1)	1 (0–2)	1 (0–2)	1 (0–2)	2 (1–3)	2 (1–4)	2 (1–5)	2 (1–5)	2 (1–4)	1 (0–3)	--
Gynecological diseases	--	--	--	--	--	--	--	--	--	--	--	--	--	--	--	--	--	--	--	--	--	--	--
Uterine fibroids	--	--	--	--	--	--	--	--	--	--	--	--	--	--	--	--	--	--	--	--	--	--	--

Endometriosis	--	--	--	--	--	--	--	--	--	--	--	--	--	--	--	--	--	--	--	--	--	--	--
Genital prolapse	--	--	--	--	--	--	--	--	--	--	--	--	--	--	--	--	--	--	--	--	--	--	--
Other gynecological diseases	--	--	--	--	--	--	--	--	--	--	--	--	--	--	--	--	--	--	--	--	--	--	--
Hemoglobinopathies and hemolytic anaemias	0 (0–1)	0 (0–1)	3 (1–6)	7 (4–13)	7 (4–11)	5 (3–8)	4 (2–7)	6 (3–9)	5 (3–9)	5 (3–9)	4 (2–7)	4 (3–7)	3 (2–6)	3 (2–6)	3 (2–4)	3 (2–5)	3 (2–4)	2 (1–4)	2 (1–3)	2 (1–4)	3 (2–5)	2 (1–2)	--
Thalassemias	0 (0–0)	0 (0–0)	0 (0–0)	0 (0–0)	0 (0–0)	0 (0–0)	0 (0–0)	0 (0–0)	0 (0–0)	0 (0–0)	0 (0–0)	0 (0–0)	0 (0–0)	0 (0–0)	0 (0–0)	0 (0–0)	0 (0–0)	0 (0–0)	0 (0–0)	0 (0–0)	0 (0–0)	0 (0–0)	--
Sickle cell disorders	0 (0–1)	0 (0–1)	3 (1–6)	7 (3–13)	7 (4–10)	5 (3–8)	4 (2–6)	4 (2–7)	4 (2–6)	3 (2–6)	2 (1–4)	2 (1–4)	1 (1–3)	1 (0–2)	0 (0–1)	0 (0–0)	0 (0–0)	0 (0–0)	0 (0–0)	0 (0–0)	0 (0–0)	0 (0–0)	--
G6PD deficiency	0 (0–0)	0 (0–0)	0 (0–0)	0 (0–1)	0 (0–1)	0 (0–1)	1 (0–1)	1 (0–2)	2 (1–3)	2 (1–3)	1 (1–3)	2 (1–3)	2 (1–3)	2 (1–3)	2 (1–3)	2 (1–3)	1 (1–2)	1 (0–2)	0 (0–1)	0 (0–1)	0 (0–0)	0 (0–0)	--
Other hemoglobinopathies and hemolytic anaemias	0 (0–0)	0 (0–0)	0 (0–0)	0 (0–0)	0 (0–0)	0 (0–0)	0 (0–0)	0 (0–0)	0 (0–0)	0 (0–0)	0 (0–0)	0 (0–0)	0 (0–0)	0 (0–1)	1 (0–1)	1 (1–2)	1 (1–2)	2 (1–3)	2 (1–2)	2 (1–3)	3 (2–5)	1 (1–2)	--
Endocrine, metabolic, blood, and immune disorders	7 (1–17)	4 (1–9)	37 (11–80)	20 (5–42)	5 (1–10)	3 (1–7)	3 (1–8)	5 (1–13)	8 (2–18)	10 (3–23)	11 (3–25)	12 (4–27)	11 (4–24)	15 (5–36)	13 (5–30)	18 (6–42)	15 (5–34)	18 (6–42)	16 (5–37)	18 (6–41)	24 (6–54)	13 (3–29)	--
Sudden infant death syndrome	--	2 (1–5)	17 (6–37)	--	--	--	--	--	--	--	--	--	--	--	--	--	--	--	--	--	--	--	--
Transport injuries	1 (0–4)	1 (0–1)	4 (2–11)	23 (10–49)	33 (19–58)	38 (24–62)	127 (78–181)	273 (172–386)	279 (179–388)	277 (182–389)	233 (153–334)	206 (128–309)	170 (104–254)	164 (101–252)	122 (74–186)	120 (73–183)	84 (52–134)	64 (41–95)	44 (29–64)	31 (21–45)	31 (17–47)	10 (5–15)	--
Road injuries	1 (0–3)	1 (0–1)	4 (1–10)	22 (9–48)	33 (19–57)	38 (23–61)	124 (77–179)	268 (169–379)	274 (174–380)	271 (178–380)	228 (149–327)	202 (125–302)	166 (103–249)	160 (99–245)	119 (72–181)	117 (71–178)	83 (50–130)	62 (40–92)	43 (28–62)	31 (21–45)	30 (17–46)	10 (5–14)	--
Pedestrian road injuries	0 (0–1)	0 (0–0)	1 (0–2)	5 (2–12)	7 (3–15)	6 (2–11)	5 (2–10)	10 (5–19)	12 (6–22)	15 (7–27)	15 (8–27)	16 (8–29)	15 (7–27)	17 (8–32)	16 (8–28)	17 (8–30)	14 (7–26)	13 (6–22)	11 (6–18)	9 (5–14)	8 (4–14)	2 (1–4)	--
Cyclist road injuries	--	--	--	0 (0–1)	0 (0–1)	1 (0–2)	2 (1–3)	3 (2–6)	4 (2–7)	4 (2–8)	4 (2–7)	4 (2–7)	3 (1–5)	3 (2–6)	2 (1–4)	3 (1–5)	2 (1–4)	1 (0–2)	0 (0–0)	0 (0–0)	0 (0–0)	0 (0–0)	--
Motorcyclist road injuries	0 (0–0)	0 (0–0)	0 (0–0)	1 (0–2)	1 (1–3)	2 (1–4)	17 (8–31)	44 (22–77)	43 (21–75)	40 (18–73)	33 (15–58)	27 (12–50)	20 (9–36)	17 (8–33)	12 (5–22)	9 (3–19)	5 (2–11)	2 (1–4)	1 (0–1)	0 (0–1)	1 (0–1)	0 (0–0)	--
Motor vehicle road injuries	1 (0–2)	0 (0–1)	3 (1–8)	16 (7–36)	24 (14–43)	29 (18–47)	100 (61–145)	210 (132–299)	215 (136–307)	212 (140–296)	176 (114–252)	155 (96–233)	129 (78–191)	122 (74–190)	89 (53–137)	89 (53–137)	61 (36–98)	47 (30–71)	31 (20–46)	22 (14–32)	21 (11–34)	7 (4–11)	--
Other road injuries	0 (0–0)	0 (0–0)	0 (0–0)	0 (0–0)	0 (0–0)	0 (0–0)	0 (0–0)	1 (0–1)	0 (0–1)	0 (0–1)	0 (0–1)	0 (0–1)	1 (0–1)	0 (0–1)	0 (0–1)	0 (0–1)	0 (0–1)	0 (0–0)	0 (0–0)	0 (0–0)	0 (0–0)	0 (0–0)	--
Other transport injuries	0 (0–0)	0 (0–0)	0 (0–1)	1 (0–2)	1 (0–1)	1 (0–2)	2 (1–4)	5 (2–9)	5 (2–9)	6 (3–11)	5 (2–10)	5 (2–10)	4 (2–8)	4 (2–7)	3 (2–6)	3 (1–5)	2 (1–3)	2 (1–4)	1 (0–2)	0 (0–1)	0 (0–0)	0 (0–0)	--
Falls	0 (0–1)	0 (0–0)	1 (0–3)	2 (1–4)	2 (1–3)	2 (1–4)	4 (2–7)	8 (4–14)	9 (4–16)	9 (5–17)	10 (5–17)	11 (6–18)	11 (6–18)	15 (9–26)	14 (8–22)	18 (10–30)	15 (9–25)	21 (12–34)	19 (12–29)	26 (16–41)	31 (18–52)	20 (11–35)	--
Drowning	1 (0–2)	0 (0–1)	3 (1–6)	16 (7–31)	16 (8–26)	19 (11–29)	26 (13–45)	31 (15–52)	26 (13–43)	25 (12–42)	21 (10–36)	18 (9–31)	14 (7–25)	13 (7–23)	10 (5–17)	10 (5–17)	7 (3–11)	5 (3–9)	4 (2–7)	3 (1–5)	4 (1–7)	2 (0–4)	--
Fire, heat, and hot substances	0 (0–1)	0 (0–1)	3 (1–7)	9 (4–19)	4 (2–8)	3 (1–5)	2 (1–4)	4 (2–7)	5 (2–8)	5 (3–10)	5 (2–8)	4 (2–8)	4 (2–8)	4 (2–7)	4 (2–6)	5 (2–8)	4 (2–6)	4 (2–7)	3 (2–6)	4 (2–7)	5 (3–8)	3 (1–4)	--
Poisonings	0 (0–1)	0 (0–2)	2 (0–5)	5 (2–10)	2 (1–3)	1 (0–3)	1 (0–2)	2 (1–5)	3 (1–5)	3 (1–5)	3 (1–5)	2 (1–4)	2 (1–4)	2 (1–3)	1 (1–2)	1 (0–2)	1 (0–1)	1 (0–1)	1 (0–1)	1 (0–1)	1 (0–1)	0 (0–1)	--
Poisoning by carbon monoxide	0 (0–0)	0 (0–0)	0 (0–0)	0 (0–2)	0 (0–1)	0 (0–1)	1 (0–1)	1 (0–2)	1 (0–2)	1 (0–2)	1 (0–2)	1 (0–2)	1 (0–2)	0 (0–1)	0 (0–1)	0 (0–1)	0 (0–0)	0 (0–0)	0 (0–0)	0 (0–0)	0 (0–0)	0 (0–0)	--
Poisoning by other means	0 (0–1)	0 (0–1)	2 (0–5)	4 (2–9)	1 (1–3)	1 (0–2)	1 (0–1)	1 (1–3)	2 (1–4)	2 (1–4)	2 (1–3)	1 (0–3)	1 (1–3)	1 (1–3)	1 (0–2)	1 (0–2)	1 (0–1)	0 (0–1)	0 (0–1)	0 (0–1)	1 (0–1)	0 (0–1)	--
Exposure to mechanical forces	0 (0–1)	1 (0–4)	1 (0–5)	2 (1–5)	3 (1–5)	3 (1–5)	10 (5–19)	21 (9–41)	20 (9–38)	19 (8–40)	16 (7–32)	15 (7–29)	11 (5–22)	10 (5–21)	8 (3–17)	6 (3–12)	4 (2–7)	3 (1–5)	2 (1–3)	1 (1–3)	2 (1–4)	1 (0–2)	--
Unintentional firearm injuries	0 (0–0)	0 (0–2)	1 (0–3)	1 (0–2)	1 (0–2)	2 (1–3)	9 (4–16)	19 (8–38)	18 (7–33)	17 (7–33)	14 (6–28)	13 (6–24)	9 (4–18)	8 (3–17)	6 (3–12)	5 (2–9)	3 (1–5)	2 (1–3)	1 (0–2)	1 (0–2)	1 (0–2)	0 (0–1)	--
Other exposure to mechanical forces	0 (0–1)	0 (0–2)	0 (0–3)	1 (0–3)	2 (1–3)	1 (0–2)	1 (0–4)	2 (1–6)	2 (0–7)	3 (1–8)	2 (0–6)	2 (1–6)	2 (1–5)	2 (1–6)	2 (1–5)	2 (1–4)	1 (0–2)	1 (0–2)	1 (0–1)	1 (0–1)	1 (0–2)	0 (0–1)	--
Adverse effects of medical treatment	2 (1–3)	1 (1–2)	4 (1–8)	3 (1–6)	2 (1–3)	2 (1–3)	2 (1–3)	4 (2–7)	4 (2–9)	4 (2–10)	5 (2–11)	6 (2–13)	6 (3–12)	9 (4–20)	9 (4–19)	12 (6–22)	12 (6–23)	12 (7–22)	12 (6–21)	11 (6–19)	10 (6–15)	5 (3–7)	--
Animal contact	0 (0–0)	0 (0–0)	0 (0–0)	0 (0–0)	0 (0–0)	0 (0–0)	0 (0–0)	0 (0–0)	0 (0–0)	0 (0–0)	0 (0–0)	0 (0–0)	0 (0–0)	0 (0–0)	0 (0–0)	0 (0–0)	0 (0–0)	0 (0–0)	0 (0–0)	0 (0–0)	0 (0–0)	0 (0–0)	--
Venomous animal contact	0 (0–0)	0 (0–0)	0 (0–0)	0 (0–0)	0 (0–0)	0 (0–0)	0 (0–0)	0 (0–0)	0 (0–0)	0 (0–0)	0 (0–0)	0 (0–0)	0 (0–0)	0 (0–0)	0 (0–0)	0 (0–0)	0 (0–0)	0 (0–0)	0 (0–0)	0 (0–0)	0 (0–0)	0 (0–0)	--
Non-venomous animal contact	0 (0–0)	0 (0–0)	0 (0–0)	0 (0–0)	0 (0–0)	0 (0–0)	0 (0–0)	0 (0–0)	0 (0–0)	0 (0–0)	0 (0–0)	0 (0–0)	0 (0–0)	0 (0–0)	0 (0–0)	0 (0–0)	0 (0–0)	0 (0–0)	0 (0–0)	0 (0–0)	0 (0–0)	0 (0–0)	--
Foreign body	5 (2–9)	3 (2–6)	22 (8–46)	18 (9–33)	9 (5–16)	11 (6–18)	6 (3–10)	12 (6–20)	14 (7–23)	14 (7–22)	14 (7–23)	15 (7–24)	14 (7–23)	15 (8–26)	14 (7–23)	16 (8–29)	15 (8–26)	16 (8–25)	12 (7–19)	11 (6–16)	6 (3–9)	3 (2–4)	--
Pulmonary aspiration and foreign body in airway	5 (2–9)	3 (2–6)	22 (8–46)	18 (9–32)	9 (5–16)	11 (6–18)	6 (3–10)	12 (5–19)	14 (7–22)	14 (7–22)	13 (6–22)	15 (7–24)	14 (7–23)	15 (8–26)	13 (7–23)	16 (8–28)	15 (8–25)	15 (7–25)	12 (6–18)	10 (5–15)	5 (3–8)	2 (1–4)	--
Foreign body in other body part	0 (0–0)	0 (0–0)	0 (0–0)	0 (0–0)	0 (0–1)	0 (0–0)	0 (0–0)	0 (0–1)	0 (0–0)	0 (0–0)	0 (0–0)	0 (0–0)	0 (0–0)	0 (0–0)	0 (0–0)	0 (0–0)	0 (0–0)	1 (0–2)	1 (0–1)	1 (0–2)	1 (0–1)	0 (0–1)	--
Environmental heat and cold exposure	0 (0–0)	0 (0–0)	0 (0–0)	0 (0–0)	0 (0–0)	0 (0–0)	0 (0–1)	0 (0–1)	1 (0–1)	1 (0–1)	0 (0–1)	1 (0–1)	0 (0–1)	1 (0–1)	1 (0–1)	1 (0–1)	0 (0–1)	1 (0–1)	1 (0–1)	1 (0–1)	1 (0–1)	0 (0–1)	--
Other unintentional injuries	1 (0–1)	0 (0–0)	2 (1–3)	6 (2–12)	7 (3–14)	9 (3–17)	16 (6–32)	30 (10–59)	35 (12–69)	34 (11–70)	31 (9–63)	28 (9–54)	23 (8–47)	17 (6–32)	12 (4–24)	9 (3–19)	6 (2–12)	4 (2–9)	3 (1–5)	2 (1–3)	1 (1–2)	1 (0–1)	--
Self-harm	--	--	--	--	--	3 (1–5)	21 (12–35)	53 (28–86)	61 (34–97)	63 (34–102)	52 (29–88)	48 (27–83)	43 (25–70)	42 (25–72)	36 (22–61)	37 (22–58)	26 (16–43)	23 (14–37)	15 (10–24)	12 (7–20)	10 (6–17)	3 (2–5)	--
Self-harm by firearm	--	--	--	--	--	0 (0–1)	4 (1–9)	11 (4–22)	12 (5–23)	11 (4–22)	9 (4–19)	9 (4–18)	7 (3–14)	7 (3–14)	5 (2–10)	5 (2–10)	3 (1–7)	3 (1–6)	2 (1–4)	1 (1–3)	1 (0–2)	0 (0–1)	--
Self-harm by other specified means	--	--	--	--	--	2 (1–4)	18 (10–30)	42 (23–69)	49 (28–80)	53 (28–84)	43 (24–72)	39 (22–66)	36 (21–60)	35 (21–60)	31 (18–52)	32 (19–50)	23 (14–37)	20 (12–32)	14 (8–22)	11 (7–18)	9 (5–15)	3 (2–5)	--
Interpersonal violence	5 (2–12)	1 (0–2)	8 (3–19)	17 (6–37)	12 (6–22)	22 (12–39)	135 (76–223)	283 (163–434)	277 (170–423)	241 (145–374)	189 (110–302)	150 (83–253)	113 (64–192)	83 (44–142)	55 (28–98)	39 (20–70)	22 (11–40)	14 (7–27)	8 (4–15)	3 (2–6)	2 (1–4)	1 (0–1)	--
Physical violence by firearm	1 (0–2)	0 (0–0)	1 (0–3)	2 (1–6)	3 (1–6)	10 (4–19)	95 (49–161)	208 (114–325)	206 (123–320)	173 (100–276)	136 (76–221)	103 (53–178)	79 (41–138)	55 (27–98)	35 (16–62)	25 (12–45)	14 (6–26)	8 (3–16)	3 (1–7)	2 (1–3)	1 (0–2)	0 (0–0)	--
Physical violence by sharp object	1 (0–1)	0 (0–0)	0 (0–1)	1 (0–2)	1 (0–2)	2 (1–5)	29 (12–59)	60 (26–110)	57 (26–106)	54 (24–102)	41 (17–80)	35 (16–70)	24 (10–48)	18 (7–36)	12 (5–26)	8 (3–15)	4 (1–9)	3 (1–7)	2 (1–5)	1 (0–2)	1 (0–1)	0 (0–1)	--
Physical violence by other means	4 (2–9)	1 (0–2)	6 (2–15)	14 (5–31)	8 (3–15)	10 (4–18)	10 (4–21)	15 (7–29)	15 (7–27)	14 (6–25)	13 (5–27)	12 (5–25)	11 (4–21)	10 (4–21)	8 (3–16)	6 (3–13)	4 (2–9)	3 (1–6)	2 (1–5)	1 (0–2)	0 (0–1)	0 (0–0)	--
Executions and police conflict	0 (0–0)	0 (0–0)	0 (0–0)	0 (0–0)	0 (0–0)	0 (0–0)	0 (0–0)	0 (0–0)	0 (0–0)	0 (0–0)	0 (0–0)	0 (0–0)	0 (0–0)	0 (0–0)	0 (0–0)	0 (0–0)	0 (0–0)	0 (0–0)	0 (0–0)	0 (0–0)	0 (0–0)	0 (0–0)	--

SR Table 12: Grenada deaths by cause and age group for males																							
Cause	Early Neonatal	Late Neonatal	Post Neonatal	1 to 4	5 to 9	10 to 14	15 to 19	20 to 24	25 to 29	30 to 34	35 to 39	40 to 44	45 to 49	50 to 54	55 to 59	60 to 64	65 to 69	70 to 74	75 to 79	80 to 84	85 to 89	90 to 94	95 plus
All causes	6 (4–8)	1 (1–1)	3 (2–4)	1 (1–2)	1 (1–1)	1 (1–1)	3 (3–4)	6 (5–7)	6 (5–7)	7 (6–8)	8 (7–9)	11 (9–13)	19 (16–22)	29 (25–33)	41 (37–45)	48 (45–52)	59 (55–63)	64 (60–68)	64 (61–68)	34 (32–35)	14 (14–15)	6 (6–7)	--
Sexually transmitted infections excluding HIV	0 (0–0)	0 (0–0)	0 (0–0)	0 (0–0)	0 (0–0)	0 (0–0)	0 (0–0)	0 (0–0)	0 (0–0)	0 (0–0)	0 (0–0)	0 (0–0)	0 (0–0)	0 (0–0)	0 (0–0)	0 (0–0)	0 (0–0)	0 (0–0)	0 (0–0)	0 (0–0)	0 (0–0)	0 (0–0)	--
Syphilis	0 (0–0)	0 (0–0)	0 (0–0)	0 (0–0)	0 (0–0)	0 (0–0)	0 (0–0)	0 (0–0)	0 (0–0)	0 (0–0)	0 (0–0)	0 (0–0)	0 (0–0)	0 (0–0)	0 (0–0)	0 (0–0)	0 (0–0)	0 (0–0)	0 (0–0)	0 (0–0)	0 (0–0)	0 (0–0)	--
Chlamydial infection	--	--	--	--	--	0 (0–0)	0 (0–0)	0 (0–0)	0 (0–0)	0 (0–0)	0 (0–0)	0 (0–0)	0 (0–0)	0 (0–0)	0 (0–0)	0 (0–0)	0 (0–0)	0 (0–0)	0 (0–0)	0 (0–0)	0 (0–0)	0 (0–0)	--
Gonococcal infection	--	--	--	--	--	0 (0–0)	0 (0–0)	0 (0–0)	0 (0–0)	0 (0–0)	0 (0–0)	0 (0–0)	0 (0–0)	0 (0–0)	0 (0–0)	0 (0–0)	0 (0–0)	0 (0–0)	0 (0–0)	0 (0–0)	0 (0–0)	0 (0–0)	--
Other sexually transmitted infections	--	--	--	--	--	0 (0–0)	0 (0–0)	0 (0–0)	0 (0–0)	0 (0–0)	0 (0–0)	0 (0–0)	0 (0–0)	0 (0–0)	0 (0–0)	0 (0–0)	0 (0–0)	0 (0–0)	0 (0–0)	0 (0–0)	0 (0–0)	0 (0–0)	--
Tuberculosis	--	--	0 (0–0)	0 (0–0)	0 (0–0)	0 (0–0)	0 (0–0)	0 (0–0)	0 (0–0)	0 (0–0)	0 (0–0)	0 (0–0)	0 (0–0)	0 (0–0)	0 (0–0)	0 (0–0)	0 (0–0)	0 (0–0)	0 (0–0)	0 (0–0)	0 (0–0)	0 (0–0)	--
Drug-susceptible tuberculosis	--	--	0 (0–0)	0 (0–0)	0 (0–0)	0 (0–0)	0 (0–0)	0 (0–0)	0 (0–0)	0 (0–0)	0 (0–0)	0 (0–0)	0 (0–0)	0 (0–0)	0 (0–0)	0 (0–0)	0 (0–0)	0 (0–0)	0 (0–0)	0 (0–0)	0 (0–0)	0 (0–0)	--
Multidrug-resistant tuberculosis without extensive drug resistance	--	--	0 (0–0)	0 (0–0)	0 (0–0)	0 (0–0)	0 (0–0)	0 (0–0)	0 (0–0)	0 (0–0)	0 (0–0)	0 (0–0)	0 (0–0)	0 (0–0)	0 (0–0)	0 (0–0)	0 (0–0)	0 (0–0)	0 (0–0)	0 (0–0)	0 (0–0)	0 (0–0)	--
Extensively drug-resistant tuberculosis	--	--	0 (0–0)	0 (0–0)	0 (0–0)	0 (0–0)	0 (0–0)	0 (0–0)	0 (0–0)	0 (0–0)	0 (0–0)	0 (0–0)	0 (0–0)	0 (0–0)	0 (0–0)	0 (0–0)	0 (0–0)	0 (0–0)	0 (0–0)	0 (0–0)	0 (0–0)	0 (0–0)	--
Lower respiratory infections	0 (0–0)	0 (0–0)	1 (0–1)	0 (0–0)	0 (0–0)	0 (0–0)	0 (0–0)	0 (0–0)	0 (0–0)	0 (0–0)	0 (0–0)	0 (0–1)	1 (0–1)	1 (1–1)	1 (1–2)	2 (1–2)	2 (2–3)	3 (2–4)	4 (3–5)	2 (2–3)	1 (1–2)	1 (1–1)	--
Upper respiratory infections	0 (0–0)	0 (0–0)	0 (0–0)	0 (0–0)	0 (0–0)	0 (0–0)	0 (0–0)	0 (0–0)	0 (0–0)	0 (0–0)	0 (0–0)	0 (0–0)	0 (0–0)	0 (0–0)	0 (0–0)	0 (0–0)	0 (0–0)	0 (0–0)	0 (0–0)	0 (0–0)	0 (0–0)	0 (0–0)	--
Otitis media	0 (0–0)	0 (0–0)	0 (0–0)	0 (0–0)	0 (0–0)	0 (0–0)	0 (0–0)	0 (0–0)	0 (0–0)	0 (0–0)	0 (0–0)	0 (0–0)	0 (0–0)	0 (0–0)	0 (0–0)	0 (0–0)	0 (0–0)	0 (0–0)	0 (0–0)	0 (0–0)	0 (0–0)	0 (0–0)	--
Diarrhoeal diseases	0 (0–0)	0 (0–0)	0 (0–0)	0 (0–0)	0 (0–0)	0 (0–0)	0 (0–0)	0 (0–0)	0 (0–0)	0 (0–0)	0 (0–0)	0 (0–0)	0 (0–0)	0 (0–0)	0 (0–0)	0 (0–0)	0 (0–0)	0 (0–0)	0 (0–0)	0 (0–0)	0 (0–0)	0 (0–0)	--
Typhoid fever	--	--	0 (0–0)	0 (0–0)	0 (0–0)	0 (0–0)	0 (0–0)	0 (0–0)	0 (0–0)	0 (0–0)	0 (0–0)	0 (0–0)	0 (0–0)	0 (0–0)	0 (0–0)	0 (0–0)	0 (0–0)	0 (0–0)	0 (0–0)	0 (0–0)	0 (0–0)	0 (0–0)	--
Paratyphoid fever	--	--	0 (0–0)	0 (0–0)	0 (0–0)	0 (0–0)	0 (0–0)	0 (0–0)	0 (0–0)	0 (0–0)	0 (0–0)	0 (0–0)	0 (0–0)	0 (0–0)	0 (0–0)	0 (0–0)	0 (0–0)	0 (0–0)	0 (0–0)	0 (0–0)	0 (0–0)	0 (0–0)	--
iNTS	--	0 (0–0)	0 (0–0)	0 (0–0)	0 (0–0)	0 (0–0)	0 (0–0)	0 (0–0)	0 (0–0)	0 (0–0)	0 (0–0)	0 (0–0)	0 (0–0)	0 (0–0)	0 (0–0)	0 (0–0)	0 (0–0)	0 (0–0)	0 (0–0)	0 (0–0)	0 (0–0)	0 (0–0)	--
Other intestinal infectious diseases	--	--	0 (0–0)	0 (0–0)	0 (0–0)	0 (0–0)	0 (0–0)	0 (0–0)	0 (0–0)	0 (0–0)	0 (0–0)	0 (0–0)	0 (0–0)	0 (0–0)	0 (0–0)	0 (0–0)	0 (0–0)	0 (0–0)	0 (0–0)	0 (0–0)	0 (0–0)	0 (0–0)	--
Malaria	0 (0–0)	0 (0–0)	0 (0–0)	0 (0–0)	0 (0–0)	0 (0–0)	0 (0–0)	0 (0–0)	0 (0–0)	0 (0–0)	0 (0–0)	0 (0–0)	0 (0–0)	0 (0–0)	0 (0–0)	0 (0–0)	0 (0–0)	0 (0–0)	0 (0–0)	0 (0–0)	0 (0–0)	0 (0–0)	--
Chagas disease	--	--	0 (0–0)	0 (0–0)	0 (0–0)	0 (0–0)	0 (0–0)	0 (0–0)	0 (0–0)	0 (0–0)	0 (0–0)	0 (0–0)	0 (0–0)	0 (0–0)	0 (0–0)	0 (0–0)	0 (0–0)	0 (0–0)	0 (0–0)	0 (0–0)	0 (0–0)	0 (0–0)	--
Visceral leishmaniasis	--	--	0 (0–0)	0 (0–0)	0 (0–0)	0 (0–0)	0 (0–0)	0 (0–0)	0 (0–0)	0 (0–0)	0 (0–0)	0 (0–0)	0 (0–0)	0 (0–0)	0 (0–0)	0 (0–0)	0 (0–0)	0 (0–0)	0 (0–0)	0 (0–0)	0 (0–0)	0 (0–0)	--
African trypanosomiasis	--	--	--	0 (0–0)	0 (0–0)	0 (0–0)	0 (0–0)	0 (0–0)	0 (0–0)	0 (0–0)	0 (0–0)	0 (0–0)	0 (0–0)	0 (0–0)	0 (0–0)	0 (0–0)	0 (0–0)	0 (0–0)	0 (0–0)	0 (0–0)	0 (0–0)	0 (0–0)	--
Schistosomiasis	--	--	0 (0–0)	0 (0–0)	0 (0–0)	0 (0–0)	0 (0–0)	0 (0–0)	0 (0–0)	0 (0–0)	0 (0–0)	0 (0–0)	0 (0–0)	0 (0–0)	0 (0–0)	0 (0–0)	0 (0–0)	0 (0–0)	0 (0–0)	0 (0–0)	0 (0–0)	0 (0–0)	--
Cysticercosis	--	--	--	0 (0–0)	0 (0–0)	0 (0–0)	0 (0–0)	0 (0–0)	0 (0–0)	0 (0–0)	0 (0–0)	0 (0–0)	0 (0–0)	0 (0–0)	0 (0–0)	0 (0–0)	0 (0–0)	0 (0–0)	0 (0–0)	0 (0–0)	0 (0–0)	0 (0–0)	--
Cystic echinococcosis	--	--	--	0 (0–0)	0 (0–0)	0 (0–0)	0 (0–0)	0 (0–0)	0 (0–0)	0 (0–0)	0 (0–0)	0 (0–0)	0 (0–0)	0 (0–0)	0 (0–0)	0 (0–0)	0 (0–0)	0 (0–0)	0 (0–0)	0 (0–0)	0 (0–0)	0 (0–0)	--
Dengue	0 (0–0)	0 (0–0)	0 (0–0)	0 (0–0)	0 (0–0)	0 (0–0)	0 (0–0)	0 (0–0)	0 (0–0)	0 (0–0)	0 (0–0)	0 (0–0)	0 (0–0)	0 (0–0)	0 (0–0)	0 (0–0)	0 (0–0)	0 (0–0)	0 (0–0)	0 (0–0)	0 (0–0)	0 (0–0)	--
Yellow fever	--	0 (0–0)	0 (0–0)	0 (0–0)	0 (0–0)	0 (0–0)	0 (0–0)	0 (0–0)	0 (0–0)	0 (0–0)	0 (0–0)	0 (0–0)	0 (0–0)	0 (0–0)	0 (0–0)	0 (0–0)	0 (0–0)	0 (0–0)	0 (0–0)	0 (0–0)	0 (0–0)	0 (0–0)	--
Rabies	--	--	0 (0–0)	0 (0–0)	0 (0–0)	0 (0–0)	0 (0–0)	0 (0–0)	0 (0–0)	0 (0–0)	0 (0–0)	0 (0–0)	0 (0–0)	0 (0–0)	0 (0–0)	0 (0–0)	0 (0–0)	0 (0–0)	0 (0–0)	0 (0–0)	0 (0–0)	0 (0–0)	--
Ascariasis	--	--	0 (0–0)	0 (0–0)	0 (0–0)	0 (0–0)	0 (0–0)	0 (0–0)	0 (0–0)	0 (0–0)	0 (0–0)	0 (0–0)	0 (0–0)	0 (0–0)	0 (0–0)	0 (0–0)	0 (0–0)	0 (0–0)	0 (0–0)	0 (0–0)	0 (0–0)	0 (0–0)	--
Zika virus disease	0 (0–0)	0 (0–0)	0 (0–0)	0 (0–0)	0 (0–0)	0 (0–0)	0 (0–0)	0 (0–0)	0 (0–0)	0 (0–0)	0 (0–0)	0 (0–0)	0 (0–0)	0 (0–0)	0 (0–0)	0 (0–0)	0 (0–0)	0 (0–0)	0 (0–0)	0 (0–0)	0 (0–0)	0 (0–0)	--
Other neglected tropical diseases	0 (0–0)	0 (0–0)	0 (0–0)	0 (0–0)	0 (0–0)	0 (0–0)	0 (0–0)	0 (0–0)	0 (0–0)	0 (0–0)	0 (0–0)	0 (0–0)	0 (0–0)	0 (0–0)	0 (0–0)	0 (0–0)	0 (0–0)	0 (0–0)	0 (0–0)	0 (0–0)	0 (0–0)	0 (0–0)	--
Meningitis	0 (0–0)	0 (0–0)	0 (0–0)	0 (0–0)	0 (0–0)	0 (0–0)	0 (0–0)	0 (0–0)	0 (0–0)	0 (0–0)	0 (0–0)	0 (0–0)	0 (0–0)	0 (0–0)	0 (0–0)	0 (0–0)	0 (0–0)	0 (0–0)	0 (0–0)	0 (0–0)	0 (0–0)	0 (0–0)	--
Encephalitis	0 (0–0)	0 (0–0)	0 (0–0)	0 (0–0)	0 (0–0)	0 (0–0)	0 (0–0)	0 (0–0)	0 (0–0)	0 (0–0)	0 (0–0)	0 (0–0)	0 (0–0)	0 (0–0)	0 (0–0)	0 (0–0)	0 (0–0)	0 (0–0)	0 (0–0)	0 (0–0)	0 (0–0)	0 (0–0)	--
Diphtheria	--	--	0 (0–0)	0 (0–0)	0 (0–0)	0 (0–0)	0 (0–0)	0 (0–0)	0 (0–0)	0 (0–0)	0 (0–0)	0 (0–0)	0 (0–0)	0 (0–0)	0 (0–0)	--	--	--	--	--	--	--	--
Whooping cough	--	--	0 (0–0)	0 (0–0)	0 (0–0)	0 (0–0)	0 (0–0)	0 (0–0)	0 (0–0)	0 (0–0)	0 (0–0)	0 (0–0)	0 (0–0)	0 (0–0)	0 (0–0)	--	--	--	--	--	--	--	--
Tetanus	0 (0–0)	0 (0–0)	0 (0–0)	0 (0–0)	0 (0–0)	0 (0–0)	0 (0–0)	0 (0–0)	0 (0–0)	0 (0–0)	0 (0–0)	0 (0–0)	0 (0–0)	0 (0–0)	0 (0–0)	0 (0–0)	0 (0–0)	0 (0–0)	0 (0–0)	0 (0–0)	0 (0–0)	0 (0–0)	--
Measles	0 (0–0)	0 (0–0)	0 (0–0)	0 (0–0)	0 (0–0)	0 (0–0)	0 (0–0)	0 (0–0)	0 (0–0)	0 (0–0)	0 (0–0)	0 (0–0)	0 (0–0)	0 (0–0)	0 (0–0)	0 (0–0)	0 (0–0)	0 (0–0)	0 (0–0)	0 (0–0)	0 (0–0)	0 (0–0)	--
[truncated: 2,156,761 more chars]
